# Quantitative Proteome Analysis of Temporally Resolved Phagosomes Following Uptake Via Key Phagocytic Receptors* [Fn FN2]

**DOI:** 10.1074/mcp.M114.044594

**Published:** 2015-05

**Authors:** Brian D. Dill, Marek Gierlinski, Anetta Härtlova, Alba González Arandilla, Manman Guo, Rosemary G. Clarke, Matthias Trost

**Affiliations:** From the ‡MRC Protein Phosphorylation and Ubiquitylation Unit,; §Division of Computational Biology and; ¶Division of Cell Signalling and Immunology, University of Dundee, Scotland, DD1 5EH, United Kingdom

## Abstract

Macrophages operate at the forefront of innate immunity and their discrimination of foreign *versus* “self” particles is critical for a number of responses including efficient pathogen killing, antigen presentation, and cytokine induction. In order to efficiently destroy the particles and detect potential threats, macrophages express an array of receptors to sense and phagocytose prey particles. In this study, we accurately quantified a proteomic time-course of isolated phagosomes from murine bone marrow-derived macrophages induced by particles conjugated to seven different ligands representing pathogen-associated molecular patterns, immune opsonins or apoptotic cell markers. We identified a clear functional differentiation over the three timepoints and detected subtle differences between certain ligand-phagosomes, indicating that triggering of receptors through a single ligand type has mild, but distinct, effects on phagosome proteome and function. Moreover, our data shows that uptake of phosphatidylserine-coated beads induces an active repression of NF-κB immune responses upon Toll-like receptor (TLR)-activation by recruitment of anti-inflammatory regulators to the phagosome. This data shows for the first time a systematic time-course analysis of bone marrow-derived macrophages phagosomes and how phagosome fate is regulated by the receptors triggered for phagocytosis.

Macrophages exist in many different tissue subsets, are extremely plastic in response to cytokines and pathogen-associated molecular patterns and perform a wide range of biological functions (1, 2). One of the most important functions of macrophages is phagocytosis, defined as the active uptake of large particles (>0.5 μm) by cells (3). Phagocytosis is an important cellular mechanism for almost all eukaryotes, highly conserved in evolution (4), and, in mammals, is a key part of the innate immune response to invading microorganisms. Moreover, during homeostasis and development, macrophages phagocytose apoptotic cells and cell debris to recycle cellular building blocks (5, 6). Phagocytosis is induced through the binding of particles as diverse as microbes, apoptotic cells, or even inert beads to cell surface receptors. After internalization, newly formed phagosomes engage in a maturation process that involves fusion with various organelles, including endosomes and ultimately lysosomes (7, 8). This leads to the formation of phagolysosomes that degrade the foreign matter. Antigens from the particle are presented via MHC class I and II molecules, bridging innate and adaptive immunity.

In order to effectively phagocytose the diverse types of particulates they can encounter, macrophages express a vast array of receptors to sense and respond to the different ligands; however, only a small subset are solely sufficient to trigger phagocytosis (9). The classic phagocytic receptors are the Fc receptor, which internalizes immunoglobulin-bound particles (10), and the complement receptors, which binds to complement-opsonized particles (11). Other well characterized ligands for phagocytic receptors include mannan, a polysaccharide common in bacterial membranes and fungal cell walls (12), that activates mannose receptors (13, 14); lipopolysaccharide (LPS)^1^, a glycolipid that constitutes the major portion of the outermost membrane of Gram-negative bacteria, that triggers CD14 as well as scavenger receptors and toll-like receptors (15–20); and phosphatidylserine (PS), a lipid normally restricted to the inner leaflet of eukaryotic plasma membranes, but exposed in the outer leaflet during apoptosis. PS provides an “eat me” signal for macrophage clearance (21, 22) and triggers a range of receptors including TIM-4, BAI1, and Stabilin-2 (23–27). Similarly, calreticulin, an endoplasmic reticulum protein that is also transported to the plasma membrane serves as a apoptotic signal has been proposed as a phagocytic ligand triggering the phagocytic receptor low-density lipoprotein receptor-related protein (LRP) (28–30).

Although it is established that phagosome function is affected by various activation states, including rate of maturation, degradative capacity, and antigen cross-presentation capabilities (31–33), controversy exists around whether phagosome activity can be controlled directly, without prior activation, by receptor engagement at the phagosome level during biogenesis (34–38). Here, we dissect the role that individual ligands play in controlling downstream phagosome maturation using a reductionist strategy of ligating single ligands to microparticles and analyzing resulting phagosomes by quantitative proteomics and fluorescent phagosome functional assays.

## MATERIALS AND METHODS

### 

#### 

##### Preparation of Ligand–Particle Conjugates for Phagosome Isolation and Functional Assays

In order to conjugate the selected ligands to particles for phagocytosis, a biotin–avidin system was used, by binding biotinylated ligands to avidinylated carboxyl-functionalized particles. Biotin-crosslinking to selected ligands was accomplished using biotin-LC-hydrazide (Pierce, Rockford, IL). IgG Fc fragment (Pierce), iC3b (Calbiochem, San Diego, CA), and calreticulin (expressed by in-house in Sf21 cells) were reacted with 1.25 mm biotin-LC-hydrazide in 100 mm MES buffer pH 5 with 6 mm 1-Ethyl-3-(-3-Dimethylaminopropyl)carbodiimide hydrochloride (EDC, Pierce) for 2 h, and unreacted reagent was removed by Zeba desalting columns (Pierce). Mannan (Sigma) and LPS (Sigma) were first oxidized in 1 or 10 mm sodium metaperiodate (Pierce), respectively, in 100 mm sodium acetate pH 5.5 on ice for 30 min. After desalting with Zeba columns (Pierce), these were incubated with 5 mm biotin-LC-hydrazide in phosphate buffered saline (PBS) for 2 h, before again desalting to remove unreacted reagent. Biotinylated ligands were stored in PBS with 0.05% sodium azide at 4 °C for no more than 3 weeks before use. Biotinylated PS, 1-oleoyl-2-(12-biotinyl(aminododecanoyl))-*sn*-glycero-3-phospho-l-serine, was purchased from Avanti Polar Lipids.

A total of 0.8 μm polystyrene particles (Estapore, Val de Fontenay, France) were used for phagosome isolation, whereas 3 μm silica particles (Kisker Biotech, Steinfurt, Germany) were used for functional assays. Carboxylated particles were conjugated to avidin by activation in EDC/NHS [250 mm 1-Ethyl-3-(3-dimethylaminopropyl)carbodiimide and 350 mm N-hydroxysuccinimide, Sigma] in 100 mm MES buffer, pH 5 for 15 min, followed by 2 h of conjugation with 1 mg/ml avidin in PBS. For functional assays, appropriate succinimide-fluorophores were conjugated to crosslinked avidin, as described below.

Biotinylated ligands were incubated with avidinylated particles overnight with shaking at 4 °C. All ligands and particles were stored in 0.05% sodium azide in PBS with 0.1% BSA and 0.05% Tween-20. Prior to inoculating macrophages, particles were washed extensively with PBS and diluted 1:50 (polystyrene particles) or 1:200 (silica particles) in complete media. Particle conjugates (with the exception of LPS) were deemed free of endotoxin using an assay with the RAW-Blue cell line (InvivoGen), as further described below.

##### Validation of Particle Coating

Flow cytometry was used to validate particle-to-particle consistency of crosslinking of avidin and coating of biotinylated ligands on avidinylated particles as well as the lack of multiparticle clumps. Fluorescent signal was measured using either biotin-conjugated R-phycoerythrin (PE), to validate avidinylation of carboxyl particles, or streptavidin-PE to validate the presence of biotinylated ligand (Life Technologies). Thus, streptavidin-PE is targeting free biotins on the ligand (those not attached to avidin on the polystyrene particle), and fluorescence signal will be directly related to how many biotins have been introduced on that ligand. Particles were labeled with a 1:10 dilution of the appropriate PE conjugate, incubated 1 h with shaking, washed extensively, and analyzed by flow cytometry using stained unconjugated particles as a negative control. Particles were analyzed on a LSR Fortessa flow cytometer (Becton Dickinson). Bead singlets were distinguished from doublets and clumps on the basis of forward scatter, side scatter, and fluorescence at 670 ± 14 nm with 640 nm excitation. Coating of beads with ligand was determined by quantifying PE fluorescence at 585 ± 40 nm with 488 nm excitation. Data analysis was performed using Flowjo software (Treestar, Ashland, OR).

To further validate attachment of each ligand type, we used specific horseradish peroxidase fused probes to confirm ligand attachment. Individual probes were used to detect ligand attachment to particles with horseradish peroxidase (HRP) development of tetramethylbenzidine (TMB, Life Technologies, Carlsbad, CA). Particles were compared against unligated control using the following HRP probes: anti-mouse IgG-HRP (ThermoFisher Scientific, Waltham, MA), anti-calreticulin (Cell Signaling, Danvers, MA) with HRP secondary antibody, iC3b: anti-iC3-HRP (Immune Systems), LPS: anti-lipid A (Abcam, Cambridge, MA) with HRP secondary, mannan: concanavalin-HRP (Sigma). PS was validated by the fluorescent probe annexin V- HRP or phycoerythrin (Life Technologies). Representative plots are shown in supplemental Fig. S1.

##### Bone Marrow Derived Macrophage (BMDM) Culture and Phagosome Isolation

BMDMs were derived from murine femurs by differentiation using growth media containing macrophage colony-stimulating factor (M-CSF), obtained from L929-conditioned media as detailed previously (39). Femurs were obtained from female wild-type C57BL/6 mice between 8 and 12 weeks of age. The use of animals in the University of Dundee is overseen by its Welfare and Ethical Use of Animals Committee. The University is a Licensed Establishment under the Animals (Scientific Procedures) Act 1986 and all animals used in this study were kept to the standards required by the UK Home Office and were euthanized by approved humane methods.

Following extraction of bone marrow and red blood cell lysis, cells were resuspended in Dulbecco's Modified Eagle Medium (DMEM; Life Technologies) supplemented with 10% heat-inactivated fetal bovine serum (FBS; Labtech, Uckfield, UK), 2 mm glutamine, 100 units/ml Penicillin-Streptomycin (Life Technologies), and 20% L929 conditioned supplement, prepared as described elsewhere (40). Cells were grown in tissue culture plasticware for 3 days, and then transferred to untreated 10 cm Petri dishes (BD Biosciences, San Jose, CA) for replication and differentiation for 7 days, yielding ∼15 dishes per mouse. Differentiation of macrophages was determined by high conversion to F4/80 positive cells.

Phagosomes were isolated as described previously (31, 41). In brief, polystyrene particles were inoculated onto macrophage monolayers for a 30 min pulse, at which point excess particles were removed and chased for a 0, 30, or 150 min incubation. Subsequently, cells were washed and broken, nuclei and cell debris removed, and phagosomes were isolated using a sucrose gradient. For each ligand and time point, phagosomes were isolated in triplicate, each from 14 dishes of macrophages.

##### Proteomic Sample Preparation and mTRAQ Labeling

Proteins from isolated phagosomes were solubilized in 1% sodium 3-[(2-methyl-2-undecyl-1,3-dioxolan-4-yl)methoxy]-1-propanesulfonate (commercially available as RapiGest, Waters, Milford, MA) in 50 mm Tris-HCl pH 8.0 with 5 mm tris(2-carboxyethyl)phosphine (TCEP, Pierce), heated at 60 °C for 5 min. Proteins were then alkylated in 10 mm iodoacetamide (Sigma) and excess reagent was quenched with 10 mm dithiothreitol (Sigma). As the extracted protein amount varied among the various ligand types, 4 μg of protein were processed for each sample, as quantified by EZQ (Life Technologies). After 10-fold dilution in 50 mm Tris pH 8.0, 5 mm calcium chloride, Trypsin Gold (Promega) was added at 1:100 for 4 h at 37 °C with shaking, and an additional dose for overnight incubation. Sodium 3-[(2-methyl-2-undecyl-1,3-dioxolan-4-yl)methoxy]-1-propanesulfonate was removed by adding 1% trifluoroacetic acid heated at 37 °C for 1 h and spinning at 1400 × *g* for 30 min. Peptides were desalted by solid phase extraction using Microspin C-18 (Nest Group), dried by SpeedVac, and labeled using mTRAQ (AB-Sciex, Framingham, MA). Each sample was labeled with the Δ8 mTRAQ reagent, and a pooled sample for a spike in reference sample (consisting of equal amounts of phagosomes from avidin, IgG, LPS, iC3b, and mannan at 30′/0, 30′/30′ and 30′/150′) was labeled with Δ0. NHS tyrosine and serine side reactions were removed using 100 mm hydroxylamine treatment for 4 h (42), and peptides were desalted as above. Peptides were checked for labeling efficiency of lysines and N termini (>97%) and side-labeling of serine, threonine, and tyrosine (<1%).

##### LC-MS/MS and Protein Identification and Quantitation

Mass spectrometric analyses were conducted similarly as previously described (43). In detail, samples of three biological replicates were analyzed on an Orbitrap Velos Pro mass spectrometer coupled to an Ultimate 3000 UHPLC system with a 50 cm Acclaim PepMap 100 analytical column (75 μm ID, 3 μm C18) in conjunction with a Pepmap trapping column (100 μm × 2 cm, 5 μm C18) (Thermo-Fisher Scientific). Acquisition settings were: lockmass of 445.120024, MS1 with 60,000 resolution, top 20 CID MS/MS using Rapid Scan, monoisotopic precursor selection, unassigned charge states and z = 1 rejected, dynamic exclusion of 60 s with repeat count 1. Six hour linear gradients were performed from 3% solvent B to 35% solvent B (solvent A: 0.1% formic acid, solvent B: 80% acetonitrile 0.08% formic acid) with a 30 min washing and re-equilibration step. For each run, 1 μg Δ0 pool and 1 μg Δ8 sample mixture was injected. The mass spectrometry raw data and the Maxquant output from this publication have been submitted to the PRIDE database (https://www.ebi.ac.uk/pride/archive/) and assigned the identifier PXD000772.

Protein identification and quantification were performed using MaxQuant Version 1.3.0.5 (44) with the following parameters: stable modification carbamidomethyl (C); variable modifications oxidation (M), acetylation (protein N terminus), deamidation (NQ); quantitation labels with mTRAQ Δ0 or Δ8 on N-terminal or lysines; maximum 5 modifications per peptide, trypsin as enzyme and 2 missed cleavages. Searches were conducted using a Uniprot-Trembl *Mus musculus* database (50,543 entries, downloaded October 18, 2012) plus common contaminants, including immunoglobulin (Ighg, Ighg1, Ighg2c, Ighg-3). Mass accuracy was set to 10 ppm for precursor ions and 0.5 Da for ion trap MS/MS data. Identifications were filtered at a 1% false-discovery rate (FDR) at the protein level, accepting a minimum peptide length of 7. Quantification used only razor and unique peptides, and required a minimum ratio count of 2. “Re-quantify” and “match between runs” were enabled. Normalized ratios were extracted for each protein/condition were extracted and used for downstream analyses.

##### Principle Component Analysis (PCA), Hierarchical Clustering, Boxplots, and GO Term Analyses

The authors collected log-ratios from seven ligands in three time points and three replicates each. This resulted in 1891 proteins with at least one valid datum across ligands, time points, and replicates. Then, we calculated the mean across all available replicates at each time point. In some cases no data were available in any of the replicates and subsequently no mean could be calculated. Data are visualized as time profiles (supplemental Fig. S3) for all 1891 proteins. For further processing, we selected only those proteins where replicate mean was available for each ligand and each time point. This resulted in 1337 proteins with full data, which were arranged either for each ligand/time point combination (resulting in a table of 21 × 1337 points) or for data averaged over three time points for each ligand (7 × 1337 points).

For clustering (Fig. 3) we selected 650 variable proteins for which the standard deviation across all ligands and time points was greater than a selected limit of 0.35, which, after iterative comparisons, provided optimal sample-dependent clustering and included known phagosomal markers. Average linkage hierarchical clustering with correlation distance was performed in two dimensions: on proteins and on ligand/timepoint combinations (Fig. 3). The protein tree was cut into three clusters based on the highest degree of divergence. As an alternative grouping method a PCA was performed both on ligand/time point combinations (Fig. 3*A*) and averaged ligand data (Fig. 3*B*).

For GO-term analysis we used GO-term/gene association file downloaded from the Mouse Genome Informatics Database (http://www.informatics.jax.org/), using all GO terms associated with the protein group members. These associations are visualized in Fig. 3. The GO-term enrichment in protein clusters was carried out with Fisher's exact test.

All bioinformatics analysis was done with custom Perl/PDL scripts. For clustering, we used Cluster 3.0 (45).

##### Phagosome Protein Network Mapping

A protein interaction map was generated in STRING v9.05 (46) using proteins found to be changing between IgG and PS phagosomes at any time point by a log_2_ ratio greater than 0.5 (*t* test *p* < 0.05). Interactions were extracted, manually curated, and visualized in Cytoscape 3.0.2 with an Organic layout (47). Nodes were colored according to the maximal log_2_ ratio across the timepoints and placed into functional groups.

##### Selected Reaction Monitoring (SRM)

Three to four peptides per protein of interest were chosen using PeptideAtlas (48), considering peptide uniqueness, adjusted sequence score, and avoiding cysteine or methionine residues when possible. Synthetic peptides (Thermo-Fisher Scientific) were used to define retention time and the optimal 6 transitions per peptide, based on optimal intensity of precursor charge state and fragment ions (transitions are defined in supplemental Table S4). Transition lists with collision energies, as well as and output peak areas were, handled using Skyline (49, 50). The SRM analysis was conducted on an ABSciex Q-Trap 5500 using identical chromatography parameters as above, except utilizing a 95 min gradient. The following settings were used for all transitions: a declustering potential (DP) of 120, an entrance potential (EP) of 10, collision cell exit potential (CXP) of 18.

##### Fluorescent Probe Phagosome Functional Assays

Fluorescent probe silica particles were prepared as above, using the following reactive dyes: BCECF for the pH assay, Alexafluor647 for the oxidative burst assay, Alexafluor405-S.E. for the proteolysis assay, and Alexafluor 647 for the uptake assay. For the oxidative burst and proteolysis assay particles, Oxyburst Green or DQ Red BSA was mixed at a 1:1 ratio with avidin during EDC/NHS conjugation (all dyes from Life Technologies).

These functional assays are adapted from methods from the Russell laboratory: DQ (32), pH (32), and OxyBurst Green (51). For each kinetic assay, day 9 BMDMs were plated onto 96-well plates at 1 E5 cells per ml 24 h prior to the experiment. Particles were diluted 1:200 in room temperature binding buffer (PBS pH 7.2 supplemented with 1 mm CaCl_2_, 2.7 mm KCl, 0.5 mm MgCl_2_, 5 mm dextrose, 10 mm hydroxyethyl piperazine ethane sulfonate [HEPES] and 5% FBS) and allowed to attach for 3 min. After removing noninternalized particles, and replacing with binding buffer, fluorescence was measured using a SpectraMax Gemini EM plate reader (Molecular Devices, Sunnyvale, CA) at 37 °C, with the maximal readings per well to allow reading time intervals of 1 min (for pH and oxidative burst) or 2 min (for proteolysis). Readouts are presented as a ratio of signal/control fluorophore for oxidative burst and proteolysis assays, whereas the pH assay is interpreted by the differential excitation ratio of the single BCECF fluorophore (490 or 450). The phagocytosis uptake assay is derived from a single time point 20 min after internalization, after quenching of noninternalized particles with Trypan blue (52); error bars indicate standard error. For each assay, eight biological replicates were used. The entire assay repeated three times to verify consistency, and representative experiments are presented. The vATPase inhibitor bafilomycin was used to inhibit maturation for a negative control in the oxidative burst, proteolysis, and pH assays. Uptake was inhibited using cytochalasin D, which inactivates actin polymerization.

##### Antigen Cross-presentation Assay

Ovalbumin (Sigma) and ligands were coupled to carboxylate silica beads at a 1:1 ratio (weight) as described above. Prior to the experiment, day 9 BMDMs were counted and seeded at 1 × 10^5^ cells per well in a 96-well plate. Ovalbumin-bead conjugates were added in 100 μl growth medium to the wells at a final concentration of 6 mg/ml and 1:50 dilution, respectively. After incubation of 4 h at 37 °C with 5% CO_2_, the cells were fixed in 1% paraformaldehyde for 15 min at room temperature and washed in PBS. B3Z cells were added to the wells at a concentration of 1 × 10^5^ cells/ml in growth medium. B3Z cells were allowed to proliferate for 24 h and were then lysed by M-PER mammalian protein extraction reagent (Thermo Scientific). After transferring the supernatants to another 96-well plate, 100 μl of luminescent β-galactosidase detection reagent (Clontech) was added in each well and incubated for 1 h, after which luminescence was measured. The experiment was repeated in three biological replicates.

##### RAW-Blue Quanti Blue Assay

The mouse macrophage cell line RAW-Blue (InvivoGen, Sunnyvale, CA), derived from RAW 264.7, was cultured in media as above, without addition of L929 supplementation. RAW-Blue (1 × 10^5^ cells/ml) were seeded in 96-wells tissue culture plates and inoculated with 3 μm avidin-conjugated silica beads, with or without coating with biotin-PS, for 30 min, and followed by replacing media with or without Pam_3_CSK_4_ (50 ng/ml, InvivoGen). Cell activation was determined after 8 h total incubation by the Quanti-Blue assay (InvivoGen), based on optical density measurement of 655 nm.

## RESULTS

### 

#### 

##### Conjugation of Macrophage Ligands to Polystyrene and Silica Particles

**Fig. 1.**
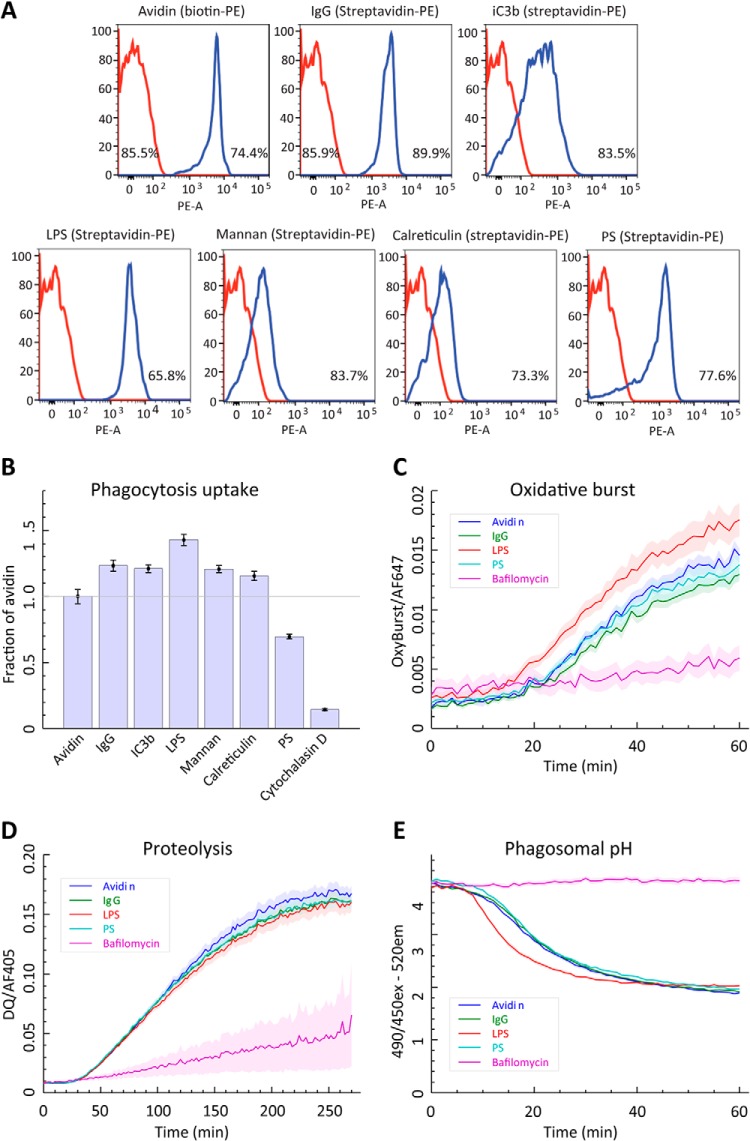
**Phagosome functional assays comparing ligand-bound particles.** *A*, Validation of particle coating. Flow cytometry analysis of particles showing ligand coating by the fluorescent marker biotin-PE (for avidin particles) or streptavidin-PE (for all ligand-conjugated particles). In each histogram, red is the negative control and blue is the indicated coated particle, and the percentage indicates the particles detectable as single particles. *B*, Phagocytic uptake. Fluorescence of ligand-particle conjugates were compared 20 min after adding particles, following Trypan blue quenching of noninternalized beads. The actin polymerization inhibitor cytochalasin D was used as a nonphagocytic negative control. Fluorescent values were normalized at 100% to avidin particles. Data shows that all particles are taken up at equal numbers except PS, which shows ∼30% reduction. *C*, Oxidative burst. The oxidation-activated fluorophore H_2_DCFDA was monitored for one hour after phagocytosis. Plotted values are a ratio of H_2_DCFDA fluorescence against a stably fluorescent control fluorophore. The vATPase inhibitor bafilomycin was included as a negative control. Data shows that except for LPS, all other tested ligands do not affect intraphagosomal oxidative burst. *D*, Proteolysis. Release of BODIPY quenching on cross-linked bovine serum albumin by proteolysis was measured for five hours after phagocytosis. Plotted values are a ratio of BODIPY fluorescence against a stably fluorescent control fluorophore. Bafilomycin was included as a negative control. Data shows that all other tested ligands do not affect phagosomal proteolysis. *E*, Phagosomal pH. The pH-dependent fluorophore BCECF was monitored for one hour after phagocytosis. The ratio of excitation wavelengths of 490 nm and 450 nm shows pH-dependent fluorescence at the excitation wavelength 520 nm, with a decreasing ratio indicating a drop in pH. Bafilomycin was included as a negative control. Data shows that except for LPS, all other tested ligands do not affect phagosomal pH.

In order to measure and interpret modulation of phagosome maturation/differential protein trafficking in response to targeted phagosome ligands, we have designed a method for ligand-to-particle attachment for phagocytosis, followed by a proteomic analysis of isolated phagosomes to quantify protein abundances across three timepoints of phagosome maturation. To represent differing categories of phagocytic bait, the following ligands were attached to polystyrene particles: immunoglobulin G Fc (IgG), complement (iC3b), lipopolysaccharide (LPS), mannan, calreticulin, and phosphatidylserine (PS) (Table I). These ligands were chosen to represent immune opsonized (IgG and iC3b), pathogen-associated molecular patterns (PAMPs; LPS and mannan), apoptotic targets (calreticulin and PS) or nontargeted phagocytosis (avidin/no ligand attached). To allow comparisons between differing ligands, it was a key consideration to ensure equivalency of attachment among the diverse ligands and among particles as well as batch-to-batch reproducibility, and stability of attachment until phagocytosis. Rather than saturating beads with ligands by adhesion, we achieved a consistent, stable coating through biotinylation of bait molecules and binding to a common pool of avidin particles. The attachment of biotinylated ligands was analyzed by flow cytometry to validate the attachment through a fluorescent streptavidin-phycoerythrin probe (Fig. 1*A*). Because a common pool of avidin particles was used, similar amounts of each ligand were attached; differing levels of biotin incorporation onto each ligand led to differences of fluorescence intensity of the probe. The relatively narrow peaks for each ligand indicated that the population of particles has a relatively similar distribution of attached ligand. Moreover, the biotinylation procedure did not cause beads to aggregate, as most analyzed particles were seen as single beads (65.8–89.9%). In order to qualitatively validate that biotinylation of ligands did not inhibit functional recognition, specific assays were conducted for selected ligands to demonstrate they still provided recognizable targets; namely recognition of biotinylated LPS by an NF-kB reporter cell line (RAW-Blue, InvivoGen), biotin-PS binding with fluorescent annexin V, and recognition of biotinylated IgG by anti-mouse with an HRP reporter (supplemental Fig. S1).

**Table I:** List of ligands used and receptors targeted in the study

	Ligand	Macrophage receptor	Reference
Opsonized particles	IgG (Fc fragment)	Fc-γ Receptor	(10)
	Complement (iC3b)	CR3 (CD11b/CD18)	(11)
		CR4 (CD11c/CD18)	
Pathogens	Mannan	Mannose receptor	(13)
	Lipopolysaccharide	TLR-4	(98)
		CD14	(19)
		MARCO	(99)
		Scavenger receptor A	(100)
Apoptotic cells	Phosphatidylserine	TIM-4	(23)
		BAI1	(24)
		Stabilin-2	(25)
	Calreticulin	LDL receptor-related protein (CD91)	(29)

Next, we conducted a set of fluorescence-based activity assays to investigate functional differences in phagosome functions for different ligand-particle combinations. These assays allow us to monitor the immediate-early timeframe of phagosome maturation. We performed fluorescence-based assays that measure particle uptake for all ligands (Fig. 1*B*), and, in real-time, oxidative burst, proteolysis and acidification using 3 μm silica particles (53) for Avidin, IgG, PS, and LPS (Fig. 1*C*–1*E*). Except for LPS that induced a quicker acidification and a stronger oxidative burst response, all ligands tested showed very similar levels for the oxidative burst, proteolysis and acidification, suggesting that receptor triggering has little effects on phagosome maturation in this setting. The moderate acceleration of acidification for LPS-bound to internalized particles matches previous data, which demonstrated this is a TLR4-independent response (37).

##### Proteomic Analysis of Isolated Phagosomes

In order to get more insights on the molecular changes on the phagosomes, we used the seven different sets of particles to isolate high purity phagosomes for proteomics experiments as described previously (31, 41). Rather than using macrophage-like cell lines, we chose to use murine BMDMs as they represent a more biologically relevant macrophage model and show overall a substantially different phagosomal proteome compared with the commonly used cell line RAW264.7 (54).

Phagosomes were isolated in biological triplicates from discrete timepoints to track the effect of given ligands during maturation. Following a 30 min “pulse” incubation with ligand-bound polystyrene particles, phagosomes were either isolated (30′/0) or incubated an additional 30 min (30′/30′) or 150 min (30′/150′). These timepoints were selected to represent an early, a middle and a late maturation state. 30′/0′ is the earliest feasible time point, based on the amount of phagosomes that can be isolated given avidin particles are taken up less efficiently than “naked” polystyrene particles. 30′/150′ was chosen as a pilot experiment demonstrated few proteome changes compared with a 30′/330′ phagosome proteome (supplemental Fig. S2). Altogether, phagosomes were isolated from ∼5 × 10^9^ BMDMs (∼900 10 cm tissue culture dishes).

**Fig. 2.**
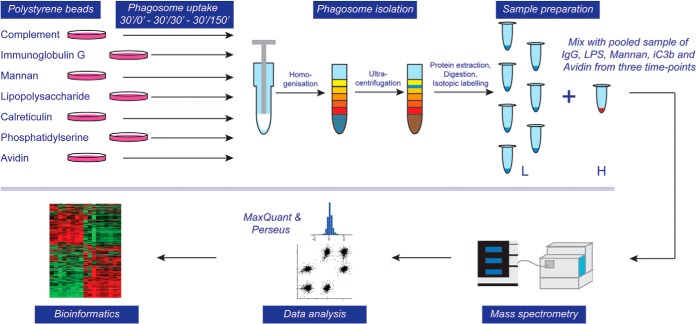
**Flow diagram of experimental approach for proteomic analysis.** Phagosomes were formed by inoculating murine bone marrow-derived macrophages (BMDMs) with polystyrene beads coated with various ligands for 30 min and a subsequent chase for 0, 30 or 150 min. Cells were homogenized and phagosomes isolated through ultracentrifugation in a sucrose gradient. Phagosomal proteins were extracted, digested, and isotopically labeled (light), whereas a mixed pool of phagosome samples that serve as a control was labeled with heavy isotopes. Combined samples were analyzed by LC-MS/MS on an Orbitrap Velos Pro mass spectrometer, data analysis was performed using MaxQuant and Perseus software suites and bioinformatic and statistical analyses were performed using in-house tools.

In order to generate quantitative data that could be compared equally among ligands and timepoints over all 63 samples, we used a strategy to spike-in a pooled sample into every LC-MS experiment (Fig. 2). This was achieved by stable isotope labeling of the pooled sample with “light” and each individual sample with “heavy” mTRAQ reagent (AB Sciex). Because the reference sample is analyzed together with each sample, the use of ion intensity ratios minimizes systematic biases in quantitation, including electrospray instability and chromatographic shifts that are inevitable during extended analysis of many samples. The pool consisted of equal mixtures of avidin, IgG, LPS, iC3b, and mannan phagosomes from all three time points. Not only did this provide an unbiased sample-to-sample ratio, but also facilitated “match between runs” during MaxQuant analysis, which enhances quantitative coverage of proteins across all samples (55).

To achieve the highest quantitative precision and sensitivity for low material amounts, we performed unfractionated 6-hour nano-LC-MS analyses in biological triplicates. In total from 63 LC-MS runs, 1891 proteins with a protein false-discovery rate (FDR) below 1% were identified. A high proportion of these, 1337 proteins, were quantified across all 21 conditions, enabled by the spike-in pool. Reproducibility was very good; the typical uncertainty of the log_2_ ratio that can be represented by the median (across all proteins and all 21 conditions) standard error was 0.13. Ninety-five percent of standard errors were between 0.02 and 0.47. The median correlation coefficient between each pair of replicates was 0.40 with 95% of the coefficients between 0.20 and 0.57. All proteins identified are listed in supplemental Table S1 with the log_2_ ratio for each replicate against the pool. Additionally, median iBAQ values (56) across ligands for each time point are presented to demonstrate the abundance ranking of phagosome proteins for each time point (supplemental Table S2). This data suggests that among the most abundant phagosomal proteins over all timepoints are Cathepsin B, Cathepsin D, LAMP1, LAMP2, Rab7a as well as Peroxiredoxin-1, NPC2, Greg1, Gpnmb and Ifitm3. Additionally, a text-searchable data file was produced to show the abundance of each protein over the three timepoints, allowing easy comparison of the various ligand-particle conjugates (supplemental Fig. S3).

##### The Dominant Feature of Proteomic Change is Time

**Fig. 3.**
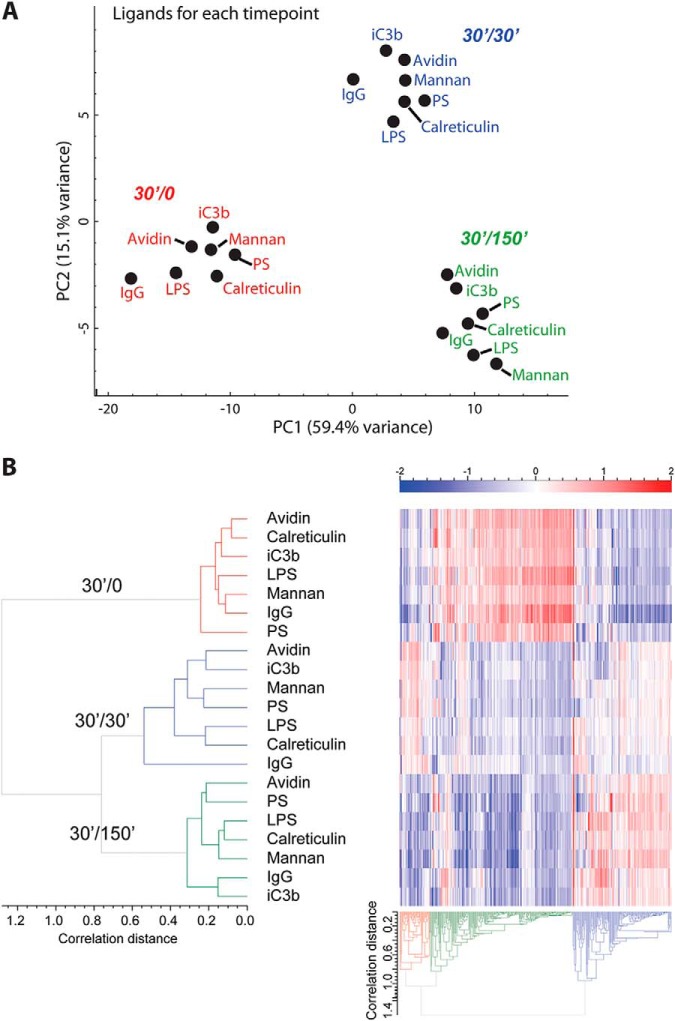
**Global phagosome proteomic changes among ligands and across time.** To evaluate high-level changes proteins showing a standard deviation greater than 0.35 were used for principal component analysis (PCA) and heat map analysis. *A*, PCA of total proteome data, analyzed for each ligand/time point shows clear separation according to time. Percentage variance for each principal component is given. *B*, Heat map of proteomic data. Values are log_2_ ratios of each ligand to the internal pool control. Average linkage hierarchical clustering with correlation distance was used. Samples have been color-coded by time point for emphasis.

To get a high-level view of the similarities and differences of phagosomes among ligands and over time, we compared the proteomic data of all ligands/timepoints by PCA (Fig. 3*A*). The clear partitioning of phagosomes into timecourse groups demonstrates that the primary feature of change is time. However, subtle systematic differences among ligand types are shown within each time-dependent cluster, such as IgG being always the most negative within each time point for component 1 and LPS tending to be more negative for component 2, suggesting distinct ligand-dependent differences in phagosome maturation.

**Fig. 4.**
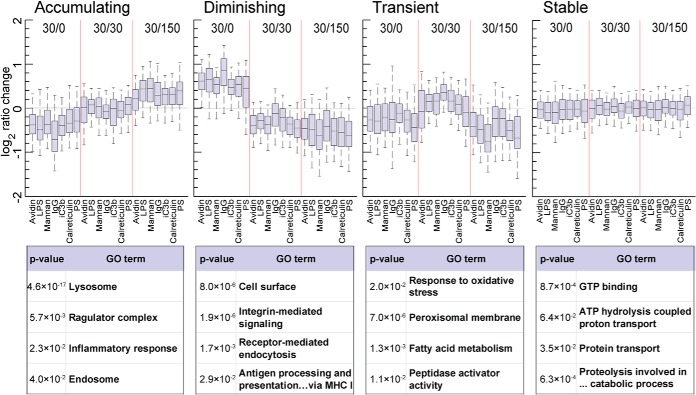
**Protein clusters exhibit a maturation-dependent profile.** The three protein cluster groups defined from hierarchical clustering (Accumulating, Diminishing, Transient), were plotted to demonstrate time point profiles. In box plots, the central lines indicate the median, boxes extend from 25th to 75th percentile and whiskers extend from 5th to 95th percentile. Selected enriched Gene Ontology (GO) groups are given for each cluster along with *p* value of enrichment against the total proteome.

To dissect interligand and intertime point differences further, we visualized protein abundance profiles by hierarchical clustering, after filtering for proteins not changing across conditions. For this, a standard deviation limit of 0.35 was chosen based on visual inspection of the resulting protein cluster profiles (Fig. 3*B*). The sample-level dendrograms demonstrated distinct branching based on timepoints, with comparably lower differences among ligands. Using box plots, we also visualized three primary clusters that followed temporal abundance patterns (Fig. 4). In these protein cluster groups, proteins were “accumulating” (239 proteins), “diminishing” (273 proteins) or exhibiting a “transient” increase at 30′/30′ (138 proteins). This classification matches that observed in a previous study, which examined phagosome membrane microdomains over a timecourse (57).

To classify proteins present in each group, we used GO term enrichment to highlight over-represented protein groups (Fig. 4). Key features of maturation are demonstrated by the acquisition of degradatory and vesicular components such as lysosomal enzymes and the lipid raft proteins flotillin 1 and 2, which have been shown to increase on phagosomes in the past (57–59), as well as the marker Rab7b (60). Moreover, we identified a constant acquisition of intracellular toll-like receptors (TLR3/7/8/9) and their complex partner unc93b1 (61, 62), whereas the surface-exposed TLR2 diminishes over time. Not surprisingly, the v-ATPase complex is increasingly more abundant on the phagosome as 12 subunits are progressively acquired over time. Two subunits, however, ATP6v0a1 and ATP6v0d2 actually decrease with time, suggesting that these proteins play regulatory roles in the acidification of the phagosome. Indeed, it has been reported that ATP6v0a1 mediates fusion of the phagosome with the lysosome in microglial cells of zebrafish (63).

Among the proteins diminishing over time, we see expectedly a large number of plasma membrane proteins that will be recycled back to the surface during maturation. In this group we also identified the actin cytoskeleton regulators ezrin and moesin, which have been shown to regulate phagocytosis and phagosome maturation (64, 65), and Rab14, which has been reported to locate to the Golgi and recycling endosomes and has been shown to be critical for phagosome maturation arrest by *Mycobacterium tuberculosis* (66–68).

Interestingly, we identified two subsets of lysosomal proteins, one of which predictably accumulates, while another is accumulating at 30′/30′ and then clearly decreases by 30′/150′. Among the lysosomal proteins that are transiently enriched are cystatin C (CST3), beta-glucuronidase (GUSB), beta-mannosidase (MANBA) and a lysosomal thioesterase (PPT2). This may indicate a unique function for this subset of lysosomal proteins in phagosome maturation. Other transient proteins are superoxide dismutase 1 (SOD1), catalase (CAT) and NCF1/p47-phox, a component of an NADPH oxidase complex, suggesting a coordinated regulation of the oxidative burst about 60 min after uptake. These protein group classifications represent a resource of time-dependent phagosome proteins in murine BMDMs.

##### Ligands Induce Subtle, but Distinct Differences to the Phagosome Proteome

**Fig. 5.**
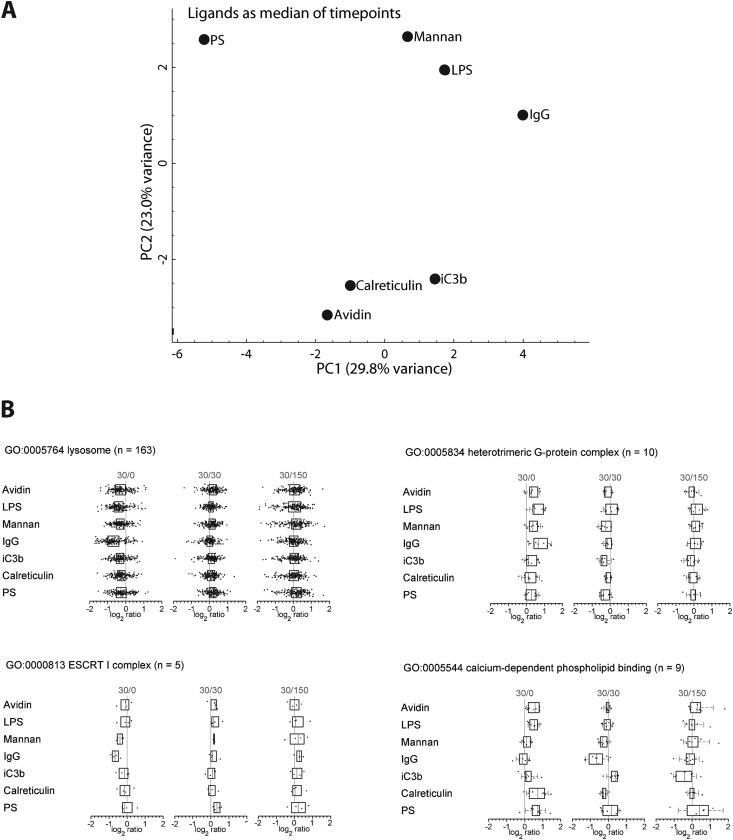
**Ligand-dependent modulation at the GO group level.** *A*, PCA of each ligand, after taking the median across the timepoints for each ligand shows separation of phagosome proteomes depending on individual ligands. *B*, The distribution of protein ratios belonging to each indicated GO group were plotted for each ligand and time point. Box plots are defined as in Fig. 4.

Next, we examined how the various ligands affected phagosome composition. For this, we calculated protein values as the median of the timepoints to consider data at the ligand level independent of time, and performed again a principal component analysis (Fig. 5*A*). Ligands clustered into three groups, separating mannan, LPS, and IgG from avidin, calreticulin, and iC3b primarily by component 2, and from PS by component 1, implying that ligands significantly affected phagosome maturation. By examining the abundance of various GO groups over time and among ligands, variations could be identified. Fig. 5*B* shows GO box plots for lysosome, calcium-dependent phospholipid binding, heterotrimeric G-protein complex, and ESCRT I complex. IgG phagosomes show a moderate retardation of lysosome acquisition, which disappears by 30′/30′. Recently, we and others showed that interferon-γ activation of macrophages similarly reduced the speed of phagosome maturation for the gain of antigen presentation (31, 32). In light of this data, we investigated if MHC class I antigen cross-presentation of IgG-triggered phagosomes was also enhanced compared with PS phagosomes, but could not identify any significant difference between LPS, IgG and PS (supplemental Fig. S4).

Moreover, there is a wide variation in calcium-dependent phospholipid binding proteins across ligands, with IgG and mannan below average and calreticulin and PS above average, suggesting significant changes in the lipid composition of phagosomes depending on the receptor triggered, potentially through the enhanced fusion of phagosomes with phosphatidylinositol-3-phosphate (PI3P)-rich endosomes or the enzymatic production of PI3P upon receptor engagement. IgG phagosomes also exhibit an increase in heterotrimeric G-protein complex proteins at 30′/0 suggesting that G-protein signaling is important for Fc-receptor triggered phagocytosis. This is interesting as G-proteins have so far only been considered important for Fc-receptor mediated phagocytosis jointly with leukotriene receptors (69, 70). Finally, the ESCRT I complex, which has been suggested to function in the formation of multivesicular bodies (MVBs) (71) is decreased at 30′/0 in IgG and mannan compared with other ligands. All GO group box plots with at least three member proteins are found in supplemental Fig. S5, and gradient plots, demonstrating change of proteins within GO groups across time, in supplemental Fig. S6.

##### Network Analysis Reveals Recruitment of Specific Protein Complexes to Phagosomes of PS- and IgG-Coated Beads

**Fig. 6.**
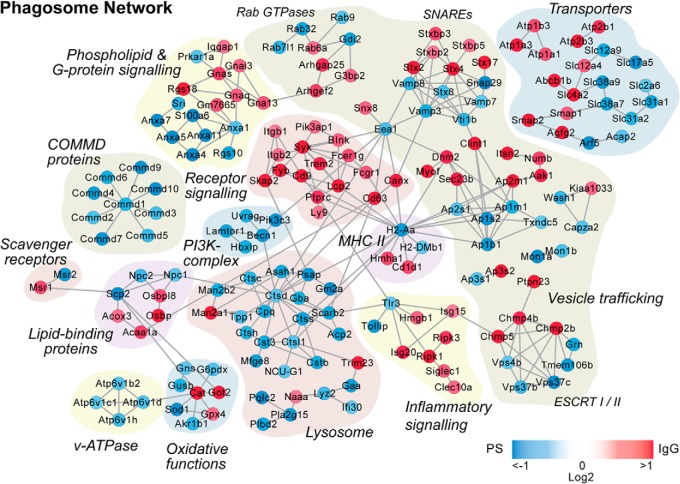
**Protein network of proteins regulated between PS and IgG phagosomes.** Proteins demonstrated to significantly change between PS and IgG phagosomes (*t* test *p* value <0.05) in at least one time point were analyzed by STRING and imported into Cytoscape for manual curation and grouping. Node color corresponds to the absolute maximum log_2_ IgG/PS ratio-of-ratios across timepoints, with increased under IgG in red and increased in PS in blue.

As demonstrated by the number of significant protein changes and PCA analysis (supplemental Table S3 and Fig. 5*A*), PS and IgG demonstrated the greatest interligand protein abundance variance. This is expected as, functionally, they are also furthest apart. While phagosomes from PS particles represent the uptake of apoptotic cells and, thus, should not trigger any immune response, IgG coated particles represent a species that the organism has already encountered and that triggered an immune response leading to antibody production. We therefore compared these two ligands against each other directly and performed a network analysis using interaction data from the STRING database (46). Fig. 6 shows the network of proteins that differ significantly between IgG and PS at any of the three timepoints. Key protein groups are highlighted, illustrating a number of interesting features. For example, a number of lysosomal proteins increase for PS, suggesting that nutrients from apoptotic cells might be more efficiently recycled than from “foreign” IgG opsonized particles. The difference in regulation is also highlighted by the differential recruitment of SNARE proteins and transporters for which the plasma membrane located proteins are more abundant in IgG phagosomes whereas the intracellular proteins are more abundant in PS phagosomes. This indicates that IgG phagosomes appear on average “younger” than phagosomes from PS particles.

Remarkably, proteins of the ESCRT-I complex are enriched on phagosomes of PS coated beads whereas the ESCRT-II complex is enriched on IgG phagosomes. As both complexes are important for the formation of multivesicular bodies (MVBs) and endosomal sorting (71) and are even thought to form one protein complex together (71–73), more work will be required to characterize their functions on the phagosome.

Finally, although several protein complexes that have been implicated in inflammatory signaling are more abundant in IgG phagosomes, PS phagosomes show a specific increase of Tollip, which is a negative regulator of Toll-like receptor (TLR) signaling (74–76). Interestingly, a complex of nine COMMD proteins, which have been shown to be involved in the inhibition of pro-inflammatory NF-κB (77–80) is also markedly increased in PS phagosomes. This might indicate that the uptake of particles via PS receptors might actively reduce inflammatory signaling, which will be beneficial to reduce immune reaction to “self” particles.

##### Validation of Individual Proteins by SRM

**Fig. 7.**
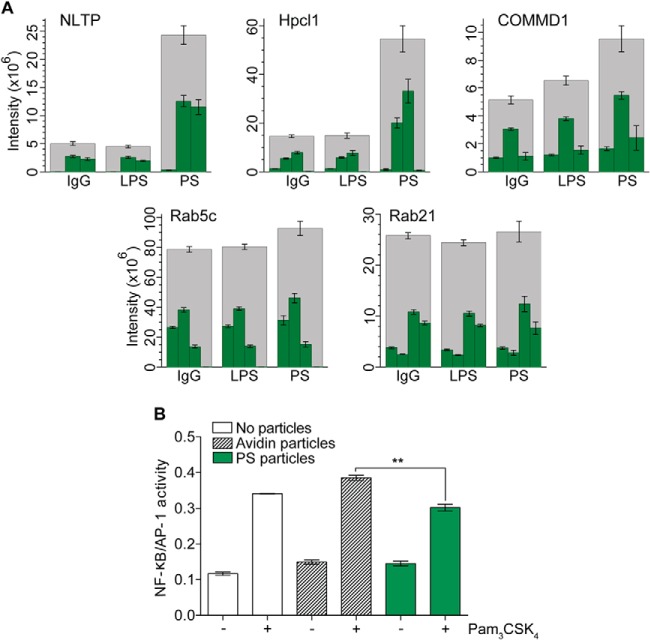
**Selected Reaction Monitoring (SRM) validation of ligand-altered abundance of selected proteins and repression of NfκB through PS-particle uptake.** *A*, NLTP, COMMD1, and Hpcal1 were measured from 30′/0 IgG, LPS, and PS phagosomes, along with the stable proteins Rab5c and Rab21 for loading control. A stacked bar graph was made using the targeted peptides for each protein. Error bars indicate standard error for each peptide. *B*, RAW-Blue cells were challenged with uncoated (gray) or PS-coated (green) beads for 30 min and then stimulated with 50 ng/ml TLR2-agonist Pam_3_CSK_4_. Data shows a 20% change of NfκB mediated inflammatory response when PS beads are internalized for the TLR stimuli.

In order to validate our proteomics results, we chose to use Selected Reaction Monitoring (SRM) mass spectrometry (81) as this is orthogonal to our proteomic discovery approach, independent of antibody availability and less starting material from the laborious phagosome isolation is required than for Western blot analysis. Three new biological replicates per condition were produced for the SRM experiment and were processed for label-free analysis. Three proteins with relative high abundance changes in PS phagosomes (NLTP, COMMD1, and Hpcal1), as well as two control proteins (Rab21 and Rab5c), which did not change among ligands or over the timecourse, were selected for analysis, with a minimum of three peptides per protein (Fig. 7). COMMD1, copper metabolism MURR1 domain-containing protein, was first implicated for a role in copper metabolism and given the name Murr1 (82). COMMD proteins have been shown to inhibit NF-κB signaling (79), thus increased abundance of COMMD1 may serve an anti-inflammatory role in the face of apoptotic cell removal. NLTP, nonspecific lipid transfer protein (also known as sterol carrier protein 2), is a protein known to be involved in transport of phospholipids, as well as cholesterol and gangliosides (83), and thus is likely involved in shuttling phospholipids or cholesterol from phagosomes after internalization of lipid-heavy targets. Hpcal1, hippocalcin-like protein 1 and also known as visinin-like protein 3, is relatively uncharacterized, but shares homology with the rhodopsin phosphorylation protein hippocalcin (84) and has a calcium-binding domain (85).

Indeed, the SRM analysis demonstrated a significant increase in the three targeted proteins in PS phagosomes compared with IgG and LPS, with a *p* value <0.05 for all three NLTP peptides, three out of four HPCL1 peptides, and two out of three COMMD1 peptides, whereas Rab21 and Rab5c showed no ligand-dependent differences (Fig. 7). The SRM transitions used are described in supplemental Table S4, and peak area data for each replicate is given in supplemental Table S5.

##### Investigating the Influence of PS Beads on Subsequent Activation Via a TLR Agonist

Based on the indications that PS-bead uptake influences inflammatory signaling, via an increase in anti-inflammatory proteins including COMMD complex proteins and TOLLIP, we investigated whether phagocytosis of PS particles could depress NF-κB signaling after subsequent activation with the TLR agonist Pam3CSK. Using the RAW-Blue cell line, we looked at quantified NF-κB activation of RAW-Blue cells that were stimulated with the TLR2 agonist PAM_3_CSK_4_ after uptake of PS-coated or uncoated particles. Internalization of PS-particles induced a highly significant 20% decrease in NF-κB activation compared with cells fed naked particles prior to addition of Pam_3_CSK_4_, with a *t* test *p* value of 0.0024 among replicate wells and repeated over 3 experiments. Supporting our proteomic data, this implicates that recognition of PS-particle uptake during phagocytosis or early phagosome maturation induces an anti-inflammatory response, probably through recruitment of COMMD proteins and TOLLIP. This mechanism has important implications on our understanding of how the immune system avoids autoimmunity after uptake of apoptotic cells.

## DISCUSSION

Phagosomes are extremely dynamic and in constant fusion and fission with other organelles, which makes them difficult to study. Consistency and many data-points are often necessary to understand the nature of this organelle. Although individual phagosomes have been shown to differ greatly in maturation even within a single cell, which led to the concept of the phagosome autonomy (38, 86), our proteomics approach identifies changes on the “average” phagosome, because protein is extracted together from all isolated phagosomes in each biological replicate. In this article, we present the time-resolved proteomes of phagosome maturation to detect differences because of differential phagocytic receptor engagement, along with functional assays to address kinetics of phagosomal enzymatic activities immediately after particle uptake.

Our time-course data provides a valuable resource for researchers in the community. Similarly to a publication analyzing phagosome microdomain/raft proteins over three timepoints in J774.1 cells (57), we also demonstrated that proteins can be separated into decreasing, increasing, transient, and stable classes. Although a large number of the proteins in the decreasing (mostly plasma membrane) and increasing (mostly lysosomal) clusters are well within our current understanding of phagosome maturation, it is of particular interest that there appears to be a group of transient proteins such as cystatin C, beta-glucuronidase or beta-mannosidase that are thought to be lysosomal. However, how could they be fully lysosomal when they are decreased in the late phagolysosome? This could mean that there is a specific intracellular pool of vesicles with these enzymes that are distinct from “classical” lysosomes and more work will be needed to characterize this phenomenon.

Similarly to our study, recent publications investigated the downstream effects following uptake of particles coated with IgG, beta-glucan, mannan, complement factors, fibronectin, mannan and LPS in BMDMs (87). In this article, Hoffmann *et al.* investigated uptake kinetics, transcriptome modulation 30 and 60 min following uptake, and a qualitative proteomic comparison of 265 proteins identified in phagosomes of IgG- and mannan-opsonized beads. Their data is very similar to ours when it comes to internalization/phagocytosis, *e.g.* showing a reduced uptake of avidinylated beads compared with beads coated with phagocytic ligands. However, their qualitative proteomics analysis showed surprisingly little overlap (40%) between mannan and IgG phagosomes and is thus very different from our results. The dramatic differences between conditions seen in their data are probably attributable to the experimental design as a qualitative rather than proteomics analysis. In their approach, the identified/not-identified result is very prone to sampling variation in the mass spectrometer, especially when rather small proportion of the analyzed proteome is identified. We believe that the quantitative proteomics approach presented here provides more reliable data, because of the vastly improved sampling depth (more than 5-fold more protein identifications) and use of MS1 based relative quantitation. Moreover, in contrast to other previous phagosome proteomics publications, we induced phagosomes in BMDMs rather than the often used macrophage/monocyte cell lines RAW267.4 and J774.1. Although these cell lines are important tools to study macrophage and phagosome functions, recent data from out lab suggests that phagosomes from BMDMs differ quite remarkably from phagosome proteomes of macrophage/monocyte cell lines (54), including in the expression of various important receptors such as the mannose receptor (MRC1).

One other difference in our work compared with previous publications is the attachment of ligands to beads. We tested various methods and chose an avidin/biotin system to ensure the molecules were attached strongly and in comparable amounts. Using flow cytometry and dot blots we could confirm equal attachment of ligands. However, as the bead surface was likely not fully saturated with ligands, it is possible that the beads used triggered not only the targeted receptors, but also additional, nonselective receptors such as scavenger receptors, which could explain why there were rather small differences between the ligands compared with experiments in which the macrophages were activated by cytokines such as interferon-γ (31), which will have a dramatically more dominant effect on phagosome maturation (37). It needs to be pointed out though, that natural prey, such as bacterial, fungal particles or apoptotic cells, will also trigger a range of different receptors, which will potentially have cumulative effects on phagosome functions and additionally affect the activation status of the phagocyte, albeit over a longer timescale.

Beads coated with mannan, calreticulin, iC3b and avidin behaved all similarly in our experiments and did not appear to have significant effects on phagosome functions in the macrophages used. So what are the reasons that these receptors did not induce significant changes to the phagosome proteome compared with avidin? It is possible that these receptors require additional signals from other receptors and/or a specific activation status of the cell. Moreover, although mannan has been shown to inhibit zymosan phagocytosis (14) and the mannose receptor has for a long time been considered phagocytic, a recent publication challenged this view as a number of different cell lines expressing the mannose receptor failed to show internalization of known ligands (88, 89). iC3b is also recognized by the class A macrophage receptor type I (MSR1/SR-A) (90), which may be why the resulting phagosome is similar to avidin beads. Finally, although calreticulin has been shown to provide a stimulating signal, it is alone insufficient to trigger phagocytosis (91) whereas interaction of calreticulin with complement factors C1q seems to be important for successful phagocytosis (92).

Our data indicates nonetheless that some ligands like LPS, IgG, and PS have effects that can cumulatively change the phagosome fate considerably. Of course, it might be that LPS-bead TLR stimulation led to an early activated phenotype of the macrophages, which would change the phagosome proteome through the activation rather than through direct down-stream signaling. This is in part one of the points raised during a controversy if TLRs regulate phagosome maturation (34–37). We therefore focused our analyses on the two “extreme” cases of prey: IgG- and PS-coated particles, which led to considerably different phagosome proteomes. For example, phagosomes from PS-coated beads accumulated MFGE8, the PS binding protein “bridging” between phagocytes and apoptotic cells (93, 94) as well as anti-inflammatory signaling molecules such as Tollip (74–76) and the COMMD-complex (77–80). Moreover, our data shows that uptake of PS-particles significantly represses NF-κB activation in RAW-Blue cells upon TLR stimulation. This indicates that triggering of PS-receptors alone is sufficient to actively down-regulate an inflammatory response. IgG-coated beads, on the other hand, induce an increased translocation of inflammatory signaling proteins to the phagosomes such as the RIP-Kinases. Interestingly, we also identified a distinct enrichment of the phosphoinositide-3 kinase (PI3K) complex around Beclin, Pik3c3/VPS34, UVRAG as well as Lamtor1 in PS-bead phagosomes. Both complexes play major roles in mTOR mediated autophagy (95, 96). As it has recently been reported that mTOR becomes activated by amino acids from digested apoptotic cell material (97), this data suggests that the mTOR complex might be recruited, but not necessarily activated down-stream of PS-receptor mediated phagocytosis, preparing the cell for the intake of amino acids. More work will need to be done to test if mammalian phagocytes can “taste” prey particles by receptor engagement and thus preparing them for appropriate responses, may it be immune activation or recycling of nutrients of apoptotic cell bodies.

In conclusion, our data indicates that triggering of phagocytic receptors can induce small, but distinct changes to the phagosome proteome. This suggests that cells are able to identify the nature of the particular particle that they are ingesting, allowing the cell to distinguish self from nonself and triggering appropriate immune responses.

## Supplementary Material

Supplemental Data

## Footnotes

^1^ The abbreviations used are:

LPS: lipopolysaccharide
PS: phosphatidylserine
LRP: low density lipoprotein receptor-related protein
PE: phycoerythrin
HRP: horseradish peroxidase
BMDM: bone marrow derived macrophage
M-CSF: macrophage-colony stimulating factor
PCA: principle component analysis
SRM: selective reaction monitoring.
