# Supplementary material for: Quantitative Proteome Analysis of Temporally Resolved Phagosomes Following Uptake Via Key Phagocytic Receptors
Source: Mol Cell Proteomics. 2015 May;14(5):1334–49. doi: 10.1074/mcp.M114.044594 (PMC4424403; doi:10.1074/mcp.M114.044594)
Supplement: Supplemental Data [file supp_14_5_1334__index.html]

Quantitative proteome analysis of temporally-resolved phagosomes following uptake via key phagocytic receptors — Quantitative Proteome Analysis of Temporally Resolved Phagosomes Following Uptake Via Key Phagocytic Receptors — Temporal and Ligand Effects on Phagosome Proteome — Supplemental Data 

# Quantitative Proteome Analysis of Temporally Resolved Phagosomes Following Uptake Via Key Phagocytic Receptors

## Supplemental Data

**Files in this Data Supplement:**

- Supplementary Figure 1: Functional validation of biotinylated ligands. - Supplementary Figure 1: Functional validation of biotinylated ligands. In order to demonstrate ligands retained functional recognition after biotinylation, the ligands were detected with the following probes: IgG Fc ? anti mouse Fc with an HRP reporter; PS ? annexin-V with an HRP reporter; and LPS using the RAW-Blue cell line with Quanti-Blue detection.
- Supplementary Figure 2: Proteomic comparison of 30?/150? and 30?/330?. - Supplementary Figure 2: Proteomic comparison of 30?/150? and 30?/330?. PCA plot of pilot proteomes from IgG, LPS, mannan, and avidin phagosomes isolated at 30?/150? or 30?/330? to evaluated degree of difference among ligands between these two late timepoints.
- Supplementary Figure 3: Protein-level timecourse plots. - Supplementary Figure 3: Protein-level timecourse plots. Log2 ratio of each protein (each ligand/timepoint to pool, average of three replicates) is plotted over time and for each ligand. Proteins are identified with Uniprot accession numbers. The median of all ligands is added as a blue line for reference. Error bars are approximately unbiased estimators of the standard error (101).
- Supplementary Figure 4: Antigen presentation assay. - Supplementary Figure 4: Antigen presentation assay. Degree of antigen presentation of ovalbumin and ligand-bead conjugates. Measured by beta-galactosidase production by B3Z T cell hybridoma cells, against a naked bead negative control. Error bars represent the standard error.
- Supplementary Figure 5: GO box plots. - Supplementary Figure 5: GO box plots. Log2 ratios of GO groups (as in Supplementary Figure 3) are examined by ligand and by timepoint, demonstrating changes in respect to GO group.
- Supplementary Figure 6: GO gradient plots. - Supplementary Figure 6: GO gradient plots. Gradient plots were produced by transforming protein abundance changes across time in to a Cartesian graph. The change from 30?/0 to 30?/30? is plotted on the x-axis, and the change from 30?/30? to 30?/150? is plotted on the y-axis. Here, the 1,891 GO groups with at least three member proteins were included. Gray dots are the total proteome background, and blue dots represent members of the given GO group.
- Supplementary Figure legends - Supplementary Figure legends
- Supplementary Table 1: Quantified proteins. - Supplementary Table 1: Quantified proteins. Protein-level log2 ratios (ligand/timepoint to pool) of all replicates against the internal pool for all identified proteins and global peptide identifications with posterior error probability.
- Supplementary Table 2: iBAQ-based quantitation of phagosome proteins. - Supplementary Table 2: iBAQ-based quantitation of phagosome proteins. iBAQ values were calculated, based on each &#x26;#916;8 mTRAQ sample, and the median of ligands were taken for each timepoint. This method, which normalises peptide intensity by the number of observable peptides, allows a semi-quantitative protein-to-protein abundance comparison.
- Supplementary Table 3: Significant protein changes between ligands. - Supplementary Table 3: Significant protein changes between ligands. Student?s t-test was used to determine proteins changing between avidin and each ligand for each timepoint. Proteins were filtered by a minimum log2 change of more than +/- 0.5 and p value &#x26;lt;0.05 (these values are provided in the first two columns of each sheet).
- Supplementary Table 4: SRM transitions. - Supplementary Table 4: SRM transitions. Transition parameters for each peptide/protein with calculated optimised collision energy.
- Supplementary Table 5: SRM area data. - Supplementary Table 5: SRM area data. Transition and peptide-level peak areas, with retention time and FWHM for each replicate.
