# Supplementary material for: Quantitative Proteome Analysis of Temporally Resolved Phagosomes Following Uptake Via Key Phagocytic Receptors
Source: Mol Cell Proteomics. 2015 May;14(5):1334–49. doi: 10.1074/mcp.M114.044594 (PMC4424403; doi:10.1074/mcp.M114.044594)
Supplement: Supplemental Data [file supp_M114.044594_mcp.M114.044594-1.pdf]

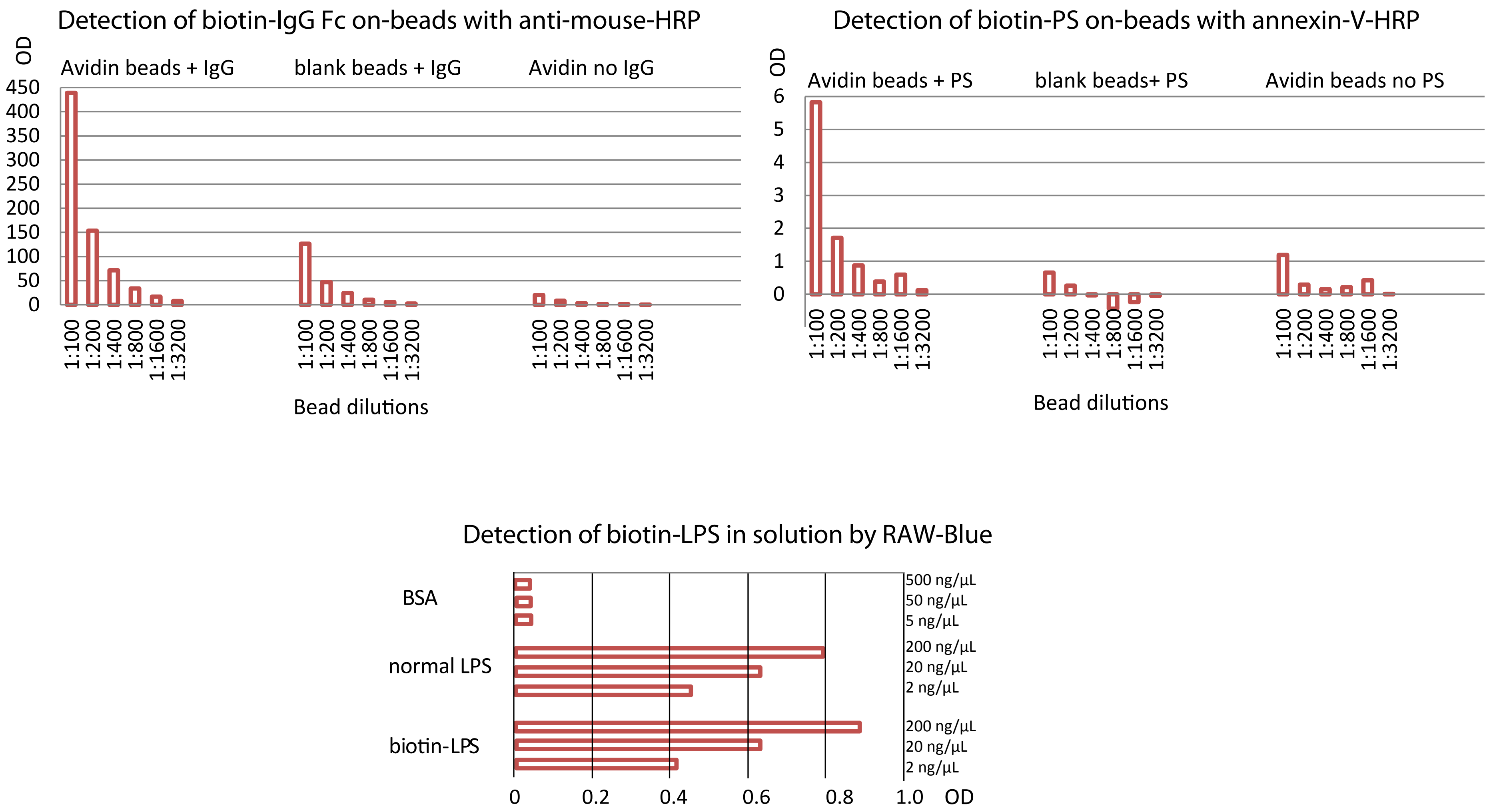

Detection of biotin-IgG Fc on-beads with anti-mouse-HRP
OD
Avidin beads + IgG
blank beads + IgG
Avidin no IgG
450
400
350
300
250
200
150
100
50
0
1:100
1:200
1:400
1:800
1:1600
1:3200
Bead dilutions
Detection of biotin-PS on-beads with annexin-V-HRP
OD
Avidin beads + PS
blank beads+ PS
Avidin beads no PS
6
5
4
3
2
1
0
1:100
1:200
1:400
1:800
1:1600
1:3200
Bead dilutions
Detection of biotin-LPS in solution by RAW-Blue
BSA
normal LPS
biotin-LPS
500 ng/µL
50 ng/µL
5 ng/µL
200 ng/µL
20 ng/µL
2 ng/µL
200 ng/µL
20 ng/µL
2 ng/µL
0
0.2
0.4
0.6
0.8
1.0
OD
