# Supplementary figures and images for: Quantitative Proteome Analysis of Temporally Resolved Phagosomes Following Uptake Via Key Phagocytic Receptors
Source: Mol Cell Proteomics. 2015 May;14(5):1334–49. doi: 10.1074/mcp.M114.044594 (PMC4424403; doi:10.1074/mcp.M114.044594)

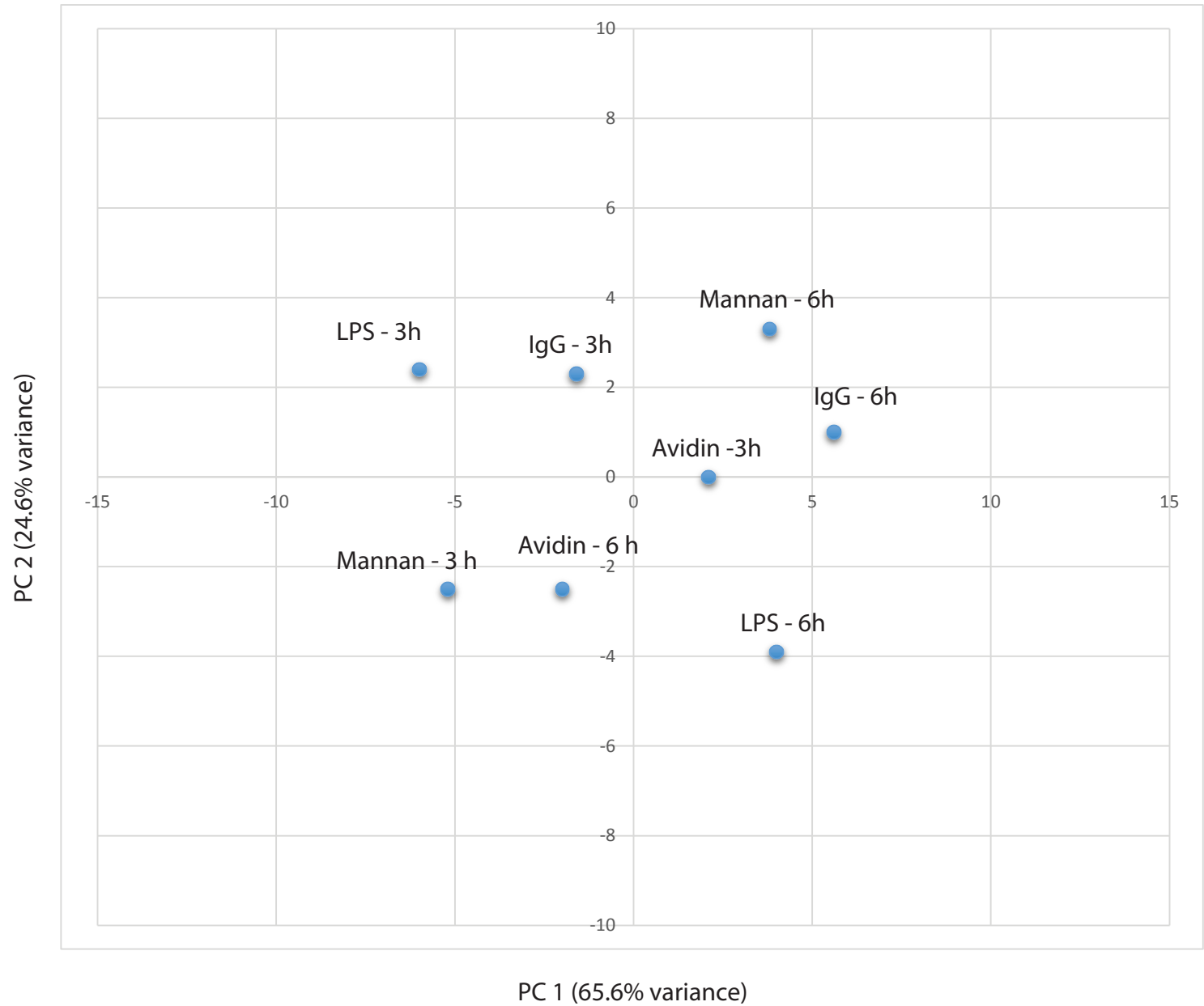

Supplement: Supplemental Data [file supp_M114.044594_mcp.M114.044594-2.pdf]

# MHC I Cross Presentation

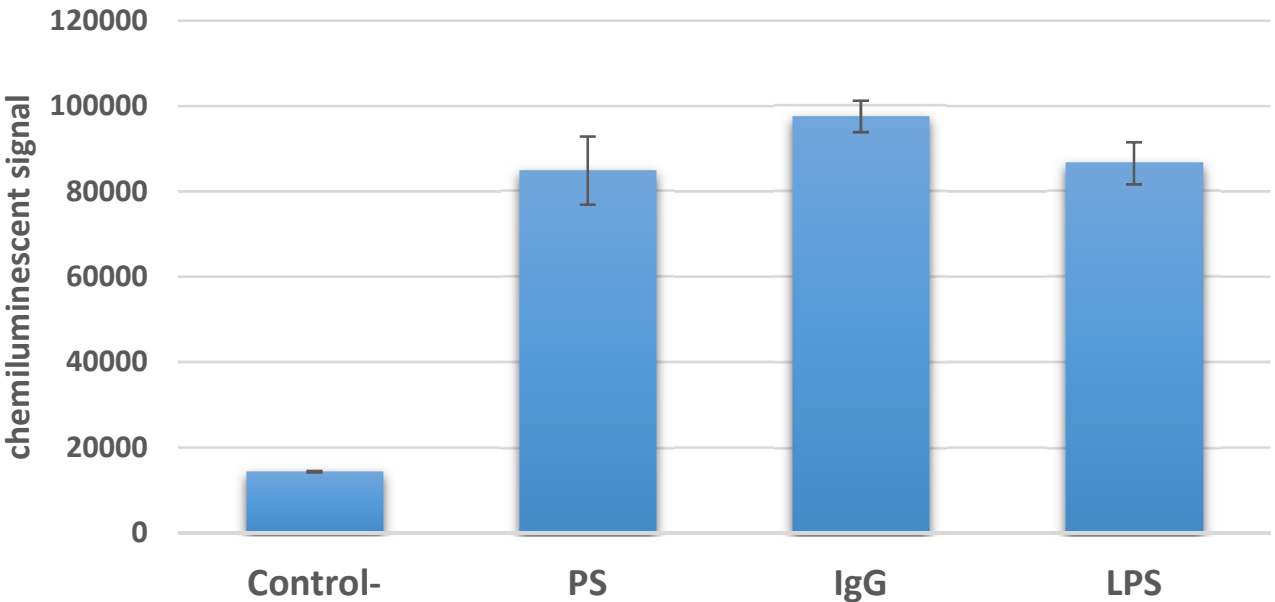

Supplement: Supplemental Data [file supp_M114.044594_mcp.M114.044594-4.pdf]
