# Supplementary material for: Quantitative Proteome Analysis of Temporally Resolved Phagosomes Following Uptake Via Key Phagocytic Receptors
Source: Mol Cell Proteomics. 2015 May;14(5):1334–49. doi: 10.1074/mcp.M114.044594 (PMC4424403; doi:10.1074/mcp.M114.044594)
Supplement: Supplemental Data [file supp_M114.044594_mcp.M114.044594-12.pdf]

Supplementary information

Supplementary Table 1: Quantified proteins. Protein-level log_2 ratios (ligand/timepoint to pool) of all replicates against the internal pool for all identified proteins and global peptide identifications with posterior error probability.

Supplementary Table 2: iBAQ-based quantitation of phagosome proteins. iBAQ values were calculated, based on each Δ8 mTRAQ sample, and the median of ligands were taken for each timepoint. This method, which normalises peptide intensity by the number of observable peptides, allows a semi-quantitative protein-to-protein abundance comparison.

Supplementary Table 3: Significant protein changes between ligands. Student's *t*-test was used to determine proteins changing between avidin and each ligand for each timepoint. Proteins were filtered by a minimum log_2 change of more than +/- 0.5 and p value <0.05 (these values are provided in the first two columns of each sheet).

Supplementary Table 4: SRM transitions. Transition parameters for each peptide/protein with calculated optimised collision energy.

Supplementary Table 5: SRM area data. Transition and peptide-level peak areas, with retention time and FWHM for each replicate.

Supplementary Figure 1: Functional validation of biotinylated ligands. In order to demonstrate ligands retained functional recognition after biotinylation, the ligands were detected with the following probes: IgG Fc – anti mouse Fc with an HRP reporter; PS – annexin-V with an HRP reporter; and LPS using the RAW-Blue cell line with Quanti-Blue detection.

Supplementary Figure 2: Proteomic comparison of 30'/150' and 30'/330'. PCA plot of pilot proteomes from IgG, LPS, mannan, and avidin phagosomes isolated at 30'/150' or 30'/330' to evaluated degree of difference among ligands between these two late timepoints.

Supplementary Figure 3: Protein-level timecourse plots. Log_2 ratio of each protein (each ligand/timepoint to pool, average of three replicates) is plotted over time and for each ligand. Proteins are identified with Uniprot accession numbers. The median of all ligands is added as a blue line for reference. Error bars are approximately unbiased estimators of the standard error (101).

Supplementary Figure 4: Antigen presentation assay. Degree of antigen presentation of ovalbumin and ligand-bead conjugates. Measured by beta-galactosidase production by B3Z T cell hybridoma cells, against a naked bead negative control. Error bars represent the standard error.

Supplementary Figure 5: GO box plots. Log_2 ratios of GO groups (as in Supplementary Figure 3) are examined by ligand and by timepoint, demonstrating changes in respect to GO group.

Supplementary Figure 6: GO gradient plots. Gradient plots were produced by transforming protein abundance changes across time in to a Cartesian graph. The change from 30'/0 to 30'/30' is plotted on the *x*-axis, and the change from 30'/30' to 30'/150' is plotted on the *y*-axis. Here, the 1,891 GO groups with at least three member proteins were included. Gray dots are the total proteome background, and blue dots represent members of the given GO group.
