# Supplementary material for: Quantitative Proteome Analysis of Temporally Resolved Phagosomes Following Uptake Via Key Phagocytic Receptors
Source: Mol Cell Proteomics. 2015 May;14(5):1334–49. doi: 10.1074/mcp.M114.044594 (PMC4424403; doi:10.1074/mcp.M114.044594)
Supplement: Supplemental Data [file supp_M114.044594_mcp.M114.044594-3.pdf]

0610031j06rik (Q9JHJ3)

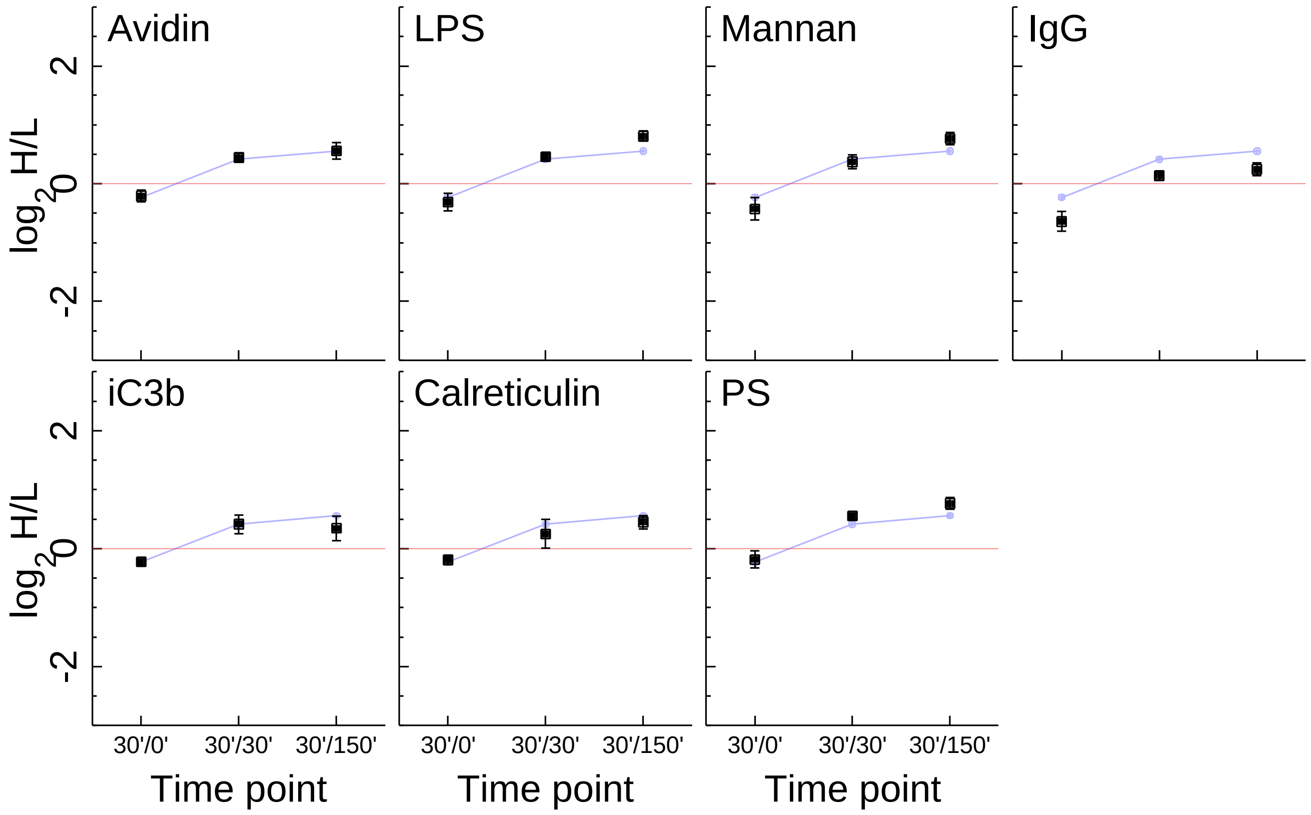

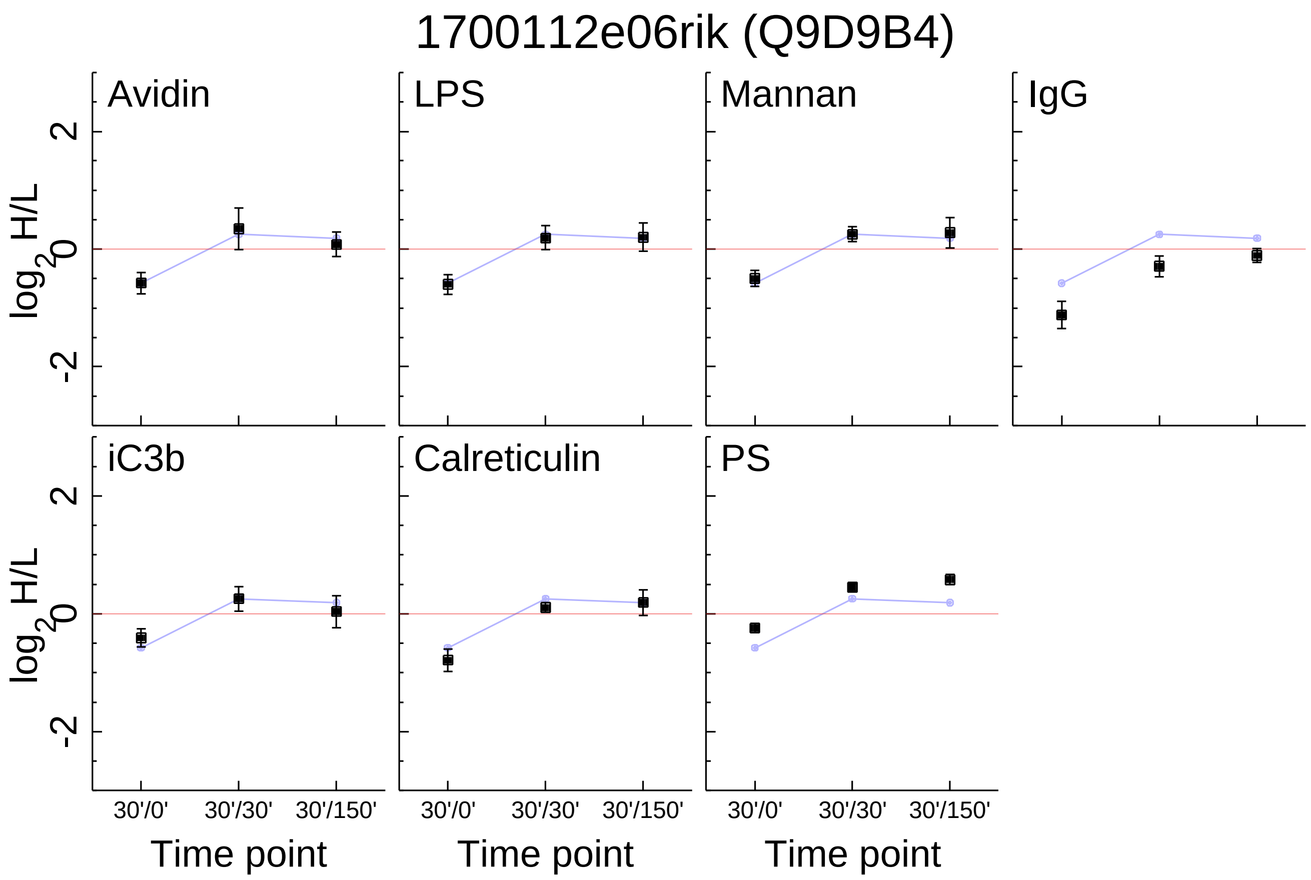
1700112e06rik (Q9D9B4)
Avidin
LPS
Mannan
IgG
iC3b
Calreticulin
PS
log2 H/L
2
0
-2
30'/0'
30'/30'
30'/150'
Time point

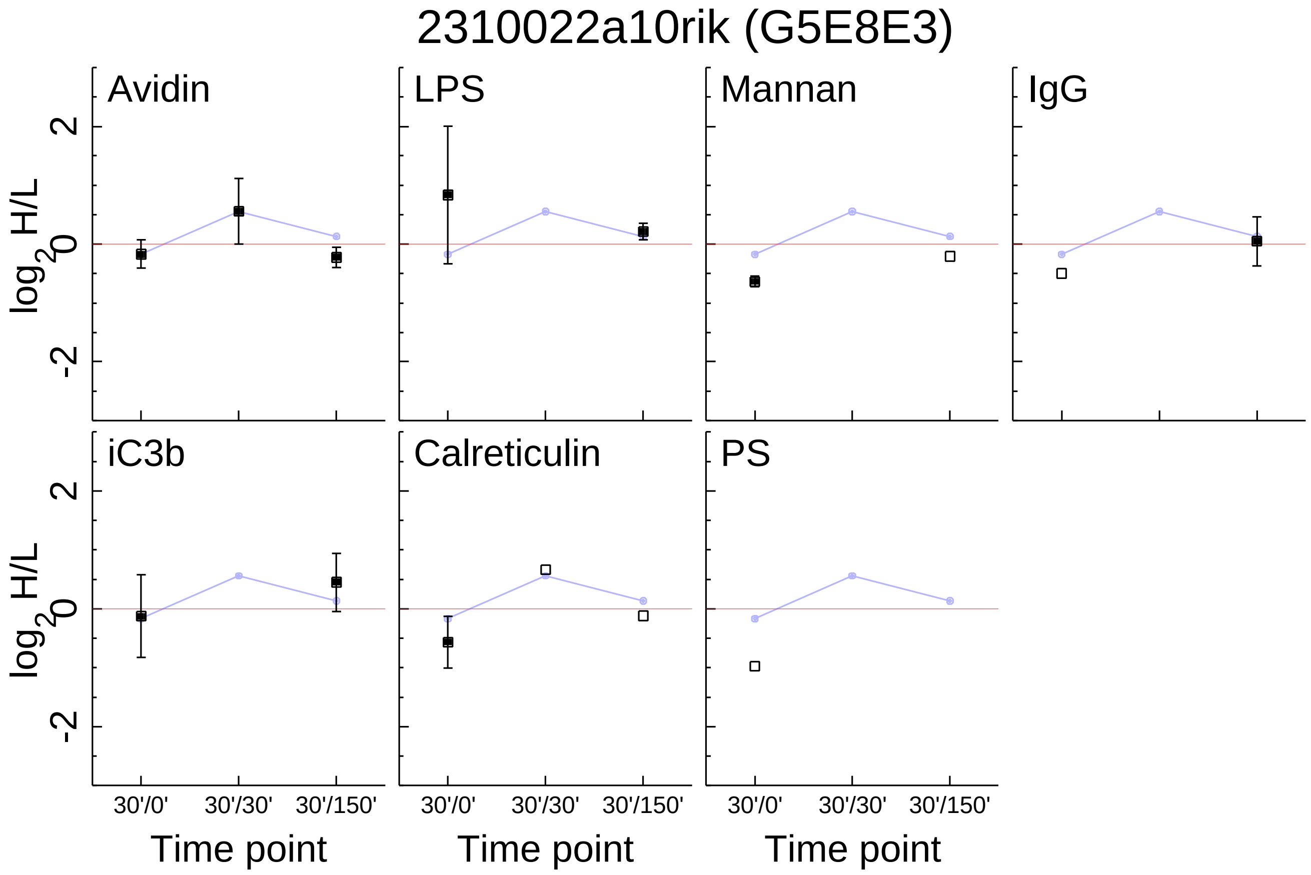

2310022a10rik (G5E8E3)
Avidin
LPS
Mannan
IgG
iC3b
Calreticulin
PS
$\log_2$ H/L
2
0
-2
30'/0'
30'/30'
30'/150'
Time point

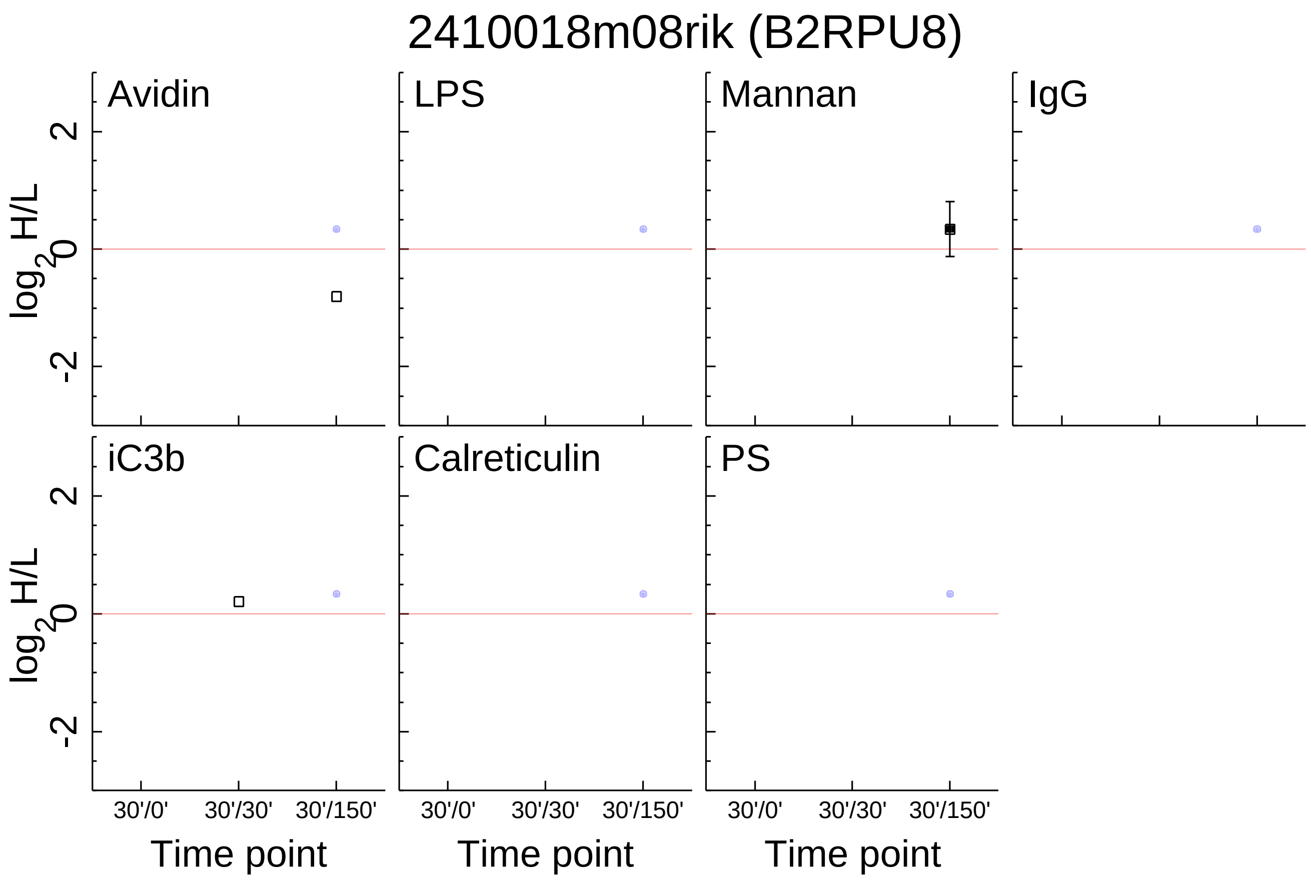
2410018m08rik (B2RPU8)
Avidin
LPS
Mannan
IgG
iC3b
Calreticulin
PS
log2 H/L
2
0
-2
30'/0'
30'/30'
30'/150'
Time point

2810422j05rik (E9QNP0)

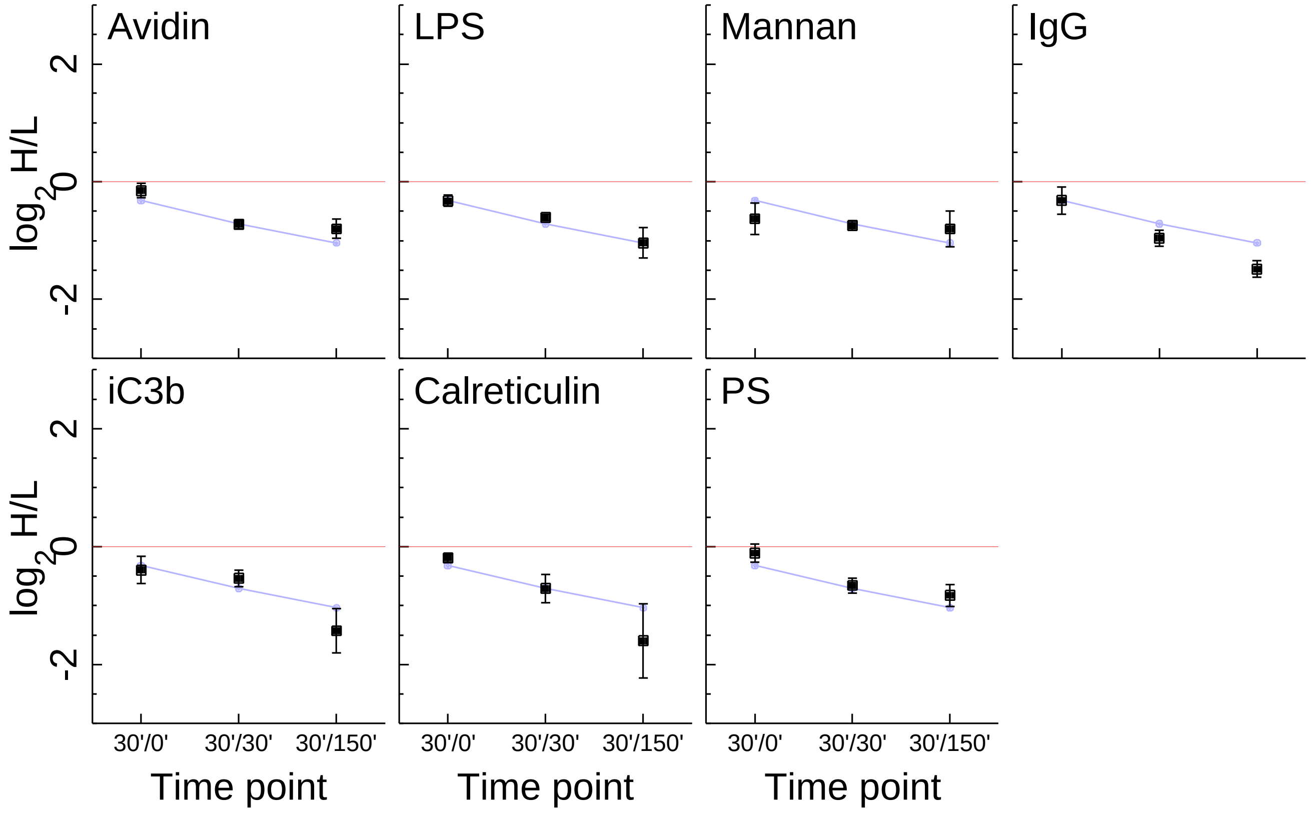

4632428n05rik (Q9D659)

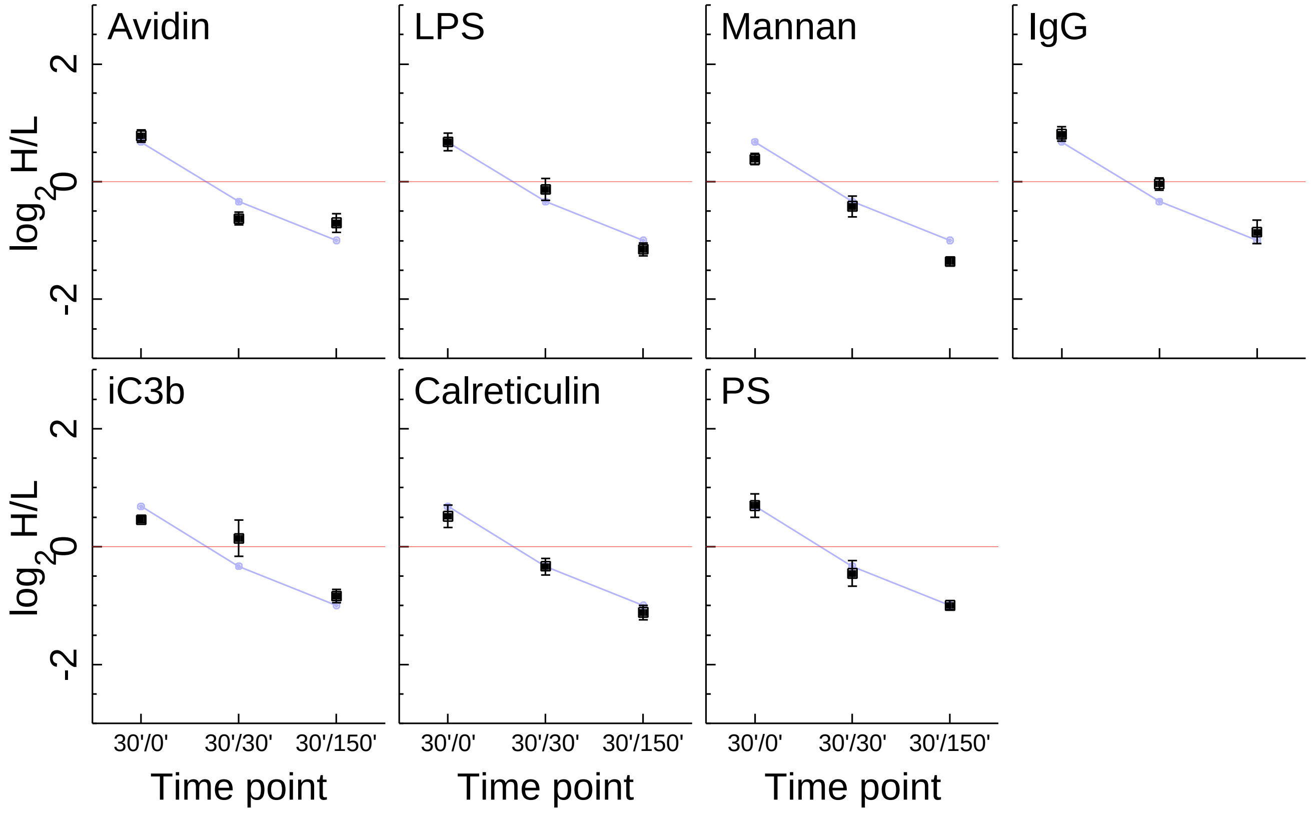

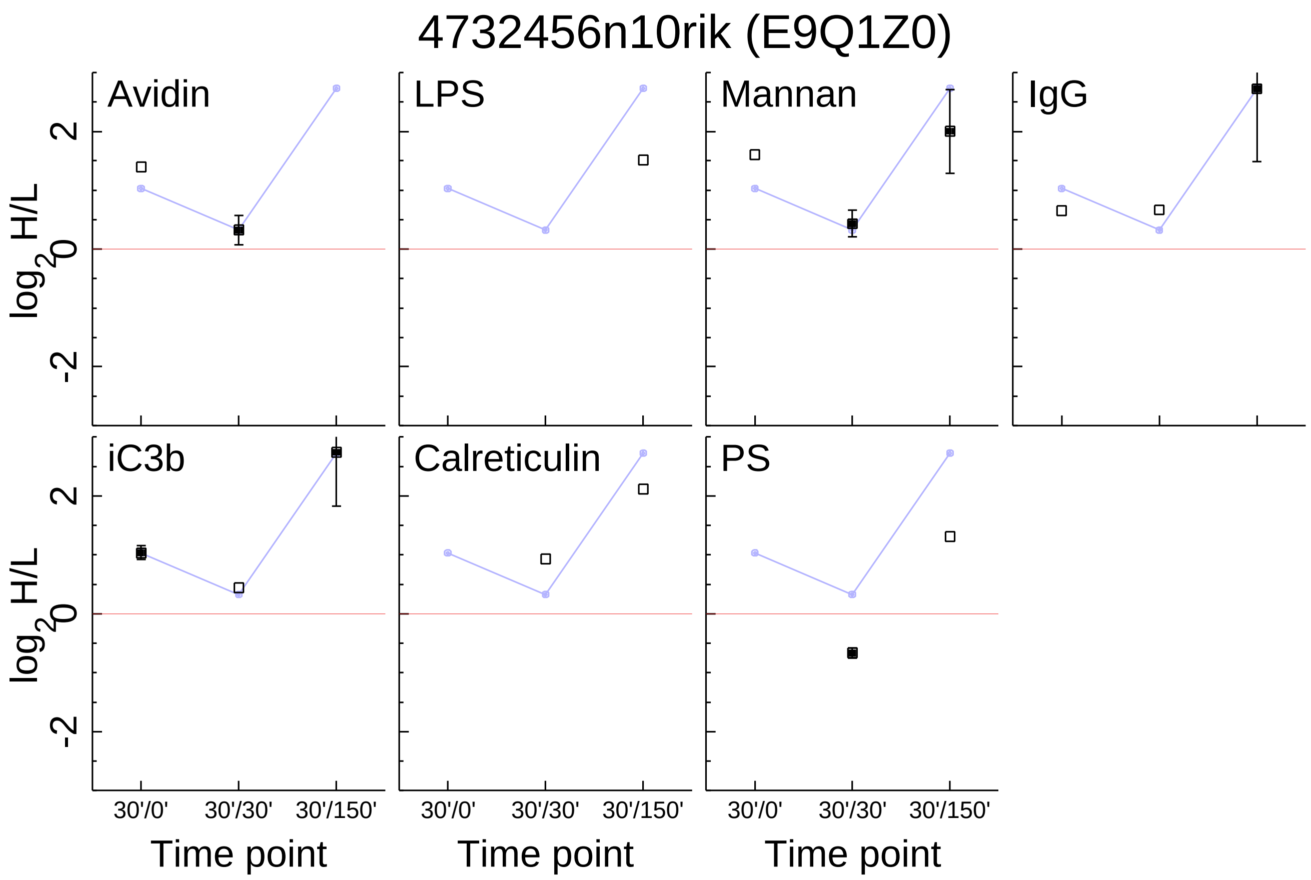

4732456n10rik (E9Q1Z0)
Avidin
LPS
Mannan
IgG
iC3b
Calreticulin
PS
log2 H/L
2
0
-2
30'/0'
30'/30'
30'/150'
Time point

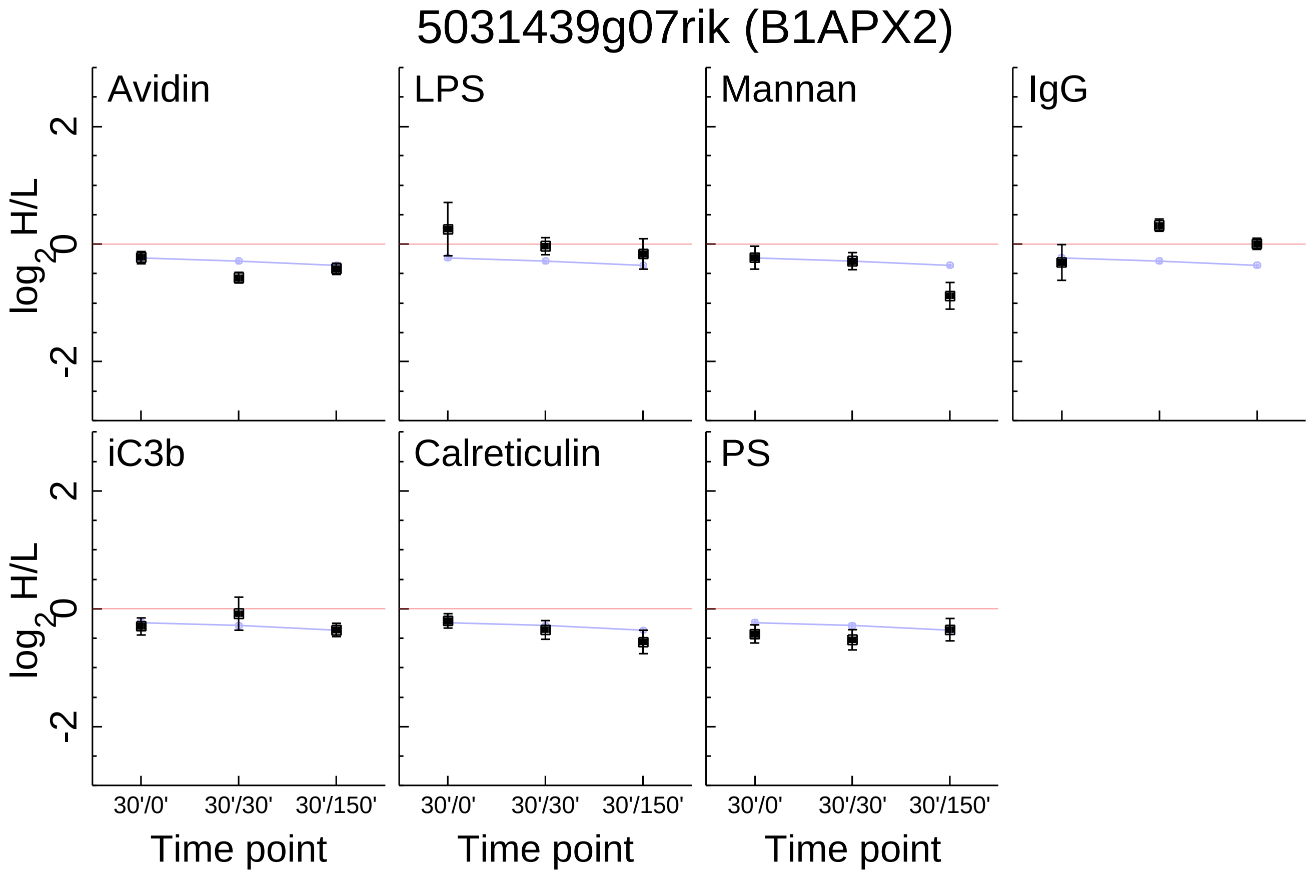
5031439g07rik (B1APX2)
Avidin
LPS
Mannan
IgG
iC3b
Calreticulin
PS
log2 H/L
2
0
-2
30'/0'
30'/30'
30'/150'
Time point

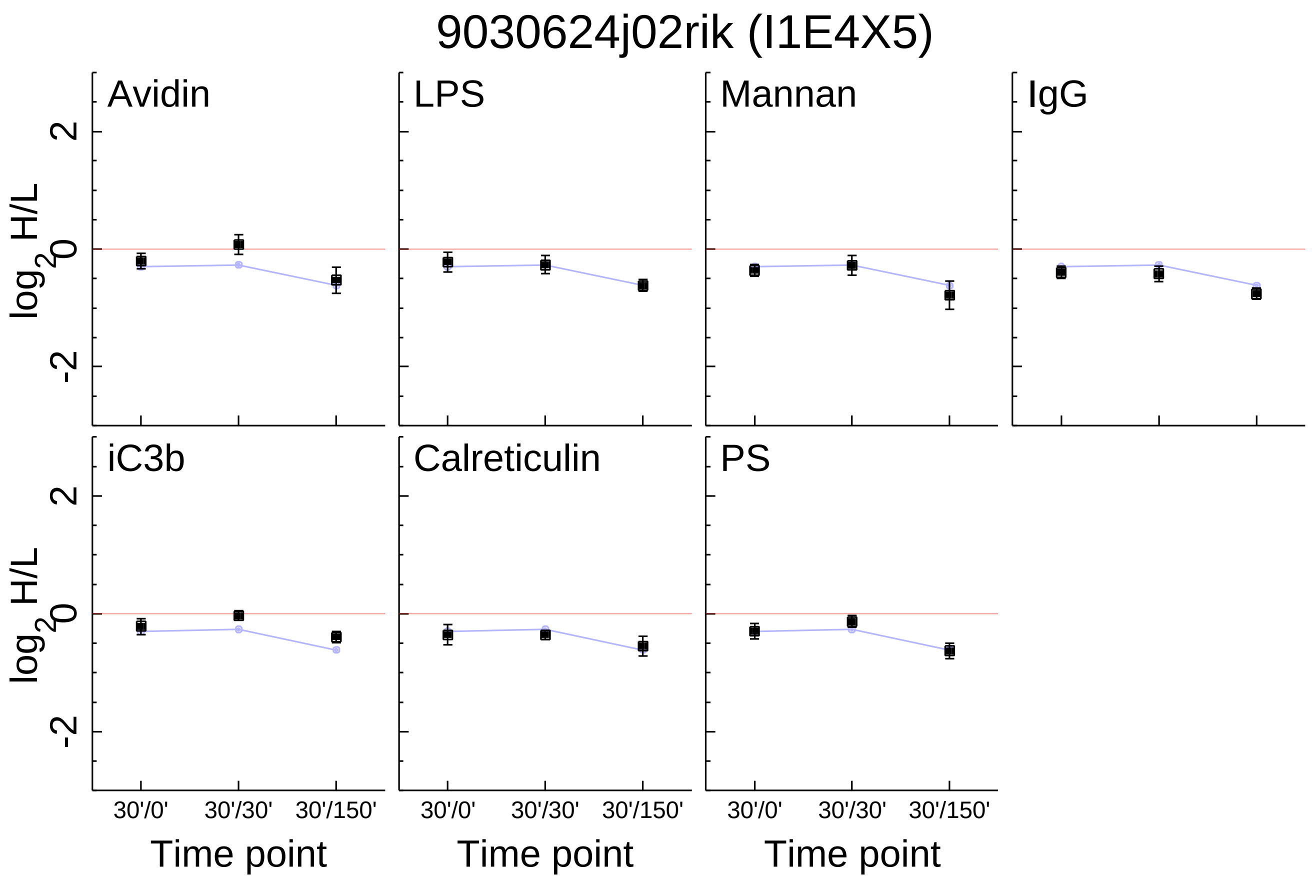
9030624j02rik (I1E4X5)
Avidin
LPS
Mannan
IgG
iC3b
Calreticulin
PS
log2 H/L
2
0
-2
30'/0'
30'/30'
30'/150'
Time point

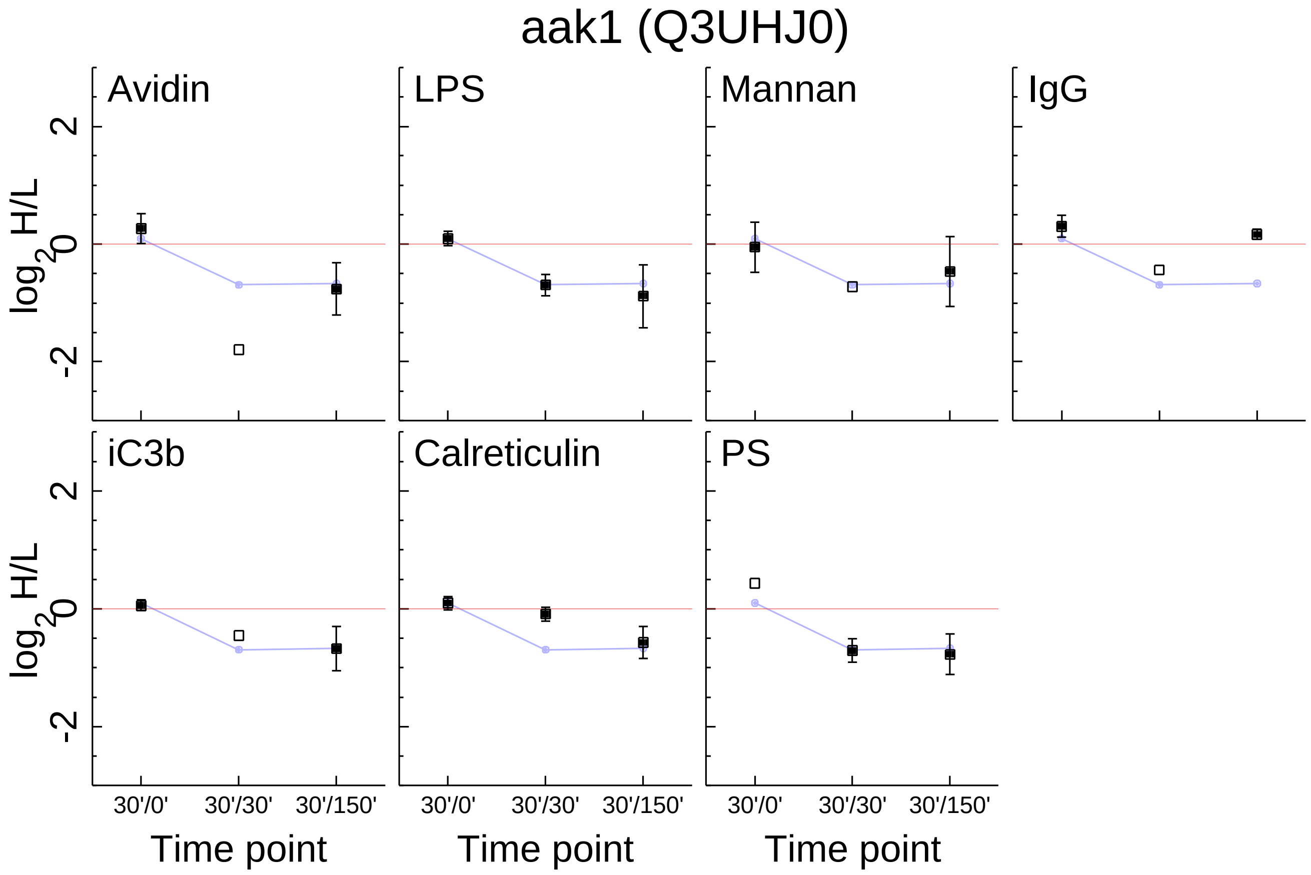
aak1 (Q3UHJ0)
Avidin
LPS
Mannan
IgG
iC3b
Calreticulin
PS
$\log_2$ H/L
2
0
-2
30'/0'
30'/30'
30'/150'
Time point

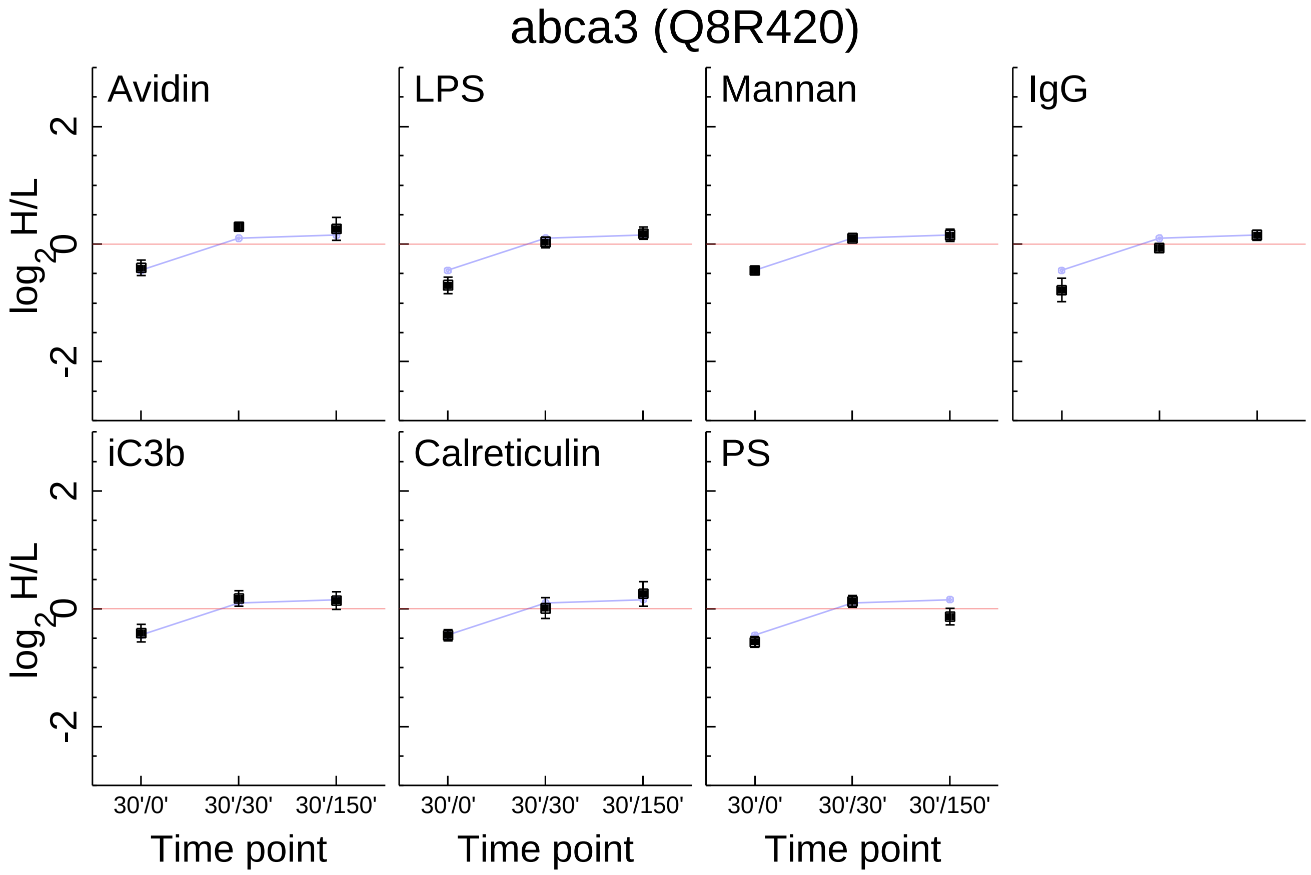

abca3 (Q8R420)
Avidin
LPS
Mannan
IgG
iC3b
Calreticulin
PS
log2 H/L
2
0
-2
30'/0'
30'/30'
30'/150'
Time point

abcb11 (J3QNY6)

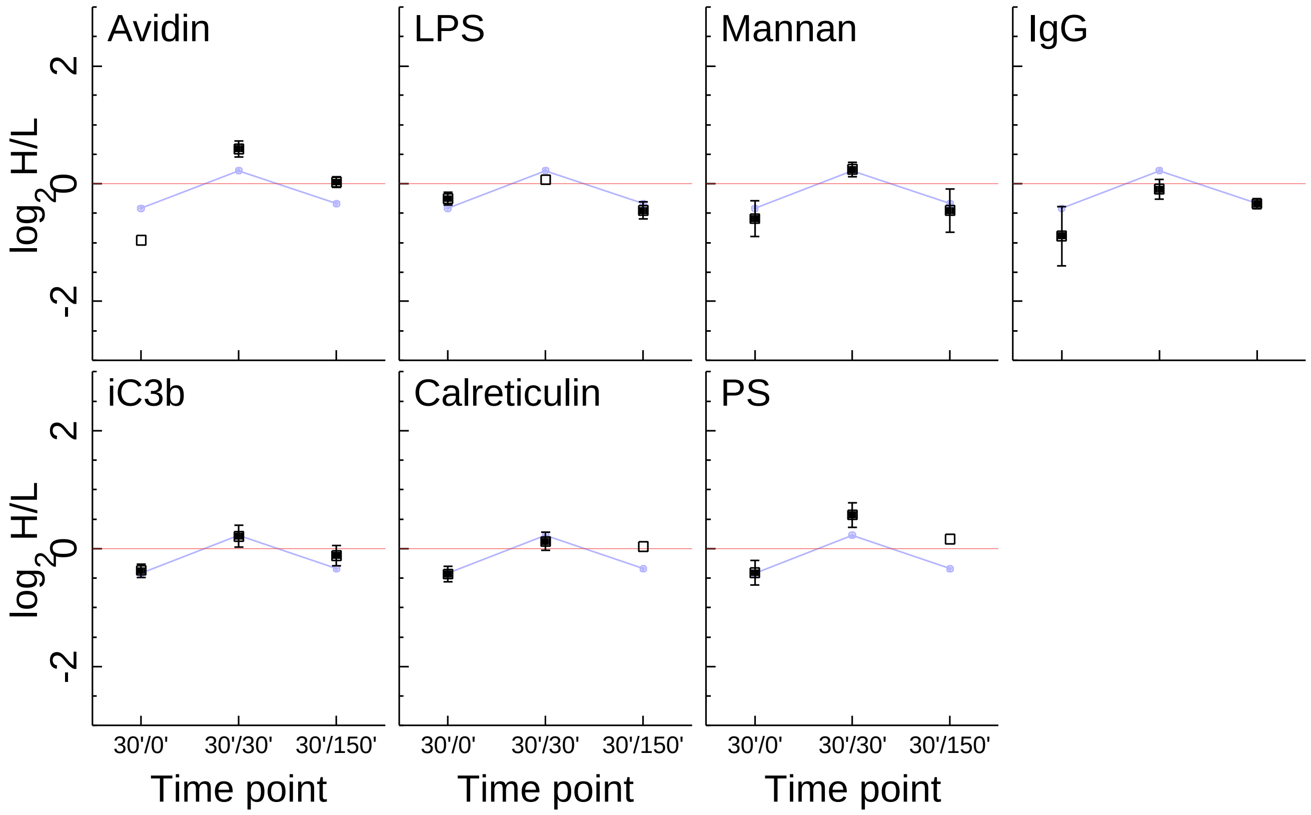

abcb1b (P06795)

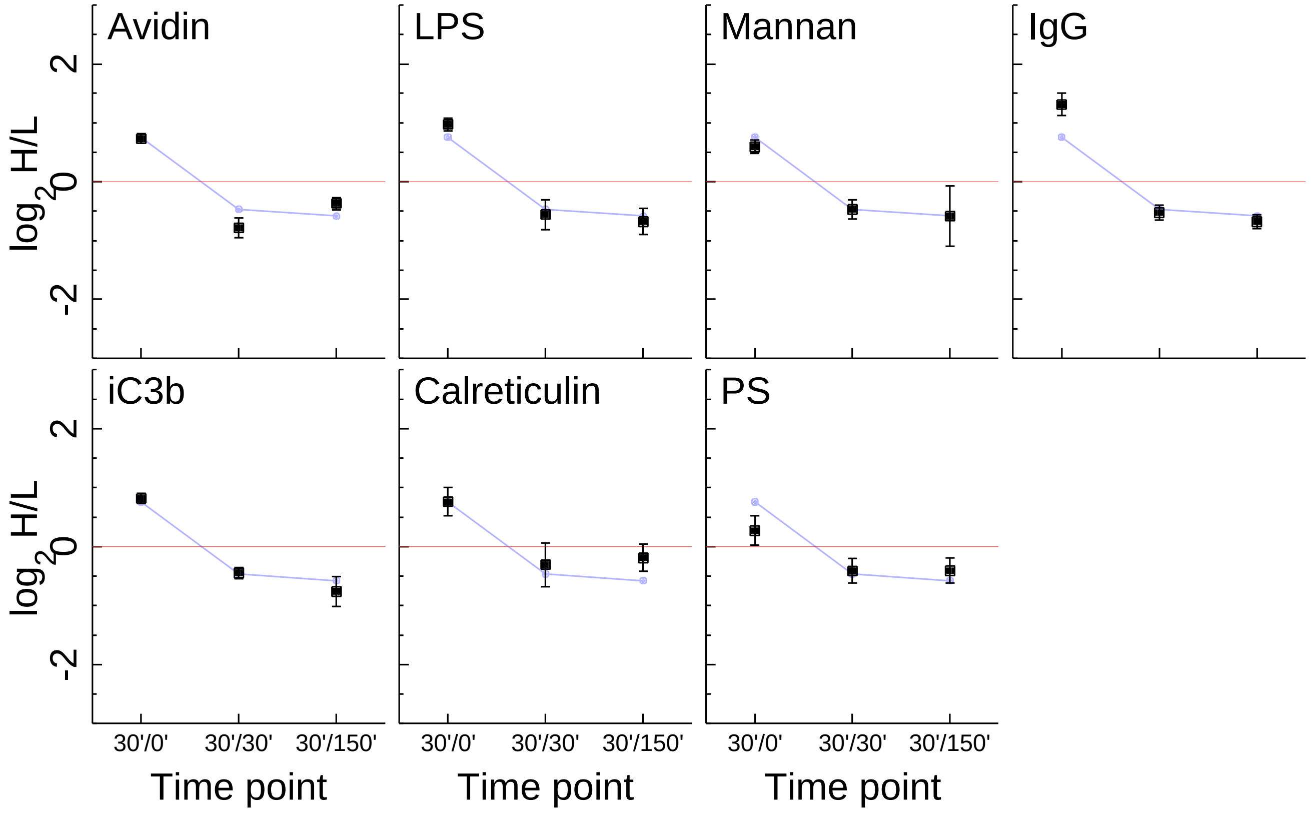

abcb6 (Q9DC29)

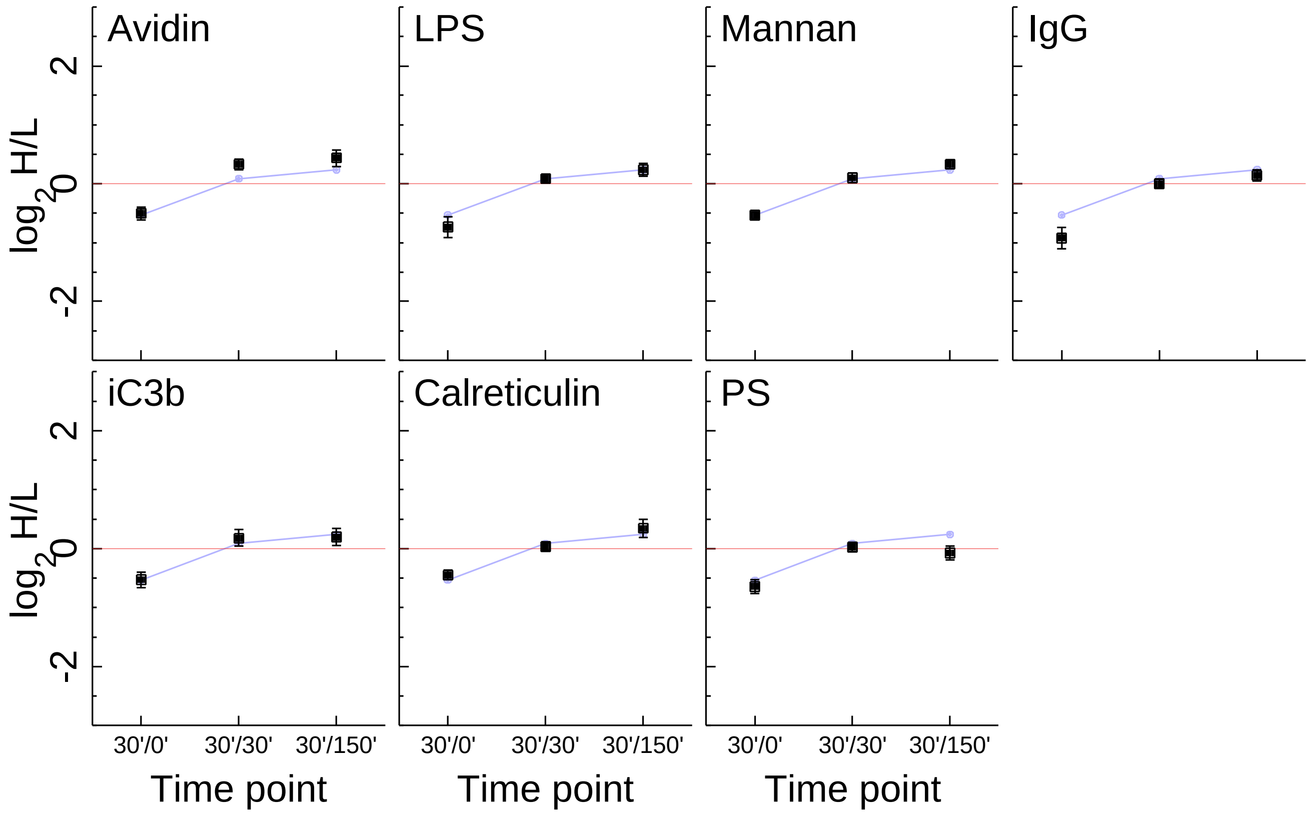

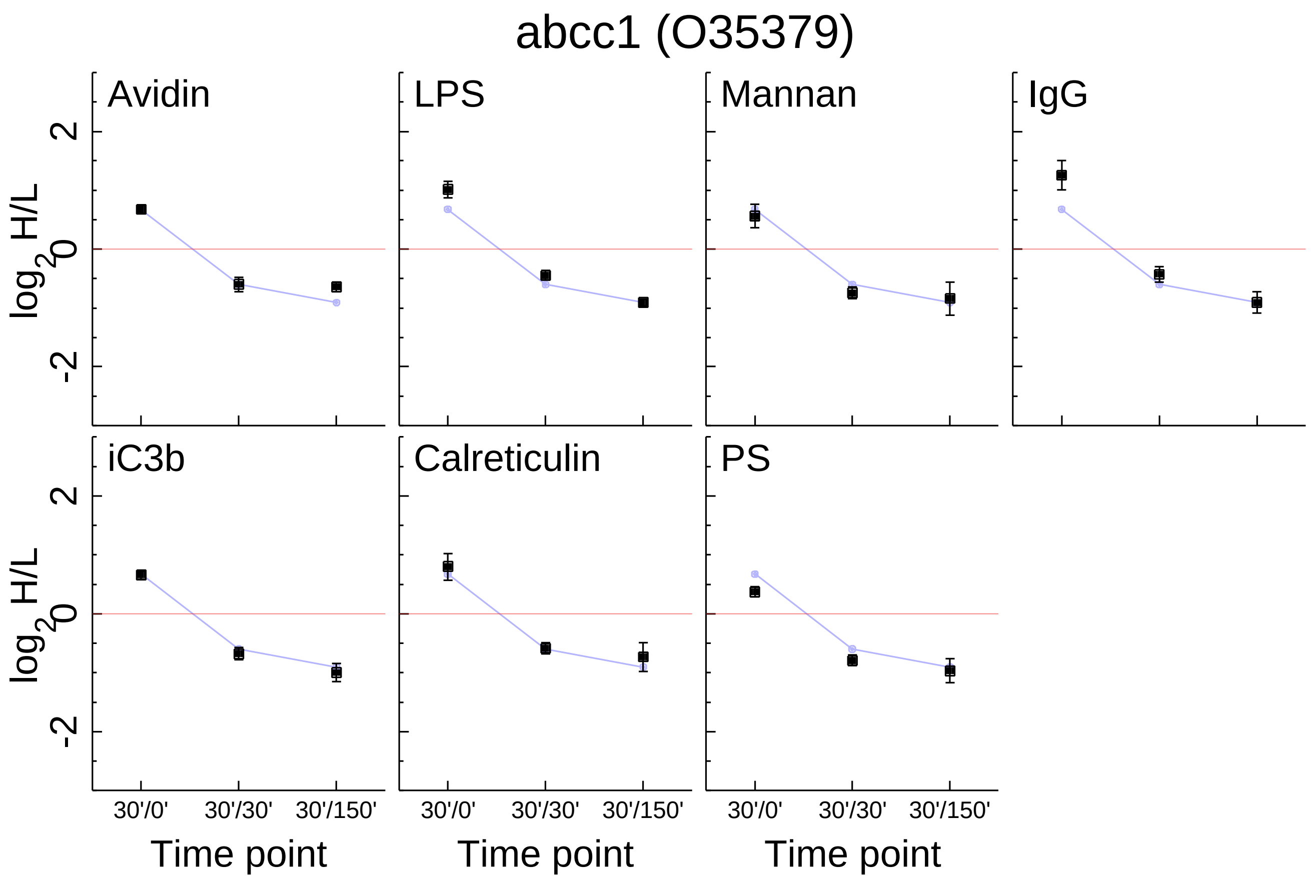
abcc1 (O35379)
Avidin
LPS
Mannan
IgG
iC3b
Calreticulin
PS
log2 H/L
2
0
-2
30'/0'
30'/30'
30'/150'
Time point

abcc3 (J3QML2)

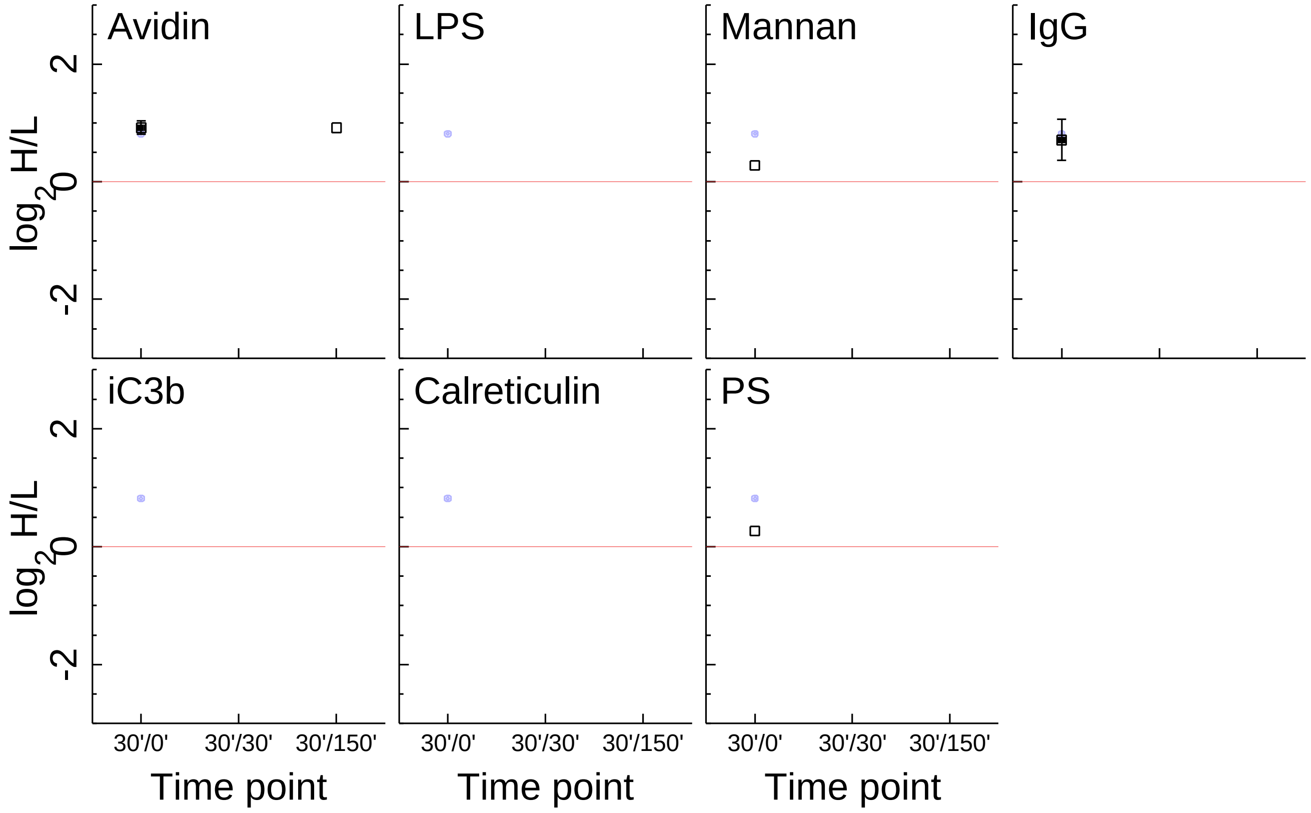

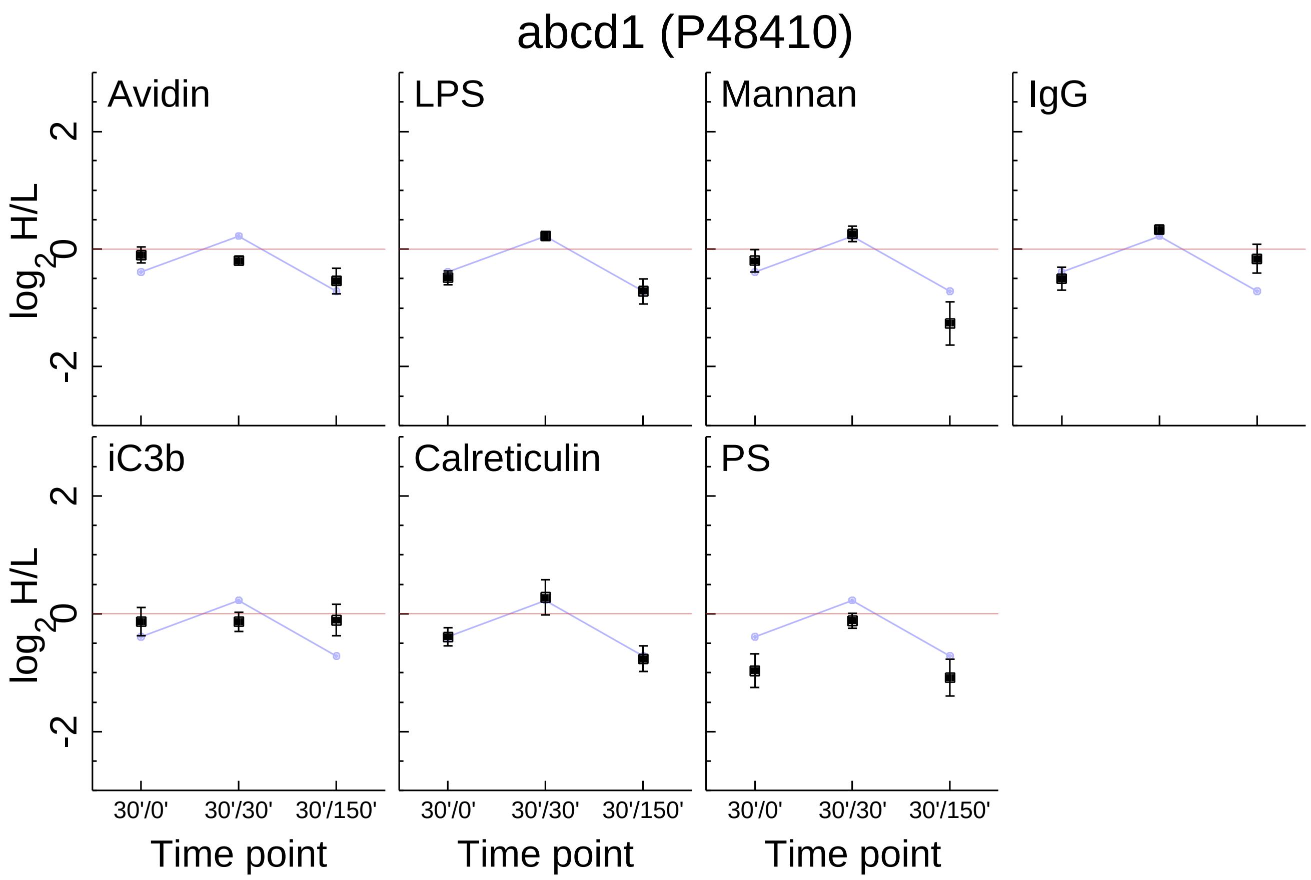
abcd1 (P48410)
Avidin
LPS
Mannan
IgG
iC3b
Calreticulin
PS
2
0
-2
log2 H/L
30'/0'
30'/30'
30'/150'
Time point

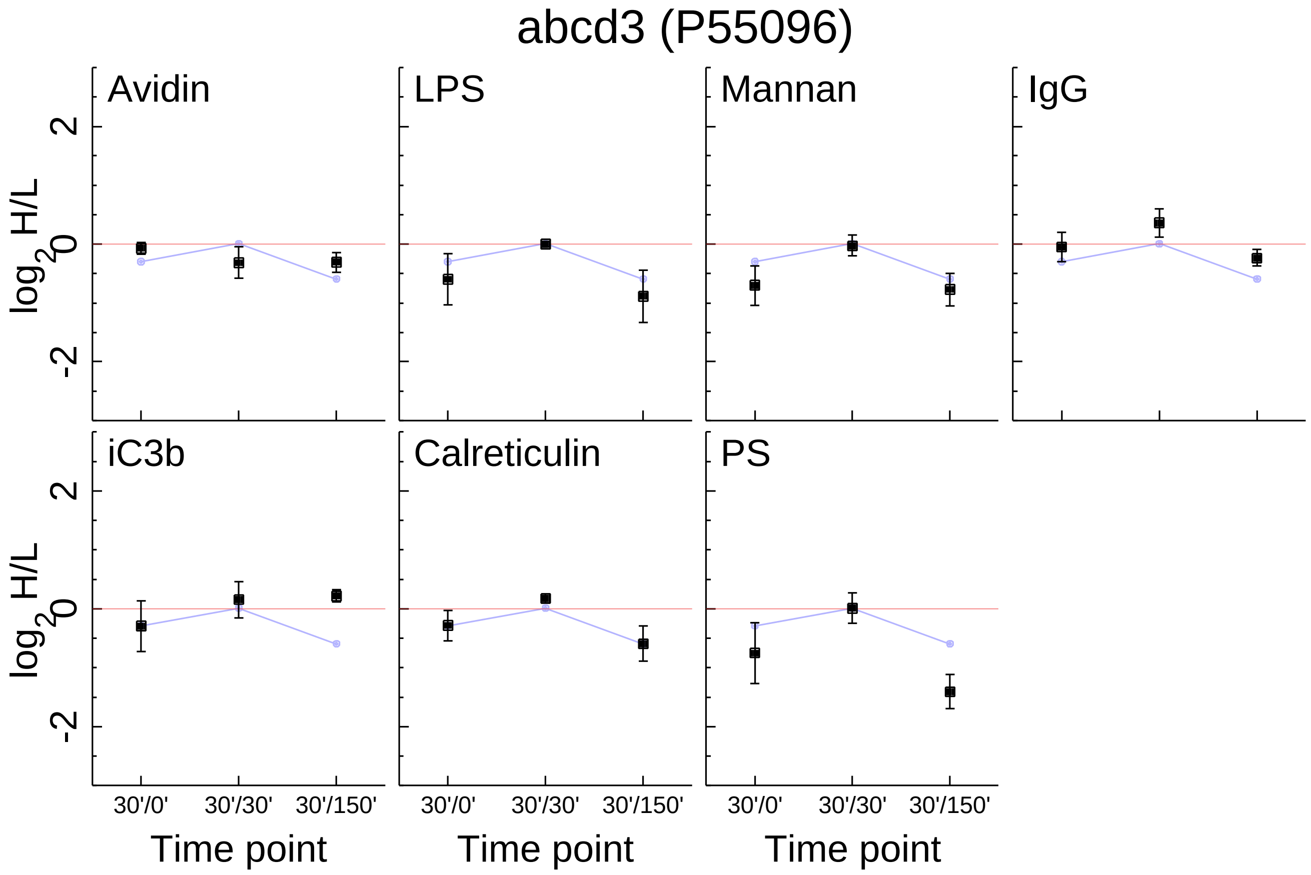
abcd3 (P55096)
Avidin
LPS
Mannan
IgG
iC3b
Calreticulin
PS
log2 H/L
2
0
-2
30'/0'
30'/30'
30'/150'
Time point

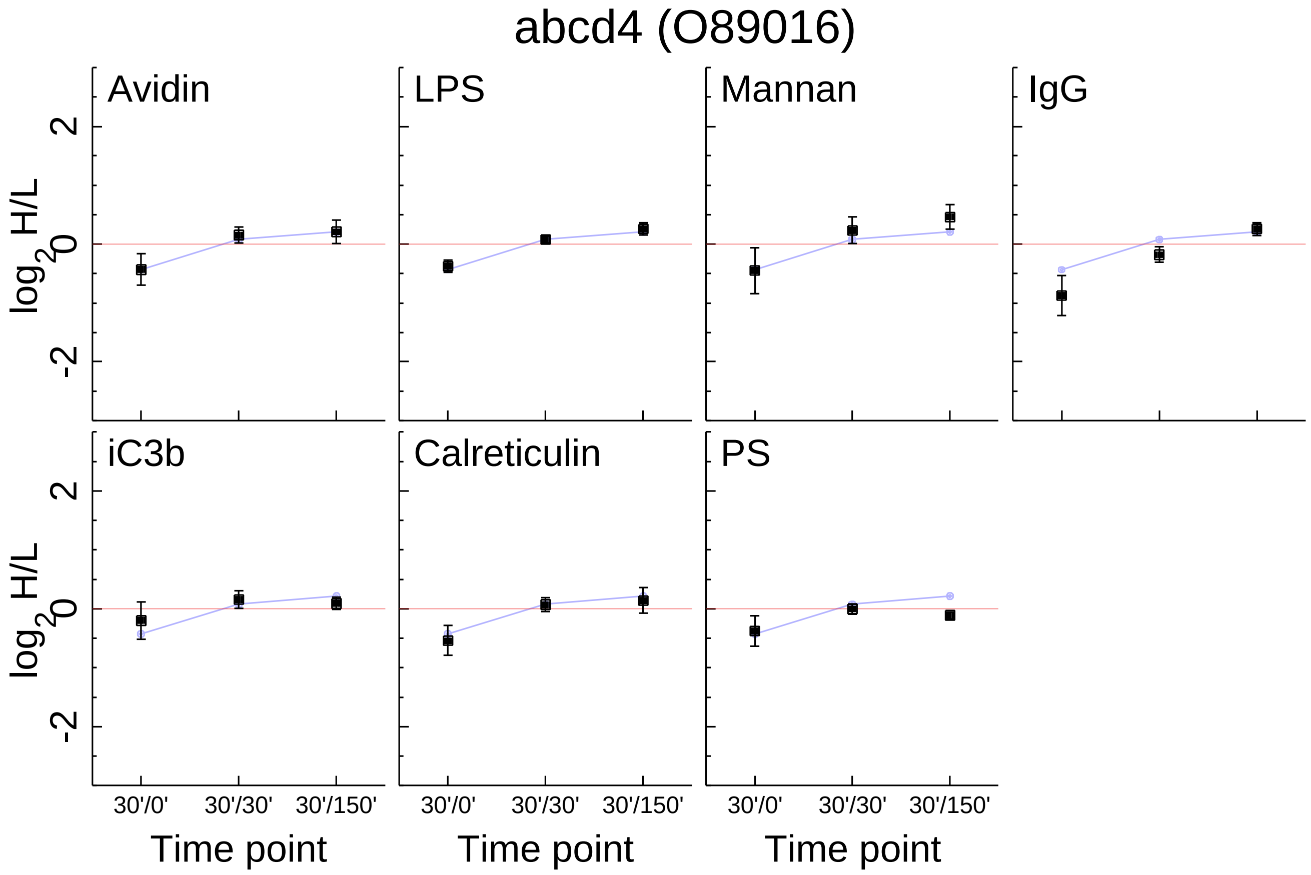
abcd4 (O89016)
Avidin
LPS
Mannan
IgG
iC3b
Calreticulin
PS
log2 H/L
2
0
-2
30'/0'
30'/30'
30'/150'
Time point

abcf2 (Q99LE6)

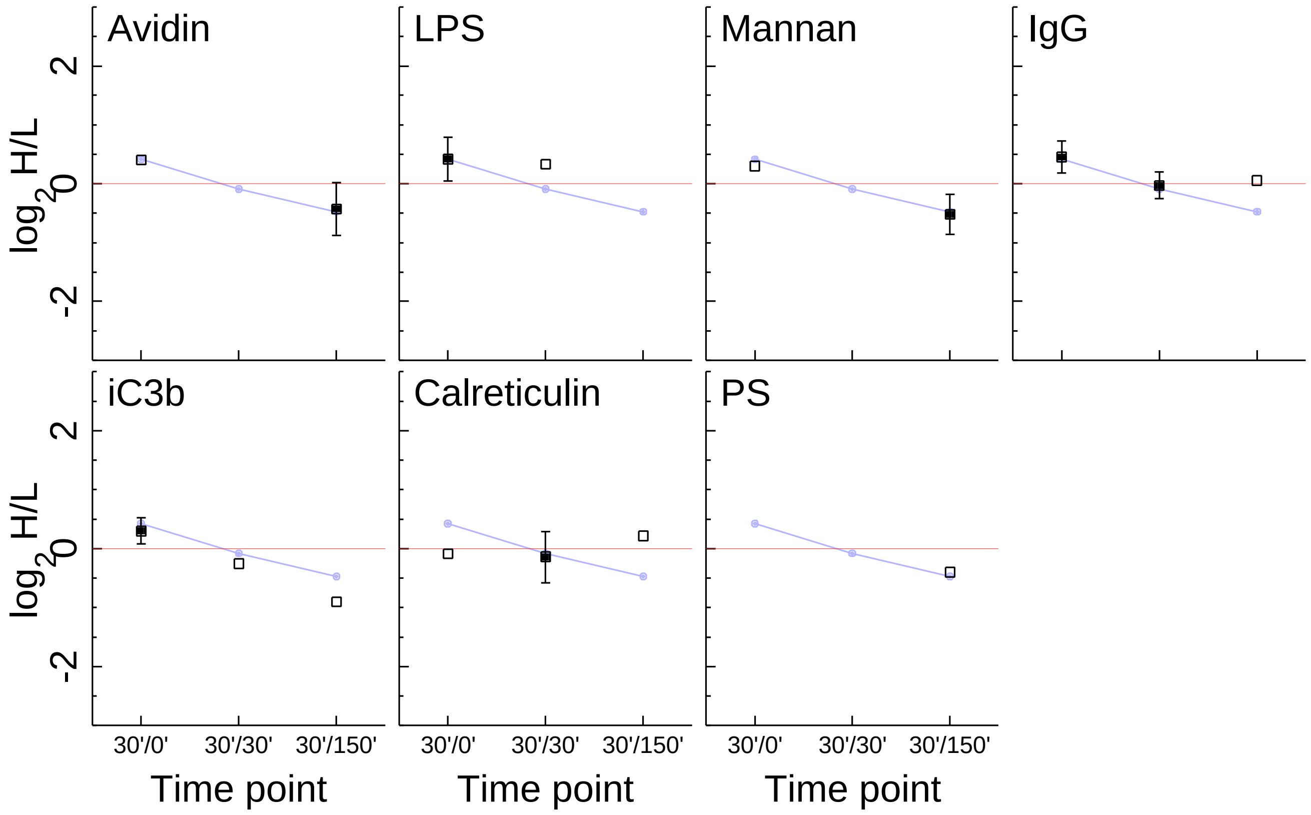

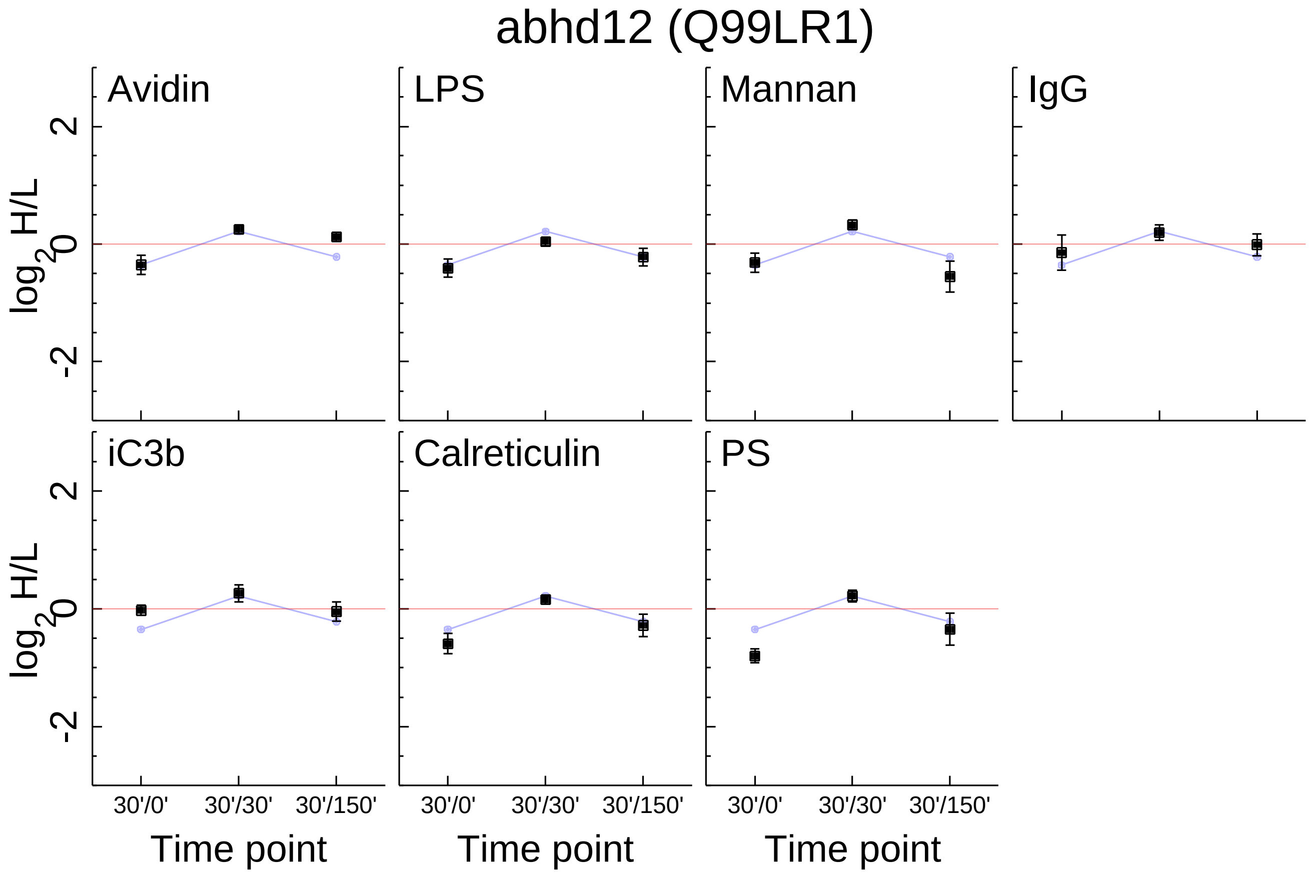
abhd12 (Q99LR1)
Avidin
LPS
Mannan
IgG
iC3b
Calreticulin
PS
log2 H/L
2
0
-2
30'/0'
30'/30'
30'/150'
Time point

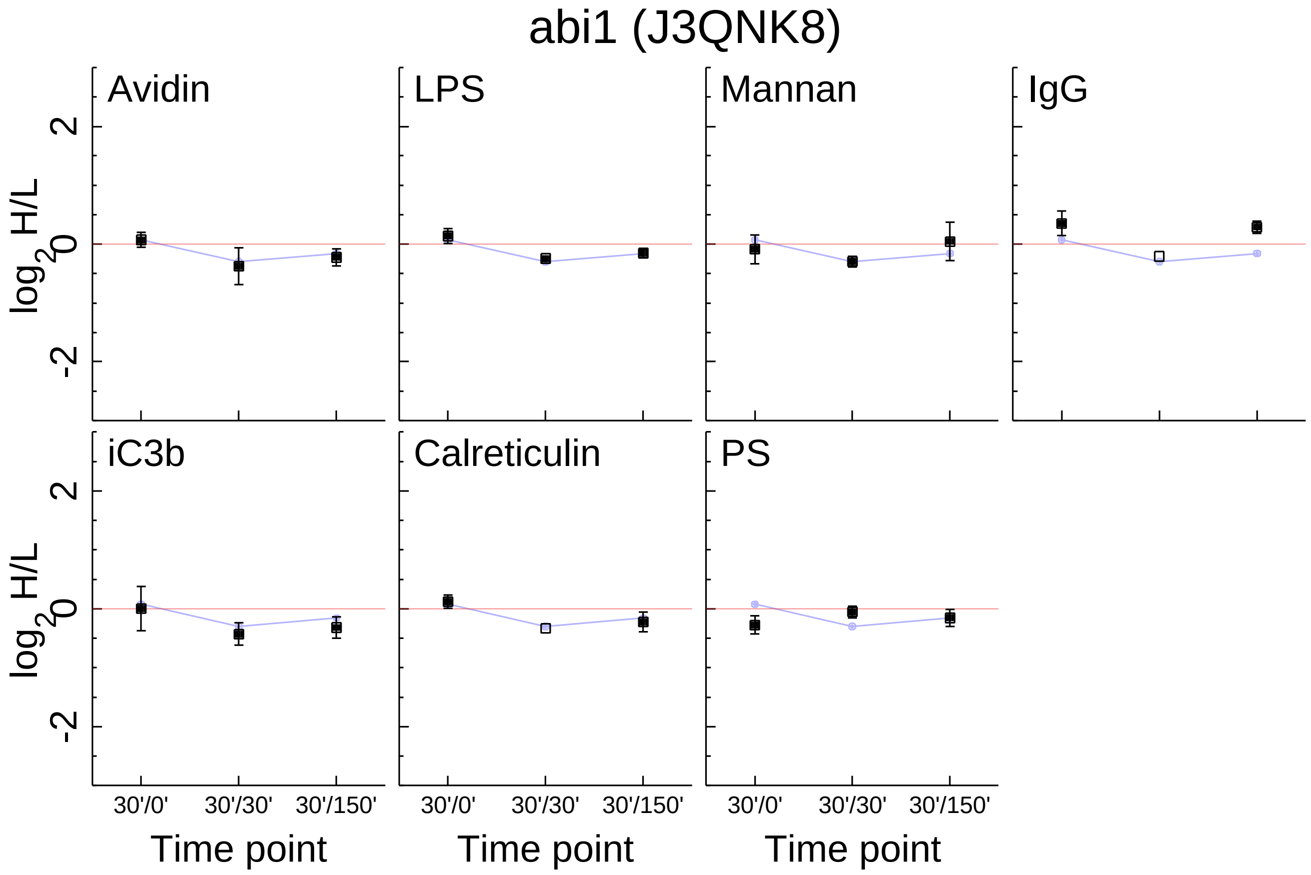
abi1 (J3QNK8)
Avidin
LPS
Mannan
IgG
iC3b
Calreticulin
PS
log2 H/L
2
0
-2
30'/0'
30'/30'
30'/150'
Time point

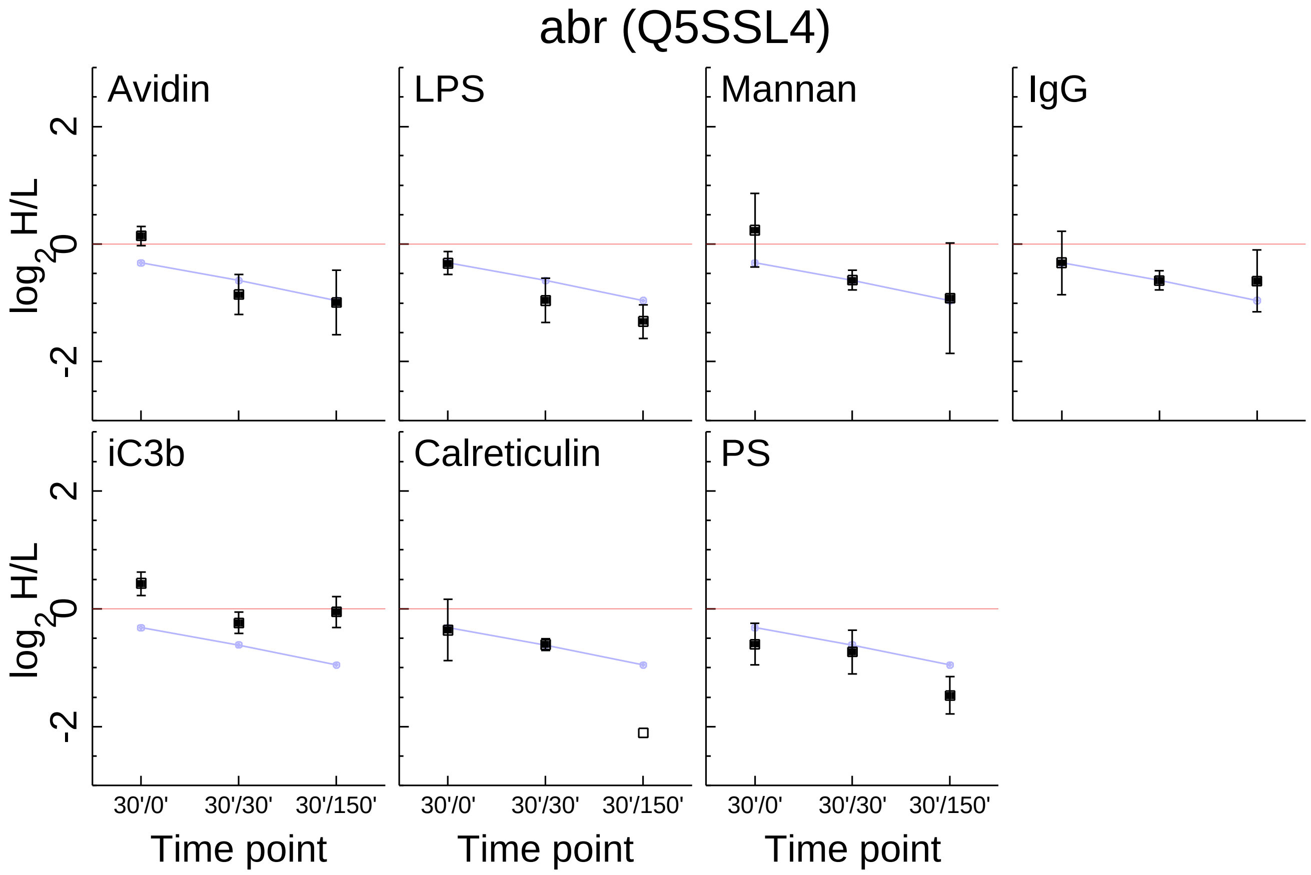
abr (Q5SSL4)
Avidin
LPS
Mannan
IgG
iC3b
Calreticulin
PS
log2 H/L
2
0
-2
30'/0'
30'/30'
30'/150'
Time point

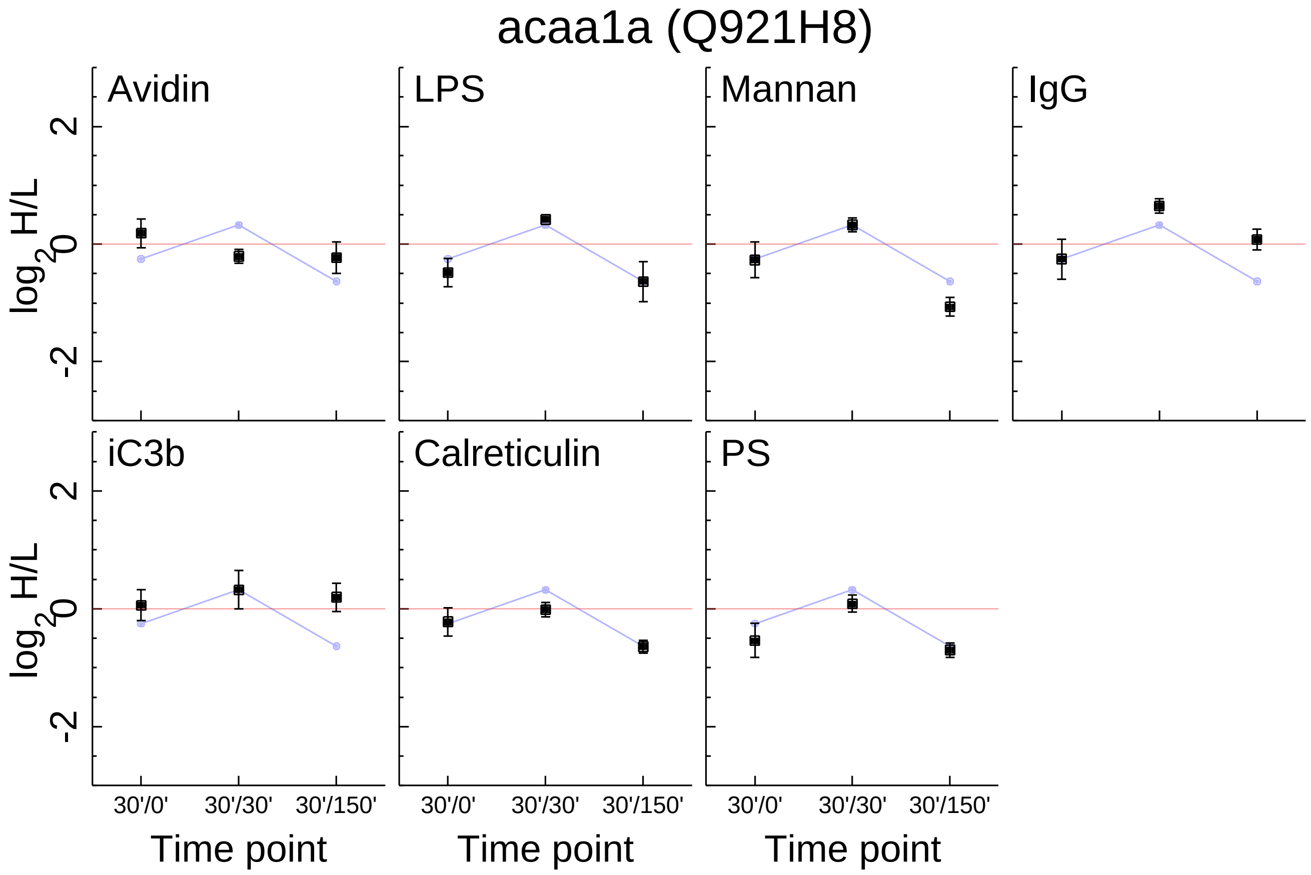
acaa1a (Q921H8)
Avidin
LPS
Mannan
IgG
iC3b
Calreticulin
PS
log2 H/L
2
0
-2
30'/0'
30'/30'
30'/150'
Time point

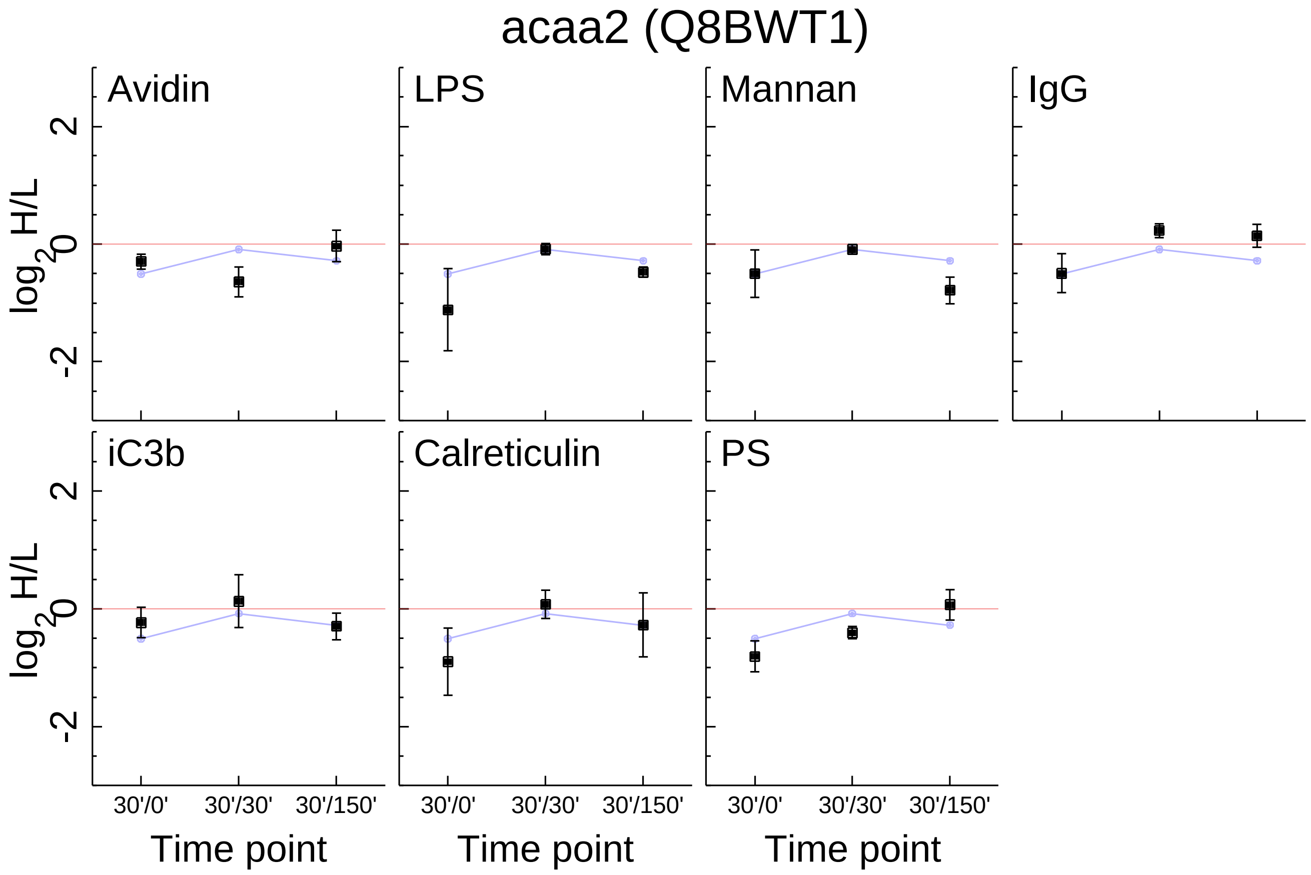
acaa2 (Q8BWT1)
Avidin
LPS
Mannan
IgG
iC3b
Calreticulin
PS
log2 H/L
2
0
-2
30'/0'
30'/30'
30'/150'
Time point

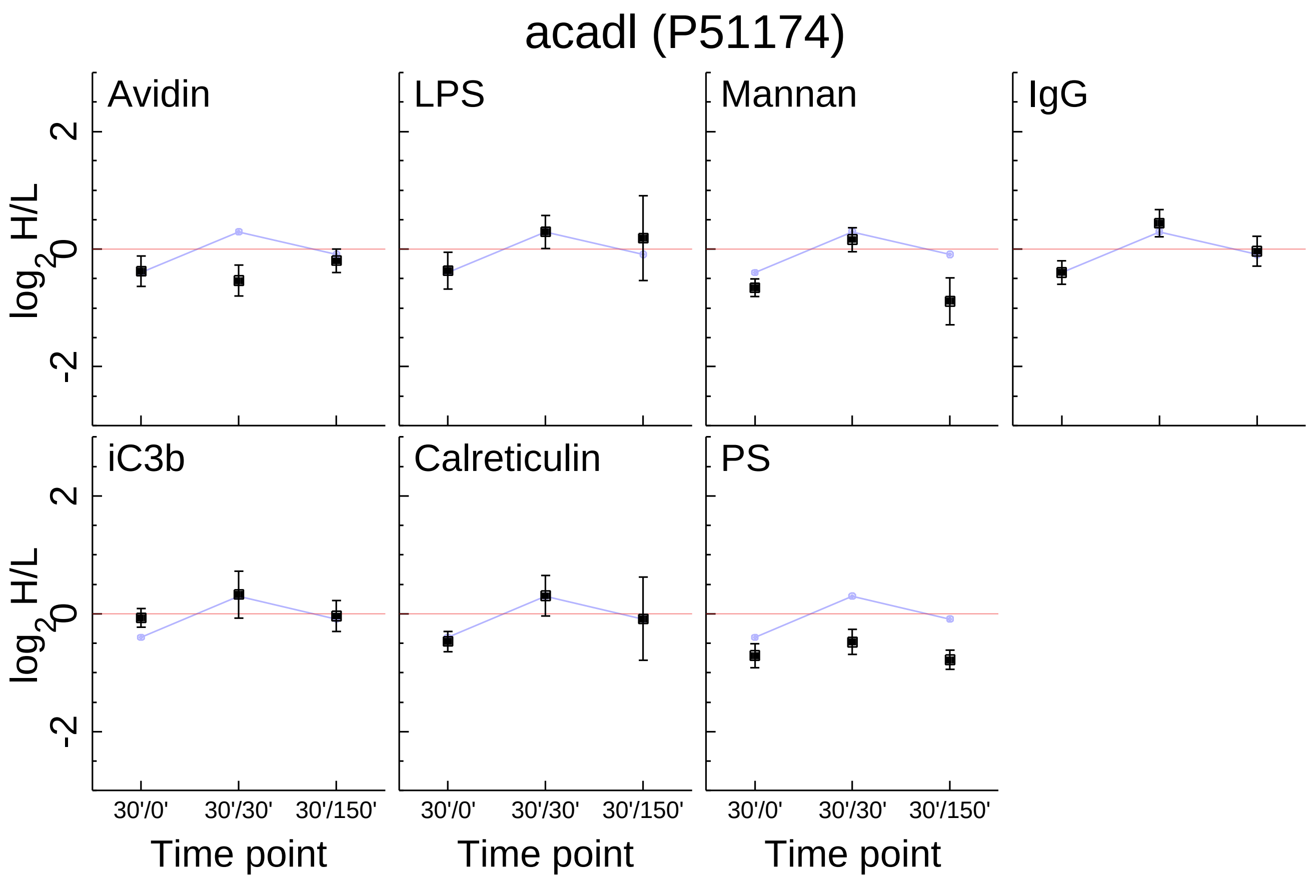
acadl (P51174)
Avidin
LPS
Mannan
IgG
iC3b
Calreticulin
PS
log2 H/L
2
0
-2
30'/0'
30'/30'
30'/150'
Time point

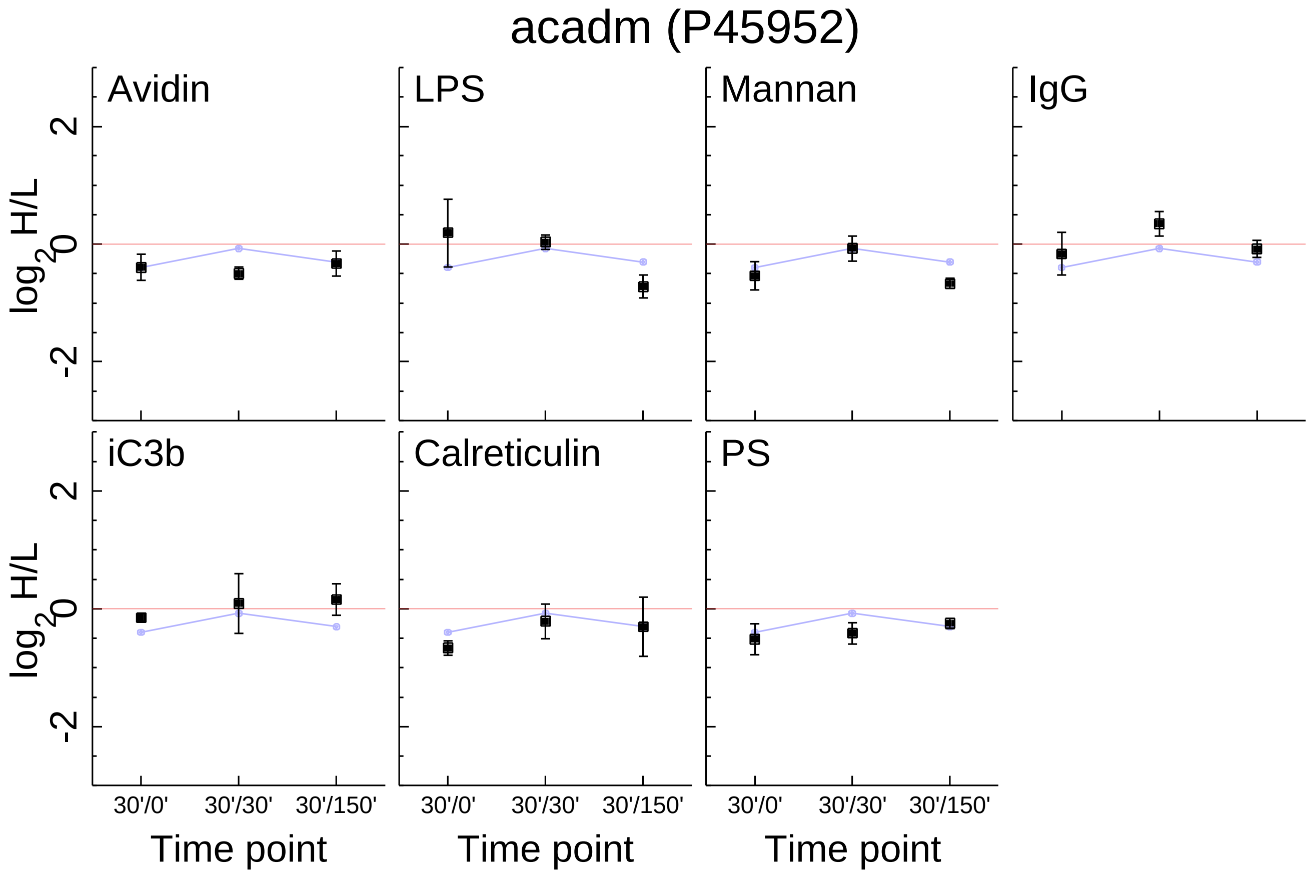
acadm (P45952)
Avidin
LPS
Mannan
IgG
iC3b
Calreticulin
PS
log2 H/L
2
0
-2
30'/0'
30'/30'
30'/150'
Time point

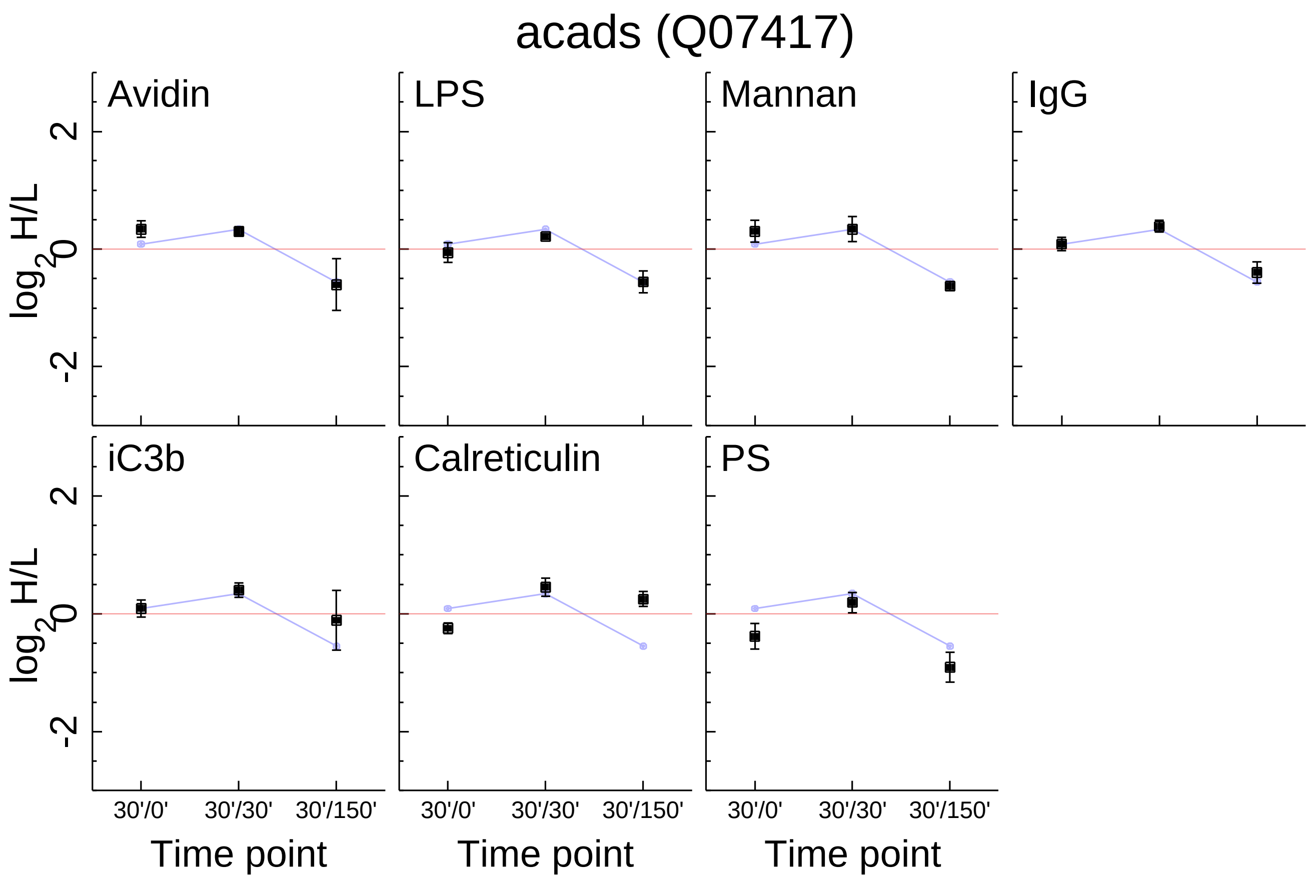
acads (Q07417)
Avidin
LPS
Mannan
IgG
iC3b
Calreticulin
PS
log2 H/L
2
0
-2
30'/0'
30'/30'
30'/150'
Time point

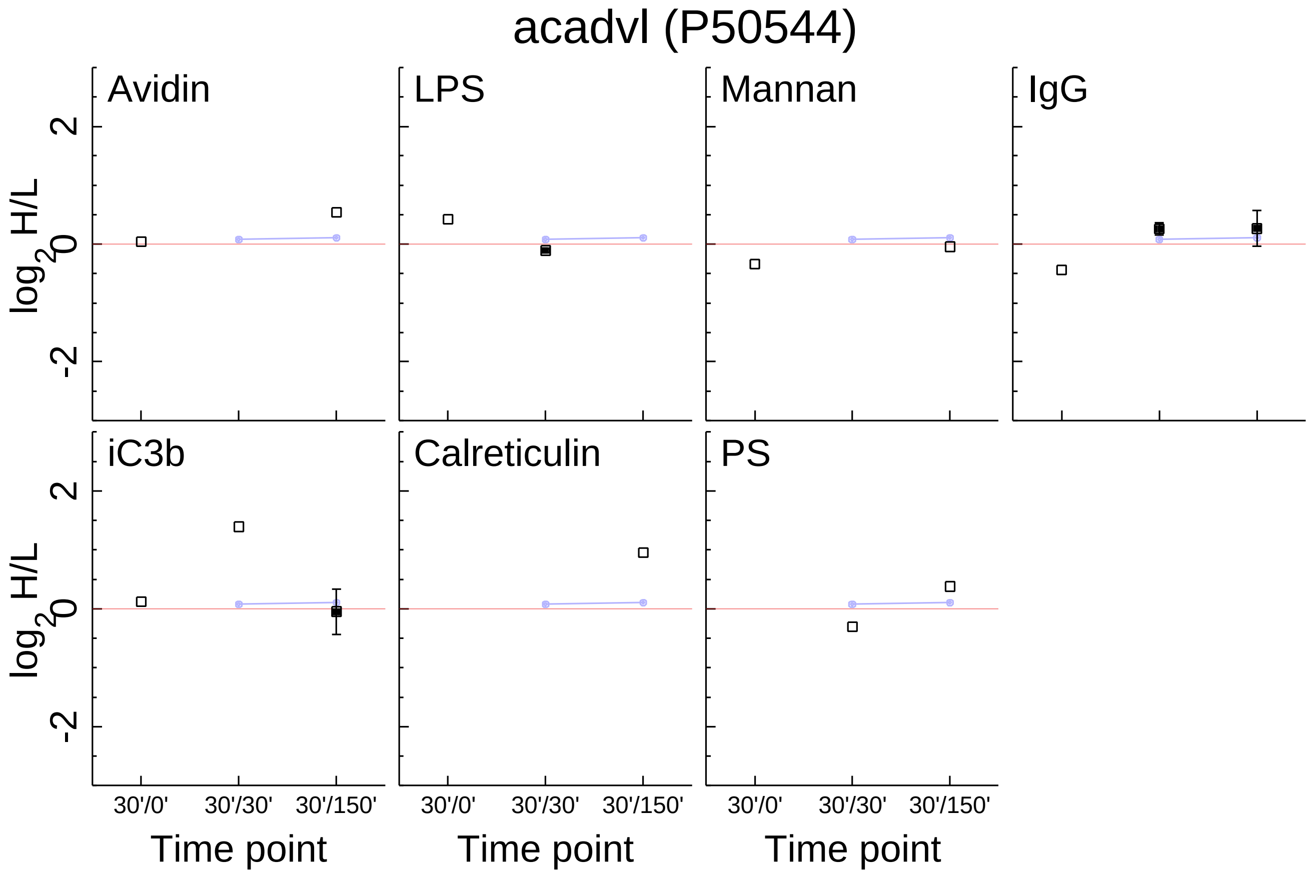
acadvl (P50544)
Avidin
LPS
Mannan
IgG
iC3b
Calreticulin
PS
log2 H/L
2
0
-2
30'/0'
30'/30'
30'/150'
Time point

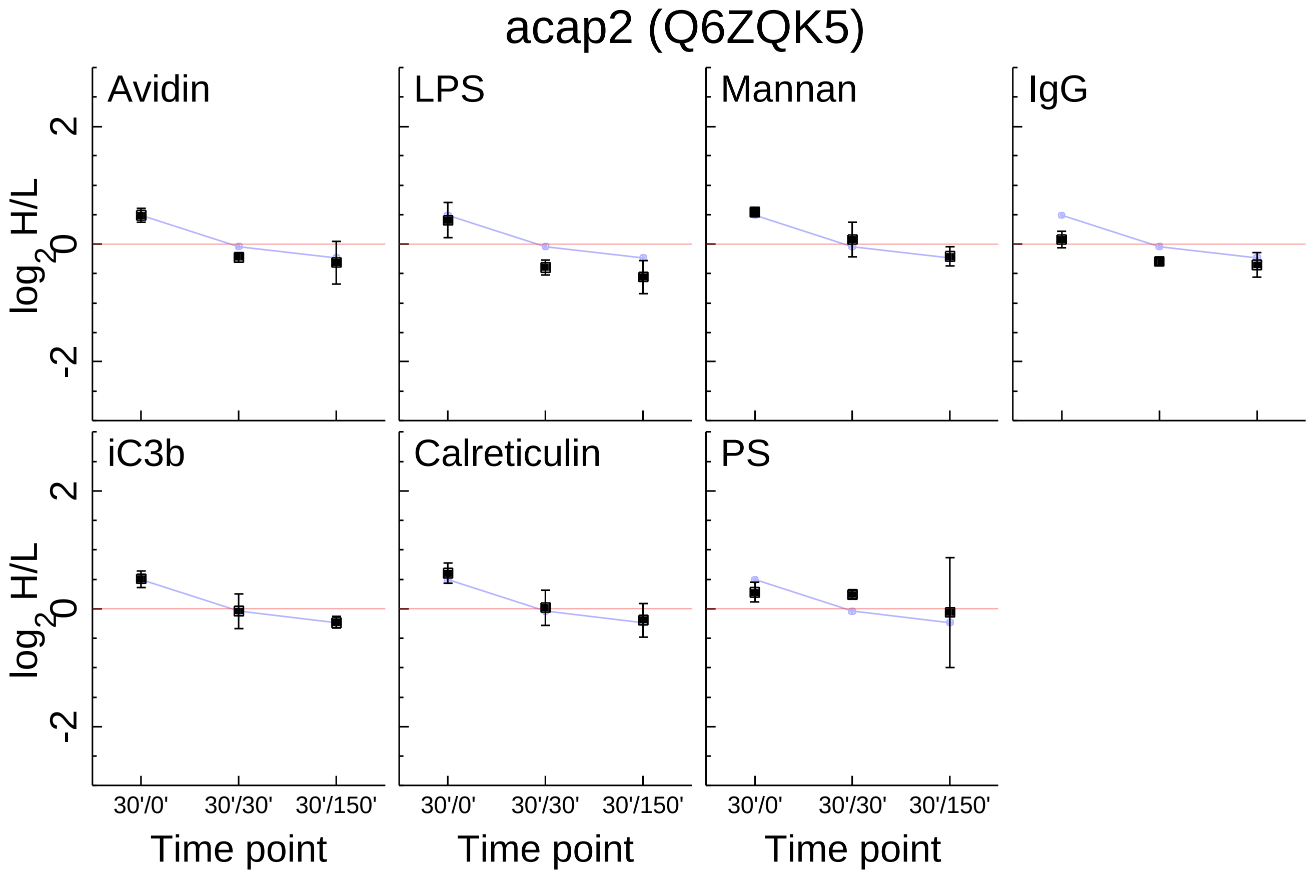

acap2 (Q6ZQK5)
Avidin
LPS
Mannan
IgG
iC3b
Calreticulin
PS
2
0
-2
log2 H/L
30'/0'
30'/30'
30'/150'
Time point

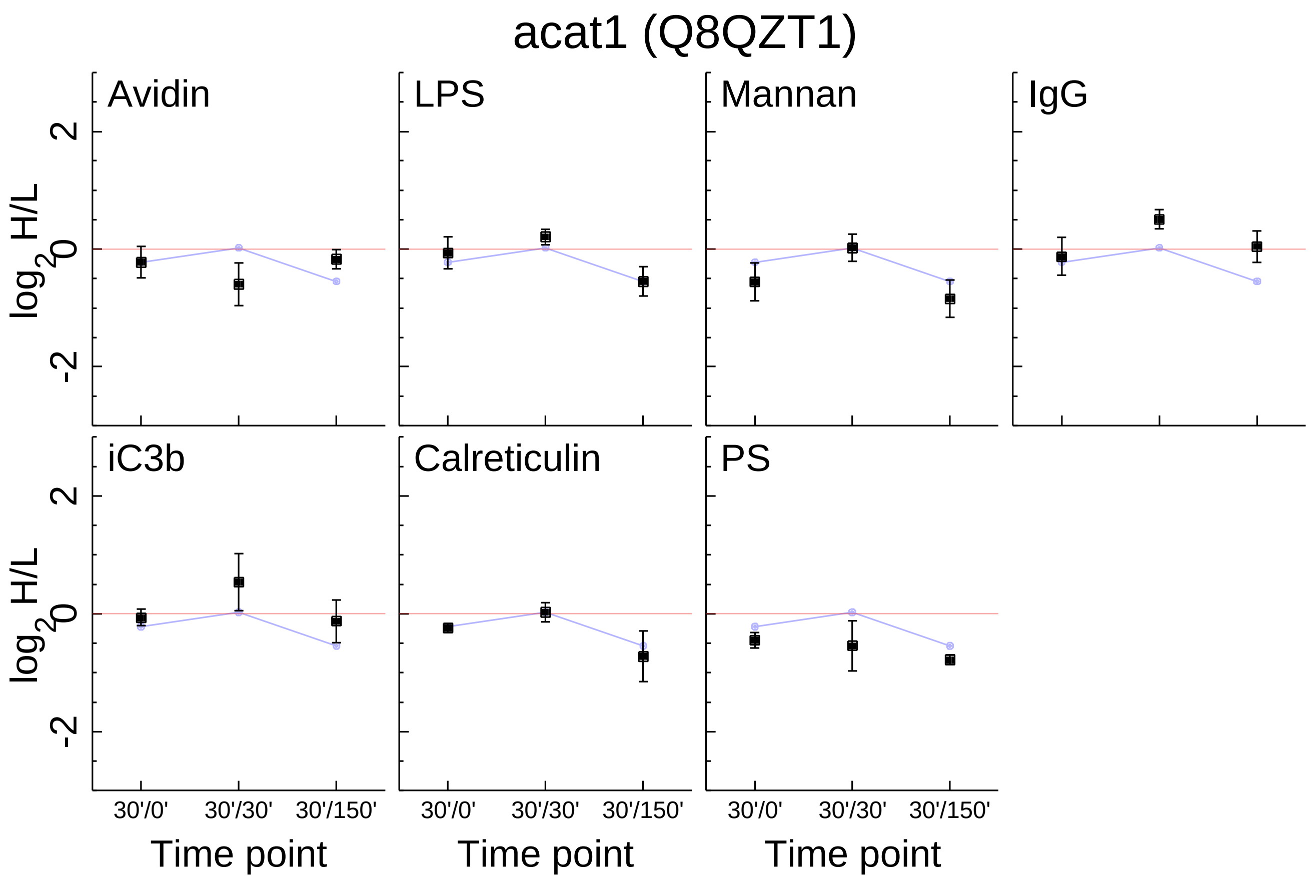
acat1 (Q8QZT1)
Avidin
LPS
Mannan
IgG
iC3b
Calreticulin
PS
log2 H/L
2
0
-2
30'/0'
30'/30'
30'/150'
Time point

acbd3 (Q8BMP6)

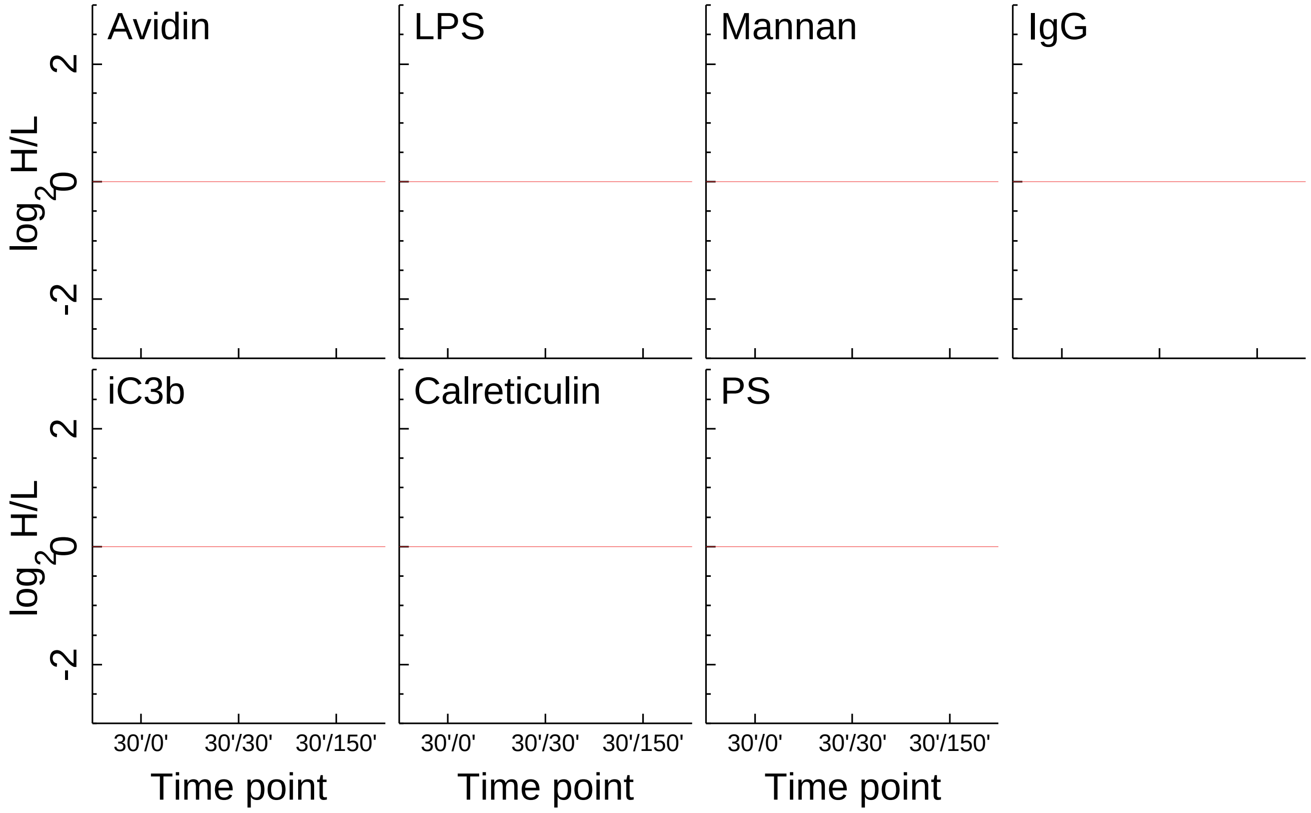

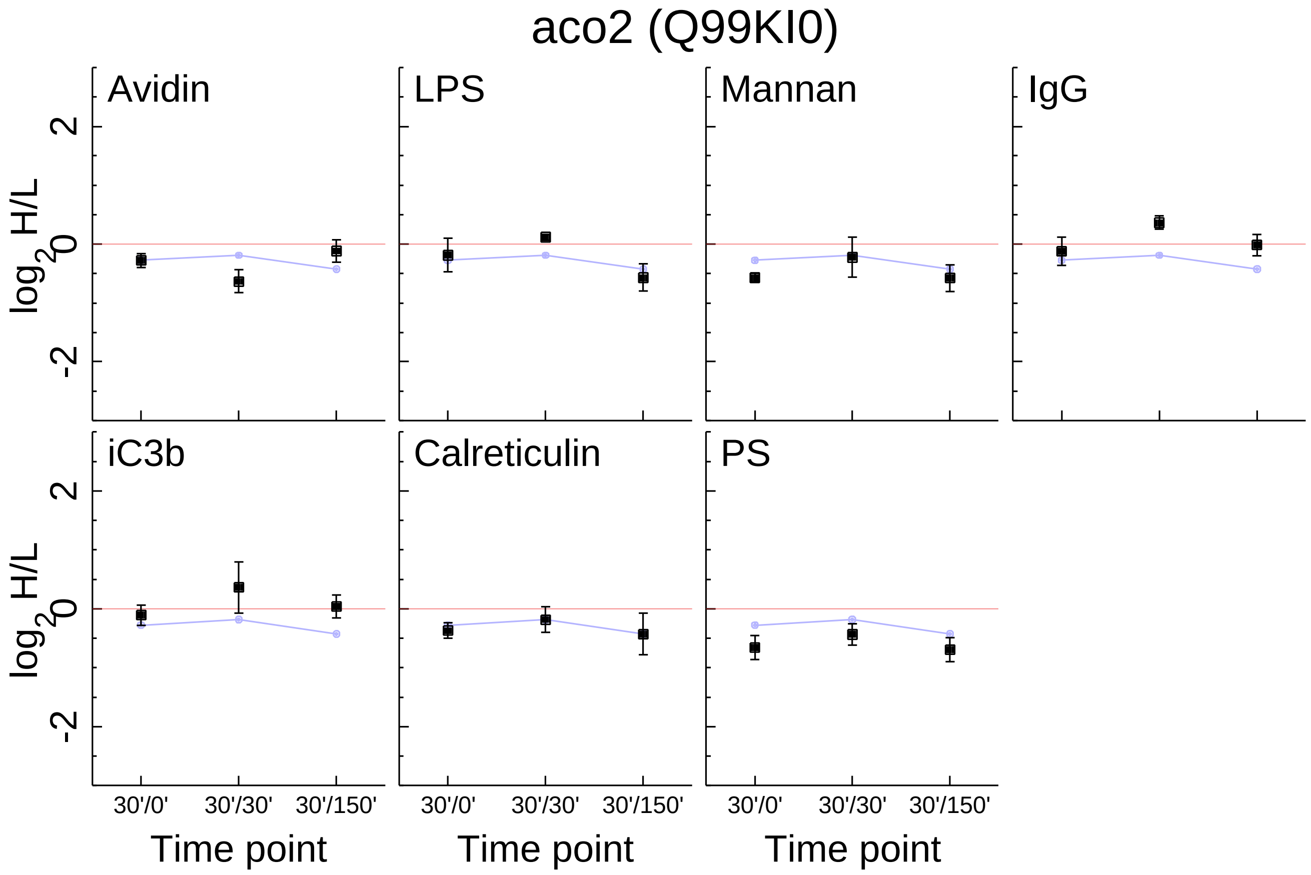

aco2 (Q99KI0)
Avidin
LPS
Mannan
IgG
iC3b
Calreticulin
PS
log2 H/L
2
0
-2
30'/0'
30'/30'
30'/150'
Time point

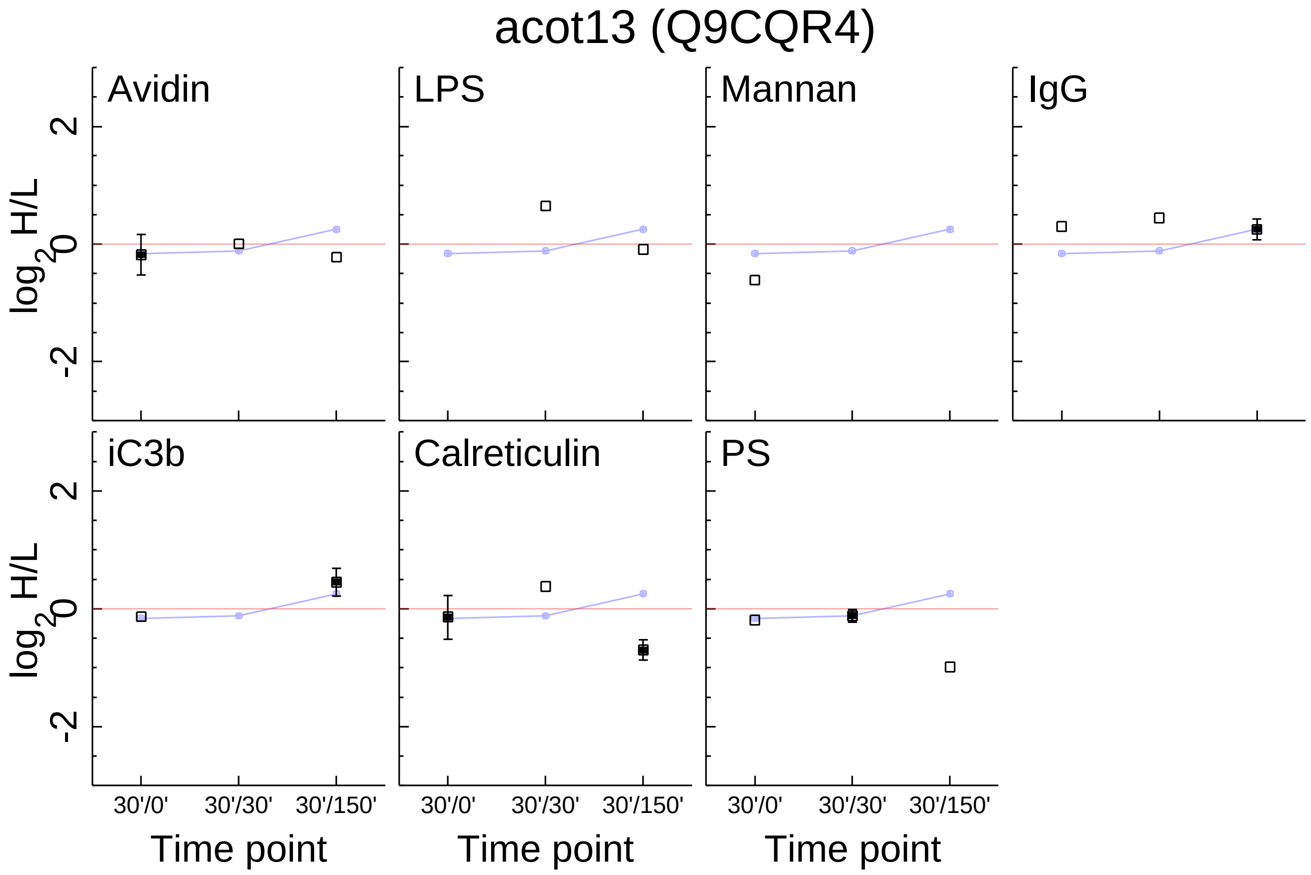

acot13 (Q9CQR4)
Avidin
LPS
Mannan
IgG
iC3b
Calreticulin
PS
log2 H/L
2
0
-2
30'/0'
30'/30'
30'/150'
Time point

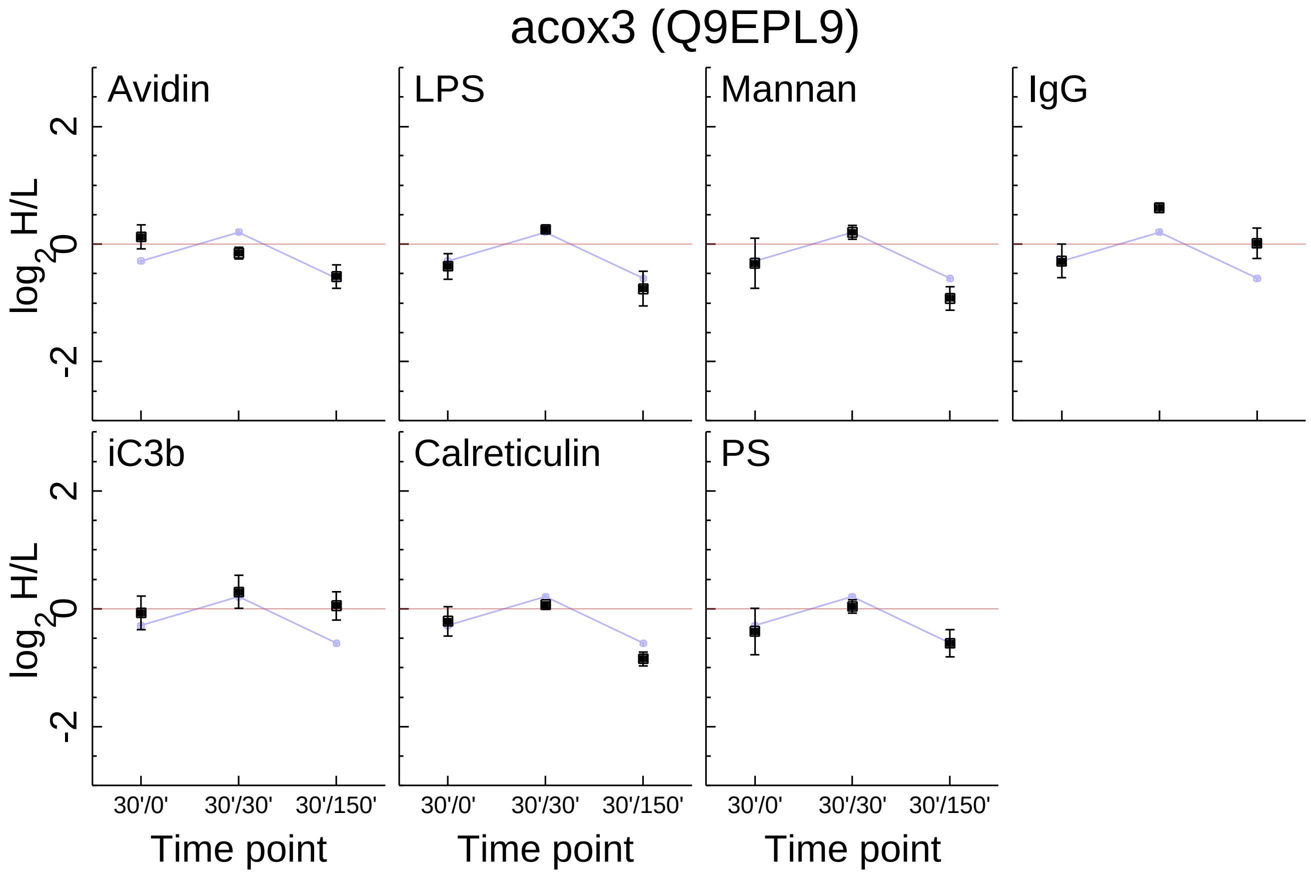
acox3 (Q9EPL9)
Avidin
LPS
Mannan
IgG
iC3b
Calreticulin
PS
log2 H/L
2
0
-2
30'/0'
30'/30'
30'/150'
Time point

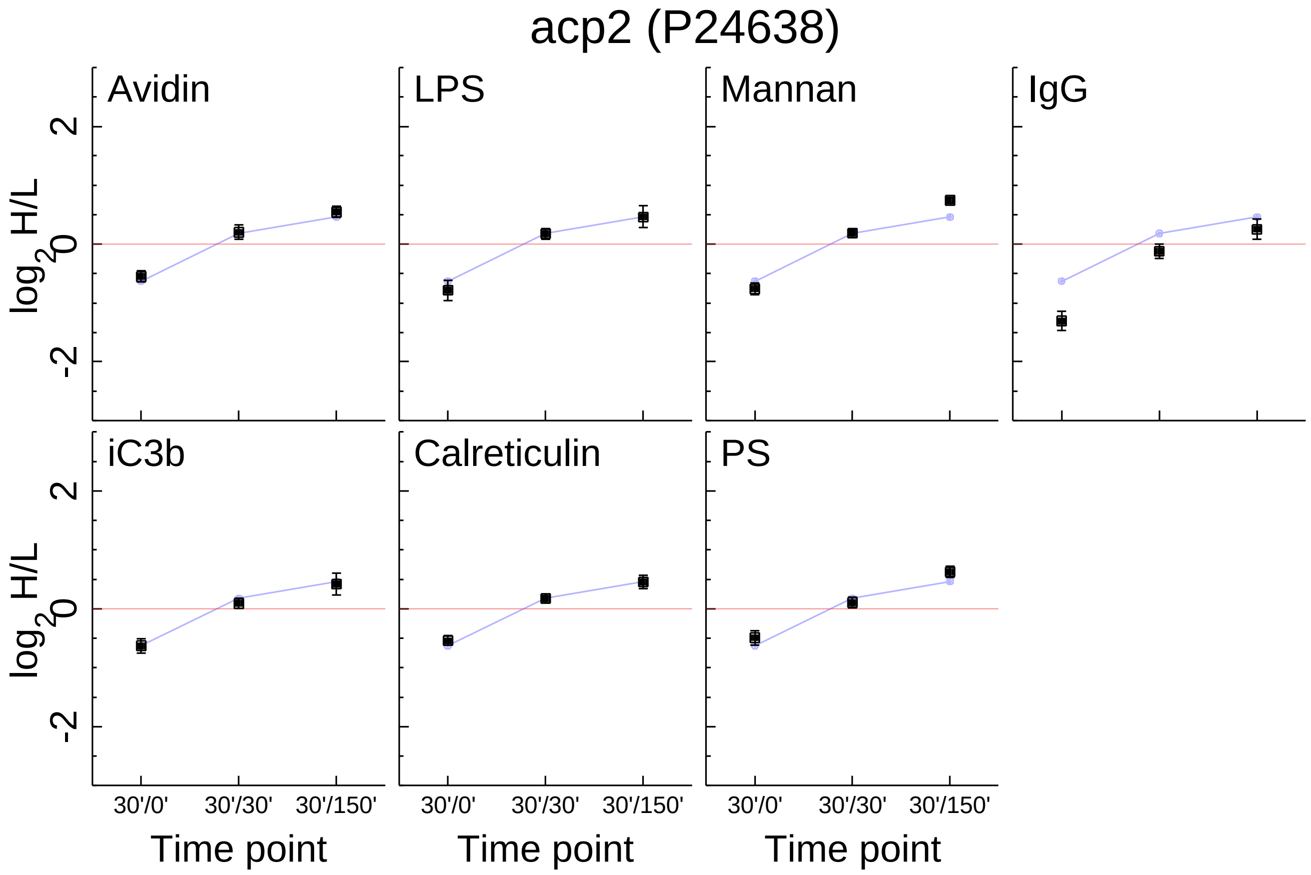

acp2 (P24638)
Avidin
LPS
Mannan
IgG
iC3b
Calreticulin
PS
log2 H/L
2
0
-2
30'/0'
30'/30'
30'/150'
Time point

acp5 (Q05117)

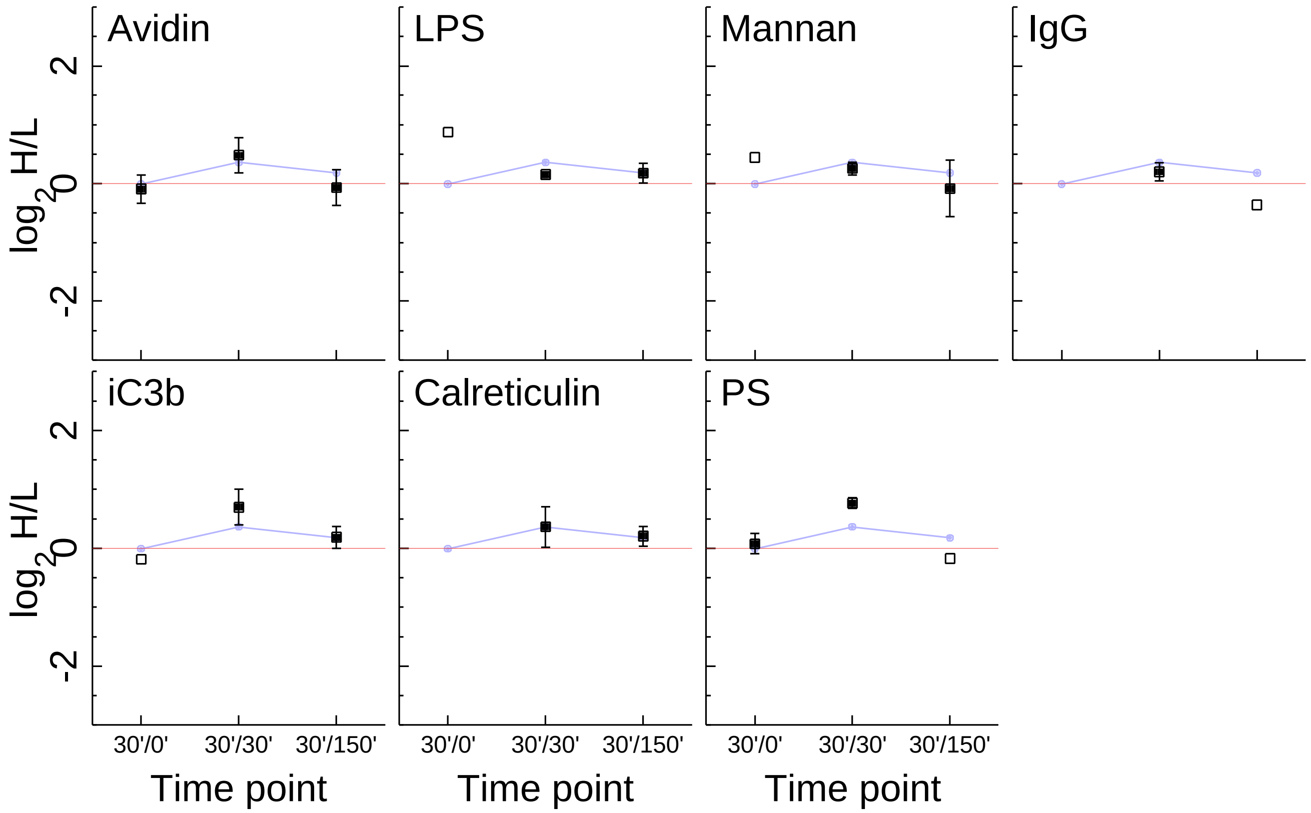

Avidin
LPS
Mannan
IgG
iC3b
Calreticulin
PS
log2 H/L
2
0
-2
30'/0'
30'/30'
30'/150'
Time point

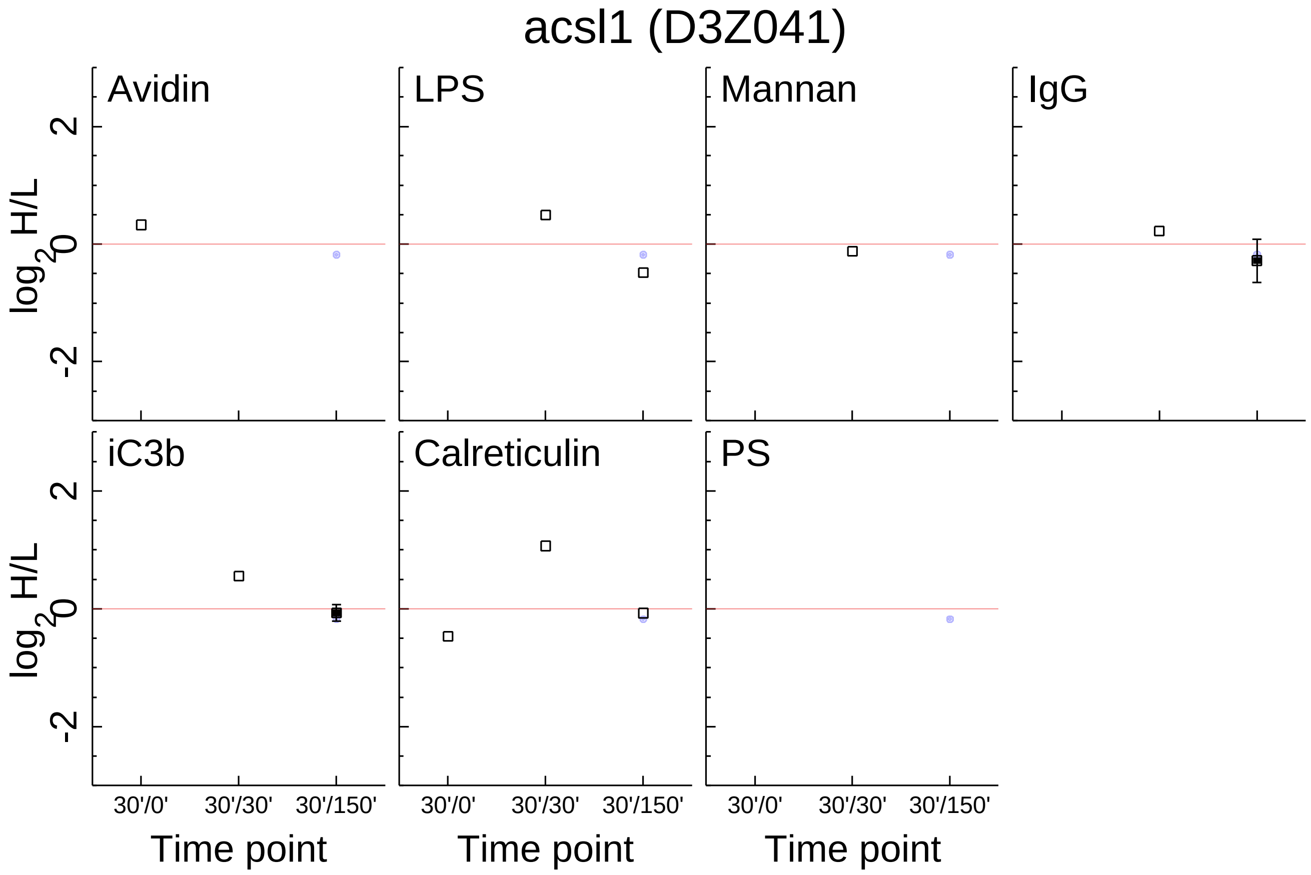
acsl1 (D3Z041)
Avidin
LPS
Mannan
IgG
iC3b
Calreticulin
PS
log2 H/L
2
0
-2
30'/0'
30'/30'
30'/150'
Time point

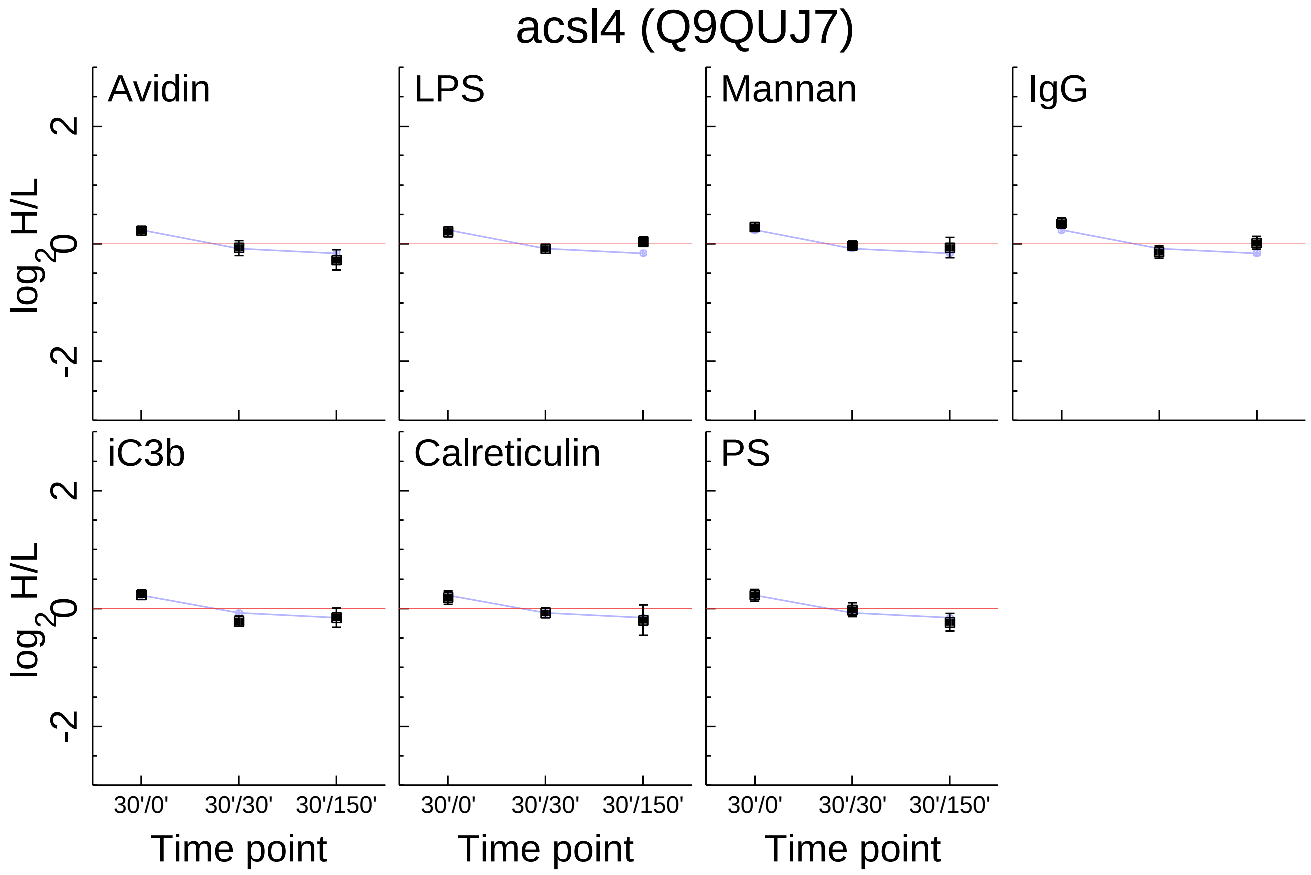

acsl4 (Q9QUJ7)
Avidin
LPS
Mannan
IgG
iC3b
Calreticulin
PS
log2 H/L
2
0
-2
30'/0'
30'/30'
30'/150'
Time point

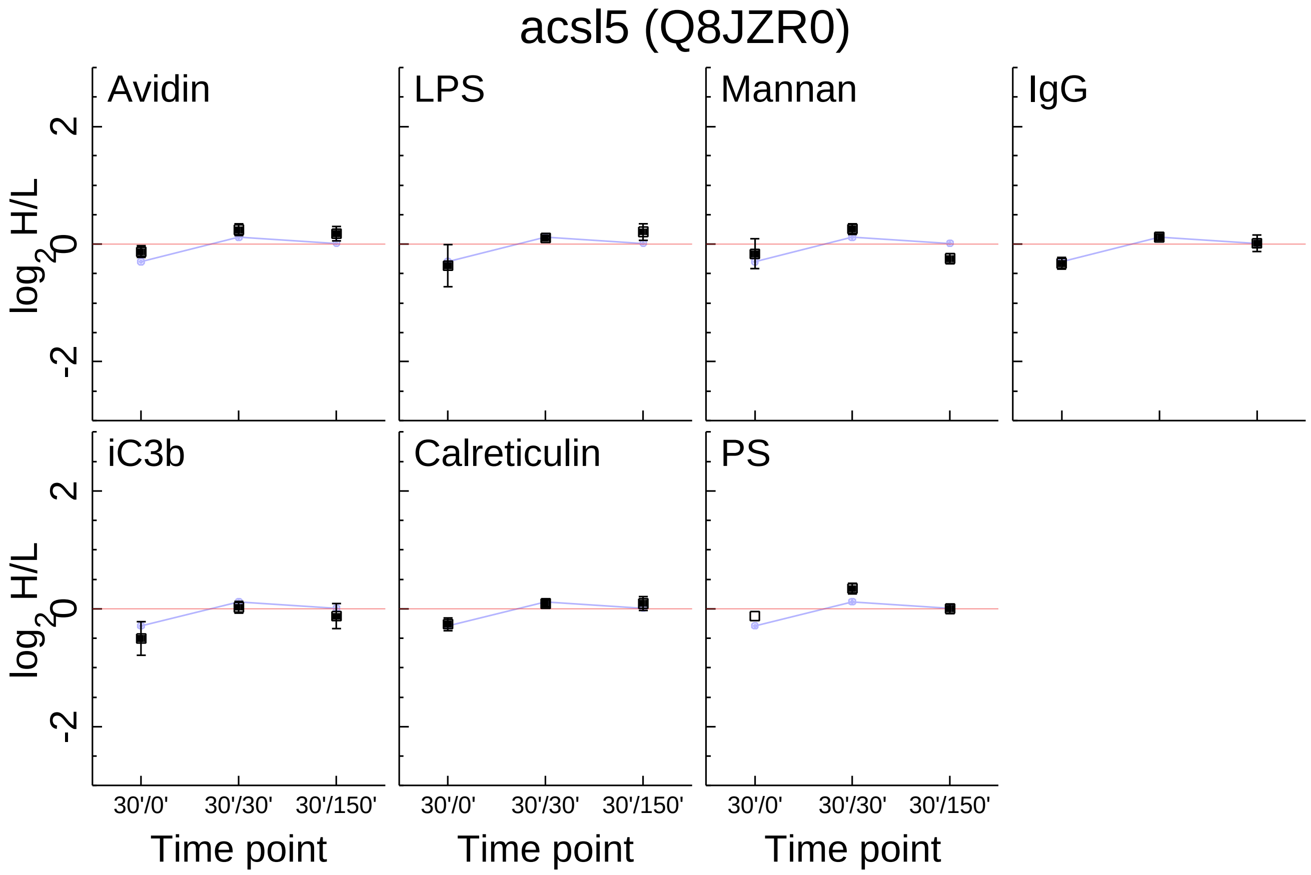

acsl5 (Q8JZR0)
Avidin
LPS
Mannan
IgG
iC3b
Calreticulin
PS
log2 H/L
2
0
-2
30'/0'
30'/30'
30'/150'
Time point

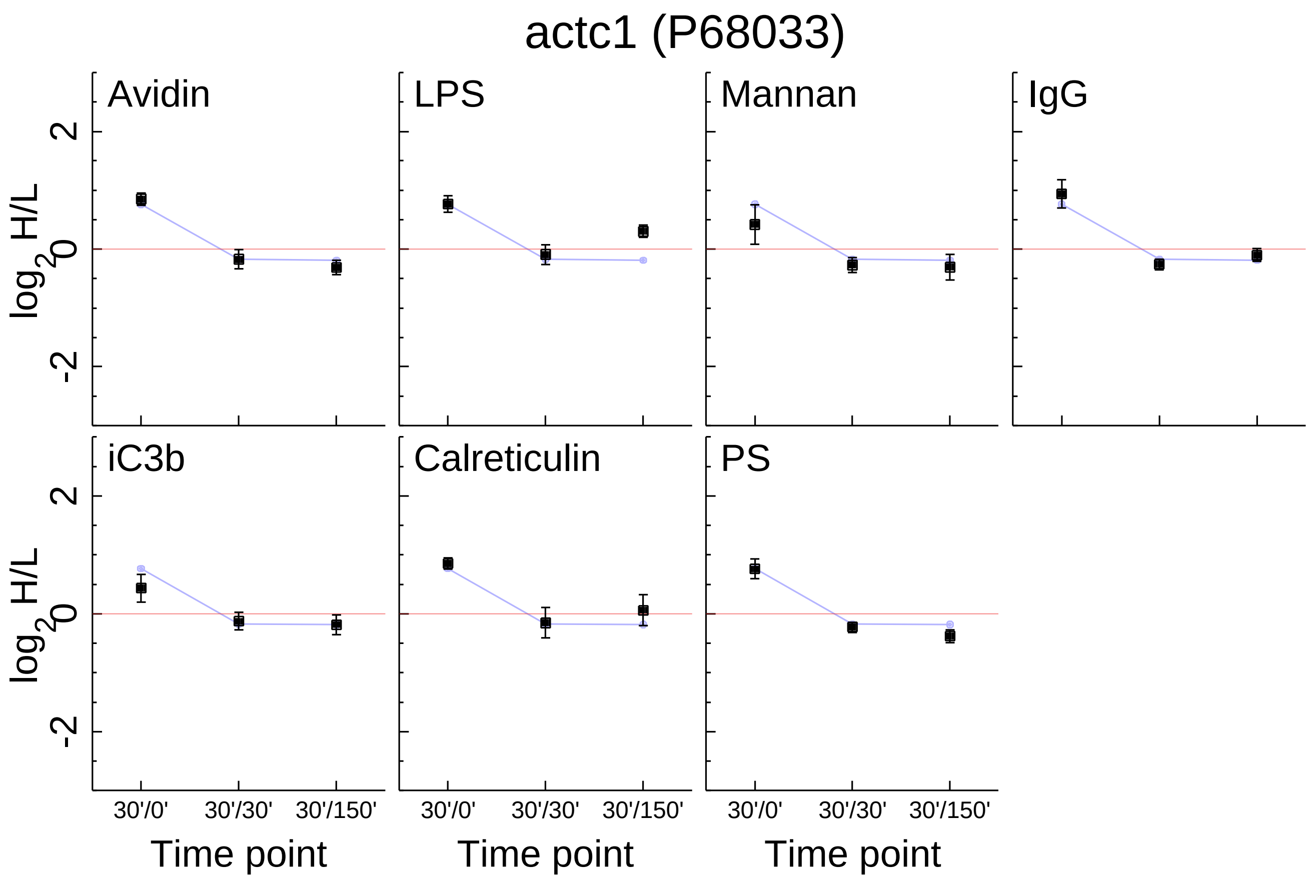
actc1 (P68033)
Avidin
LPS
Mannan
IgG
iC3b
Calreticulin
PS
log2 H/L
2
0
-2
30'/0'
30'/30'
30'/150'
Time point

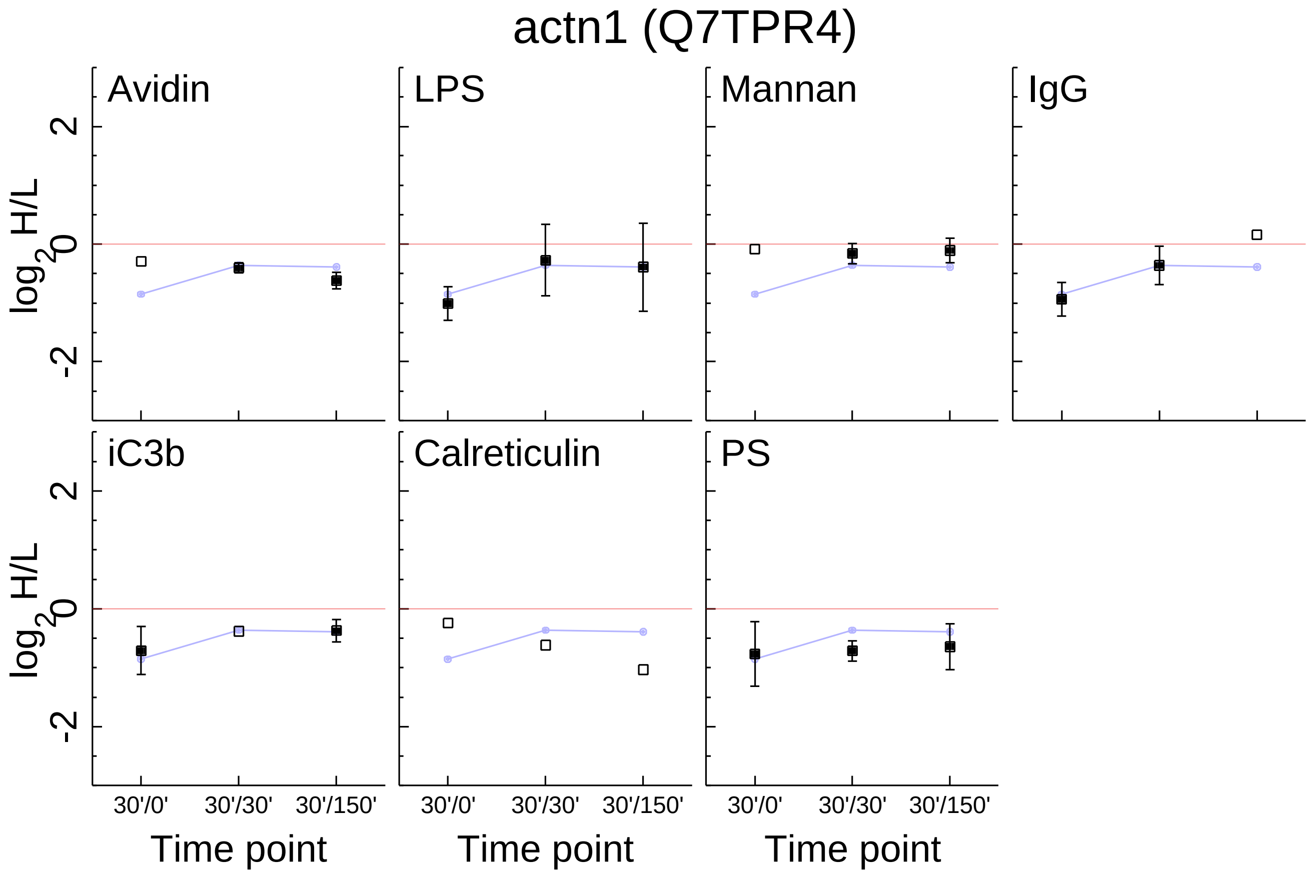
actn1 (Q7TPR4)
Avidin
LPS
Mannan
IgG
iC3b
Calreticulin
PS
log2 H/L
2
0
-2
30'/0'
30'/30'
30'/150'
Time point

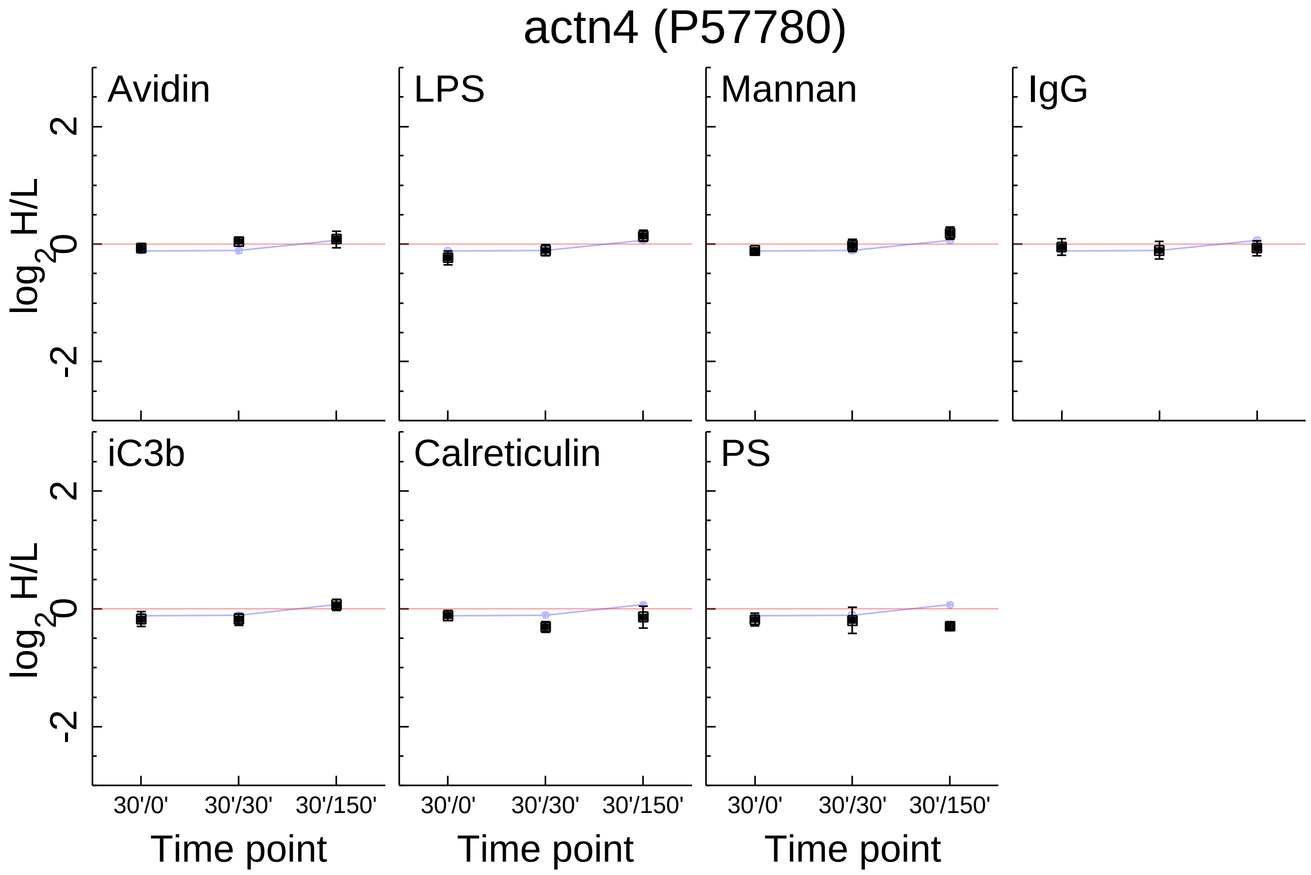
actn4 (P57780)
Avidin
LPS
Mannan
IgG
iC3b
Calreticulin
PS
log2 H/L
2
0
-2
30'/0'
30'/30'
30'/150'
Time point

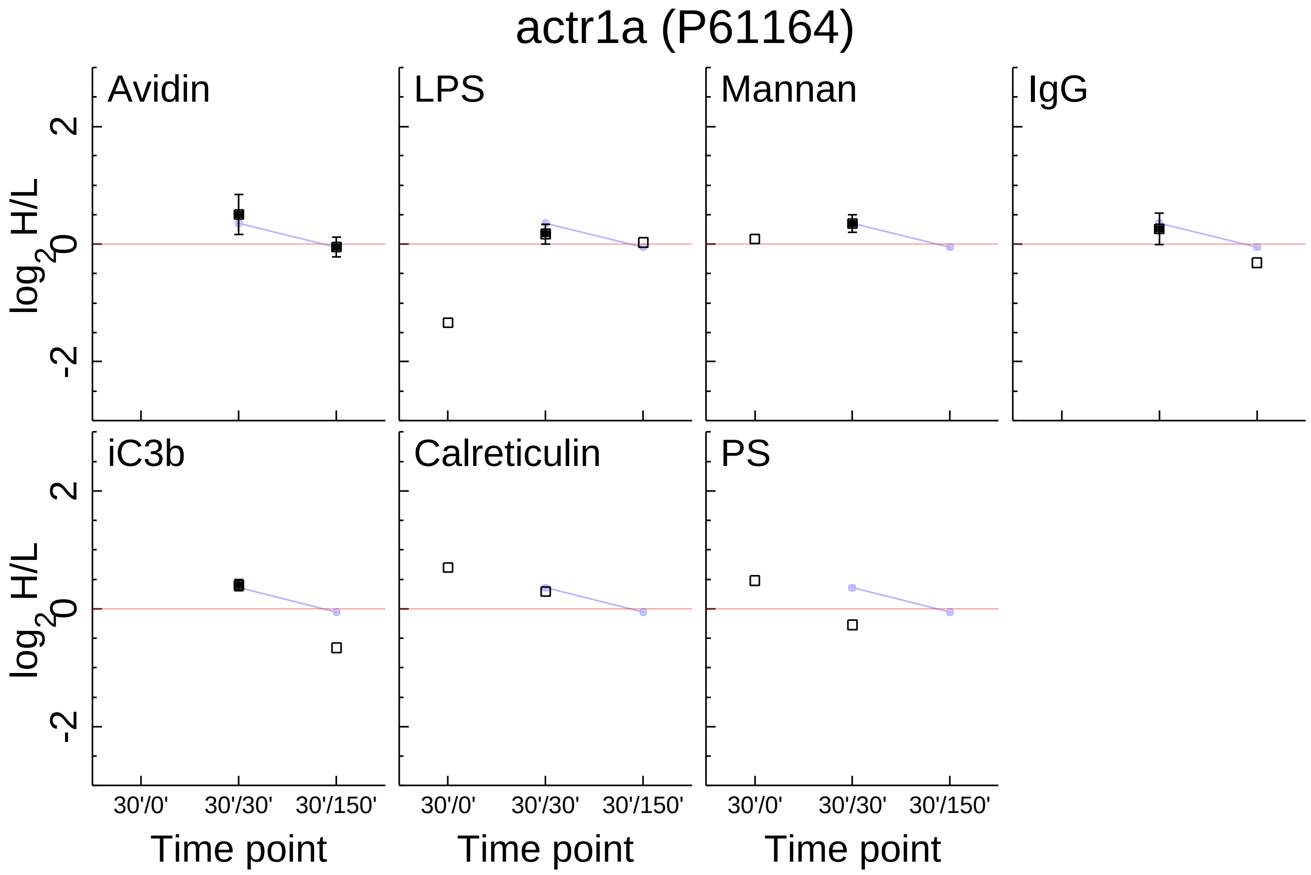

actr1a (P61164)
Avidin
LPS
Mannan
IgG
iC3b
Calreticulin
PS
log2 H/L
2
0
-2
30'/0'
30'/30'
30'/150'
Time point

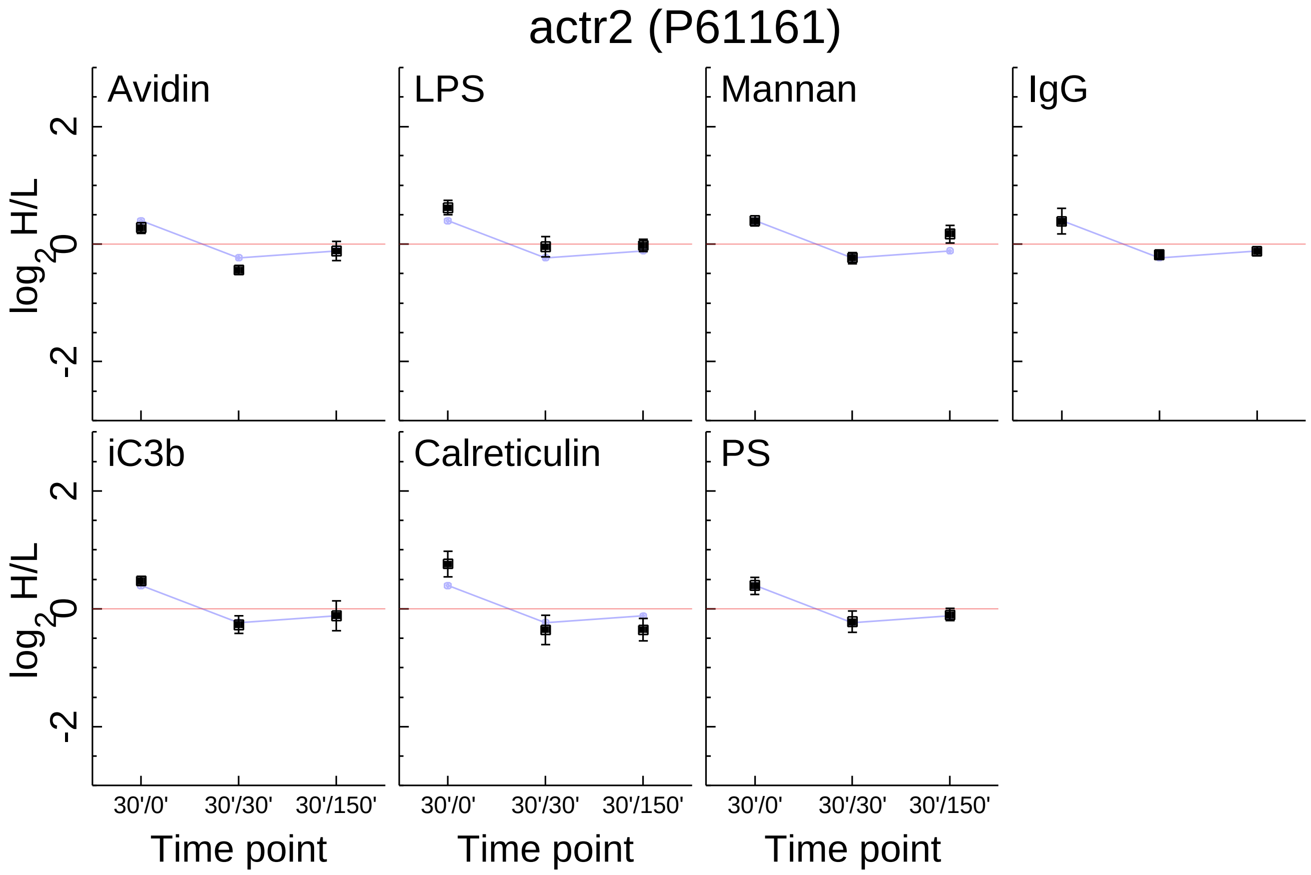
actr2 (P61161)
Avidin
LPS
Mannan
IgG
iC3b
Calreticulin
PS
log2 H/L
2
0
-2
30'/0'
30'/30'
30'/150'
Time point

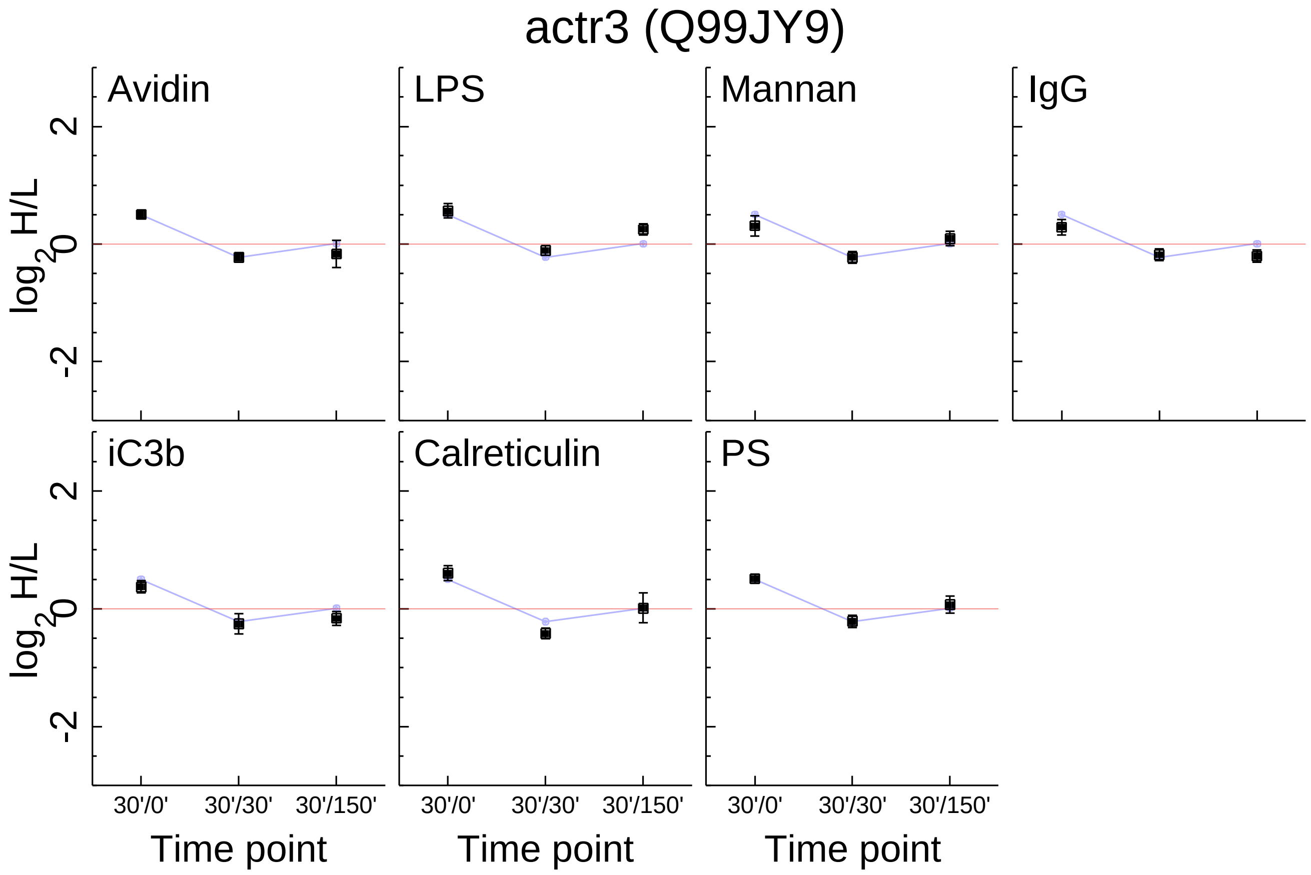

actr3 (Q99JY9)
Avidin
LPS
Mannan
IgG
iC3b
Calreticulin
PS
log2 H/L
2
0
-2
30'/0'
30'/30'
30'/150'
Time point

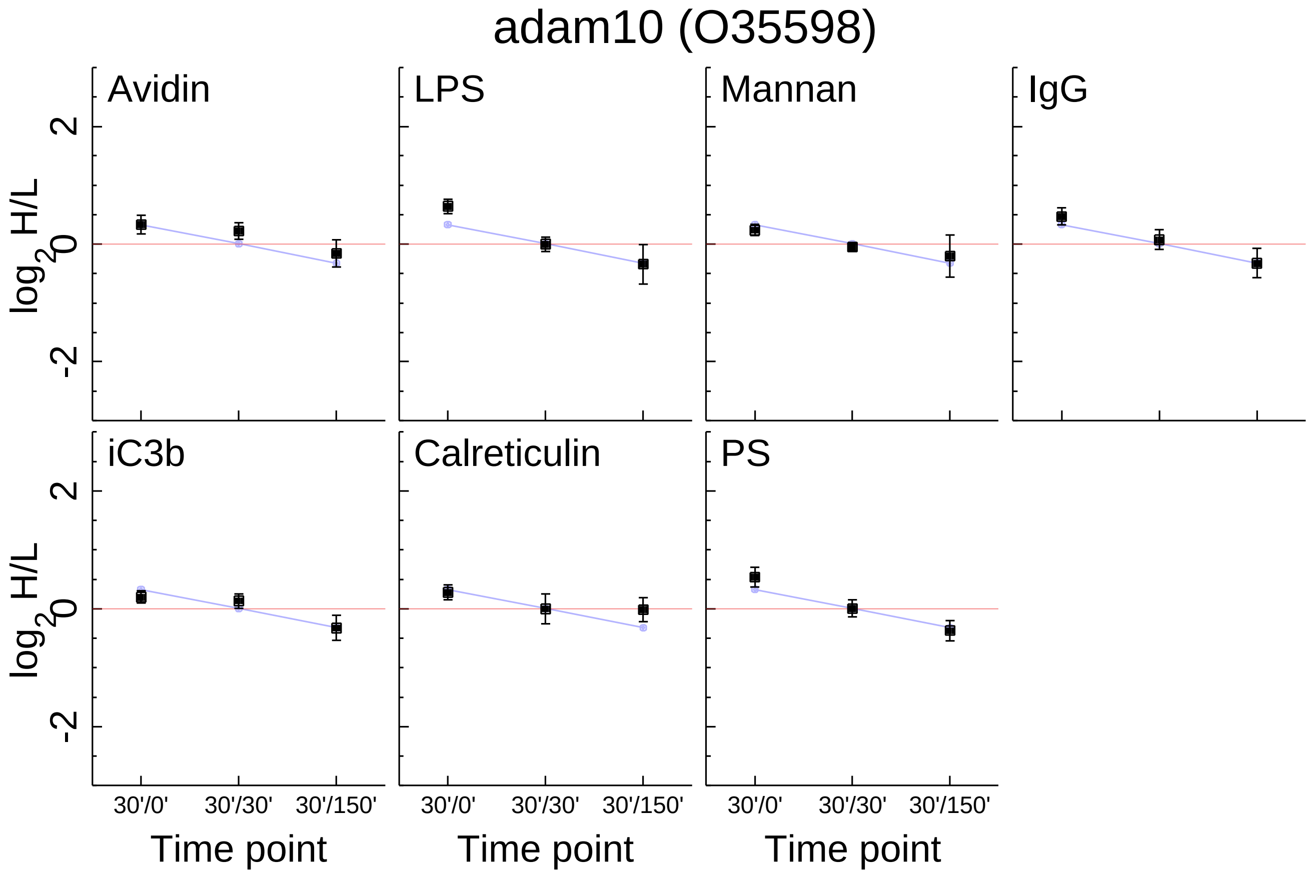
adam10 (O35598)
Avidin
LPS
Mannan
IgG
iC3b
Calreticulin
PS
log2 H/L
2
0
-2
30'/0'
30'/30'
30'/150'
Time point

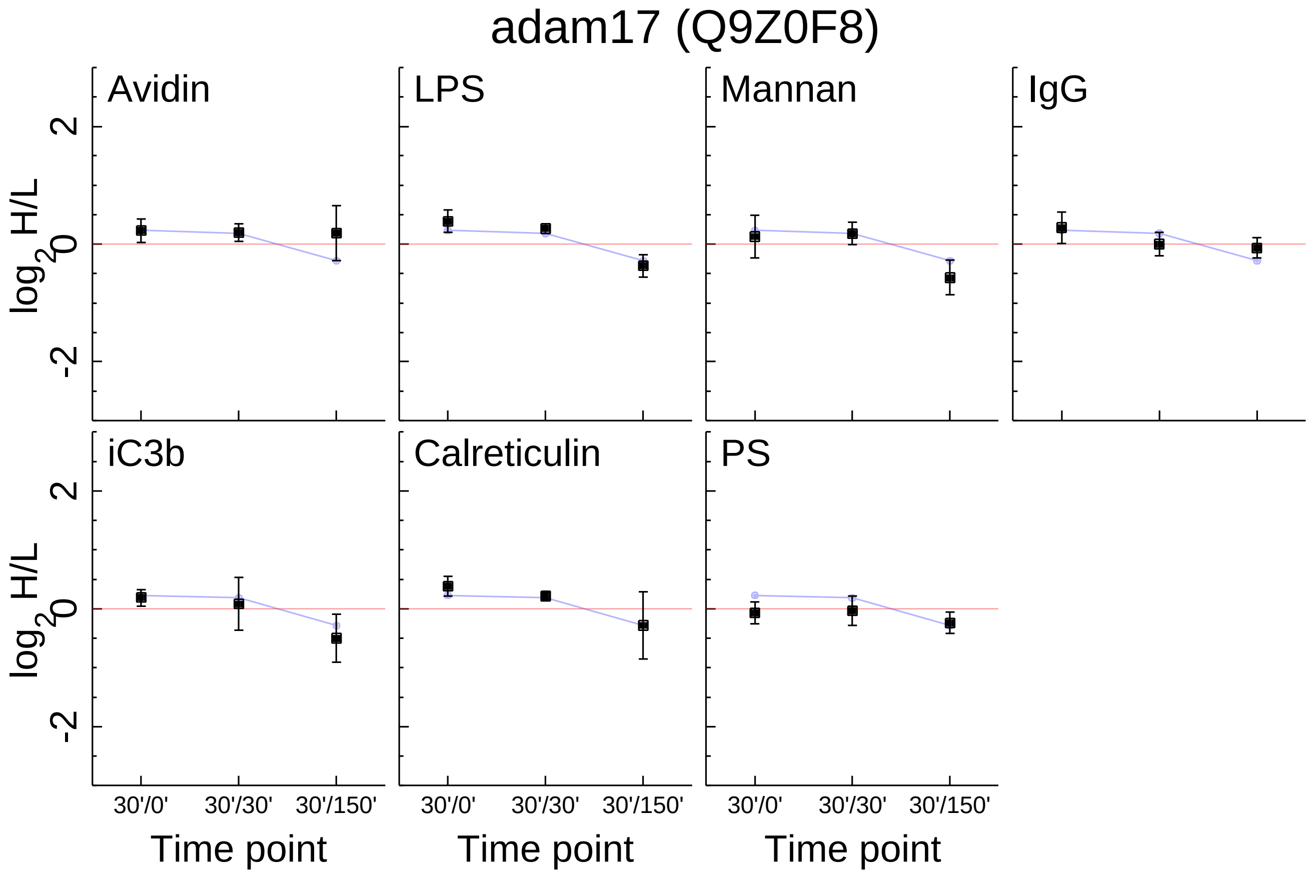
adam17 (Q9Z0F8)
Avidin
LPS
Mannan
IgG
iC3b
Calreticulin
PS
log2 H/L
2
0
-2
30'/0'
30'/30'
30'/150'
Time point

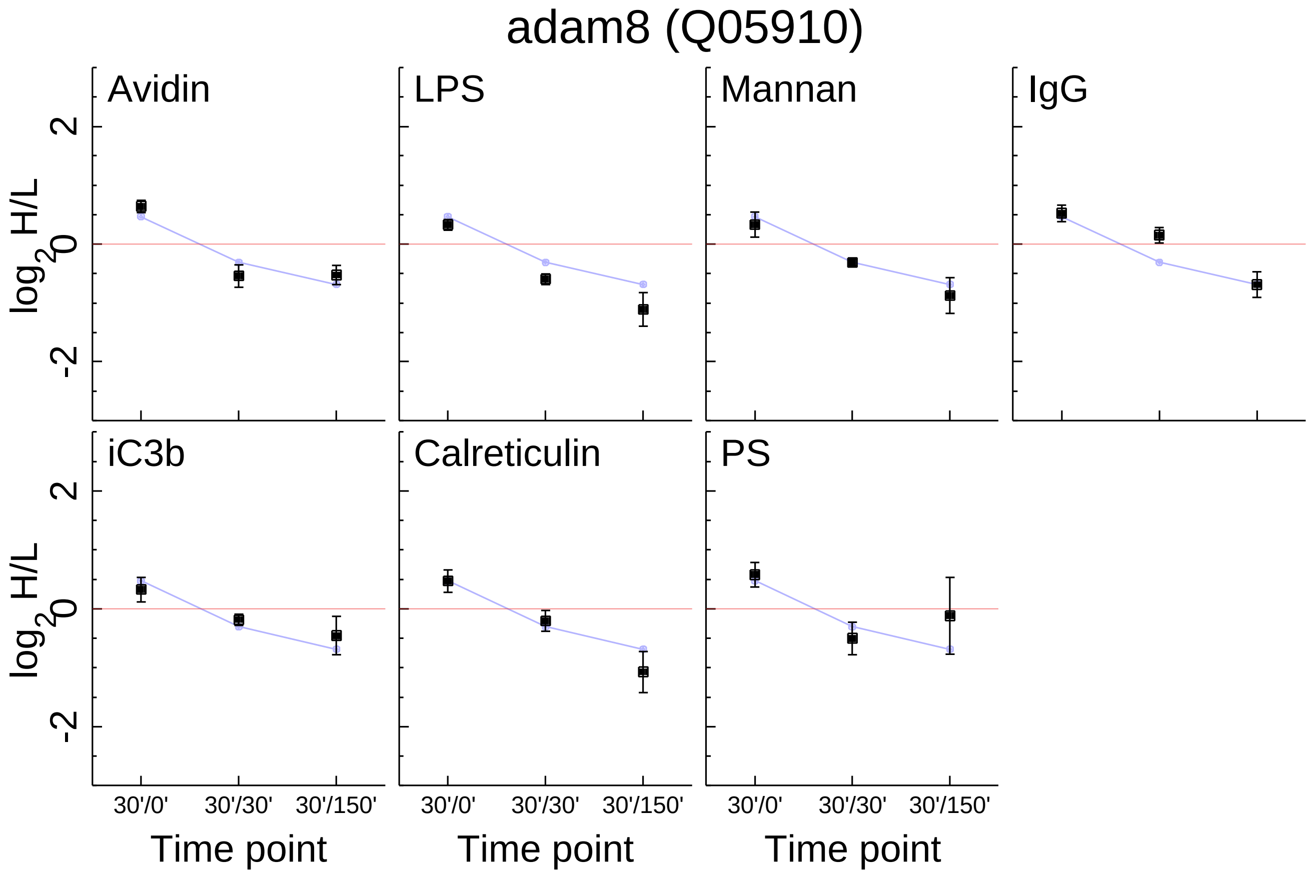
adam8 (Q05910)
Avidin
LPS
Mannan
IgG
iC3b
Calreticulin
PS
log2 H/L
2
0
-2
30'/0'
30'/30'
30'/150'
Time point

adam9 (Q61072)

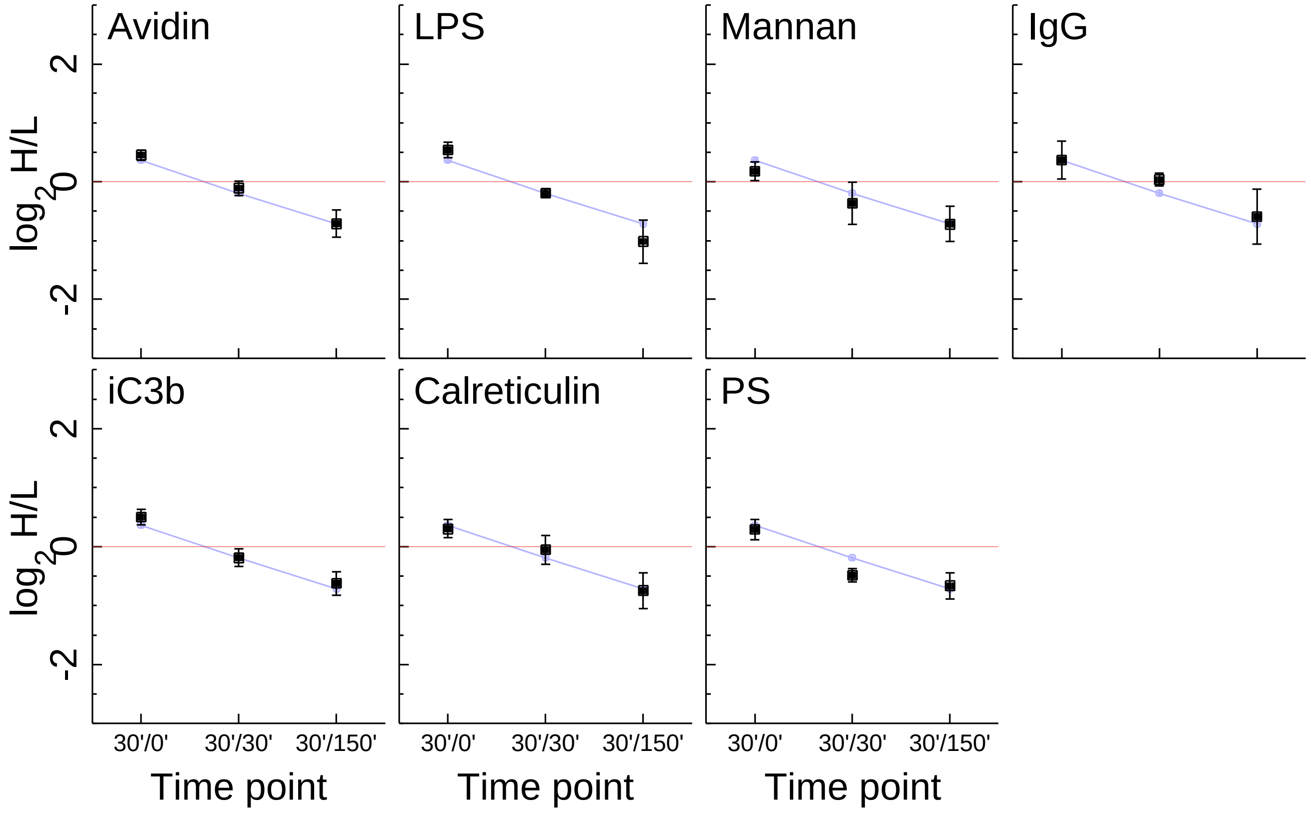

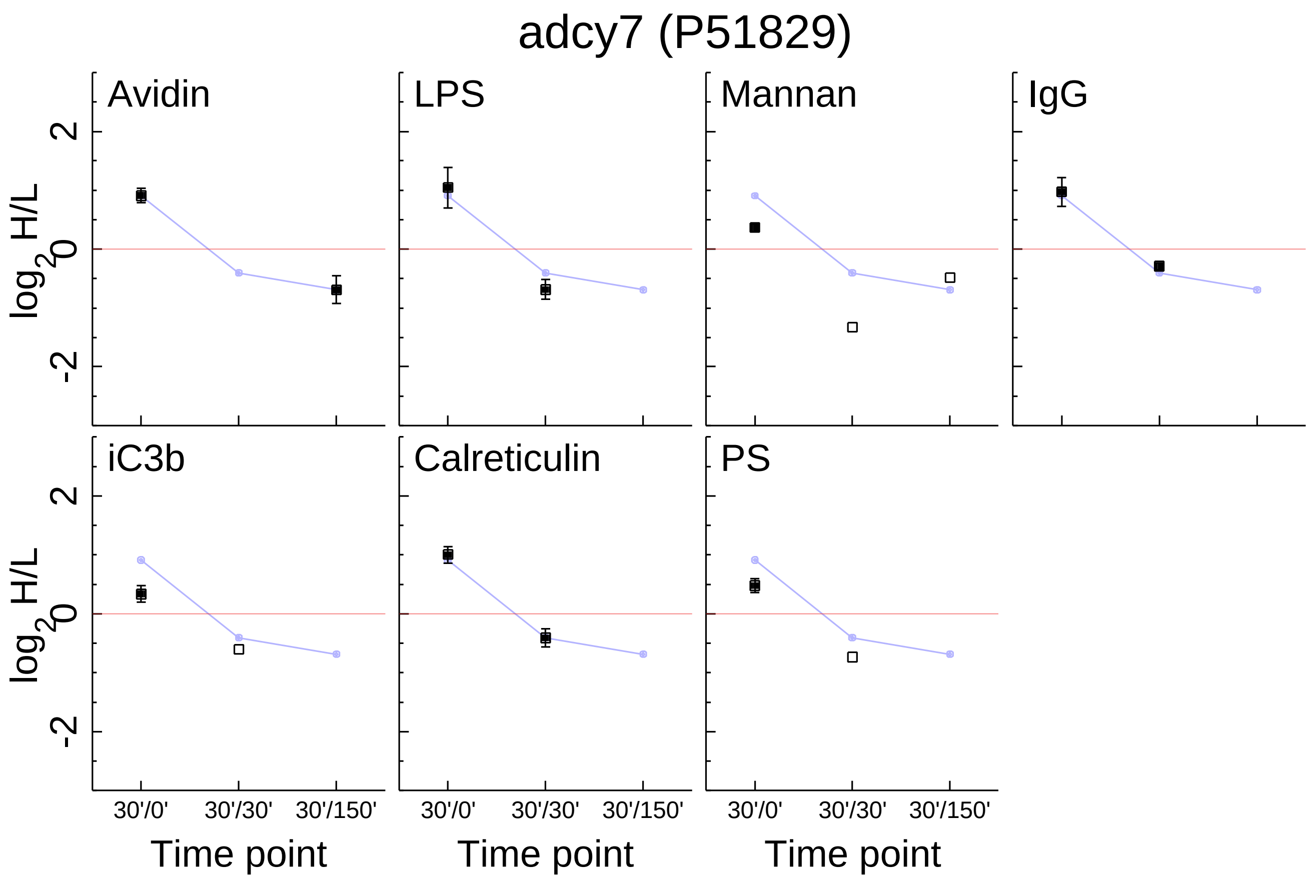

adcy7 (P51829)
Avidin
LPS
Mannan
IgG
iC3b
Calreticulin
PS
log2 H/L
2
0
-2
30'/0'
30'/30'
30'/150'
Time point

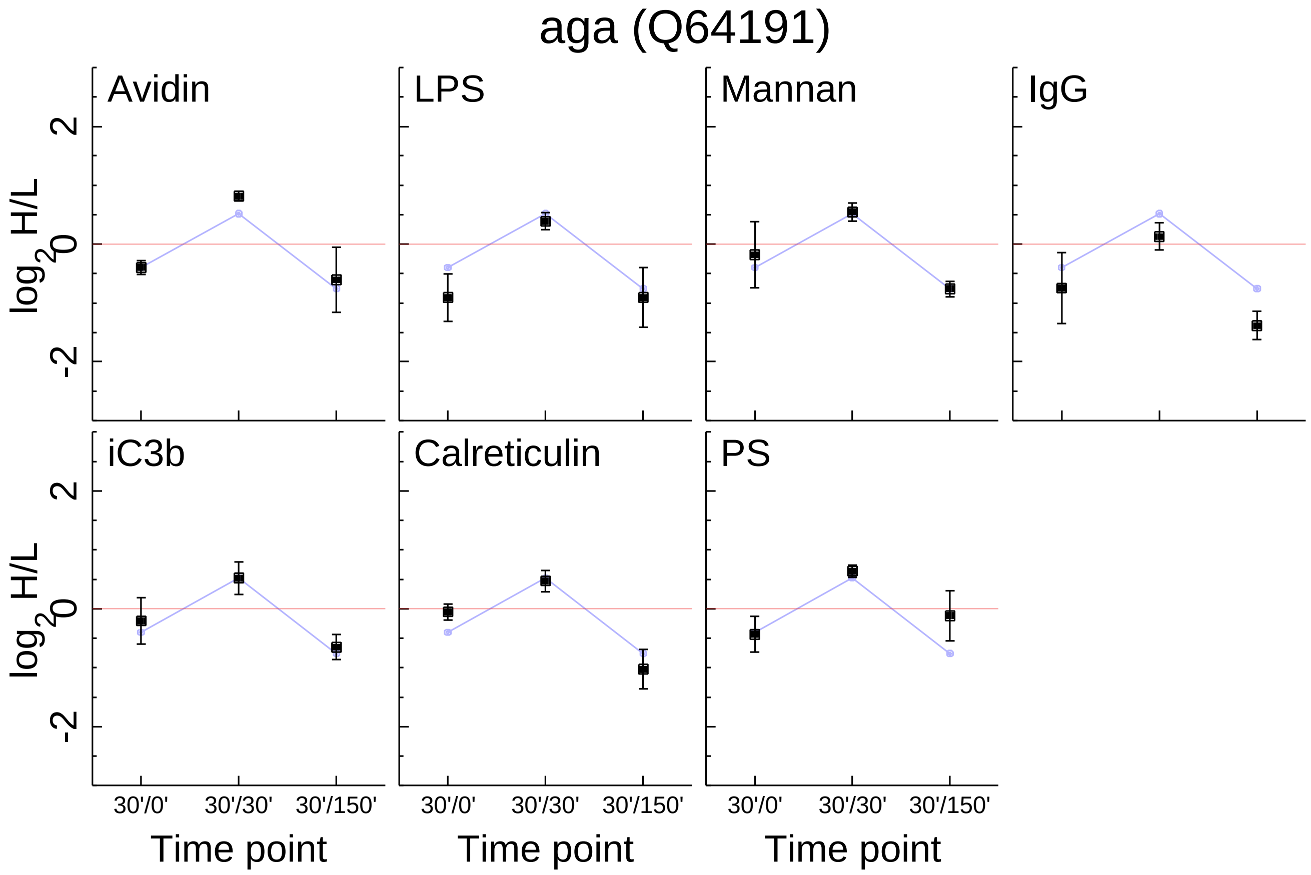
aga (Q64191)
Avidin
LPS
Mannan
IgG
iC3b
Calreticulin
PS
log2 H/L
2
0
-2
30'/0'
30'/30'
30'/150'
Time point

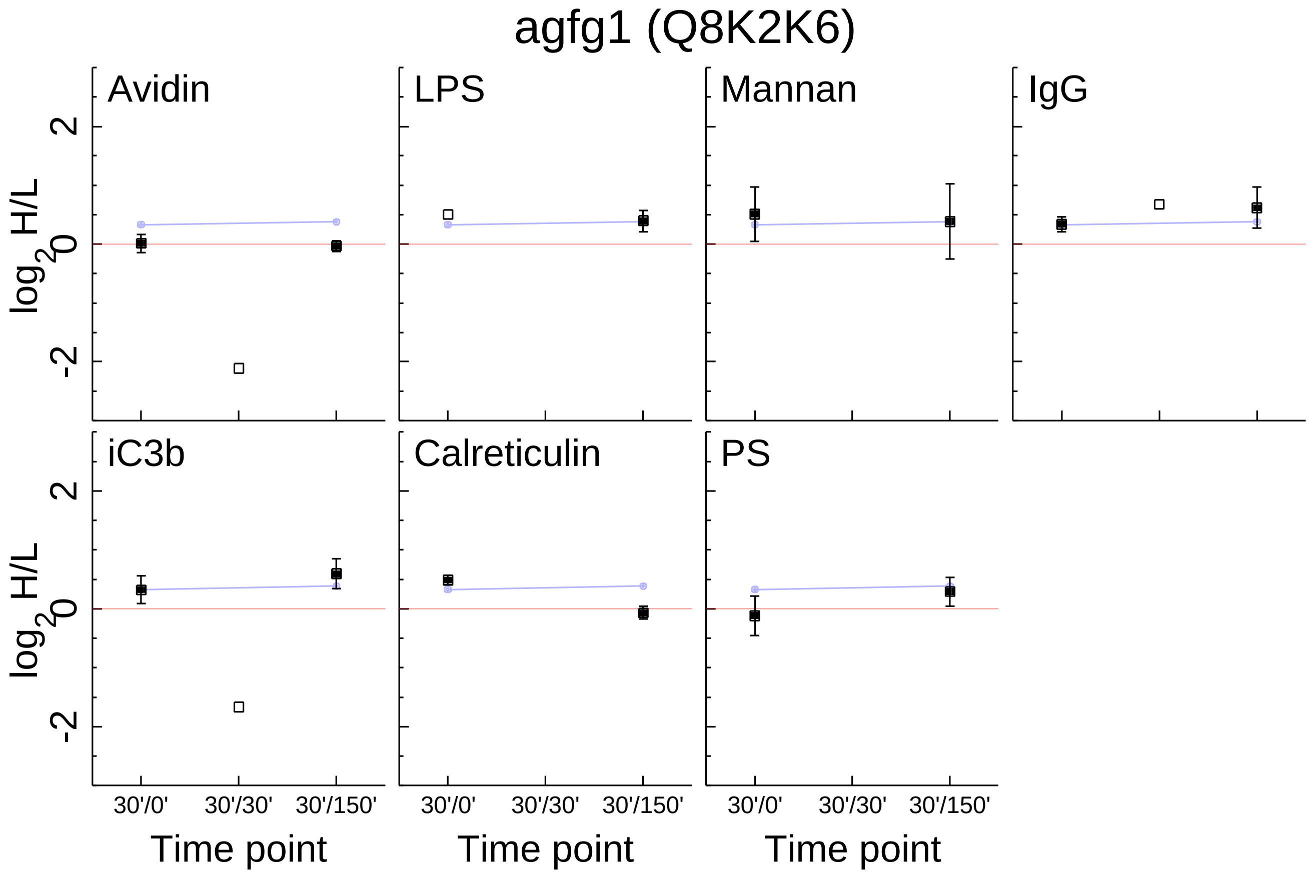
agfg1 (Q8K2K6)
Avidin
LPS
Mannan
IgG
iC3b
Calreticulin
PS
log2 H/L
2
0
-2
30'/0'
30'/30'
30'/150'
Time point

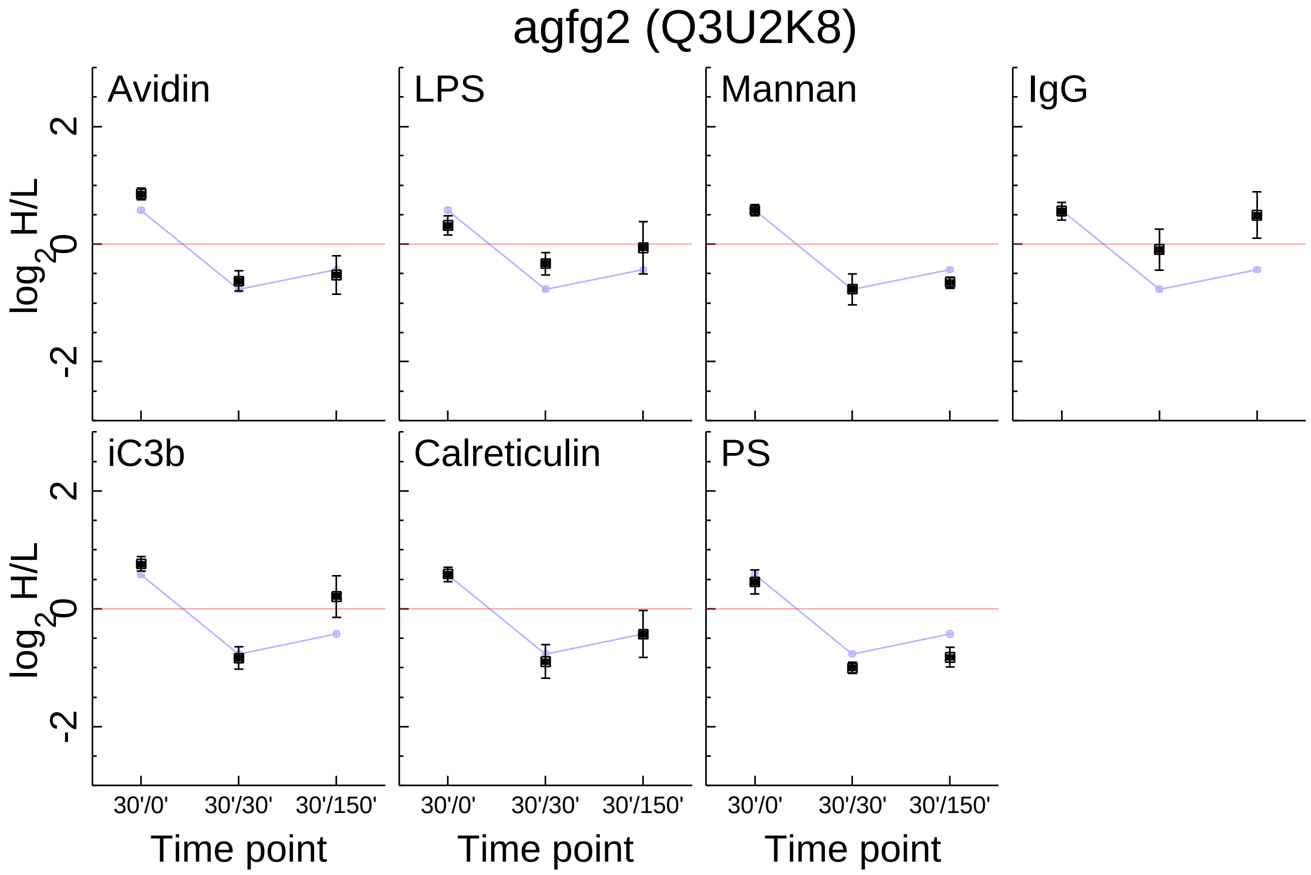
agfg2 (Q3U2K8)
Avidin
LPS
Mannan
IgG
iC3b
Calreticulin
PS
log2 H/L
2
0
-2
30'/0'
30'/30'
30'/150'
Time point

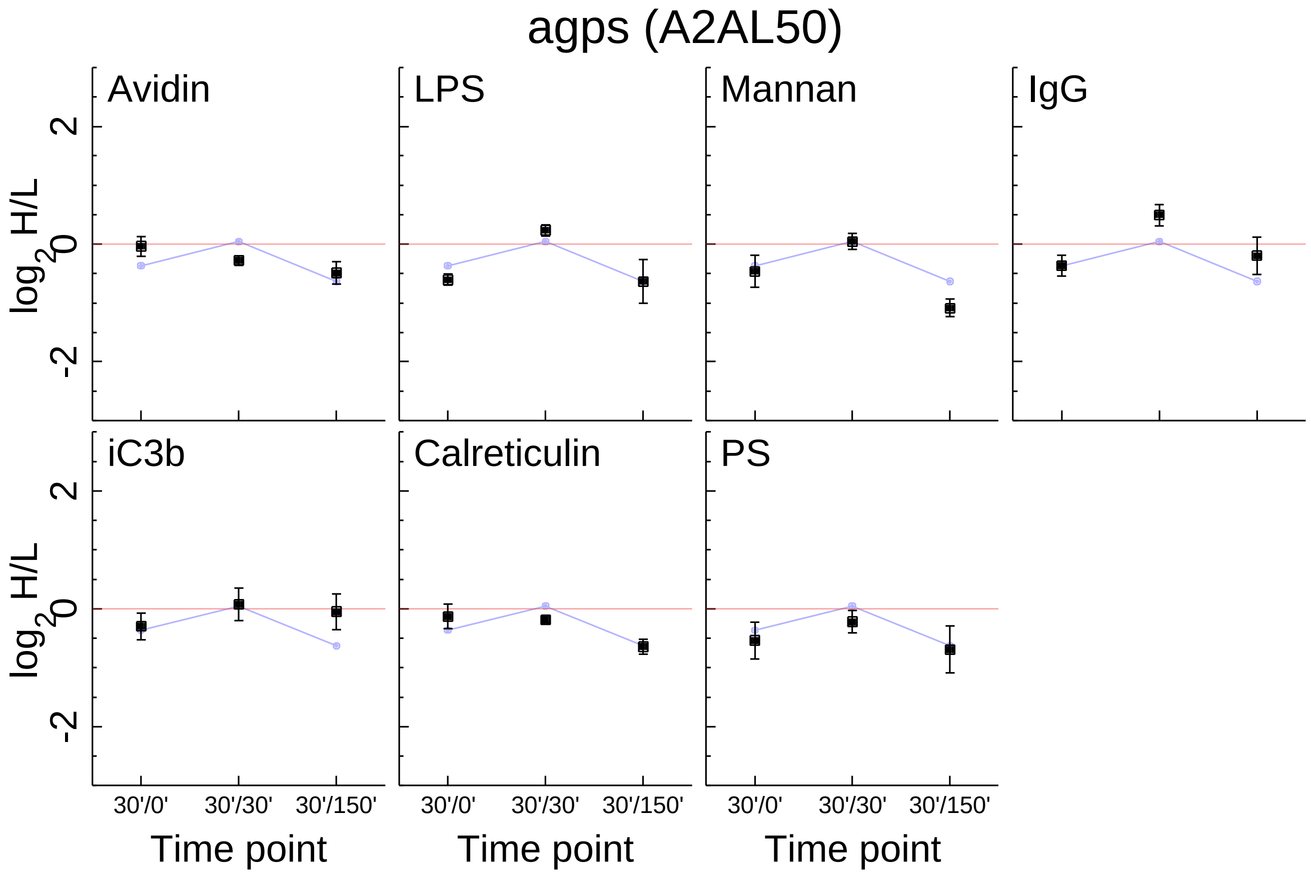
agps (A2AL50)
Avidin
LPS
Mannan
IgG
iC3b
Calreticulin
PS
log2 H/L
2
0
-2
30'/0'
30'/30'
30'/150'
Time point

agtrap (Q9WVK0)

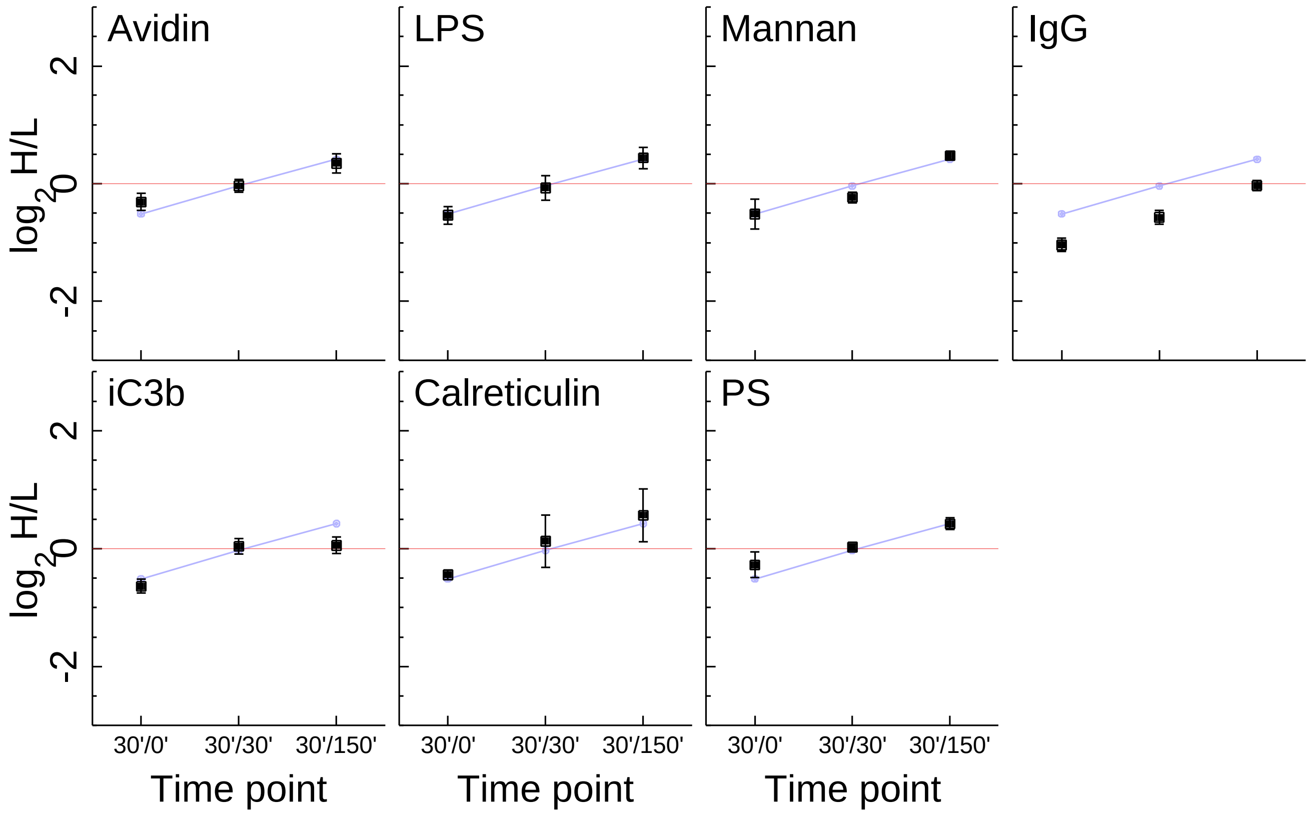

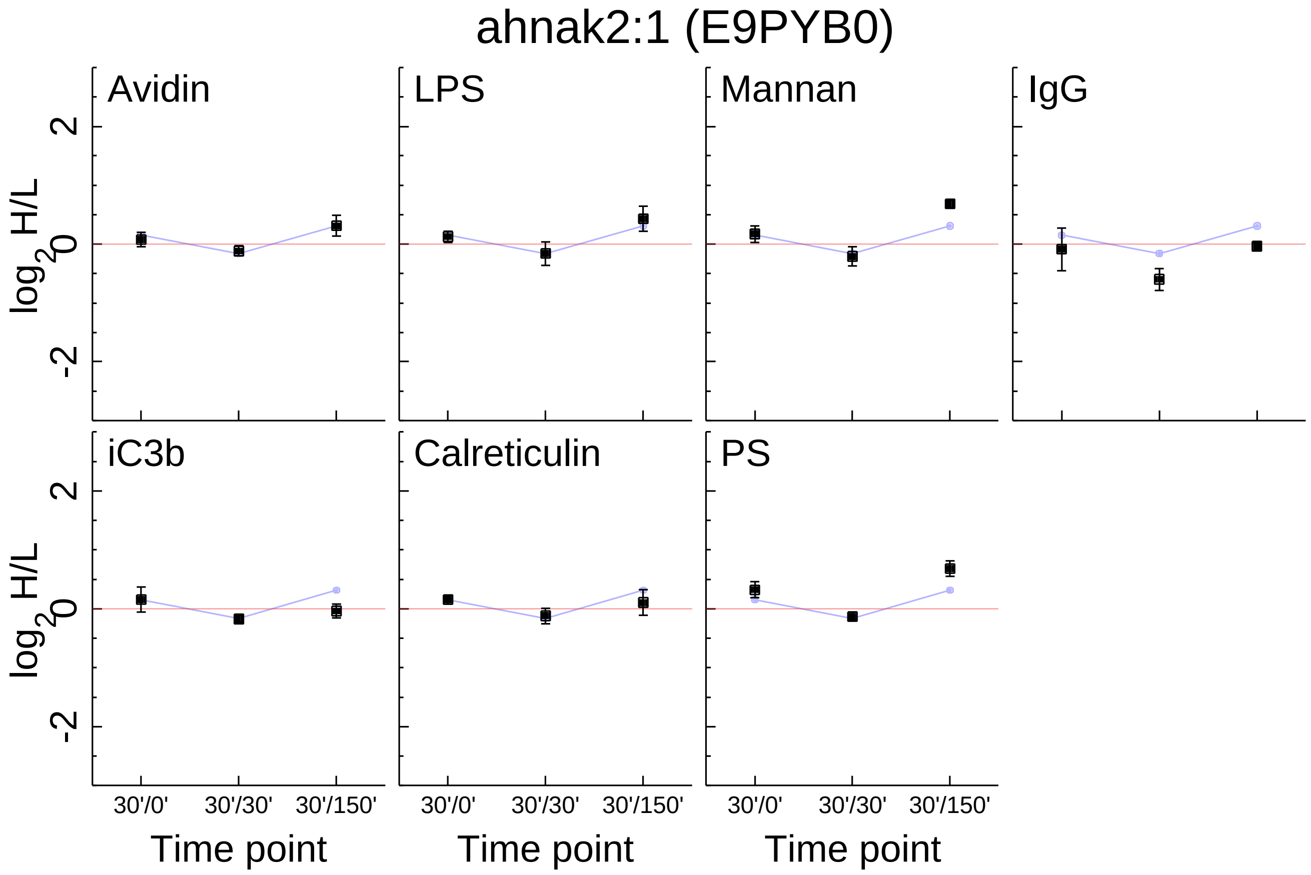
ahnak2:1 (E9PYB0)
Avidin
LPS
Mannan
IgG
iC3b
Calreticulin
PS
log2 H/L
2
0
-2
30'/0'
30'/30'
30'/150'
Time point

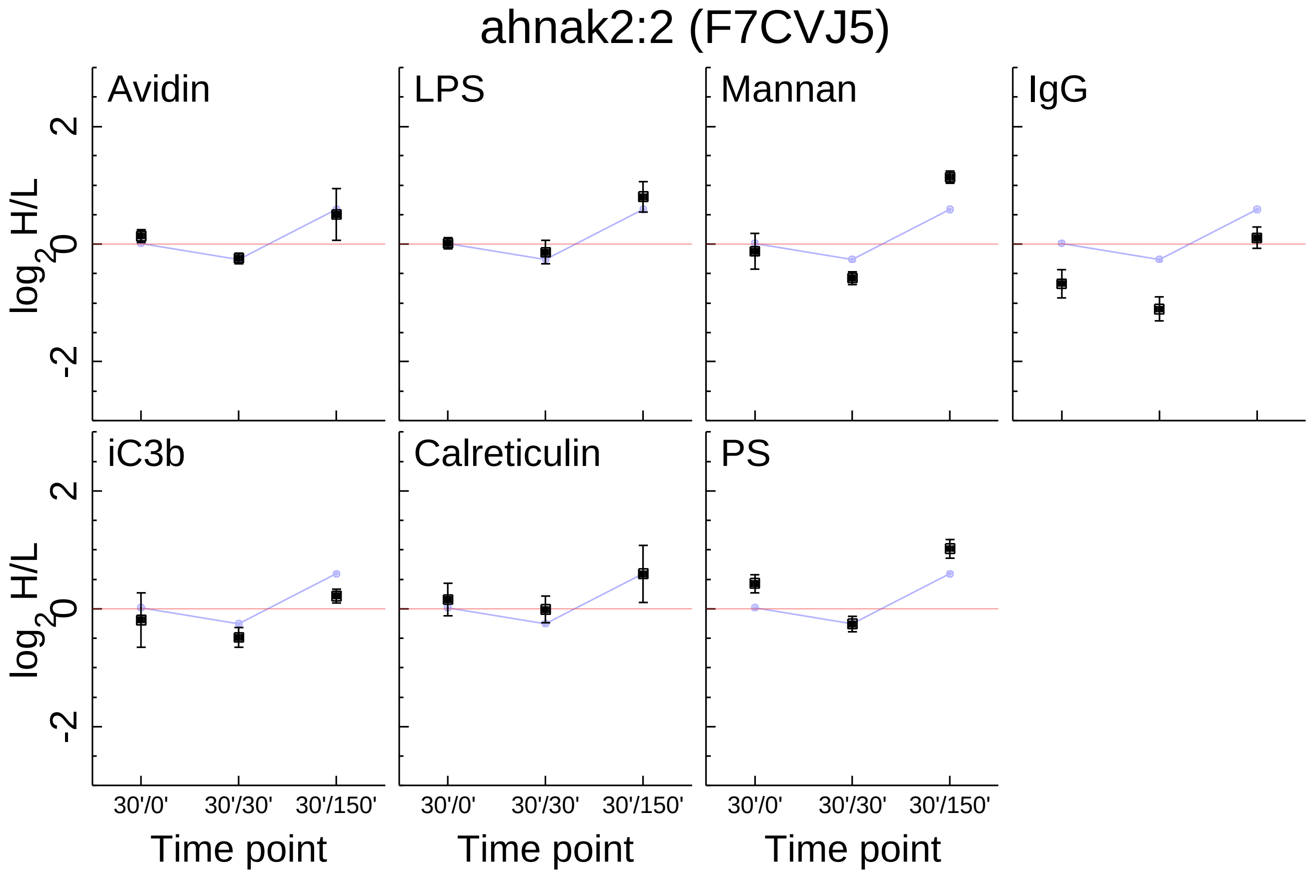

ahnak2:2 (F7CVJ5)
Avidin
LPS
Mannan
IgG
iC3b
Calreticulin
PS
log2 H/L
2
0
-2
30'/0'
30'/30'
30'/150'
Time point

ahnak2:3 (F7DBB3)

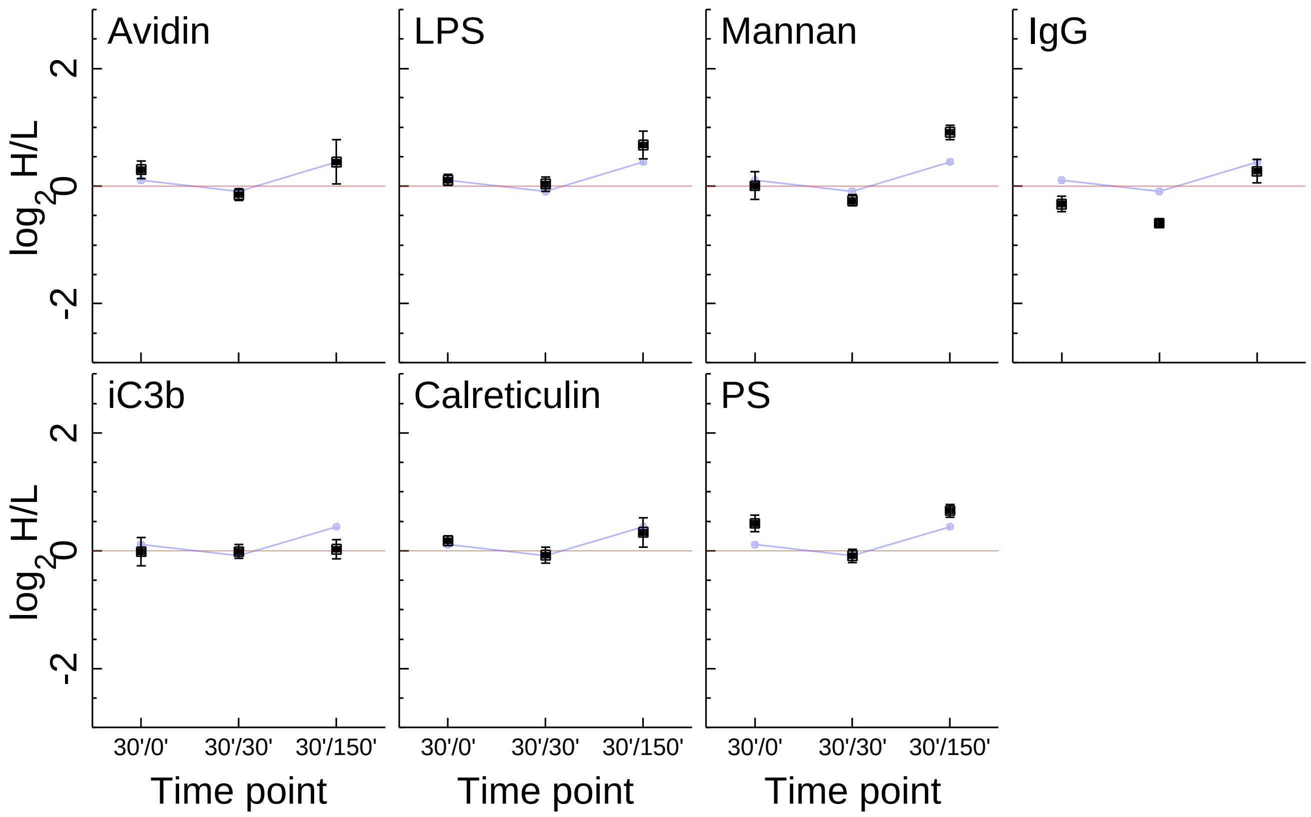

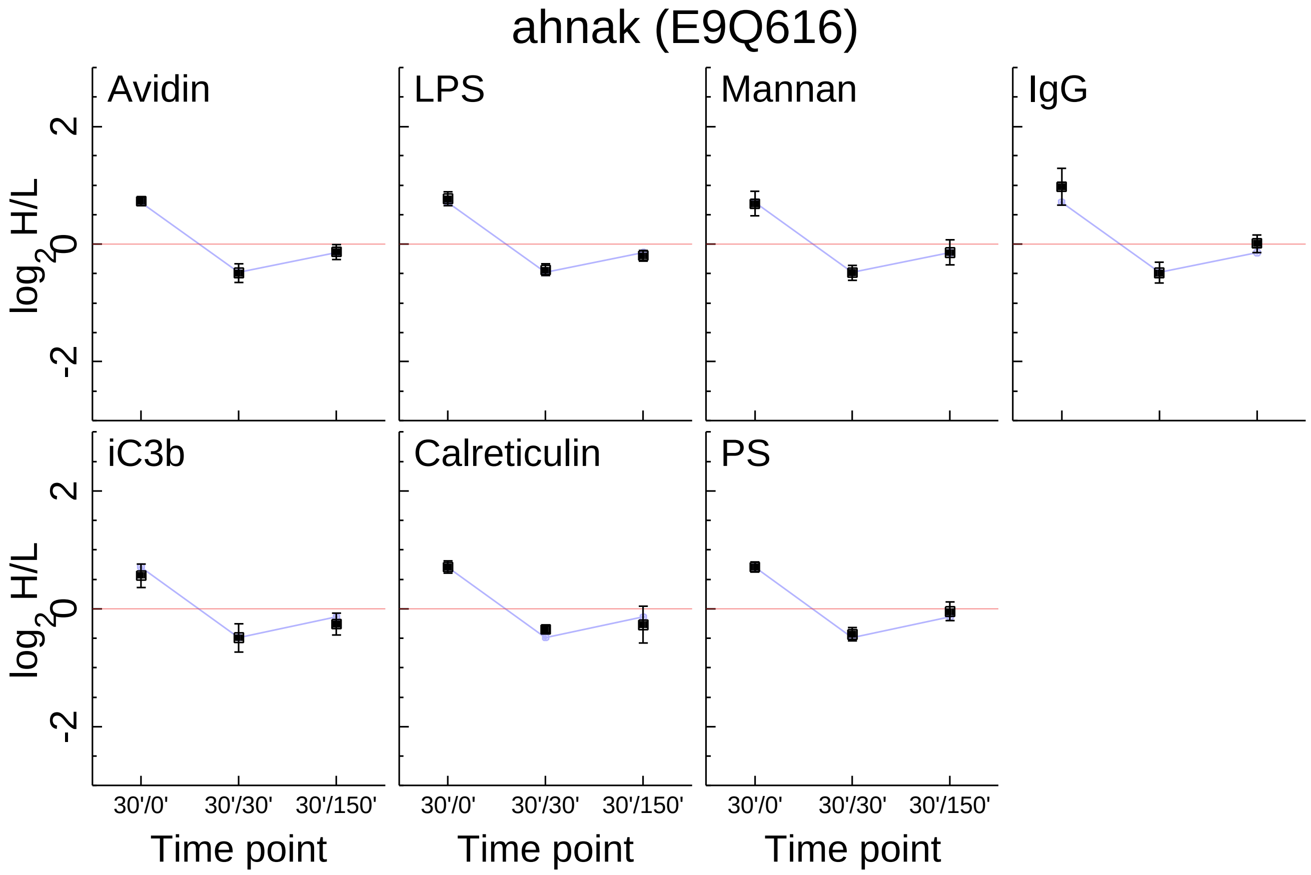
ahnak (E9Q616)
Avidin
LPS
Mannan
IgG
iC3b
Calreticulin
PS
log2 H/L
2
0
-2
30'/0'
30'/30'
30'/150'
Time point

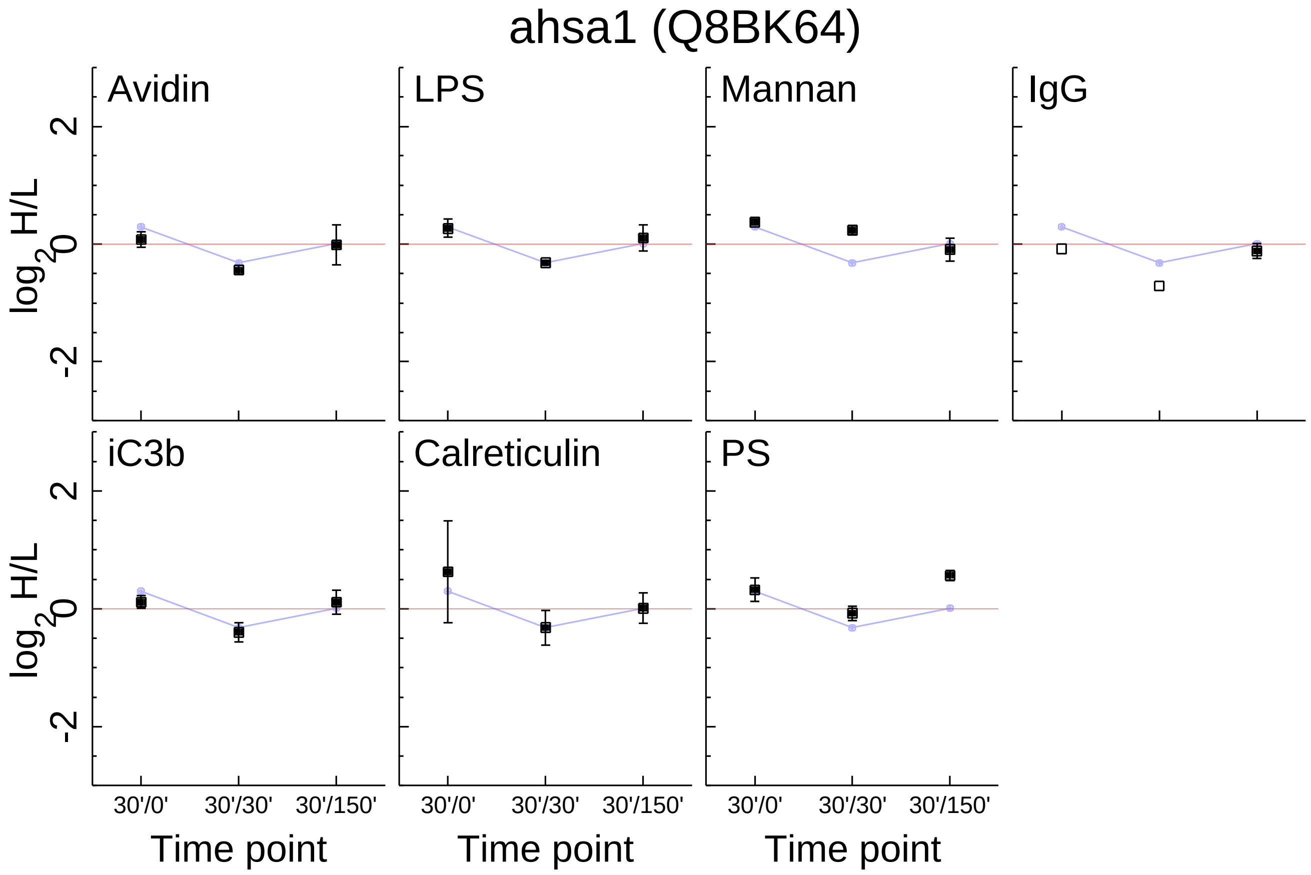
ahsa1 (Q8BK64)
Avidin
LPS
Mannan
IgG
iC3b
Calreticulin
PS
log2 H/L
2
0
-2
30'/0'
30'/30'
30'/150'
Time point

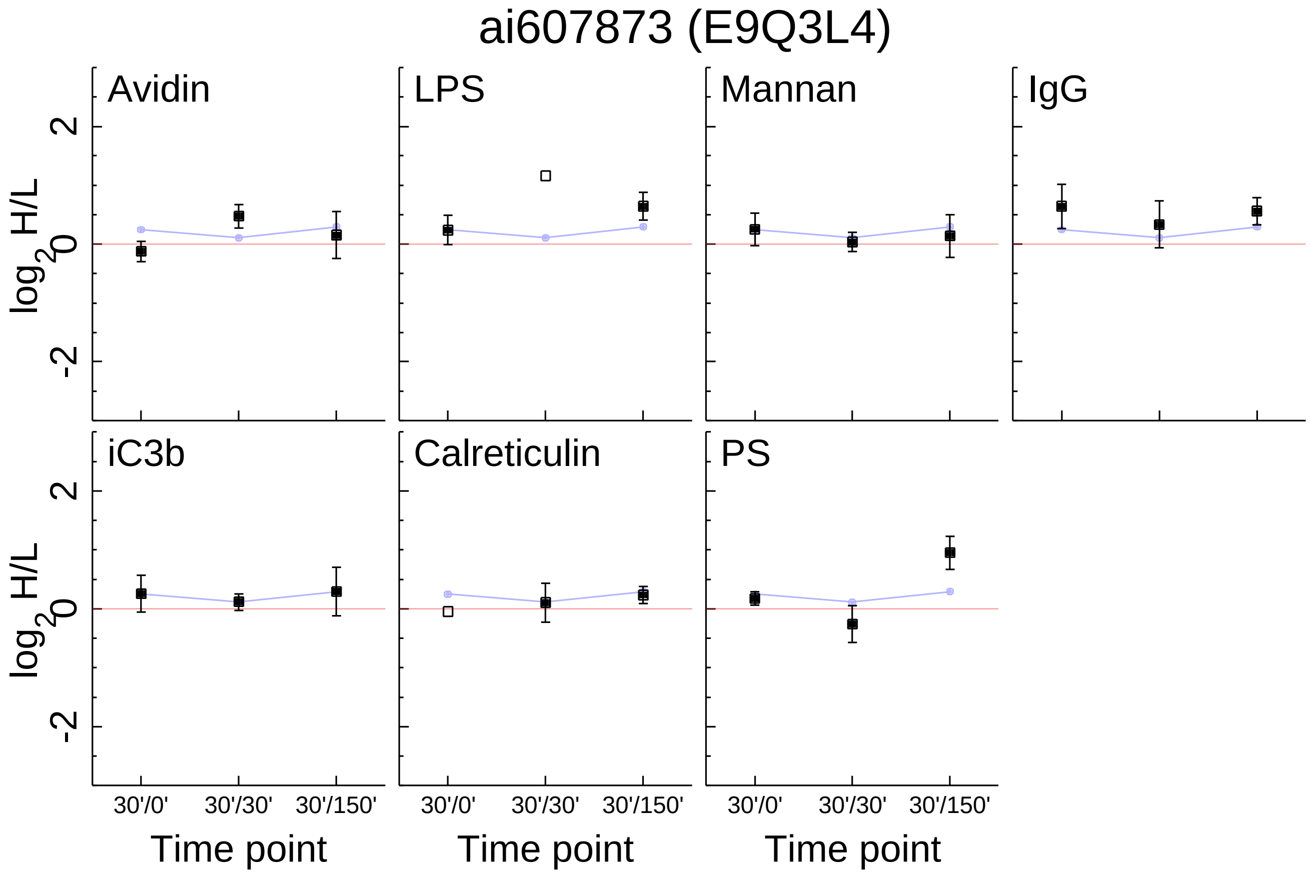
ai607873 (E9Q3L4)
Avidin
LPS
Mannan
IgG
iC3b
Calreticulin
PS
log2 H/L
2
0
-2
30'/0'
30'/30'
30'/150'
Time point

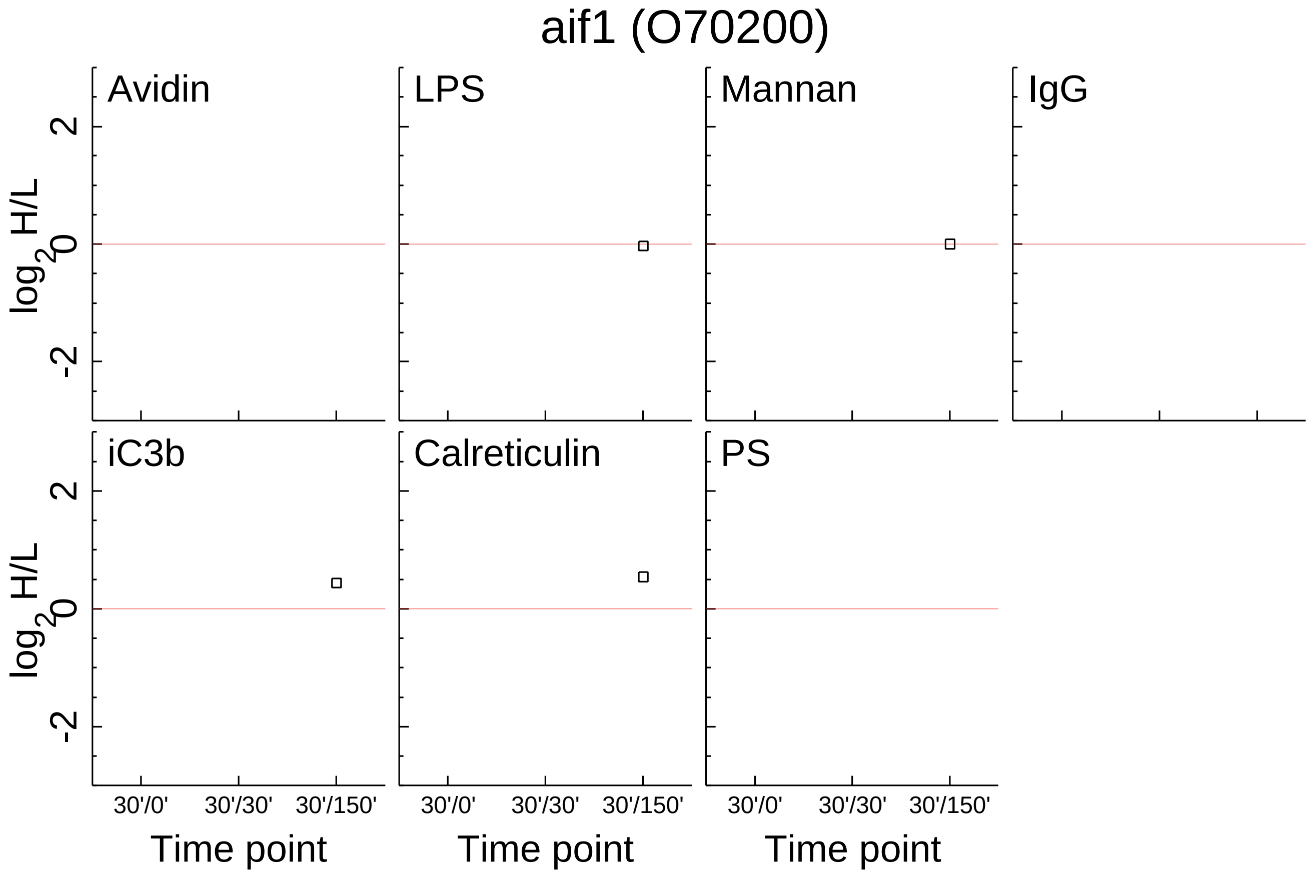
aif1 (O70200)
Avidin
LPS
Mannan
IgG
iC3b
Calreticulin
PS
log2 H/L
2
0
-2
30'/0'
30'/30'
30'/150'
Time point

aifm1 (Q9Z0X1)

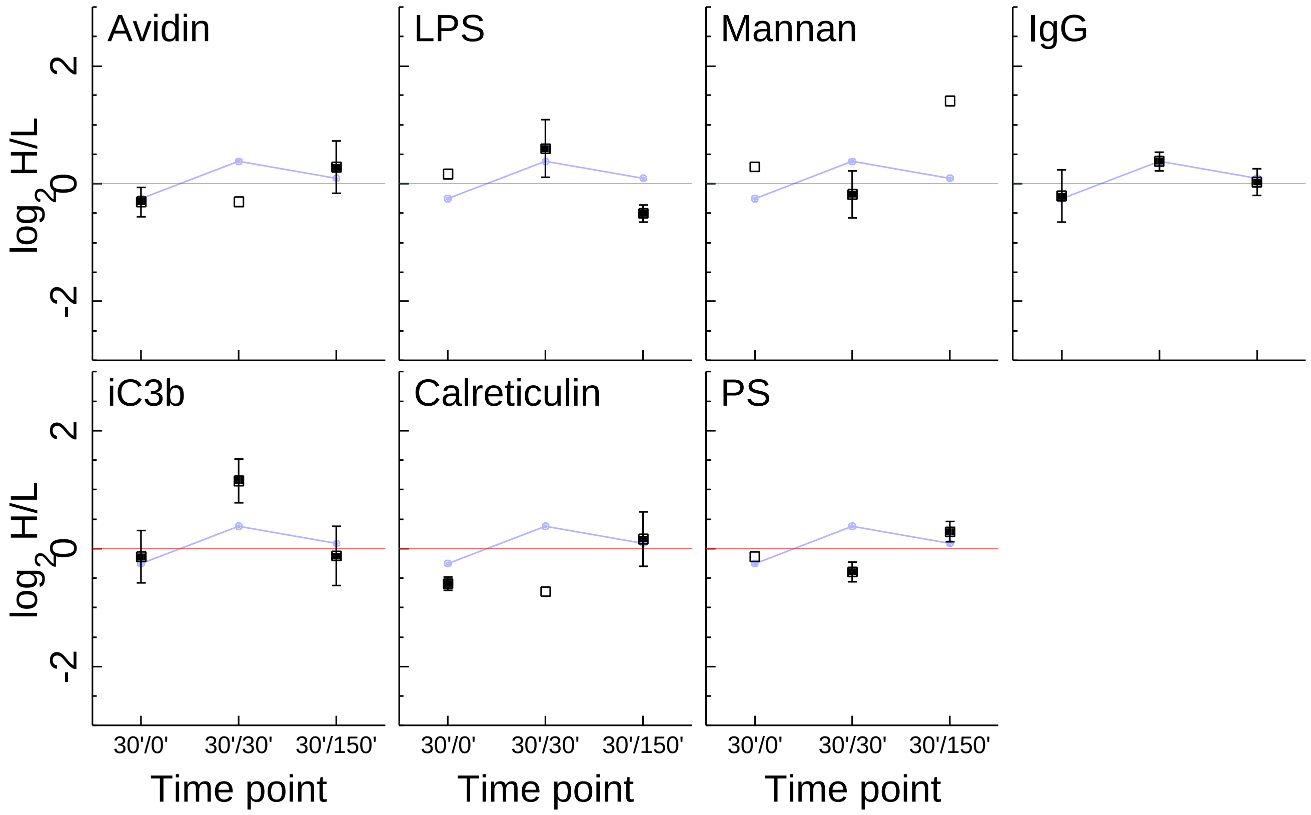

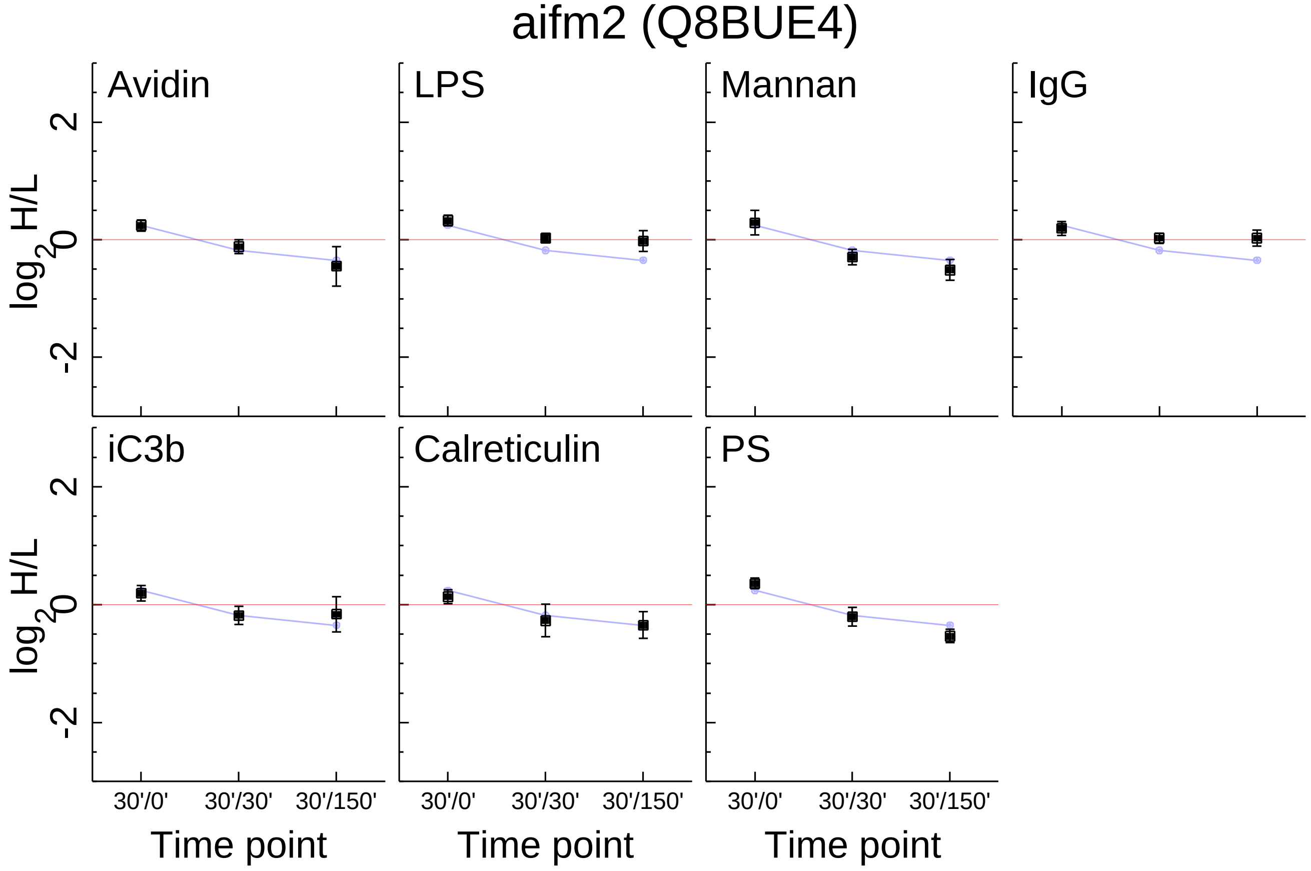

aifm2 (Q8BUE4)
Avidin
LPS
Mannan
IgG
iC3b
Calreticulin
PS
$\log_2$ H/L
2
0
-2
30'/0'
30'/30'
30'/150'
Time point

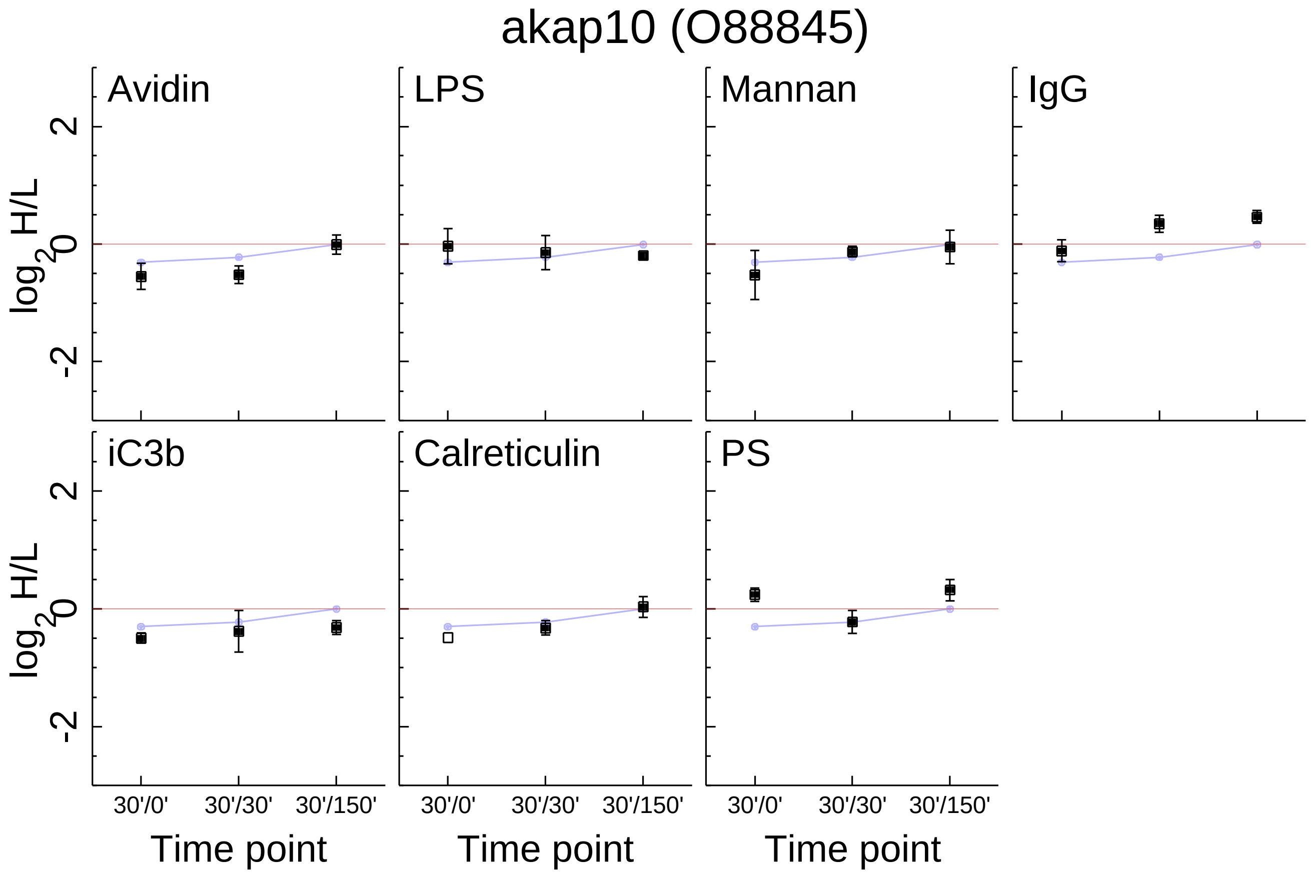

akap10 (O88845)
Avidin
LPS
Mannan
IgG
iC3b
Calreticulin
PS
log2 H/L
2
0
-2
30'/0'
30'/30'
30'/150'
Time point

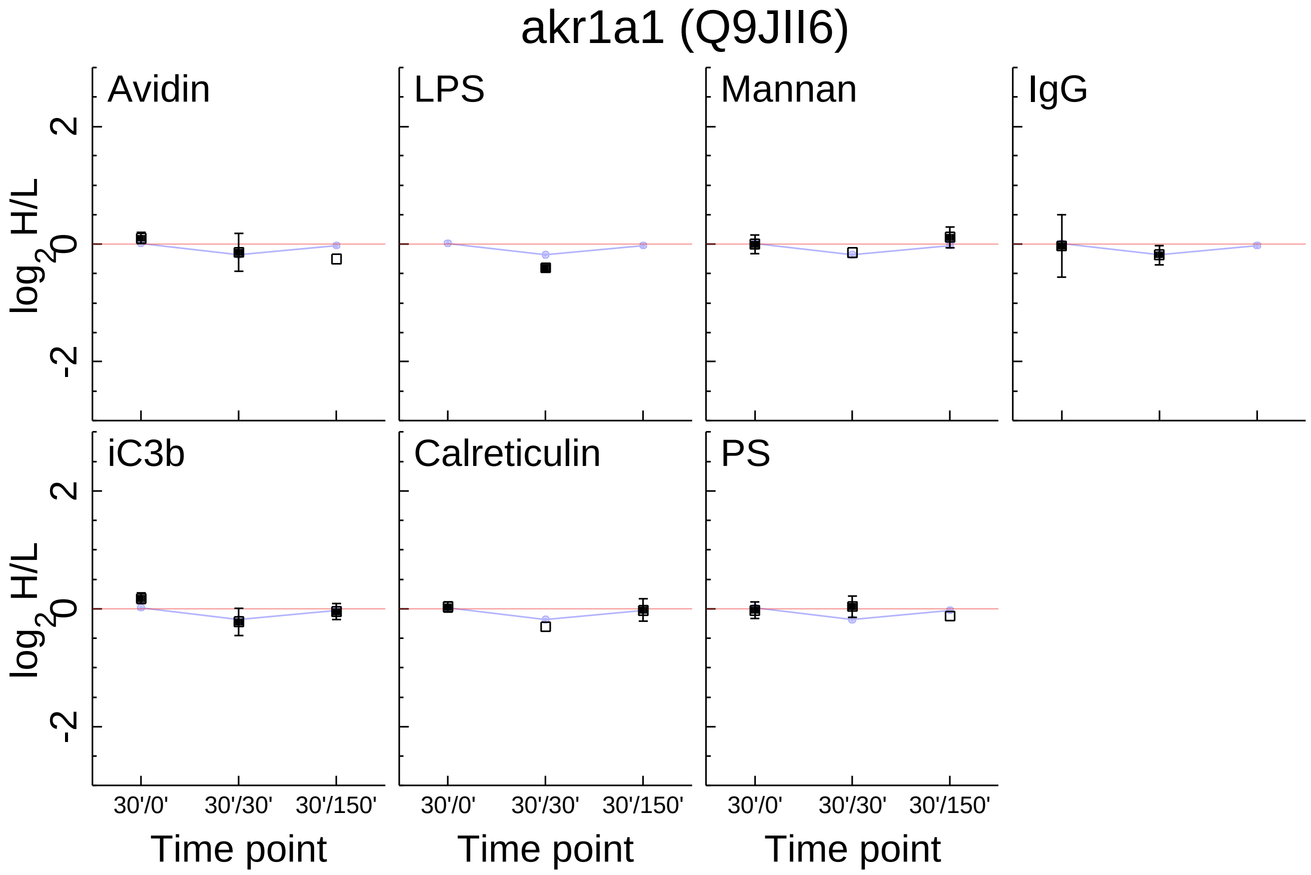

akr1a1 (Q9JII6)
Avidin
LPS
Mannan
IgG
iC3b
Calreticulin
PS
log2 H/L
2
0
-2
30'/0'
30'/30'
30'/150'
Time point

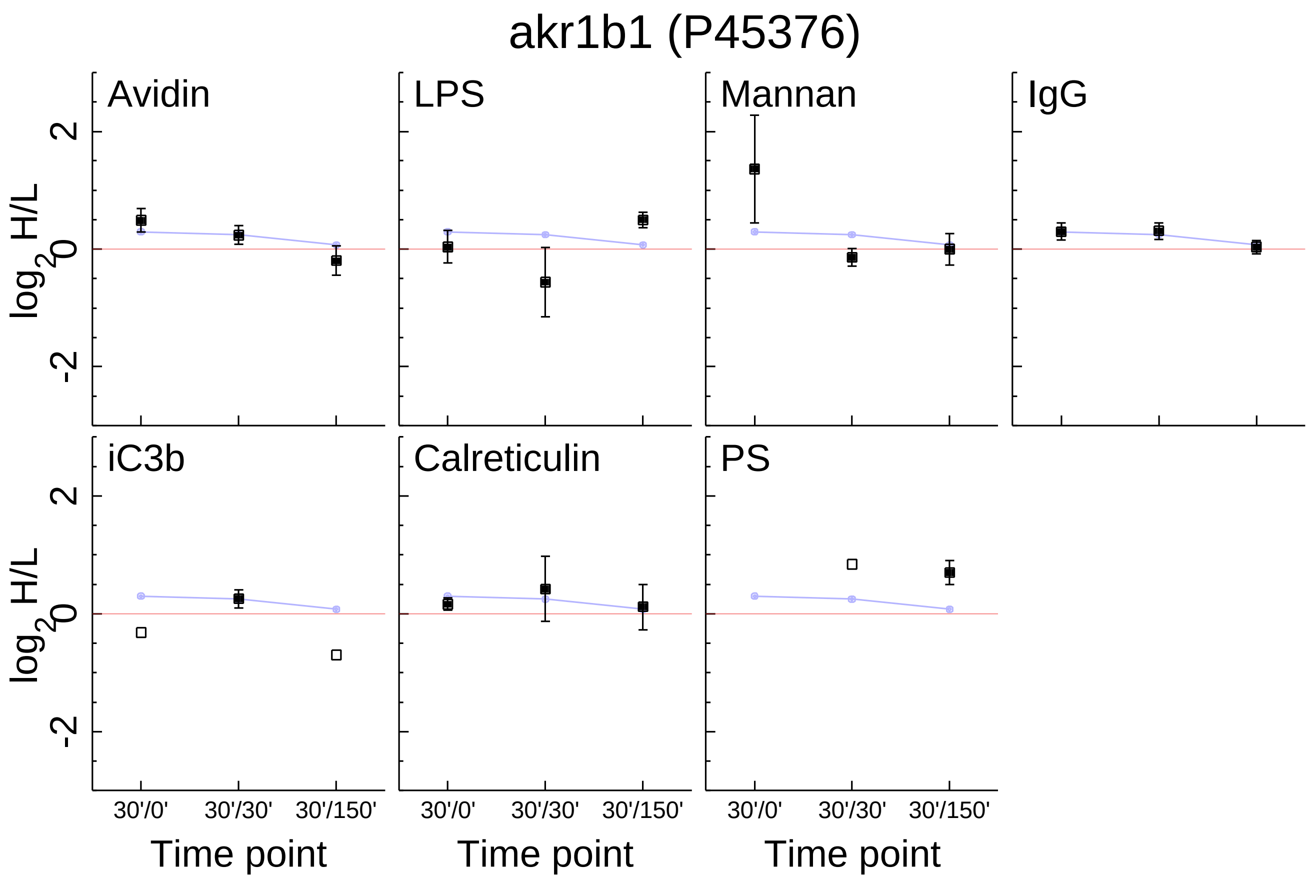
akr1b1 (P45376)
Avidin
LPS
Mannan
IgG
iC3b
Calreticulin
PS
log2 H/L
2
0
-2
30'/0'
30'/30'
30'/150'
Time point

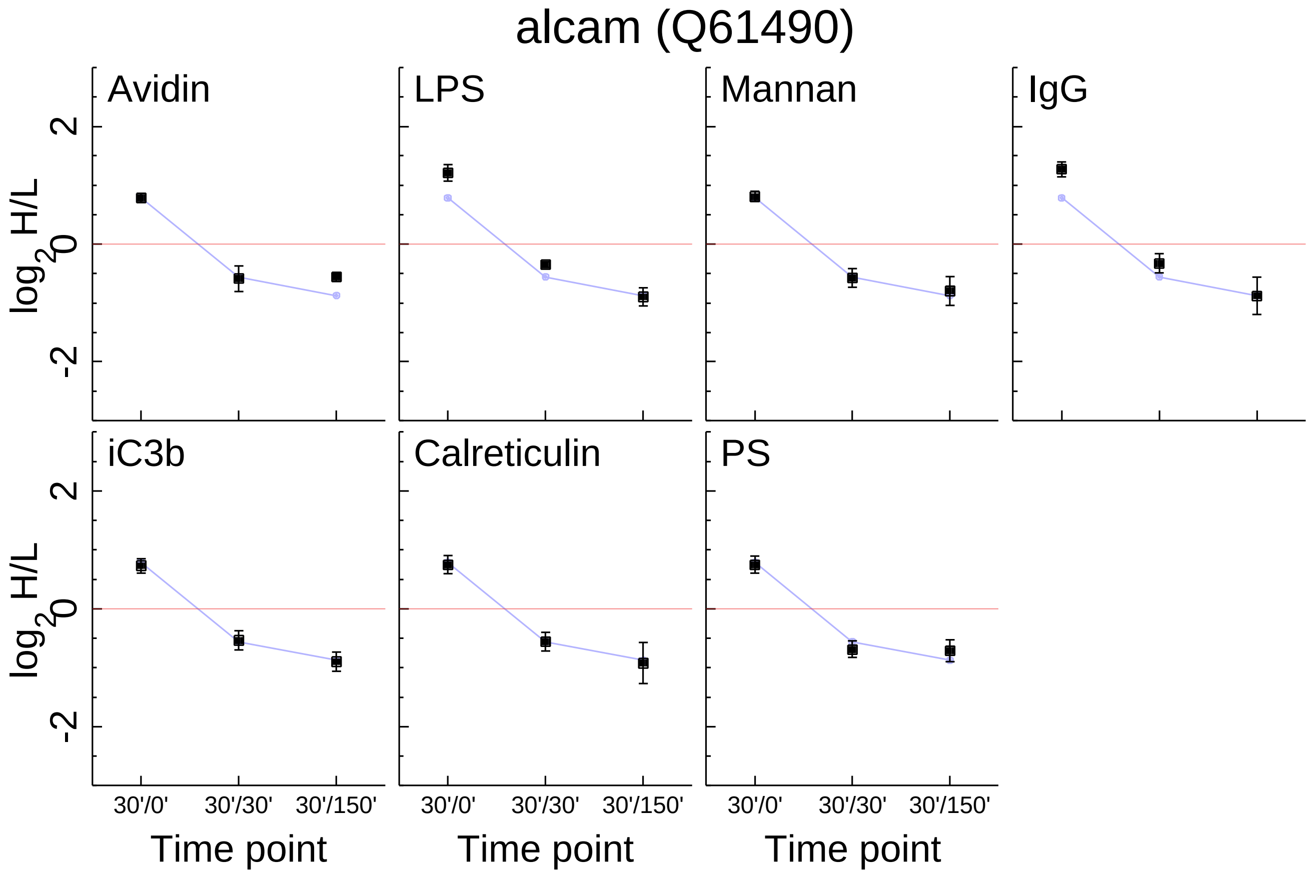
alcam (Q61490)
Avidin
LPS
Mannan
IgG
iC3b
Calreticulin
PS
log2 H/L
2
0
-2
30'/0'
30'/30'
30'/150'
Time point

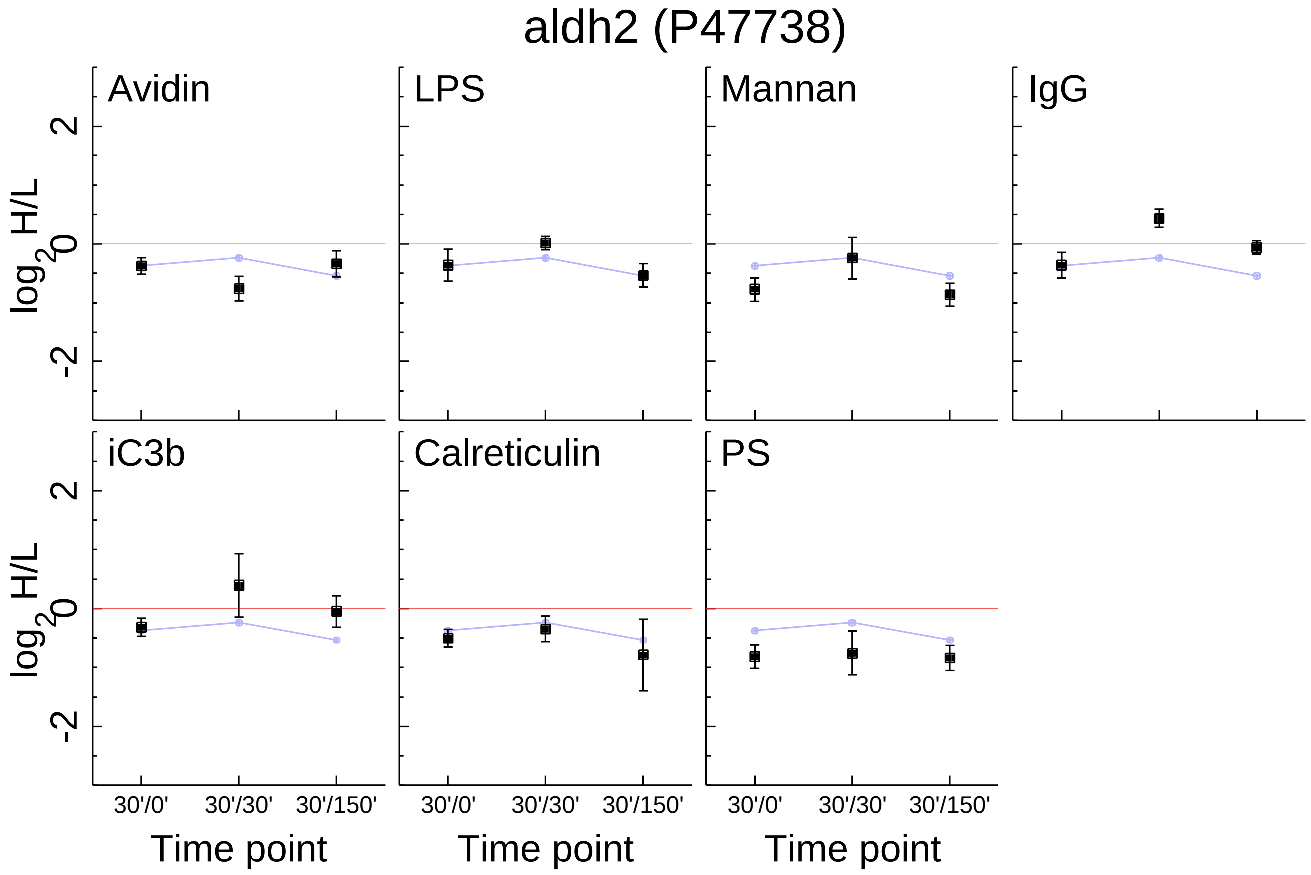
aldh2 (P47738)
Avidin
LPS
Mannan
IgG
iC3b
Calreticulin
PS
log2 H/L
2
0
-2
30'/0'
30'/30'
30'/150'
Time point

aldh3a2 (B1ATI0)

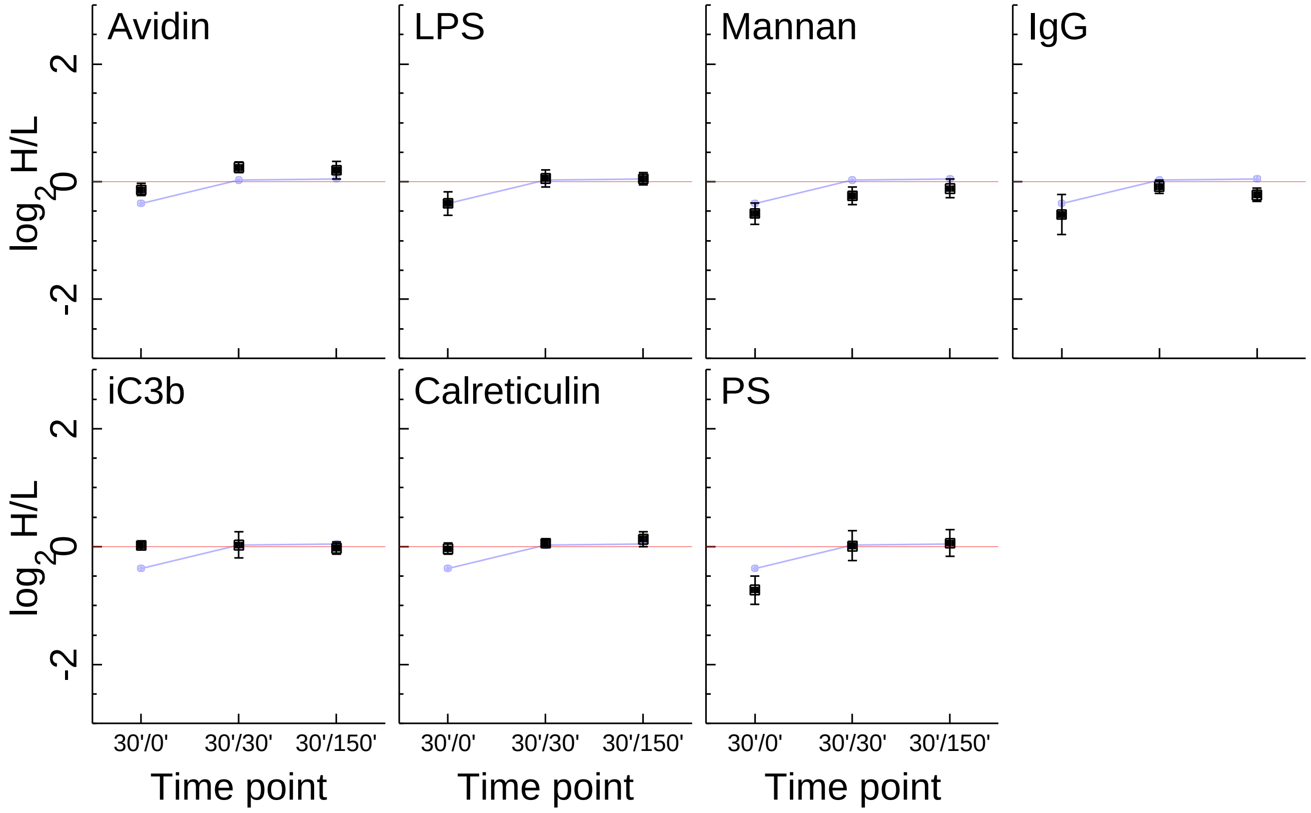

aldh3b1 (Q80VQ0)

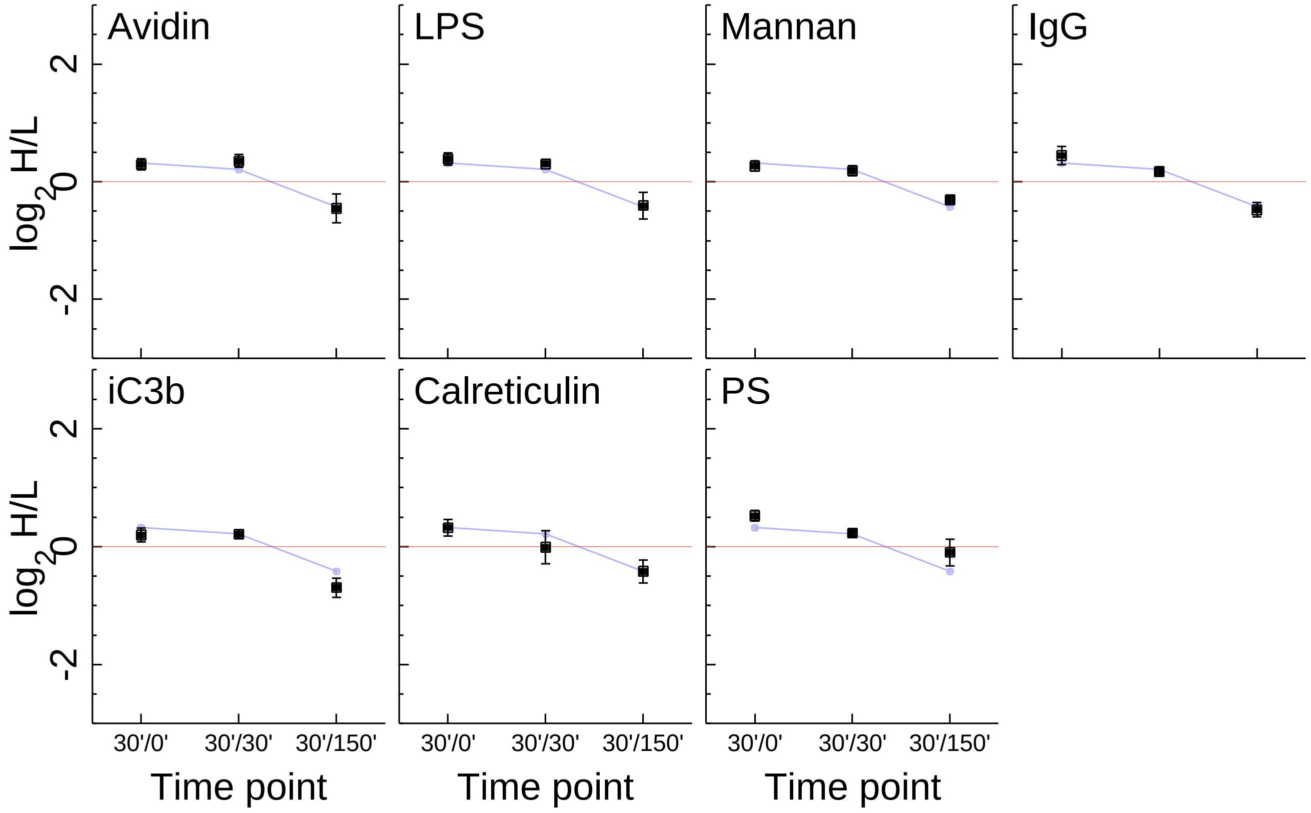

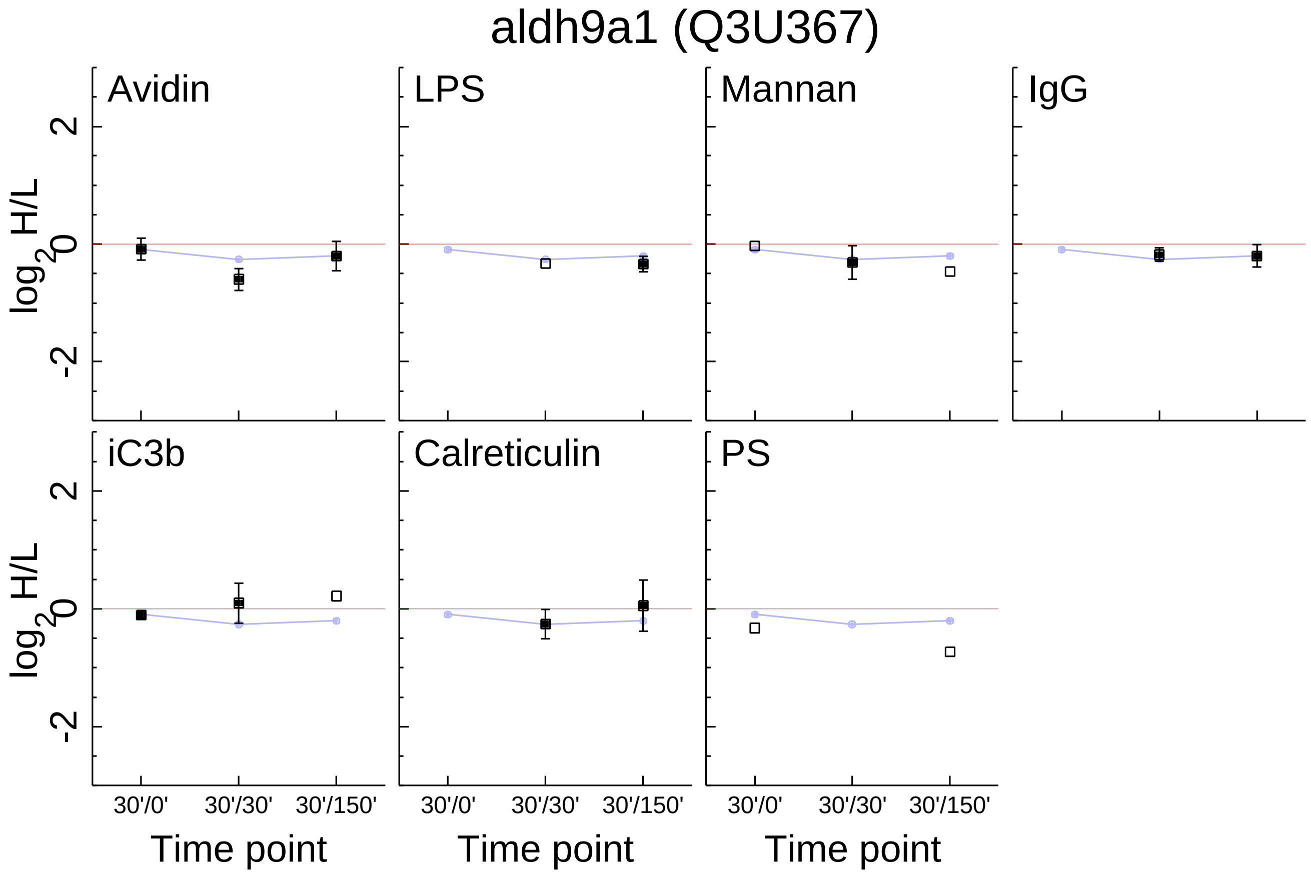
aldh9a1 (Q3U367)
Avidin
LPS
Mannan
IgG
iC3b
Calreticulin
PS
log2 H/L
2
0
-2
30'/0'
30'/30'
30'/150'
Time point

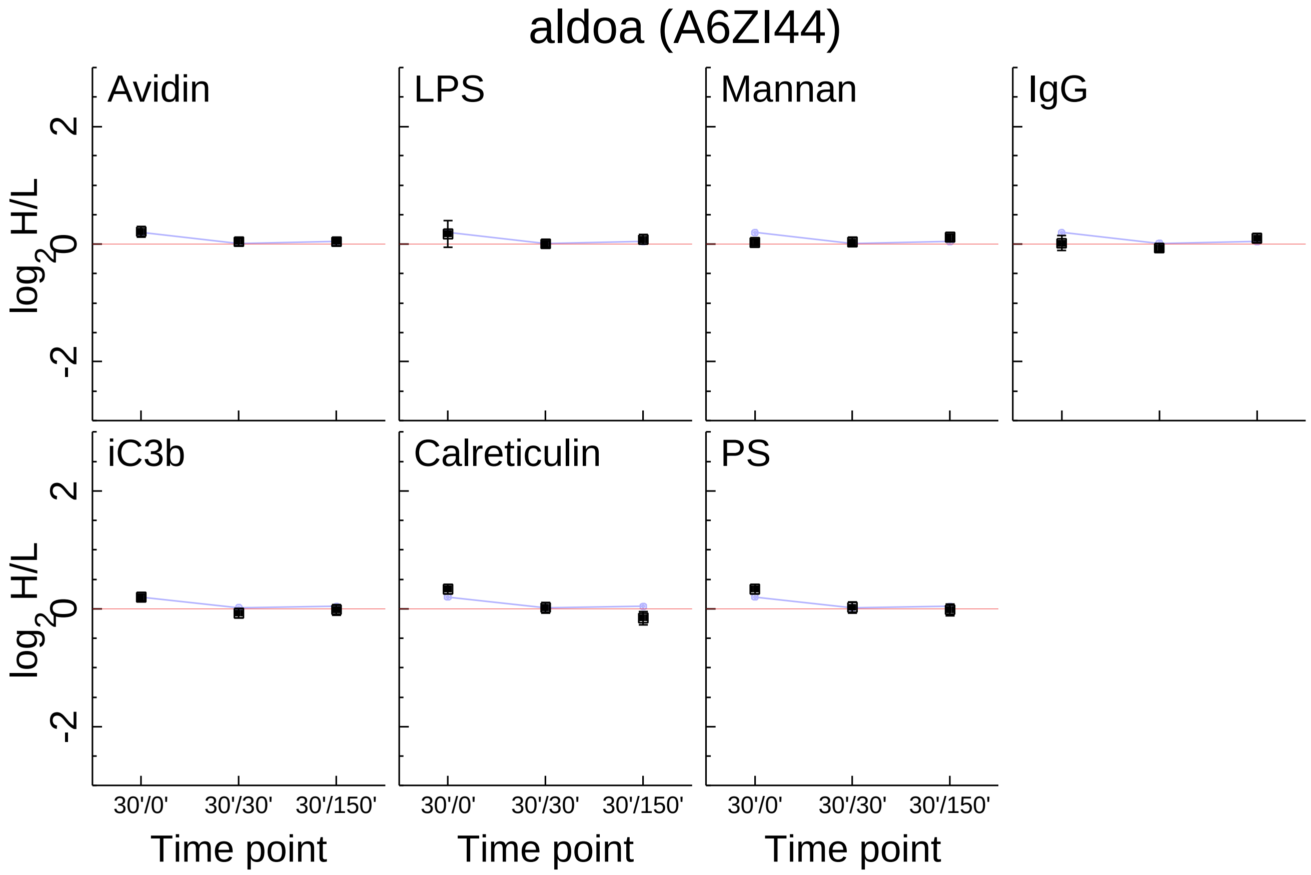
aldoa (A6ZI44)
Avidin
LPS
Mannan
IgG
iC3b
Calreticulin
PS
log2 H/L
2
0
-2
30'/0'
30'/30'
30'/150'
Time point

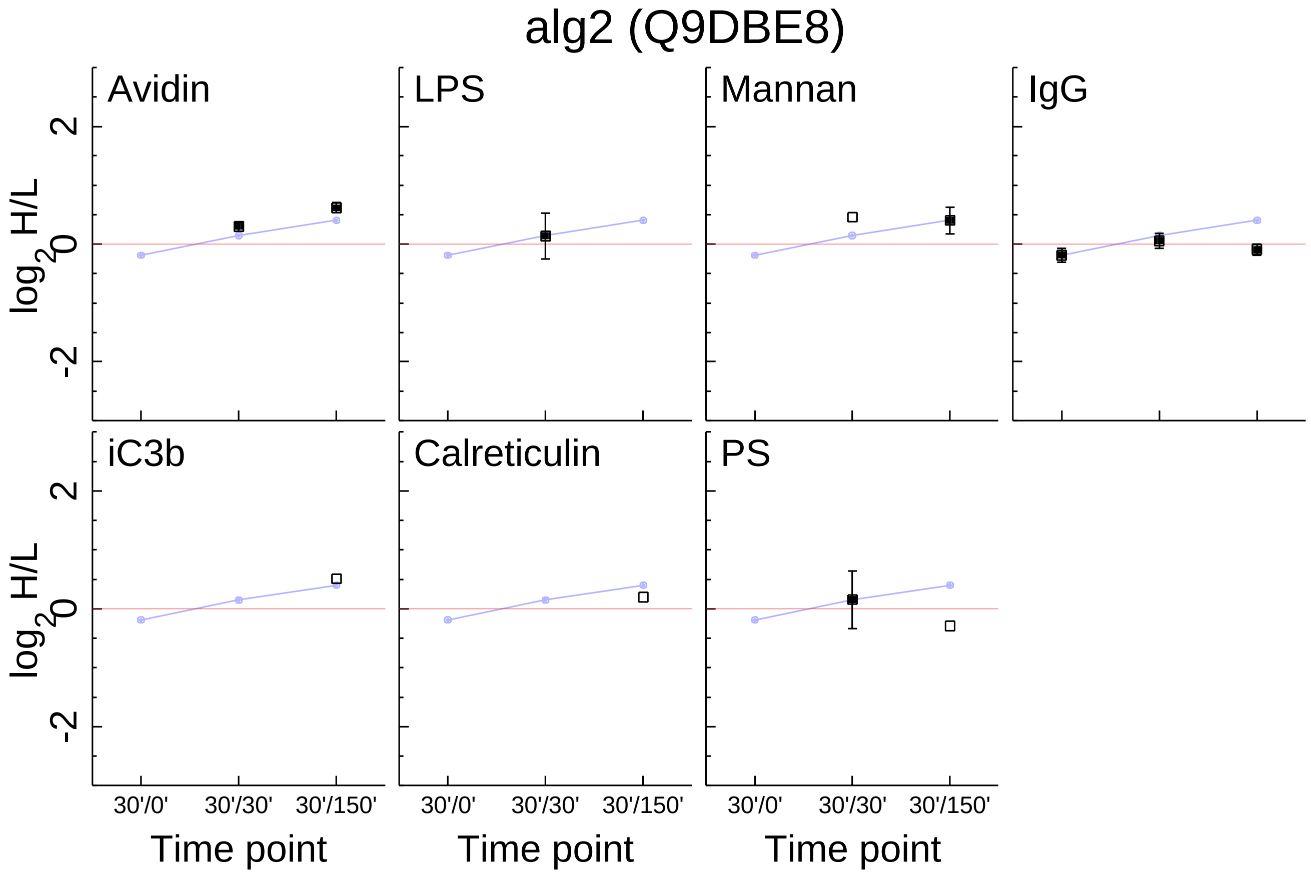

alg2 (Q9DBE8)
Avidin
LPS
Mannan
IgG
iC3b
Calreticulin
PS
log2 H/L
2
0
-2
30'/0'
30'/30'
30'/150'
Time point

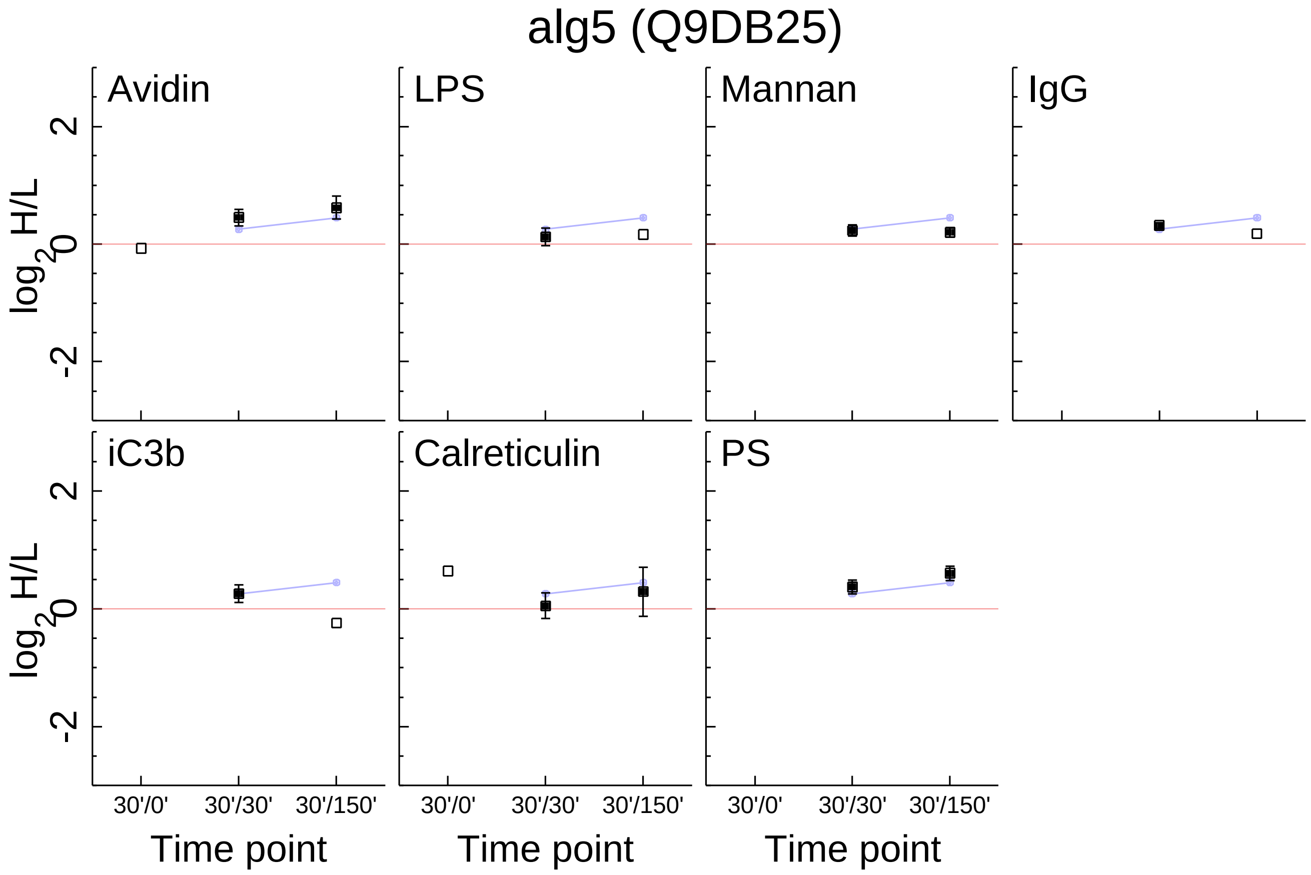

alg5 (Q9DB25)
Avidin
LPS
Mannan
IgG
iC3b
Calreticulin
PS
log2 H/L
2
0
-2
30'/0'
30'/30'
30'/150'
Time point

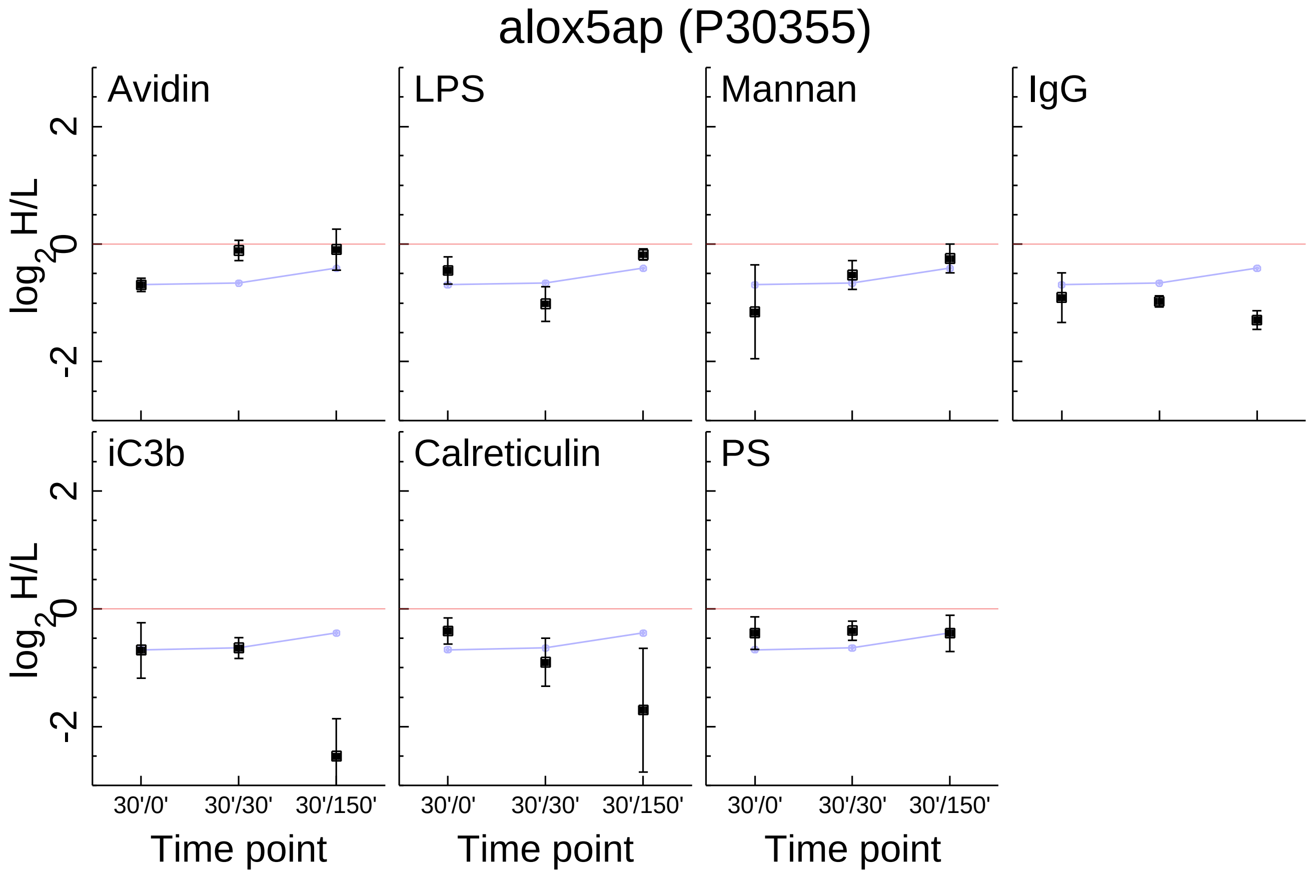

alox5ap (P30355)
Avidin
LPS
Mannan
IgG
iC3b
Calreticulin
PS
log2 H/L
2
0
-2
30'/0'
30'/30'
30'/150'
Time point

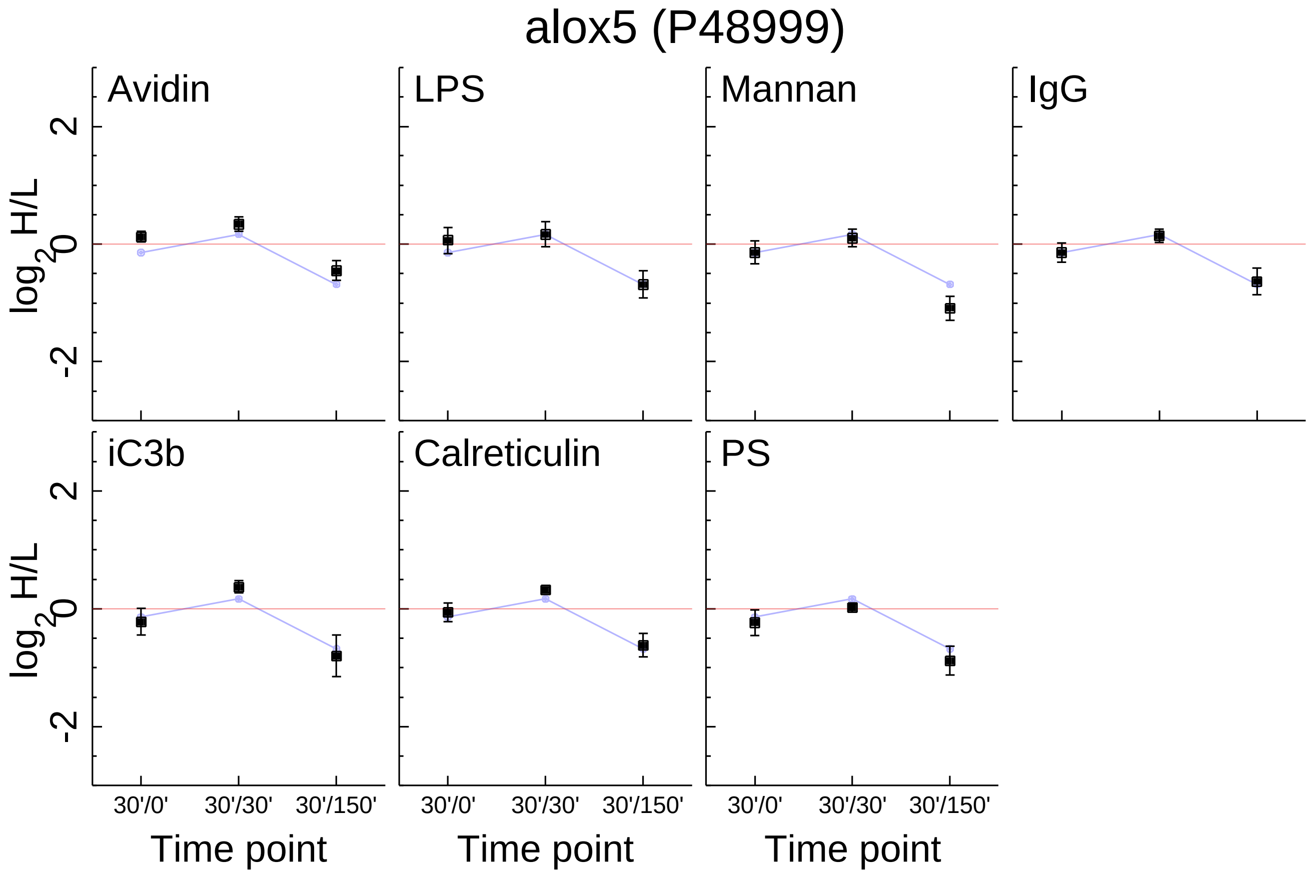

alox5 (P48999)
Avidin
LPS
Mannan
IgG
iC3b
Calreticulin
PS
log2 H/L
2
0
-2
30'/0'
30'/30'
30'/150'
Time point

alyref (O08583)

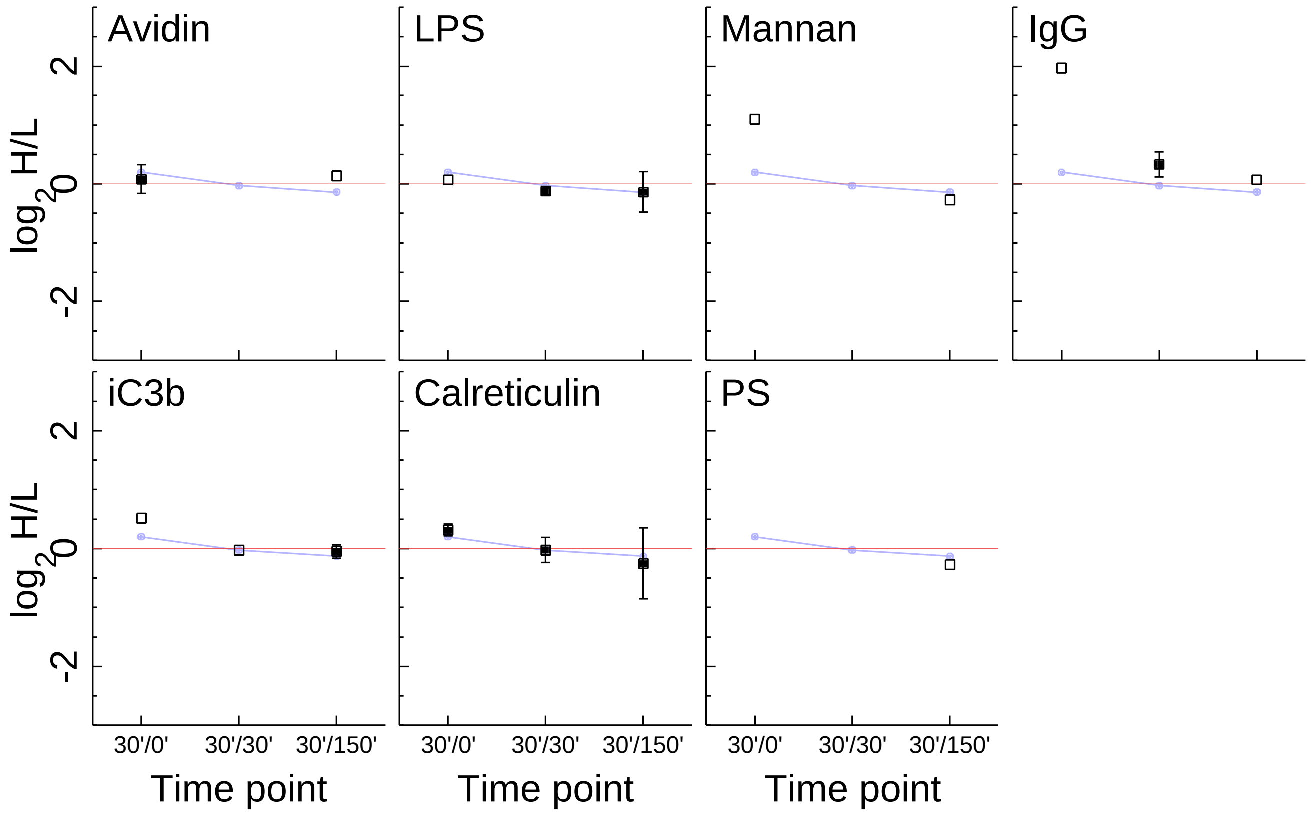

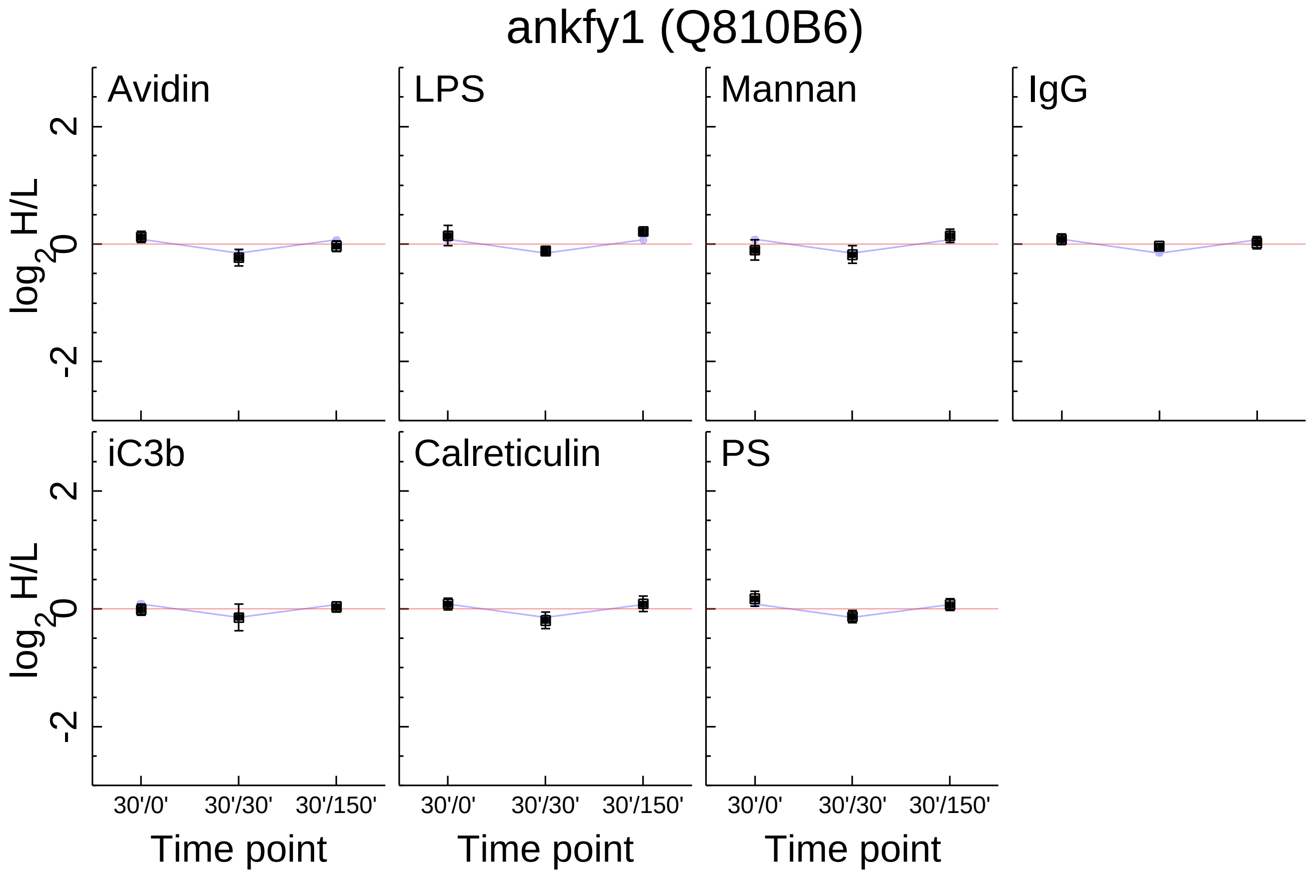
ankfy1 (Q810B6)
Avidin
LPS
Mannan
IgG
iC3b
Calreticulin
PS
log2 H/L
2
0
-2
30'/0'
30'/30'
30'/150'
Time point

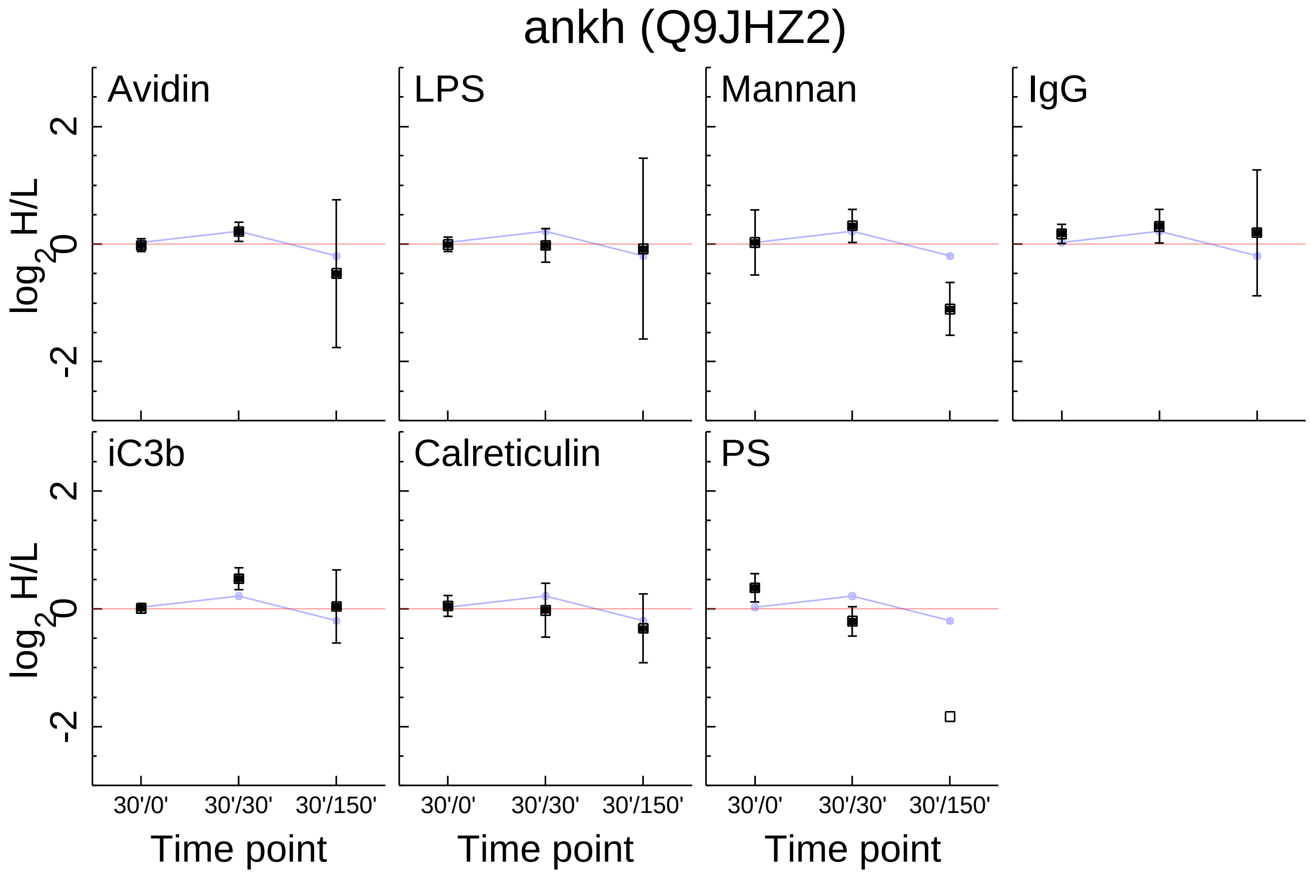
ankh (Q9JHZ2)
Avidin
LPS
Mannan
IgG
iC3b
Calreticulin
PS
log2 H/L
2
0
-2
30'/0'
30'/30'
30'/150'
Time point

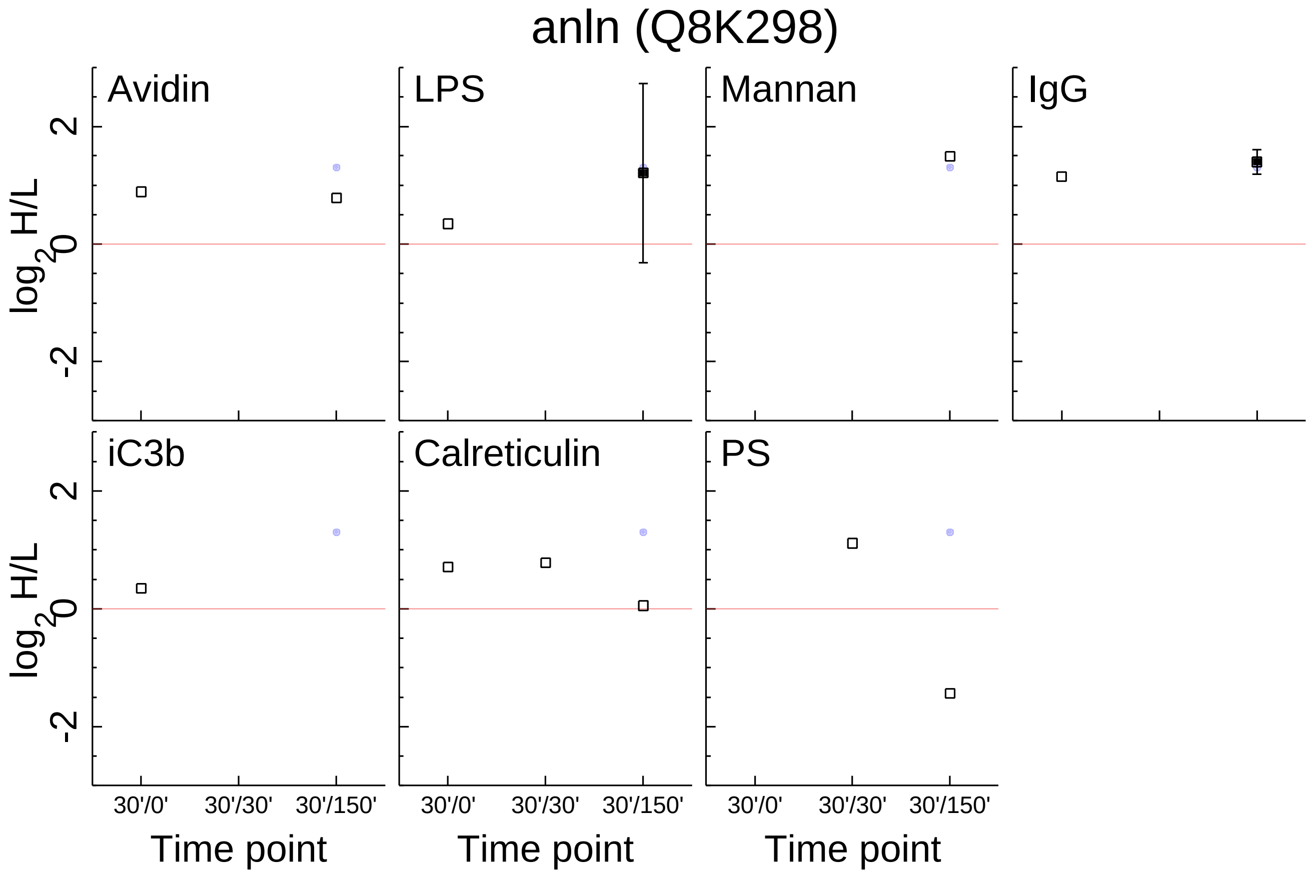
anln (Q8K298)
Avidin
LPS
Mannan
IgG
iC3b
Calreticulin
PS
log2 H/L
2
0
-2
30'/0'
30'/30'
30'/150'
Time point

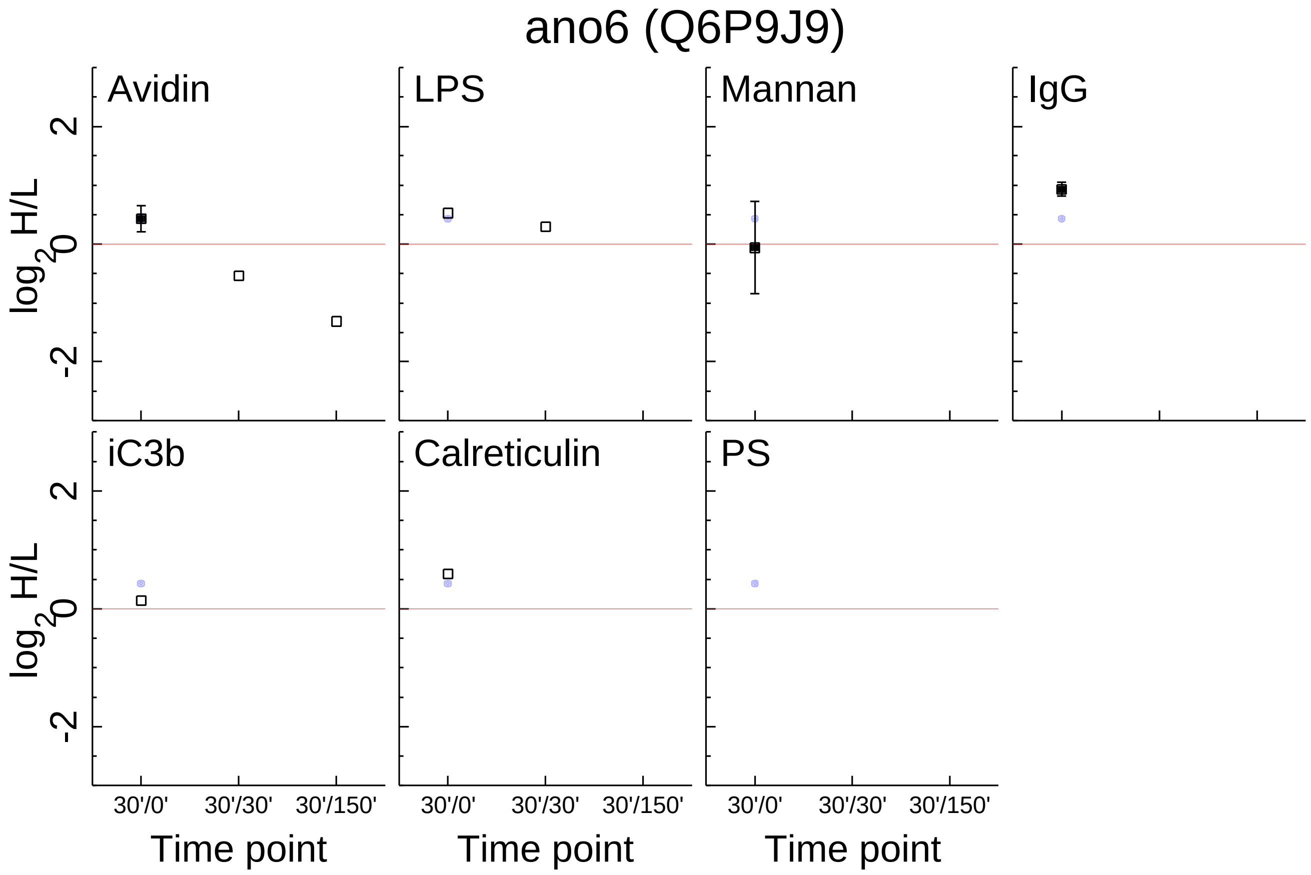
ano6 (Q6P9J9)
Avidin
LPS
Mannan
IgG
iC3b
Calreticulin
PS
log2 H/L
2
0
-2
30'/0'
30'/30'
30'/150'
Time point

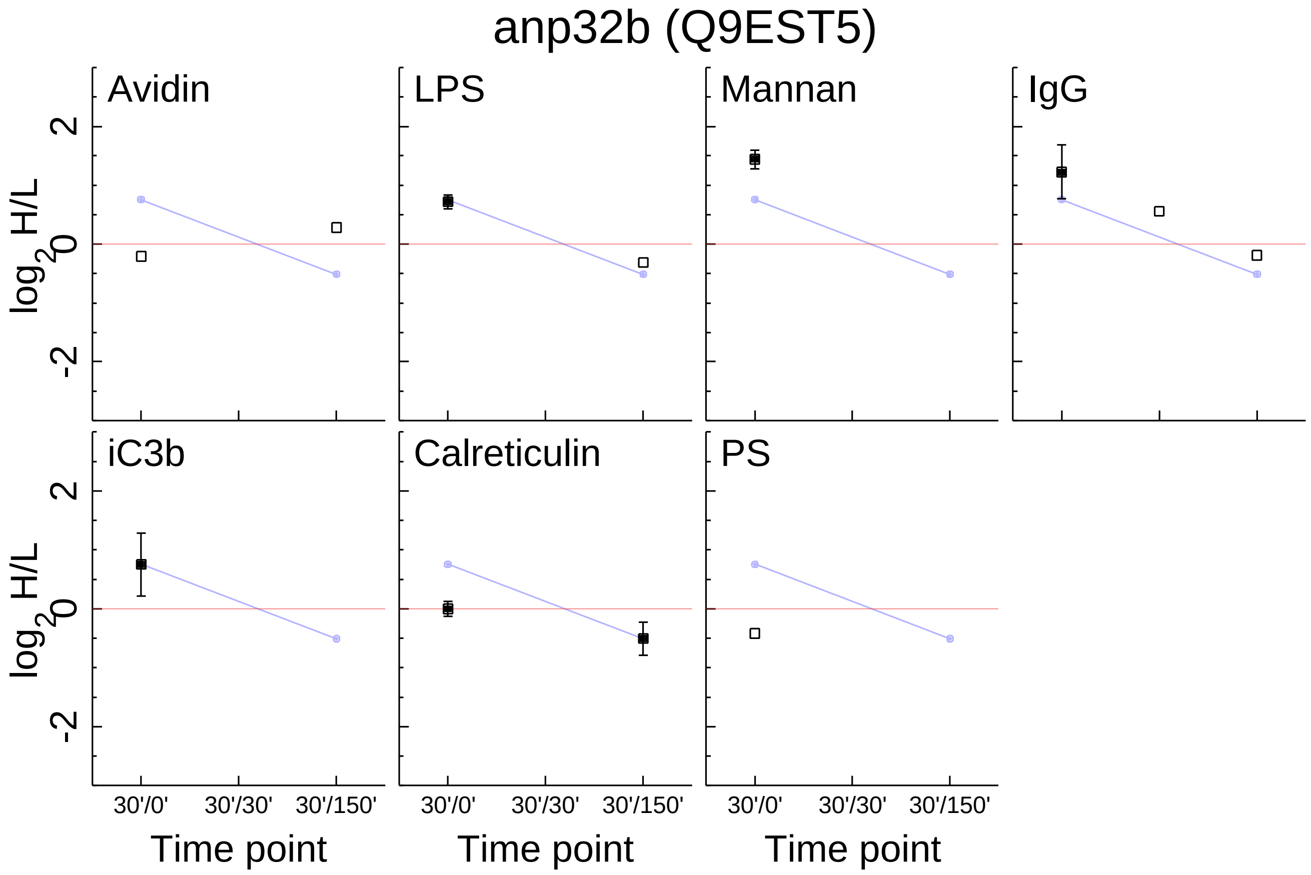

anp32b (Q9EST5)
Avidin
LPS
Mannan
IgG
iC3b
Calreticulin
PS
log2 H/L
2
0
-2
30'/0'
30'/30'
30'/150'
Time point

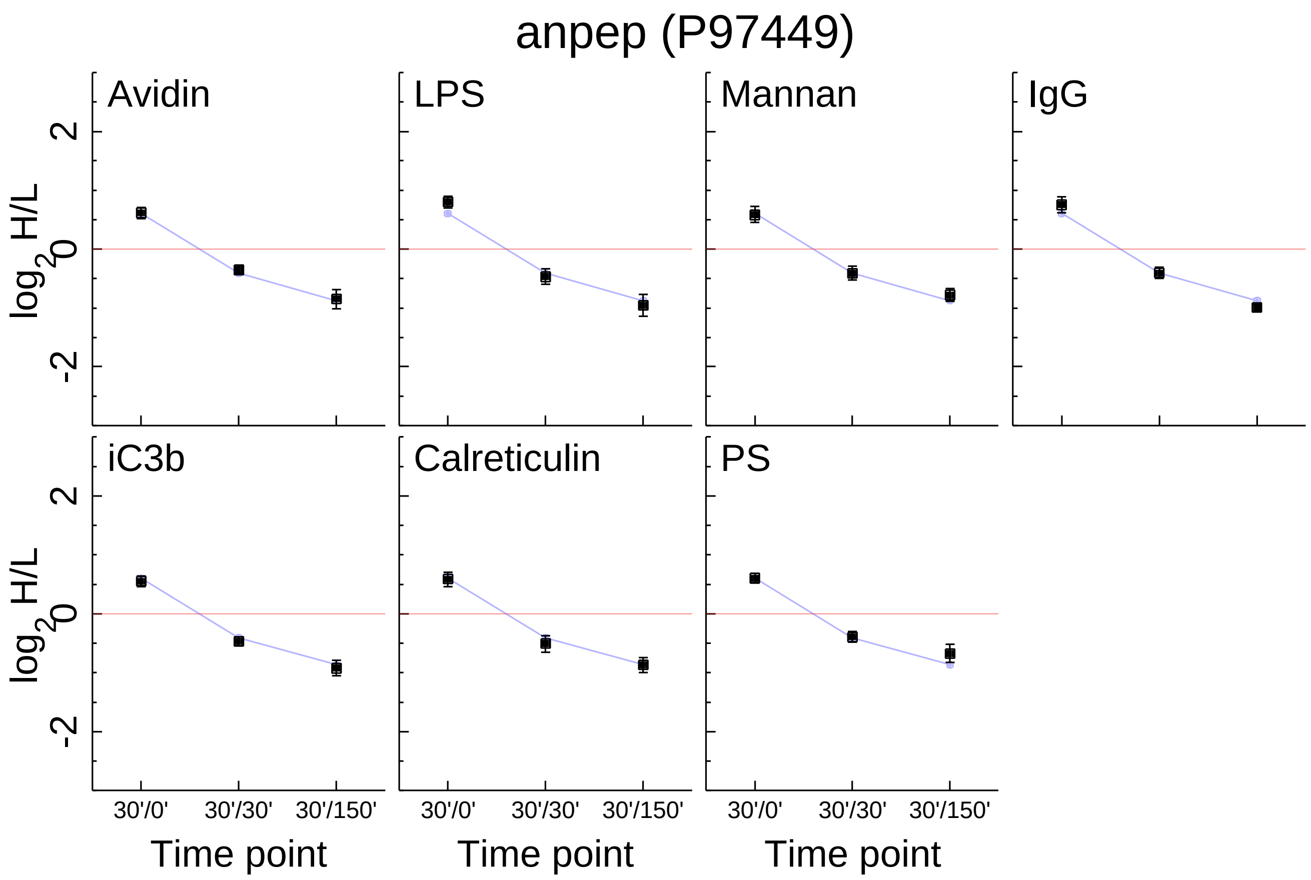
anpep (P97449)
Avidin
LPS
Mannan
IgG
iC3b
Calreticulin
PS
log2 H/L
2
0
-2
30'/0'
30'/30'
30'/150'
Time point

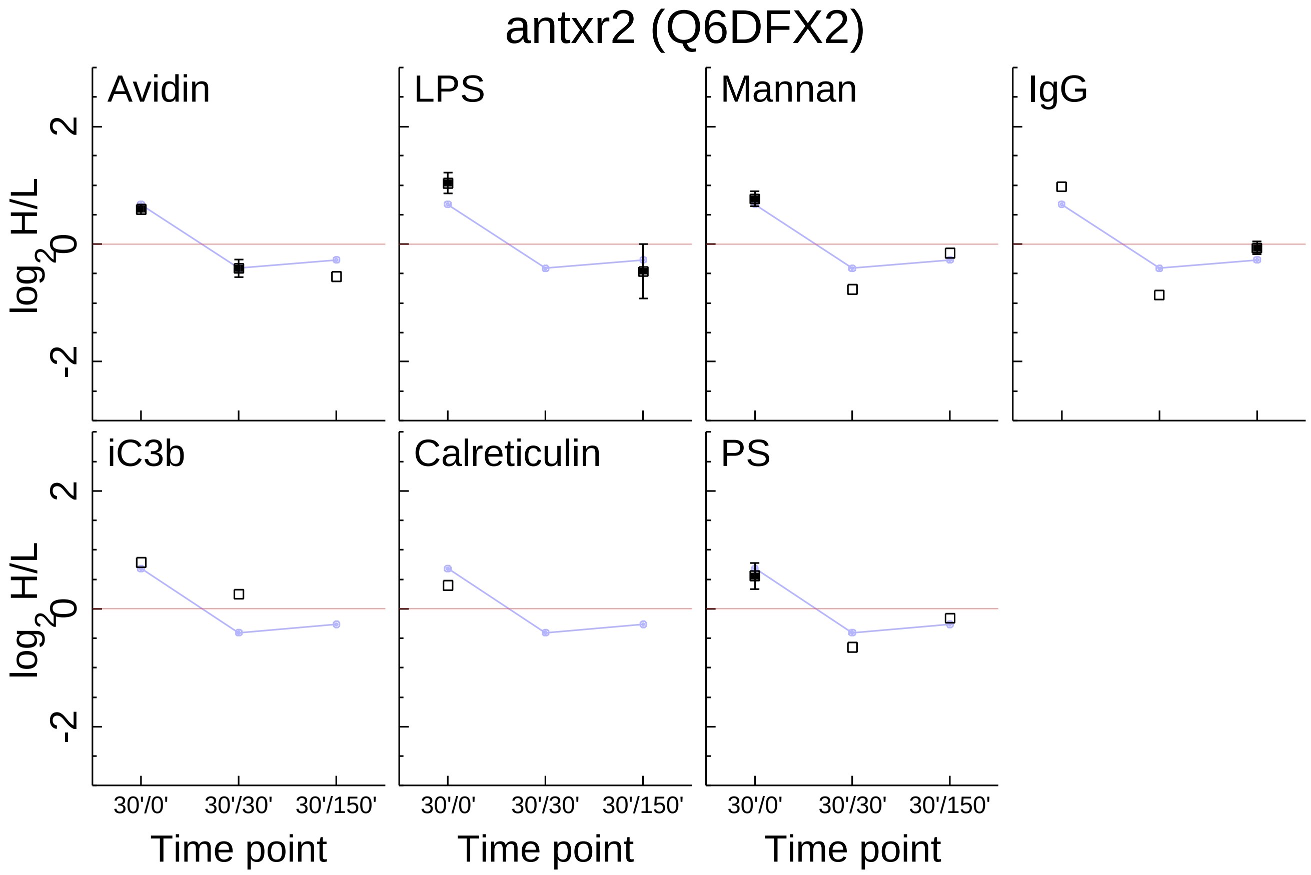
antxr2 (Q6DFX2)
Avidin
LPS
Mannan
IgG
iC3b
Calreticulin
PS
log2 H/L
2
0
-2
30'/0'
30'/30'
30'/150'
Time point

anxa11 (P97384)

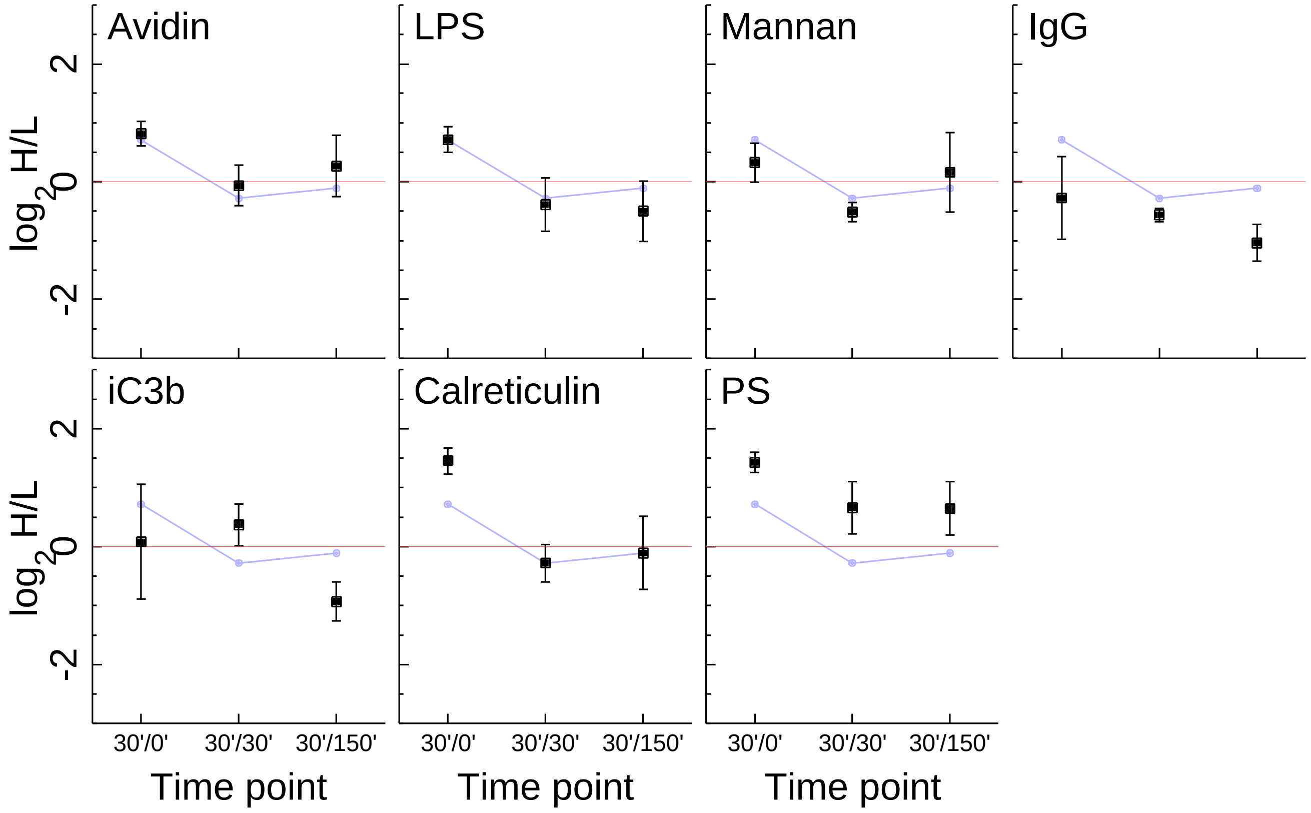

anxa1 (P10107)

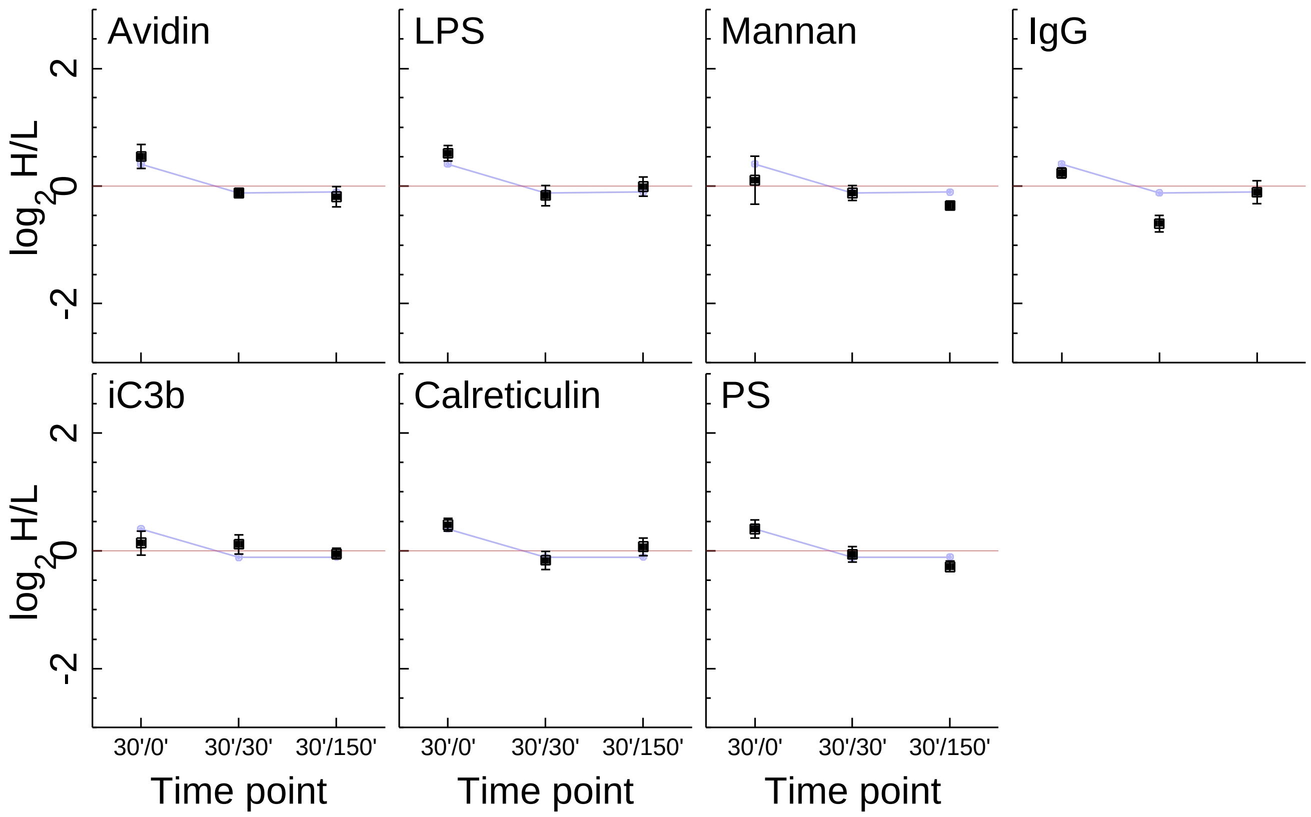

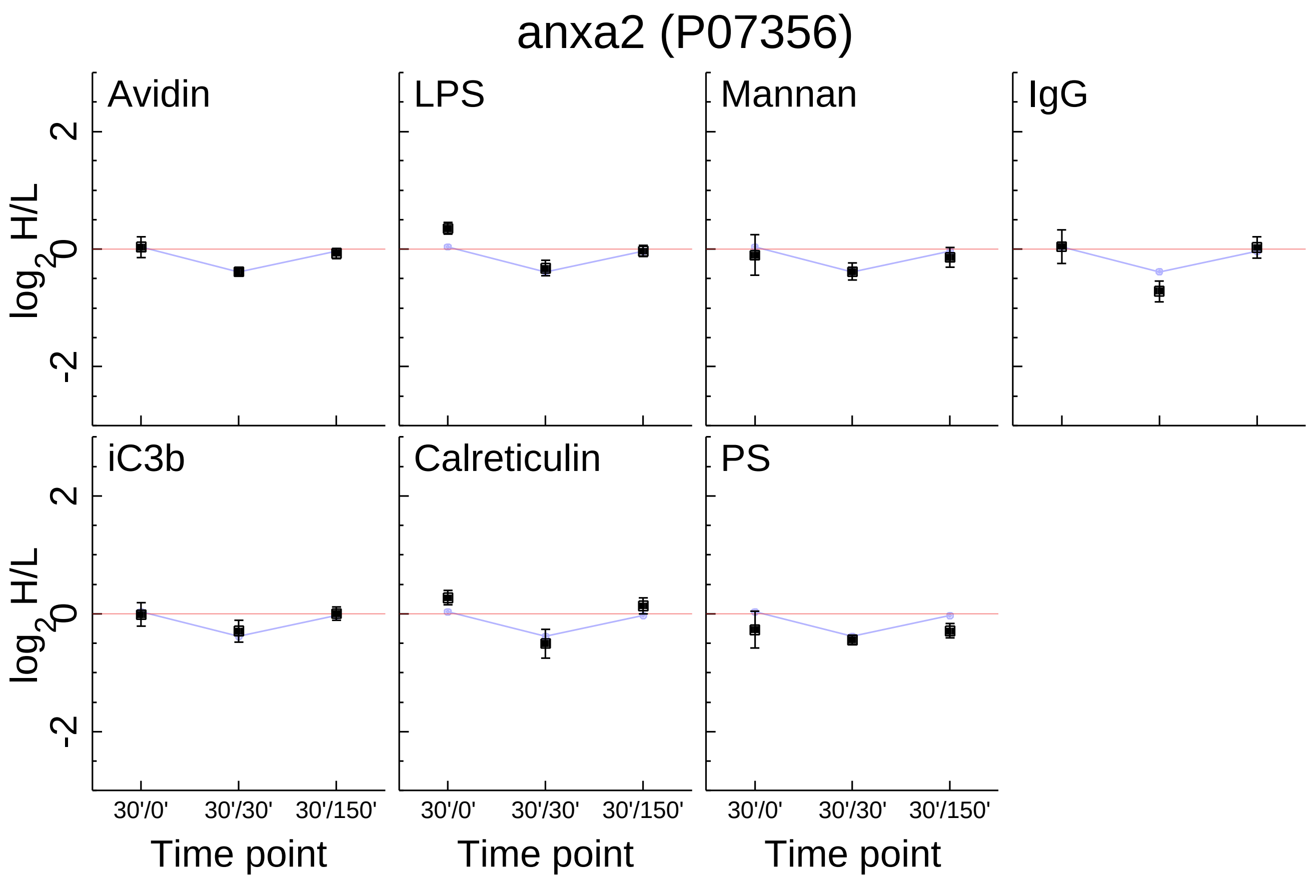
anxa2 (P07356)
Avidin
LPS
Mannan
IgG
iC3b
Calreticulin
PS
log2 H/L
2
0
-2
30'/0'
30'/30'
30'/150'
Time point

anxa3 (O35639)

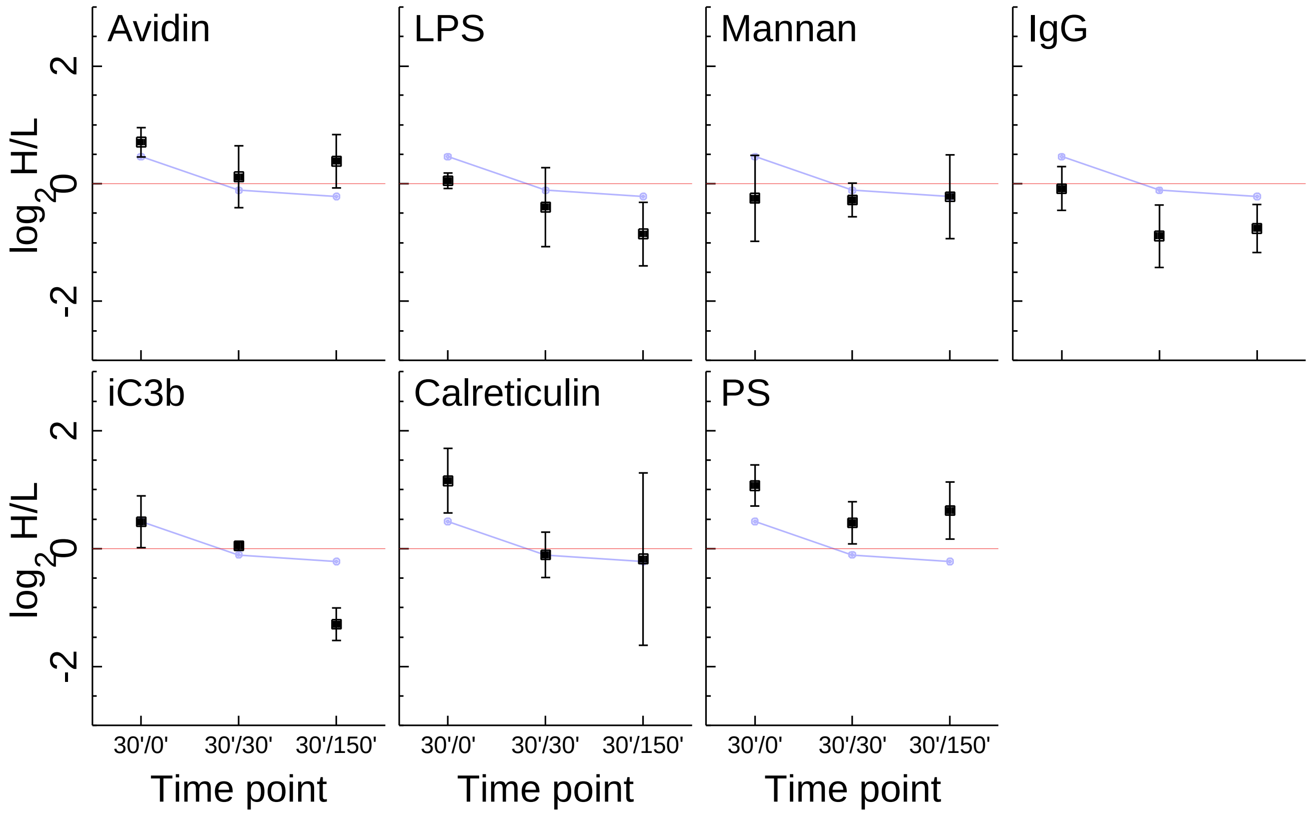

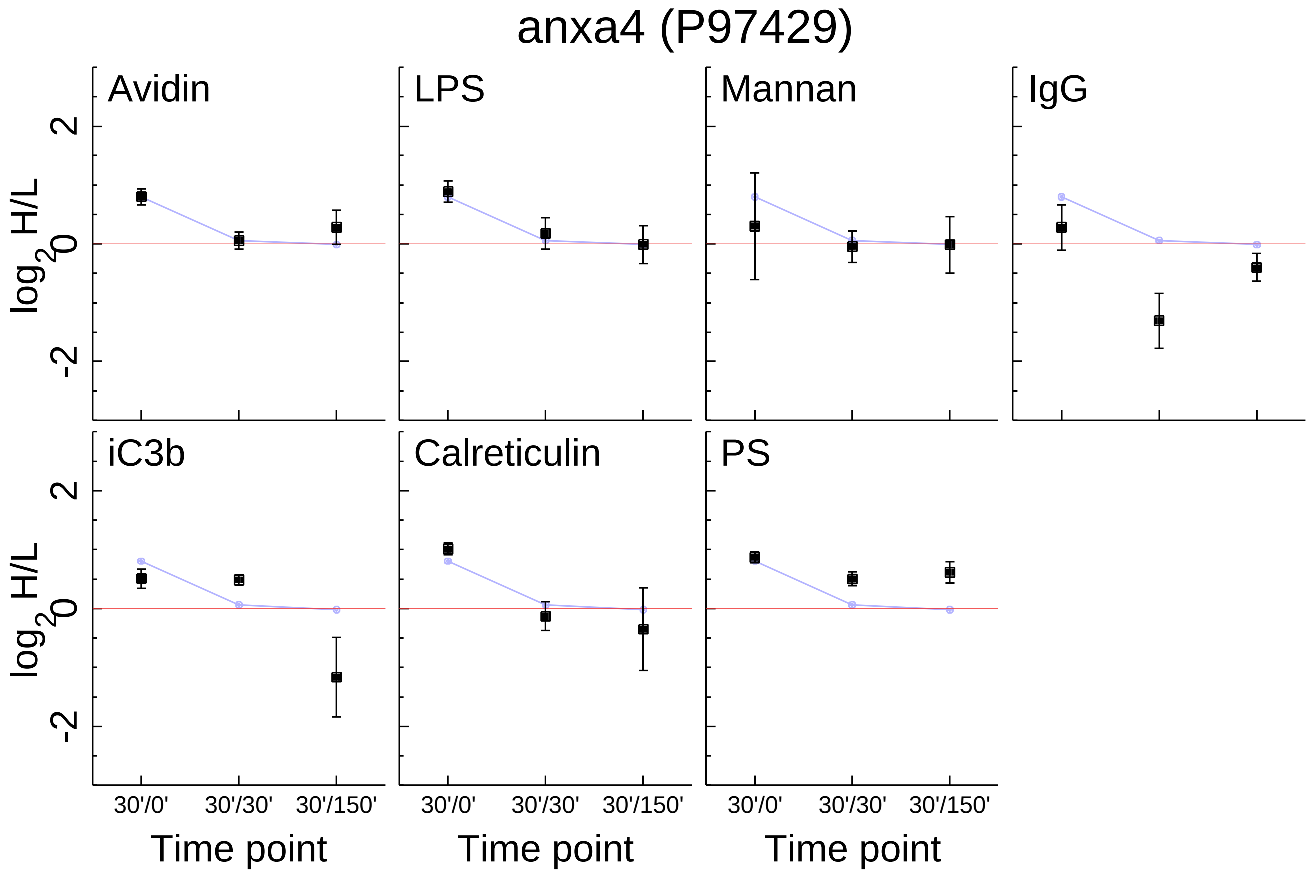
anxa4 (P97429)
Avidin
LPS
Mannan
IgG
iC3b
Calreticulin
PS
log2 H/L
2
0
-2
30'/0'
30'/30'
30'/150'
Time point

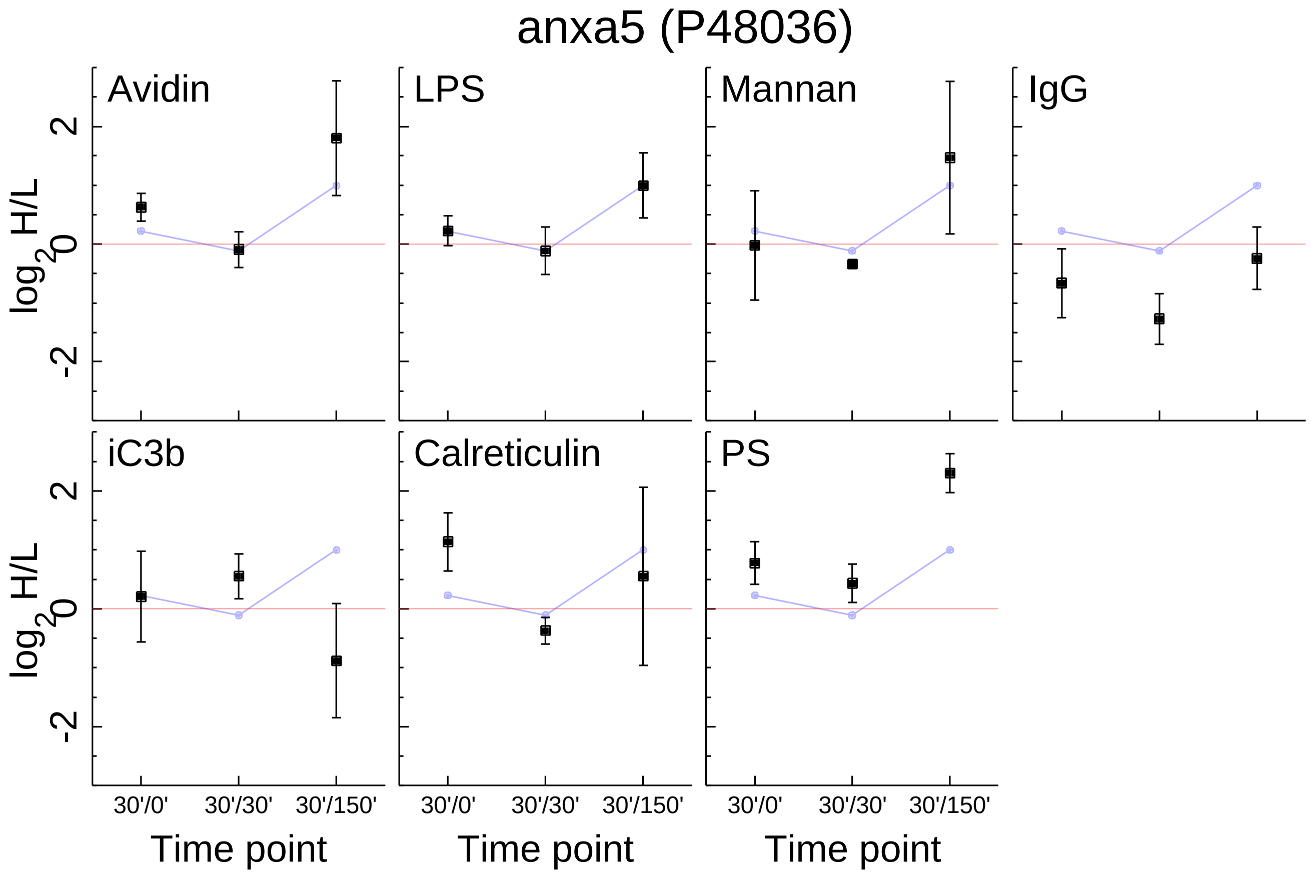
anxa5 (P48036)
Avidin
LPS
Mannan
IgG
iC3b
Calreticulin
PS
log2 H/L
2
0
-2
30'/0'
30'/30'
30'/150'
Time point

anxa6:1 (F8WIT2)

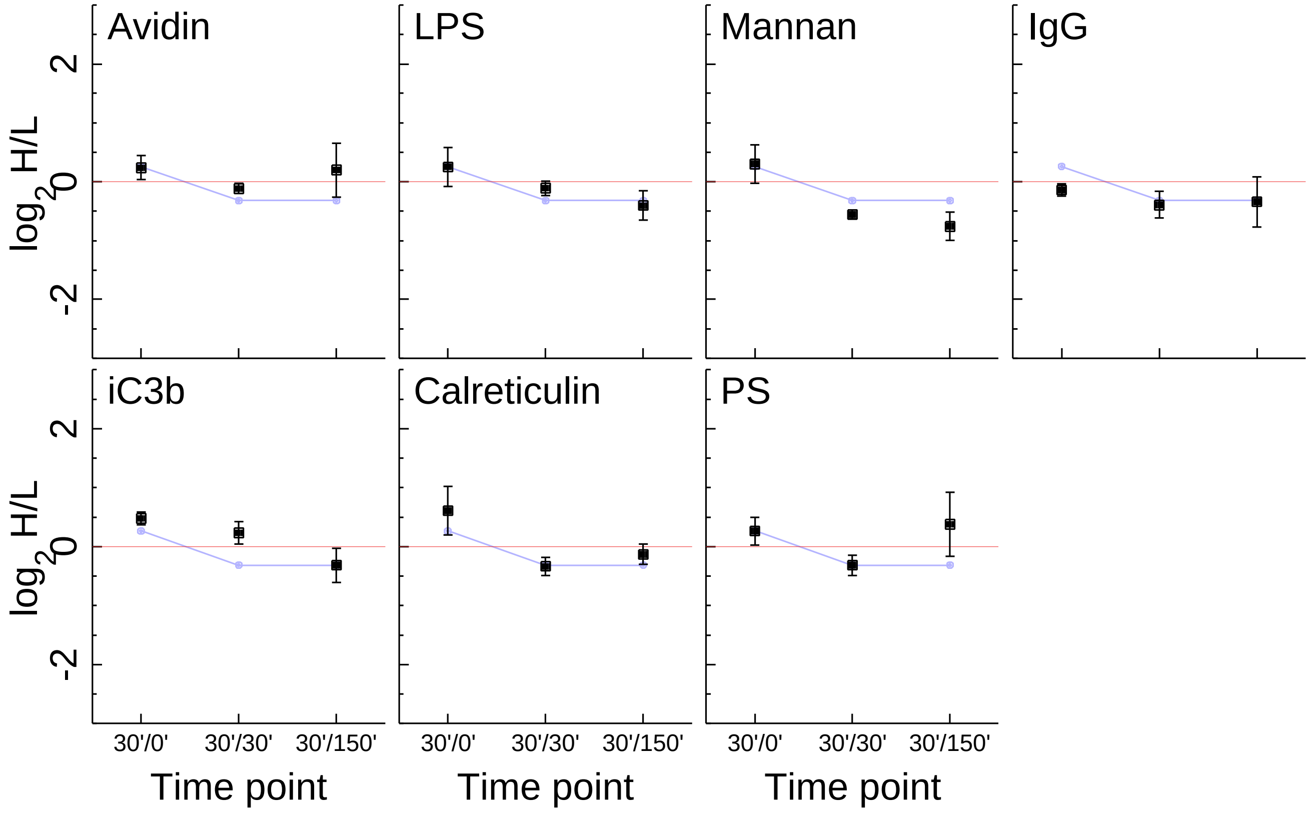

anxa6:2 (P14824)

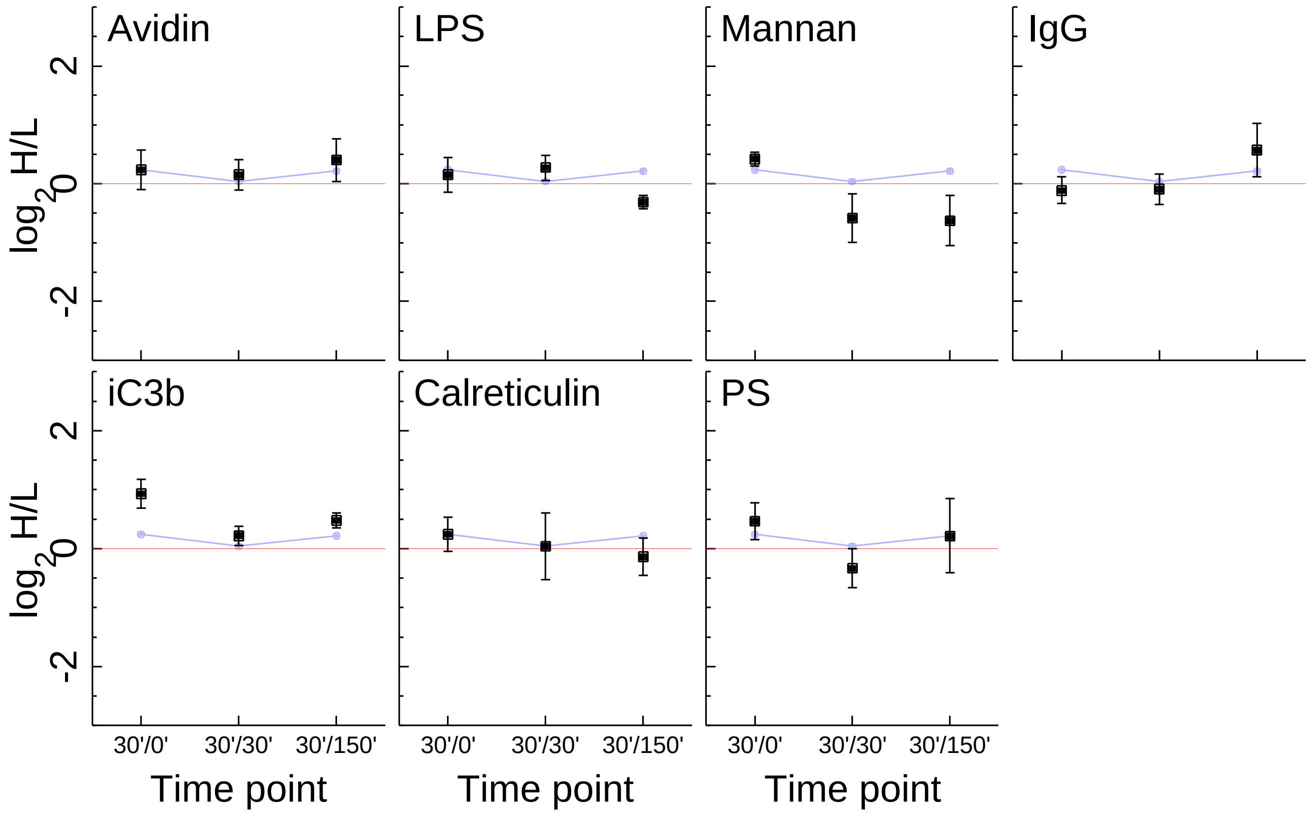

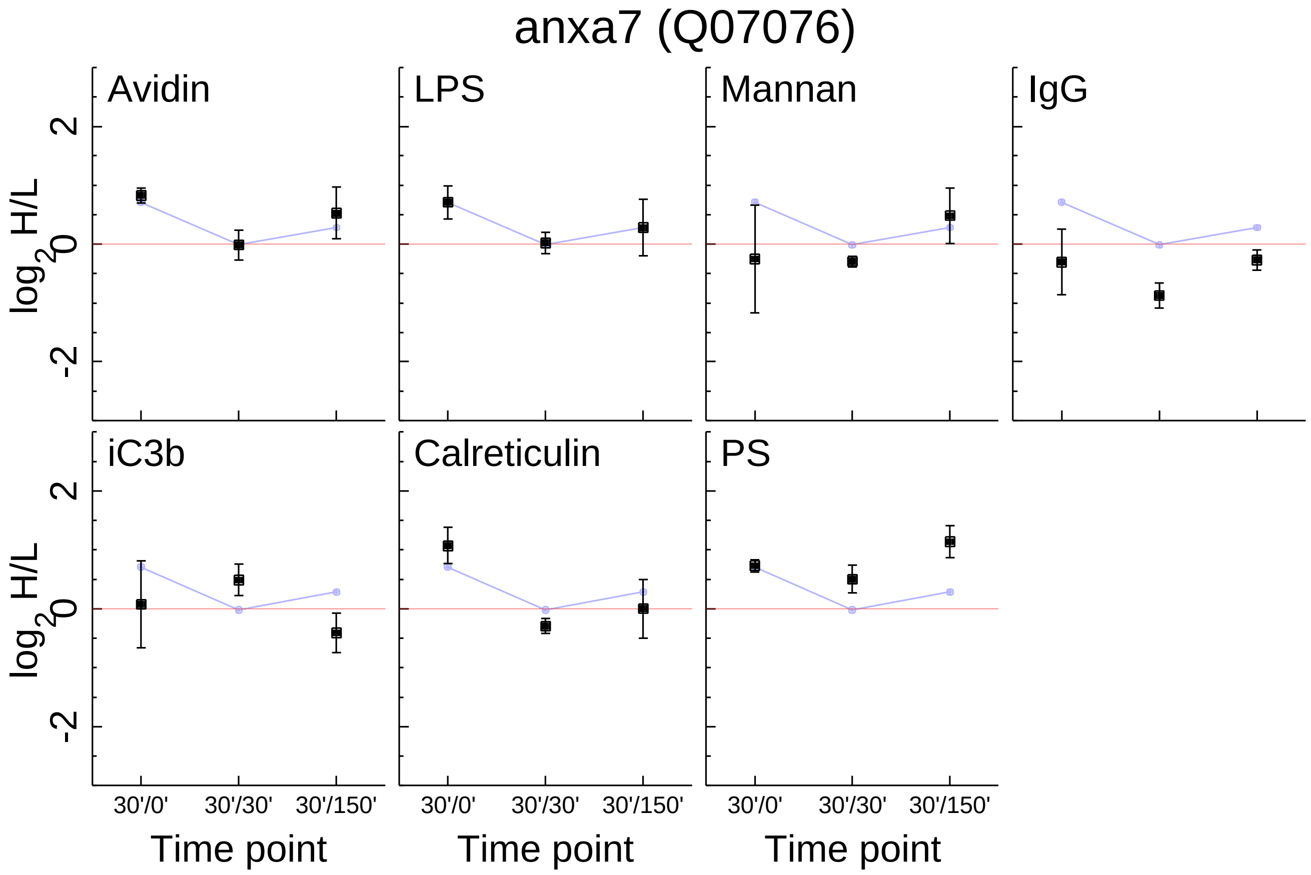
anxa7 (Q07076)
Avidin
LPS
Mannan
IgG
iC3b
Calreticulin
PS
log2 H/L
2
0
-2
30'/0'
30'/30'
30'/150'
Time point

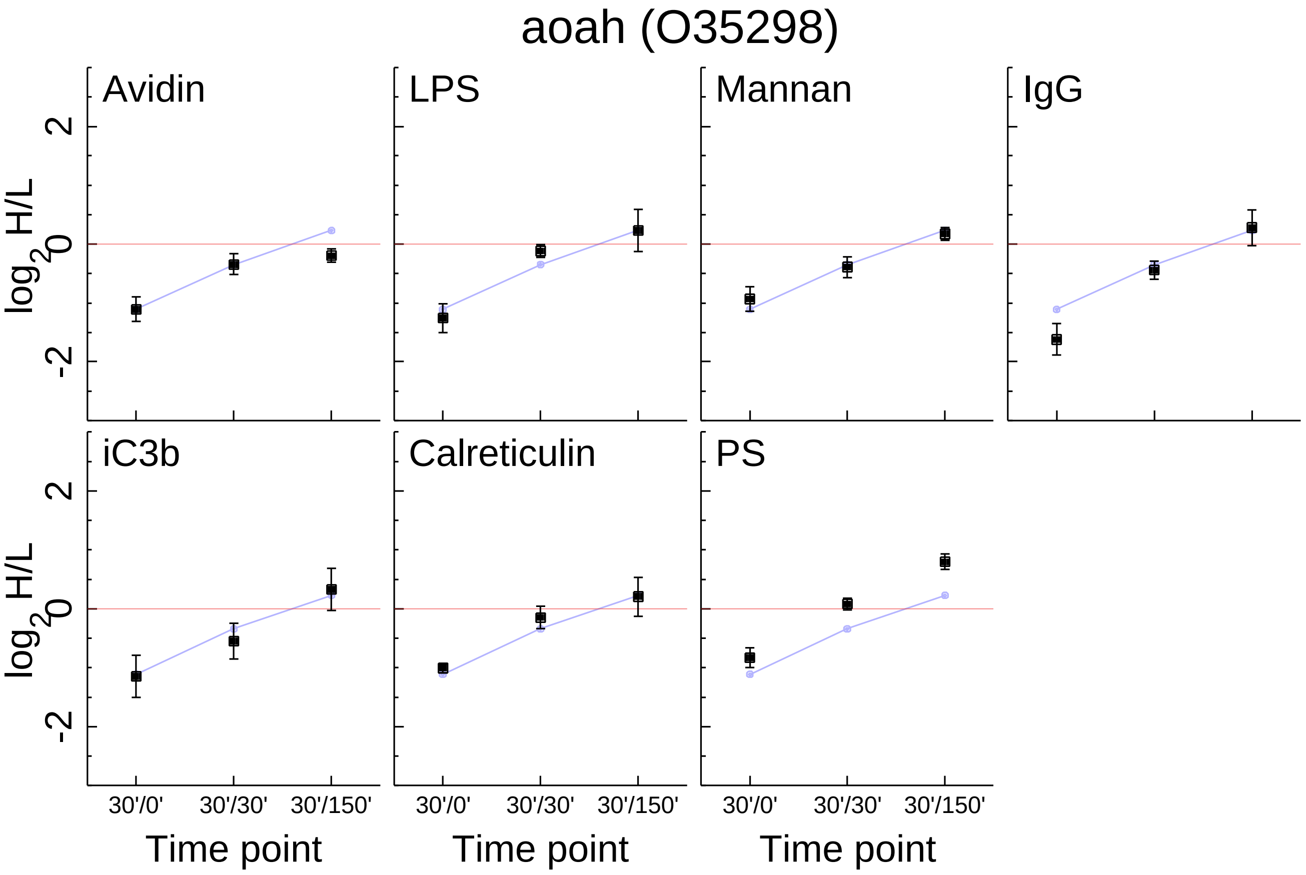

aoah (O35298)
Avidin
LPS
Mannan
IgG
iC3b
Calreticulin
PS
log2 H/L
2
0
-2
30'/0'
30'/30'
30'/150'
Time point

ap1b1 (O35643)

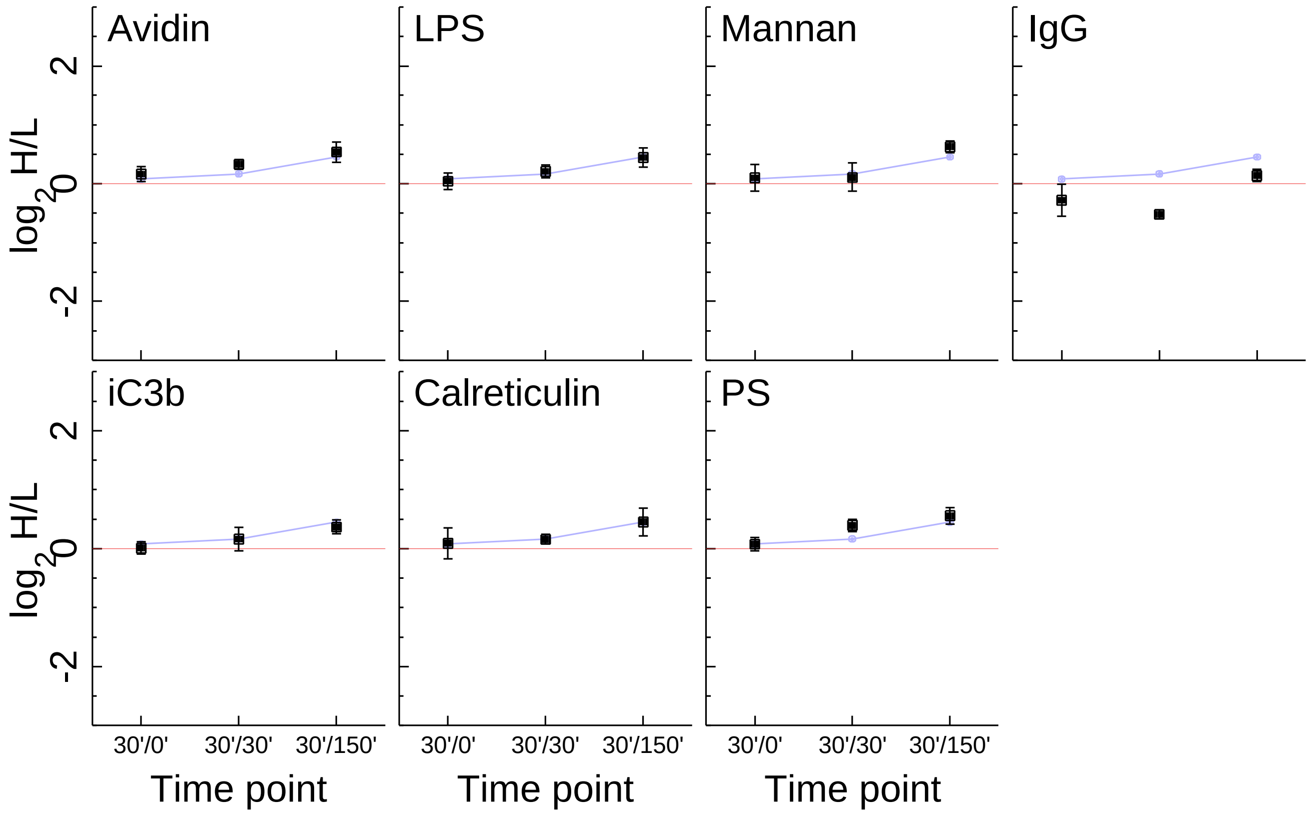

ap1g1 (Q8CBB7)

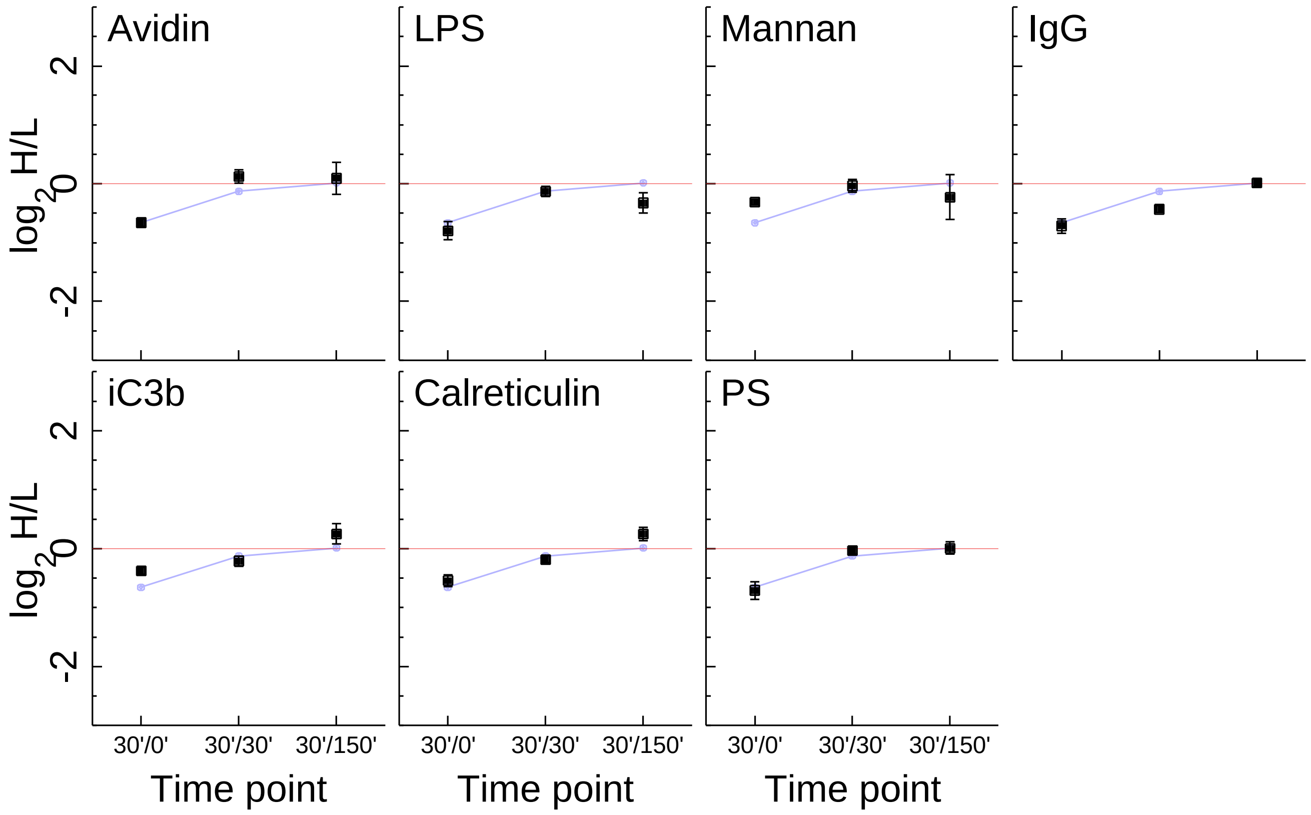

ap1g2 (O88512)

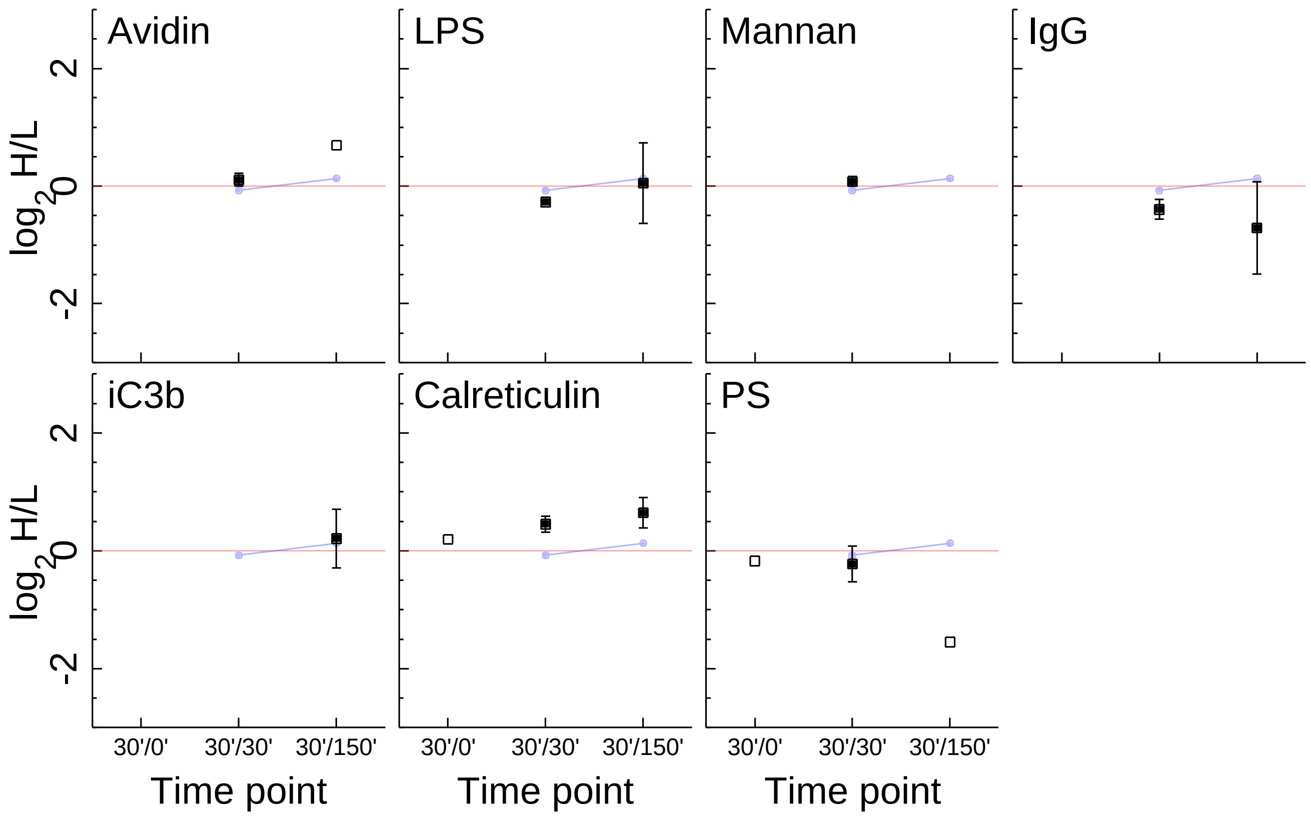

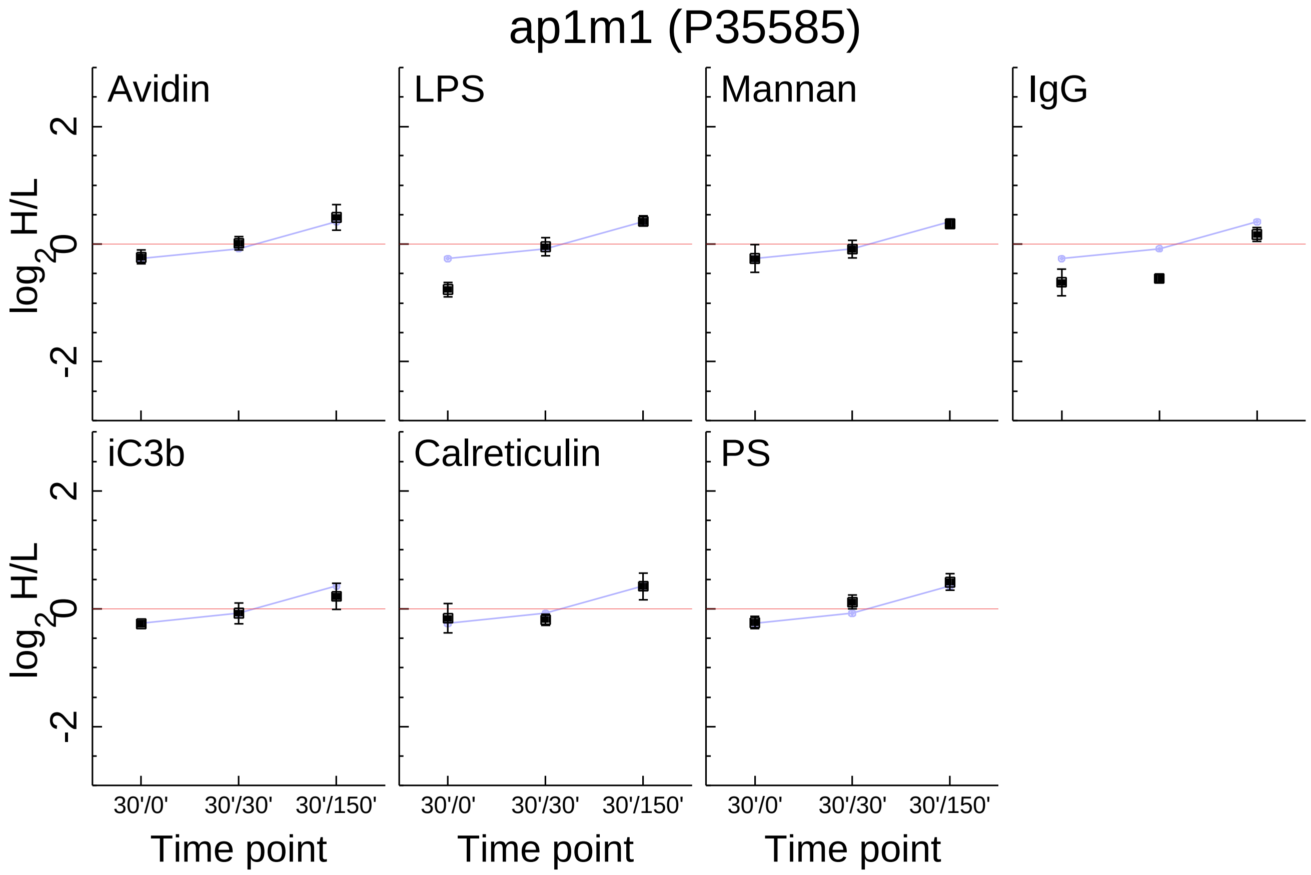

ap1m1 (P35585)
Avidin
LPS
Mannan
IgG
iC3b
Calreticulin
PS
log2 H/L
2
0
-2
30'/0'
30'/30'
30'/150'
Time point

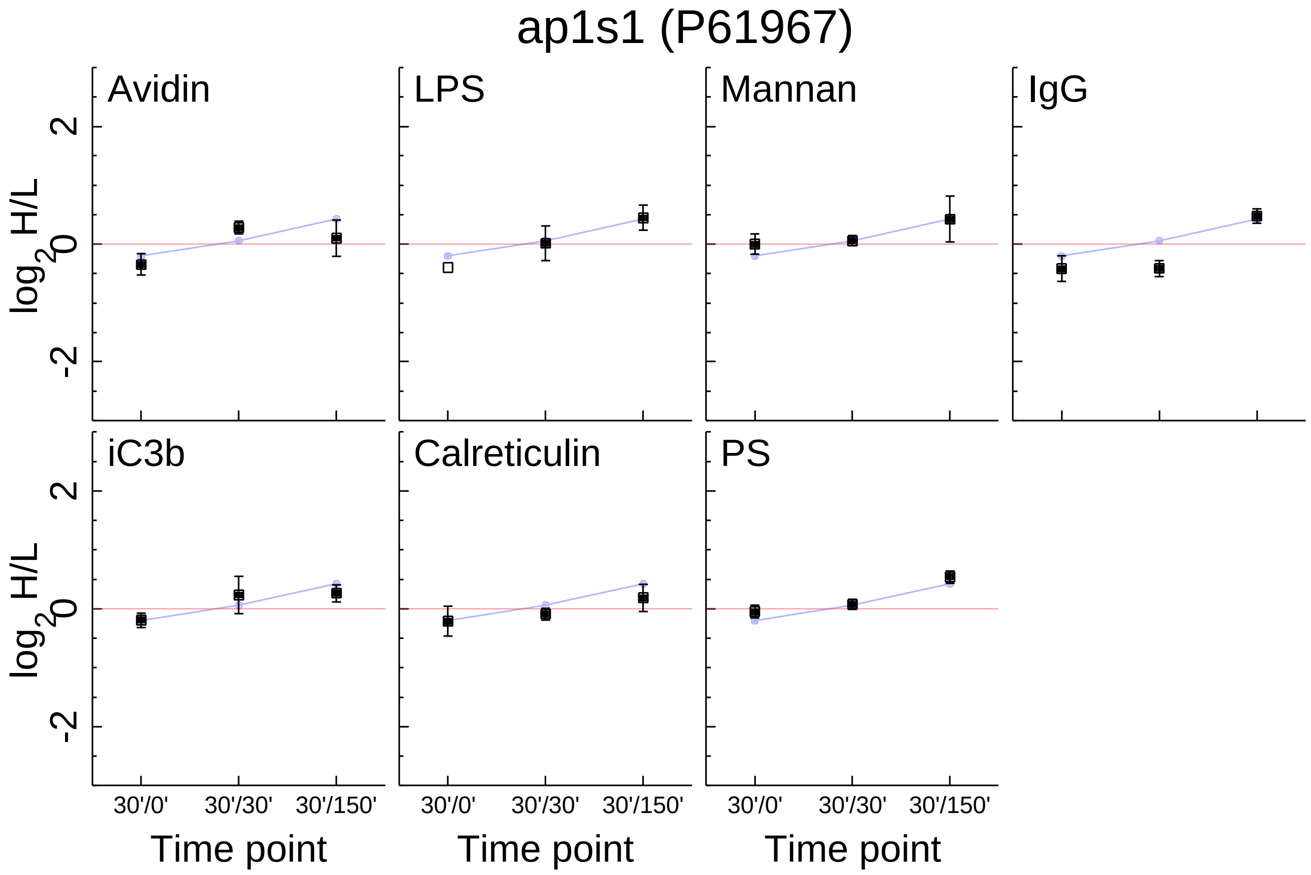
ap1s1 (P61967)
Avidin
LPS
Mannan
IgG
iC3b
Calreticulin
PS
log2 H/L
2
0
-2
30'/0'
30'/30'
30'/150'
Time point

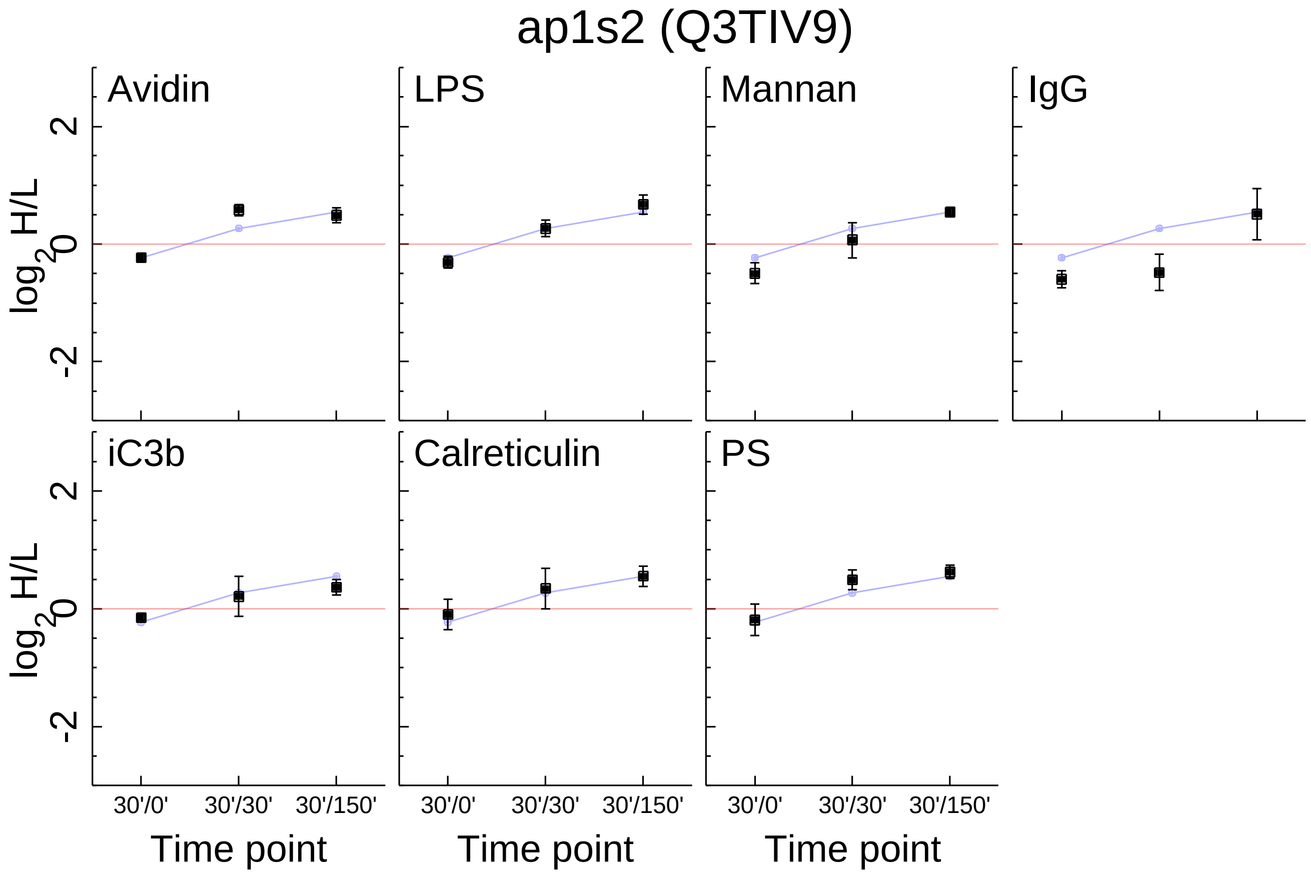

ap1s2 (Q3TIV9)
Avidin
LPS
Mannan
IgG
iC3b
Calreticulin
PS
log2 H/L
2
0
-2
30'/0'
30'/30'
30'/150'
Time point

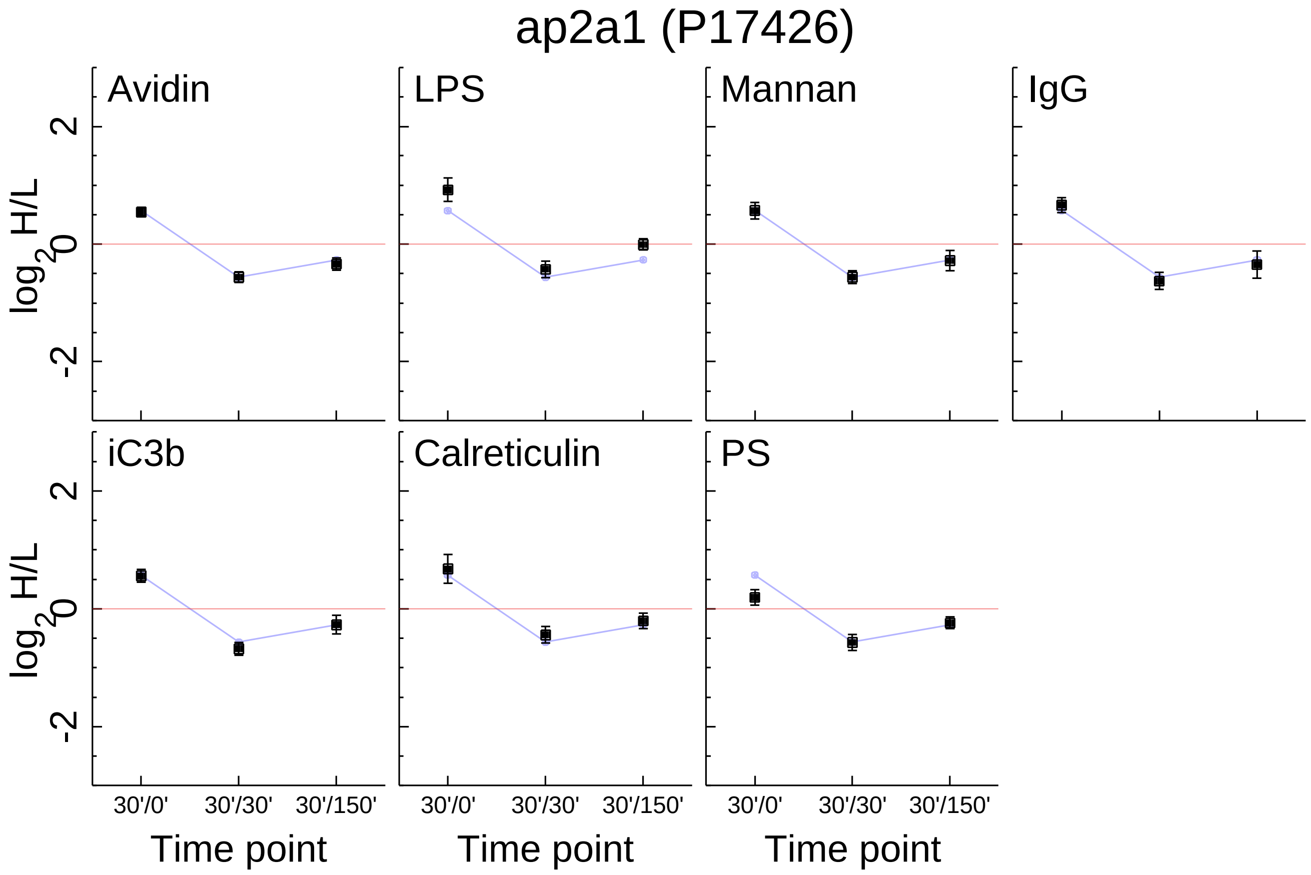

ap2a1 (P17426)
Avidin
LPS
Mannan
IgG
iC3b
Calreticulin
PS
log2 H/L
2
0
-2
30'/0'
30'/30'
30'/150'
Time point

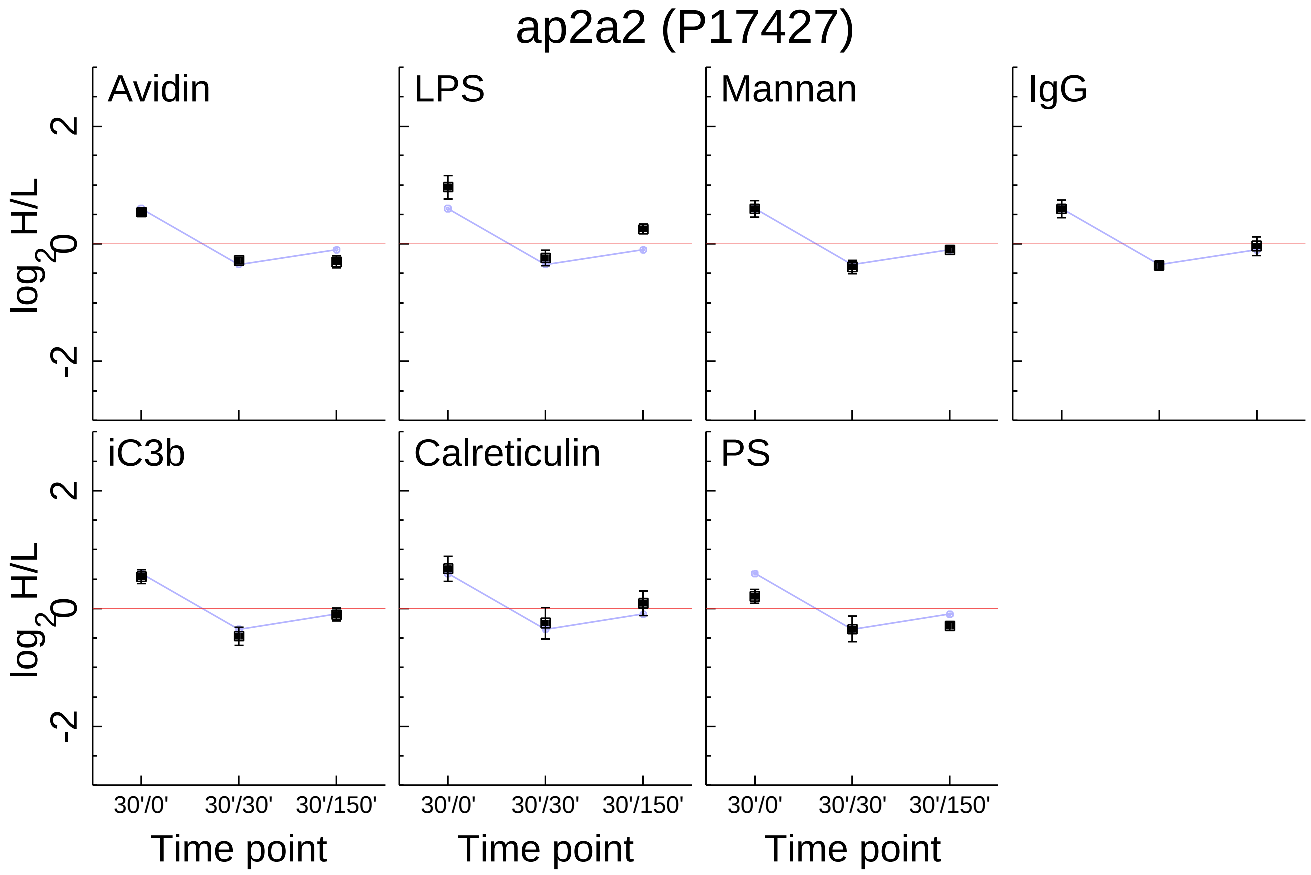
ap2a2 (P17427)
Avidin
LPS
Mannan
IgG
iC3b
Calreticulin
PS
log2 H/L
2
0
-2
30'/0'
30'/30'
30'/150'
Time point

ap2b1 (Q9DBG3)

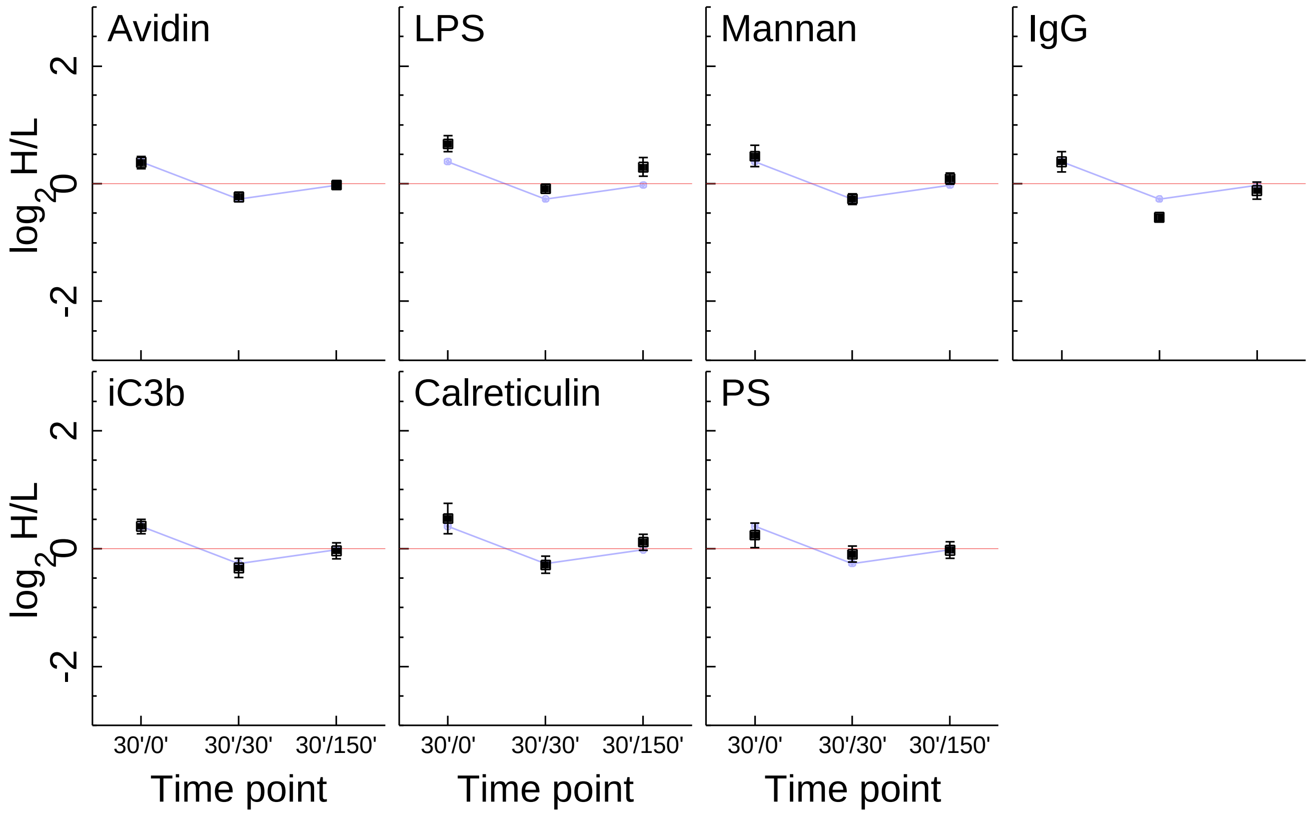

ap2m1 (Q3TWV4)

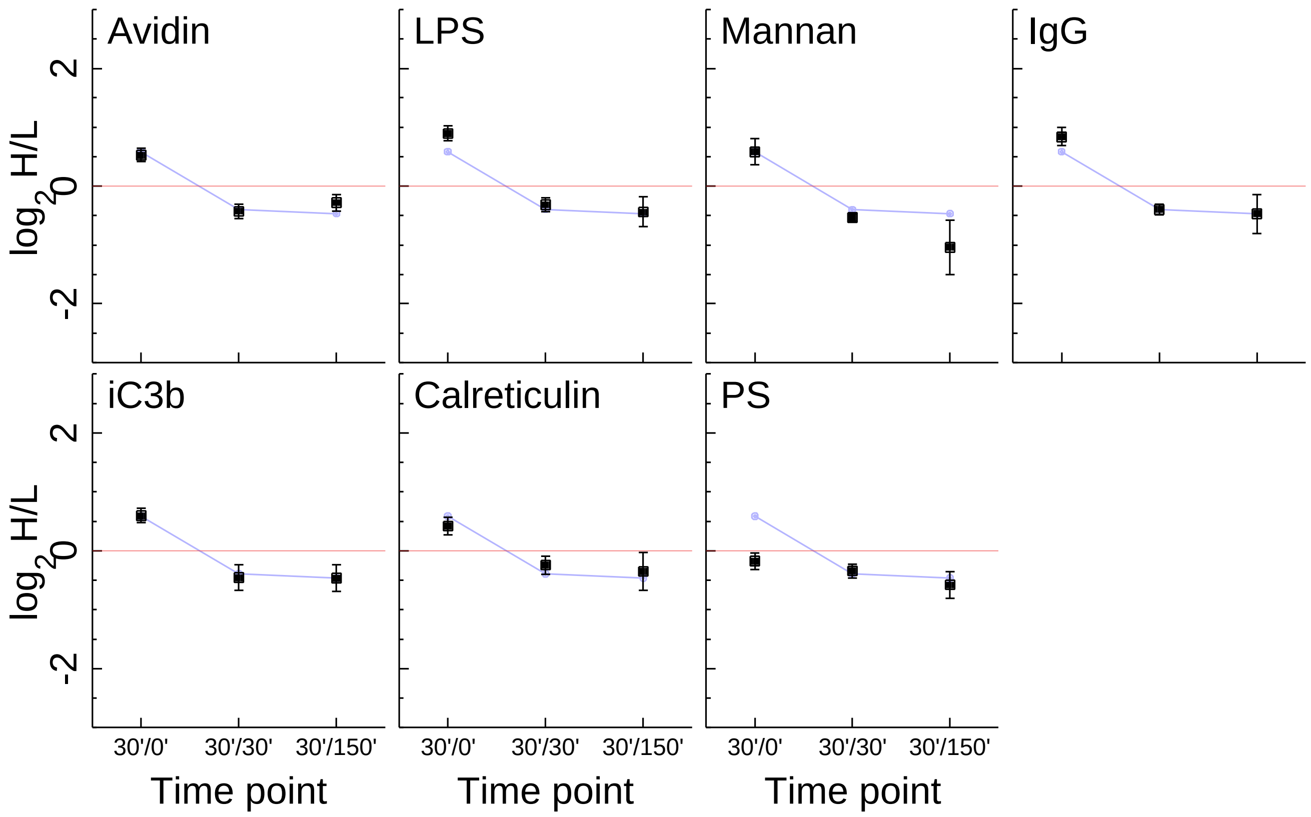

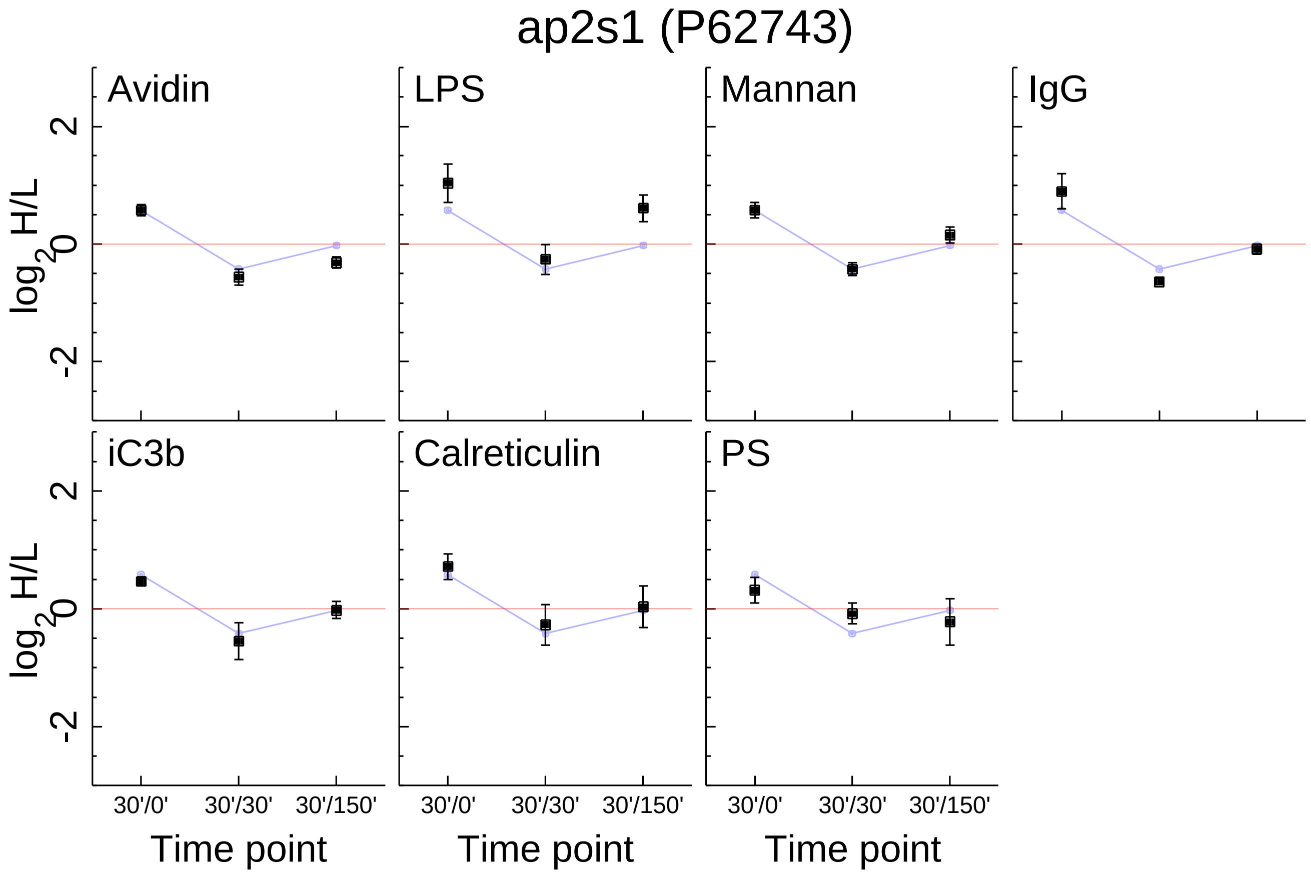

ap2s1 (P62743)
Avidin
LPS
Mannan
IgG
iC3b
Calreticulin
PS
log2 H/L
2
0
-2
30'/0'
30'/30'
30'/150'
Time point

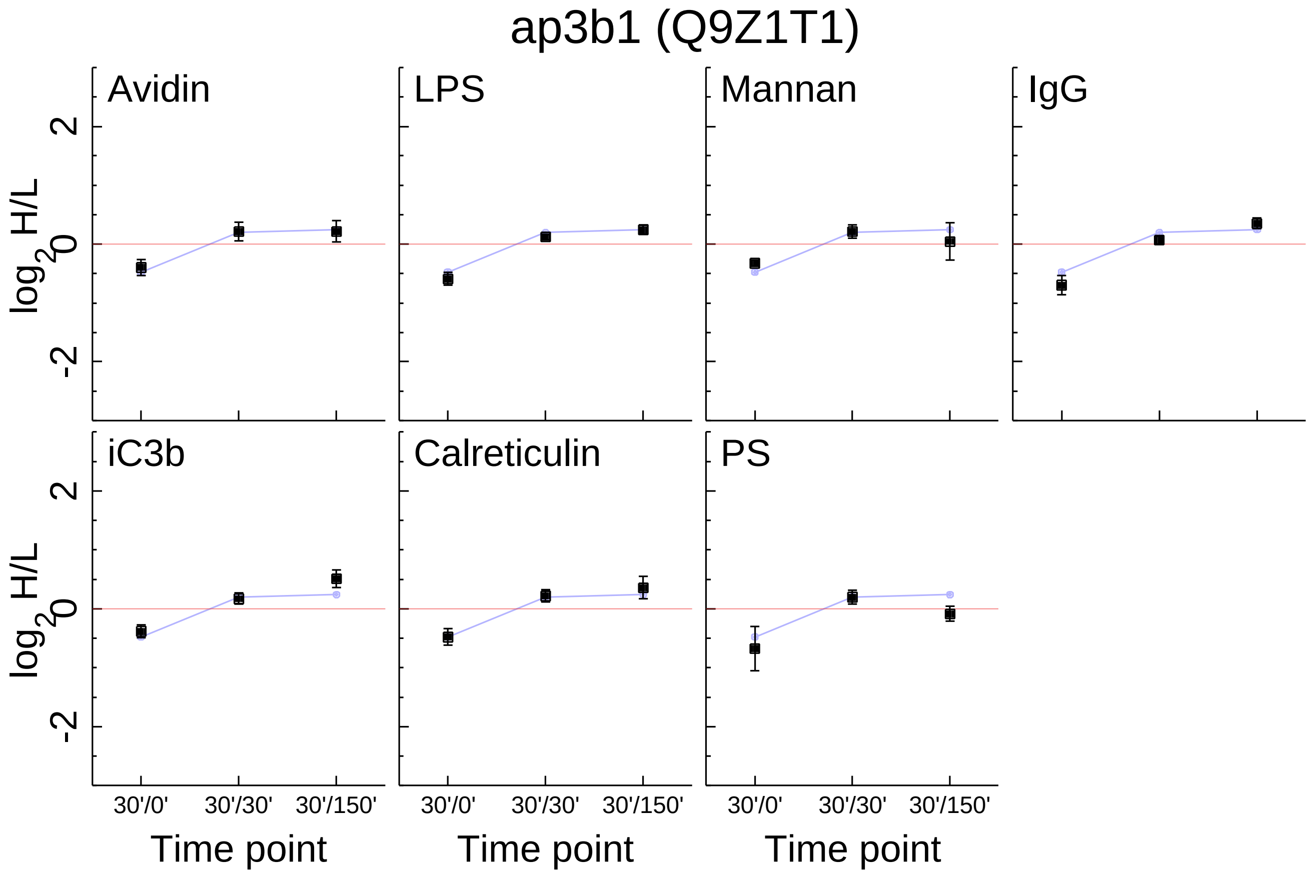
ap3b1 (Q9Z1T1)
Avidin
LPS
Mannan
IgG
iC3b
Calreticulin
PS
log2 H/L
2
0
-2
30'/0'
30'/30'
30'/150'
Time point

ap3d1 (O54774)

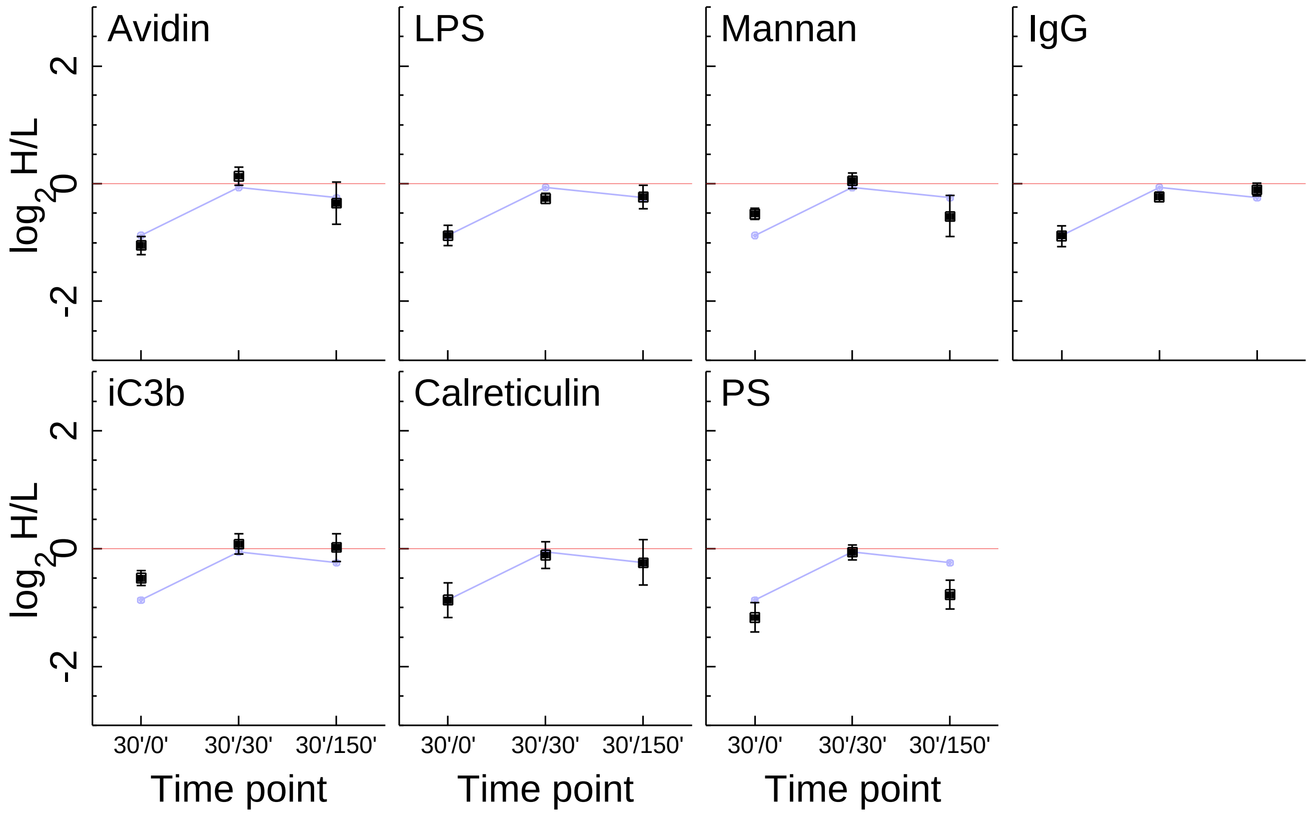

ap3m1 (Q9JKC8)

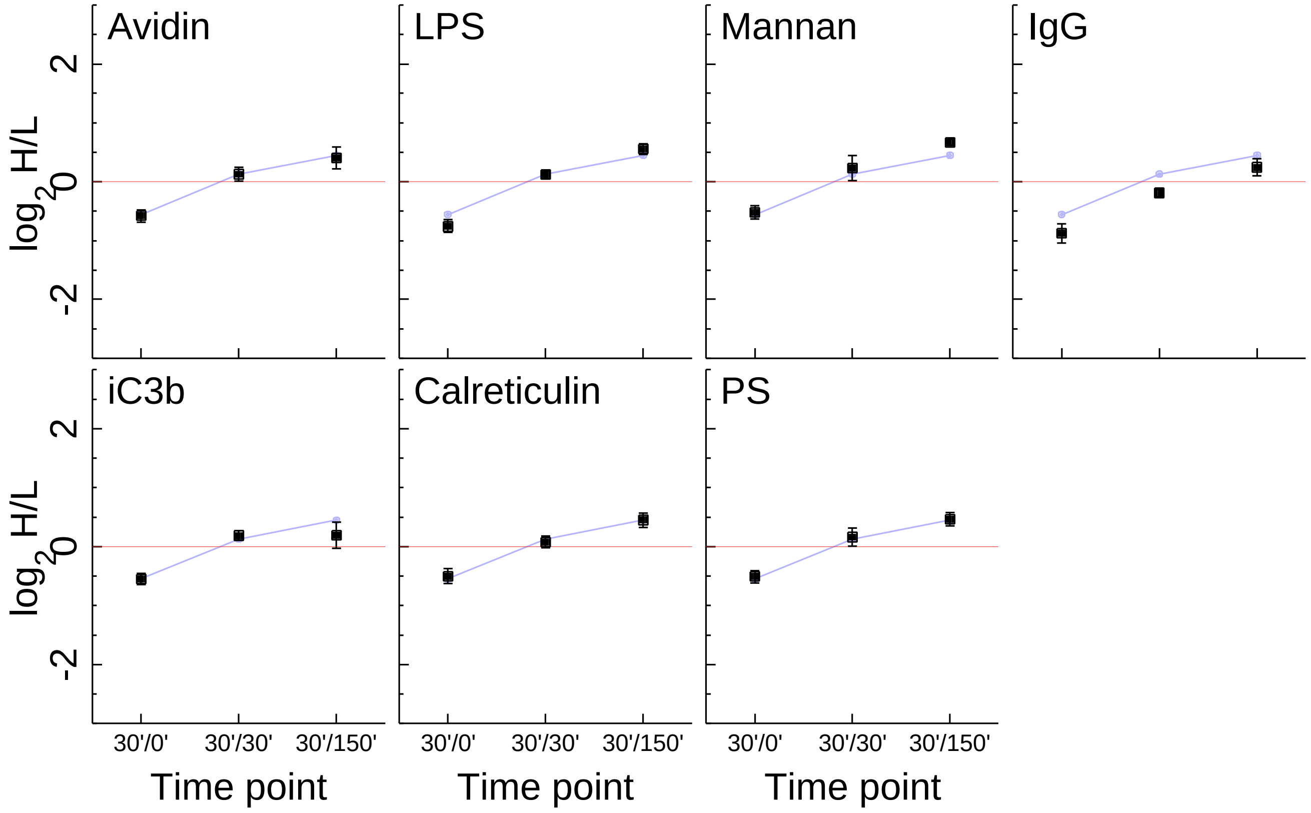

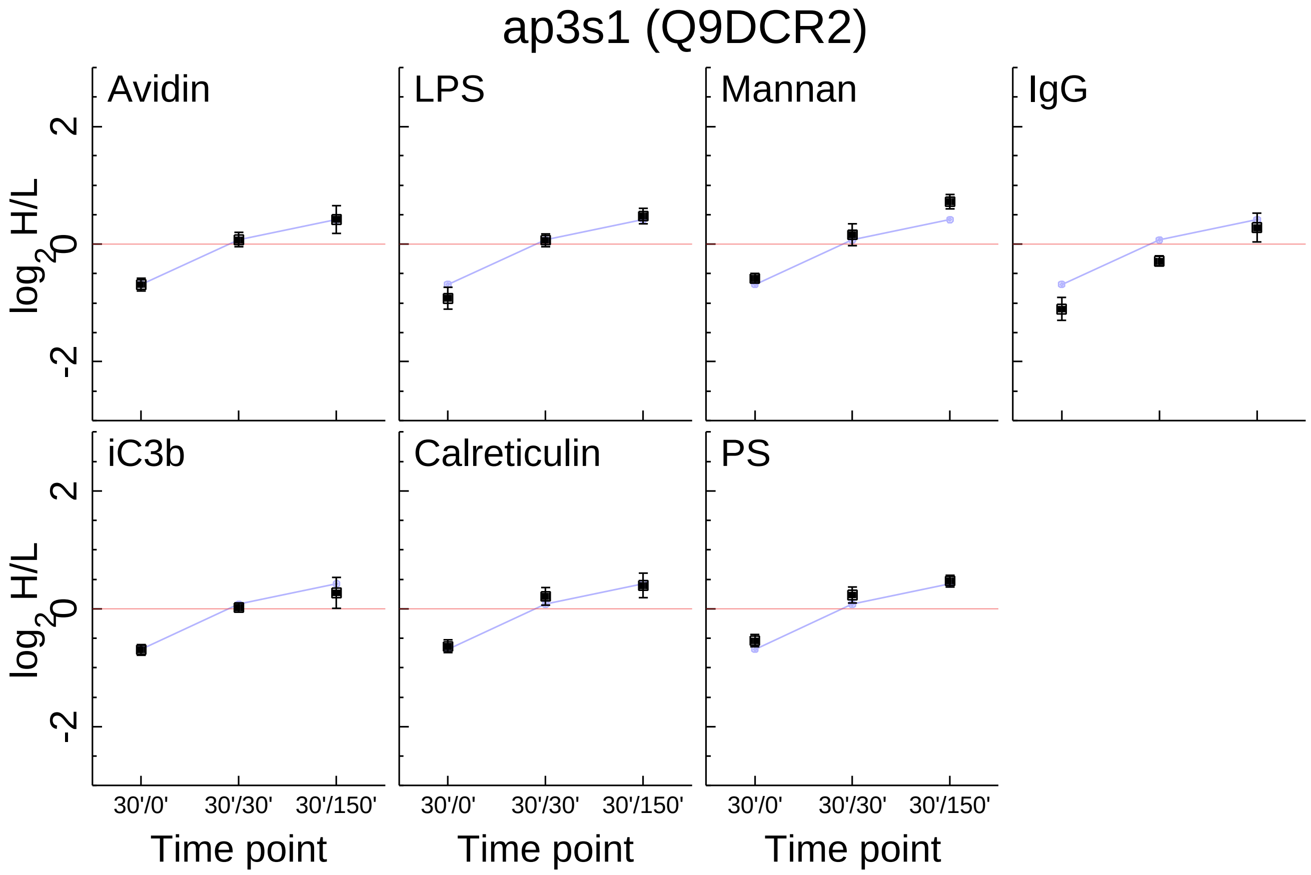

ap3s1 (Q9DCR2)
Avidin
LPS
Mannan
IgG
iC3b
Calreticulin
PS
log2 H/L
2
0
-2
30'/0'
30'/30'
30'/150'
Time point

ap3s2 (Q8BSZ2)

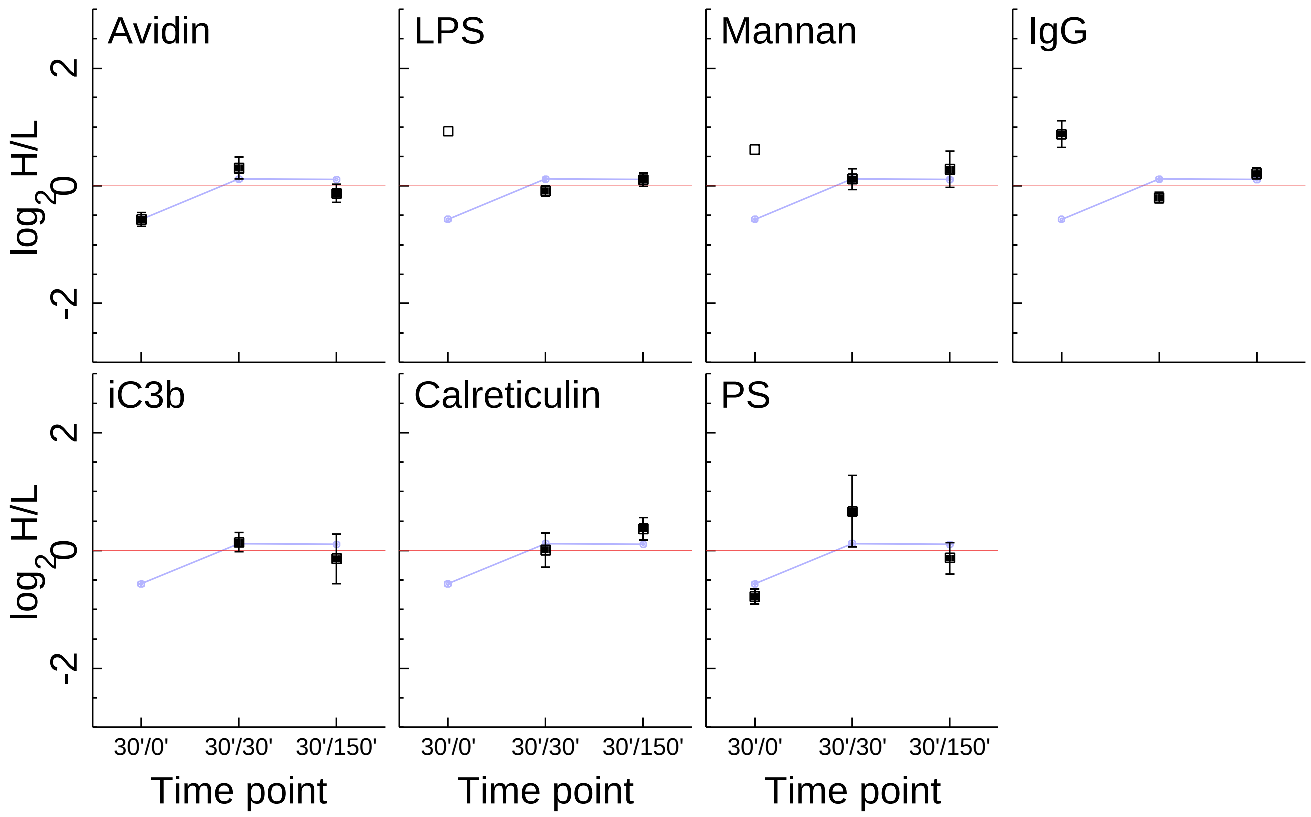

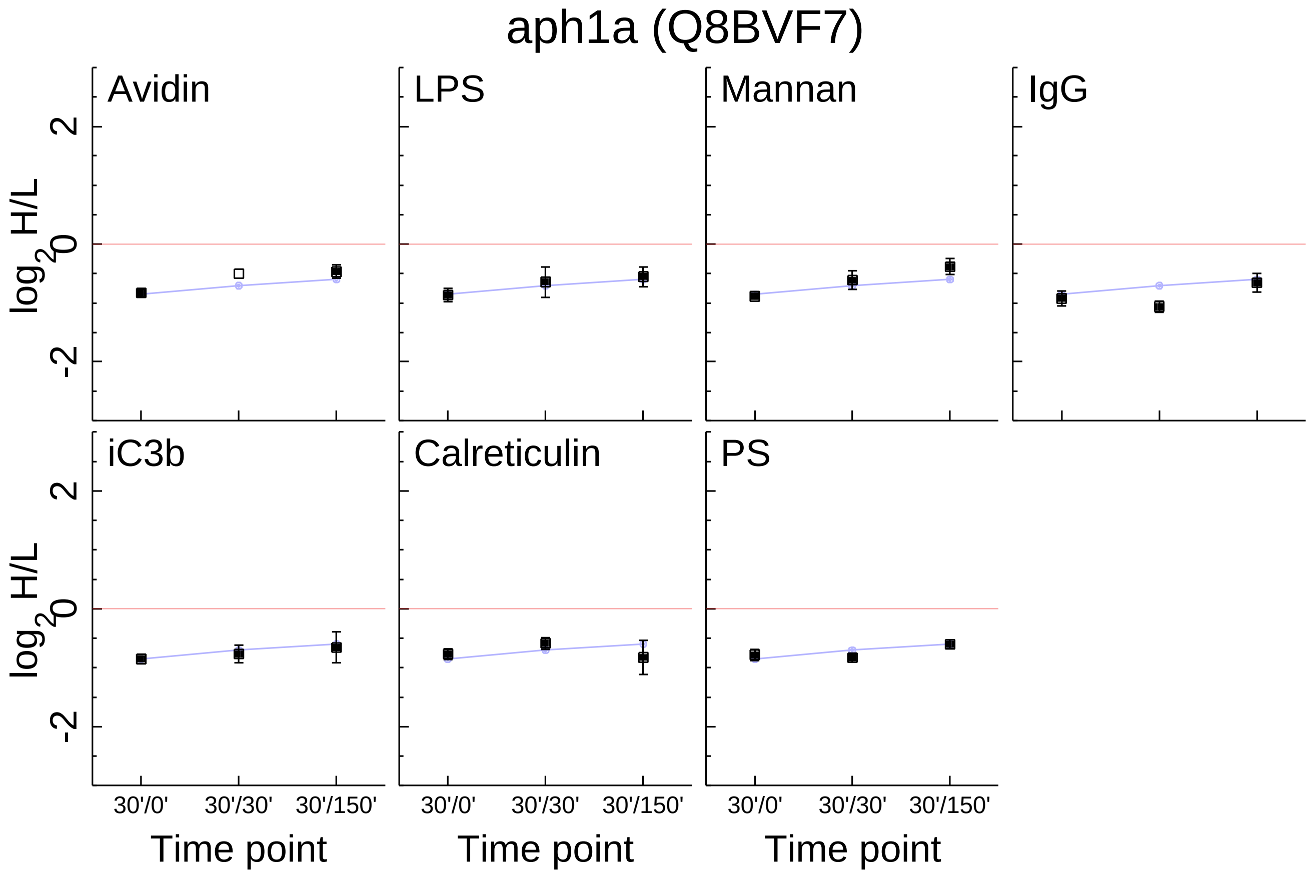

aph1a (Q8BVF7)
Avidin
LPS
Mannan
IgG
iC3b
Calreticulin
PS
log2 H/L
2
0
-2
30'/0'
30'/30'
30'/150'
Time point

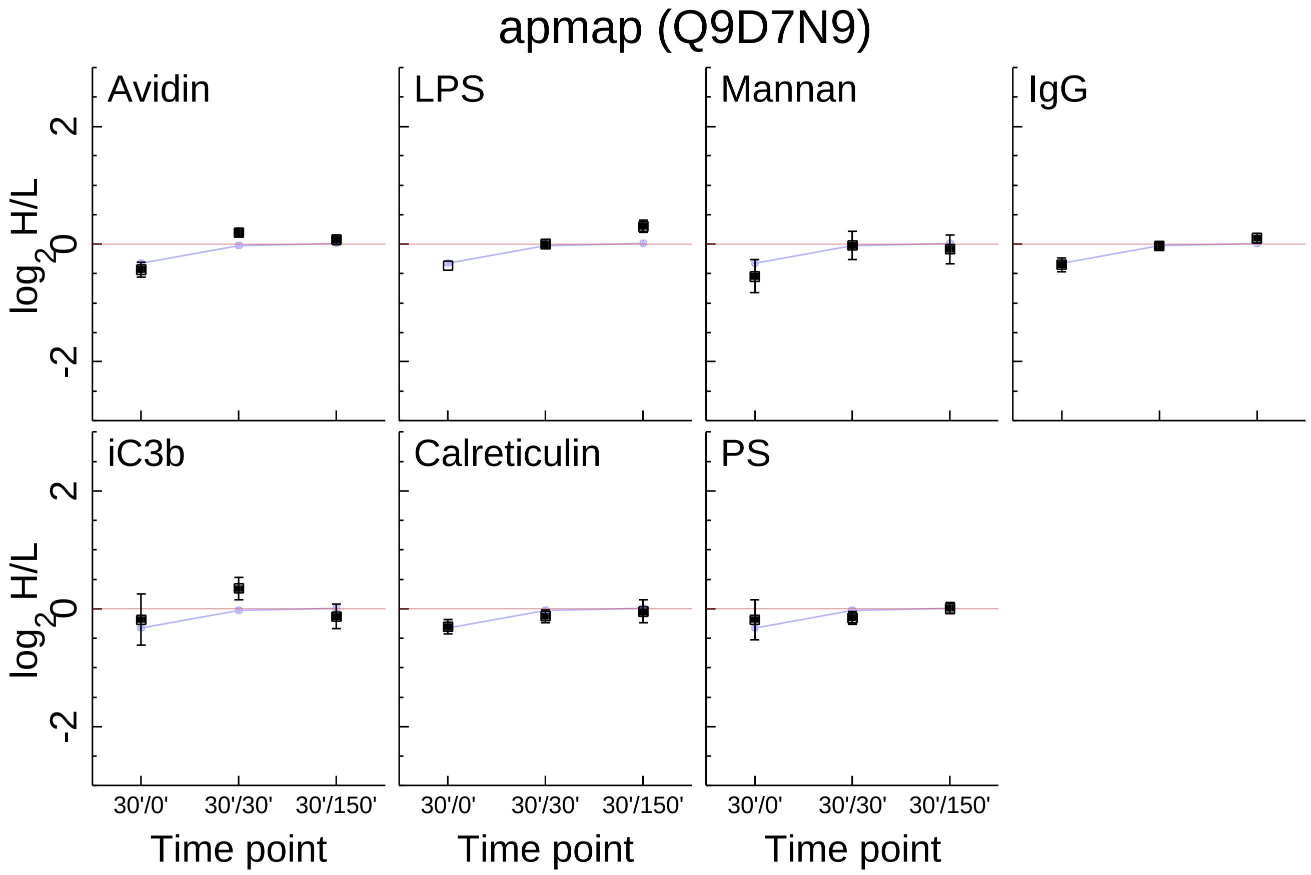
apmap (Q9D7N9)
Avidin
LPS
Mannan
IgG
iC3b
Calreticulin
PS
log2 H/L
2
0
-2
30'/0'
30'/30'
30'/150'
Time point

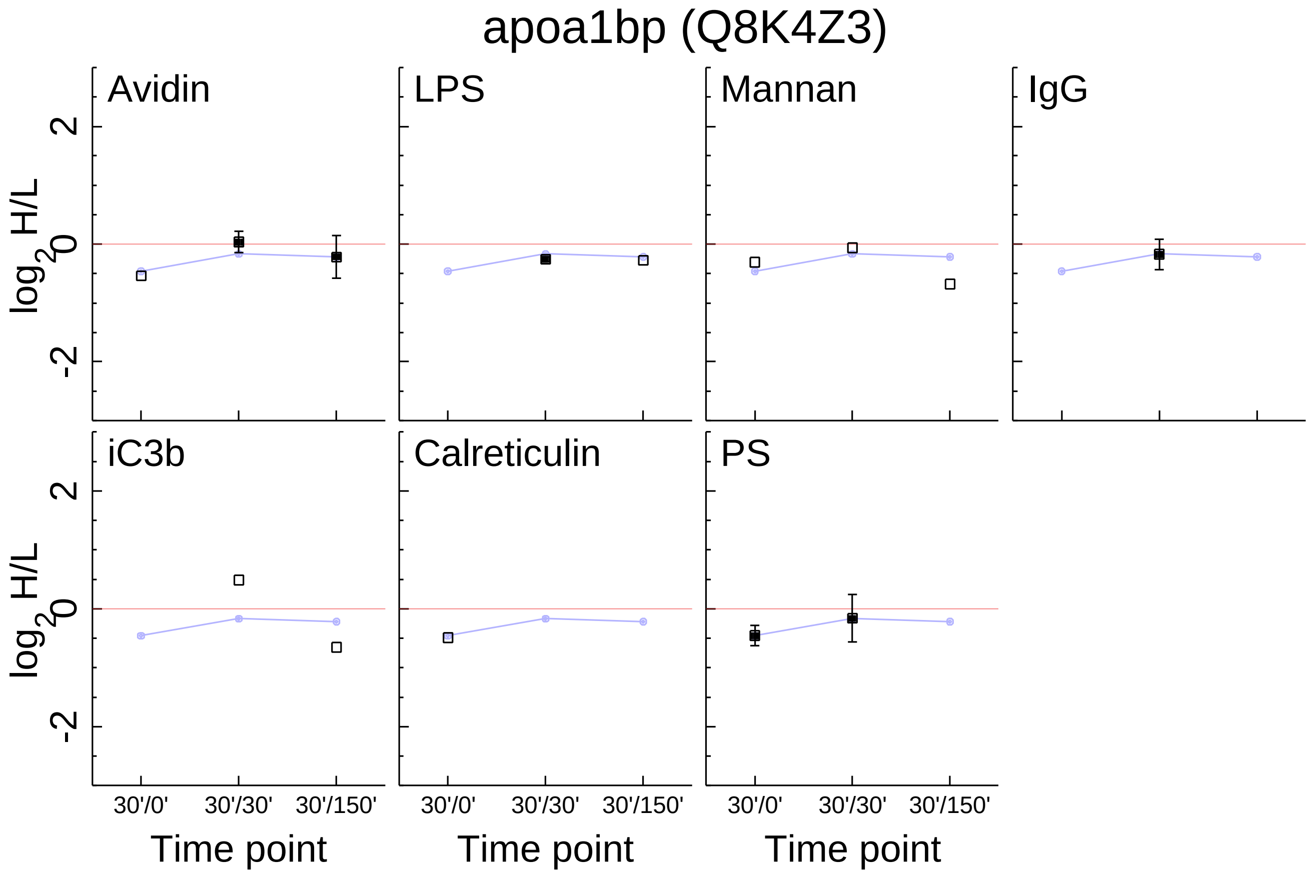

apoa1bp (Q8K4Z3)
Avidin
LPS
Mannan
IgG
iC3b
Calreticulin
PS
log2 H/L
2
0
-2
30'/0'
30'/30'
30'/150'
Time point

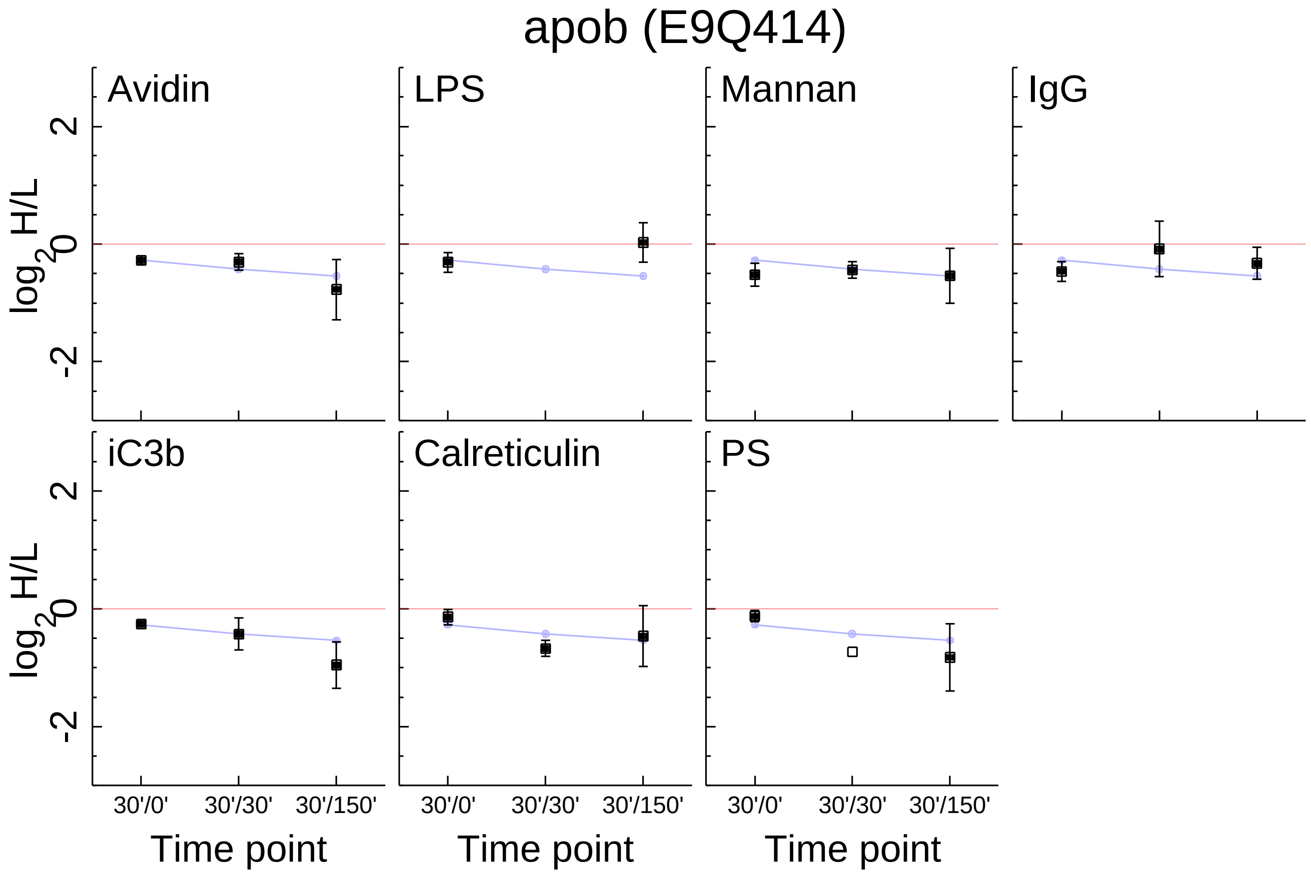
apob (E9Q414)
Avidin
LPS
Mannan
IgG
iC3b
Calreticulin
PS
log2 H/L
2
0
-2
30'/0'
30'/30'
30'/150'
Time point

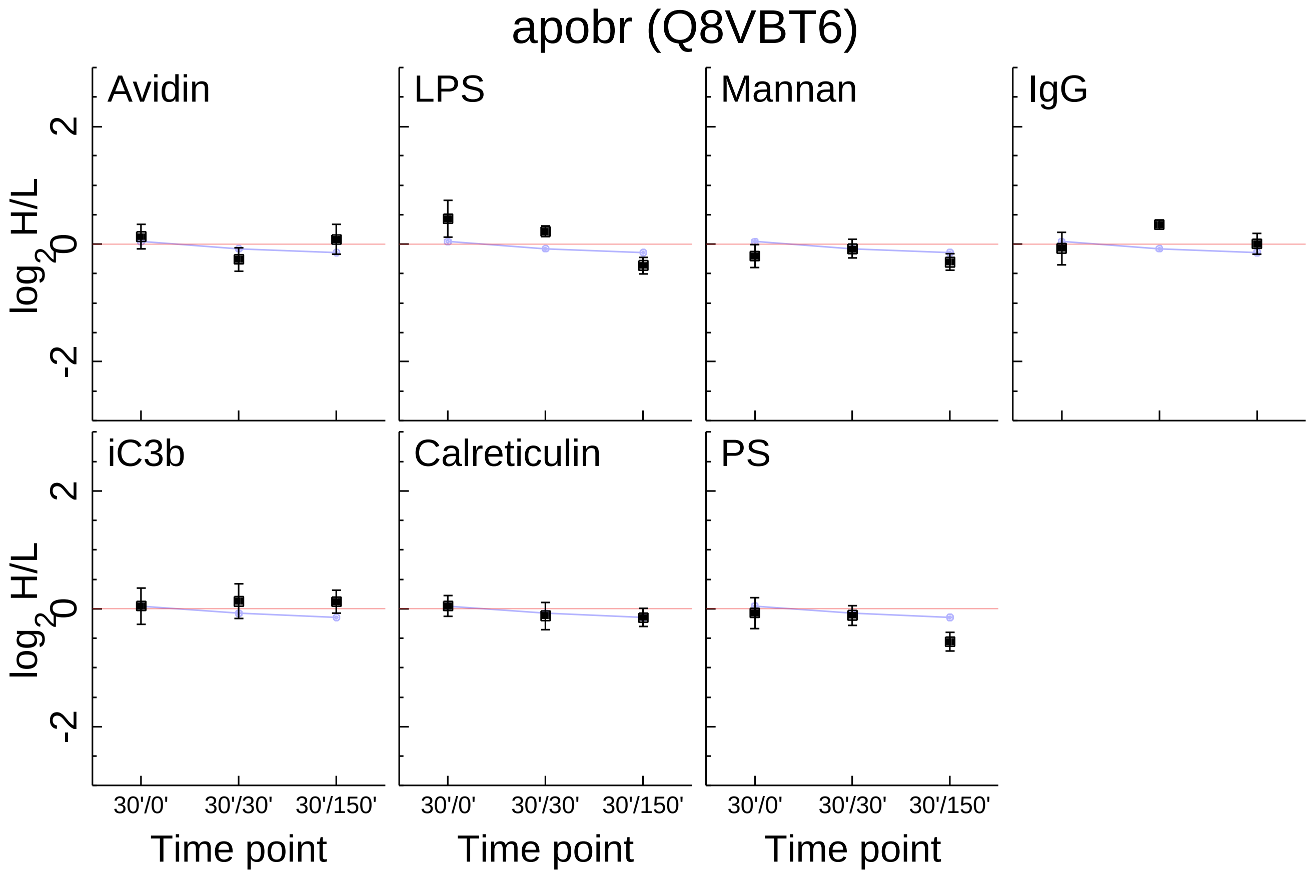

apobr (Q8VBT6)
Avidin
LPS
Mannan
IgG
iC3b
Calreticulin
PS
log2 H/L
2
0
-2
30'/0'
30'/30'
30'/150'
Time point

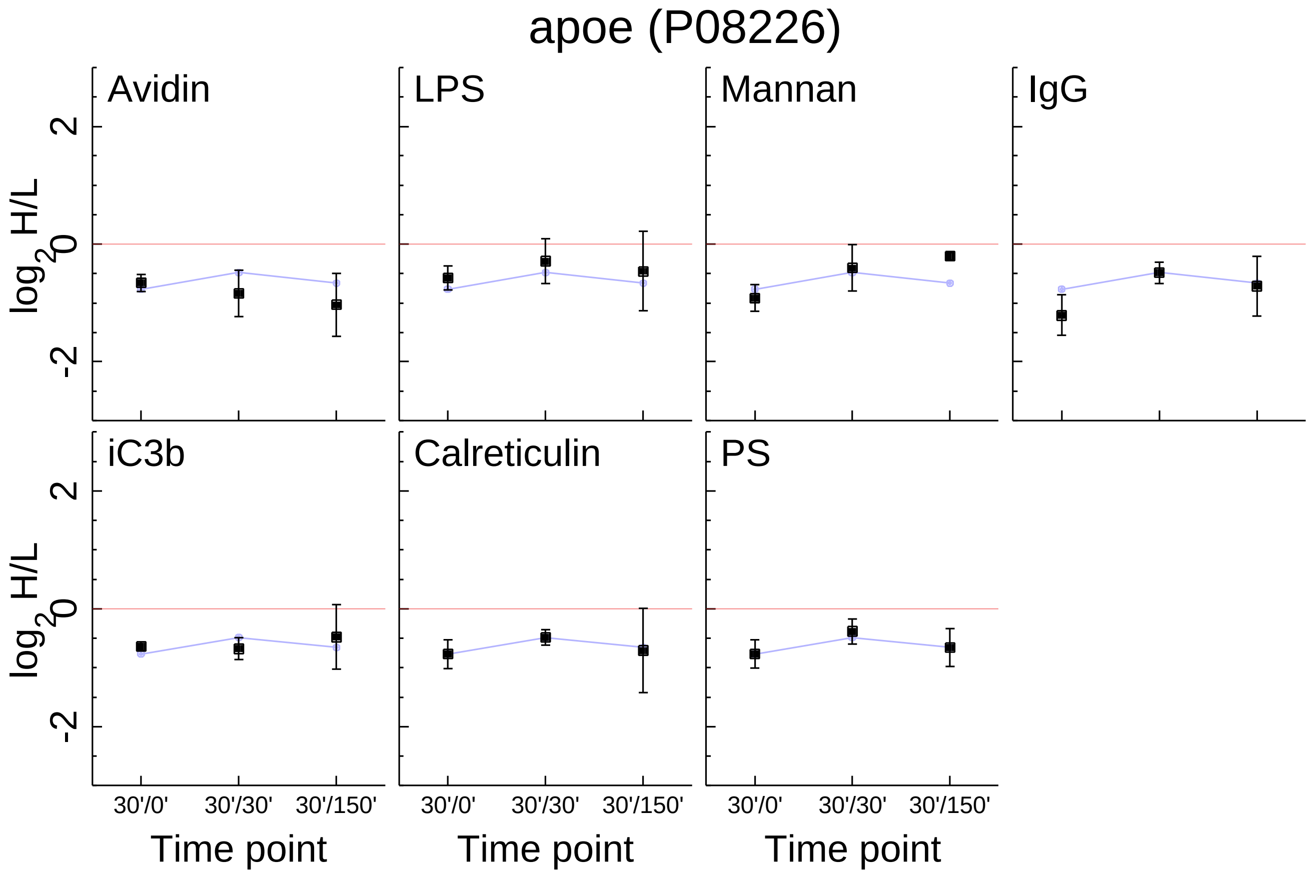

apoe (P08226)
Avidin
LPS
Mannan
IgG
iC3b
Calreticulin
PS
log2 H/L
2
0
-2
30'/0'
30'/30'
30'/150'
Time point

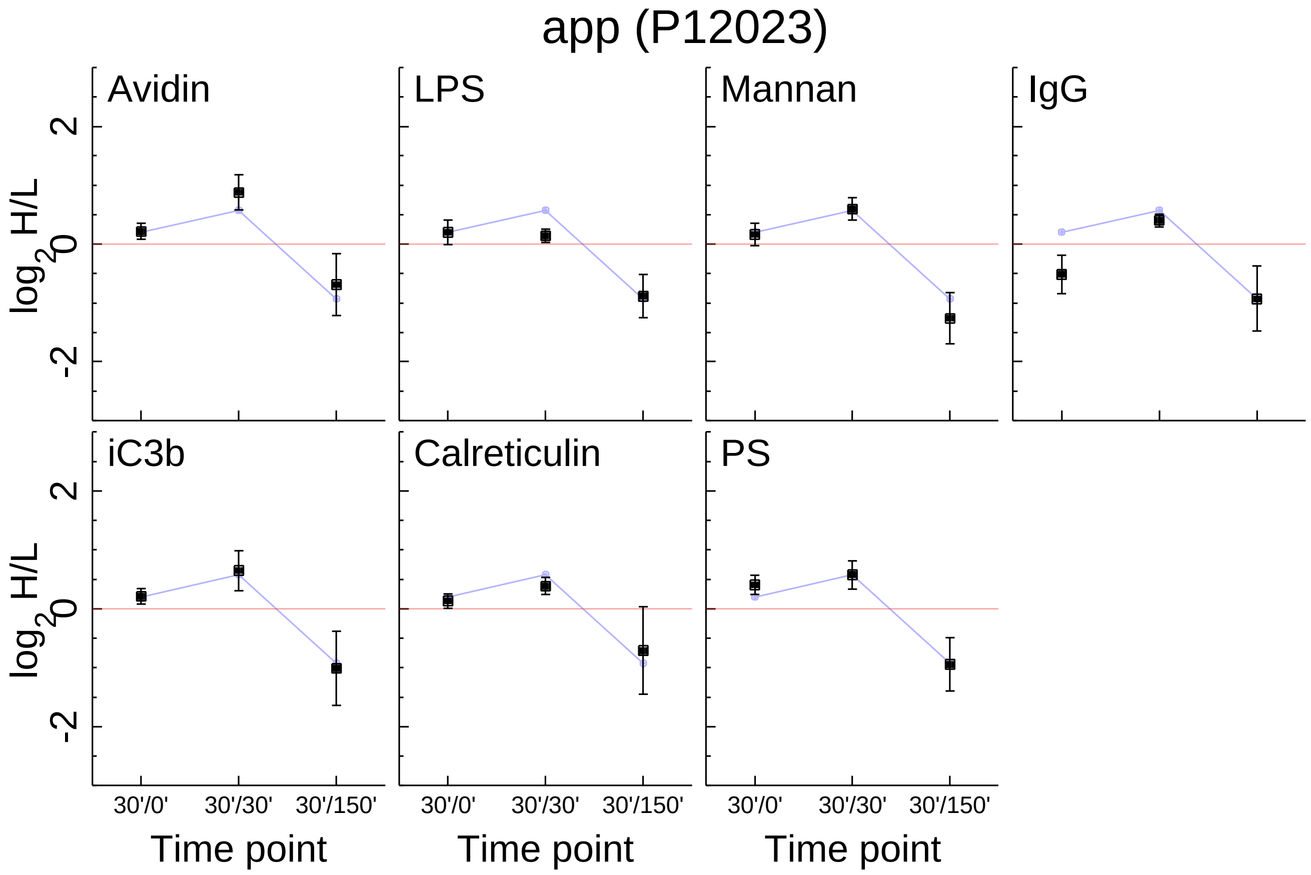
app (P12023)
Avidin
LPS
Mannan
IgG
iC3b
Calreticulin
PS
log2 H/L
2
0
-2
30'/0'
30'/30'
30'/150'
Time point

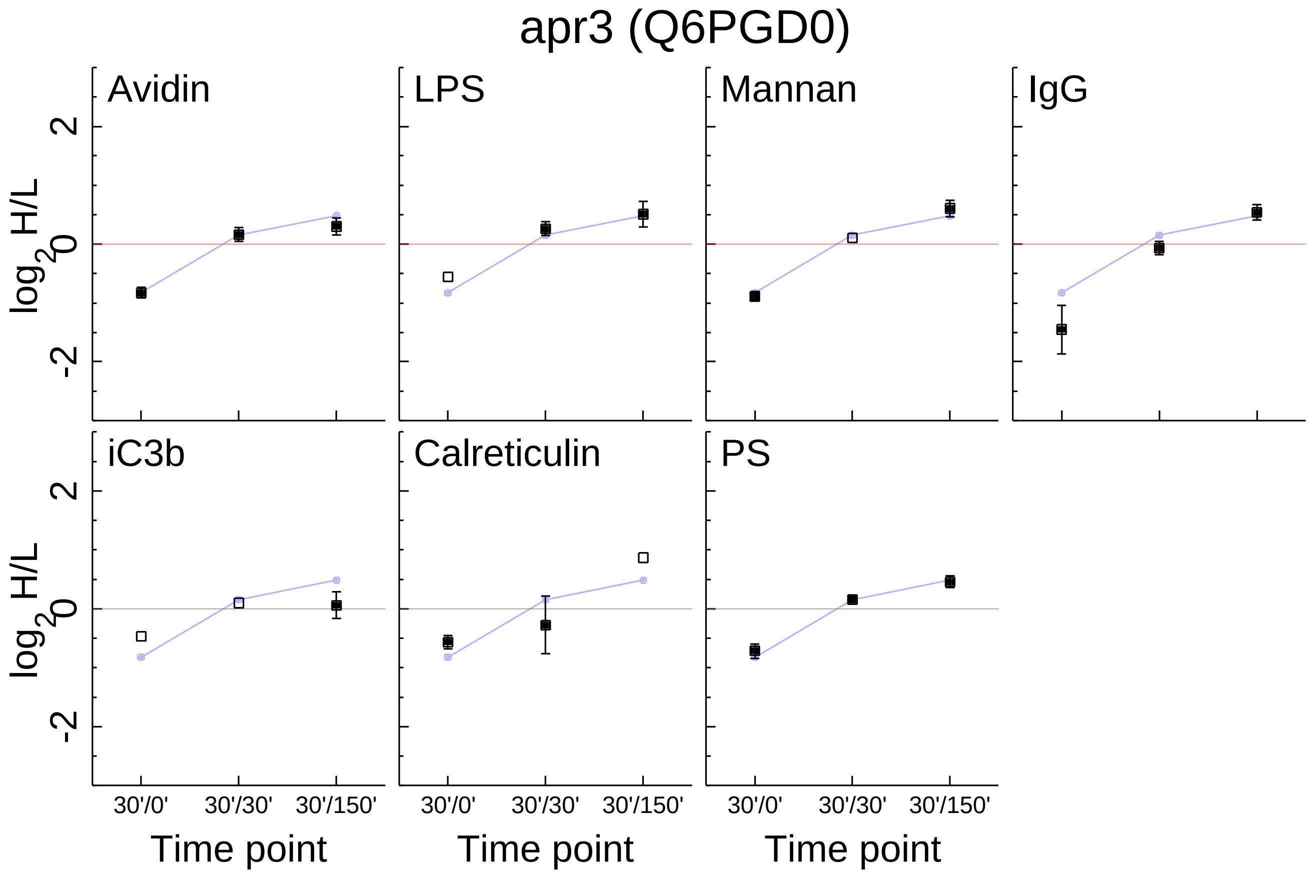
apr3 (Q6PGD0)
Avidin
LPS
Mannan
IgG
iC3b
Calreticulin
PS
log2 H/L
2
0
-2
30'/0'
30'/30'
30'/150'
Time point

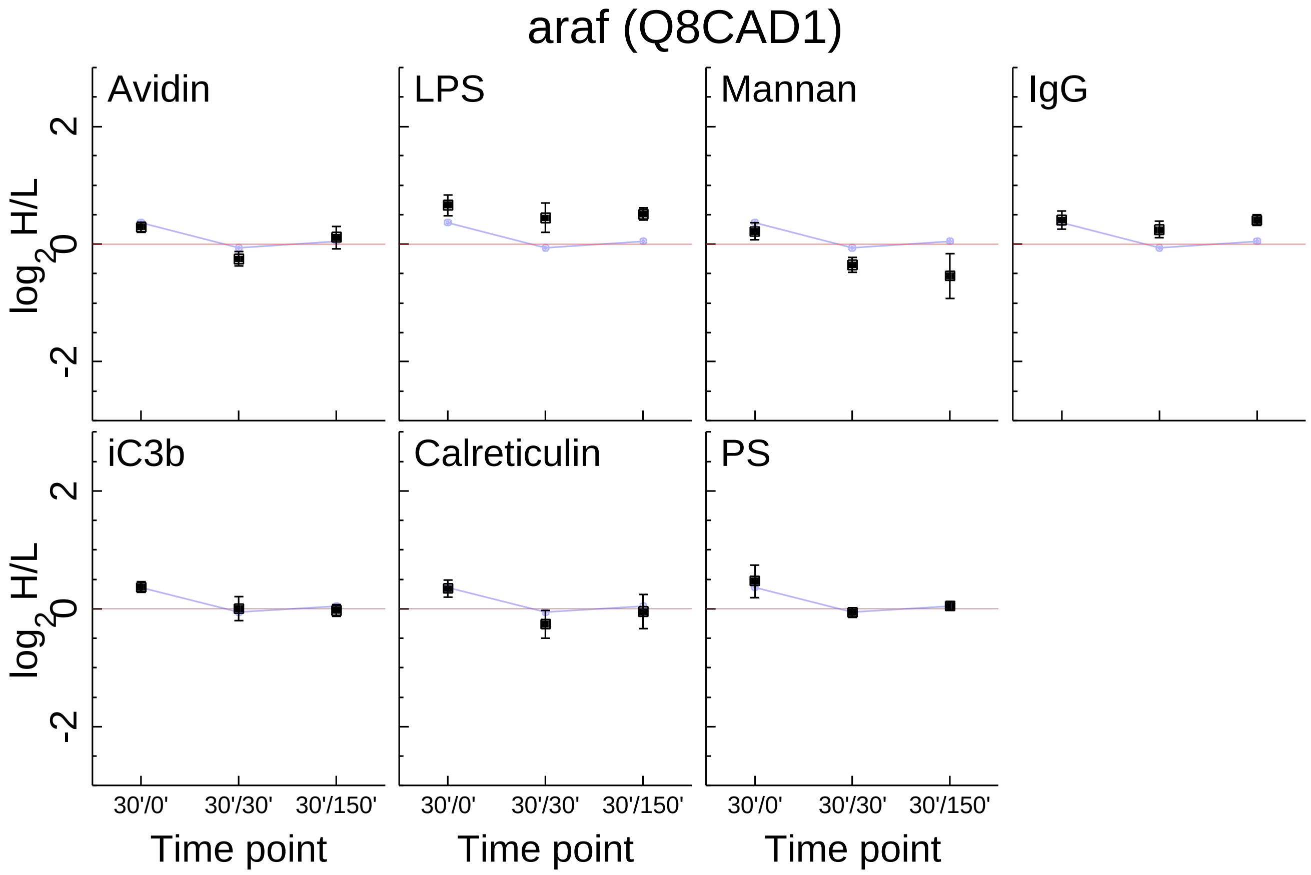
araf (Q8CAD1)
Avidin
LPS
Mannan
IgG
iC3b
Calreticulin
PS
log2 H/L
2
0
-2
30'/0'
30'/30'
30'/150'
Time point

arap1 (Q4LDD4)

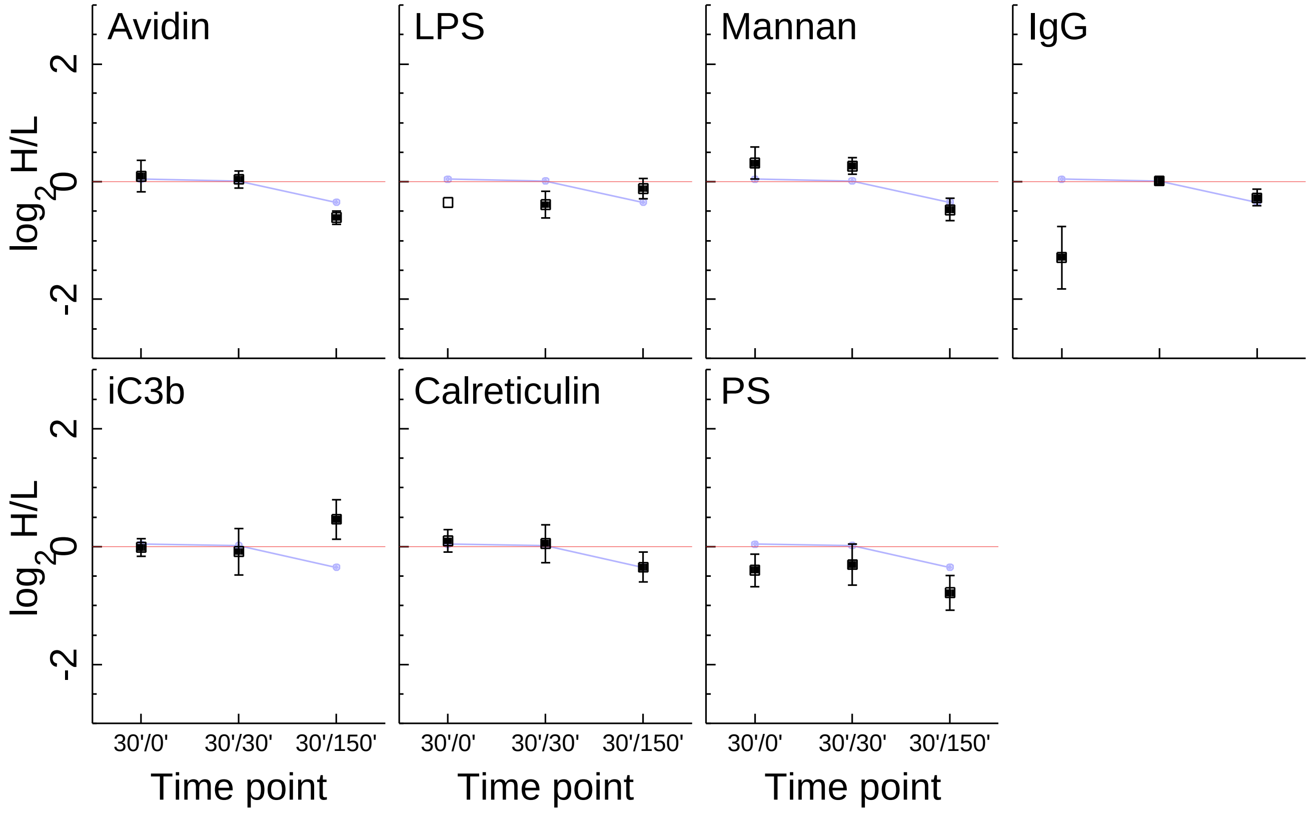

arf3 (P61205)

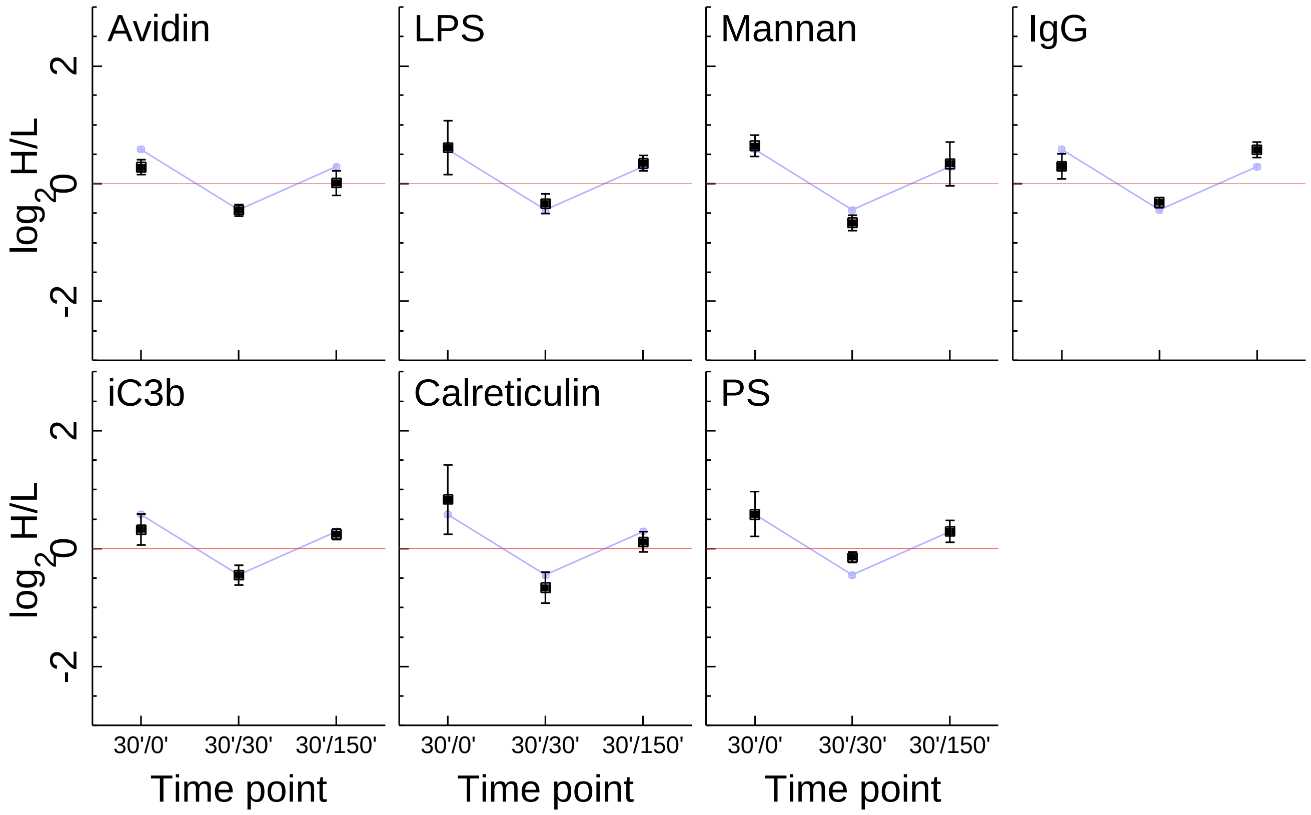

Avidin
LPS
Mannan
IgG
iC3b
Calreticulin
PS
log2 H/L
2
0
-2
30'/0'
30'/30'
30'/150'
Time point

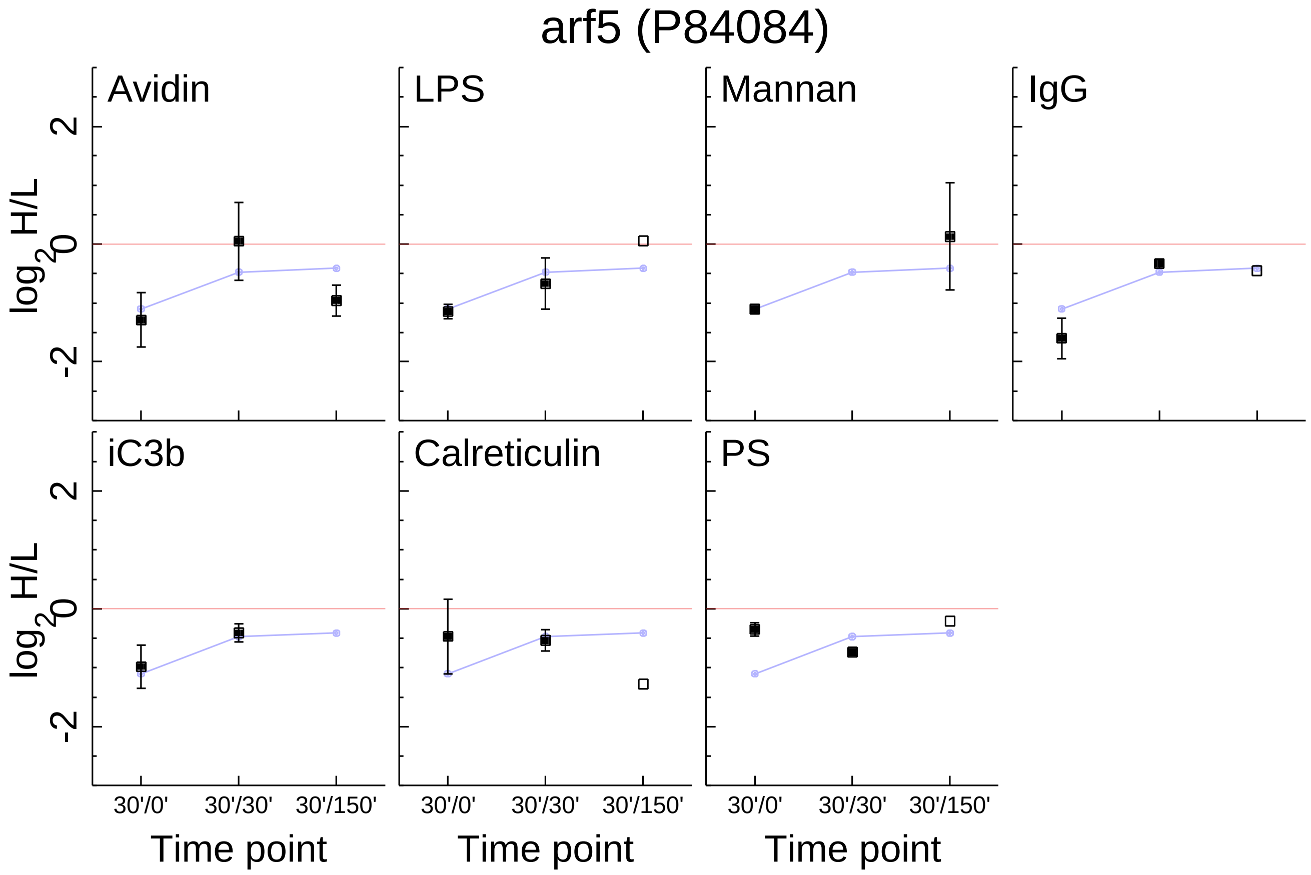

arf5 (P84084)
Avidin
LPS
Mannan
IgG
iC3b
Calreticulin
PS
log2 H/L
2
0
-2
30'/0'
30'/30'
30'/150'
Time point

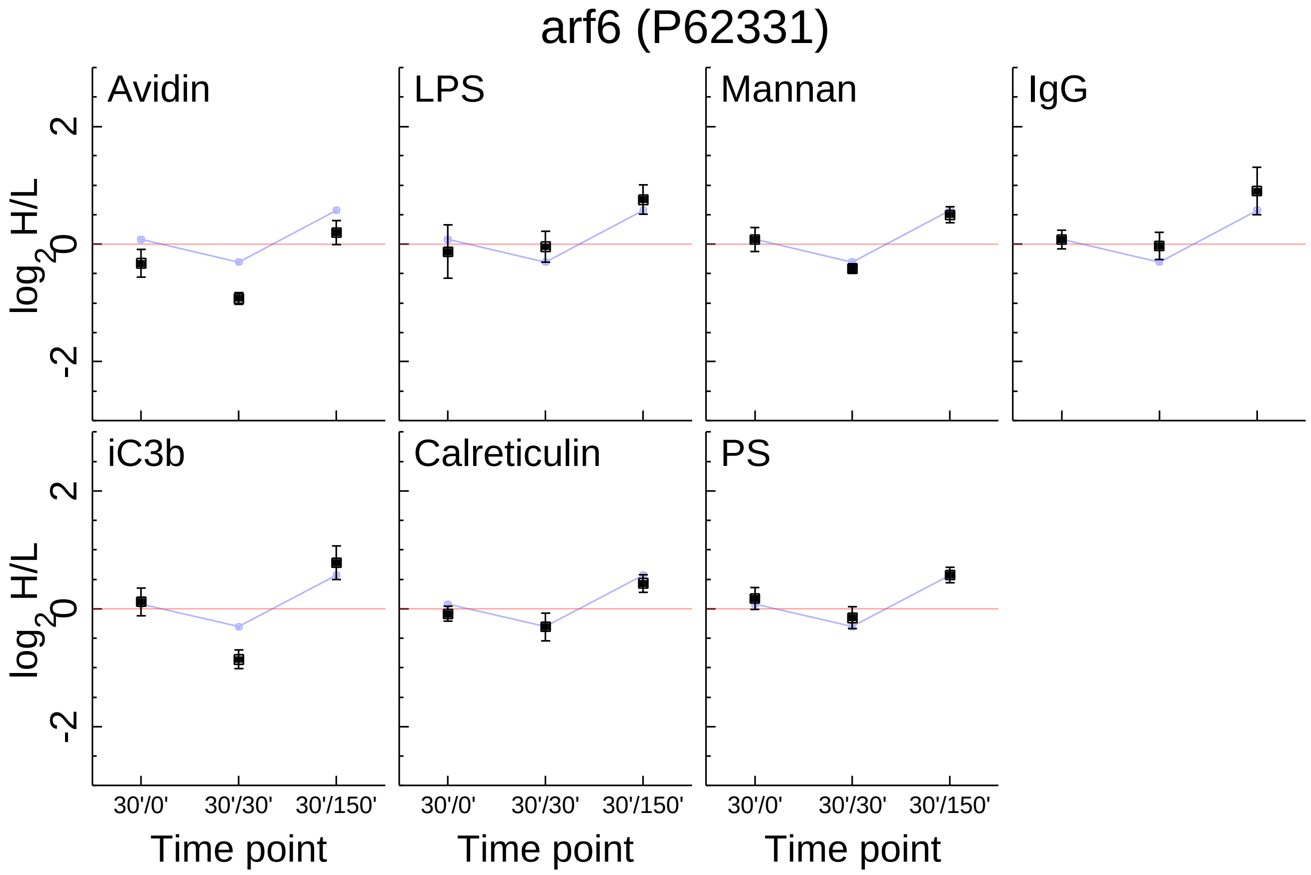
arf6 (P62331)
Avidin
LPS
Mannan
IgG
iC3b
Calreticulin
PS
$\log_2$ H/L
2
0
-2
30'/0'
30'/30'
30'/150'
Time point

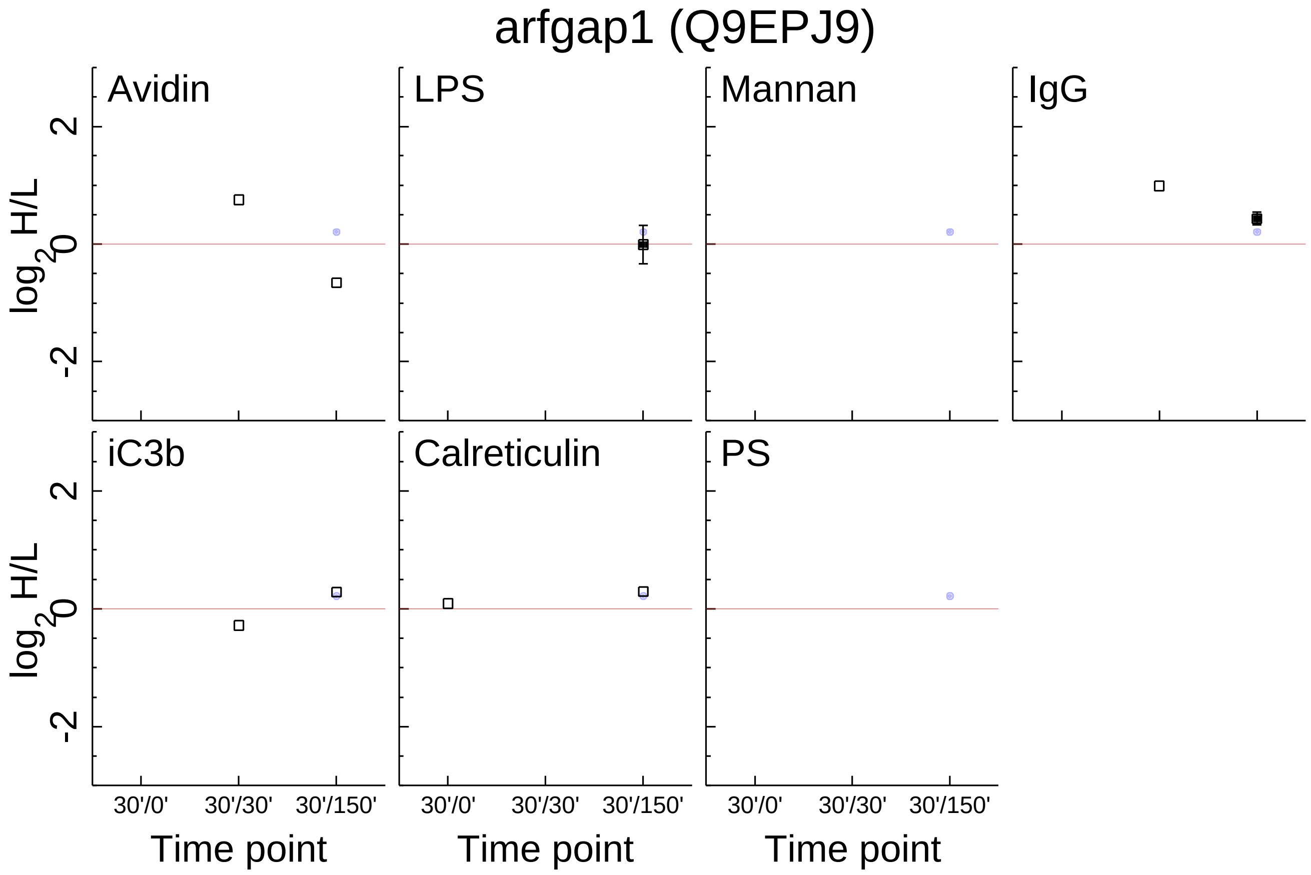
arfgap1 (Q9EPJ9)
Avidin
LPS
Mannan
IgG
iC3b
Calreticulin
PS
2
0
-2
log2 H/L
30'/0'
30'/30'
30'/150'
Time point

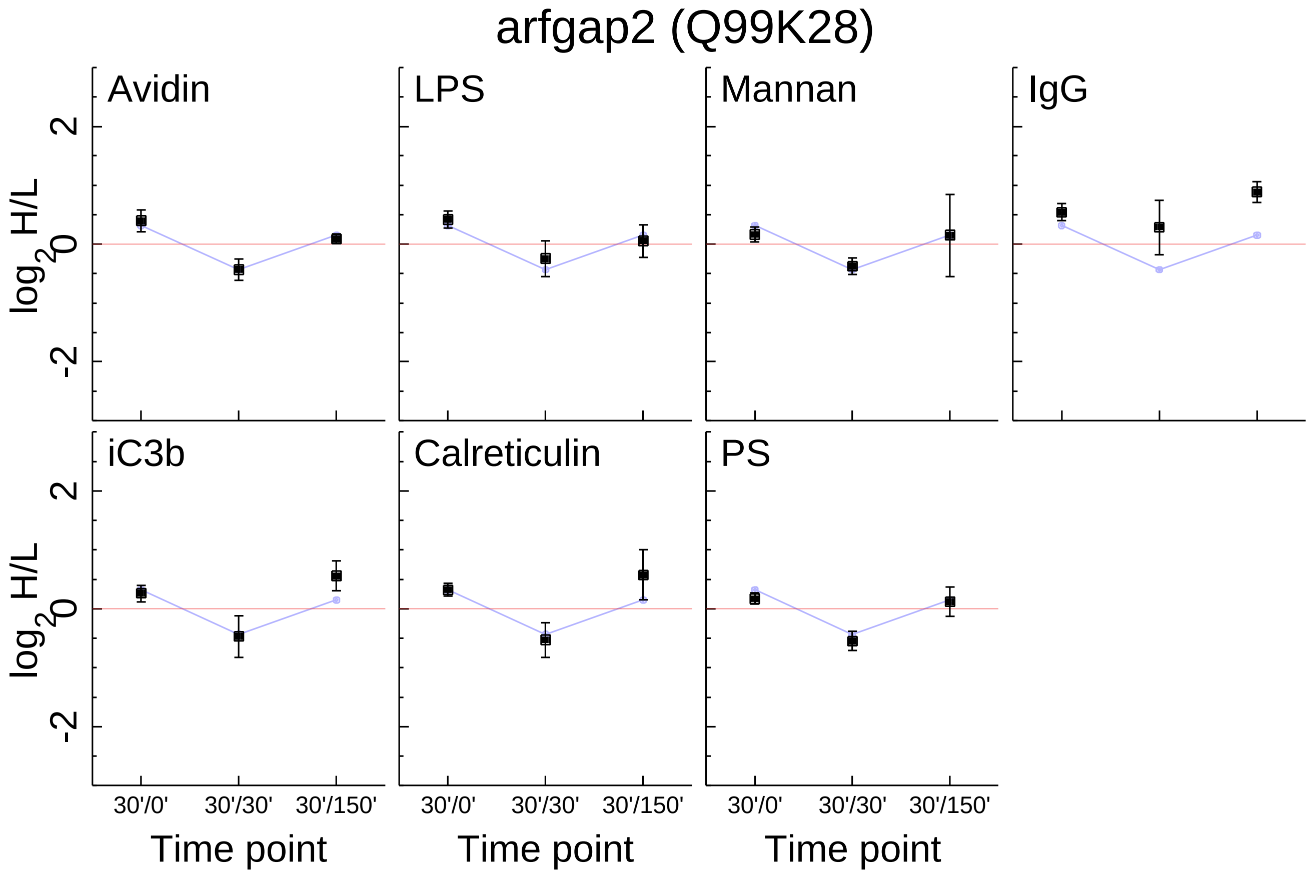
arfgap2 (Q99K28)
Avidin
LPS
Mannan
IgG
iC3b
Calreticulin
PS
log2 H/L
2
0
-2
30'/0'
30'/30'
30'/150'
Time point

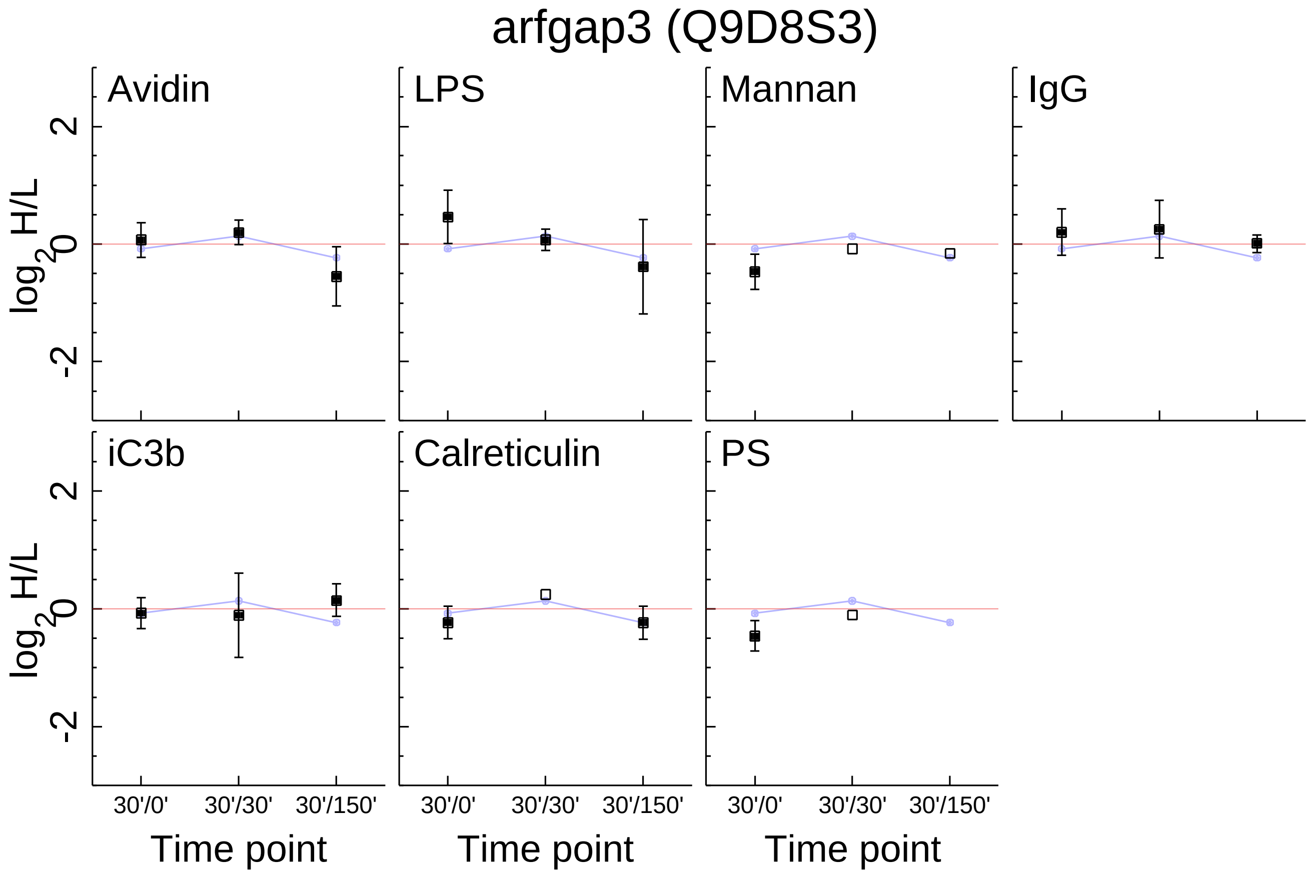
arfgap3 (Q9D8S3)
Avidin
LPS
Mannan
IgG
iC3b
Calreticulin
PS
log2 H/L
2
0
-2
30'/0'
30'/30'
30'/150'
Time point

arfip1 (G5E8V9)

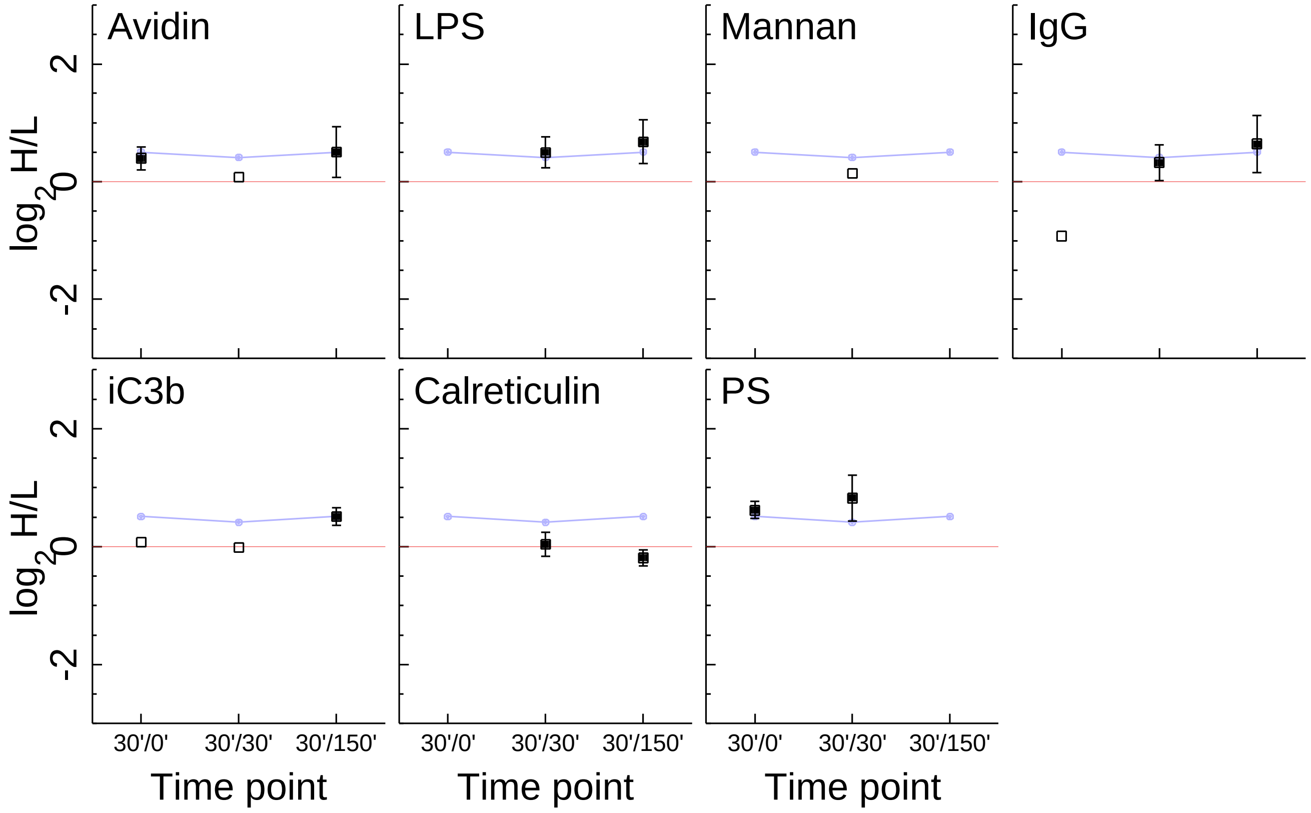

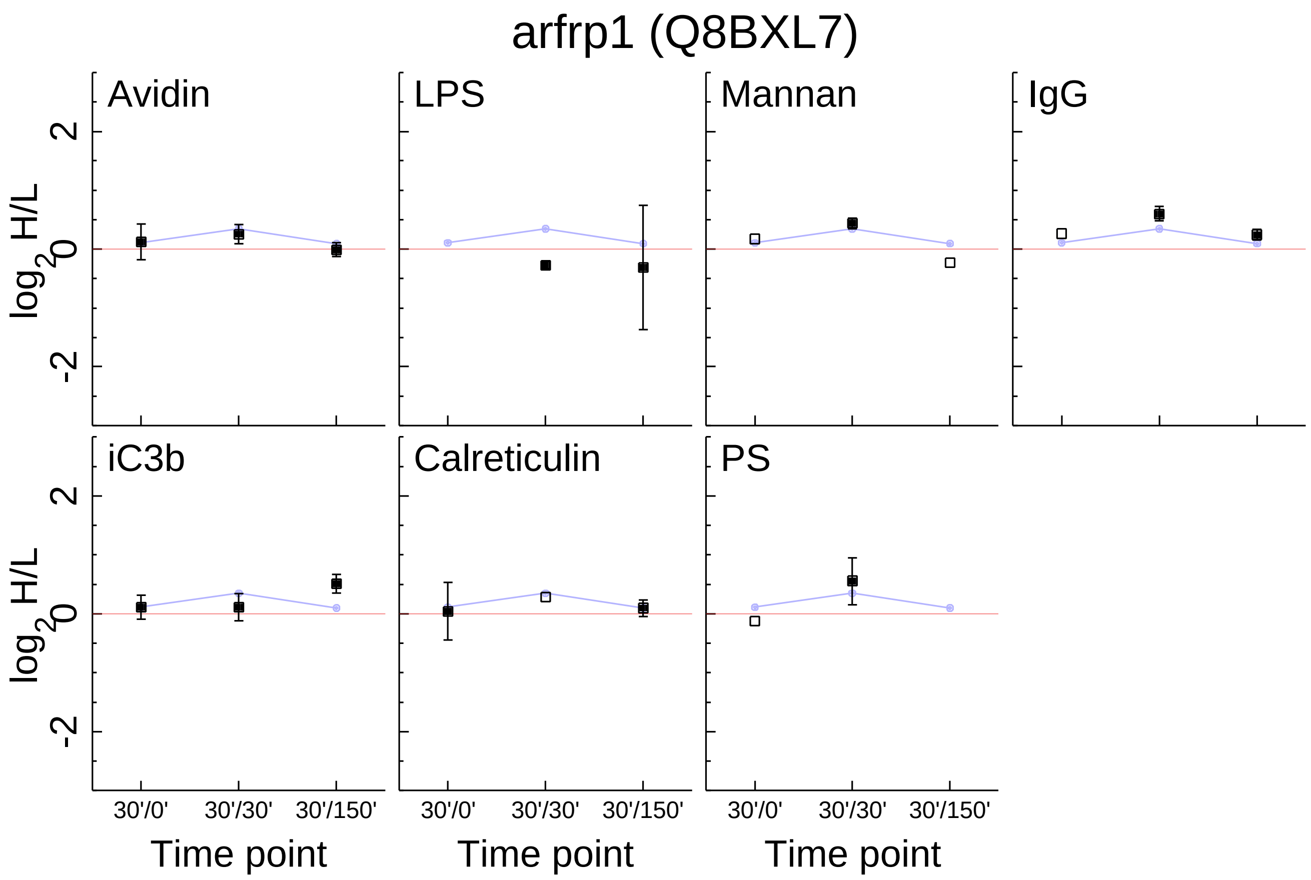
arfrp1 (Q8BXL7)
Avidin
LPS
Mannan
IgG
iC3b
Calreticulin
PS
log2 H/L
2
0
-2
30'/0'
30'/30'
30'/150'
Time point

arhgap17 (Q3UIA2)

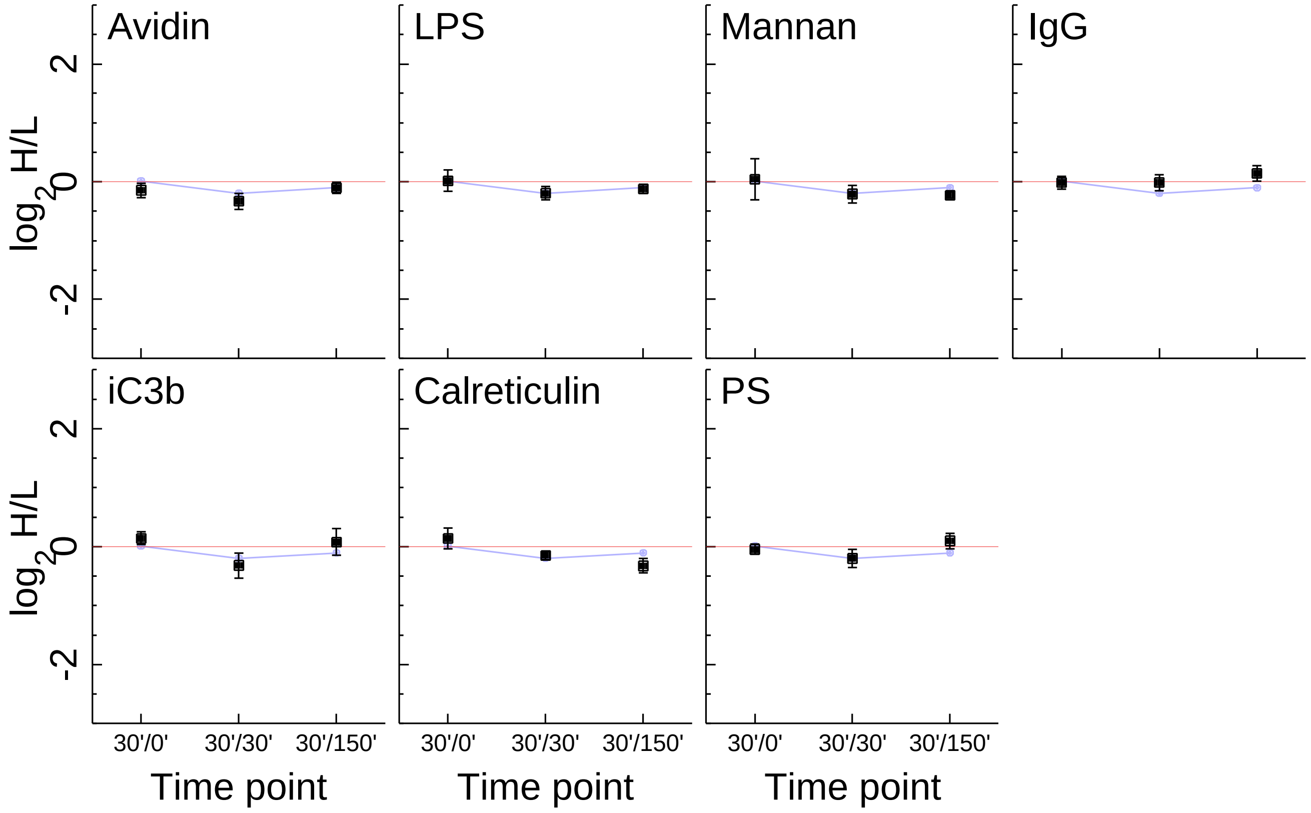

arhgap1 (A2AH25)

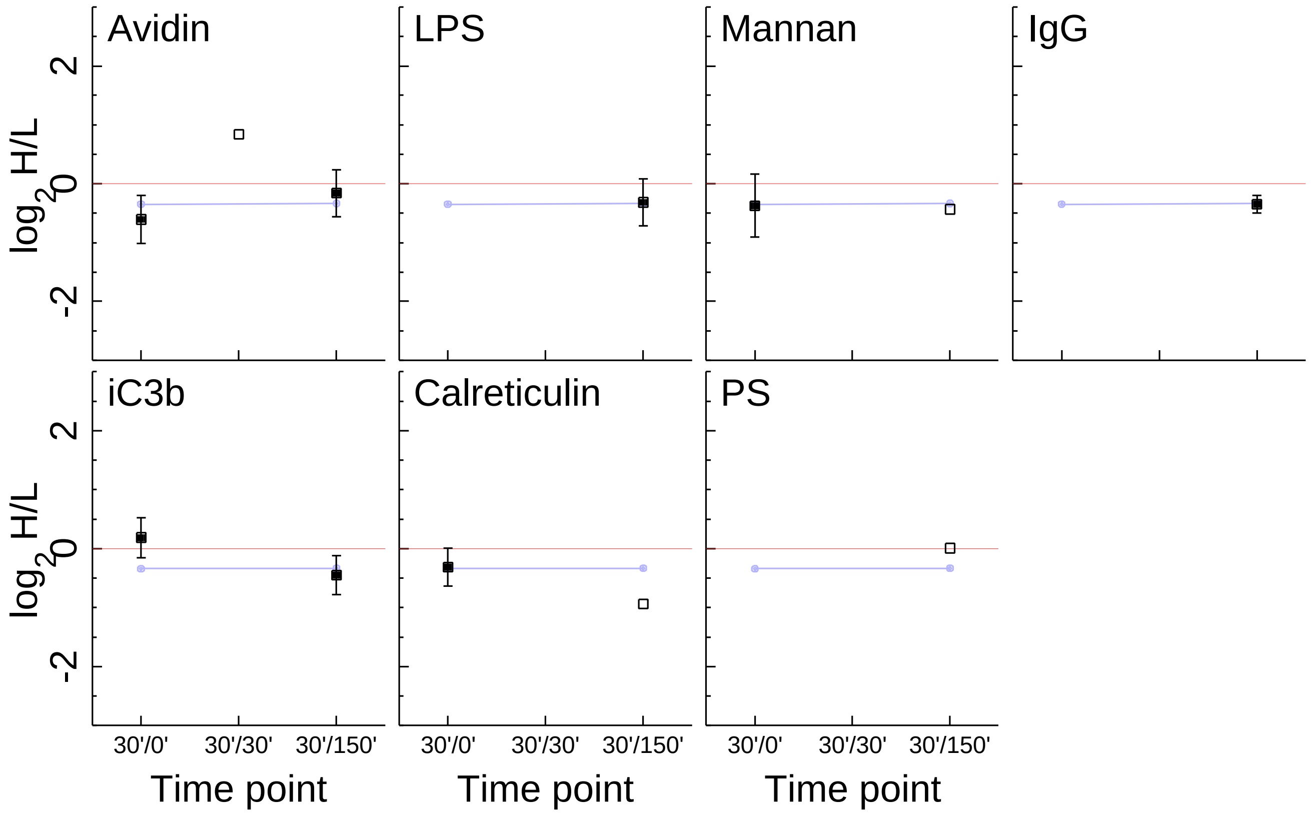

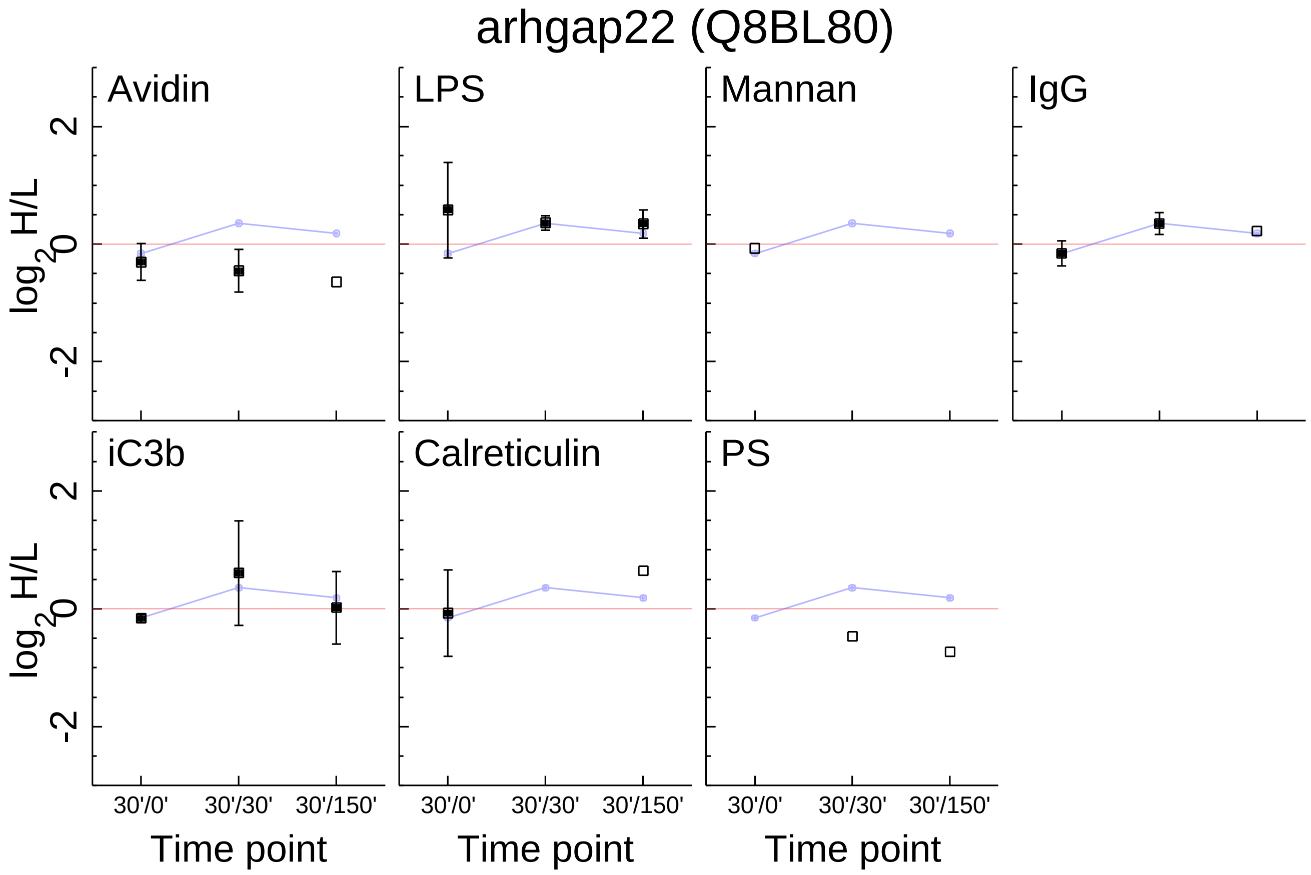
arhgap22 (Q8BL80)
Avidin
LPS
Mannan
IgG
iC3b
Calreticulin
PS
log2 H/L
2
0
-2
30'/0'
30'/30'
30'/150'
Time point

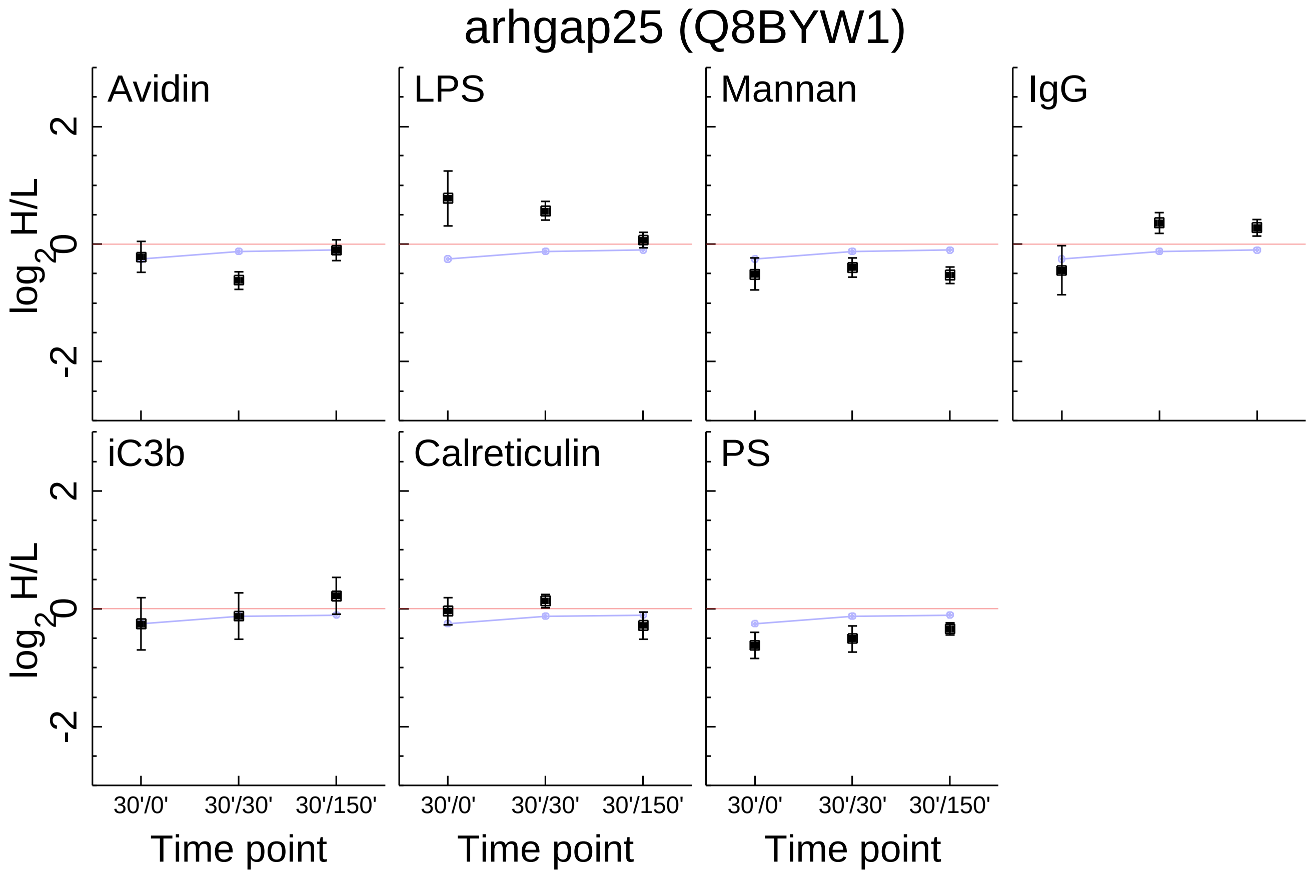
arhgap25 (Q8BYW1)
Avidin
LPS
Mannan
IgG
iC3b
Calreticulin
PS
log2 H/L
2
0
-2
30'/0'
30'/30'
30'/150'
Time point

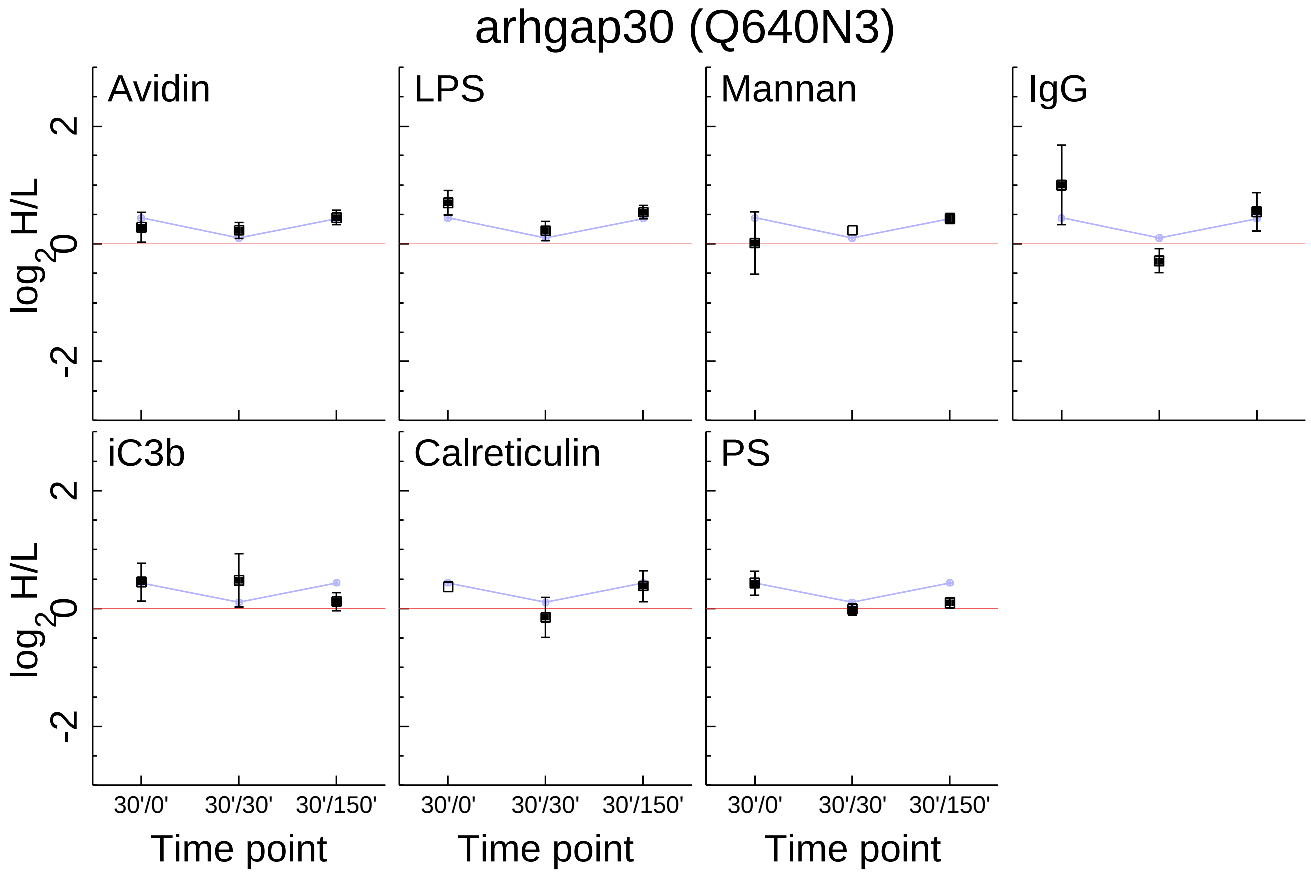

arhgap30 (Q640N3)
Avidin
LPS
Mannan
IgG
iC3b
Calreticulin
PS
log2 H/L
2
0
-2
30'/0'
30'/30'
30'/150'
Time point

arhgap4 (B1AUY3)

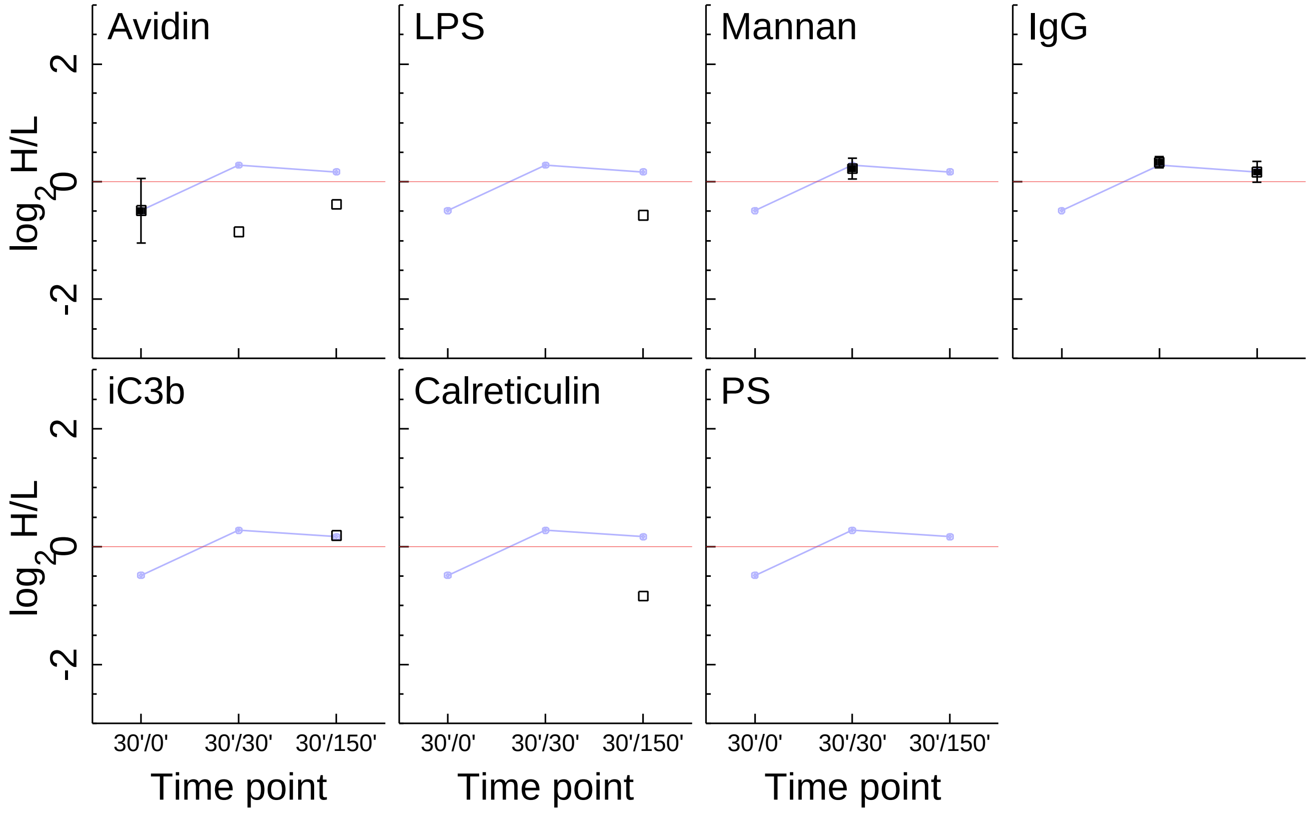

arhgap9 (Q8QZW8)

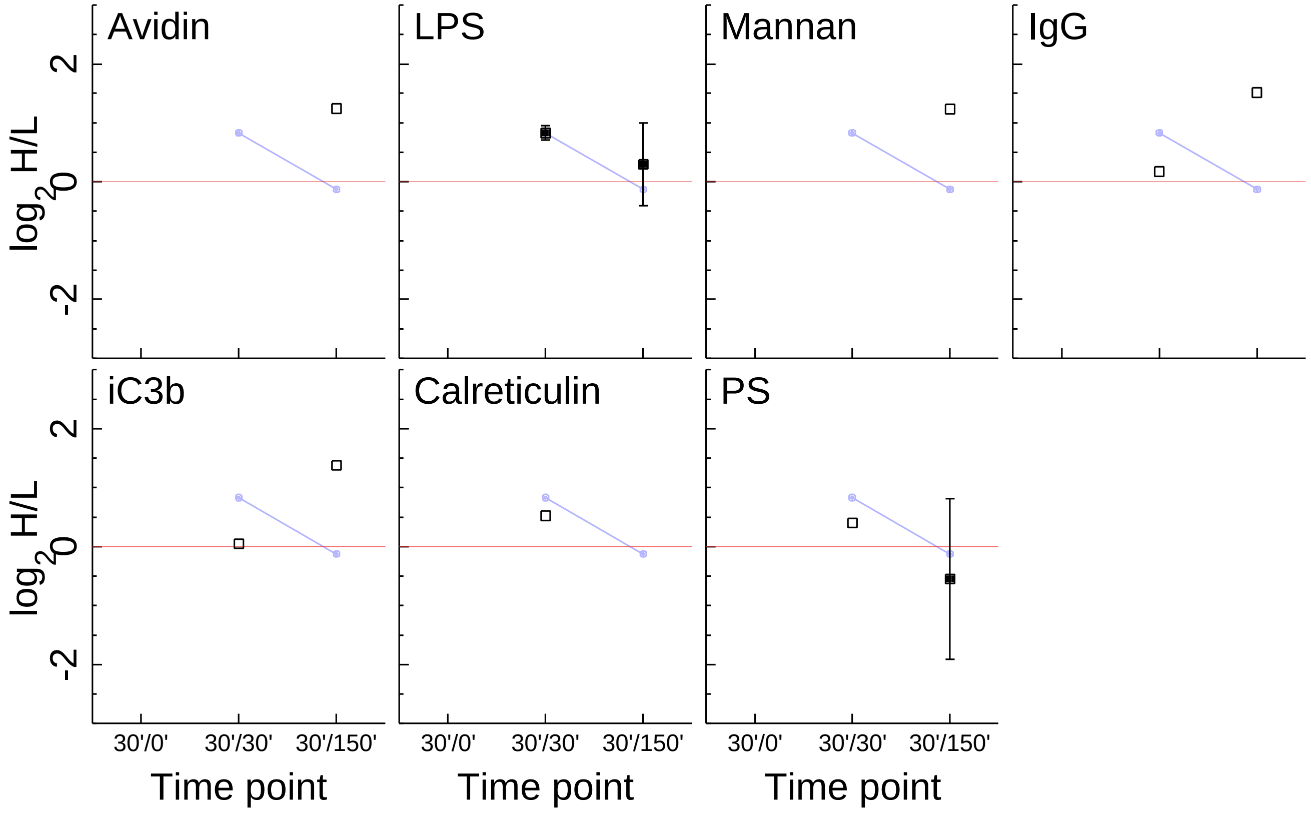

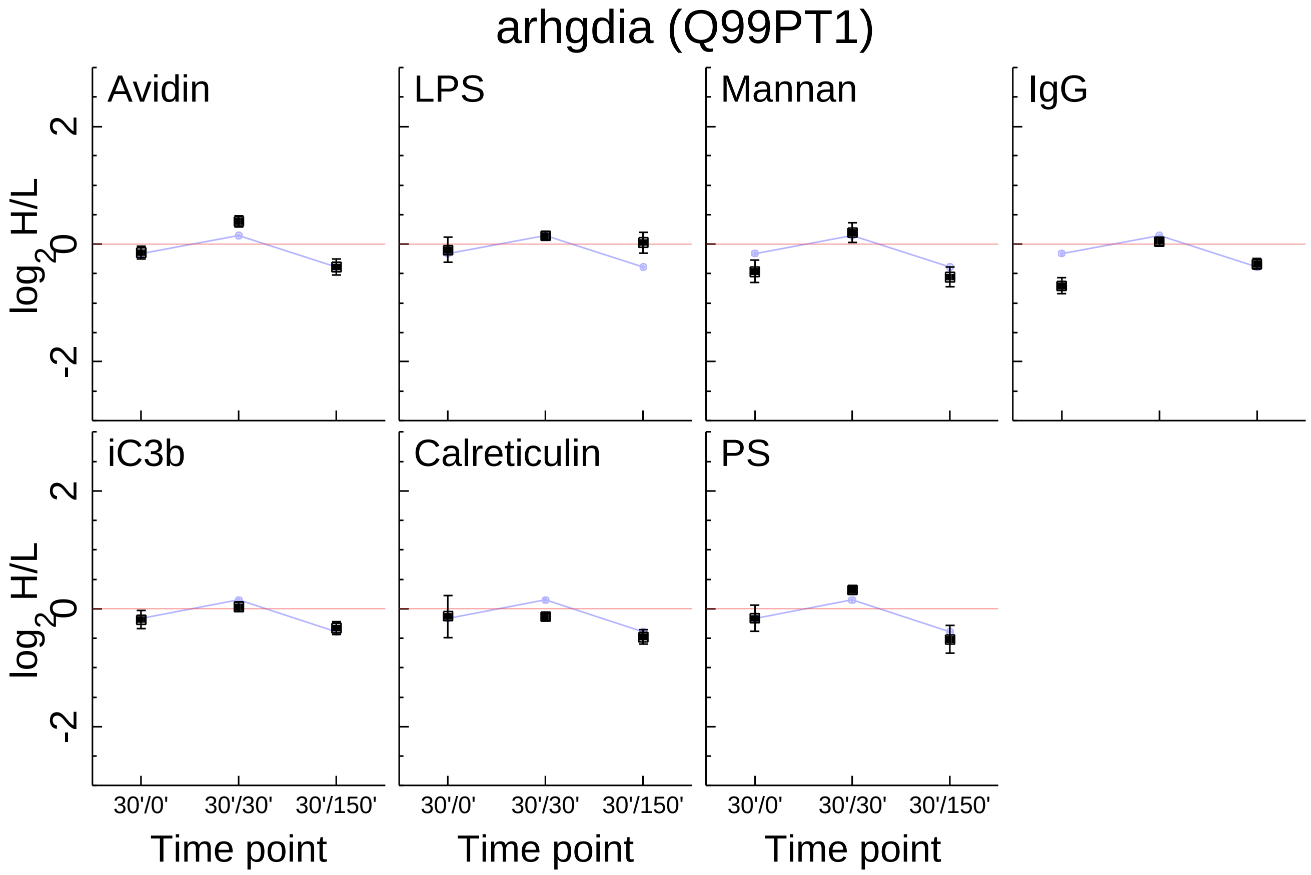
arhgdia (Q99PT1)
Avidin
LPS
Mannan
IgG
iC3b
Calreticulin
PS
log2 H/L
2
0
-2
30'/0'
30'/30'
30'/150'
Time point

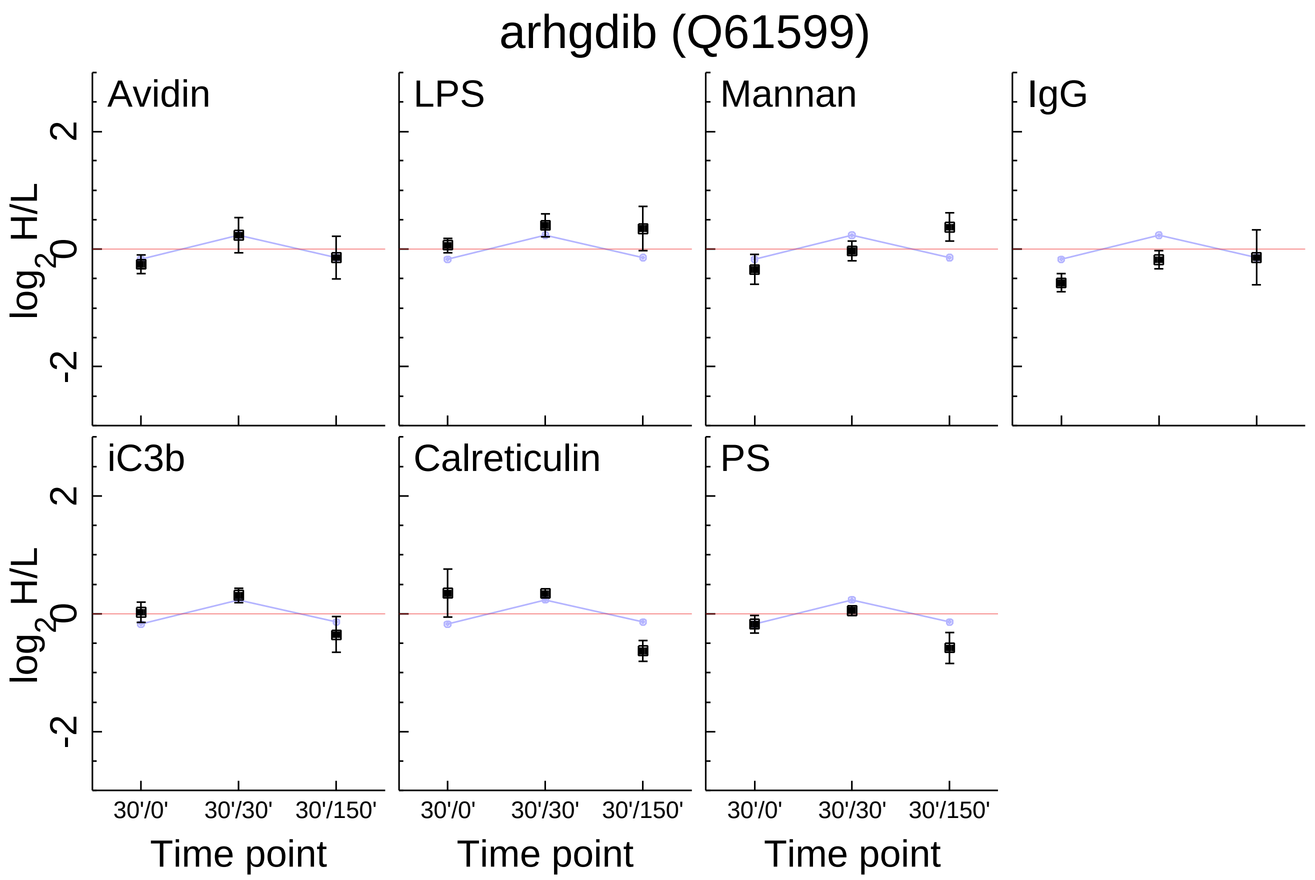

arhgdib (Q61599)
Avidin
LPS
Mannan
IgG
iC3b
Calreticulin
PS
log2 H/L
2
0
-2
30'/0'
30'/30'
30'/150'
Time point

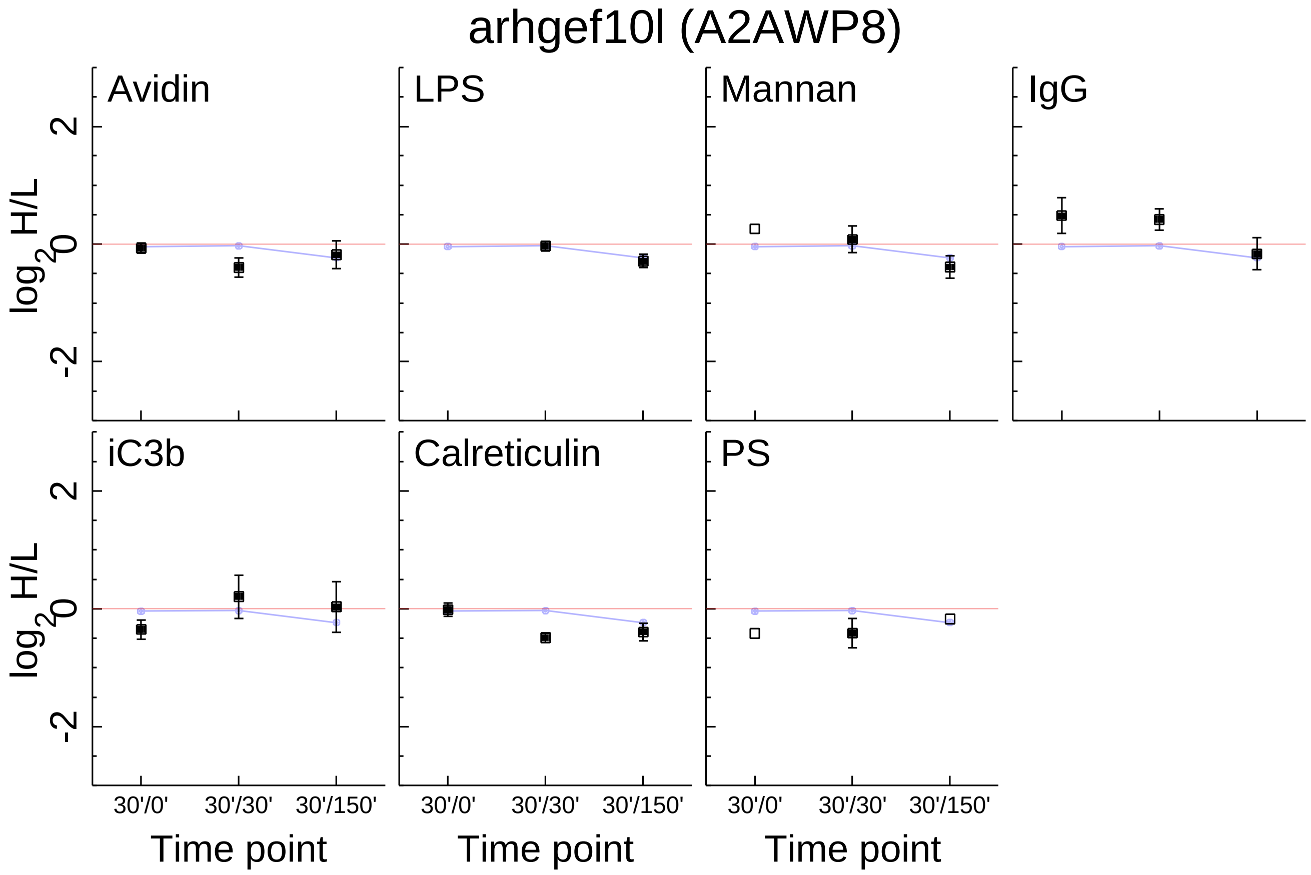
arhgef10l (A2AWP8)
Avidin
LPS
Mannan
IgG
iC3b
Calreticulin
PS
log2 H/L
2
0
-2
30'/0'
30'/30'
30'/150'
Time point

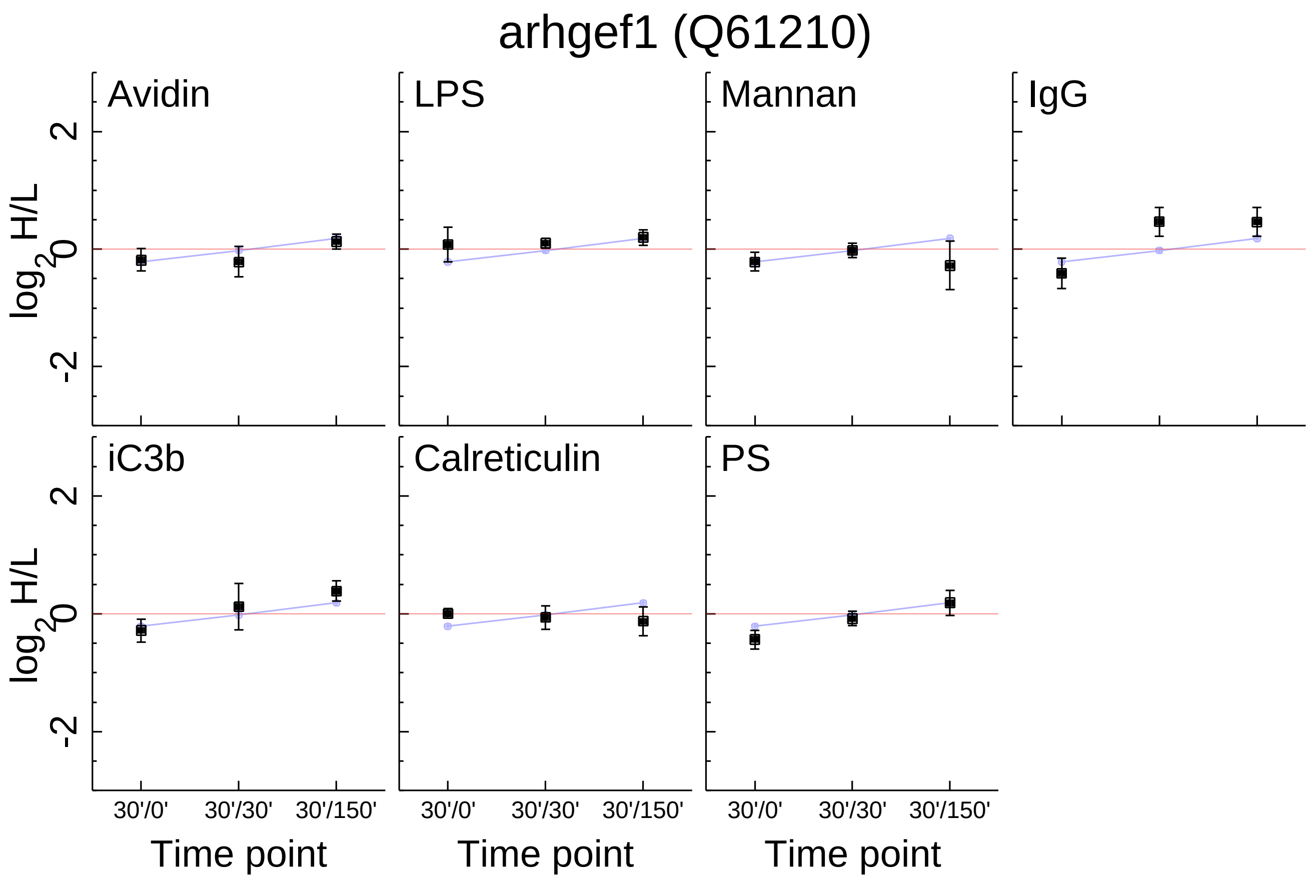

arhgef1 (Q61210)
Avidin
LPS
Mannan
IgG
iC3b
Calreticulin
PS
log2 H/L
2
0
-2
30'/0'
30'/30'
30'/150'
Time point

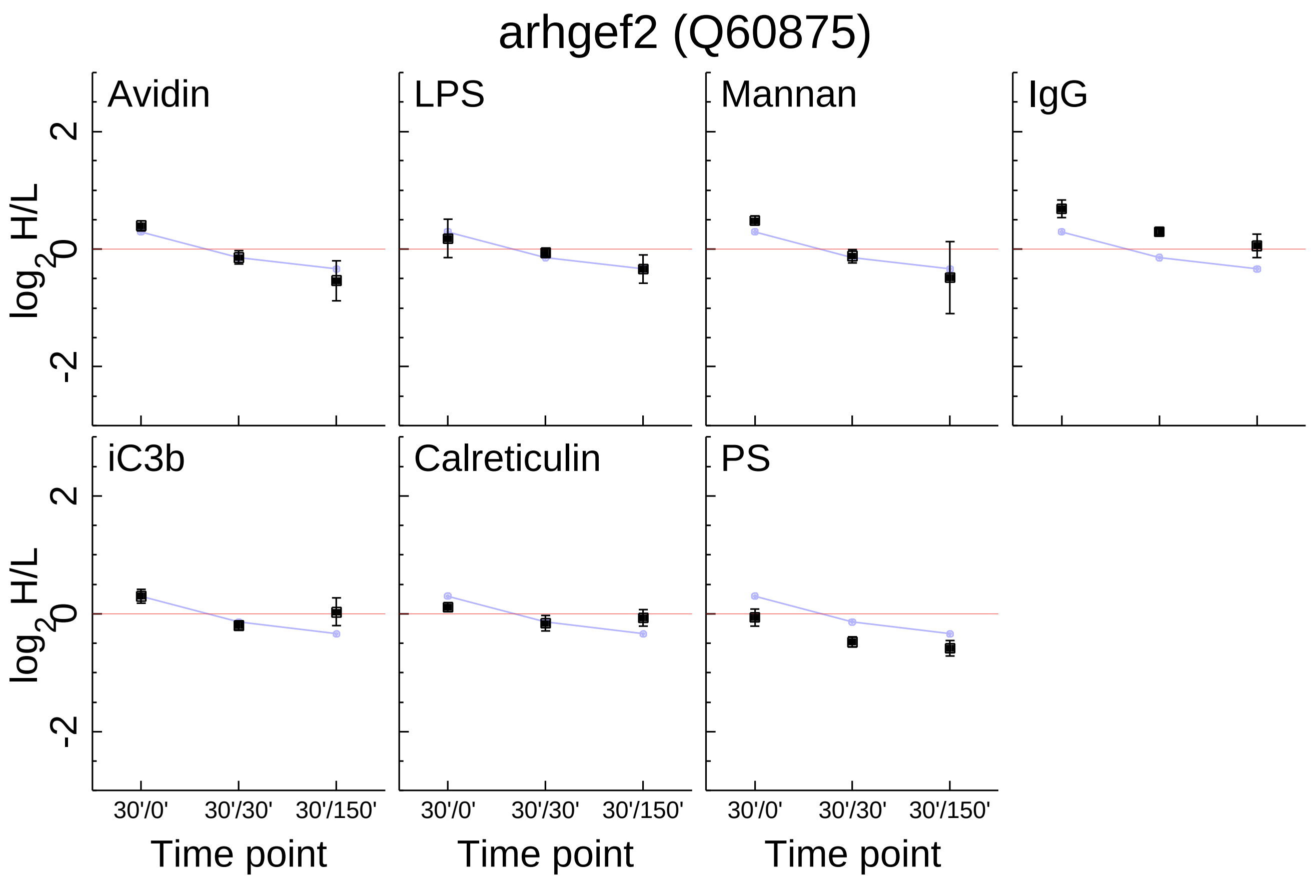
arhgef2 (Q60875)
Avidin
LPS
Mannan
IgG
iC3b
Calreticulin
PS
log2 H/L
2
0
-2
30'/0'
30'/30'
30'/150'
Time point

arhgef7 (Q9ES28)

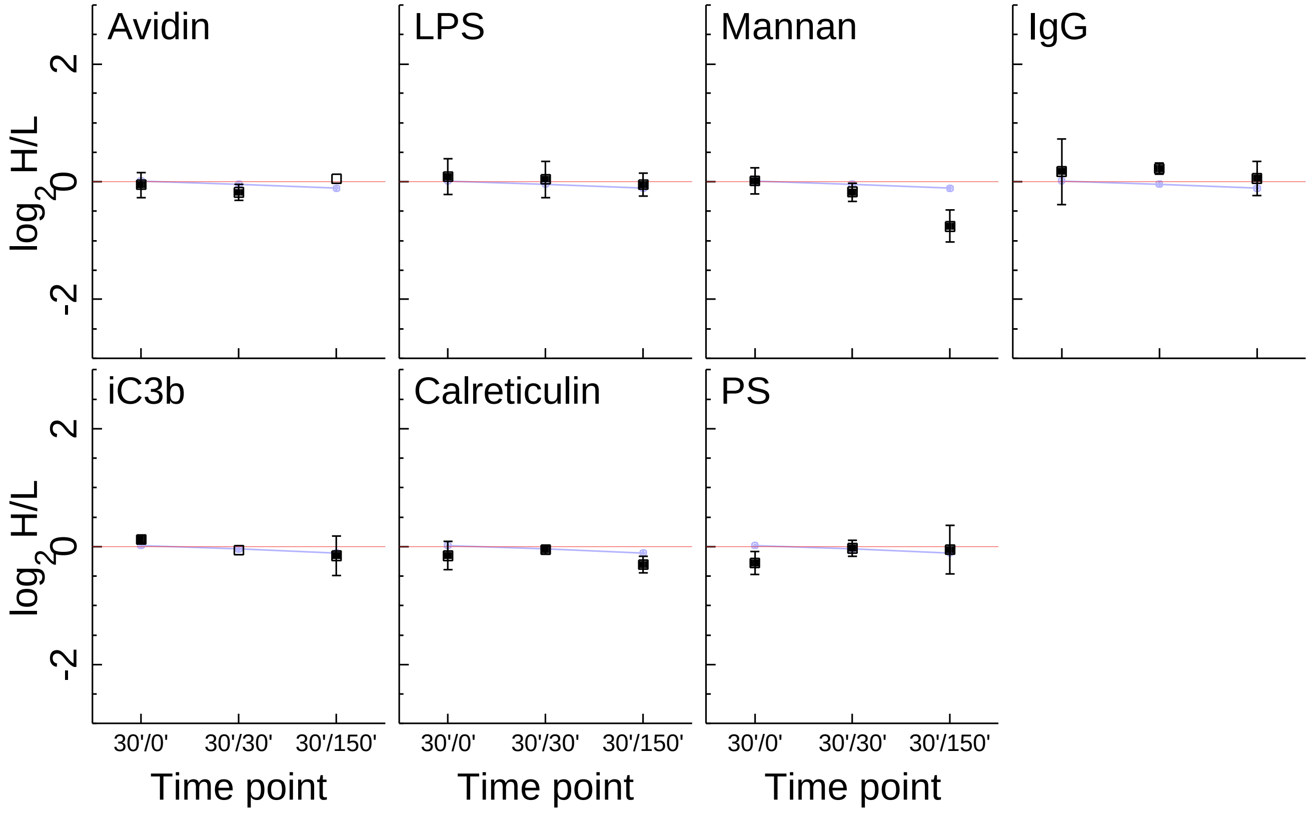

arl1 (P61211)

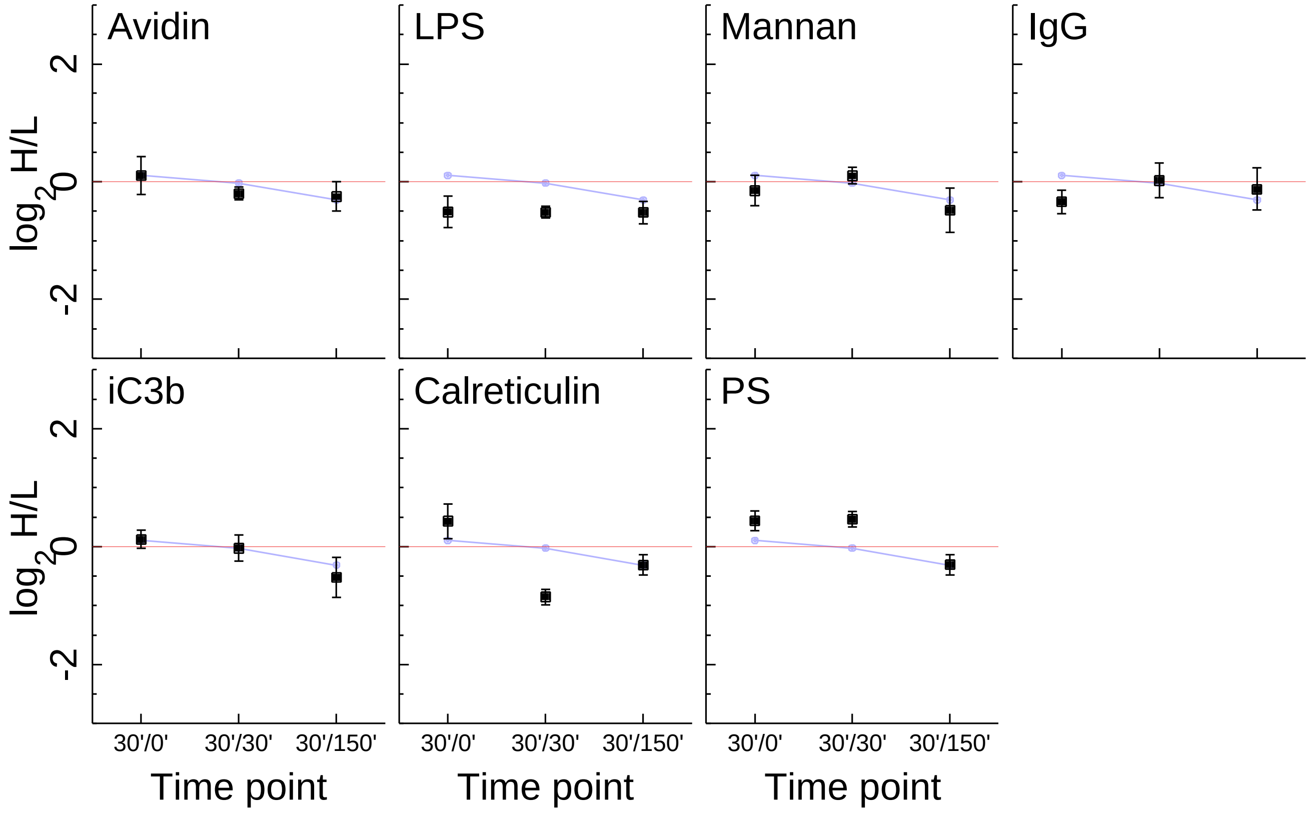

Avidin
LPS
Mannan
IgG
iC3b
Calreticulin
PS
log2 H/L
2
0
-2
30'/0'
30'/30'
30'/150'
Time point

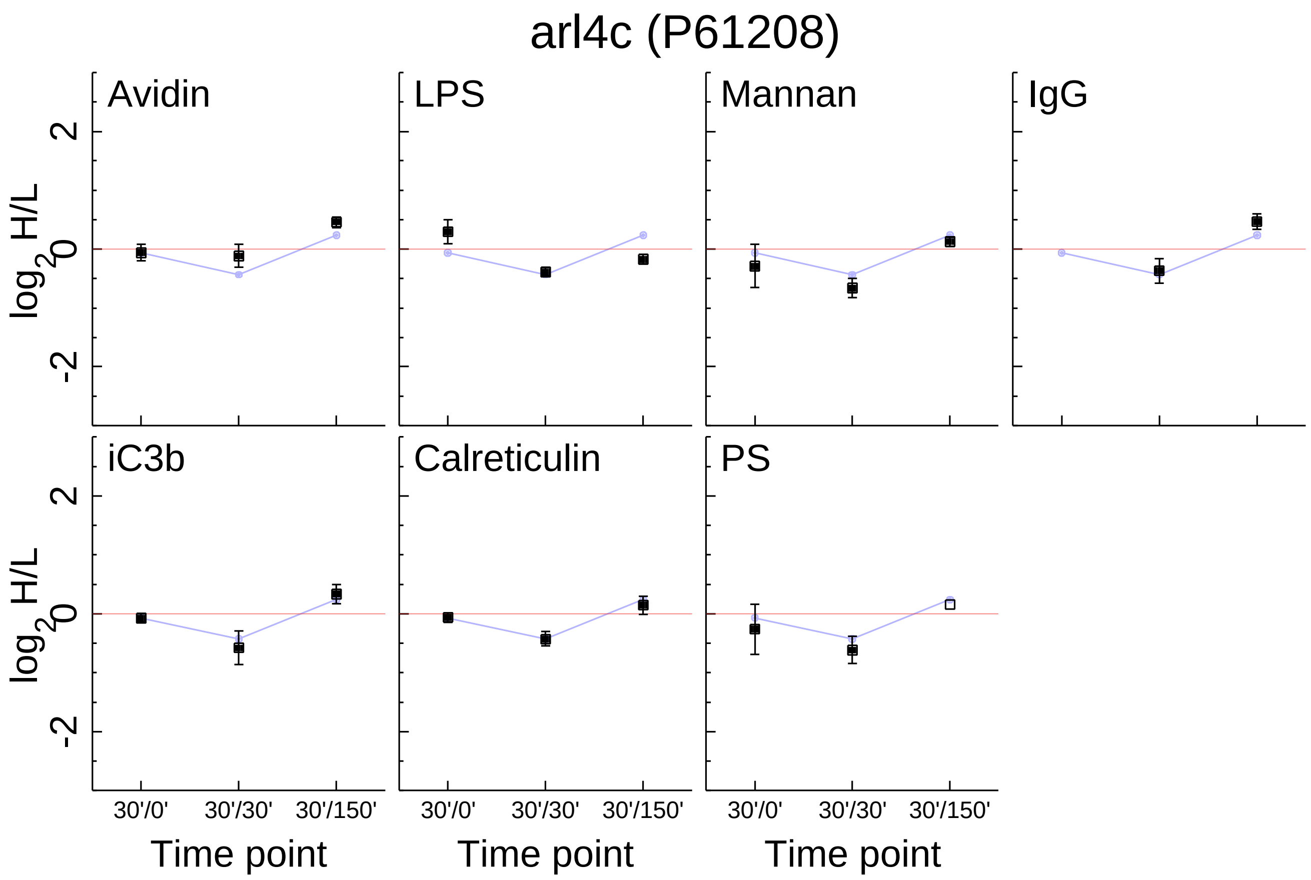
arl4c (P61208)
Avidin
LPS
Mannan
IgG
iC3b
Calreticulin
PS
log2 H/L
2
0
-2
30'/0'
30'/30'
30'/150'
Time point

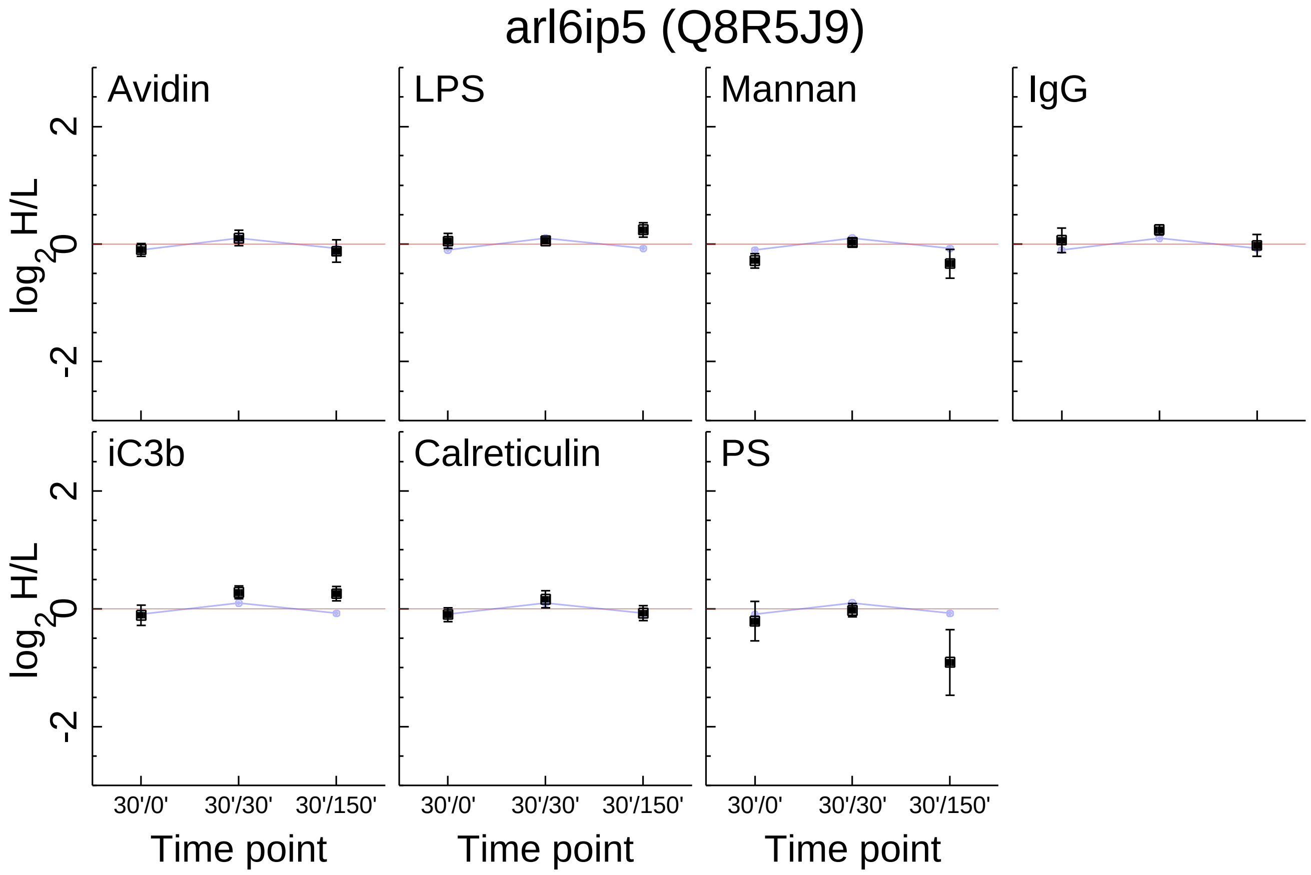

arl6ip5 (Q8R5J9)
Avidin
LPS
Mannan
IgG
iC3b
Calreticulin
PS
log2 H/L
2
0
-2
30'/0'
30'/30'
30'/150'
Time point

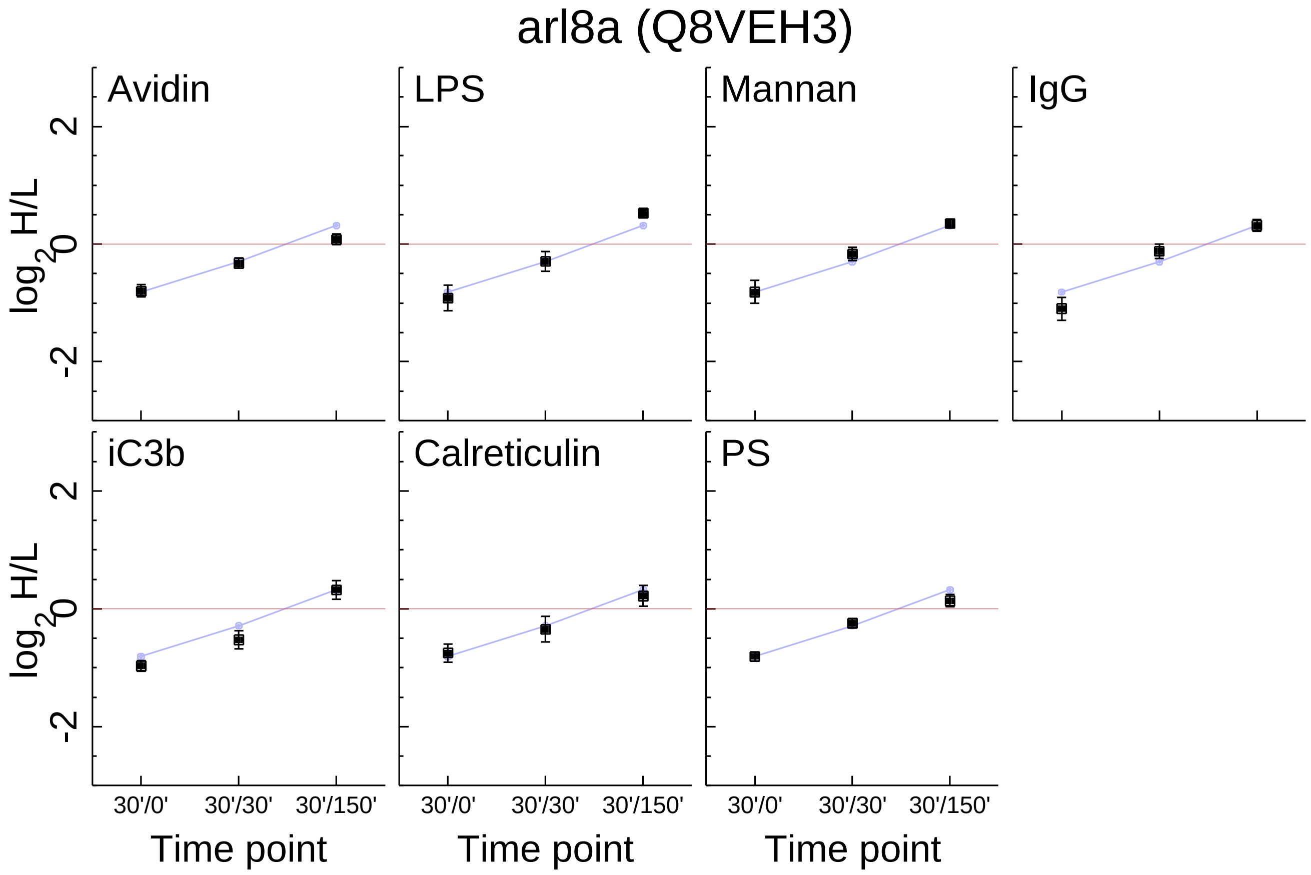
arl8a (Q8VEH3)
Avidin
LPS
Mannan
IgG
iC3b
Calreticulin
PS
log2 H/L
2
0
-2
30'/0'
30'/30'
30'/150'
Time point

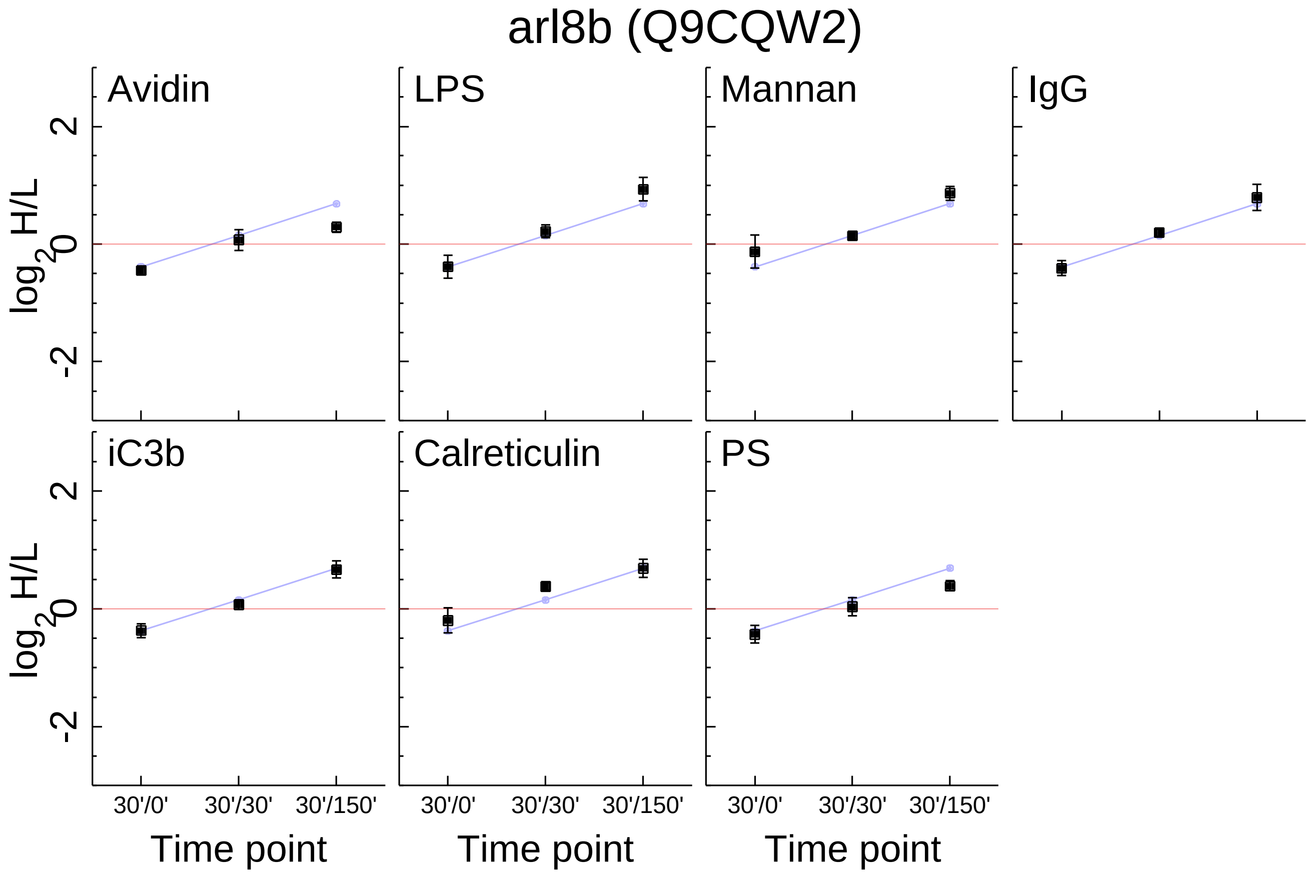
arl8b (Q9CQW2)
Avidin
LPS
Mannan
IgG
iC3b
Calreticulin
PS
log2 H/L
2
0
-2
30'/0'
30'/30'
30'/150'
Time point

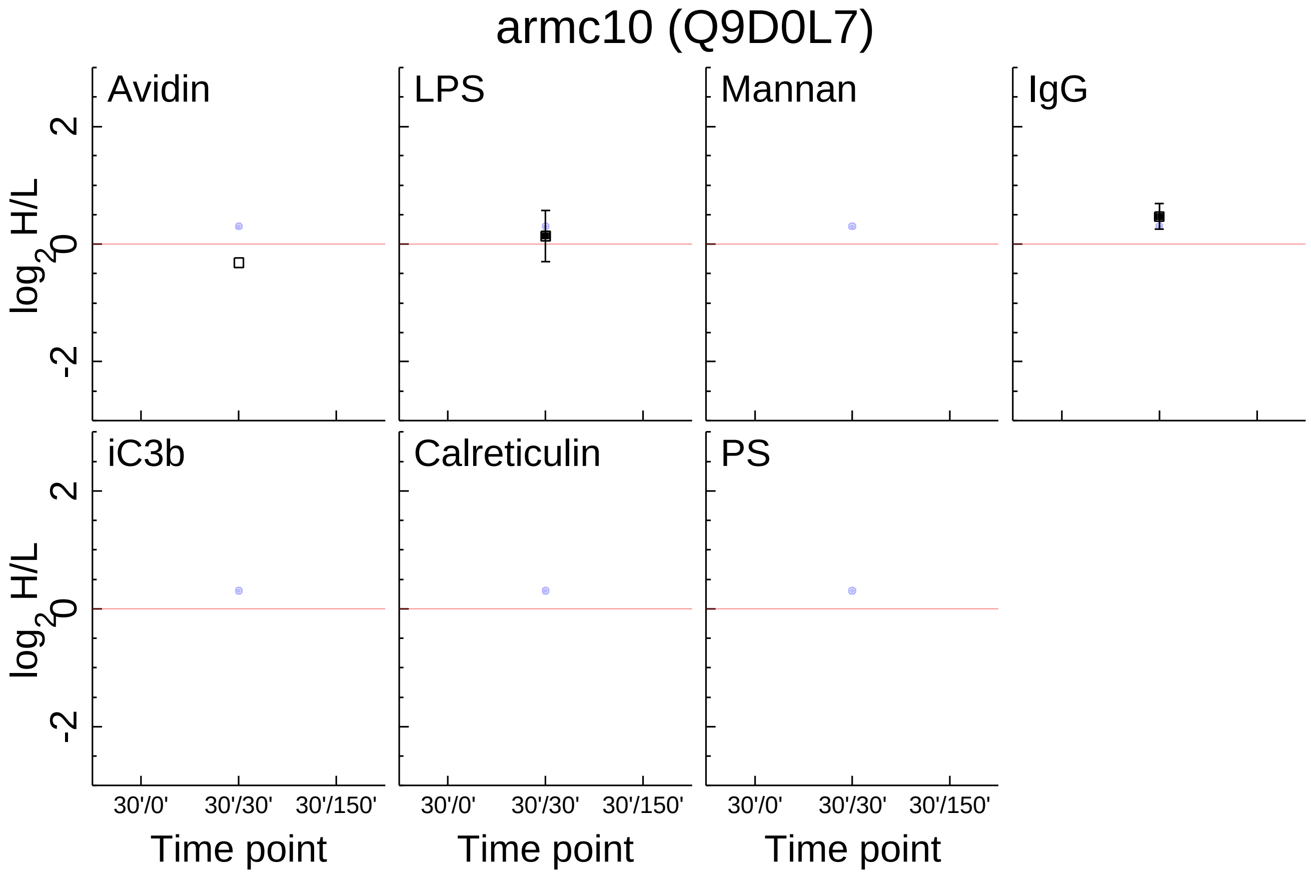
armc10 (Q9D0L7)
Avidin
LPS
Mannan
IgG
iC3b
Calreticulin
PS
log2 H/L
2
0
-2
30'/0'
30'/30'
30'/150'
Time point

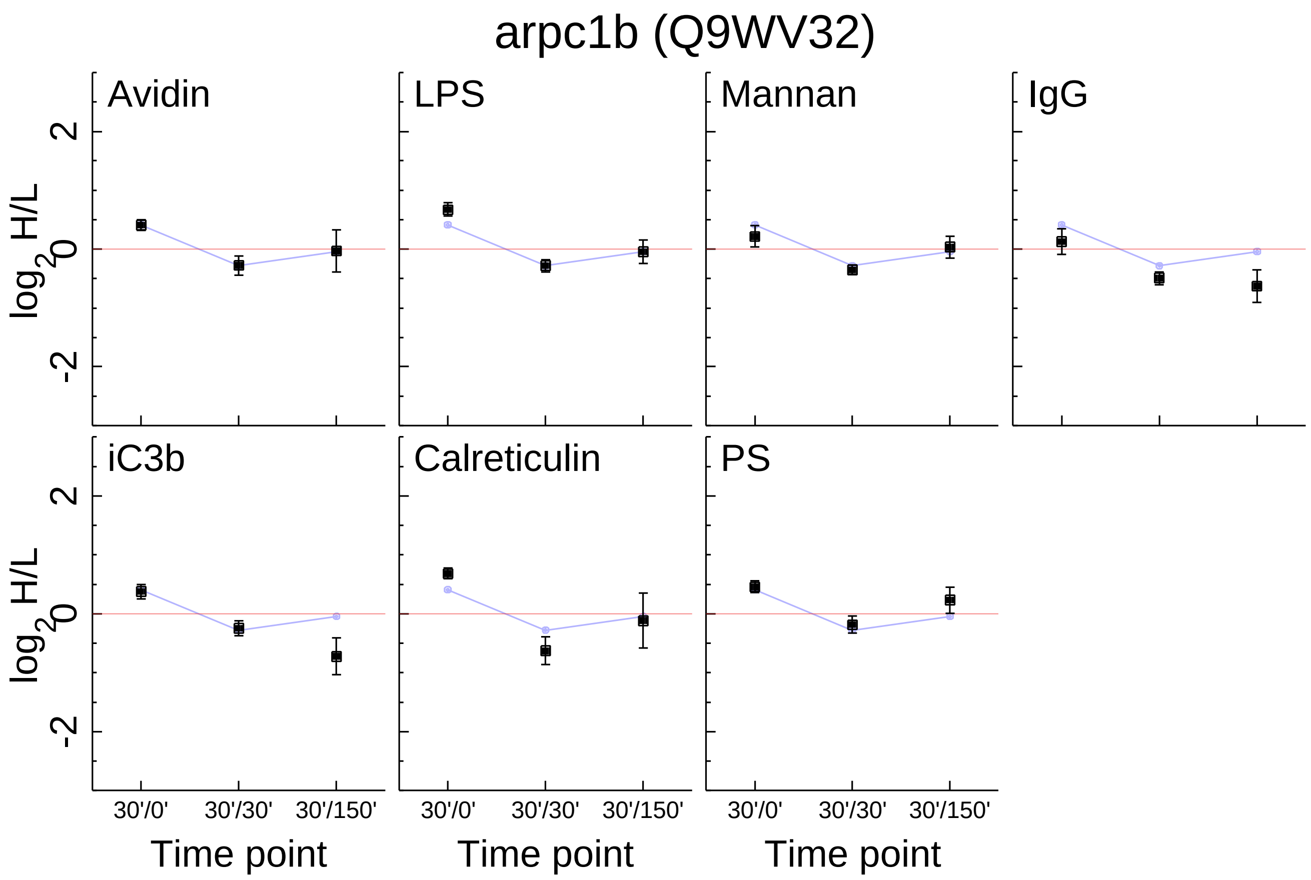
arpc1b (Q9WV32)
Avidin
LPS
Mannan
IgG
iC3b
Calreticulin
PS
log2 H/L
2
0
-2
30'/0'
30'/30'
30'/150'
Time point

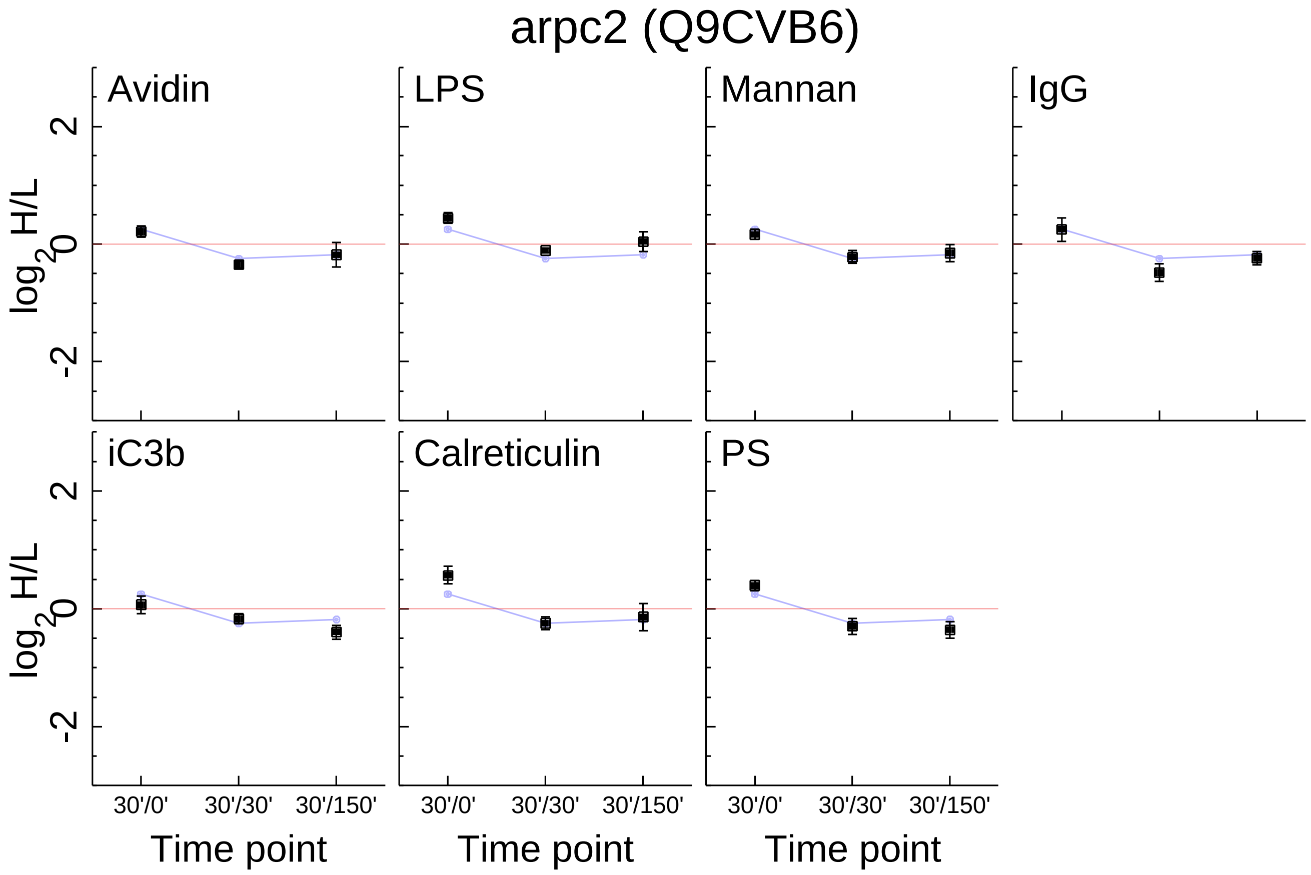
arpc2 (Q9CVB6)
Avidin
LPS
Mannan
IgG
iC3b
Calreticulin
PS
log2 H/L
2
0
-2
30'/0'
30'/30'
30'/150'
Time point

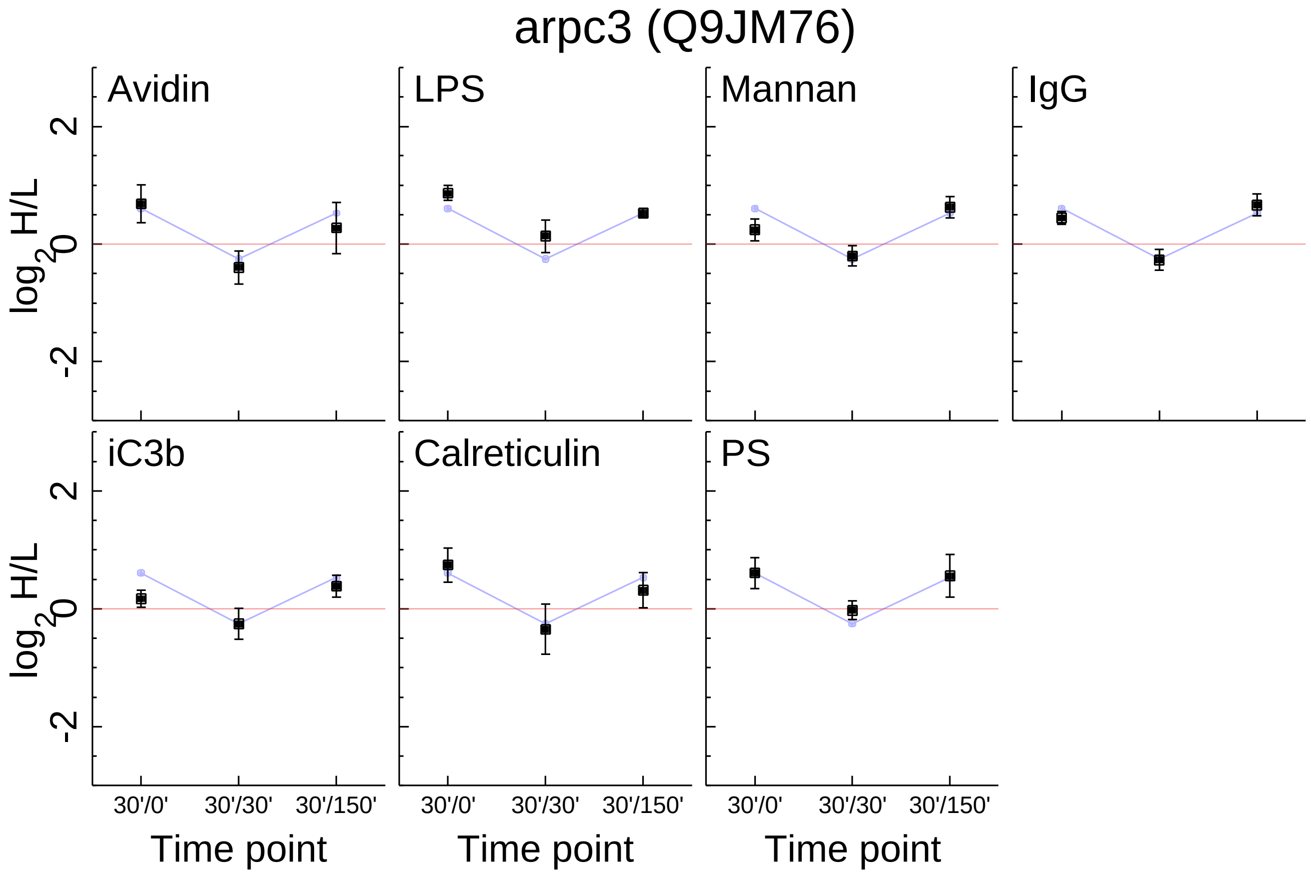
arpc3 (Q9JM76)
Avidin
LPS
Mannan
IgG
iC3b
Calreticulin
PS
log2 H/L
2
0
-2
30'/0'
30'/30'
30'/150'
Time point

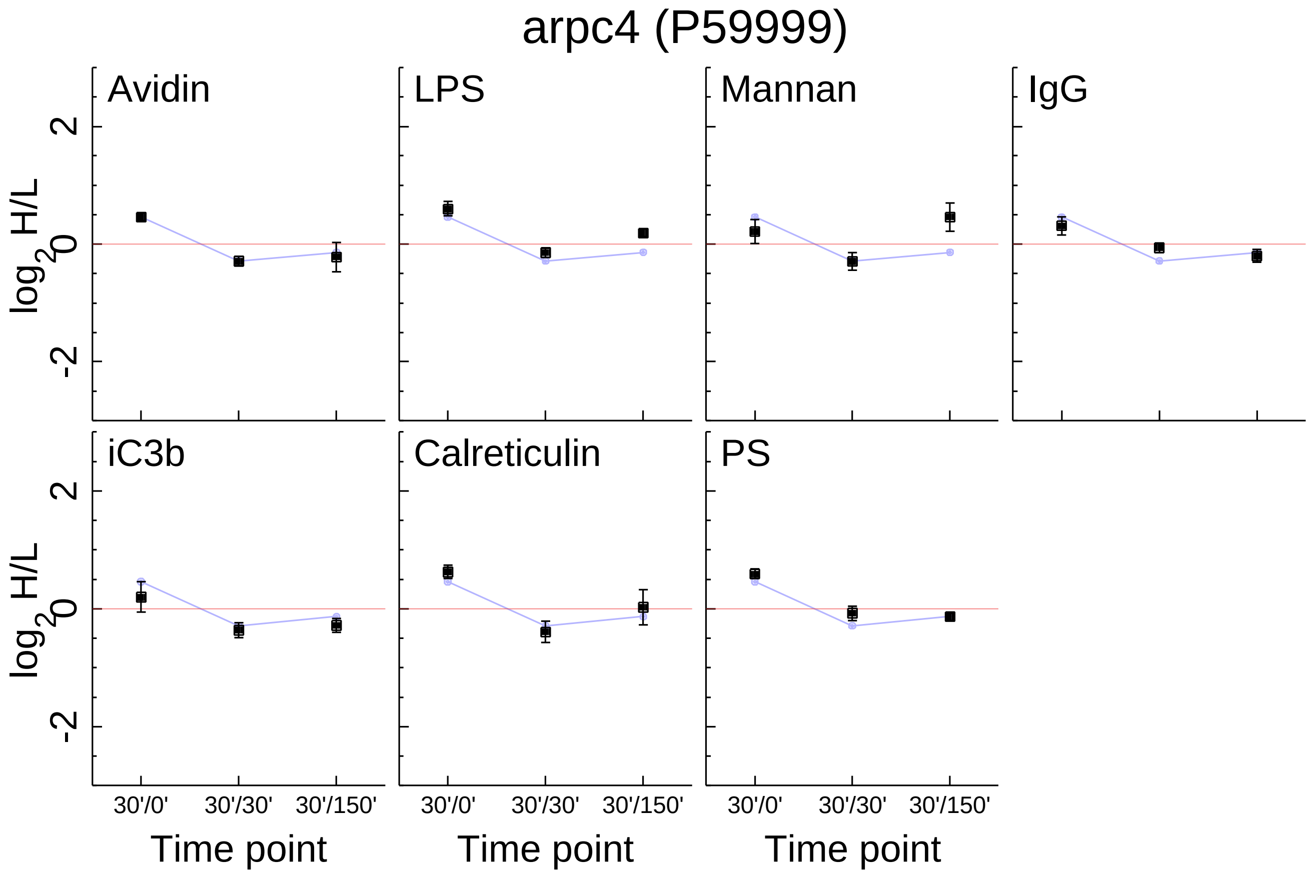
arpc4 (P59999)
Avidin
LPS
Mannan
IgG
iC3b
Calreticulin
PS
log2 H/L
2
0
-2
30'/0'
30'/30'
30'/150'
Time point

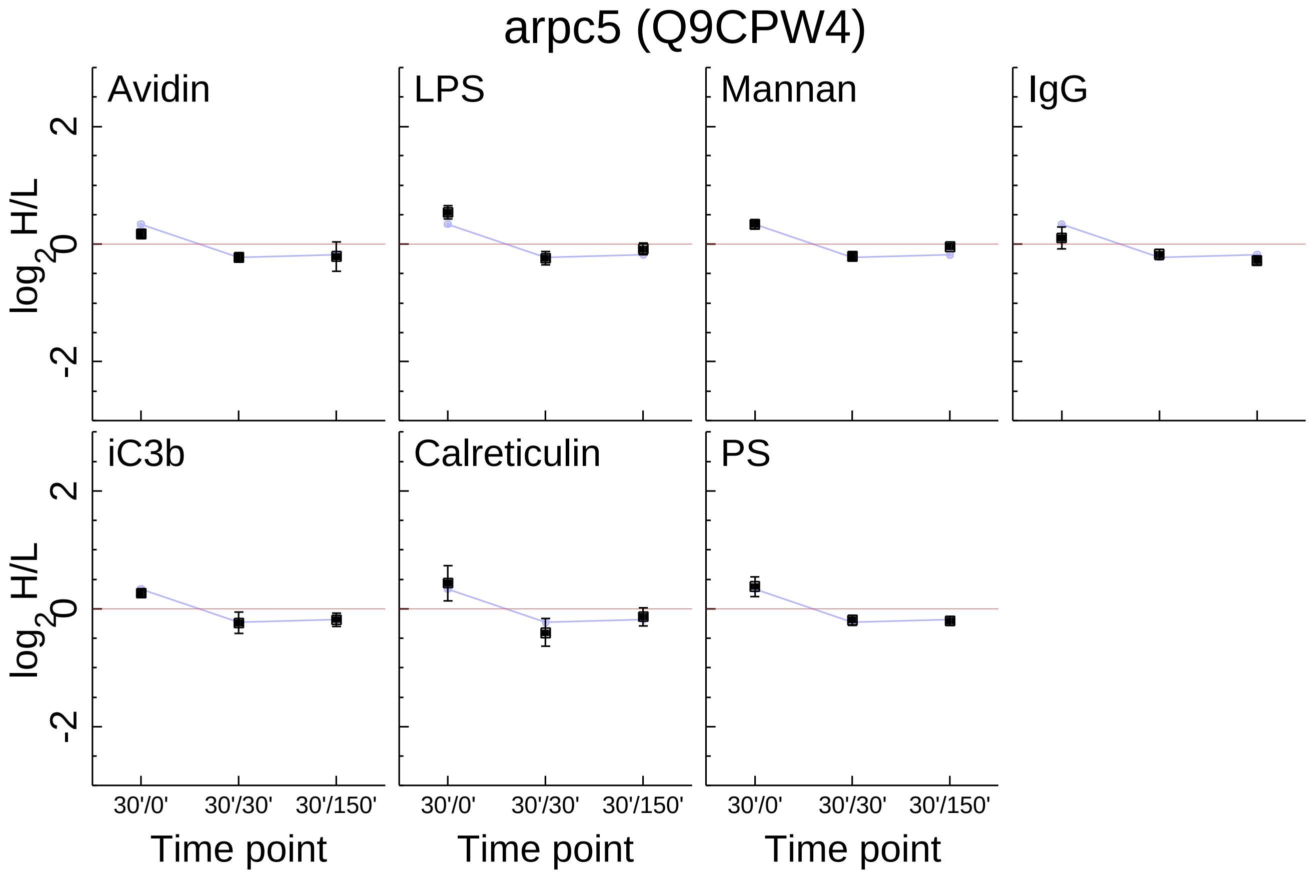

arpc5 (Q9CPW4)
Avidin
LPS
Mannan
IgG
iC3b
Calreticulin
PS
log2 H/L
2
0
-2
30'/0'
30'/30'
30'/150'
Time point

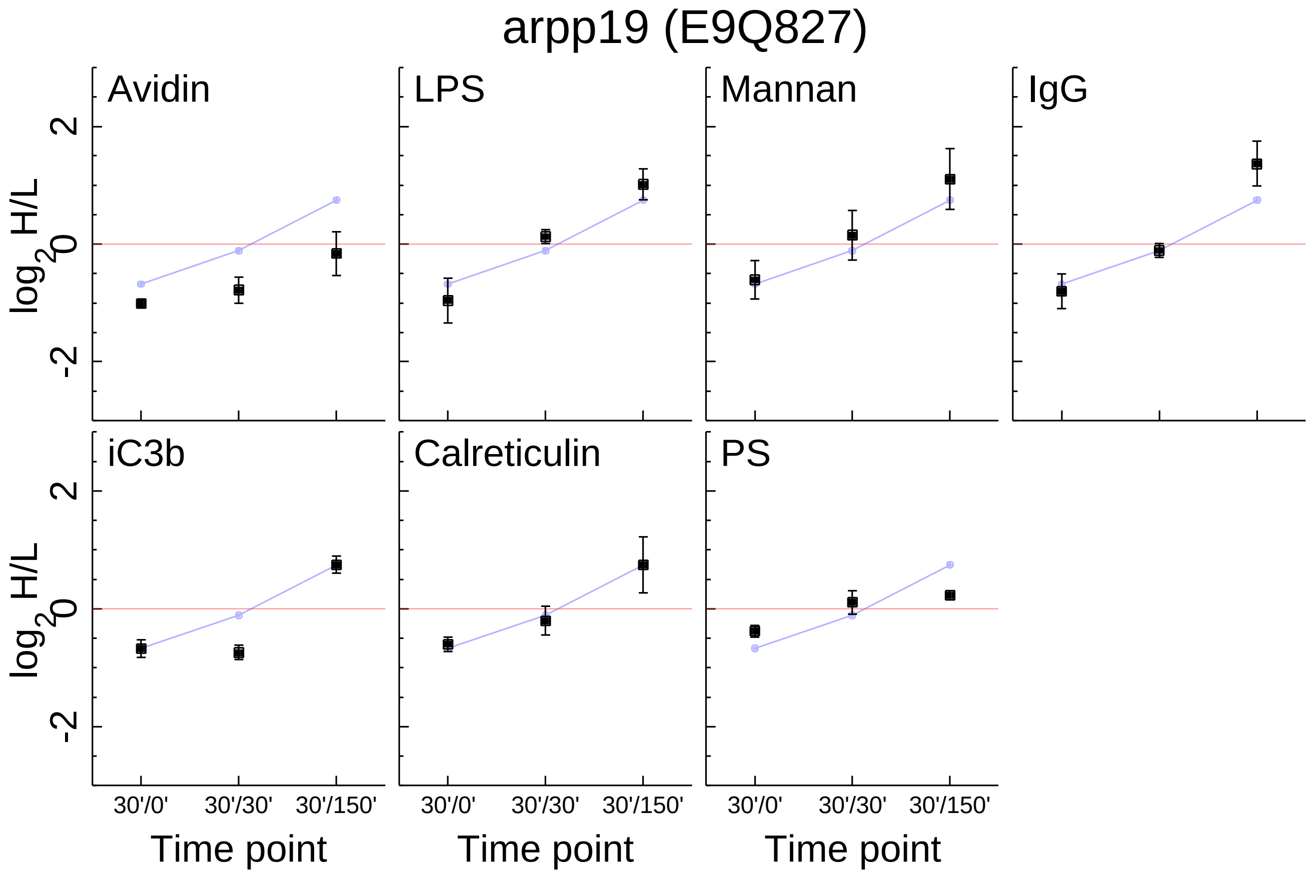

arpp19 (E9Q827)
Avidin
LPS
Mannan
IgG
iC3b
Calreticulin
PS
log2 H/L
2
0
-2
30'/0'
30'/30'
30'/150'
Time point

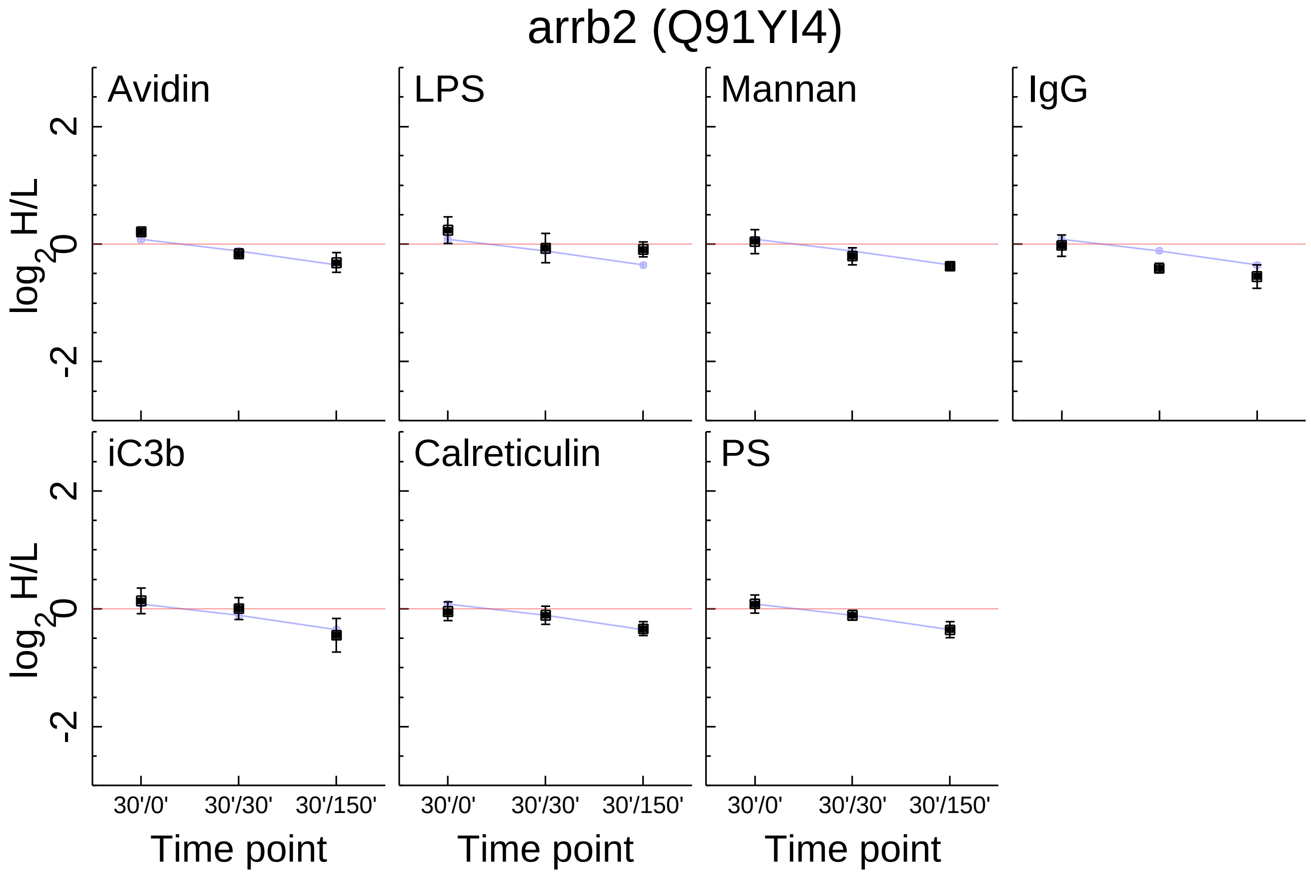

arrb2 (Q91YI4)
Avidin
LPS
Mannan
IgG
iC3b
Calreticulin
PS
log2 H/L
2
0
-2
30'/0'
30'/30'
30'/150'
Time point

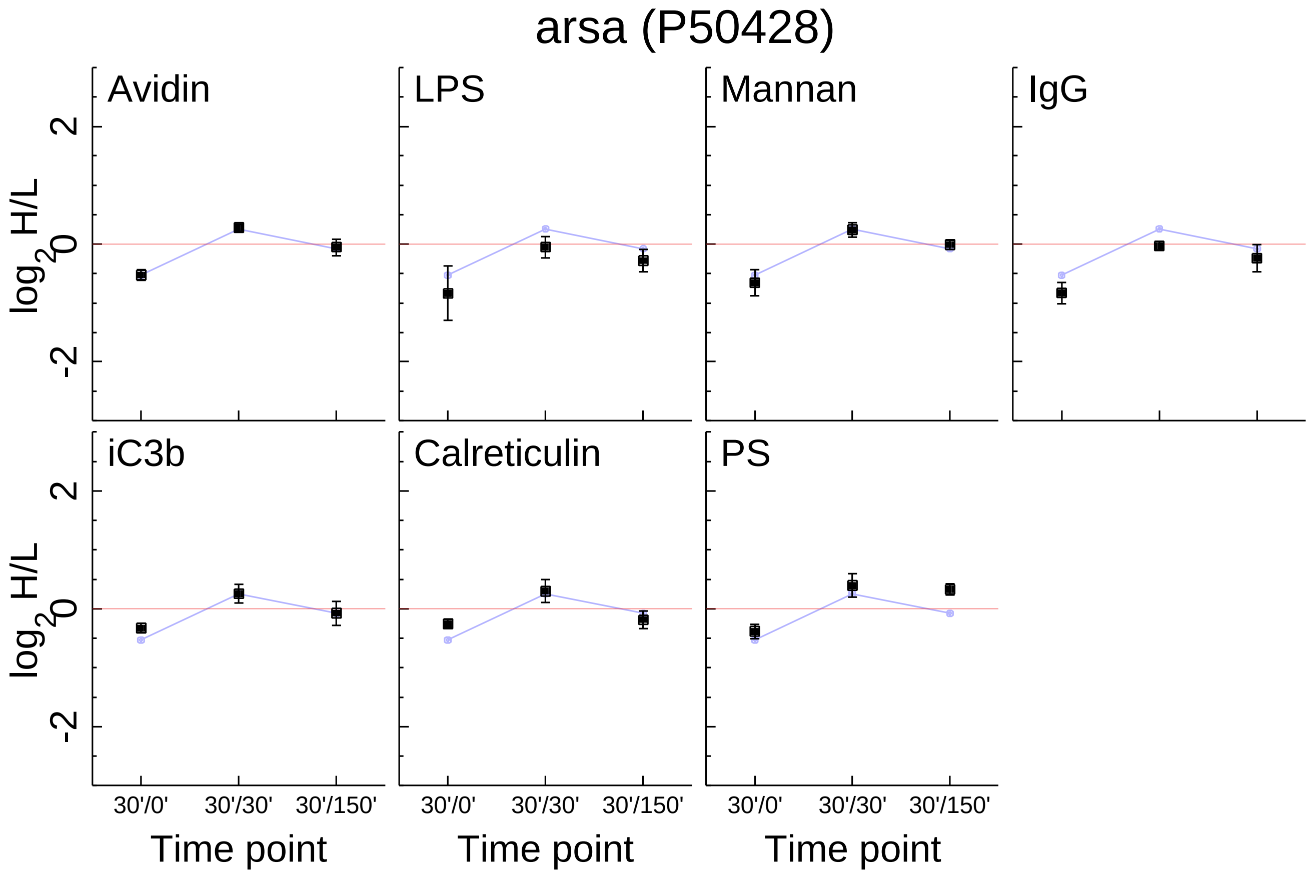

arsa (P50428)
Avidin
LPS
Mannan
IgG
iC3b
Calreticulin
PS
log2 H/L
2
0
-2
30'/0'
30'/30'
30'/150'
Time point

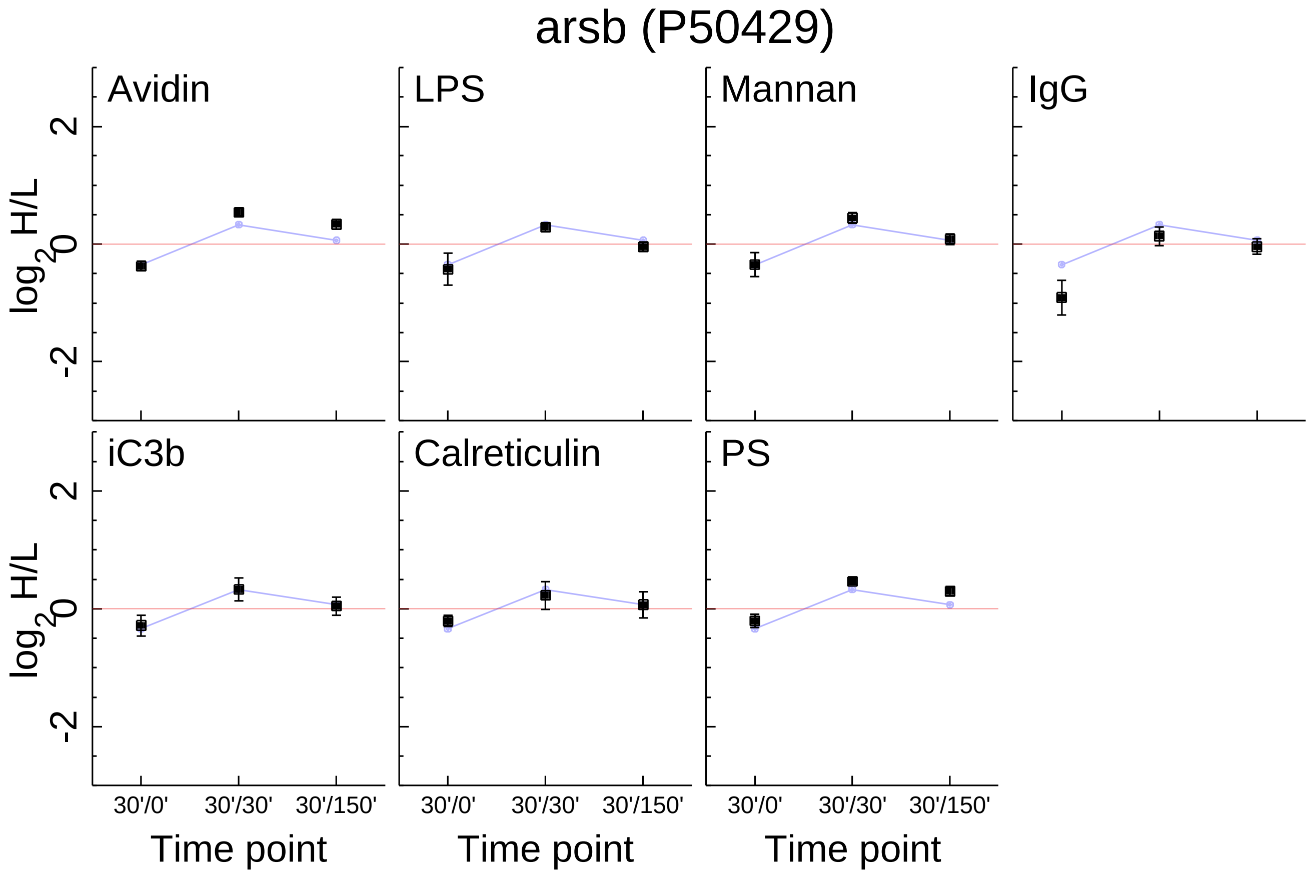

arsb (P50429)
Avidin
LPS
Mannan
IgG
iC3b
Calreticulin
PS
log2 H/L
2
0
-2
30'/0'
30'/30'
30'/150'
Time point

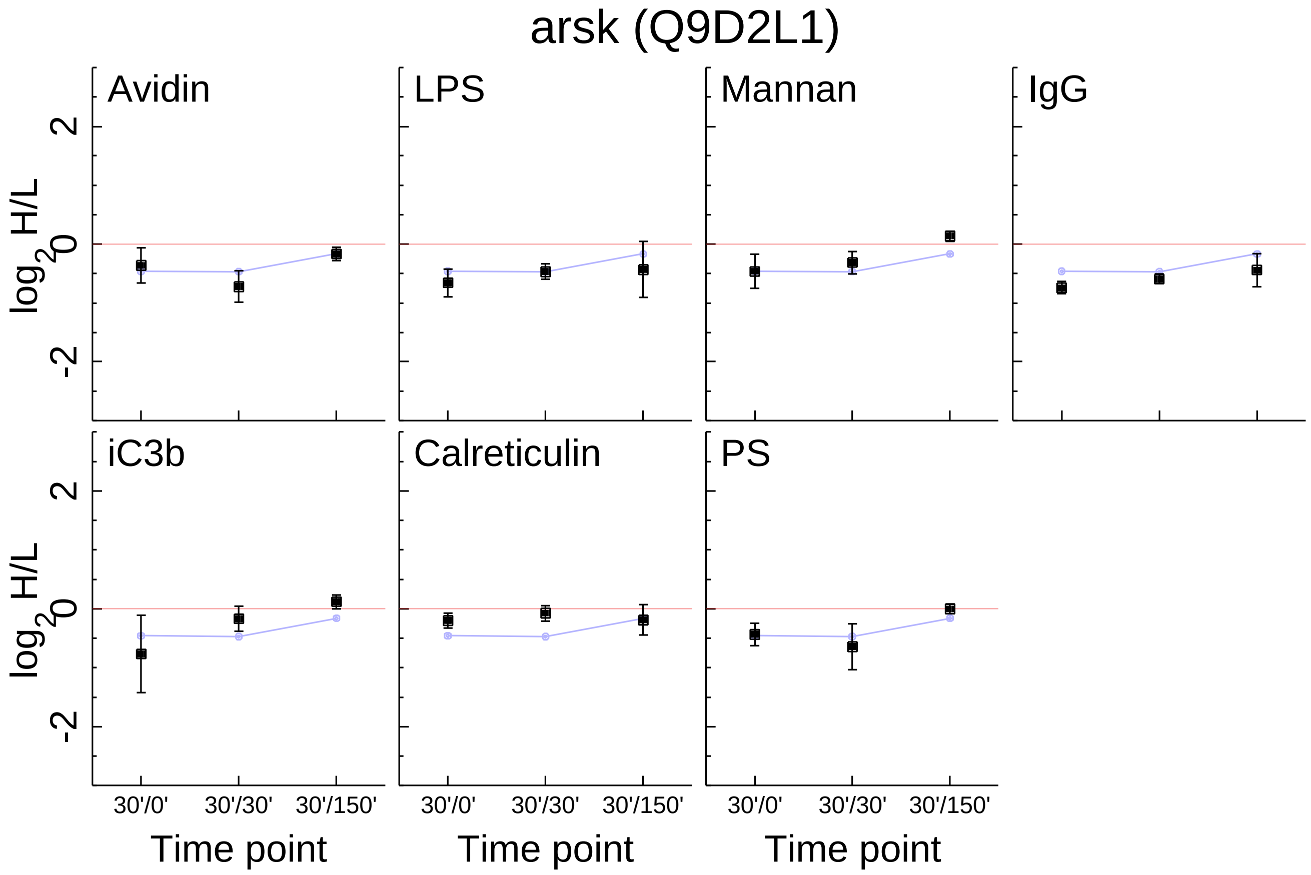
arsk (Q9D2L1)
Avidin
LPS
Mannan
IgG
iC3b
Calreticulin
PS
log2 H/L
2
0
-2
30'/0'
30'/30'
30'/150'
Time point

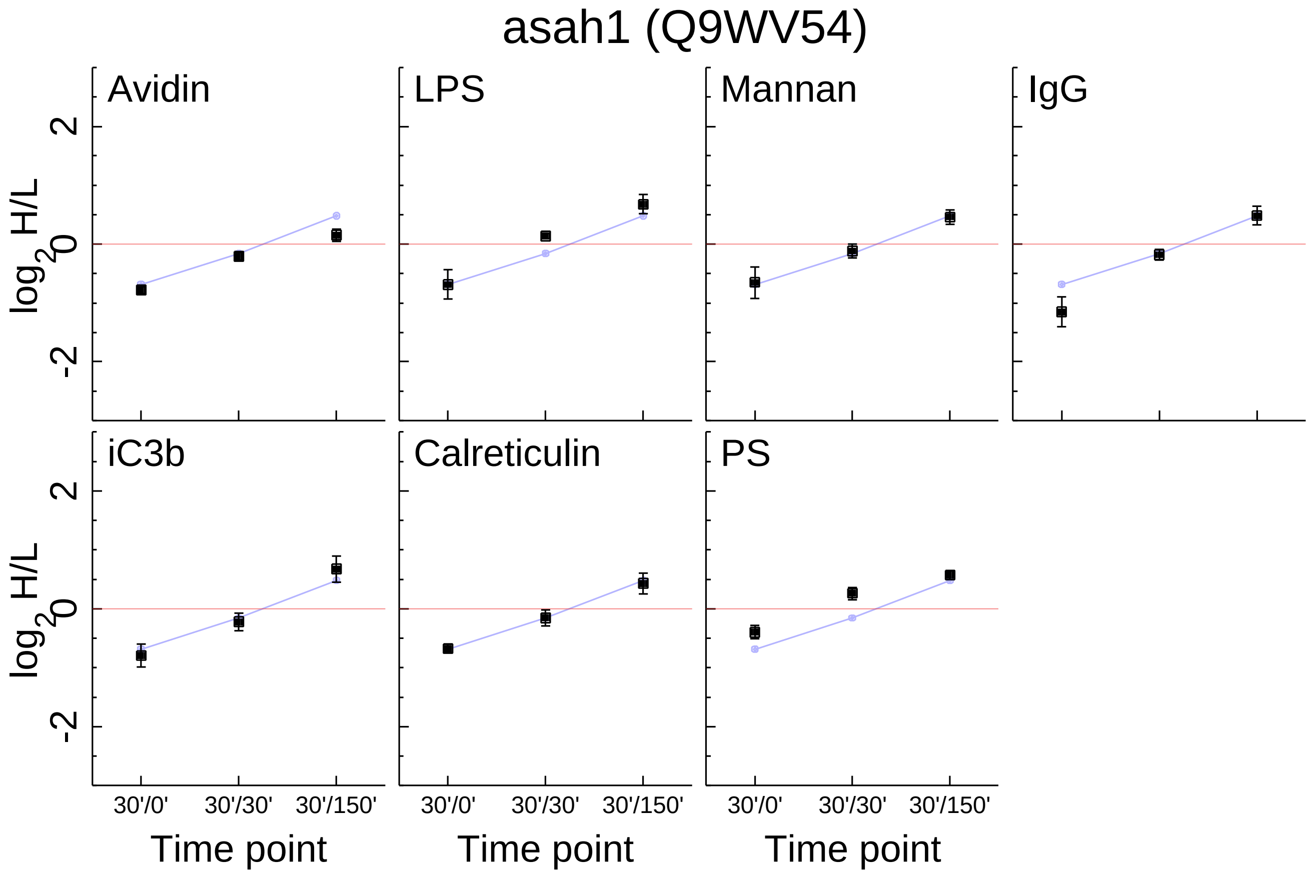
asah1 (Q9WV54)
Avidin
LPS
Mannan
IgG
iC3b
Calreticulin
PS
log2 H/L
2
0
-2
30'/0'
30'/30'
30'/150'
Time point

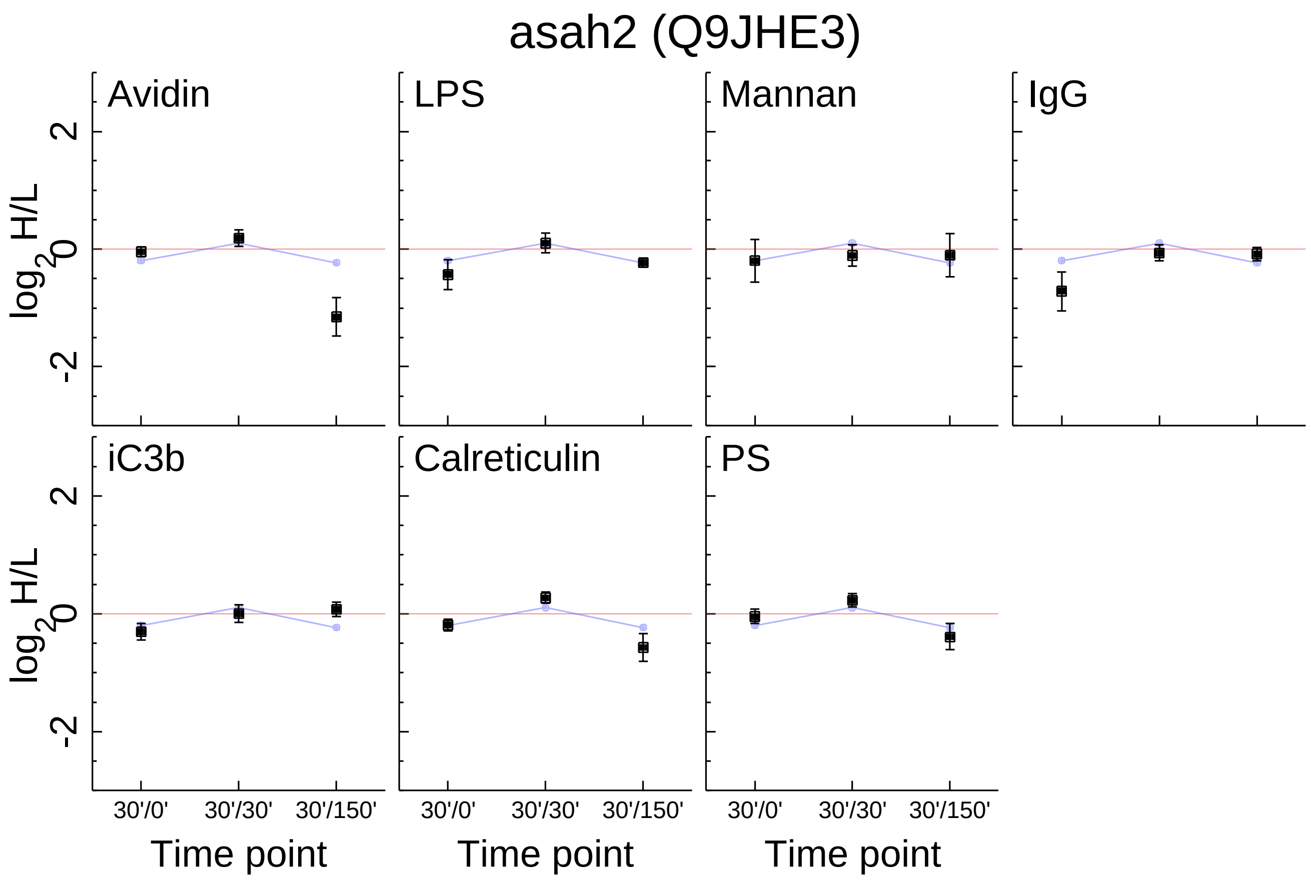

asah2 (Q9JHE3)
Avidin
LPS
Mannan
IgG
iC3b
Calreticulin
PS
log2 H/L
2
0
-2
30'/0'
30'/30'
30'/150'
Time point

asph (Q8BSY0)

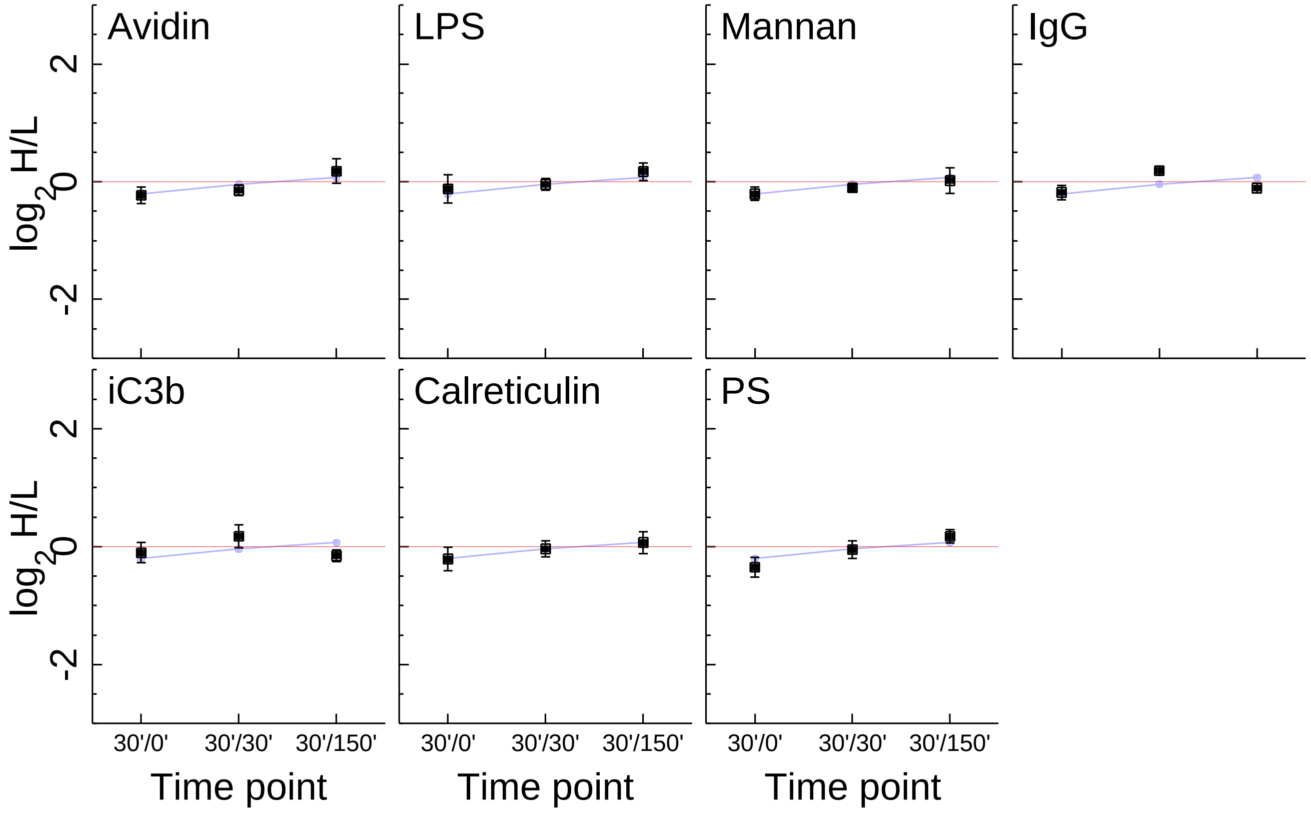

Avidin
LPS
Mannan
IgG
iC3b
Calreticulin
PS
log2 H/L
2
0
-2
30'/0'
30'/30'
30'/150'
Time point

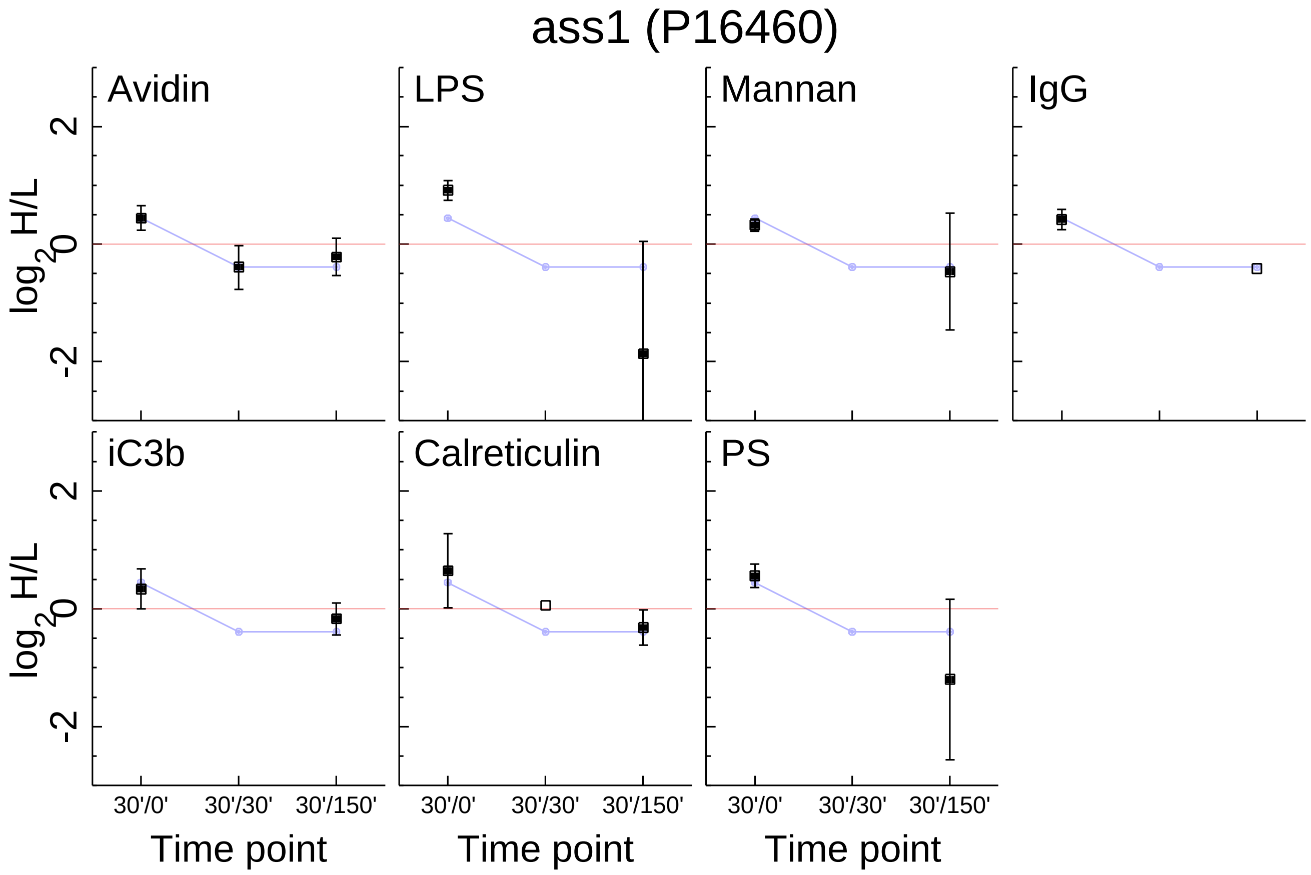
ass1 (P16460)
Avidin
LPS
Mannan
IgG
iC3b
Calreticulin
PS
log2 H/L
2
0
-2
30'/0'
30'/30'
30'/150'
Time point

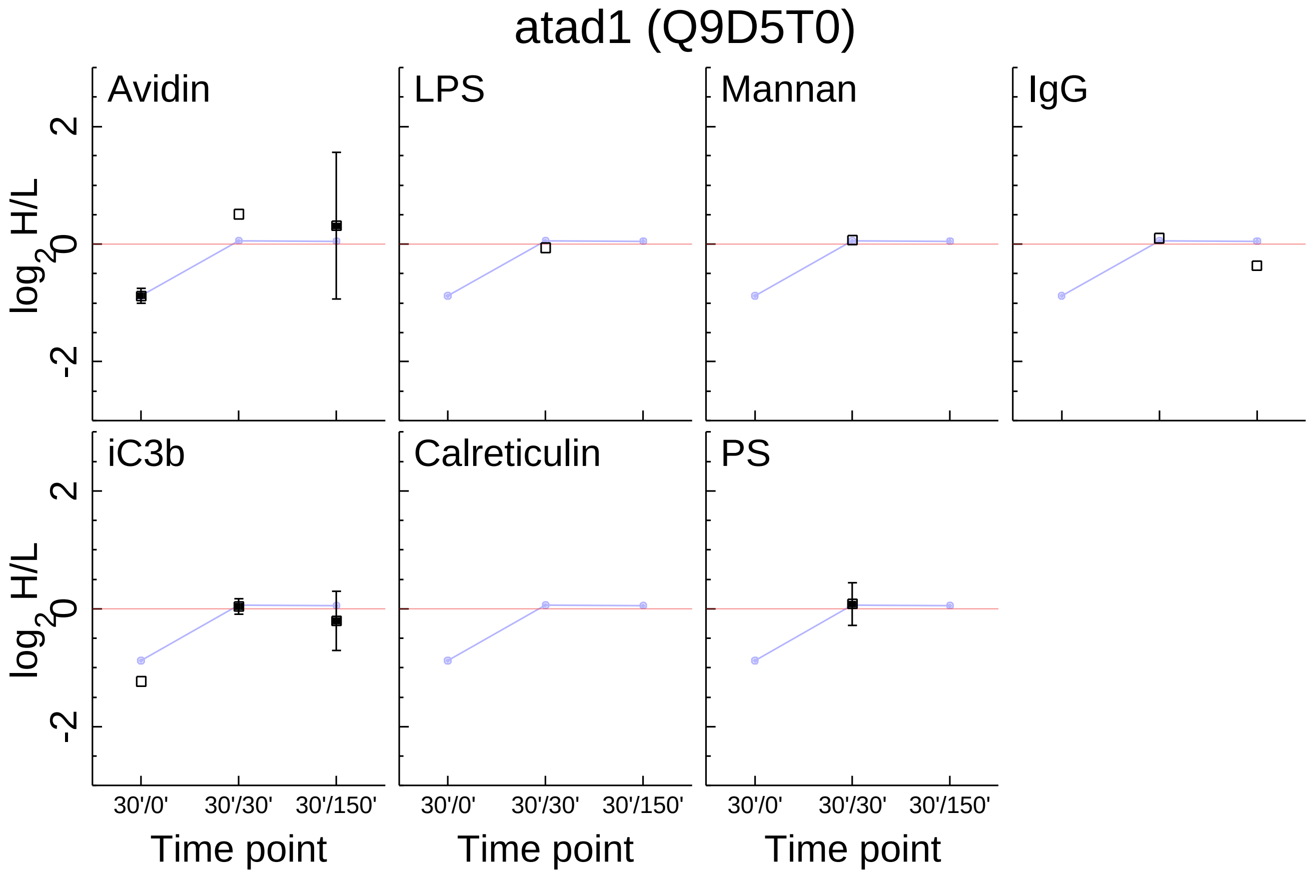
atad1 (Q9D5T0)
Avidin
LPS
Mannan
IgG
iC3b
Calreticulin
PS
log2 H/L
2
0
-2
30'/0'
30'/30'
30'/150'
Time point

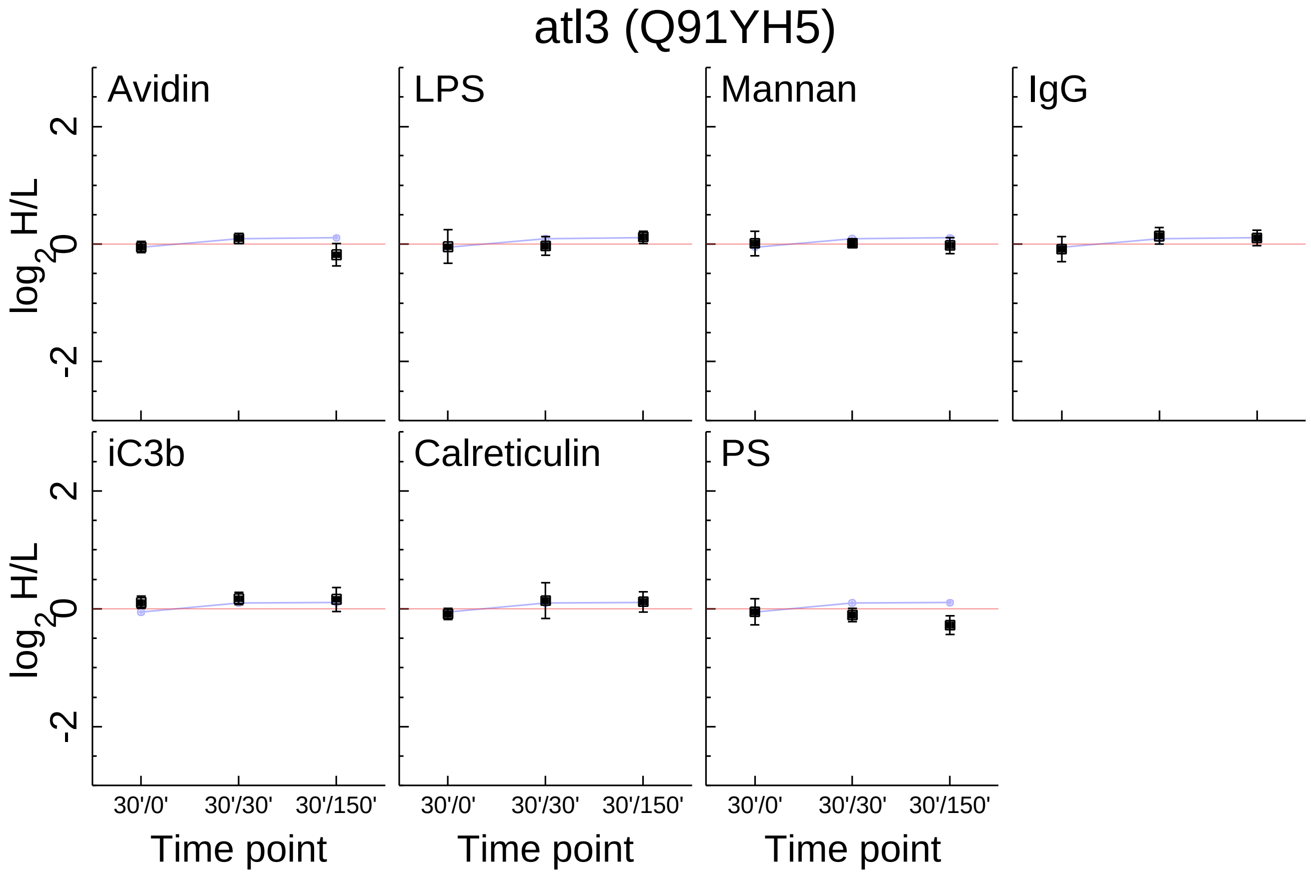

atl3 (Q91YH5)
Avidin
LPS
Mannan
IgG
iC3b
Calreticulin
PS
2
0
-2
log2 H/L
30'/0'
30'/30'
30'/150'
Time point

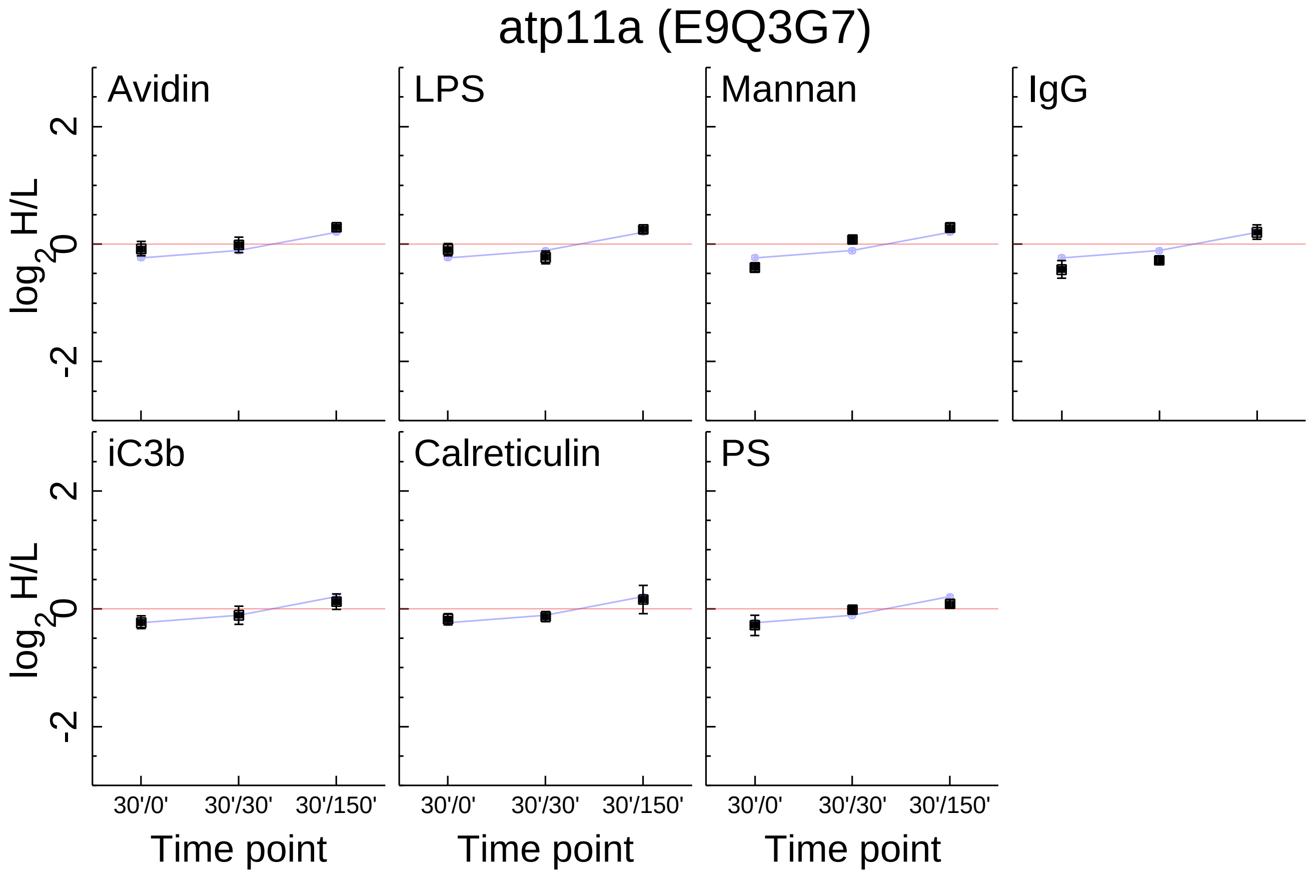

atp11a (E9Q3G7)
Avidin
LPS
Mannan
IgG
iC3b
Calreticulin
PS
log2 H/L
2
0
-2
30'/0'
30'/30'
30'/150'
Time point

atp11c (F6Q8D3)

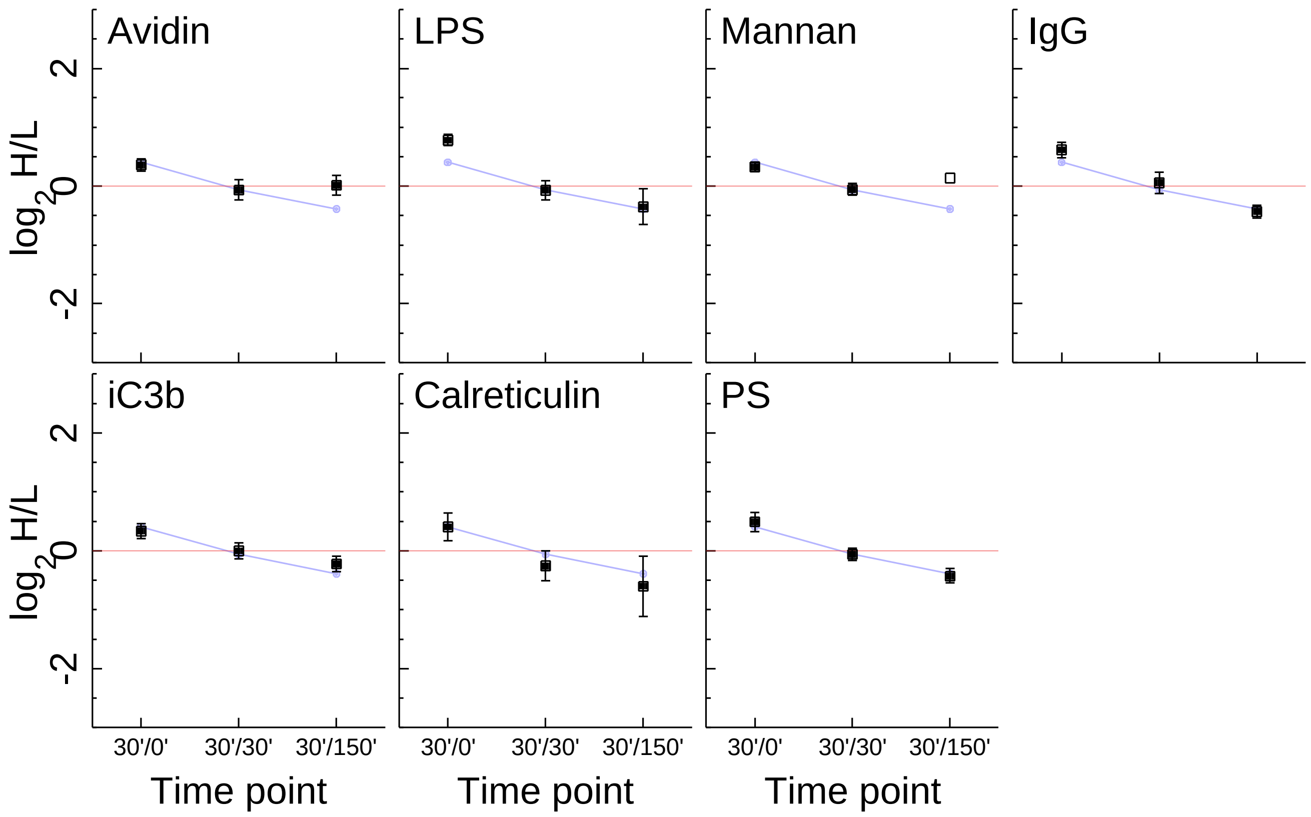

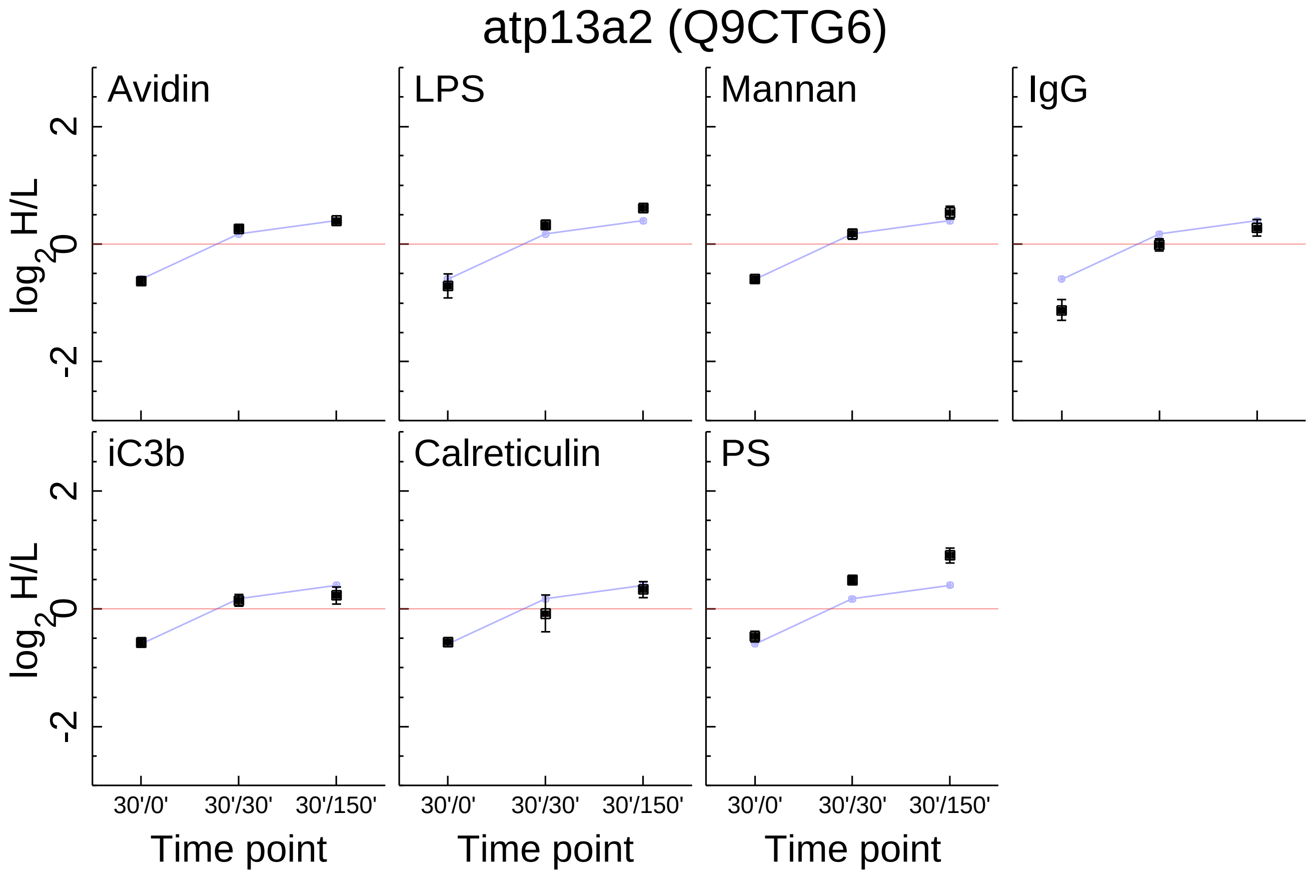
atp13a2 (Q9CTG6)
Avidin
LPS
Mannan
IgG
iC3b
Calreticulin
PS
log2 H/L
2
0
-2
30'/0'
30'/30'
30'/150'
Time point

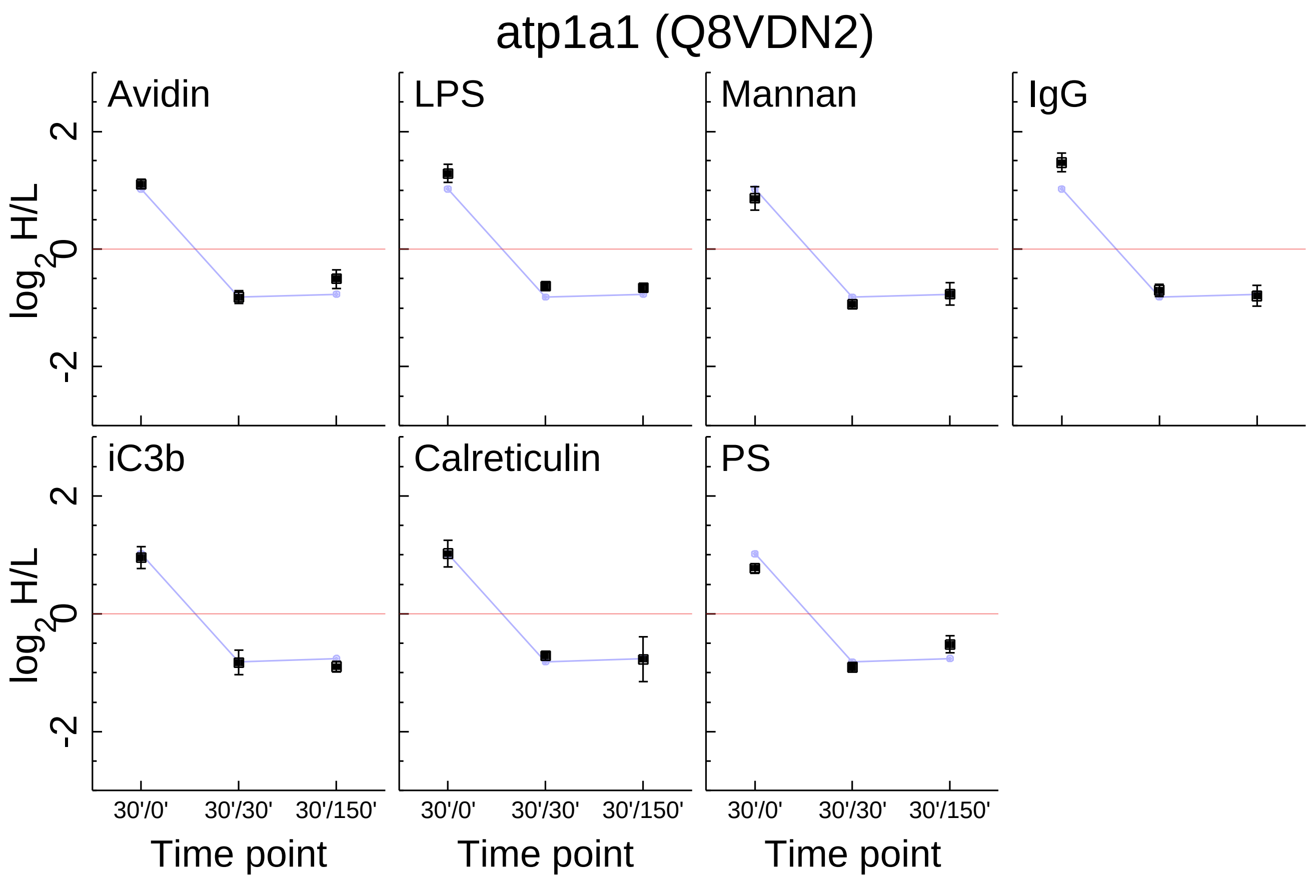
atp1a1 (Q8VDN2)
Avidin
LPS
Mannan
IgG
iC3b
Calreticulin
PS
$\log_2$ H/L
2
0
-2
30'/0'
30'/30'
30'/150'
Time point

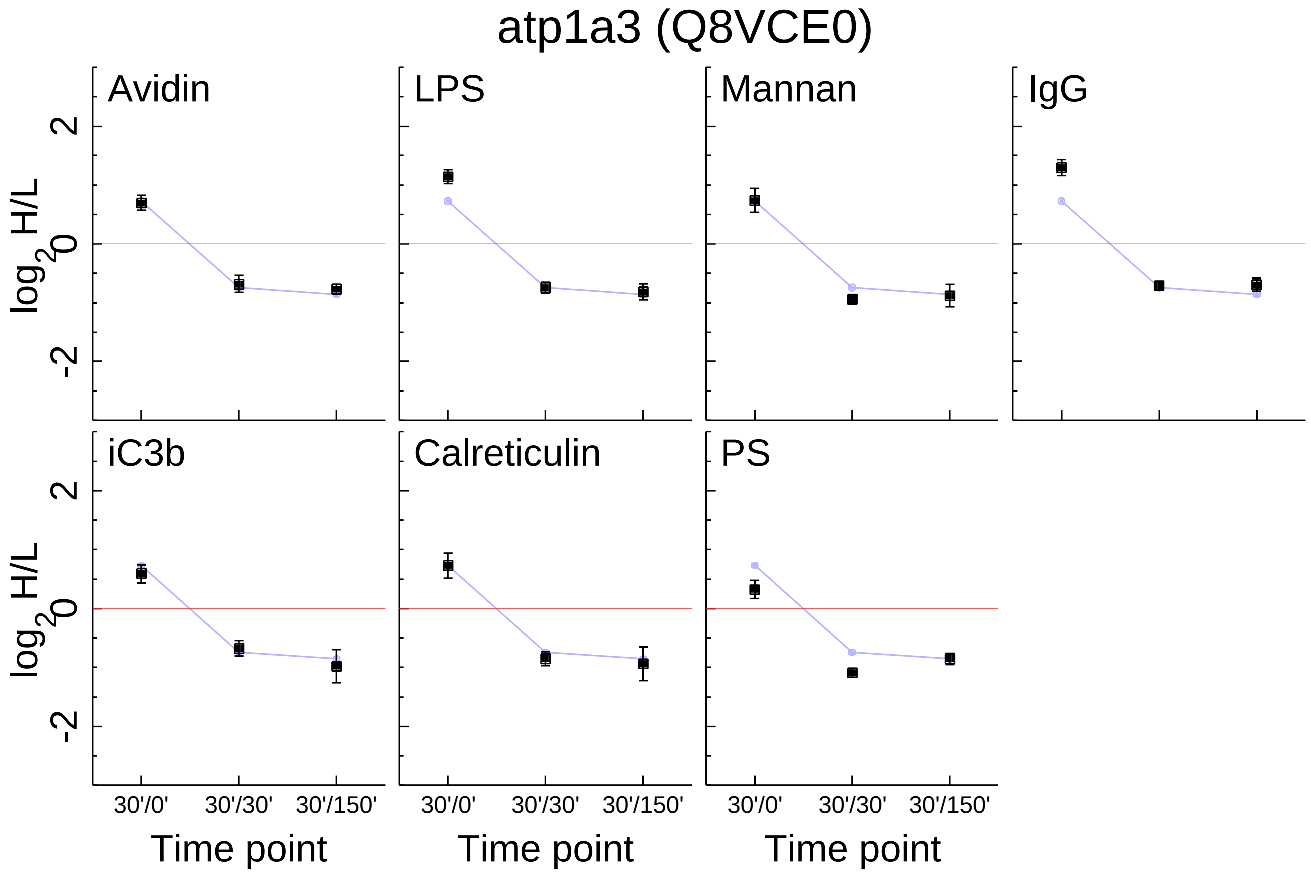

atp1a3 (Q8VCE0)
Avidin
LPS
Mannan
IgG
iC3b
Calreticulin
PS
log2 H/L
2
0
-2
30'/0'
30'/30'
30'/150'
Time point

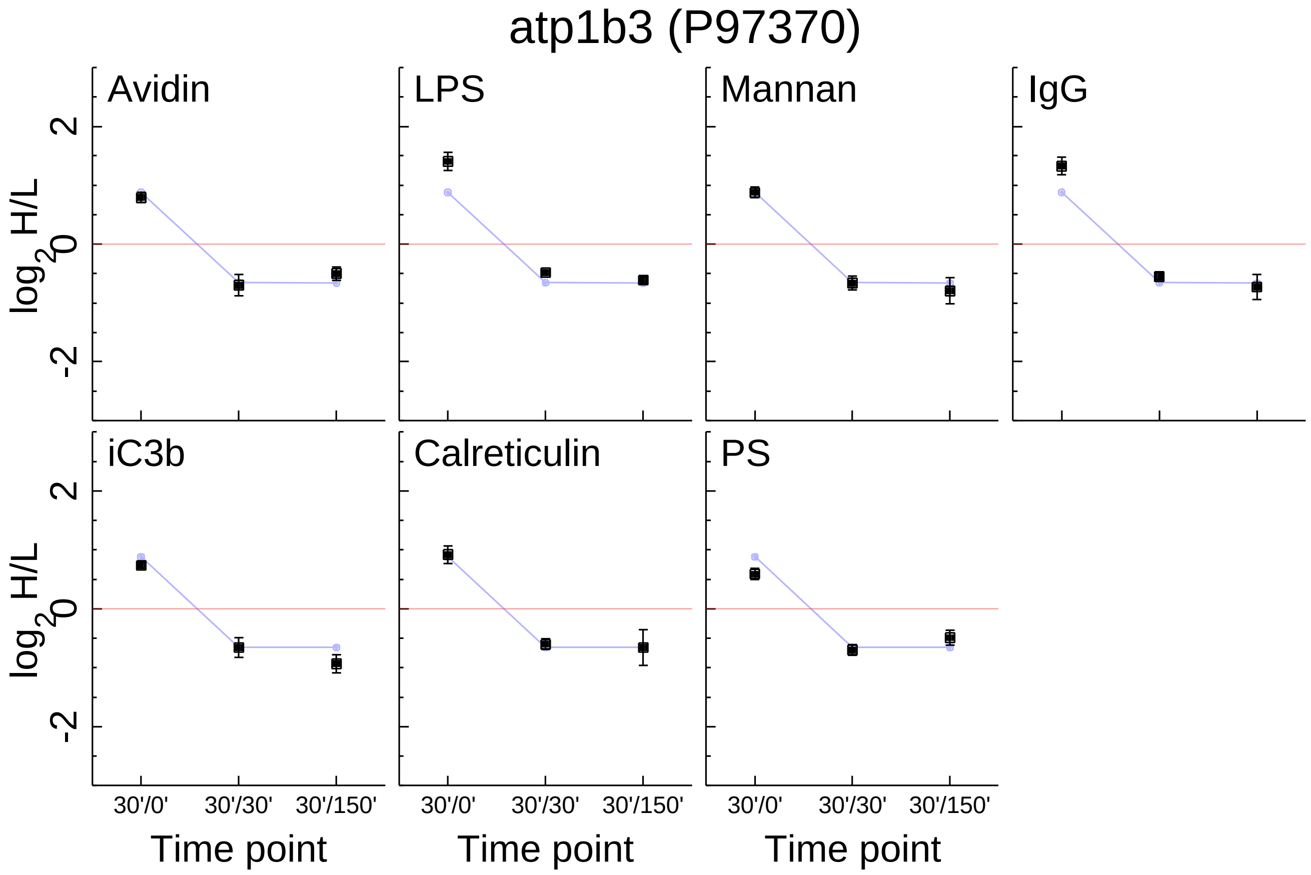

atp1b3 (P97370)
Avidin
LPS
Mannan
IgG
iC3b
Calreticulin
PS
log2 H/L
2
0
-2
30'/0'
30'/30'
30'/150'
Time point

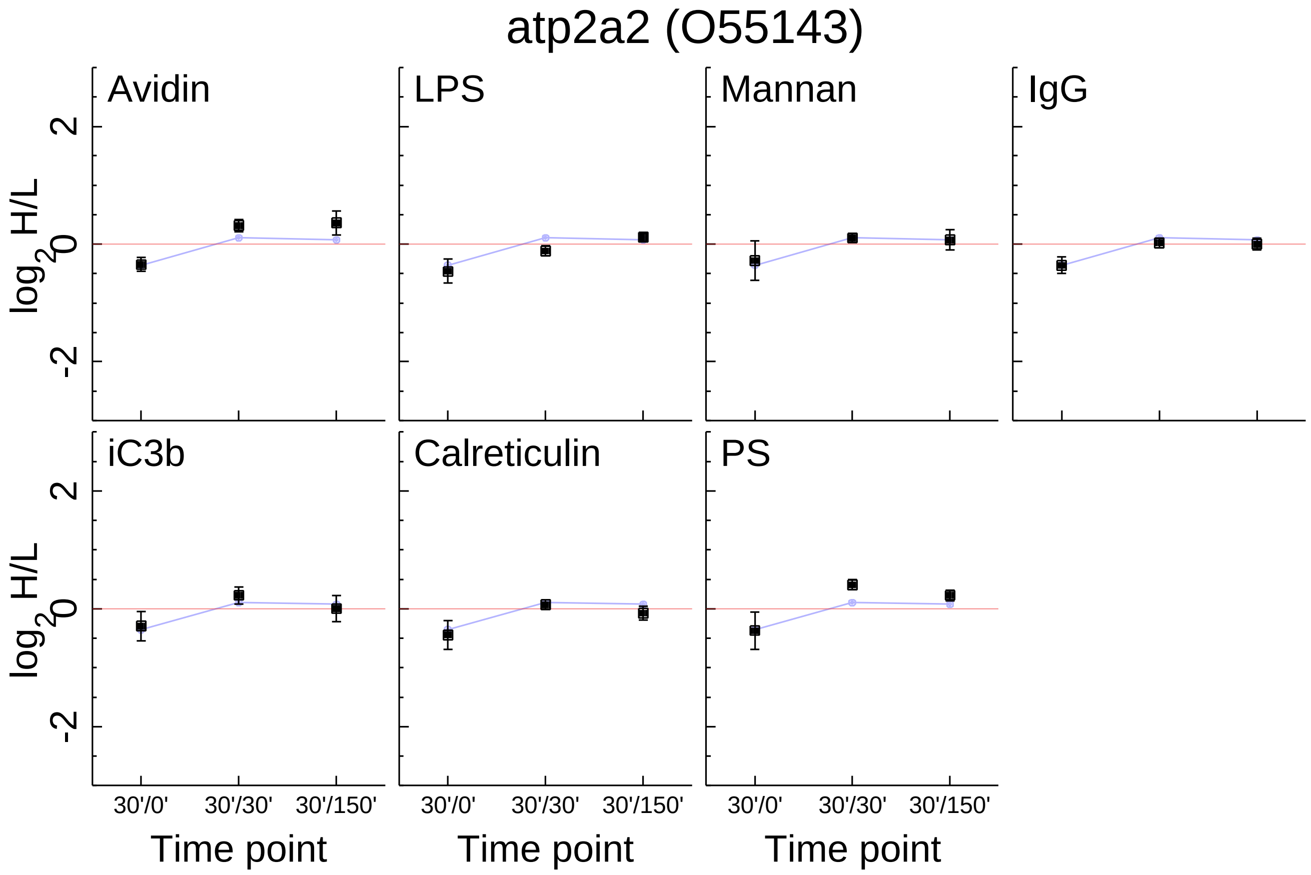
atp2a2 (O55143)
Avidin
LPS
Mannan
IgG
iC3b
Calreticulin
PS
log2 H/L
2
0
-2
30'/0'
30'/30'
30'/150'
Time point

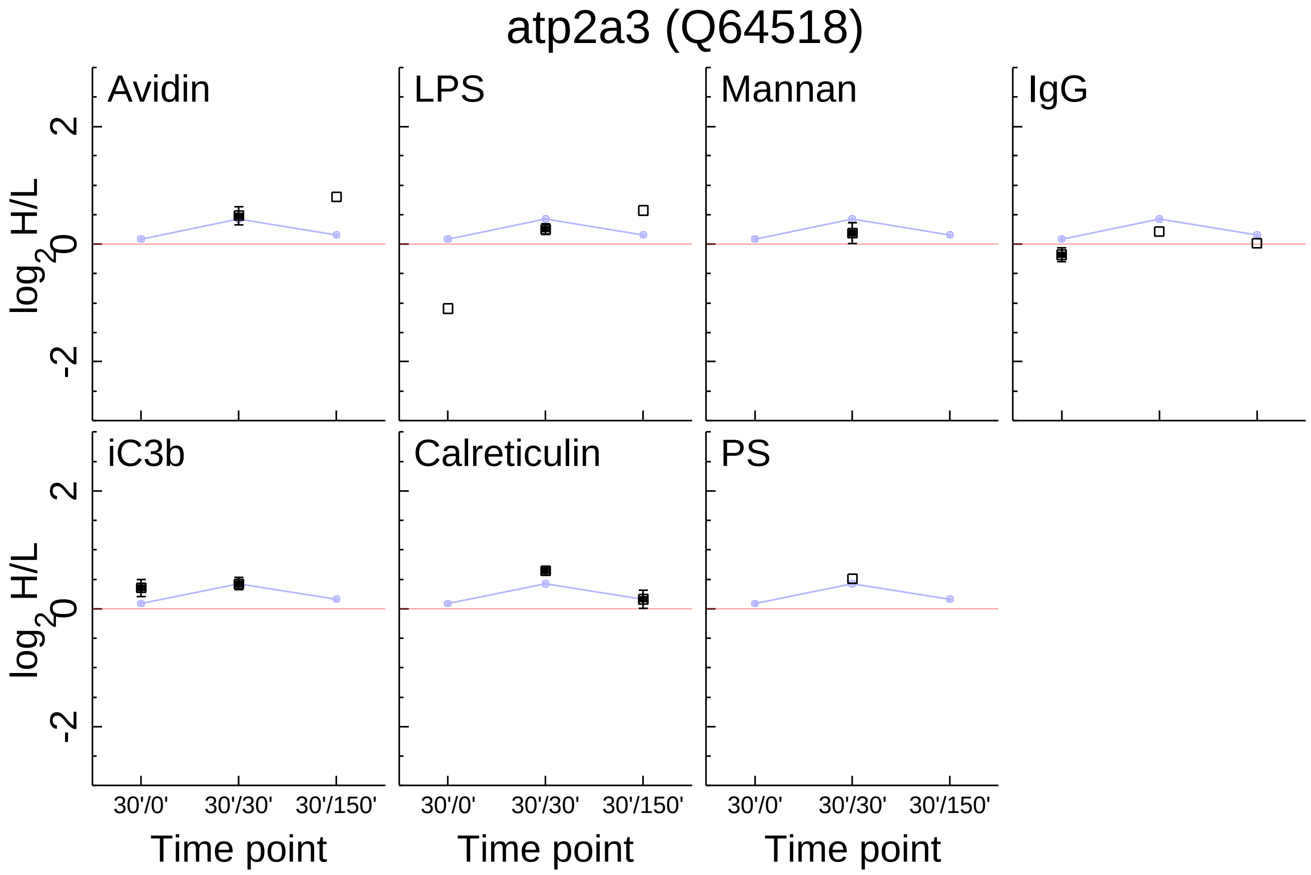
atp2a3 (Q64518)
Avidin
LPS
Mannan
IgG
iC3b
Calreticulin
PS
log2 H/L
2
0
-2
30'/0'
30'/30'
30'/150'
Time point

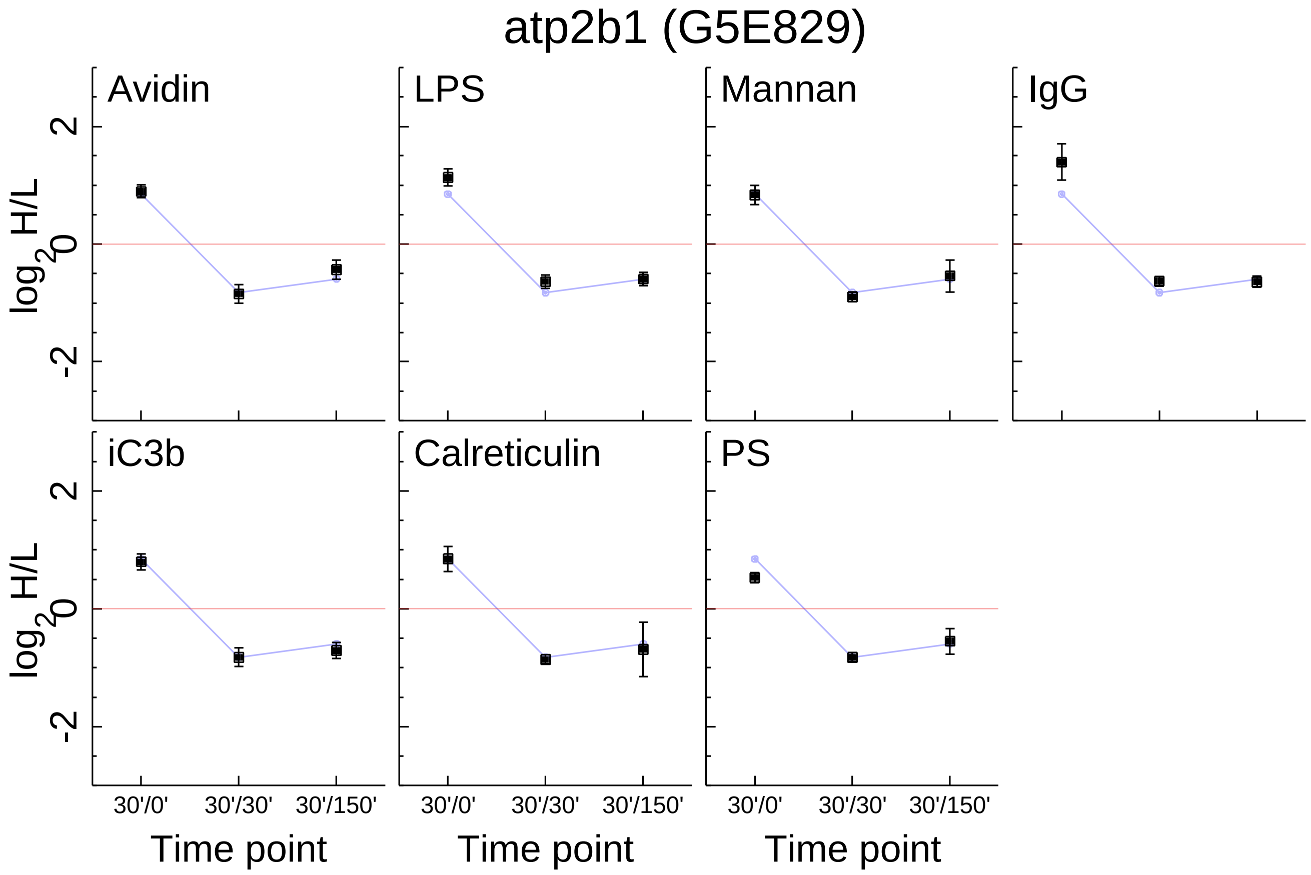
atp2b1 (G5E829)
Avidin
LPS
Mannan
IgG
iC3b
Calreticulin
PS
log2 H/L
2
0
-2
30'/0'
30'/30'
30'/150'
Time point

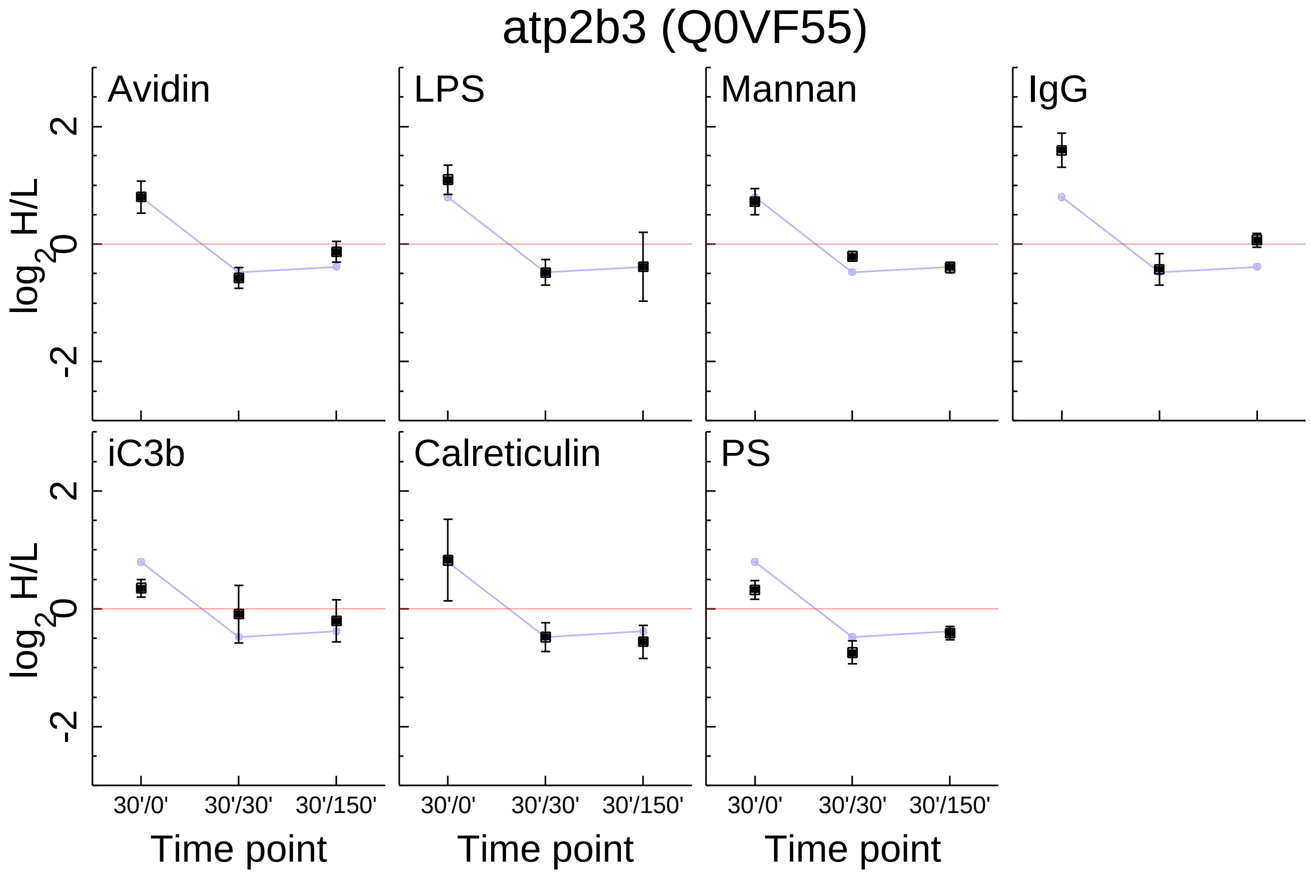

atp2b3 (Q0VF55)
Avidin
LPS
Mannan
IgG
iC3b
Calreticulin
PS
log2 H/L
2
0
-2
30'/0'
30'/30'
30'/150'
Time point

atp5a1 (Q03265)

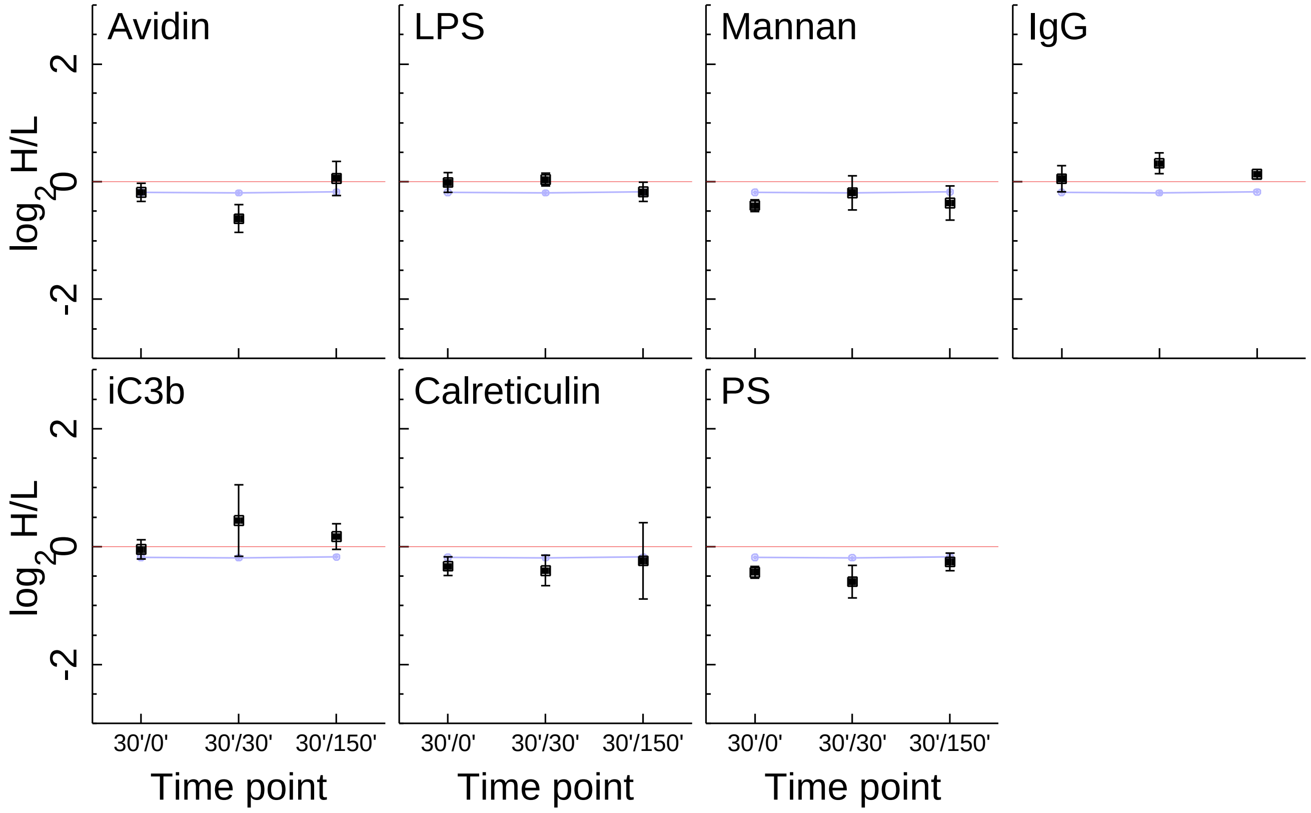

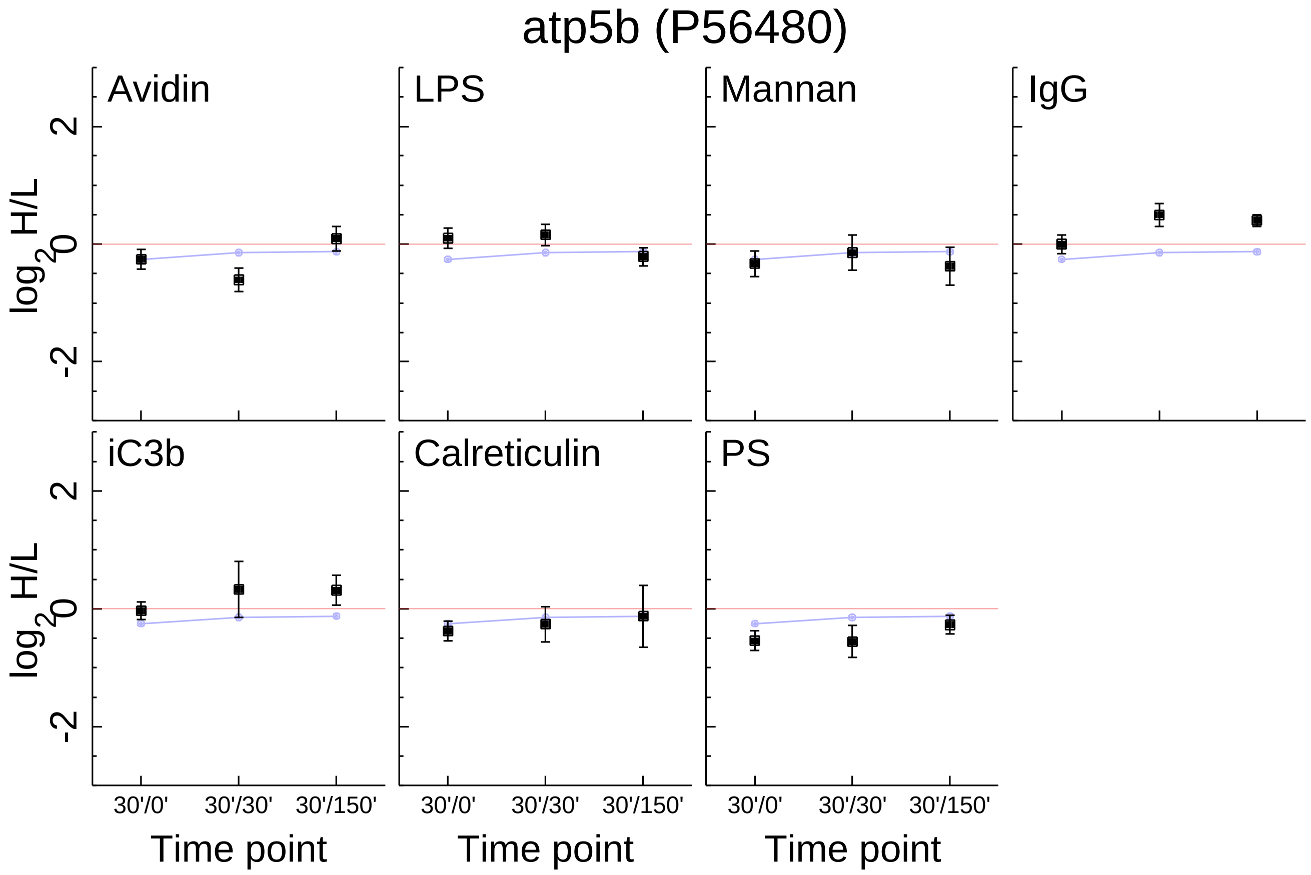

atp5b (P56480)
Avidin
LPS
Mannan
IgG
iC3b
Calreticulin
PS
log2 H/L
2
0
-2
30'/0'
30'/30'
30'/150'
Time point

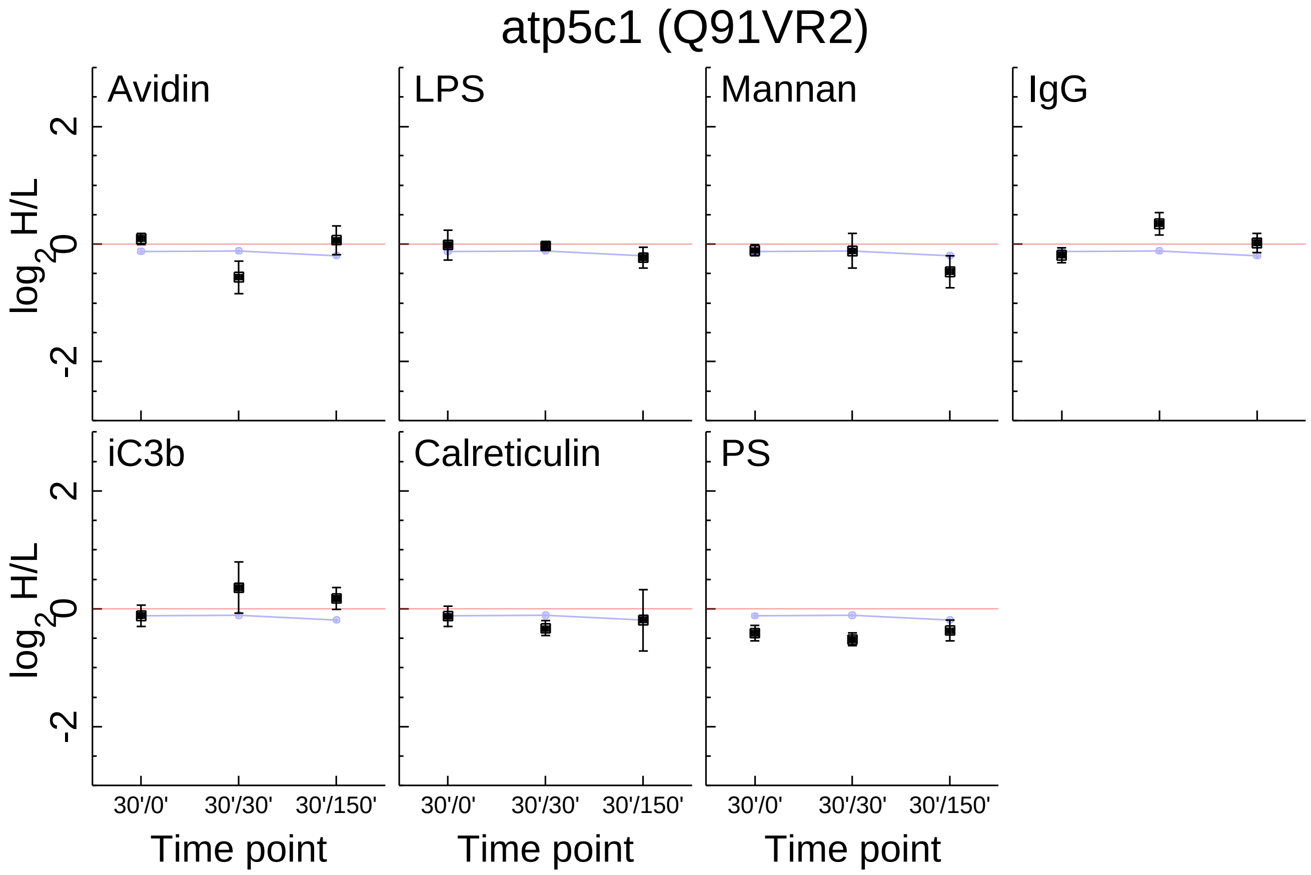

atp5c1 (Q91VR2)
Avidin
LPS
Mannan
IgG
iC3b
Calreticulin
PS
log2 H/L
2
0
-2
30'/0'
30'/30'
30'/150'
Time point

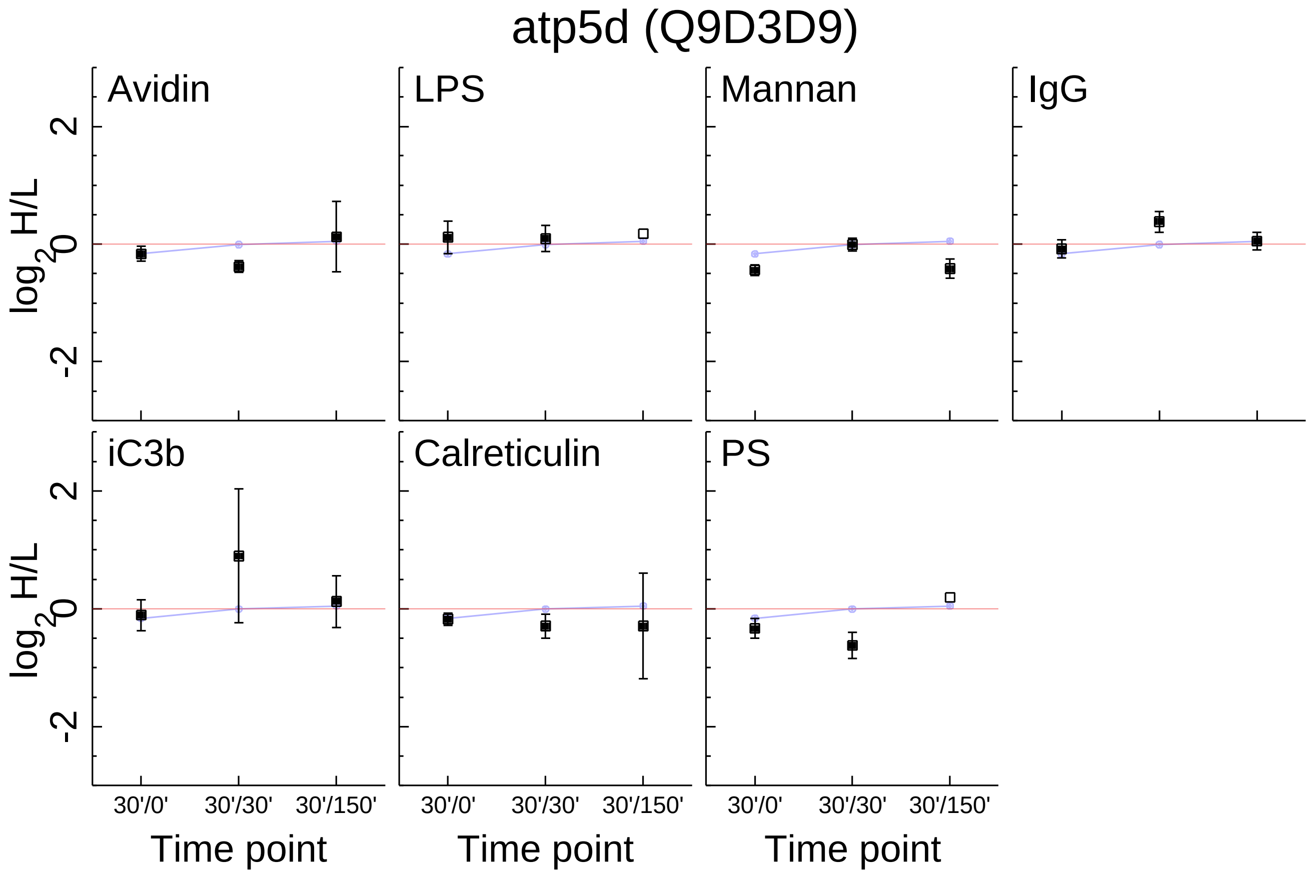
atp5d (Q9D3D9)
Avidin
LPS
Mannan
IgG
iC3b
Calreticulin
PS
log2 H/L
2
0
-2
30'/0'
30'/30'
30'/150'
Time point

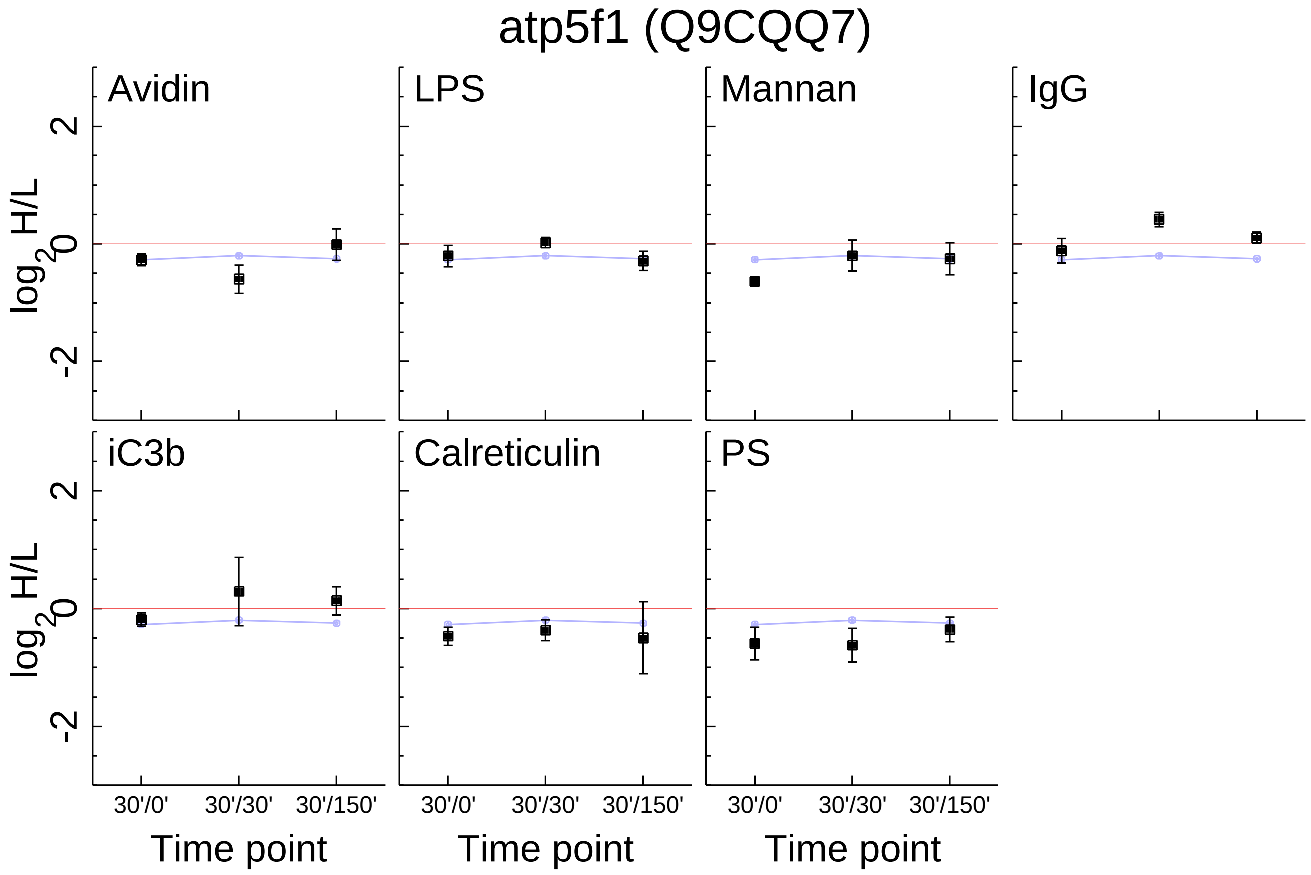
atp5f1 (Q9CQQ7)
Avidin
LPS
Mannan
IgG
iC3b
Calreticulin
PS
log2 H/L
2
0
-2
30'/0'
30'/30'
30'/150'
Time point

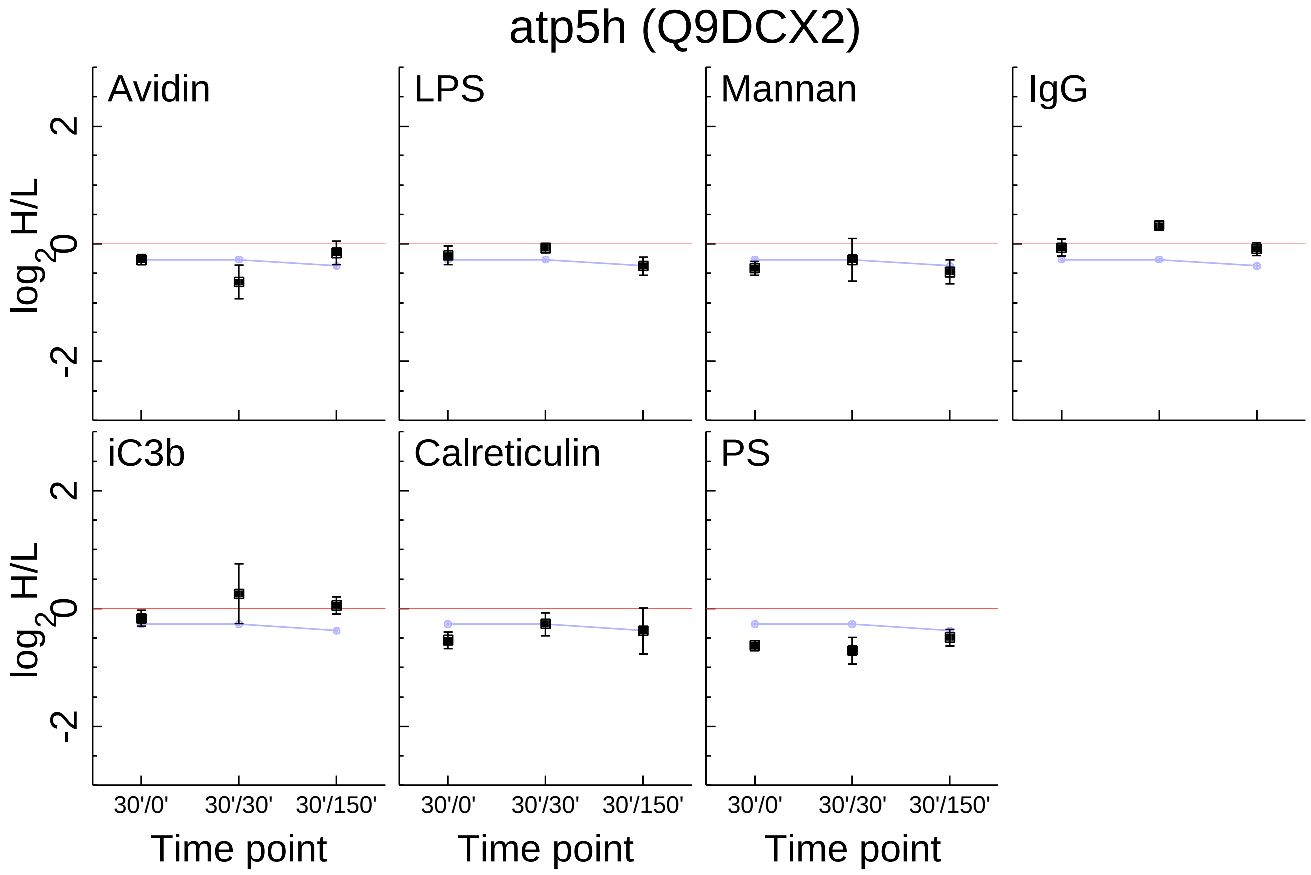
atp5h (Q9DCX2)
Avidin
LPS
Mannan
IgG
iC3b
Calreticulin
PS
log2 H/L
2
0
-2
30'/0'
30'/30'
30'/150'
Time point

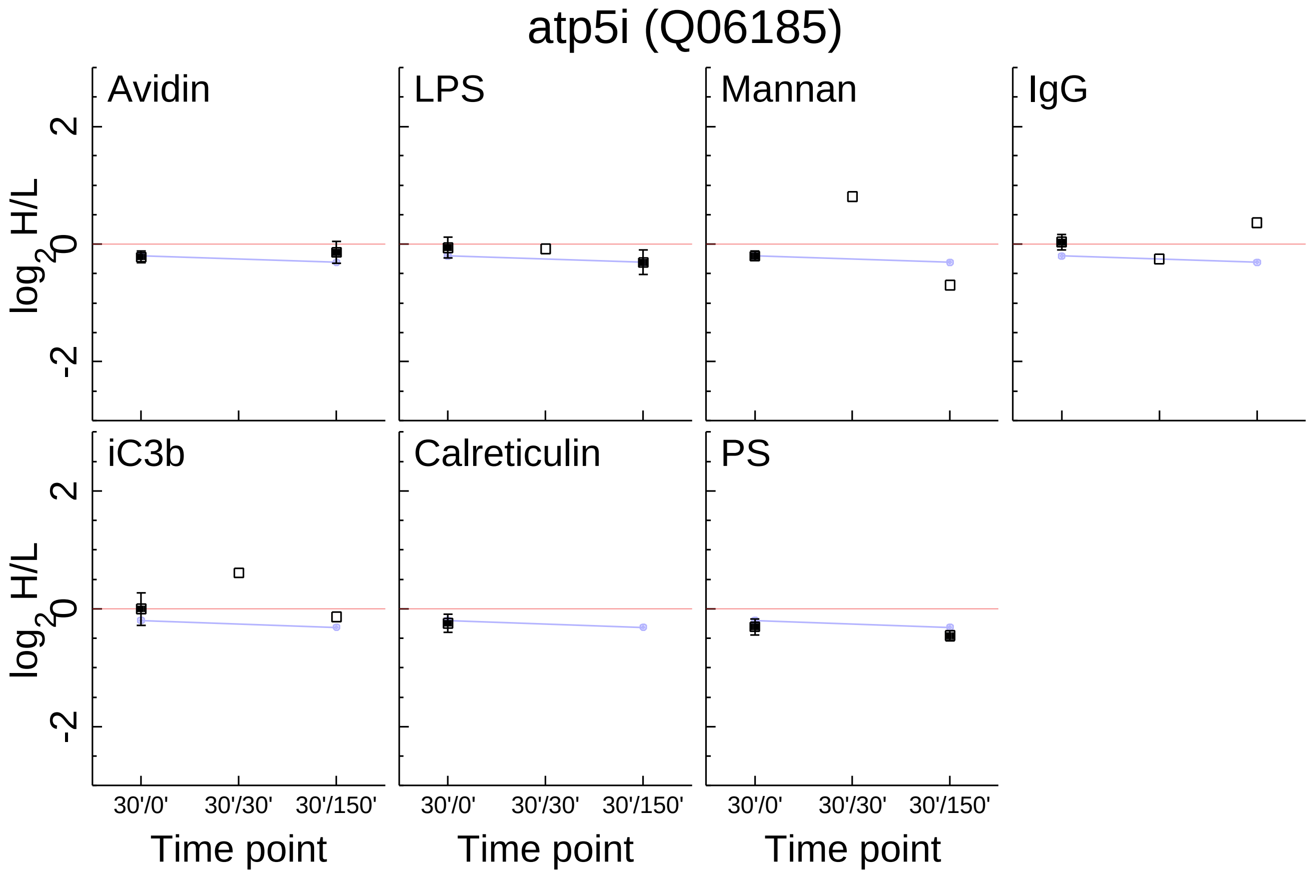
atp5i (Q06185)
Avidin
LPS
Mannan
IgG
iC3b
Calreticulin
PS
log2 H/L
2
0
-2
30'/0'
30'/30'
30'/150'
Time point

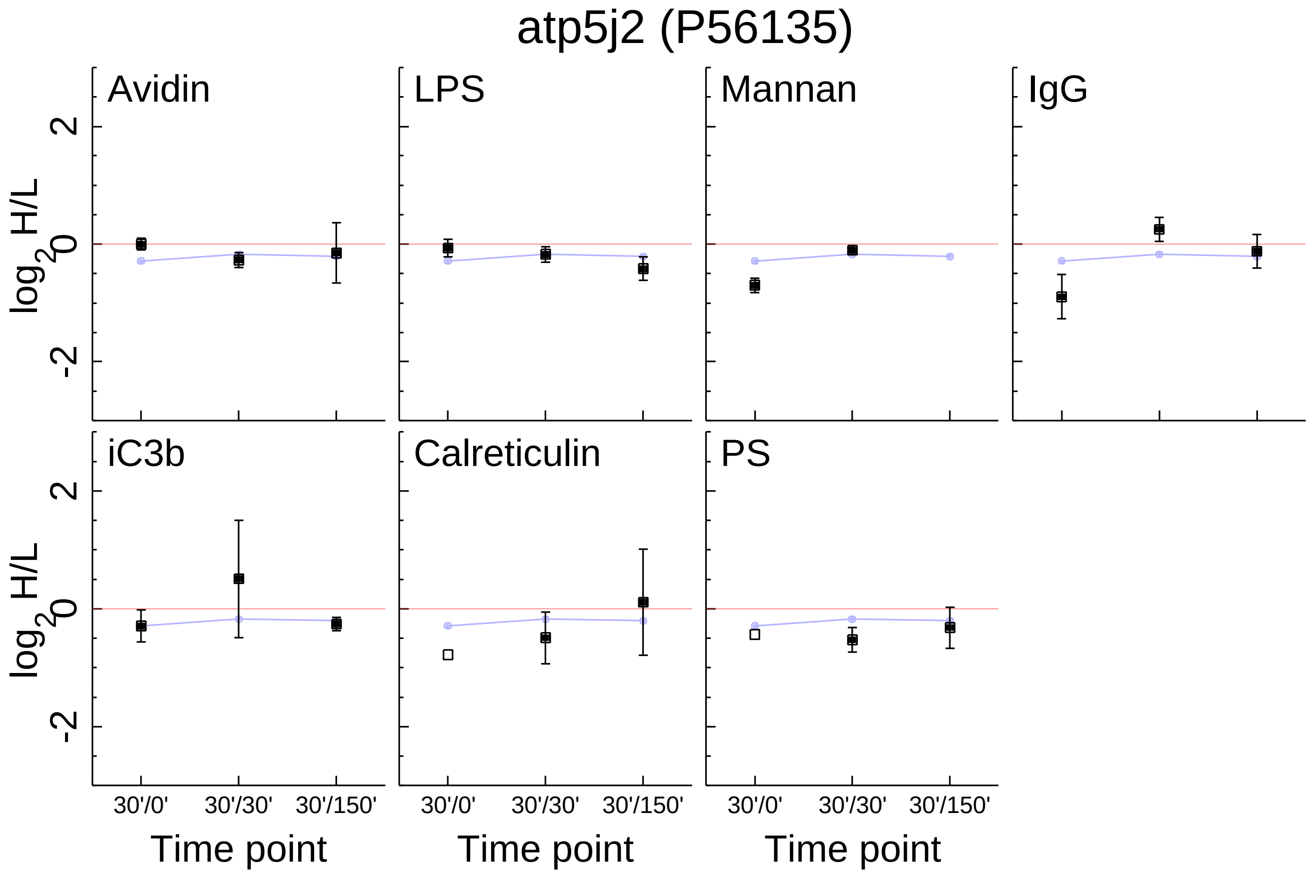

atp5j2 (P56135)
Avidin
LPS
Mannan
IgG
iC3b
Calreticulin
PS
log2 H/L
2
0
-2
30'/0'
30'/30'
30'/150'
Time point

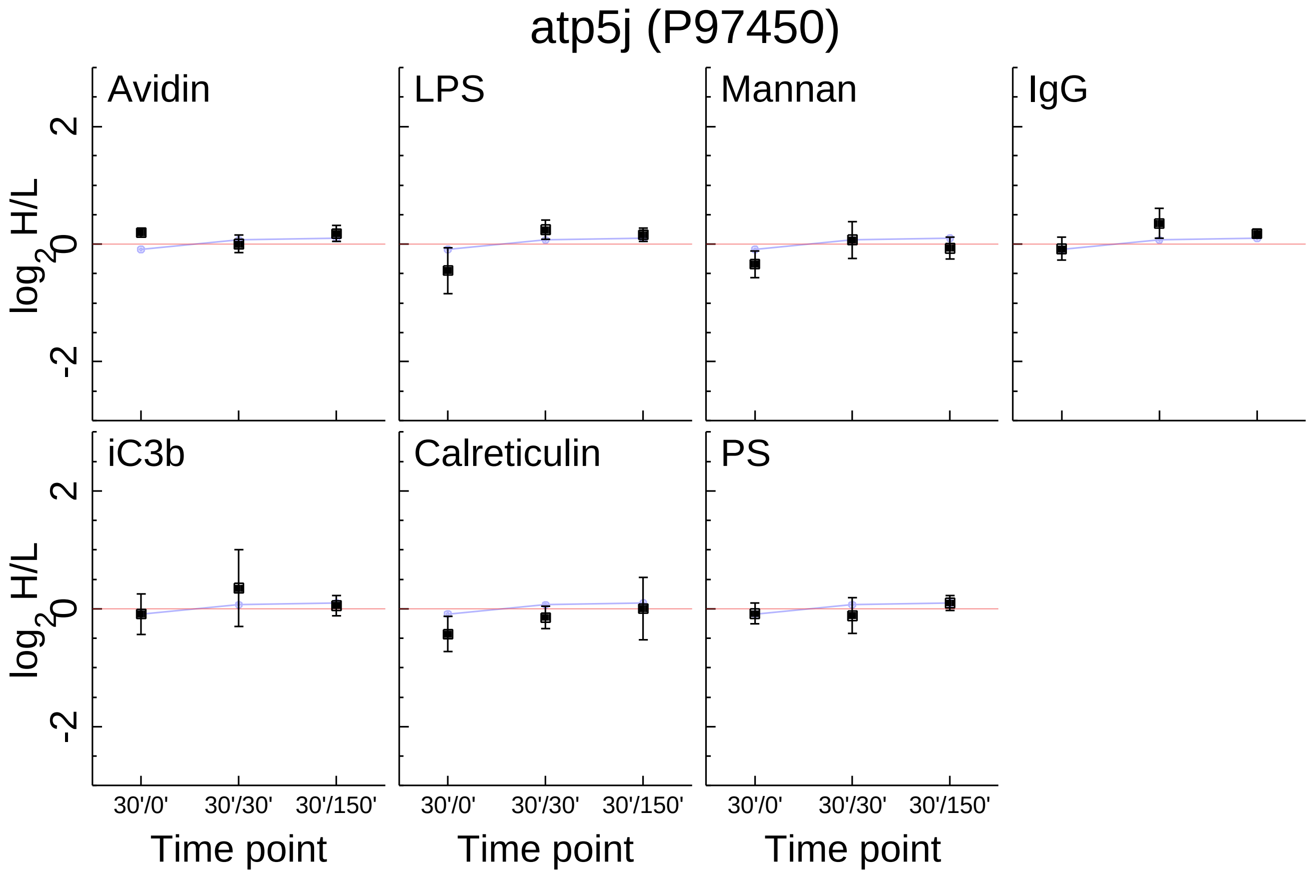

atp5j (P97450)
Avidin
LPS
Mannan
IgG
iC3b
Calreticulin
PS
log2 H/L
2
0
-2
30'/0'
30'/30'
30'/150'
Time point

atp5l (Q9CPQ8)

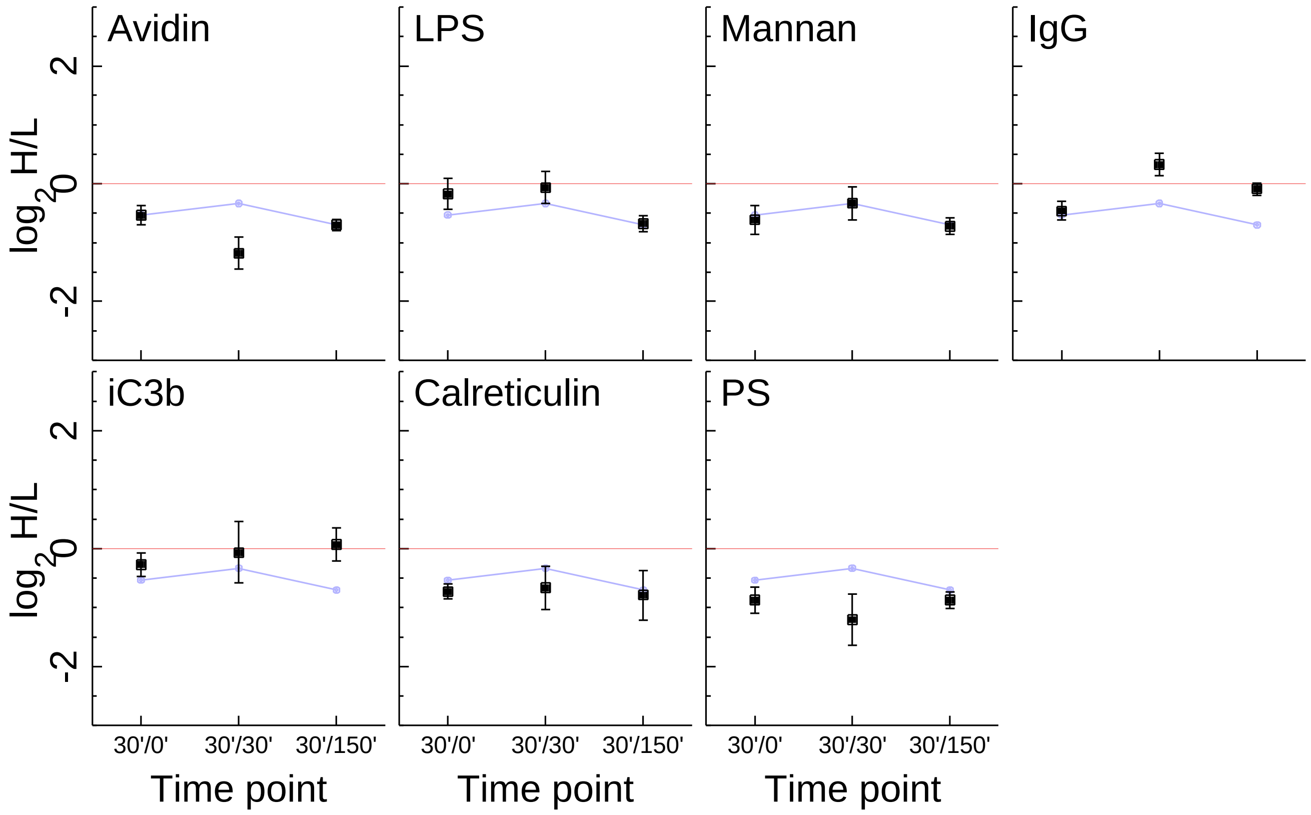

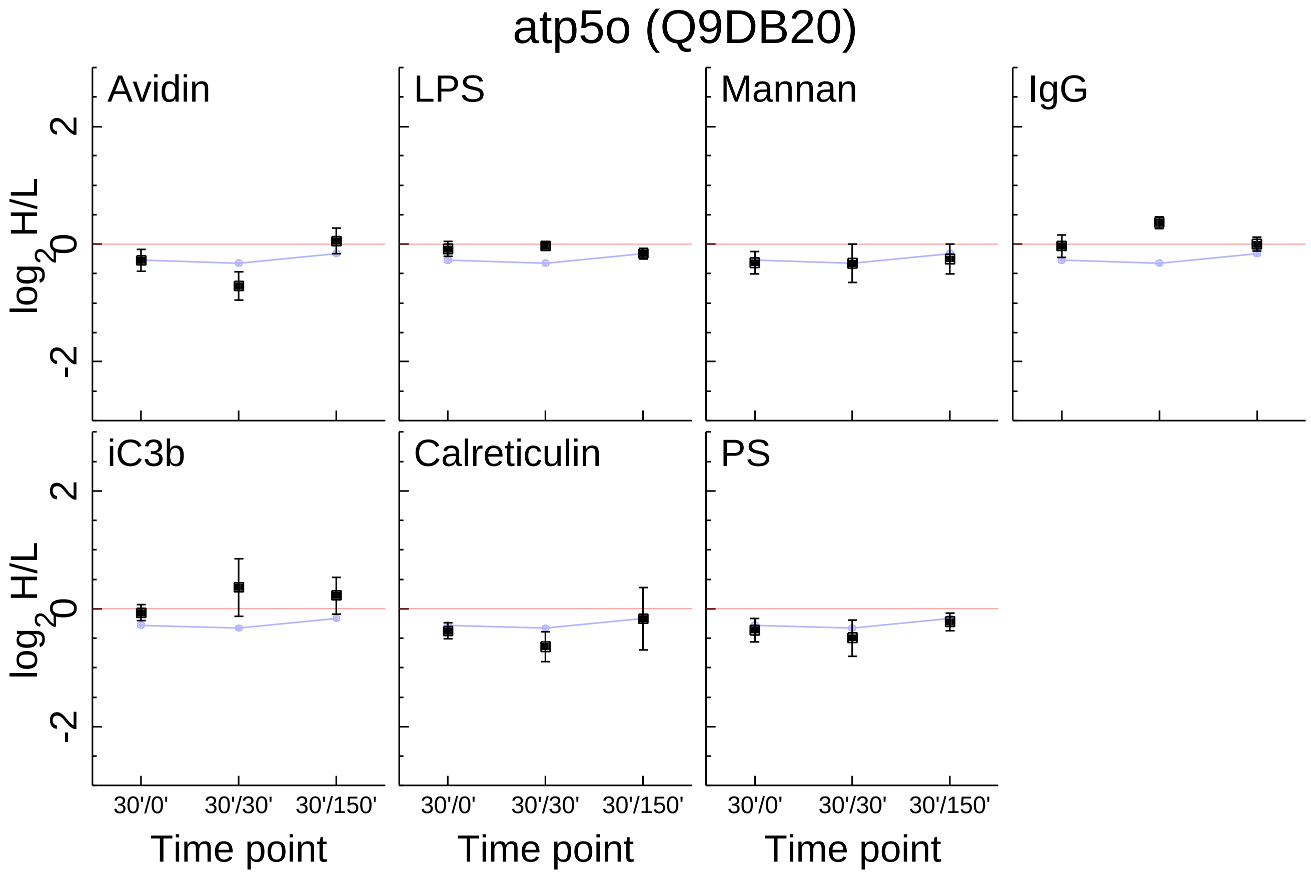
atp5o (Q9DB20)
Avidin
LPS
Mannan
IgG
iC3b
Calreticulin
PS
log2 H/L
2
0
-2
30'/0'
30'/30'
30'/150'
Time point

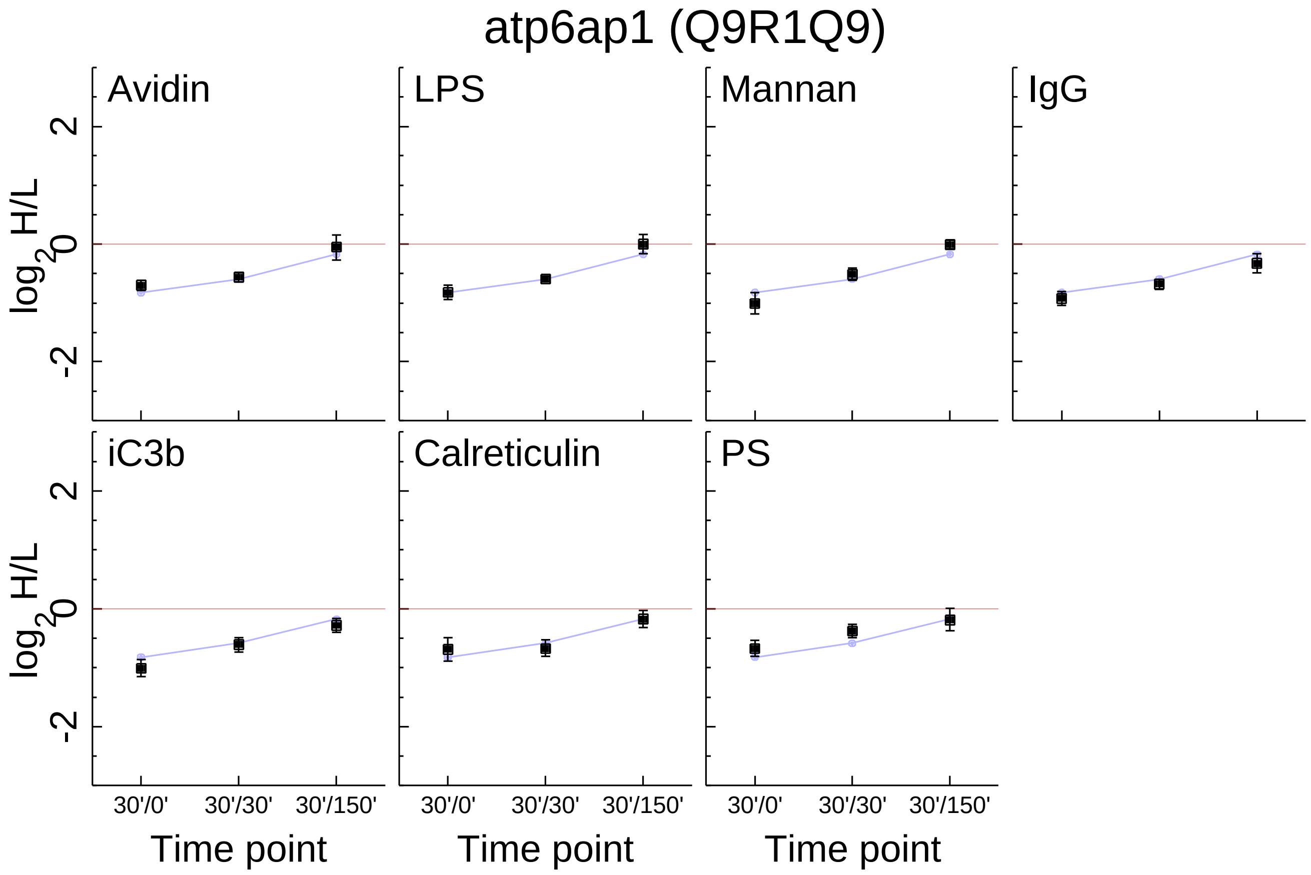
atp6ap1 (Q9R1Q9)
Avidin
LPS
Mannan
IgG
iC3b
Calreticulin
PS
log2 H/L
2
0
-2
30'/0'
30'/30'
30'/150'
Time point

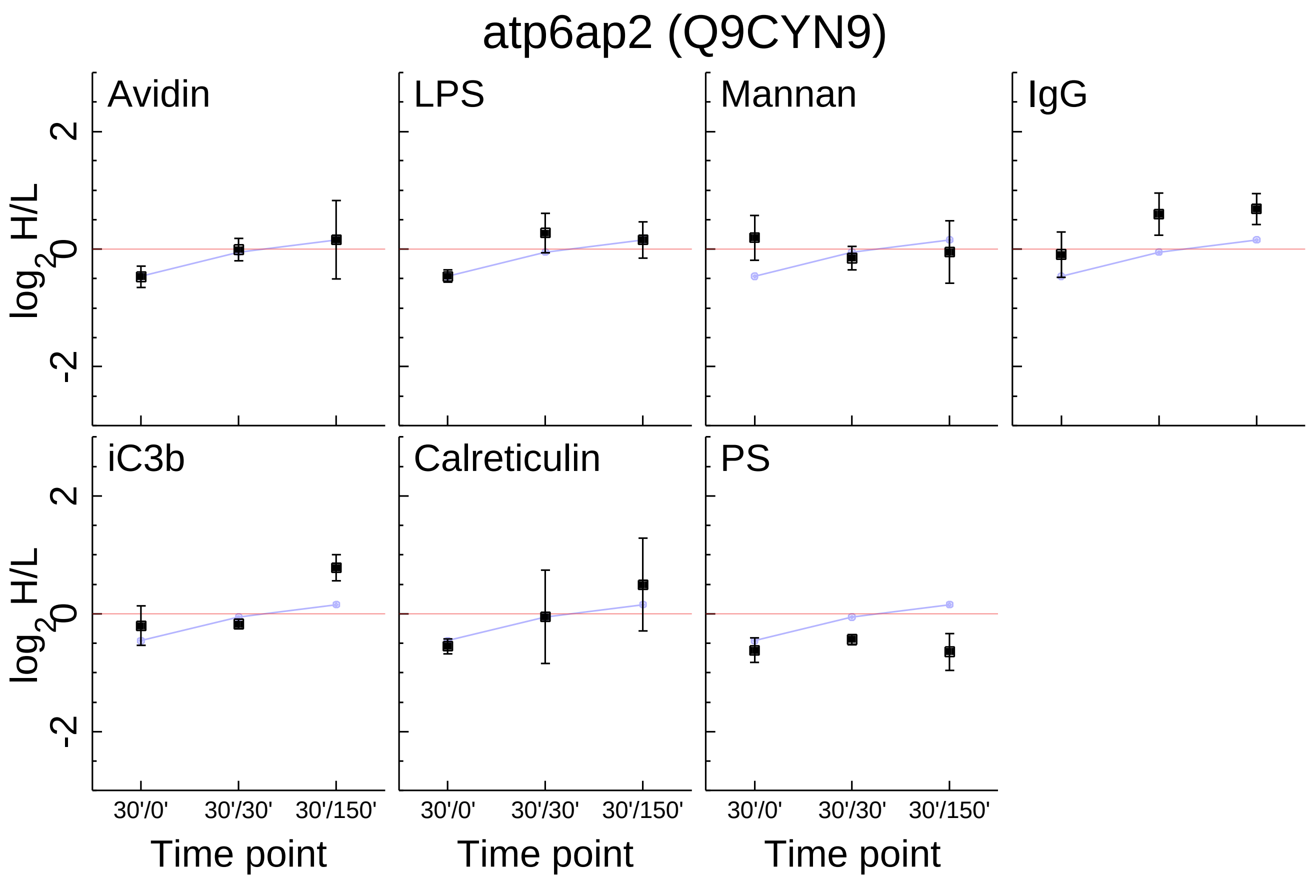

atp6ap2 (Q9CYN9)
Avidin
LPS
Mannan
IgG
iC3b
Calreticulin
PS
log2 H/L
2
0
-2
30'/0'
30'/30'
30'/150'
Time point

atp6v0a1 (Q9Z1G4)

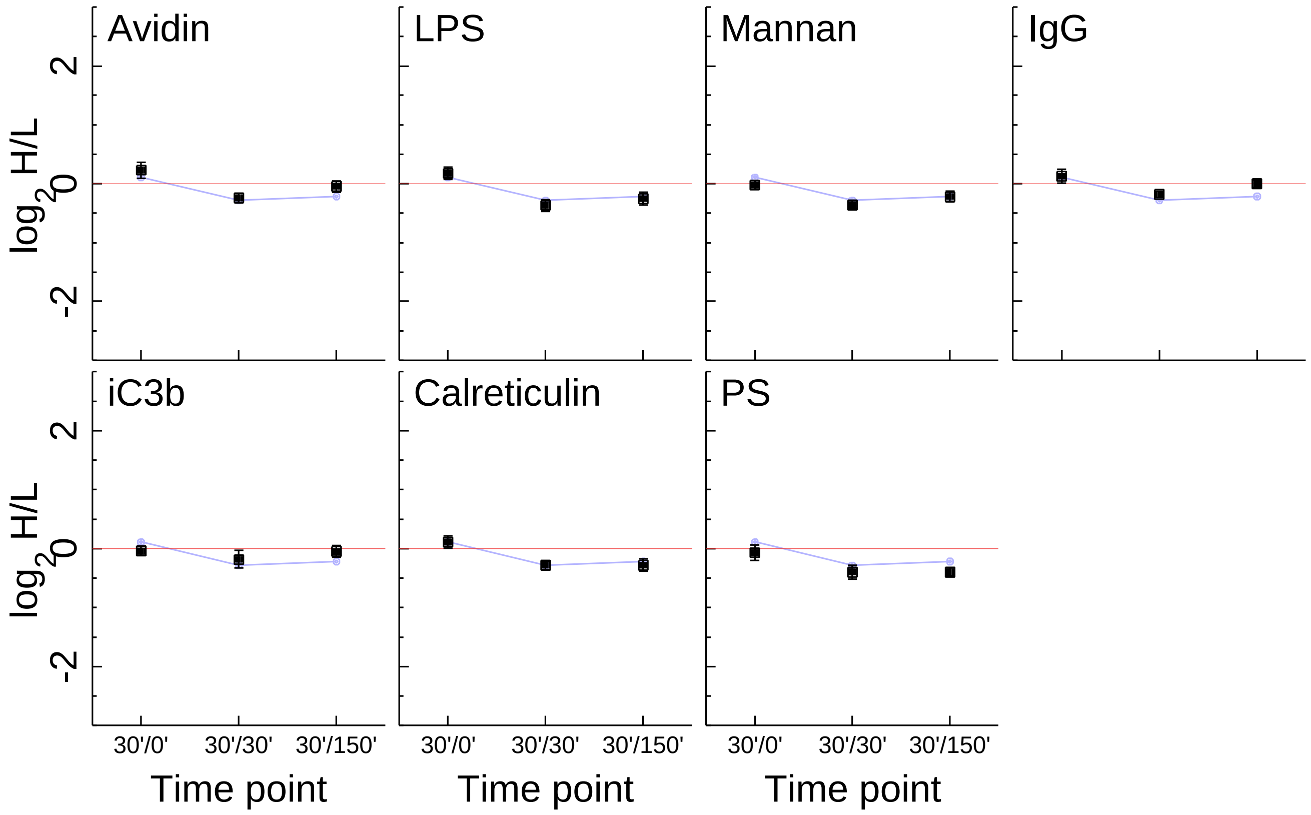

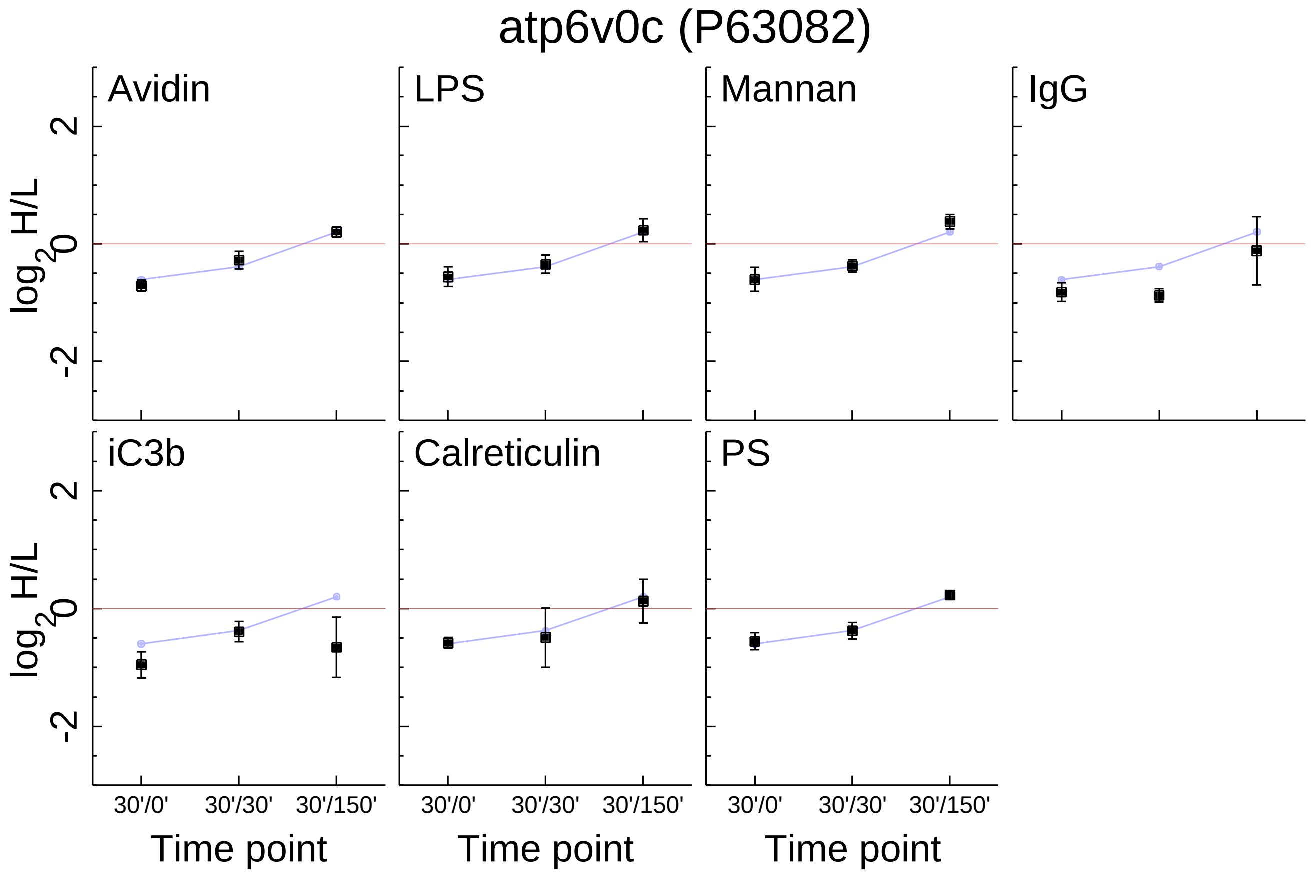
atp6v0c (P63082)
Avidin
LPS
Mannan
IgG
iC3b
Calreticulin
PS
log2 H/L
2
0
-2
30'/0'
30'/30'
30'/150'
Time point

atp6v0d1 (P51863)

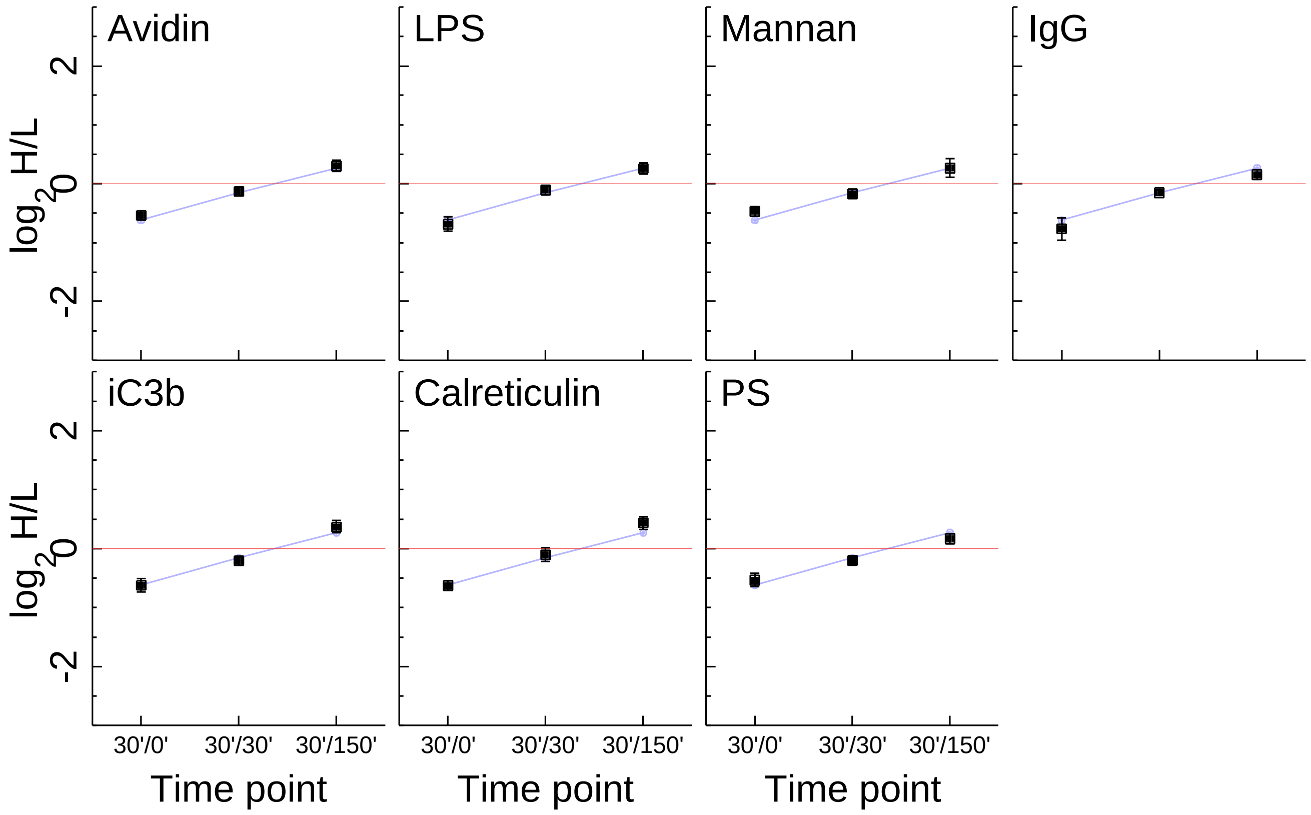

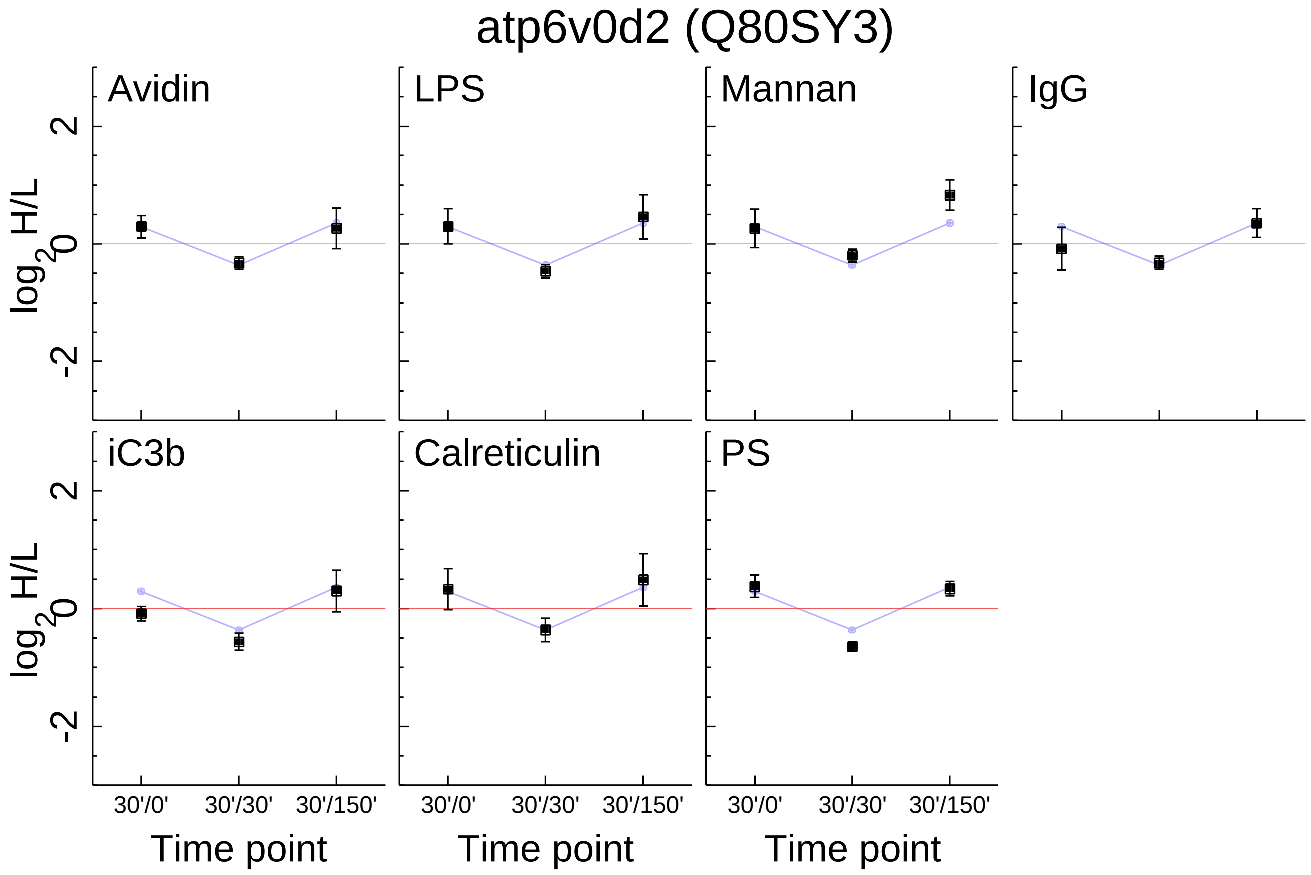
atp6v0d2 (Q80SY3)
Avidin
LPS
Mannan
IgG
iC3b
Calreticulin
PS
log2 H/L
2
0
-2
30'/0'
30'/30'
30'/150'
Time point

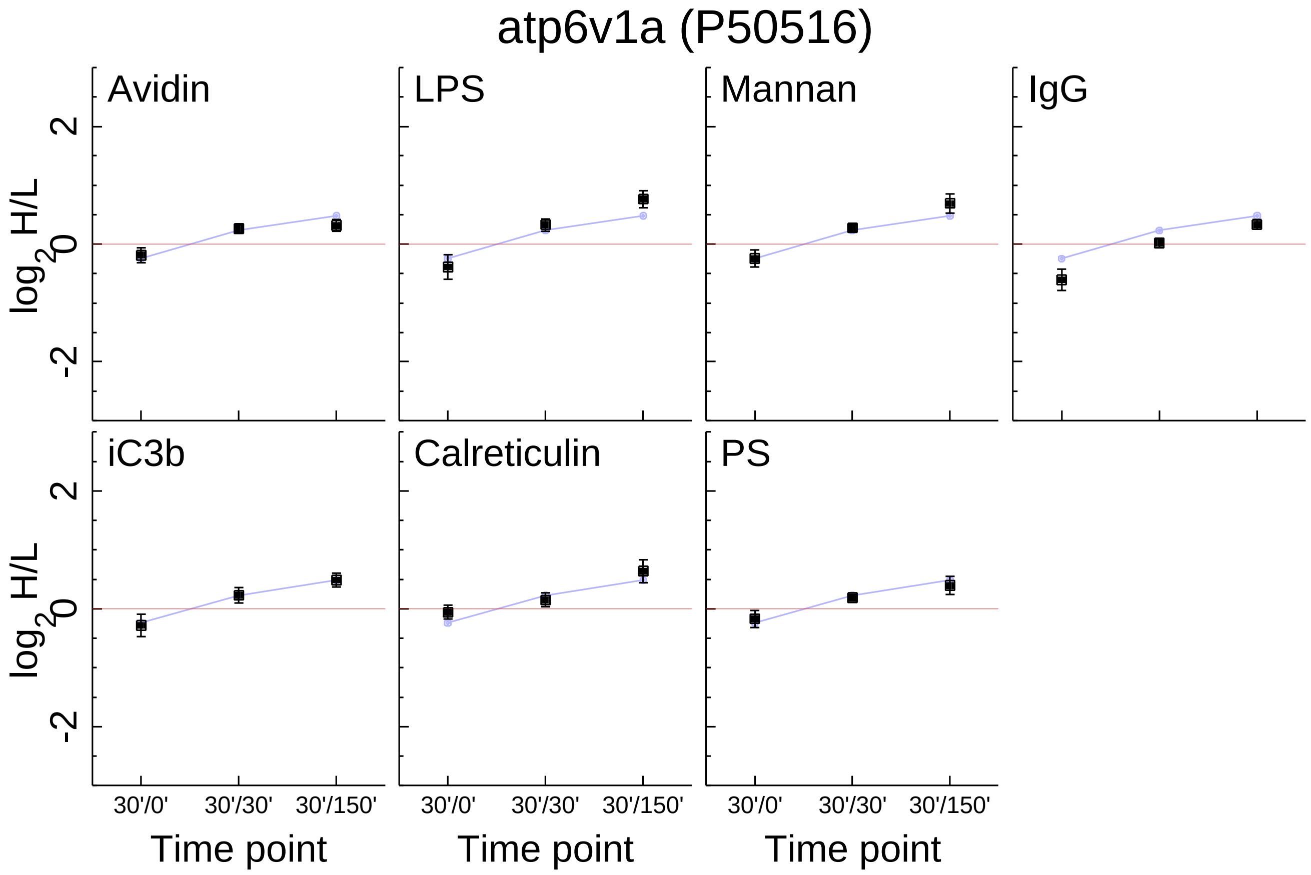
atp6v1a (P50516)
Avidin
LPS
Mannan
IgG
iC3b
Calreticulin
PS
log2 H/L
2
0
-2
30'/0'
30'/30'
30'/150'
Time point

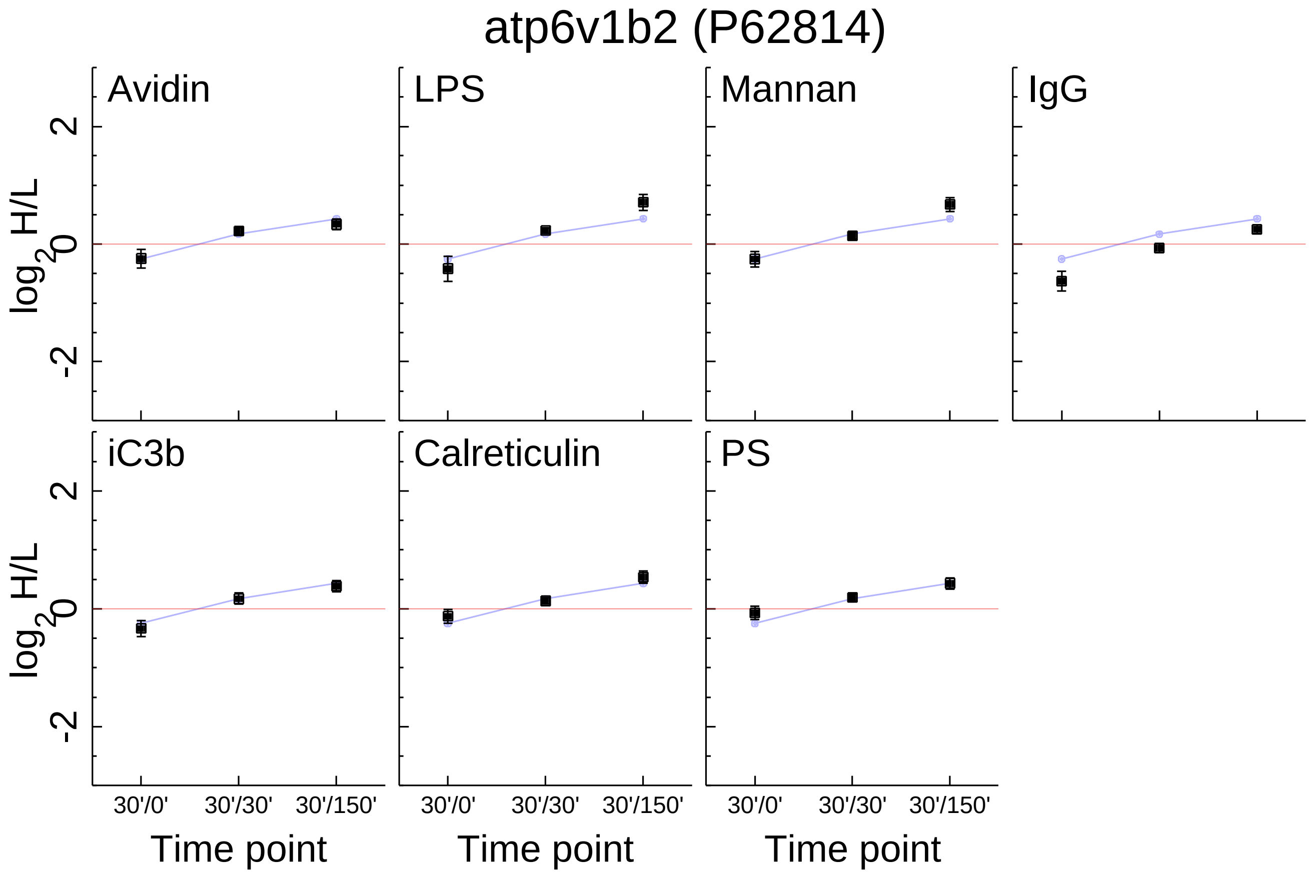
atp6v1b2 (P62814)
Avidin
LPS
Mannan
IgG
iC3b
Calreticulin
PS
log2 H/L
2
0
-2
30'/0'
30'/30'
30'/150'
Time point

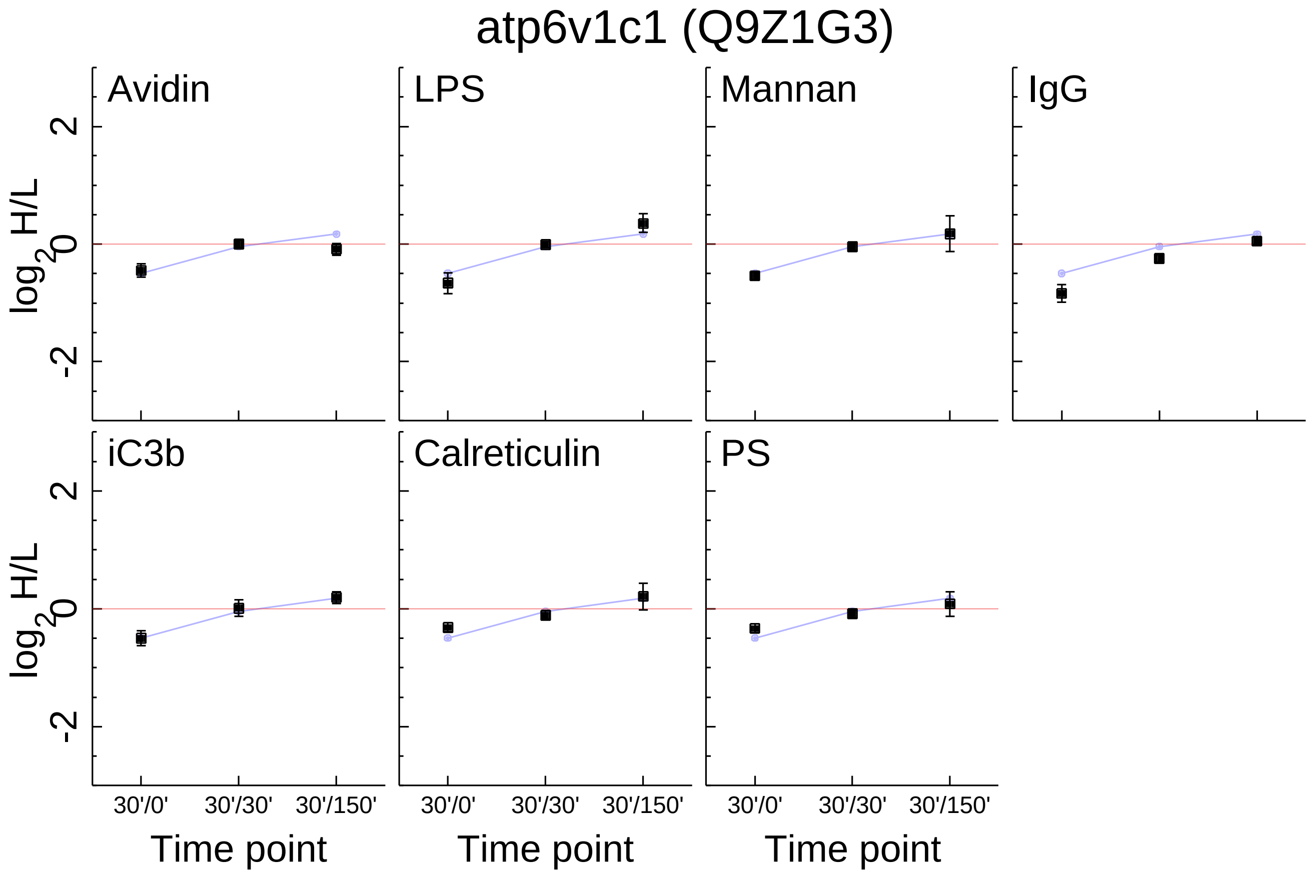
atp6v1c1 (Q9Z1G3)
Avidin
LPS
Mannan
IgG
iC3b
Calreticulin
PS
log2 H/L
2
0
-2
30'/0'
30'/30'
30'/150'
Time point

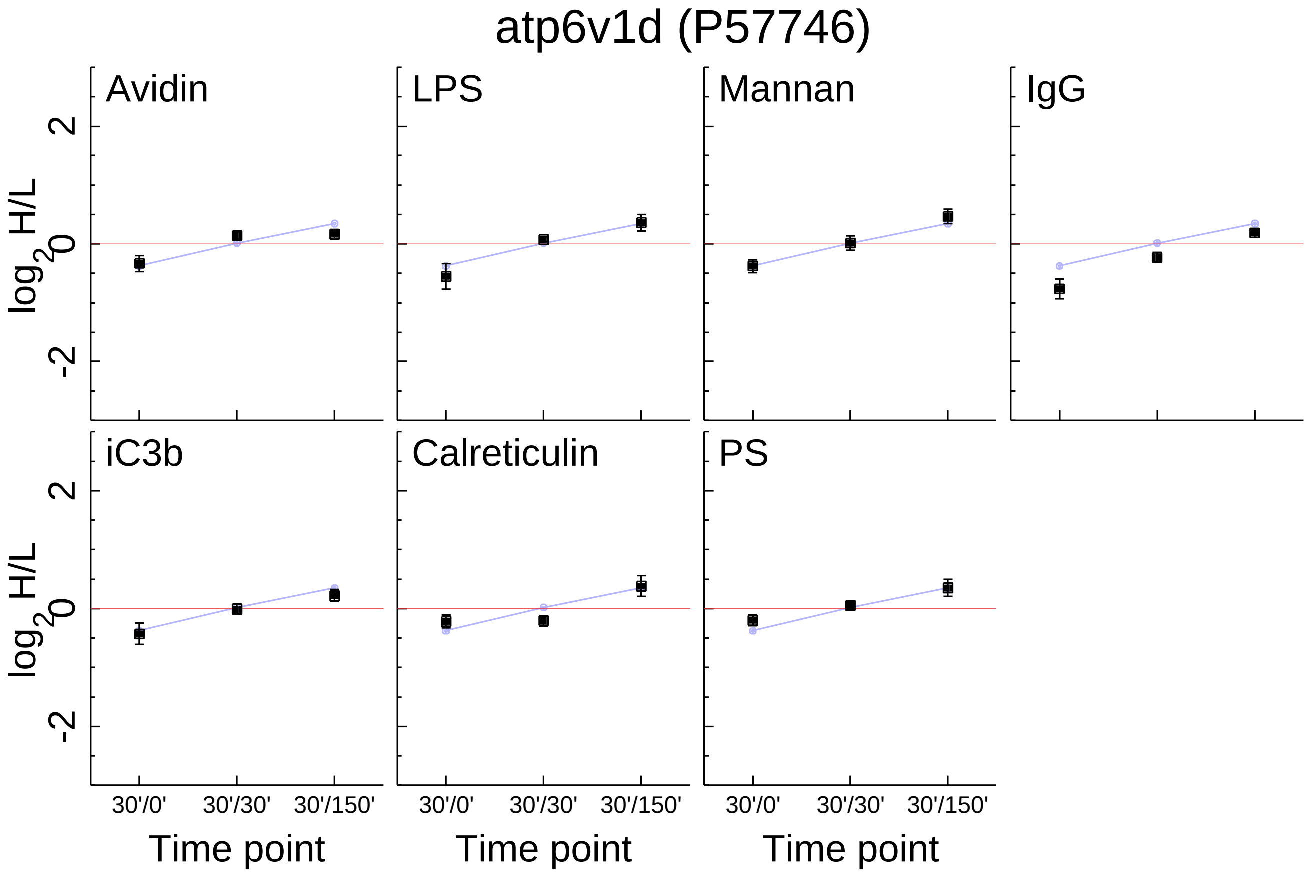
atp6v1d (P57746)
Avidin
LPS
Mannan
IgG
iC3b
Calreticulin
PS
log2 H/L
2
0
-2
30'/0'
30'/30'
30'/150'
Time point

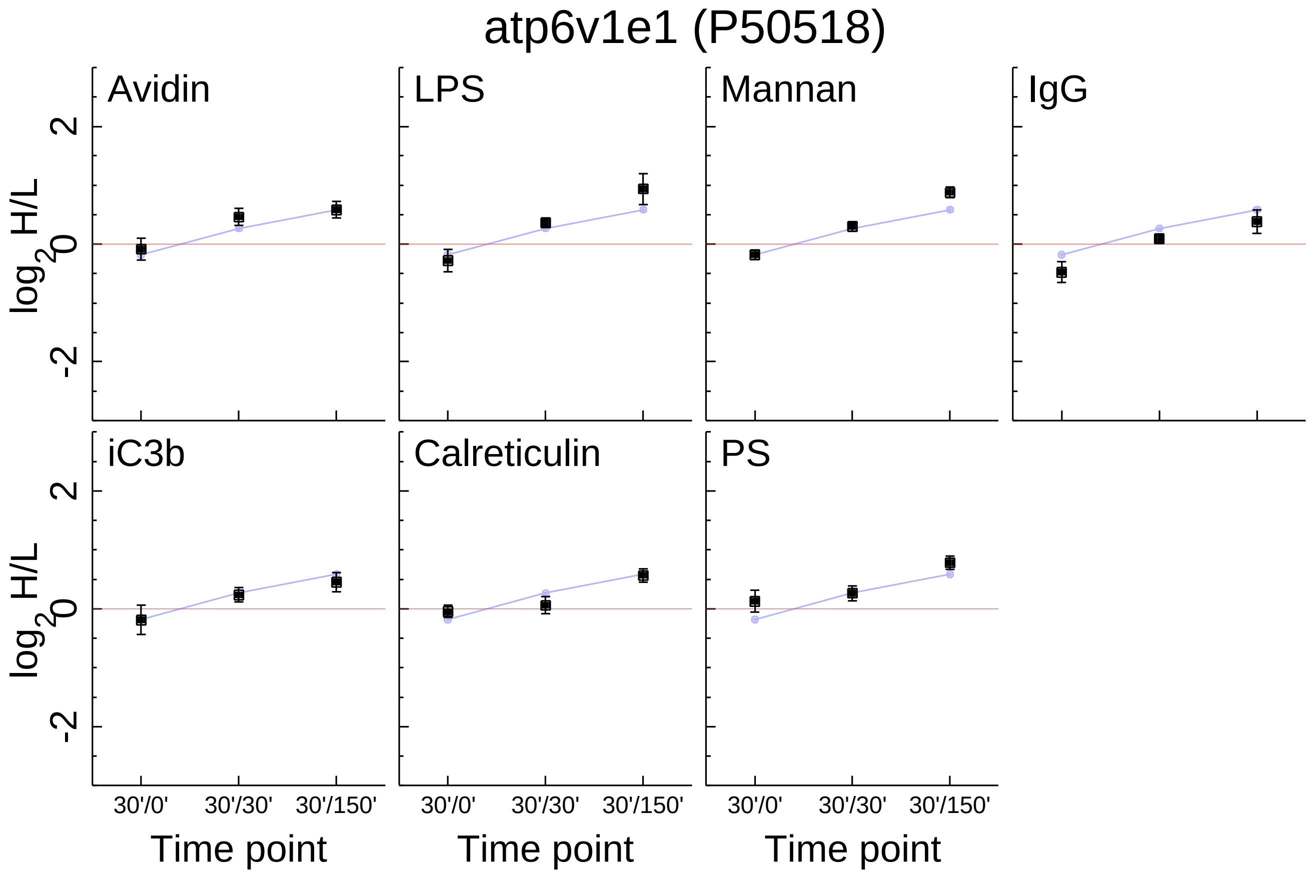
atp6v1e1 (P50518)
Avidin
LPS
Mannan
IgG
iC3b
Calreticulin
PS
log2 H/L
2
0
-2
30'/0'
30'/30'
30'/150'
Time point

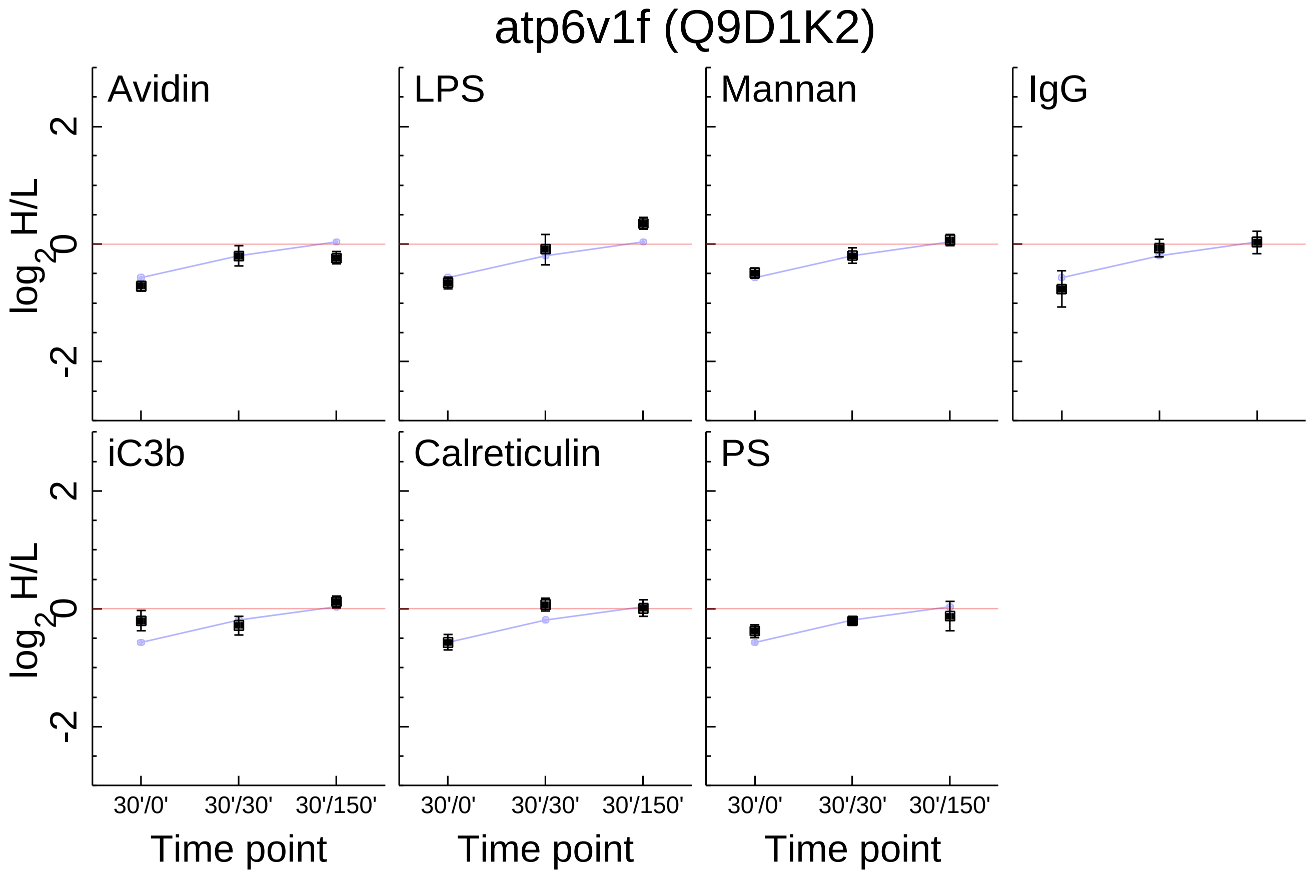

atp6v1f (Q9D1K2)
Avidin
LPS
Mannan
IgG
iC3b
Calreticulin
PS
log2 H/L
2
0
-2
30'/0'
30'/30'
30'/150'
Time point

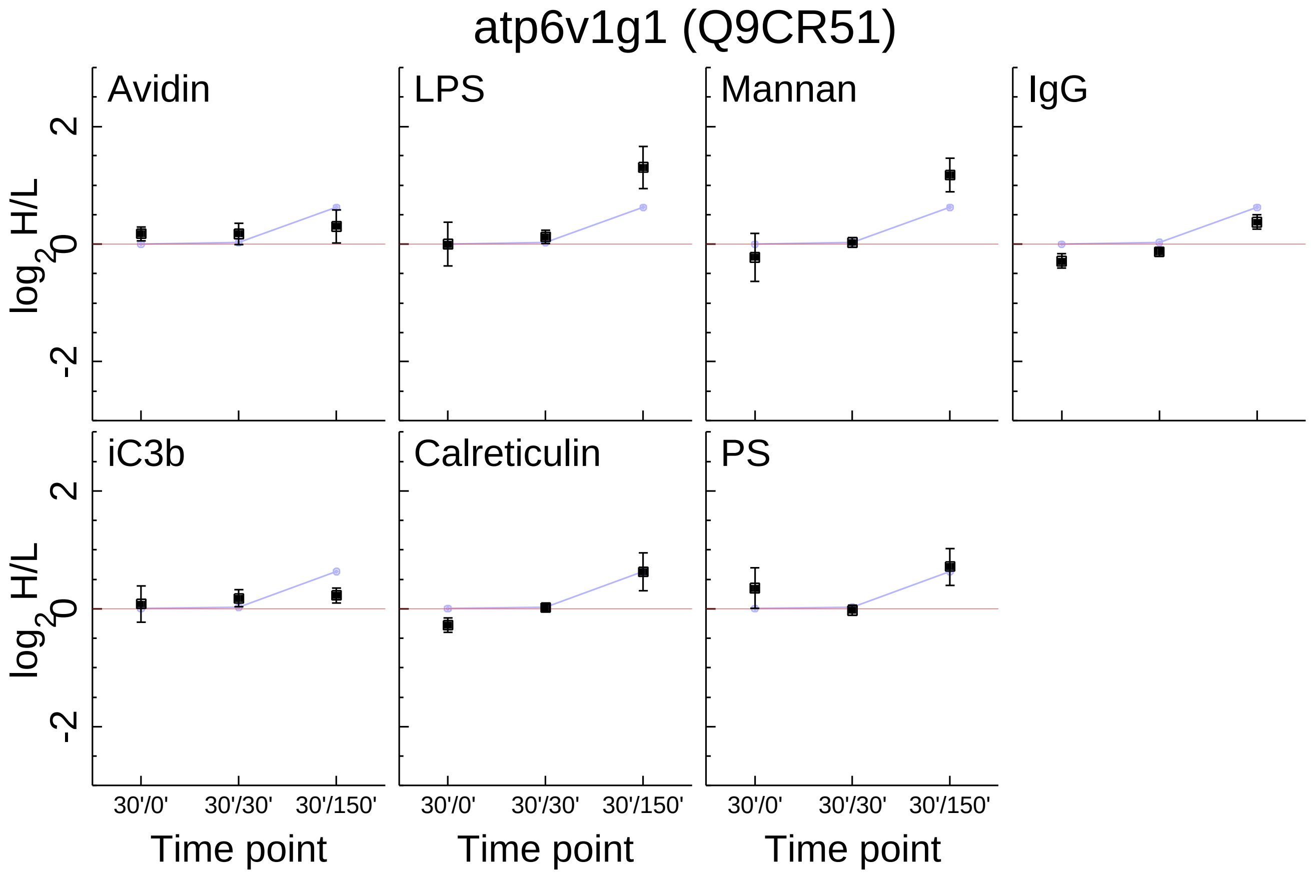
atp6v1g1 (Q9CR51)
Avidin
LPS
Mannan
IgG
iC3b
Calreticulin
PS
log2 H/L
2
0
-2
30'/0'
30'/30'
30'/150'
Time point

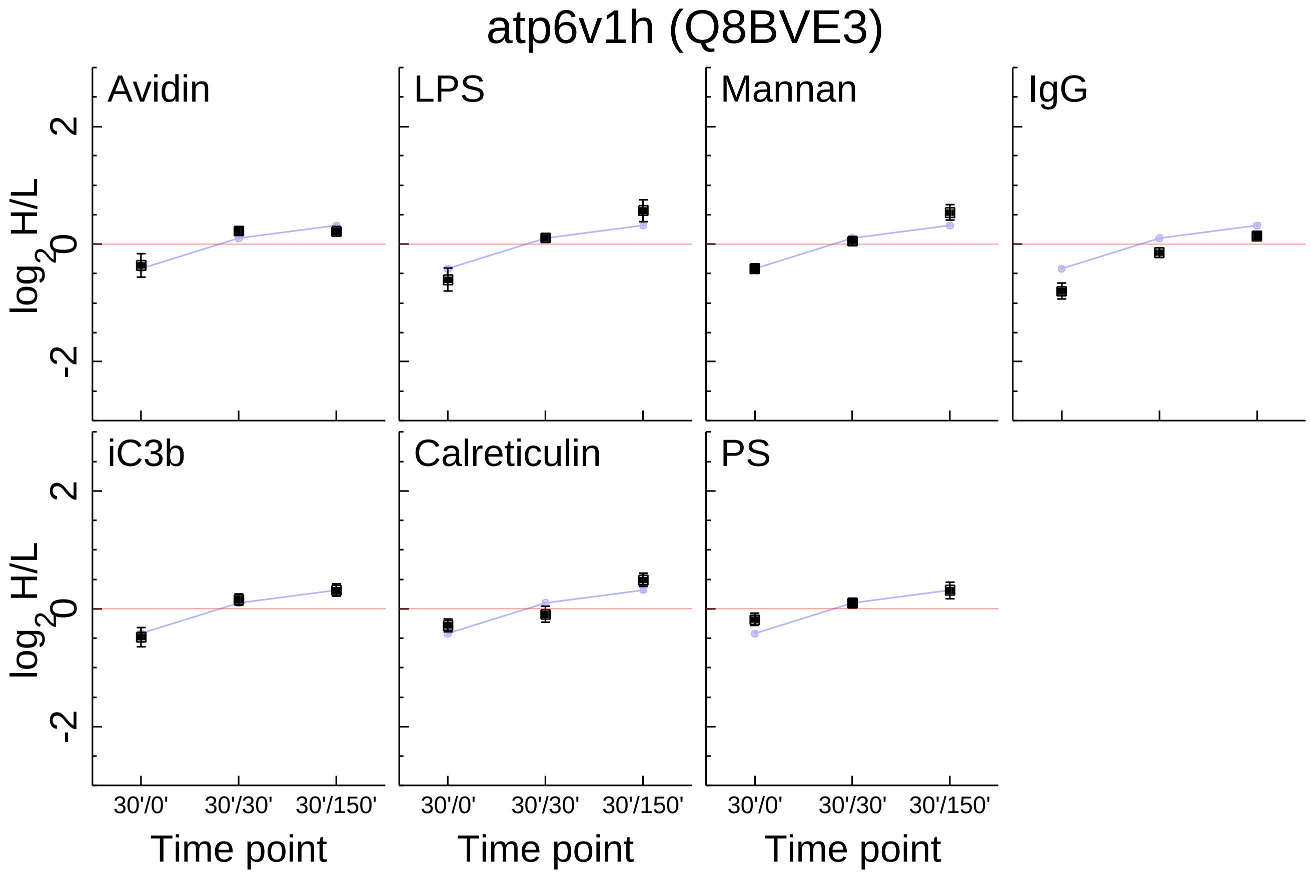
atp6v1h (Q8BVE3)
Avidin
LPS
Mannan
IgG
iC3b
Calreticulin
PS
log2 H/L
2
0
-2
30'/0'
30'/30'
30'/150'
Time point

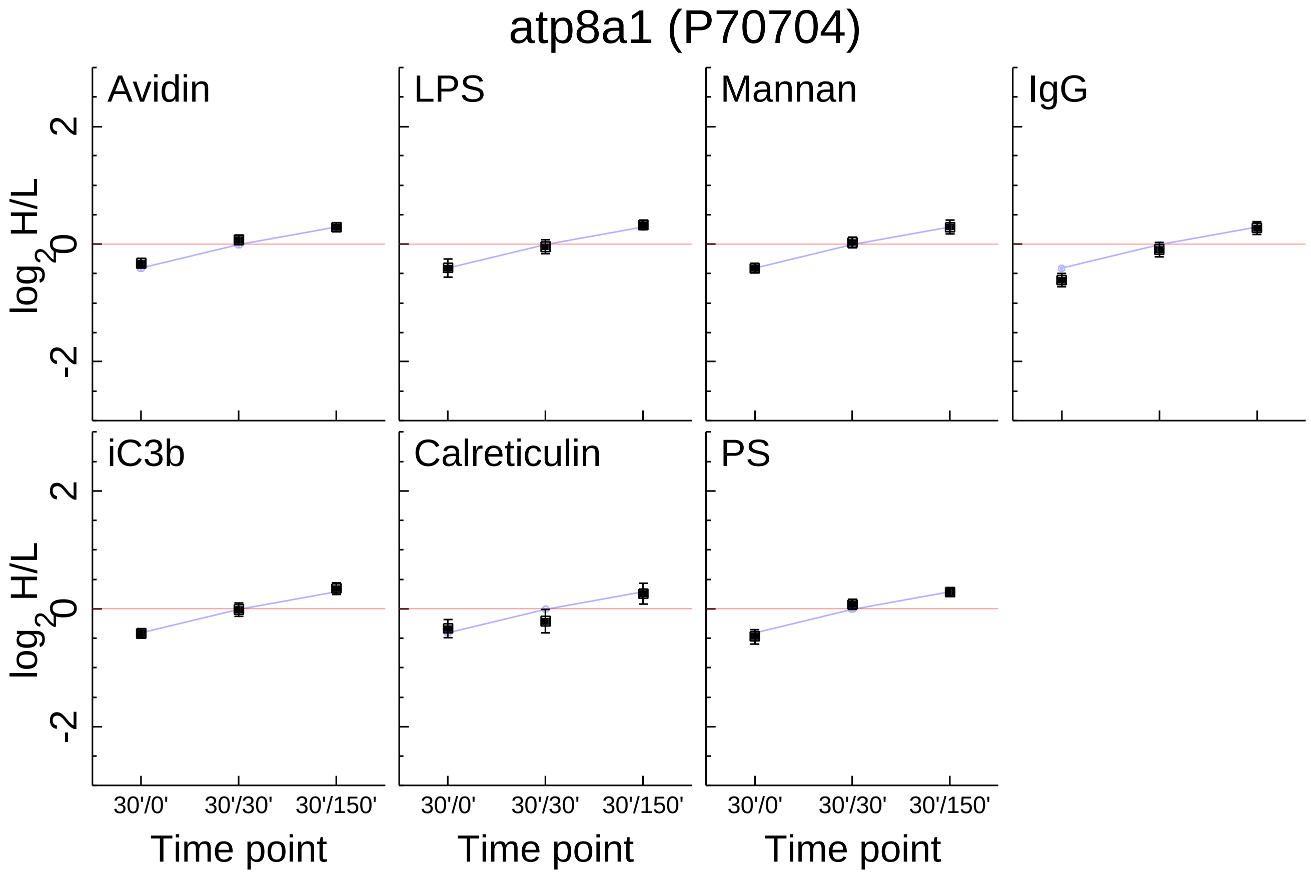
atp8a1 (P70704)
Avidin
LPS
Mannan
IgG
iC3b
Calreticulin
PS
log2 H/L
2
0
-2
30'/0'
30'/30'
30'/150'
Time point

atp9b (D3YV00)

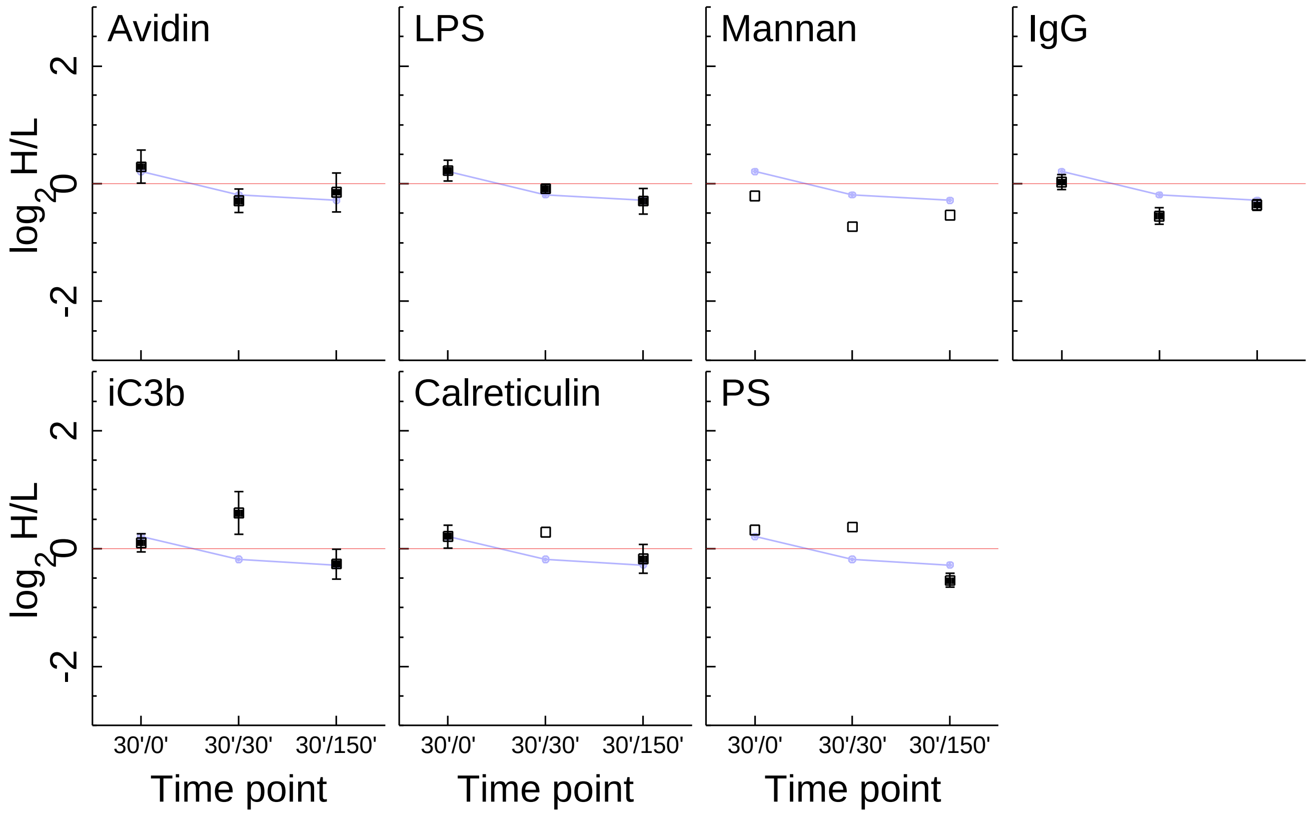

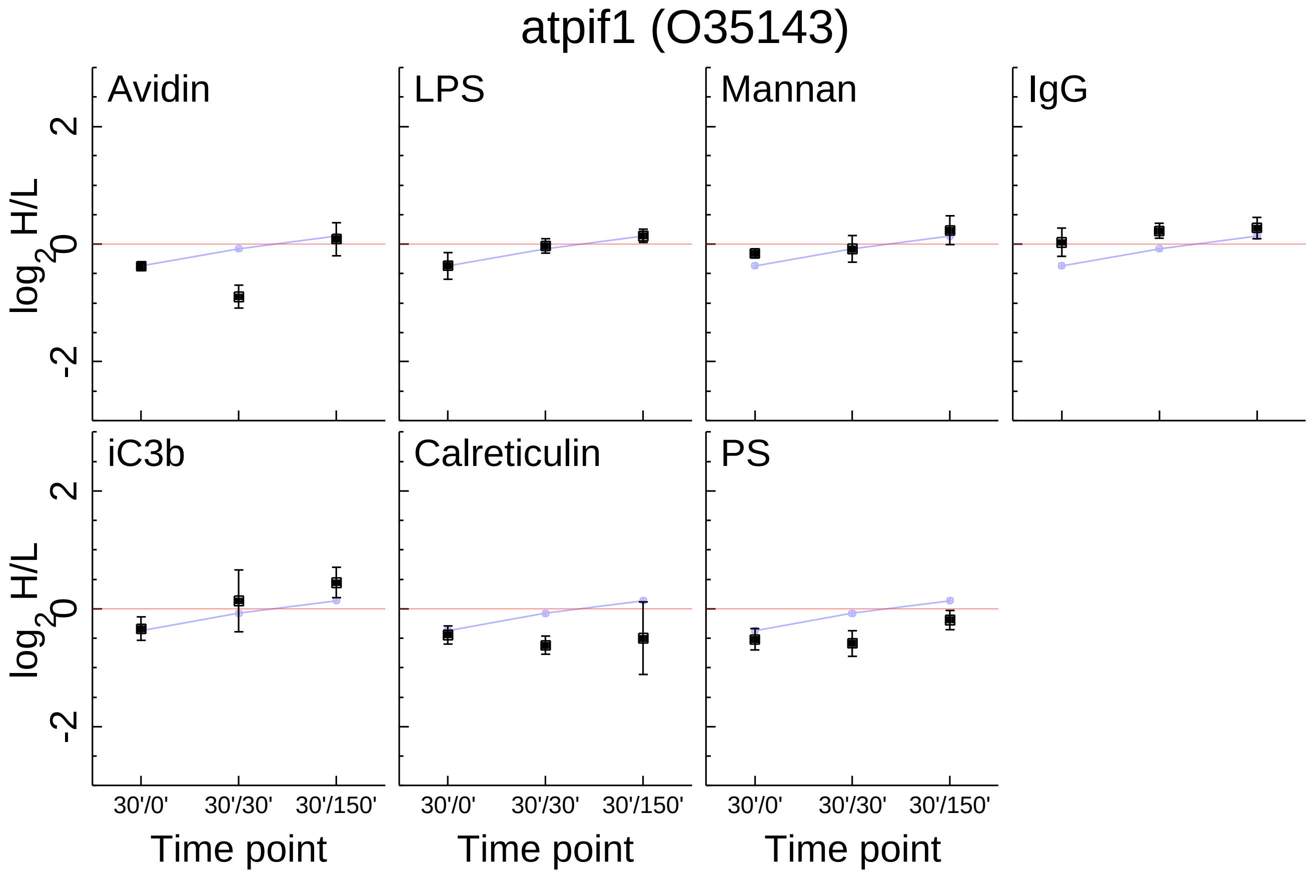

atpif1 (O35143)
Avidin
LPS
Mannan
IgG
iC3b
Calreticulin
PS
log2 H/L
2
0
-2
30'/0'
30'/30'
30'/150'
Time point

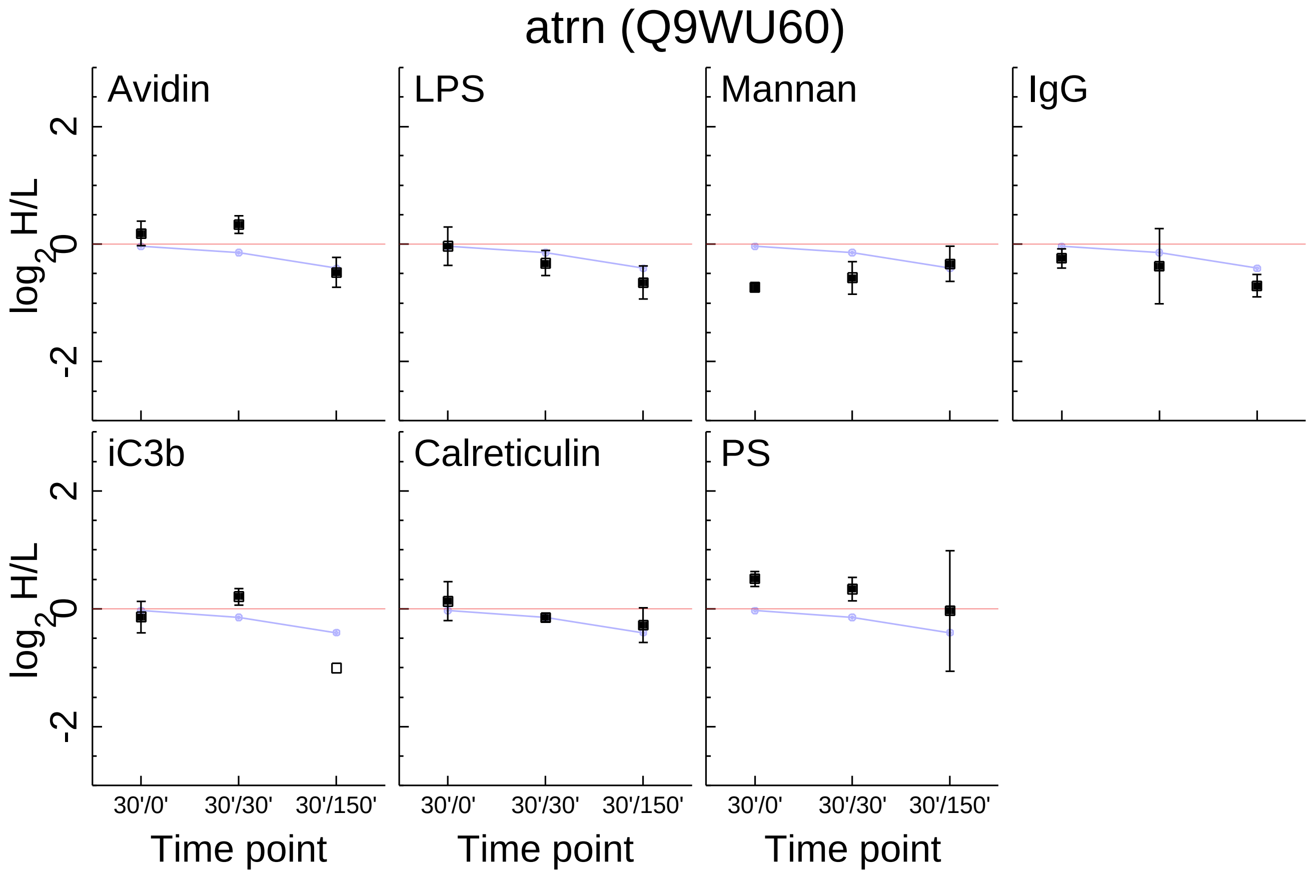

atrn (Q9WU60)
Avidin
LPS
Mannan
IgG
iC3b
Calreticulin
PS
log2 H/L
2
0
-2
30'/0'
30'/30'
30'/150'
Time point

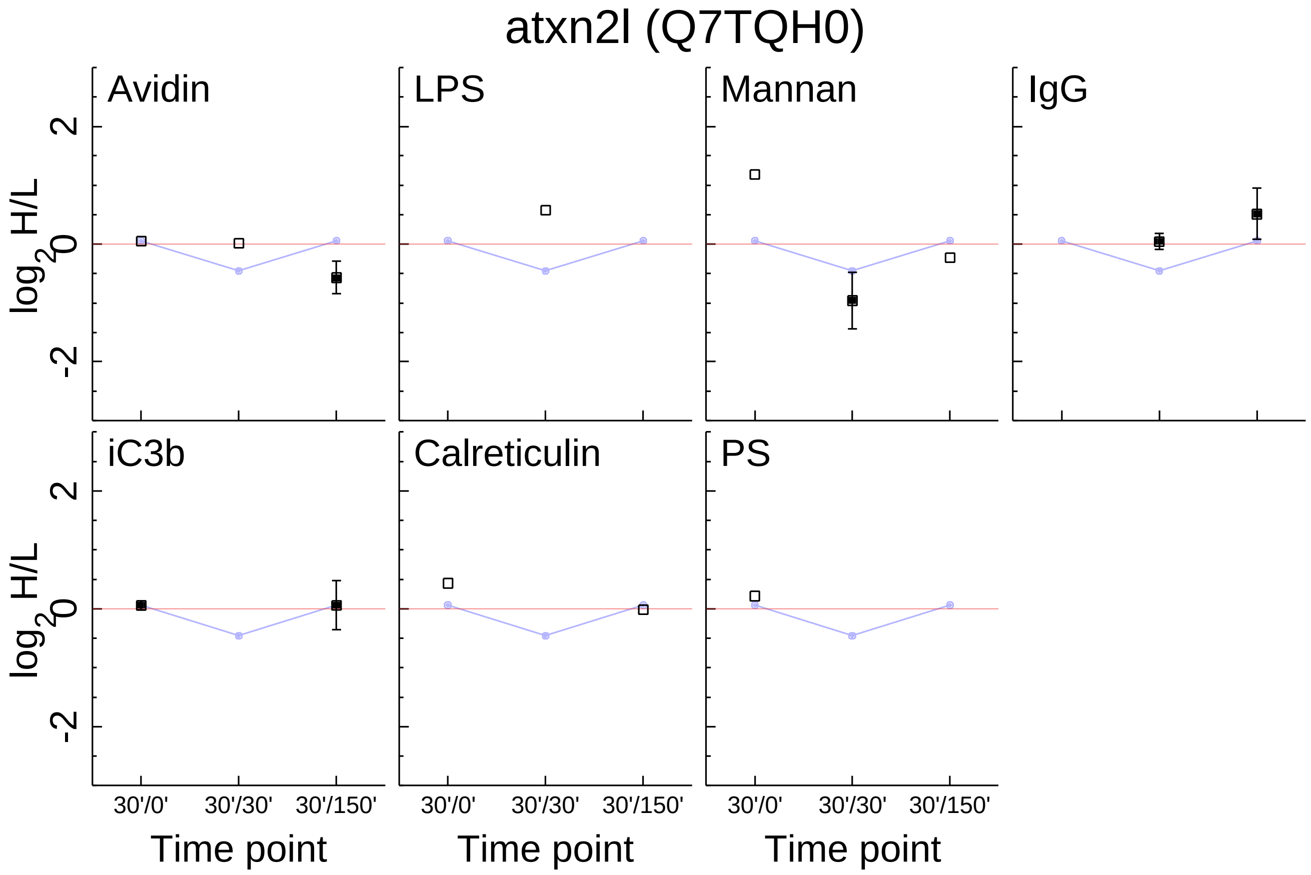
atxn2l (Q7TQH0)
Avidin
LPS
Mannan
IgG
iC3b
Calreticulin
PS
log2 H/L
2
0
-2
30'/0'
30'/30'
30'/150'
Time point

aup1 (Q3U3K9)

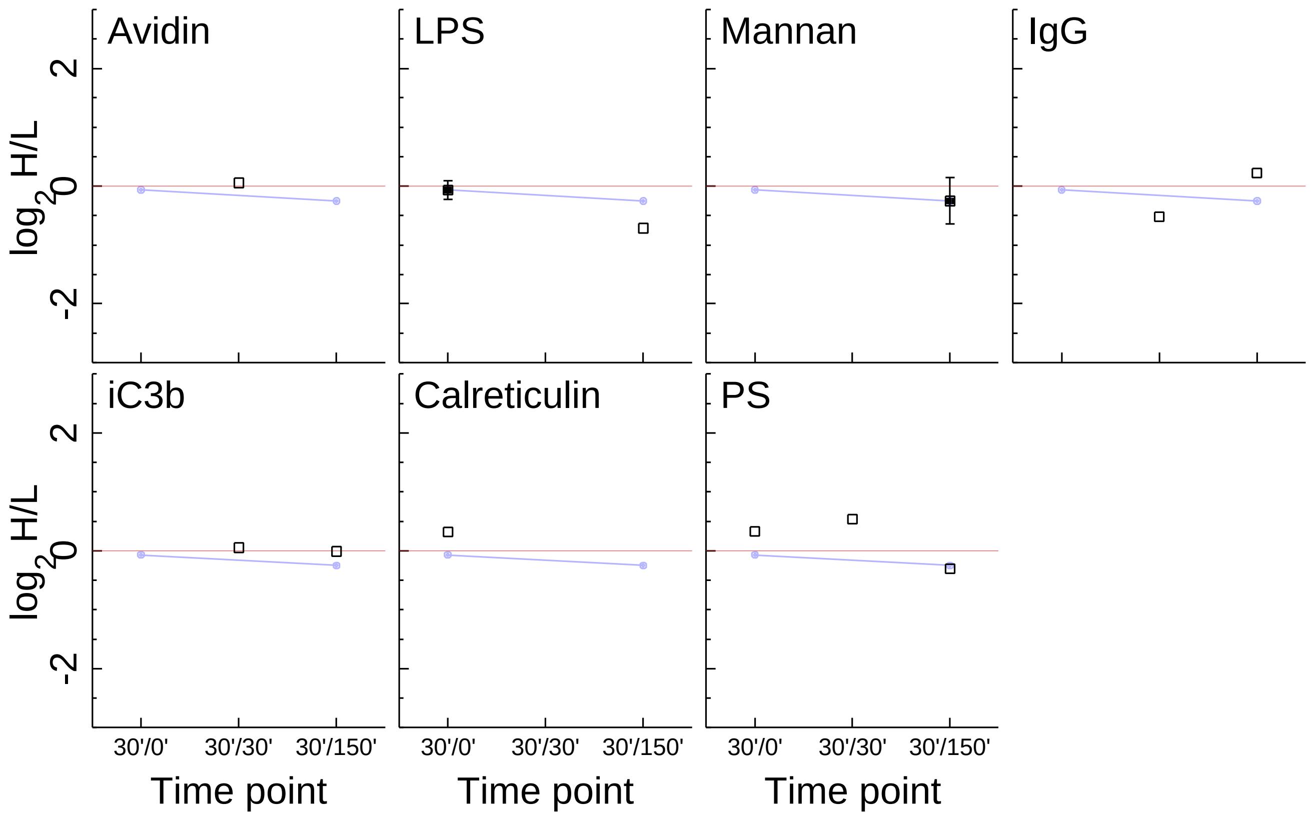

Avidin
LPS
Mannan
IgG
iC3b
Calreticulin
PS
log2 H/L
2
0
-2
30'/0'
30'/30'
30'/150'
Time point

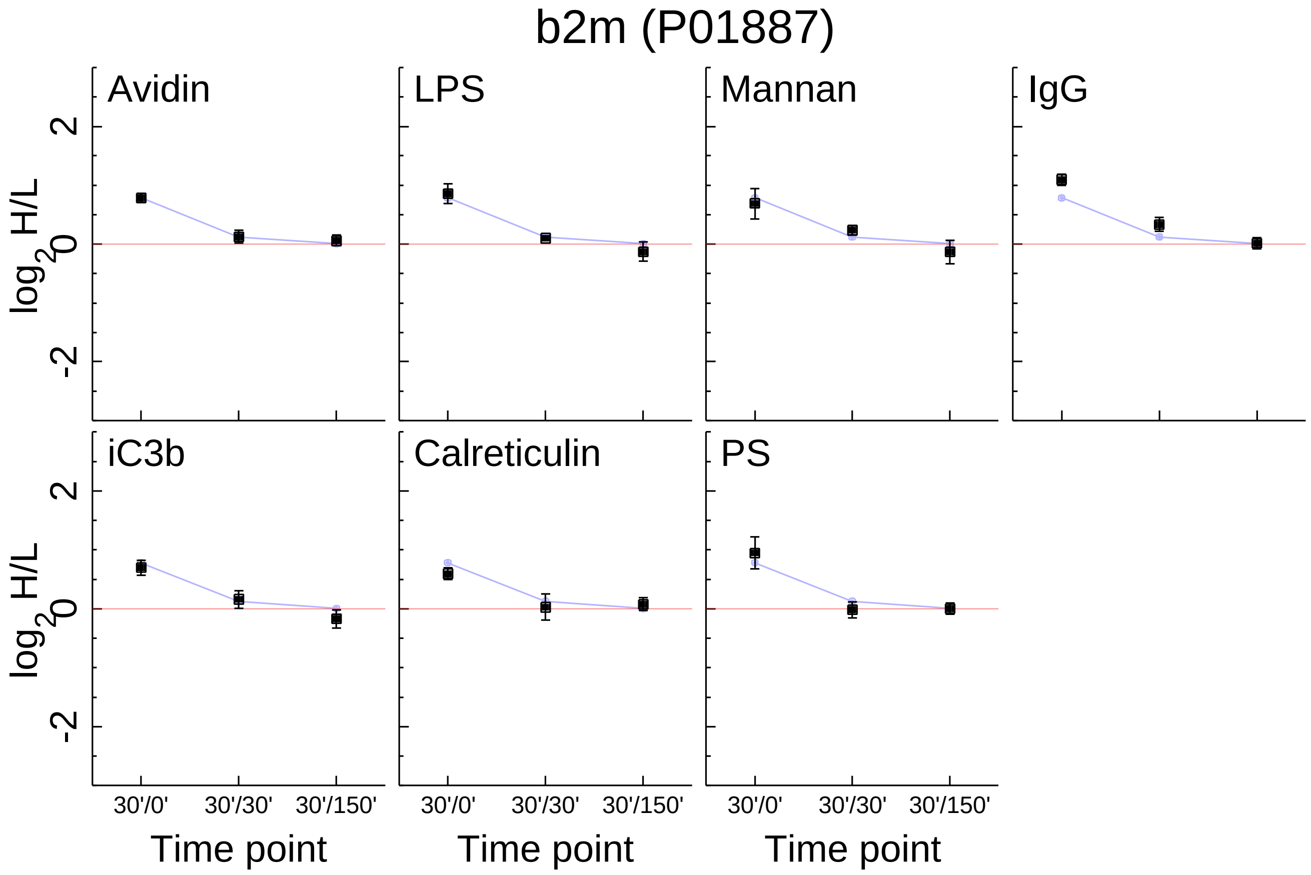
b2m (P01887)
Avidin
LPS
Mannan
IgG
iC3b
Calreticulin
PS
log2 H/L
2
0
-2
30'/0'
30'/30'
30'/150'
Time point

b4galnt1 (Q09200)

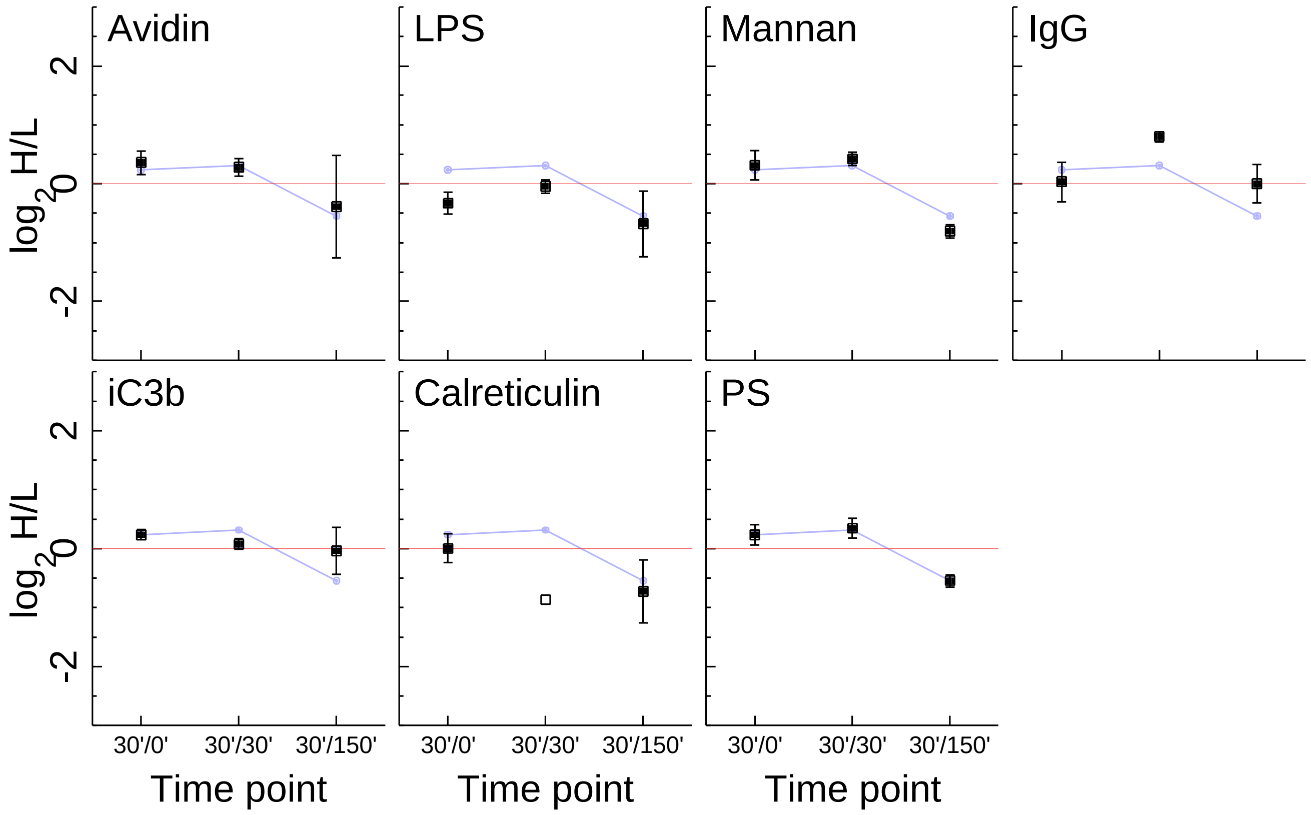

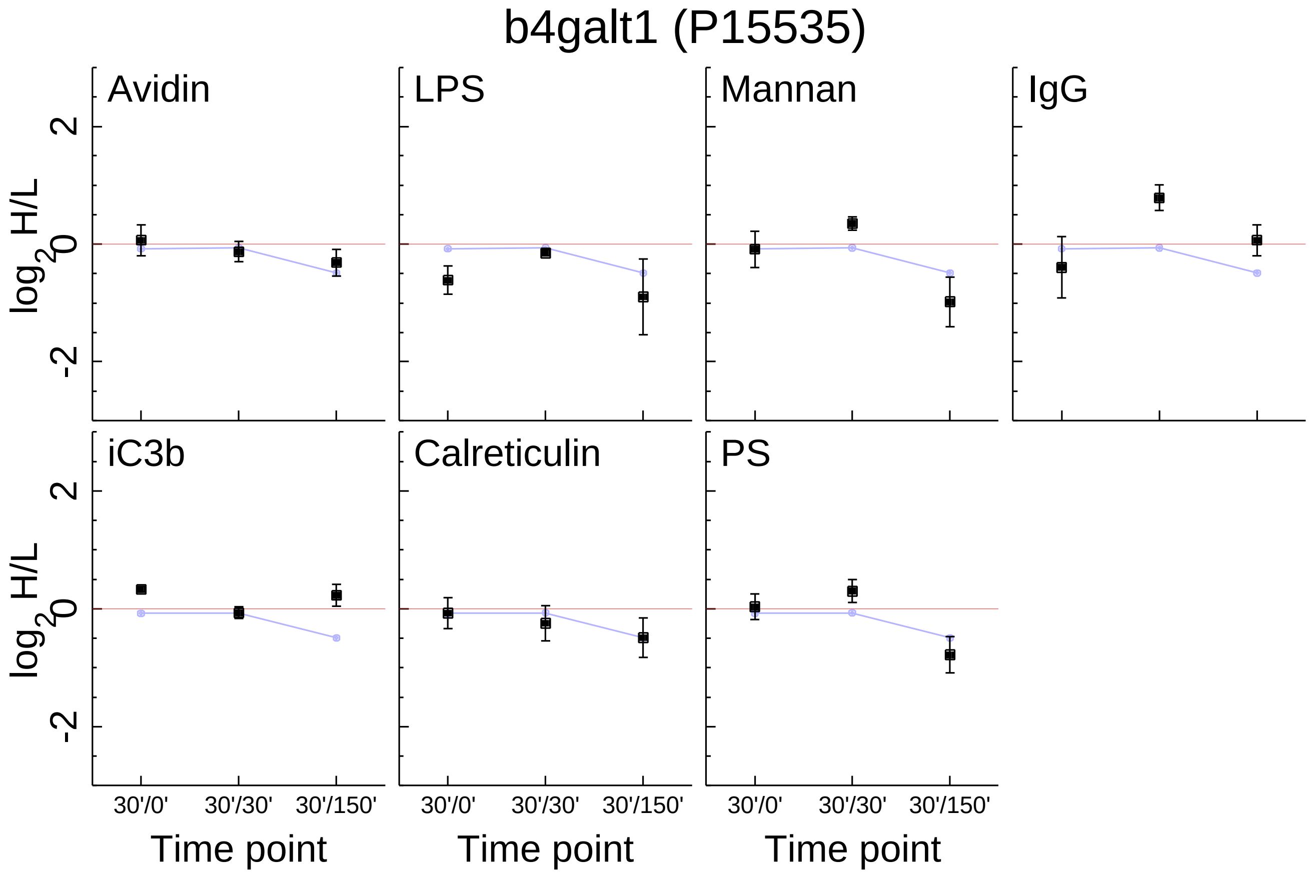

b4galt1 (P15535)
Avidin
LPS
Mannan
IgG
iC3b
Calreticulin
PS
log2 H/L
2
0
-2
30'/0'
30'/30'
30'/150'
Time point

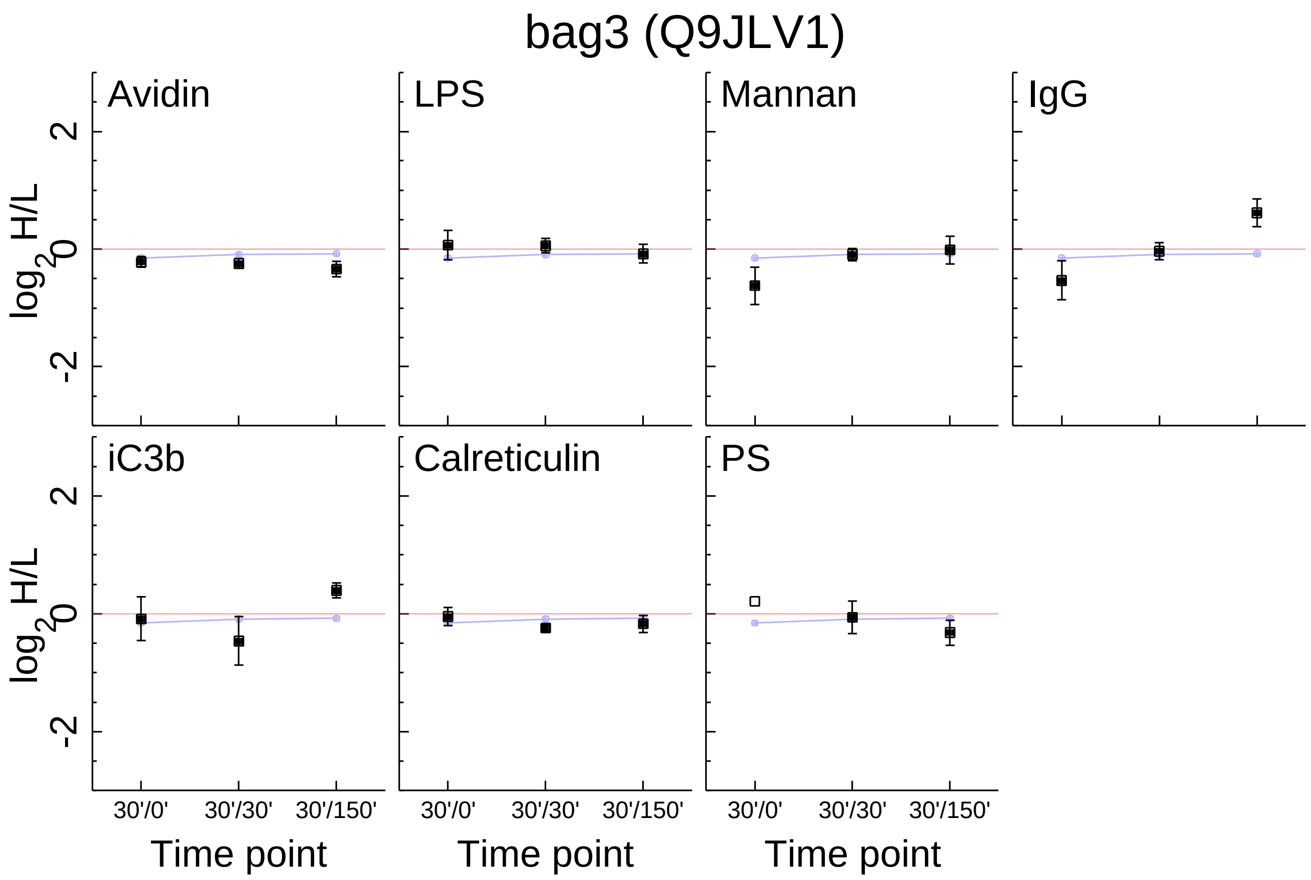

bag3 (Q9JLV1)
Avidin
LPS
Mannan
IgG
iC3b
Calreticulin
PS
log2 H/L
2
0
-2
30'/0'
30'/30'
30'/150'
Time point

bak1 (E9QM21)

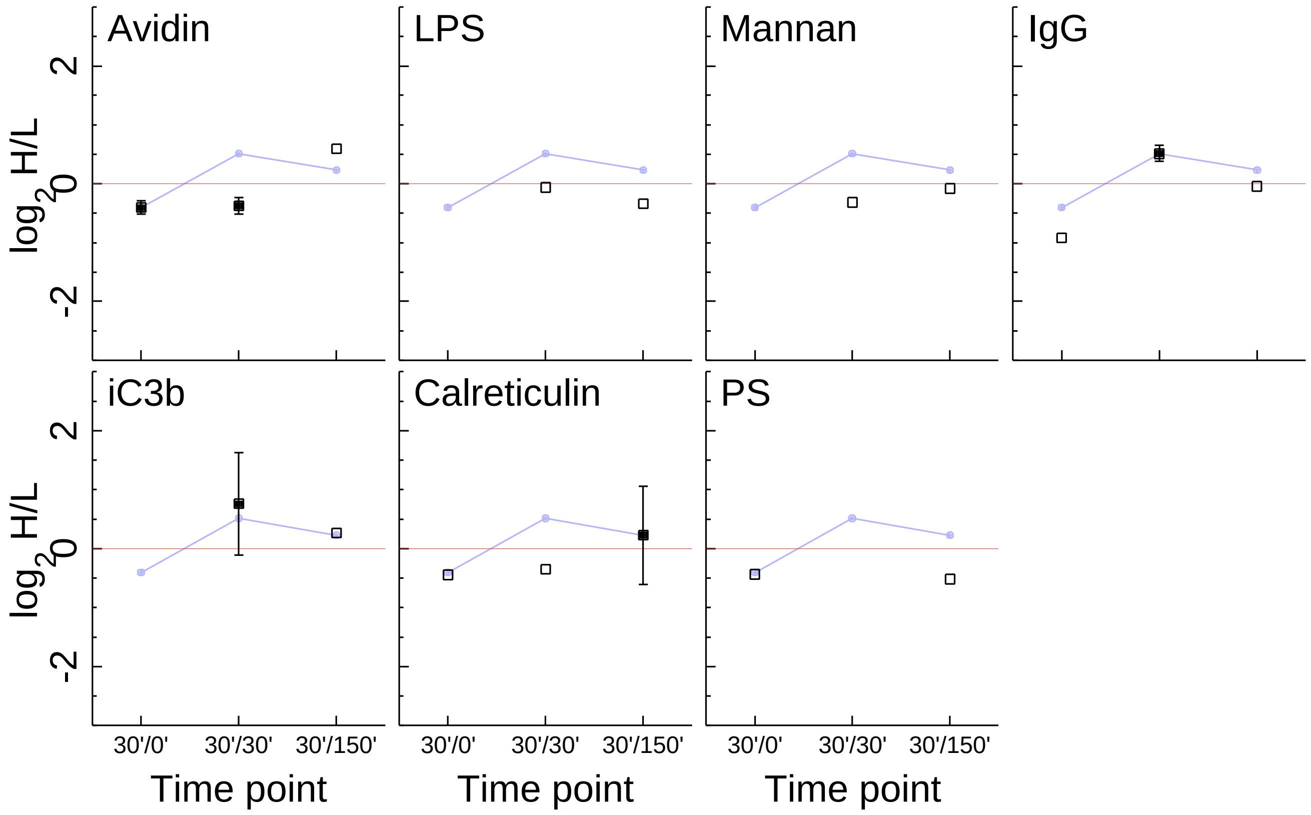

Avidin
LPS
Mannan
IgG
iC3b
Calreticulin
PS
log2 H/L
2
0
-2
30'/0'
30'/30'
30'/150'
Time point

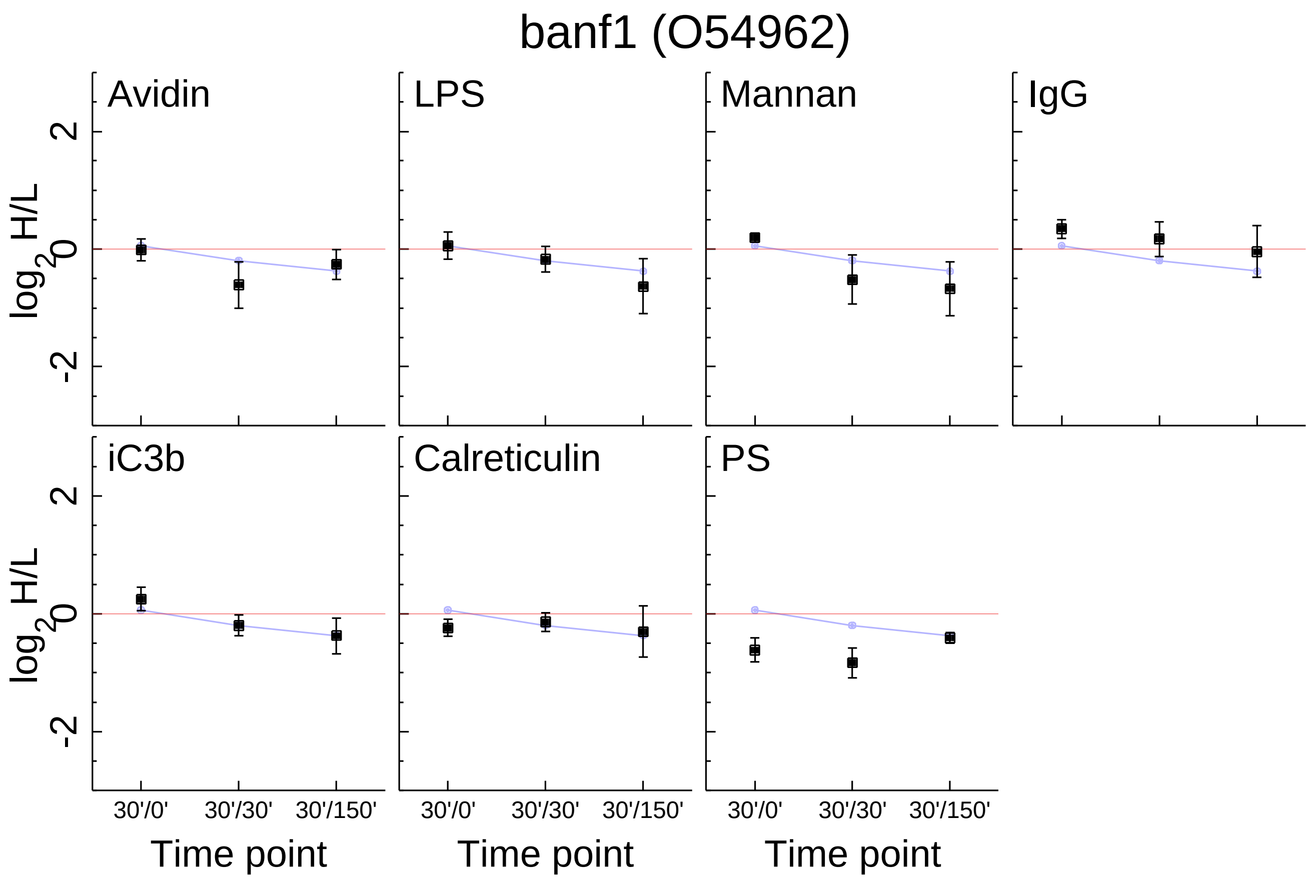
banf1 (O54962)
Avidin
LPS
Mannan
IgG
iC3b
Calreticulin
PS
log2 H/L
2
0
-2
30'/0'
30'/30'
30'/150'
Time point

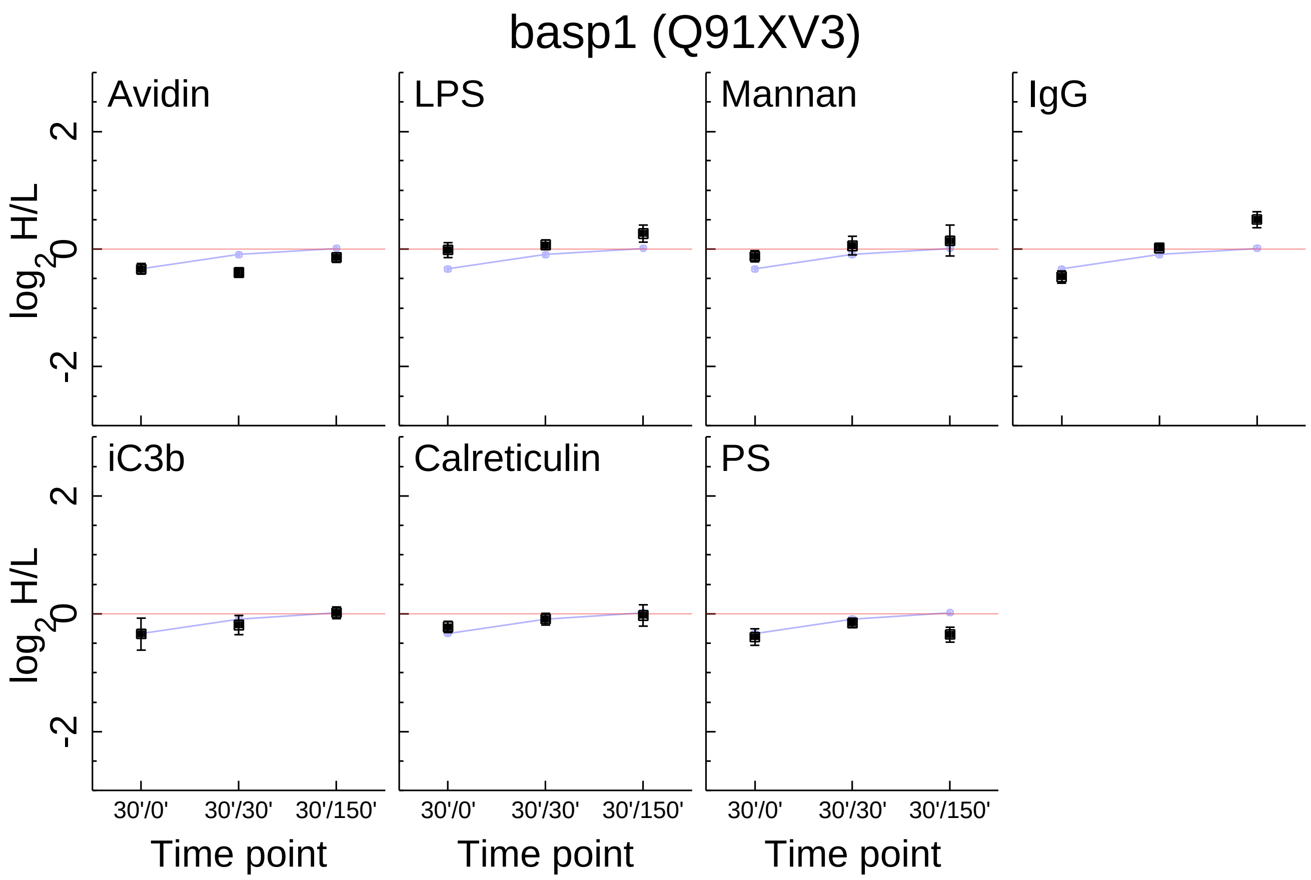

basp1 (Q91XV3)
Avidin
LPS
Mannan
IgG
iC3b
Calreticulin
PS
log2 H/L
2
0
-2
30'/0'
30'/30'
30'/150'
Time point

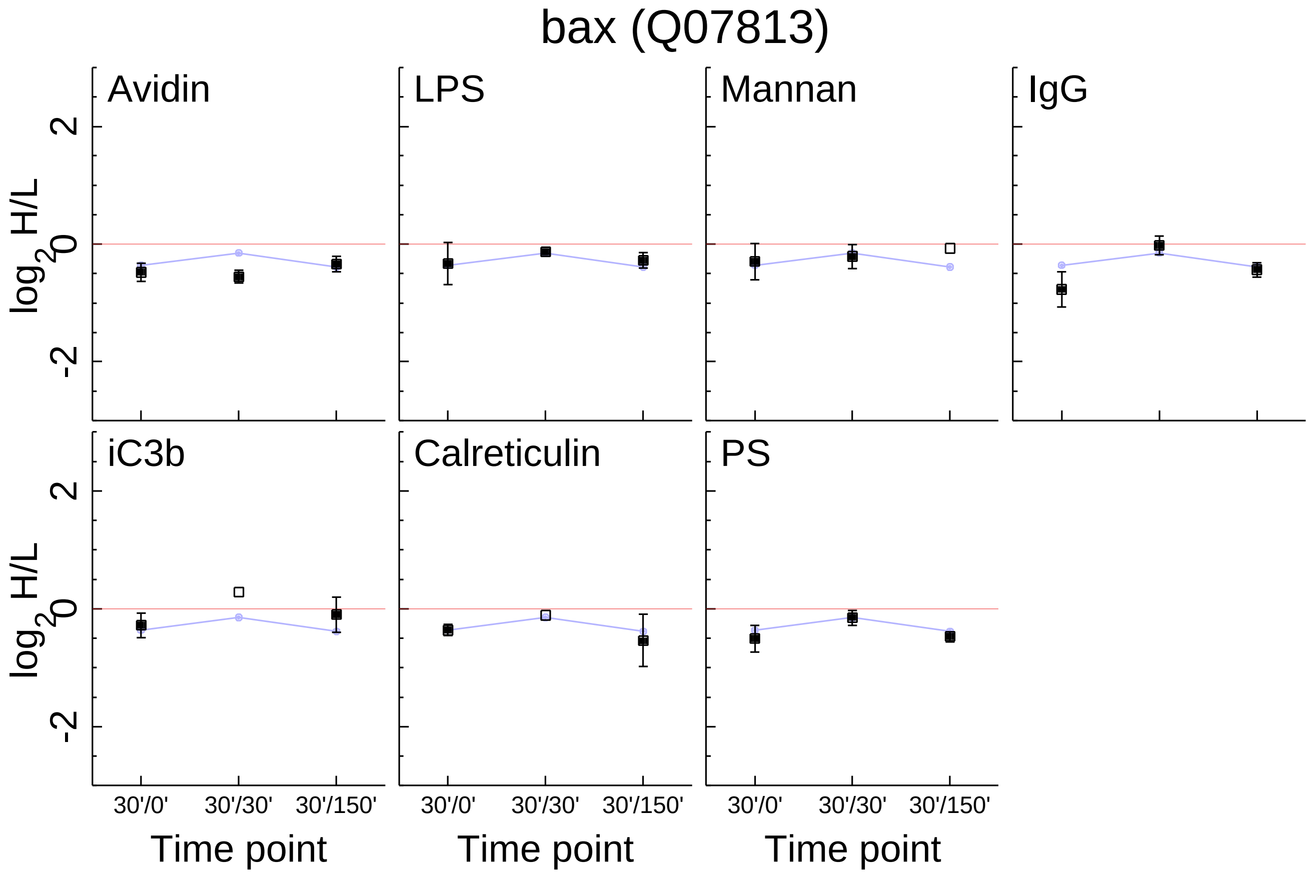

bax (Q07813)
Avidin
LPS
Mannan
IgG
iC3b
Calreticulin
PS
log2 H/L
2
0
-2
30'/0'
30'/30'
30'/150'
Time point

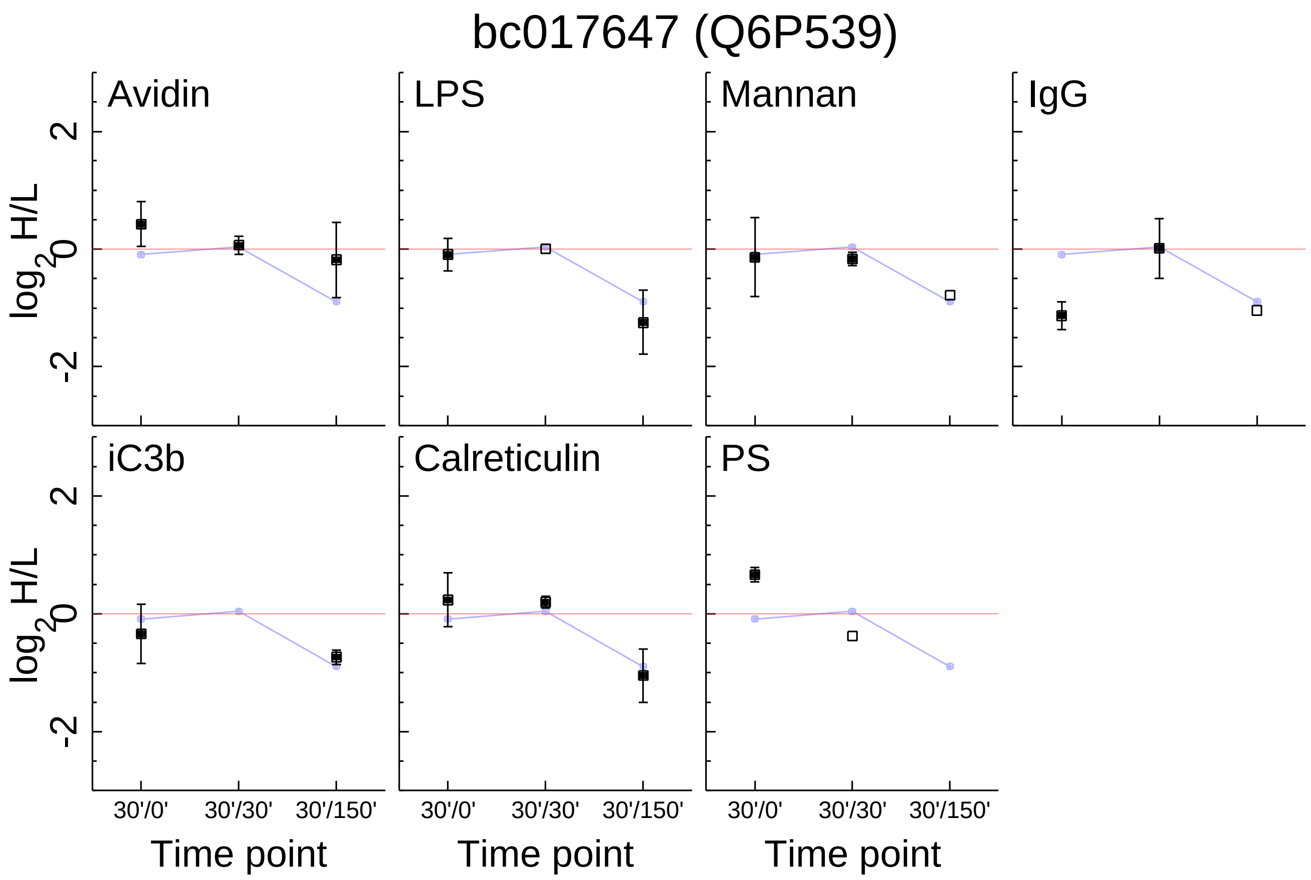

bc017647 (Q6P539)
Avidin
LPS
Mannan
IgG
iC3b
Calreticulin
PS
log2 H/L
2
0
-2
30'/0'
30'/30'
30'/150'
Time point

bcap31 (Q61335)

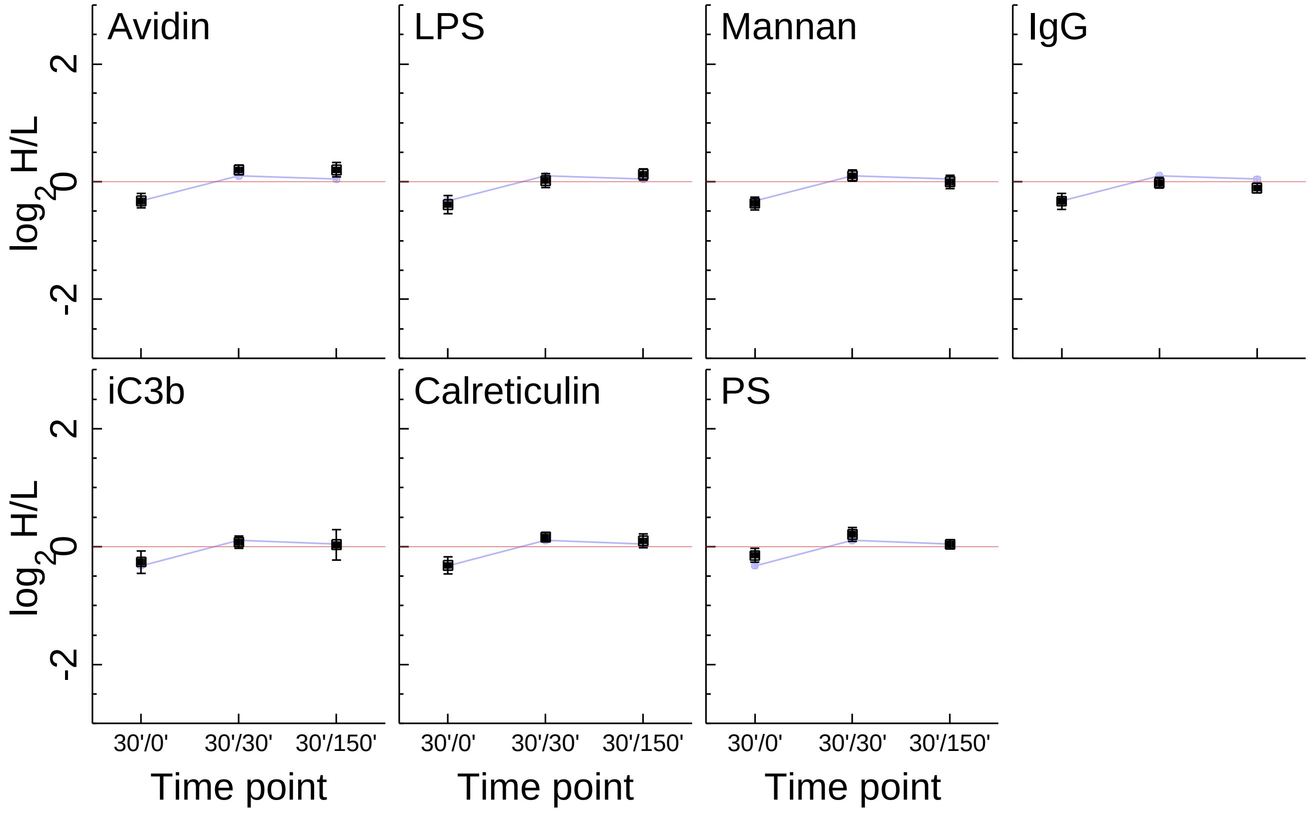

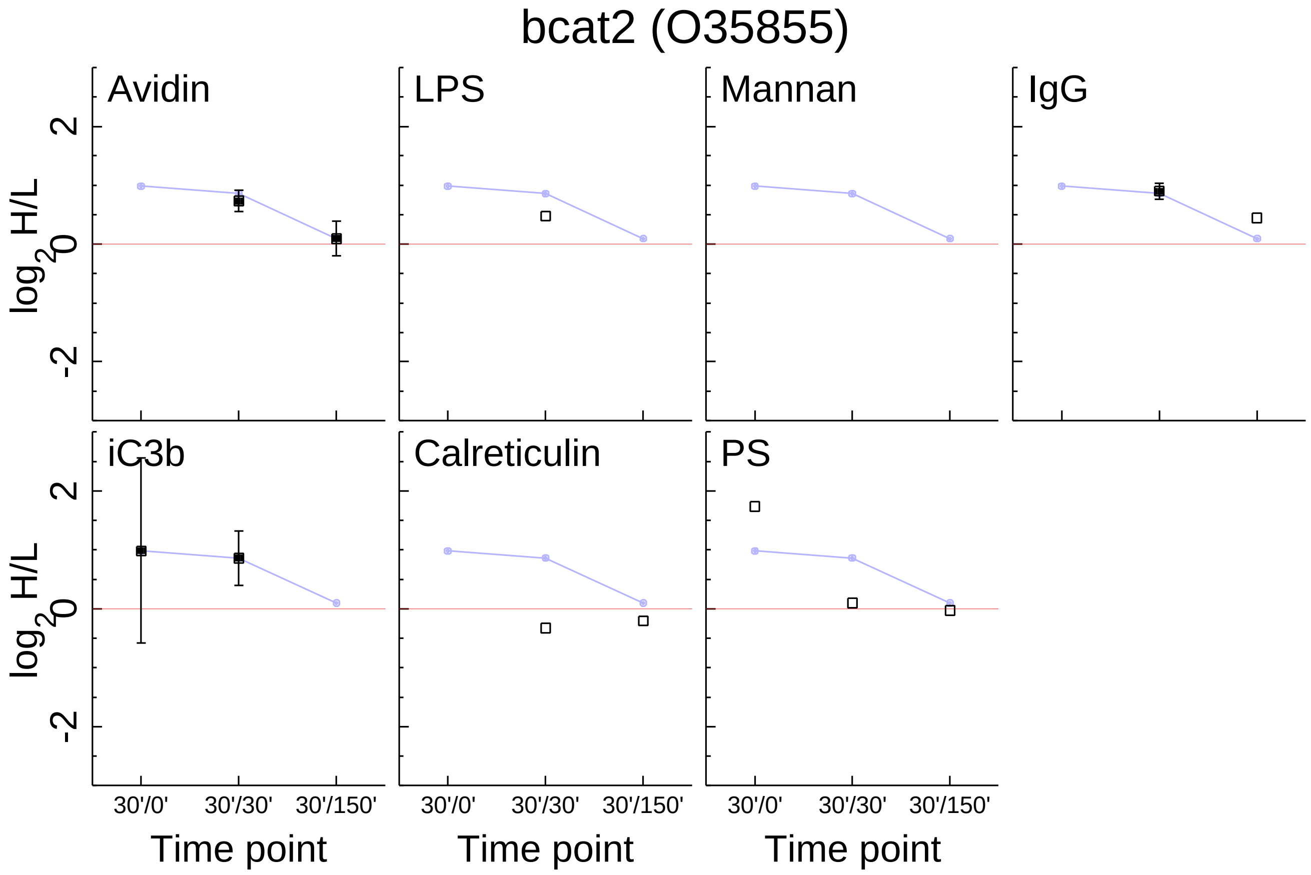

bcat2 (O35855)
Avidin
LPS
Mannan
IgG
iC3b
Calreticulin
PS
log2 H/L
2
0
-2
30'/0'
30'/30'
30'/150'
Time point

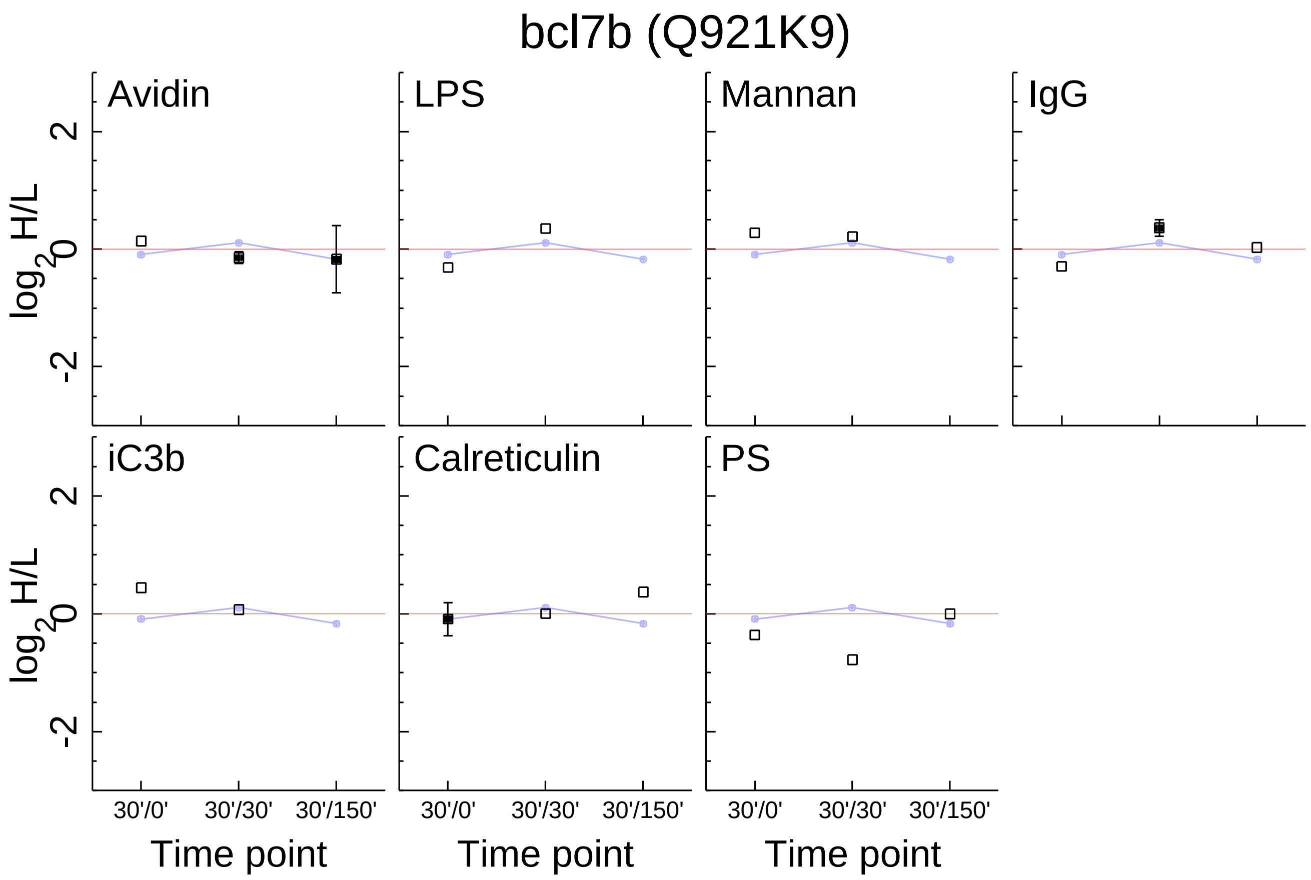

bcl7b (Q921K9)
Avidin
LPS
Mannan
IgG
iC3b
Calreticulin
PS
log2 H/L
2
0
-2
30'/0'
30'/30'
30'/150'
Time point

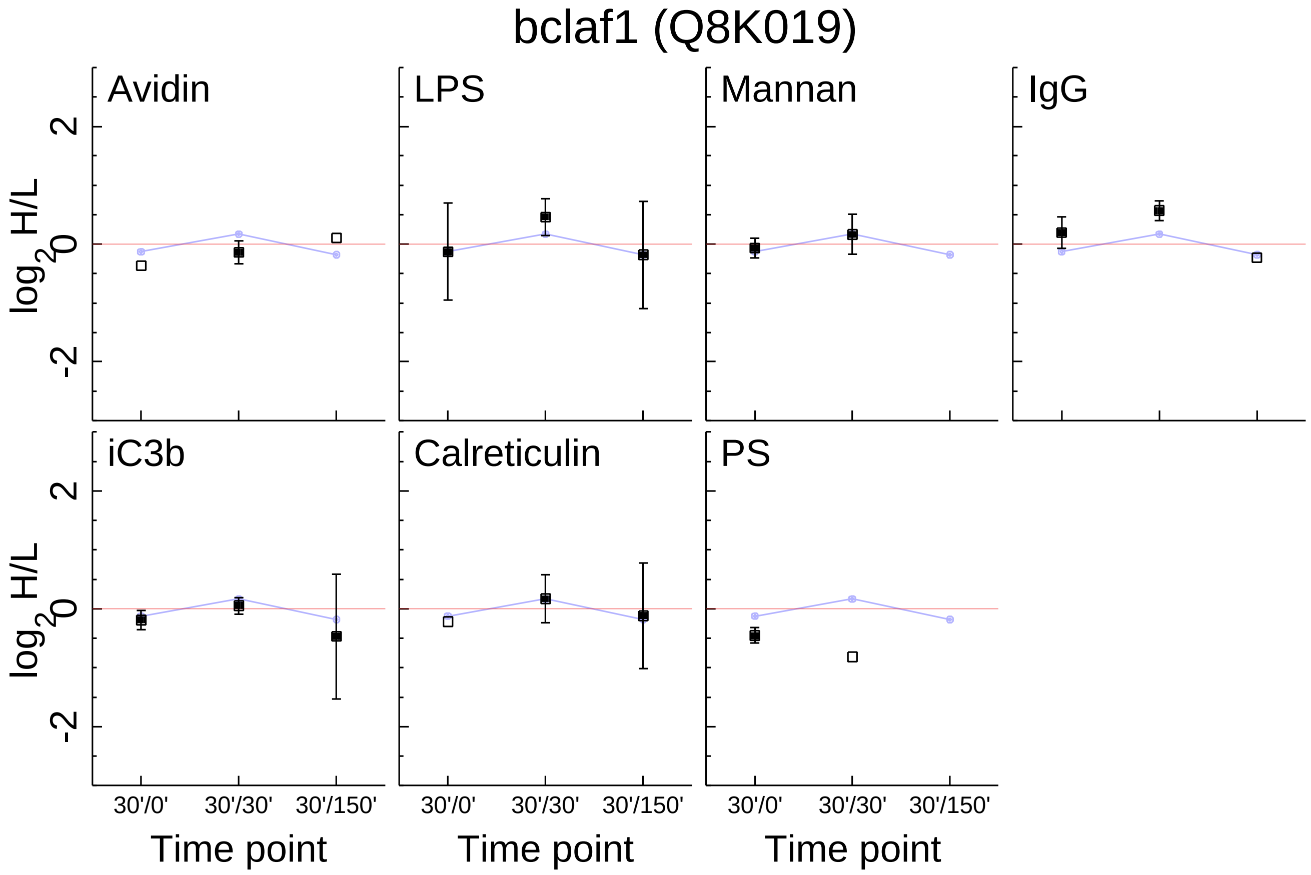

bclaf1 (Q8K019)
Avidin
LPS
Mannan
IgG
iC3b
Calreticulin
PS
log2 H/L
2
0
-2
30'/0'
30'/30'
30'/150'
Time point

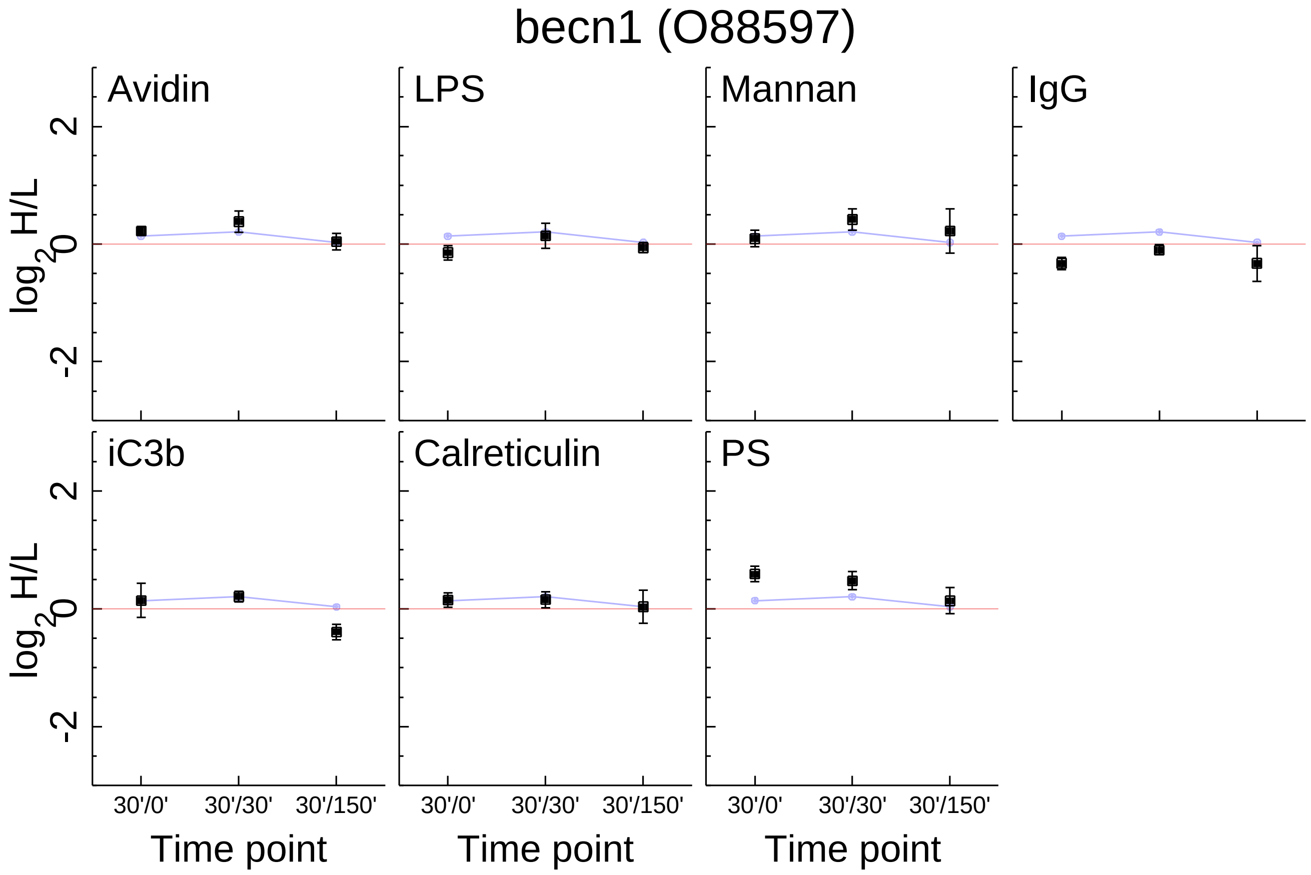
becn1 (O88597)
Avidin
LPS
Mannan
IgG
iC3b
Calreticulin
PS
log2 H/L
2
0
-2
30'/0'
30'/30'
30'/150'
Time point

bin1 (O08539)

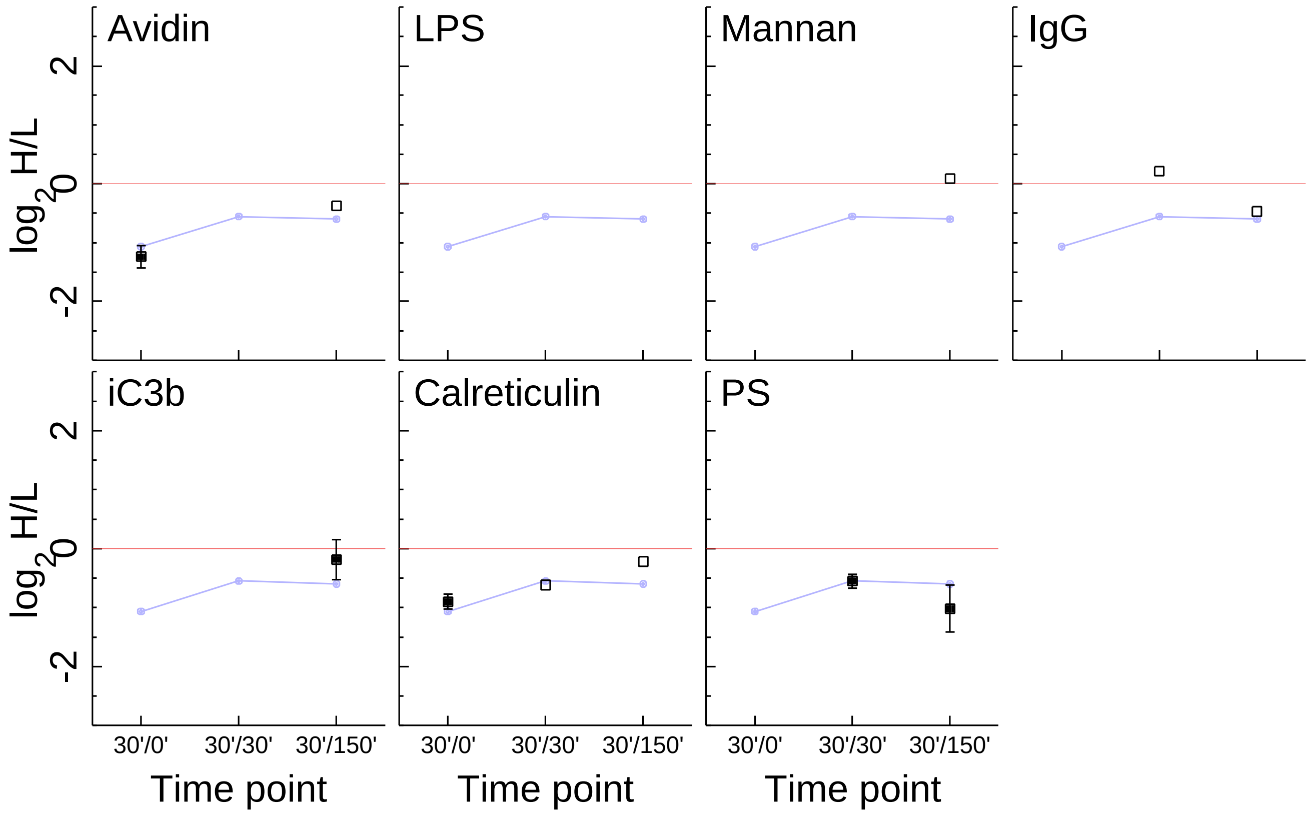

Avidin
LPS
Mannan
IgG
iC3b
Calreticulin
PS
log2 H/L
2
0
-2
30'/0'
30'/30'
30'/150'
Time point

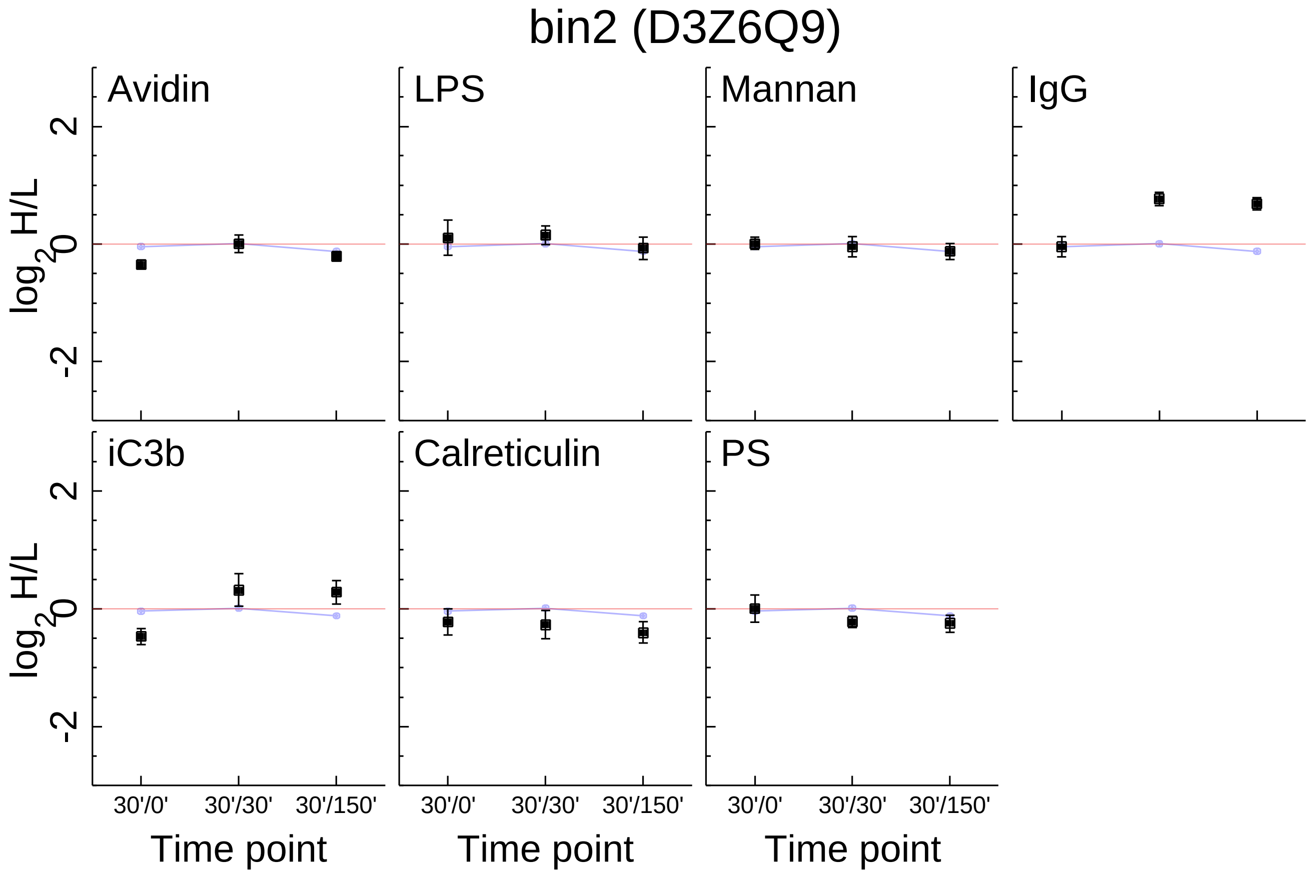
bin2 (D3Z6Q9)
Avidin
LPS
Mannan
IgG
iC3b
Calreticulin
PS
log2 H/L
2
0
-2
30'/0'
30'/30'
30'/150'
Time point

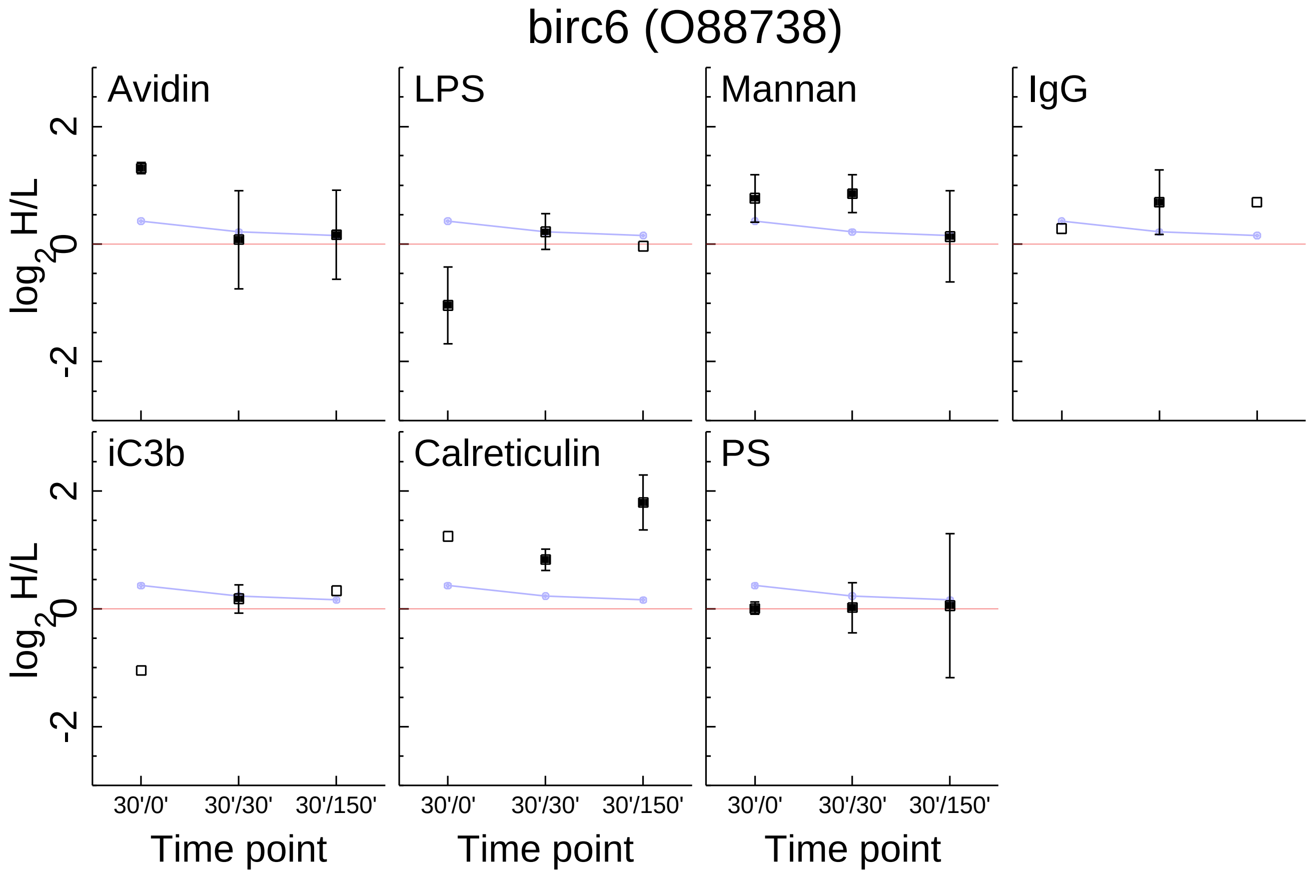

birc6 (O88738)
Avidin
LPS
Mannan
IgG
iC3b
Calreticulin
PS
log2 H/L
2
0
-2
30'/0'
30'/30'
30'/150'
Time point

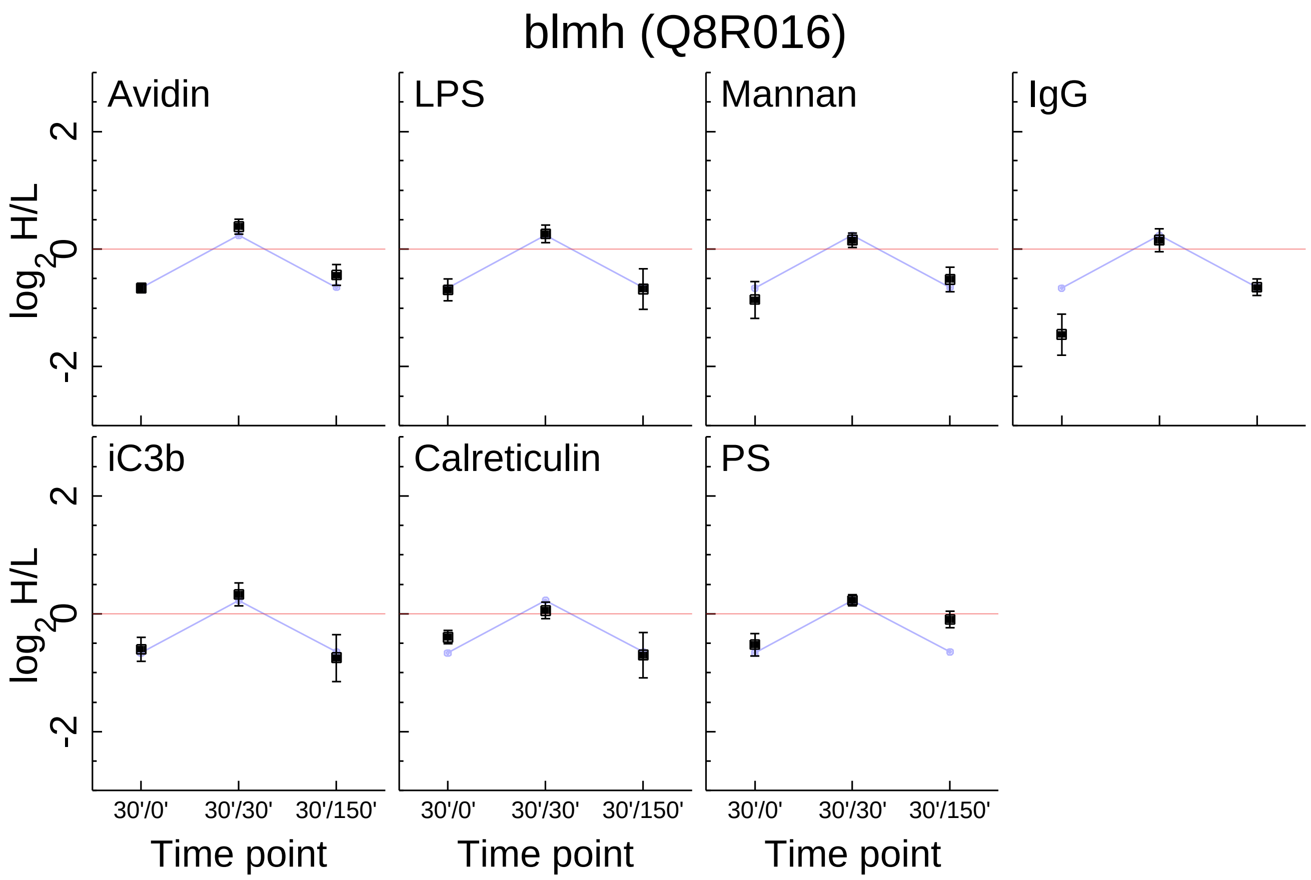

blmh (Q8R016)
Avidin
LPS
Mannan
IgG
iC3b
Calreticulin
PS
log2 H/L
2
0
-2
30'/0'
30'/30'
30'/150'
Time point

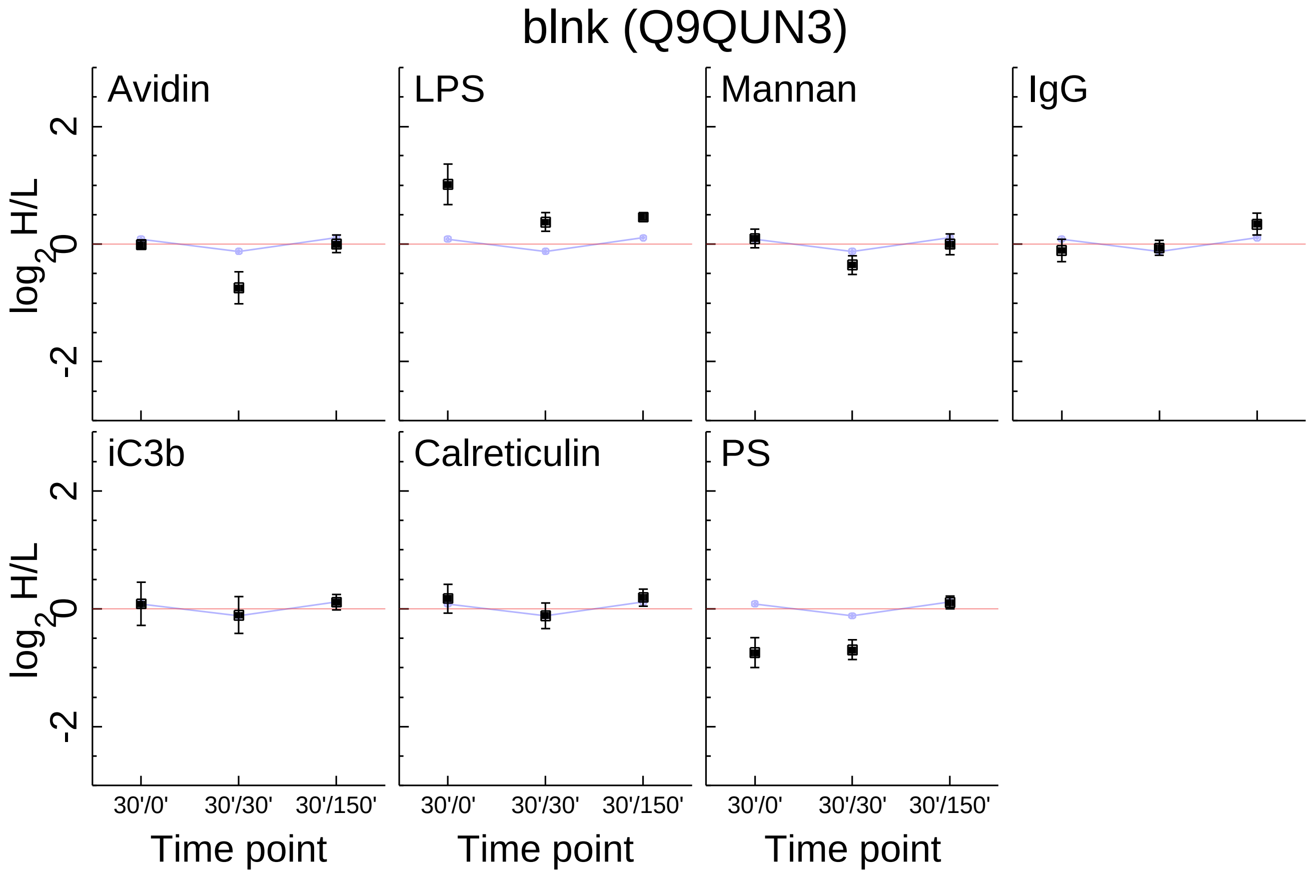

blnk (Q9QUN3)
Avidin
LPS
Mannan
IgG
iC3b
Calreticulin
PS
log2 H/L
2
0
-2
30'/0'
30'/30'
30'/150'
Time point

bloc1s1 (O55102)

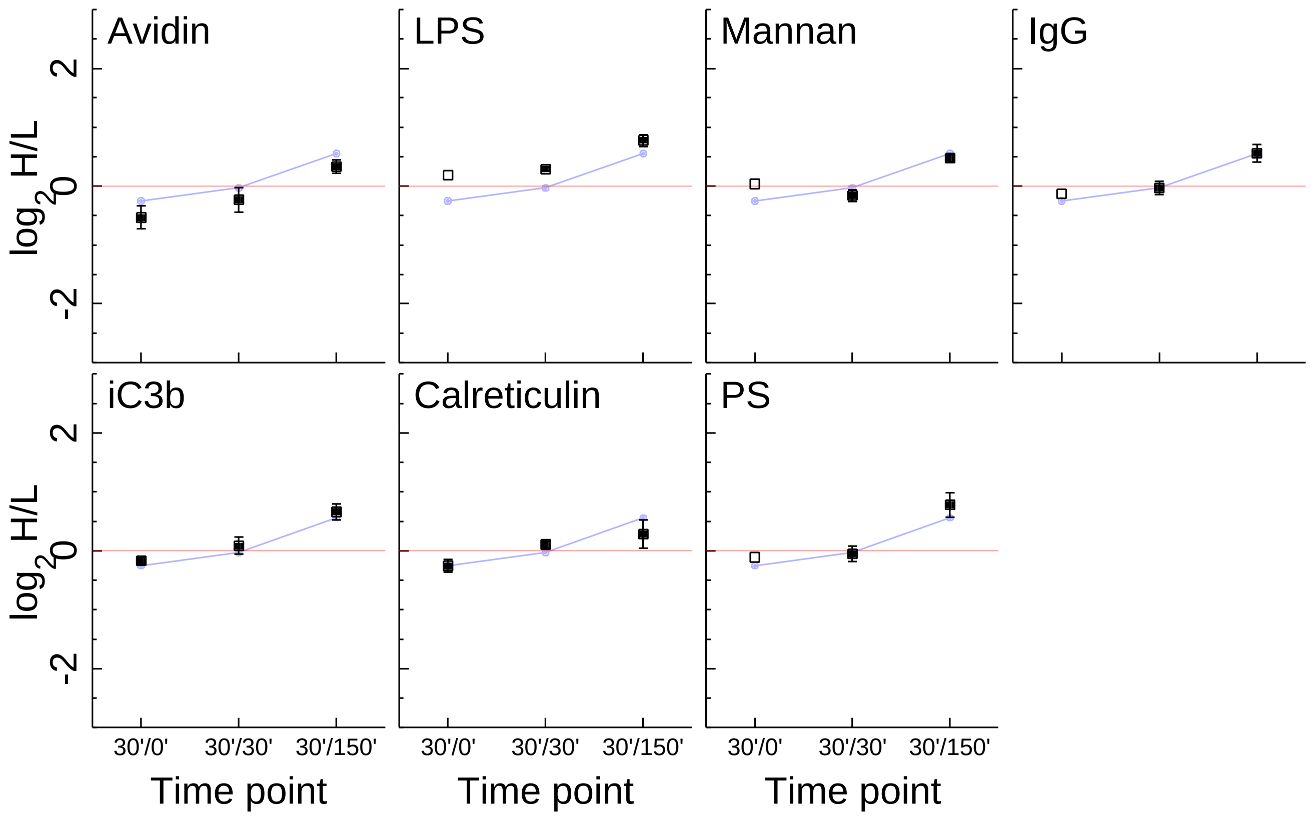

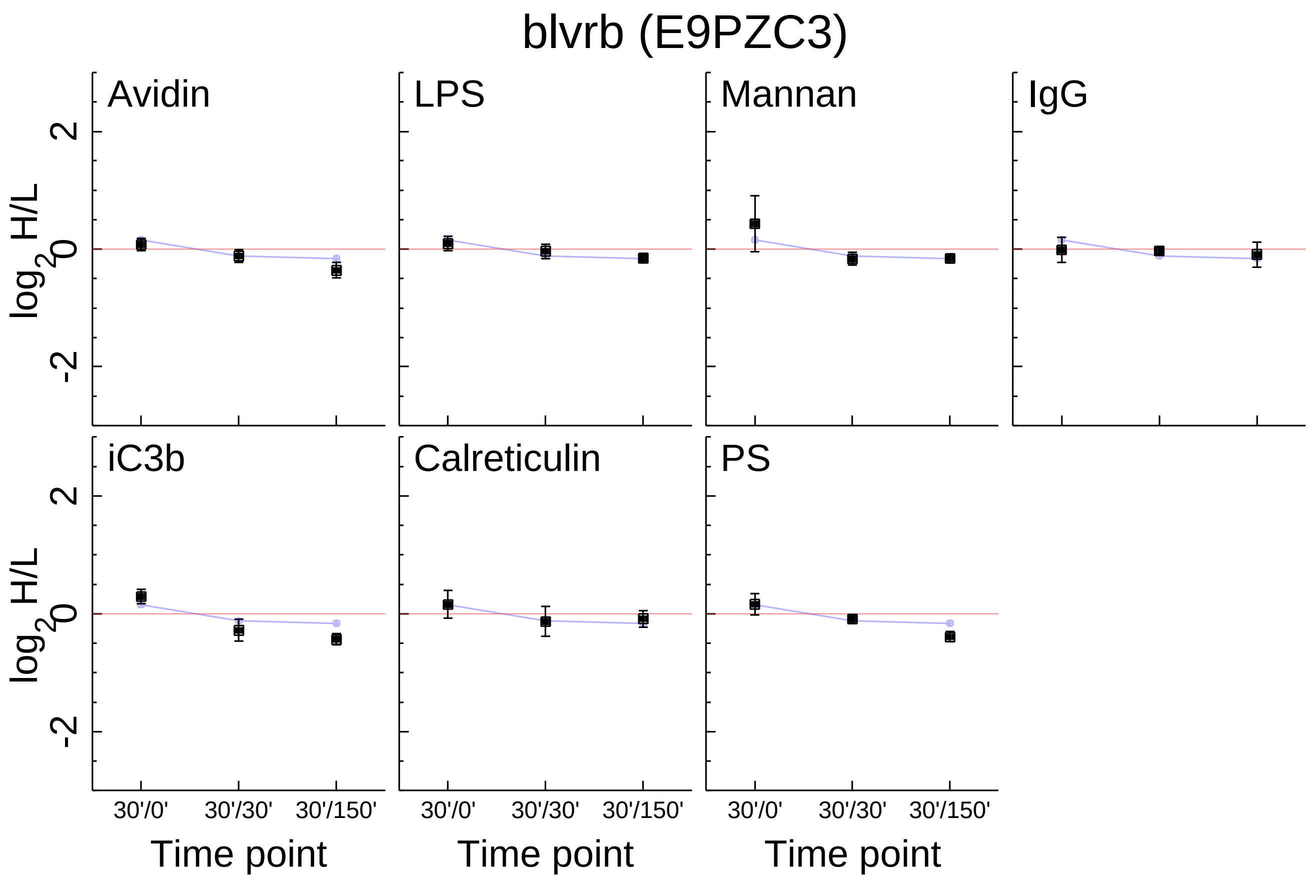

blvrb (E9PZC3)
Avidin
LPS
Mannan
IgG
iC3b
Calreticulin
PS
log2 H/L
2
0
-2
30'/0'
30'/30'
30'/150'
Time point

bmp2k (Q91Z96)

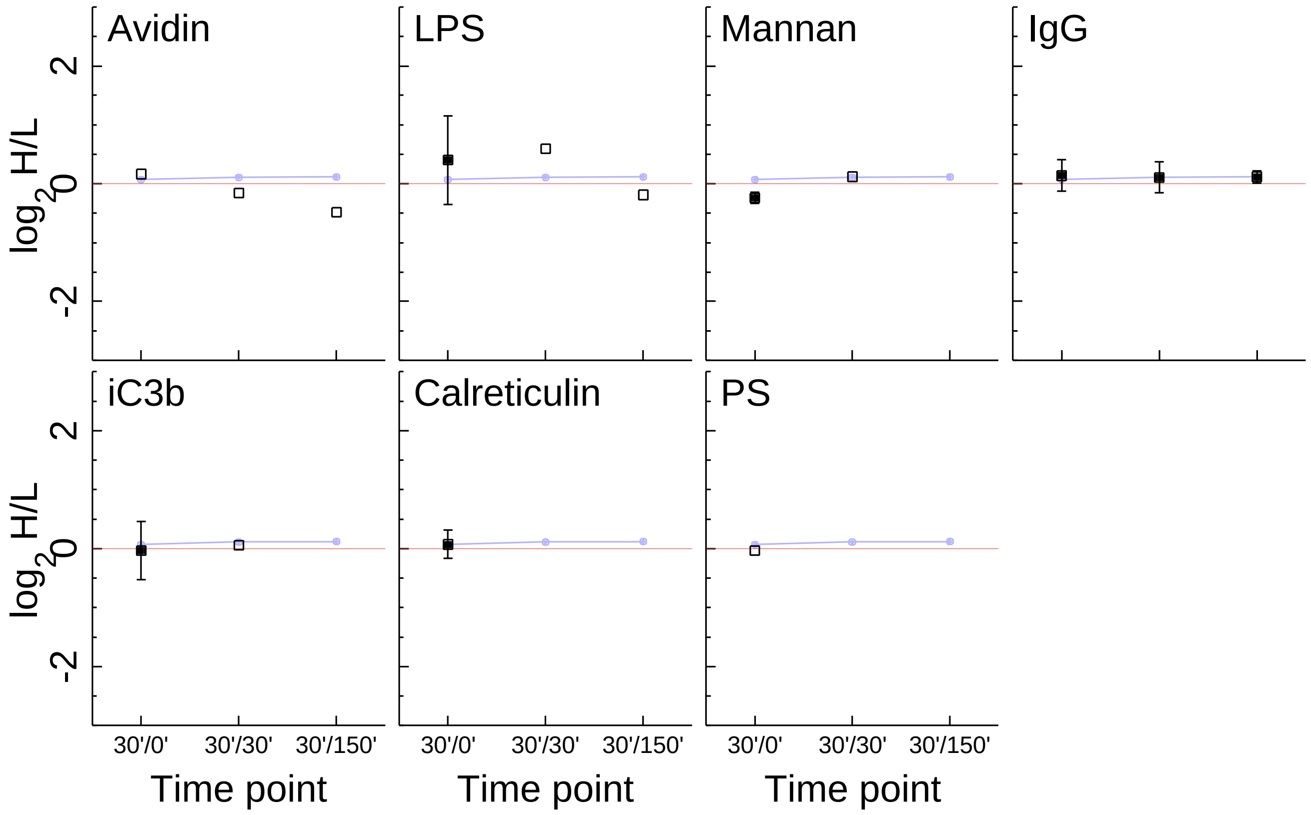

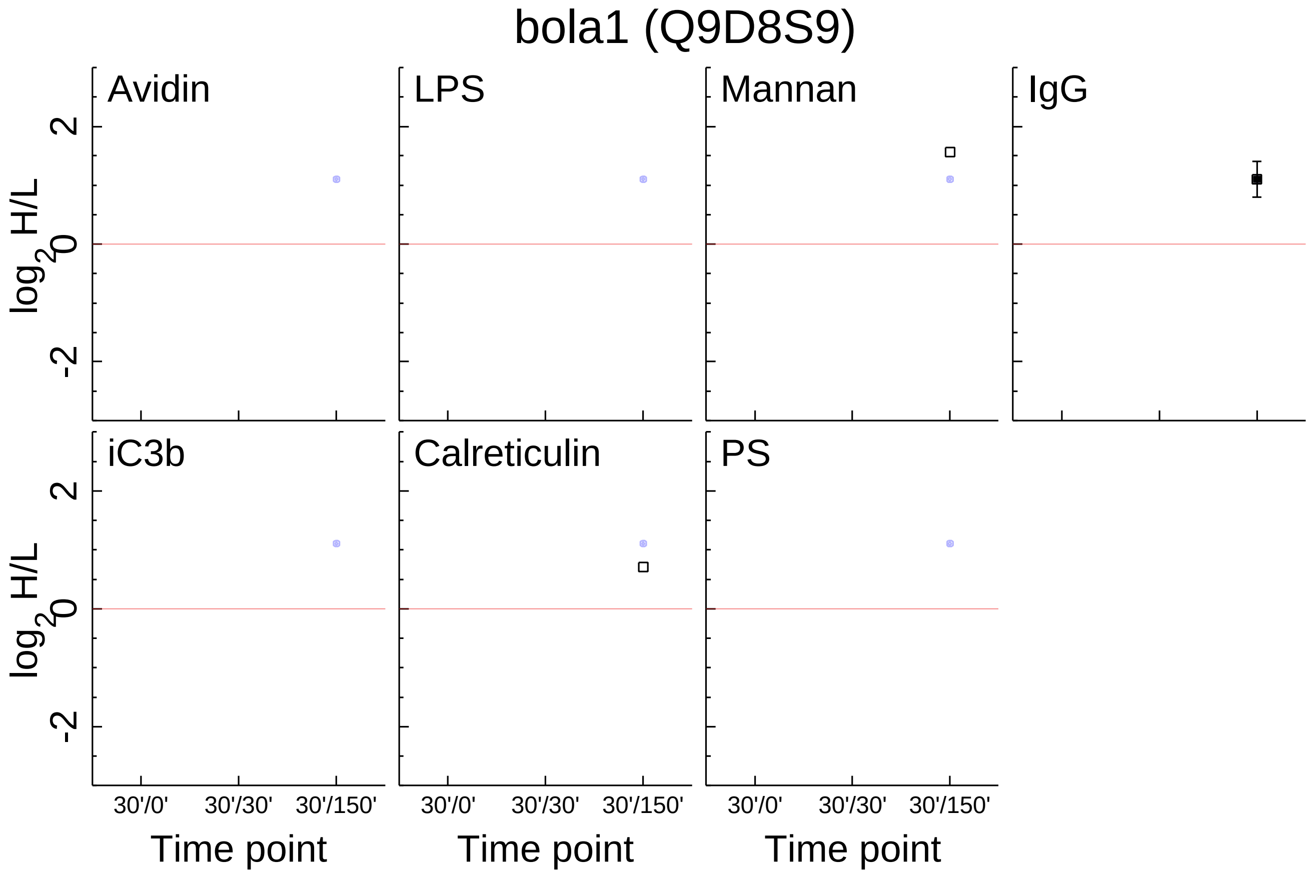
bola1 (Q9D8S9)
Avidin
LPS
Mannan
IgG
iC3b
Calreticulin
PS
log2 H/L
2
0
-2
30'/0'
30'/30'
30'/150'
Time point

bphl (Q8R164)

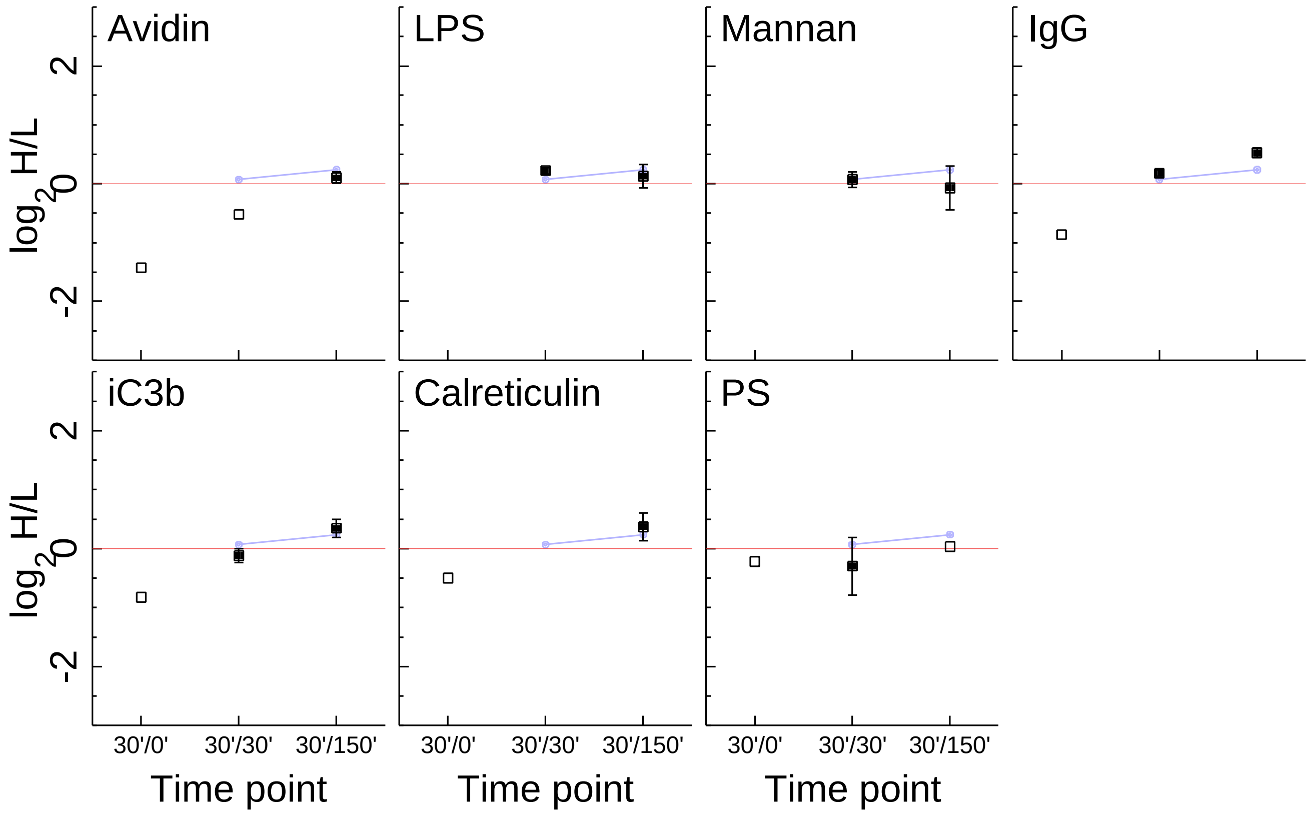

Avidin
LPS
Mannan
IgG
iC3b
Calreticulin
PS
log2 H/L
2
0
-2
30'/0'
30'/30'
30'/150'
Time point

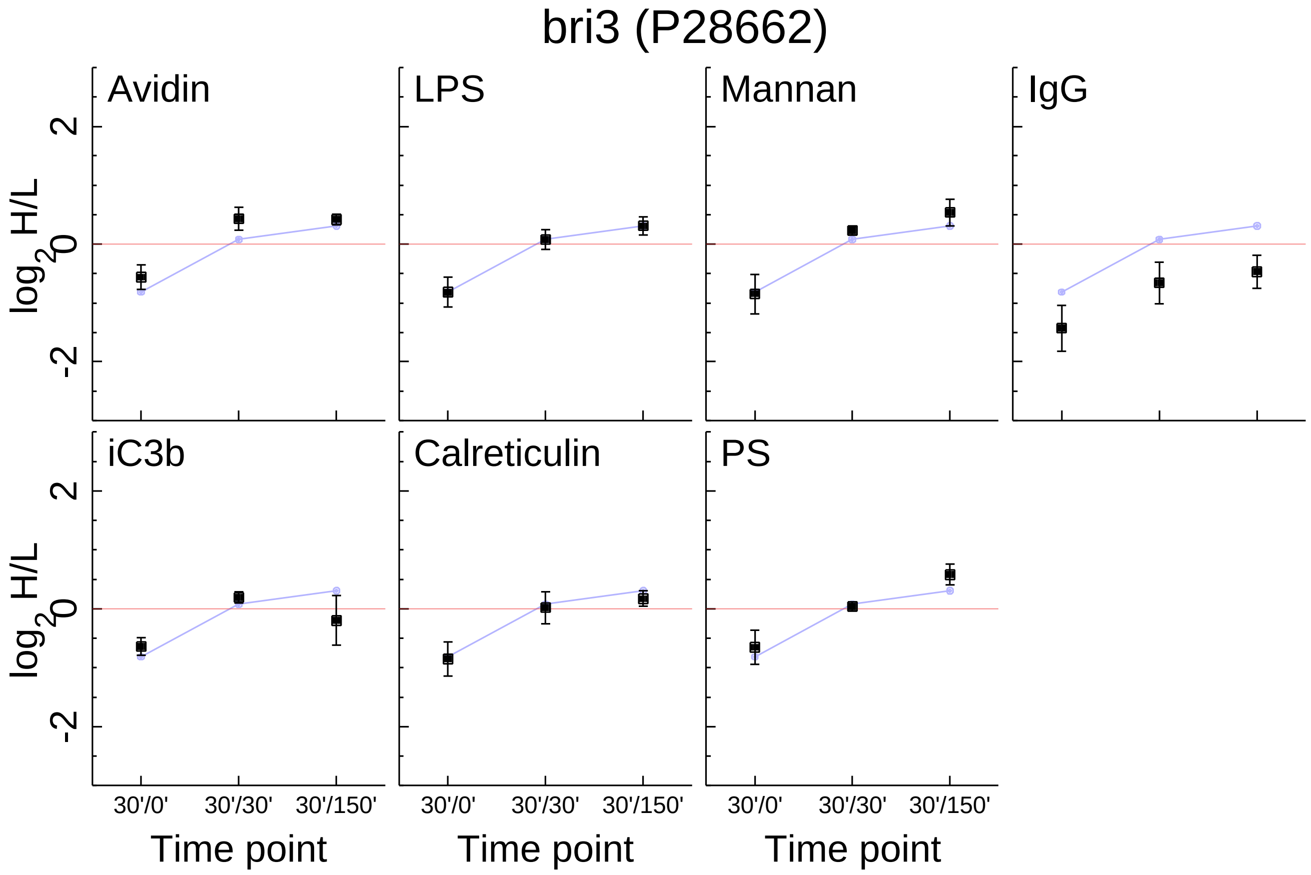
bri3 (P28662)
Avidin
LPS
Mannan
IgG
iC3b
Calreticulin
PS
log2 H/L
2
0
-2
30'/0'
30'/30'
30'/150'
Time point

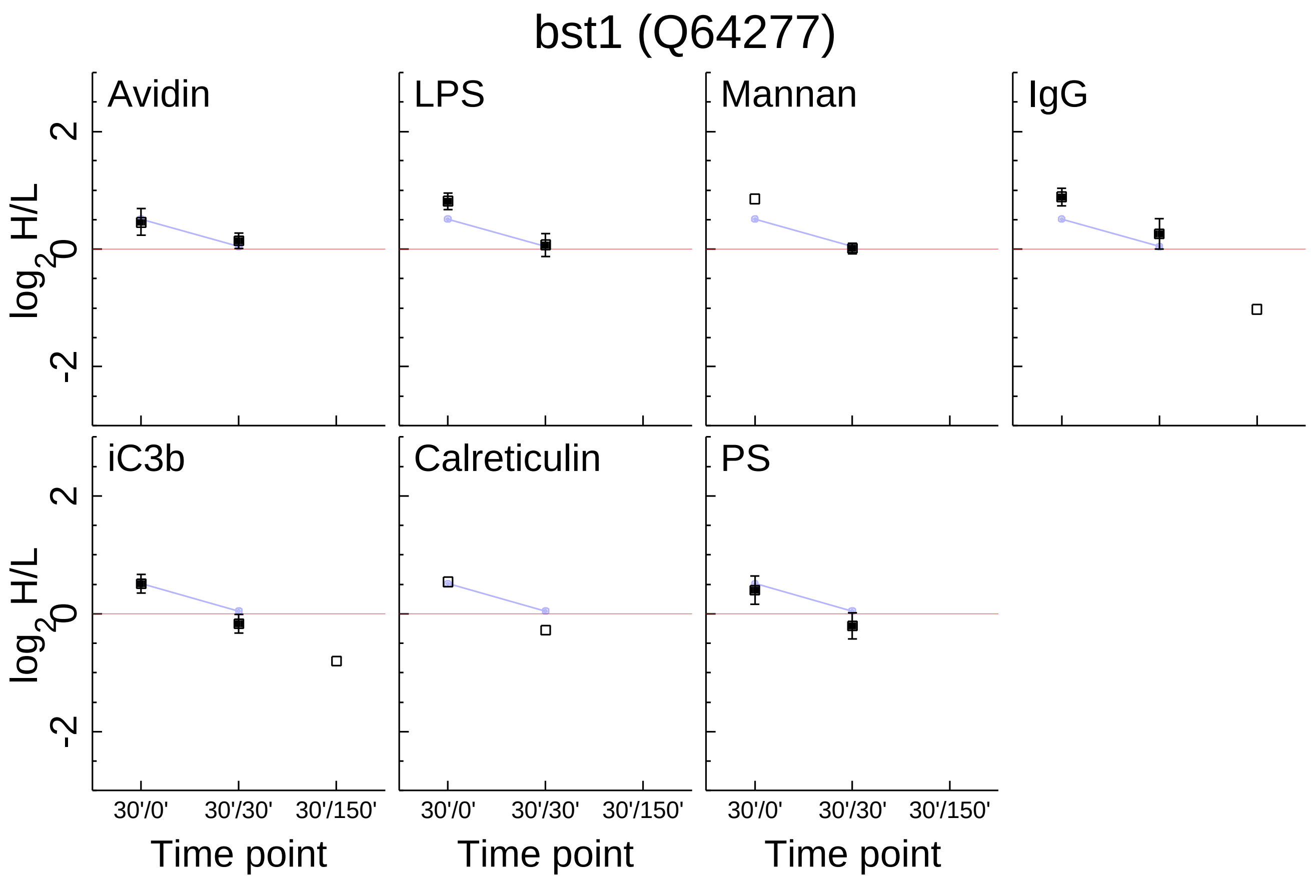
bst1 (Q64277)
Avidin
LPS
Mannan
IgG
iC3b
Calreticulin
PS
log2 H/L
2
0
-2
30'/0'
30'/30'
30'/150'
Time point

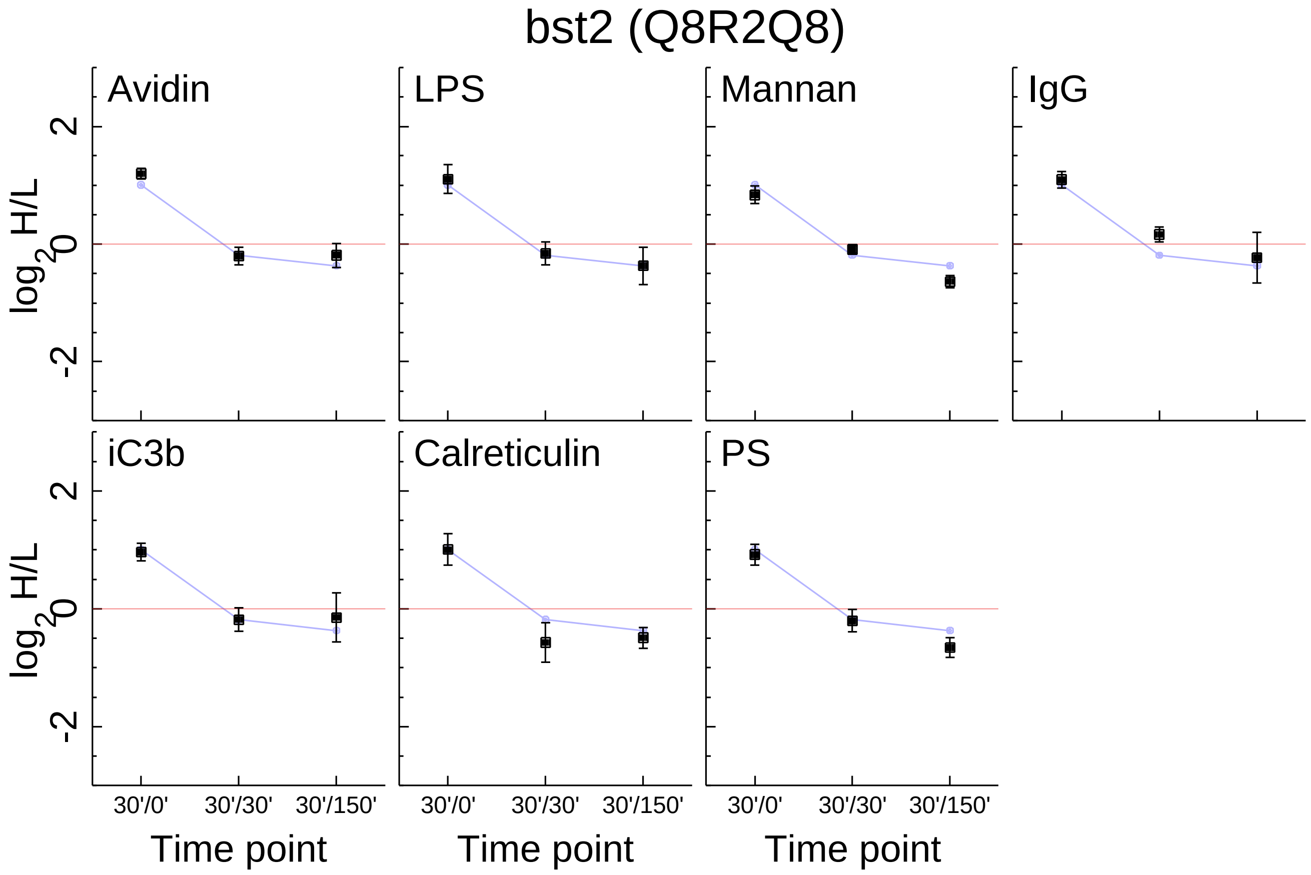
bst2 (Q8R2Q8)
Avidin
LPS
Mannan
IgG
iC3b
Calreticulin
PS
log2 H/L
2
0
-2
30'/0'
30'/30'
30'/150'
Time point

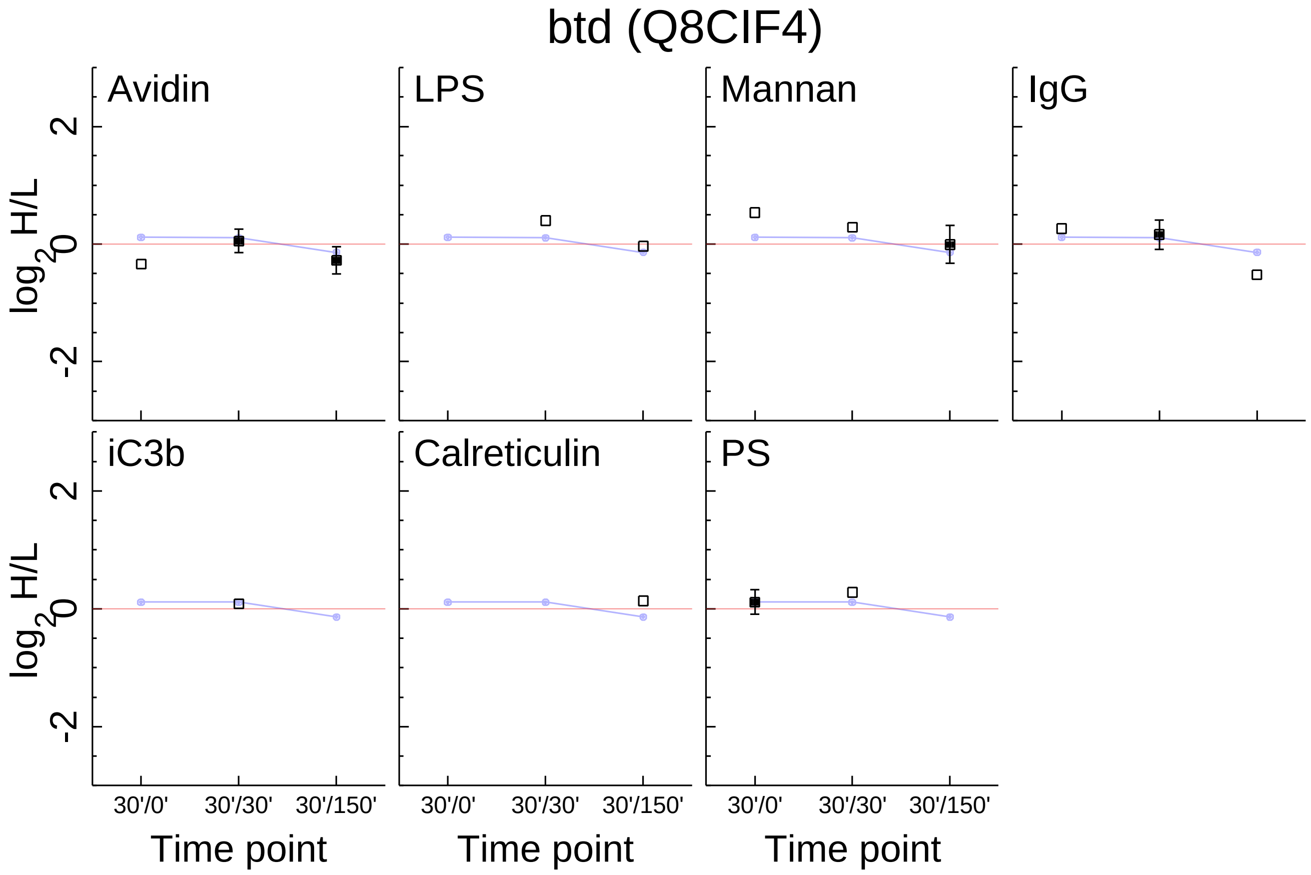

btd (Q8CIF4)
Avidin
LPS
Mannan
IgG
iC3b
Calreticulin
PS
log2 H/L
2
0
-2
30'/0'
30'/30'
30'/150'
Time point

btf3 (Q64152)

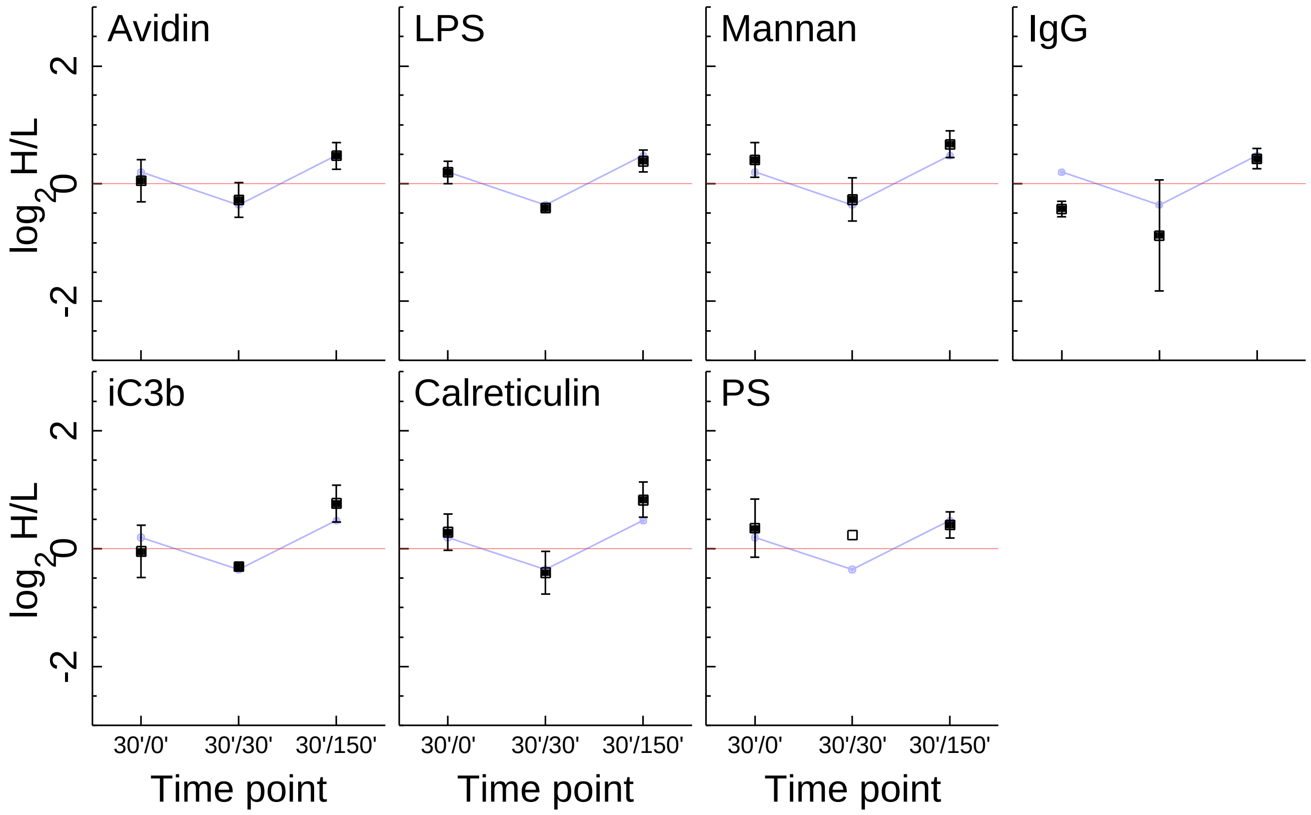

Avidin
LPS
Mannan
IgG
iC3b
Calreticulin
PS
log2 H/L
2
0
-2
30'/0'
30'/30'
30'/150'
Time point

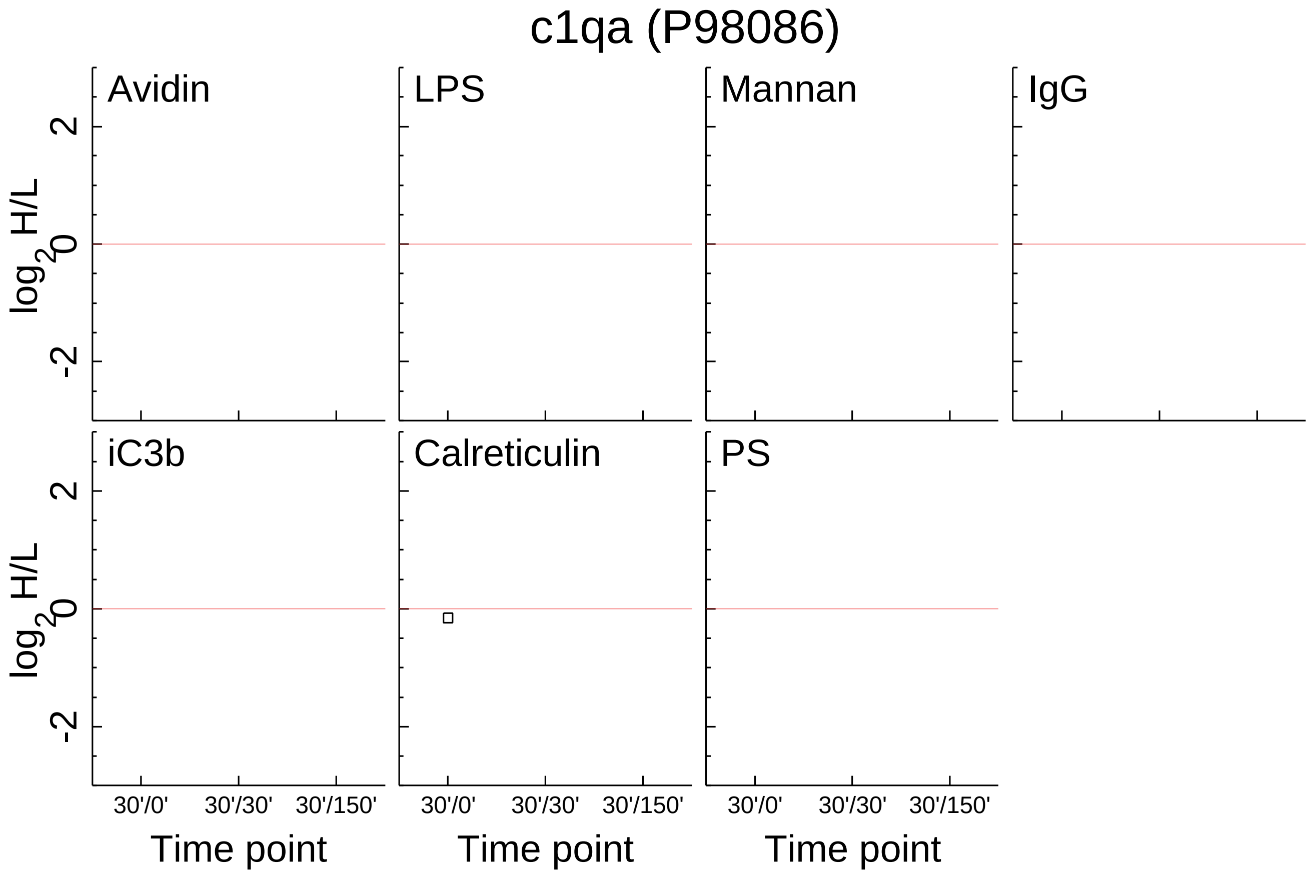
c1qa (P98086)
Avidin
LPS
Mannan
IgG
iC3b
Calreticulin
PS
2
0
-2
log2 H/L
30'/0'
30'/30'
30'/150'
Time point

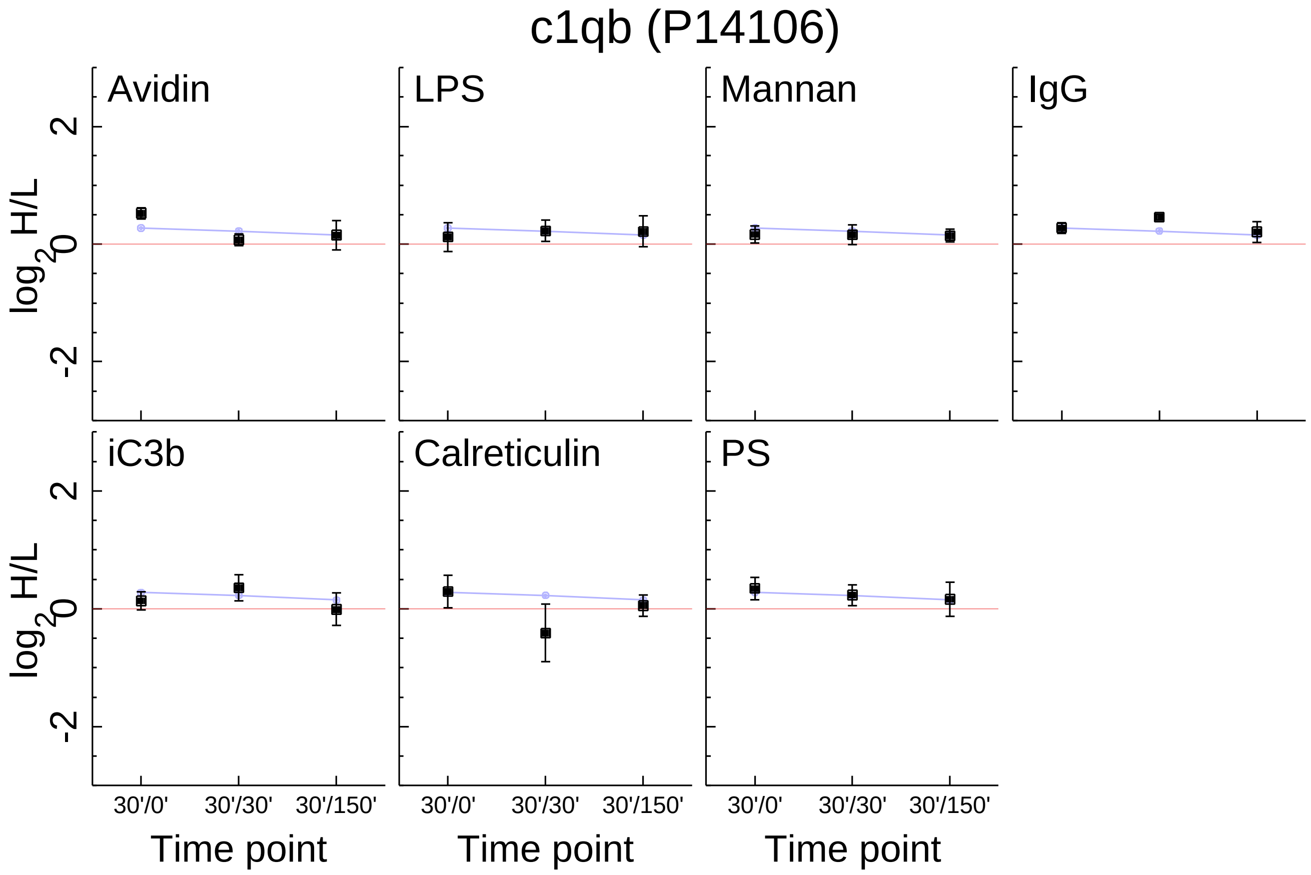
c1qb (P14106)
Avidin
LPS
Mannan
IgG
iC3b
Calreticulin
PS
log2 H/L
2
0
-2
30'/0'
30'/30'
30'/150'
Time point

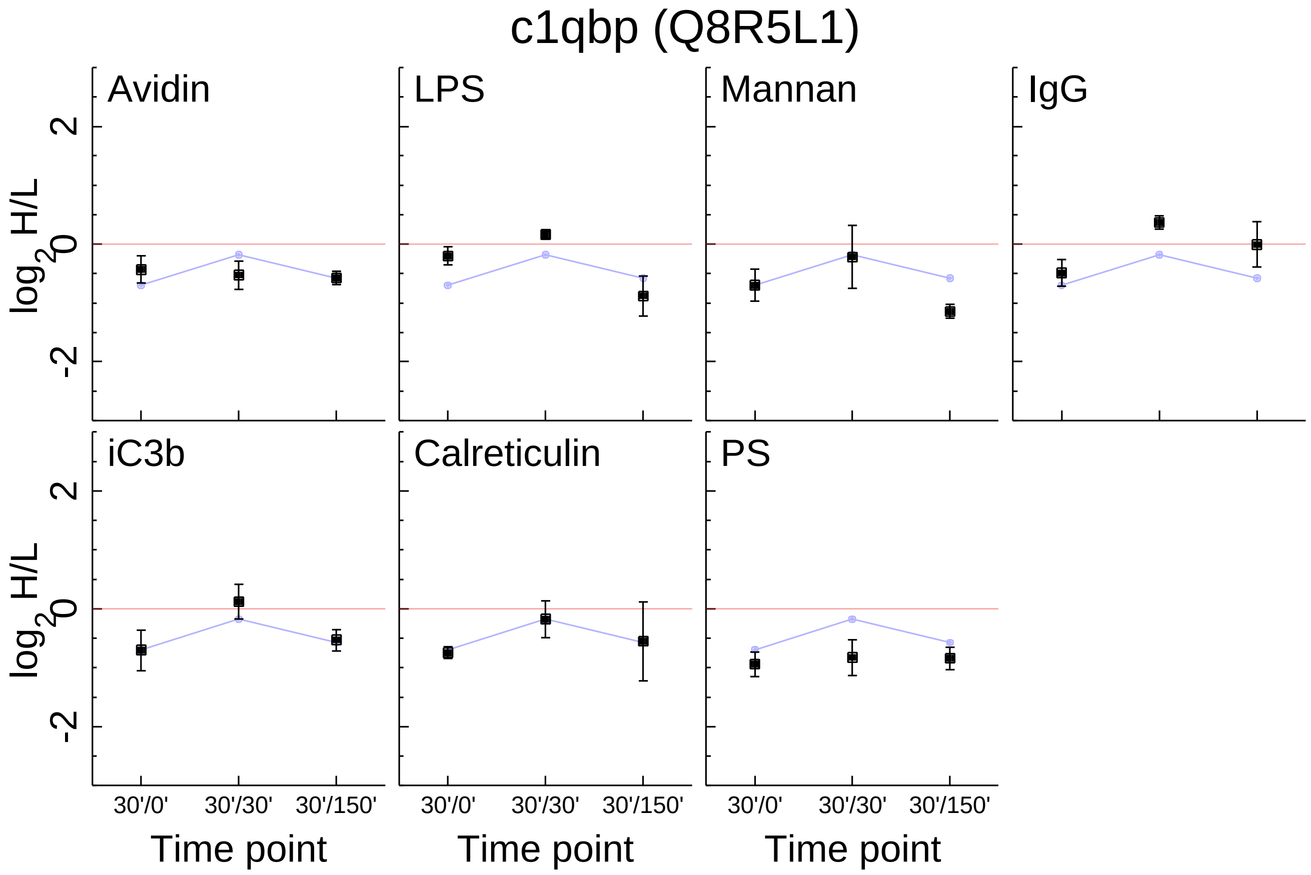

c1qbp (Q8R5L1)
Avidin
LPS
Mannan
IgG
iC3b
Calreticulin
PS
$\log_2$ H/L
2
0
-2
30'/0'
30'/30'
30'/150'
Time point

c1qc (Q02105)

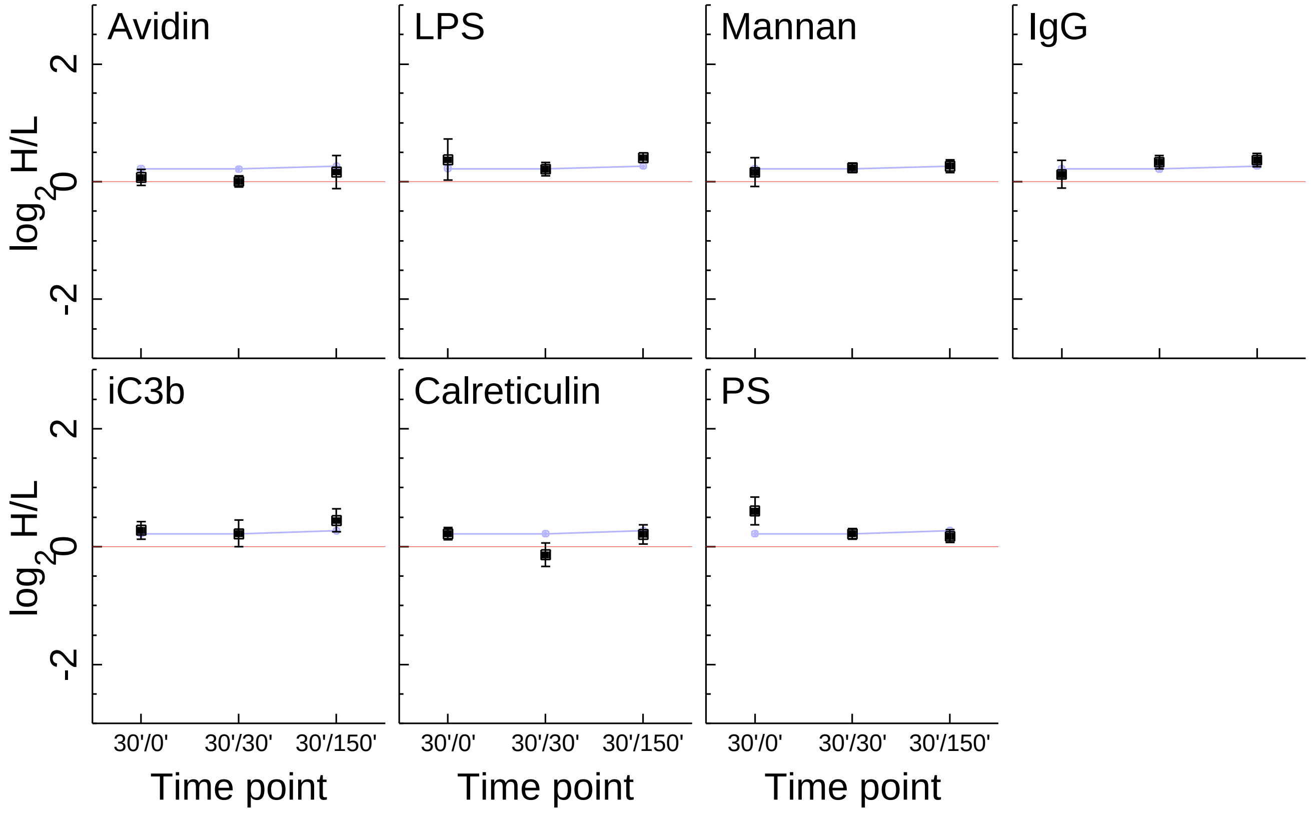
Avidin
LPS
Mannan
IgG
iC3b
Calreticulin
PS
log2 H/L
2
0
-2
30'/0'
30'/30'
30'/150'
Time point

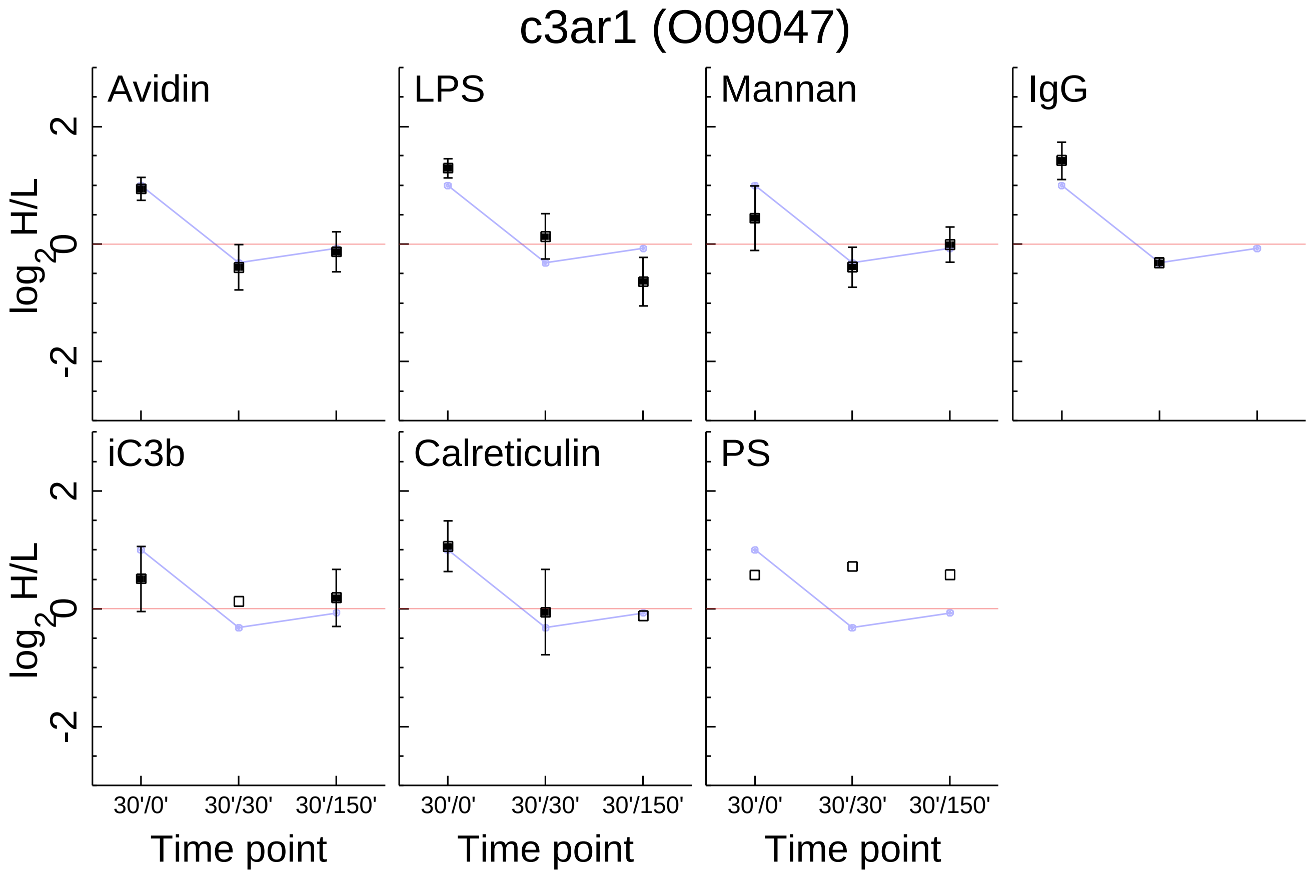

c3ar1 (O09047)
Avidin
LPS
Mannan
IgG
iC3b
Calreticulin
PS
log2 H/L
2
0
-2
30'/0'
30'/30'
30'/150'
Time point

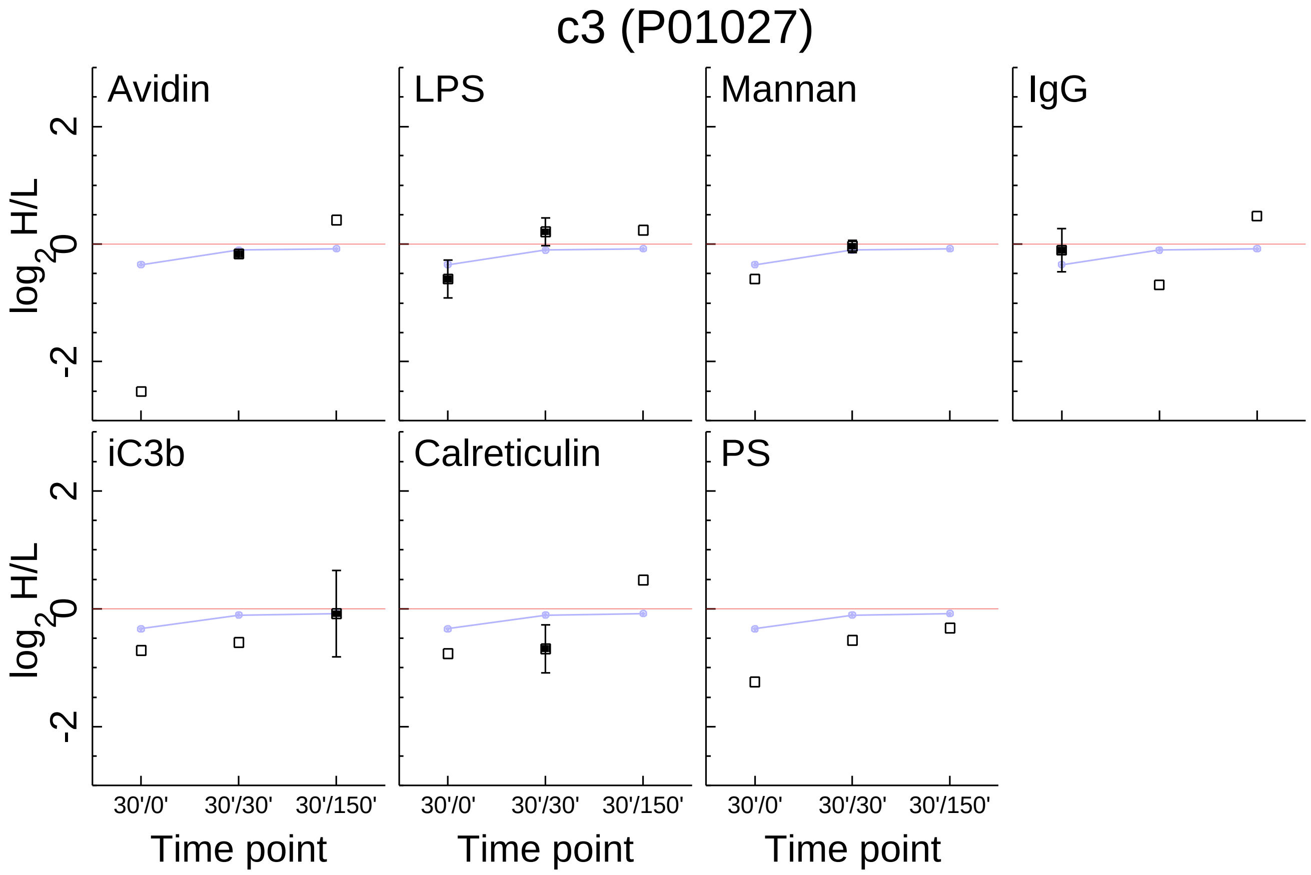
c3 (P01027)
Avidin
LPS
Mannan
IgG
iC3b
Calreticulin
PS
log2 H/L
2
0
-2
30'/0'
30'/30'
30'/150'
Time point

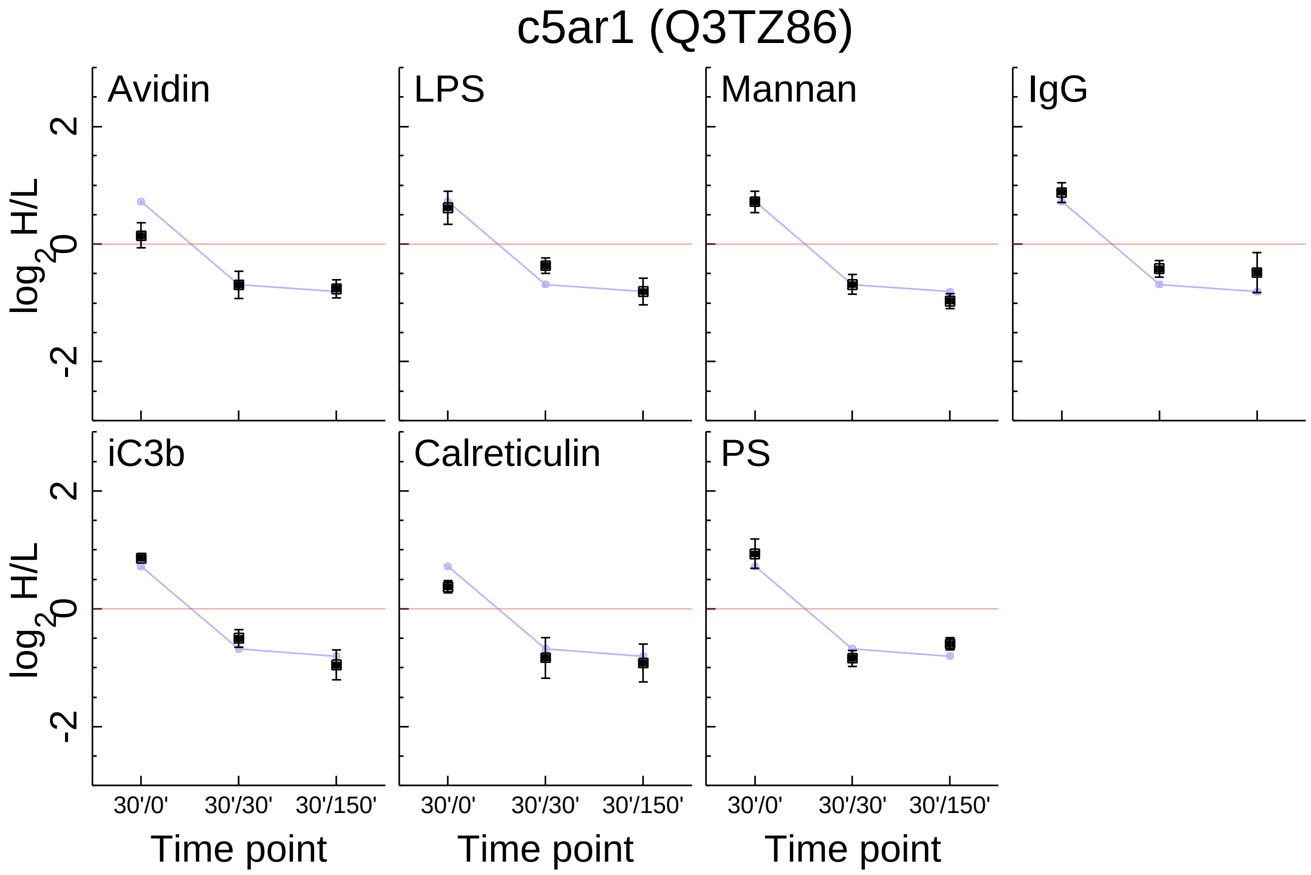

c5ar1 (Q3TZ86)
Avidin
LPS
Mannan
IgG
iC3b
Calreticulin
PS
log2 H/L
2
0
-2
30'/0'
30'/30'
30'/150'
Time point

cab39 (Q06138)

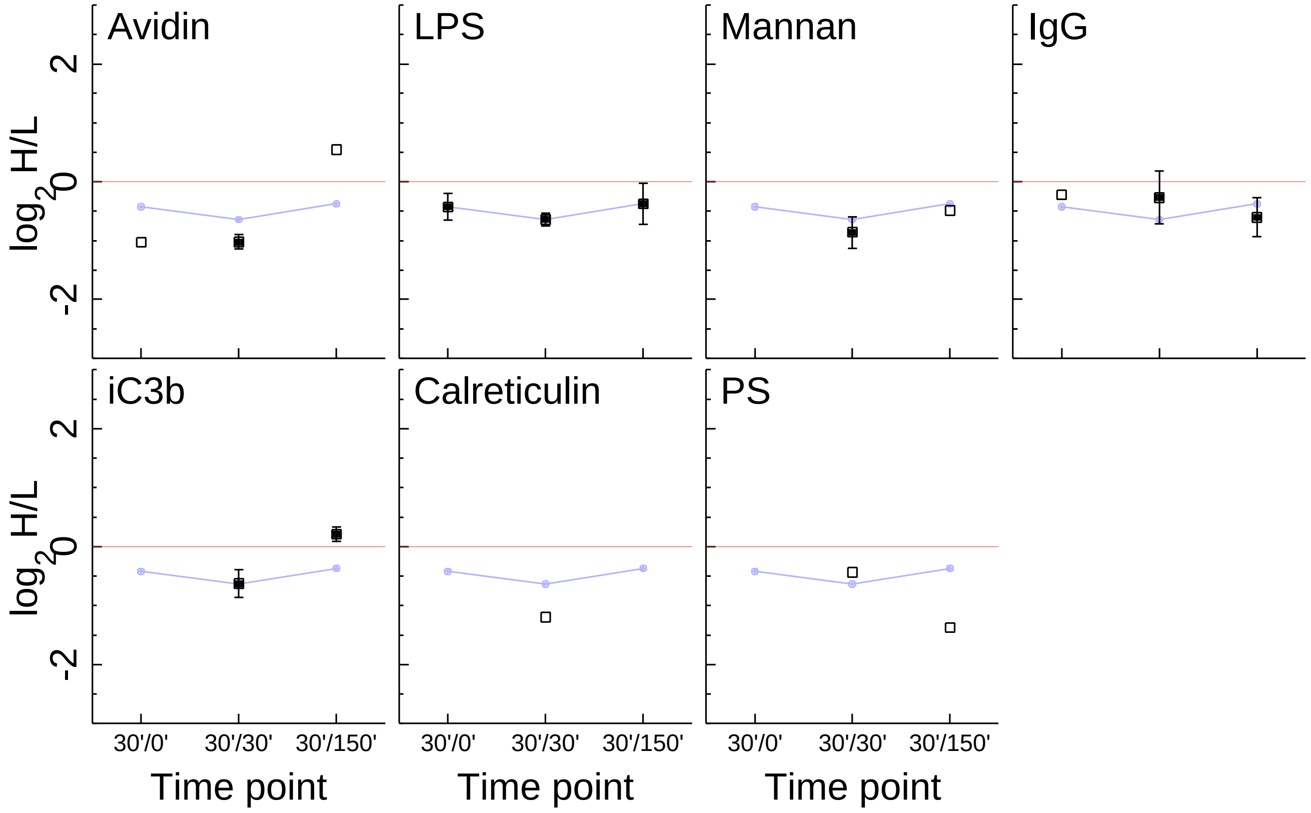

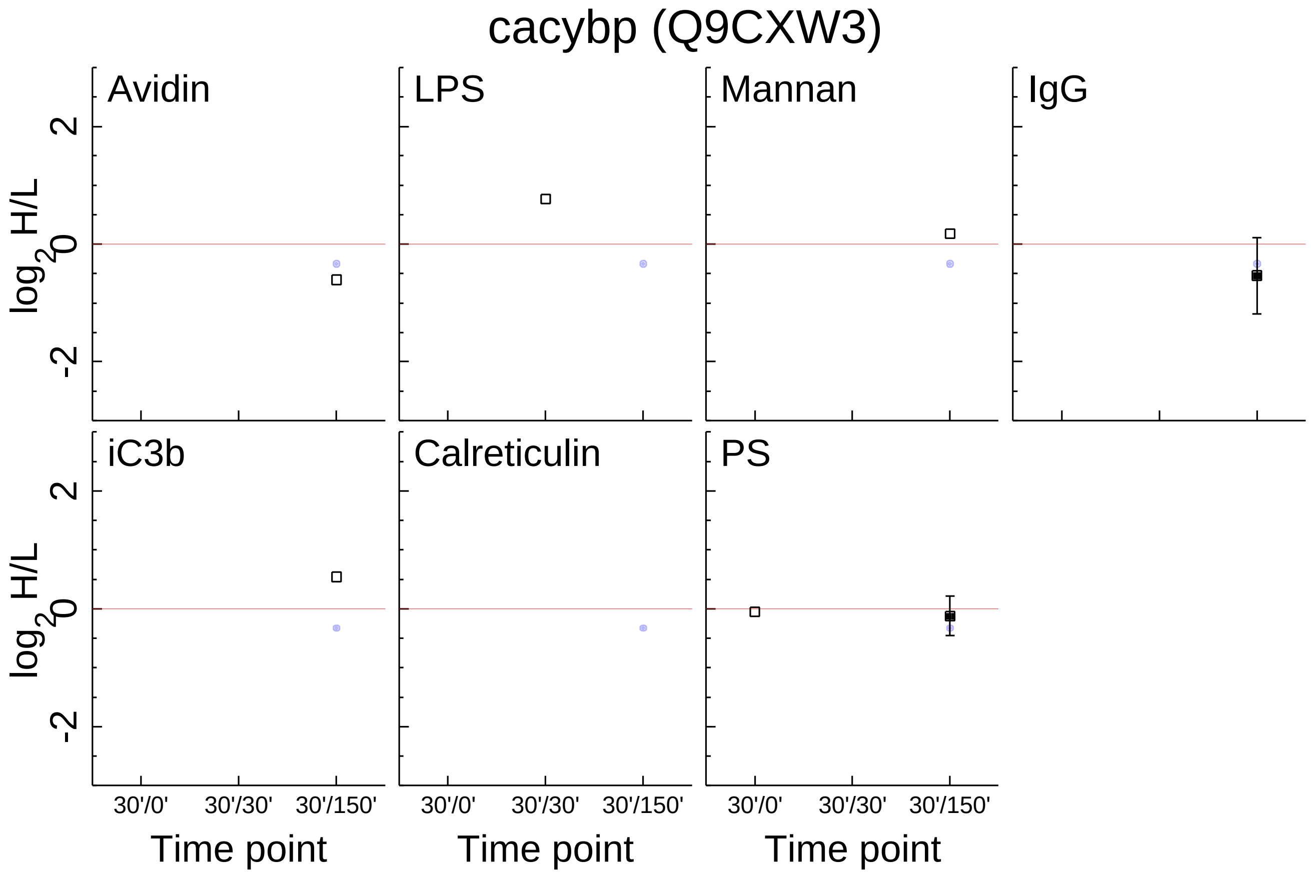

cacybp (Q9CXW3)
Avidin
LPS
Mannan
IgG
iC3b
Calreticulin
PS
log2 H/L
2
0
-2
30'/0'
30'/30'
30'/150'
Time point

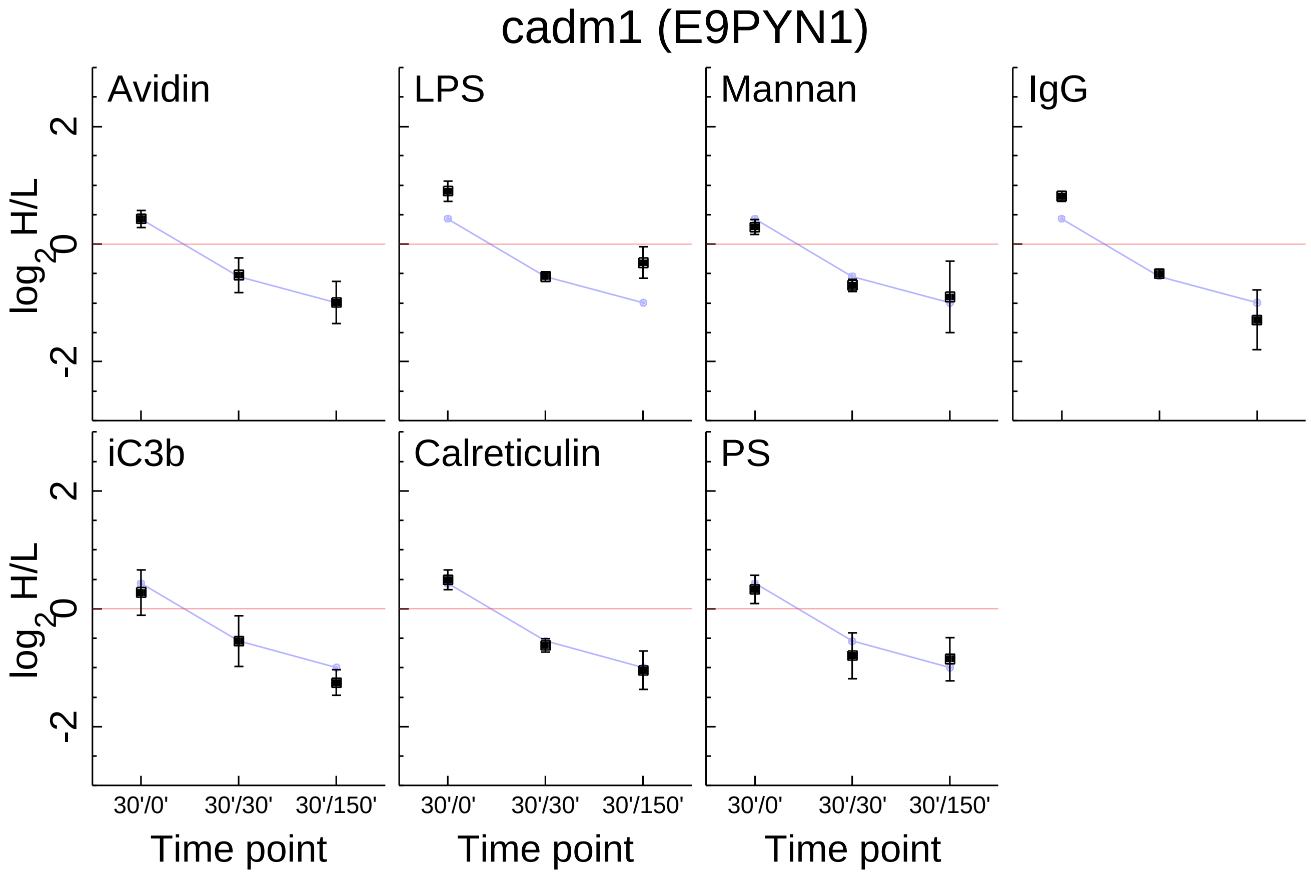
cadm1 (E9PYN1)
Avidin
LPS
Mannan
IgG
iC3b
Calreticulin
PS
log2 H/L
2
0
-2
30'/0'
30'/30'
30'/150'
Time point

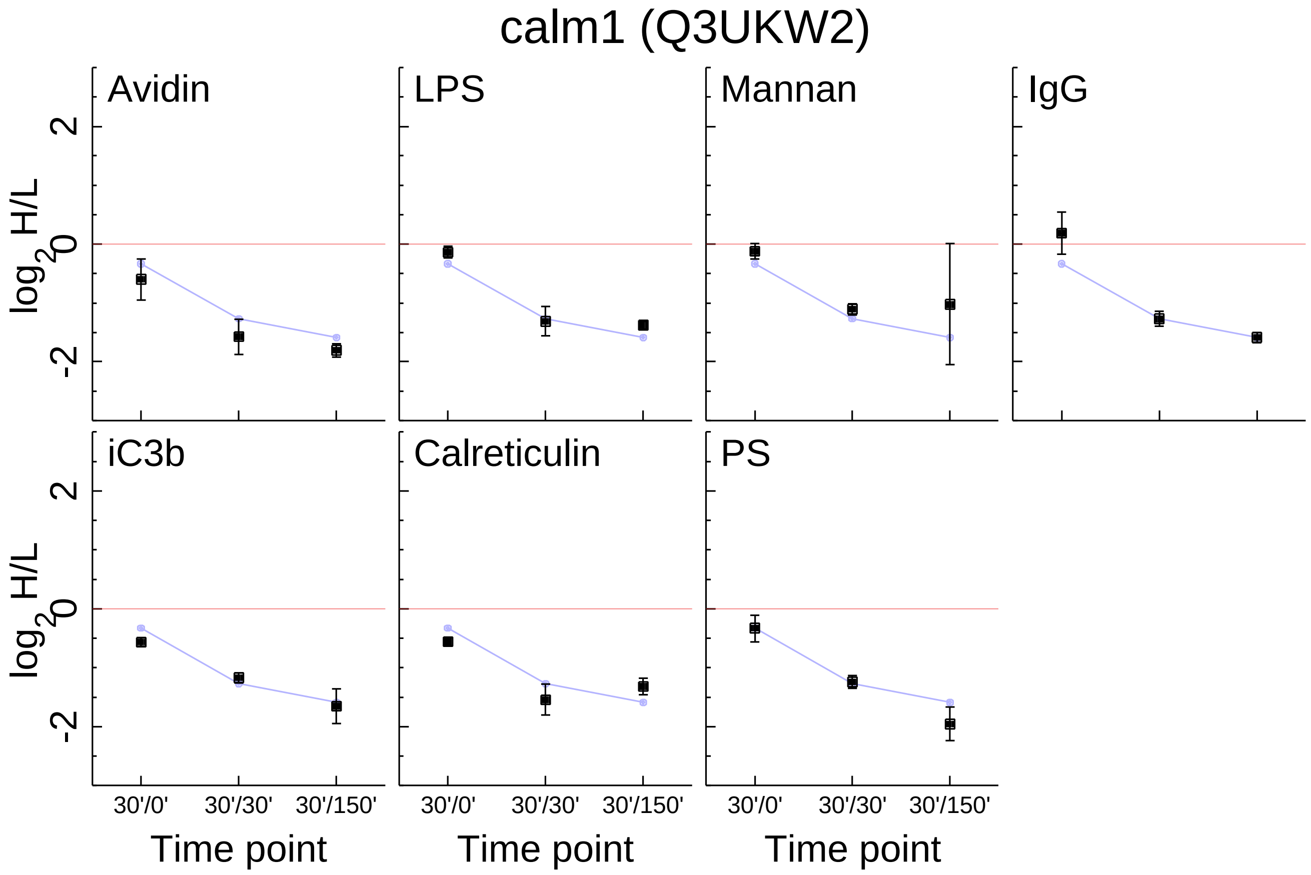

calm1 (Q3UKW2)
Avidin
LPS
Mannan
IgG
iC3b
Calreticulin
PS
log_2 H/L
2
0
-2
30'/0'
30'/30'
30'/150'
Time point

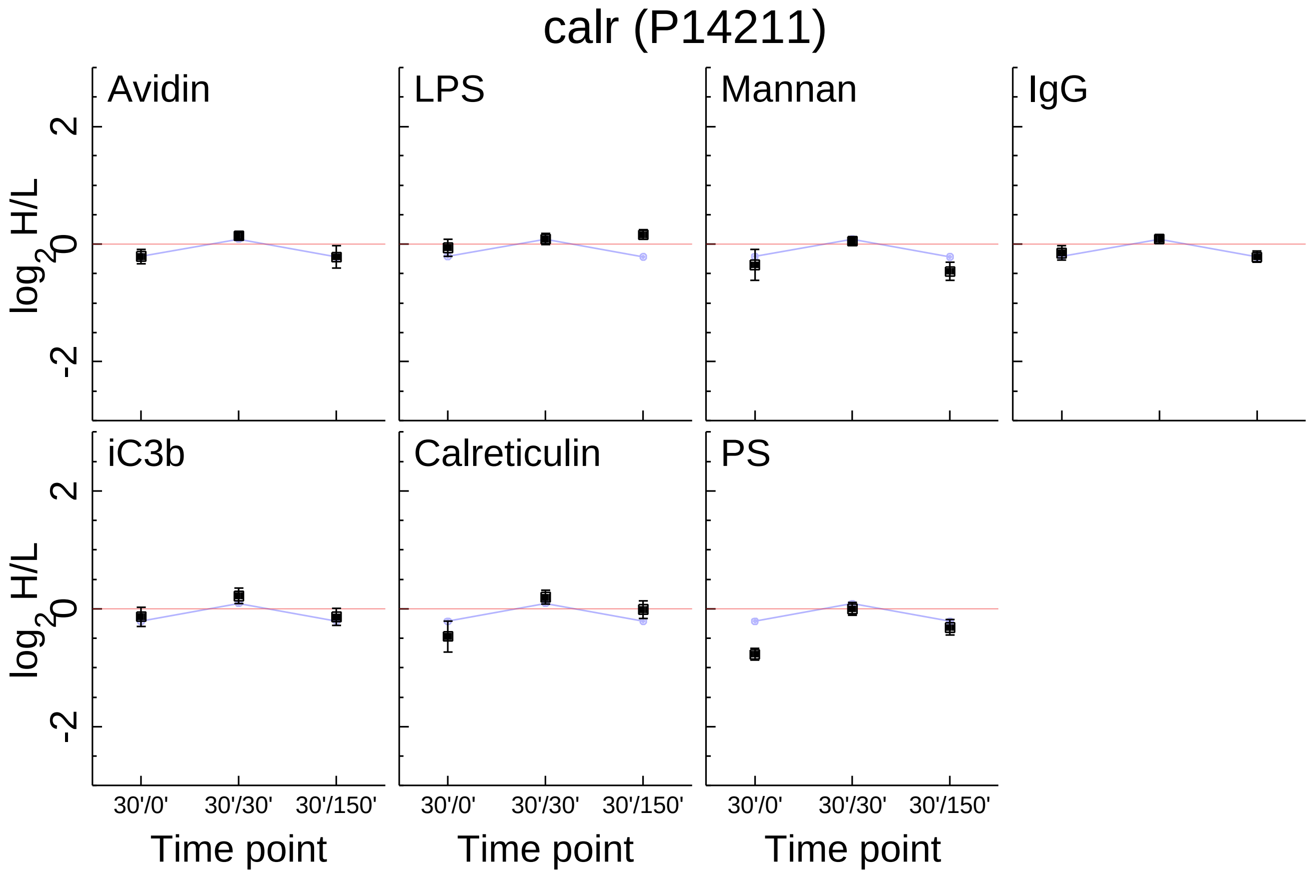
calr (P14211)
Avidin
LPS
Mannan
IgG
iC3b
Calreticulin
PS
log2 H/L
2
0
-2
30'/0'
30'/30'
30'/150'
Time point

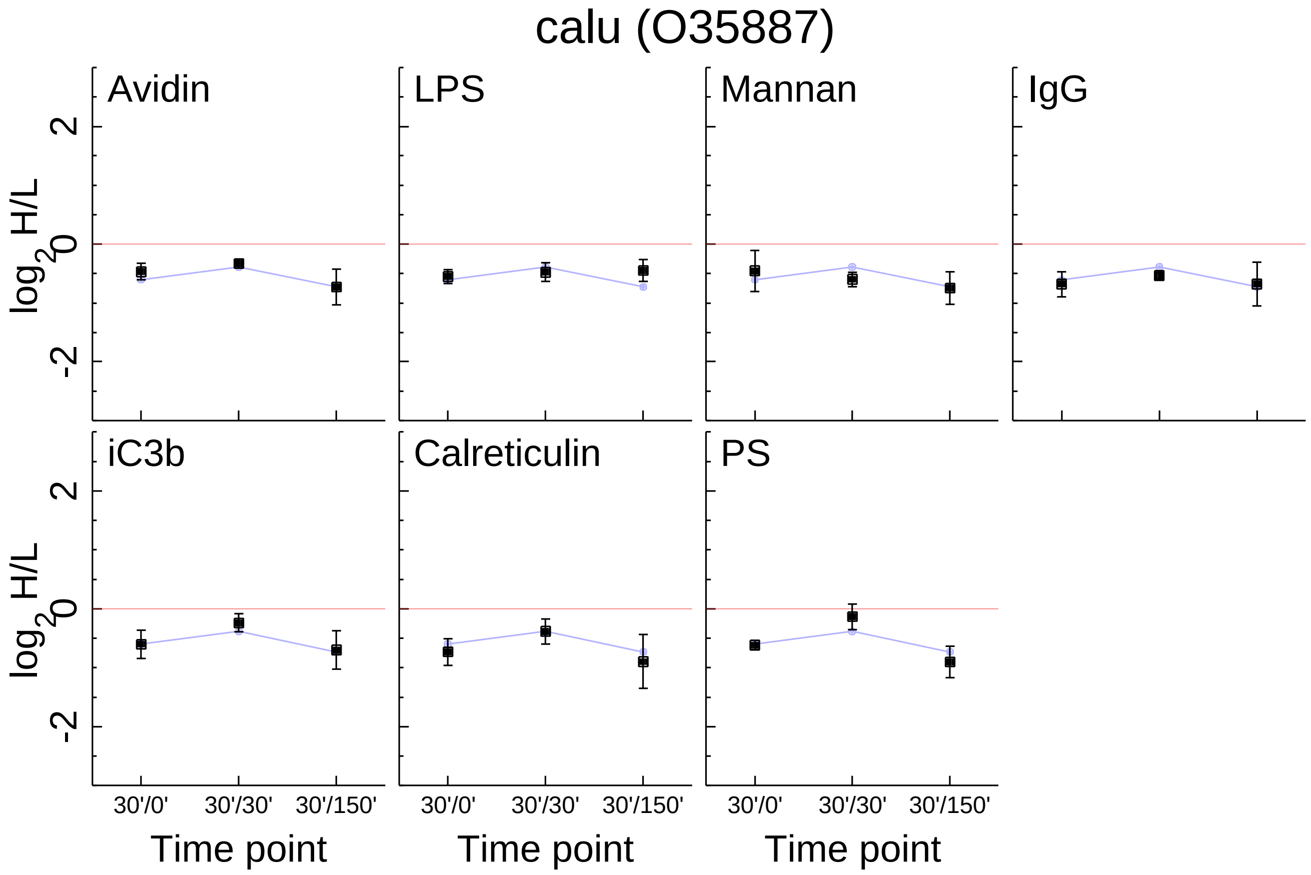

calu (O35887)
Avidin
LPS
Mannan
IgG
iC3b
Calreticulin
PS
log2 H/L
2
0
-2
30'/0'
30'/30'
30'/150'
Time point

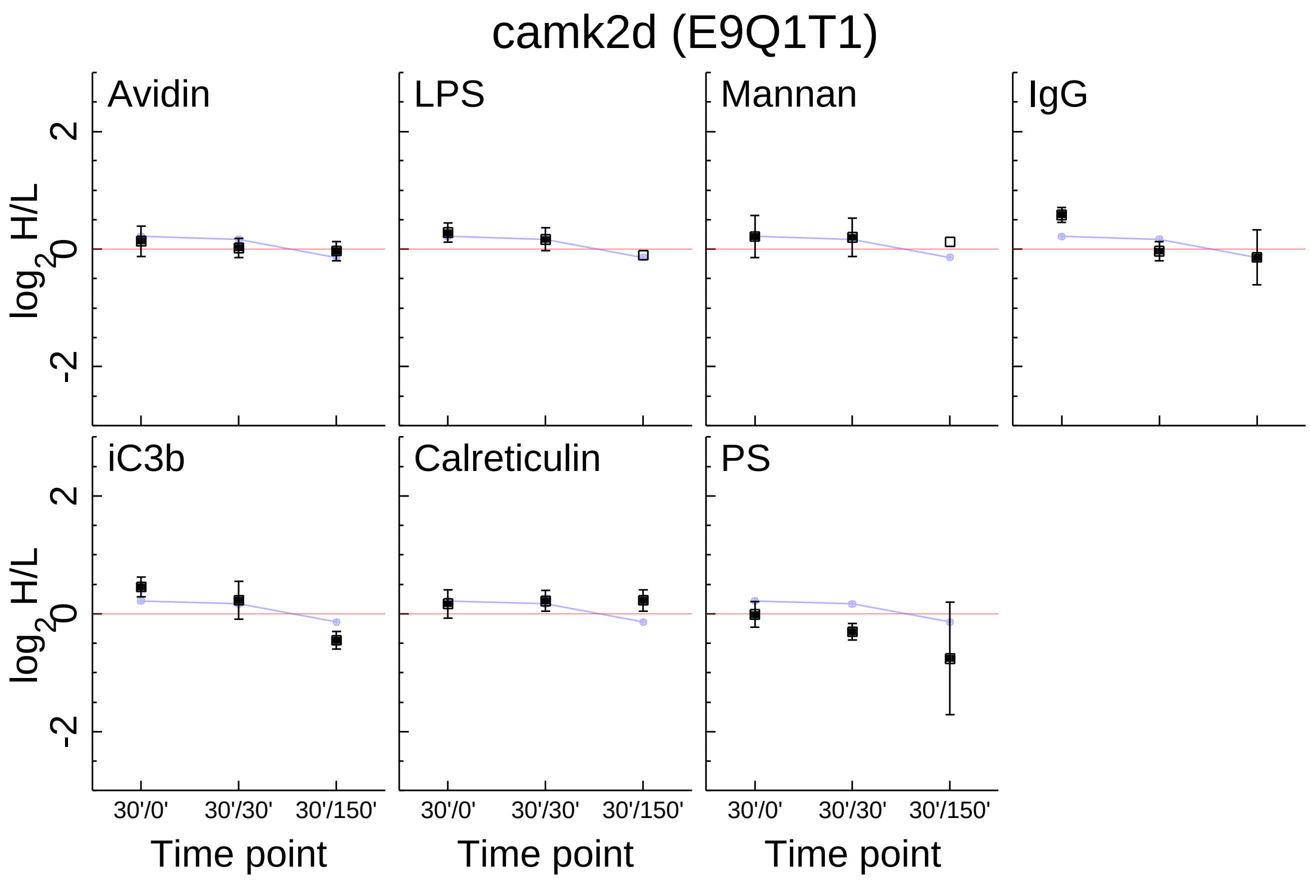

camk2d (E9Q1T1)
Avidin
LPS
Mannan
IgG
iC3b
Calreticulin
PS
log2 H/L
2
0
-2
30'/0'
30'/30'
30'/150'
Time point

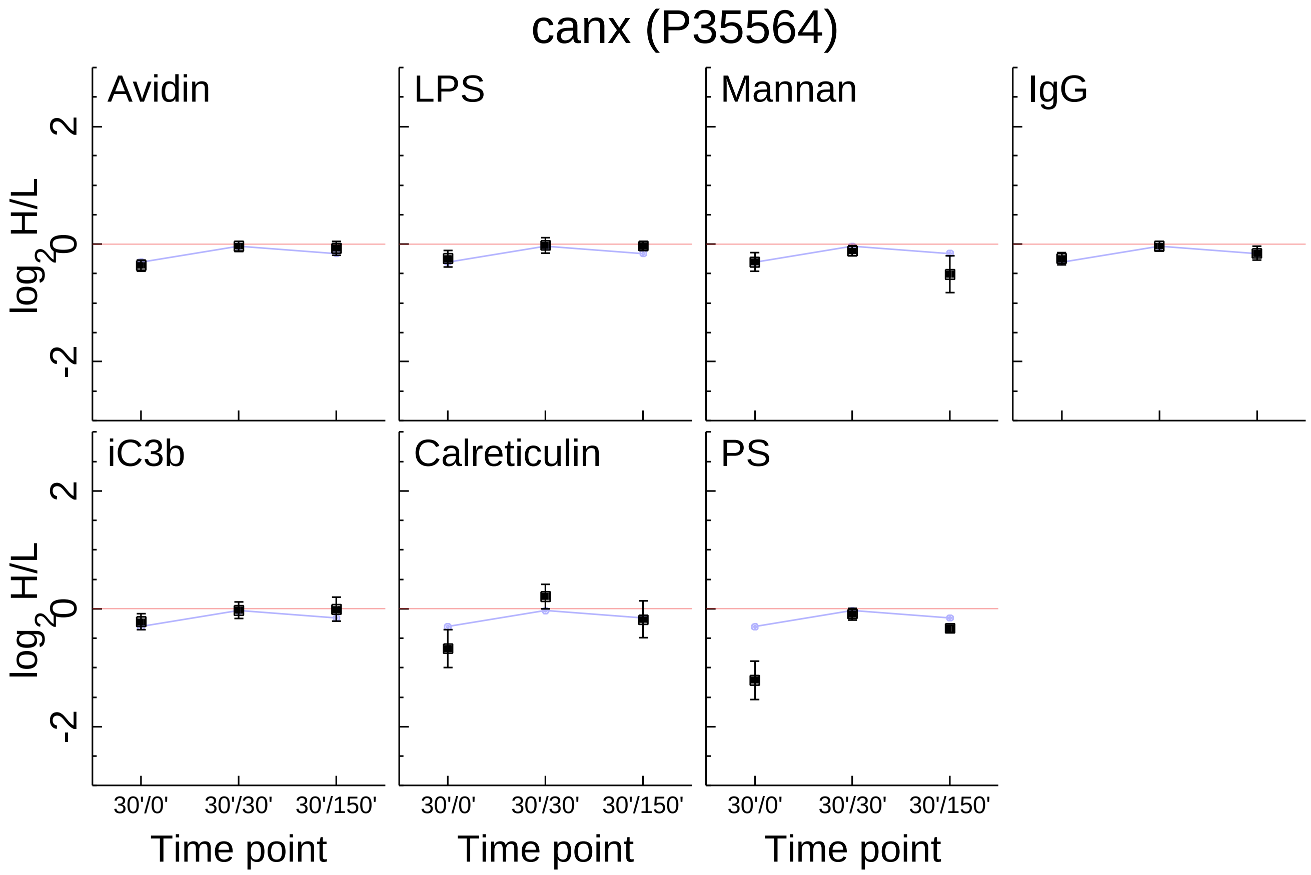

canx (P35564)
Avidin
LPS
Mannan
IgG
iC3b
Calreticulin
PS
log2 H/L
2
0
-2
30'/0'
30'/30'
30'/150'
Time point

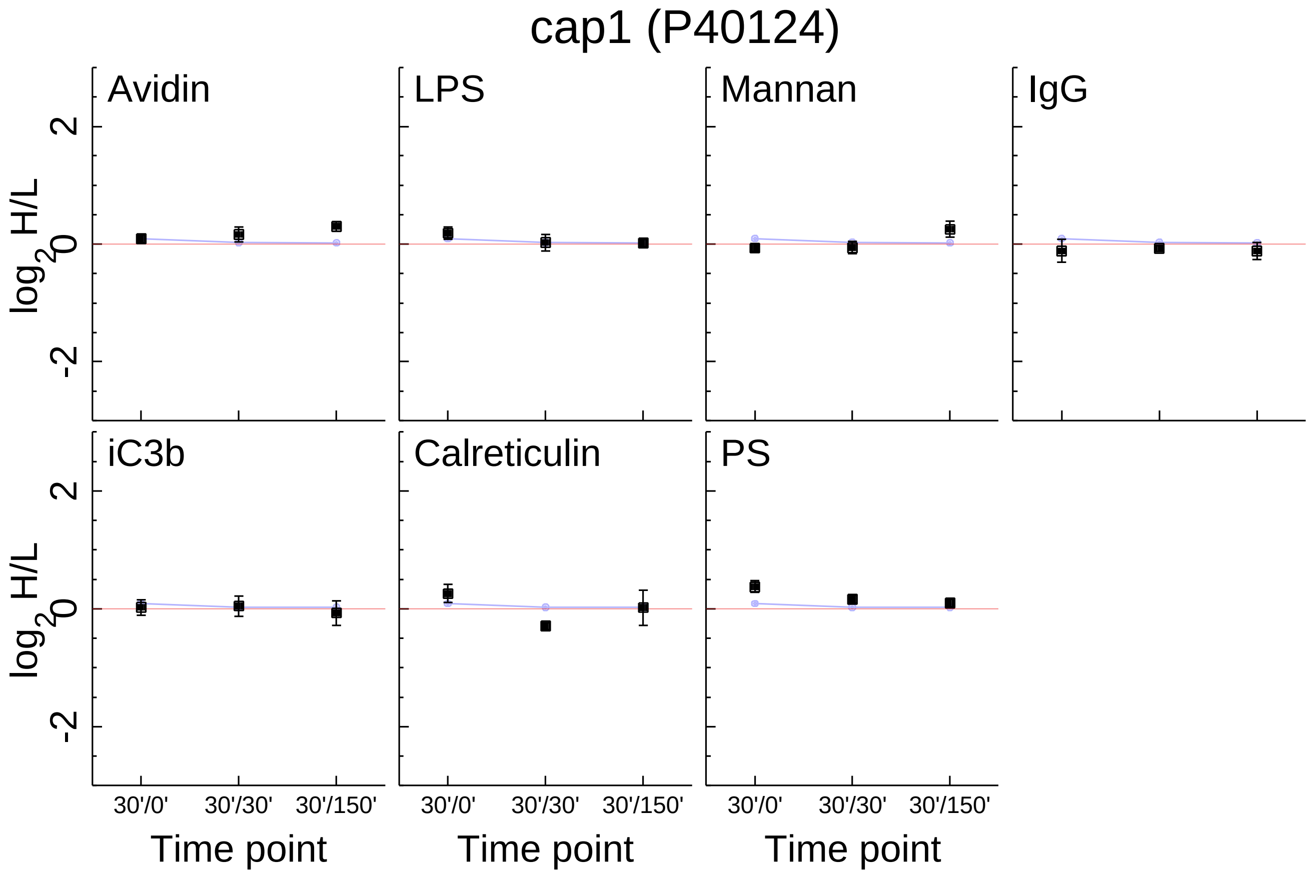
cap1 (P40124)
Avidin
LPS
Mannan
IgG
iC3b
Calreticulin
PS
log2 H/L
2
0
-2
30'/0'
30'/30'
30'/150'
Time point

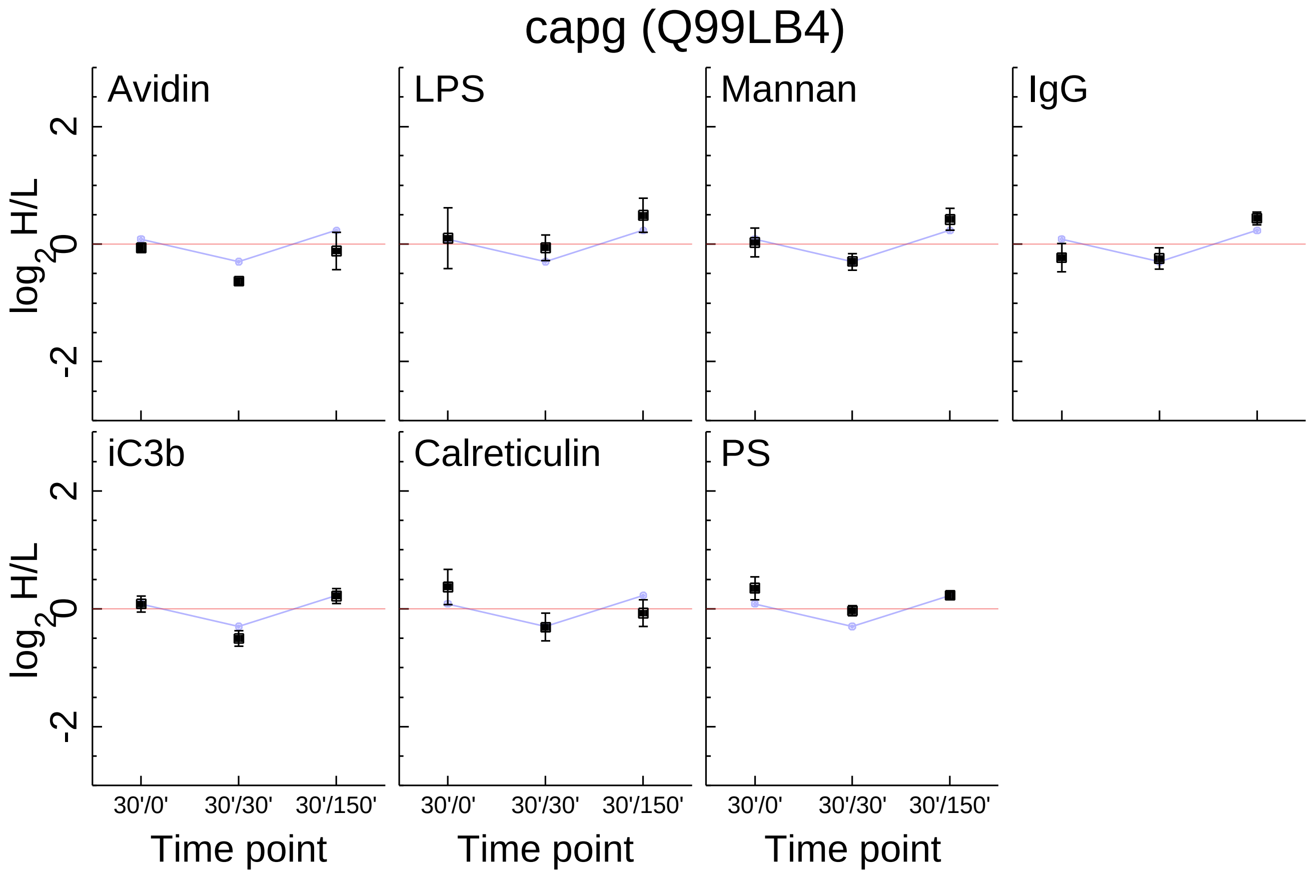
capg (Q99LB4)
Avidin
LPS
Mannan
IgG
iC3b
Calreticulin
PS
2
0
-2
log2 H/L
30'/0'
30'/30'
30'/150'
Time point

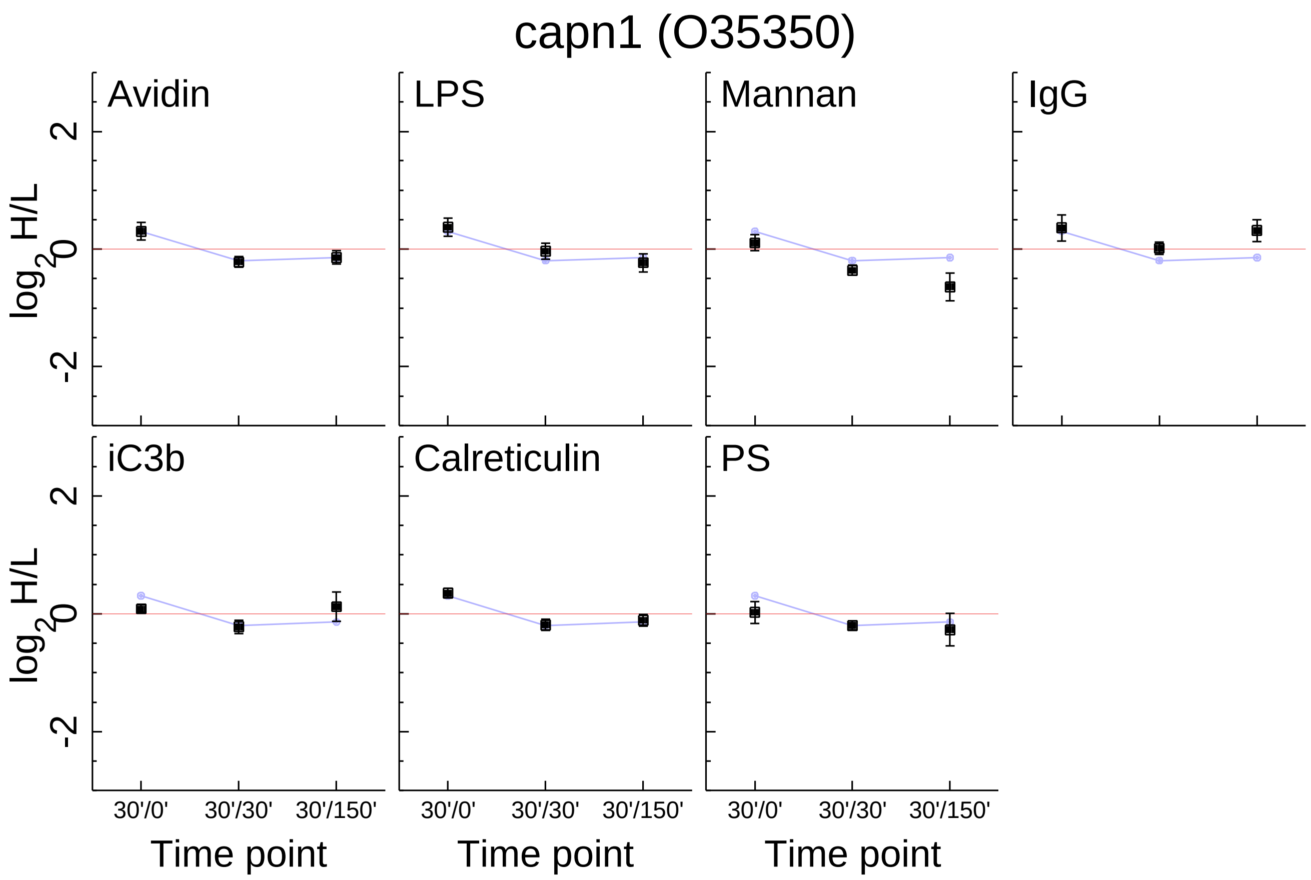
capn1 (O35350)
Avidin
LPS
Mannan
IgG
iC3b
Calreticulin
PS
log2 H/L
2
0
-2
30'/0'
30'/30'
30'/150'
Time point

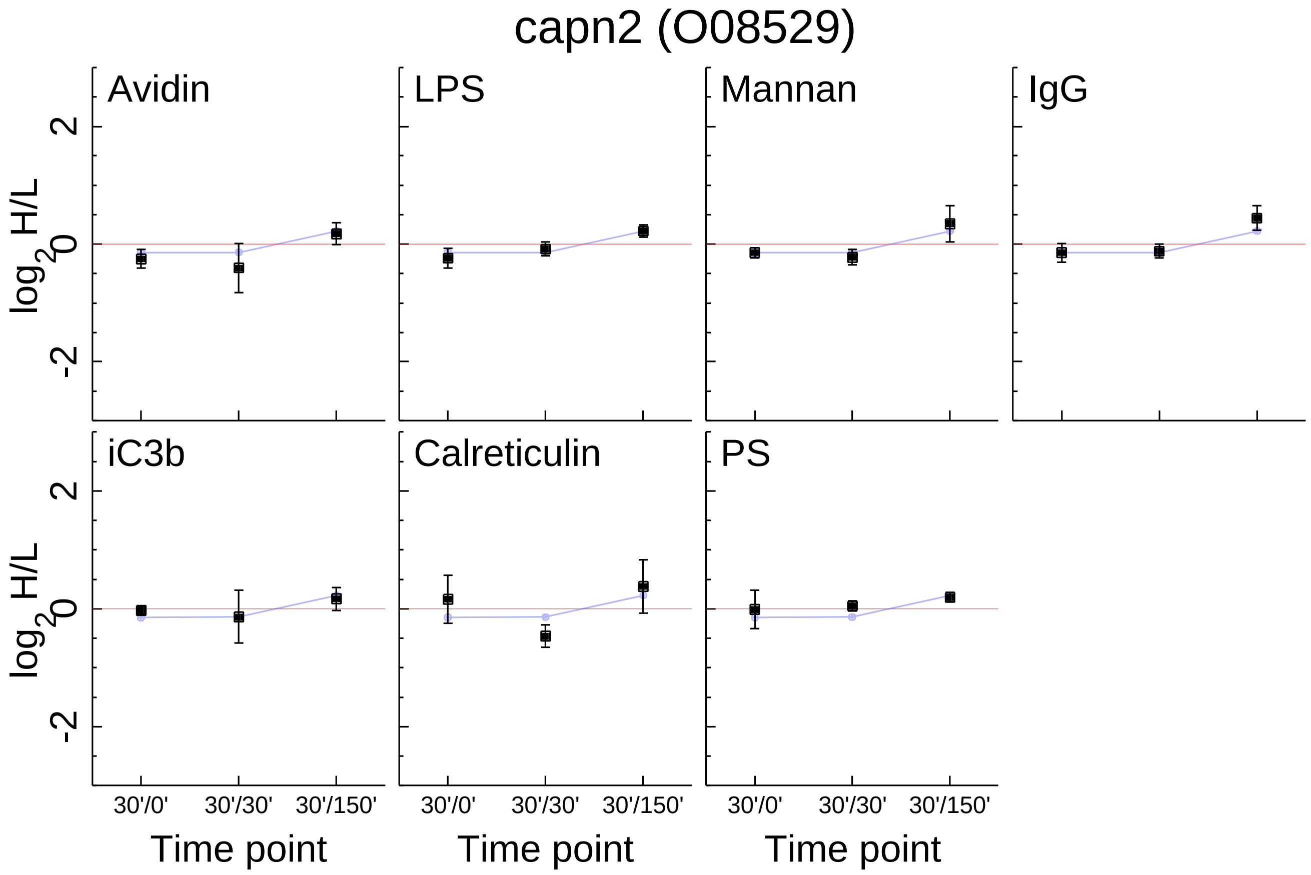

capn2 (O08529)
Avidin
LPS
Mannan
IgG
iC3b
Calreticulin
PS
log2 H/L
2
0
-2
30'/0'
30'/30'
30'/150'
Time point

capn5 (O08688)

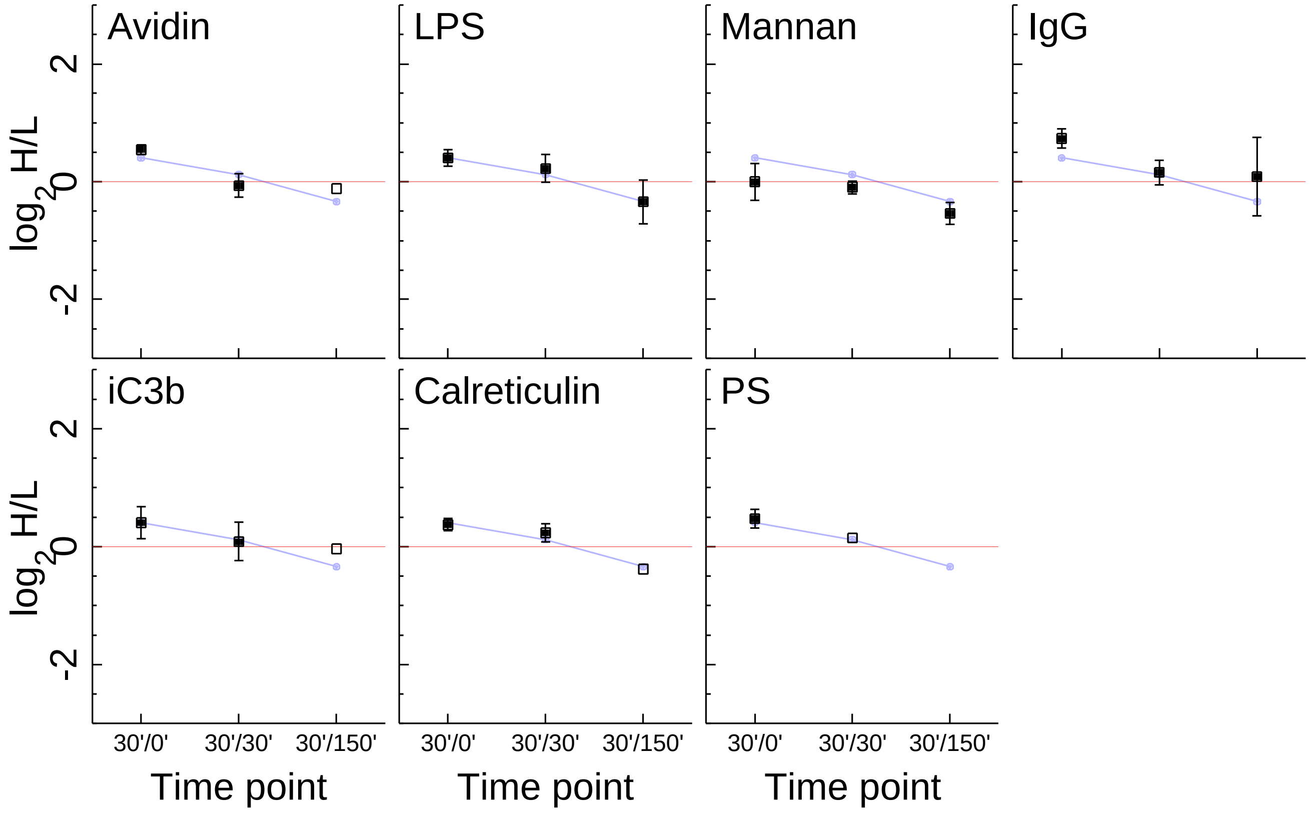

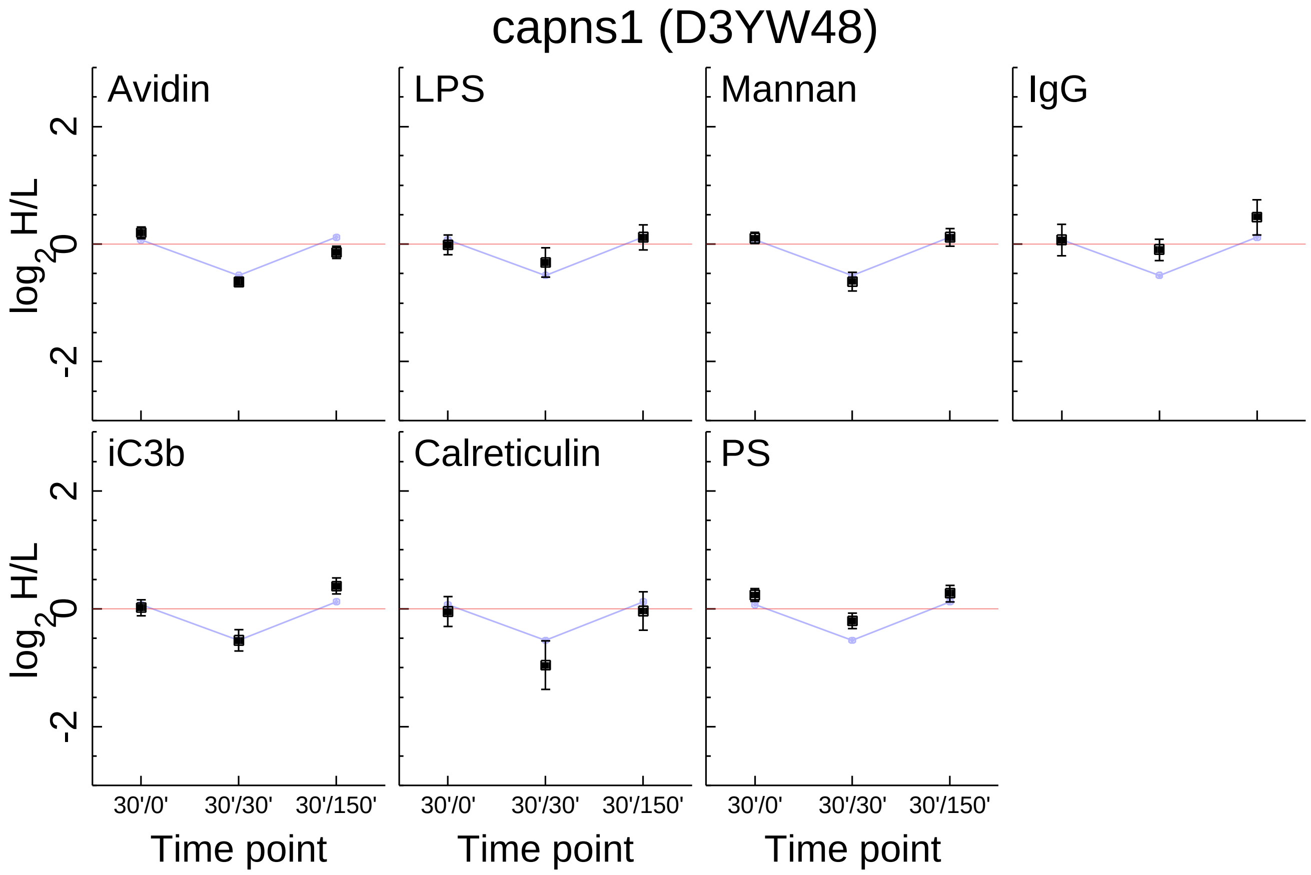
capns1 (D3YW48)
Avidin
LPS
Mannan
IgG
iC3b
Calreticulin
PS
log2 H/L
2
0
-2
30'/0'
30'/30'
30'/150'
Time point

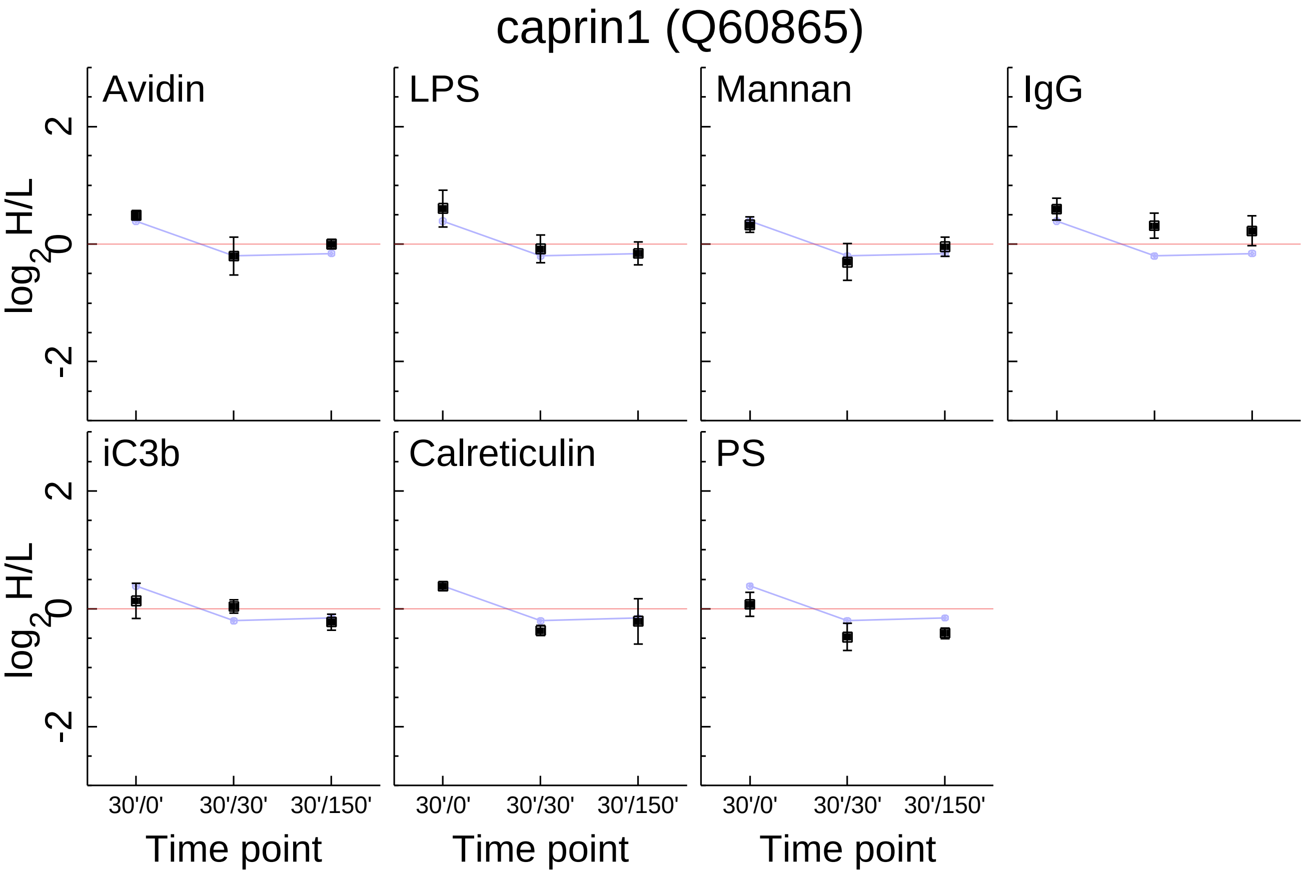
caprin1 (Q60865)
Avidin
LPS
Mannan
IgG
iC3b
Calreticulin
PS
log2 H/L
2
0
-2
30'/0'
30'/30'
30'/150'
Time point

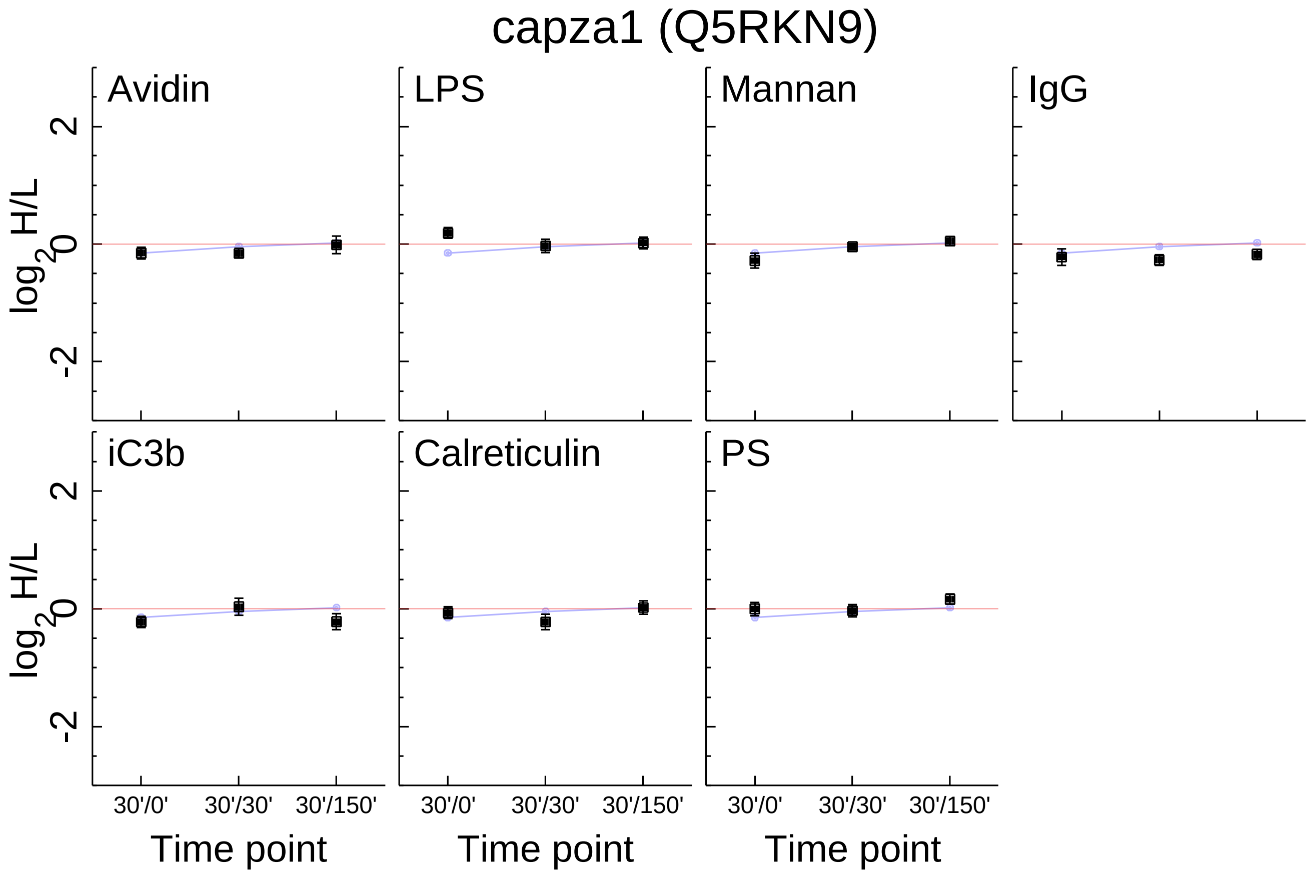
capza1 (Q5RKN9)
Avidin
LPS
Mannan
IgG
iC3b
Calreticulin
PS
log2 H/L
2
0
-2
30'/0'
30'/30'
30'/150'
Time point

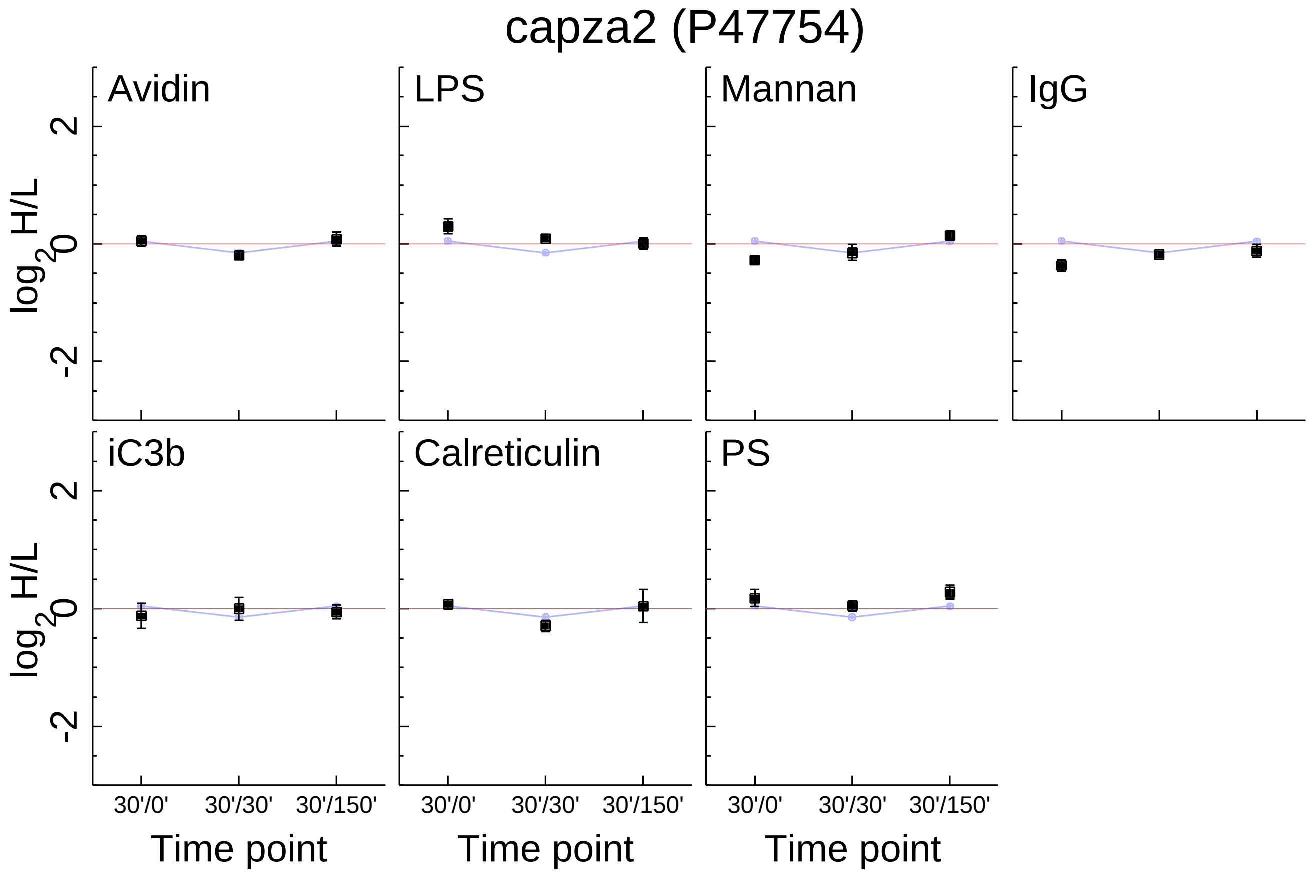

capza2 (P47754)
Avidin
LPS
Mannan
IgG
iC3b
Calreticulin
PS
log2 H/L
2
0
-2
30'/0'
30'/30'
30'/150'
Time point

capzb (A2AMV7)

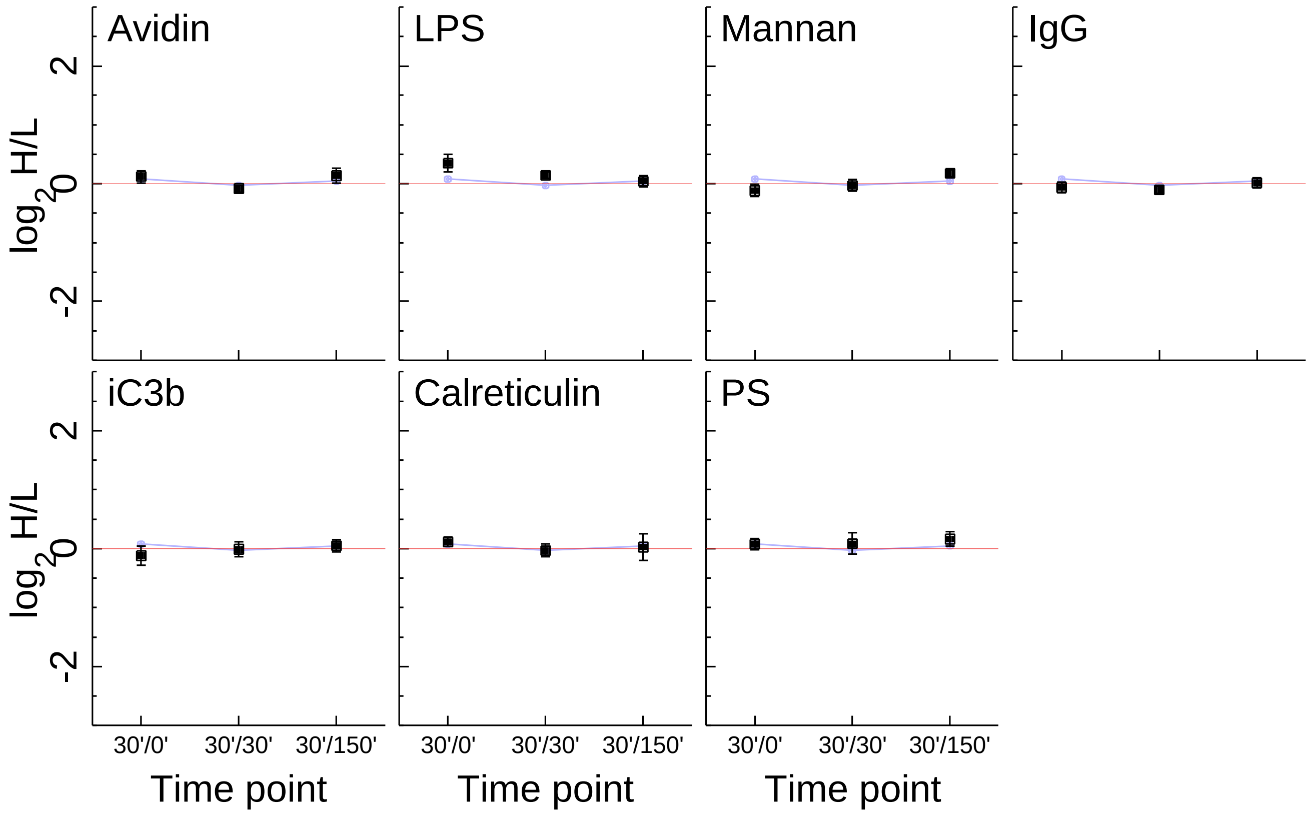

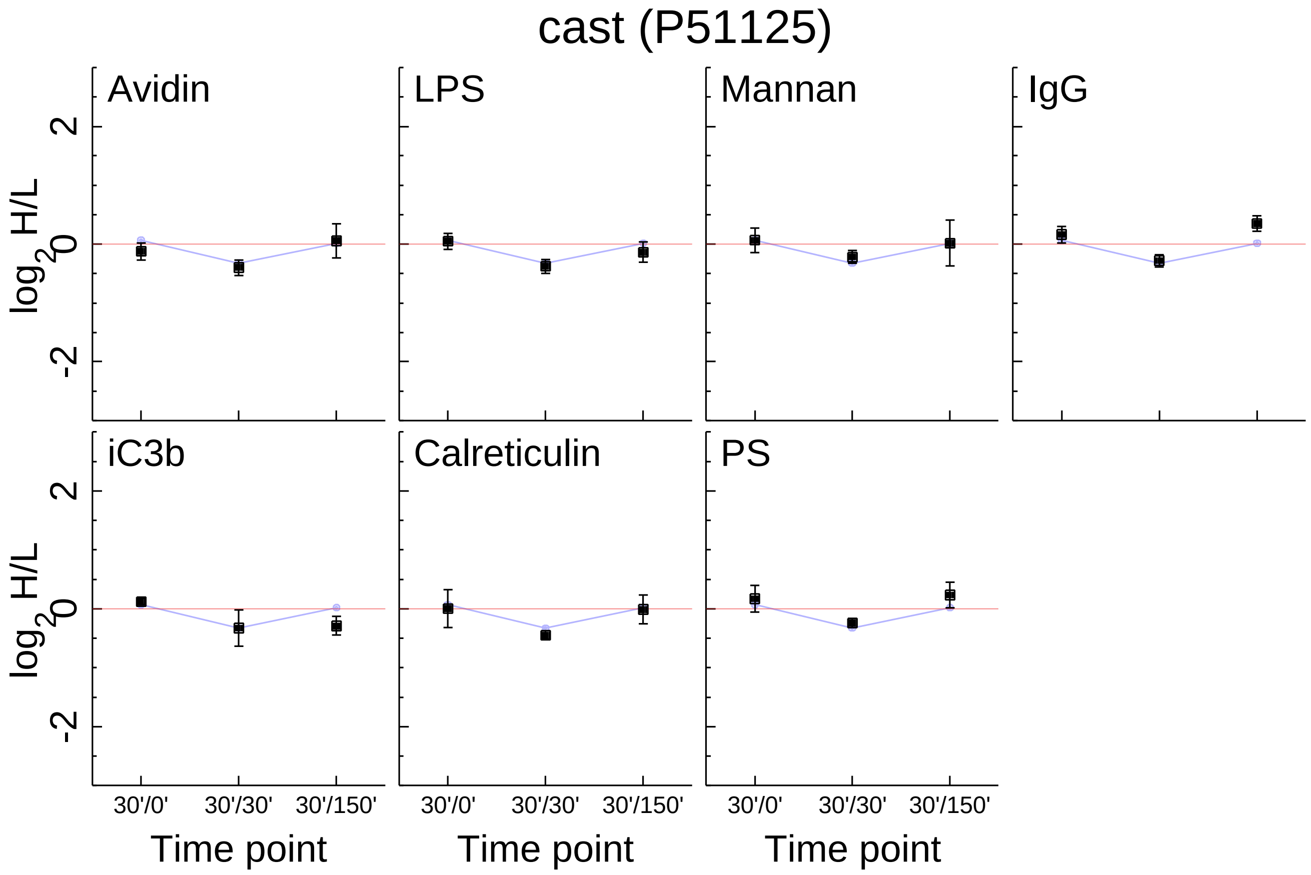
cast (P51125)
Avidin
LPS
Mannan
IgG
iC3b
Calreticulin
PS
log2 H/L
2
0
-2
30'/0'
30'/30'
30'/150'
Time point

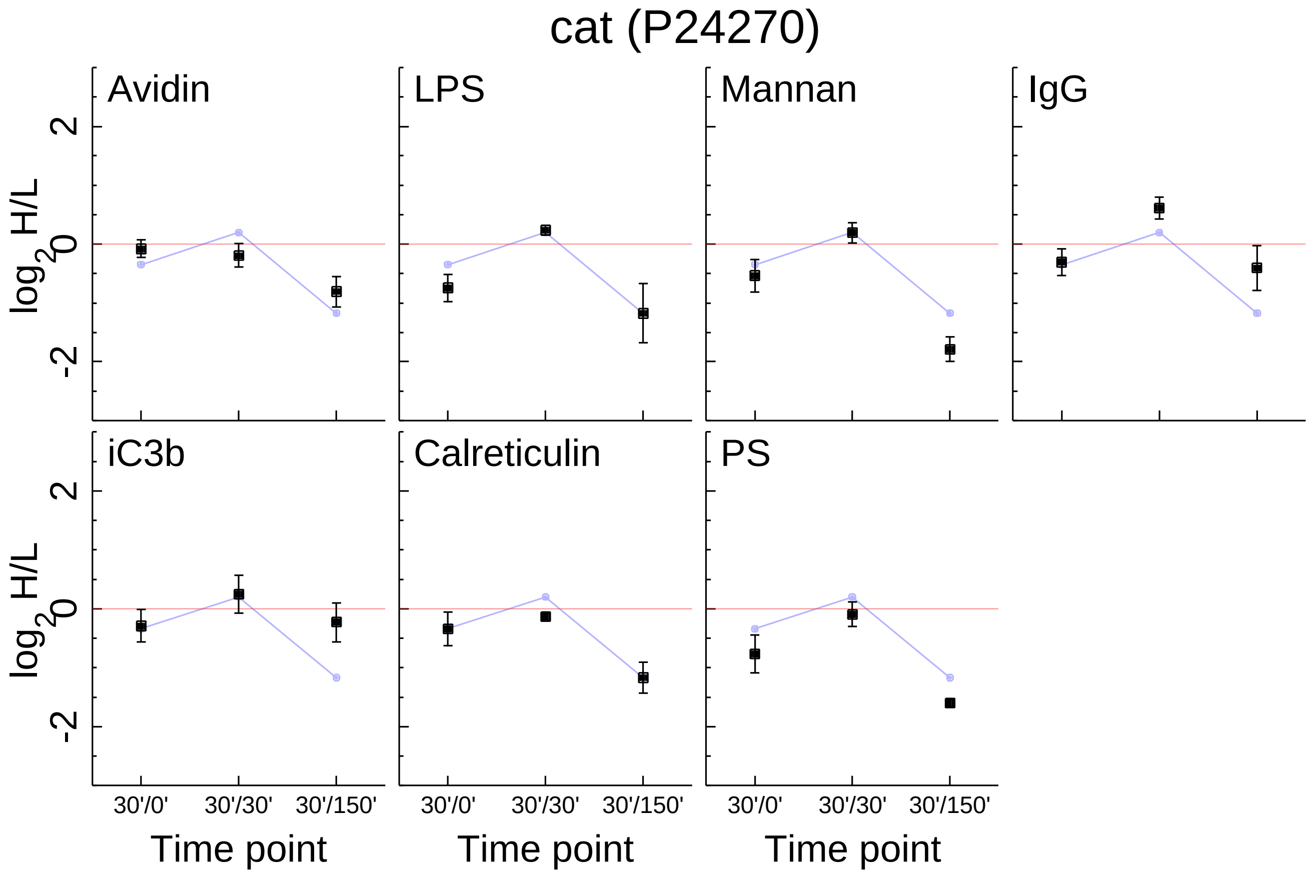

cat (P24270)
Avidin
LPS
Mannan
IgG
iC3b
Calreticulin
PS
log_2 H/L
2
0
-2
30'/0'
30'/30'
30'/150'
Time point

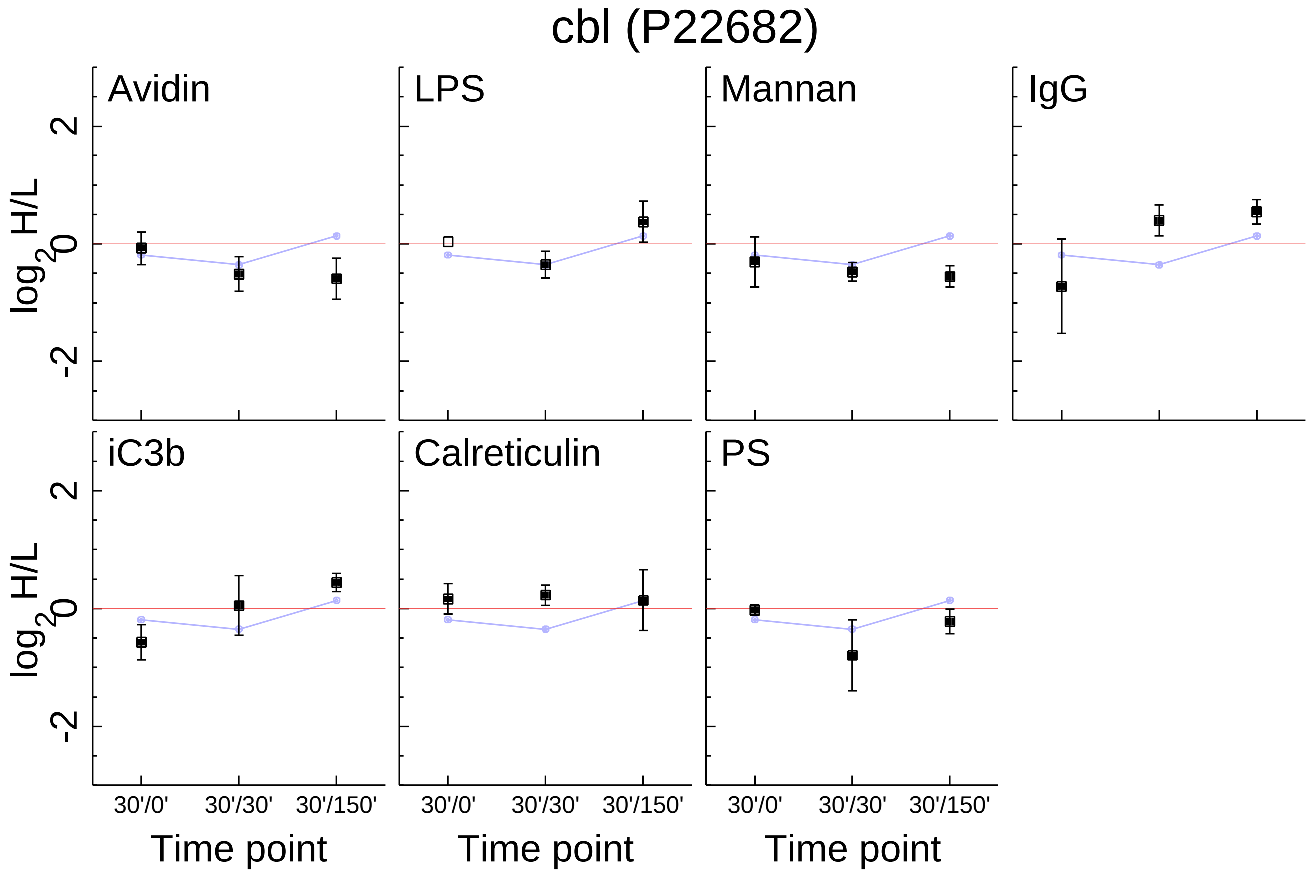
cbl (P22682)
Avidin
LPS
Mannan
IgG
iC3b
Calreticulin
PS
log2 H/L
2
0
-2
30'/0'
30'/30'
30'/150'
Time point

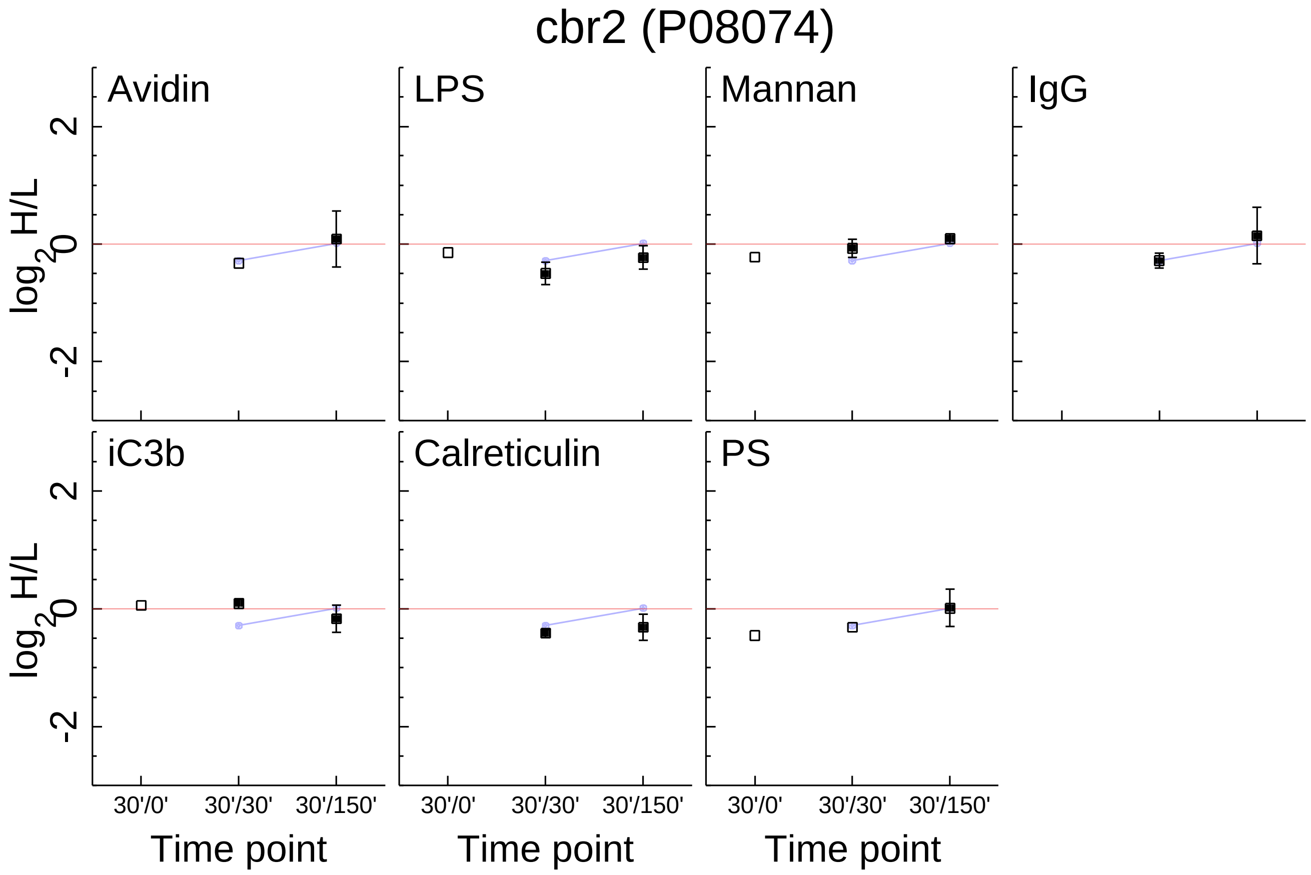
cbr2 (P08074)
Avidin
LPS
Mannan
IgG
iC3b
Calreticulin
PS
log2 H/L
2
0
-2
30'/0'
30'/30'
30'/150'
Time point

cbx3 (P23198)

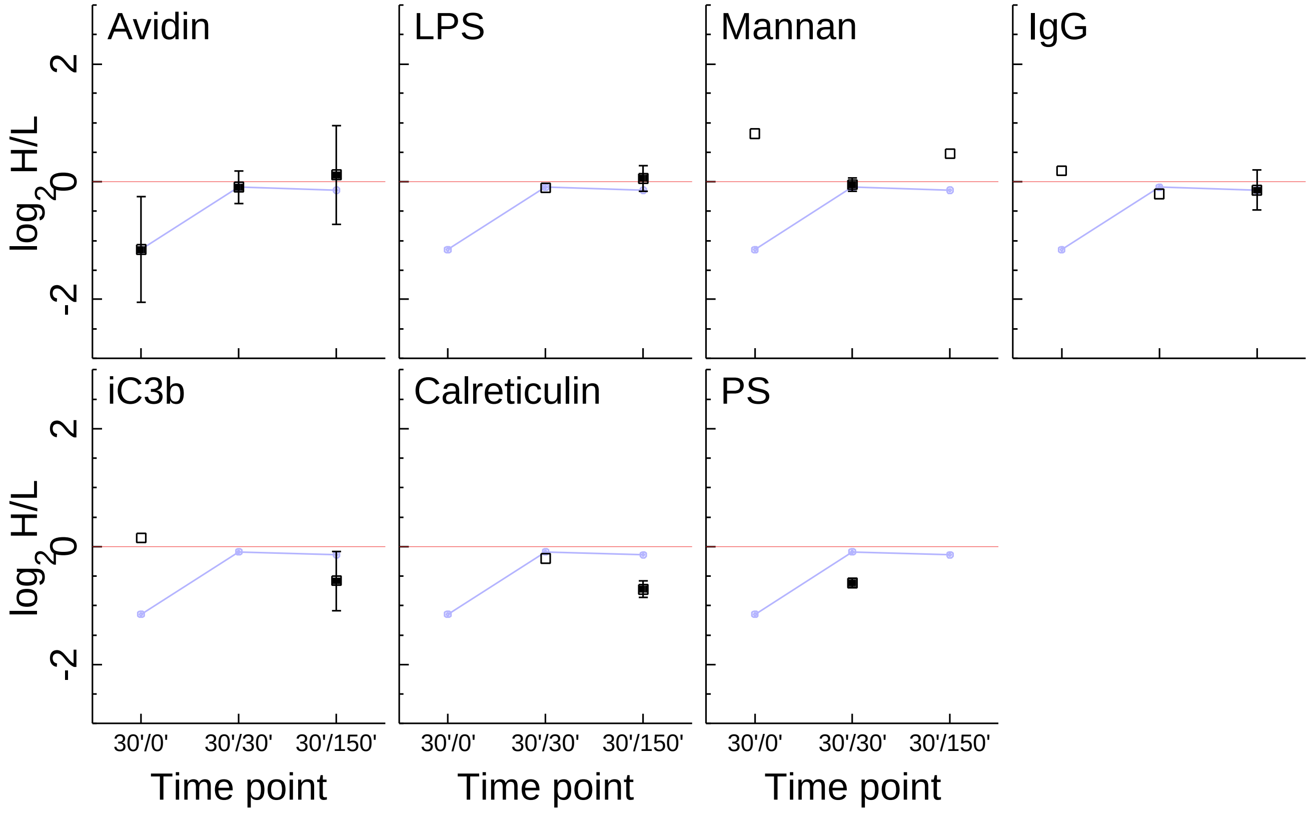
Avidin
LPS
Mannan
IgG
iC3b
Calreticulin
PS
log2 H/L
2
0
-2
30'/0'
30'/30'
30'/150'
Time point

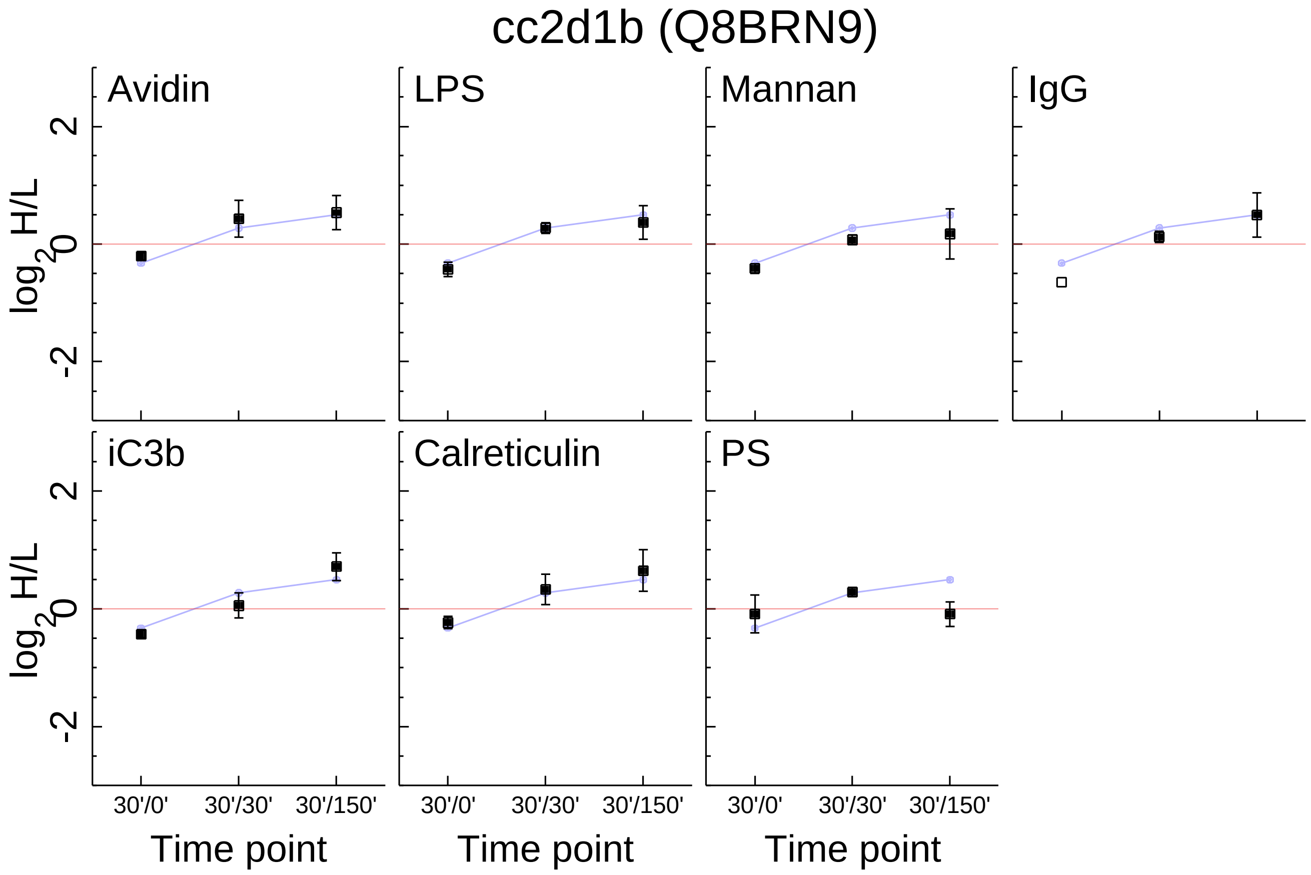

cc2d1b (Q8BRN9)
Avidin
LPS
Mannan
IgG
iC3b
Calreticulin
PS
log2 H/L
2
0
-2
30'/0'
30'/30'
30'/150'
Time point

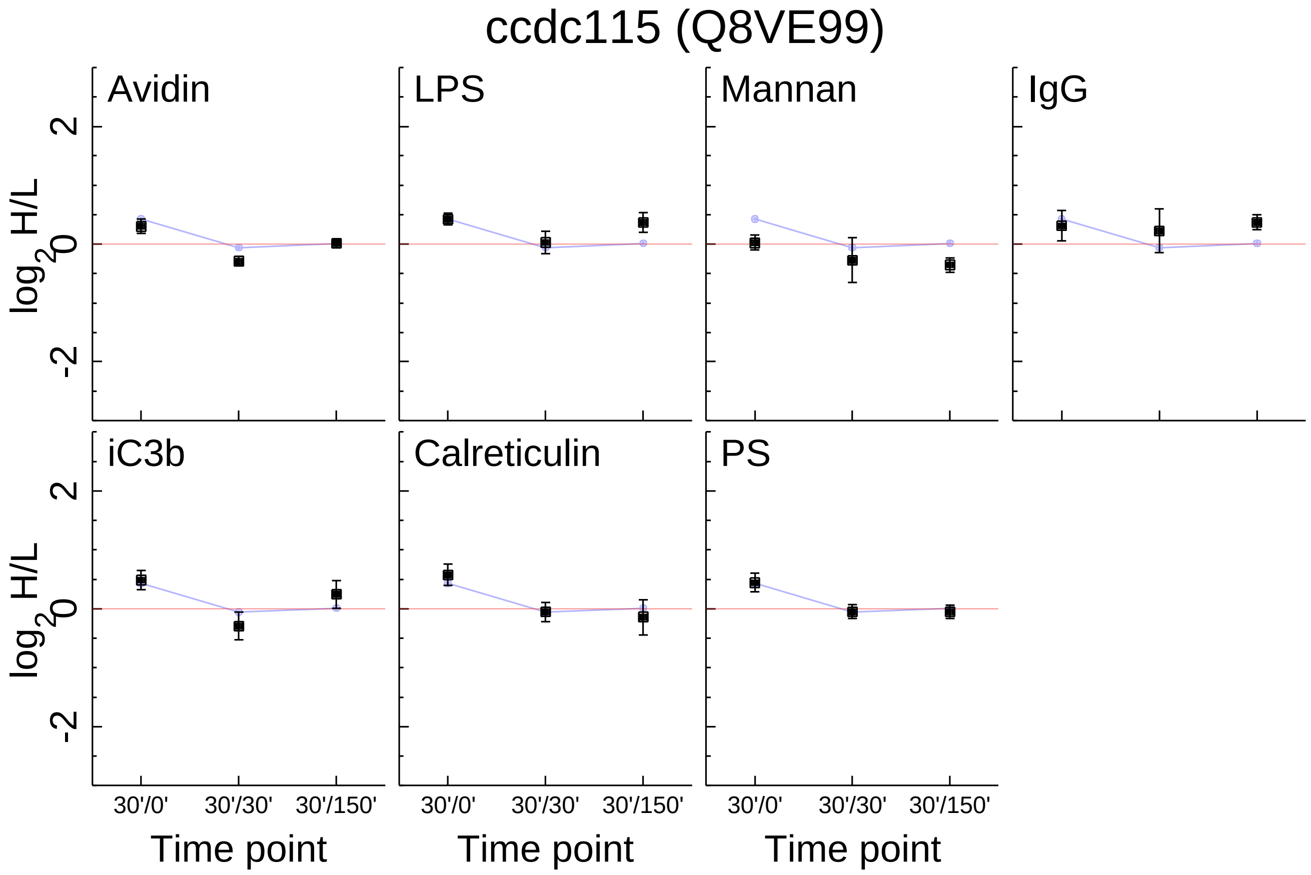
ccdc115 (Q8VE99)
Avidin
LPS
Mannan
IgG
iC3b
Calreticulin
PS
log2 H/L
2
0
-2
30'/0'
30'/30'
30'/150'
Time point

ccdc12 (Q8R344)

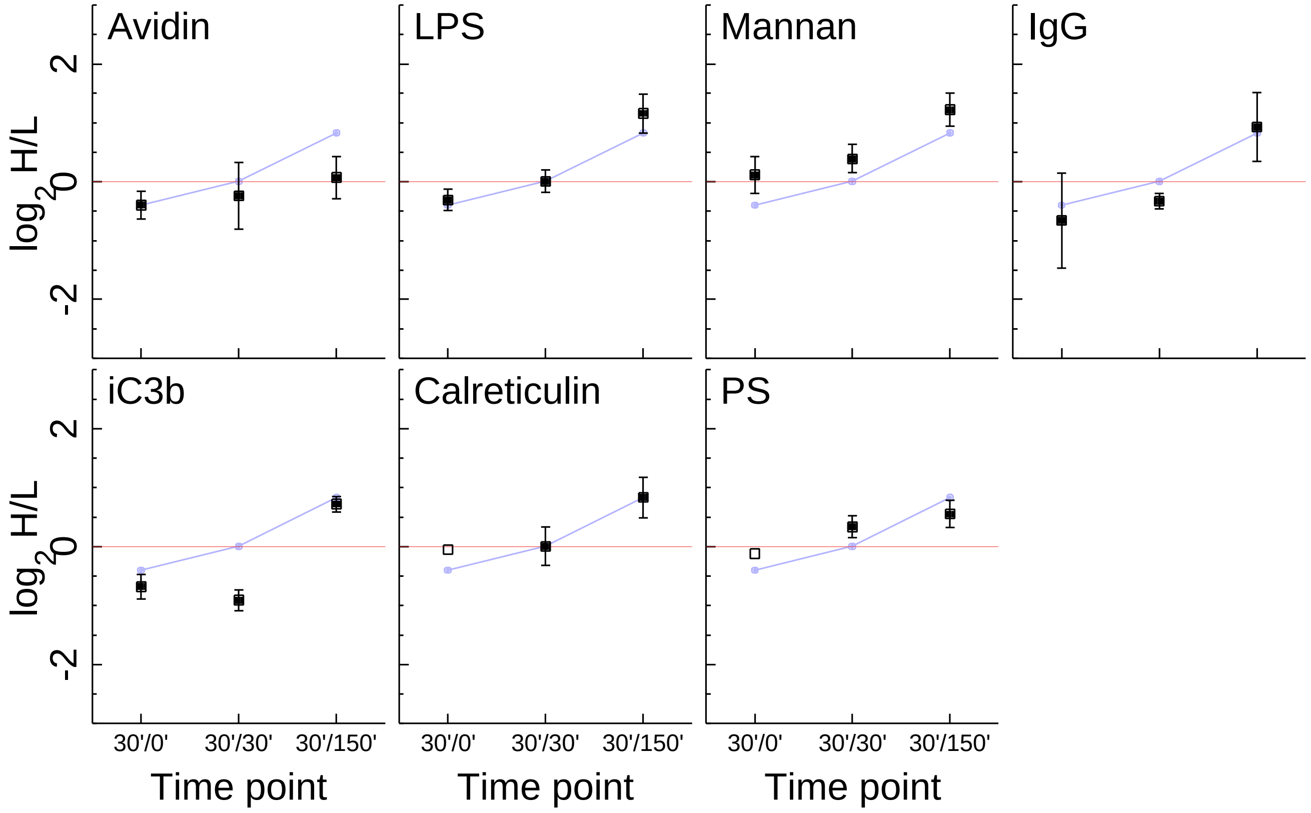

ccdc22 (Q9JIG7)

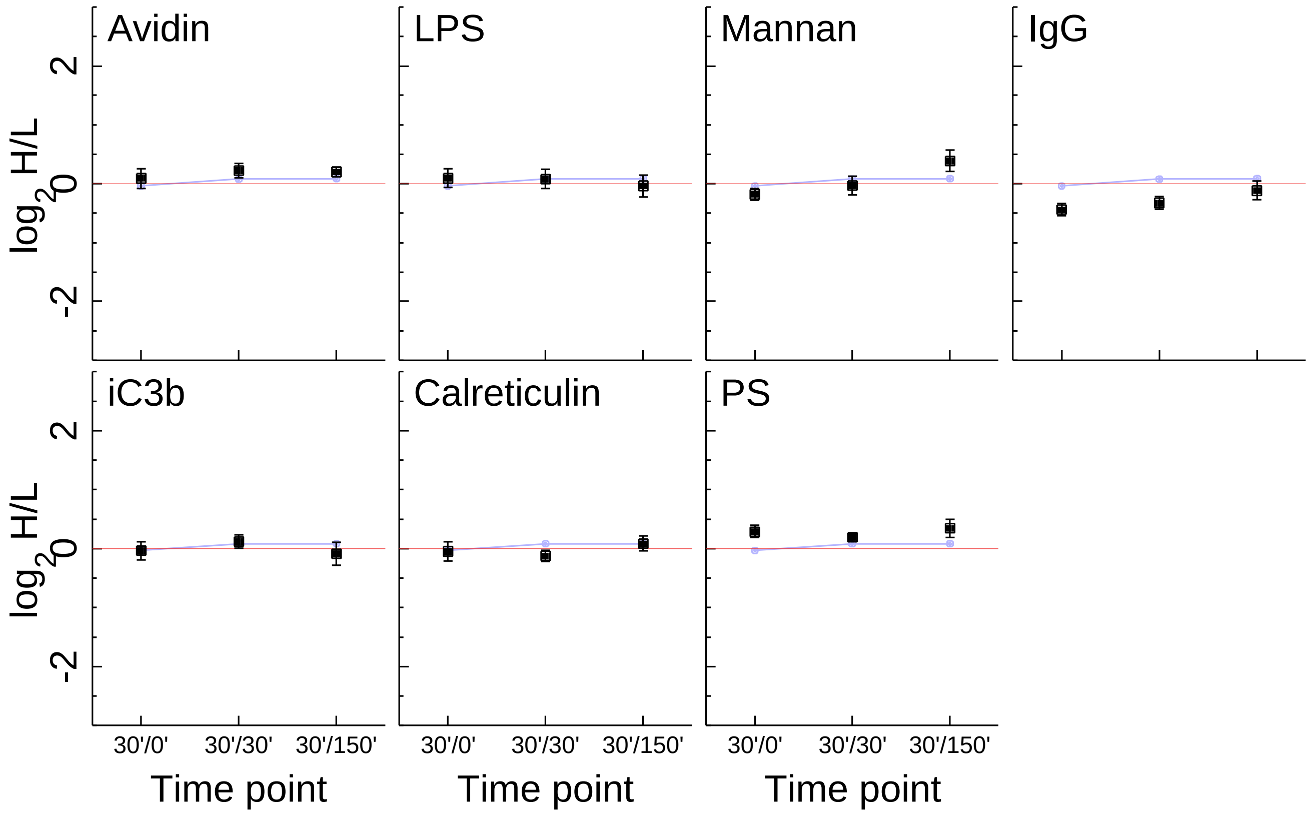

ccdc50 (Q810U5)

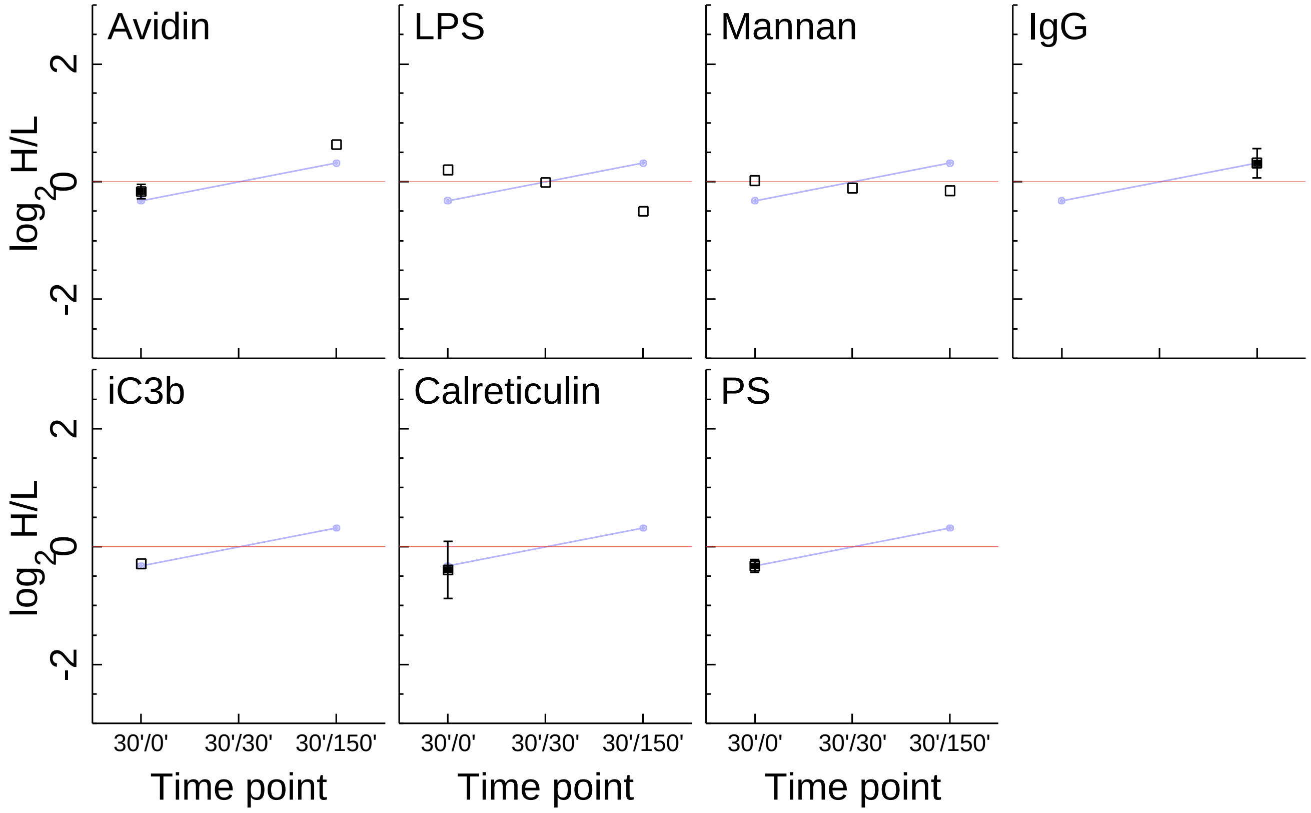

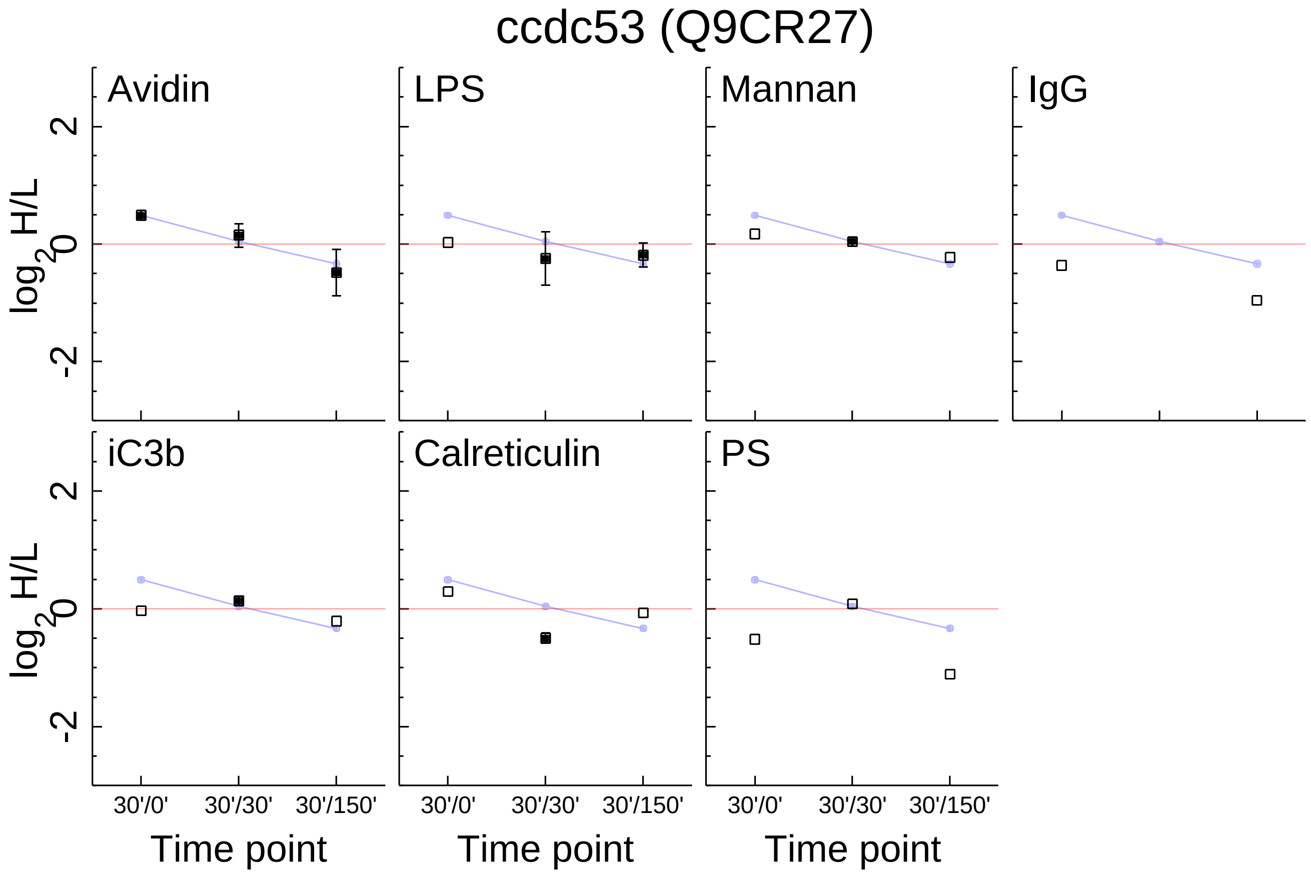
ccdc53 (Q9CR27)
Avidin
LPS
Mannan
IgG
iC3b
Calreticulin
PS
log2 H/L
2
0
-2
30'/0'
30'/30'
30'/150'
Time point

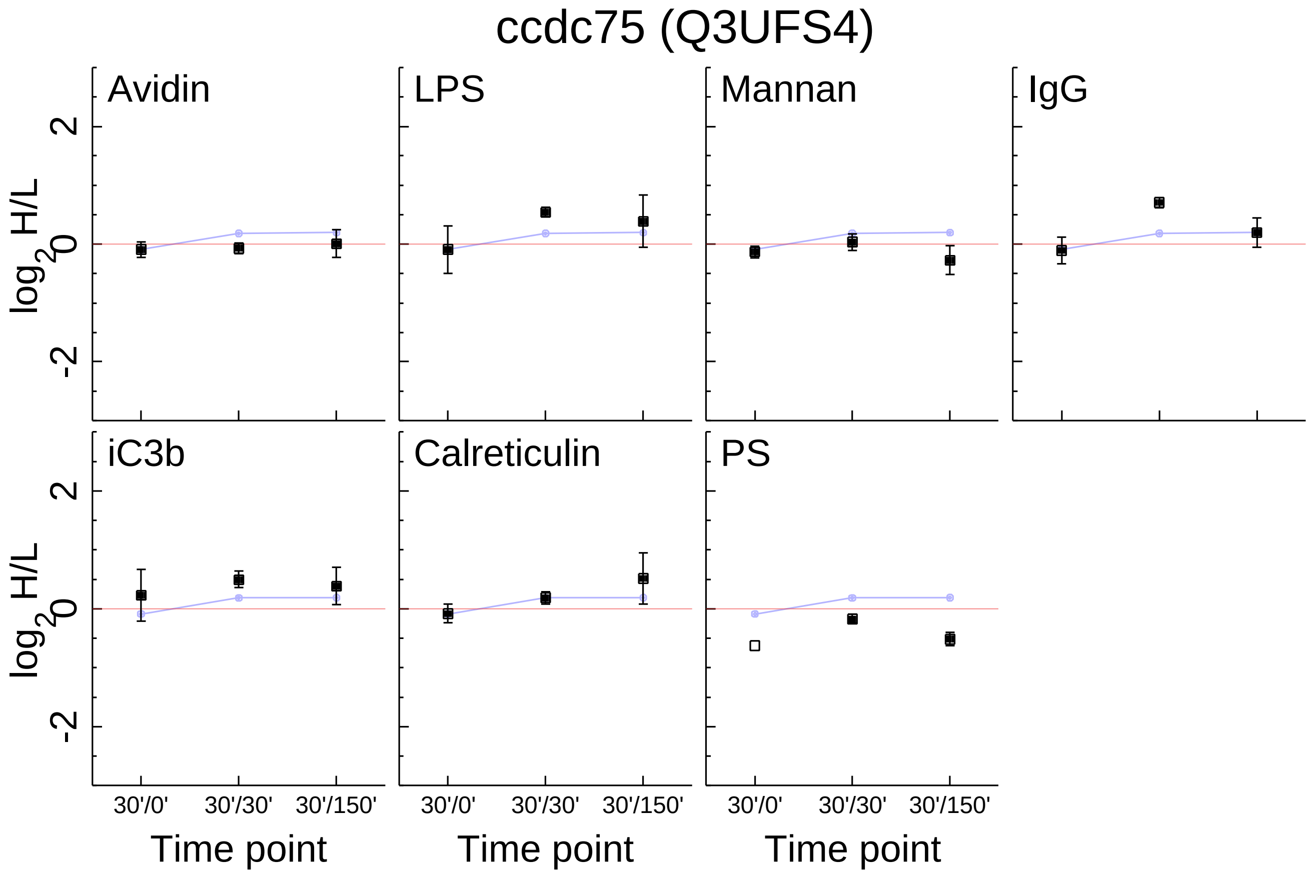
ccdc75 (Q3UFS4)
Avidin
LPS
Mannan
IgG
iC3b
Calreticulin
PS
log2 H/L
2
0
-2
30'/0'
30'/30'
30'/150'
Time point

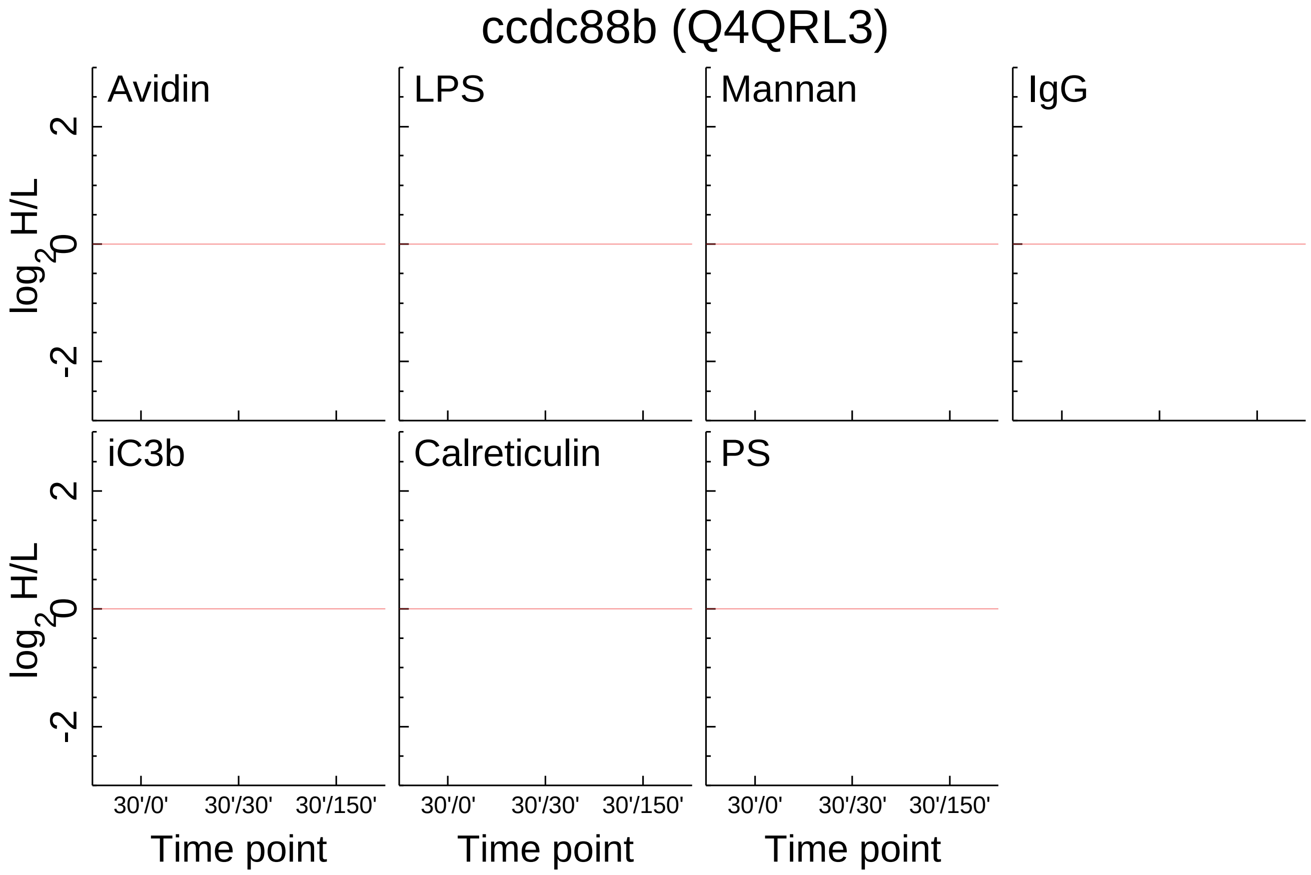

ccdc88b (Q4QRL3)
Avidin
LPS
Mannan
IgG
iC3b
Calreticulin
PS
log2 H/L
2
0
-2
30'/0'
30'/30'
30'/150'
Time point

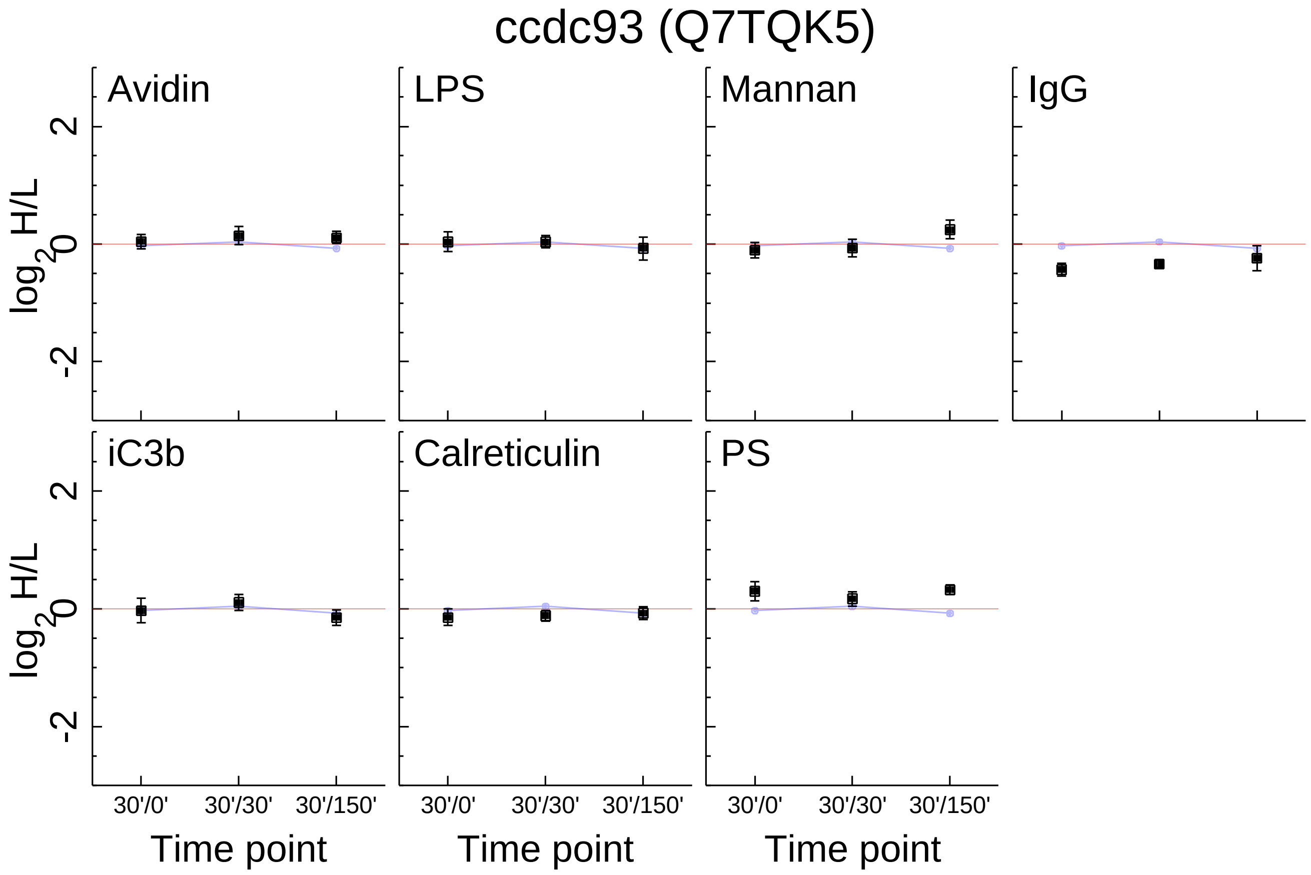
ccdc93 (Q7TQK5)
Avidin
LPS
Mannan
IgG
iC3b
Calreticulin
PS
log2 H/L
2
0
-2
30'/0'
30'/30'
30'/150'
Time point

cct2 (P80314)

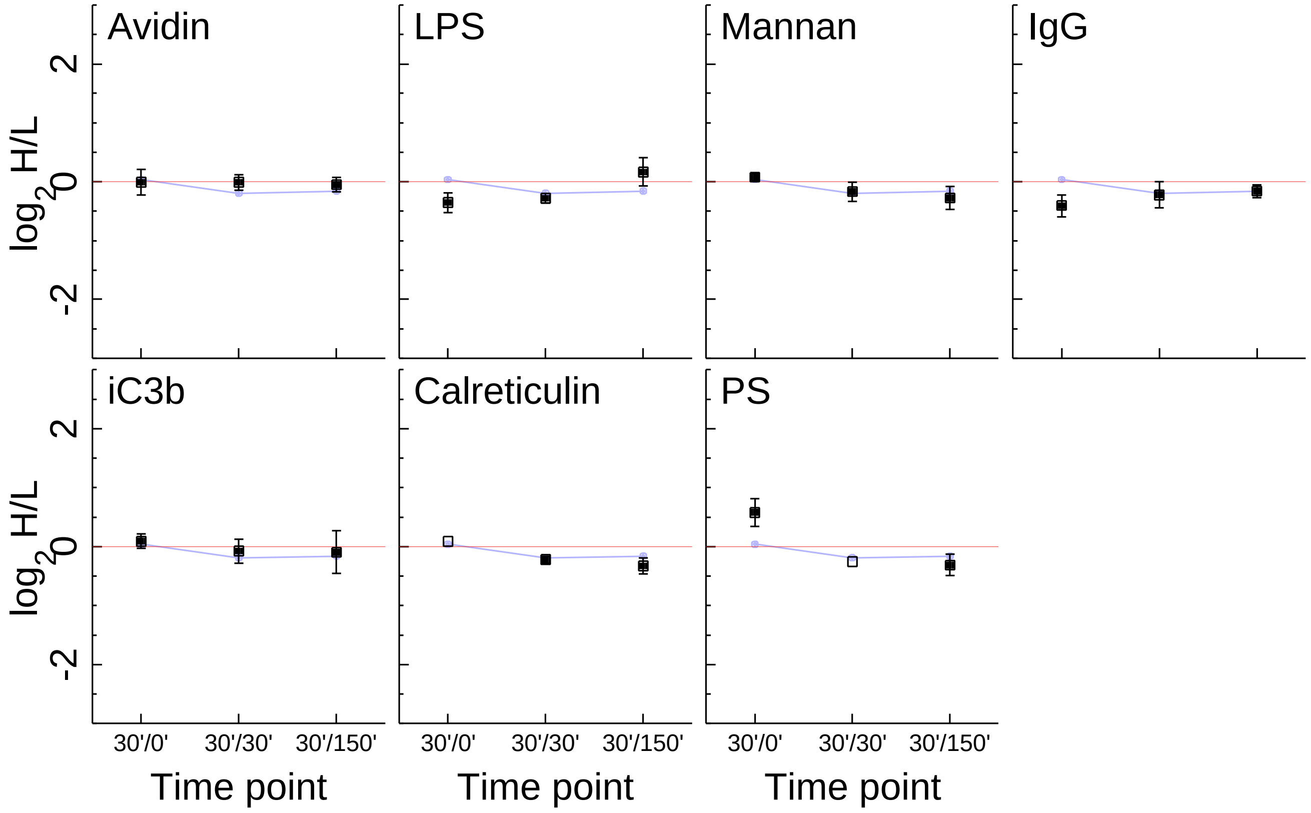

Avidin
LPS
Mannan
IgG
iC3b
Calreticulin
PS
log2 H/L
2
0
-2
30'/0'
30'/30'
30'/150'
Time point

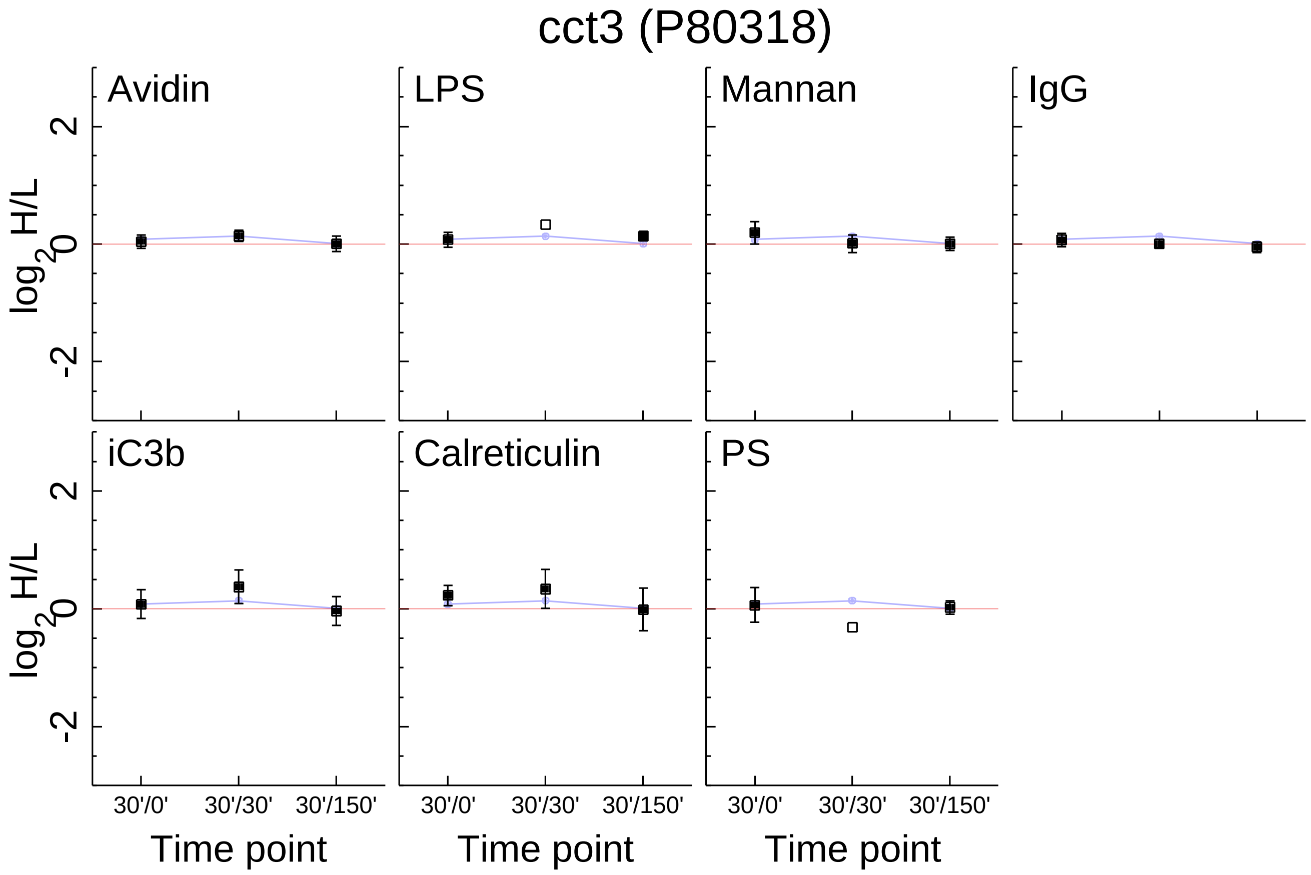
cct3 (P80318)
Avidin
LPS
Mannan
IgG
iC3b
Calreticulin
PS
log2 H/L
2
0
-2
30'/0'
30'/30'
30'/150'
Time point

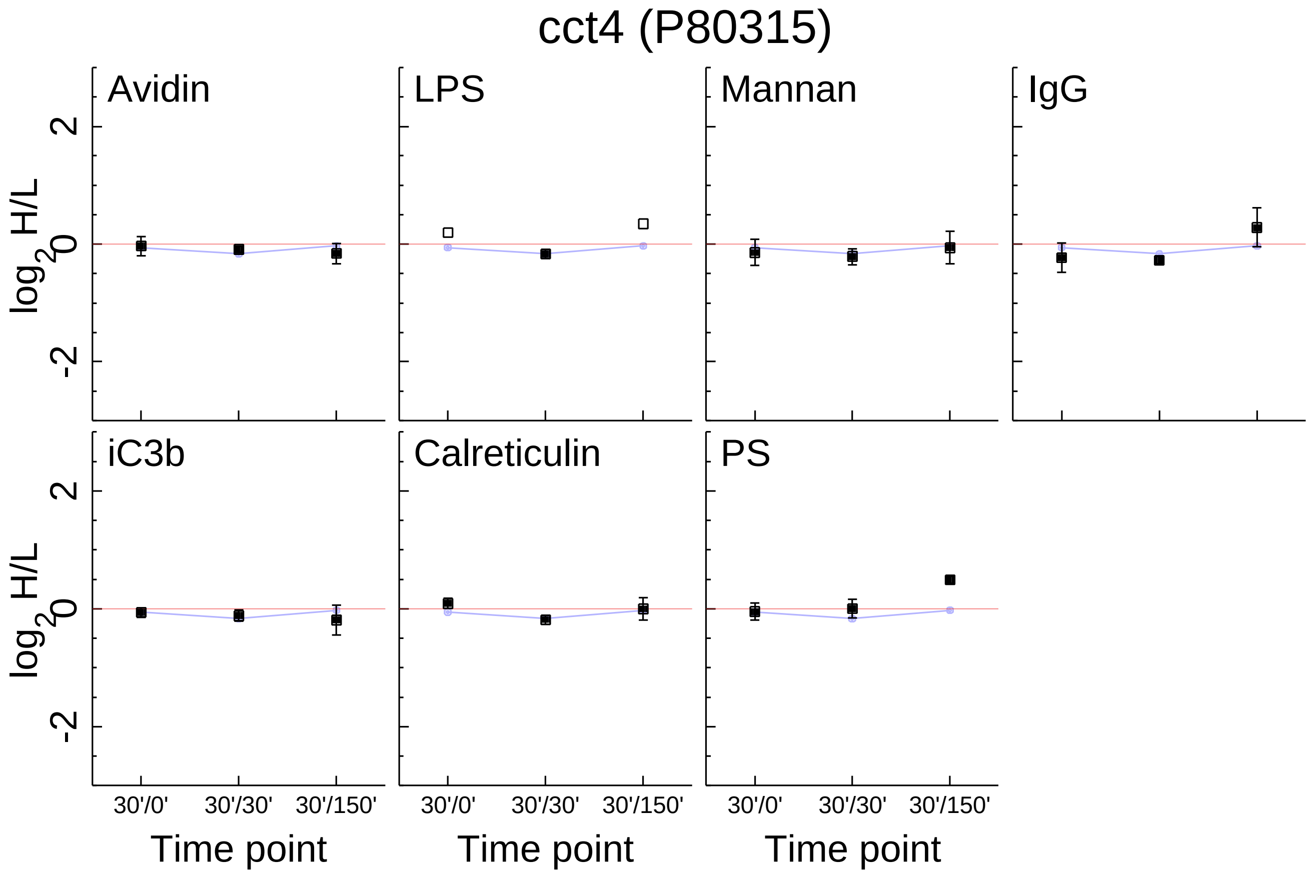
cct4 (P80315)
Avidin
LPS
Mannan
IgG
iC3b
Calreticulin
PS
log2 H/L
2
0
-2
30'/0'
30'/30'
30'/150'
Time point

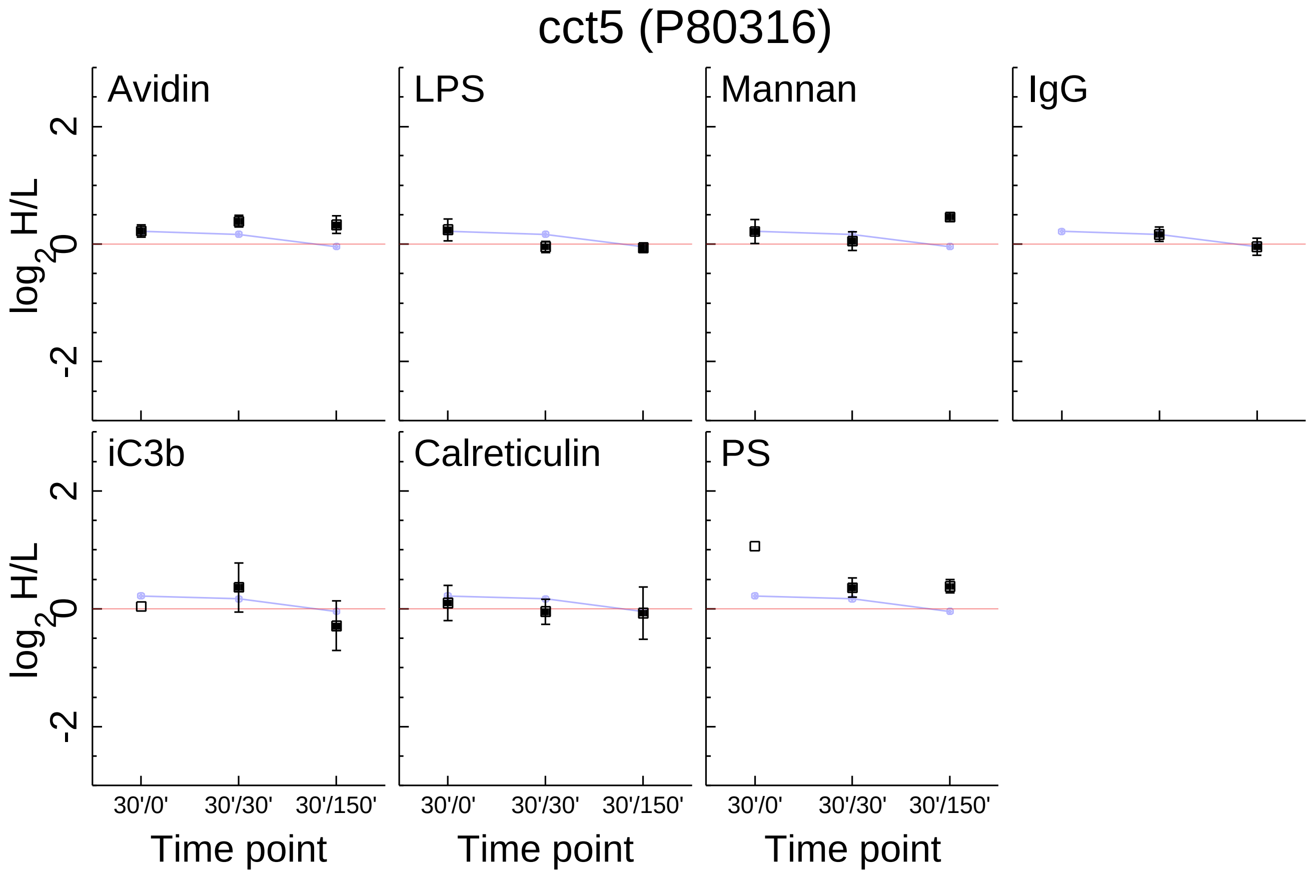
cct5 (P80316)
Avidin
LPS
Mannan
IgG
iC3b
Calreticulin
PS
log2 H/L
2
0
-2
30'/0'
30'/30'
30'/150'
Time point

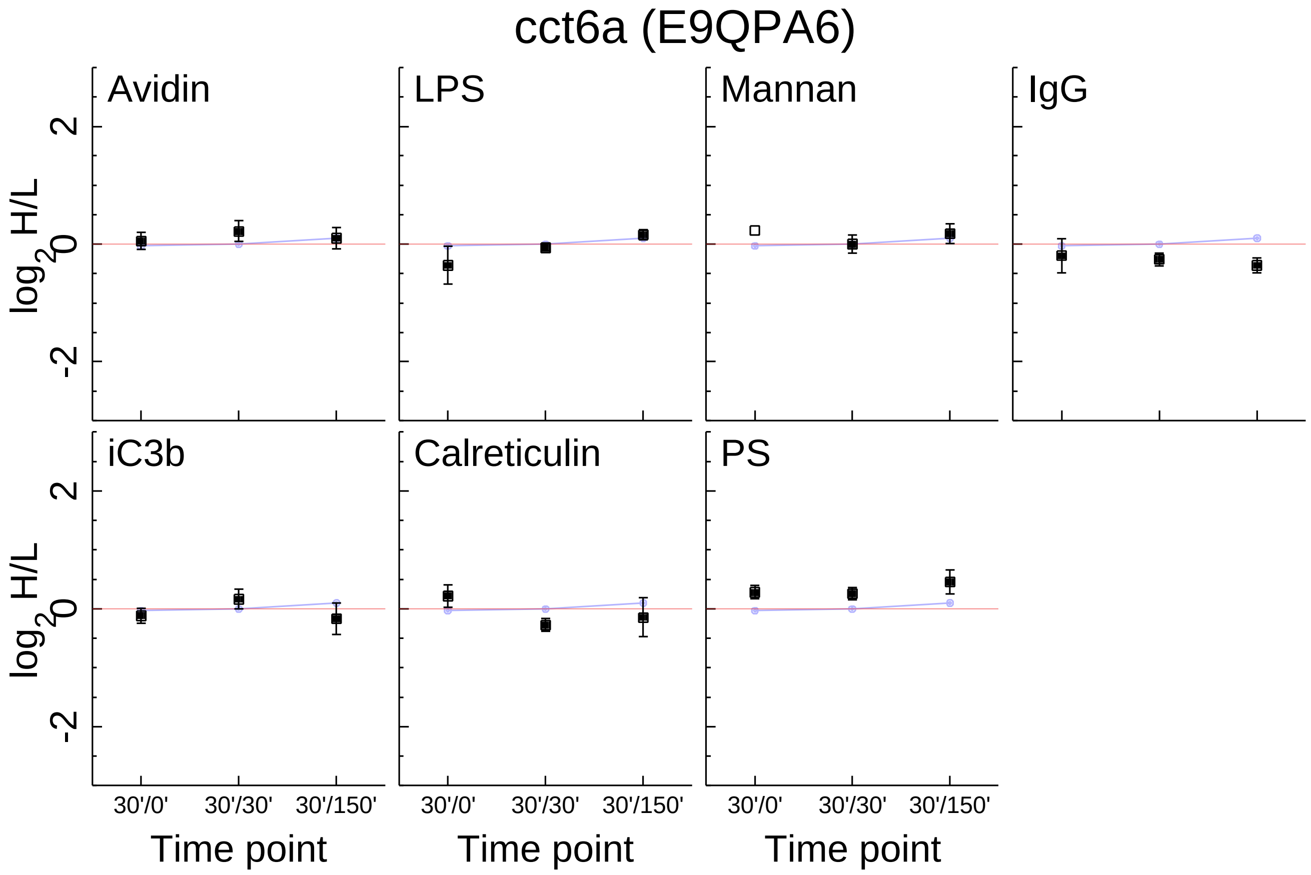
cct6a (E9QPA6)
Avidin
LPS
Mannan
IgG
iC3b
Calreticulin
PS
log2 H/L
2
0
-2
30'/0'
30'/30'
30'/150'
Time point

cct7 (P80313)

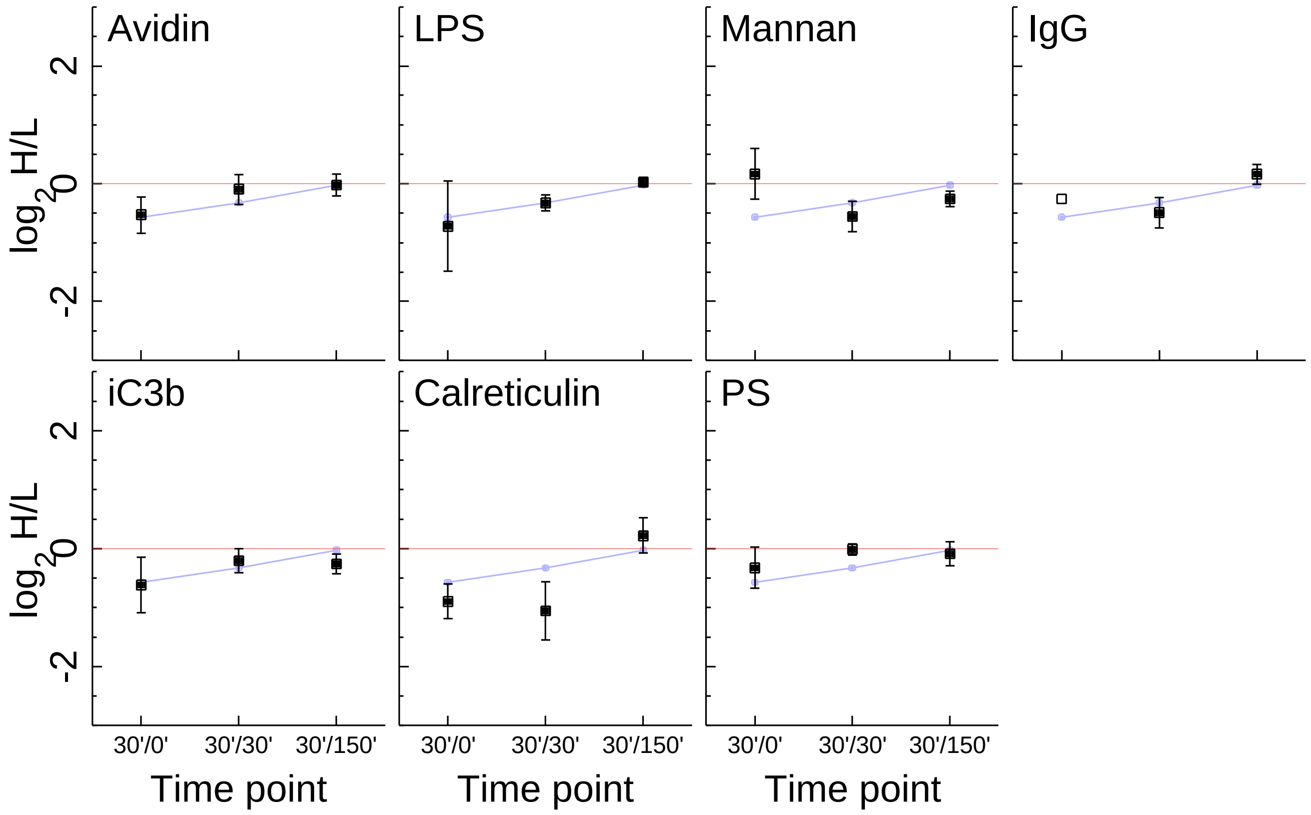

Avidin
LPS
Mannan
IgG
iC3b
Calreticulin
PS
log2 H/L
2
0
-2
30'/0'
30'/30'
30'/150'
Time point

cct8 (P42932)

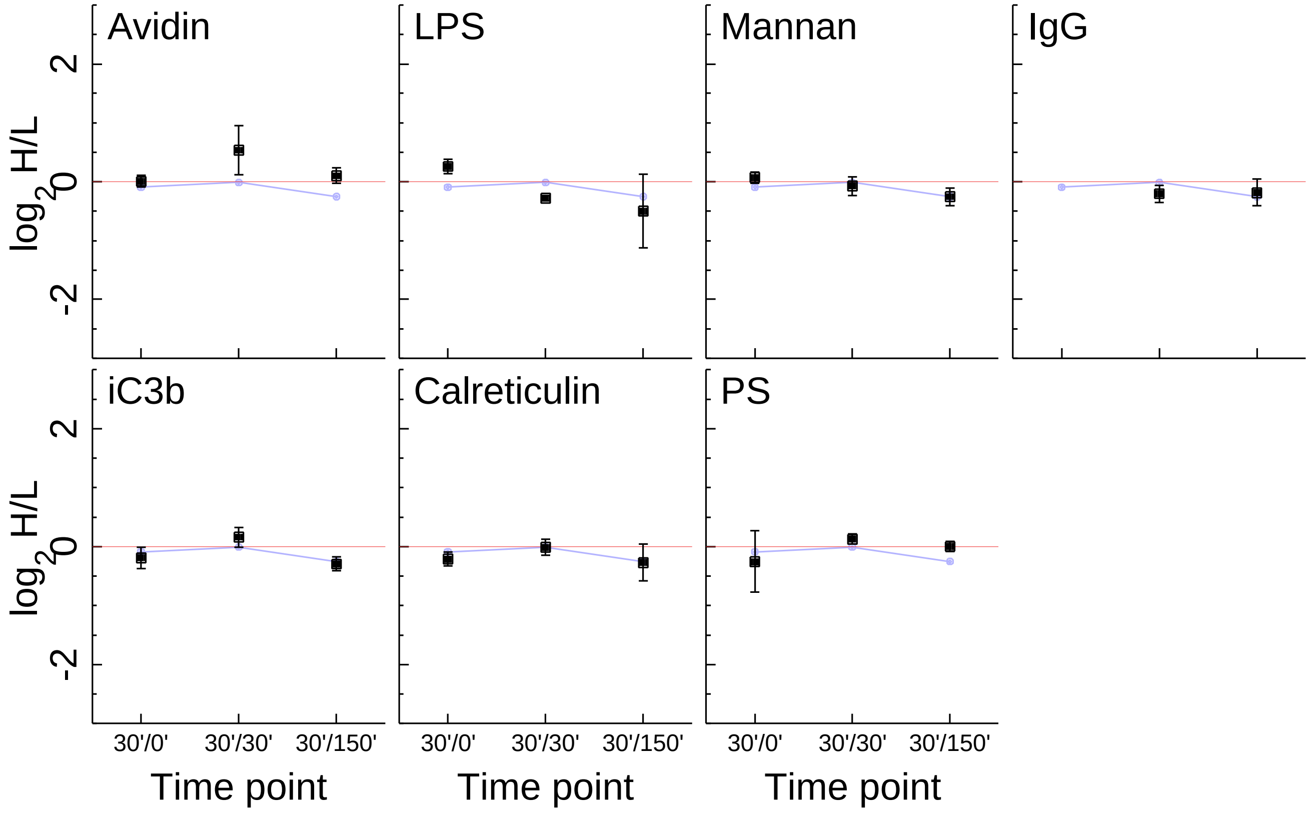
Avidin
LPS
Mannan
IgG
iC3b
Calreticulin
PS
log2 H/L
2
0
-2
30'/0'
30'/30'
30'/150'
Time point

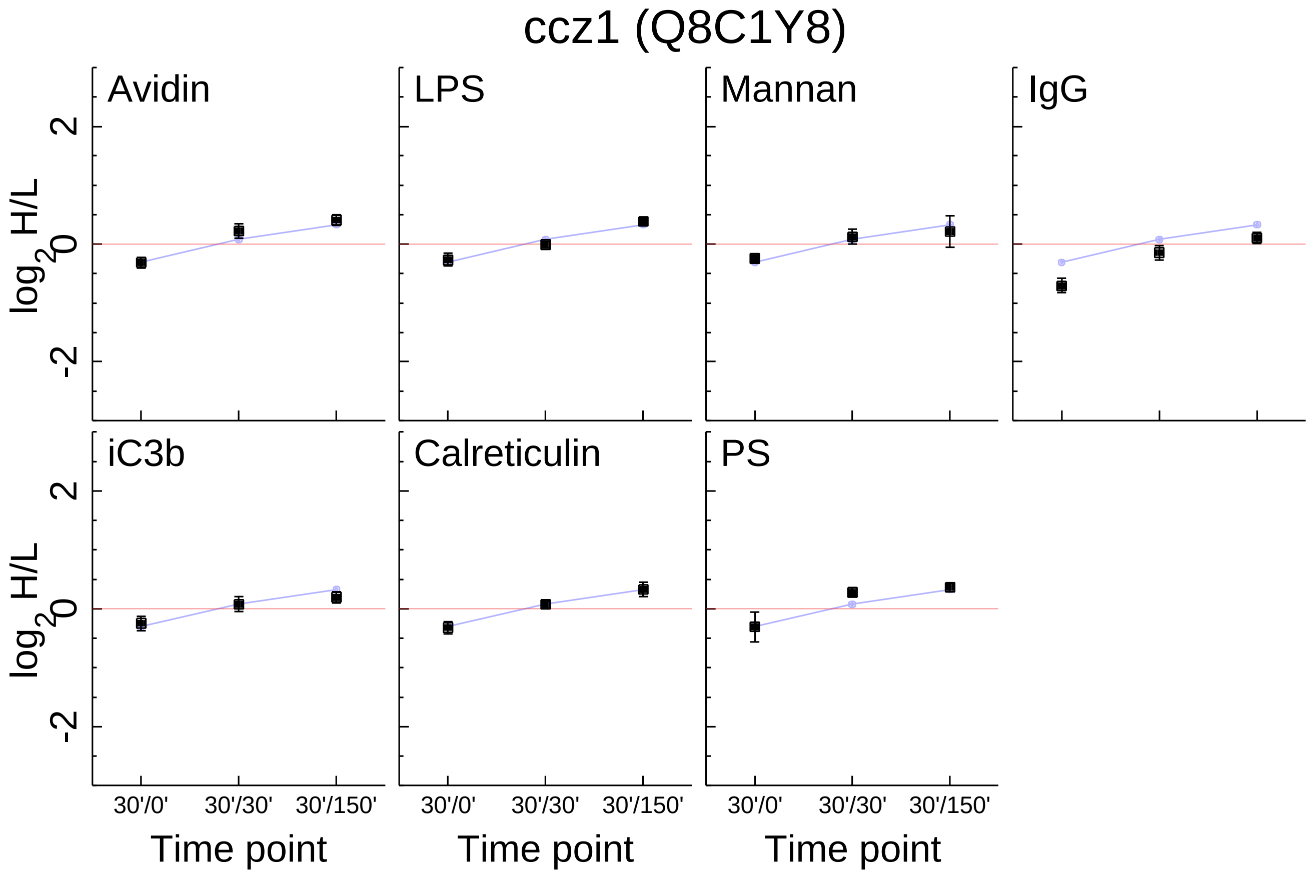

ccz1 (Q8C1Y8)
Avidin
LPS
Mannan
IgG
iC3b
Calreticulin
PS
2
0
-2
log2 H/L
30'/0'
30'/30'
30'/150'
Time point

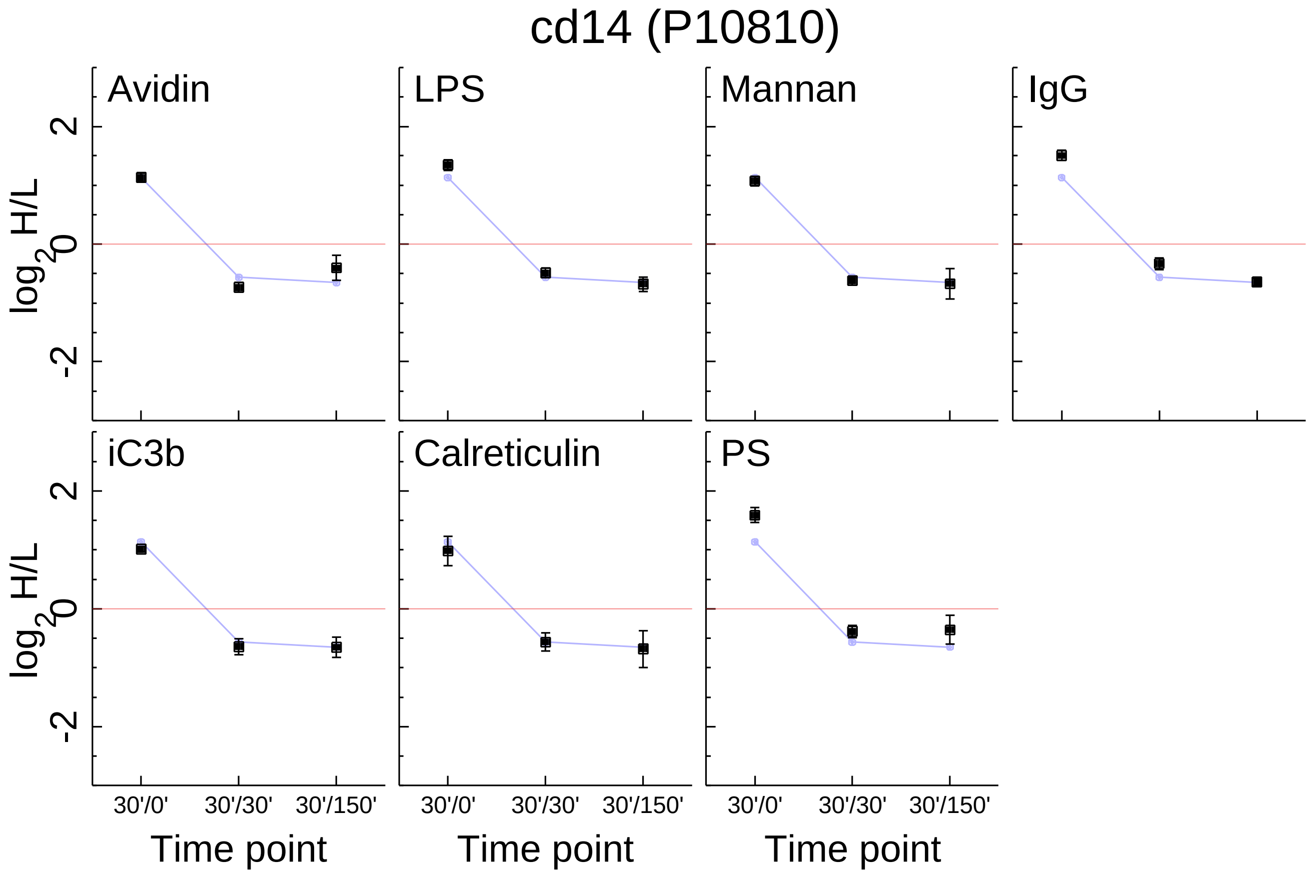
cd14 (P10810)
Avidin
LPS
Mannan
IgG
iC3b
Calreticulin
PS
log2 H/L
2
0
-2
30'/0'
30'/30'
30'/150'
Time point

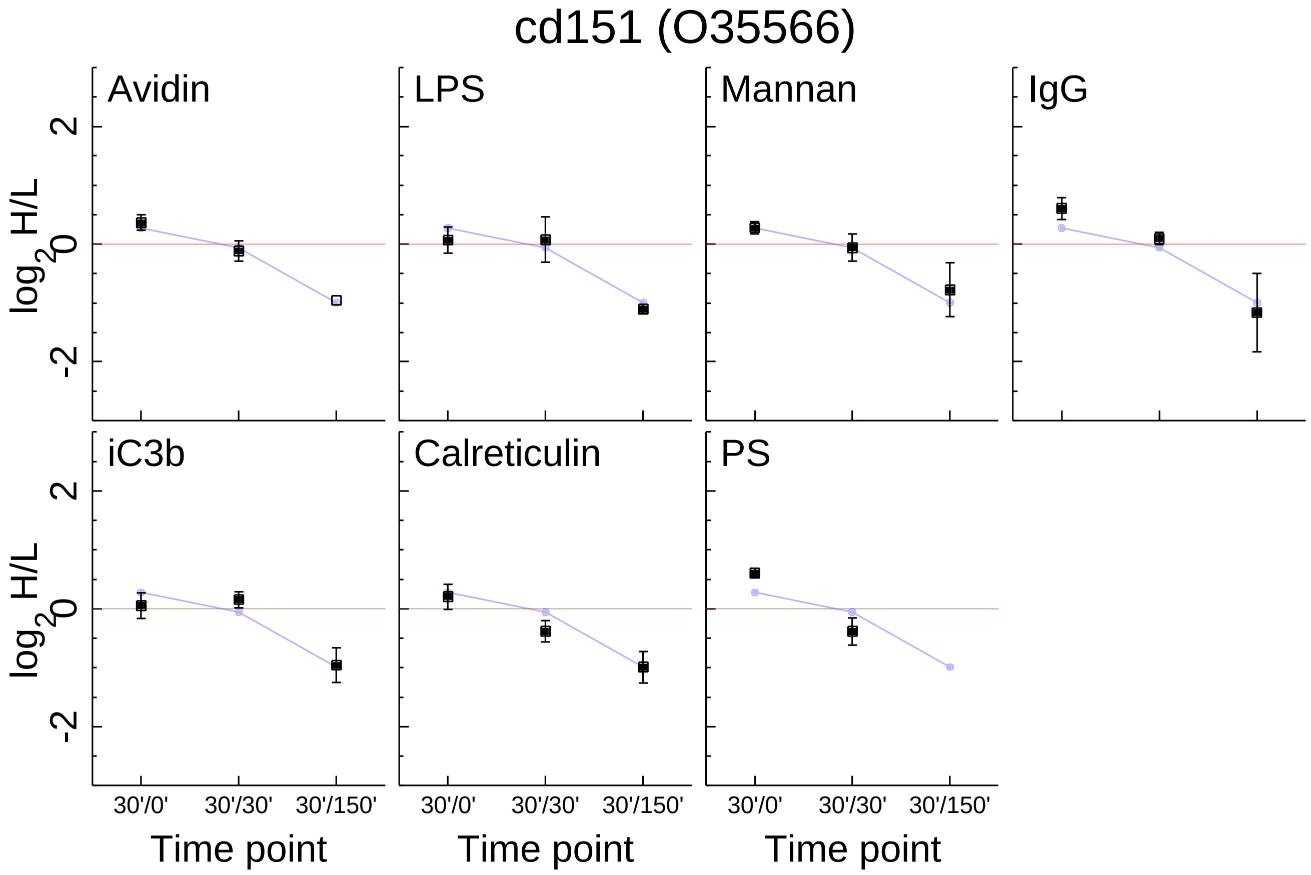
cd151 (O35566)
Avidin
LPS
Mannan
IgG
iC3b
Calreticulin
PS
log2 H/L
2
0
-2
30'/0'
30'/30'
30'/150'
Time point

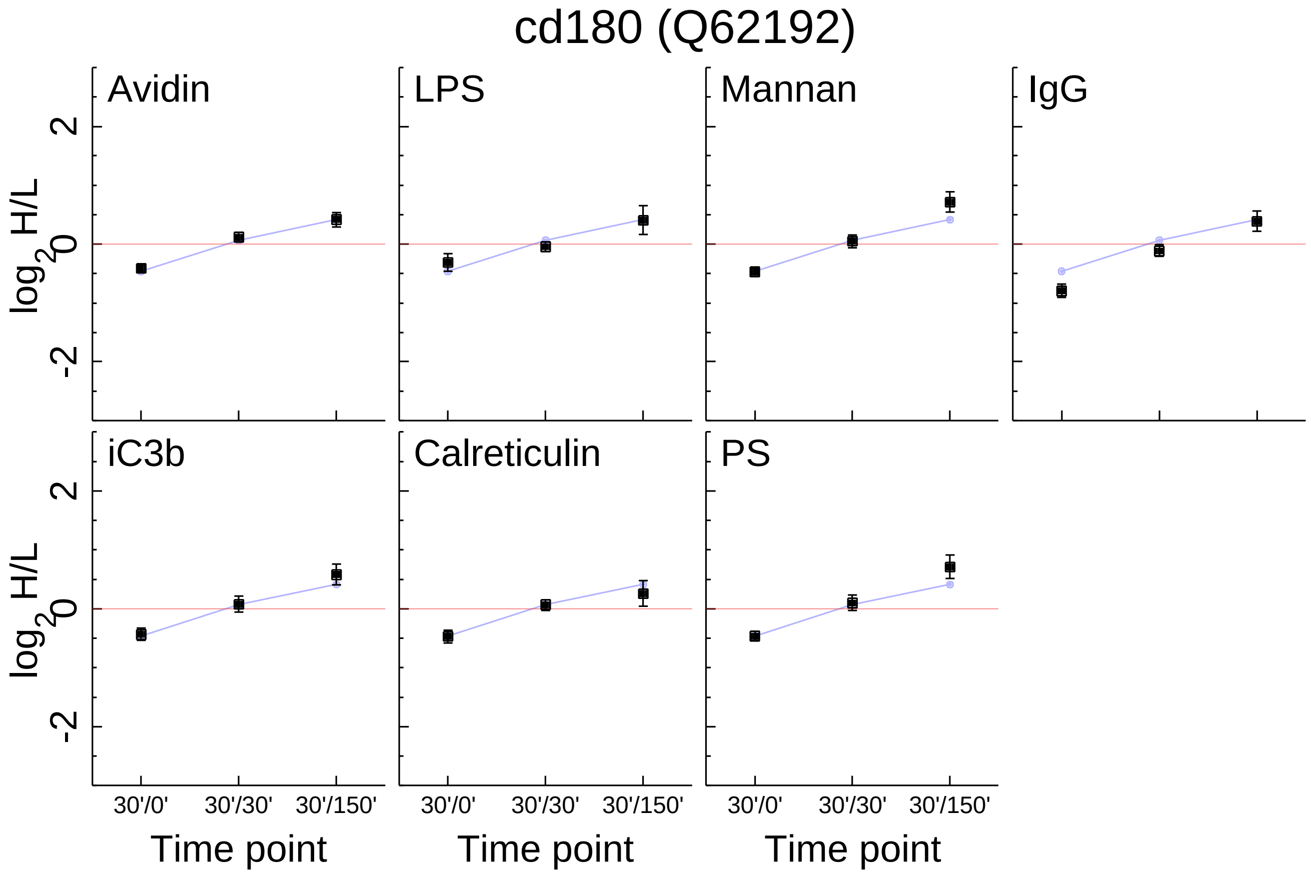

cd180 (Q62192)
Avidin
LPS
Mannan
IgG
iC3b
Calreticulin
PS
log2 H/L
2
0
-2
30'/0'
30'/30'
30'/150'
Time point

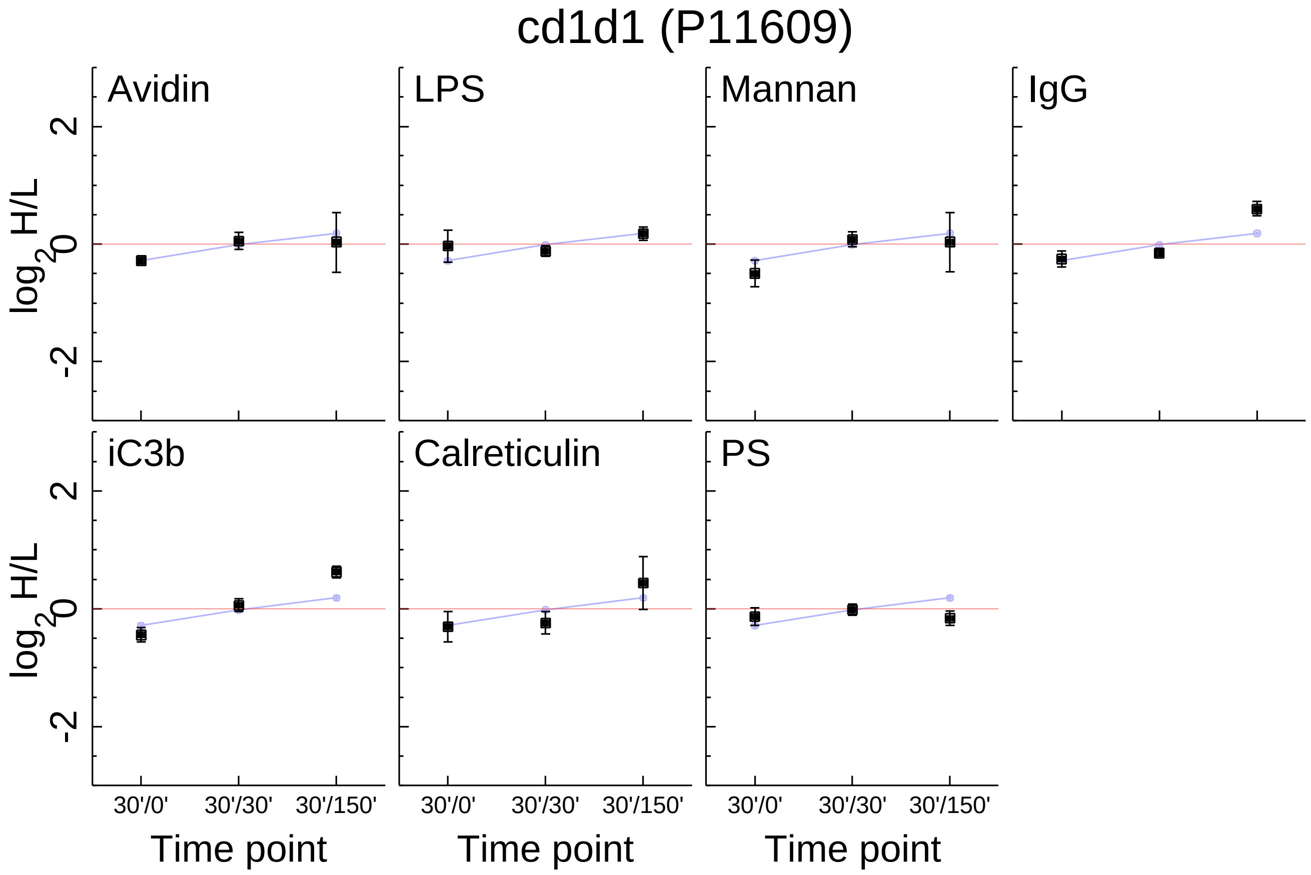

cd1d1 (P11609)
Avidin
LPS
Mannan
IgG
iC3b
Calreticulin
PS
log2 H/L
2
0
-2
30'/0'
30'/30'
30'/150'
Time point

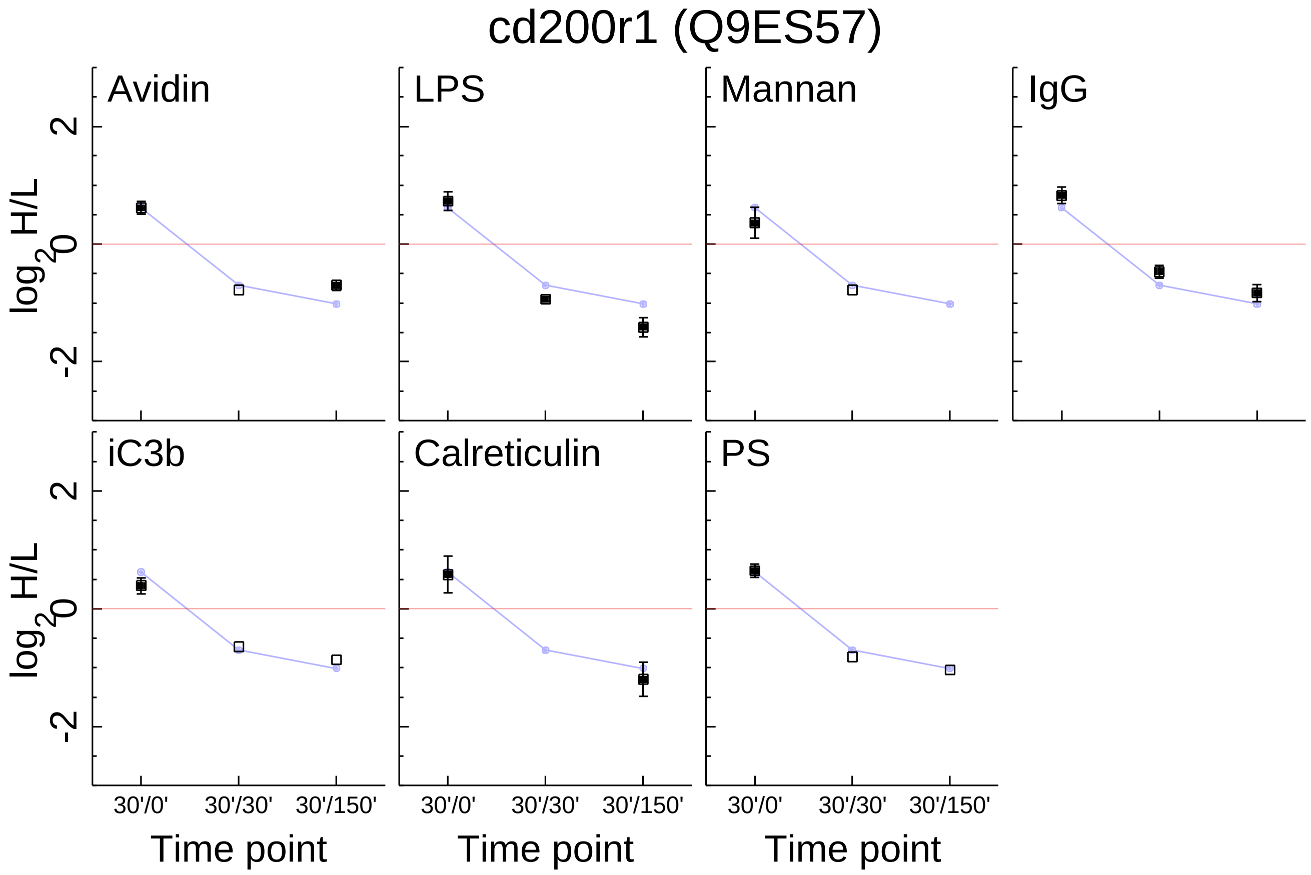
cd200r1 (Q9ES57)
Avidin
LPS
Mannan
IgG
iC3b
Calreticulin
PS
log2 H/L
2
0
-2
30'/0'
30'/30'
30'/150'
Time point

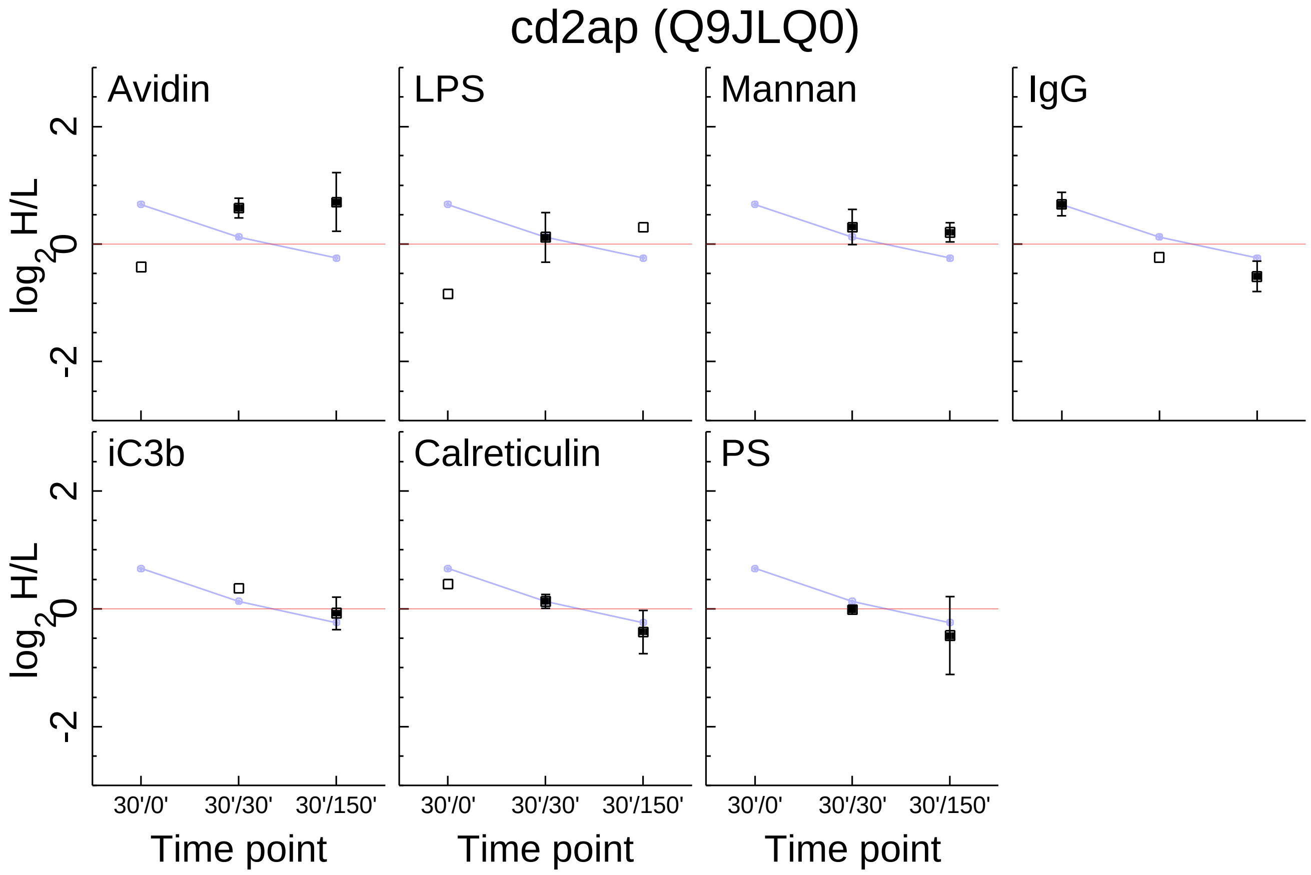
cd2ap (Q9JLQ0)
Avidin
LPS
Mannan
IgG
iC3b
Calreticulin
PS
$\log_2$ H/L
2
0
-2
30'/0'
30'/30'
30'/150'
Time point

cd300a (Q6SJQ0)

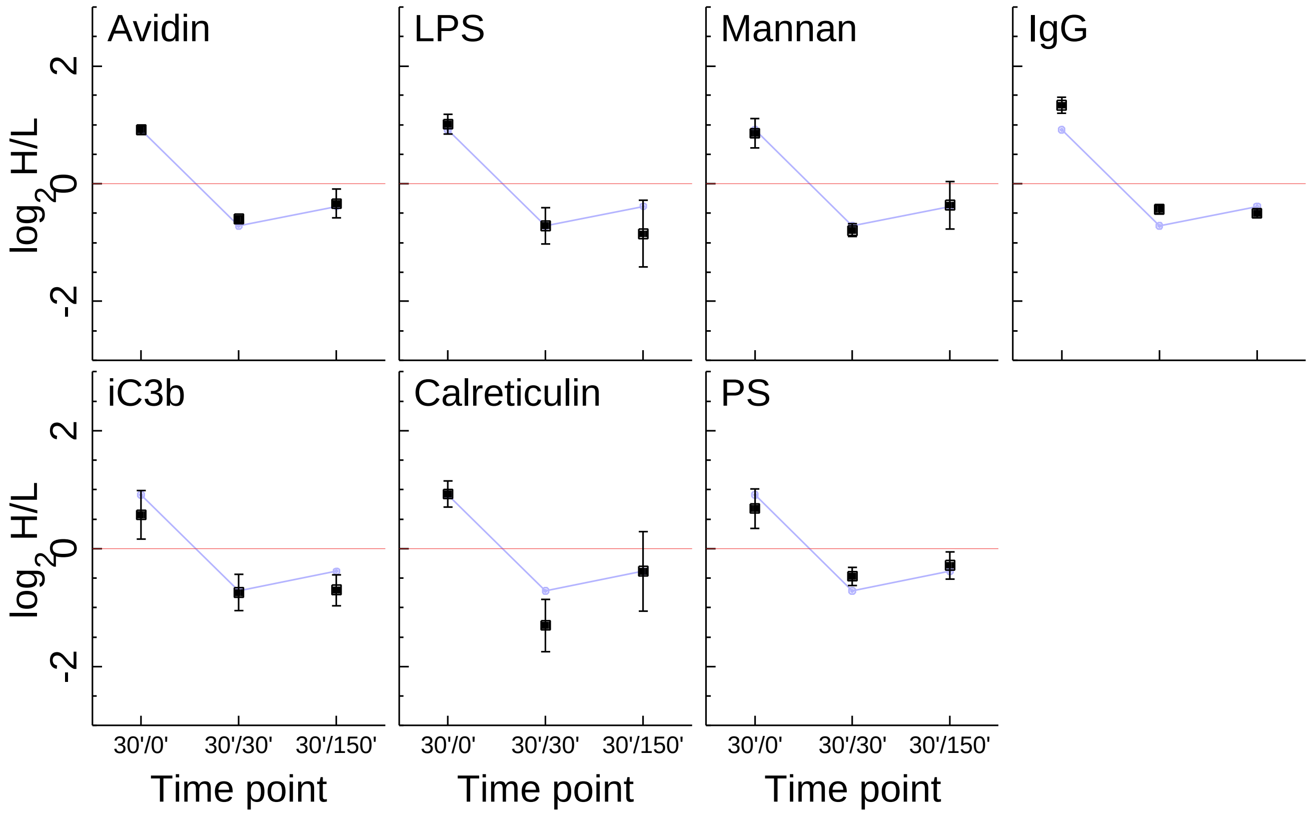

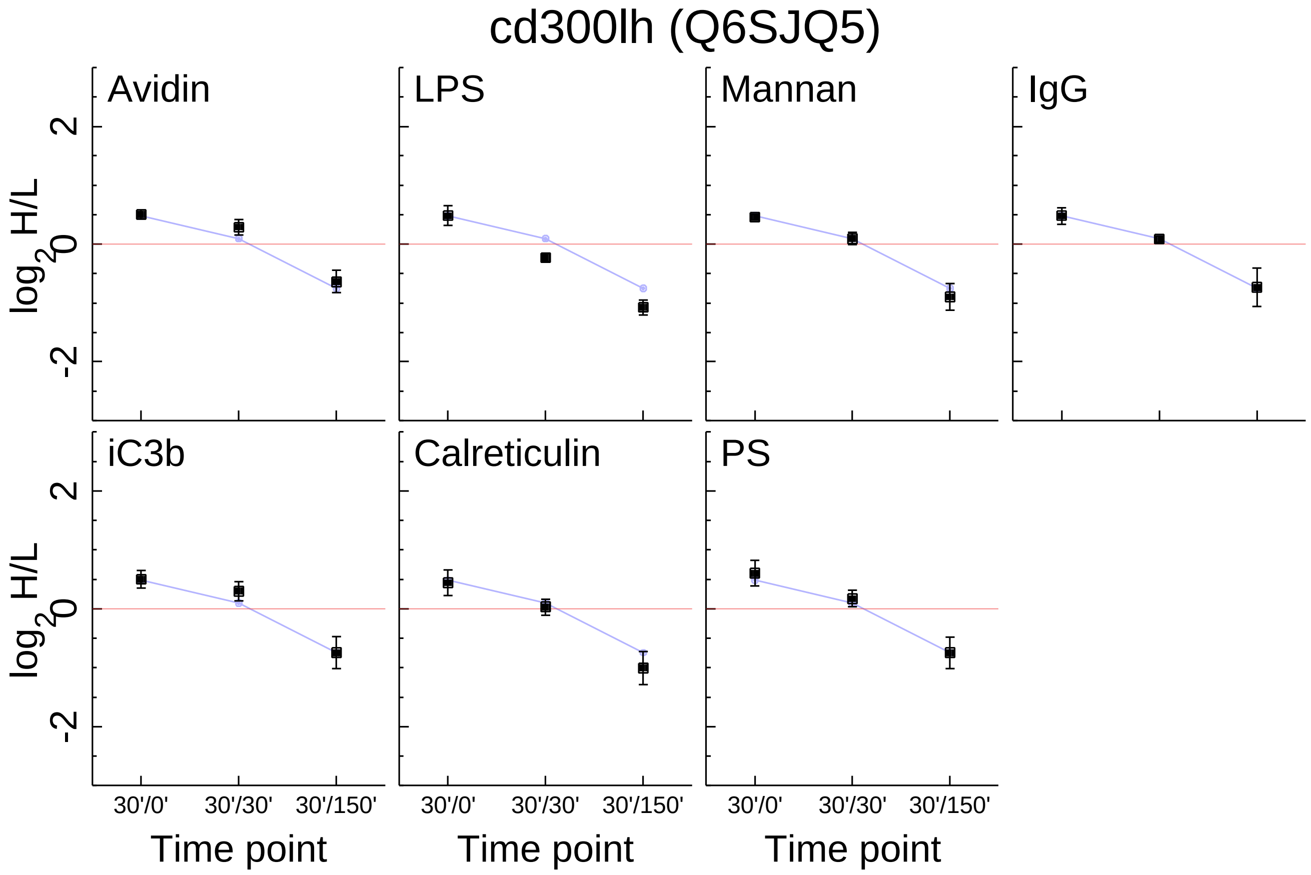

cd300lh (Q6SJQ5)
Avidin
LPS
Mannan
IgG
iC3b
Calreticulin
PS
2
0
-2
log2 H/L
30'/0'
30'/30'
30'/150'
Time point

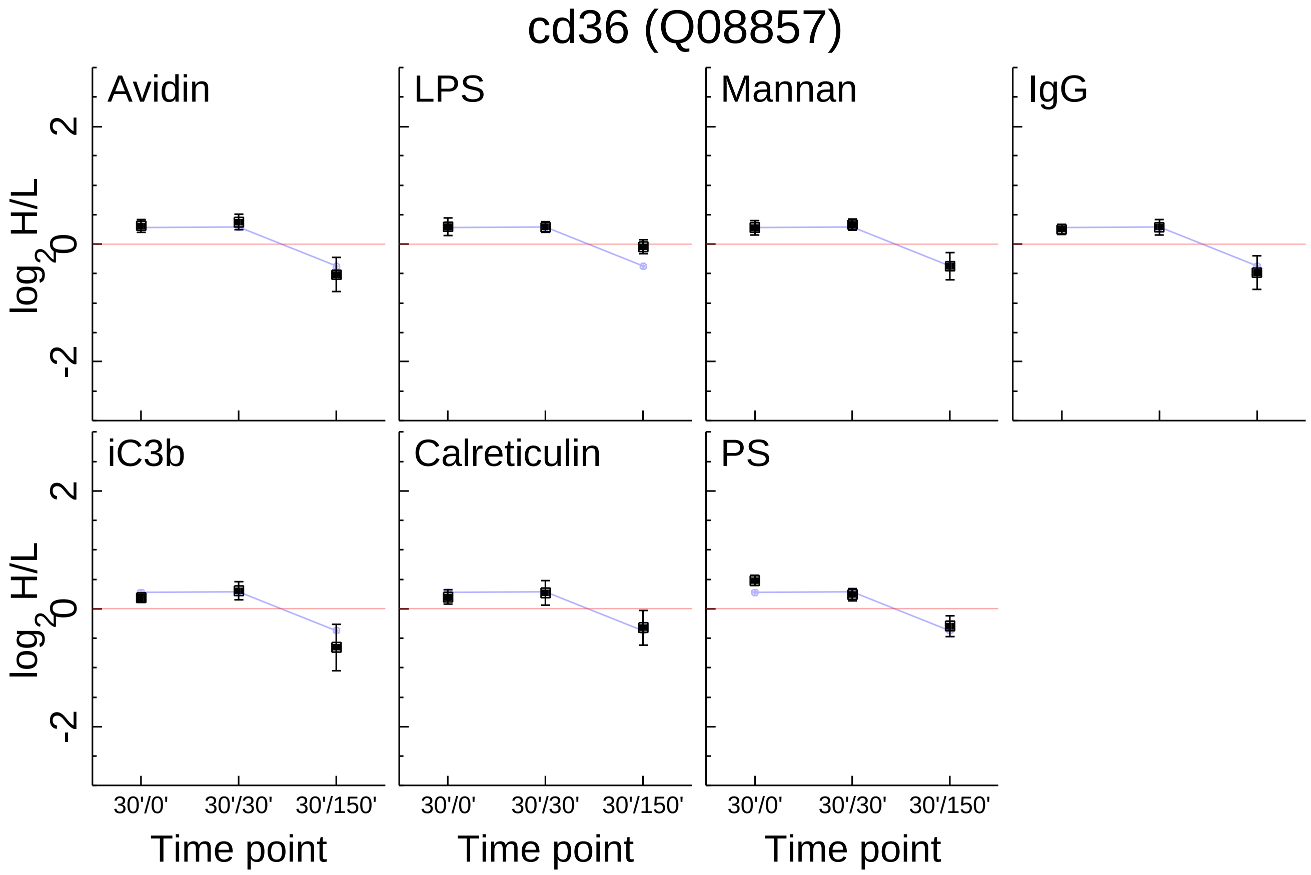
cd36 (Q08857)
Avidin
LPS
Mannan
IgG
iC3b
Calreticulin
PS
log2 H/L
2
0
-2
30'/0'
30'/30'
30'/150'
Time point

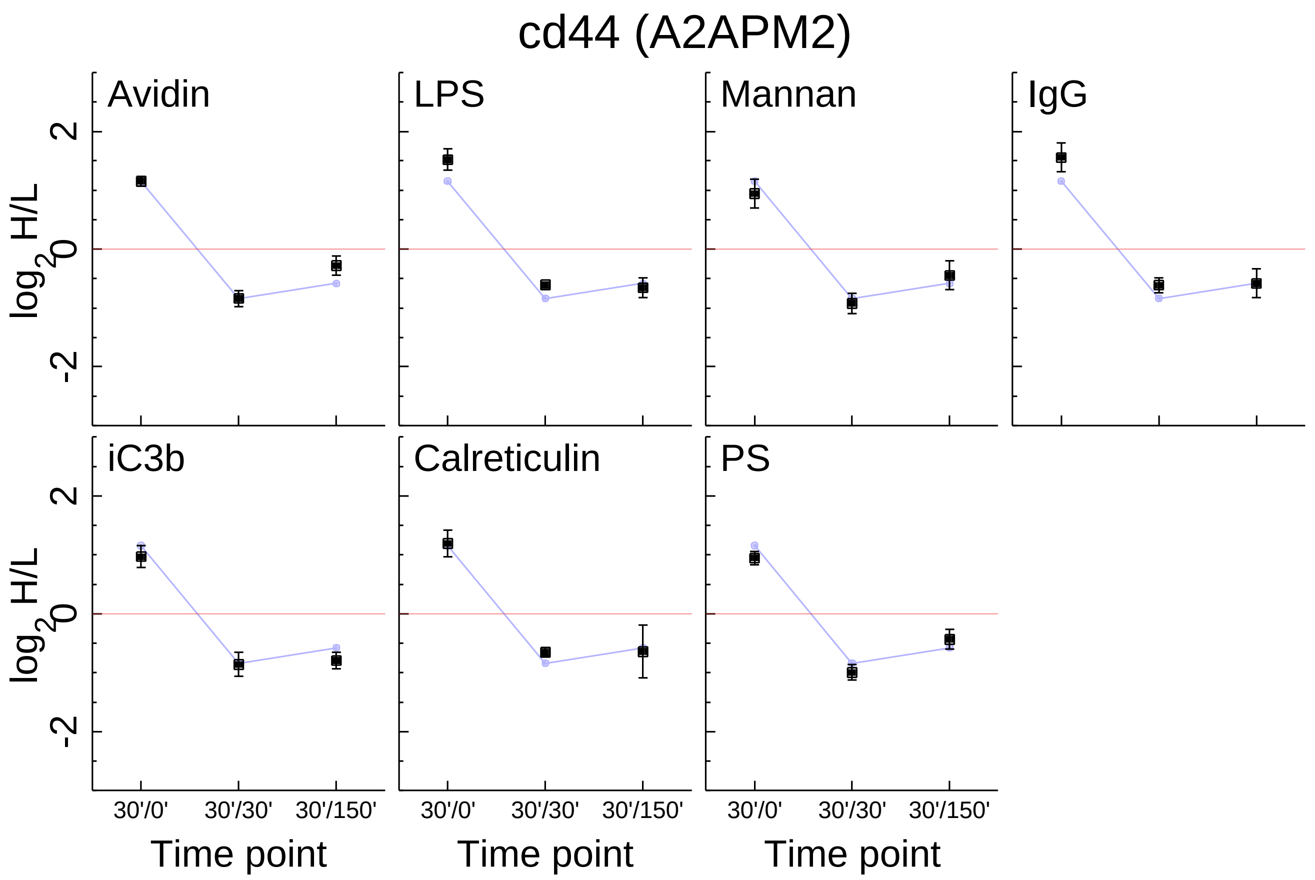

cd44 (A2APM2)
Avidin
LPS
Mannan
IgG
iC3b
Calreticulin
PS
$\log_2$ H/L
2
0
-2
30'/0'
30'/30'
30'/150'
Time point

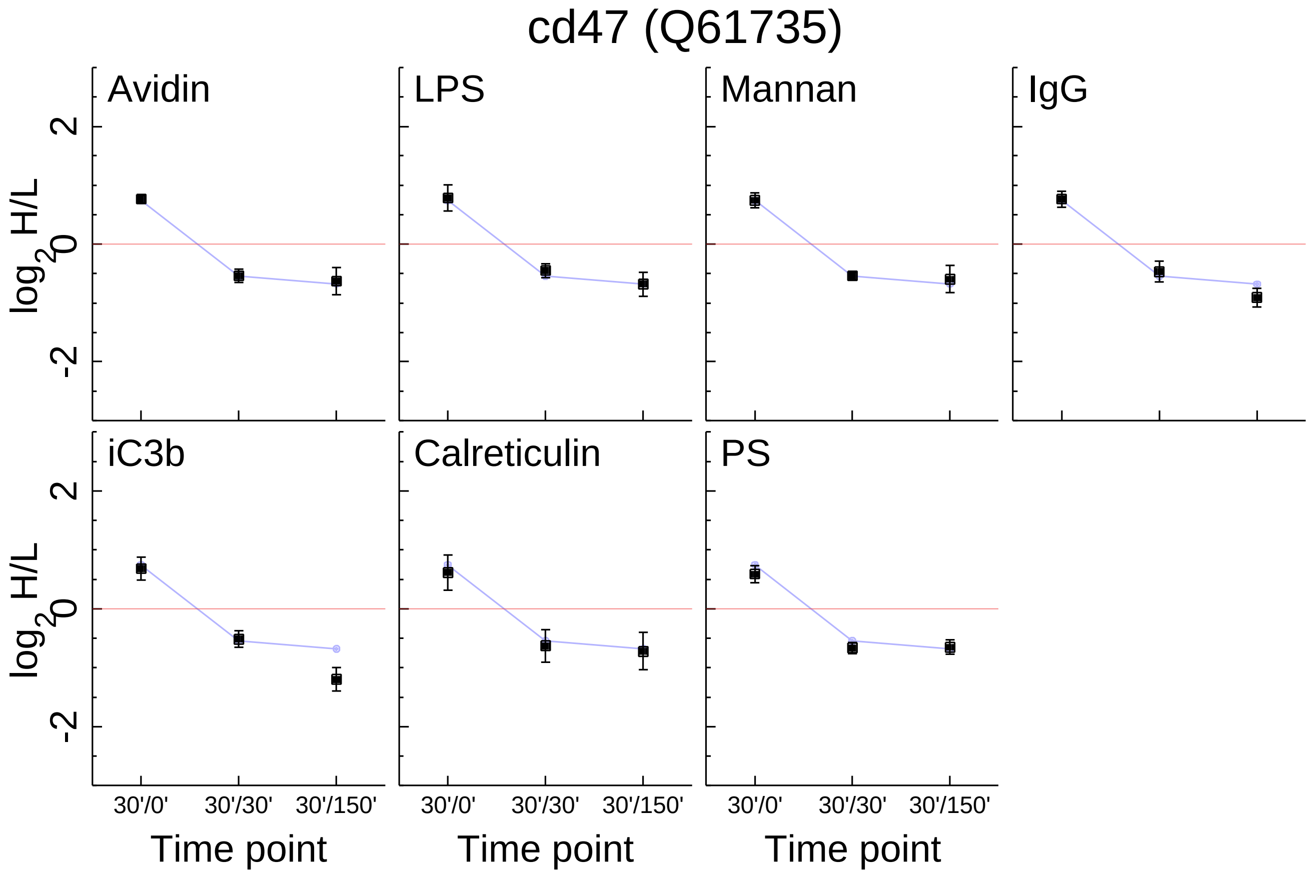
cd47 (Q61735)
Avidin
LPS
Mannan
IgG
iC3b
Calreticulin
PS
log2 H/L
2
0
-2
30'/0'
30'/30'
30'/150'
Time point

cd48 (P18181)

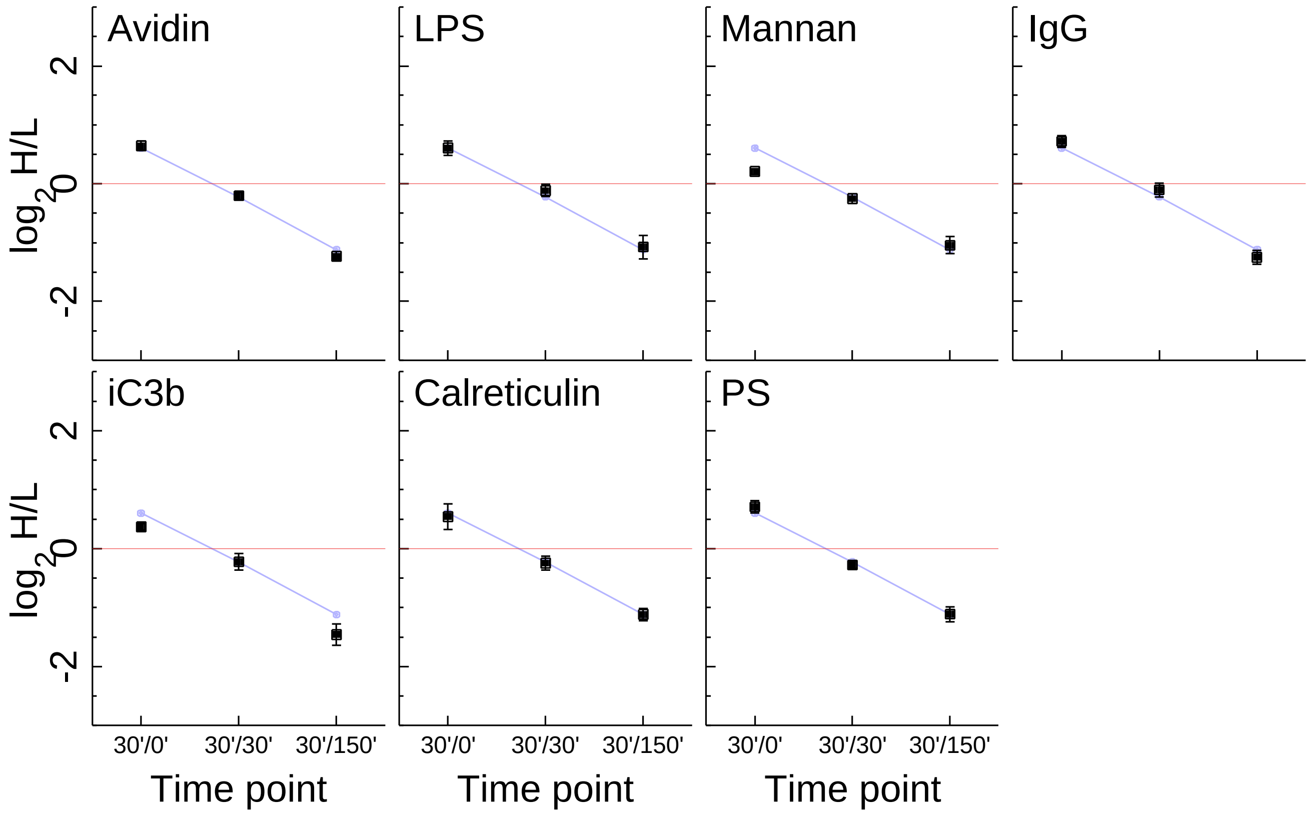
Avidin
LPS
Mannan
IgG
iC3b
Calreticulin
PS
2
0
-2
log2 H/L
30'/0'
30'/30'
30'/150'
Time point

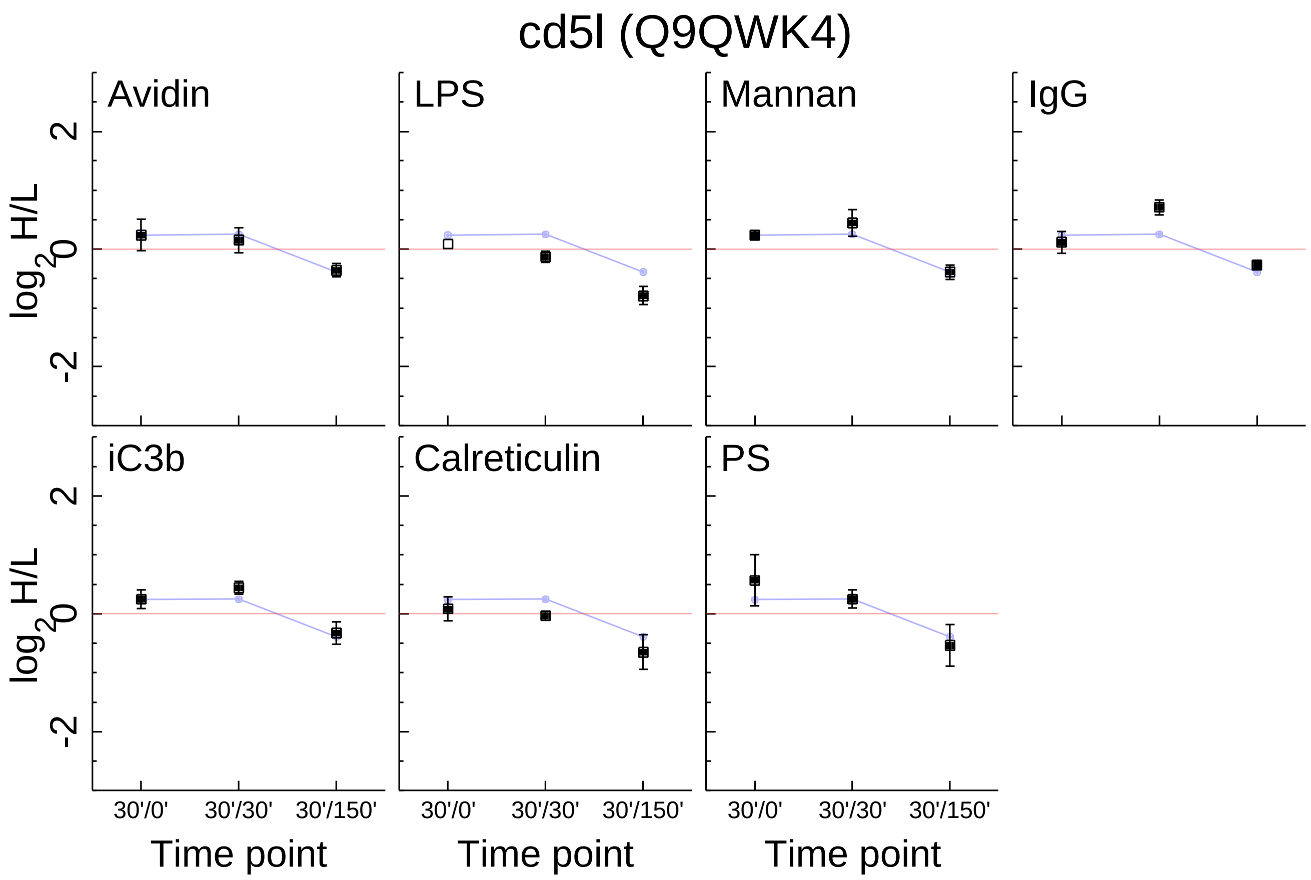

cd5l (Q9QWK4)
Avidin
LPS
Mannan
IgG
iC3b
Calreticulin
PS
log2 H/L
2
0
-2
30'/0'
30'/30'
30'/150'
Time point

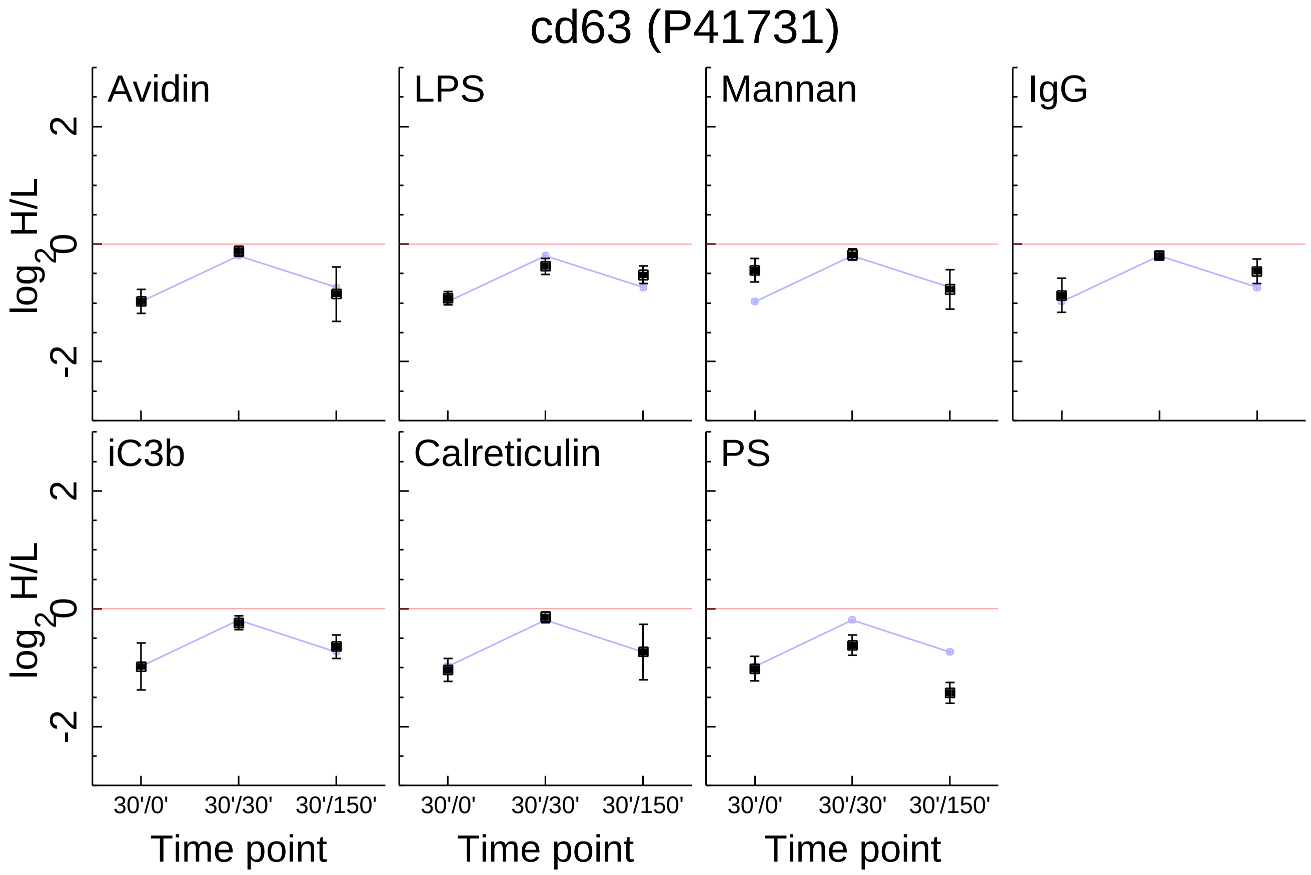
cd63 (P41731)
Avidin
LPS
Mannan
IgG
iC3b
Calreticulin
PS
log2 H/L
2
0
-2
30'/0'
30'/30'
30'/150'
Time point

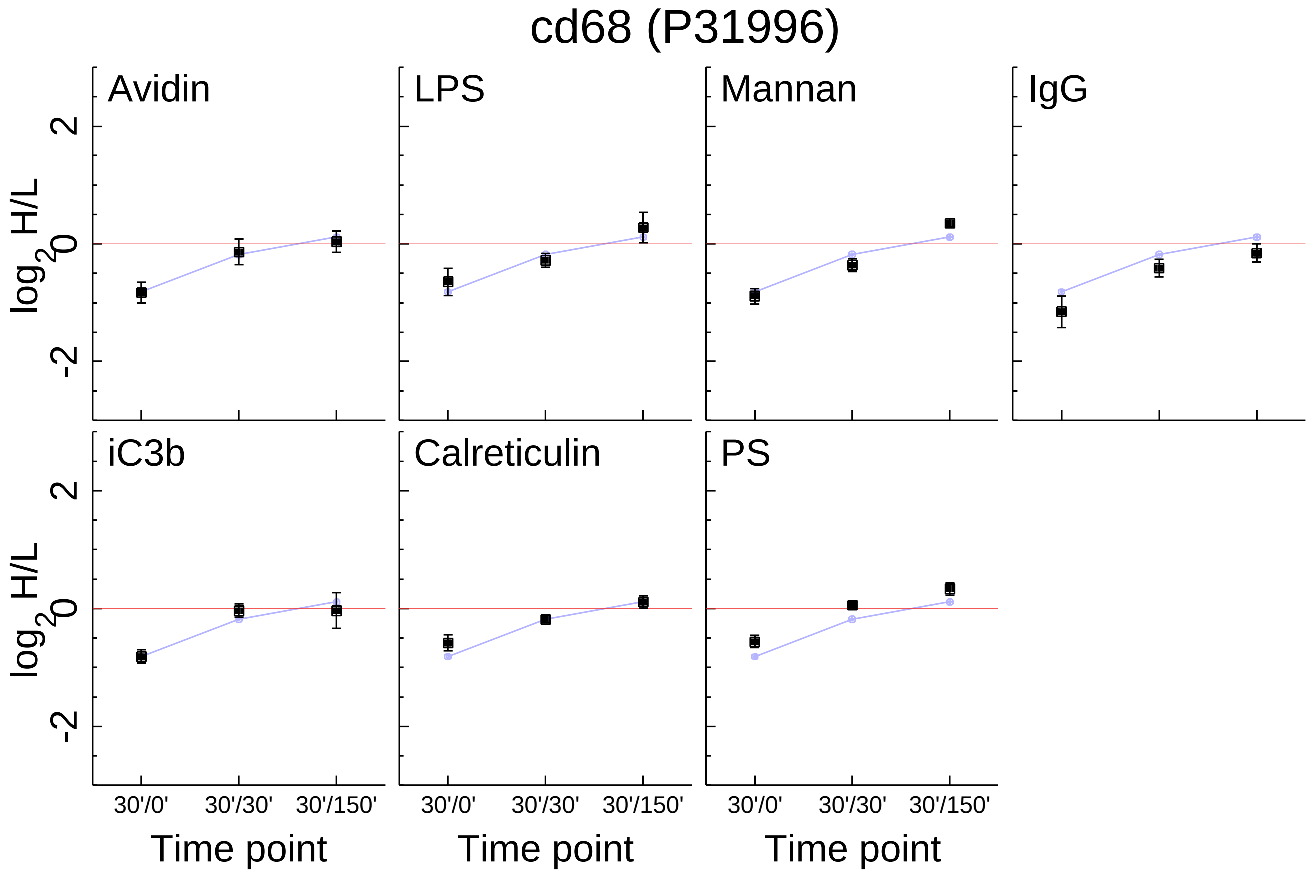

cd68 (P31996)
Avidin
LPS
Mannan
IgG
iC3b
Calreticulin
PS
log2 H/L
2
0
-2
30'/0'
30'/30'
30'/150'
Time point

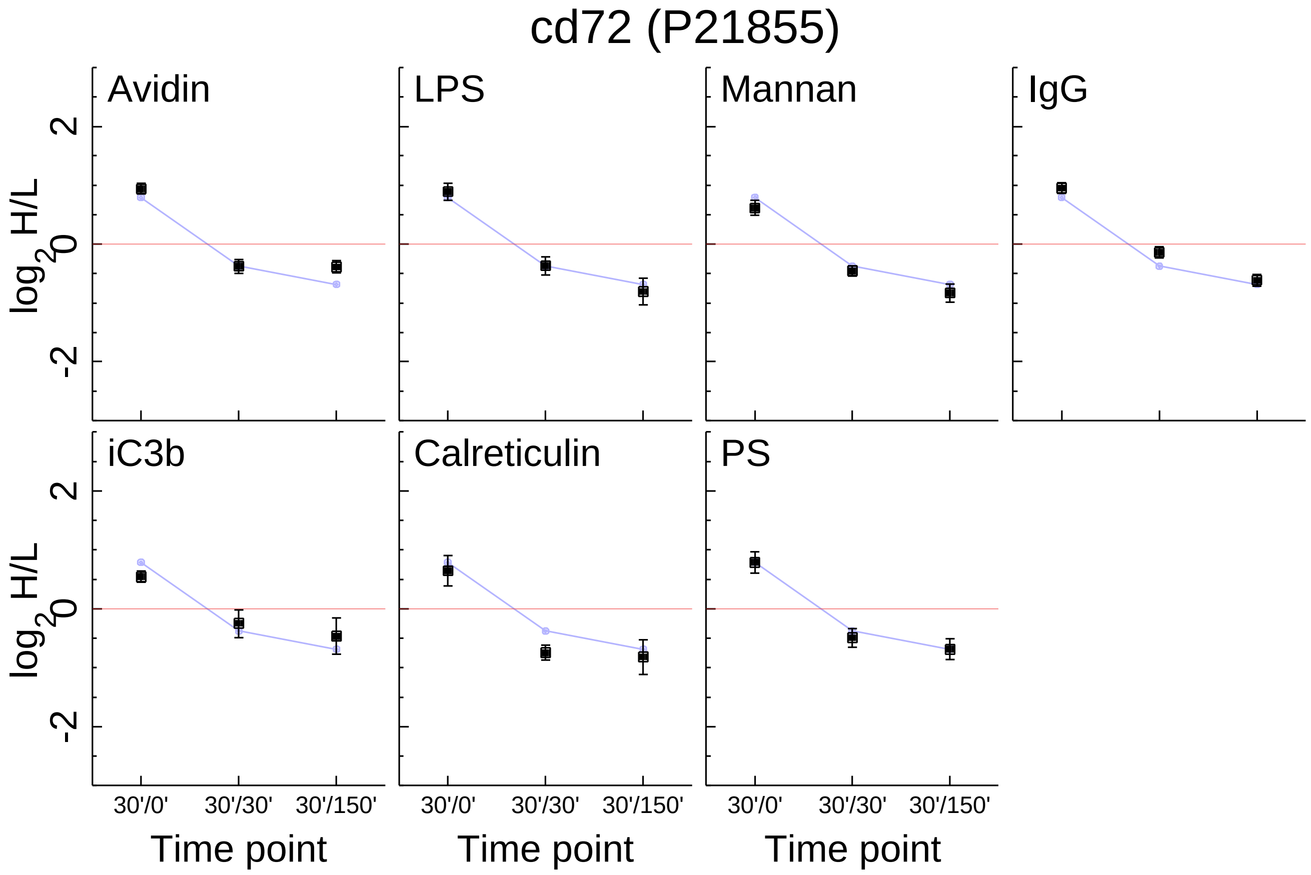
cd72 (P21855)
Avidin
LPS
Mannan
IgG
iC3b
Calreticulin
PS
log2 H/L
2
0
-2
30'/0'
30'/30'
30'/150'
Time point

cd74 (P04441)

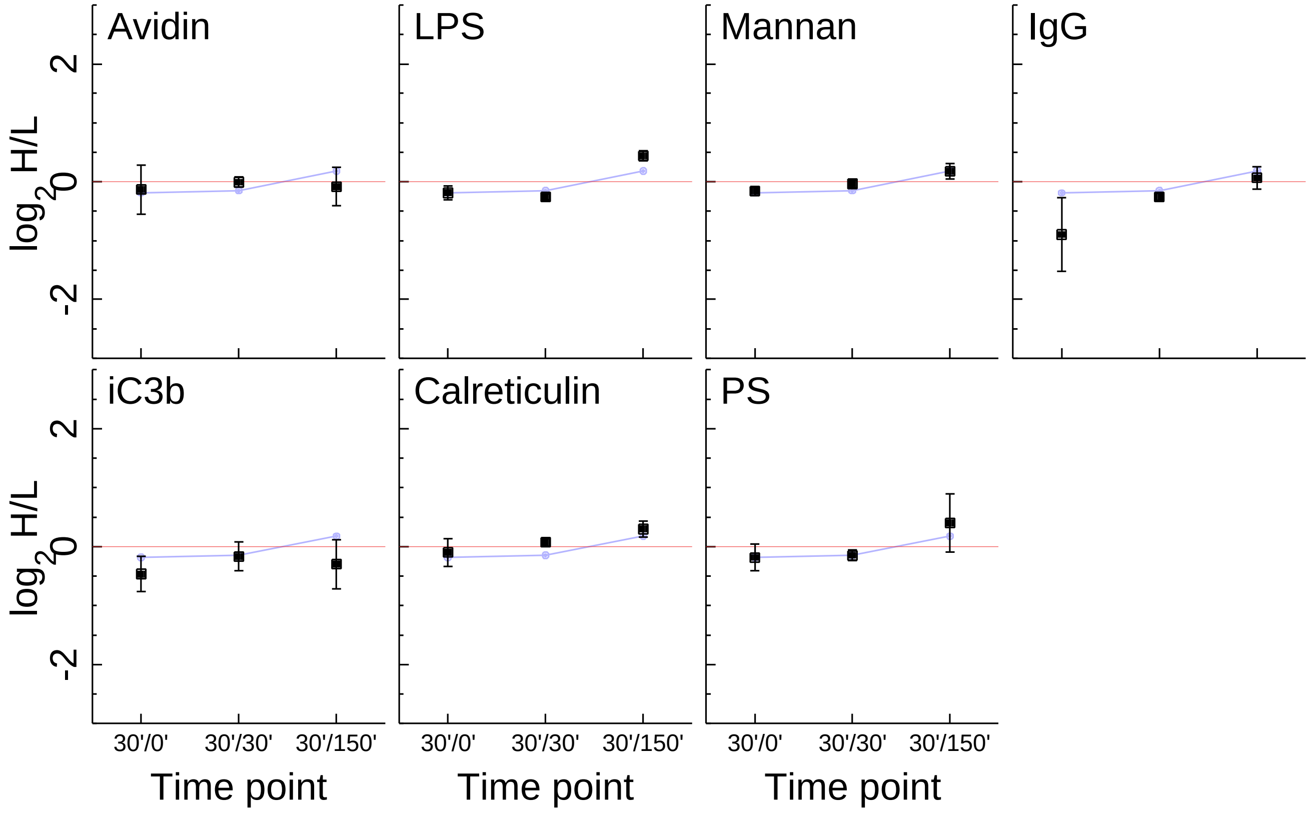

Avidin
LPS
Mannan
IgG
iC3b
Calreticulin
PS
log2 H/L
2
0
-2
30'/0'
30'/30'
30'/150'
Time point

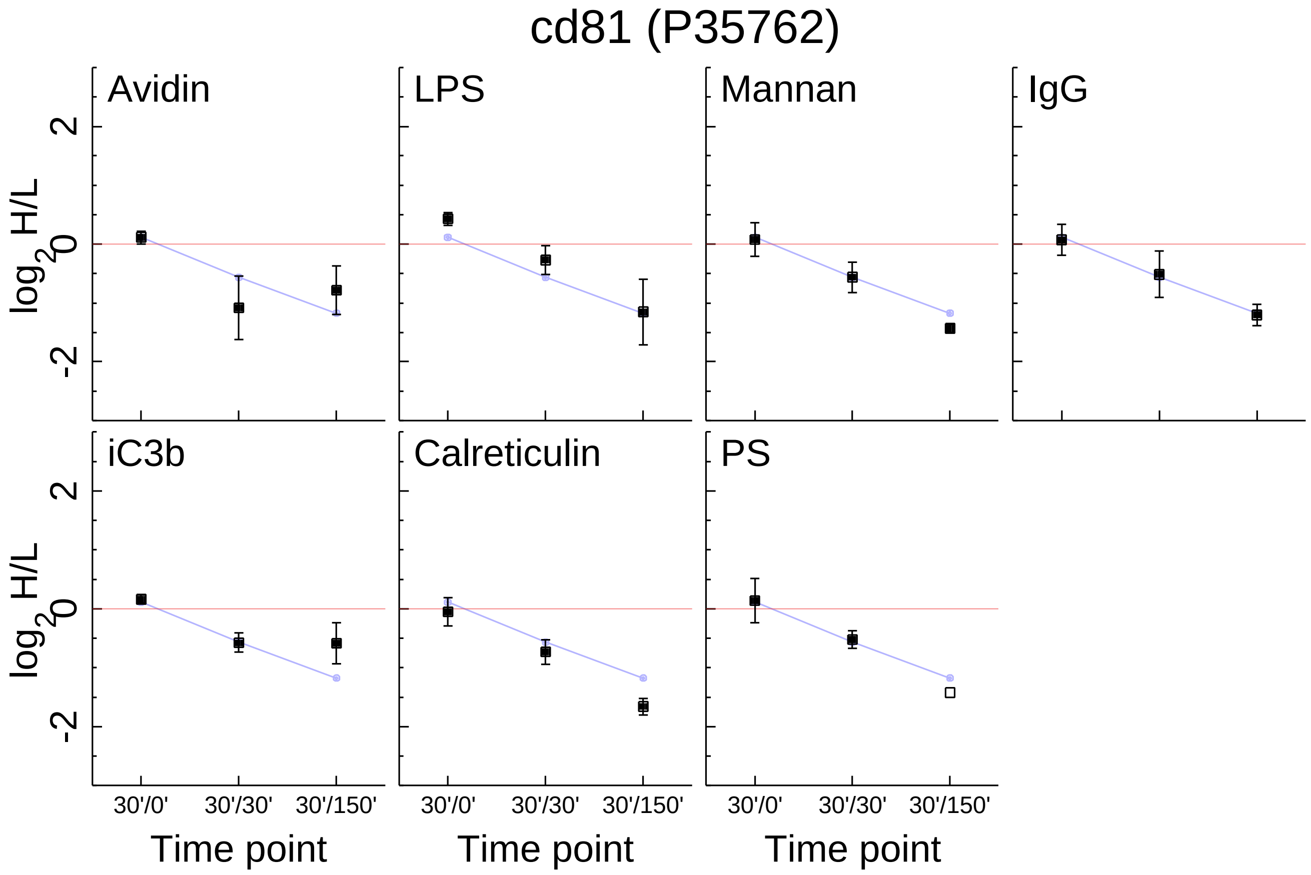

cd81 (P35762)
Avidin
LPS
Mannan
IgG
iC3b
Calreticulin
PS
log2 H/L
2
0
-2
30'/0'
30'/30'
30'/150'
Time point

cd82 (P40237)

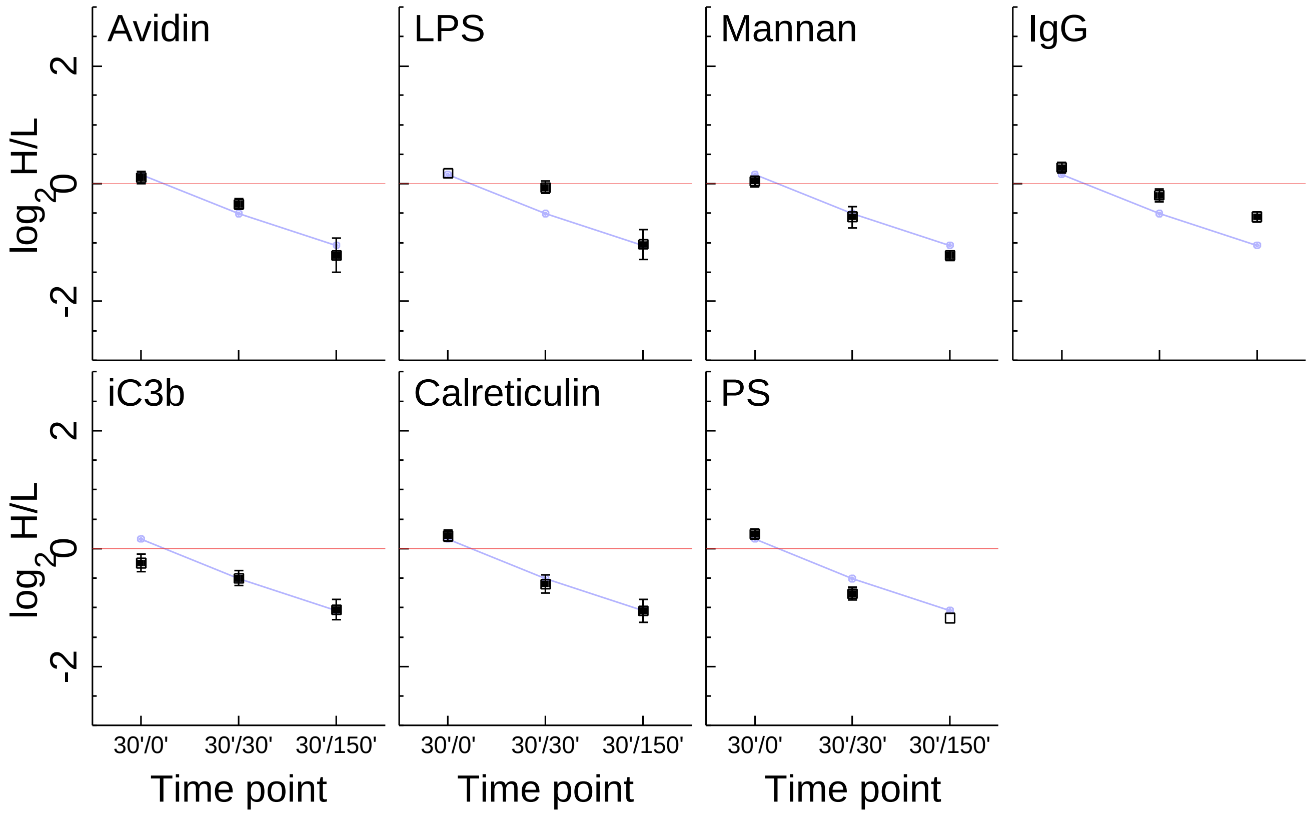
Avidin
LPS
Mannan
IgG
iC3b
Calreticulin
PS
log2 H/L
2
0
-2
30'/0'
30'/30'
30'/150'
Time point

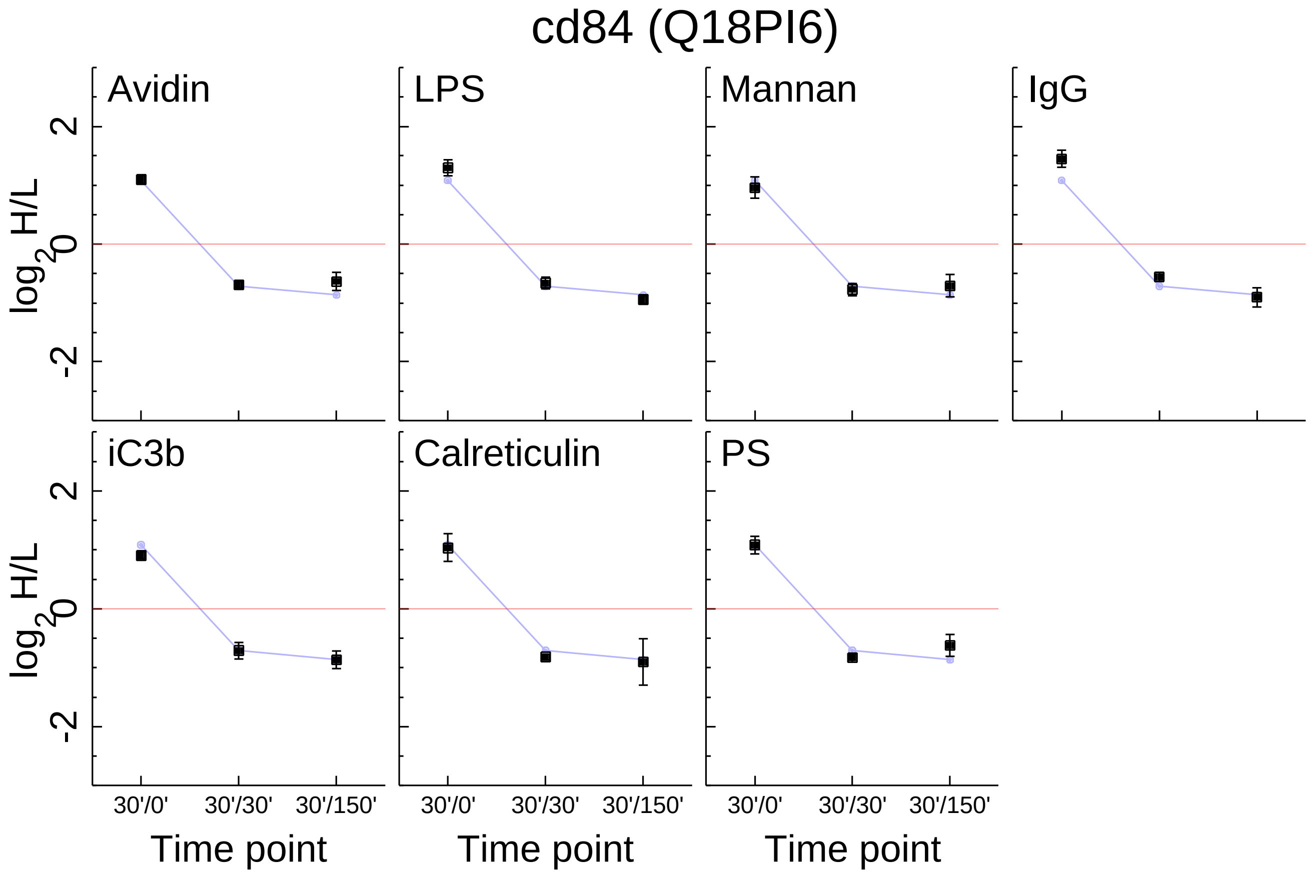

cd84 (Q18PI6)
Avidin
LPS
Mannan
IgG
iC3b
Calreticulin
PS
log_2 H/L
2
0
-2
30'/0'
30'/30'
30'/150'
Time point

cd93 (O89103)

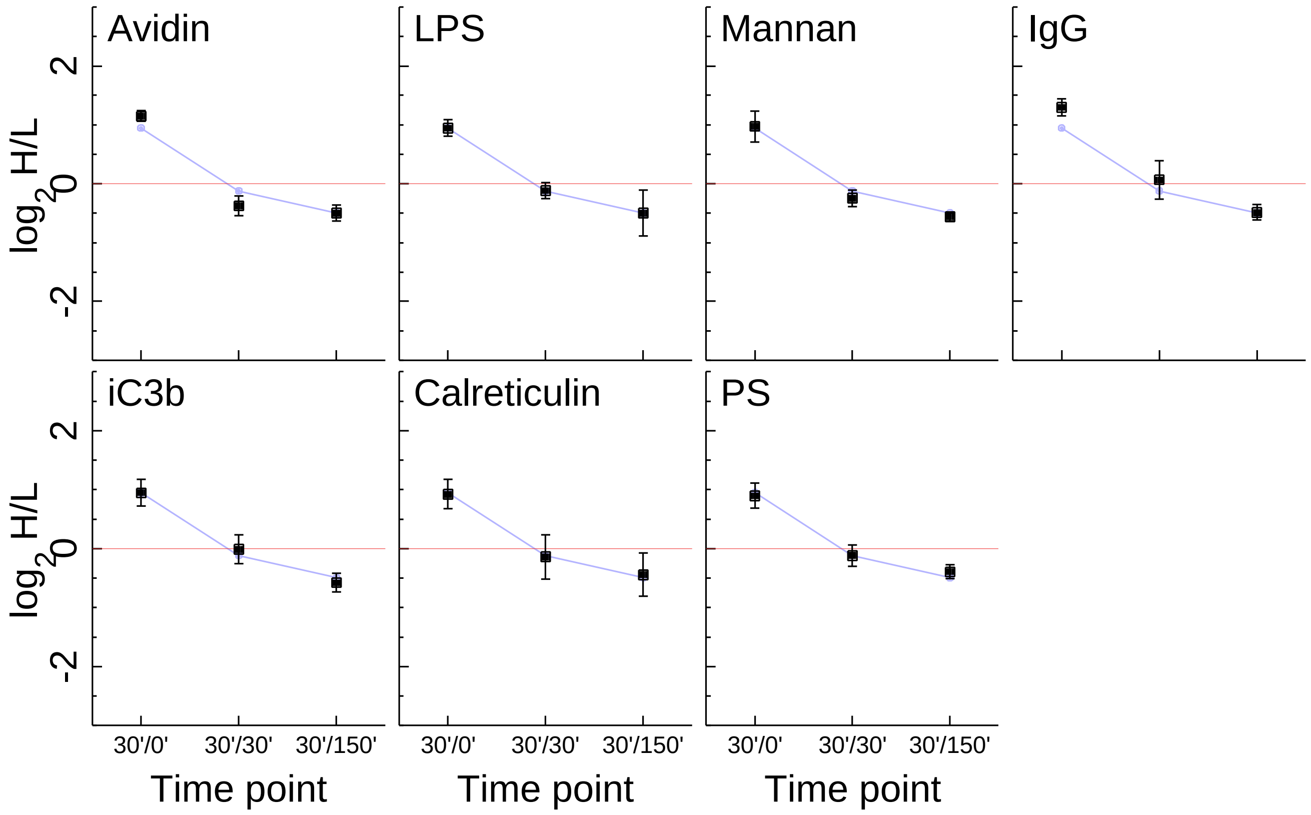

Avidin
LPS
Mannan
IgG
iC3b
Calreticulin
PS
log2 H/L
2
0
-2
30'/0'
30'/30'
30'/150'
Time point

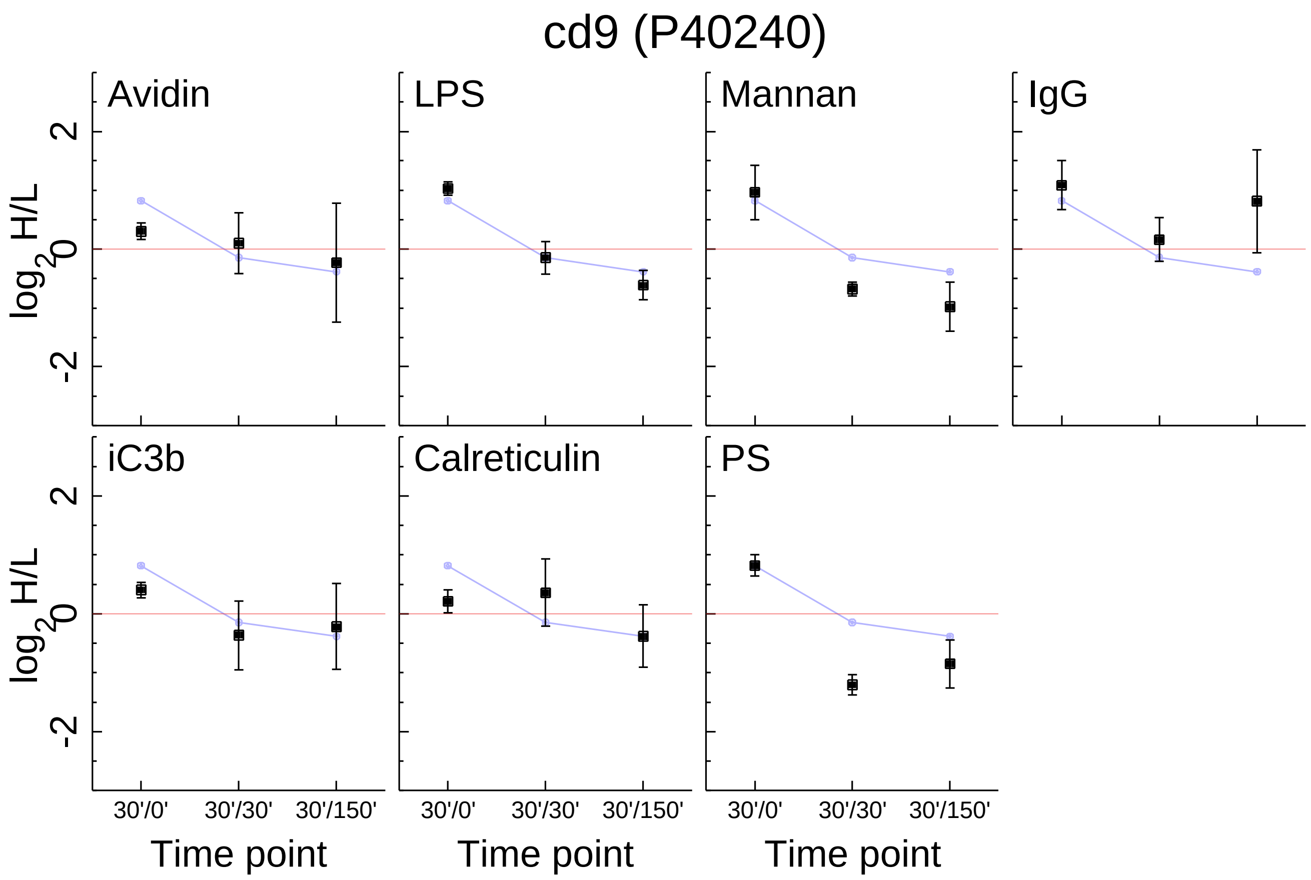
cd9 (P40240)
Avidin
LPS
Mannan
IgG
iC3b
Calreticulin
PS
log2 H/L
2
0
-2
30'/0'
30'/30'
30'/150'
Time point

cdc42bpb (Q7TT50)

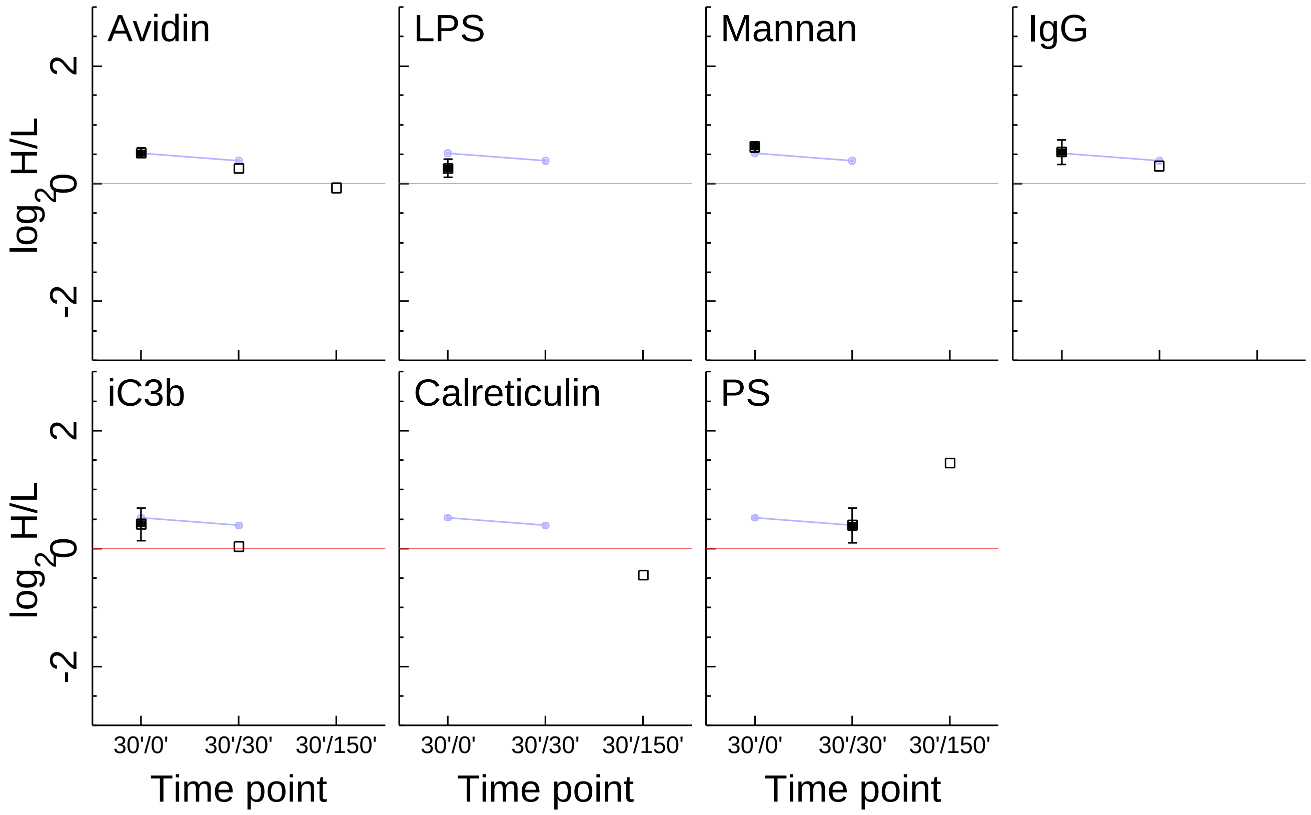

cdc42ep4 (Q9JM96)

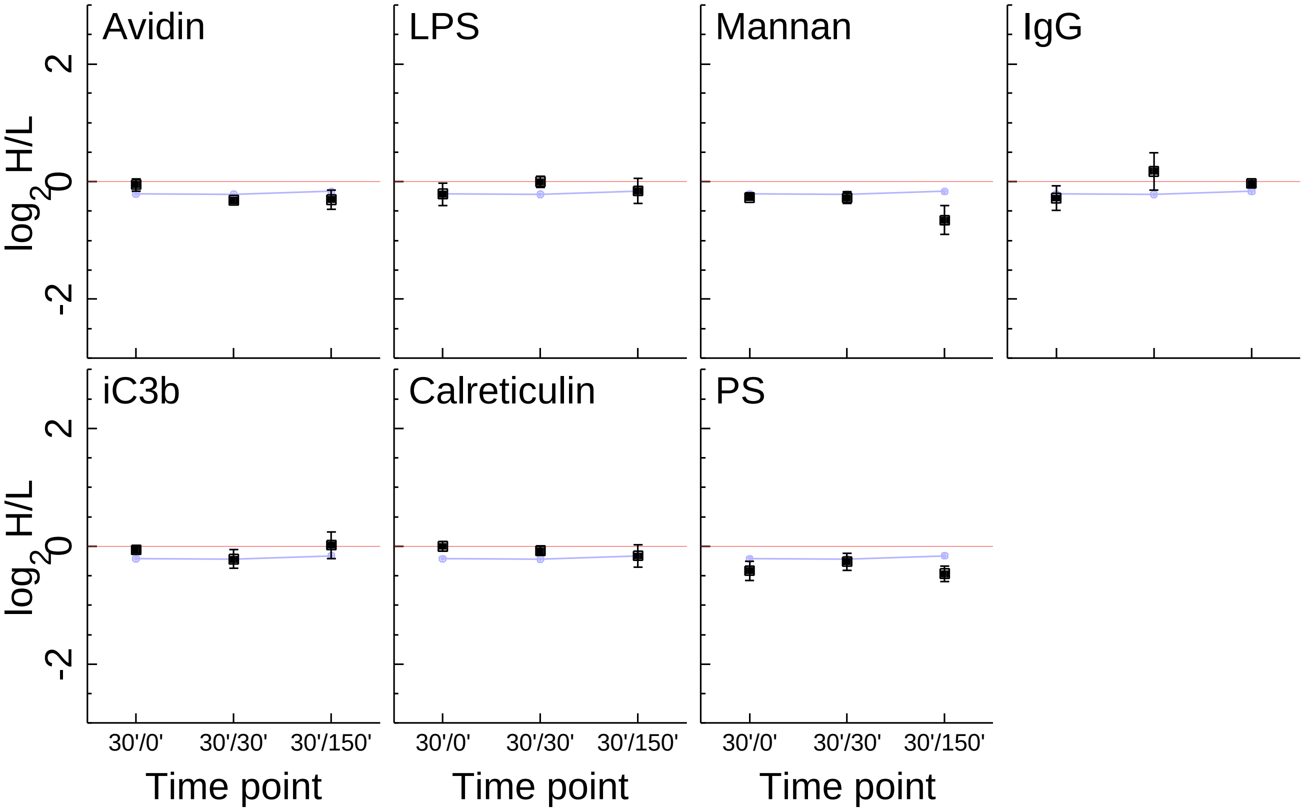

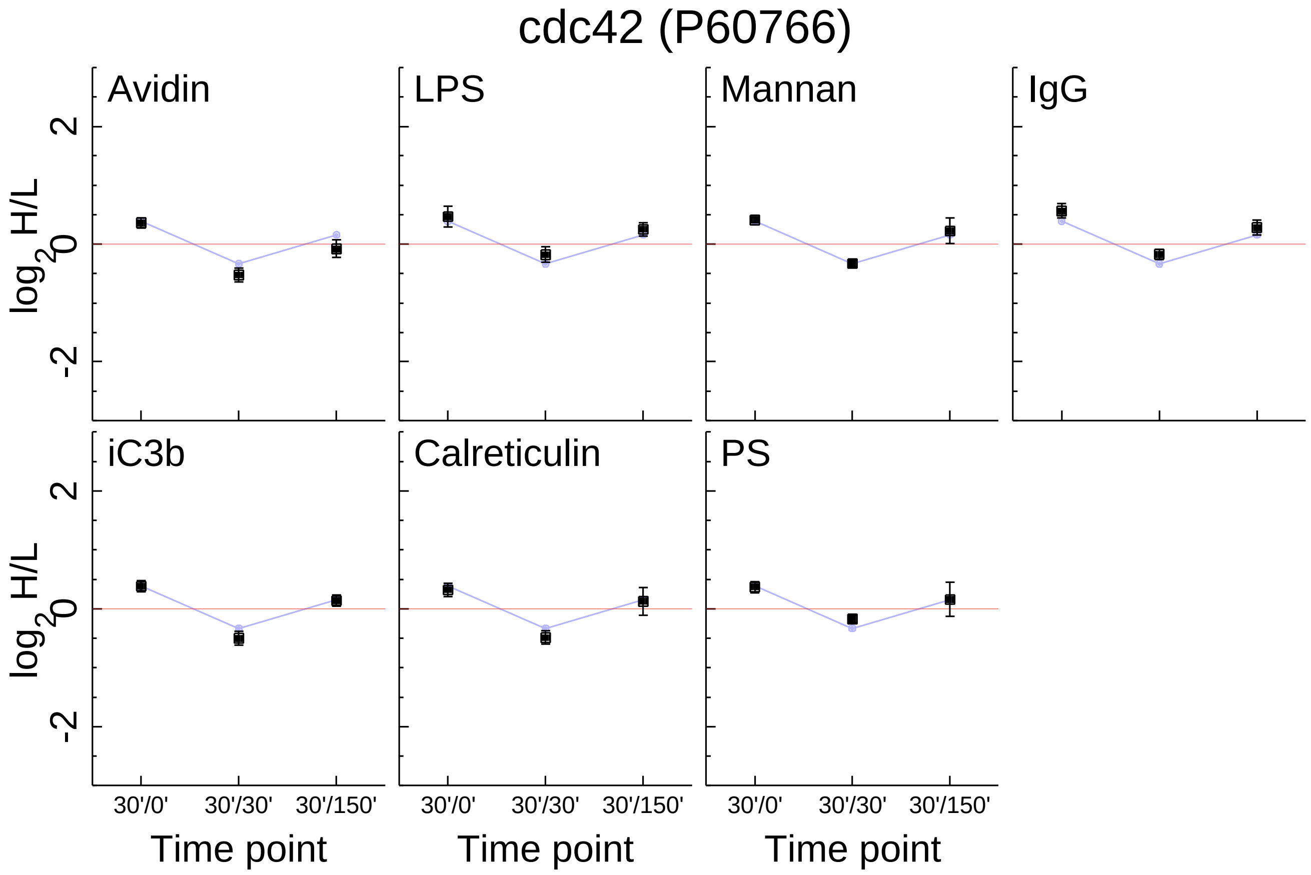
cdc42 (P60766)
Avidin
LPS
Mannan
IgG
iC3b
Calreticulin
PS
log2 H/L
2
0
-2
30'/0'
30'/30'
30'/150'
Time point

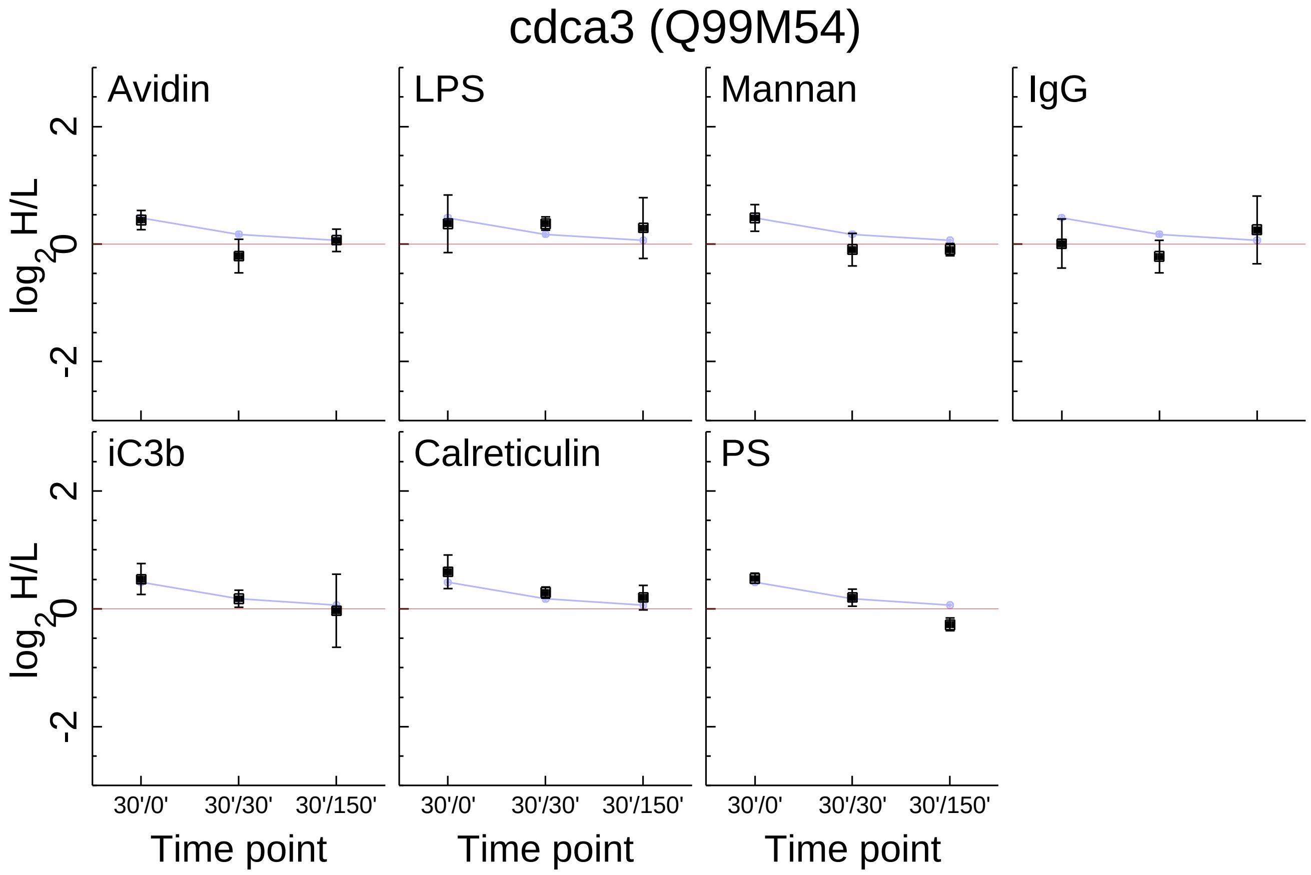
cdca3 (Q99M54)
Avidin
LPS
Mannan
IgG
iC3b
Calreticulin
PS
log2 H/L
2
0
-2
30'/0'
30'/30'
30'/150'
Time point

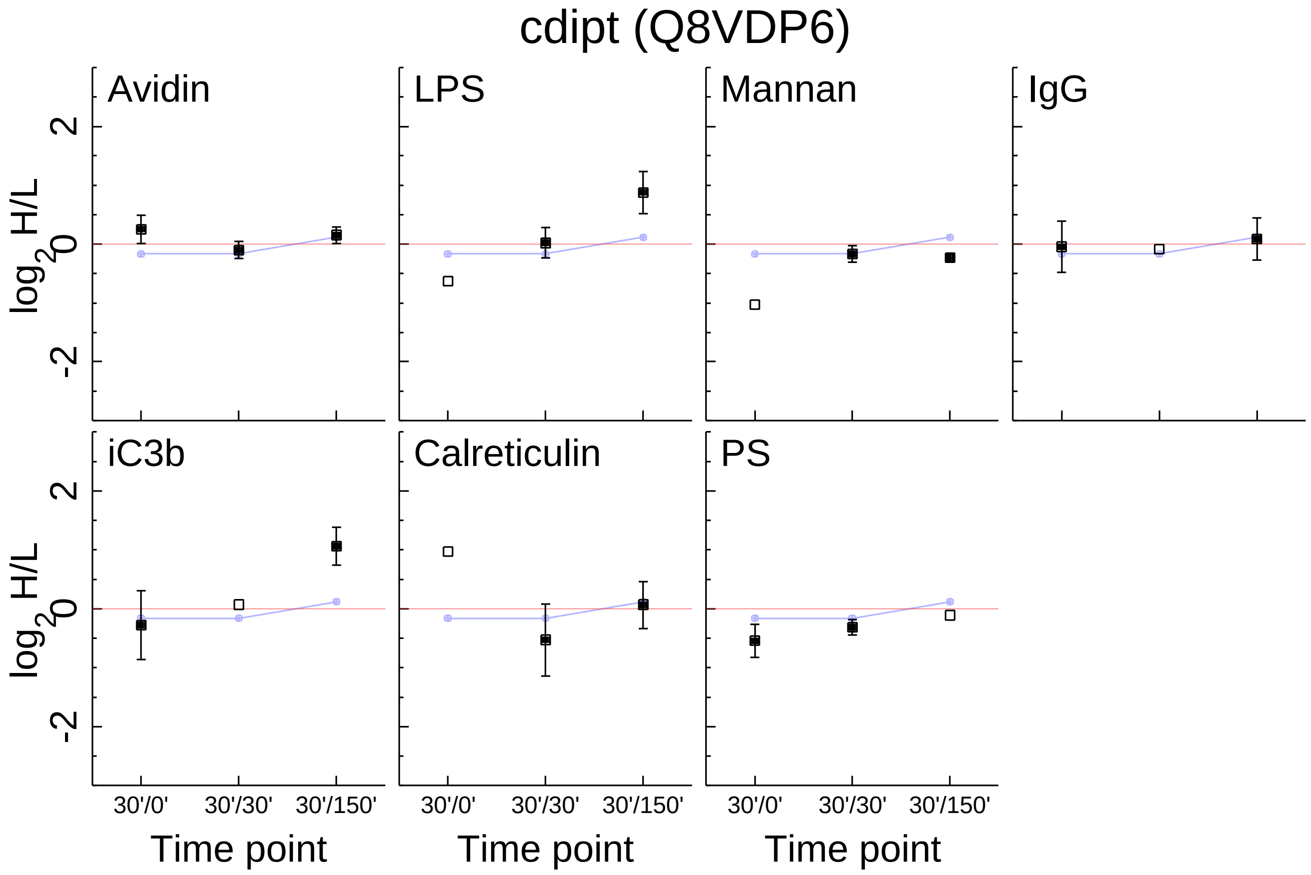
cdipt (Q8VDP6)
Avidin
LPS
Mannan
IgG
iC3b
Calreticulin
PS
log2 H/L
2
0
-2
30'/0'
30'/30'
30'/150'
Time point

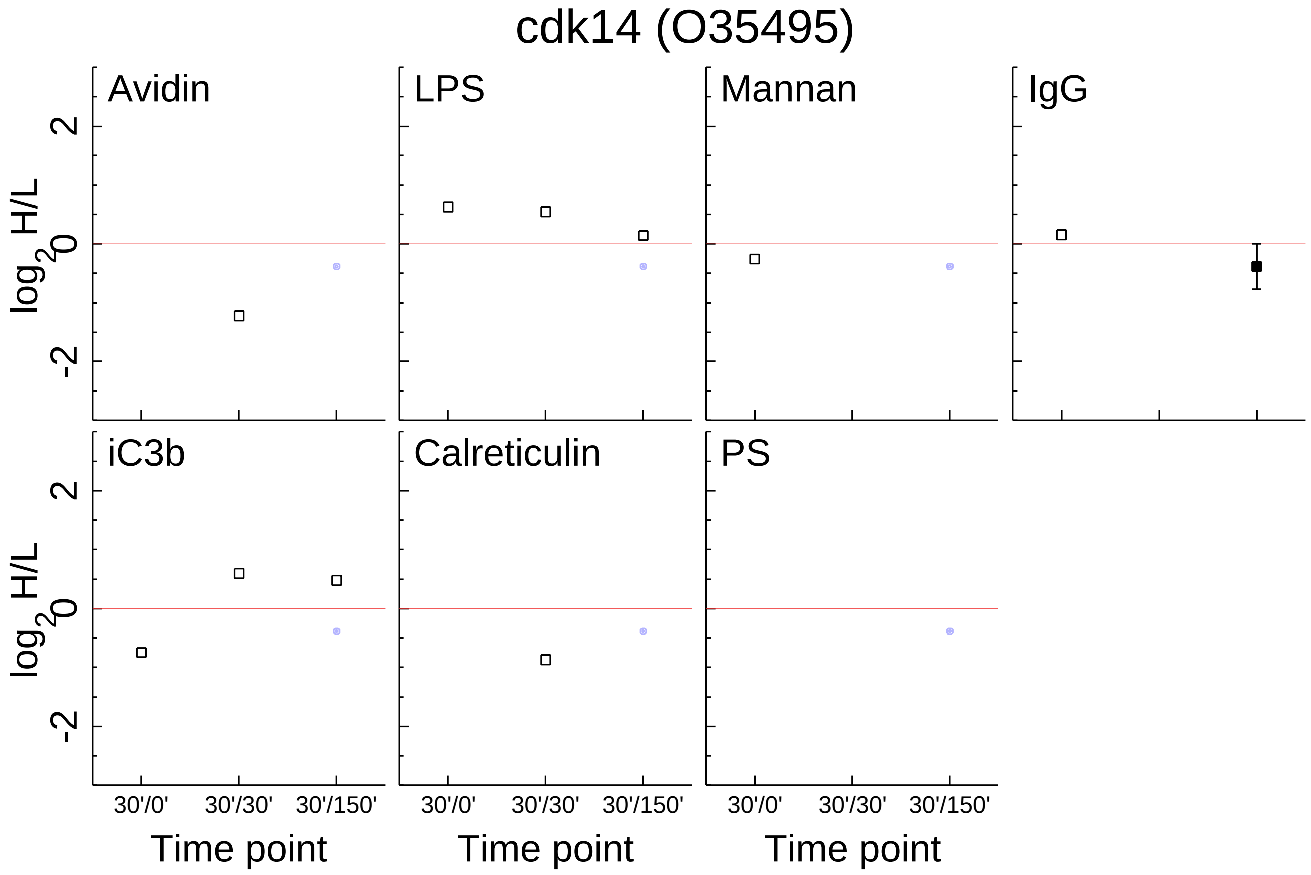
cdk14 (O35495)
Avidin
LPS
Mannan
IgG
iC3b
Calreticulin
PS
log2 H/L
2
0
-2
30'/0'
30'/30'
30'/150'
Time point

cdk18 (Q04899)

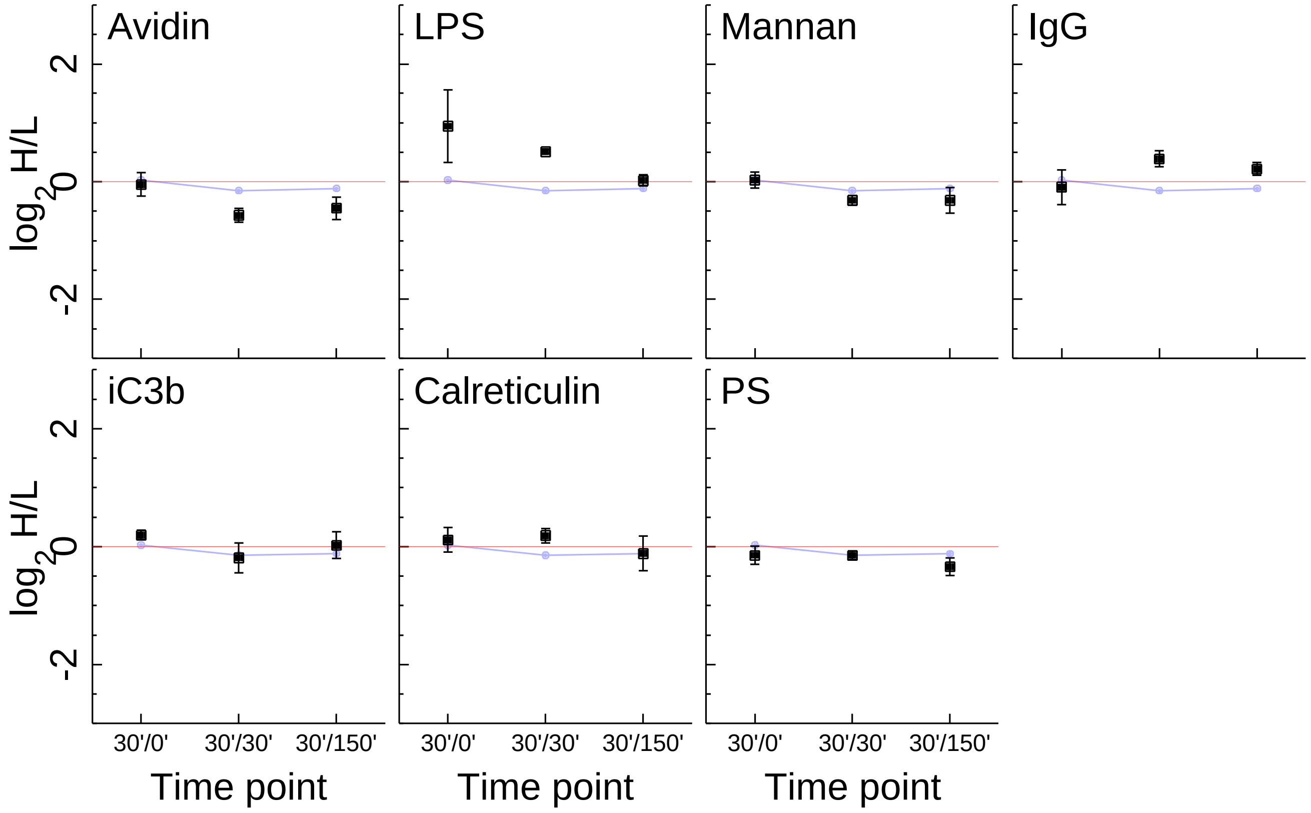

cdk1 (P11440)

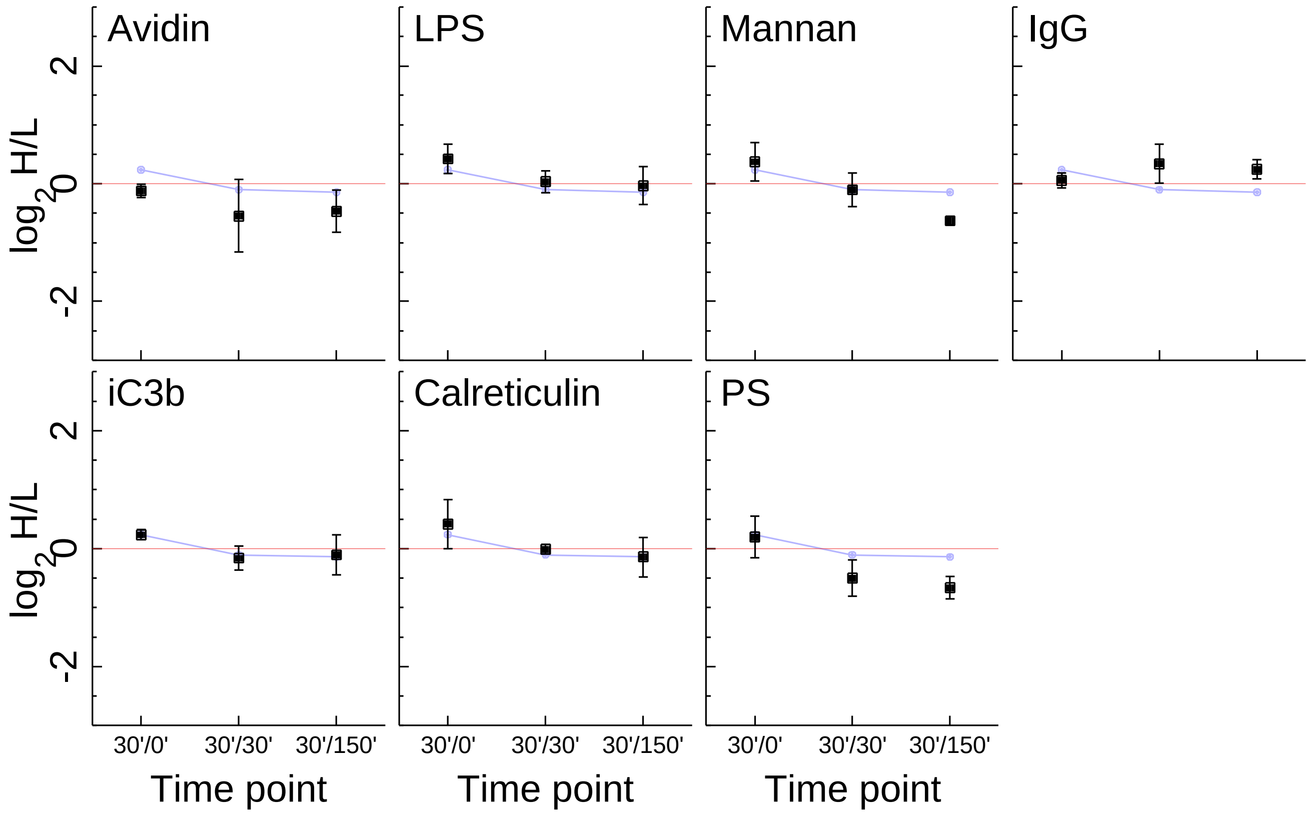

Avidin
LPS
Mannan
IgG
iC3b
Calreticulin
PS
log2 H/L
2
0
-2
30'/0'
30'/30'
30'/150'
Time point

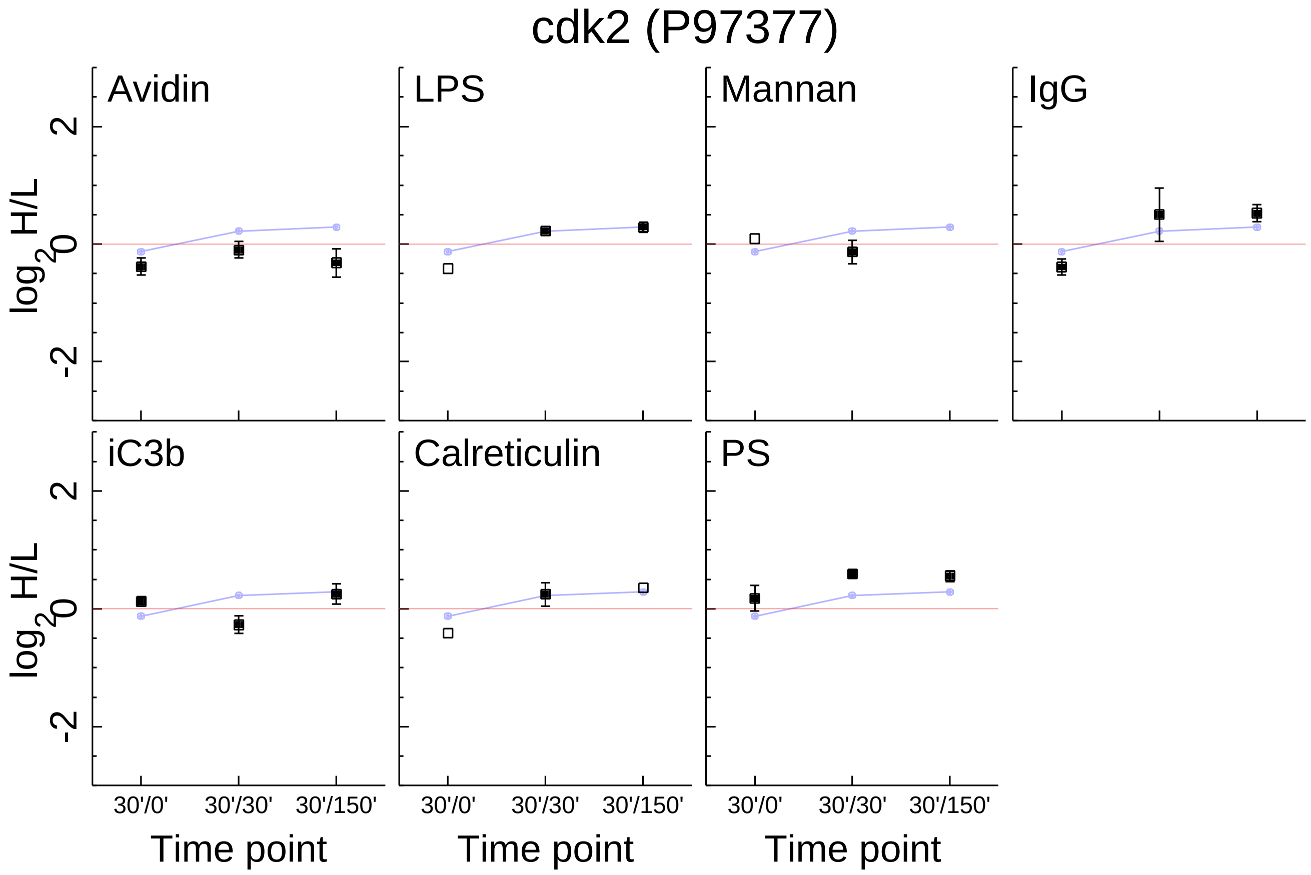

cdk2 (P97377)
Avidin
LPS
Mannan
IgG
iC3b
Calreticulin
PS
log2 H/L
2
0
-2
30'/0'
30'/30'
30'/150'
Time point

cdk5 (P49615)

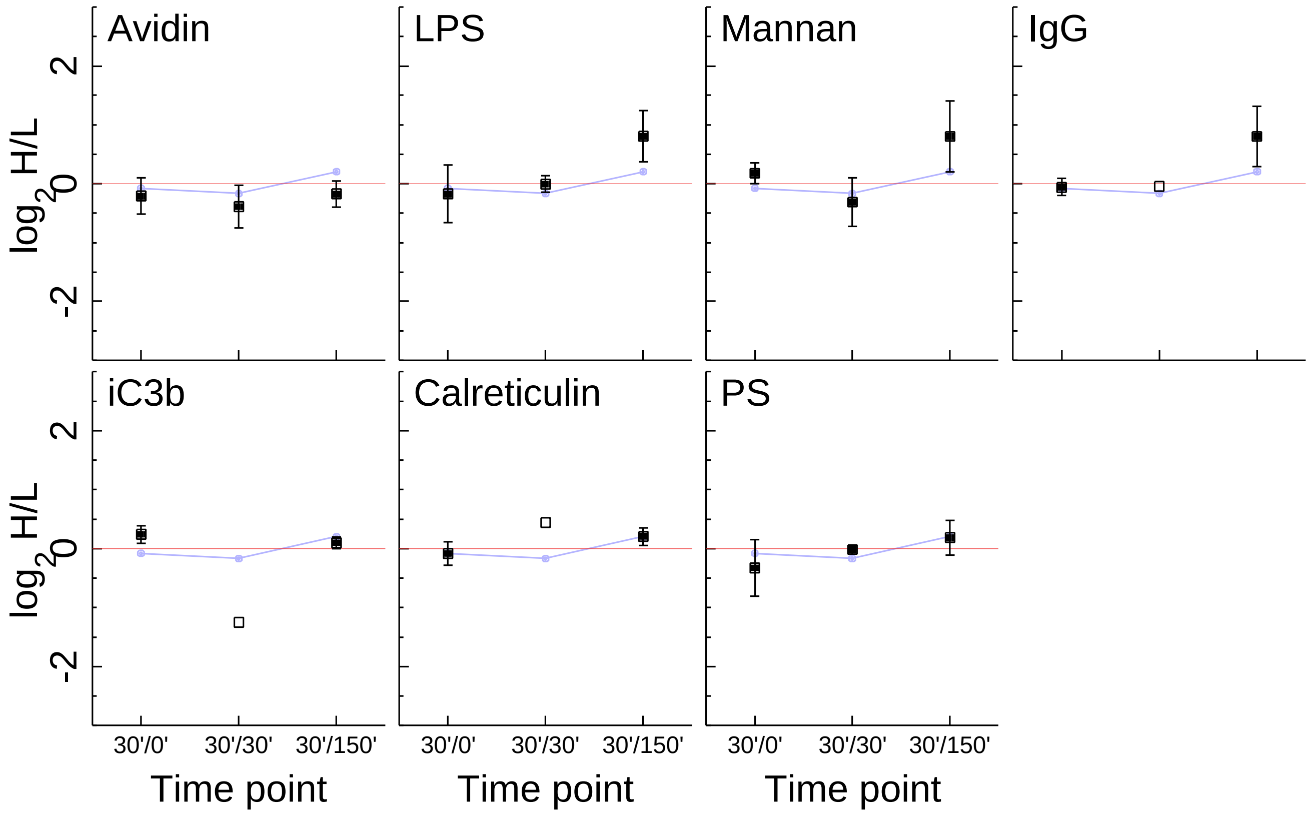

Avidin
LPS
Mannan
IgG
iC3b
Calreticulin
PS
log2 H/L
2
0
-2
30'/0'
30'/30'
30'/150'
Time point

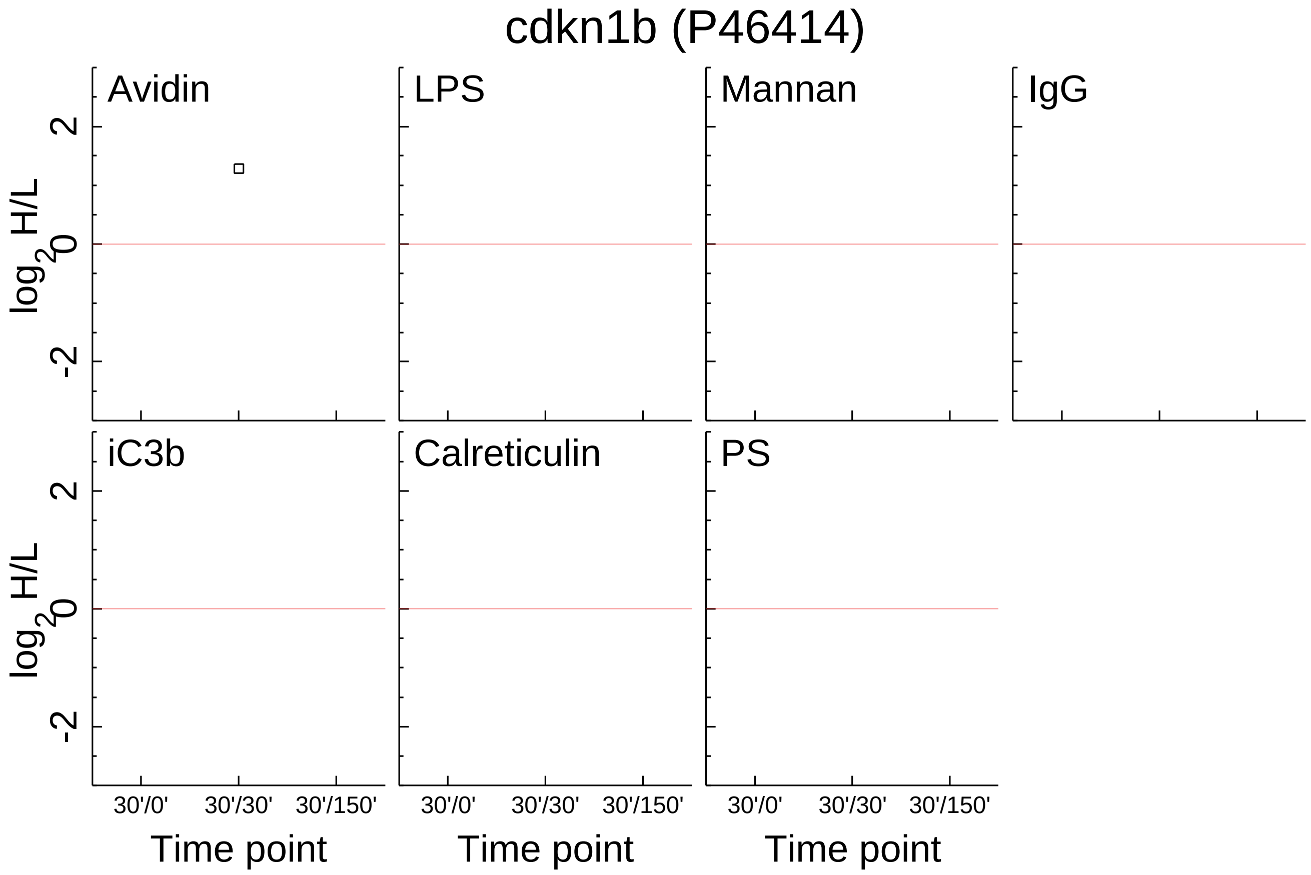
cdkn1b (P46414)
Avidin
LPS
Mannan
IgG
iC3b
Calreticulin
PS
log2 H/L
2
0
-2
30'/0'
30'/30'
30'/150'
Time point

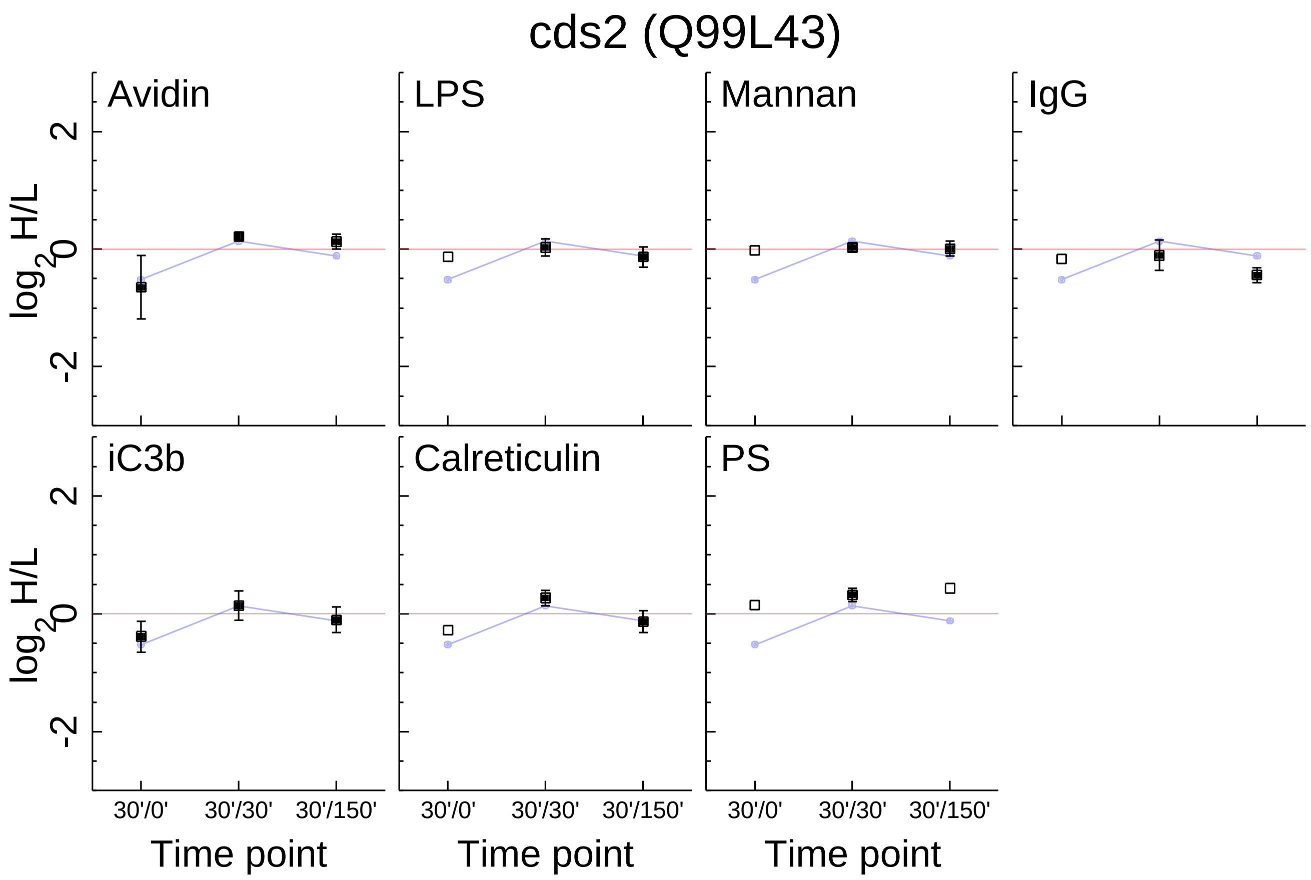
cds2 (Q99L43)
Avidin
LPS
Mannan
IgG
iC3b
Calreticulin
PS
log2 H/L
2
0
-2
30'/0'
30'/30'
30'/150'
Time point

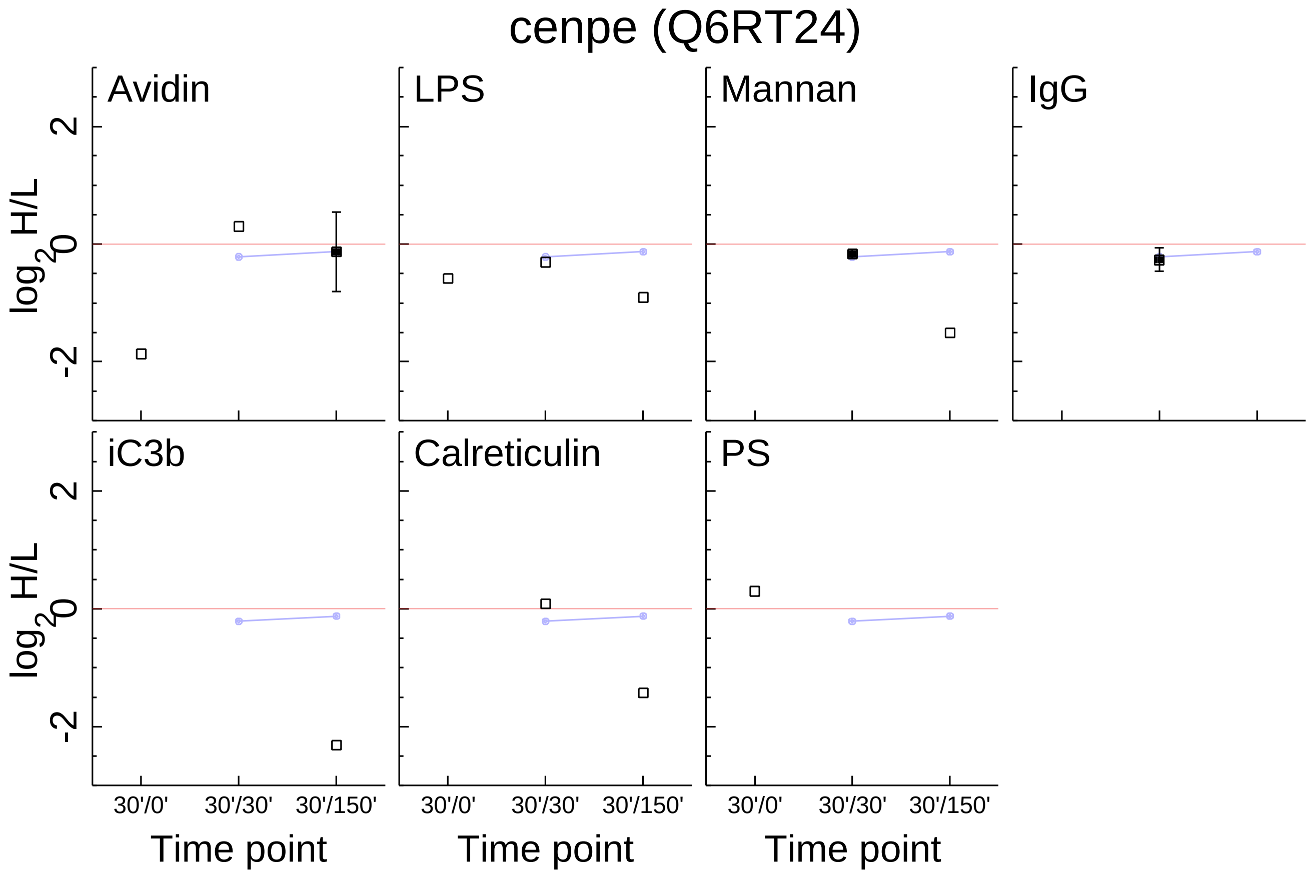

cenpe (Q6RT24)
Avidin
LPS
Mannan
IgG
iC3b
Calreticulin
PS
log2 H/L
2
0
-2
30'/0'
30'/30'
30'/150'
Time point

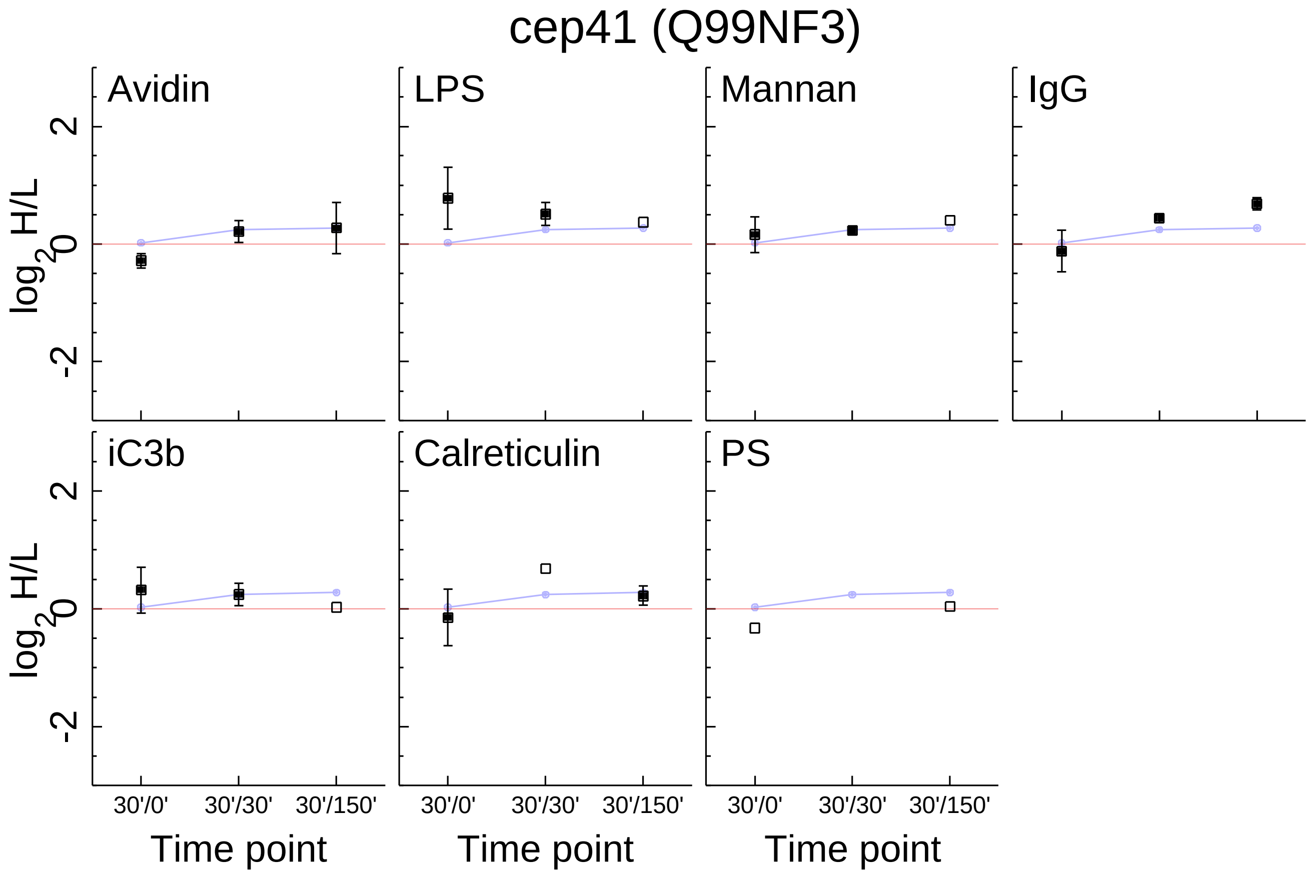

cep41 (Q99NF3)
Avidin
LPS
Mannan
IgG
iC3b
Calreticulin
PS
log2 H/L
2
0
-2
30'/0'
30'/30'
30'/150'
Time point

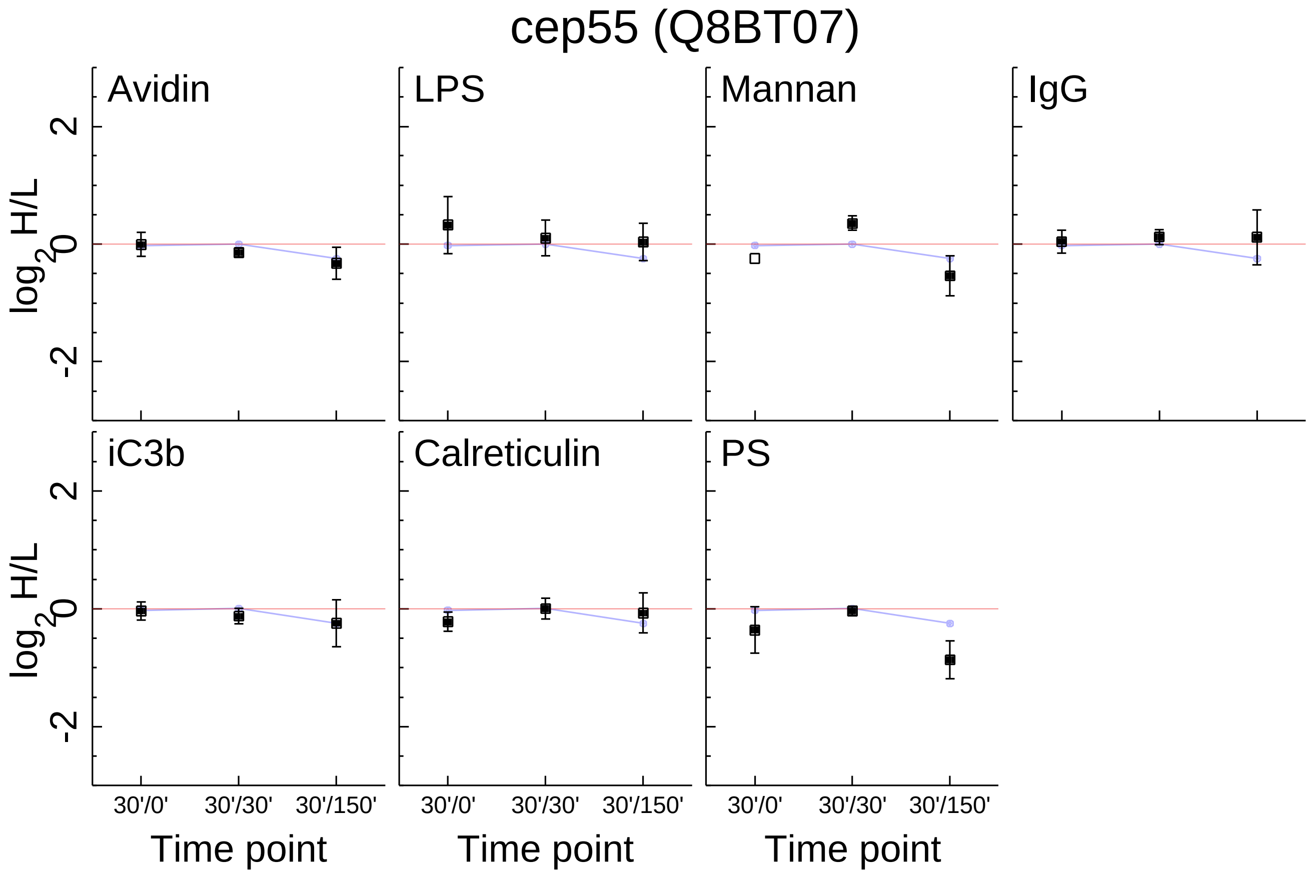
cep55 (Q8BT07)
Avidin
LPS
Mannan
IgG
iC3b
Calreticulin
PS
log2 H/L
2
0
-2
30'/0'
30'/30'
30'/150'
Time point

cers2 (Q924Z4)

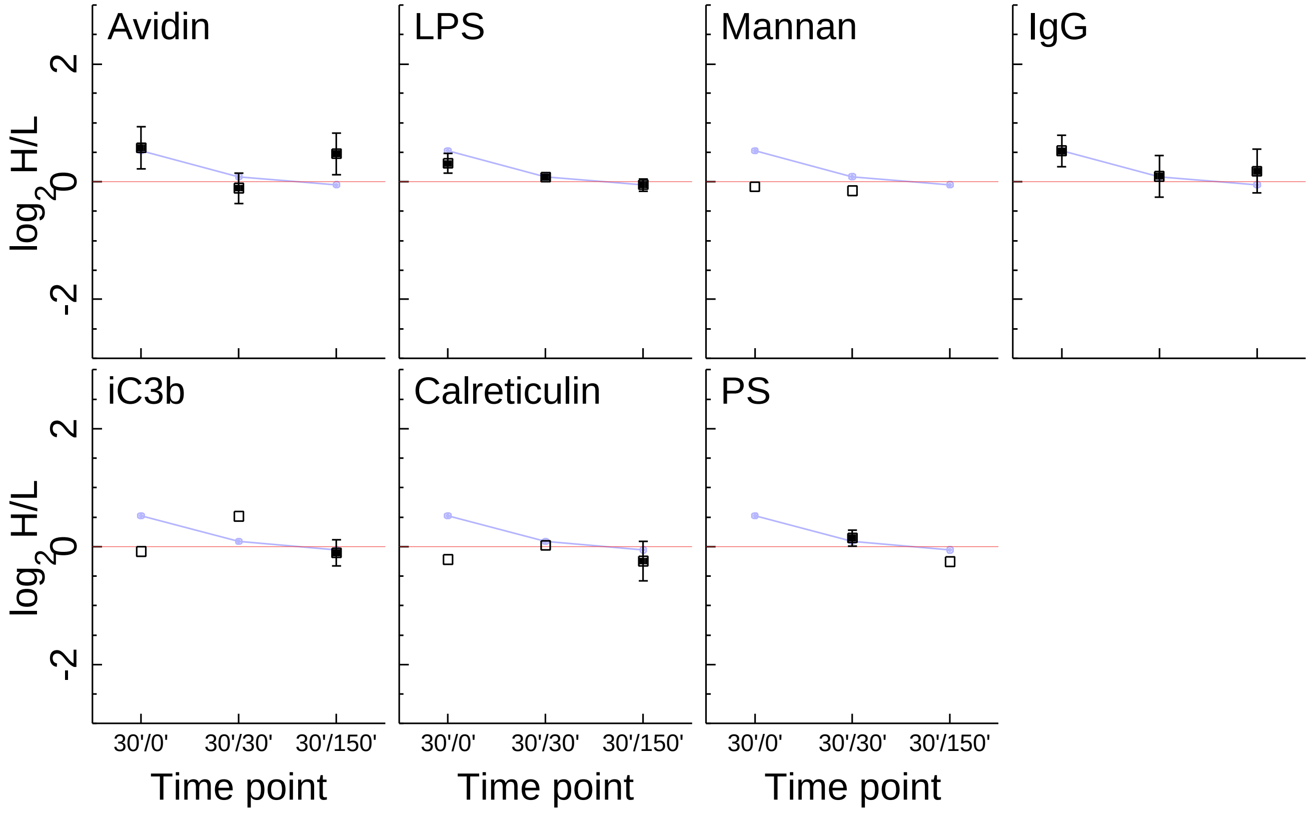

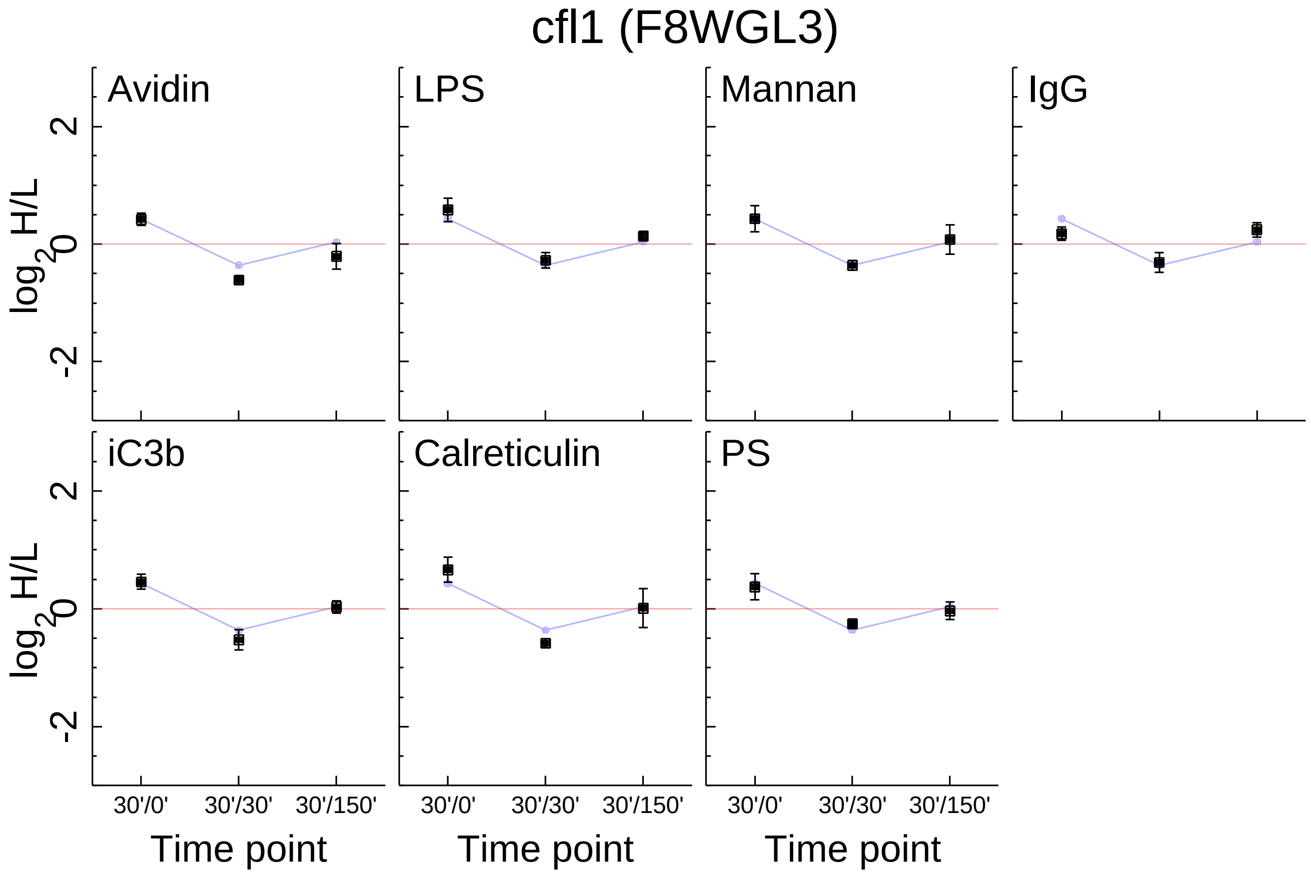
cfl1 (F8WGL3)
Avidin
LPS
Mannan
IgG
iC3b
Calreticulin
PS
2
0
-2
log2 H/L
30'/0'
30'/30'
30'/150'
Time point

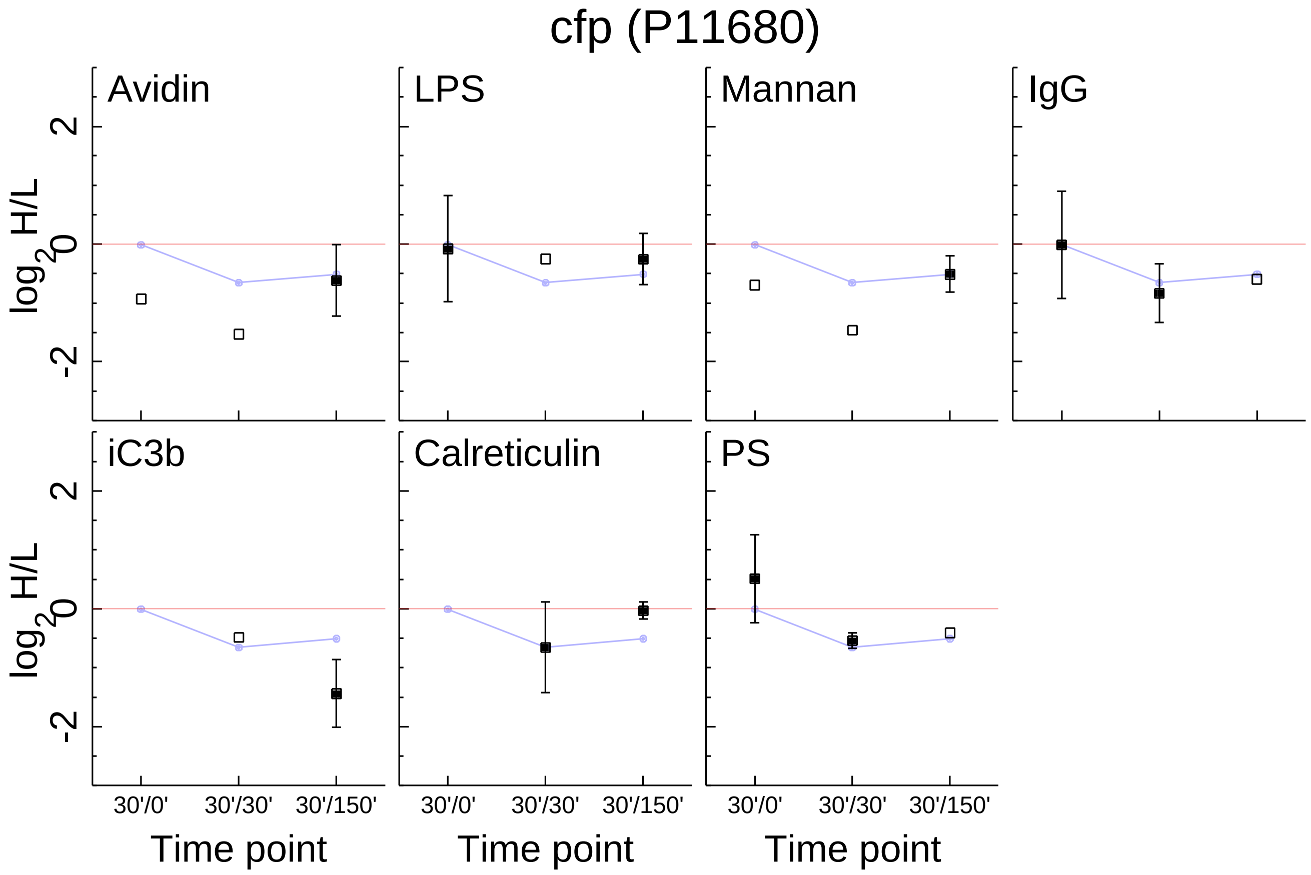
cfp (P11680)
Avidin
LPS
Mannan
IgG
iC3b
Calreticulin
PS
log2 H/L
2
0
-2
30'/0'
30'/30'
30'/150'
Time point

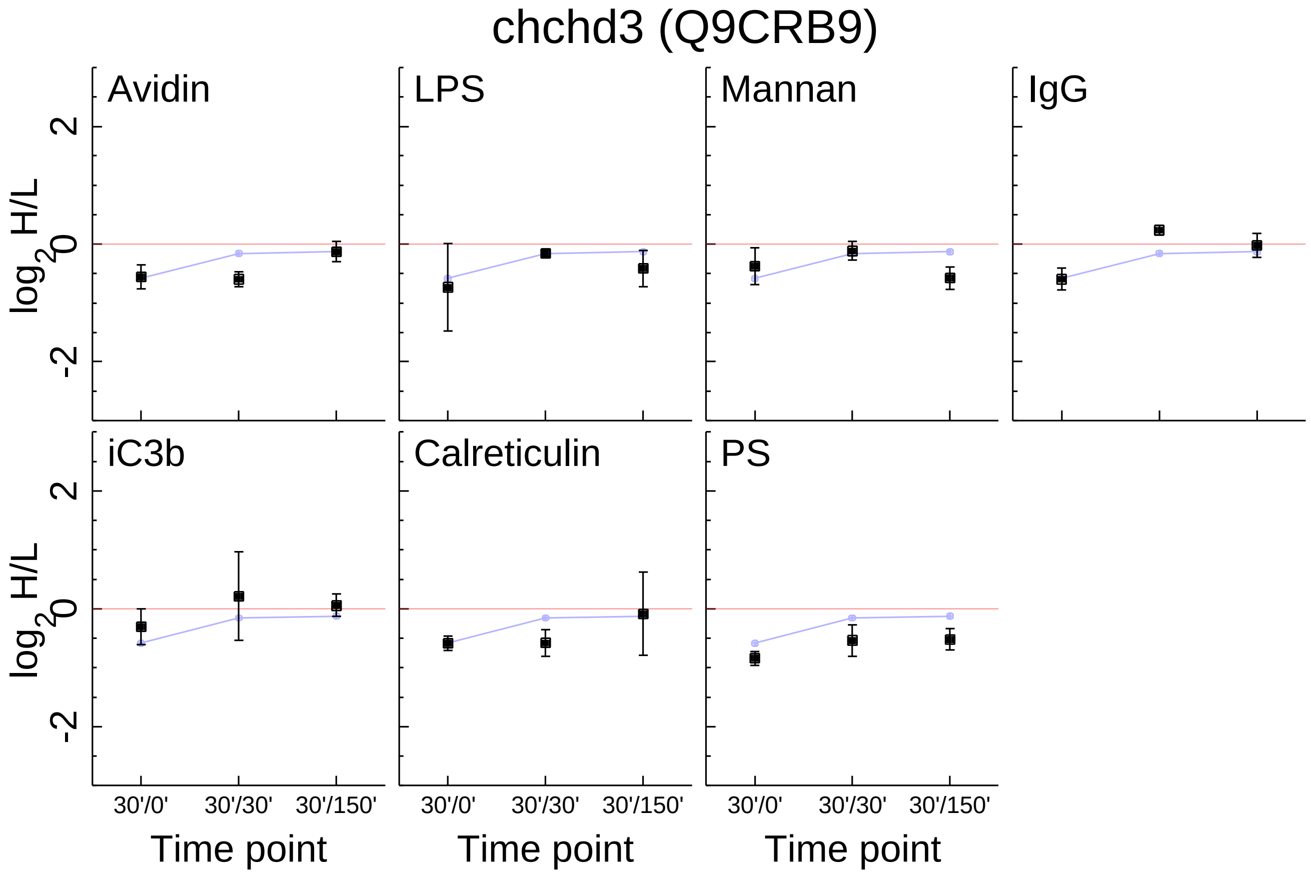
chchd3 (Q9CRB9)
Avidin
LPS
Mannan
IgG
iC3b
Calreticulin
PS
log2 H/L
2
0
-2
30'/0'
30'/30'
30'/150'
Time point

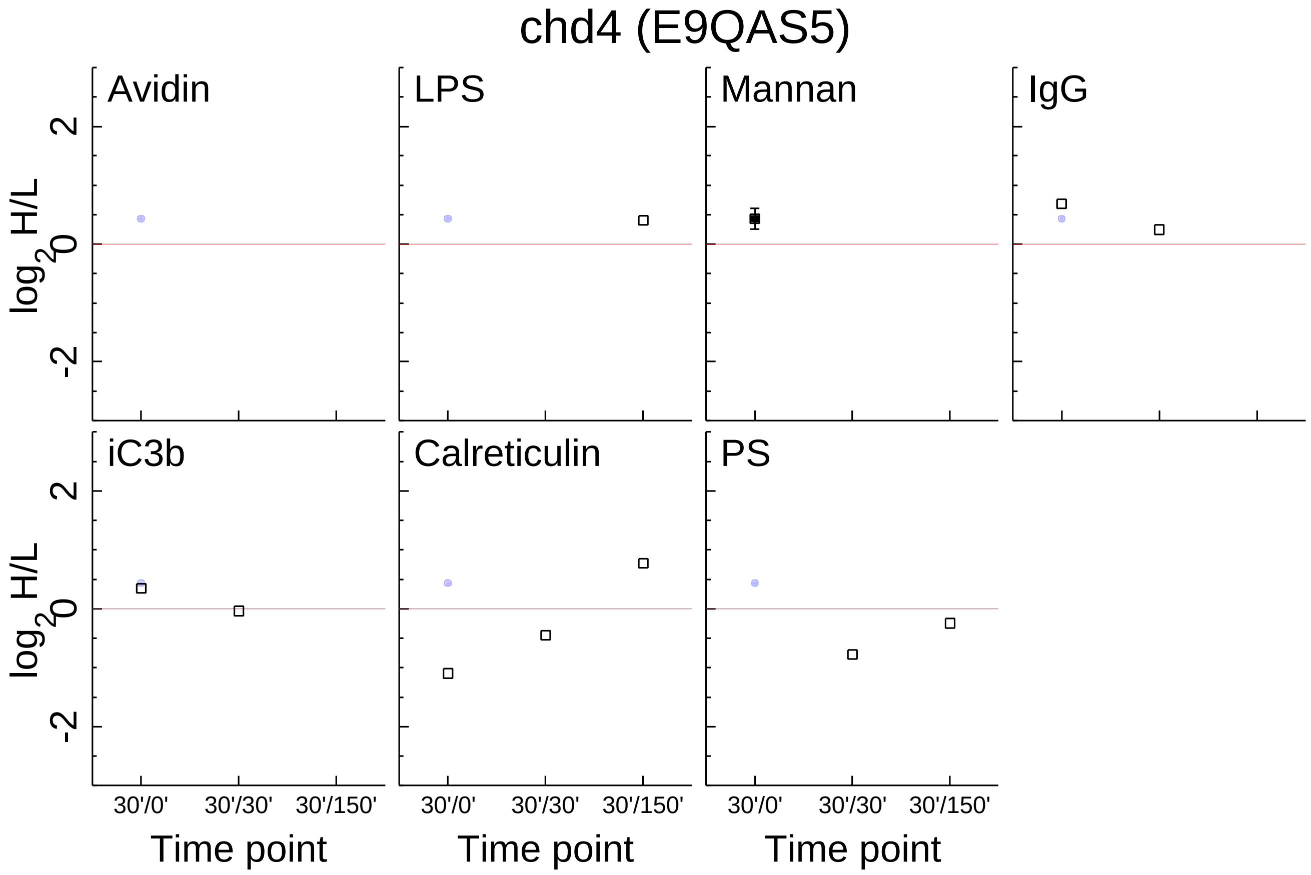
chd4 (E9QAS5)
Avidin
LPS
Mannan
IgG
iC3b
Calreticulin
PS
log2 H/L
2
0
-2
30'/0'
30'/30'
30'/150'
Time point

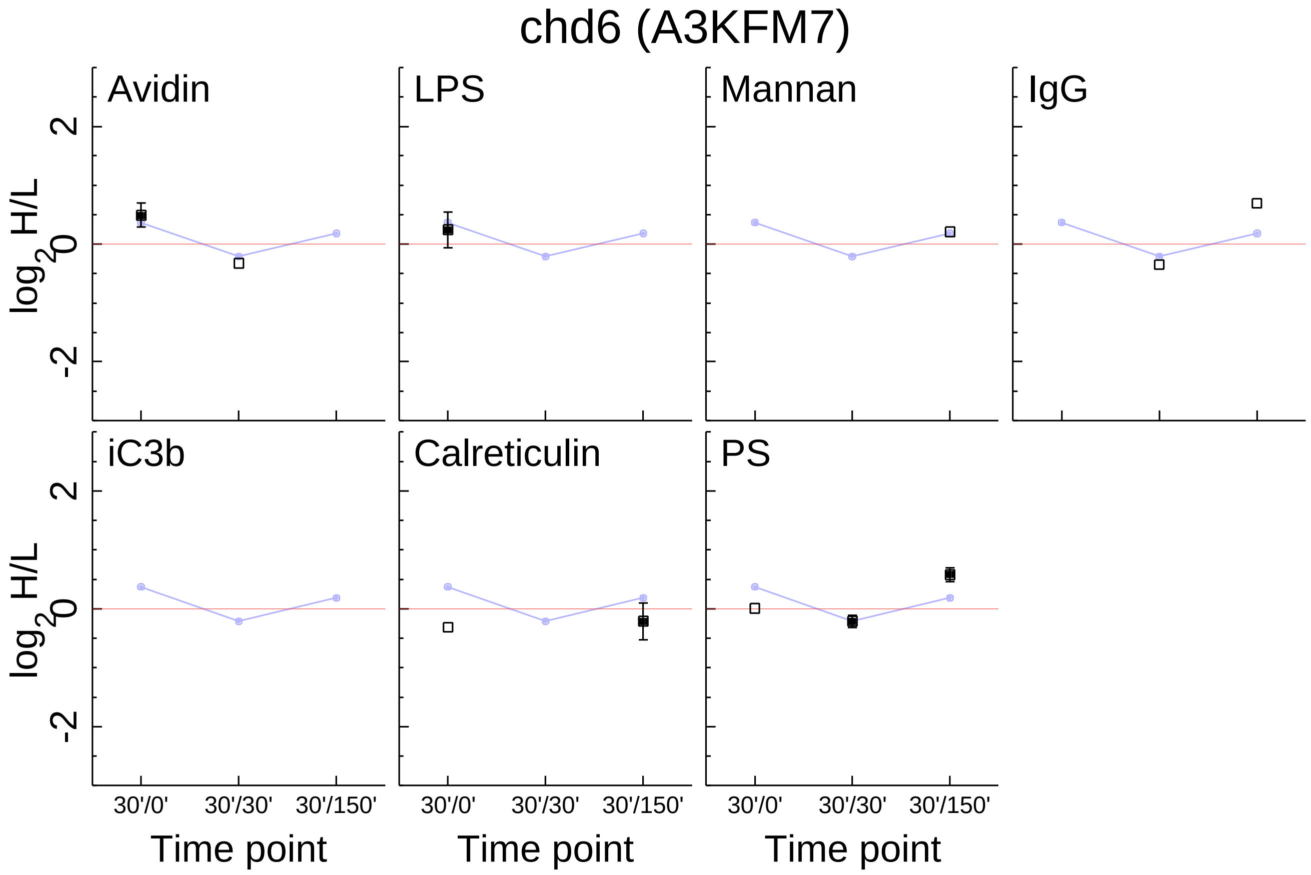

chd6 (A3KFM7)
Avidin
LPS
Mannan
IgG
iC3b
Calreticulin
PS
log2 H/L
2
0
-2
30'/0'
30'/30'
30'/150'
Time point

chi3l3 (O35744)

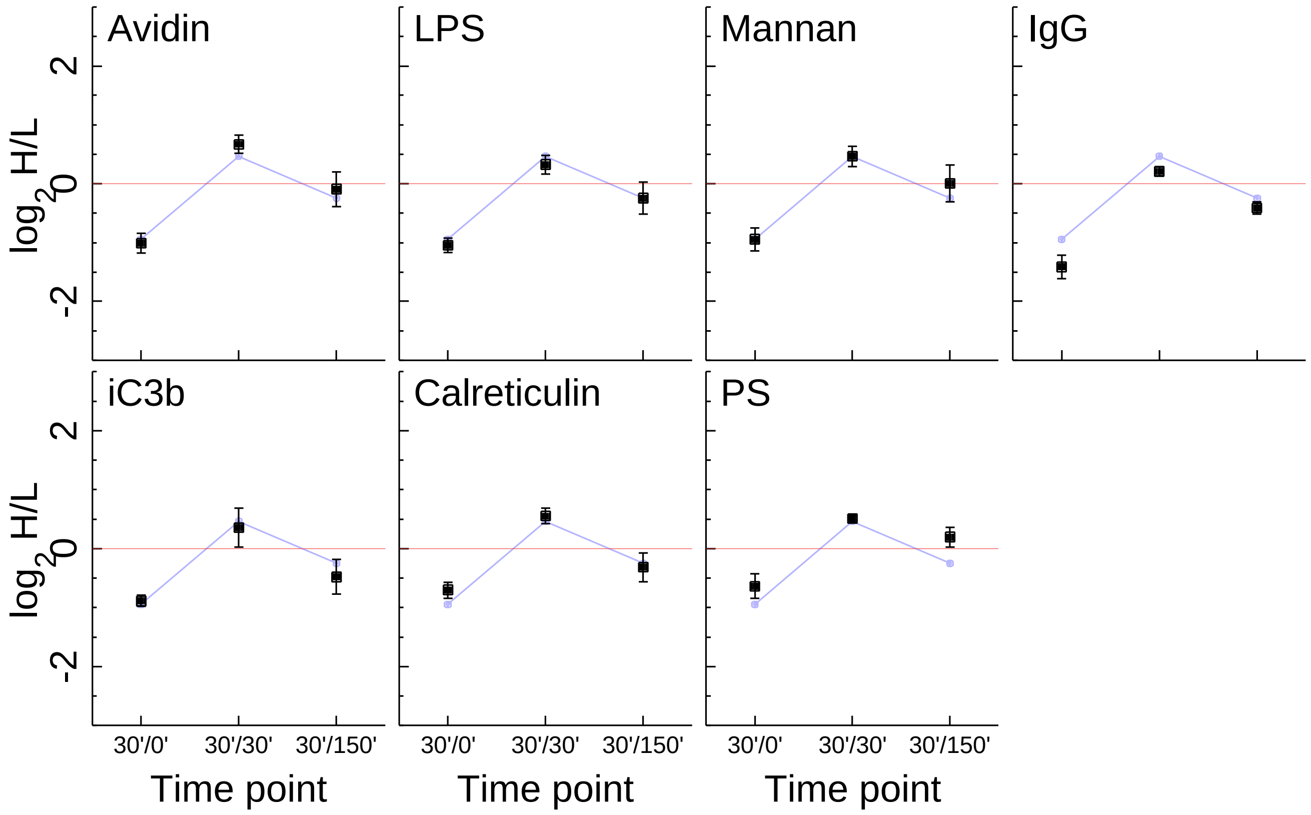

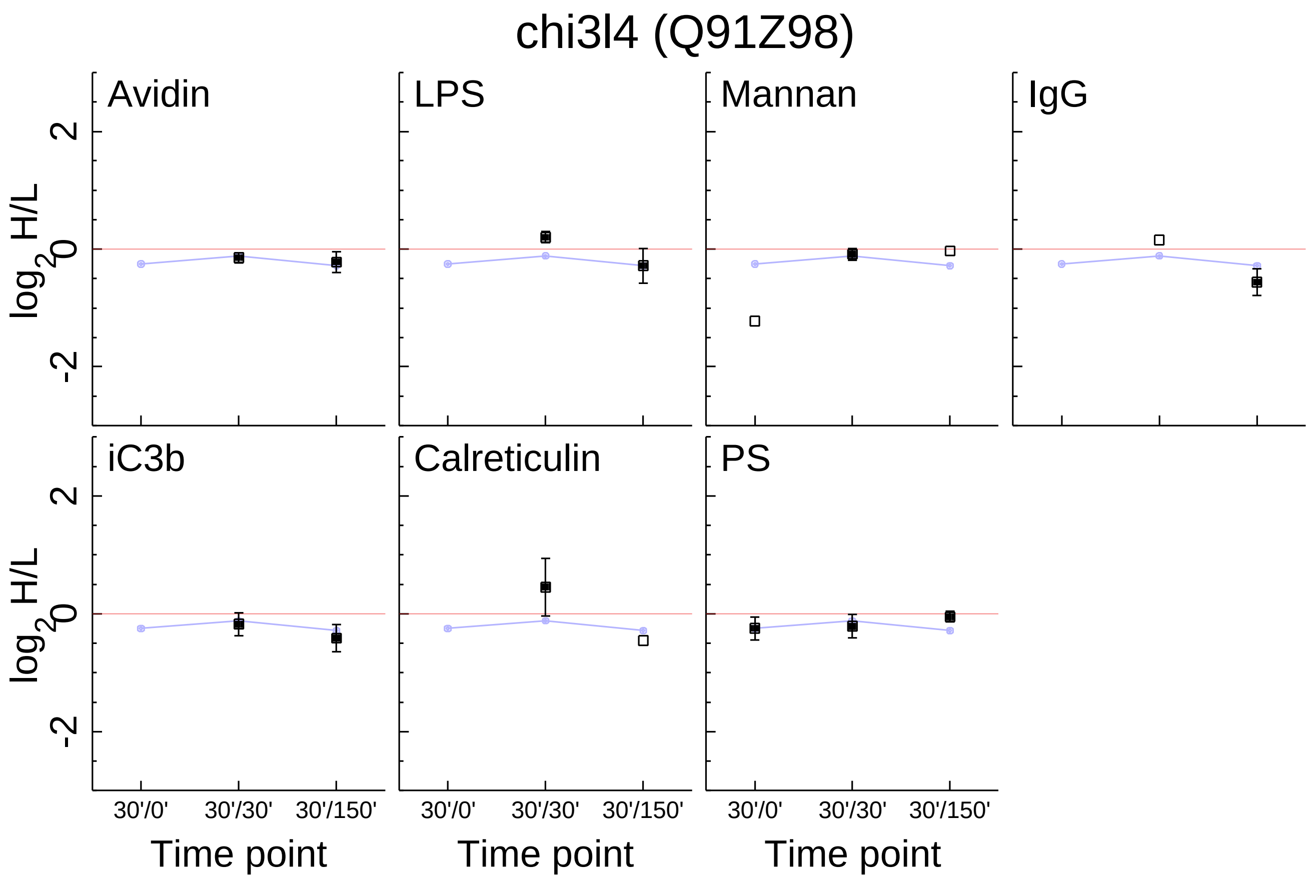

chi3l4 (Q91Z98)
Avidin
LPS
Mannan
IgG
iC3b
Calreticulin
PS
log2 H/L
2
0
-2
30'/0'
30'/30'
30'/150'
Time point

chmp1a (Q921W0)

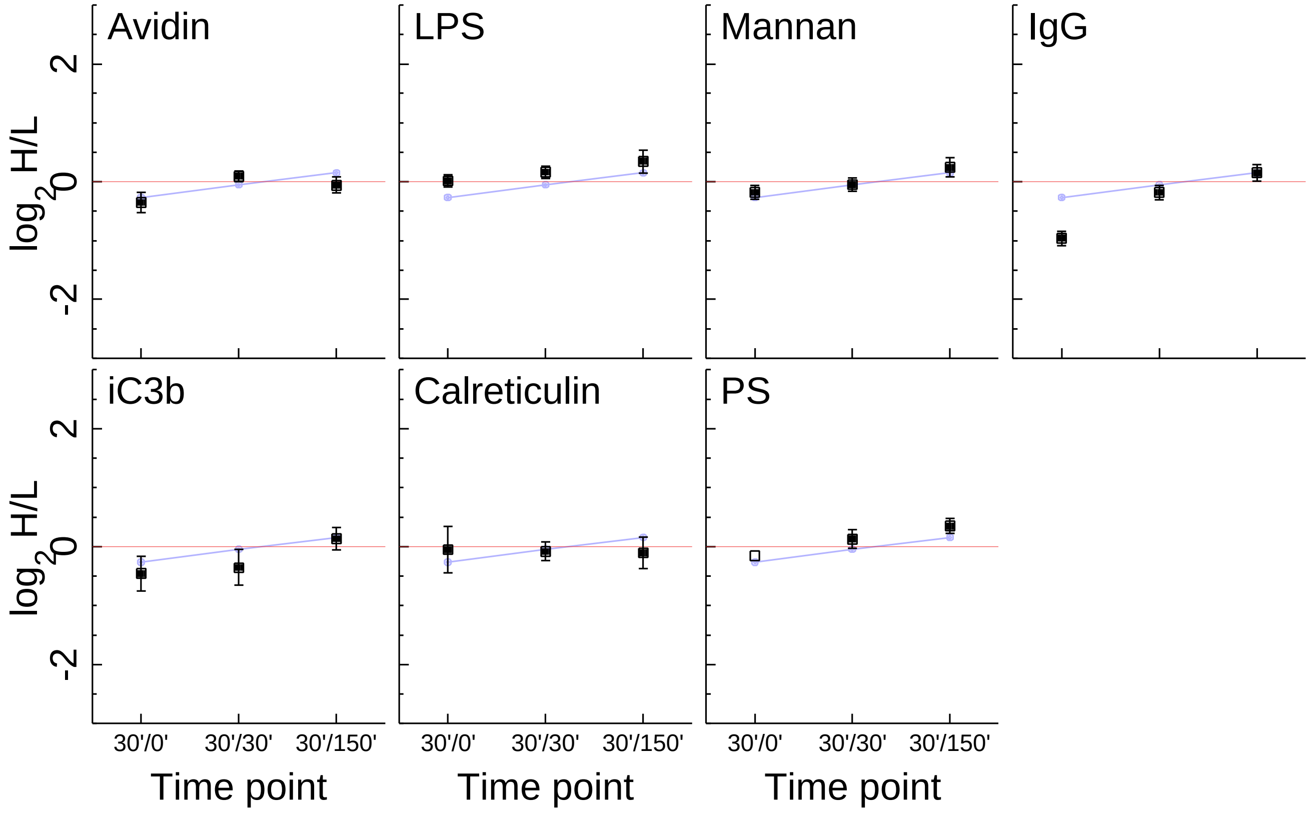

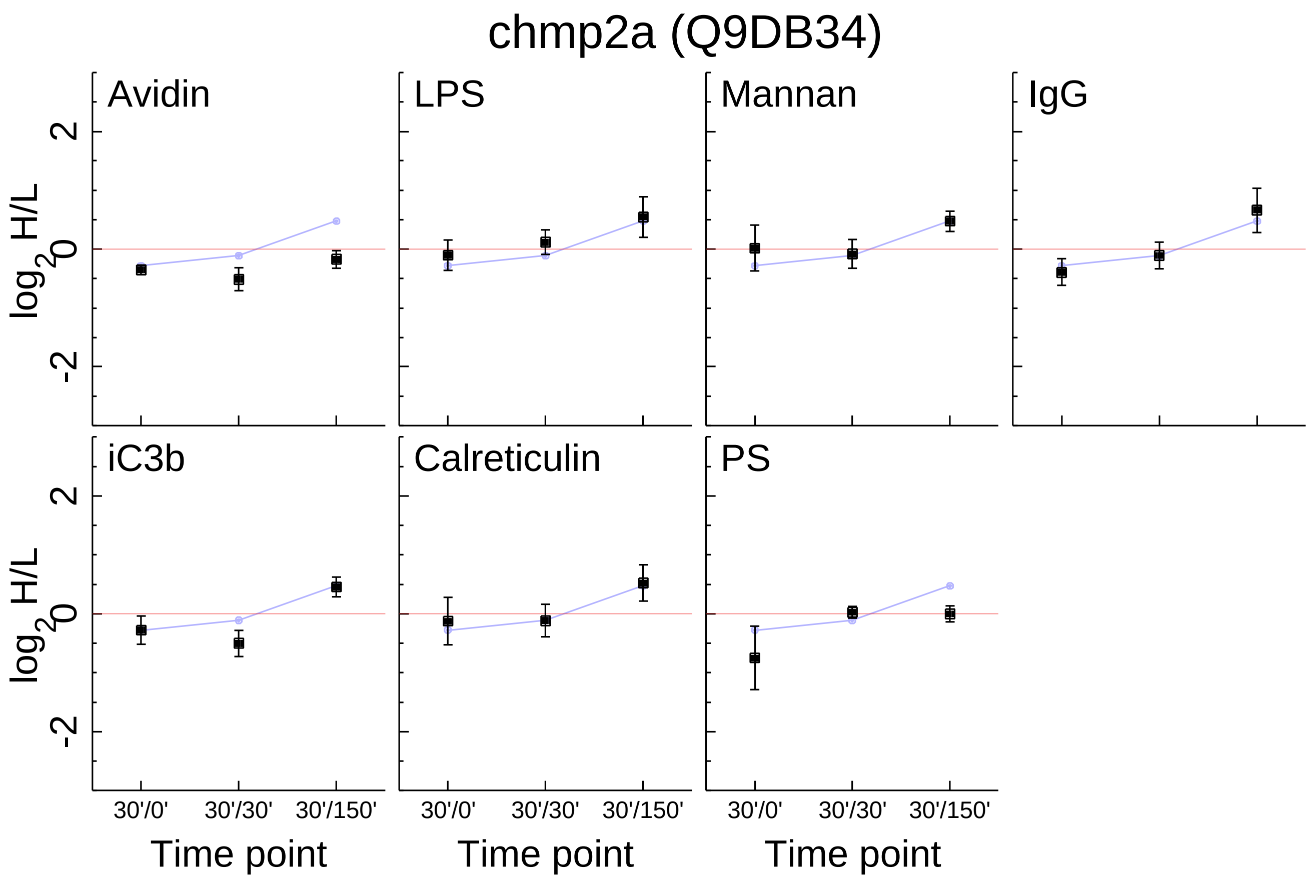

chmp2a (Q9DB34)
Avidin
LPS
Mannan
IgG
iC3b
Calreticulin
PS
log2 H/L
2
0
-2
30'/0'
30'/30'
30'/150'
Time point

chmp2b (Q8BJF9)

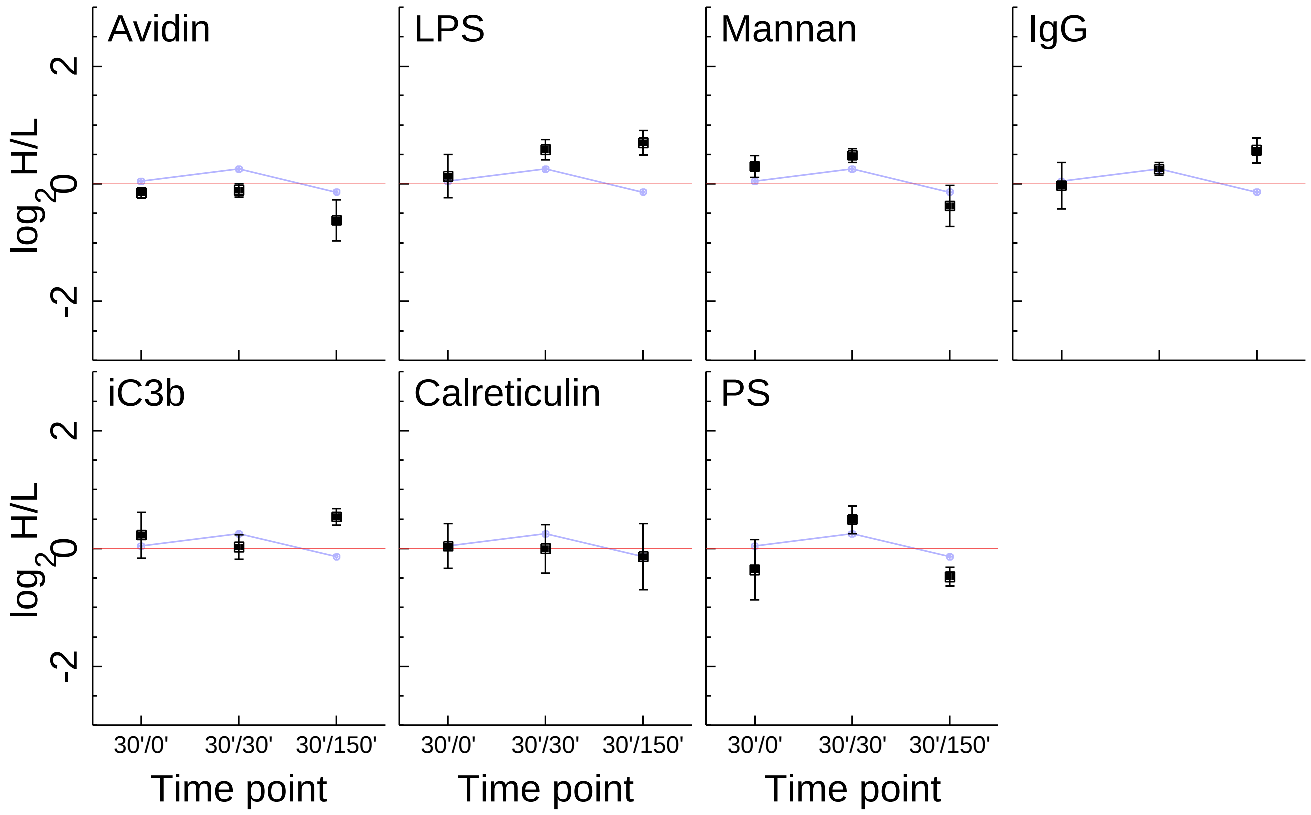

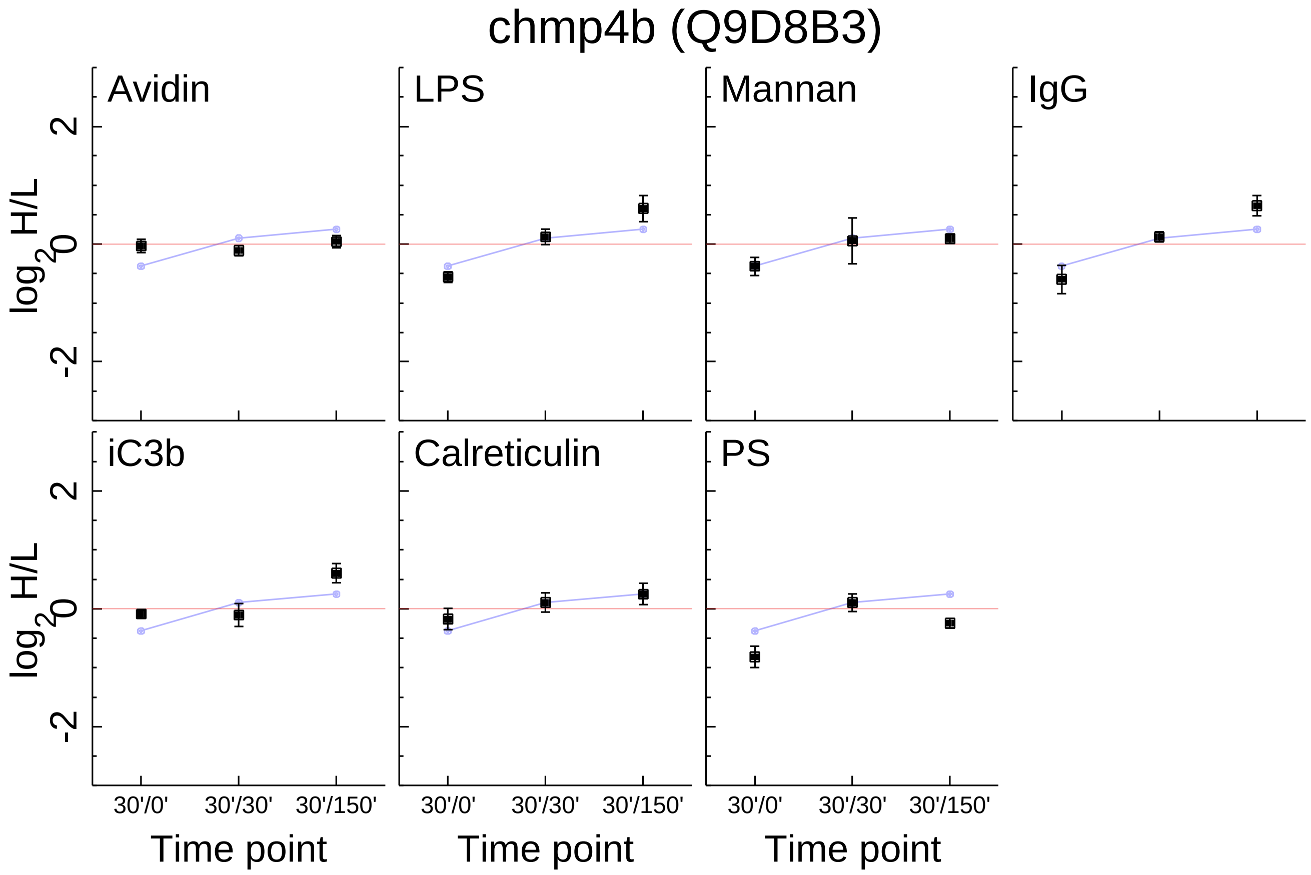

chmp4b (Q9D8B3)
Avidin
LPS
Mannan
IgG
iC3b
Calreticulin
PS
log2 H/L
2
0
-2
30'/0'
30'/30'
30'/150'
Time point

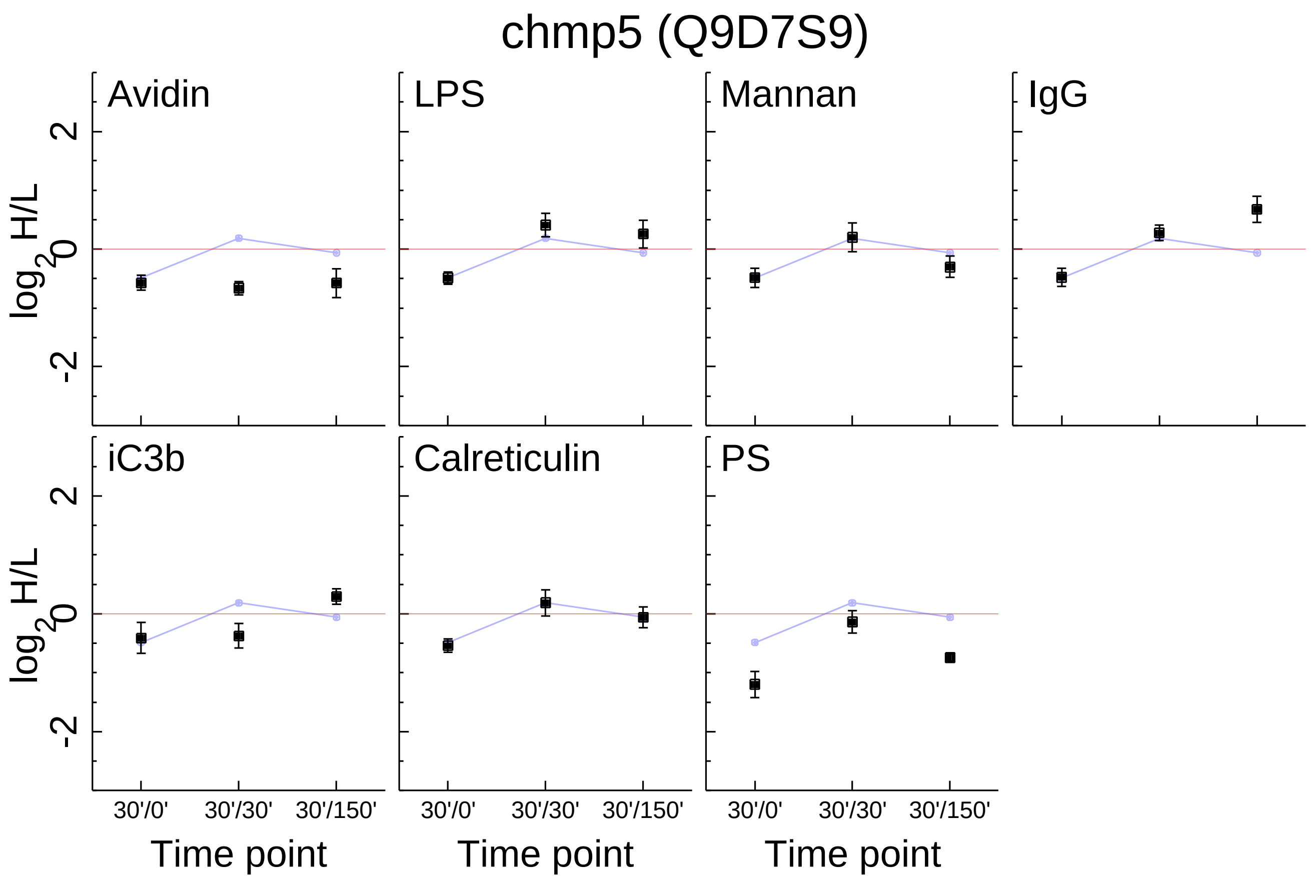
chmp5 (Q9D7S9)
Avidin
LPS
Mannan
IgG
iC3b
Calreticulin
PS
log_2 H/L
2
0
-2
30'/0'
30'/30'
30'/150'
Time point

chmp6 (P0C0A3)

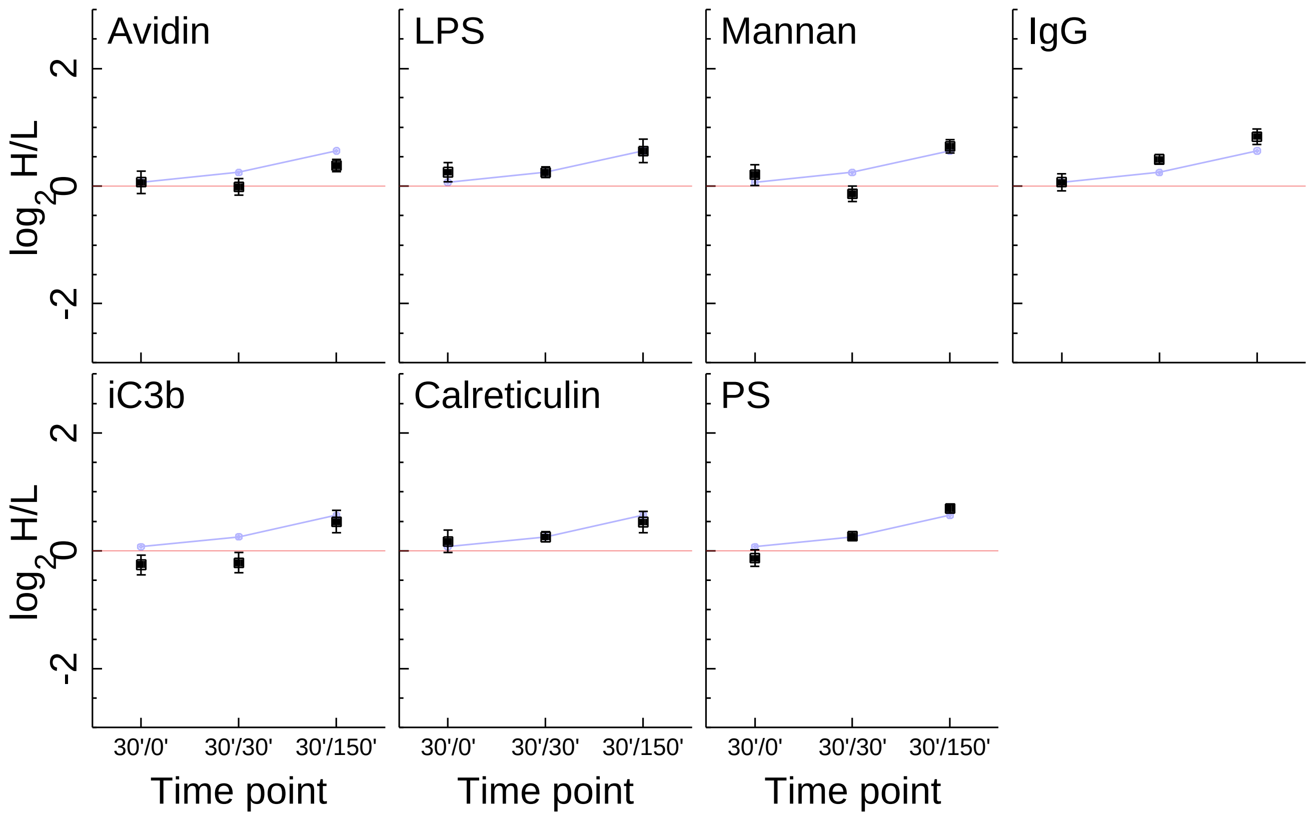

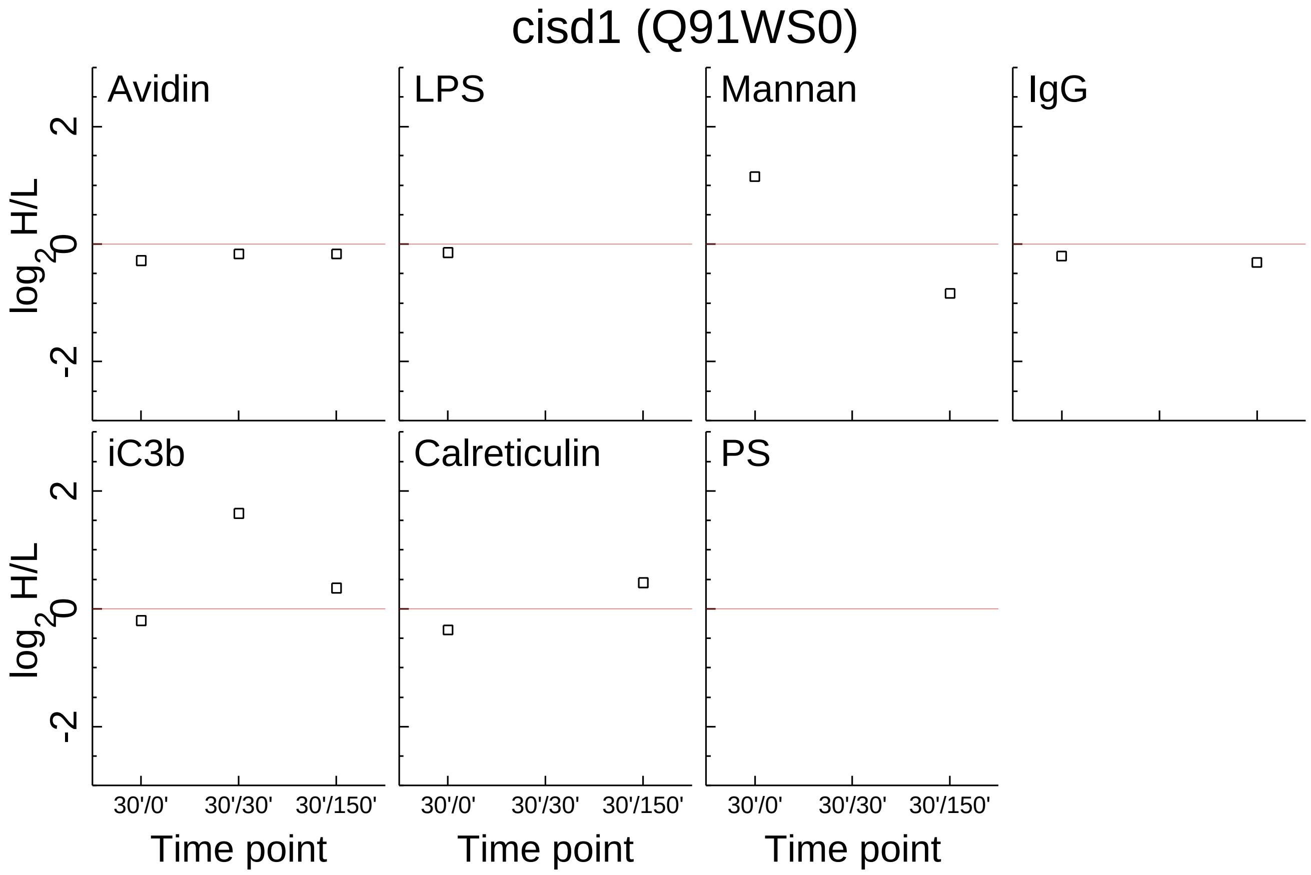

cisd1 (Q91WS0)
Avidin
LPS
Mannan
IgG
iC3b
Calreticulin
PS
log2 H/L
2
0
-2
30'/0'
30'/30'
30'/150'
Time point

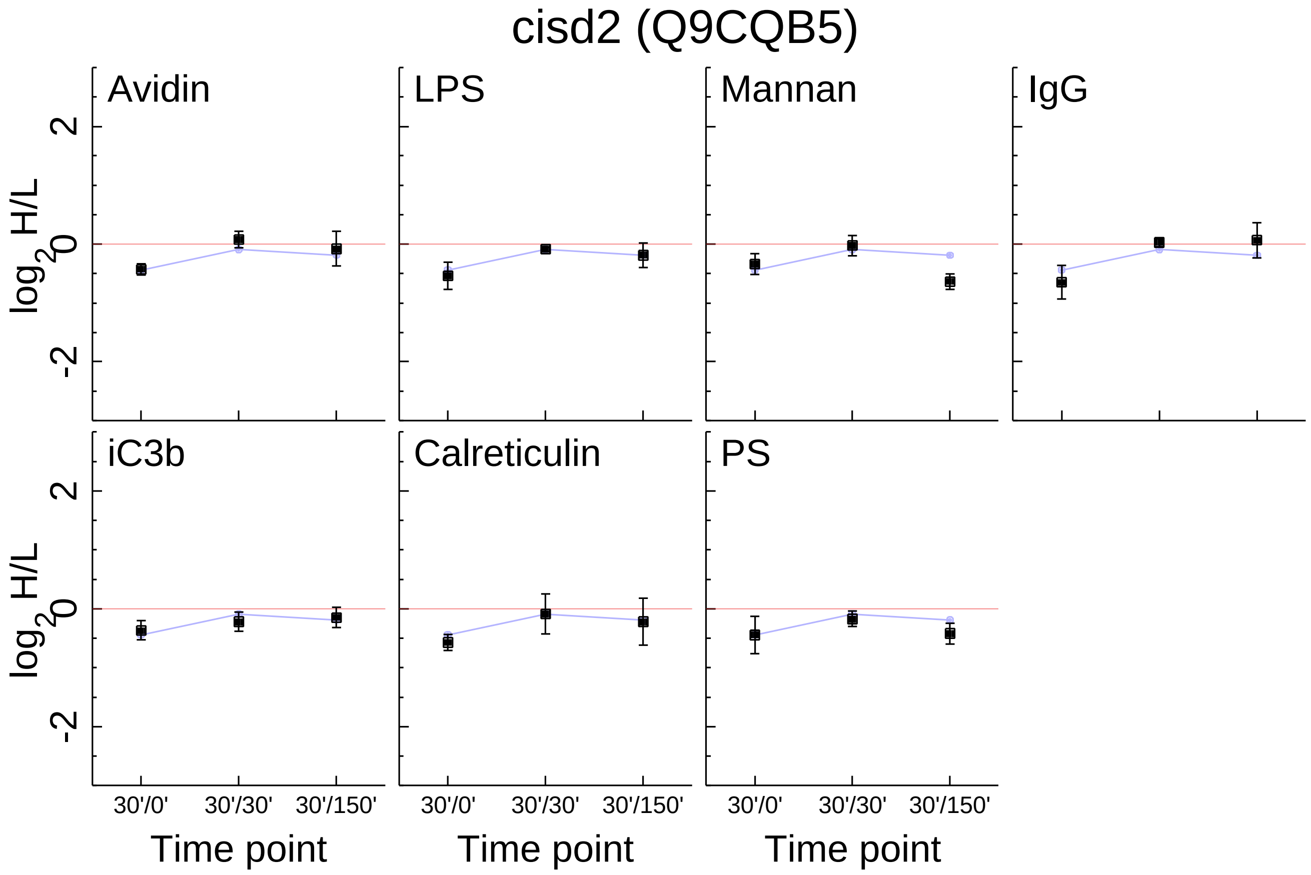
cisd2 (Q9CQB5)
Avidin
LPS
Mannan
IgG
iC3b
Calreticulin
PS
log2 H/L
2
0
-2
30'/0'
30'/30'
30'/150'
Time point

ckap4 (Q8BMK4)

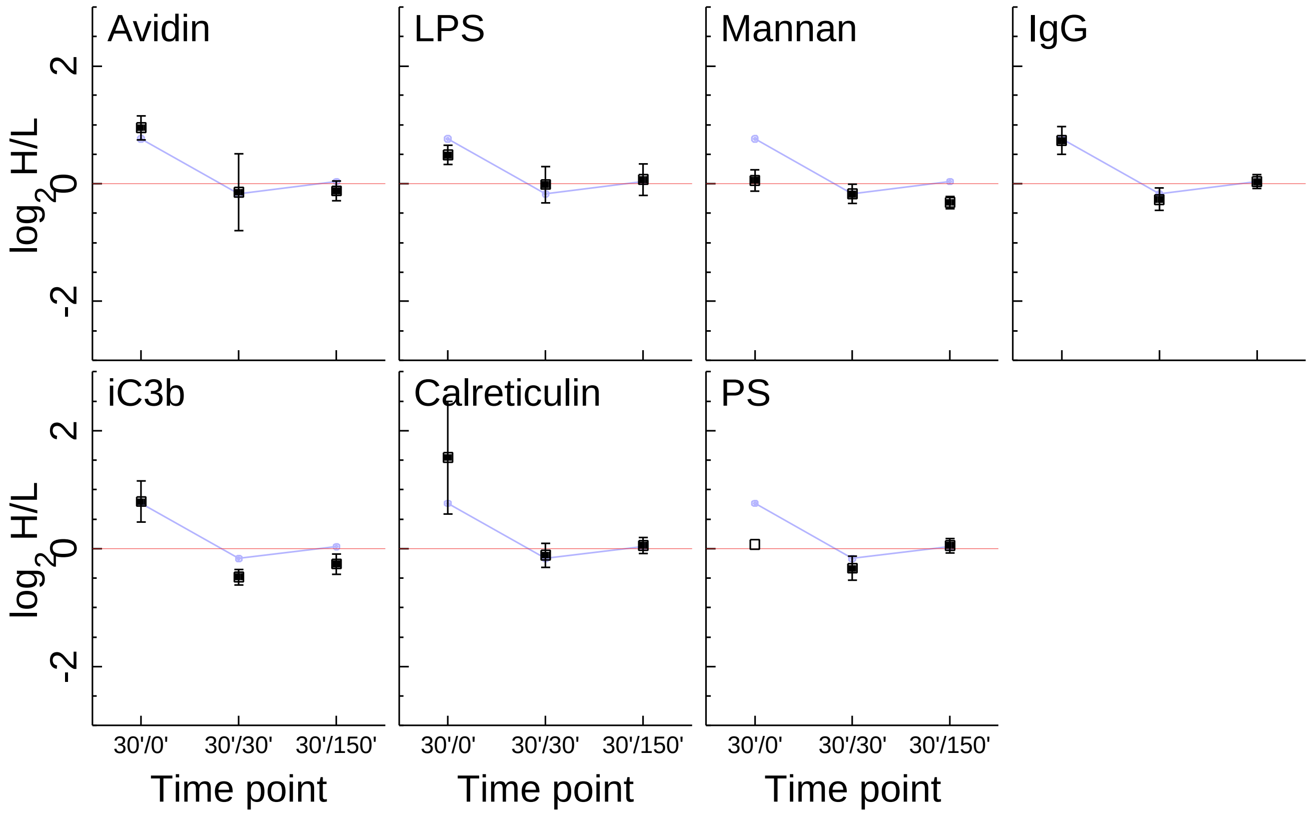

ckap5 (A2AGT5)

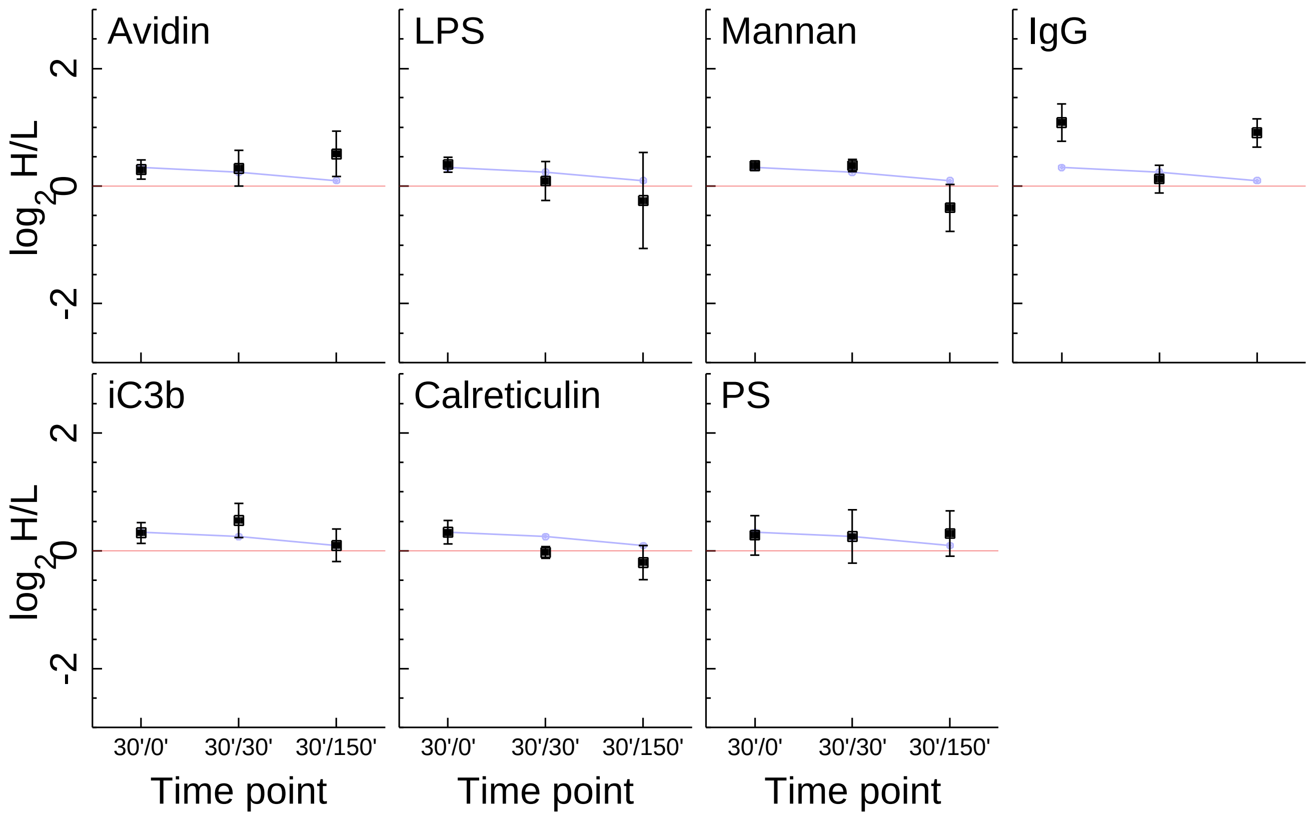

ckb (Q04447)

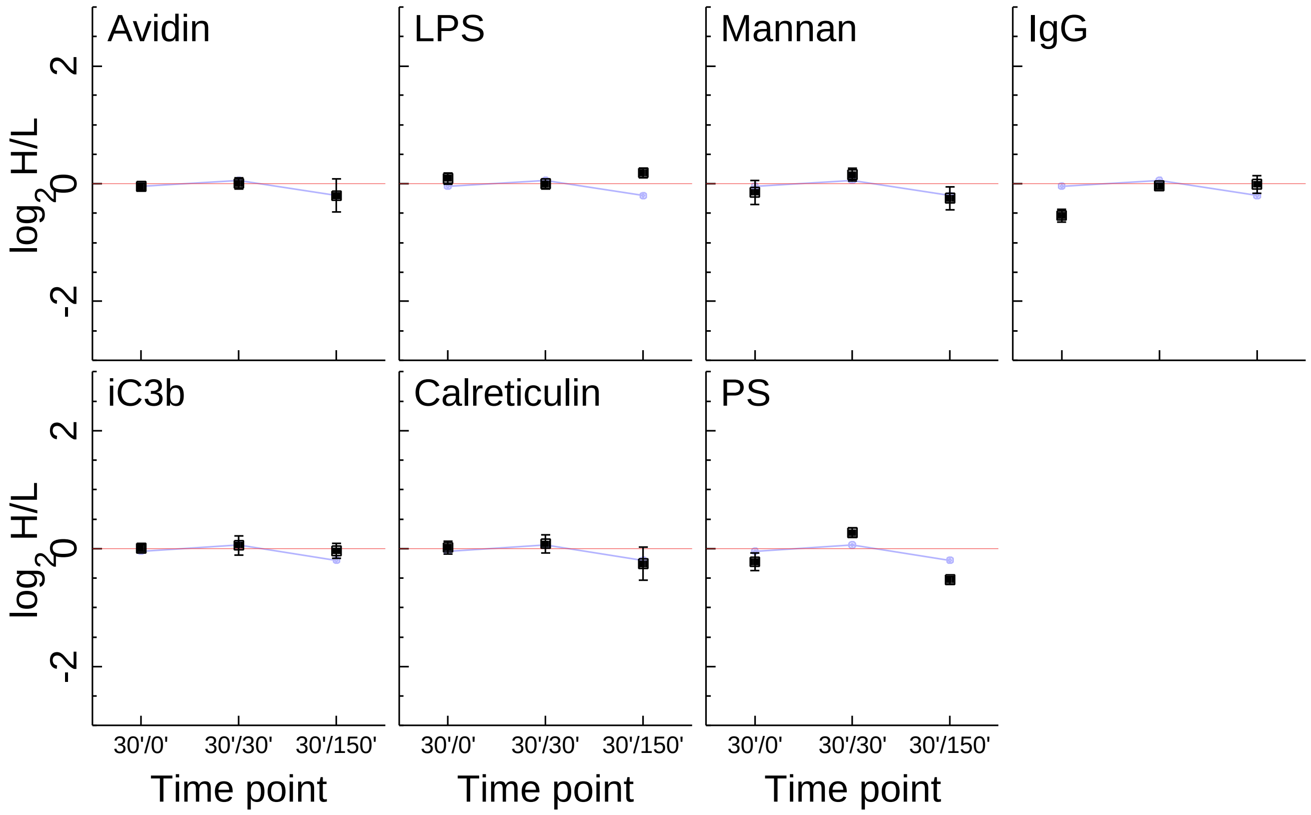
Avidin
LPS
Mannan
IgG
iC3b
Calreticulin
PS
log2 H/L
2
0
-2
30'/0'
30'/30'
30'/150'
Time point

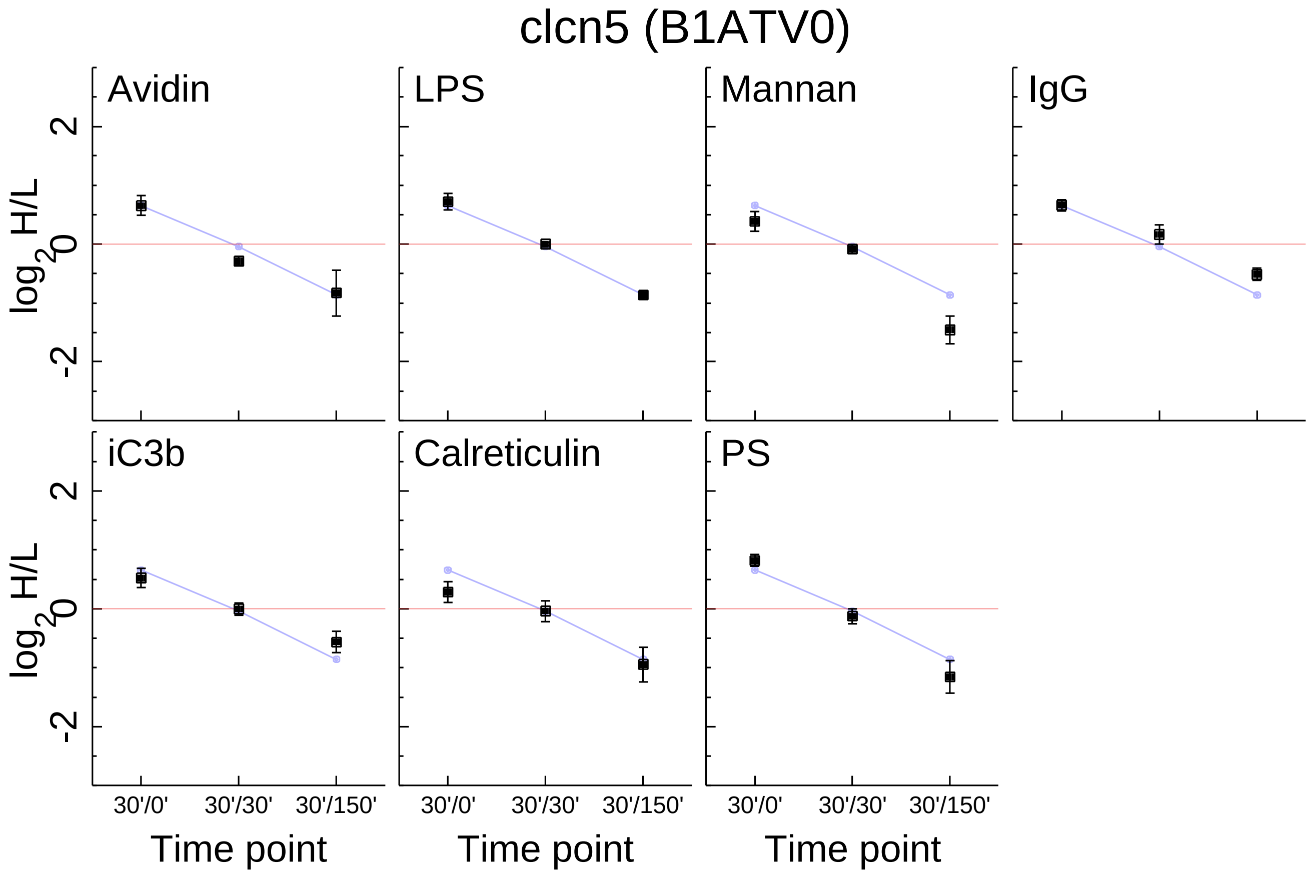
clcn5 (B1ATV0)
Avidin
LPS
Mannan
IgG
iC3b
Calreticulin
PS
log2 H/L
2
0
-2
30'/0'
30'/30'
30'/150'
Time point

clcn6 (A2A7F6)

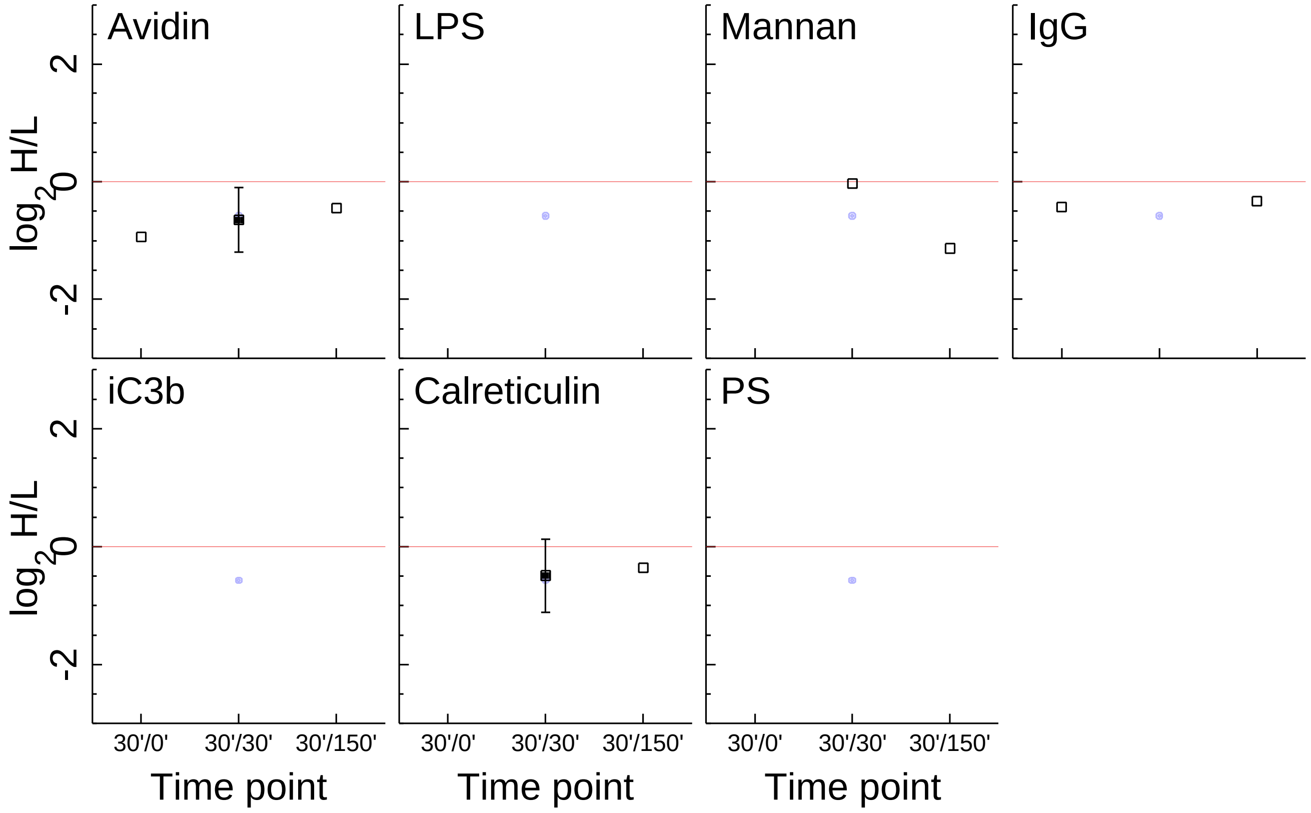

clcn7 (O70496)

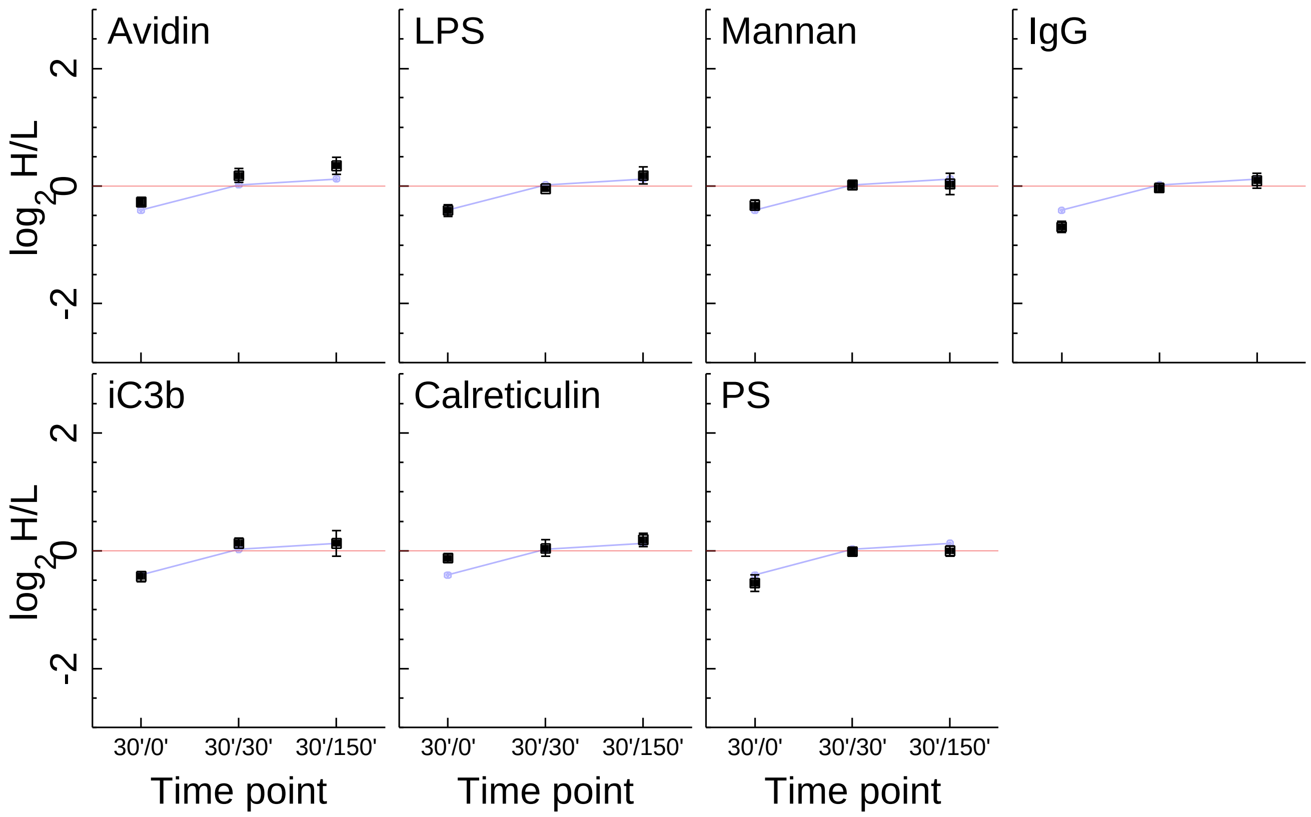

clec10a (F8WHB7)

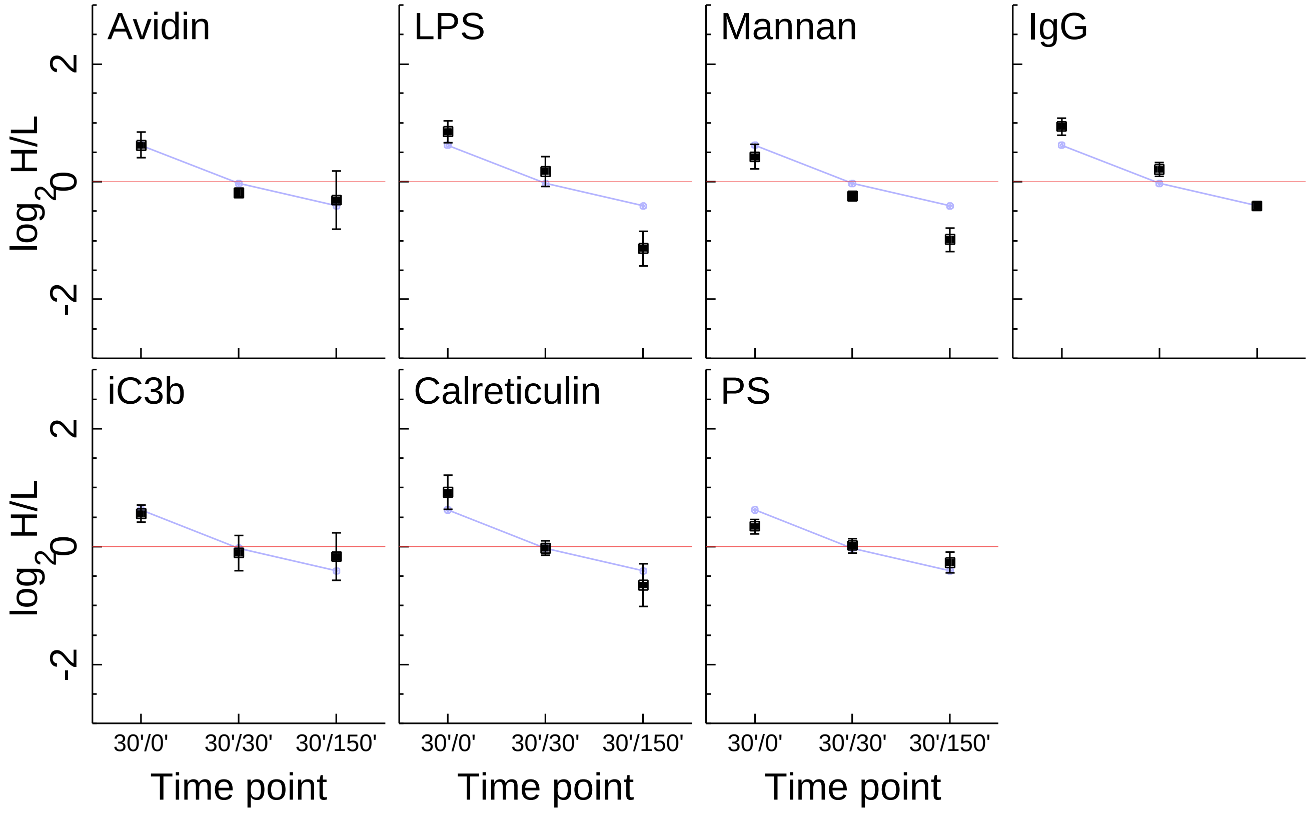

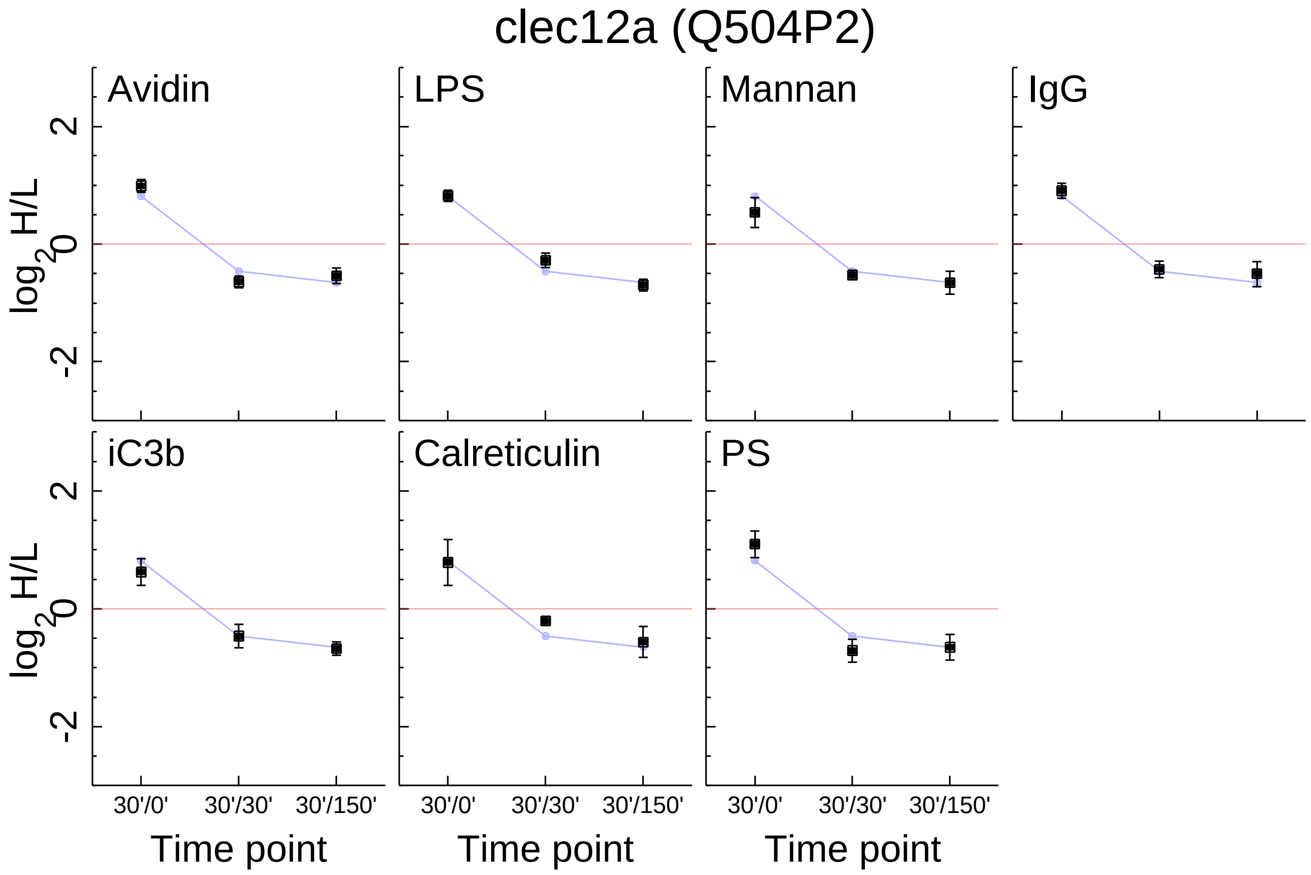
clec12a (Q504P2)
Avidin
LPS
Mannan
IgG
iC3b
Calreticulin
PS
log2 H/L
2
0
-2
30'/0'
30'/30'
30'/150'
Time point

clec4e (Q9R0Q8)

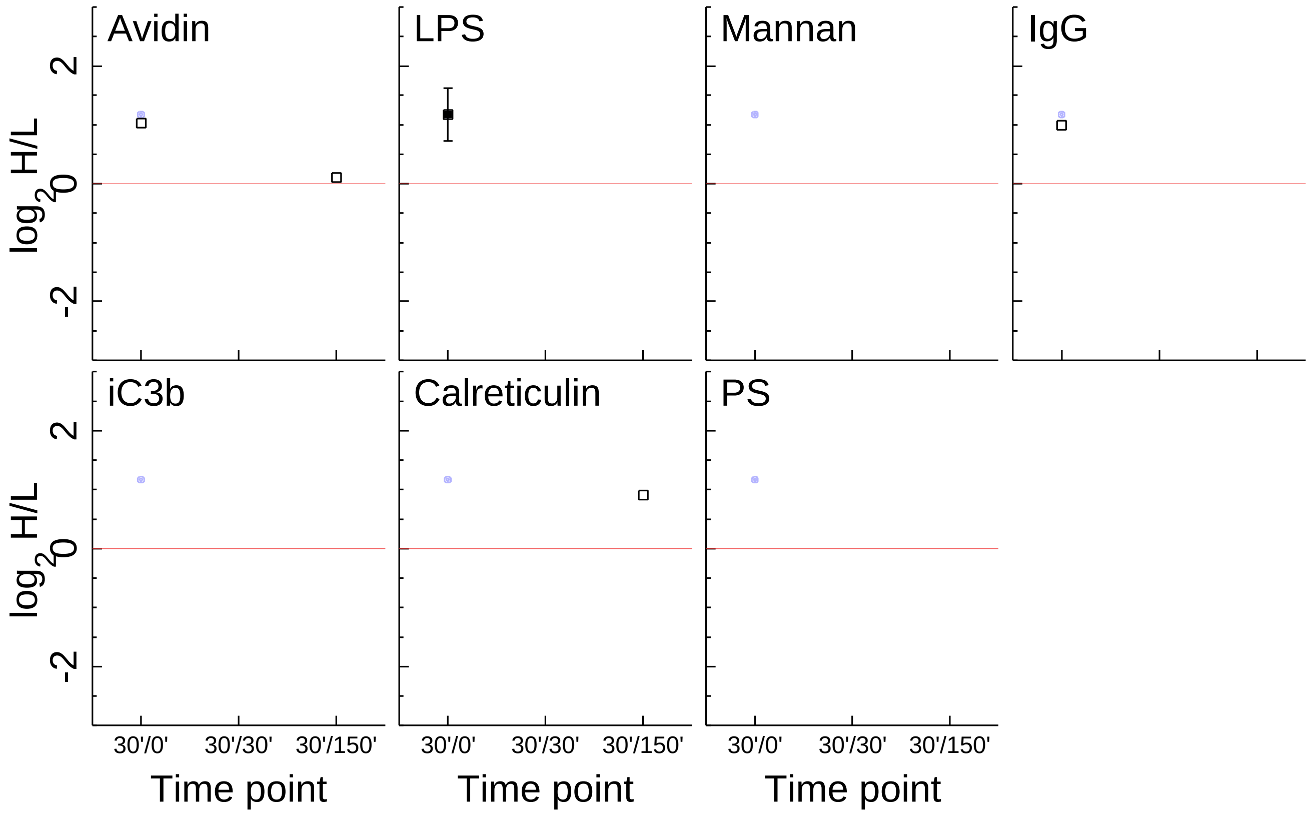

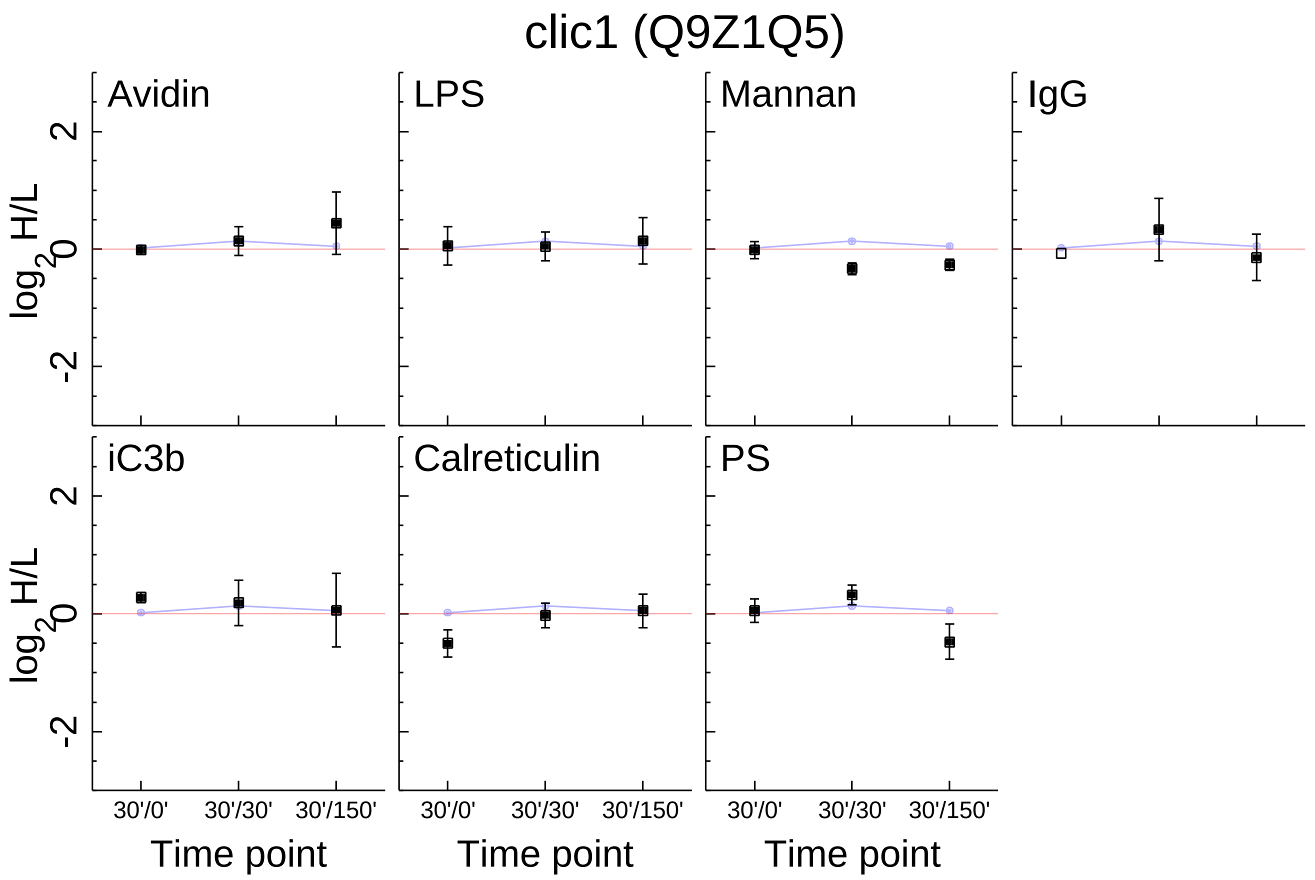
clic1 (Q9Z1Q5)
Avidin
LPS
Mannan
IgG
iC3b
Calreticulin
PS
log2 H/L
2
0
-2
30'/0'
30'/30'
30'/150'
Time point

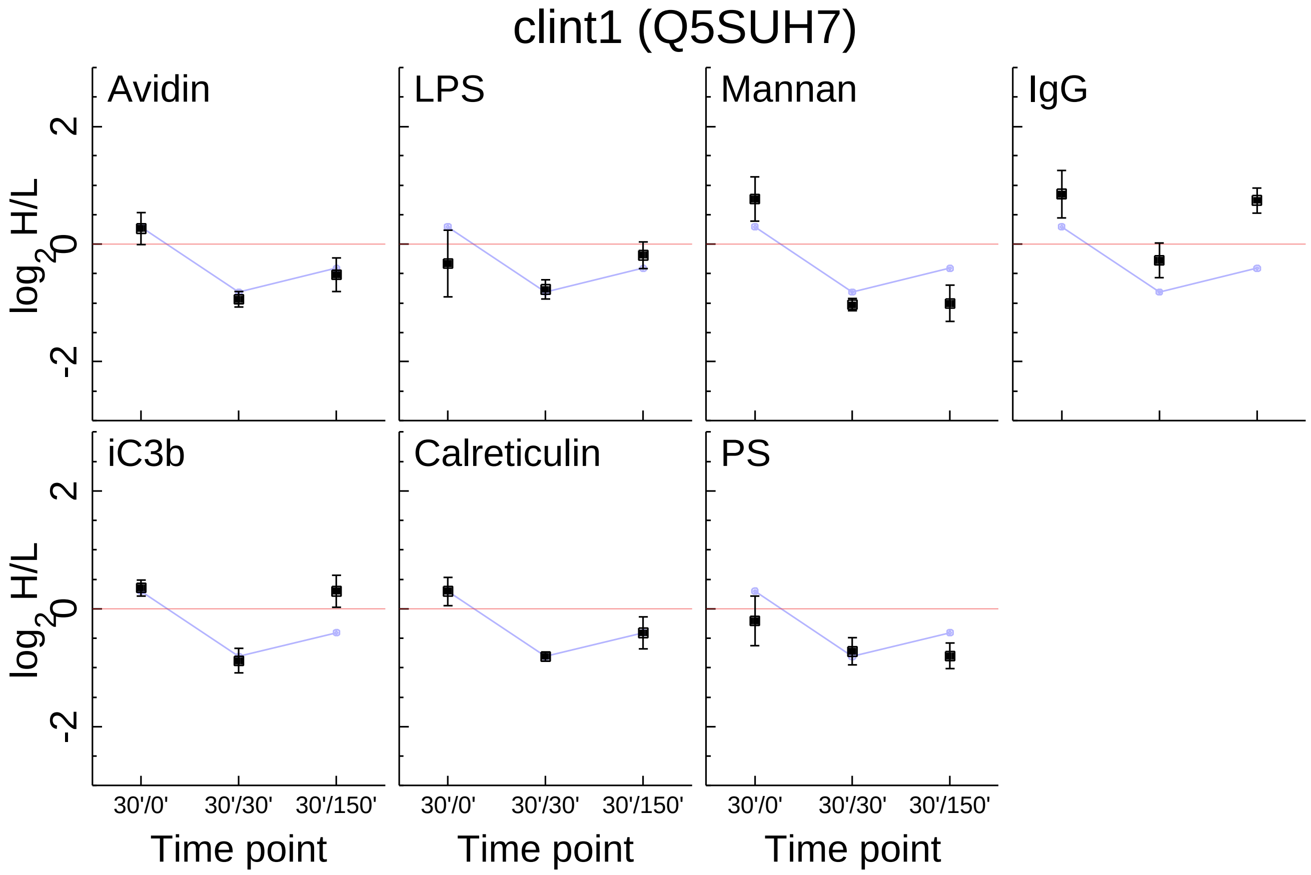
clint1 (Q5SUH7)
Avidin
LPS
Mannan
IgG
iC3b
Calreticulin
PS
log2 H/L
2
0
-2
30'/0'
30'/30'
30'/150'
Time point

cln3 (Q61124)

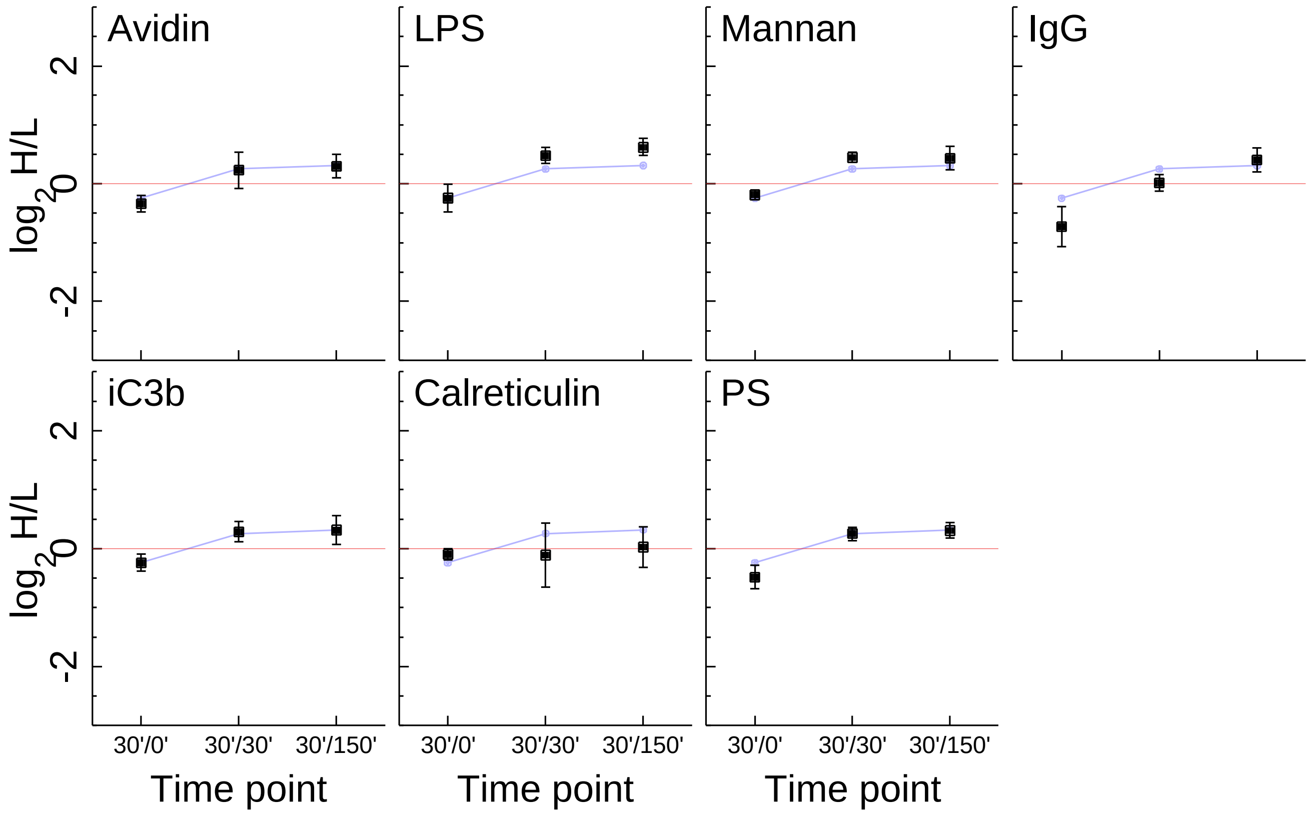

Avidin
LPS
Mannan
IgG
iC3b
Calreticulin
PS
log2 H/L
2
0
-2
30'/0'
30'/30'
30'/150'
Time point

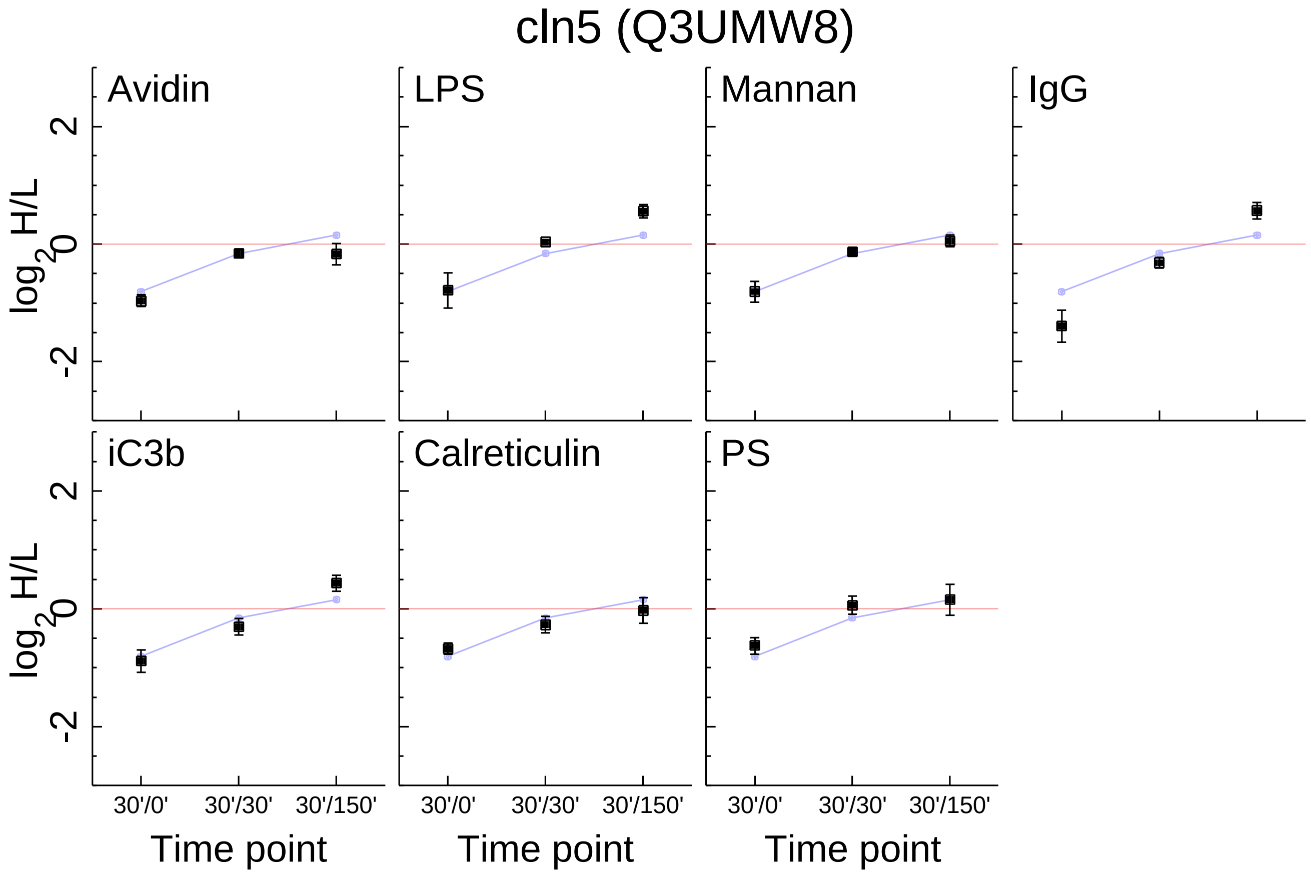

cln5 (Q3UMW8)
Avidin
LPS
Mannan
IgG
iC3b
Calreticulin
PS
log2 H/L
2
0
-2
30'/0'
30'/30'
30'/150'
Time point

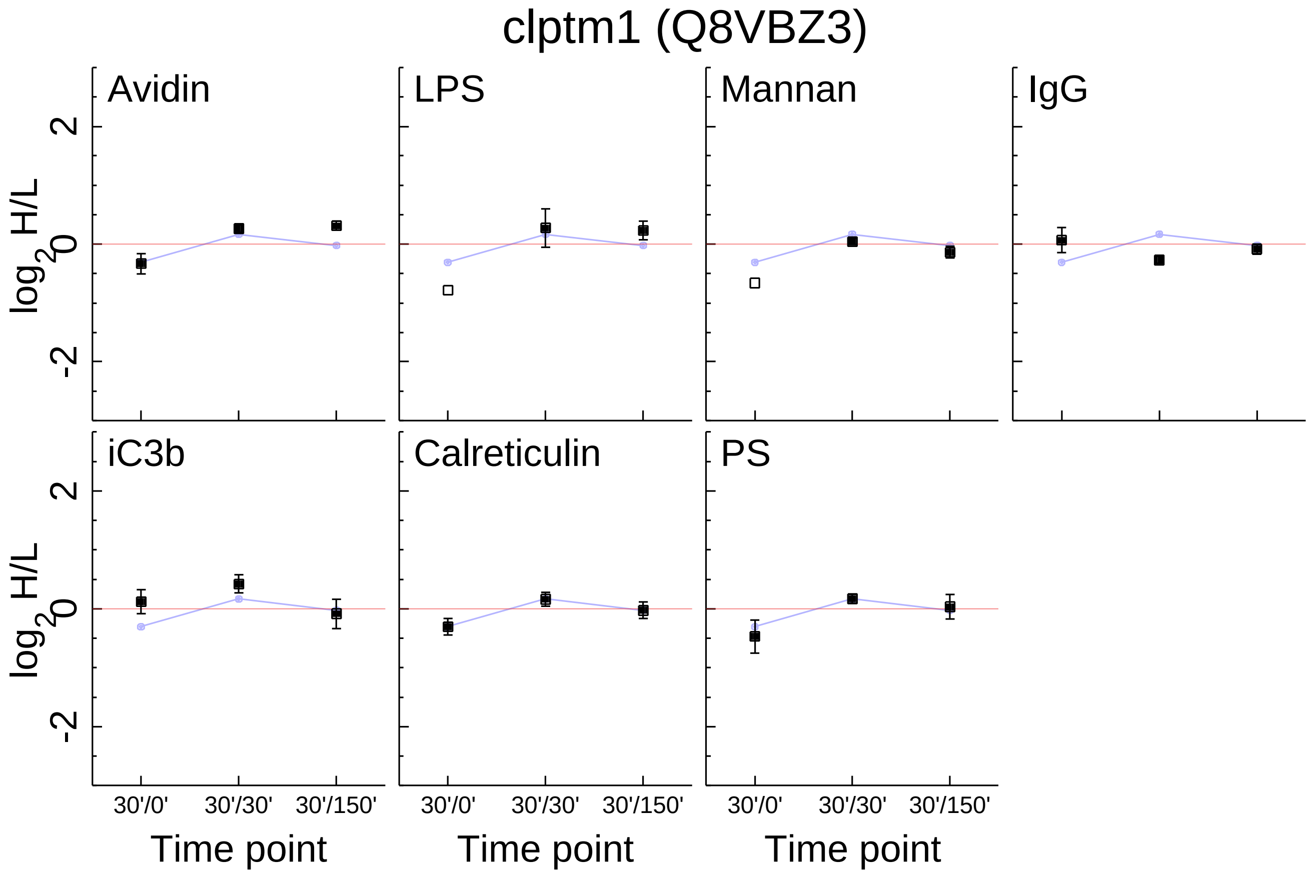
clptm1 (Q8VBZ3)
Avidin
LPS
Mannan
IgG
iC3b
Calreticulin
PS
log2 H/L
2
0
-2
30'/0'
30'/30'
30'/150'
Time point

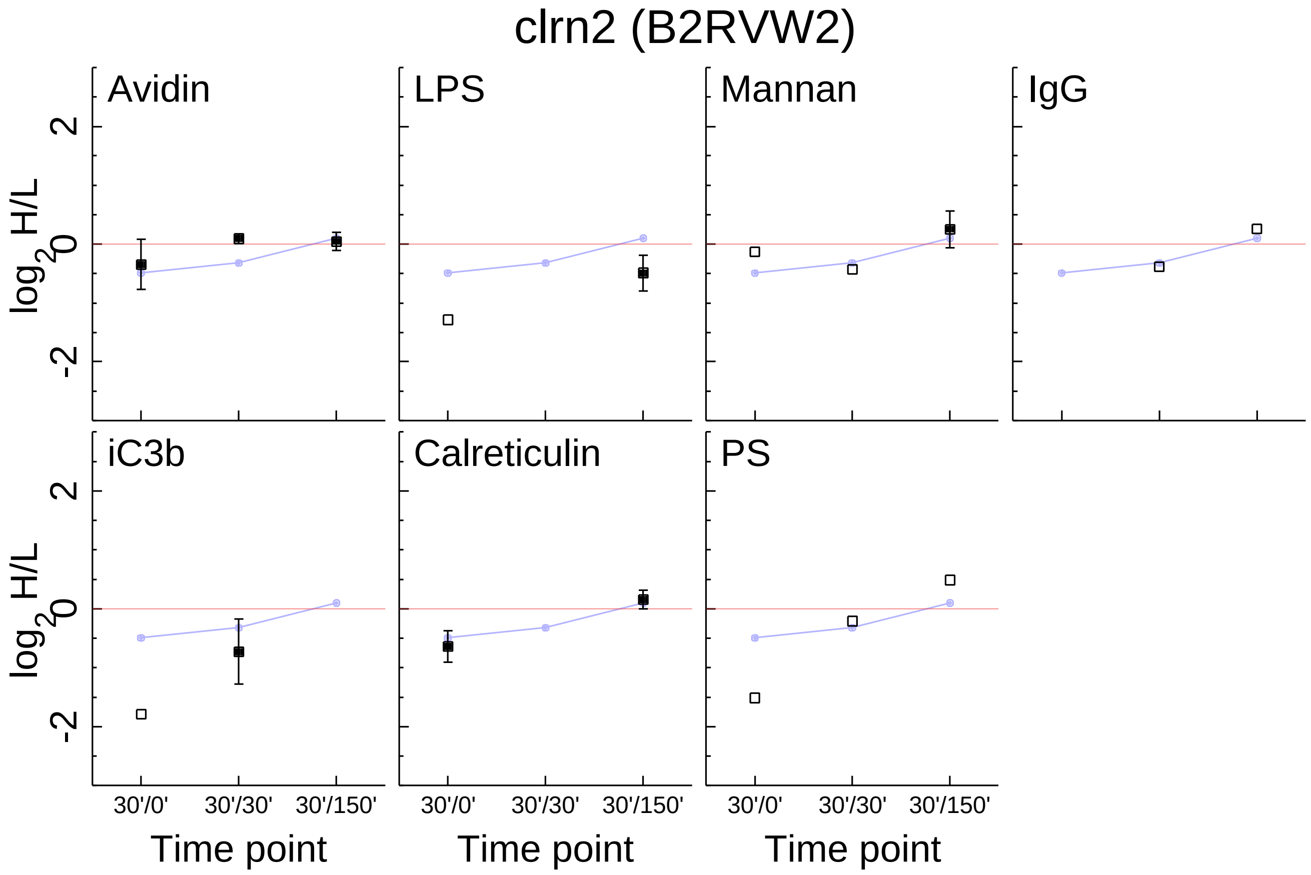

clrn2 (B2RVW2)
Avidin
LPS
Mannan
IgG
iC3b
Calreticulin
PS
log2 H/L
2
0
-2
30'/0'
30'/30'
30'/150'
Time point

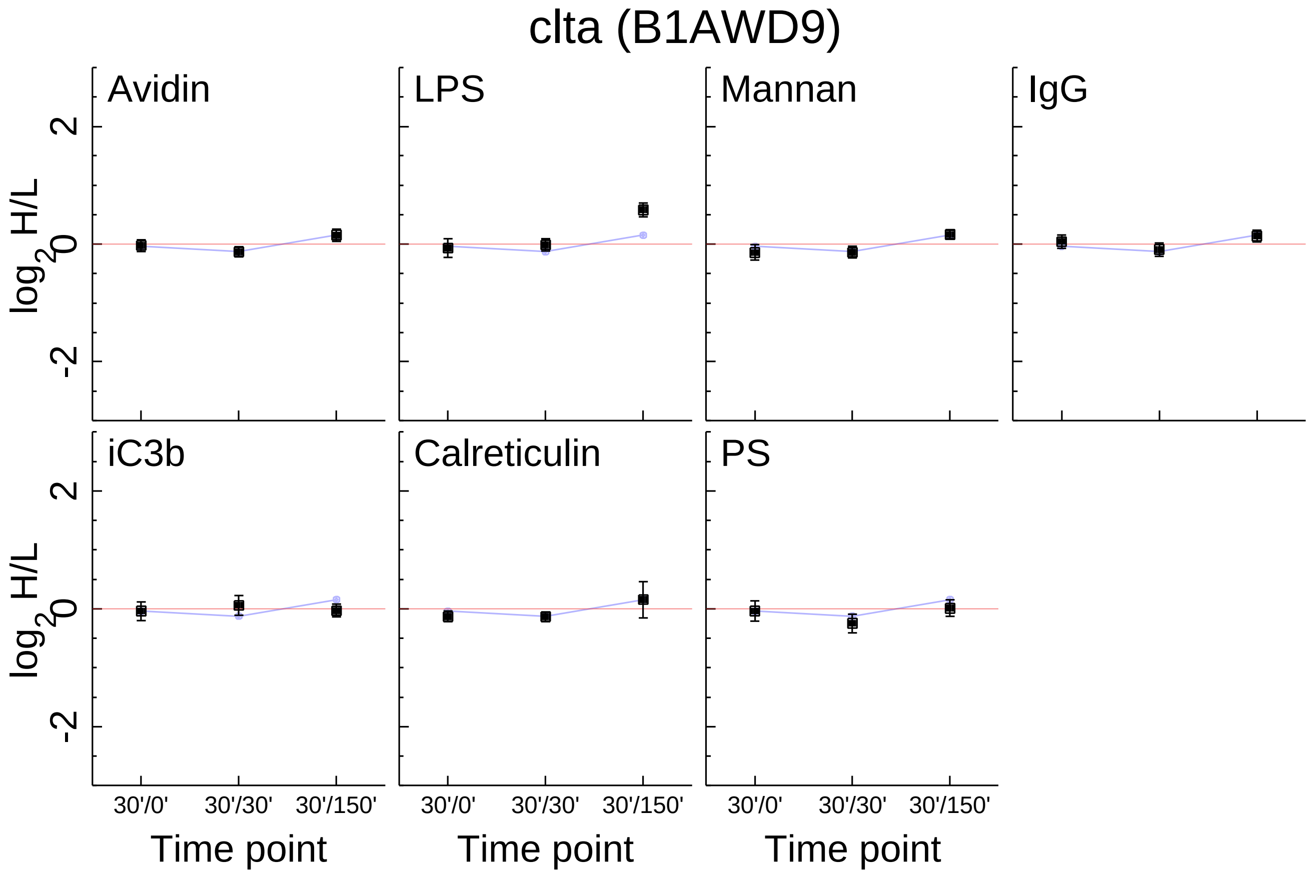
clta (B1AWD9)
Avidin
LPS
Mannan
IgG
iC3b
Calreticulin
PS
log2 H/L
2
0
-2
30'/0'
30'/30'
30'/150'
Time point

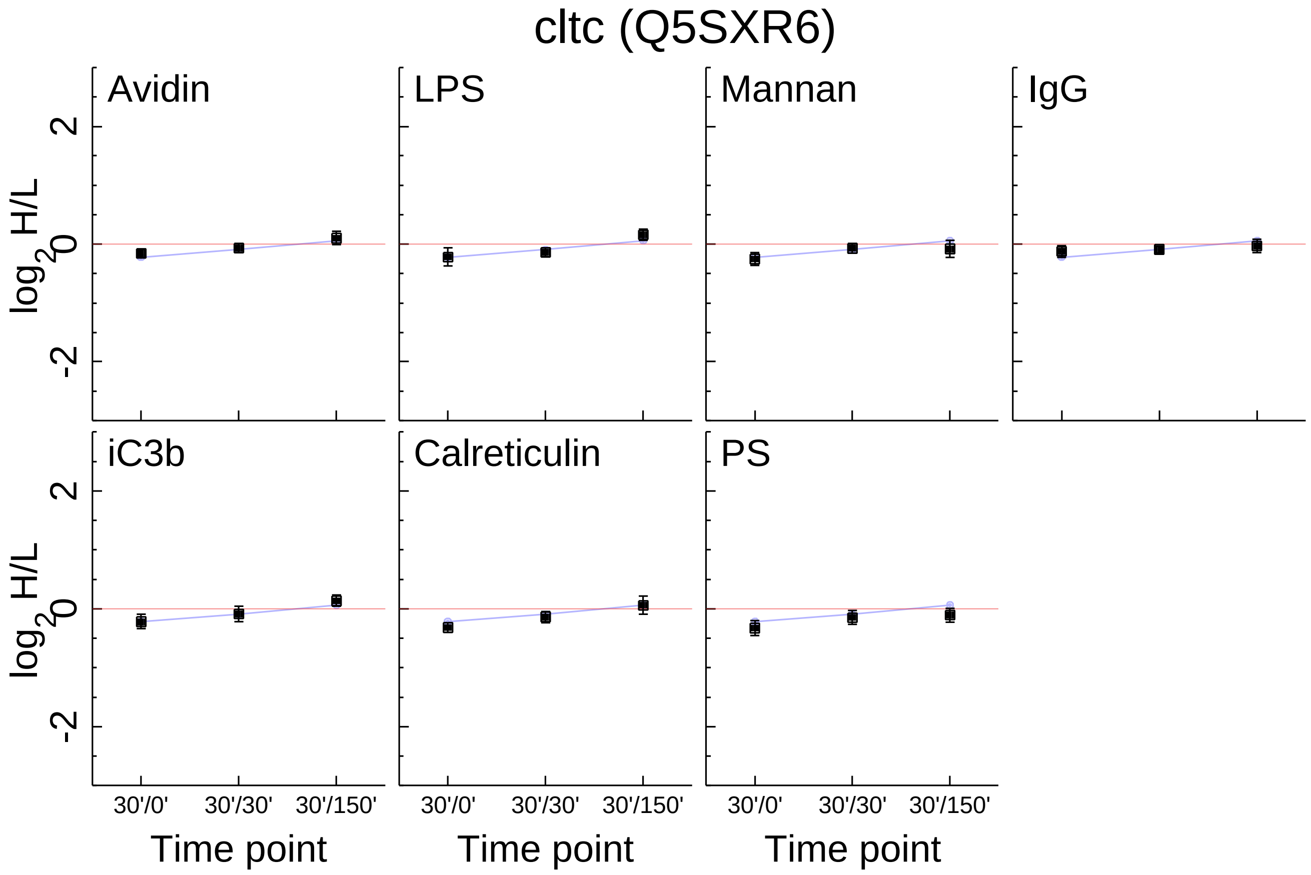
cltc (Q5SXR6)
Avidin
LPS
Mannan
IgG
iC3b
Calreticulin
PS
log2 H/L
2
0
-2
30'/0'
30'/30'
30'/150'
Time point

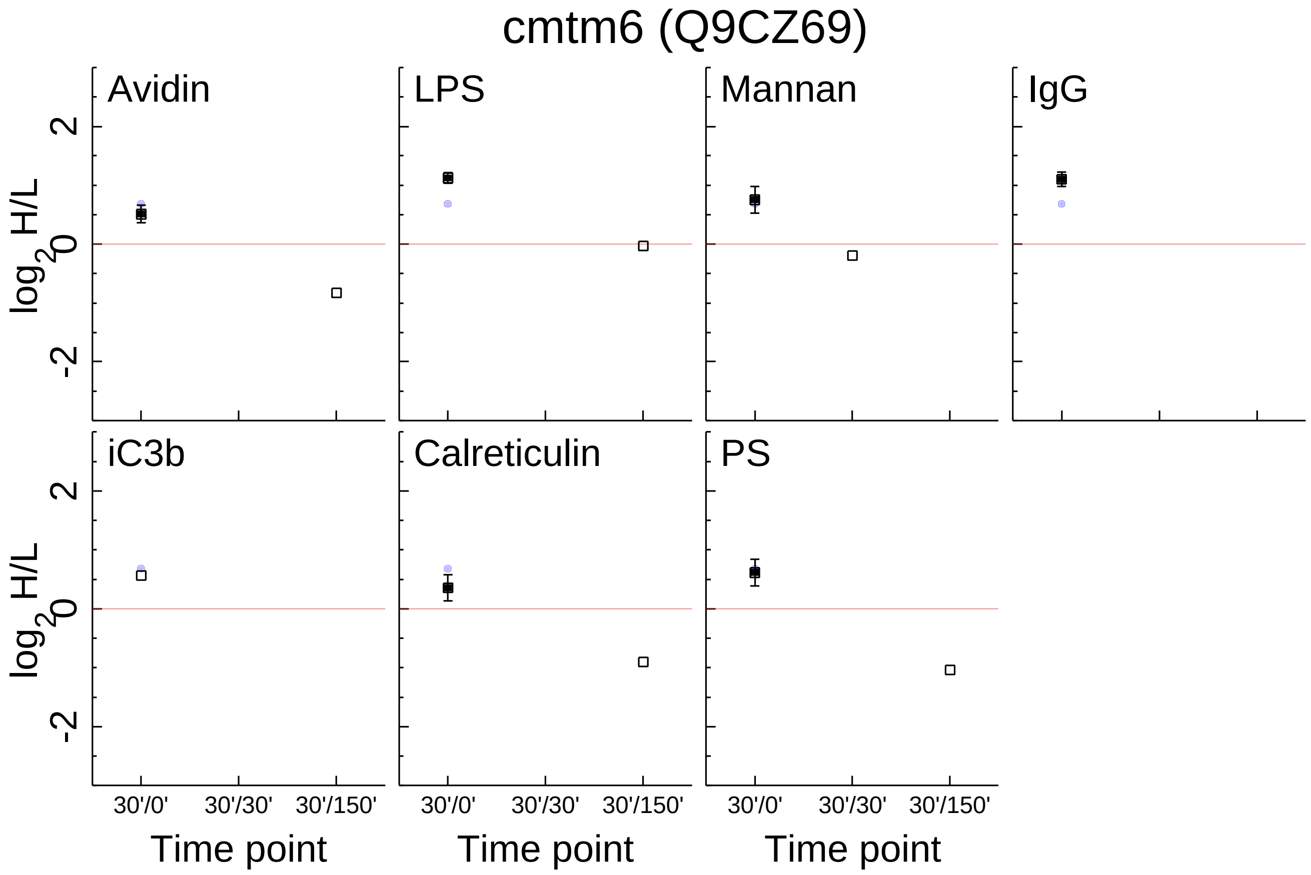
cmtm6 (Q9CZ69)
Avidin
LPS
Mannan
IgG
iC3b
Calreticulin
PS
log2 H/L
2
0
-2
30'/0'
30'/30'
30'/150'
Time point

cndp2 (Q9D1A2)

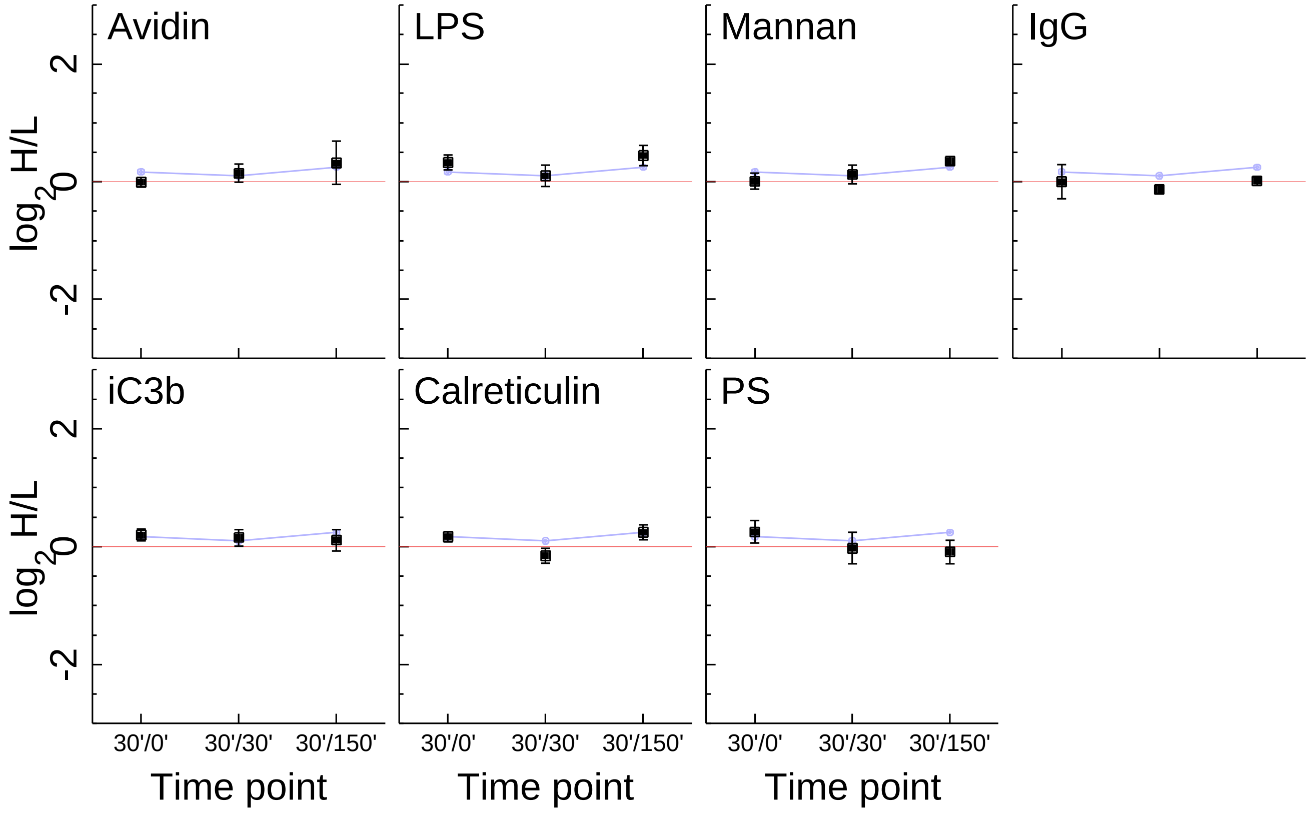

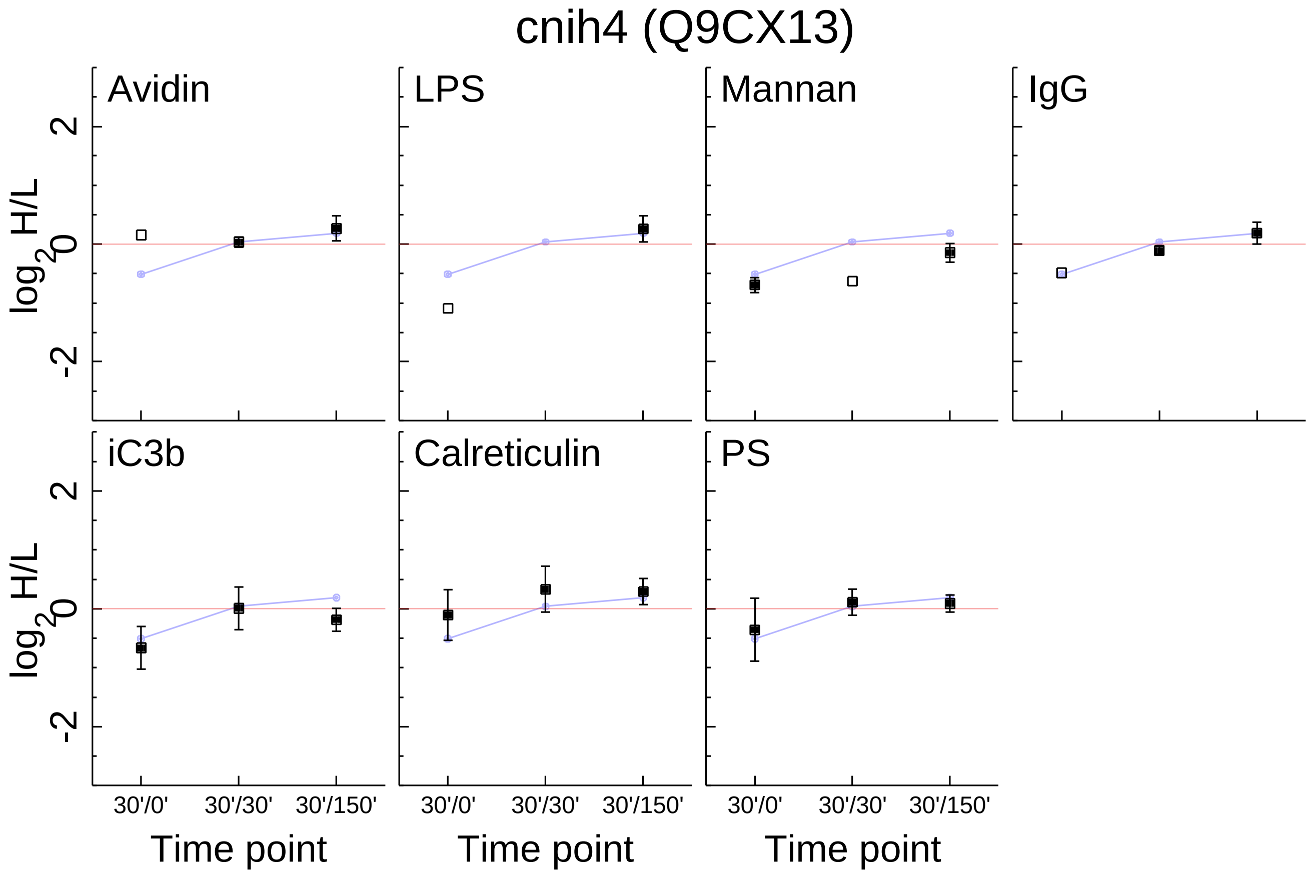

cnih4 (Q9CX13)
Avidin
LPS
Mannan
IgG
iC3b
Calreticulin
PS
log2 H/L
2
0
-2
30'/0'
30'/30'
30'/150'
Time point

cnn2 (Q08093)

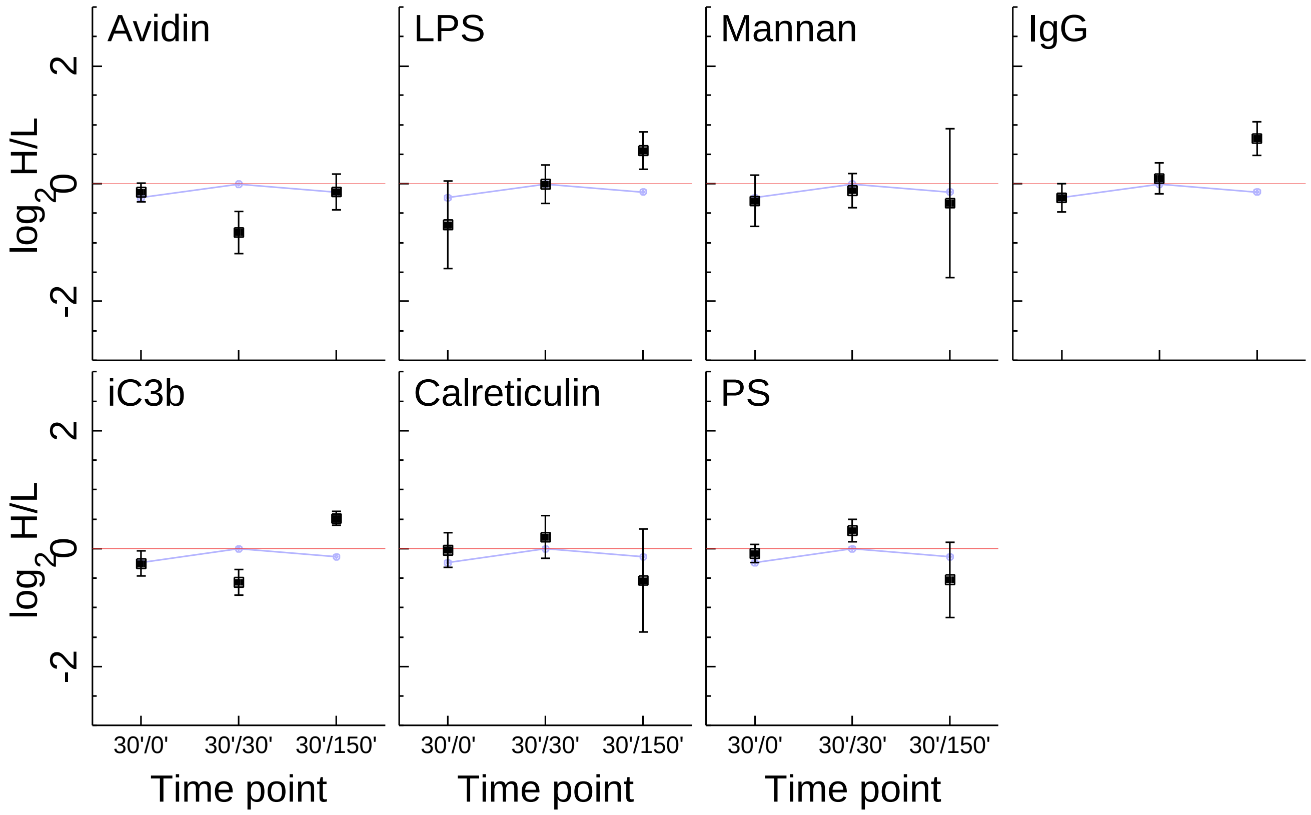
Avidin
LPS
Mannan
IgG
iC3b
Calreticulin
PS
log2 H/L
2
0
-2
30'/0'
30'/30'
30'/150'
Time point

cnot4 (Q8BT14)

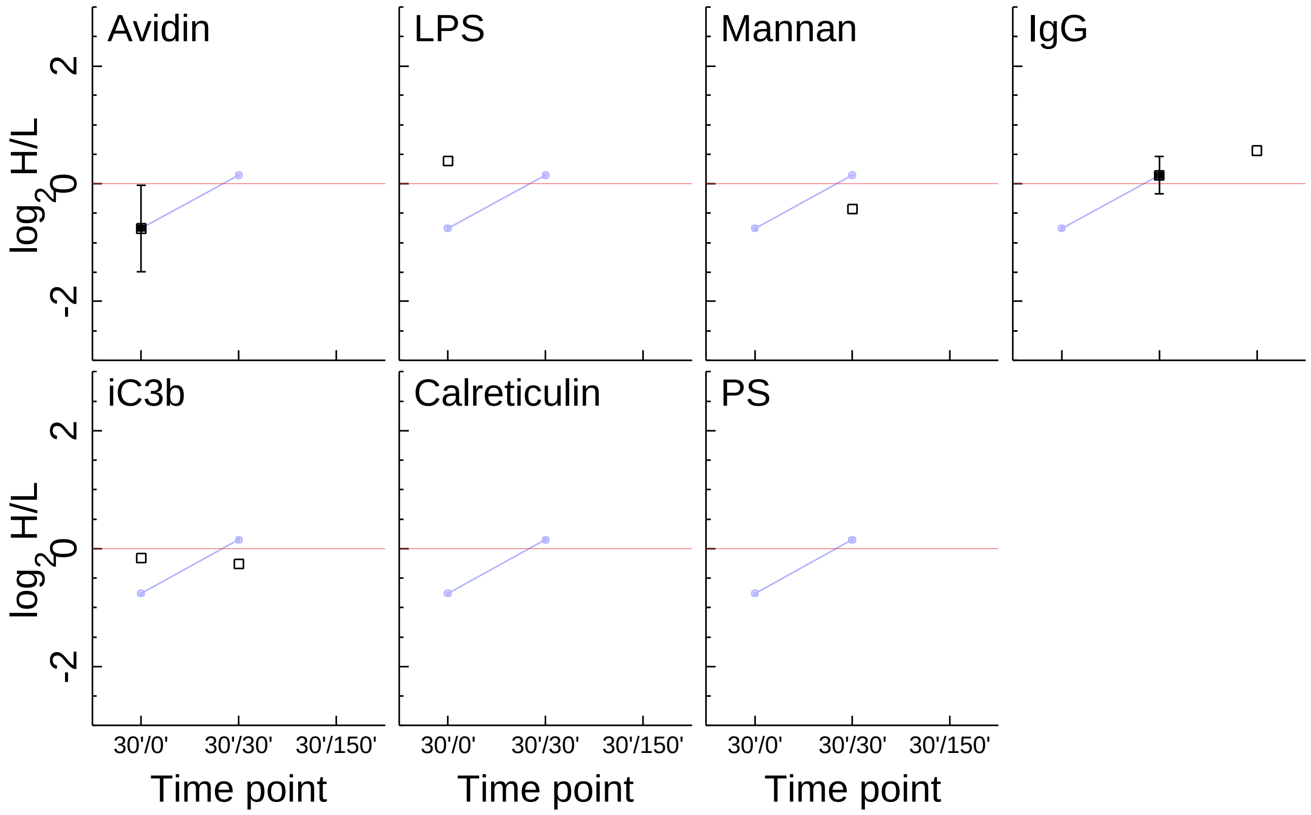

cnp (P16330)

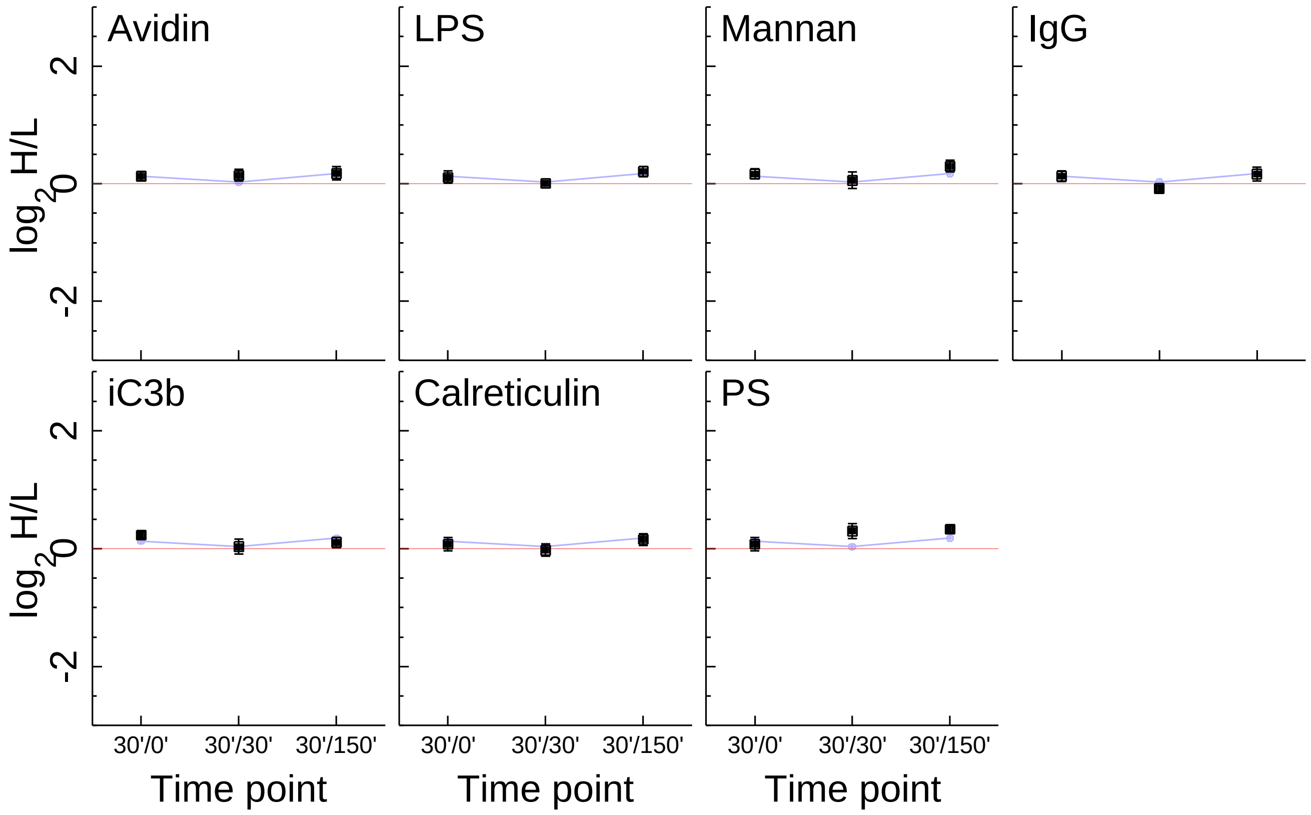

Avidin
LPS
Mannan
IgG
iC3b
Calreticulin
PS
log2 H/L
2
0
-2
30'/0'
30'/30'
30'/150'
Time point

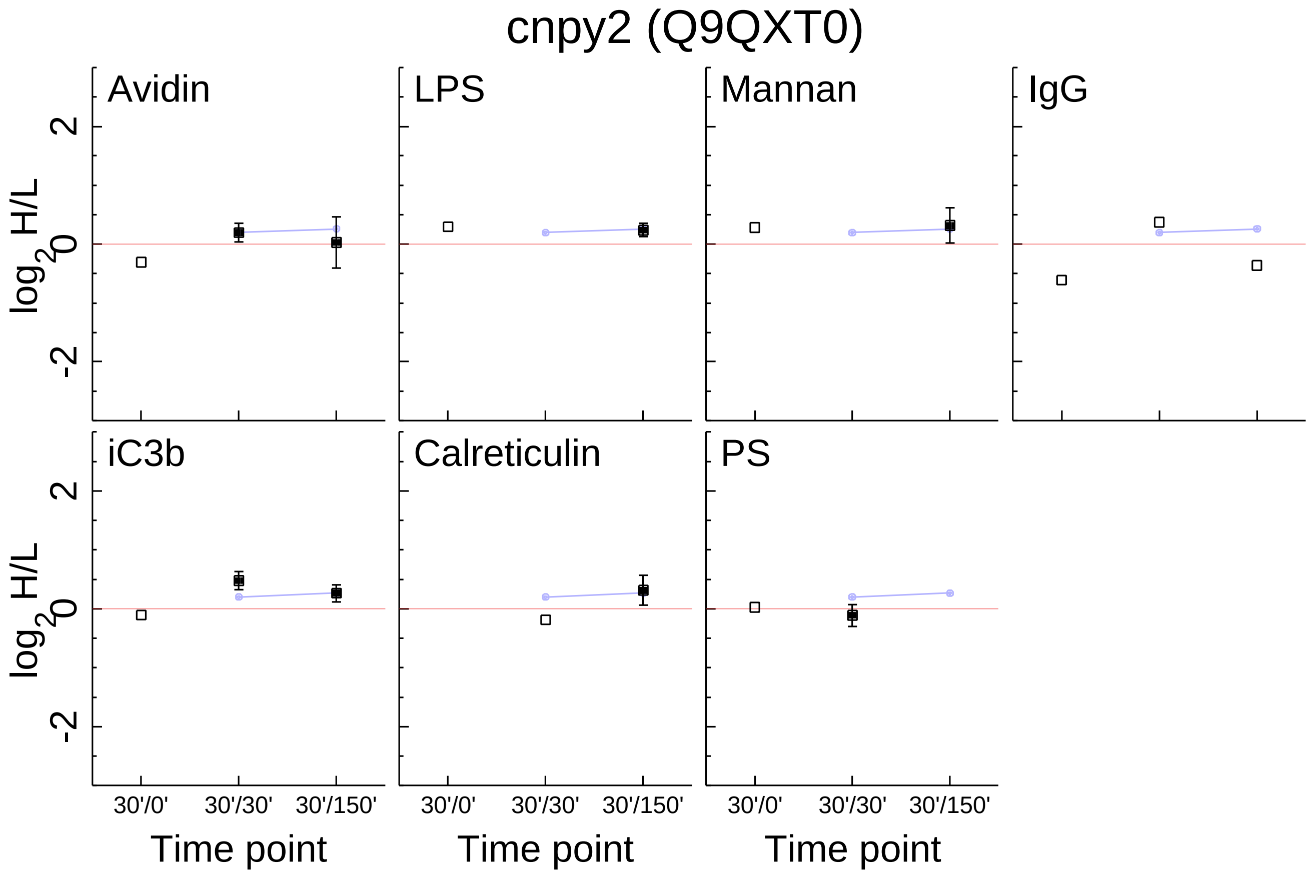

cnpy2 (Q9QXT0)
Avidin
LPS
Mannan
IgG
iC3b
Calreticulin
PS
log2 H/L
2
0
-2
30'/0'
30'/30'
30'/150'
Time point

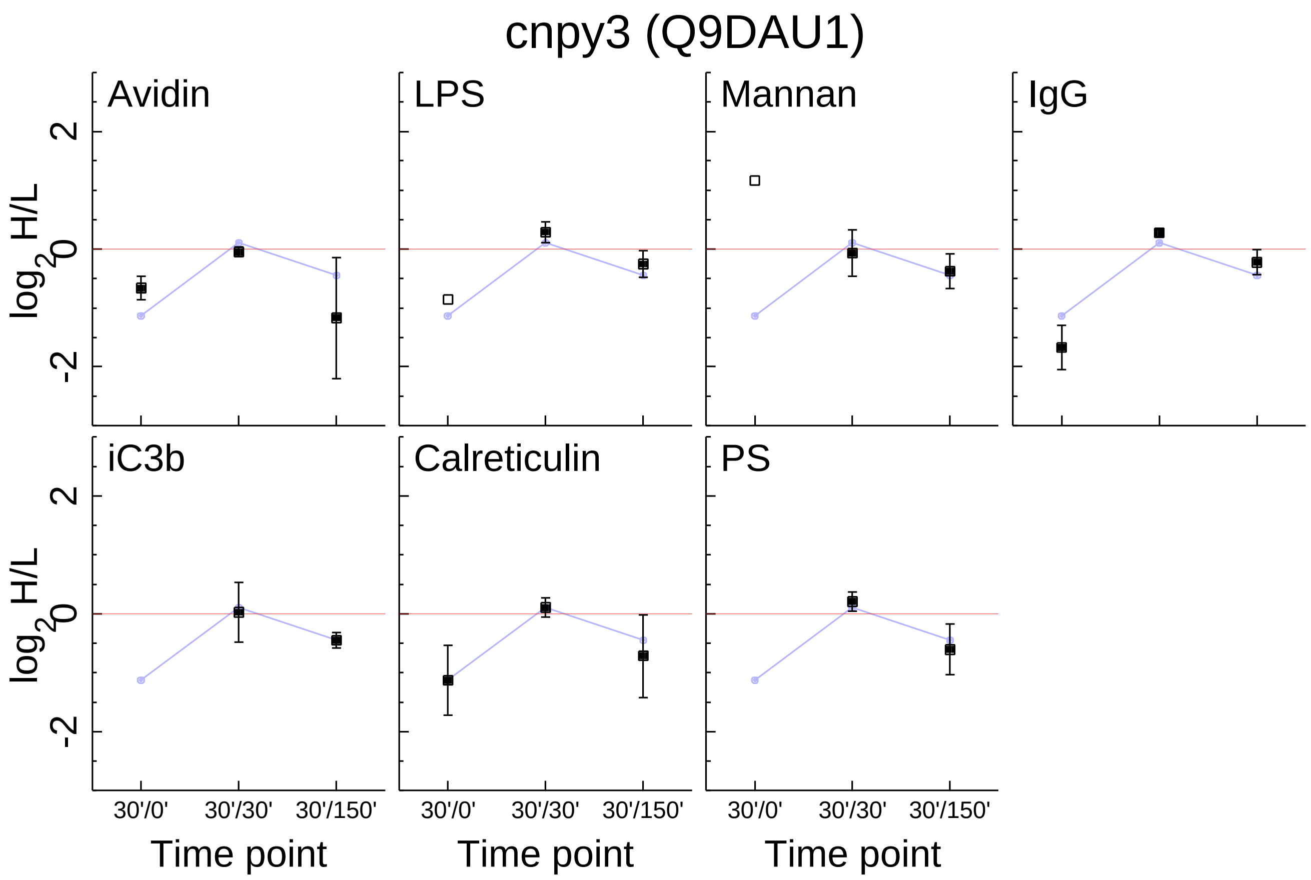
cnpy3 (Q9DAU1)
Avidin
LPS
Mannan
IgG
iC3b
Calreticulin
PS
log2 H/L
2
0
-2
30'/0'
30'/30'
30'/150'
Time point

cnpy4 (Q8BQ47)

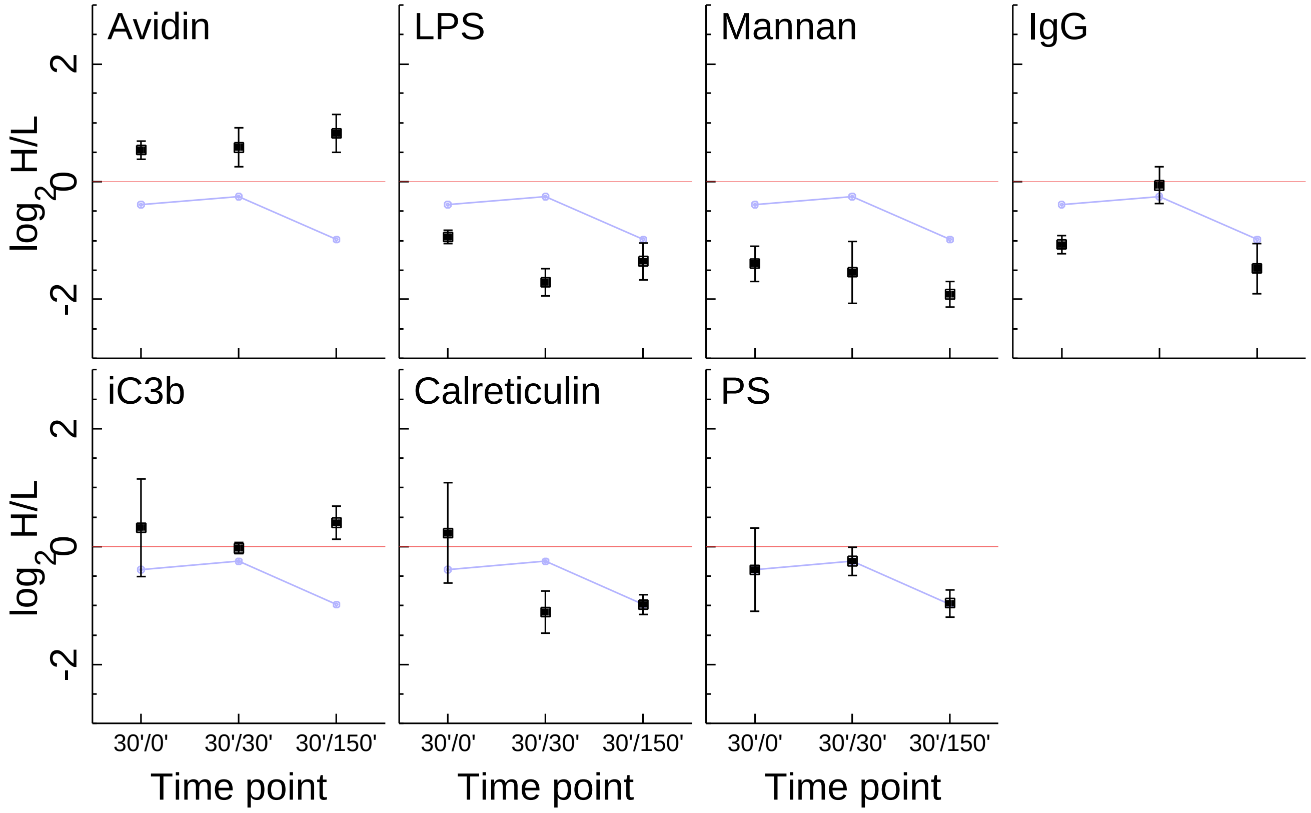

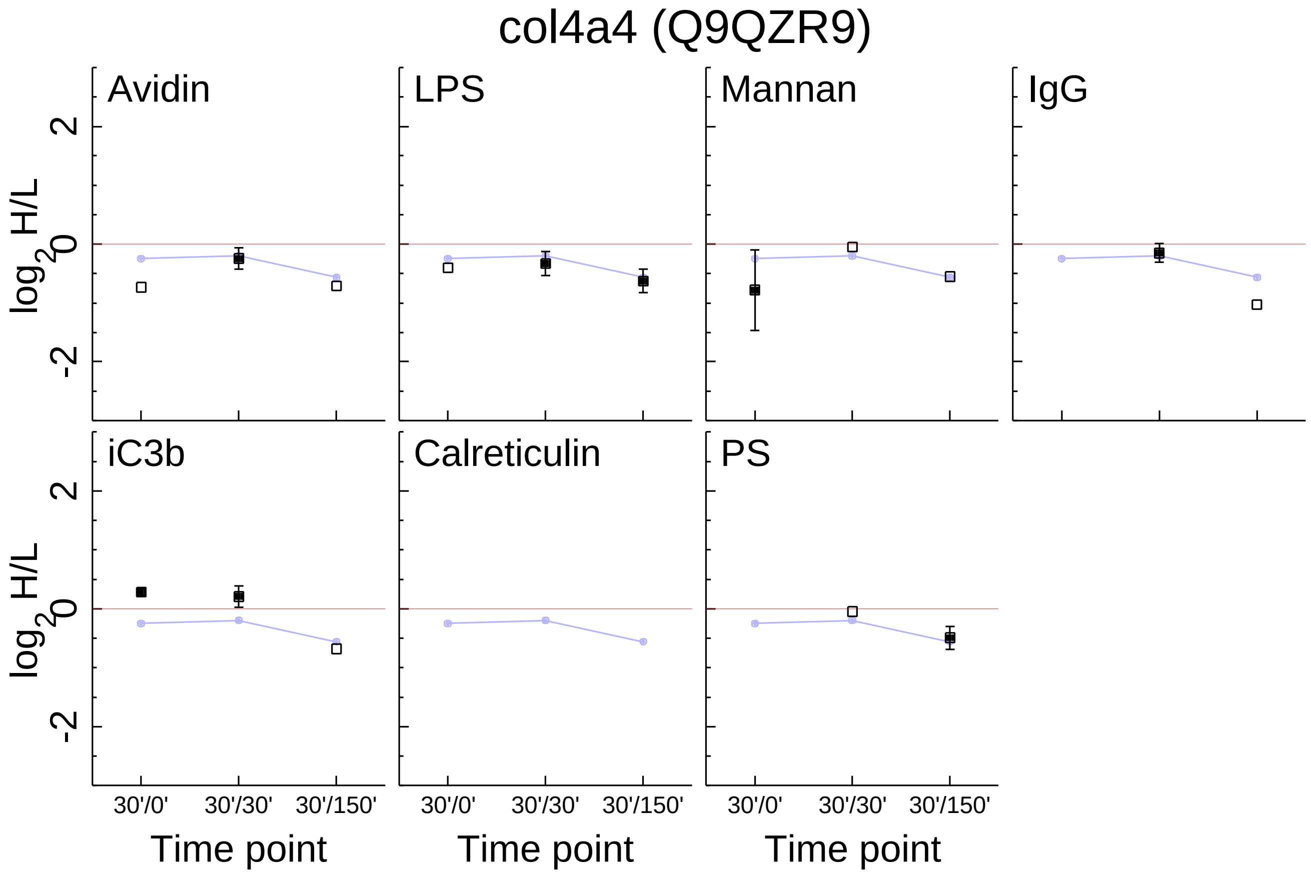

col4a4 (Q9QZR9)
Avidin
LPS
Mannan
IgG
iC3b
Calreticulin
PS
log2 H/L
2
0
-2
30'/0'
30'/30'
30'/150'
Time point

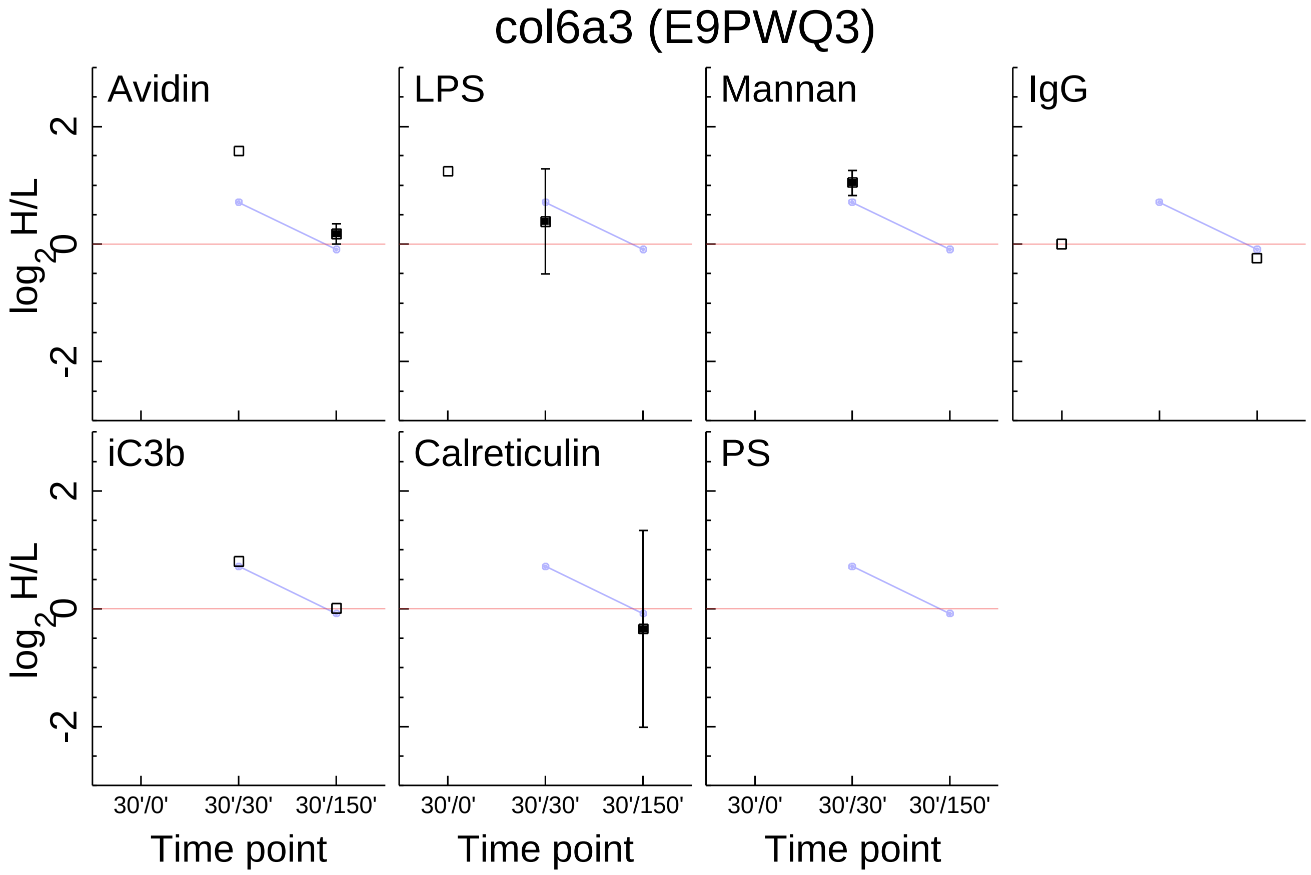
col6a3 (E9PWQ3)
Avidin
LPS
Mannan
IgG
iC3b
Calreticulin
PS
log2 H/L
2
0
-2
30'/0'
30'/30'
30'/150'
Time point

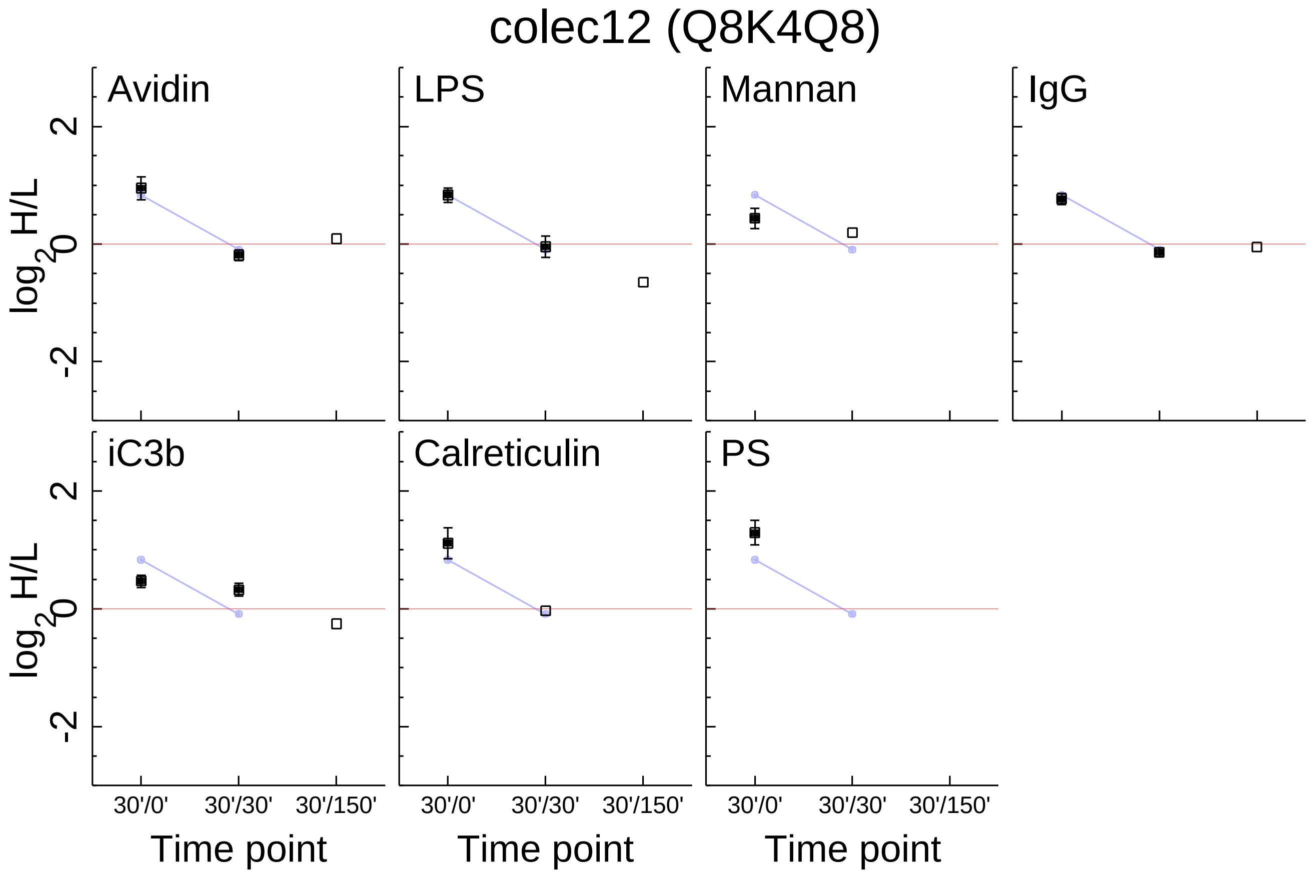

colec12 (Q8K4Q8)
Avidin
LPS
Mannan
IgG
iC3b
Calreticulin
PS
log2 H/L
2
0
-2
30'/0'
30'/30'
30'/150'
Time point

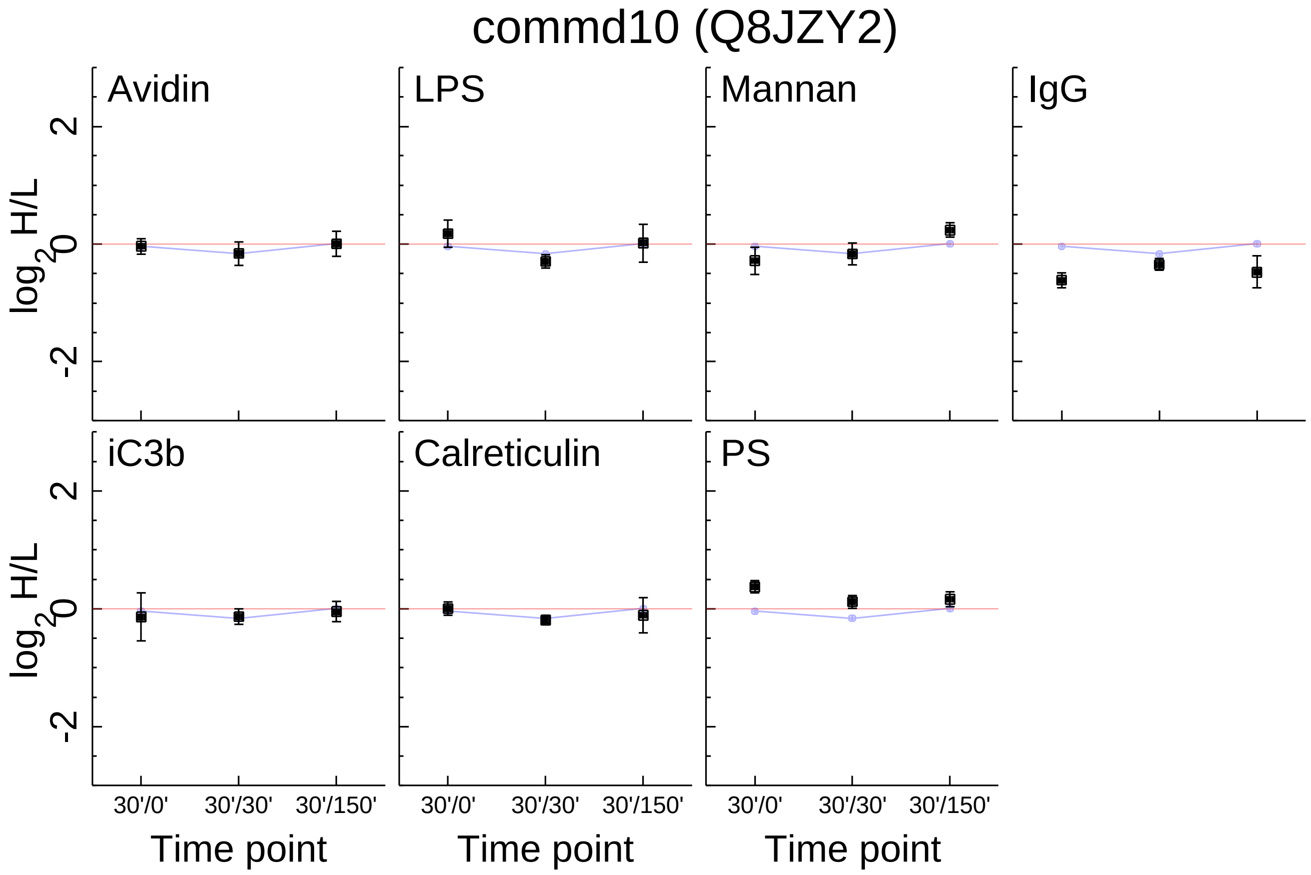
commd10 (Q8JZY2)
Avidin
LPS
Mannan
IgG
iC3b
Calreticulin
PS
log2 H/L
2
0
-2
30'/0'
30'/30'
30'/150'
Time point

commd1 (Q8VI86)

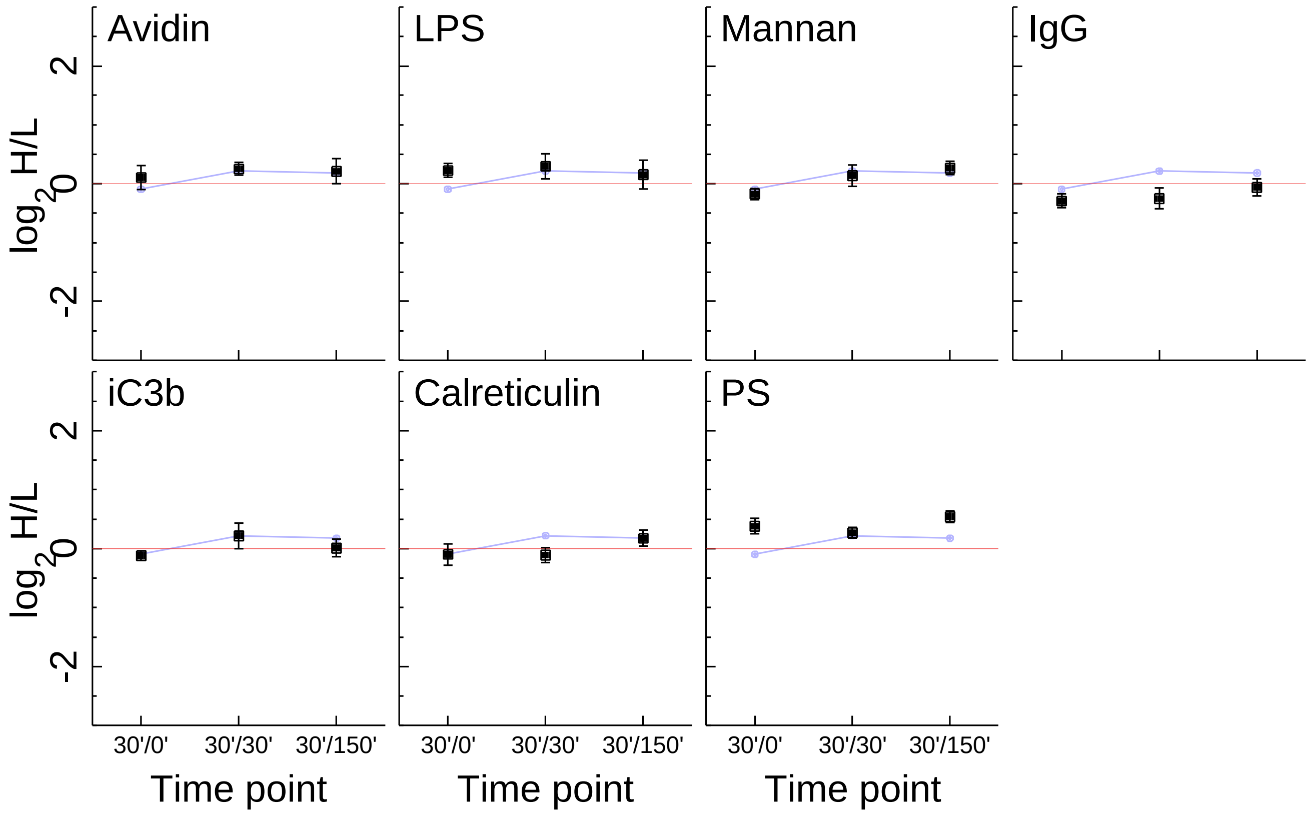

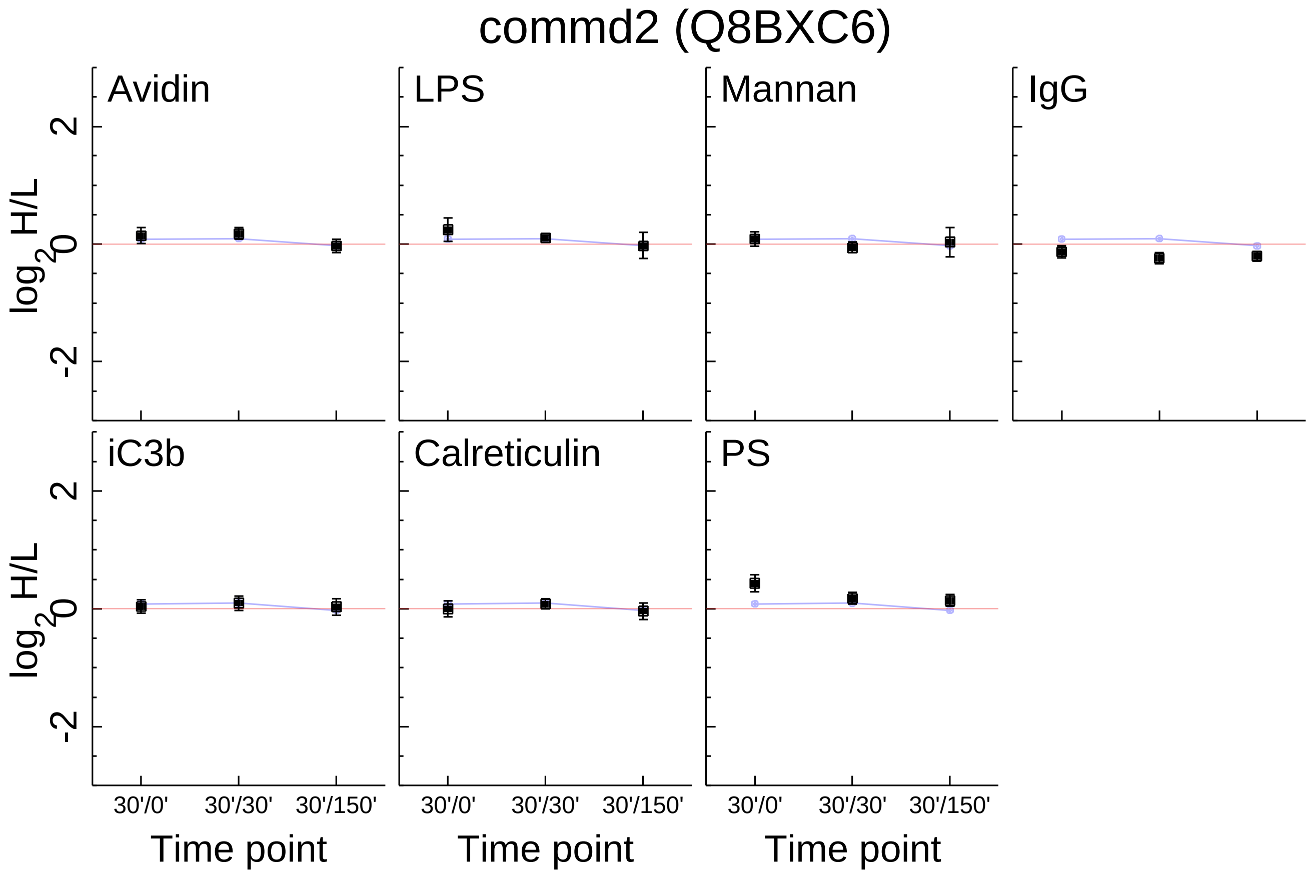
commd2 (Q8BXC6)
Avidin
LPS
Mannan
IgG
iC3b
Calreticulin
PS
log2 H/L
2
0
-2
30'/0'
30'/30'
30'/150'
Time point

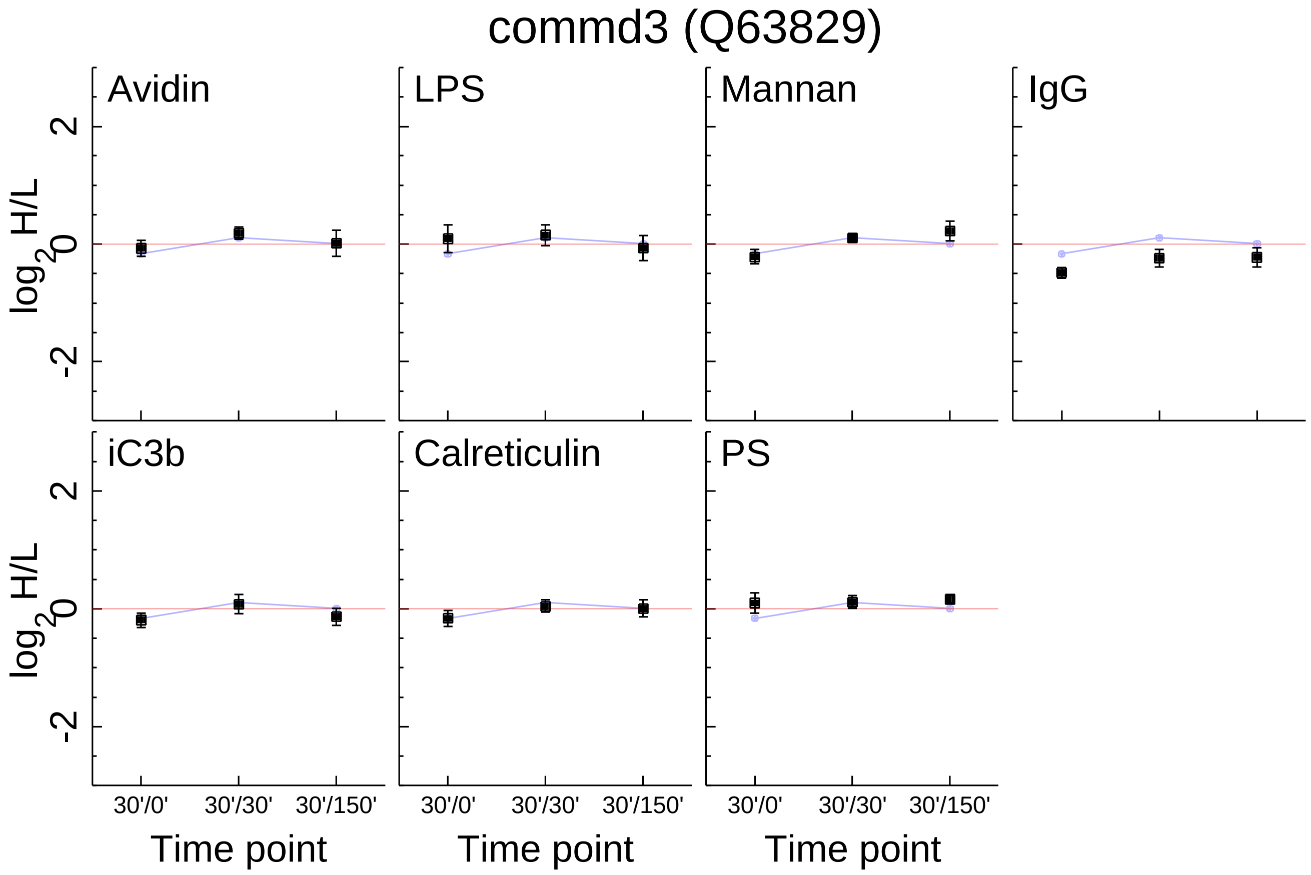
commd3 (Q63829)
Avidin
LPS
Mannan
IgG
iC3b
Calreticulin
PS
log2 H/L
2
0
-2
30'/0'
30'/30'
30'/150'
Time point

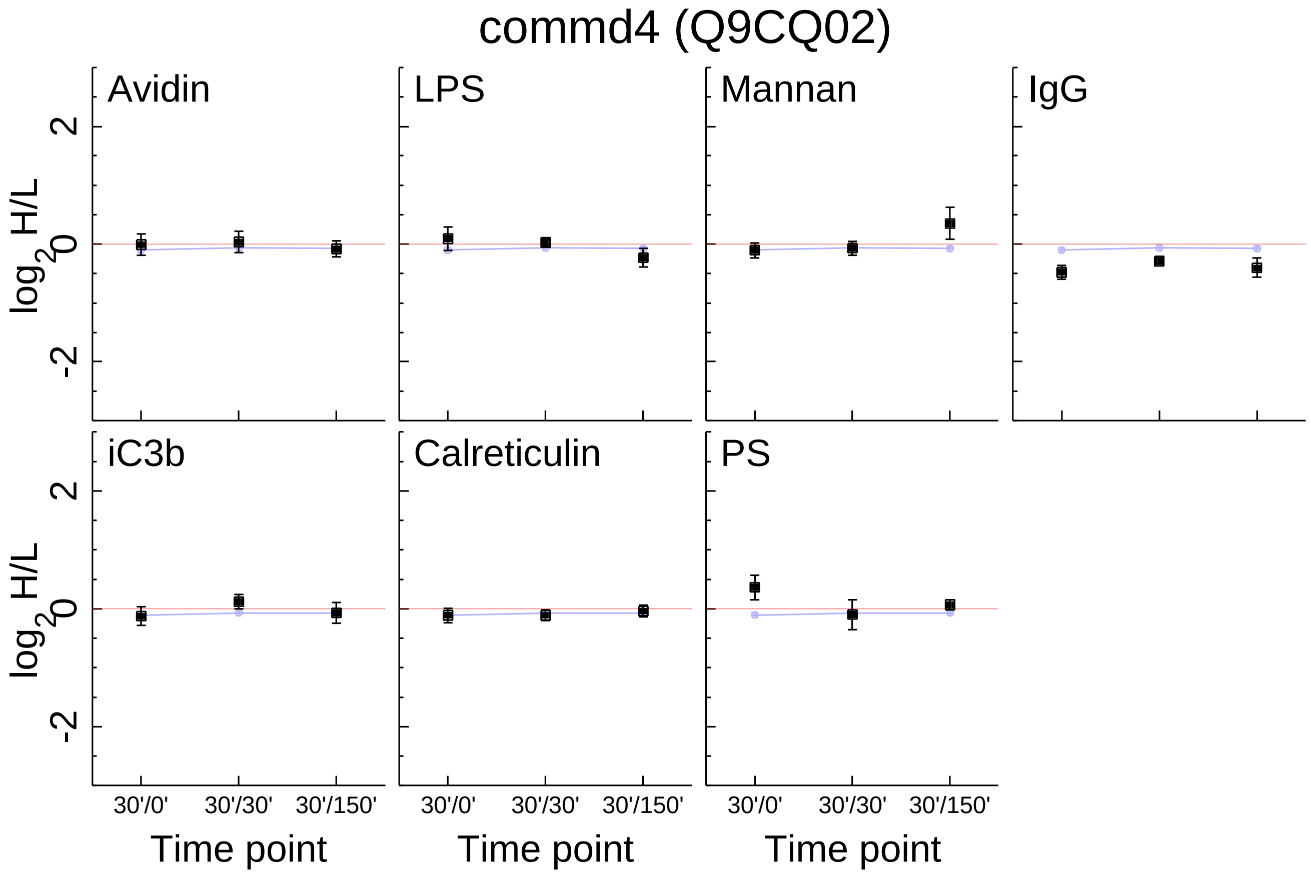
commd4 (Q9CQ02)
Avidin
LPS
Mannan
IgG
iC3b
Calreticulin
PS
$\log_2$ H/L
2
0
-2
30'/0'
30'/30'
30'/150'
Time point

commd5 (Q8R395)

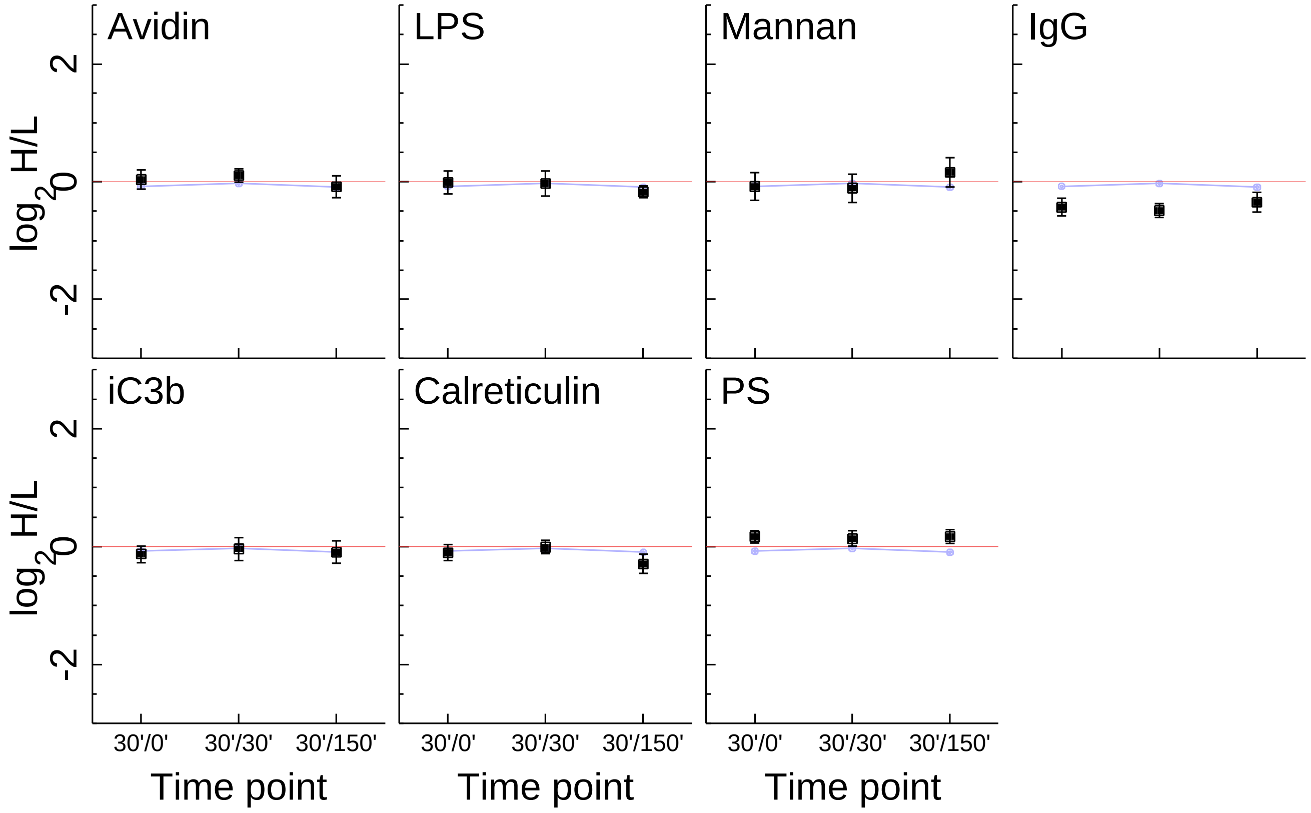

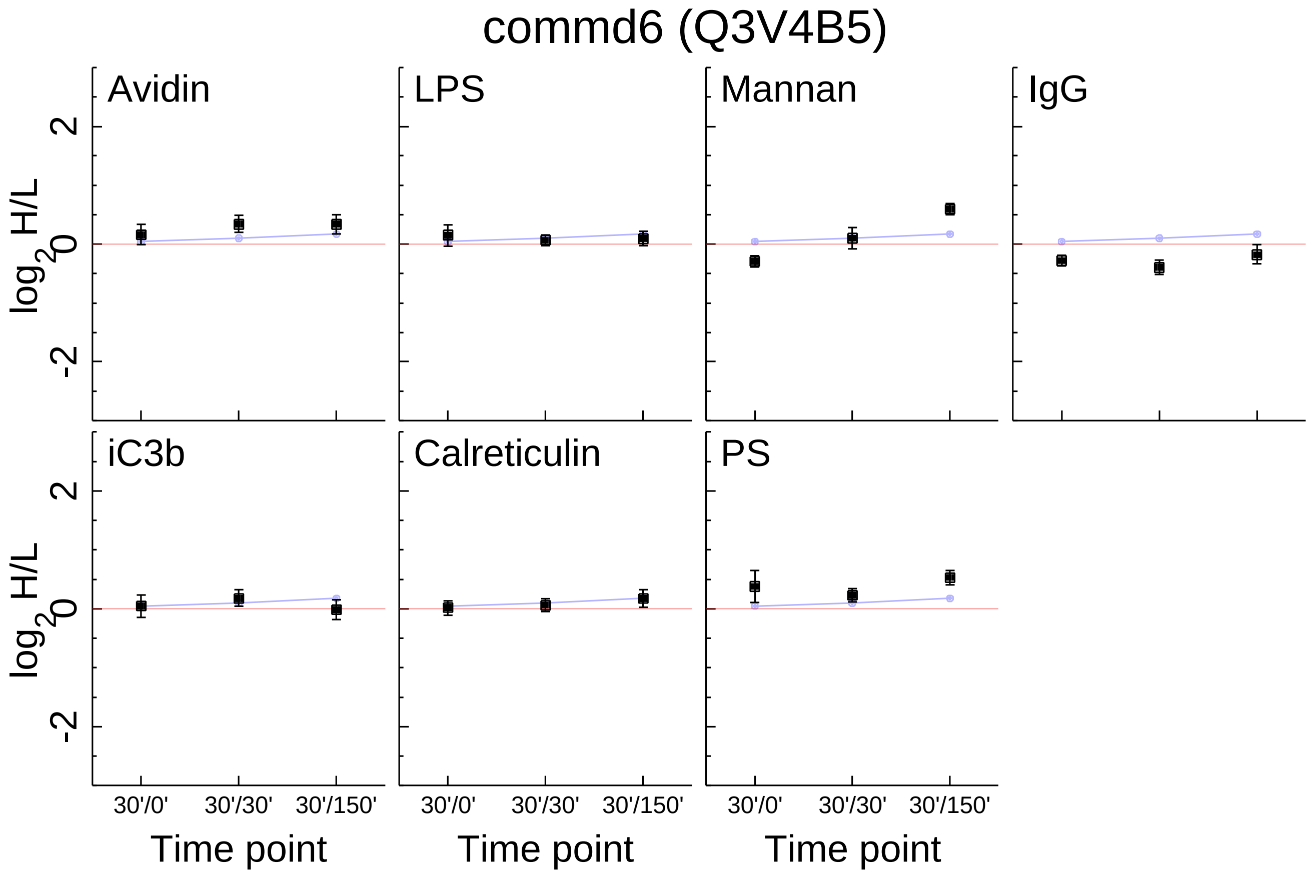
commd6 (Q3V4B5)
Avidin
LPS
Mannan
IgG
iC3b
Calreticulin
PS
log_2 H/L
2
0
-2
30'/0'
30'/30'
30'/150'
Time point

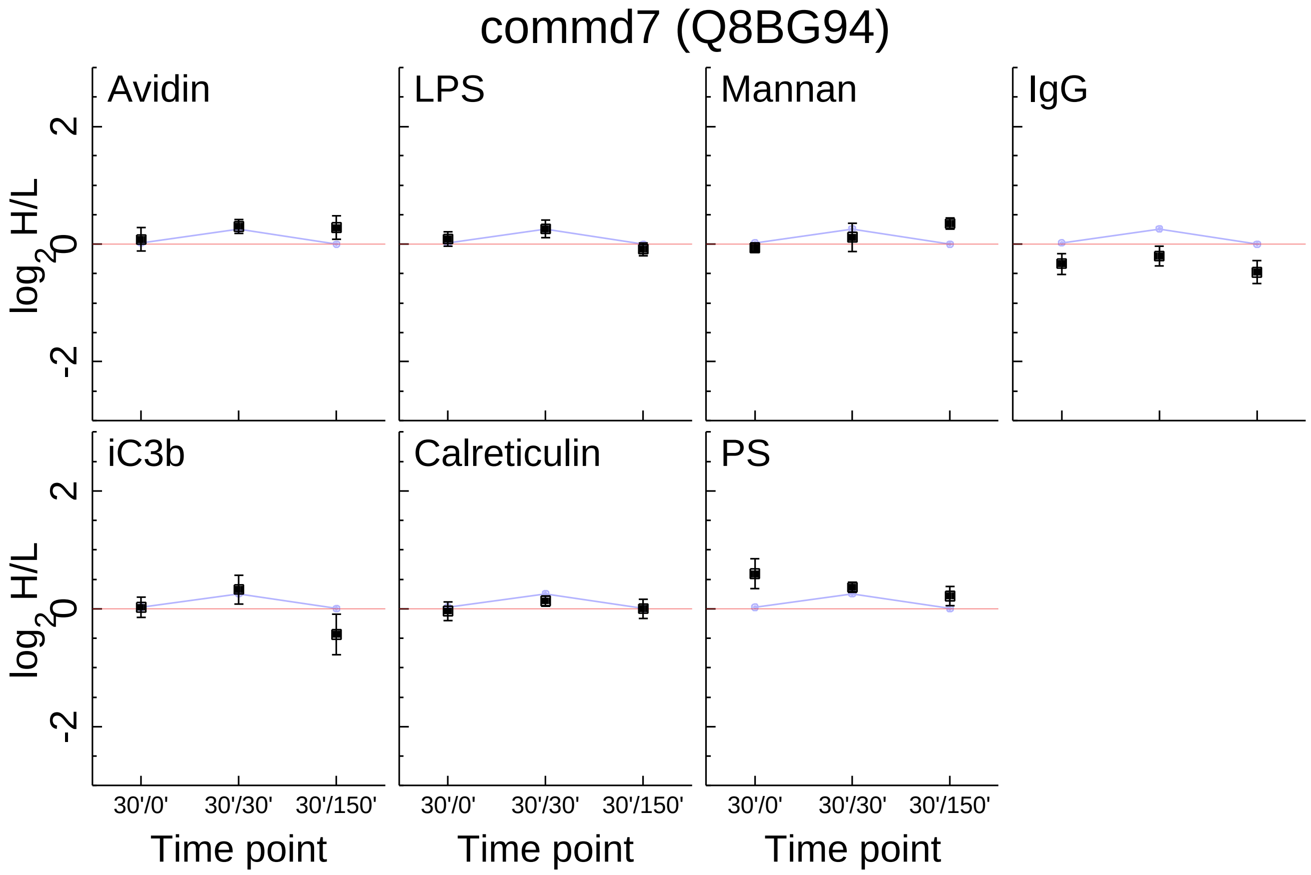
commd7 (Q8BG94)
Avidin
LPS
Mannan
IgG
iC3b
Calreticulin
PS
log2 H/L
2
0
-2
30'/0'
30'/30'
30'/150'
Time point

commd8 (Q9CZG3)

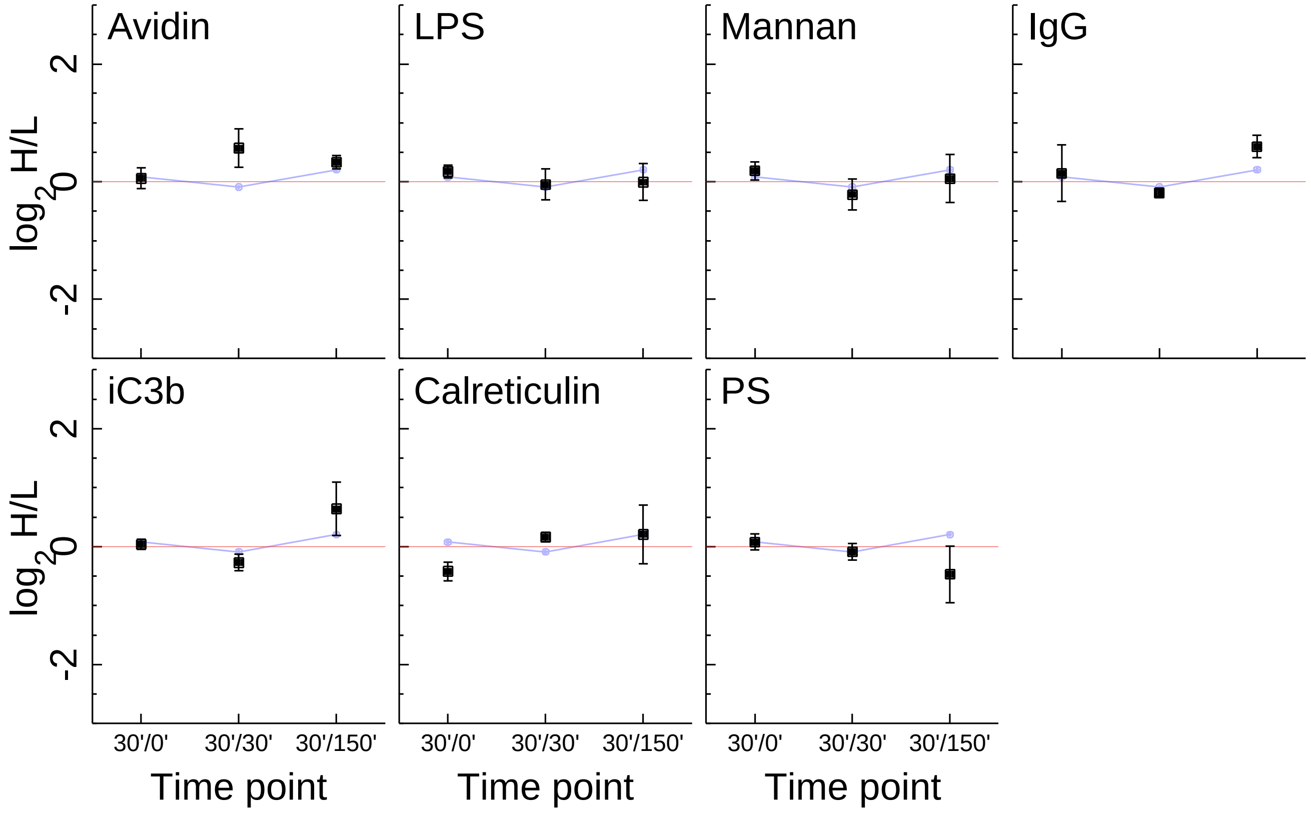

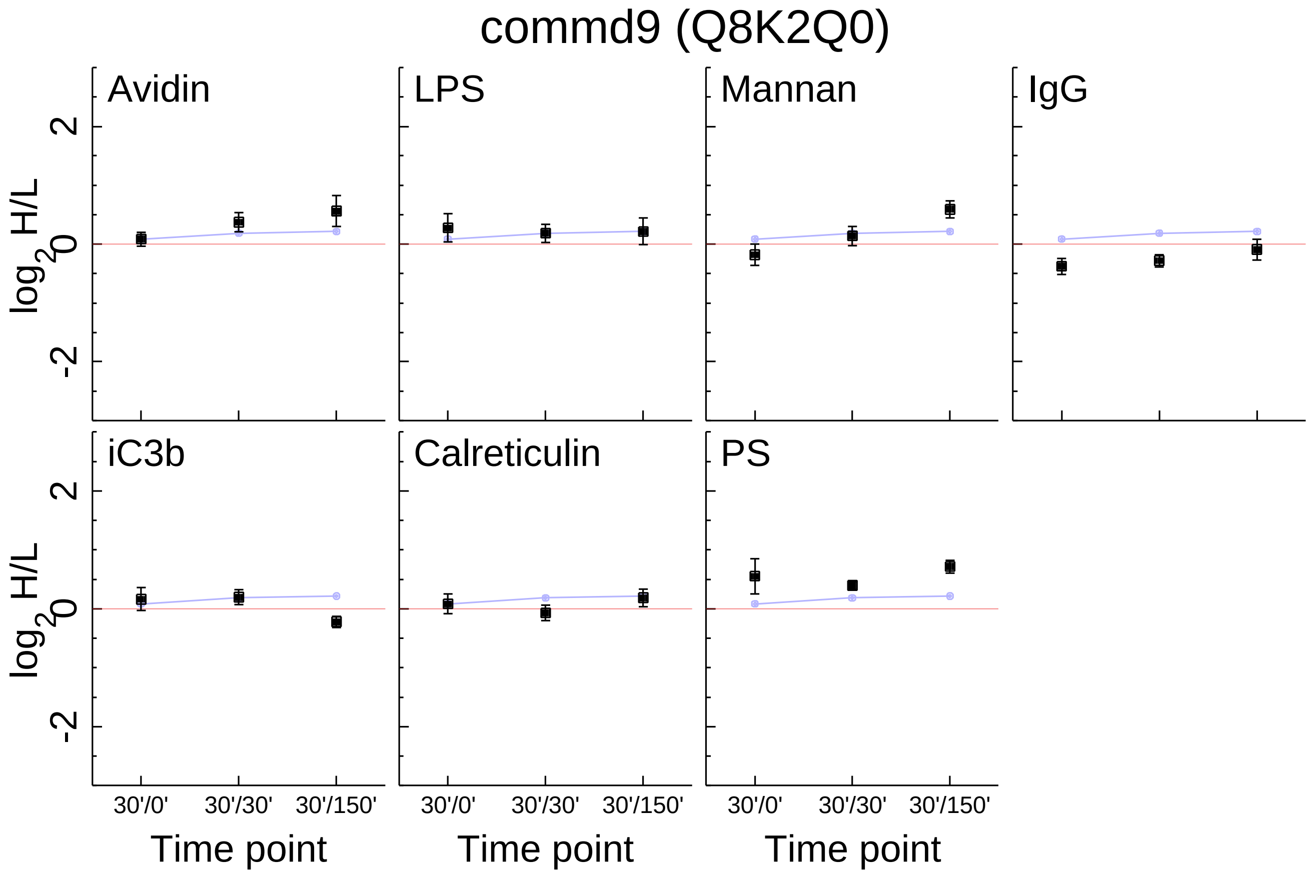

commd9 (Q8K2Q0)
Avidin
LPS
Mannan
IgG
iC3b
Calreticulin
PS
log2 H/L
2
0
-2
30'/0'
30'/30'
30'/150'
Time point

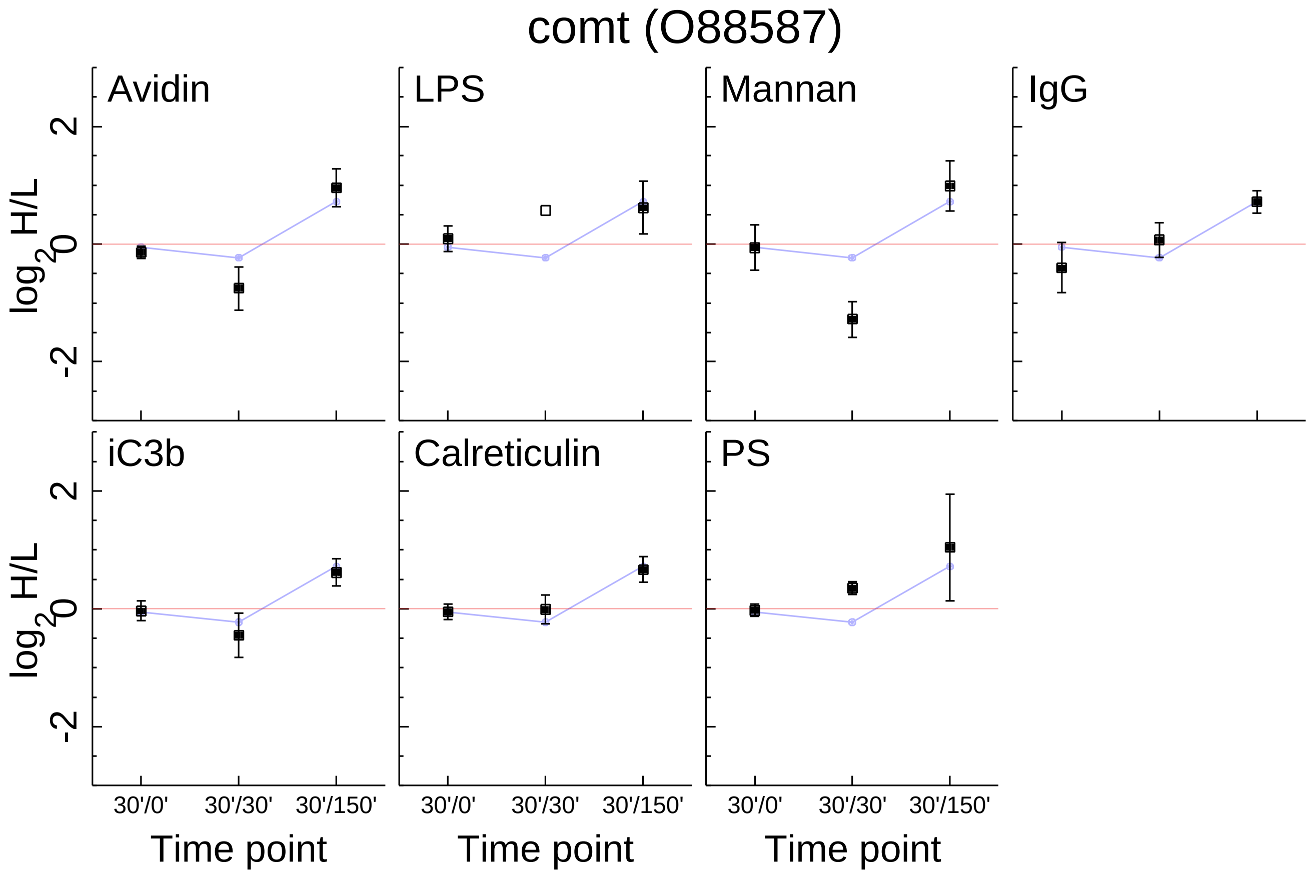

comt (O88587)
Avidin
LPS
Mannan
IgG
iC3b
Calreticulin
PS
log2 H/L
2
0
-2
30'/0'
30'/30'
30'/150'
Time point

copb2 (O55029)

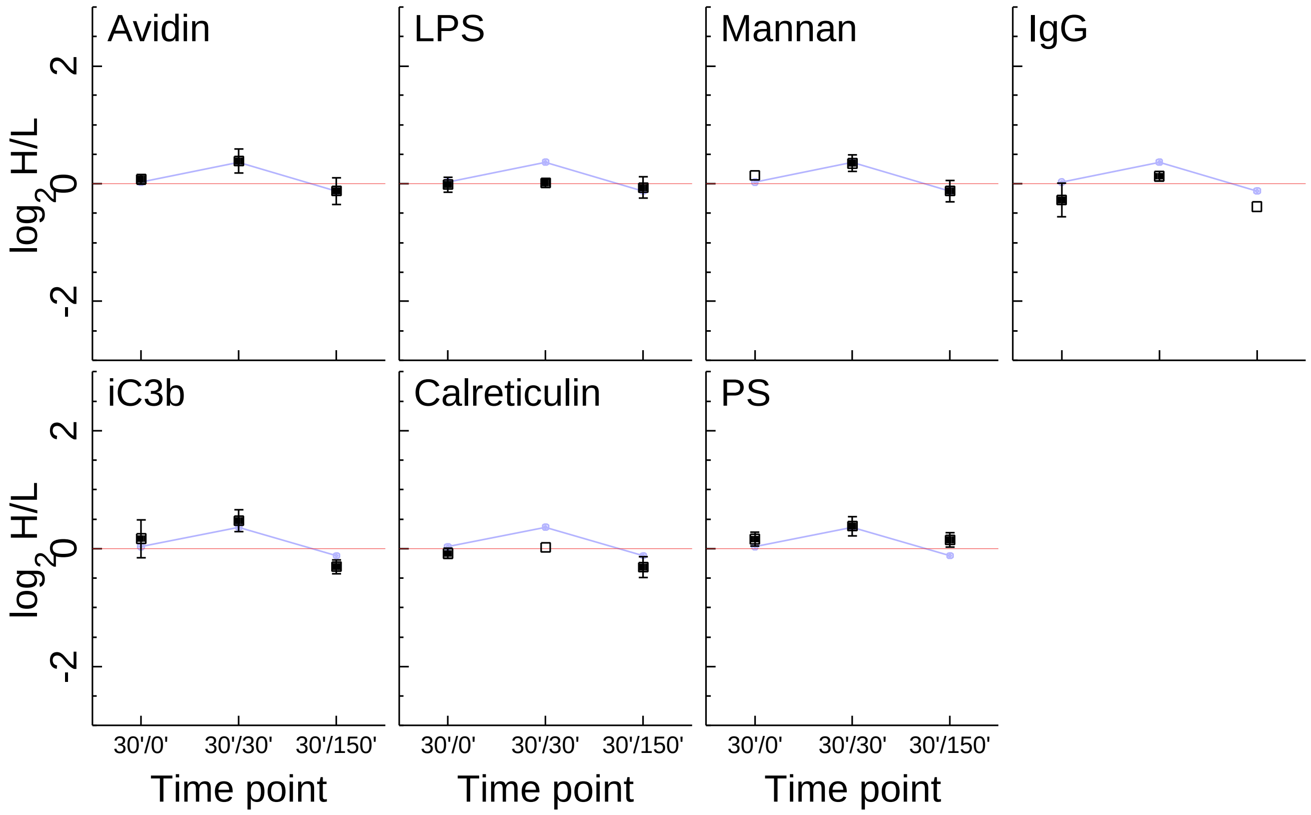

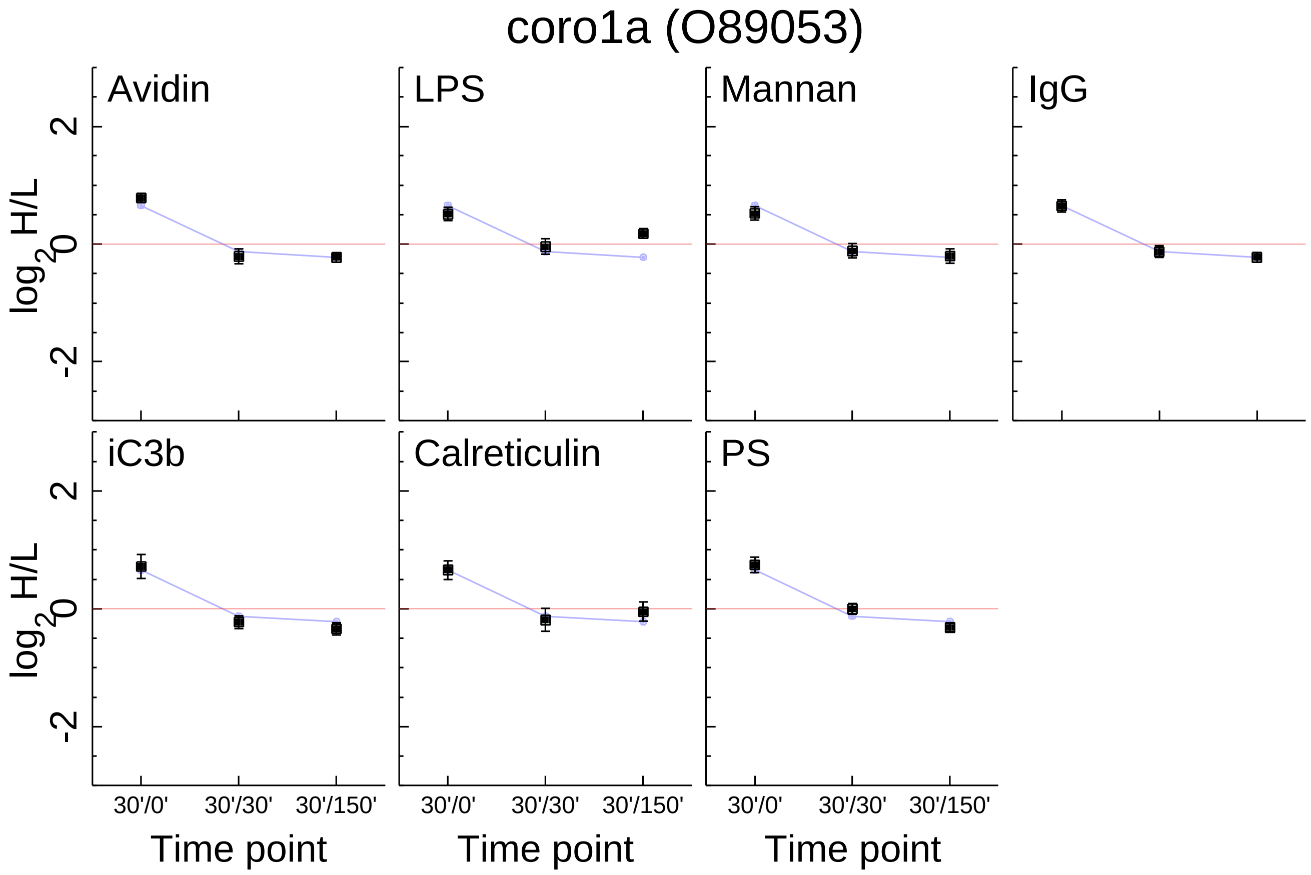

coro1a (O89053)
Avidin
LPS
Mannan
IgG
iC3b
Calreticulin
PS
log2 H/L
2
0
-2
30'/0'
30'/30'
30'/150'
Time point

coro1c (Q9WUM4)

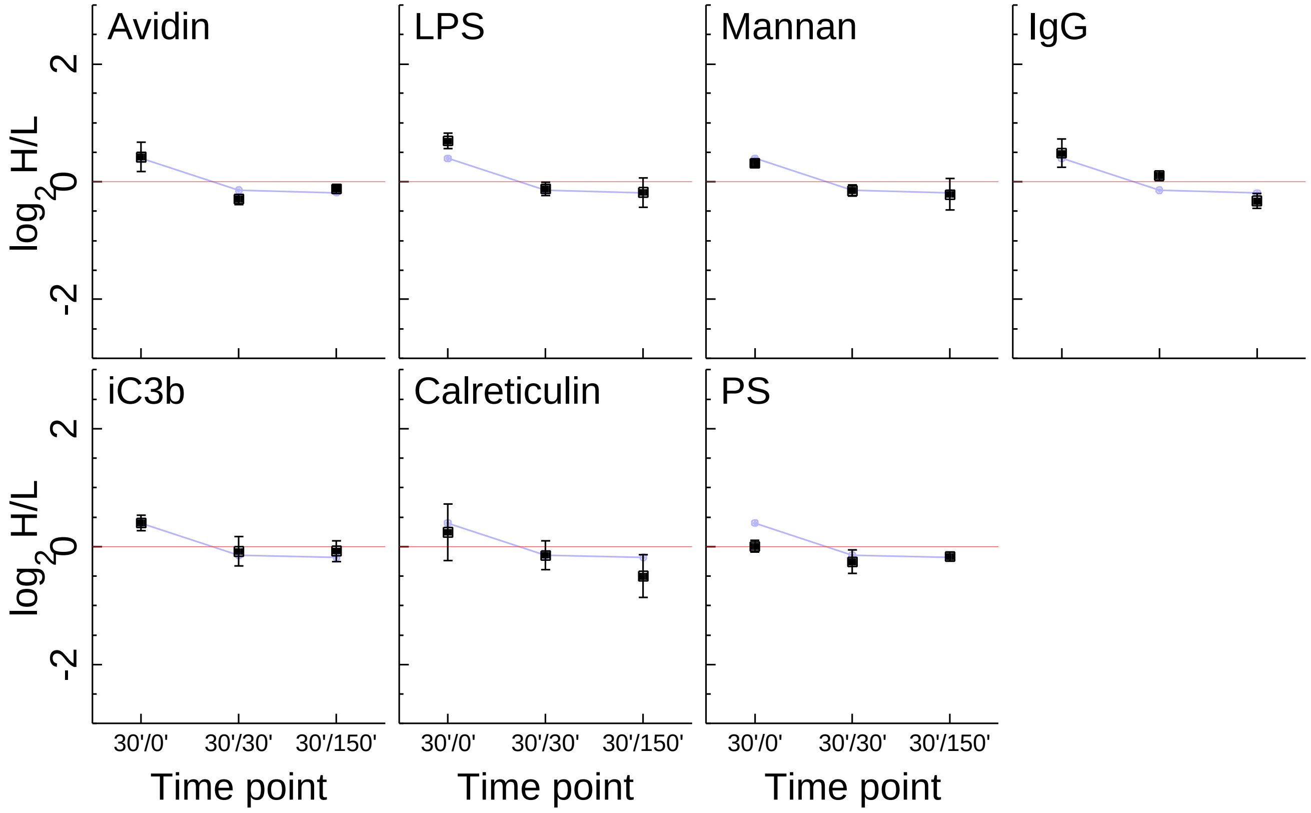

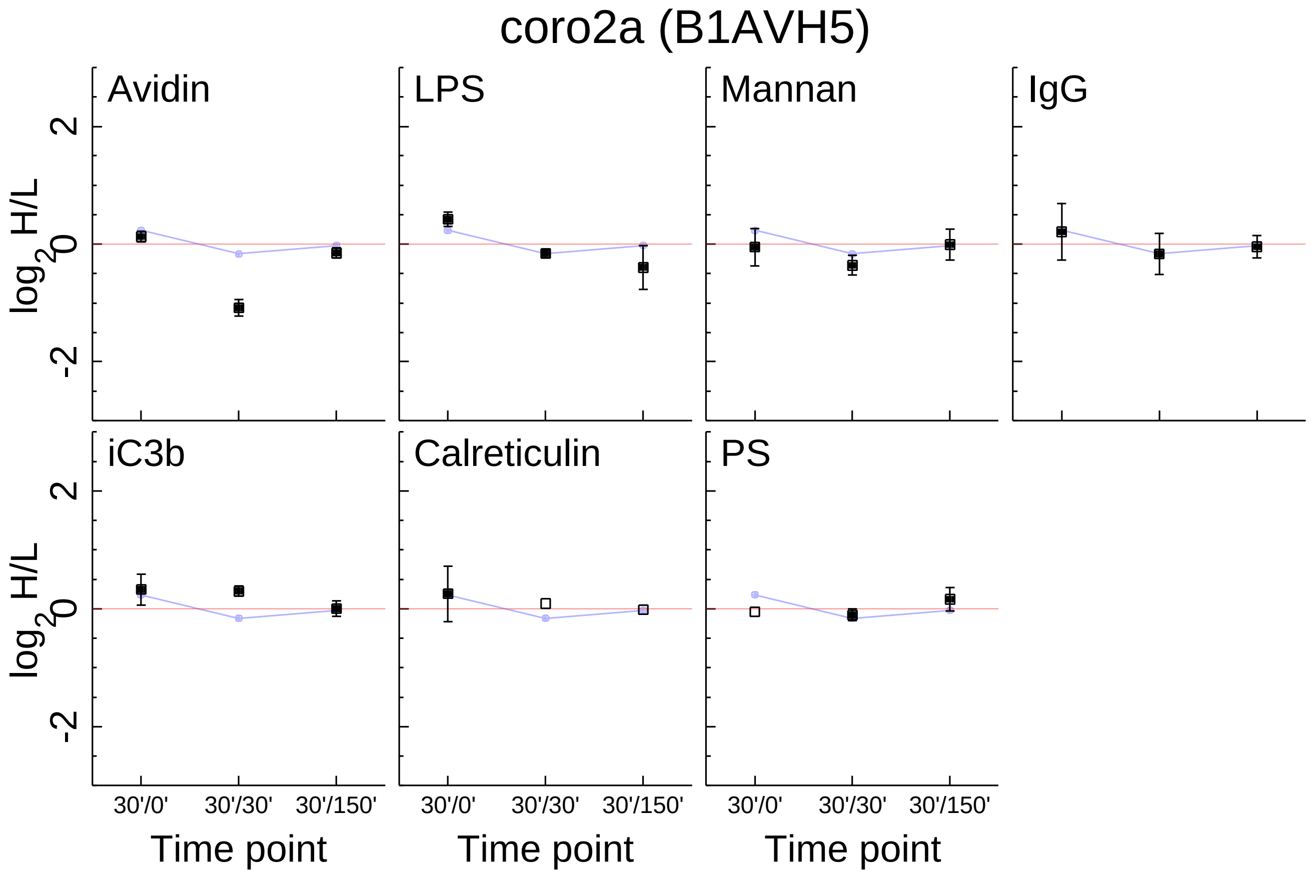

coro2a (B1AVH5)
Avidin
LPS
Mannan
IgG
iC3b
Calreticulin
PS
log2 H/L
2
0
-2
30'/0'
30'/30'
30'/150'
Time point

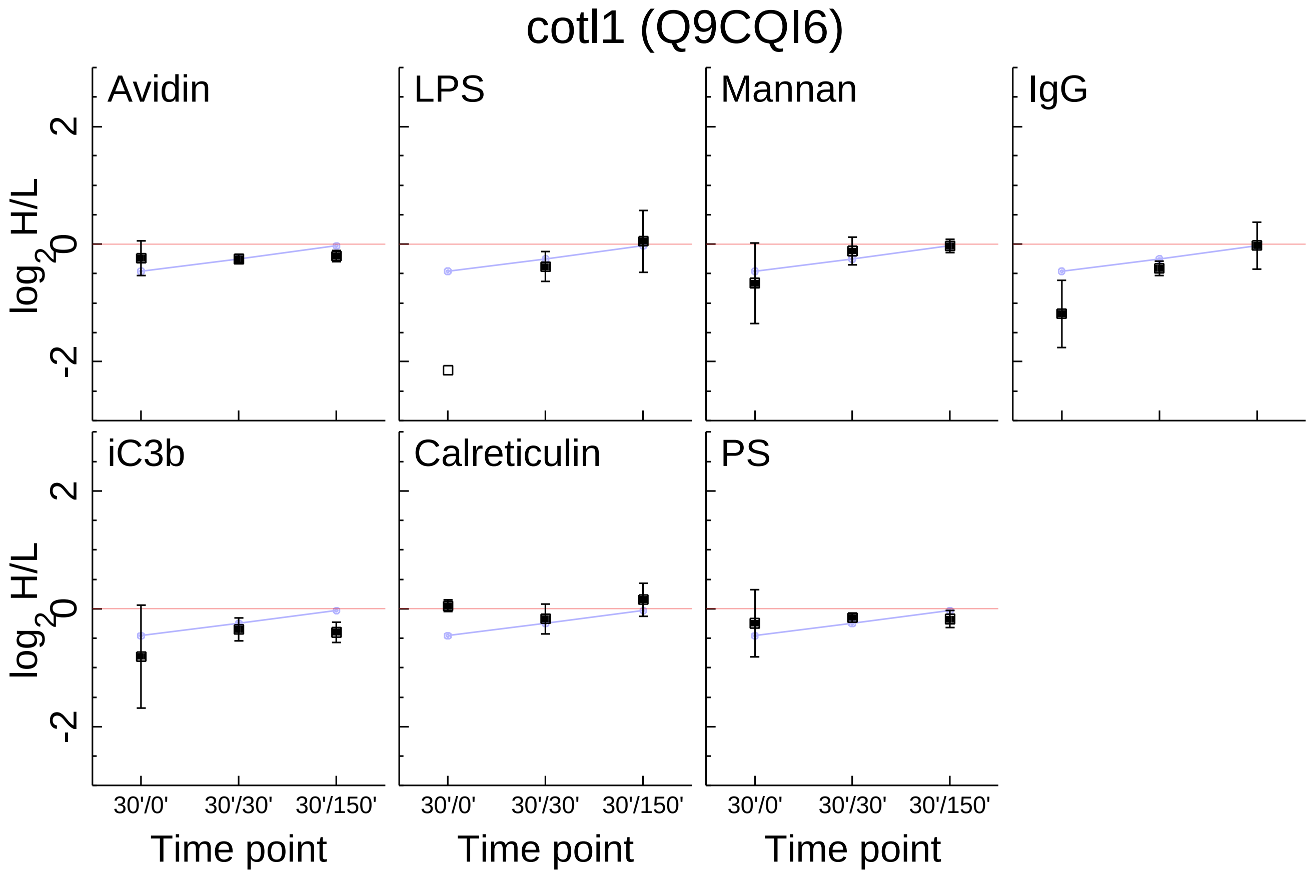
cotl1 (Q9CQI6)
Avidin
LPS
Mannan
IgG
iC3b
Calreticulin
PS
log2 H/L
2
0
-2
30'/0'
30'/30'
30'/150'
Time point

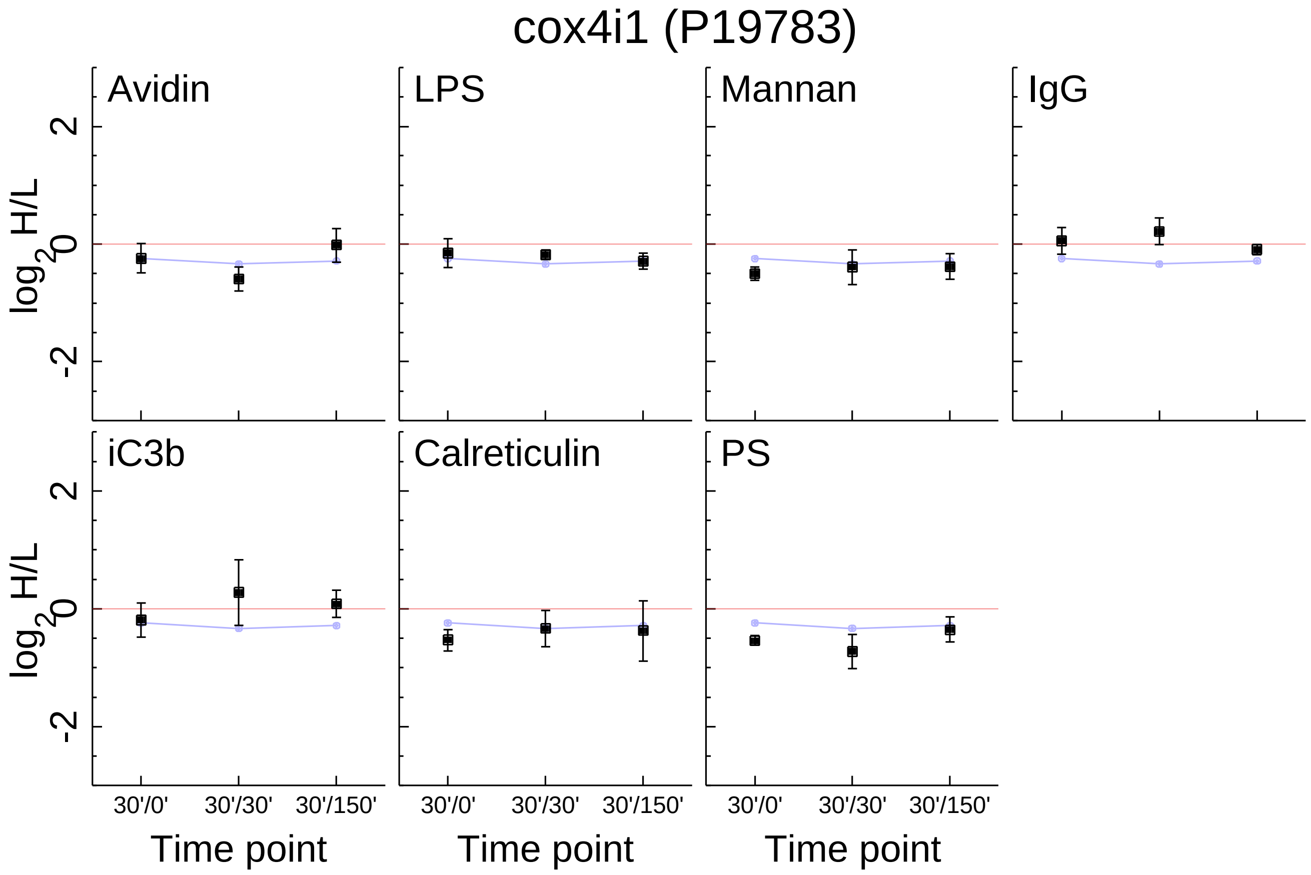
cox4i1 (P19783)
Avidin
LPS
Mannan
IgG
iC3b
Calreticulin
PS
log2 H/L
2
0
-2
30'/0'
30'/30'
30'/150'
Time point

cox4nb (O70378)

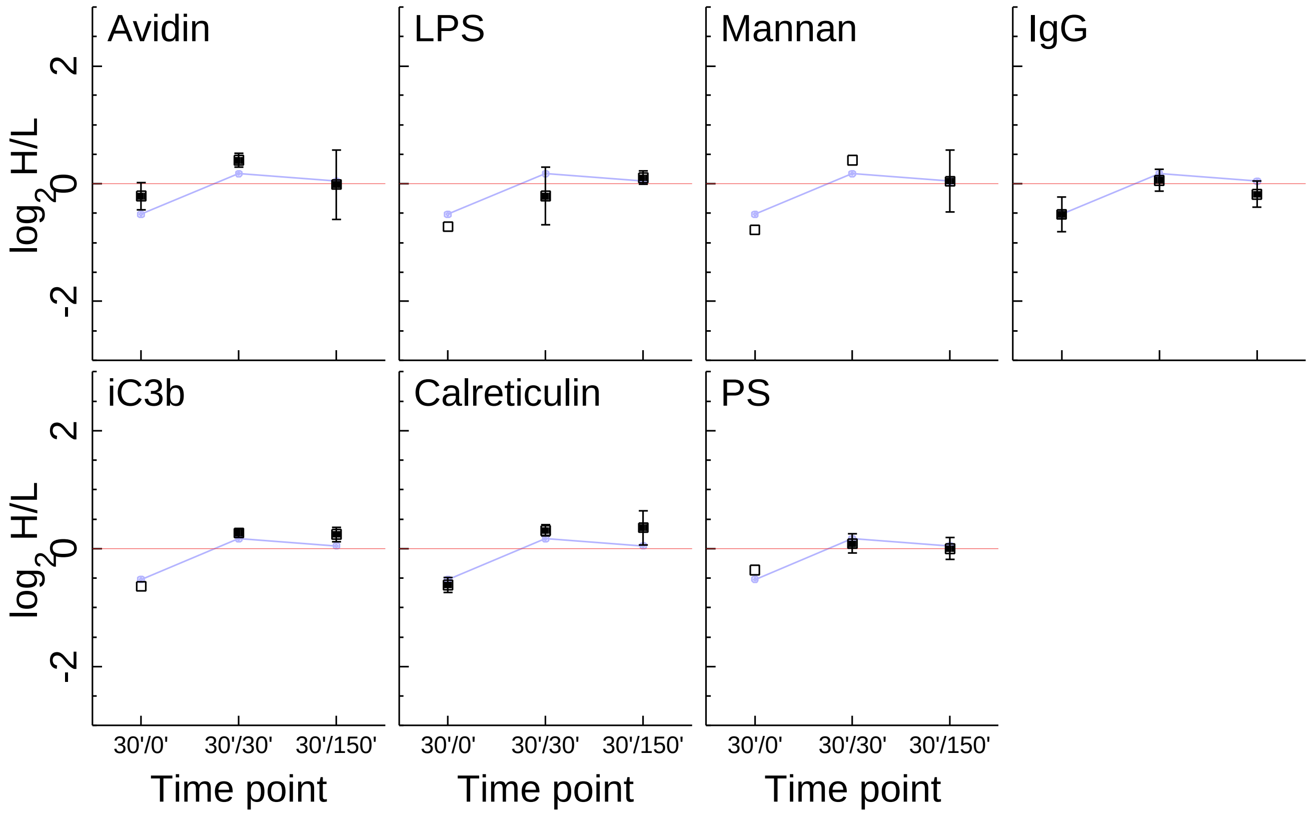

cox5a (P12787)

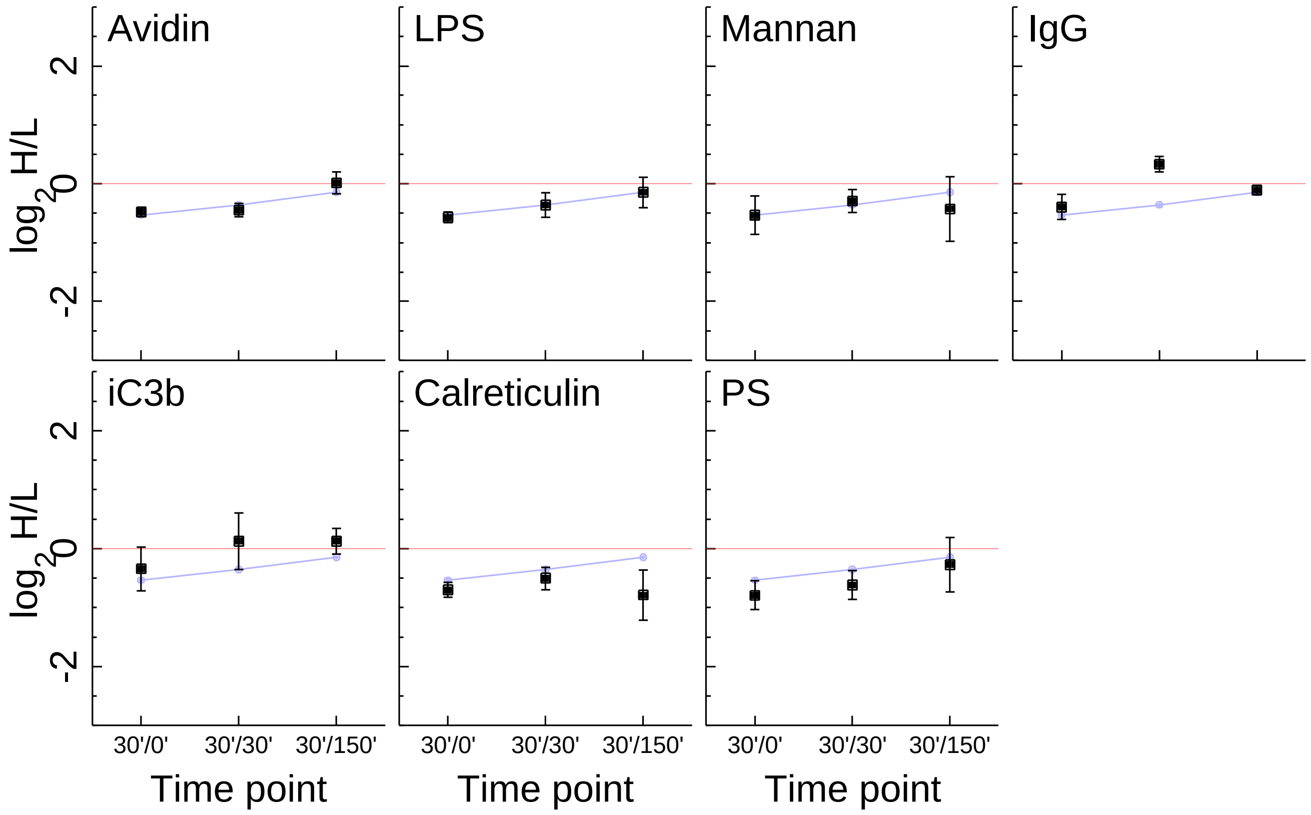

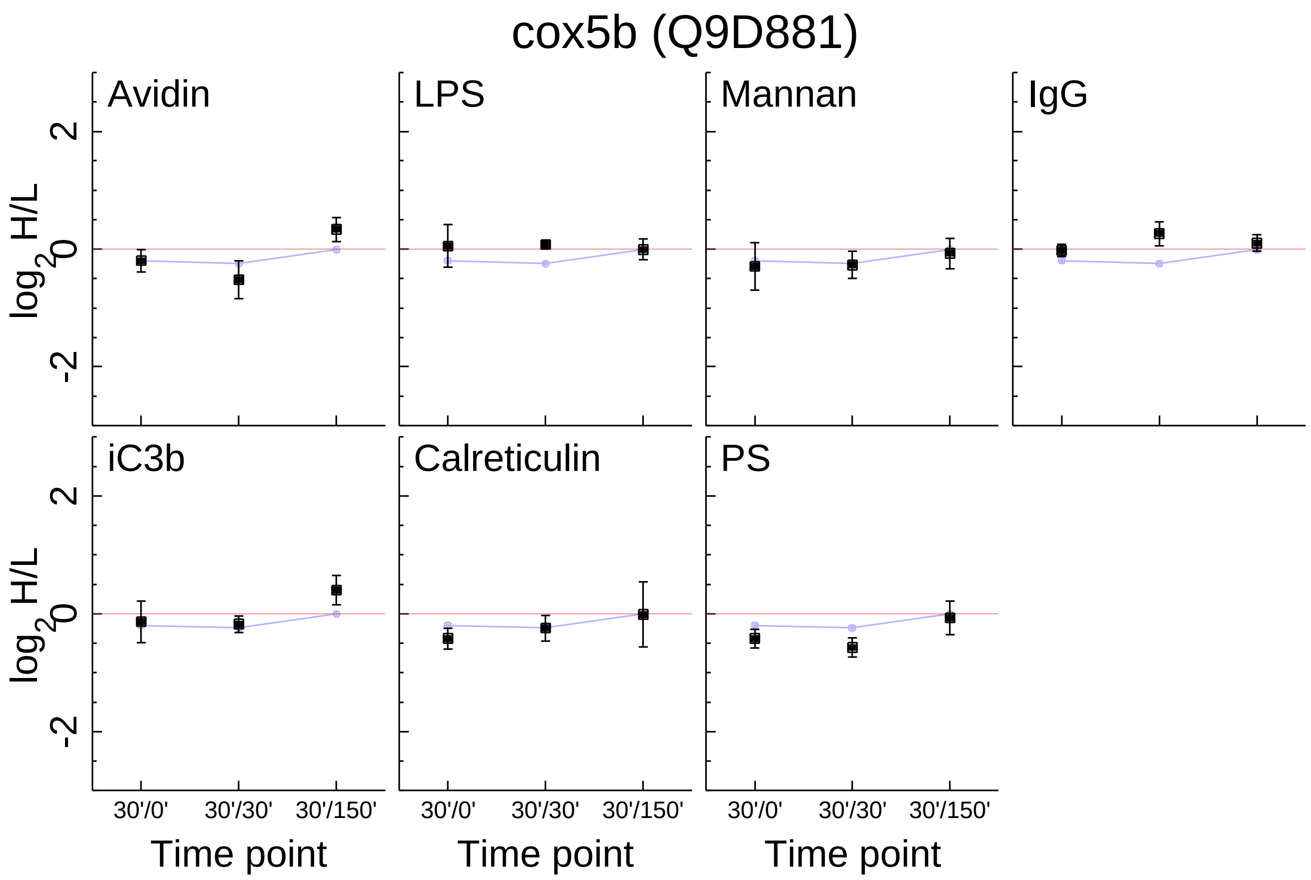
cox5b (Q9D881)
Avidin
LPS
Mannan
IgG
iC3b
Calreticulin
PS
log2 H/L
2
0
-2
30'/0'
30'/30'
30'/150'
Time point

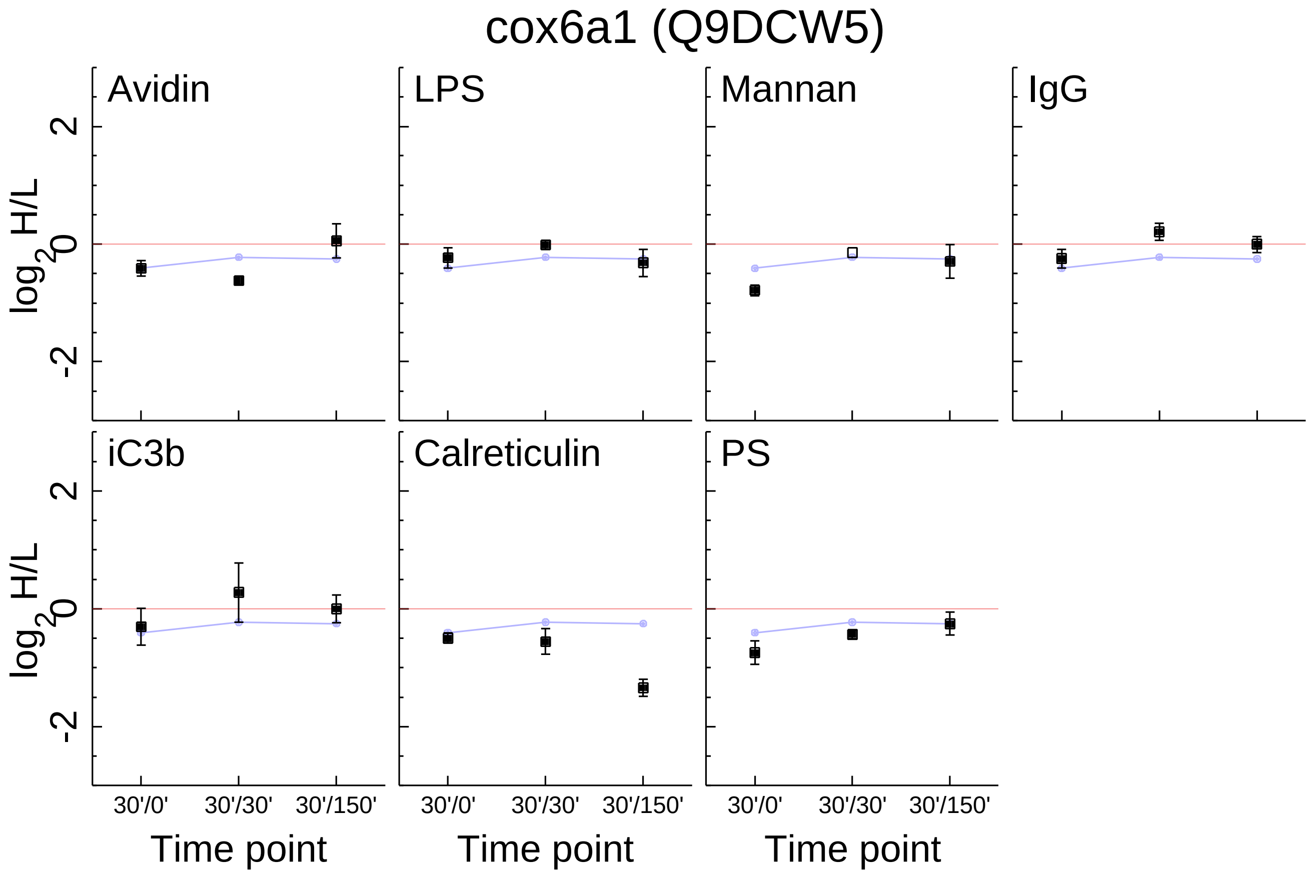
cox6a1 (Q9DCW5)
Avidin
LPS
Mannan
IgG
iC3b
Calreticulin
PS
log2 H/L
2
0
-2
30'/0'
30'/30'
30'/150'
Time point

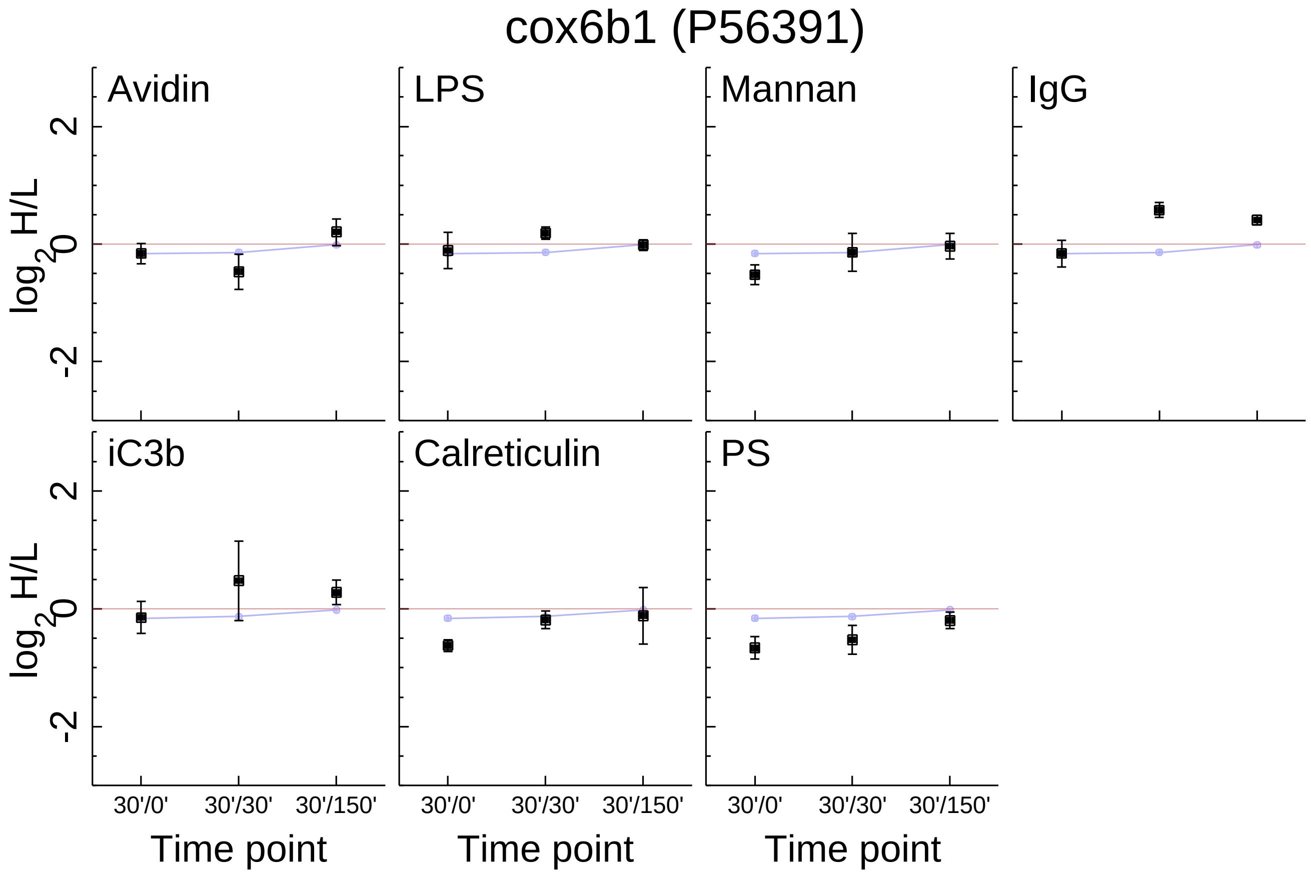

cox6b1 (P56391)
Avidin
LPS
Mannan
IgG
iC3b
Calreticulin
PS
log2 H/L
2
0
-2
30'/0'
30'/30'
30'/150'
Time point

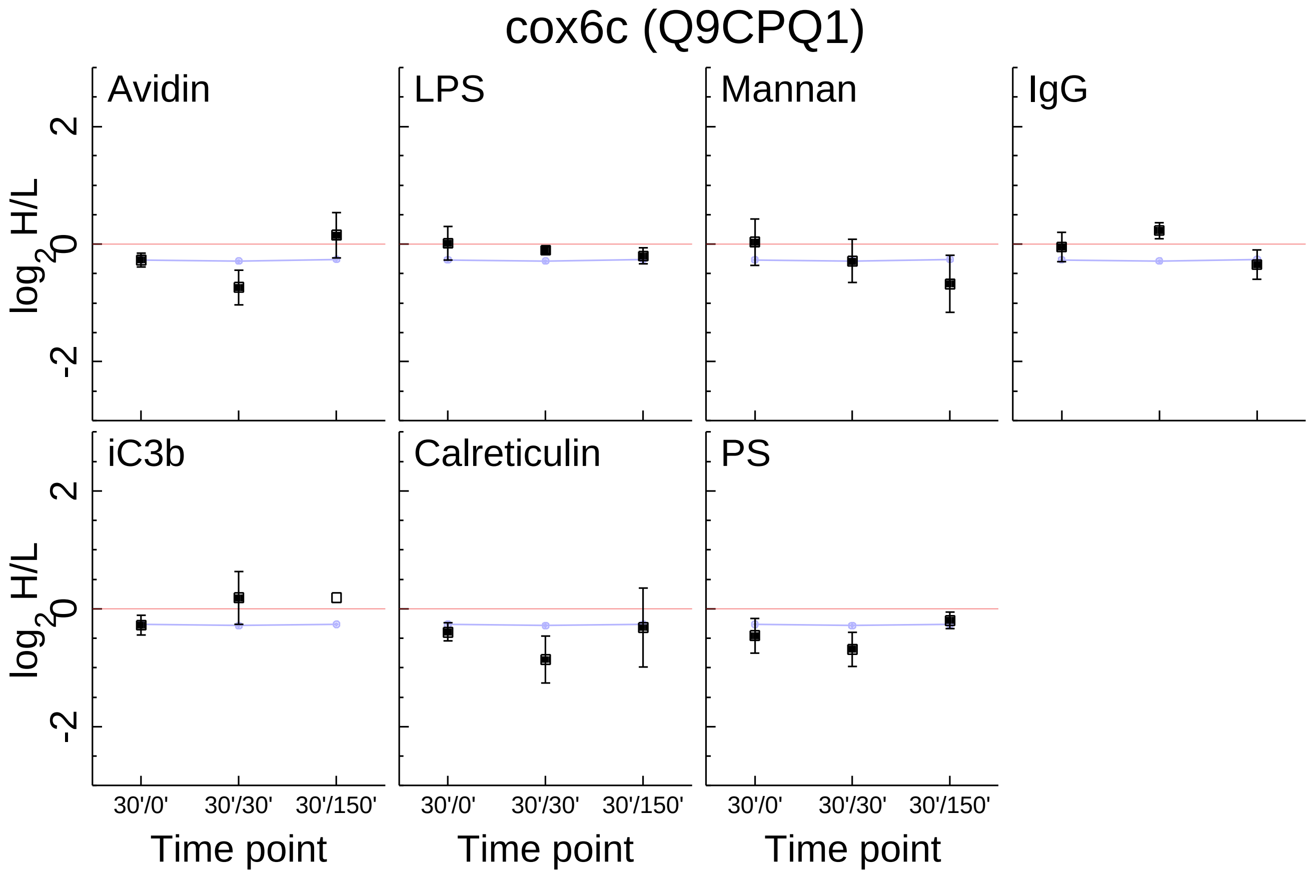
cox6c (Q9CPQ1)
Avidin
LPS
Mannan
IgG
iC3b
Calreticulin
PS
log2 H/L
2
0
-2
30'/0'
30'/30'
30'/150'
Time point

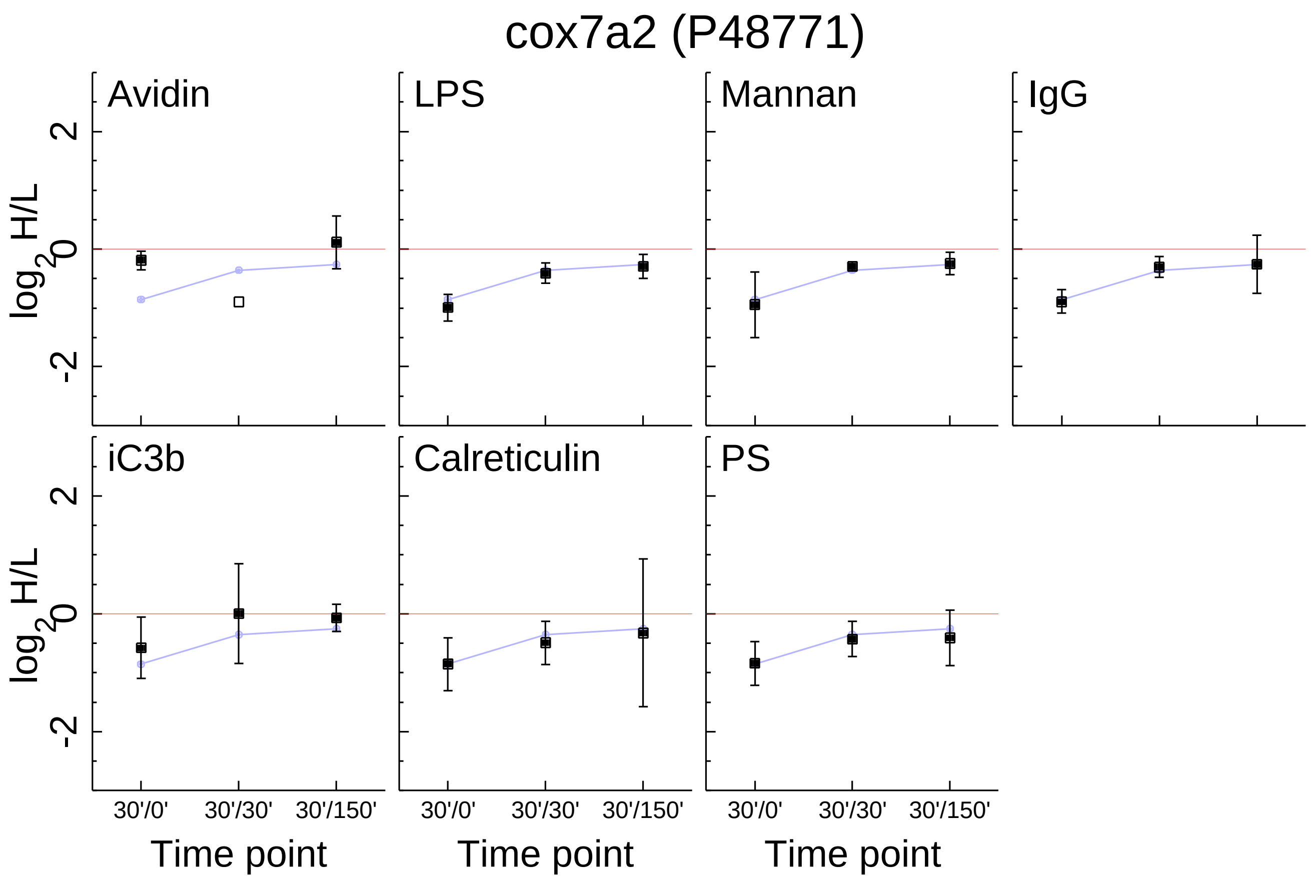
cox7a2 (P48771)
Avidin
LPS
Mannan
IgG
iC3b
Calreticulin
PS
log2 H/L
2
0
-2
30'/0'
30'/30'
30'/150'
Time point

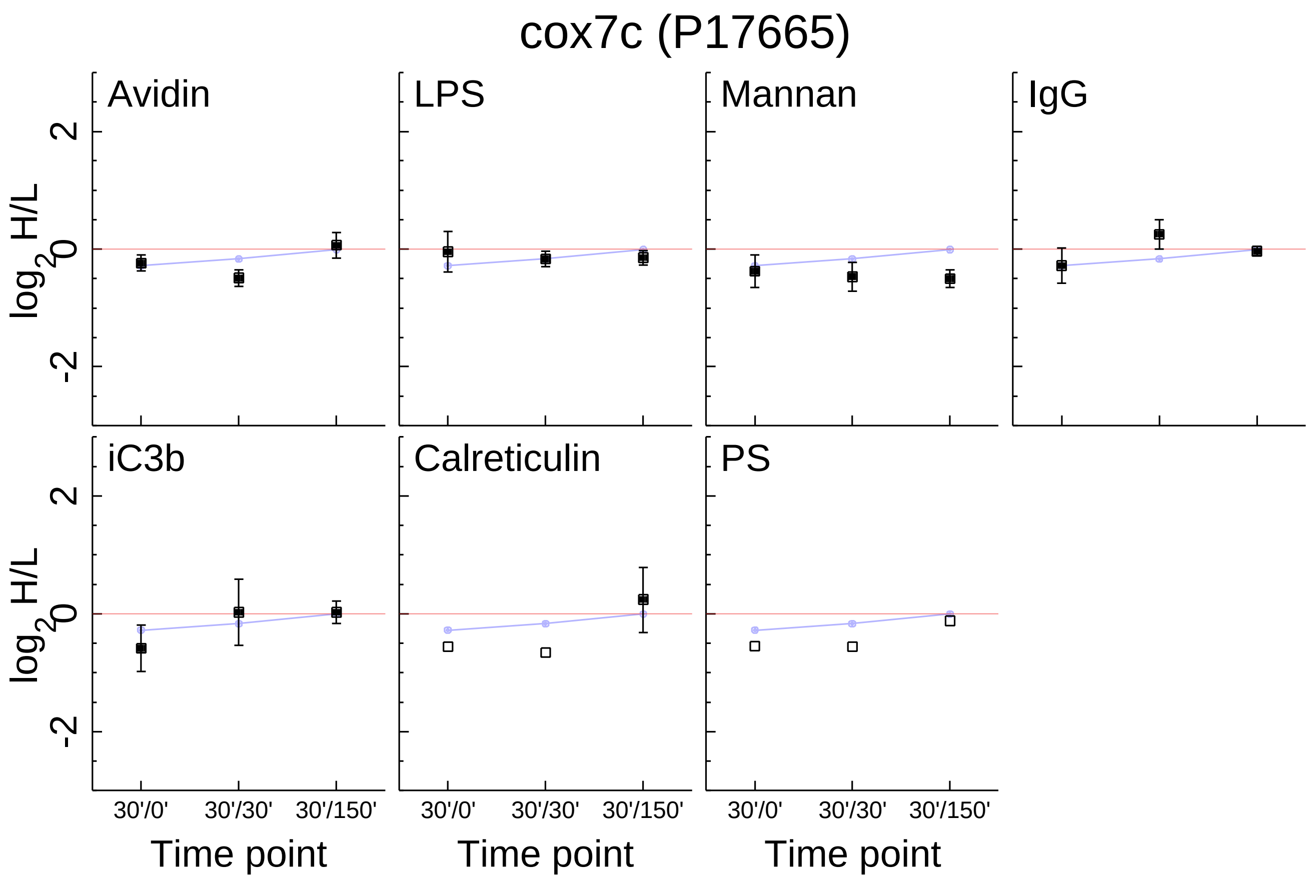

cox7c (P17665)
Avidin
LPS
Mannan
IgG
iC3b
Calreticulin
PS
log2 H/L
2
0
-2
30'/0'
30'/30'
30'/150'
Time point

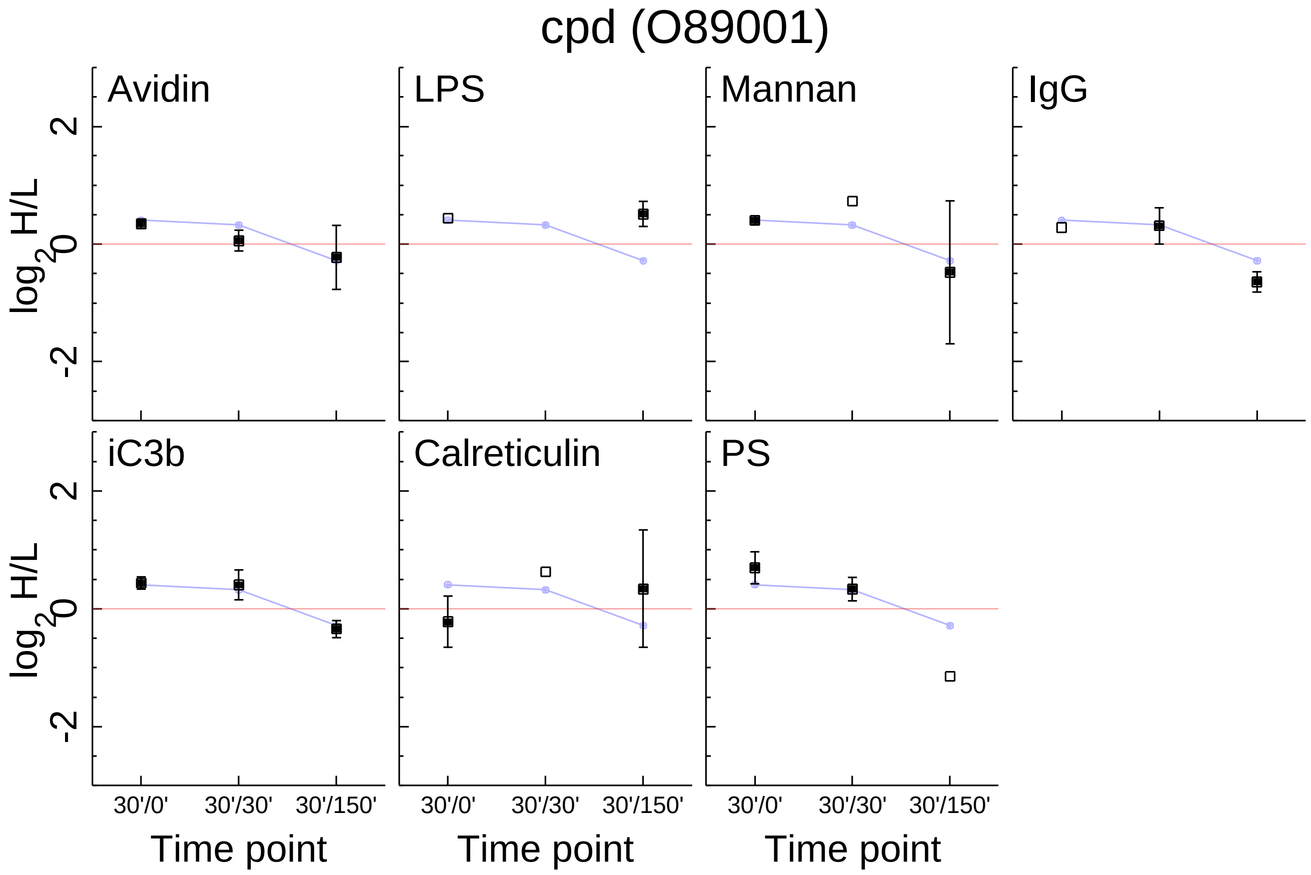
cpd (O89001)
Avidin
LPS
Mannan
IgG
iC3b
Calreticulin
PS
log2 H/L
2
0
-2
30'/0'
30'/30'
30'/150'
Time point

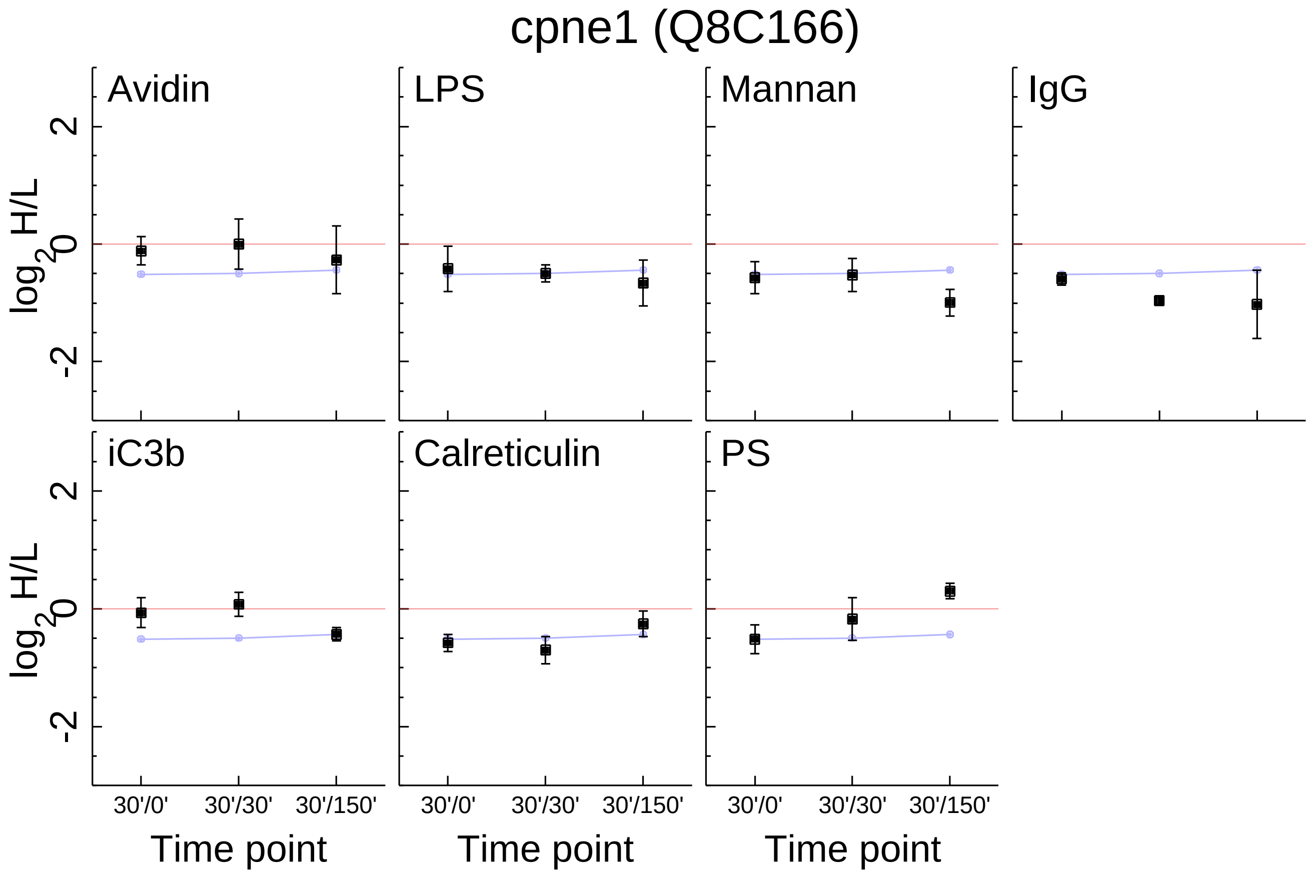

cpne1 (Q8C166)
Avidin
LPS
Mannan
IgG
iC3b
Calreticulin
PS
log2 H/L
2
0
-2
30'/0'
30'/30'
30'/150'
Time point

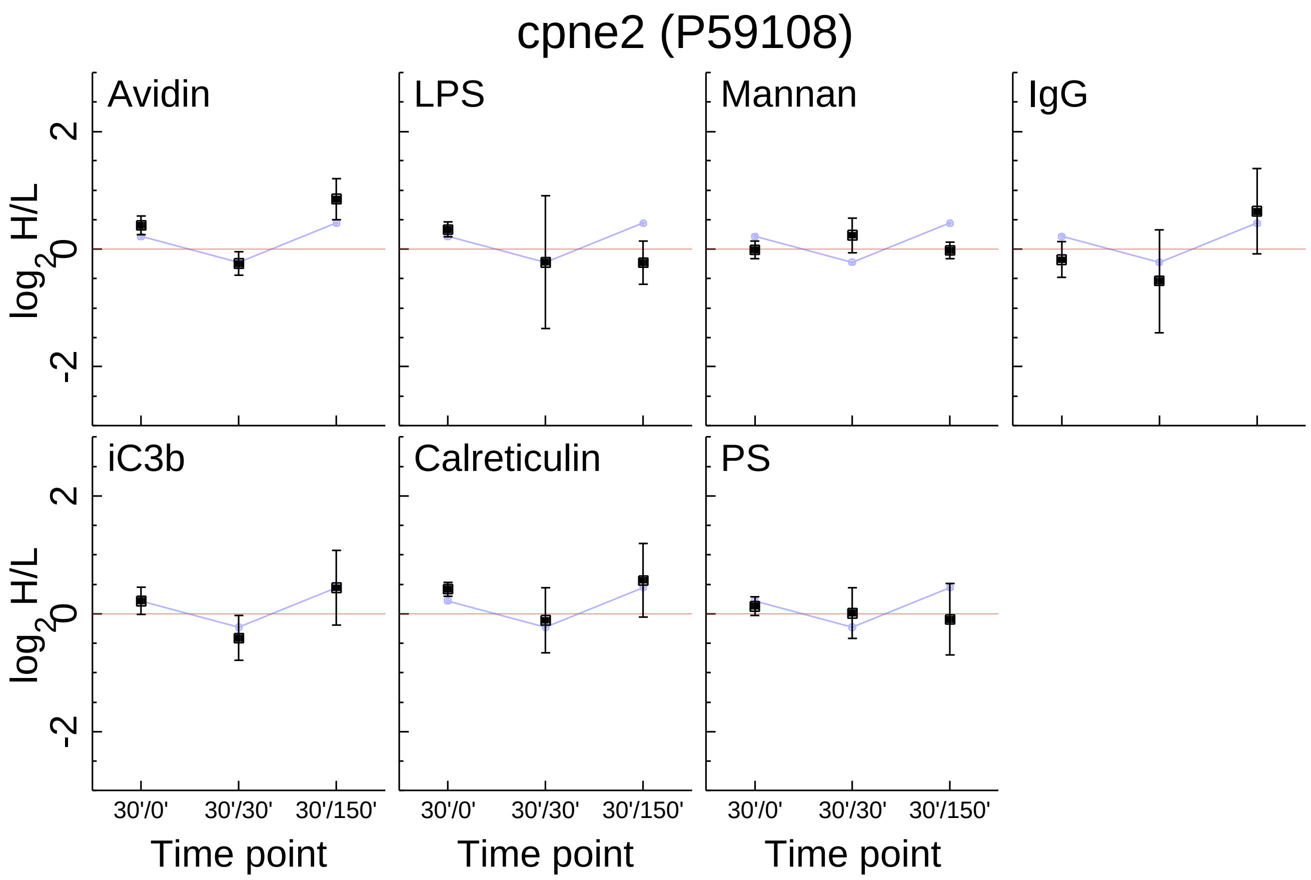
cpne2 (P59108)
Avidin
LPS
Mannan
IgG
iC3b
Calreticulin
PS
log2 H/L
2
0
-2
30'/0'
30'/30'
30'/150'
Time point

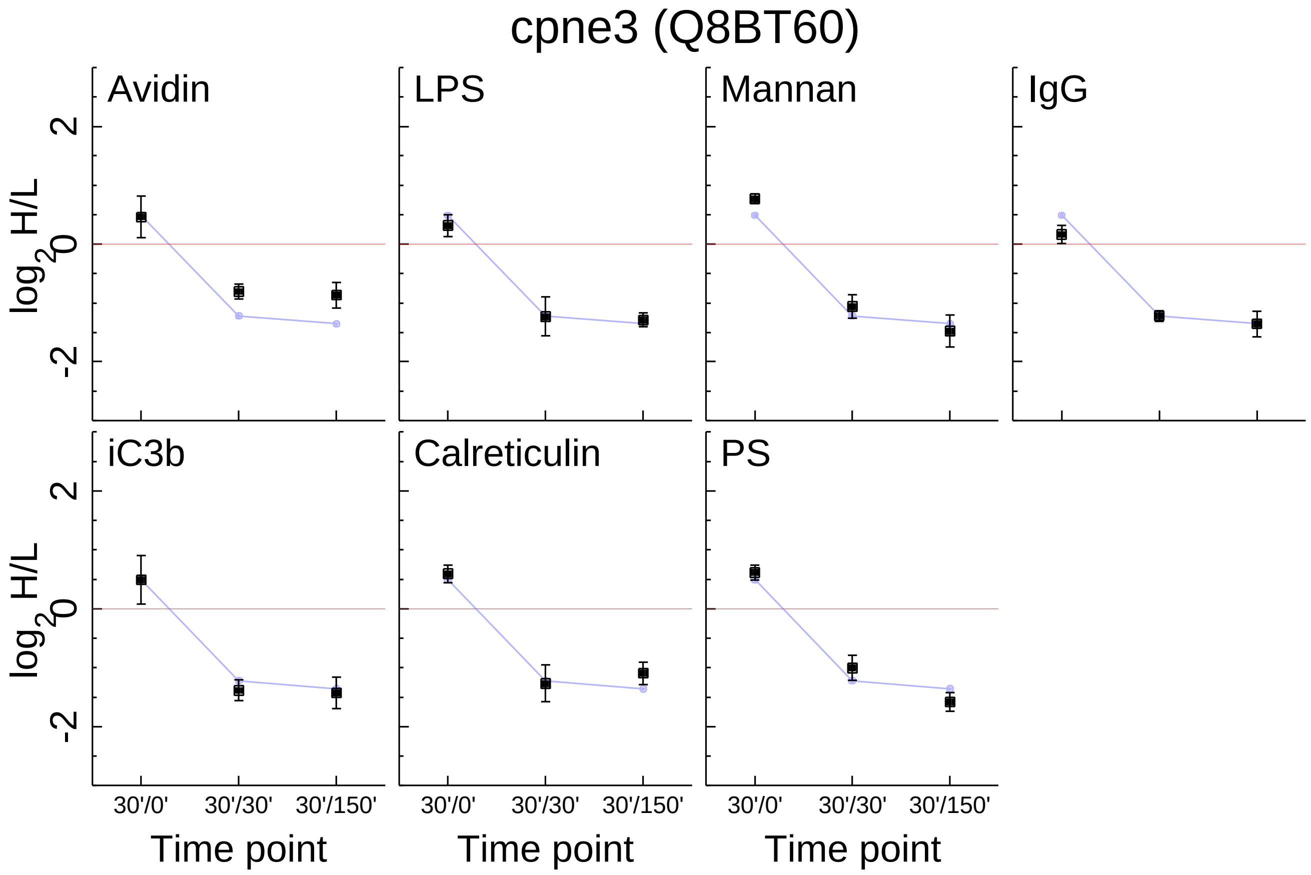
cpne3 (Q8BT60)
Avidin
LPS
Mannan
IgG
iC3b
Calreticulin
PS
log2 H/L
2
0
-2
30'/0'
30'/30'
30'/150'
Time point

cpq (Q9WVJ3)

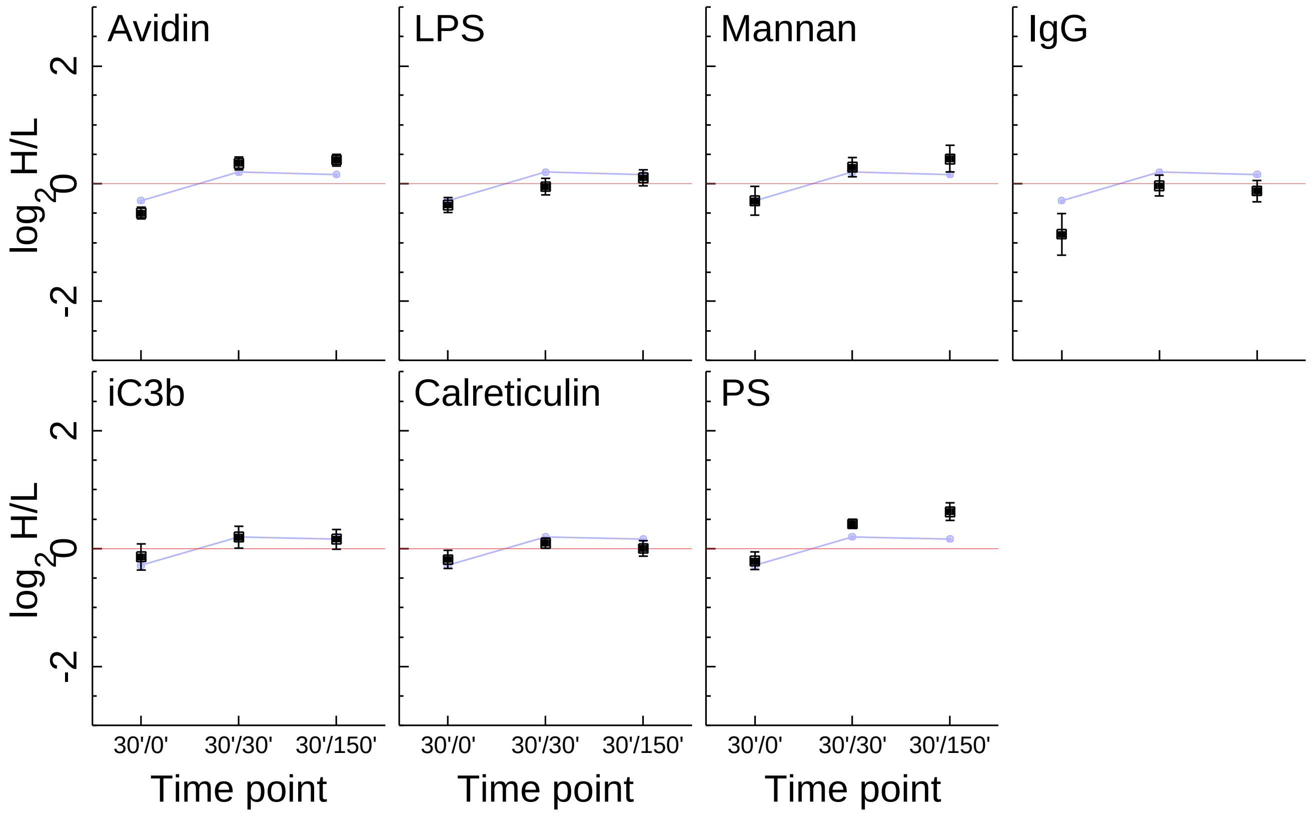
Avidin
LPS
Mannan
IgG
iC3b
Calreticulin
PS
2
0
-2
$\log_2$ H/L
30'/0'
30'/30'
30'/150'
Time point

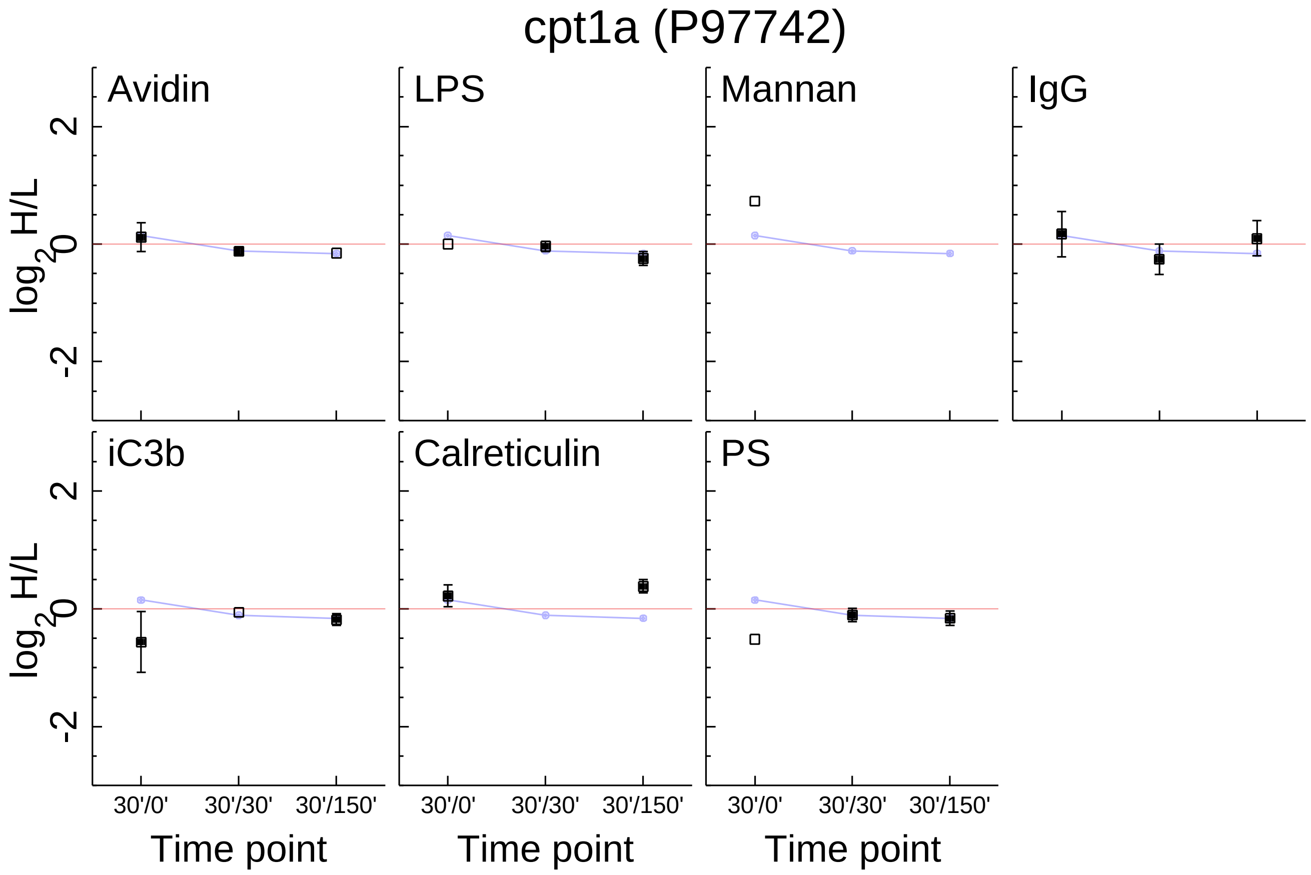
cpt1a (P97742)
Avidin
LPS
Mannan
IgG
iC3b
Calreticulin
PS
log2 H/L
2
0
-2
30'/0'
30'/30'
30'/150'
Time point

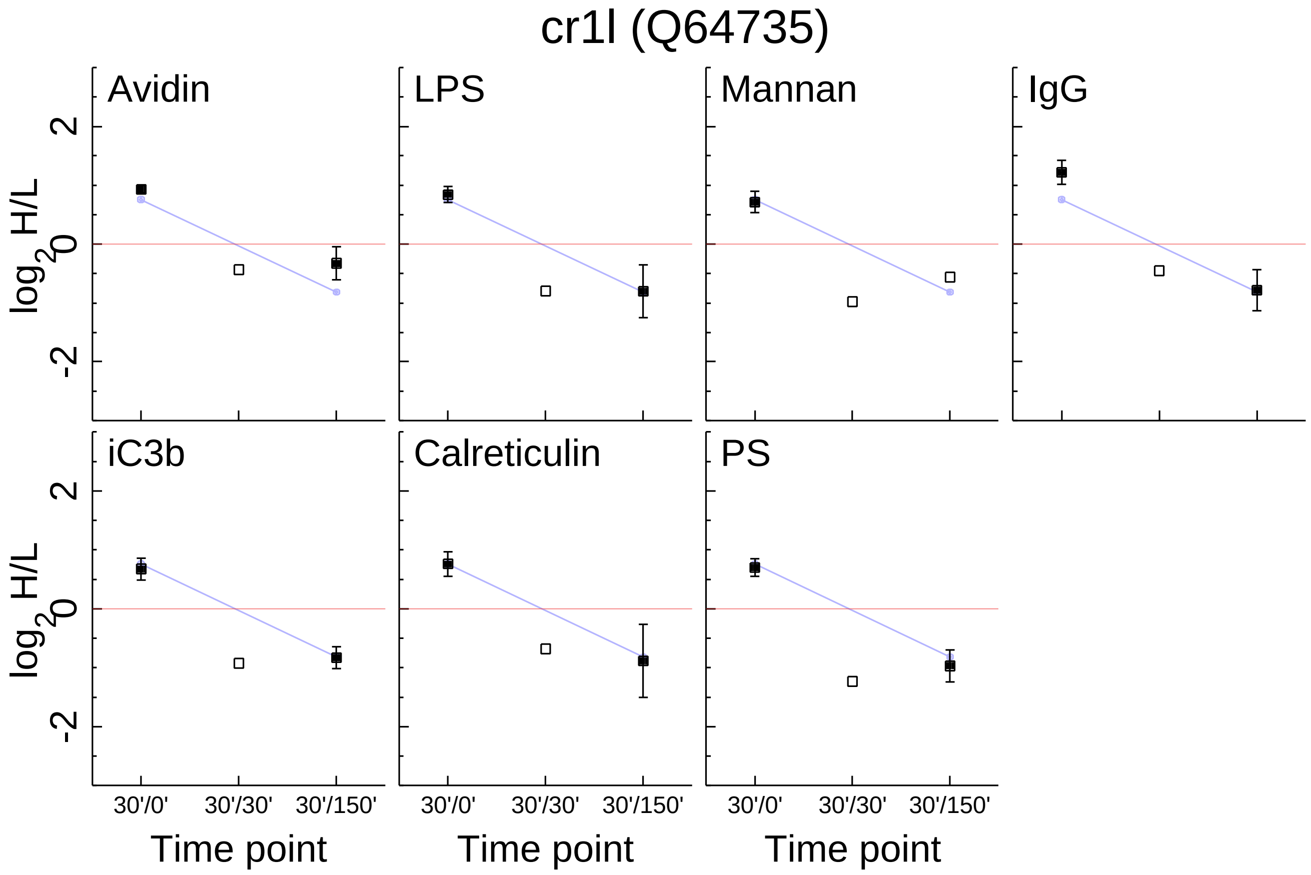

cr1l (Q64735)
Avidin
LPS
Mannan
IgG
iC3b
Calreticulin
PS
log2 H/L
2
0
-2
30'/0'
30'/30'
30'/150'
Time point

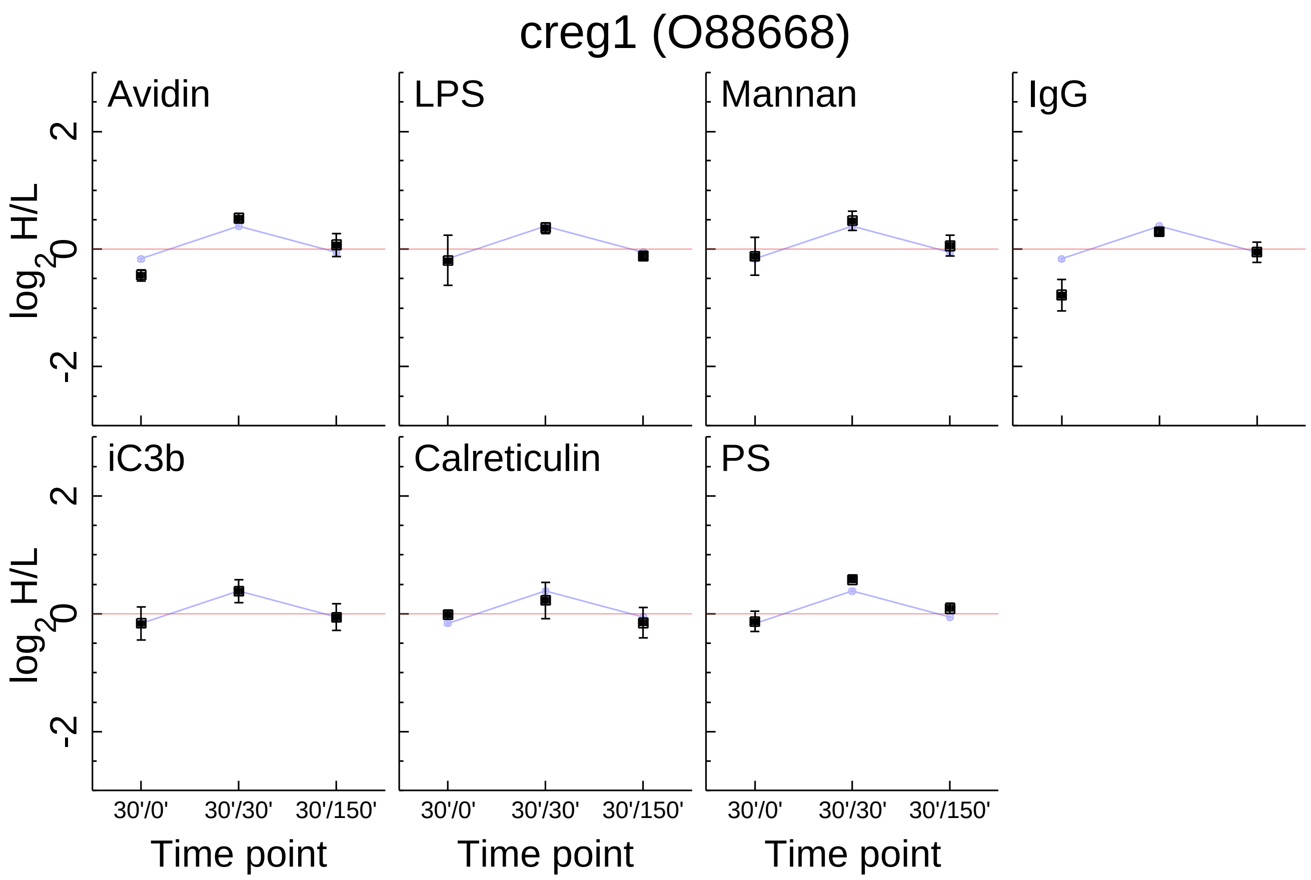

creg1 (O88668)
Avidin
LPS
Mannan
IgG
iC3b
Calreticulin
PS
log2 H/L
2
0
-2
30'/0'
30'/30'
30'/150'
Time point

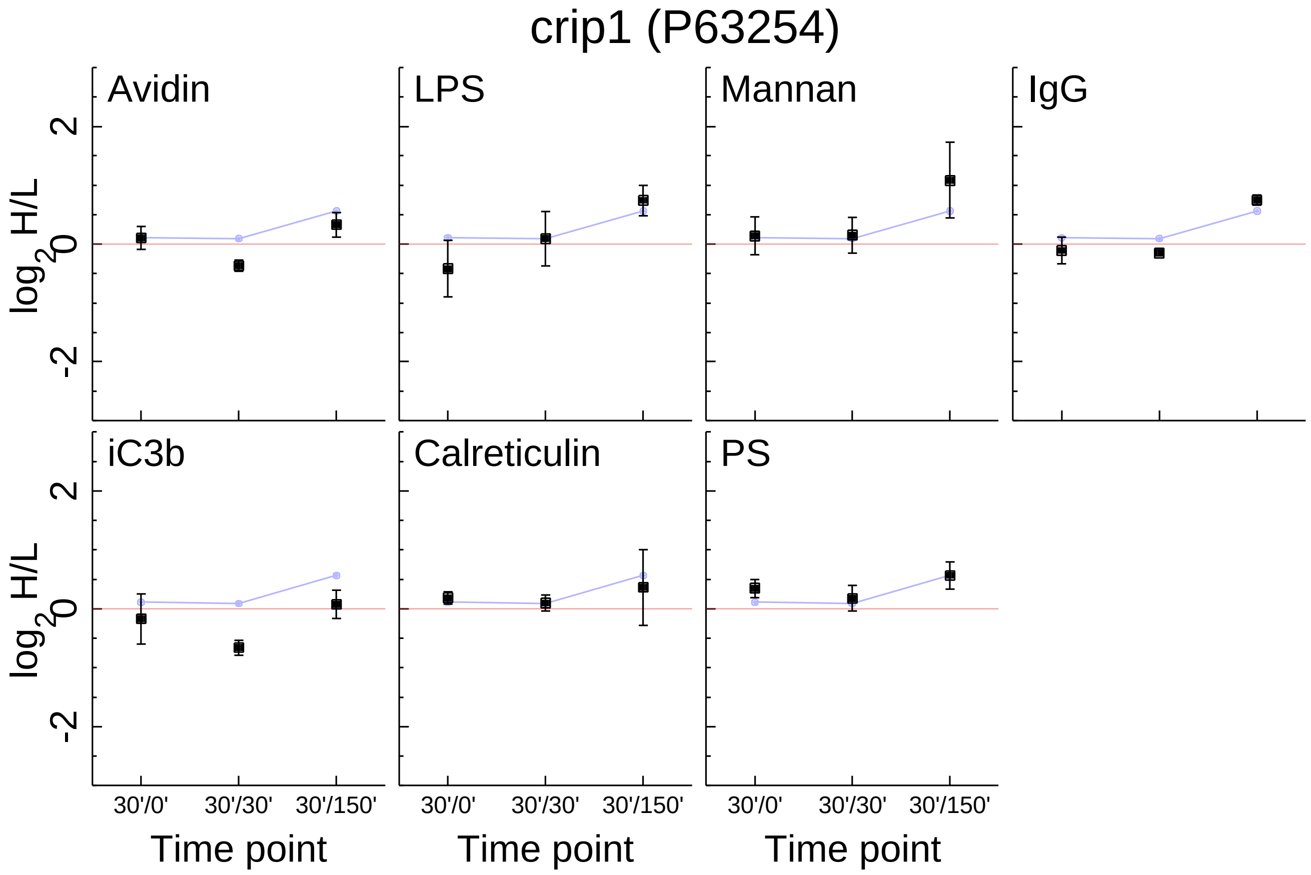

crip1 (P63254)
Avidin
LPS
Mannan
IgG
iC3b
Calreticulin
PS
log2 H/L
2
0
-2
30'/0'
30'/30'
30'/150'
Time point

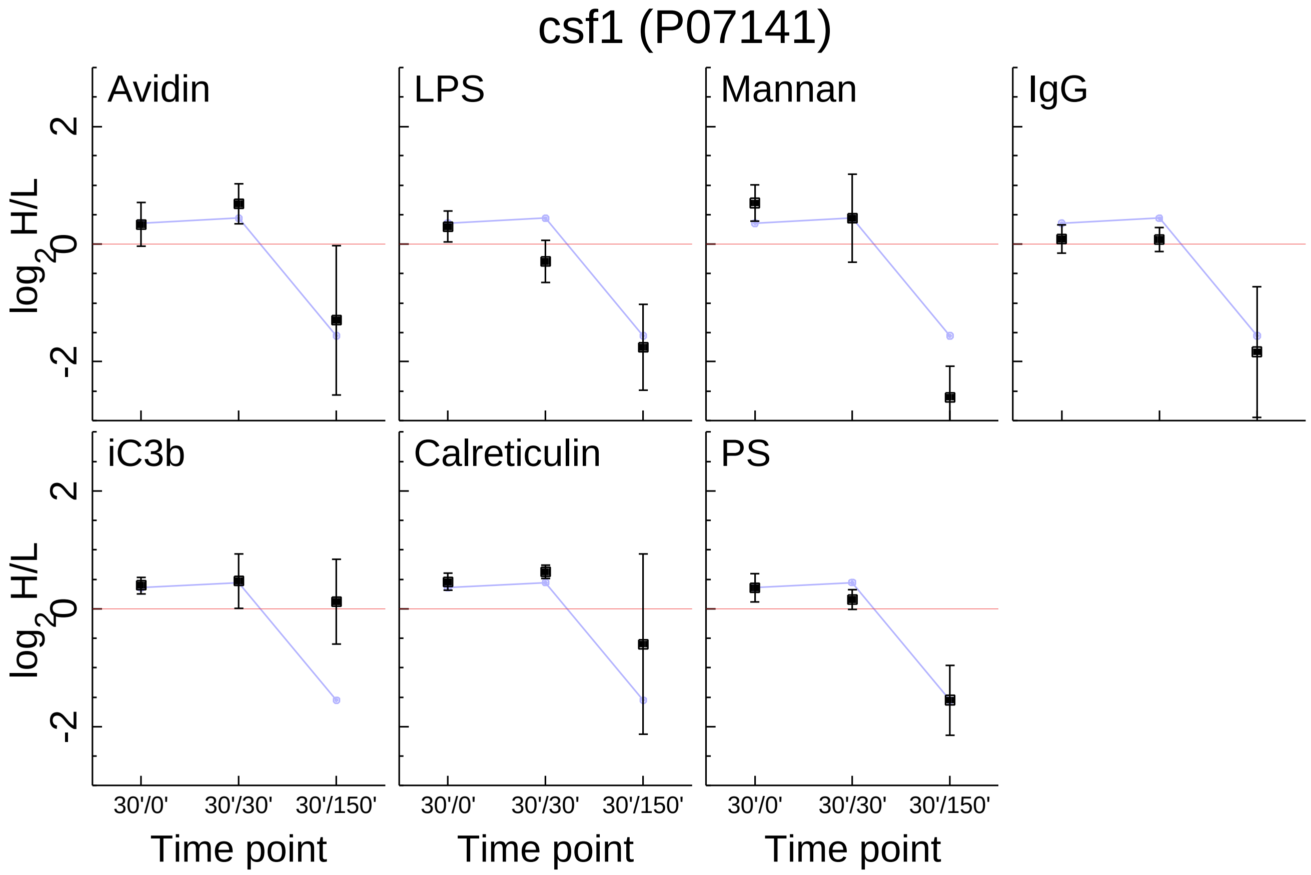

csf1 (P07141)
Avidin
LPS
Mannan
IgG
iC3b
Calreticulin
PS
log2 H/L
2
0
-2
30'/0'
30'/30'
30'/150'
Time point

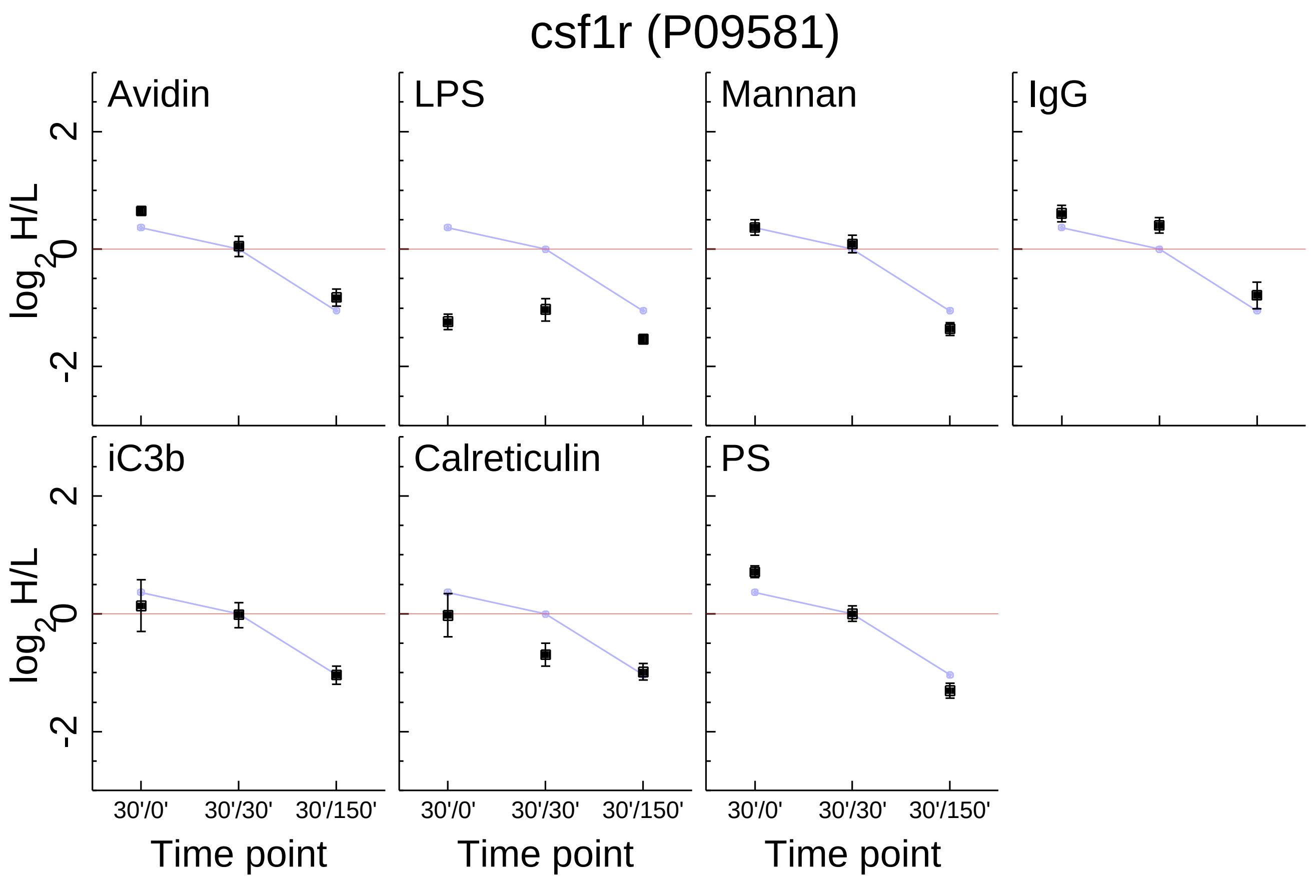

csf1r (P09581)
Avidin
LPS
Mannan
IgG
iC3b
Calreticulin
PS
log2 H/L
2
0
-2
30'/0'
30'/30'
30'/150'
Time point

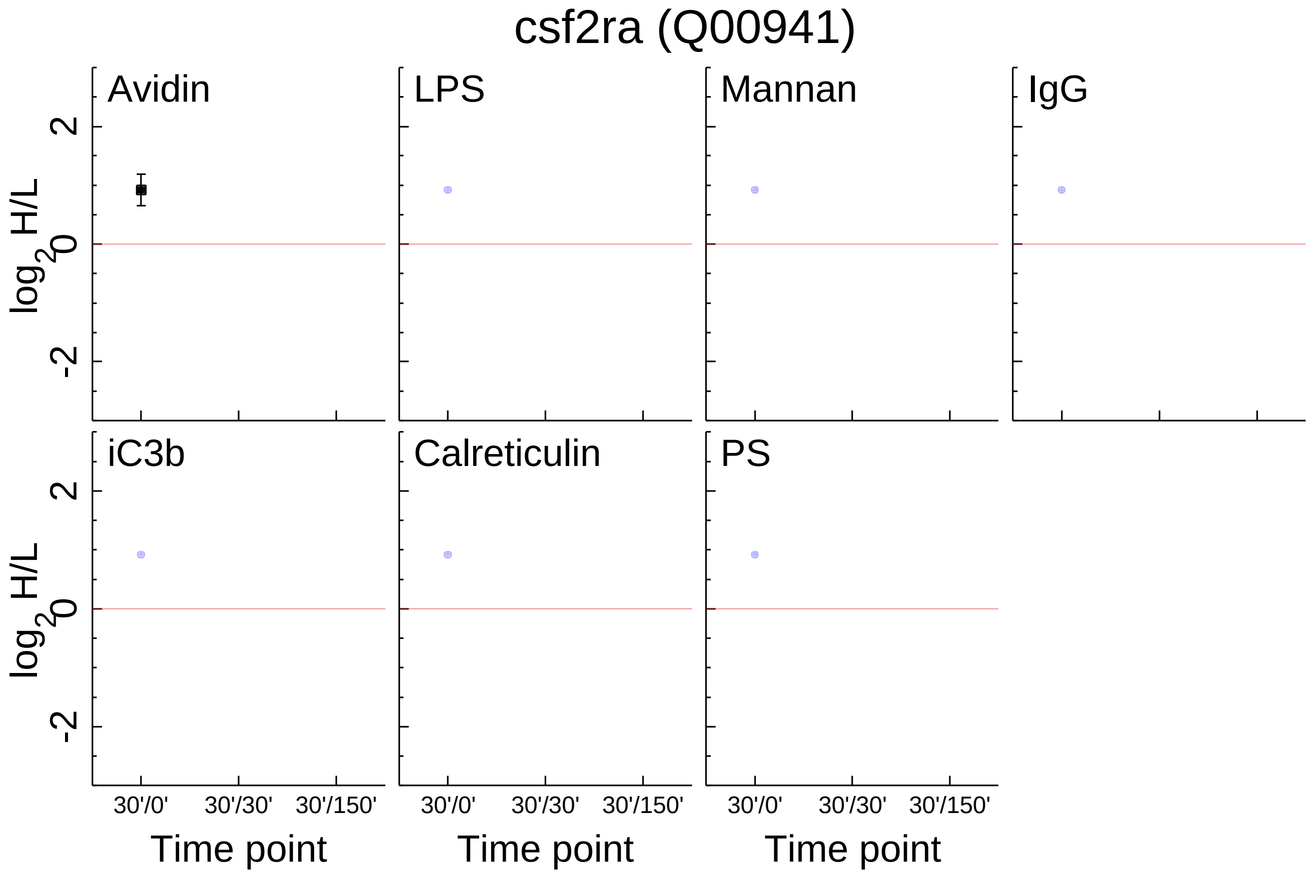

csf2ra (Q00941)
Avidin
LPS
Mannan
IgG
iC3b
Calreticulin
PS
log2 H/L
2
0
-2
30'/0'
30'/30'
30'/150'
Time point

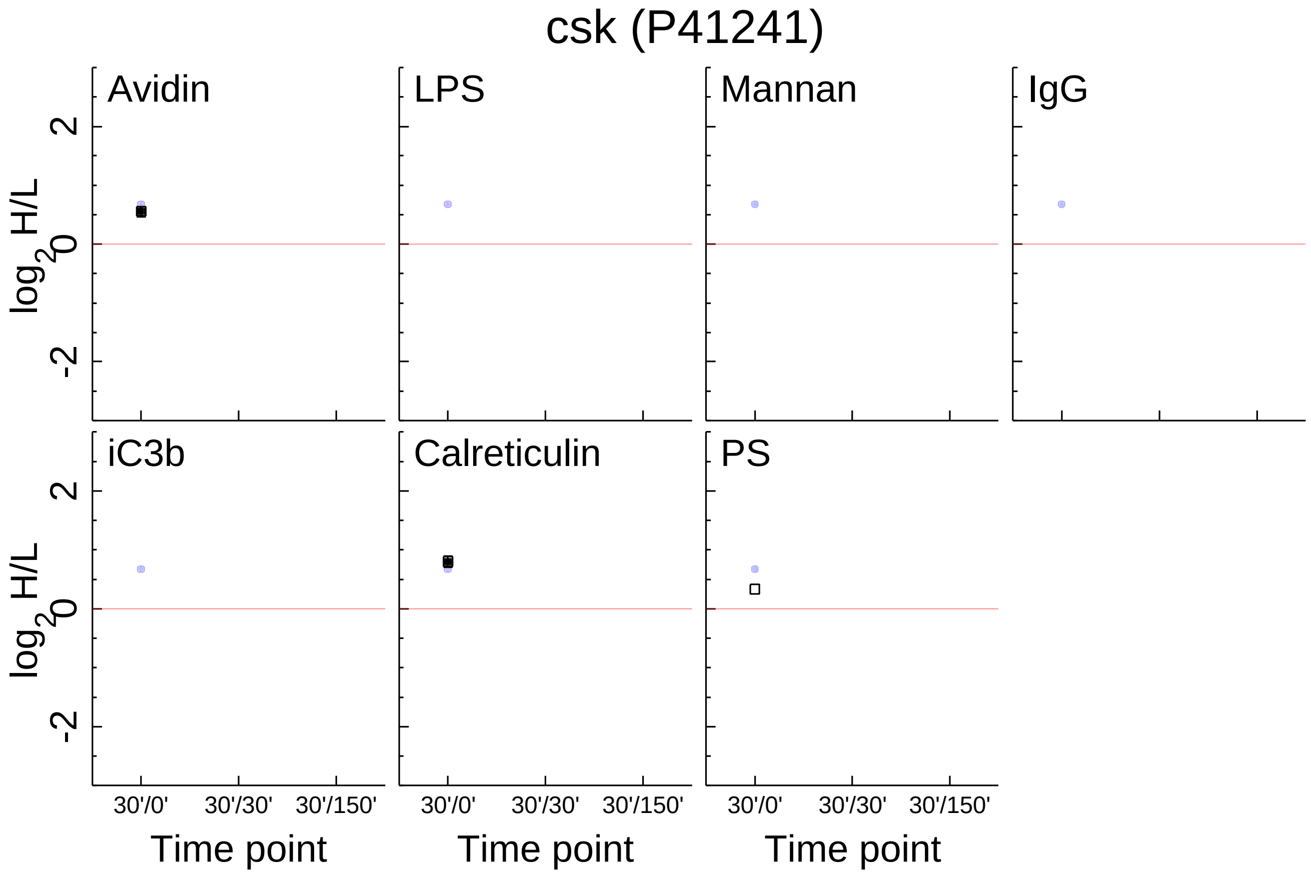
csk (P41241)
Avidin
LPS
Mannan
IgG
iC3b
Calreticulin
PS
log2 H/L
2
0
-2
30'/0'
30'/30'
30'/150'
Time point

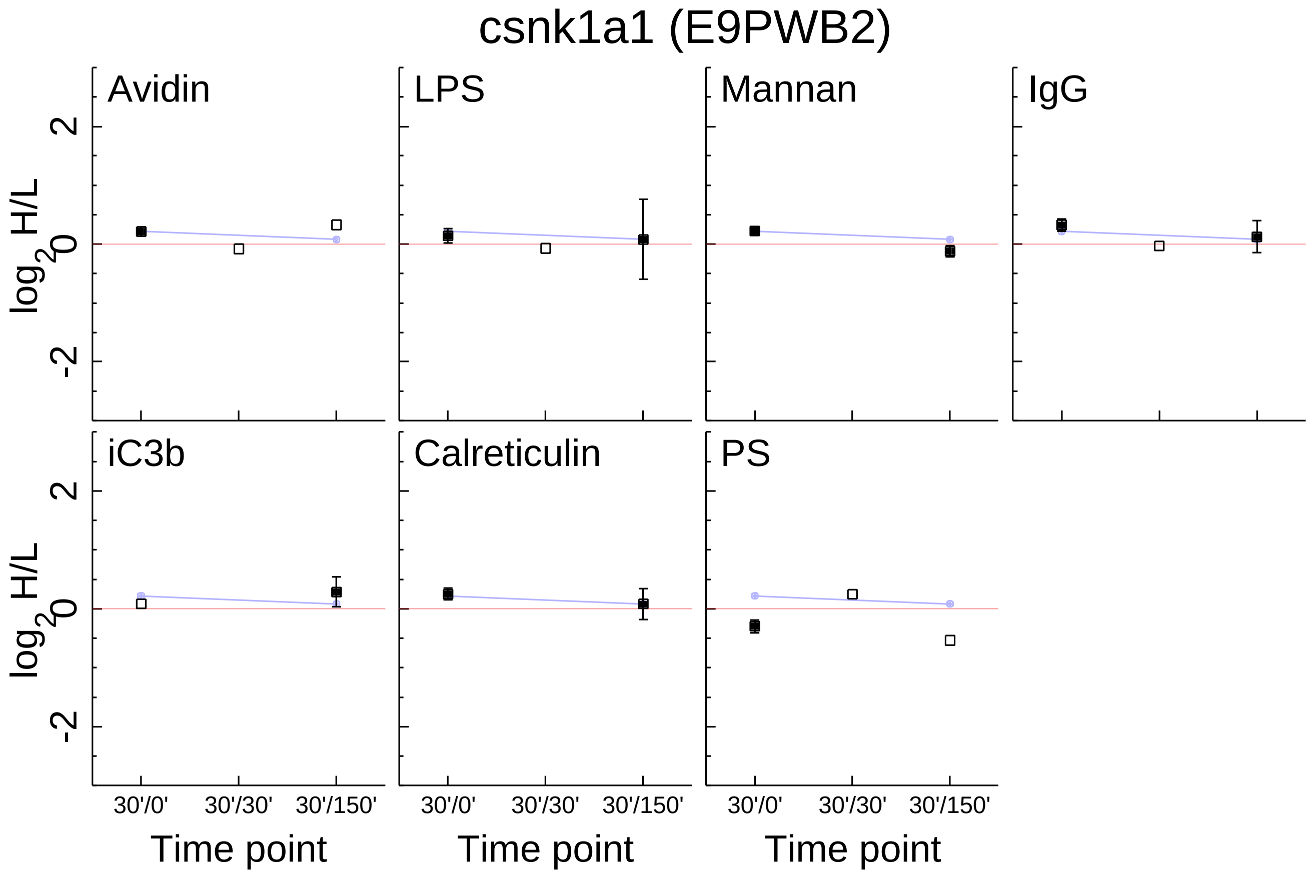

csnk1a1 (E9PWB2)
Avidin
LPS
Mannan
IgG
iC3b
Calreticulin
PS
log2 H/L
2
0
-2
30'/0'
30'/30'
30'/150'
Time point

csnk1g3 (Q8C4X2)

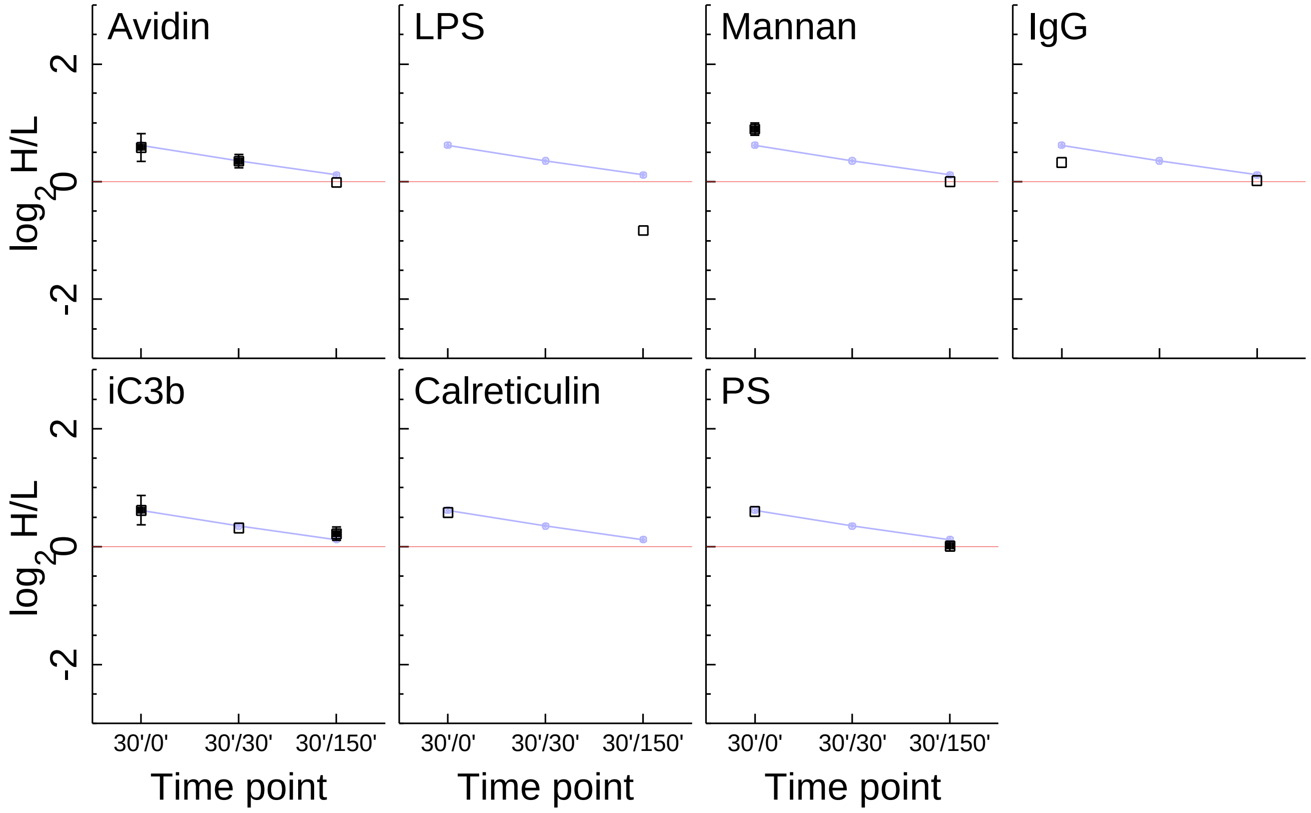

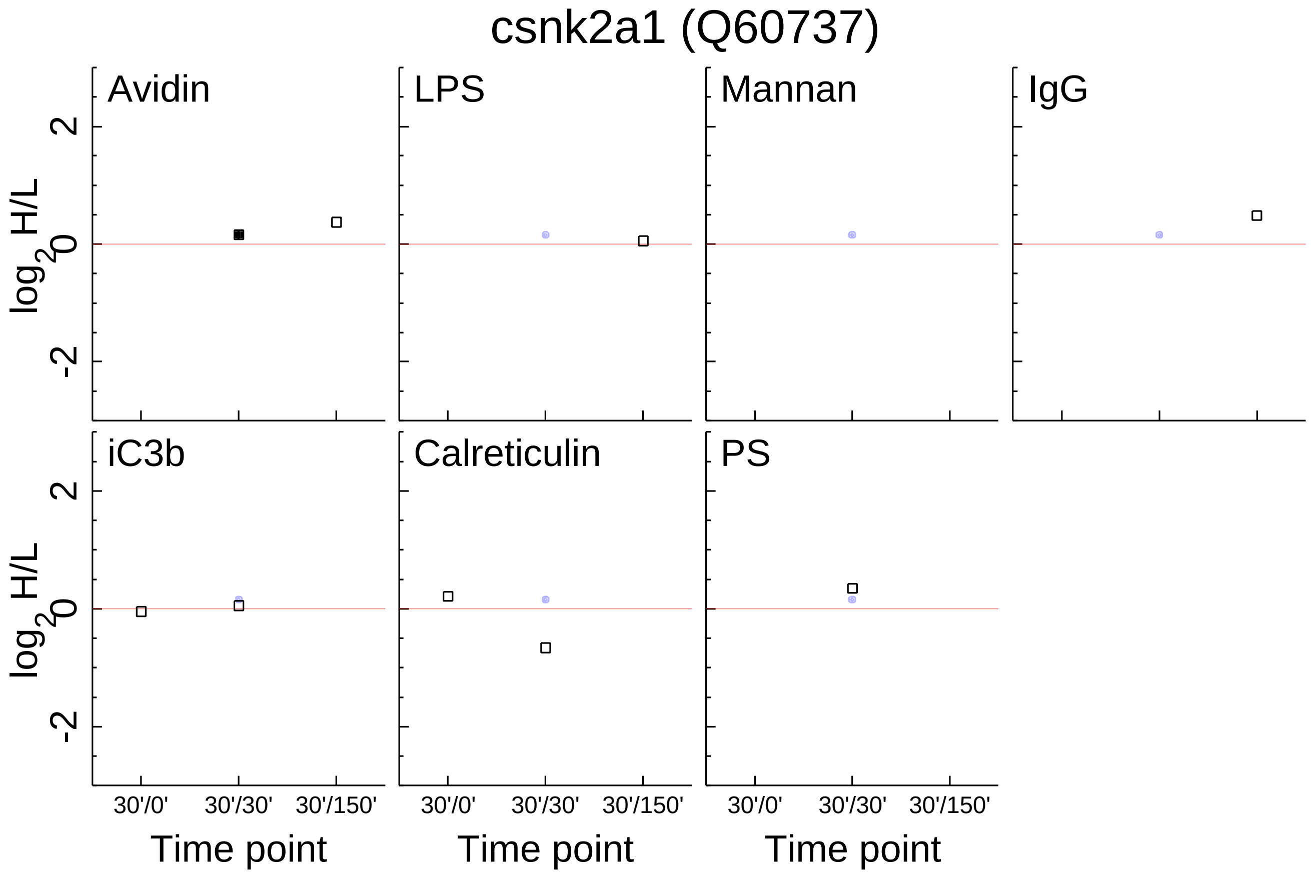

csnk2a1 (Q60737)
Avidin
LPS
Mannan
IgG
iC3b
Calreticulin
PS
log2 H/L
2
0
-2
30'/0'
30'/30'
30'/150'
Time point

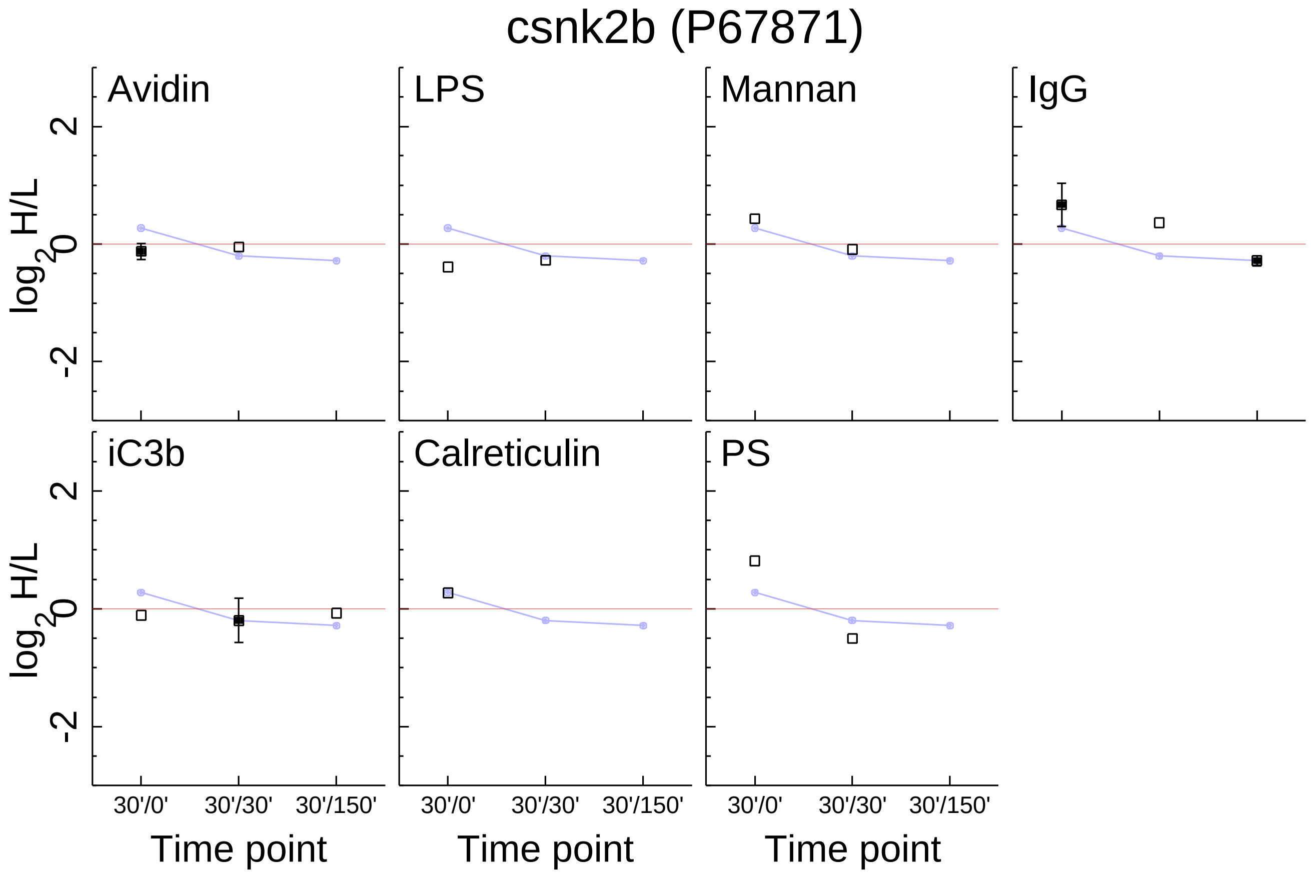

csnk2b (P67871)
Avidin
LPS
Mannan
IgG
iC3b
Calreticulin
PS
log2 H/L
2
0
-2
30'/0'
30'/30'
30'/150'
Time point

cs (Q9CZU6)

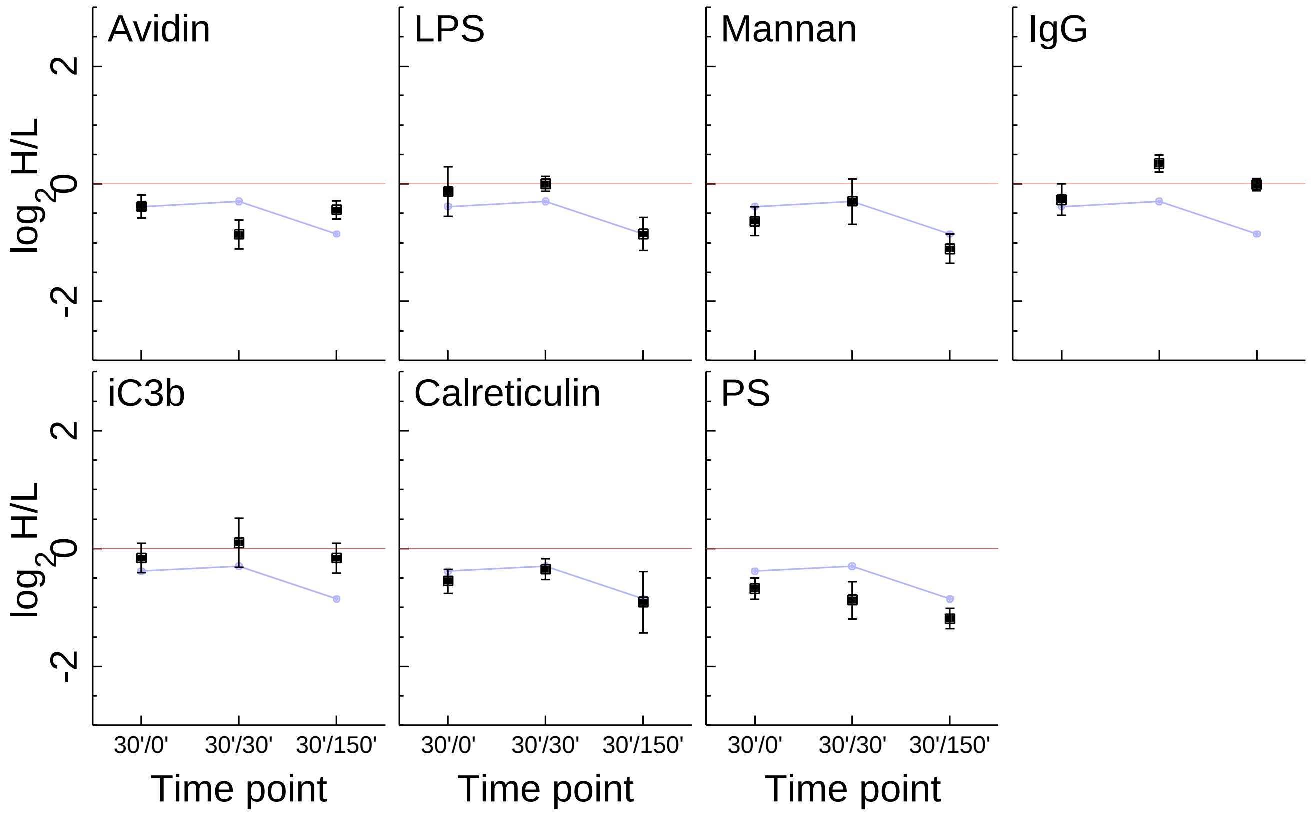

Avidin
LPS
Mannan
IgG
iC3b
Calreticulin
PS
log2 H/L
2
0
-2
30'/0'
30'/30'
30'/150'
Time point

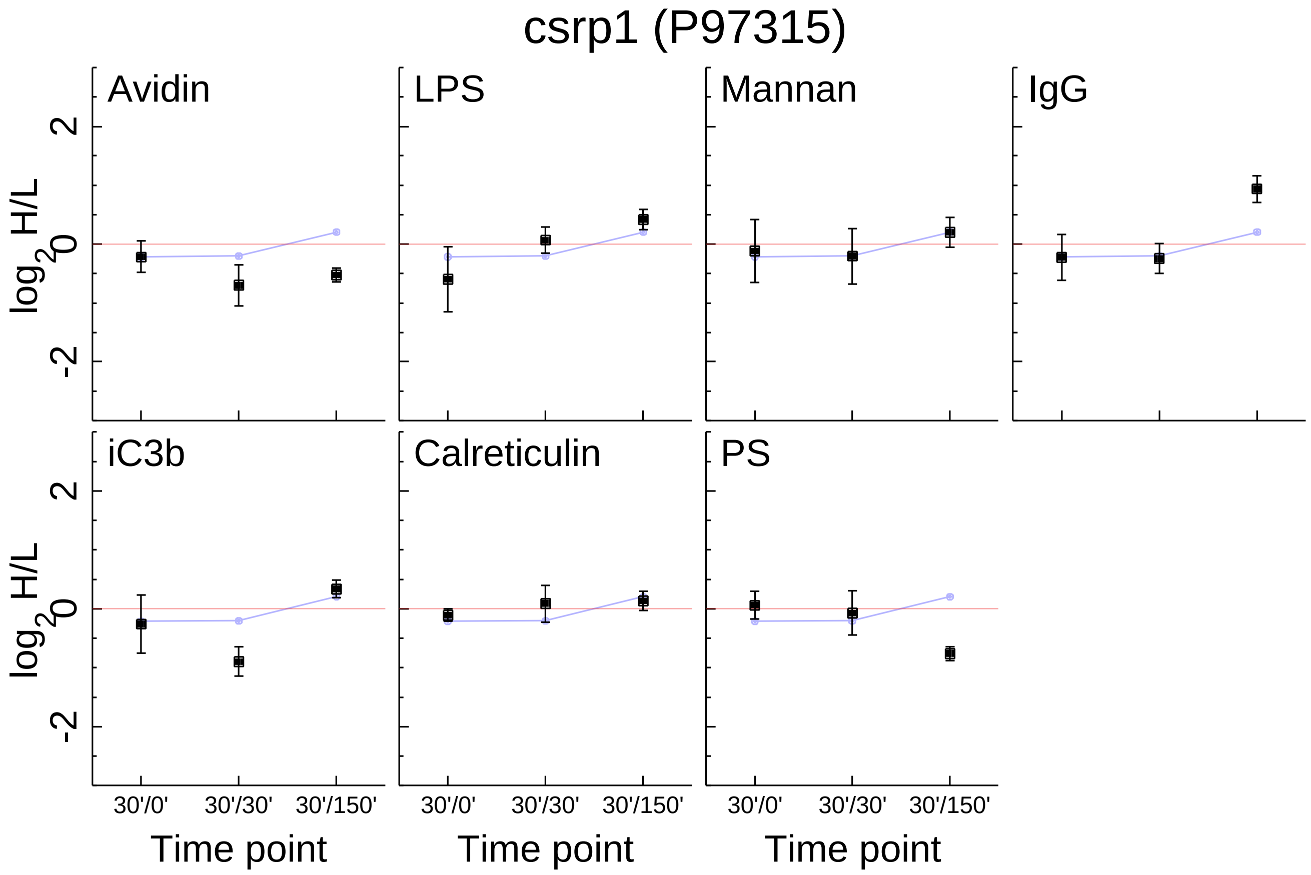
csrp1 (P97315)
Avidin
LPS
Mannan
IgG
iC3b
Calreticulin
PS
log2 H/L
2
0
-2
30'/0'
30'/30'
30'/150'
Time point

cst3 (P21460)

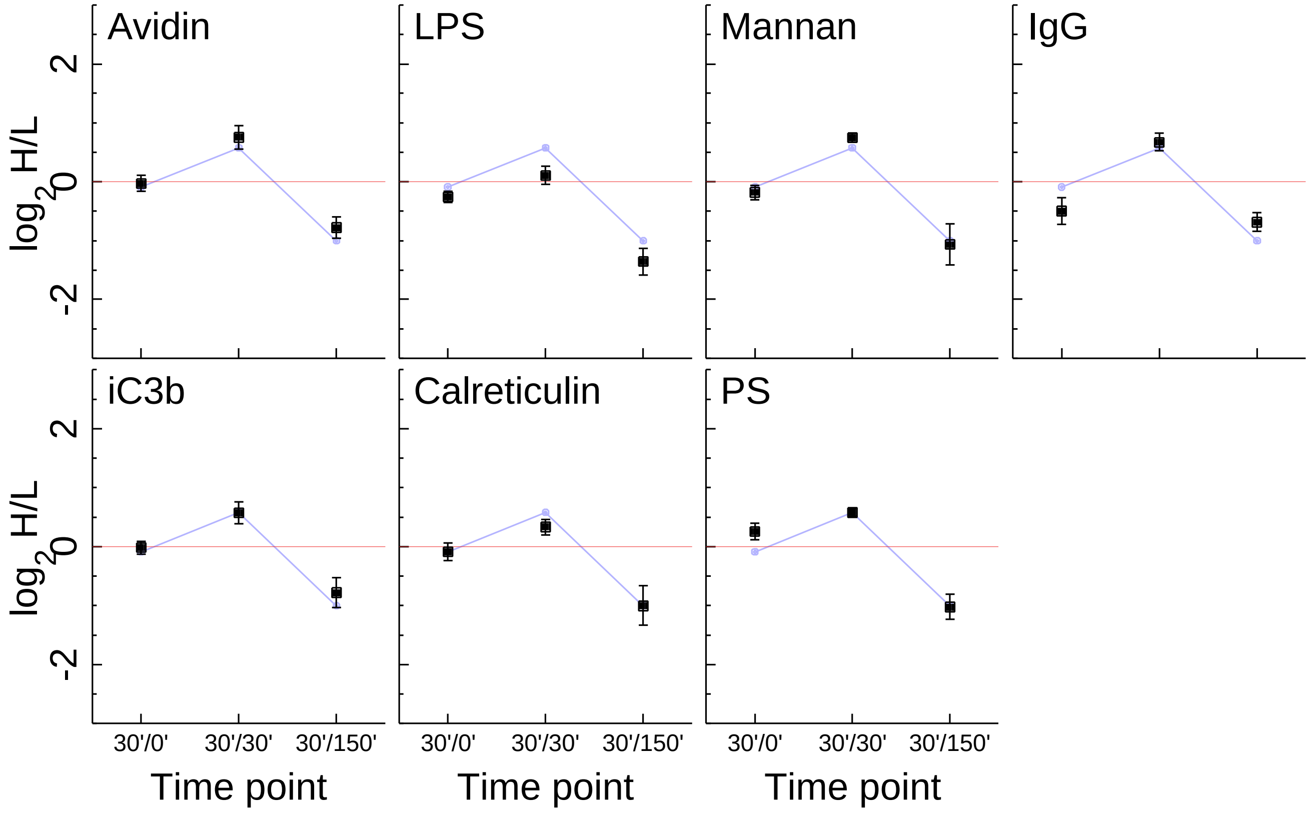

Avidin
LPS
Mannan
IgG
iC3b
Calreticulin
PS
log2 H/L
2
0
-2
30'/0'
30'/30'
30'/150'
Time point

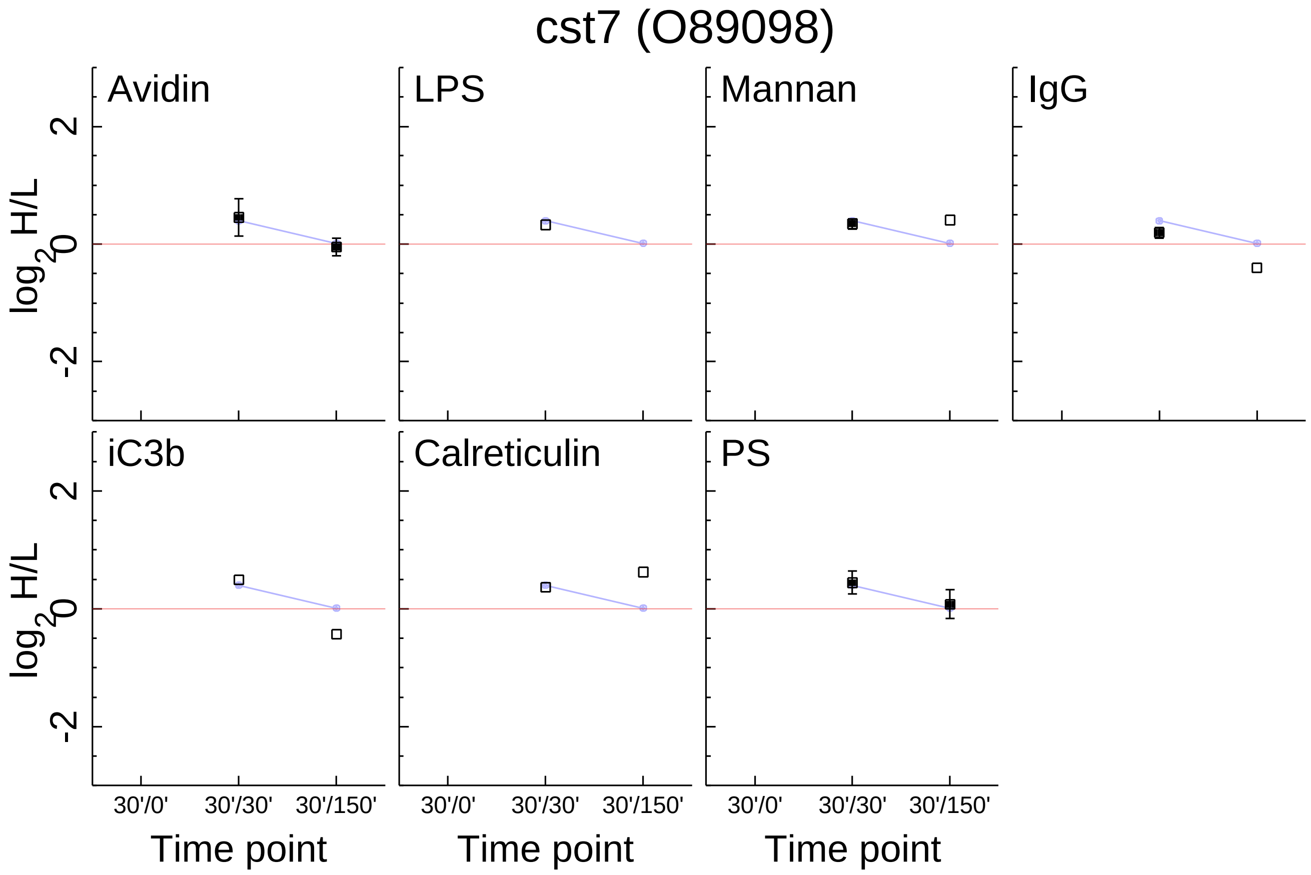

cst7 (O89098)
Avidin
LPS
Mannan
IgG
iC3b
Calreticulin
PS
log2 H/L
2
0
-2
30'/0'
30'/30'
30'/150'
Time point

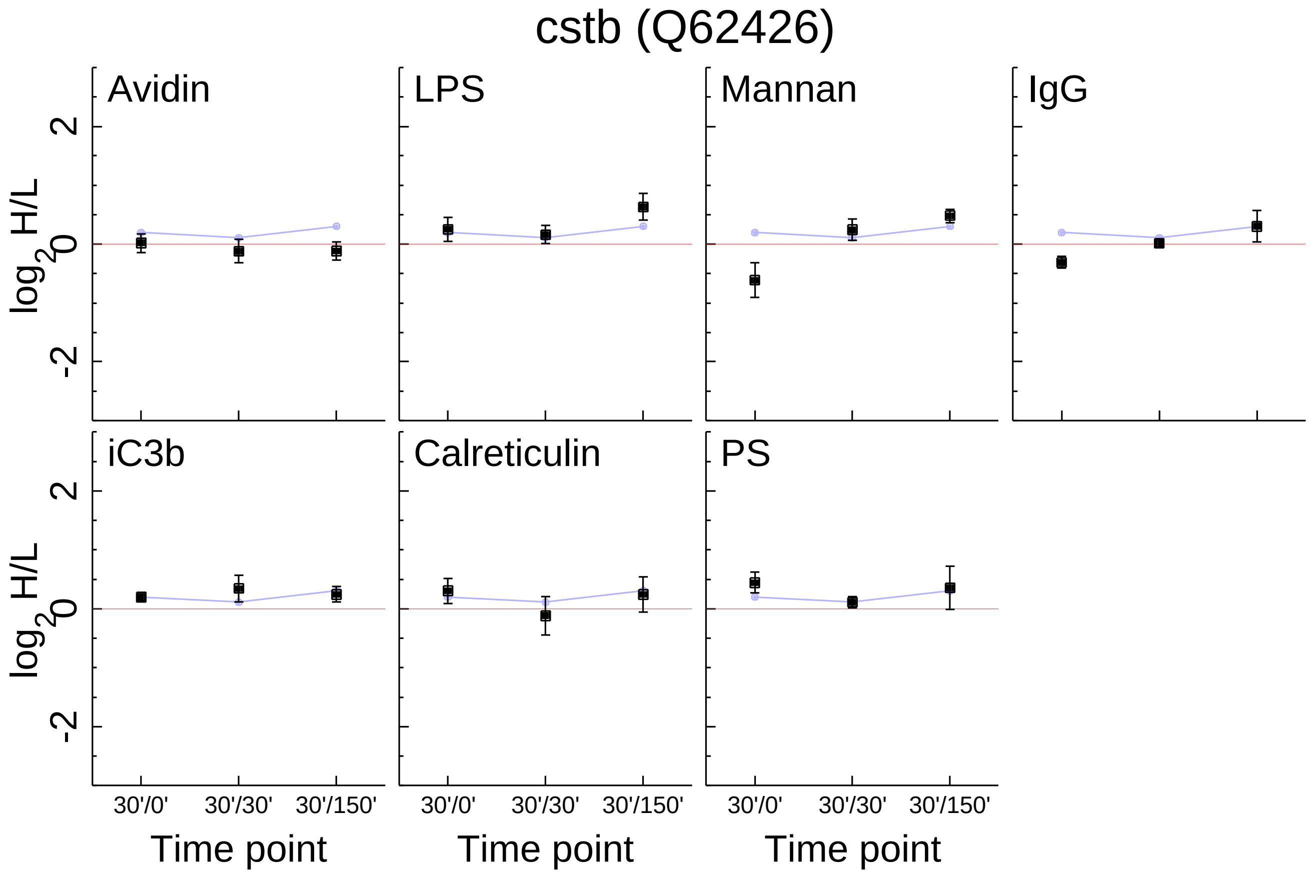

cstb (Q62426)
Avidin
LPS
Mannan
IgG
iC3b
Calreticulin
PS
log2 H/L
2
0
-2
30'/0'
30'/30'
30'/150'
Time point

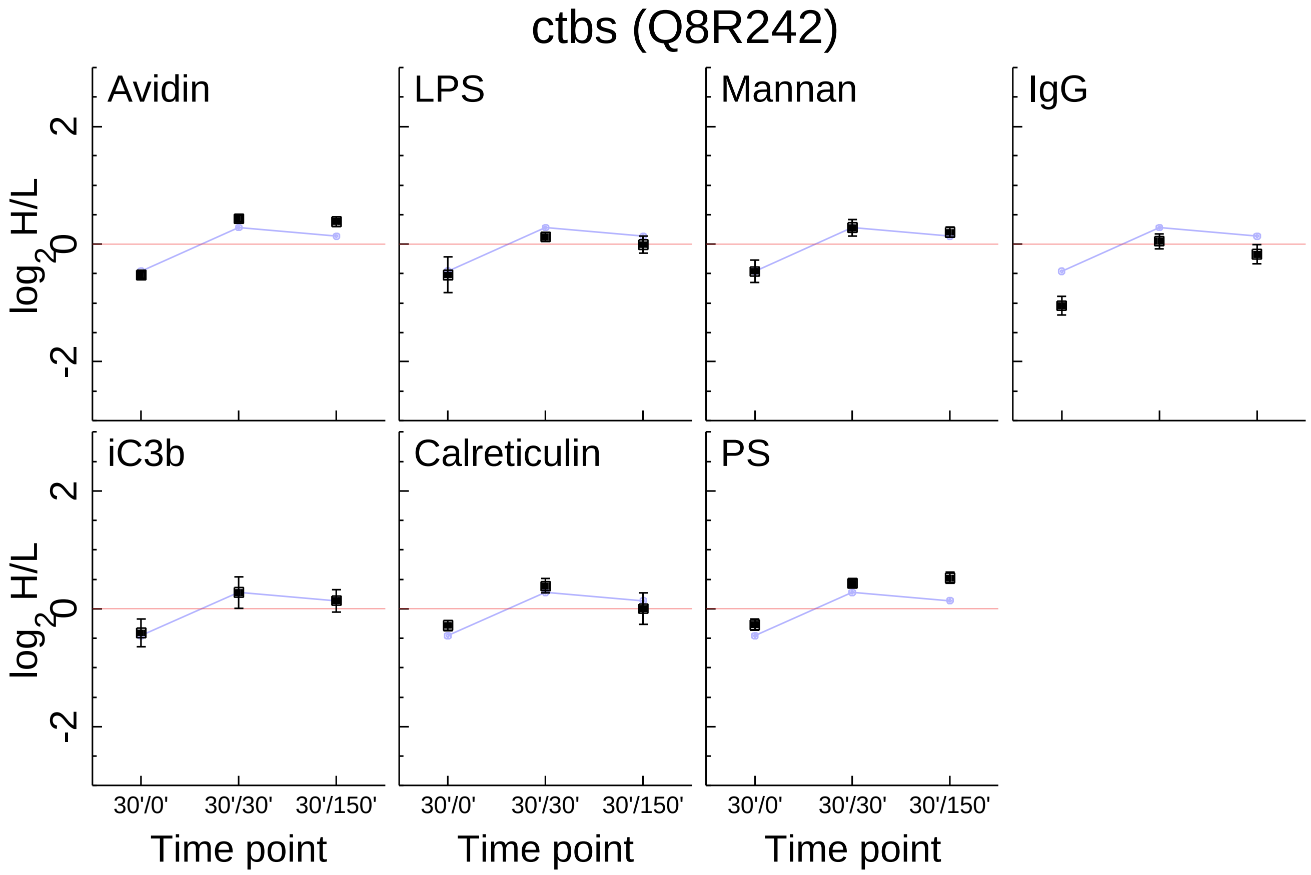
ctbs (Q8R242)
Avidin
LPS
Mannan
IgG
iC3b
Calreticulin
PS
log2 H/L
2
0
-2
30'/0'
30'/30'
30'/150'
Time point

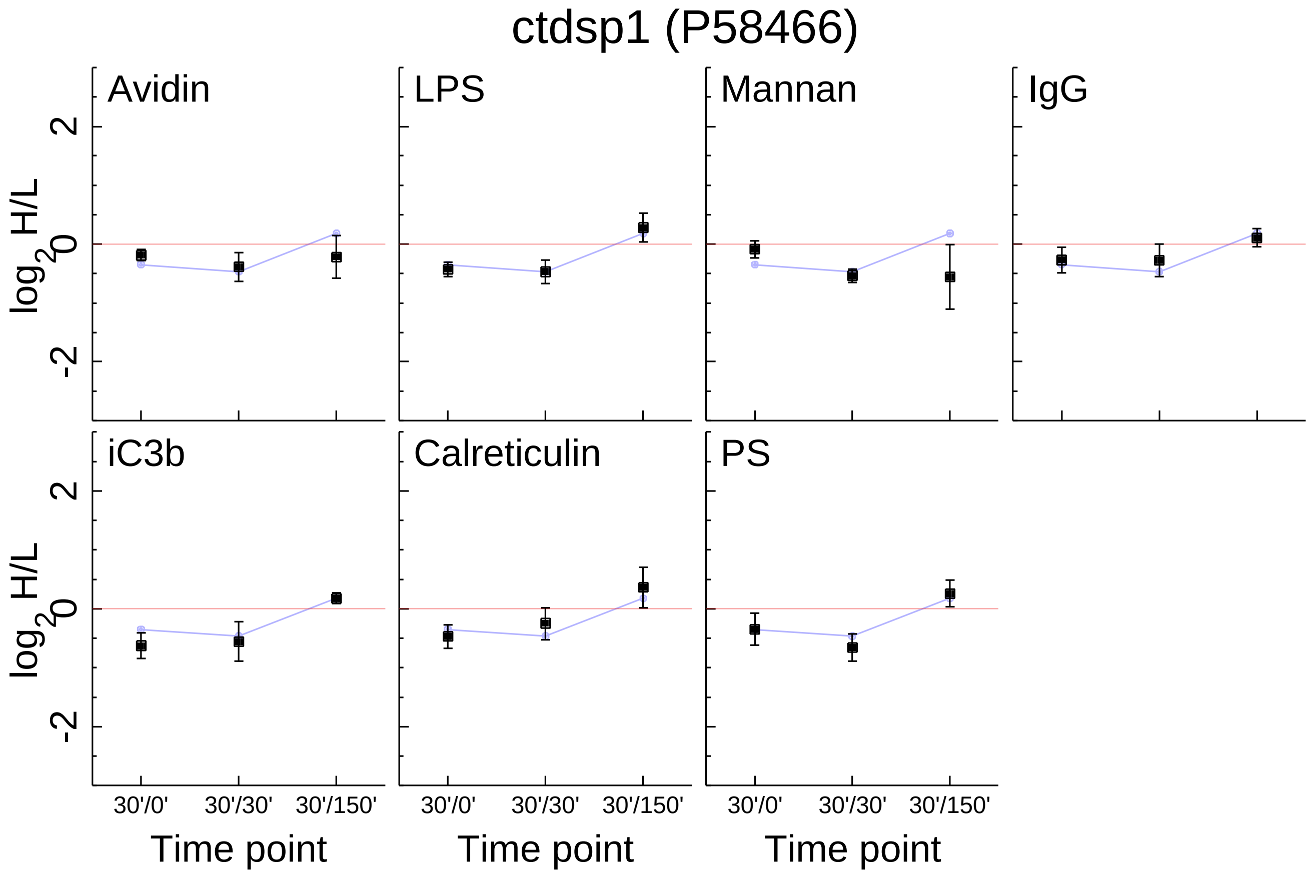
ctdsp1 (P58466)
Avidin
LPS
Mannan
IgG
iC3b
Calreticulin
PS
log2 H/L
2
0
-2
30'/0'
30'/30'
30'/150'
Time point

ctnnd1 (E9Q8Z8)

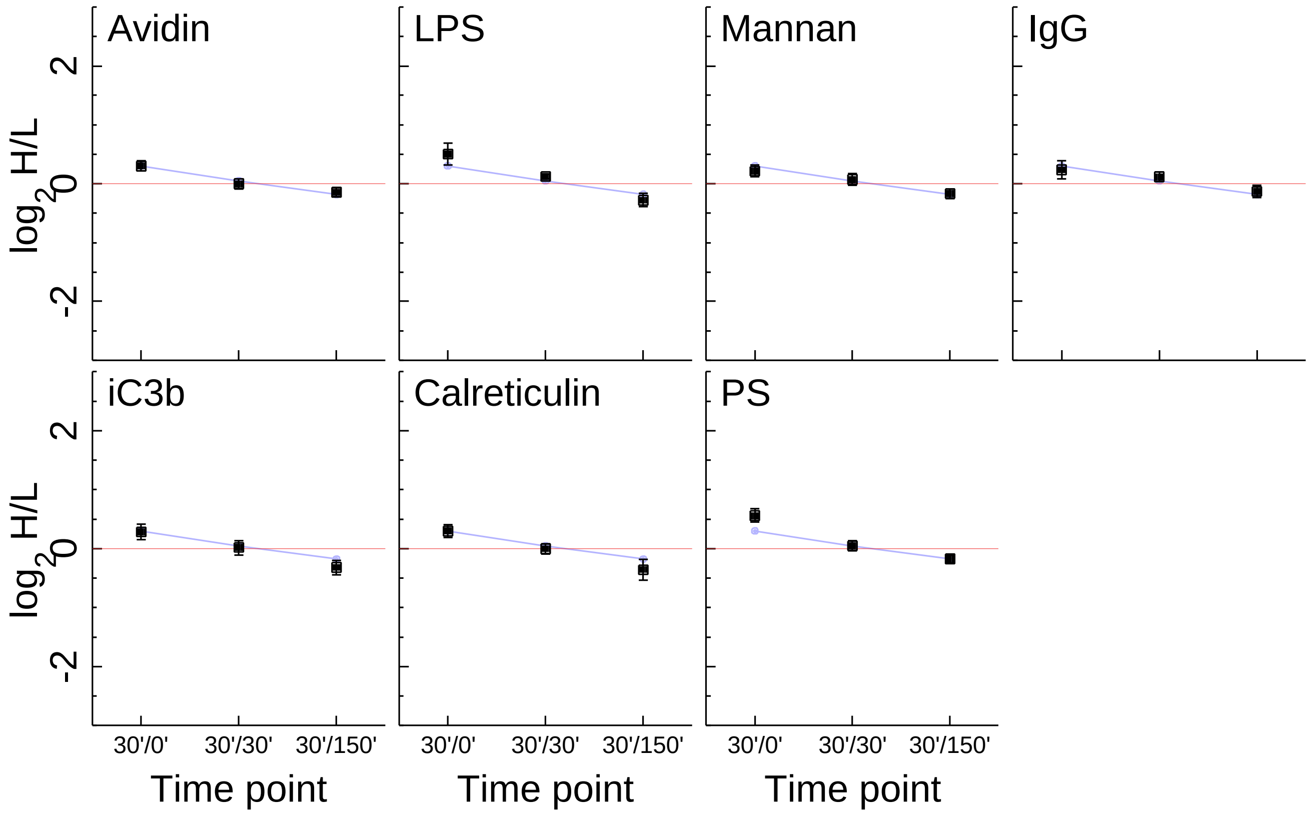

ctsa (G3X8T3)

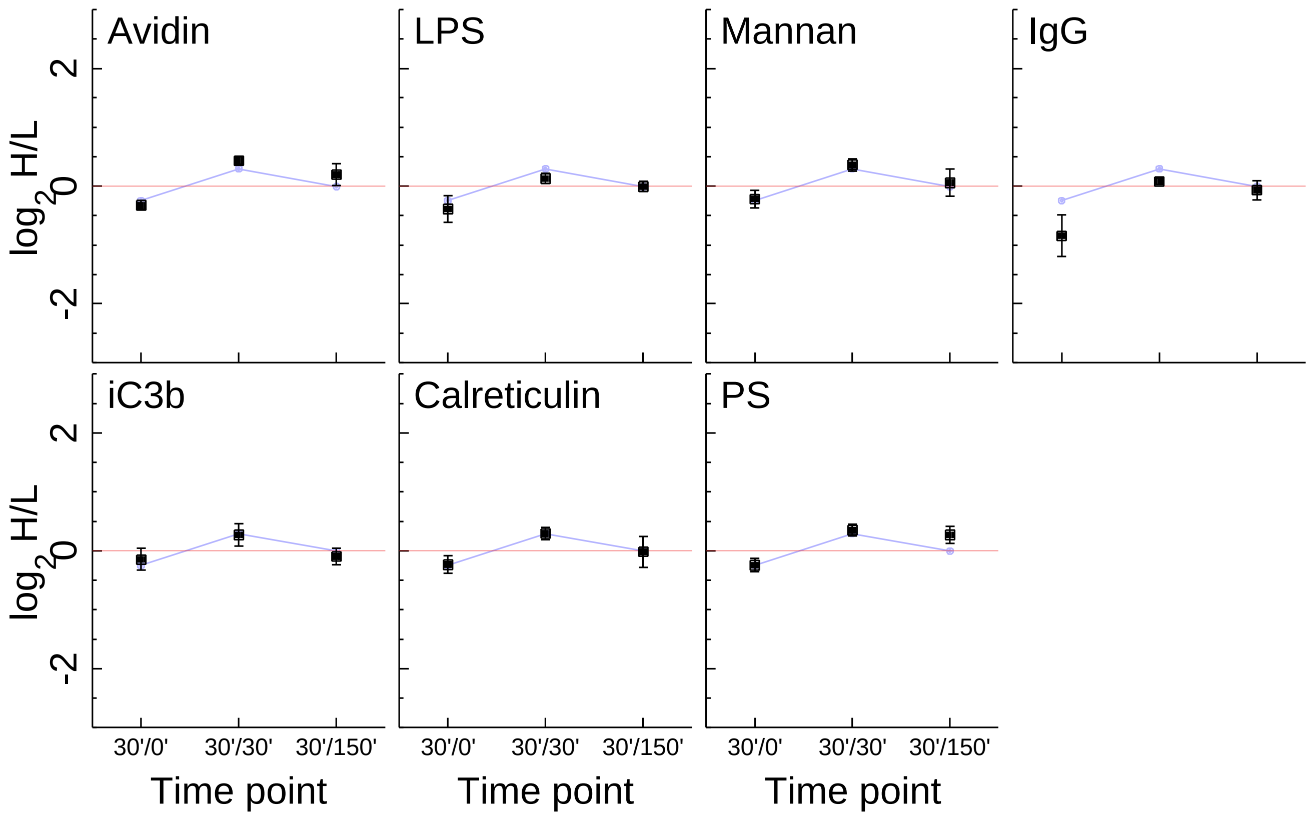
Avidin
LPS
Mannan
IgG
iC3b
Calreticulin
PS
log2 H/L
2
0
-2
30'/0'
30'/30'
30'/150'
Time point

ctsb (P10605)

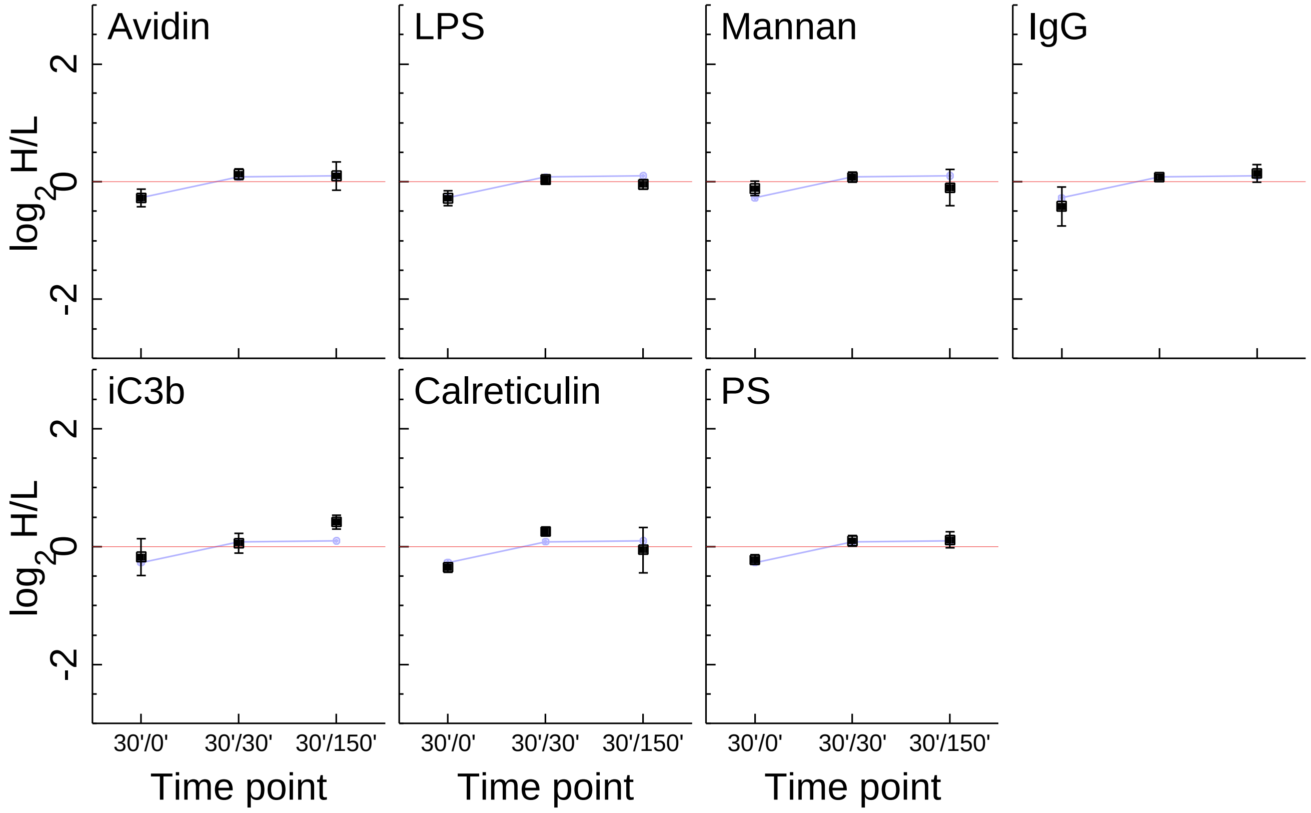

Avidin
LPS
Mannan
IgG
iC3b
Calreticulin
PS
log2 H/L
2
0
-2
30'/0'
30'/30'
30'/150'
Time point

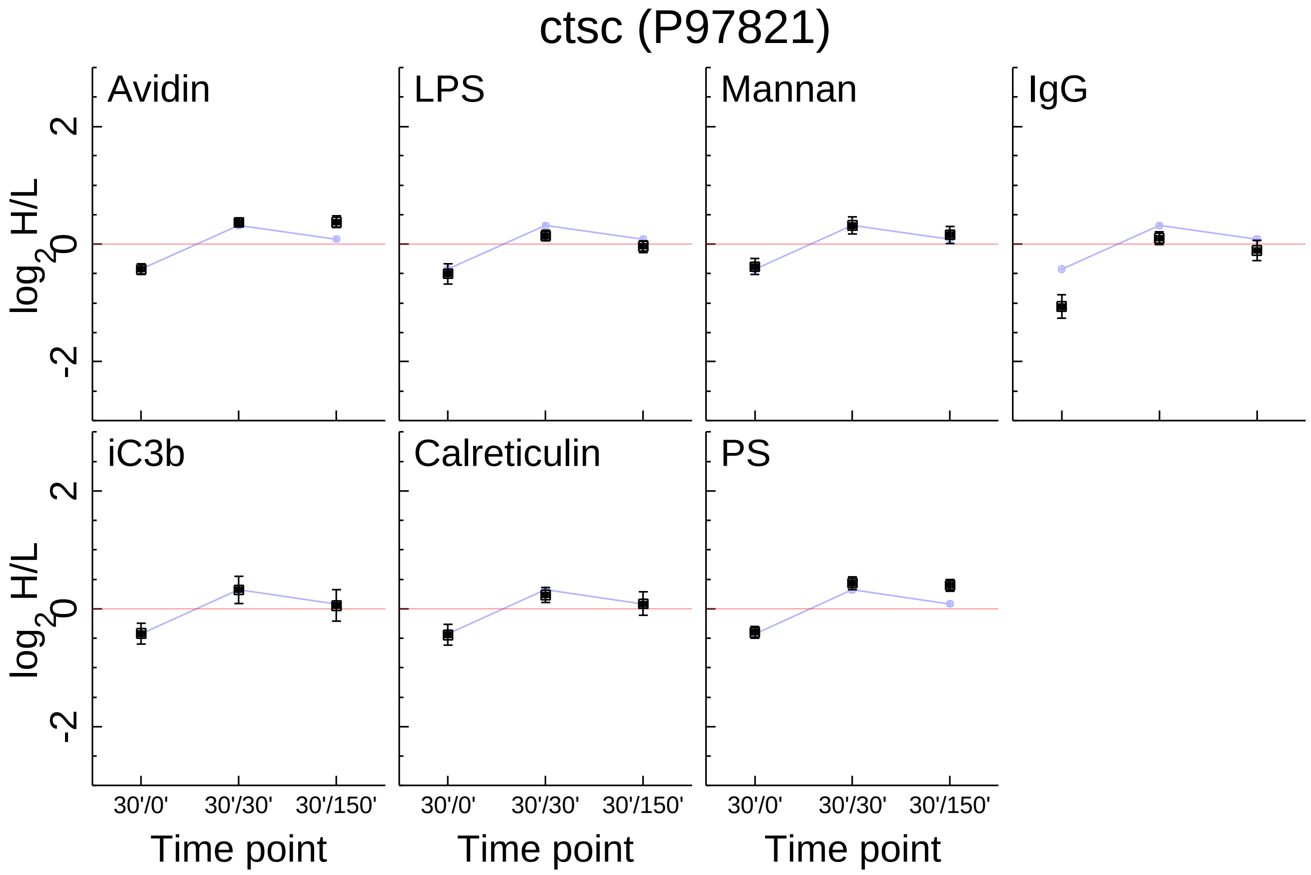

ctsc (P97821)
Avidin
LPS
Mannan
IgG
iC3b
Calreticulin
PS
log2 H/L
2
0
-2
30'/0'
30'/30'
30'/150'
Time point

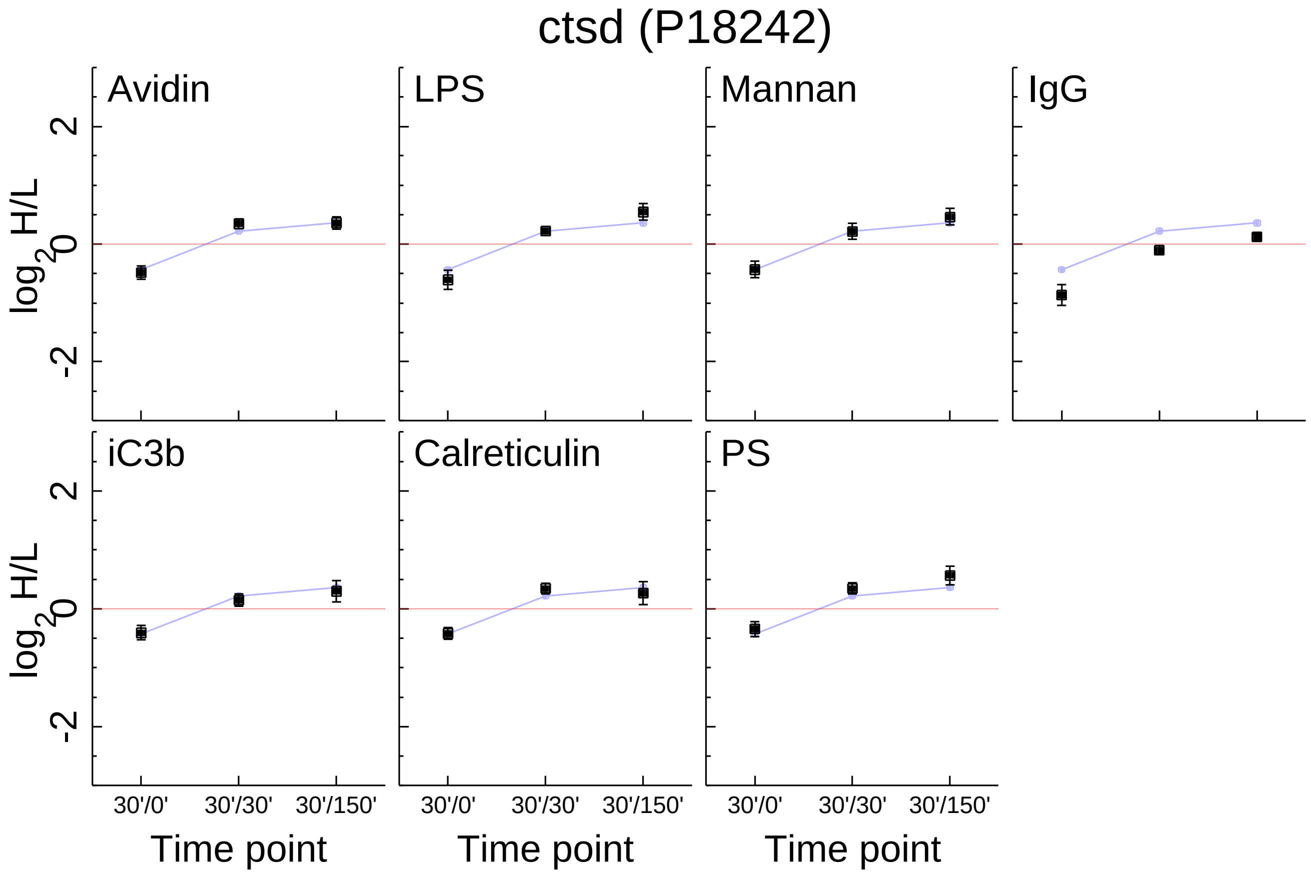
ctsd (P18242)
Avidin
LPS
Mannan
IgG
iC3b
Calreticulin
PS
log2 H/L
2
0
-2
30'/0'
30'/30'
30'/150'
Time point

ctsf (Q9R013)

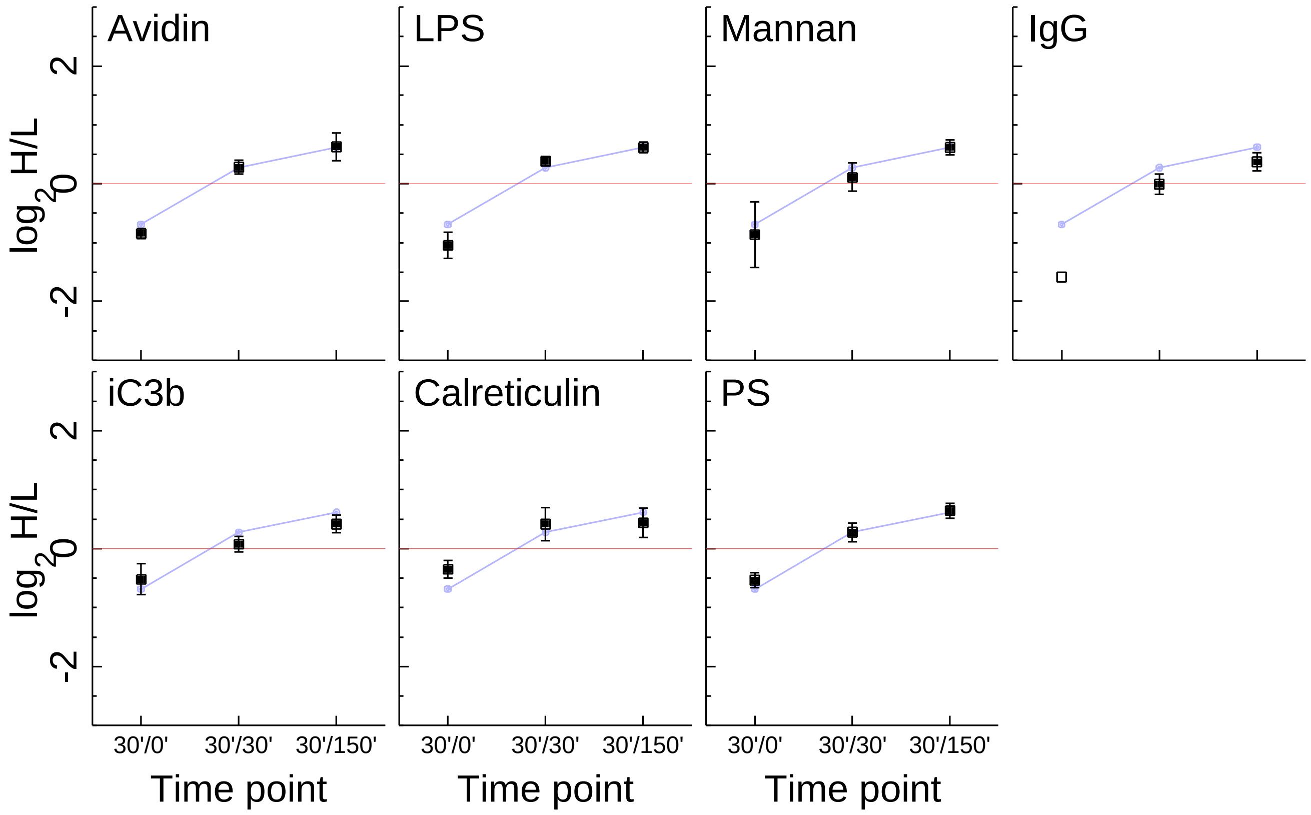

Avidin
LPS
Mannan
IgG
iC3b
Calreticulin
PS
log2 H/L
2
0
-2
30'/0'
30'/30'
30'/150'
Time point

ctsh (P49935)

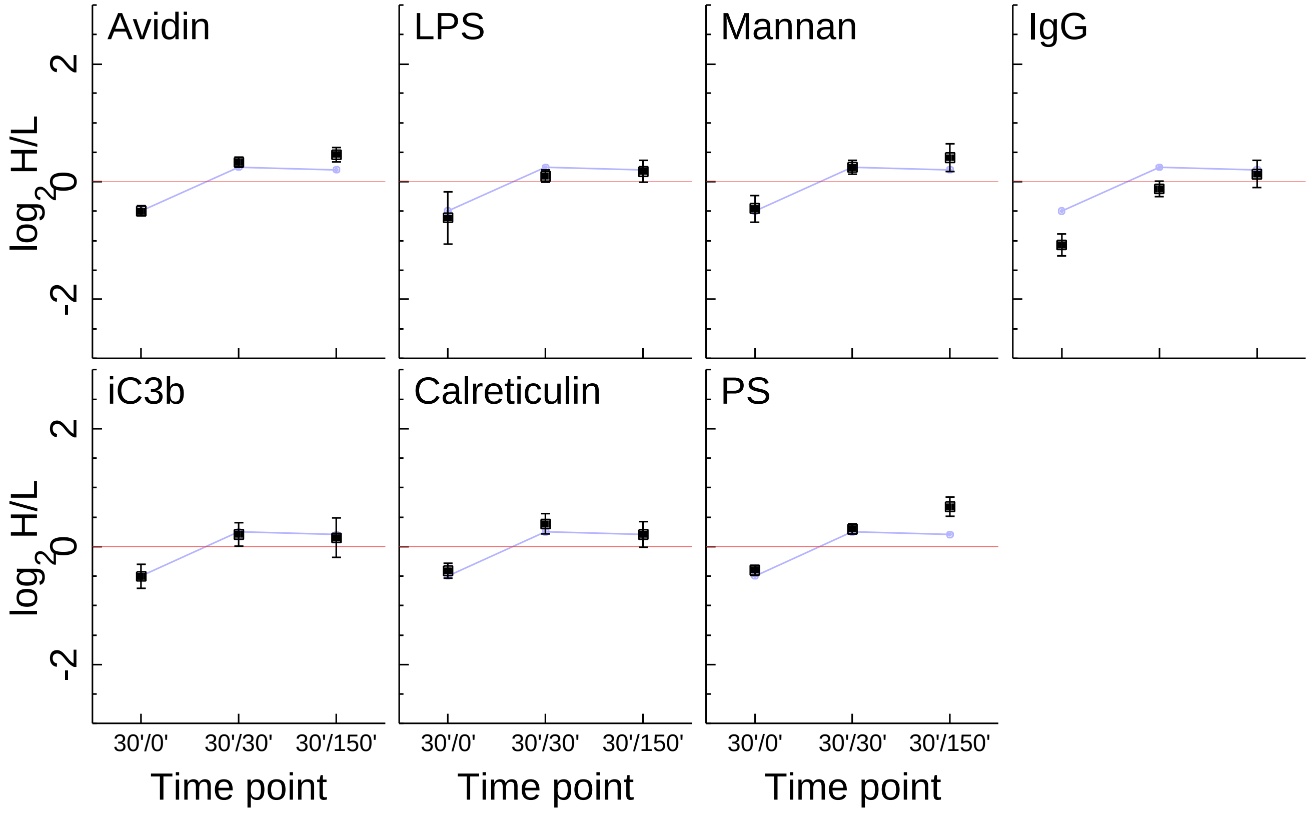

Avidin
LPS
Mannan
IgG
iC3b
Calreticulin
PS
log2 H/L
2
0
-2
30'/0'
30'/30'
30'/150'
Time point

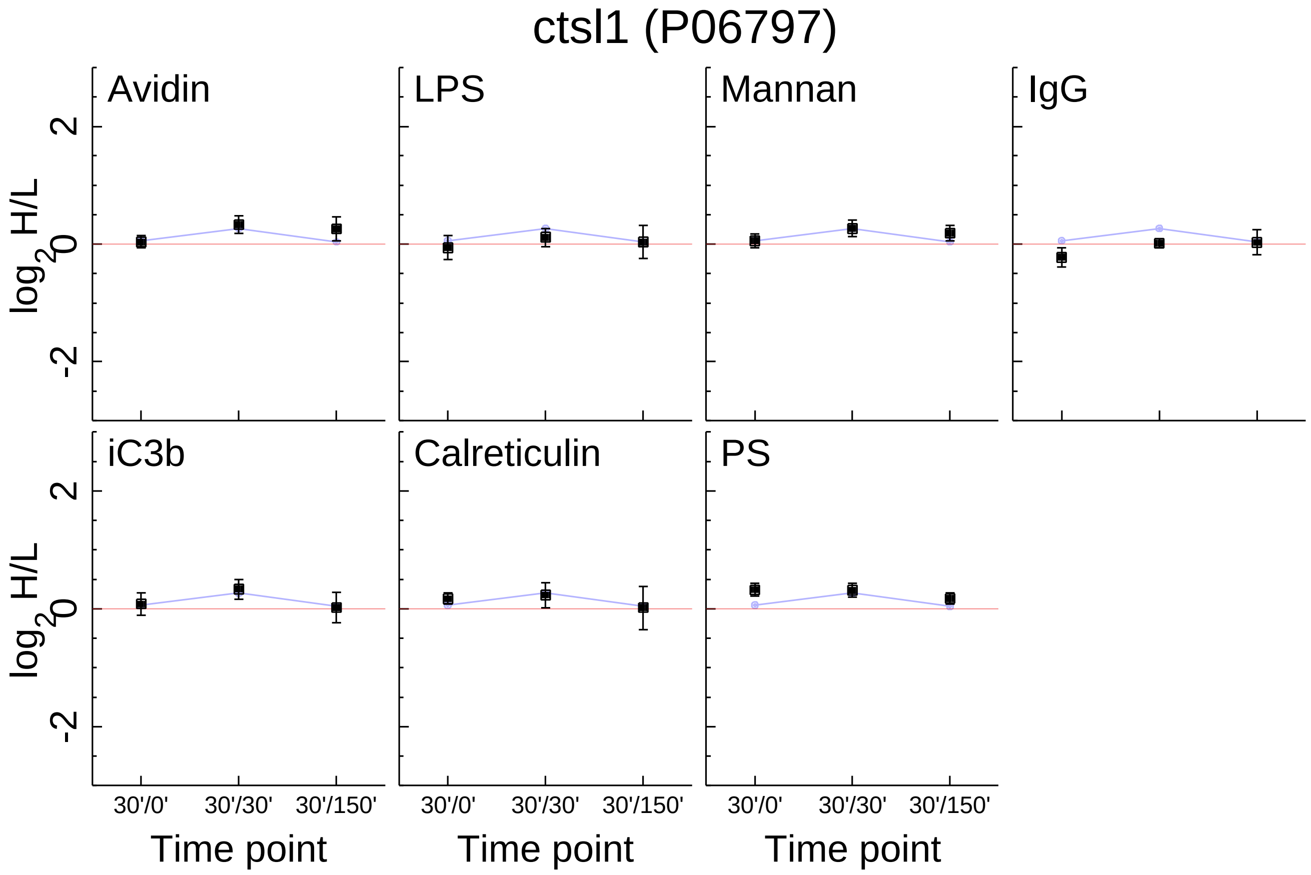
ctsl1 (P06797)
Avidin
LPS
Mannan
IgG
iC3b
Calreticulin
PS
log2 H/L
2
0
-2
30'/0'
30'/30'
30'/150'
Time point

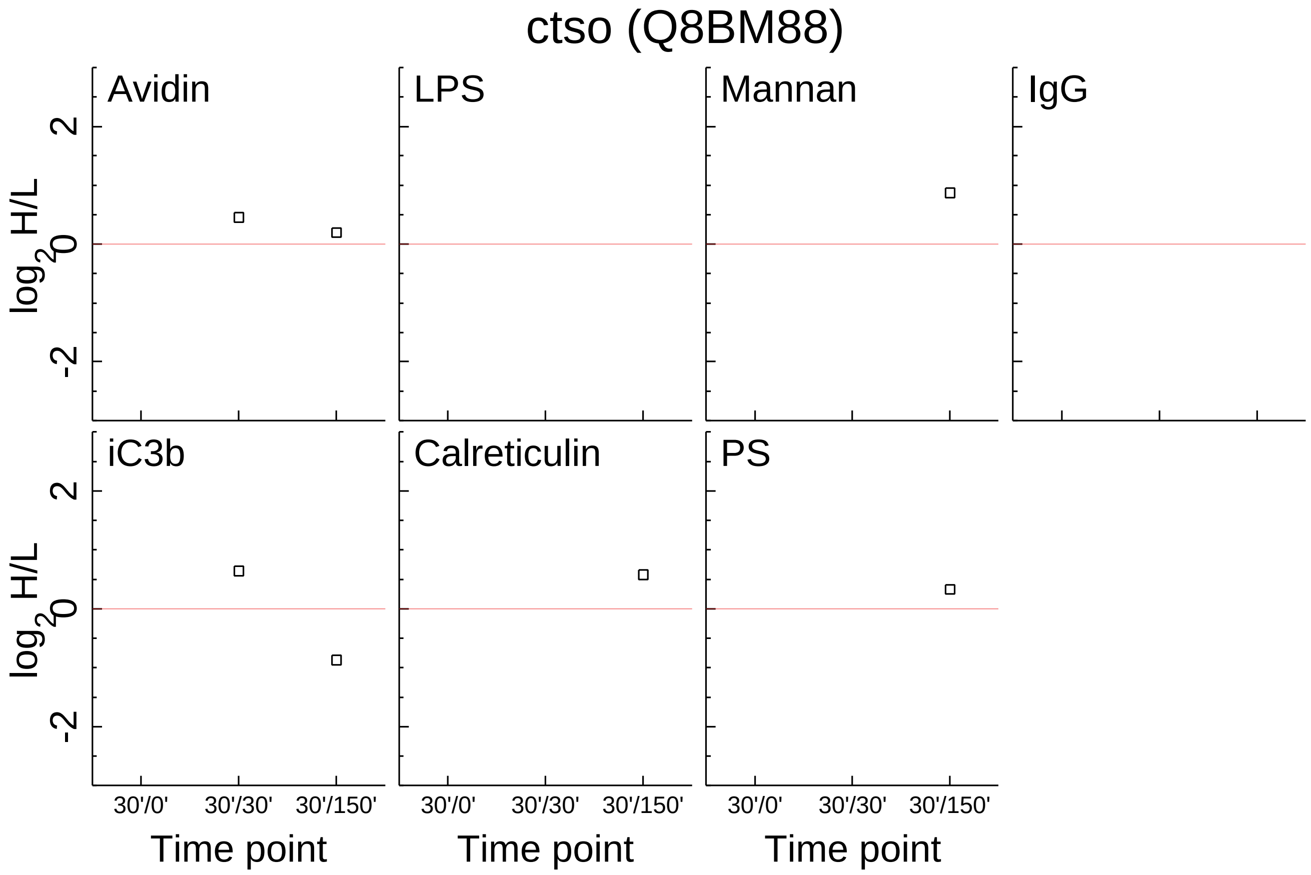
ctso (Q8BM88)
Avidin
LPS
Mannan
IgG
iC3b
Calreticulin
PS
log2 H/L
2
0
-2
30'/0'
30'/30'
30'/150'
Time point

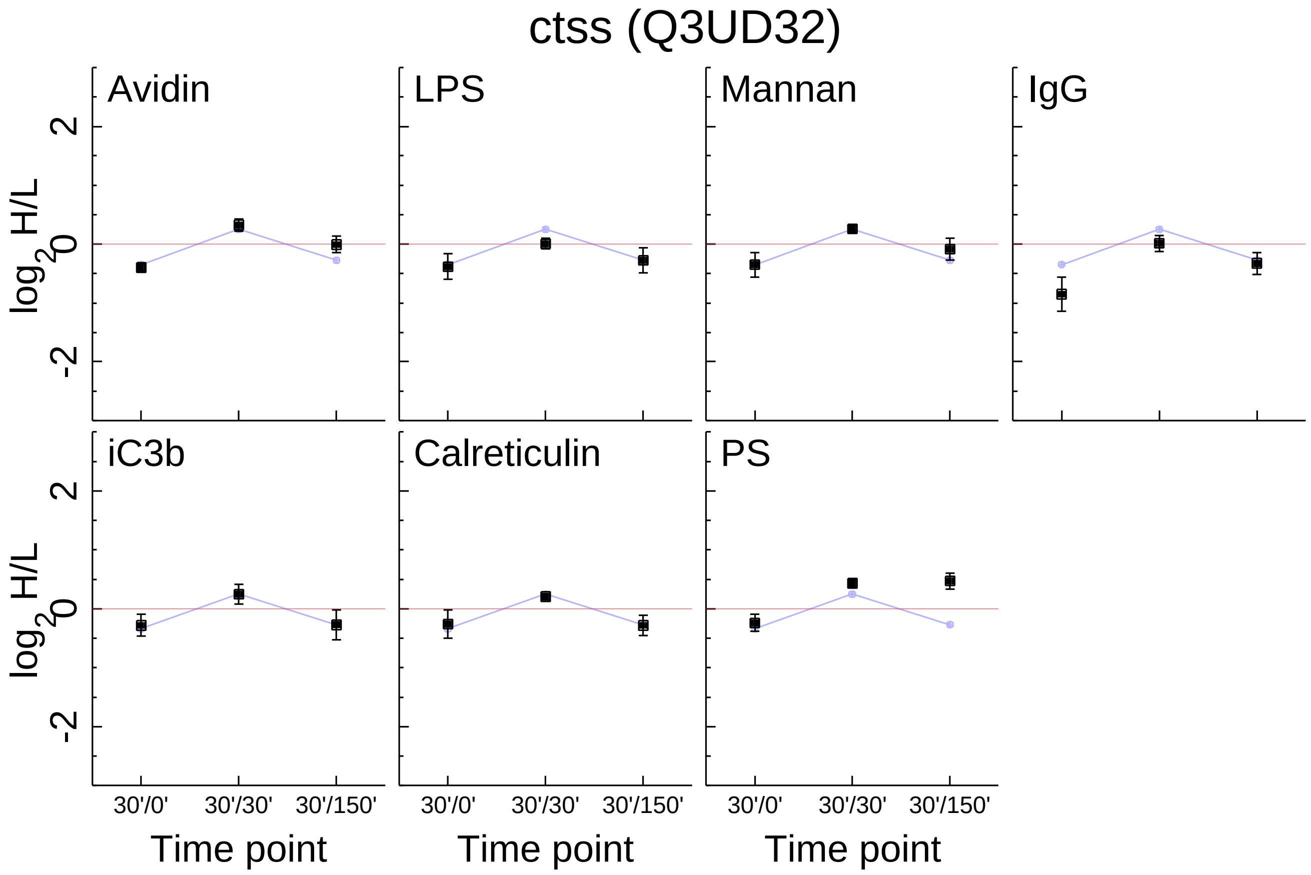

ctss (Q3UD32)
Avidin
LPS
Mannan
IgG
iC3b
Calreticulin
PS
log2 H/L
2
0
-2
30'/0'
30'/30'
30'/150'
Time point

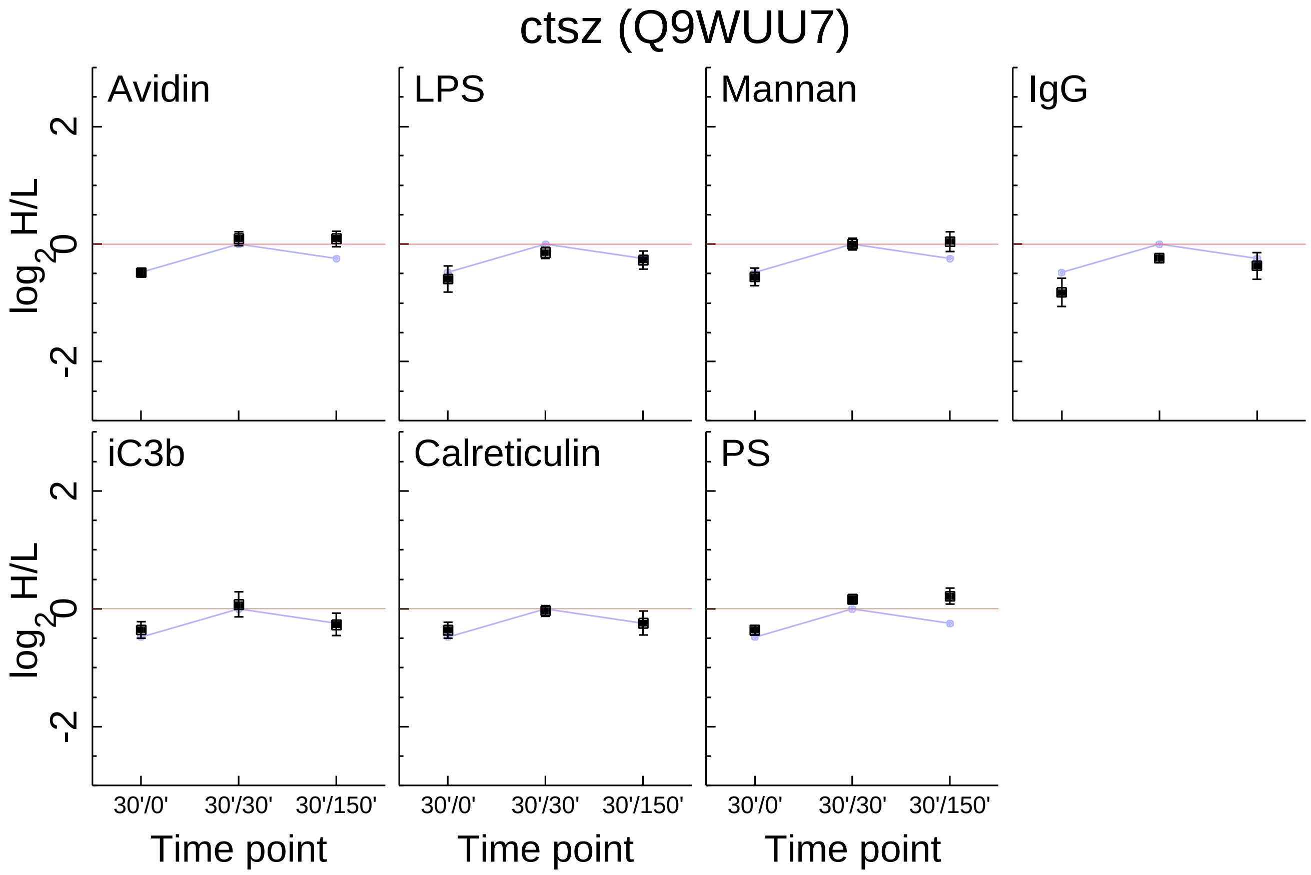

ctsz (Q9WUU7)
Avidin
LPS
Mannan
IgG
iC3b
Calreticulin
PS
log2 H/L
2
0
-2
30'/0'
30'/30'
30'/150'
Time point

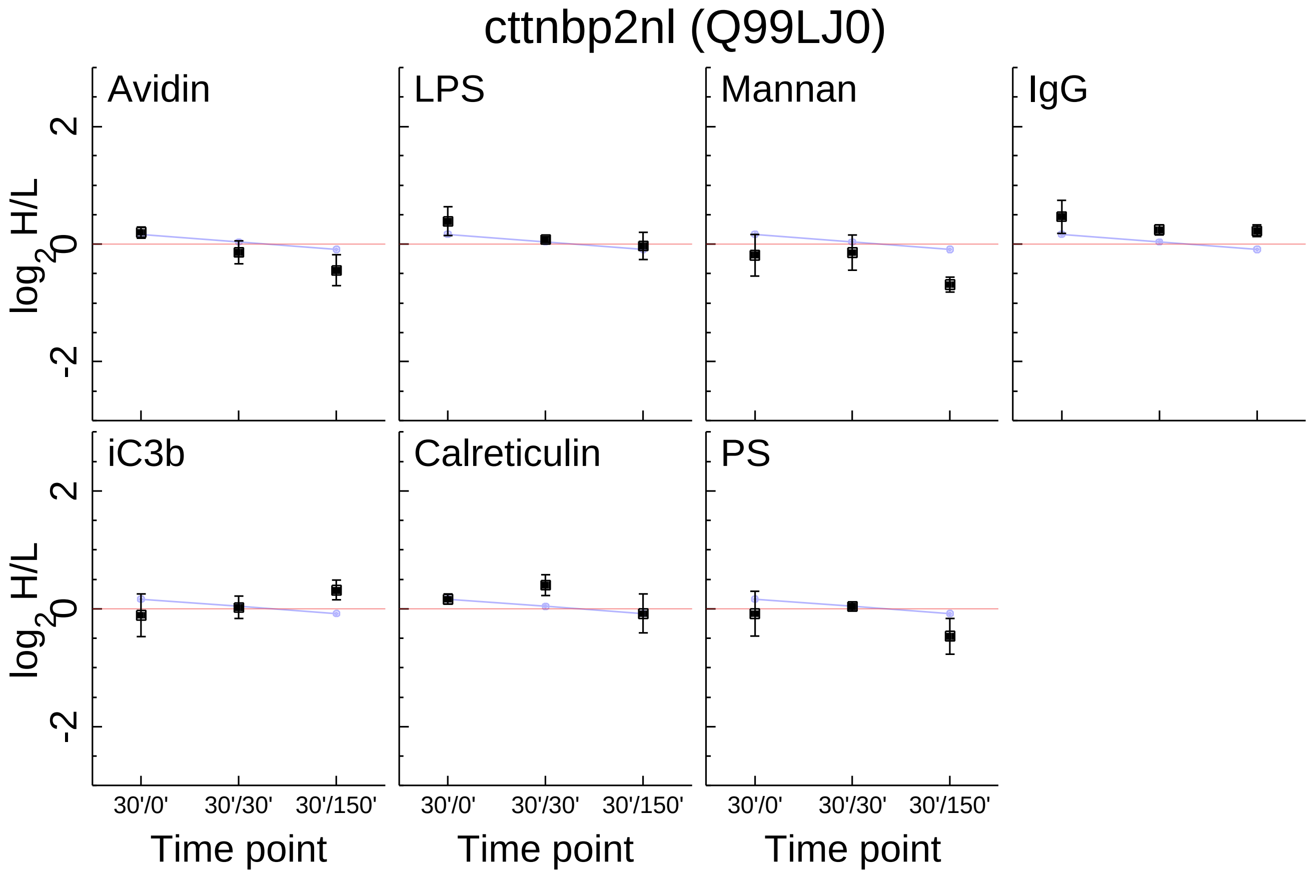
cttnbp2nl (Q99LJ0)
Avidin
LPS
Mannan
IgG
iC3b
Calreticulin
PS
log2 H/L
2
0
-2
30'/0'
30'/30'
30'/150'
Time point

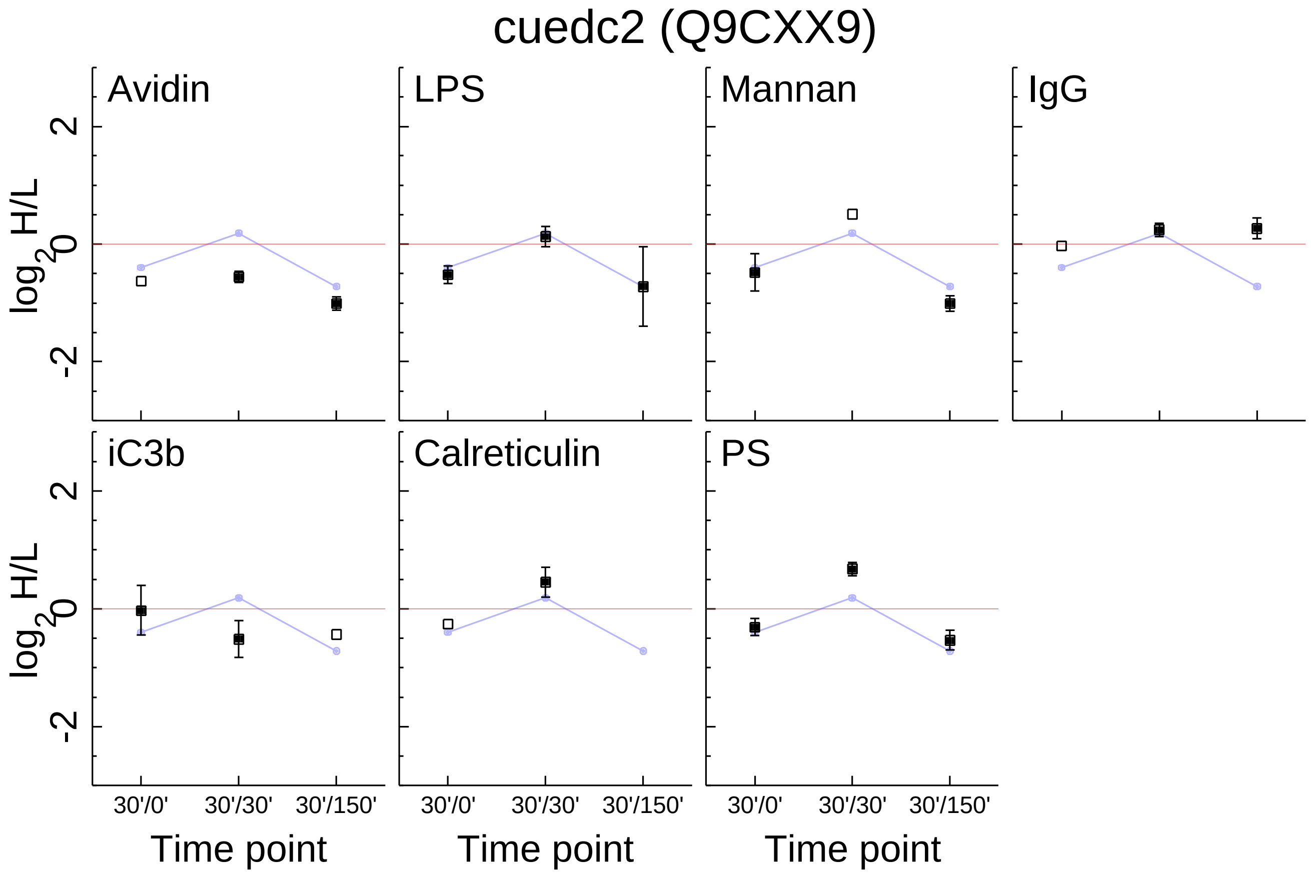

cuedc2 (Q9CXX9)
Avidin
LPS
Mannan
IgG
iC3b
Calreticulin
PS
log2 H/L
2
0
-2
30'/0'
30'/30'
30'/150'
Time point

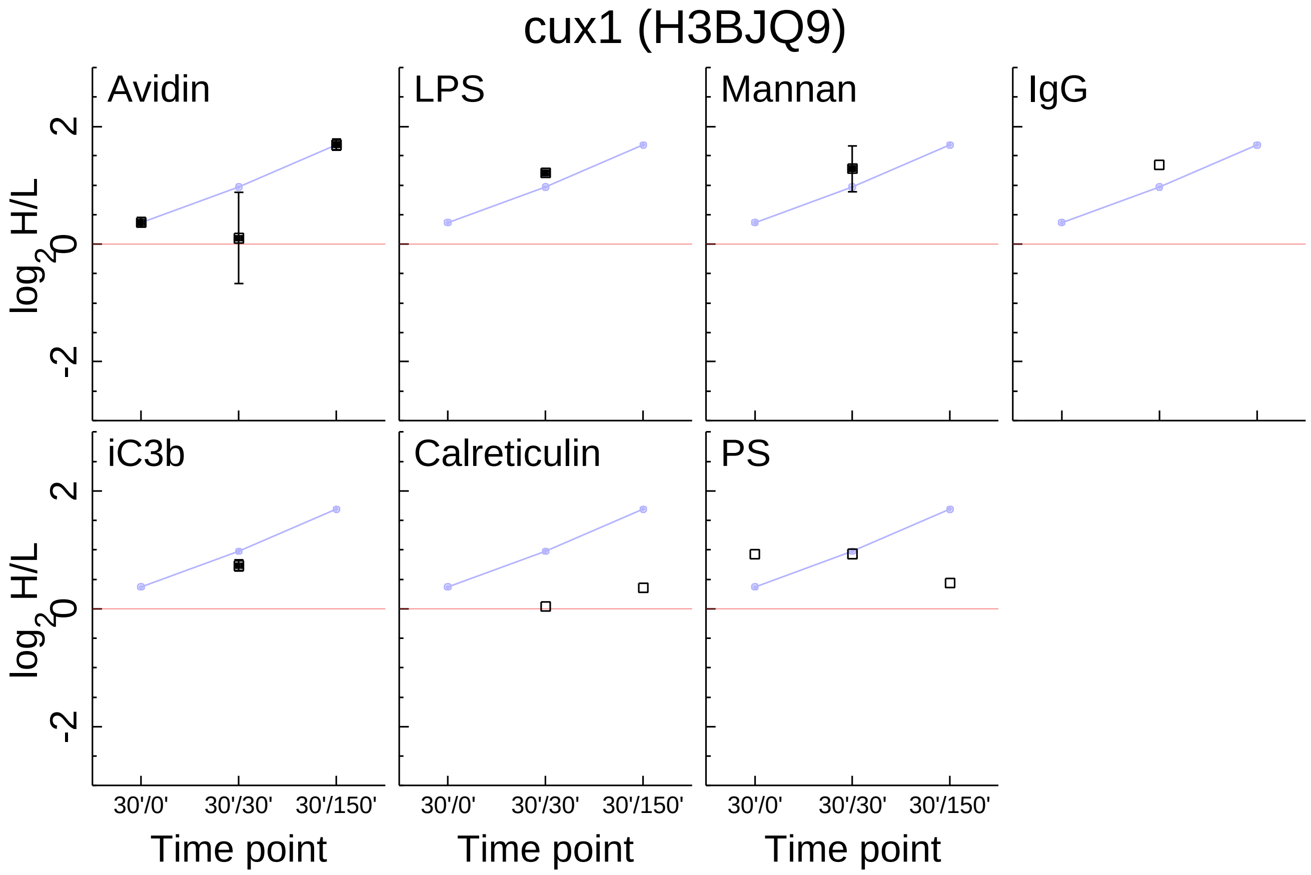
cux1 (H3BJQ9)
Avidin
LPS
Mannan
IgG
iC3b
Calreticulin
PS
log2 H/L
2
0
-2
30'/0'
30'/30'
30'/150'
Time point

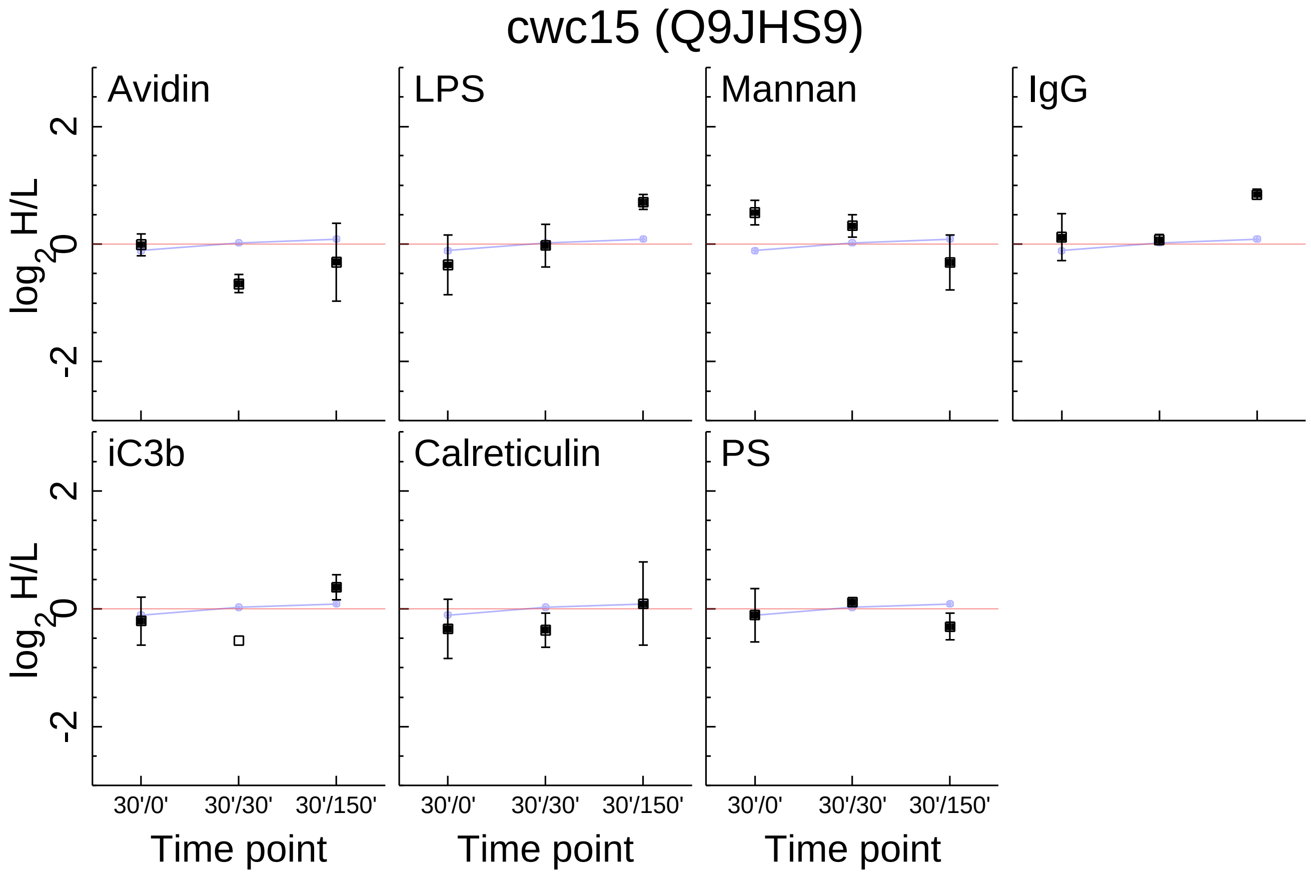

cwc15 (Q9JHS9)
Avidin
LPS
Mannan
IgG
iC3b
Calreticulin
PS
log2 H/L
2
0
-2
30'/0'
30'/30'
30'/150'
Time point

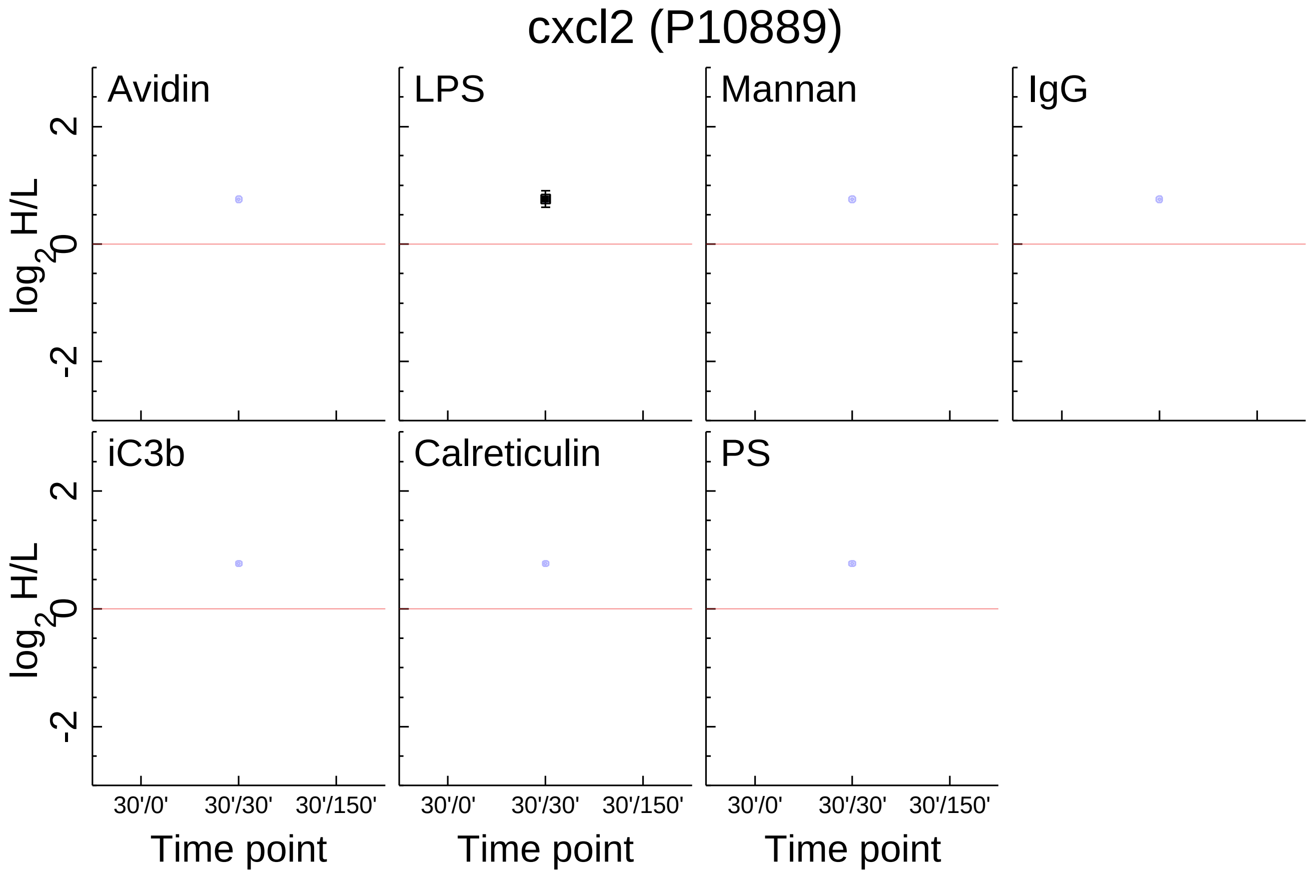
cxcl2 (P10889)
Avidin
LPS
Mannan
IgG
iC3b
Calreticulin
PS
log2 H/L
2
0
-2
30'/0'
30'/30'
30'/150'
Time point

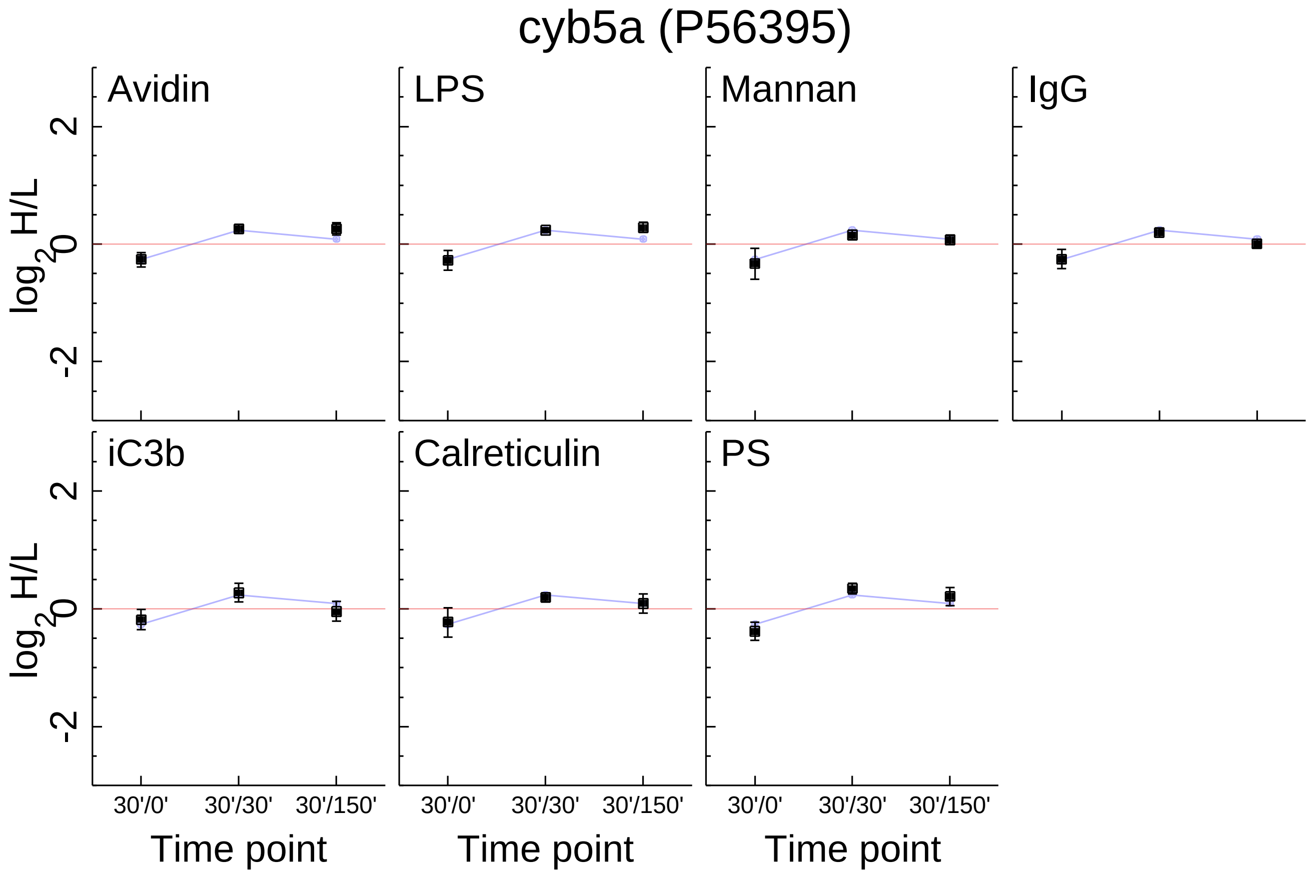

cyb5a (P56395)
Avidin
LPS
Mannan
IgG
iC3b
Calreticulin
PS
log2 H/L
2
0
-2
30'/0'
30'/30'
30'/150'
Time point

cyb5b (Q9CQX2)

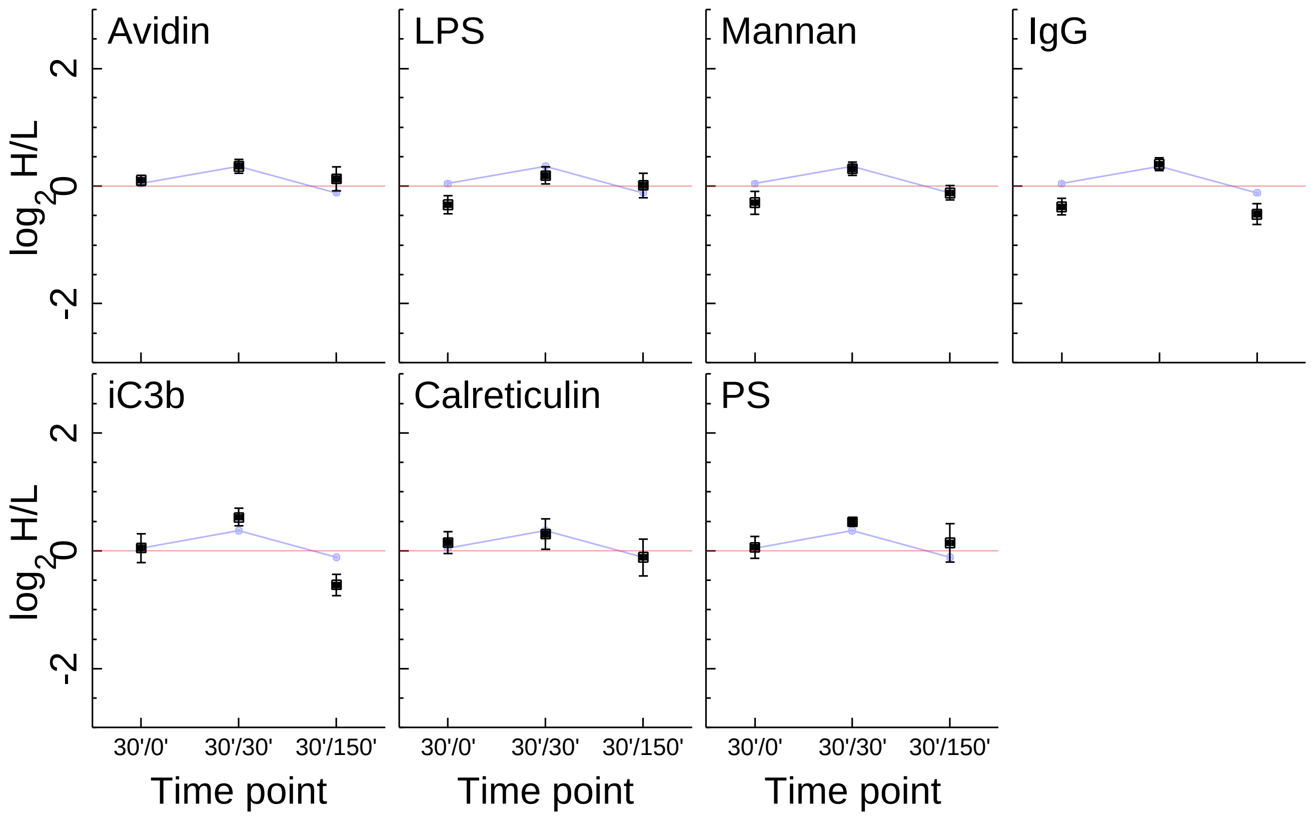

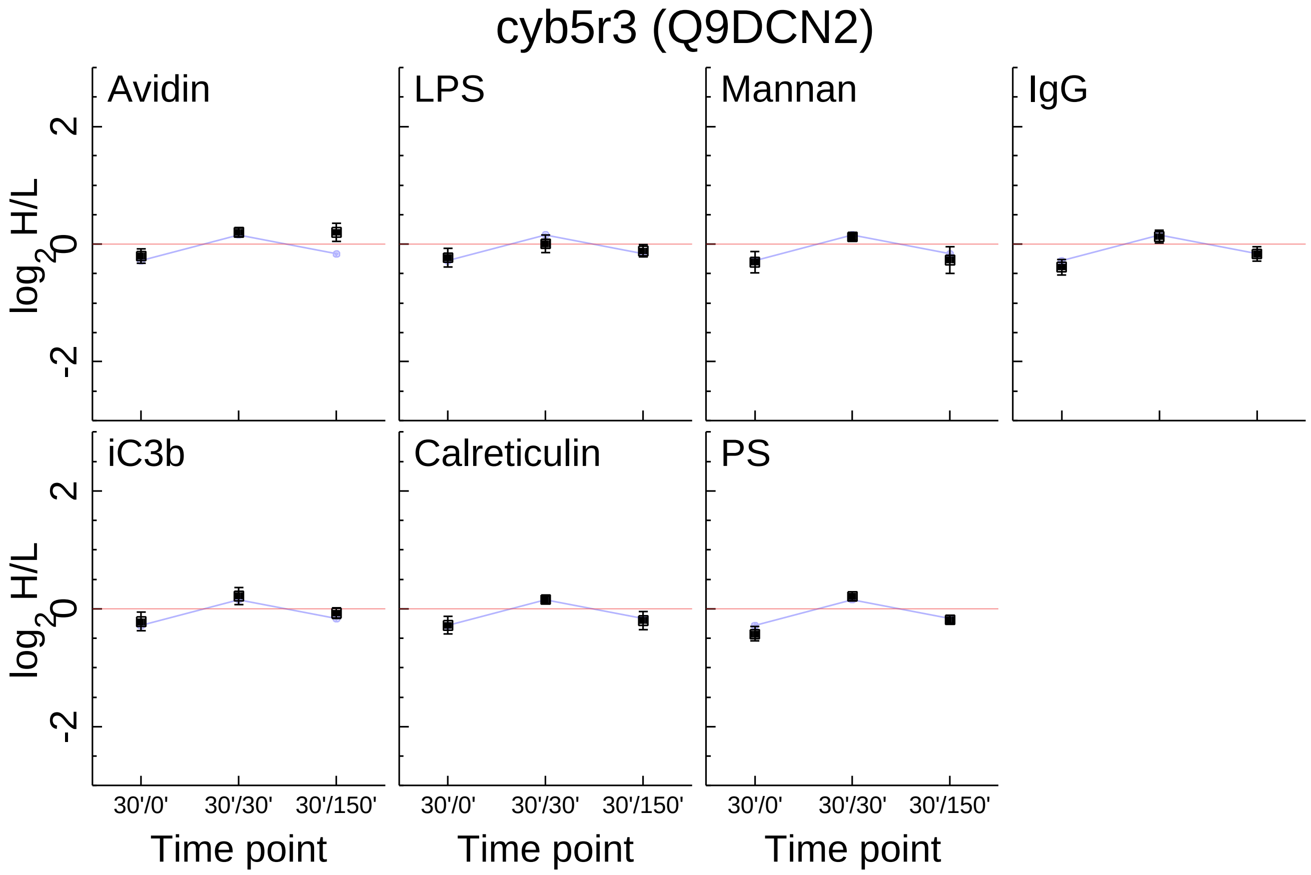

cyb5r3 (Q9DCN2)
Avidin
LPS
Mannan
IgG
iC3b
Calreticulin
PS
log2 H/L
2
0
-2
30'/0'
30'/30'
30'/150'
Time point

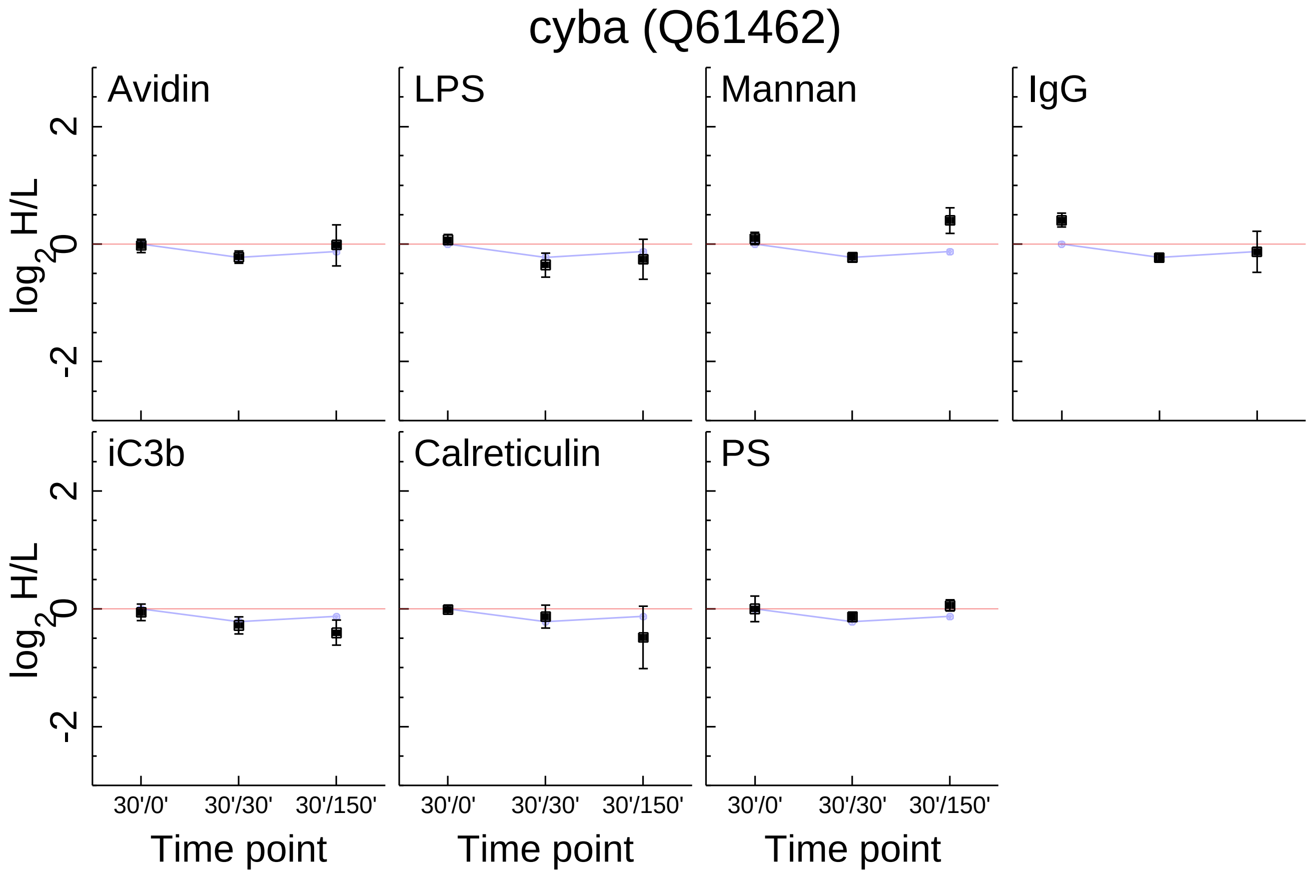
cyba (Q61462)
Avidin
LPS
Mannan
IgG
iC3b
Calreticulin
PS
log2 H/L
2
0
-2
30'/0'
30'/30'
30'/150'
Time point

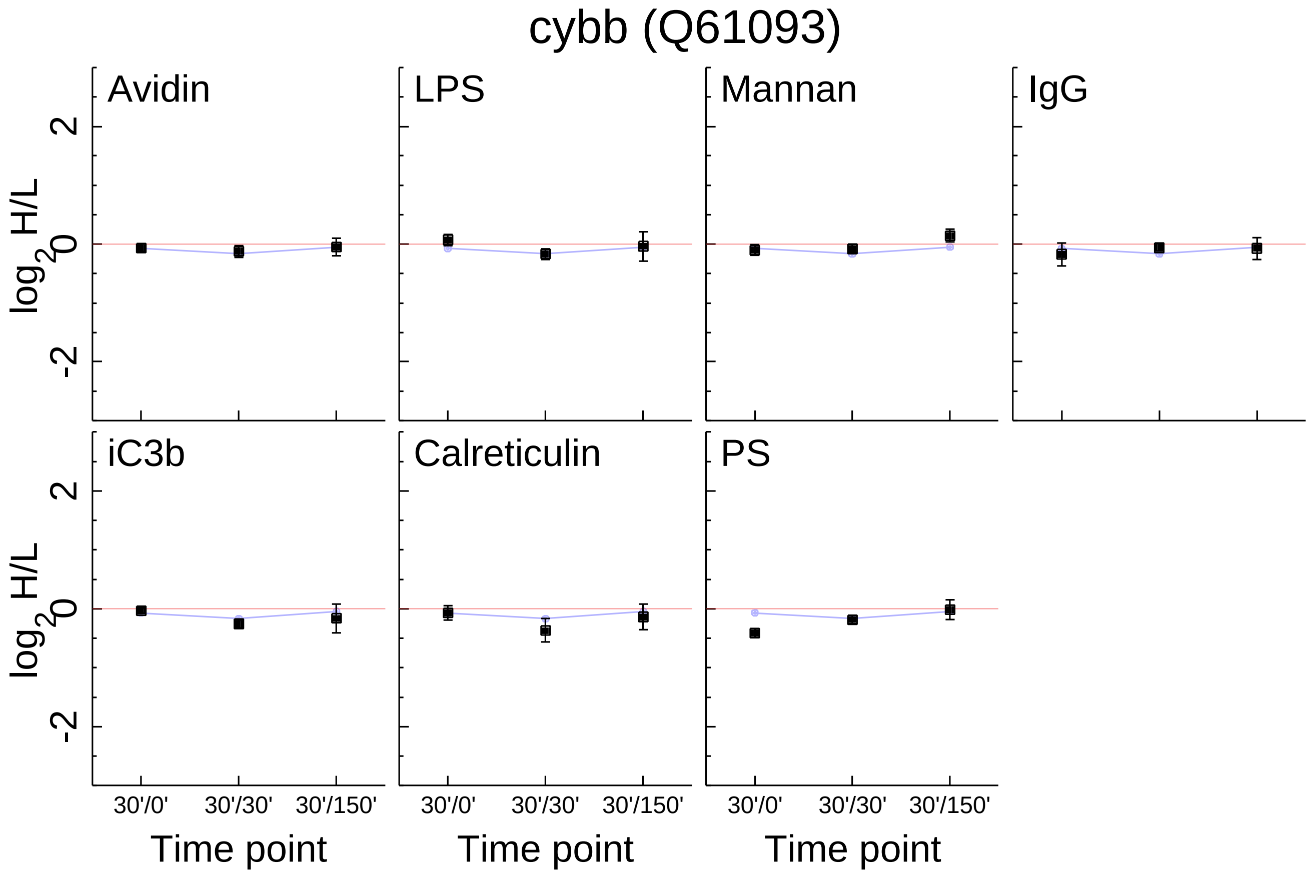
cybb (Q61093)
Avidin
LPS
Mannan
IgG
iC3b
Calreticulin
PS
log2 H/L
2
0
-2
30'/0'
30'/30'
30'/150'
Time point

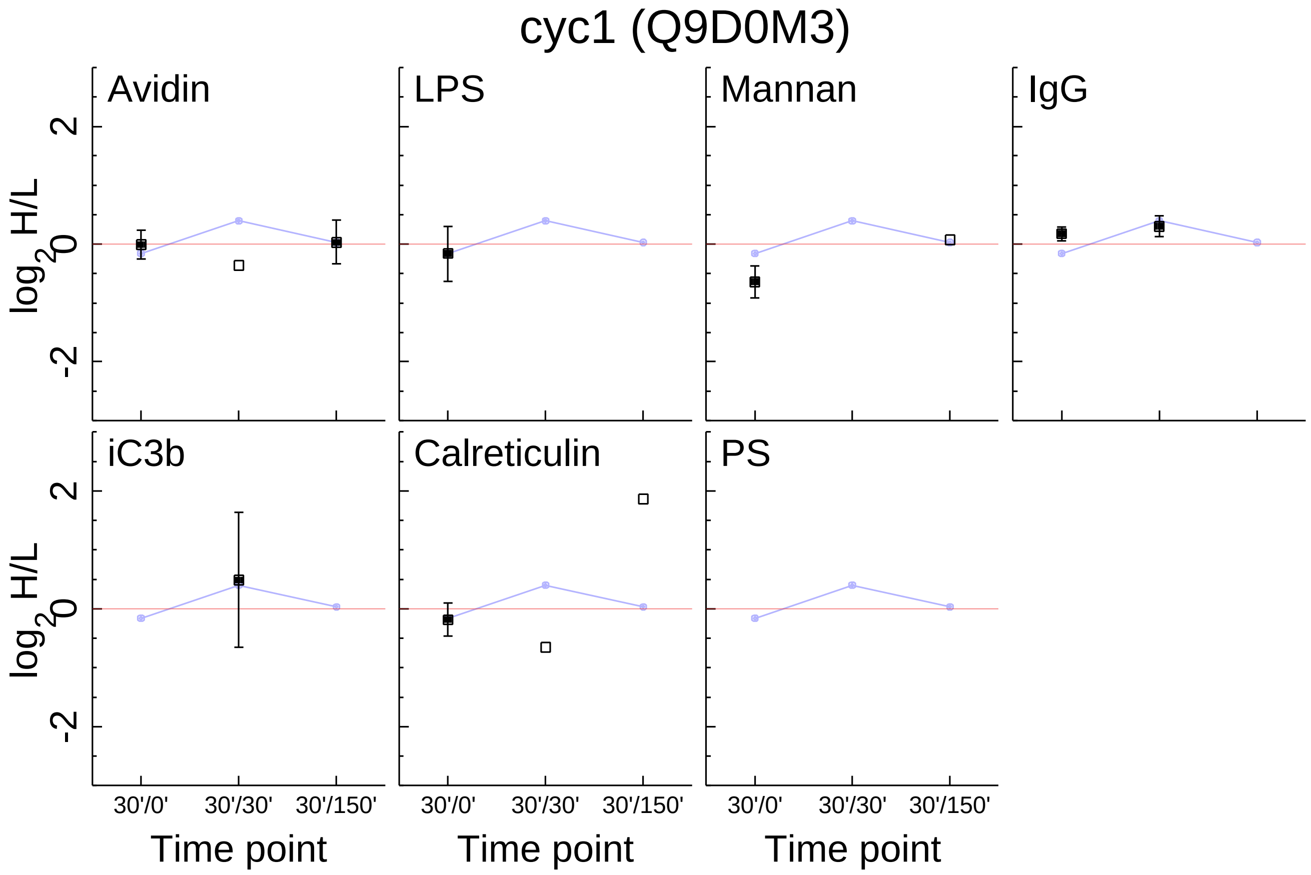

cyc1 (Q9D0M3)
Avidin
LPS
Mannan
IgG
iC3b
Calreticulin
PS
log2 H/L
2
0
-2
30'/0'
30'/30'
30'/150'
Time point

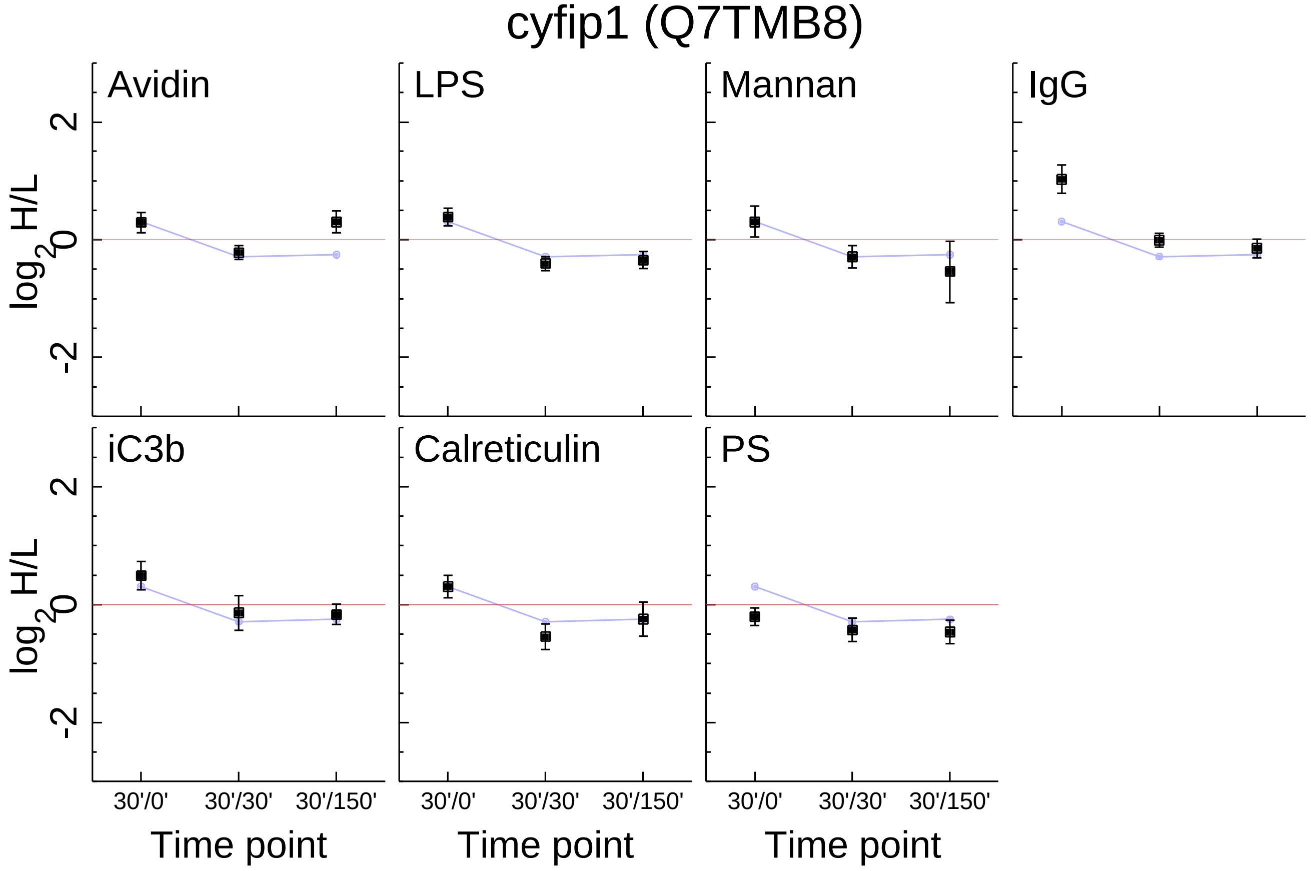
cyfip1 (Q7TMB8)
Avidin
LPS
Mannan
IgG
iC3b
Calreticulin
PS
log2 H/L
2
0
-2
30'/0'
30'/30'
30'/150'
Time point

cyp20a1 (Q8BKE6)

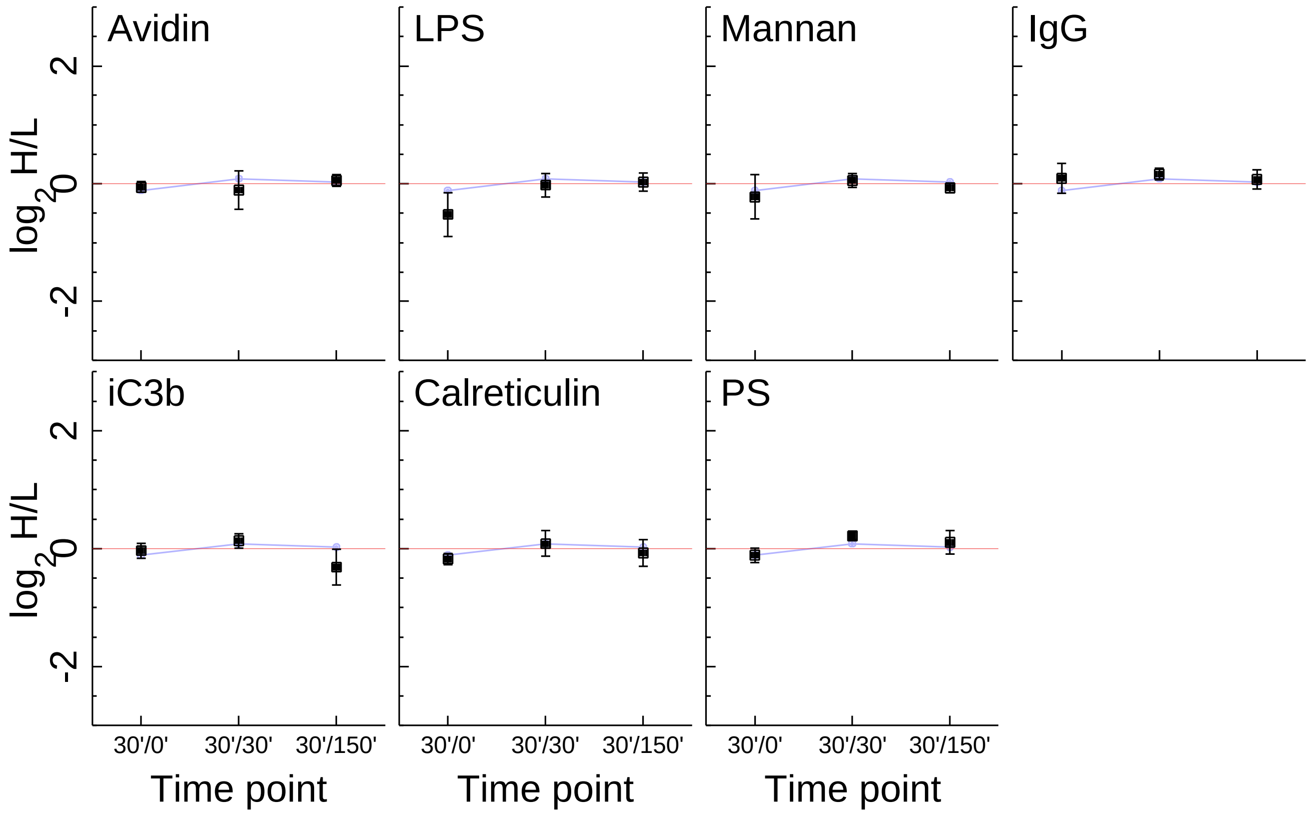

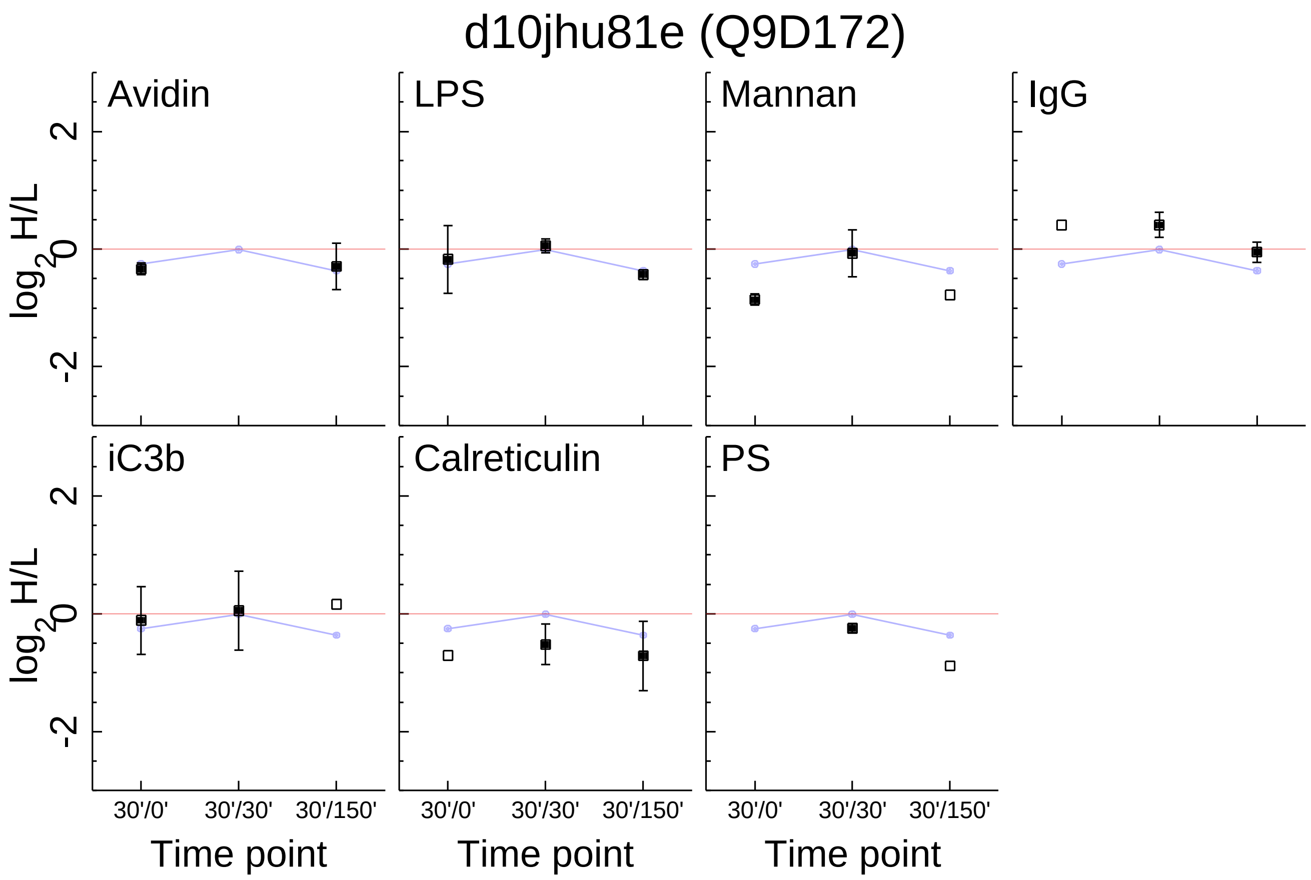
d10jhu81e (Q9D172)
Avidin
LPS
Mannan
IgG
iC3b
Calreticulin
PS
log2 H/L
2
0
-2
30'/0'
30'/30'
30'/150'
Time point

d10wsu52e (Q99LF4)

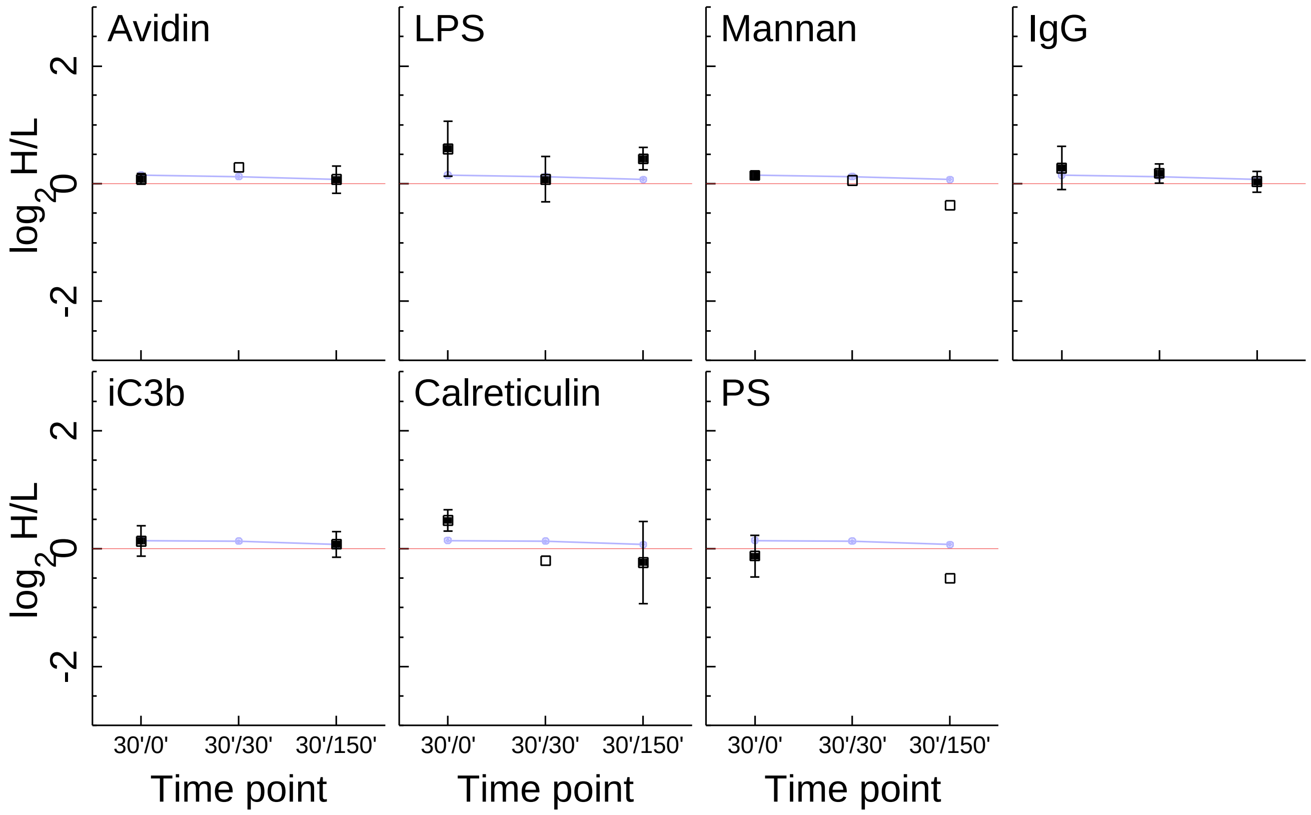

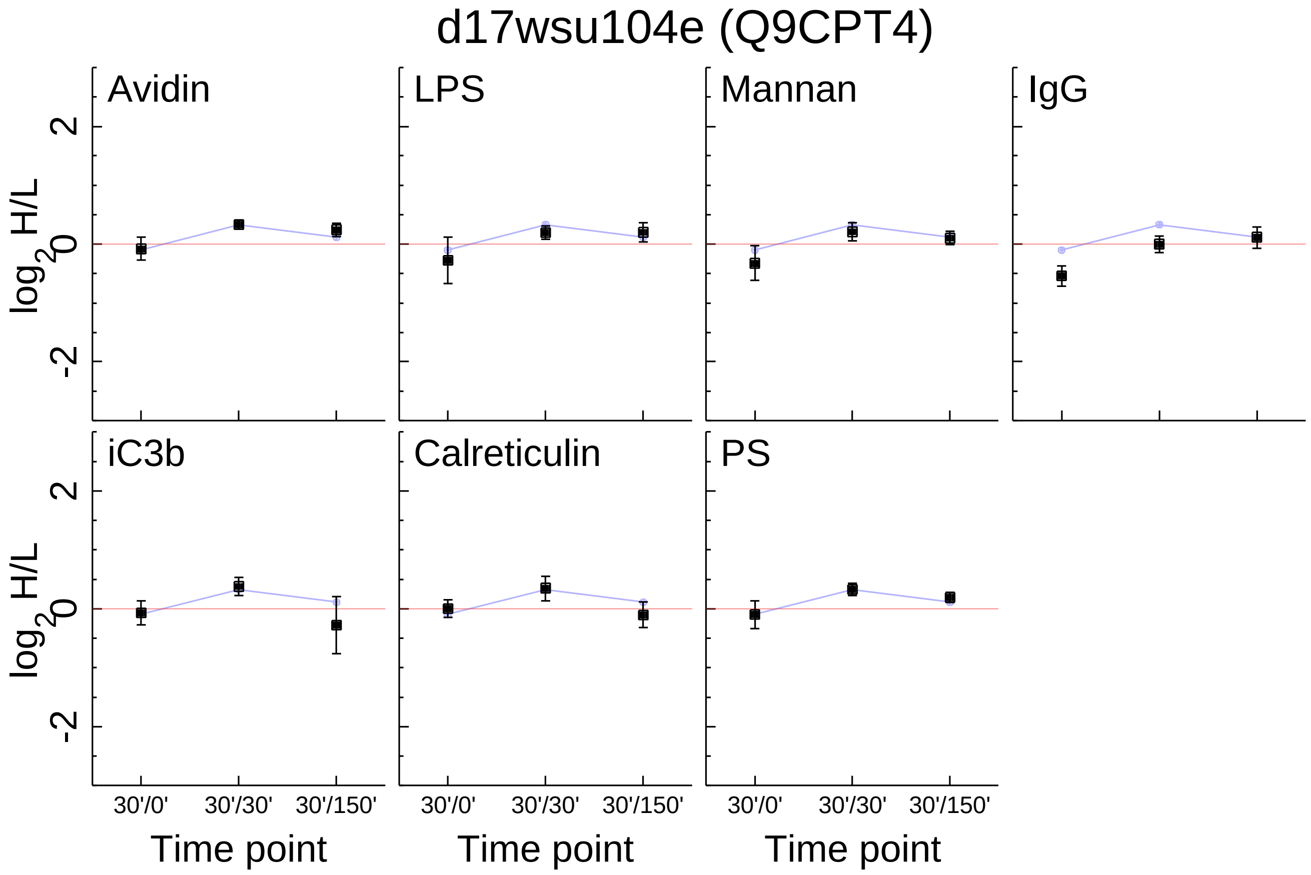
d17wsu104e (Q9CPT4)
Avidin
LPS
Mannan
IgG
iC3b
Calreticulin
PS
log2 H/L
2
0
-2
30'/0'
30'/30'
30'/150'
Time point

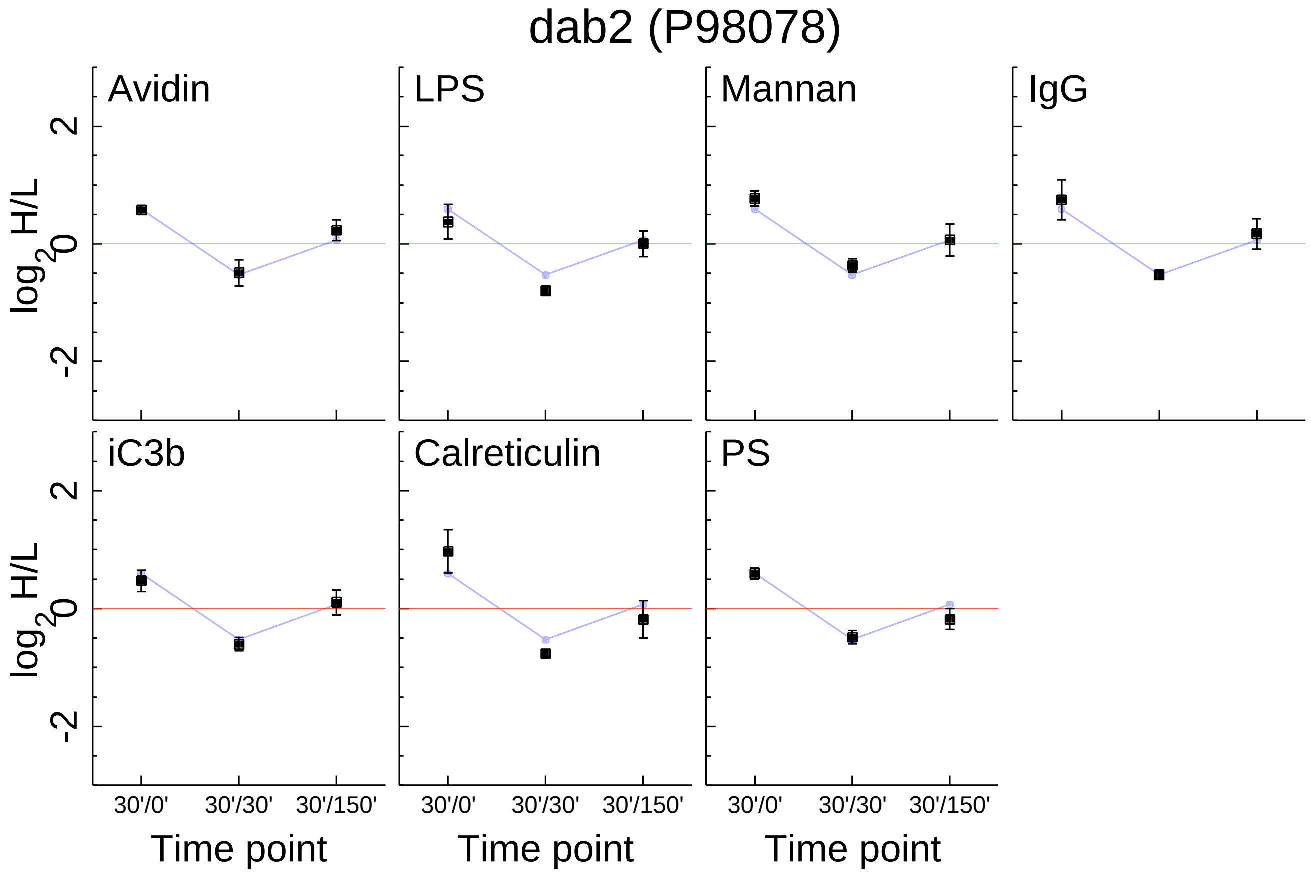

dab2 (P98078)
Avidin
LPS
Mannan
IgG
iC3b
Calreticulin
PS
log2 H/L
2
0
-2
30'/0'
30'/30'
30'/150'
Time point

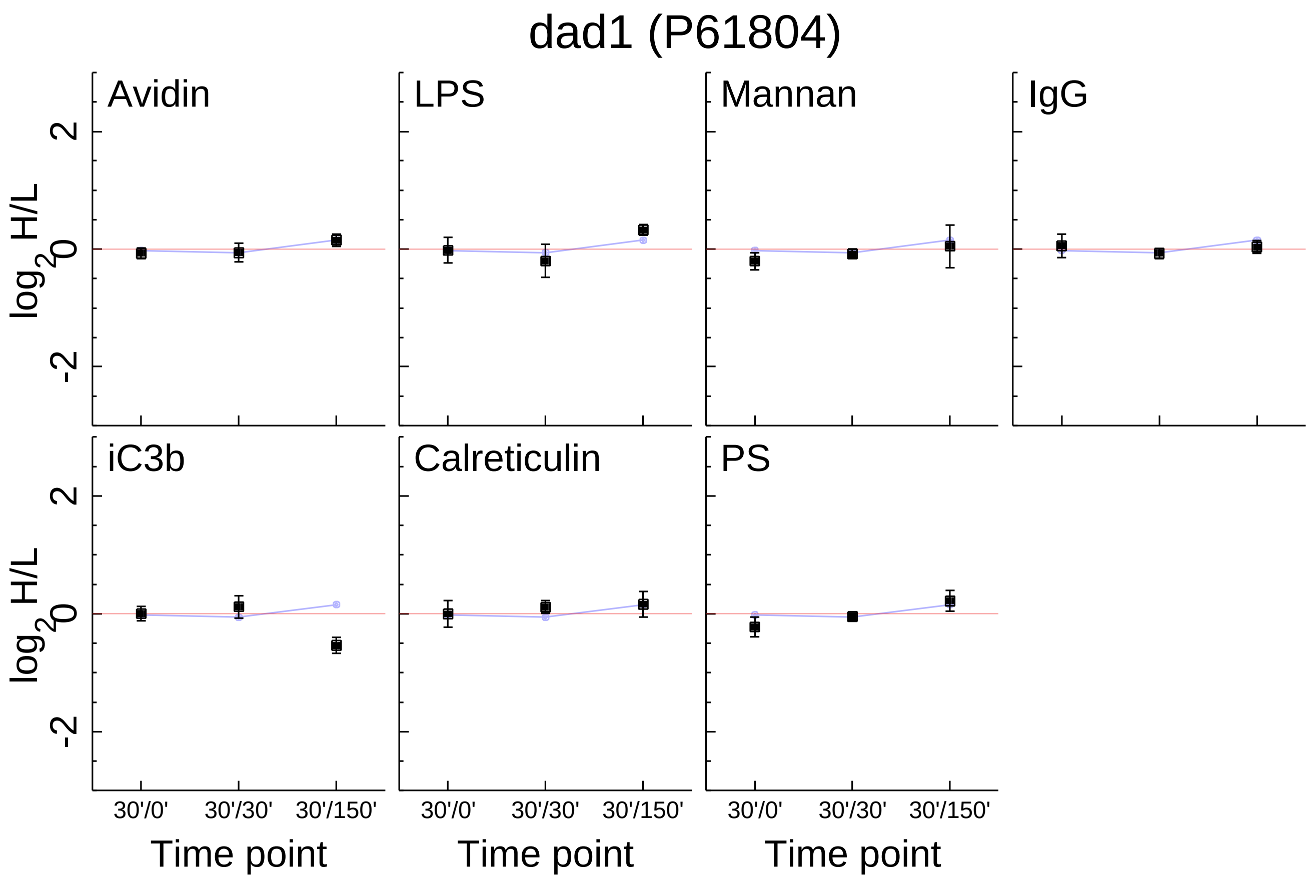
dad1 (P61804)
Avidin
LPS
Mannan
IgG
iC3b
Calreticulin
PS
log2 H/L
2
0
-2
30'/0'
30'/30'
30'/150'
Time point

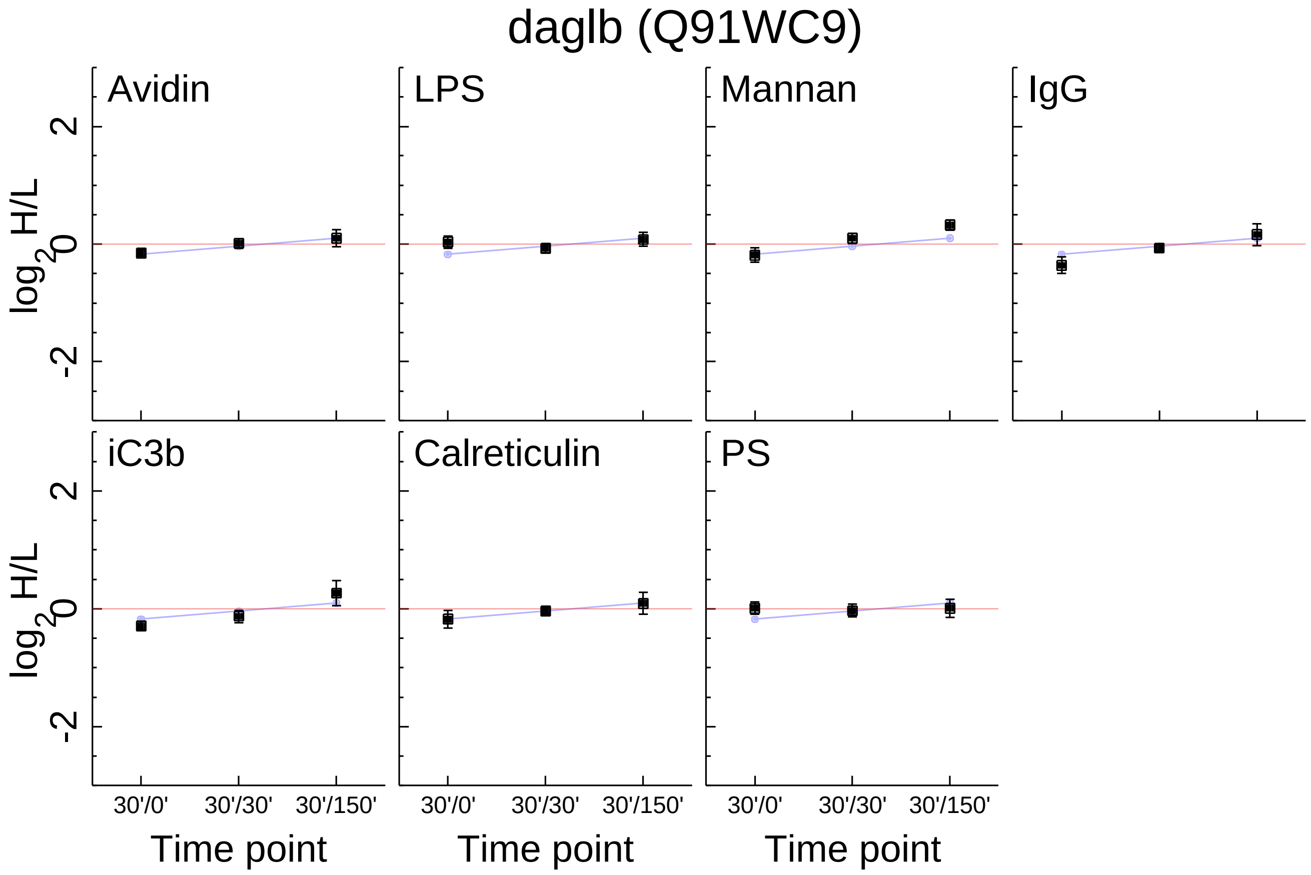

daglb (Q91WC9)
Avidin
LPS
Mannan
IgG
iC3b
Calreticulin
PS
log2 H/L
2
0
-2
30'/0'
30'/30'
30'/150'
Time point

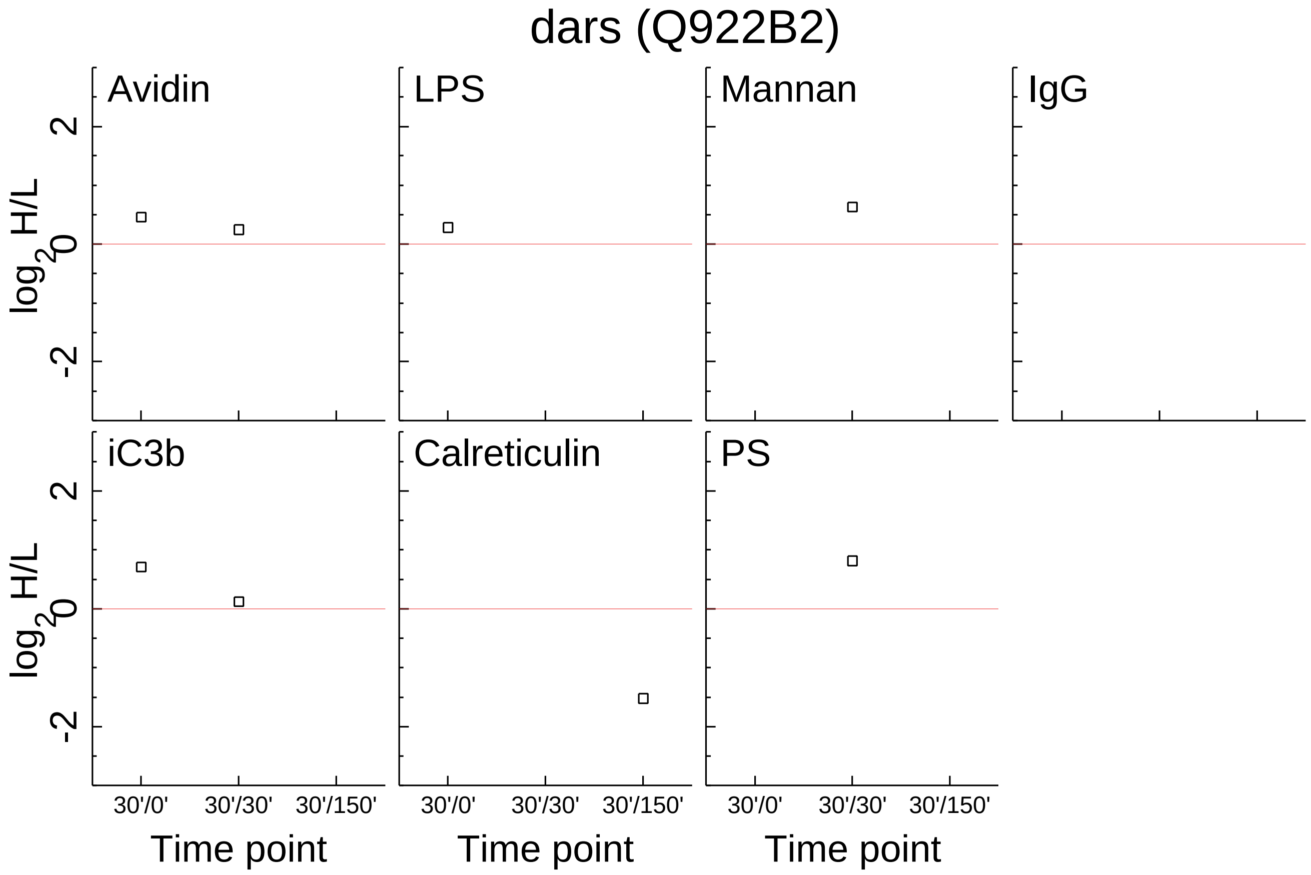

dars (Q922B2)
Avidin
LPS
Mannan
IgG
iC3b
Calreticulin
PS
log2 H/L
2
0
-2
30'/0'
30'/30'
30'/150'
Time point

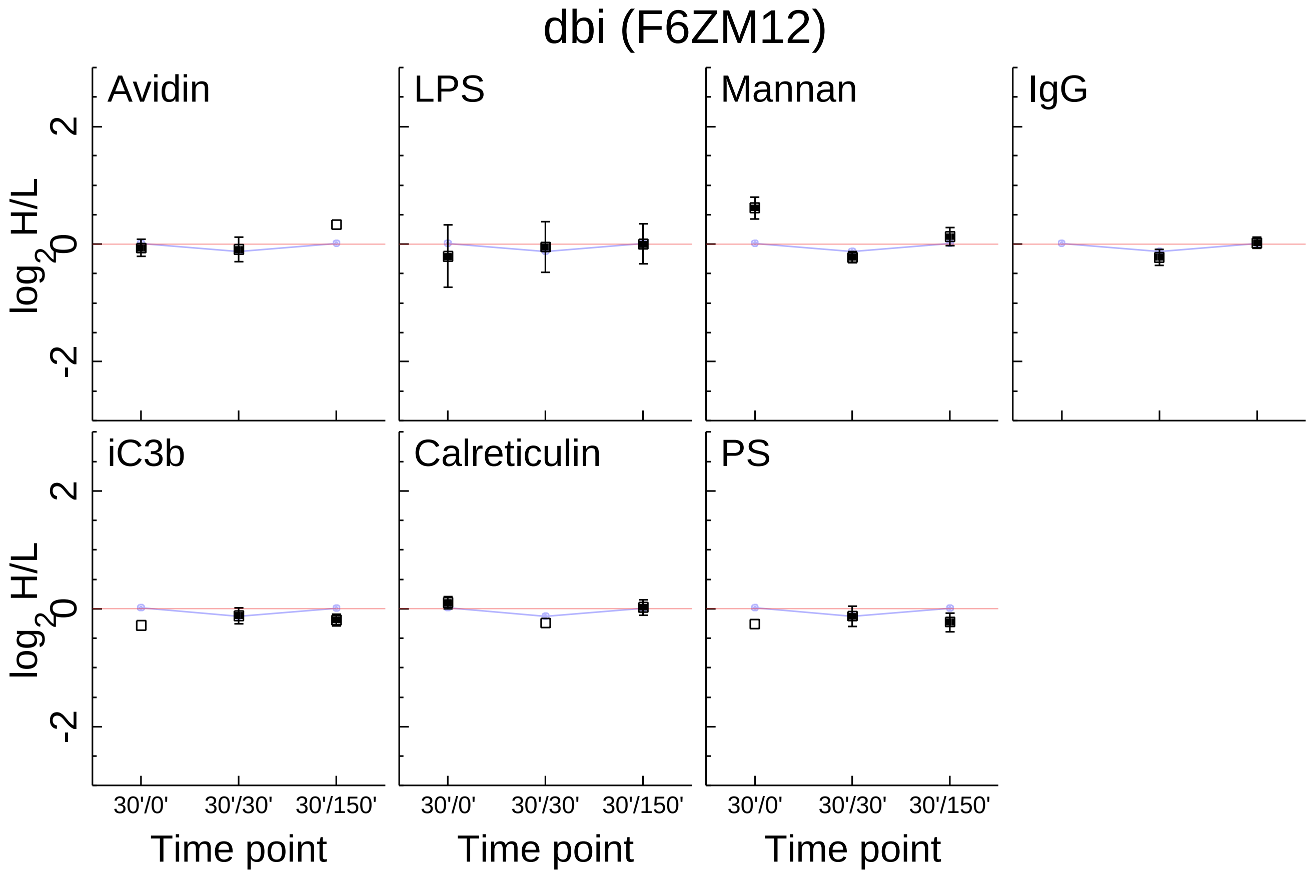
dbi (F6ZM12)
Avidin
LPS
Mannan
IgG
iC3b
Calreticulin
PS
log2 H/L
2
0
-2
30'/0'
30'/30'
30'/150'
Time point

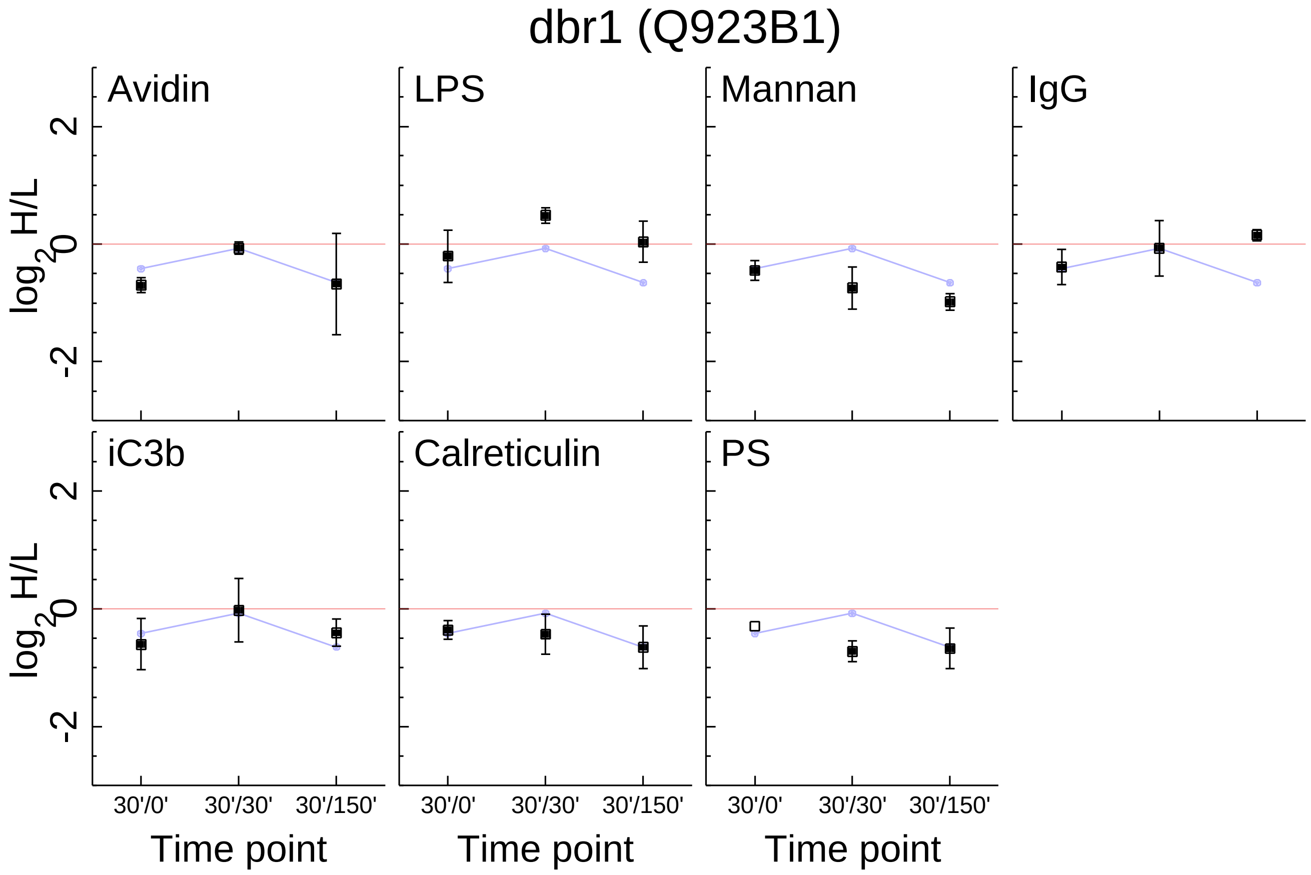
dbr1 (Q923B1)
Avidin
LPS
Mannan
IgG
iC3b
Calreticulin
PS
2
0
-2
log2 H/L
30'/0'
30'/30'
30'/150'
Time point

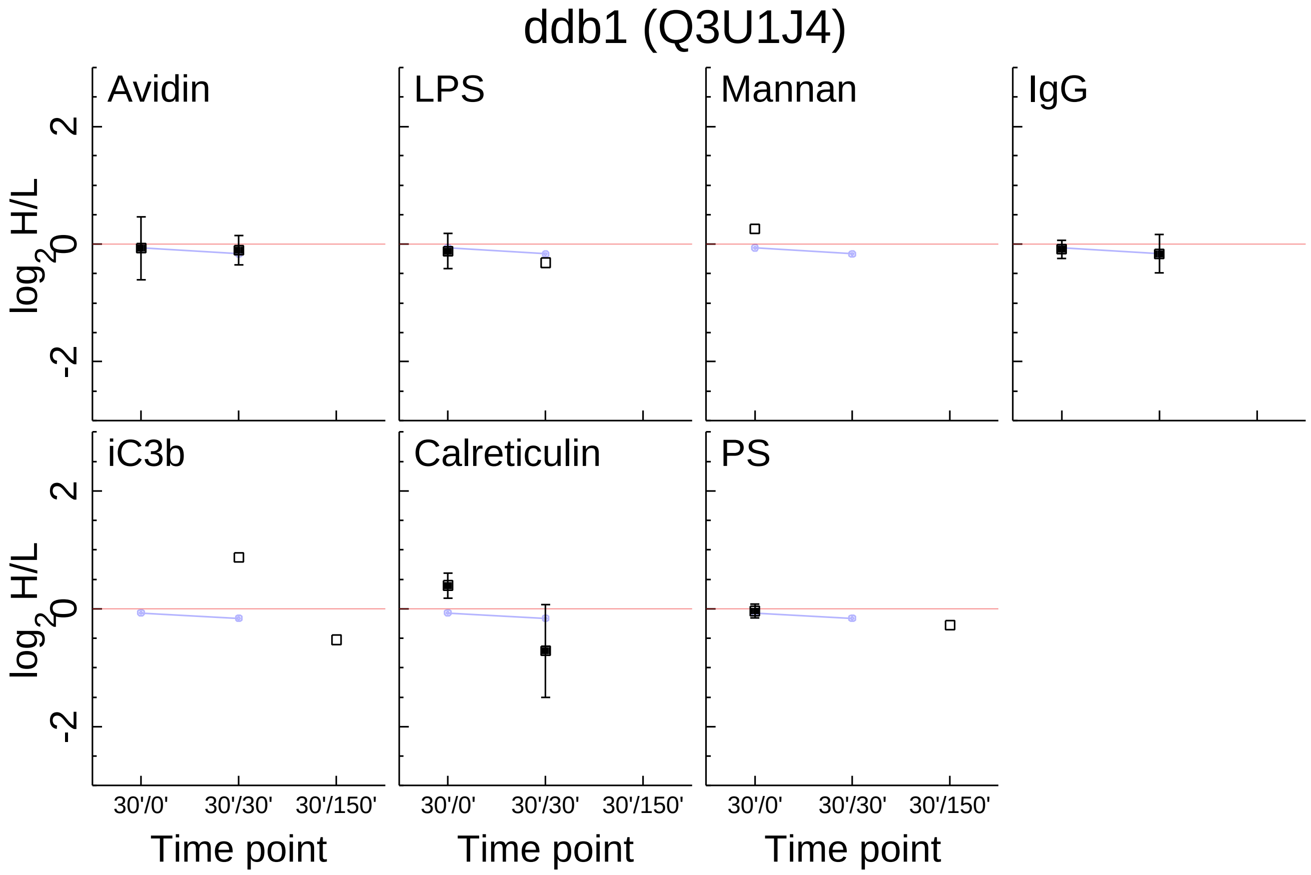

ddb1 (Q3U1J4)
Avidin
LPS
Mannan
IgG
iC3b
Calreticulin
PS
log2 H/L
2
0
-2
30'/0'
30'/30'
30'/150'
Time point

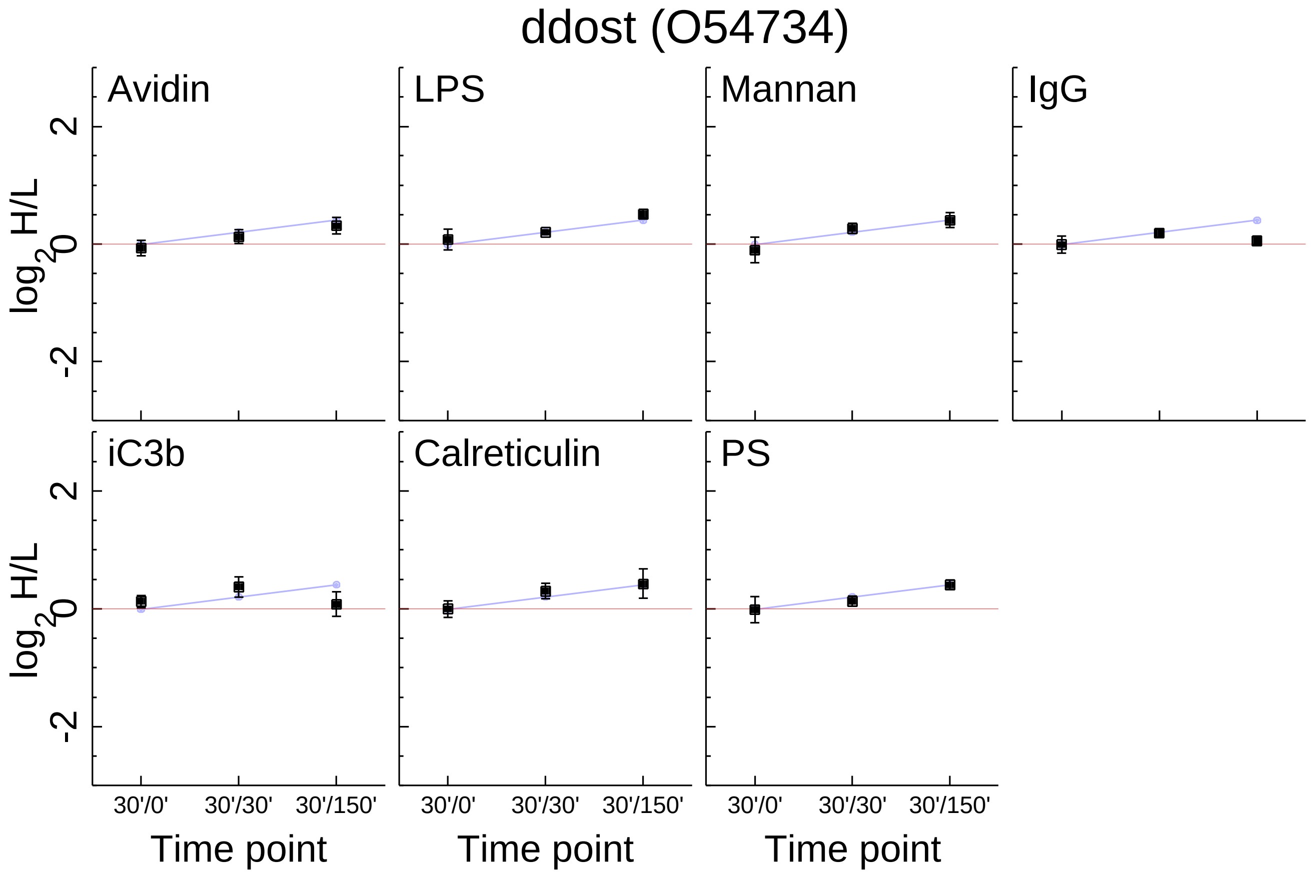

ddost (O54734)
Avidin
LPS
Mannan
IgG
iC3b
Calreticulin
PS
log2 H/L
2
0
-2
30'/0'
30'/30'
30'/150'
Time point

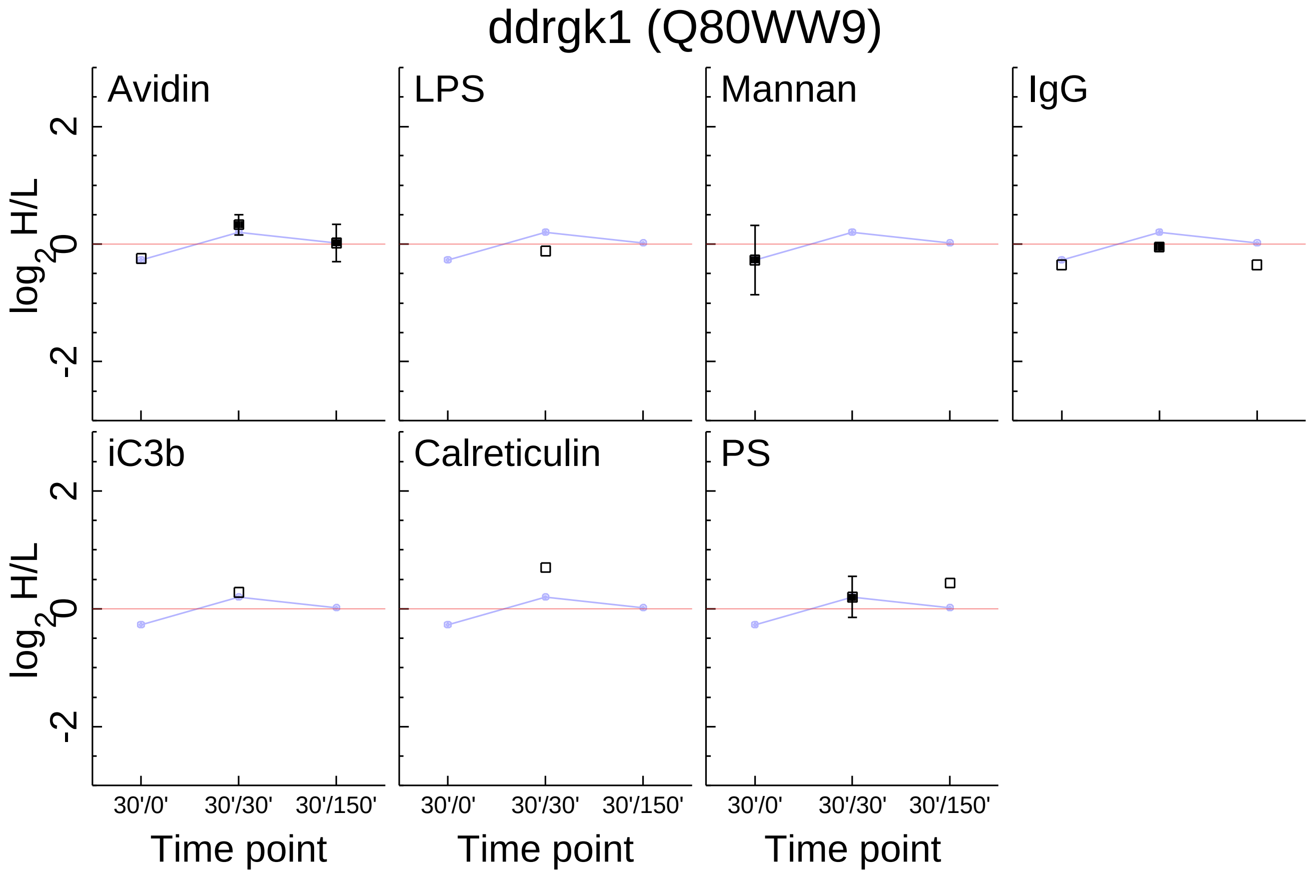

ddrgk1 (Q80WW9)
Avidin
LPS
Mannan
IgG
iC3b
Calreticulin
PS
log2 H/L
2
0
-2
30'/0'
30'/30'
30'/150'
Time point

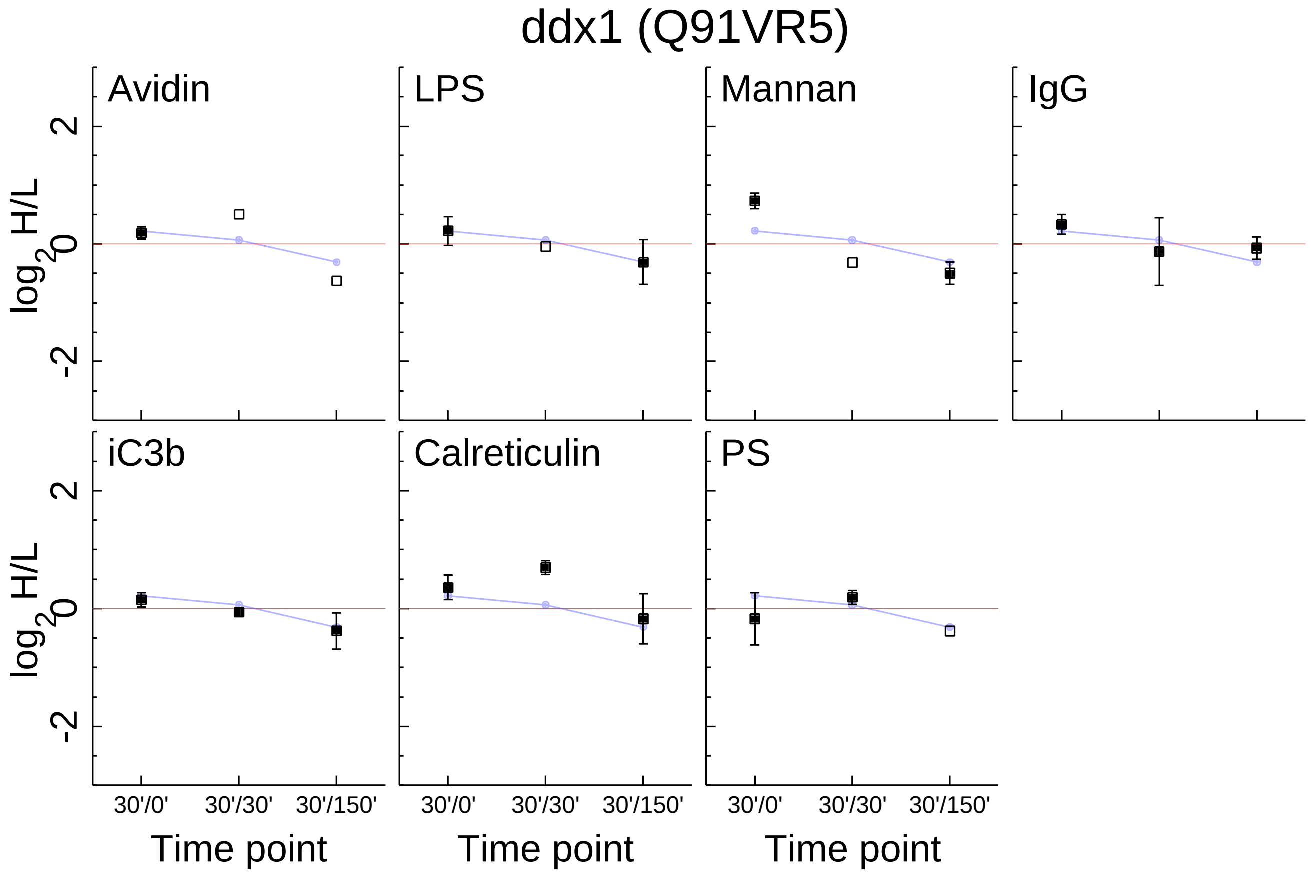
ddx1 (Q91VR5)
Avidin
LPS
Mannan
IgG
iC3b
Calreticulin
PS
log2 H/L
2
0
-2
30'/0'
30'/30'
30'/150'
Time point

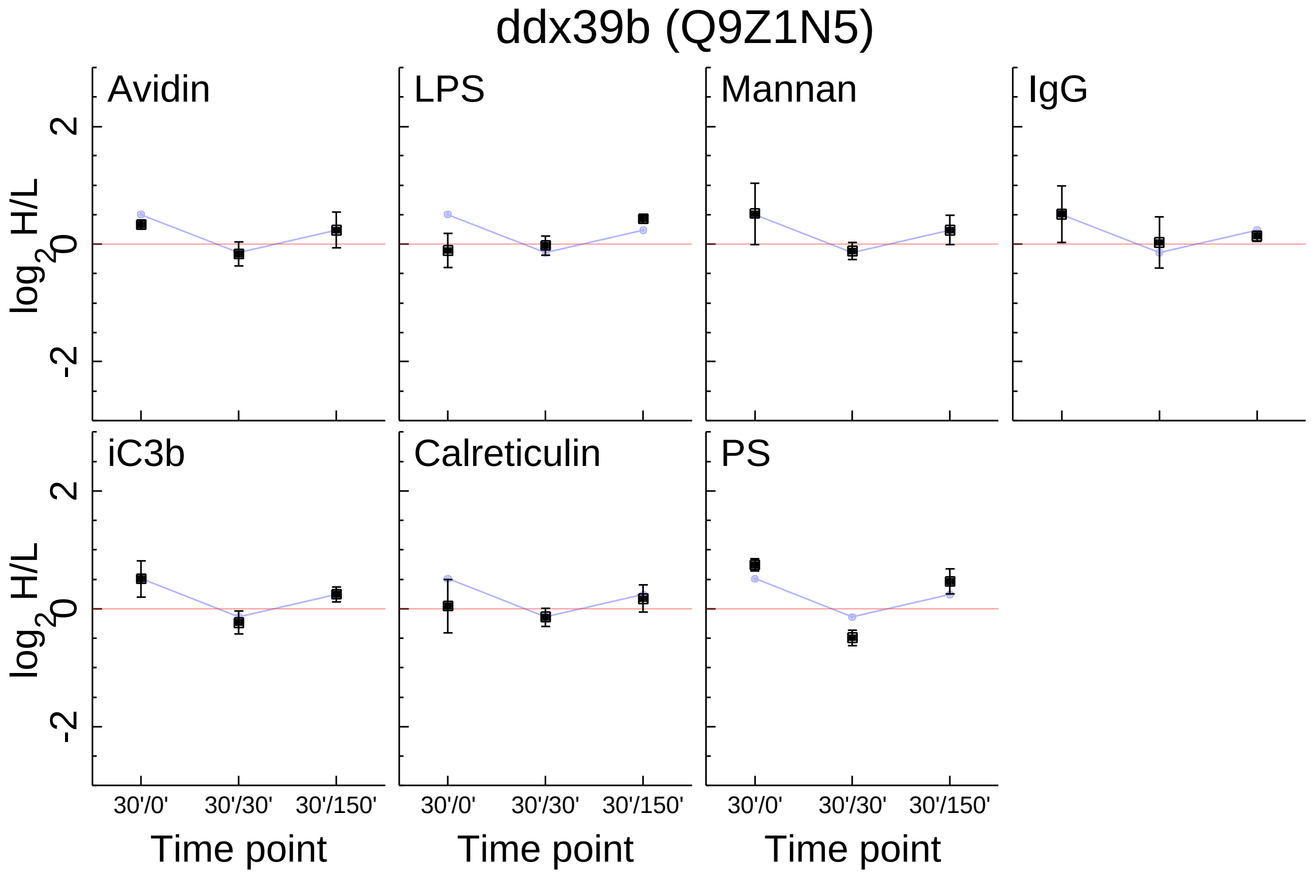
ddx39b (Q9Z1N5)
Avidin
LPS
Mannan
IgG
iC3b
Calreticulin
PS
2
0
-2
log2 H/L
30'/0'
30'/30'
30'/150'
Time point

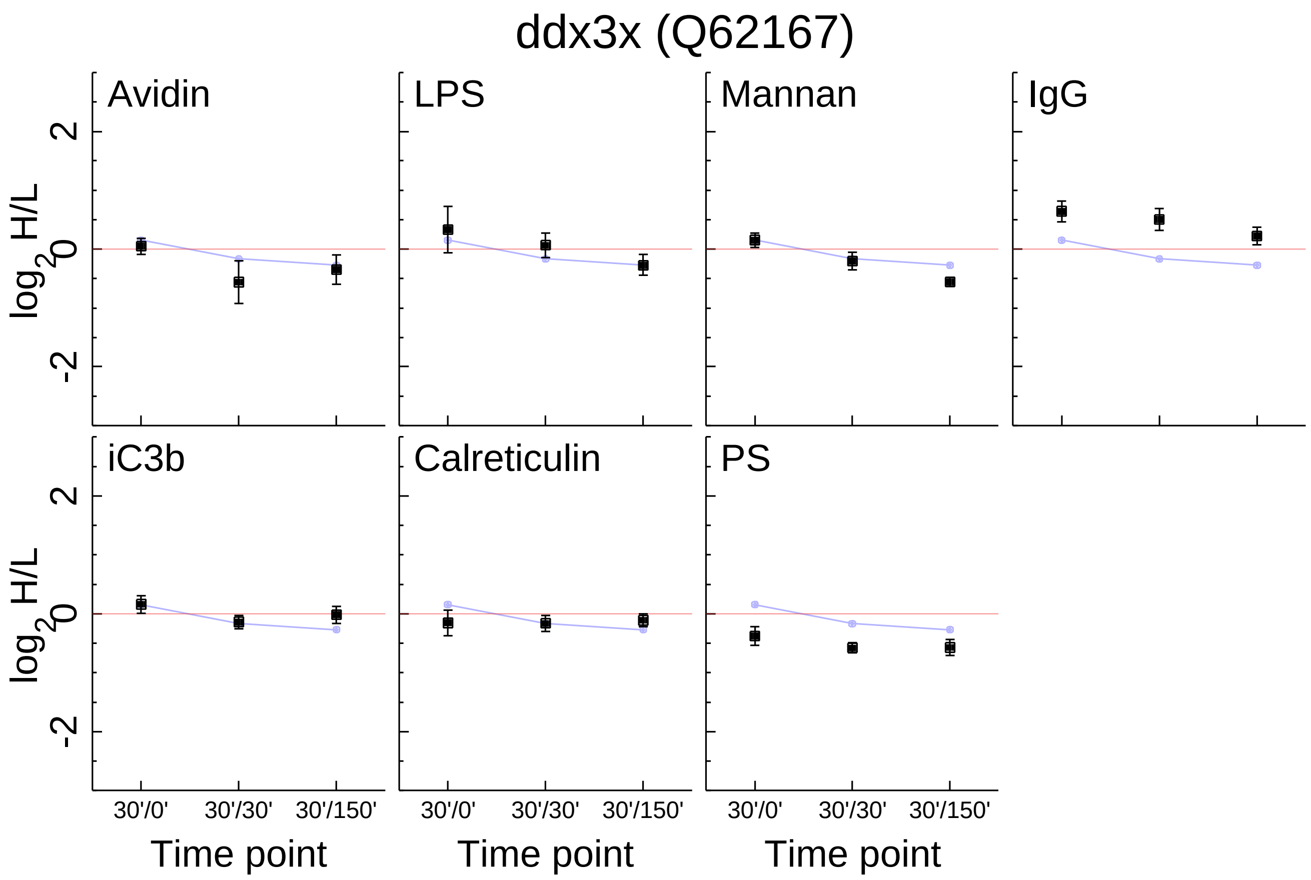

ddx3x (Q62167)
Avidin
LPS
Mannan
IgG
iC3b
Calreticulin
PS
log2 H/L
2
0
-2
30'/0'
30'/30'
30'/150'
Time point

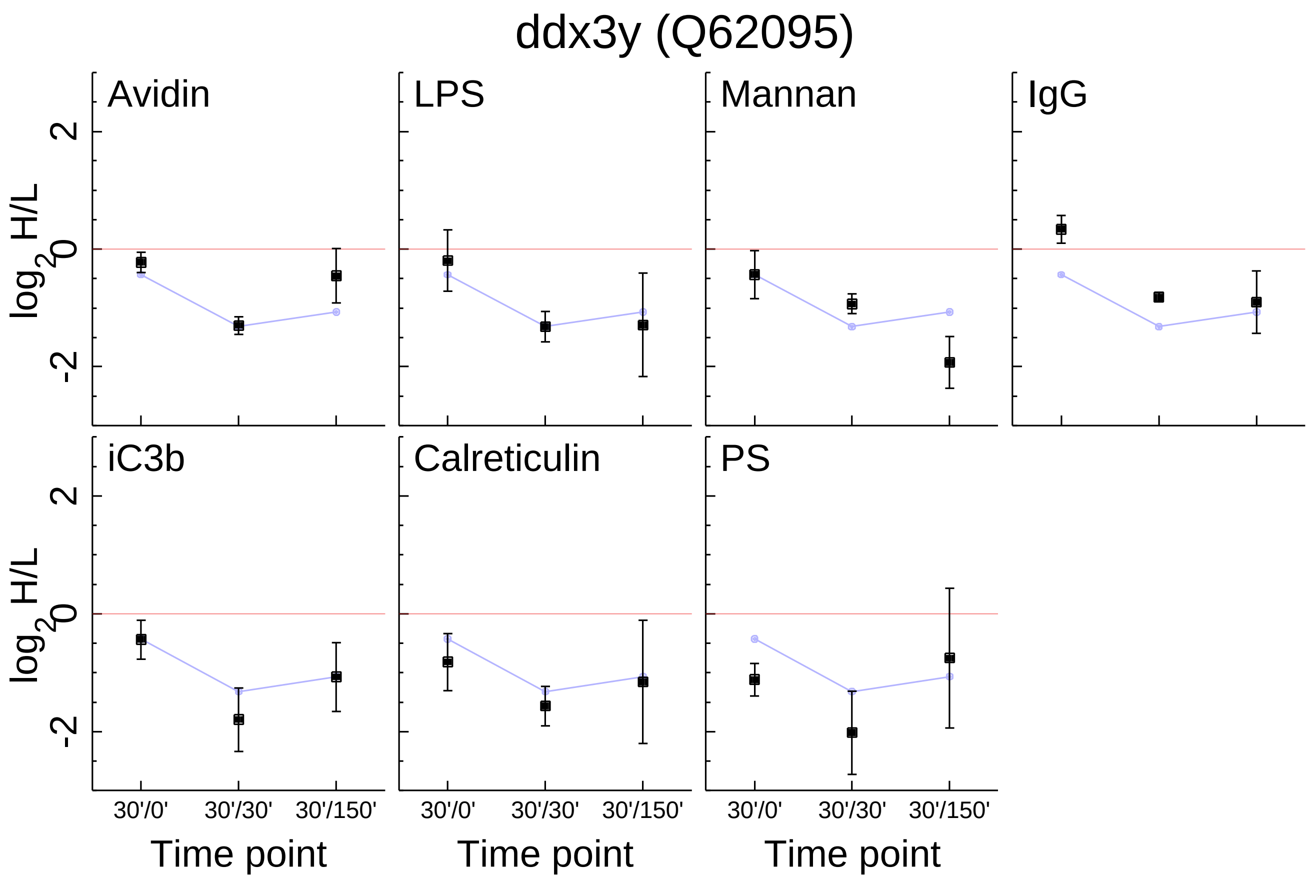

ddx3y (Q62095)
Avidin
LPS
Mannan
IgG
iC3b
Calreticulin
PS
log2 H/L
2
0
-2
30'/0'
30'/30'
30'/150'
Time point

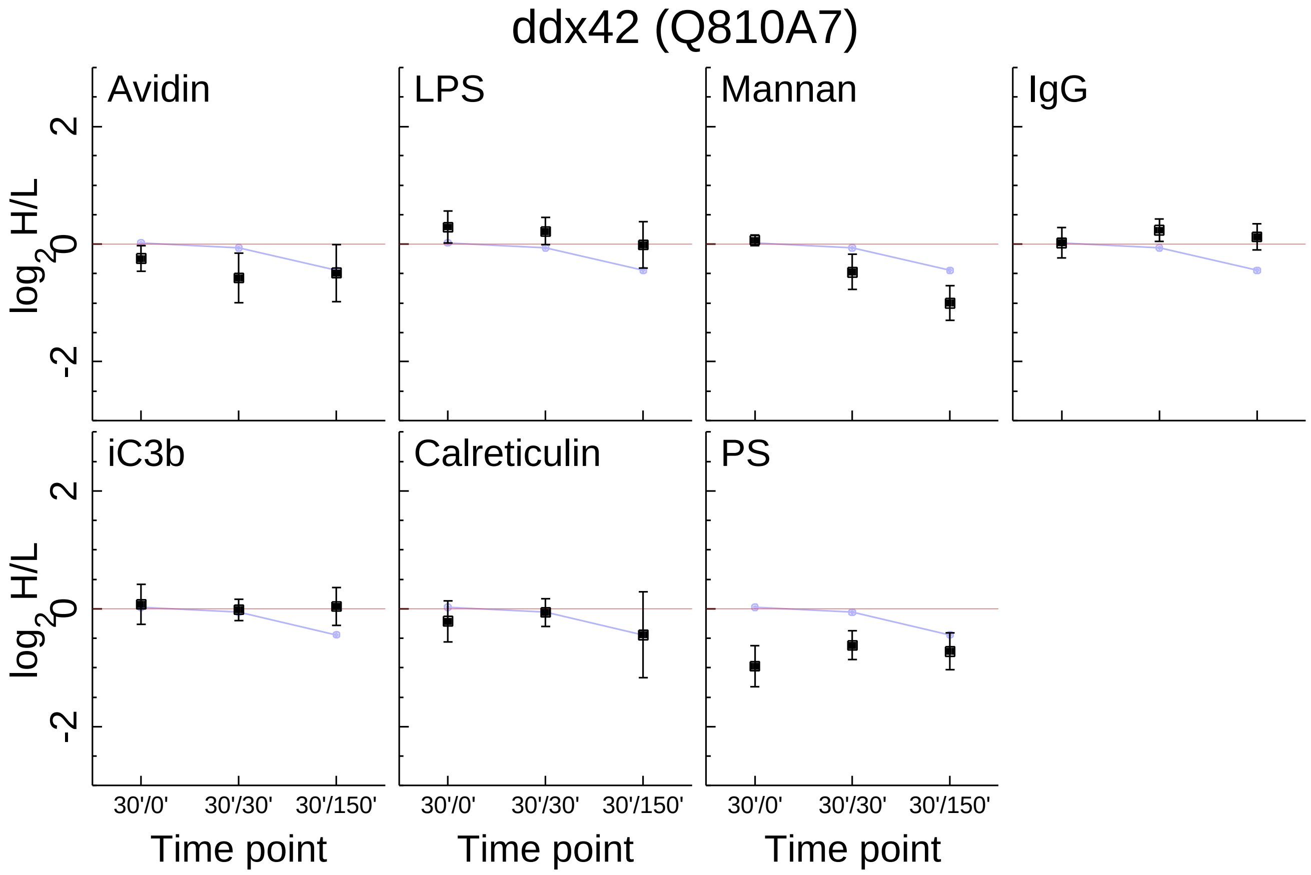
ddx42 (Q810A7)
Avidin
LPS
Mannan
IgG
iC3b
Calreticulin
PS
log2 H/L
2
0
-2
30'/0'
30'/30'
30'/150'
Time point

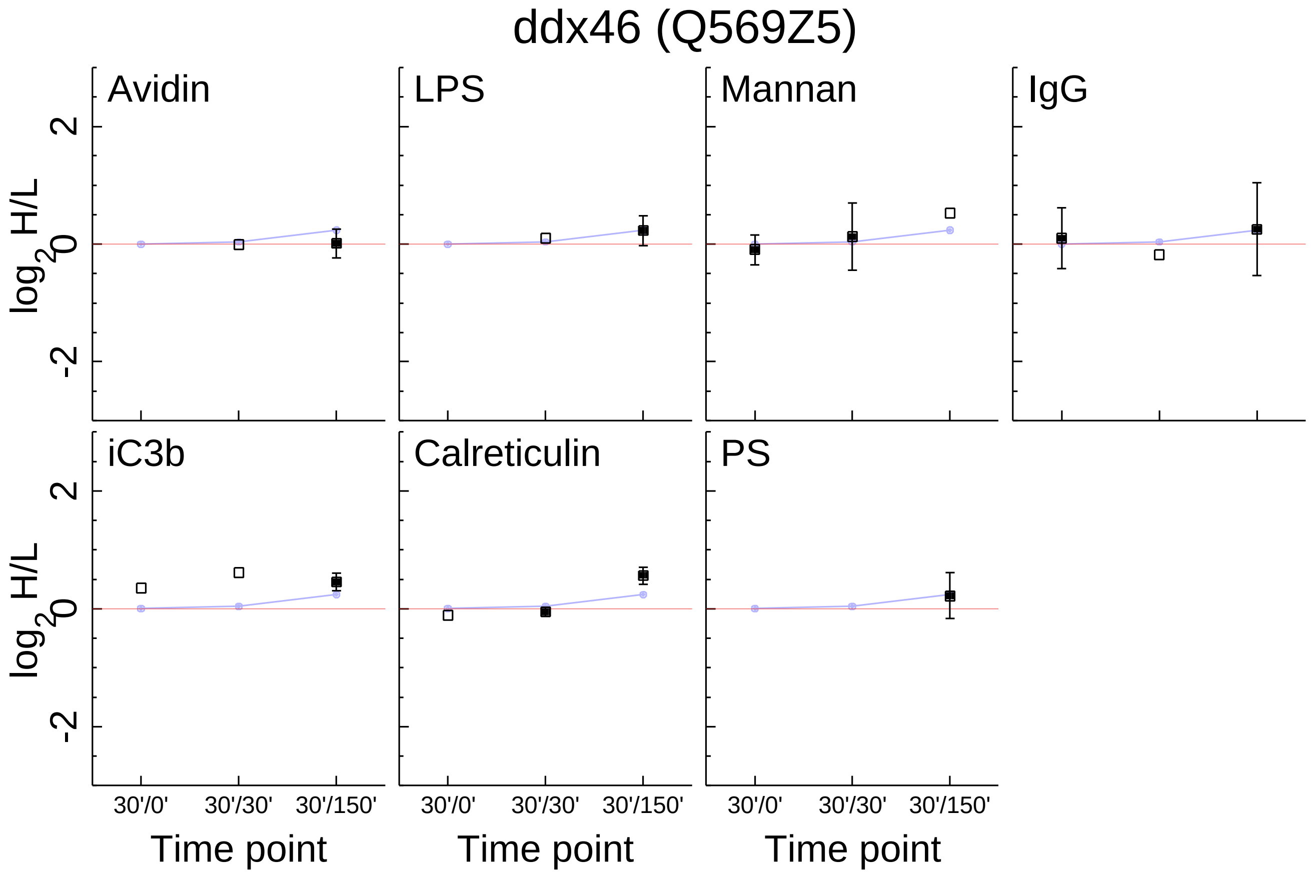
ddx46 (Q569Z5)
Avidin
LPS
Mannan
IgG
iC3b
Calreticulin
PS
log2 H/L
2
0
-2
30'/0'
30'/30'
30'/150'
Time point

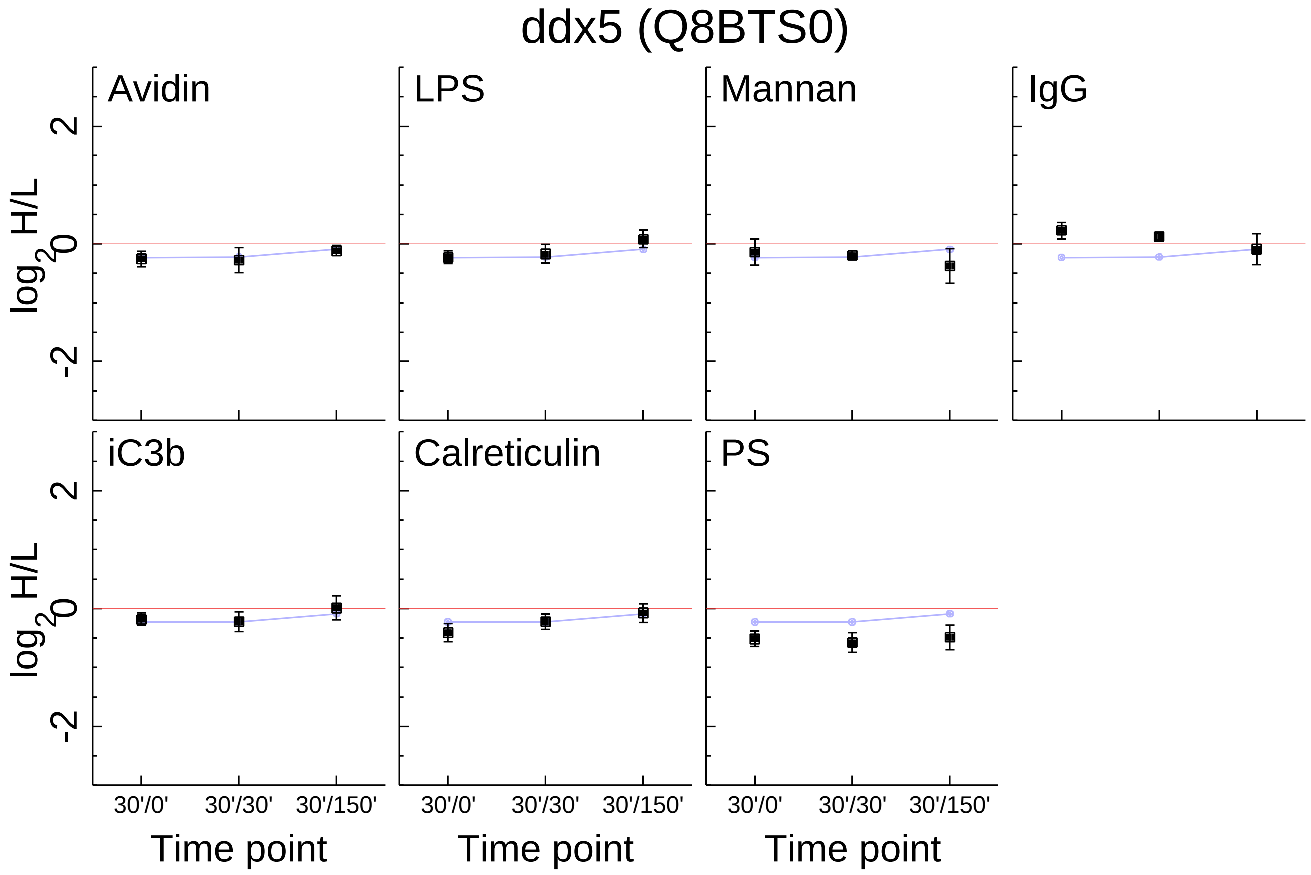

ddx5 (Q8BTS0)
Avidin
LPS
Mannan
IgG
iC3b
Calreticulin
PS
log2 H/L
2
0
-2
30'/0'
30'/30'
30'/150'
Time point

decr1 (Q9CQ62)

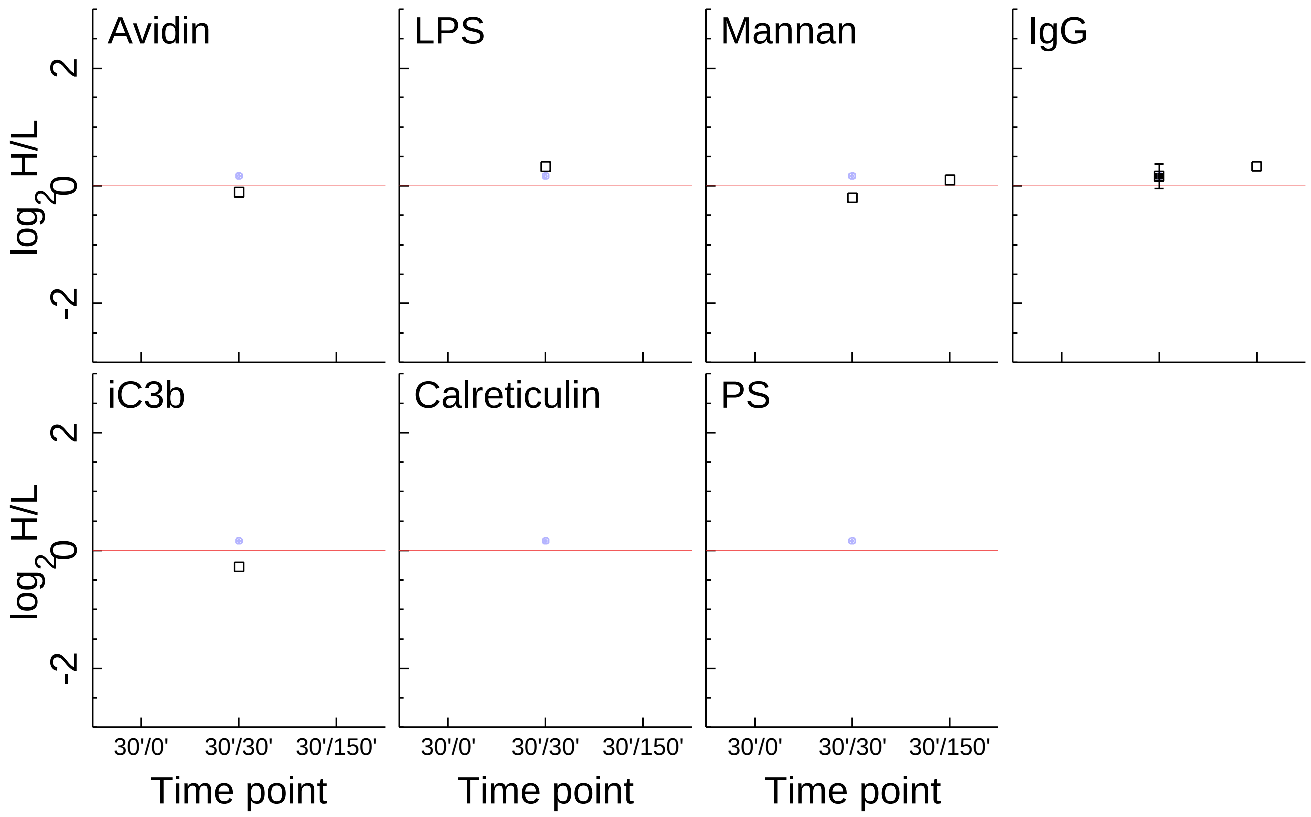

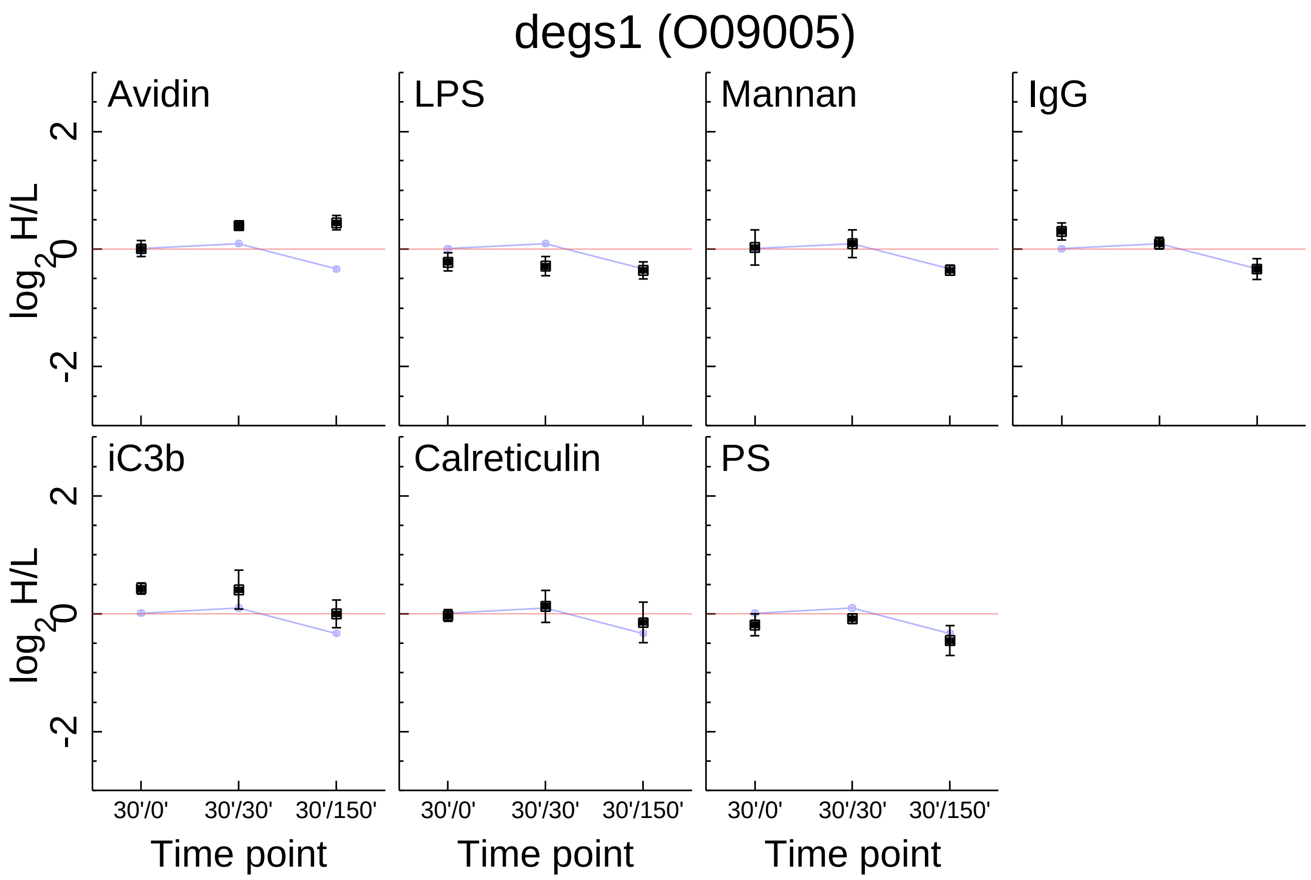
degs1 (O09005)
Avidin
LPS
Mannan
IgG
iC3b
Calreticulin
PS
log2 H/L
2
0
-2
30'/0'
30'/30'
30'/150'
Time point

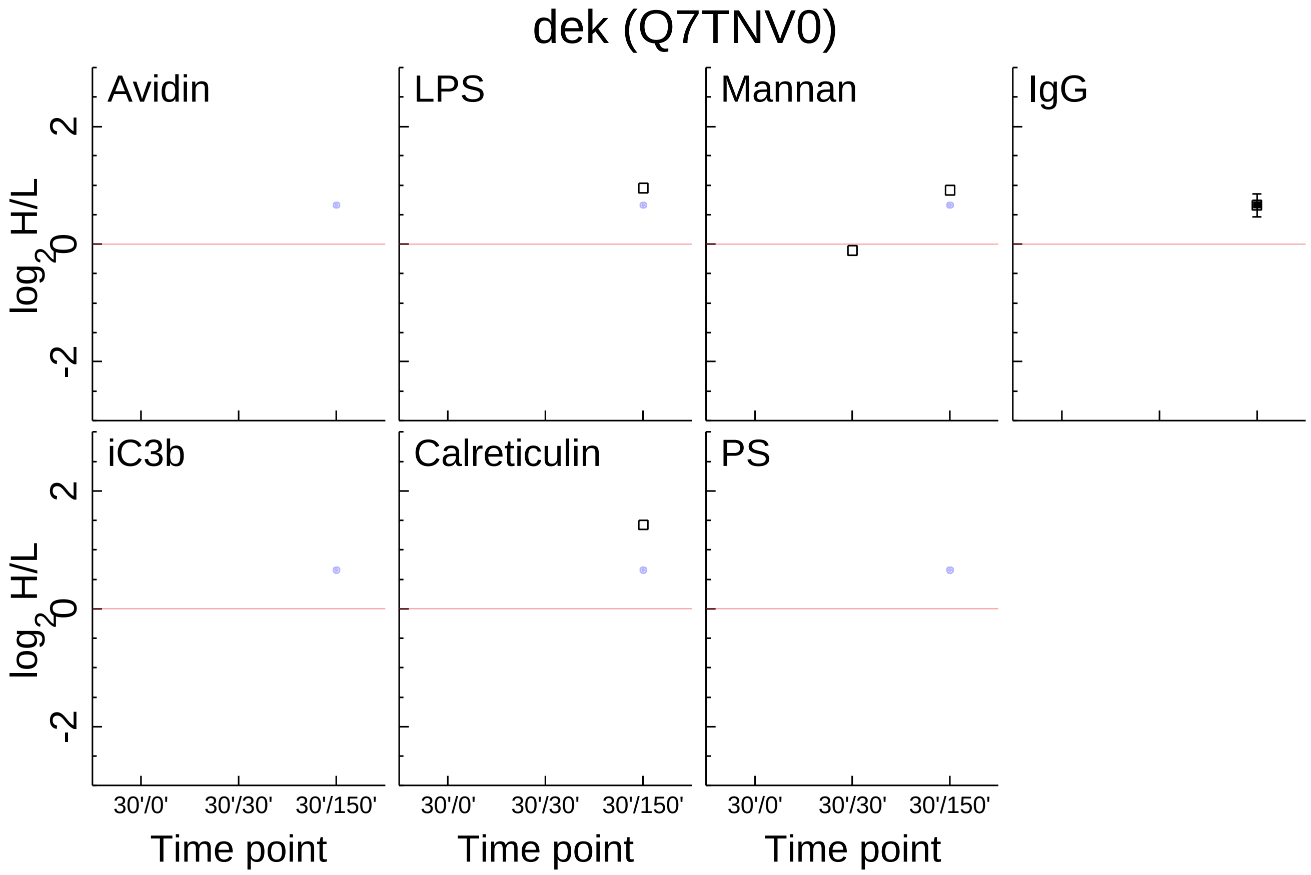
dek (Q7TNV0)
Avidin
LPS
Mannan
IgG
iC3b
Calreticulin
PS
log2 H/L
2
0
-2
30'/0'
30'/30'
30'/150'
Time point

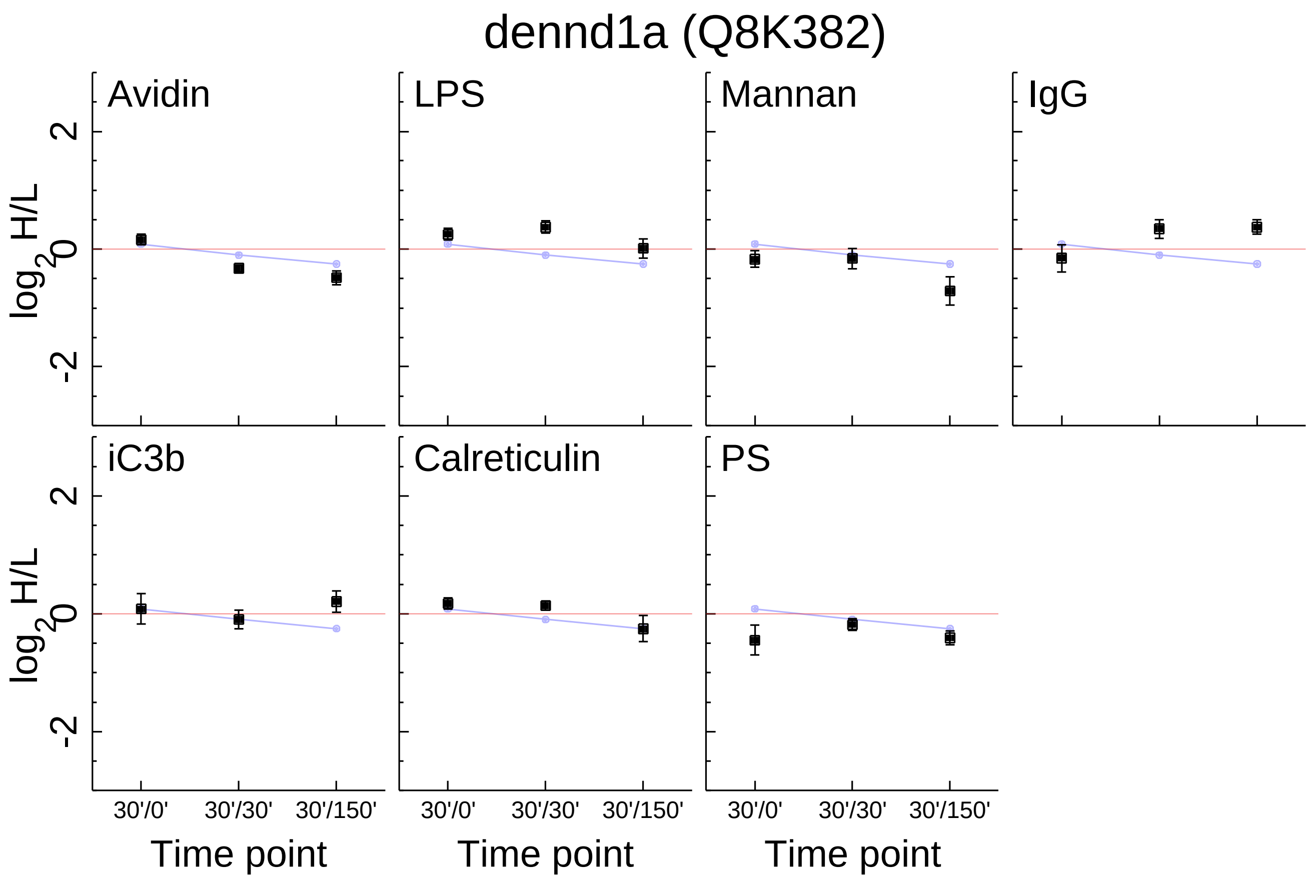
dennd1a (Q8K382)
Avidin
LPS
Mannan
IgG
iC3b
Calreticulin
PS
log2 H/L
2
0
-2
30'/0'
30'/30'
30'/150'
Time point

dhrs1 (Q99L04)

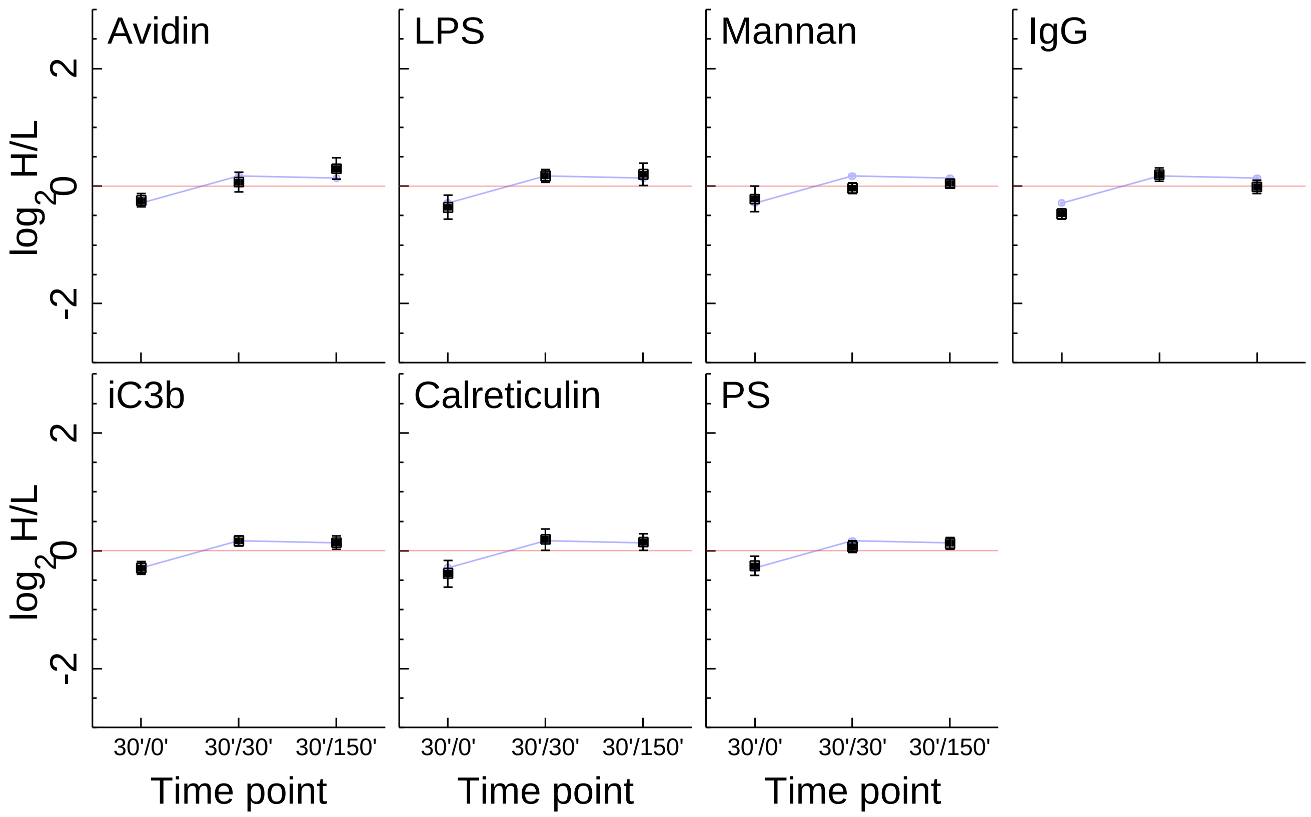

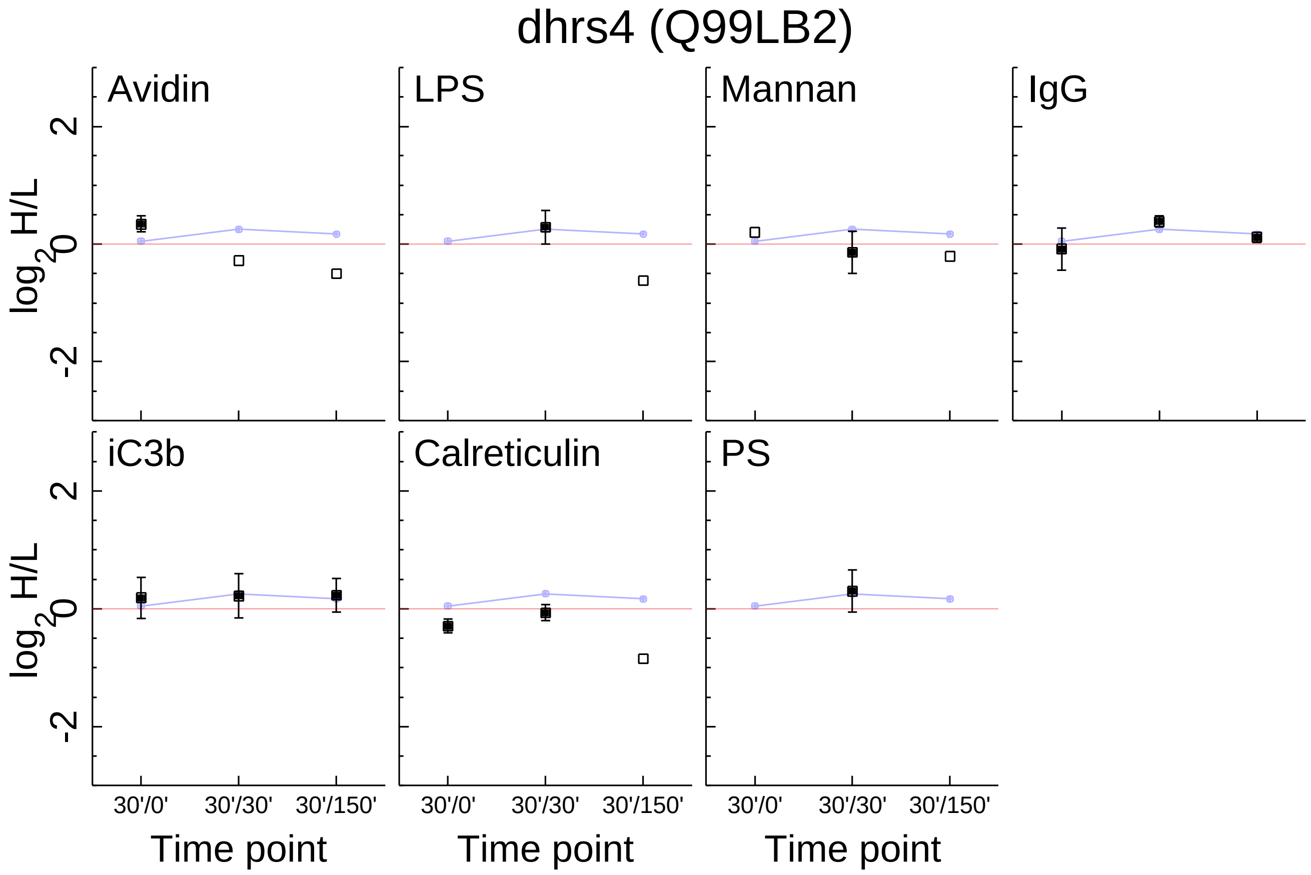
dhrs4 (Q99LB2)
Avidin
LPS
Mannan
IgG
iC3b
Calreticulin
PS
log2 H/L
2
0
-2
30'/0'
30'/30'
30'/150'
Time point

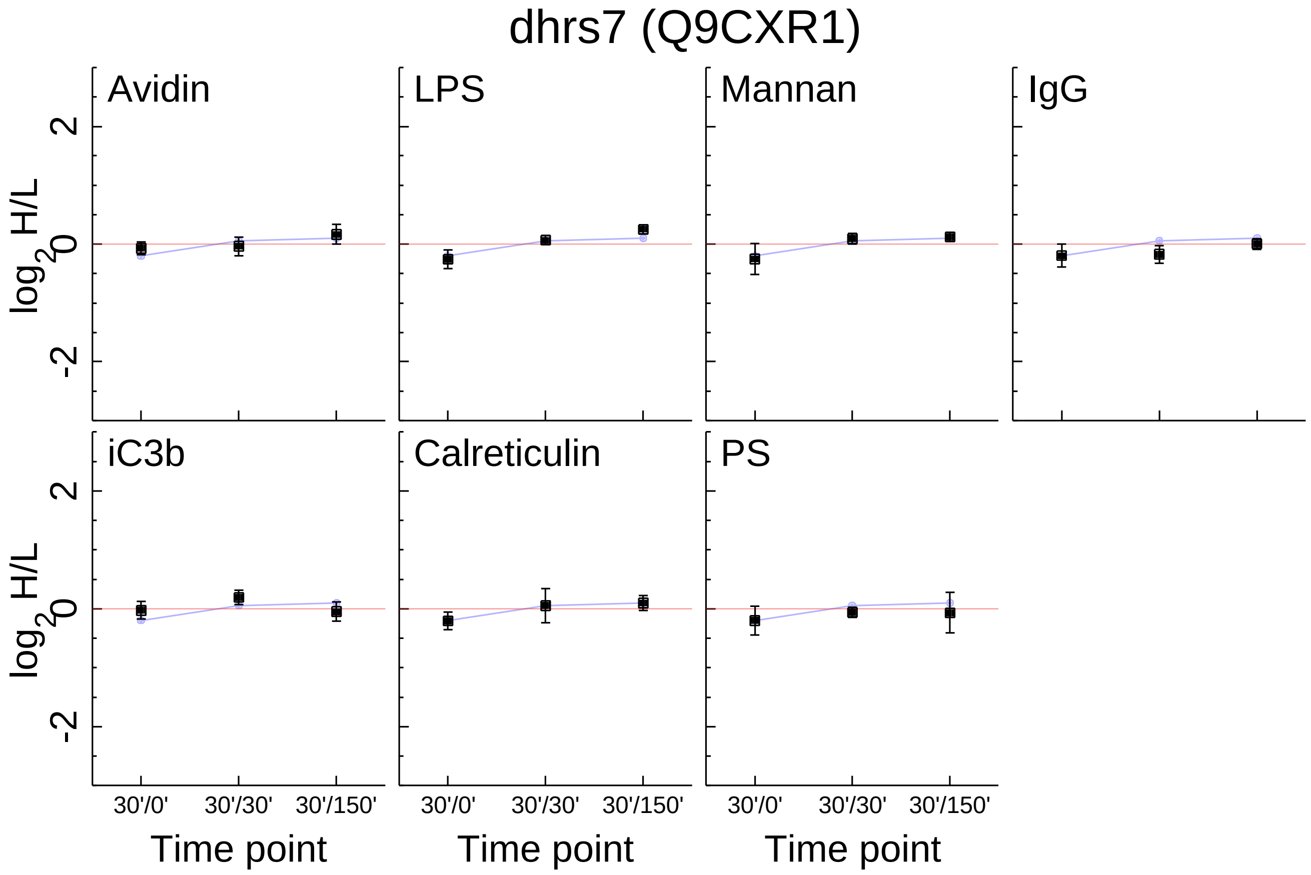
dhrs7 (Q9CXR1)
Avidin
LPS
Mannan
IgG
iC3b
Calreticulin
PS
log2 H/L
2
0
-2
30'/0'
30'/30'
30'/150'
Time point

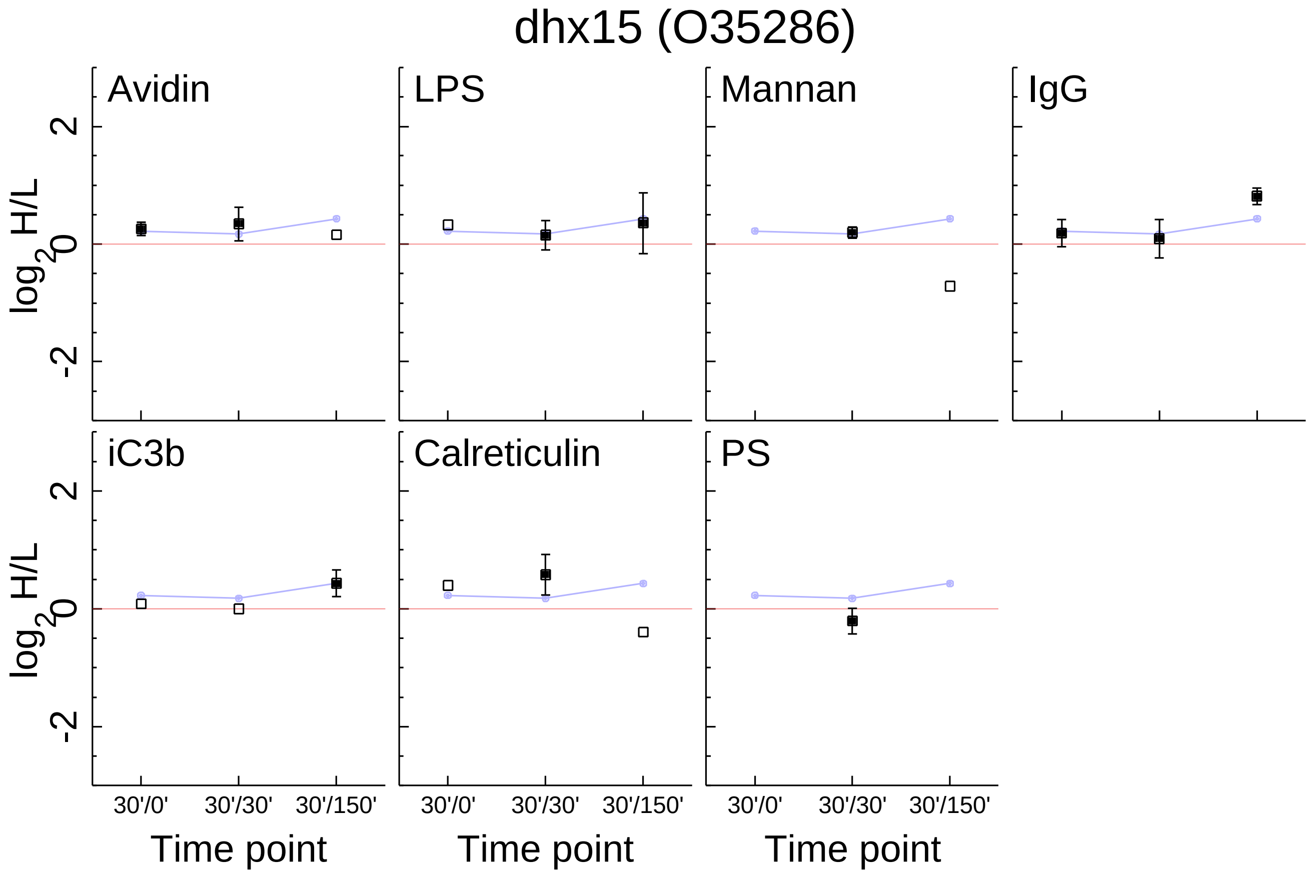
dhx15 (O35286)
Avidin
LPS
Mannan
IgG
iC3b
Calreticulin
PS
log2 H/L
2
0
-2
30'/0'
30'/30'
30'/150'
Time point

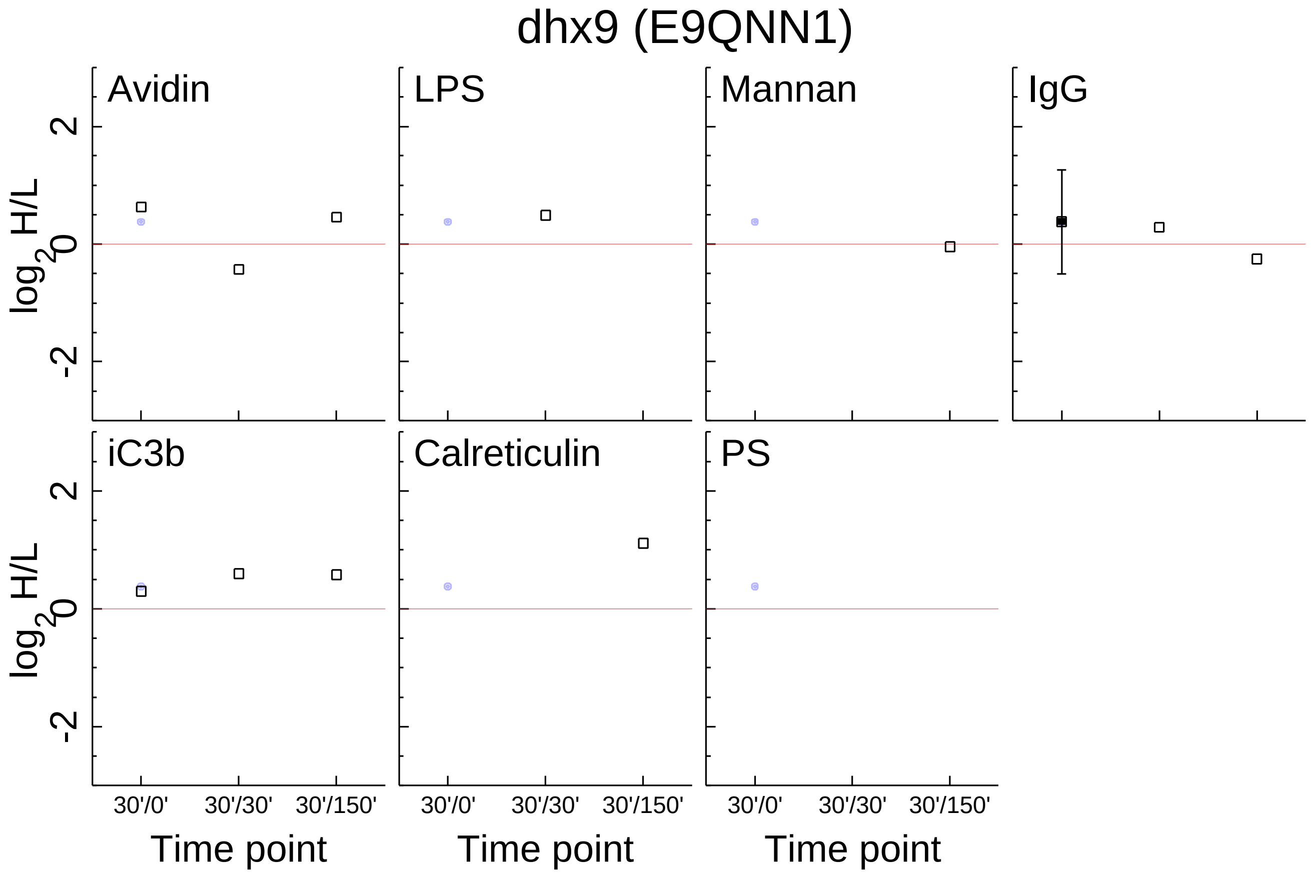

dhx9 (E9QNN1)
Avidin
LPS
Mannan
IgG
iC3b
Calreticulin
PS
log2 H/L
2
0
-2
30'/0'
30'/30'
30'/150'
Time point

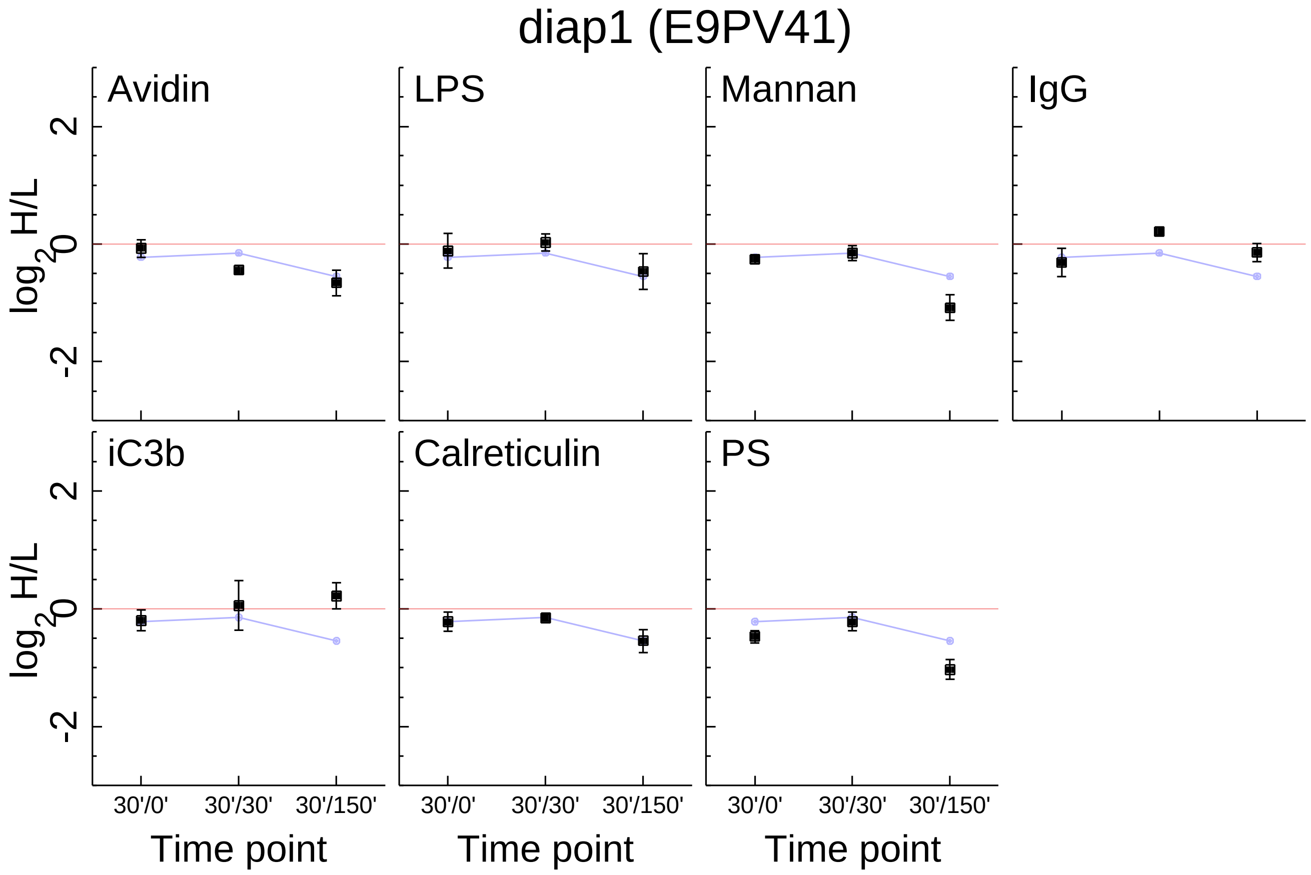
diap1 (E9PV41)
Avidin
LPS
Mannan
IgG
iC3b
Calreticulin
PS
log2 H/L
2
0
-2
30'/0'
30'/30'
30'/150'
Time point

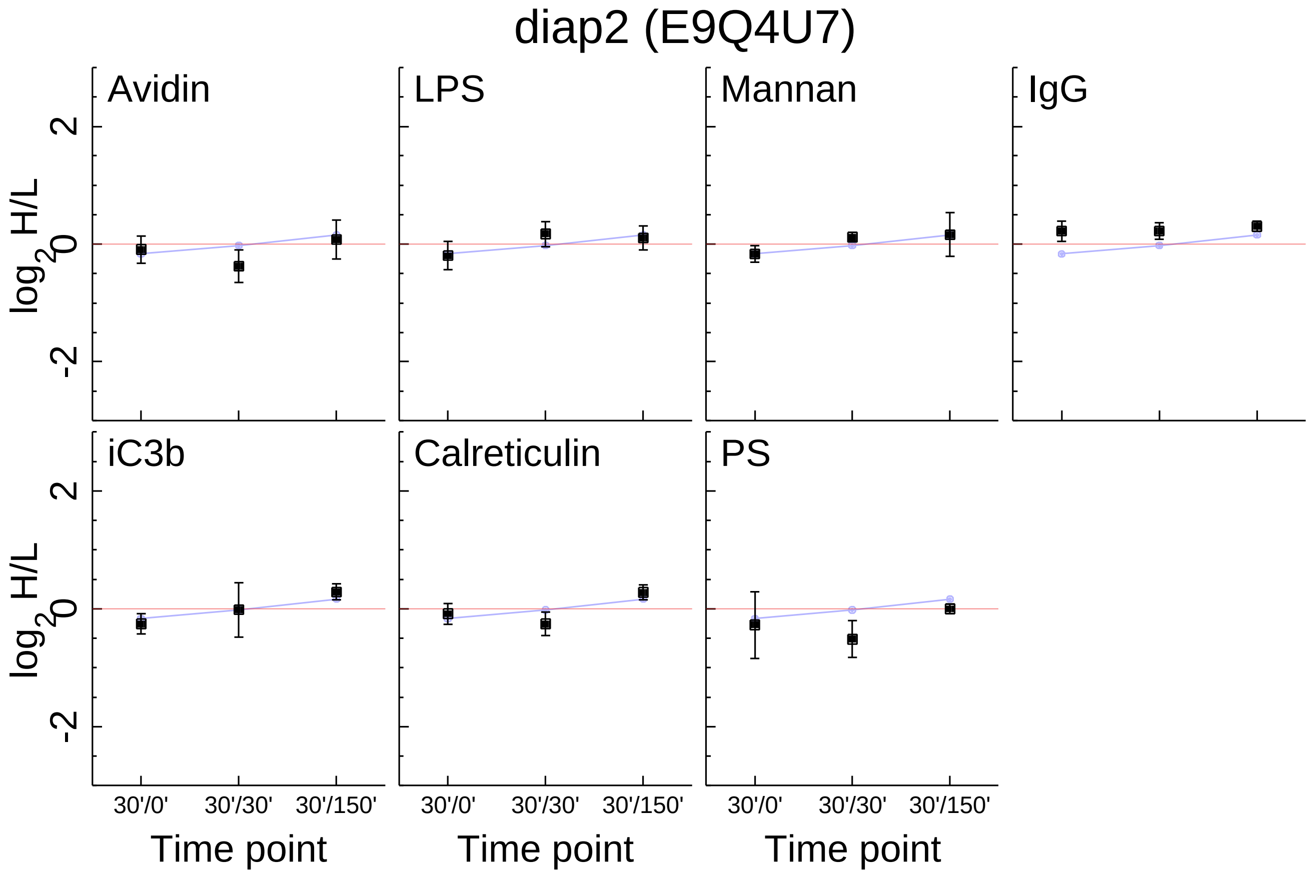

diap2 (E9Q4U7)
Avidin
LPS
Mannan
IgG
iC3b
Calreticulin
PS
log2 H/L
2
0
-2
30'/0'
30'/30'
30'/150'
Time point

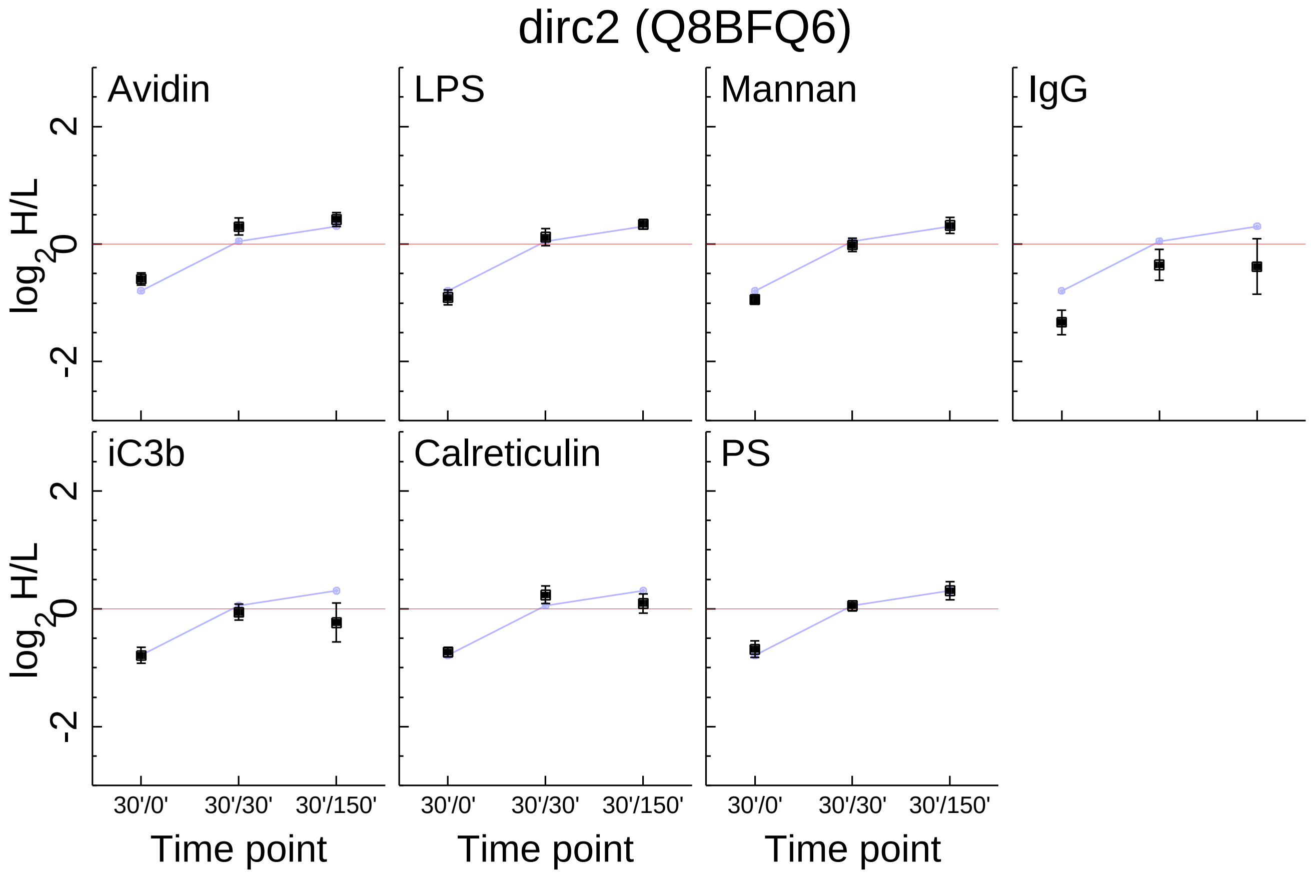

dirc2 (Q8BFQ6)
Avidin
LPS
Mannan
IgG
iC3b
Calreticulin
PS
$\log_2$ H/L
2
0
-2
30'/0'
30'/30'
30'/150'
Time point

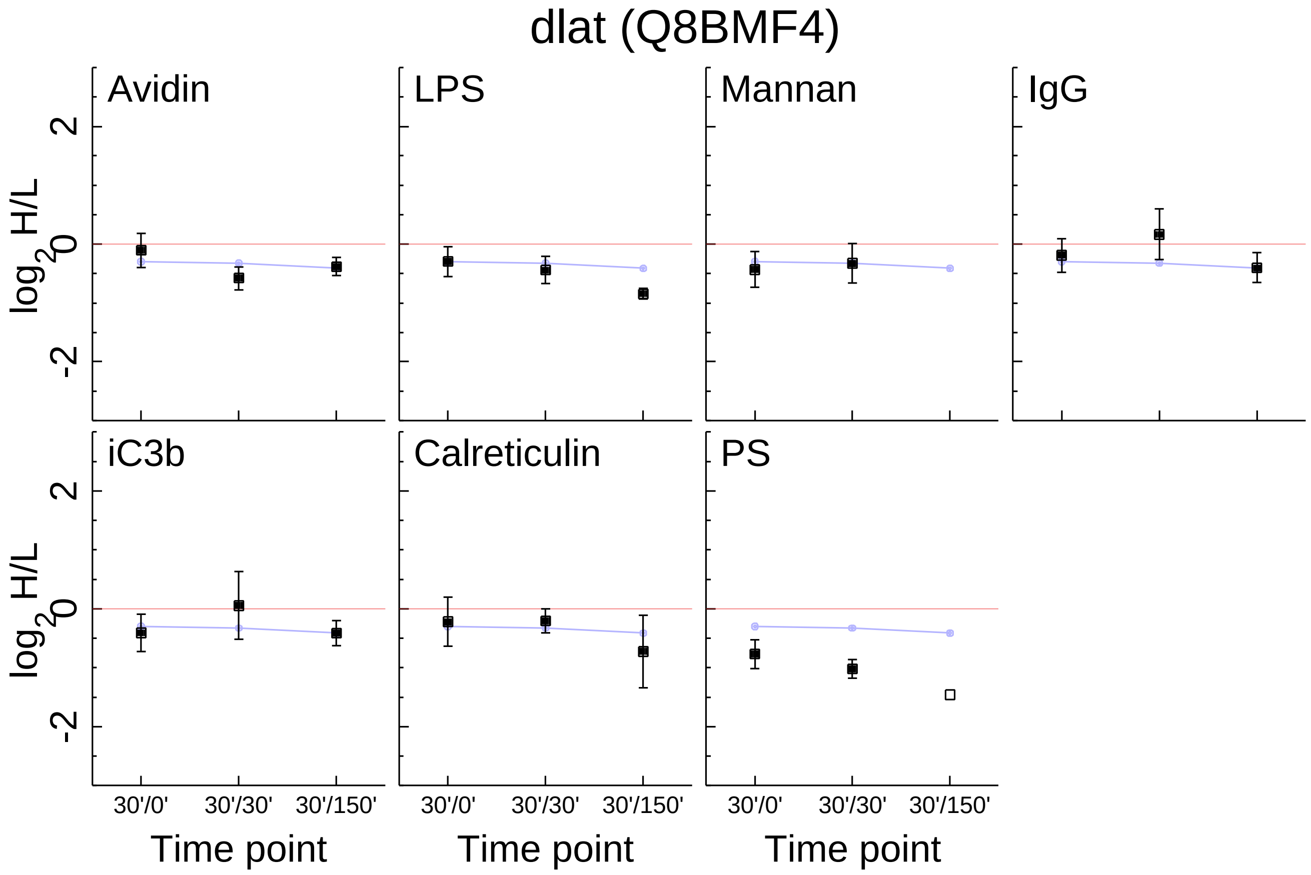
dlat (Q8BMF4)
Avidin
LPS
Mannan
IgG
iC3b
Calreticulin
PS
$\log_2$ H/L
2
0
-2
30'/0'
30'/30'
30'/150'
Time point

dld (O08749)

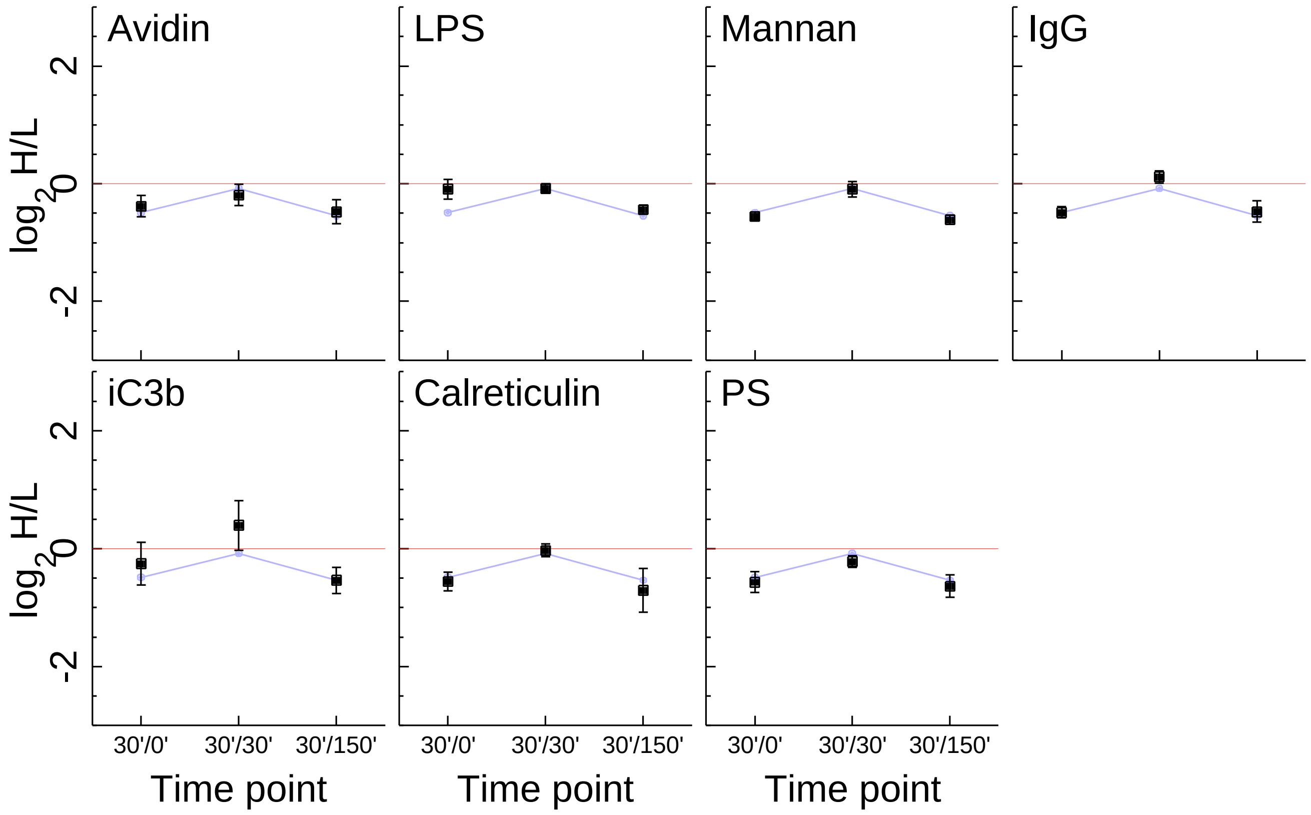
Avidin
LPS
Mannan
IgG
iC3b
Calreticulin
PS
log2 H/L
2
0
-2
30'/0'
30'/30'
30'/150'
Time point

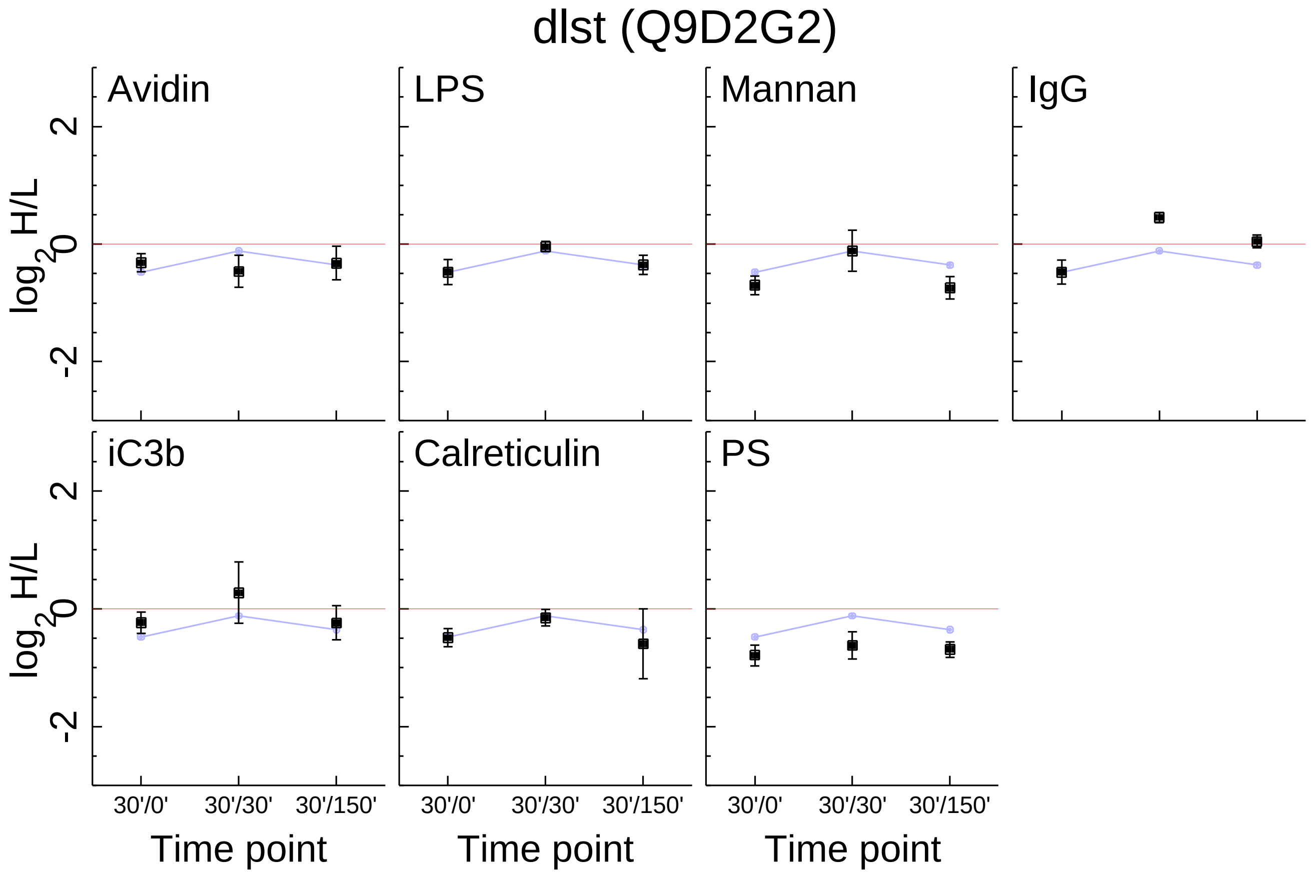
dlst (Q9D2G2)
Avidin
LPS
Mannan
IgG
iC3b
Calreticulin
PS
log2 H/L
2
0
-2
30'/0'
30'/30'
30'/150'
Time point

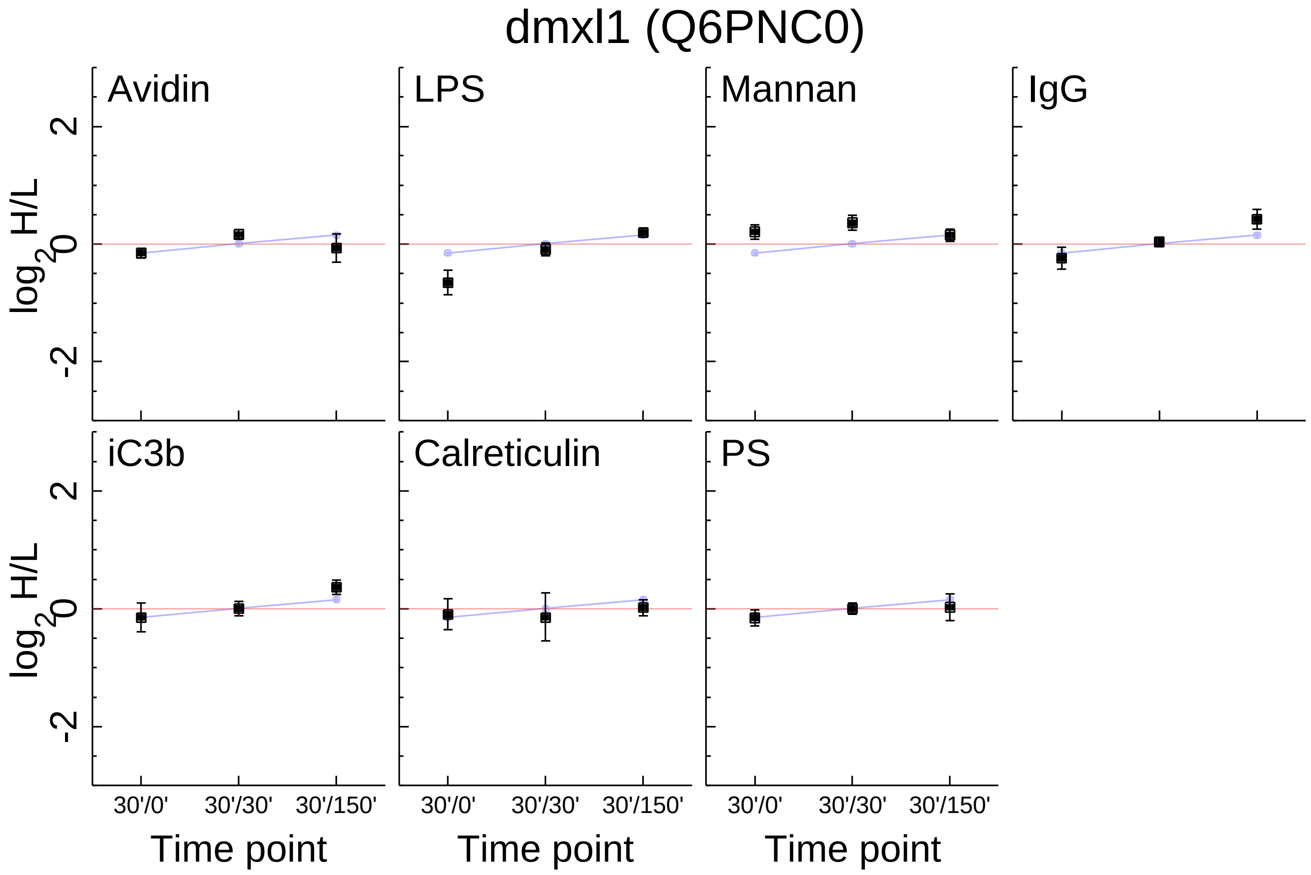
dmxl1 (Q6PNC0)
Avidin
LPS
Mannan
IgG
iC3b
Calreticulin
PS
log2 H/L
2
0
-2
30'/0'
30'/30'
30'/150'
Time point

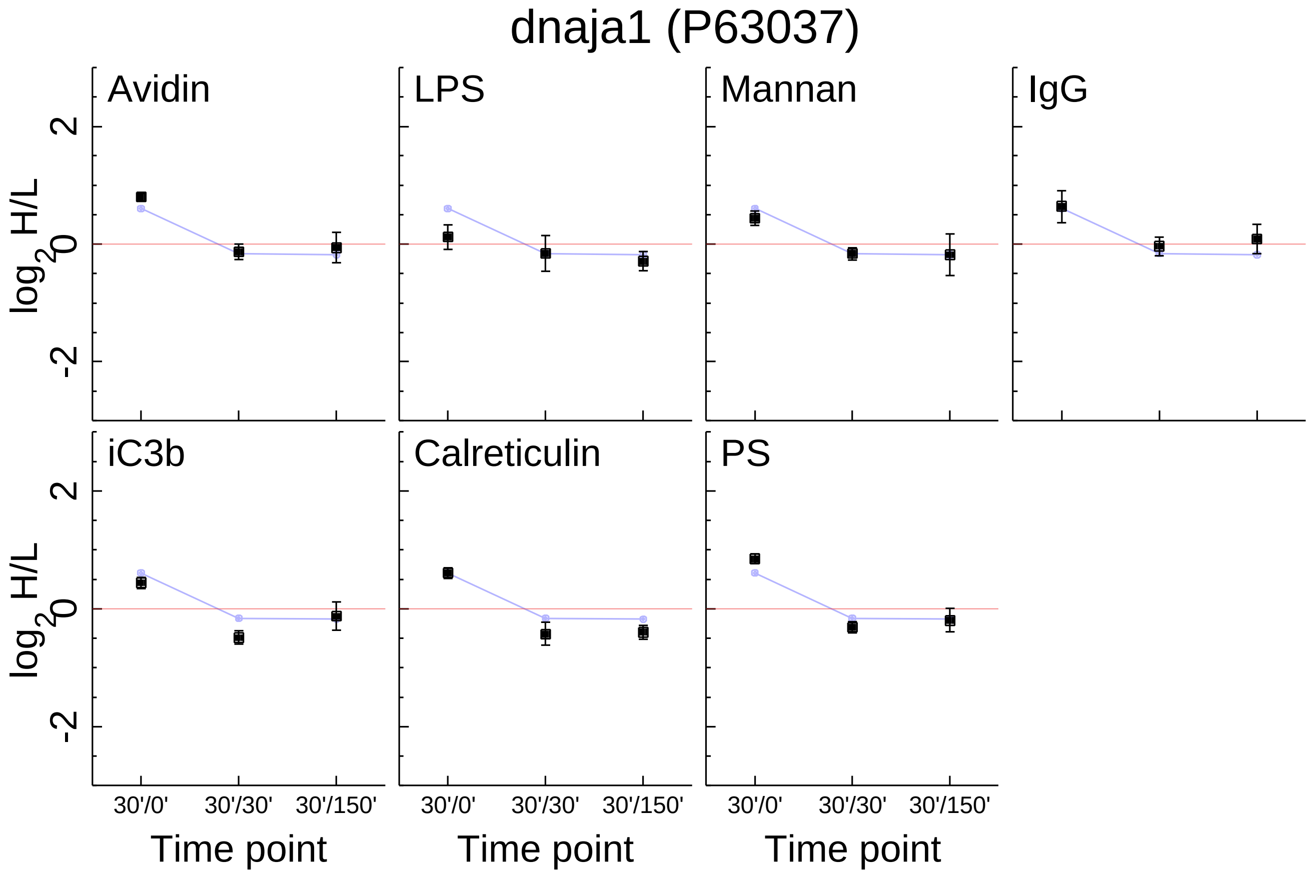
dnaja1 (P63037)
Avidin
LPS
Mannan
IgG
iC3b
Calreticulin
PS
log2 H/L
2
0
-2
30'/0'
30'/30'
30'/150'
Time point

dnaja2 (Q9QYJ0)

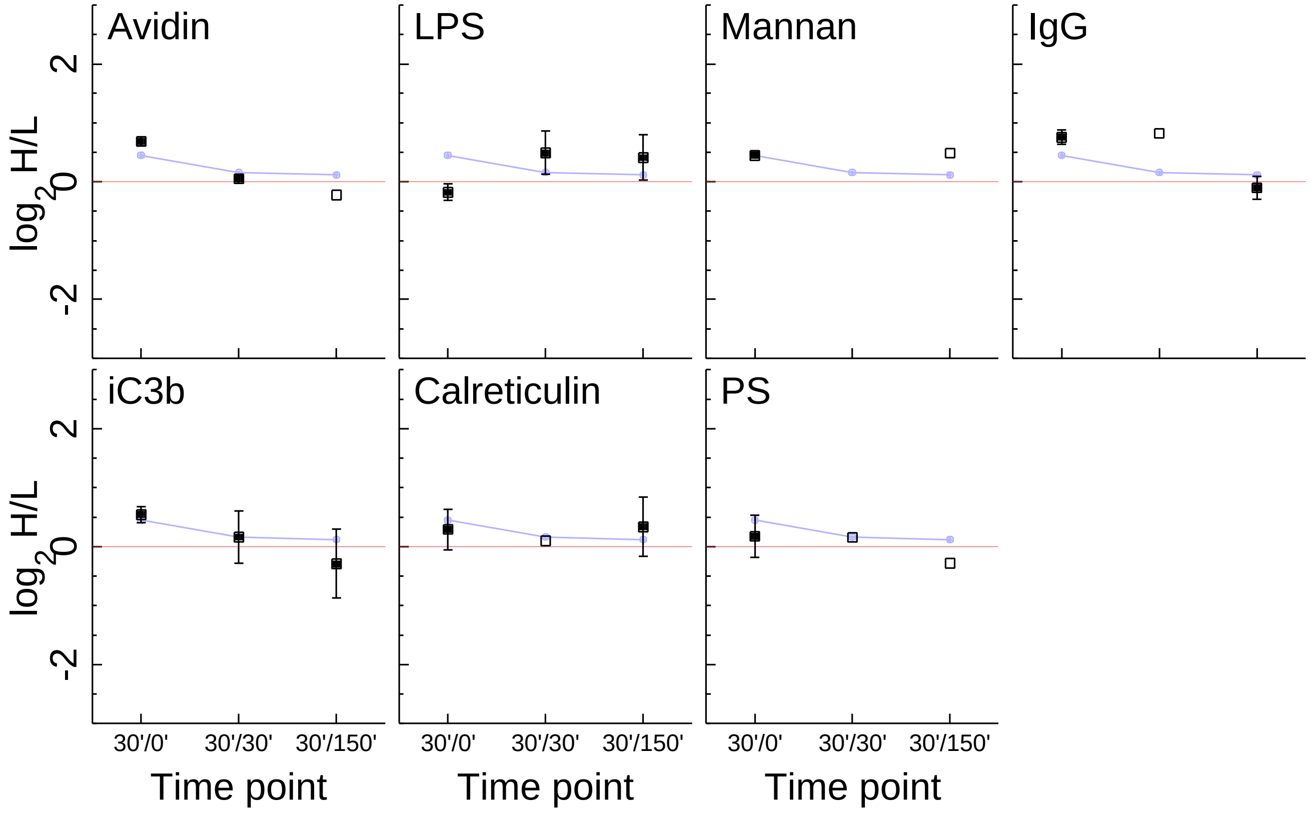

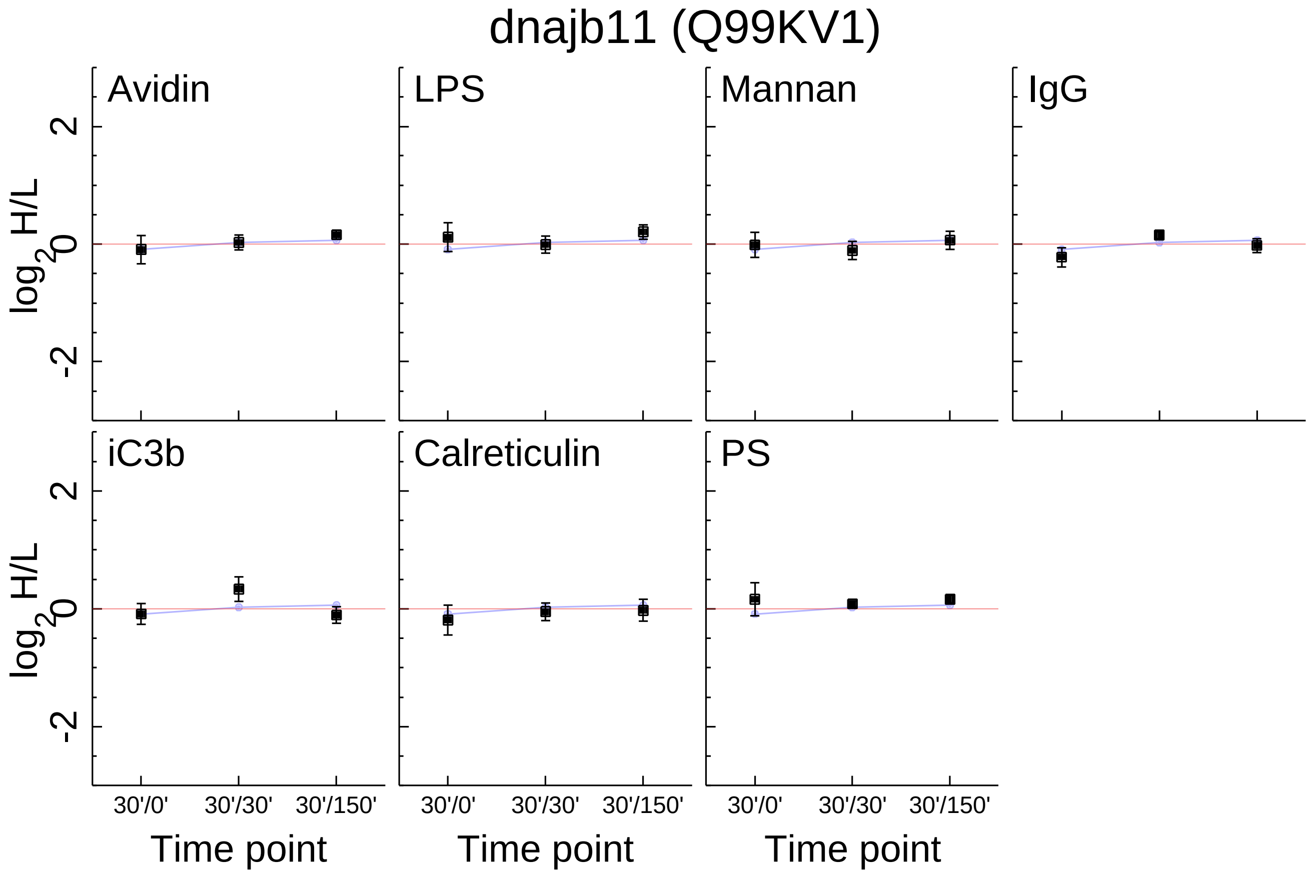
dnajb11 (Q99KV1)
Avidin
LPS
Mannan
IgG
iC3b
Calreticulin
PS
log2 H/L
2
0
-2
30'/0'
30'/30'
30'/150'
Time point

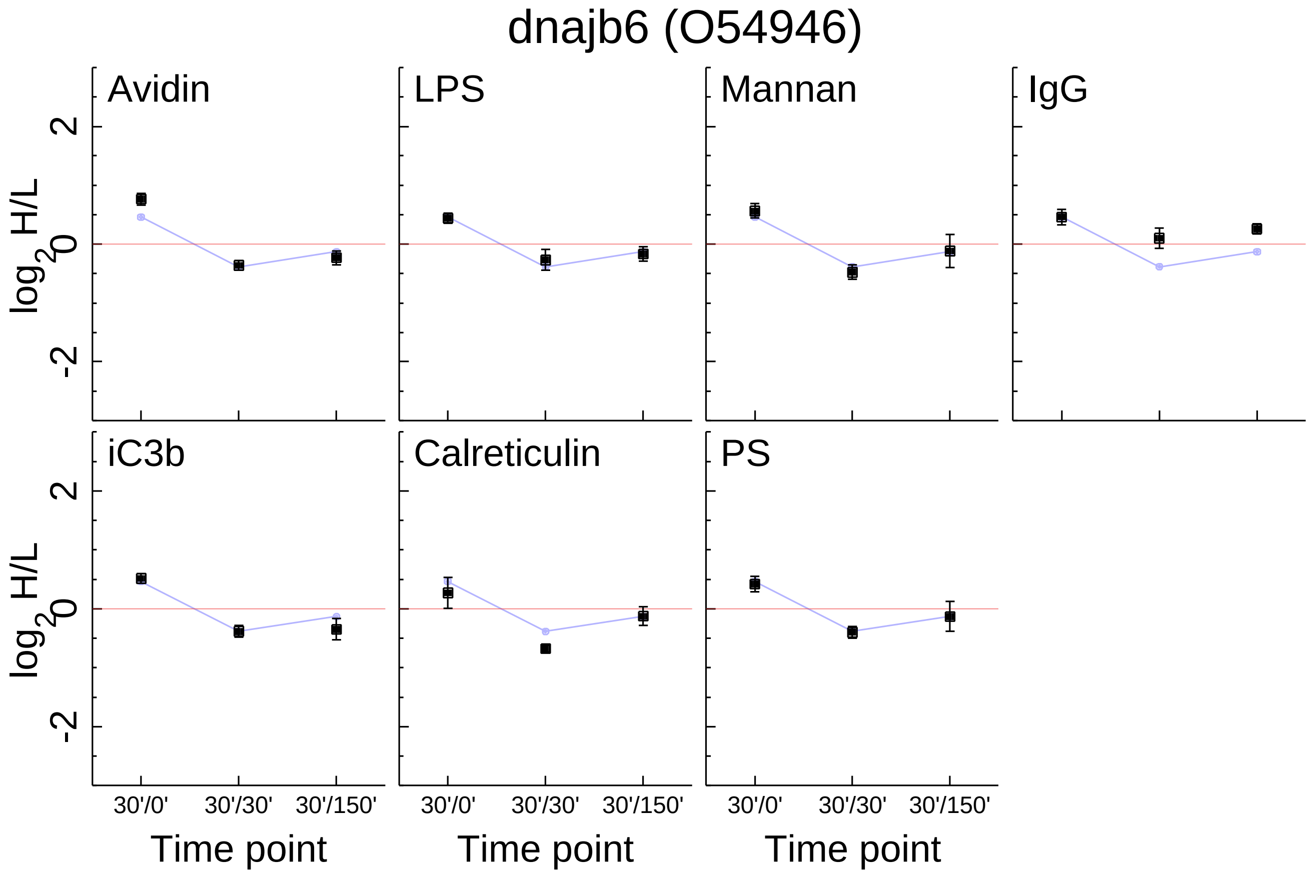
dnajb6 (O54946)
Avidin
LPS
Mannan
IgG
iC3b
Calreticulin
PS
log2 H/L
2
0
-2
30'/0'
30'/30'
30'/150'
Time point

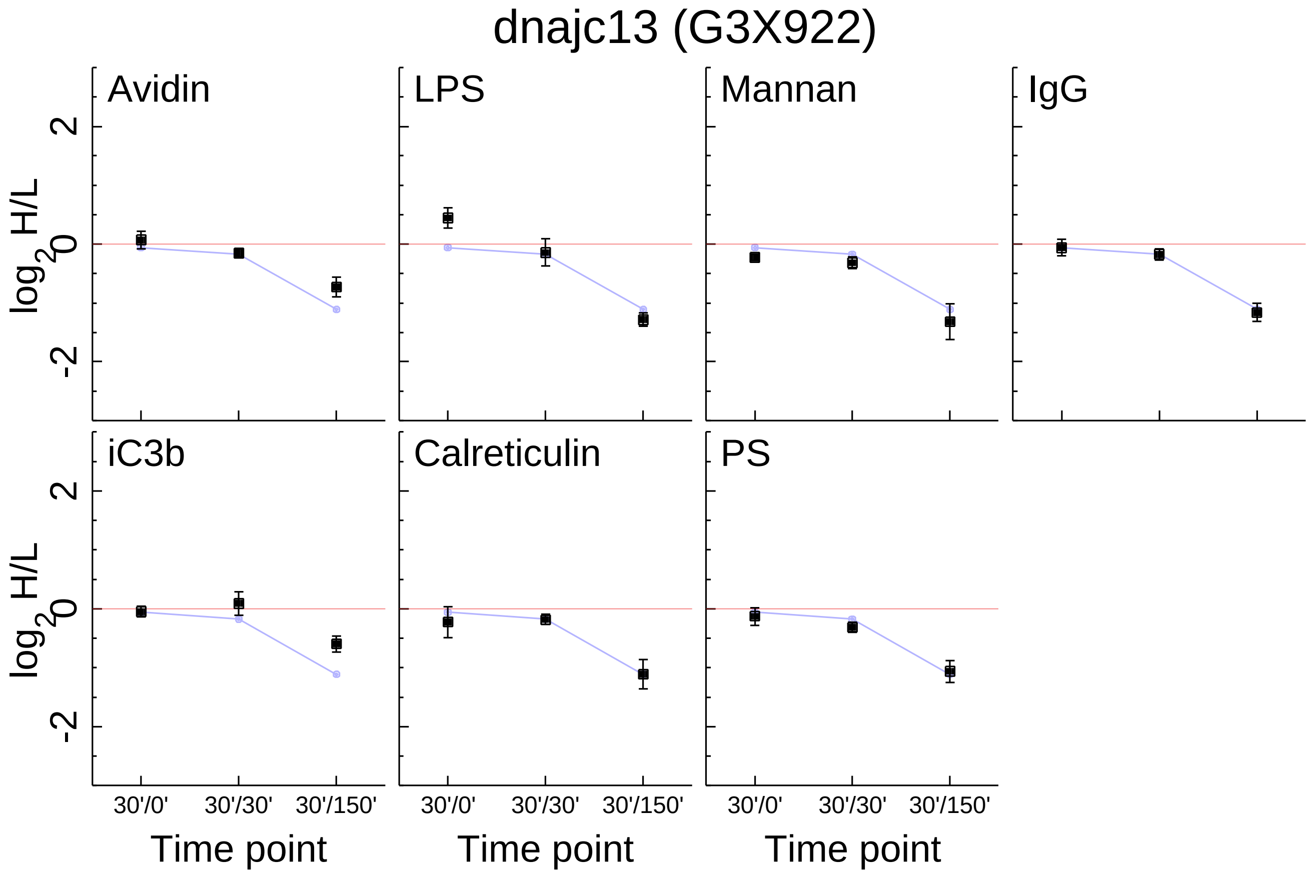

dnajc13 (G3X922)
Avidin
LPS
Mannan
IgG
iC3b
Calreticulin
PS
log2 H/L
2
0
-2
30'/0'
30'/30'
30'/150'
Time point

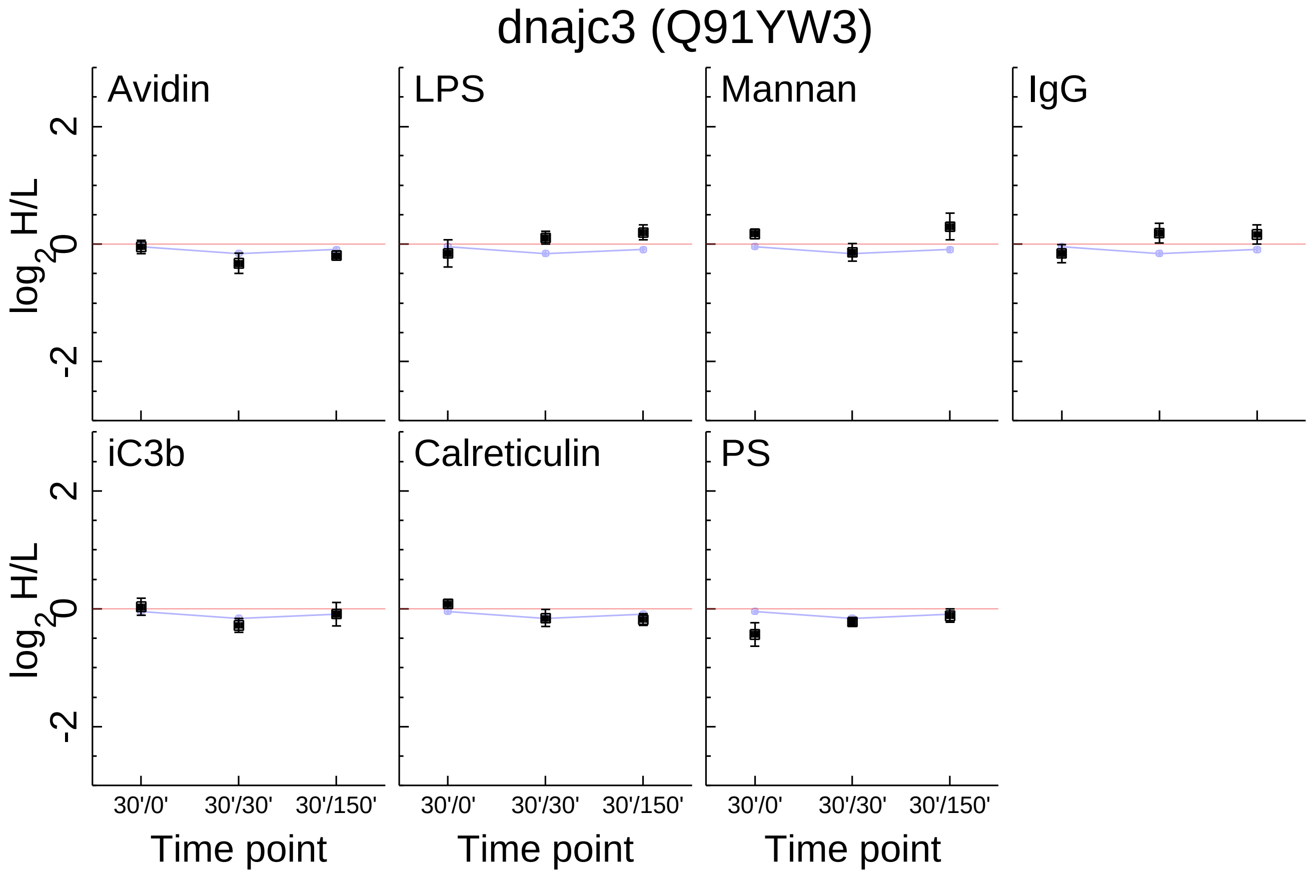

dnajc3 (Q91YW3)
Avidin
LPS
Mannan
IgG
iC3b
Calreticulin
PS
log2 H/L
2
0
-2
30'/0'
30'/30'
30'/150'
Time point

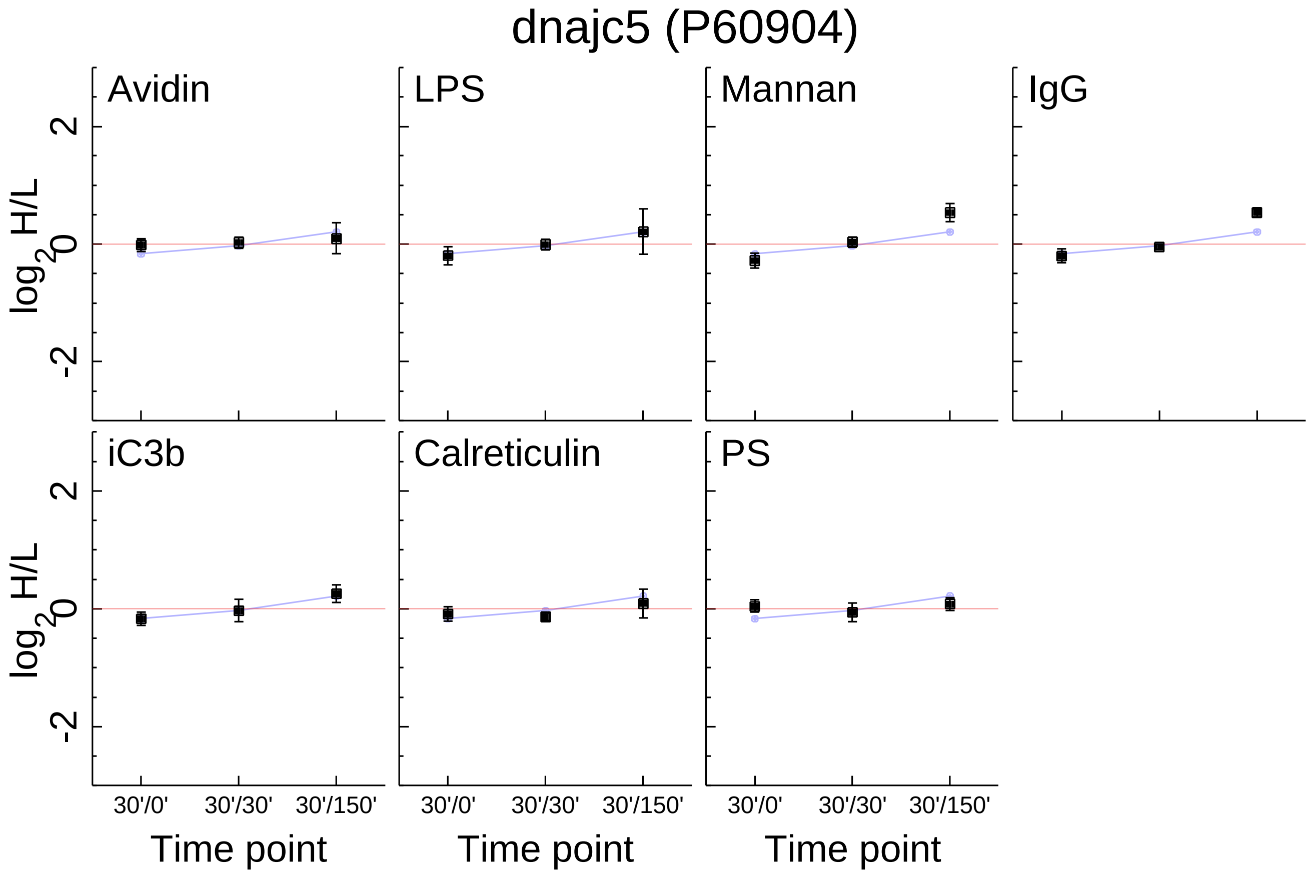
dnajc5 (P60904)
Avidin
LPS
Mannan
IgG
iC3b
Calreticulin
PS
log2 H/L
2
0
-2
30'/0'
30'/30'
30'/150'
Time point

dnajc8 (Q6NZB0)

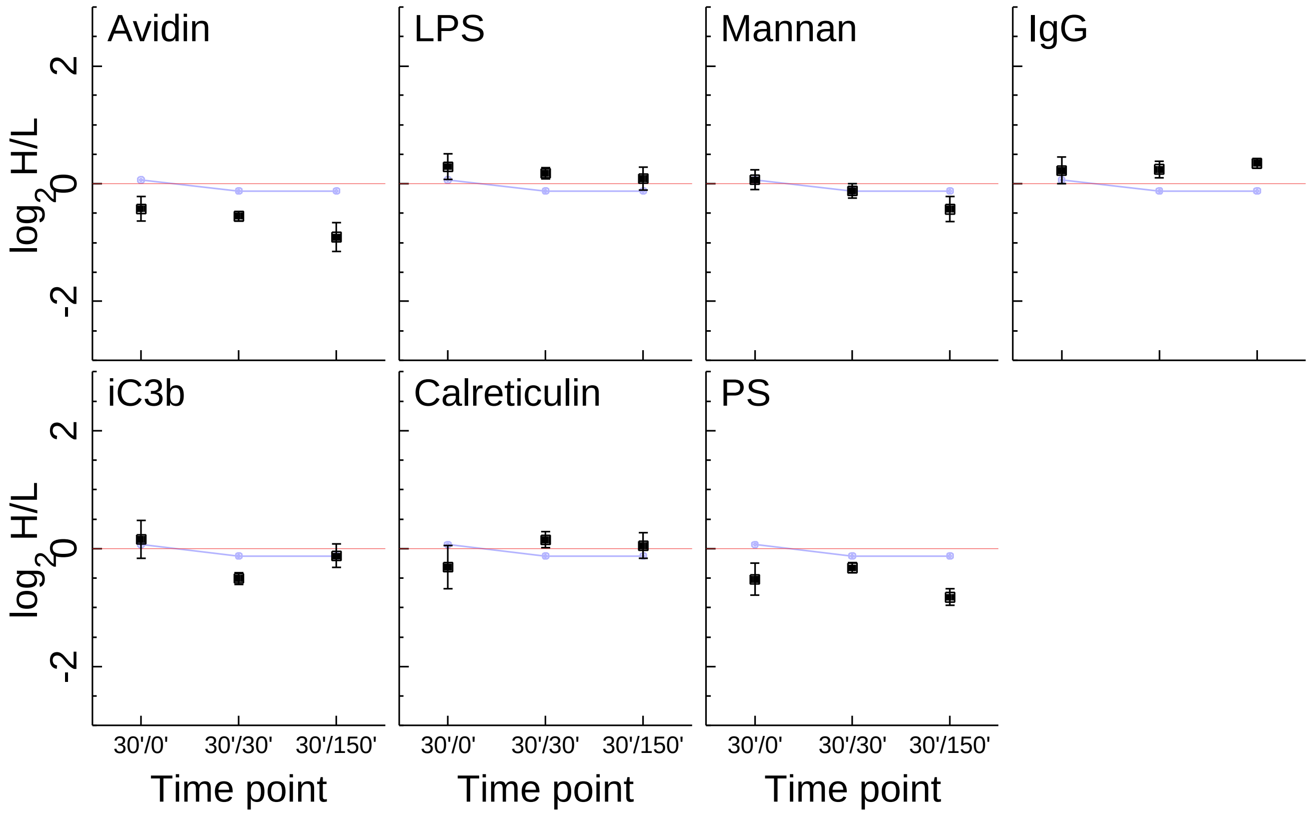

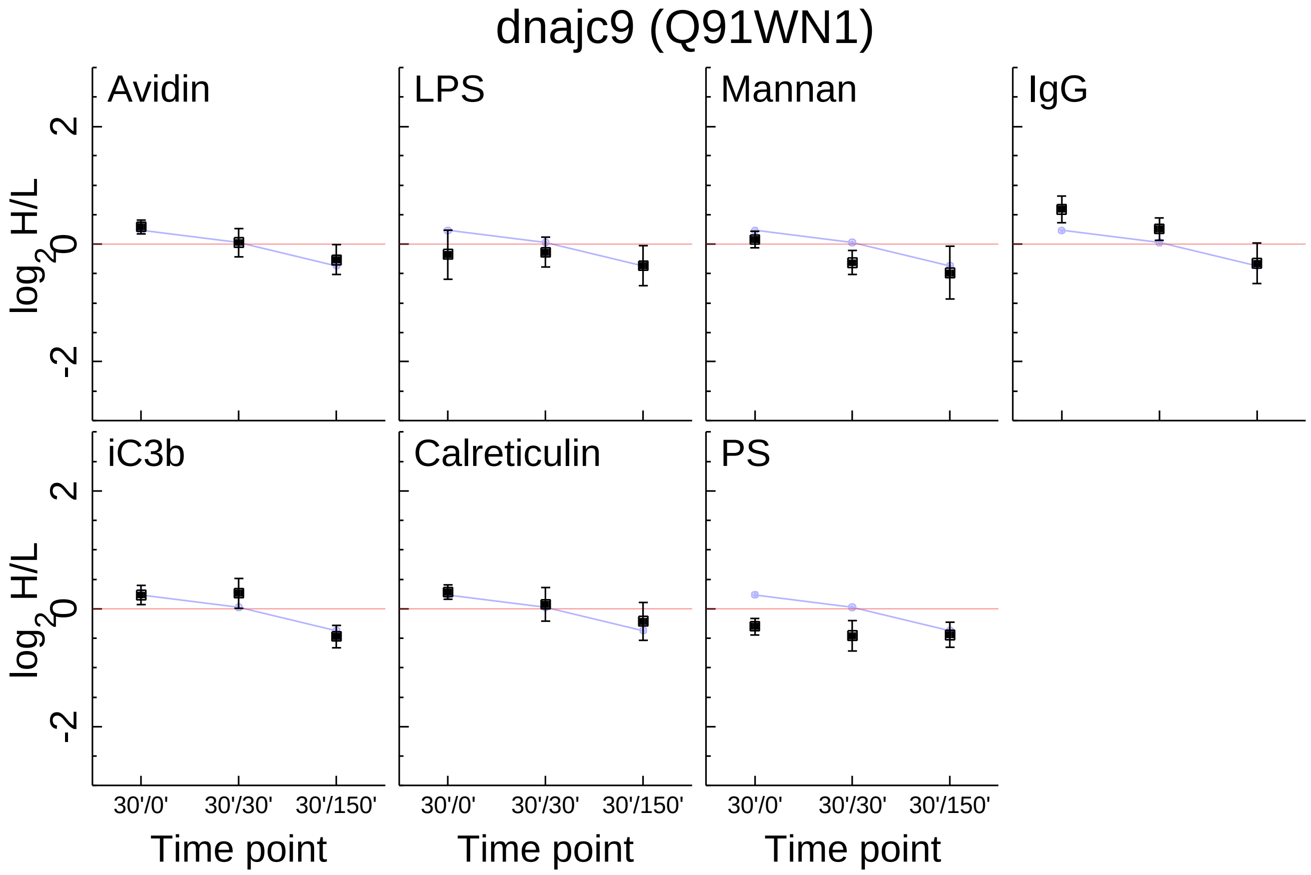

dnajc9 (Q91WN1)
Avidin
LPS
Mannan
IgG
iC3b
Calreticulin
PS
log2 H/L
2
0
-2
30'/0'
30'/30'
30'/150'
Time point

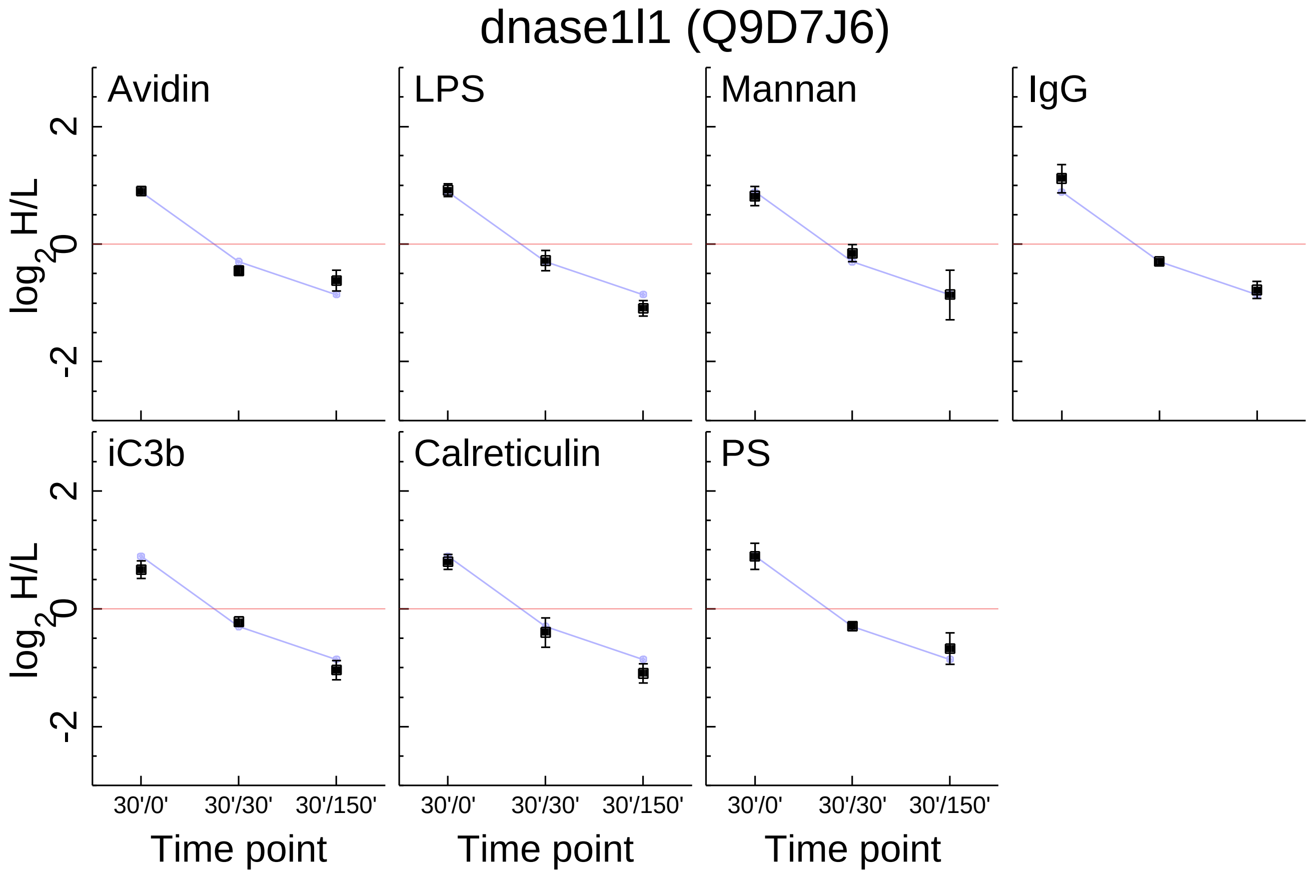

dnase1l1 (Q9D7J6)
Avidin
LPS
Mannan
IgG
iC3b
Calreticulin
PS
log2 H/L
2
0
-2
30'/0'
30'/30'
30'/150'
Time point

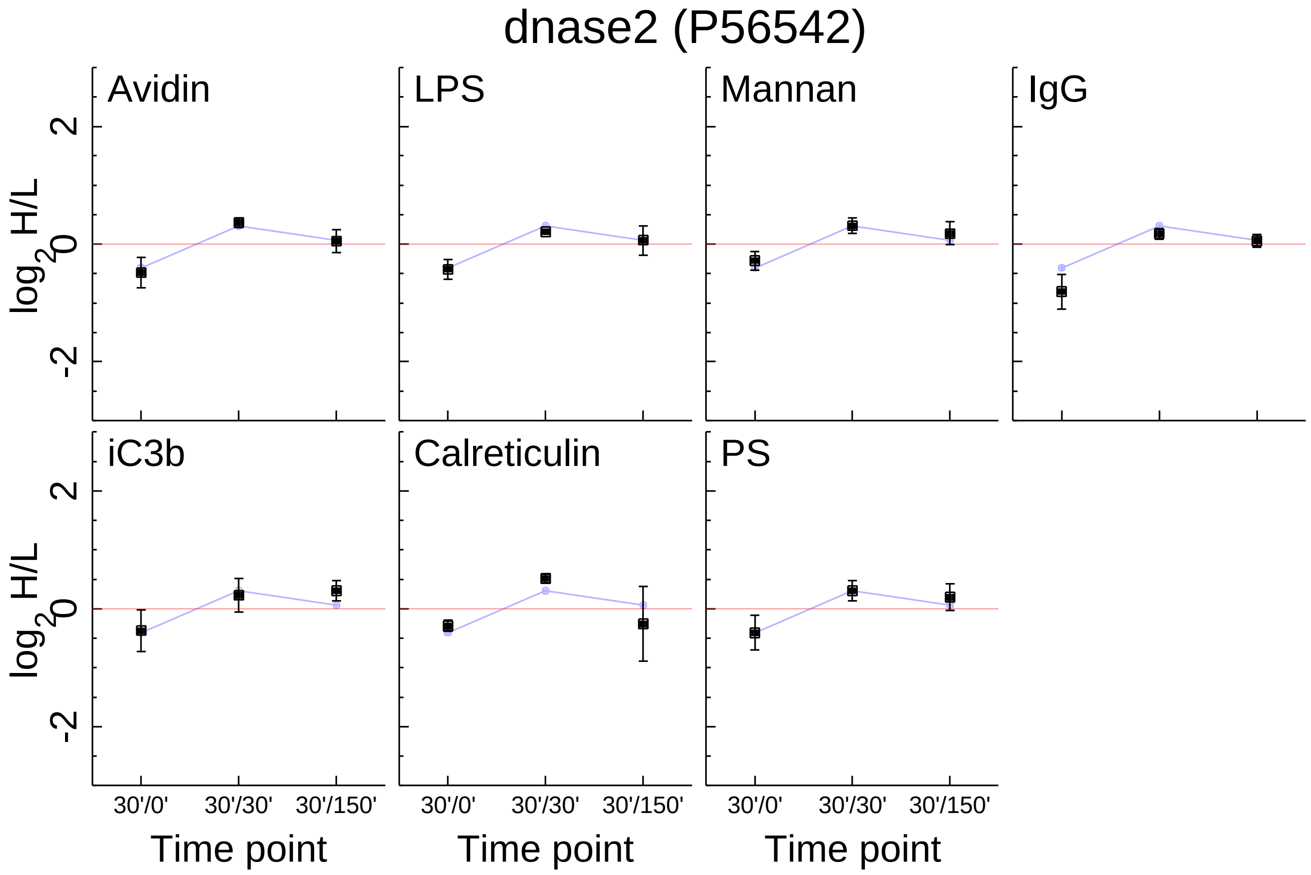

dnase2 (P56542)
Avidin
LPS
Mannan
IgG
iC3b
Calreticulin
PS
log2 H/L
2
0
-2
30'/0'
30'/30'
30'/150'
Time point

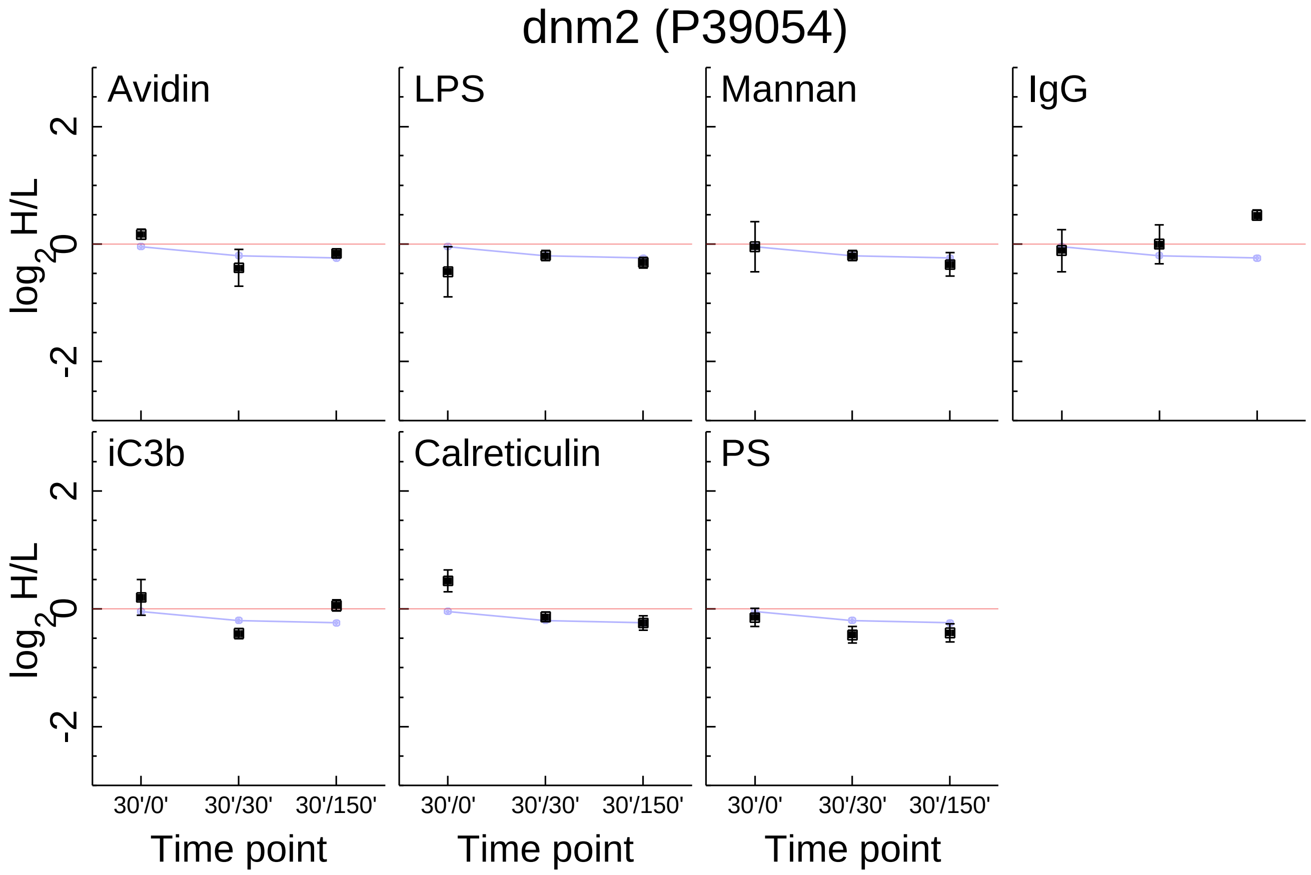

dnm2 (P39054)
Avidin
LPS
Mannan
IgG
iC3b
Calreticulin
PS
log2 H/L
2
0
-2
30'/0'
30'/30'
30'/150'
Time point

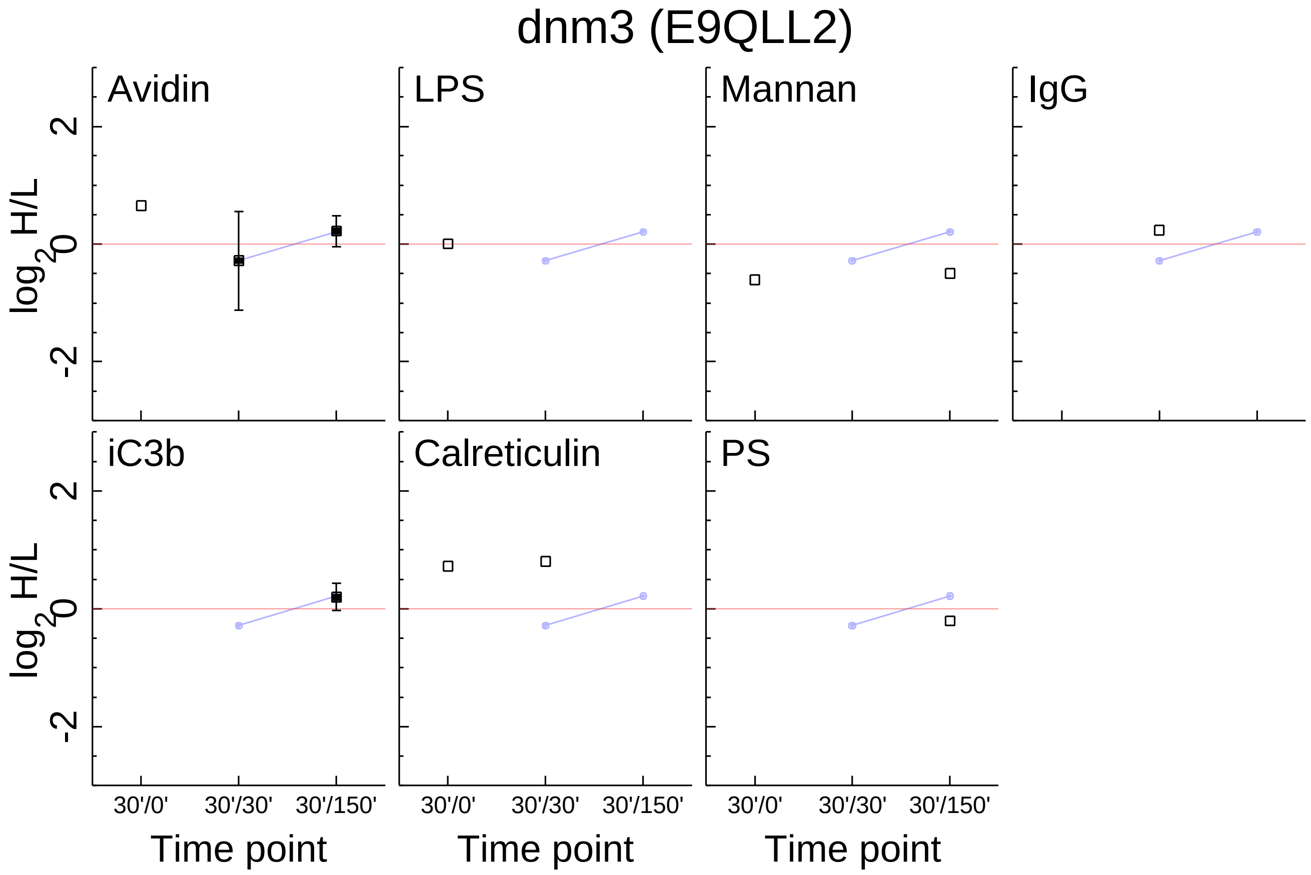
dnm3 (E9QLL2)
Avidin
LPS
Mannan
IgG
iC3b
Calreticulin
PS
log2 H/L
2
0
-2
30'/0'
30'/30'
30'/150'
Time point

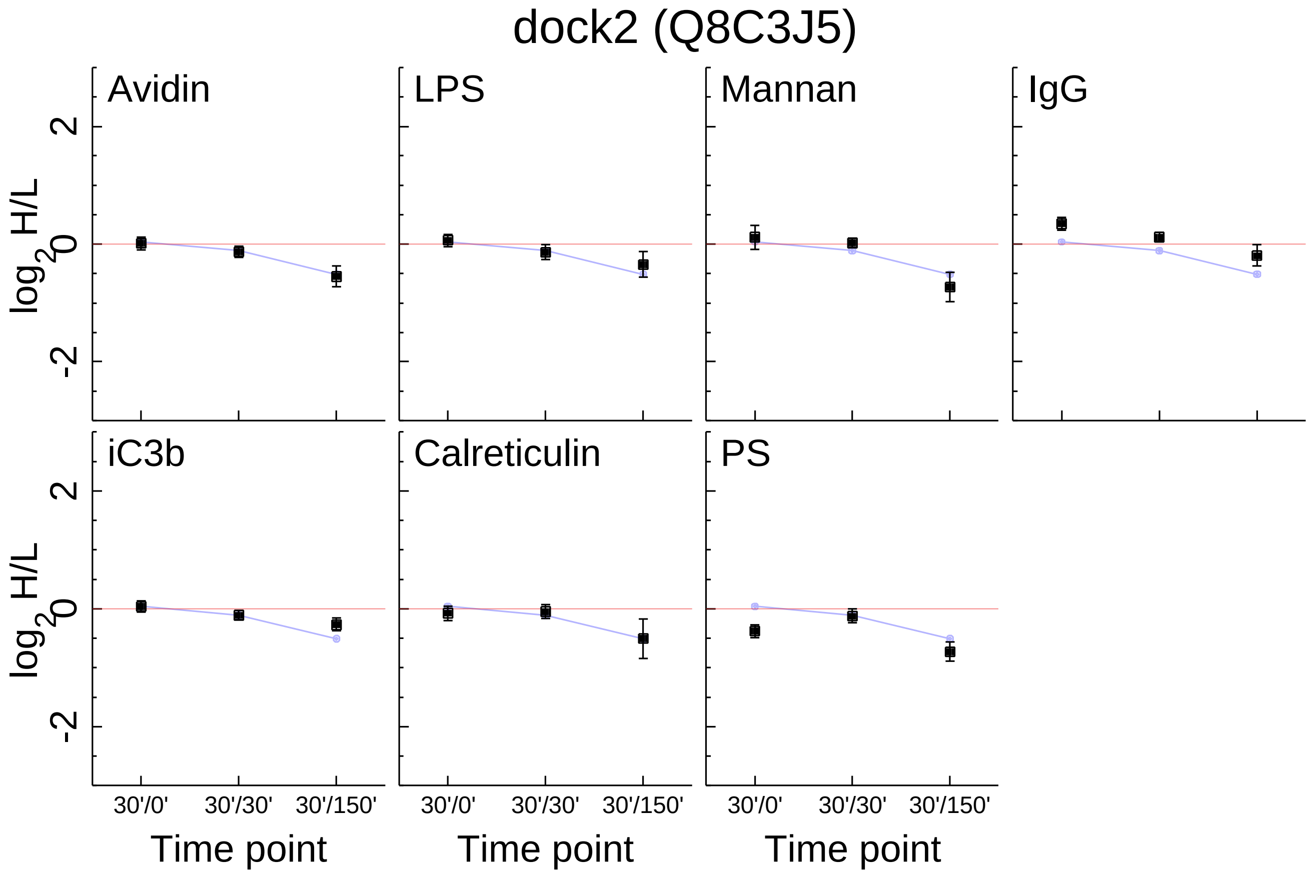
dock2 (Q8C3J5)
Avidin
LPS
Mannan
IgG
iC3b
Calreticulin
PS
log2 H/L
2
0
-2
30'/0'
30'/30'
30'/150'
Time point

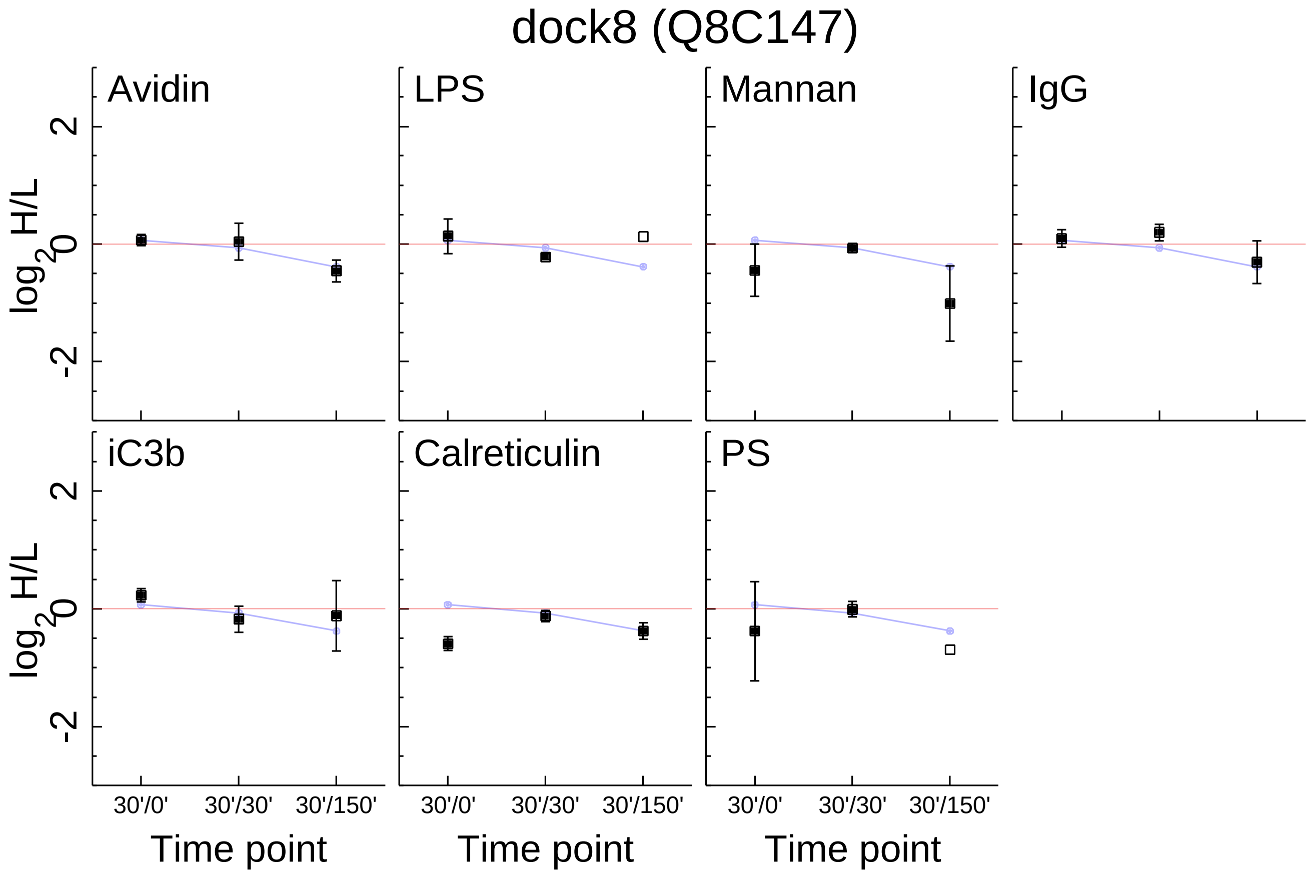
dock8 (Q8C147)
Avidin
LPS
Mannan
IgG
iC3b
Calreticulin
PS
log2 H/L
2
0
-2
30'/0'
30'/30'
30'/150'
Time point

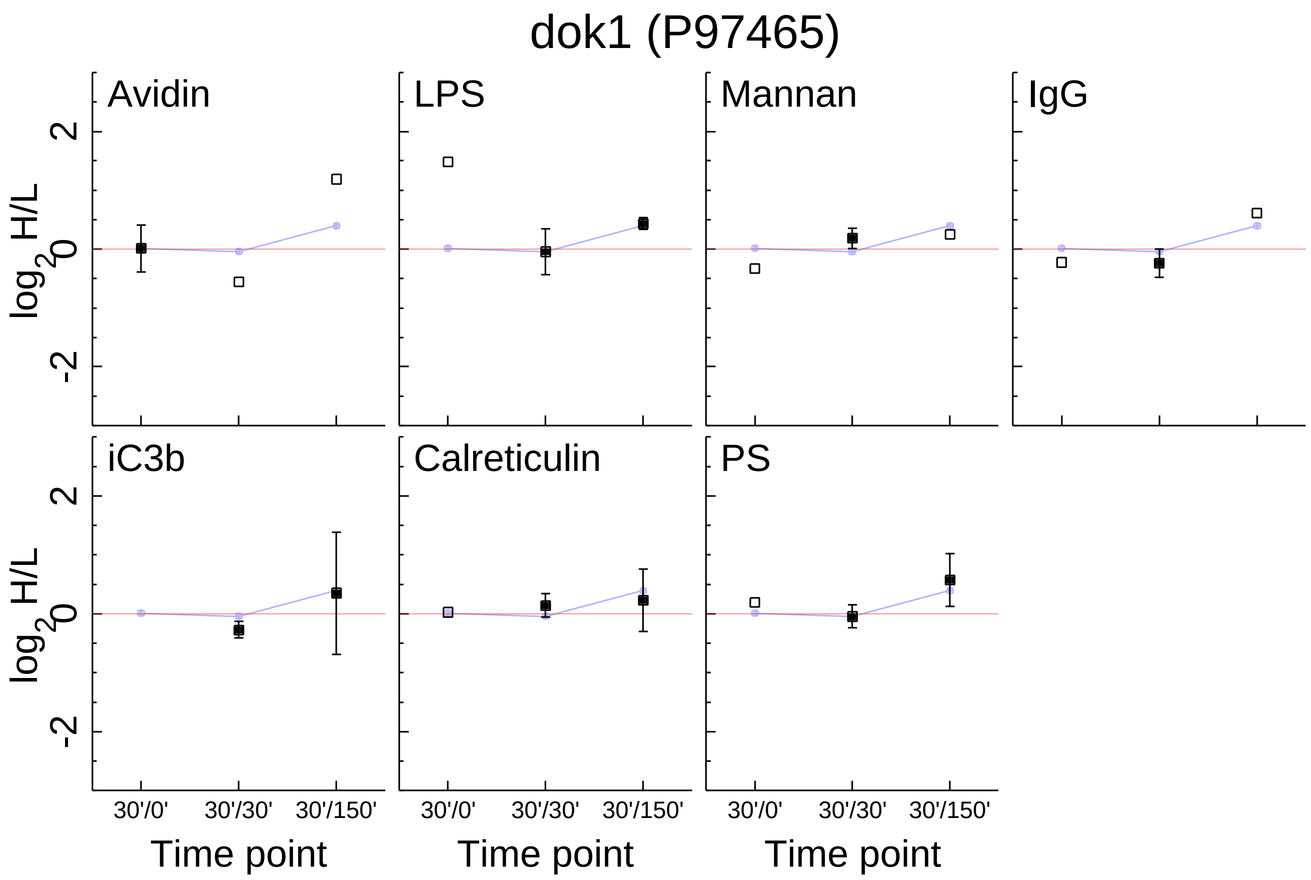
dok1 (P97465)
Avidin
LPS
Mannan
IgG
iC3b
Calreticulin
PS
log2 H/L
2
0
-2
30'/0'
30'/30'
30'/150'
Time point

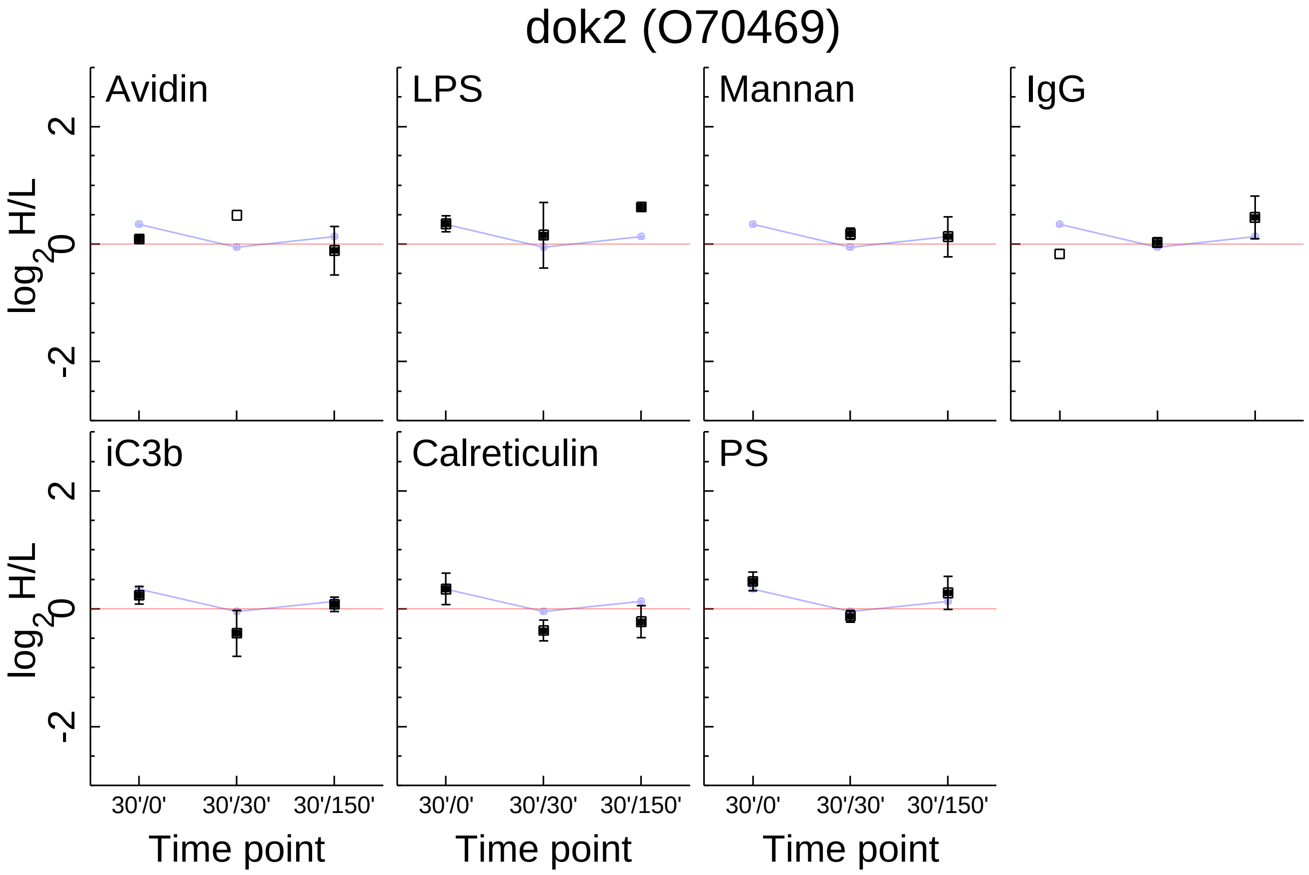
dok2 (O70469)
Avidin
LPS
Mannan
IgG
iC3b
Calreticulin
PS
log2 H/L
2
0
-2
30'/0'
30'/30'
30'/150'
Time point

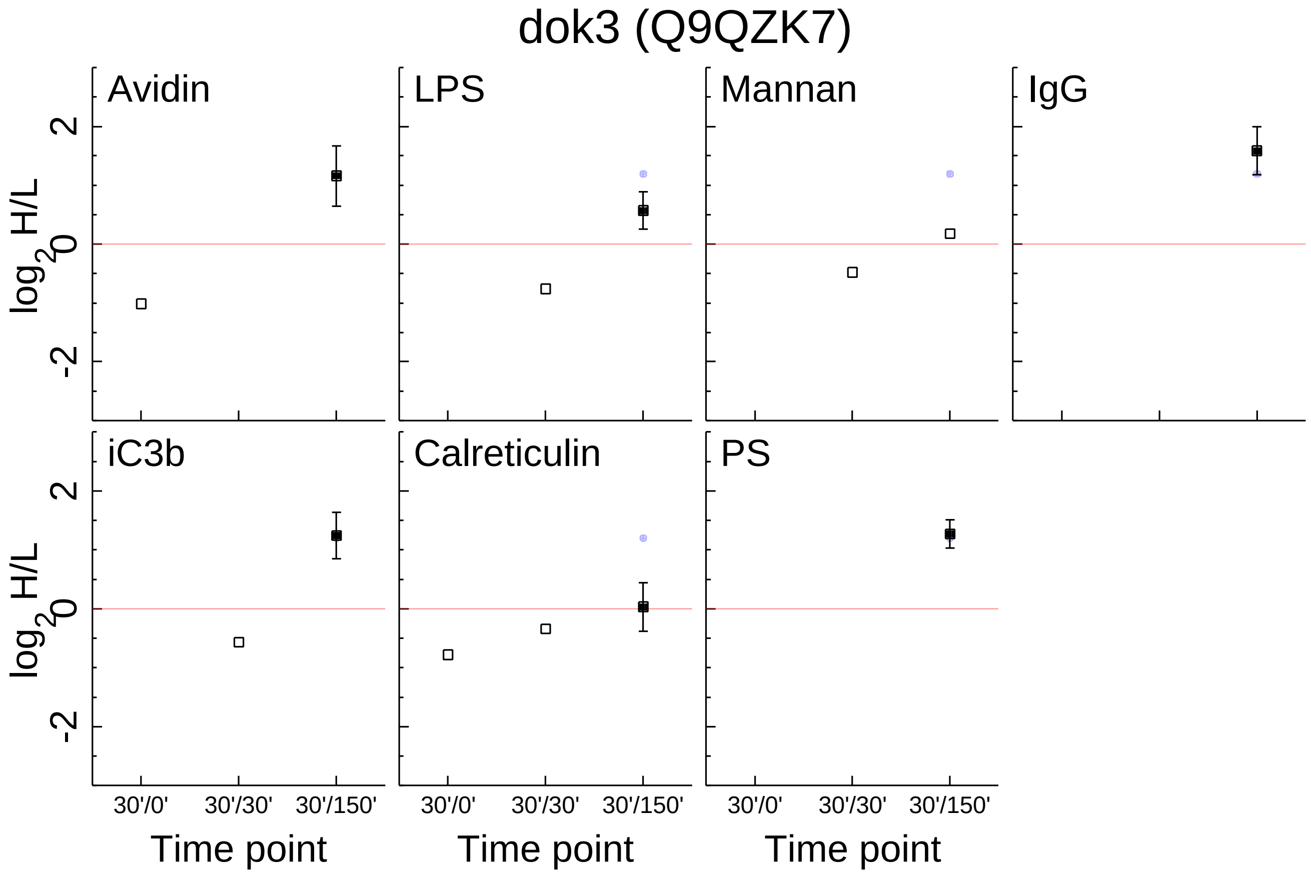
dok3 (Q9QZK7)
Avidin
LPS
Mannan
IgG
iC3b
Calreticulin
PS
log2 H/L
2
0
-2
30'/0'
30'/30'
30'/150'
Time point

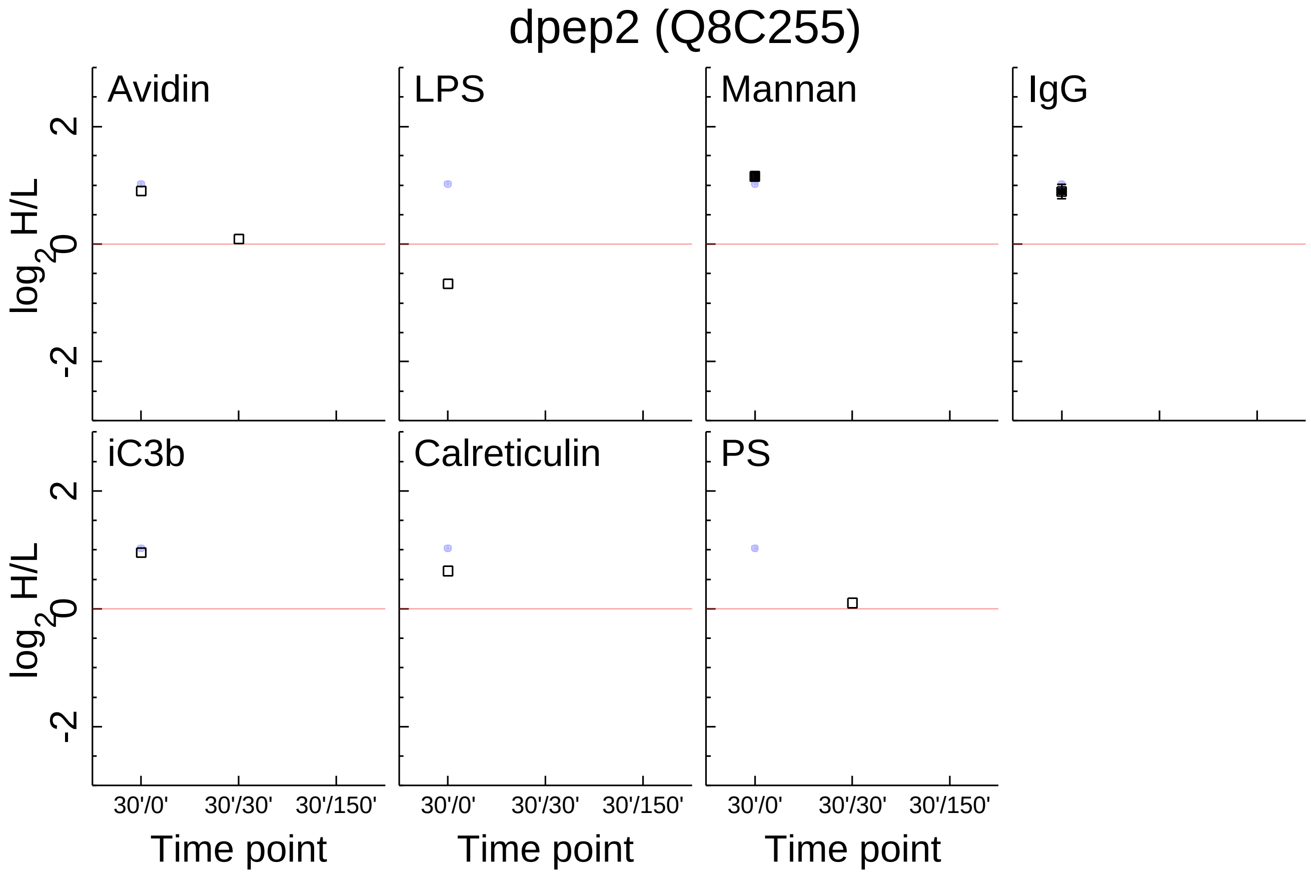
dpep2 (Q8C255)
Avidin
LPS
Mannan
IgG
iC3b
Calreticulin
PS
log2 H/L
2
0
-2
30'/0'
30'/30'
30'/150'
Time point

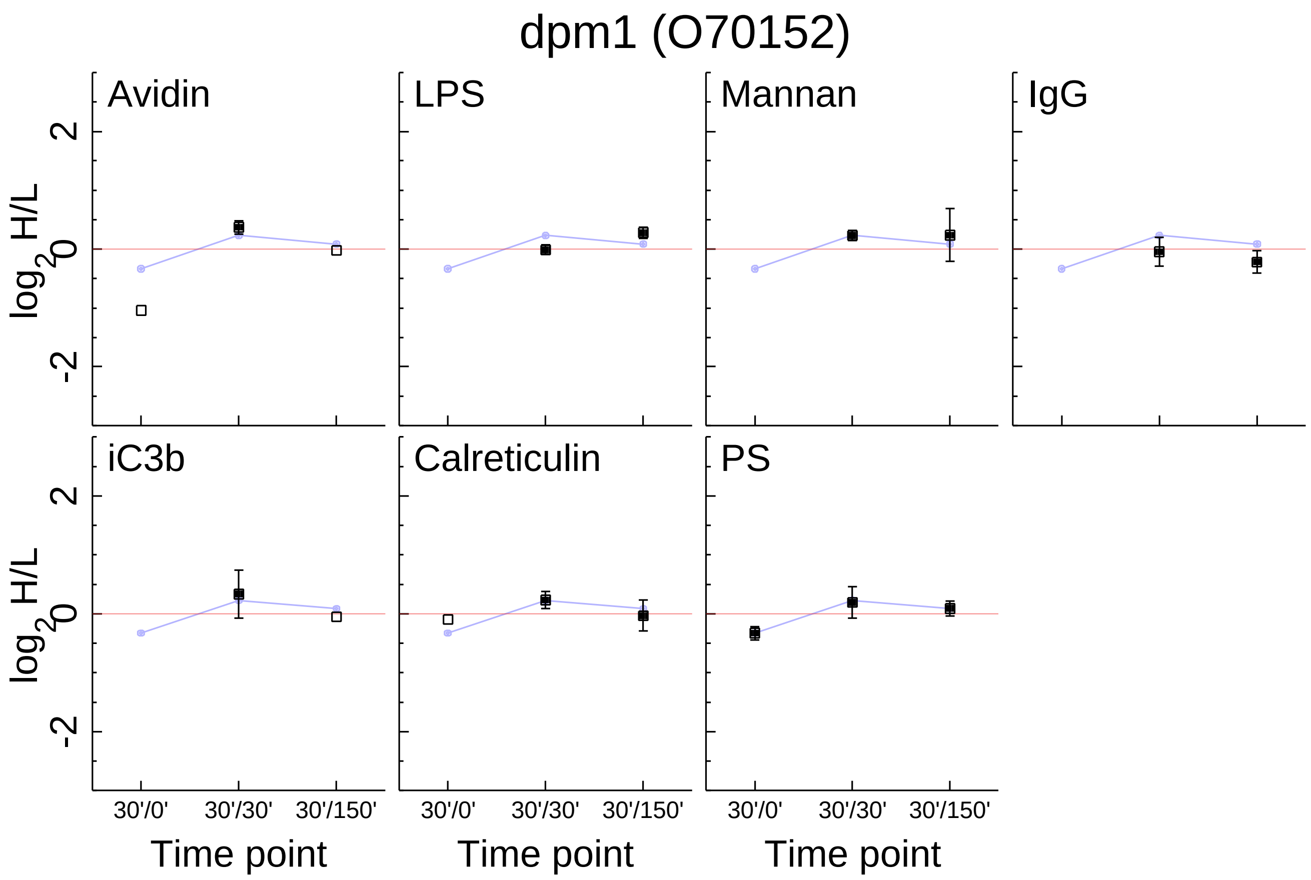
dpm1 (O70152)
Avidin
LPS
Mannan
IgG
iC3b
Calreticulin
PS
log2 H/L
2
0
-2
30'/0'
30'/30'
30'/150'
Time point

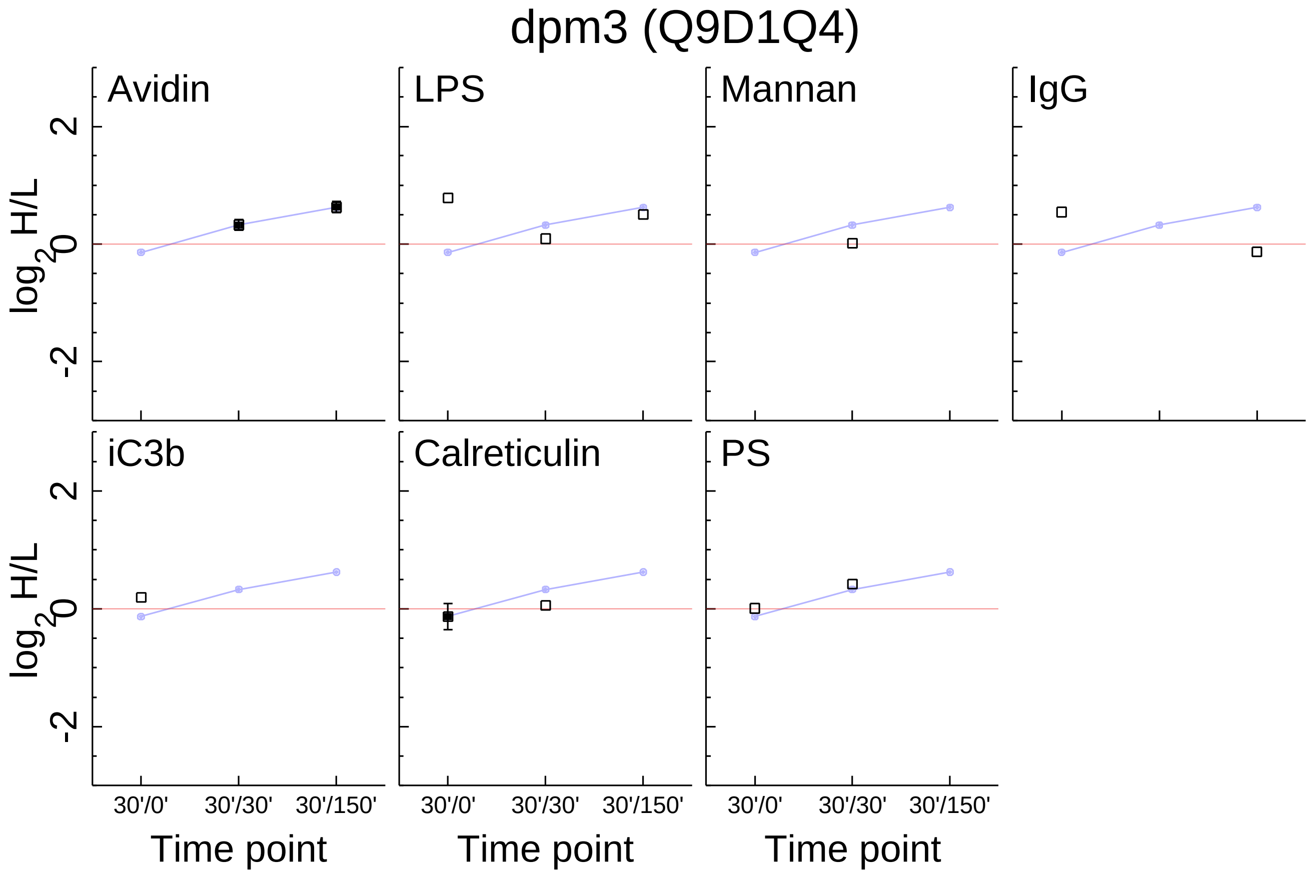

dpm3 (Q9D1Q4)
Avidin
LPS
Mannan
IgG
iC3b
Calreticulin
PS
log2 H/L
2
0
-2
30'/0'
30'/30'
30'/150'
Time point

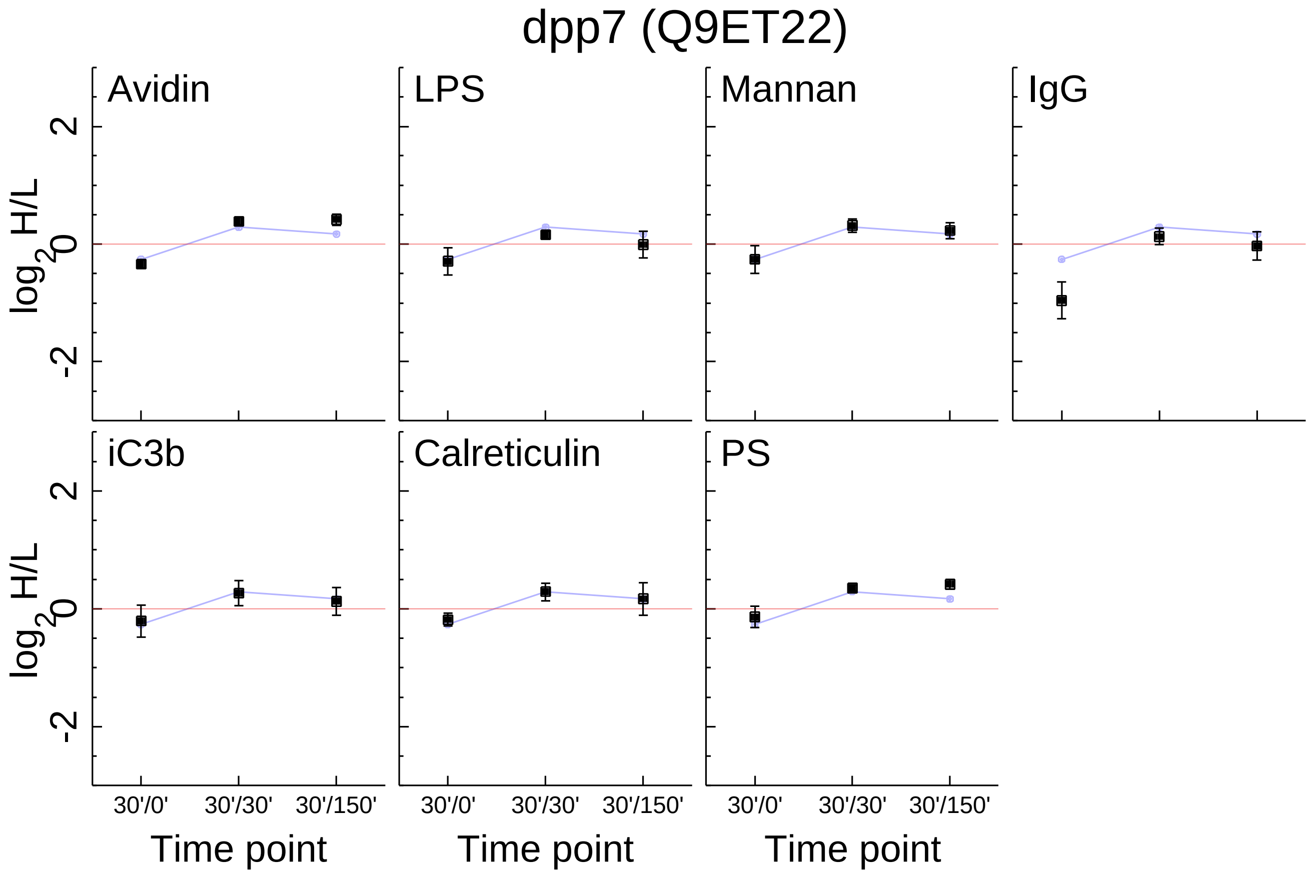
dpp7 (Q9ET22)
Avidin
LPS
Mannan
IgG
iC3b
Calreticulin
PS
log2 H/L
2
0
-2
30'/0'
30'/30'
30'/150'
Time point

dpysl2 (O08553)

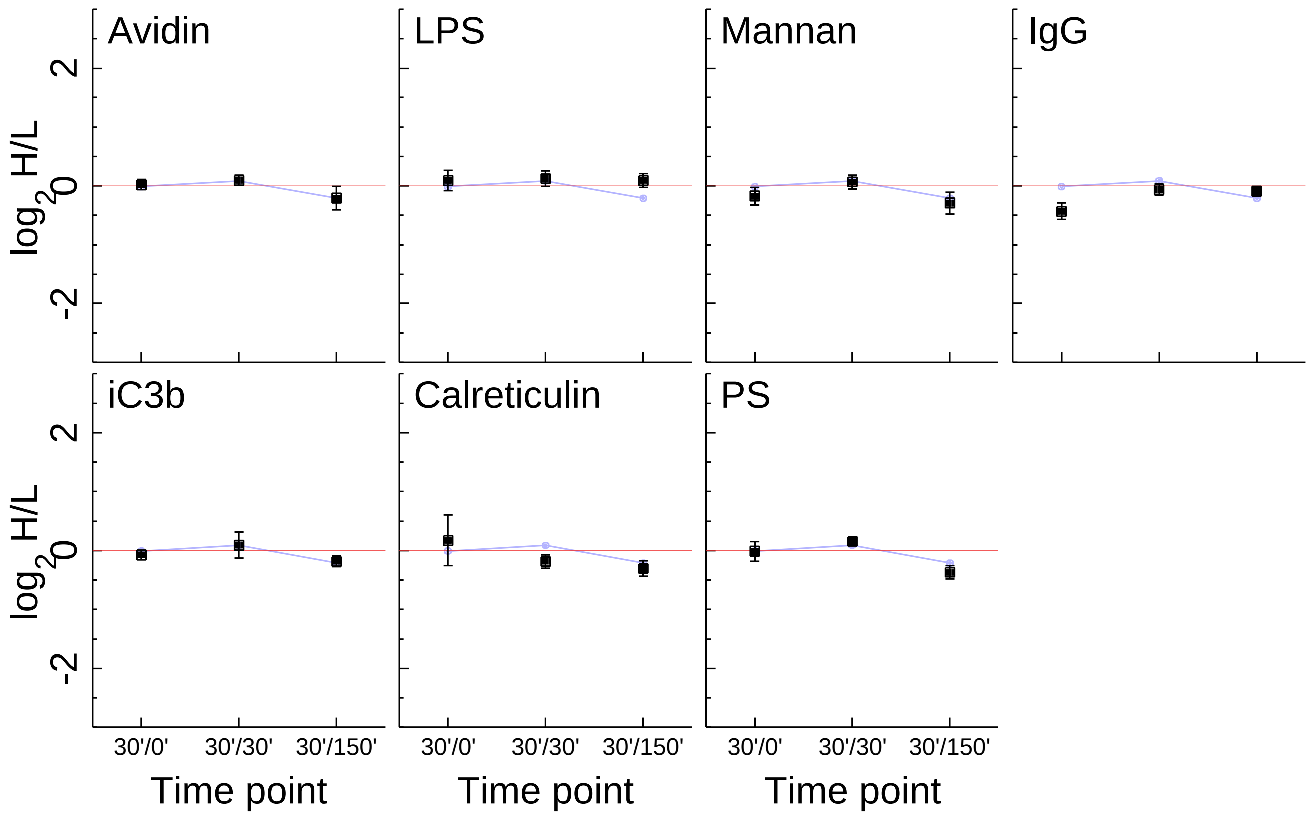

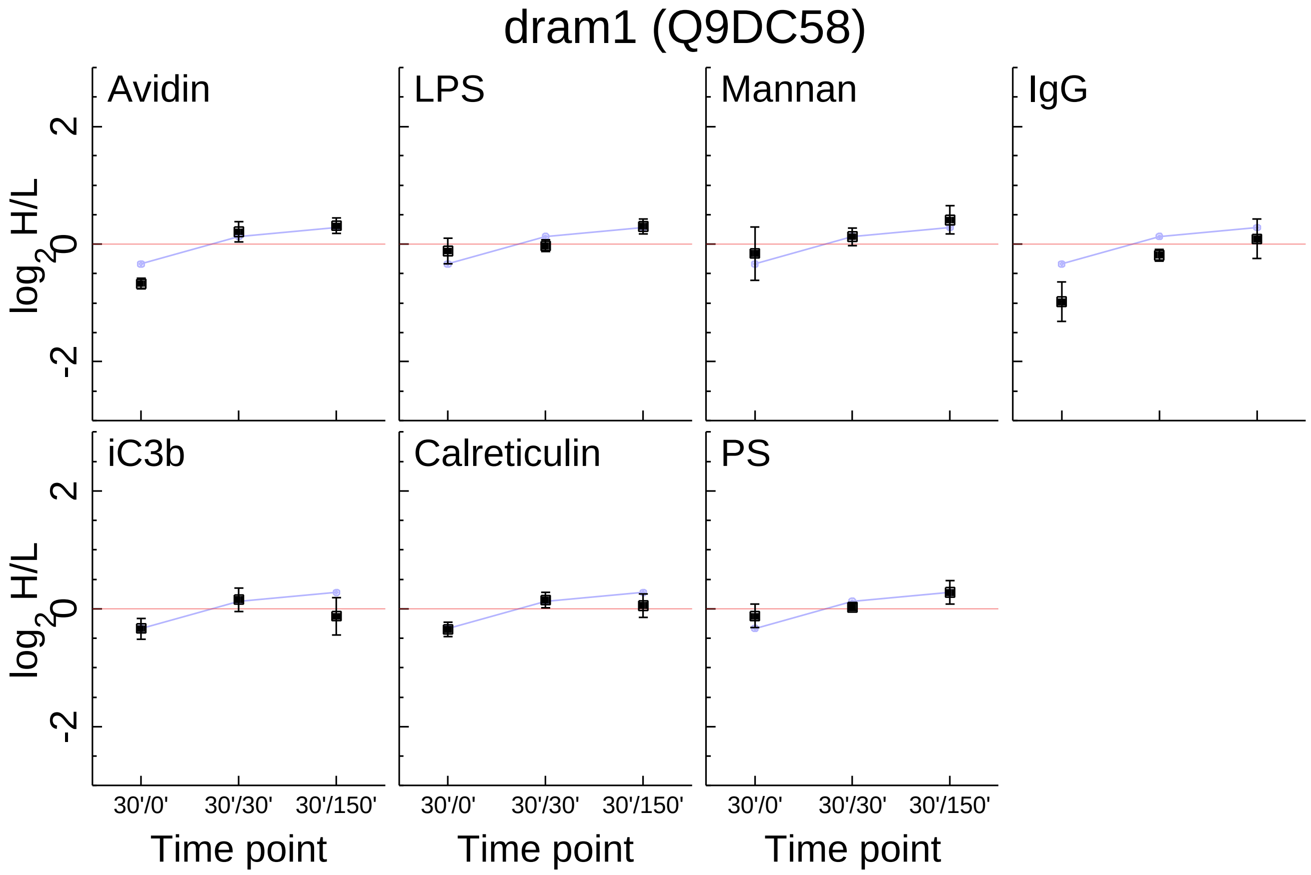
dram1 (Q9DC58)
Avidin
LPS
Mannan
IgG
iC3b
Calreticulin
PS
log2 H/L
2
0
-2
30'/0'
30'/30'
30'/150'
Time point

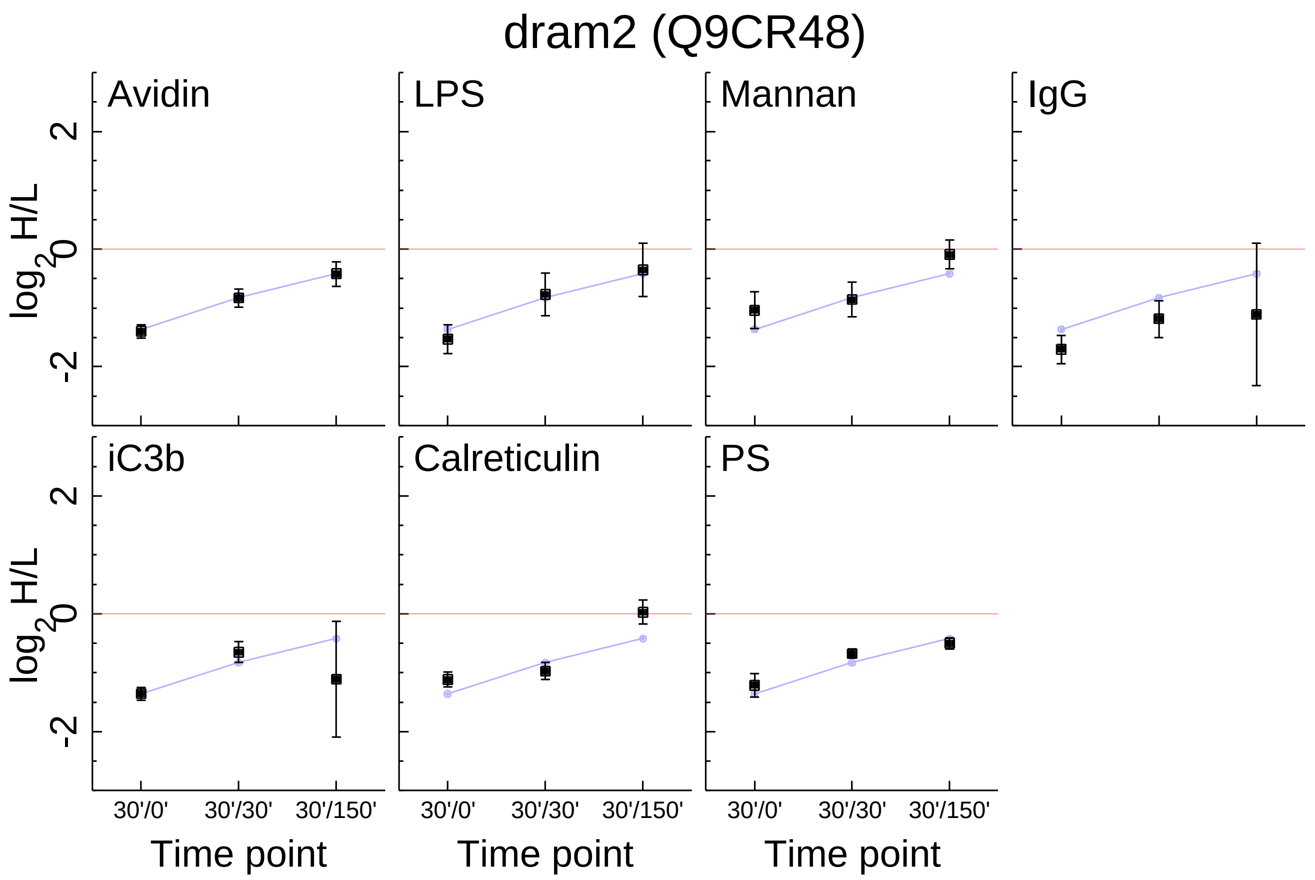
dram2 (Q9CR48)
Avidin
LPS
Mannan
IgG
iC3b
Calreticulin
PS
log2 H/L
2
0
-2
30'/0'
30'/30'
30'/150'
Time point

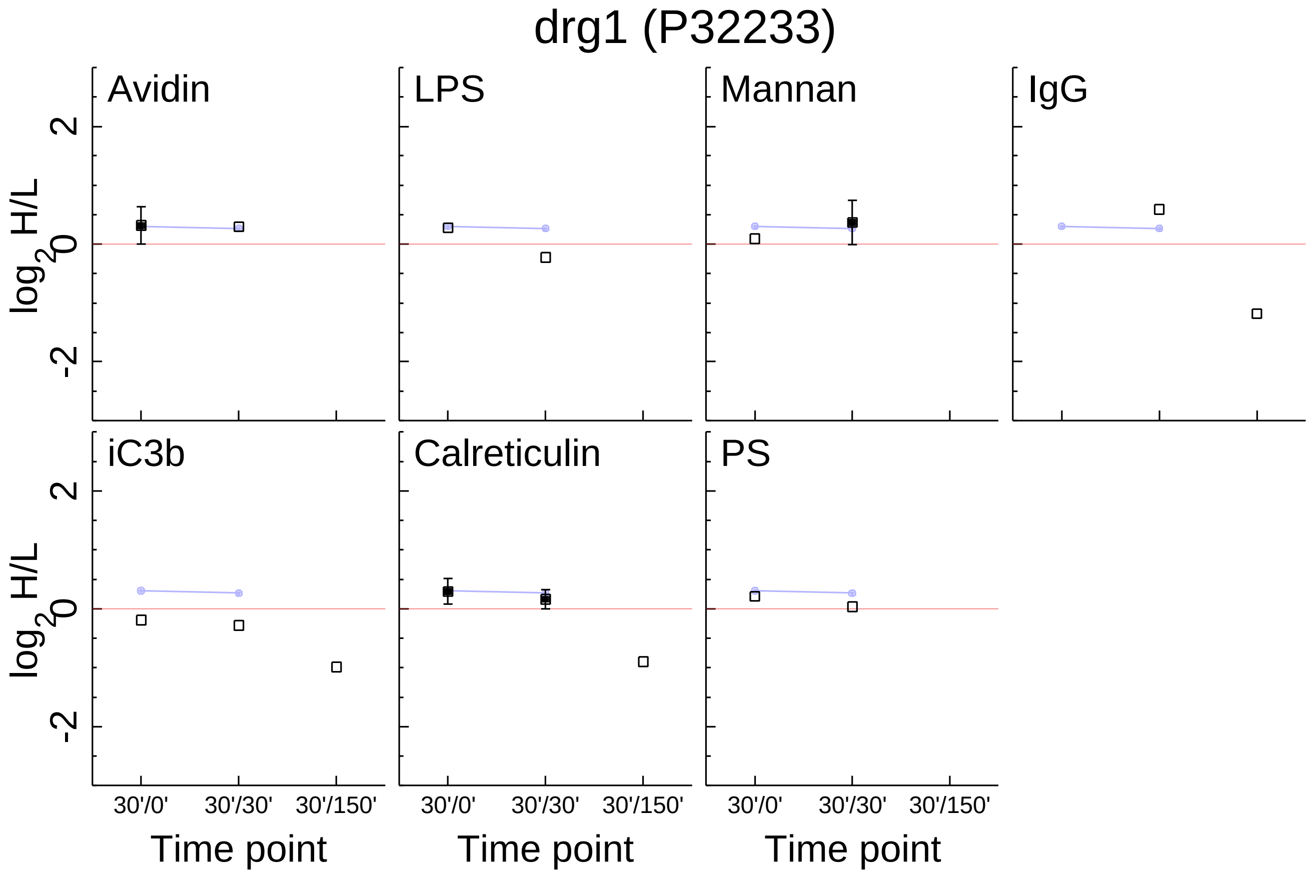
drg1 (P32233)
Avidin
LPS
Mannan
IgG
iC3b
Calreticulin
PS
log2 H/L
2
0
-2
30'/0'
30'/30'
30'/150'
Time point

dscr3 (O35075)

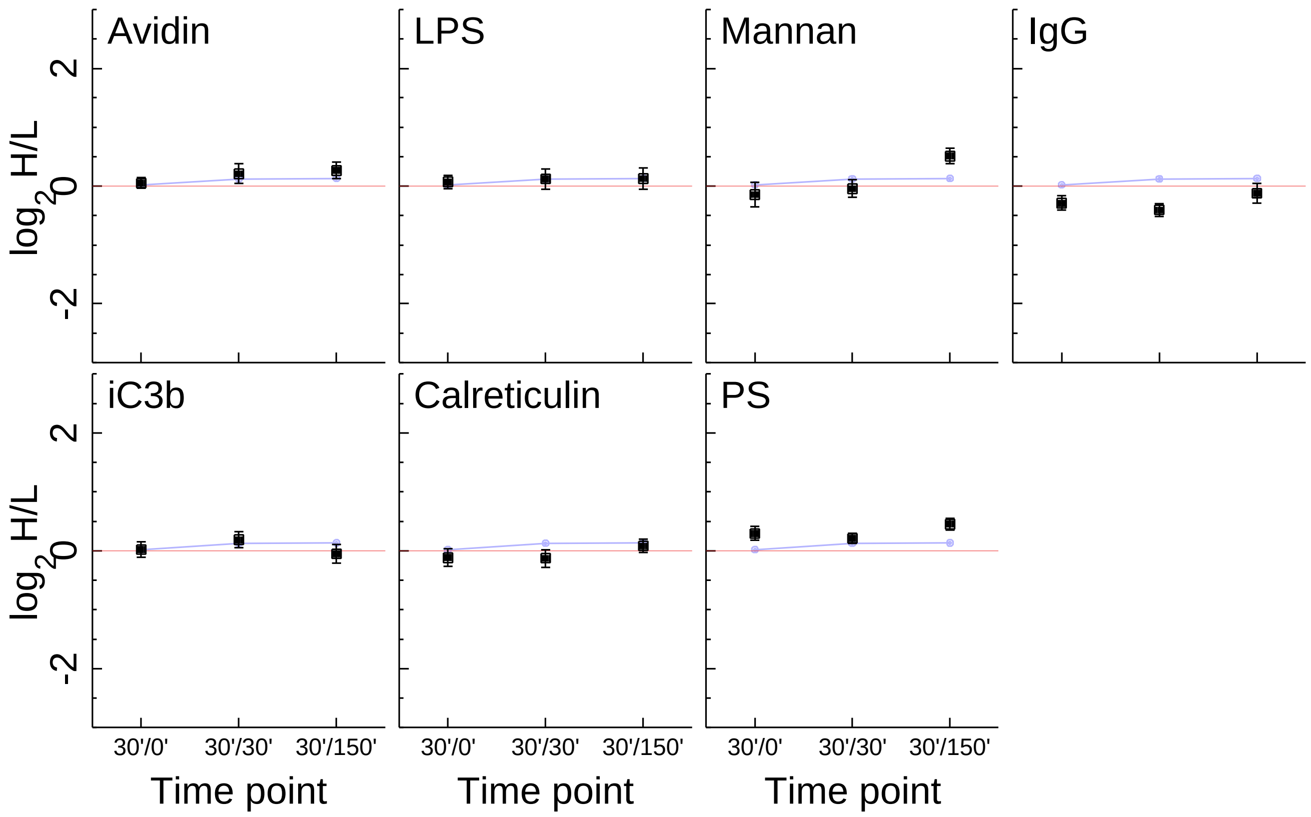

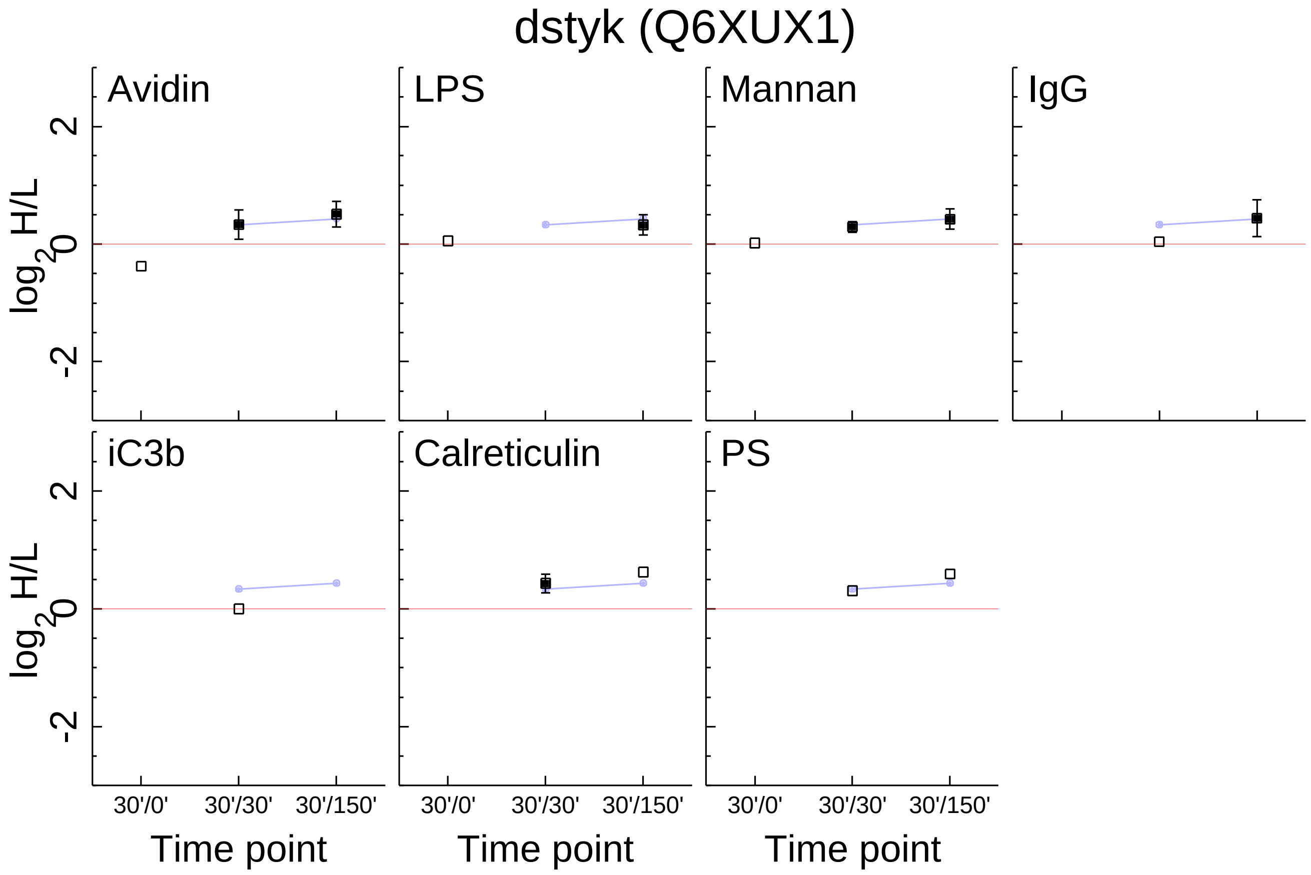
dstyk (Q6XUX1)
Avidin
LPS
Mannan
IgG
iC3b
Calreticulin
PS
log2 H/L
2
0
-2
30'/0'
30'/30'
30'/150'
Time point

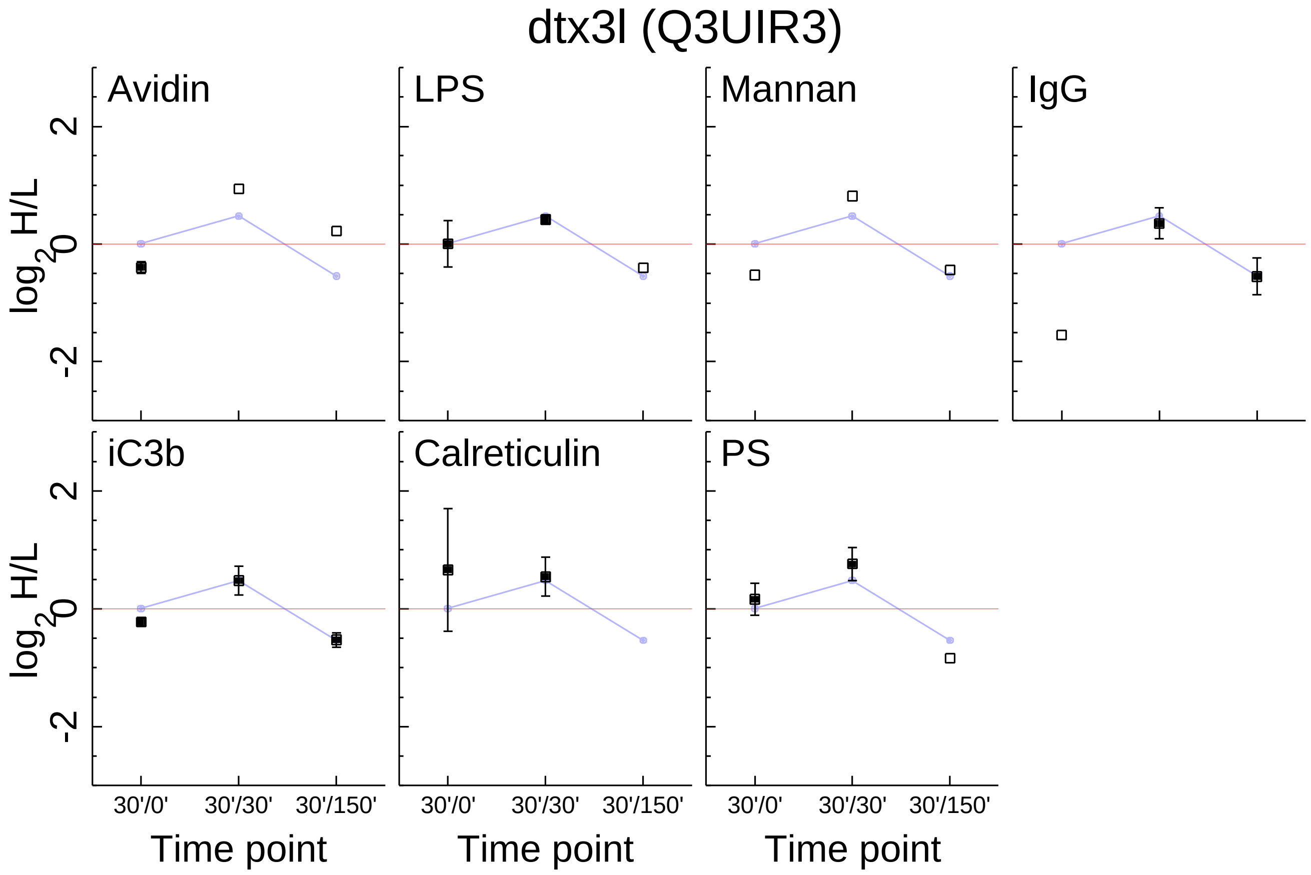

dtx3l (Q3UIR3)
Avidin
LPS
Mannan
IgG
iC3b
Calreticulin
PS
log2 H/L
2
0
-2
30'/0'
30'/30'
30'/150'
Time point

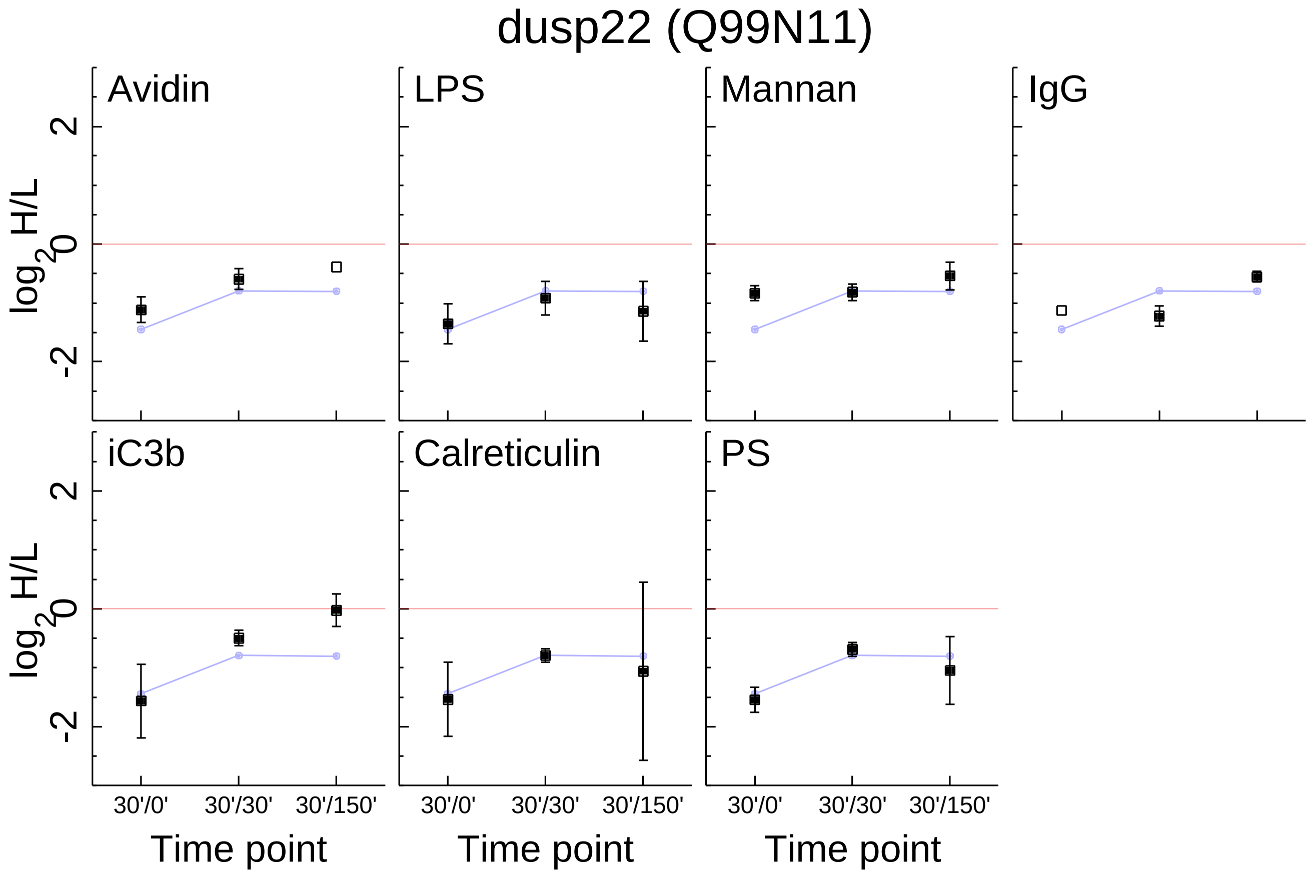
dusp22 (Q99N11)
Avidin
LPS
Mannan
IgG
iC3b
Calreticulin
PS
log2 H/L
2
0
-2
30'/0'
30'/30'
30'/150'
Time point

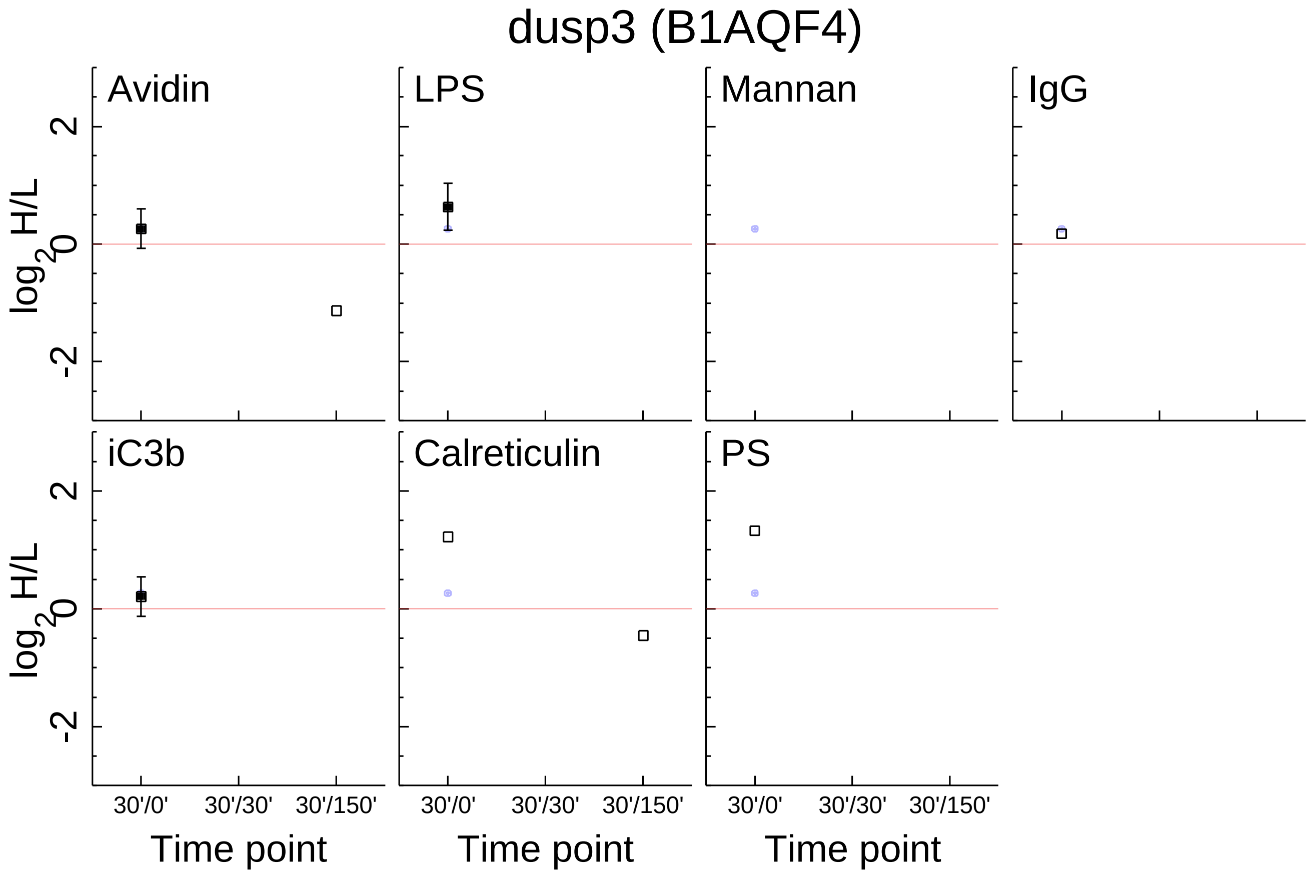

dusp3 (B1AQF4)
Avidin
LPS
Mannan
IgG
iC3b
Calreticulin
PS
log2 H/L
2
0
-2
30'/0'
30'/30'
30'/150'
Time point

dync1h1 (Q9JHU4)

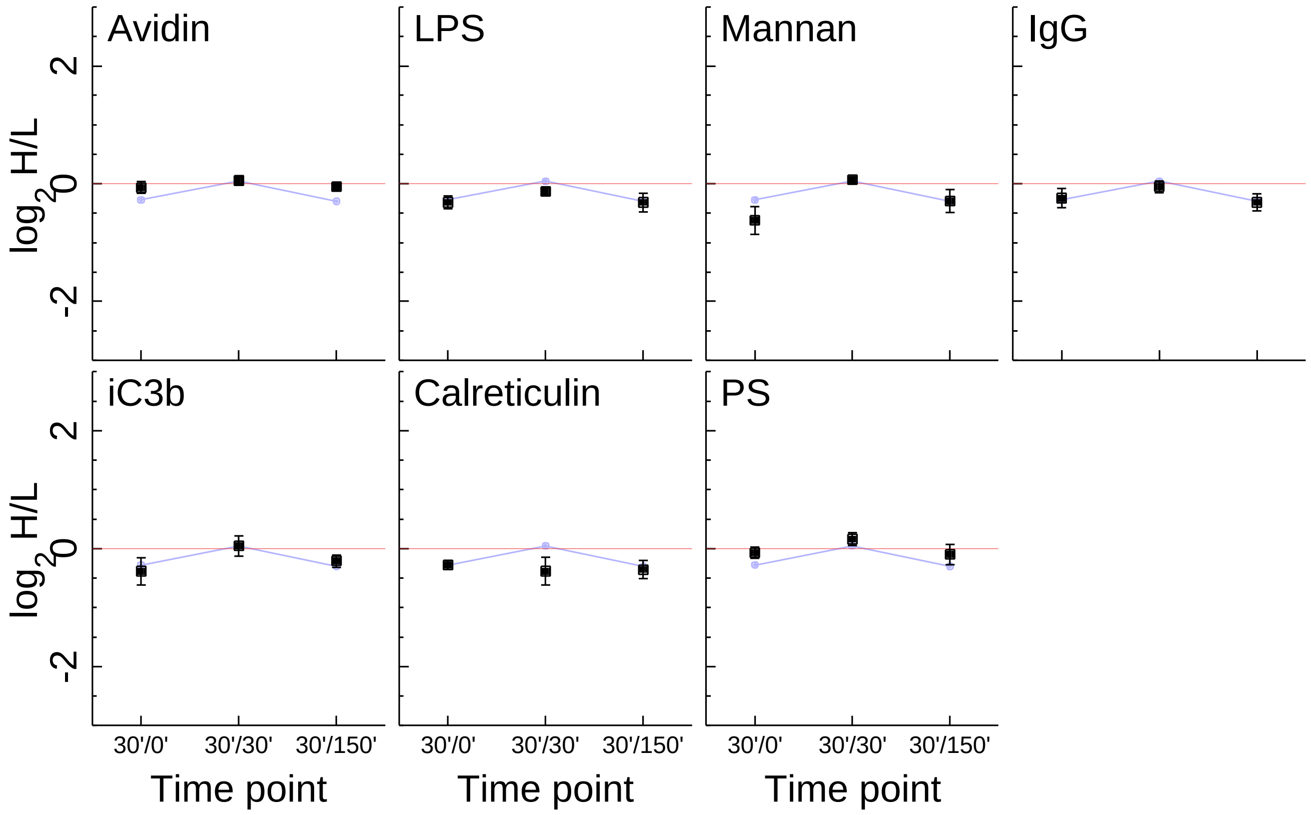

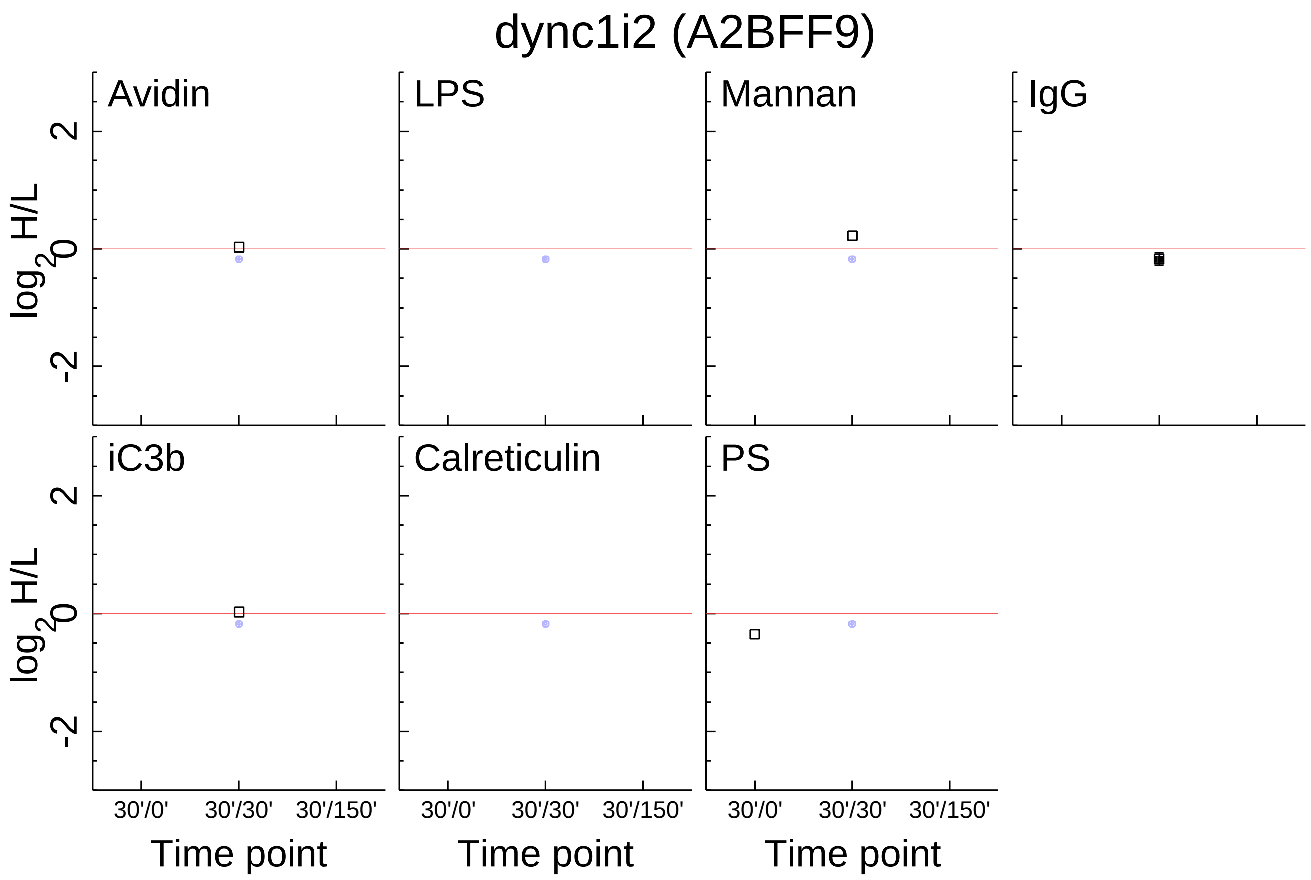
dync1i2 (A2BFF9)
Avidin
LPS
Mannan
IgG
iC3b
Calreticulin
PS
log2 H/L
2
0
-2
30'/0'
30'/30'
30'/150'
Time point

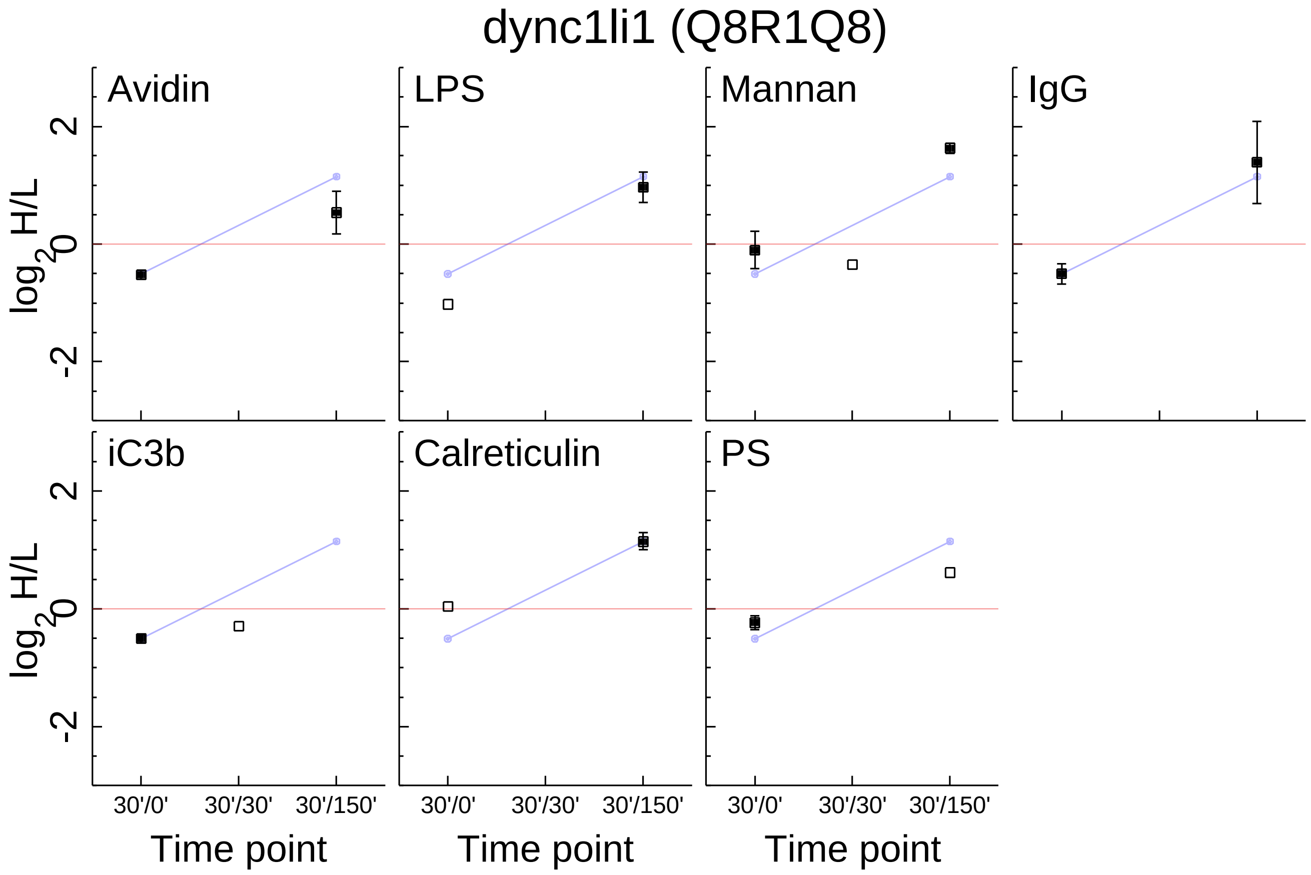
dync1li1 (Q8R1Q8)
Avidin
LPS
Mannan
IgG
iC3b
Calreticulin
PS
log2 H/L
2
0
-2
30'/0'
30'/30'
30'/150'
Time point

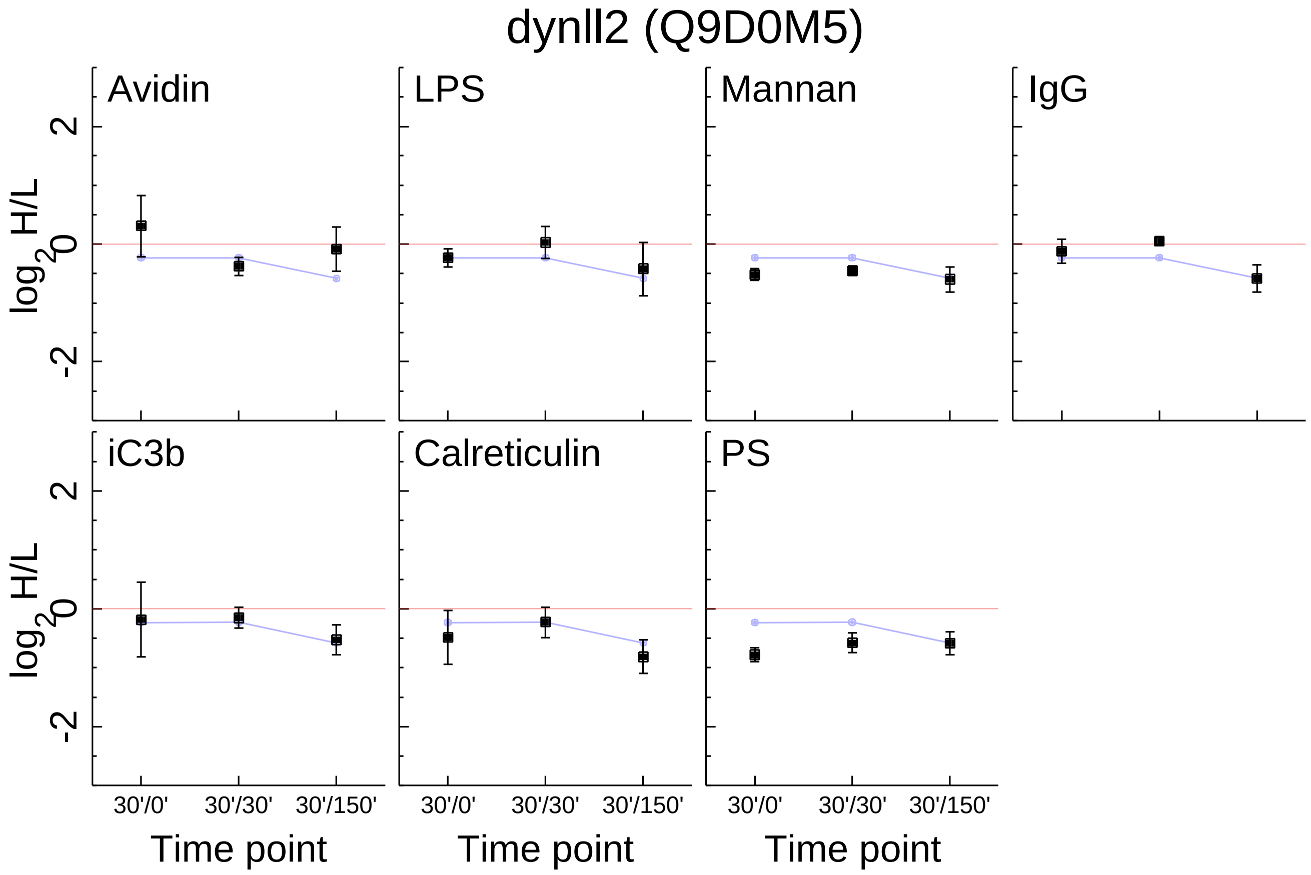
dynll2 (Q9D0M5)
Avidin
LPS
Mannan
IgG
iC3b
Calreticulin
PS
log2 H/L
2
0
-2
30'/0'
30'/30'
30'/150'
Time point

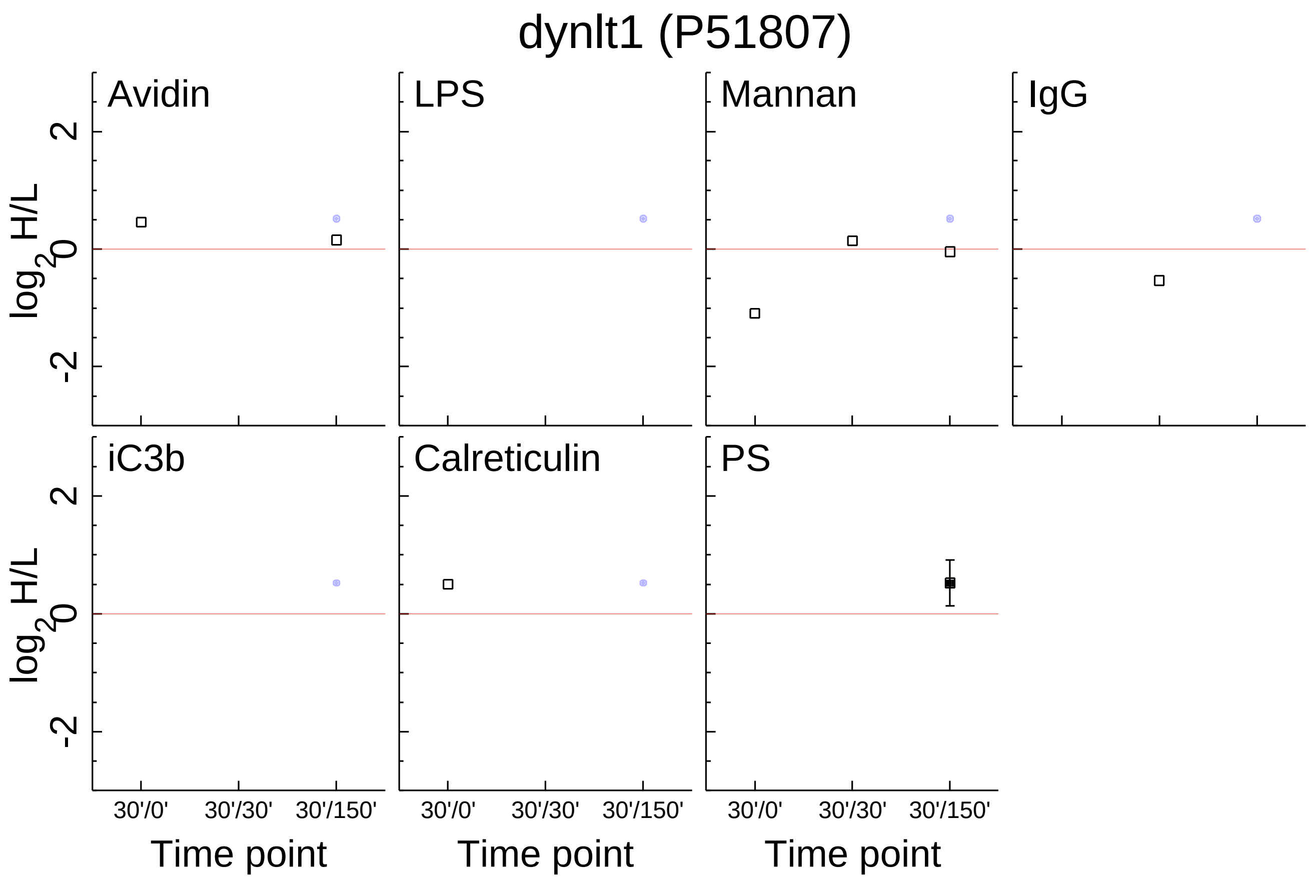

dynlt1 (P51807)
Avidin
LPS
Mannan
IgG
iC3b
Calreticulin
PS
log2 H/L
2
0
-2
30'/0'
30'/30'
30'/150'
Time point

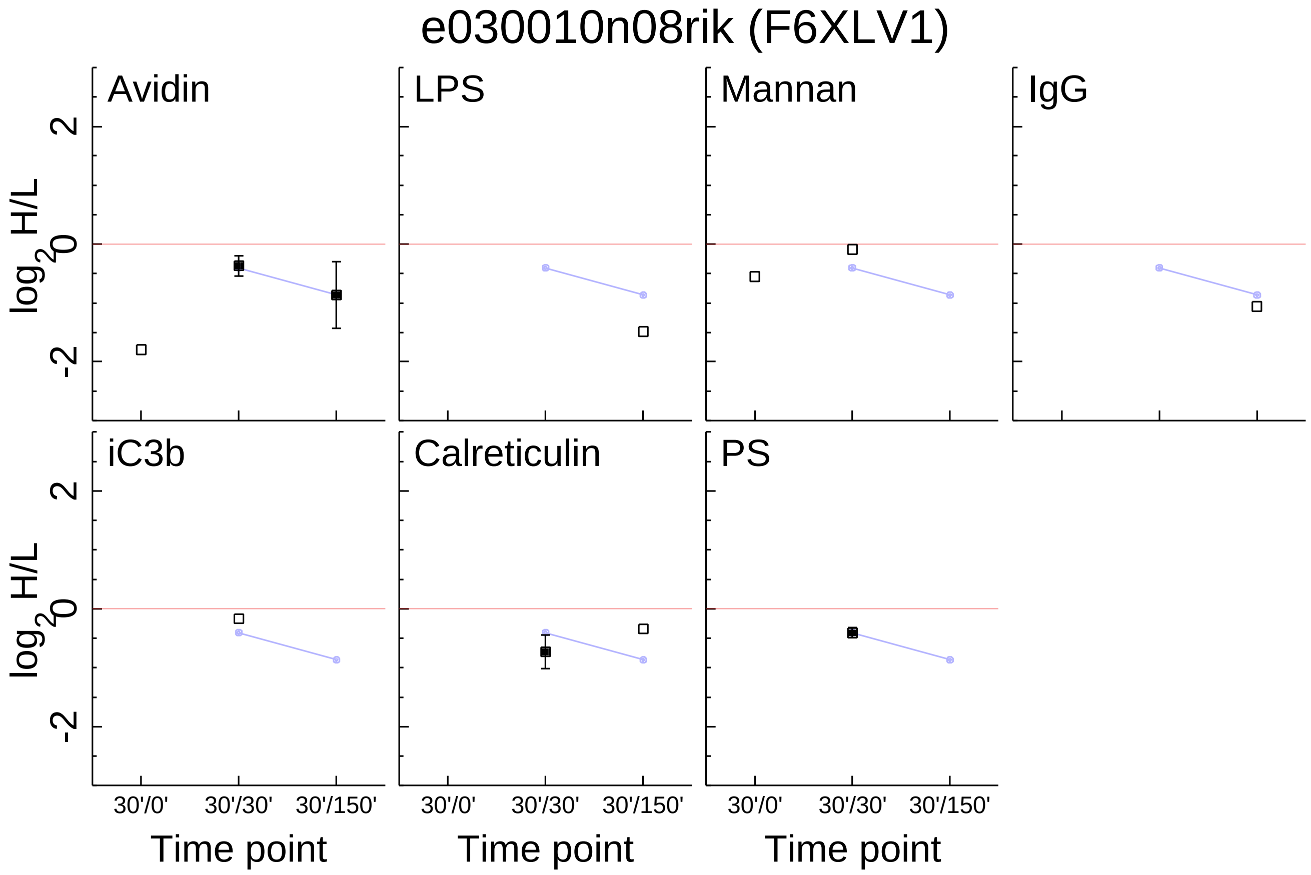
e030010n08rik (F6XLV1)
Avidin
LPS
Mannan
IgG
iC3b
Calreticulin
PS
log2 H/L
2
0
-2
30'/0'
30'/30'
30'/150'
Time point

ear2 (P97425)

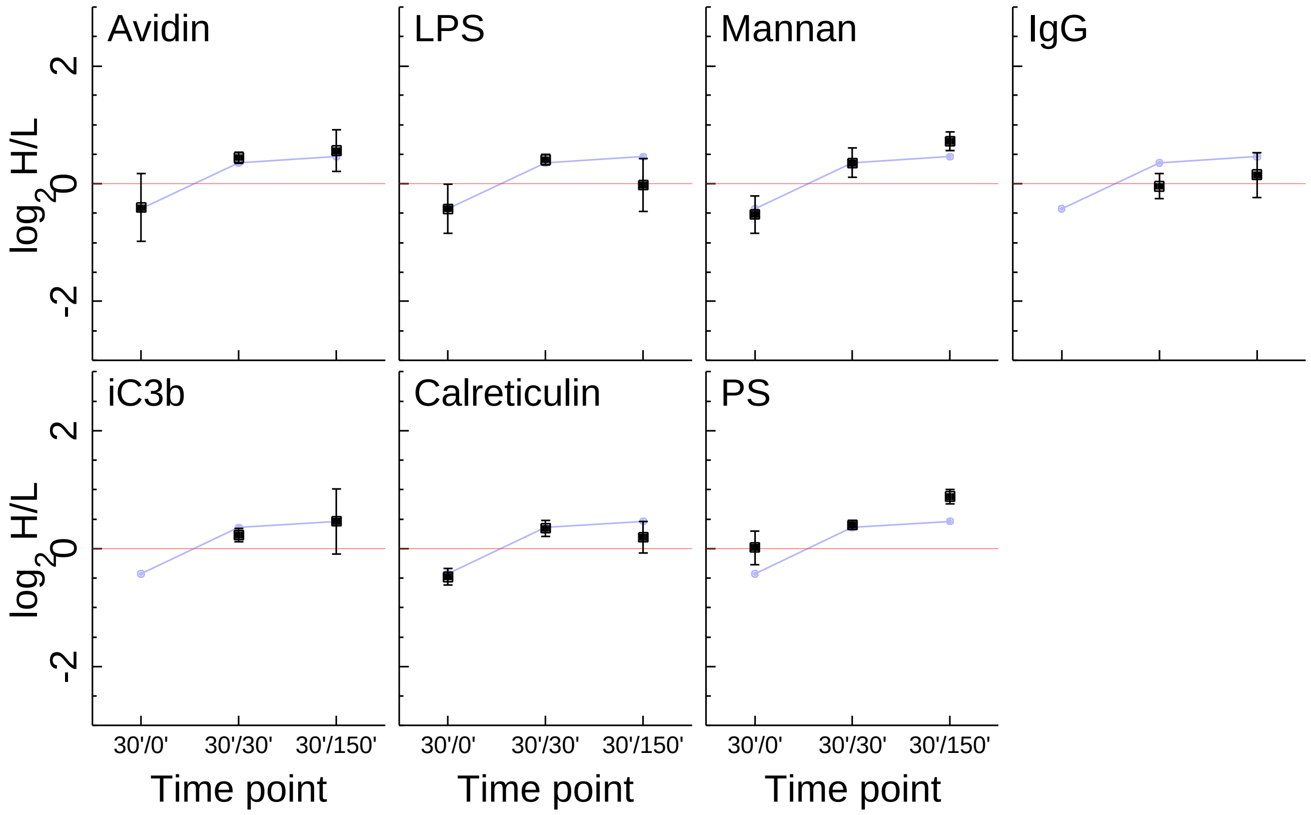

Avidin
LPS
Mannan
IgG
iC3b
Calreticulin
PS
2
0
-2
log_2 H/L
30'/0'
30'/30'
30'/150'
Time point

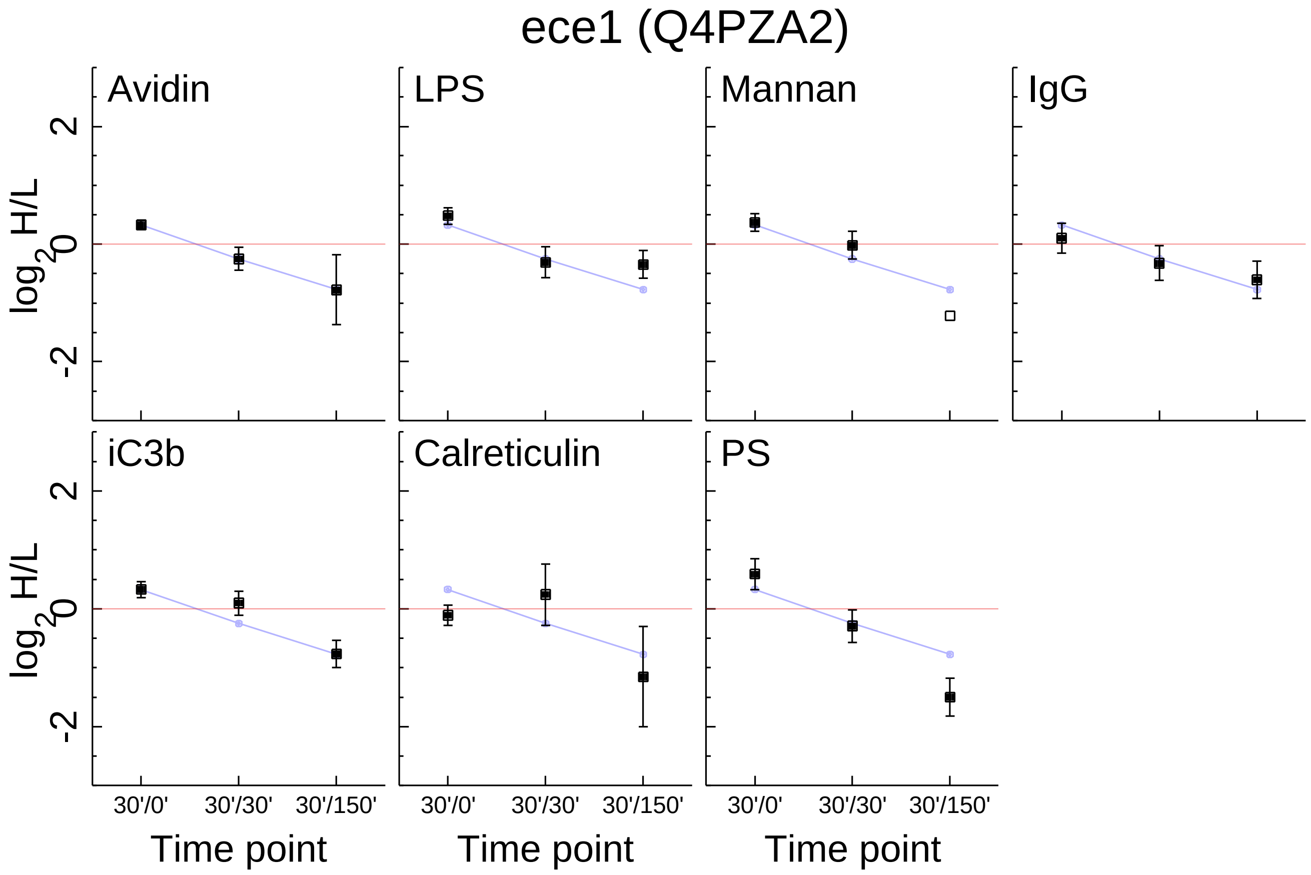
ece1 (Q4PZA2)
Avidin
LPS
Mannan
IgG
iC3b
Calreticulin
PS
log2 H/L
2
0
-2
30'/0'
30'/30'
30'/150'
Time point

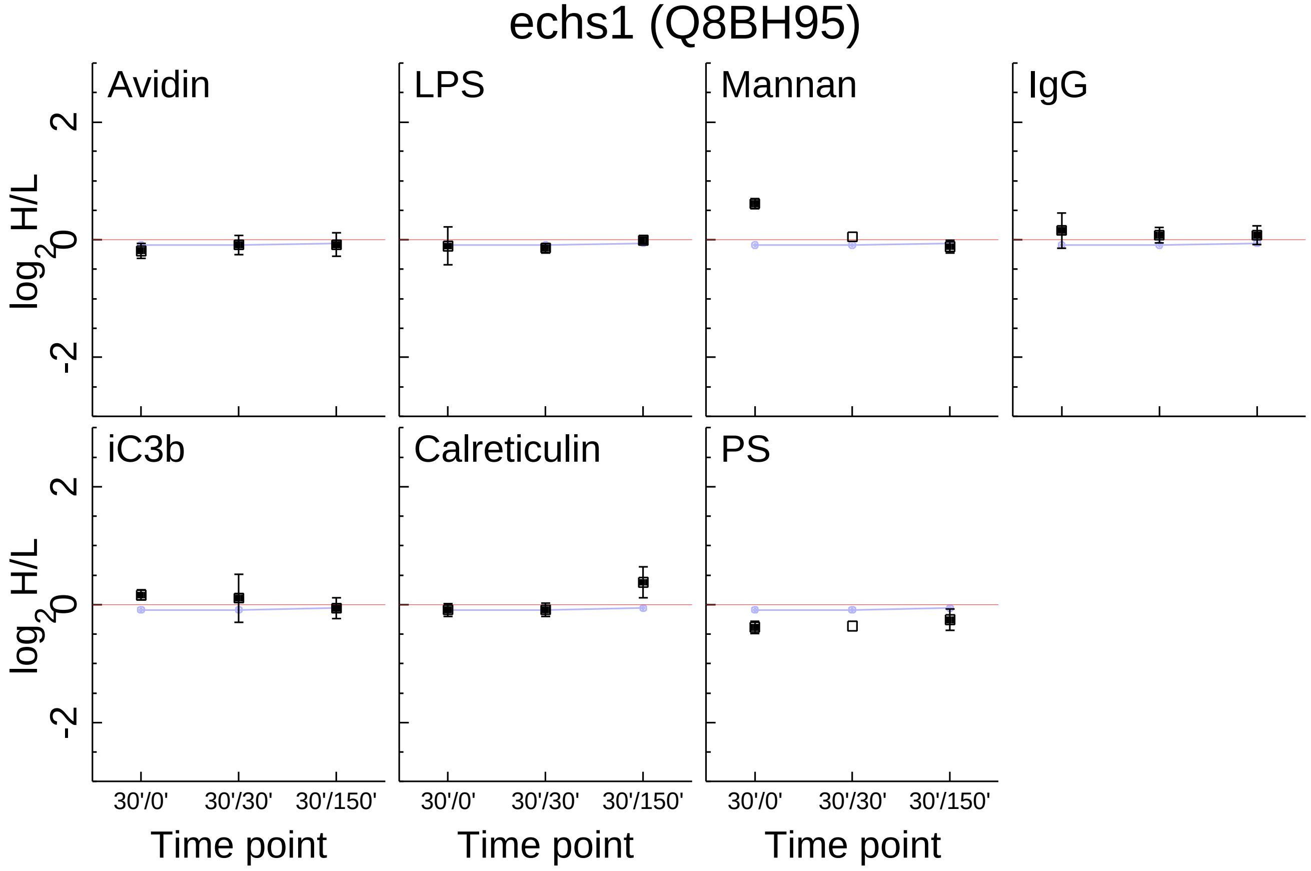

echs1 (Q8BH95)
Avidin
LPS
Mannan
IgG
iC3b
Calreticulin
PS
log2 H/L
2
0
-2
30'/0'
30'/30'
30'/150'
Time point

edil3 (O35474)

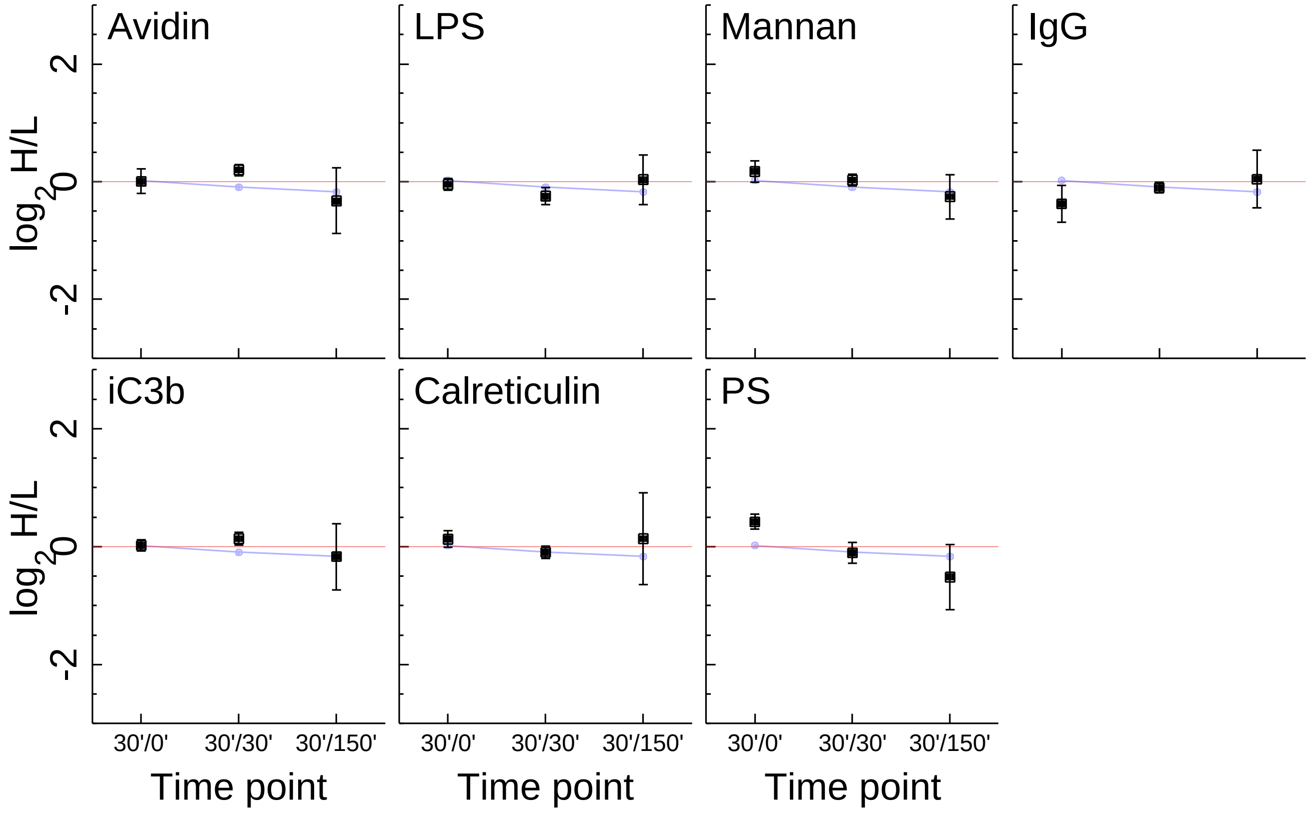

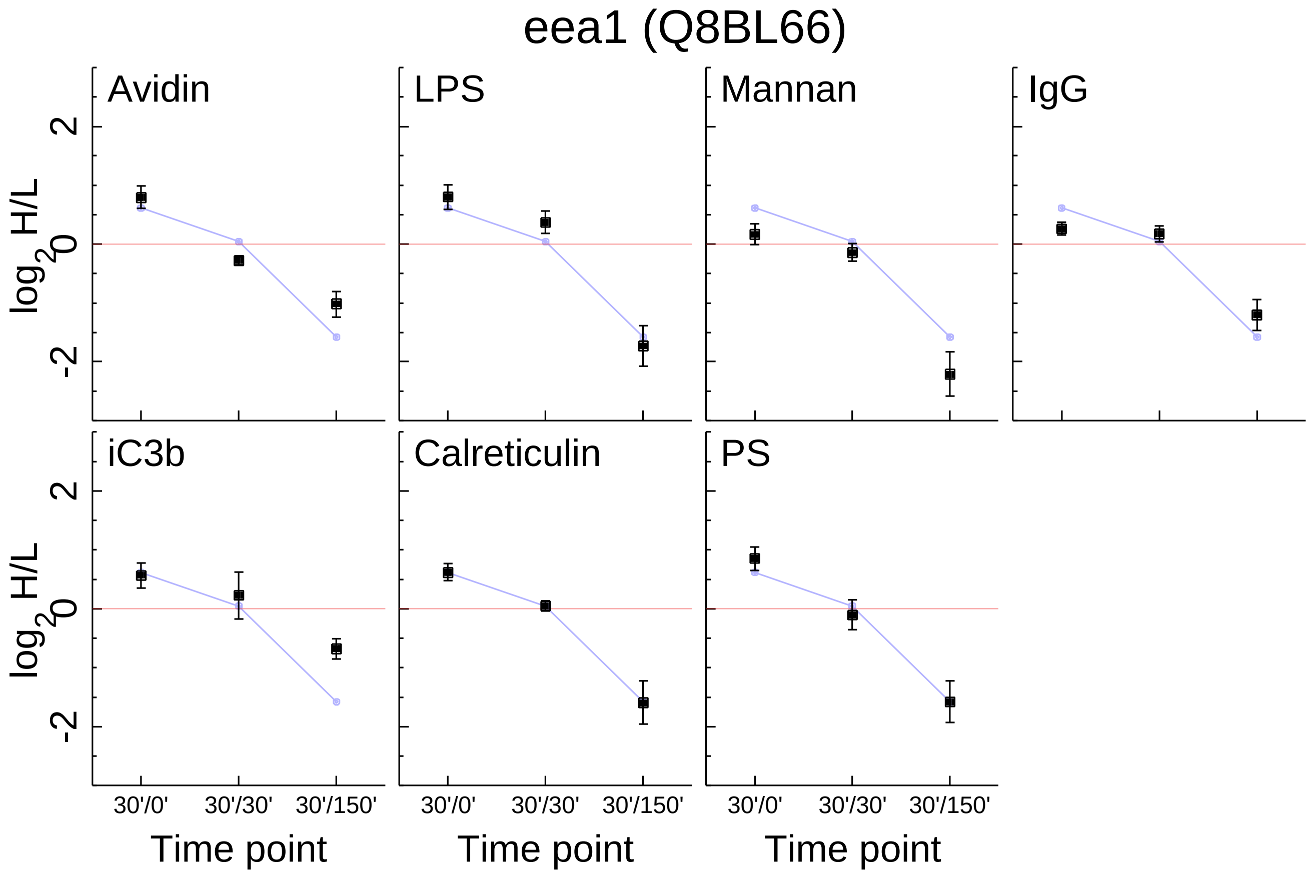

eea1 (Q8BL66)
Avidin
LPS
Mannan
IgG
iC3b
Calreticulin
PS
log2 H/L
2
0
-2
30'/0'
30'/30'
30'/150'
Time point

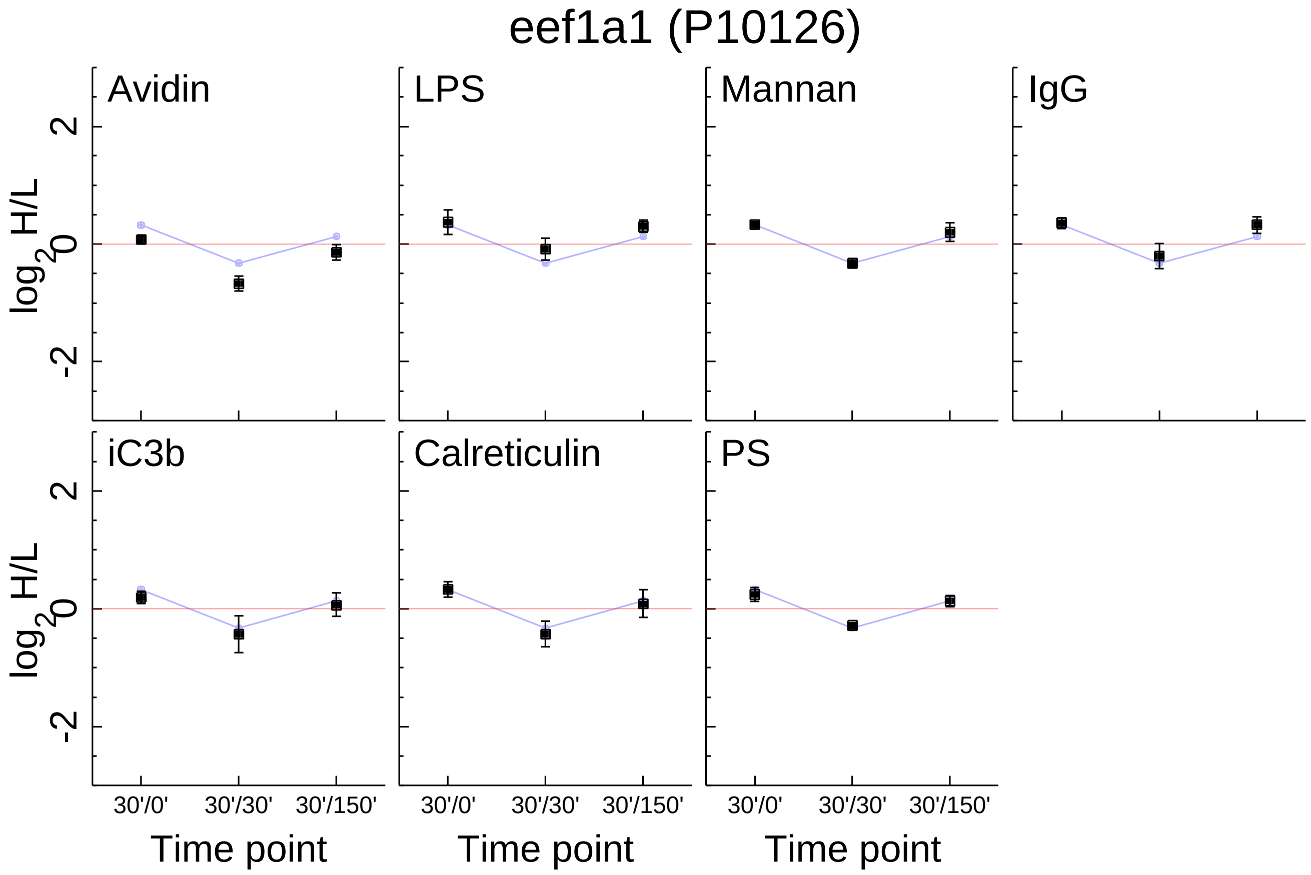
eef1a1 (P10126)
Avidin
LPS
Mannan
IgG
iC3b
Calreticulin
PS
log2 H/L
2
0
-2
30'/0'
30'/30'
30'/150'
Time point

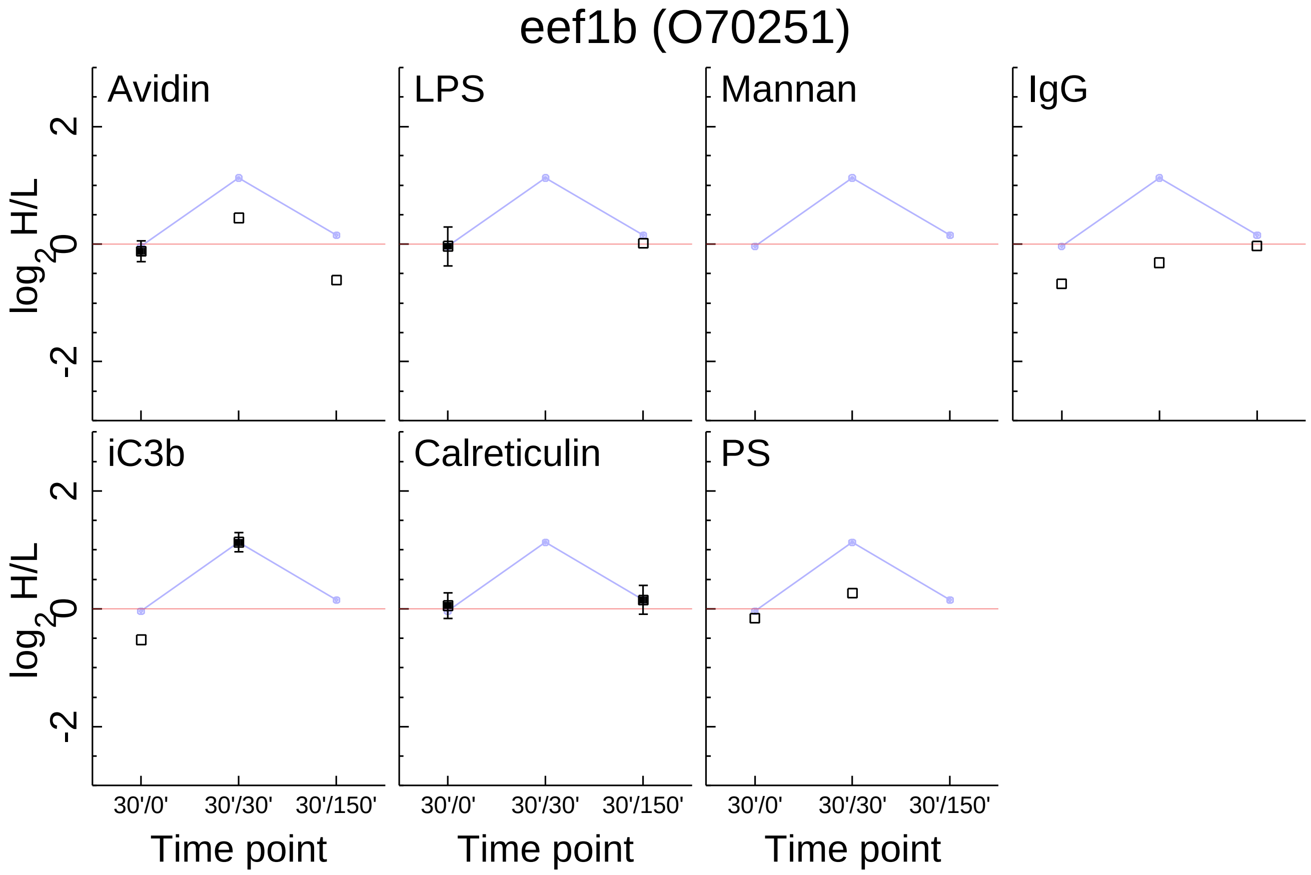

eef1b (O70251)
Avidin
LPS
Mannan
IgG
iC3b
Calreticulin
PS
log2 H/L
2
0
-2
30'/0'
30'/30'
30'/150'
Time point

eef1d (P57776)

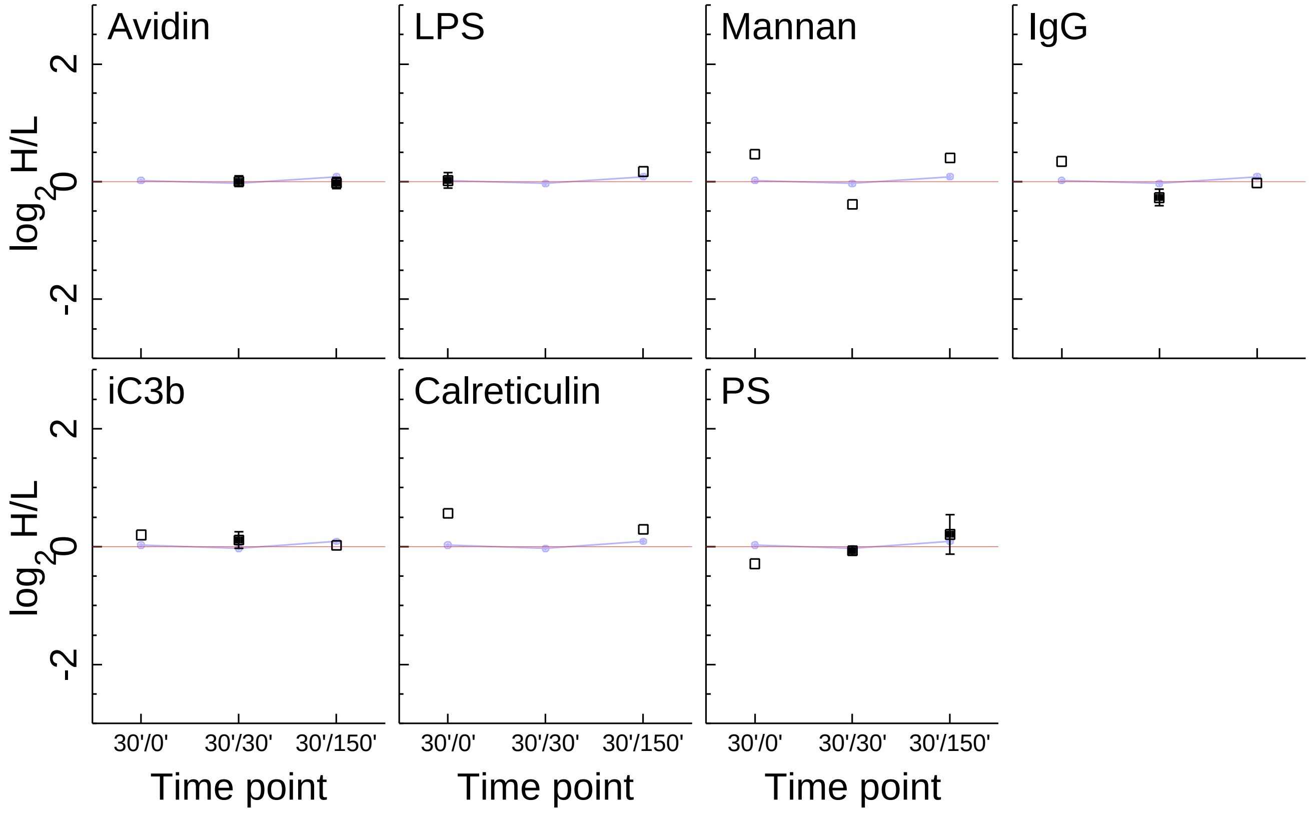

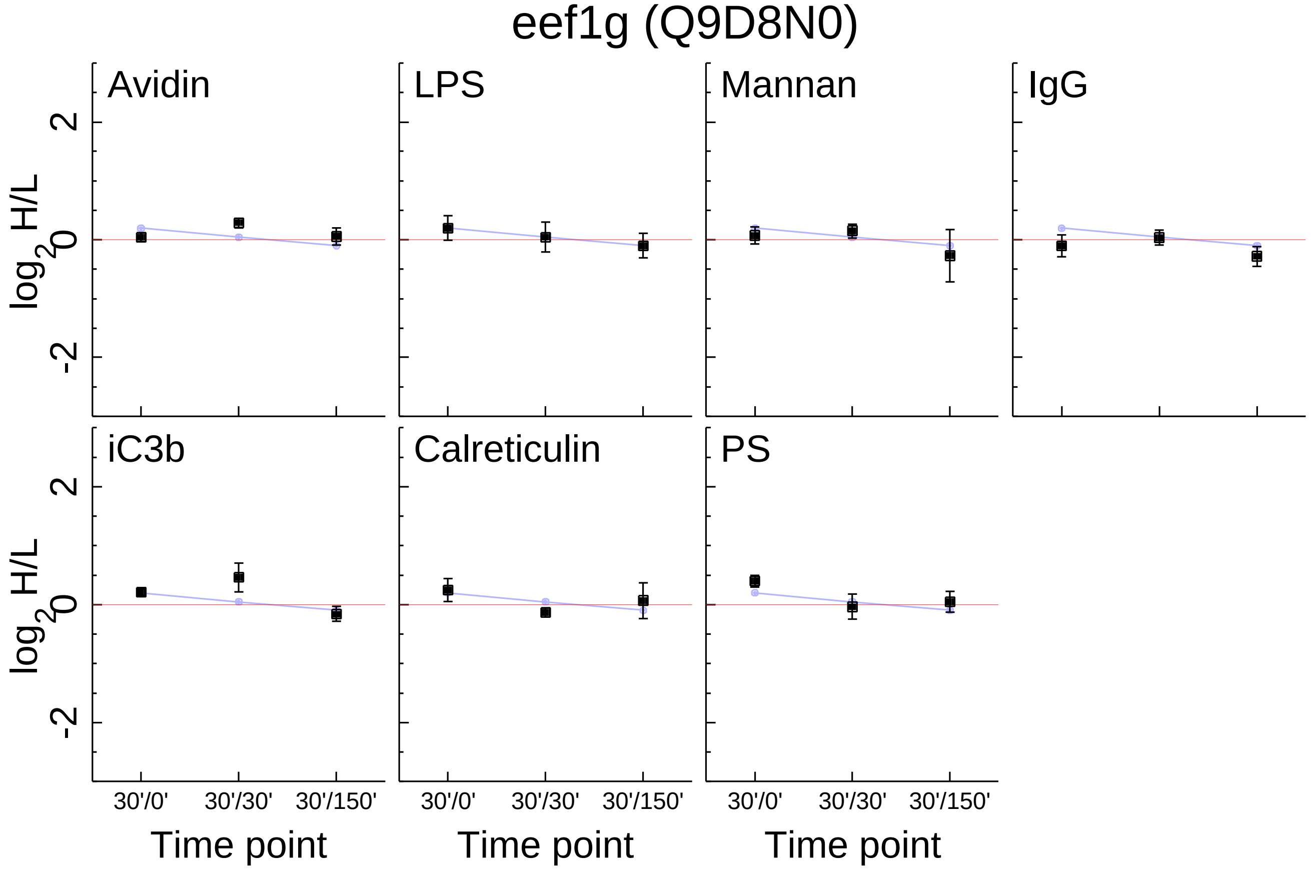
eef1g (Q9D8N0)
Avidin
LPS
Mannan
IgG
iC3b
Calreticulin
PS
log2 H/L
2
0
-2
30'/0'
30'/30'
30'/150'
Time point

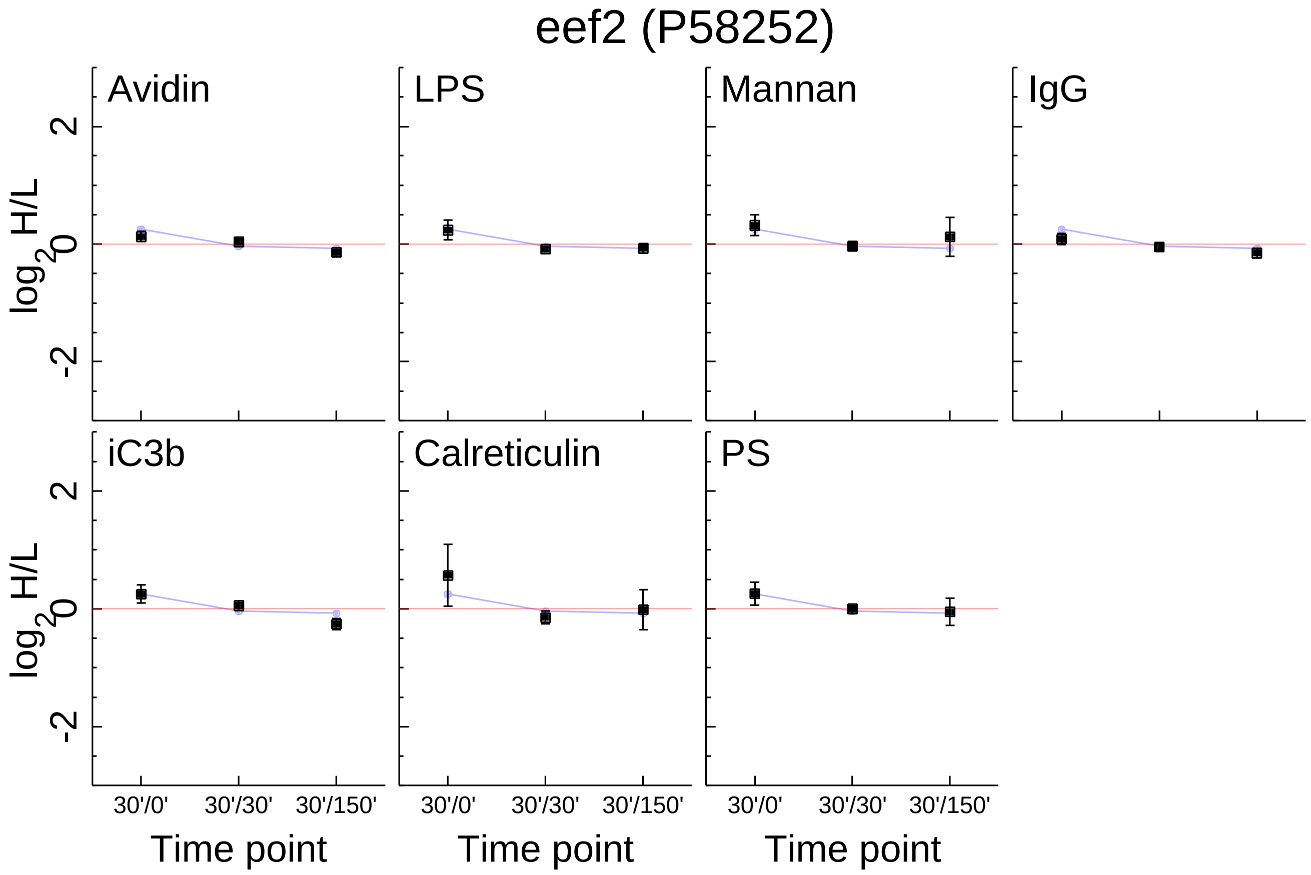
eef2 (P58252)
Avidin
LPS
Mannan
IgG
iC3b
Calreticulin
PS
log2 H/L
2
0
-2
30'/0'
30'/30'
30'/150'
Time point

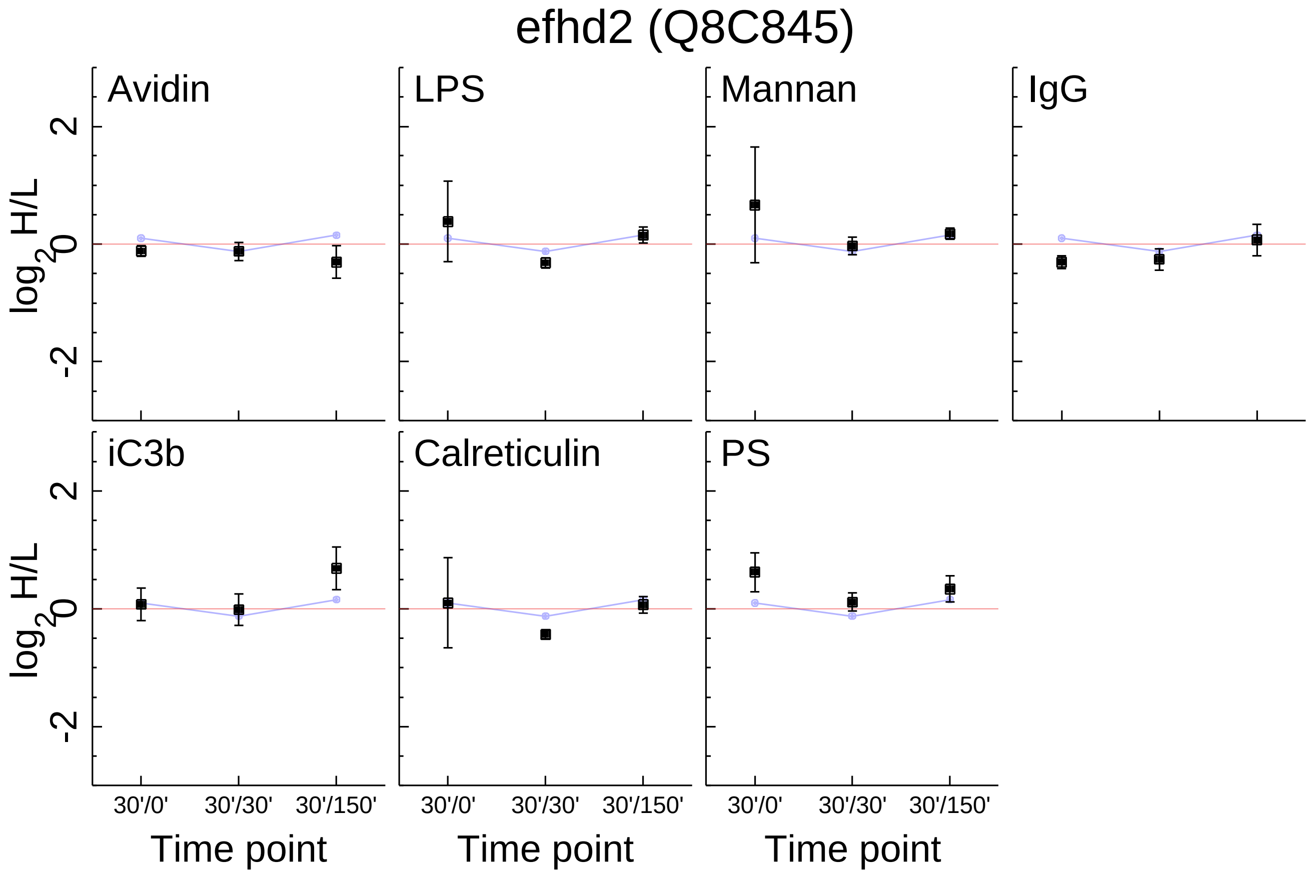
efhd2 (Q8C845)
Avidin
LPS
Mannan
IgG
iC3b
Calreticulin
PS
log2 H/L
2
0
-2
30'/0'
30'/30'
30'/150'
Time point

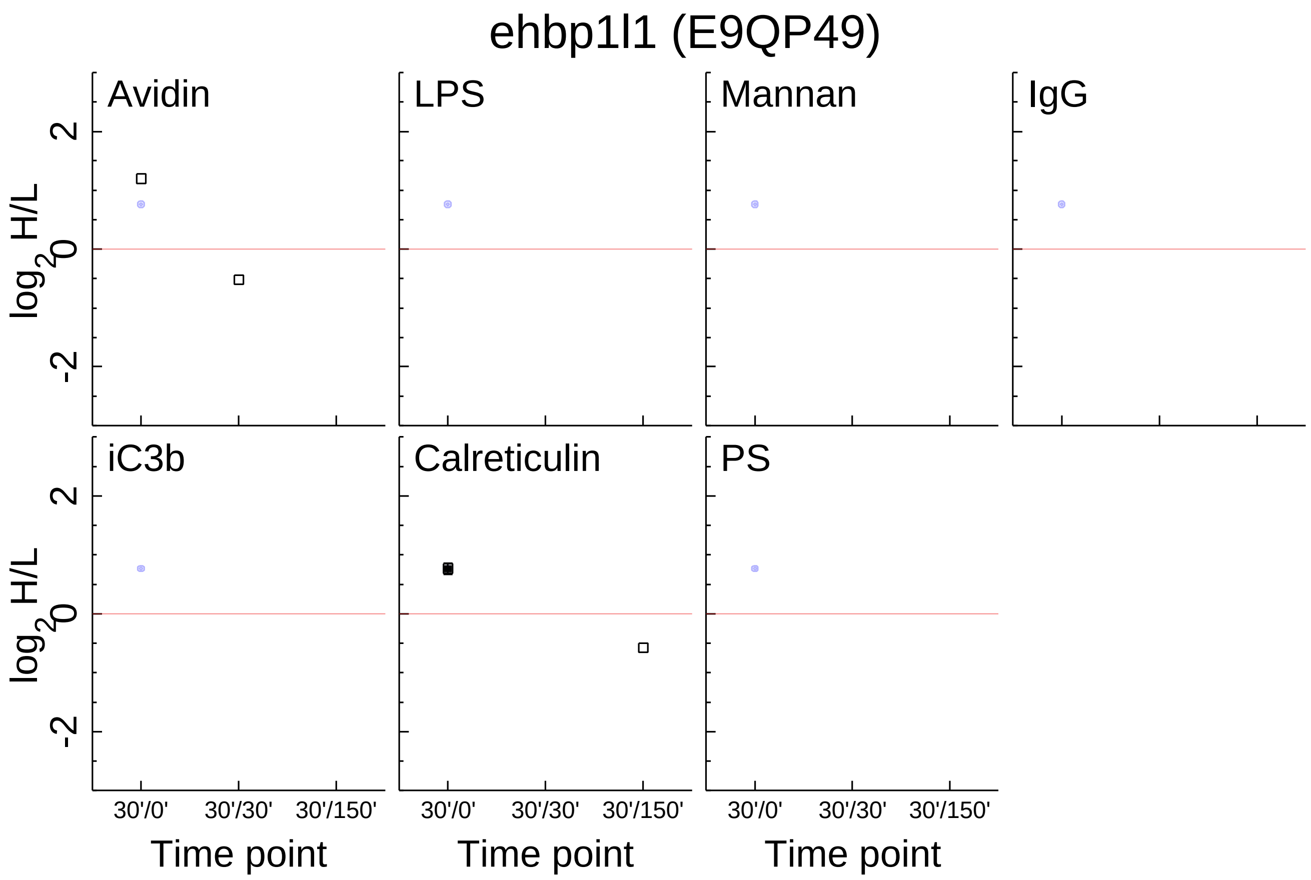

ehbp1l1 (E9QP49)
Avidin
LPS
Mannan
IgG
iC3b
Calreticulin
PS
log2 H/L
2
0
-2
30'/0'
30'/30'
30'/150'
Time point

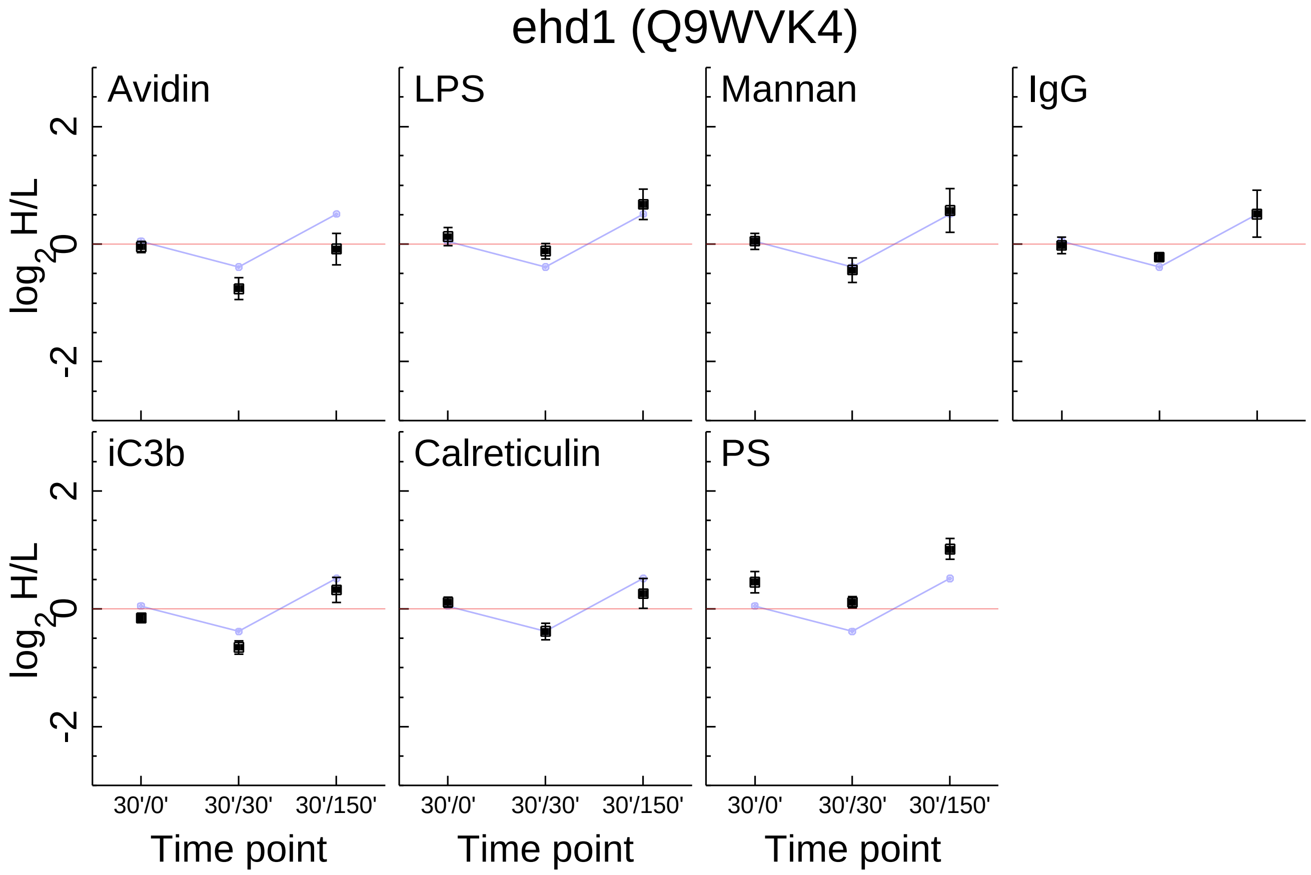

ehd1 (Q9WVK4)
Avidin
LPS
Mannan
IgG
iC3b
Calreticulin
PS
log2 H/L
2
0
-2
30'/0'
30'/30'
30'/150'
Time point

ehd4 (Q9EQP2)

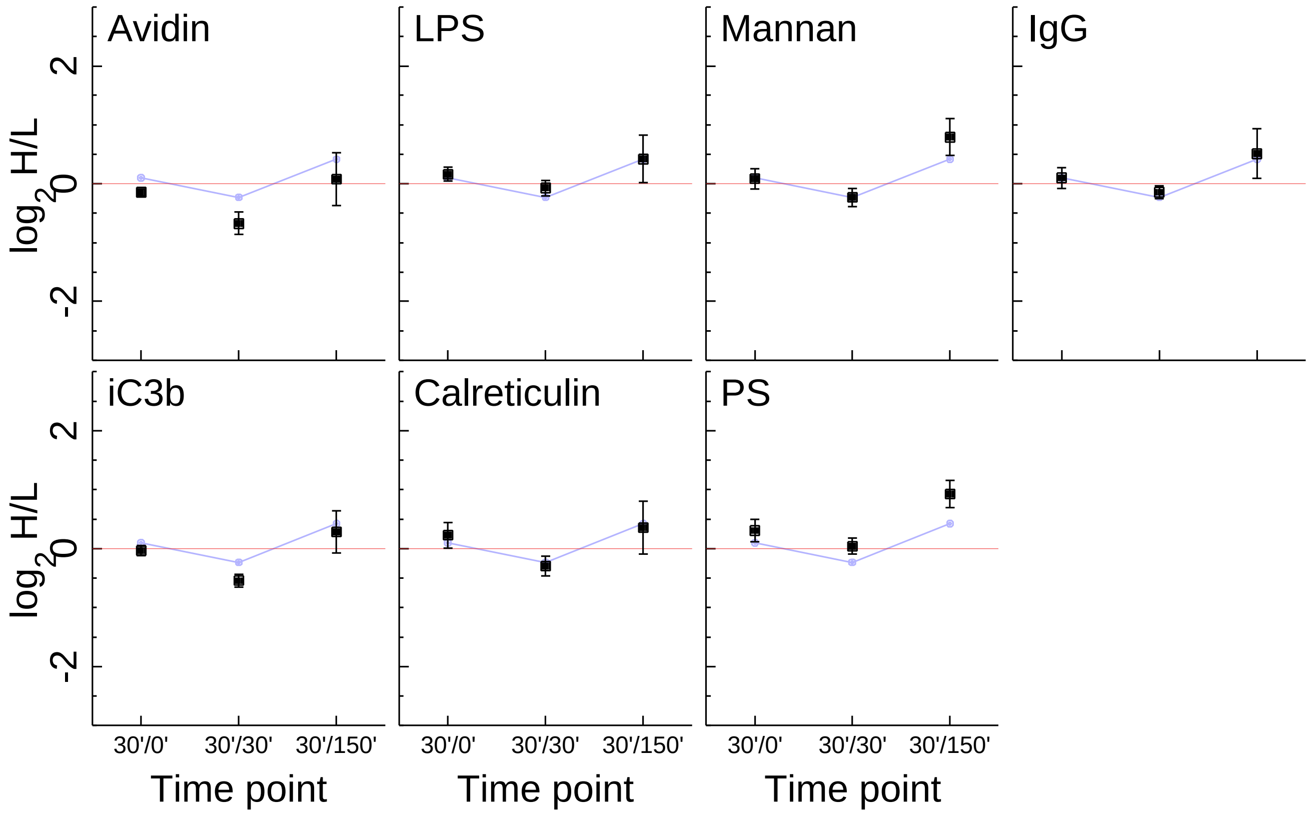

Avidin
LPS
Mannan
IgG
iC3b
Calreticulin
PS
log2 H/L
2
0
-2
30'/0'
30'/30'
30'/150'
Time point

eif1a (Q60872)

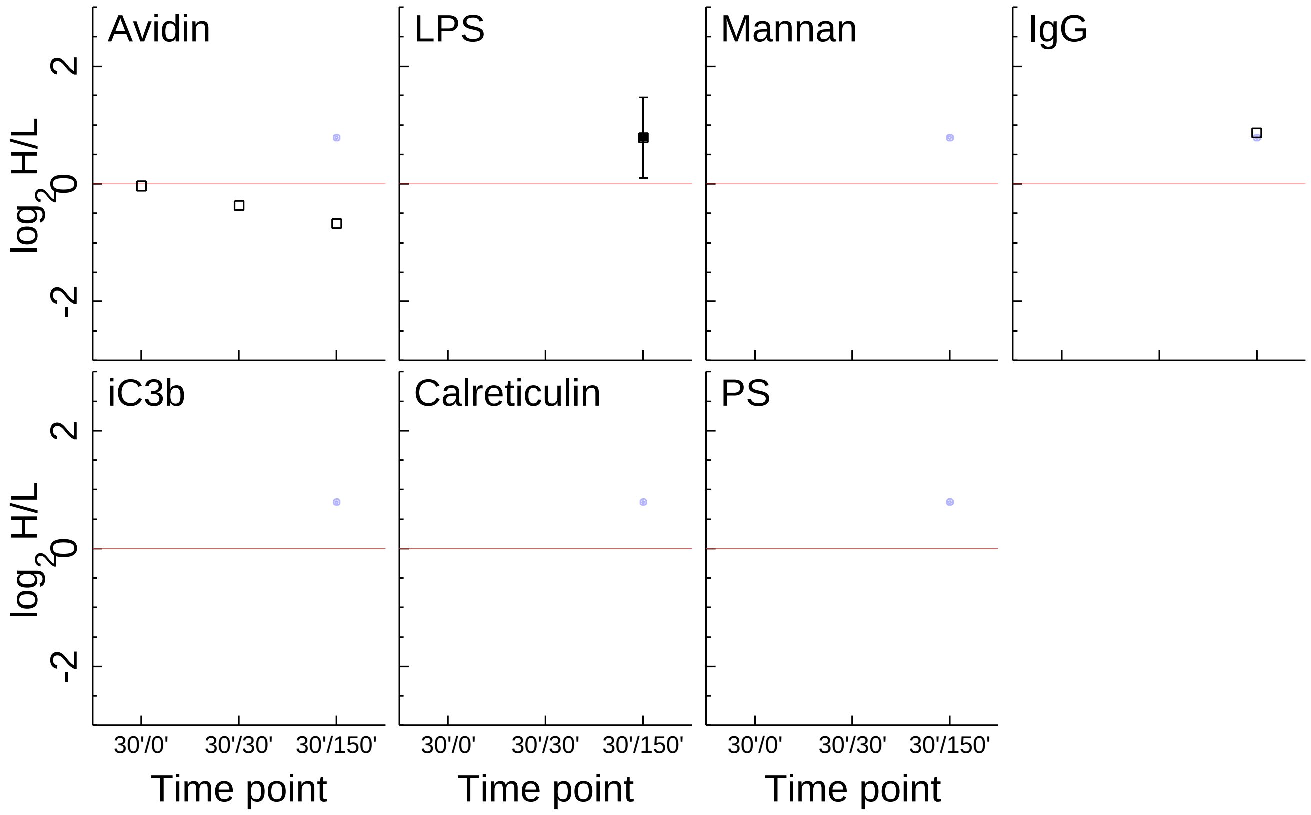

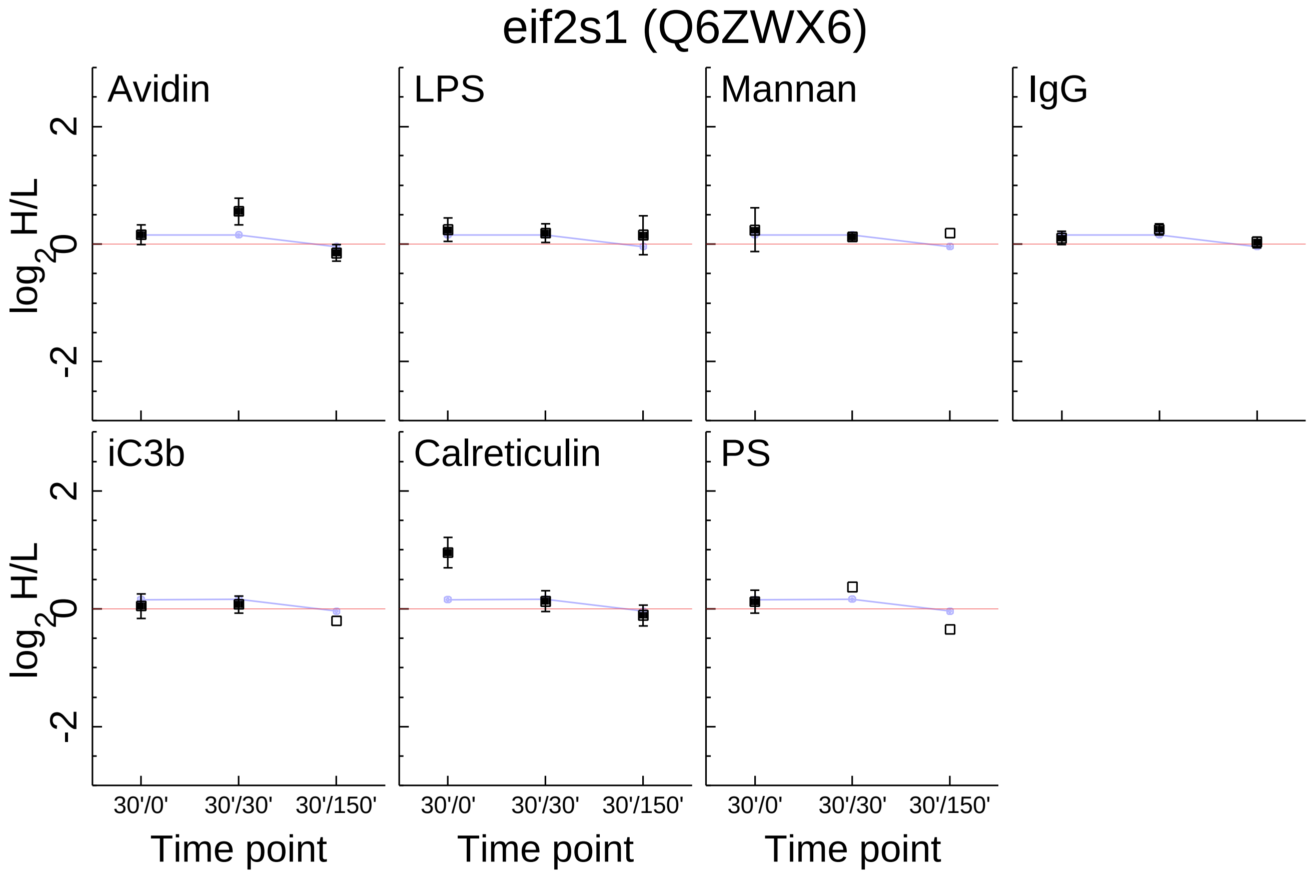

eif2s1 (Q6ZWX6)
Avidin
LPS
Mannan
IgG
iC3b
Calreticulin
PS
log2 H/L
2
0
-2
30'/0'
30'/30'
30'/150'
Time point

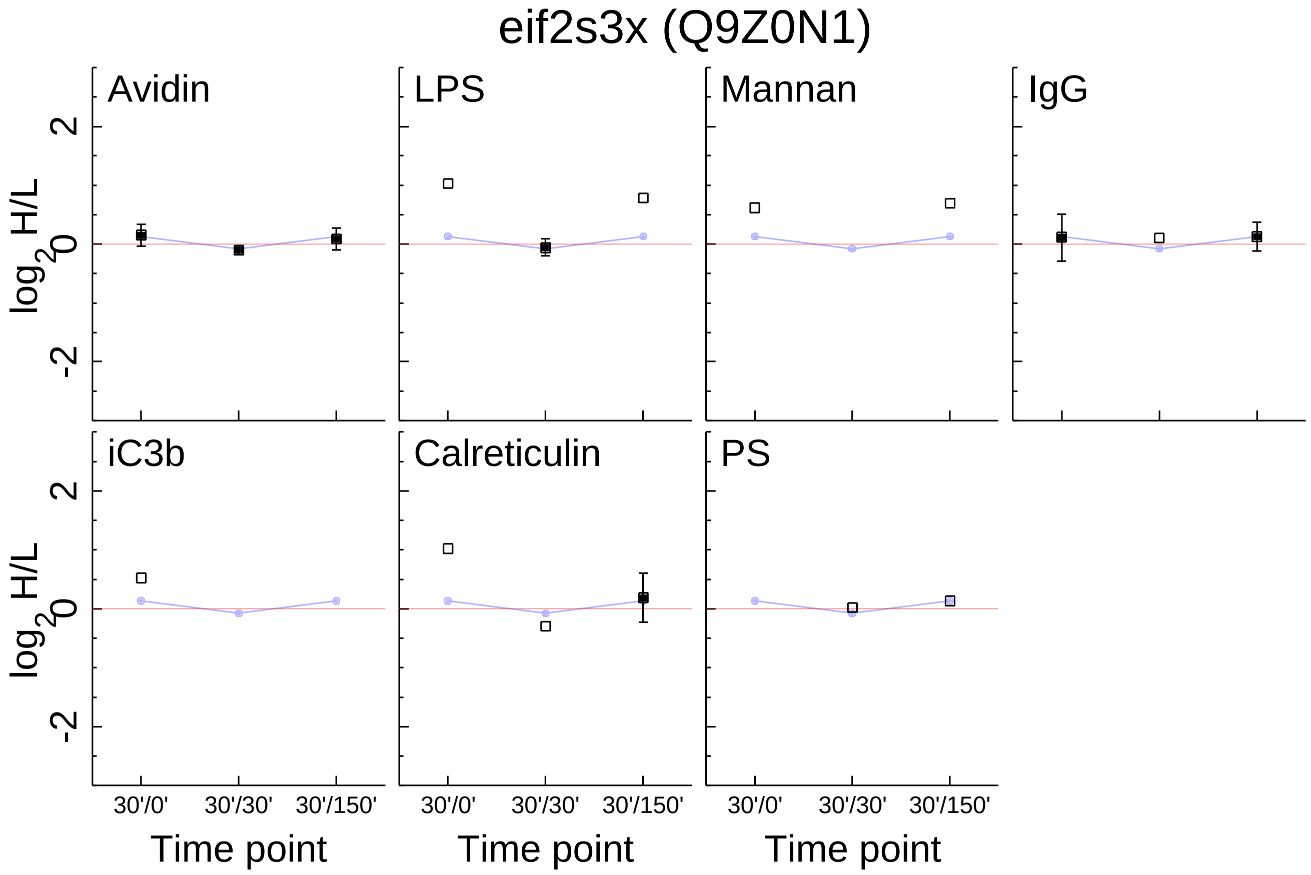

eif2s3x (Q9Z0N1)
Avidin
LPS
Mannan
IgG
iC3b
Calreticulin
PS
log2 H/L
2
0
-2
30'/0'
30'/30'
30'/150'
Time point

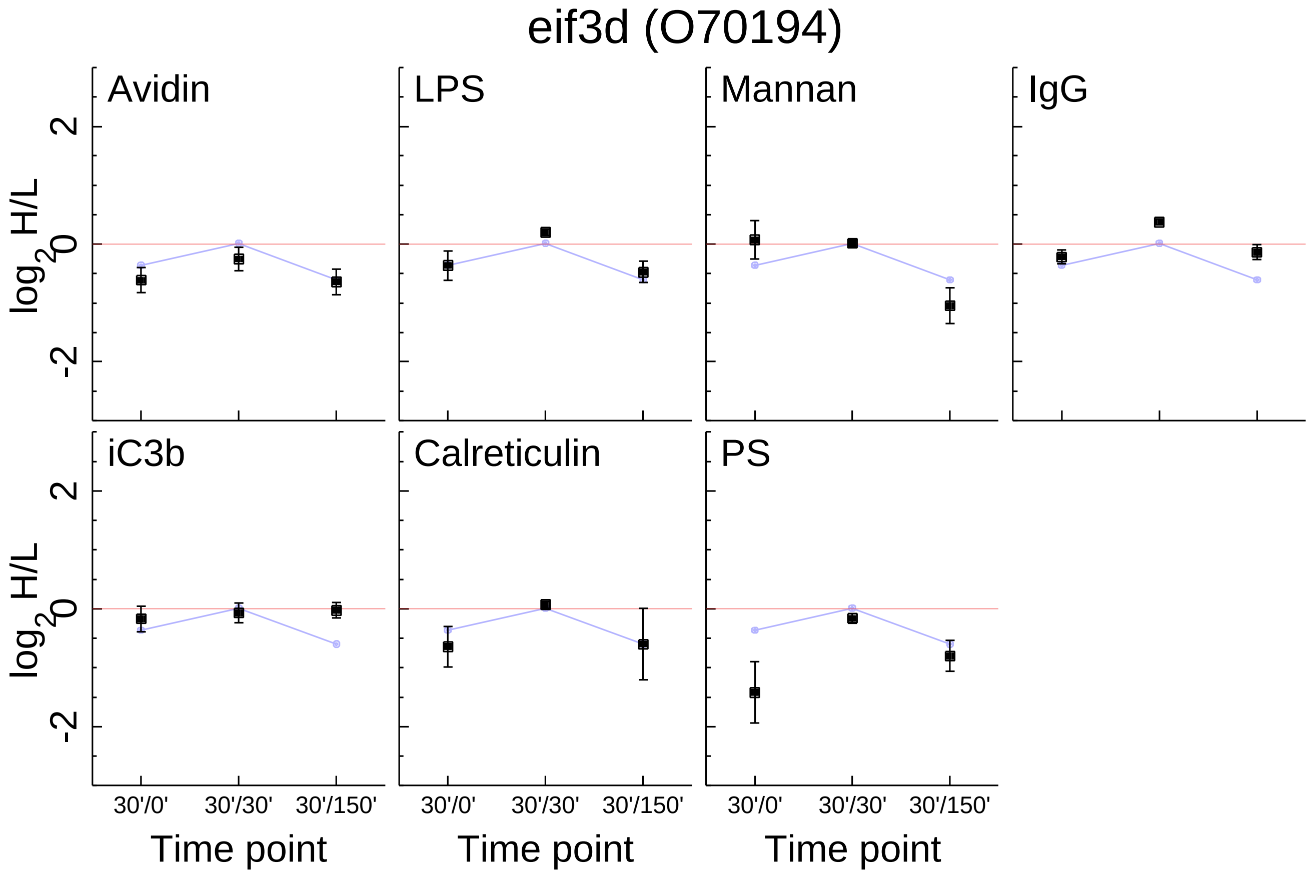
eif3d (O70194)
Avidin
LPS
Mannan
IgG
iC3b
Calreticulin
PS
log2 H/L
2
0
-2
30'/0'
30'/30'
30'/150'
Time point

eif3l (Q8QZY1)

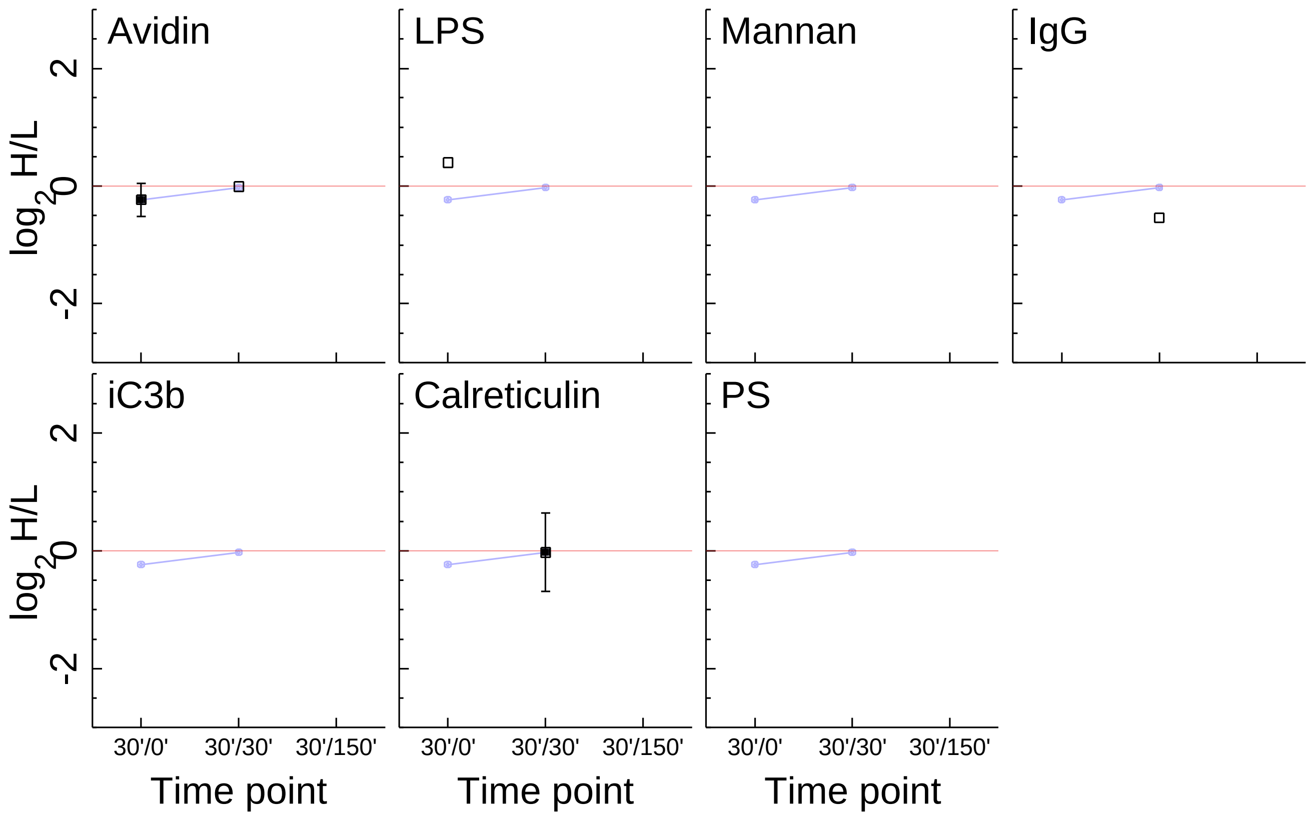

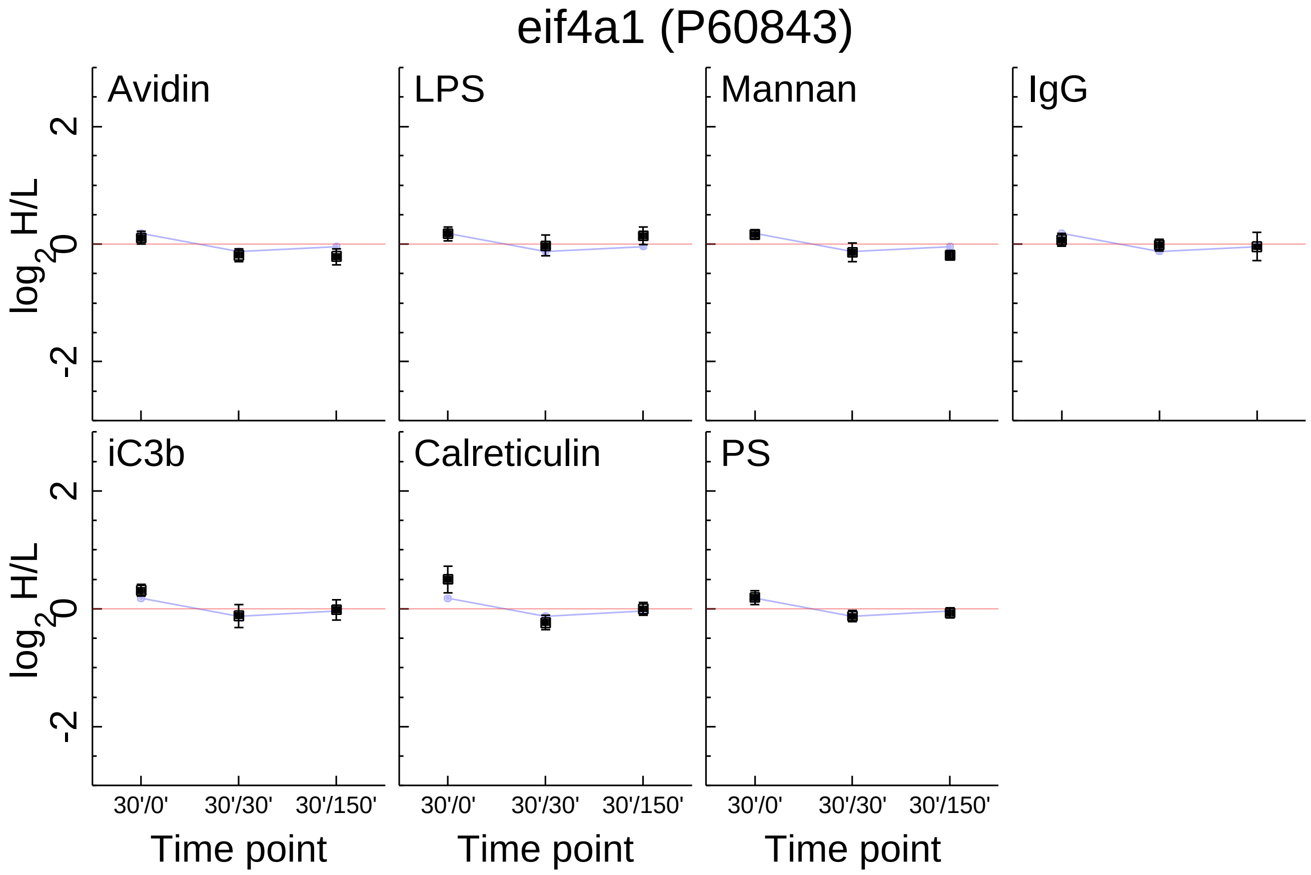

eif4a1 (P60843)
Avidin
LPS
Mannan
IgG
iC3b
Calreticulin
PS
log2 H/L
2
0
-2
30'/0'
30'/30'
30'/150'
Time point

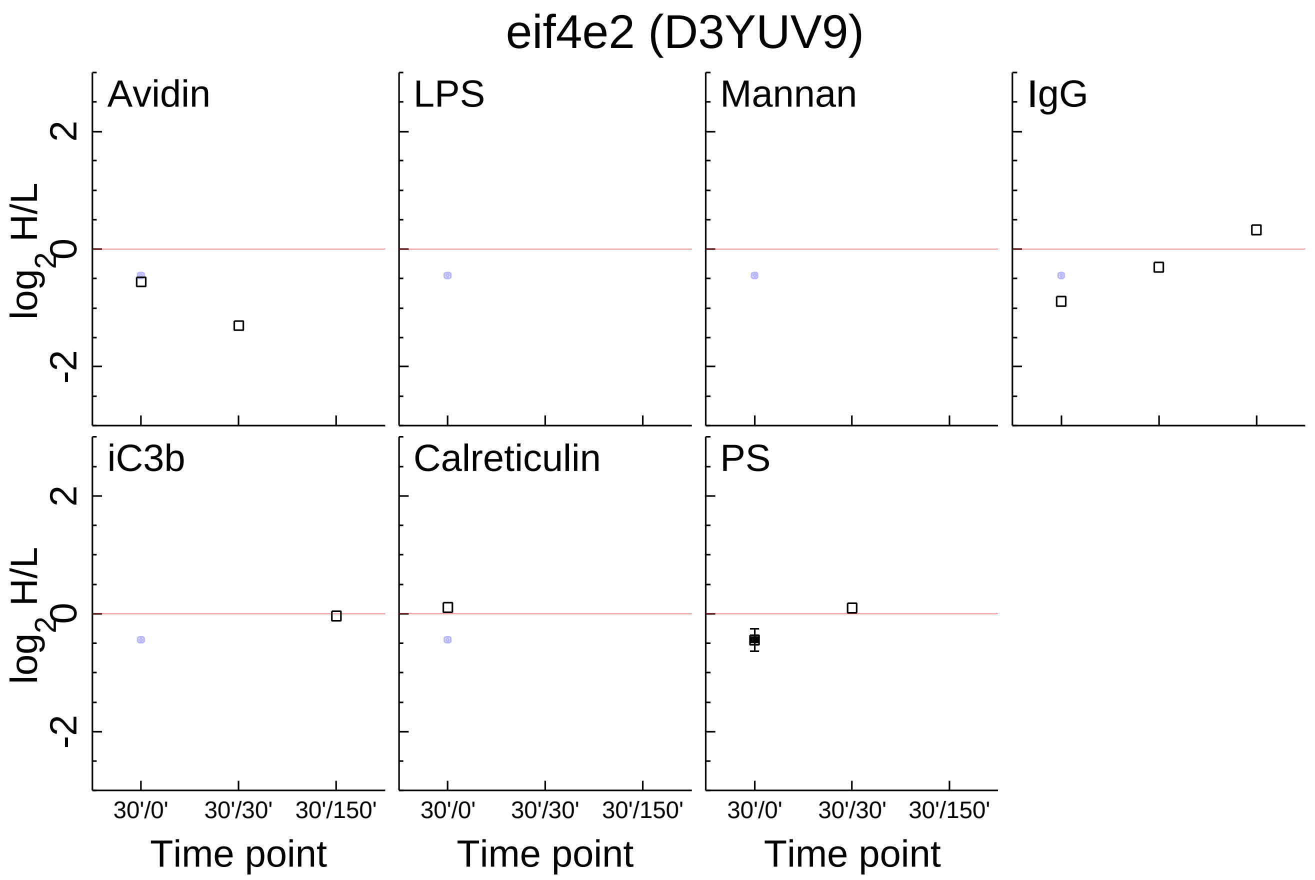

eif4e2 (D3YUV9)
Avidin
LPS
Mannan
IgG
iC3b
Calreticulin
PS
log2 H/L
2
0
-2
30'/0'
30'/30'
30'/150'
Time point

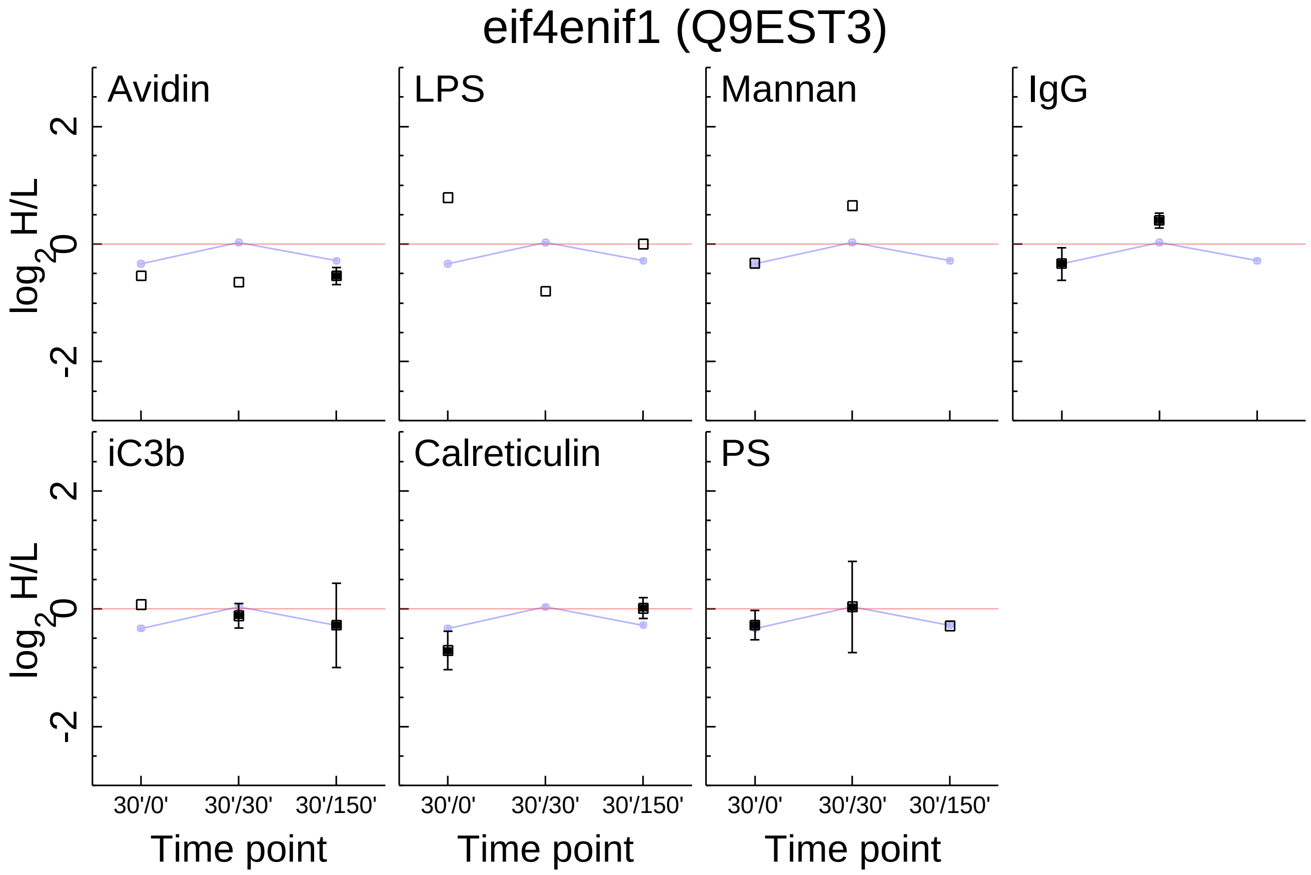

eif4enif1 (Q9EST3)
Avidin
LPS
Mannan
IgG
iC3b
Calreticulin
PS
log2 H/L
2
0
-2
30'/0'
30'/30'
30'/150'
Time point

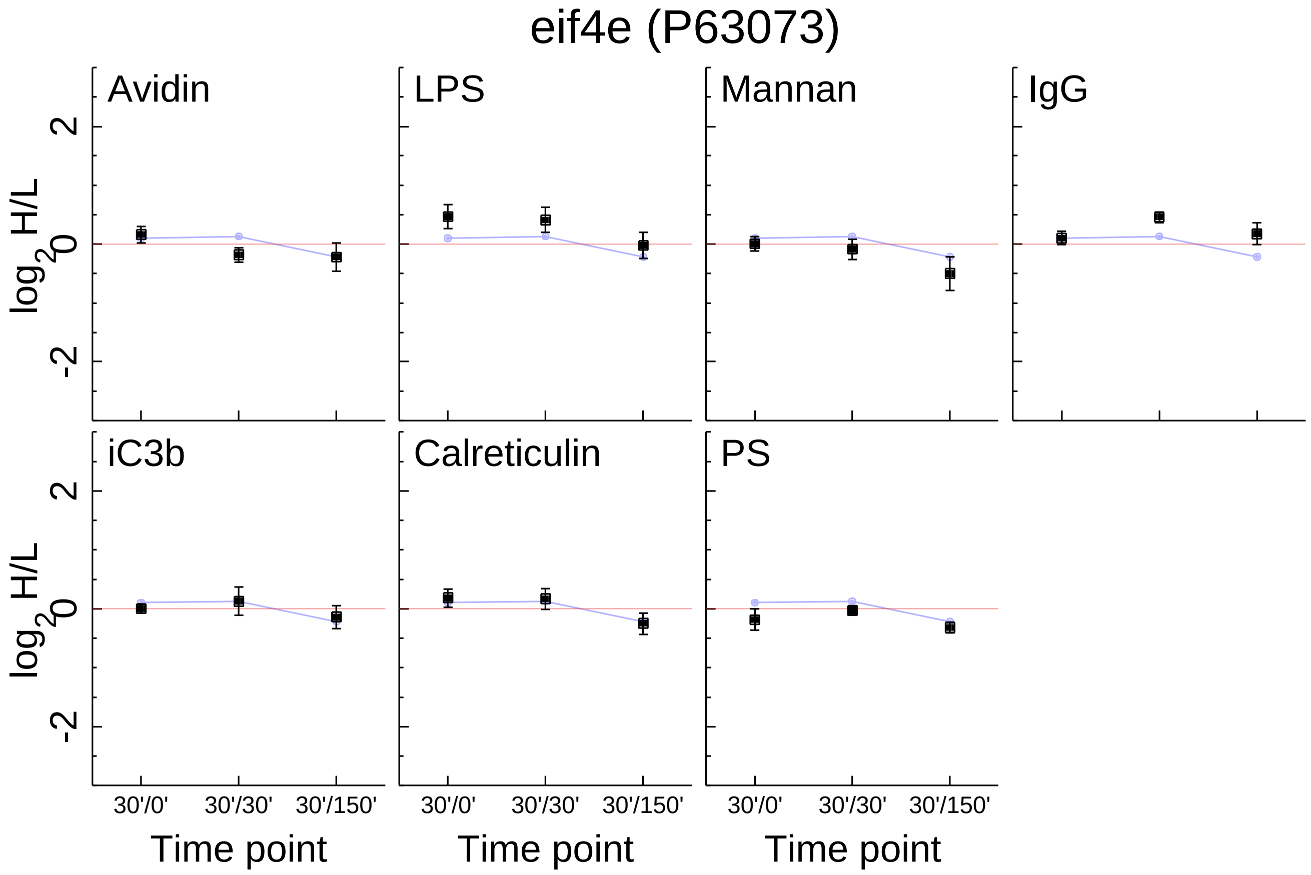
eif4e (P63073)
Avidin
LPS
Mannan
IgG
iC3b
Calreticulin
PS
log2 H/L
2
0
-2
30'/0'
30'/30'
30'/150'
Time point

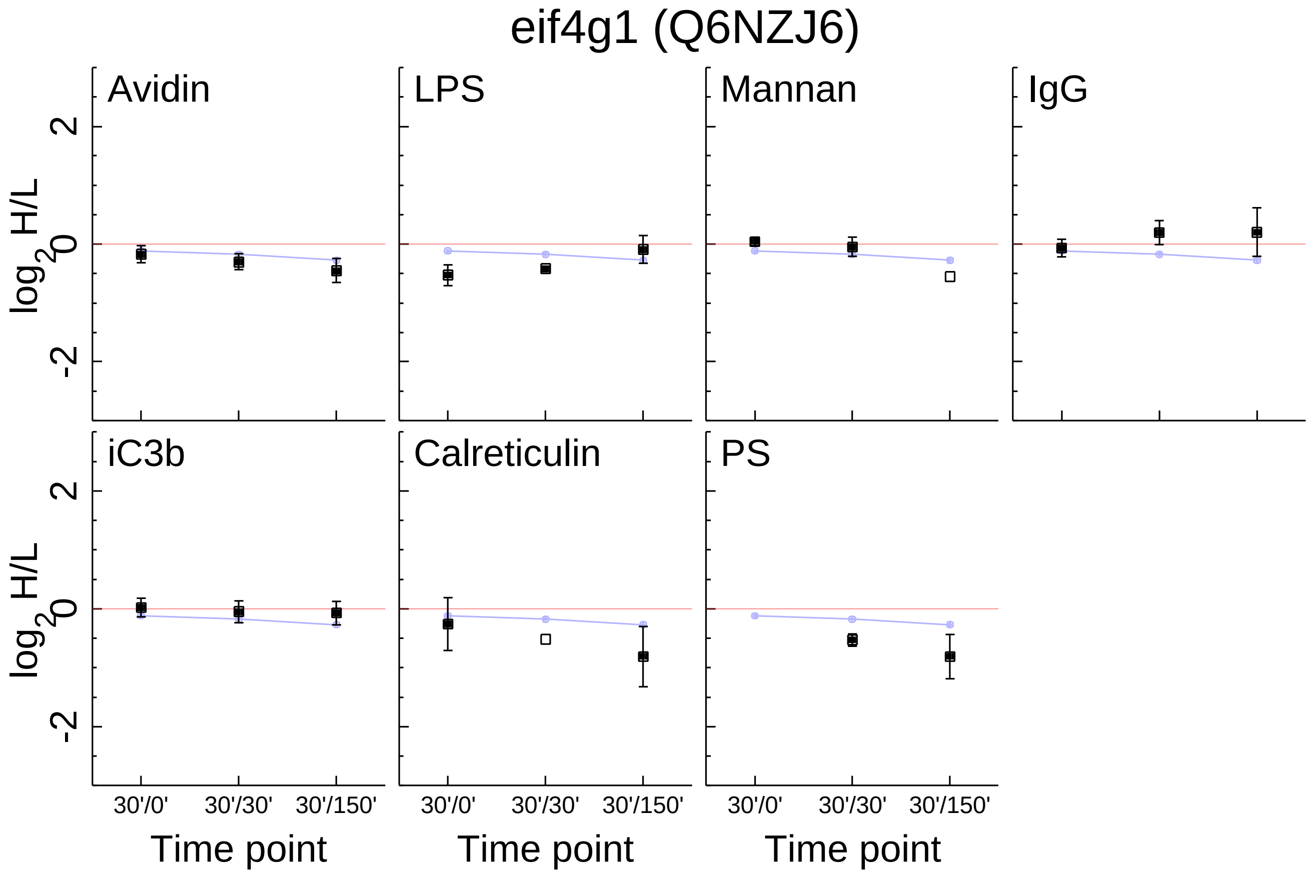
eif4g1 (Q6NZJ6)
Avidin
LPS
Mannan
IgG
iC3b
Calreticulin
PS
log2 H/L
2
0
-2
30'/0'
30'/30'
30'/150'
Time point

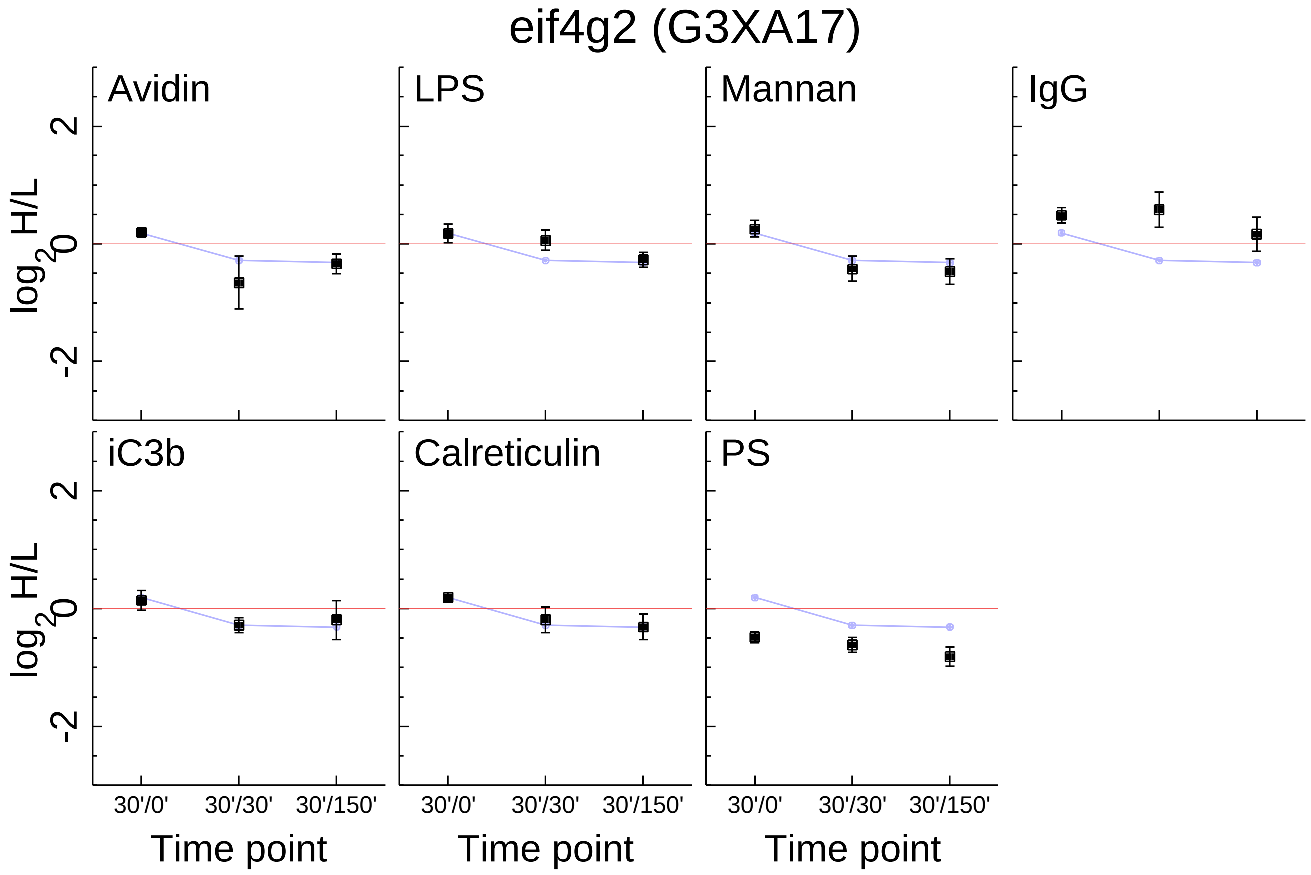
eif4g2 (G3XA17)
Avidin
LPS
Mannan
IgG
iC3b
Calreticulin
PS
log2 H/L
2
0
-2
30'/0'
30'/30'
30'/150'
Time point

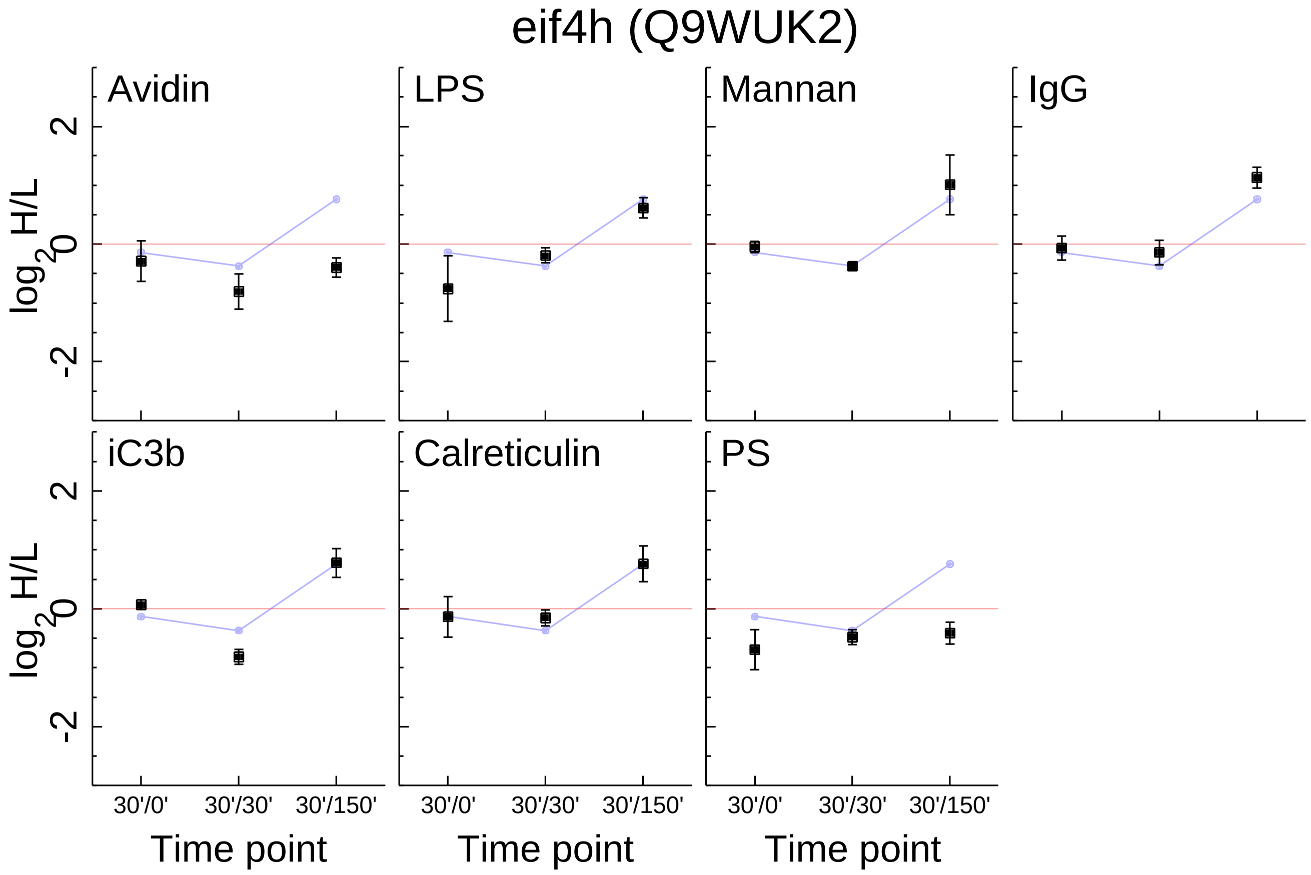
eif4h (Q9WUK2)
Avidin
LPS
Mannan
IgG
iC3b
Calreticulin
PS
log2 H/L
2
0
-2
30'/0'
30'/30'
30'/150'
Time point

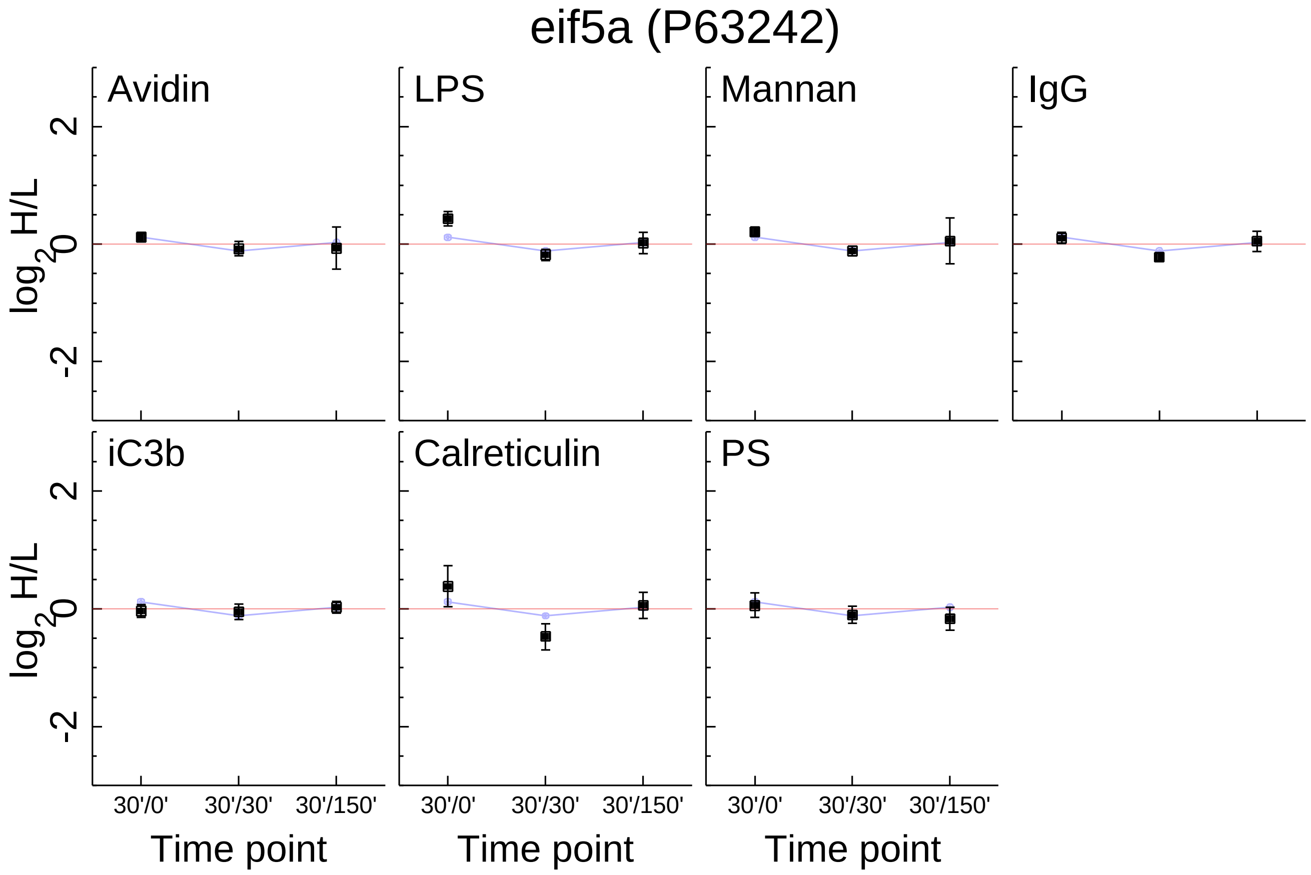

eif5a (P63242)
Avidin
LPS
Mannan
IgG
iC3b
Calreticulin
PS
log2 H/L
2
0
-2
30'/0'
30'/30'
30'/150'
Time point

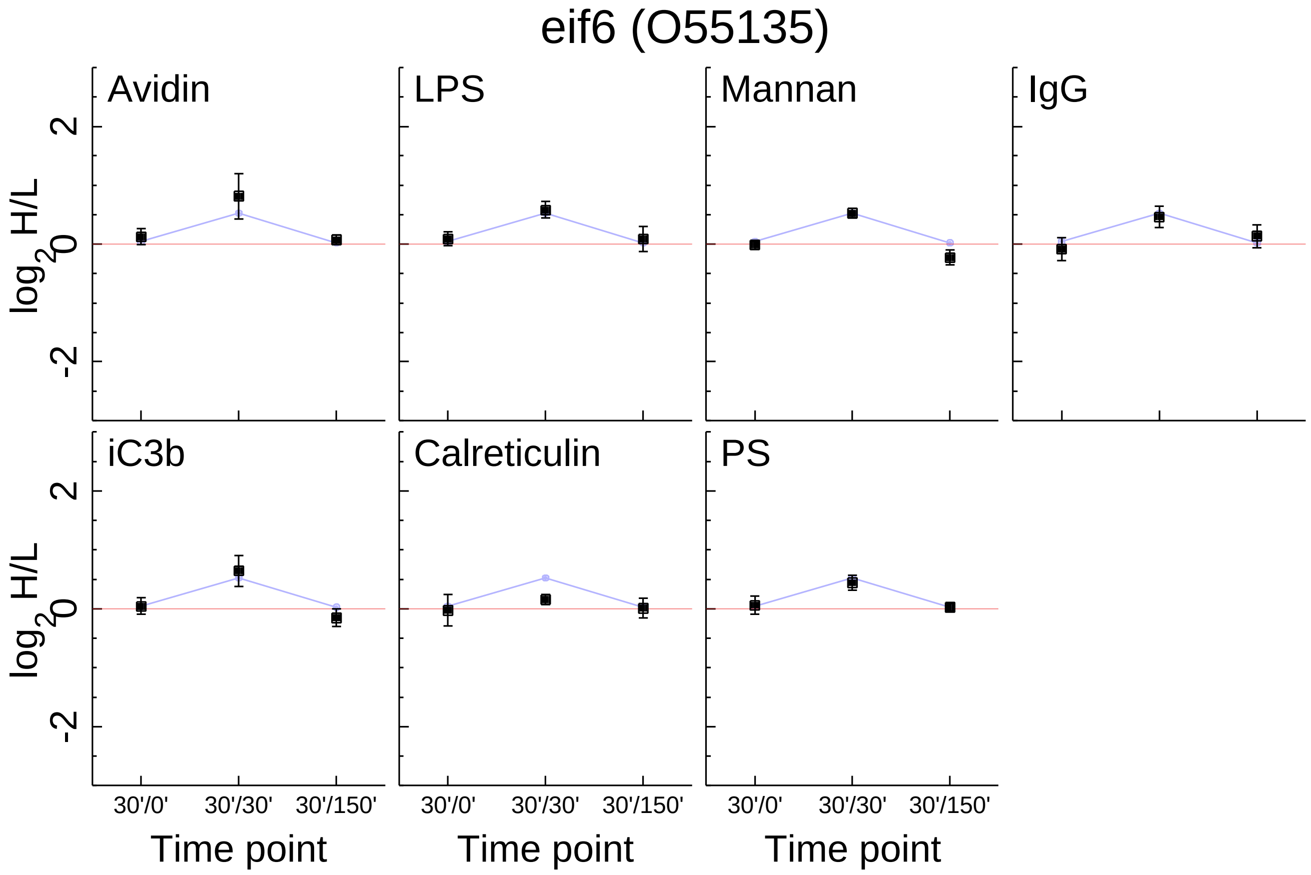
eif6 (O55135)
Avidin
LPS
Mannan
IgG
iC3b
Calreticulin
PS
log2 H/L
2
0
-2
30'/0'
30'/30'
30'/150'
Time point

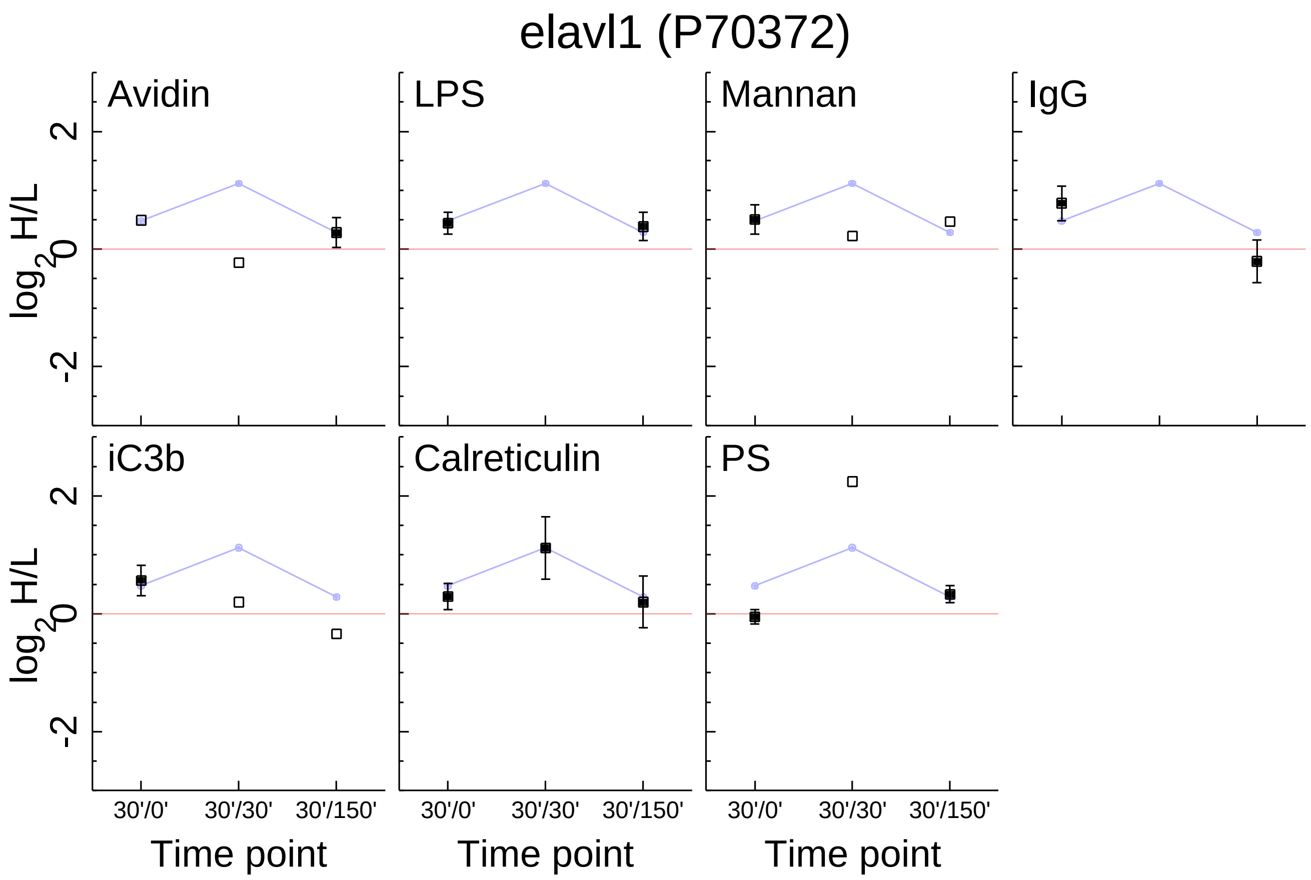

elavl1 (P70372)
Avidin
LPS
Mannan
IgG
iC3b
Calreticulin
PS
log2 H/L
2
0
-2
30'/0'
30'/30'
30'/150'
Time point

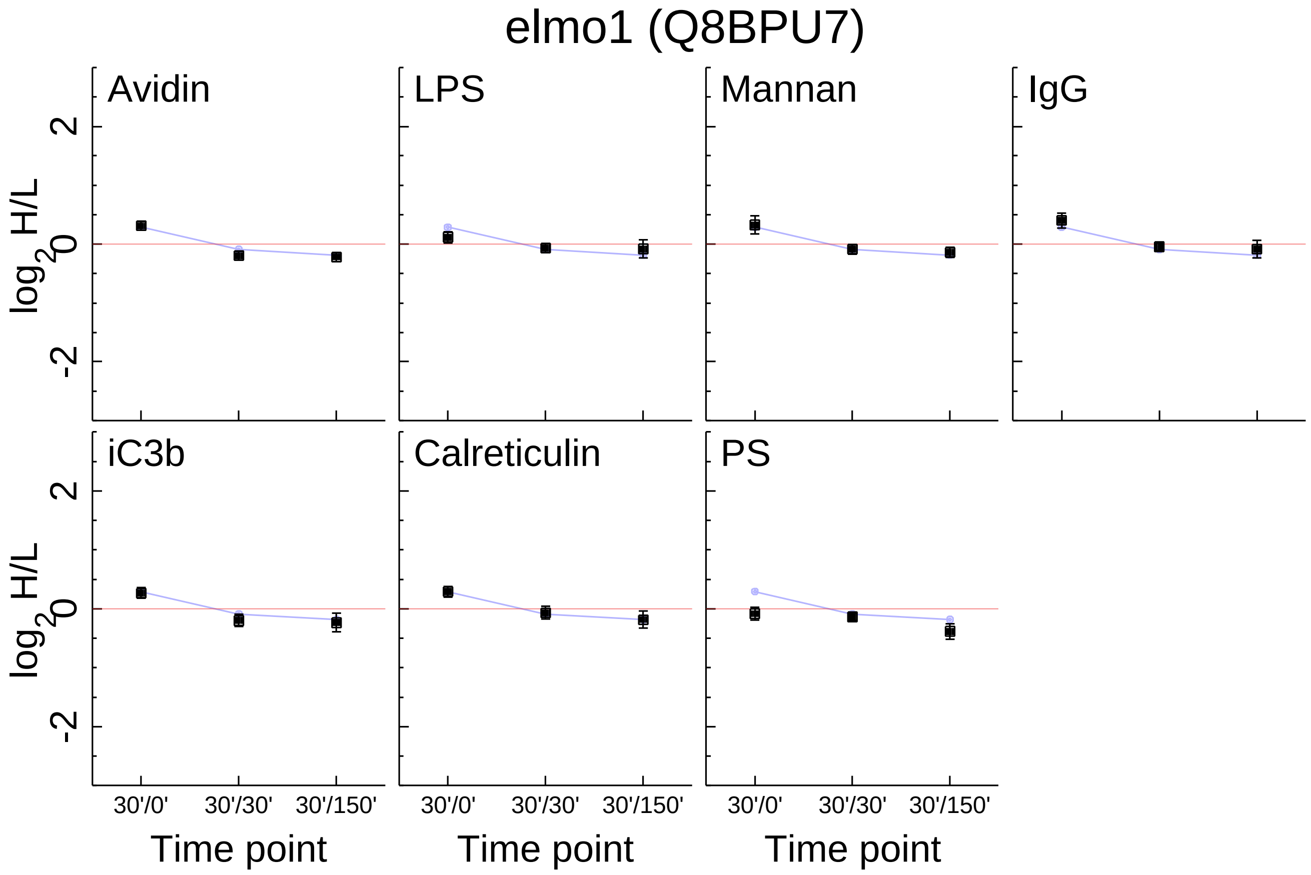

elmo1 (Q8BPU7)
Avidin
LPS
Mannan
IgG
iC3b
Calreticulin
PS
log2 H/L
2
0
-2
30'/0'
30'/30'
30'/150'
Time point

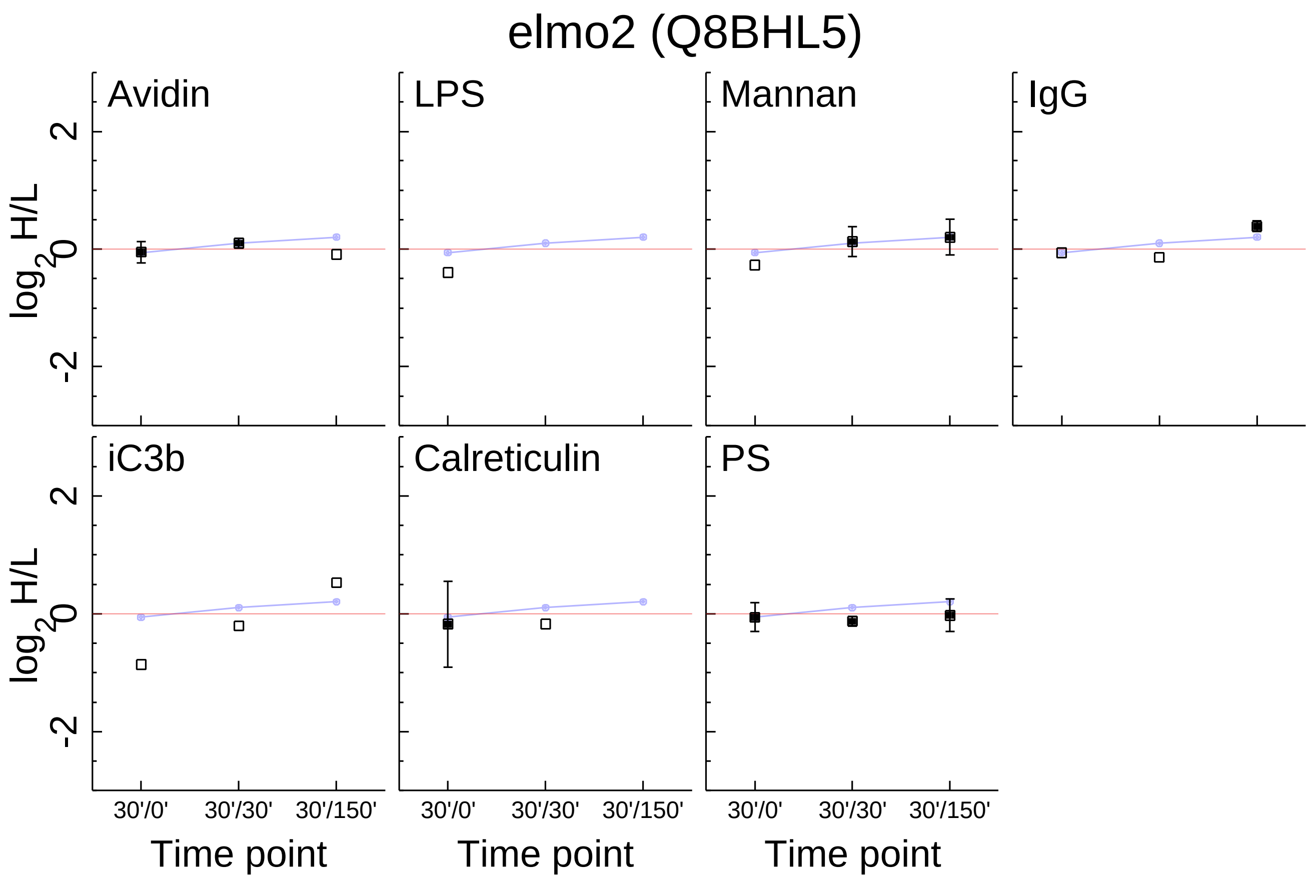
elmo2 (Q8BHL5)
Avidin
LPS
Mannan
IgG
iC3b
Calreticulin
PS
log2 H/L
2
0
-2
30'/0'
30'/30'
30'/150'
Time point

elovl1 (Q9JLJ5)

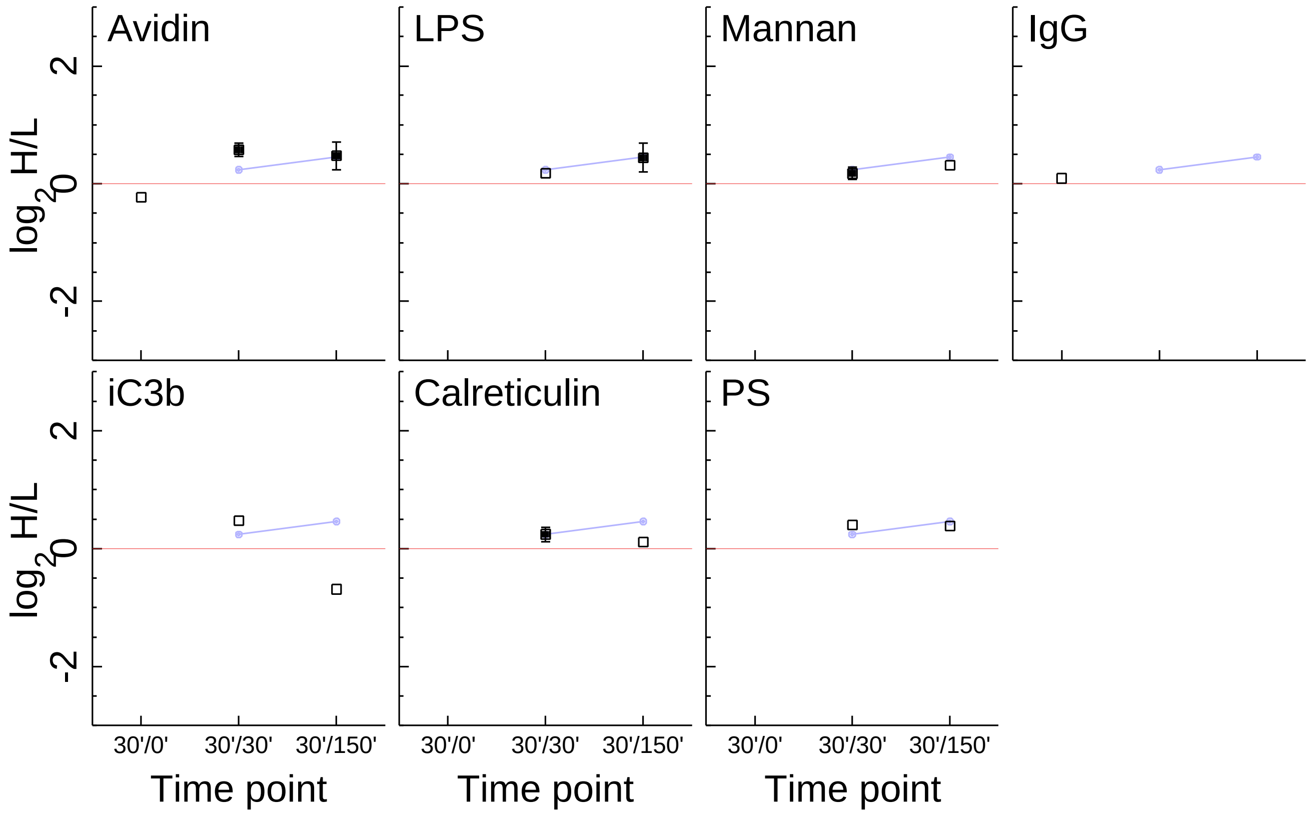

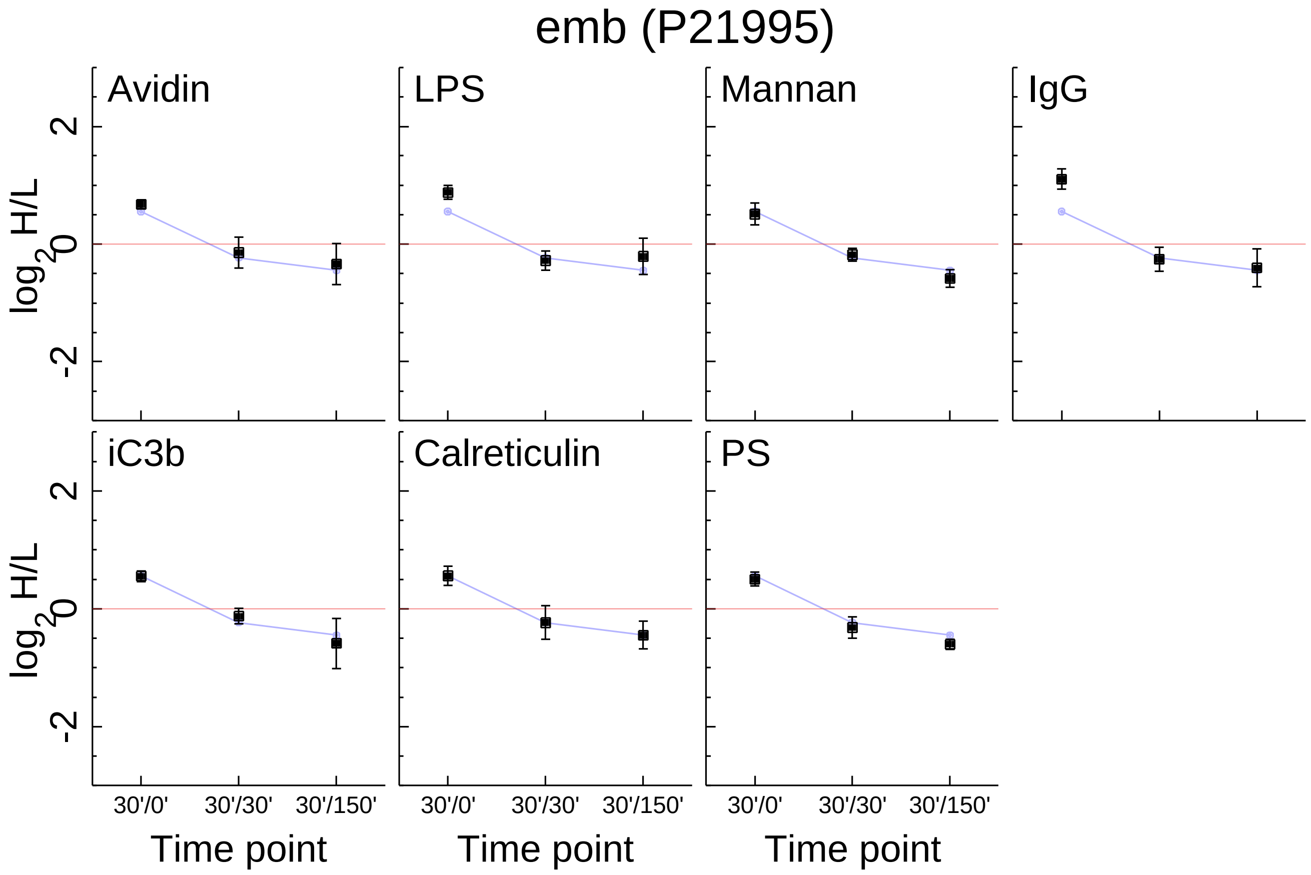
emb (P21995)
Avidin
LPS
Mannan
IgG
iC3b
Calreticulin
PS
log2 H/L
2
0
-2
30'/0'
30'/30'
30'/150'
Time point

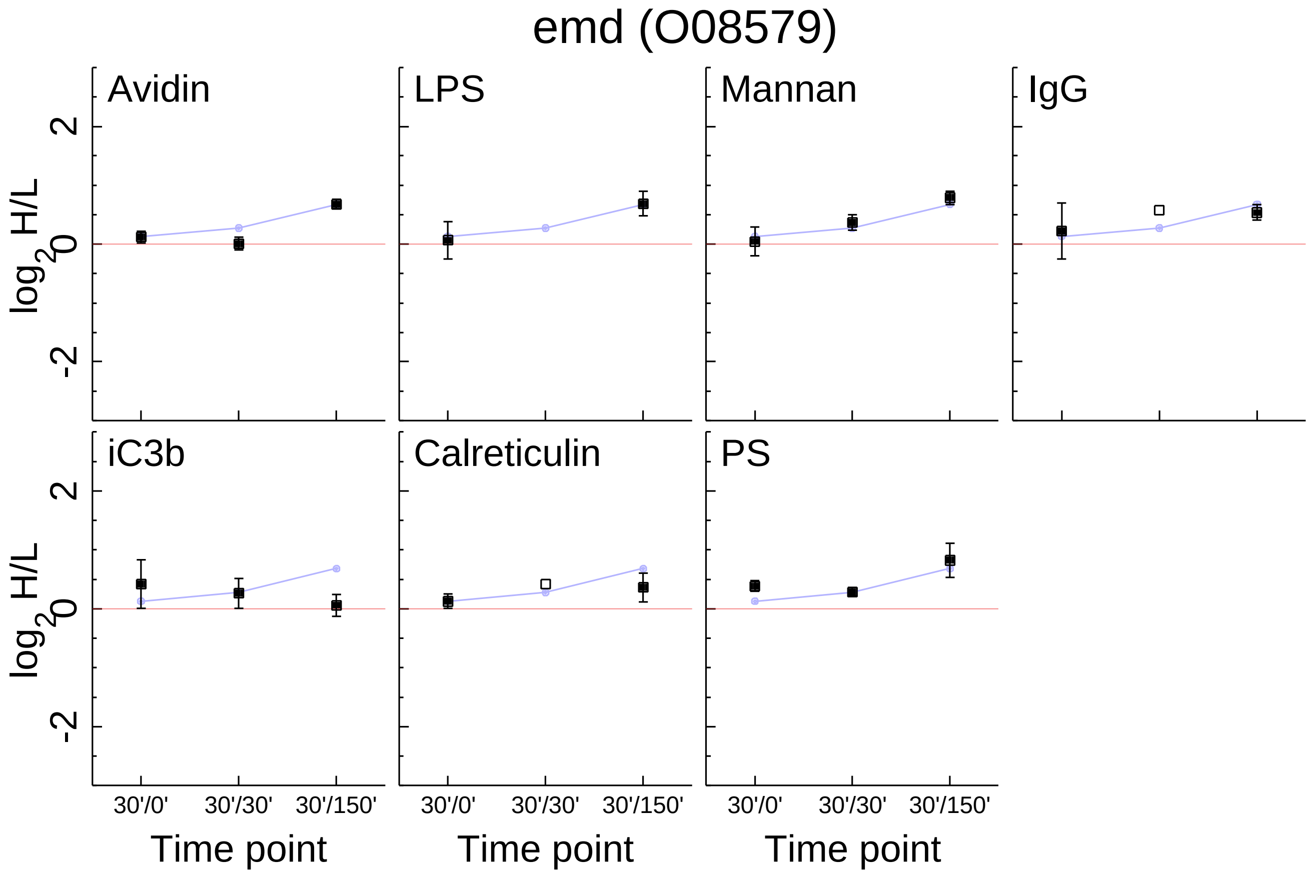
emd (O08579)
Avidin
LPS
Mannan
IgG
iC3b
Calreticulin
PS
log2 H/L
2
0
-2
30'/0'
30'/30'
30'/150'
Time point

emp2 (O88662)

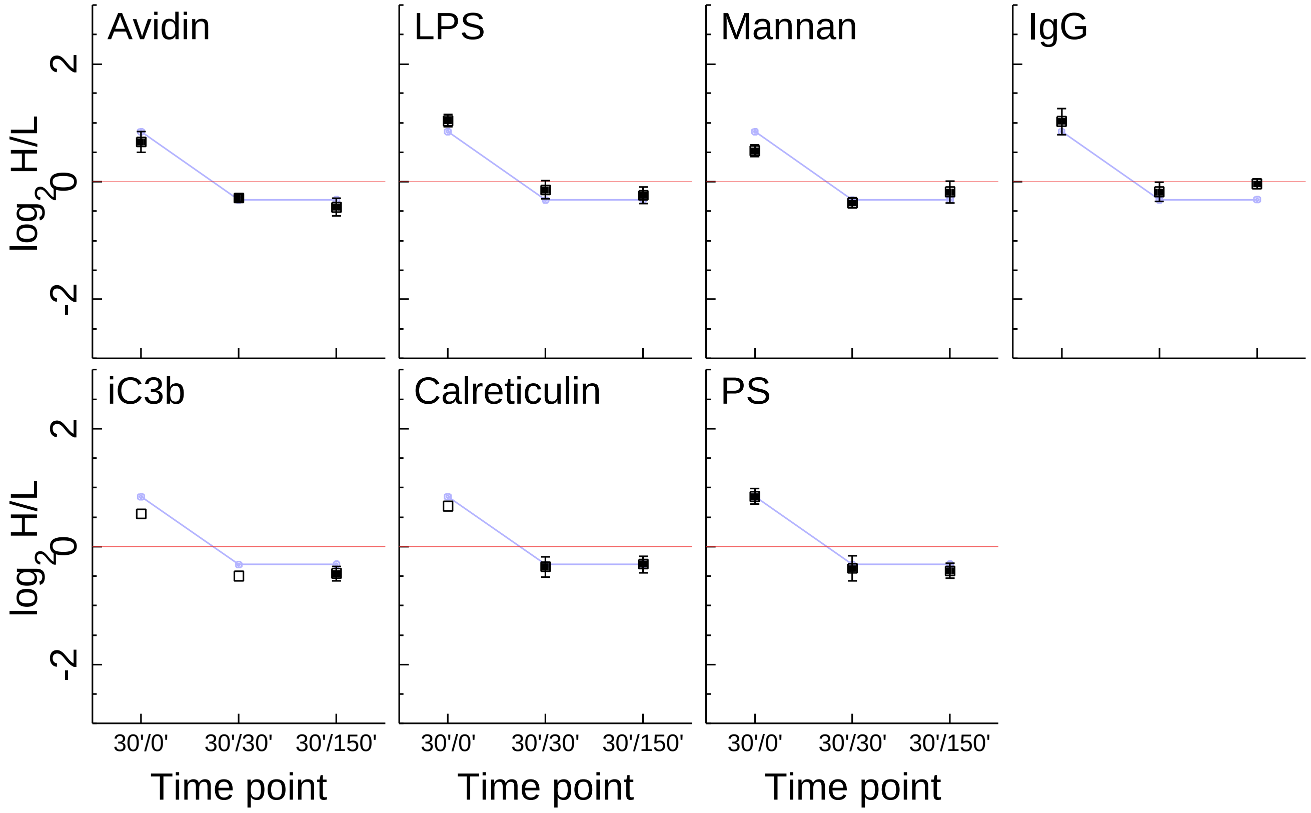

Avidin
LPS
Mannan
IgG
iC3b
Calreticulin
PS
log2 H/L
2
0
-2
30'/0'
30'/30'
30'/150'
Time point

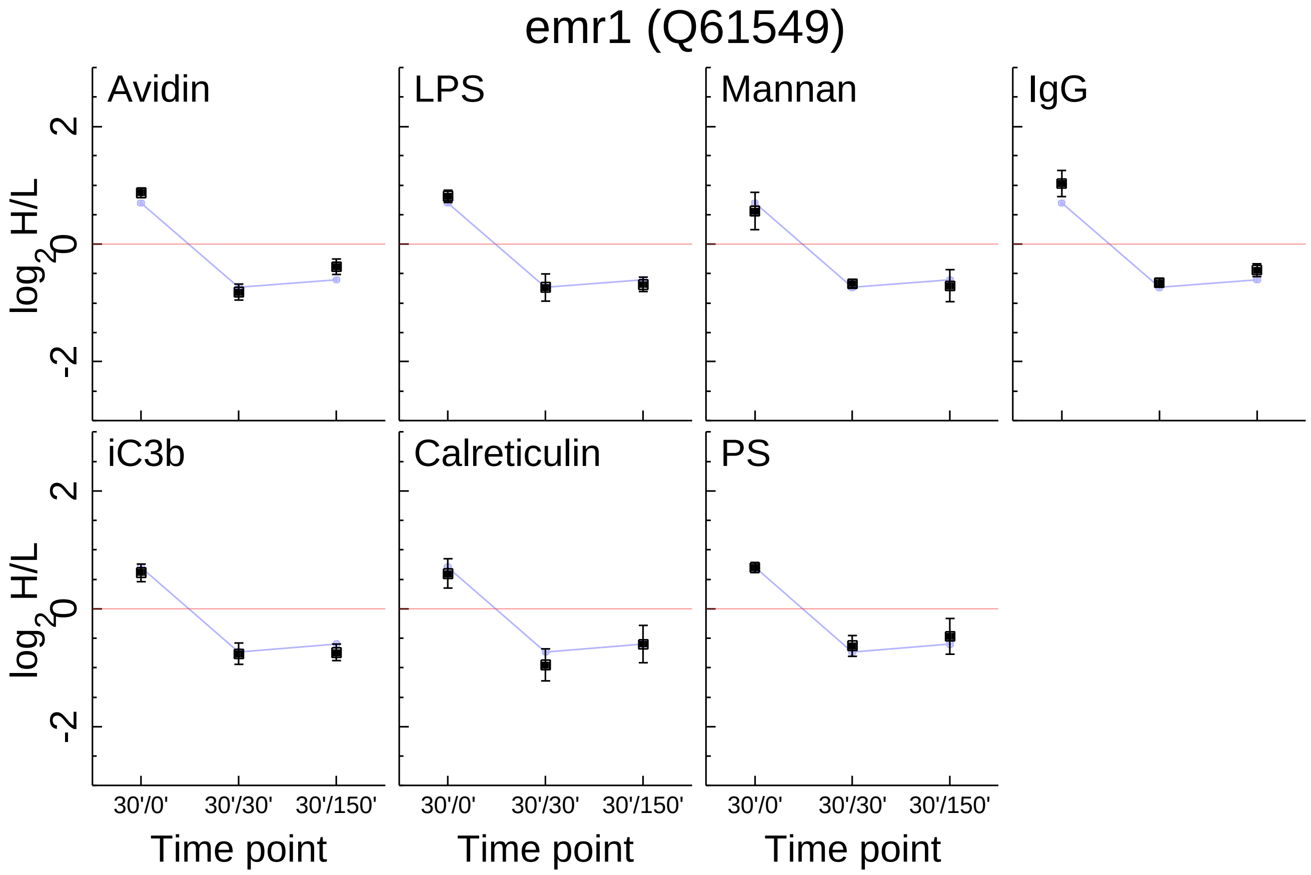

emr1 (Q61549)
Avidin
LPS
Mannan
IgG
iC3b
Calreticulin
PS
log2 H/L
2
0
-2
30'/0'
30'/30'
30'/150'
Time point

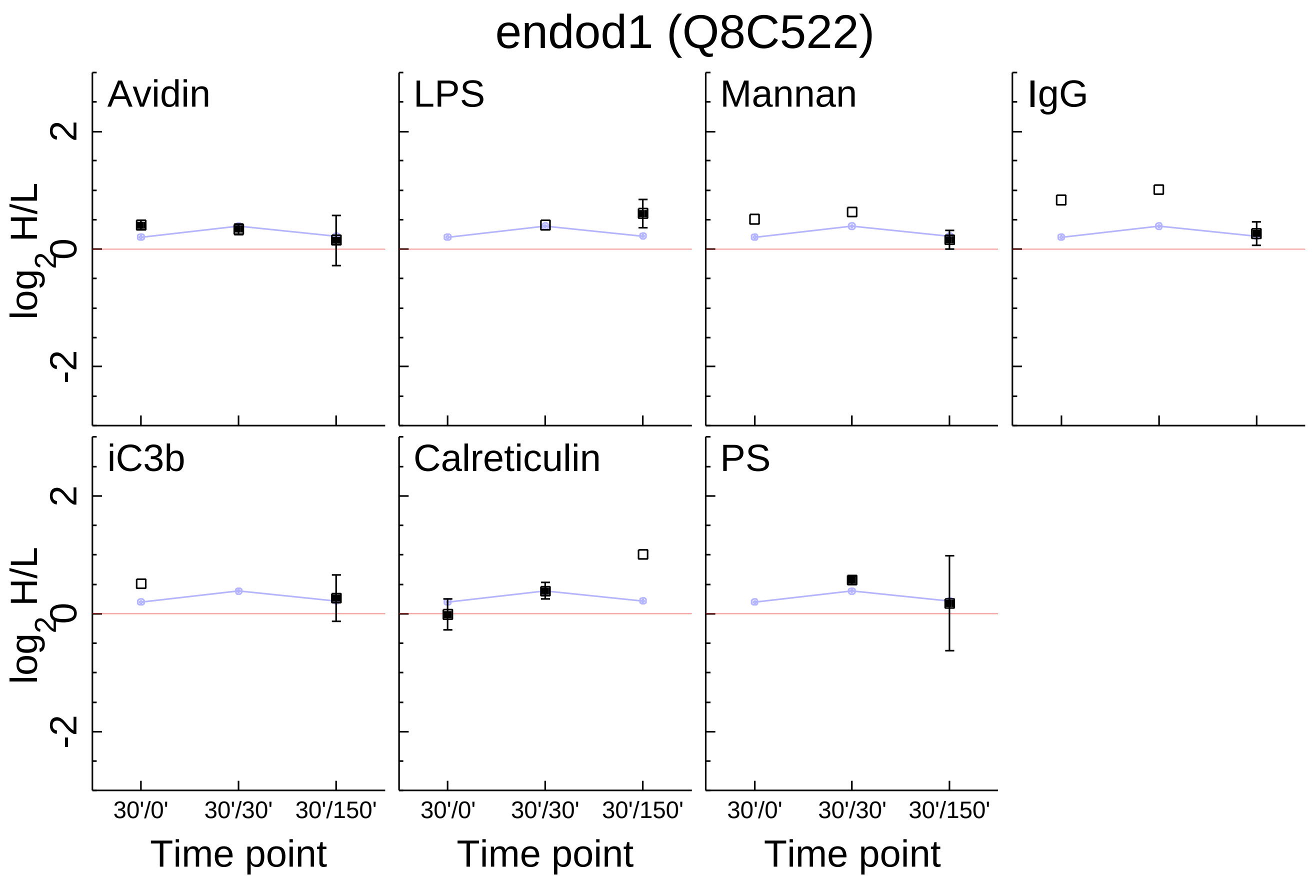
endod1 (Q8C522)
Avidin
LPS
Mannan
IgG
iC3b
Calreticulin
PS
log2 H/L
2
0
-2
30'/0'
30'/30'
30'/150'
Time point

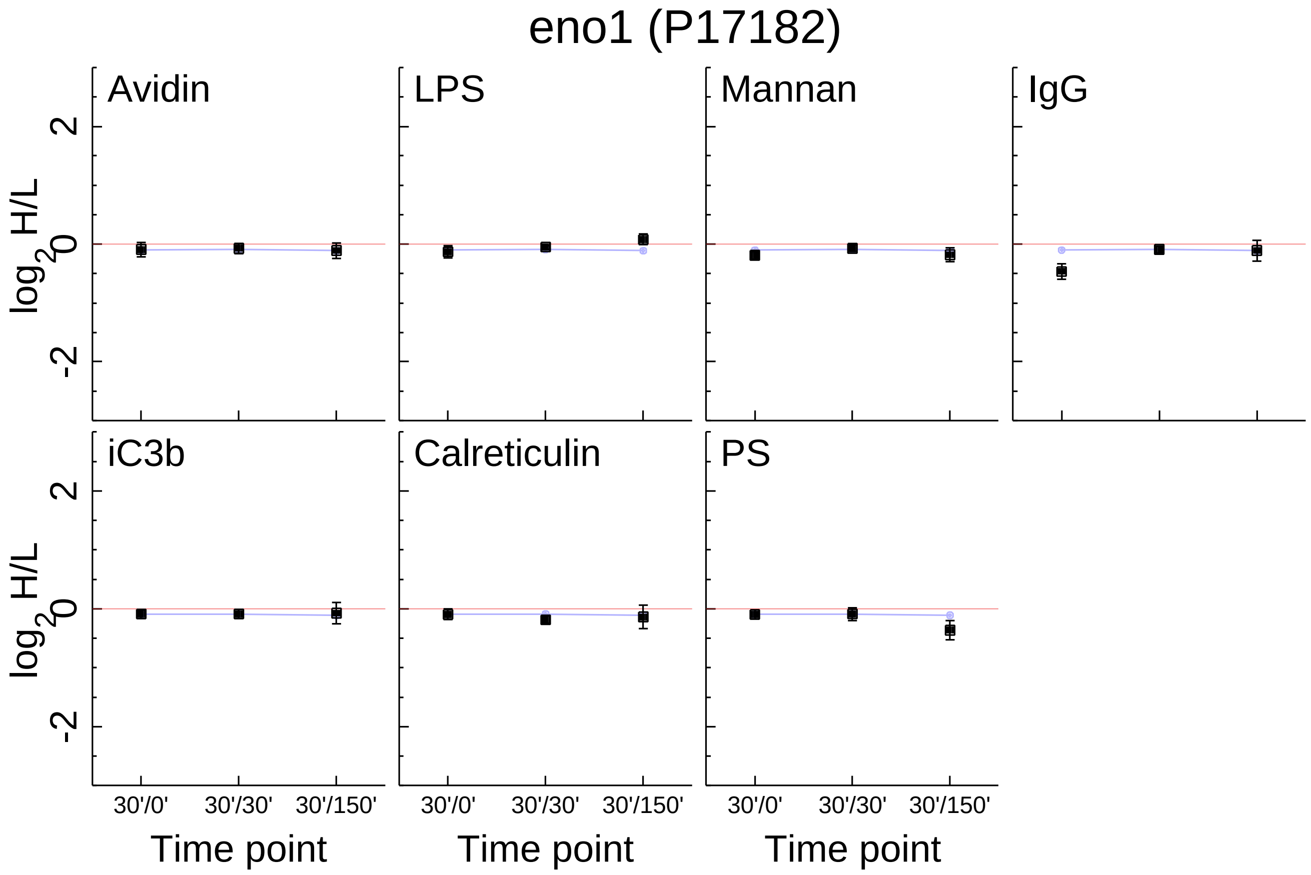

eno1 (P17182)
Avidin
LPS
Mannan
IgG
iC3b
Calreticulin
PS
log2 H/L
2
0
-2
30'/0'
30'/30'
30'/150'
Time point

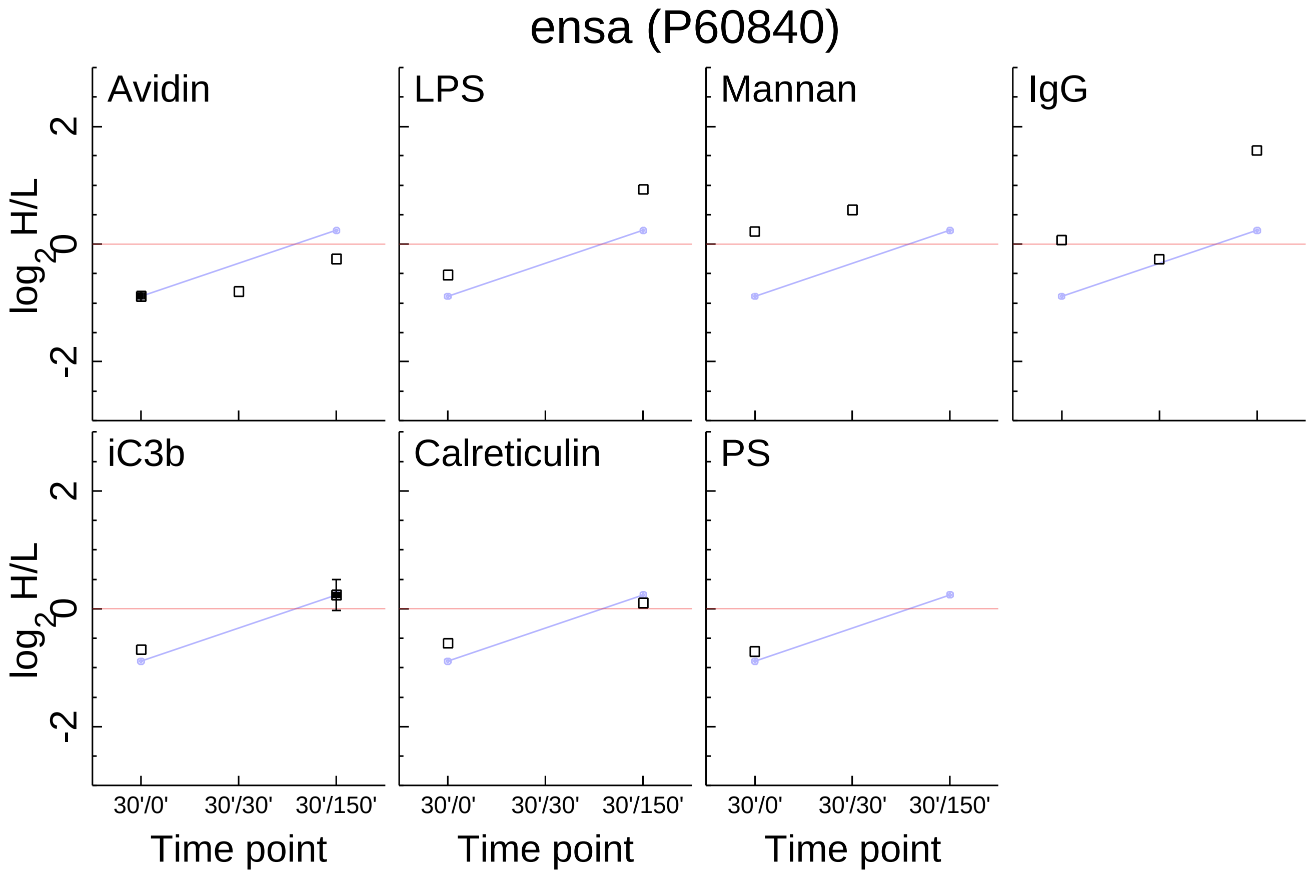
ensa (P60840)
Avidin
LPS
Mannan
IgG
iC3b
Calreticulin
PS
log2 H/L
2
0
-2
30'/0'
30'/30'
30'/150'
Time point

entpd1 (Q8CDV7)

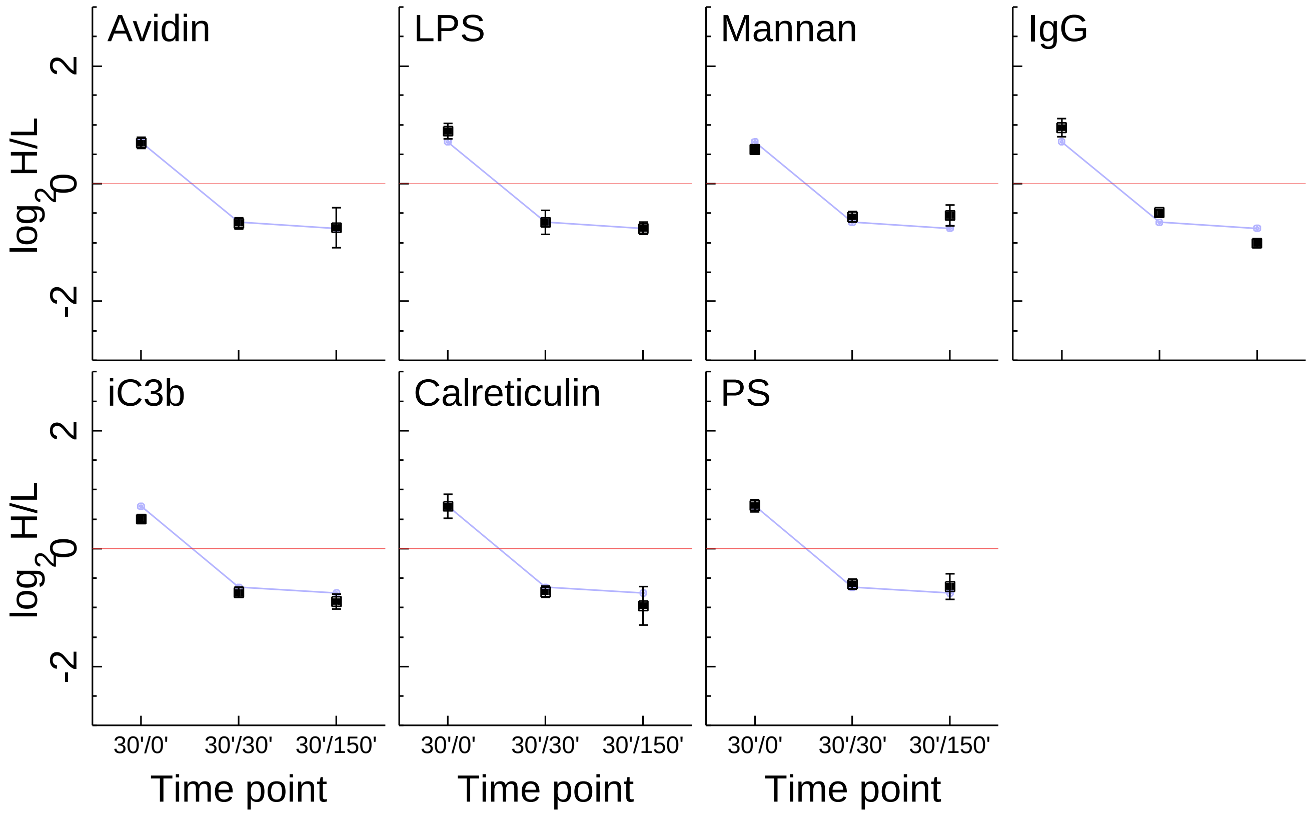

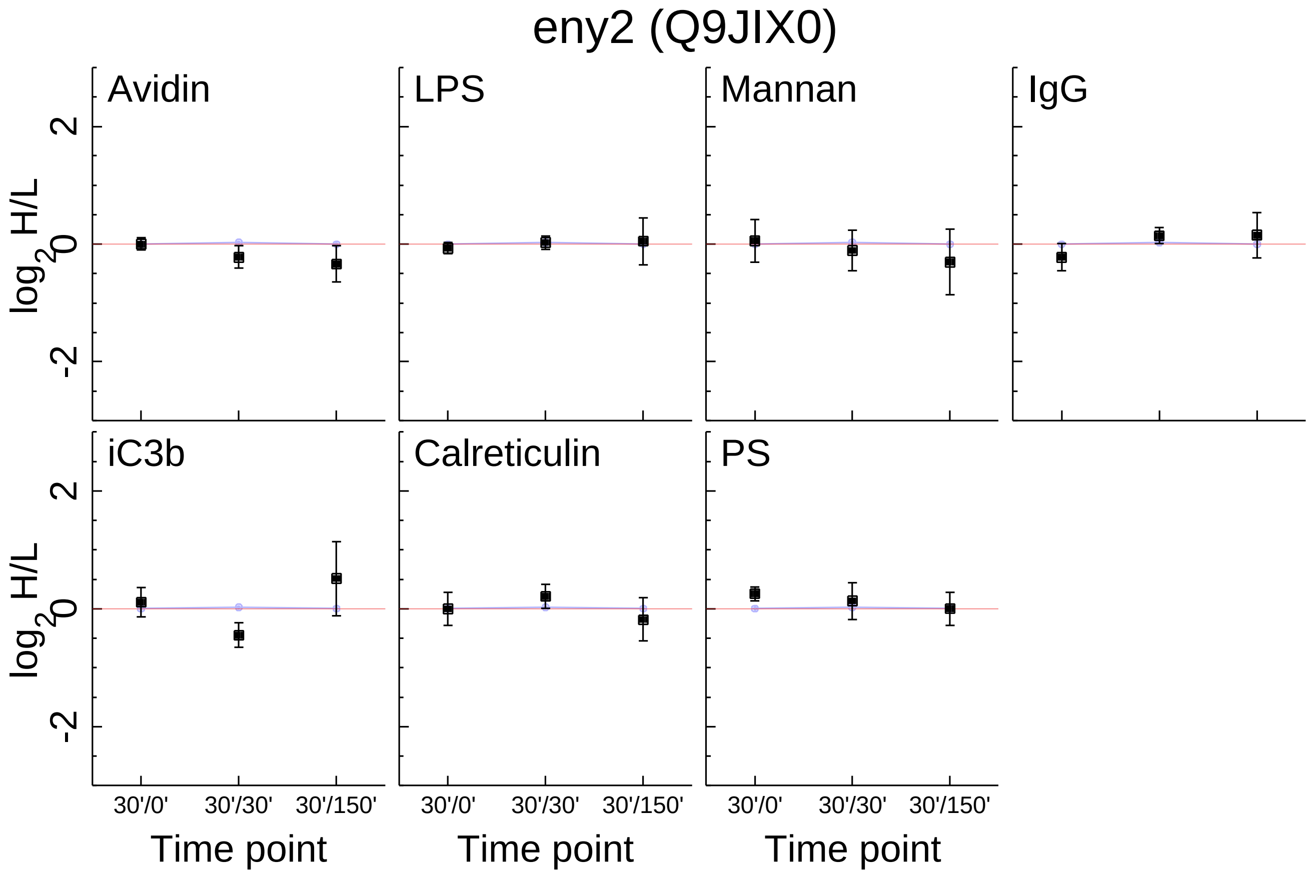
eny2 (Q9JIX0)
Avidin
LPS
Mannan
IgG
iC3b
Calreticulin
PS
log2 H/L
2
0
-2
30'/0'
30'/30'
30'/150'
Time point

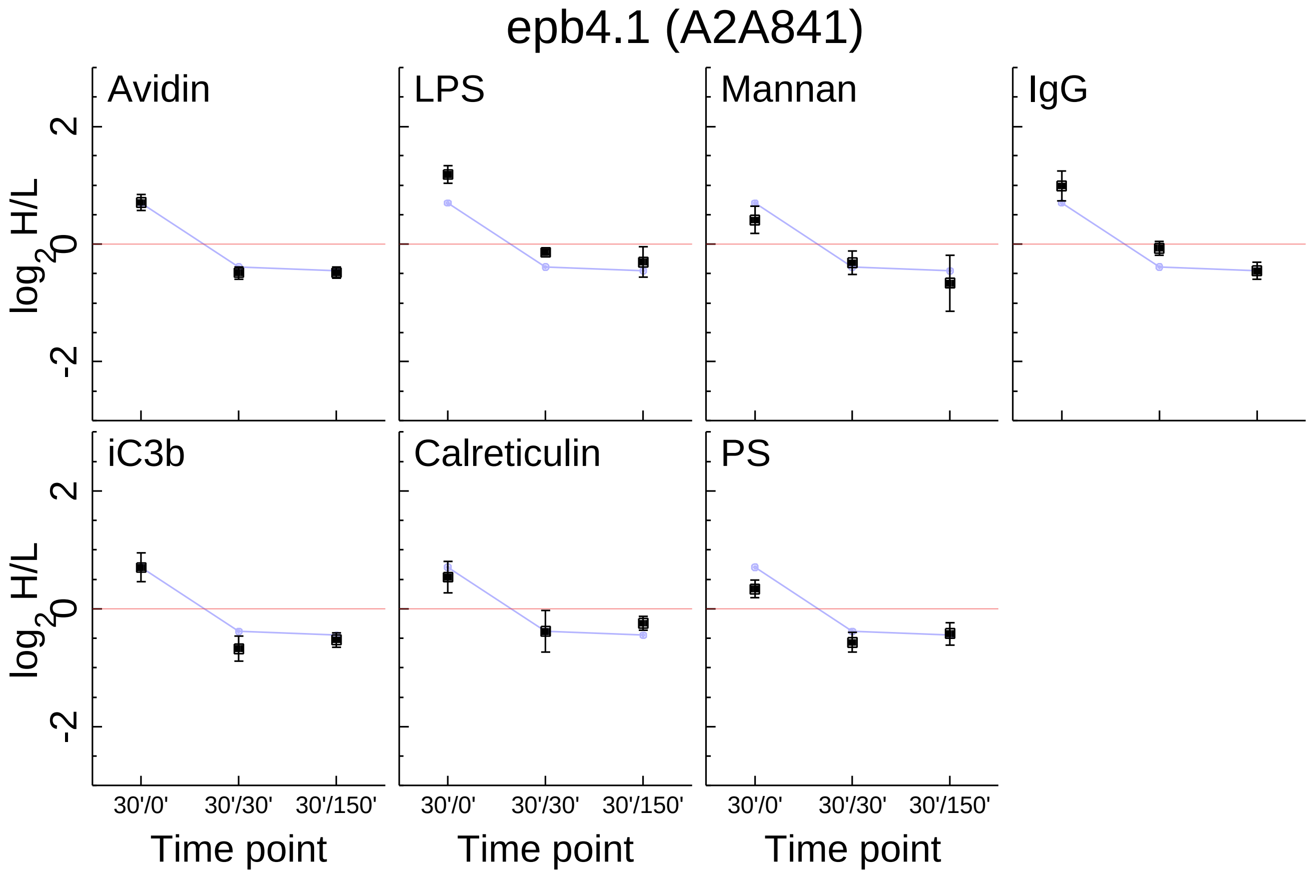

epb4.1 (A2A841)
Avidin
LPS
Mannan
IgG
iC3b
Calreticulin
PS
log2 H/L
2
0
-2
30'/0'
30'/30'
30'/150'
Time point

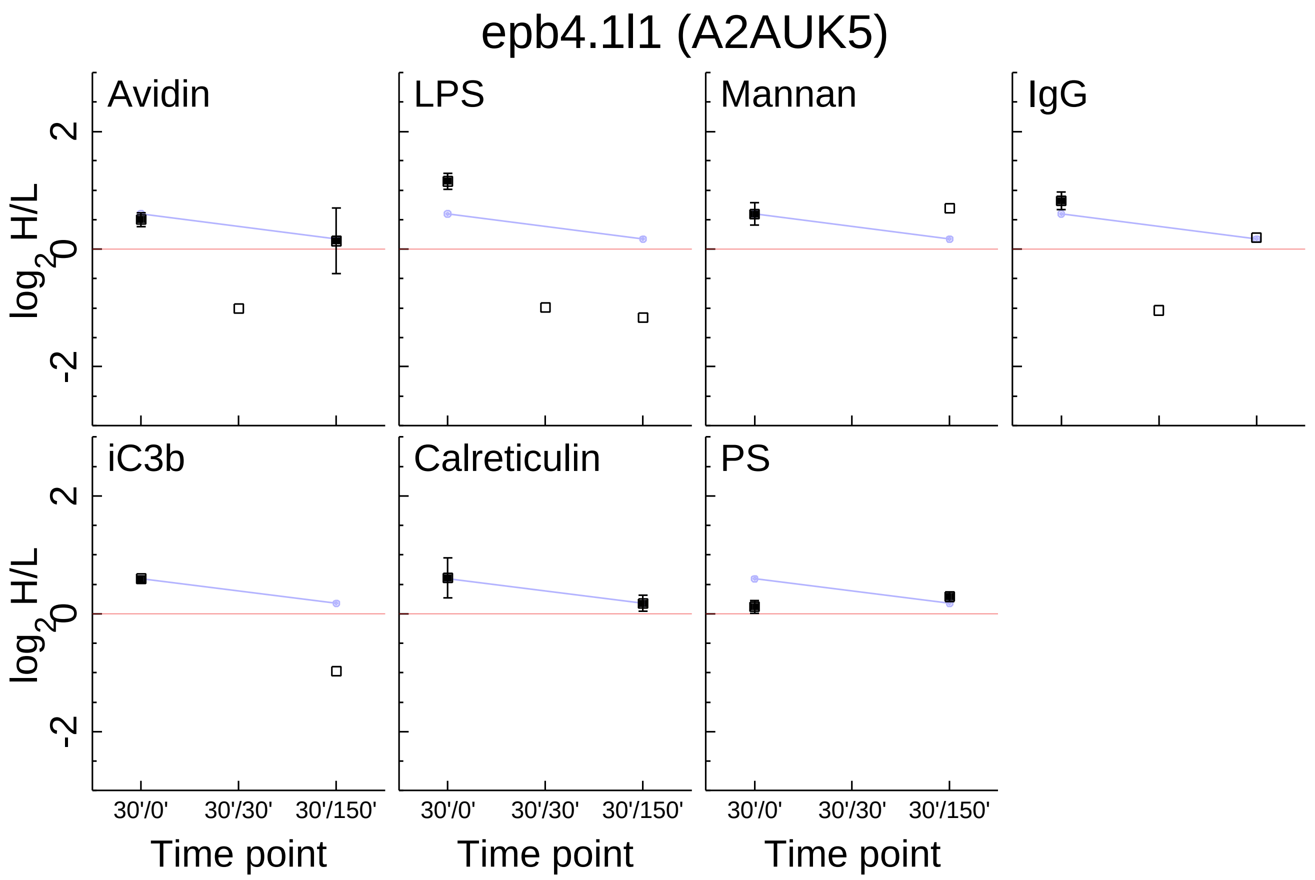

epb4.1l1 (A2AUK5)
Avidin
LPS
Mannan
IgG
iC3b
Calreticulin
PS
log2 H/L
2
0
-2
30'/0'
30'/30'
30'/150'
Time point

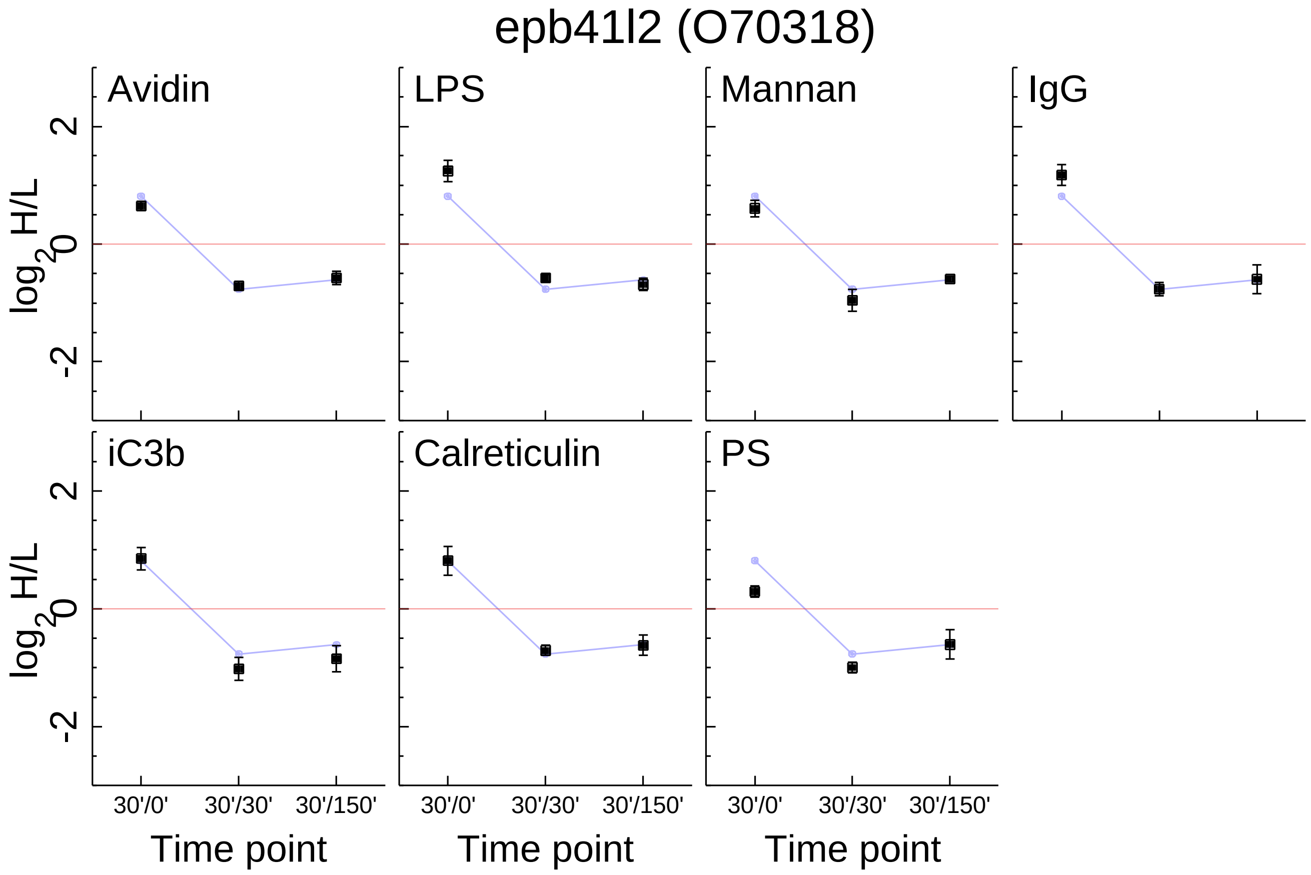
epb41l2 (O70318)
Avidin
LPS
Mannan
IgG
iC3b
Calreticulin
PS
log2 H/L
2
0
-2
30'/0'
30'/30'
30'/150'
Time point

epdr1 (Q99M71)

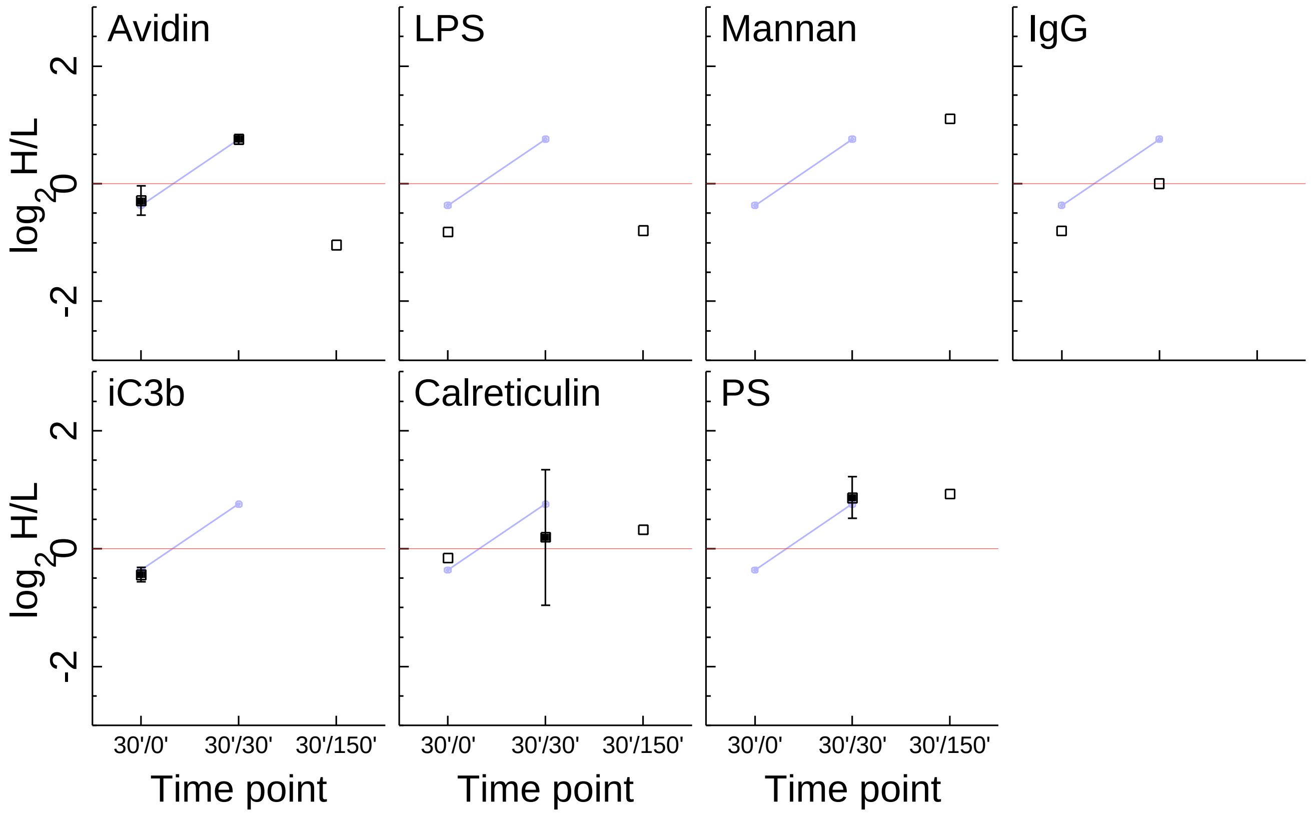

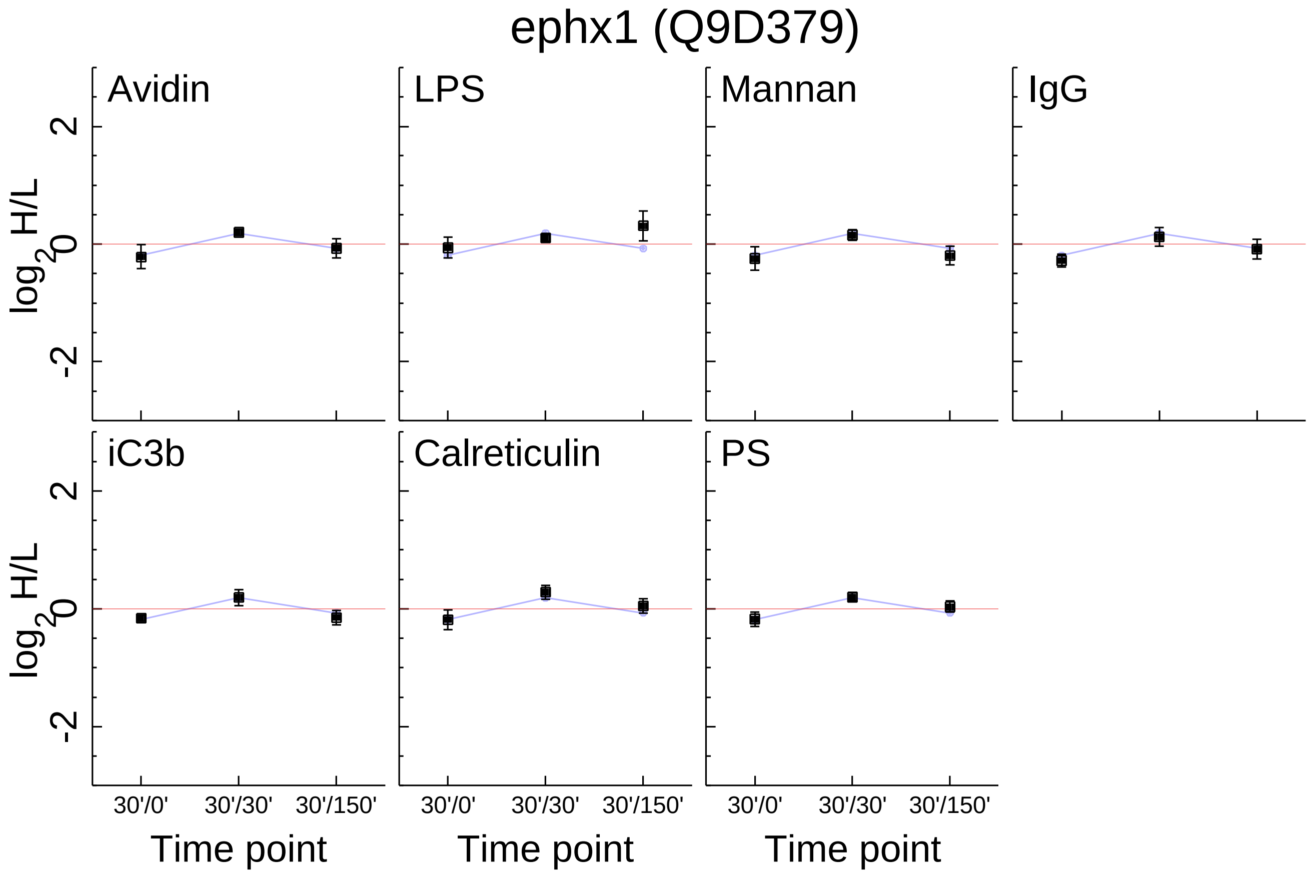
ephx1 (Q9D379)
Avidin
LPS
Mannan
IgG
iC3b
Calreticulin
PS
log2 H/L
2
0
-2
30'/0'
30'/30'
30'/150'
Time point

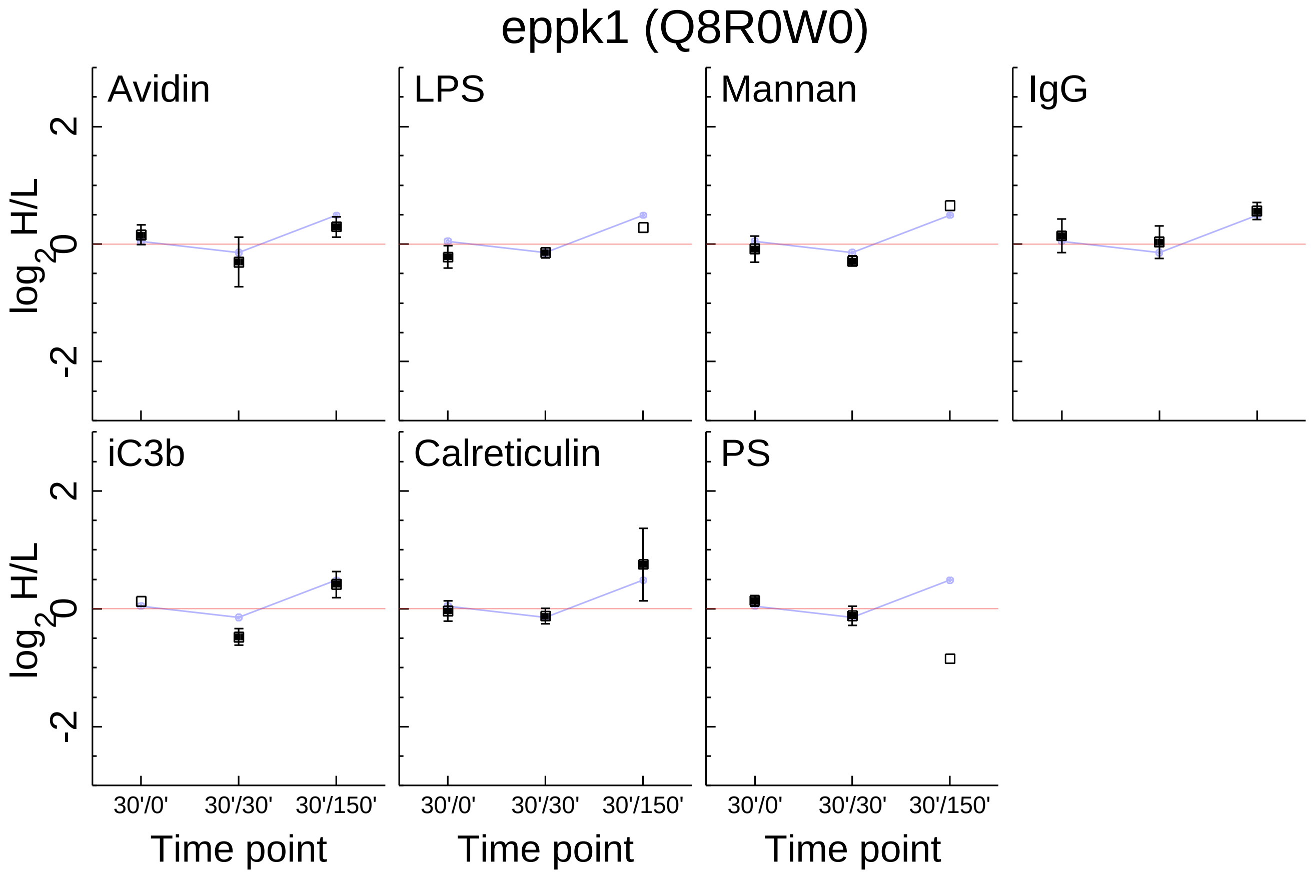
eppk1 (Q8R0W0)
Avidin
LPS
Mannan
IgG
iC3b
Calreticulin
PS
log2 H/L
2
0
-2
30'/0'
30'/30'
30'/150'
Time point

eps15l1 (Q60902)

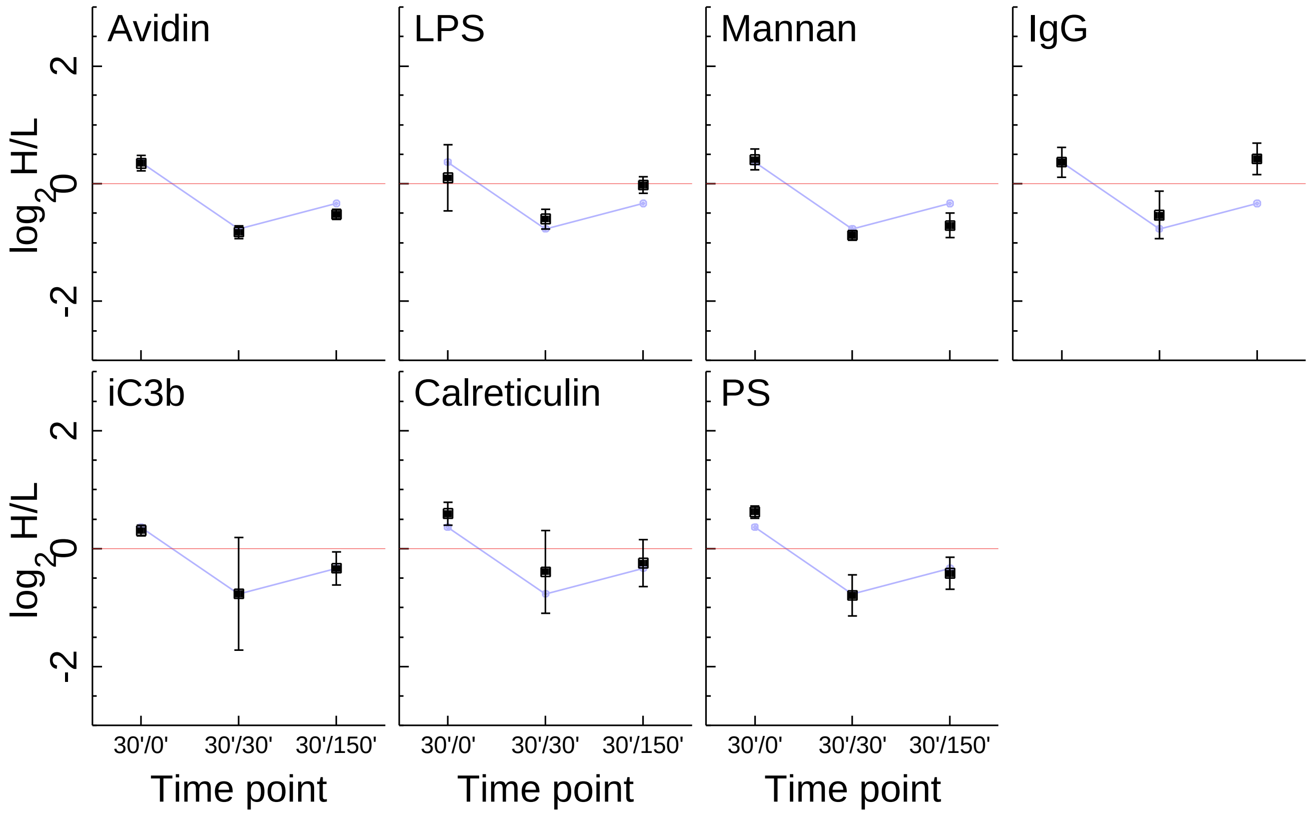

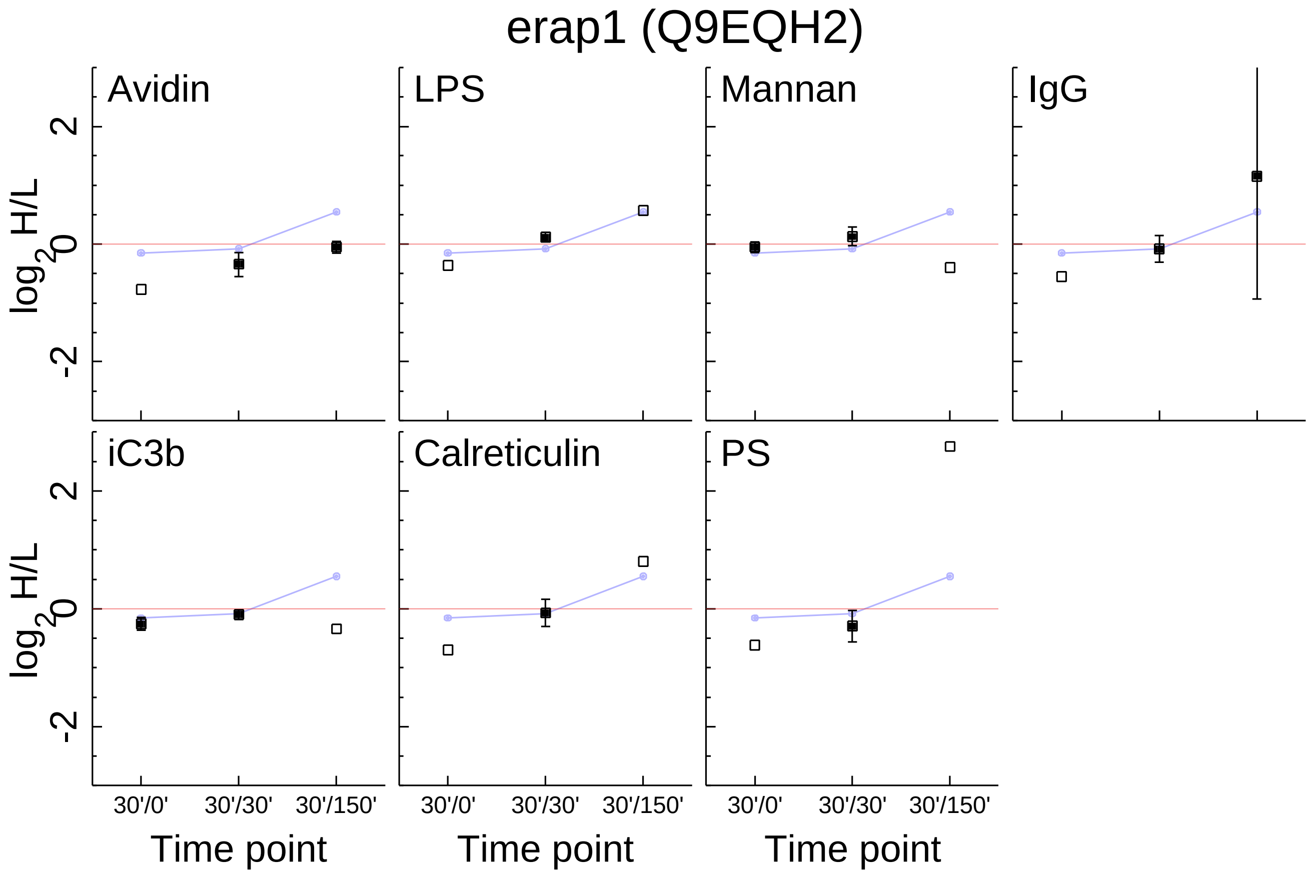
erap1 (Q9EQH2)
Avidin
LPS
Mannan
IgG
iC3b
Calreticulin
PS
log2 H/L
2
0
-2
30'/0'
30'/30'
30'/150'
Time point

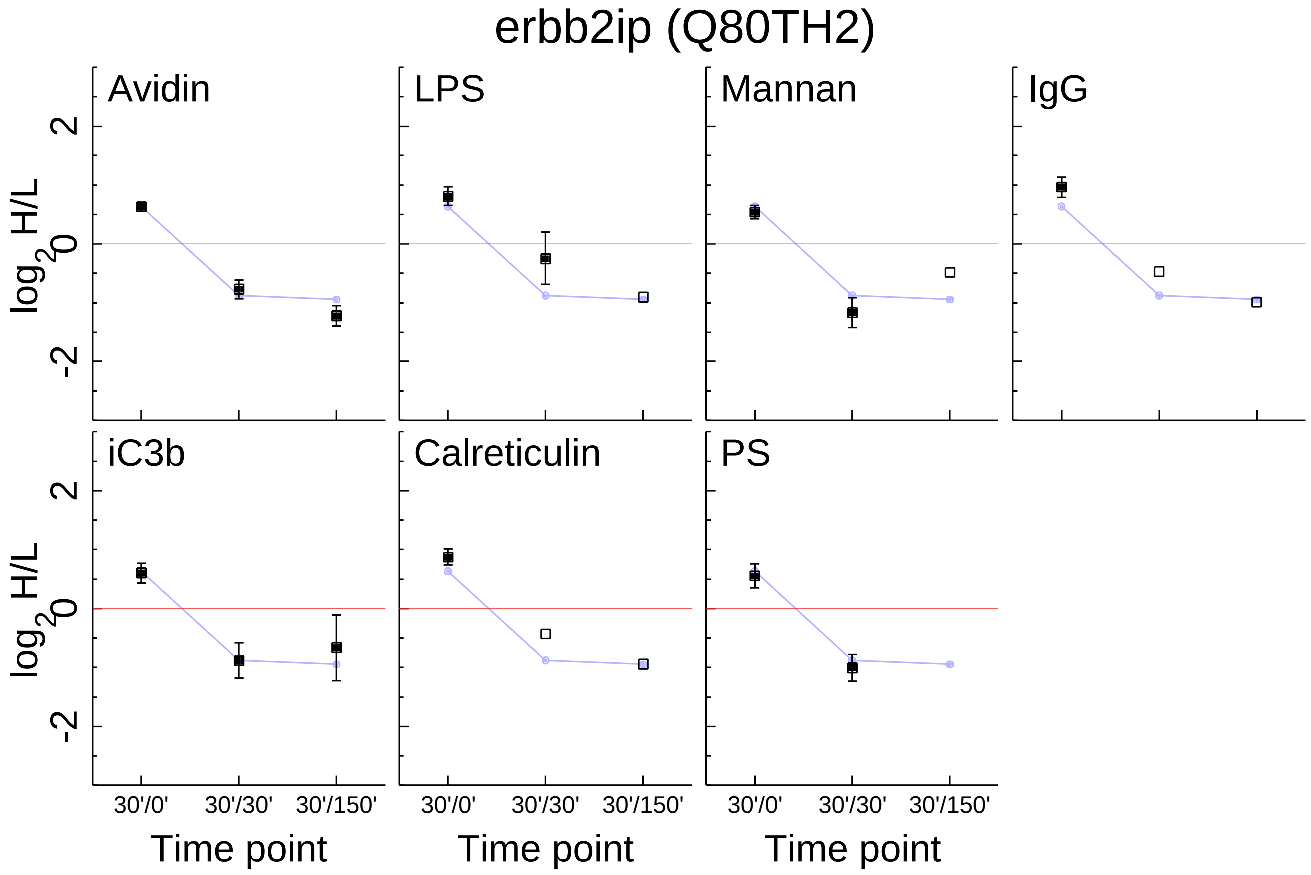
erbb2ip (Q80TH2)
Avidin
LPS
Mannan
IgG
iC3b
Calreticulin
PS
log2 H/L
2
0
-2
30'/0'
30'/30'
30'/150'
Time point

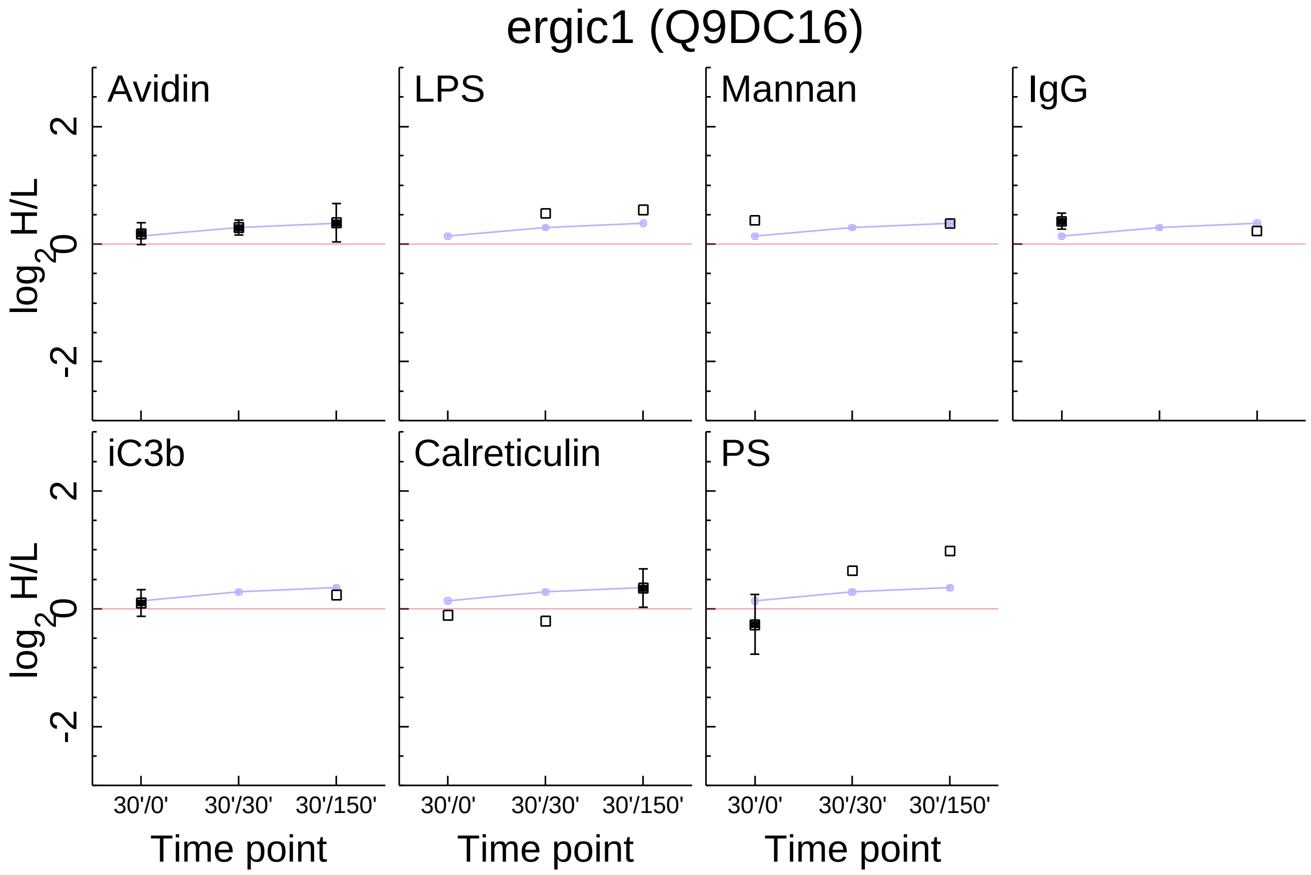

ergic1 (Q9DC16)
Avidin
LPS
Mannan
IgG
iC3b
Calreticulin
PS
log2 H/L
2
0
-2
30'/0'
30'/30'
30'/150'
Time point

erh (G3UW85)

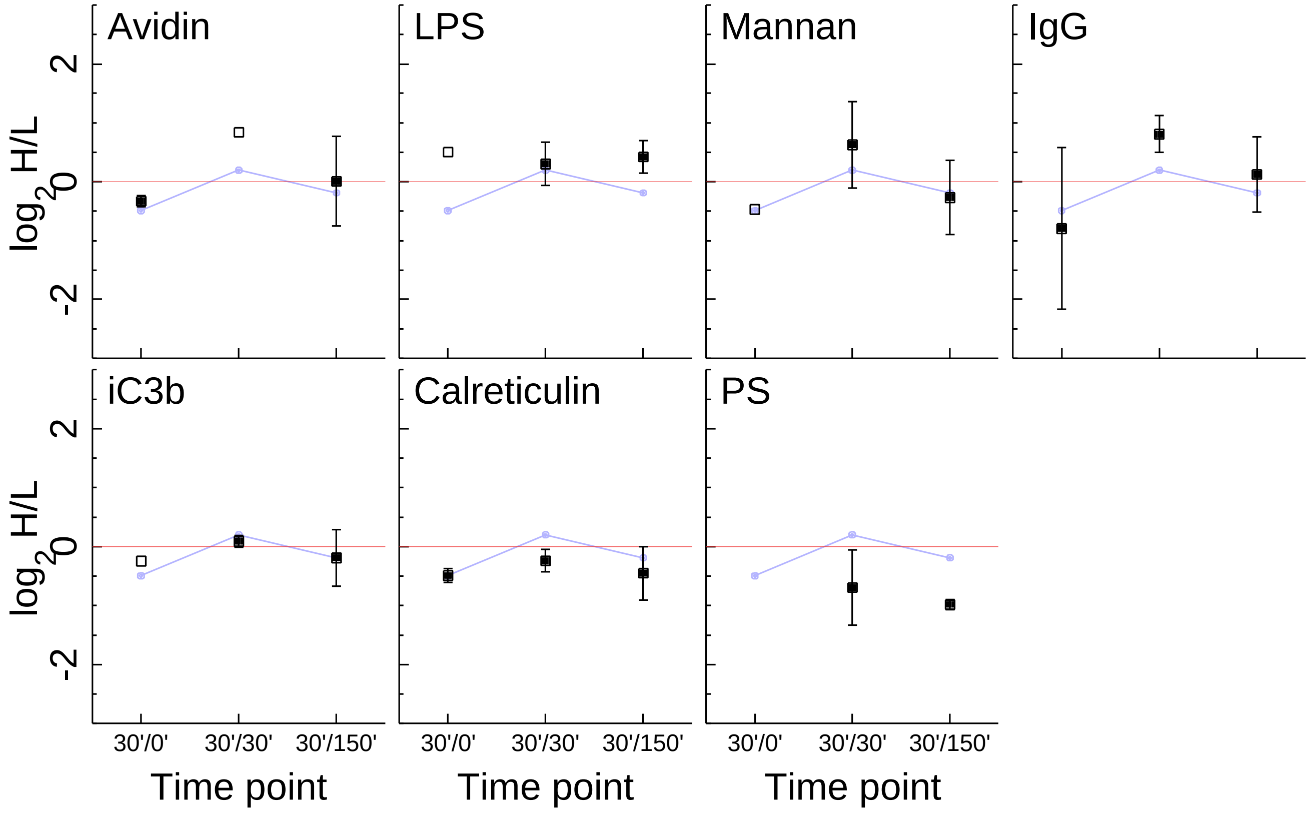

Avidin
LPS
Mannan
IgG
iC3b
Calreticulin
PS
log2 H/L
2
0
-2
30'/0'
30'/30'
30'/150'
Time point

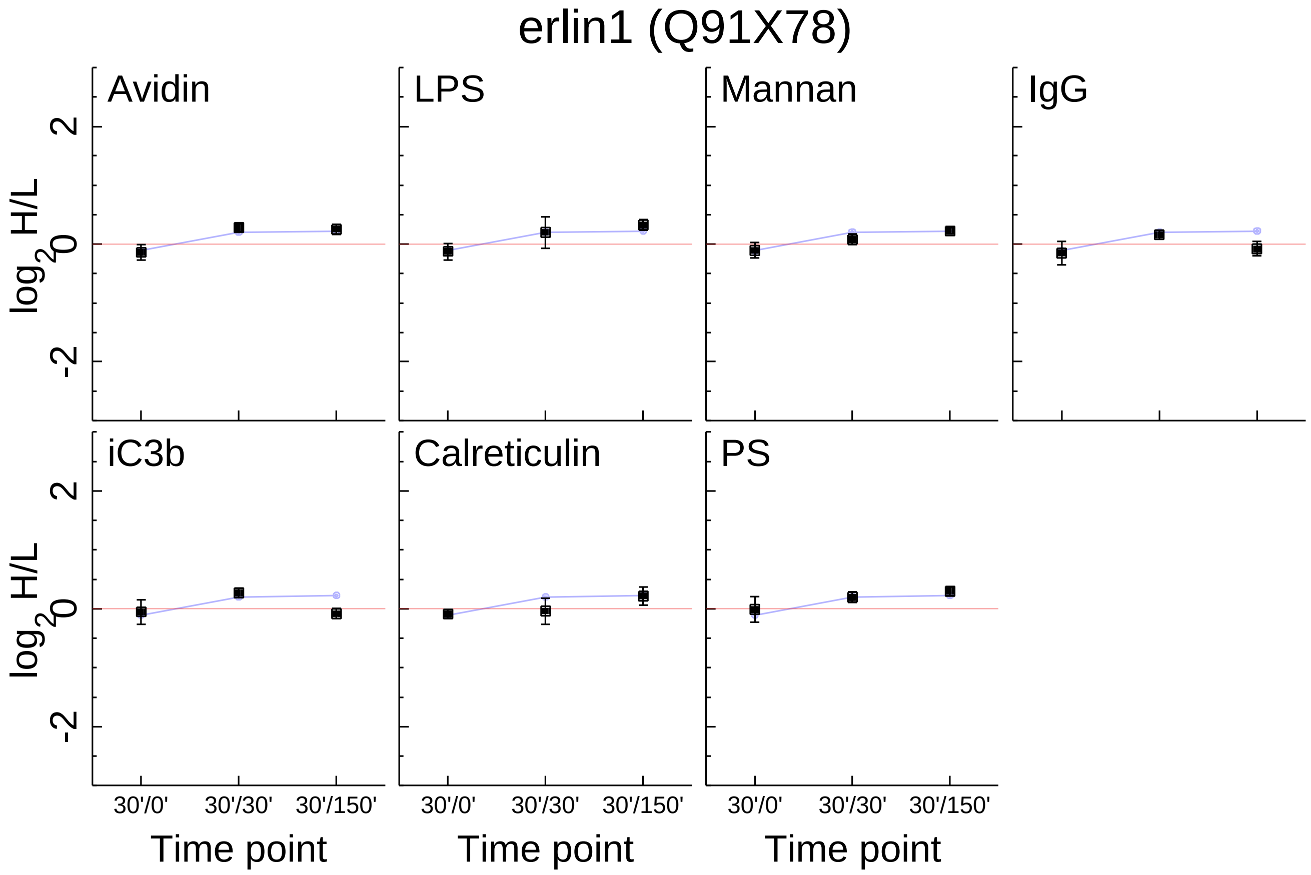

erlin1 (Q91X78)
Avidin
LPS
Mannan
IgG
iC3b
Calreticulin
PS
log2 H/L
2
0
-2
30'/0'
30'/30'
30'/150'
Time point

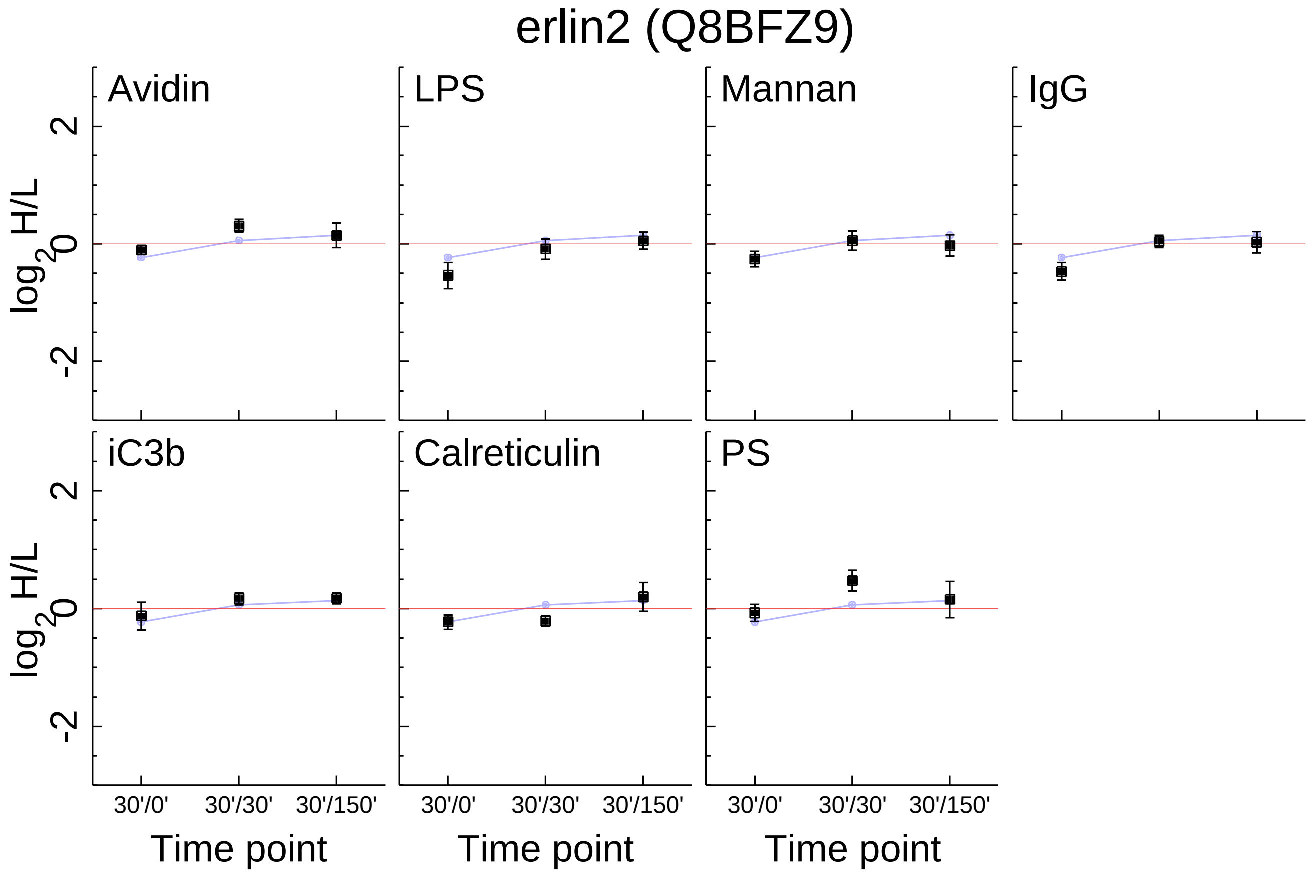
erlin2 (Q8BFZ9)
Avidin
LPS
Mannan
IgG
iC3b
Calreticulin
PS
log2 H/L
2
0
-2
30'/0'
30'/30'
30'/150'
Time point

erp29 (P57759)

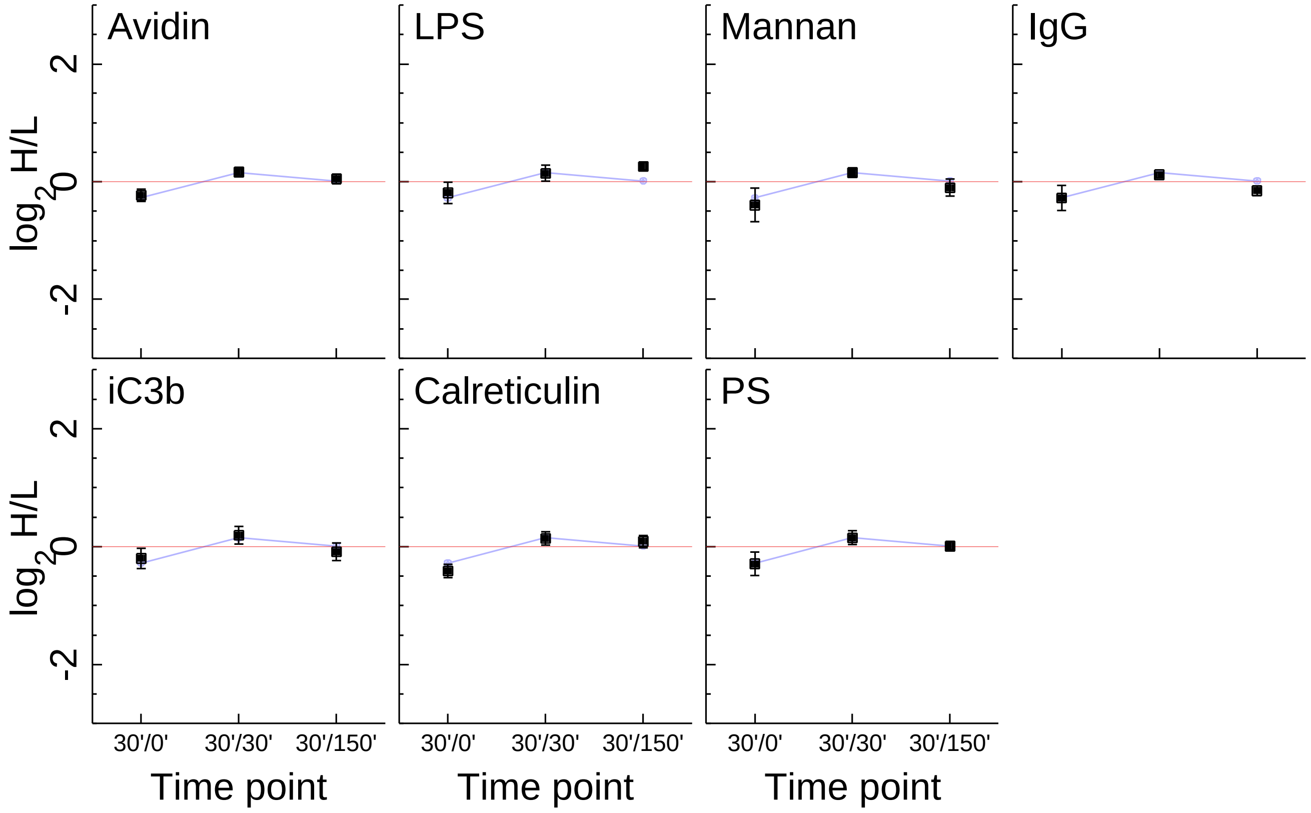

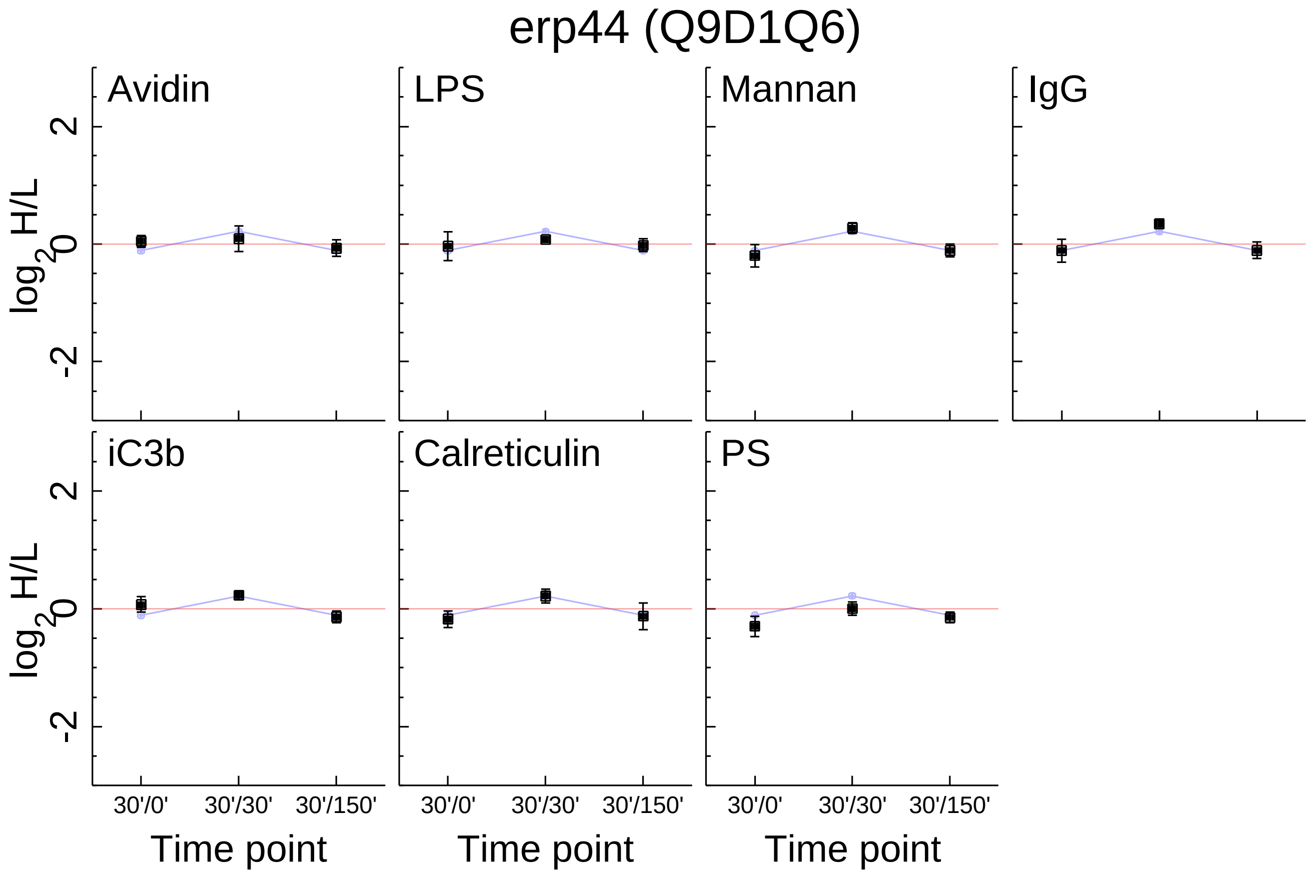

erp44 (Q9D1Q6)
Avidin
LPS
Mannan
IgG
iC3b
Calreticulin
PS
log2 H/L
2
0
-2
30'/0'
30'/30'
30'/150'
Time point

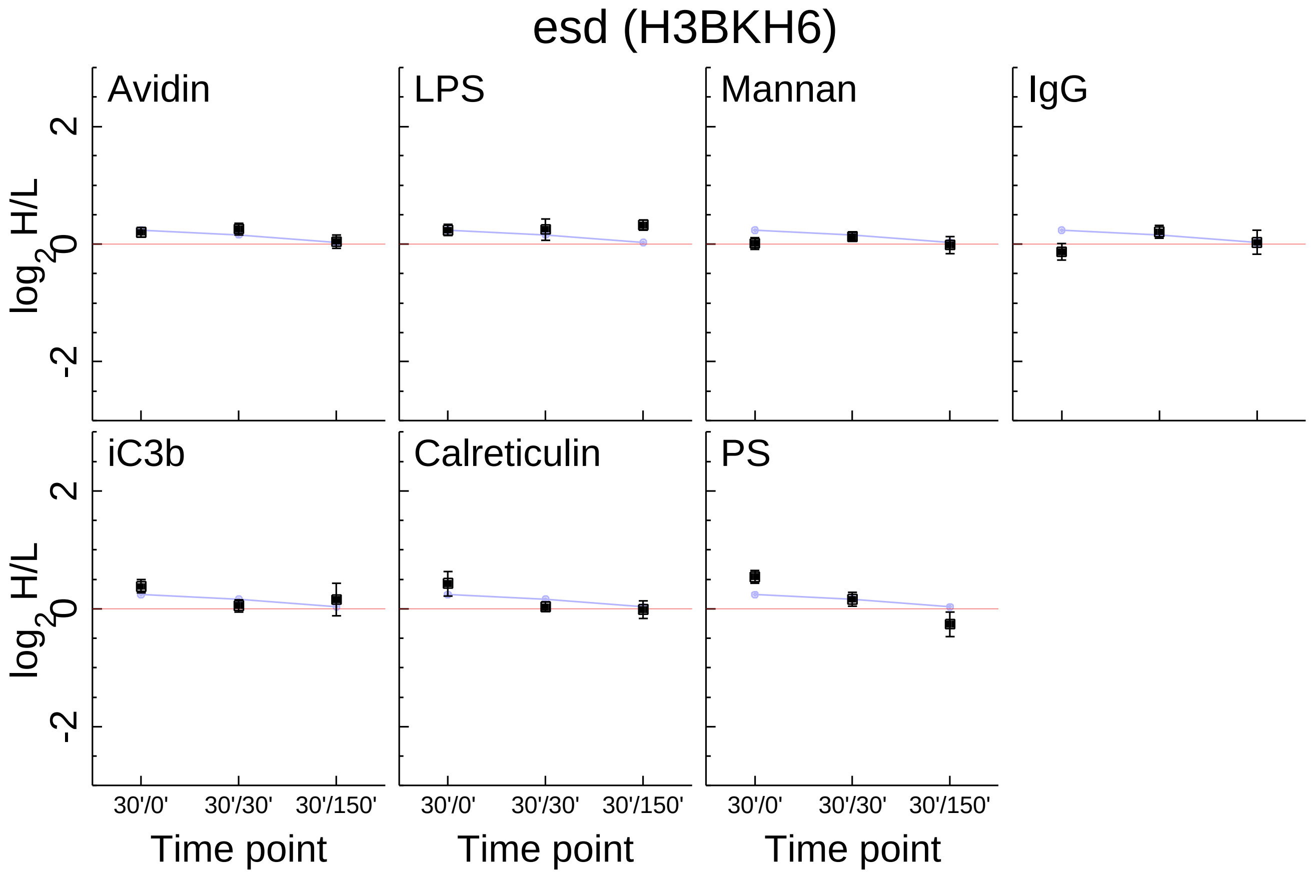
esd (H3BKH6)
Avidin
LPS
Mannan
IgG
iC3b
Calreticulin
PS
log2 H/L
2
0
-2
30'/0'
30'/30'
30'/150'
Time point

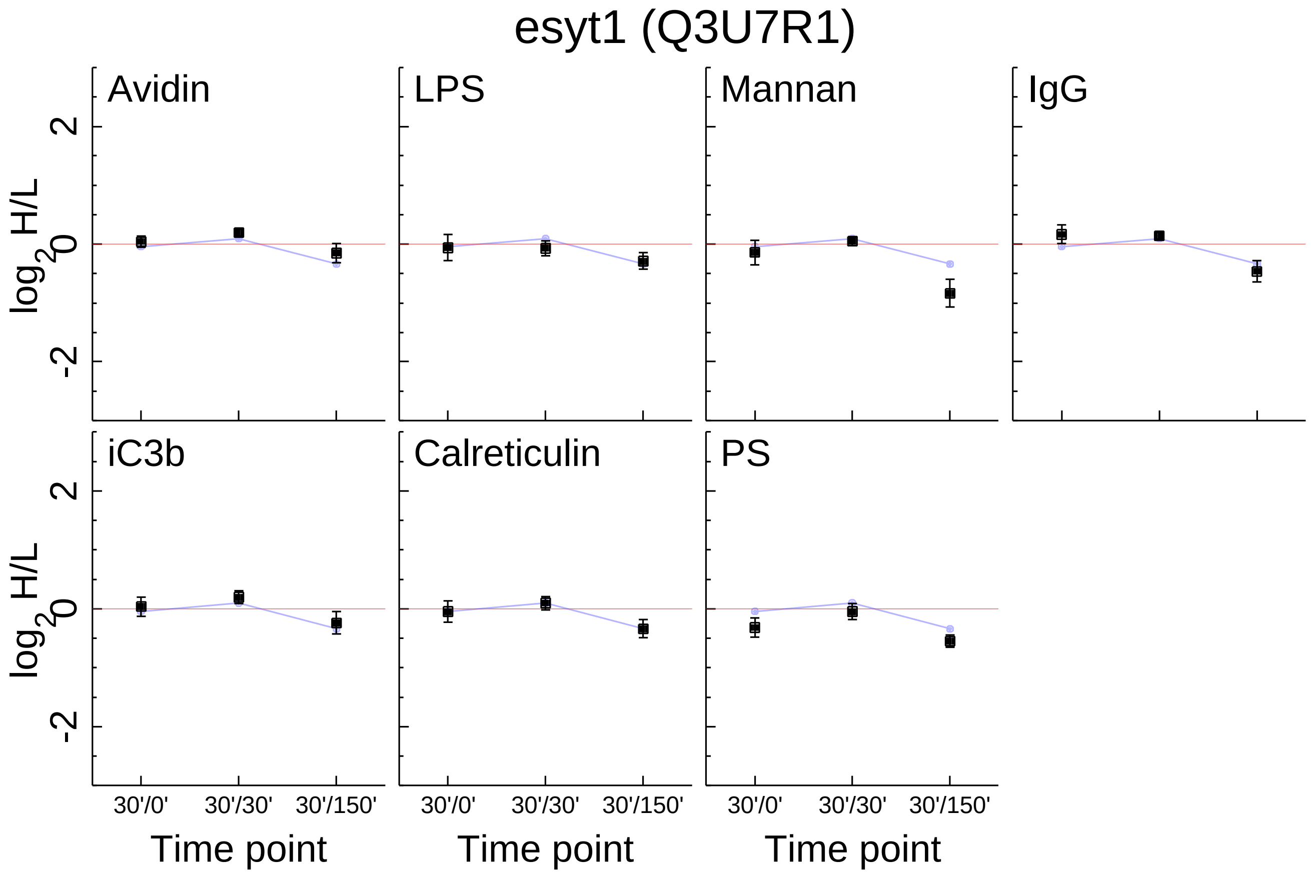
esyt1 (Q3U7R1)
Avidin
LPS
Mannan
IgG
iC3b
Calreticulin
PS
log2 H/L
2
0
-2
30'/0'
30'/30'
30'/150'
Time point

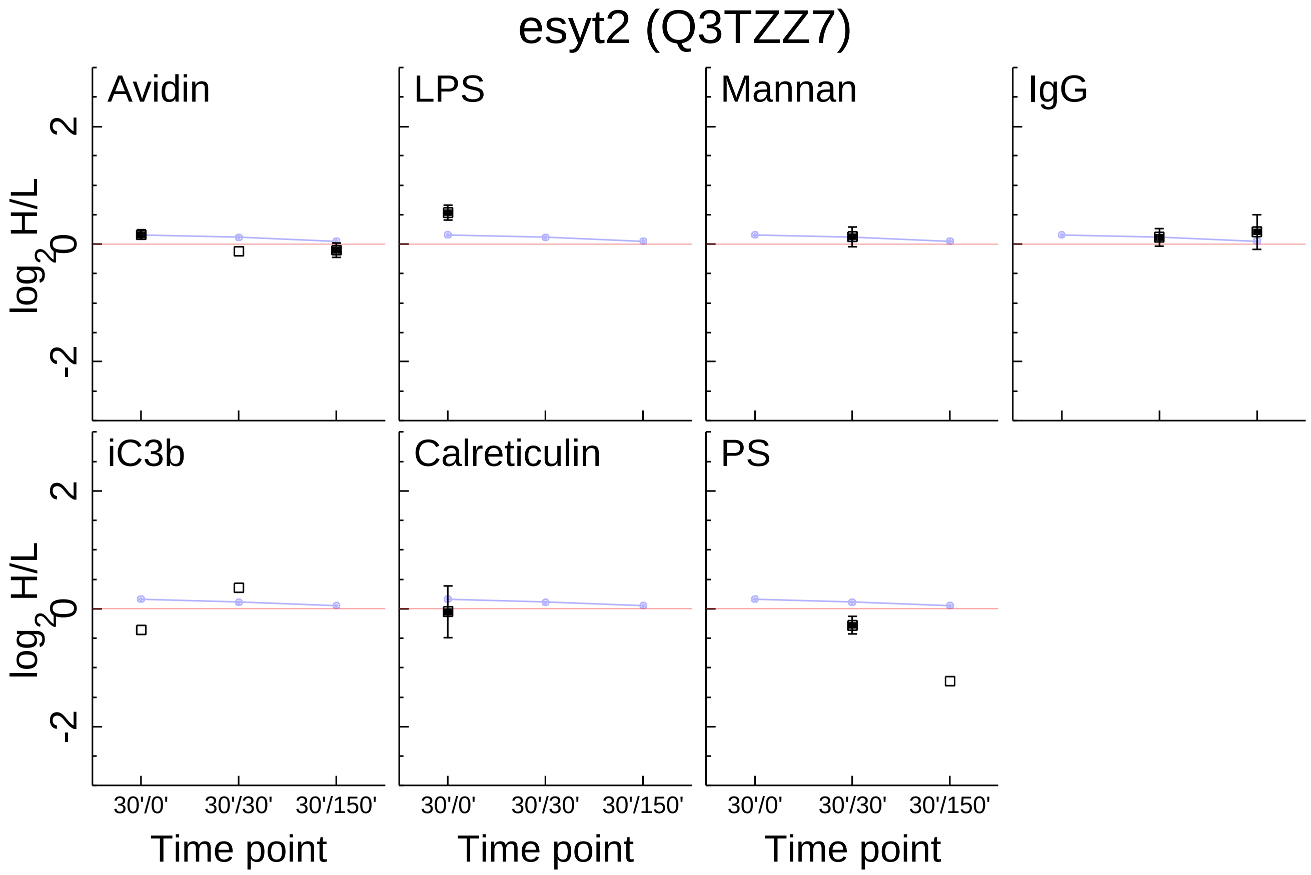

esyt2 (Q3TZZ7)
Avidin
LPS
Mannan
IgG
iC3b
Calreticulin
PS
log2 H/L
2
0
-2
30'/0'
30'/30'
30'/150'
Time point

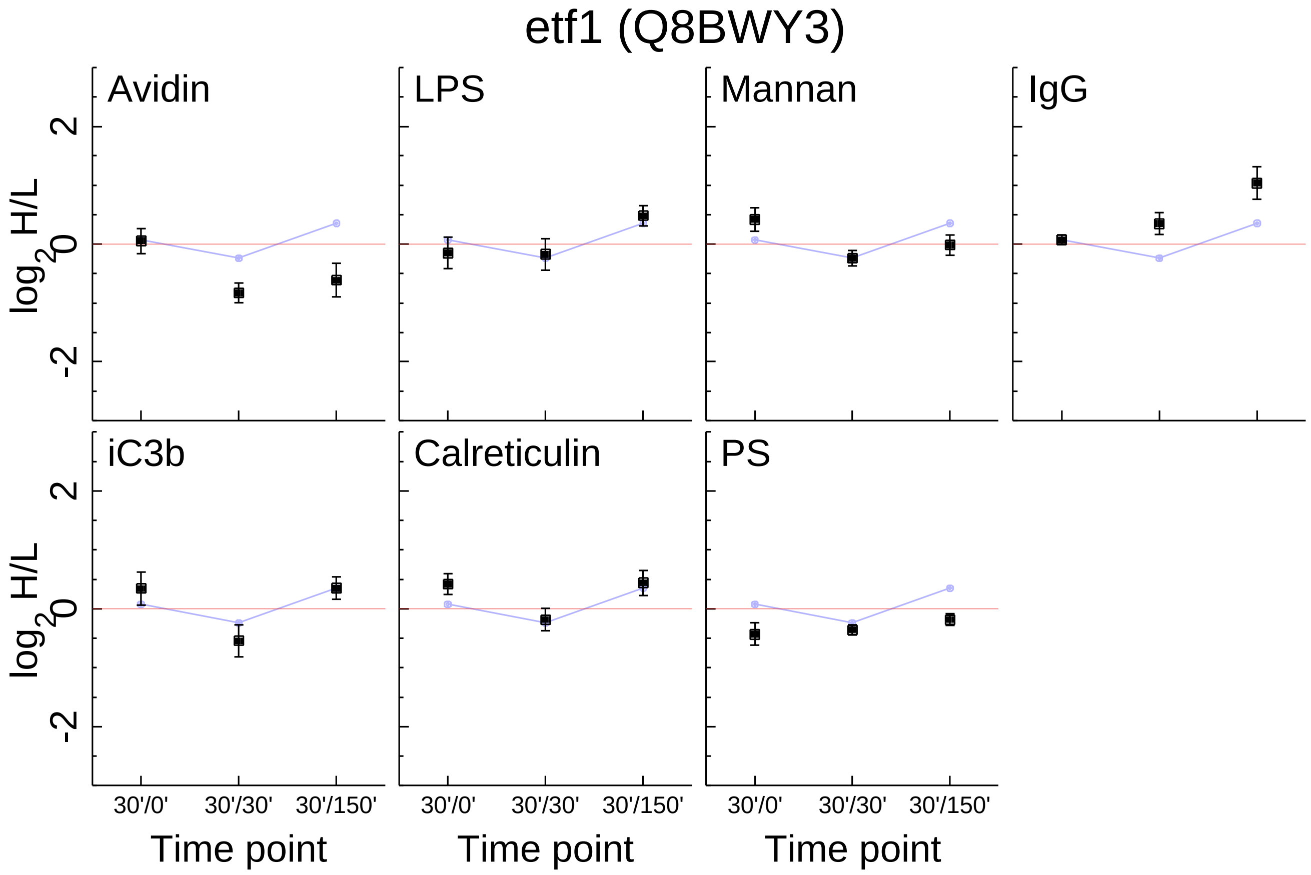

etf1 (Q8BWY3)
Avidin
LPS
Mannan
IgG
iC3b
Calreticulin
PS
log2 H/L
2
0
-2
30'/0'
30'/30'
30'/150'
Time point

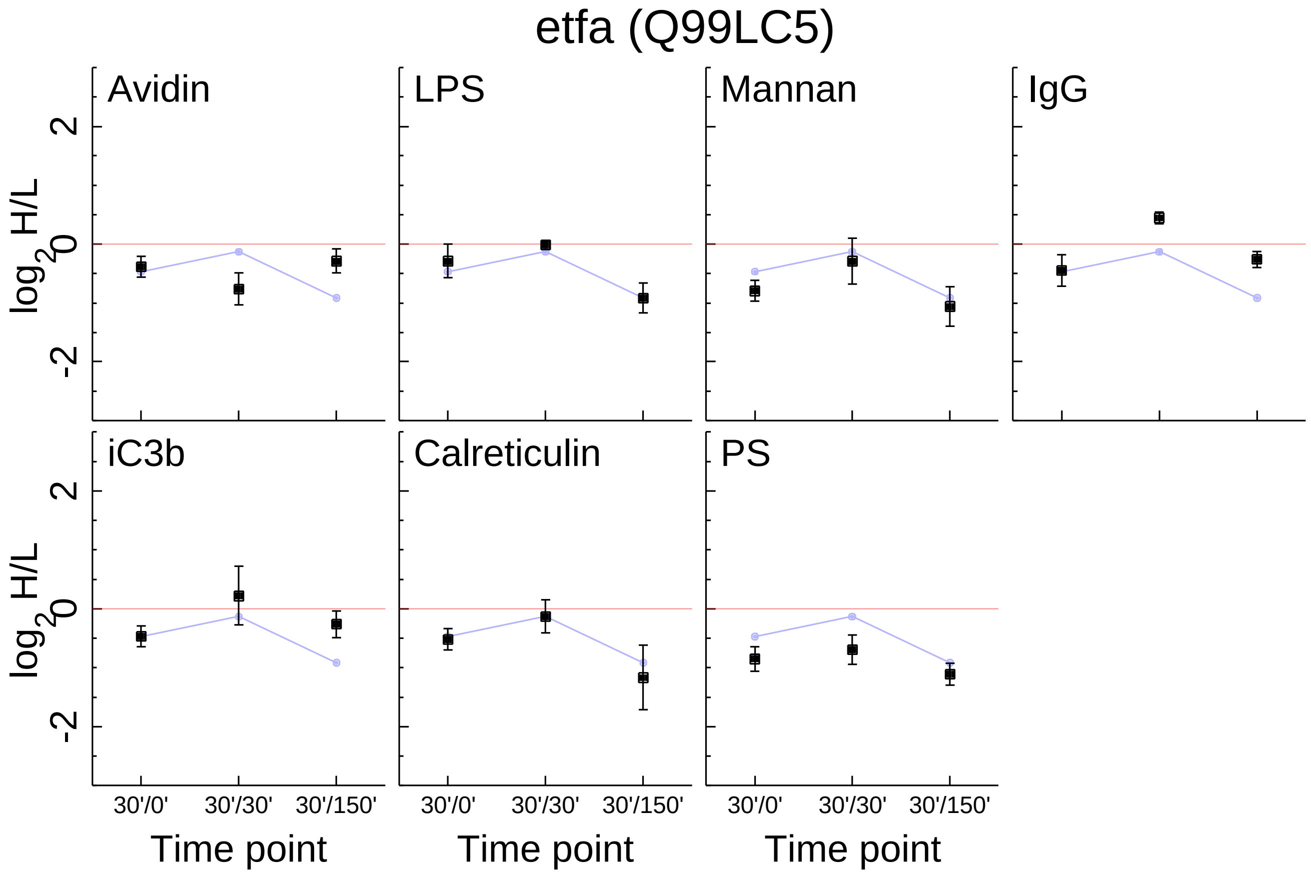
etfa (Q99LC5)
Avidin
LPS
Mannan
IgG
iC3b
Calreticulin
PS
log2 H/L
2
0
-2
30'/0'
30'/30'
30'/150'
Time point

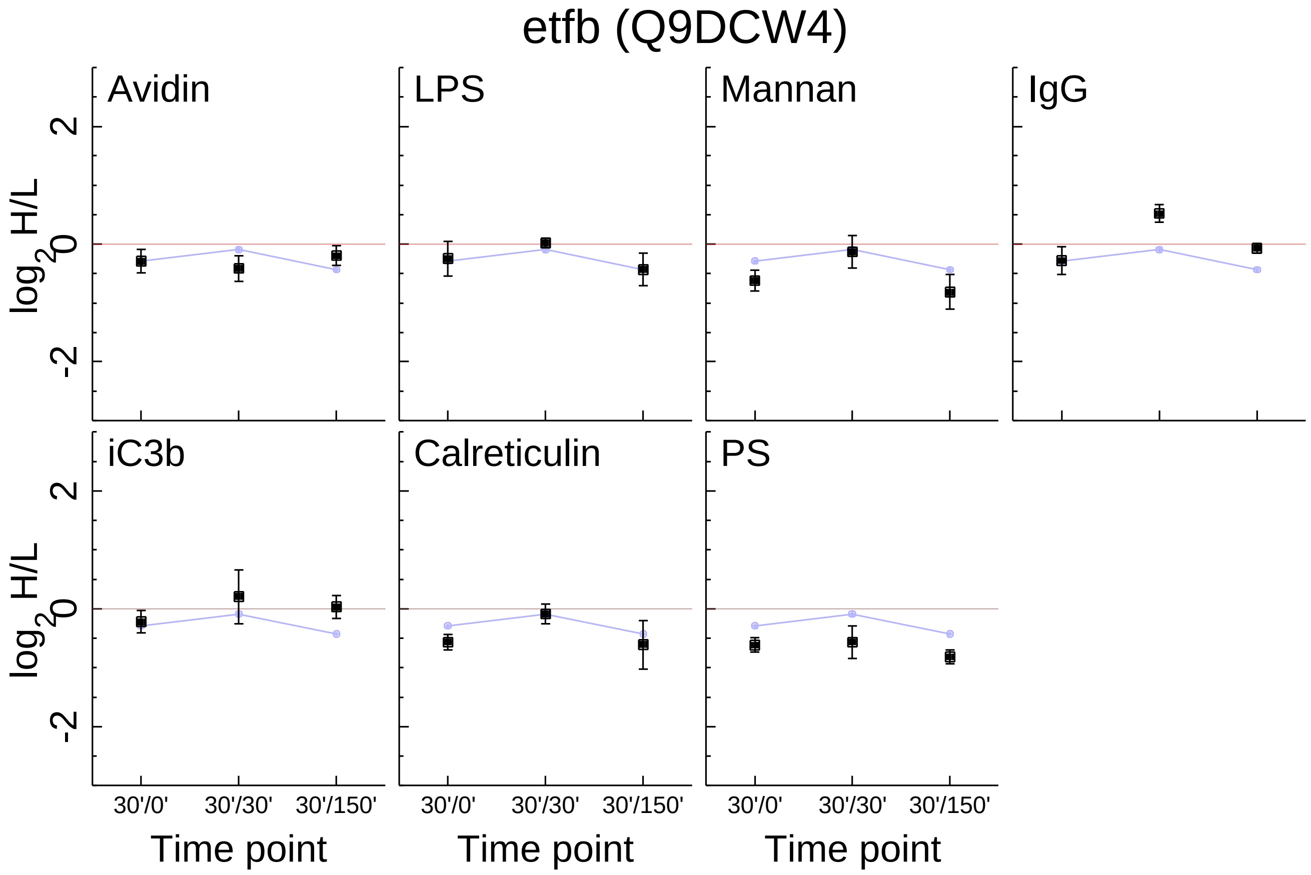
etfb (Q9DCW4)
Avidin
LPS
Mannan
IgG
iC3b
Calreticulin
PS
log2 H/L
2
0
-2
30'/0'
30'/30'
30'/150'
Time point

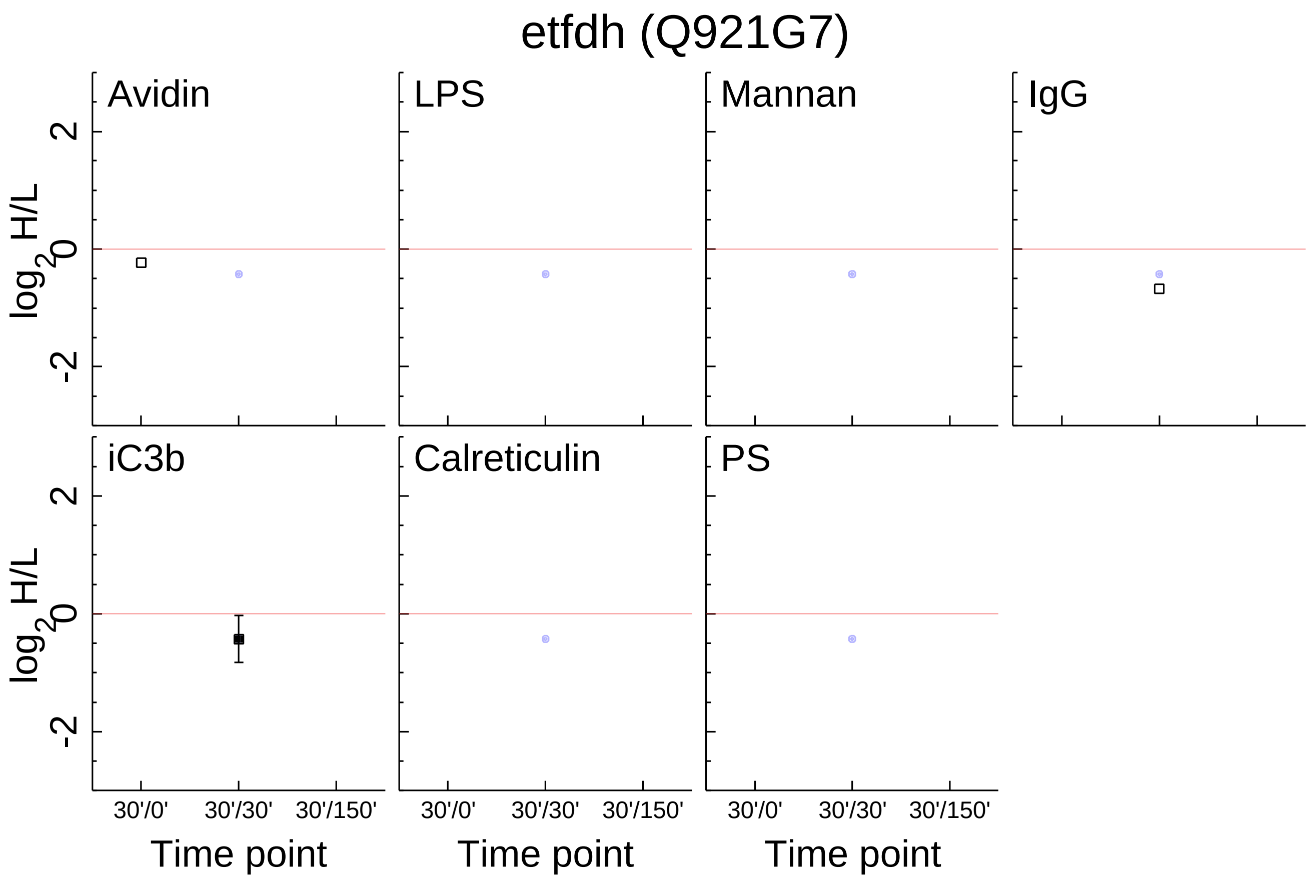
etfdh (Q921G7)
Avidin
LPS
Mannan
IgG
iC3b
Calreticulin
PS
log2 H/L
2
0
-2
30'/0'
30'/30'
30'/150'
Time point

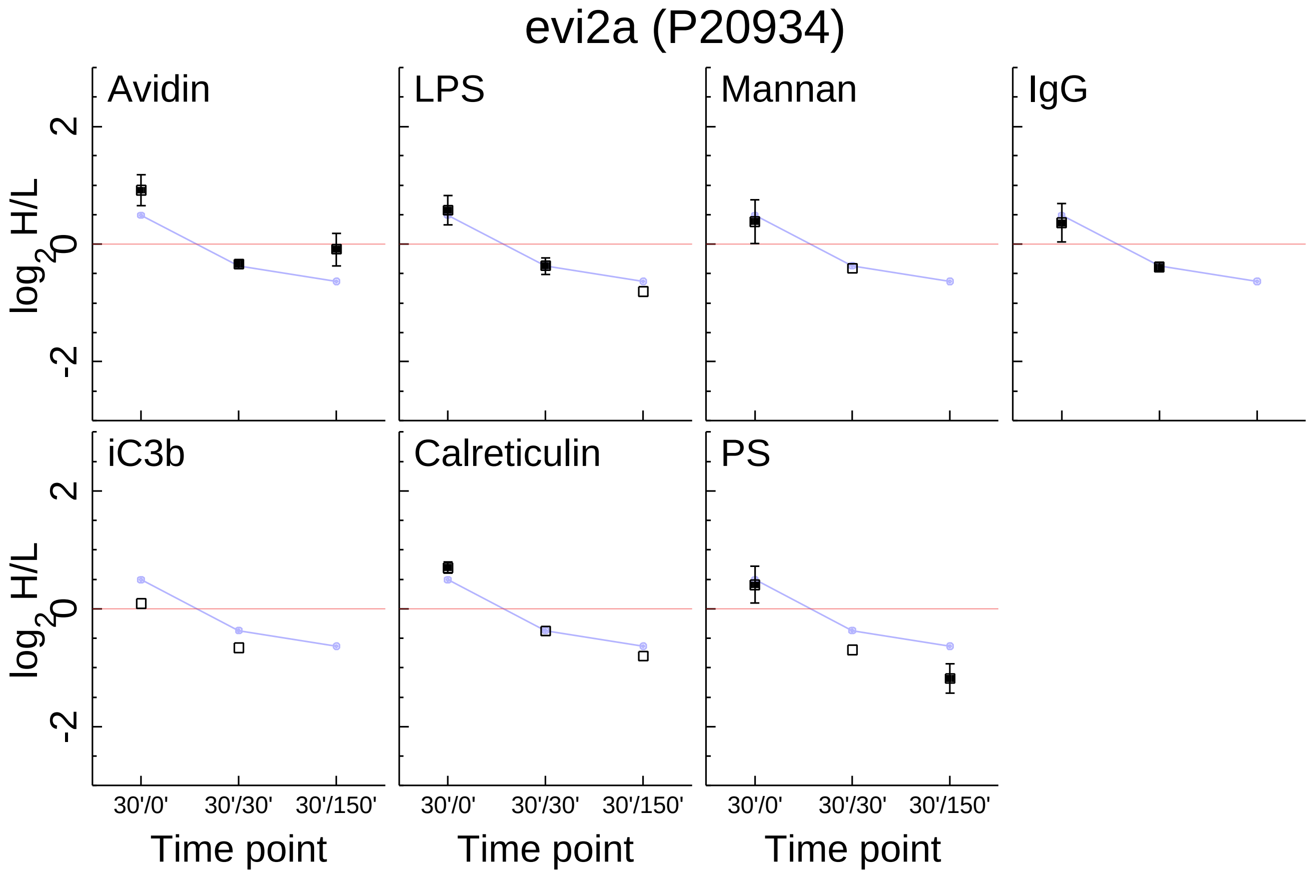

evi2a (P20934)
Avidin
LPS
Mannan
IgG
iC3b
Calreticulin
PS
log2 H/L
2
0
-2
30'/0'
30'/30'
30'/150'
Time point

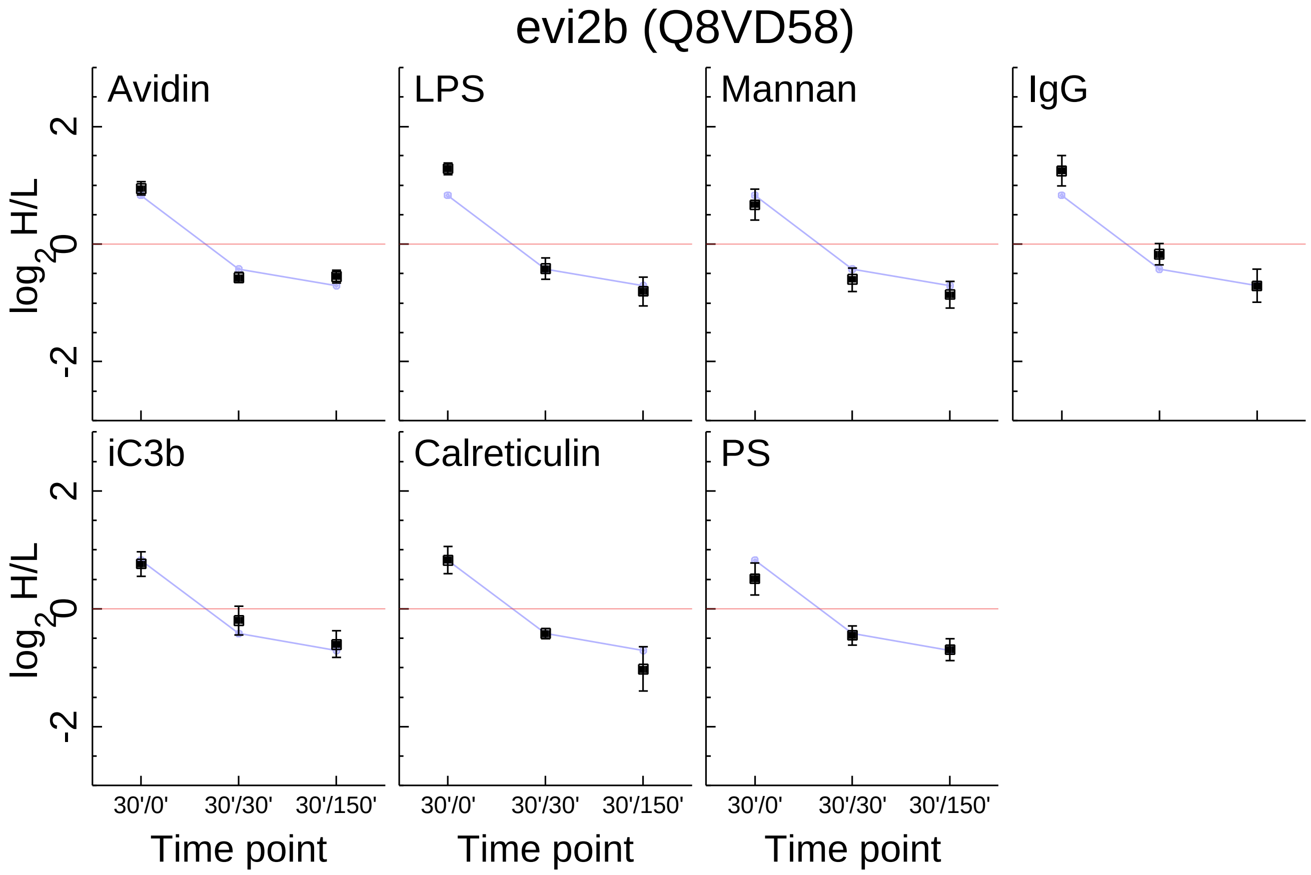

evi2b (Q8VD58)
Avidin
LPS
Mannan
IgG
iC3b
Calreticulin
PS
2
0
-2
log2 H/L
30'/0'
30'/30'
30'/150'
Time point

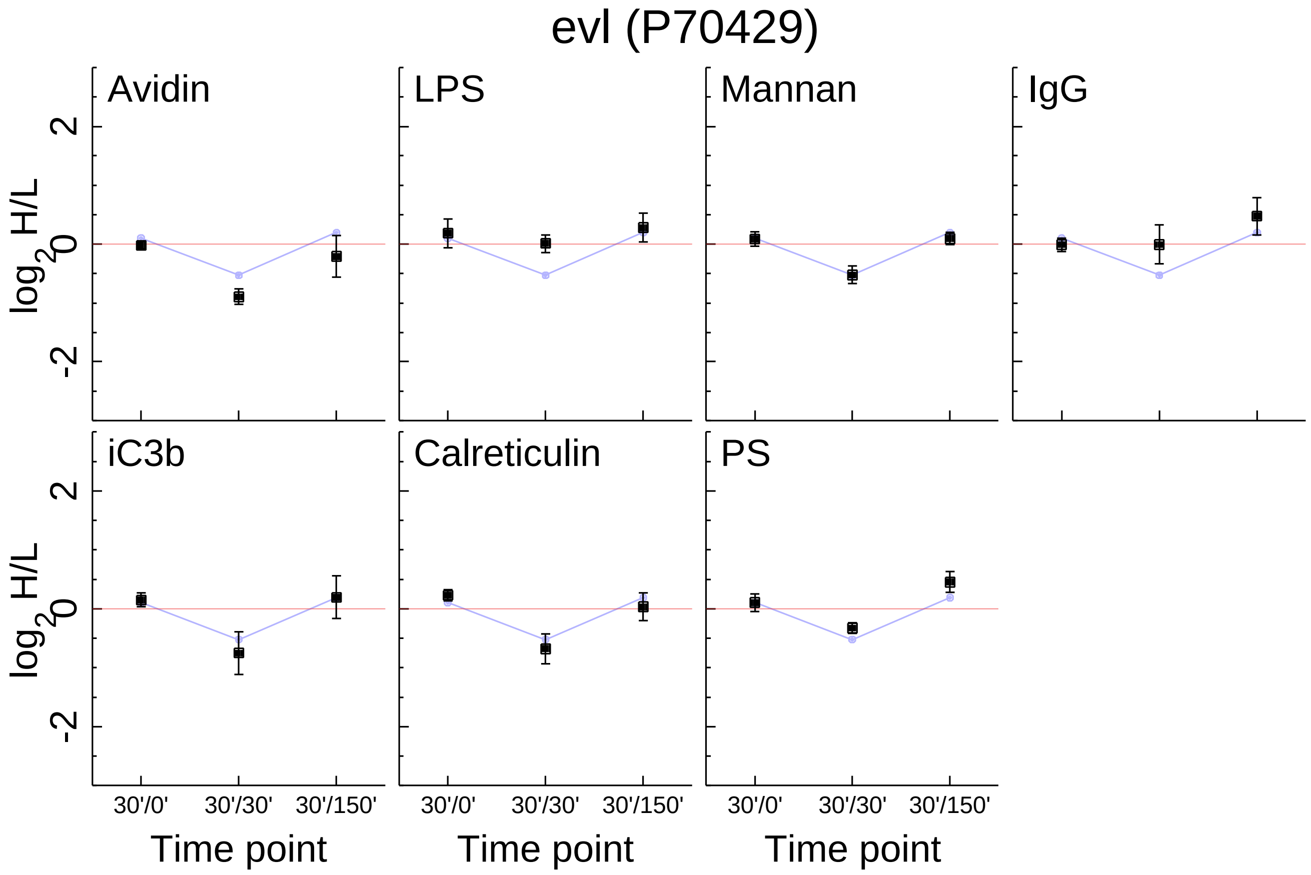

evl (P70429)
Avidin
LPS
Mannan
IgG
iC3b
Calreticulin
PS
2
0
-2
log2 H/L
30'/0'
30'/30'
30'/150'
Time point

ewsr1 (Q5SUS9)

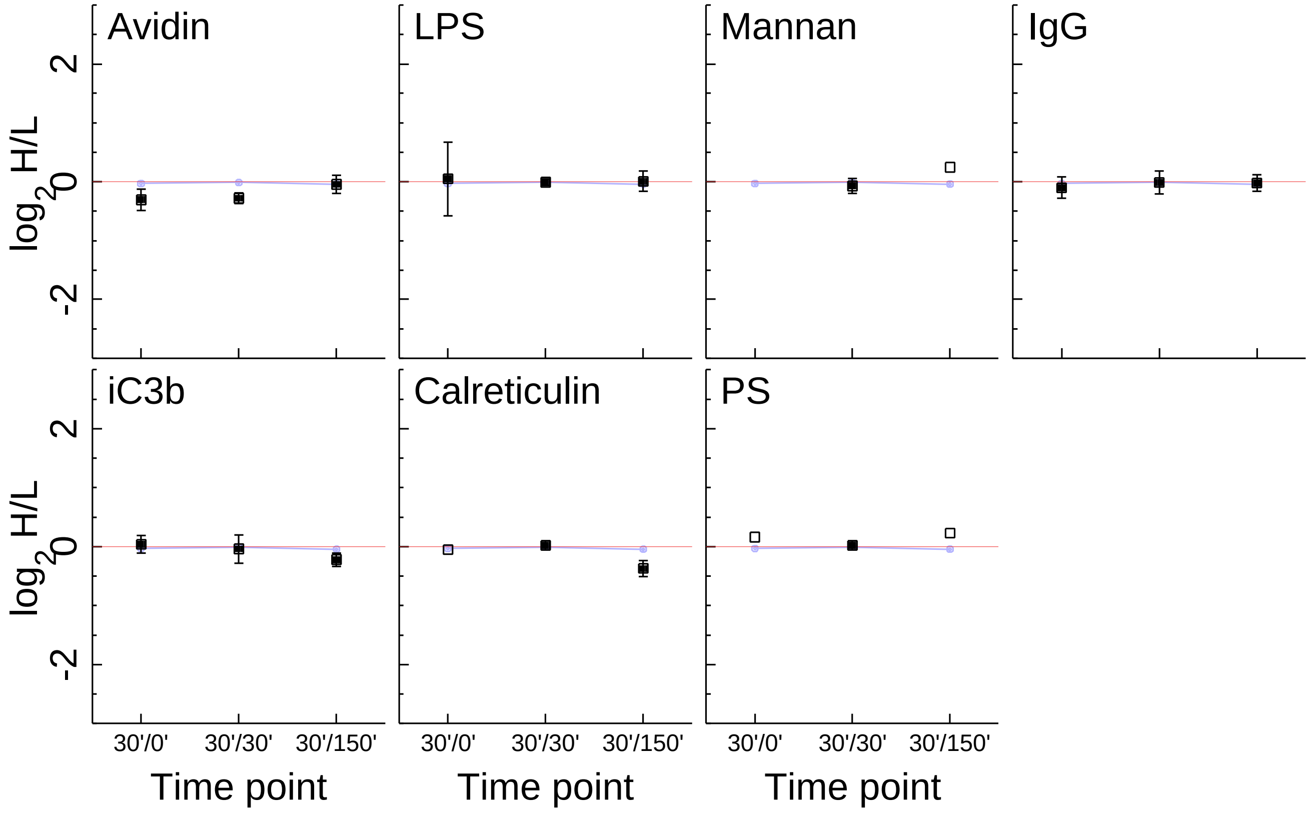

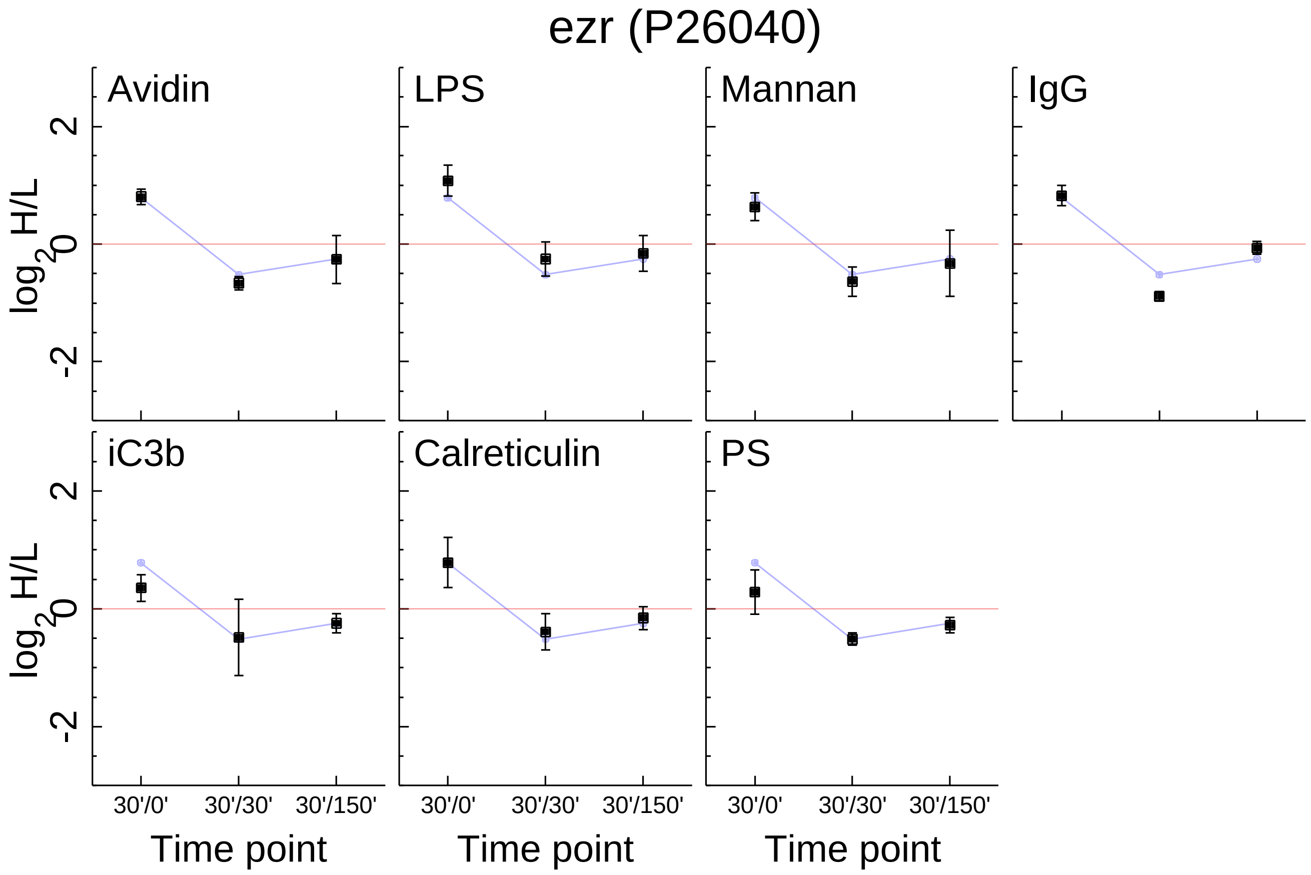

ezr (P26040)
Avidin
LPS
Mannan
IgG
iC3b
Calreticulin
PS
log2 H/L
2
0
-2
30'/0'
30'/30'
30'/150'
Time point

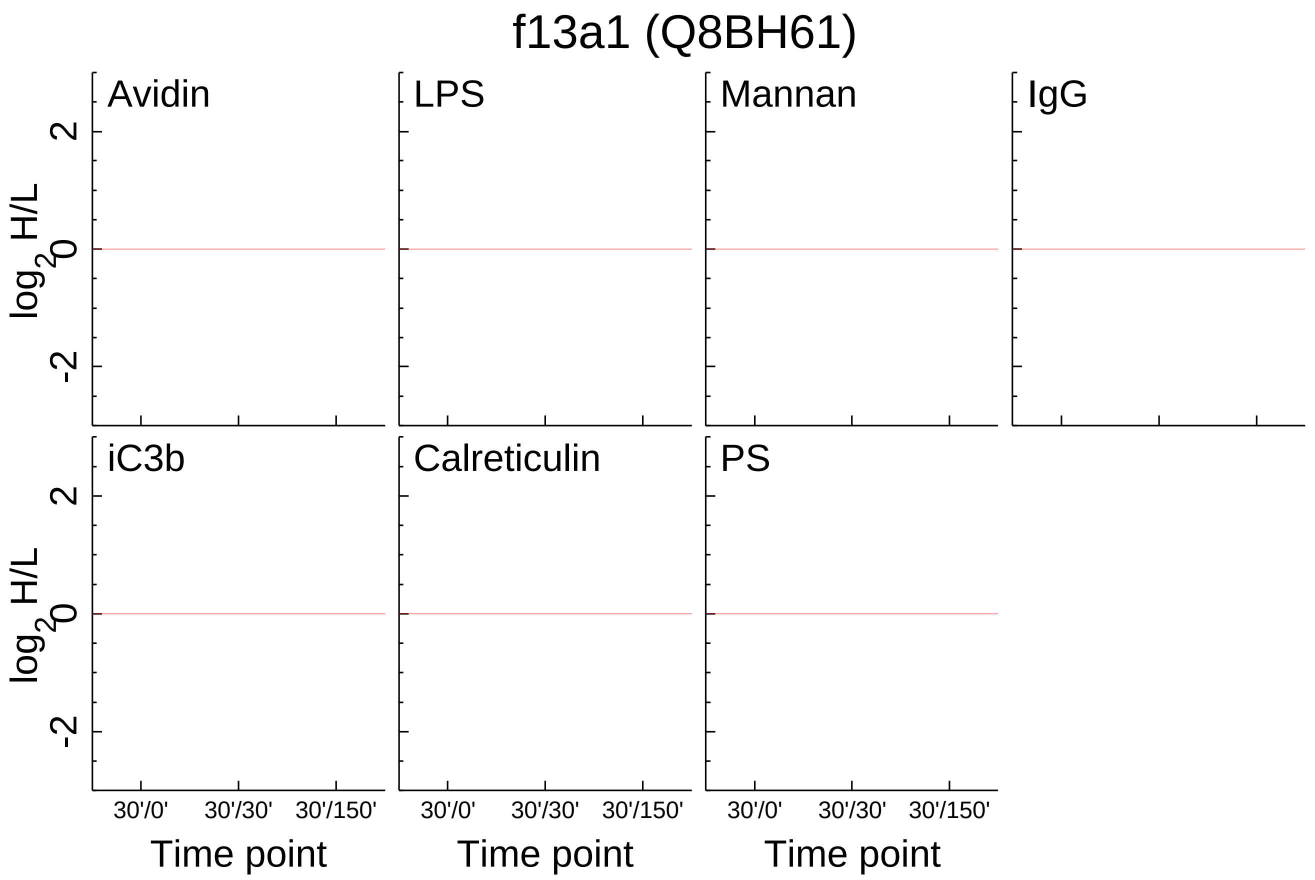
f13a1 (Q8BH61)
Avidin
LPS
Mannan
IgG
iC3b
Calreticulin
PS
log2 H/L
2
0
-2
30'/0'
30'/30'
30'/150'
Time point

faf2 (Q3TDN2)

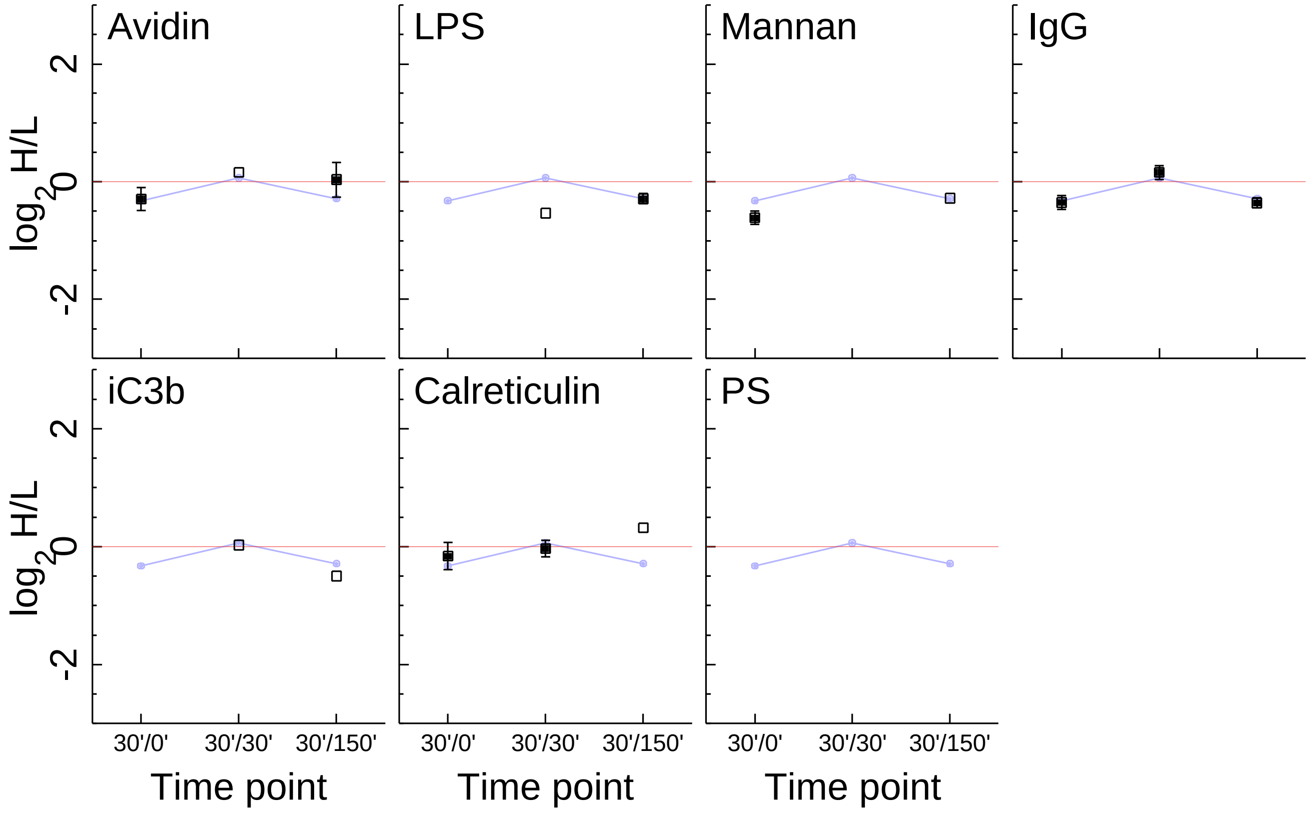
Avidin
LPS
Mannan
IgG
iC3b
Calreticulin
PS
log2 H/L
2
0
-2
30'/0'
30'/30'
30'/150'
Time point

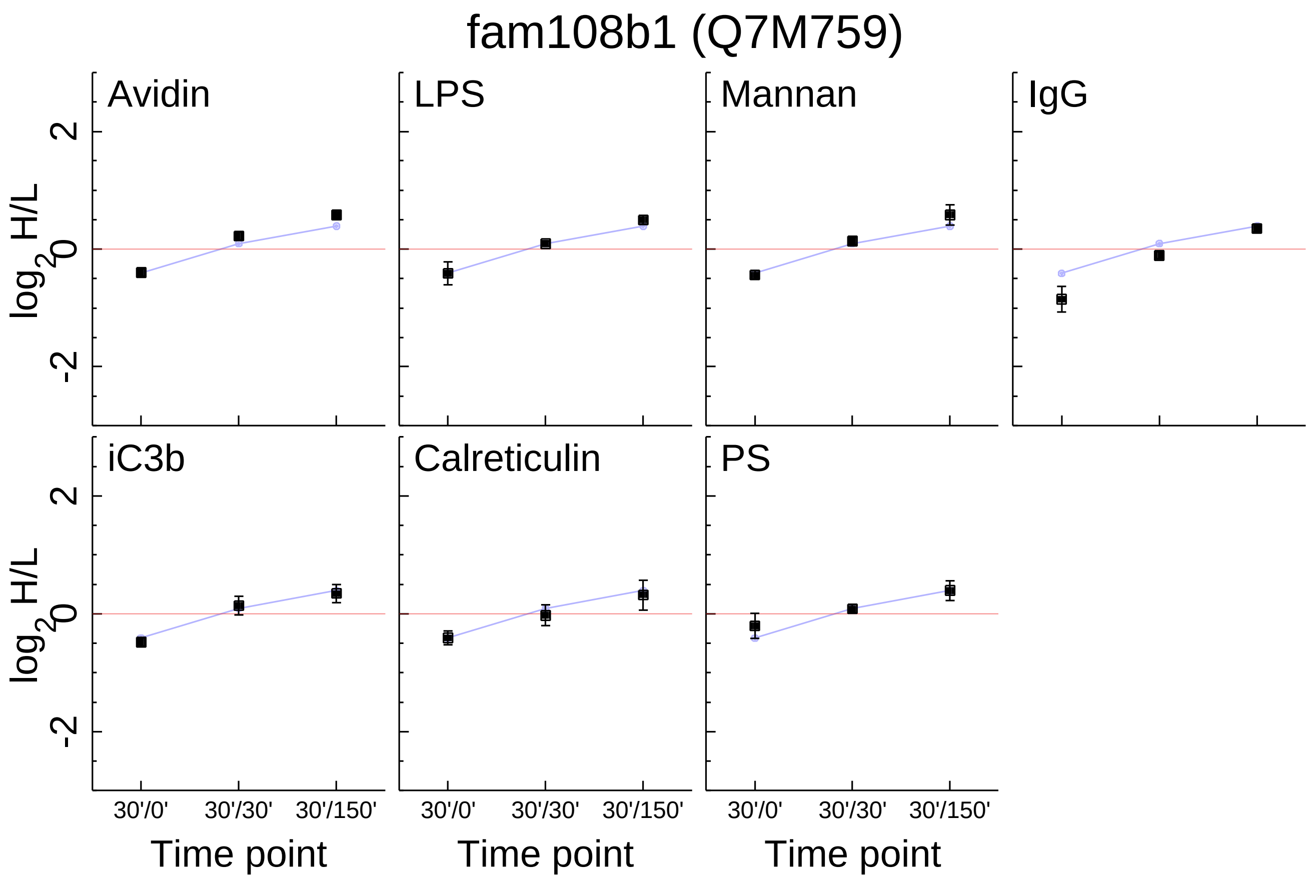
fam108b1 (Q7M759)
Avidin
LPS
Mannan
IgG
iC3b
Calreticulin
PS
log2 H/L
2
0
-2
30'/0'
30'/30'
30'/150'
Time point

fam120a (Q6A0A9)

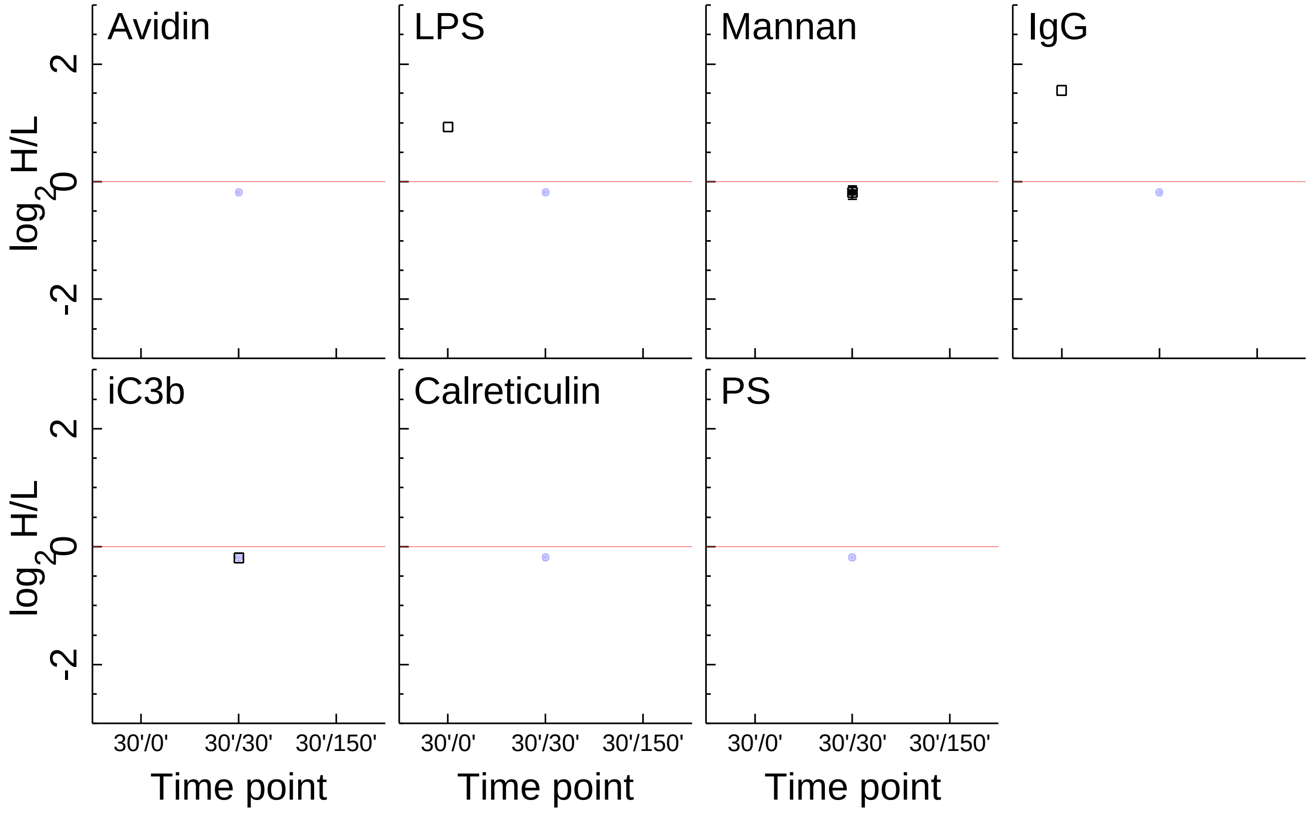

fam125a (Q78HU3)

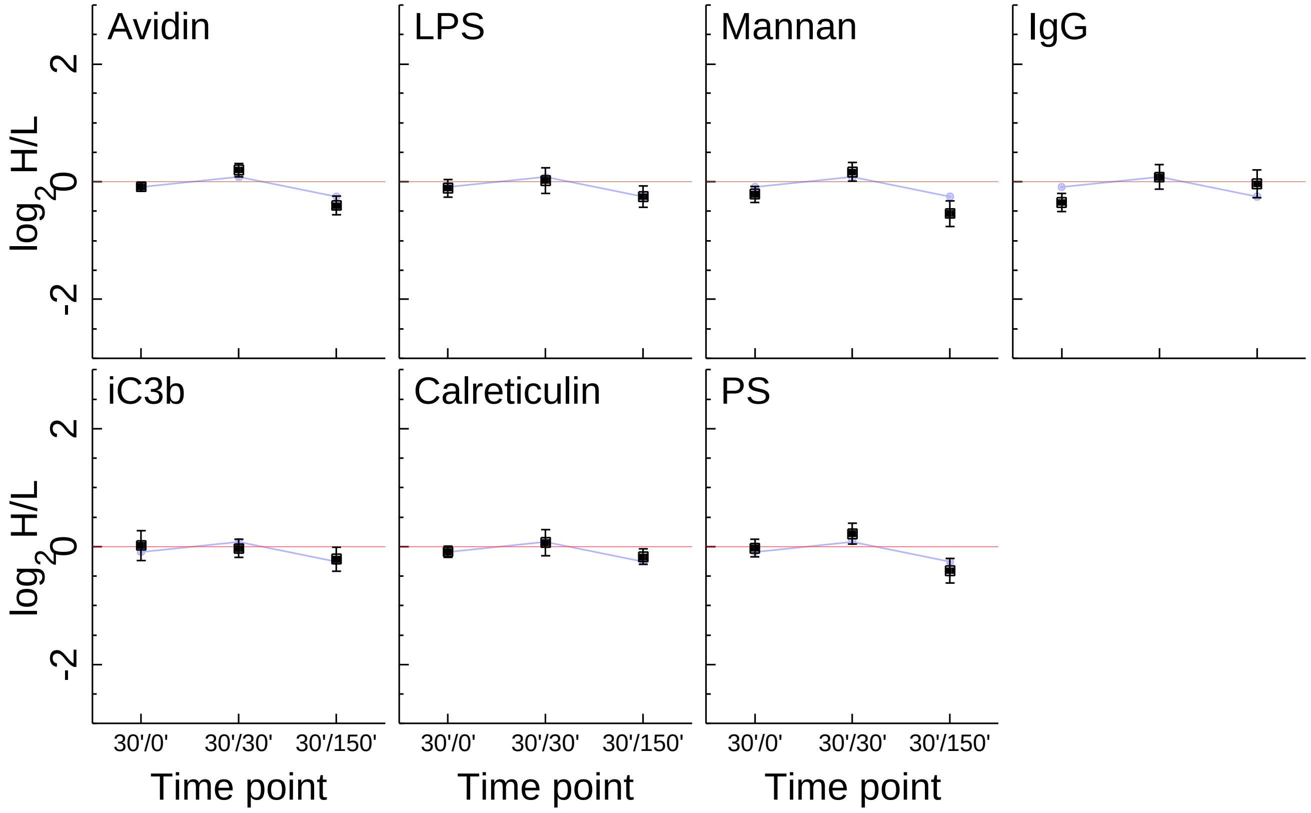

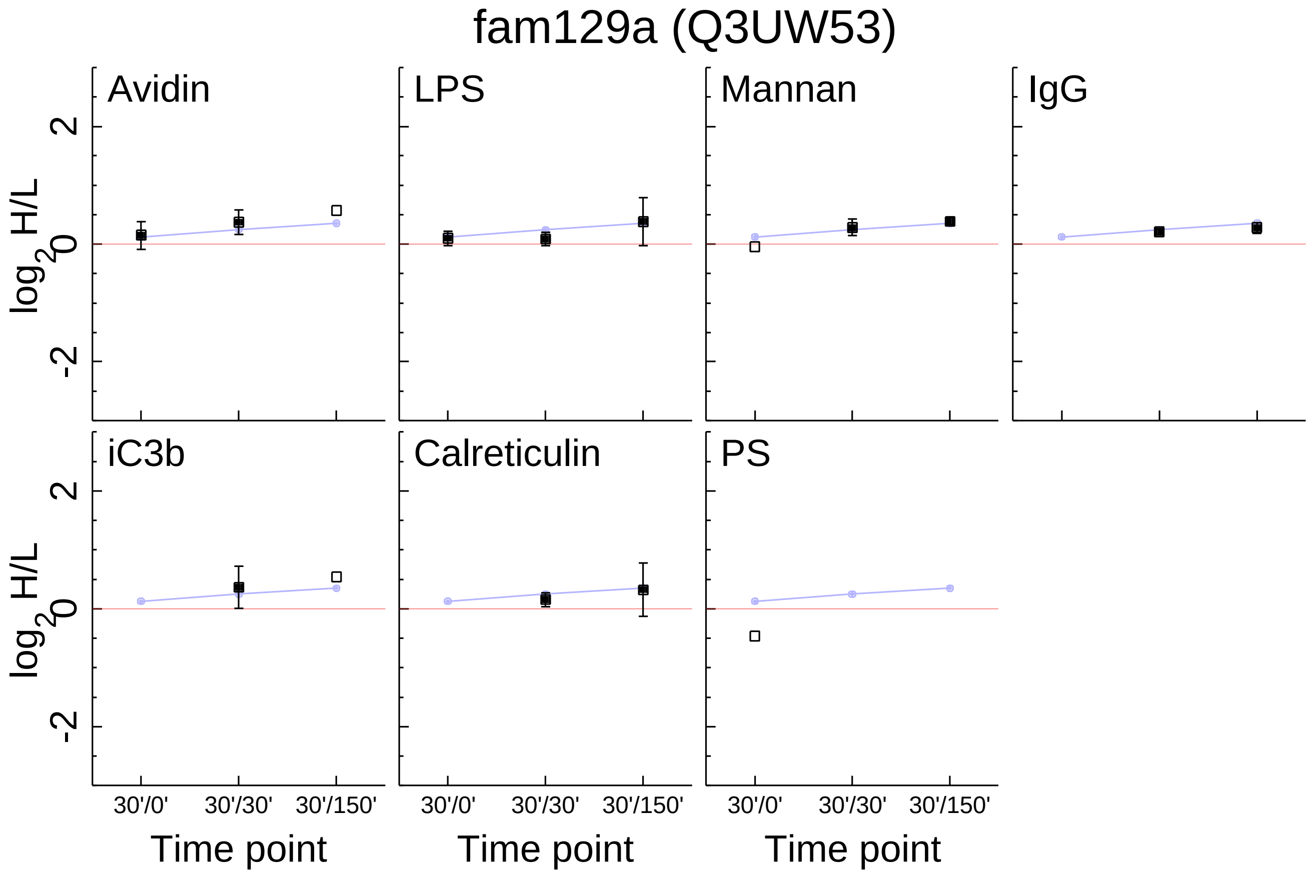
fam129a (Q3UW53)
Avidin
LPS
Mannan
IgG
iC3b
Calreticulin
PS
log2 H/L
2
0
-2
30'/0'
30'/30'
30'/150'
Time point

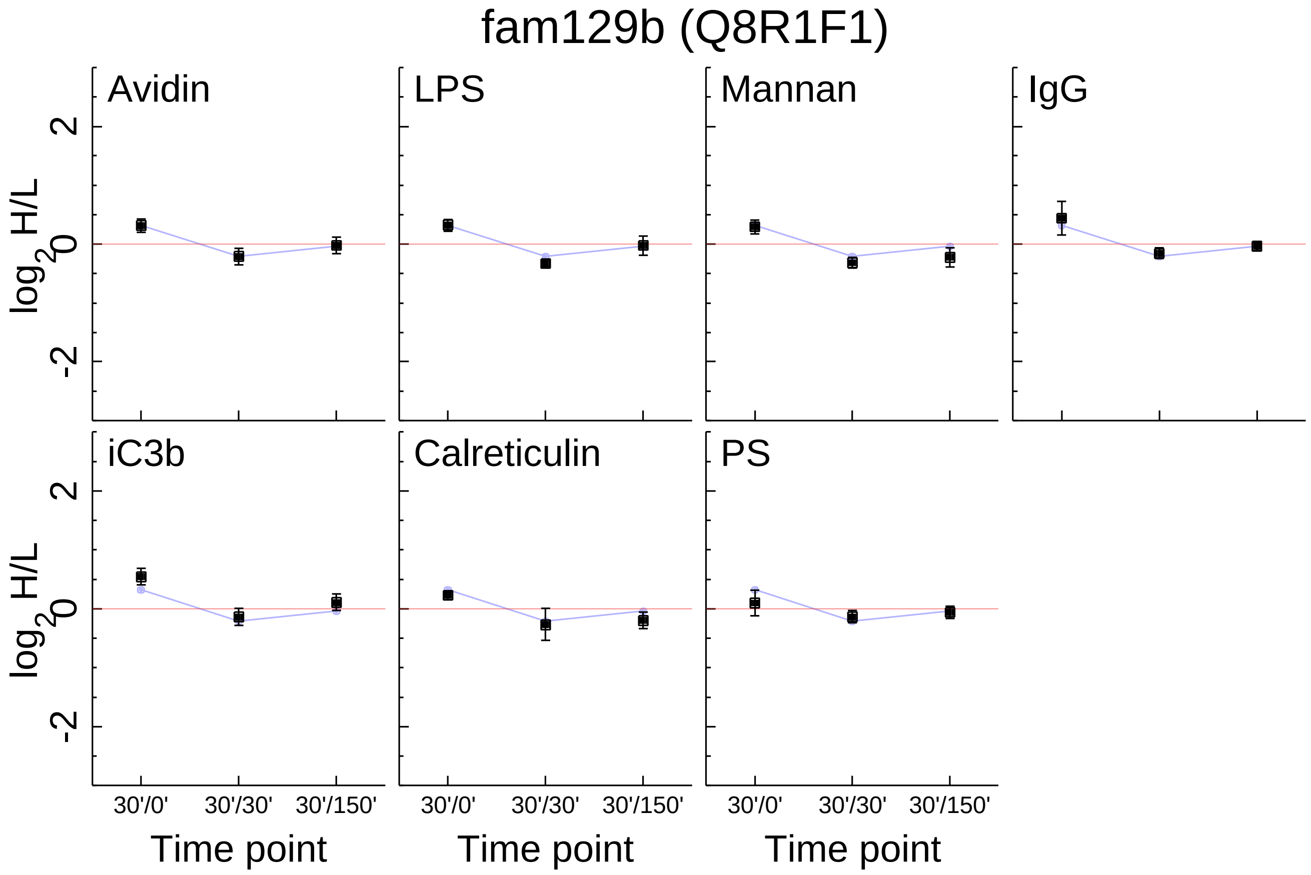

fam129b (Q8R1F1)
Avidin
LPS
Mannan
IgG
iC3b
Calreticulin
PS
log2 H/L
2
0
-2
30'/0'
30'/30'
30'/150'
Time point

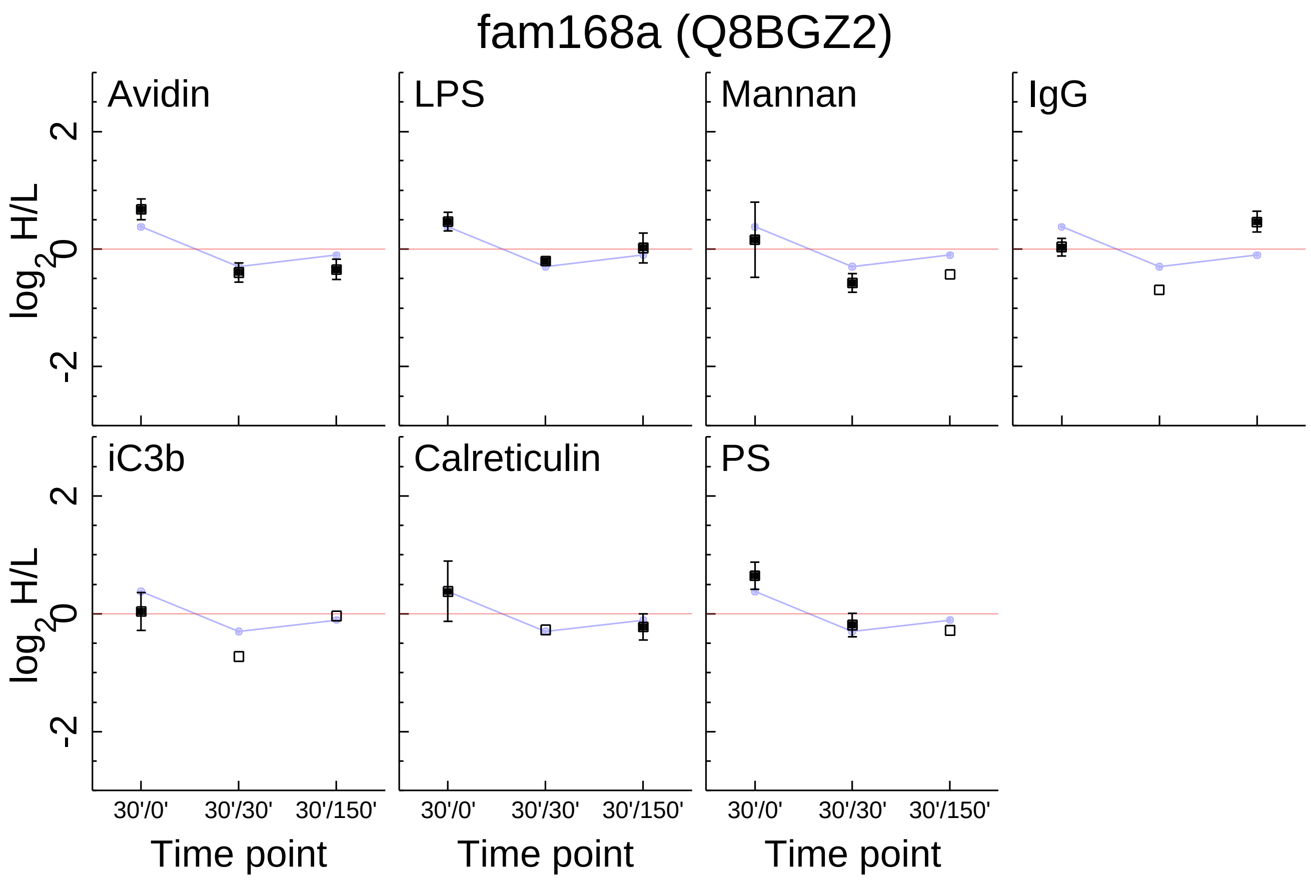
fam168a (Q8BGZ2)
Avidin
LPS
Mannan
IgG
iC3b
Calreticulin
PS
log2 H/L
2
0
-2
30'/0'
30'/30'
30'/150'
Time point

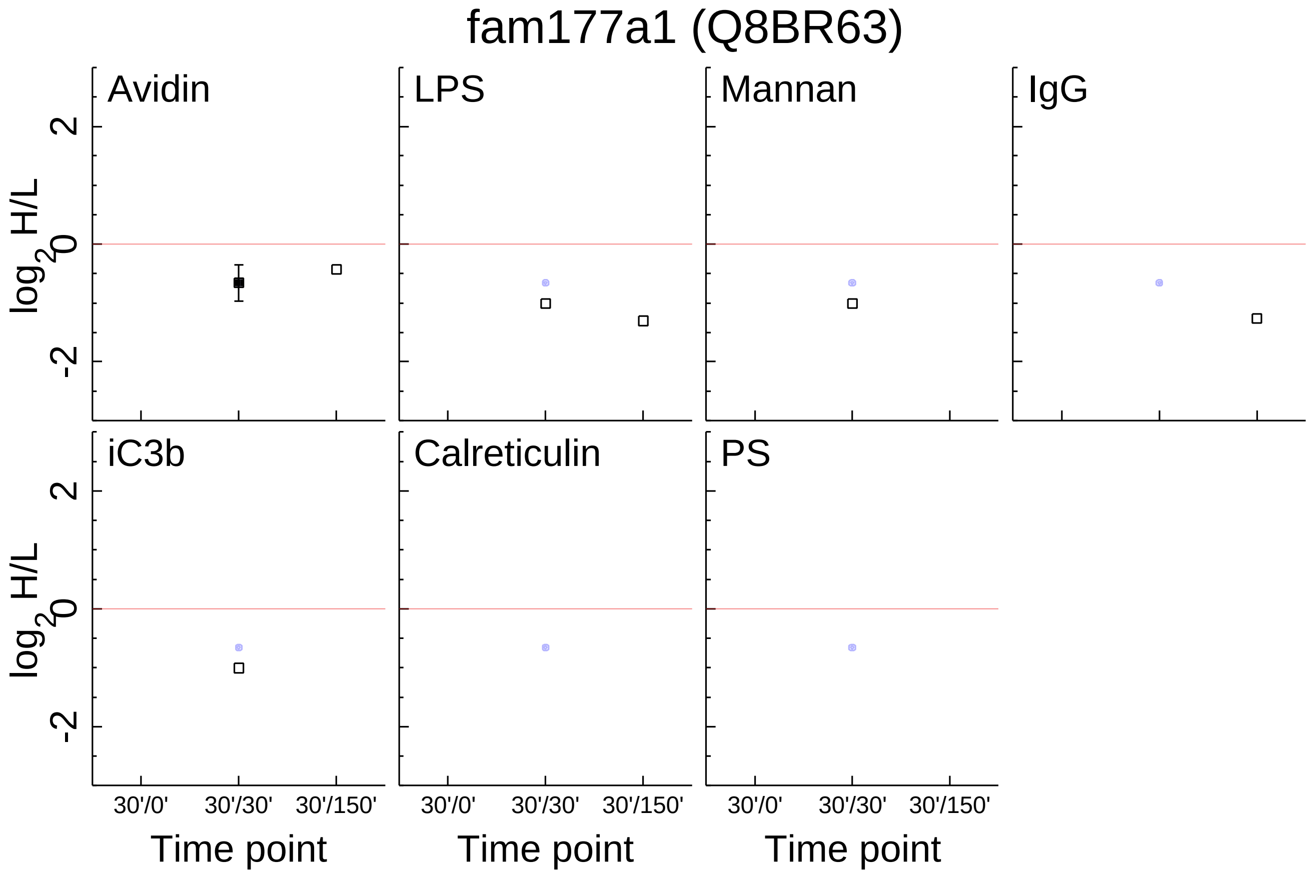
fam177a1 (Q8BR63)
Avidin
LPS
Mannan
IgG
iC3b
Calreticulin
PS
log2 H/L
2
0
-2
30'/0'
30'/30'
30'/150'
Time point

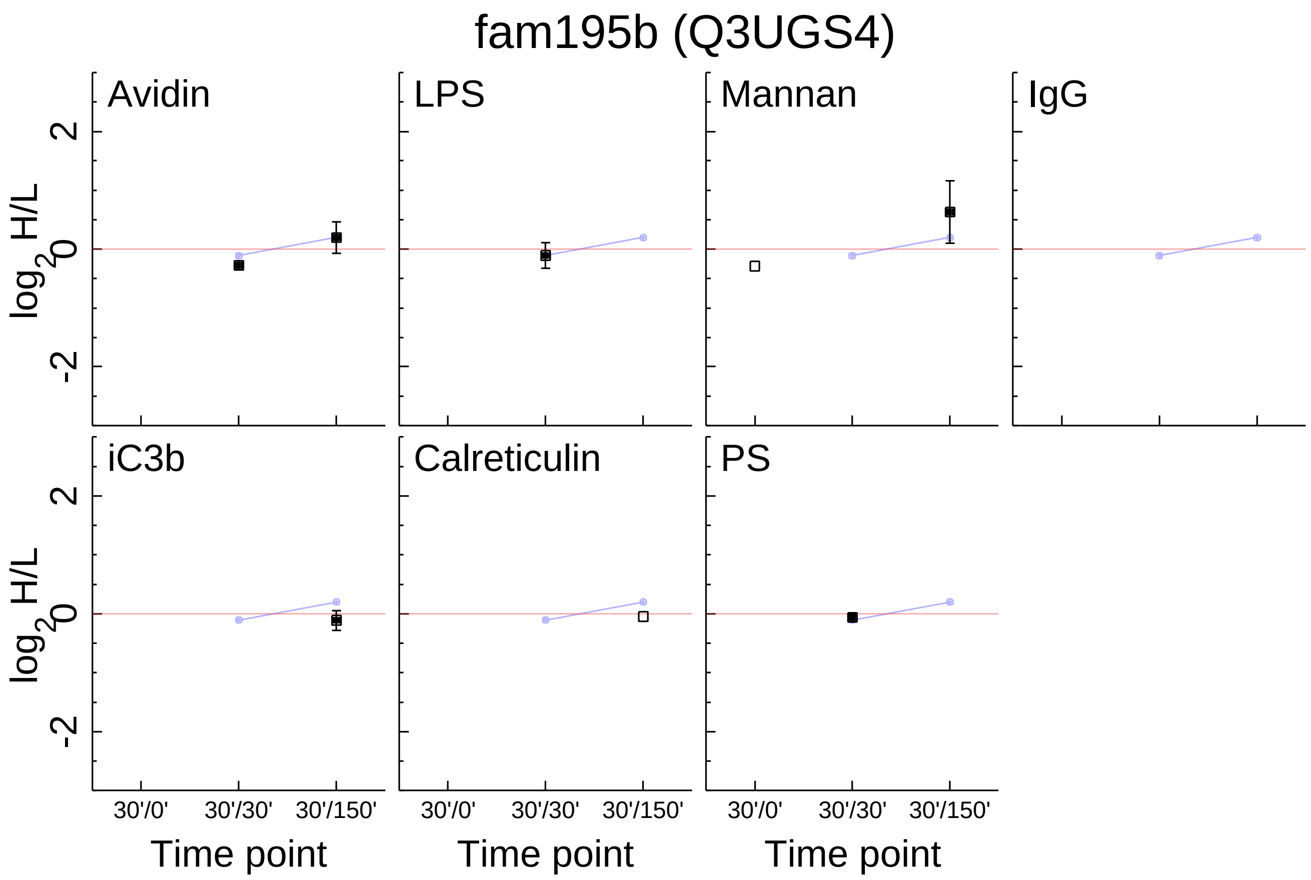

fam195b (Q3UGS4)
Avidin
LPS
Mannan
IgG
iC3b
Calreticulin
PS
2
0
-2
log2 H/L
30'/0'
30'/30'
30'/150'
Time point

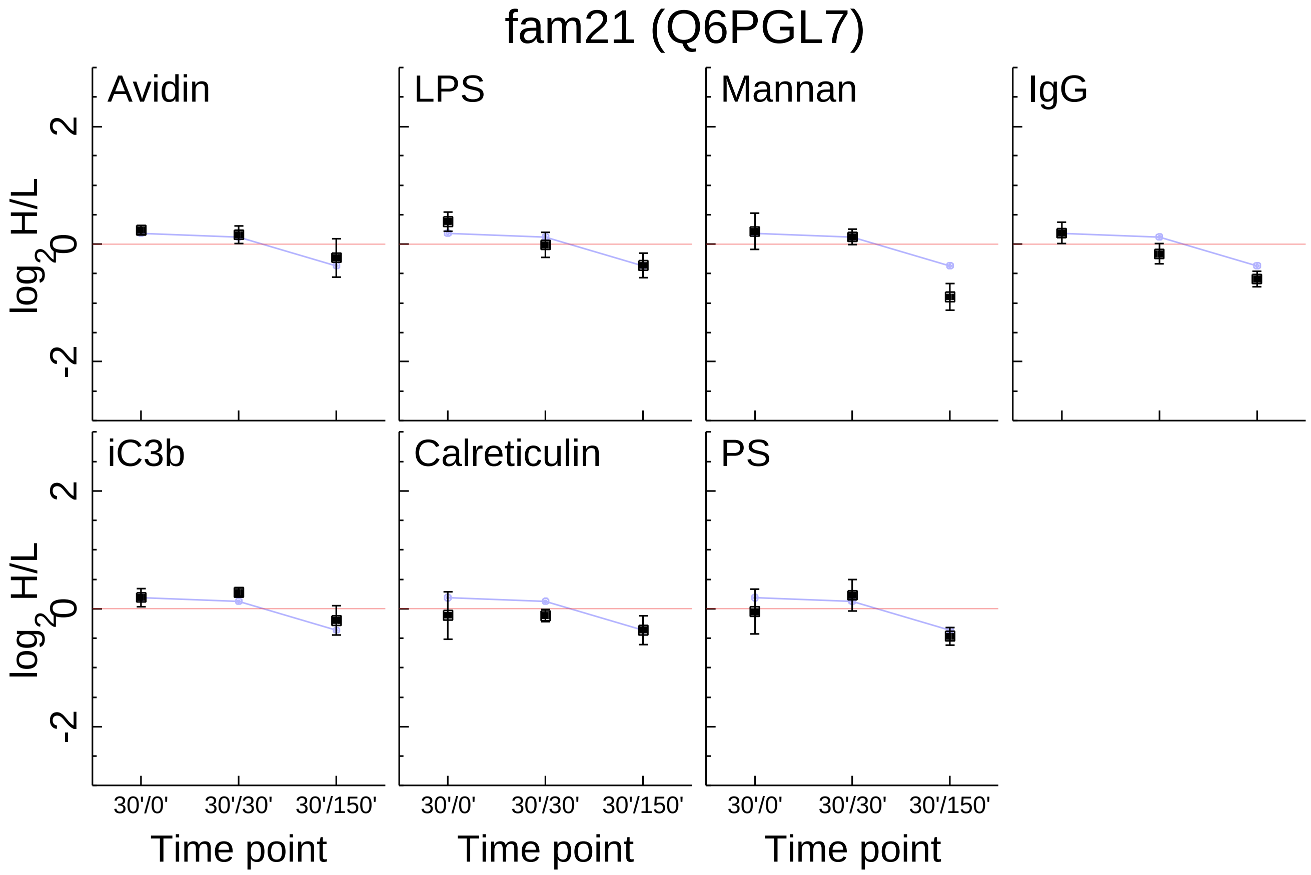
fam21 (Q6PGL7)
Avidin
LPS
Mannan
IgG
iC3b
Calreticulin
PS
log2 H/L
2
0
-2
30'/0'
30'/30'
30'/150'
Time point

fam26f (Q8C9E8)

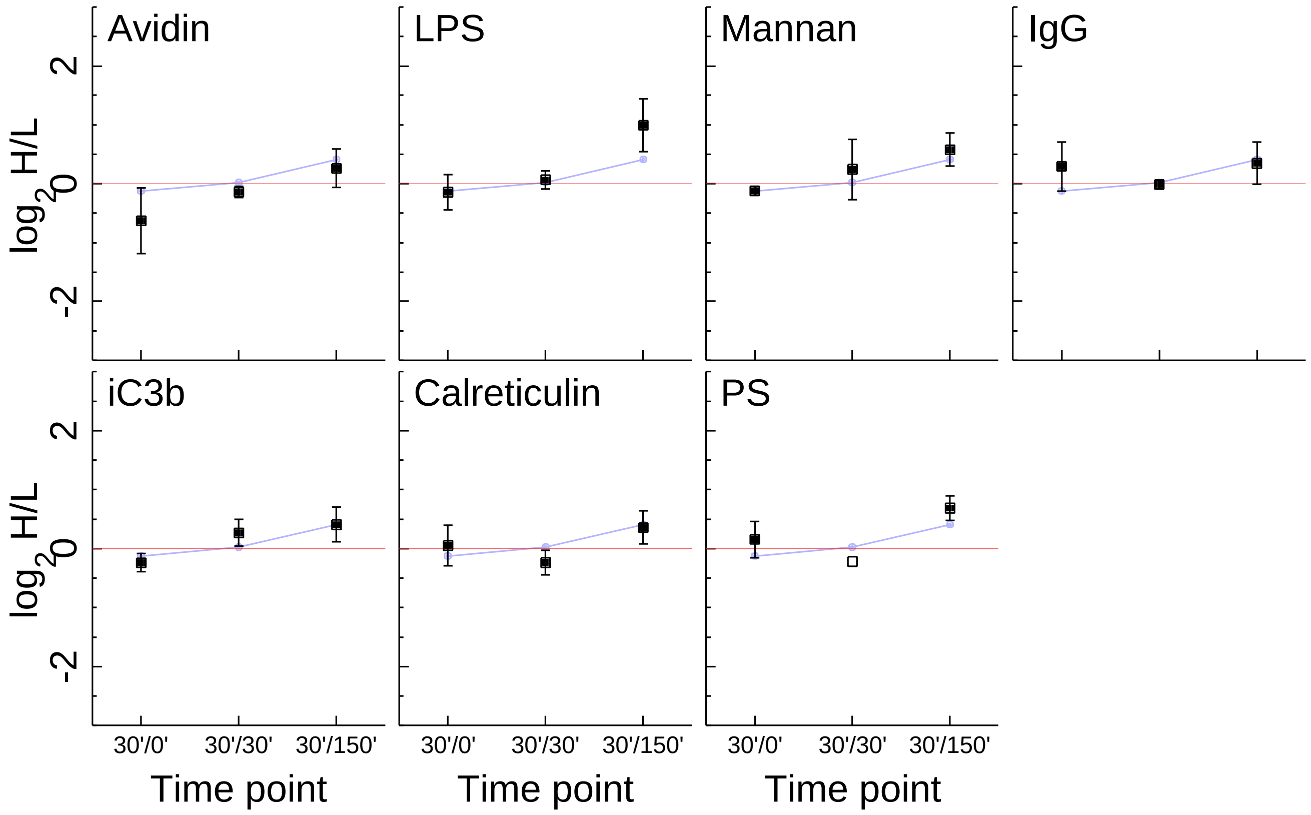

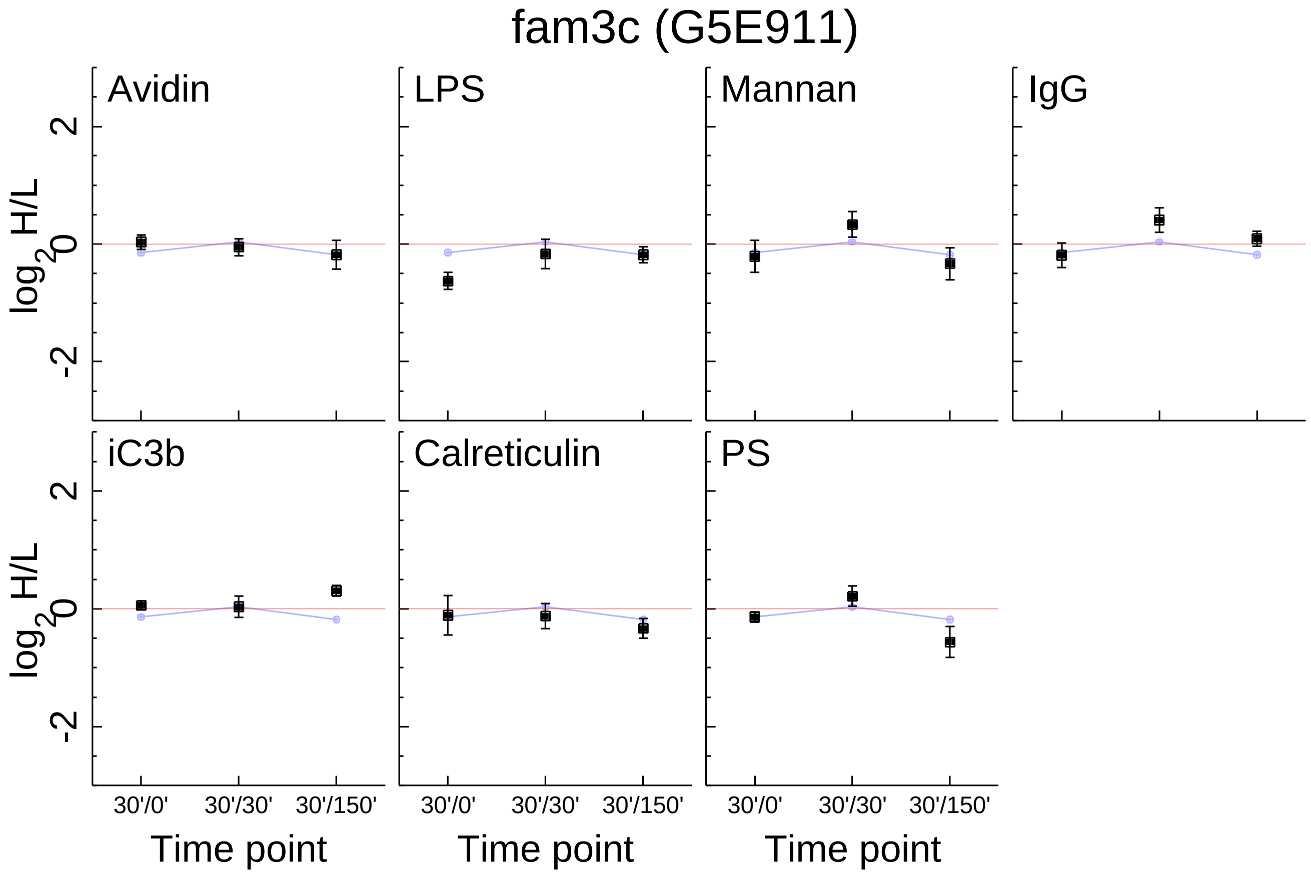
fam3c (G5E911)
Avidin
LPS
Mannan
IgG
iC3b
Calreticulin
PS
log2 H/L
2
0
-2
30'/0'
30'/30'
30'/150'
Time point

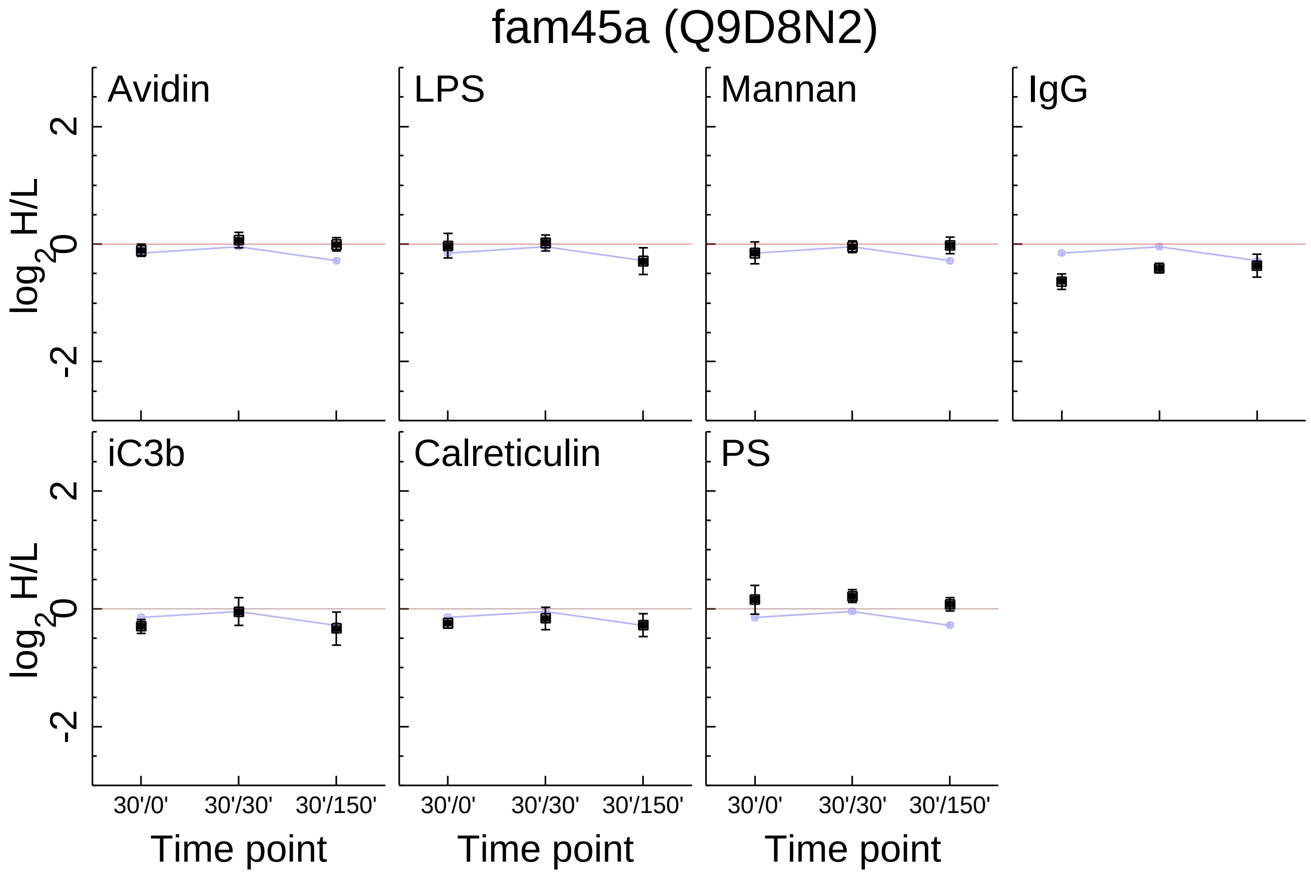
fam45a (Q9D8N2)
Avidin
LPS
Mannan
IgG
iC3b
Calreticulin
PS
log2 H/L
2
0
-2
30'/0'
30'/30'
30'/150'
Time point

fam49b (Q921M7)

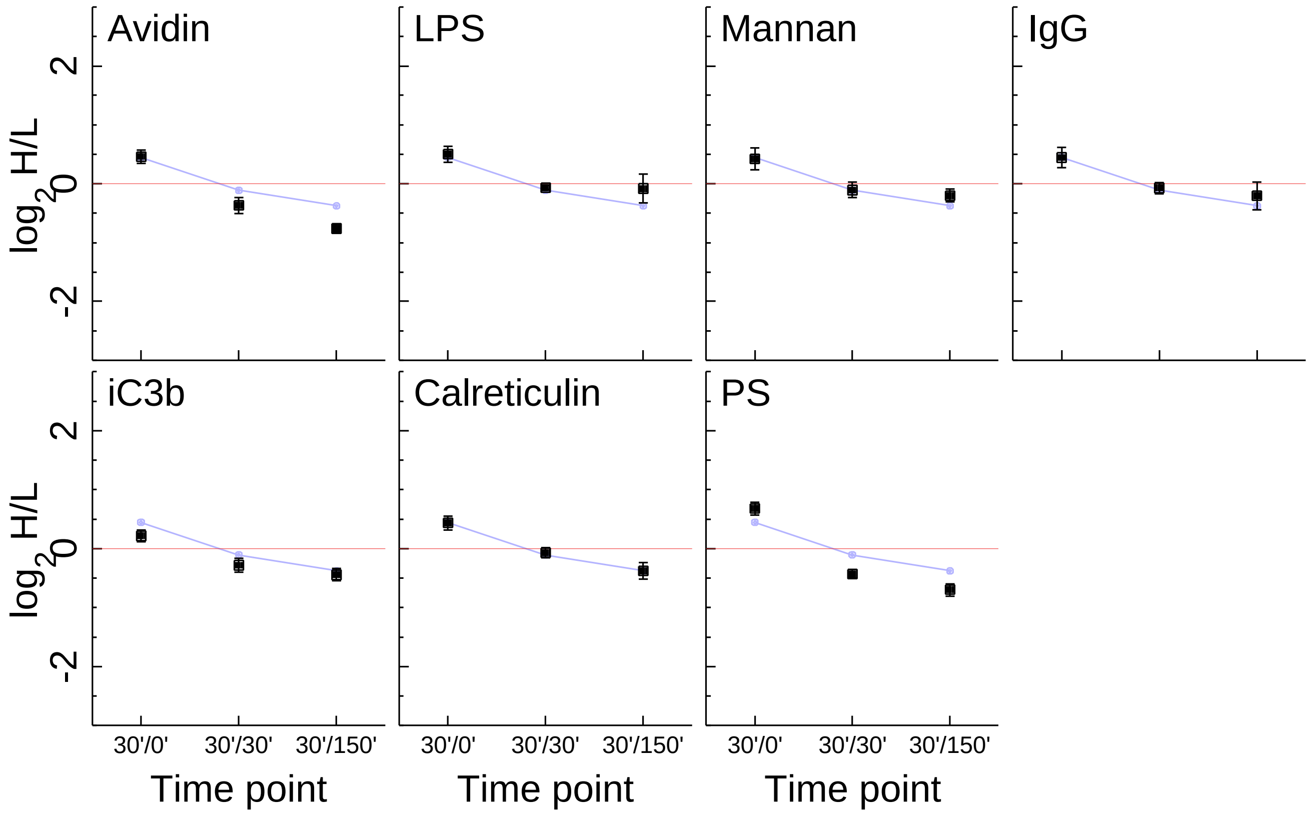

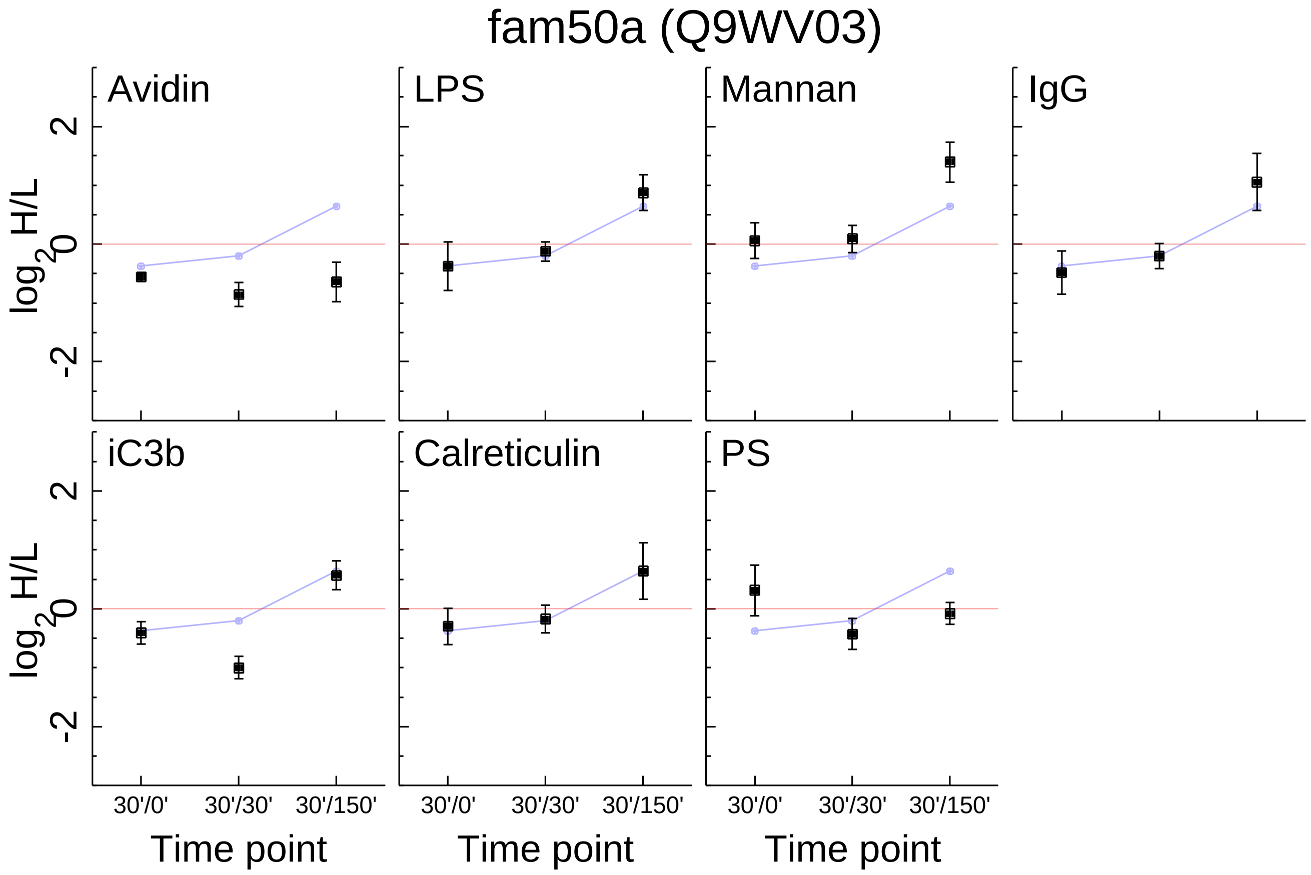
fam50a (Q9WV03)
Avidin
LPS
Mannan
IgG
iC3b
Calreticulin
PS
log2 H/L
2
0
-2
30'/0'
30'/30'
30'/150'
Time point

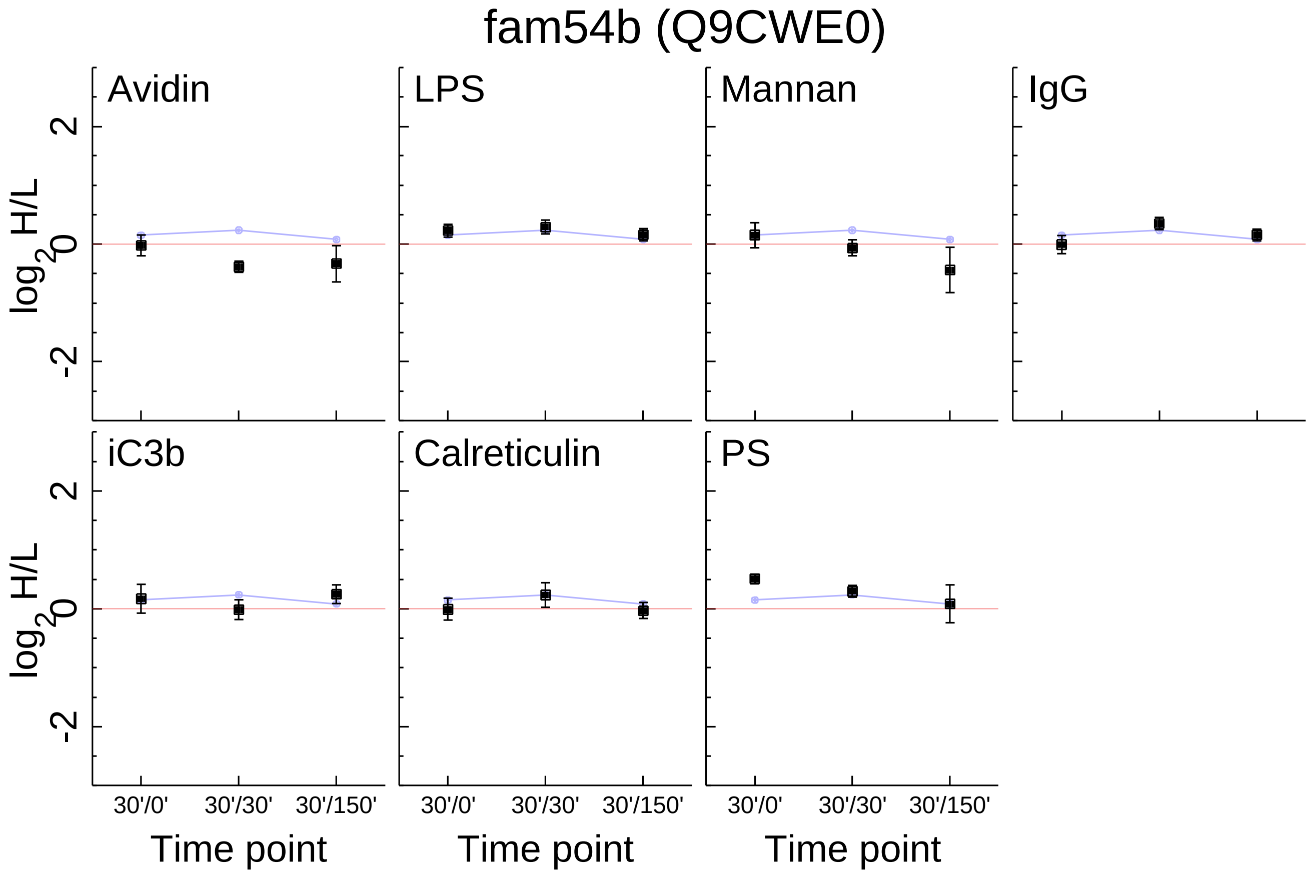
fam54b (Q9CWE0)
Avidin
LPS
Mannan
IgG
iC3b
Calreticulin
PS
log2 H/L
2
0
-2
30'/0'
30'/30'
30'/150'
Time point

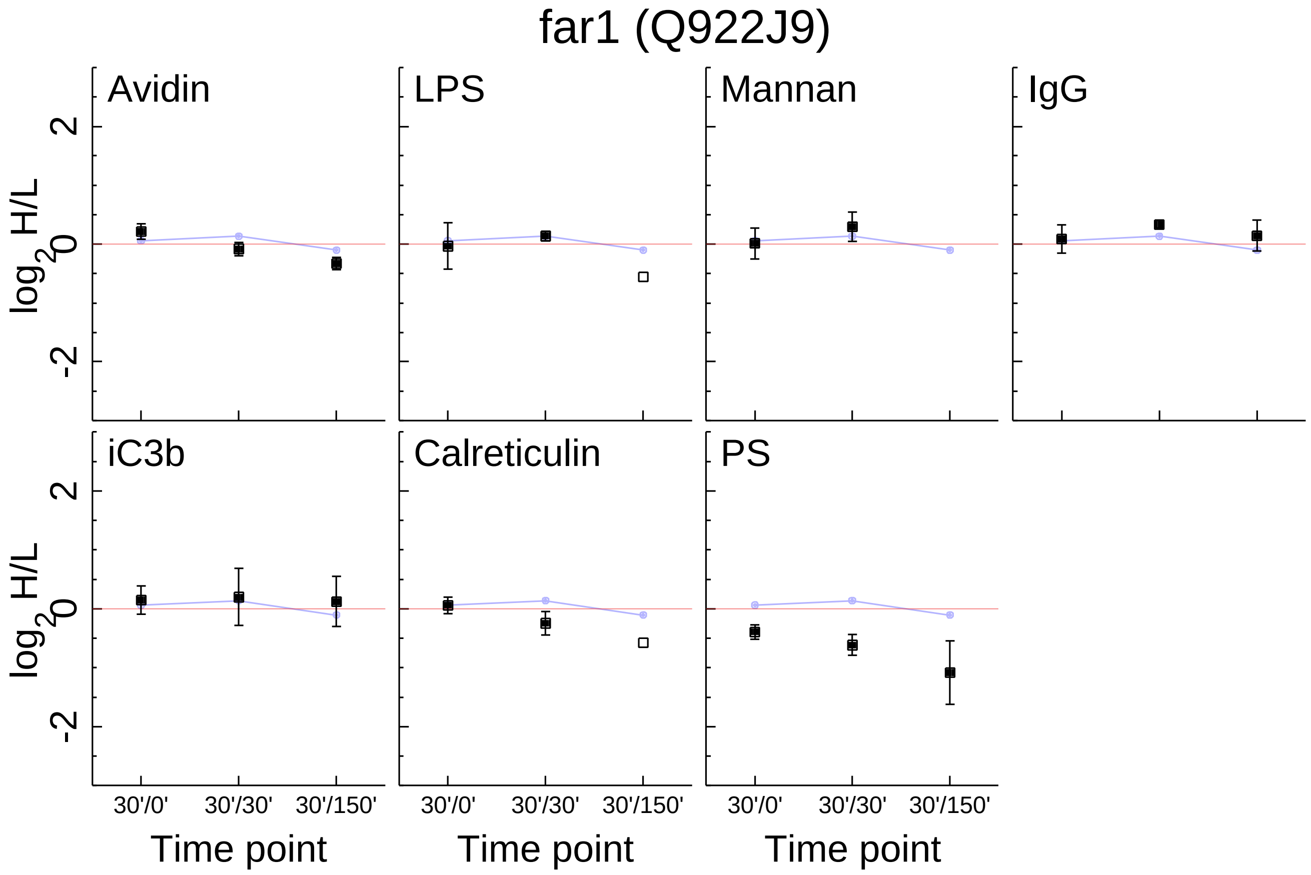
far1 (Q922J9)
Avidin
LPS
Mannan
IgG
iC3b
Calreticulin
PS
log2 H/L
2
0
-2
30'/0'
30'/30'
30'/150'
Time point

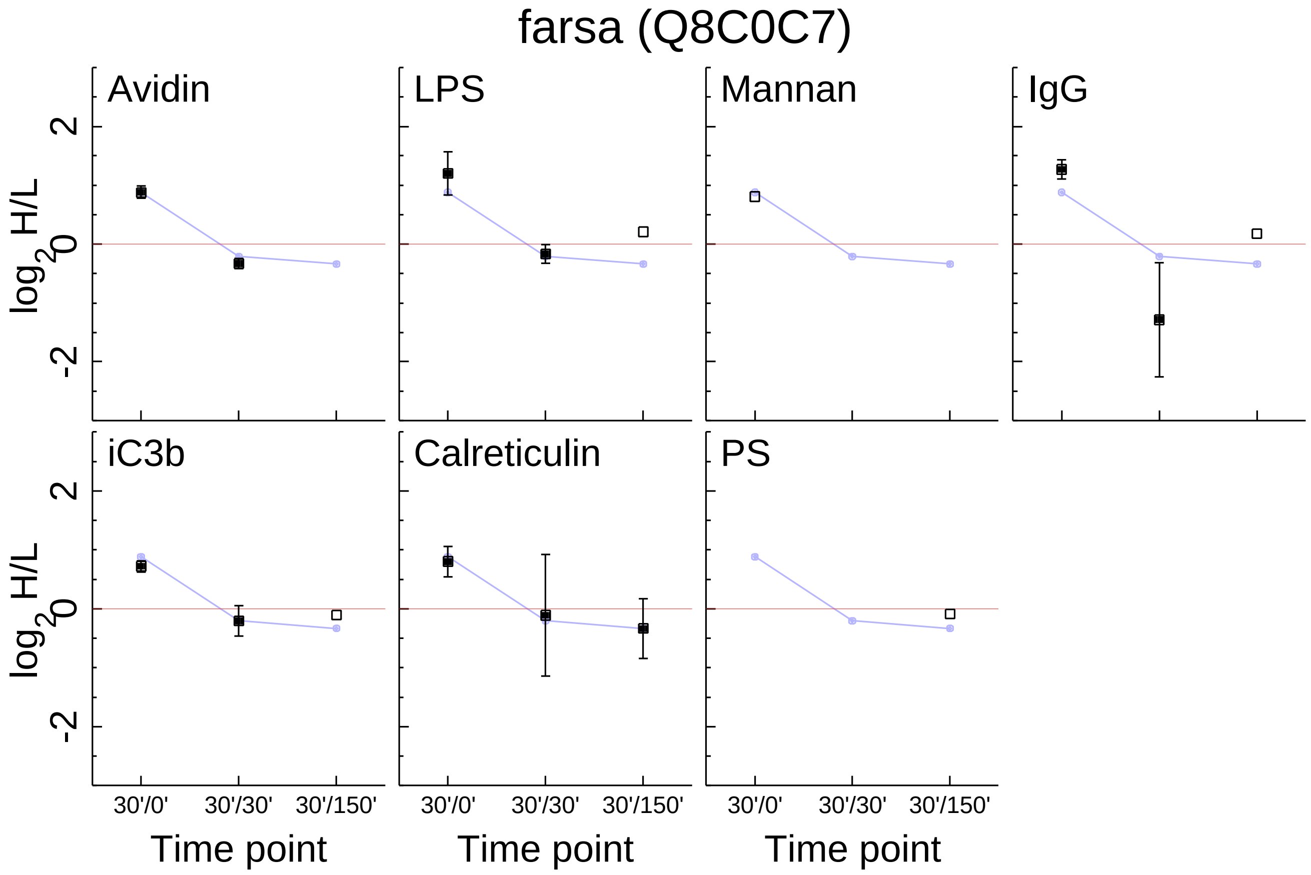
farsa (Q8C0C7)
Avidin
LPS
Mannan
IgG
iC3b
Calreticulin
PS
log2 H/L
2
0
-2
30'/0'
30'/30'
30'/150'
Time point

farsb (Q9WUA2)

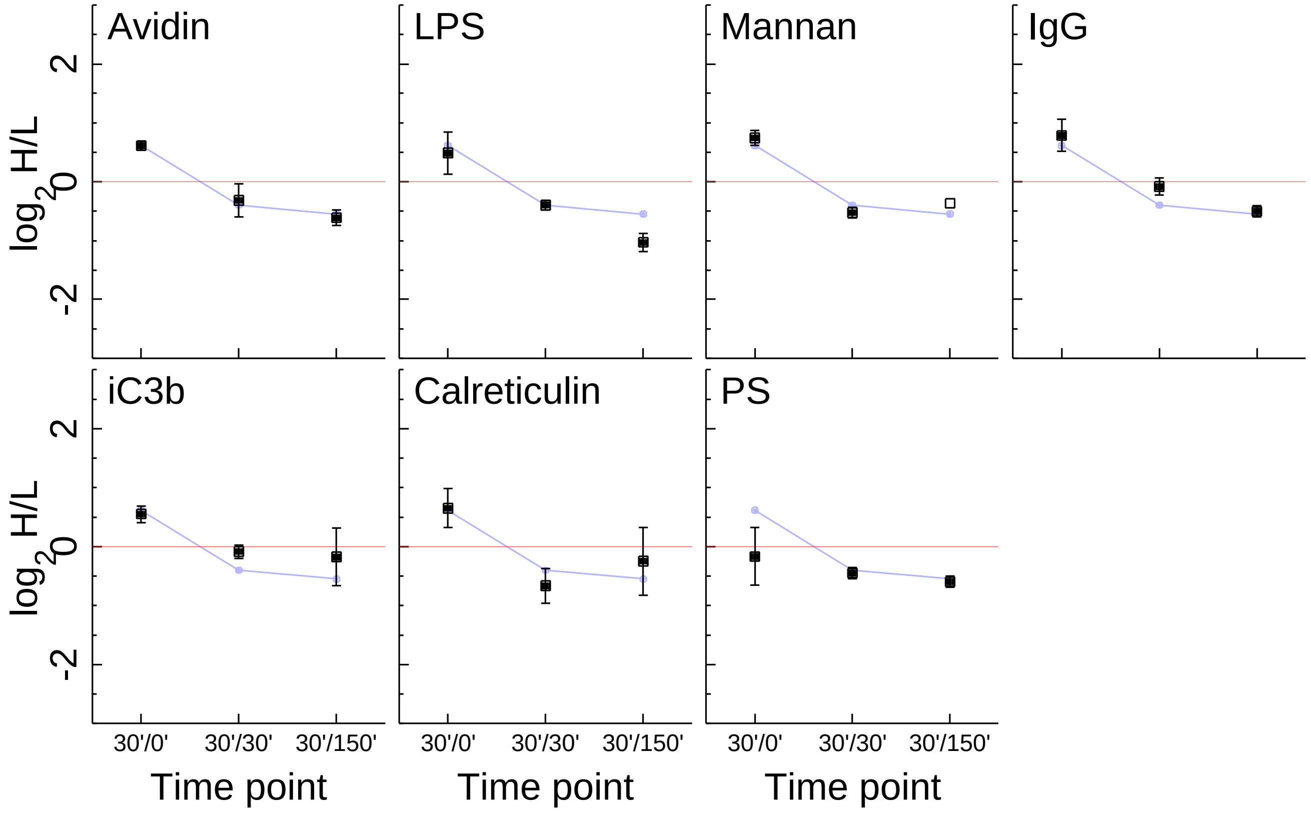

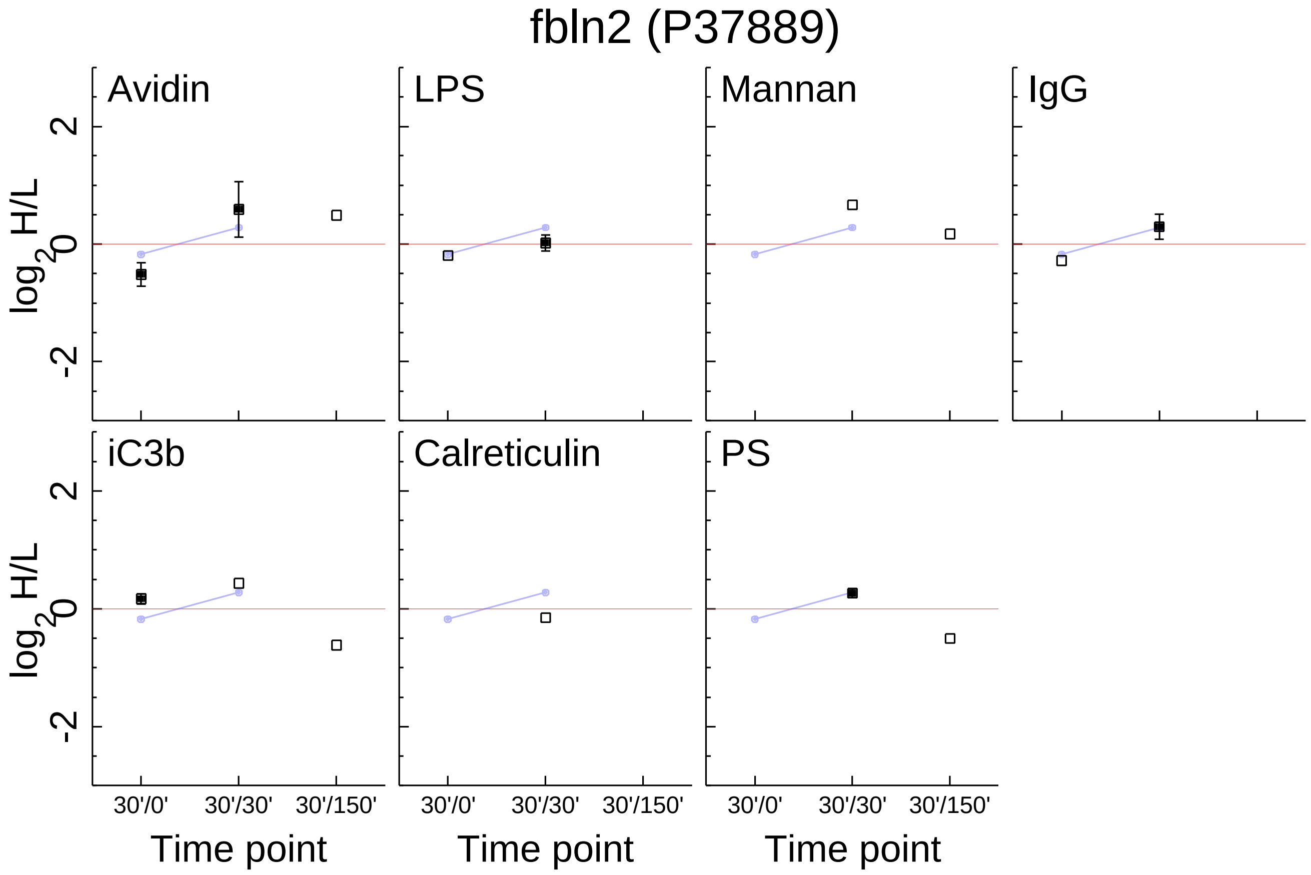

fbln2 (P37889)
Avidin
LPS
Mannan
IgG
iC3b
Calreticulin
PS
log2 H/L
2
0
-2
30'/0'
30'/30'
30'/150'
Time point

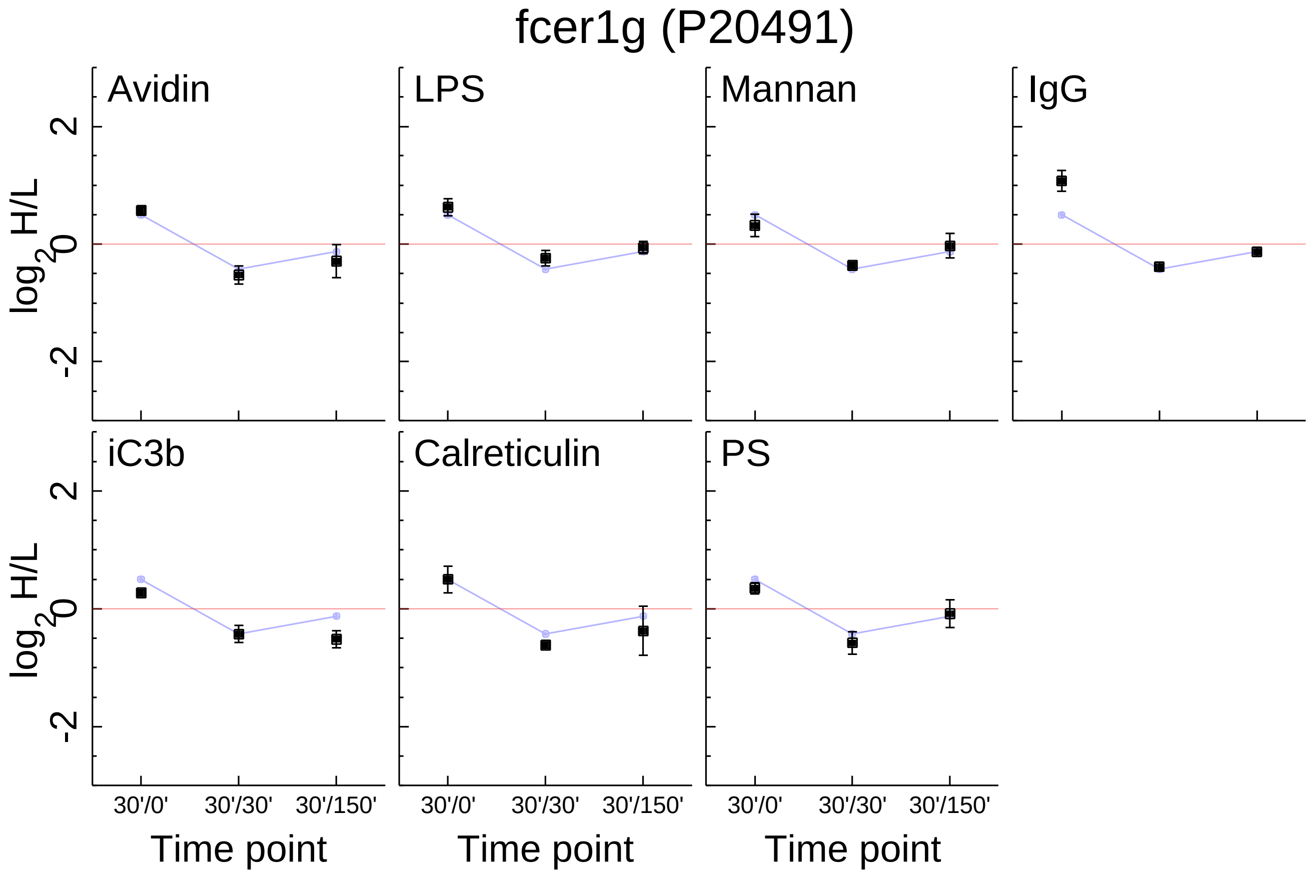
fcer1g (P20491)
Avidin
LPS
Mannan
IgG
iC3b
Calreticulin
PS
log2 H/L
2
0
-2
30'/0'
30'/30'
30'/150'
Time point

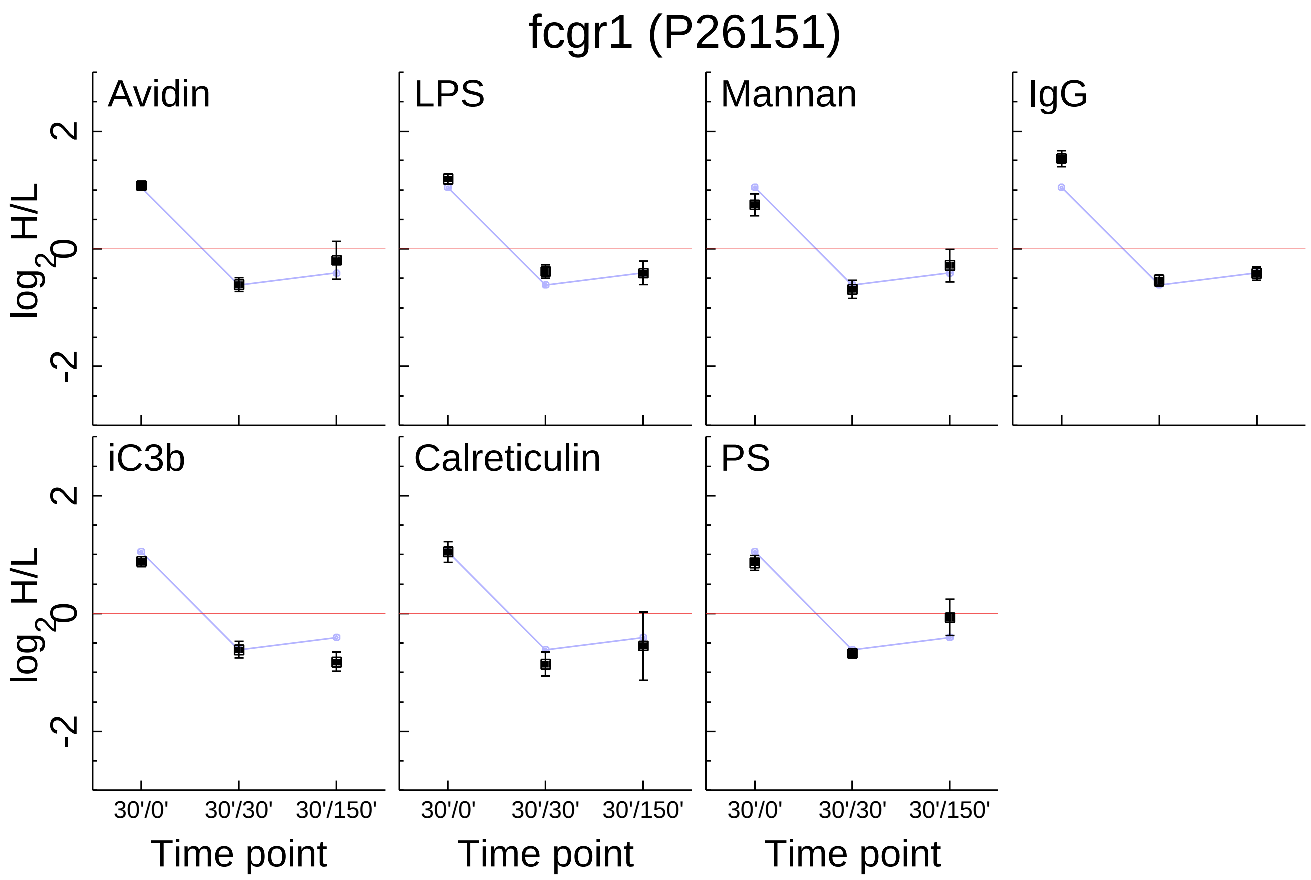

fcgr1 (P26151)
Avidin
LPS
Mannan
IgG
iC3b
Calreticulin
PS
log2 H/L
2
0
-2
30'/0'
30'/30'
30'/150'
Time point

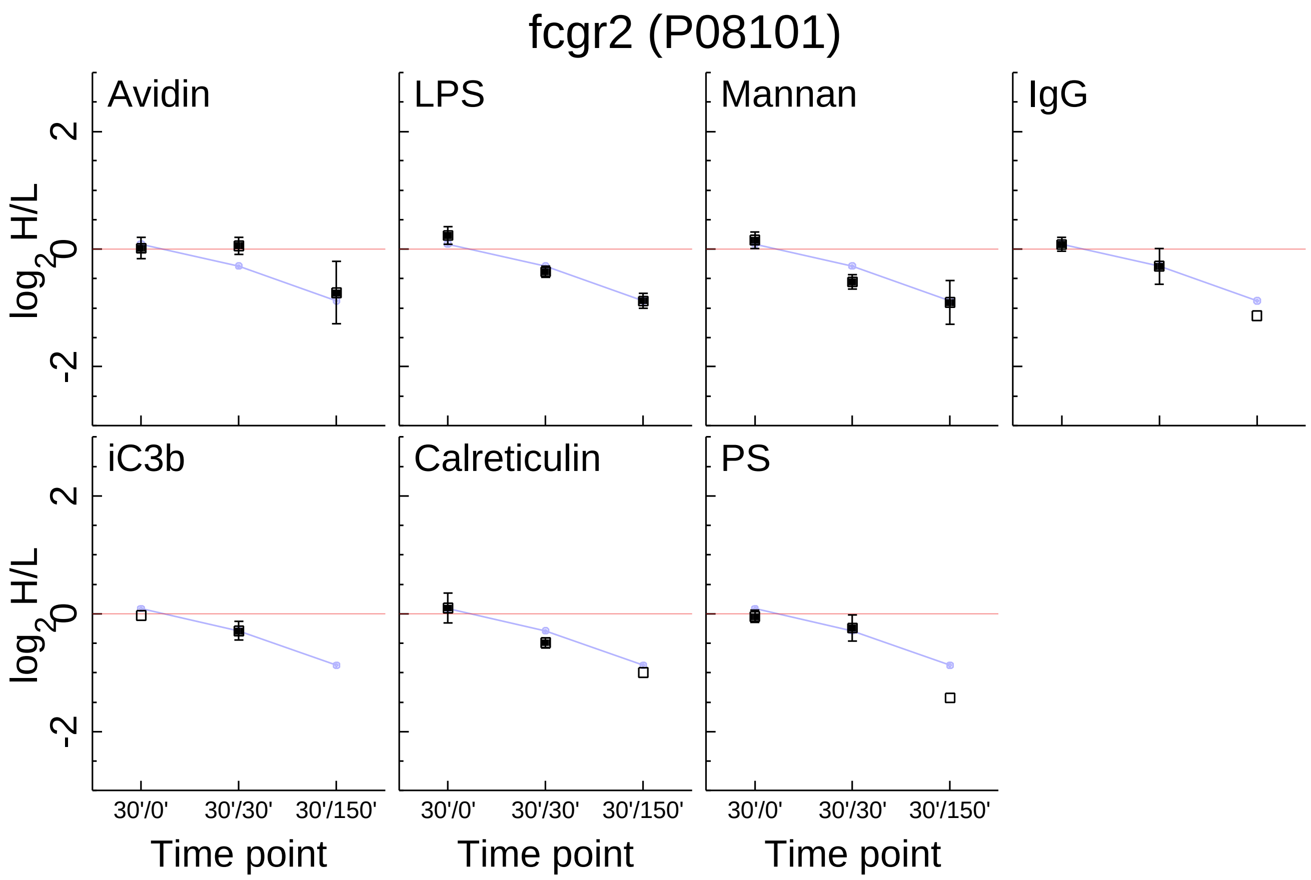
fcgr2 (P08101)
Avidin
LPS
Mannan
IgG
iC3b
Calreticulin
PS
log2 H/L
2
0
-2
30'/0'
30'/30'
30'/150'
Time point

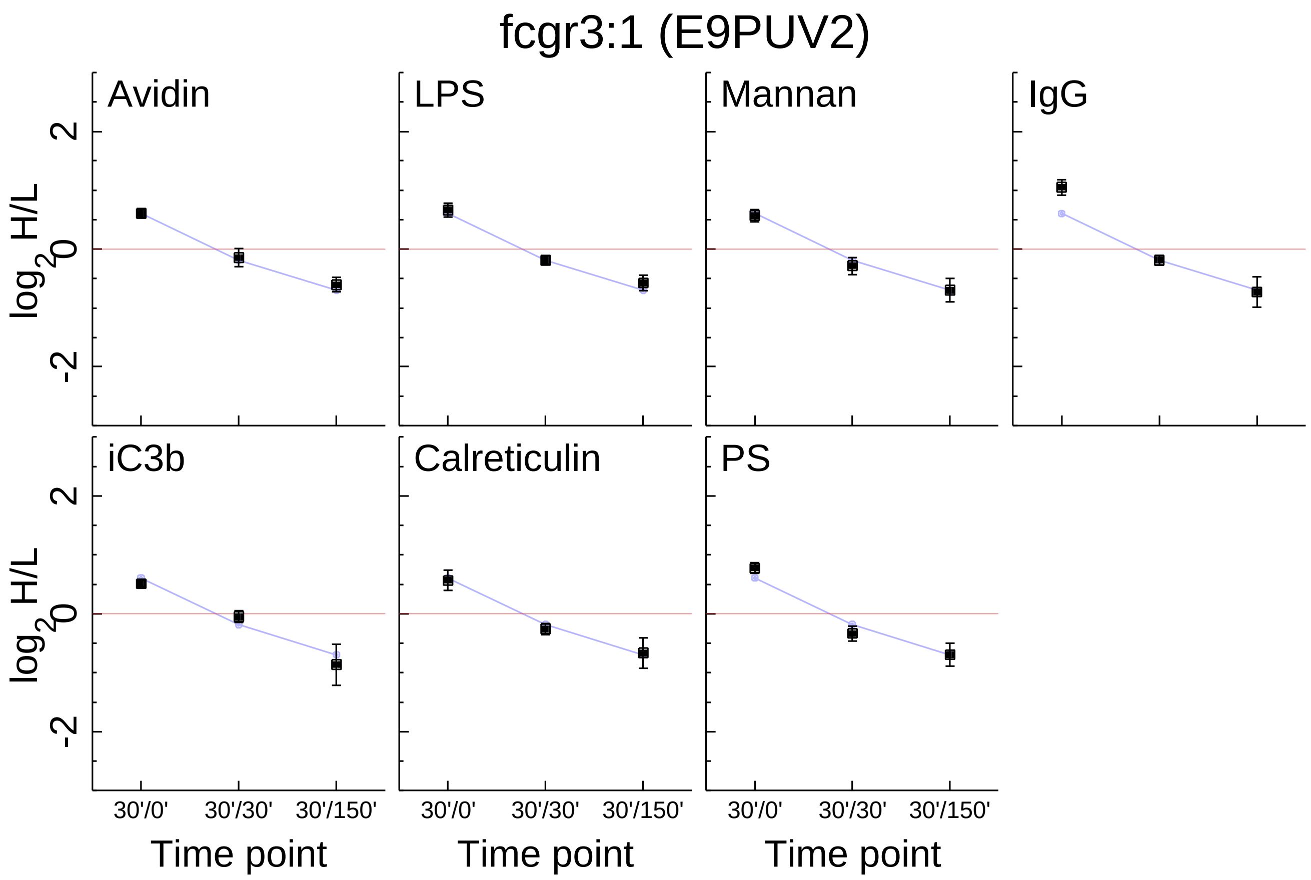

fcgr3:1 (E9PUV2)
Avidin
LPS
Mannan
IgG
iC3b
Calreticulin
PS
log2 H/L
2
0
-2
30'/0'
30'/30'
30'/150'
Time point

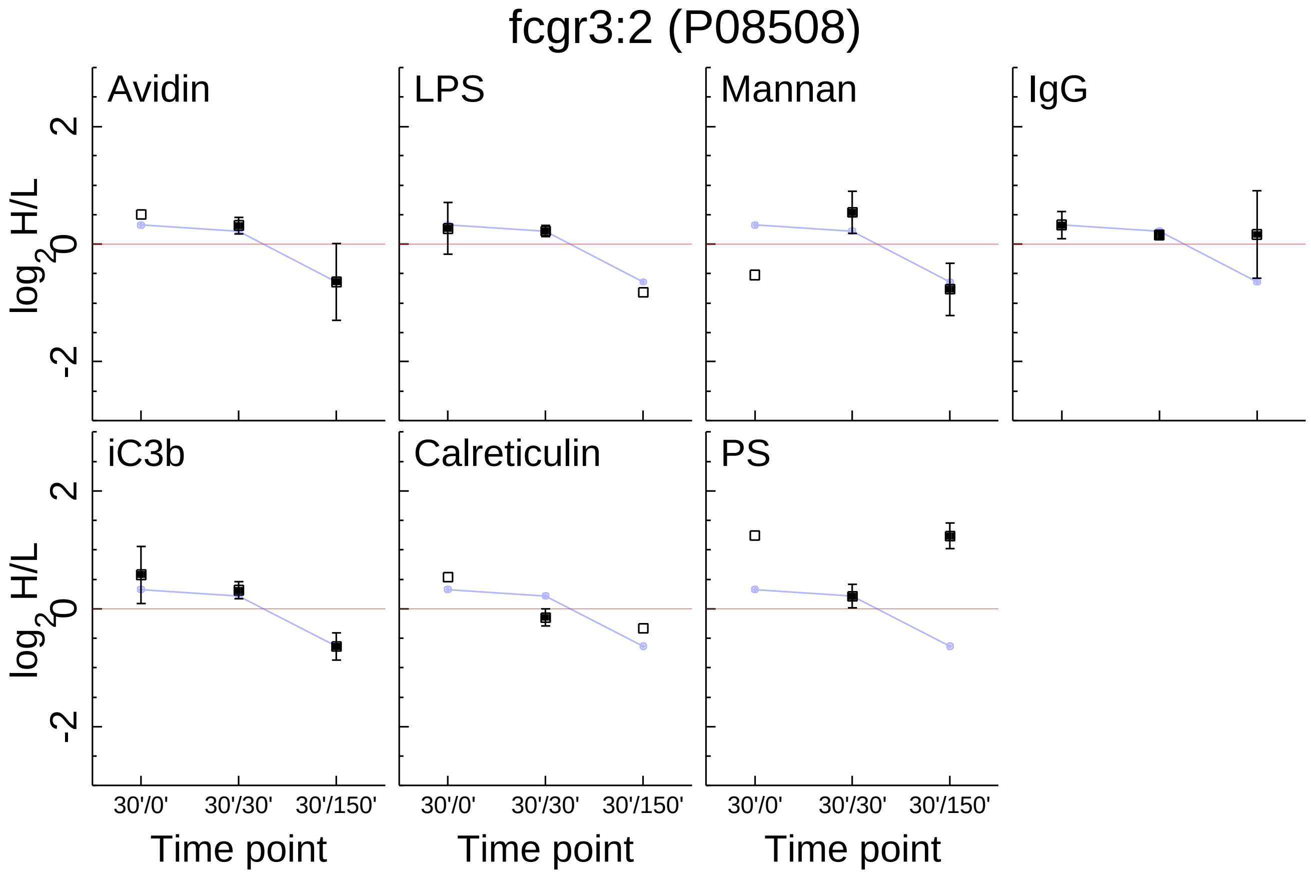
fcgr3:2 (P08508)
Avidin
LPS
Mannan
IgG
iC3b
Calreticulin
PS
log2 H/L
2
0
-2
30'/0'
30'/30'
30'/150'
Time point

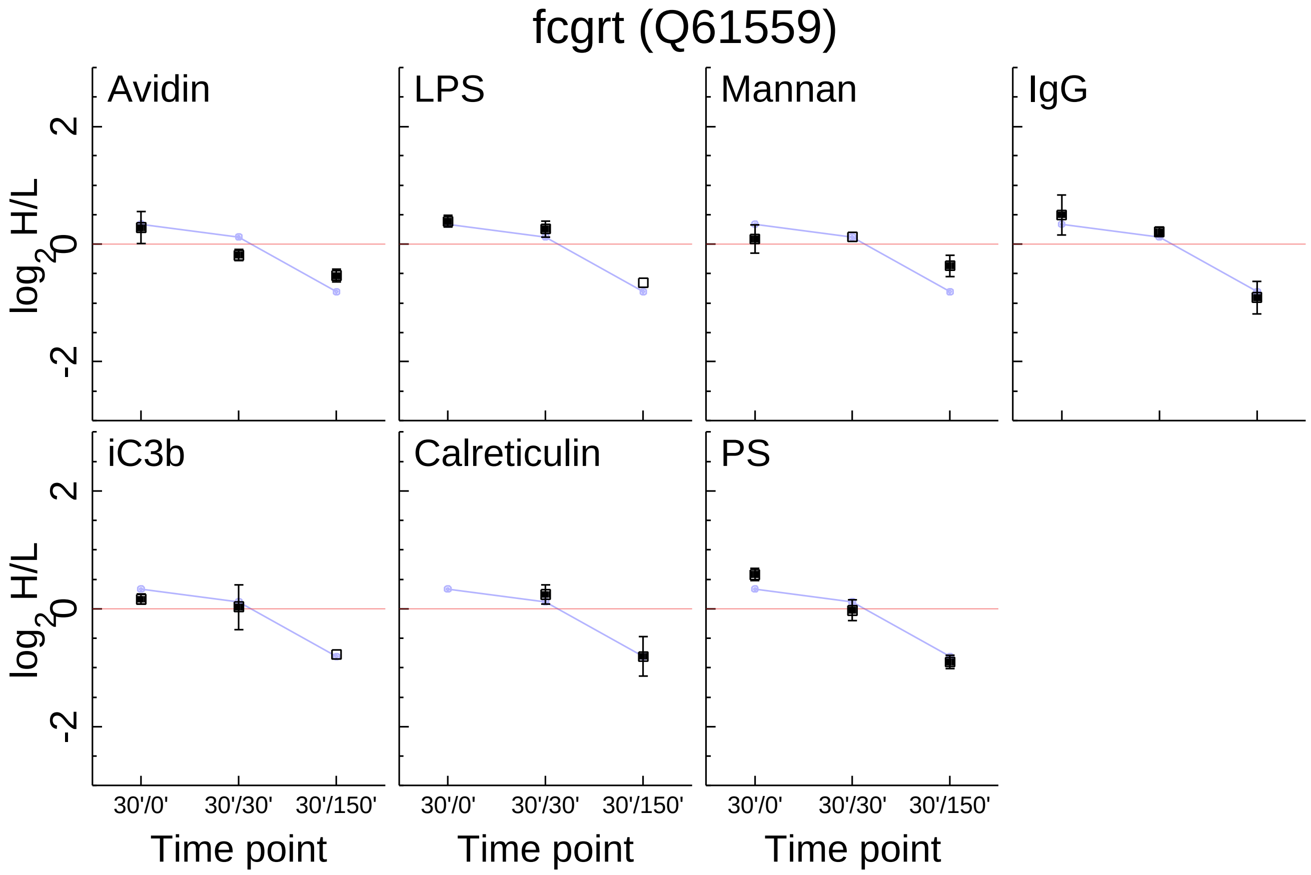

fcgrt (Q61559)
Avidin
LPS
Mannan
IgG
iC3b
Calreticulin
PS
log2 H/L
2
0
-2
30'/0'
30'/30'
30'/150'
Time point

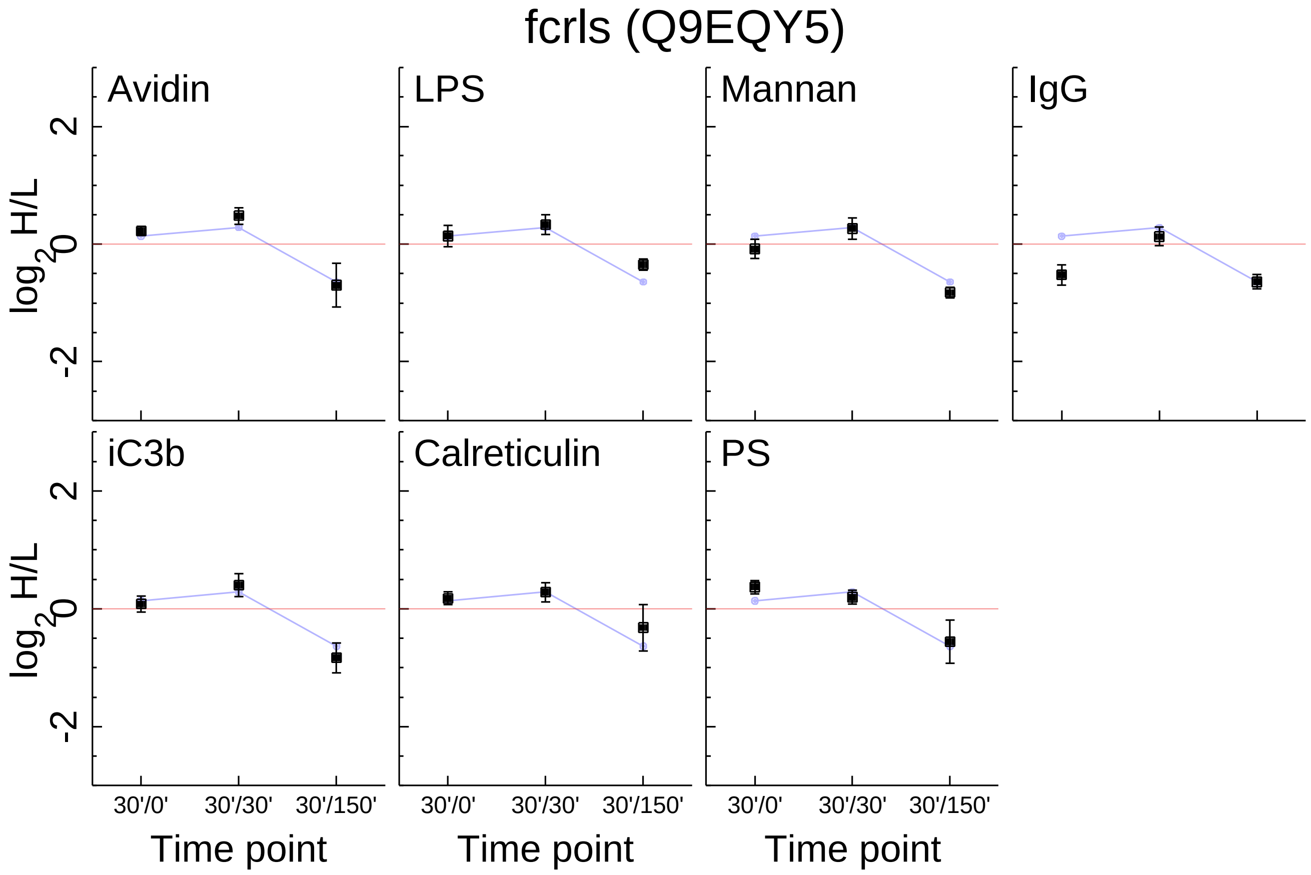
fcrls (Q9EQY5)
Avidin
LPS
Mannan
IgG
iC3b
Calreticulin
PS
$\log_2$ H/L
2
0
-2
30'/0'
30'/30'
30'/150'
Time point

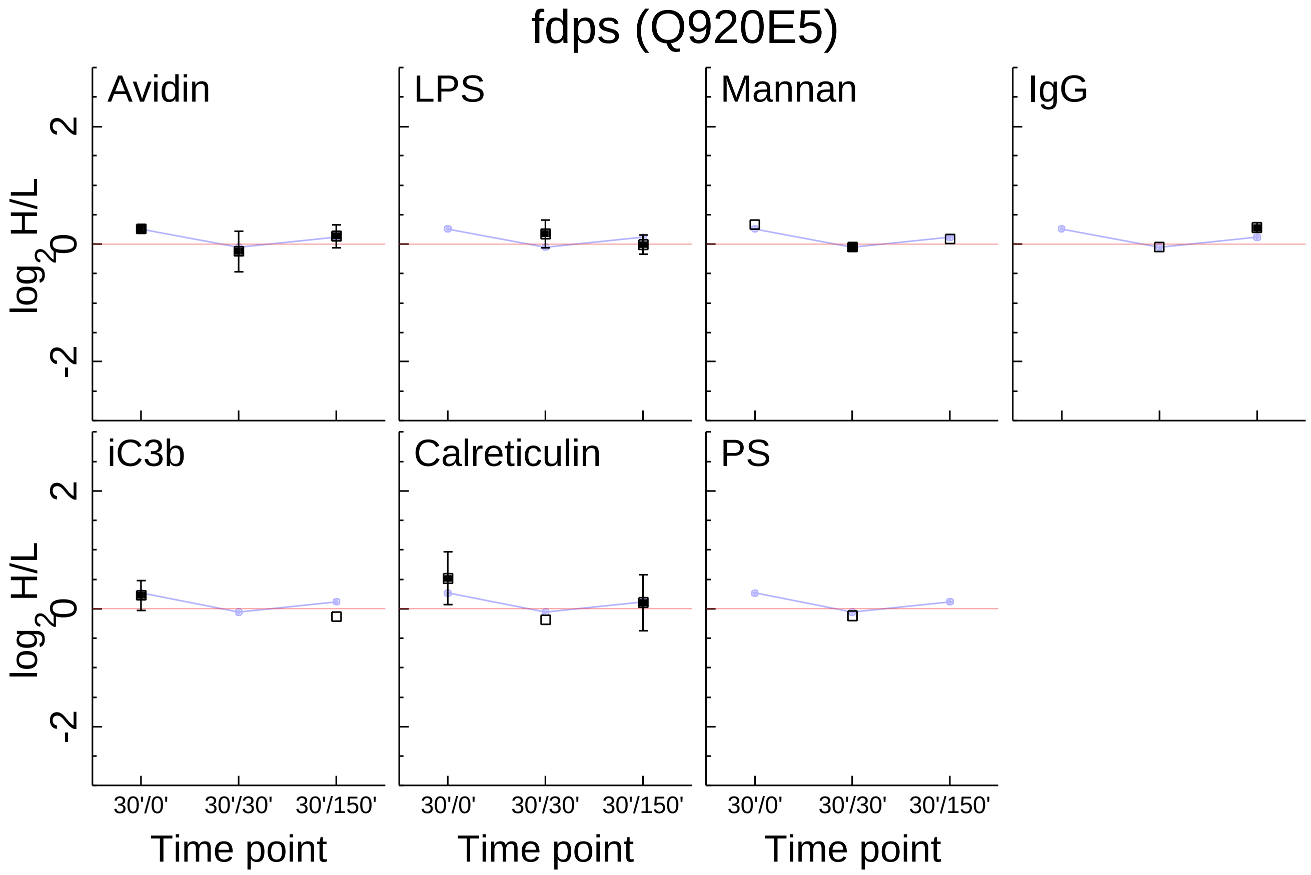

fdps (Q920E5)
Avidin
LPS
Mannan
IgG
iC3b
Calreticulin
PS
log2 H/L
2
0
-2
30'/0'
30'/30'
30'/150'
Time point

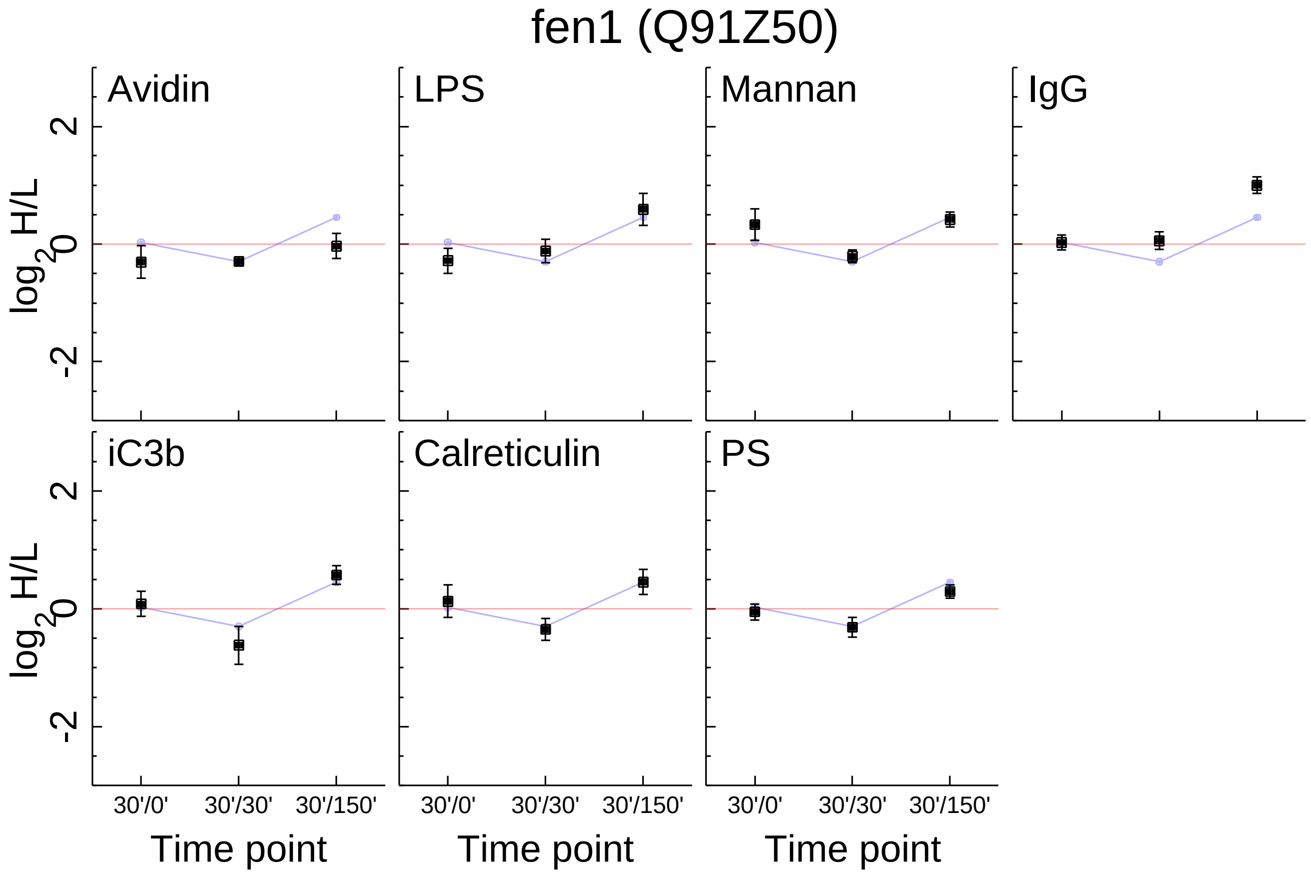
fen1 (Q91Z50)
Avidin
LPS
Mannan
IgG
iC3b
Calreticulin
PS
log2 H/L
2
0
-2
30'/0'
30'/30'
30'/150'
Time point

fermt3 (Q8K1B8)

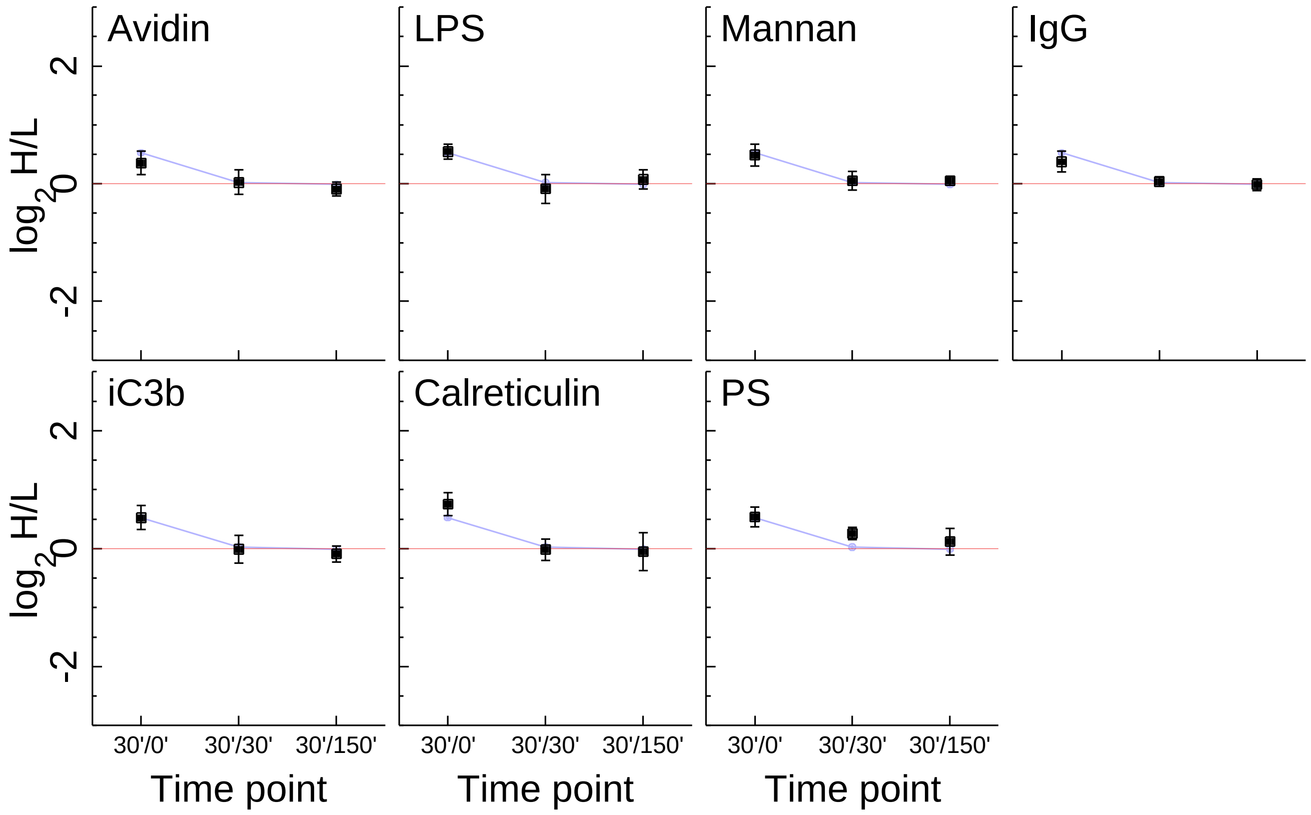

fes (P16879)

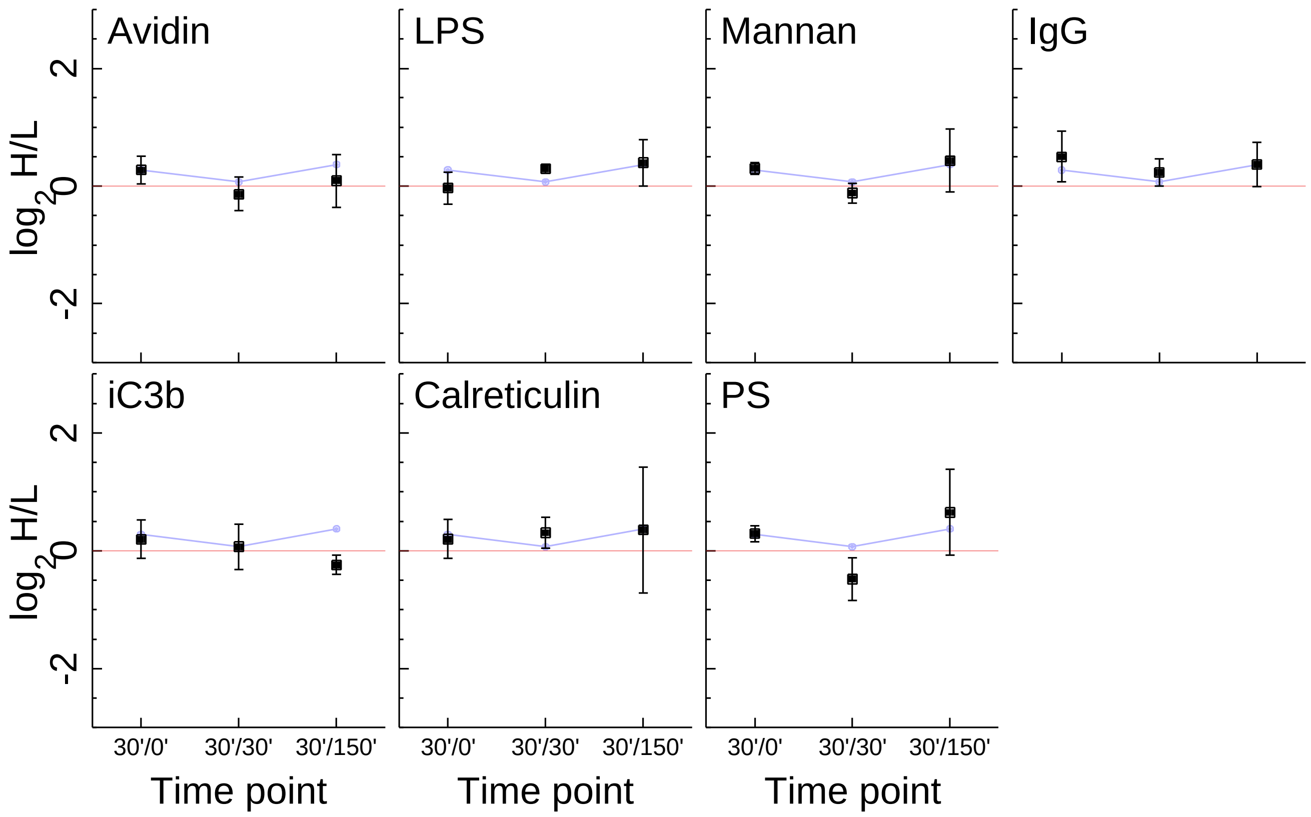
Avidin
LPS
Mannan
IgG
iC3b
Calreticulin
PS
2
0
-2
log2 H/L
30'/0'
30'/30'
30'/150'
Time point

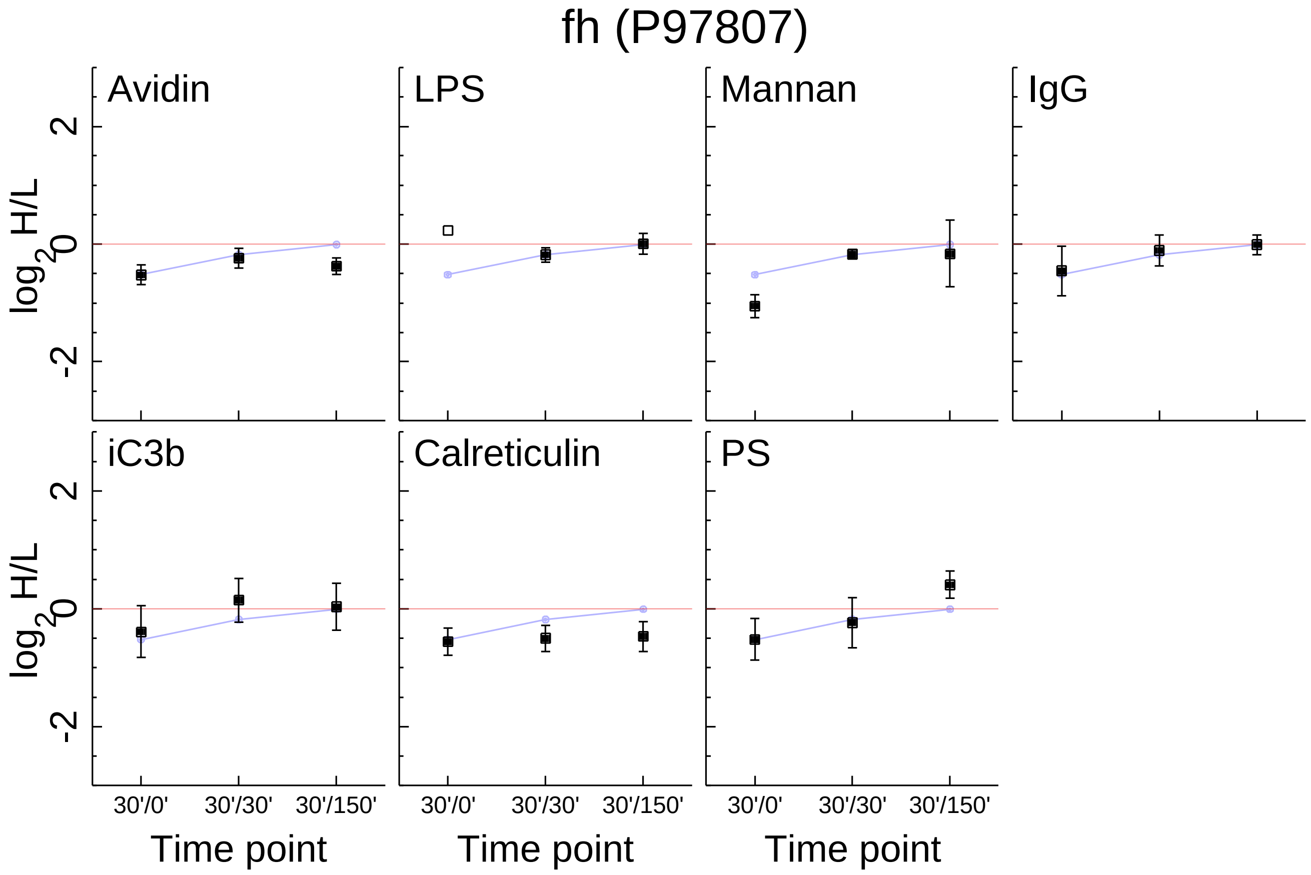
fh (P97807)
Avidin
LPS
Mannan
IgG
iC3b
Calreticulin
PS
log2 H/L
2
0
-2
30'/0'
30'/30'
30'/150'
Time point

fis1 (Q9CQ92)

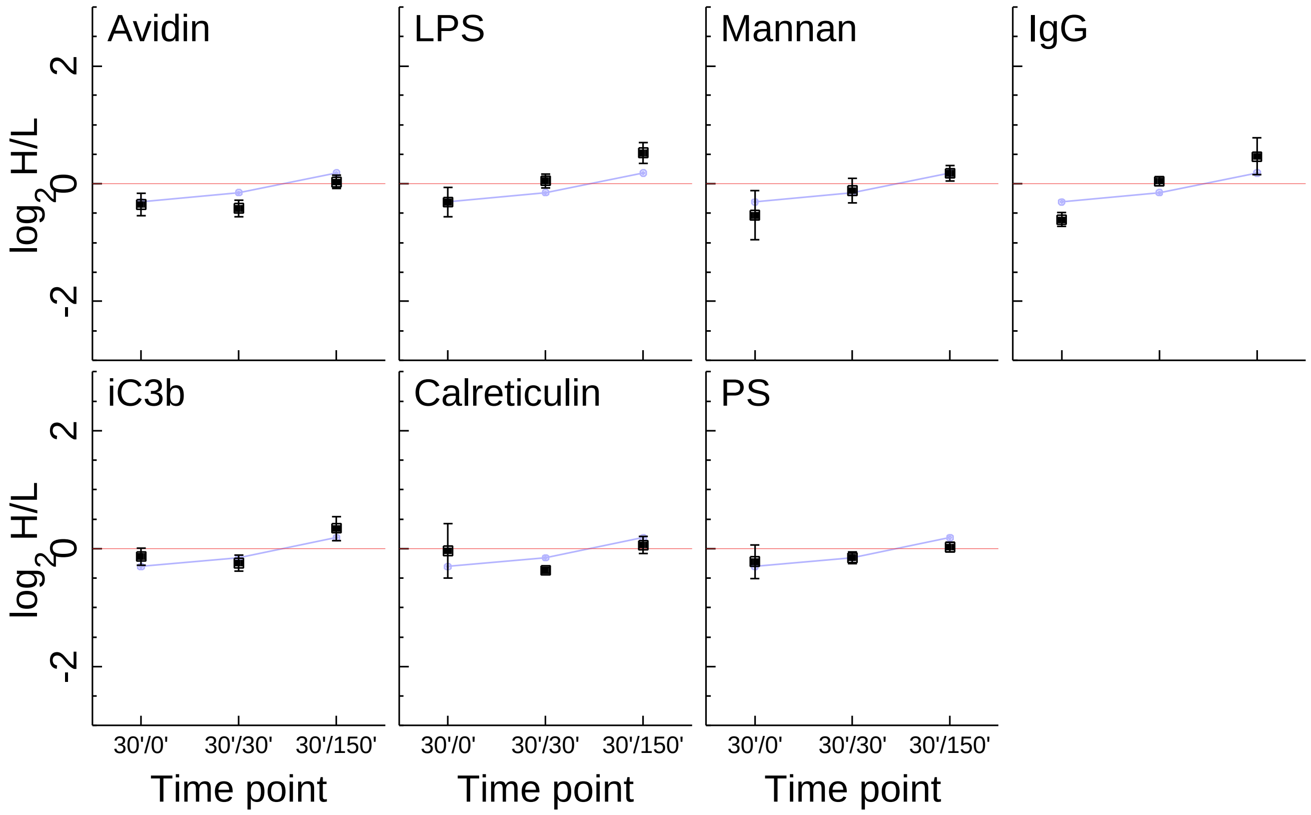
Avidin
LPS
Mannan
IgG
iC3b
Calreticulin
PS
$\log_2$ H/L
2
0
-2
30'/0'
30'/30'
30'/150'
Time point

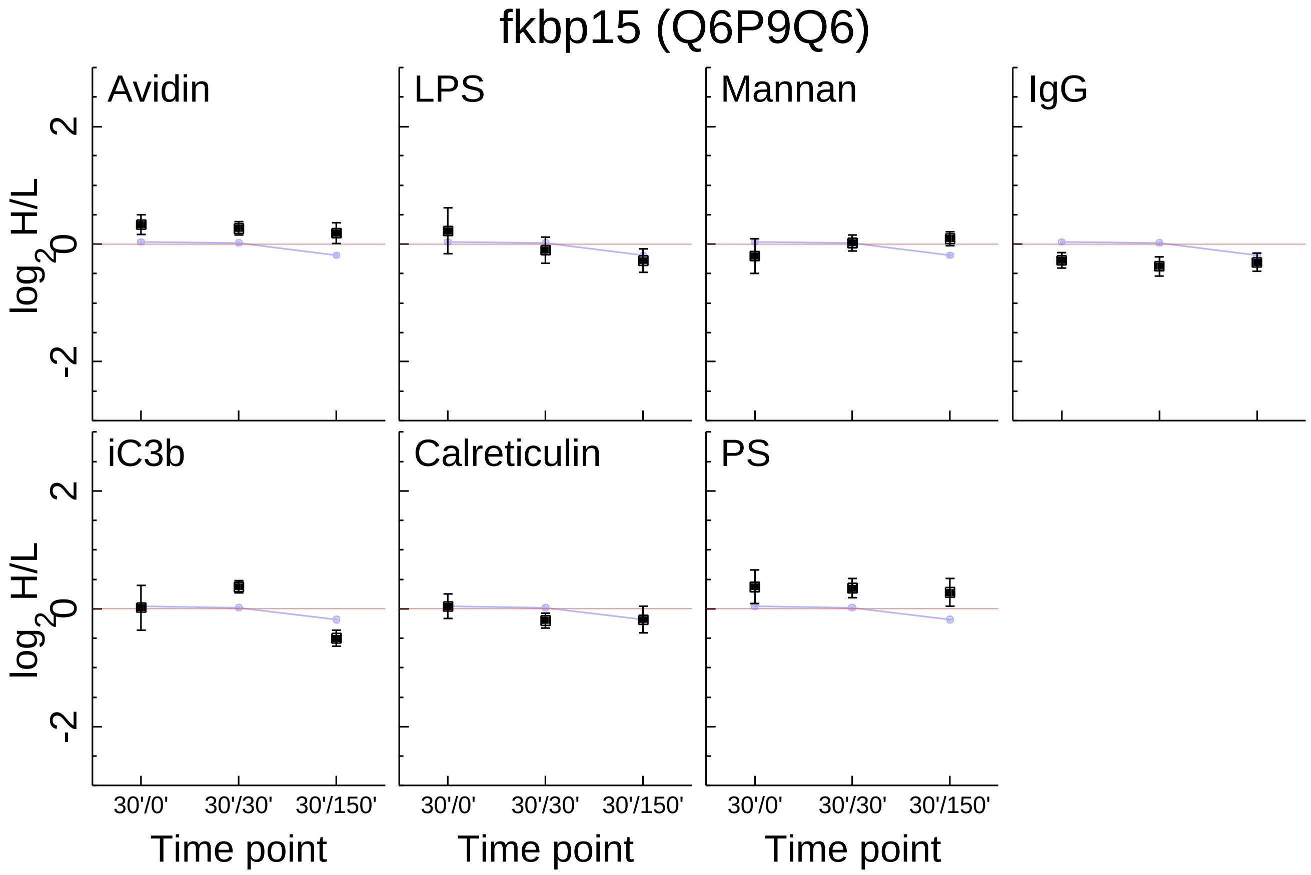
fkbp15 (Q6P9Q6)
Avidin
LPS
Mannan
IgG
iC3b
Calreticulin
PS
log2 H/L
2
0
-2
30'/0'
30'/30'
30'/150'
Time point

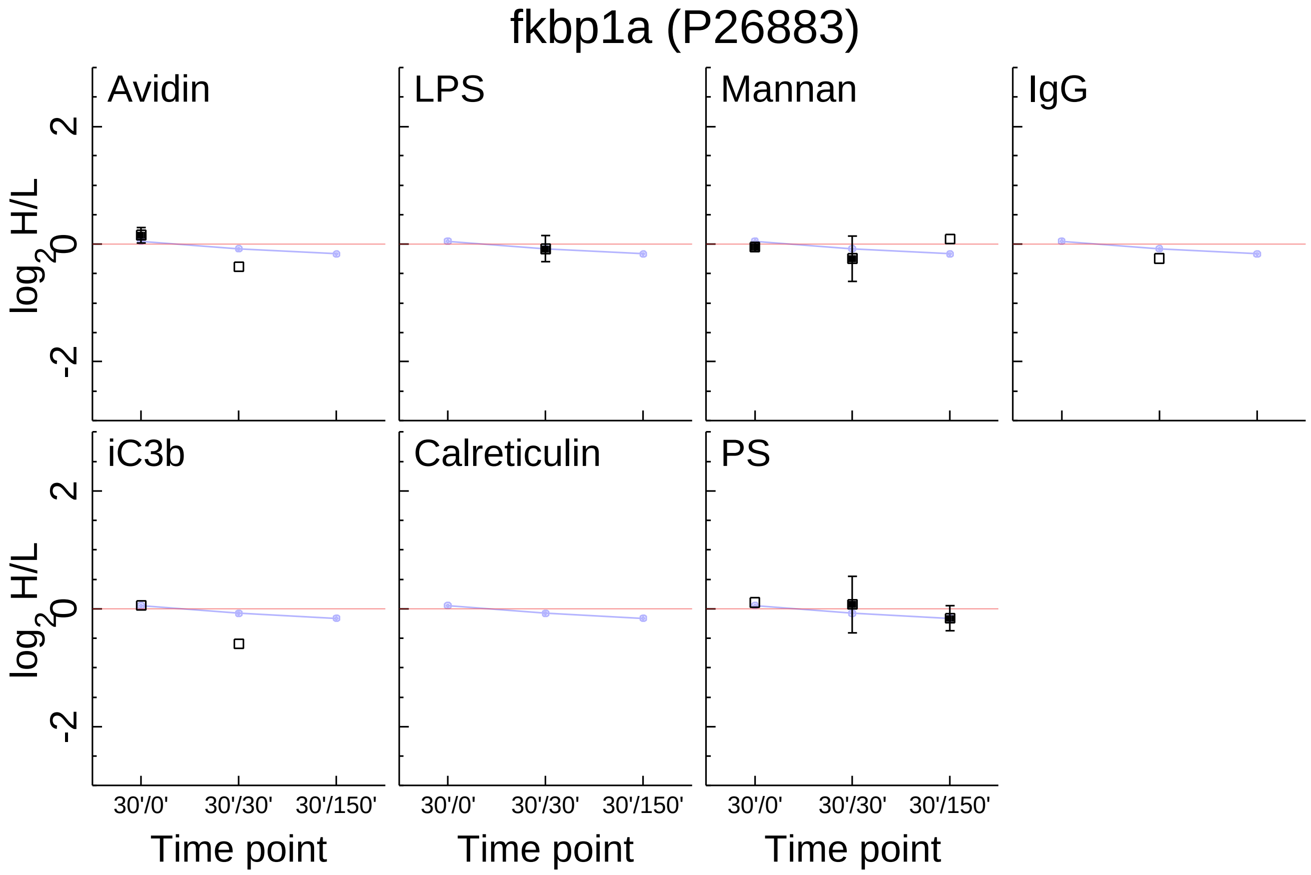
fkbp1a (P26883)
Avidin
LPS
Mannan
IgG
iC3b
Calreticulin
PS
log2 H/L
2
0
-2
30'/0'
30'/30'
30'/150'
Time point

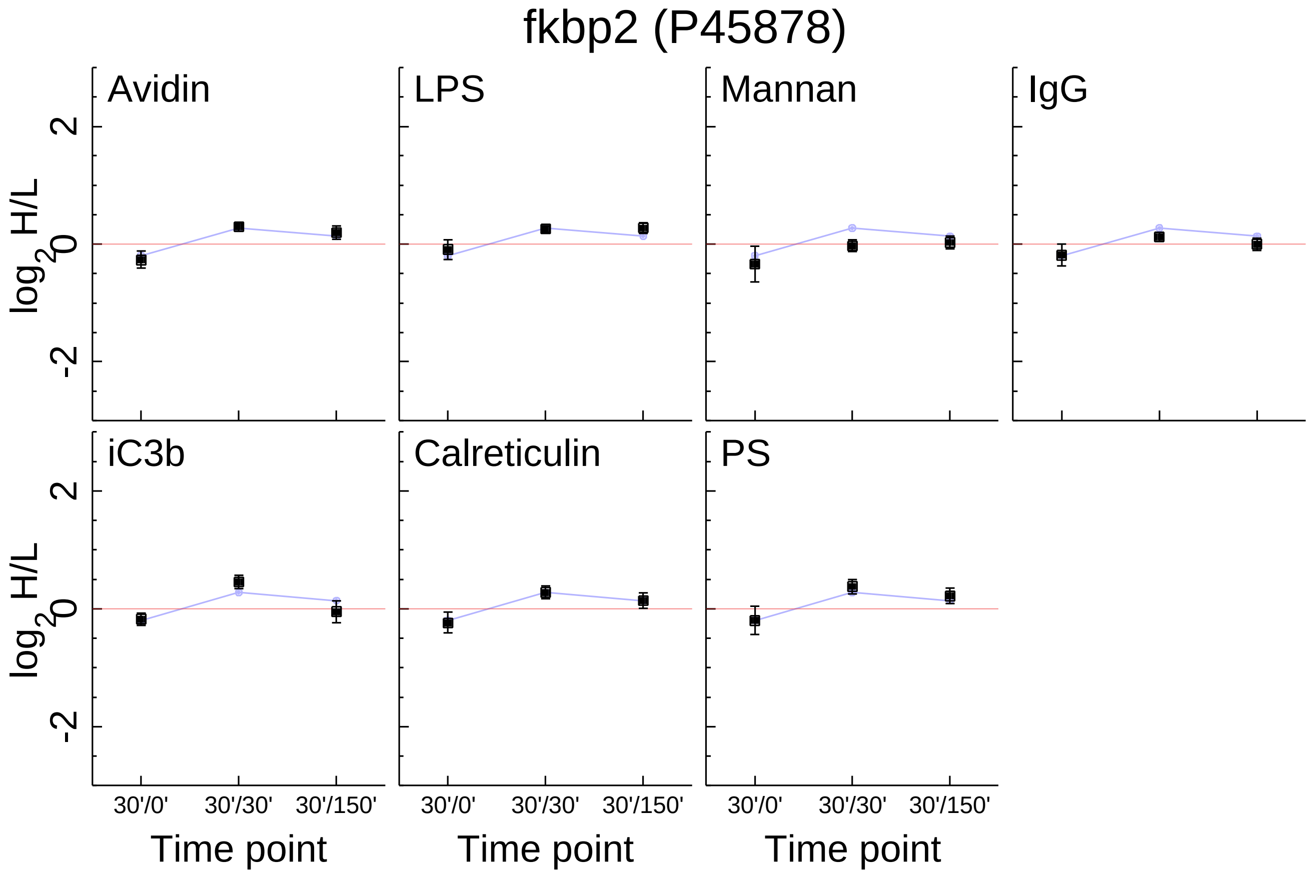
fkbp2 (P45878)
Avidin
LPS
Mannan
IgG
iC3b
Calreticulin
PS
log2 H/L
2
0
-2
30'/0'
30'/30'
30'/150'
Time point

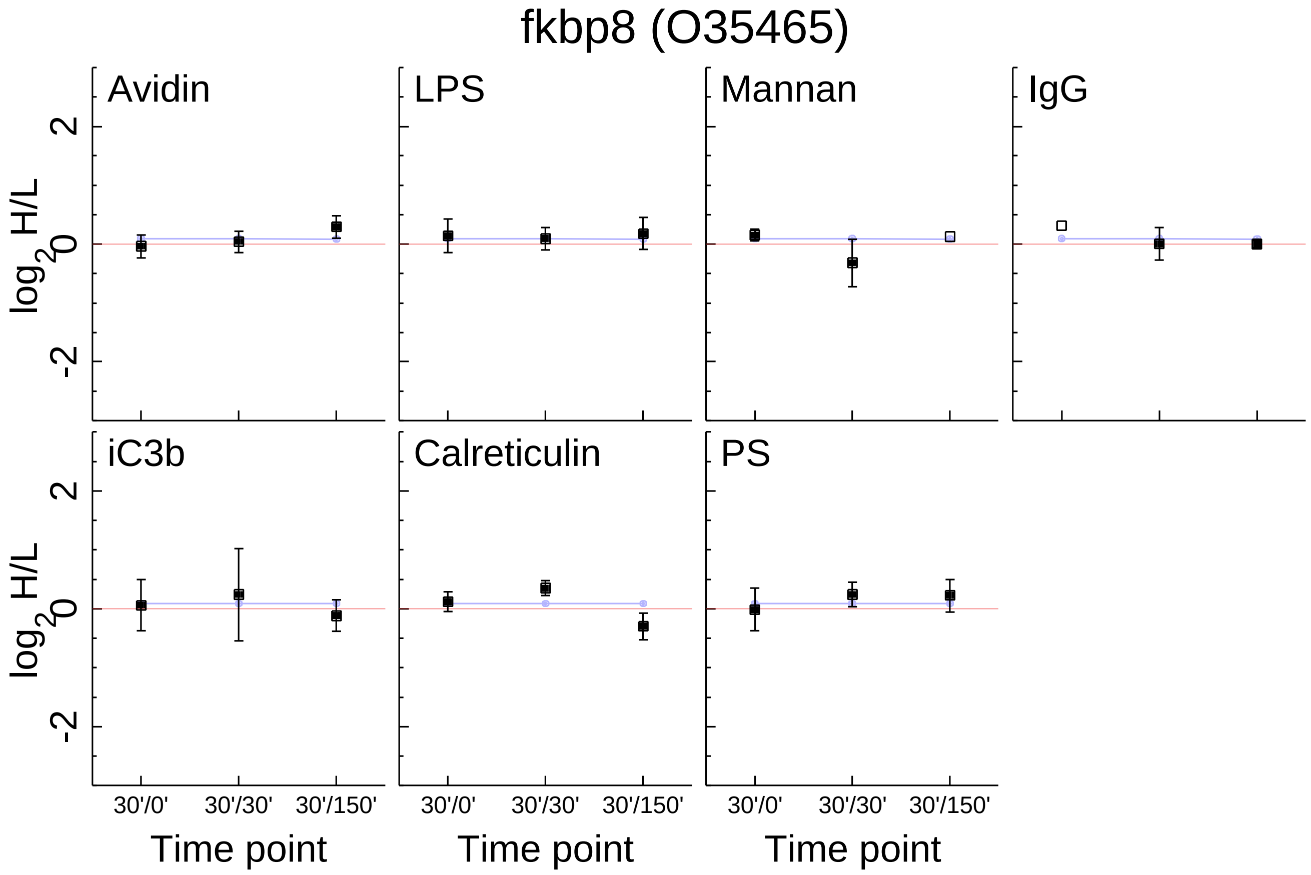

fkbp8 (O35465)
Avidin
LPS
Mannan
IgG
iC3b
Calreticulin
PS
log2 H/L
2
0
-2
30'/0'
30'/30'
30'/150'
Time point

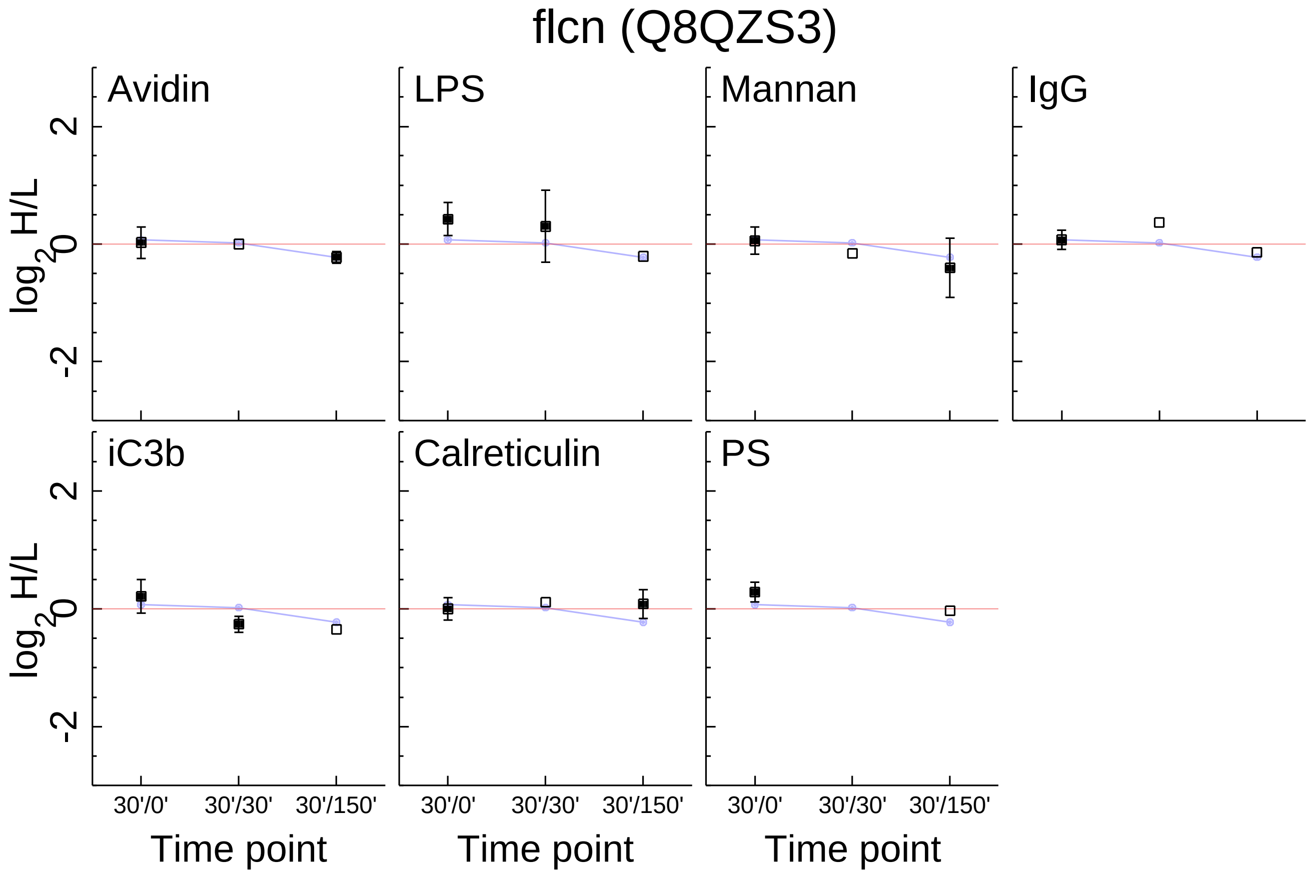
flcn (Q8QZS3)
Avidin
LPS
Mannan
IgG
iC3b
Calreticulin
PS
log2 H/L
2
0
-2
30'/0'
30'/30'
30'/150'
Time point

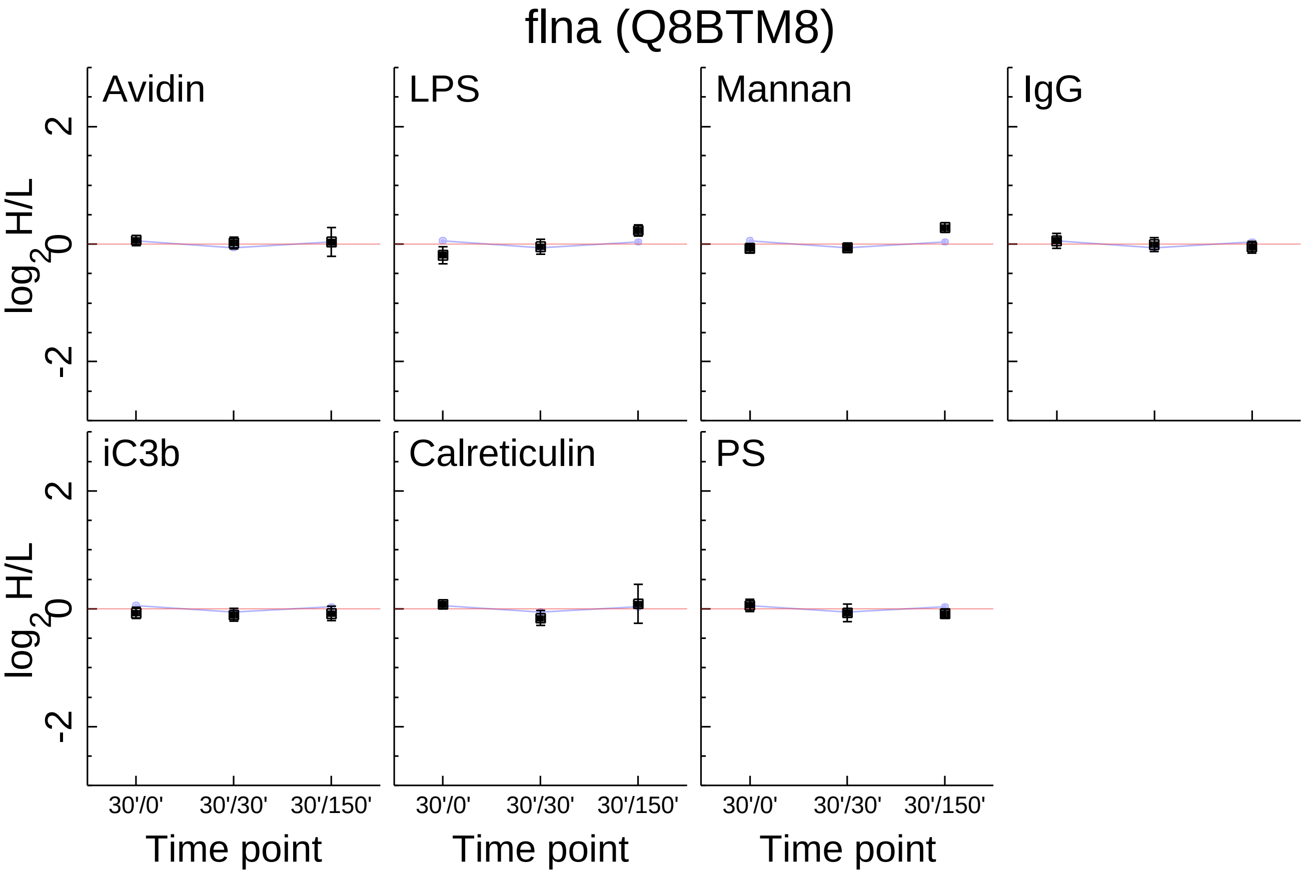
flna (Q8BTM8)
Avidin
LPS
Mannan
IgG
iC3b
Calreticulin
PS
log2 H/L
2
0
-2
30'/0'
30'/30'
30'/150'
Time point

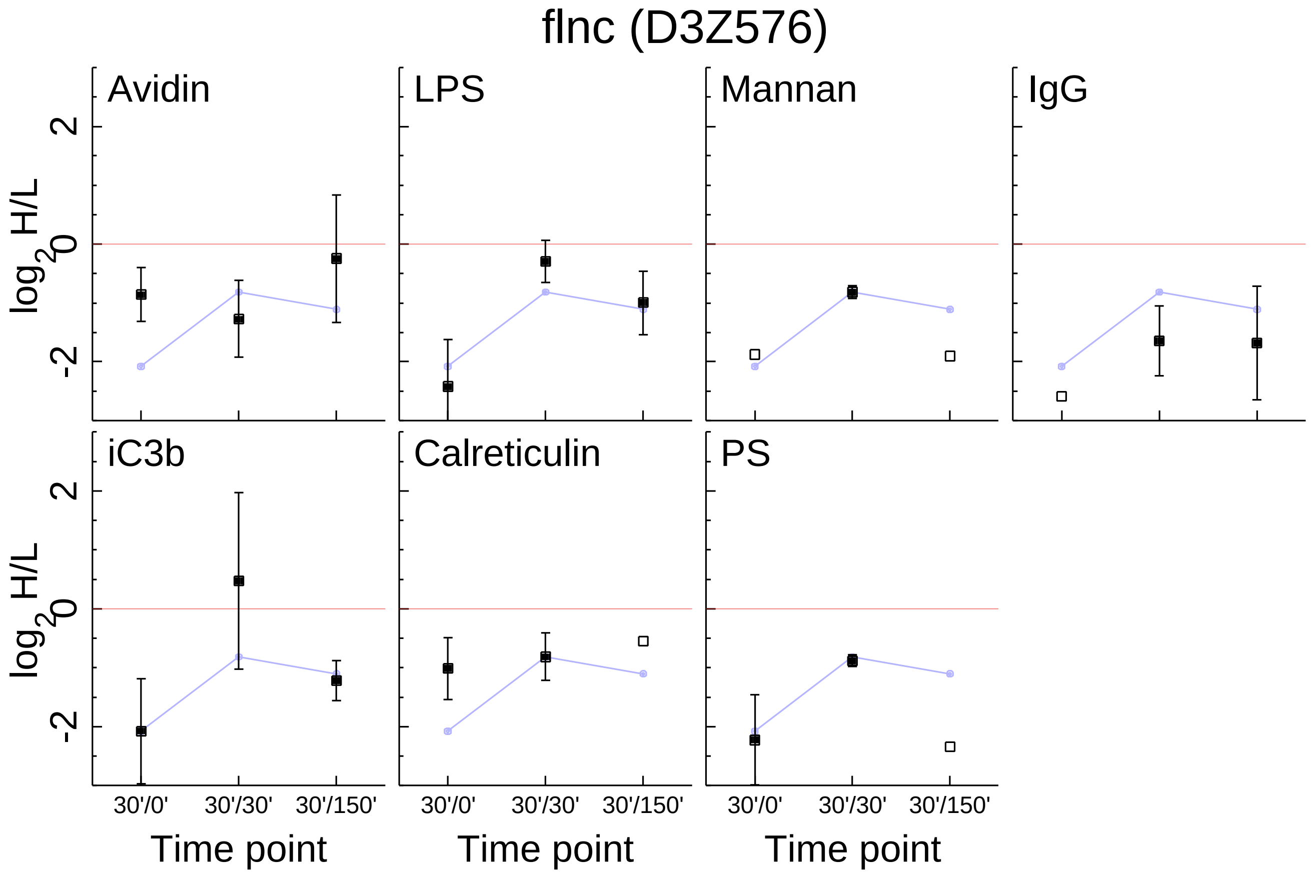
flnc (D3Z576)
Avidin
LPS
Mannan
IgG
iC3b
Calreticulin
PS
log2 H/L
2
0
-2
30'/0'
30'/30'
30'/150'
Time point

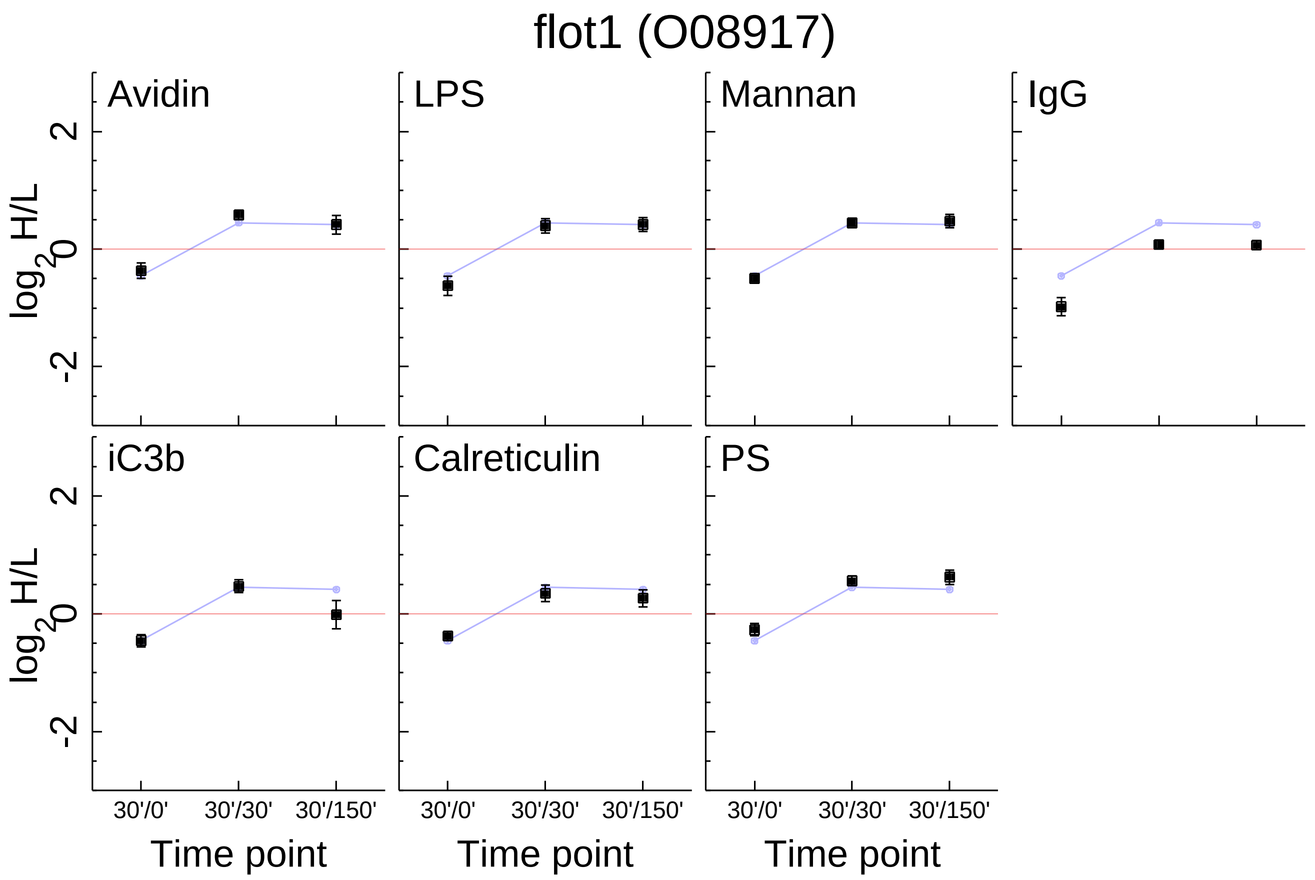
flot1 (O08917)
Avidin
LPS
Mannan
IgG
iC3b
Calreticulin
PS
log2 H/L
2
0
-2
30'/0'
30'/30'
30'/150'
Time point

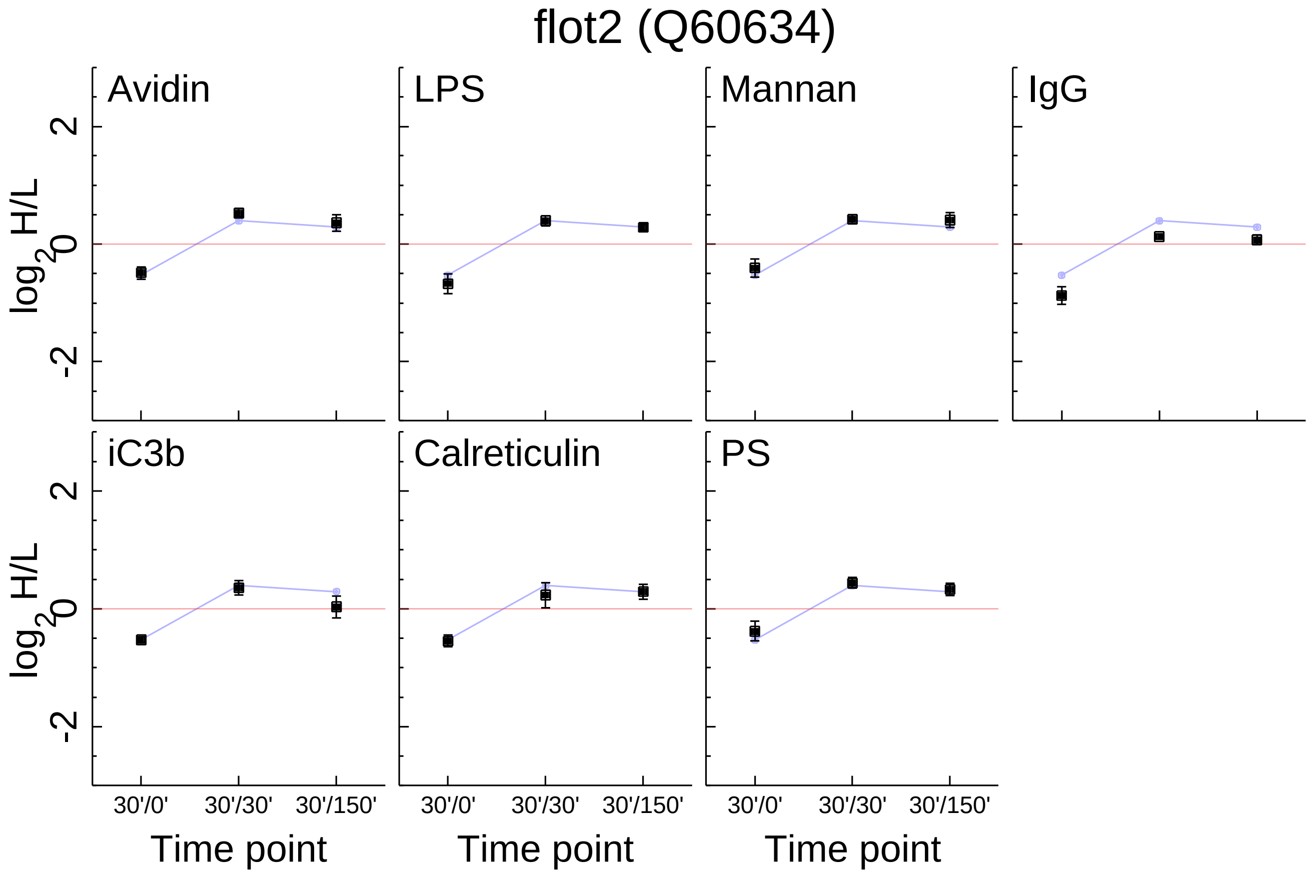

flot2 (Q60634)
Avidin
LPS
Mannan
IgG
iC3b
Calreticulin
PS
log2 H/L
2
0
-2
30'/0'
30'/30'
30'/150'
Time point

fmnl1 (Q9JL26)

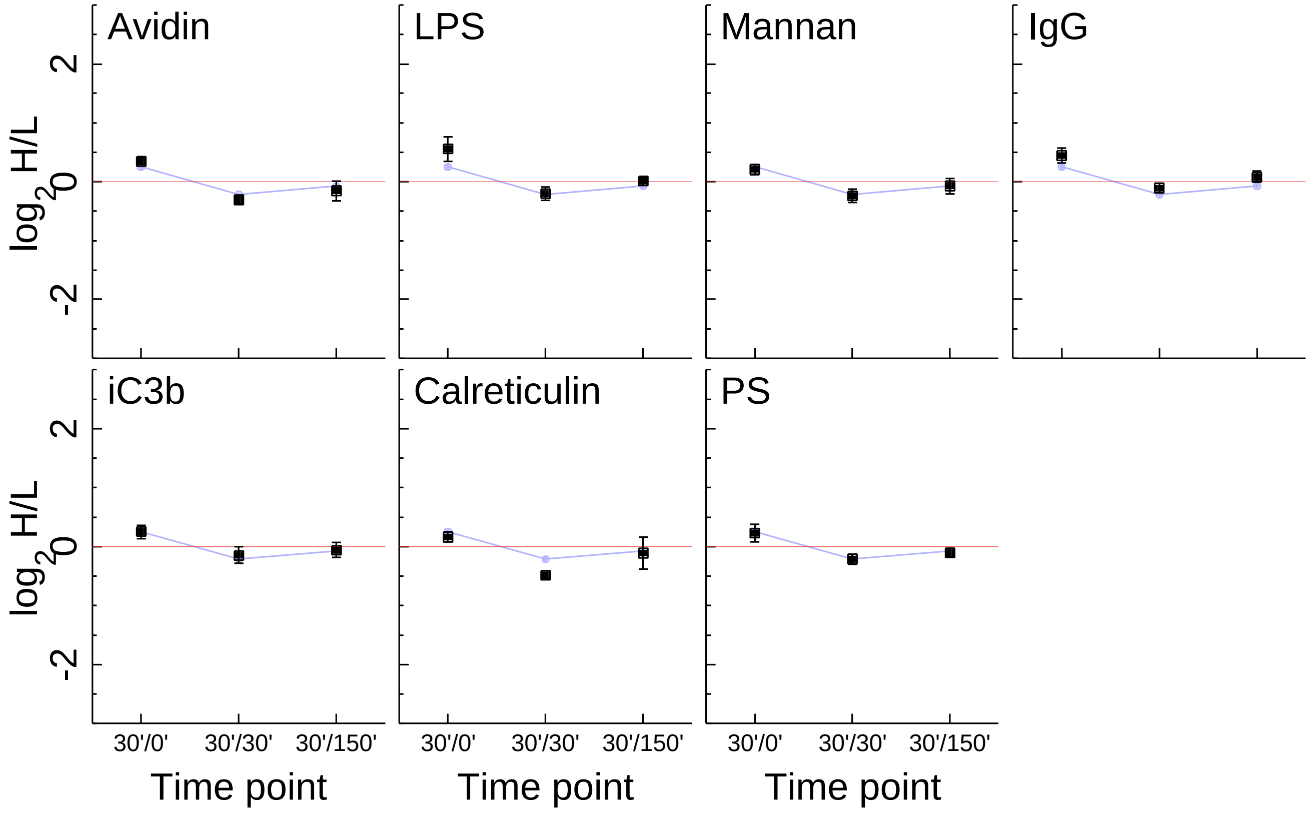

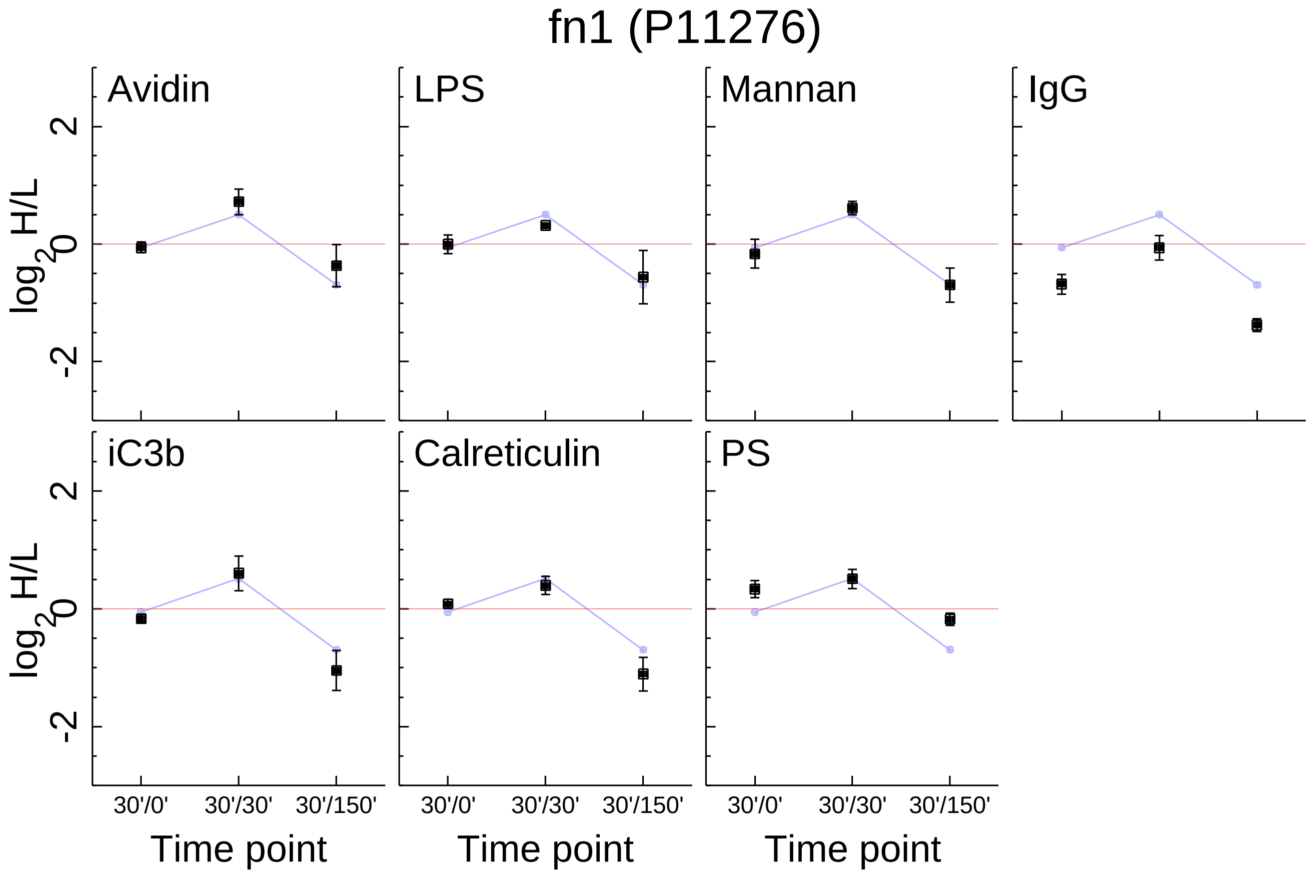
fn1 (P11276)
Avidin
LPS
Mannan
IgG
iC3b
Calreticulin
PS
log2 H/L
2
0
-2
30'/0'
30'/30'
30'/150'
Time point

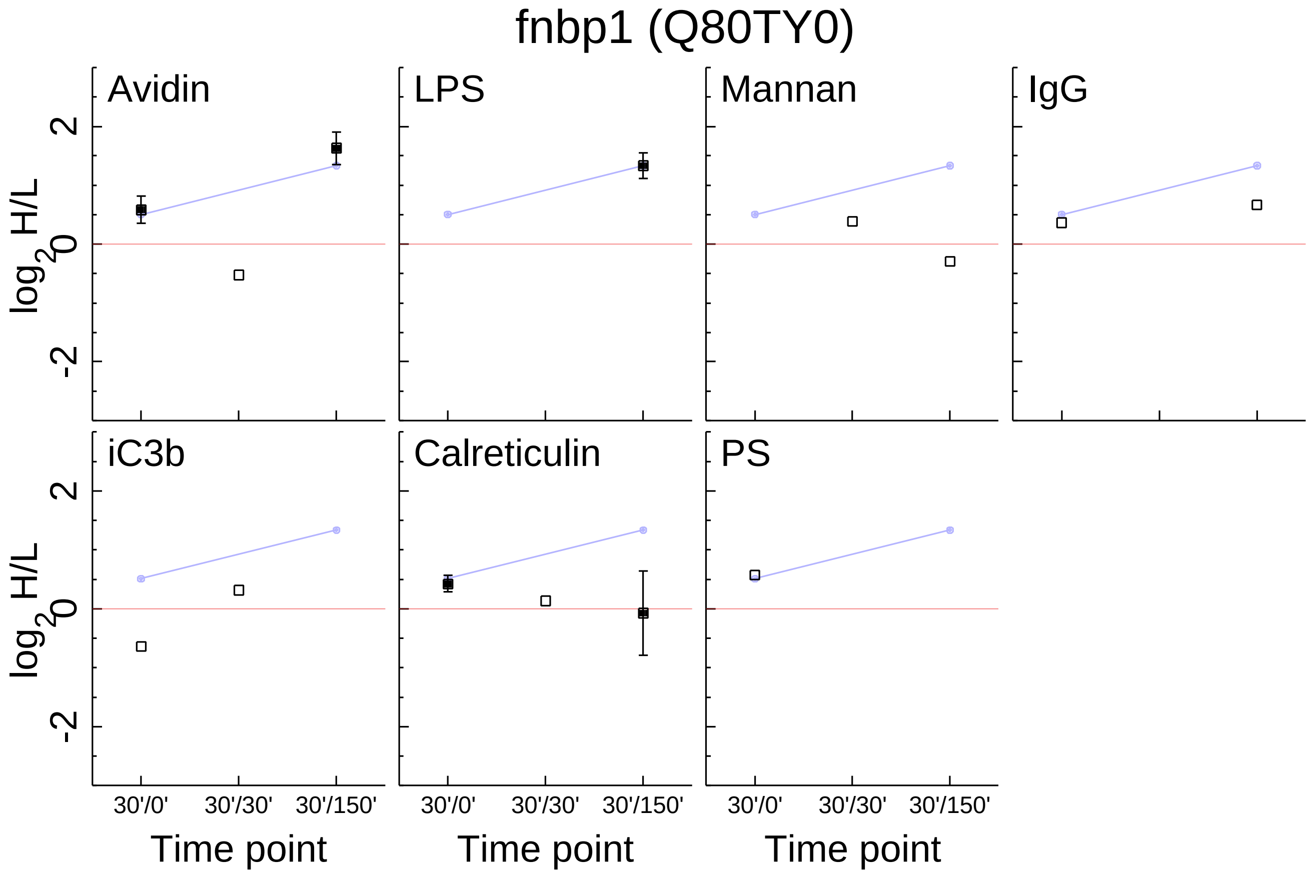
fnbp1 (Q80TY0)
Avidin
LPS
Mannan
IgG
iC3b
Calreticulin
PS
log2 H/L
2
0
-2
30'/0'
30'/30'
30'/150'
Time point

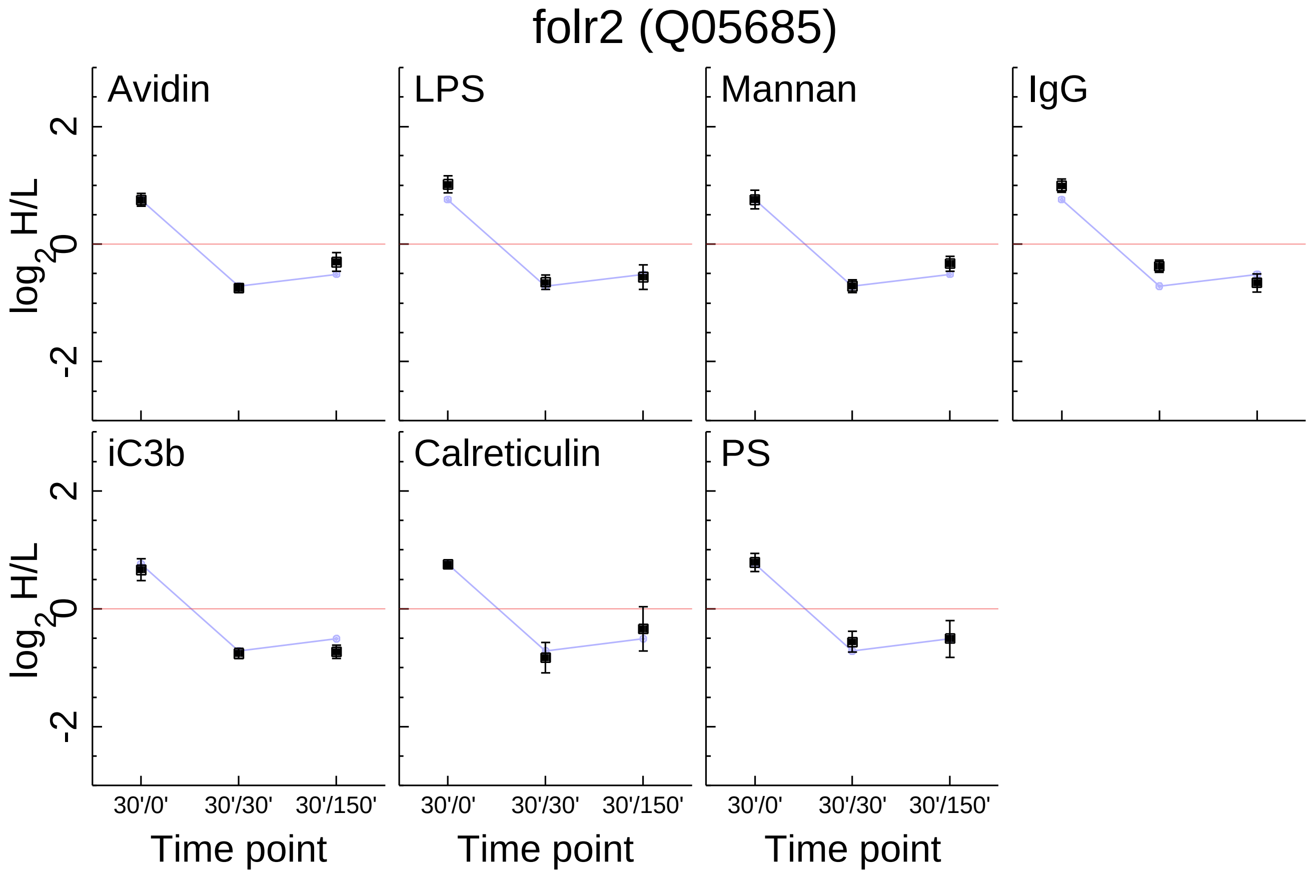
folr2 (Q05685)
Avidin
LPS
Mannan
IgG
iC3b
Calreticulin
PS
log2 H/L
2
0
-2
30'/0'
30'/30'
30'/150'
Time point

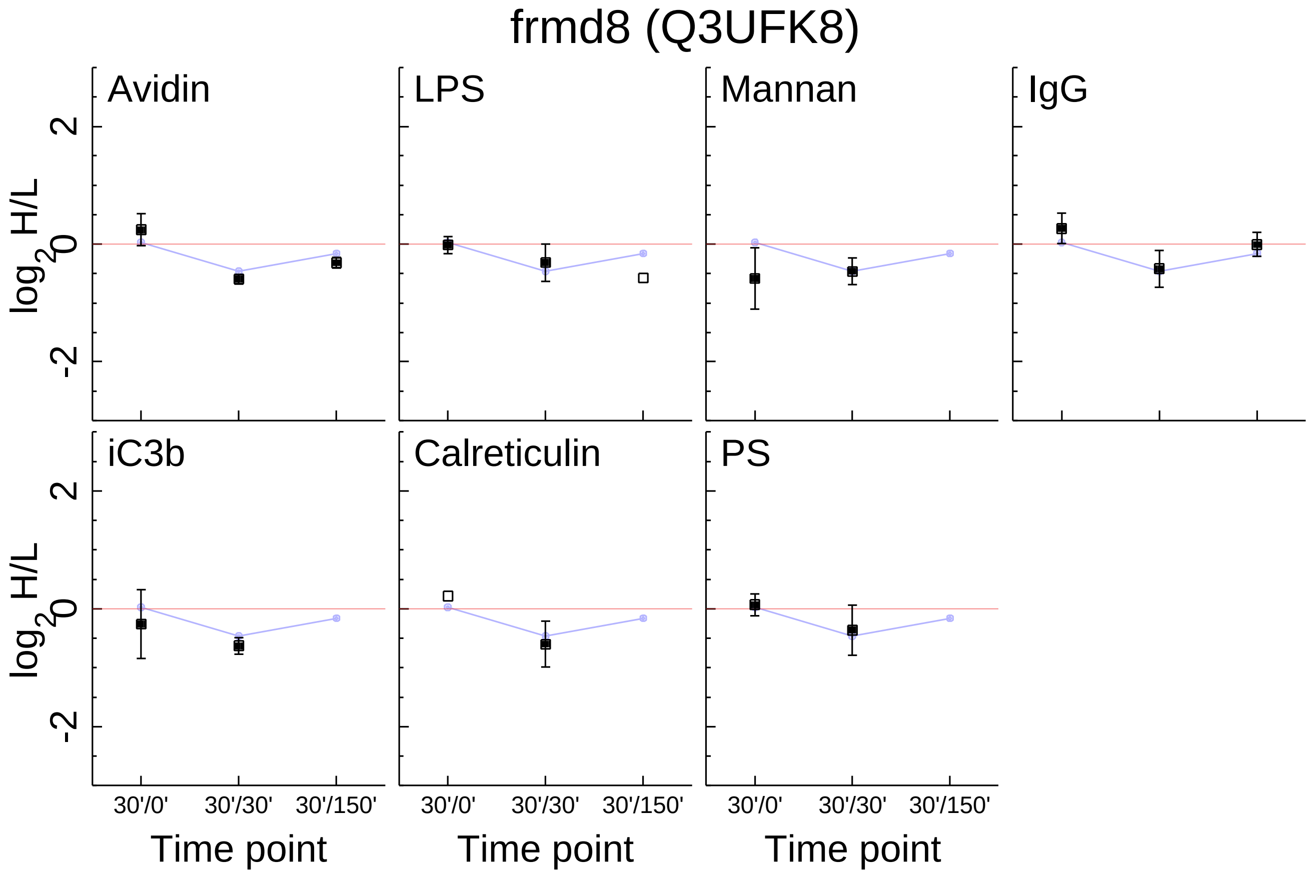
frmd8 (Q3UFK8)
Avidin
LPS
Mannan
IgG
iC3b
Calreticulin
PS
log2 H/L
2
0
-2
30'/0'
30'/30'
30'/150'
Time point

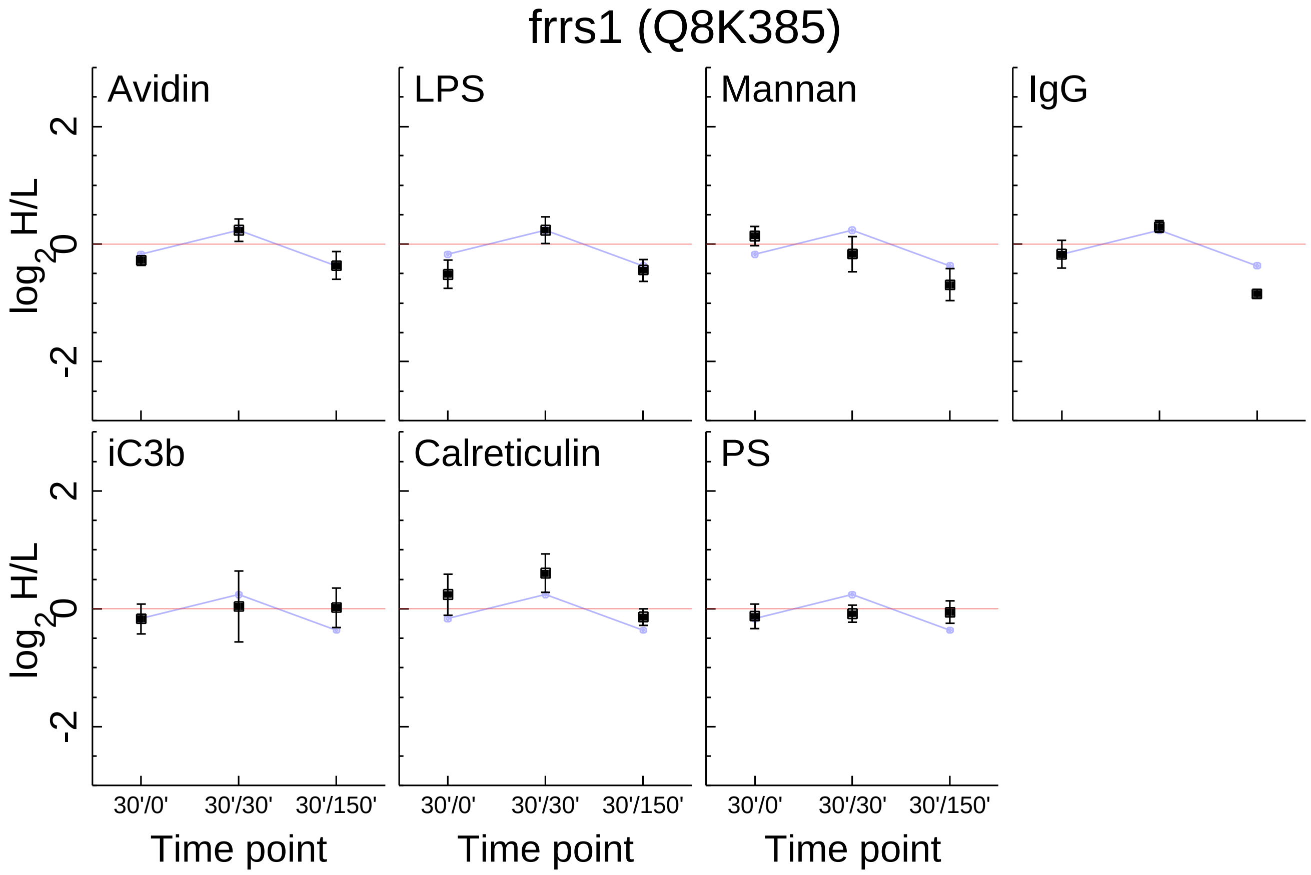

frrs1 (Q8K385)
Avidin
LPS
Mannan
IgG
iC3b
Calreticulin
PS
log2 H/L
2
0
-2
30'/0'
30'/30'
30'/150'
Time point

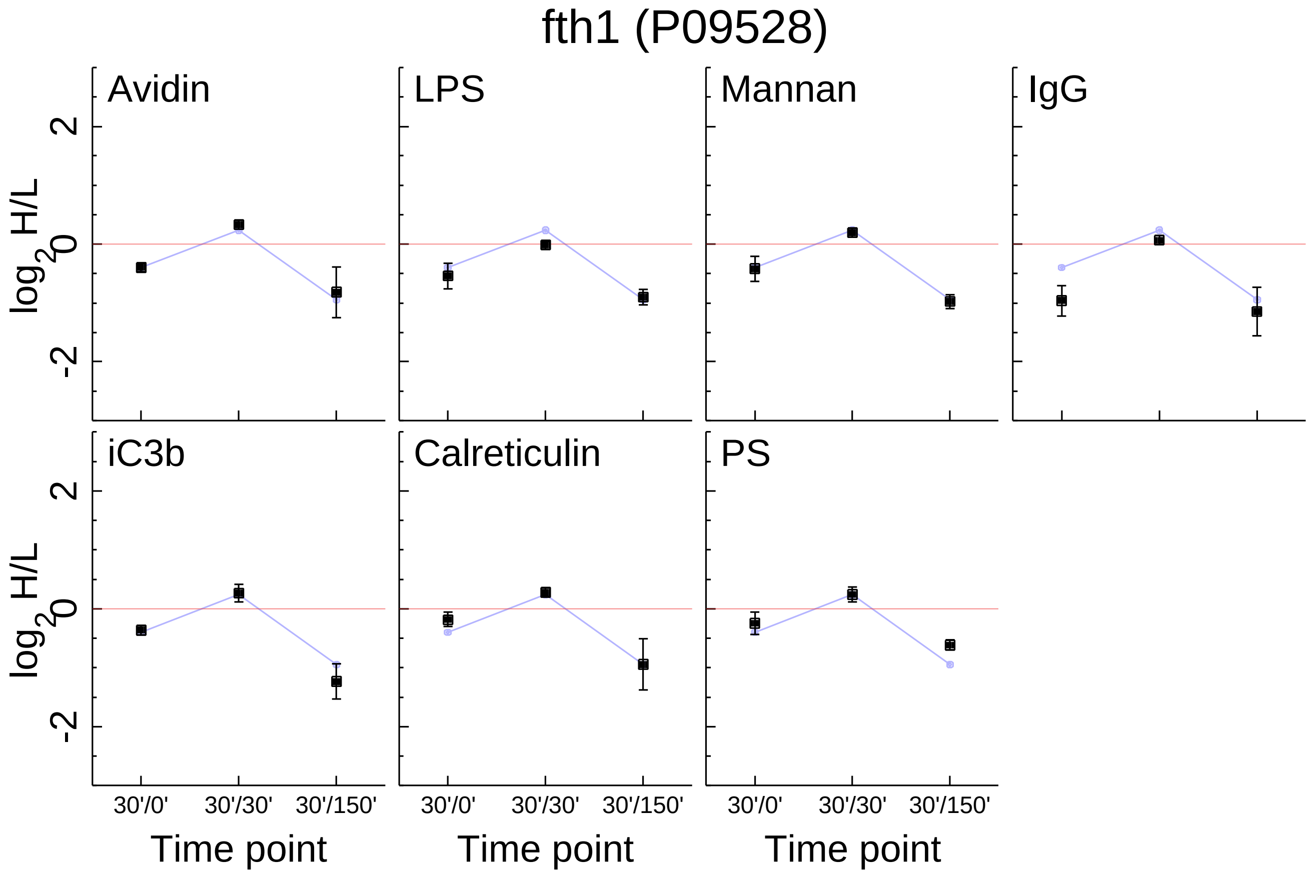
fth1 (P09528)
Avidin
LPS
Mannan
IgG
iC3b
Calreticulin
PS
log2 H/L
2
0
-2
30'/0'
30'/30'
30'/150'
Time point

ftl1 (Q9CPX4)

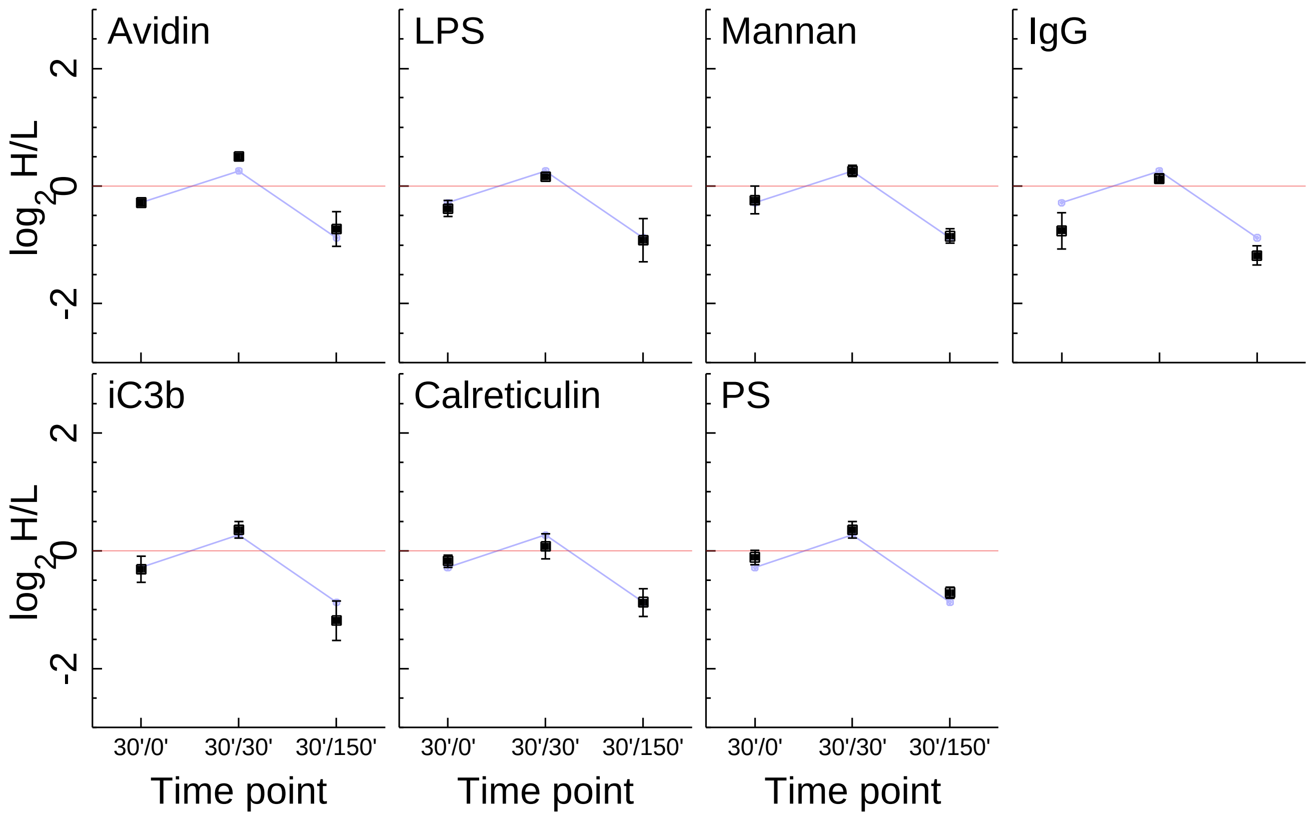

Avidin
LPS
Mannan
IgG
iC3b
Calreticulin
PS
log2 H/L
2
0
-2
30'/0'
30'/30'
30'/150'
Time point

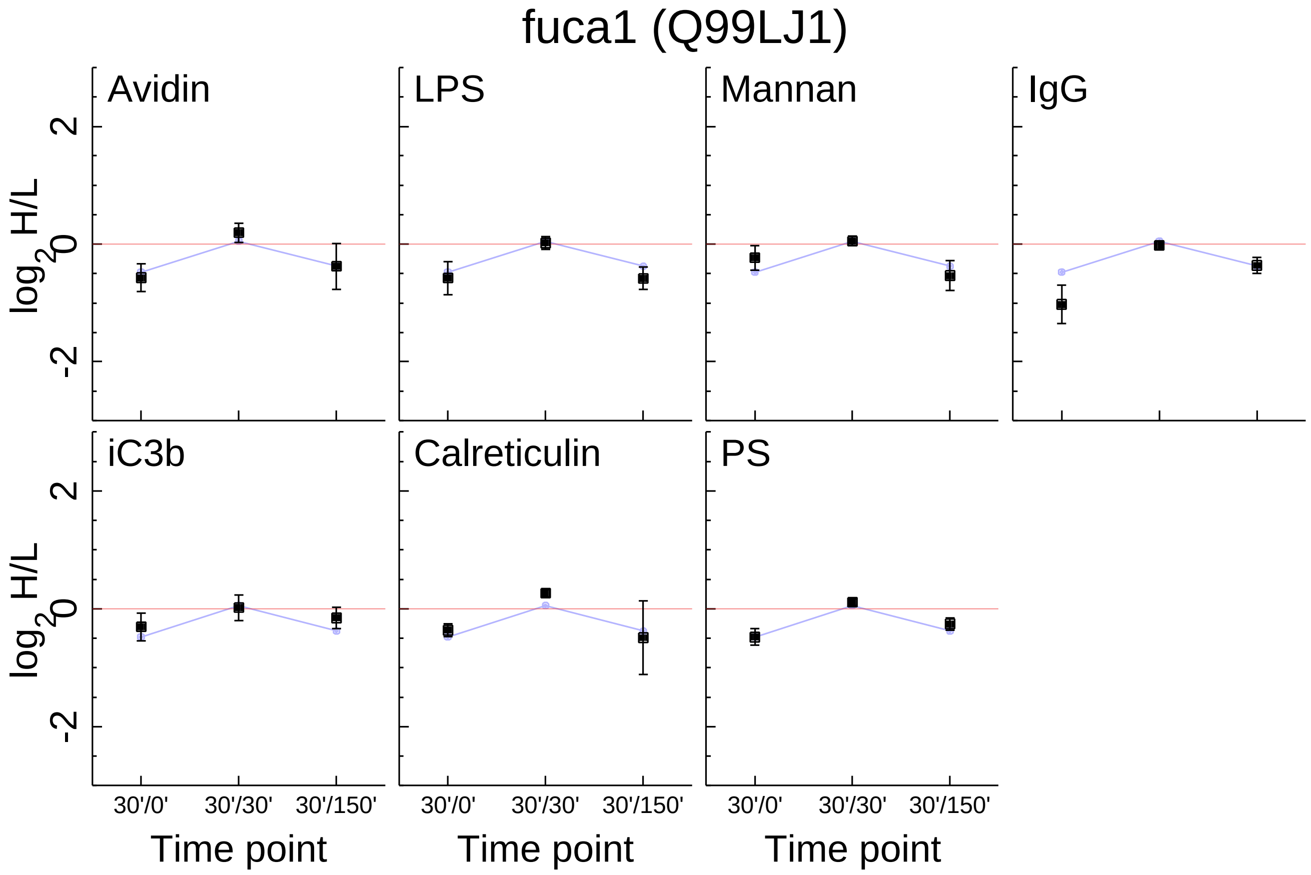

fuca1 (Q99LJ1)
Avidin
LPS
Mannan
IgG
iC3b
Calreticulin
PS
log2 H/L
2
0
-2
30'/0'
30'/30'
30'/150'
Time point

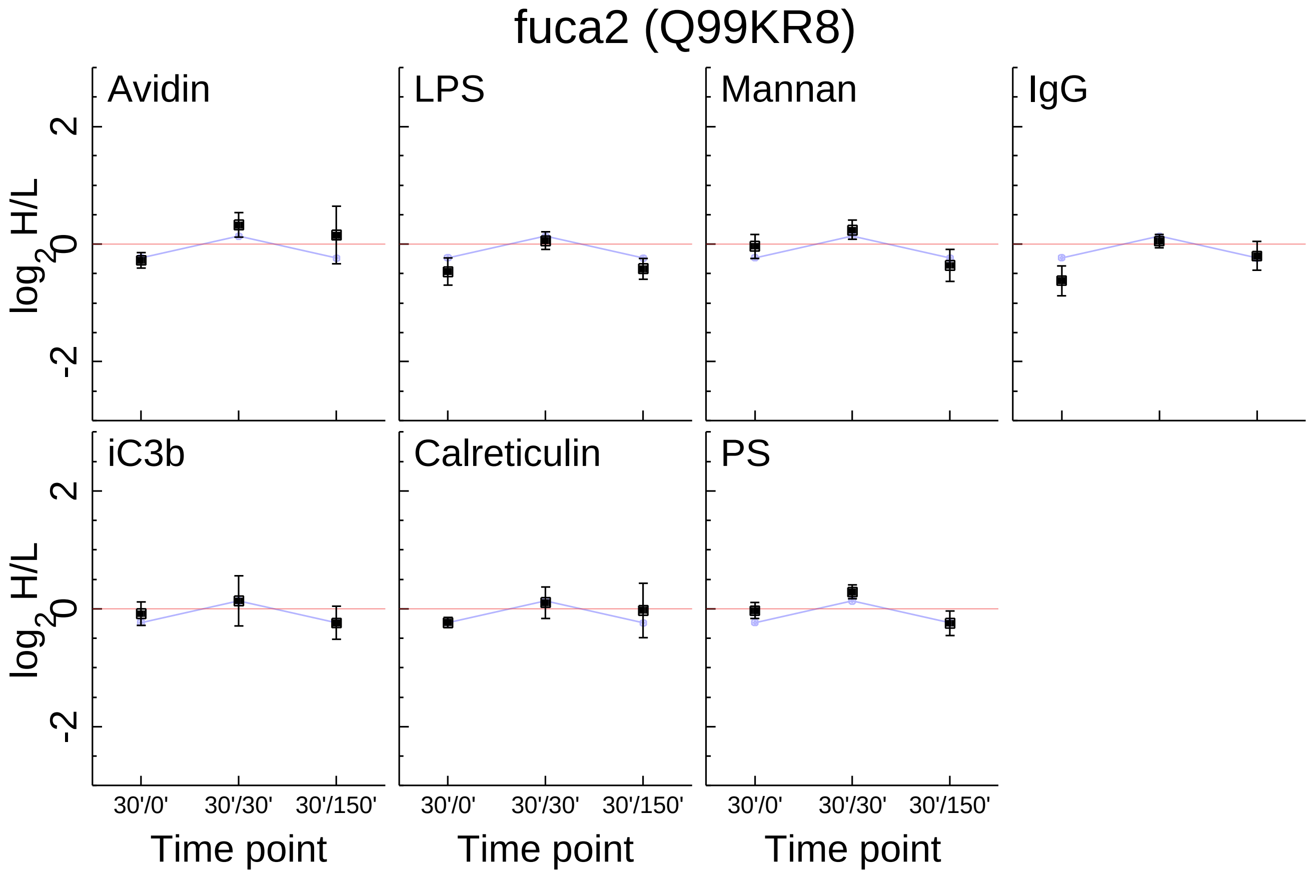

fuca2 (Q99KR8)
Avidin
LPS
Mannan
IgG
iC3b
Calreticulin
PS
log2 H/L
2
0
-2
30'/0'
30'/30'
30'/150'
Time point

fus (P56959)

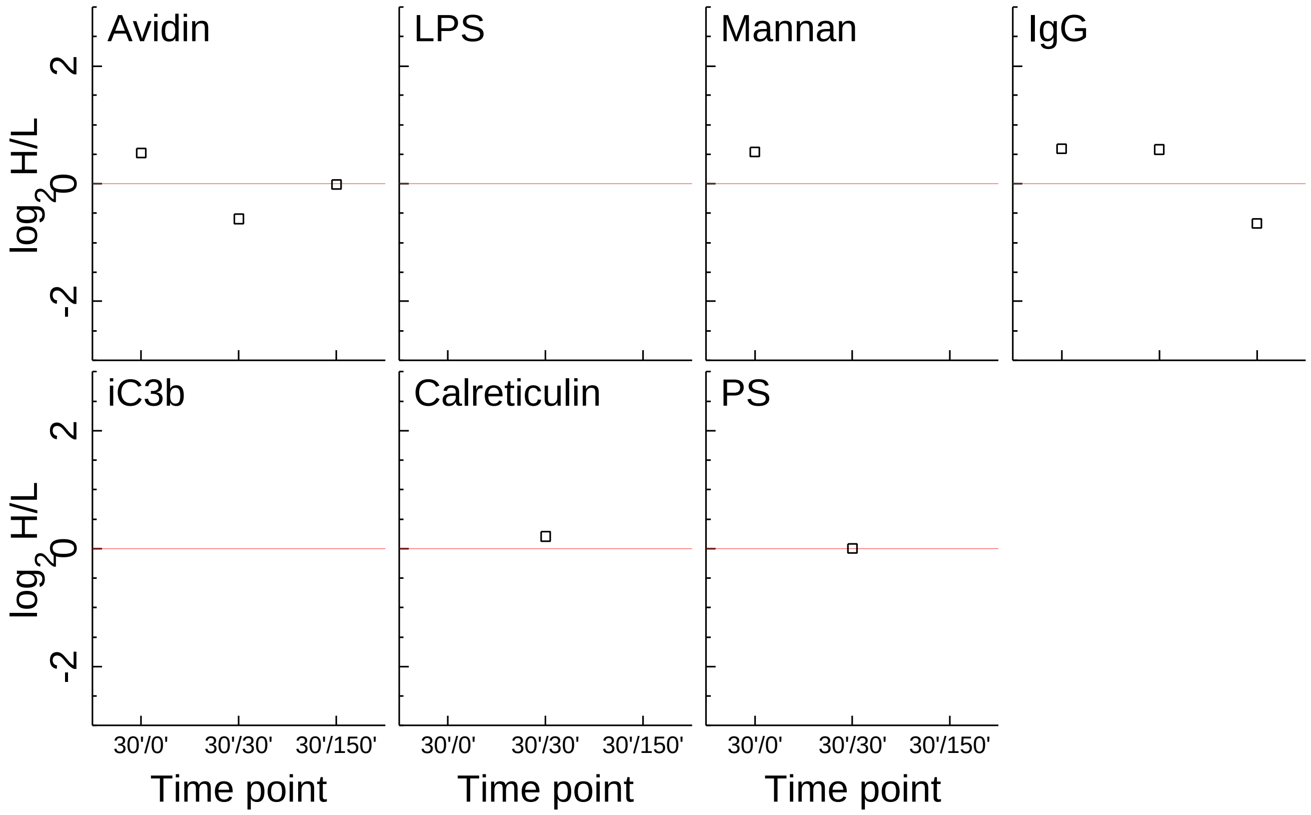

Avidin
LPS
Mannan
IgG
iC3b
Calreticulin
PS
log2 H/L
2
0
-2
30'/0'
30'/30'
30'/150'
Time point

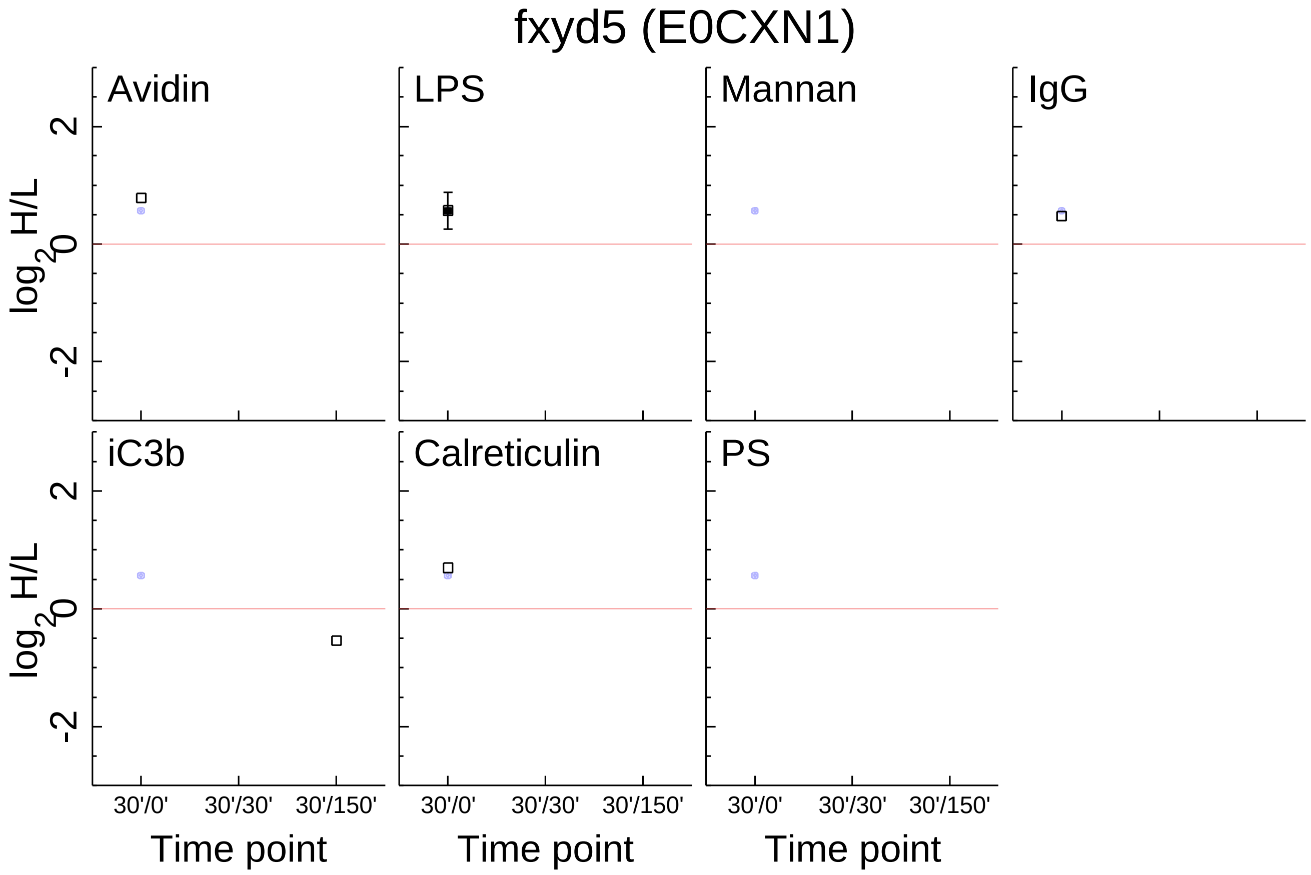

fxyd5 (E0CXN1)
Avidin
LPS
Mannan
IgG
iC3b
Calreticulin
PS
log2 H/L
2
0
-2
30'/0'
30'/30'
30'/150'
Time point

fyb (O35601)

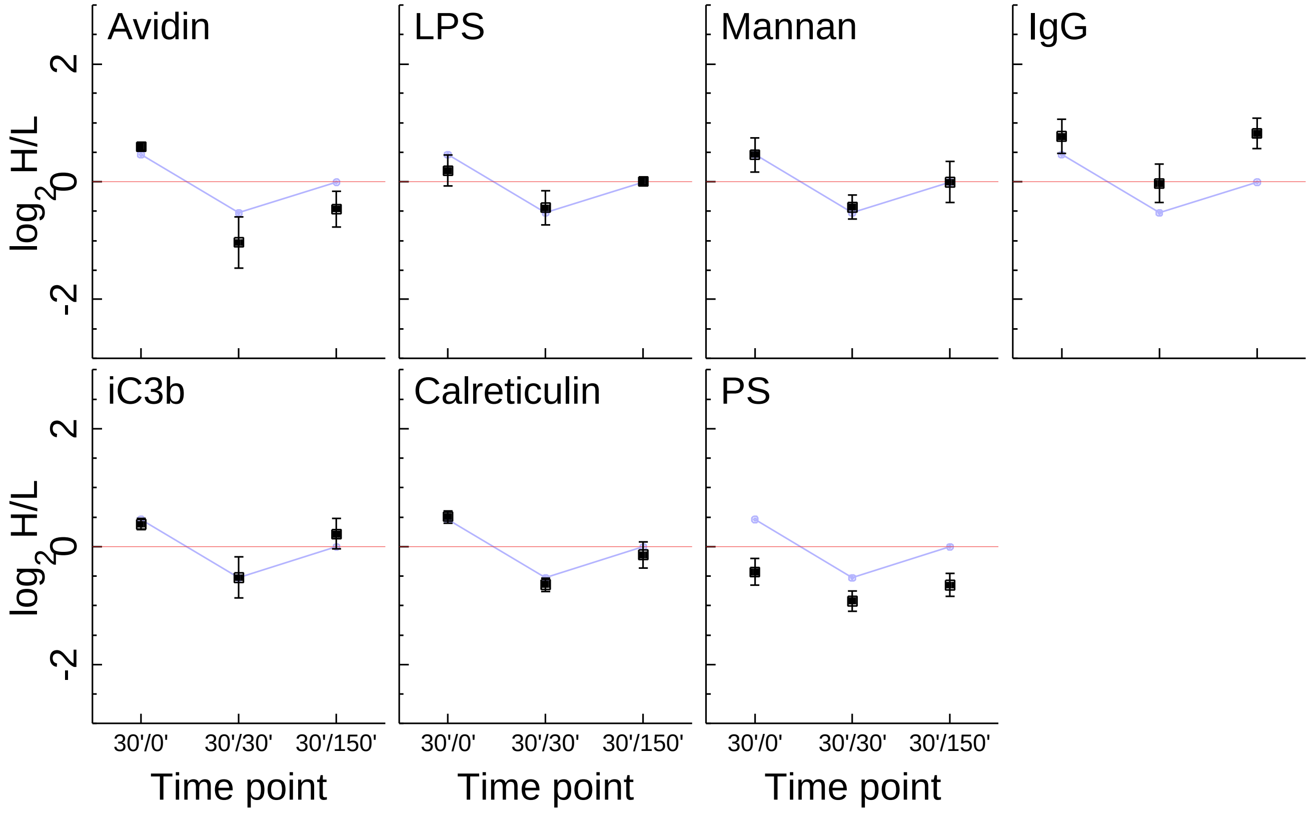

Avidin
LPS
Mannan
IgG
iC3b
Calreticulin
PS
log2 H/L
2
0
-2
30'/0'
30'/30'
30'/150'
Time point

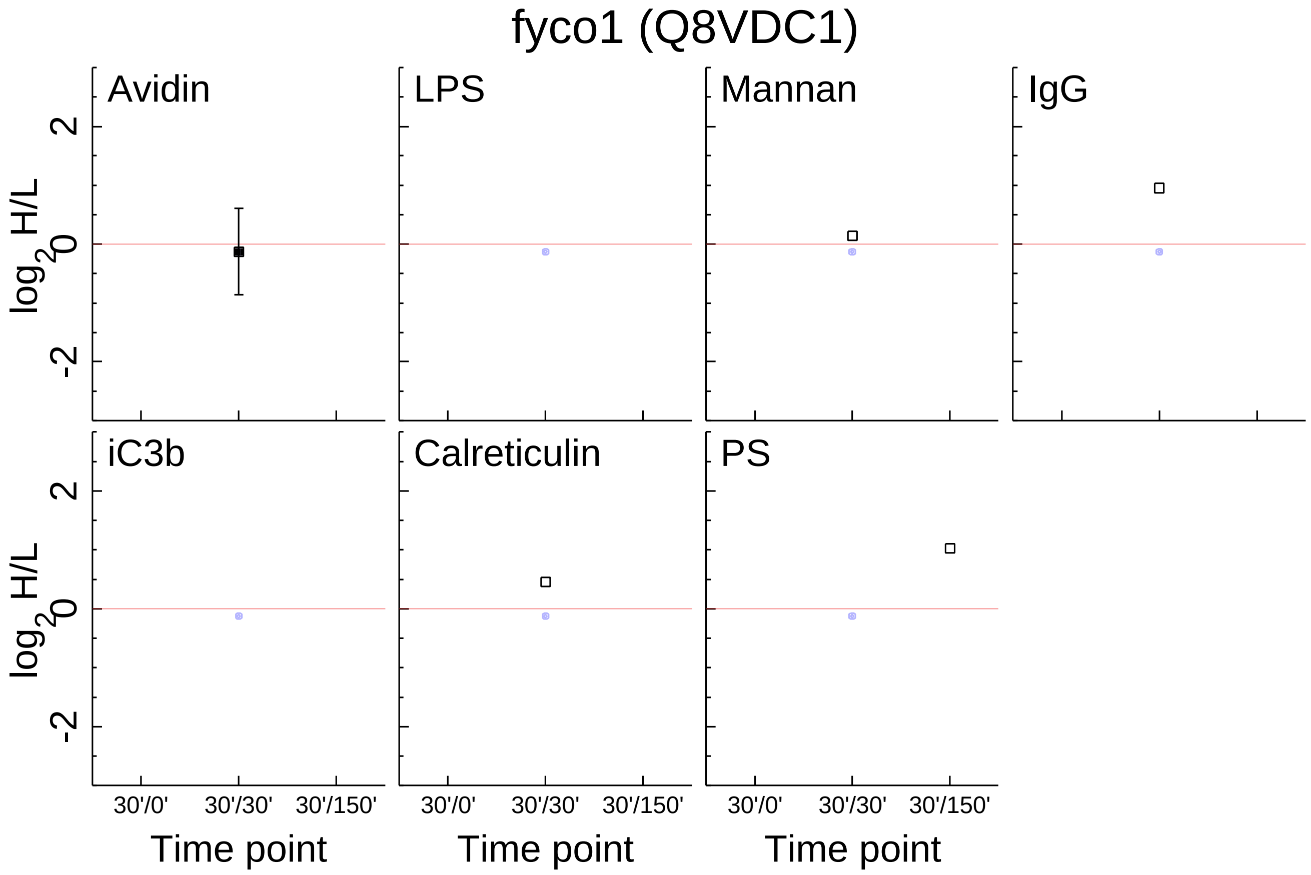

fyco1 (Q8VDC1)
Avidin
LPS
Mannan
IgG
iC3b
Calreticulin
PS
log2 H/L
2
0
-2
30'/0'
30'/30'
30'/150'
Time point

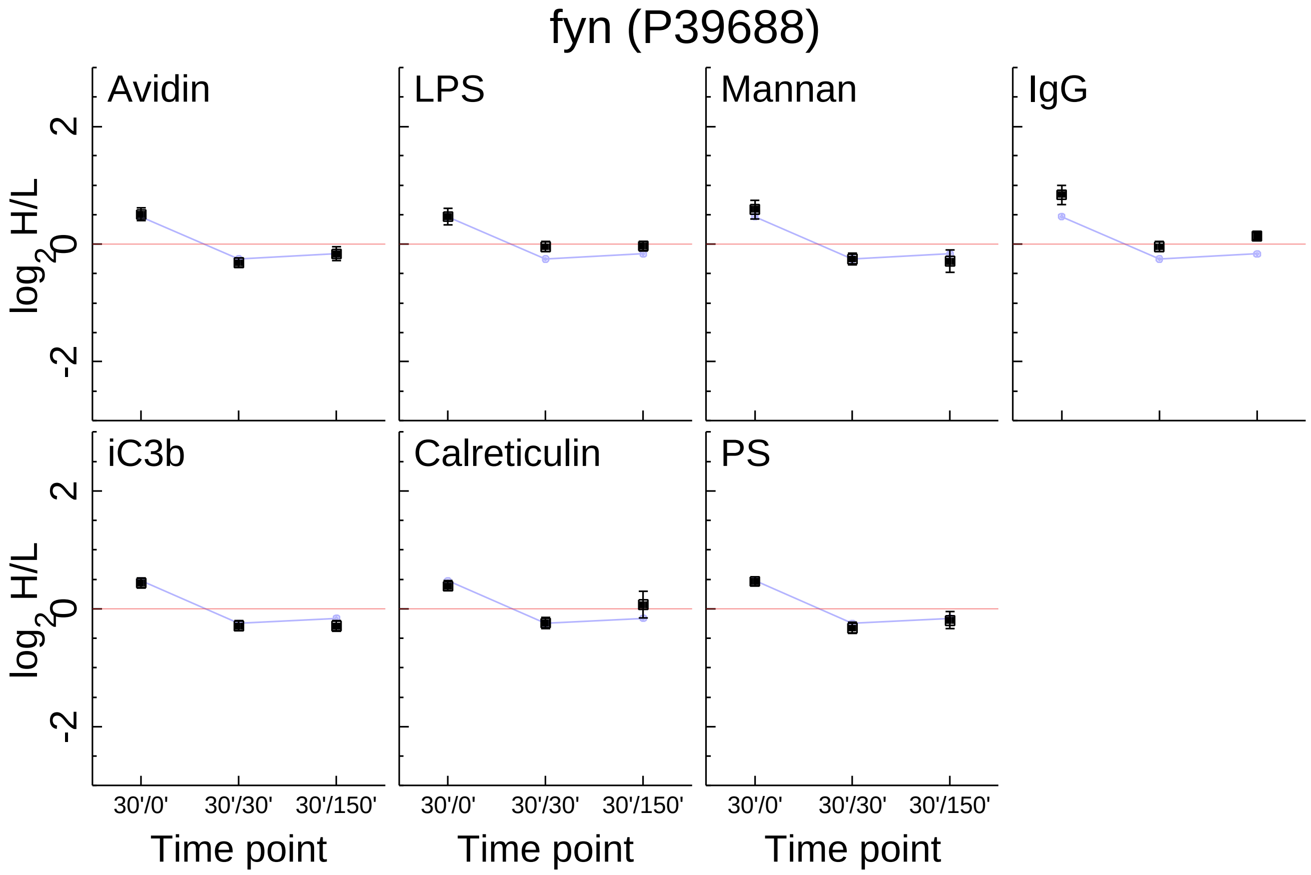

fyn (P39688)
Avidin
LPS
Mannan
IgG
iC3b
Calreticulin
PS
log2 H/L
2
0
-2
30'/0'
30'/30'
30'/150'
Time point

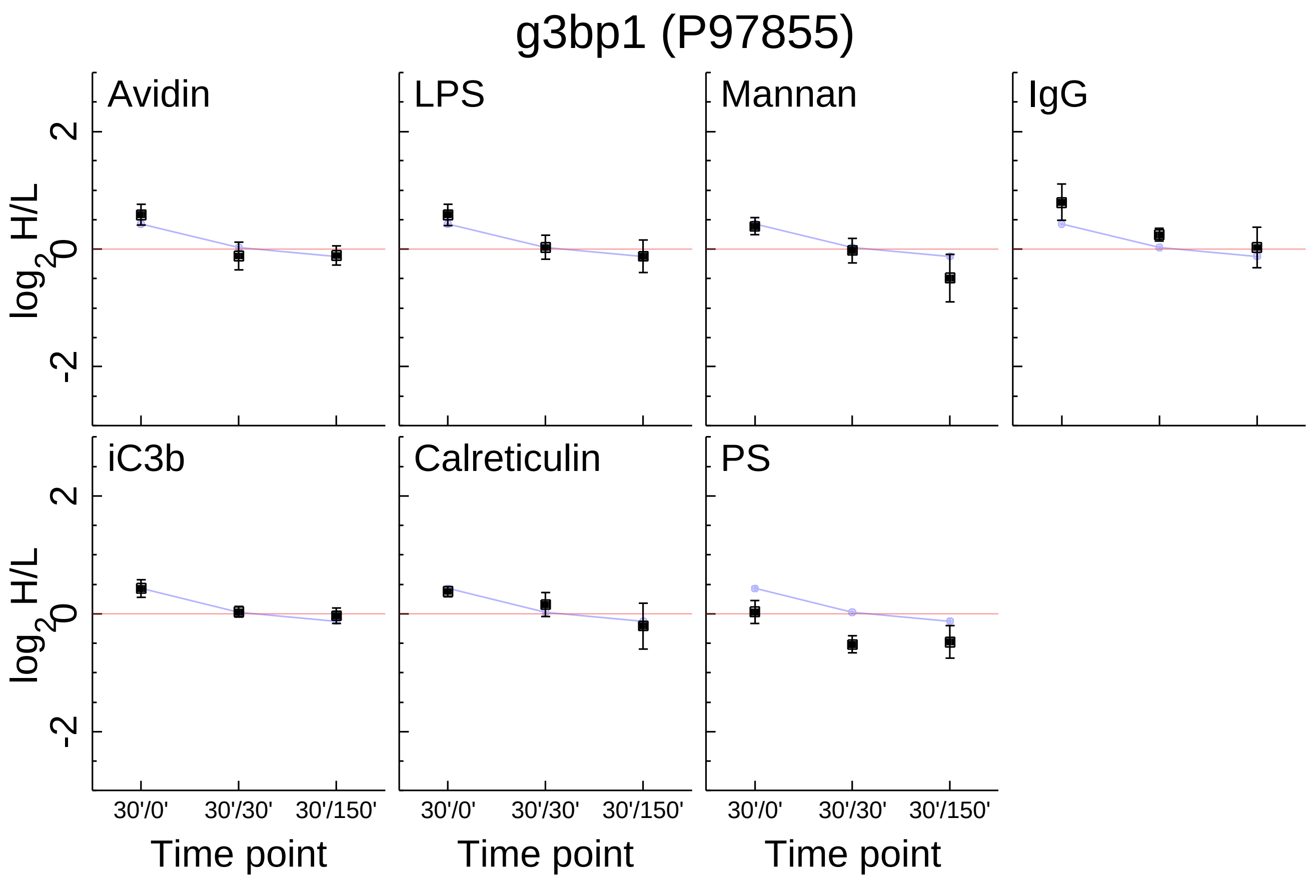

g3bp1 (P97855)
Avidin
LPS
Mannan
IgG
iC3b
Calreticulin
PS
log2 H/L
2
0
-2
30'/0'
30'/30'
30'/150'
Time point

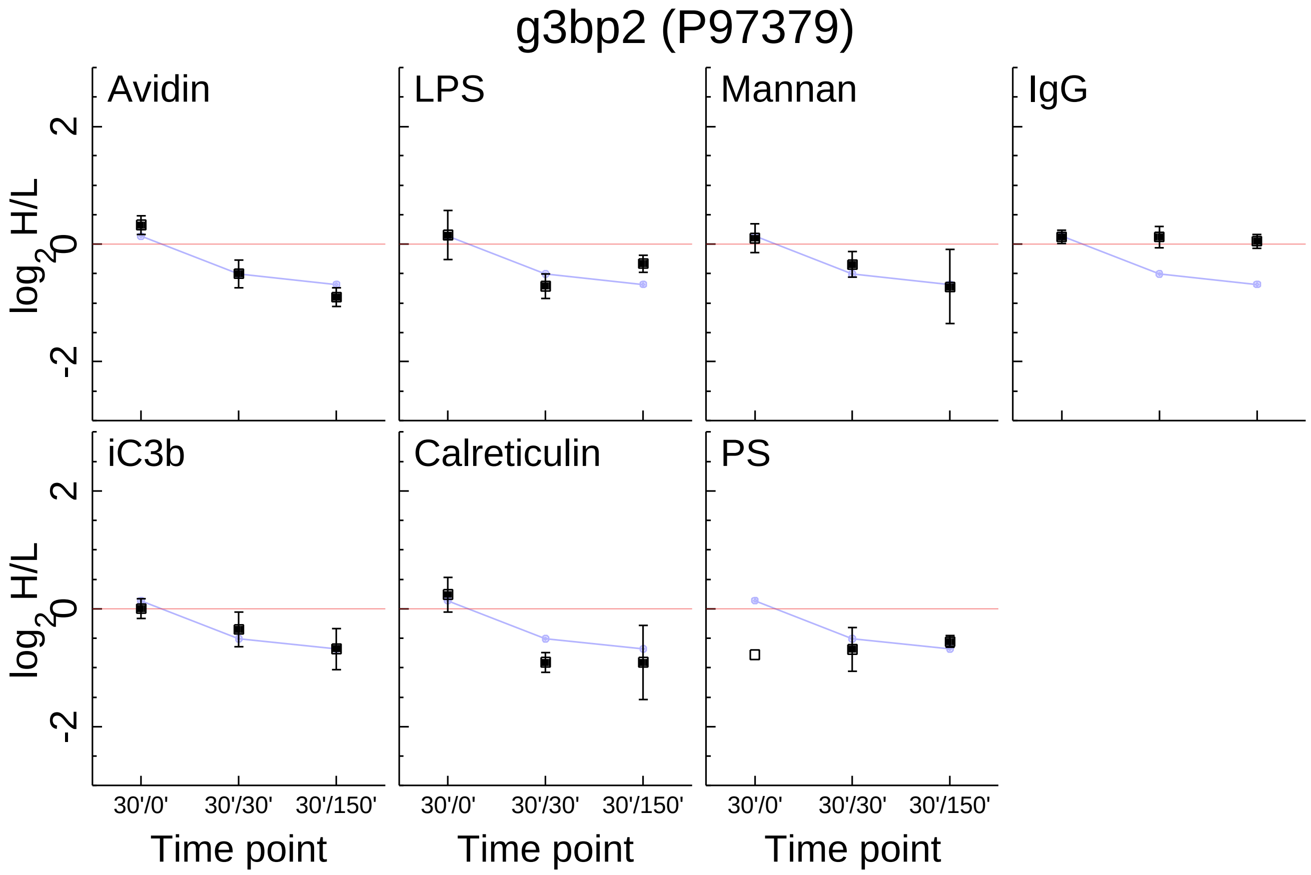
g3bp2 (P97379)
Avidin
LPS
Mannan
IgG
iC3b
Calreticulin
PS
log2 H/L
2
0
-2
30'/0'
30'/30'
30'/150'
Time point

g6pdx (Q00612)

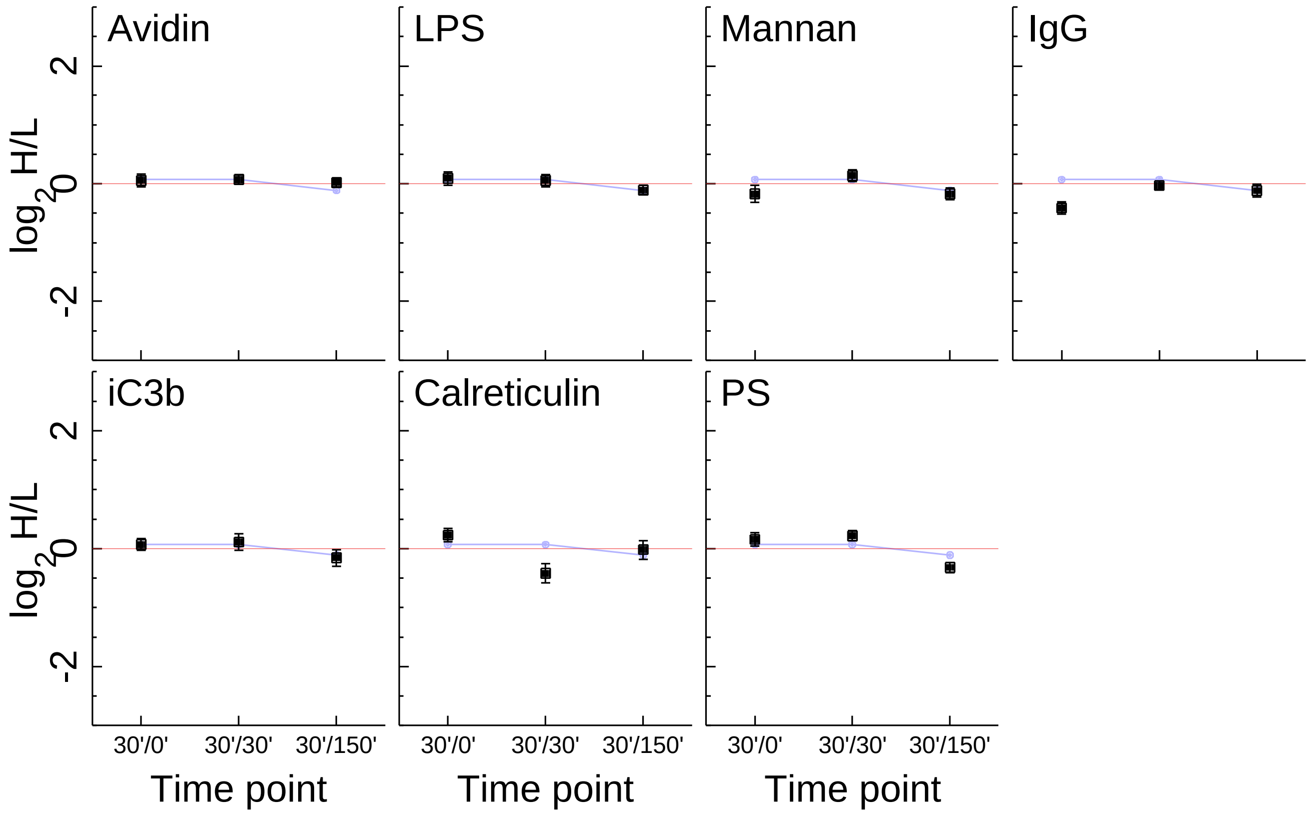

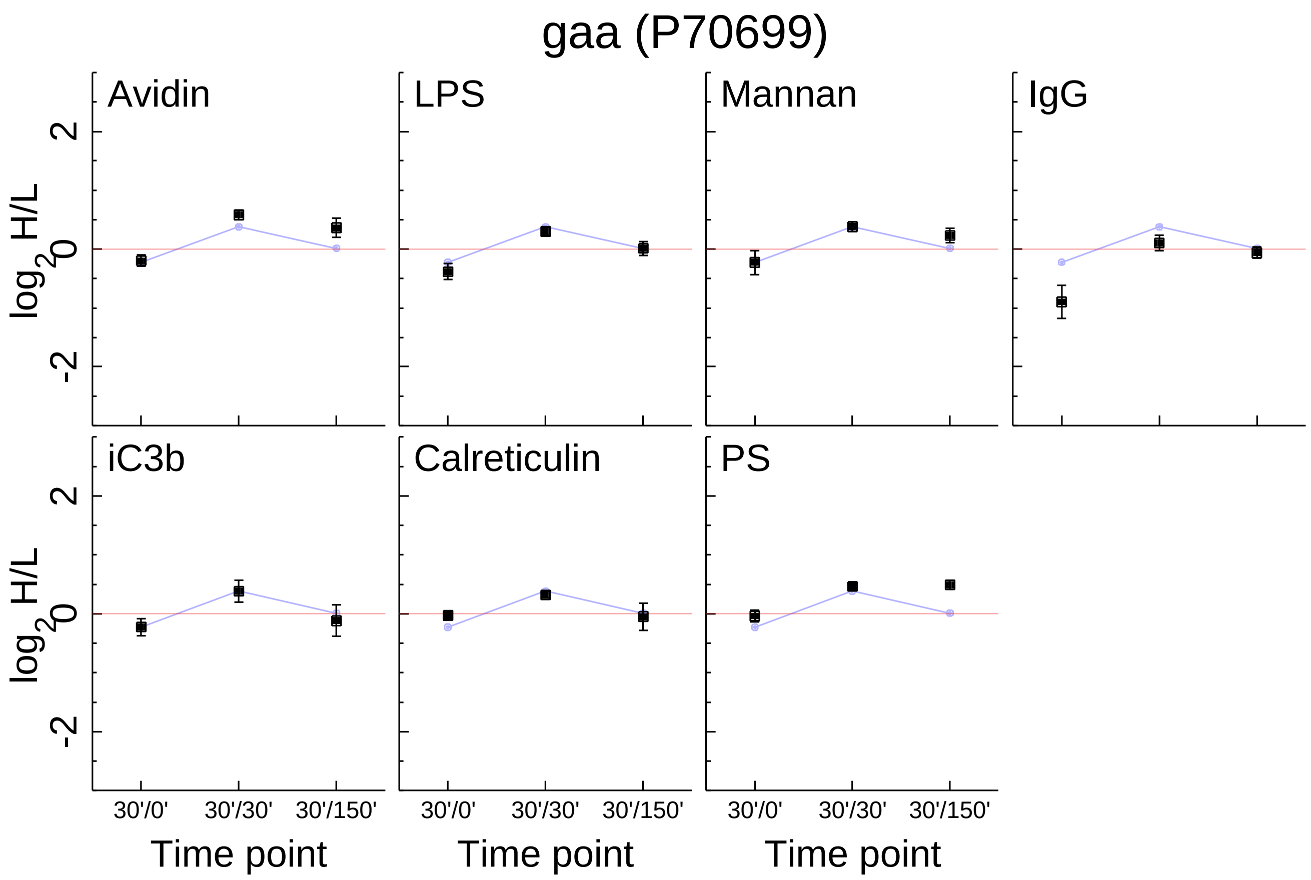
gaa (P70699)
Avidin
LPS
Mannan
IgG
iC3b
Calreticulin
PS
log2 H/L
2
0
-2
30'/0'
30'/30'
30'/150'
Time point

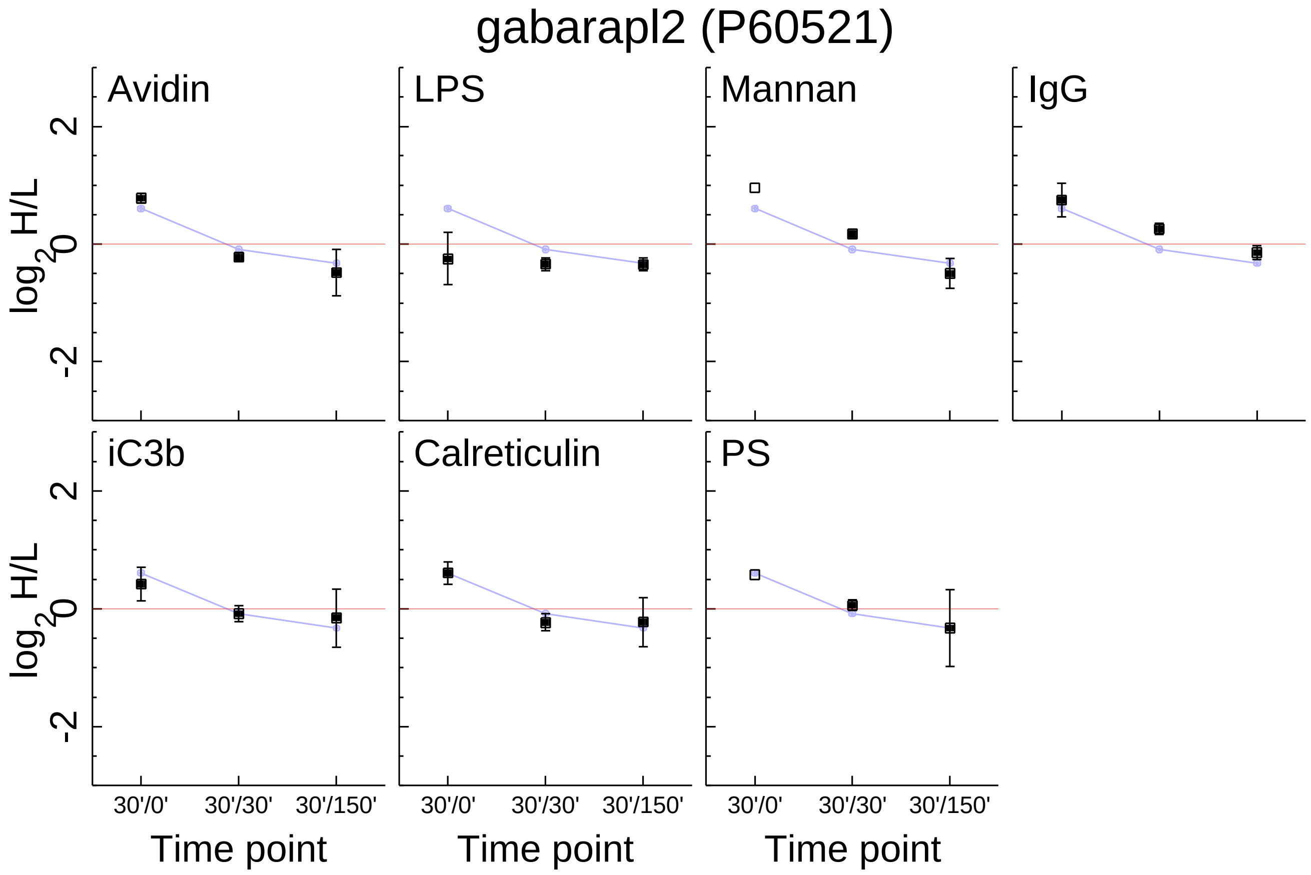
gabarapl2 (P60521)
Avidin
LPS
Mannan
IgG
iC3b
Calreticulin
PS
log2 H/L
2
0
-2
30'/0'
30'/30'
30'/150'
Time point

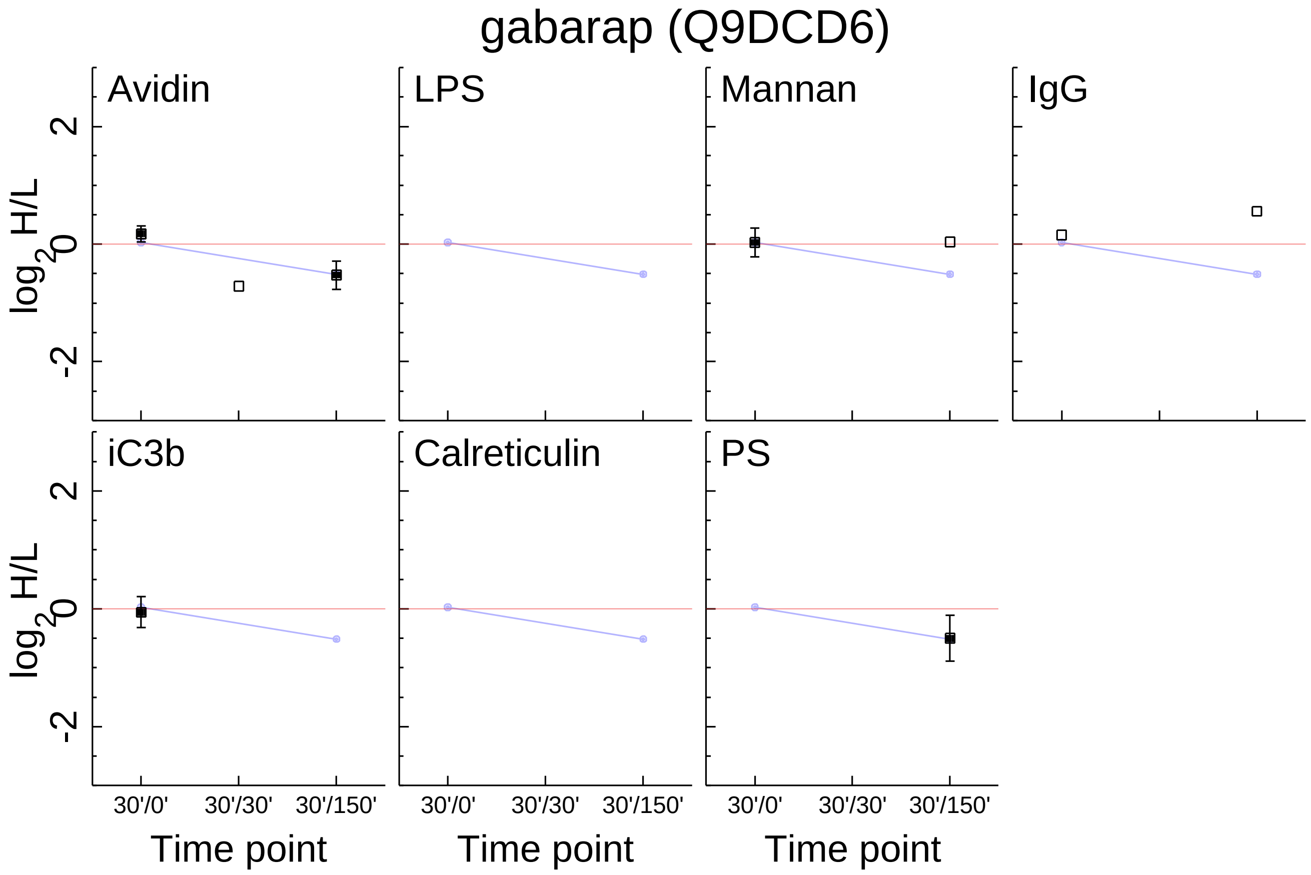

gabarap (Q9DCD6)
Avidin
LPS
Mannan
IgG
iC3b
Calreticulin
PS
$\log_2$ H/L
2
0
-2
30'/0'
30'/30'
30'/150'
Time point

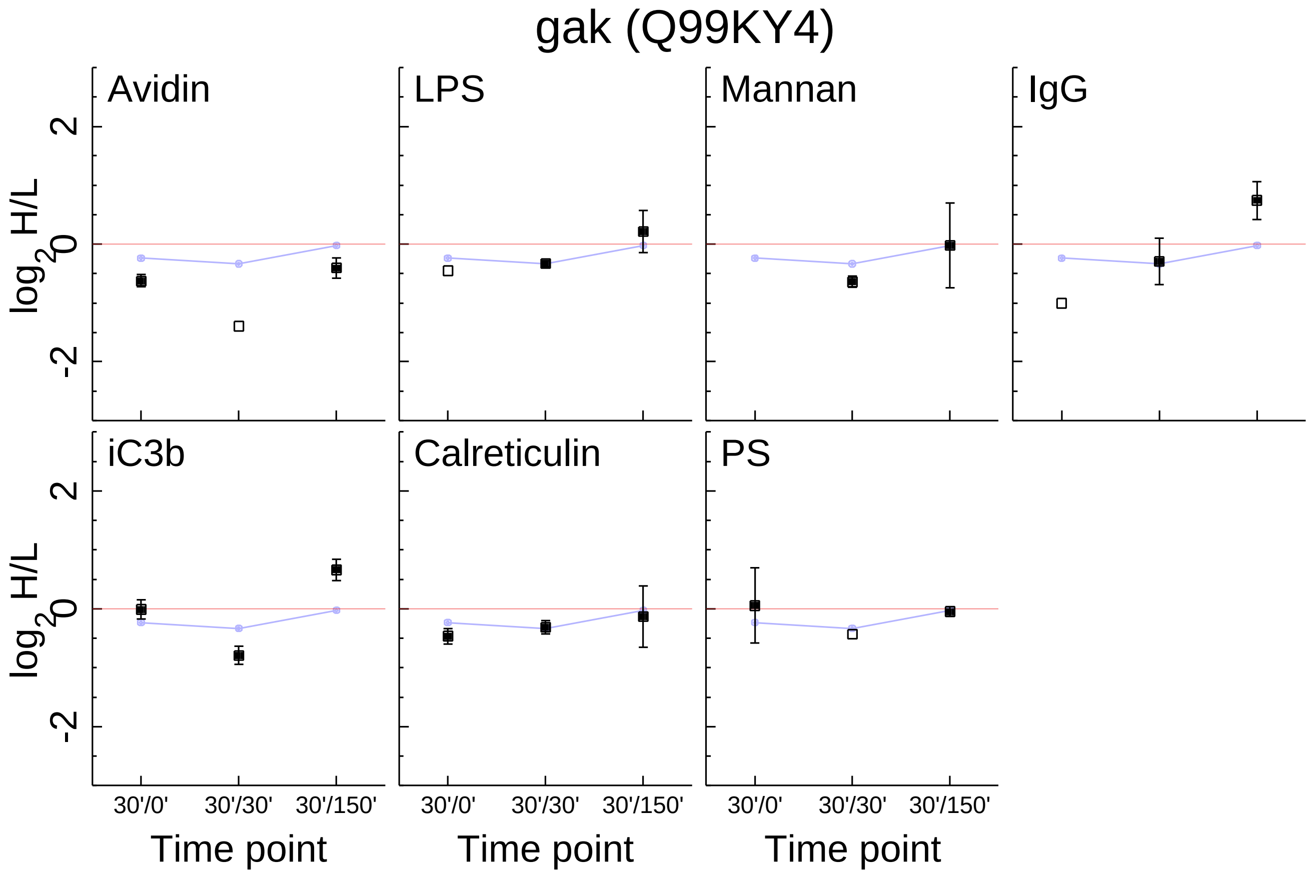

gak (Q99KY4)
Avidin
LPS
Mannan
IgG
iC3b
Calreticulin
PS
log2 H/L
2
0
-2
30'/0'
30'/30'
30'/150'
Time point

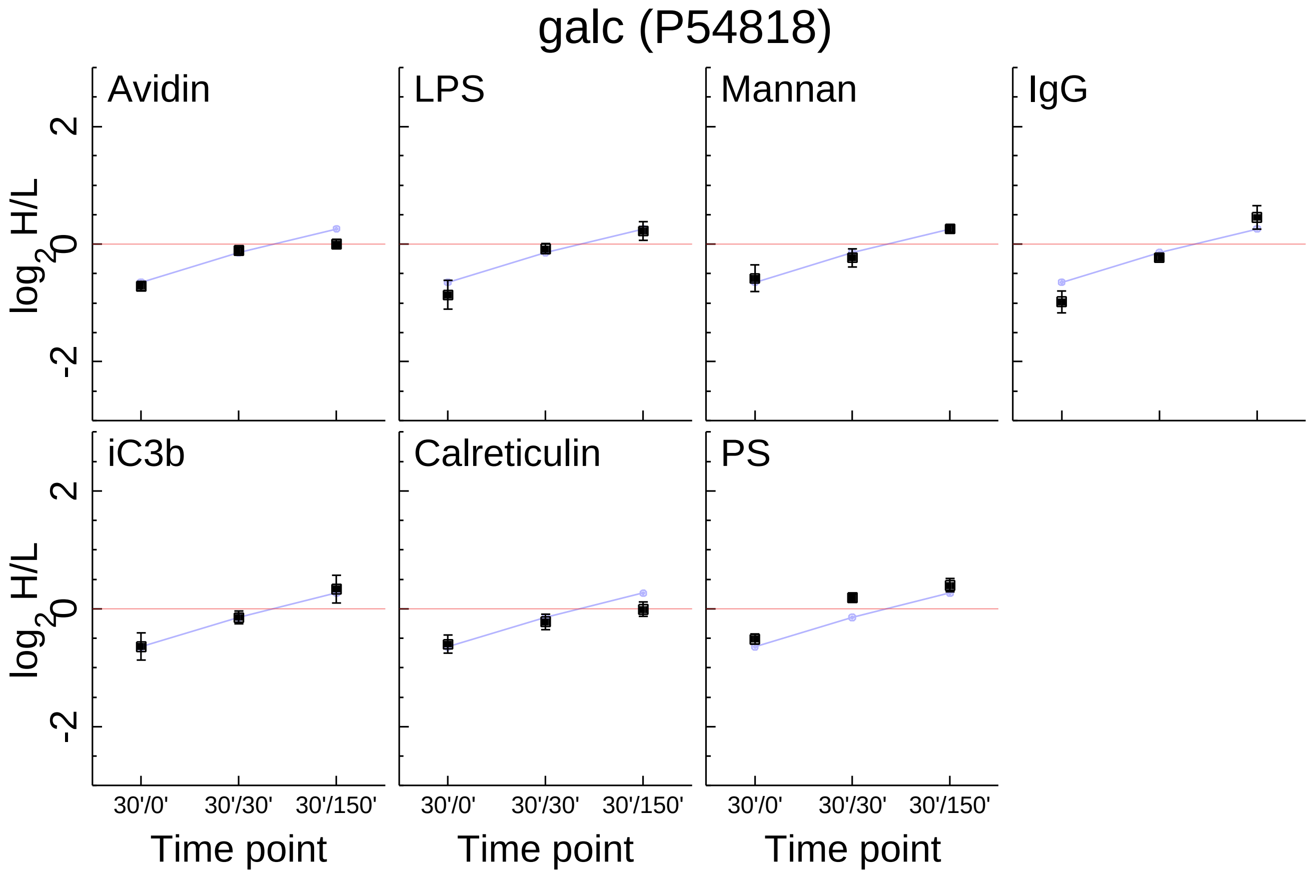
galc (P54818)
Avidin
LPS
Mannan
IgG
iC3b
Calreticulin
PS
log2 H/L
2
0
-2
30'/0'
30'/30'
30'/150'
Time point

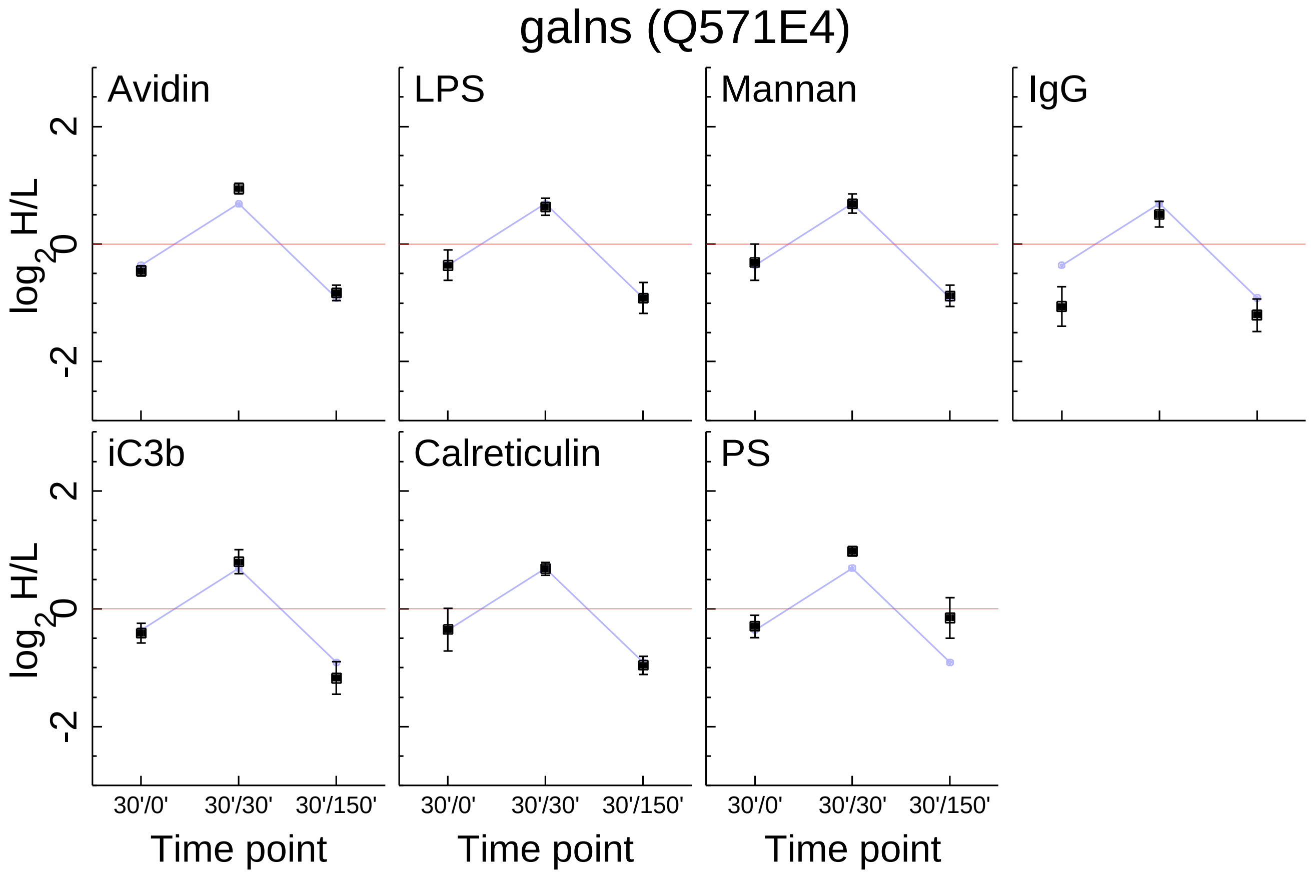
galns (Q571E4)
Avidin
LPS
Mannan
IgG
iC3b
Calreticulin
PS
log2 H/L
2
0
-2
30'/0'
30'/30'
30'/150'
Time point

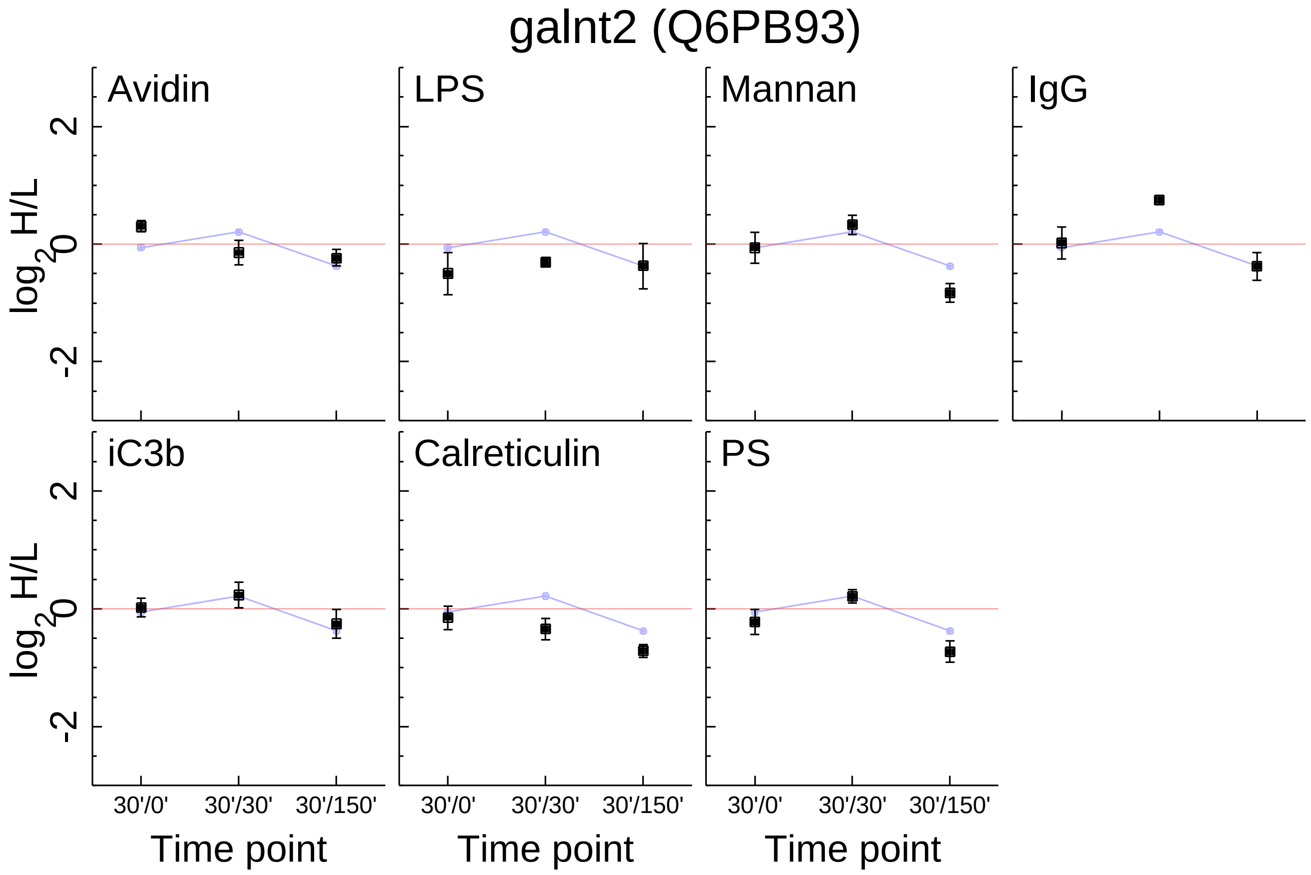
galnt2 (Q6PB93)
Avidin
LPS
Mannan
IgG
iC3b
Calreticulin
PS
log2 H/L
2
0
-2
30'/0'
30'/30'
30'/150'
Time point

galnt7 (Q80VA0)

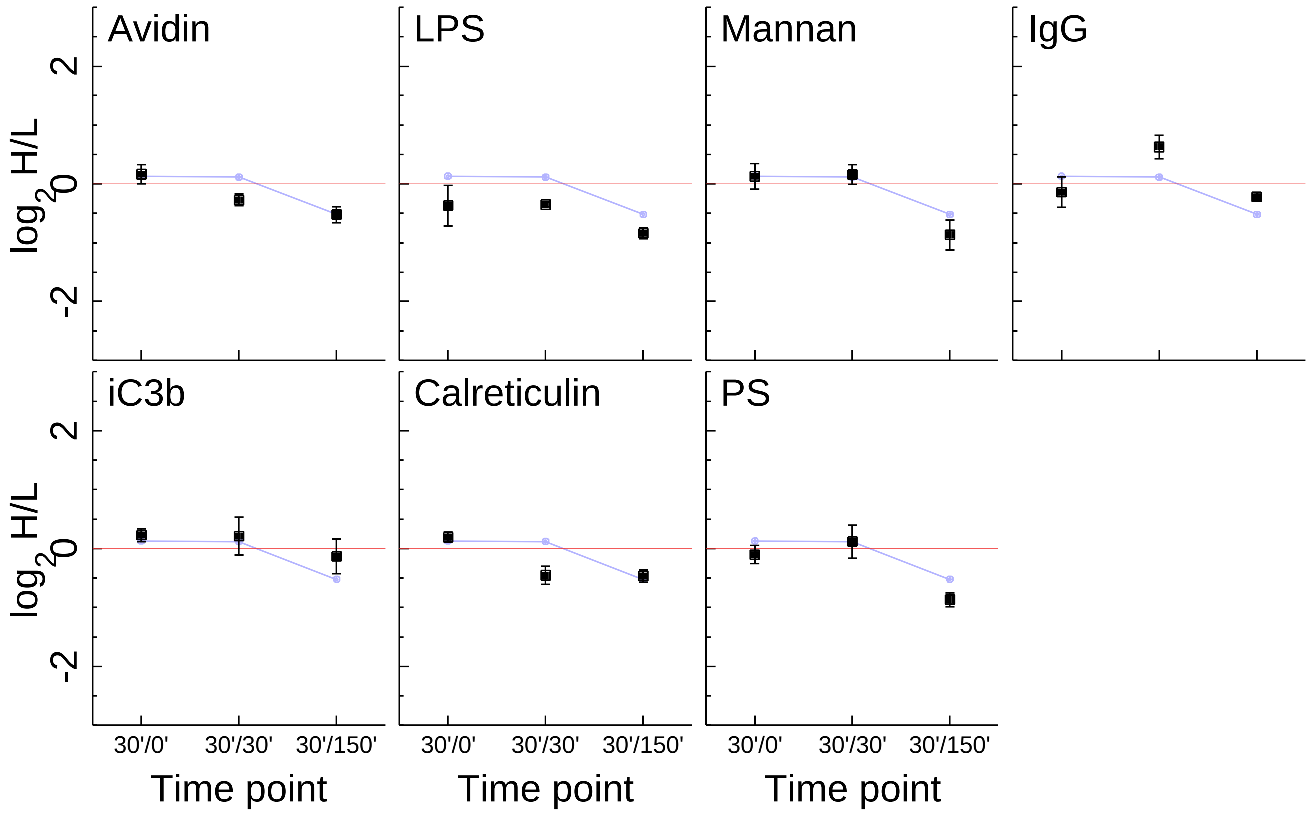

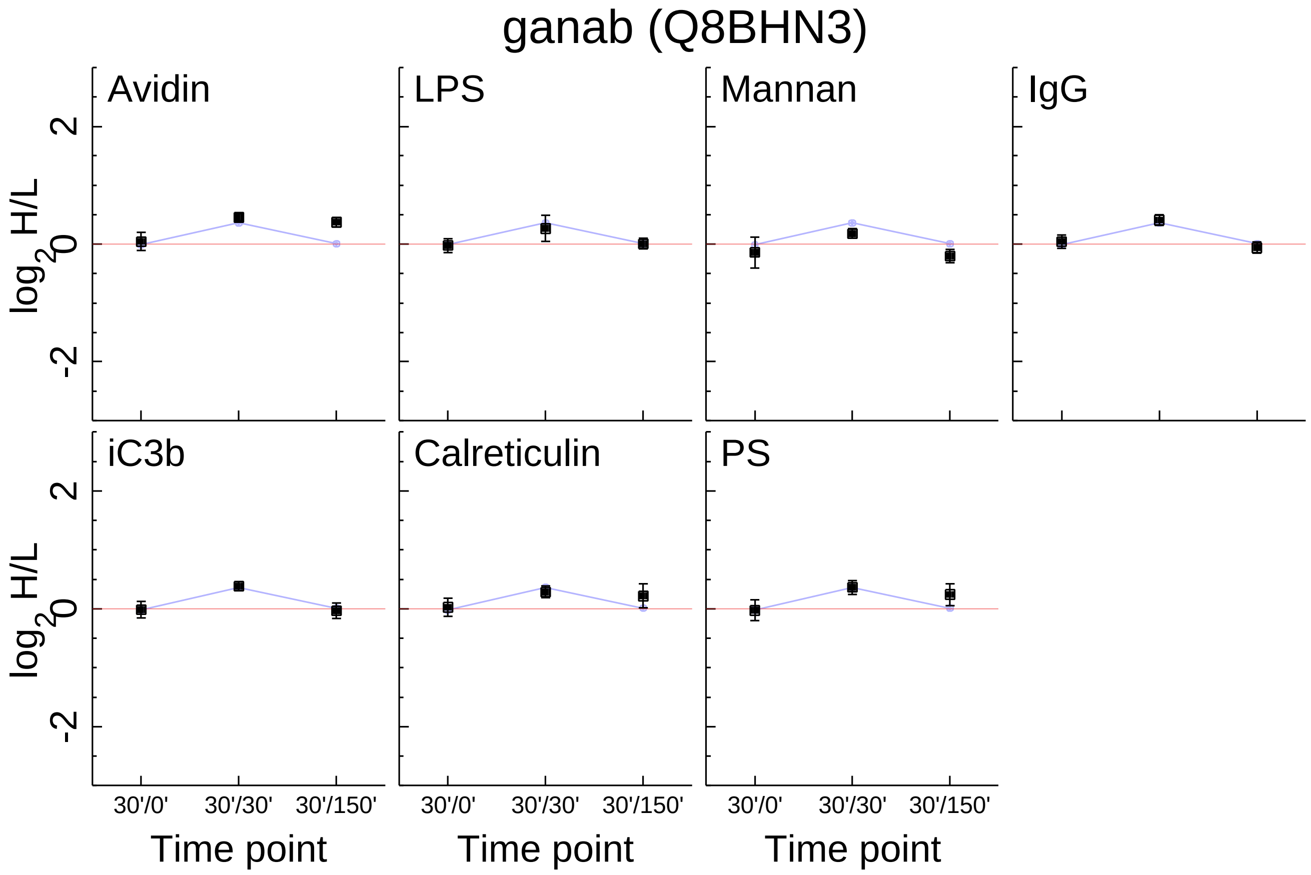
ganab (Q8BHN3)
Avidin
LPS
Mannan
IgG
iC3b
Calreticulin
PS
log2 H/L
2
0
-2
30'/0'
30'/30'
30'/150'
Time point

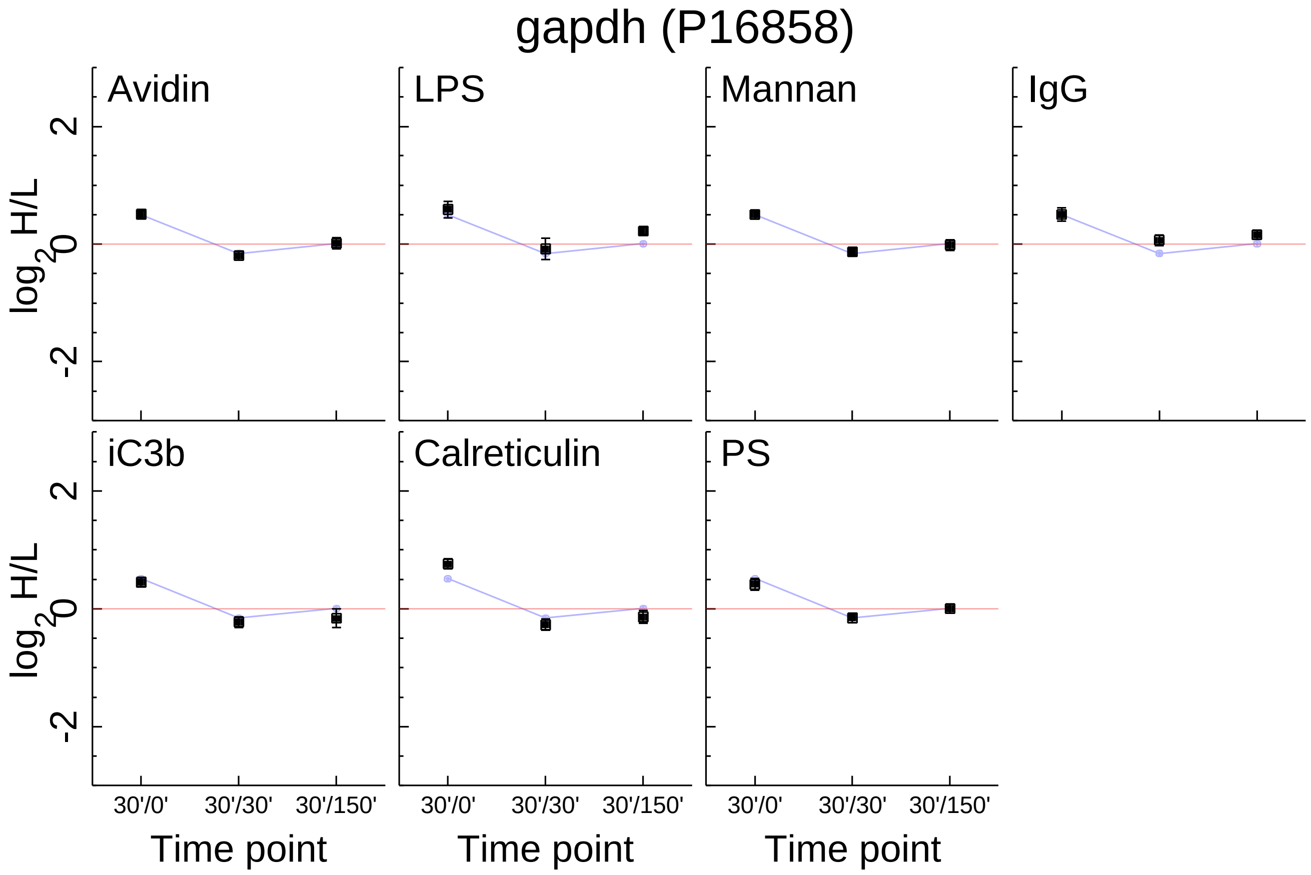
gapdh (P16858)
Avidin
LPS
Mannan
IgG
iC3b
Calreticulin
PS
log2 H/L
2
0
-2
30'/0'
30'/30'
30'/150'
Time point

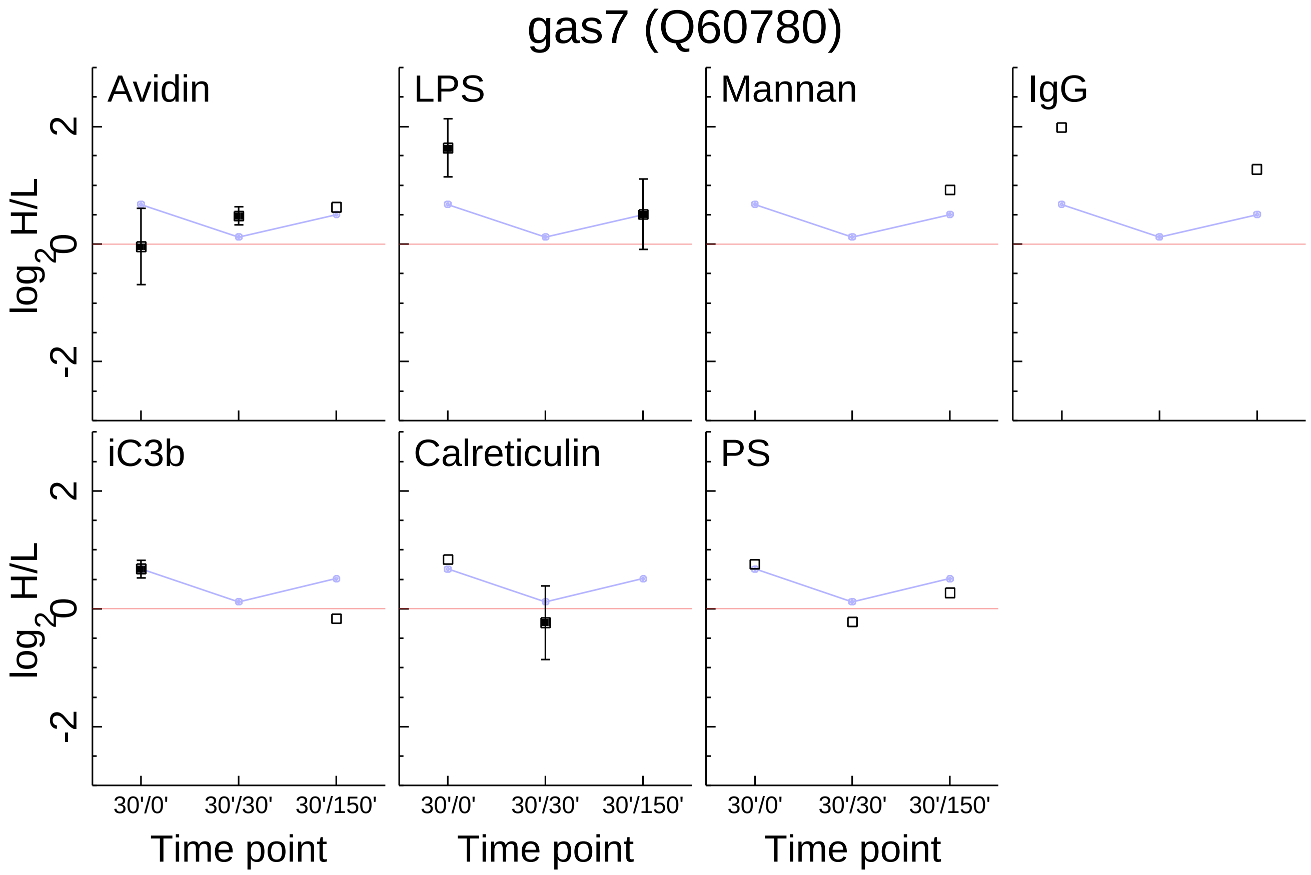

gas7 (Q60780)
Avidin
LPS
Mannan
IgG
iC3b
Calreticulin
PS
log2 H/L
2
0
-2
30'/0'
30'/30'
30'/150'
Time point

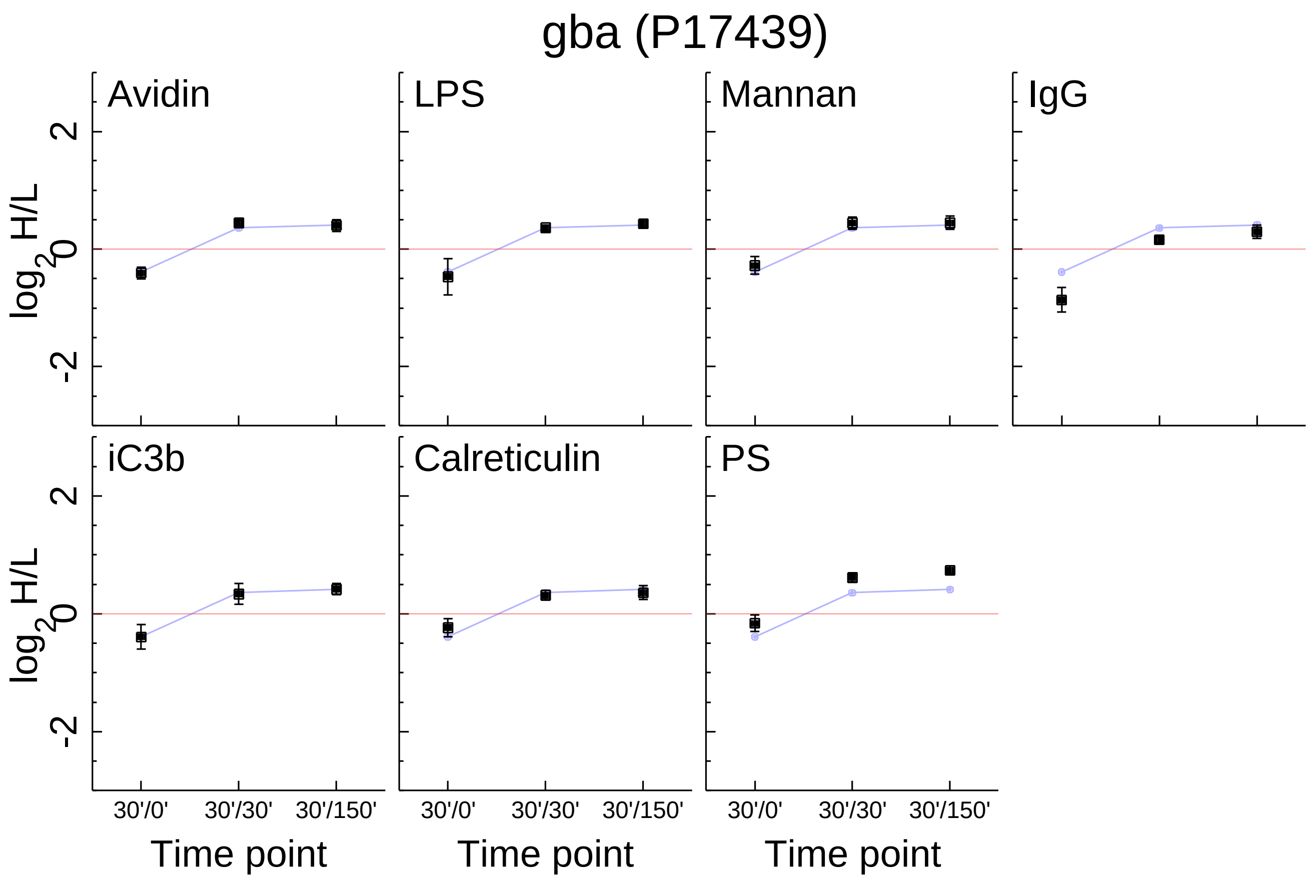
gba (P17439)
Avidin
LPS
Mannan
IgG
iC3b
Calreticulin
PS
log2 H/L
2
0
-2
30'/0'
30'/30'
30'/150'
Time point

gbp5 (Q8CFB4)

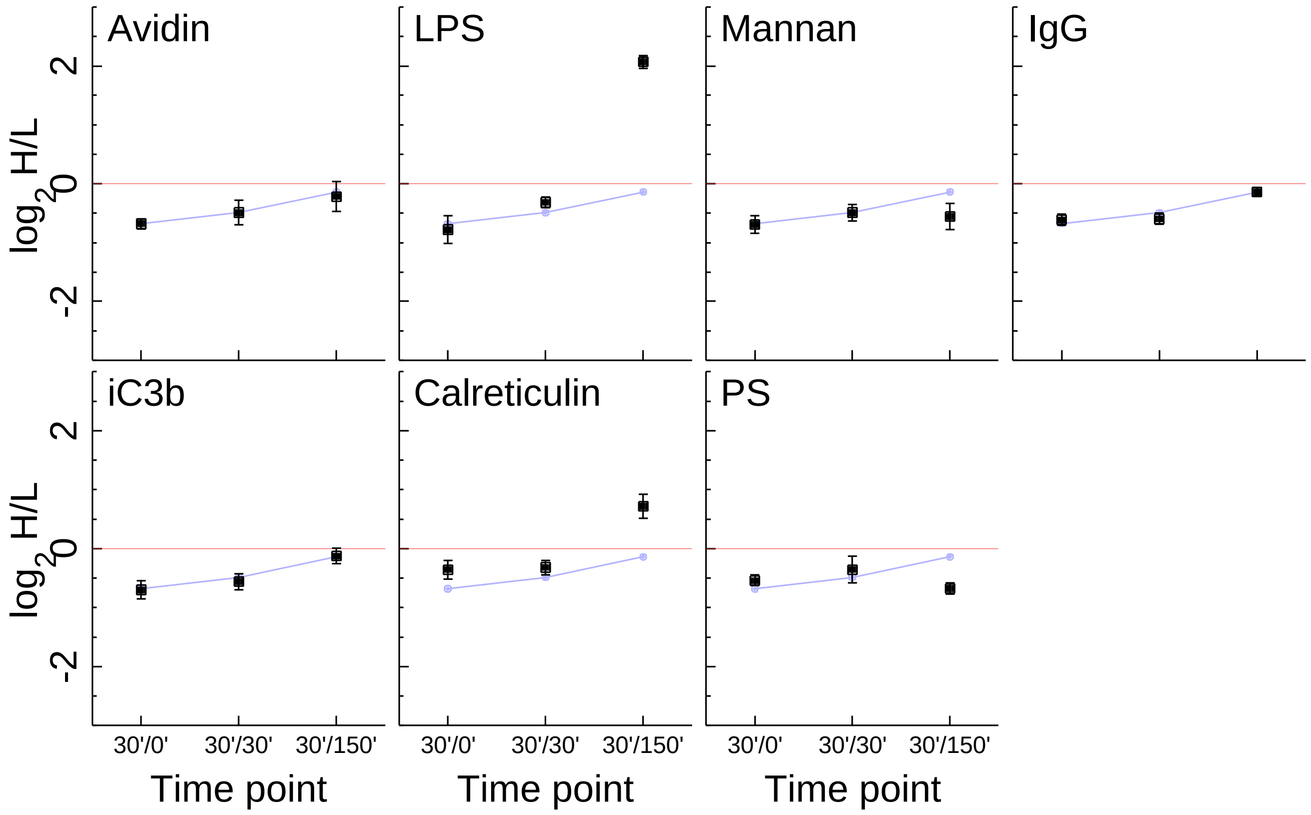
Avidin
LPS
Mannan
IgG
iC3b
Calreticulin
PS
log_2 H/L
2
0
-2
30'/0'
30'/30'
30'/150'
Time point

gclm (O09172)

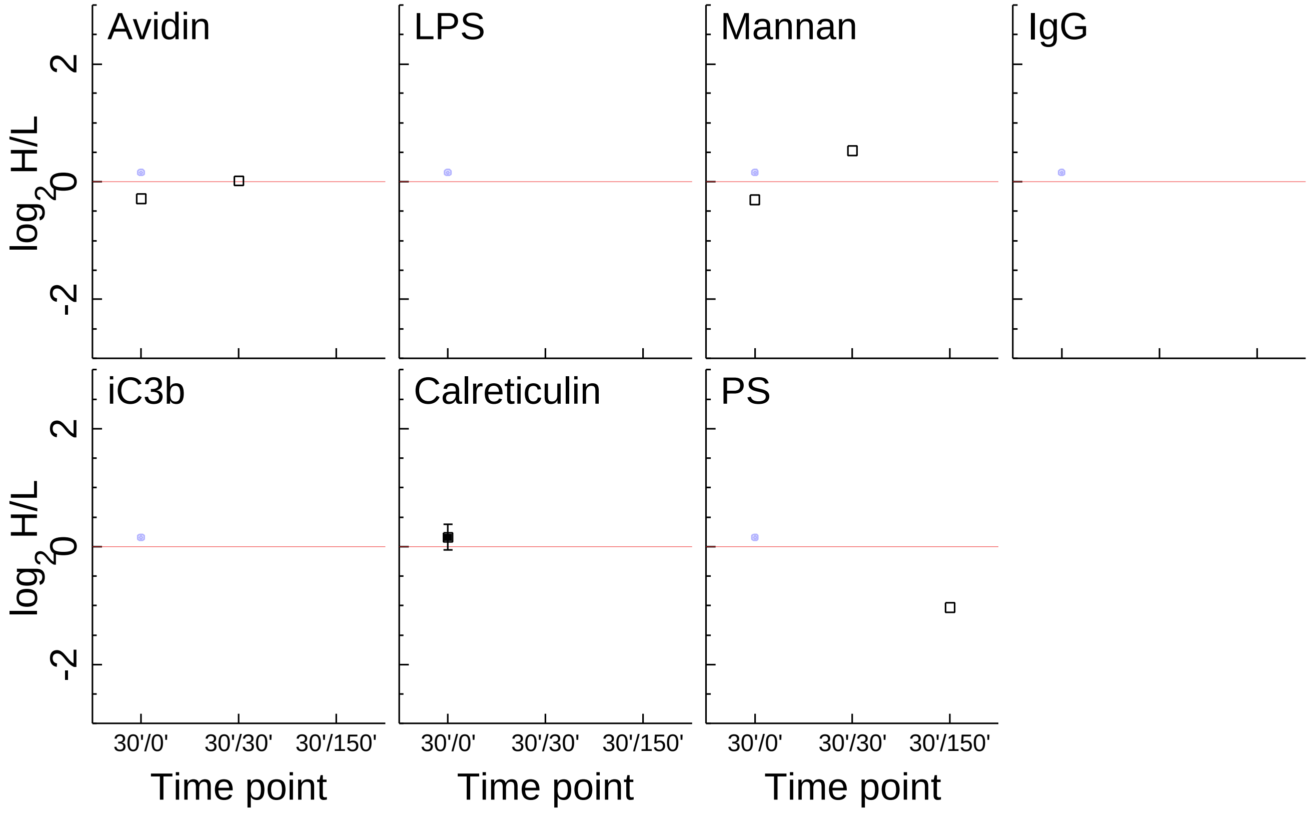

Avidin
LPS
Mannan
IgG
iC3b
Calreticulin
PS
2
0
-2
log_2 H/L
30'/0'
30'/30'
30'/150'
Time point

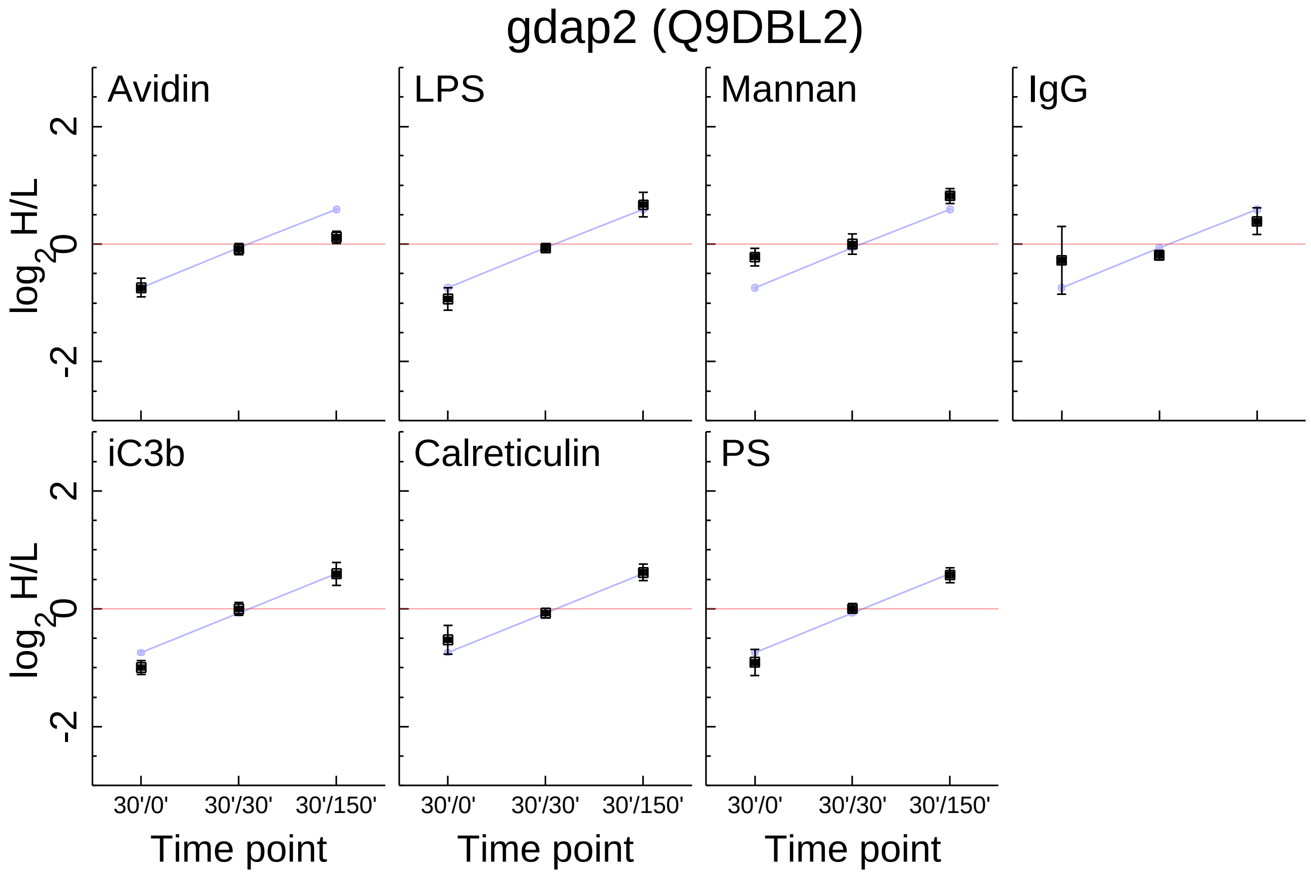
gdap2 (Q9DBL2)
Avidin
LPS
Mannan
IgG
iC3b
Calreticulin
PS
log2 H/L
2
0
-2
30'/0'
30'/30'
30'/150'
Time point

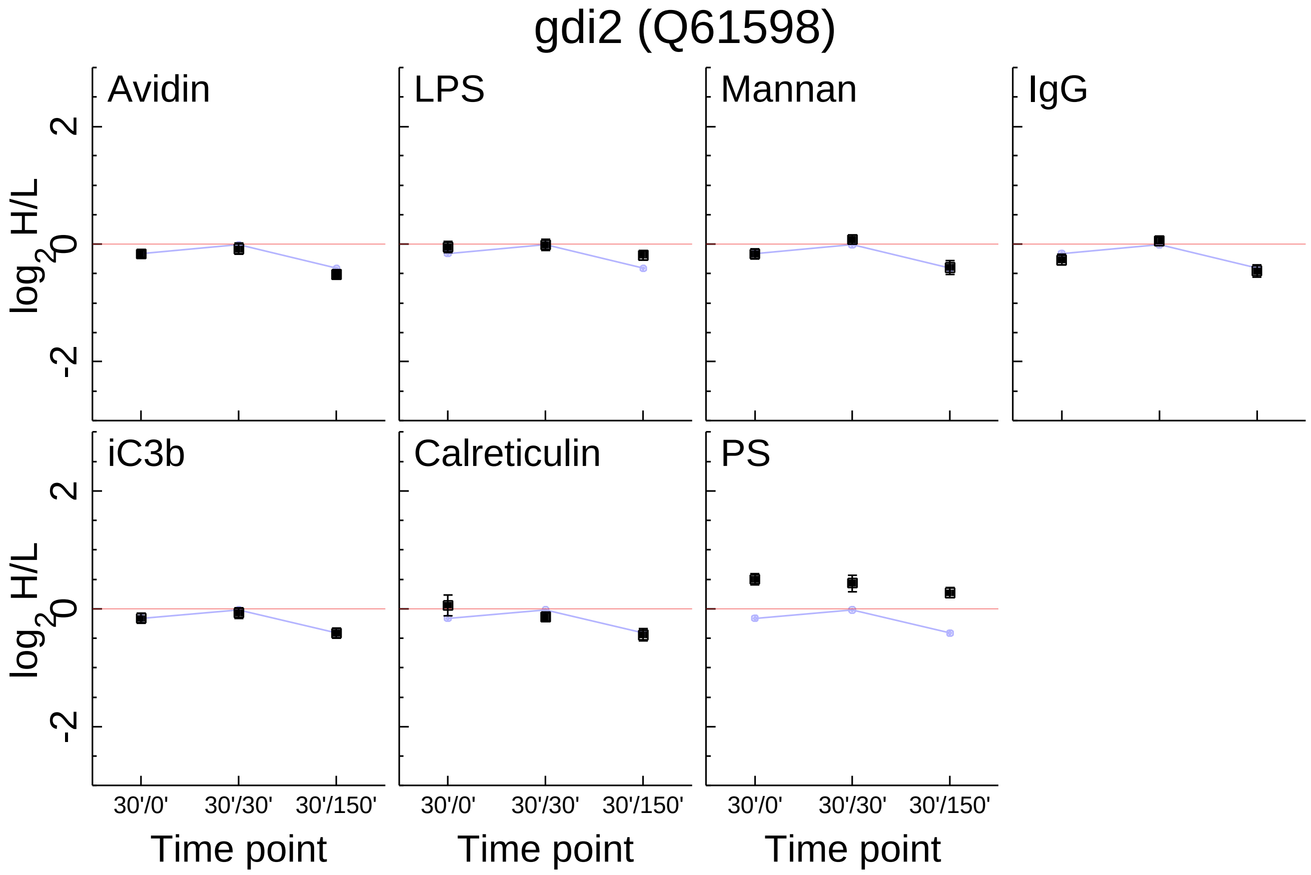
gdi2 (Q61598)
Avidin
LPS
Mannan
IgG
iC3b
Calreticulin
PS
log2 H/L
2
0
-2
30'/0'
30'/30'
30'/150'
Time point

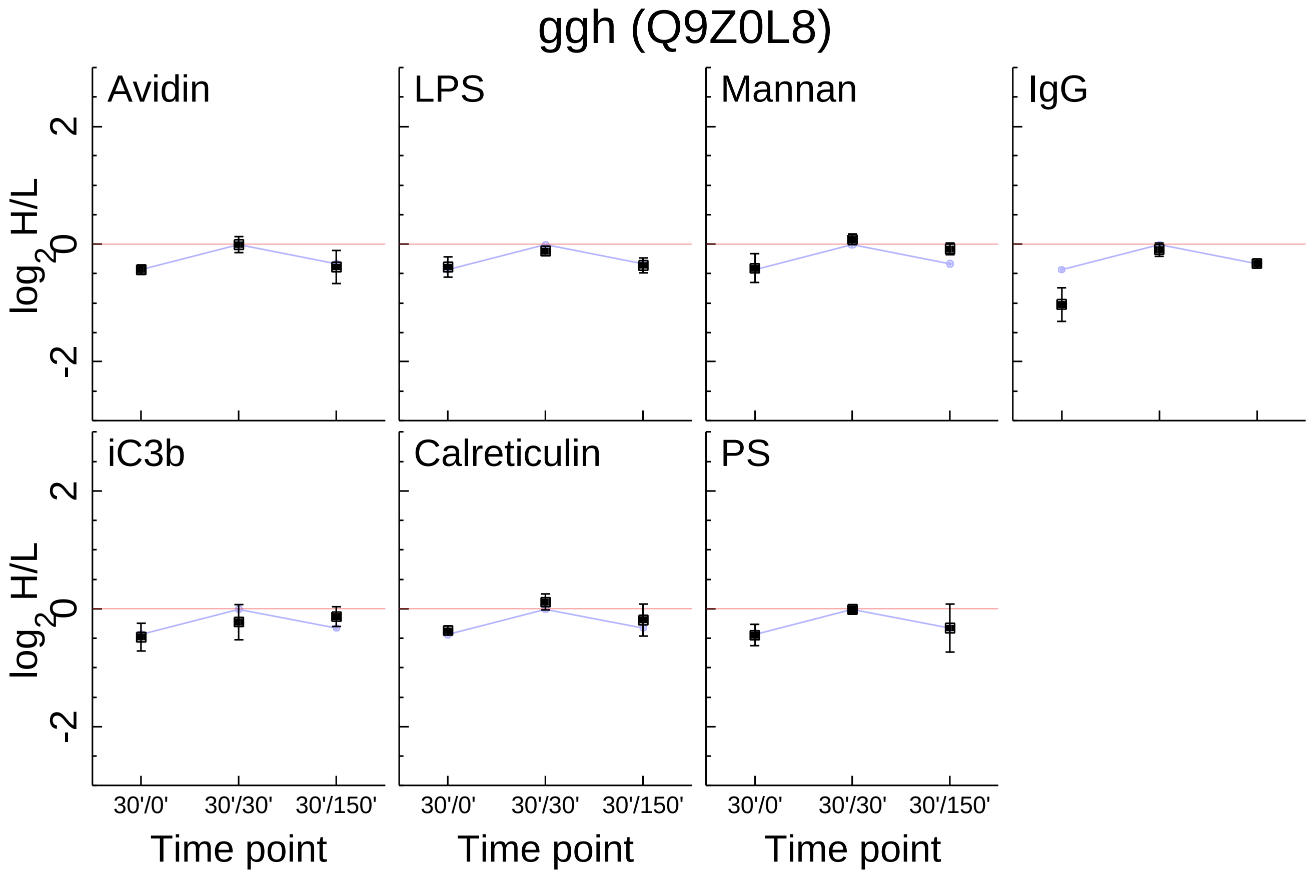
ggh (Q9Z0L8)
Avidin
LPS
Mannan
IgG
iC3b
Calreticulin
PS
log2 H/L
2
0
-2
30'/0'
30'/30'
30'/150'
Time point

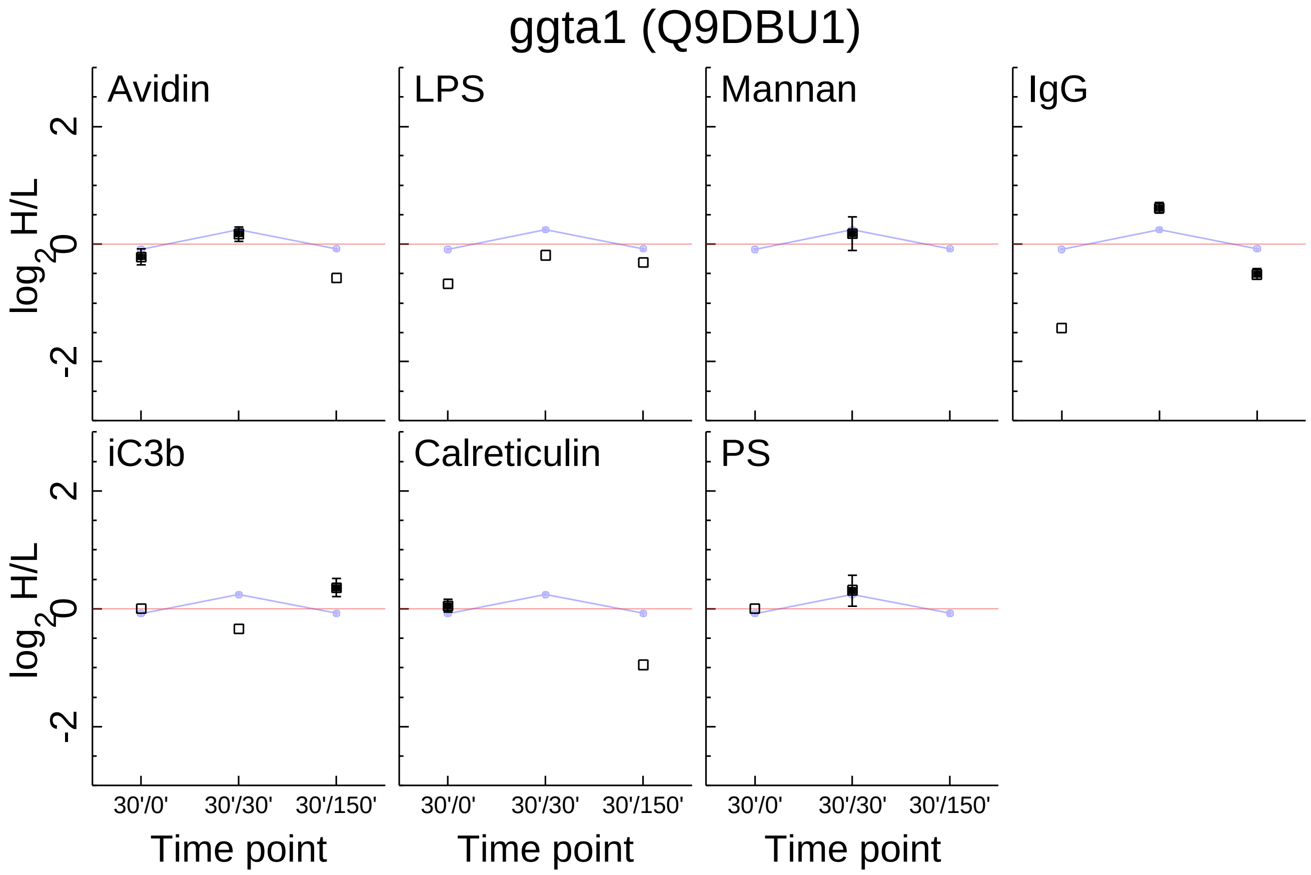

ggta1 (Q9DBU1)
Avidin
LPS
Mannan
IgG
iC3b
Calreticulin
PS
log2 H/L
2
0
-2
30'/0'
30'/30'
30'/150'
Time point

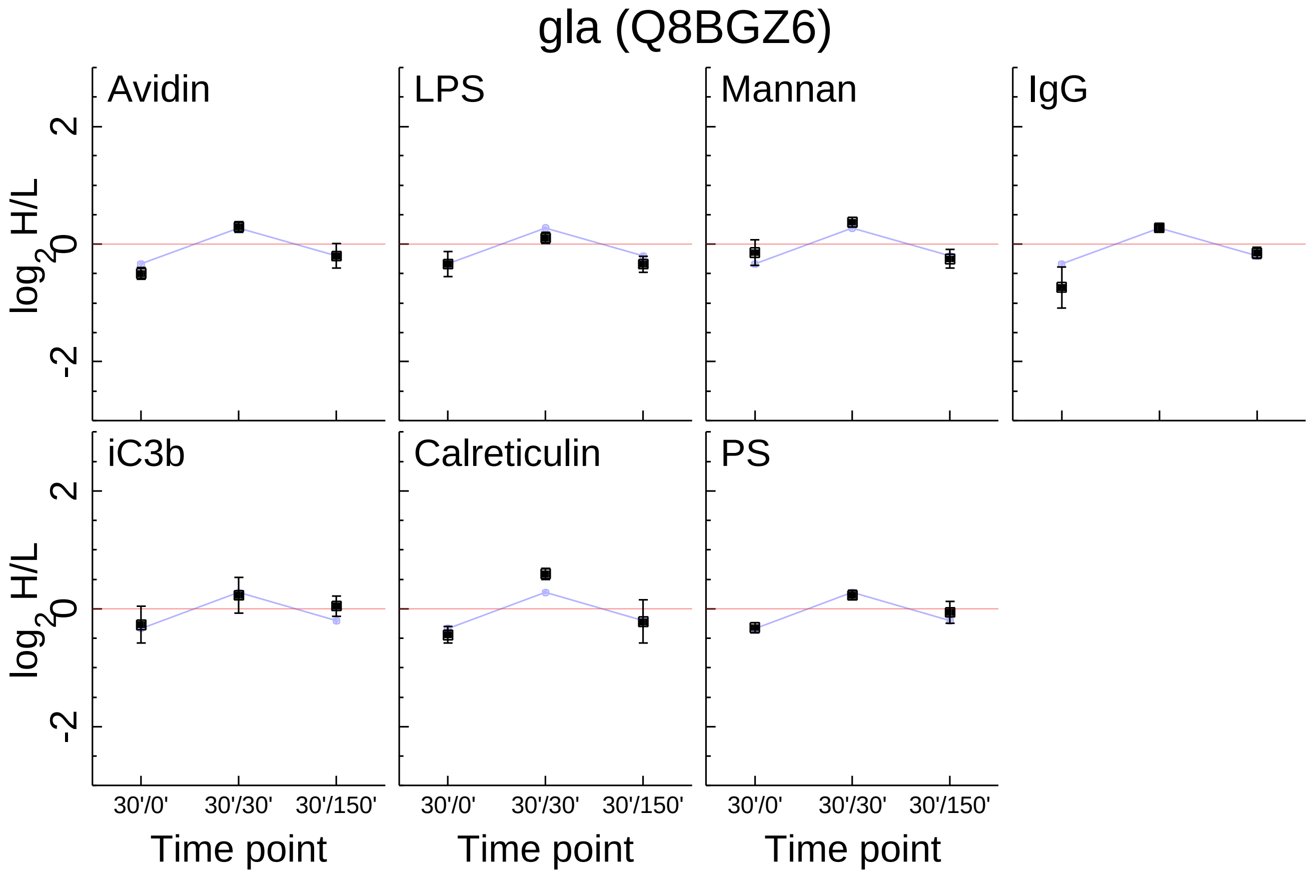

gla (Q8BGZ6)
Avidin
LPS
Mannan
IgG
iC3b
Calreticulin
PS
log2 H/L
2
0
-2
30'/0'
30'/30'
30'/150'
Time point

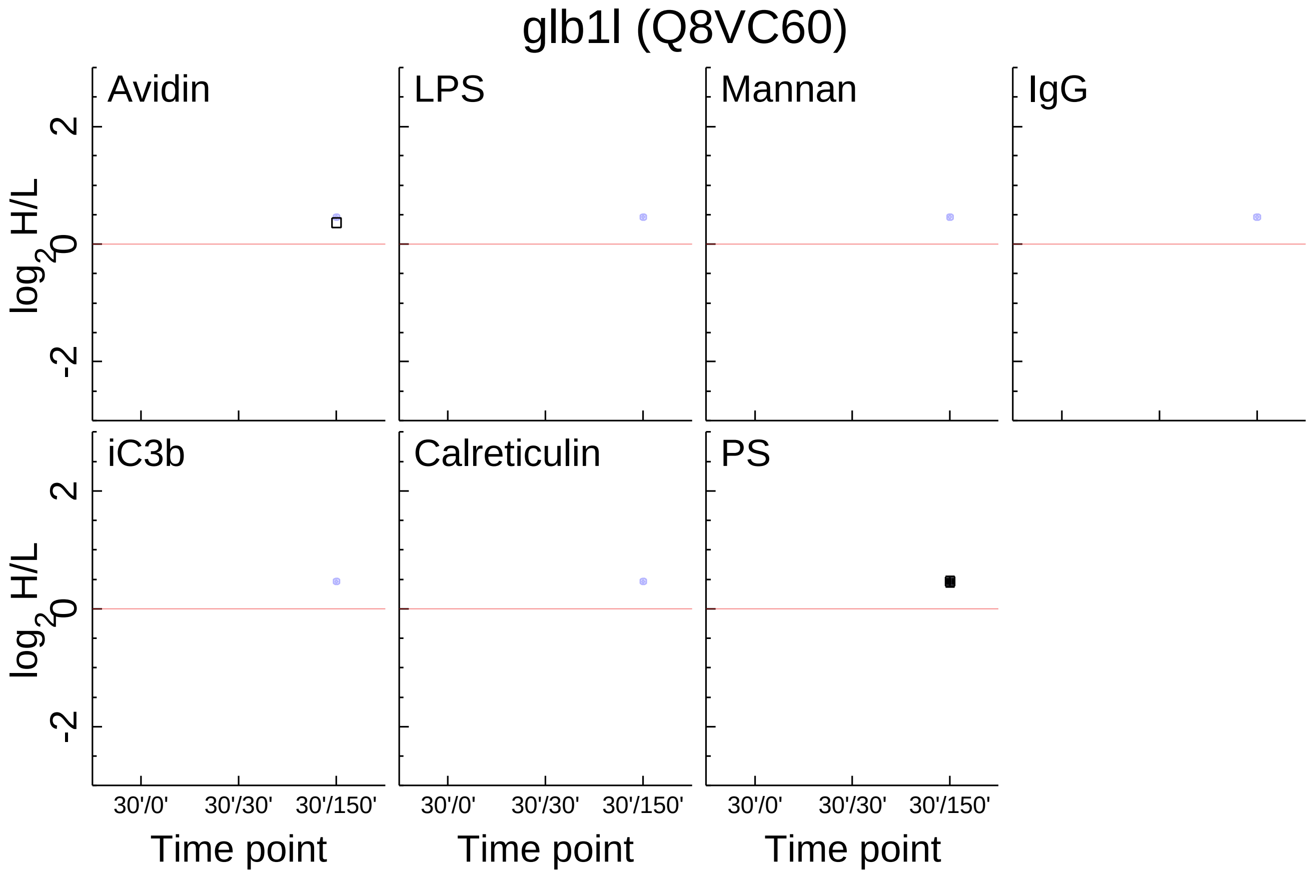
glb1l (Q8VC60)
Avidin
LPS
Mannan
IgG
iC3b
Calreticulin
PS
log2 H/L
2
0
-2
30'/0'
30'/30'
30'/150'
Time point

glb1 (P23780)

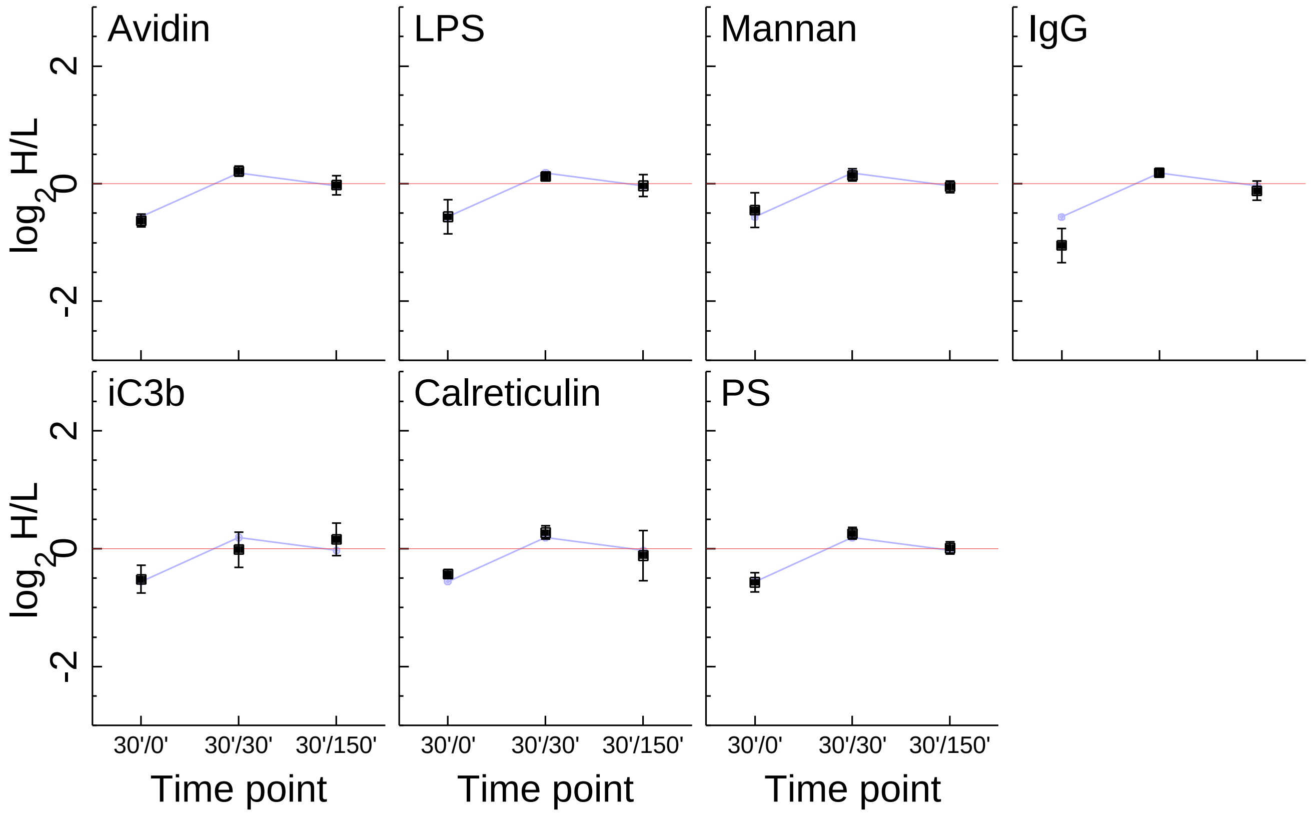
Avidin
LPS
Mannan
IgG
iC3b
Calreticulin
PS
log2 H/L
2
0
-2
30'/0'
30'/30'
30'/150'
Time point

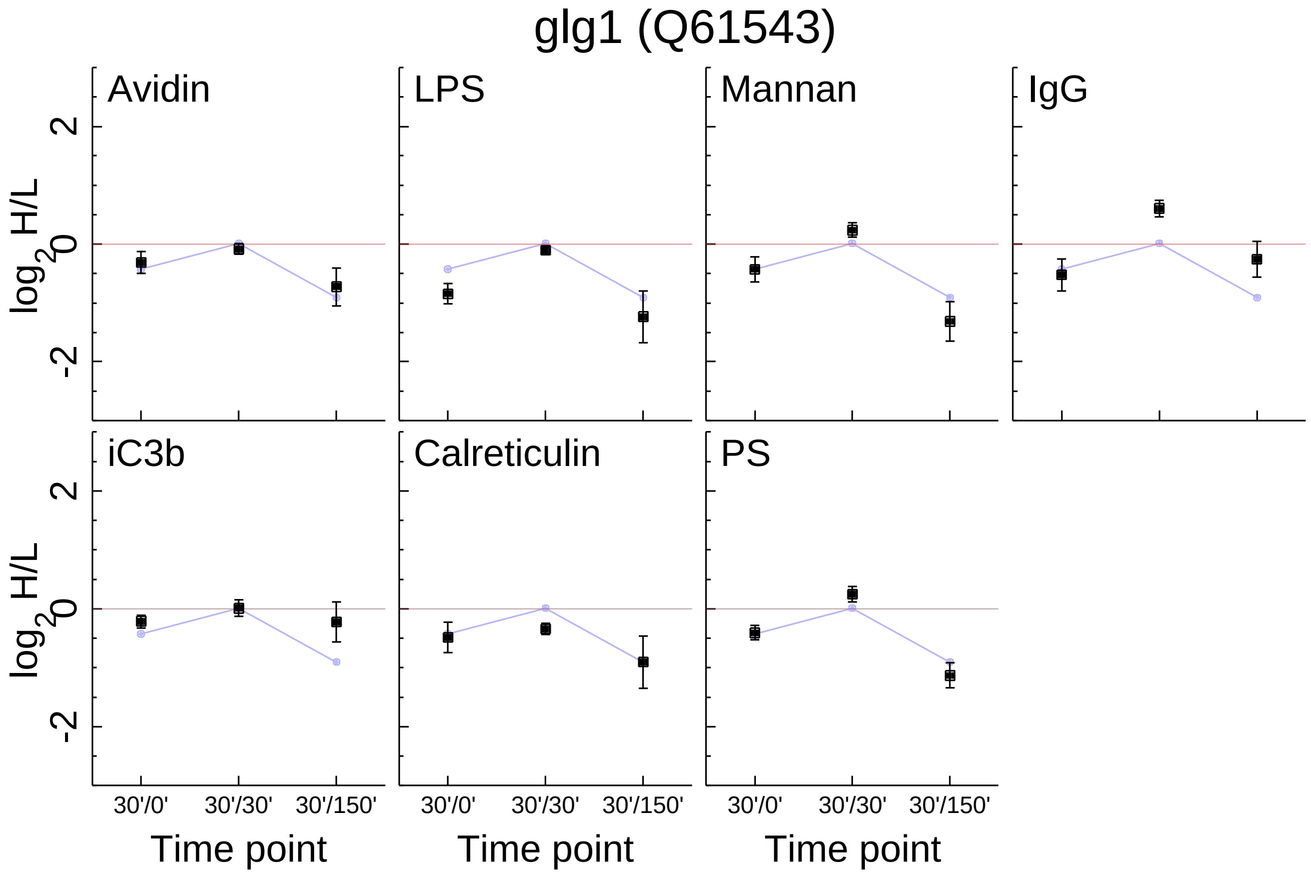

glg1 (Q61543)
Avidin
LPS
Mannan
IgG
iC3b
Calreticulin
PS
log2 H/L
2
0
-2
30'/0'
30'/30'
30'/150'
Time point

glipr1 (Q9CWG1)

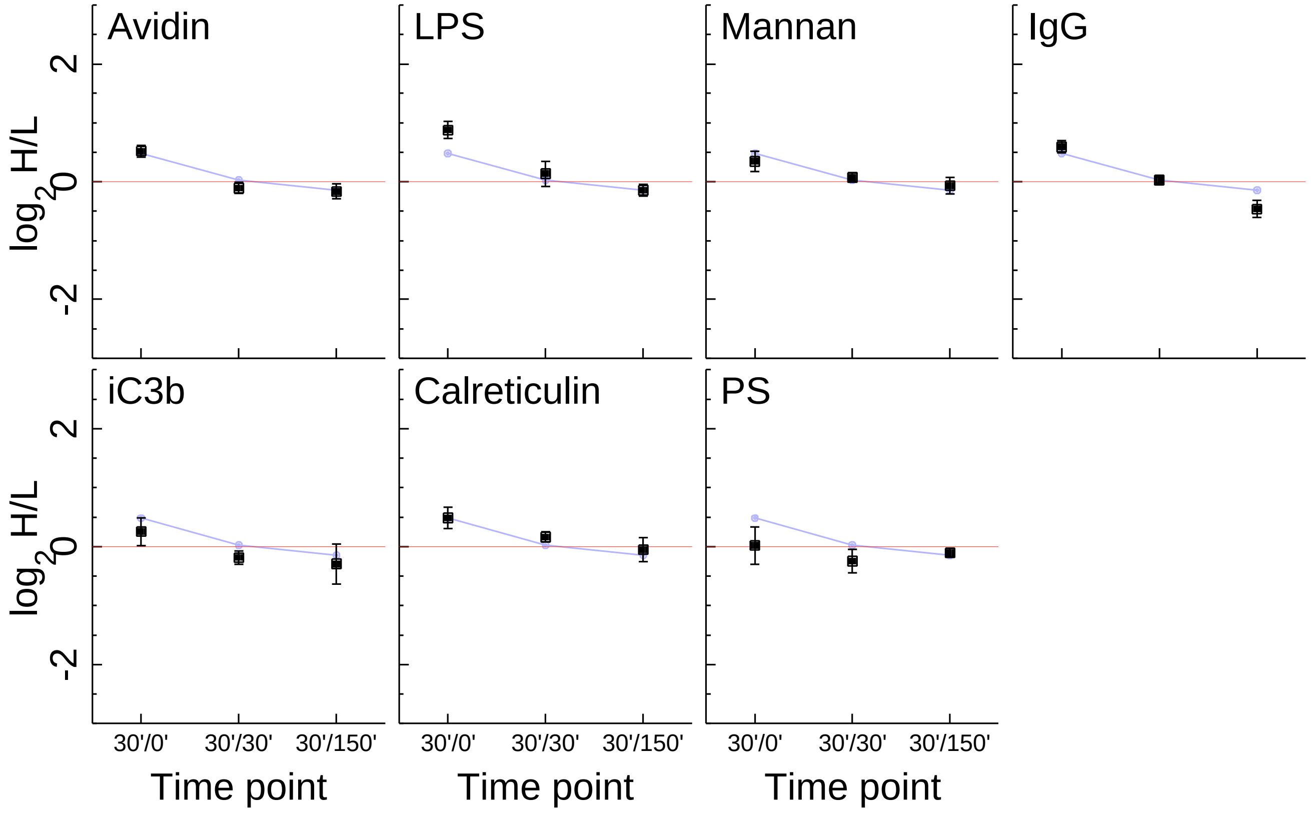

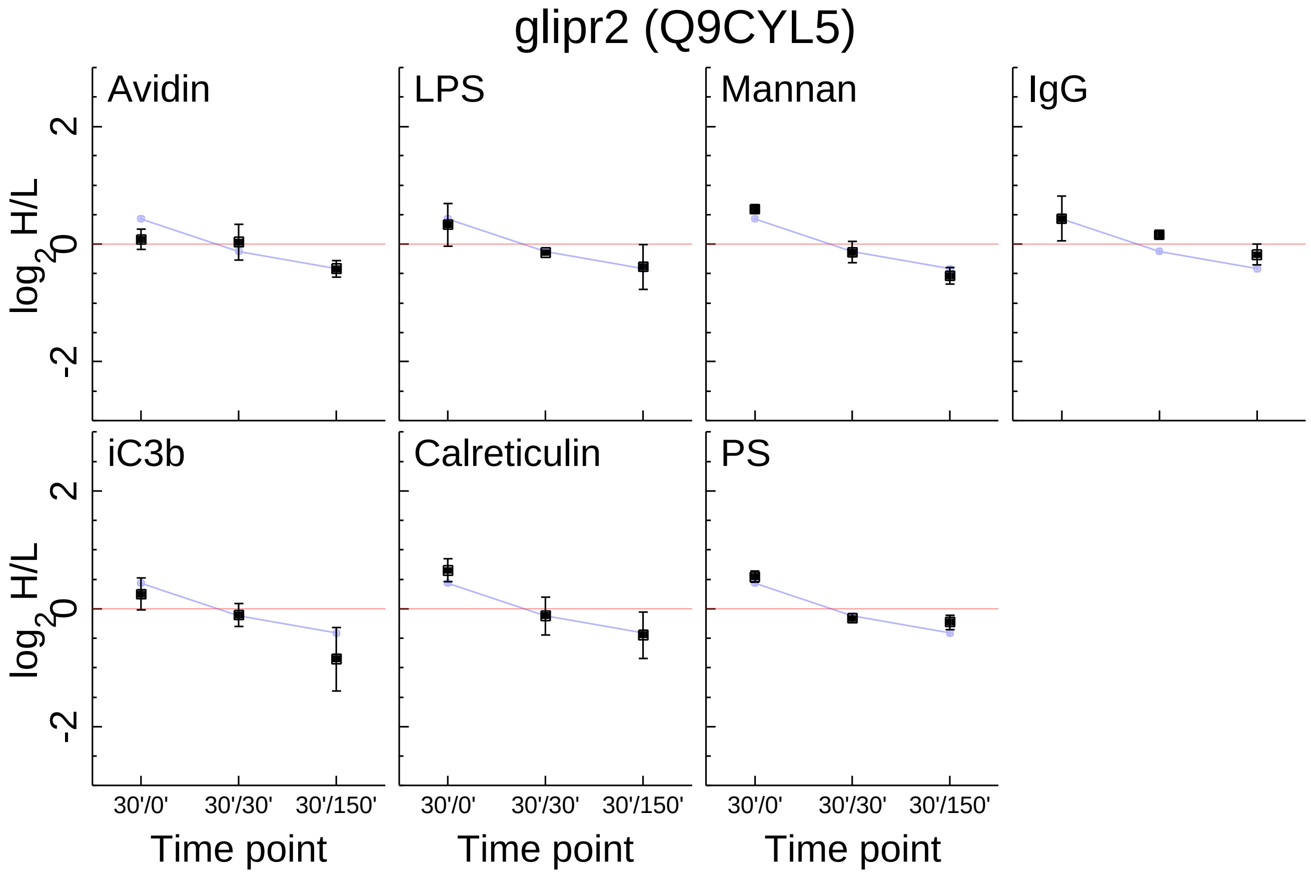
glipr2 (Q9CYL5)
Avidin
LPS
Mannan
IgG
iC3b
Calreticulin
PS
log2 H/L
2
0
-2
30'/0'
30'/30'
30'/150'
Time point

gls (D3Z7P3)

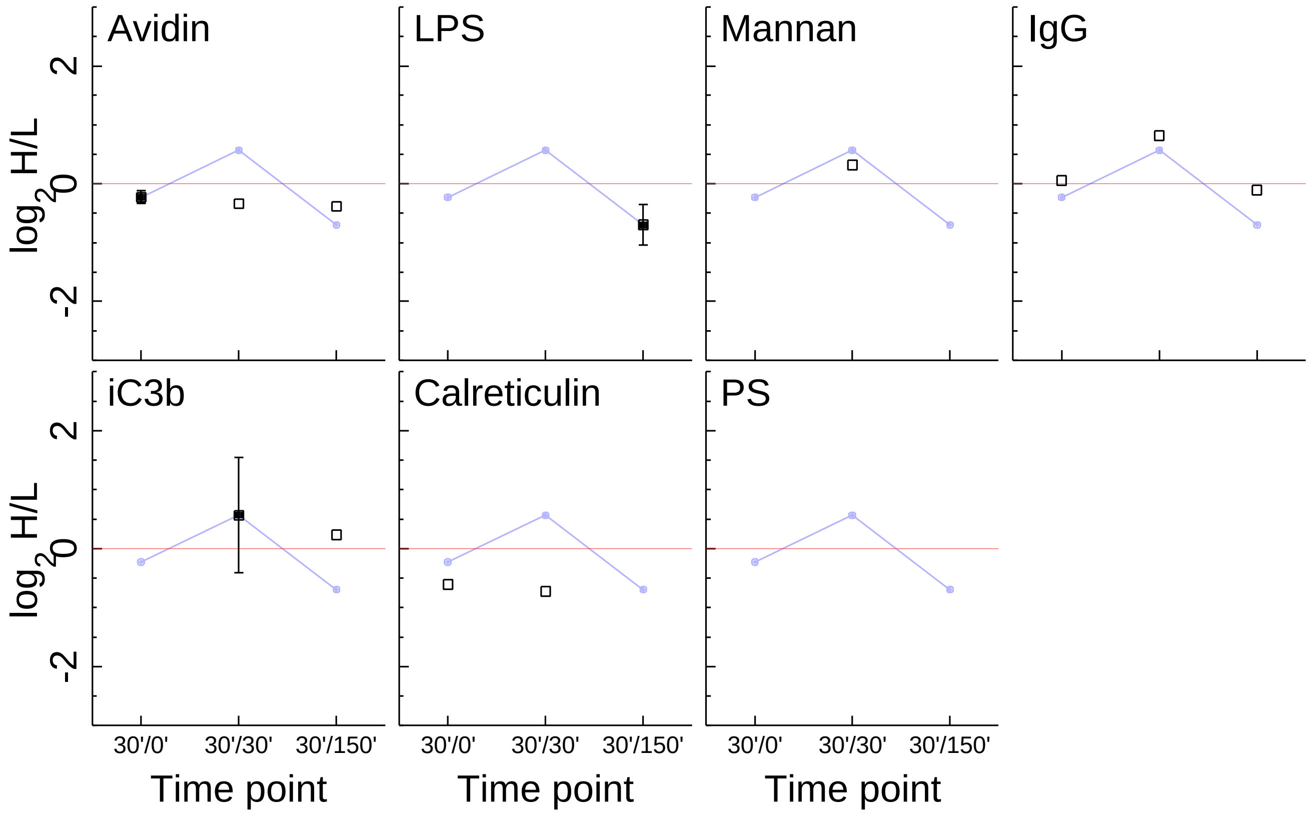

Avidin
LPS
Mannan
IgG
iC3b
Calreticulin
PS
log2 H/L
2
0
-2
30'/0'
30'/30'
30'/150'
Time point

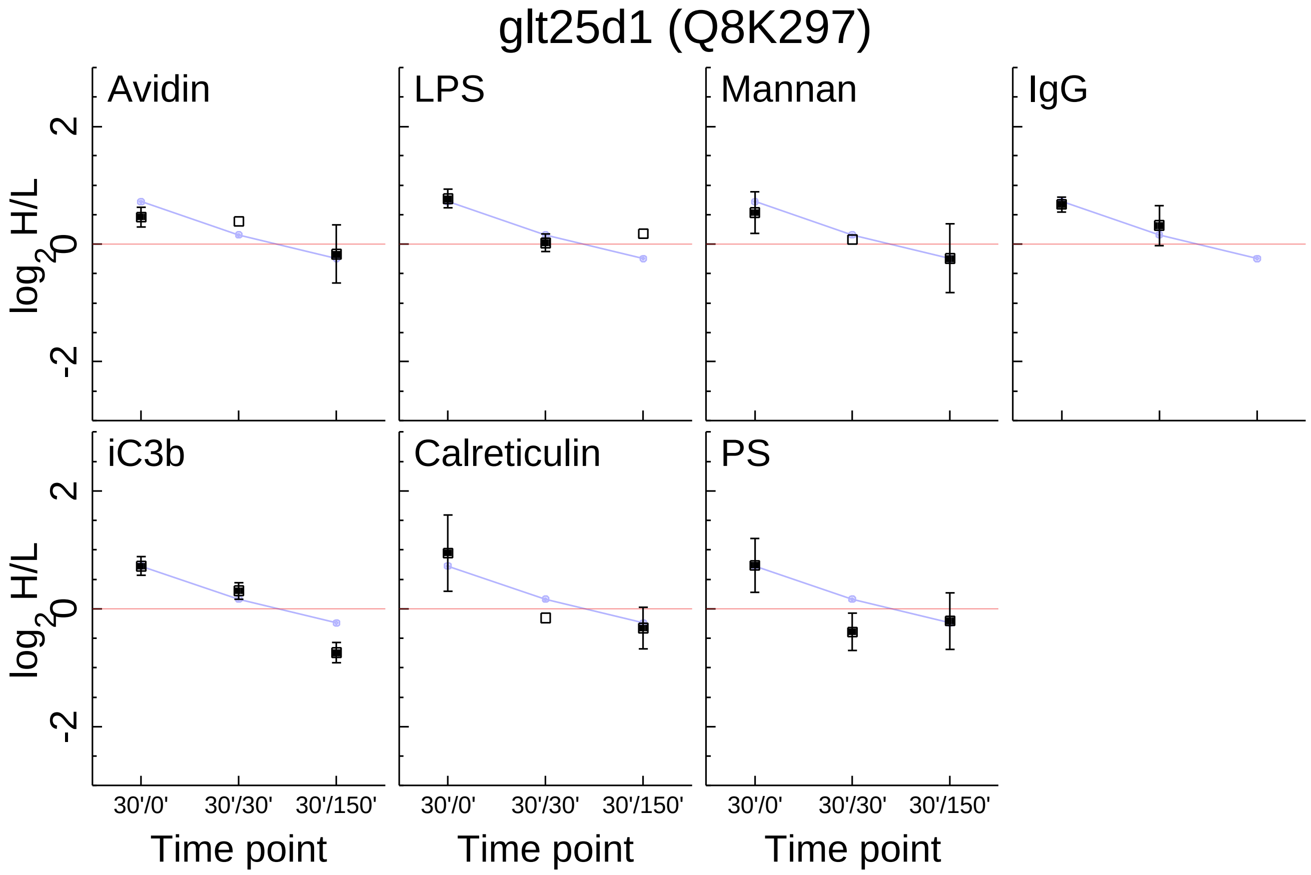

glt25d1 (Q8K297)
Avidin
LPS
Mannan
IgG
iC3b
Calreticulin
PS
log2 H/L
2
0
-2
30'/0'
30'/30'
30'/150'
Time point

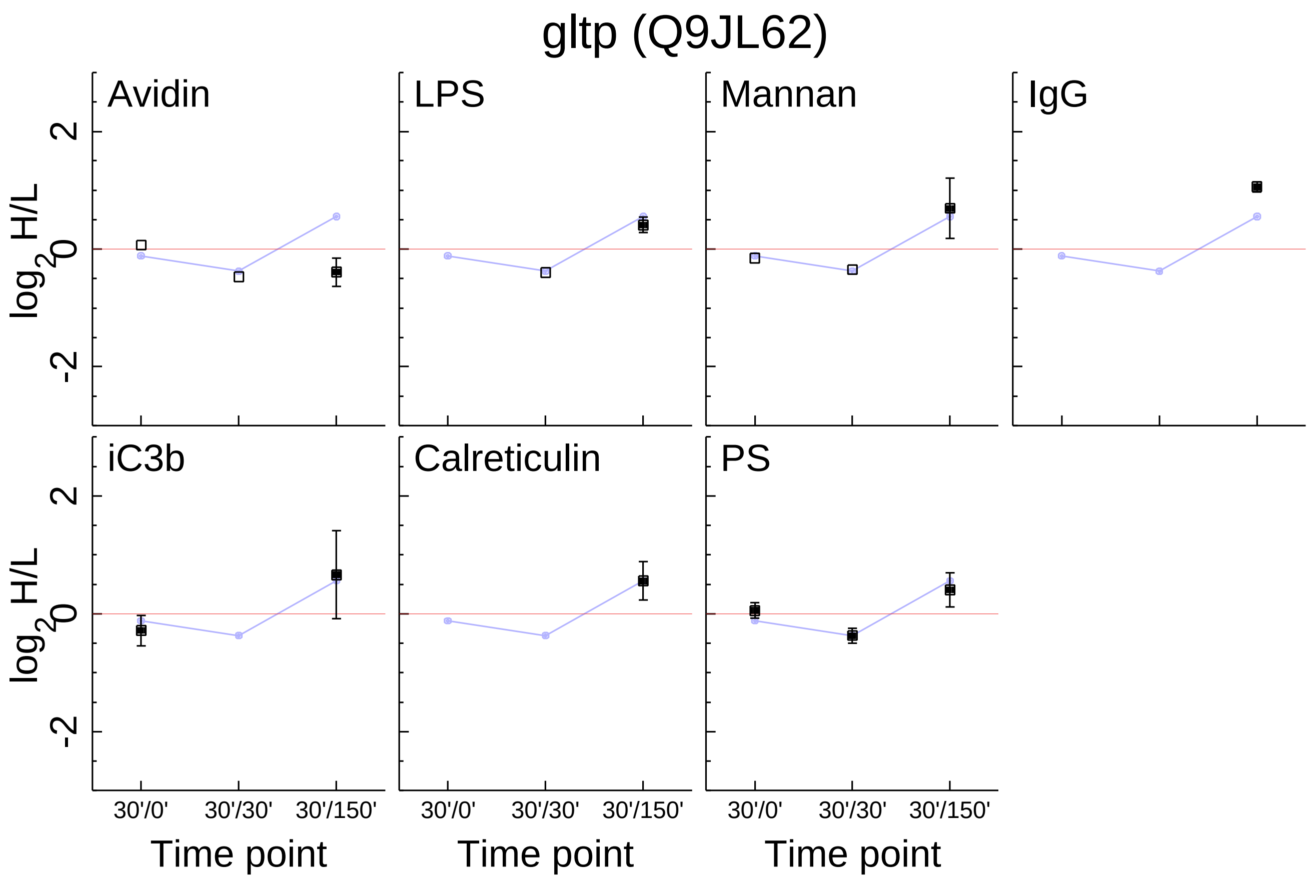
gltp (Q9JL62)
Avidin
LPS
Mannan
IgG
iC3b
Calreticulin
PS
2
0
-2
log2 H/L
30'/0'
30'/30'
30'/150'
Time point

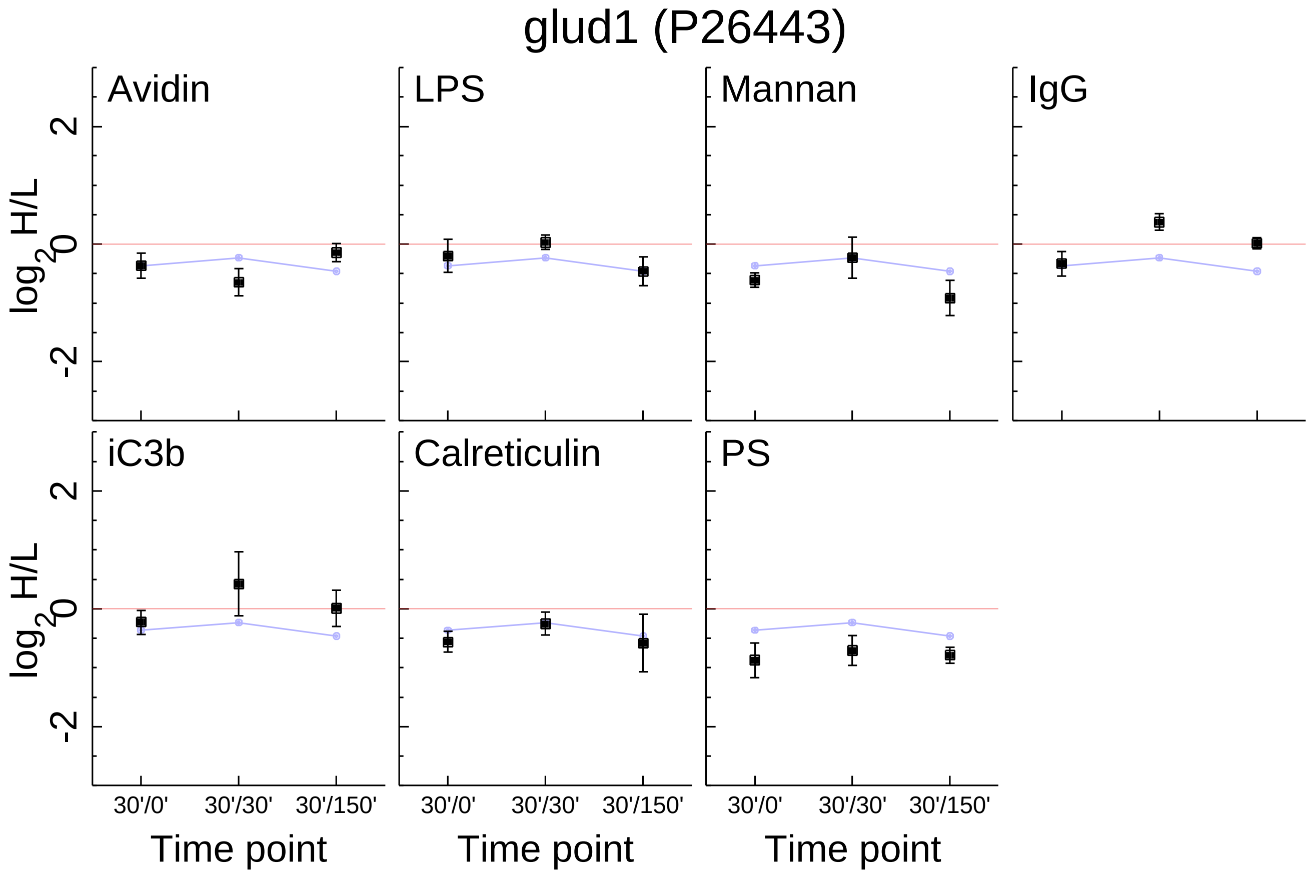
glud1 (P26443)
Avidin
LPS
Mannan
IgG
iC3b
Calreticulin
PS
log2 H/L
2
0
-2
30'/0'
30'/30'
30'/150'
Time point

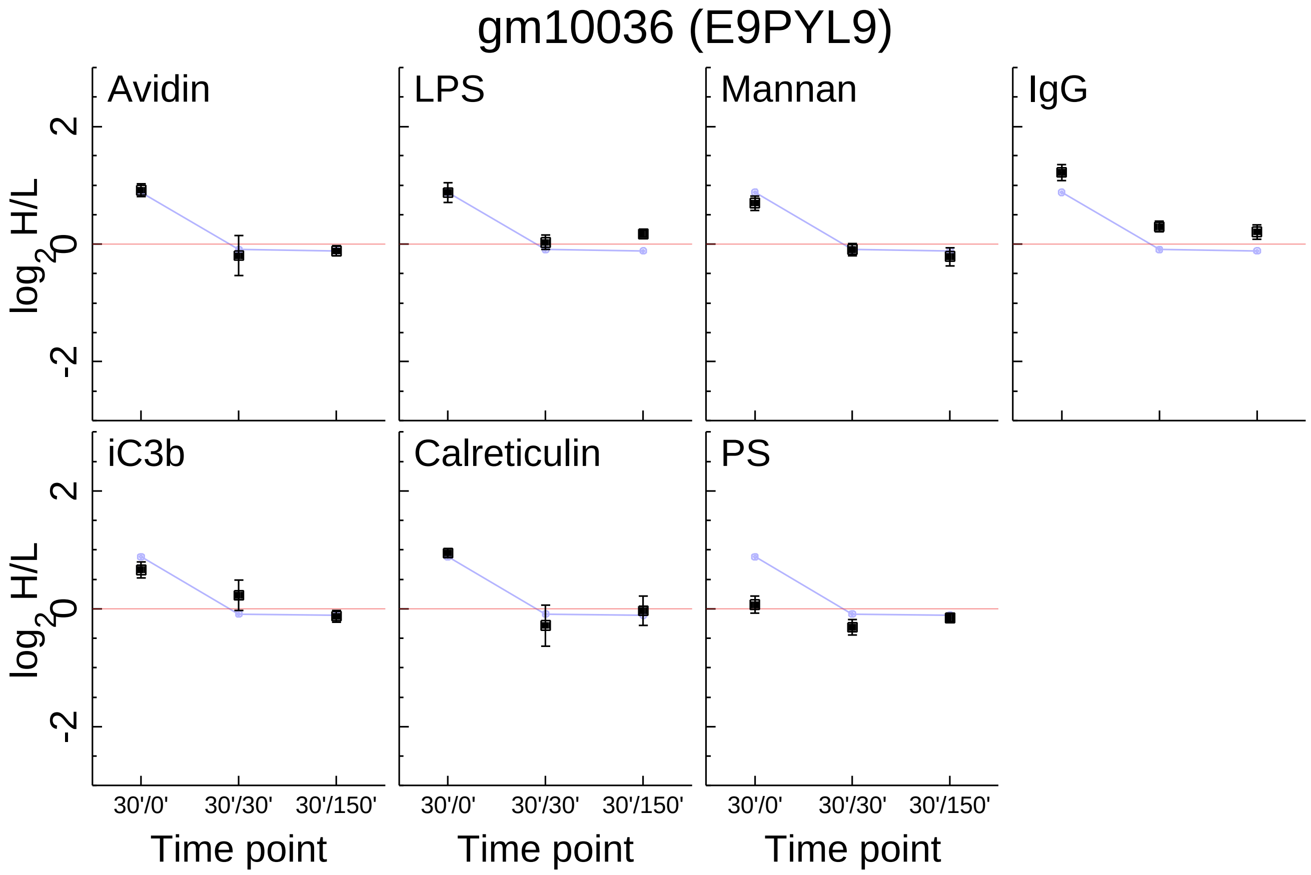
gm10036 (E9PYL9)
Avidin
LPS
Mannan
IgG
iC3b
Calreticulin
PS
log2 H/L
2
0
-2
30'/0'
30'/30'
30'/150'
Time point

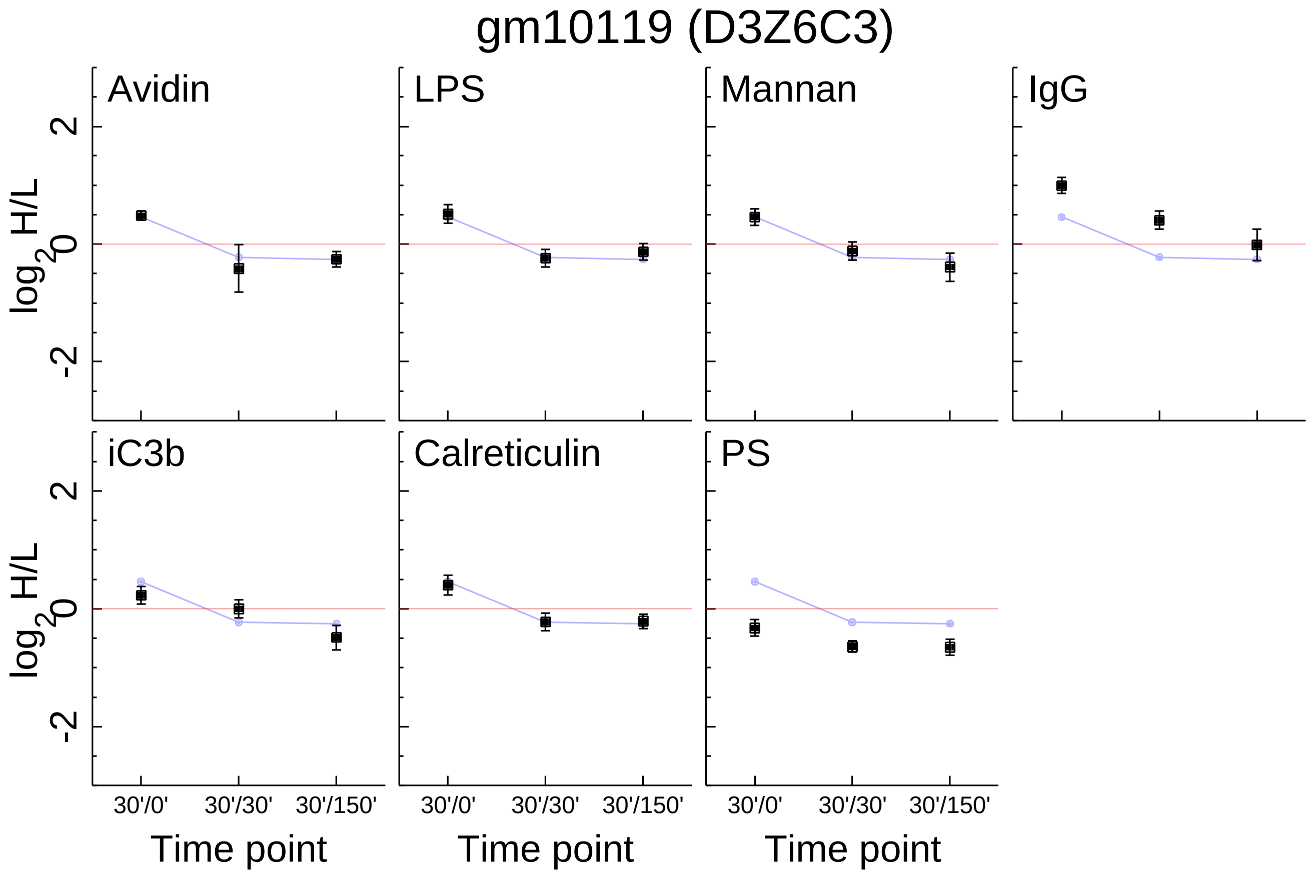
gm10119 (D3Z6C3)
Avidin
LPS
Mannan
IgG
iC3b
Calreticulin
PS
log2 H/L
2
0
-2
30'/0'
30'/30'
30'/150'
Time point

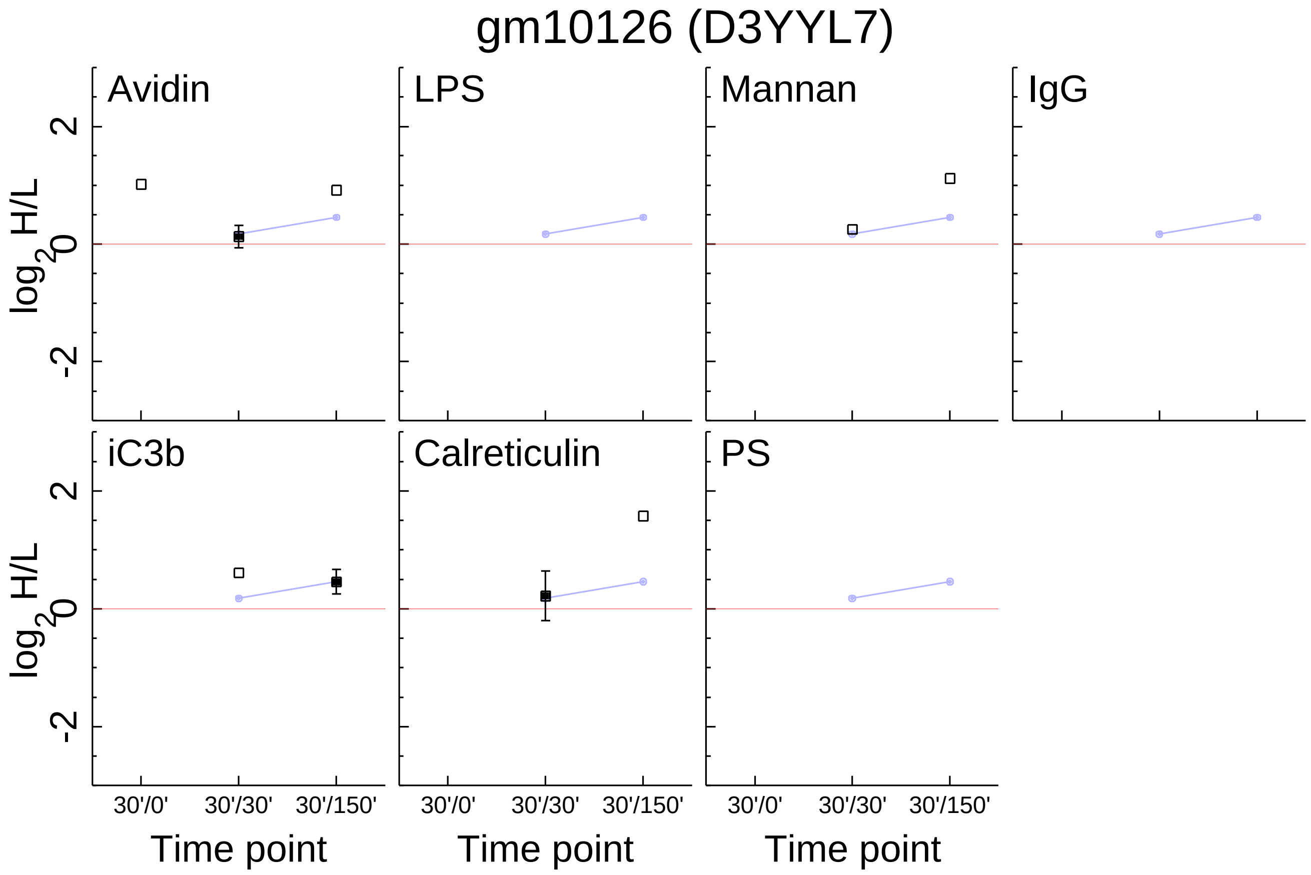

gm10126 (D3YYL7)
Avidin
LPS
Mannan
IgG
iC3b
Calreticulin
PS
log2 H/L
2
0
-2
30'/0'
30'/30'
30'/150'
Time point

gm10320 (E9PW43)

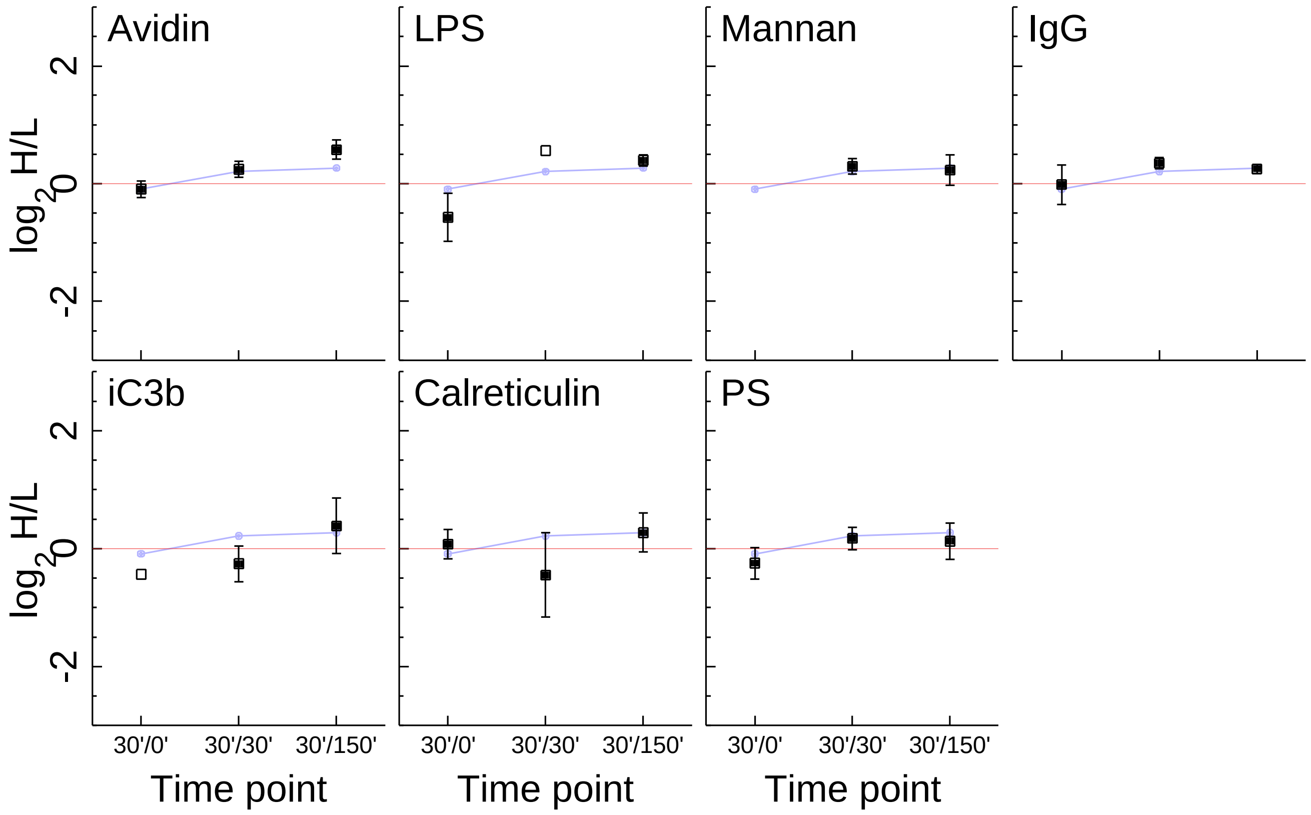

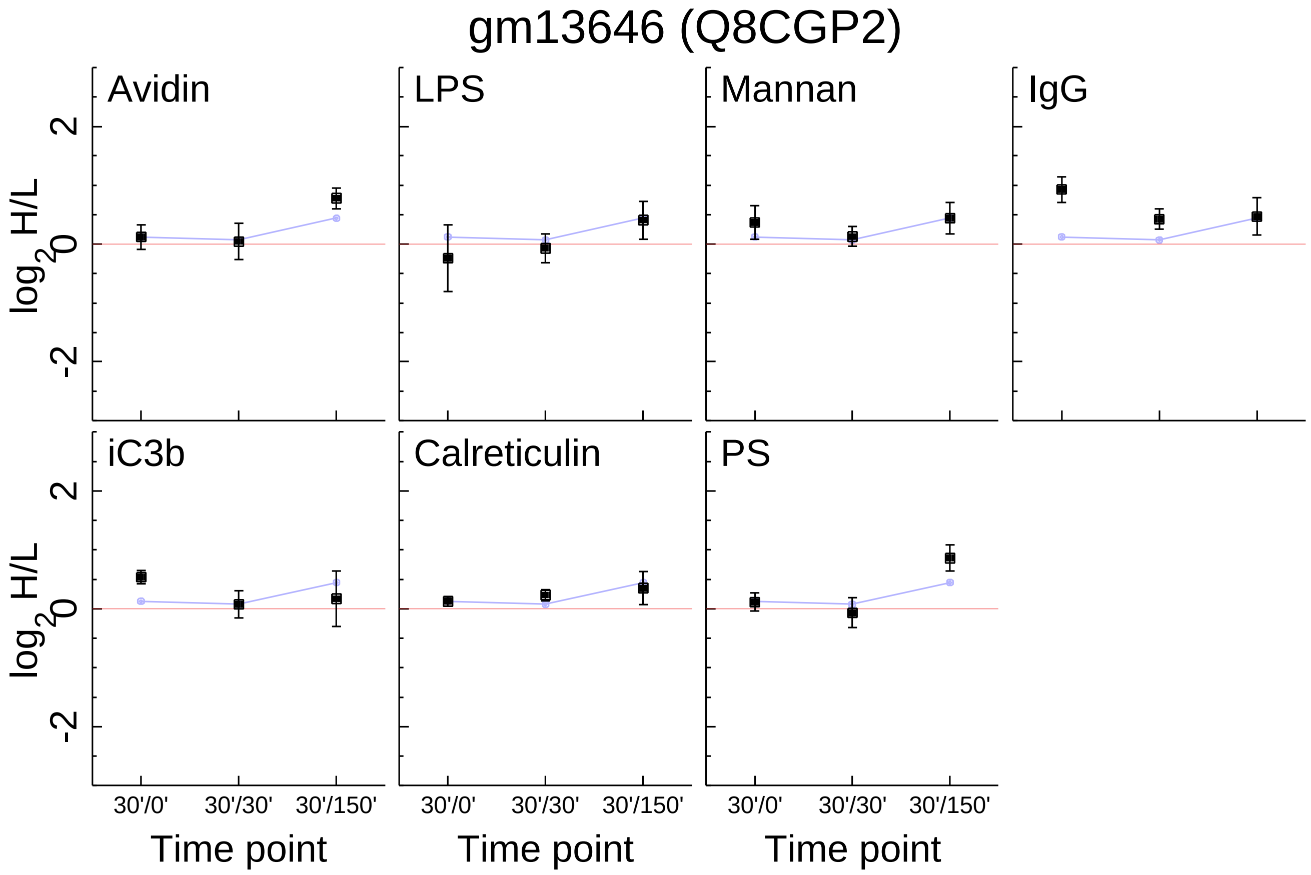
gm13646 (Q8CGP2)
Avidin
LPS
Mannan
IgG
iC3b
Calreticulin
PS
log2 H/L
2
0
-2
30'/0'
30'/30'
30'/150'
Time point

gm14548 (E9Q1Z6)

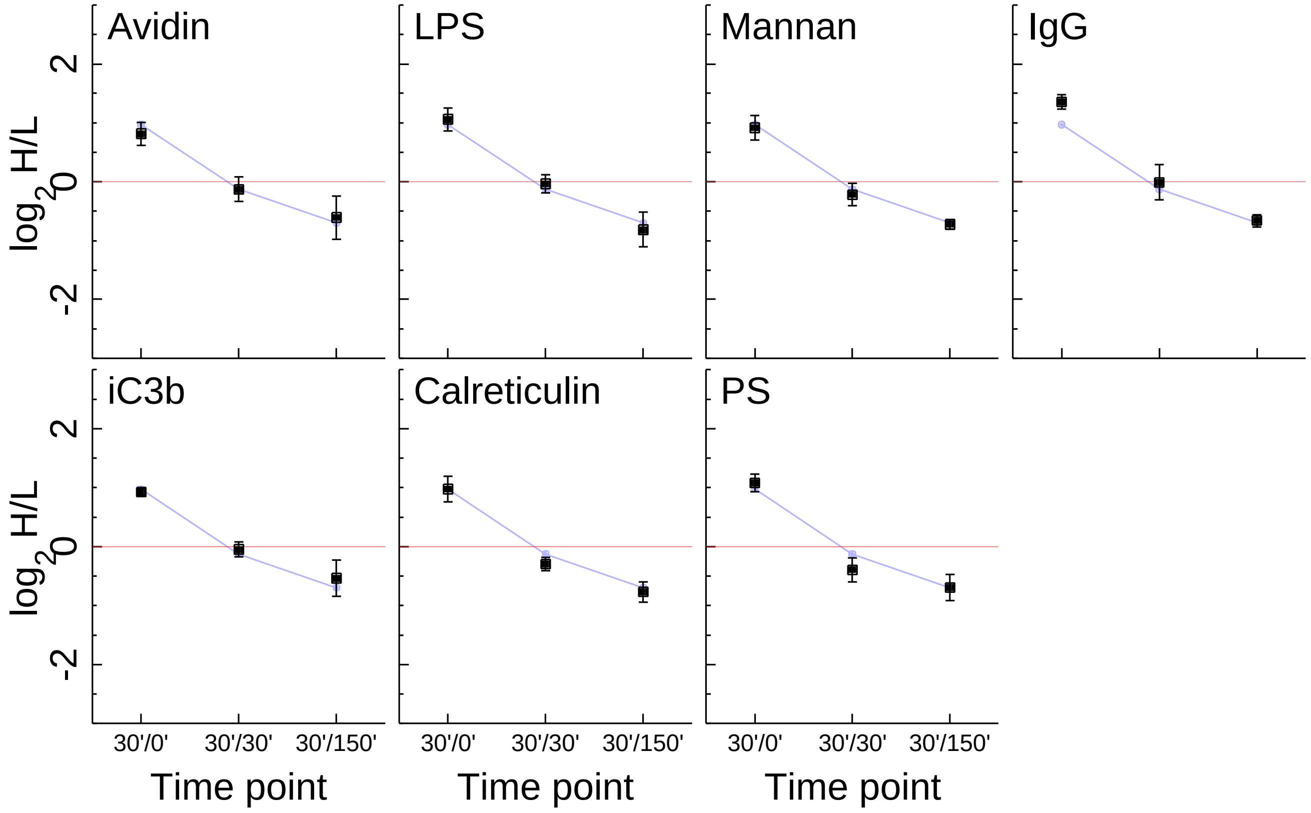

gm16477 (D3Z3R0)

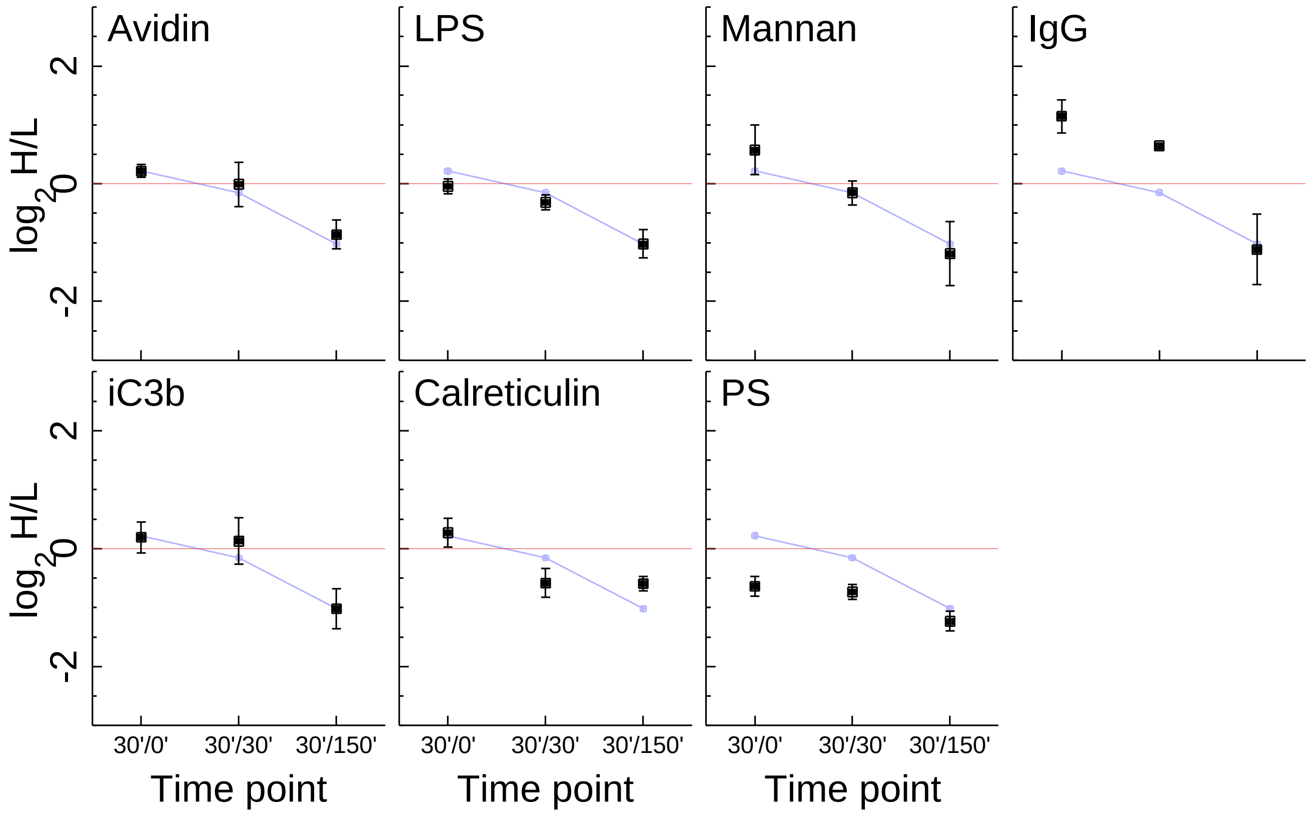

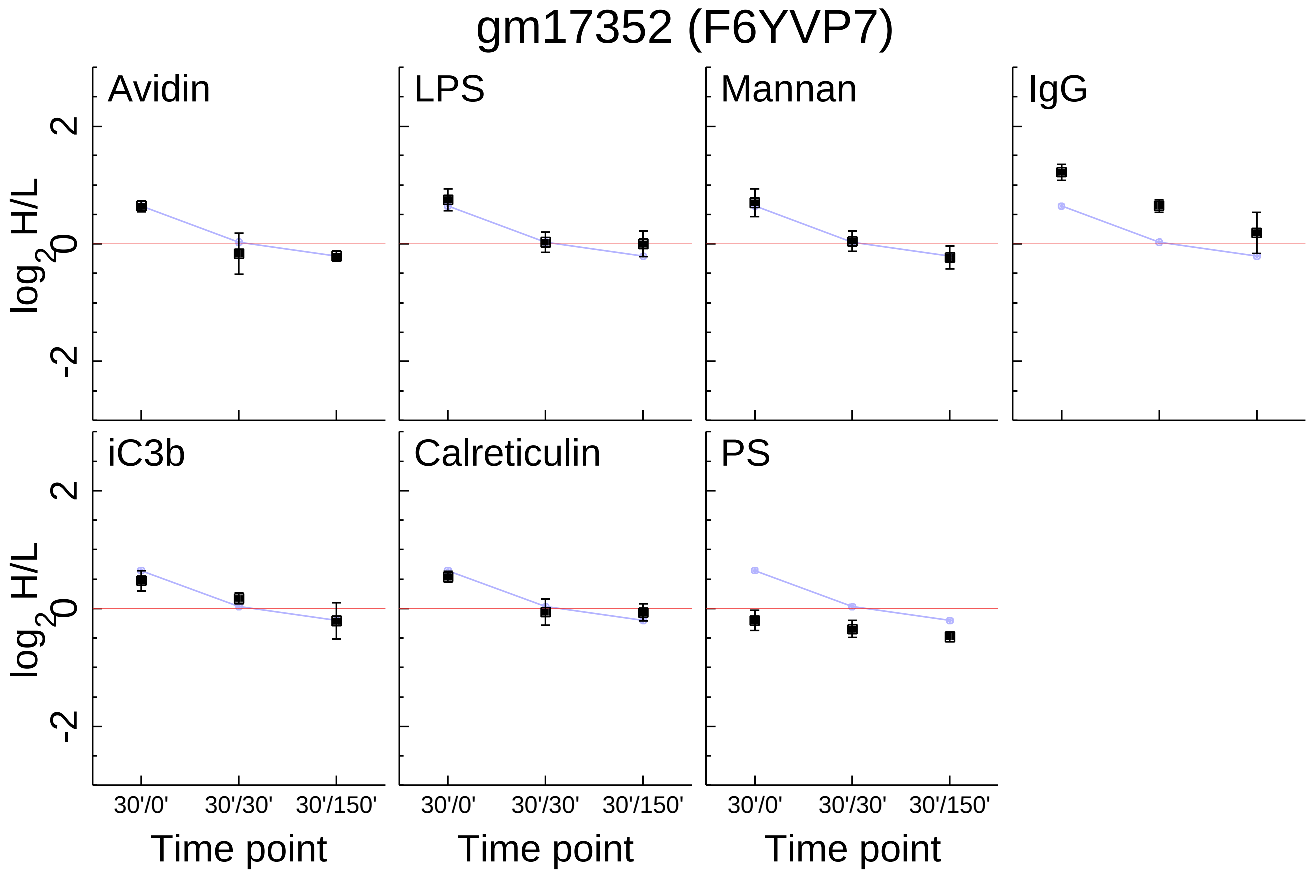

gm17352 (F6YVP7)
Avidin
LPS
Mannan
IgG
iC3b
Calreticulin
PS
log2 H/L
2
0
-2
30'/0'
30'/30'
30'/150'
Time point

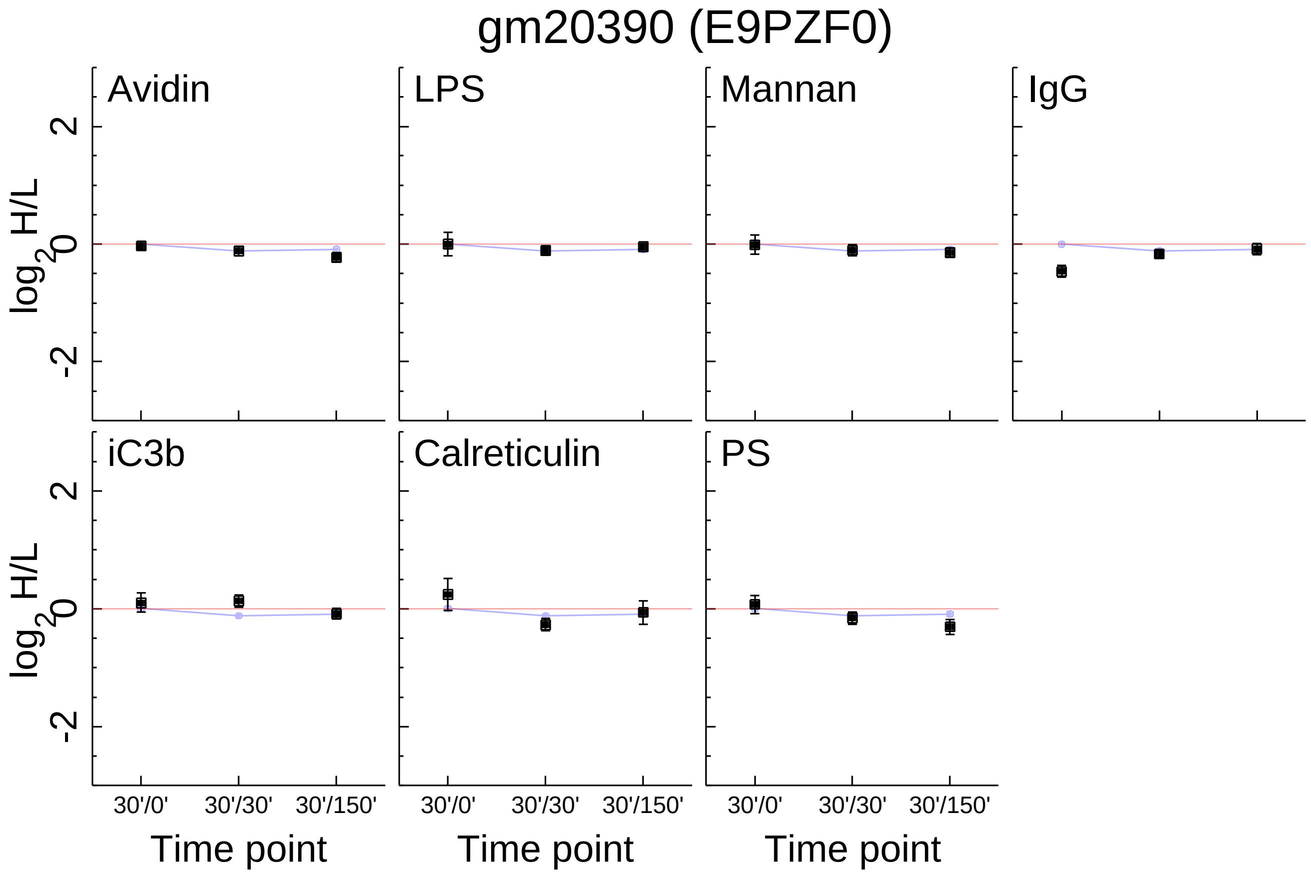

gm20390 (E9PZF0)
Avidin
LPS
Mannan
IgG
iC3b
Calreticulin
PS
log2 H/L
2
0
-2
30'/0'
30'/30'
30'/150'
Time point

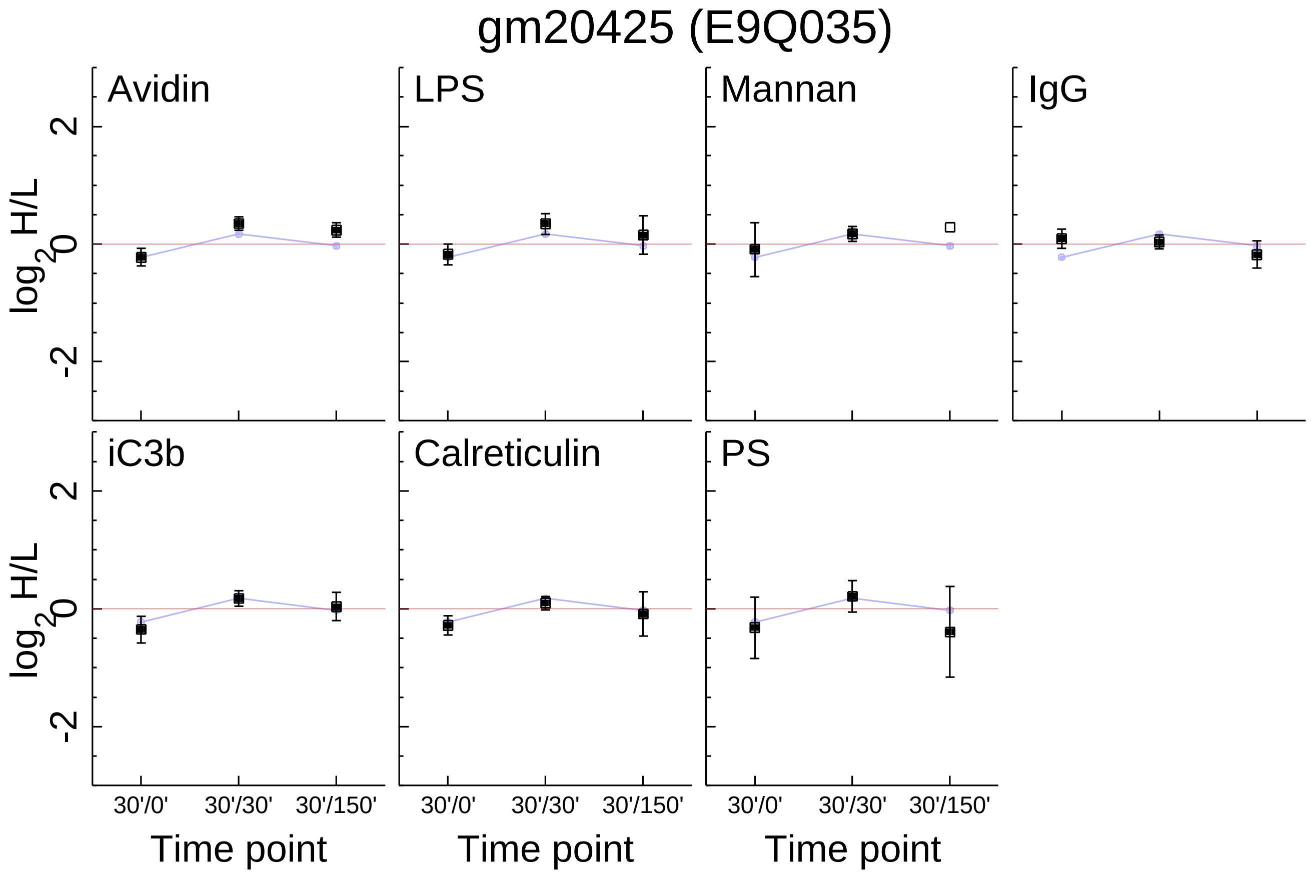

gm20425 (E9Q035)
Avidin
LPS
Mannan
IgG
iC3b
Calreticulin
PS
log2 H/L
2
0
-2
30'/0'
30'/30'
30'/150'
Time point

gm20431 (E9PY39)

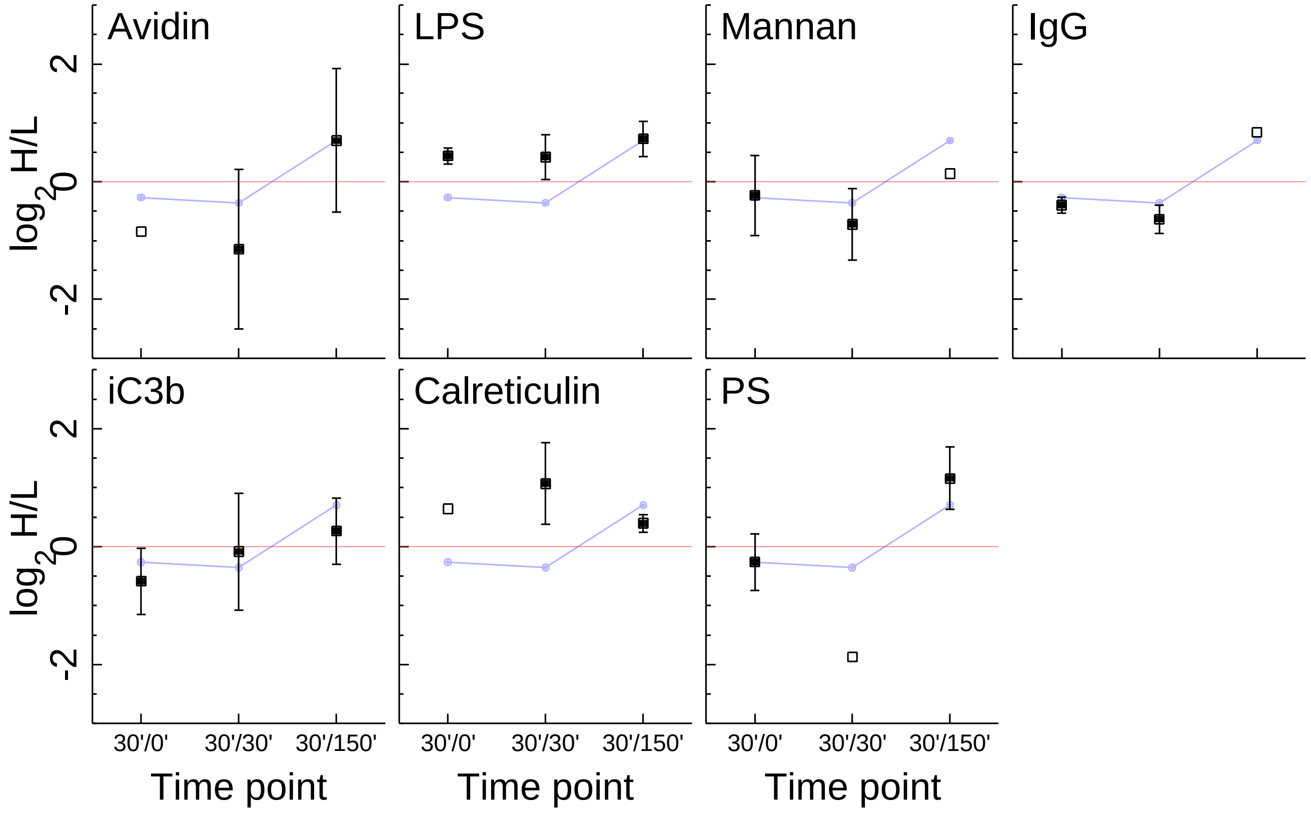

gm20489 (A2AGH5)

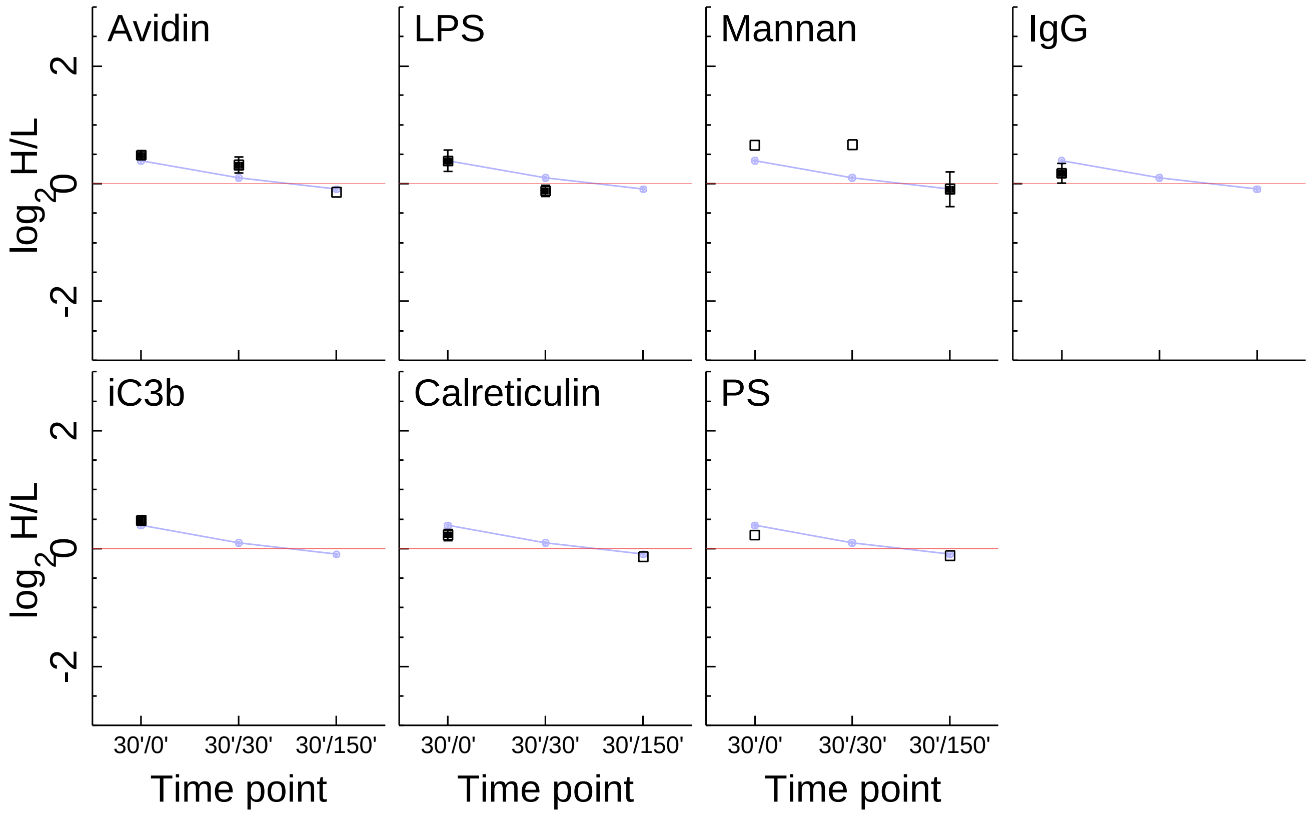

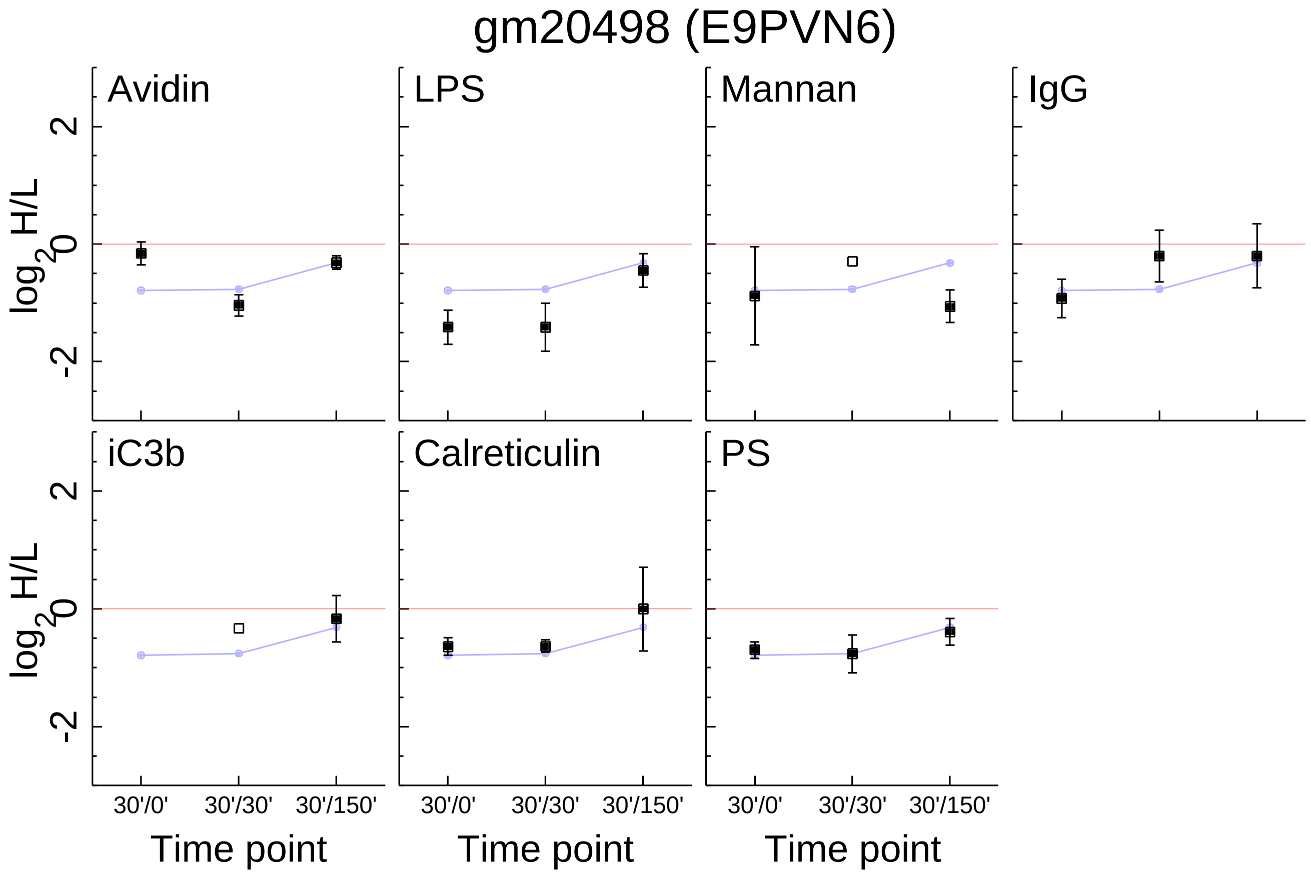
gm20498 (E9PVN6)
Avidin
LPS
Mannan
IgG
iC3b
Calreticulin
PS
log2 H/L
2
0
-2
30'/0'
30'/30'
30'/150'
Time point

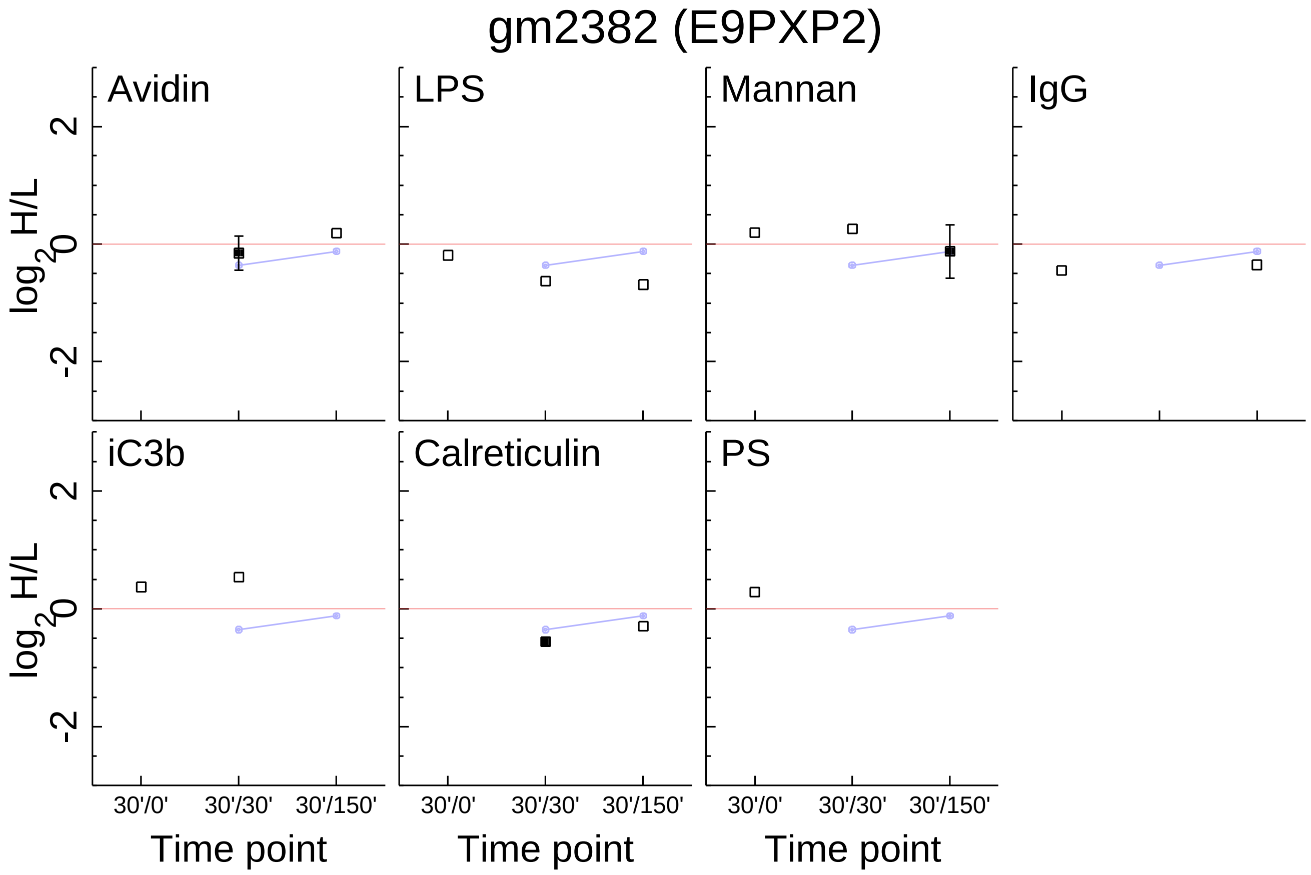
gm2382 (E9PXP2)
Avidin
LPS
Mannan
IgG
iC3b
Calreticulin
PS
log2 H/L
2
0
-2
30'/0'
30'/30'
30'/150'
Time point

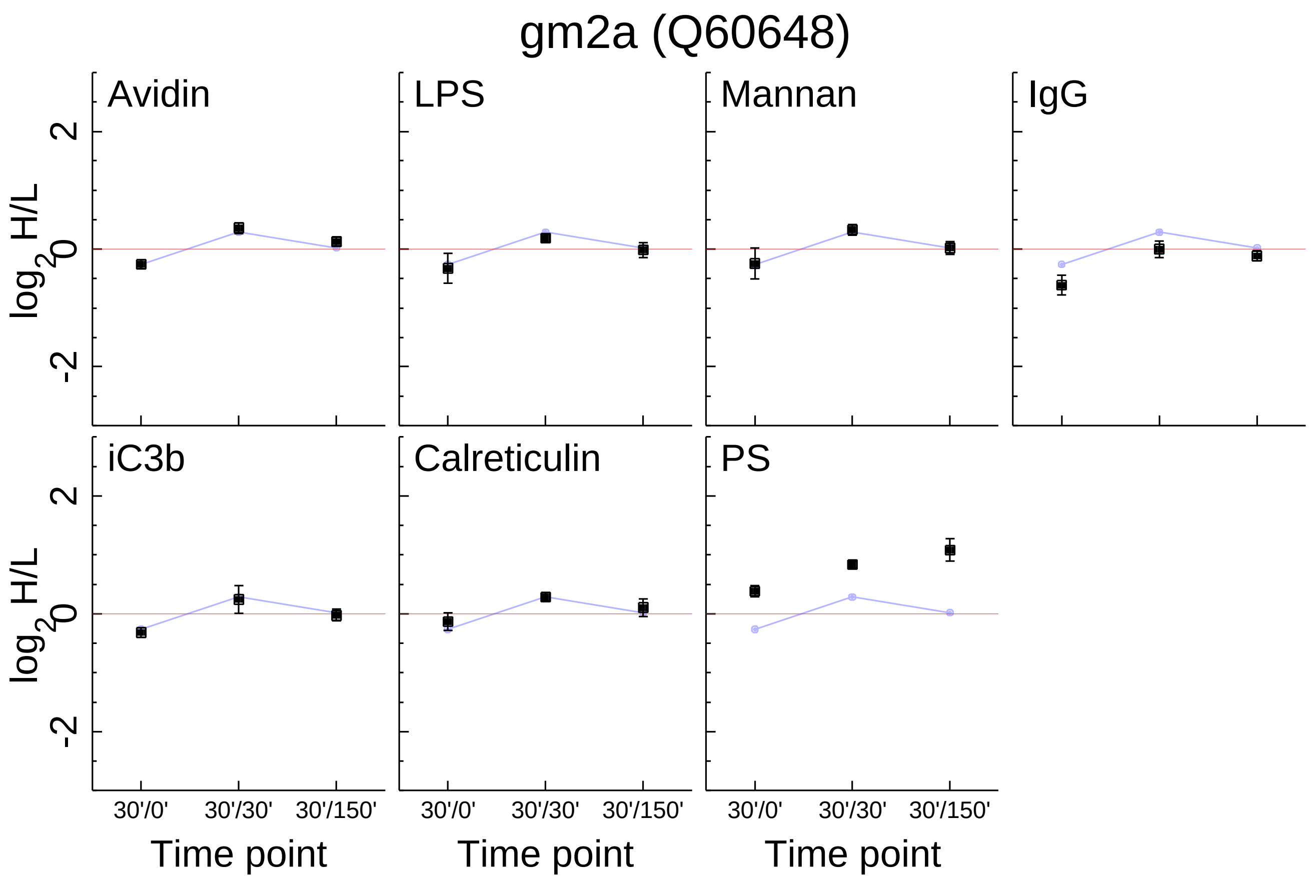
gm2a (Q60648)
Avidin
LPS
Mannan
IgG
iC3b
Calreticulin
PS
log2 H/L
2
0
-2
30'/0'
30'/30'
30'/150'
Time point

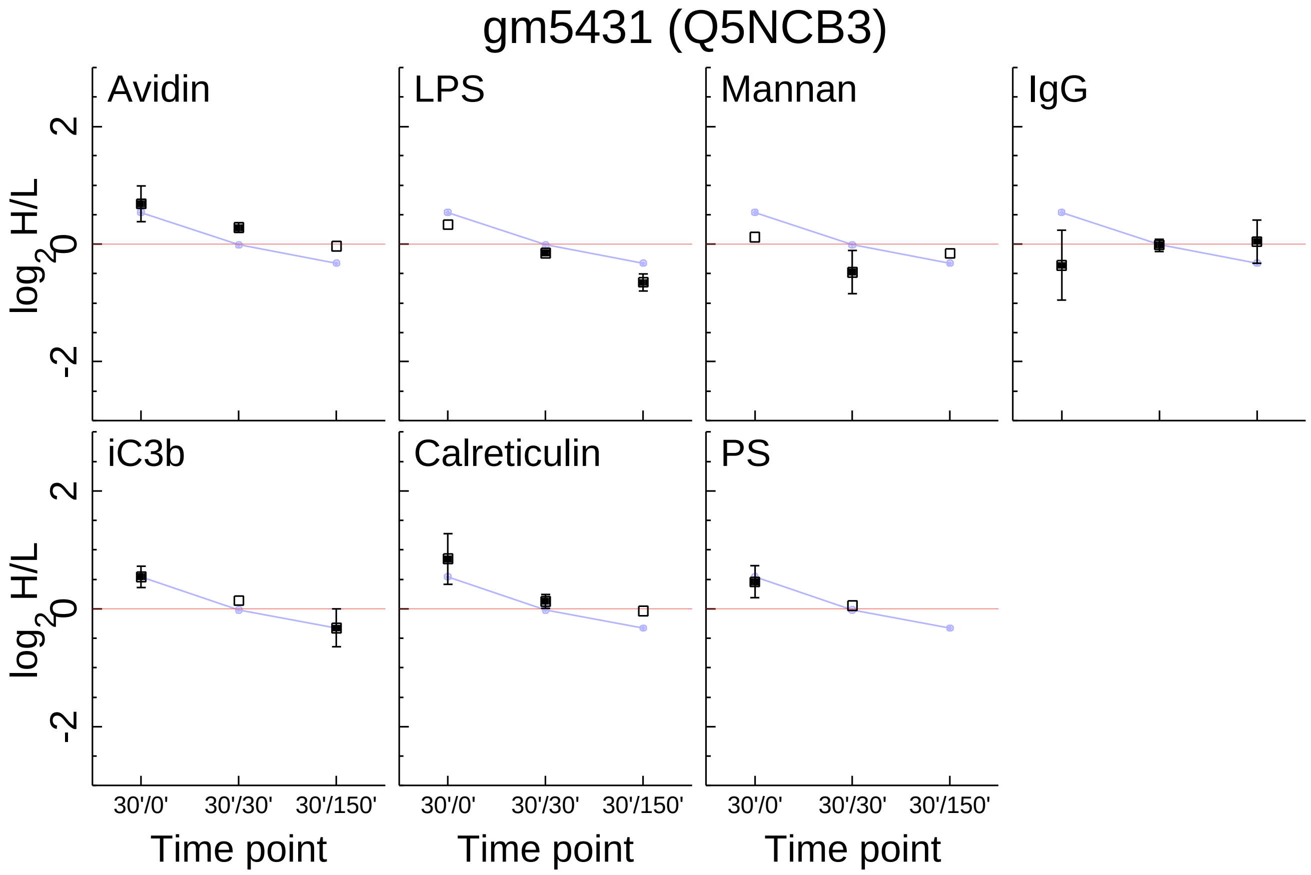
gm5431 (Q5NCB3)
Avidin
LPS
Mannan
IgG
iC3b
Calreticulin
PS
log2 H/L
2
0
-2
30'/0'
30'/30'
30'/150'
Time point

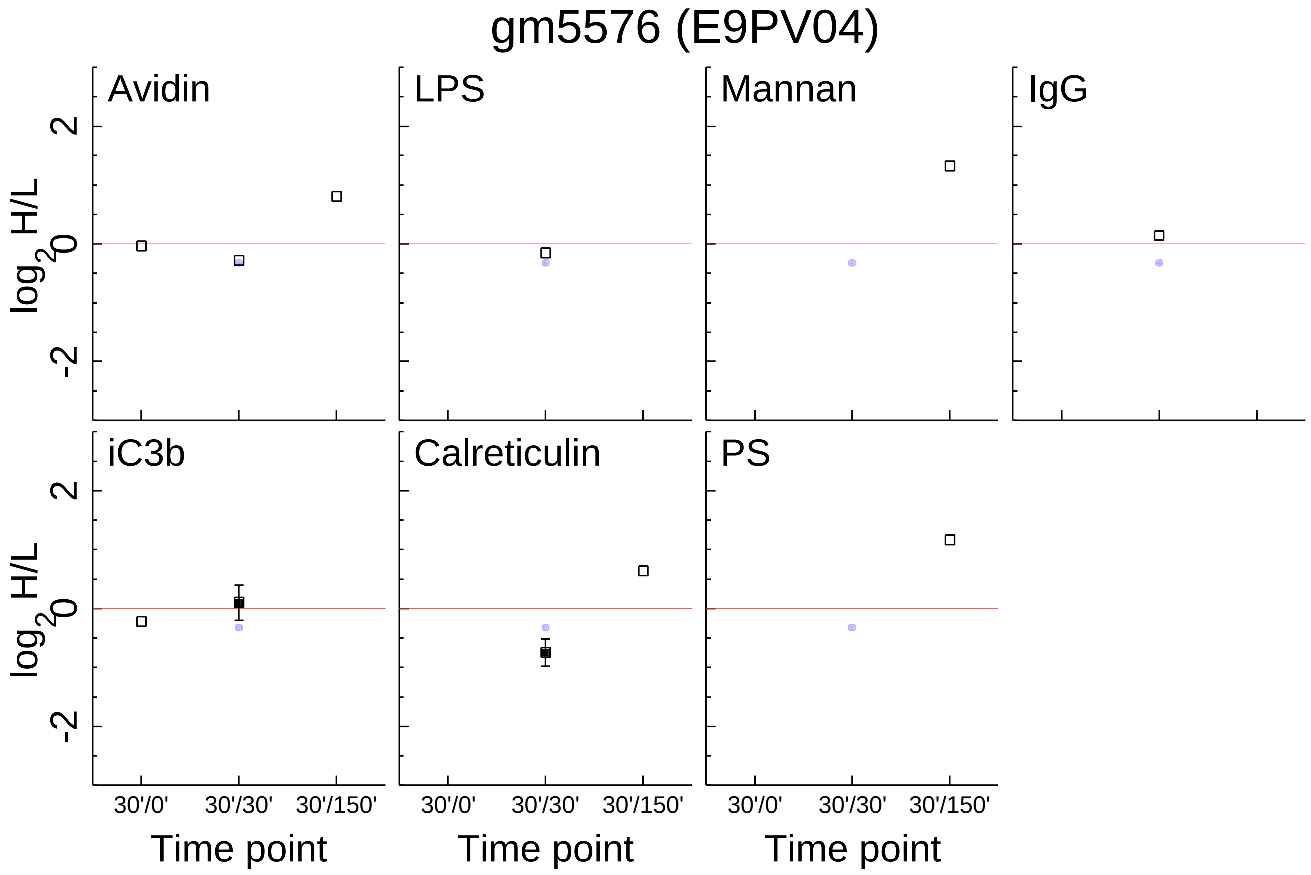

gm5576 (E9PV04)
Avidin
LPS
Mannan
IgG
iC3b
Calreticulin
PS
log2 H/L
2
0
-2
30'/0'
30'/30'
30'/150'
Time point

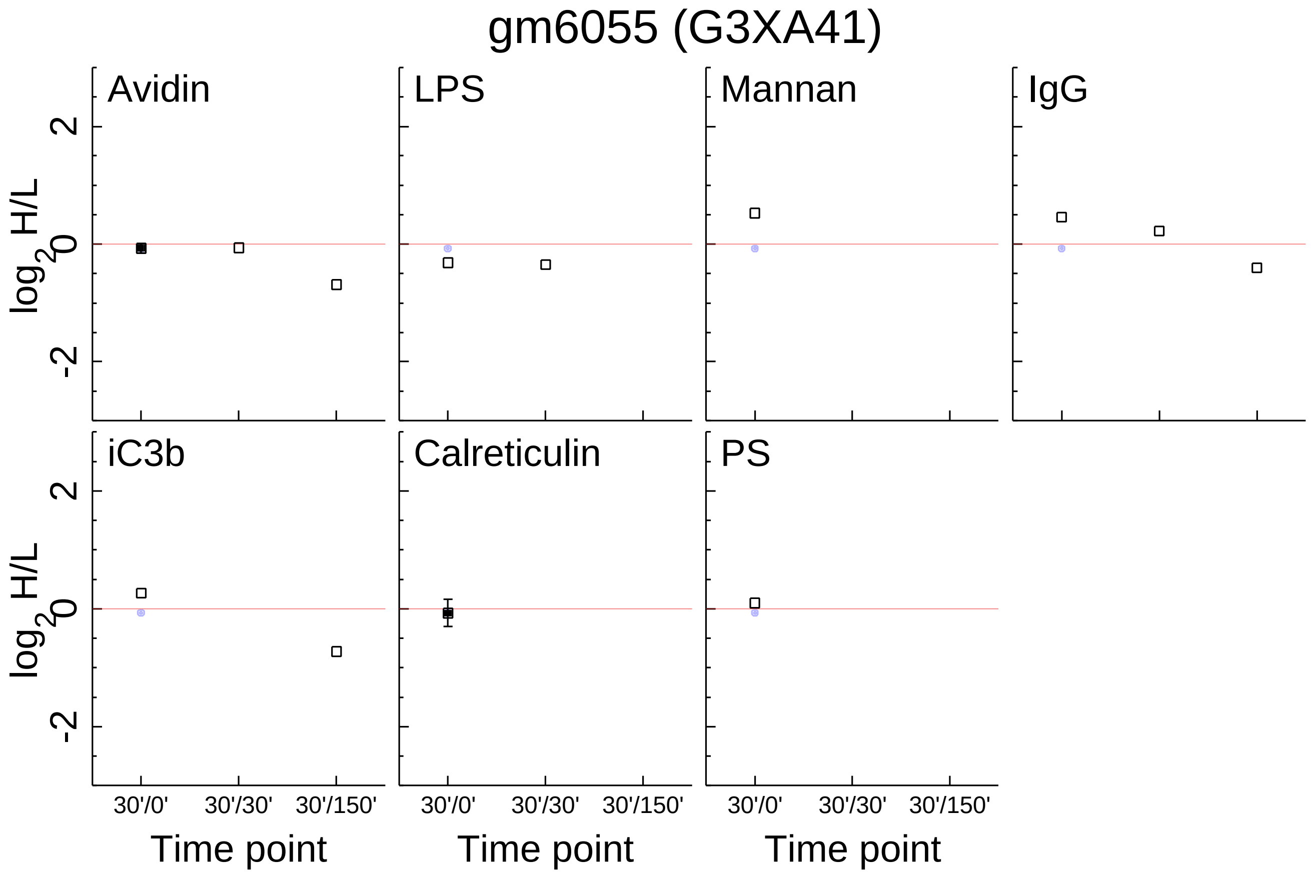

gm6055 (G3XA41)
Avidin
LPS
Mannan
IgG
iC3b
Calreticulin
PS
log2 H/L
2
0
-2
30'/0'
30'/30'
30'/150'
Time point

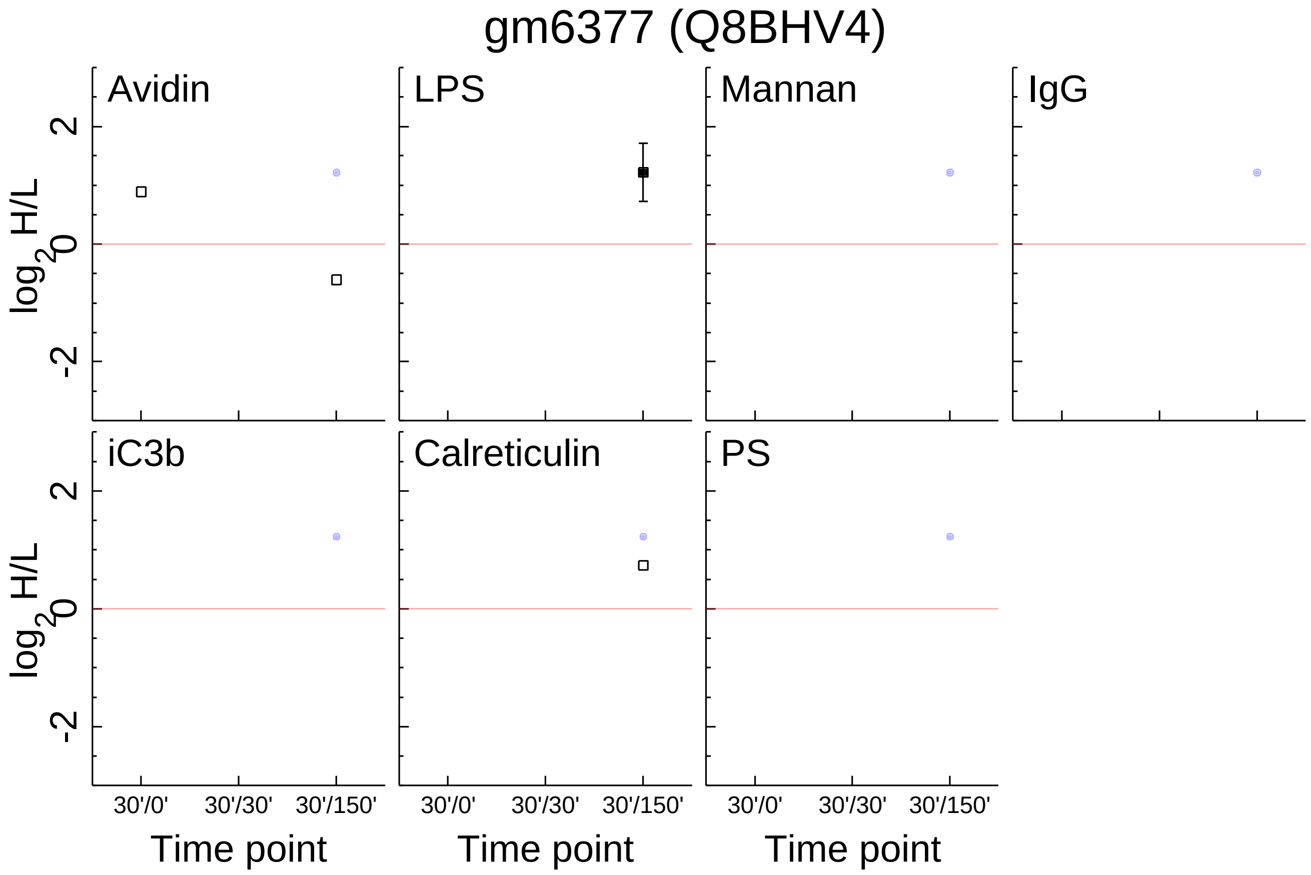

gm6377 (Q8BHV4)
Avidin
LPS
Mannan
IgG
iC3b
Calreticulin
PS
log2 H/L
2
0
-2
30'/0'
30'/30'
30'/150'
Time point

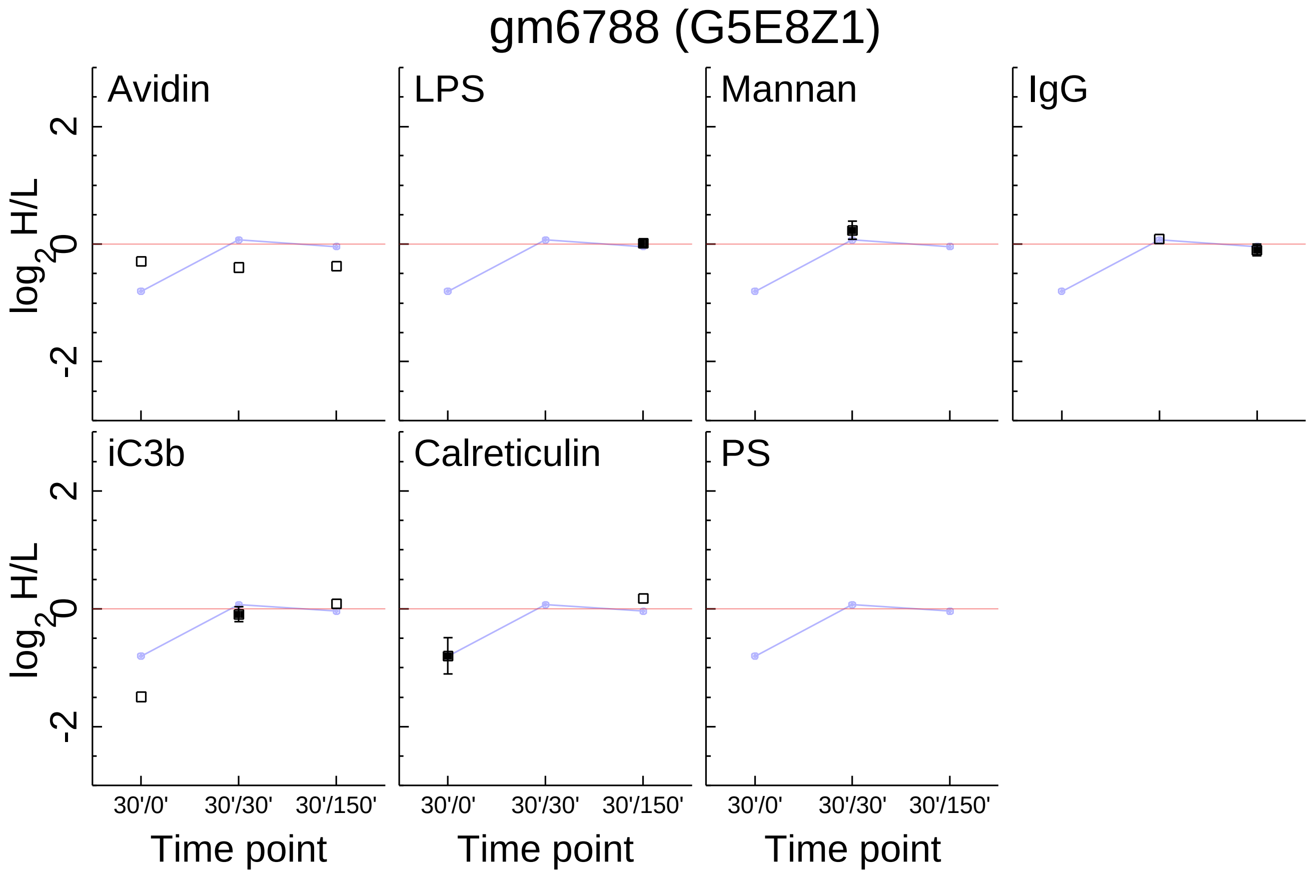

gm6788 (G5E8Z1)
Avidin
LPS
Mannan
IgG
iC3b
Calreticulin
PS
log2 H/L
2
0
-2
30'/0'
30'/30'
30'/150'
Time point

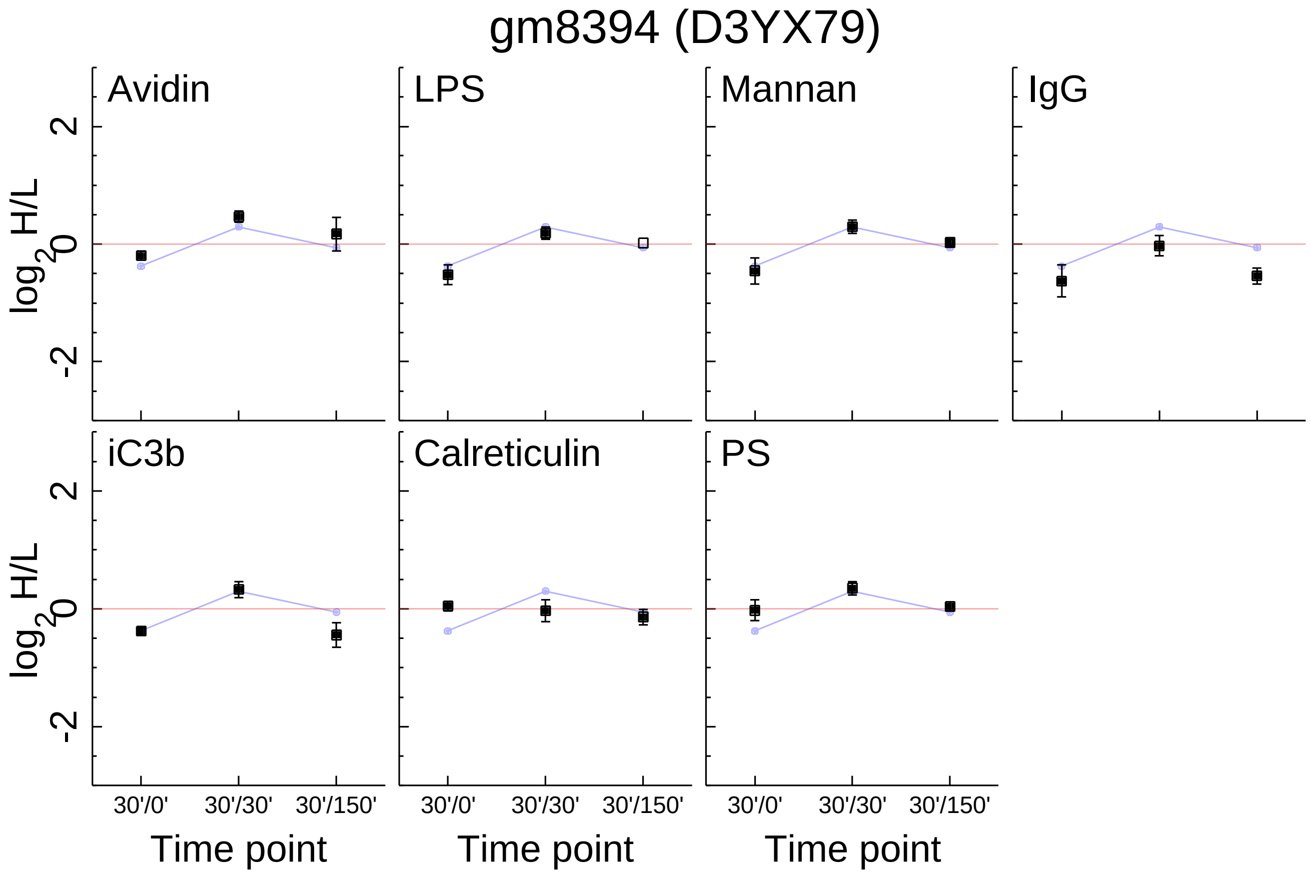

gm8394 (D3YX79)
Avidin
LPS
Mannan
IgG
iC3b
Calreticulin
PS
log2 H/L
2
0
-2
30'/0'
30'/30'
30'/150'
Time point

gm9755 (D3YVN7)

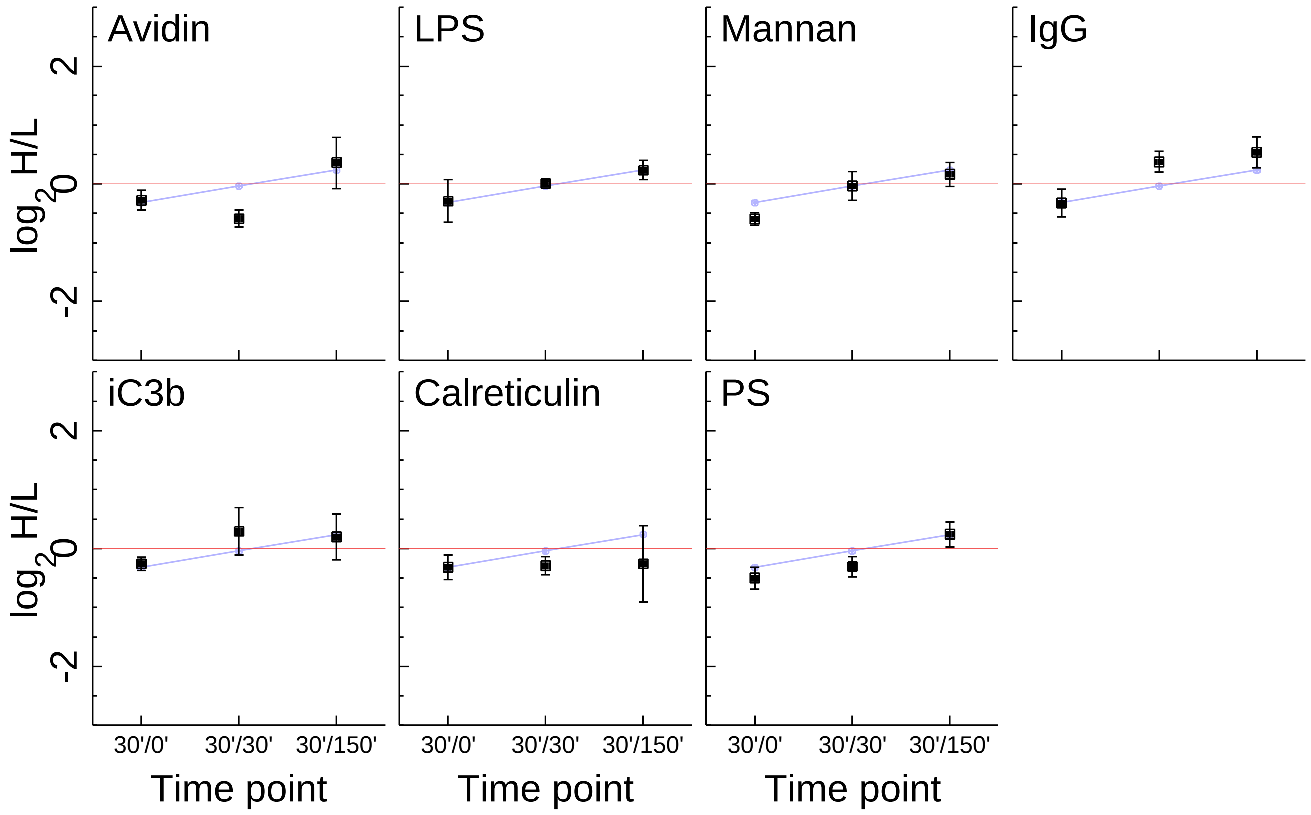

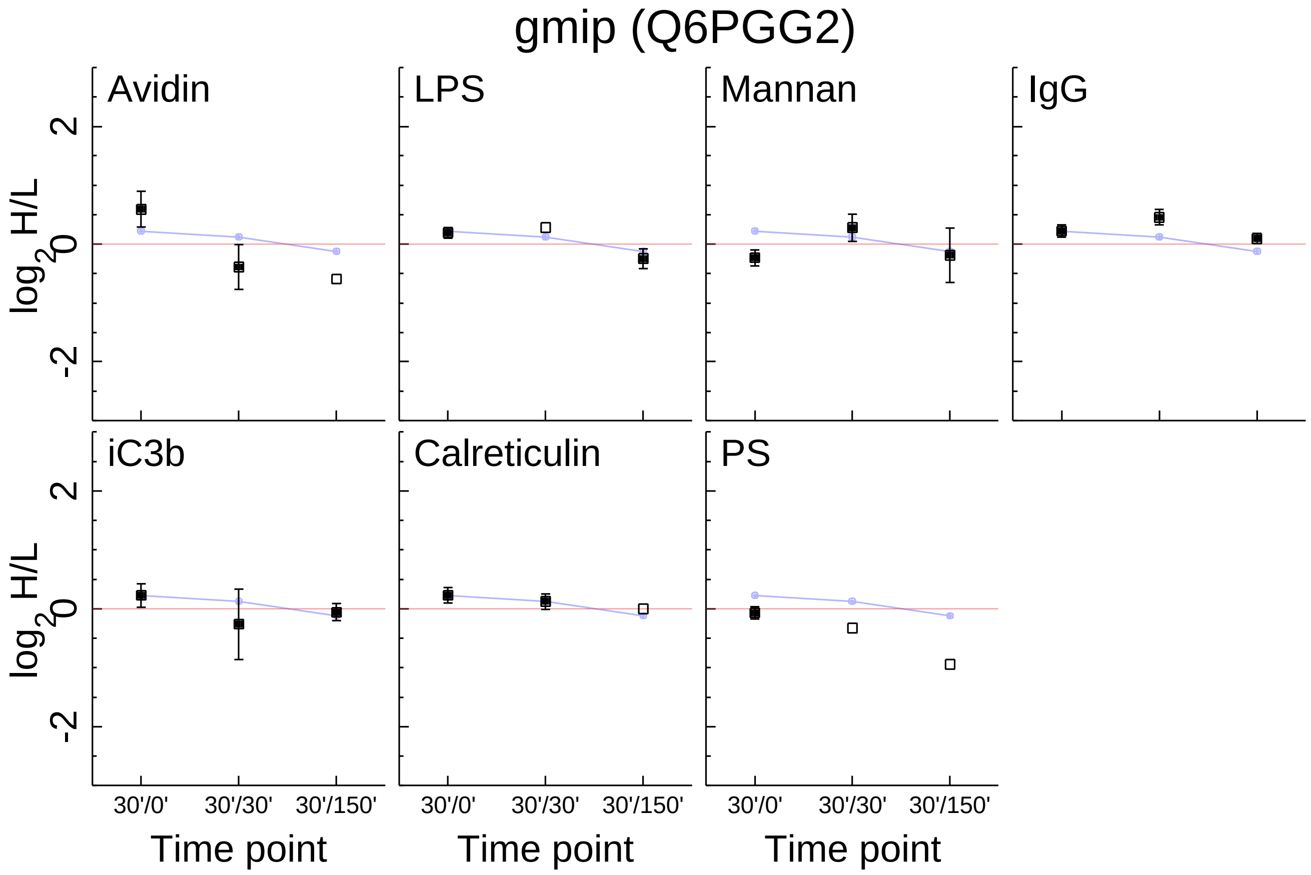
gmip (Q6PGG2)
Avidin
LPS
Mannan
IgG
iC3b
Calreticulin
PS
log2 H/L
2
0
-2
30'/0'
30'/30'
30'/150'
Time point

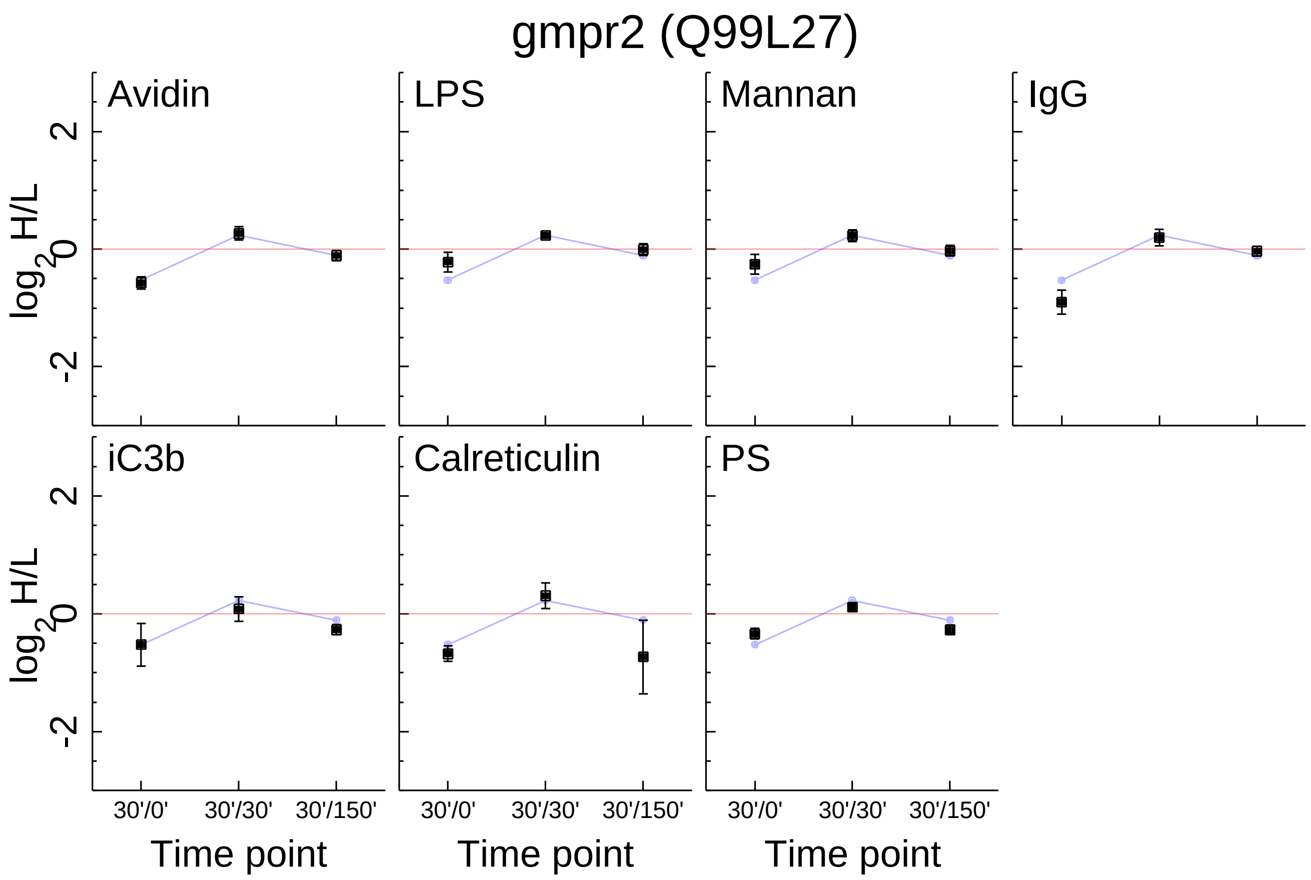
gmpr2 (Q99L27)
Avidin
LPS
Mannan
IgG
iC3b
Calreticulin
PS
log2 H/L
2
0
-2
30'/0'
30'/30'
30'/150'
Time point

gna13 (P27601)

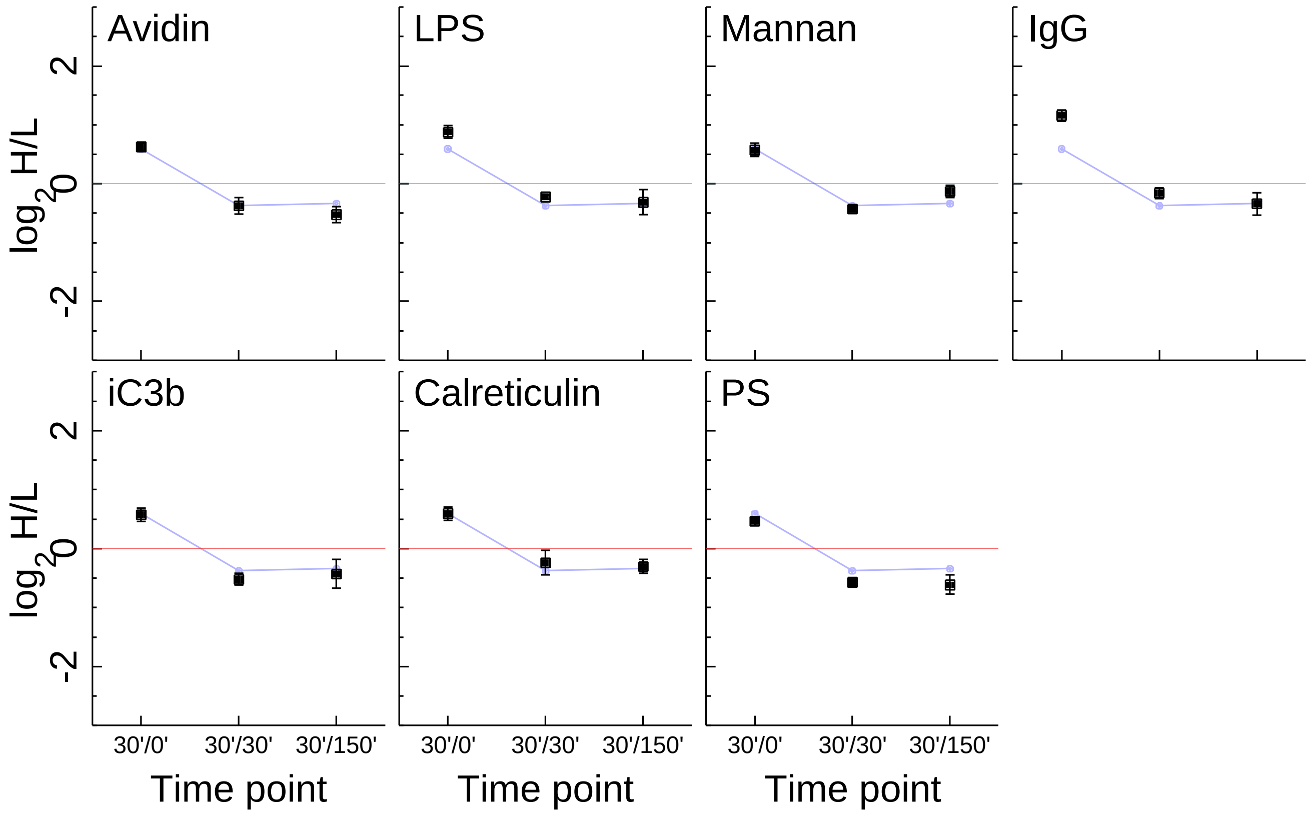

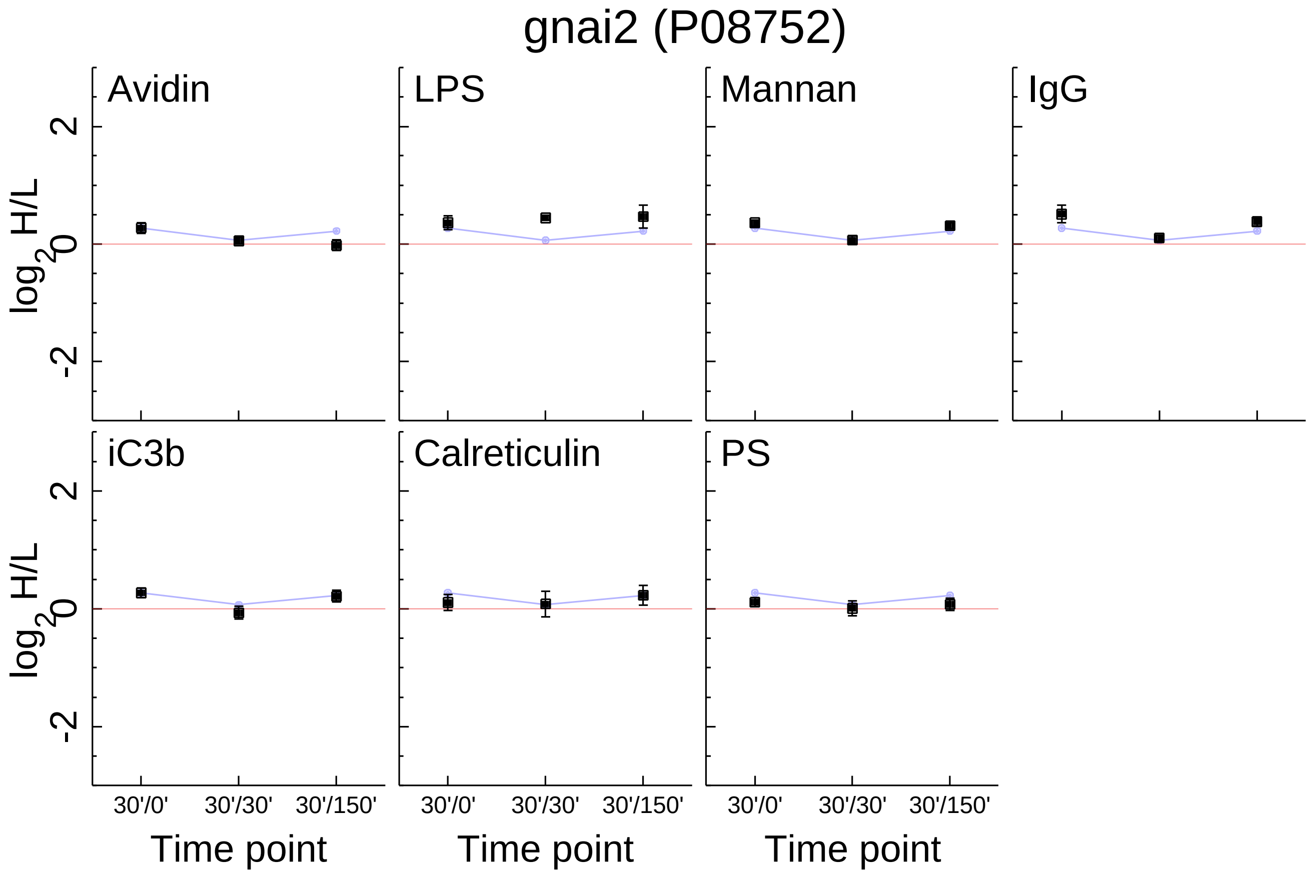
gnai2 (P08752)
Avidin
LPS
Mannan
IgG
iC3b
Calreticulin
PS
log2 H/L
2
0
-2
30'/0'
30'/30'
30'/150'
Time point

gnai3 (Q9DC51)

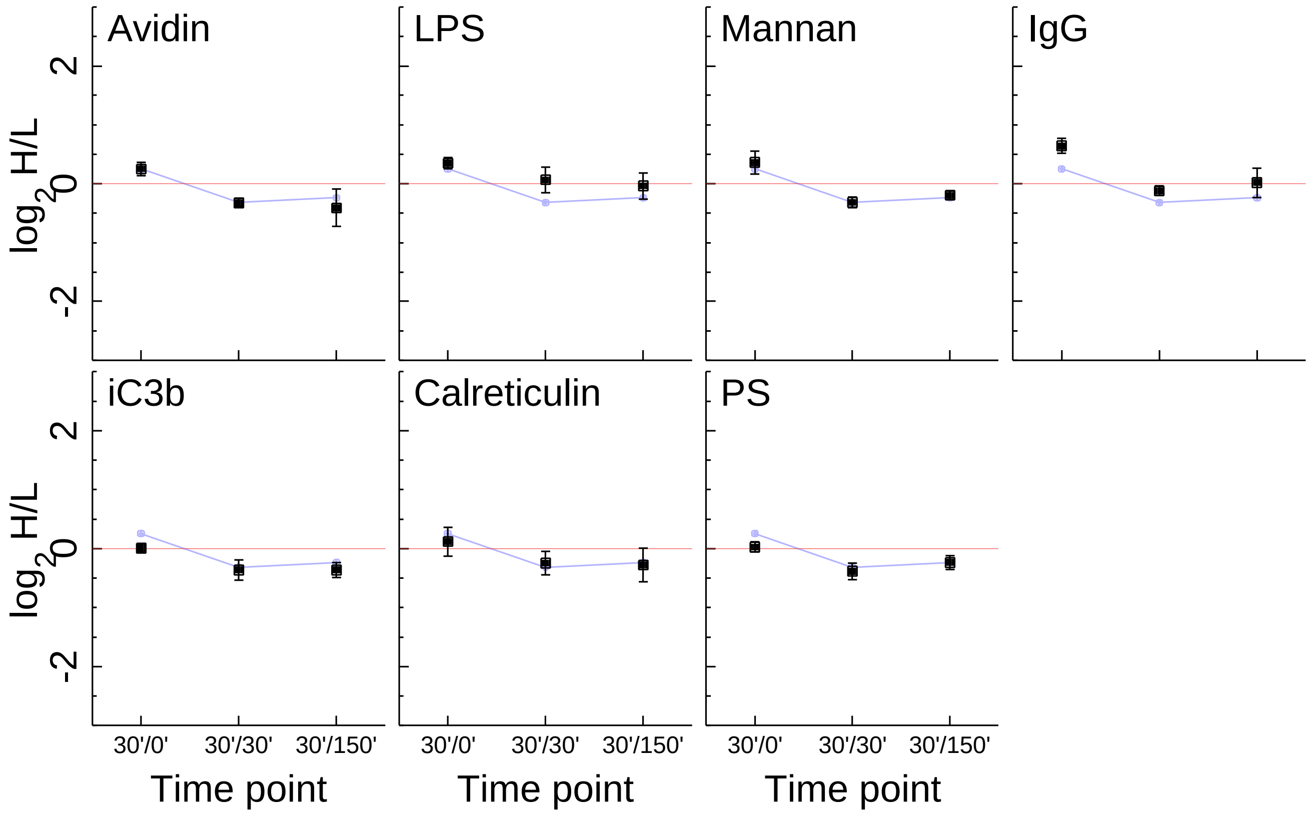

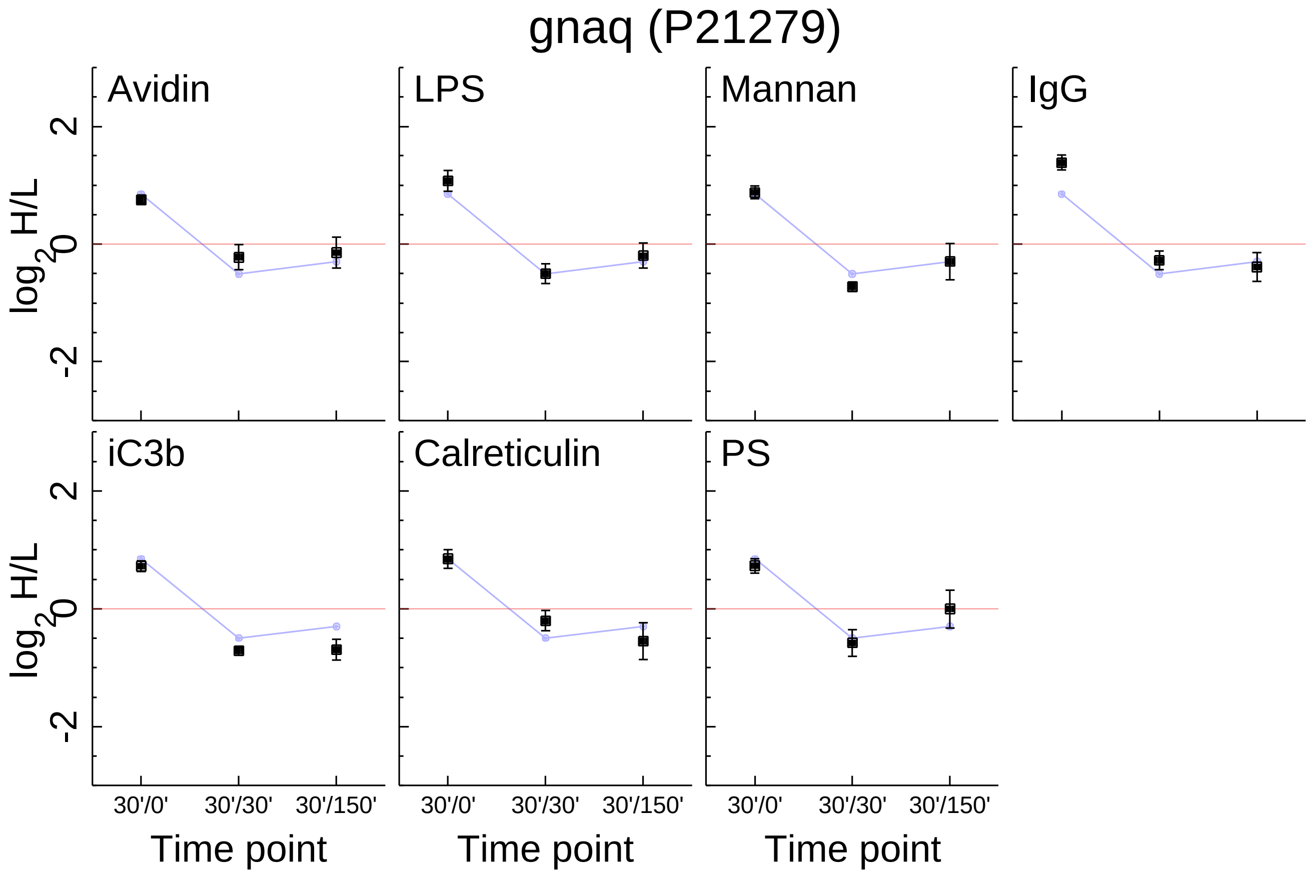

gnaq (P21279)
Avidin
LPS
Mannan
IgG
iC3b
Calreticulin
PS
log2 H/L
2
0
-2
30'/0'
30'/30'
30'/150'
Time point

gnas (P63094)

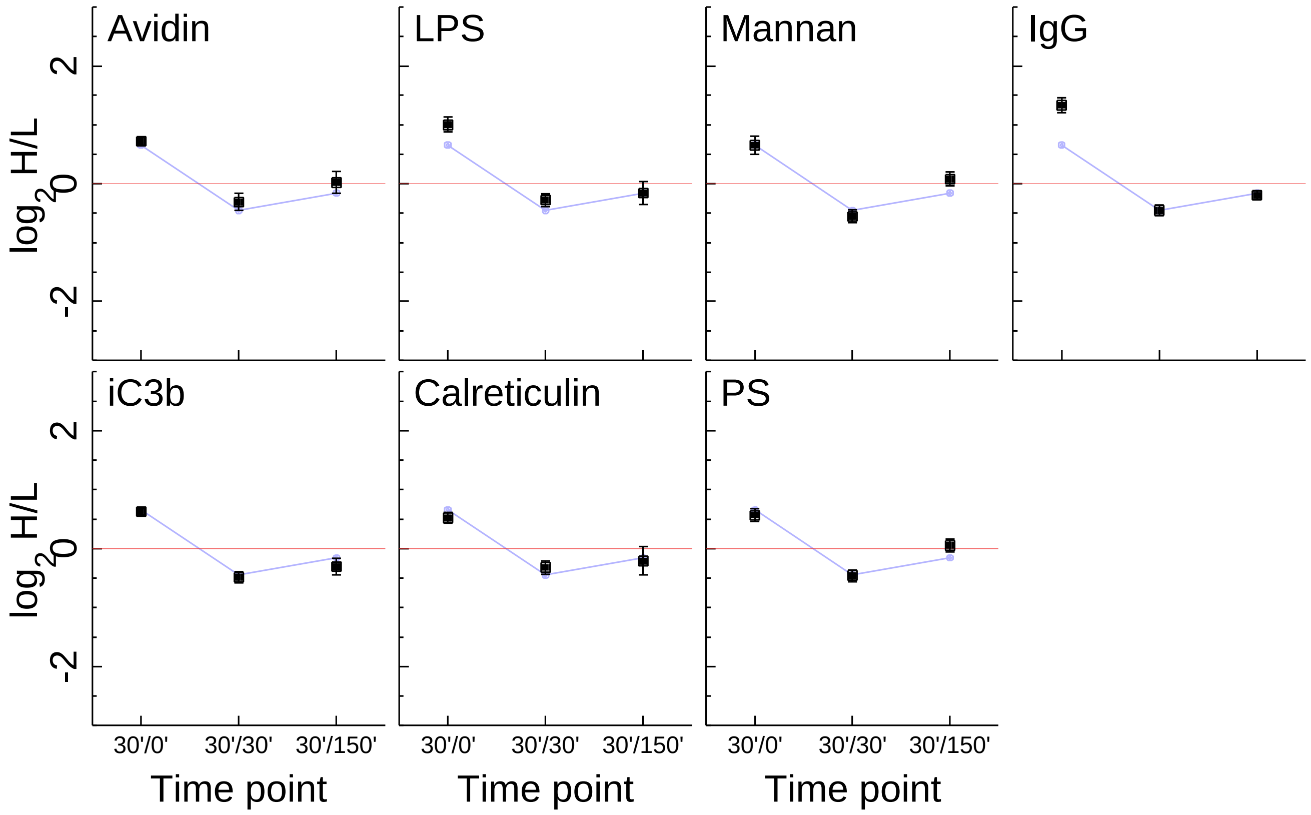

Avidin
LPS
Mannan
IgG
iC3b
Calreticulin
PS
log2 H/L
2
0
-2
30'/0'
30'/30'
30'/150'
Time point

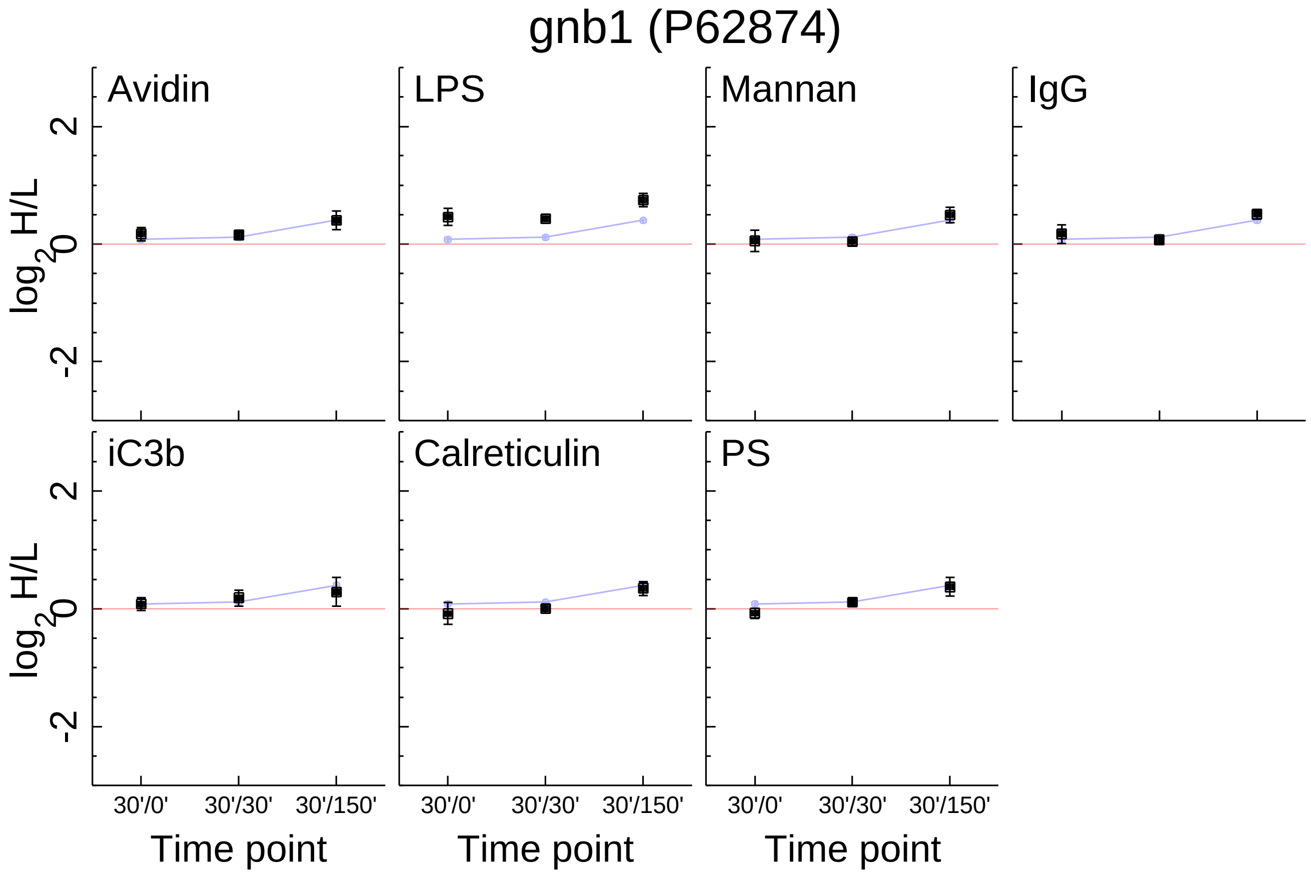

gnb1 (P62874)
Avidin
LPS
Mannan
IgG
iC3b
Calreticulin
PS
2
0
-2
log_2 H/L
30'/0'
30'/30'
30'/150'
Time point

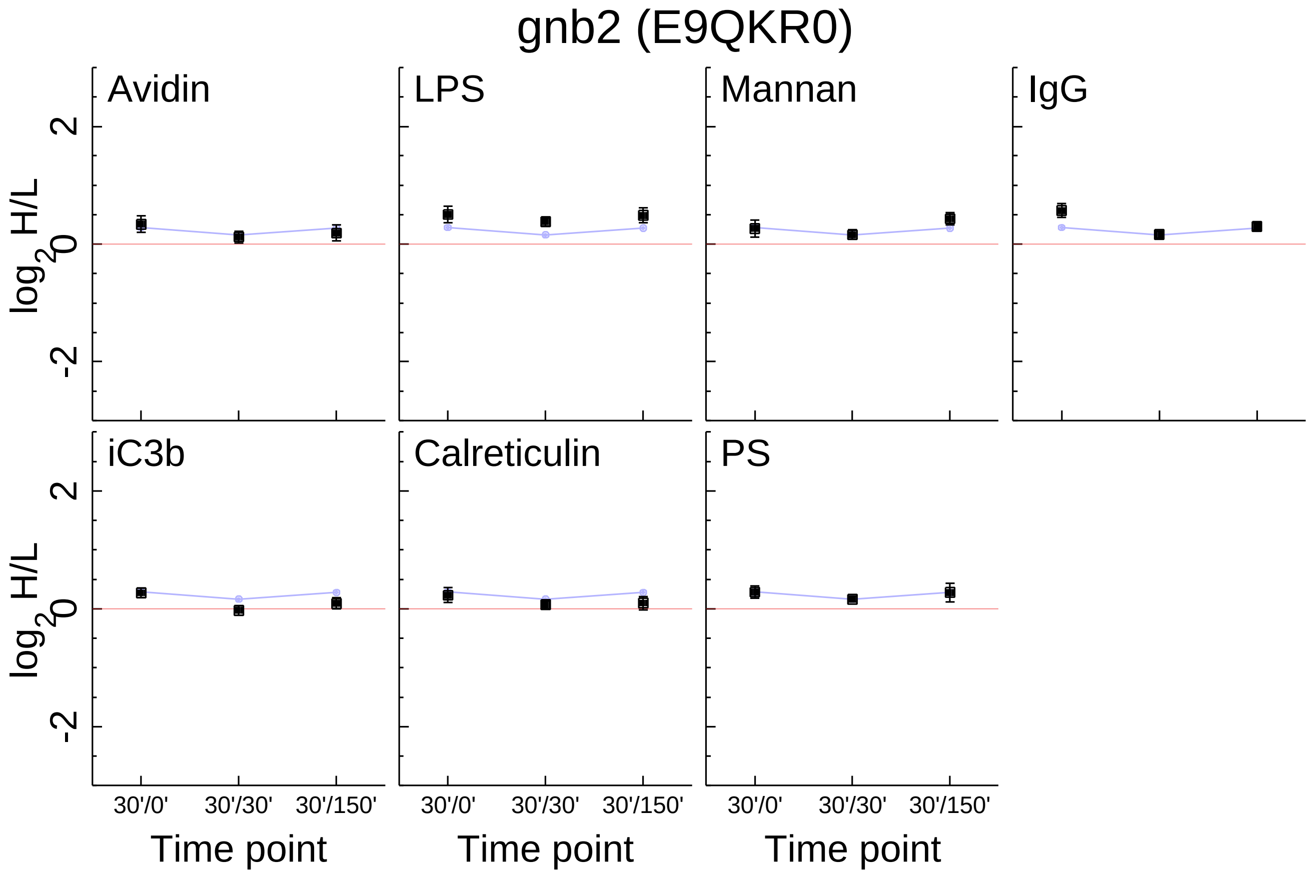
gnb2 (E9QKR0)
Avidin
LPS
Mannan
IgG
iC3b
Calreticulin
PS
log2 H/L
2
0
-2
30'/0'
30'/30'
30'/150'
Time point

gnb2l1 (P68040)

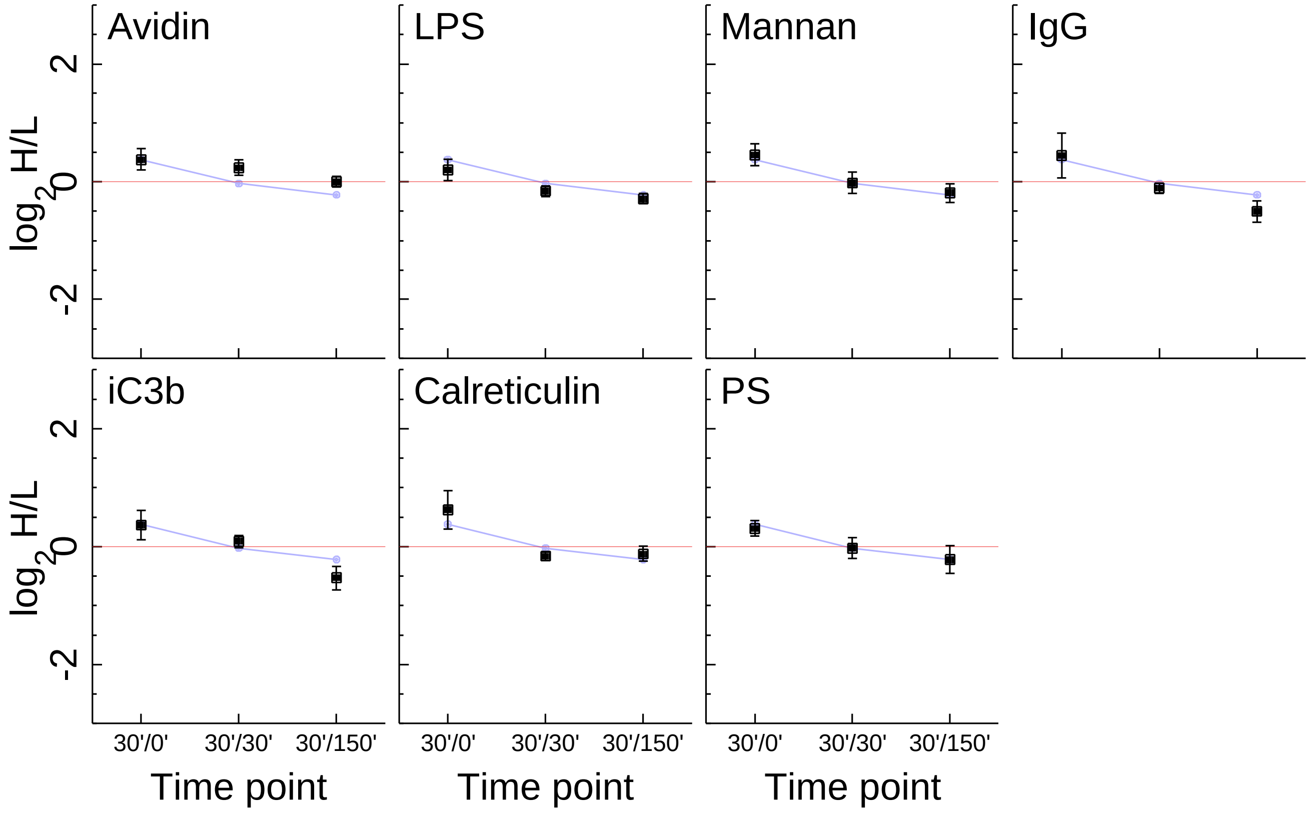

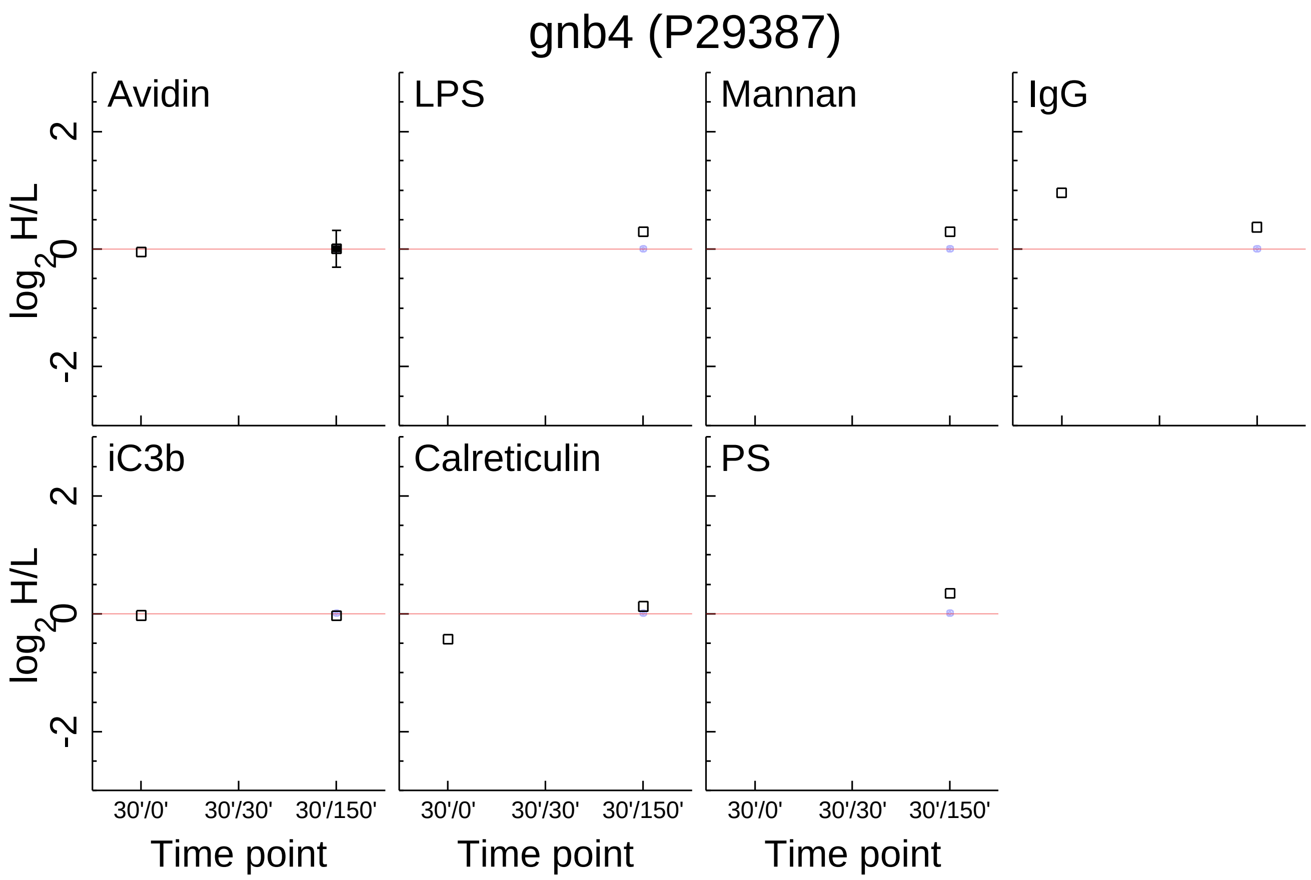
gnb4 (P29387)
Avidin
LPS
Mannan
IgG
iC3b
Calreticulin
PS
log2 H/L
2
0
-2
30'/0'
30'/30'
30'/150'
Time point

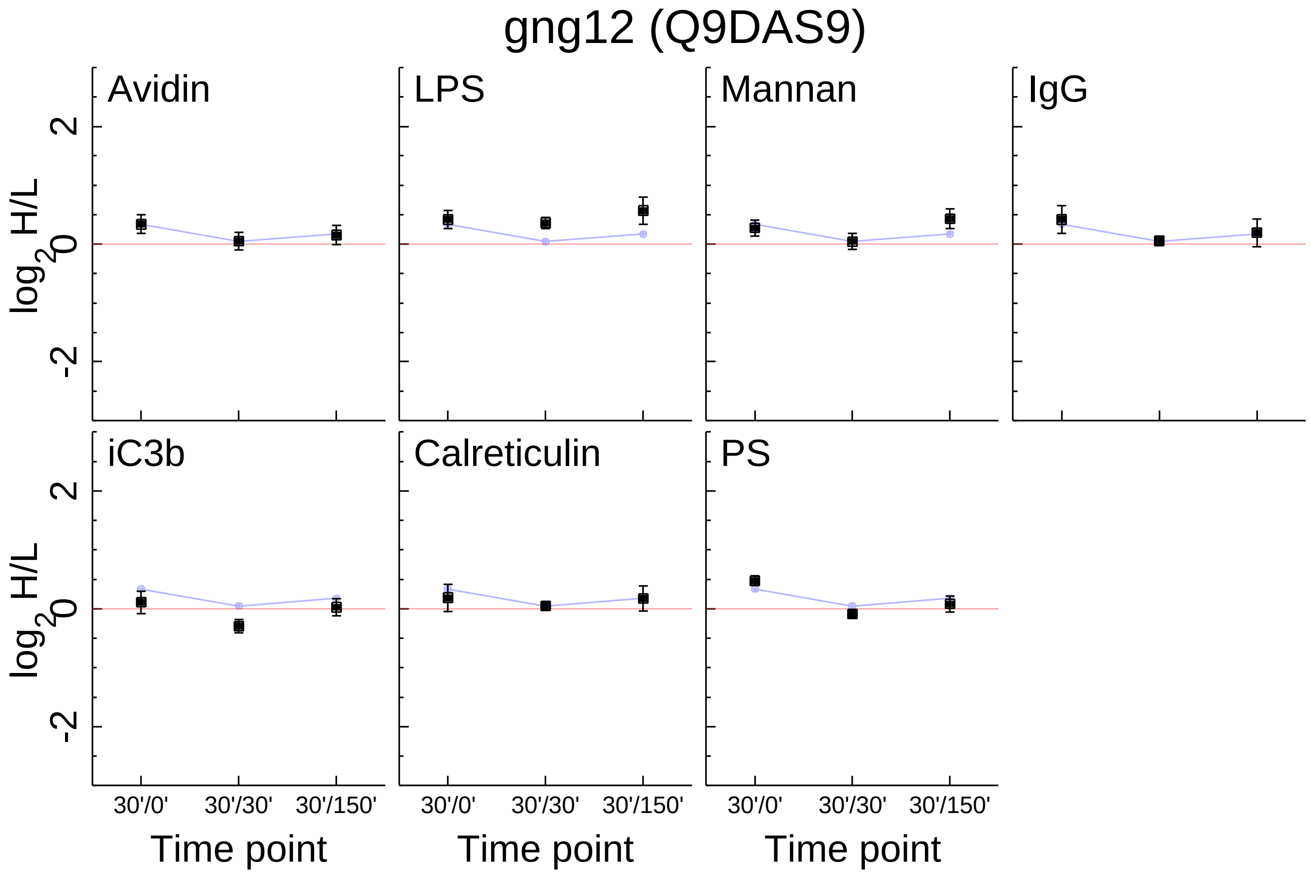

gng12 (Q9DAS9)
Avidin
LPS
Mannan
IgG
iC3b
Calreticulin
PS
log2 H/L
2
0
-2
30'/0'
30'/30'
30'/150'
Time point

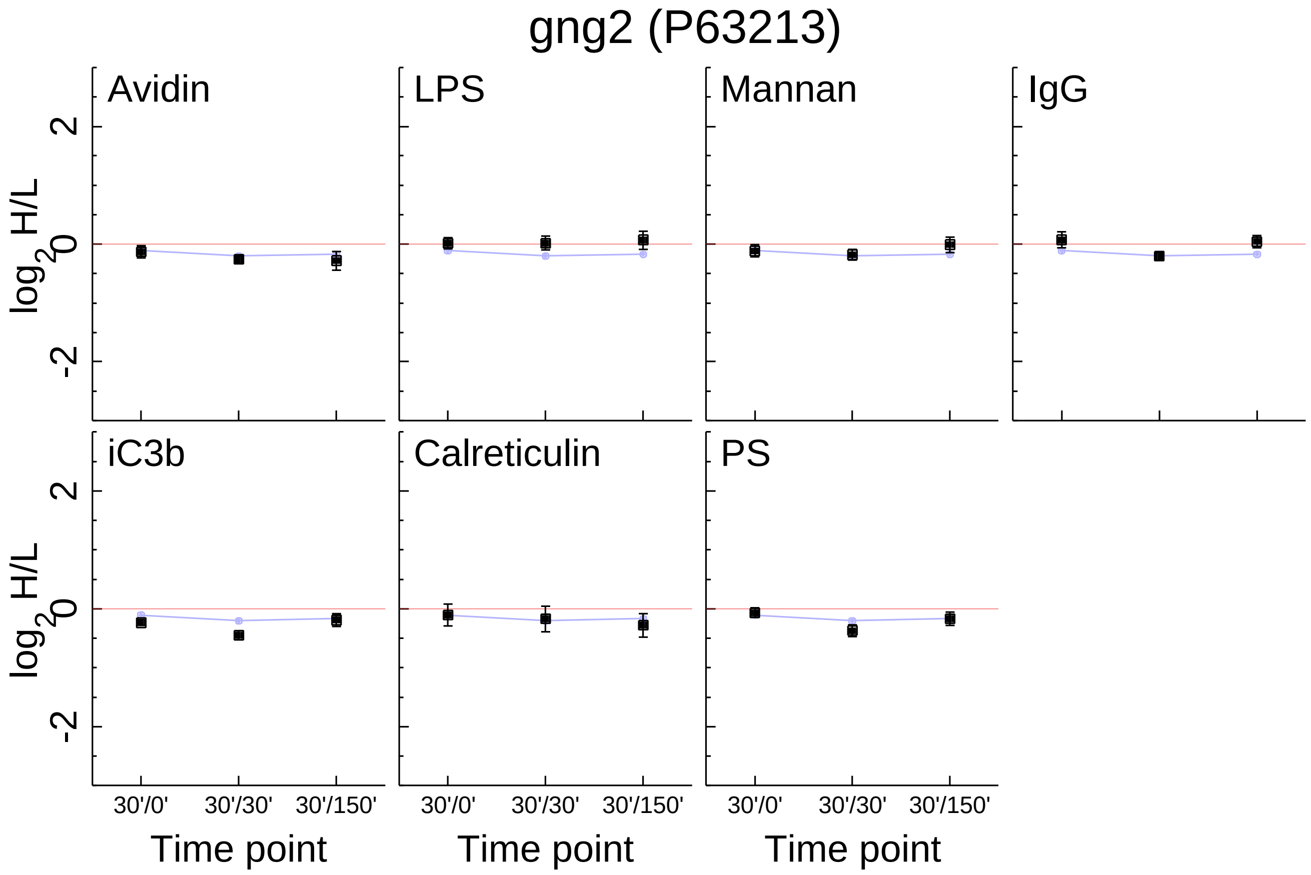
gng2 (P63213)
Avidin
LPS
Mannan
IgG
iC3b
Calreticulin
PS
log2 H/L
2
0
-2
30'/0'
30'/30'
30'/150'
Time point

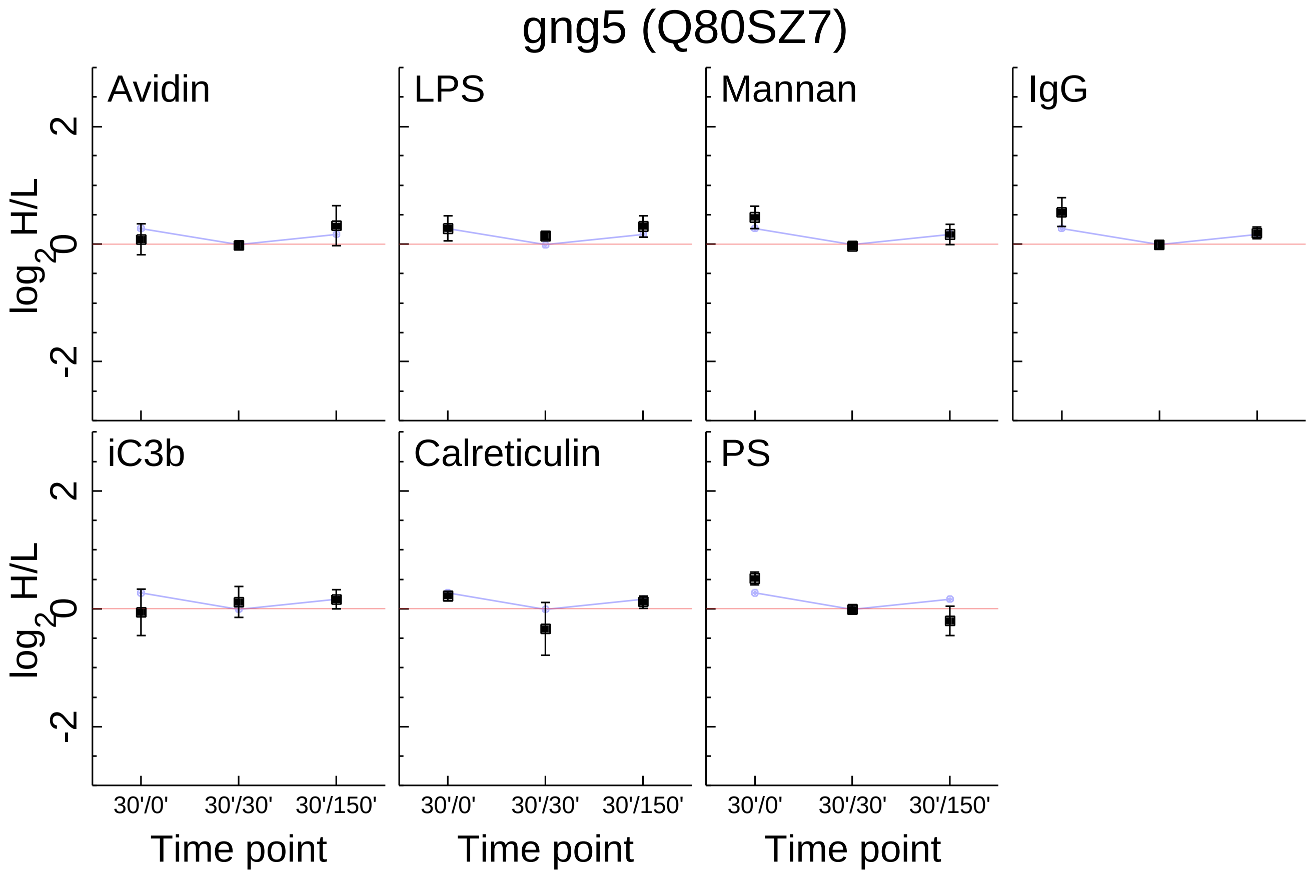
gng5 (Q80SZ7)
Avidin
LPS
Mannan
IgG
iC3b
Calreticulin
PS
log2 H/L
2
0
-2
30'/0'
30'/30'
30'/150'
Time point

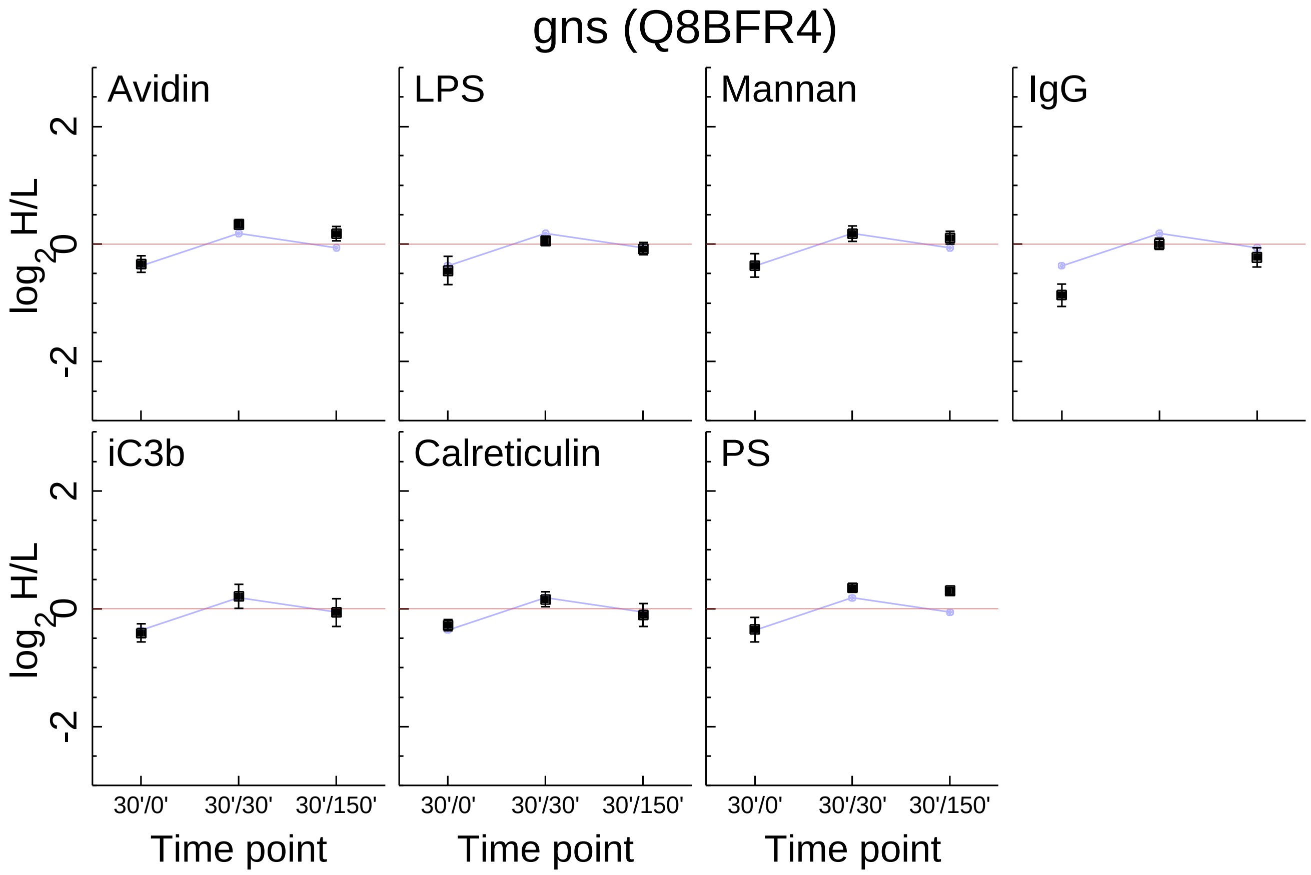
gns (Q8BFR4)
Avidin
LPS
Mannan
IgG
iC3b
Calreticulin
PS
log2 H/L
2
0
-2
30'/0'
30'/30'
30'/150'
Time point

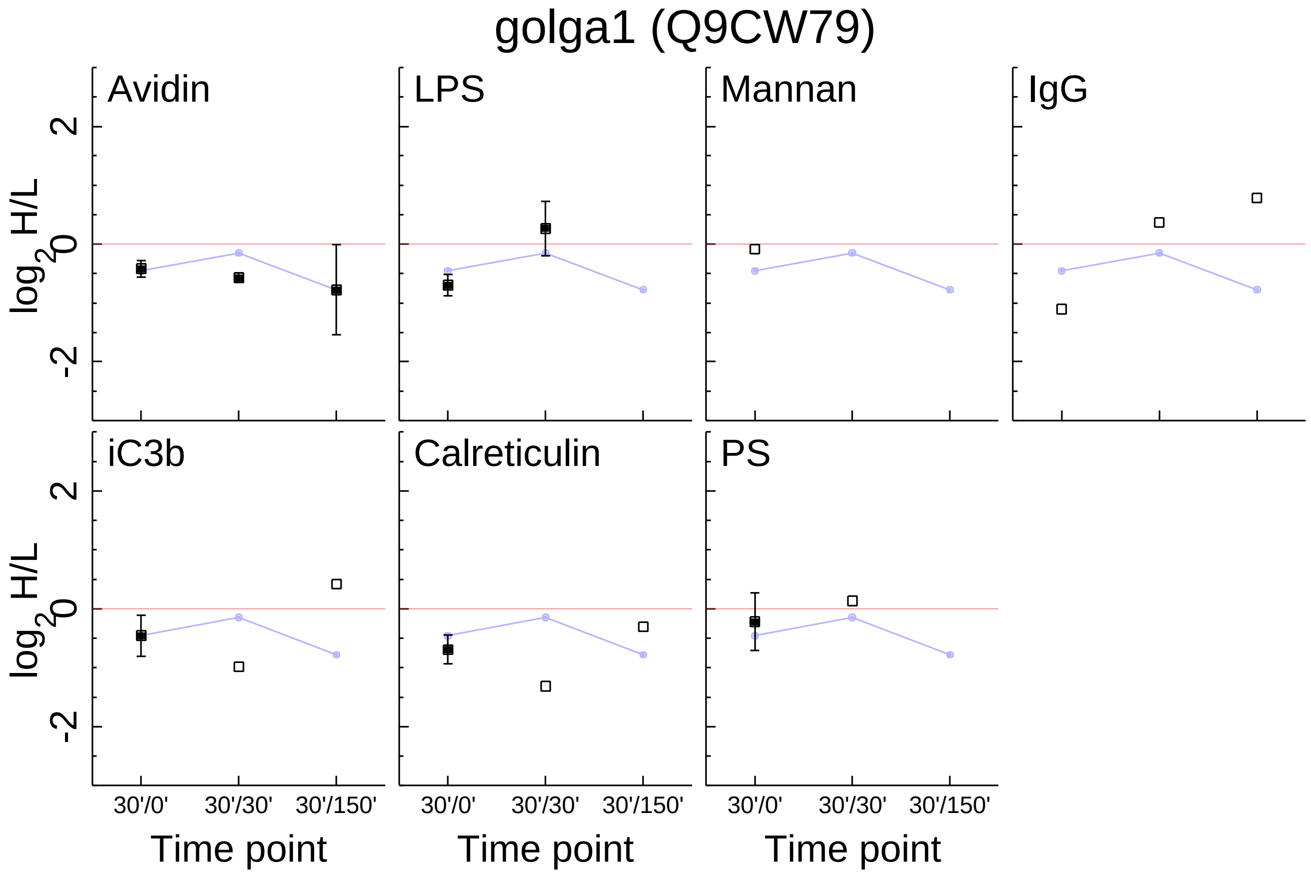
golga1 (Q9CW79)
Avidin
LPS
Mannan
IgG
iC3b
Calreticulin
PS
$\log_2$ H/L
2
0
-2
30'/0'
30'/30'
30'/150'
Time point

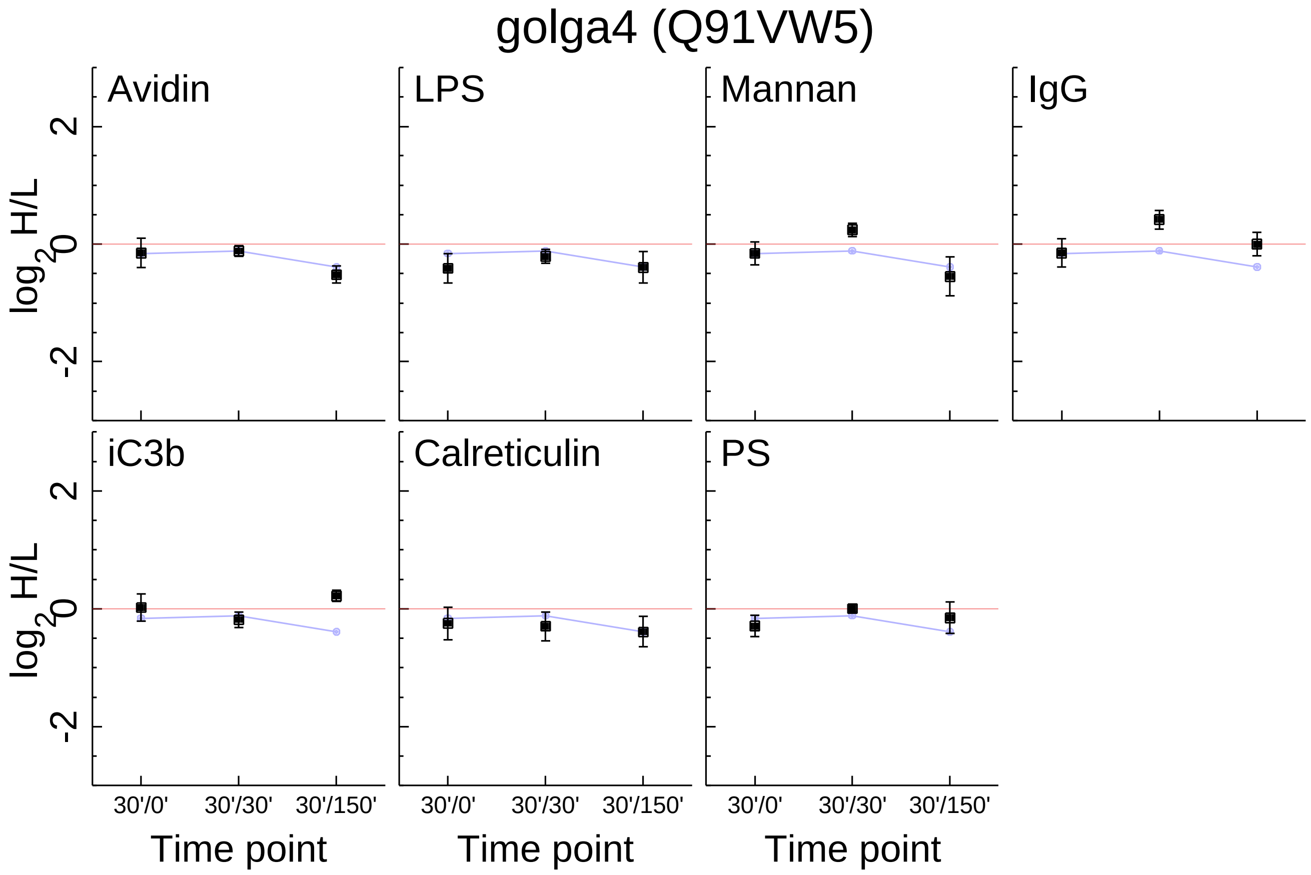

golga4 (Q91VW5)
Avidin
LPS
Mannan
IgG
iC3b
Calreticulin
PS
log2 H/L
2
0
-2
30'/0'
30'/30'
30'/150'
Time point

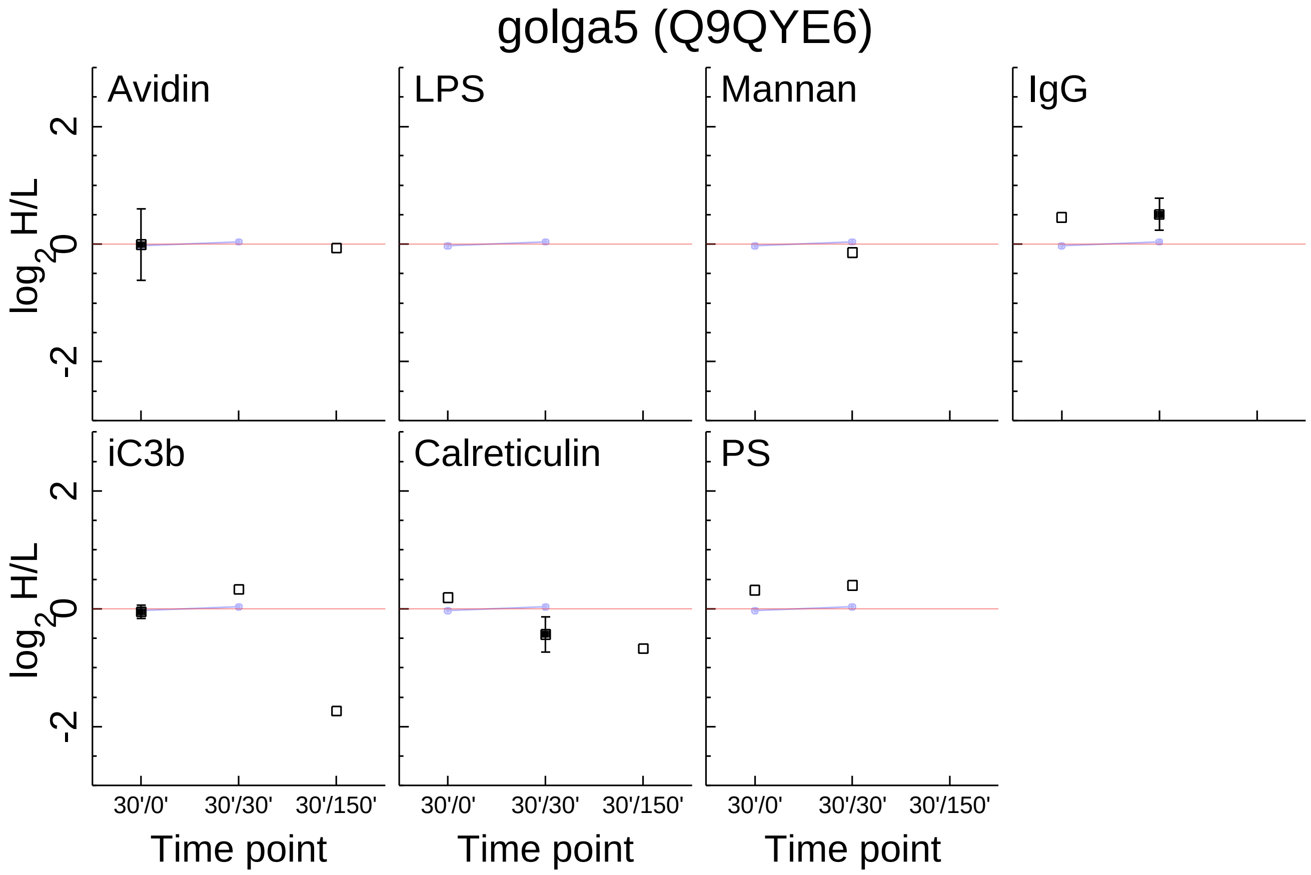
golga5 (Q9QYE6)
Avidin
LPS
Mannan
IgG
iC3b
Calreticulin
PS
log2 H/L
2
0
-2
30'/0'
30'/30'
30'/150'
Time point

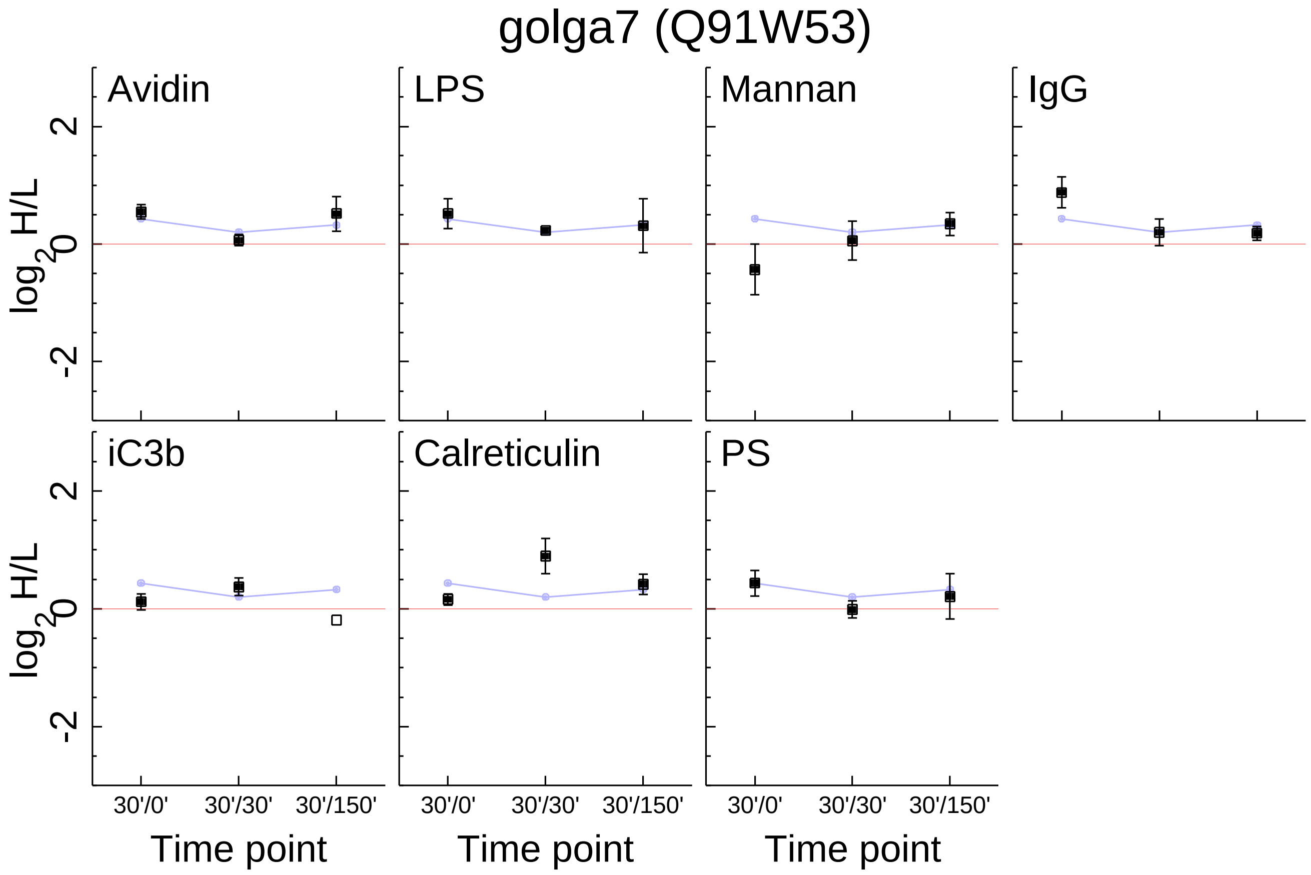
golga7 (Q91W53)
Avidin
LPS
Mannan
IgG
iC3b
Calreticulin
PS
log2 H/L
2
0
-2
30'/0'
30'/30'
30'/150'
Time point

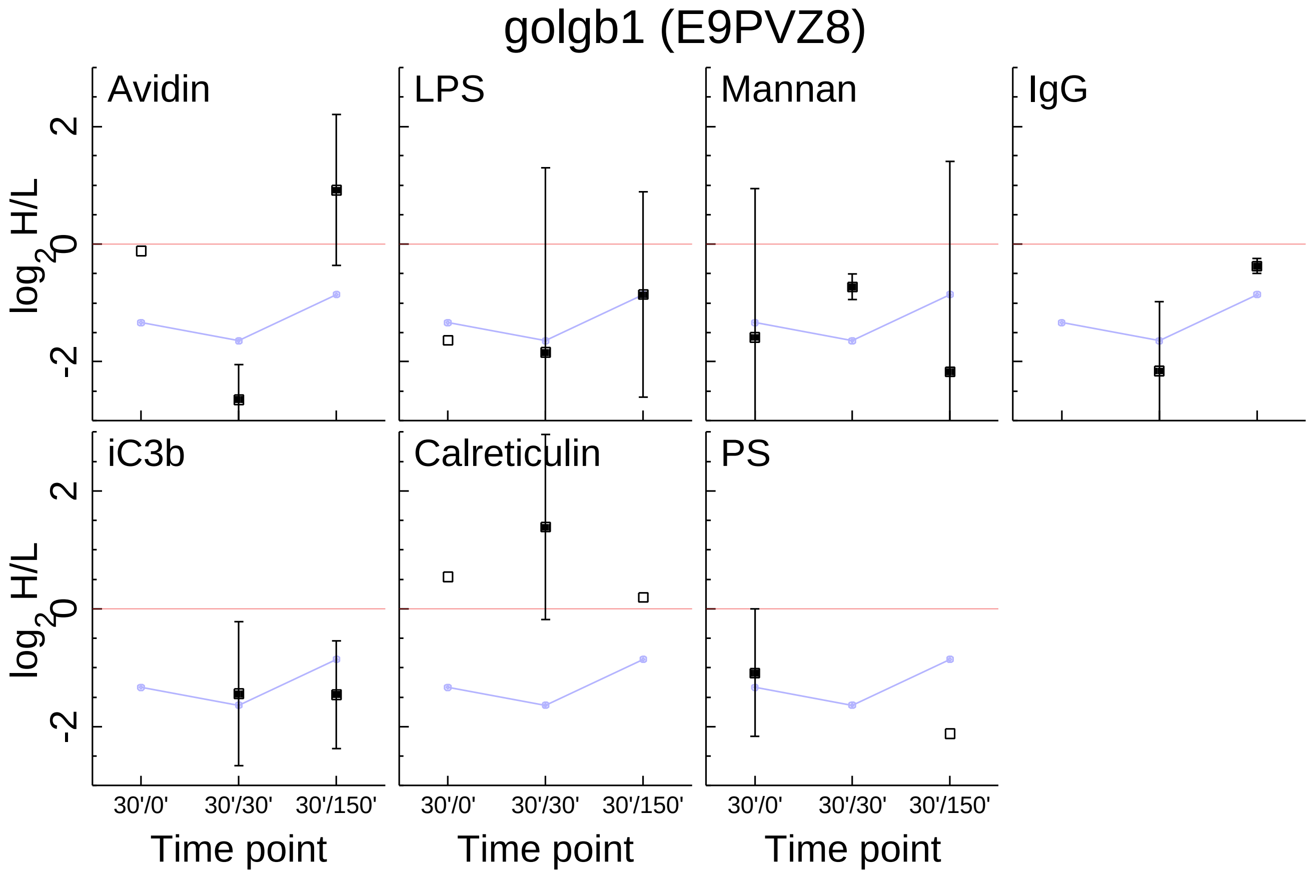
golgb1 (E9PVZ8)
Avidin
LPS
Mannan
IgG
iC3b
Calreticulin
PS
log2 H/L
2
0
-2
30'/0'
30'/30'
30'/150'
Time point

gosr1 (O88630)

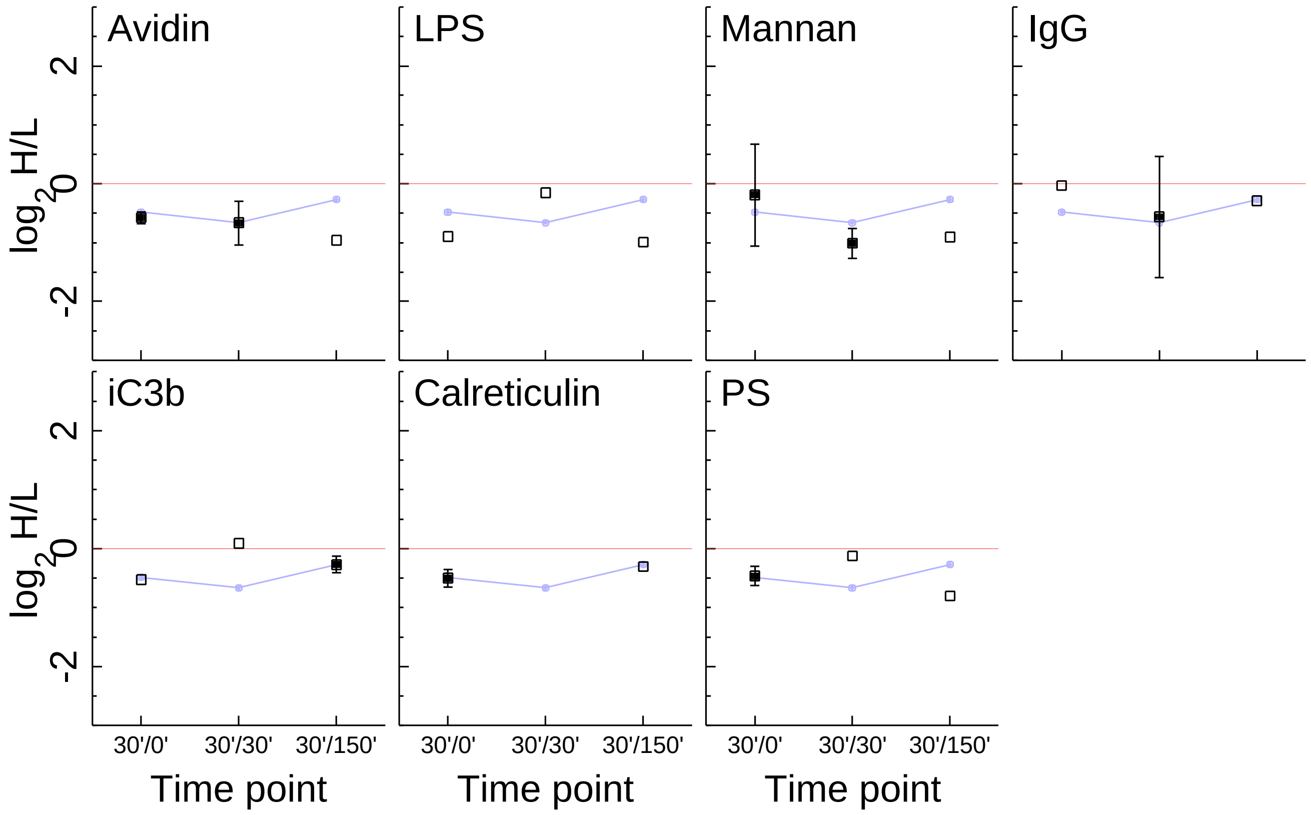

got2 (P05202)

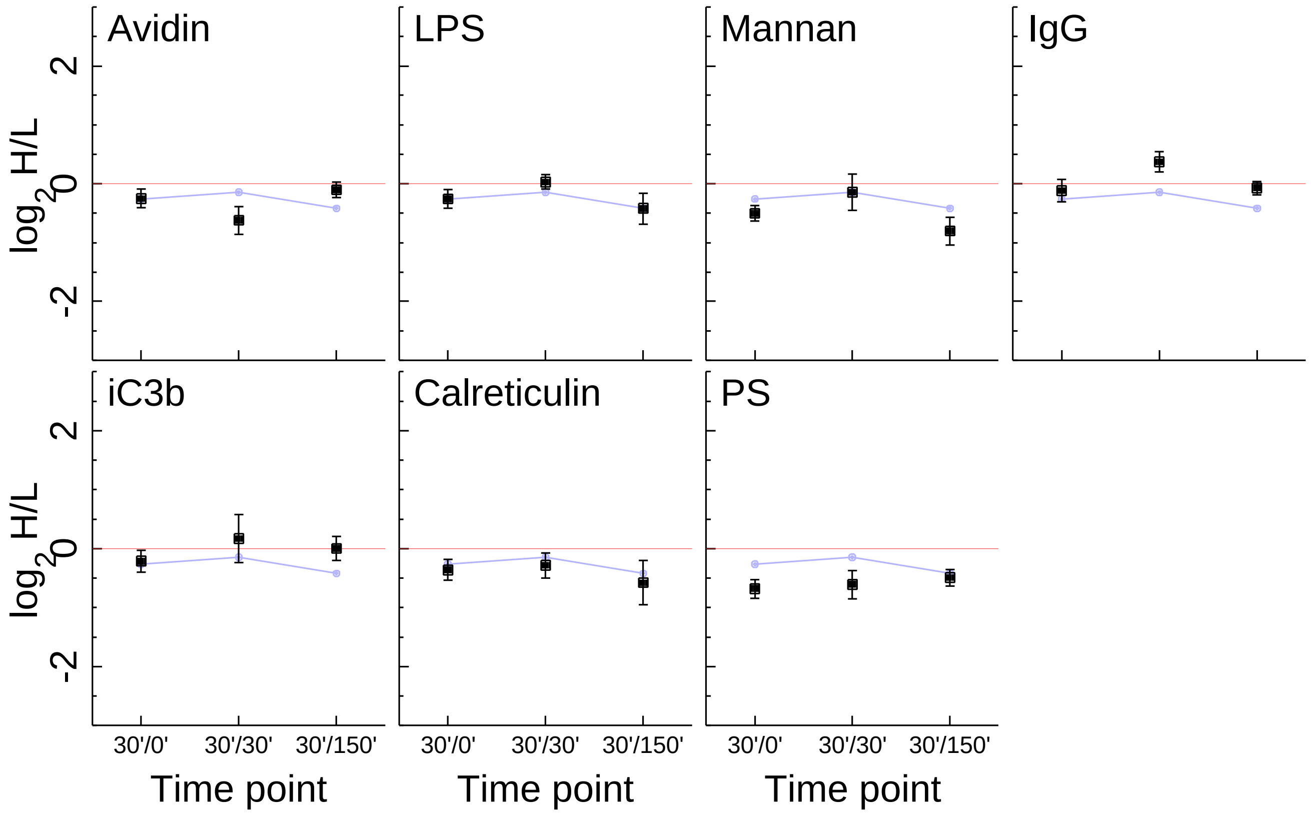

Avidin
LPS
Mannan
IgG
iC3b
Calreticulin
PS
log2 H/L
2
0
-2
30'/0'
30'/30'
30'/150'
Time point

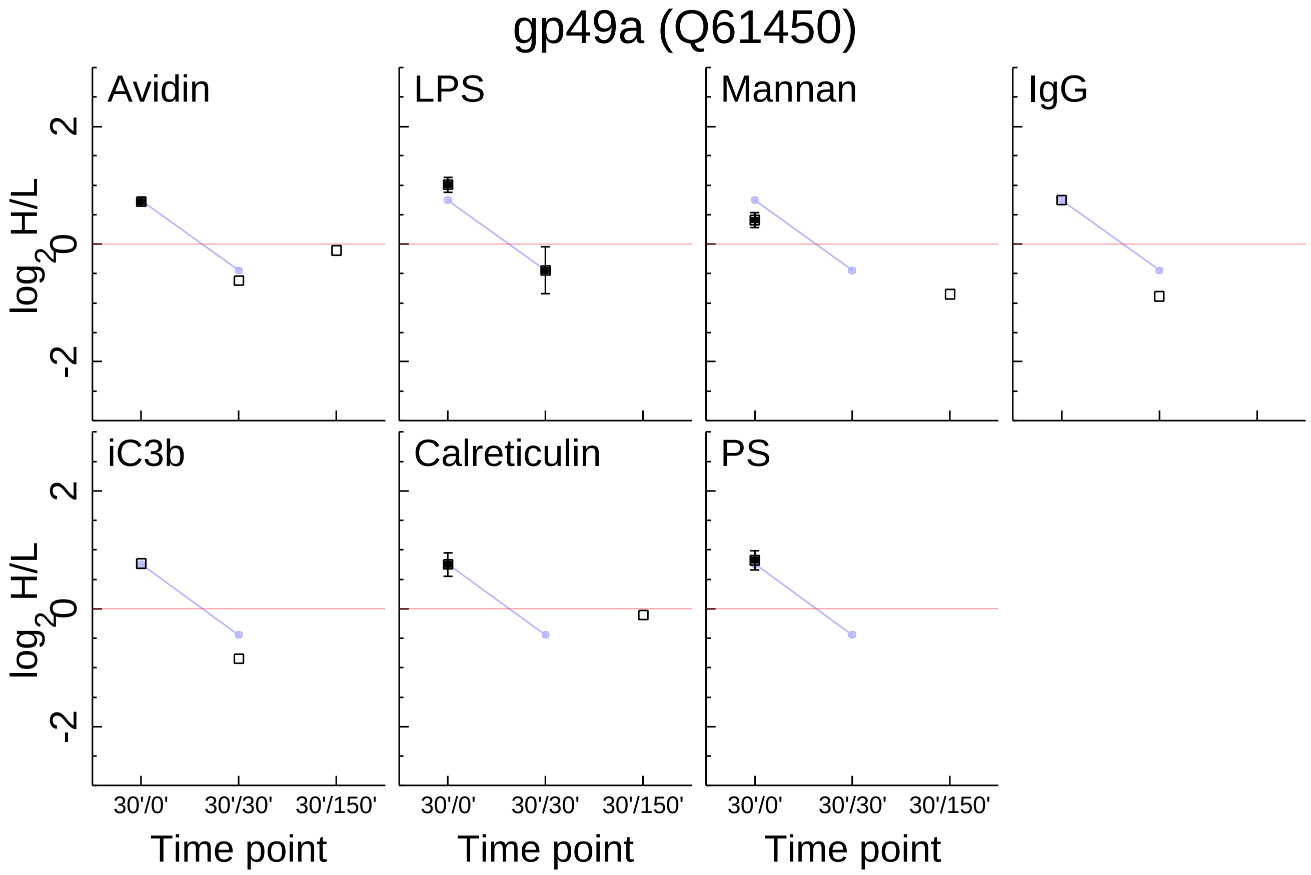

gp49a (Q61450)
Avidin
LPS
Mannan
IgG
iC3b
Calreticulin
PS
log2 H/L
2
0
-2
30'/0'
30'/30'
30'/150'
Time point

gpatch8 (A2A6A1)

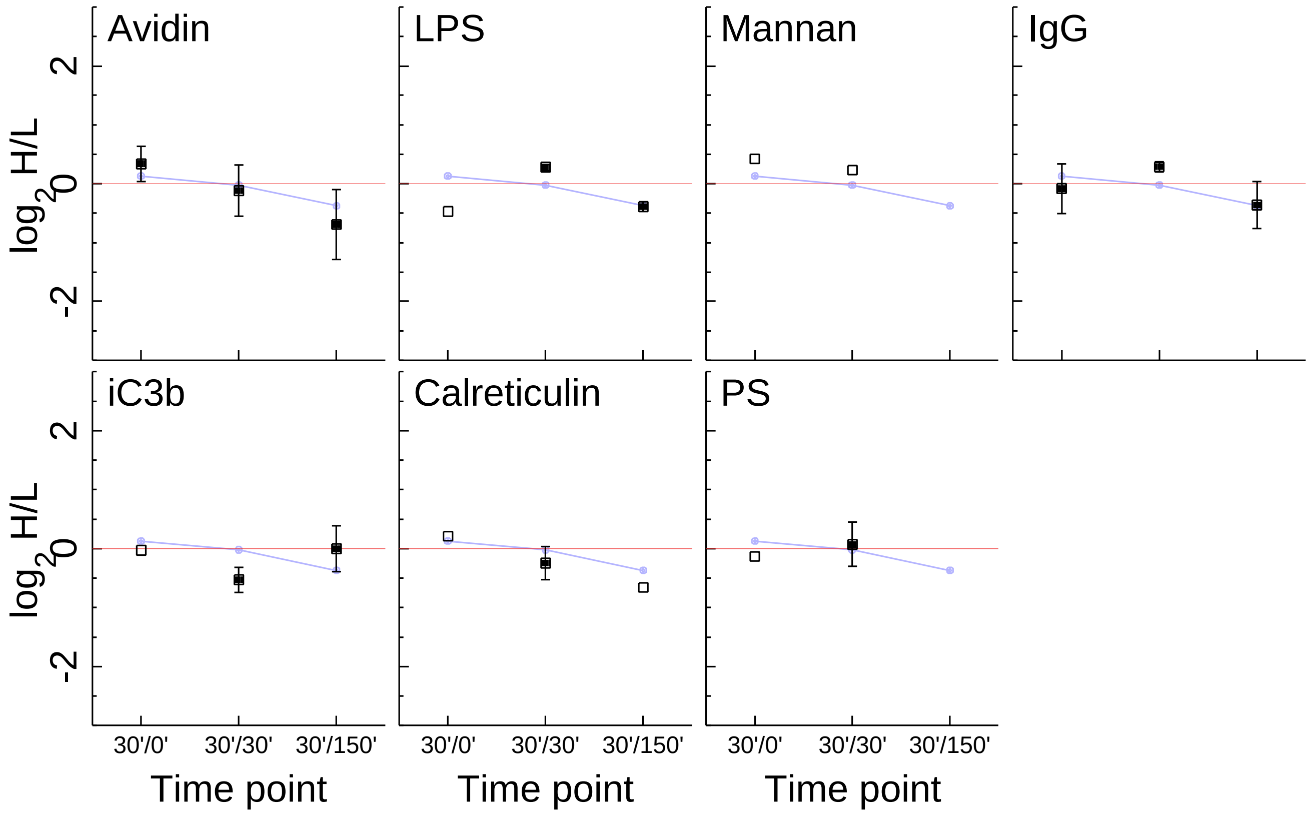

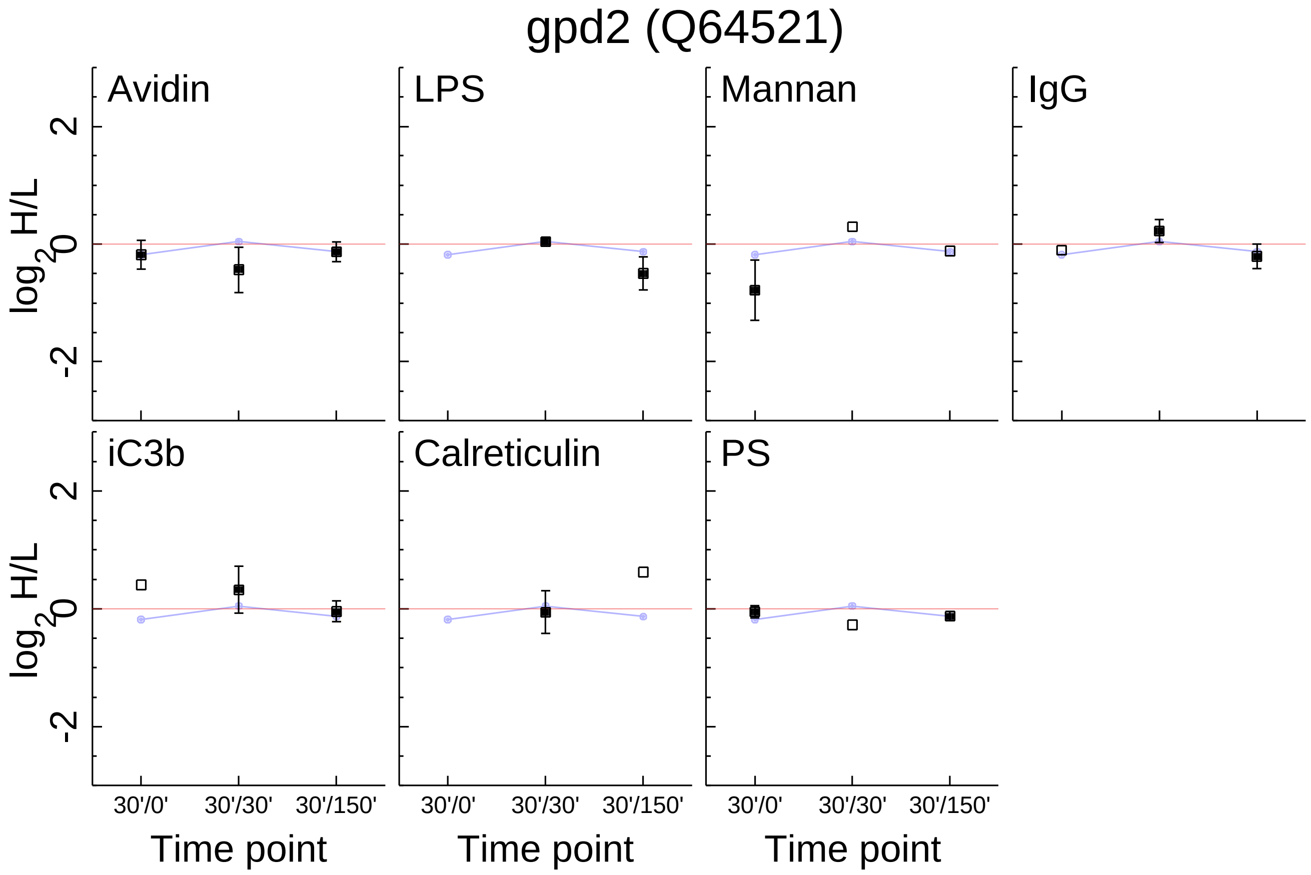
gpd2 (Q64521)
Avidin
LPS
Mannan
IgG
iC3b
Calreticulin
PS
log2 H/L
2
0
-2
30'/0'
30'/30'
30'/150'
Time point

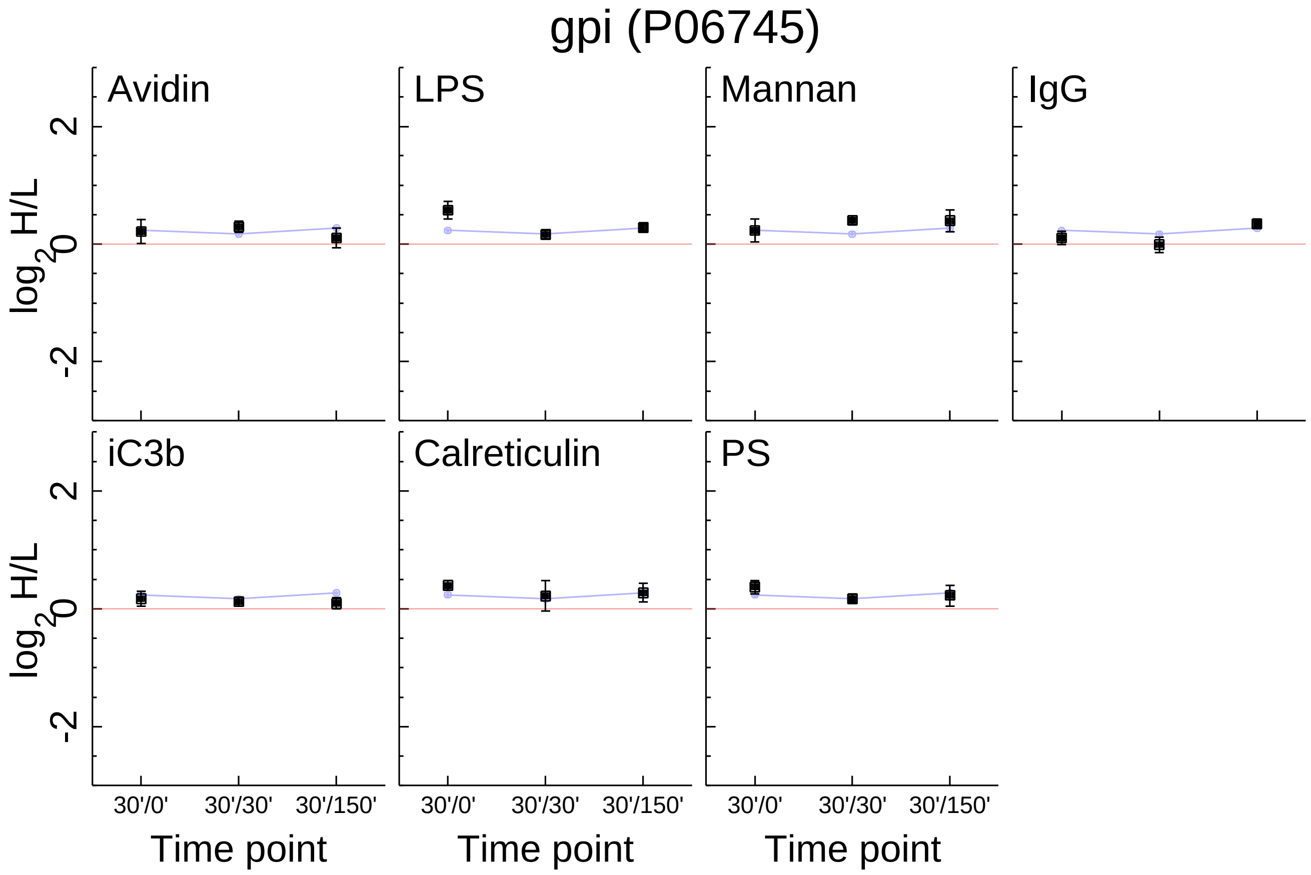

gpi (P06745)
Avidin
LPS
Mannan
IgG
iC3b
Calreticulin
PS
log2 H/L
2
0
-2
30'/0'
30'/30'
30'/150'
Time point

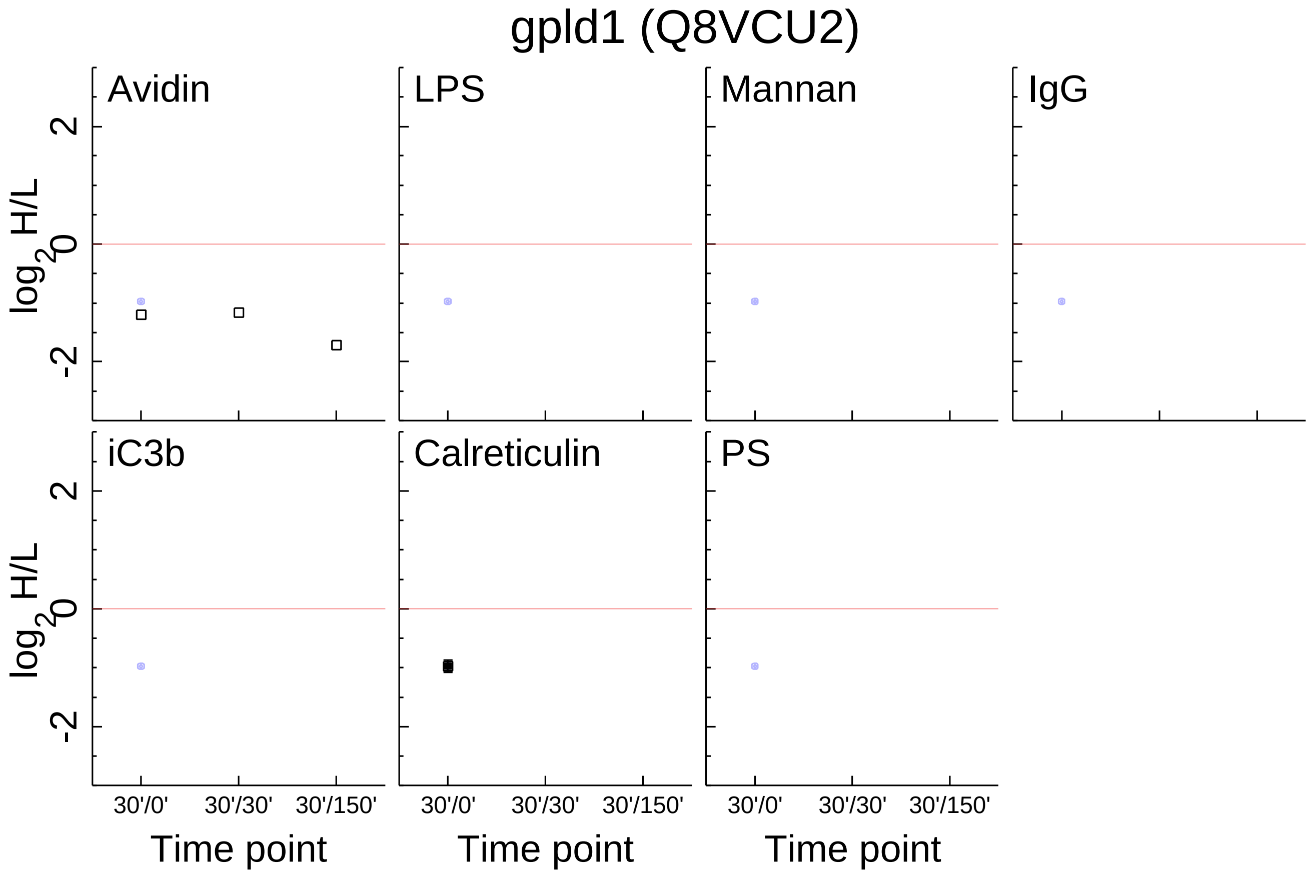
gpld1 (Q8VCU2)
Avidin
LPS
Mannan
IgG
iC3b
Calreticulin
PS
log2 H/L
2
0
-2
30'/0'
30'/30'
30'/150'
Time point

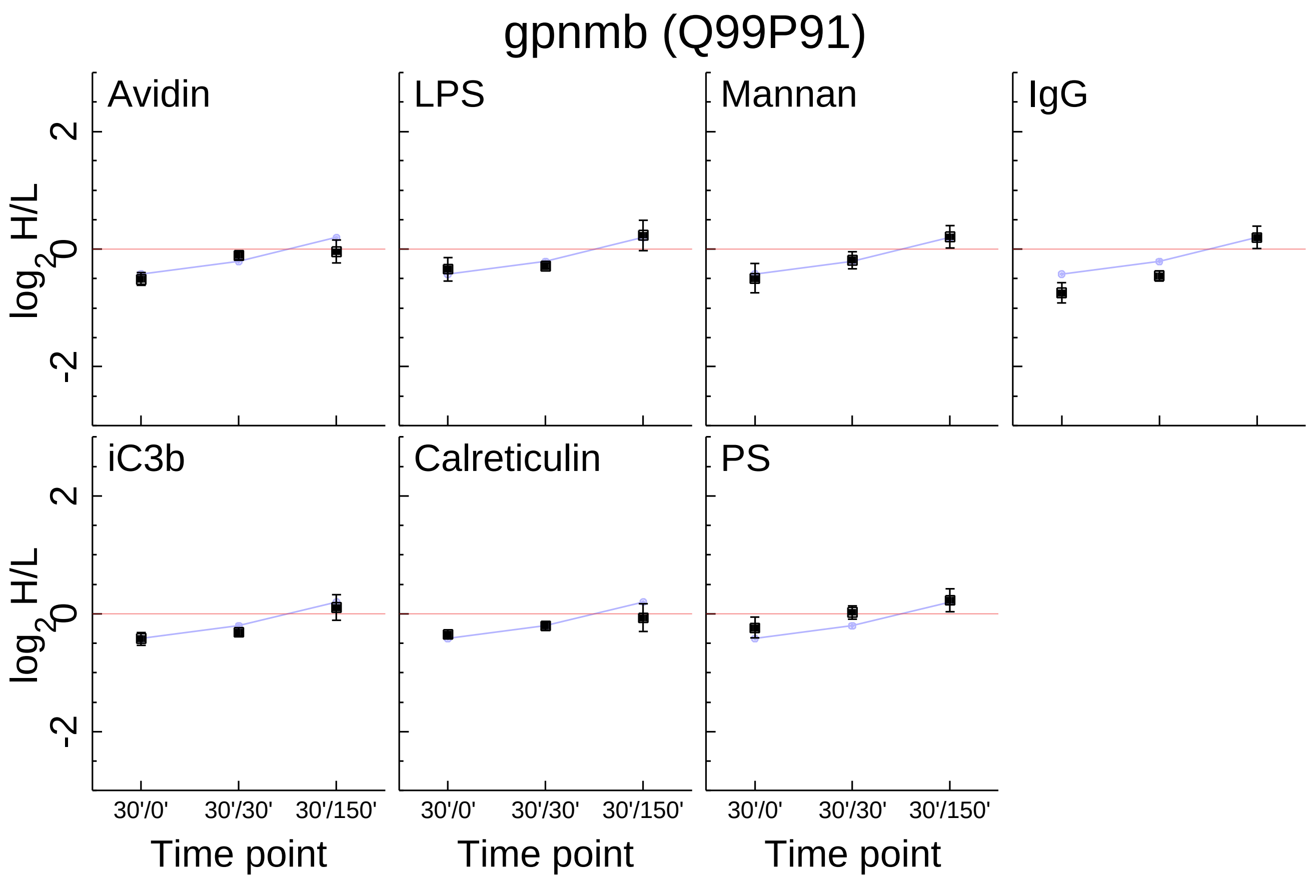
gpnmb (Q99P91)
Avidin
LPS
Mannan
IgG
iC3b
Calreticulin
PS
log2 H/L
2
0
-2
30'/0'
30'/30'
30'/150'
Time point

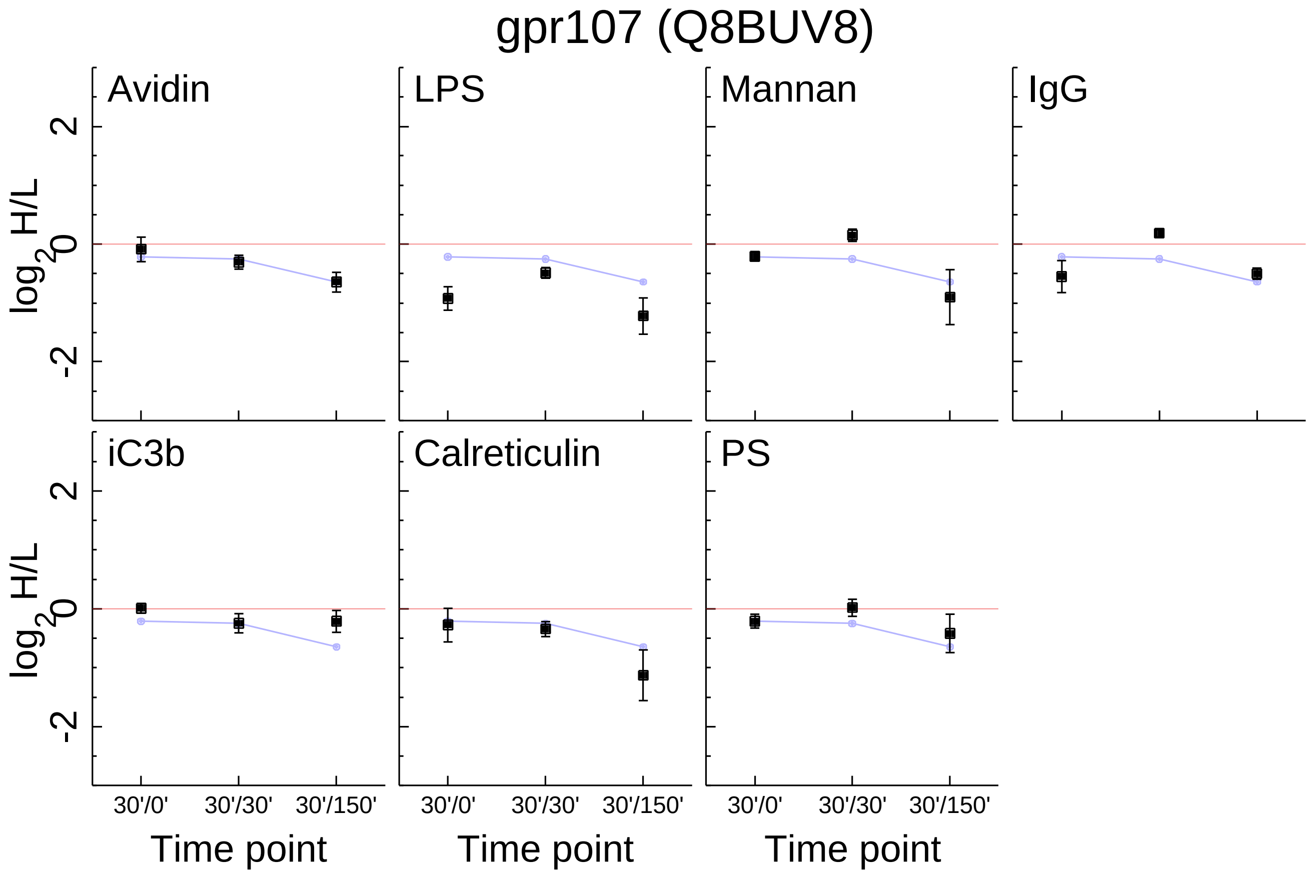

gpr107 (Q8BUV8)
Avidin
LPS
Mannan
IgG
iC3b
Calreticulin
PS
log2 H/L
2
0
-2
30'/0'
30'/30'
30'/150'
Time point

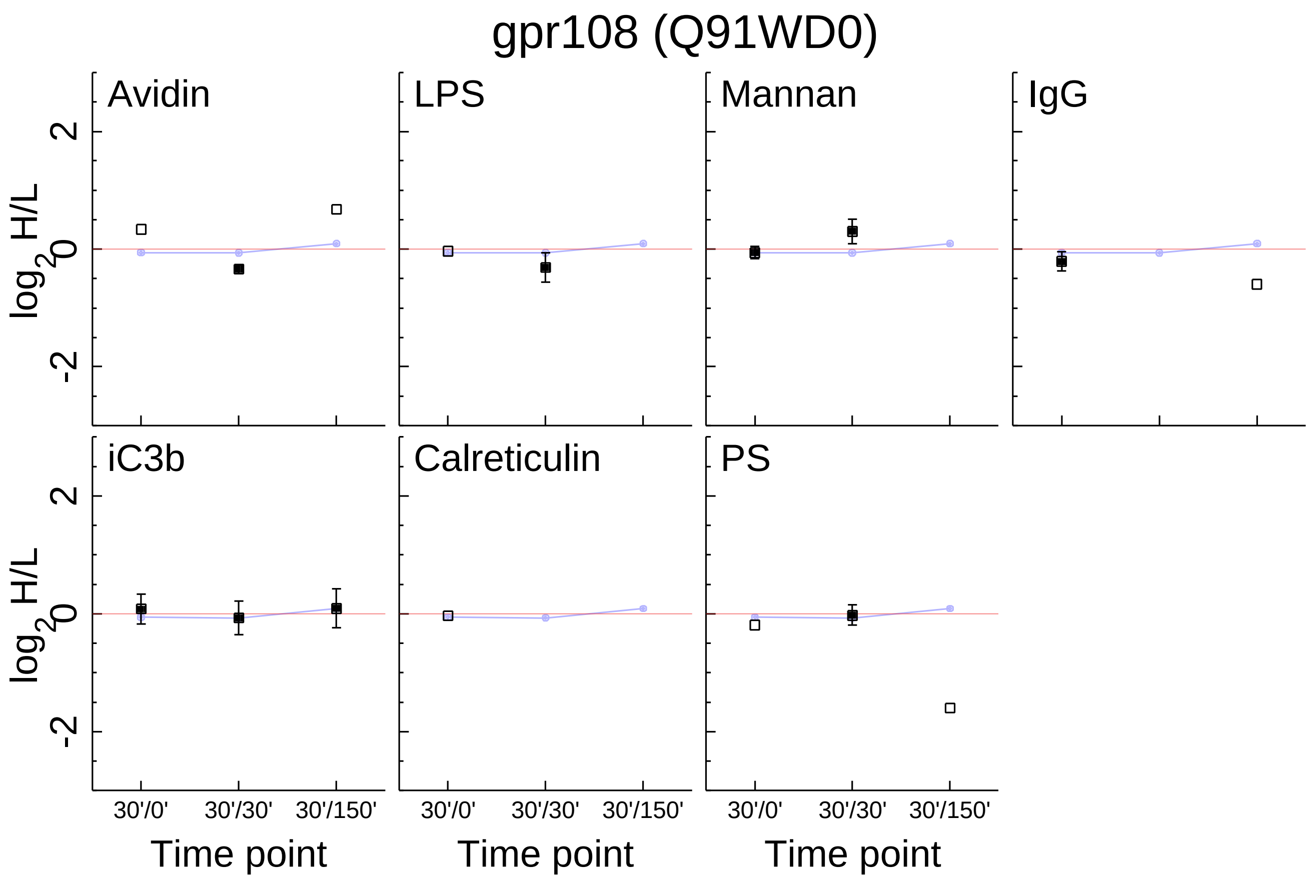
gpr108 (Q91WD0)
Avidin
LPS
Mannan
IgG
iC3b
Calreticulin
PS
log2 H/L
2
0
-2
30'/0'
30'/30'
30'/150'
Time point

gpr137b (Q8BNQ3)

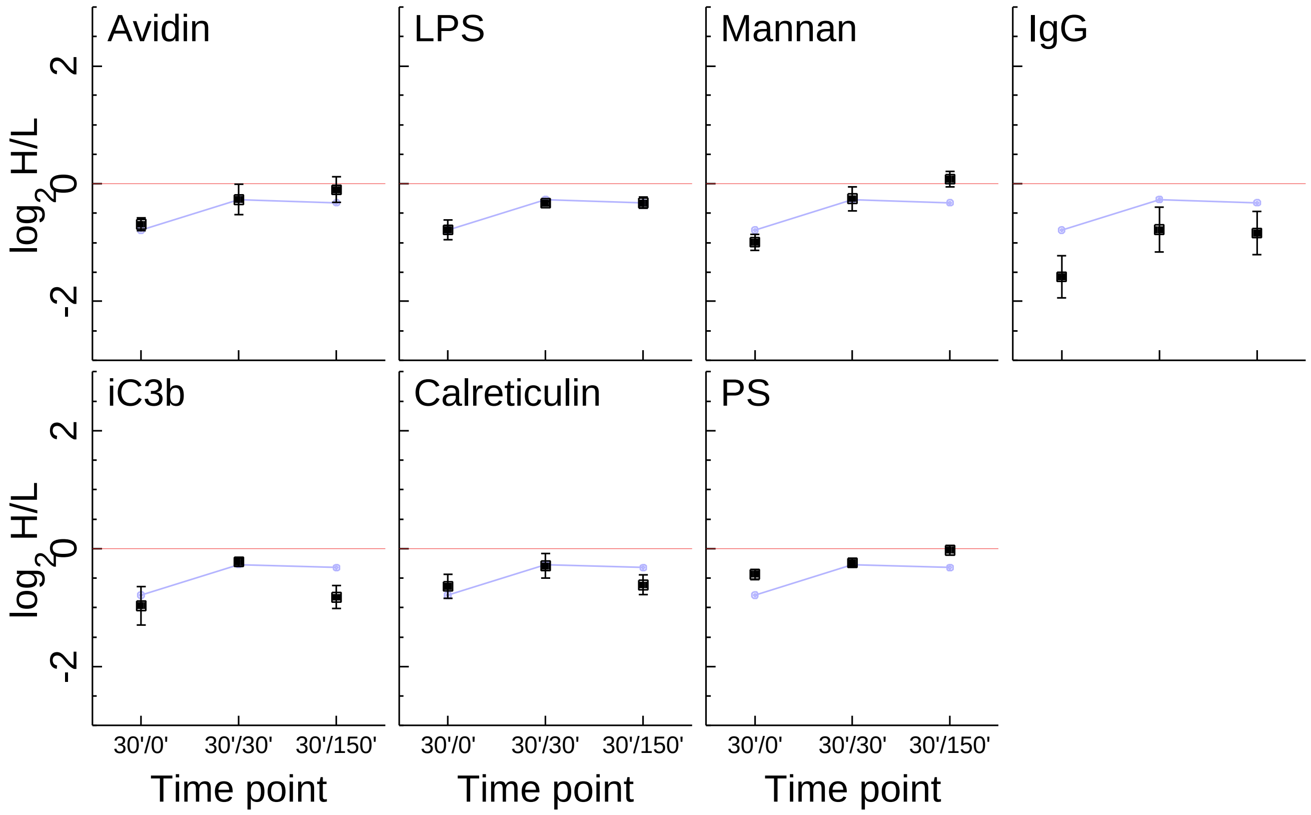

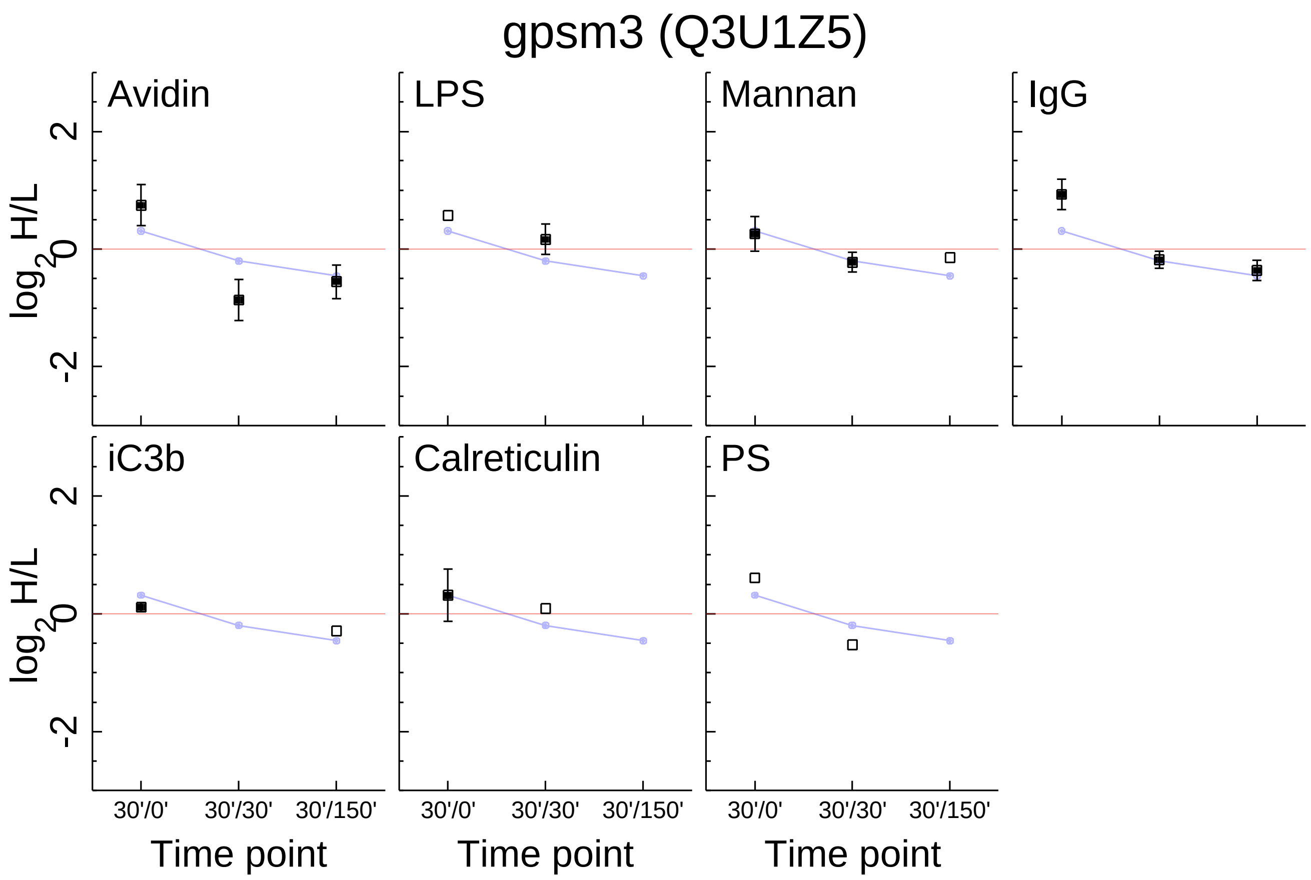
gpsm3 (Q3U1Z5)
Avidin
LPS
Mannan
IgG
iC3b
Calreticulin
PS
$\log_2$ H/L
2
0
-2
30'/0'
30'/30'
30'/150'
Time point

gpx1 (P11352)

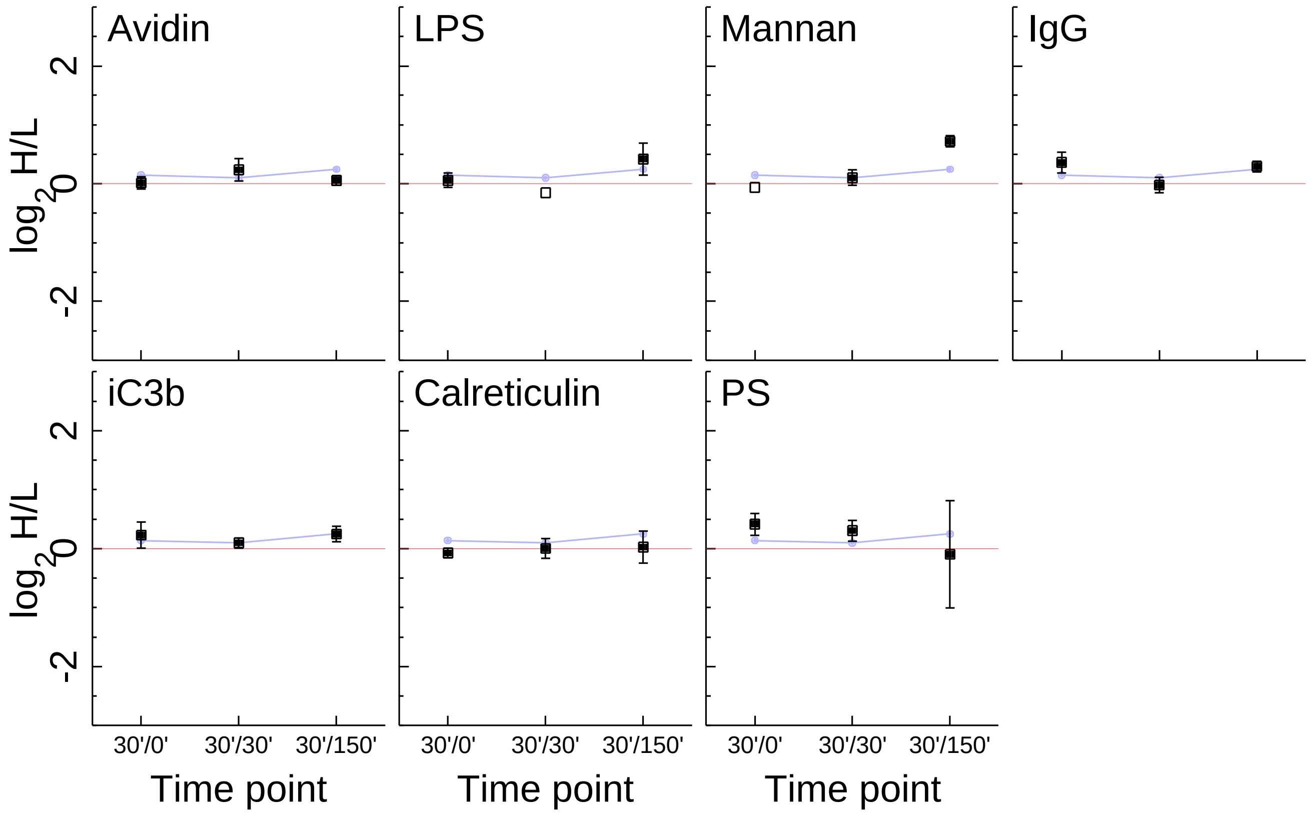
Avidin
LPS
Mannan
IgG
iC3b
Calreticulin
PS
log2 H/L
2
0
-2
30'/0'
30'/30'
30'/150'
Time point

gpx4 (Q76LV0)

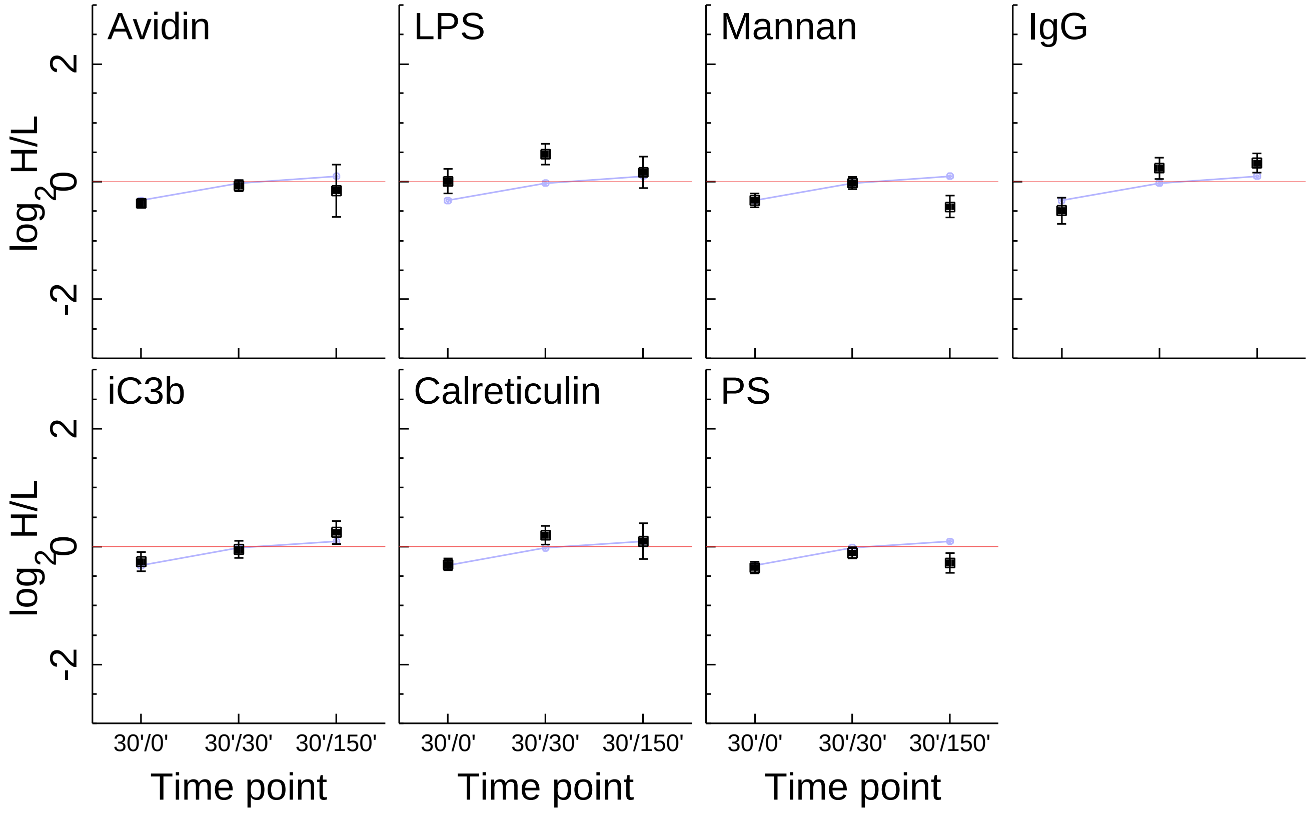
Avidin
LPS
Mannan
IgG
iC3b
Calreticulin
PS
2
0
-2
log2 H/L
30'/0'
30'/30'
30'/150'
Time point

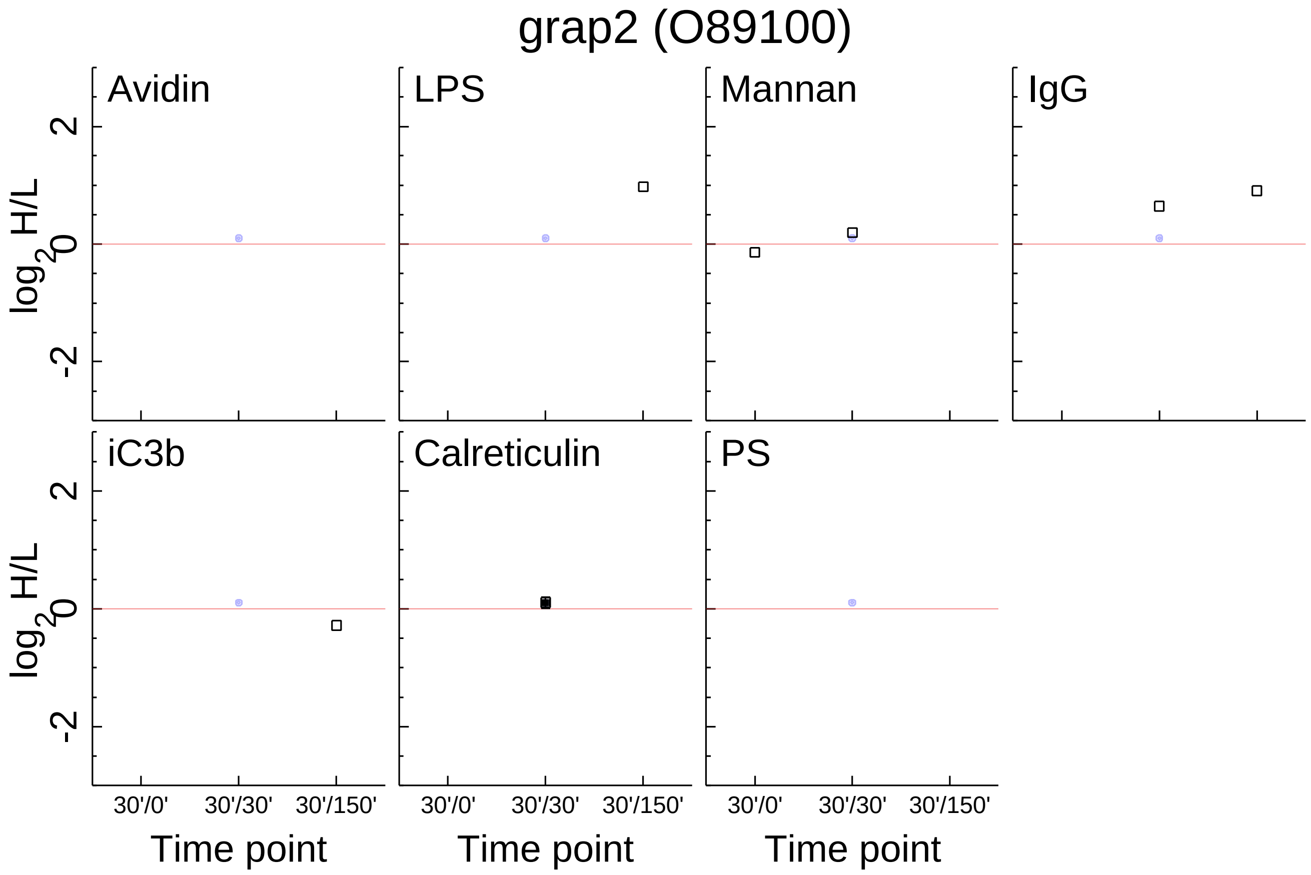
grap2 (O89100)
Avidin
LPS
Mannan
IgG
iC3b
Calreticulin
PS
log2 H/L
2
0
-2
30'/0'
30'/30'
30'/150'
Time point

grb2 (Q60631)

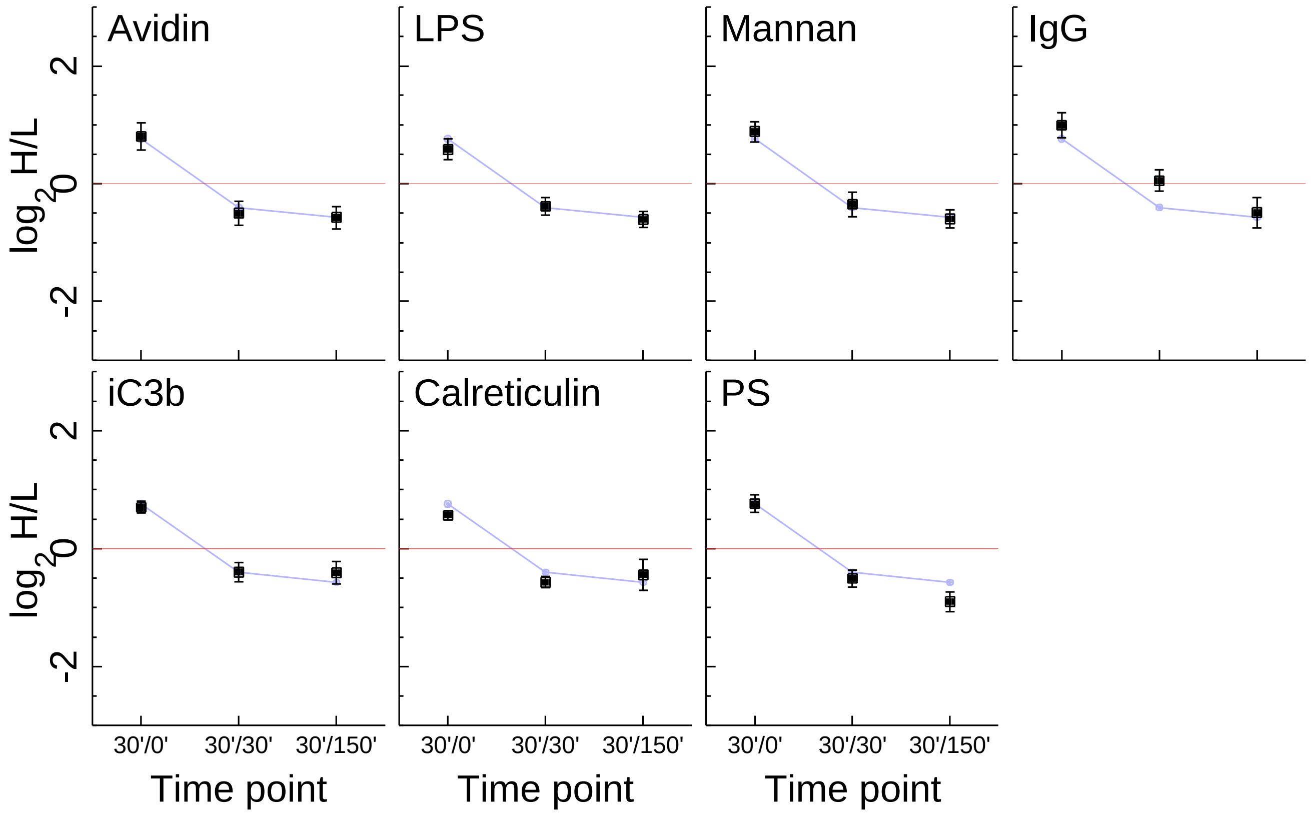
Avidin
LPS
Mannan
IgG
iC3b
Calreticulin
PS
2
0
-2
log2 H/L
30'/0'
30'/30'
30'/150'
Time point

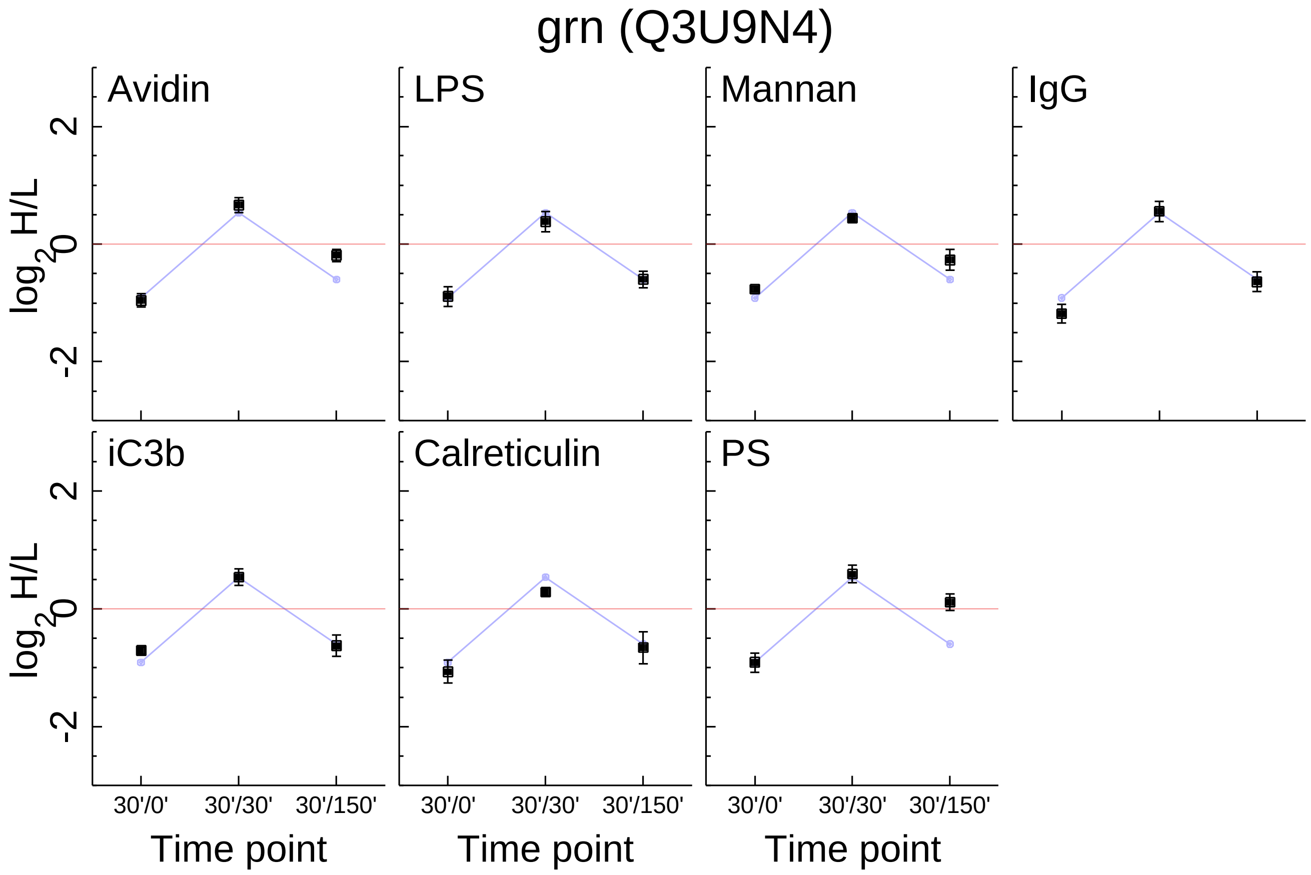
grn (Q3U9N4)
Avidin
LPS
Mannan
IgG
iC3b
Calreticulin
PS
log2 H/L
2
0
-2
30'/0'
30'/30'
30'/150'
Time point

grpel1 (Q99LP6)

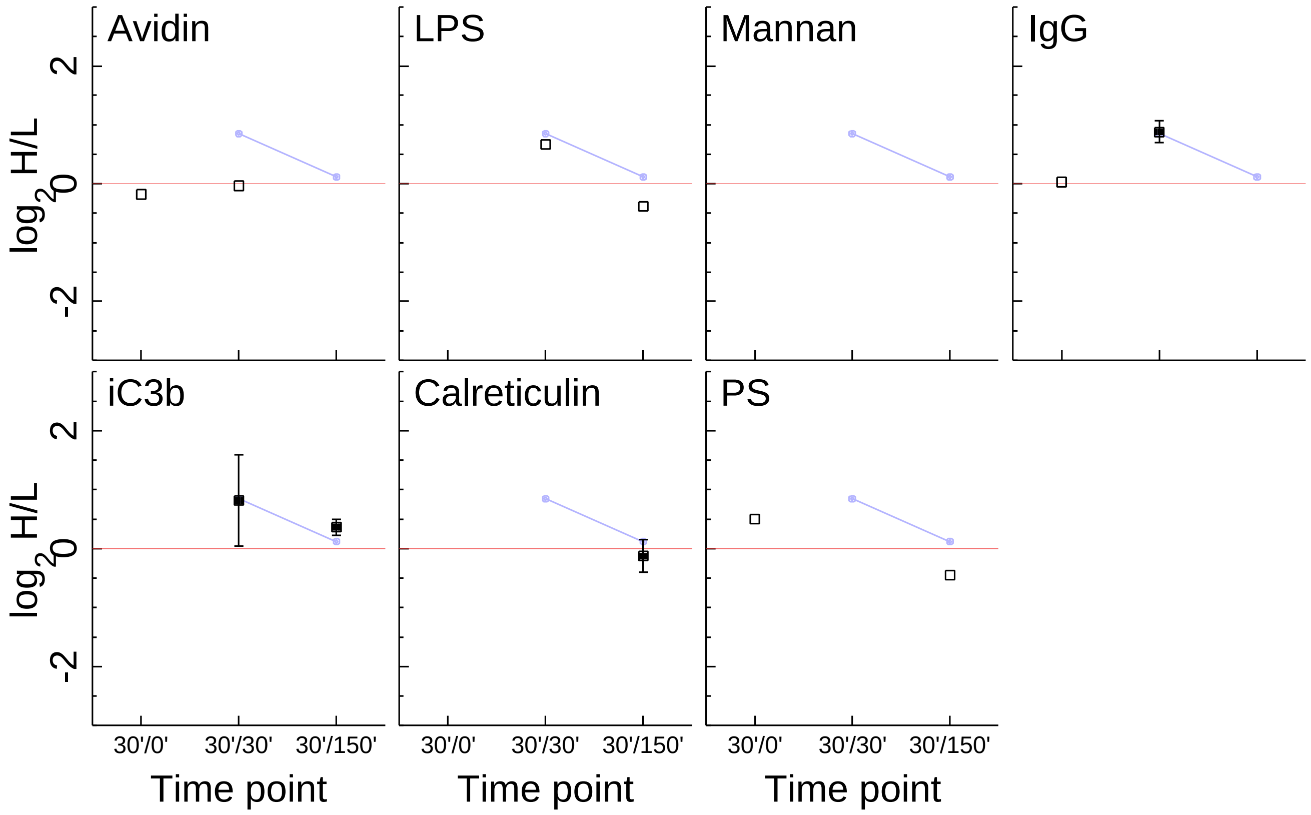

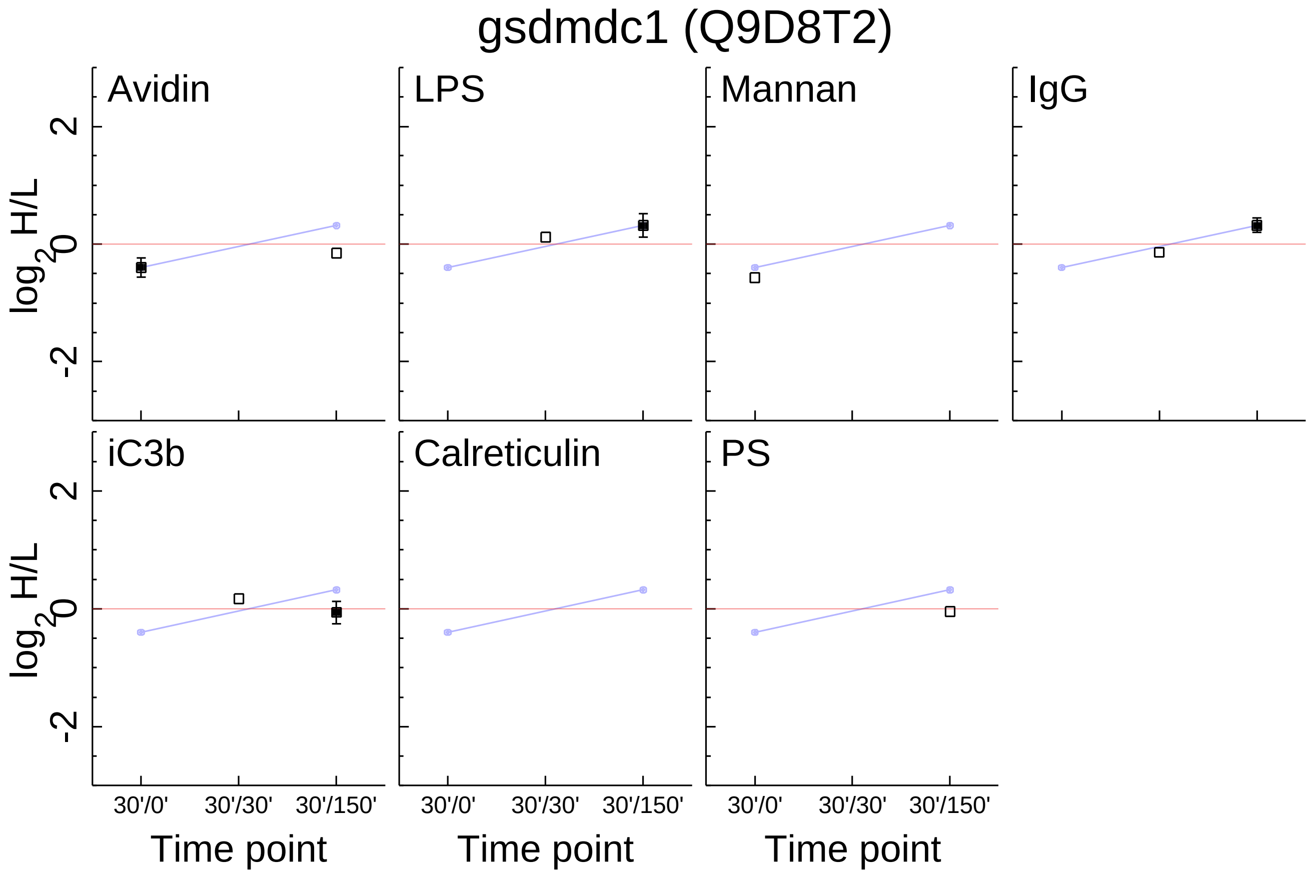

gsdmdc1 (Q9D8T2)
Avidin
LPS
Mannan
IgG
iC3b
Calreticulin
PS
log2 H/L
2
0
-2
30'/0'
30'/30'
30'/150'
Time point

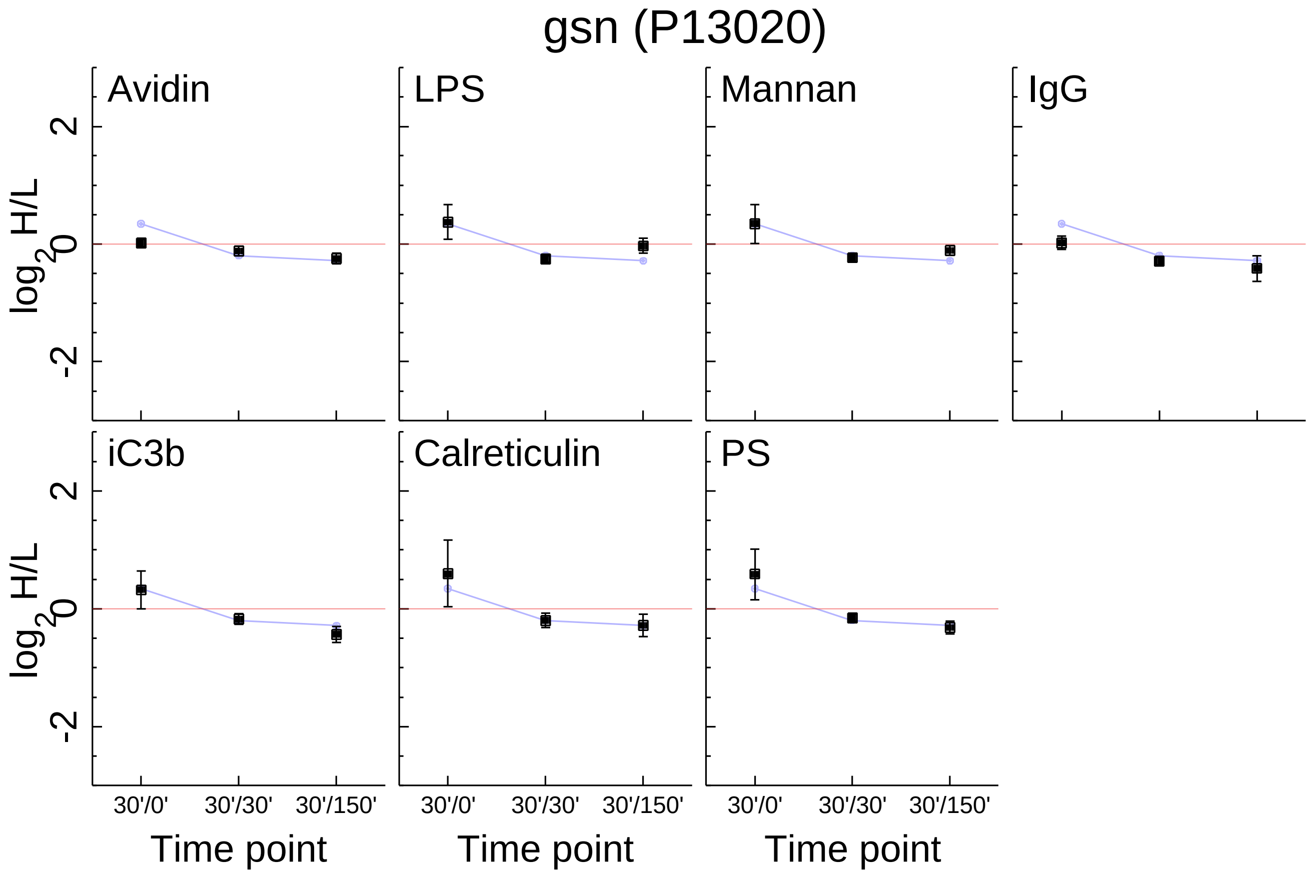

gsn (P13020)
Avidin
LPS
Mannan
IgG
iC3b
Calreticulin
PS
log2 H/L
2
0
-2
30'/0'
30'/30'
30'/150'
Time point

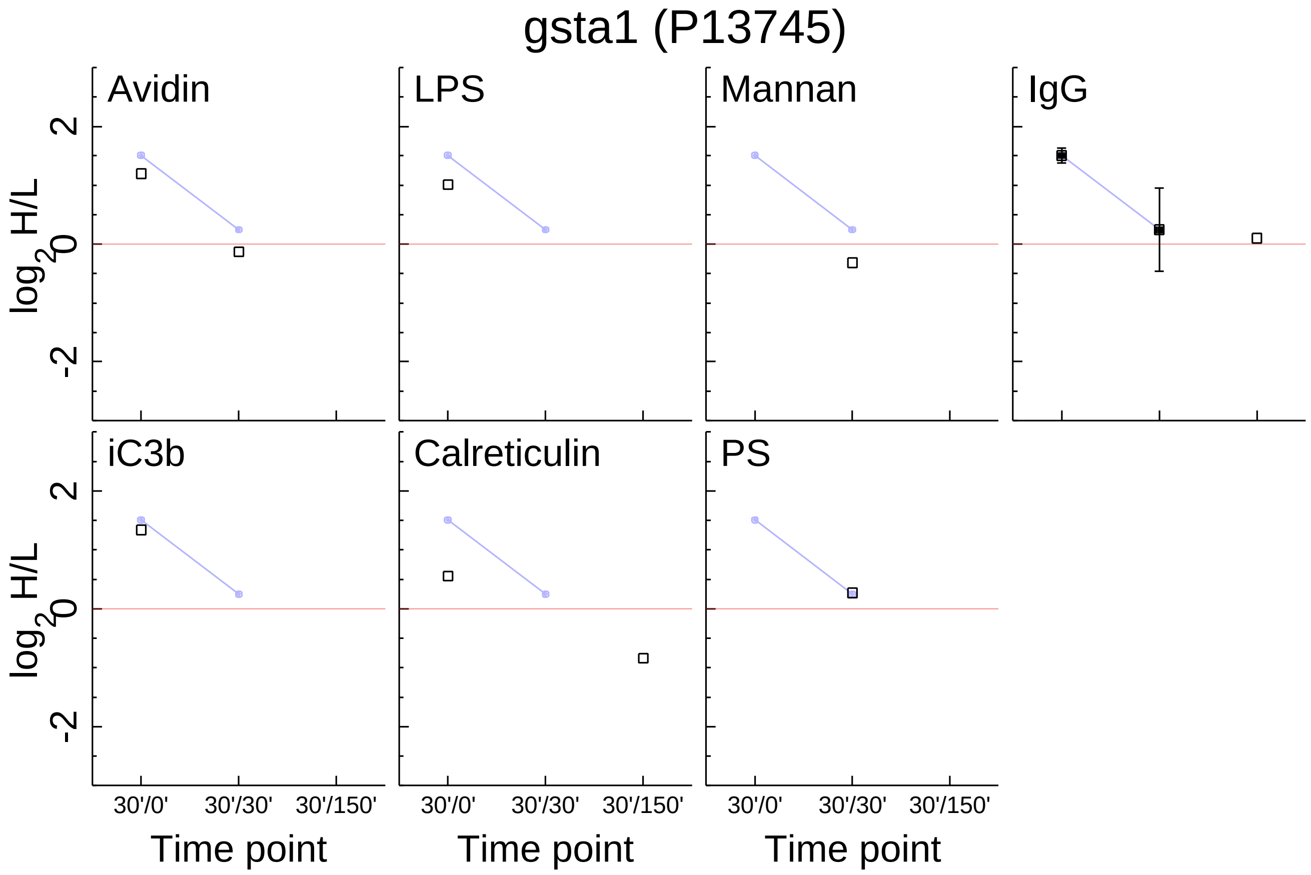

gsta1 (P13745)
Avidin
LPS
Mannan
IgG
iC3b
Calreticulin
PS
log2 H/L
2
0
-2
30'/0'
30'/30'
30'/150'
Time point

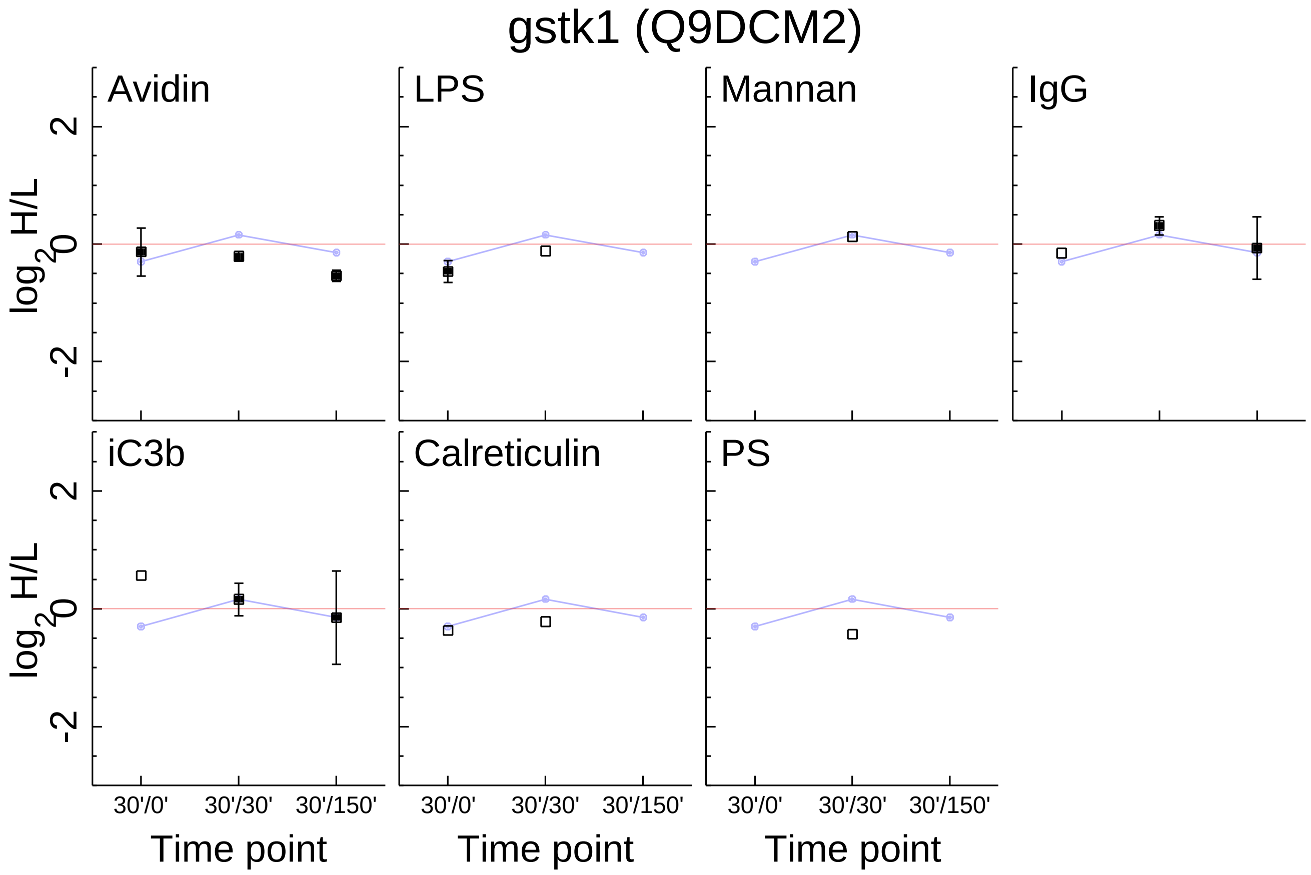

gstk1 (Q9DCM2)
Avidin
LPS
Mannan
IgG
iC3b
Calreticulin
PS
log2 H/L
2
0
-2
30'/0'
30'/30'
30'/150'
Time point

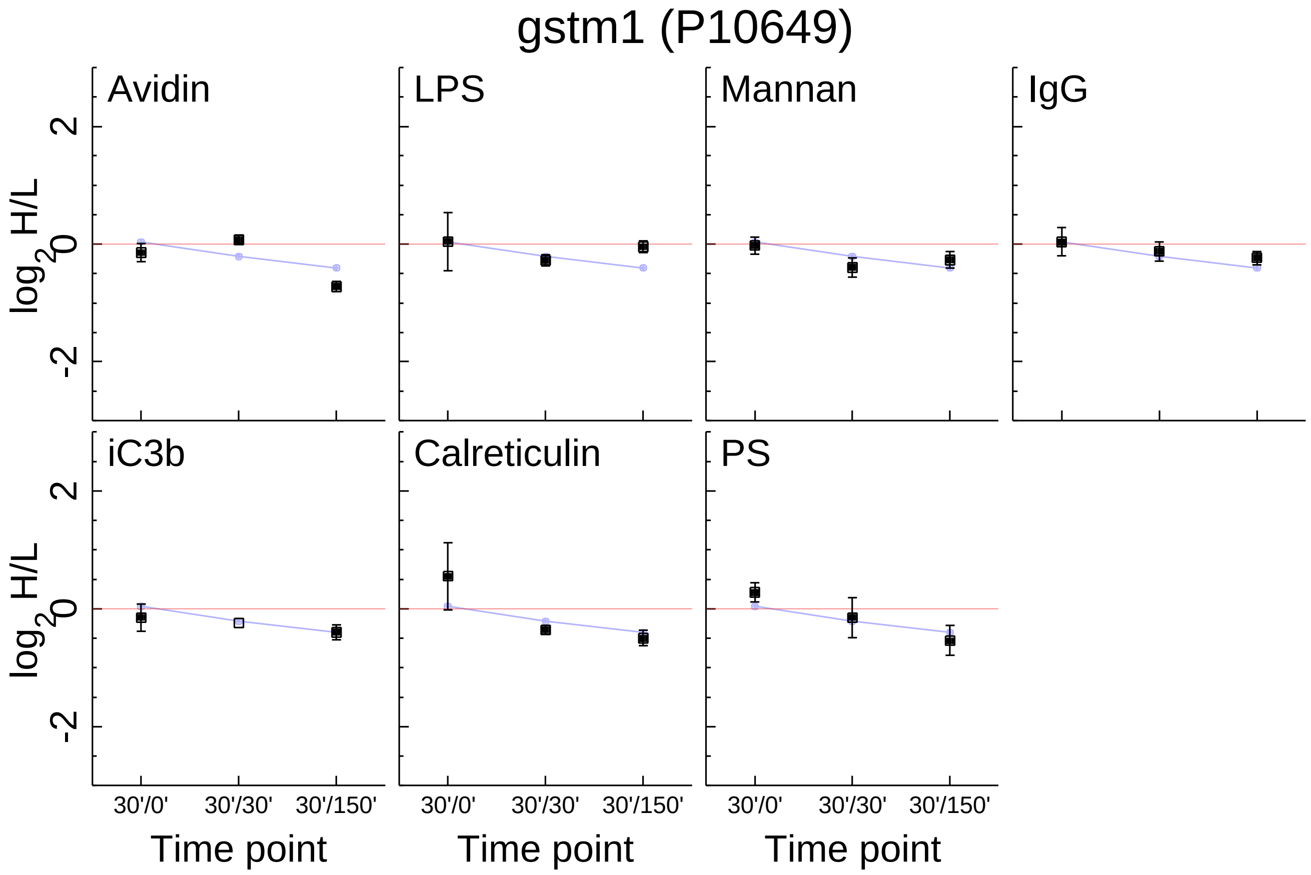

gstm1 (P10649)
Avidin
LPS
Mannan
IgG
iC3b
Calreticulin
PS
log2 H/L
2
0
-2
30'/0'
30'/30'
30'/150'
Time point

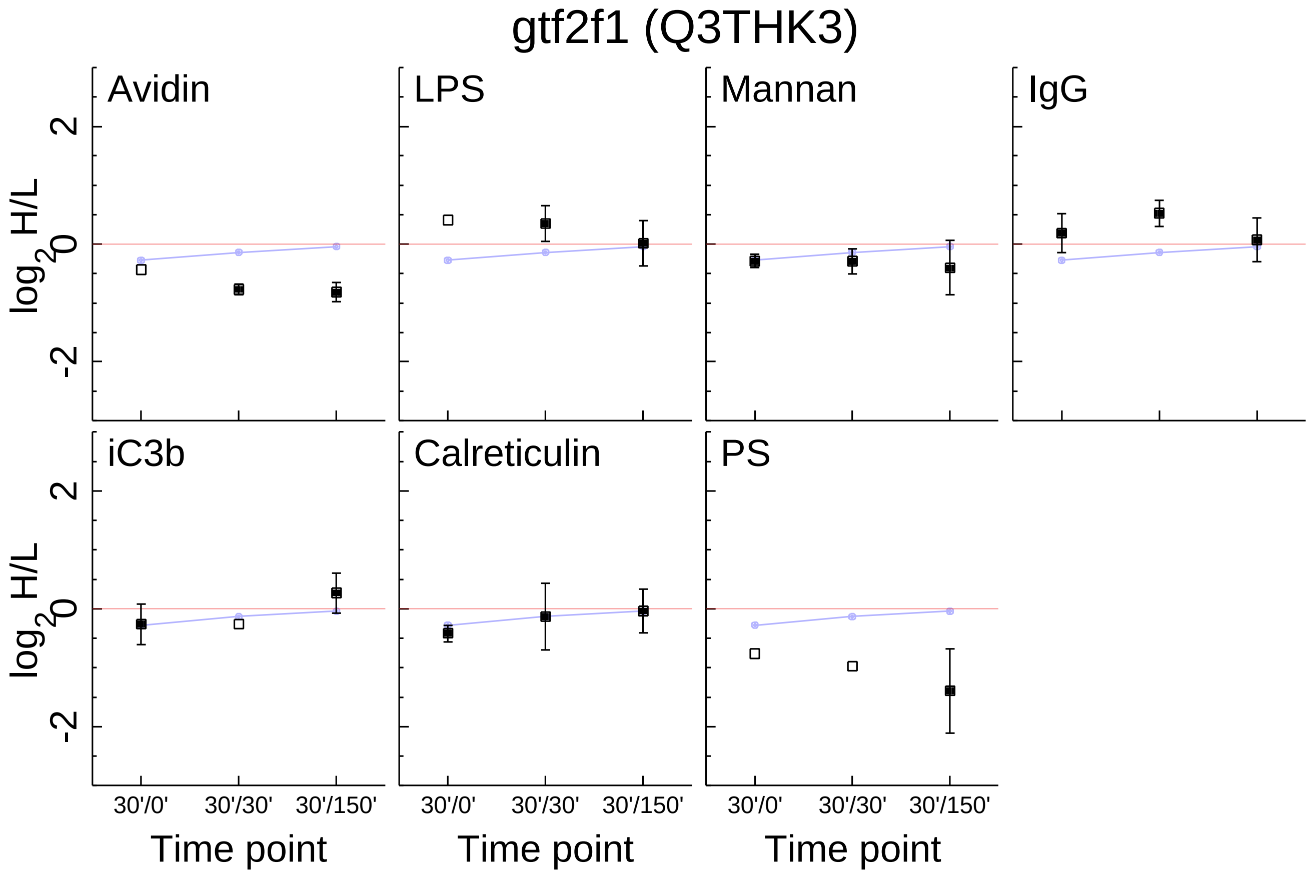
gtf2f1 (Q3THK3)
Avidin
LPS
Mannan
IgG
iC3b
Calreticulin
PS
log2 H/L
2
0
-2
30'/0'
30'/30'
30'/150'
Time point

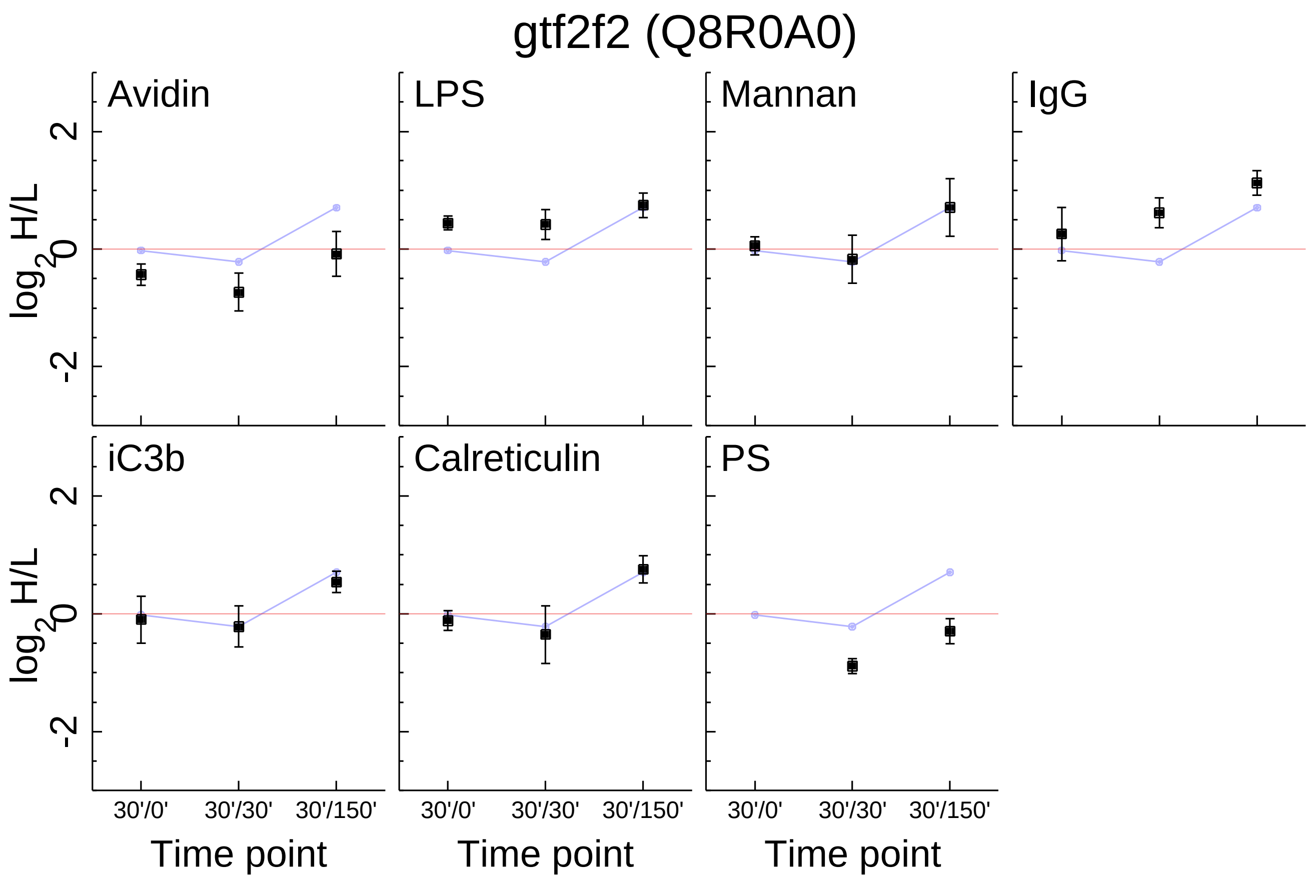
gtf2f2 (Q8R0A0)
Avidin
LPS
Mannan
IgG
iC3b
Calreticulin
PS
log2 H/L
2
0
-2
30'/0'
30'/30'
30'/150'
Time point

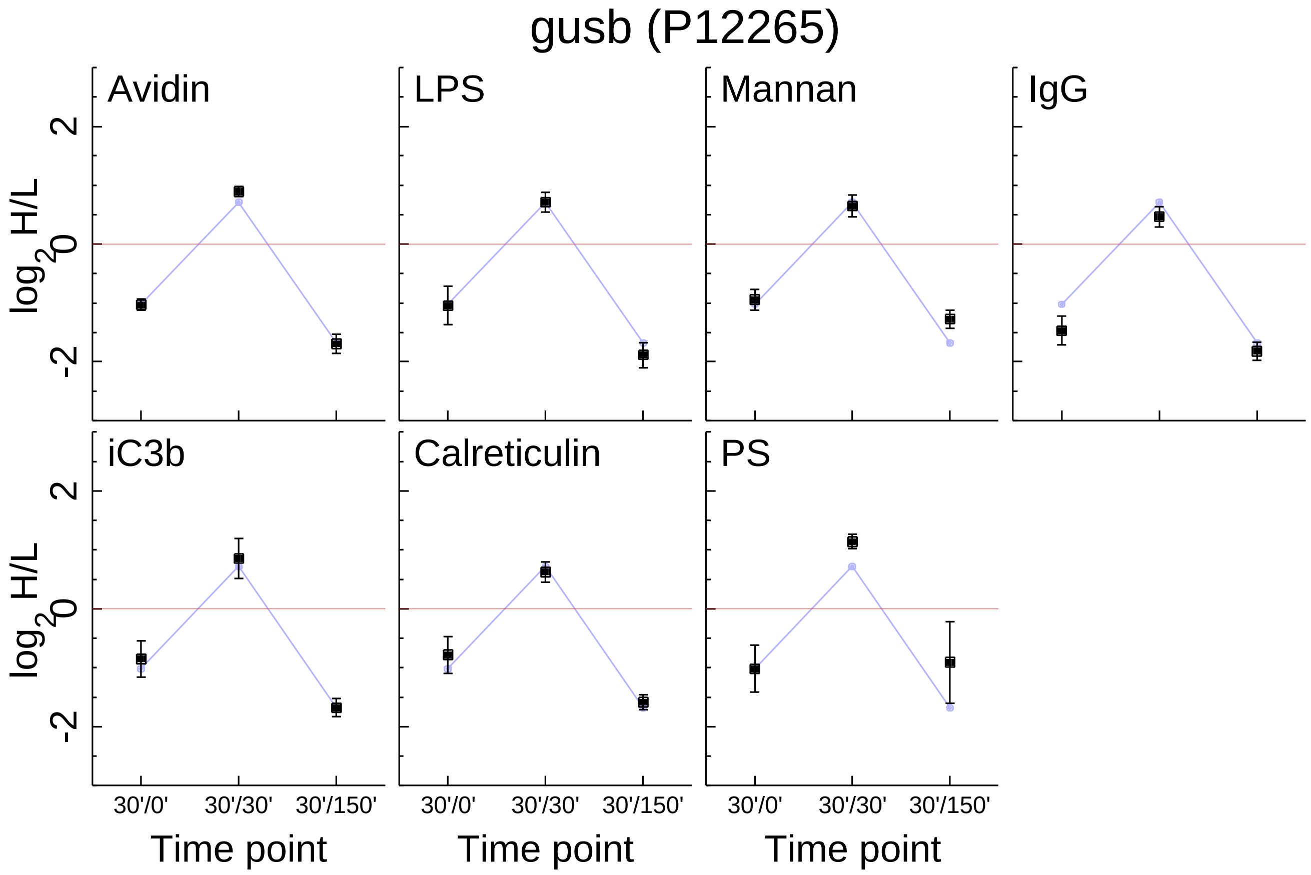
gusb (P12265)
Avidin
LPS
Mannan
IgG
iC3b
Calreticulin
PS
log2 H/L
2
0
-2
30'/0'
30'/30'
30'/150'
Time point

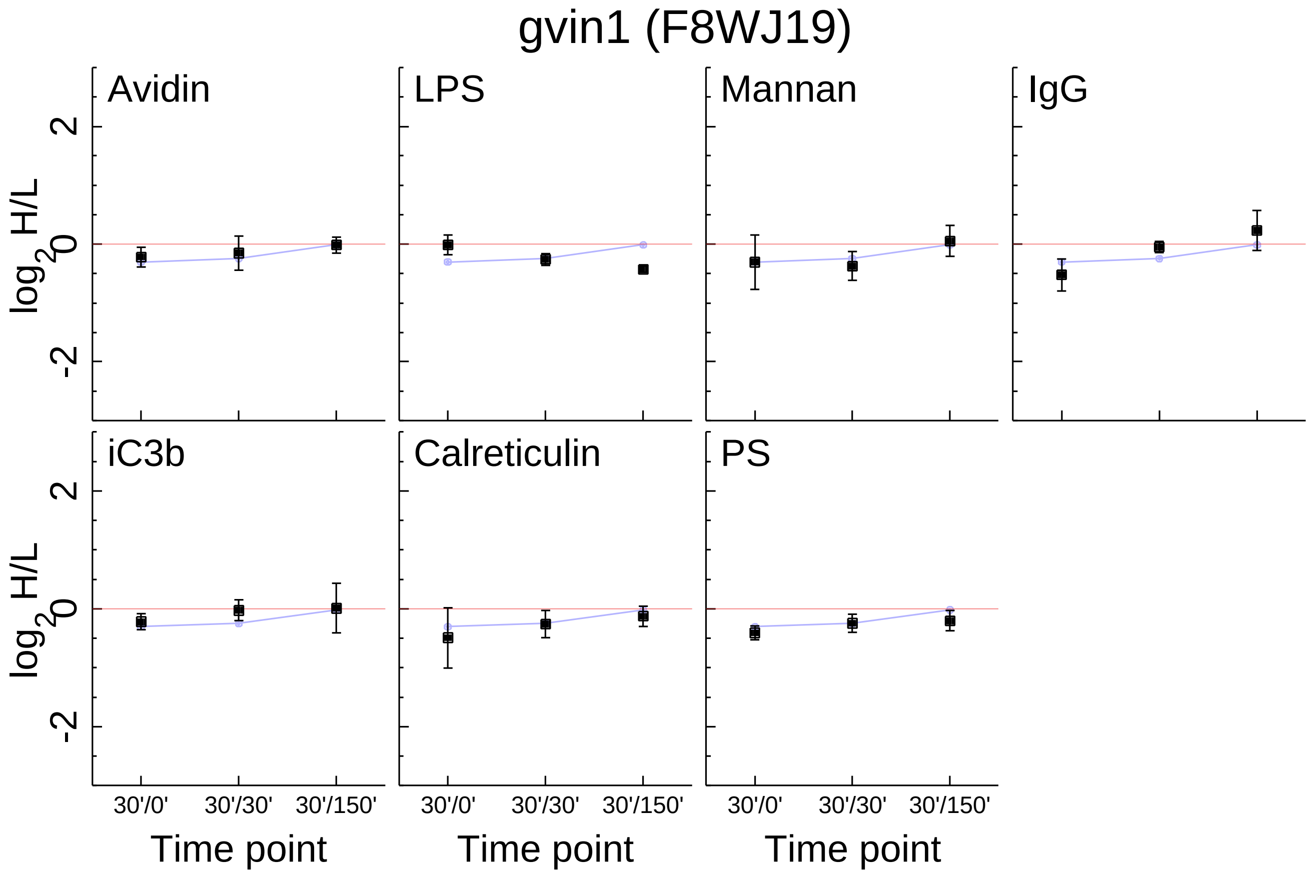

gvin1 (F8WJ19)
Avidin
LPS
Mannan
IgG
iC3b
Calreticulin
PS
log2 H/L
2
0
-2
30'/0'
30'/30'
30'/150'
Time point

h1f0 (P10922)

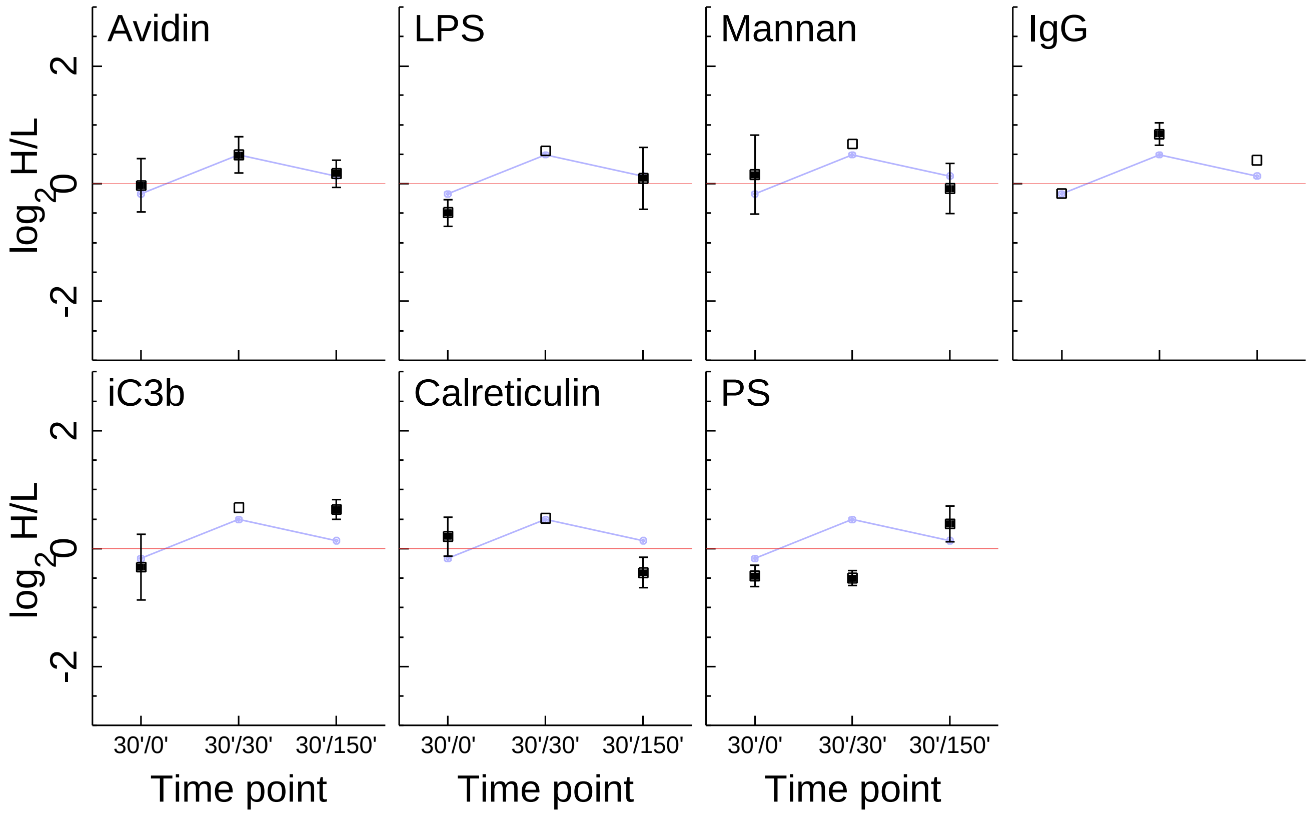

Avidin
LPS
Mannan
IgG
iC3b
Calreticulin
PS
log2 H/L
2
0
-2
30'/0'
30'/30'
30'/150'
Time point

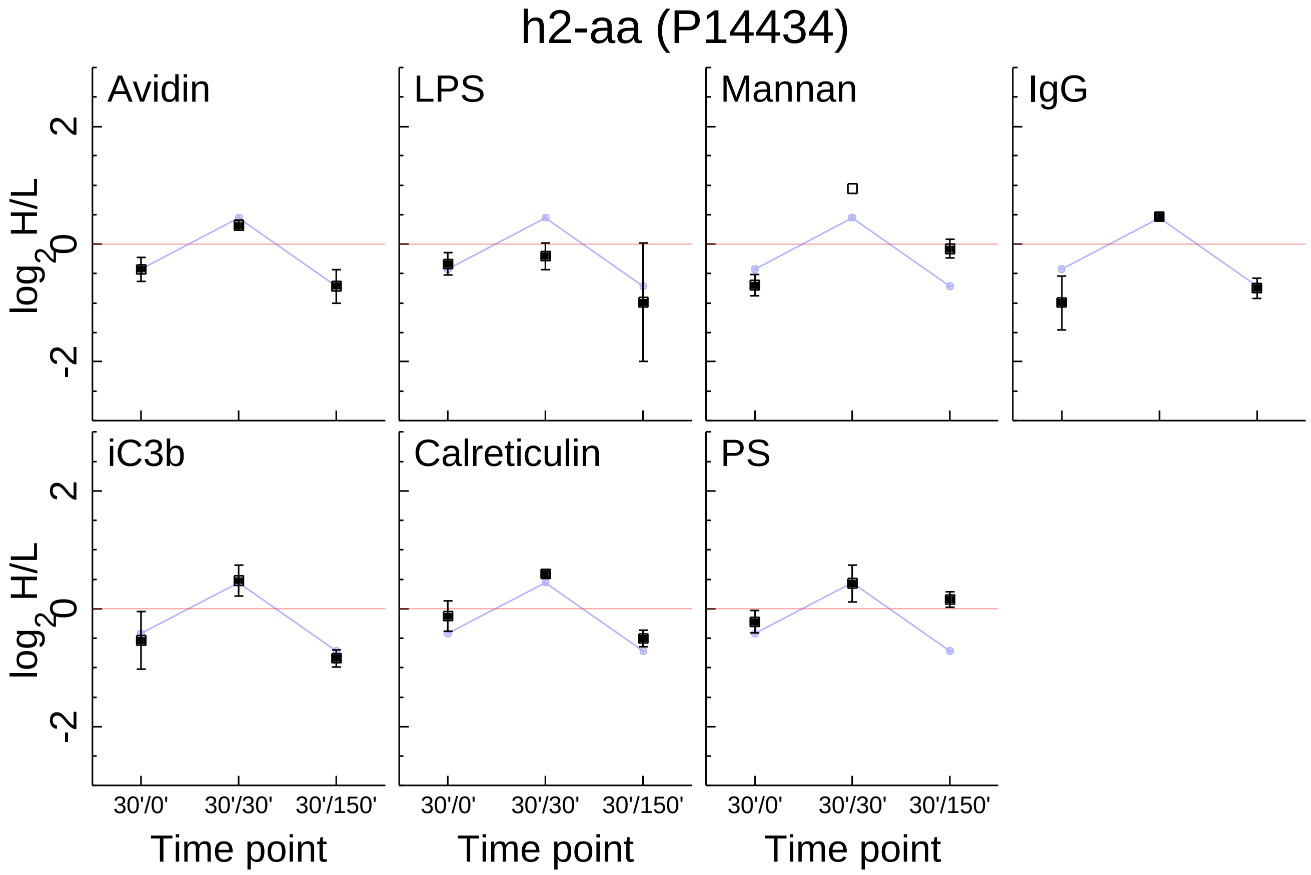

h2-aa (P14434)
Avidin
LPS
Mannan
IgG
iC3b
Calreticulin
PS
log2 H/L
2
0
-2
30'/0'
30'/30'
30'/150'
Time point

h2-ab1 (P14483)

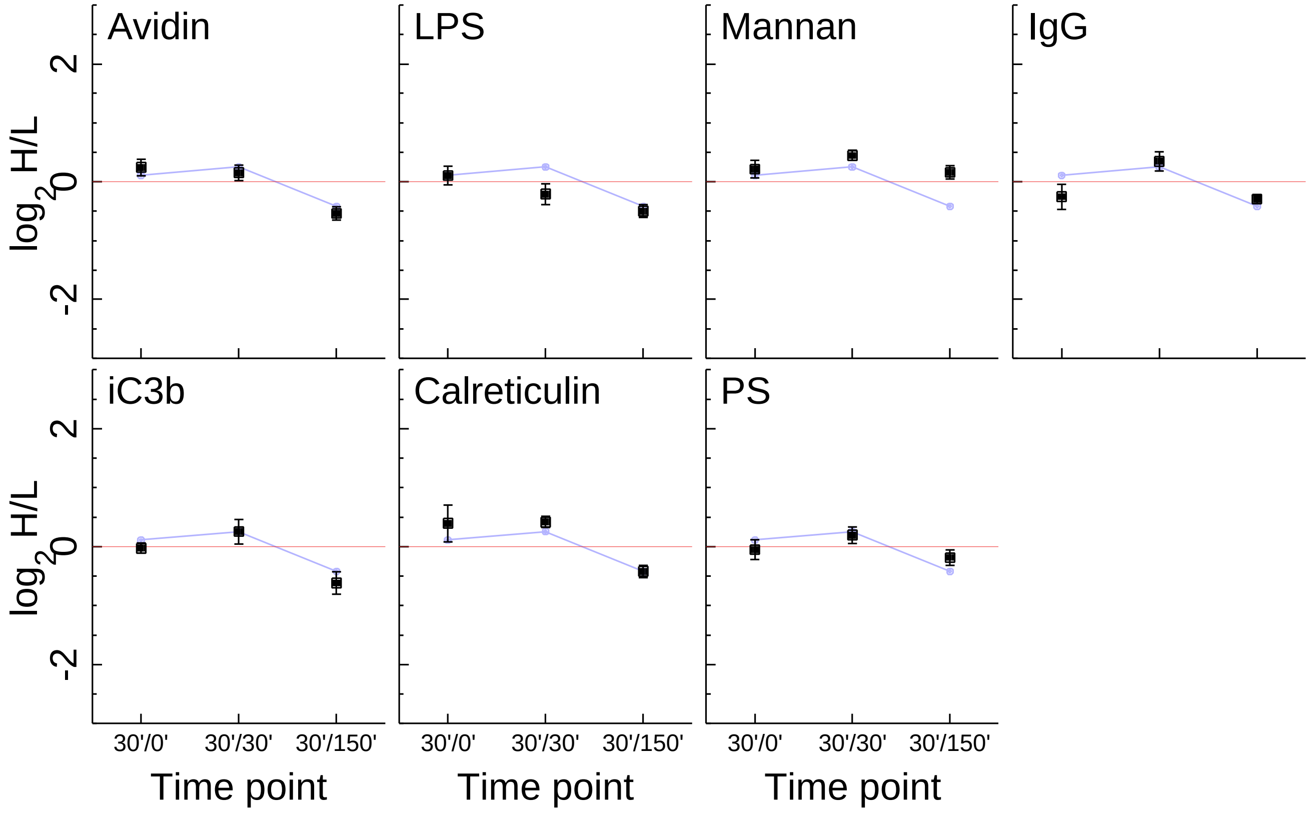

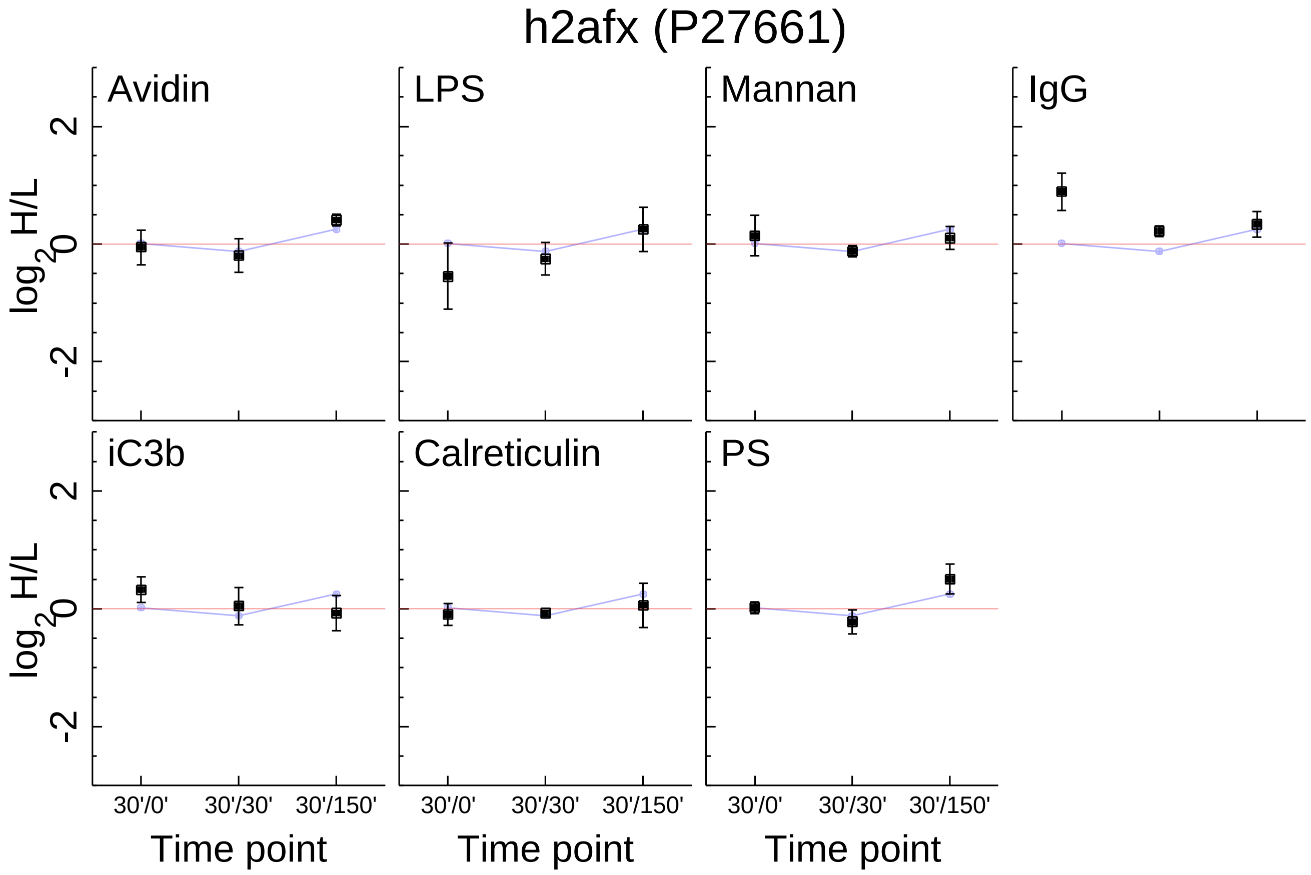
h2afx (P27661)
Avidin
LPS
Mannan
IgG
iC3b
Calreticulin
PS
log2 H/L
2
0
-2
30'/0'
30'/30'
30'/150'
Time point

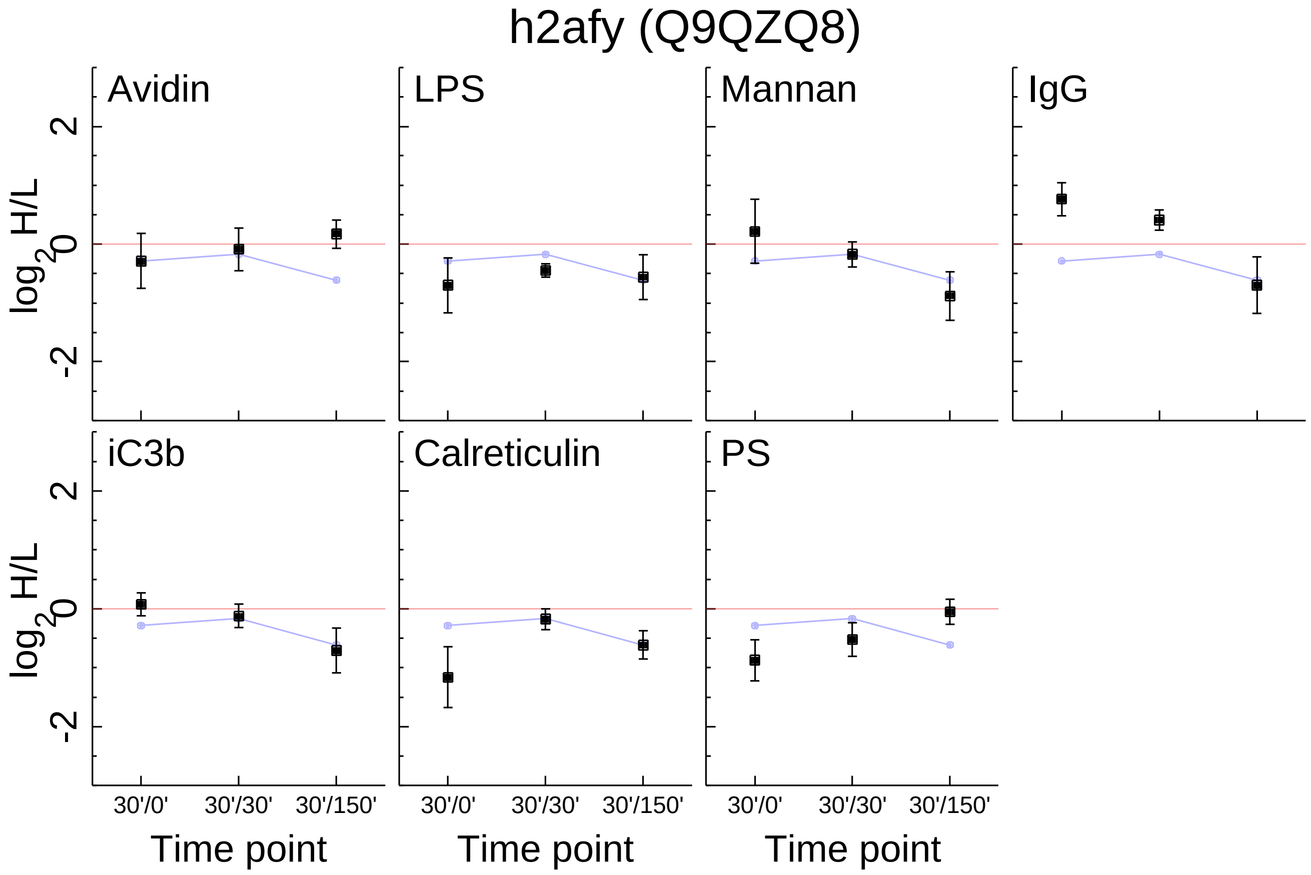
h2afy (Q9QZQ8)
Avidin
LPS
Mannan
IgG
iC3b
Calreticulin
PS
log2 H/L
2
0
-2
30'/0'
30'/30'
30'/150'
Time point

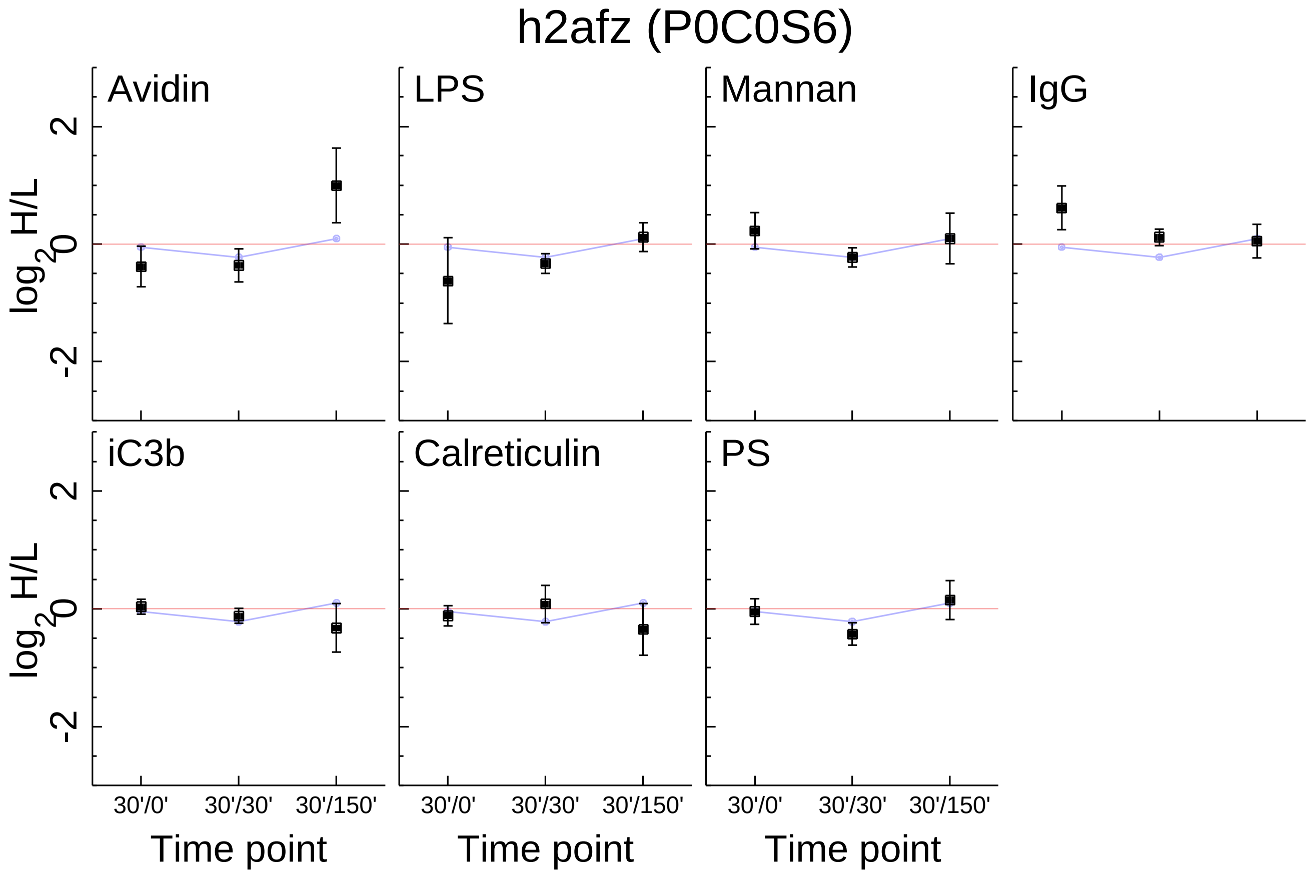
h2afz (P0C0S6)
Avidin
LPS
Mannan
IgG
iC3b
Calreticulin
PS
log2 H/L
2
0
-2
30'/0'
30'/30'
30'/150'
Time point

h2-d1:1 (P01899)

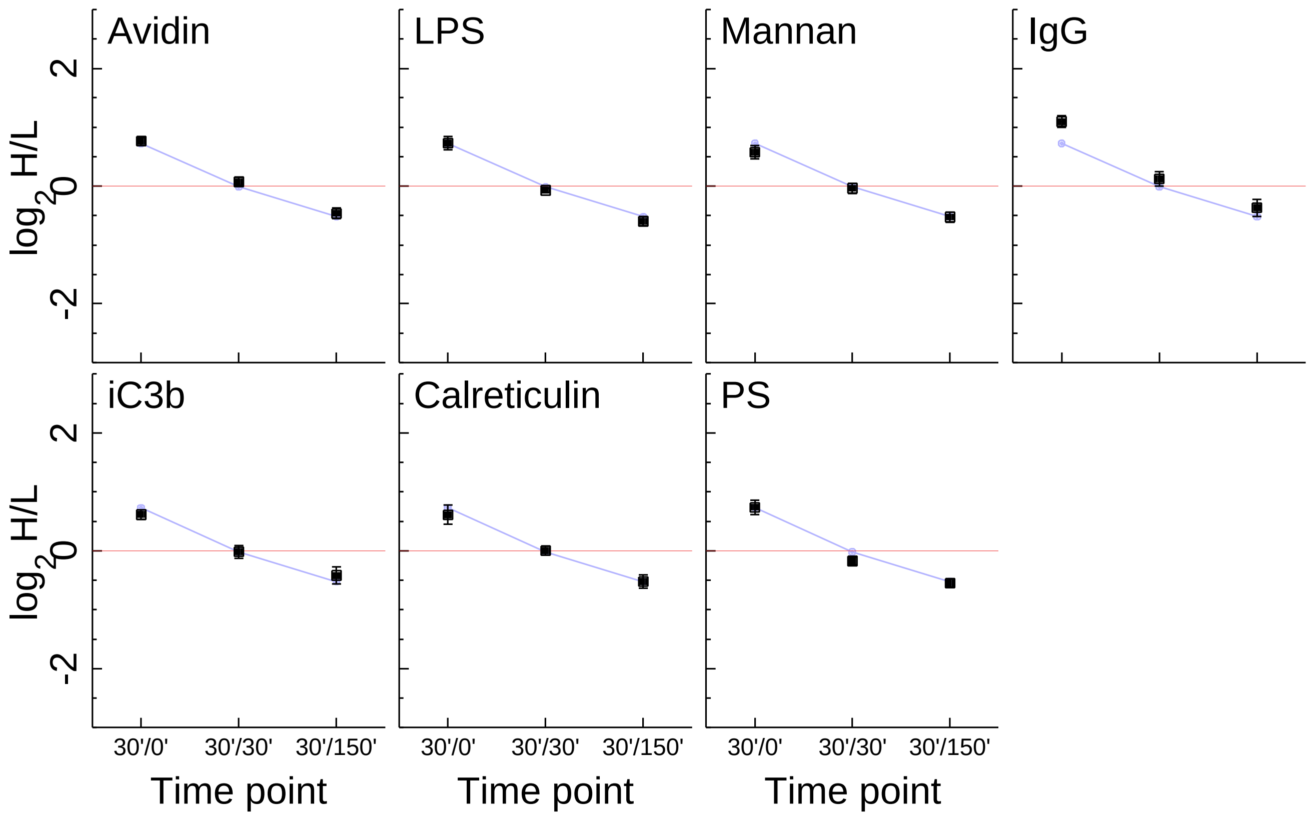

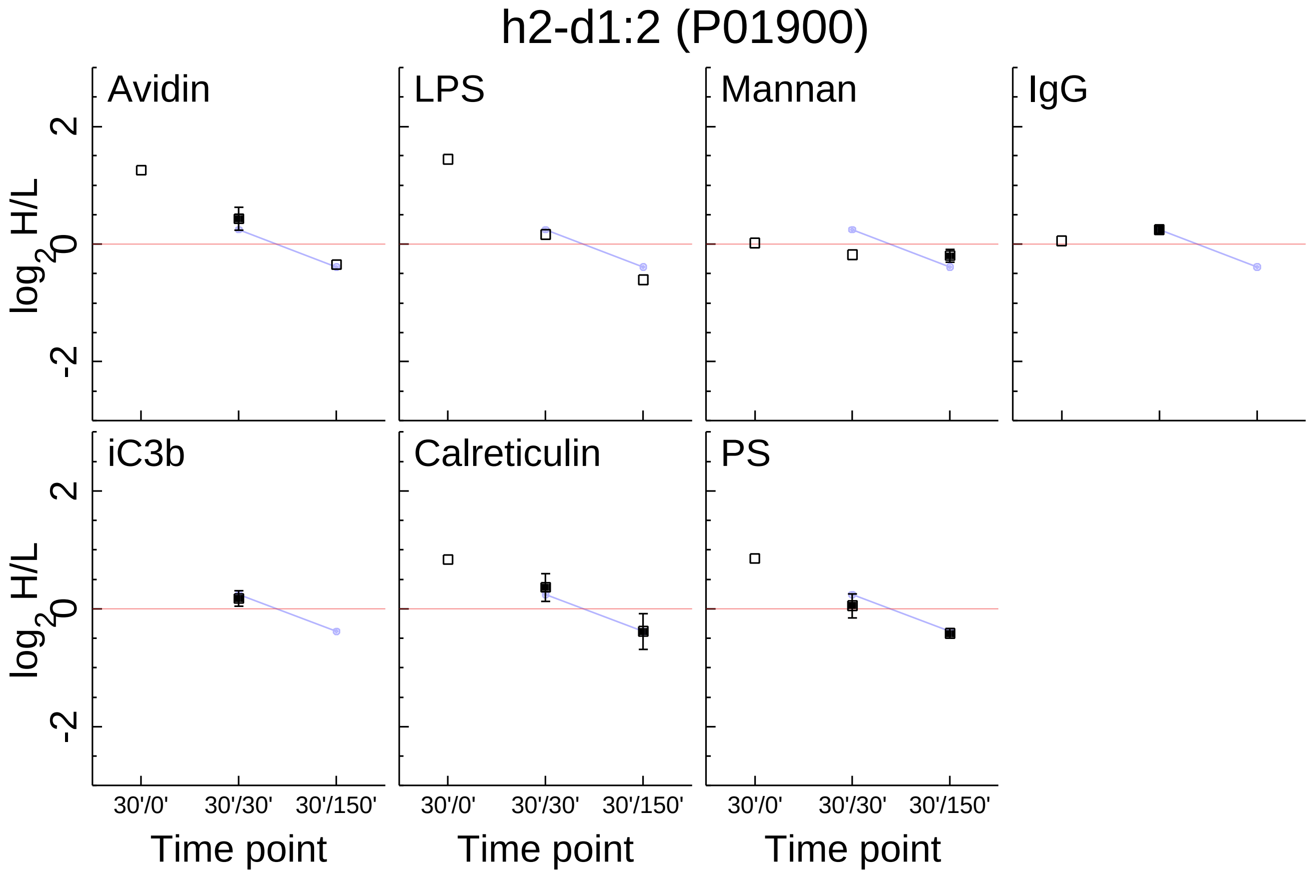
h2-d1:2 (P01900)
Avidin
LPS
Mannan
IgG
iC3b
Calreticulin
PS
2
0
-2
log2 H/L
30'/0'
30'/30'
30'/150'
Time point

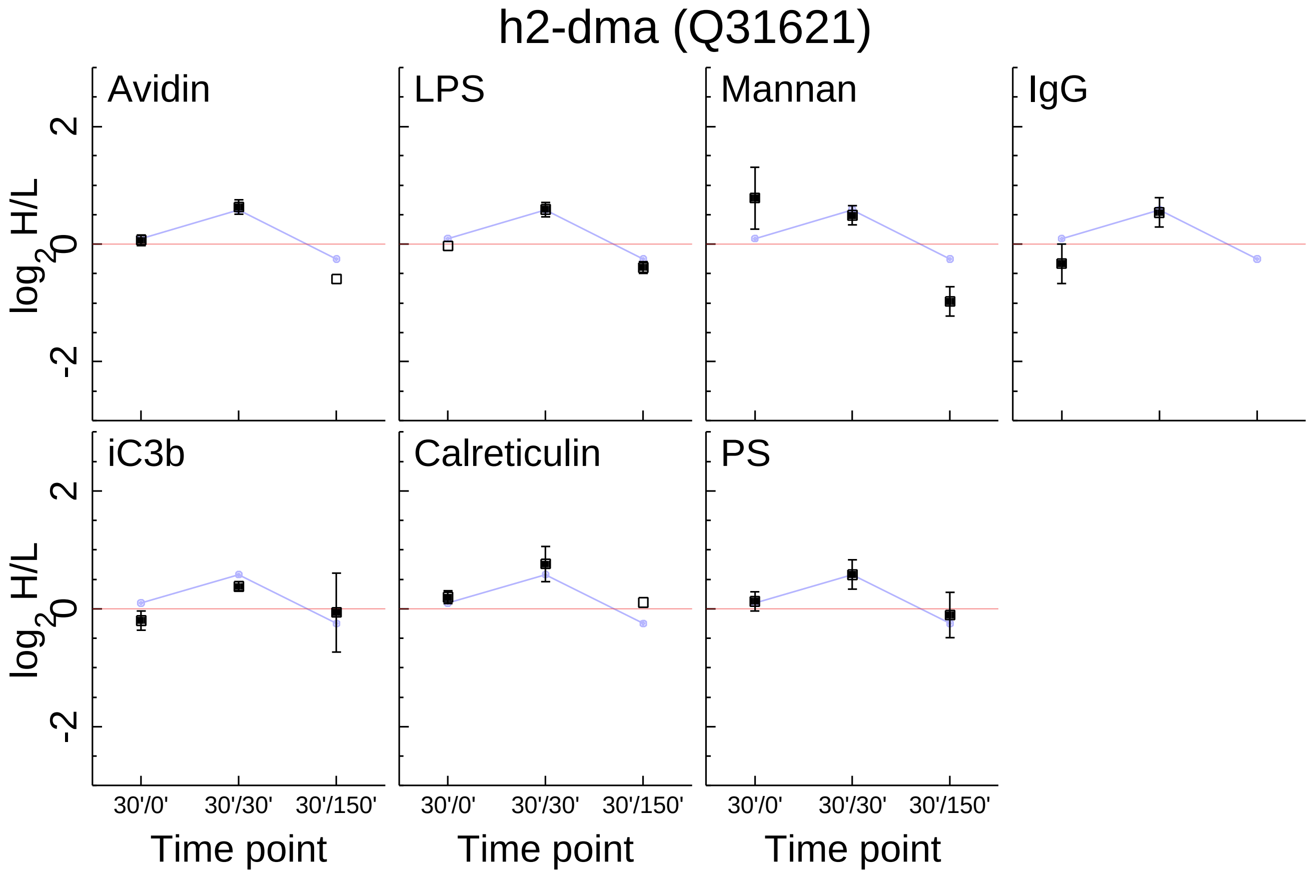
h2-dma (Q31621)
Avidin
LPS
Mannan
IgG
iC3b
Calreticulin
PS
$\log_2$ H/L
2
0
-2
30'/0'
30'/30'
30'/150'
Time point

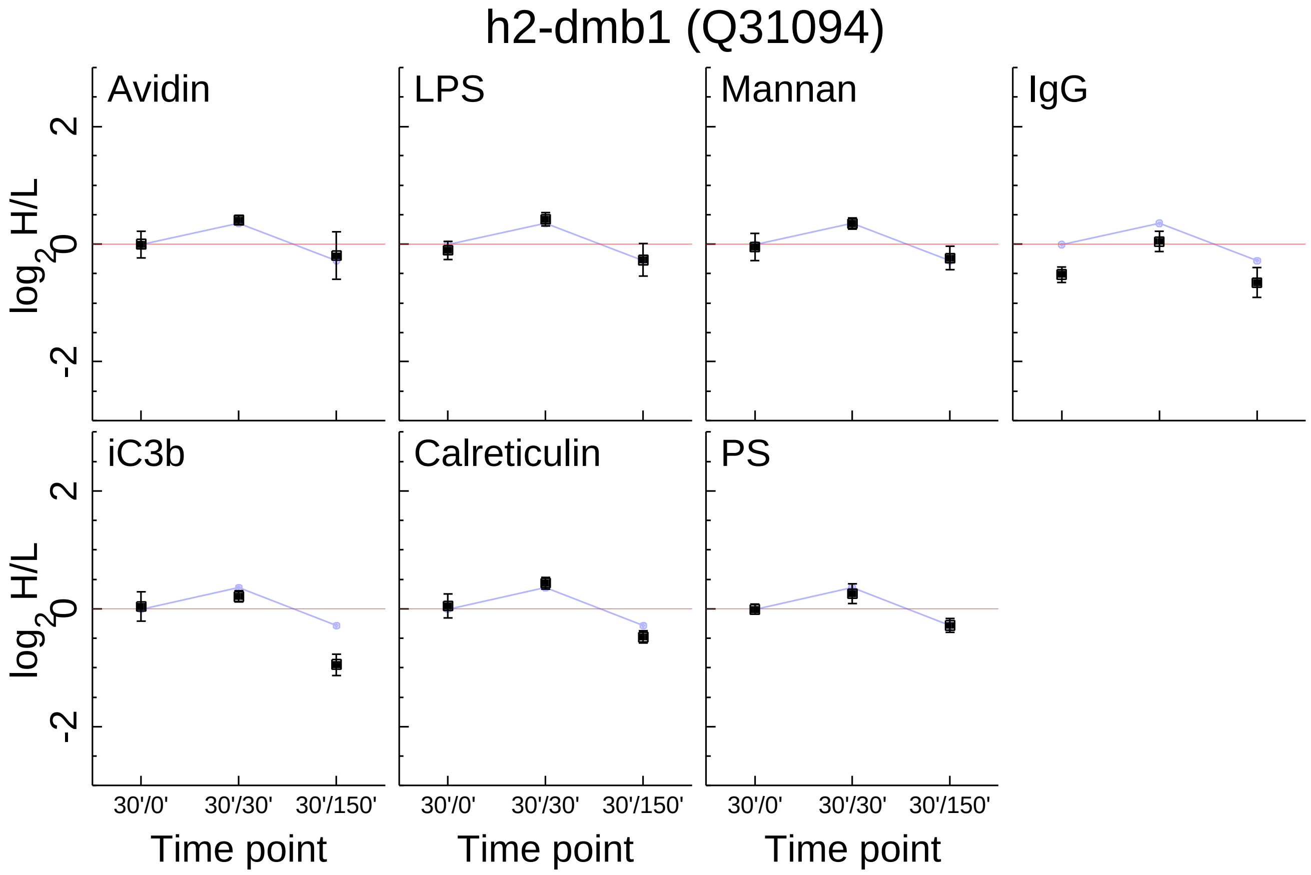
h2-dmb1 (Q31094)
Avidin
LPS
Mannan
IgG
iC3b
Calreticulin
PS
log2 H/L
2
0
-2
30'/0'
30'/30'
30'/150'
Time point

h2-k1 (P01901)

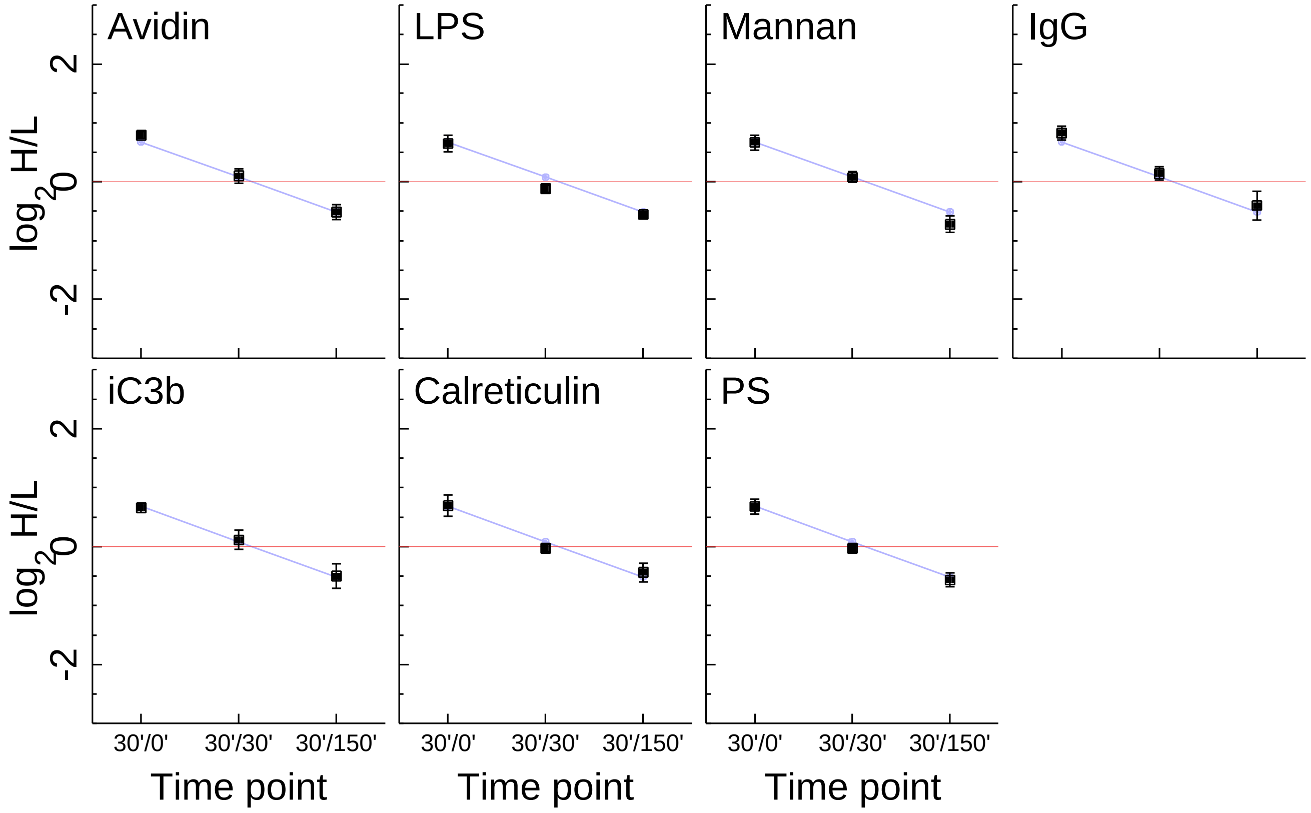

Avidin
LPS
Mannan
IgG
iC3b
Calreticulin
PS
log2 H/L
2
0
-2
30'/0'
30'/30'
30'/150'
Time point

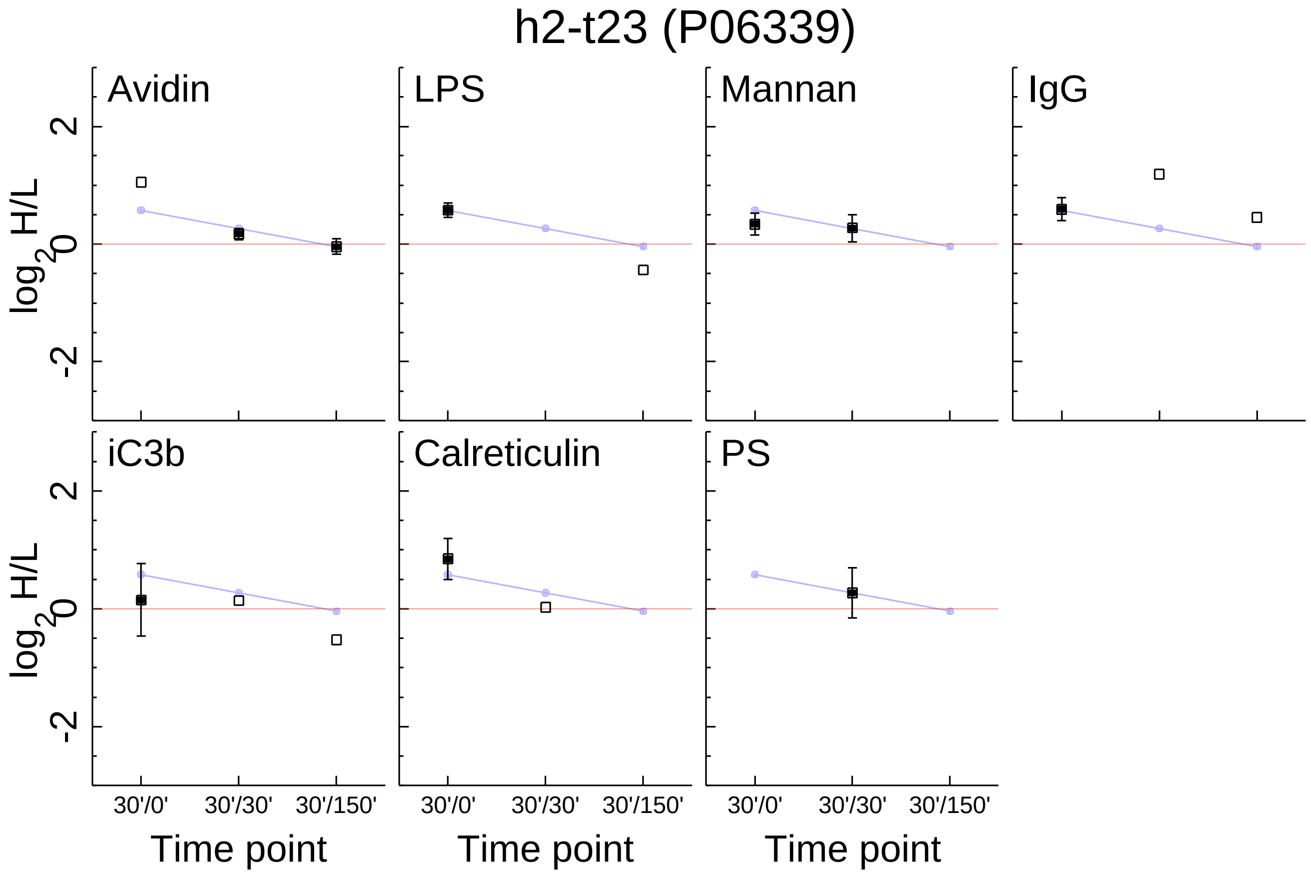
h2-t23 (P06339)
Avidin
LPS
Mannan
IgG
iC3b
Calreticulin
PS
log2 H/L
2
0
-2
30'/0'
30'/30'
30'/150'
Time point

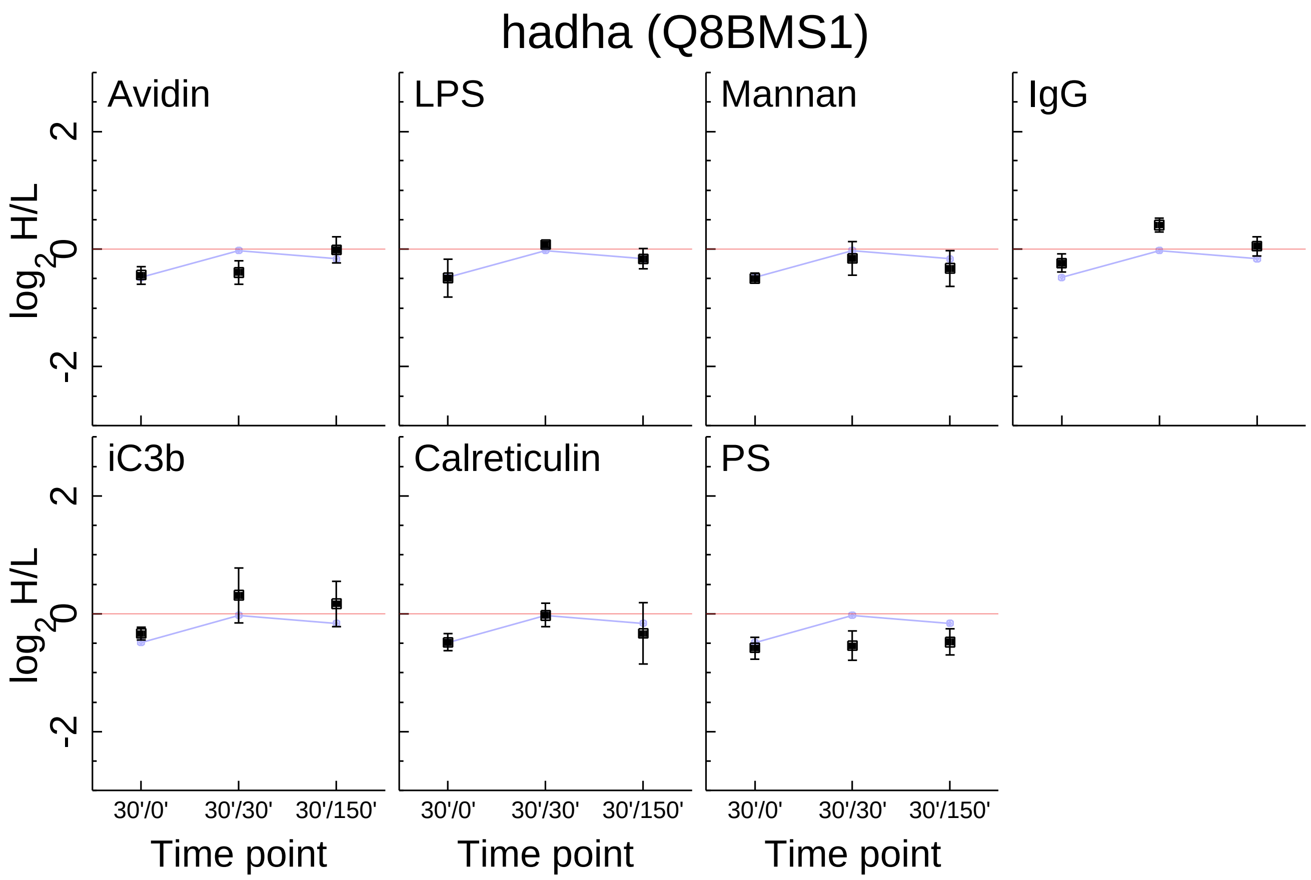
hadha (Q8BMS1)
Avidin
LPS
Mannan
IgG
iC3b
Calreticulin
PS
log2 H/L
2
0
-2
30'/0'
30'/30'
30'/150'
Time point

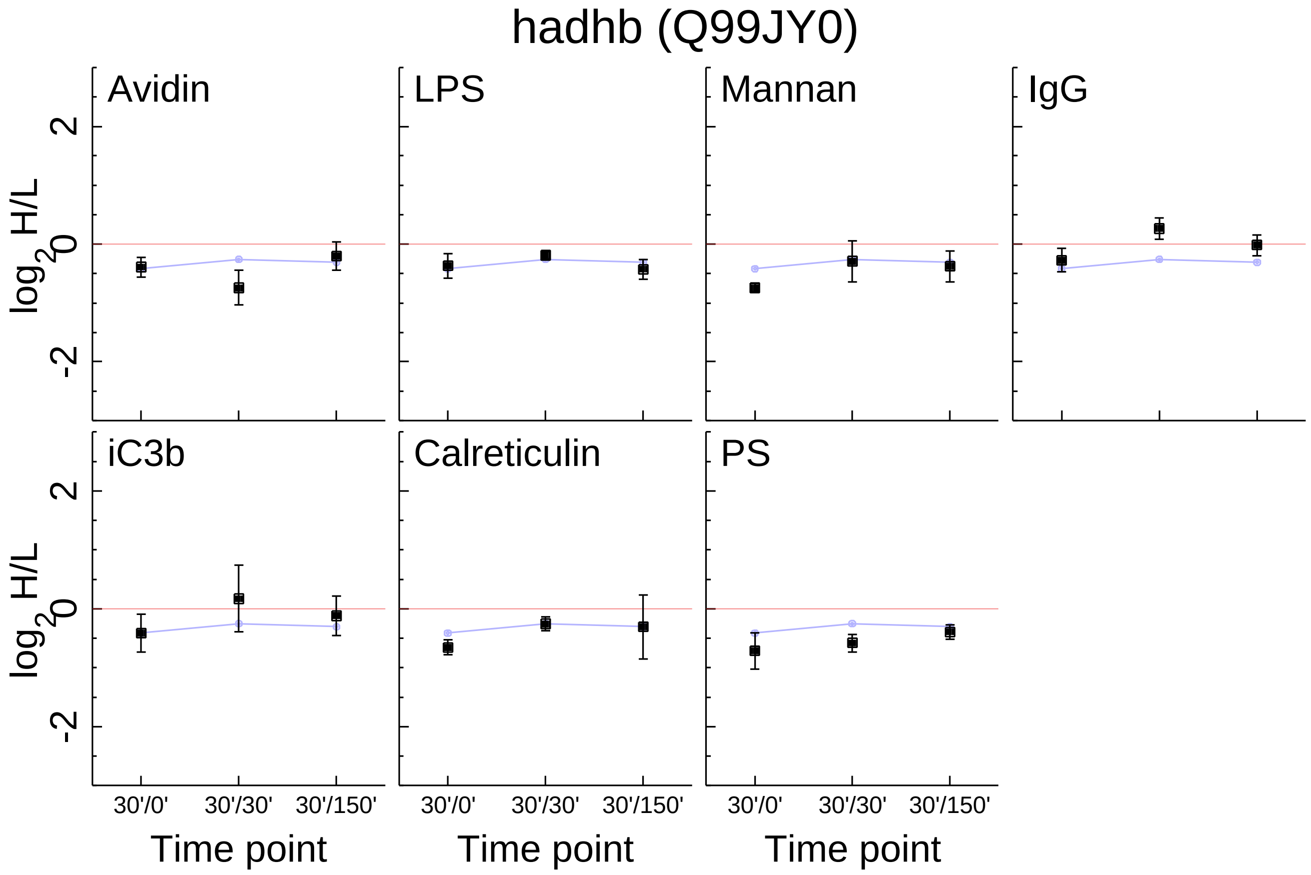
hadhb (Q99JY0)
Avidin
LPS
Mannan
IgG
iC3b
Calreticulin
PS
log2 H/L
2
0
-2
30'/0'
30'/30'
30'/150'
Time point

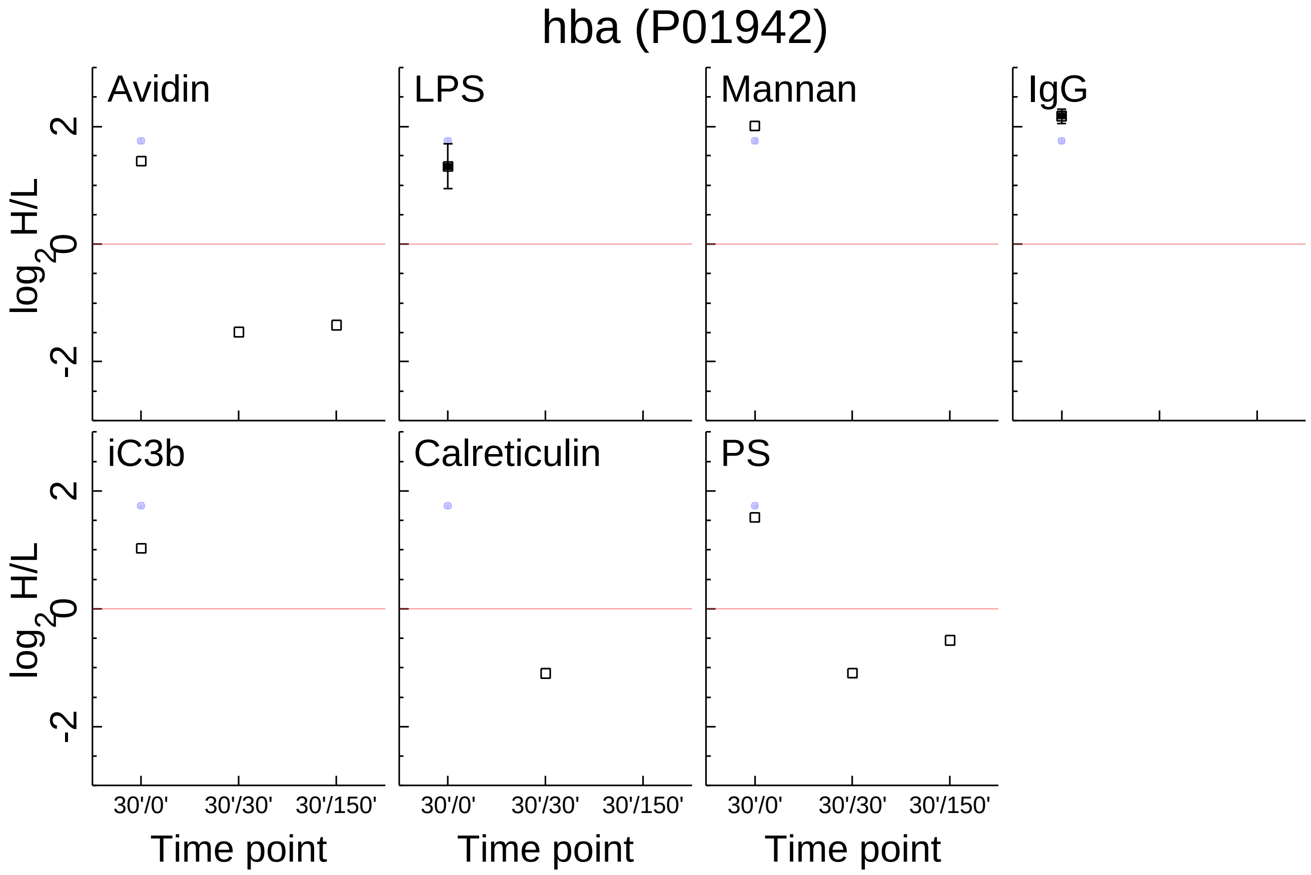
hba (P01942)
Avidin
LPS
Mannan
IgG
iC3b
Calreticulin
PS
log2 H/L
2
0
-2
30'/0'
30'/30'
30'/150'
Time point

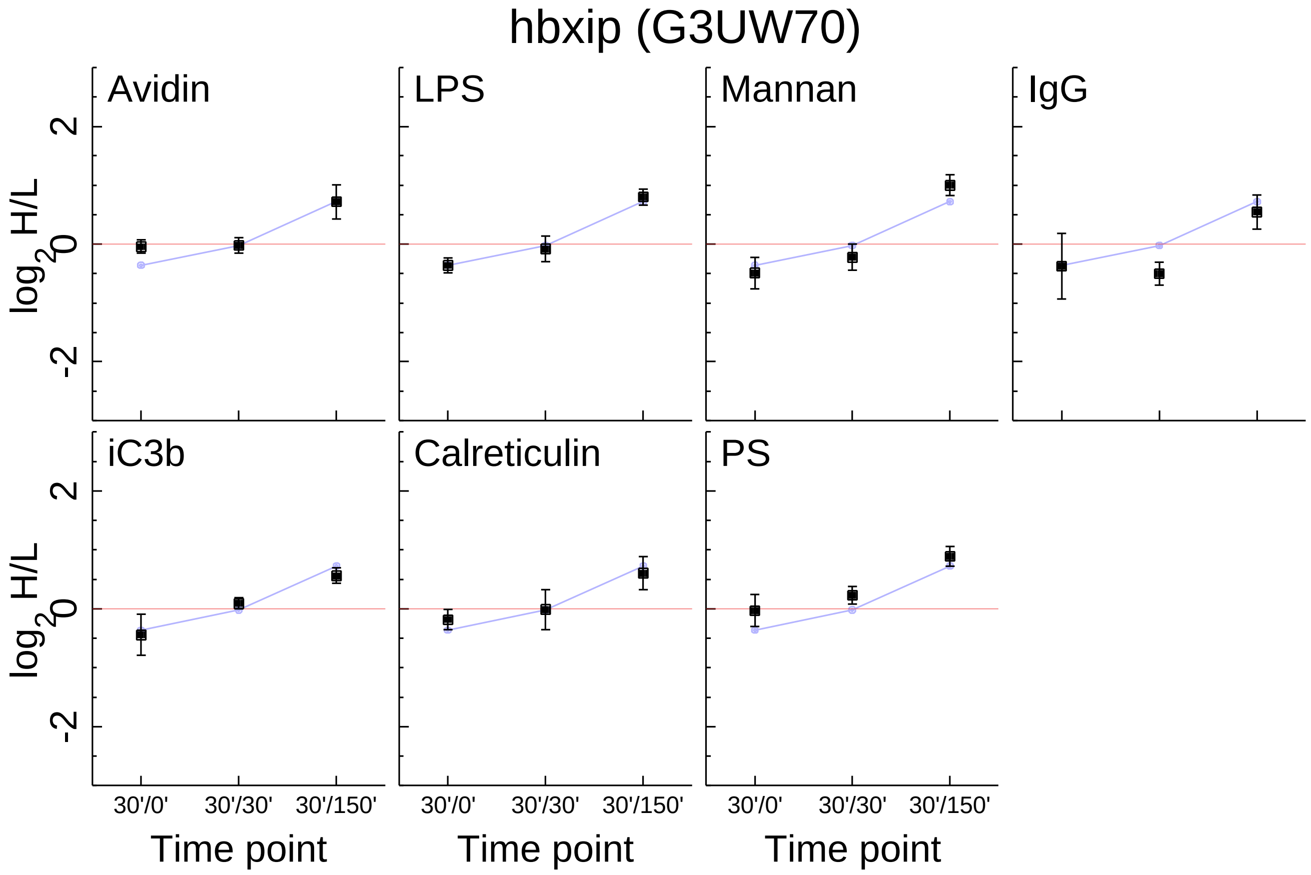
hbxip (G3UW70)
Avidin
LPS
Mannan
IgG
iC3b
Calreticulin
PS
log2 H/L
2
0
-2
30'/0'
30'/30'
30'/150'
Time point

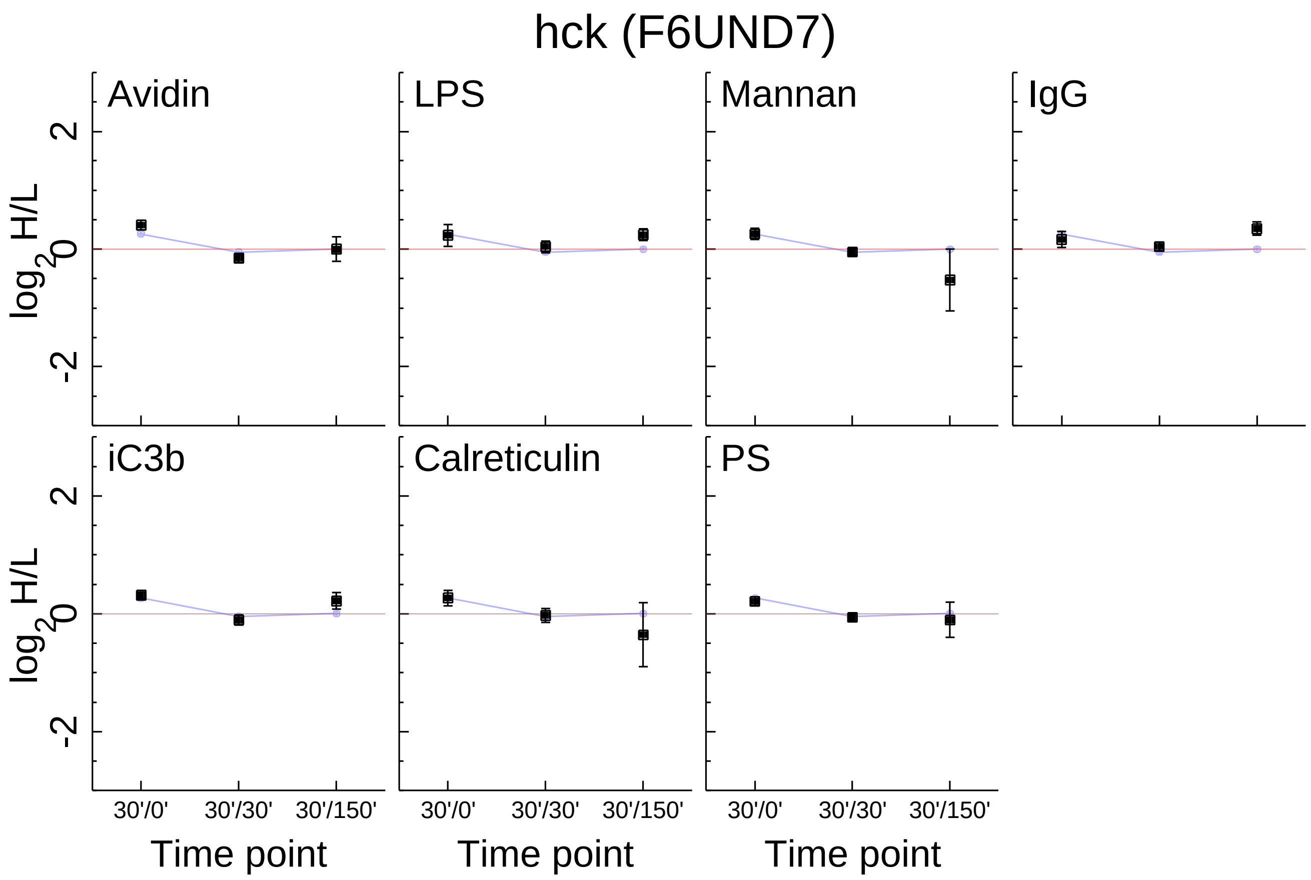

hck (F6UND7)
Avidin
LPS
Mannan
IgG
iC3b
Calreticulin
PS
log2 H/L
2
0
-2
30'/0'
30'/30'
30'/150'
Time point

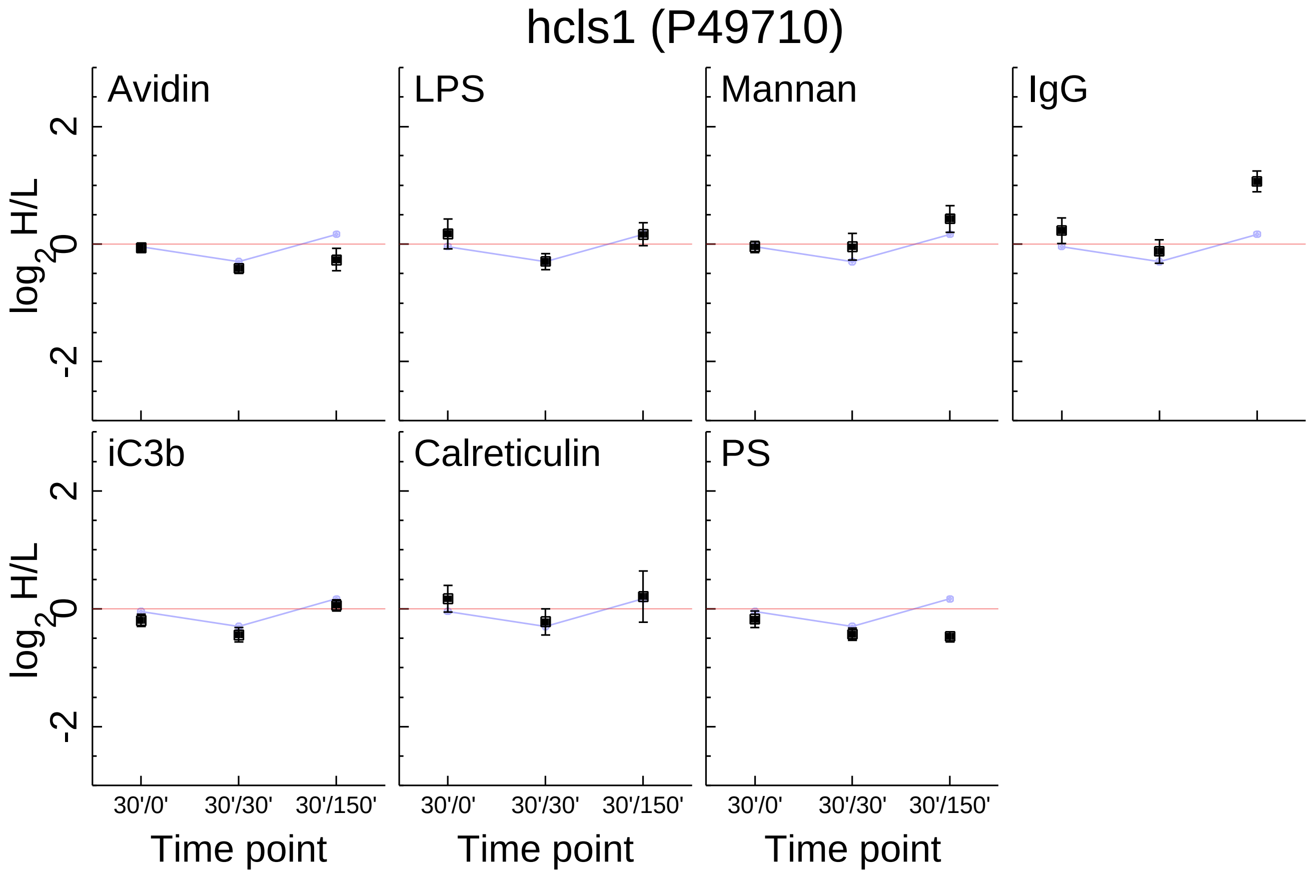
hcls1 (P49710)
Avidin
LPS
Mannan
IgG
iC3b
Calreticulin
PS
log2 H/L
2
0
-2
30'/0'
30'/30'
30'/150'
Time point

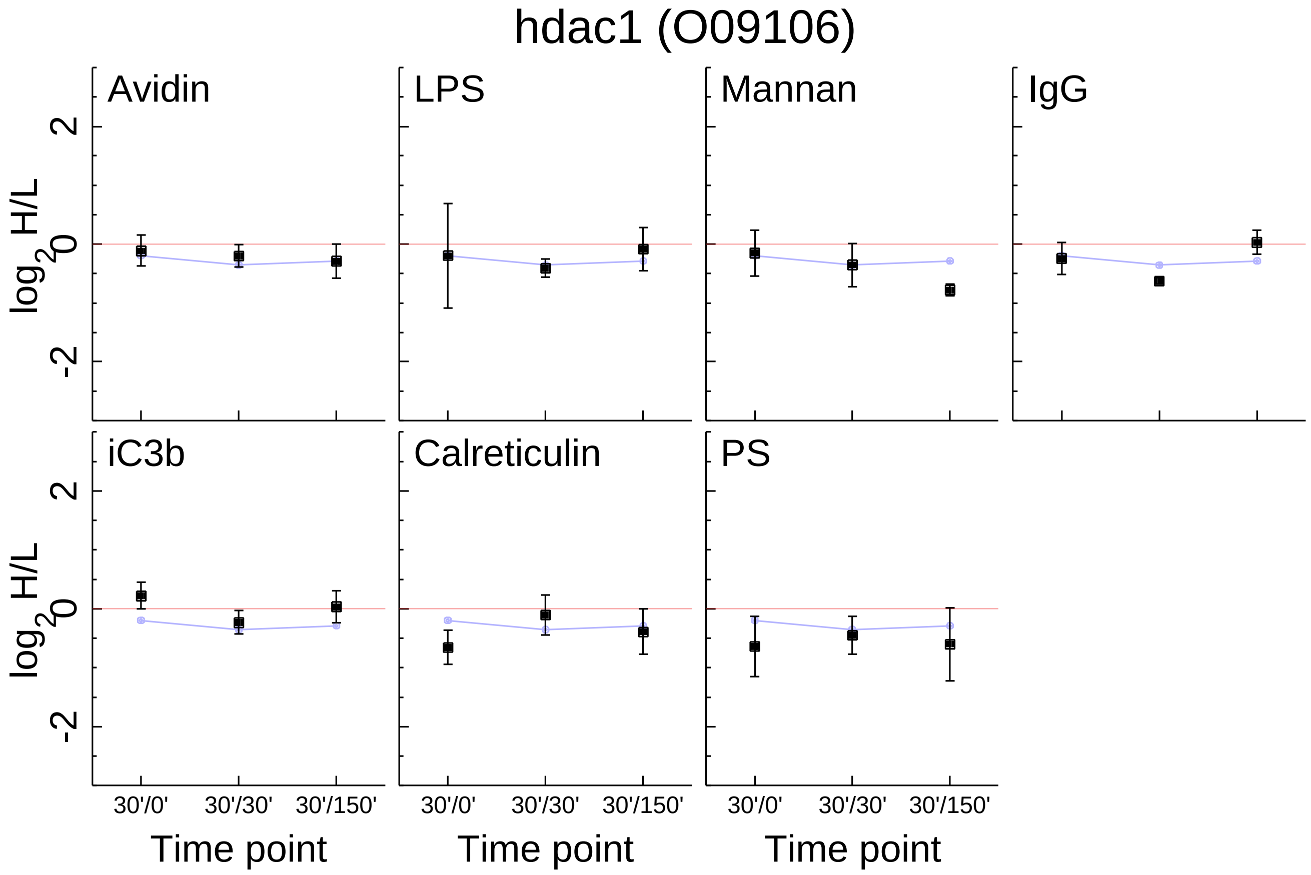
hdac1 (O09106)
Avidin
LPS
Mannan
IgG
iC3b
Calreticulin
PS
log2 H/L
2
0
-2
30'/0'
30'/30'
30'/150'
Time point

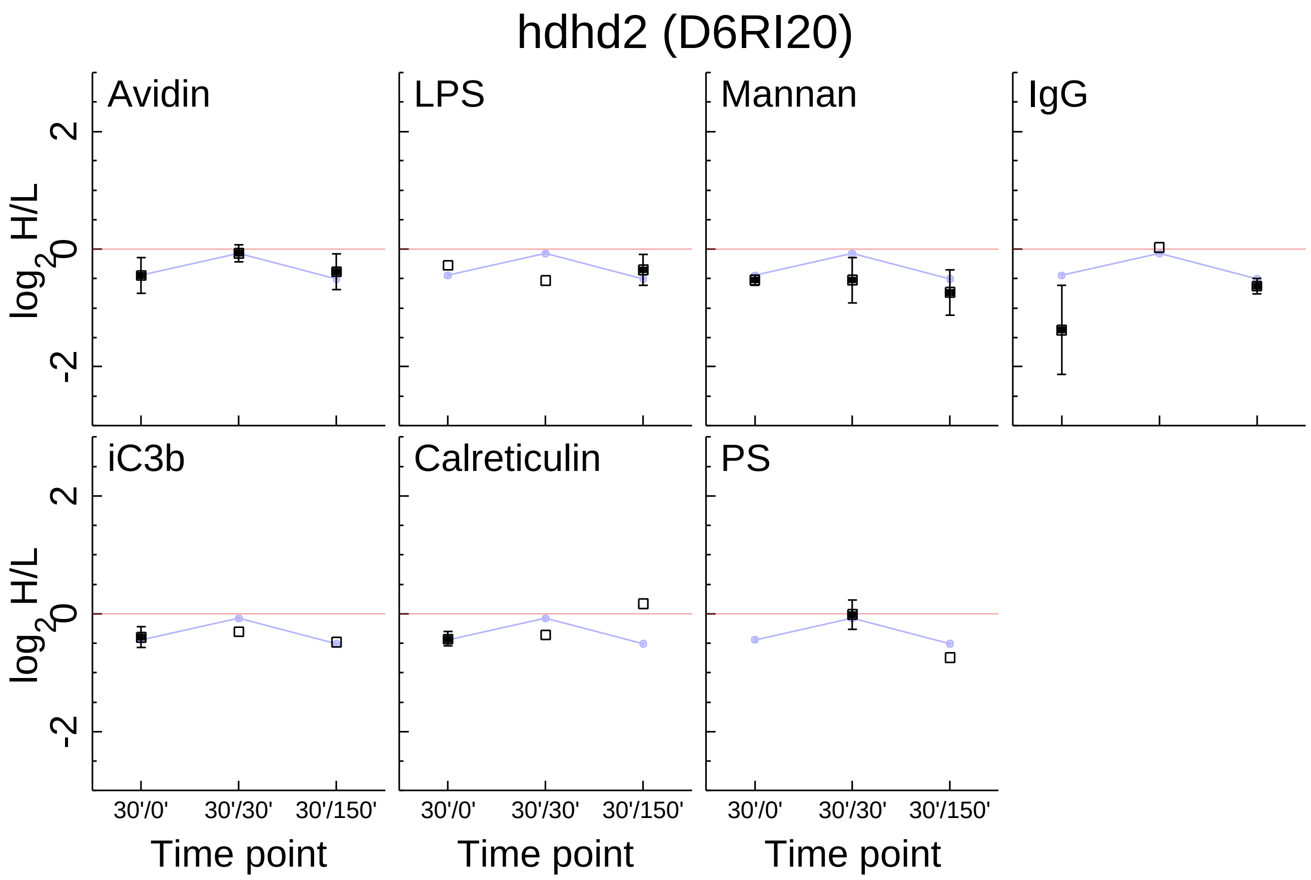

hdhd2 (D6RI20)
Avidin
LPS
Mannan
IgG
iC3b
Calreticulin
PS
log2 H/L
2
0
-2
30'/0'
30'/30'
30'/150'
Time point

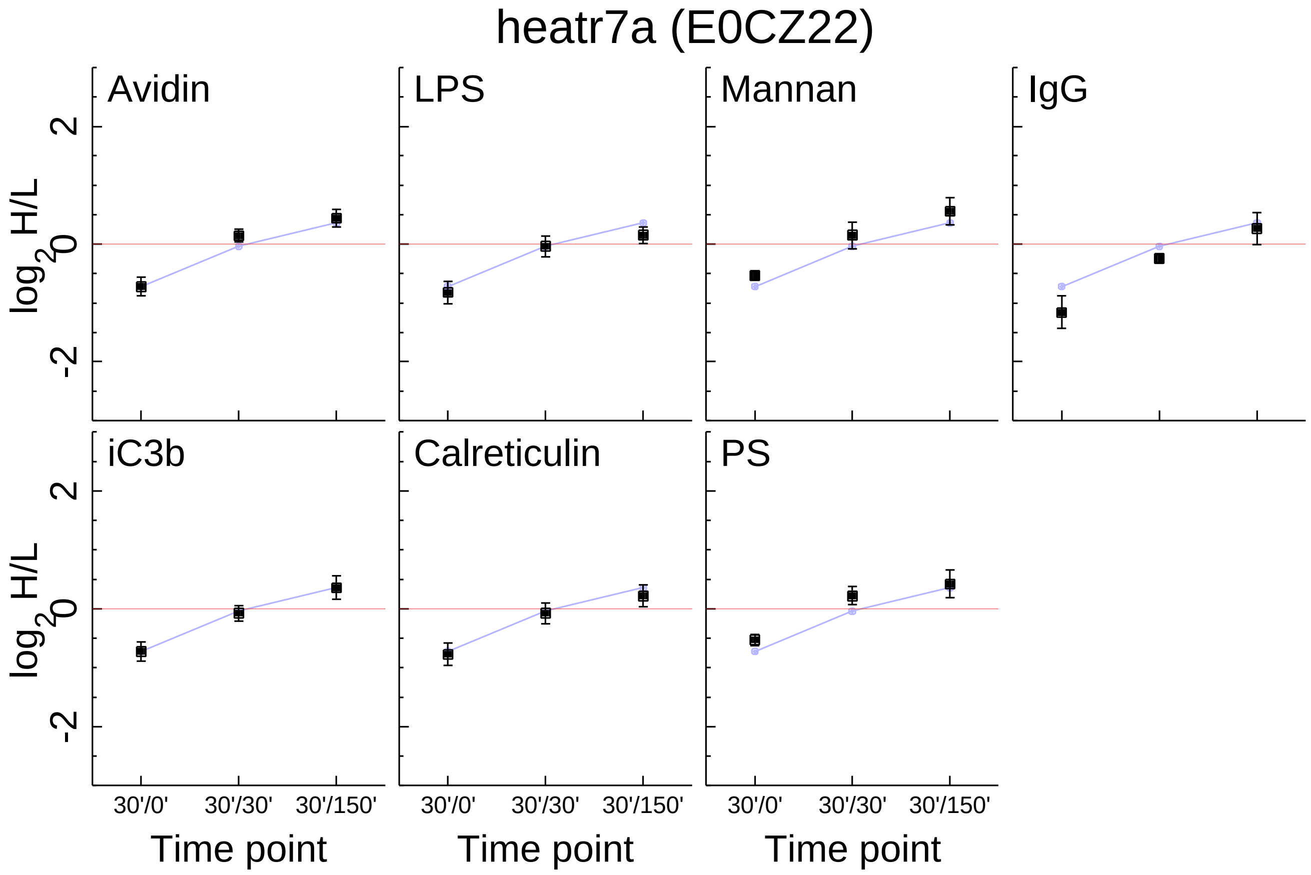
heatr7a (E0CZ22)
Avidin
LPS
Mannan
IgG
iC3b
Calreticulin
PS
log2 H/L
2
0
-2
30'/0'
30'/30'
30'/150'
Time point

hexa (P29416)

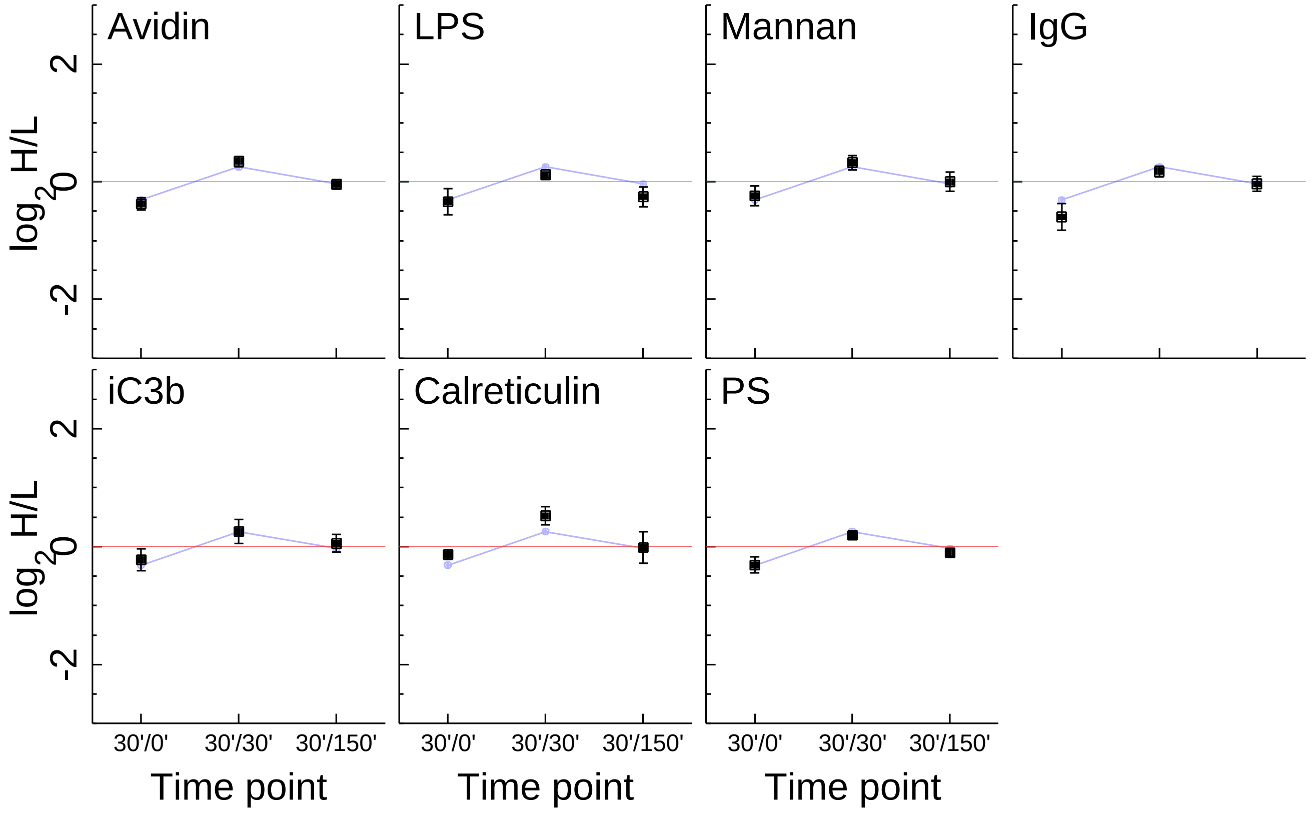

Avidin
LPS
Mannan
IgG
iC3b
Calreticulin
PS
log2 H/L
2
0
-2
30'/0'
30'/30'
30'/150'
Time point

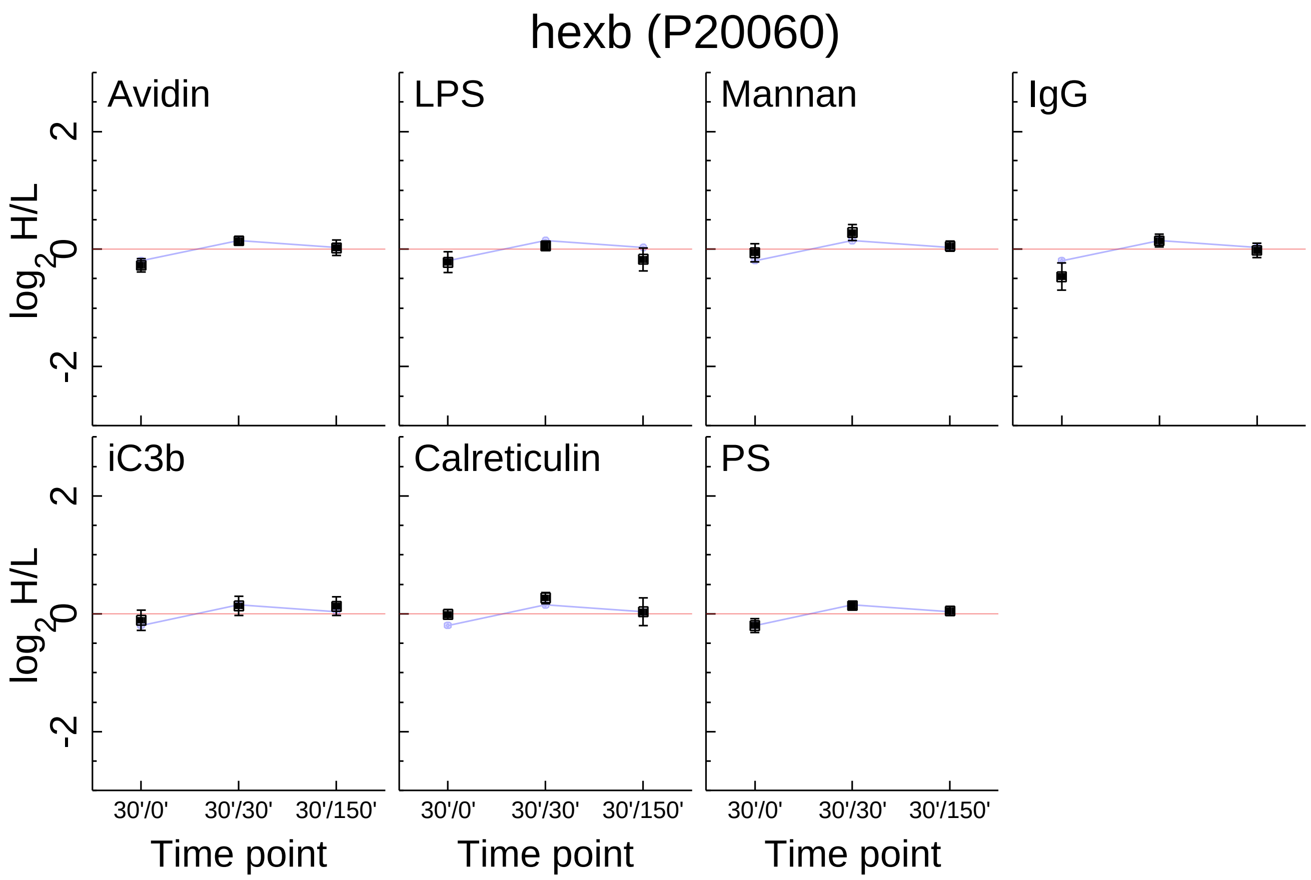

hexb (P20060)
Avidin
LPS
Mannan
IgG
iC3b
Calreticulin
PS
log2 H/L
2
0
-2
30'/0'
30'/30'
30'/150'
Time point

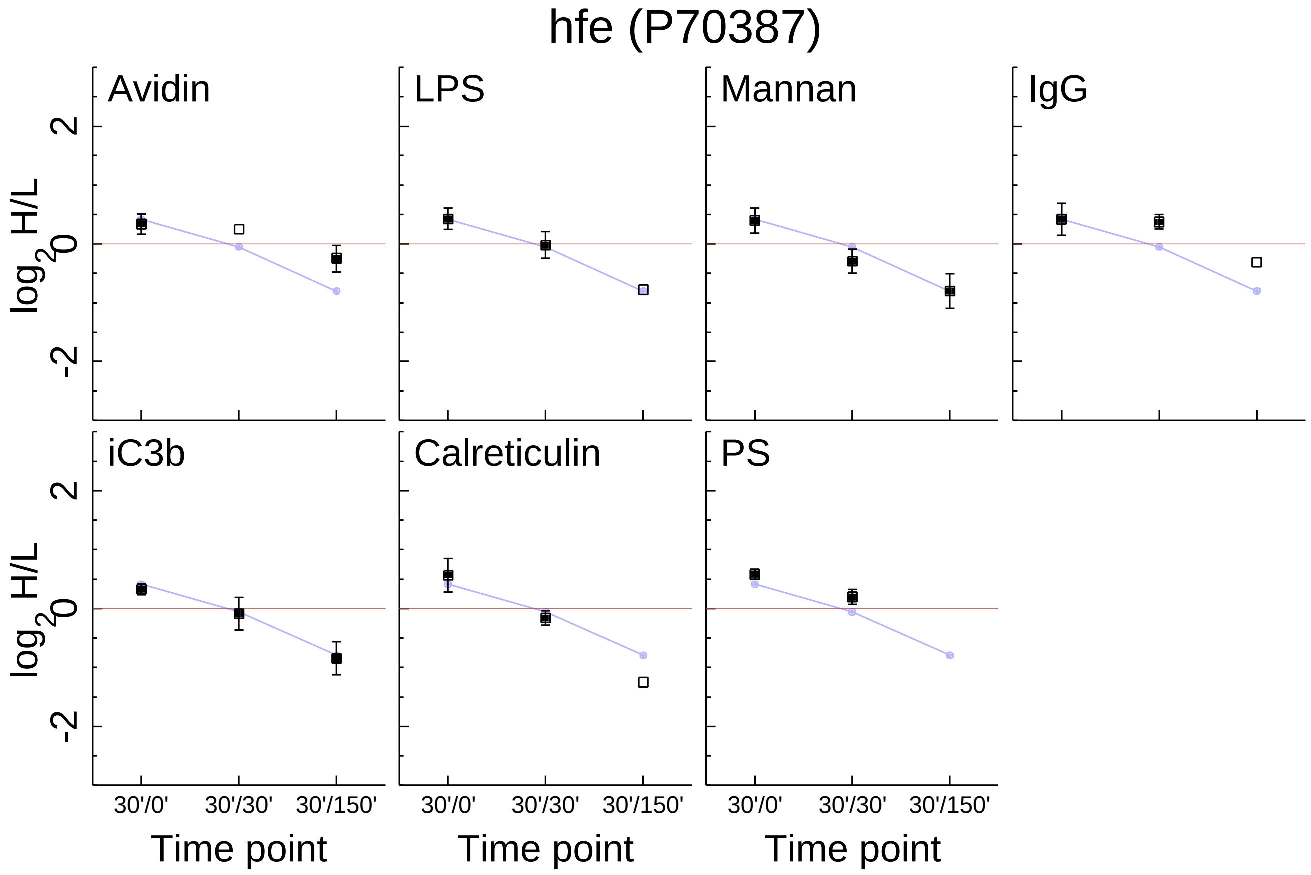
hfe (P70387)
Avidin
LPS
Mannan
IgG
iC3b
Calreticulin
PS
log2 H/L
2
0
-2
30'/0'
30'/30'
30'/150'
Time point

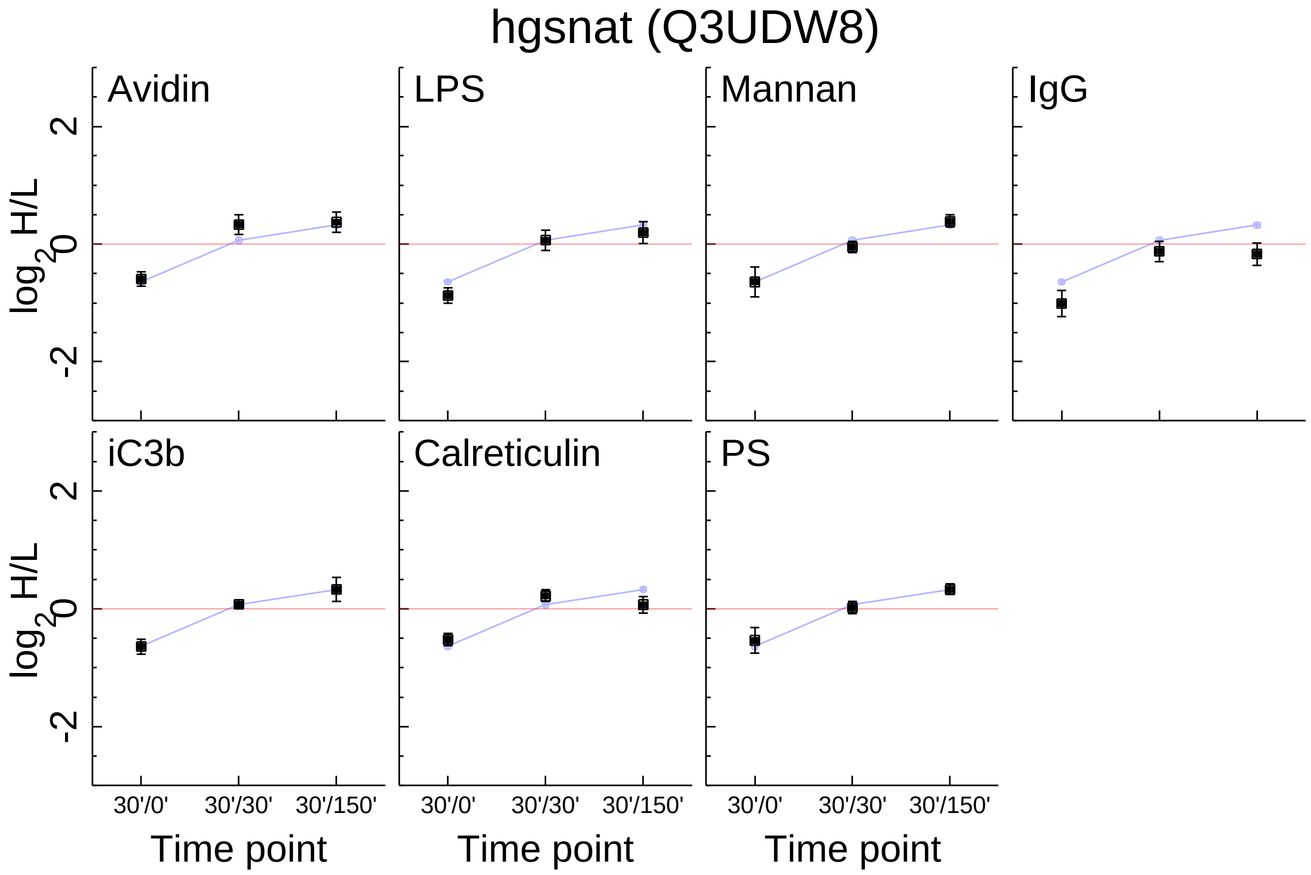
hgsnat (Q3UDW8)
Avidin
LPS
Mannan
IgG
iC3b
Calreticulin
PS
log2 H/L
2
0
-2
30'/0'
30'/30'
30'/150'
Time point

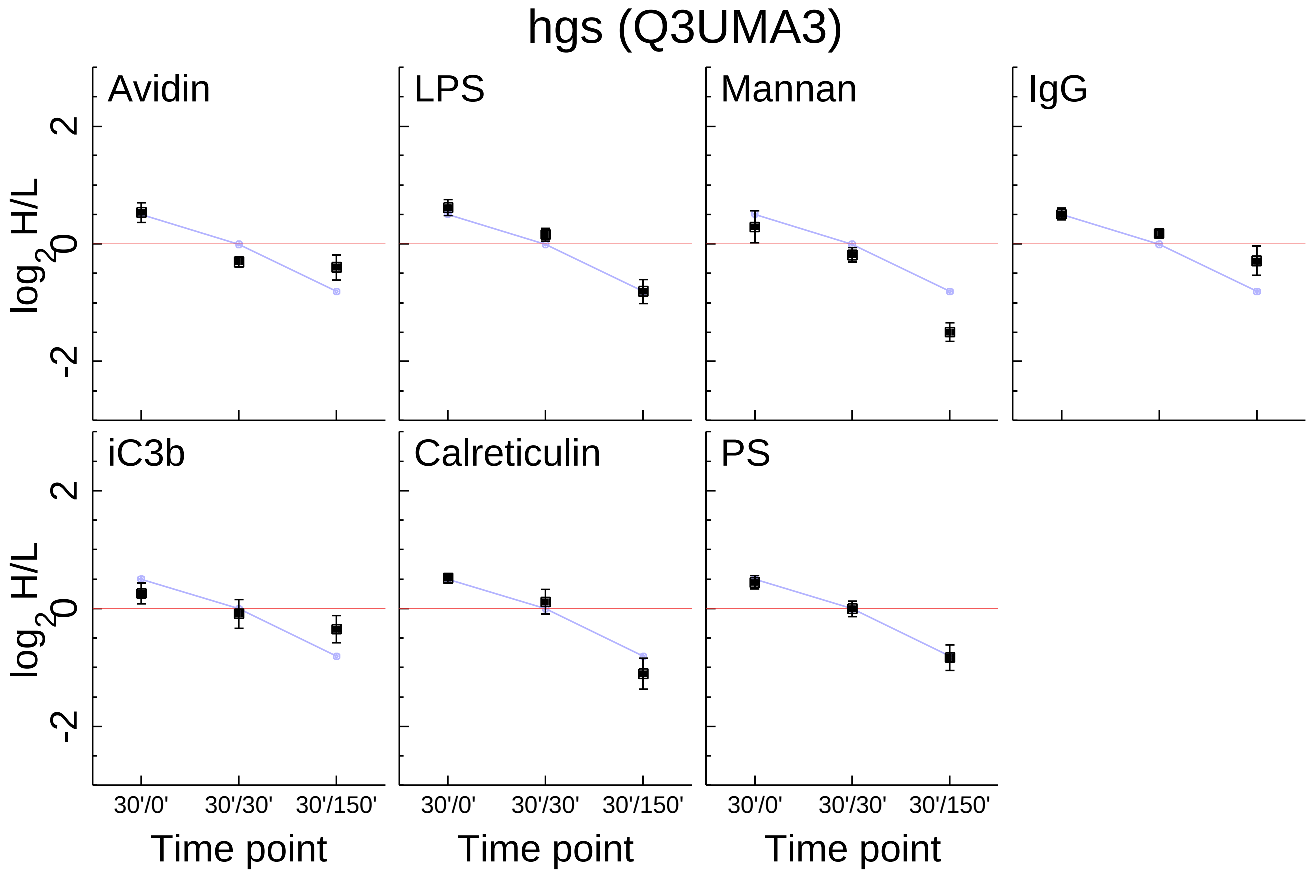

hgs (Q3UMA3)
Avidin
LPS
Mannan
IgG
iC3b
Calreticulin
PS
log2 H/L
2
0
-2
30'/0'
30'/30'
30'/150'
Time point

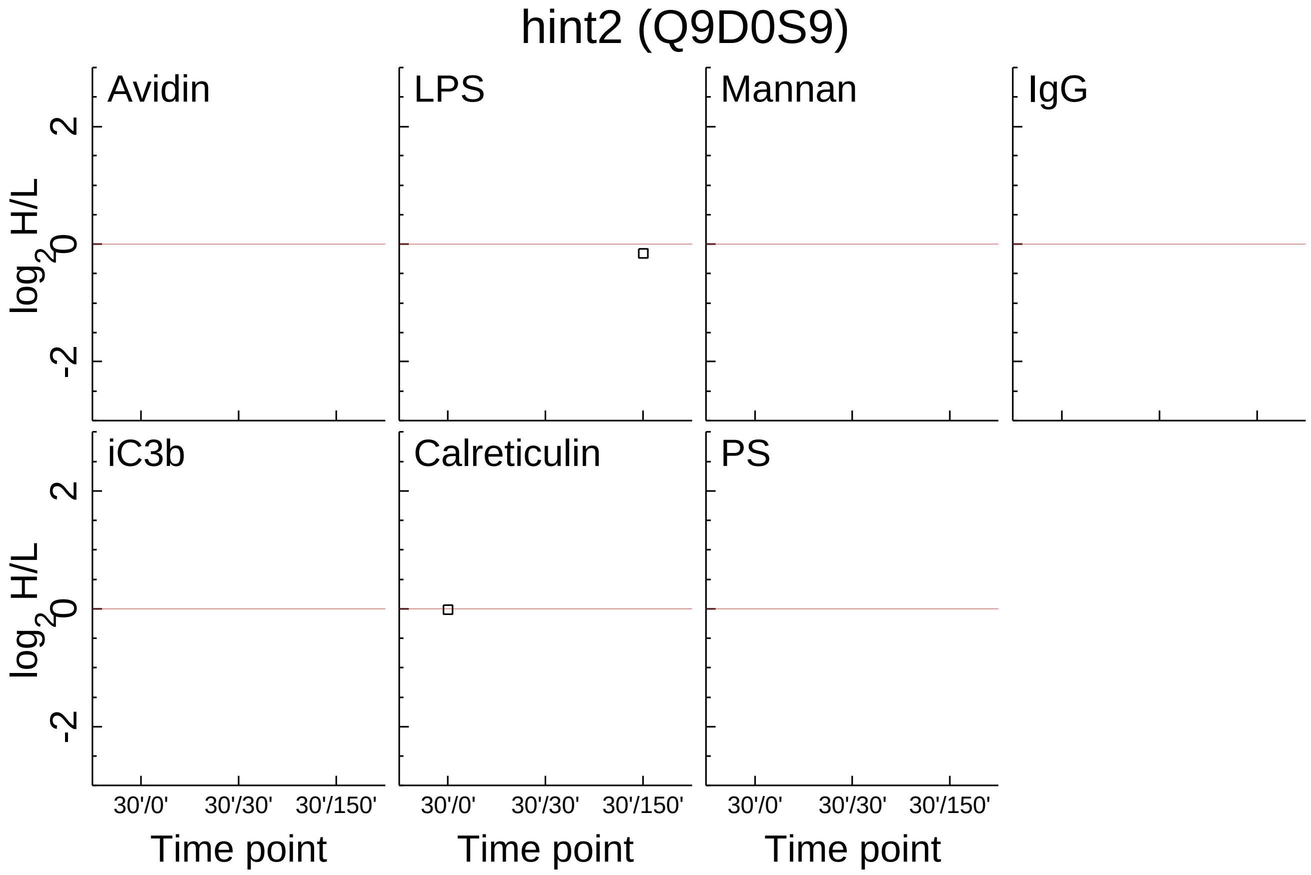

hint2 (Q9D0S9)
Avidin
LPS
Mannan
IgG
iC3b
Calreticulin
PS
log2 H/L
2
0
-2
30'/0'
30'/30'
30'/150'
Time point

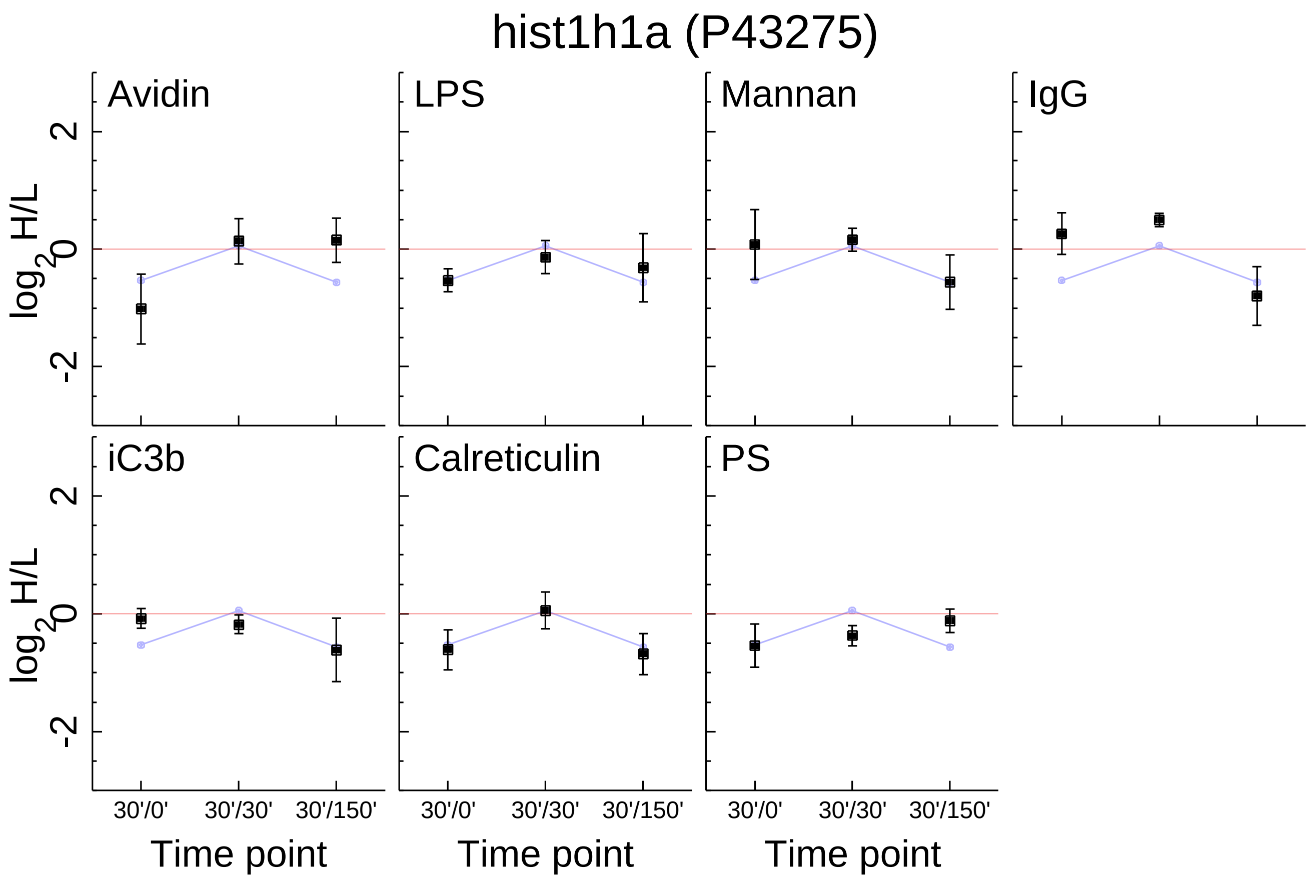
hist1h1a (P43275)
Avidin
LPS
Mannan
IgG
iC3b
Calreticulin
PS
log2 H/L
2
0
-2
30'/0'
30'/30'
30'/150'
Time point

hist1h1b (P43276)

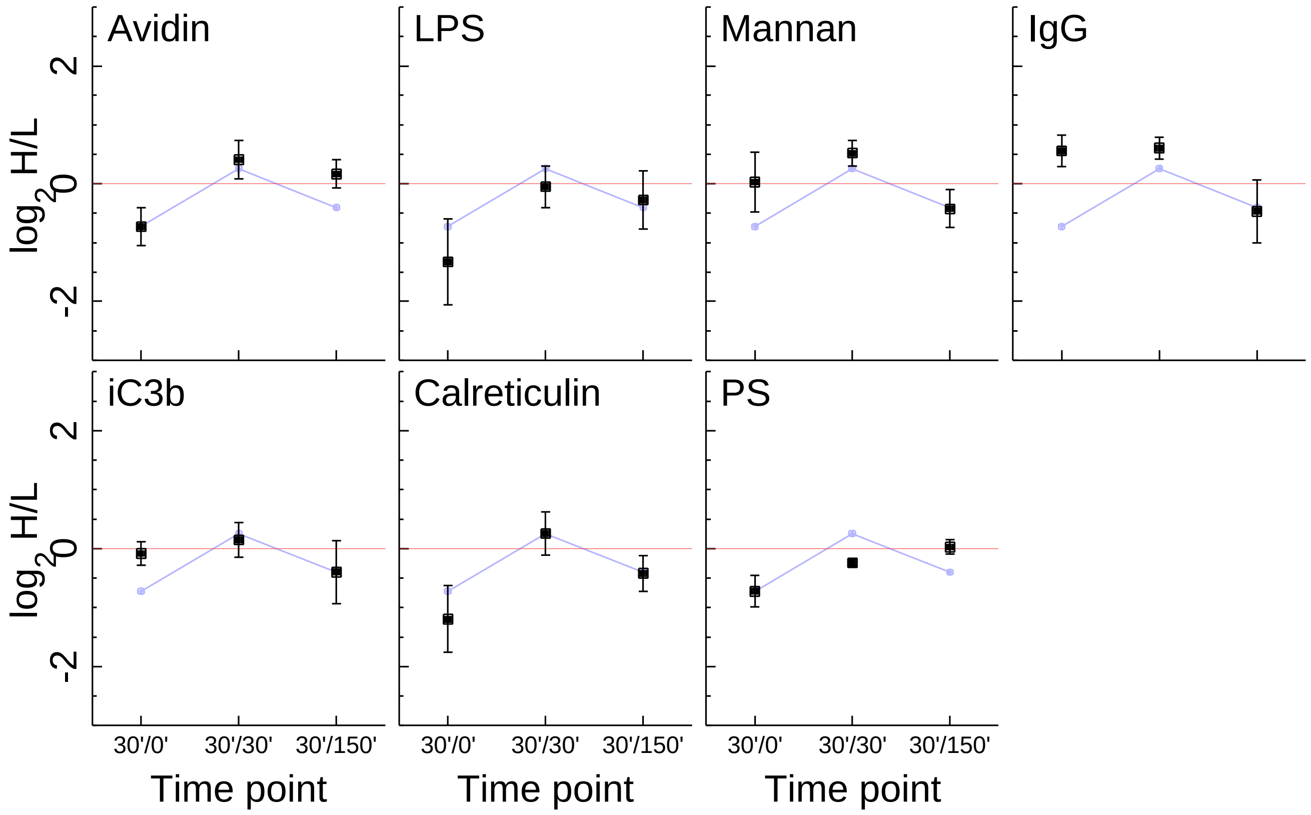

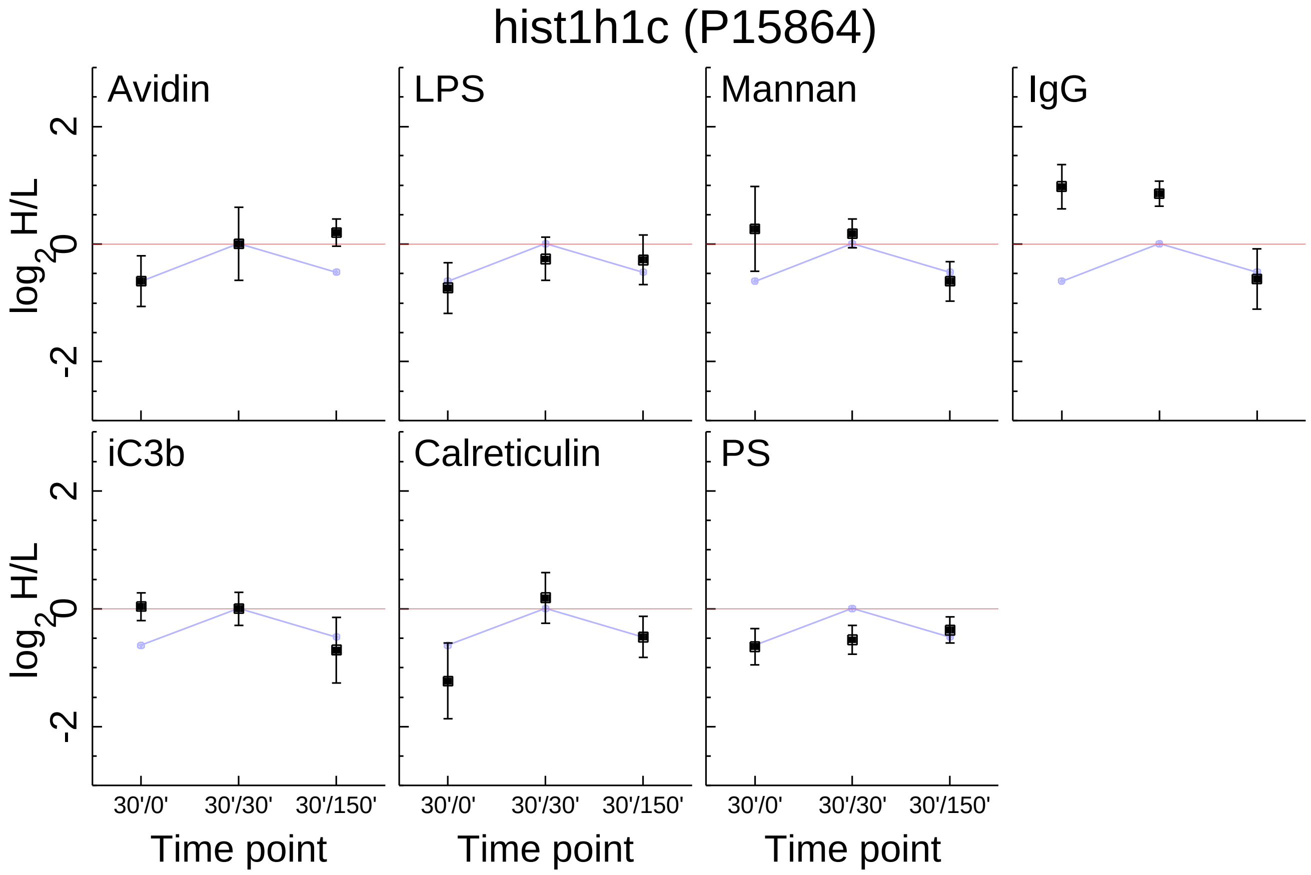
hist1h1c (P15864)
Avidin
LPS
Mannan
IgG
iC3b
Calreticulin
PS
log2 H/L
2
0
-2
30'/0'
30'/30'
30'/150'
Time point

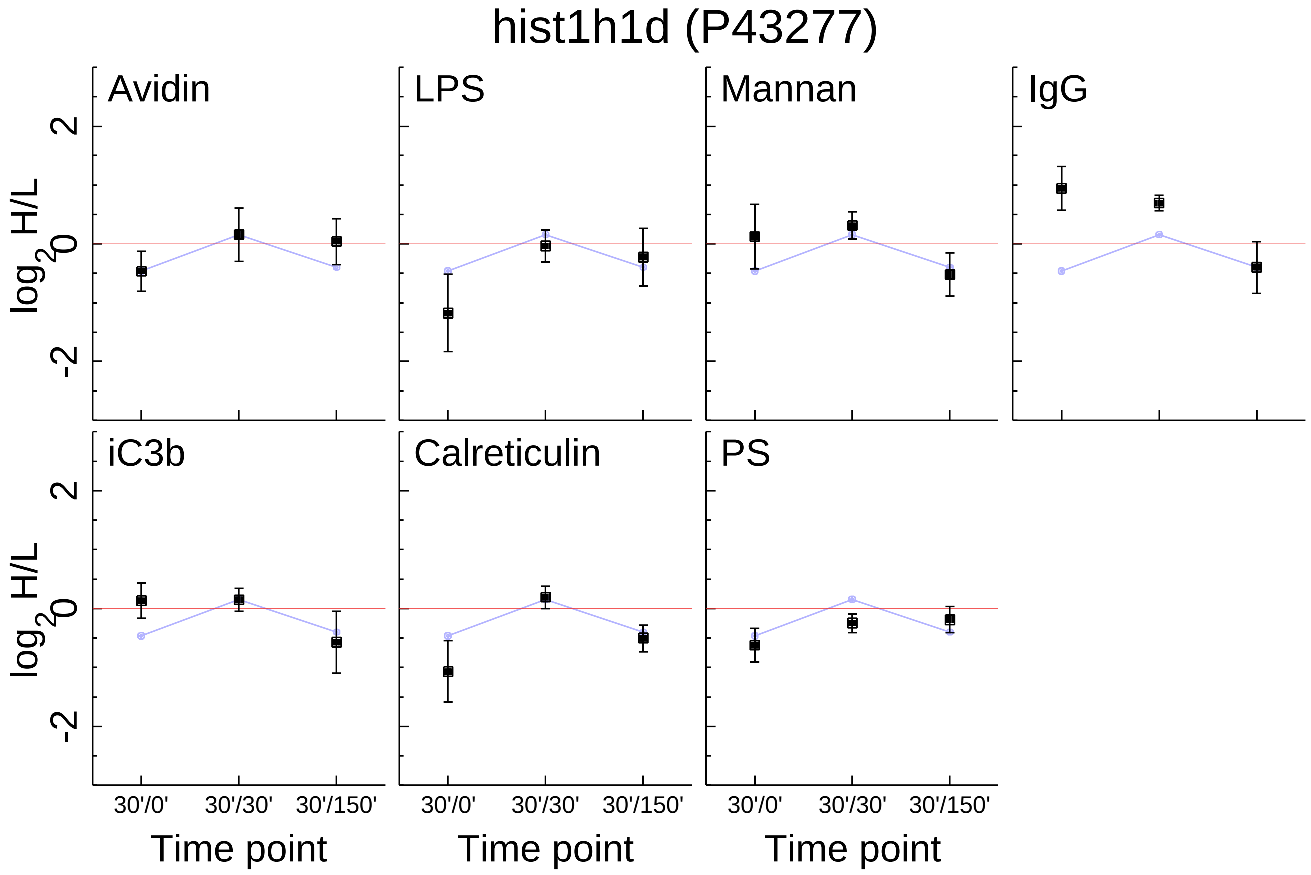

hist1h1d (P43277)
Avidin
LPS
Mannan
IgG
iC3b
Calreticulin
PS
log2 H/L
2
0
-2
30'/0'
30'/30'
30'/150'
Time point

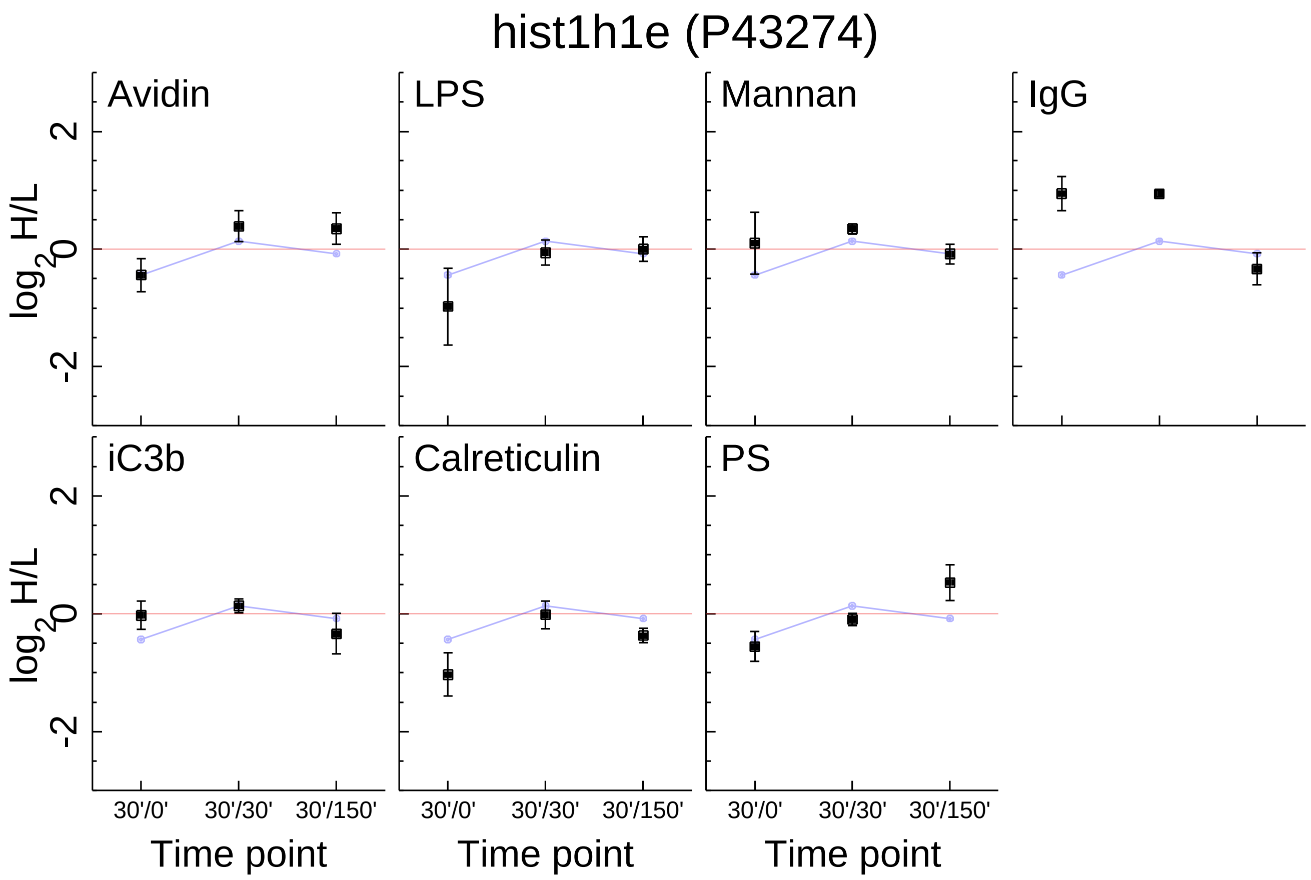
hist1h1e (P43274)
Avidin
LPS
Mannan
IgG
iC3b
Calreticulin
PS
log2 H/L
2
0
-2
30'/0'
30'/30'
30'/150'
Time point

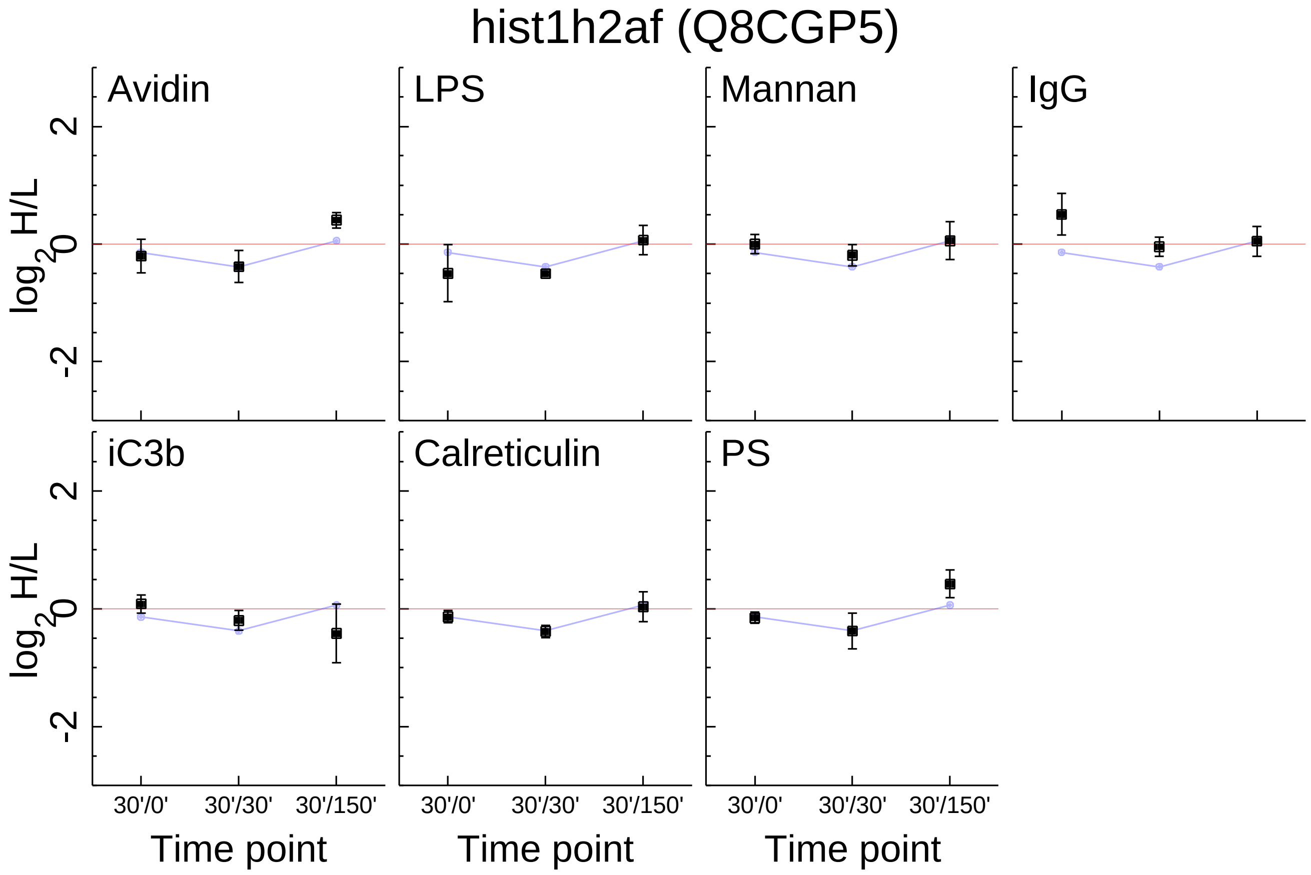

hist1h2af (Q8CGP5)
Avidin
LPS
Mannan
IgG
iC3b
Calreticulin
PS
log2 H/L
2
0
-2
30'/0'
30'/30'
30'/150'
Time point

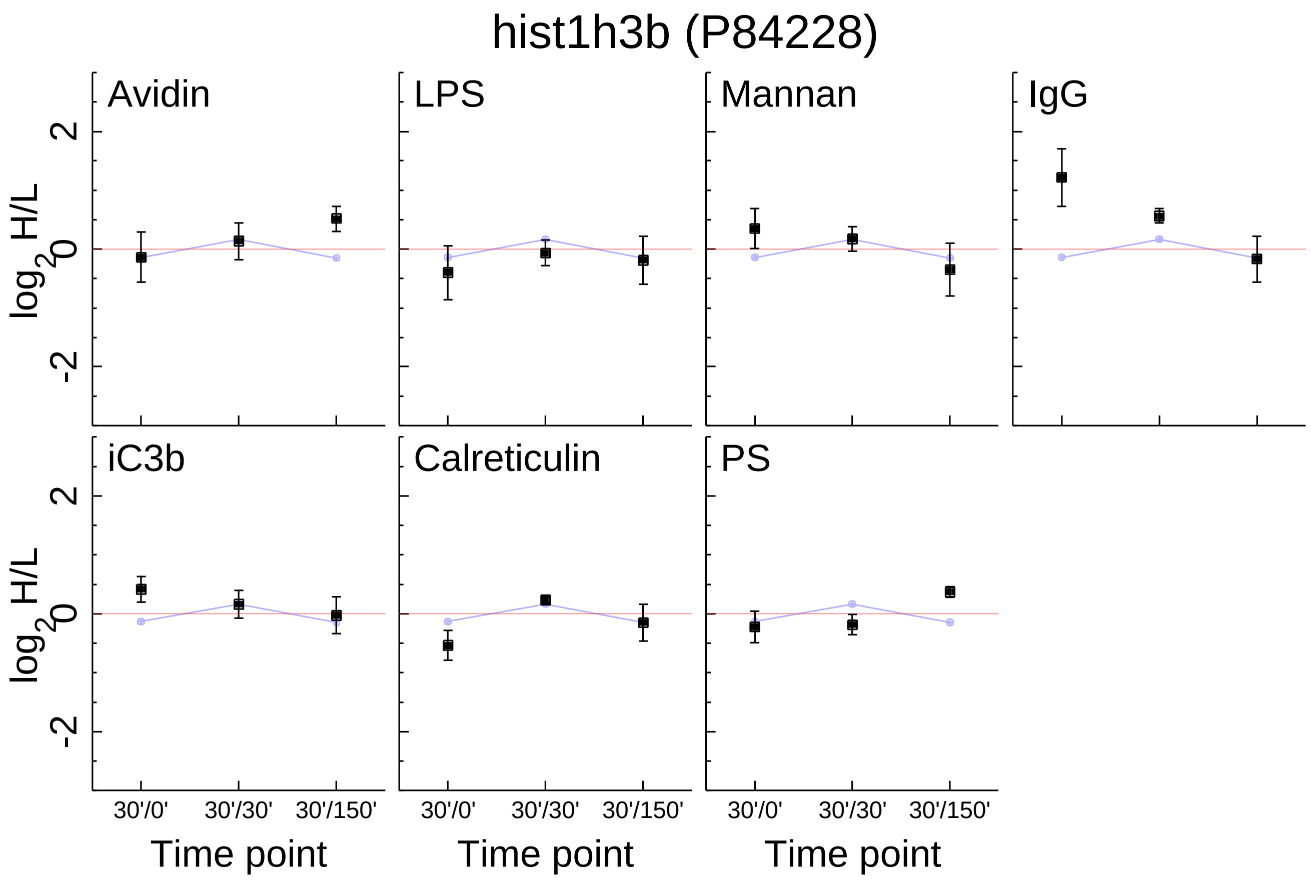
hist1h3b (P84228)
Avidin
LPS
Mannan
IgG
iC3b
Calreticulin
PS
log2 H/L
2
0
-2
30'/0'
30'/30'
30'/150'
Time point

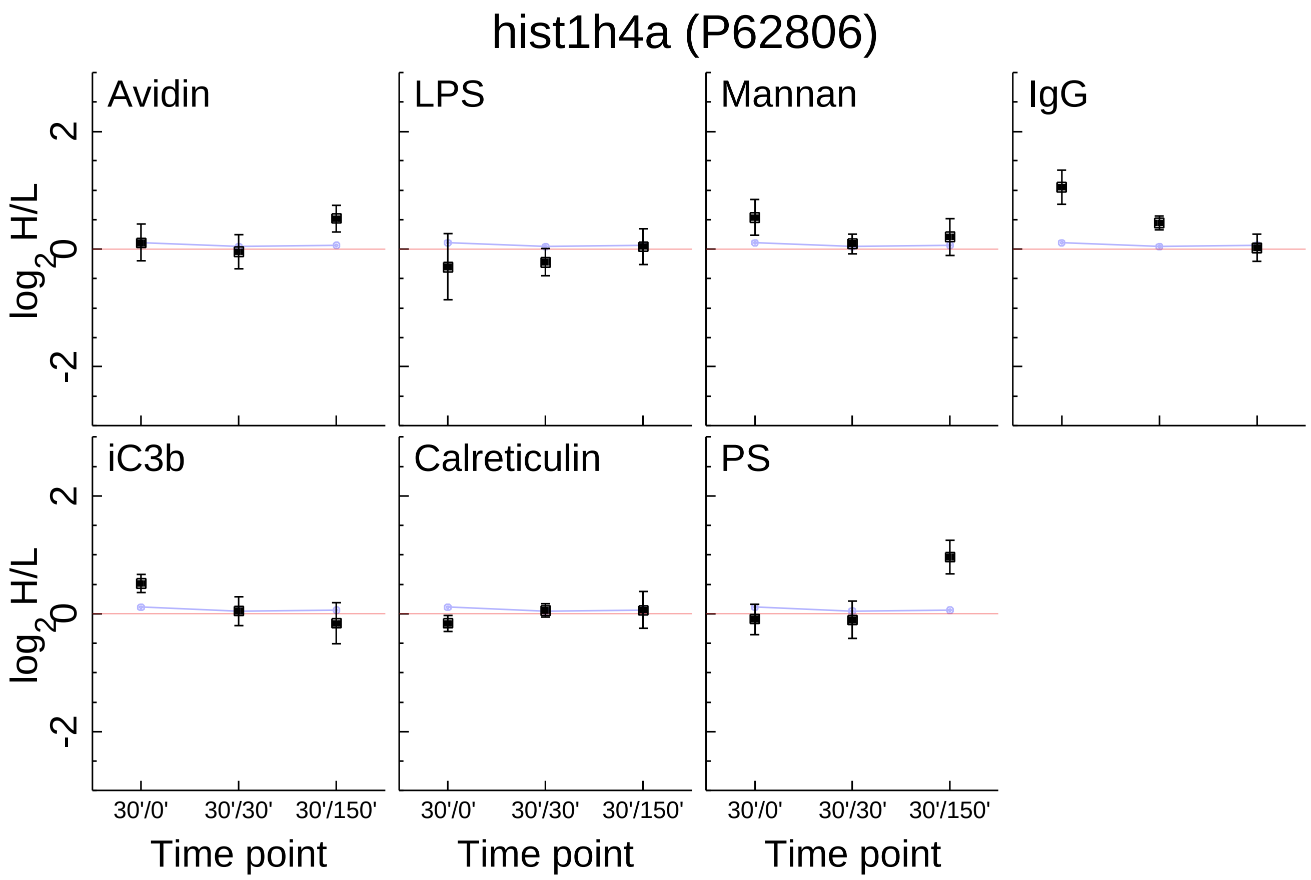
hist1h4a (P62806)
Avidin
LPS
Mannan
IgG
iC3b
Calreticulin
PS
log2 H/L
2
0
-2
30'/0'
30'/30'
30'/150'
Time point

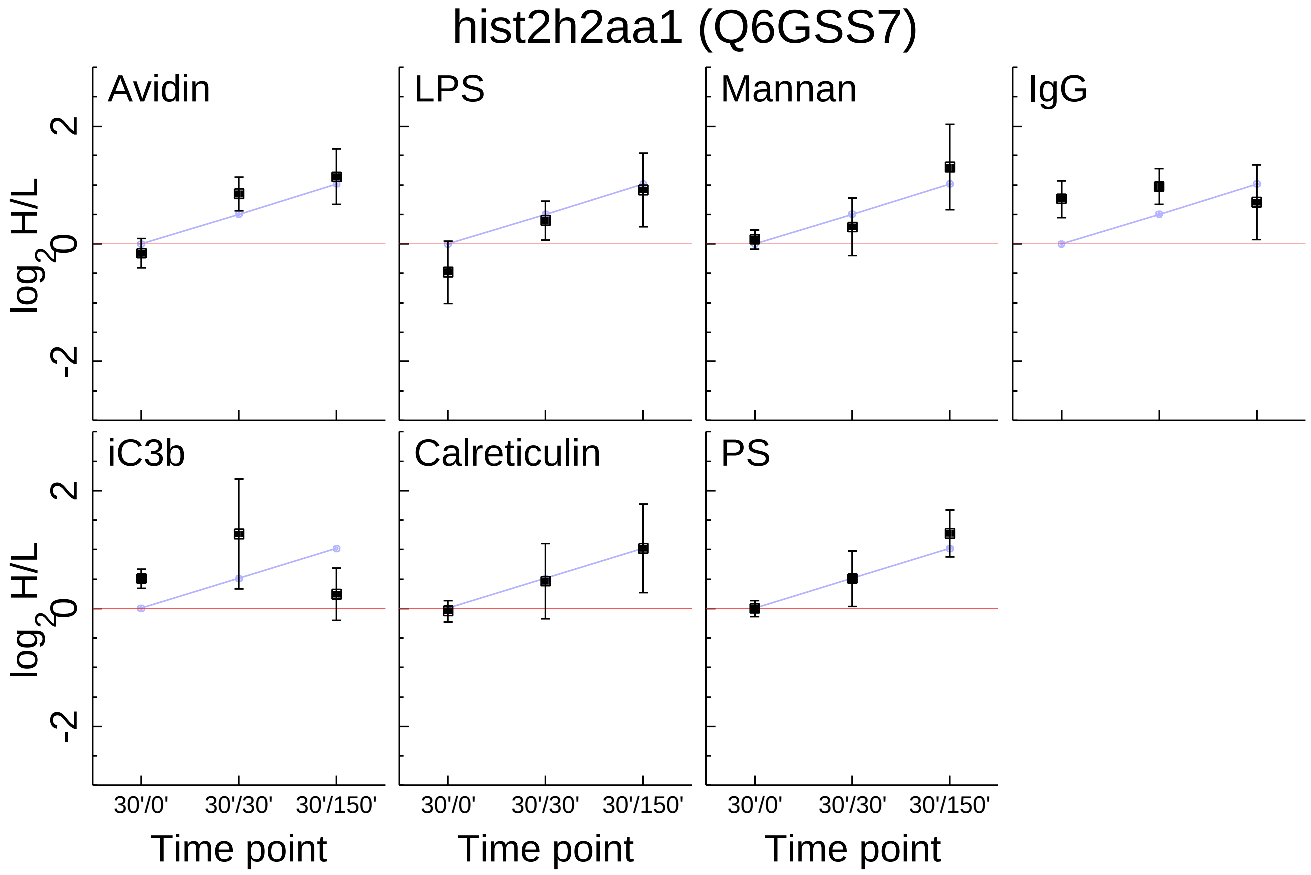
hist2h2aa1 (Q6GSS7)
Avidin
LPS
Mannan
IgG
iC3b
Calreticulin
PS
log2 H/L
2
0
-2
30'/0'
30'/30'
30'/150'
Time point

hist3h2bb (Q8CGP0)

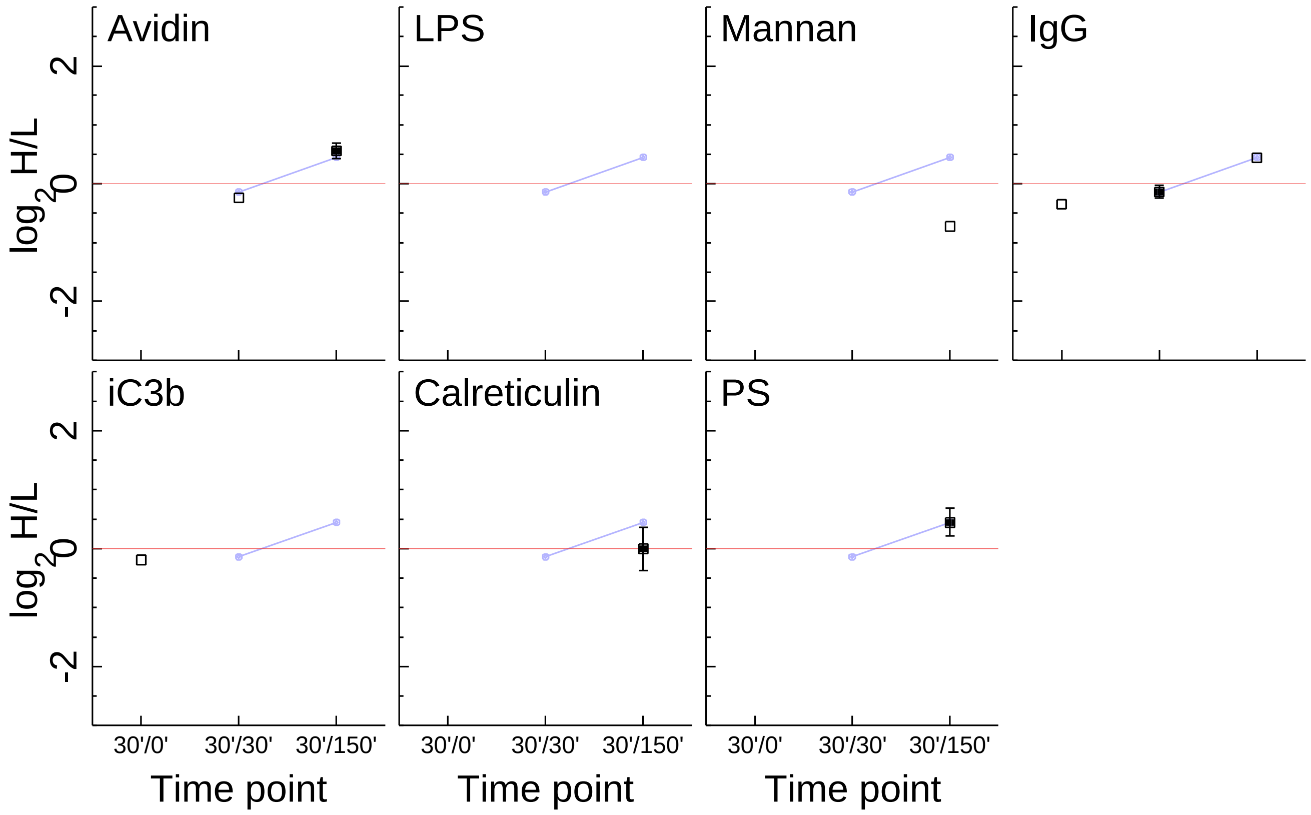

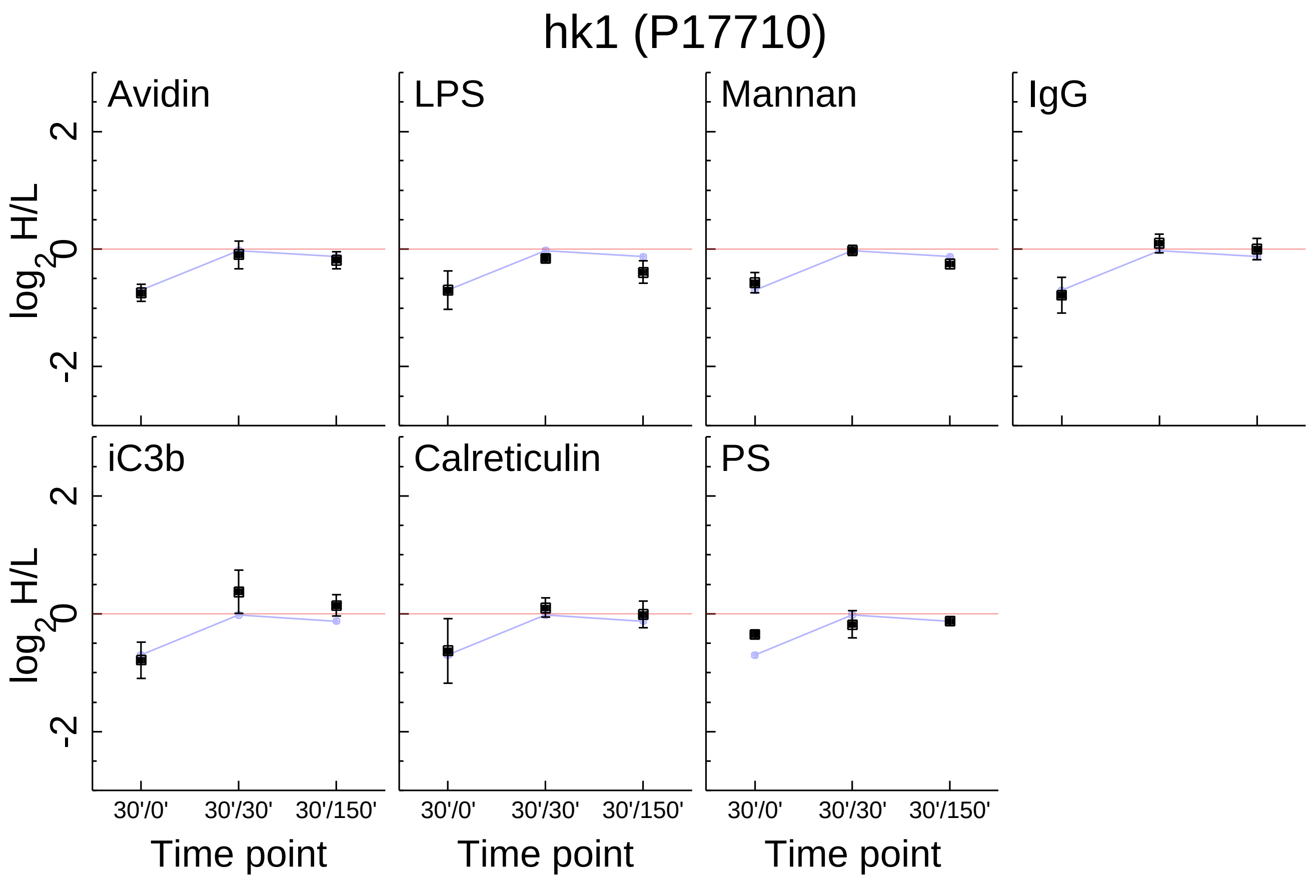
hk1 (P17710)
Avidin
LPS
Mannan
IgG
iC3b
Calreticulin
PS
log2 H/L
2
0
-2
30'/0'
30'/30'
30'/150'
Time point

hk2 (O08528)

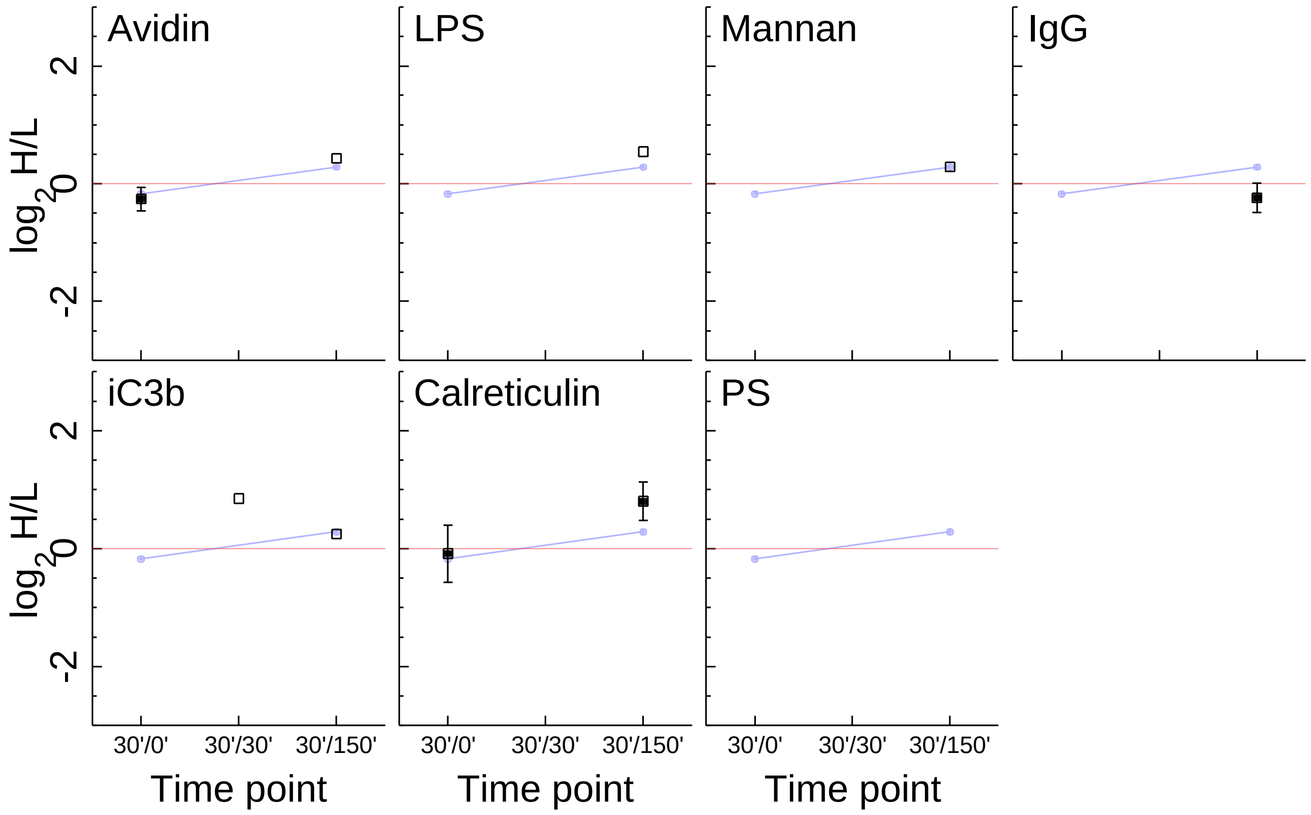

Avidin
LPS
Mannan
IgG
iC3b
Calreticulin
PS
log2 H/L
2
0
-2
30'/0'
30'/30'
30'/150'
Time point

hm13 (Q9D8V0)

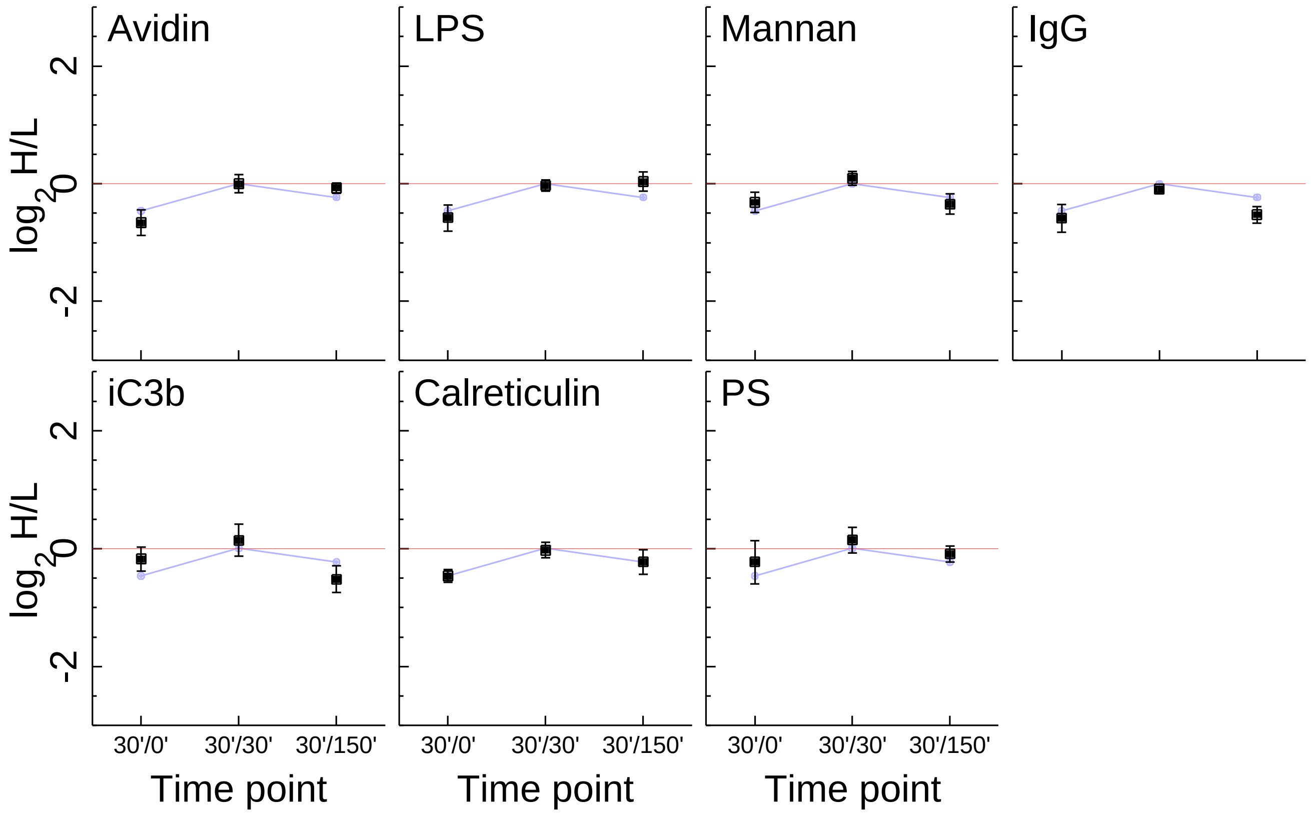

Avidin
LPS
Mannan
IgG
iC3b
Calreticulin
PS
2
0
-2
log2 H/L
30'/0'
30'/30'
30'/150'
Time point

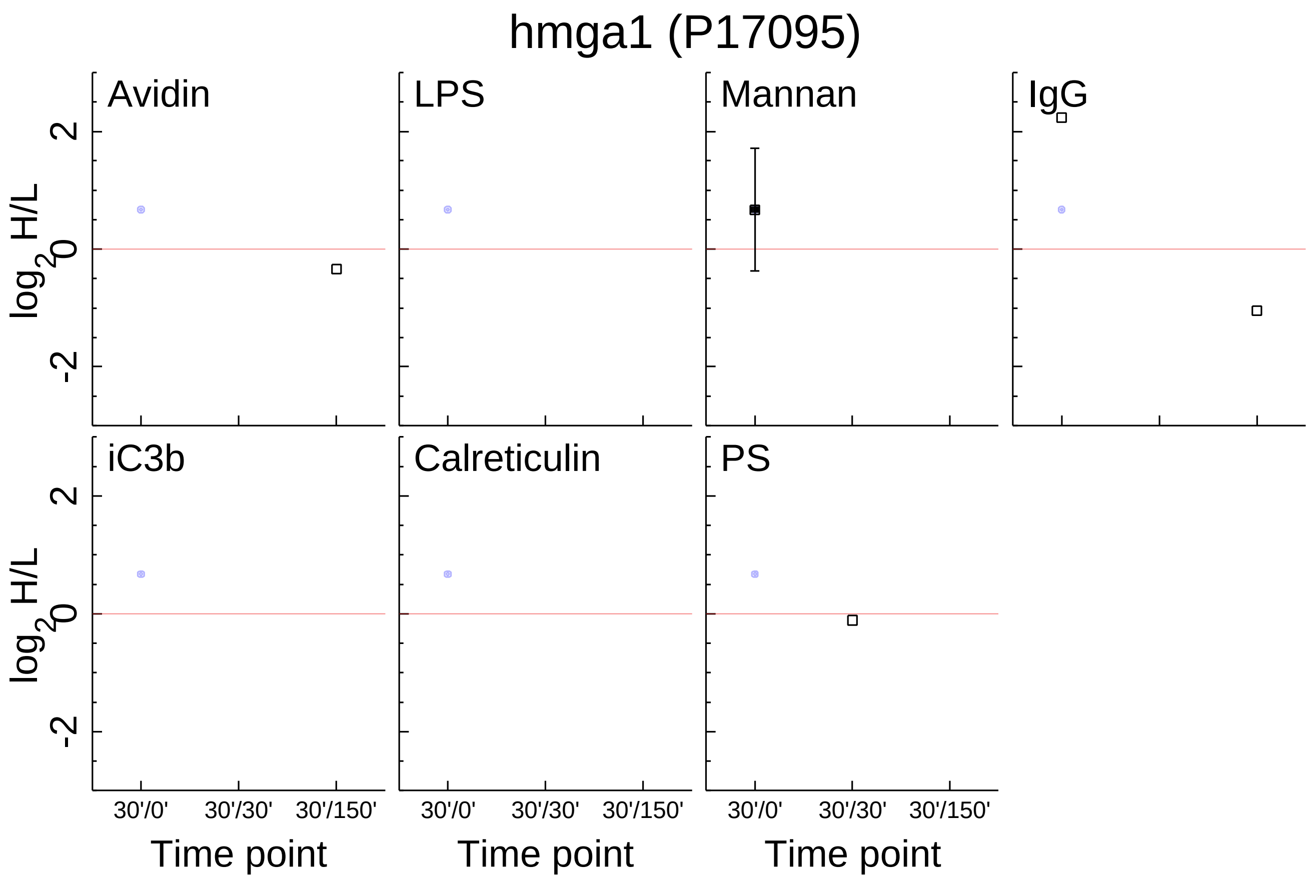
hmga1 (P17095)
Avidin
LPS
Mannan
IgG
iC3b
Calreticulin
PS
log2 H/L
2
0
-2
30'/0'
30'/30'
30'/150'
Time point

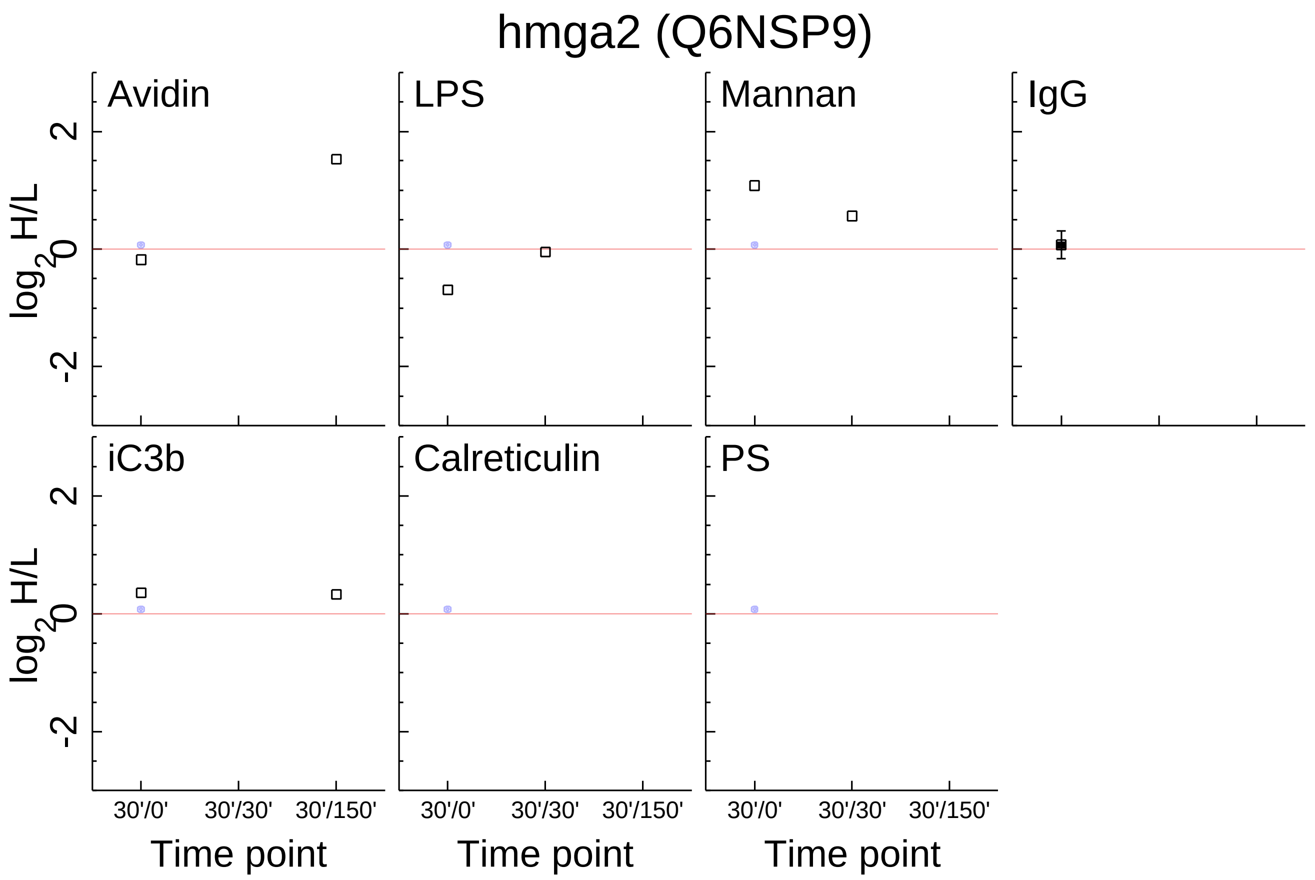
hmga2 (Q6NSP9)
Avidin
LPS
Mannan
IgG
iC3b
Calreticulin
PS
log2 H/L
2
0
-2
30'/0'
30'/30'
30'/150'
Time point

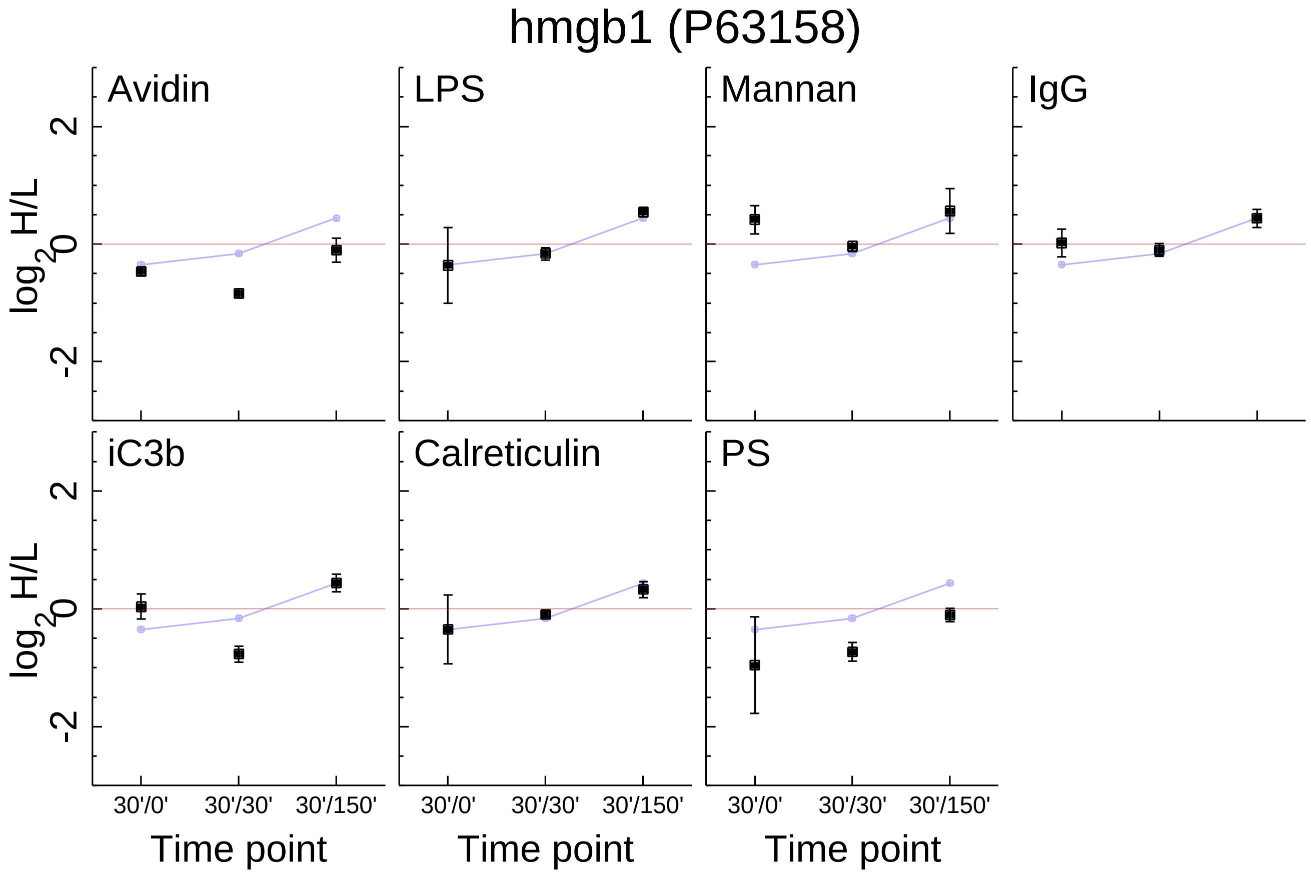
hmgb1 (P63158)
Avidin
LPS
Mannan
IgG
iC3b
Calreticulin
PS
log2 H/L
2
0
-2
30'/0'
30'/30'
30'/150'
Time point

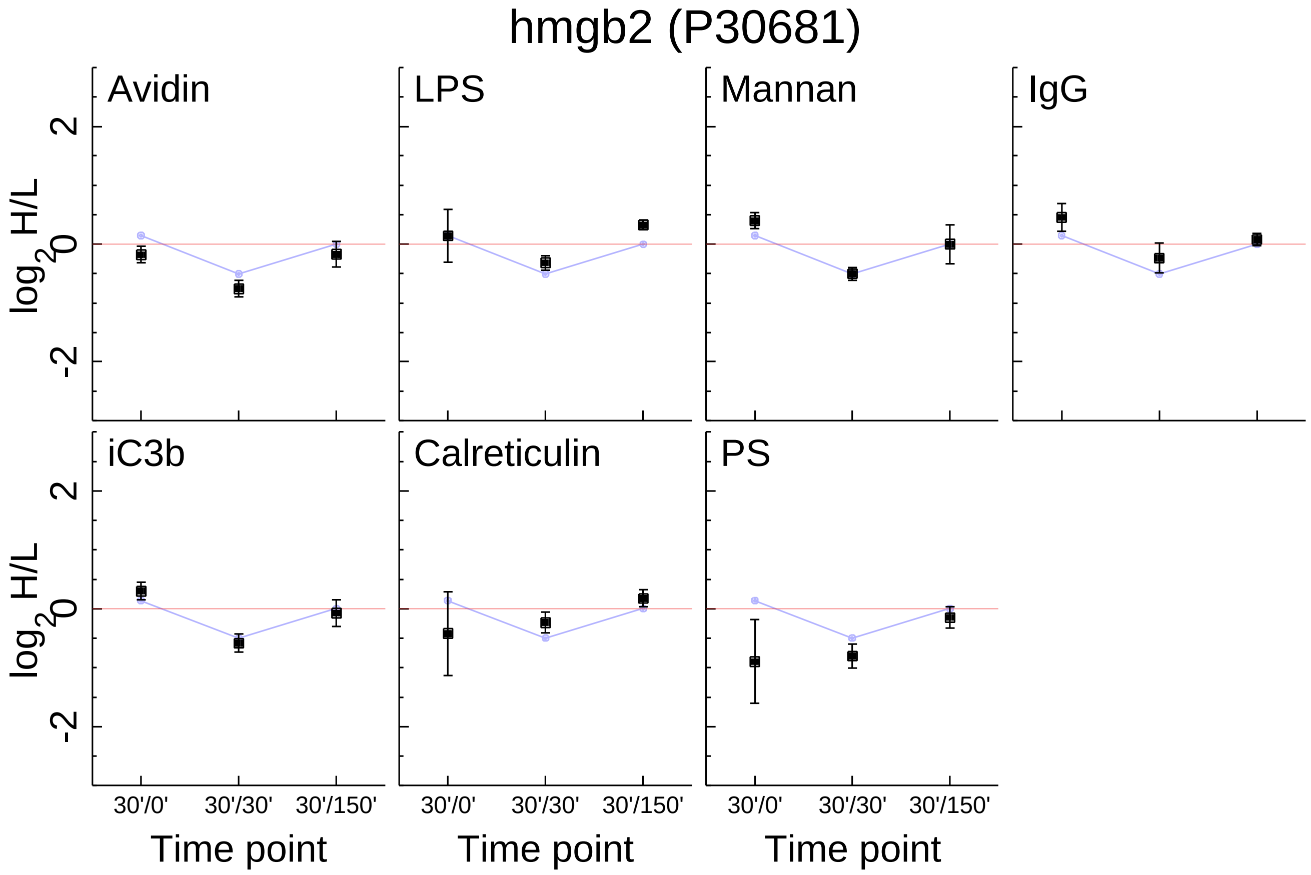

hmgb2 (P30681)
Avidin
LPS
Mannan
IgG
iC3b
Calreticulin
PS
log2 H/L
2
0
-2
30'/0'
30'/30'
30'/150'
Time point

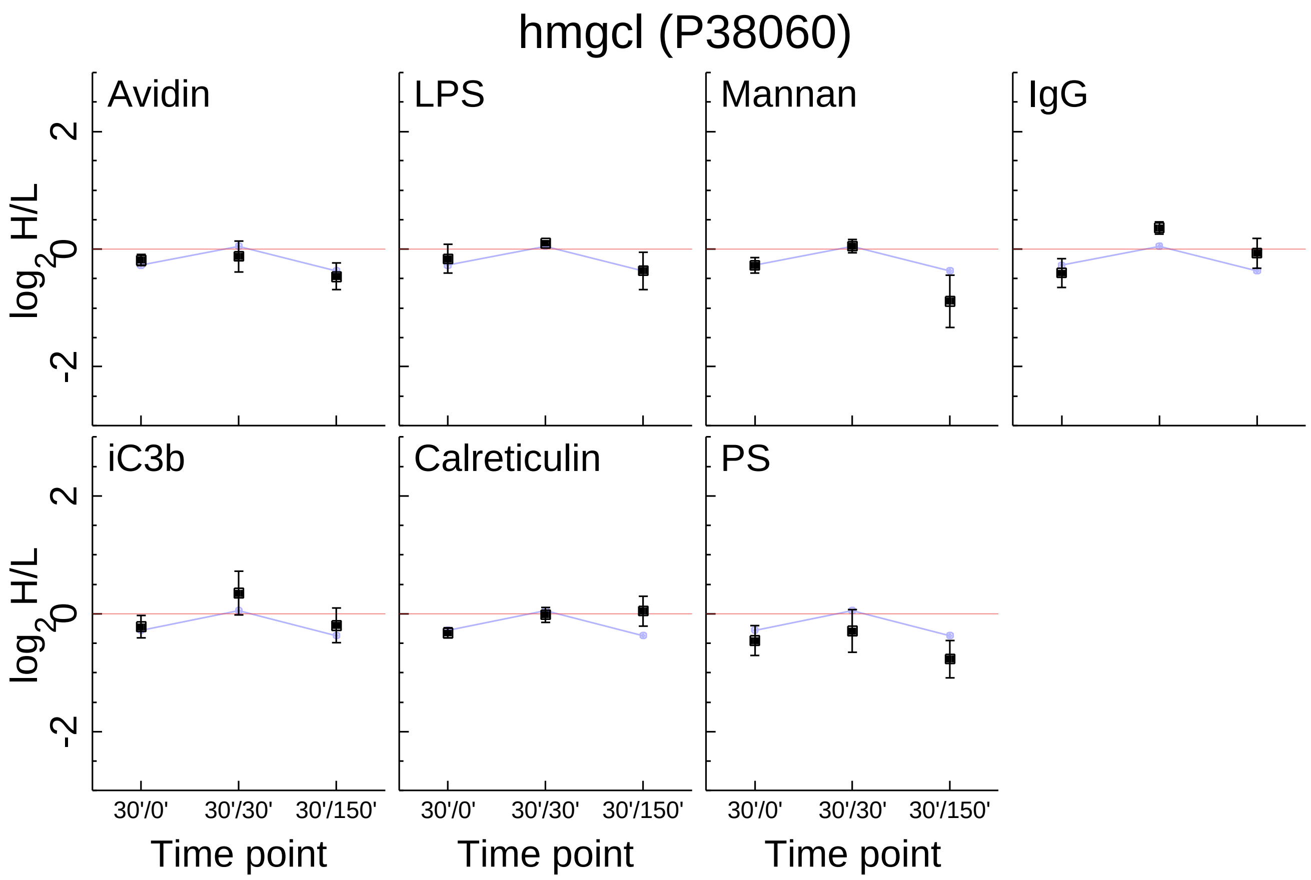

hmgcl (P38060)
Avidin
LPS
Mannan
IgG
iC3b
Calreticulin
PS
log2 H/L
2
0
-2
30'/0'
30'/30'
30'/150'
Time point

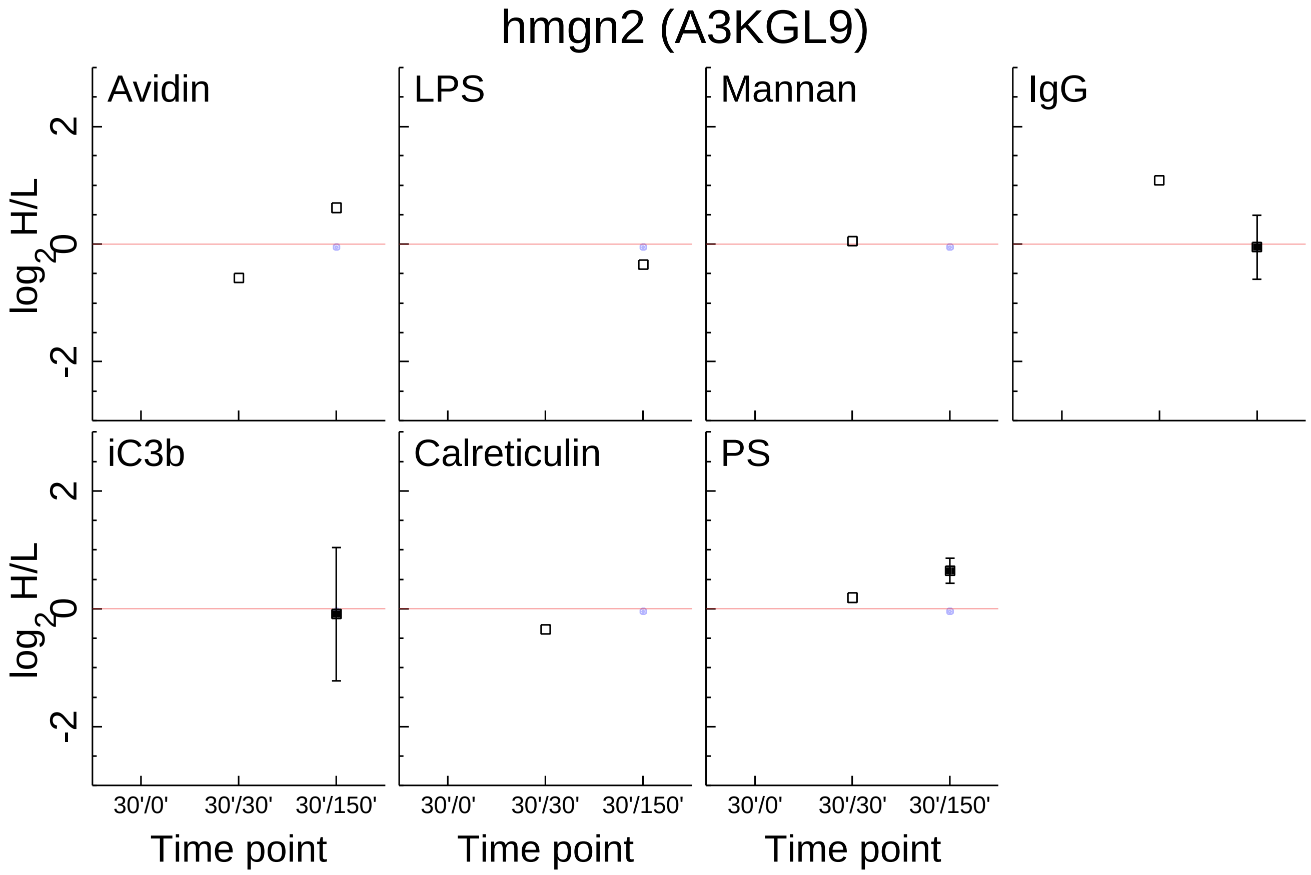
hmgn2 (A3KGL9)
Avidin
LPS
Mannan
IgG
iC3b
Calreticulin
PS
log2 H/L
2
0
-2
30'/0'
30'/30'
30'/150'
Time point

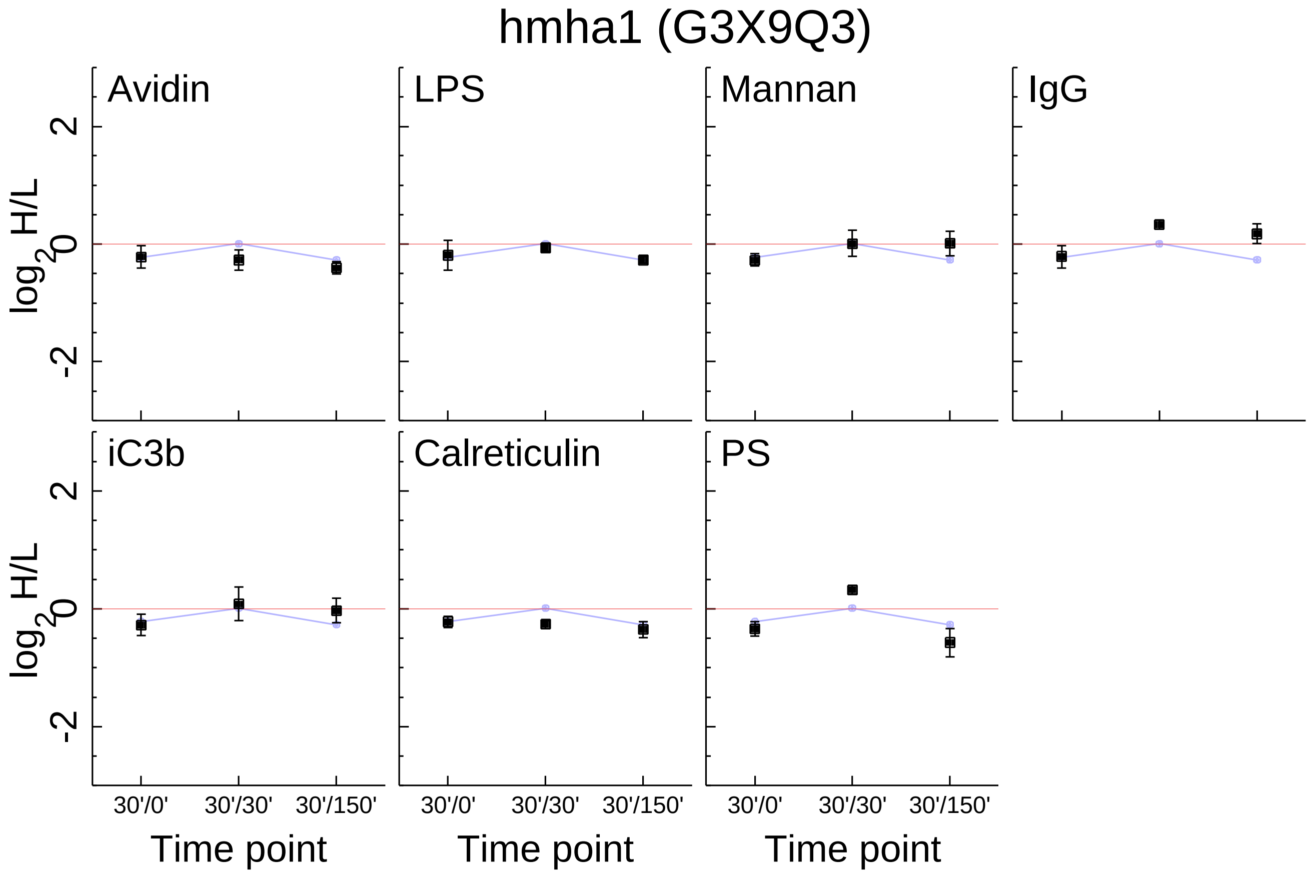
hmha1 (G3X9Q3)
Avidin
LPS
Mannan
IgG
iC3b
Calreticulin
PS
log2 H/L
2
0
-2
30'/0'
30'/30'
30'/150'
Time point

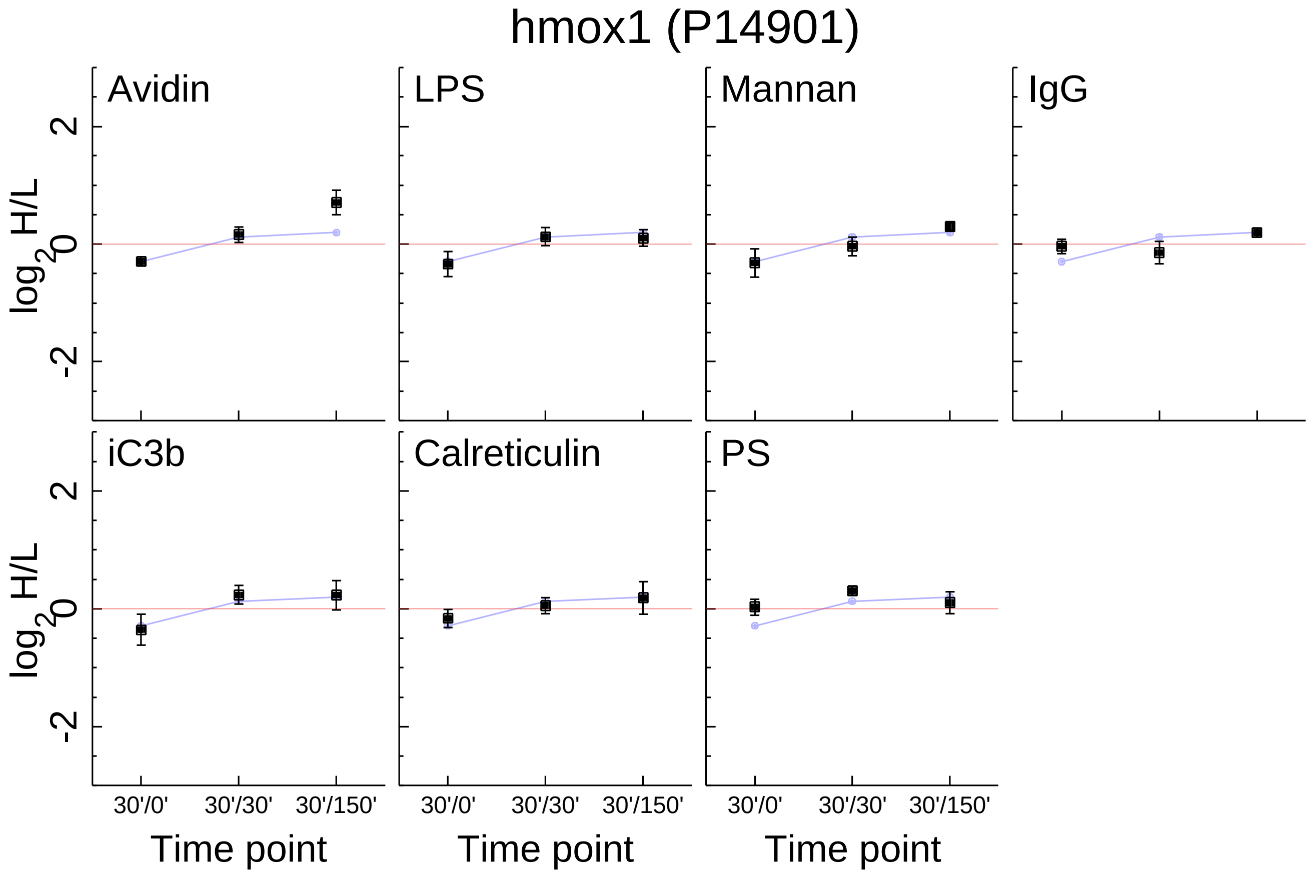

hmox1 (P14901)
Avidin
LPS
Mannan
IgG
iC3b
Calreticulin
PS
log2 H/L
2
0
-2
30'/0'
30'/30'
30'/150'
Time point

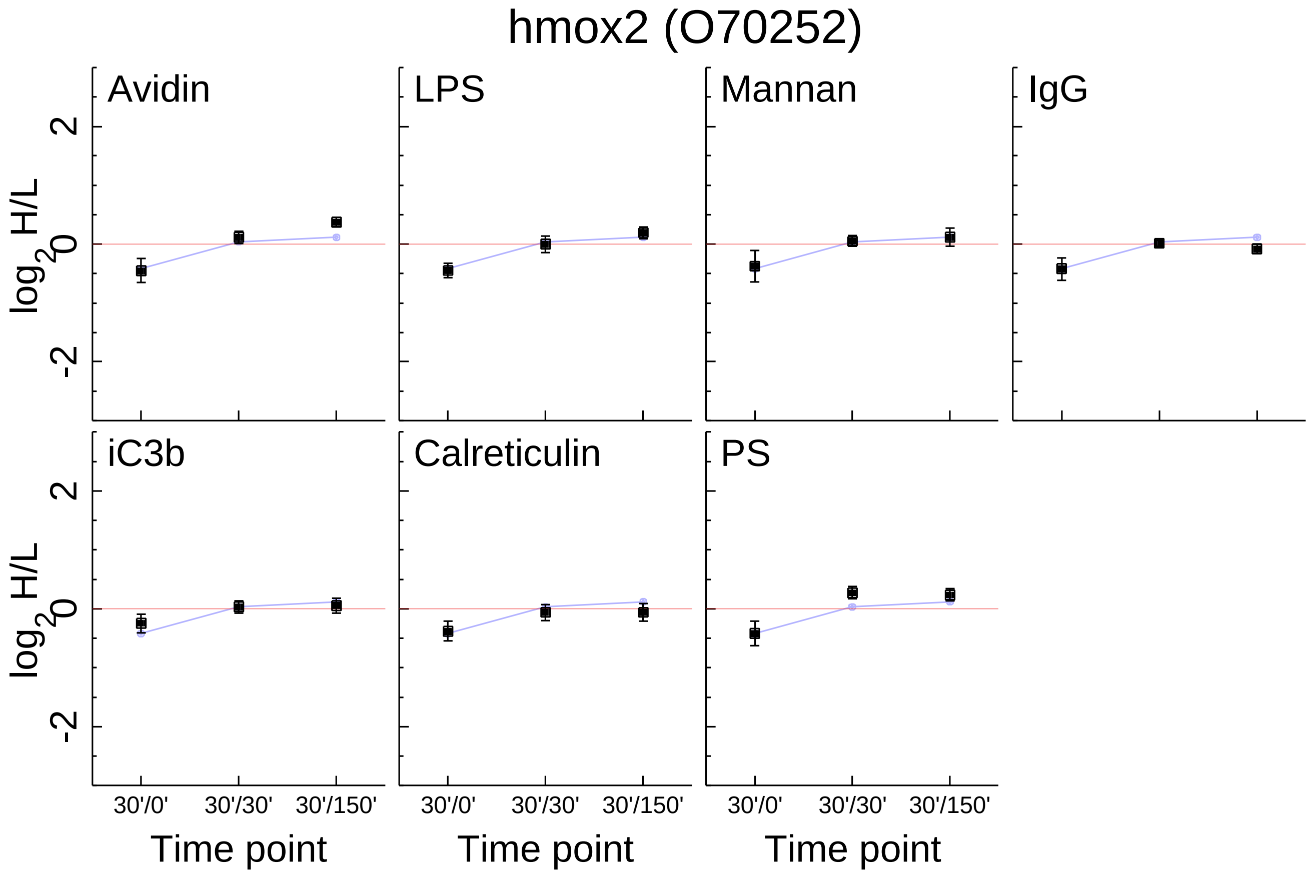
hmox2 (O70252)
Avidin
LPS
Mannan
IgG
iC3b
Calreticulin
PS
log2 H/L
2
0
-2
30'/0'
30'/30'
30'/150'
Time point

hn1l (Q6PGH2)

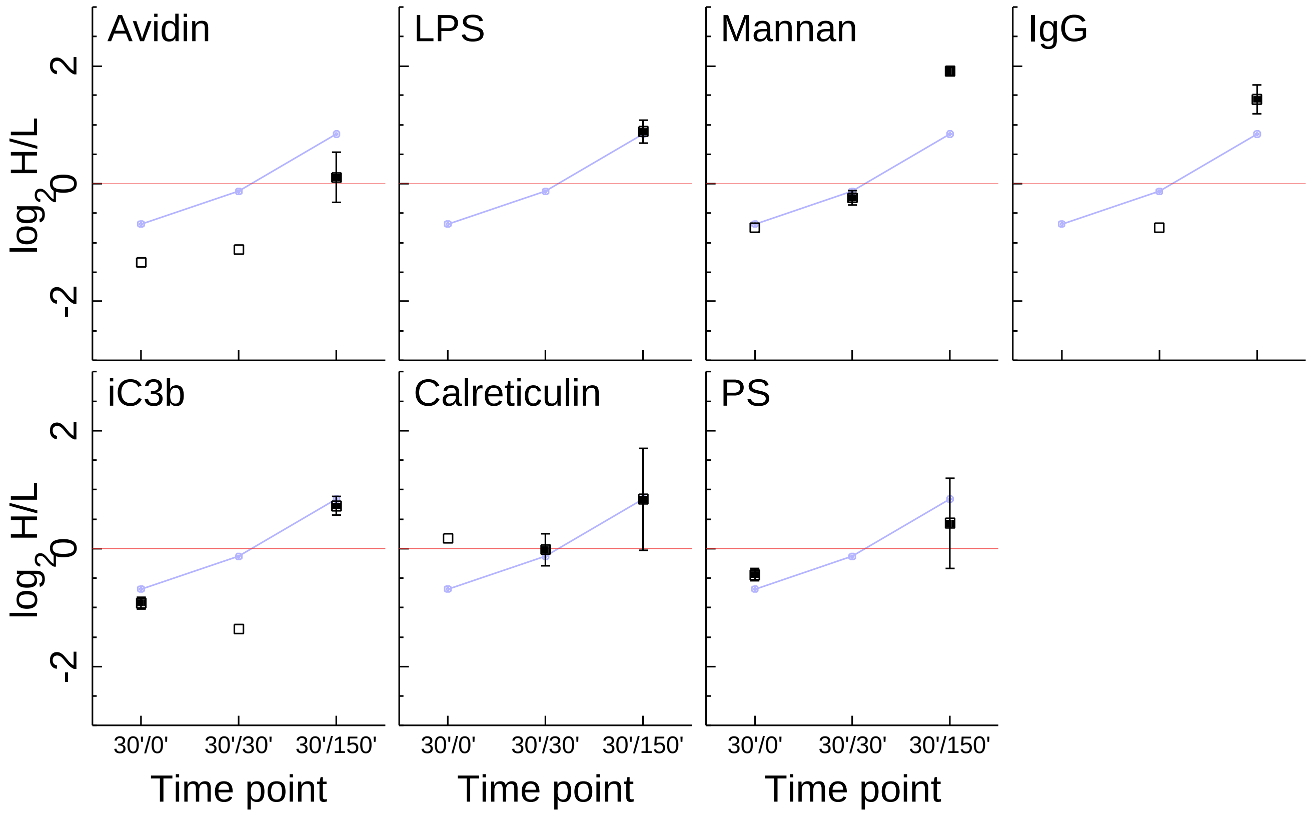

Avidin
LPS
Mannan
IgG
iC3b
Calreticulin
PS
2
0
-2
log2 H/L
30'/0'
30'/30'
30'/150'
Time point

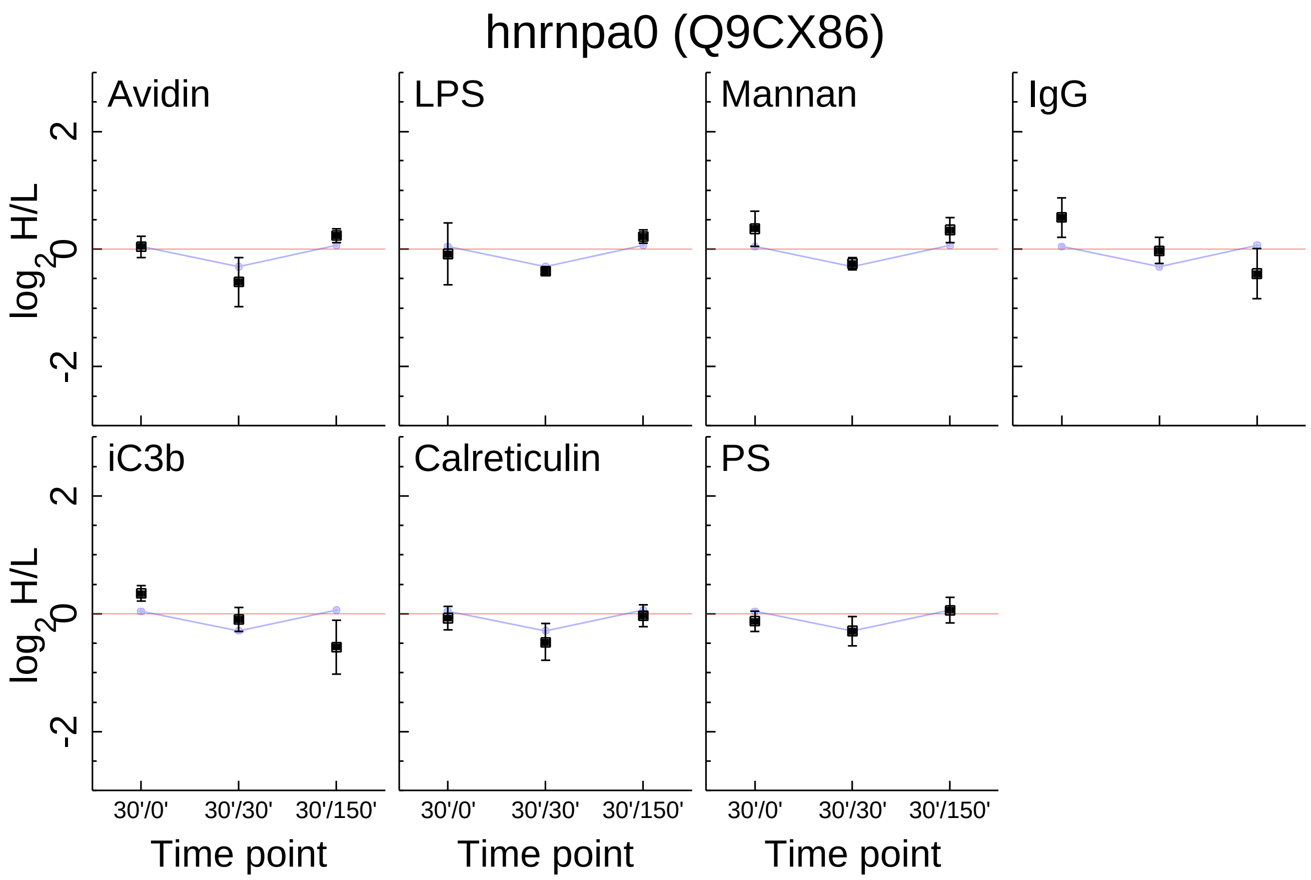

hnrnpa0 (Q9CX86)
Avidin
LPS
Mannan
IgG
iC3b
Calreticulin
PS
$\log_2$ H/L
2
0
-2
30'/0'
30'/30'
30'/150'
Time point

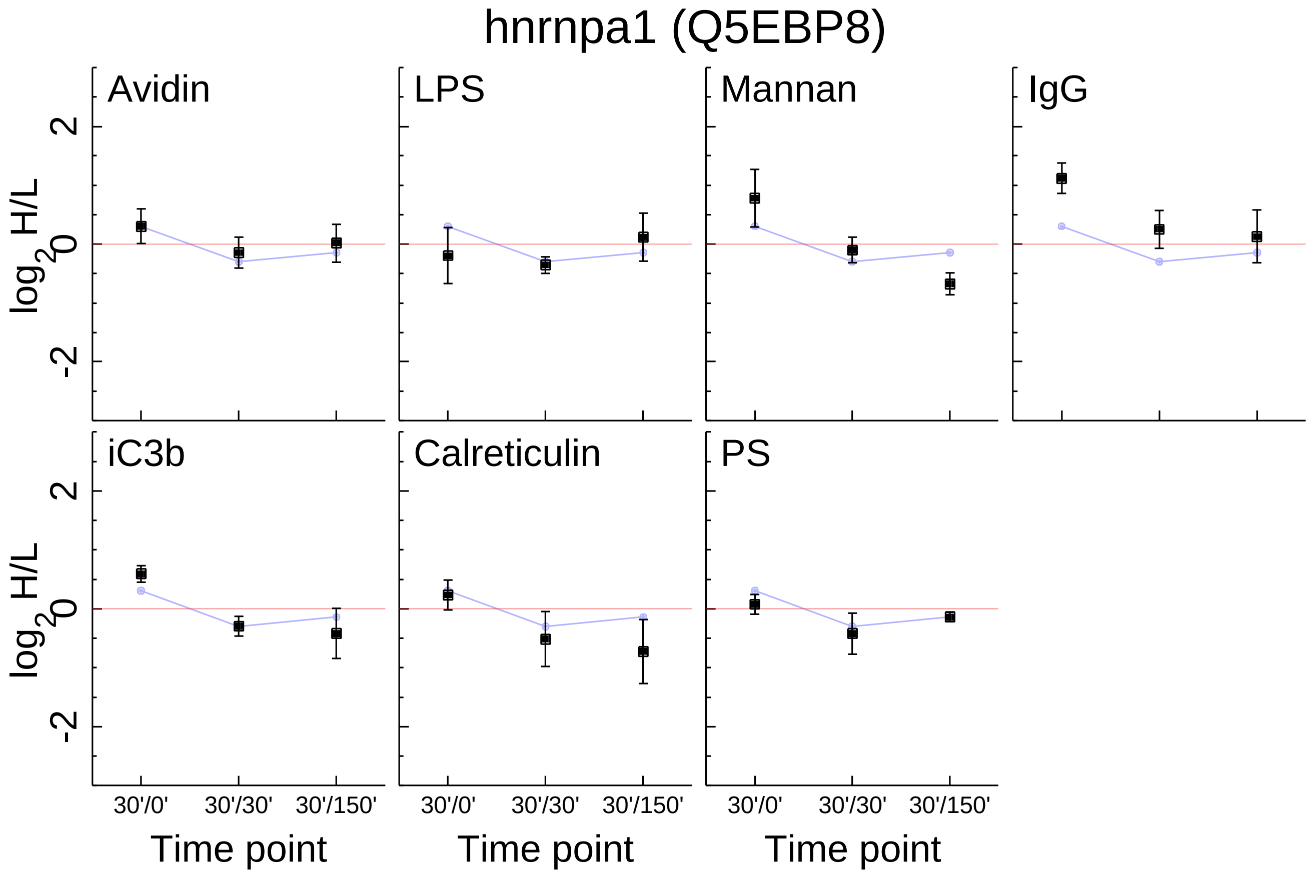
hnrnpa1 (Q5EBP8)
Avidin
LPS
Mannan
IgG
iC3b
Calreticulin
PS
log2 H/L
2
0
-2
30'/0'
30'/30'
30'/150'
Time point

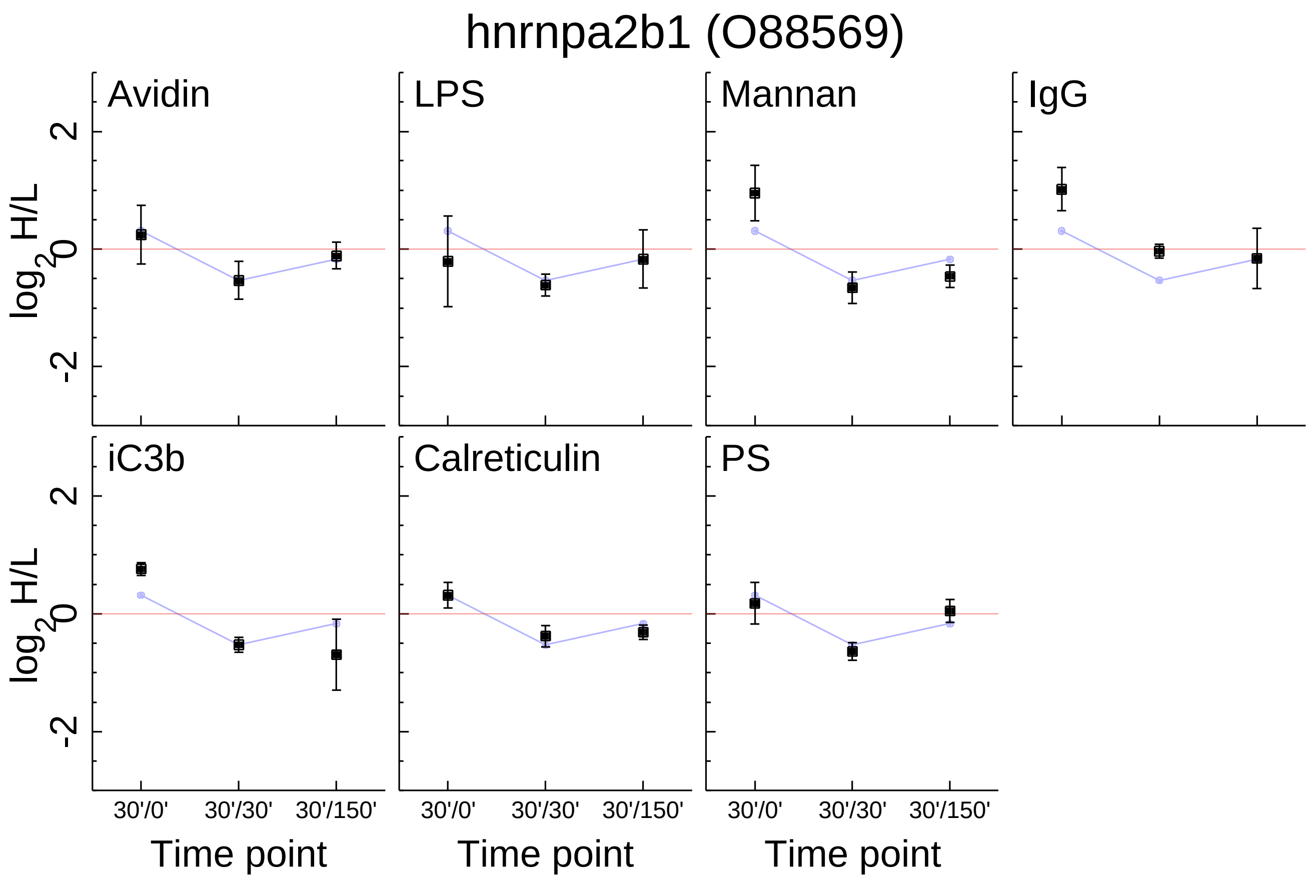
hnrnpa2b1 (O88569)
Avidin
LPS
Mannan
IgG
iC3b
Calreticulin
PS
log2 H/L
2
0
-2
30'/0'
30'/30'
30'/150'
Time point

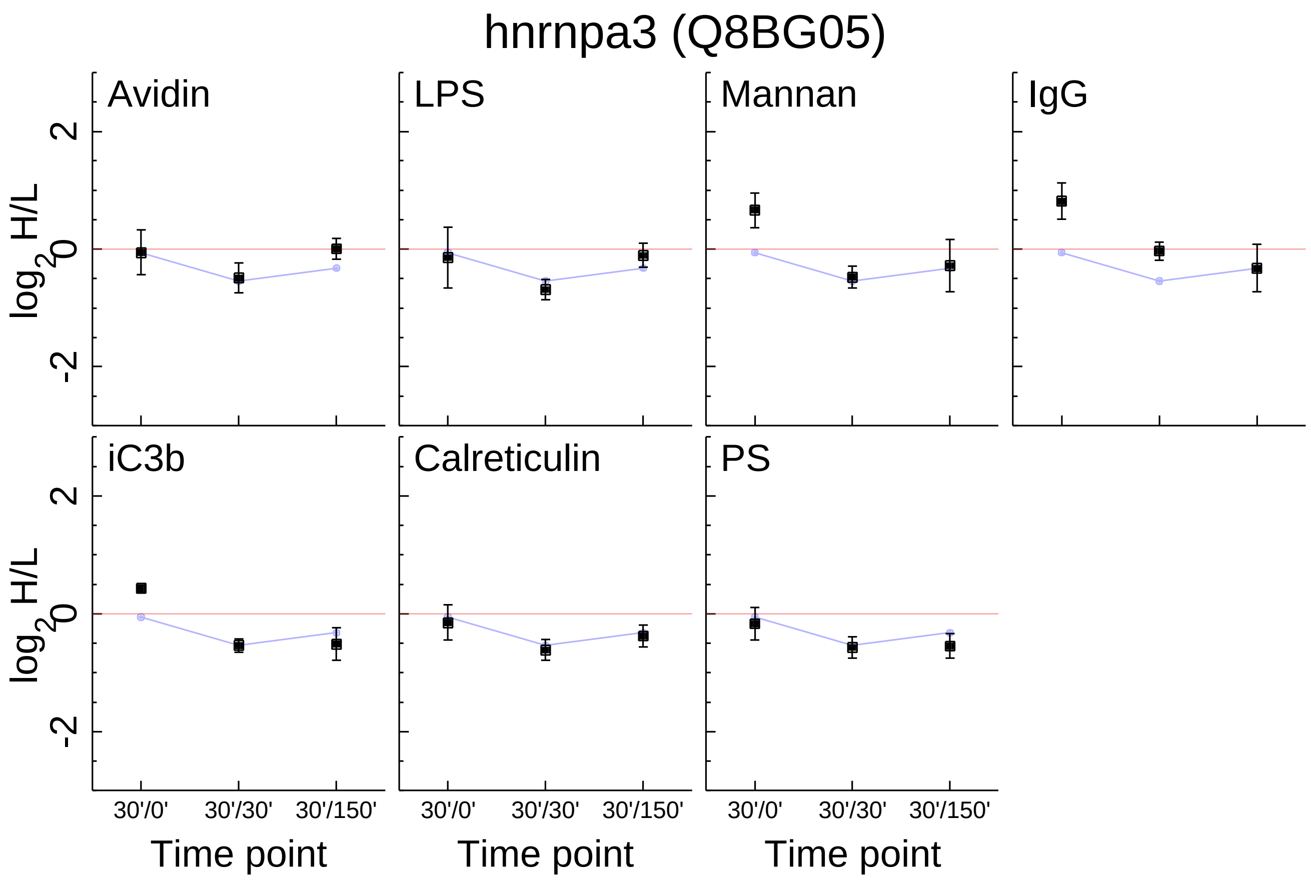

hnrnpa3 (Q8BG05)
Avidin
LPS
Mannan
IgG
iC3b
Calreticulin
PS
log2 H/L
2
0
-2
30'/0'
30'/30'
30'/150'
Time point

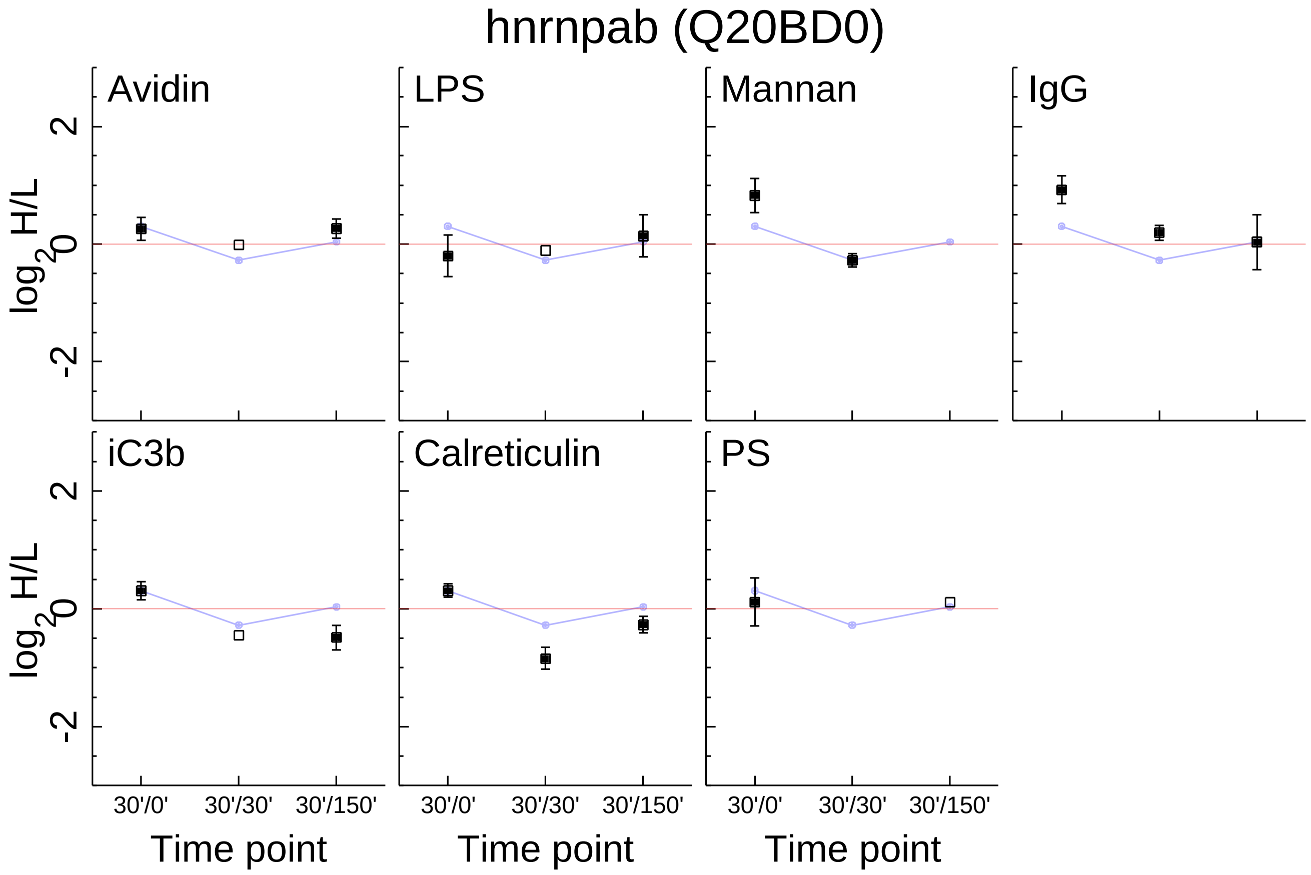
hnrnpab (Q20BD0)
Avidin
LPS
Mannan
IgG
iC3b
Calreticulin
PS
log2 H/L
2
0
-2
30'/0'
30'/30'
30'/150'
Time point

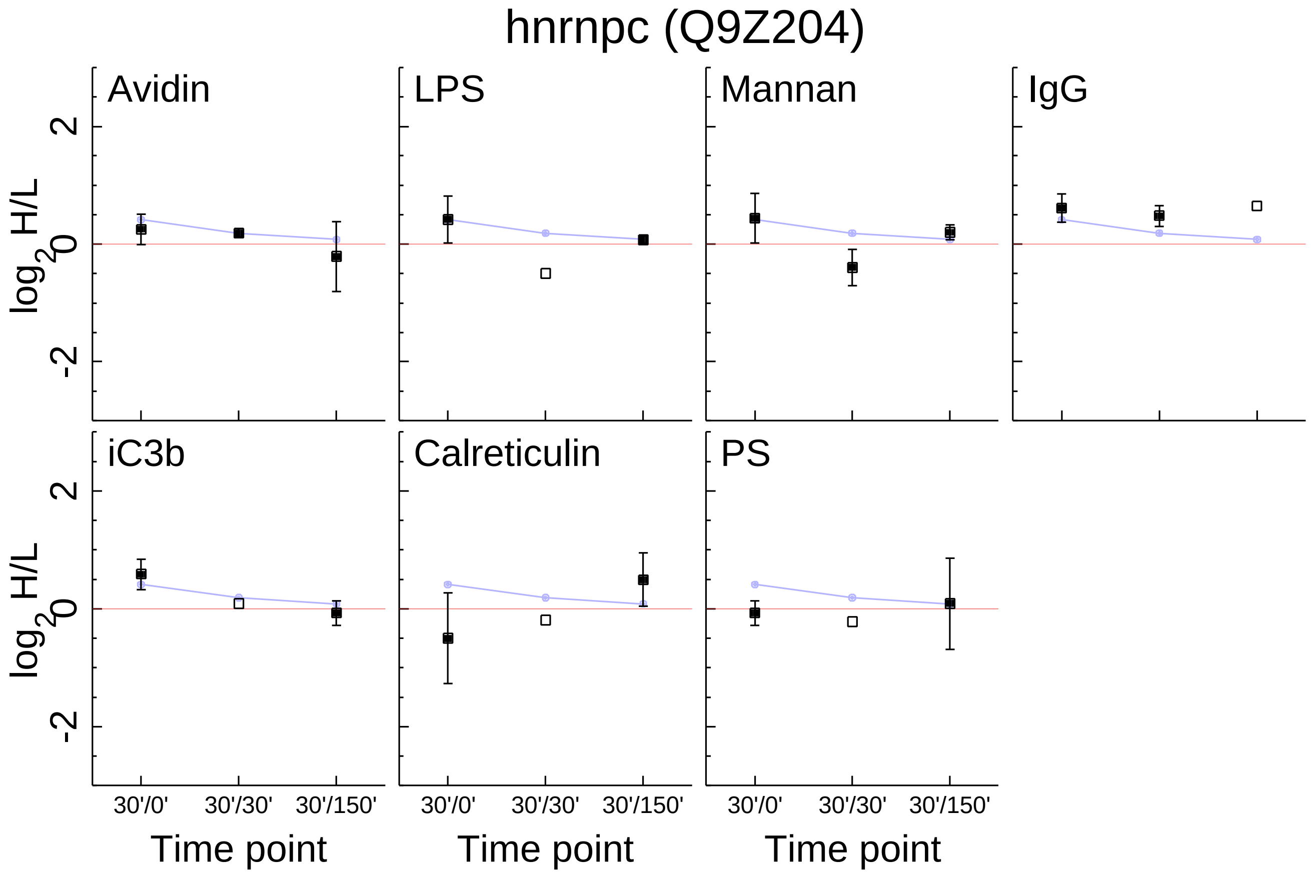

hnrnpc (Q9Z204)
Avidin
LPS
Mannan
IgG
iC3b
Calreticulin
PS
log2 H/L
2
0
-2
30'/0'
30'/30'
30'/150'
Time point

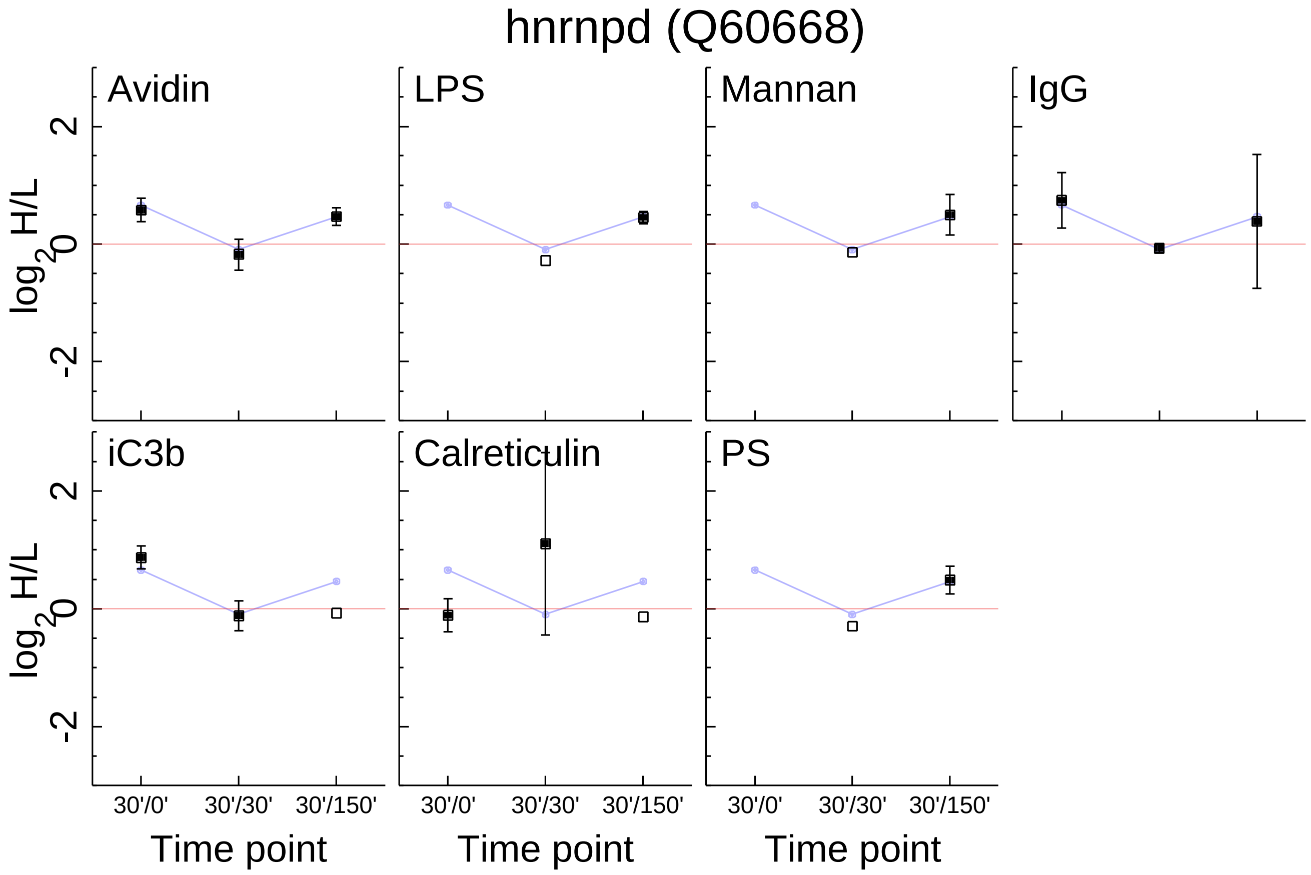

hnrnpd (Q60668)
Avidin
LPS
Mannan
IgG
iC3b
Calreticulin
PS
log2 H/L
2
0
-2
30'/0'
30'/30'
30'/150'
Time point

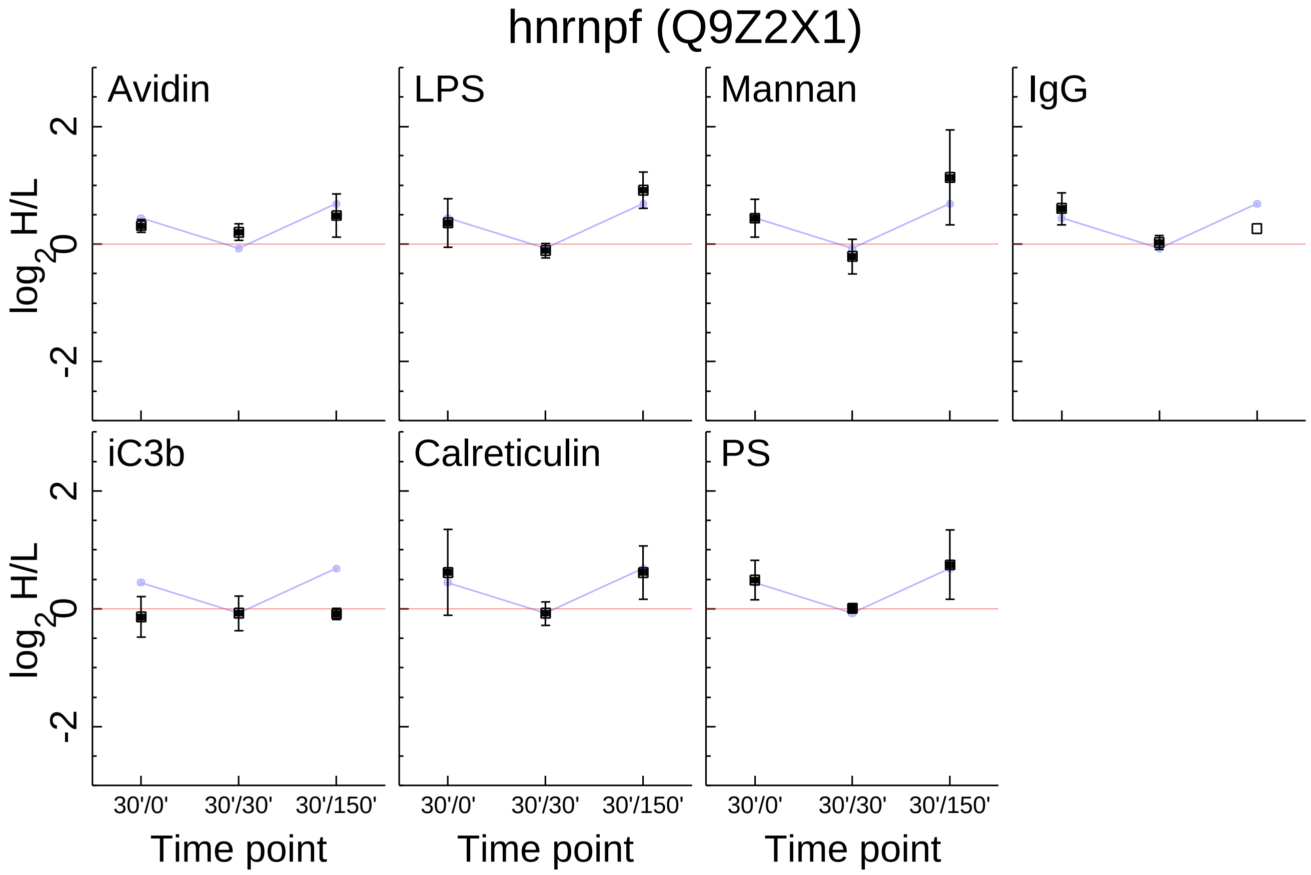

hnrnpf (Q9Z2X1)
Avidin
LPS
Mannan
IgG
iC3b
Calreticulin
PS
log2 H/L
2
0
-2
30'/0'
30'/30'
30'/150'
Time point

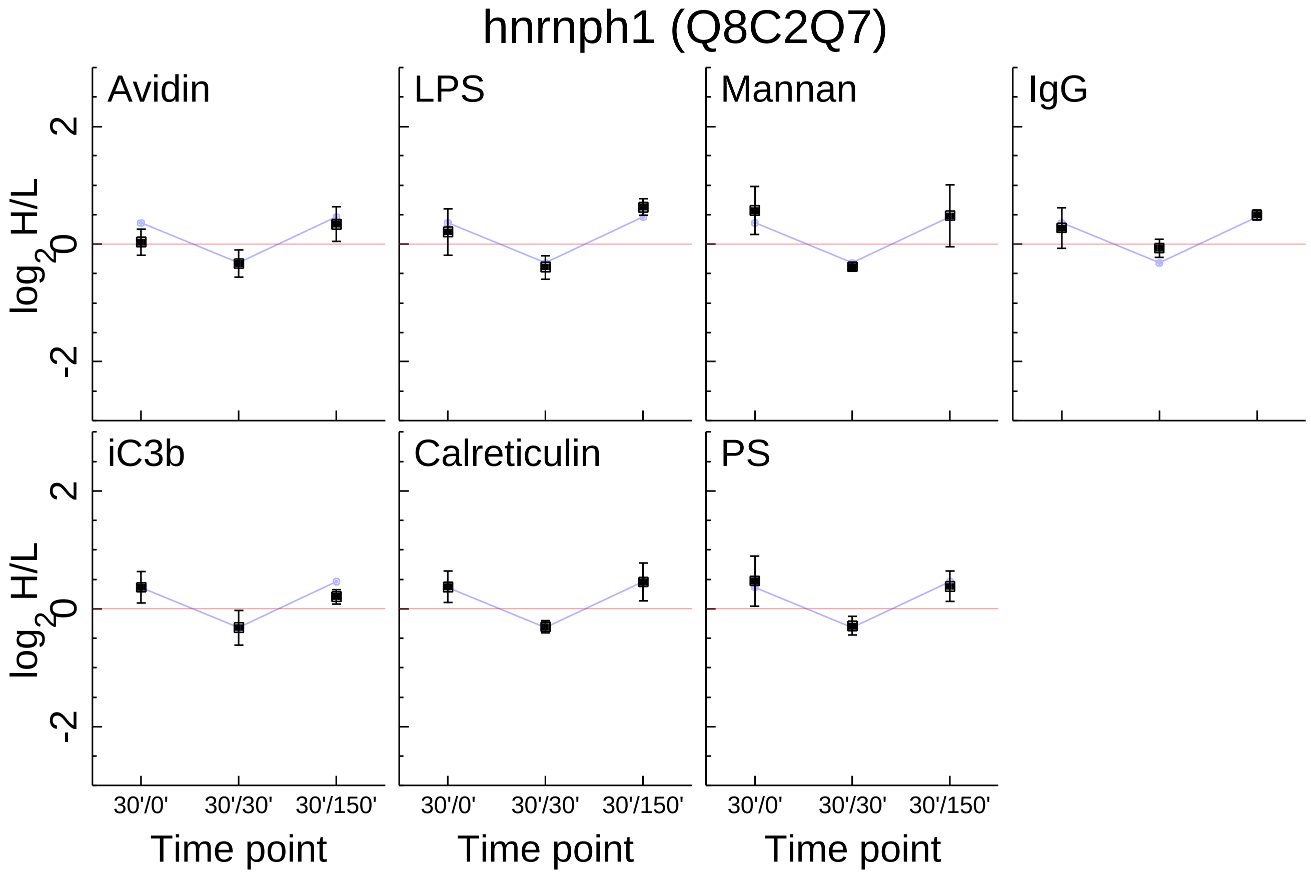
hnrnph1 (Q8C2Q7)
Avidin
LPS
Mannan
IgG
iC3b
Calreticulin
PS
log2 H/L
2
0
-2
30'/0'
30'/30'
30'/150'
Time point

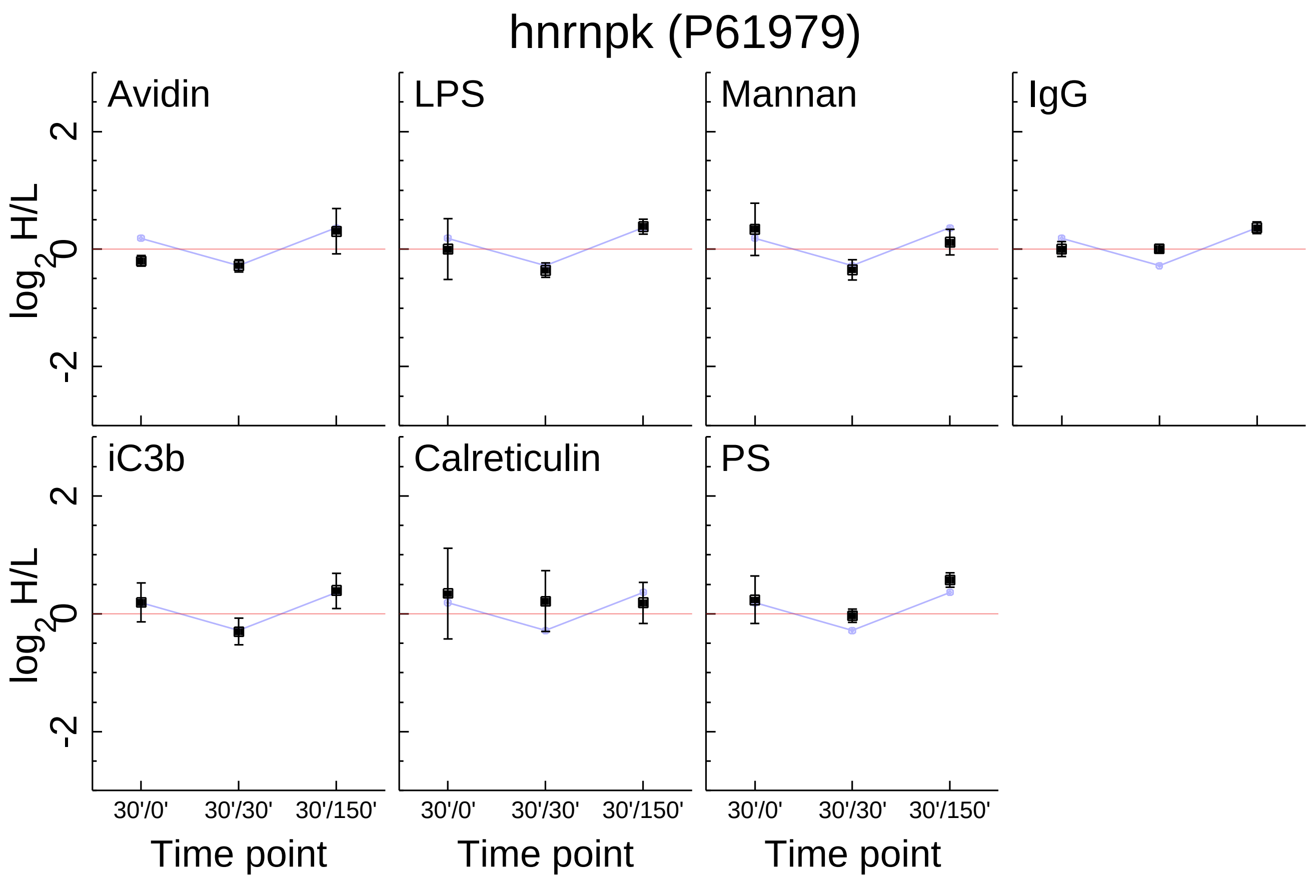
hnrnpk (P61979)
Avidin
LPS
Mannan
IgG
iC3b
Calreticulin
PS
log2 H/L
2
0
-2
30'/0'
30'/30'
30'/150'
Time point

hnrnpl (G5E924)

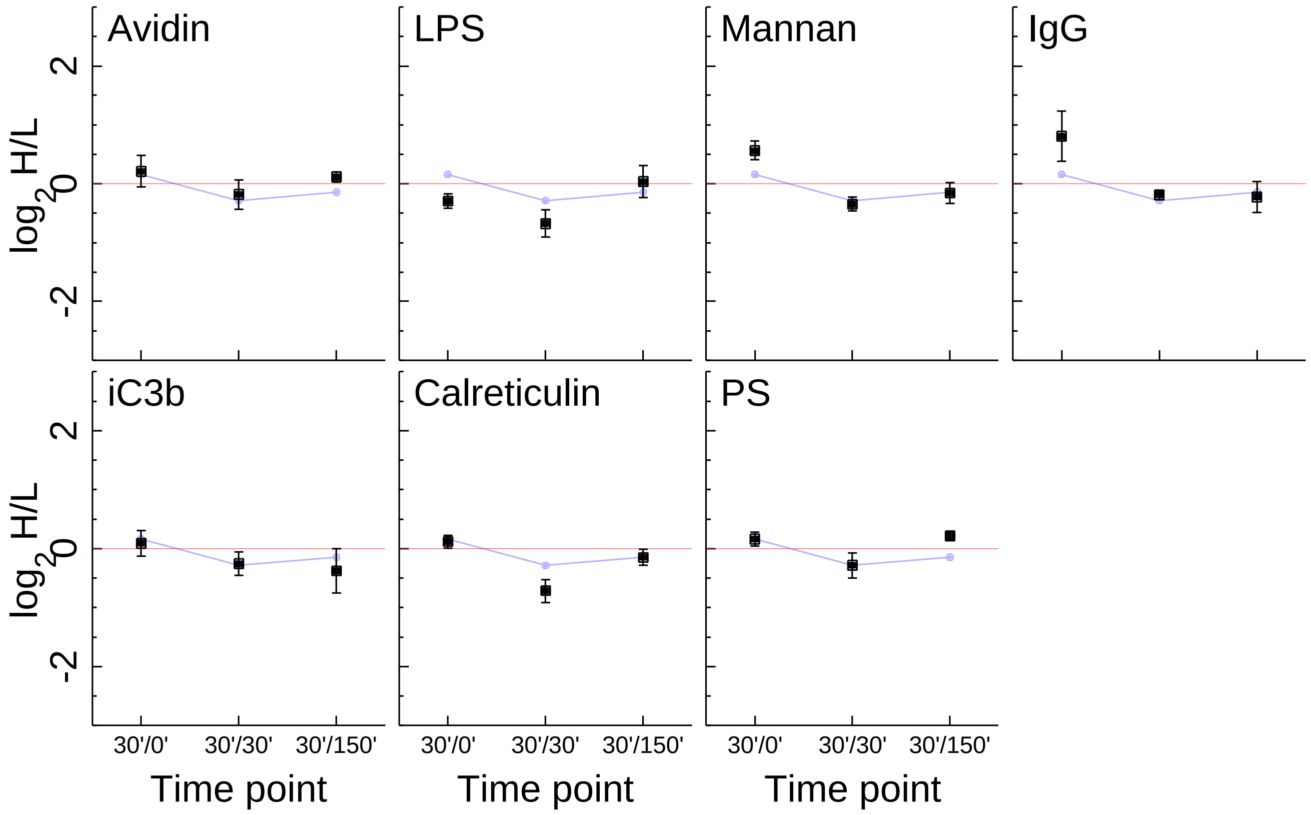

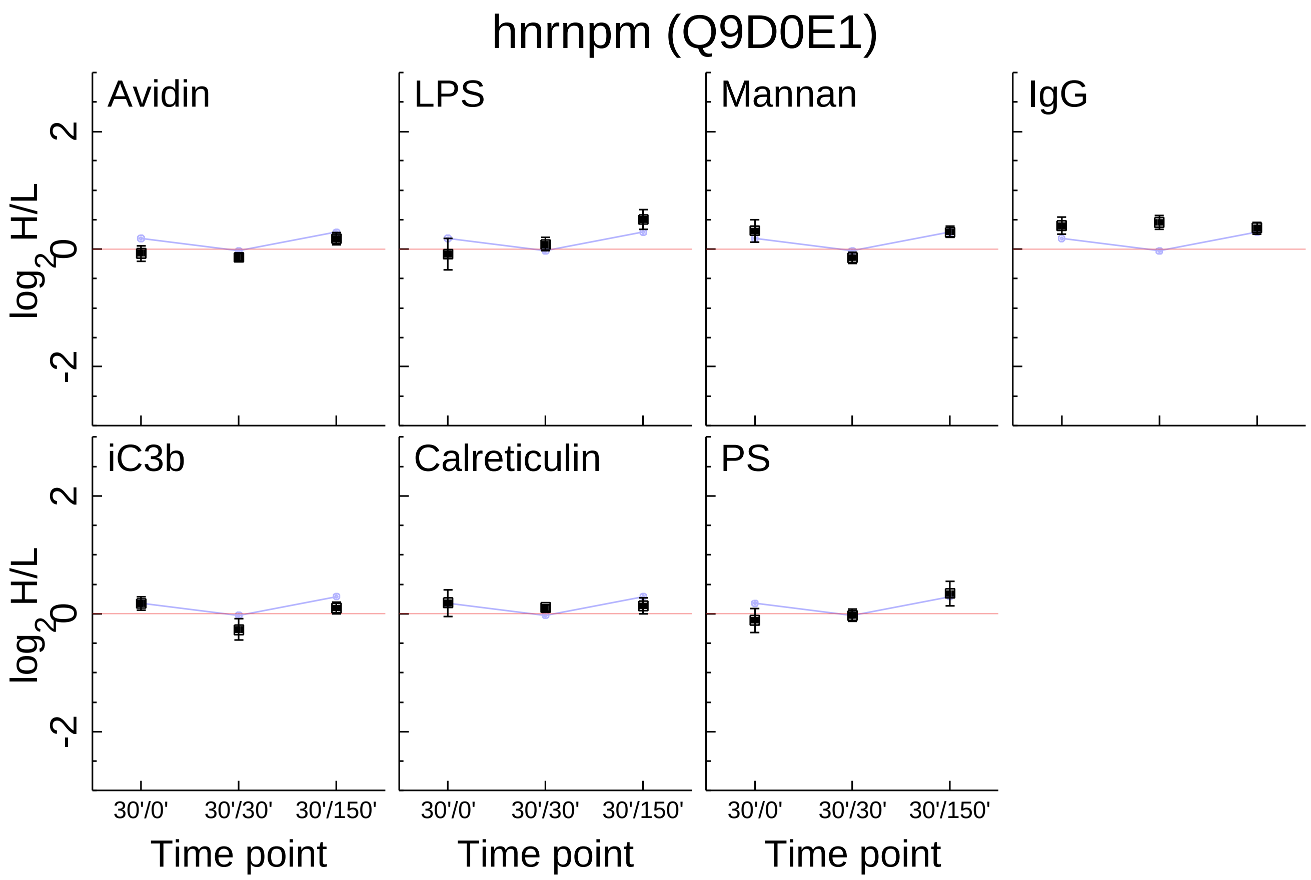
hnrnpm (Q9D0E1)
Avidin
LPS
Mannan
IgG
iC3b
Calreticulin
PS
log2 H/L
2
0
-2
30'/0'
30'/30'
30'/150'
Time point

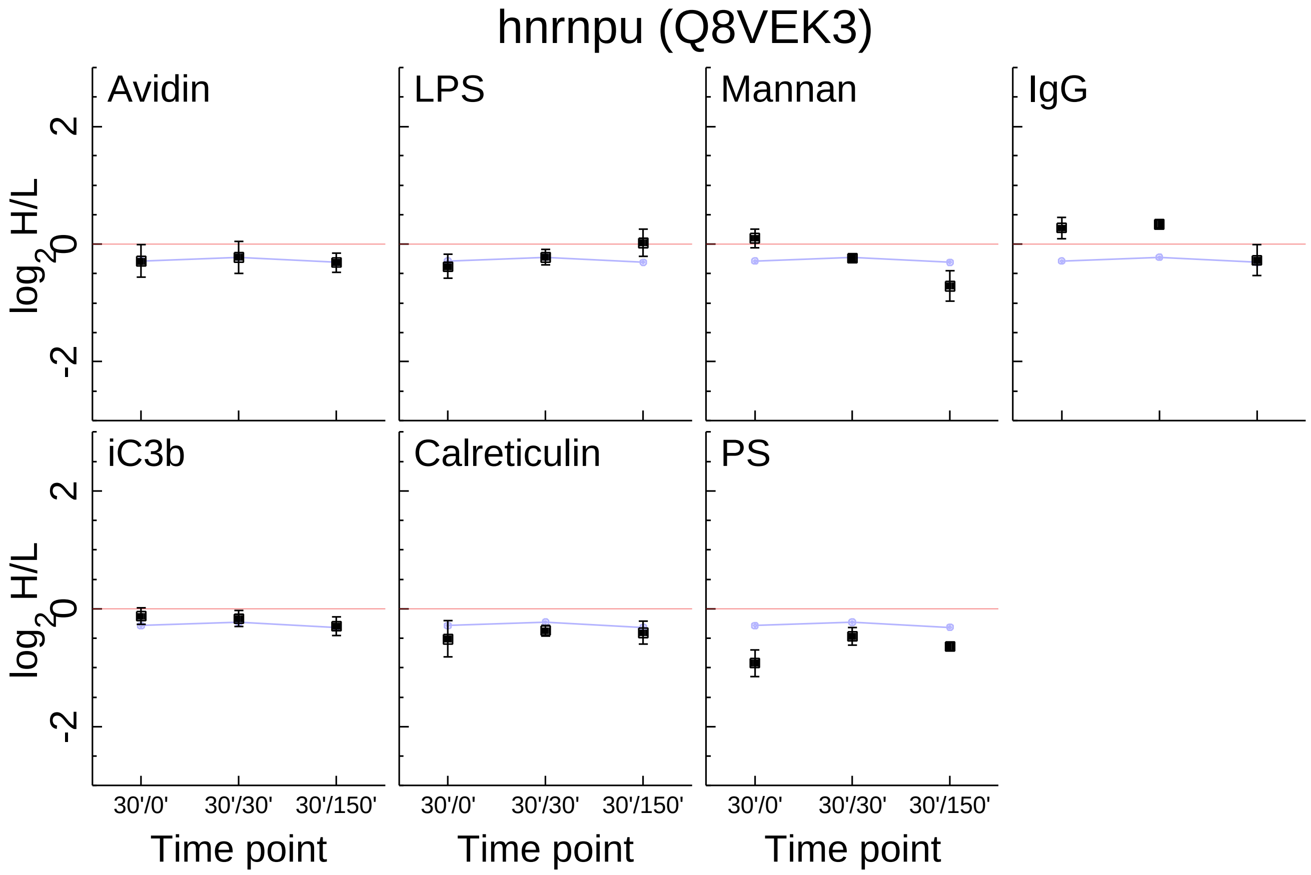
hnrnpu (Q8VEK3)
Avidin
LPS
Mannan
IgG
iC3b
Calreticulin
PS
log2 H/L
2
0
-2
30'/0'
30'/30'
30'/150'
Time point

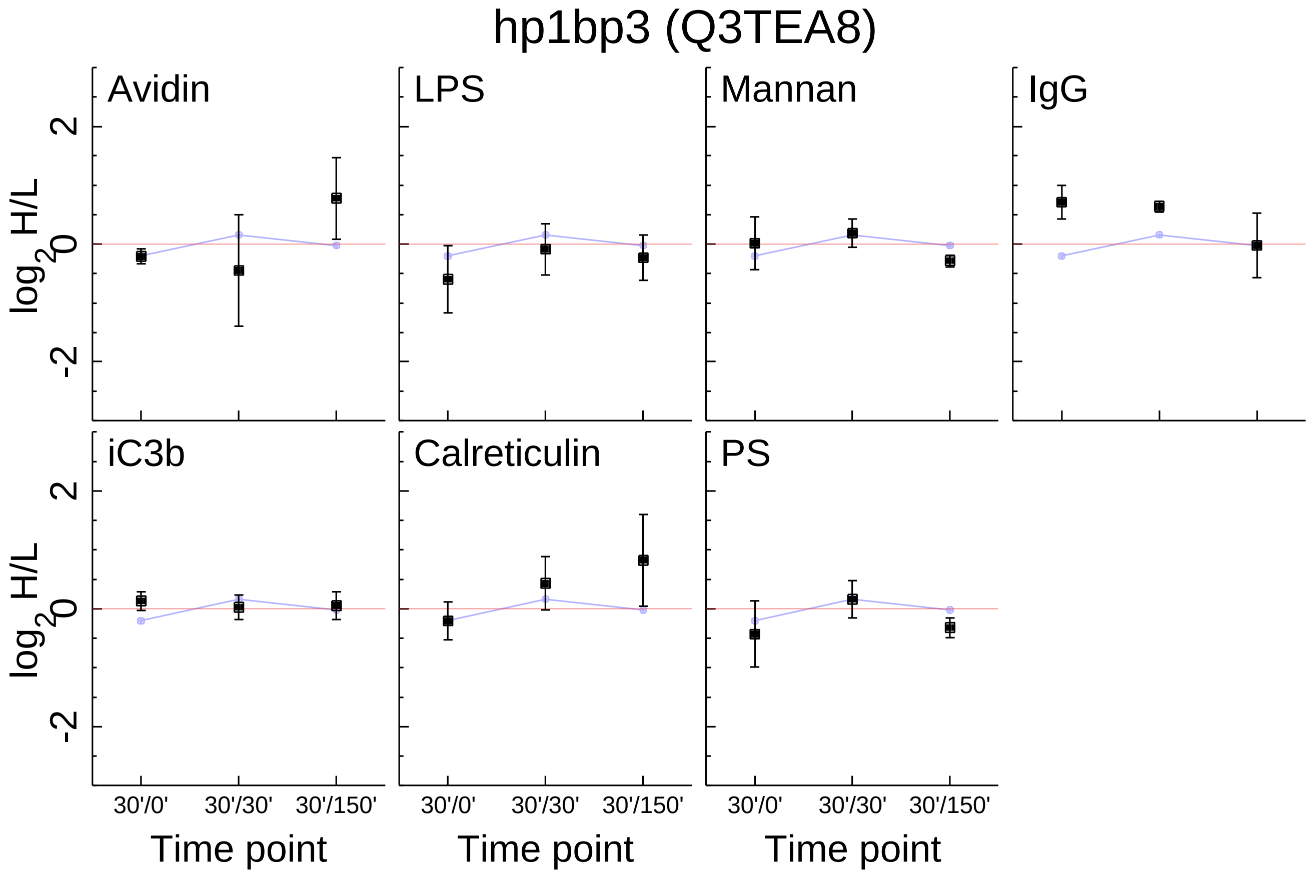

hp1bp3 (Q3TEA8)
Avidin
LPS
Mannan
IgG
iC3b
Calreticulin
PS
log2 H/L
2
0
-2
30'/0'
30'/30'
30'/150'
Time point

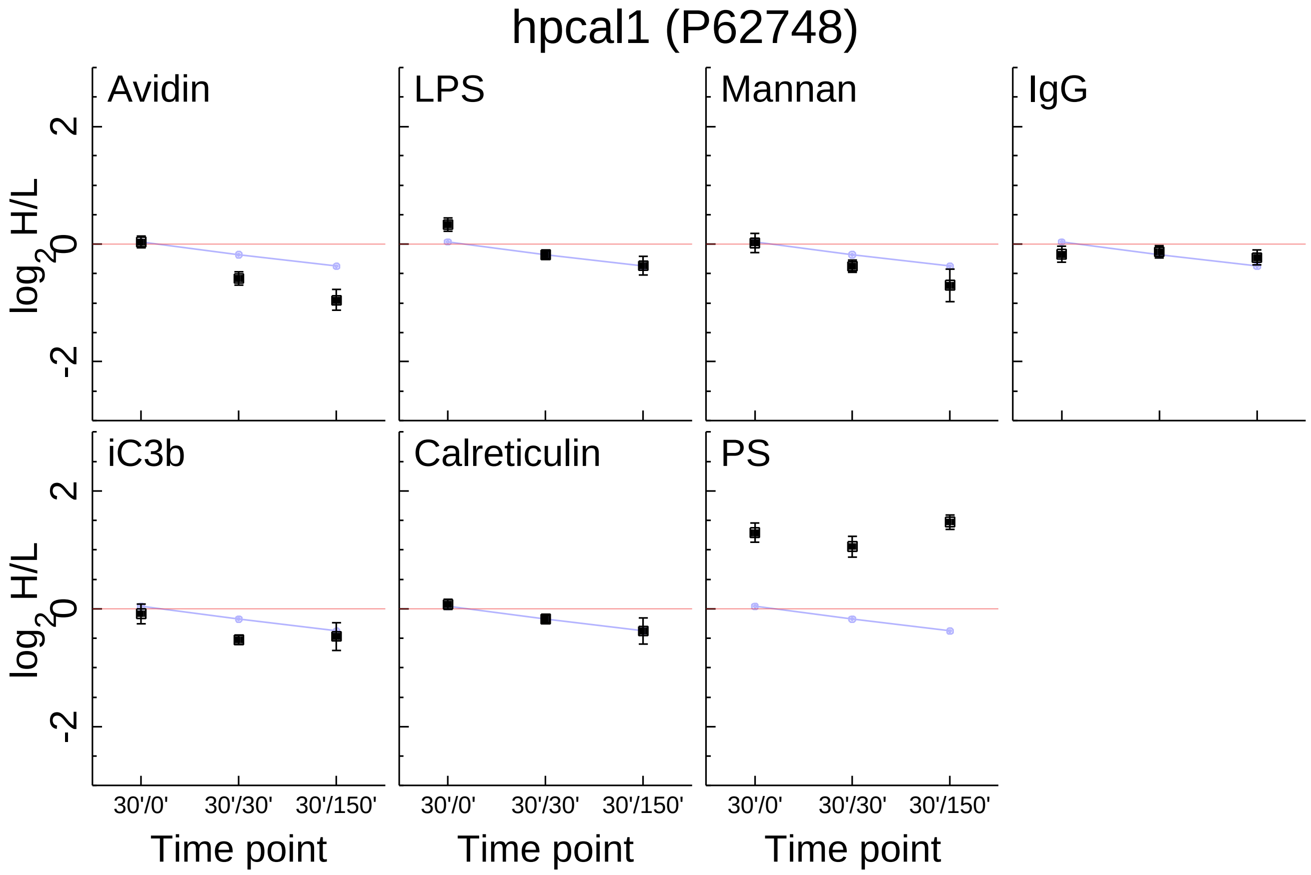
hpcal1 (P62748)
Avidin
LPS
Mannan
IgG
iC3b
Calreticulin
PS
log2 H/L
2
0
-2
30'/0'
30'/30'
30'/150'
Time point

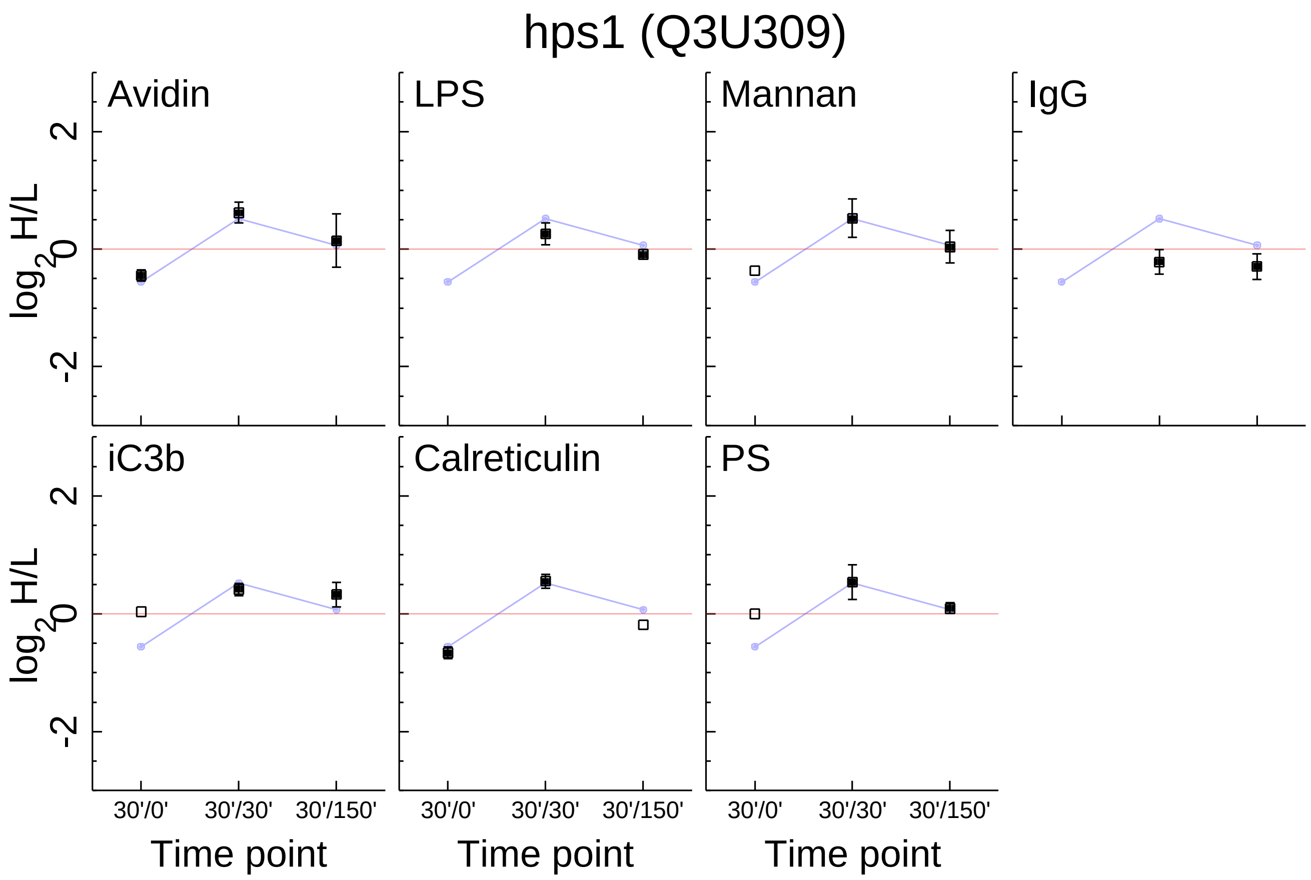
hps1 (Q3U309)
Avidin
LPS
Mannan
IgG
iC3b
Calreticulin
PS
log2 H/L
2
0
-2
30'/0'
30'/30'
30'/150'
Time point

hps3 (Q91VB4)

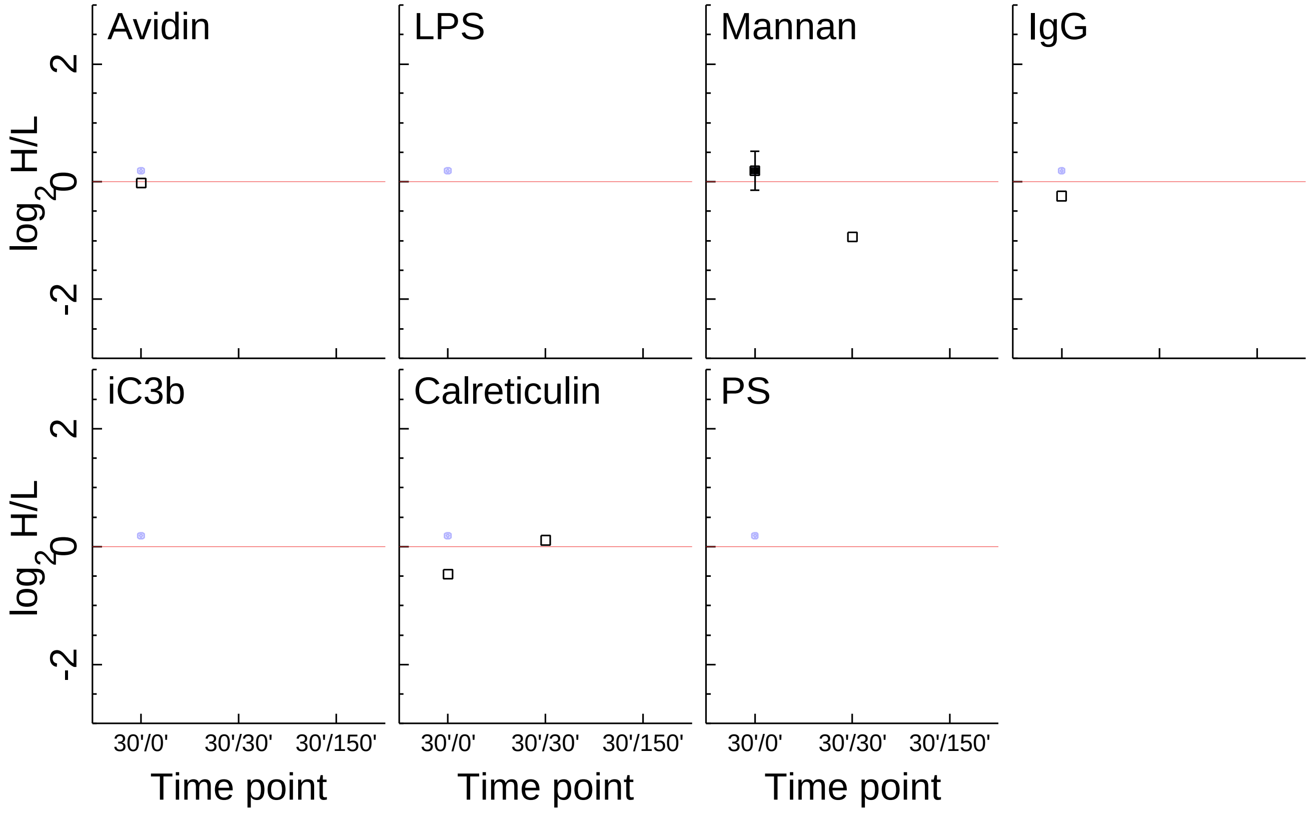

Avidin
LPS
Mannan
IgG
iC3b
Calreticulin
PS
log2 H/L
2
0
-2
30'/0'
30'/30'
30'/150'
Time point

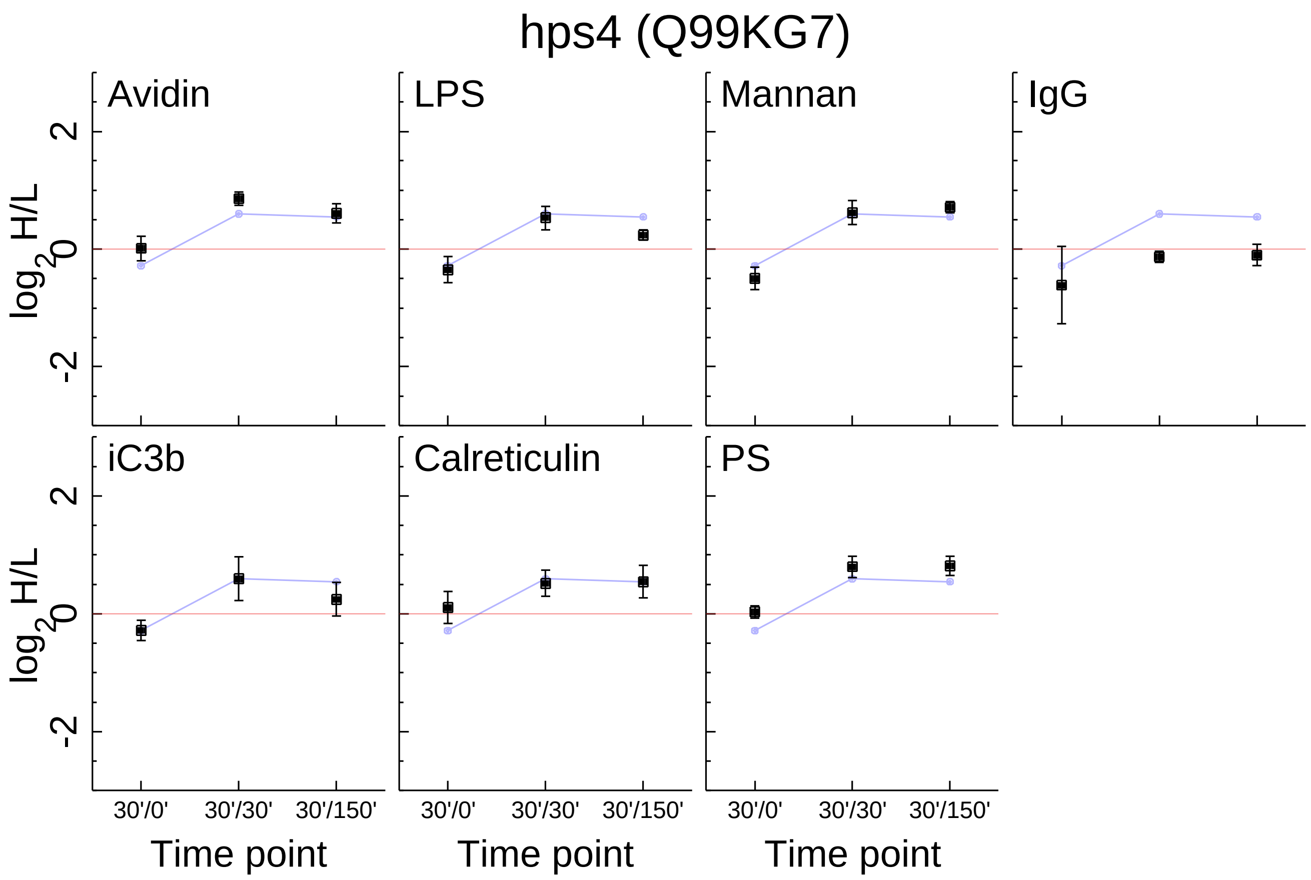

hps4 (Q99KG7)
Avidin
LPS
Mannan
IgG
iC3b
Calreticulin
PS
log2 H/L
2
0
-2
30'/0'
30'/30'
30'/150'
Time point

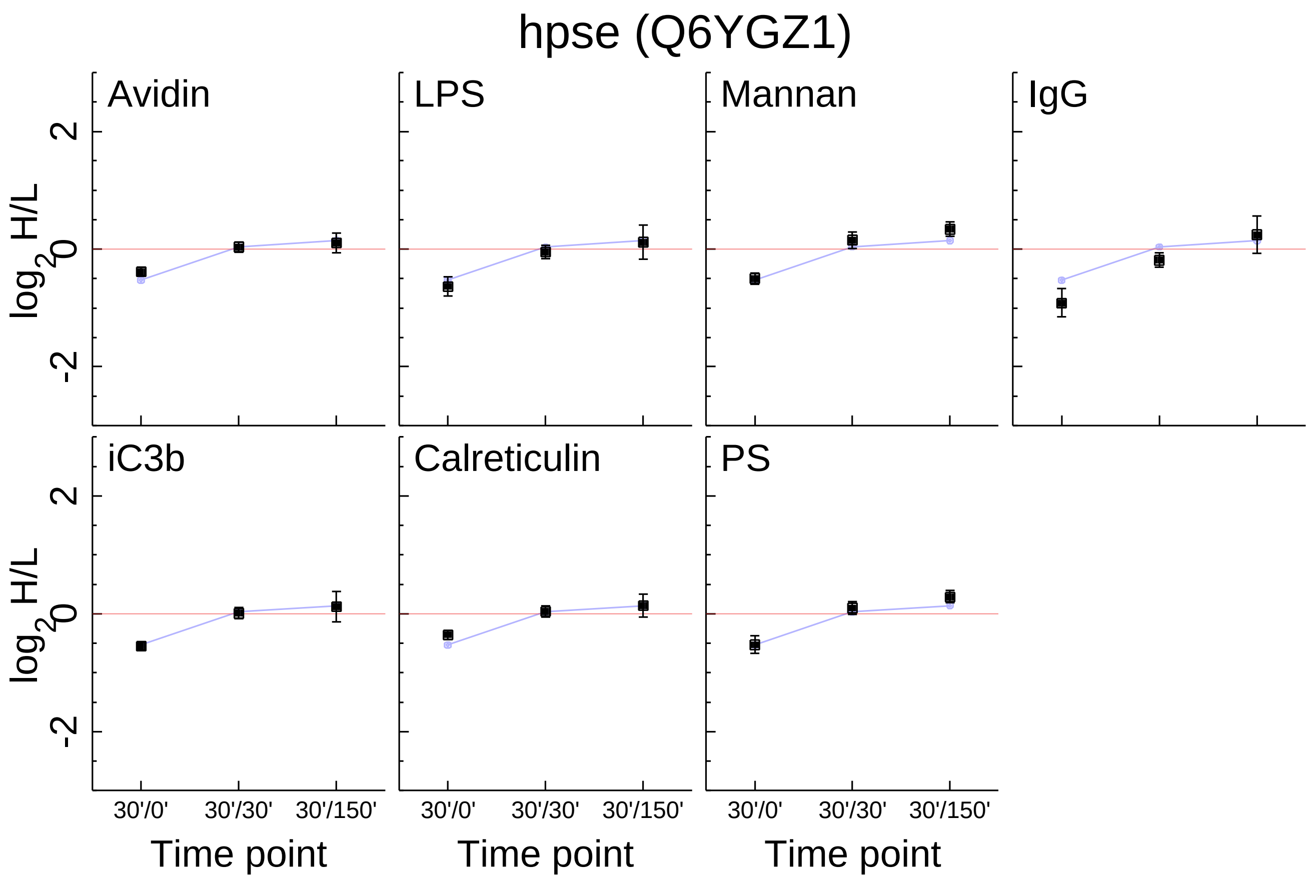

hpse (Q6YGZ1)
Avidin
LPS
Mannan
IgG
iC3b
Calreticulin
PS
log2 H/L
2
0
-2
30'/0'
30'/30'
30'/150'
Time point

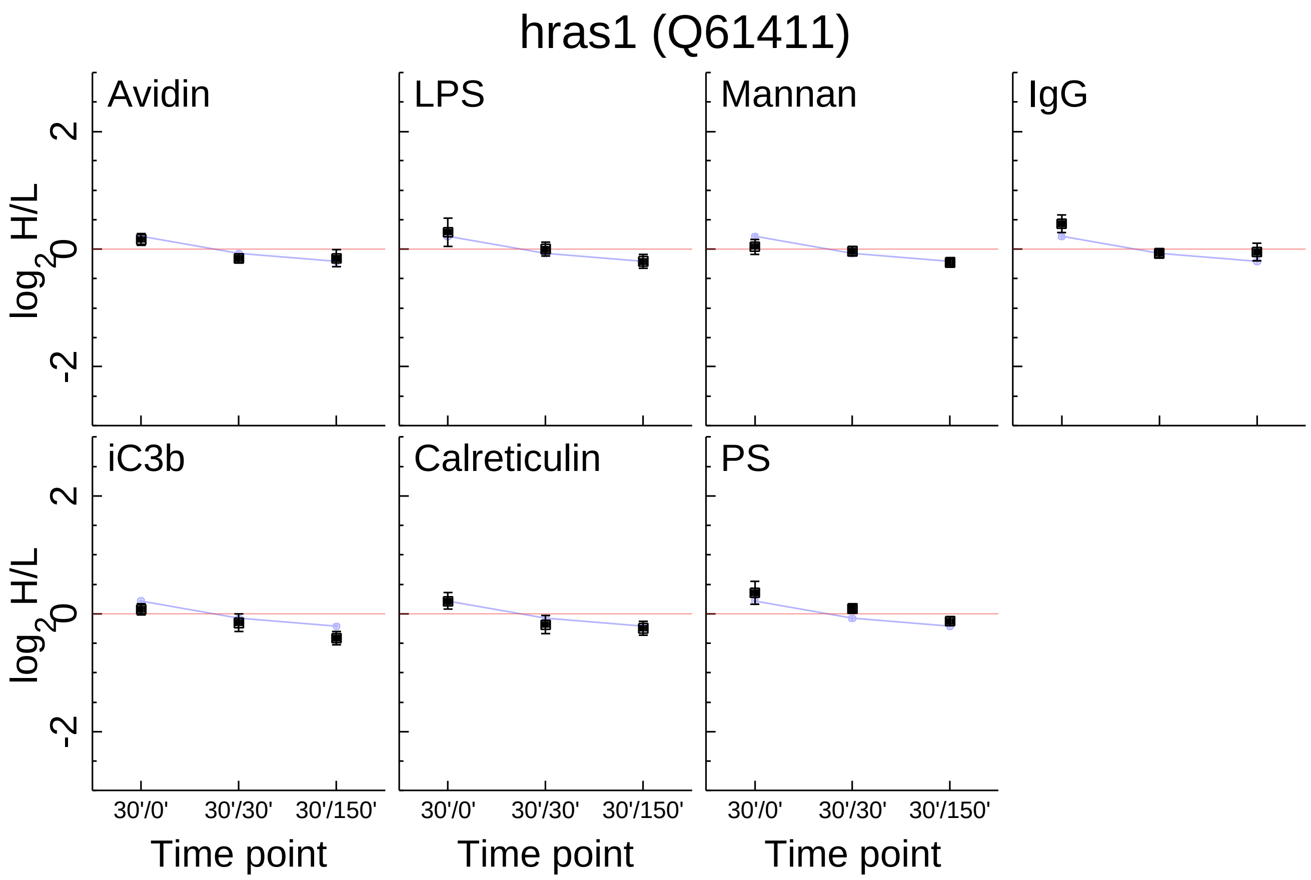

hras1 (Q61411)
Avidin
LPS
Mannan
IgG
iC3b
Calreticulin
PS
log2 H/L
2
0
-2
30'/0'
30'/30'
30'/150'
Time point

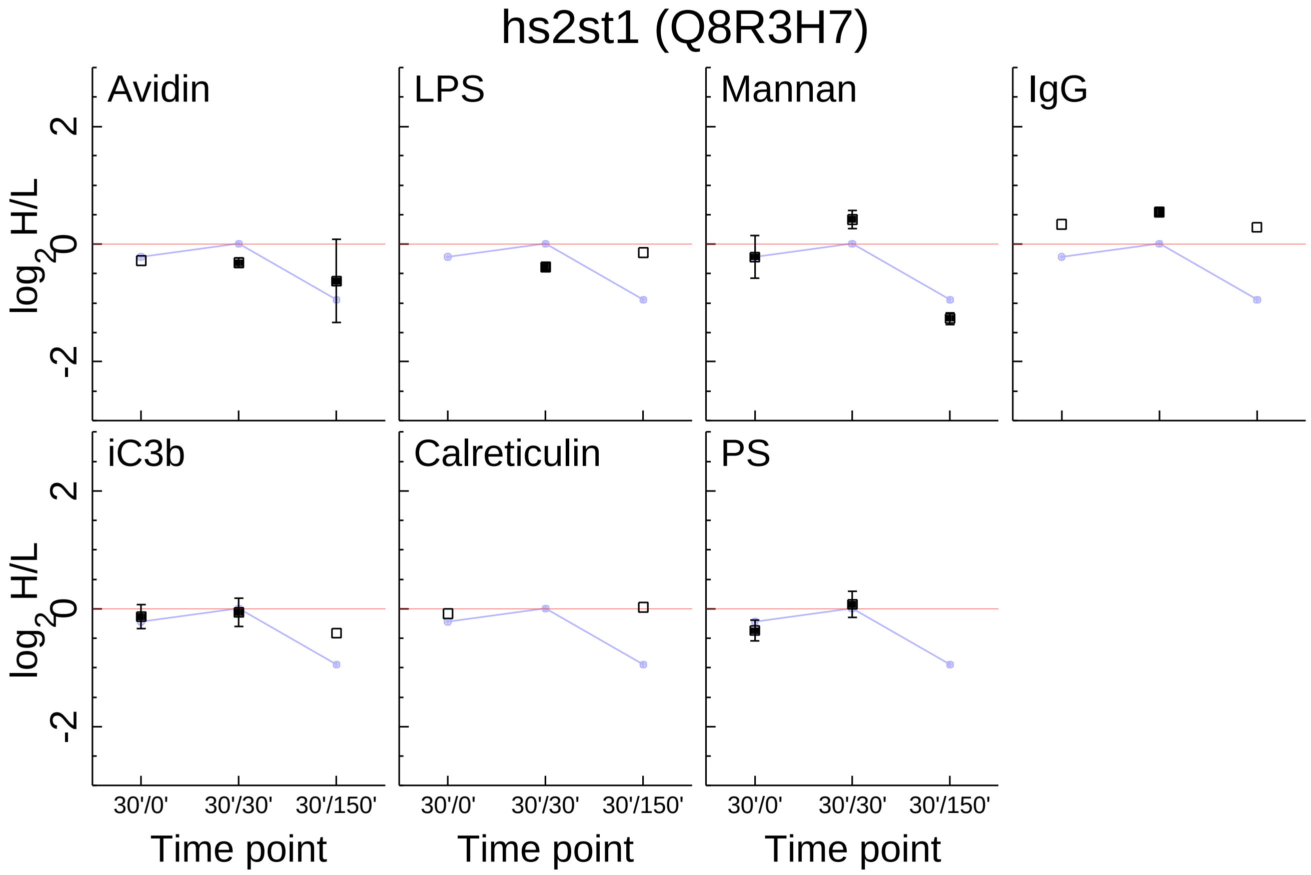

hs2st1 (Q8R3H7)
Avidin
LPS
Mannan
IgG
iC3b
Calreticulin
PS
log2 H/L
2
0
-2
30'/0'
30'/30'
30'/150'
Time point

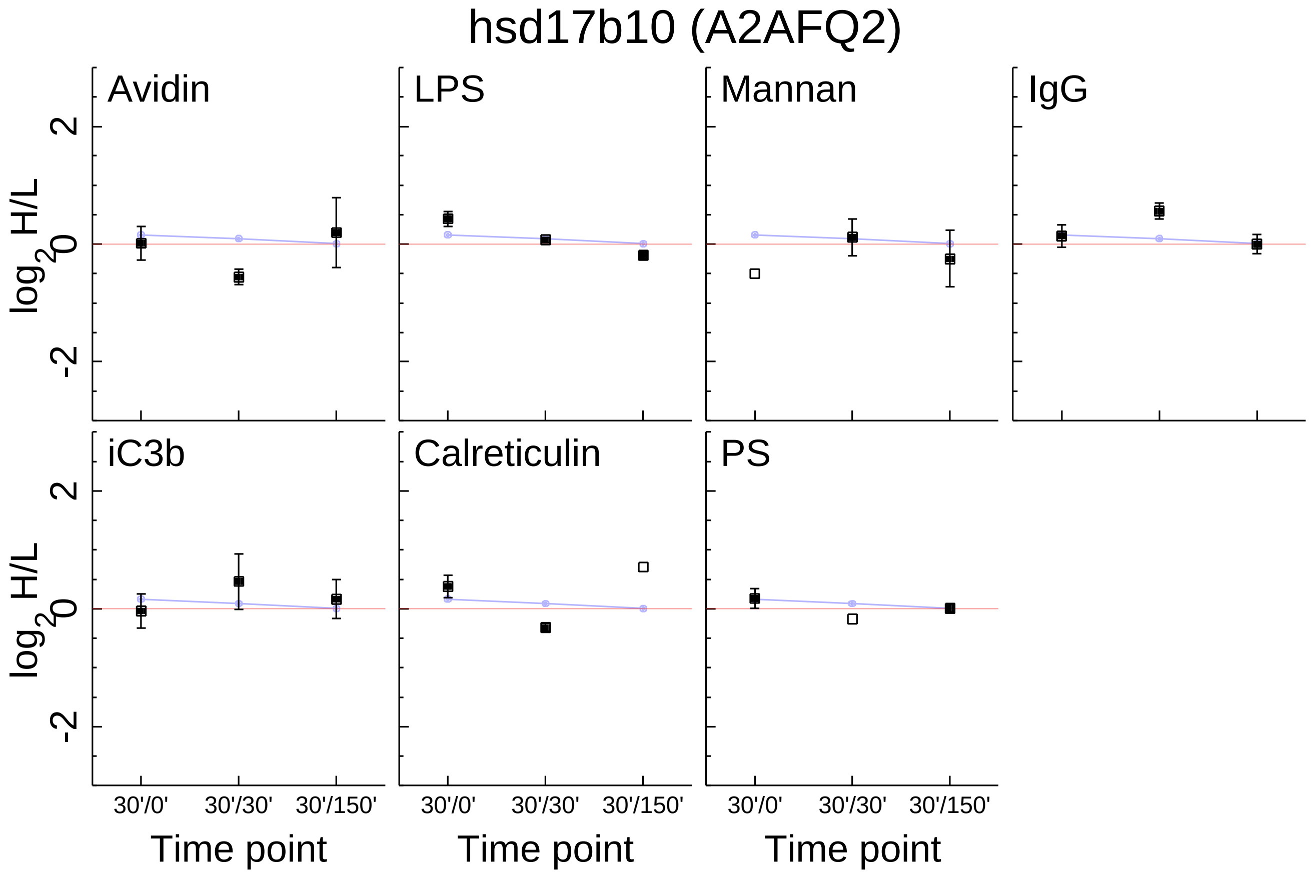
hsd17b10 (A2AFQ2)
Avidin
LPS
Mannan
IgG
iC3b
Calreticulin
PS
log2 H/L
2
0
-2
30'/0'
30'/30'
30'/150'
Time point

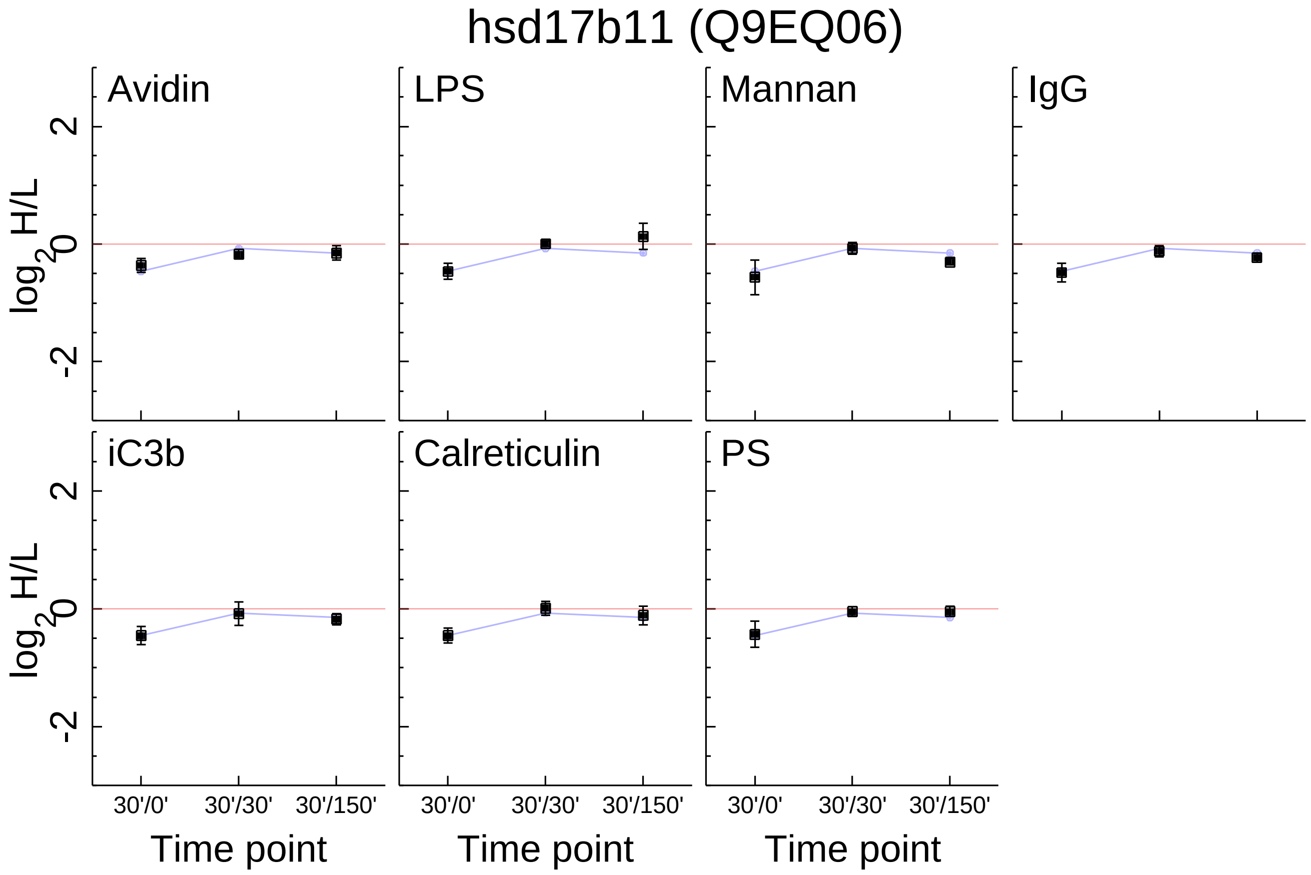
hsd17b11 (Q9EQ06)
Avidin
LPS
Mannan
IgG
iC3b
Calreticulin
PS
log2 H/L
2
0
-2
30'/0'
30'/30'
30'/150'
Time point

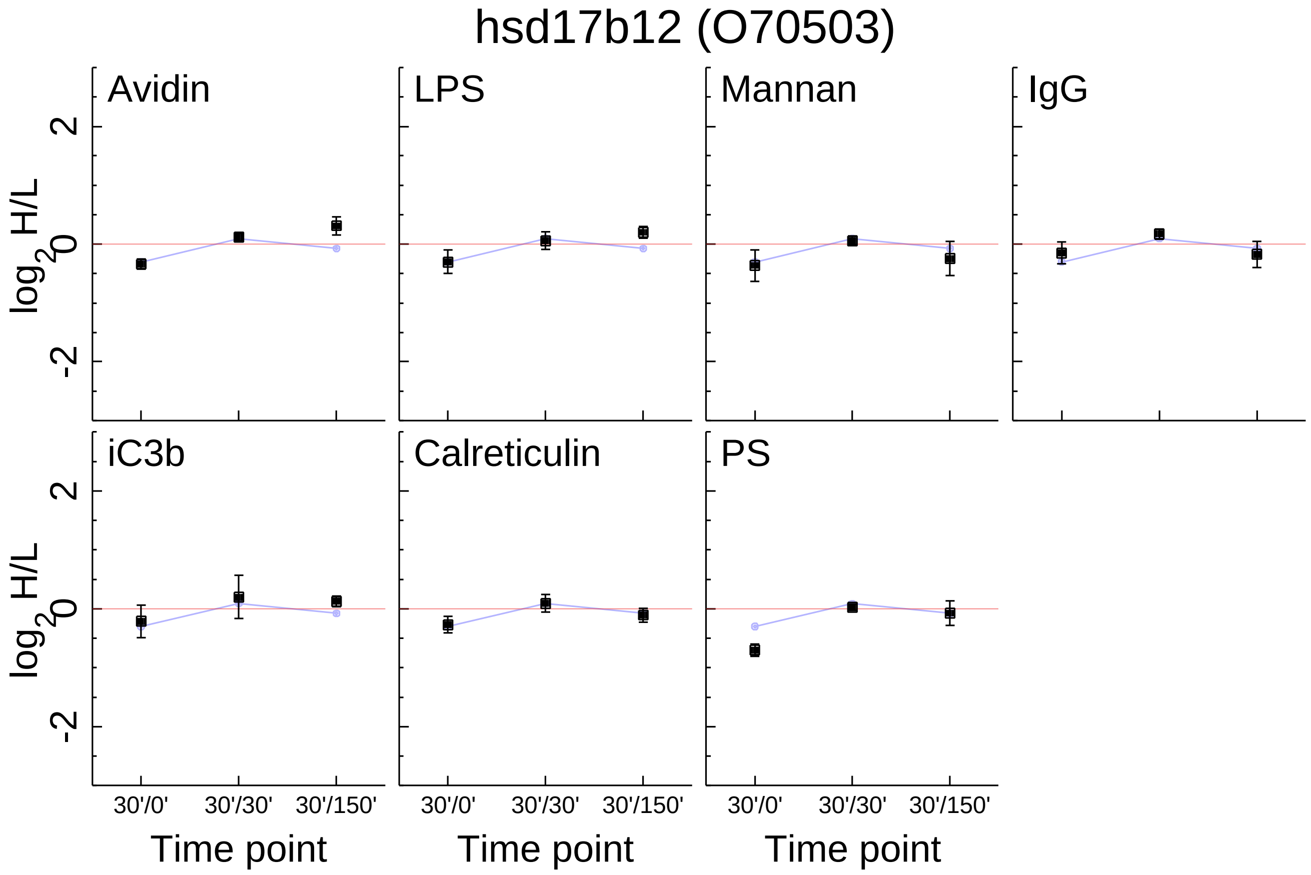

hsd17b12 (O70503)
Avidin
LPS
Mannan
IgG
iC3b
Calreticulin
PS
log2 H/L
2
0
-2
30'/0'
30'/30'
30'/150'
Time point

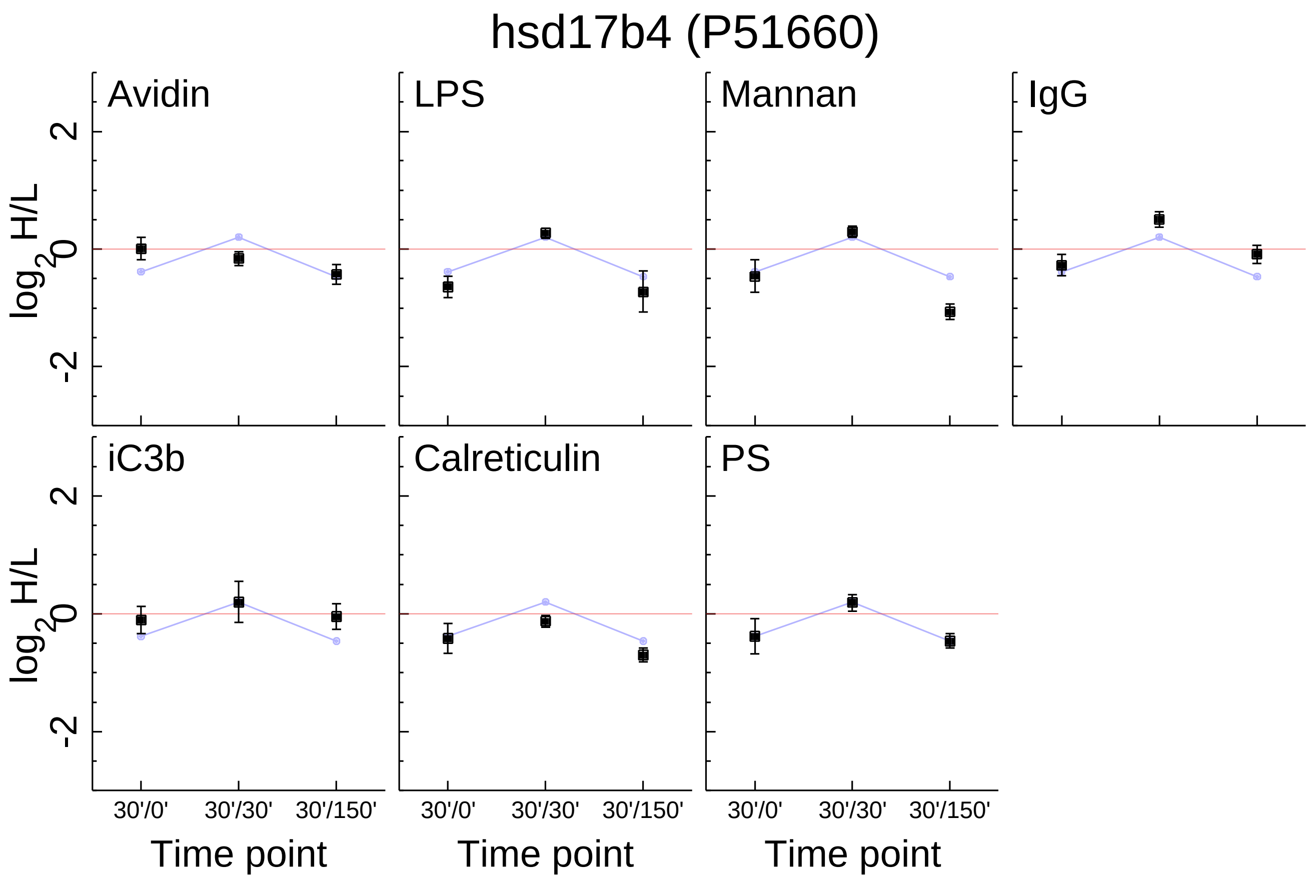
hsd17b4 (P51660)
Avidin
LPS
Mannan
IgG
iC3b
Calreticulin
PS
log2 H/L
2
0
-2
30'/0'
30'/30'
30'/150'
Time point

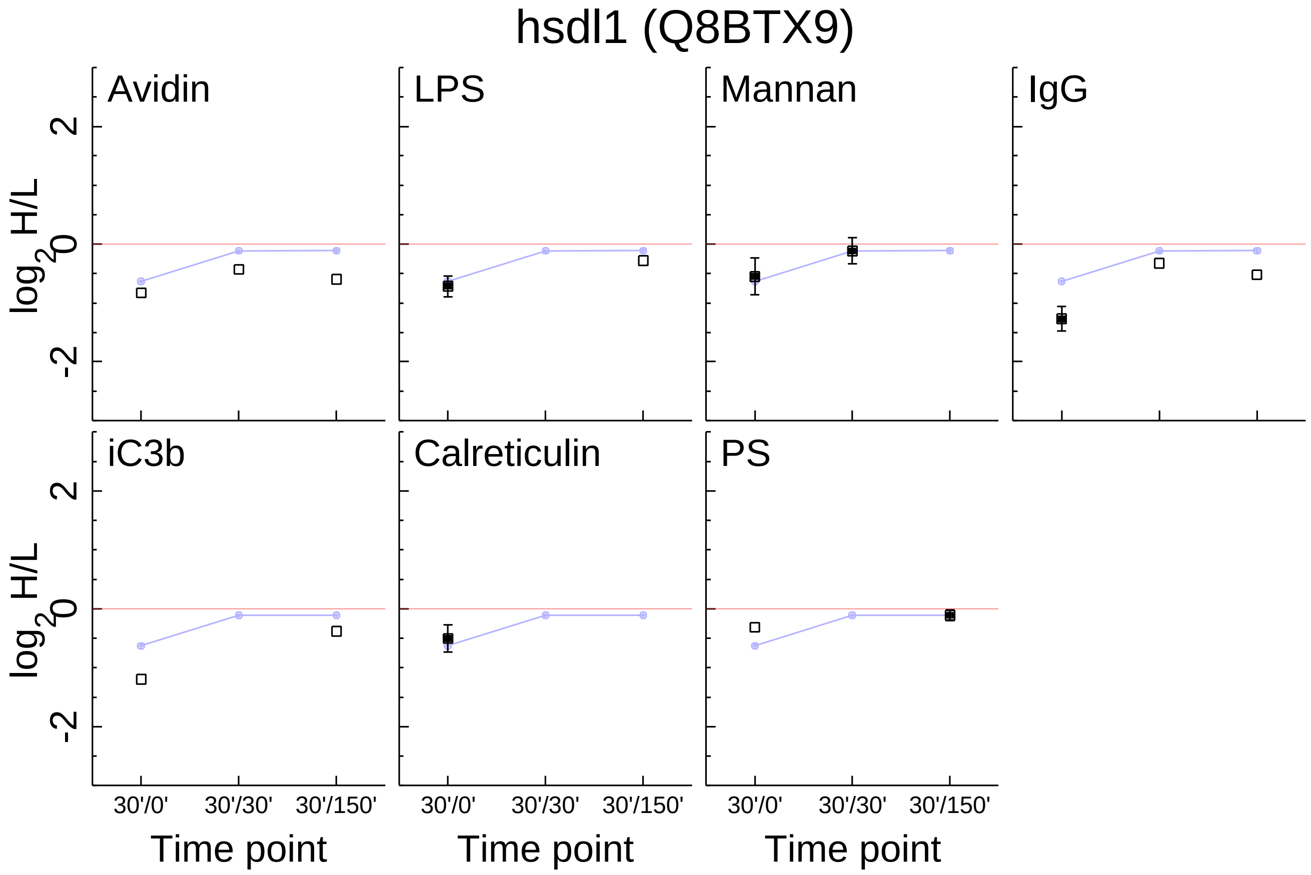
hsdl1 (Q8BTX9)
Avidin
LPS
Mannan
IgG
iC3b
Calreticulin
PS
log2 H/L
2
0
-2
30'/0'
30'/30'
30'/150'
Time point

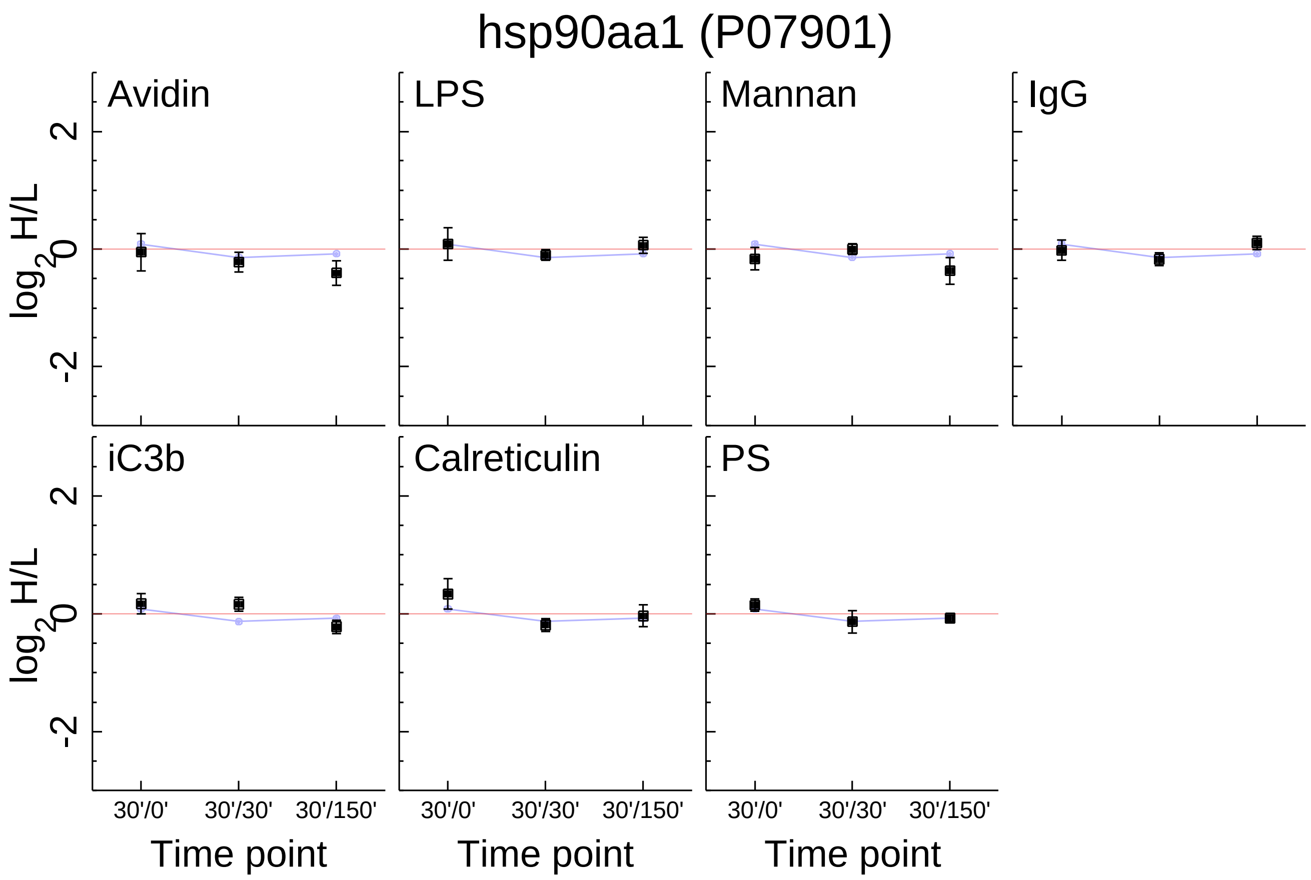

hsp90aa1 (P07901)
Avidin
LPS
Mannan
IgG
iC3b
Calreticulin
PS
log2 H/L
2
0
-2
30'/0'
30'/30'
30'/150'
Time point

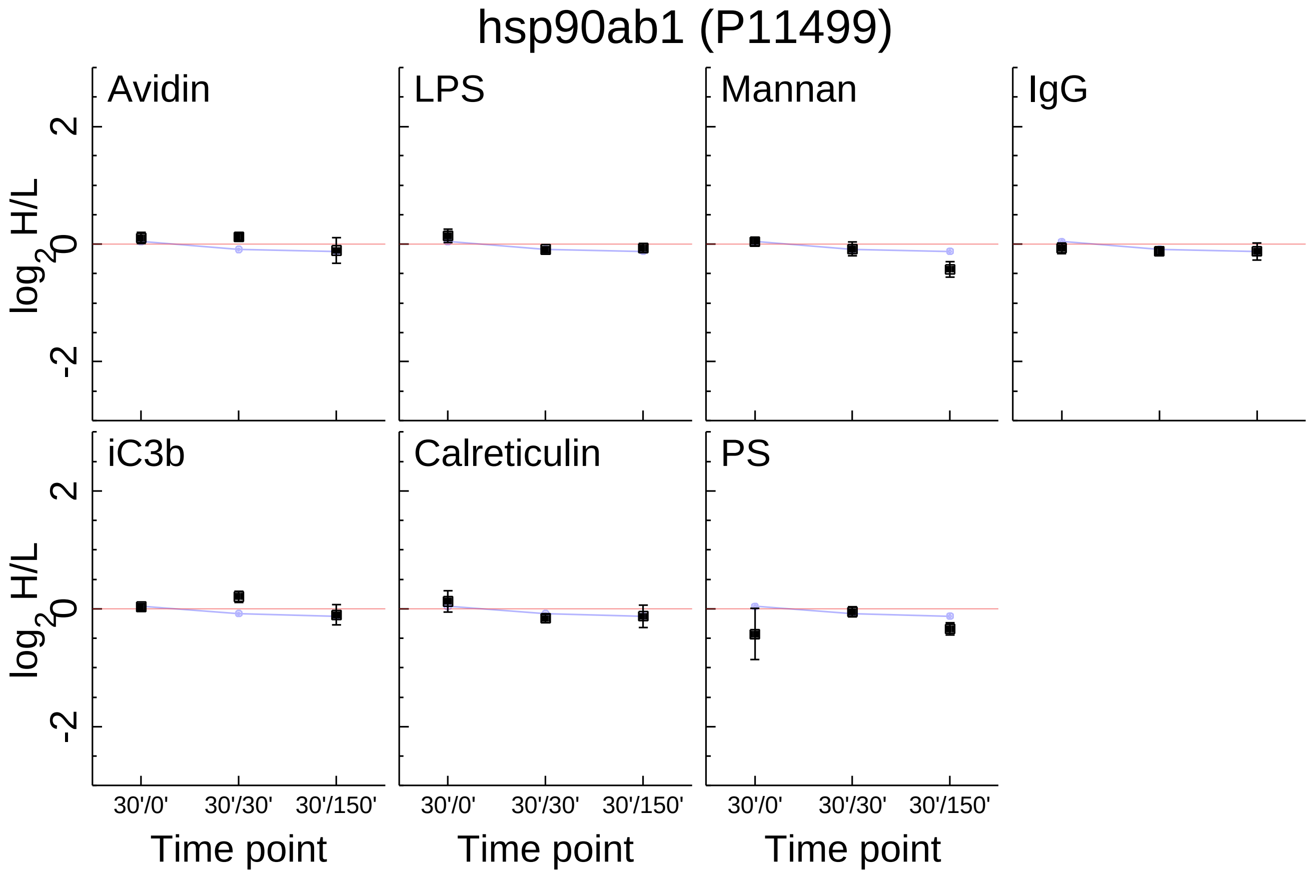
hsp90ab1 (P11499)
Avidin
LPS
Mannan
IgG
iC3b
Calreticulin
PS
log2 H/L
2
0
-2
30'/0'
30'/30'
30'/150'
Time point

hsp90b1 (P08113)

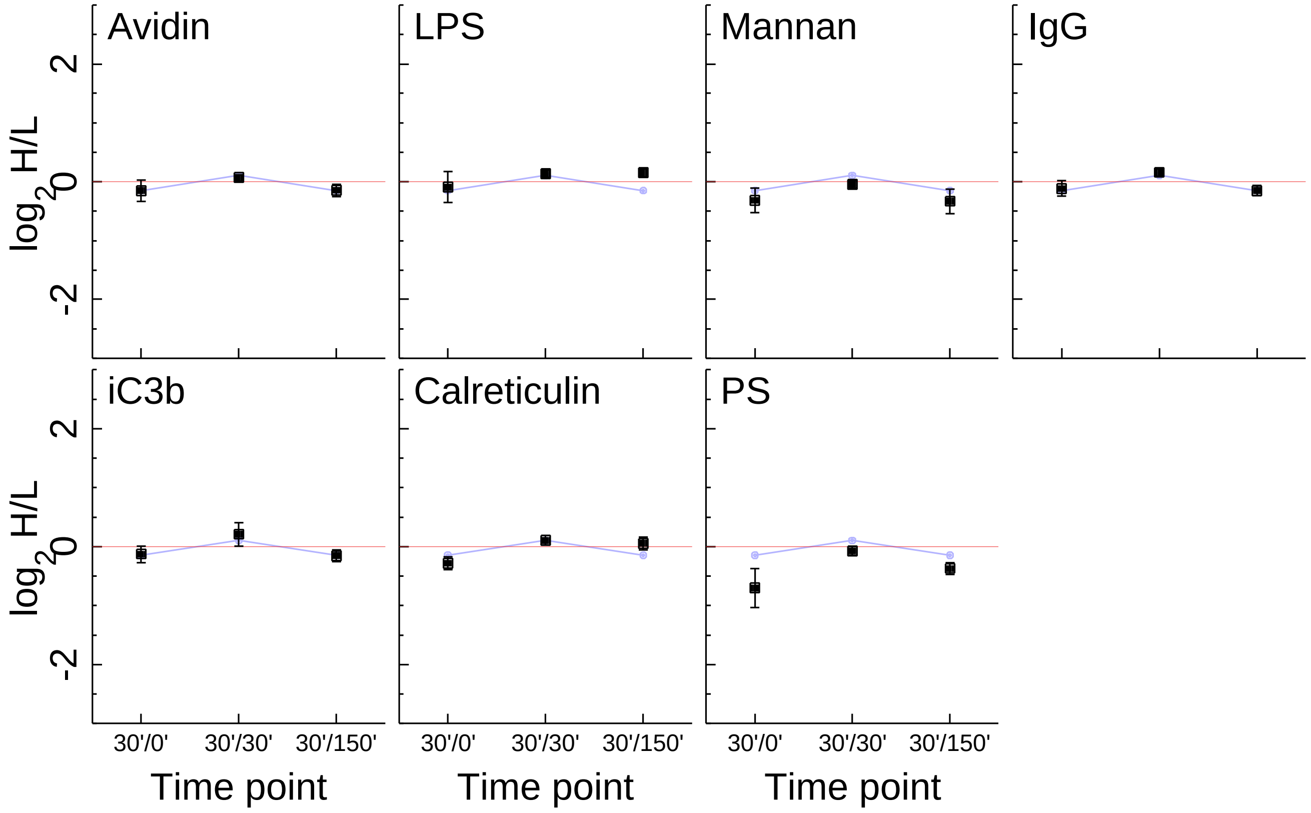

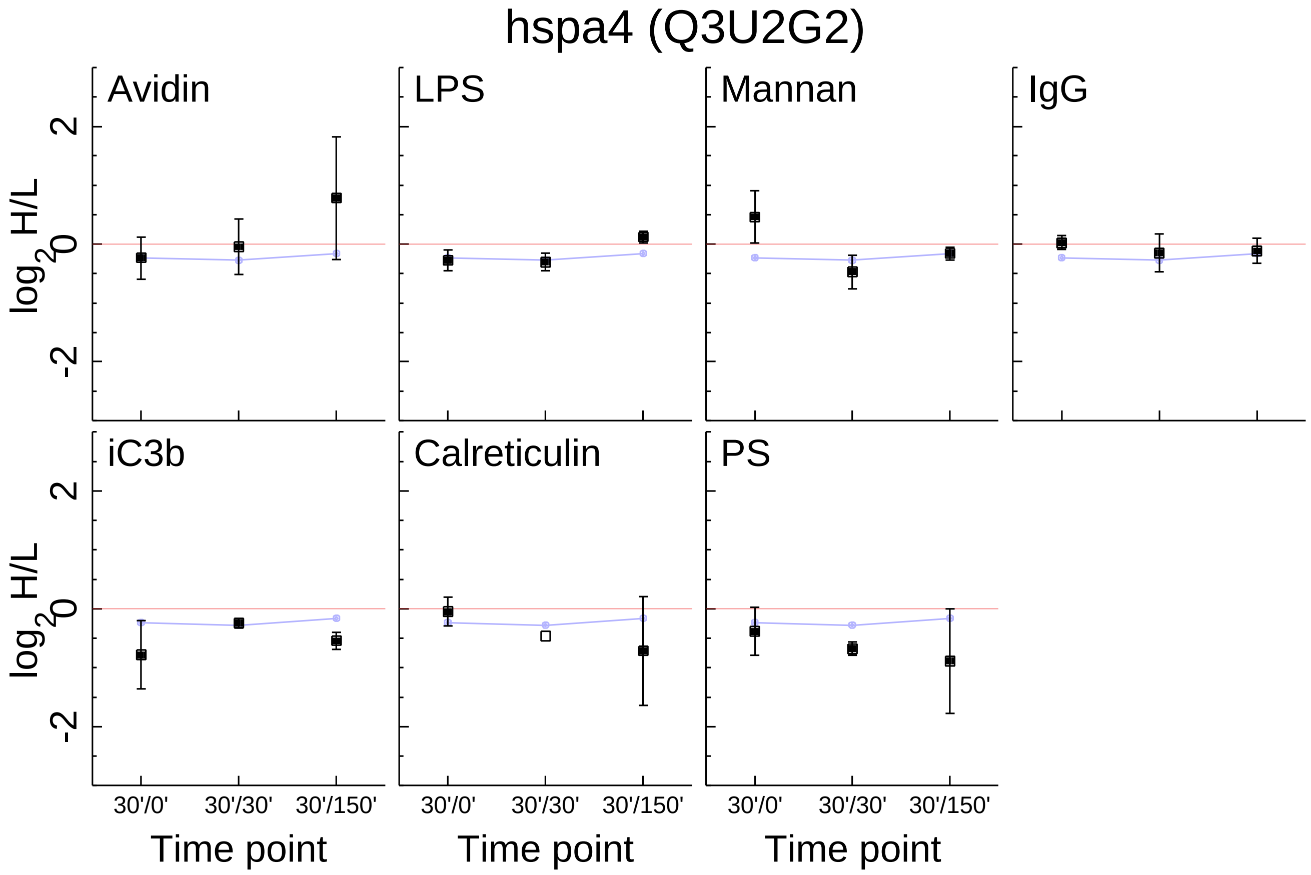
hspa4 (Q3U2G2)
Avidin
LPS
Mannan
IgG
iC3b
Calreticulin
PS
log2 H/L
2
0
-2
30'/0'
30'/30'
30'/150'
Time point

hspa5 (P20029)

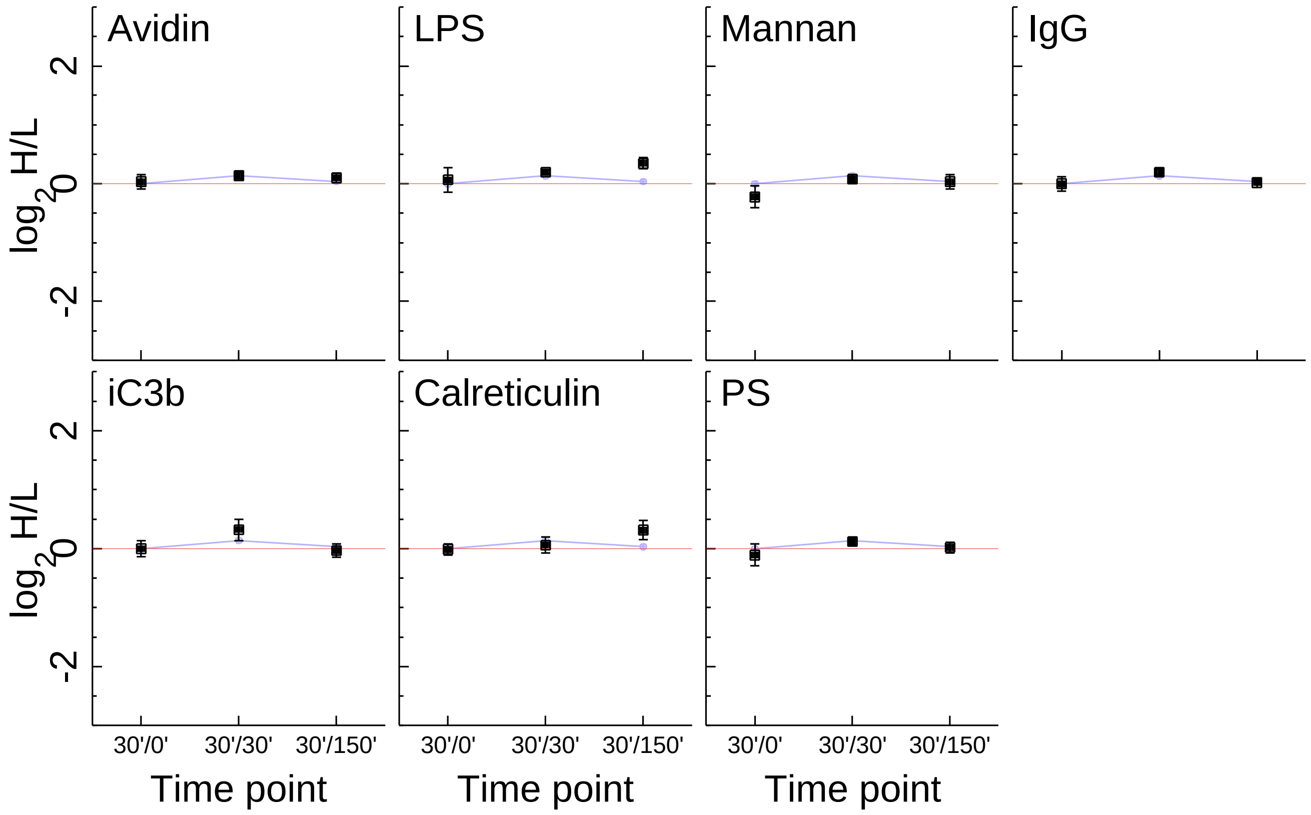

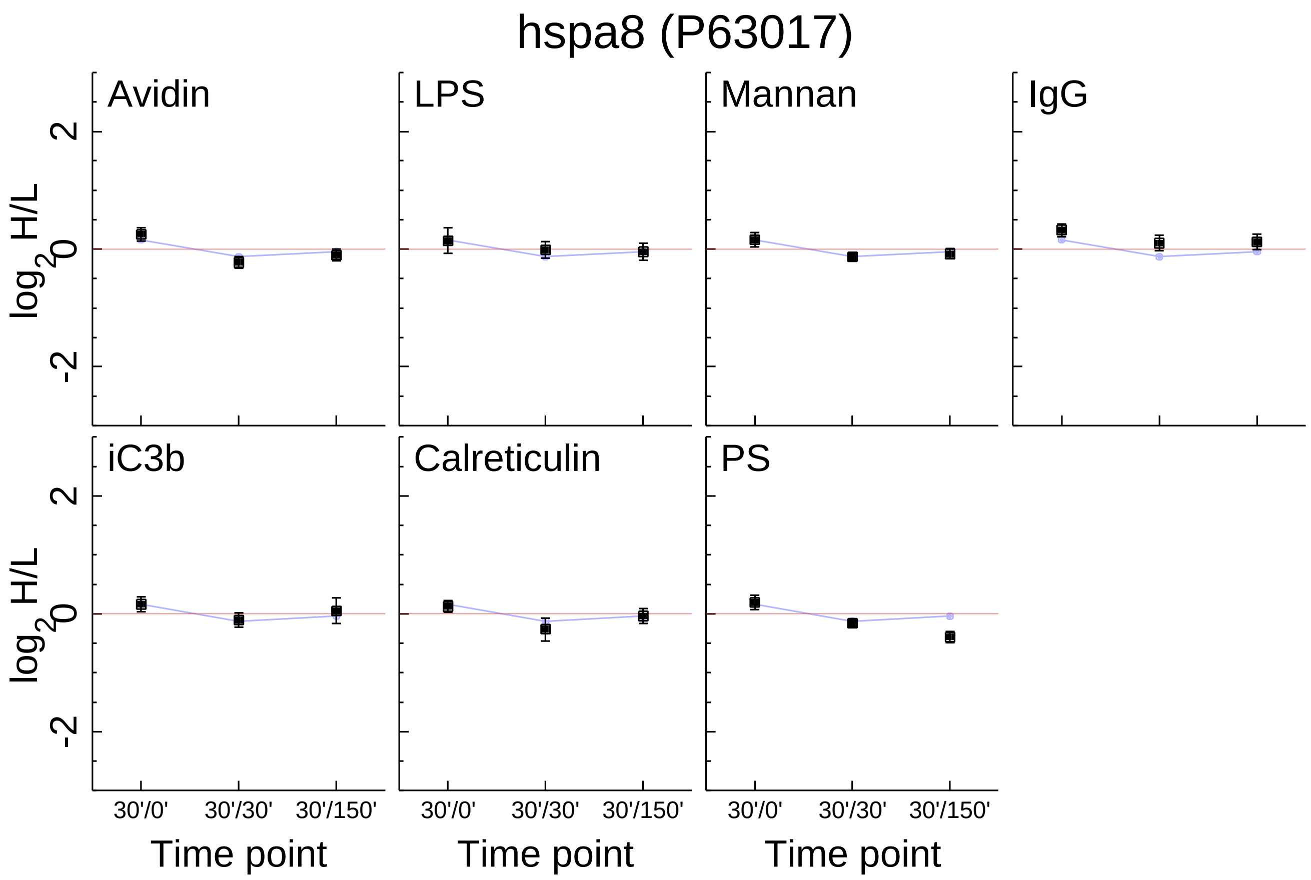
hspa8 (P63017)
Avidin
LPS
Mannan
IgG
iC3b
Calreticulin
PS
2
0
-2
log2 H/L
30'/0'
30'/30'
30'/150'
Time point

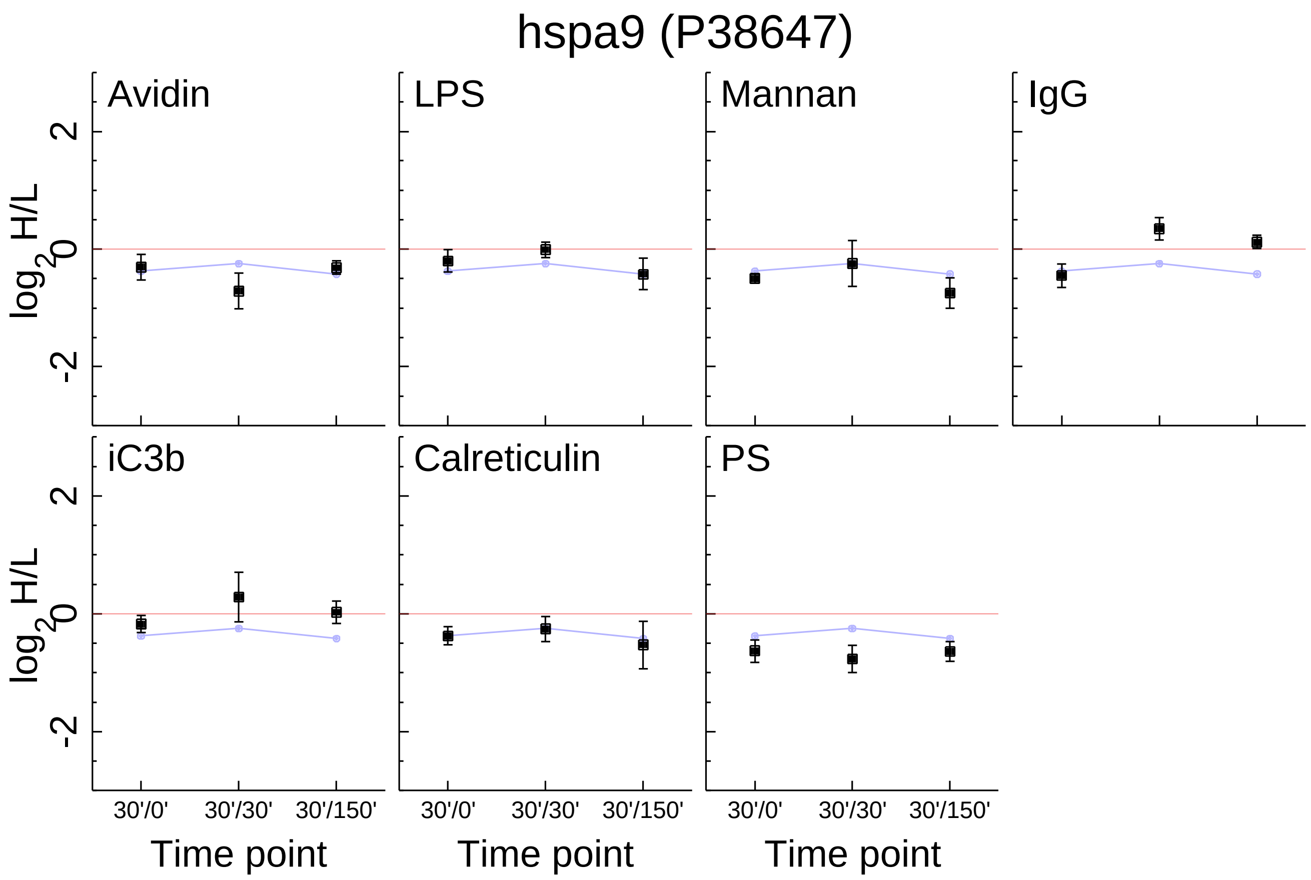
hspa9 (P38647)
Avidin
LPS
Mannan
IgG
iC3b
Calreticulin
PS
log2 H/L
2
0
-2
30'/0'
30'/30'
30'/150'
Time point

hspd1 (P63038)

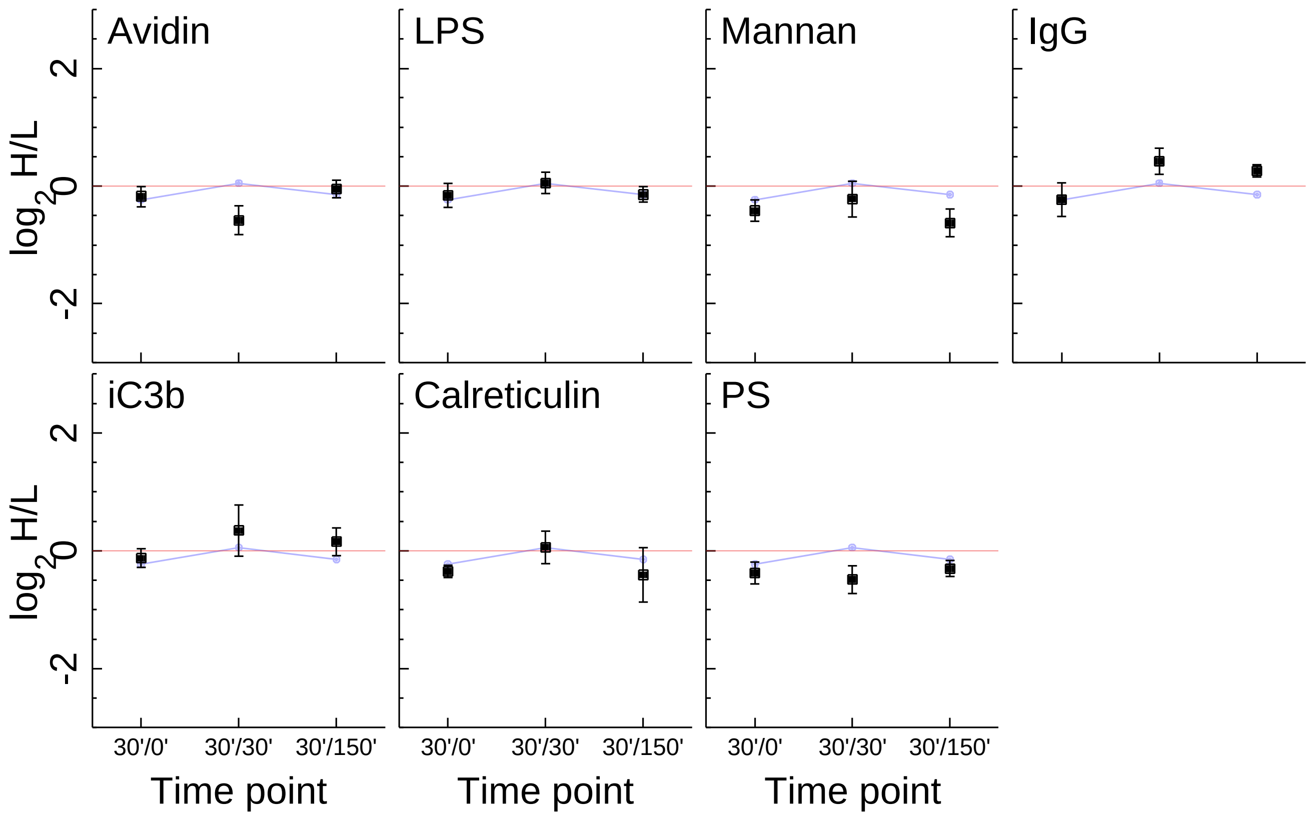

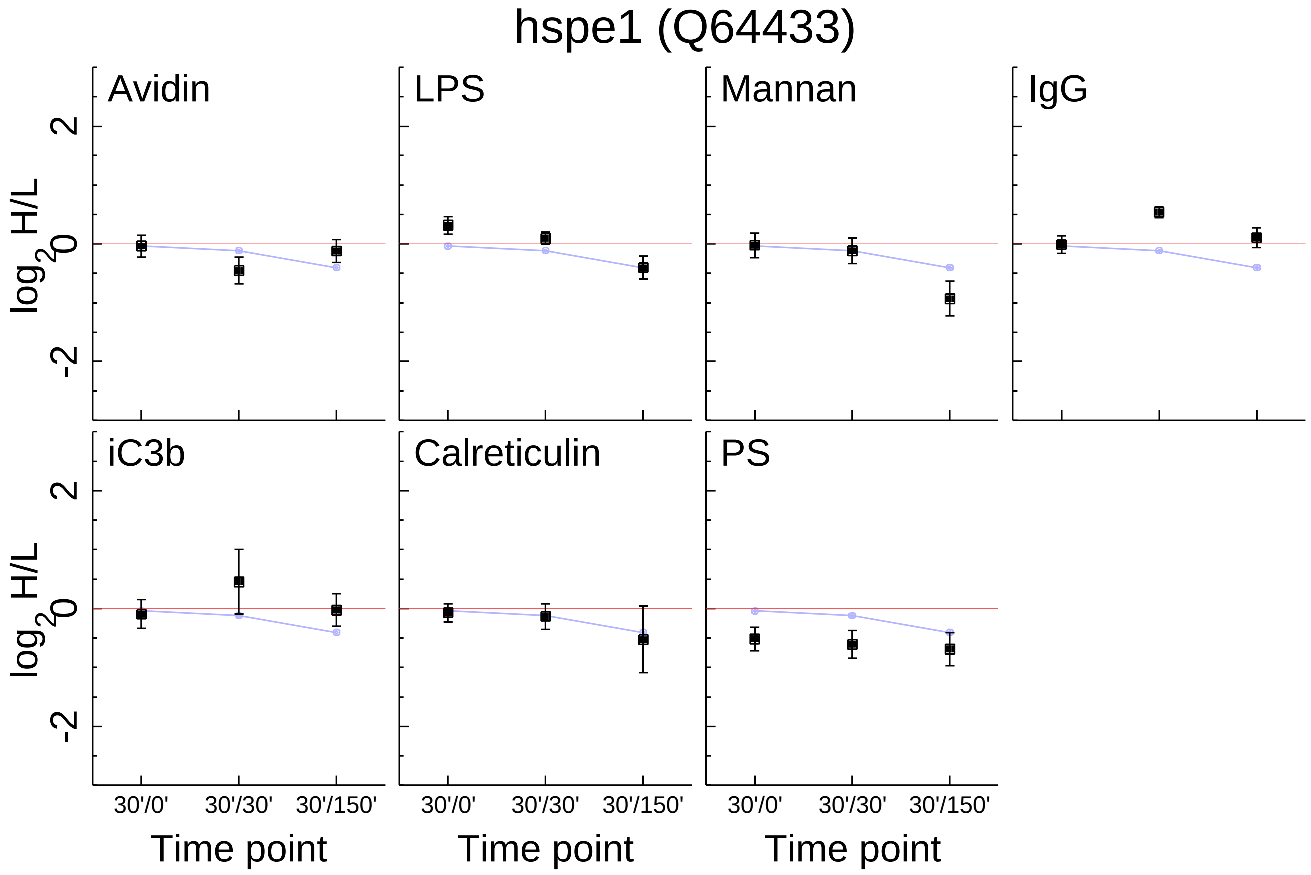
hspe1 (Q64433)
Avidin
LPS
Mannan
IgG
iC3b
Calreticulin
PS
log2 H/L
2
0
-2
30'/0'
30'/30'
30'/150'
Time point

hspg2 (E9PZ16)

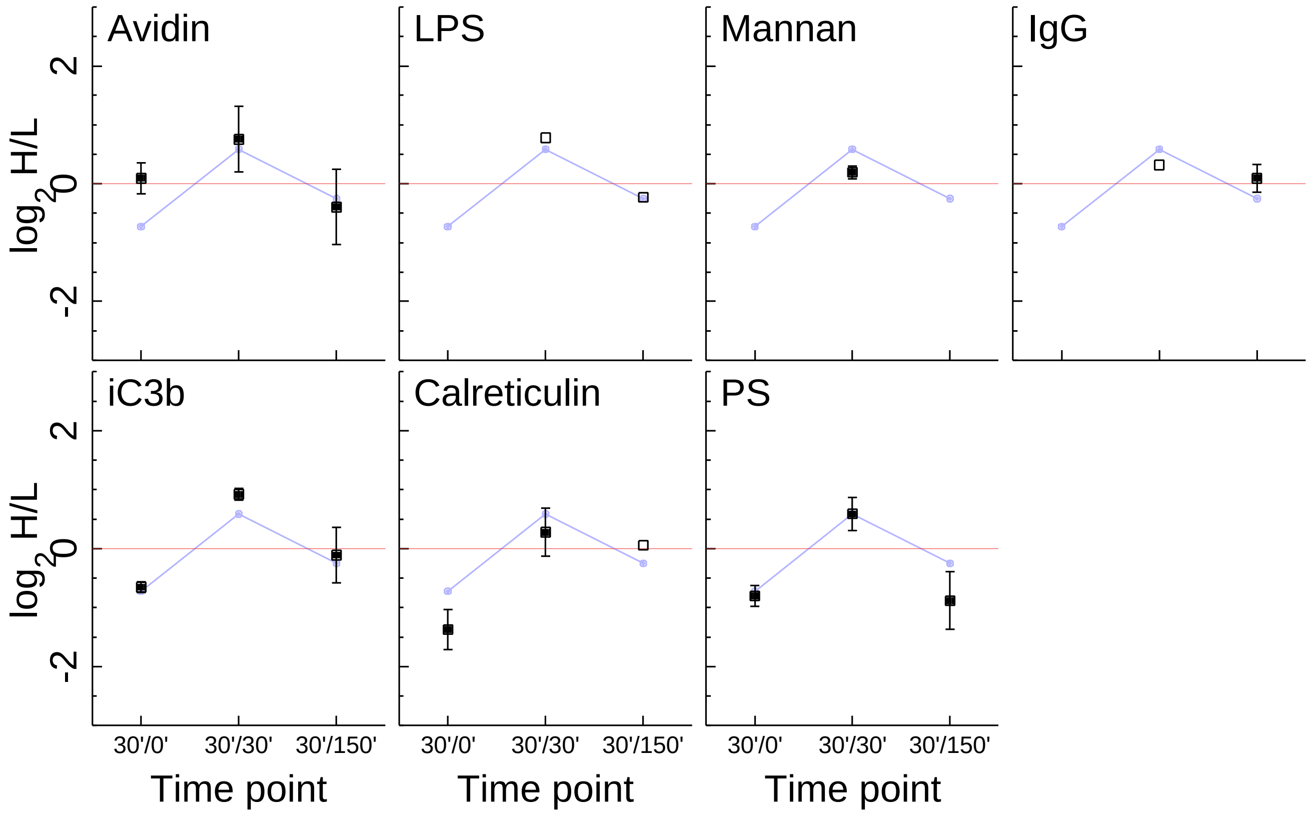

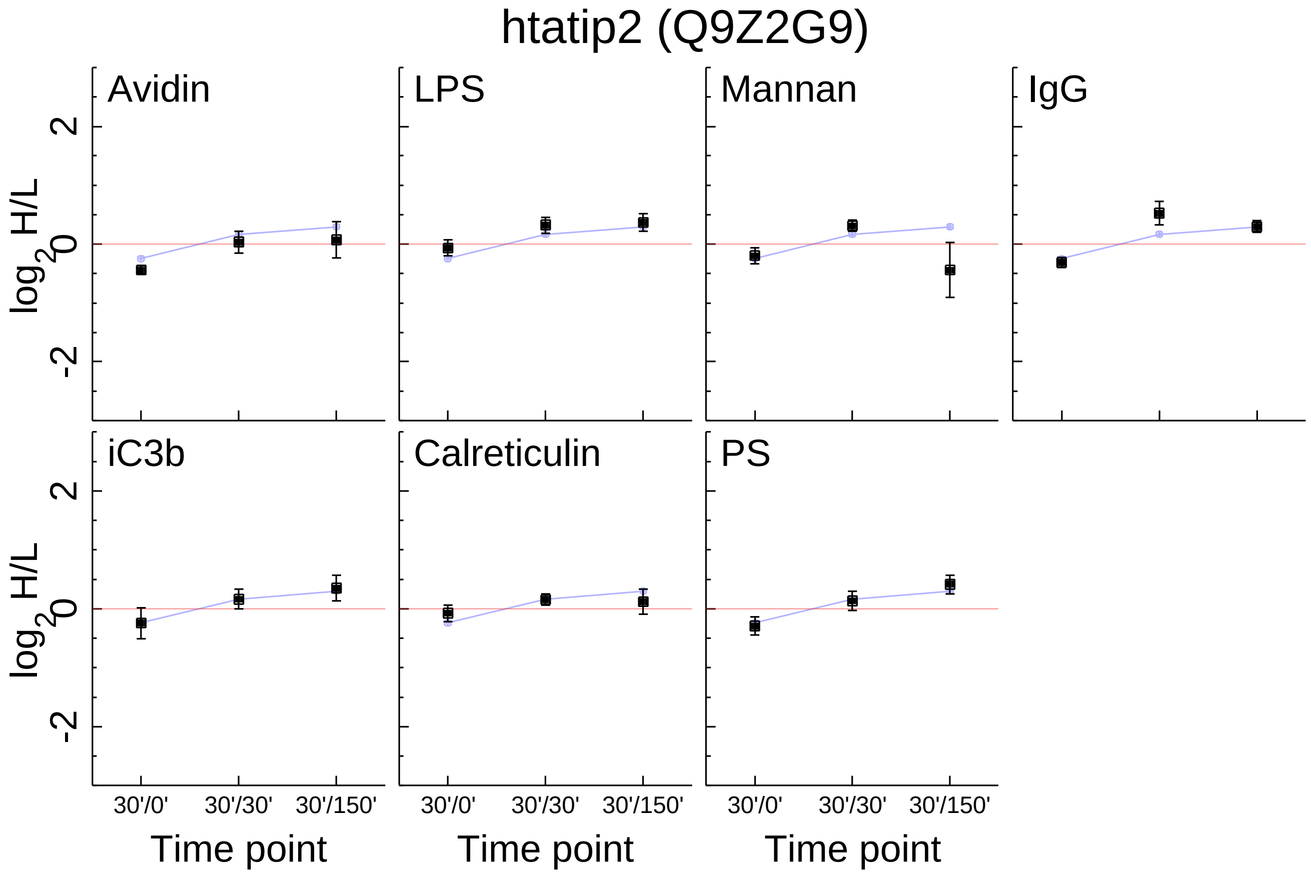

htatip2 (Q9Z2G9)
Avidin
LPS
Mannan
IgG
iC3b
Calreticulin
PS
log2 H/L
2
0
-2
30'/0'
30'/30'
30'/150'
Time point

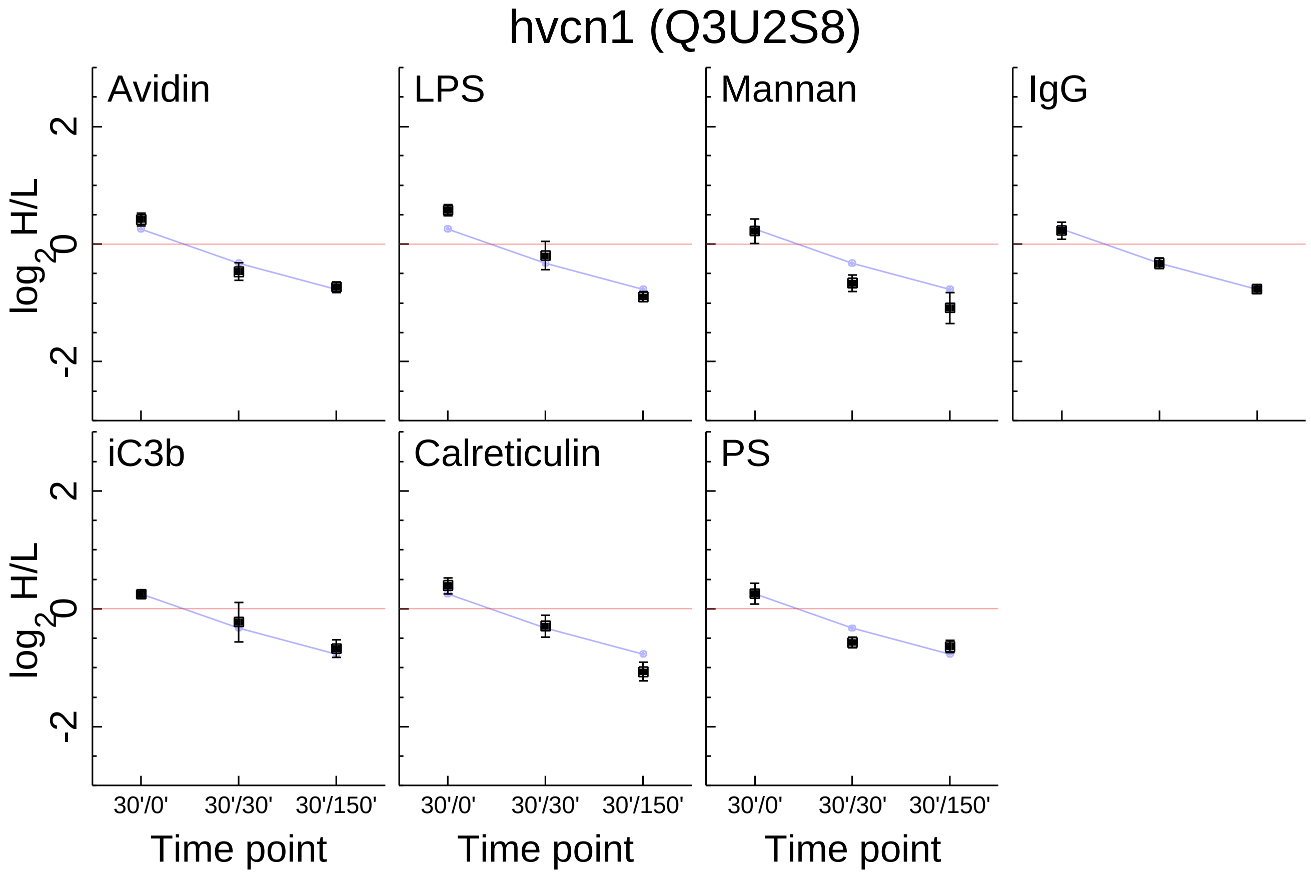

hvcn1 (Q3U2S8)
Avidin
LPS
Mannan
IgG
iC3b
Calreticulin
PS
log2 H/L
2
0
-2
30'/0'
30'/30'
30'/150'
Time point

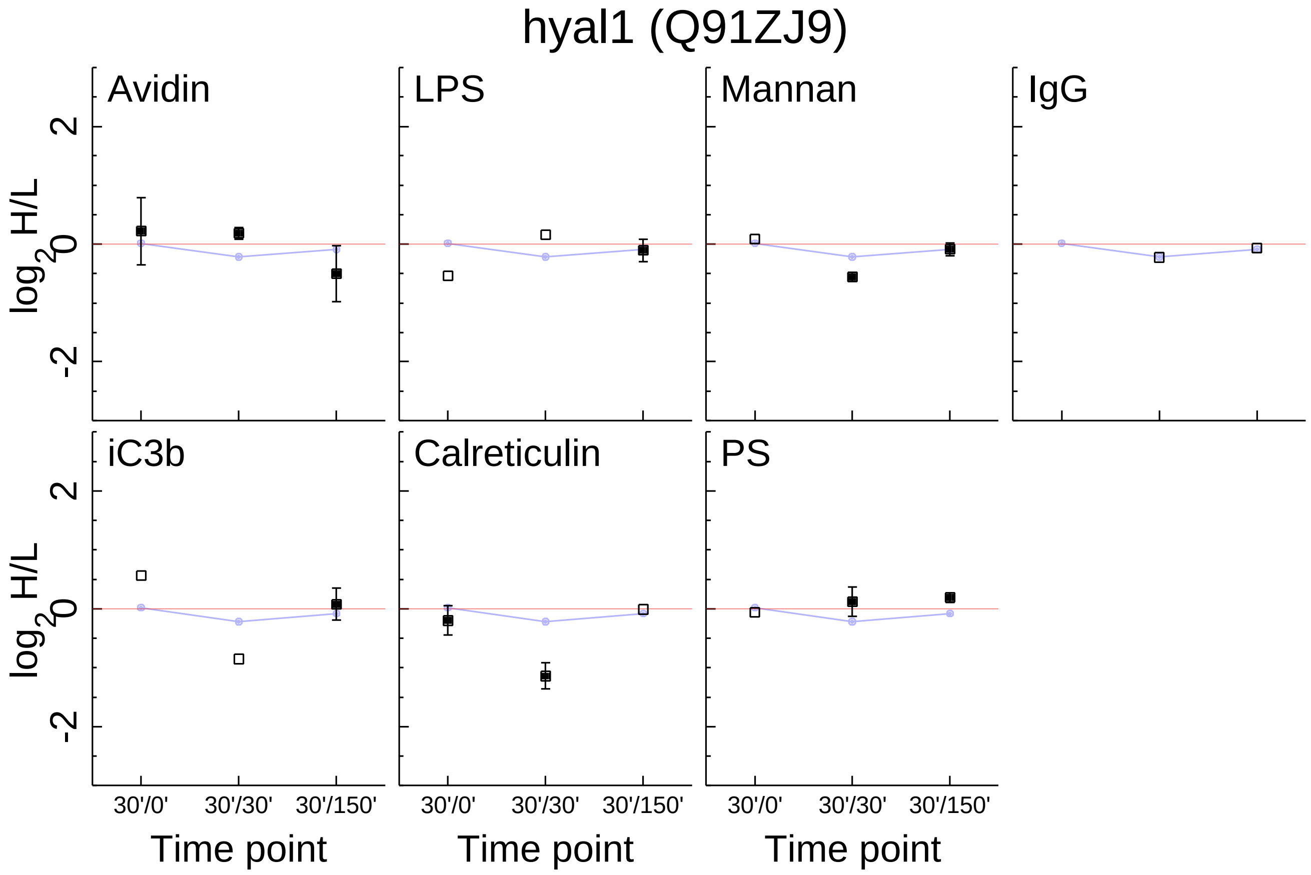

hyal1 (Q91ZJ9)
Avidin
LPS
Mannan
IgG
iC3b
Calreticulin
PS
log_2 H/L
2
0
-2
30'/0'
30'/30'
30'/150'
Time point

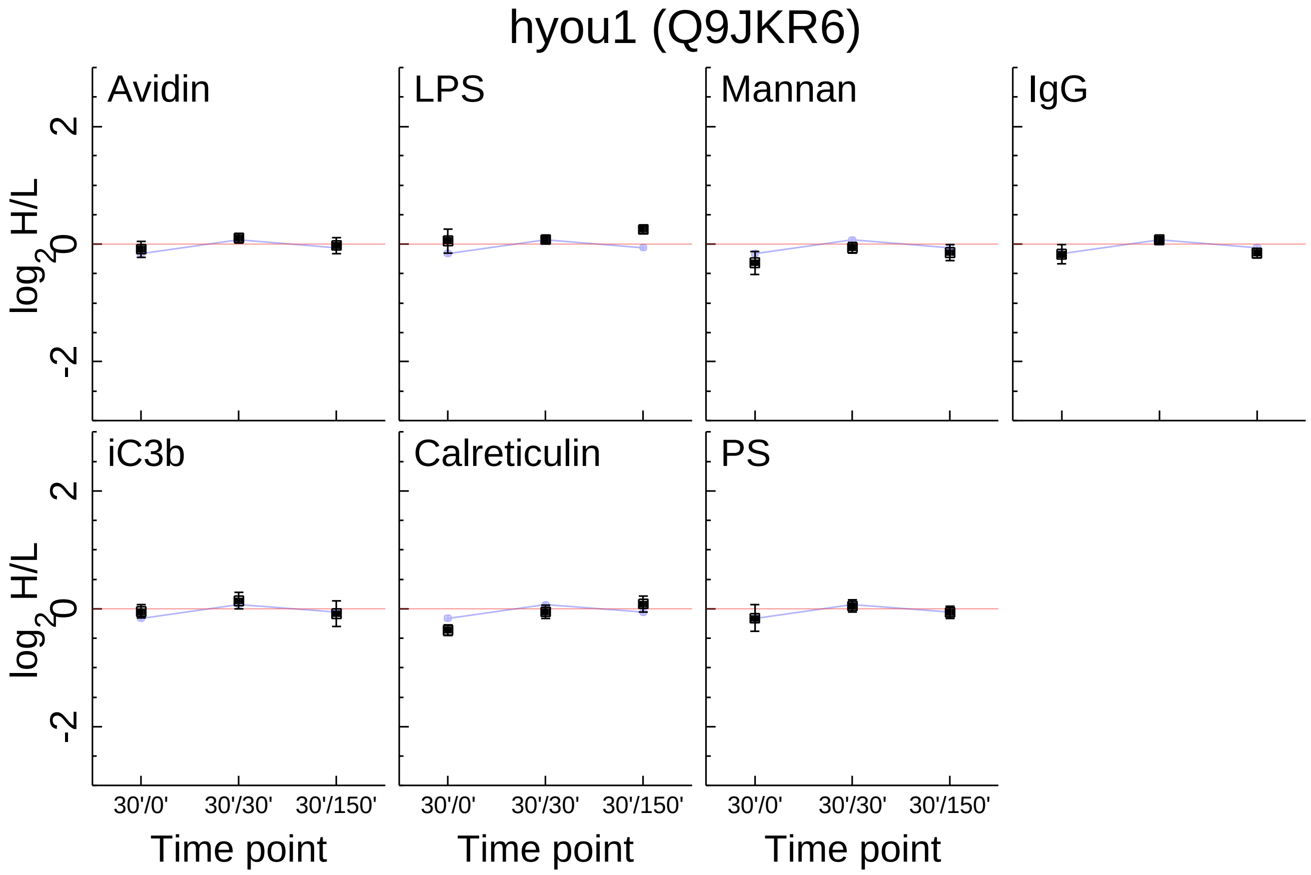
hyou1 (Q9JKR6)
Avidin
LPS
Mannan
IgG
iC3b
Calreticulin
PS
log2 H/L
2
0
-2
30'/0'
30'/30'
30'/150'
Time point

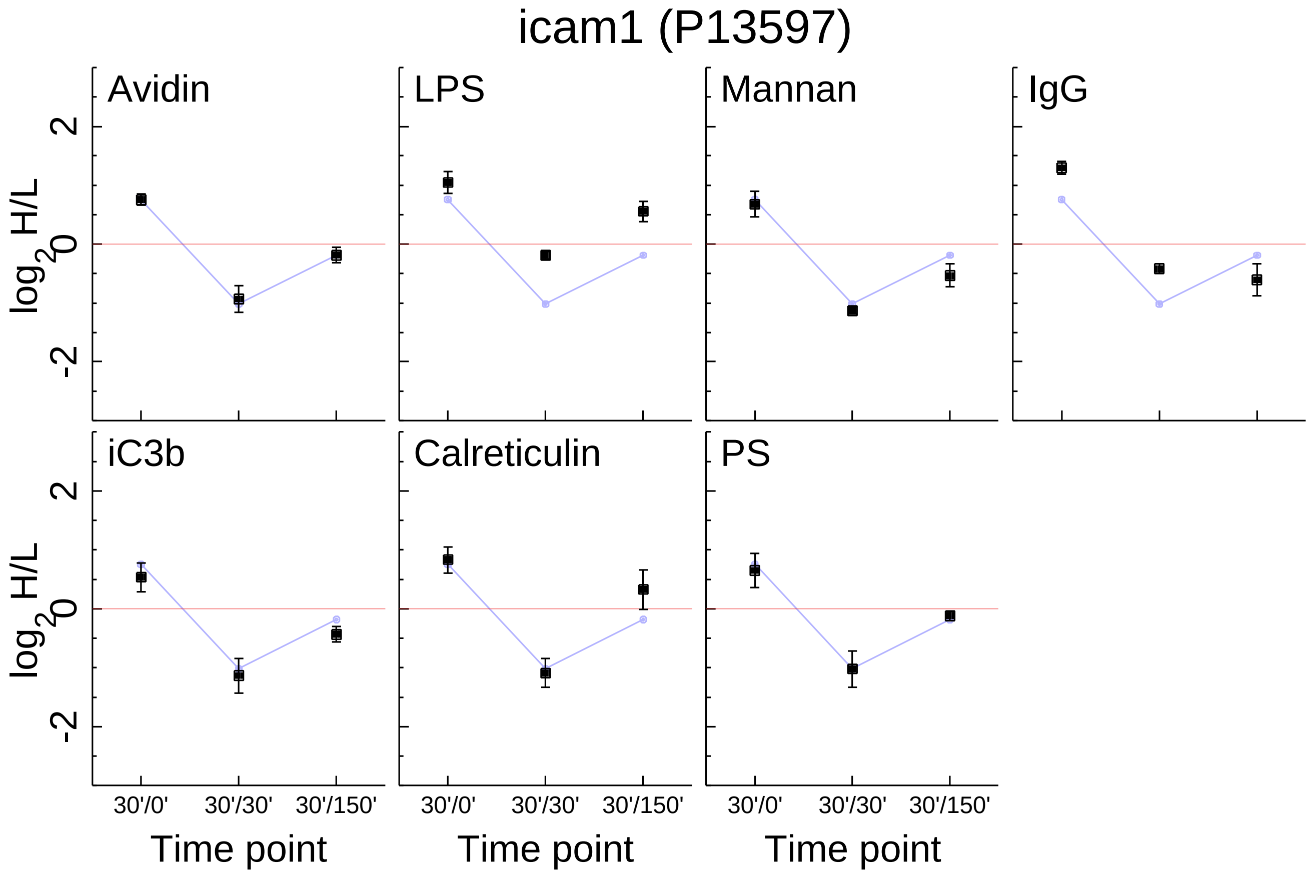

icam1 (P13597)
Avidin
LPS
Mannan
IgG
iC3b
Calreticulin
PS
log2 H/L
2
0
-2
30'/0'
30'/30'
30'/150'
Time point

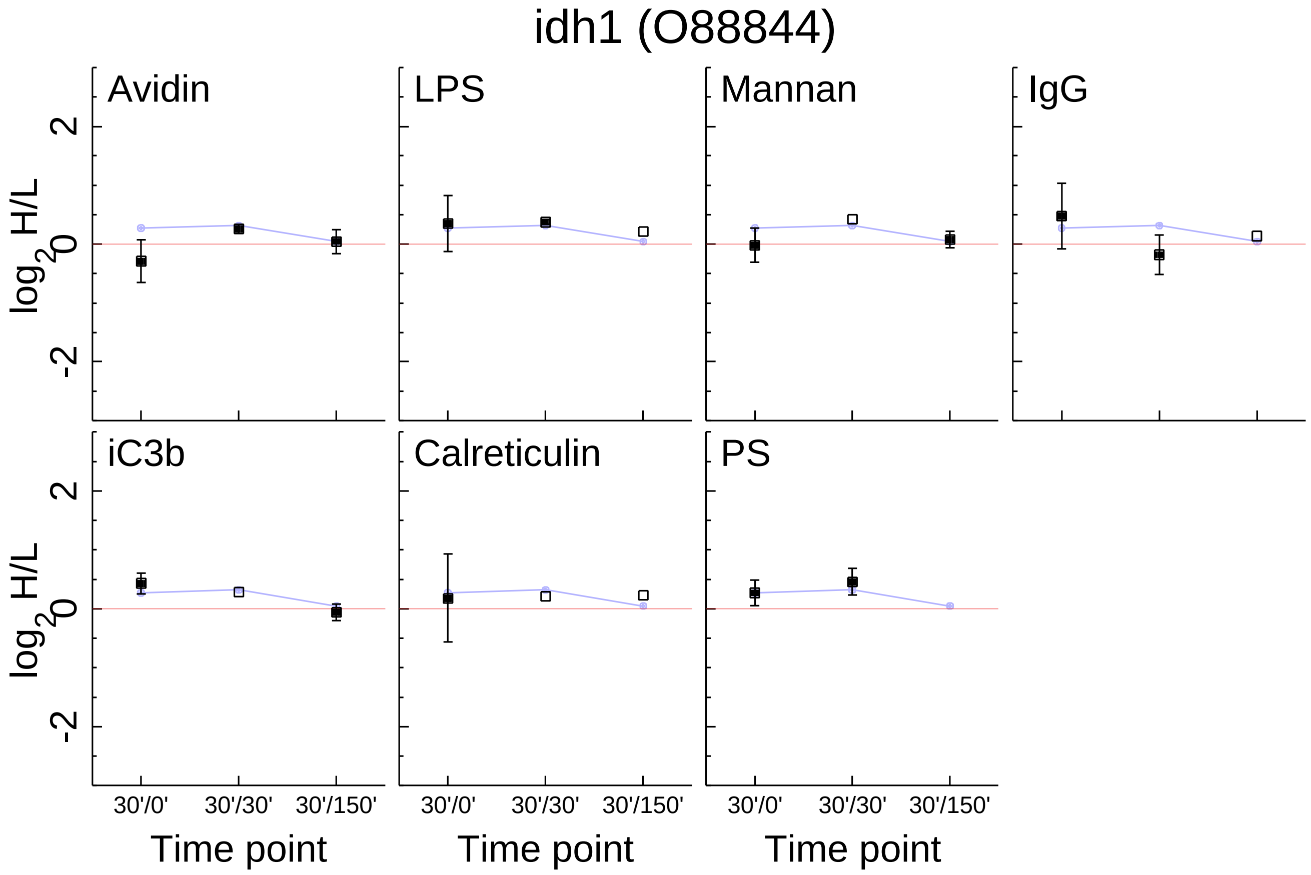
idh1 (O88844)
Avidin
LPS
Mannan
IgG
iC3b
Calreticulin
PS
log2 H/L
2
0
-2
30'/0'
30'/30'
30'/150'
Time point

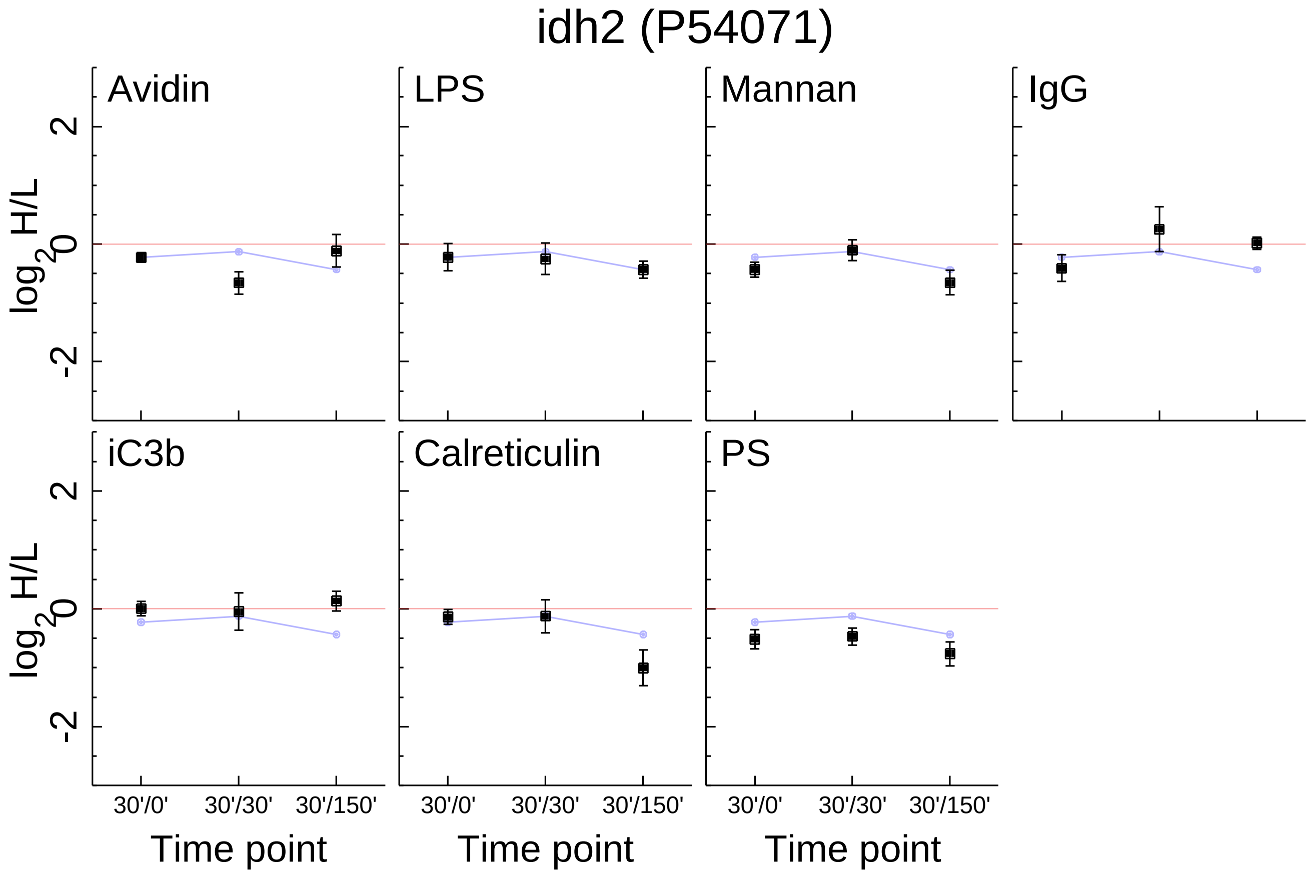

idh2 (P54071)
Avidin
LPS
Mannan
IgG
iC3b
Calreticulin
PS
log2 H/L
2
0
-2
30'/0'
30'/30'
30'/150'
Time point

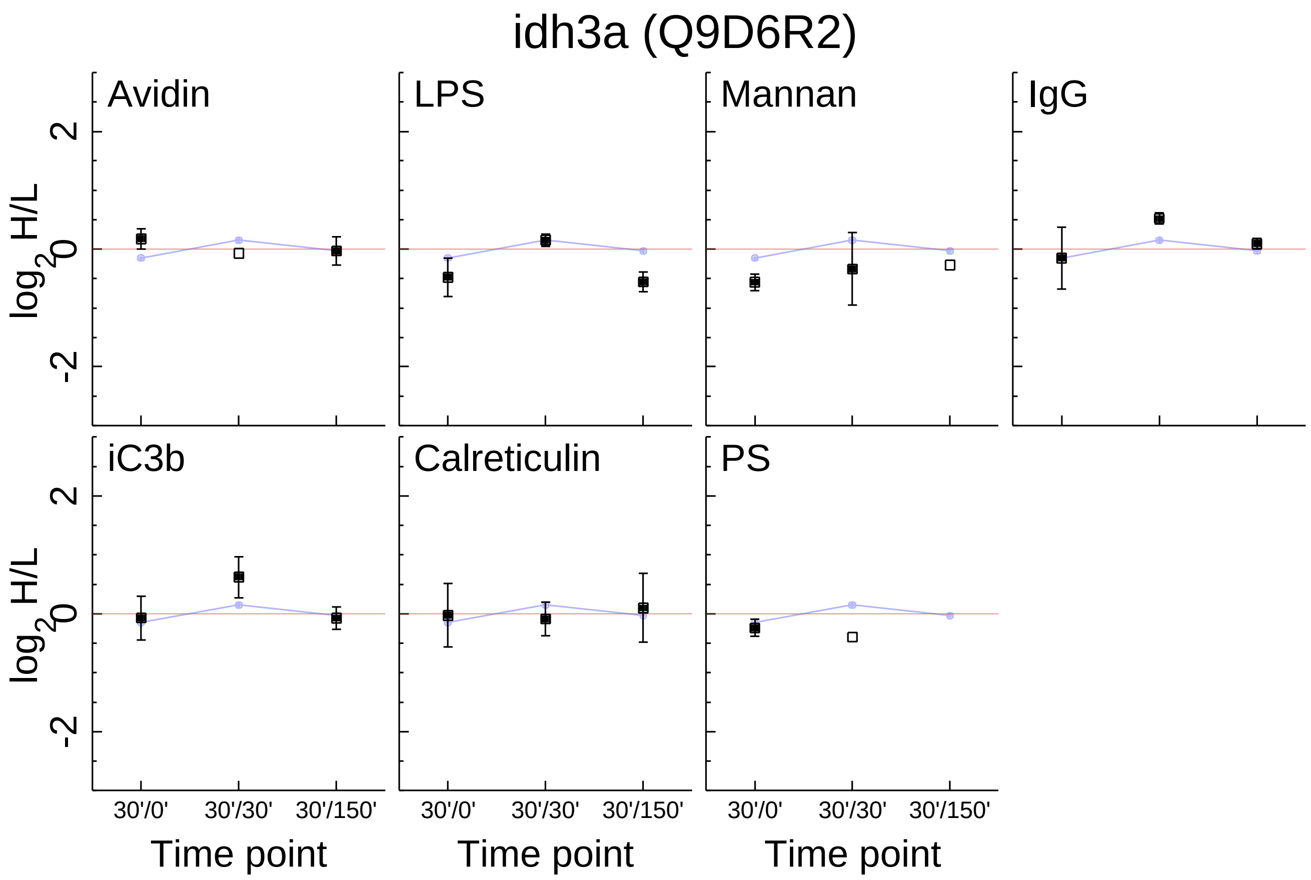

idh3a (Q9D6R2)
Avidin
LPS
Mannan
IgG
iC3b
Calreticulin
PS
log2 H/L
2
0
-2
30'/0'
30'/30'
30'/150'
Time point

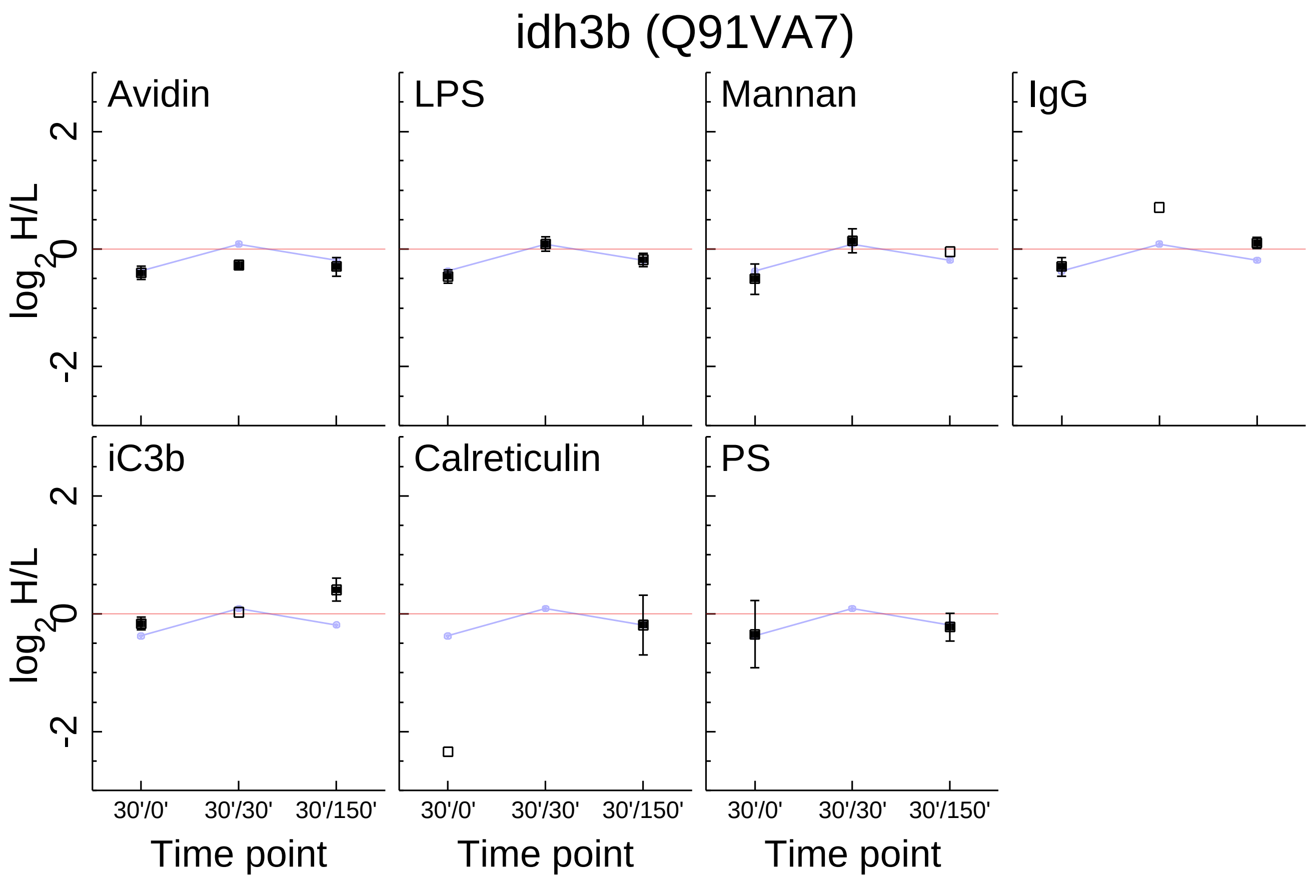
idh3b (Q91VA7)
Avidin
LPS
Mannan
IgG
iC3b
Calreticulin
PS
log2 H/L
2
0
-2
30'/0'
30'/30'
30'/150'
Time point

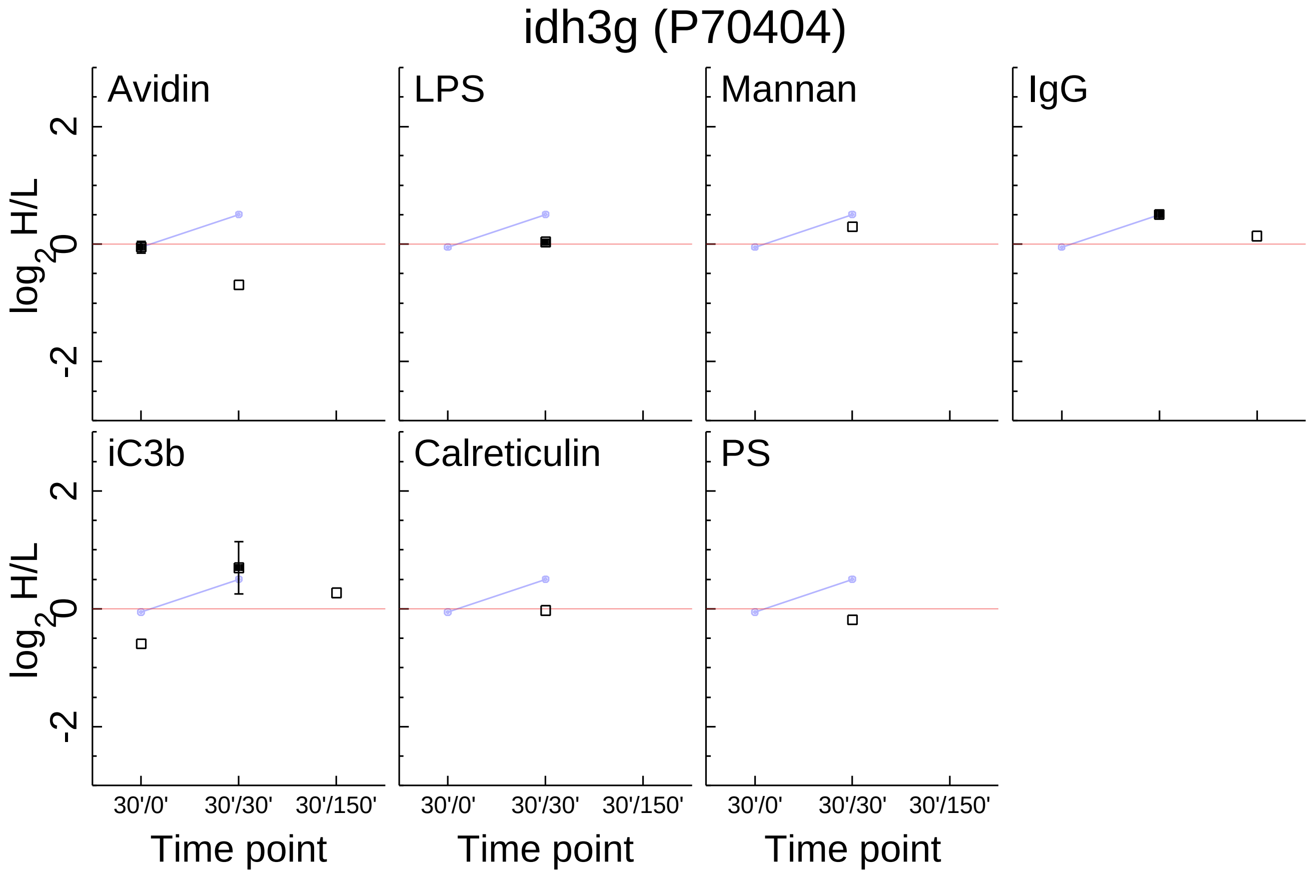
idh3g (P70404)
Avidin
LPS
Mannan
IgG
iC3b
Calreticulin
PS
log2 H/L
2
0
-2
30'/0'
30'/30'
30'/150'
Time point

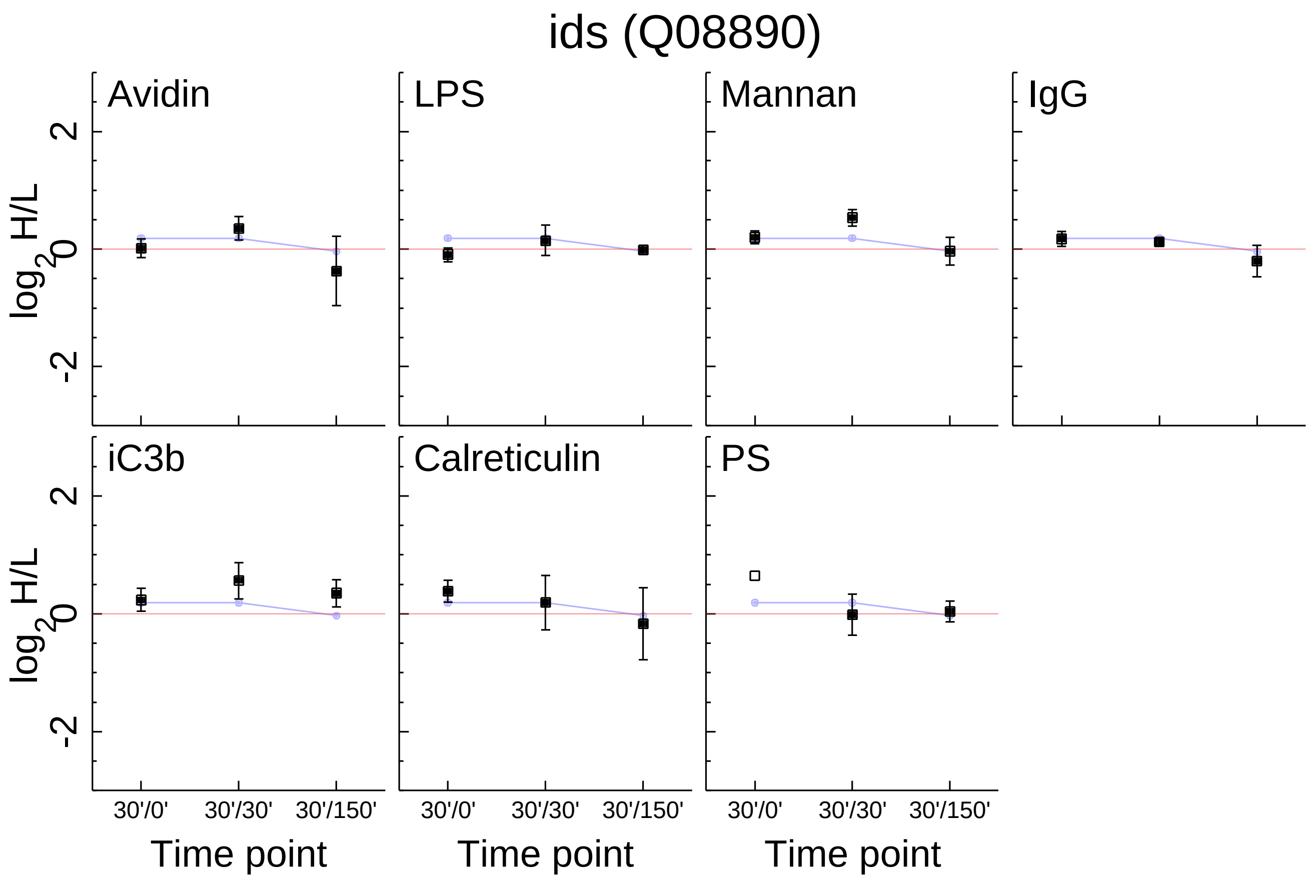
ids (Q08890)
Avidin
LPS
Mannan
IgG
iC3b
Calreticulin
PS
log2 H/L
2
0
-2
30'/0'
30'/30'
30'/150'
Time point

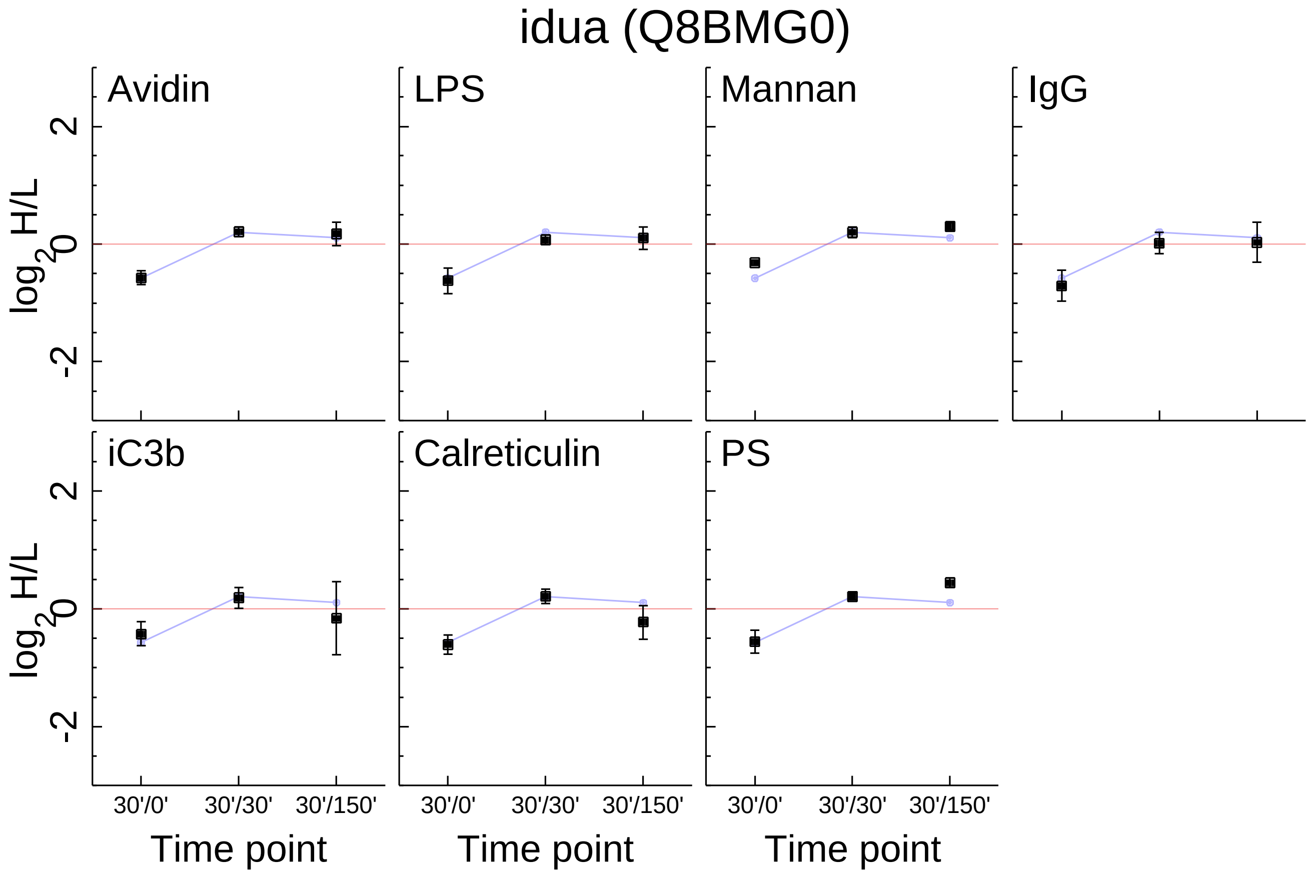
idua (Q8BMG0)
Avidin
LPS
Mannan
IgG
iC3b
Calreticulin
PS
log2 H/L
2
0
-2
30'/0'
30'/30'
30'/150'
Time point

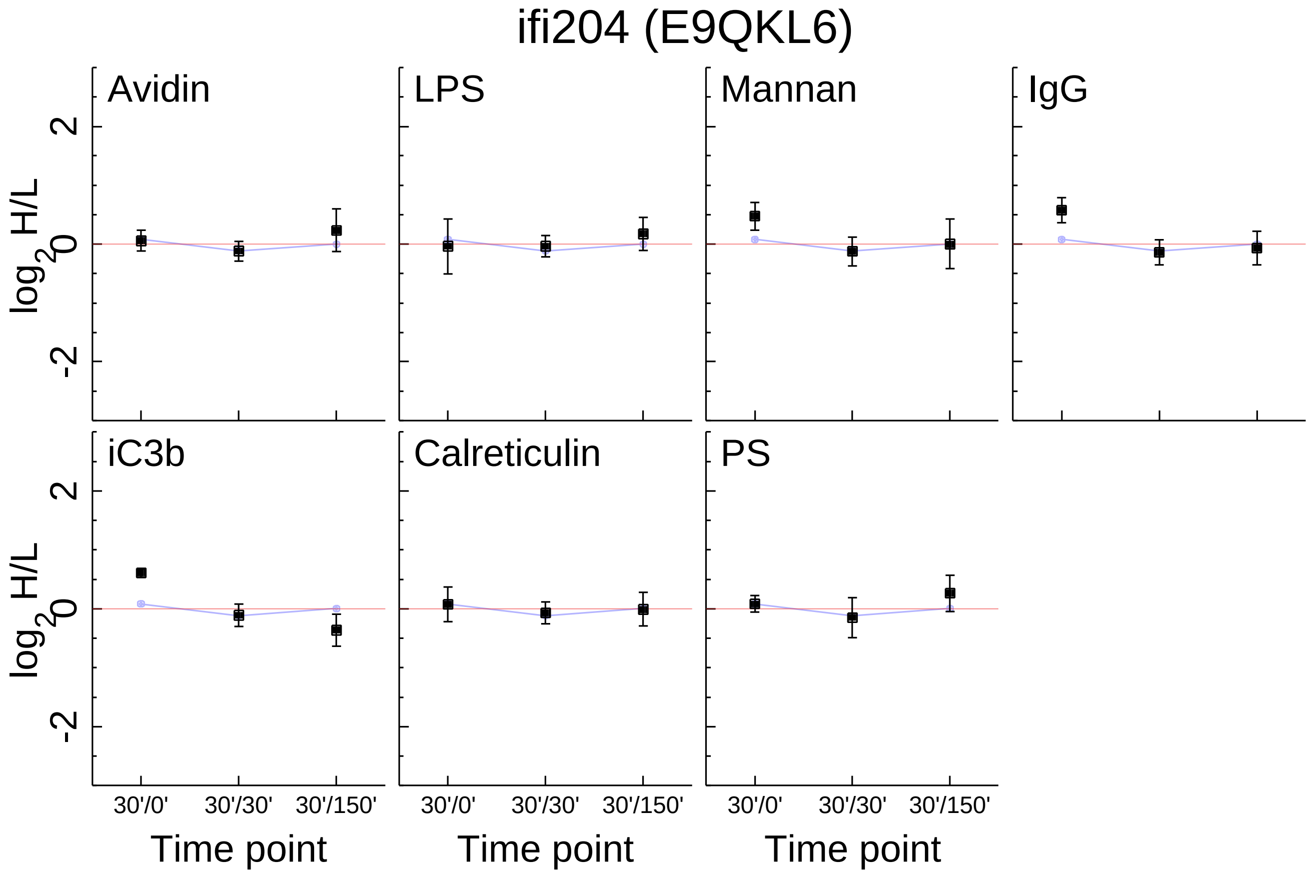
ifi204 (E9QKL6)
Avidin
LPS
Mannan
IgG
iC3b
Calreticulin
PS
$\log_2$ H/L
2
0
-2
30'/0'
30'/30'
30'/150'
Time point

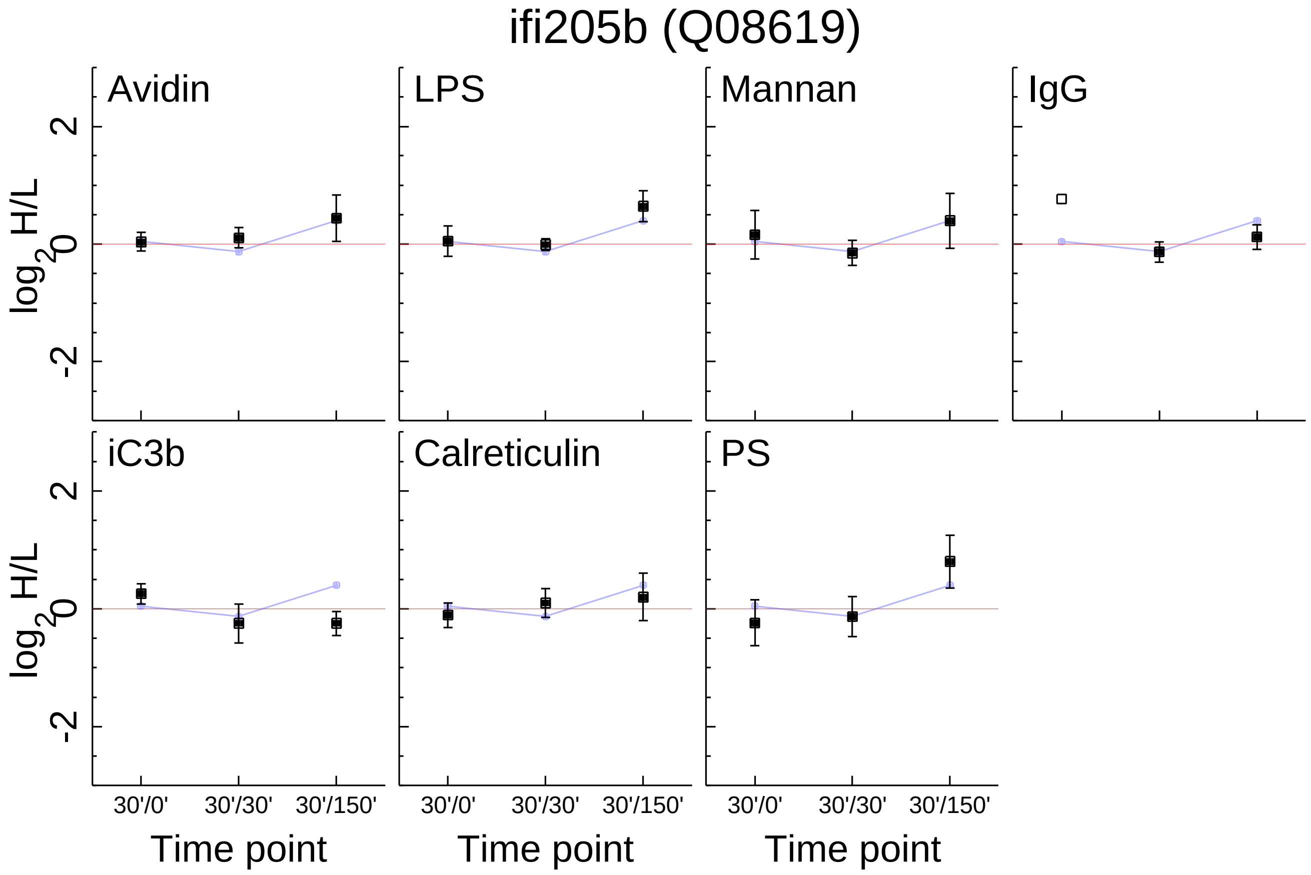

ifi205b (Q08619)
Avidin
LPS
Mannan
IgG
iC3b
Calreticulin
PS
log2 H/L
2
0
-2
30'/0'
30'/30'
30'/150'
Time point

ifi30 (Q9ESY9)

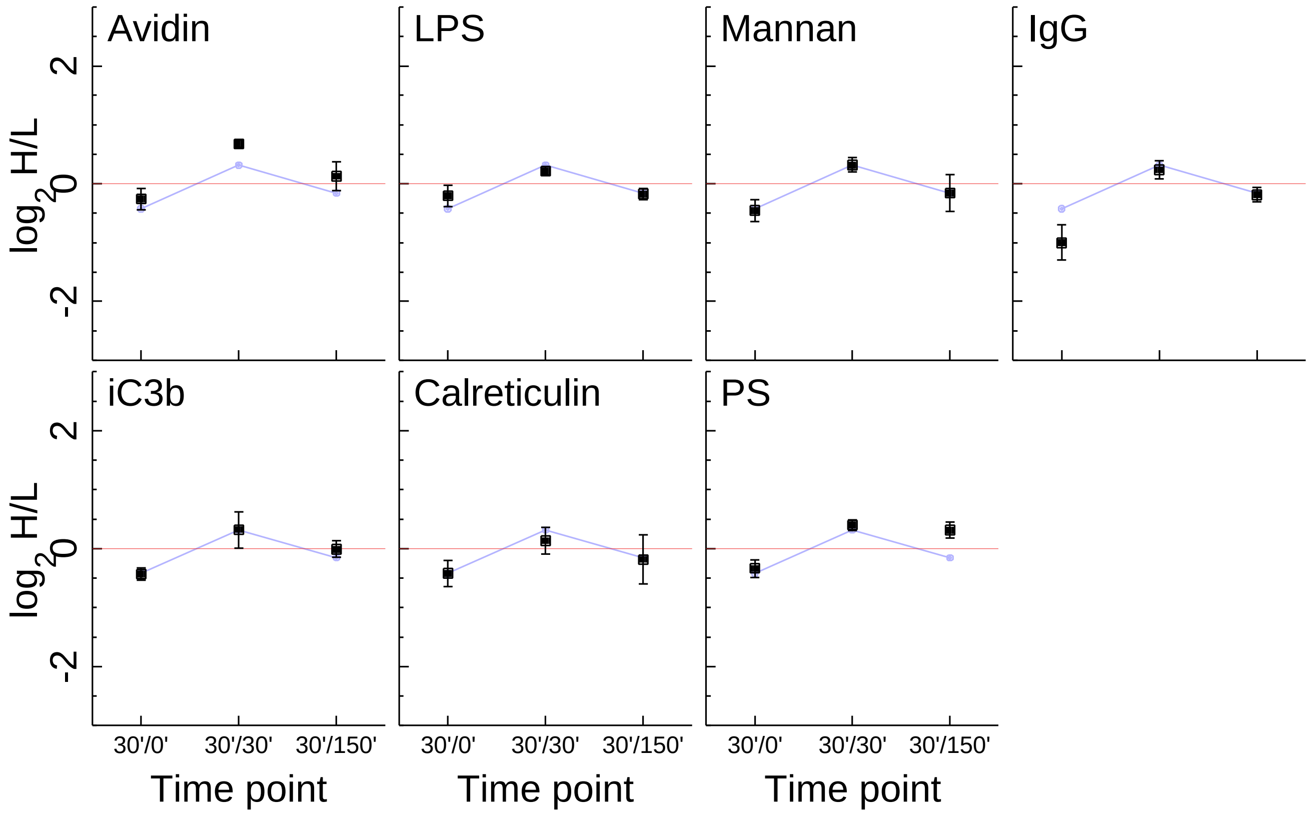

ifi35 (Q9D8C4)

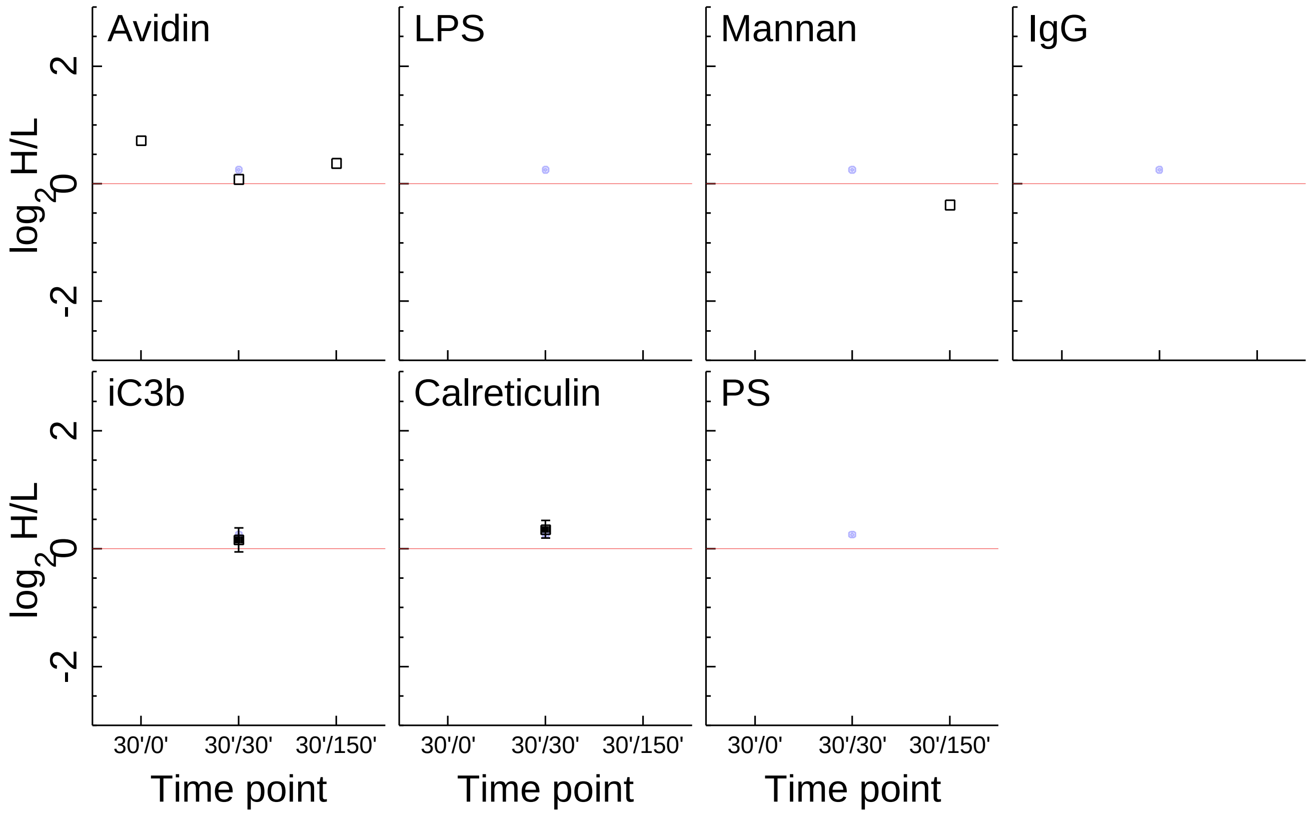

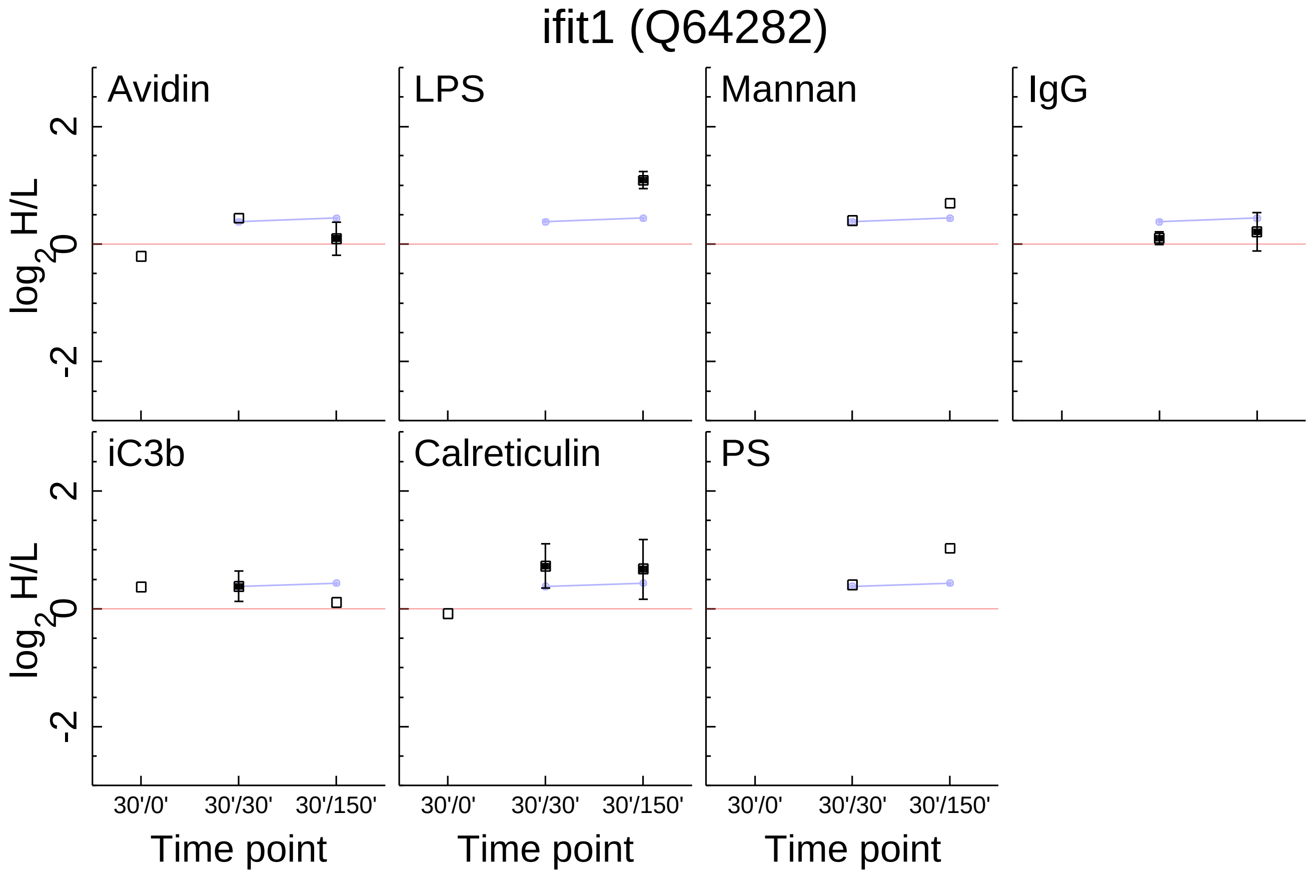
ifit1 (Q64282)
Avidin
LPS
Mannan
IgG
iC3b
Calreticulin
PS
log2 H/L
2
0
-2
30'/0'
30'/30'
30'/150'
Time point

ifitm2 (Q99J93)

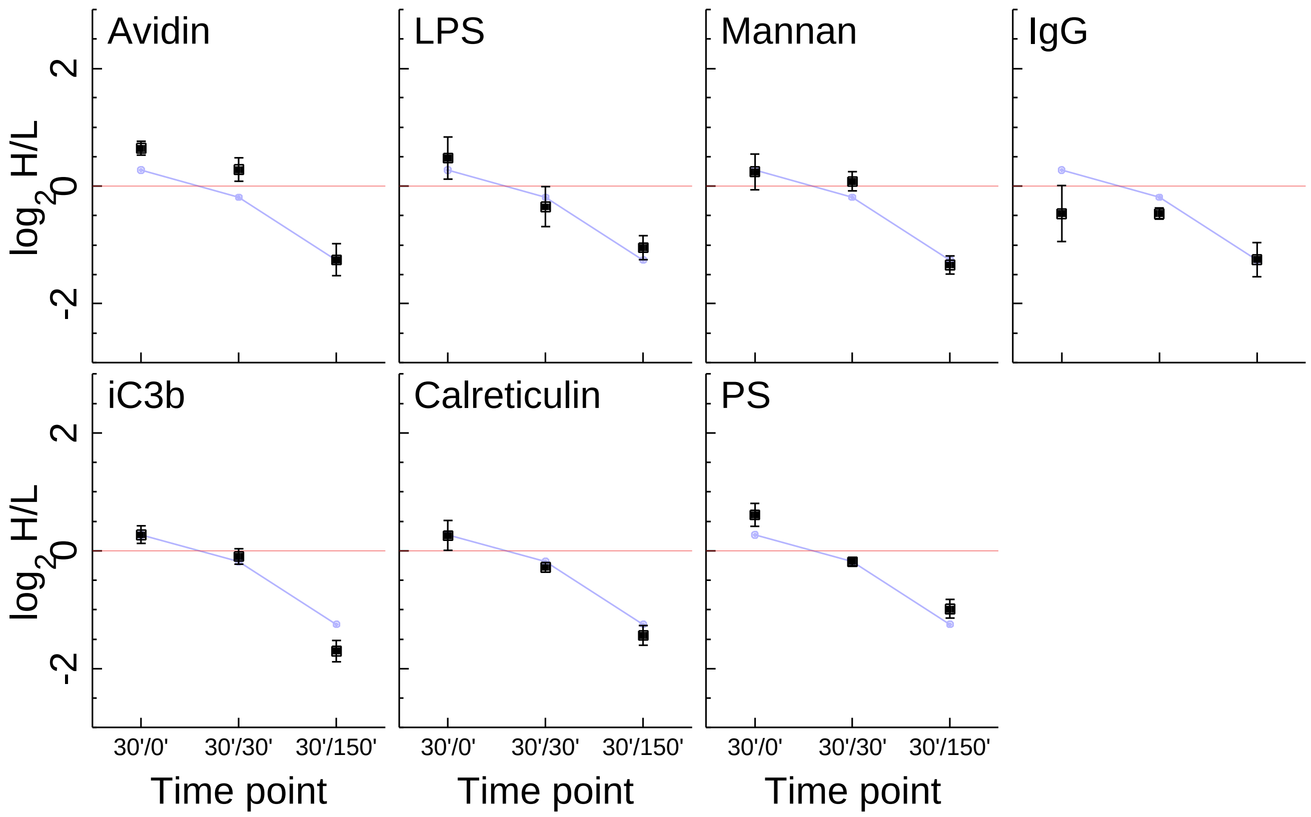

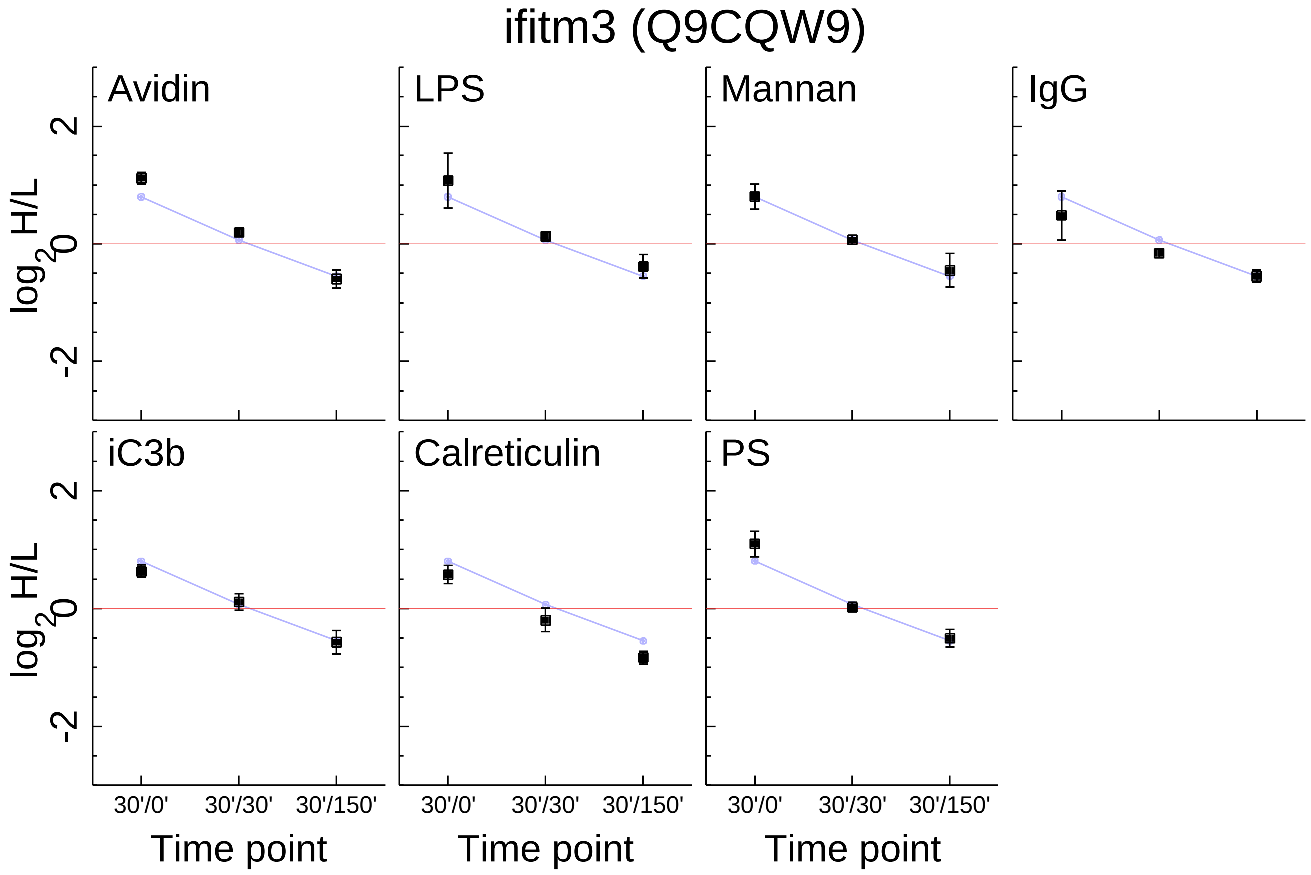

ifitm3 (Q9CQW9)
Avidin
LPS
Mannan
IgG
iC3b
Calreticulin
PS
$\log_2$ H/L
2
0
-2
30'/0'
30'/30'
30'/150'
Time point

ifngr1 (P15261)

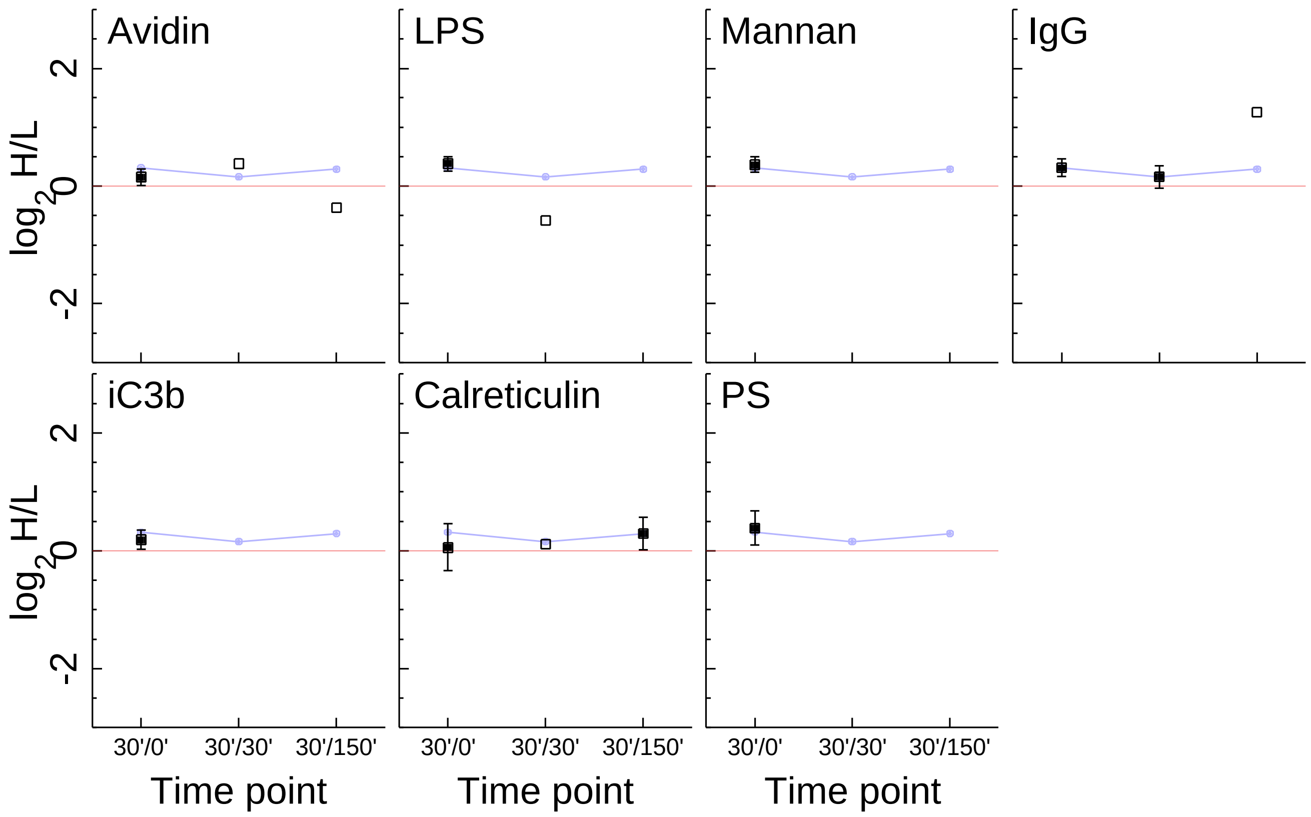

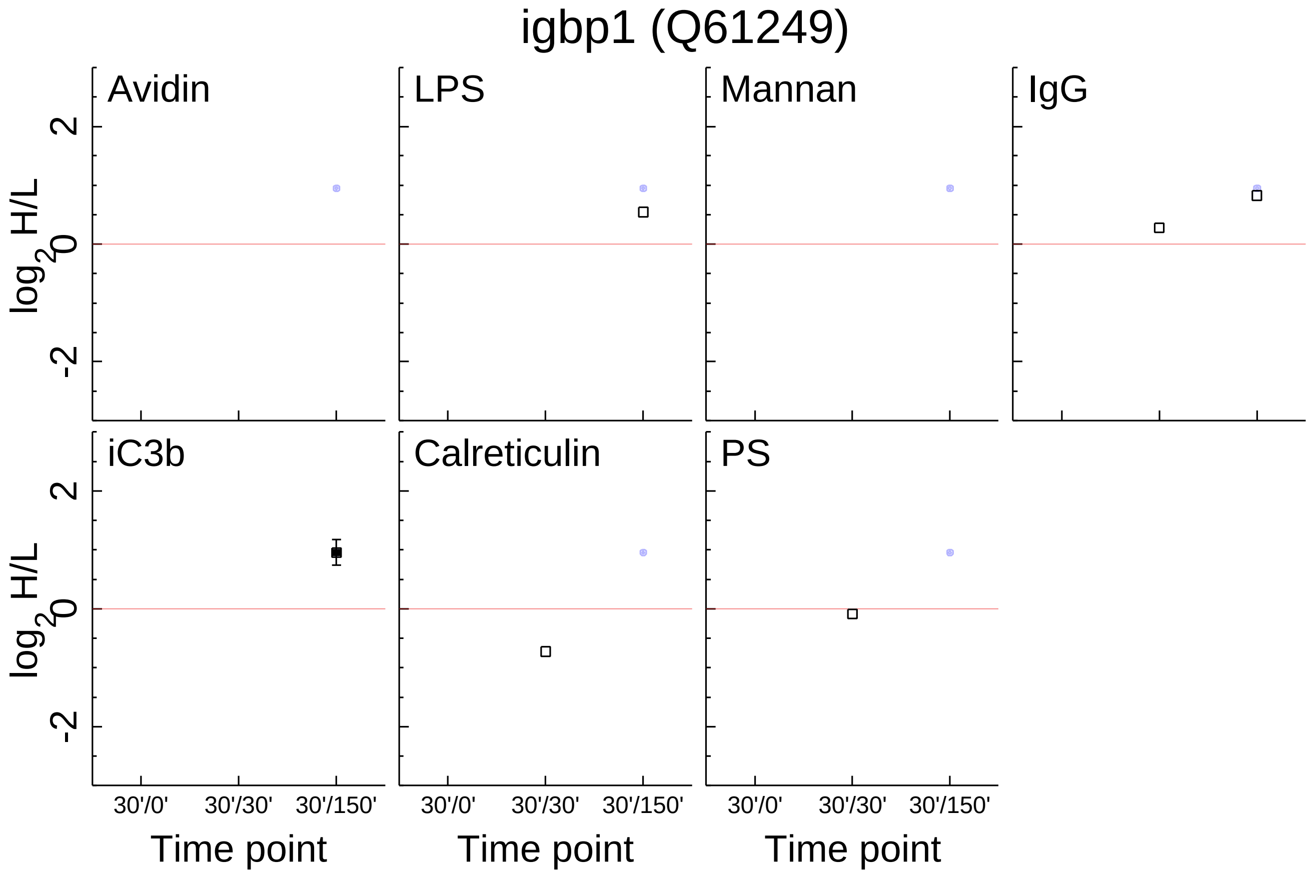

igbp1 (Q61249)
Avidin
LPS
Mannan
IgG
iC3b
Calreticulin
PS
log2 H/L
2
0
-2
30'/0'
30'/30'
30'/150'
Time point

igf2r (Q07113)

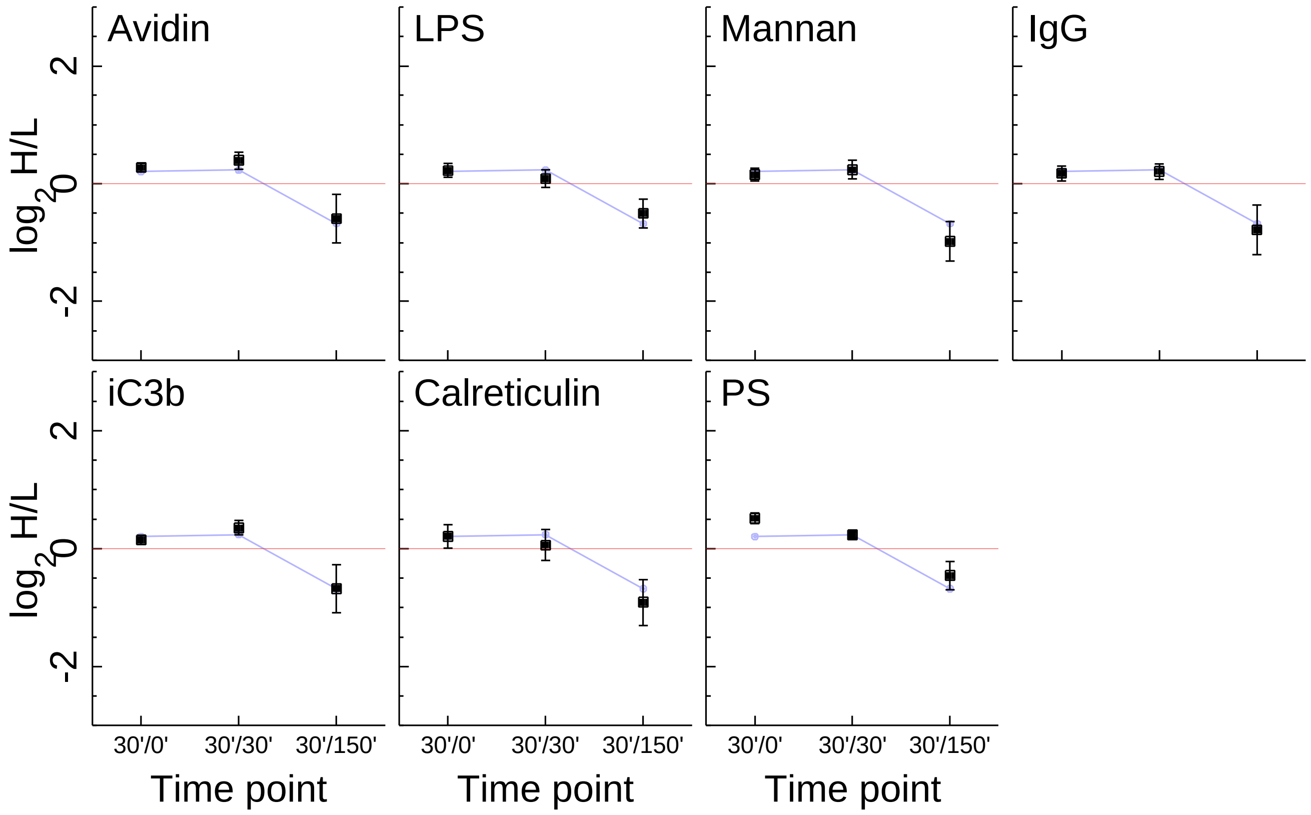

igsf6 (P0C6B7)

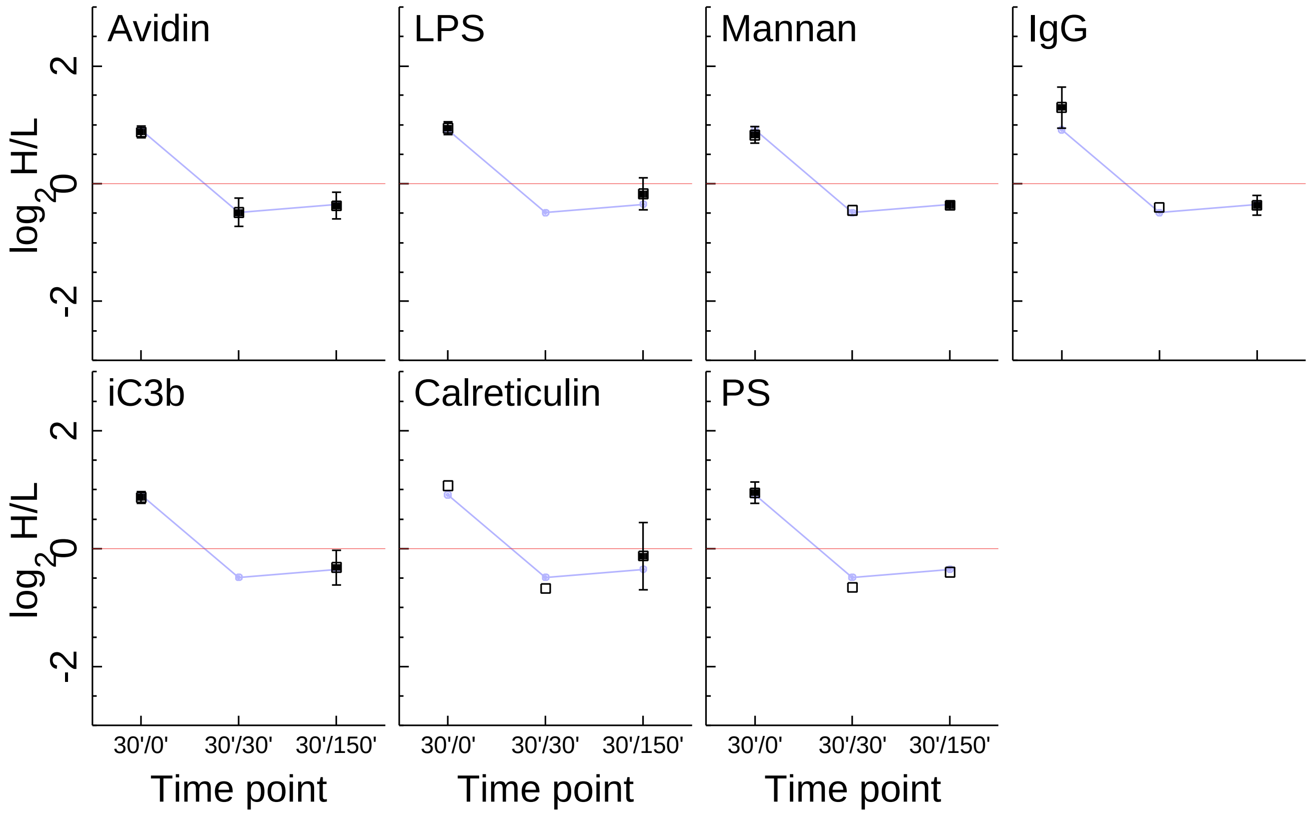

igtp (Q9DCE9)

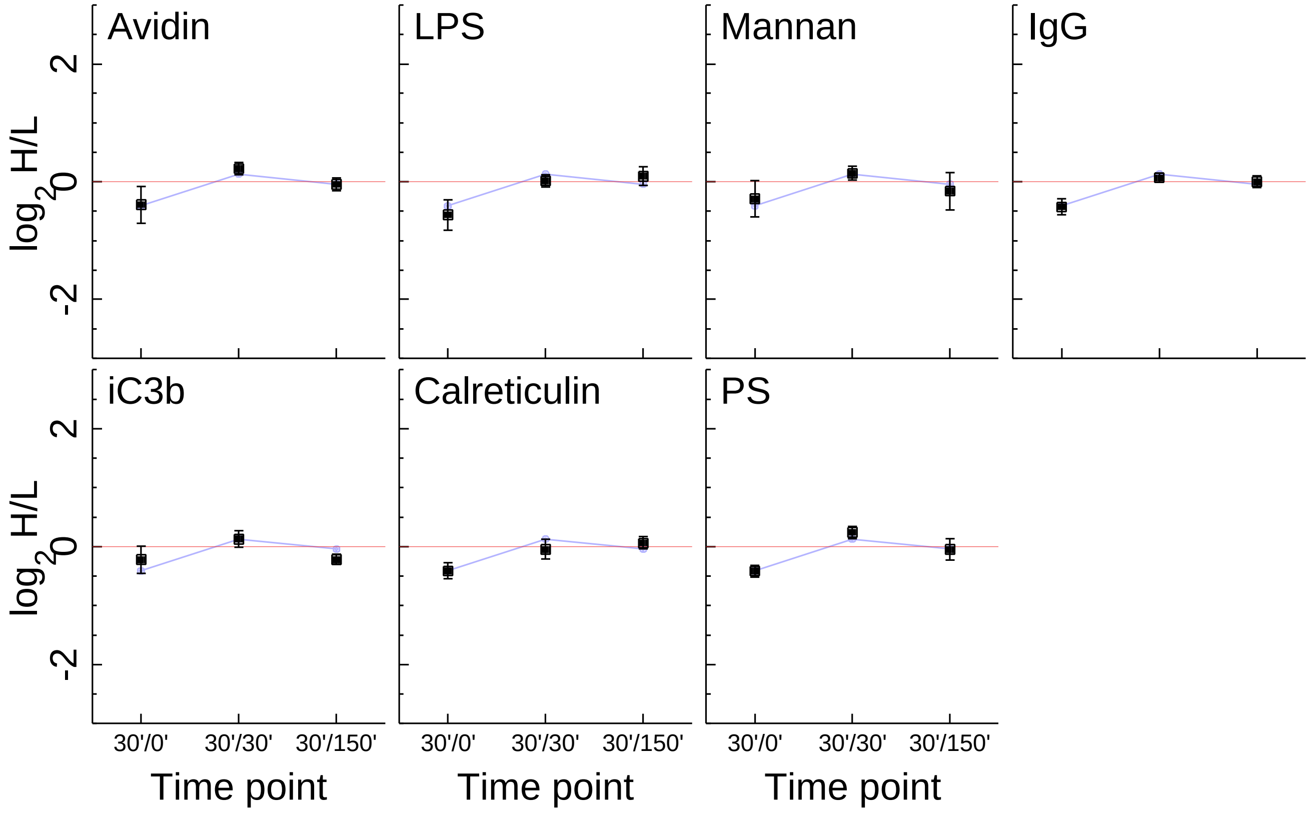

Avidin
LPS
Mannan
IgG
iC3b
Calreticulin
PS
log2 H/L
2
0
-2
30'/0'
30'/30'
30'/150'
Time point

il16 (O54824)

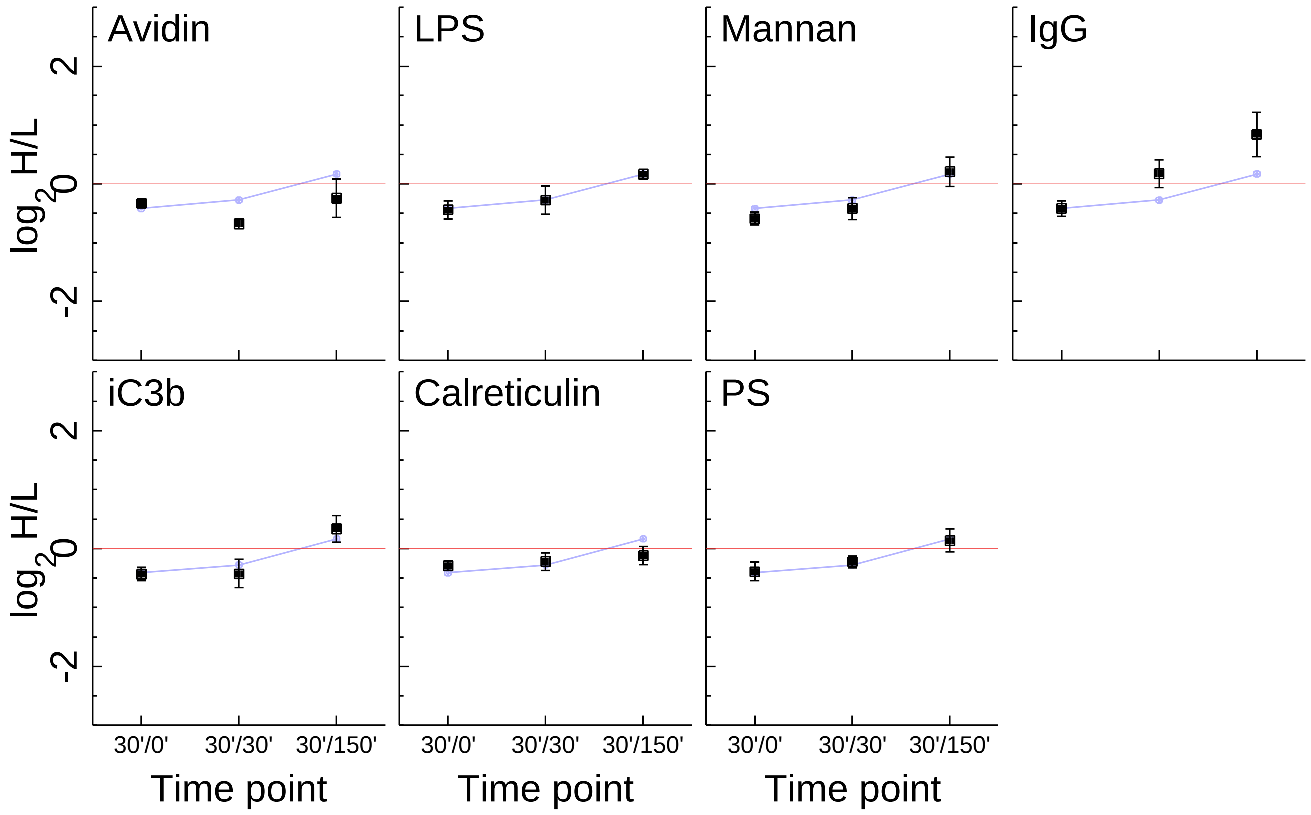

Avidin
LPS
Mannan
IgG
iC3b
Calreticulin
PS
log2 H/L
2
0
-2
30'/0'
30'/30'
30'/150'
Time point

ilk (O55222)

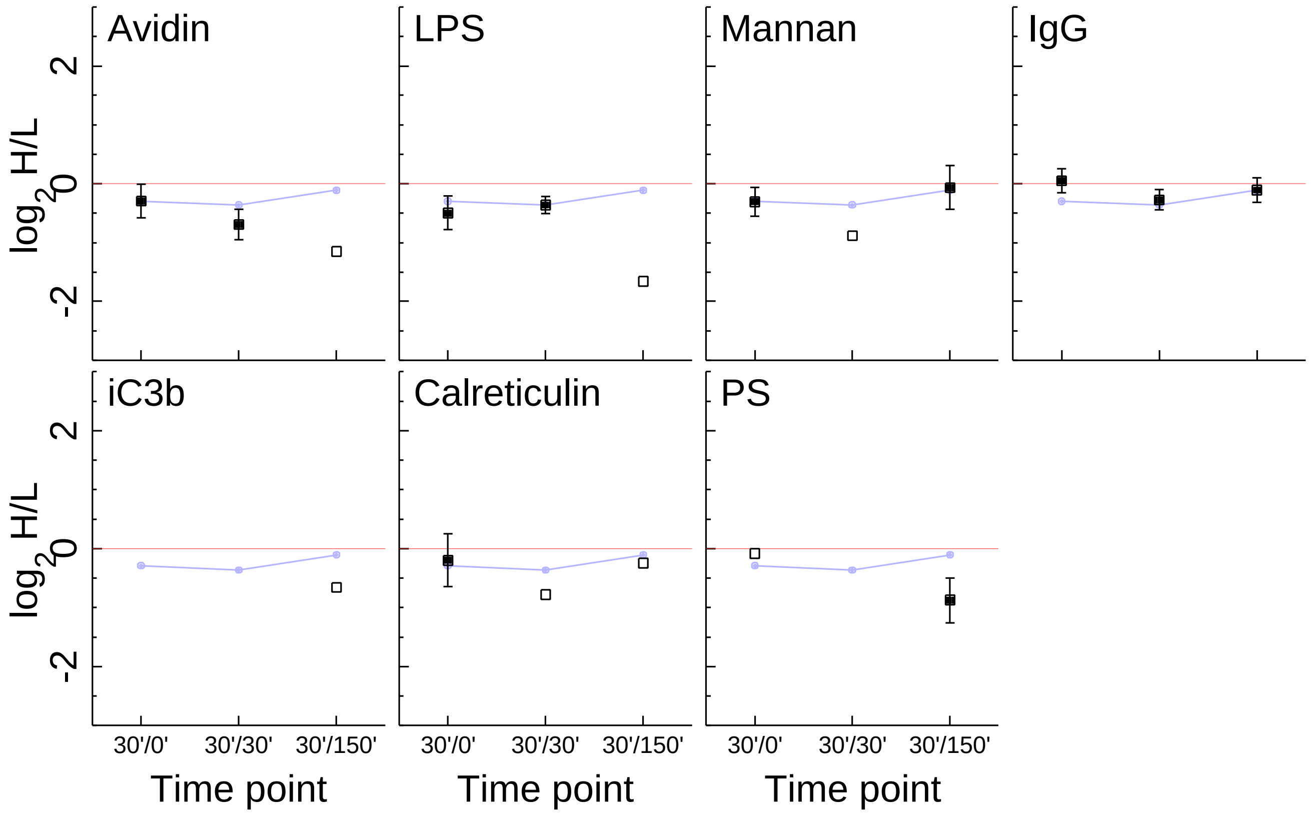

Avidin
LPS
Mannan
IgG
iC3b
Calreticulin
PS
log2 H/L
2
0
-2
30'/0'
30'/30'
30'/150'
Time point

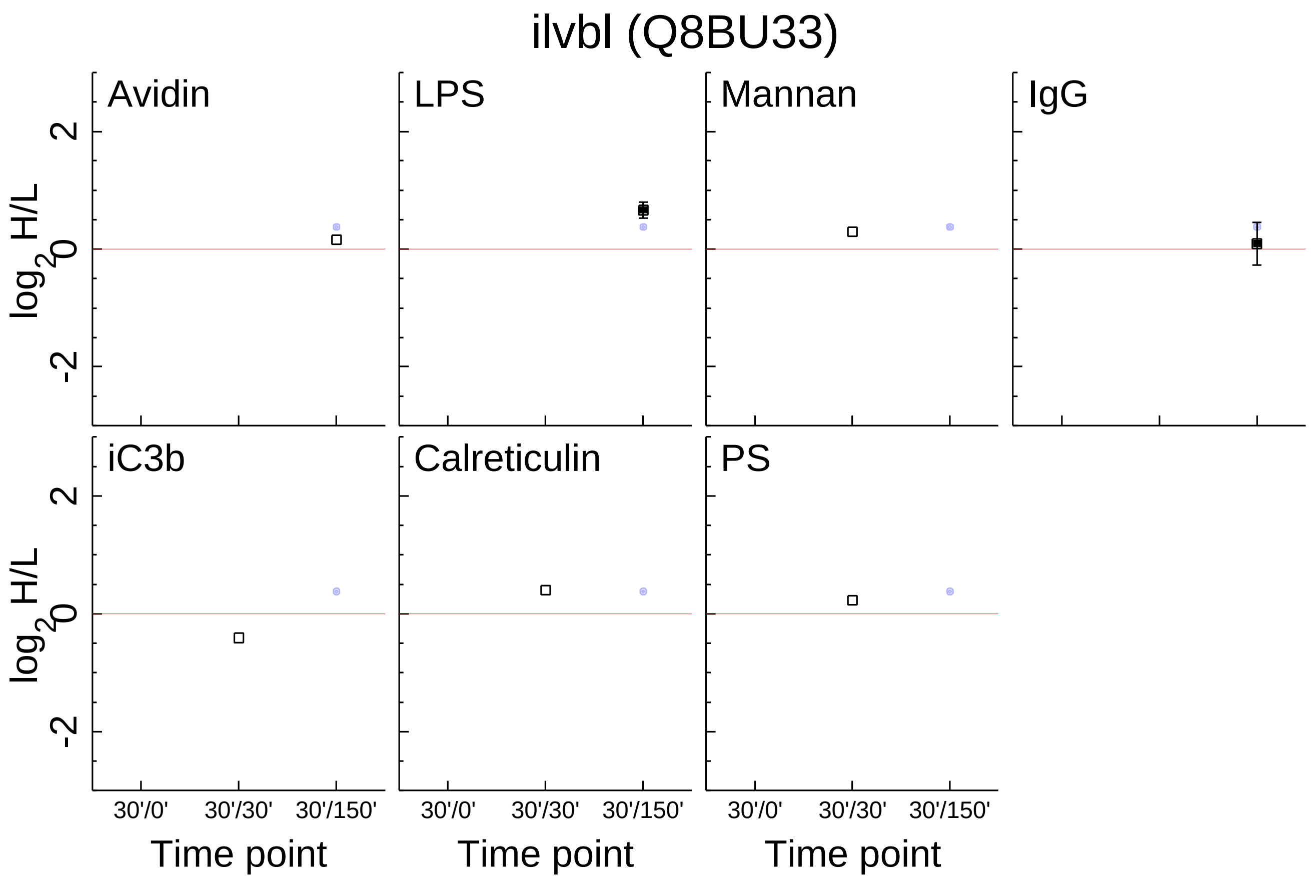
ilvbl (Q8BU33)
Avidin
LPS
Mannan
IgG
iC3b
Calreticulin
PS
log2 H/L
2
0
-2
30'/0'
30'/30'
30'/150'
Time point

inf2 (E9QLA5)

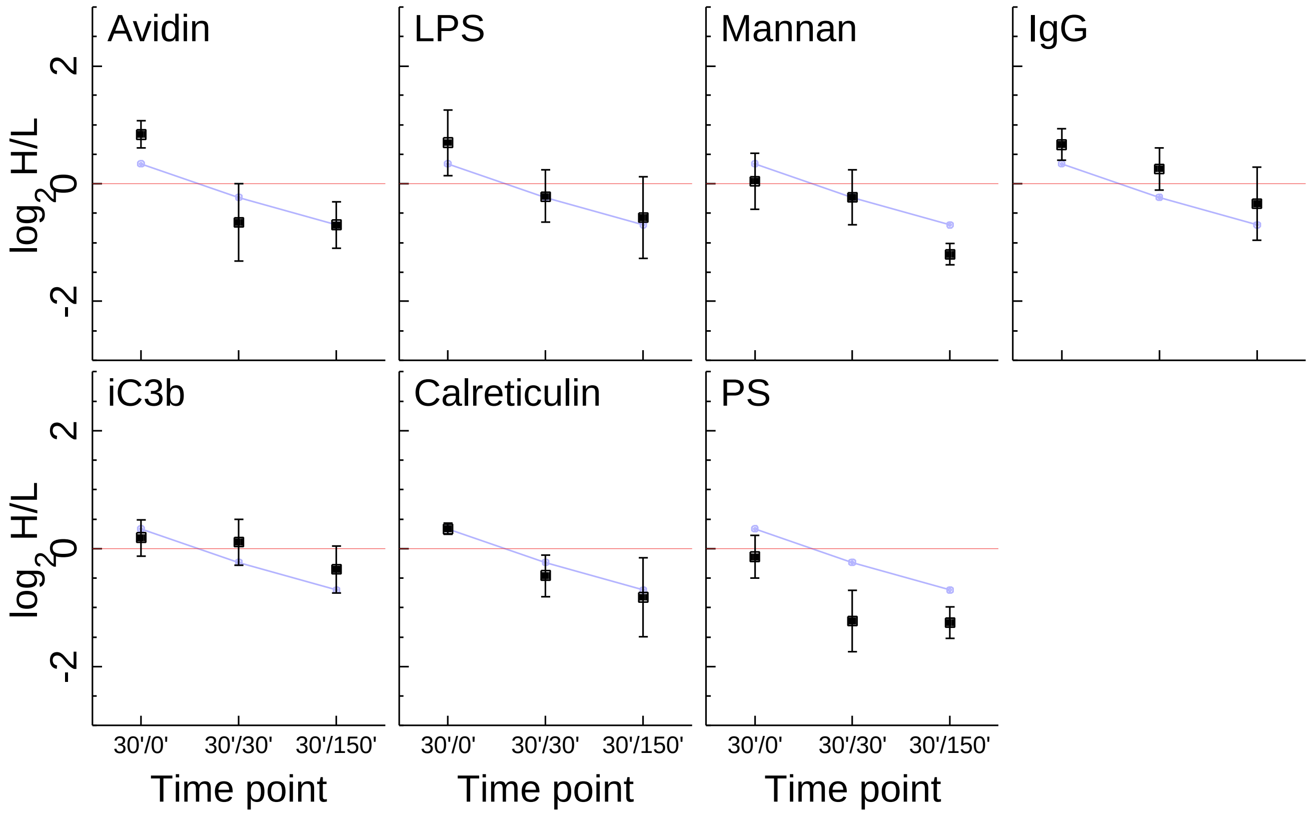

Avidin
LPS
Mannan
IgG
iC3b
Calreticulin
PS
log2 H/L
2
0
-2
30'/0'
30'/30'
30'/150'
Time point

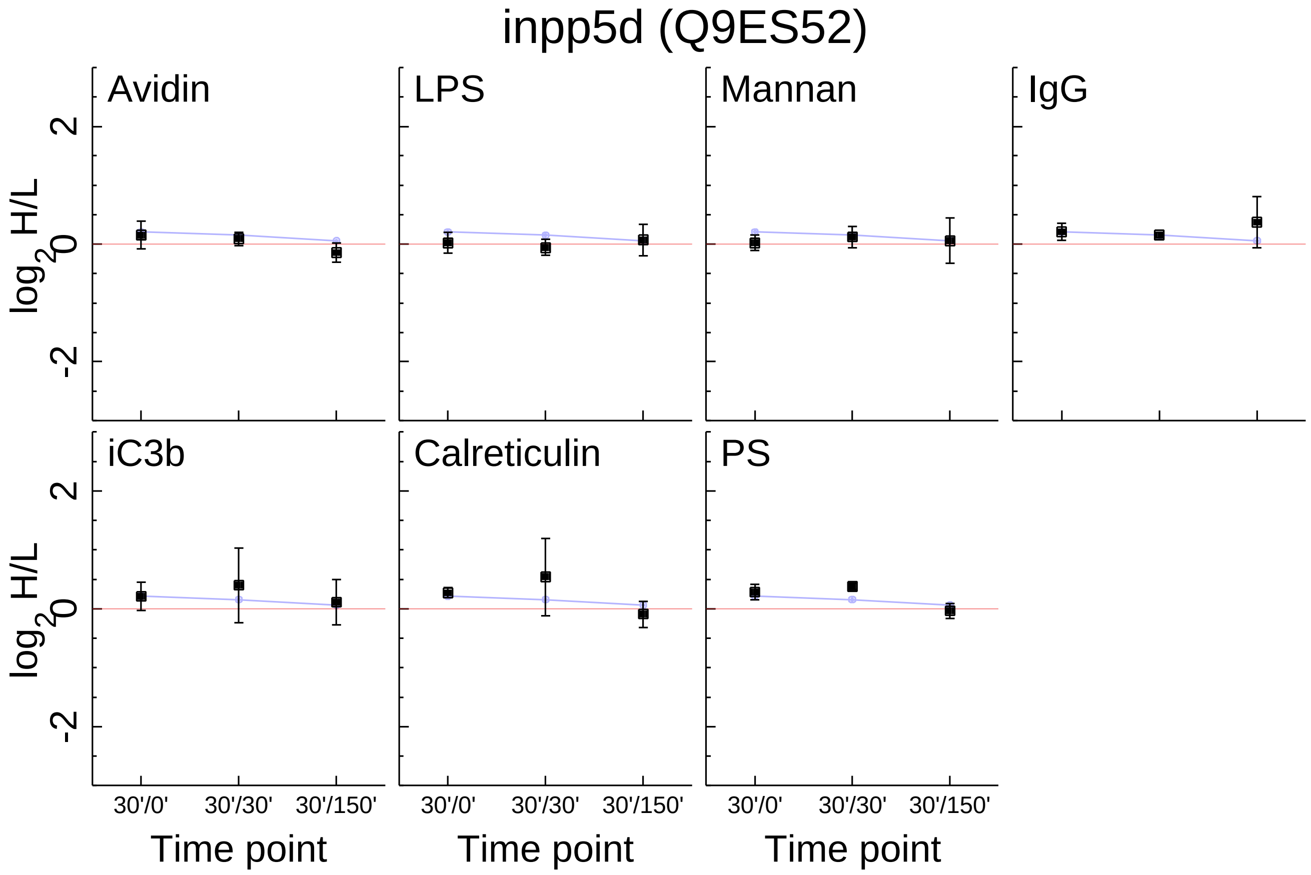

inpp5d (Q9ES52)
Avidin
LPS
Mannan
IgG
iC3b
Calreticulin
PS
log2 H/L
2
0
-2
30'/0'
30'/30'
30'/150'
Time point

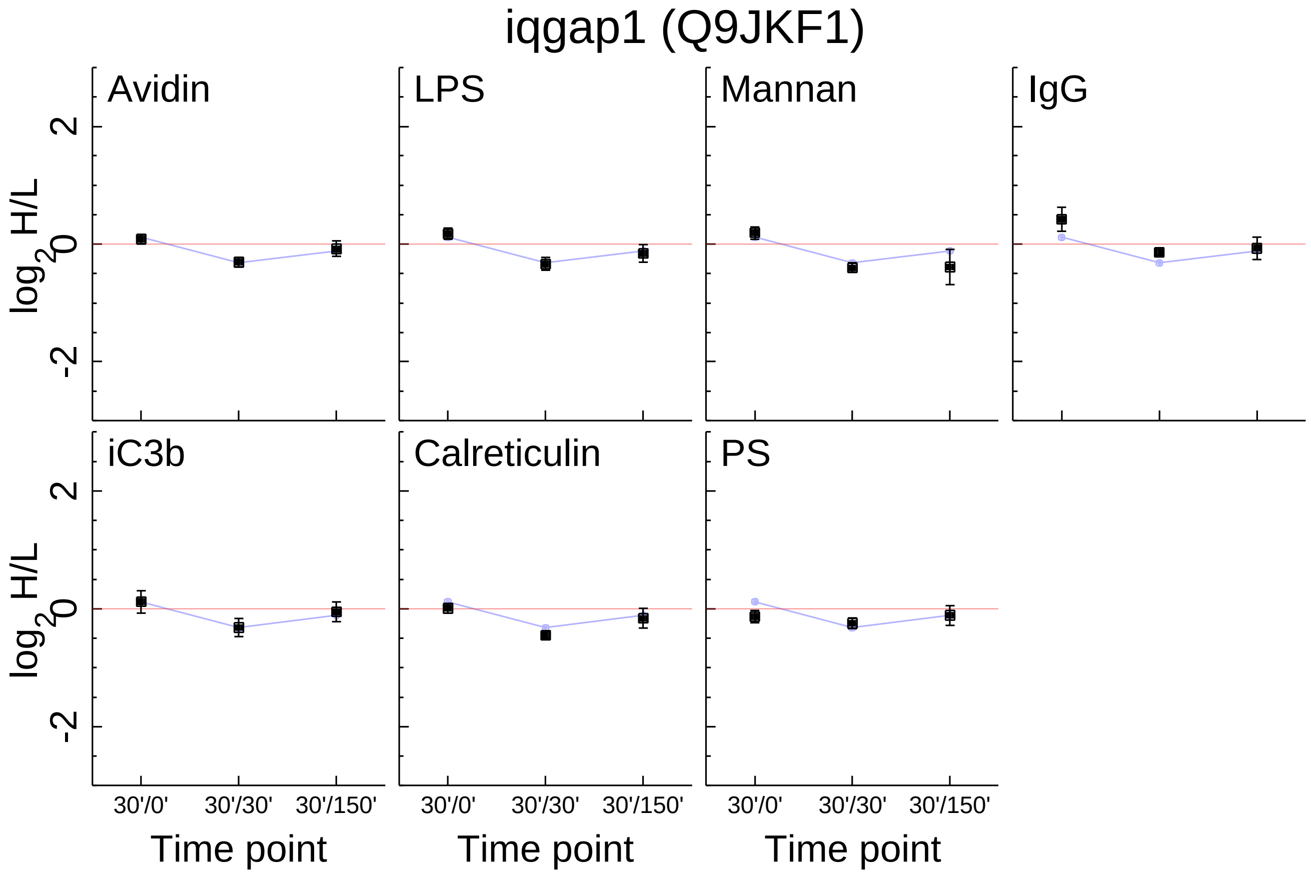

iqgap1 (Q9JKF1)
Avidin
LPS
Mannan
IgG
iC3b
Calreticulin
PS
$\log_2$ H/L
2
0
-2
30'/0'
30'/30'
30'/150'
Time point

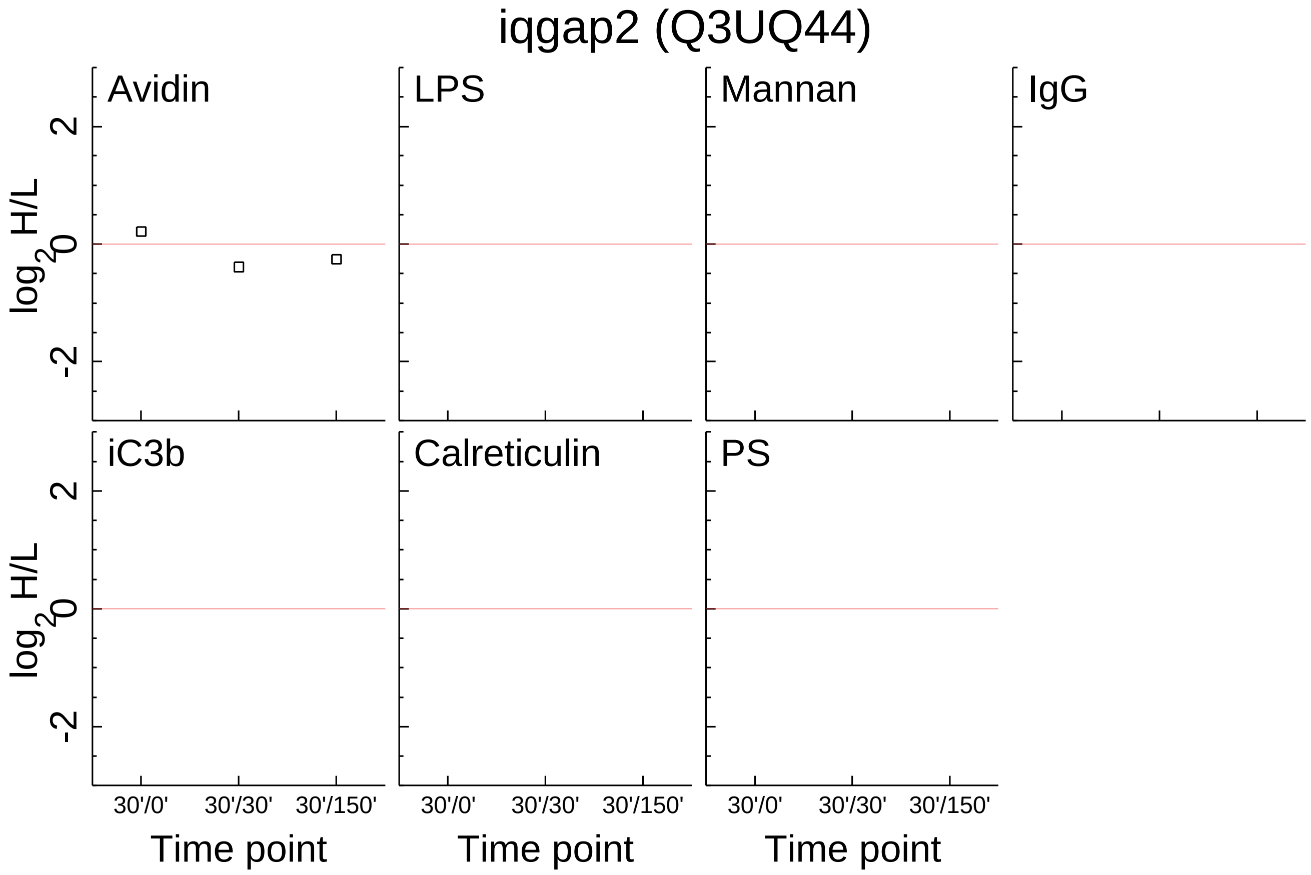
iqgap2 (Q3UQ44)
Avidin
LPS
Mannan
IgG
iC3b
Calreticulin
PS
2
0
-2
log2 H/L
30'/0'
30'/30'
30'/150'
Time point

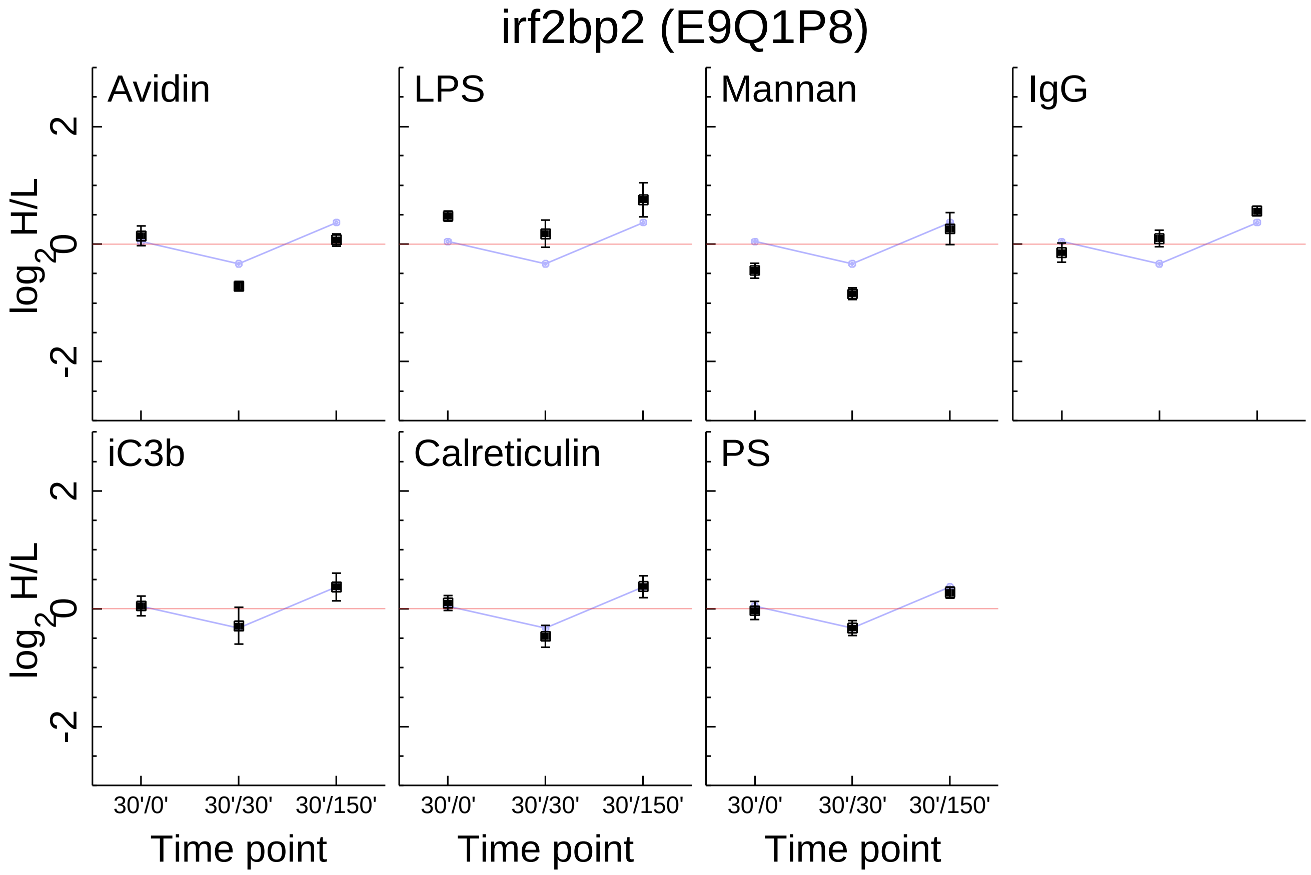

irf2bp2 (E9Q1P8)
Avidin
LPS
Mannan
IgG
iC3b
Calreticulin
PS
log2 H/L
2
0
-2
30'/0'
30'/30'
30'/150'
Time point

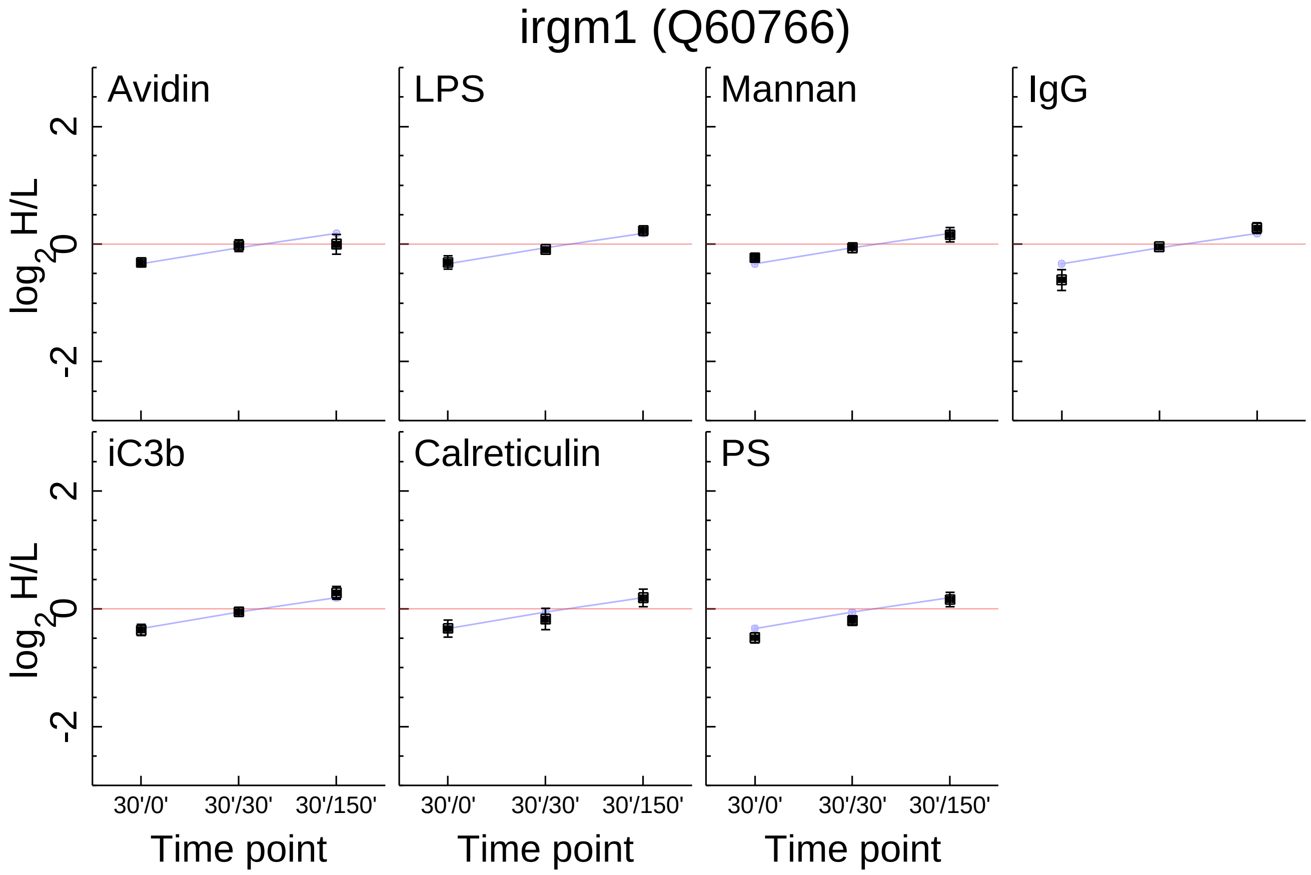
irgm1 (Q60766)
Avidin
LPS
Mannan
IgG
iC3b
Calreticulin
PS
log2 H/L
2
0
-2
30'/0'
30'/30'
30'/150'
Time point

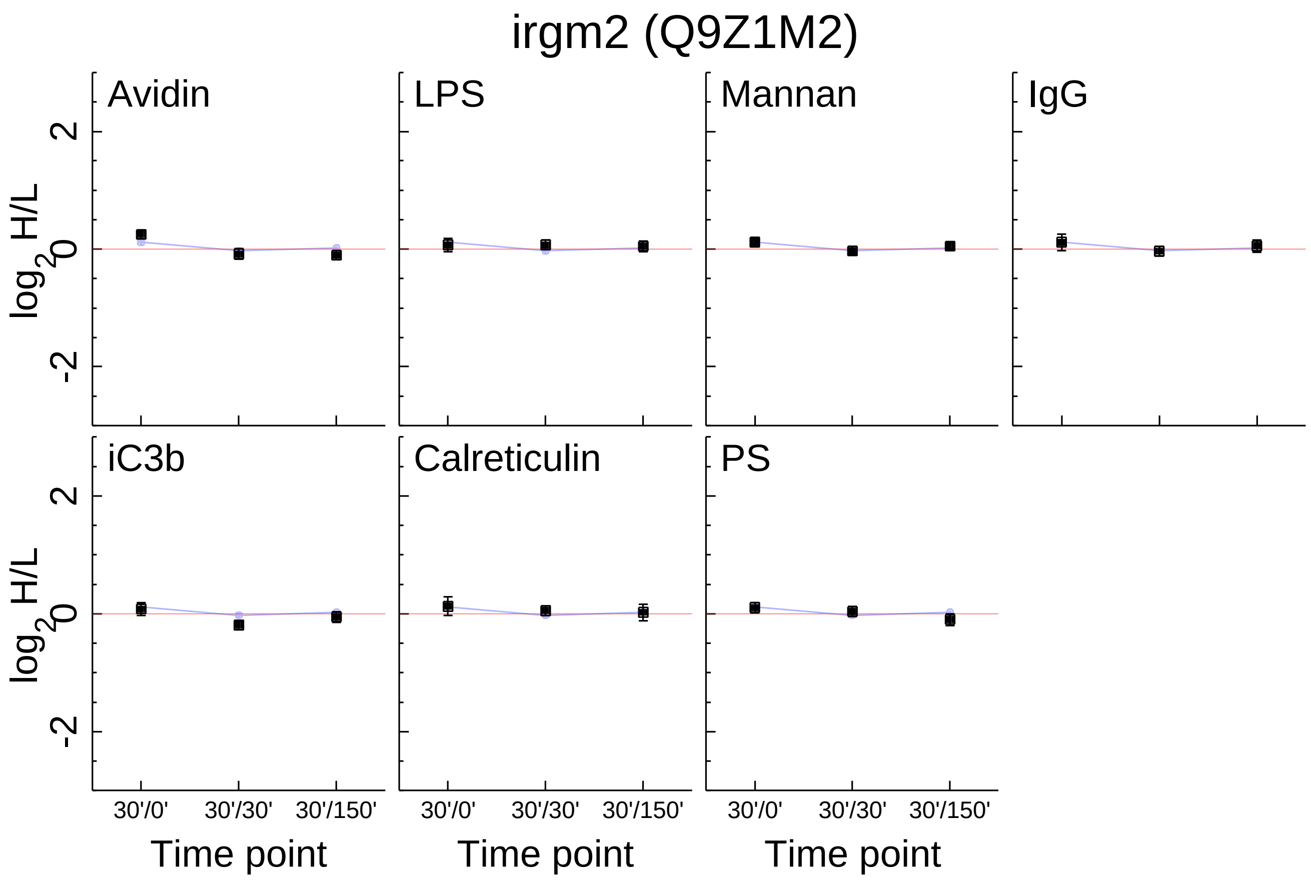

irgm2 (Q9Z1M2)
Avidin
LPS
Mannan
IgG
iC3b
Calreticulin
PS
log2 H/L
2
0
-2
30'/0'
30'/30'
30'/150'
Time point

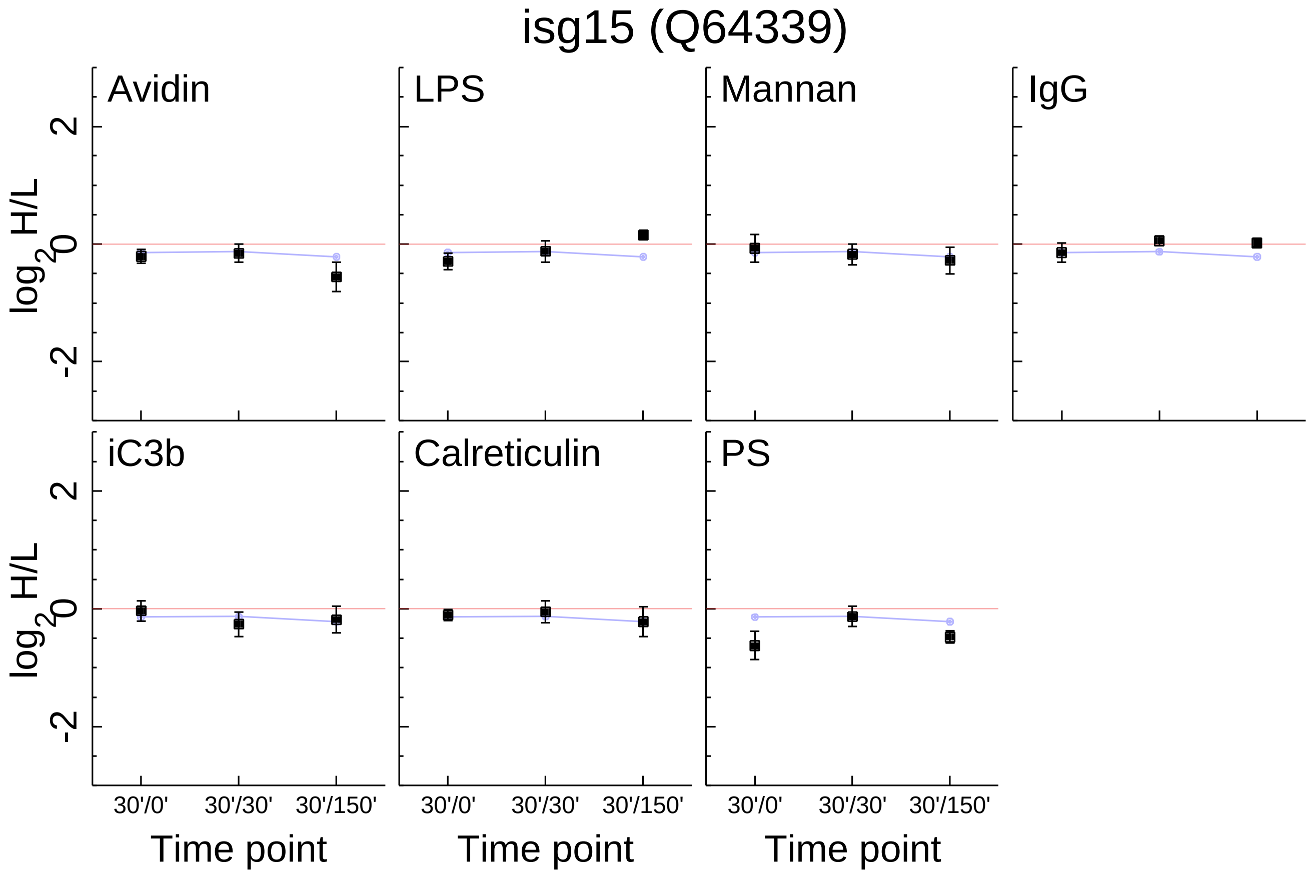

isg15 (Q64339)
Avidin
LPS
Mannan
IgG
iC3b
Calreticulin
PS
log2 H/L
2
0
-2
30'/0'
30'/30'
30'/150'
Time point

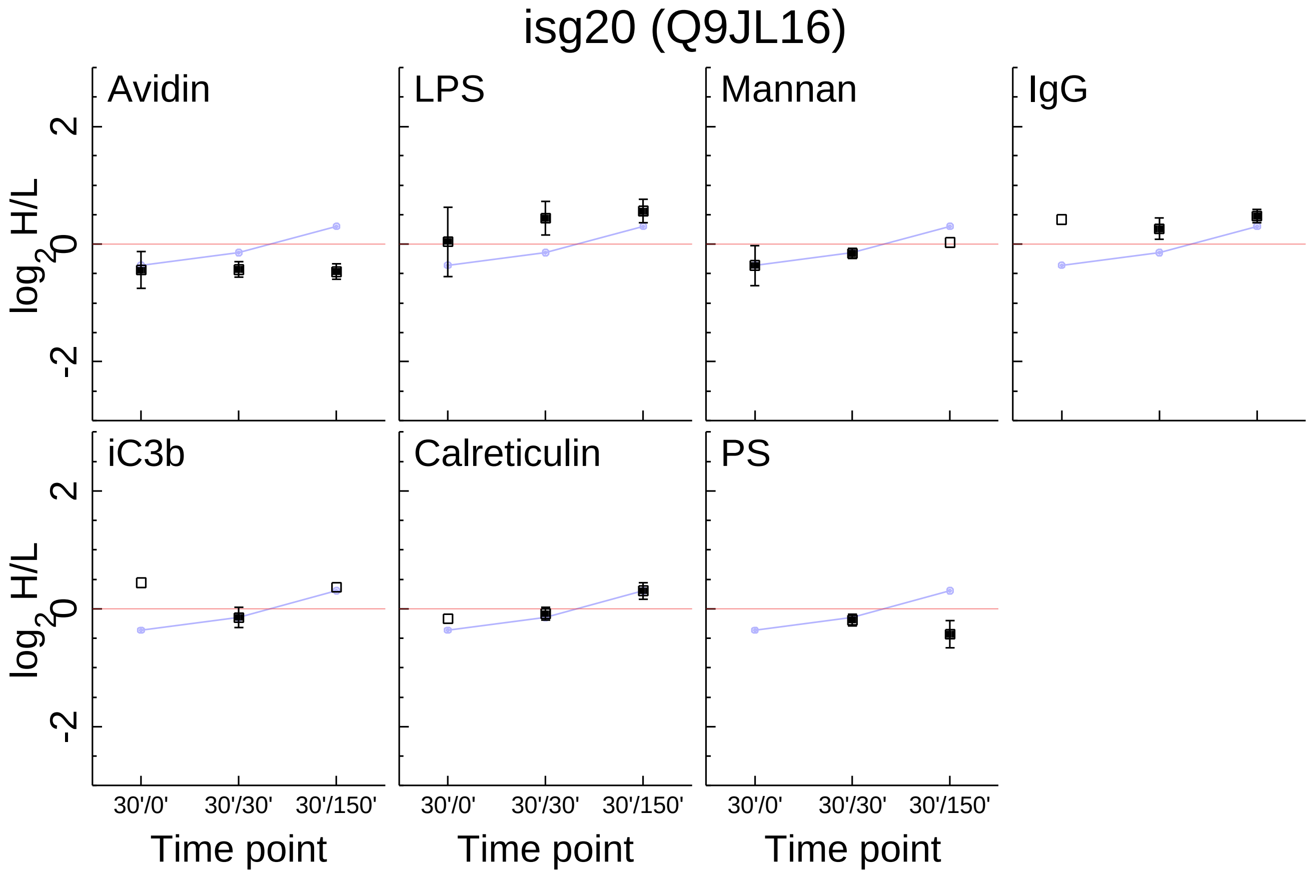
isg20 (Q9JL16)
Avidin
LPS
Mannan
IgG
iC3b
Calreticulin
PS
log2 H/L
2
0
-2
30'/0'
30'/30'
30'/150'
Time point

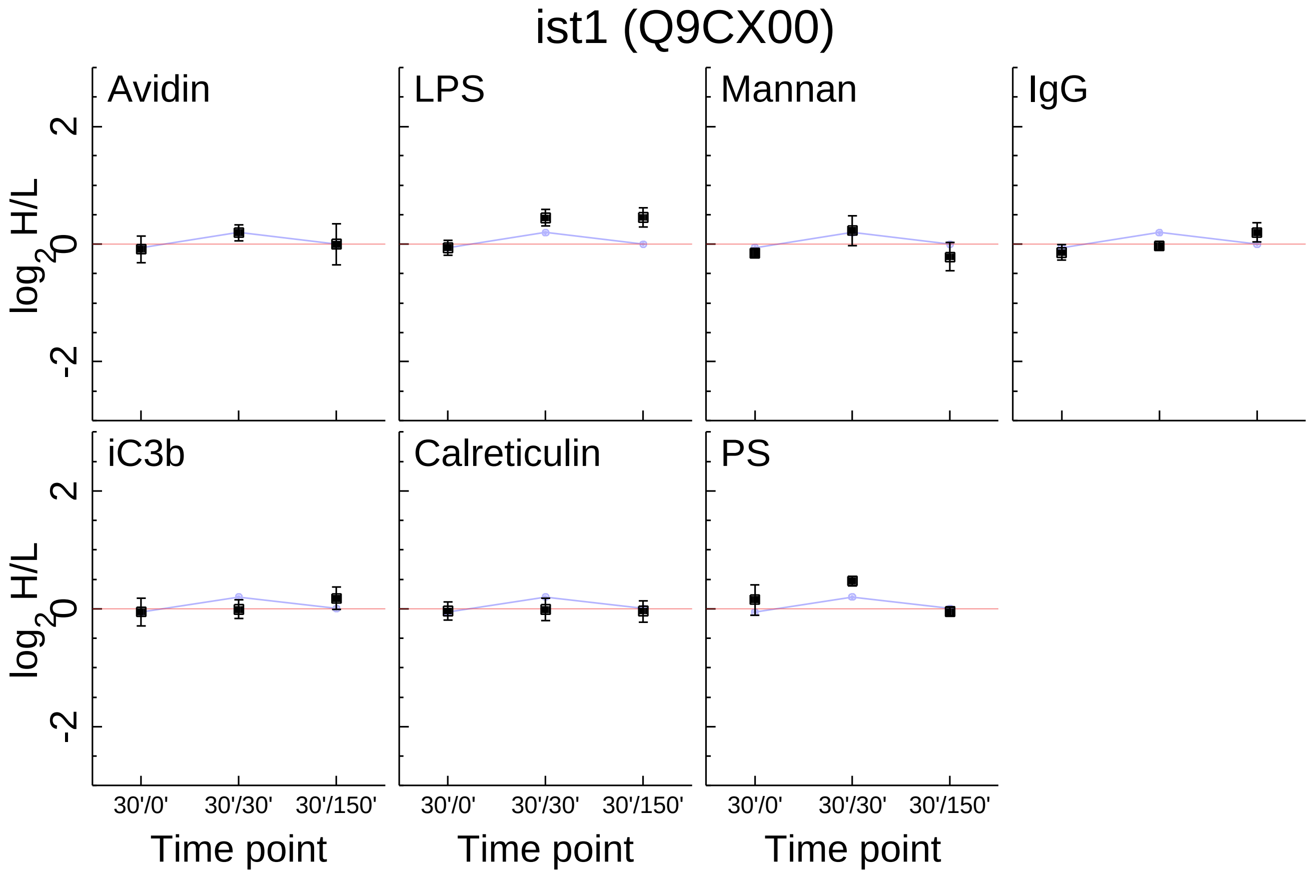

ist1 (Q9CX00)
Avidin
LPS
Mannan
IgG
iC3b
Calreticulin
PS
log2 H/L
2
0
-2
30'/0'
30'/30'
30'/150'
Time point

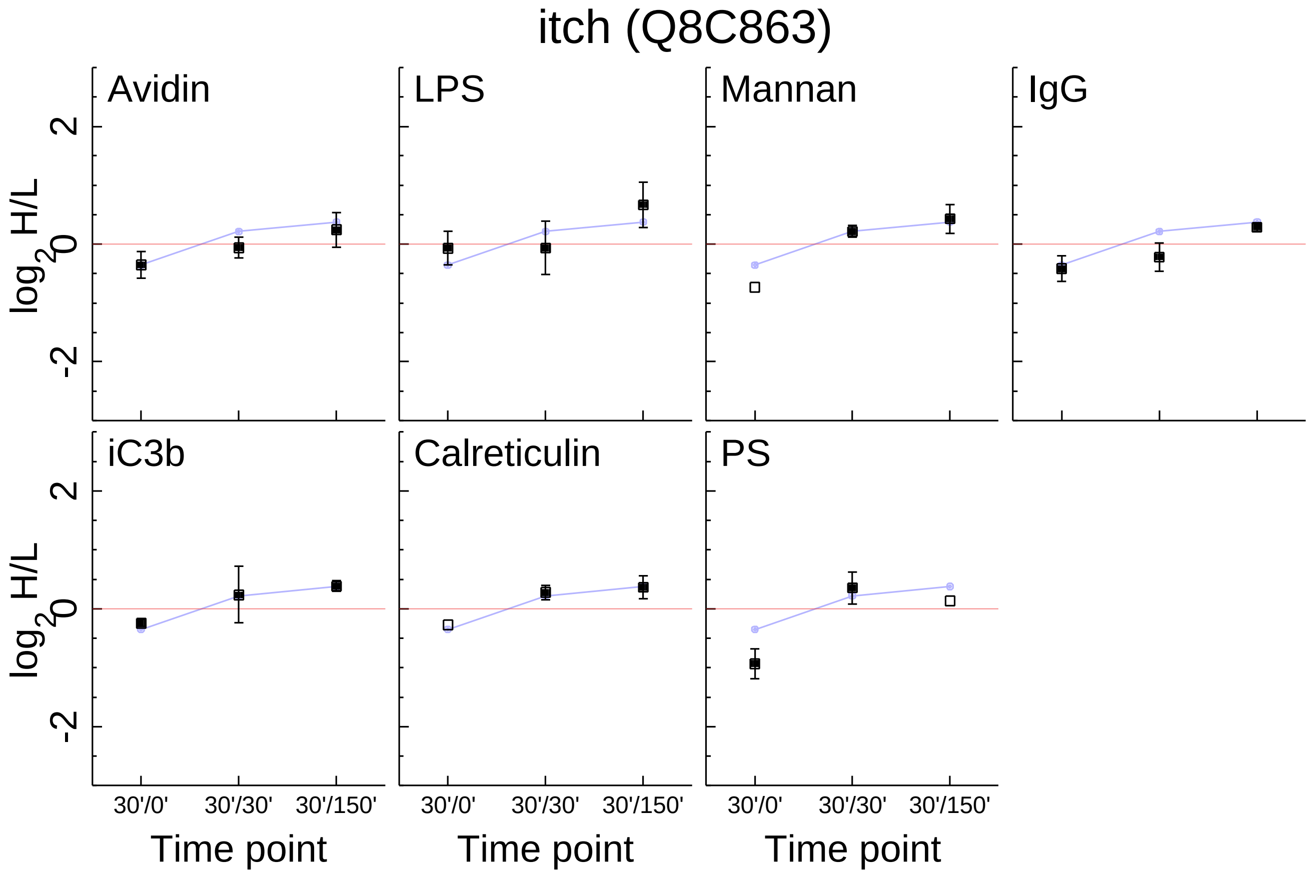
itch (Q8C863)
Avidin
LPS
Mannan
IgG
iC3b
Calreticulin
PS
log2 H/L
2
0
-2
30'/0'
30'/30'
30'/150'
Time point

itga4 (Q00651)

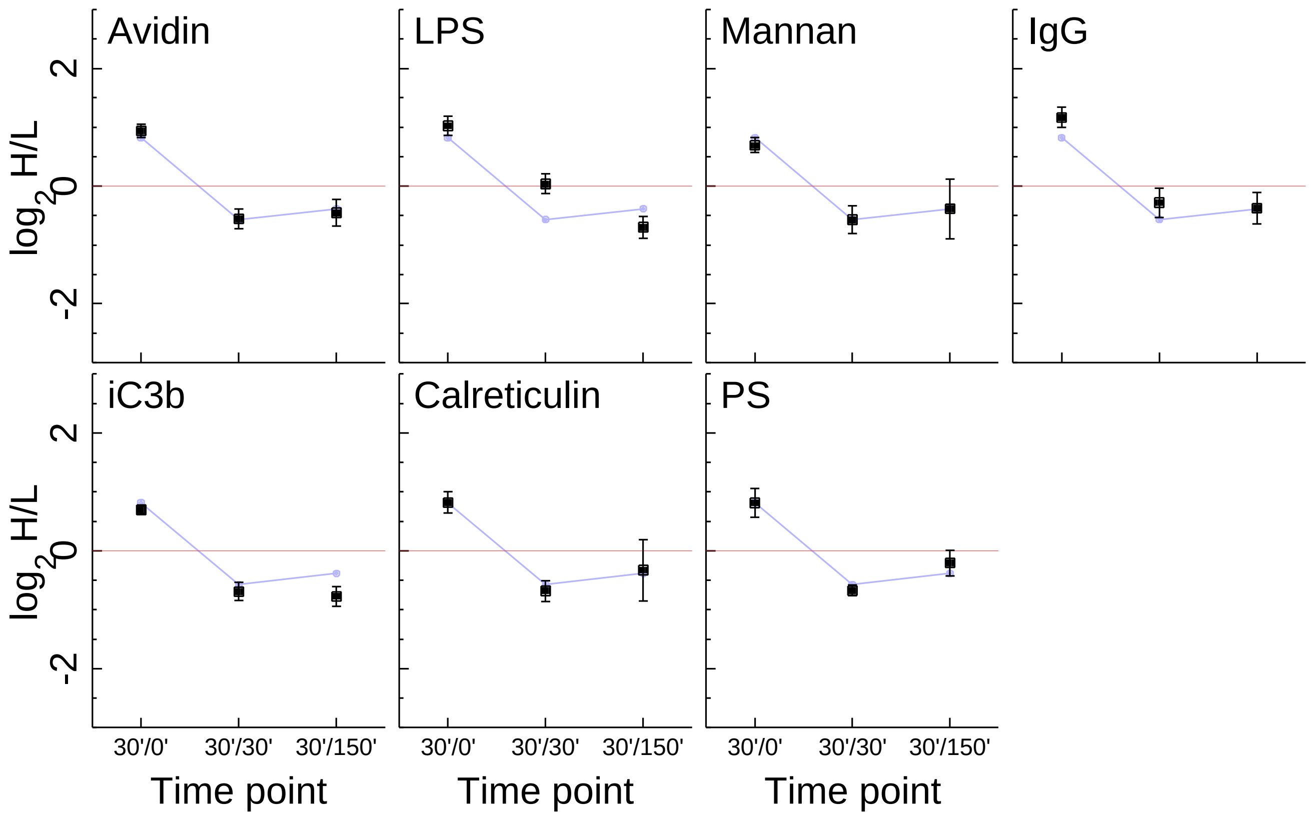

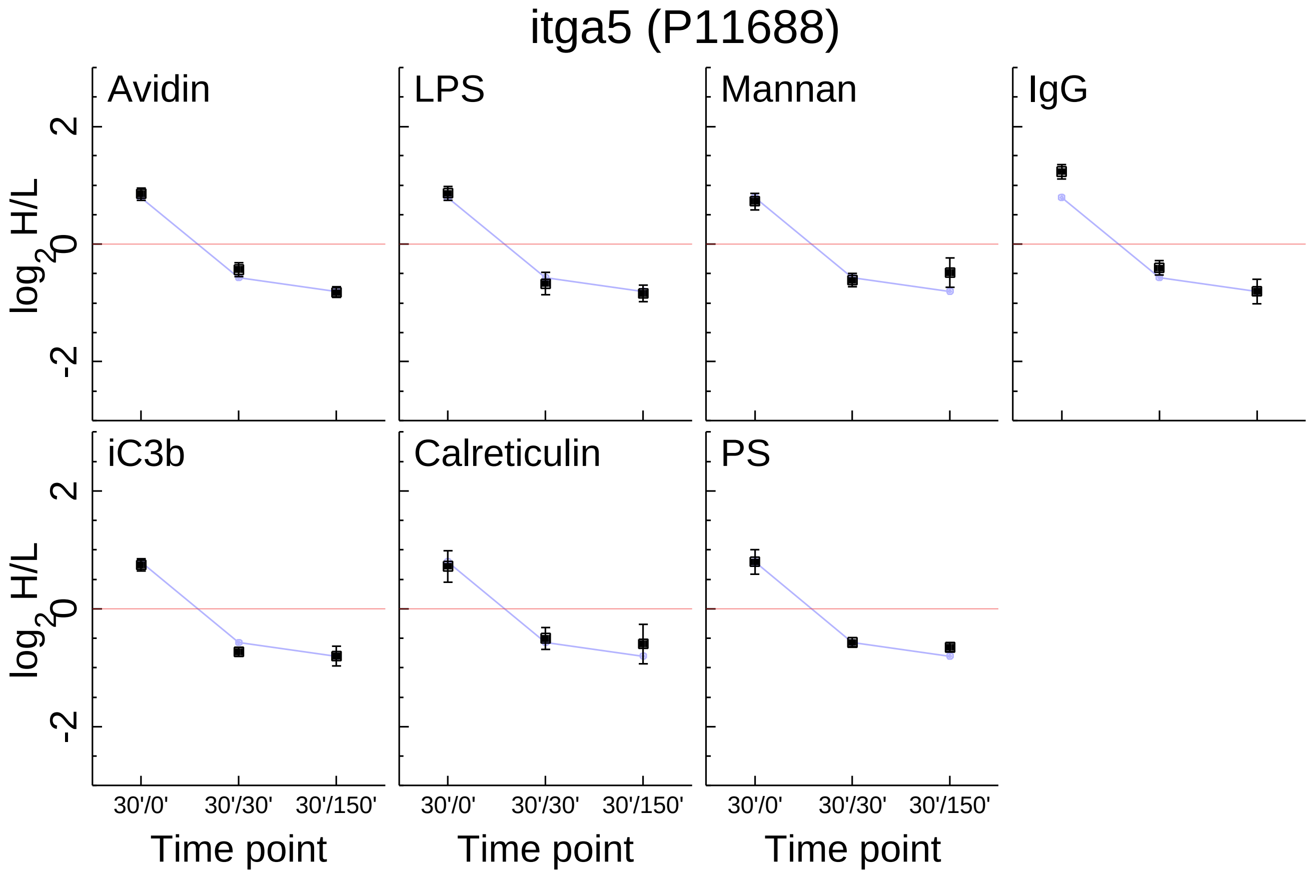
itga5 (P11688)
Avidin
LPS
Mannan
IgG
iC3b
Calreticulin
PS
log2 H/L
2
0
-2
30'/0'
30'/30'
30'/150'
Time point

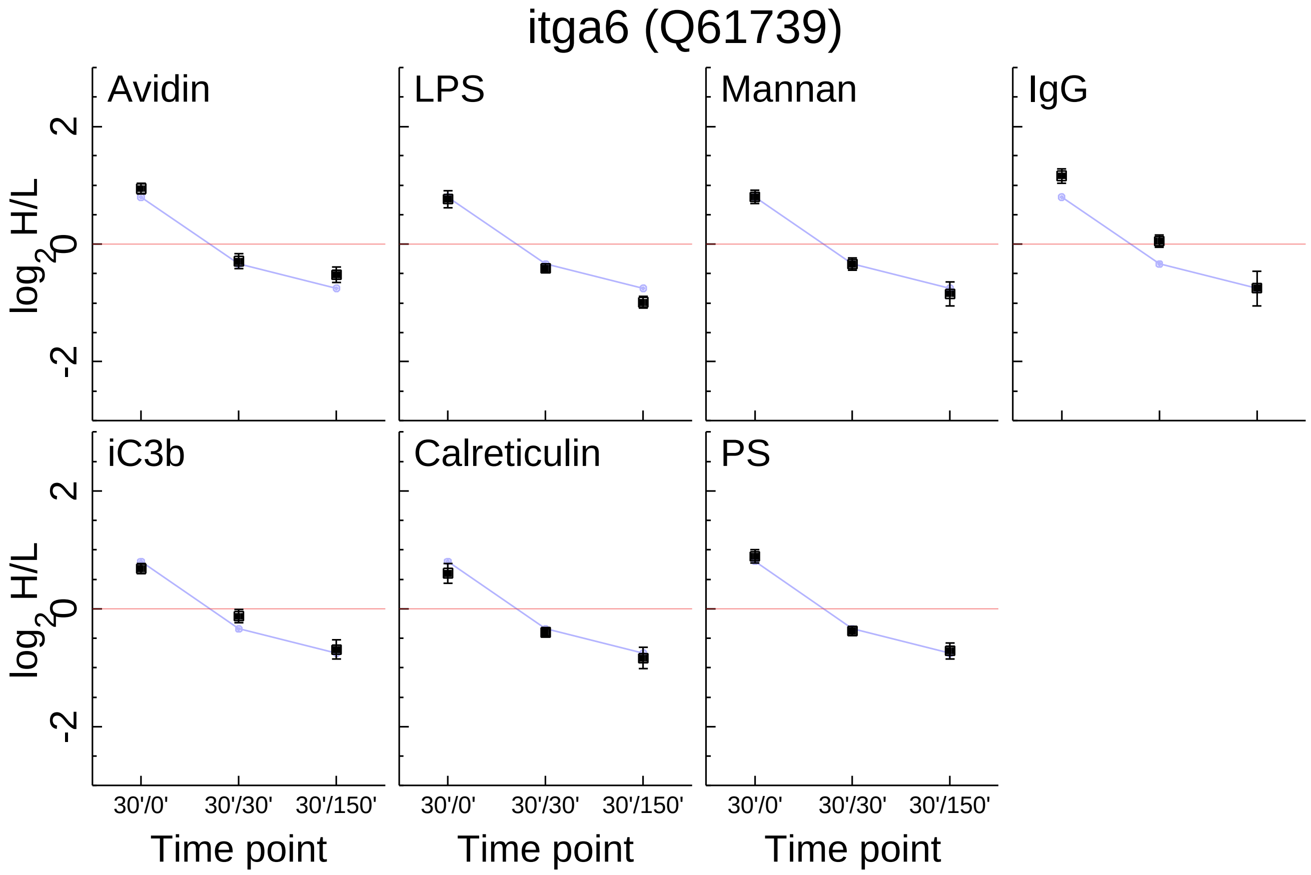
itga6 (Q61739)
Avidin
LPS
Mannan
IgG
iC3b
Calreticulin
PS
log2 H/L
2
0
-2
30'/0'
30'/30'
30'/150'
Time point

itgam (E9Q604)

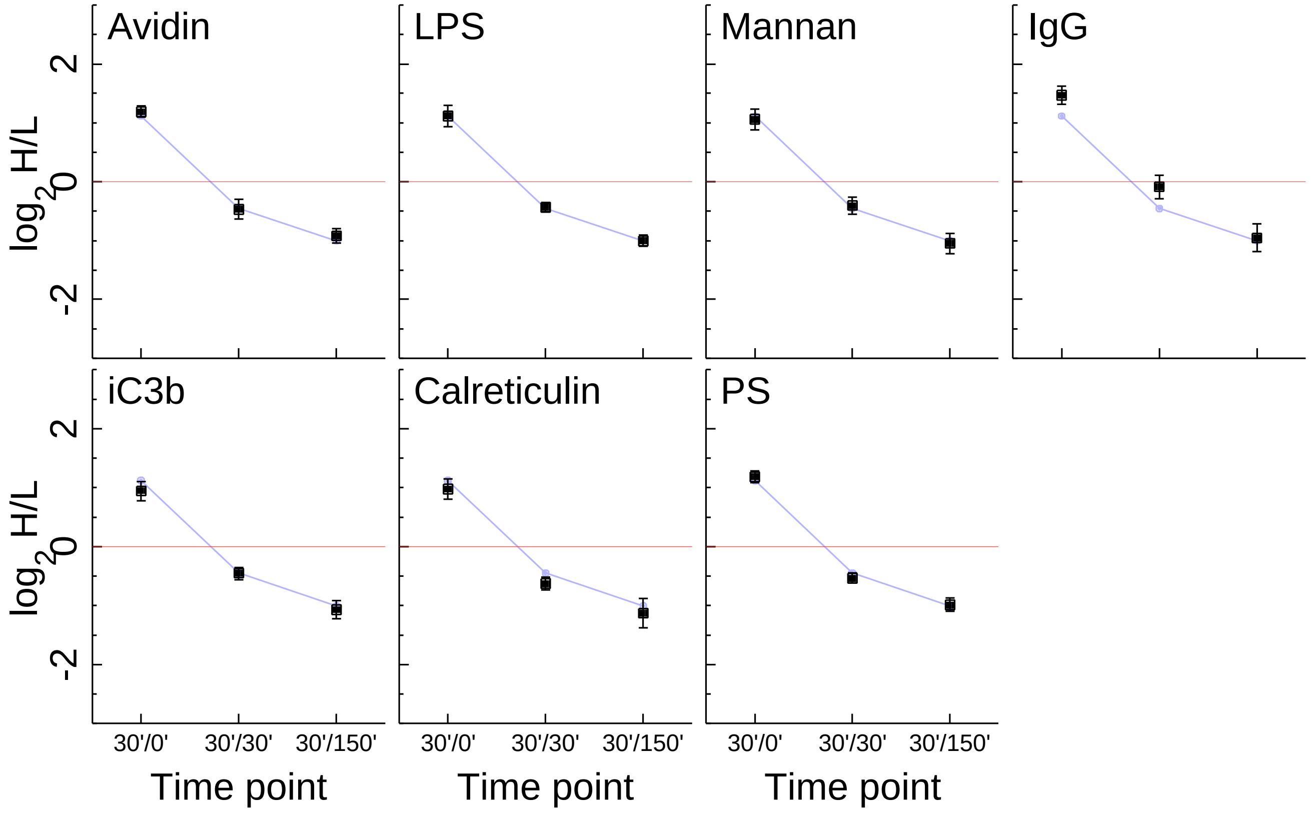

itgav (P43406)

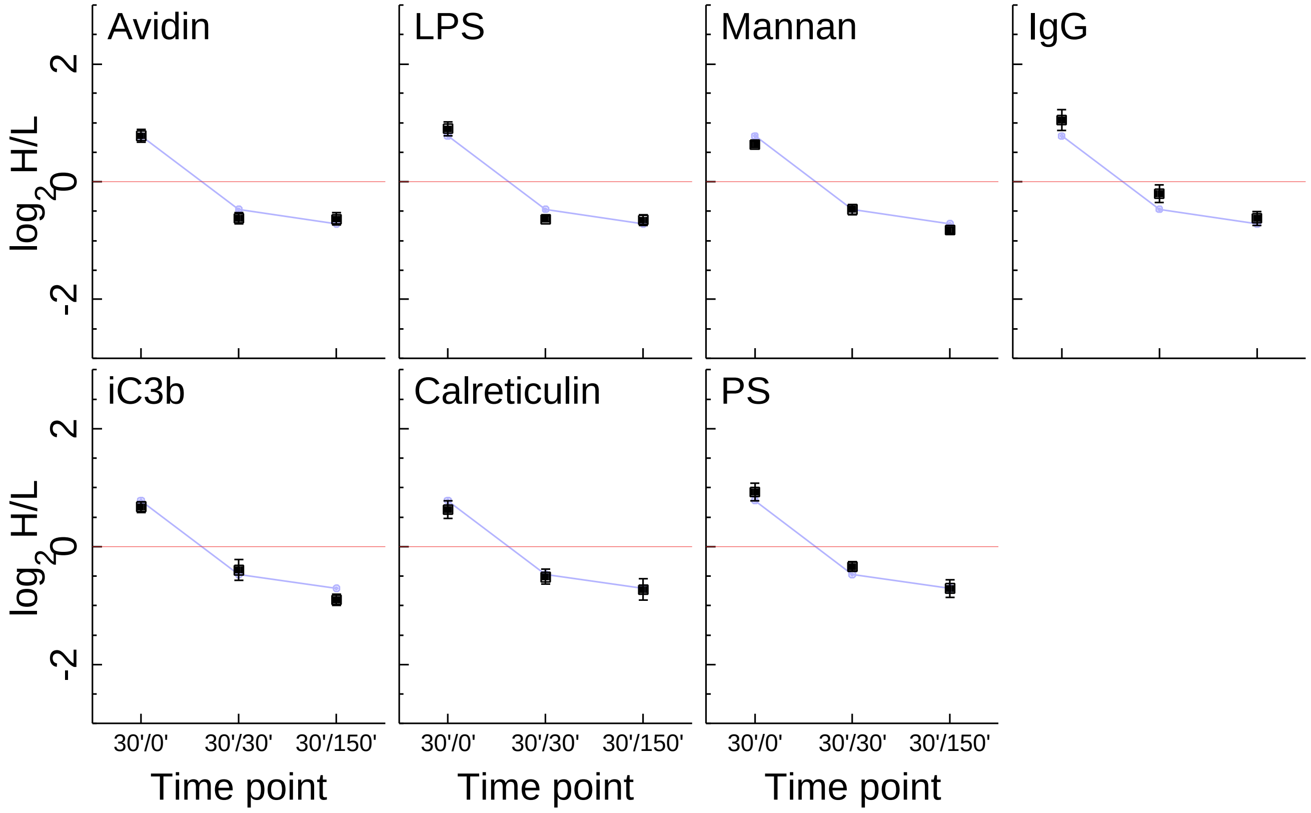

itgax (Q9QXH4)

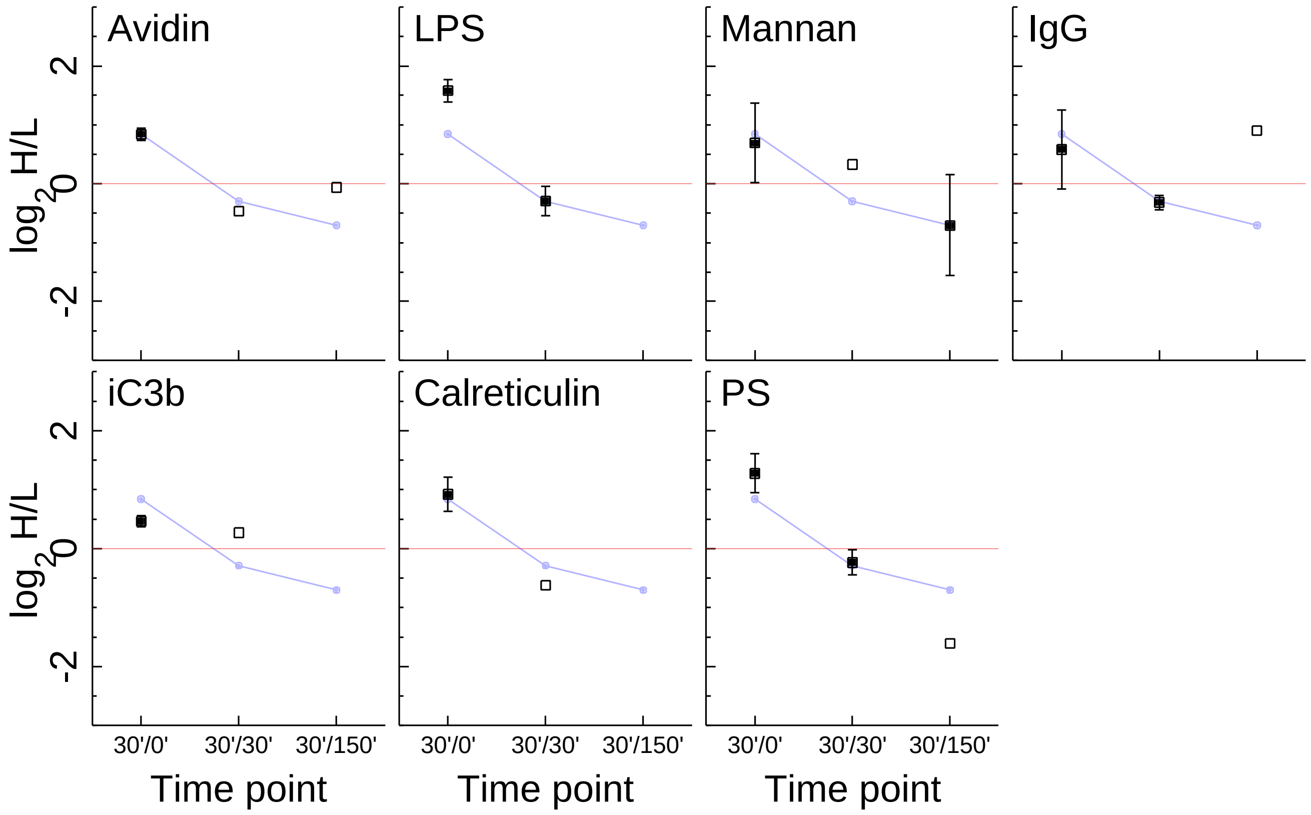

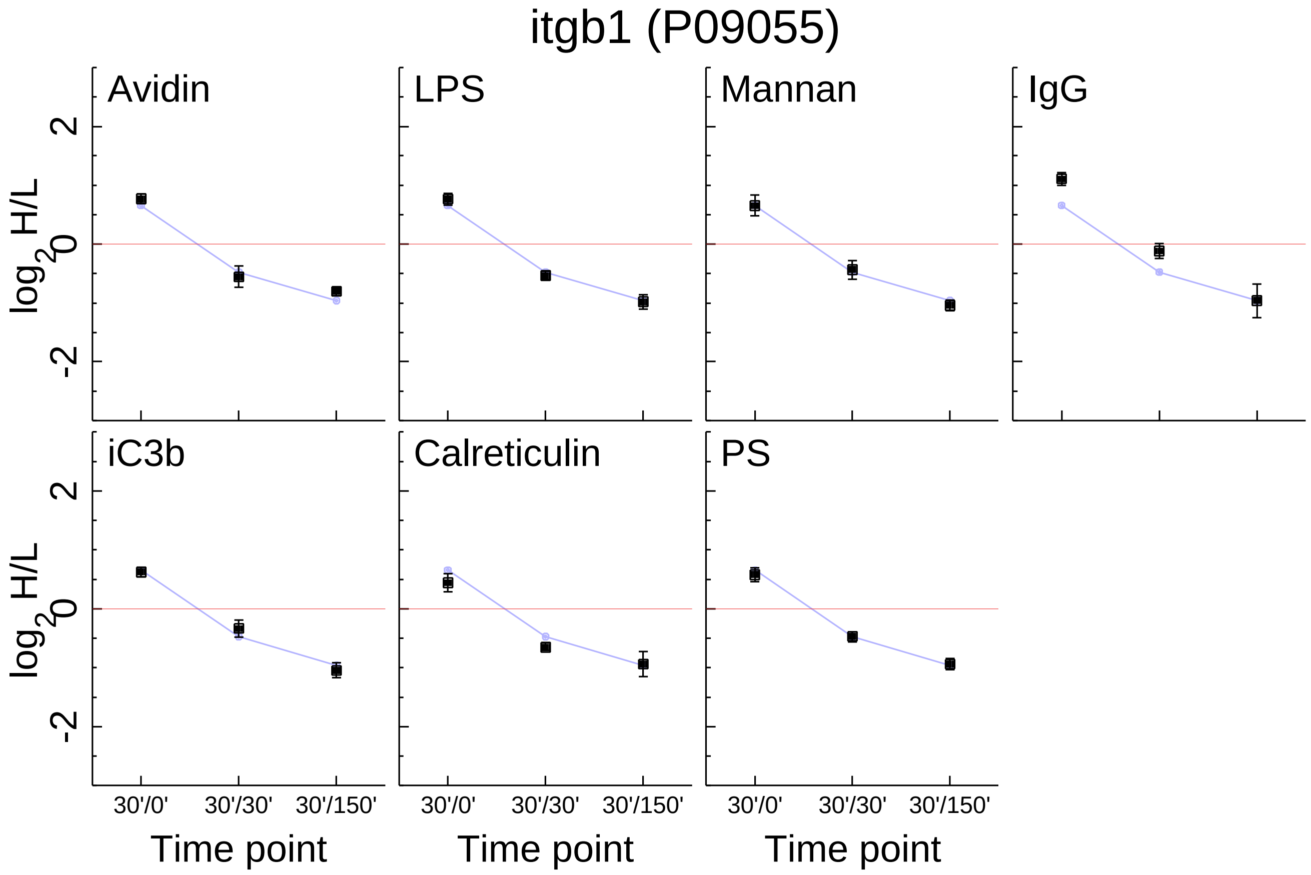

itgb1 (P09055)
Avidin
LPS
Mannan
IgG
iC3b
Calreticulin
PS
$\log_2$ H/L
2
0
-2
30'/0'
30'/30'
30'/150'
Time point

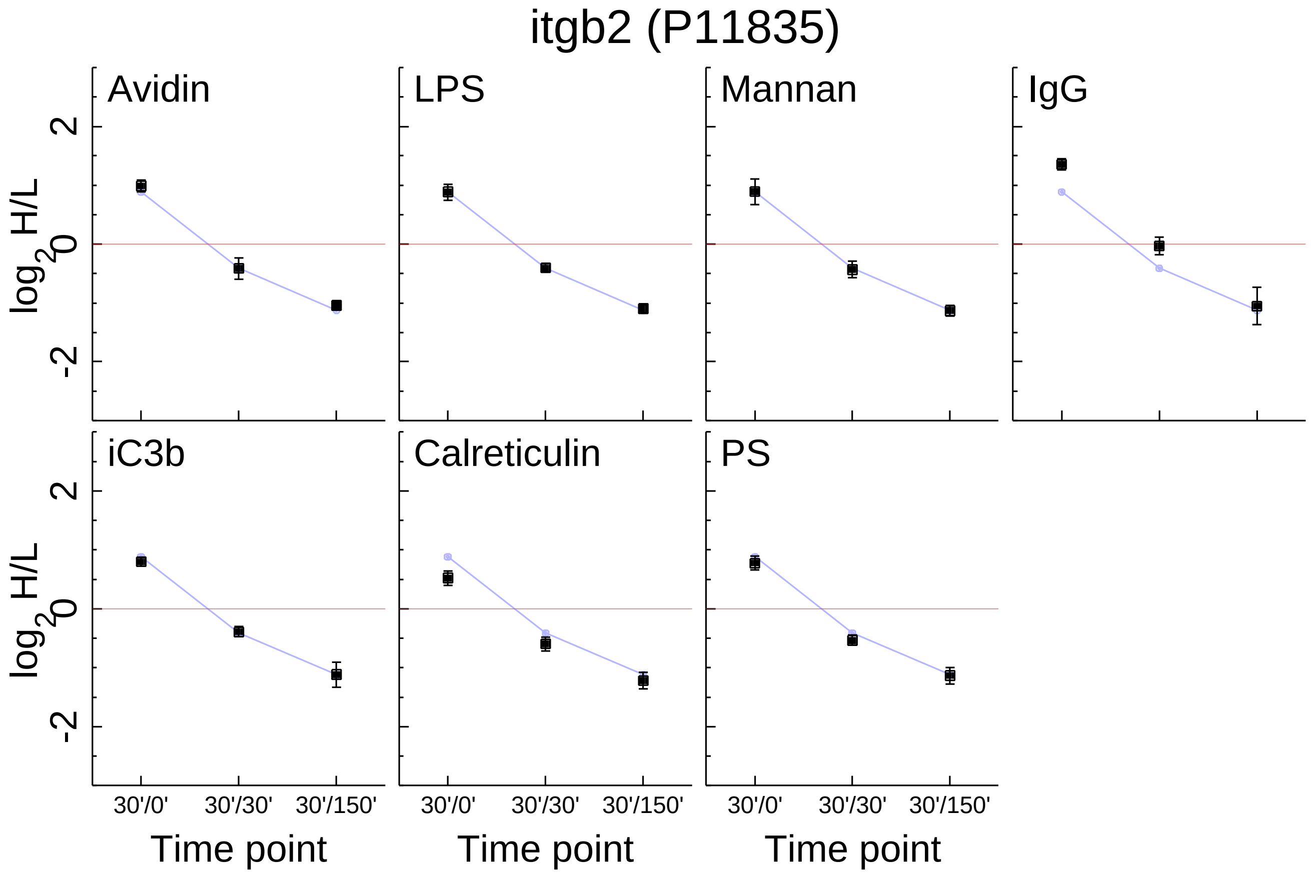
itgb2 (P11835)
Avidin
LPS
Mannan
IgG
iC3b
Calreticulin
PS
log2 H/L
2
0
-2
30'/0'
30'/30'
30'/150'
Time point

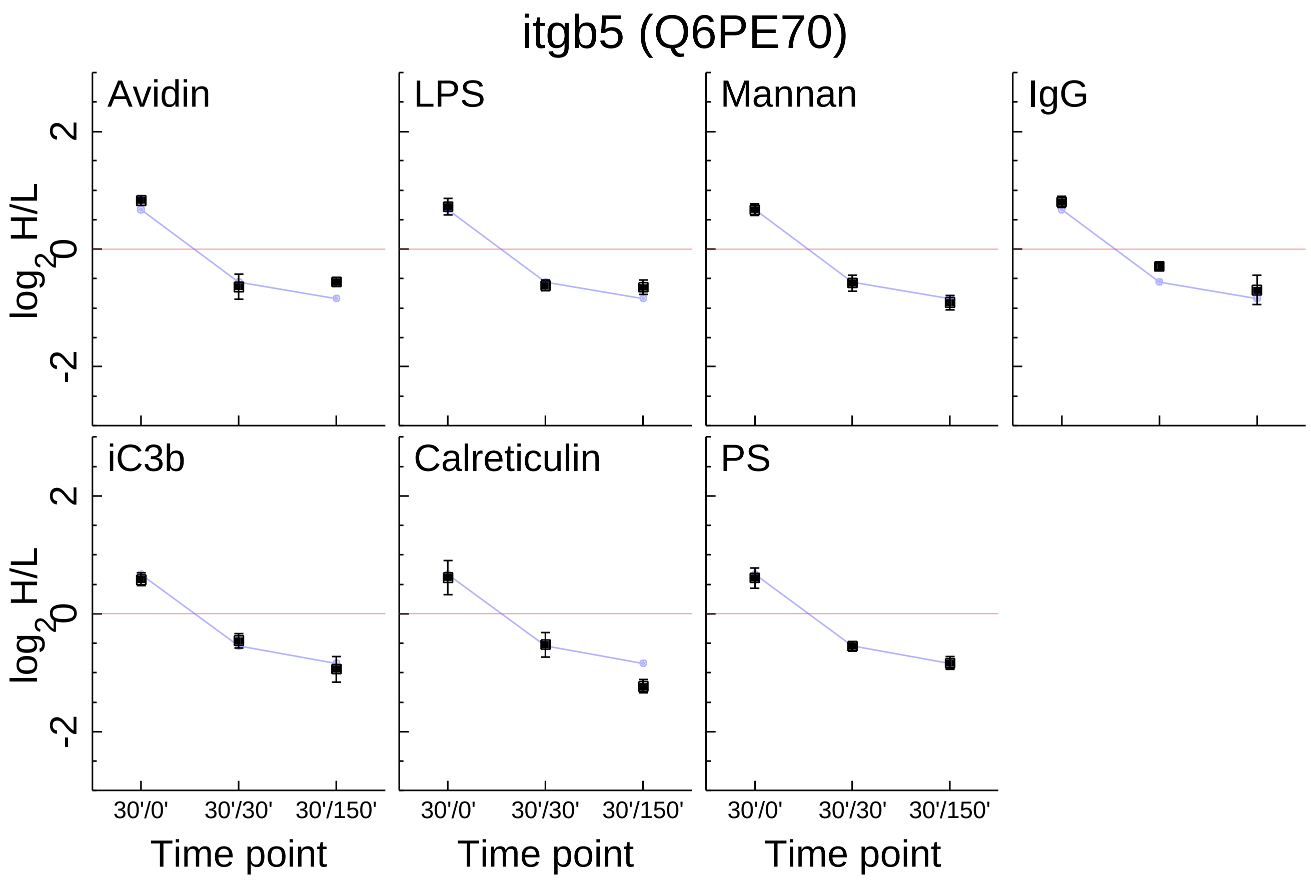
itgb5 (Q6PE70)
Avidin
LPS
Mannan
IgG
iC3b
Calreticulin
PS
log2 H/L
2
0
-2
30'/0'
30'/30'
30'/150'
Time point

itm2b (O89051)

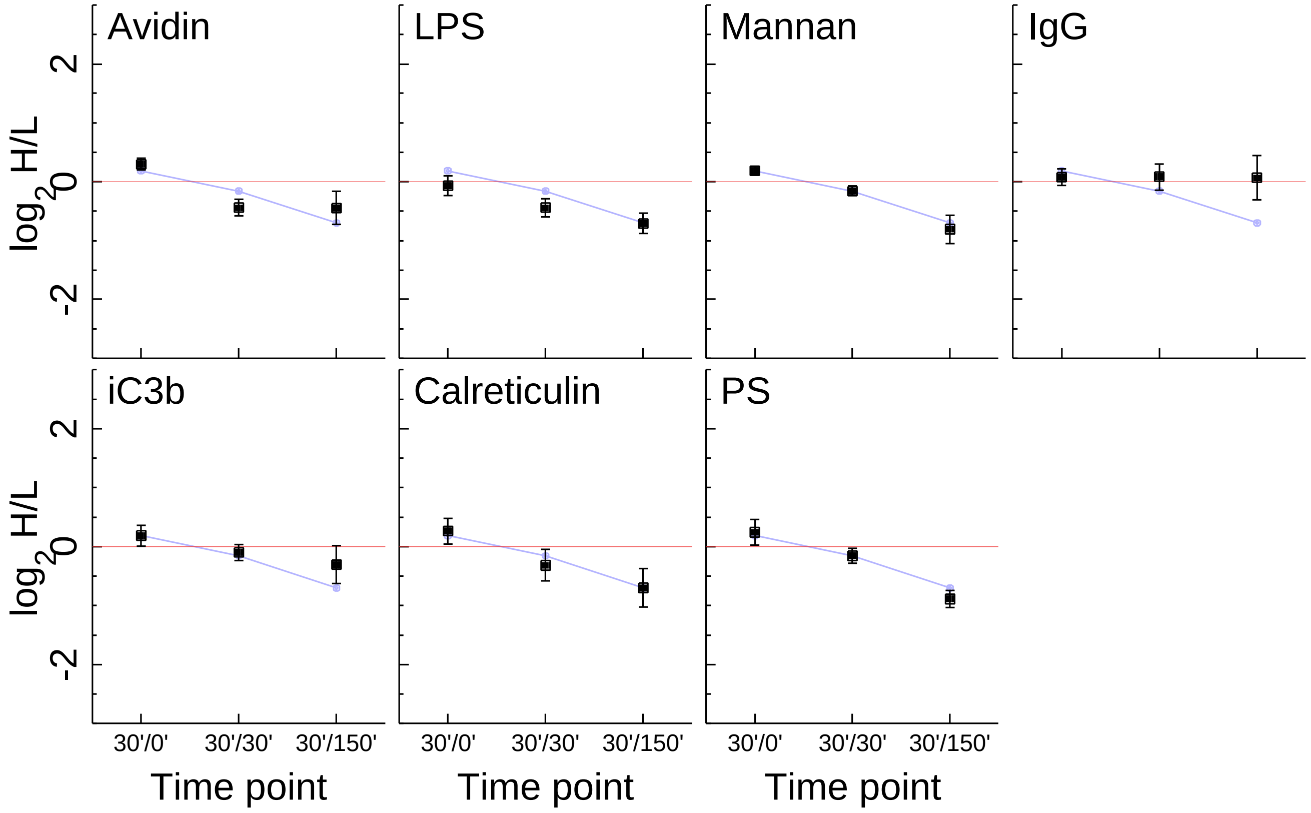

itsn2 (Q9Z0R6)

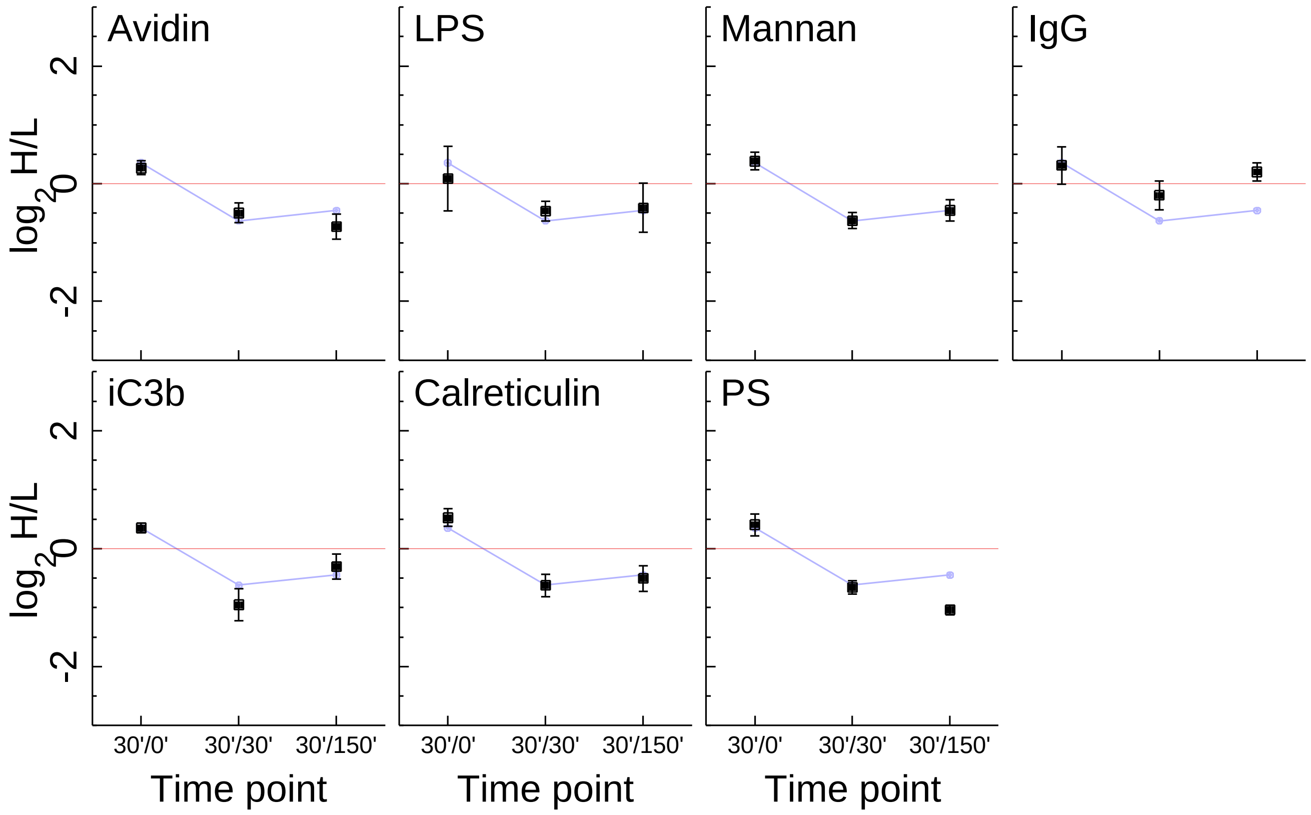

j3qnb0 (J3QNB0)

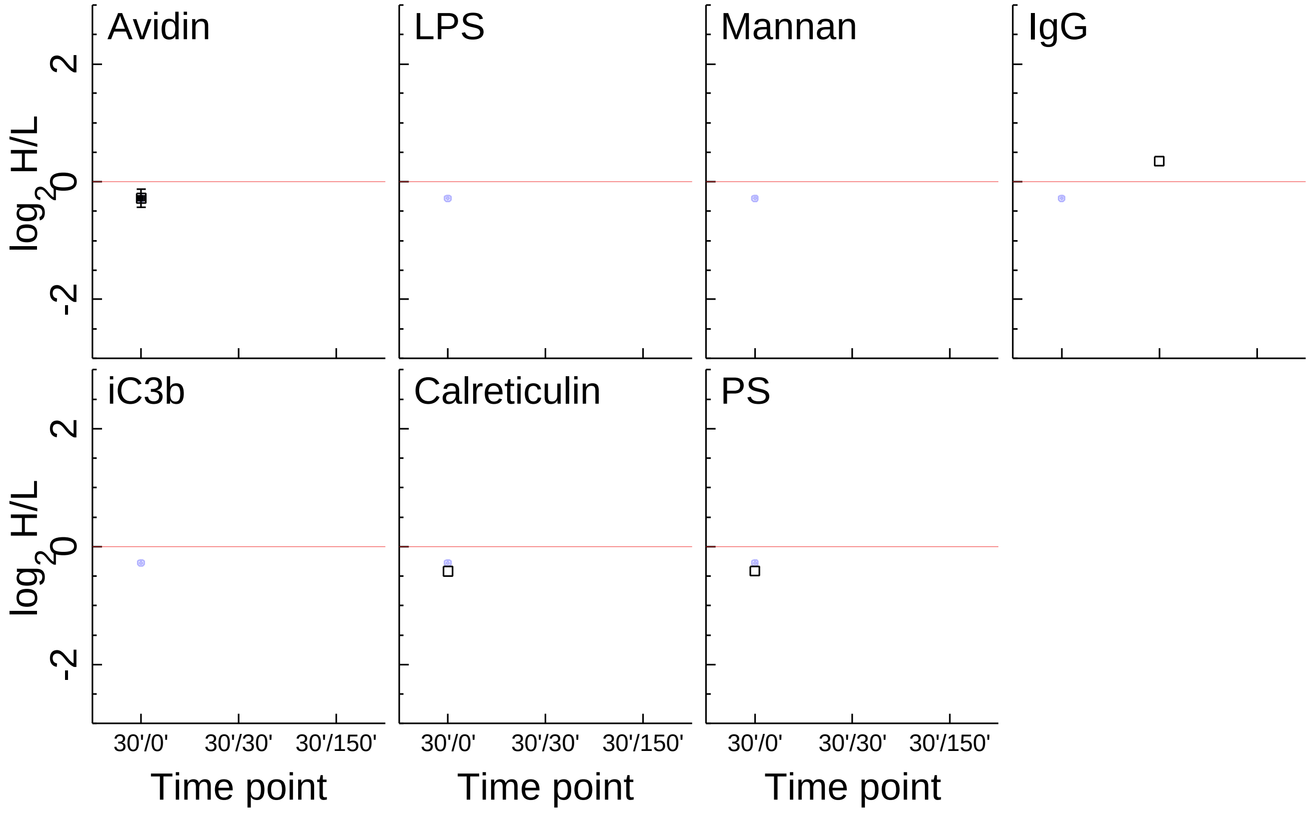

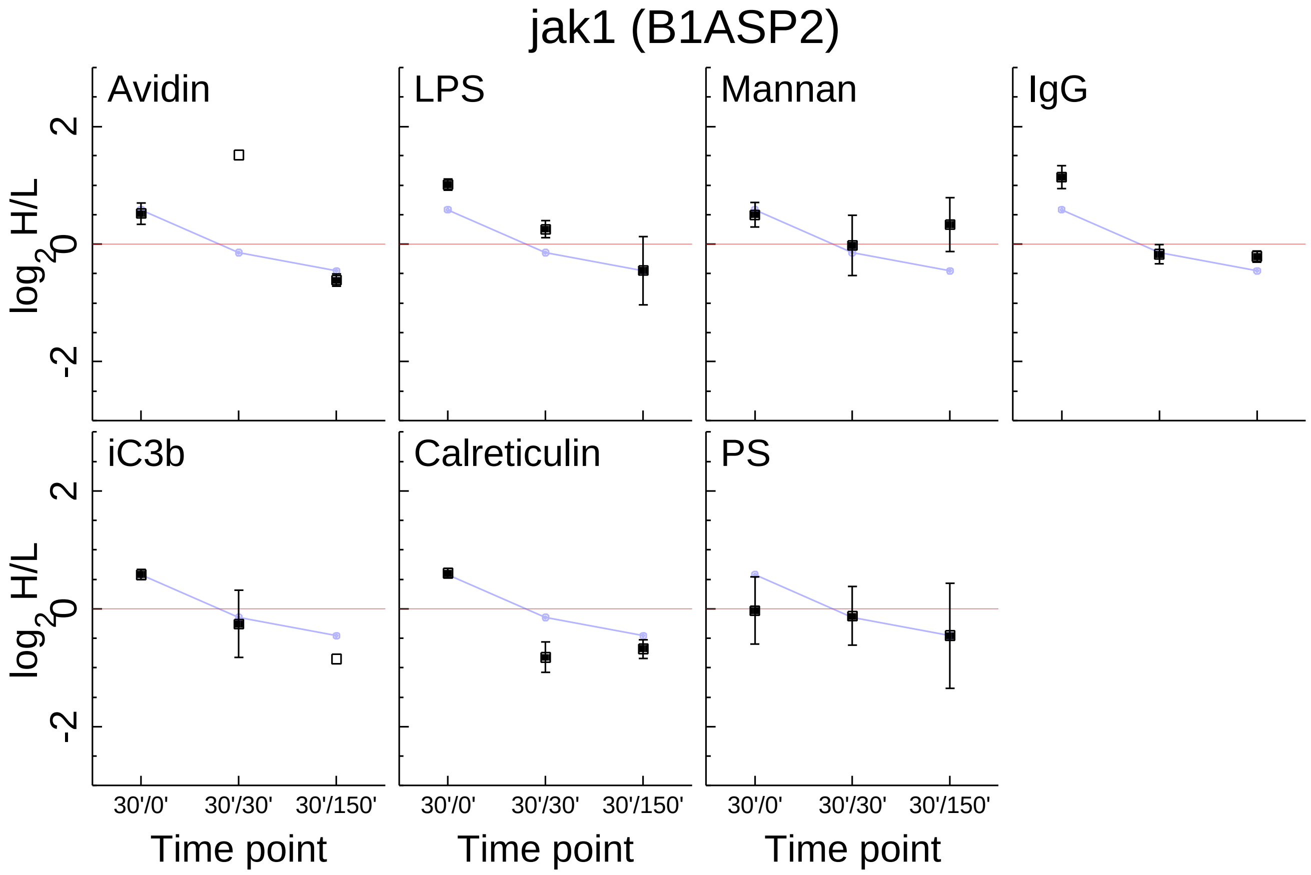
jak1 (B1ASP2)
Avidin
LPS
Mannan
IgG
iC3b
Calreticulin
PS
log2 H/L
2
0
-2
30'/0'
30'/30'
30'/150'
Time point

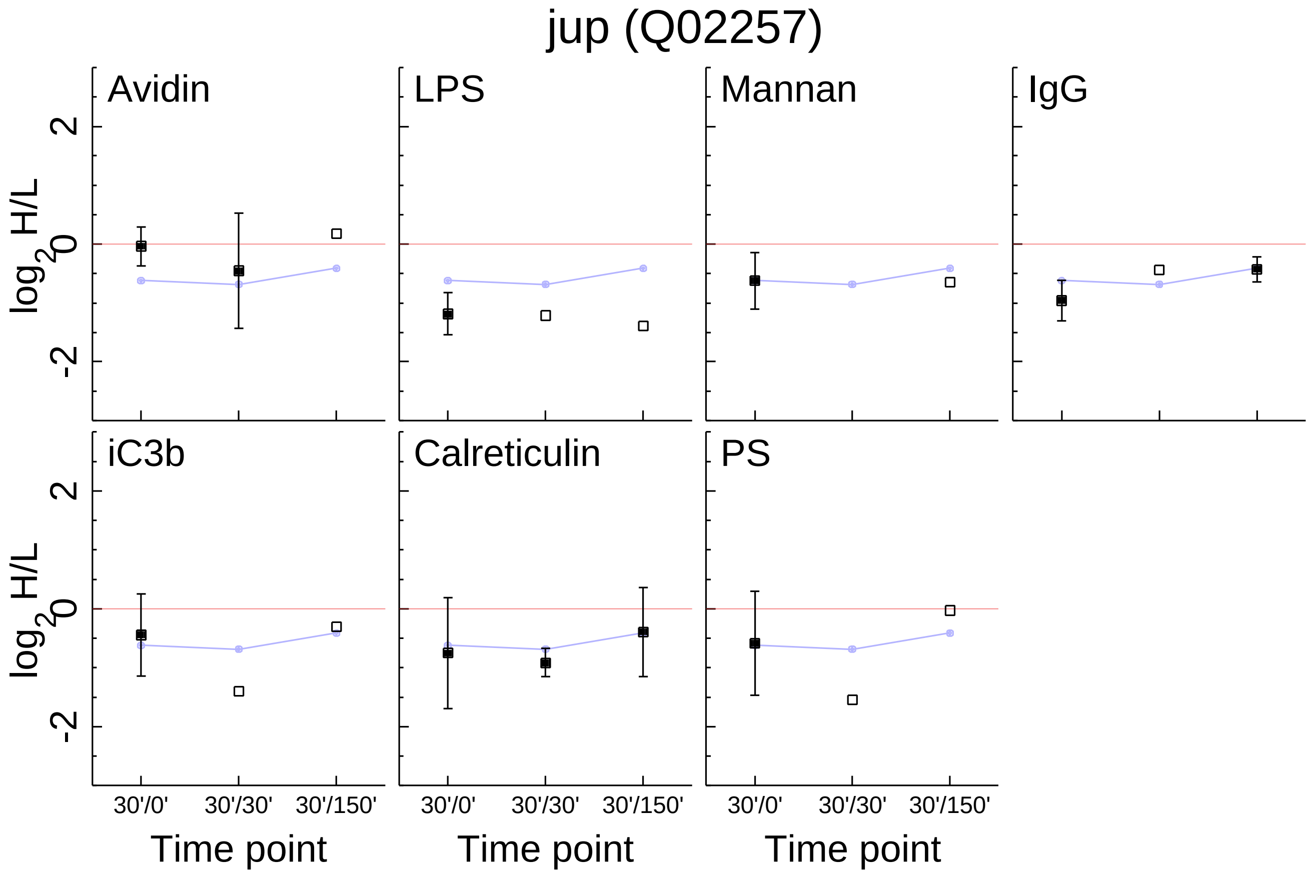
jup (Q02257)
Avidin
LPS
Mannan
IgG
iC3b
Calreticulin
PS
log2 H/L
2
0
-2
30'/0'
30'/30'
30'/150'
Time point

kat2a (Q9JHD2)

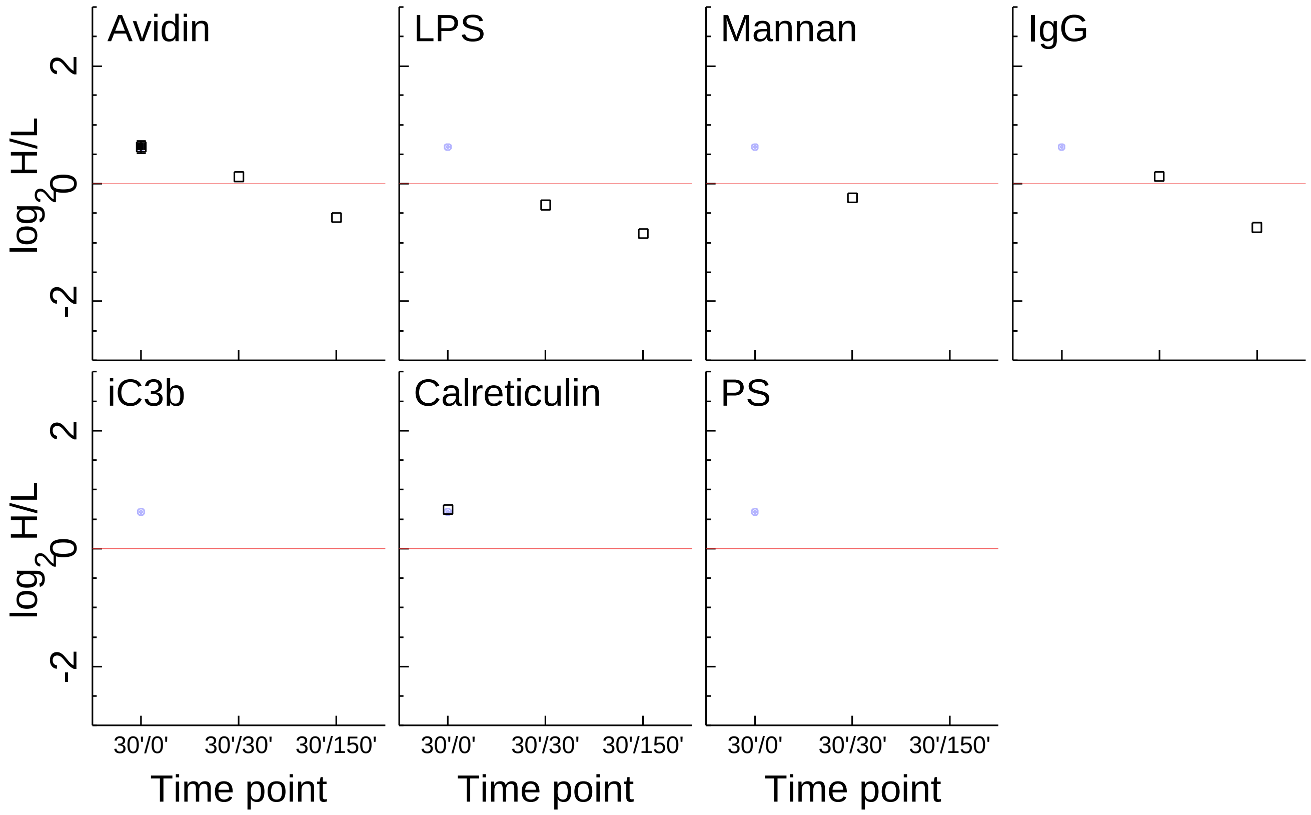

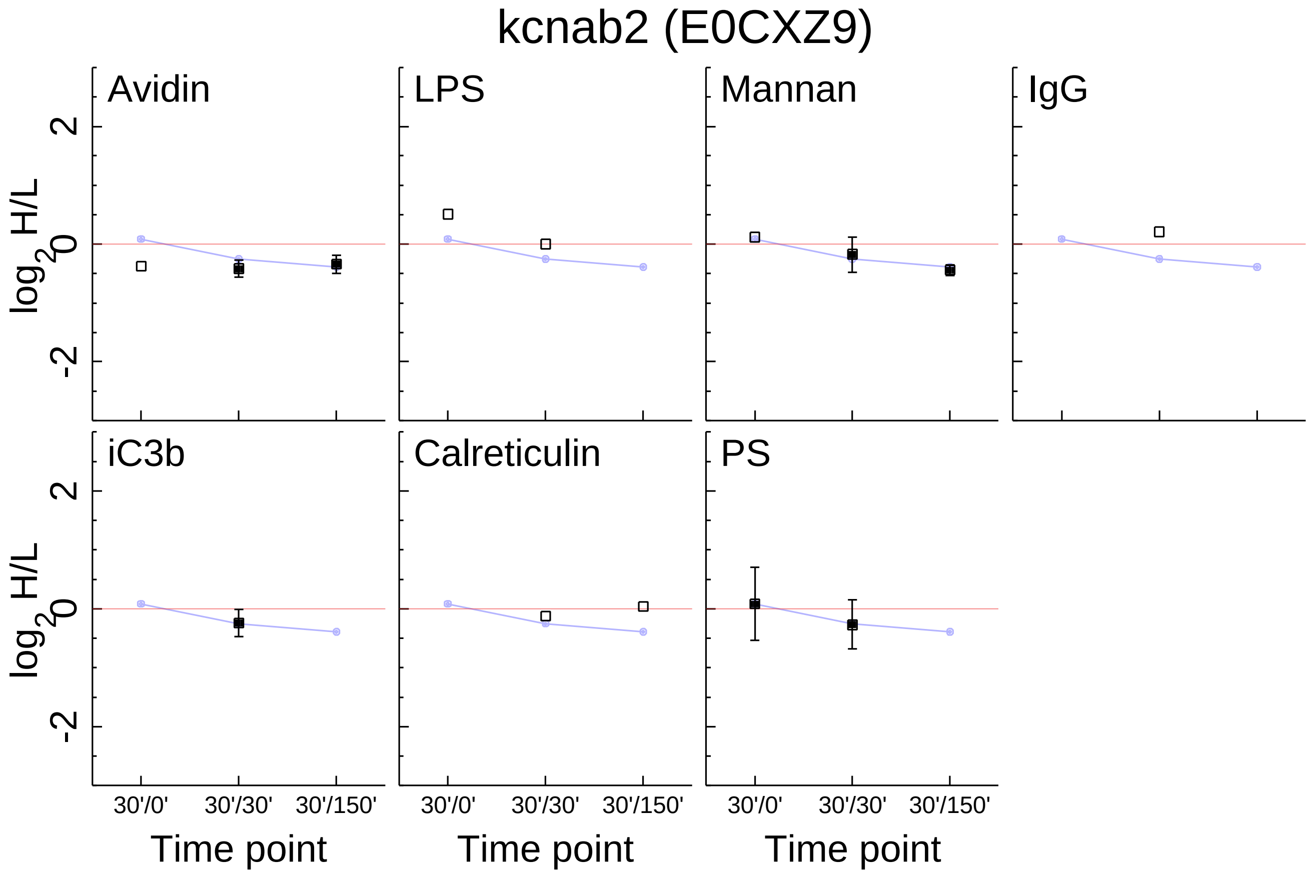
kcnab2 (E0CXZ9)
Avidin
LPS
Mannan
IgG
iC3b
Calreticulin
PS
log2 H/L
2
0
-2
30'/0'
30'/30'
30'/150'
Time point

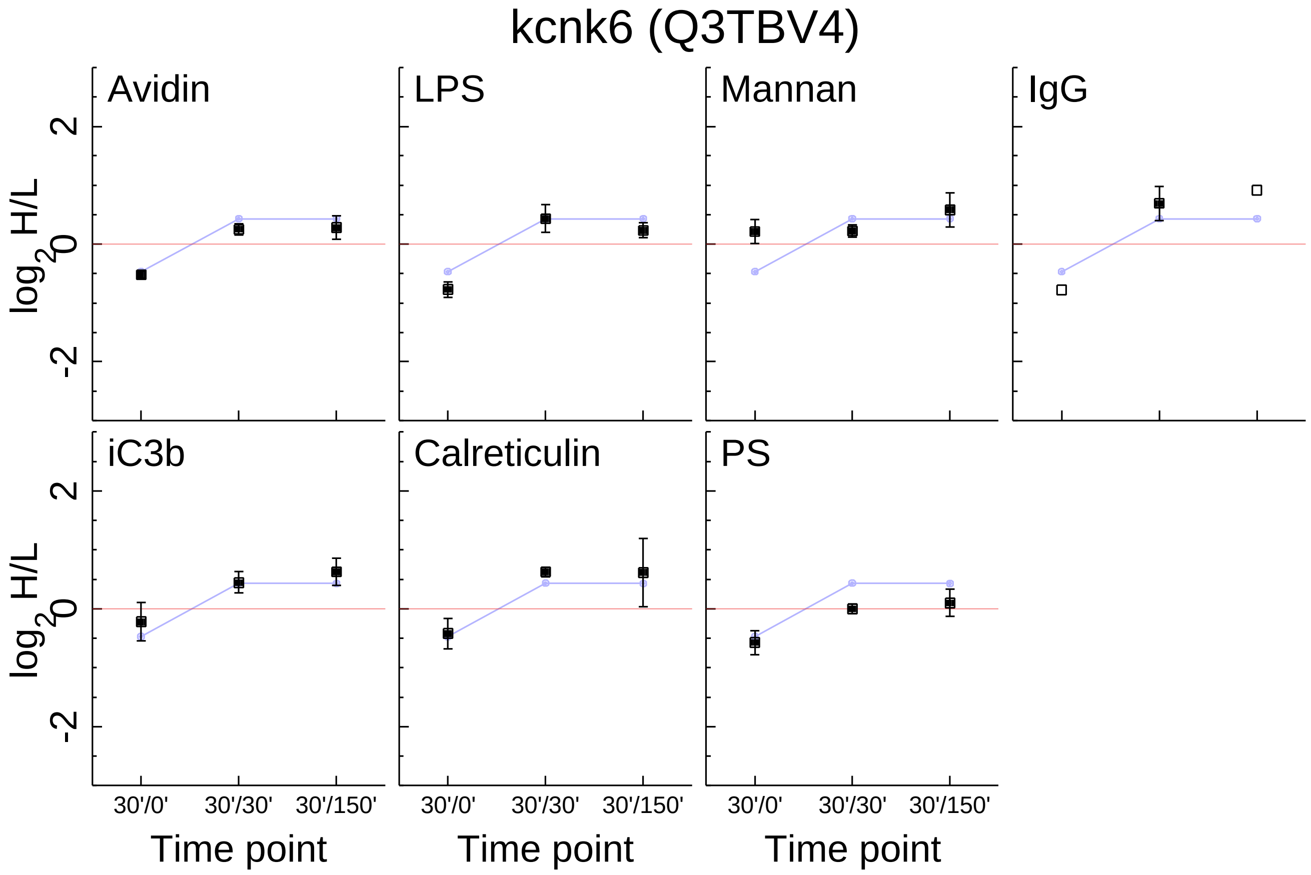
kcnk6 (Q3TBV4)
Avidin
LPS
Mannan
IgG
iC3b
Calreticulin
PS
log2 H/L
2
0
-2
30'/0'
30'/30'
30'/150'
Time point

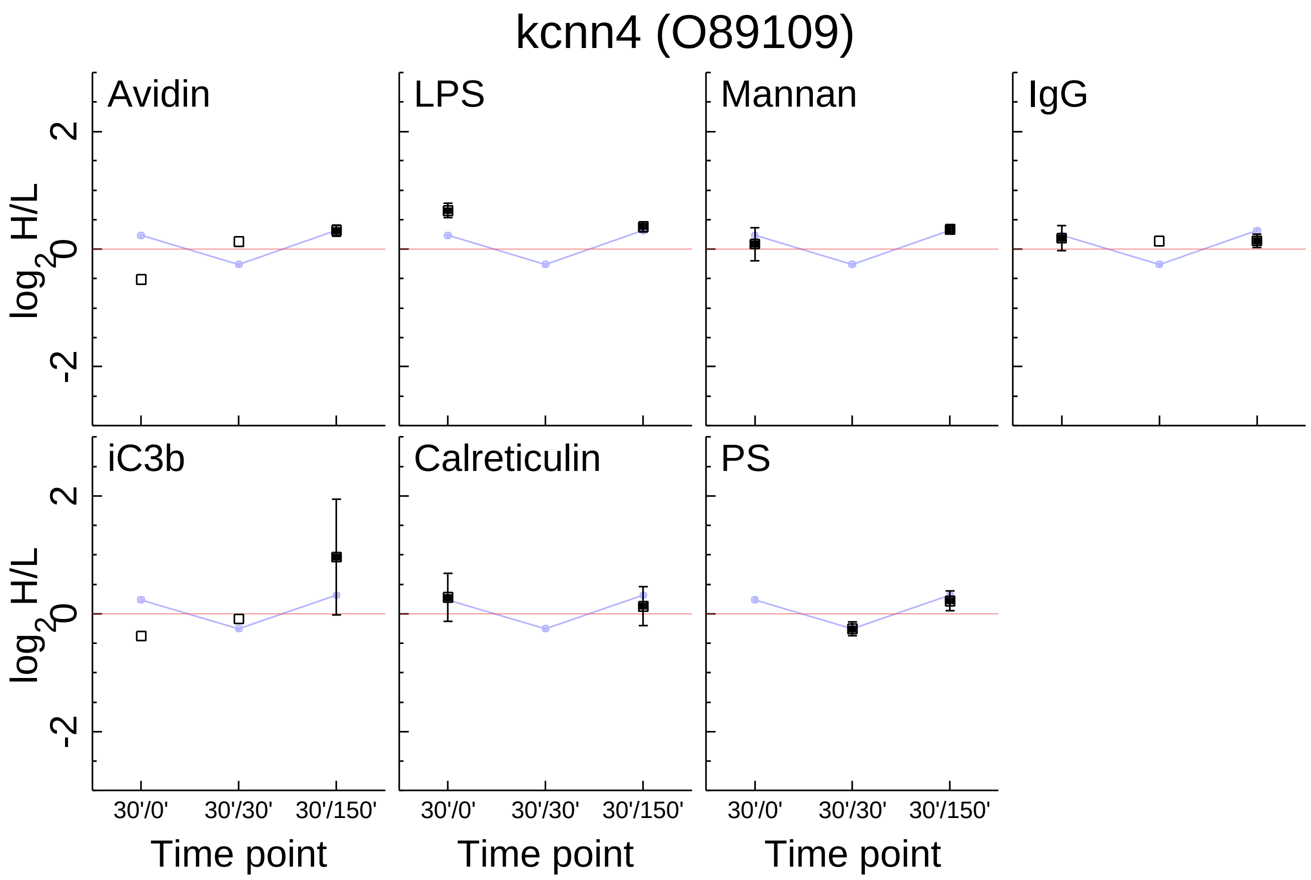
kcnn4 (O89109)
Avidin
LPS
Mannan
IgG
iC3b
Calreticulin
PS
log2 H/L
2
0
-2
30'/0'
30'/30'
30'/150'
Time point

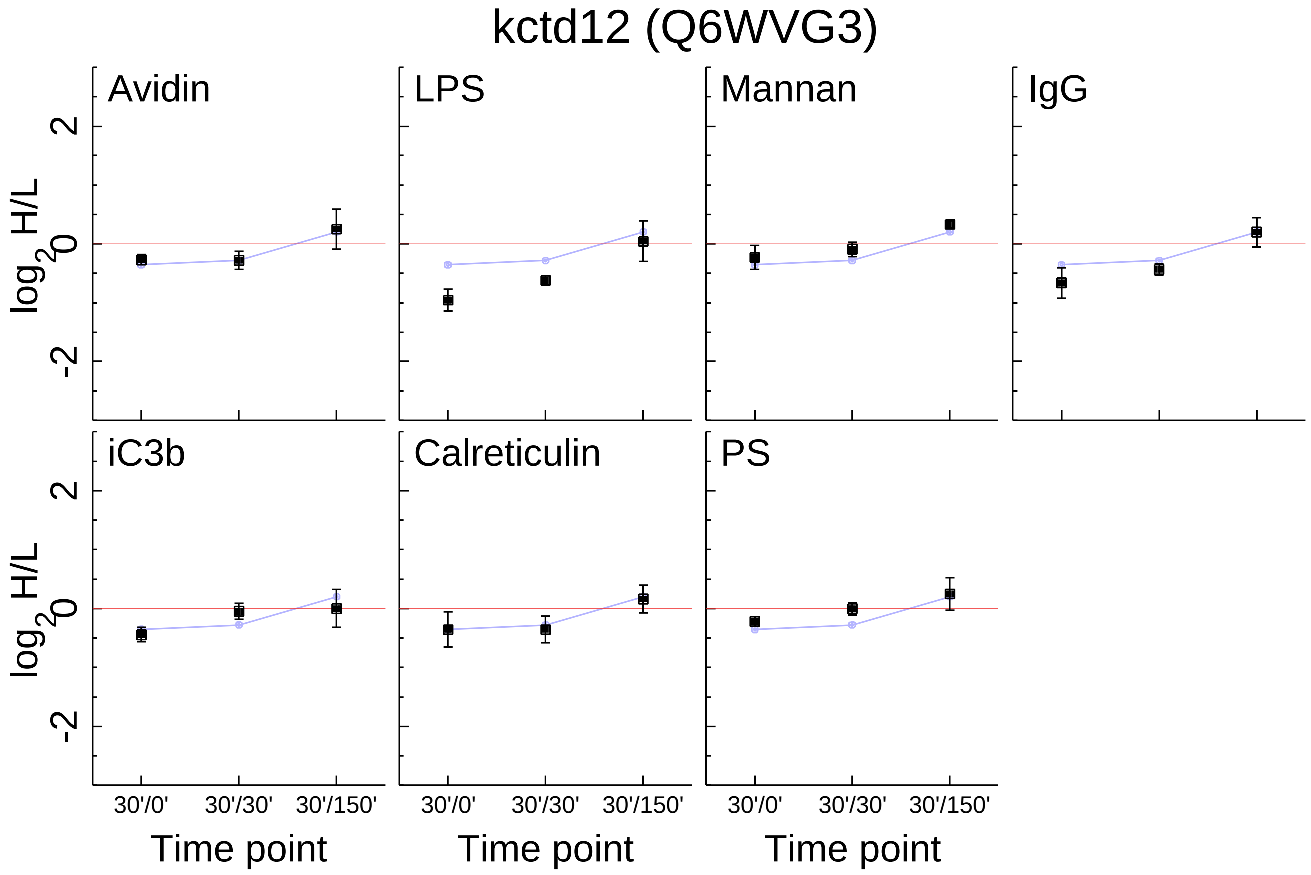
kctd12 (Q6WVG3)
Avidin
LPS
Mannan
IgG
iC3b
Calreticulin
PS
log2 H/L
2
0
-2
30'/0'
30'/30'
30'/150'
Time point

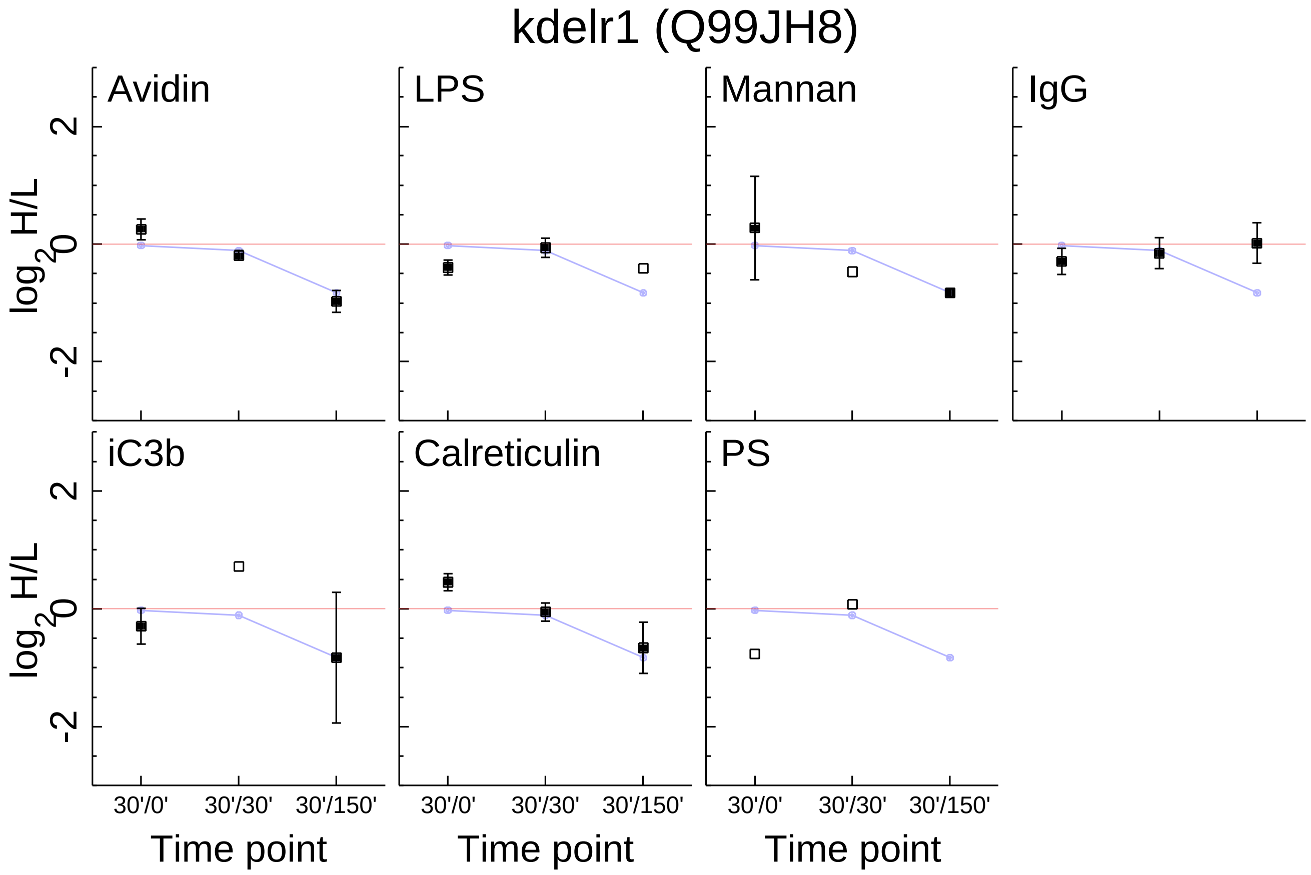
kdelr1 (Q99JH8)
Avidin
LPS
Mannan
IgG
iC3b
Calreticulin
PS
$\log_2$ H/L
2
0
-2
30'/0'
30'/30'
30'/150'
Time point

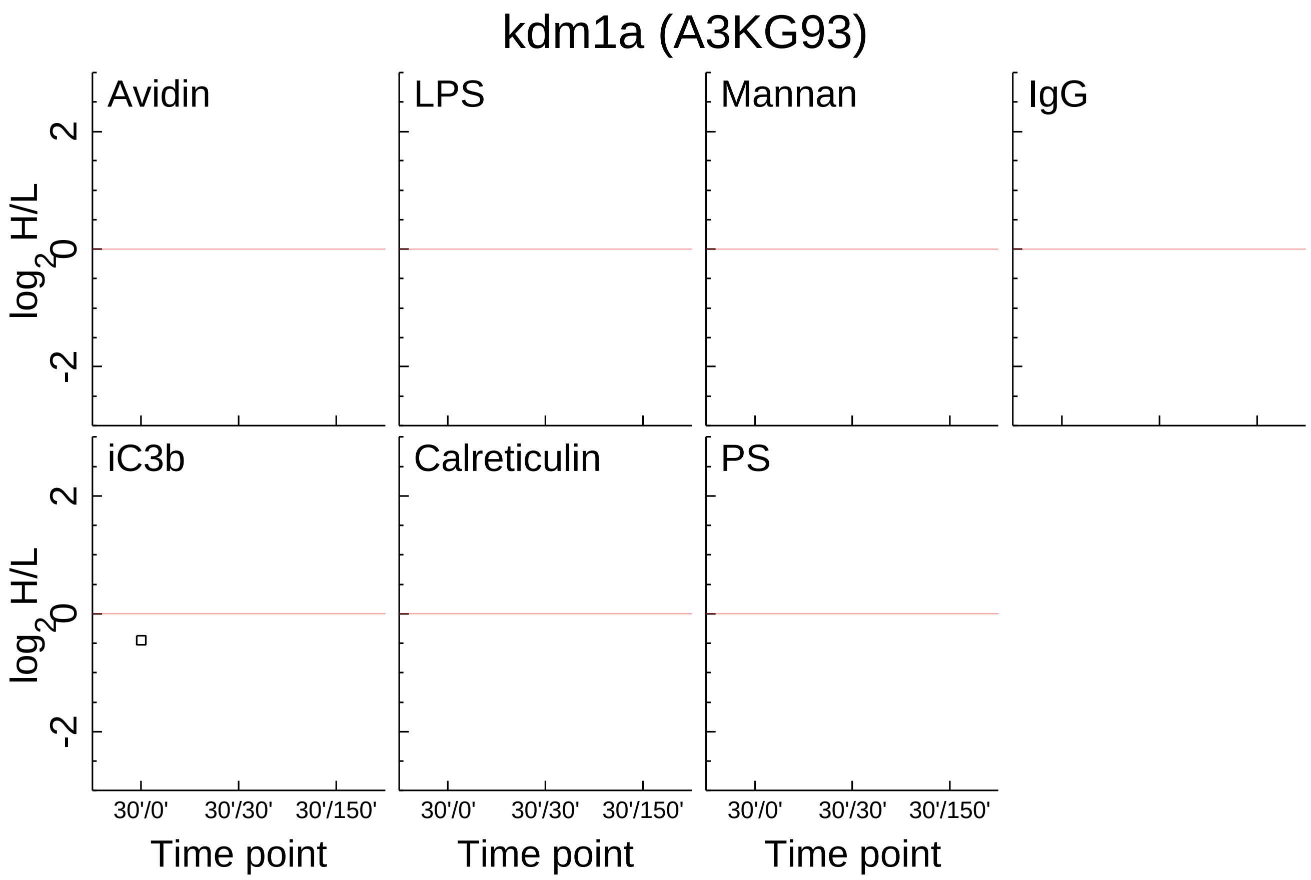
kdm1a (A3KG93)
Avidin
LPS
Mannan
IgG
iC3b
Calreticulin
PS
log2 H/L
2
0
-2
30'/0'
30'/30'
30'/150'
Time point

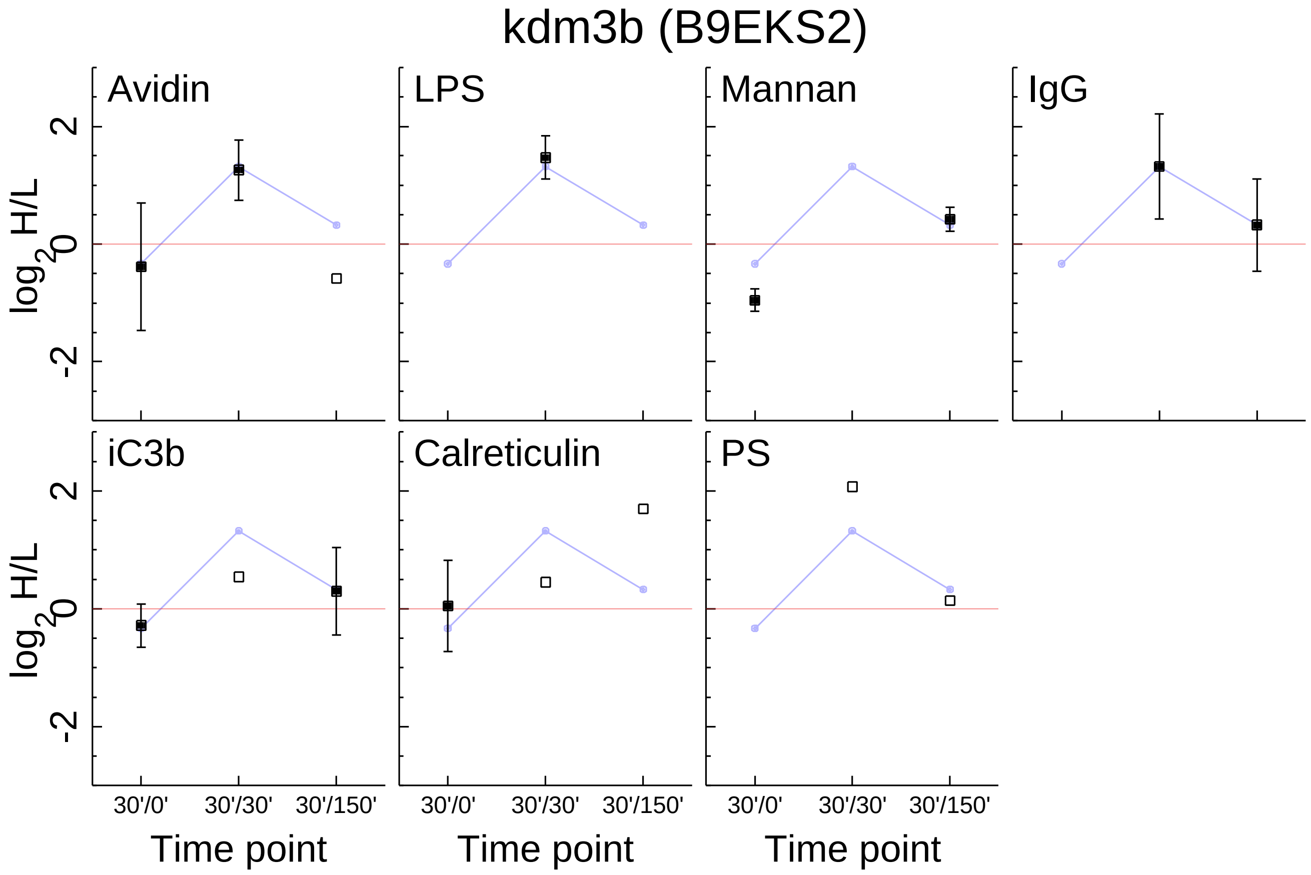
kdm3b (B9EKS2)
Avidin
LPS
Mannan
IgG
iC3b
Calreticulin
PS
log2 H/L
2
0
-2
30'/0'
30'/30'
30'/150'
Time point

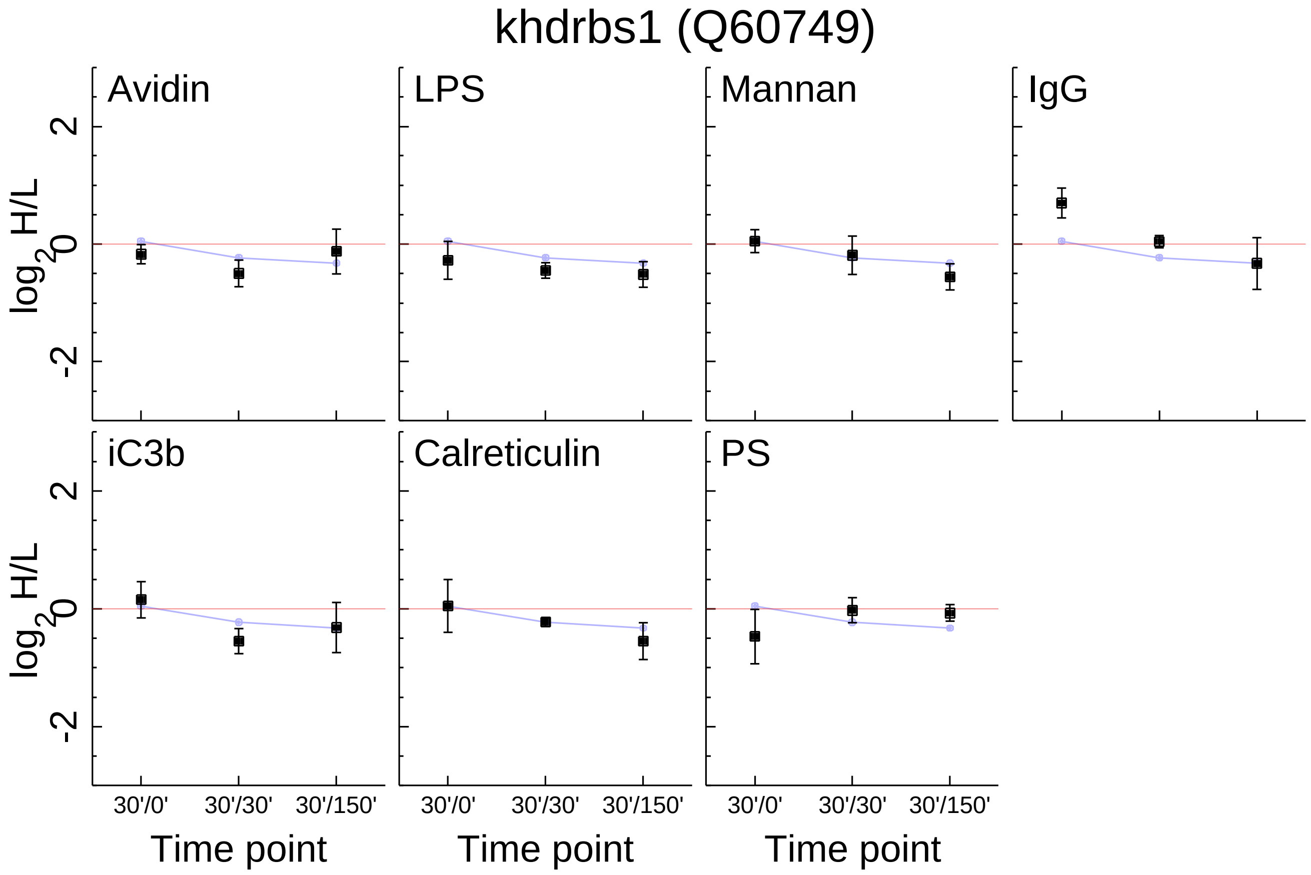

khdrbs1 (Q60749)
Avidin
LPS
Mannan
IgG
iC3b
Calreticulin
PS
log2 H/L
2
0
-2
30'/0'
30'/30'
30'/150'
Time point

khsrp (Q3U0V1)

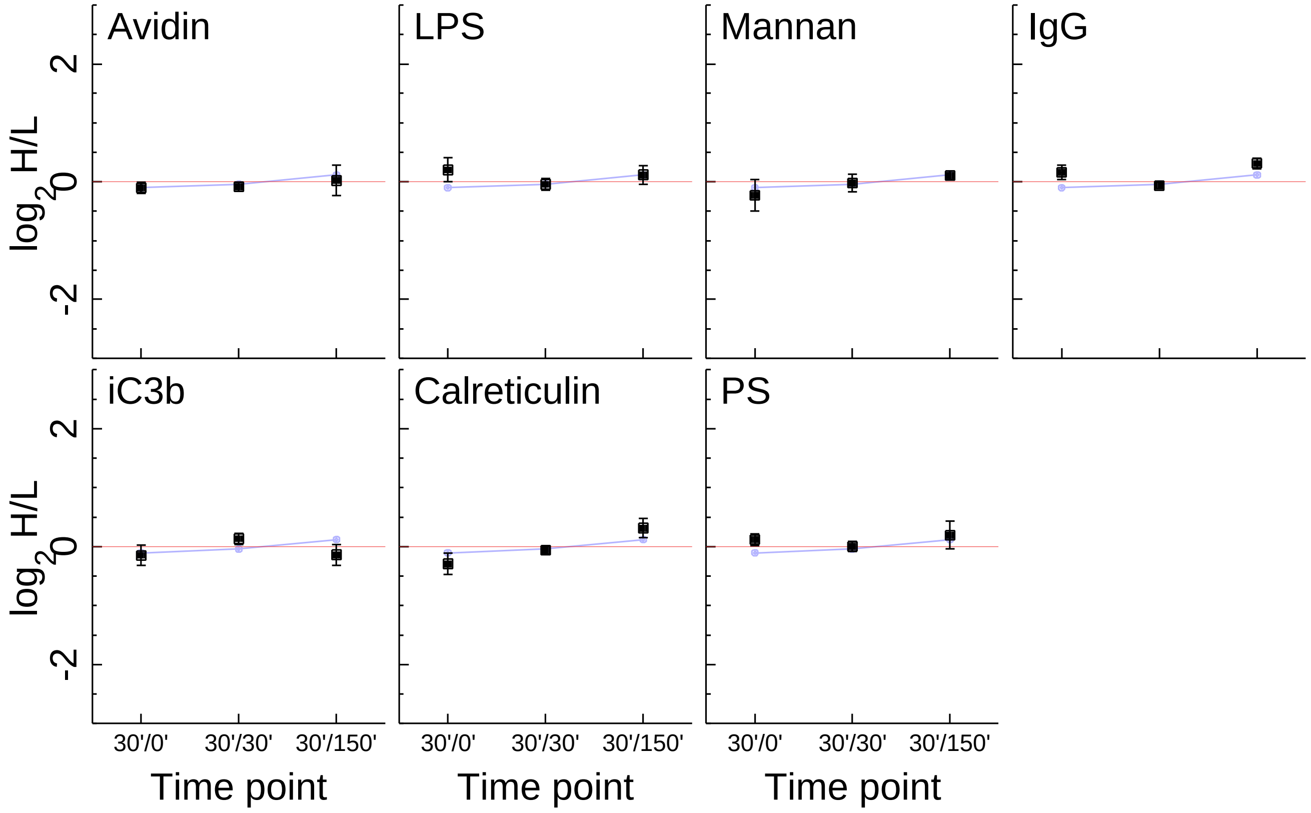

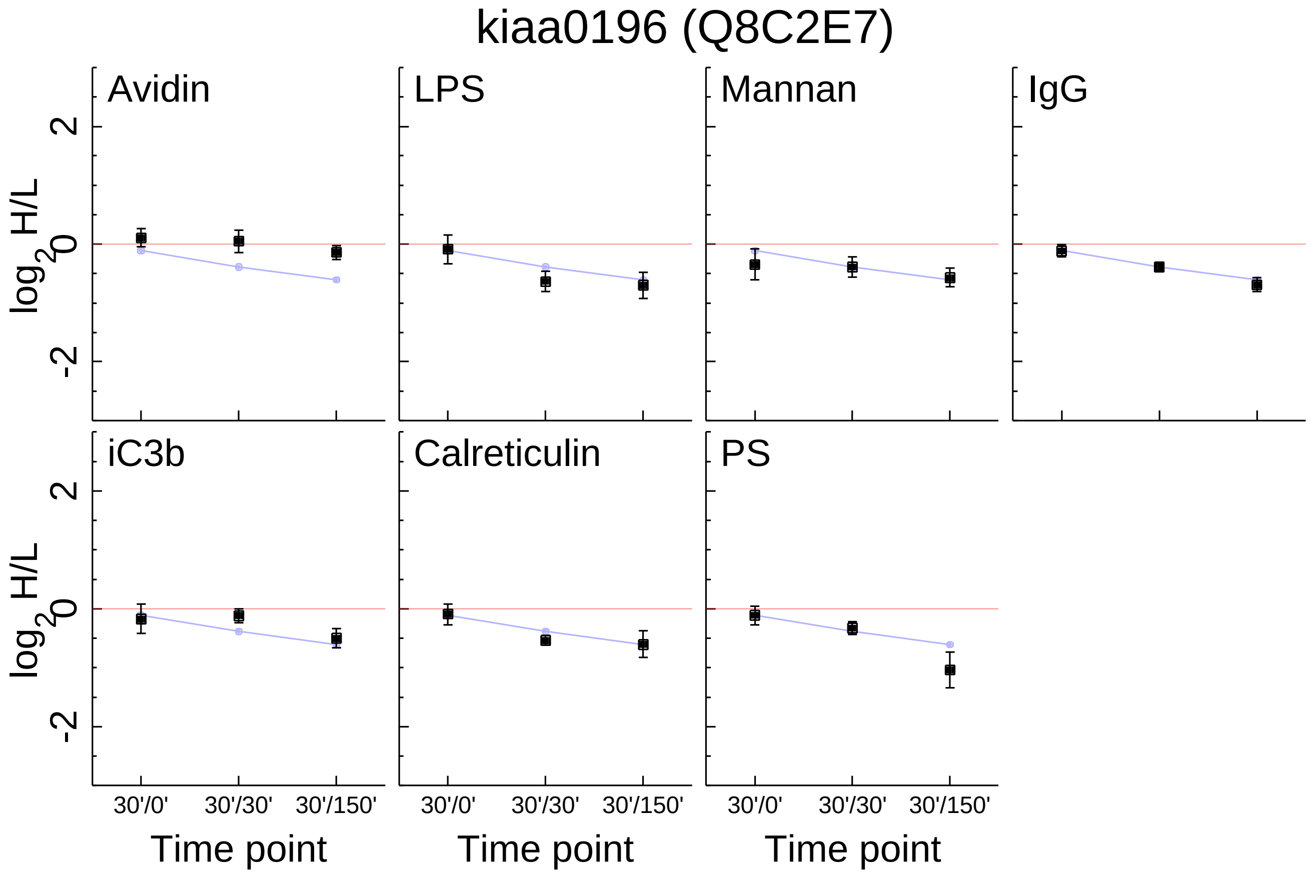

kiaa0196 (Q8C2E7)
Avidin
LPS
Mannan
IgG
iC3b
Calreticulin
PS
log2 H/L
2
0
-2
30'/0'
30'/30'
30'/150'
Time point

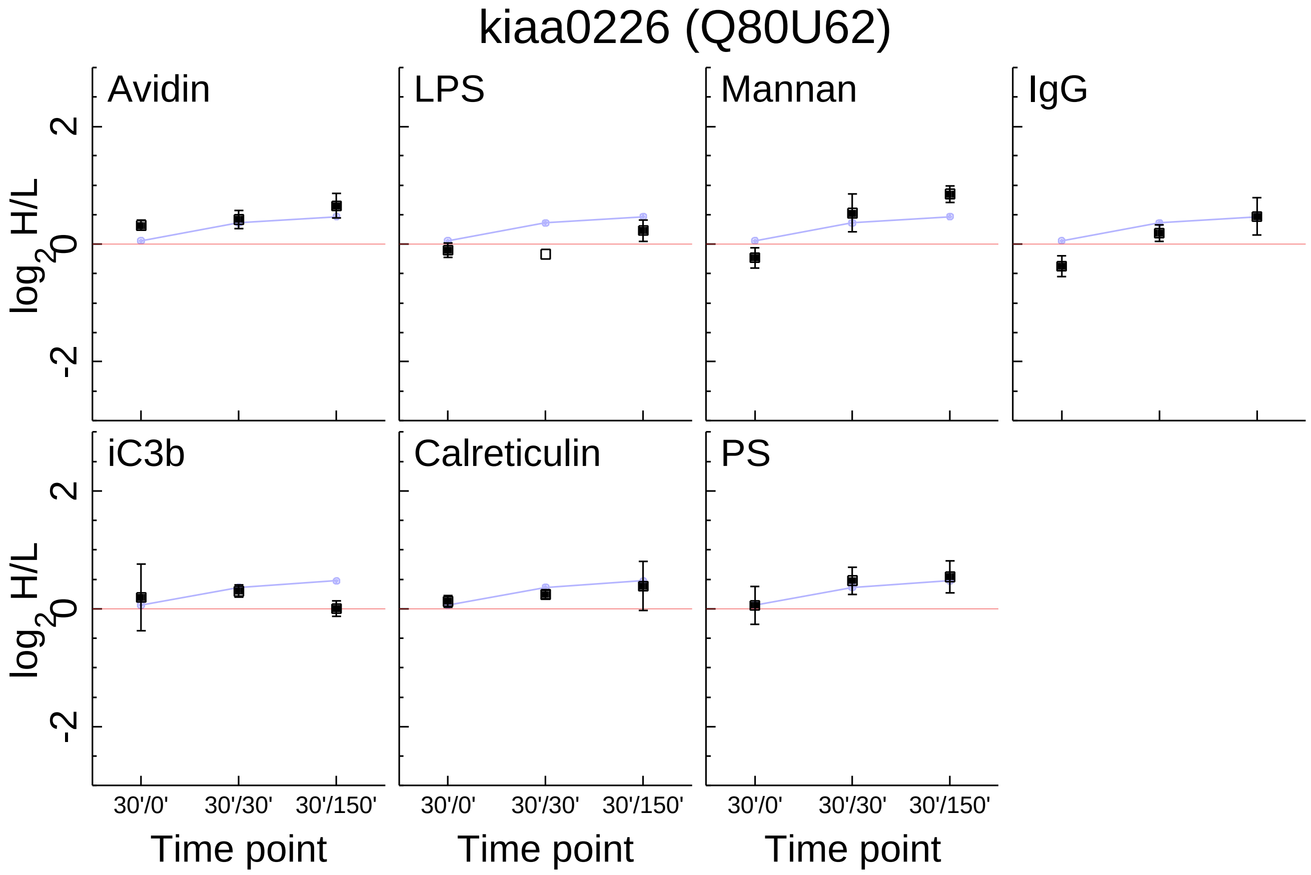
kiaa0226 (Q80U62)
Avidin
LPS
Mannan
IgG
iC3b
Calreticulin
PS
log2 H/L
2
0
-2
30'/0'
30'/30'
30'/150'
Time point

kiaa0907 (Q3TCX3)

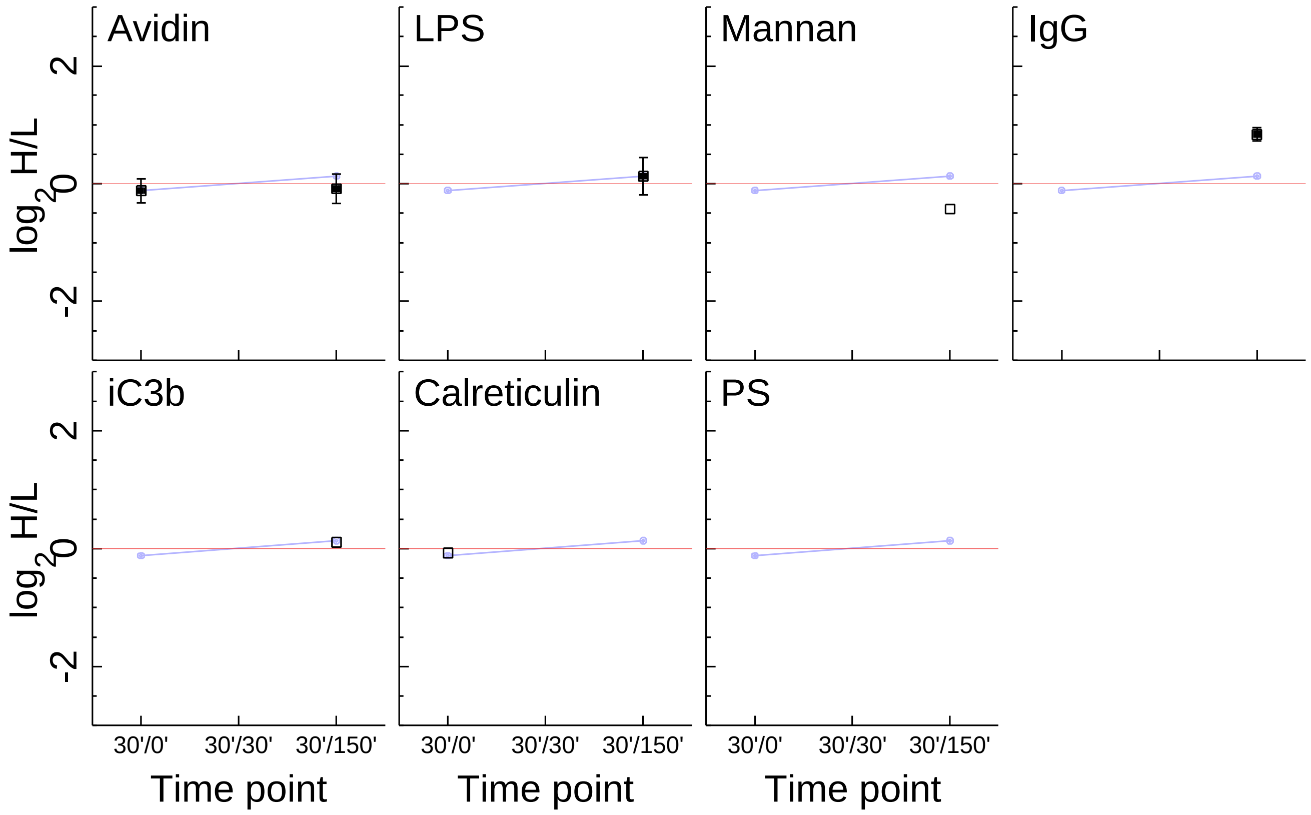

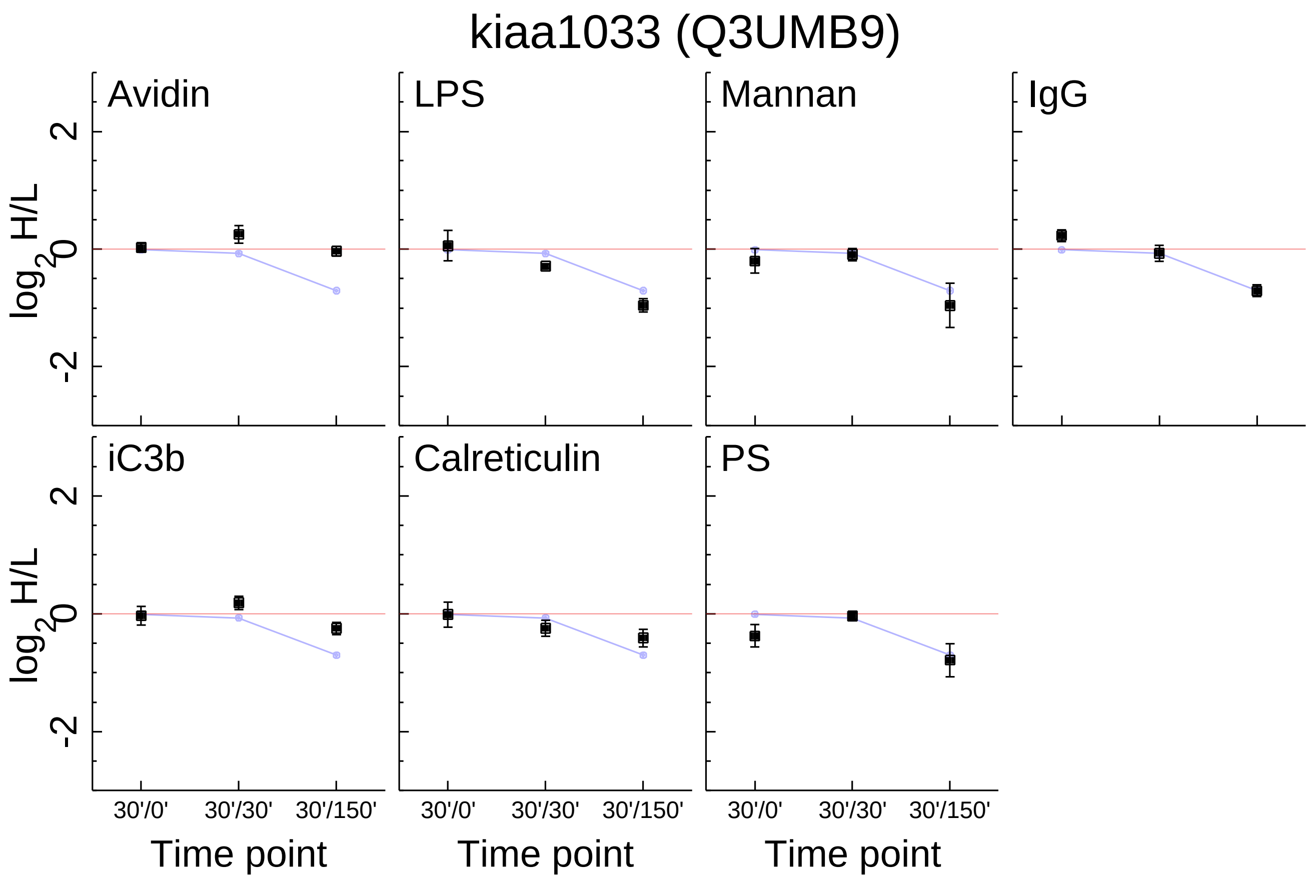

kiaa1033 (Q3UMB9)
Avidin
LPS
Mannan
IgG
iC3b
Calreticulin
PS
log2 H/L
2
0
-2
30'/0'
30'/30'
30'/150'
Time point

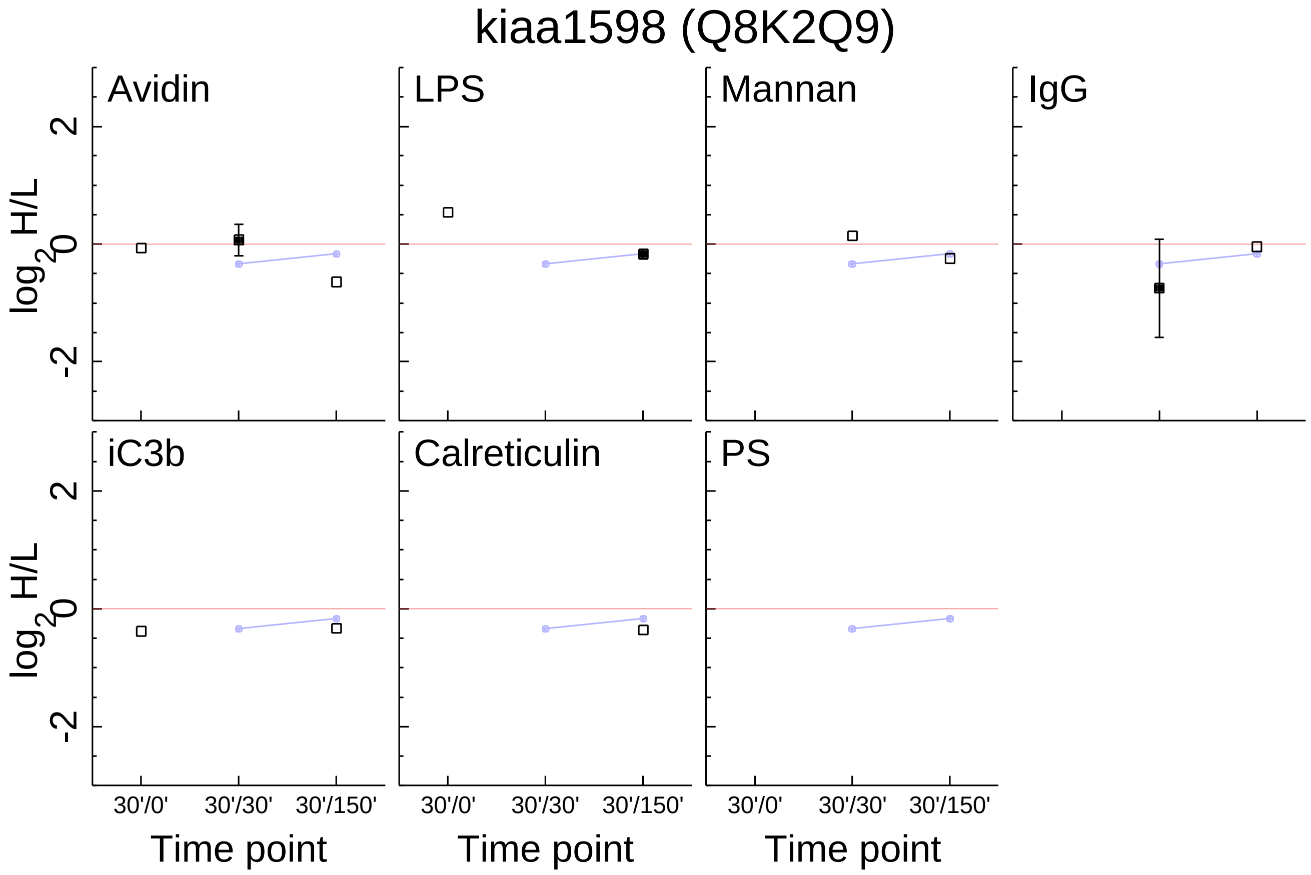

kiaa1598 (Q8K2Q9)
Avidin
LPS
Mannan
IgG
iC3b
Calreticulin
PS
log2 H/L
2
0
-2
30'/0'
30'/30'
30'/150'
Time point

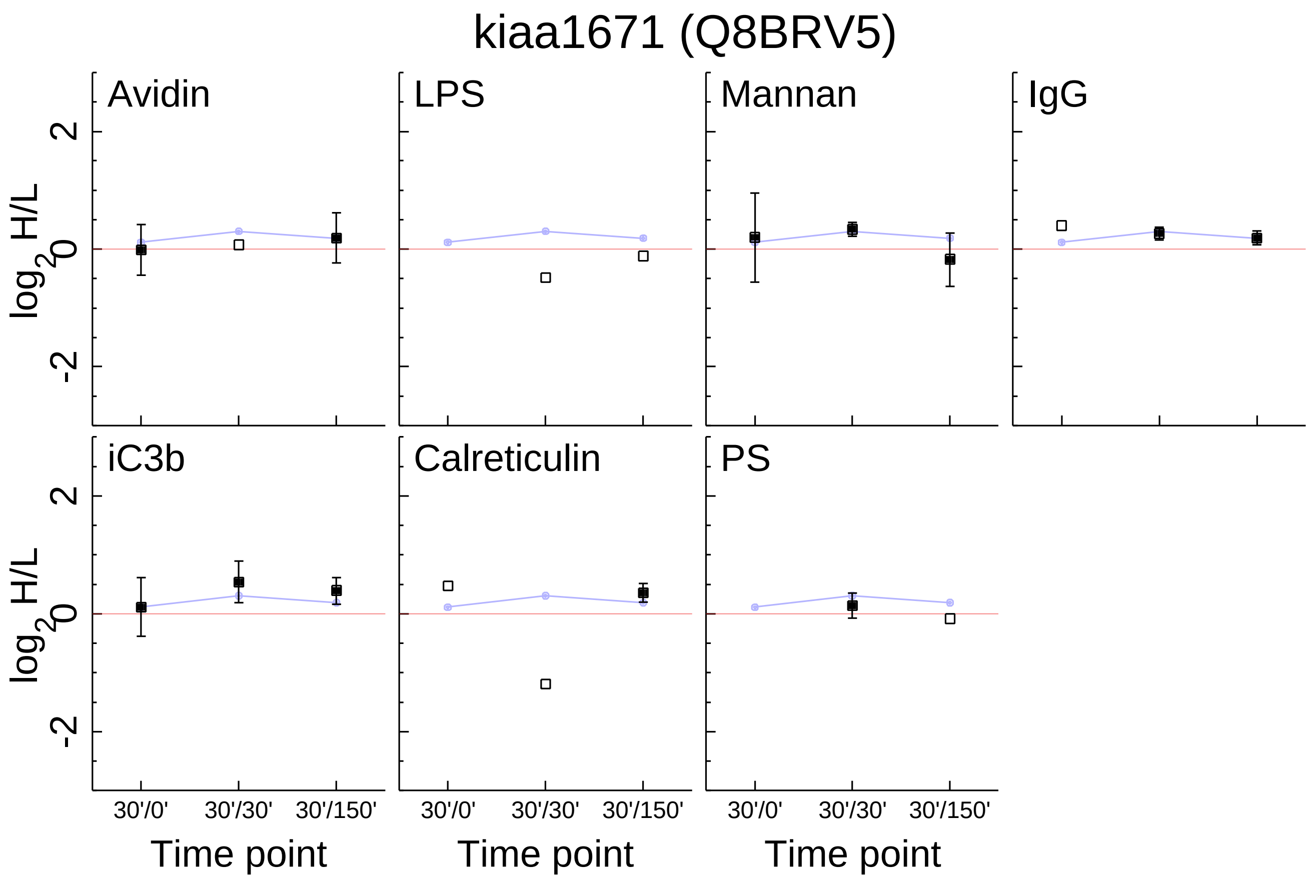

kiaa1671 (Q8BRV5)
Avidin
LPS
Mannan
IgG
iC3b
Calreticulin
PS
log2 H/L
2
0
-2
30'/0'
30'/30'
30'/150'
Time point

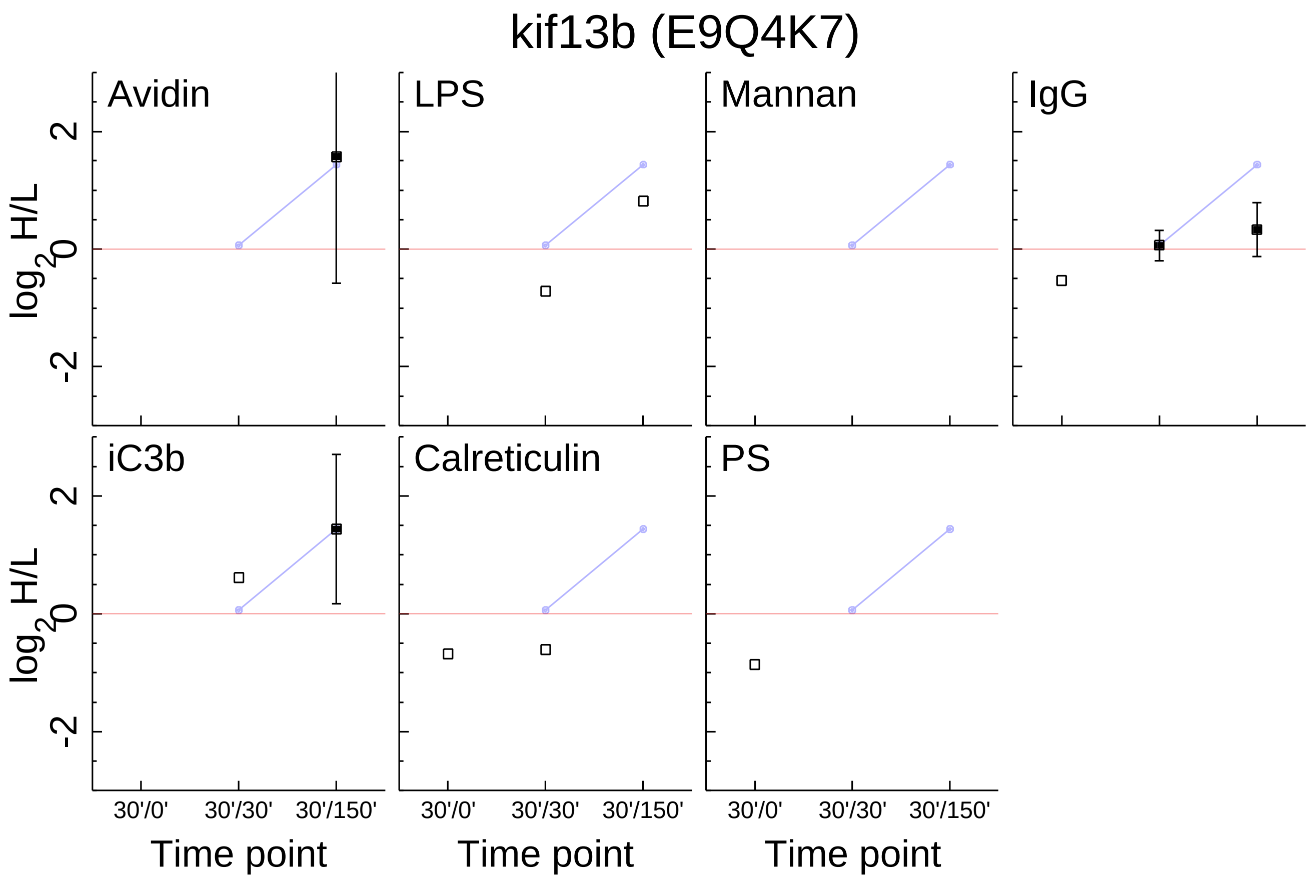
kif13b (E9Q4K7)
Avidin
LPS
Mannan
IgG
iC3b
Calreticulin
PS
log2 H/L
2
0
-2
30'/0'
30'/30'
30'/150'
Time point

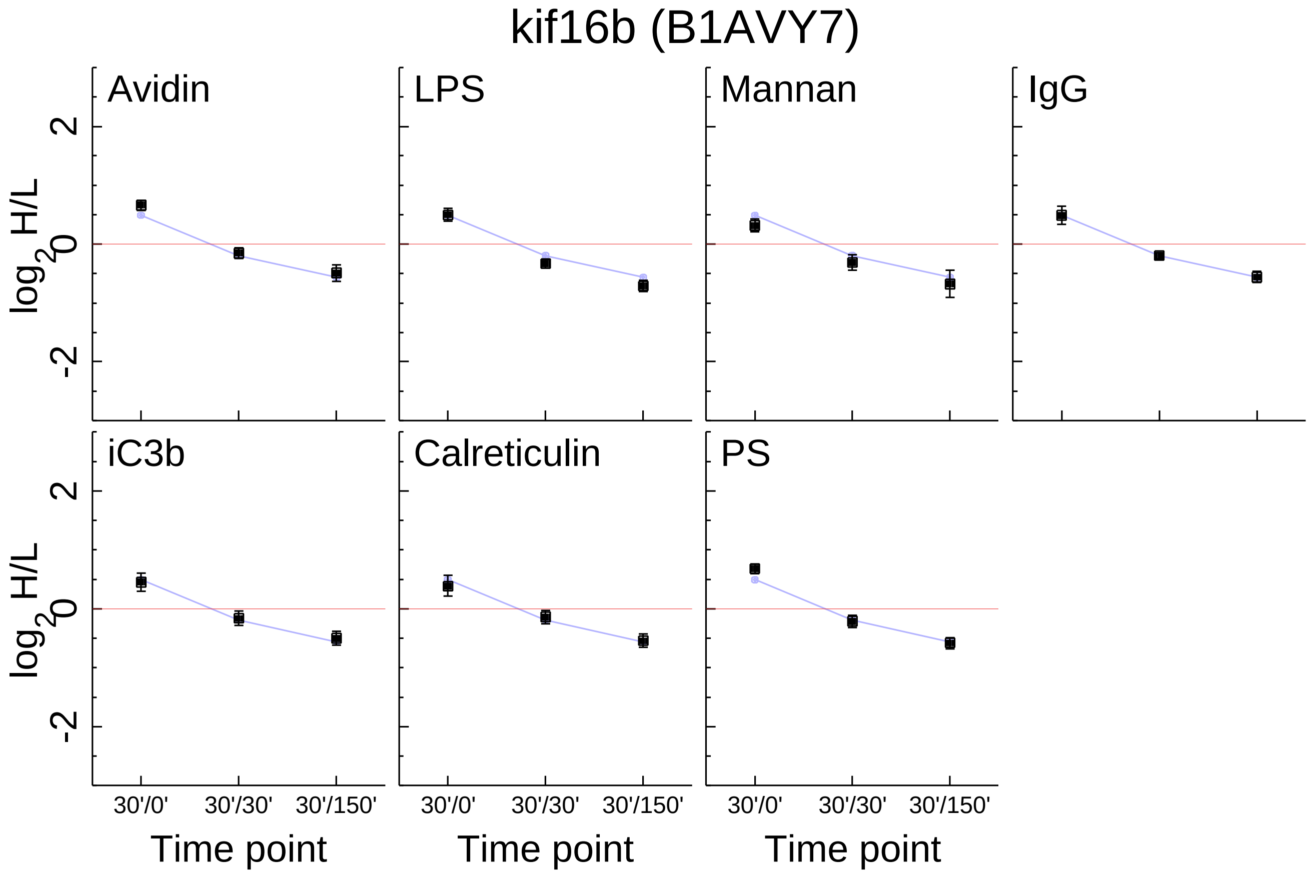
kif16b (B1AVY7)
Avidin
LPS
Mannan
IgG
iC3b
Calreticulin
PS
log2 H/L
2
0
-2
30'/0'
30'/30'
30'/150'
Time point

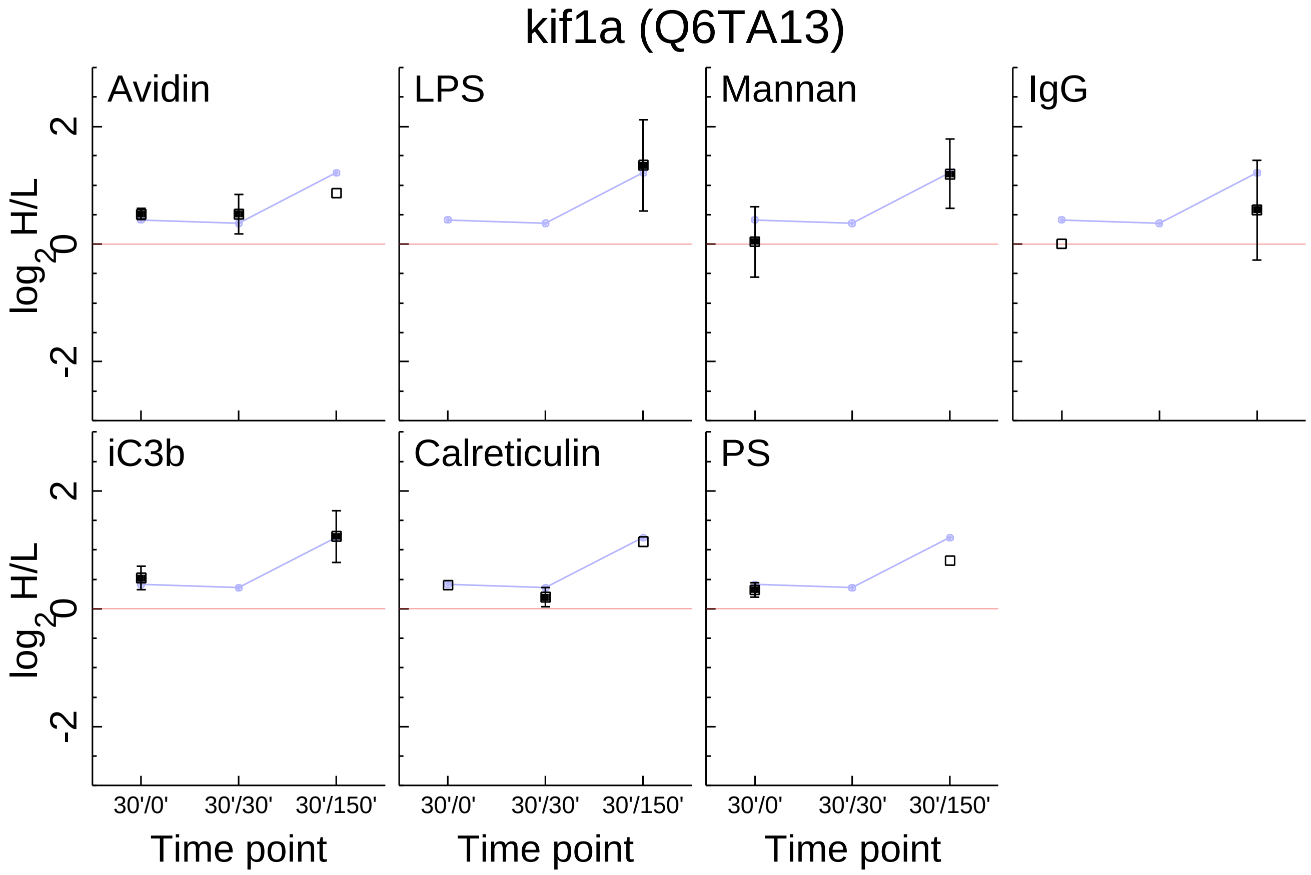
kif1a (Q6TA13)
Avidin
LPS
Mannan
IgG
iC3b
Calreticulin
PS
log2 H/L
2
0
-2
30'/0'
30'/30'
30'/150'
Time point

kif5b (Q61768)

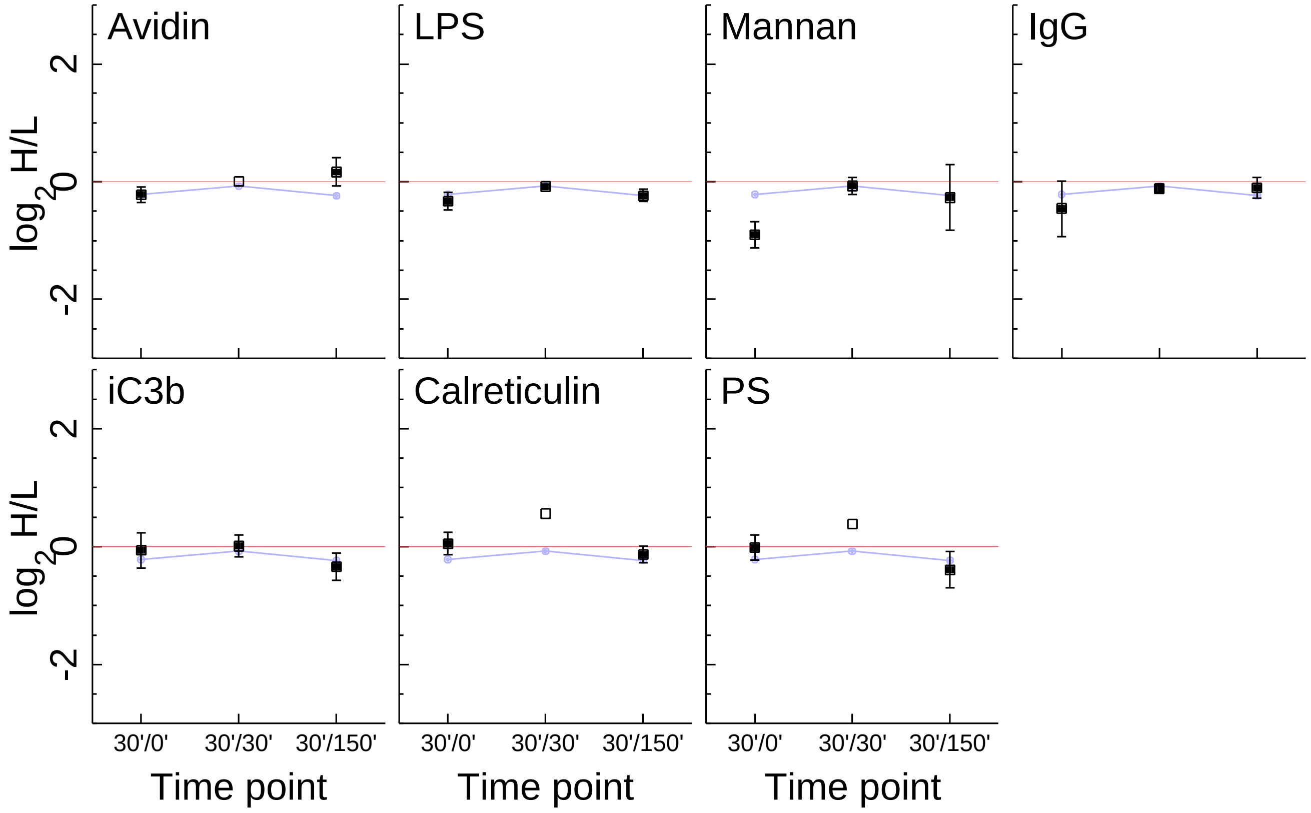

kpnb1 (P70168)

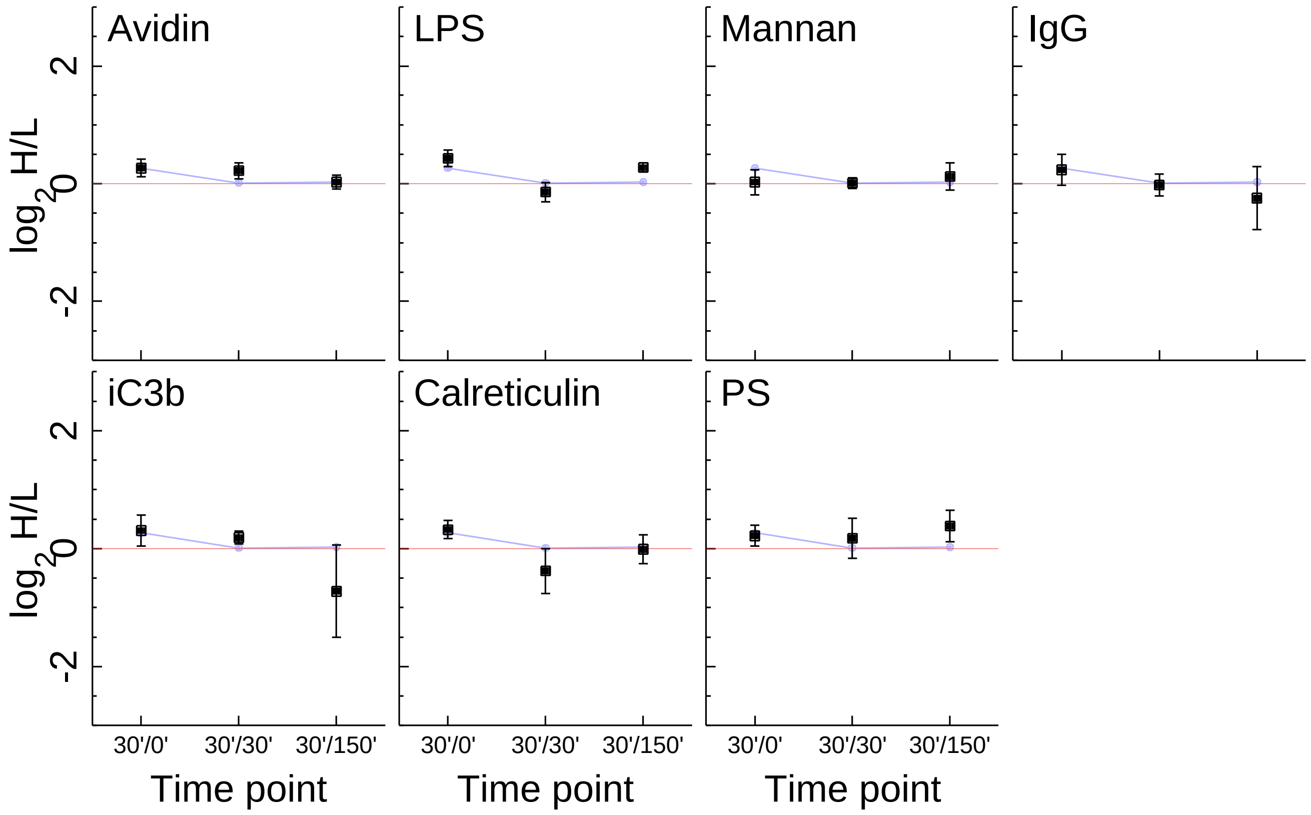

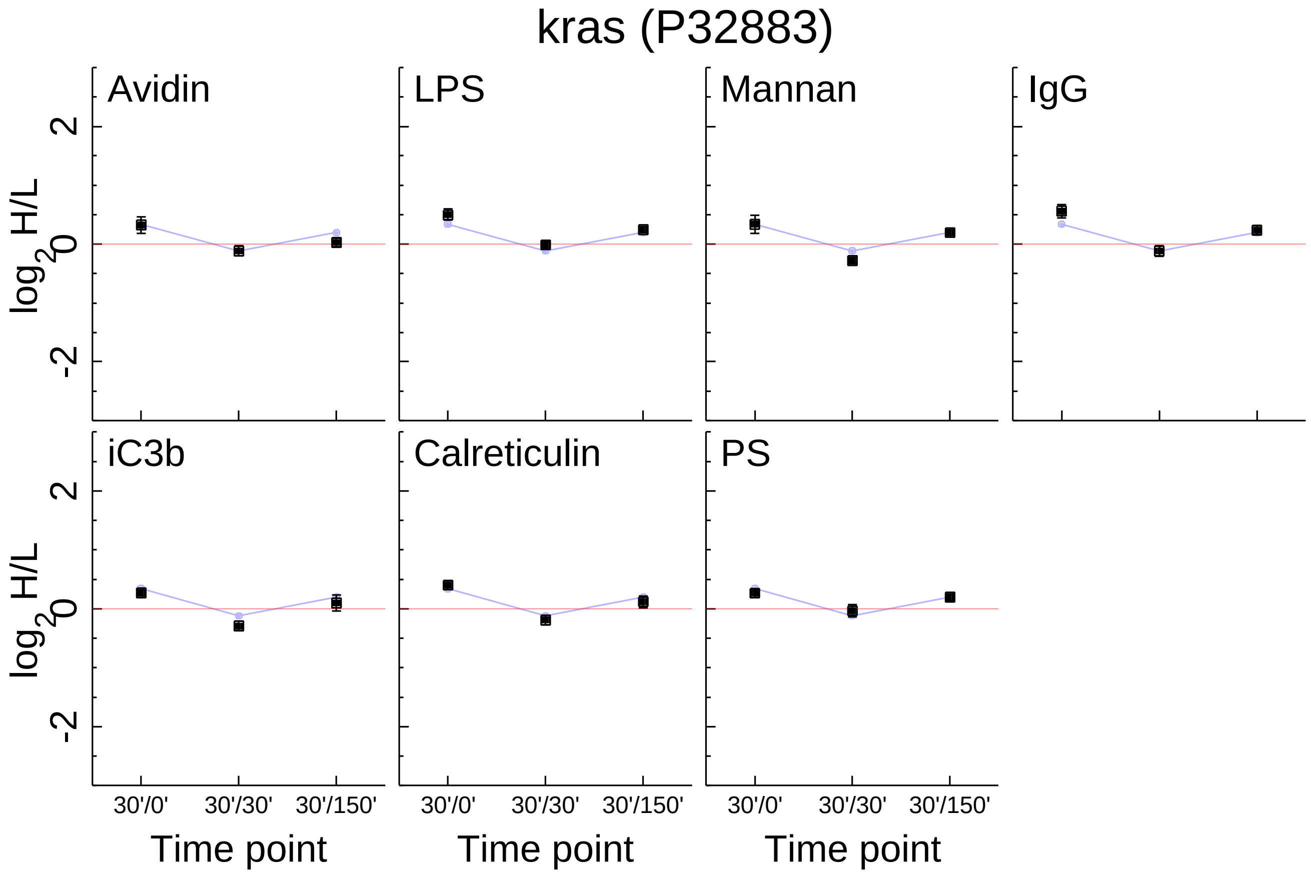

kras (P32883)
Avidin
LPS
Mannan
IgG
iC3b
Calreticulin
PS
log2 H/L
2
0
-2
30'/0'
30'/30'
30'/150'
Time point

krt28 (A6BLY7)

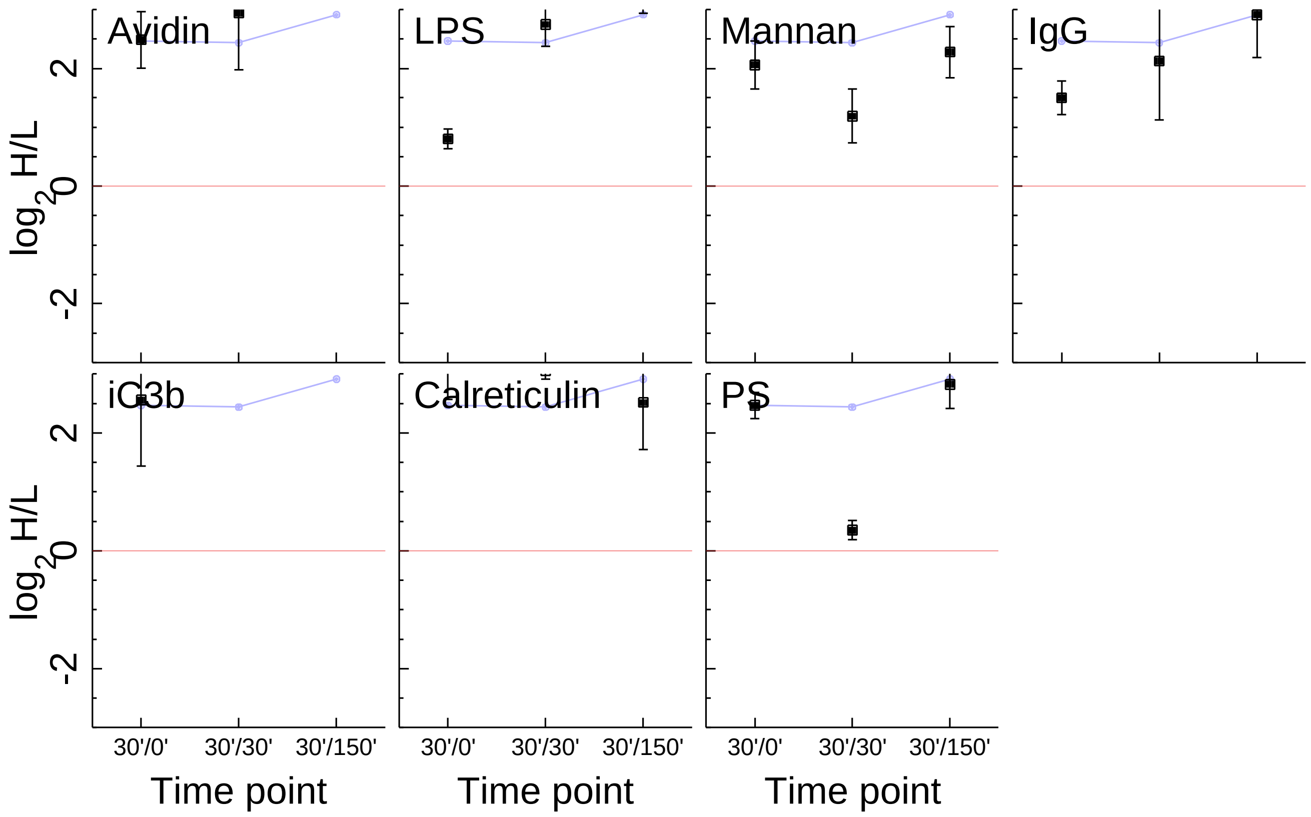

krt76 (Q3UV17)

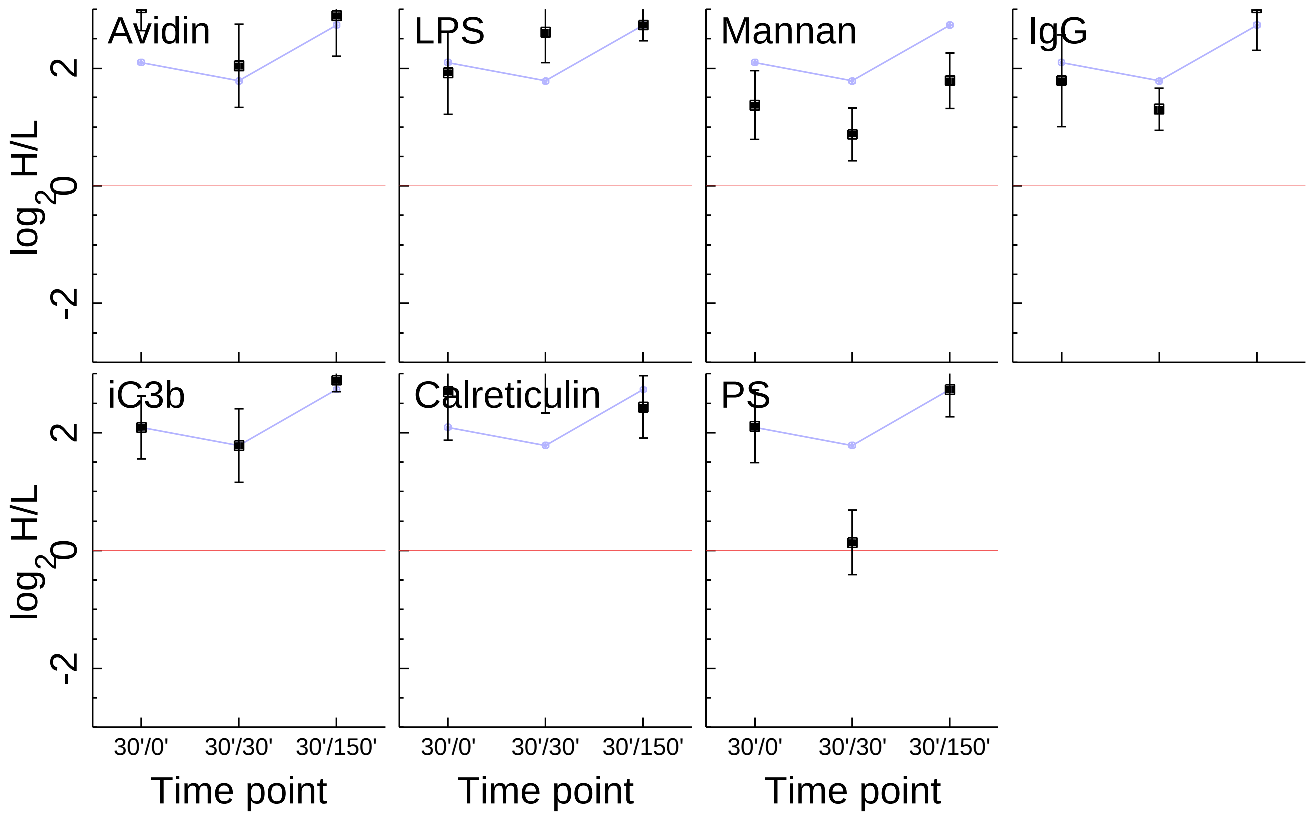

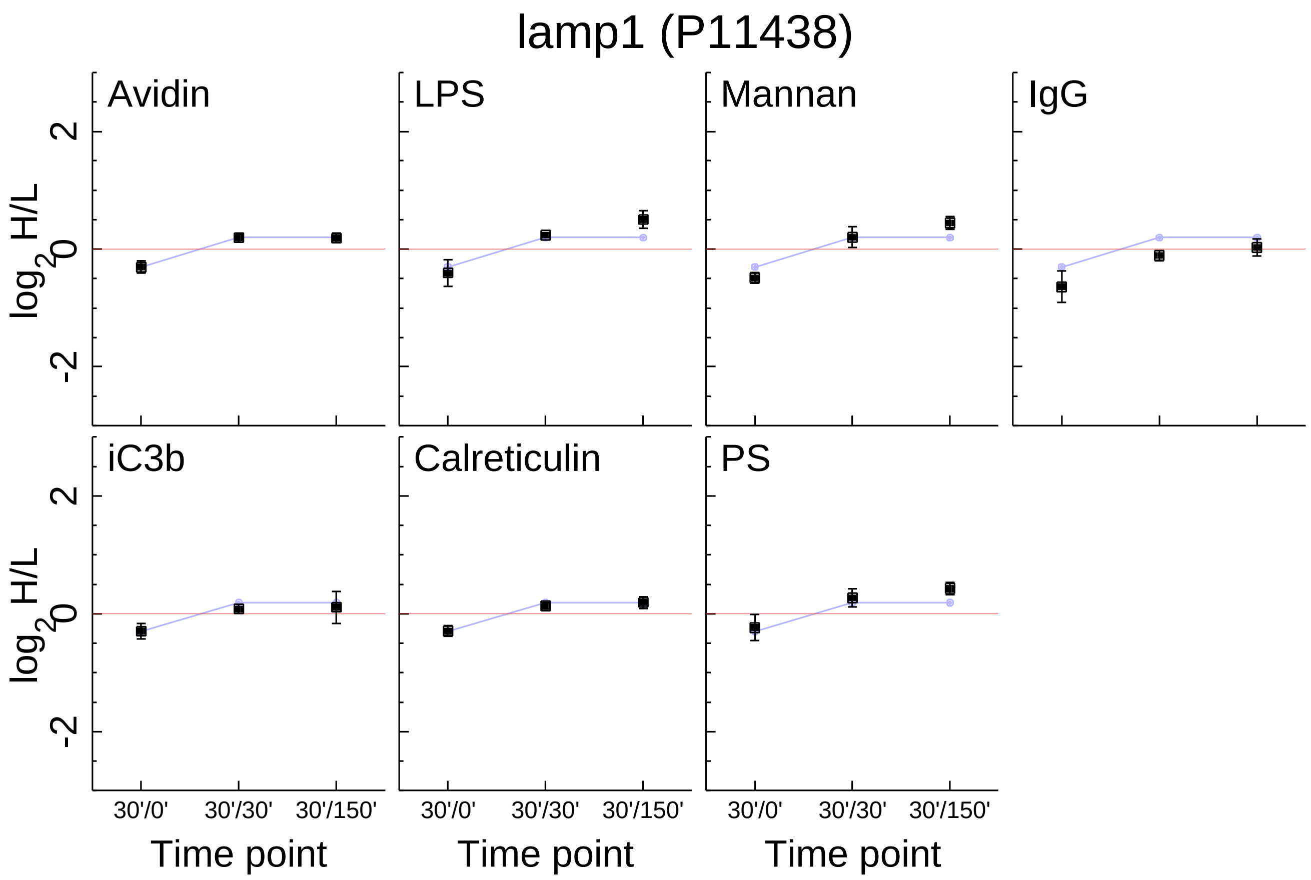
lamp1 (P11438)
Avidin
LPS
Mannan
IgG
iC3b
Calreticulin
PS
log2 H/L
2
0
-2
30'/0'
30'/30'
30'/150'
Time point

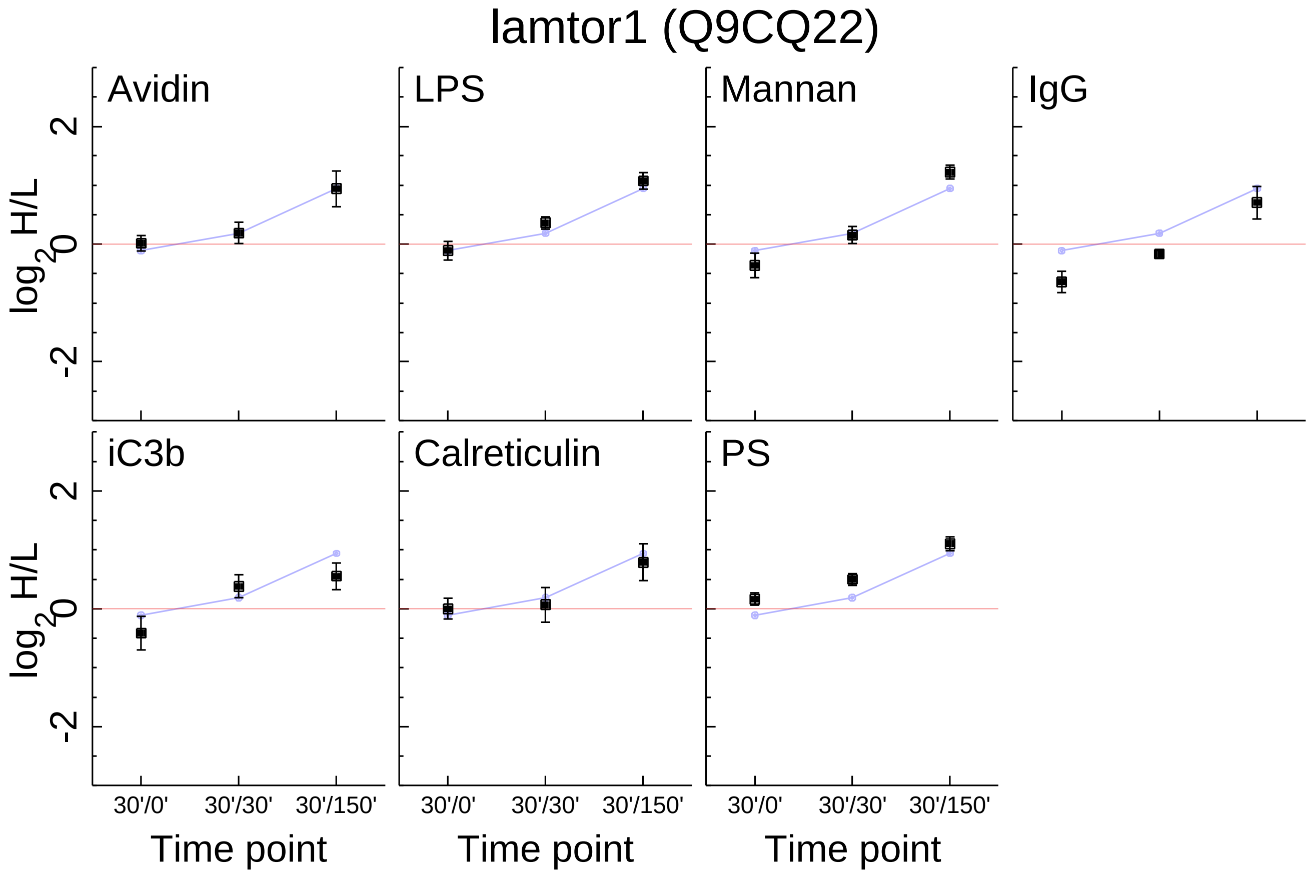

lamtor1 (Q9CQ22)
Avidin
LPS
Mannan
IgG
iC3b
Calreticulin
PS
log2 H/L
2
0
-2
30'/0'
30'/30'
30'/150'
Time point

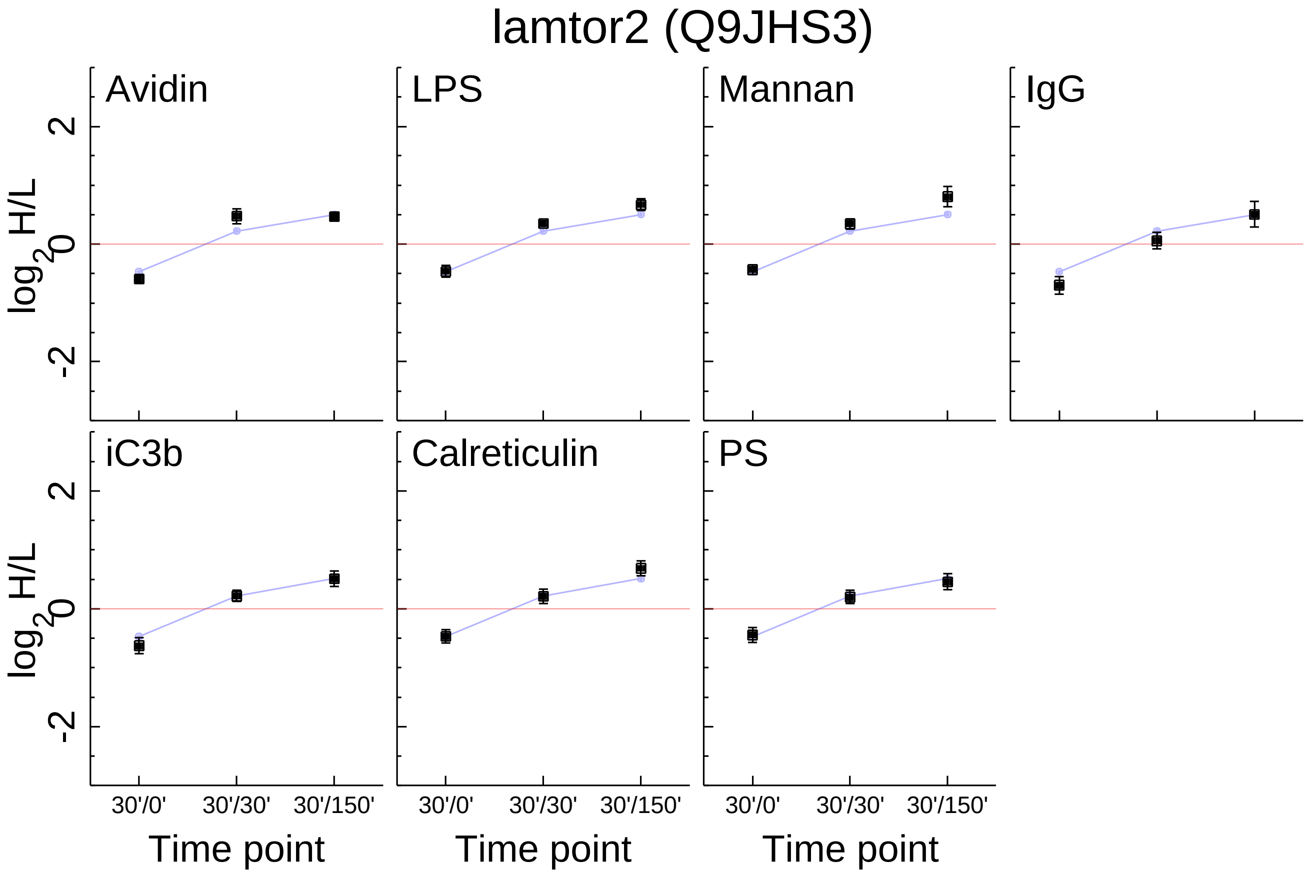

lamtor2 (Q9JHS3)
Avidin
LPS
Mannan
IgG
iC3b
Calreticulin
PS
2
0
-2
log2 H/L
30'/0'
30'/30'
30'/150'
Time point

lamtor3 (O88653)

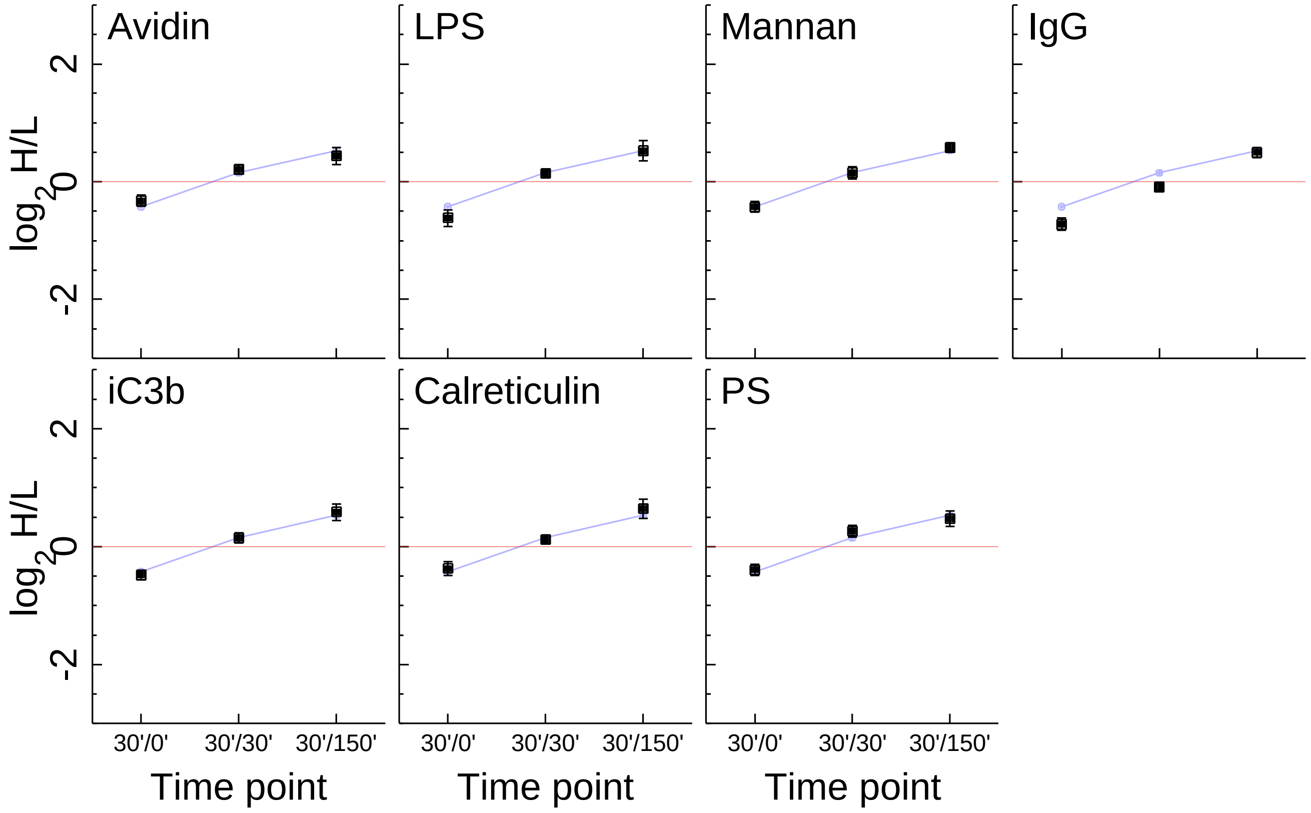

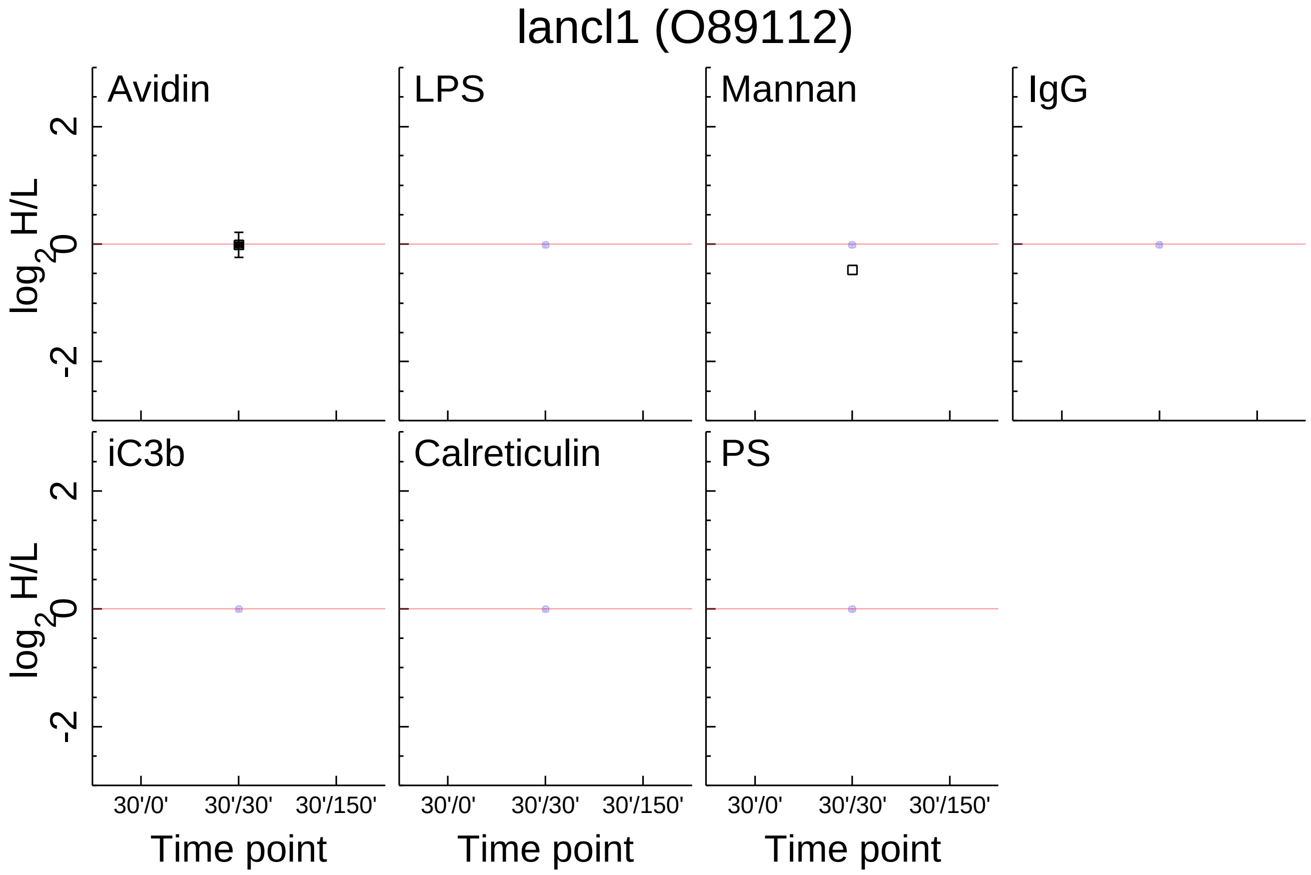

lancl1 (O89112)
Avidin
LPS
Mannan
IgG
iC3b
Calreticulin
PS
log2 H/L
2
0
-2
30'/0'
30'/30'
30'/150'
Time point

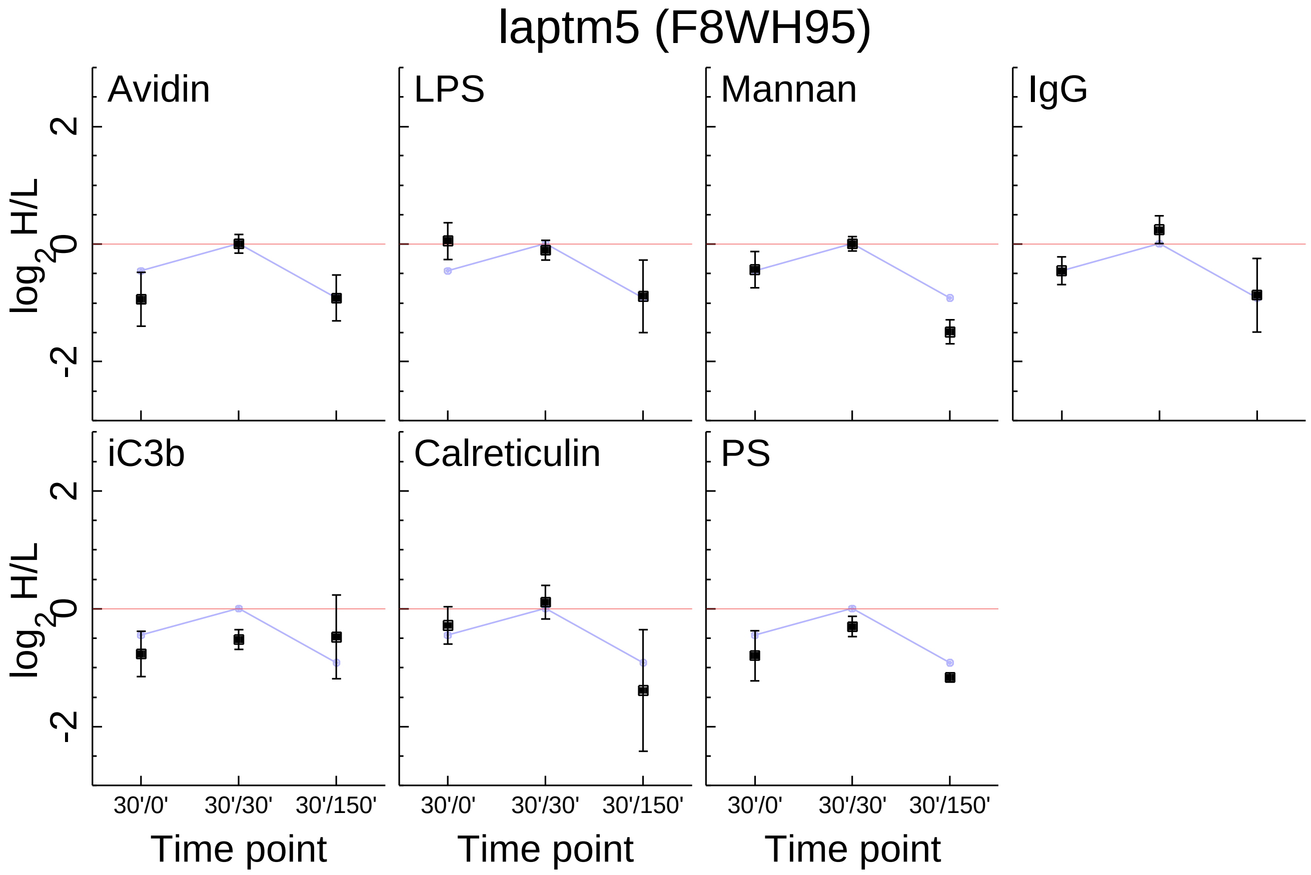

laptm5 (F8WH95)
Avidin
LPS
Mannan
IgG
iC3b
Calreticulin
PS
log2 H/L
2
0
-2
30'/0'
30'/30'
30'/150'
Time point

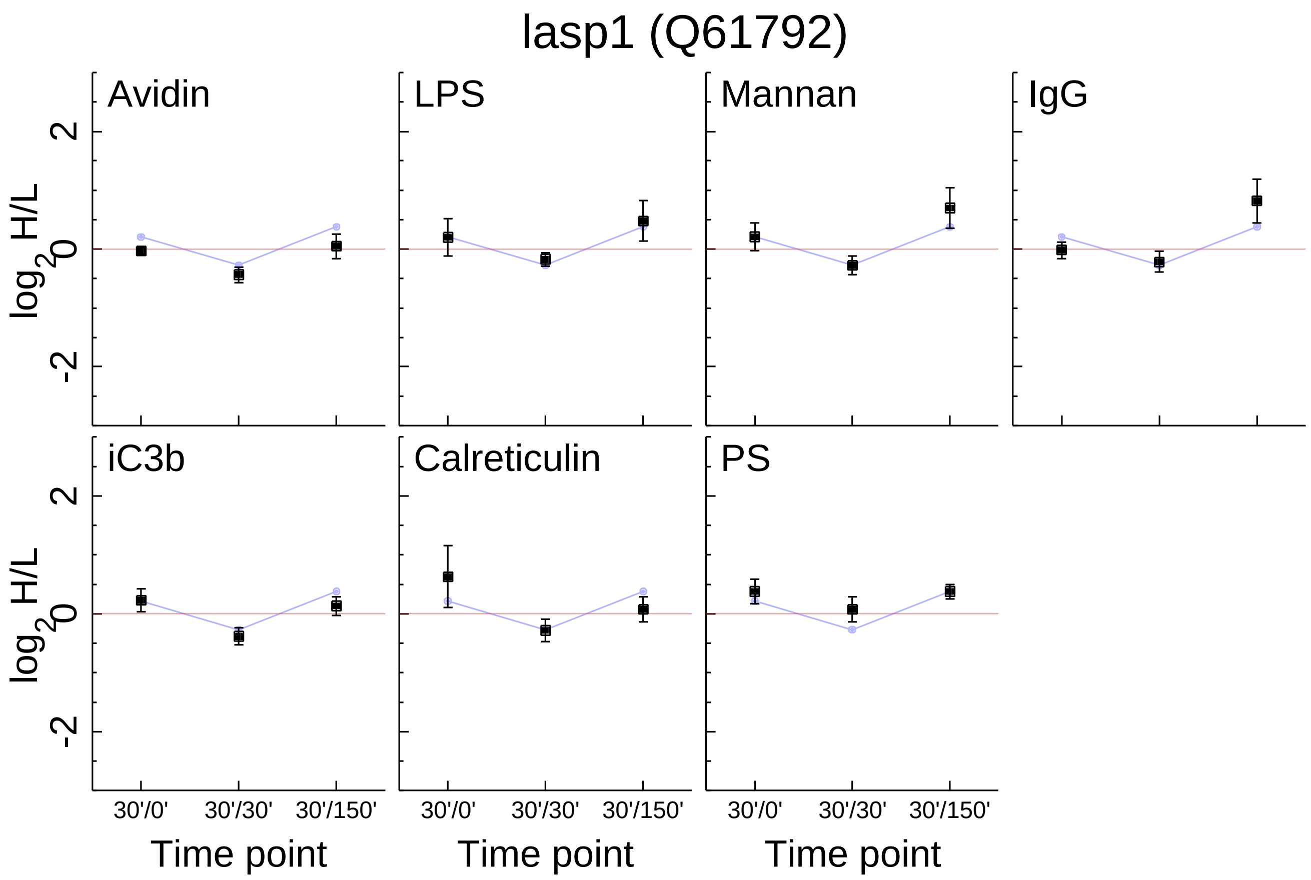
lasp1 (Q61792)
Avidin
LPS
Mannan
IgG
iC3b
Calreticulin
PS
log2 H/L
2
0
-2
30'/0'
30'/30'
30'/150'
Time point

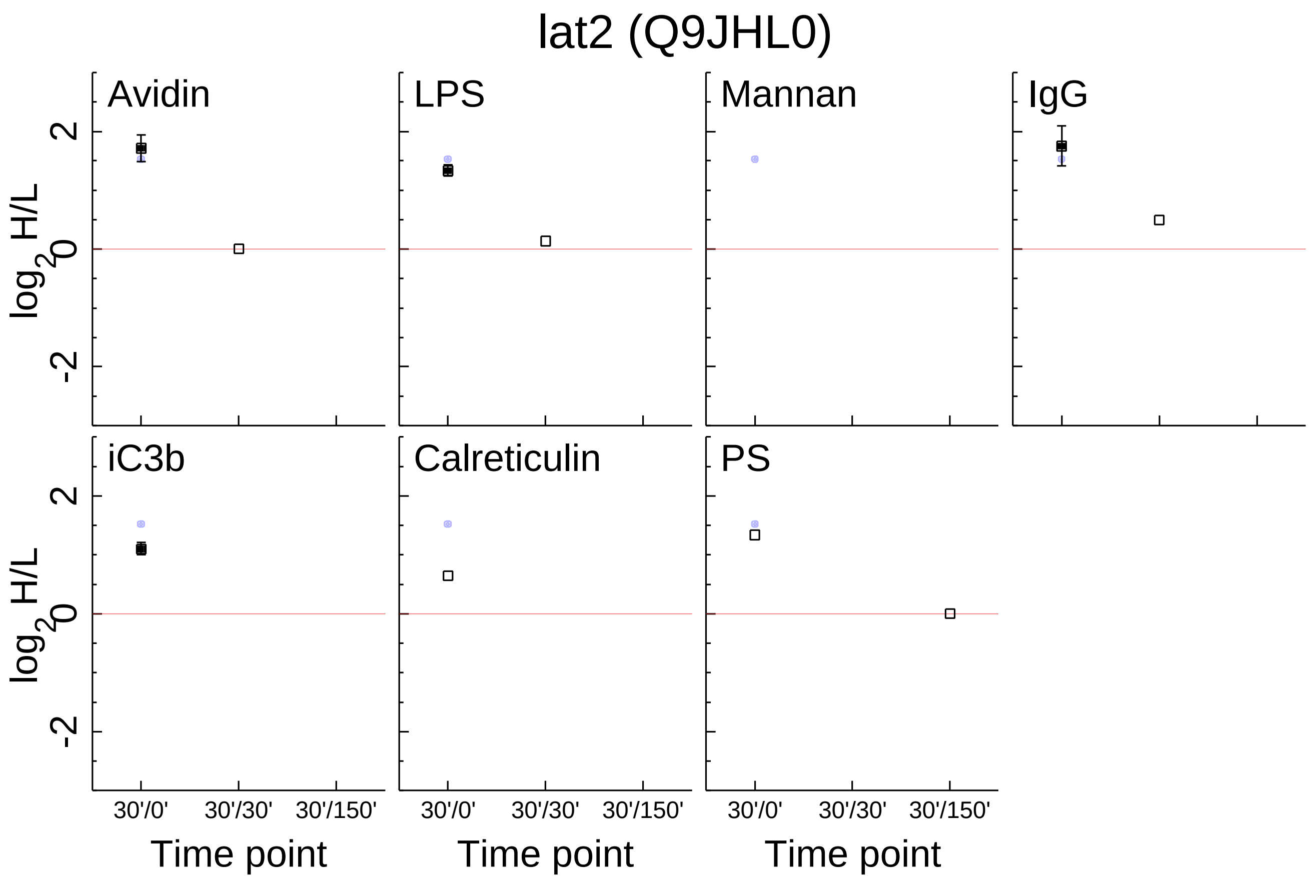
lat2 (Q9JHL0)
Avidin
LPS
Mannan
IgG
iC3b
Calreticulin
PS
log2 H/L
2
0
-2
30'/0'
30'/30'
30'/150'
Time point

lbr (Q3U9G9)

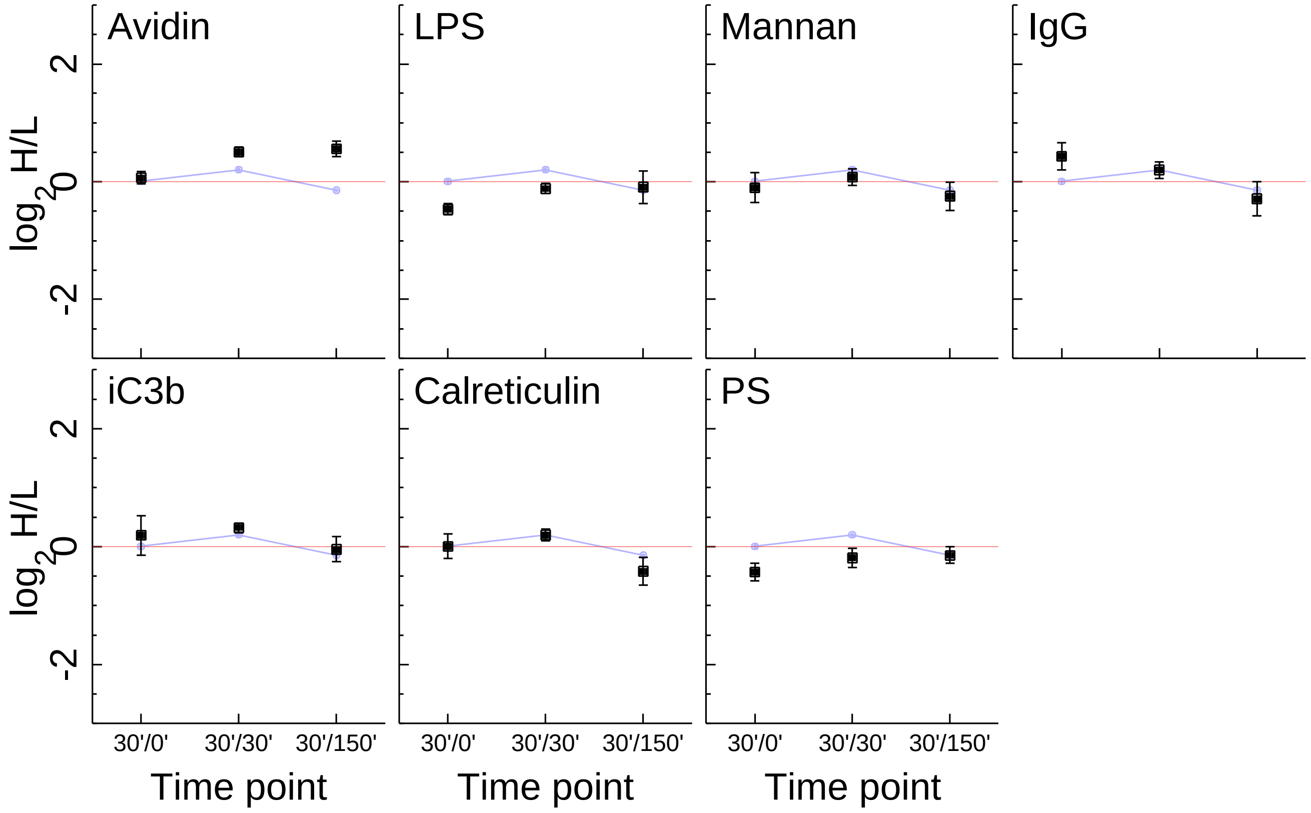

Avidin
LPS
Mannan
IgG
iC3b
Calreticulin
PS
log2 H/L
2
0
-2
30'/0'
30'/30'
30'/150'
Time point

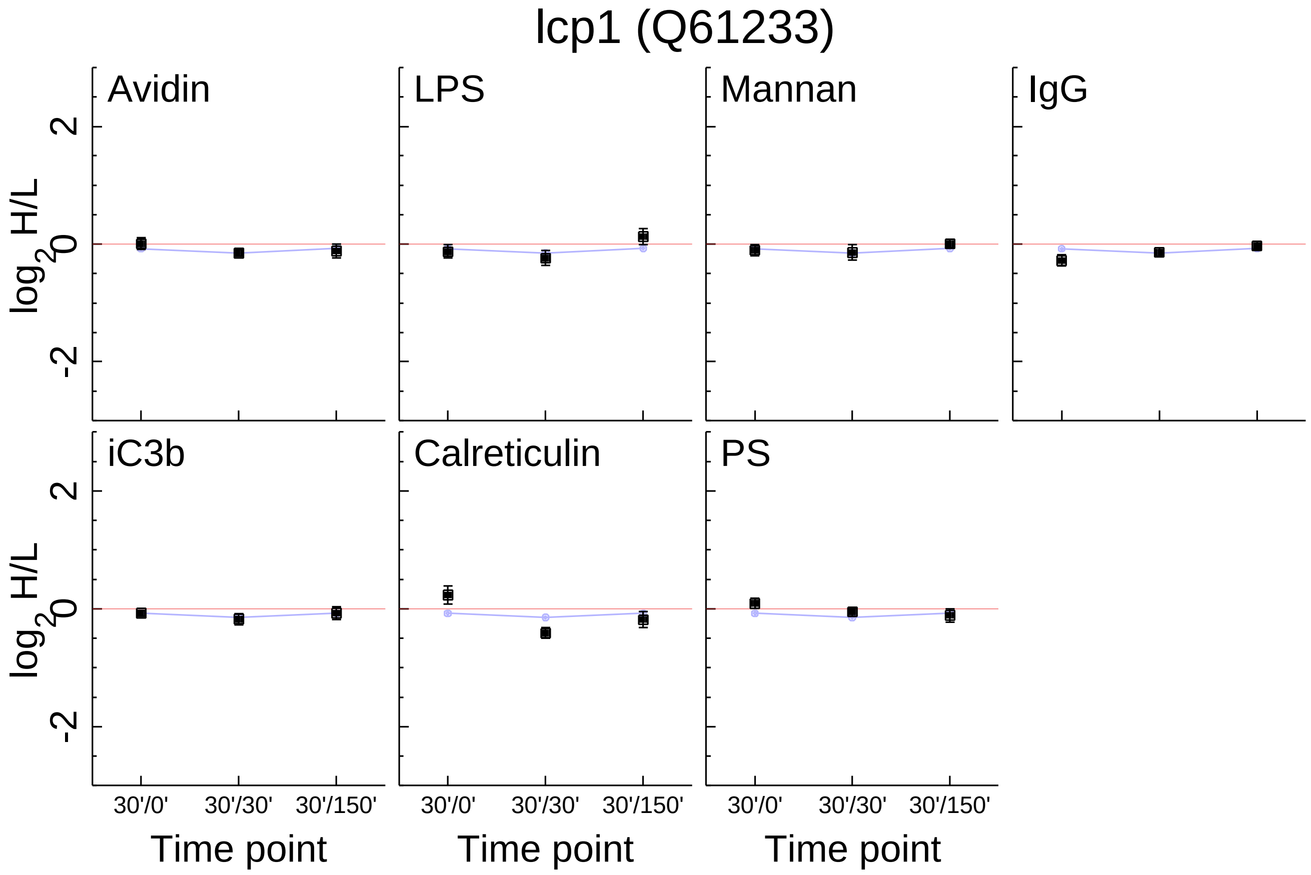

lcp1 (Q61233)
Avidin
LPS
Mannan
IgG
iC3b
Calreticulin
PS
log2 H/L
2
0
-2
30'/0'
30'/30'
30'/150'
Time point

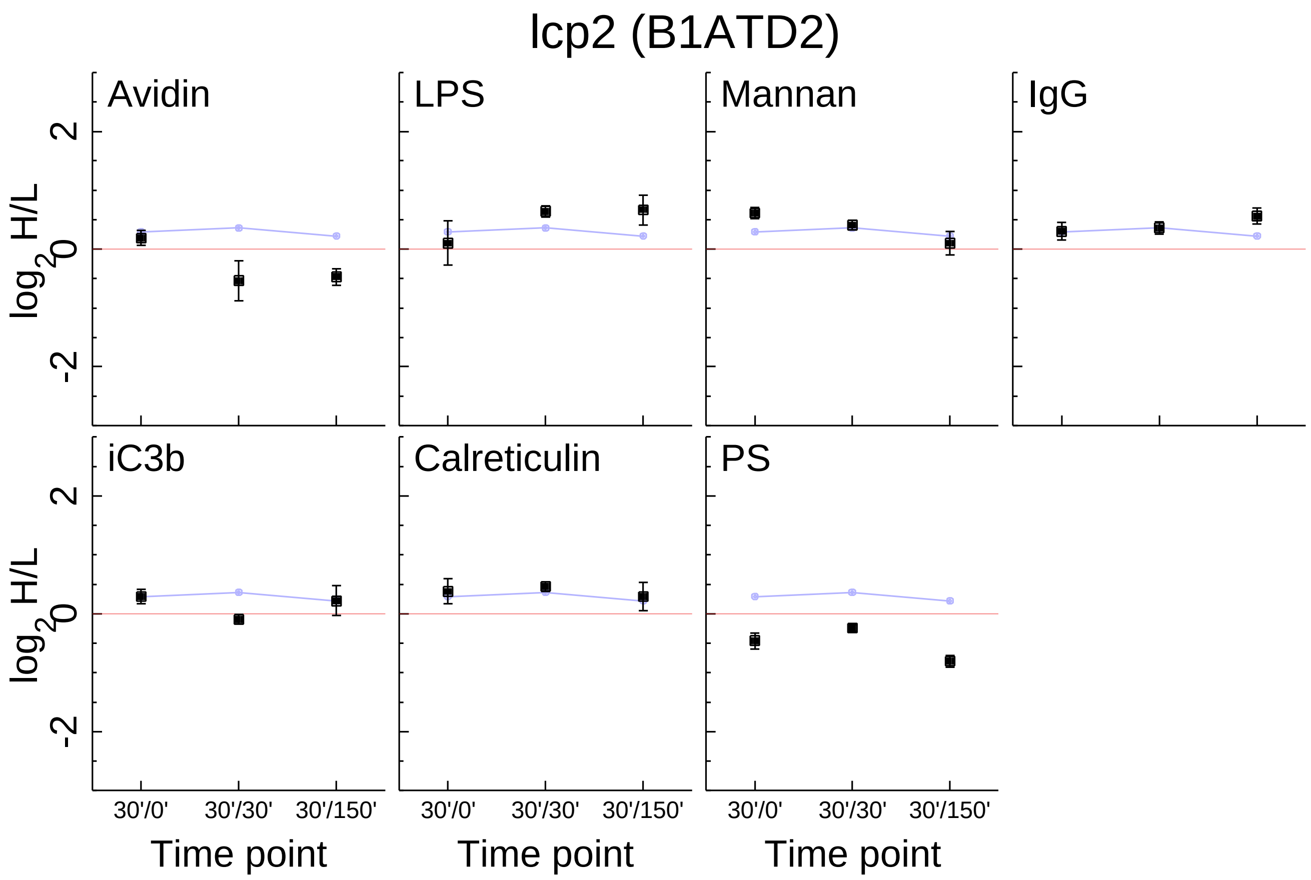
lcp2 (B1ATD2)
Avidin
LPS
Mannan
IgG
iC3b
Calreticulin
PS
log2 H/L
2
0
-2
30'/0'
30'/30'
30'/150'
Time point

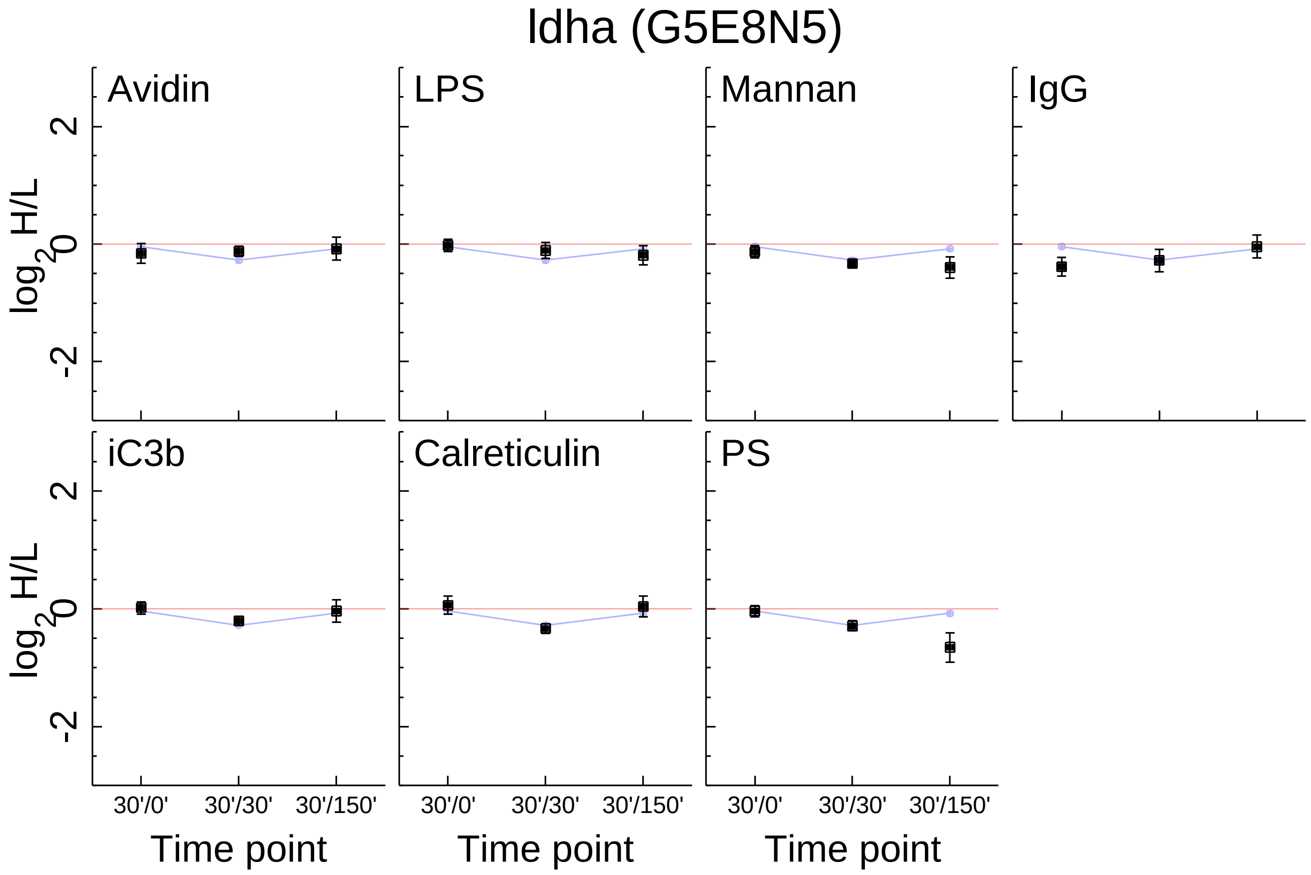

Ldha (G5E8N5)
Avidin
LPS
Mannan
IgG
iC3b
Calreticulin
PS
log2 H/L
2
0
-2
30'/0'
30'/30'
30'/150'
Time point

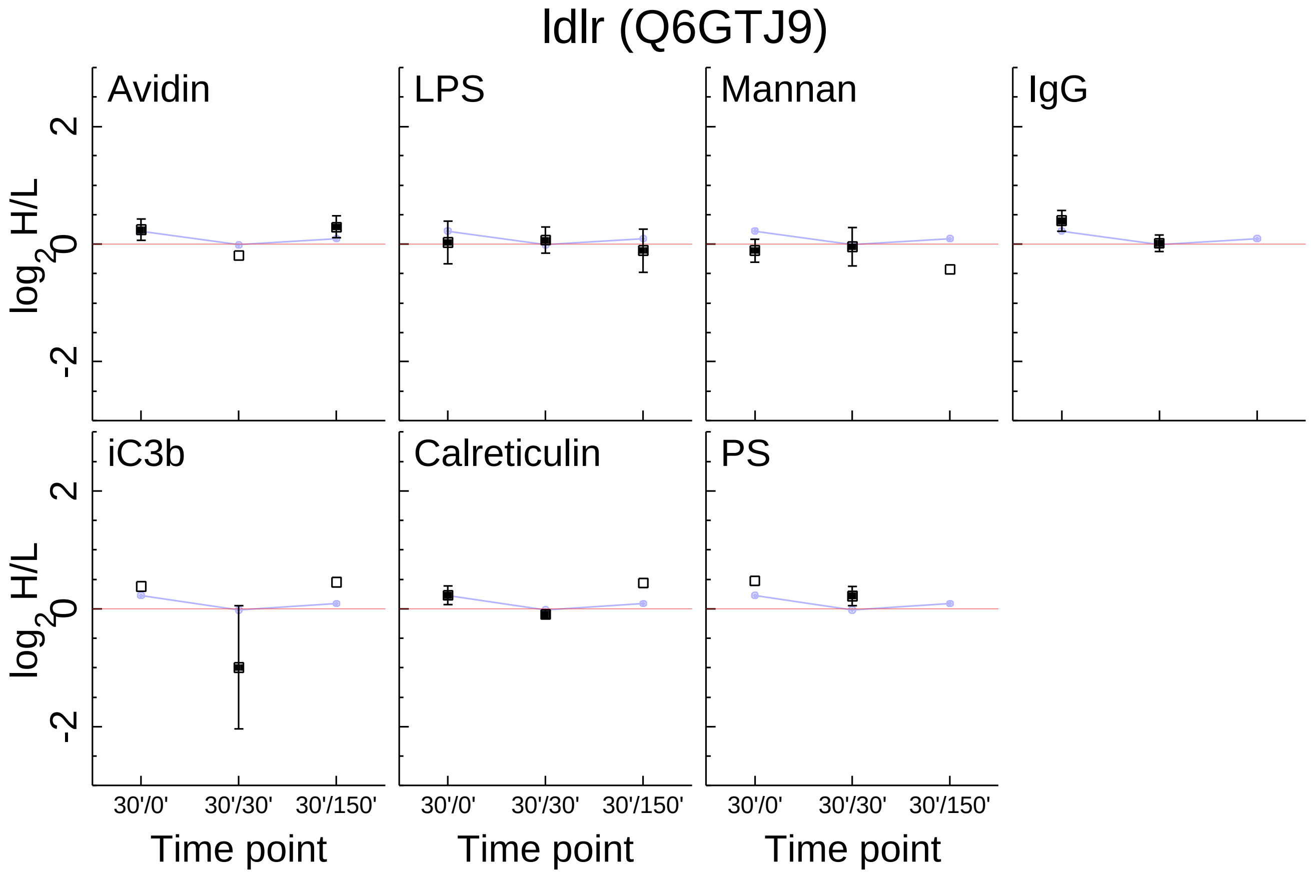

ldlr (Q6GTJ9)
Avidin
LPS
Mannan
IgG
iC3b
Calreticulin
PS
log2 H/L
2
0
-2
30'/0'
30'/30'
30'/150'
Time point

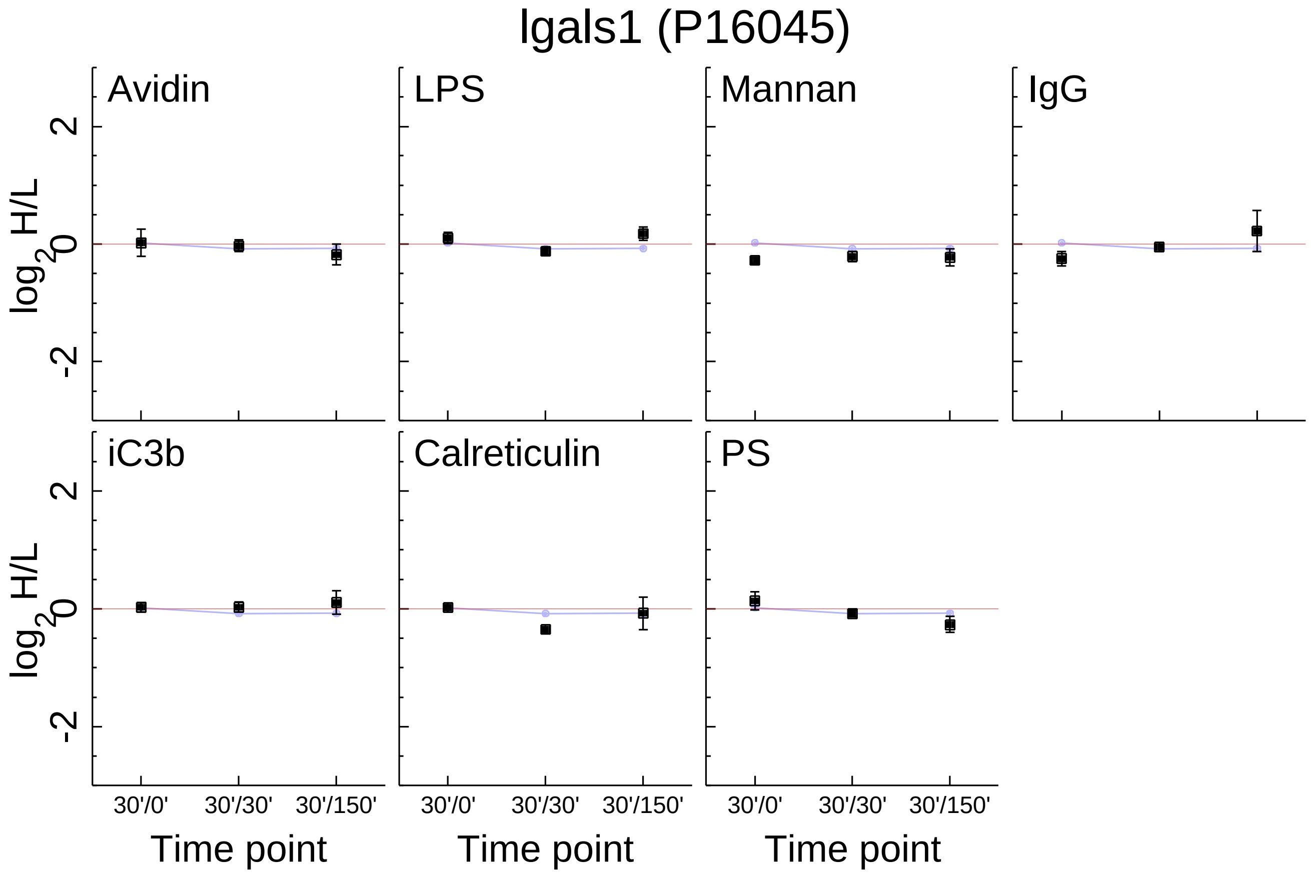
lgals1 (P16045)
Avidin
LPS
Mannan
IgG
iC3b
Calreticulin
PS
log2 H/L
2
0
-2
30'/0'
30'/30'
30'/150'
Time point

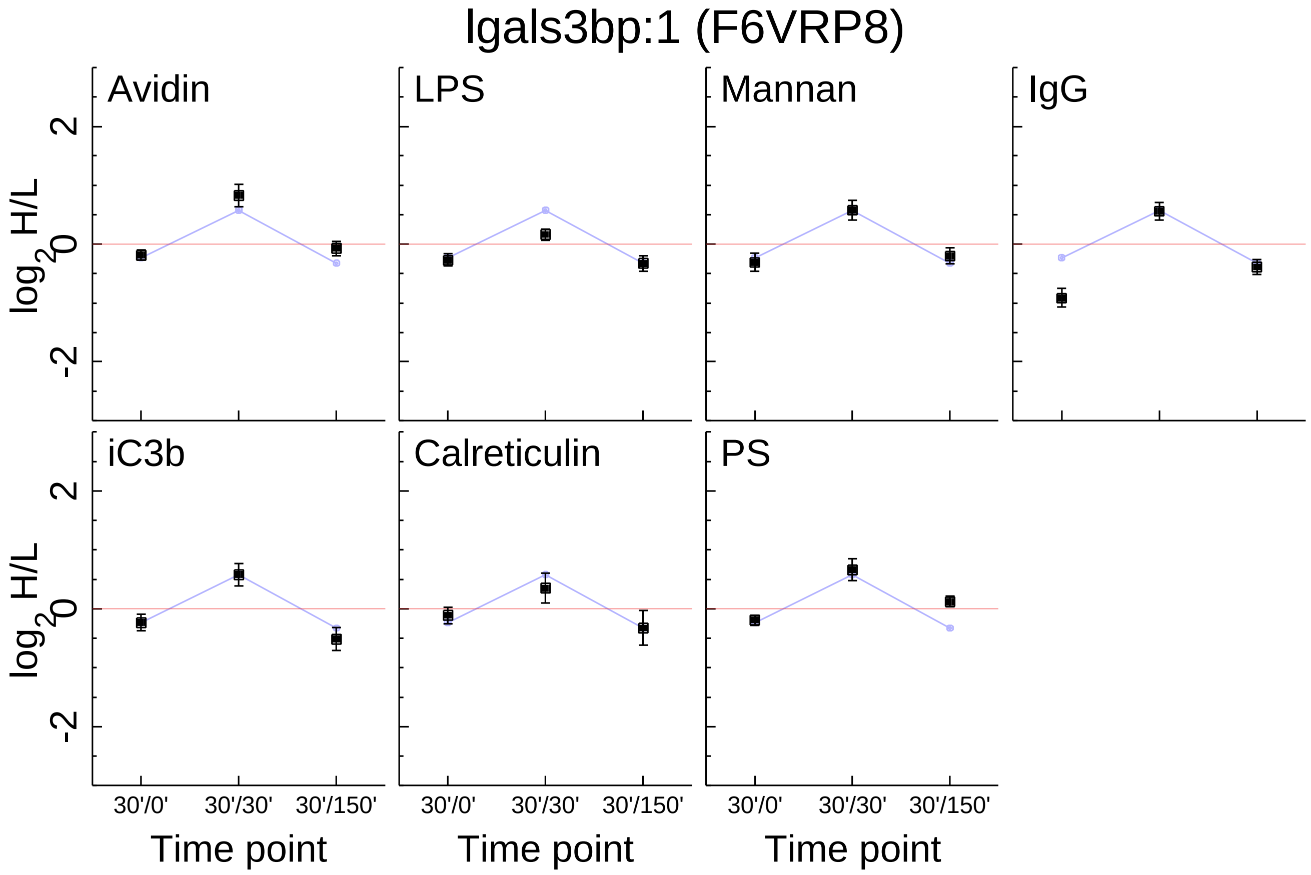

lgals3bp:1 (F6VRP8)
Avidin
LPS
Mannan
IgG
iC3b
Calreticulin
PS
log2 H/L
2
0
-2
30'/0'
30'/30'
30'/150'
Time point

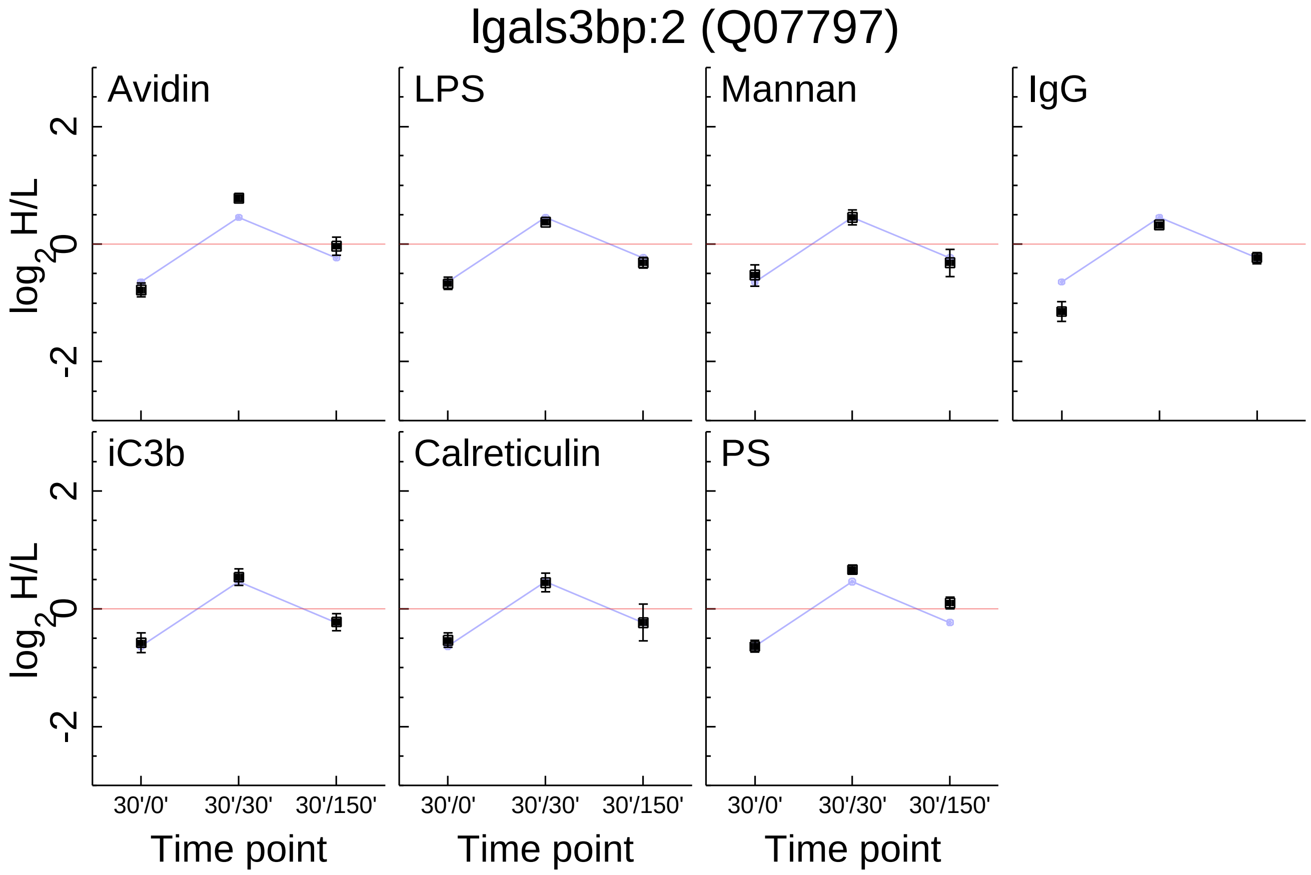

lgals3bp:2 (Q07797)
Avidin
LPS
Mannan
IgG
iC3b
Calreticulin
PS
log2 H/L
2
0
-2
30'/0'
30'/30'
30'/150'
Time point

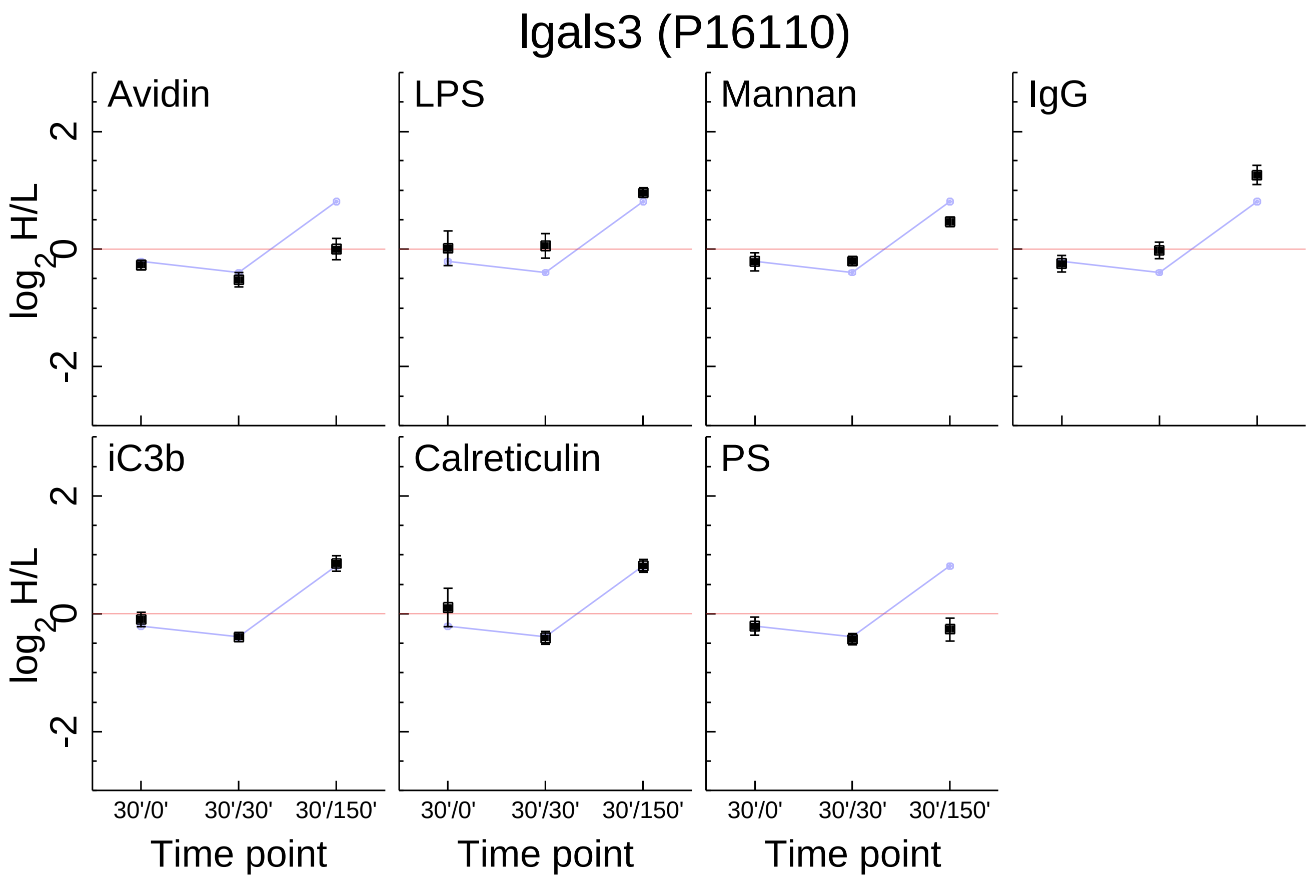
Lgals3 (P16110)
Avidin
LPS
Mannan
IgG
iC3b
Calreticulin
PS
log2 H/L
2
0
-2
30'/0'
30'/30'
30'/150'
Time point

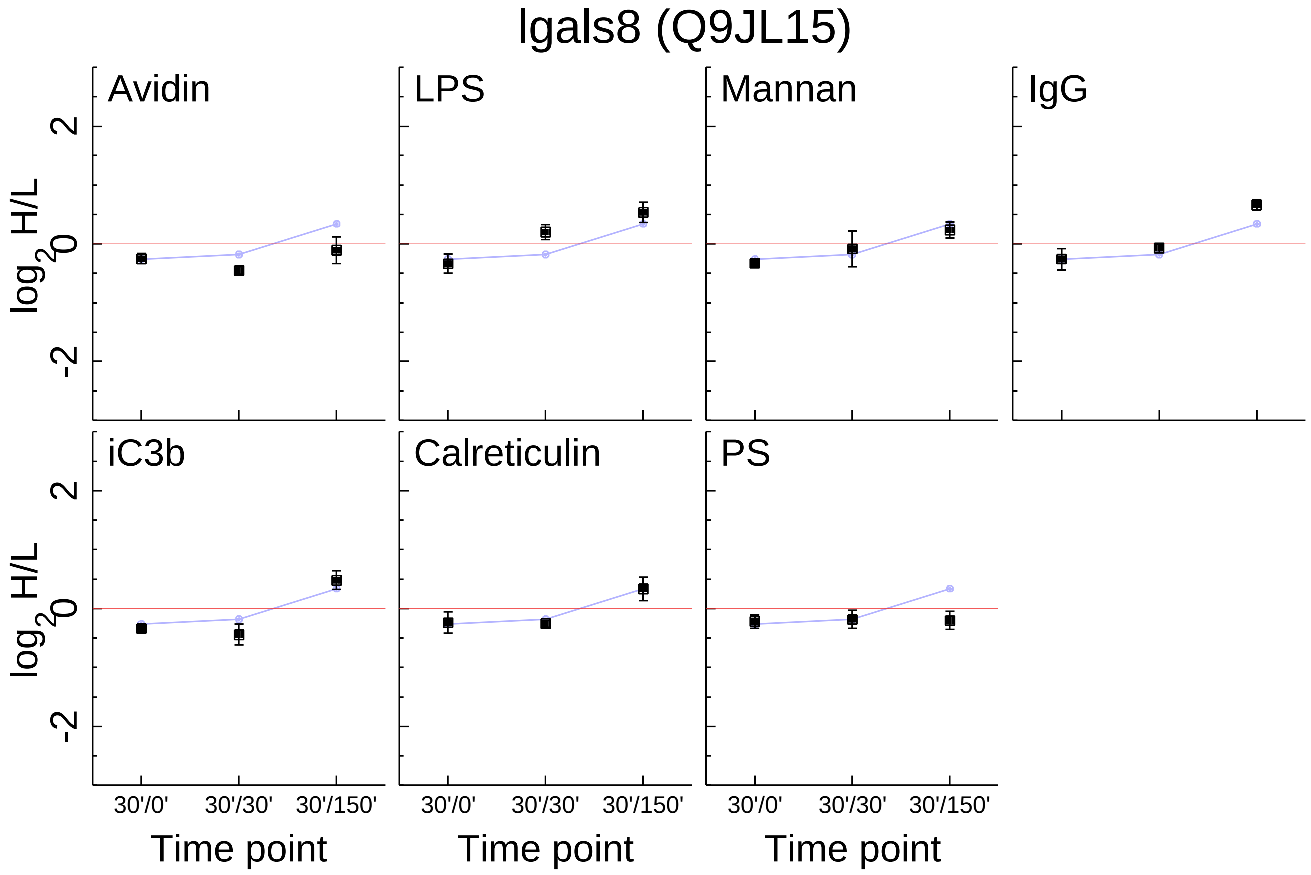
Igals8 (Q9JL15)
Avidin
LPS
Mannan
IgG
iC3b
Calreticulin
PS
log2 H/L
2
0
-2
30'/0'
30'/30'
30'/150'
Time point

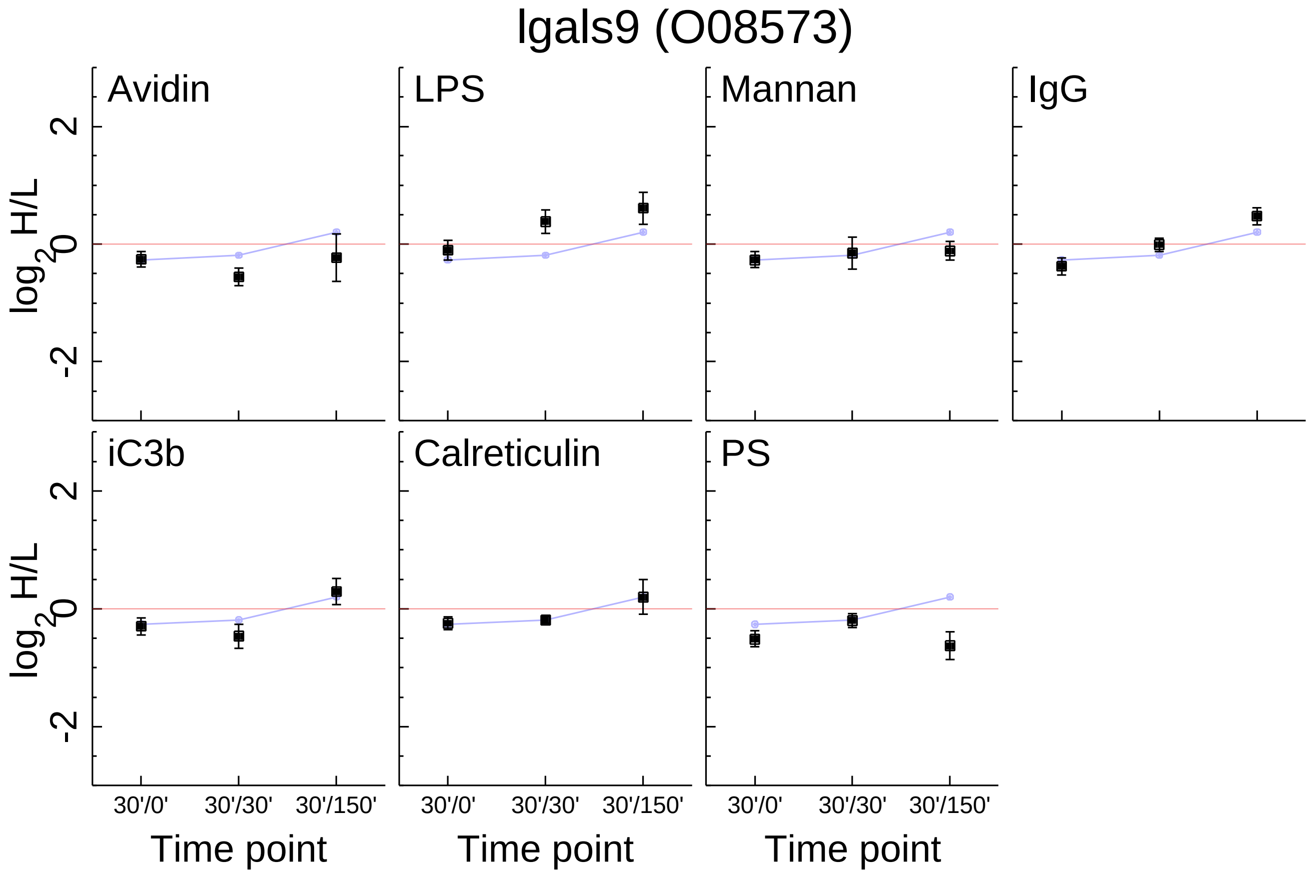

lgals9 (O08573)
Avidin
LPS
Mannan
IgG
iC3b
Calreticulin
PS
log2 H/L
2
0
-2
30'/0'
30'/30'
30'/150'
Time point

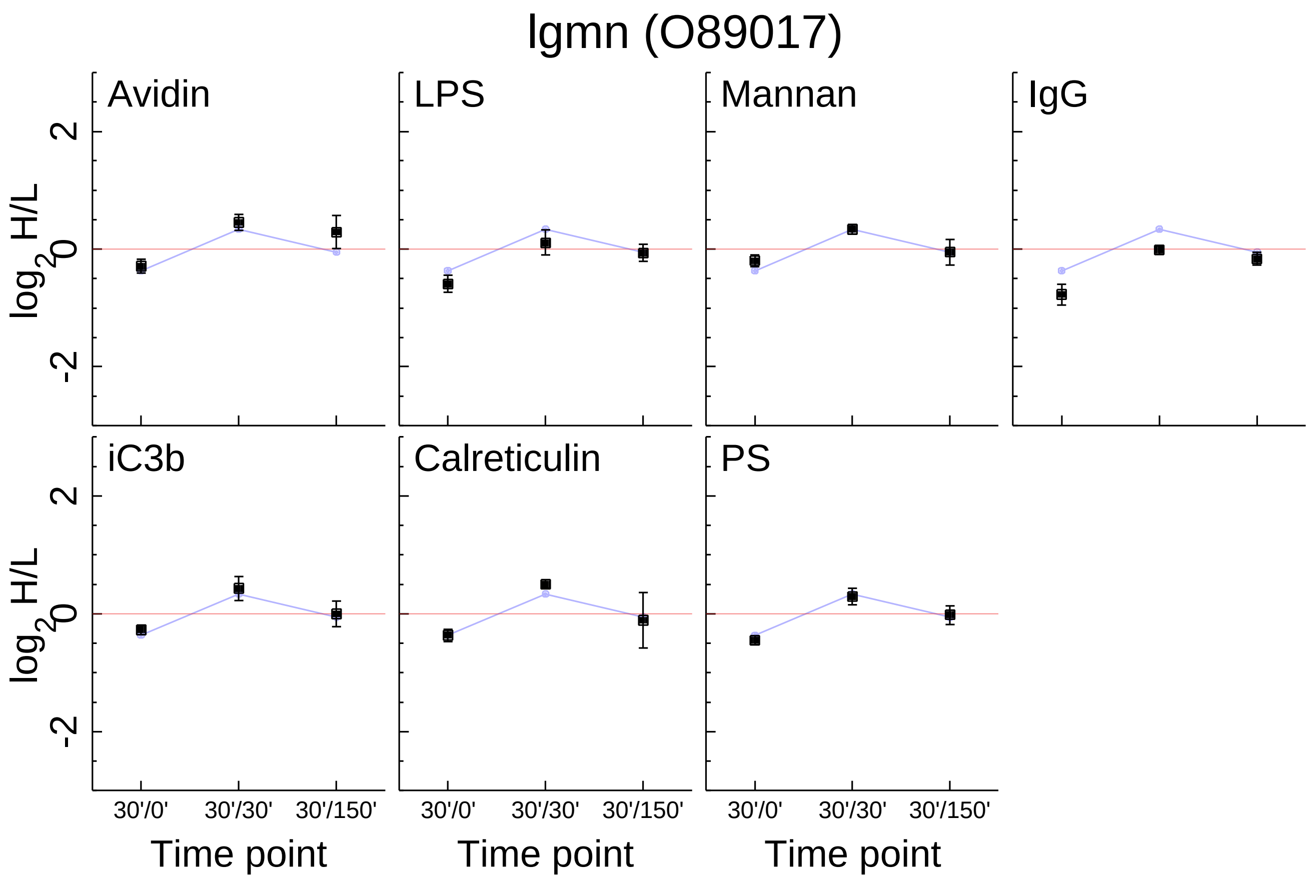

lgmn (O89017)
Avidin
LPS
Mannan
IgG
iC3b
Calreticulin
PS
log2 H/L
2
0
-2
30'/0'
30'/30'
30'/150'
Time point

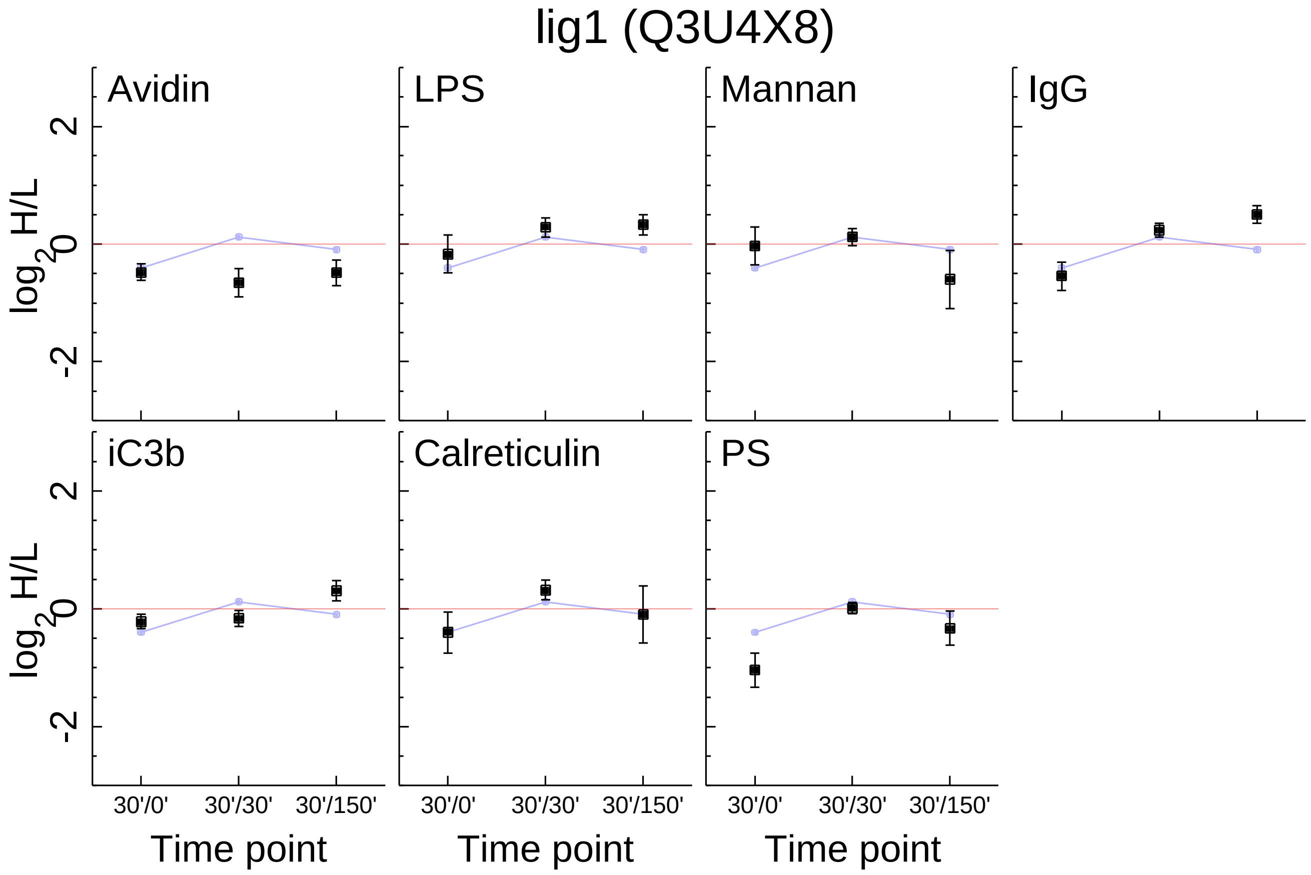
lig1 (Q3U4X8)
Avidin
LPS
Mannan
IgG
iC3b
Calreticulin
PS
log2 H/L
2
0
-2
30'/0'
30'/30'
30'/150'
Time point

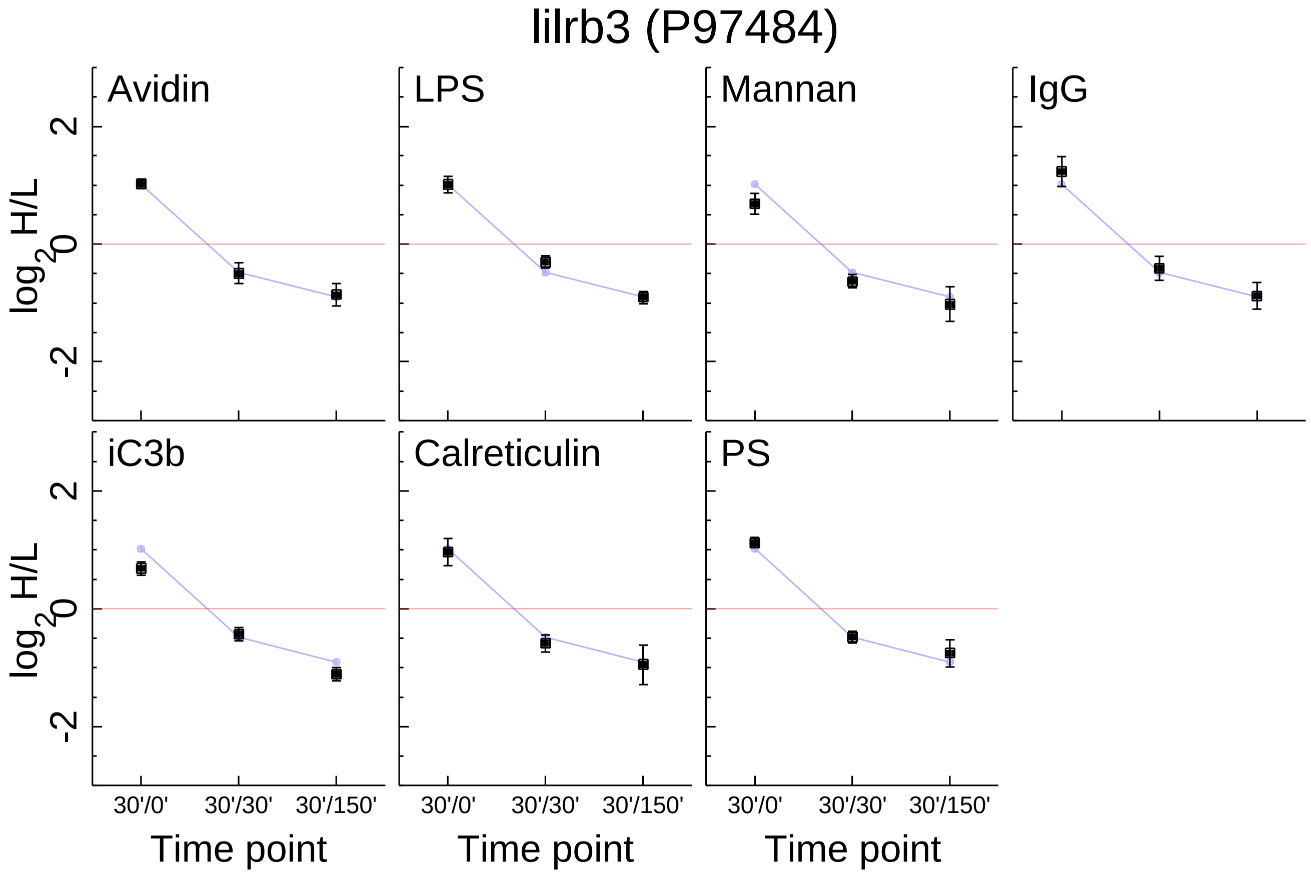

lilrb3 (P97484)
Avidin
LPS
Mannan
IgG
iC3b
Calreticulin
PS
log2 H/L
2
0
-2
30'/0'
30'/30'
30'/150'
Time point

lilrb4 (Q64281)

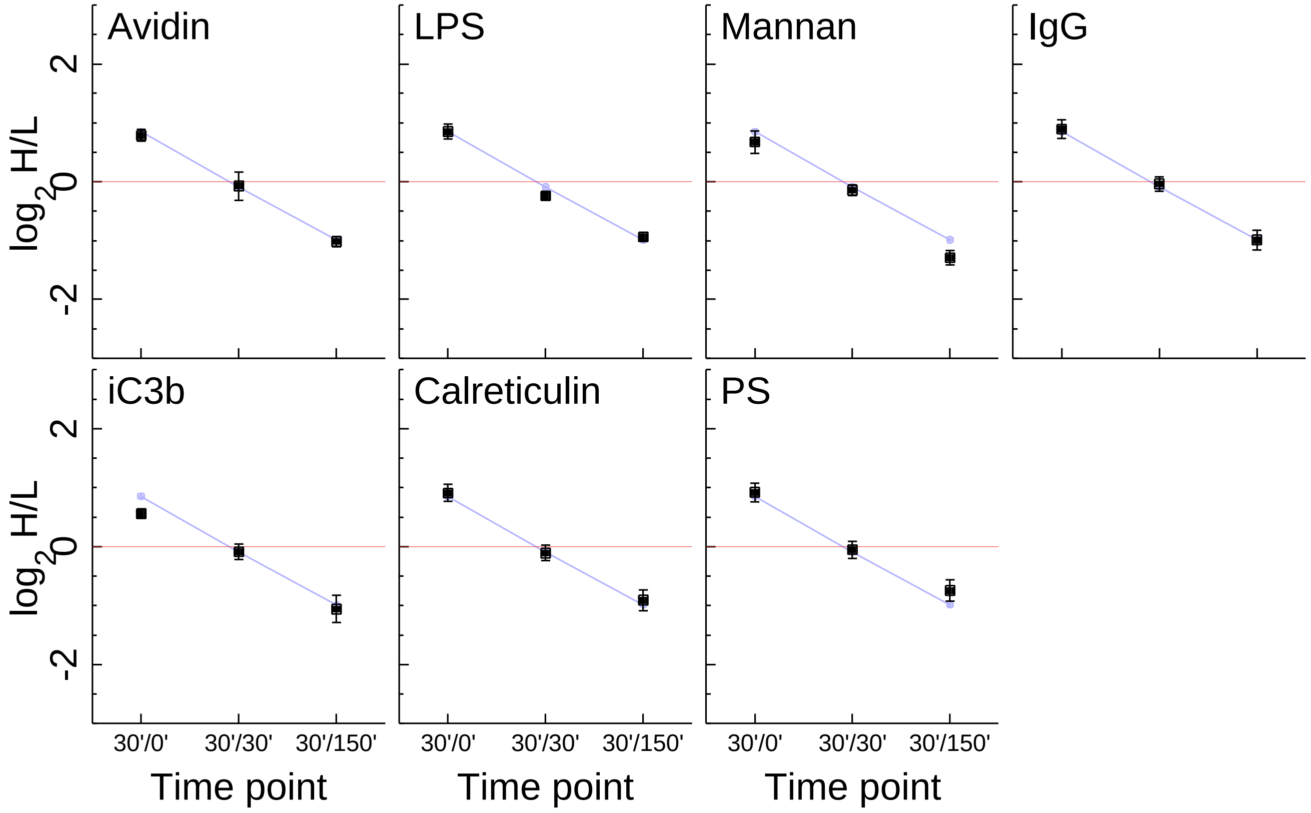

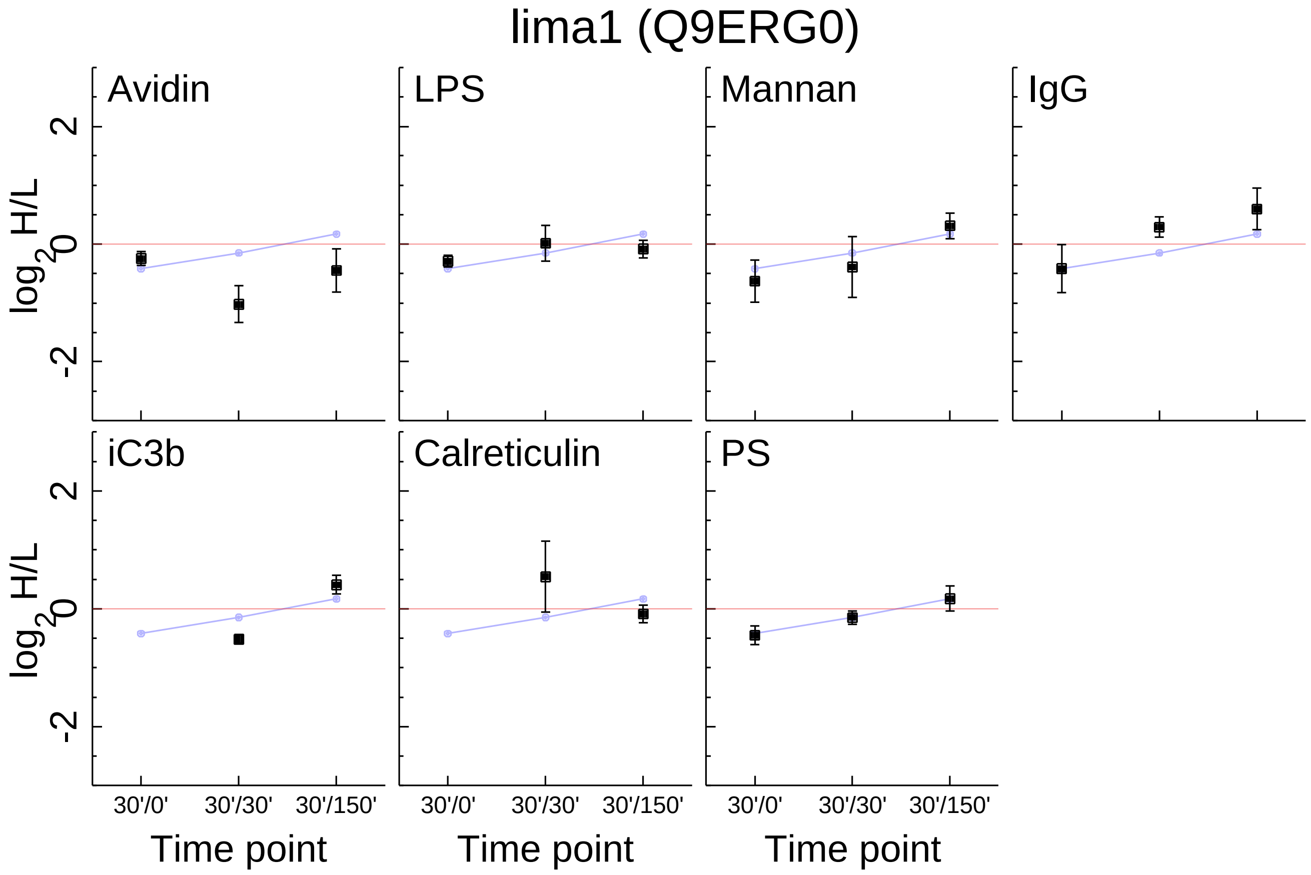
lima1 (Q9ERG0)
Avidin
LPS
Mannan
IgG
iC3b
Calreticulin
PS
log2 H/L
2
0
-2
30'/0'
30'/30'
30'/150'
Time point

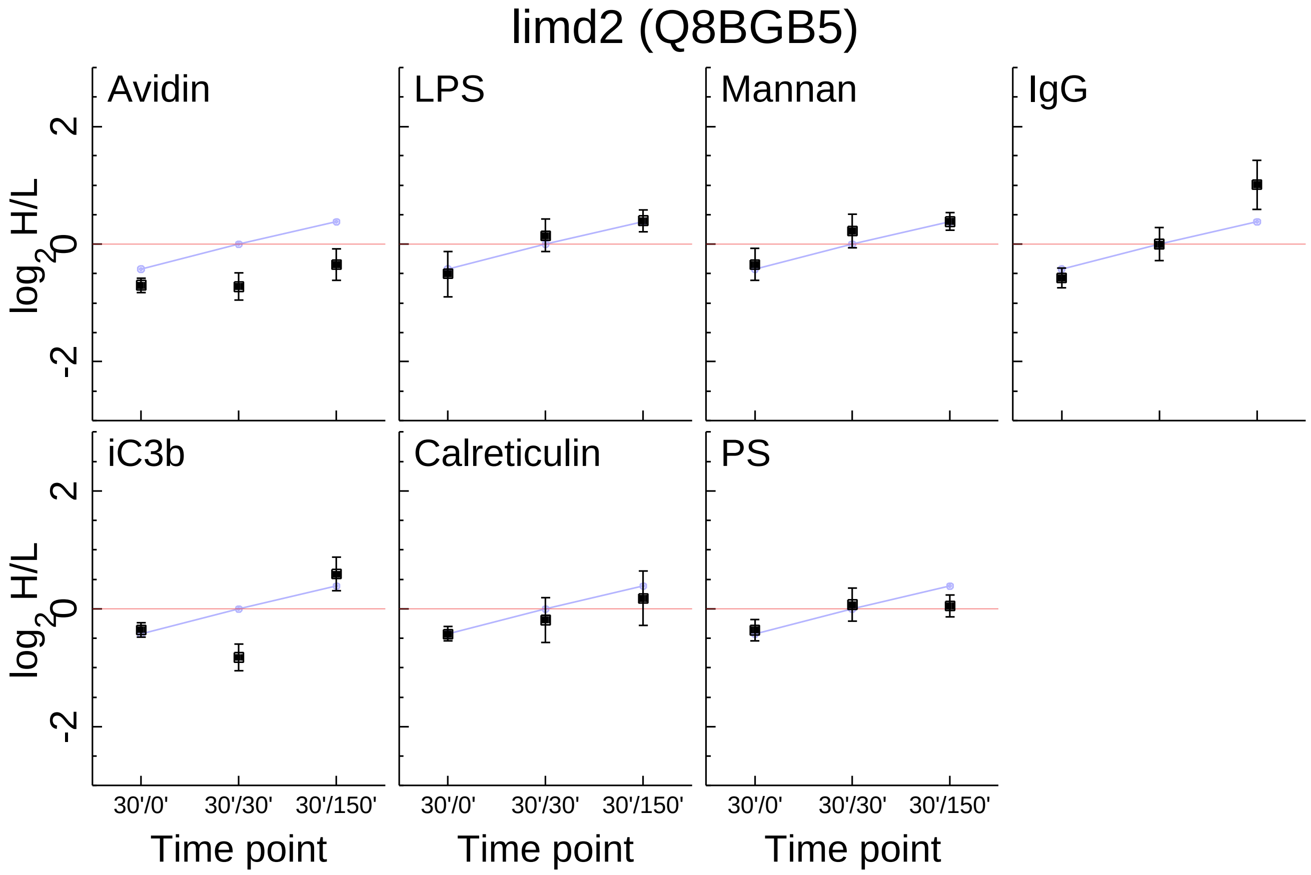
limd2 (Q8BGB5)
Avidin
LPS
Mannan
IgG
iC3b
Calreticulin
PS
log2 H/L
2
0
-2
30'/0'
30'/30'
30'/150'
Time point

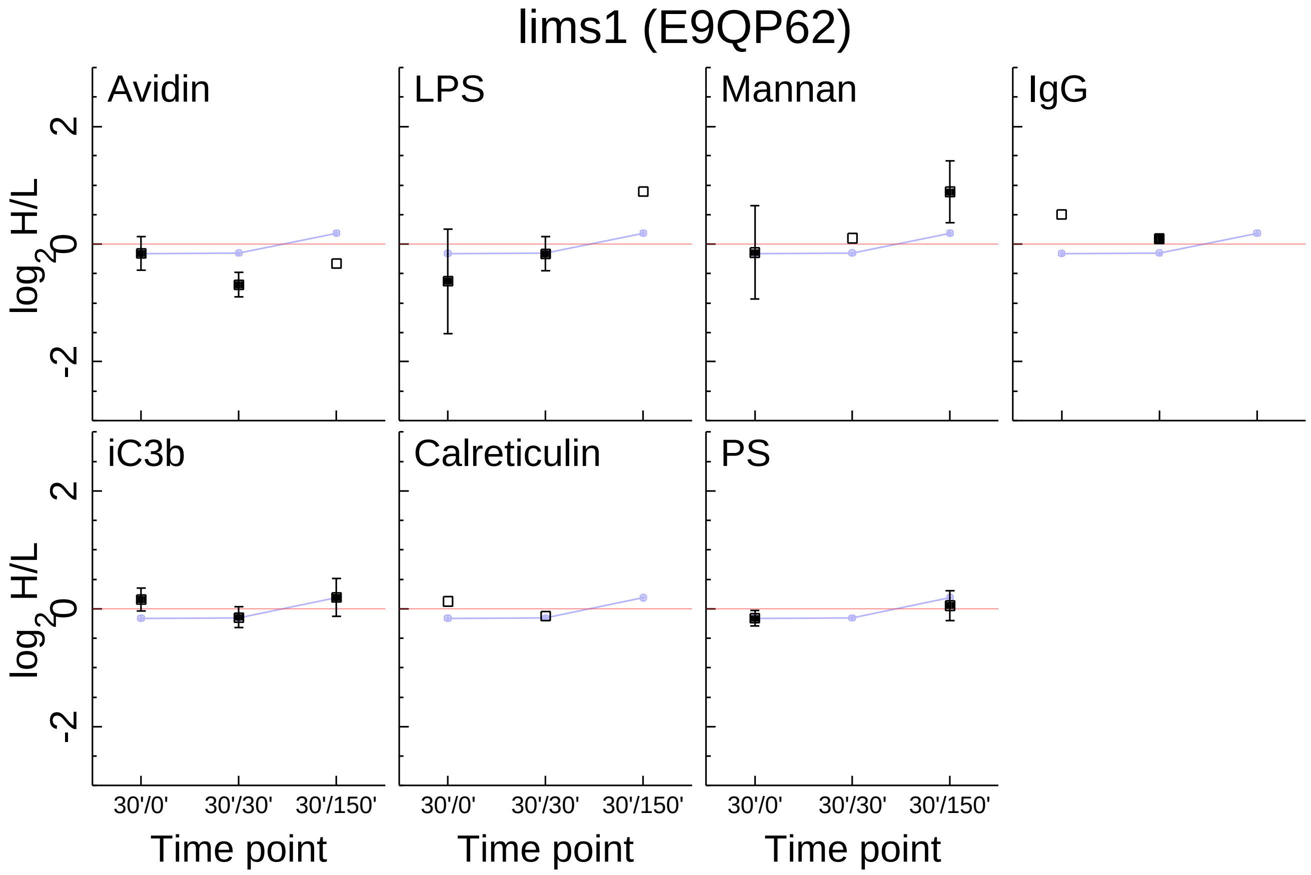

lims1 (E9QP62)
Avidin
LPS
Mannan
IgG
iC3b
Calreticulin
PS
$\log_2$ H/L
2
0
-2
30'/0'
30'/30'
30'/150'
Time point

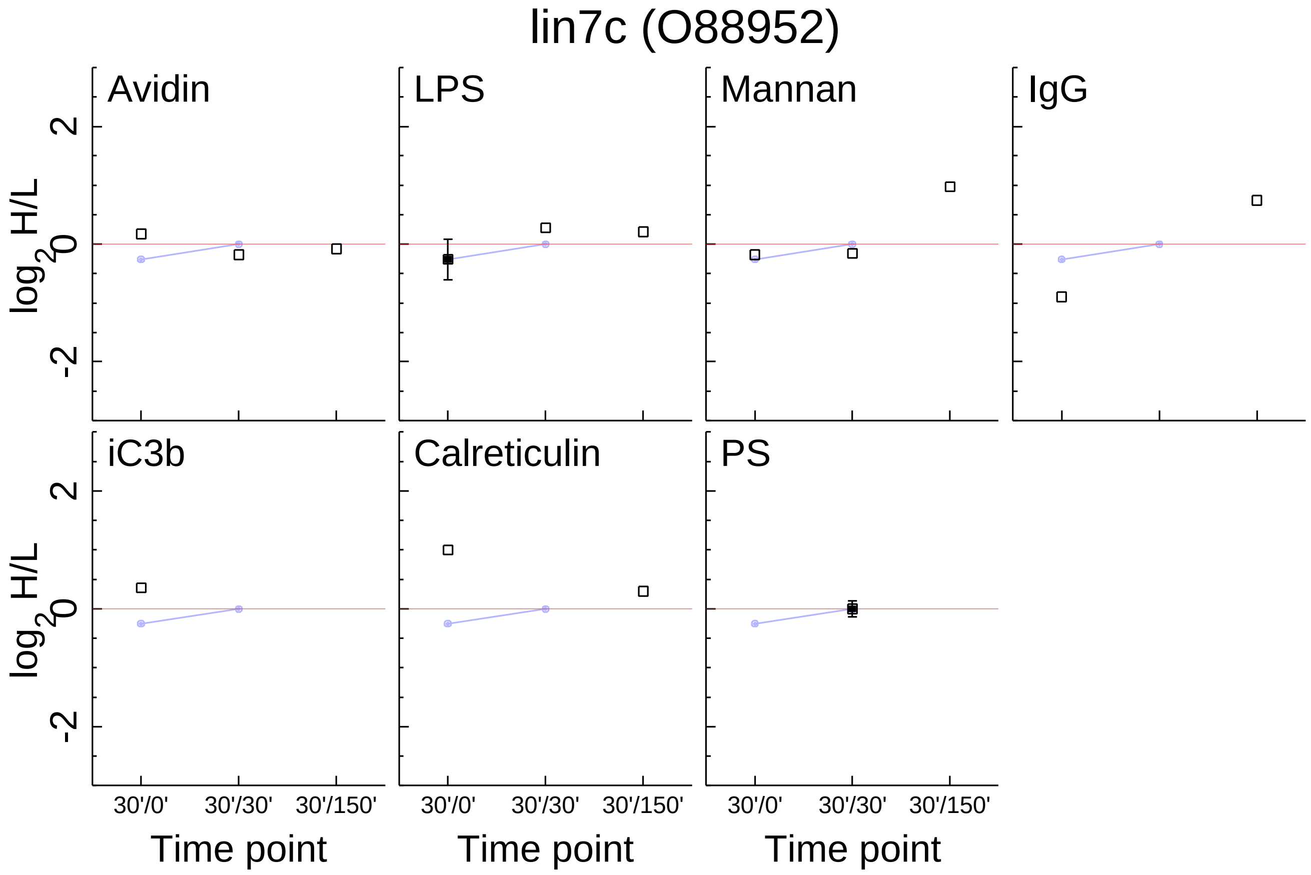
lin7c (O88952)
Avidin
LPS
Mannan
IgG
iC3b
Calreticulin
PS
log2 H/L
2
0
-2
30'/0'
30'/30'
30'/150'
Time point

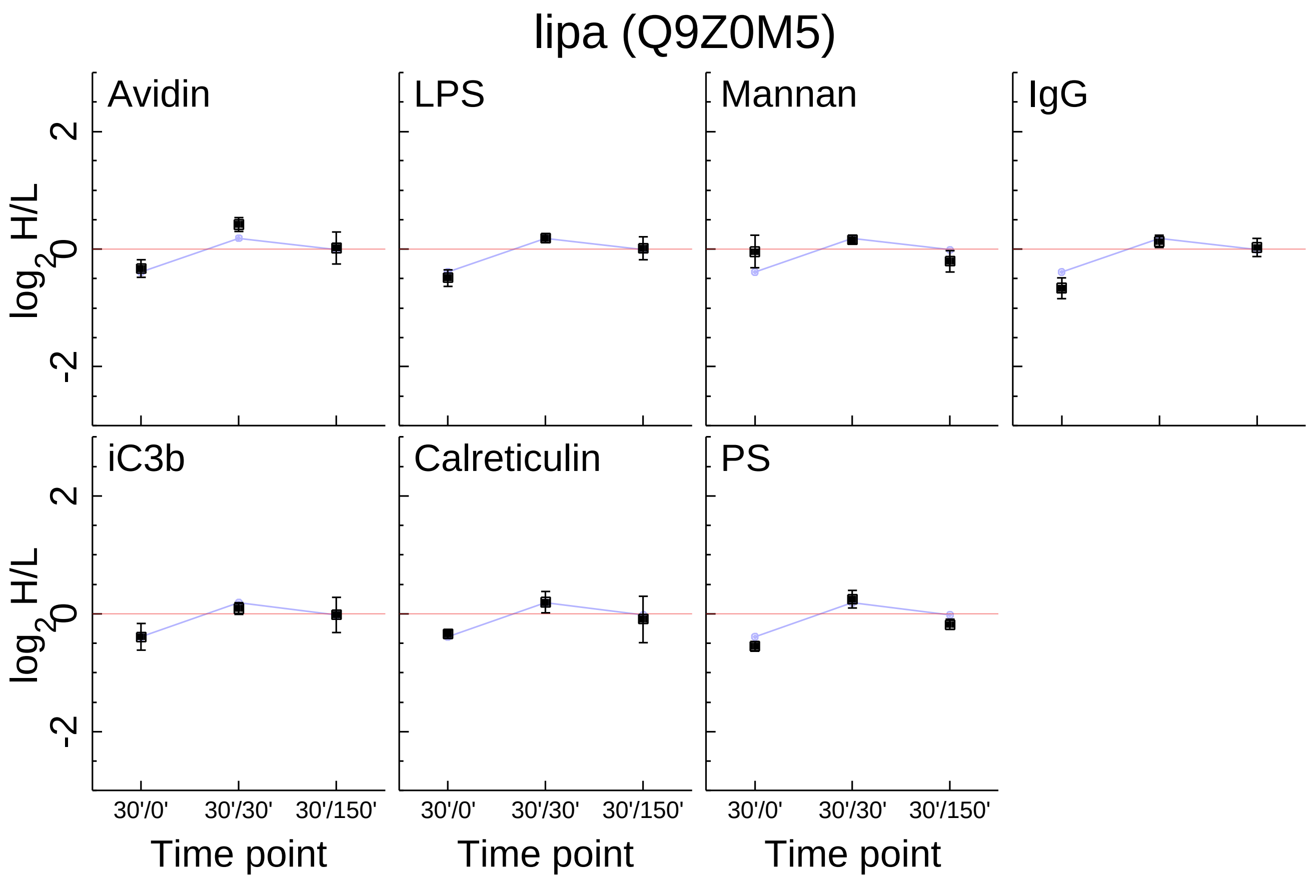

lipa (Q9Z0M5)
Avidin
LPS
Mannan
IgG
iC3b
Calreticulin
PS
log2 H/L
2
0
-2
30'/0'
30'/30'
30'/150'
Time point

Iman1 (Q9D0F3)

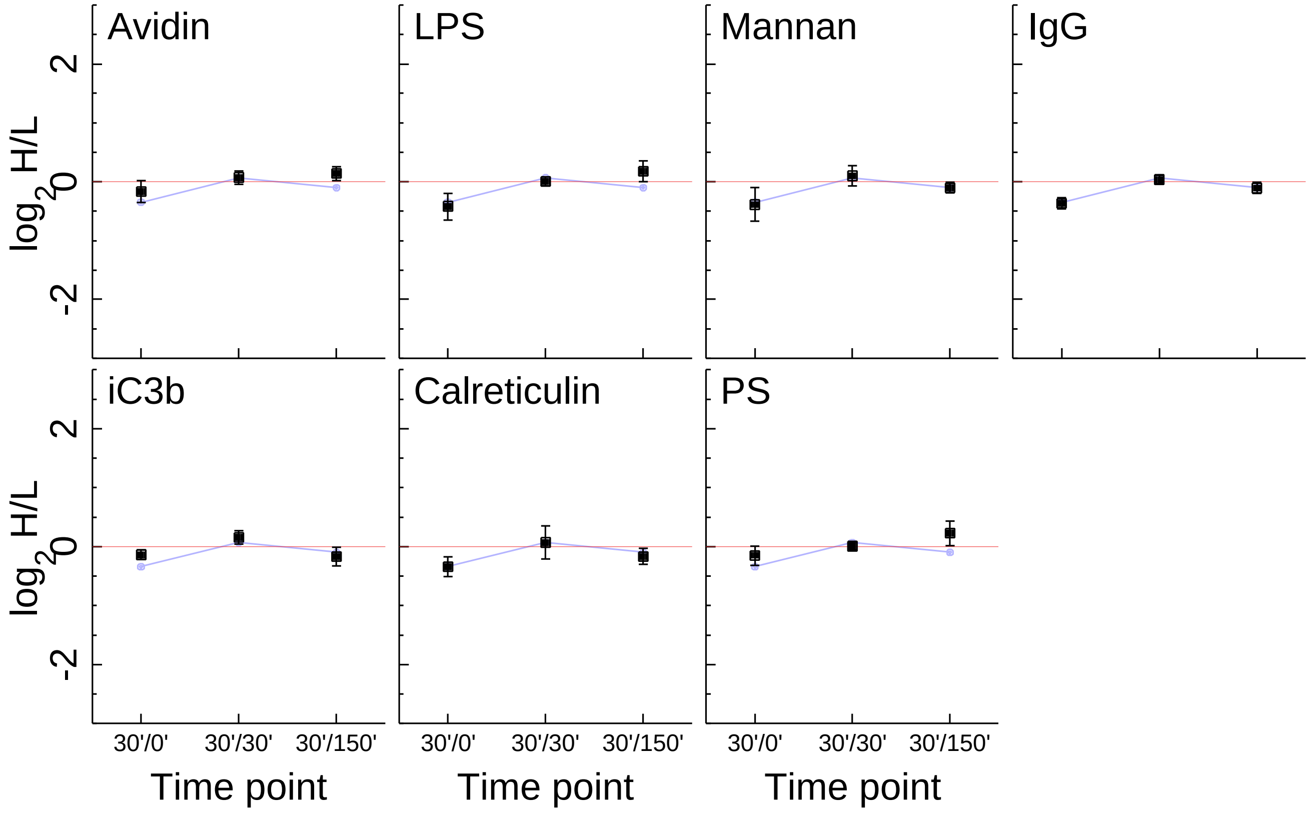

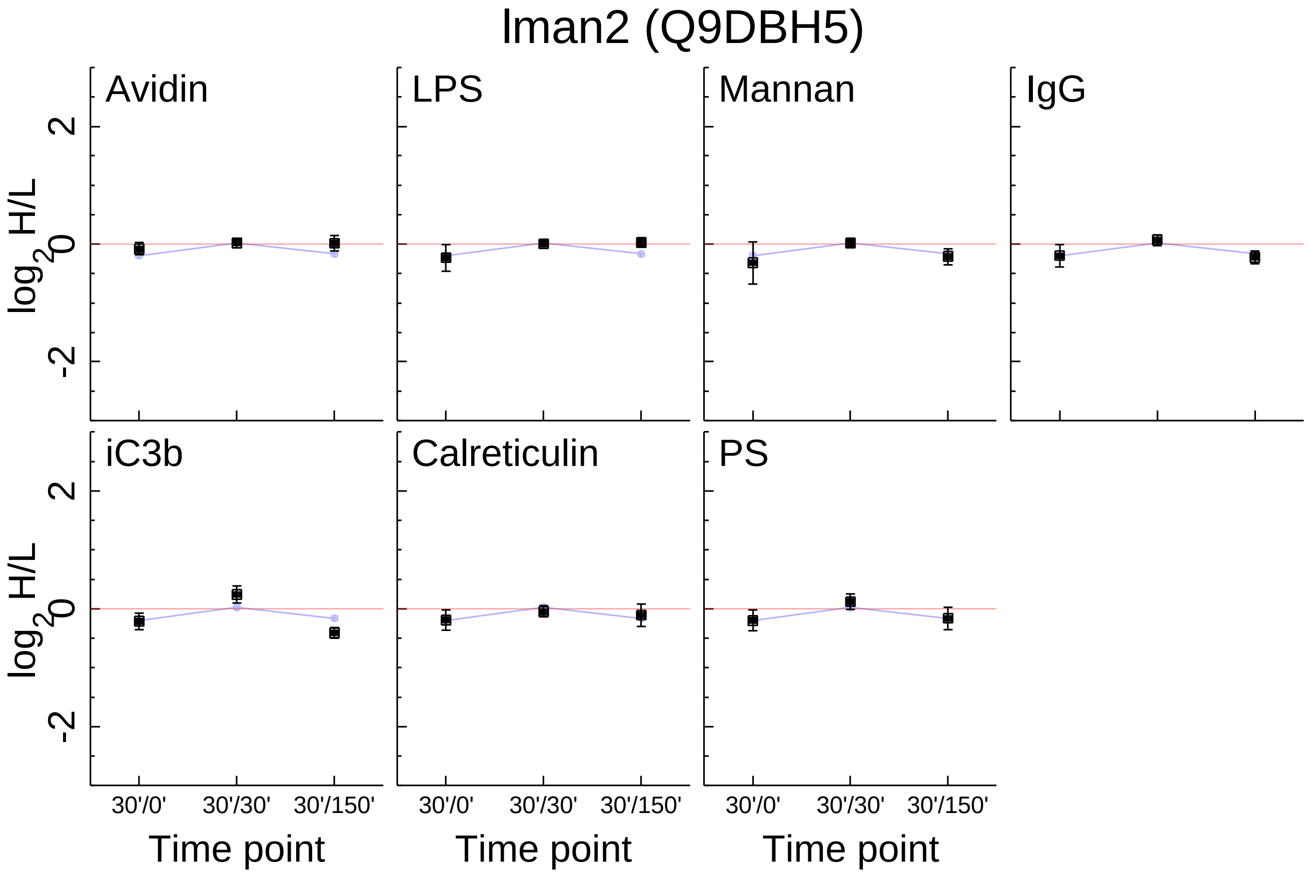
lman2 (Q9DBH5)
Avidin
LPS
Mannan
IgG
iC3b
Calreticulin
PS
log2 H/L
2
0
-2
30'/0'
30'/30'
30'/150'
Time point

lmbrd1 (Q8K0B2)

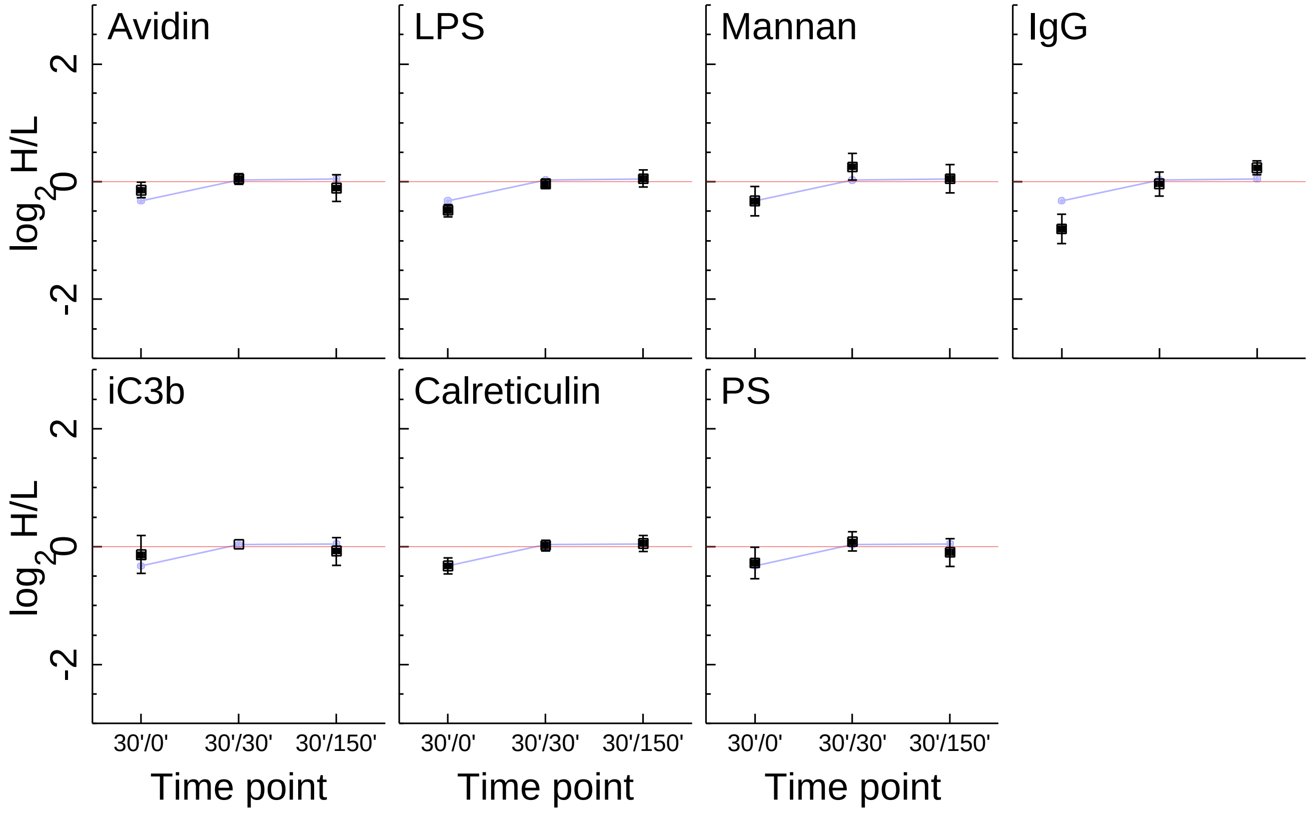

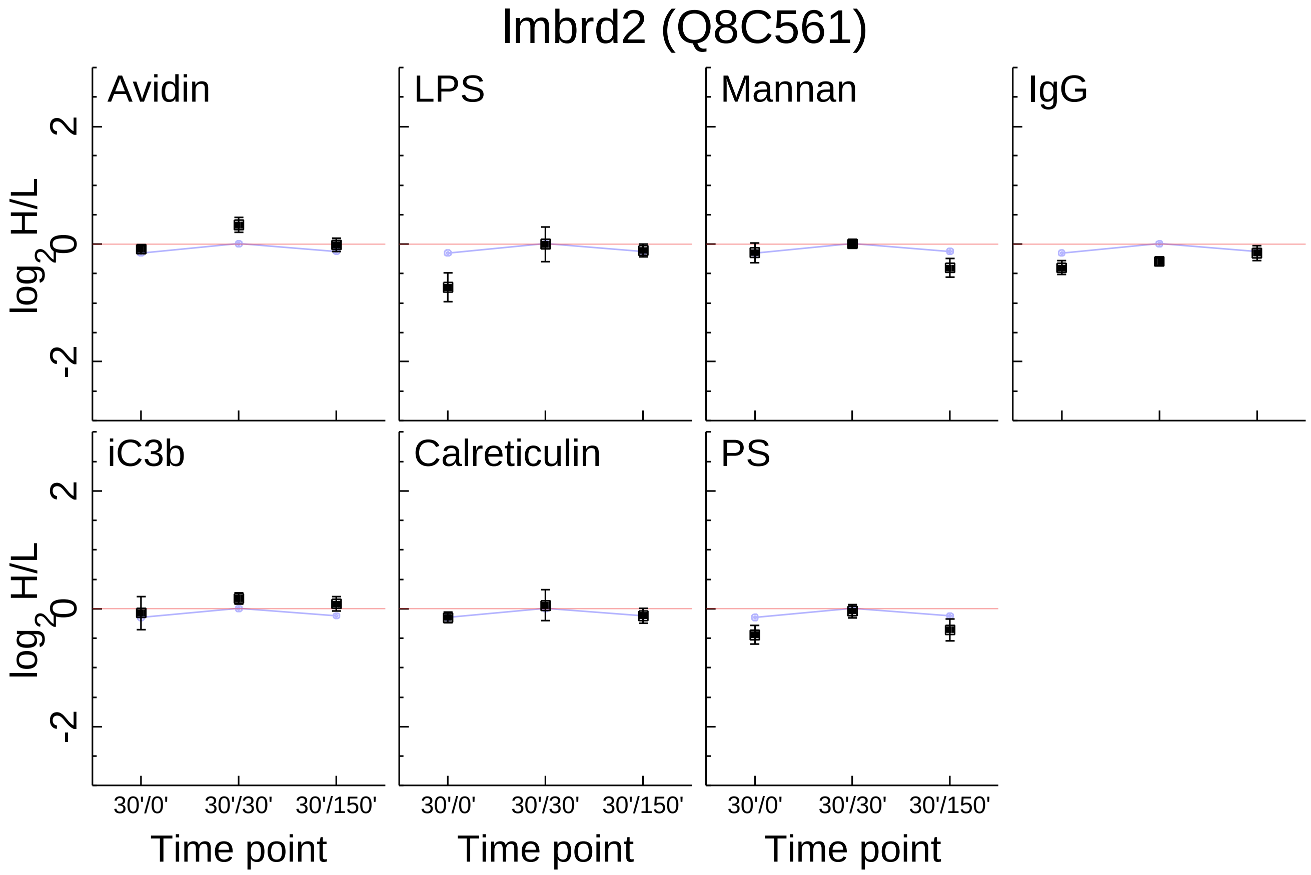

lmbrd2 (Q8C561)
Avidin
LPS
Mannan
IgG
iC3b
Calreticulin
PS
log2 H/L
2
0
-2
30'/0'
30'/30'
30'/150'
Time point

lmna (P48678)

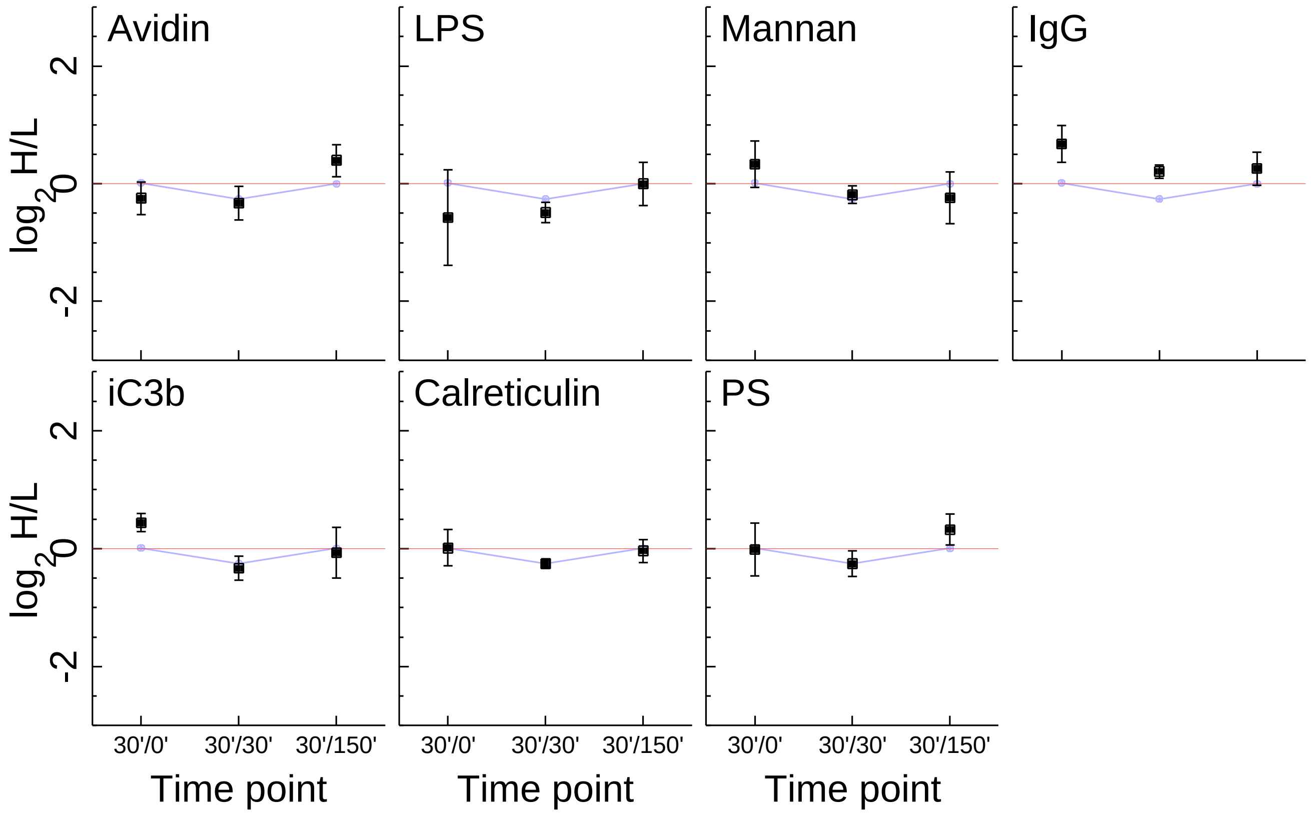
Avidin
LPS
Mannan
IgG
iC3b
Calreticulin
PS
log2 H/L
2
0
-2
30'/0'
30'/30'
30'/150'
Time point

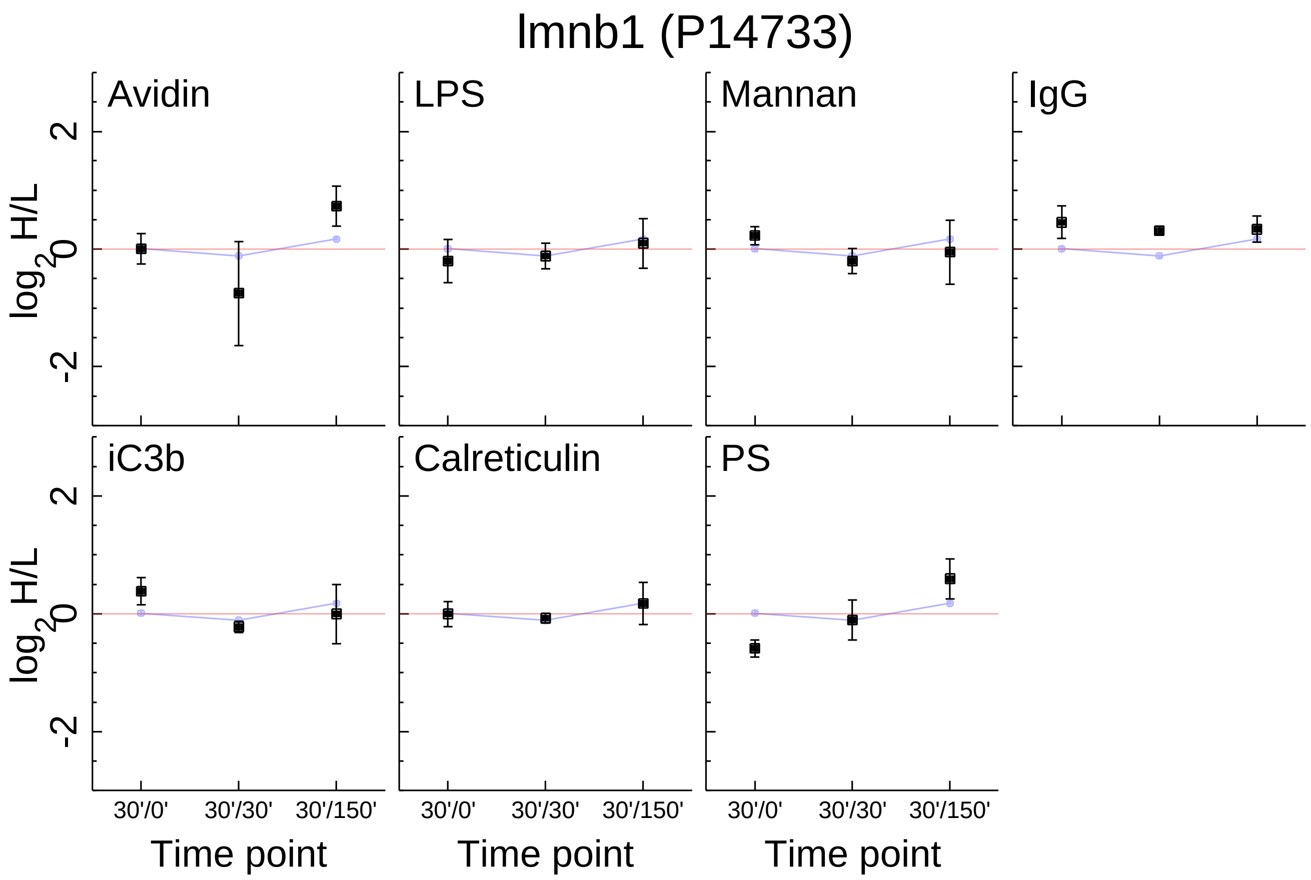

lmnb1 (P14733)
Avidin
LPS
Mannan
IgG
iC3b
Calreticulin
PS
log2 H/L
2
0
-2
30'/0'
30'/30'
30'/150'
Time point

lmnb2 (P21619)

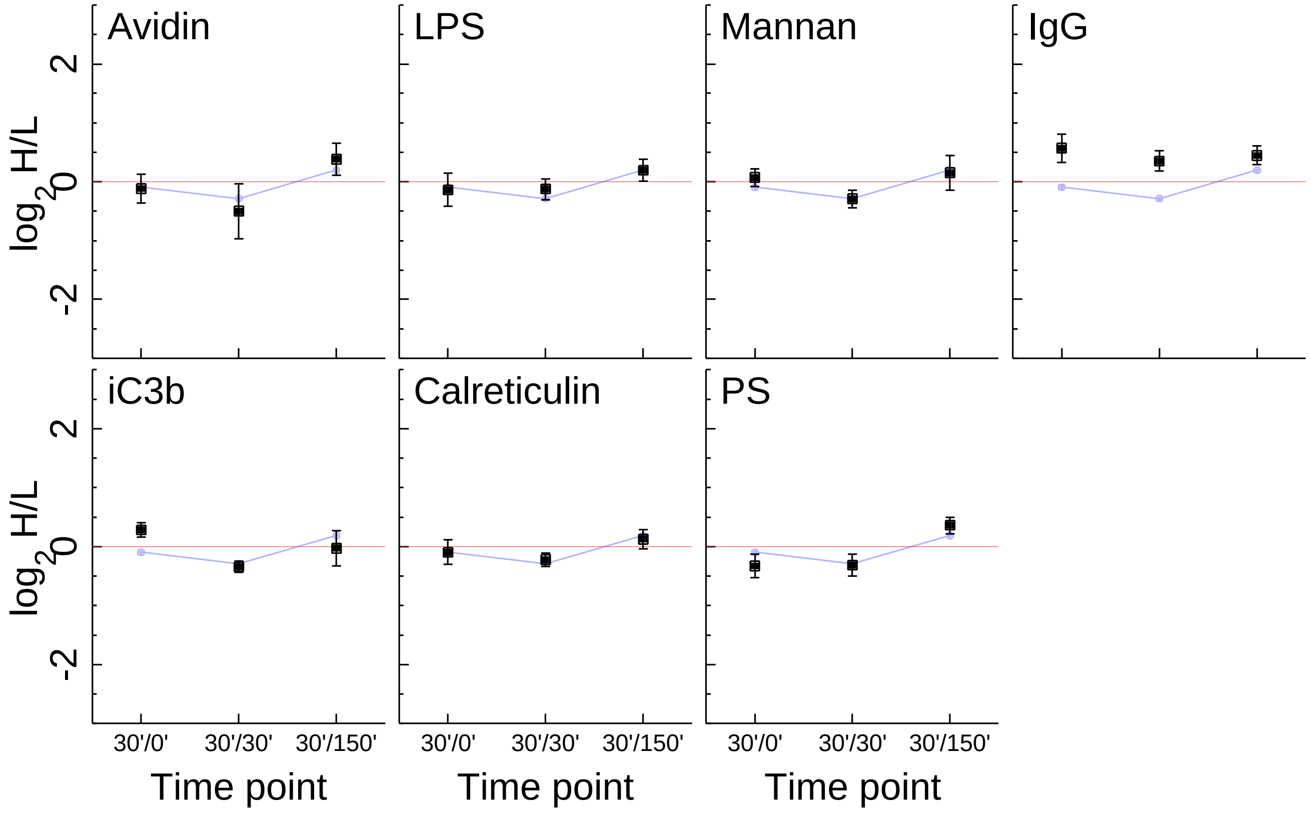

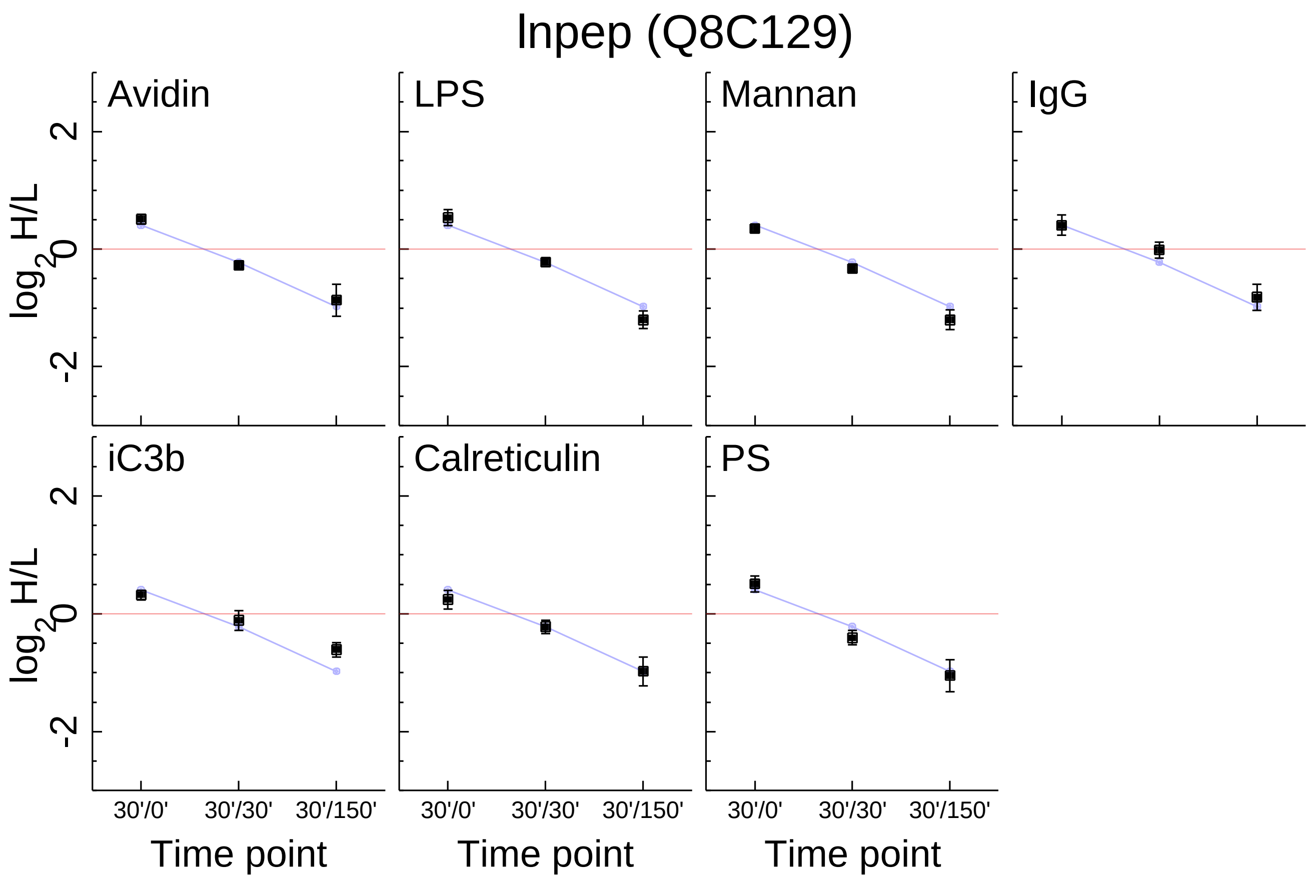
Inpep (Q8C129)
Avidin
LPS
Mannan
IgG
iC3b
Calreticulin
PS
log2 H/L
2
0
-2
30'/0'
30'/30'
30'/150'
Time point

lob12cr1 (E9Q2W8)

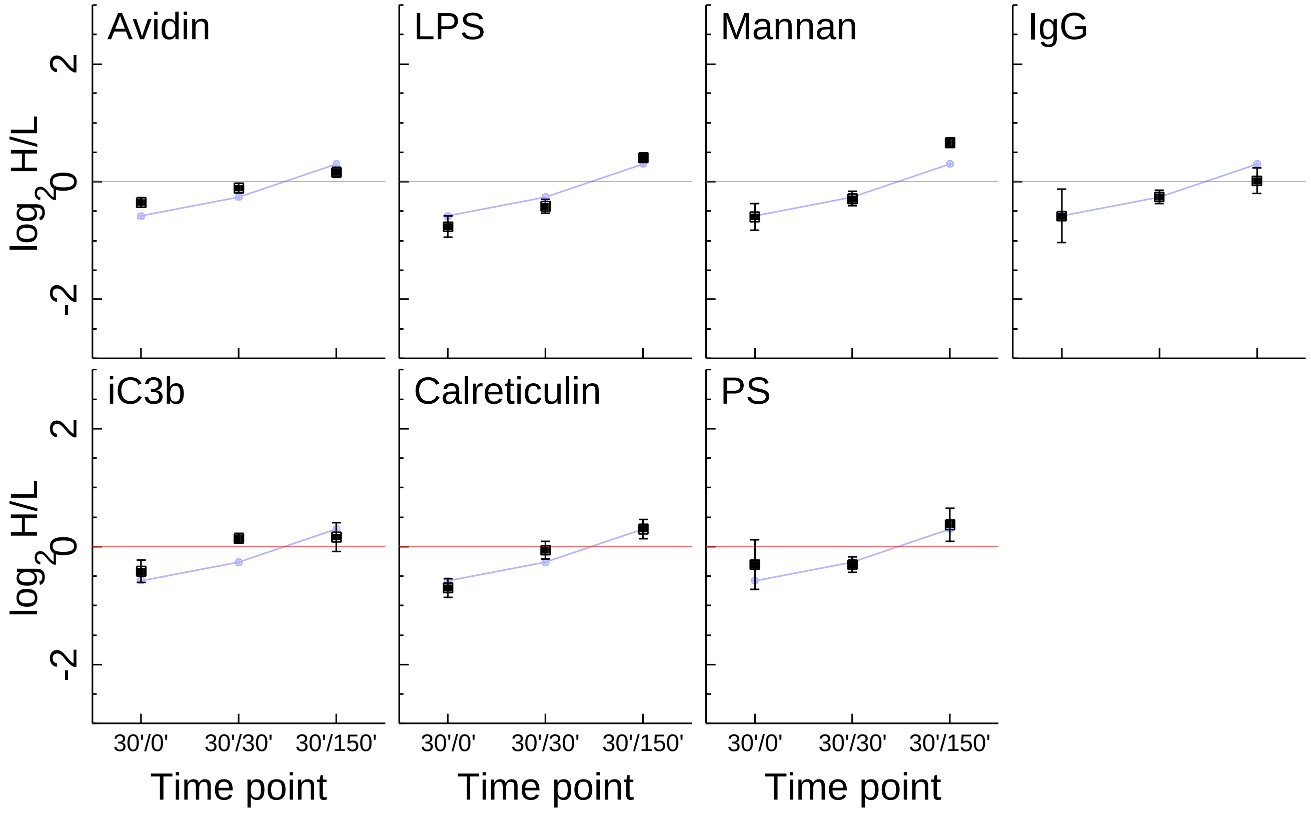

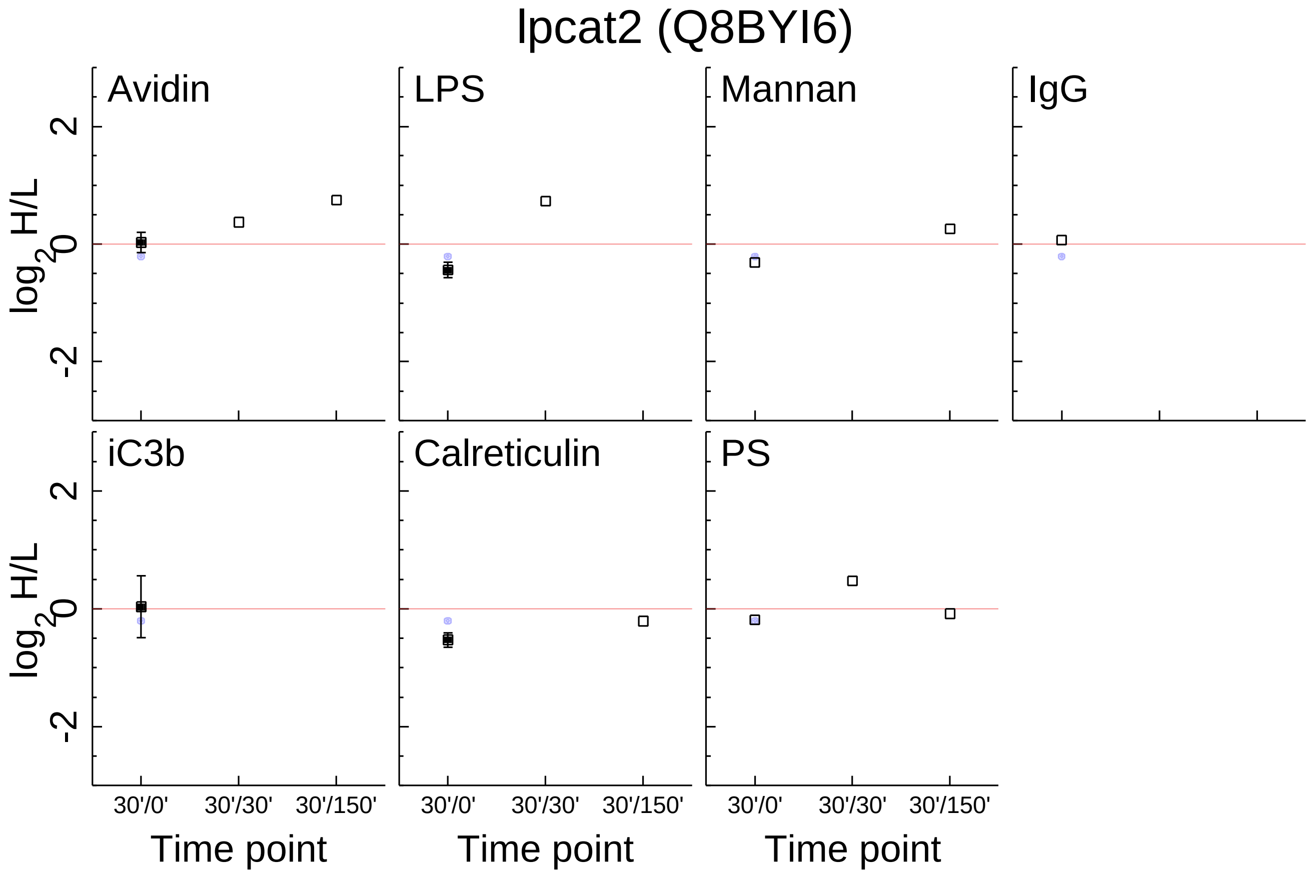

lpcat2 (Q8BYI6)
Avidin
LPS
Mannan
IgG
iC3b
Calreticulin
PS
$\log_2$ H/L
2
0
-2
30'/0'
30'/30'
30'/150'
Time point

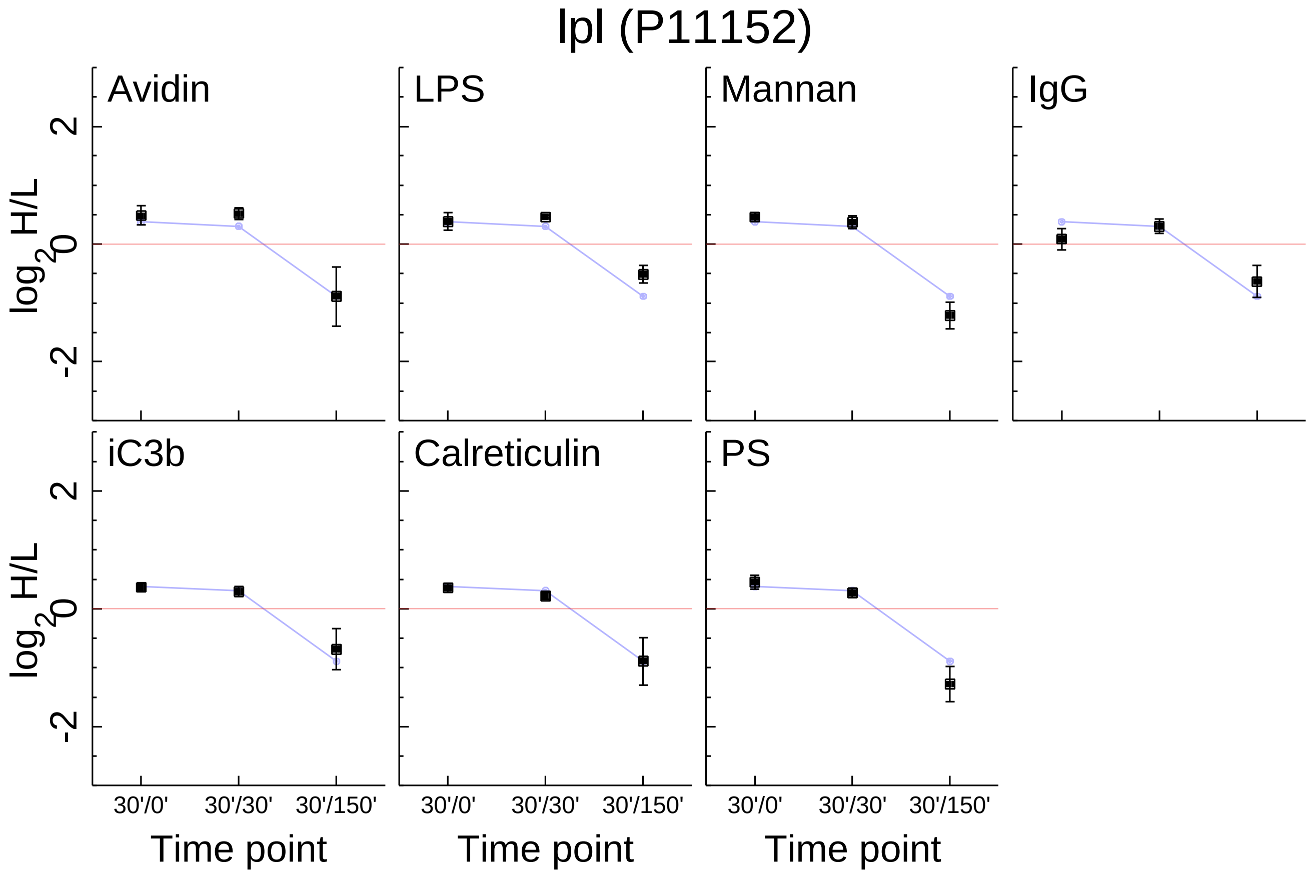
lpl (P11152)
Avidin
LPS
Mannan
IgG
iC3b
Calreticulin
PS
log2 H/L
2
0
-2
30'/0'
30'/30'
30'/150'
Time point

lrp1b (A2API5)

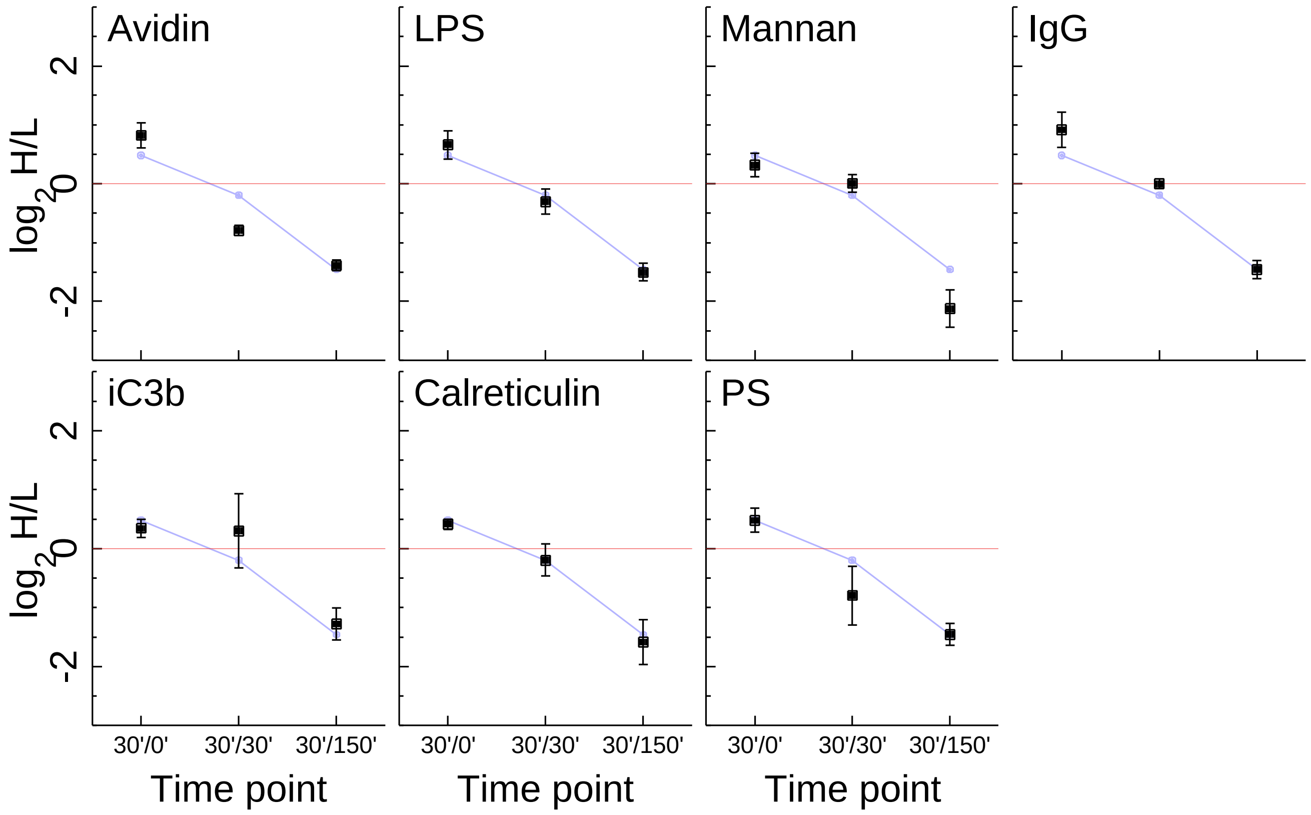

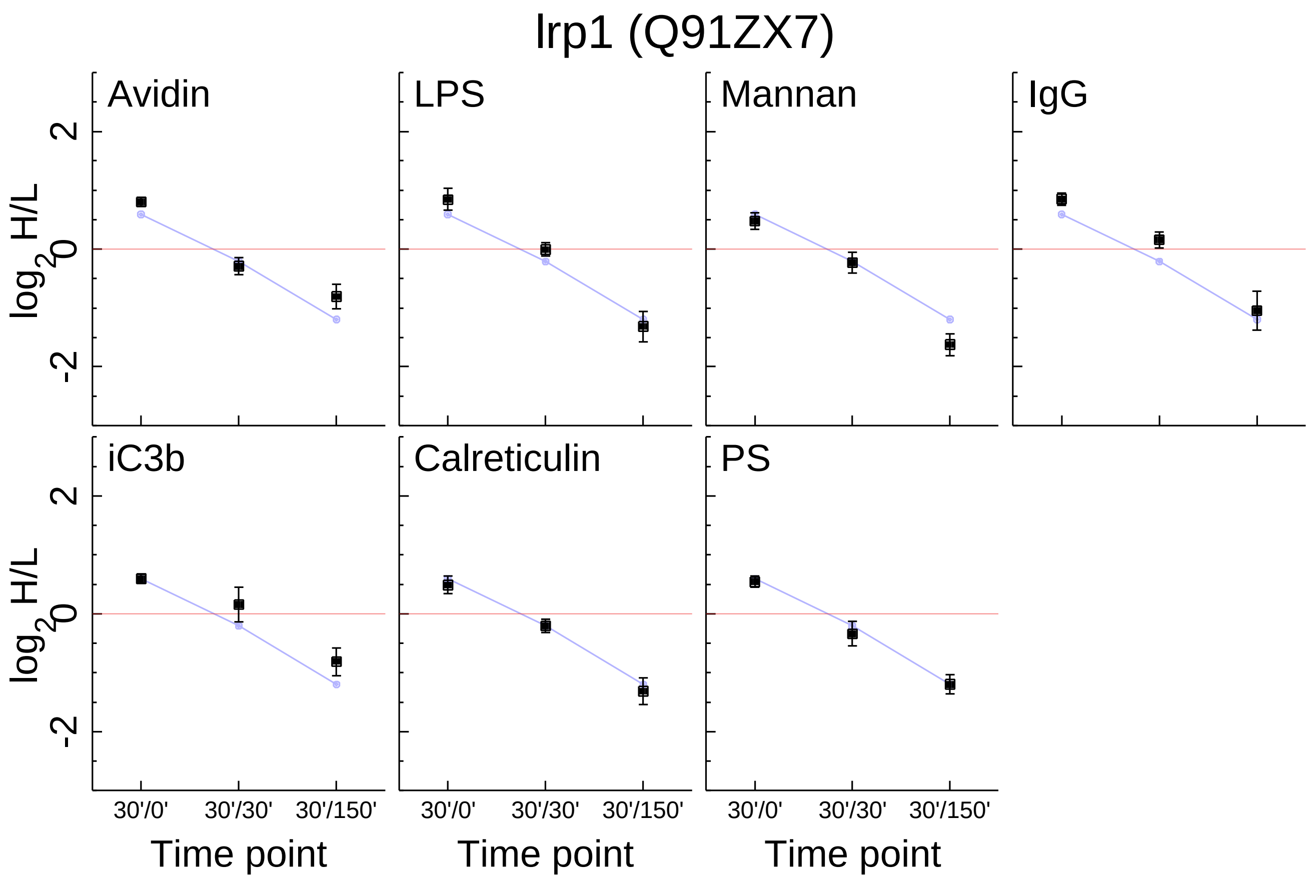
lrp1 (Q91ZX7)
Avidin
LPS
Mannan
IgG
iC3b
Calreticulin
PS
log2 H/L
2
0
-2
30'/0'
30'/30'
30'/150'
Time point

lrpap1 (P55302)

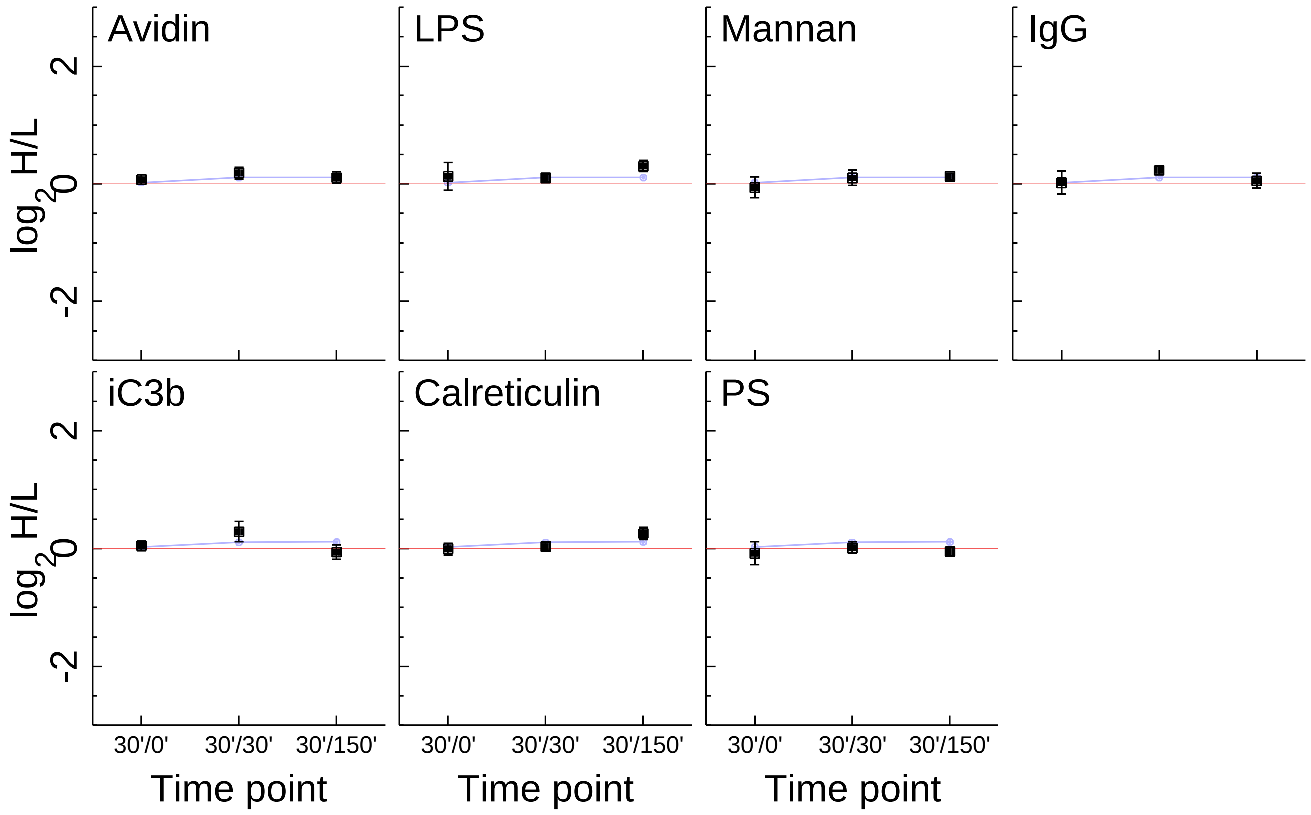

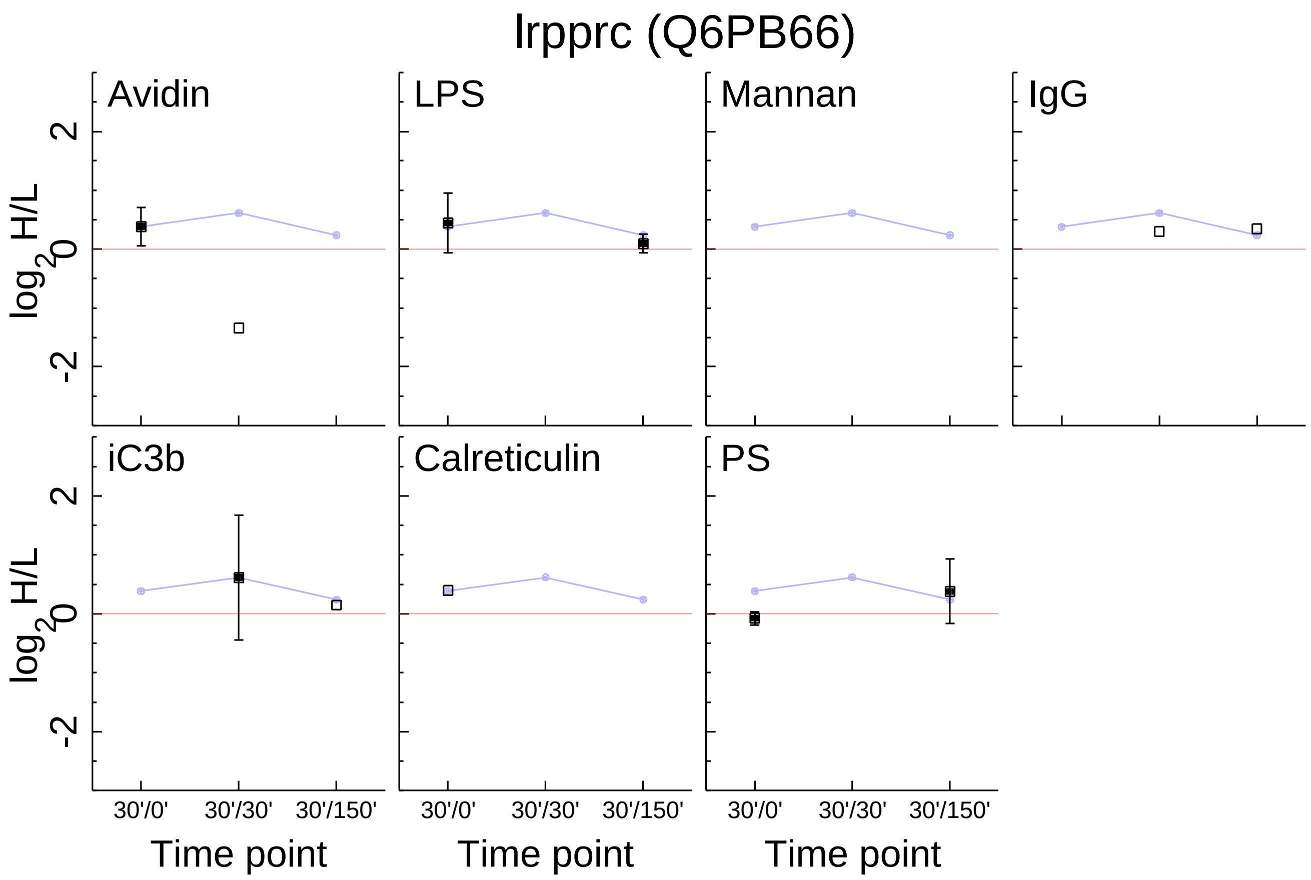

lrpprc (Q6PB66)
Avidin
LPS
Mannan
IgG
iC3b
Calreticulin
PS
log2 H/L
2
0
-2
30'/0'
30'/30'
30'/150'
Time point

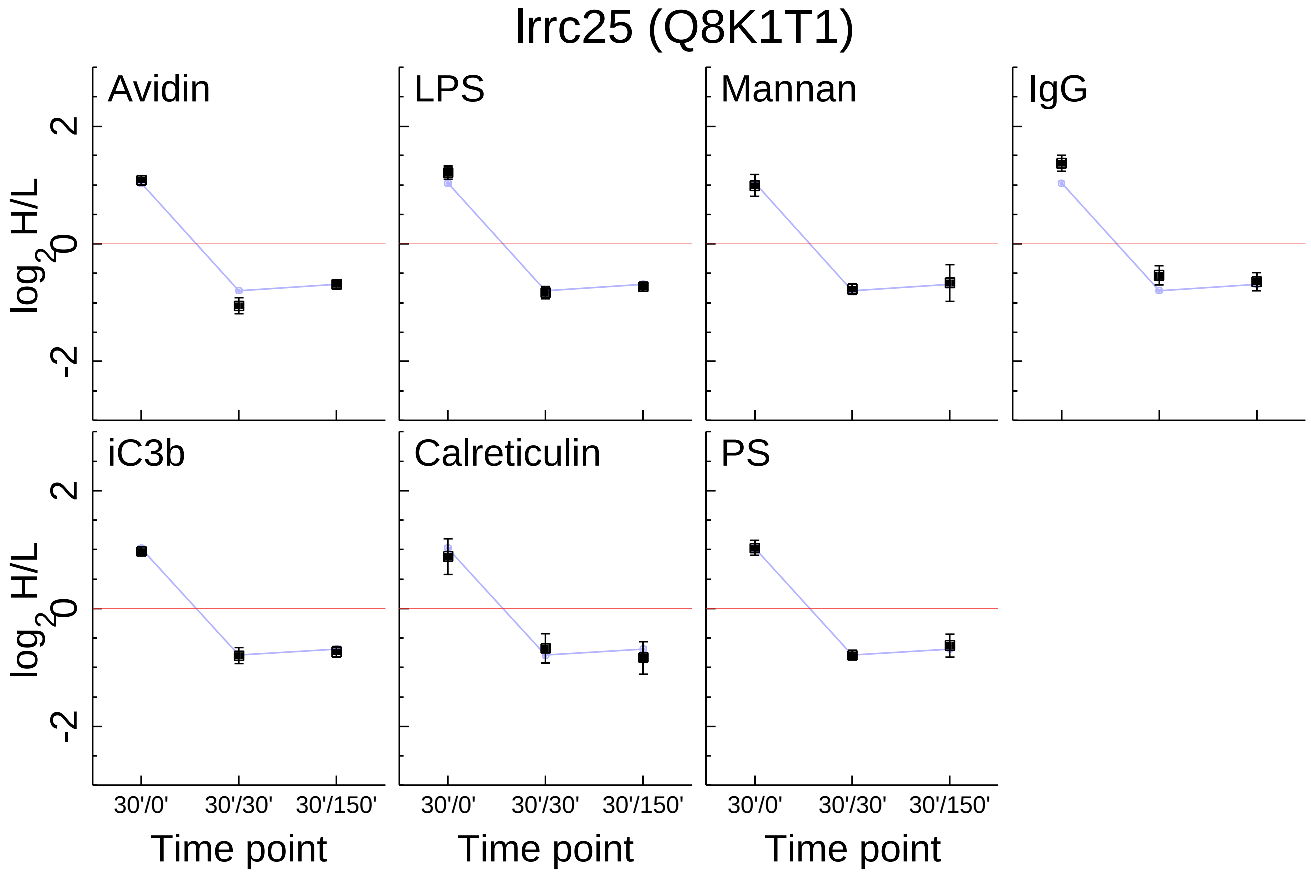

lrrc25 (Q8K1T1)
Avidin
LPS
Mannan
IgG
iC3b
Calreticulin
PS
log2 H/L
2
0
-2
30'/0'
30'/30'
30'/150'
Time point

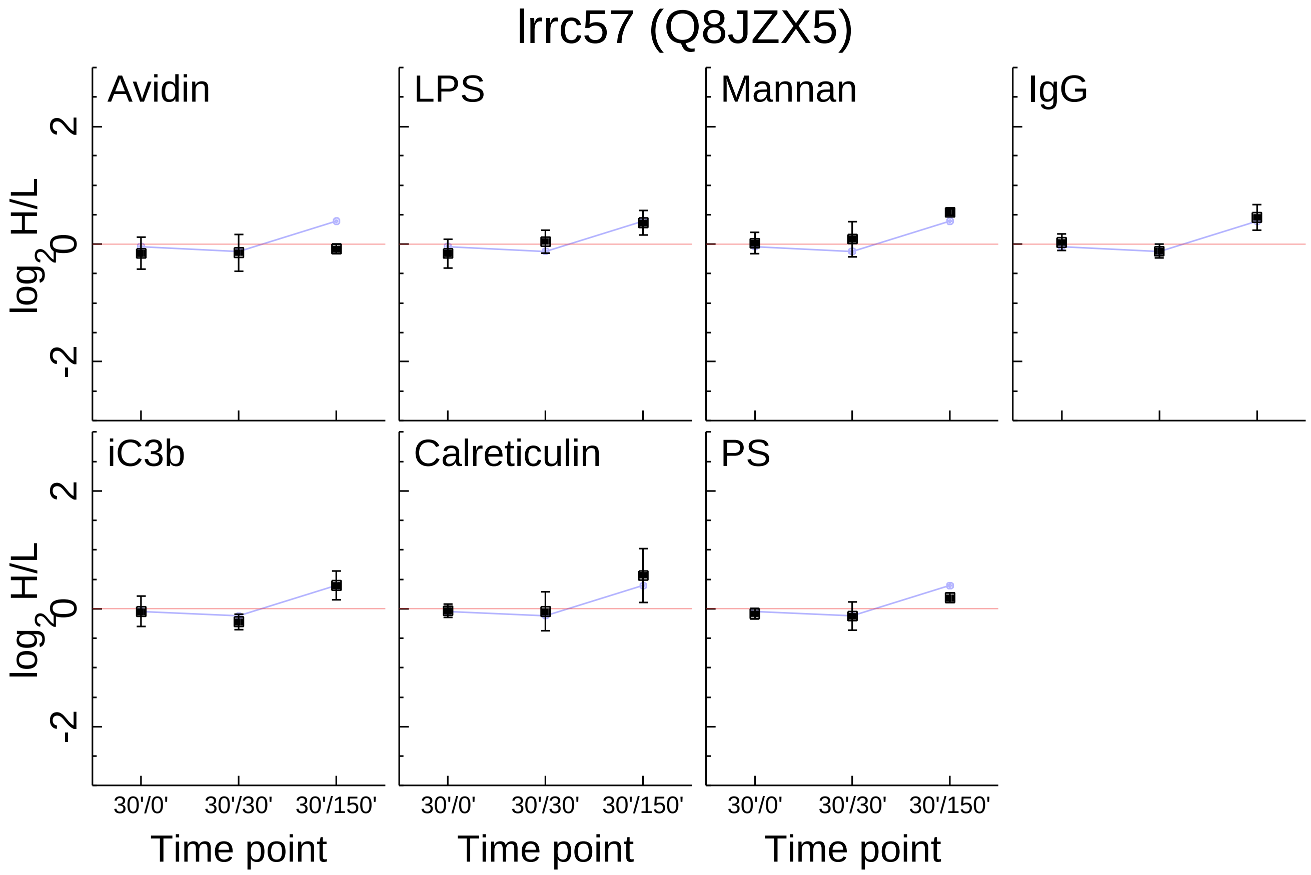

lrrc57 (Q8JZX5)
Avidin
LPS
Mannan
IgG
iC3b
Calreticulin
PS
log2 H/L
2
0
-2
30'/0'
30'/30'
30'/150'
Time point

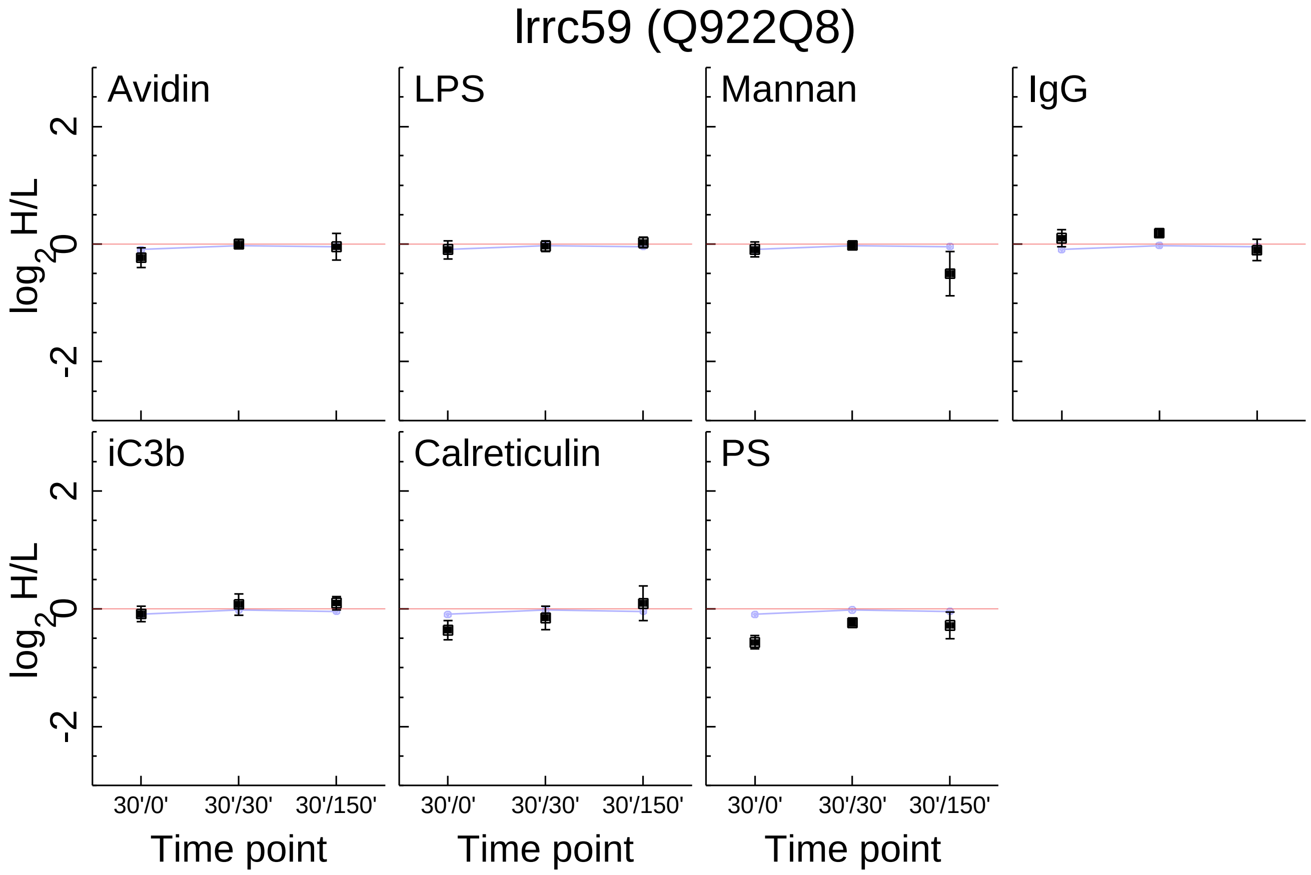

lrrc59 (Q922Q8)
Avidin
LPS
Mannan
IgG
iC3b
Calreticulin
PS
log2 H/L
2
0
-2
30'/0'
30'/30'
30'/150'
Time point

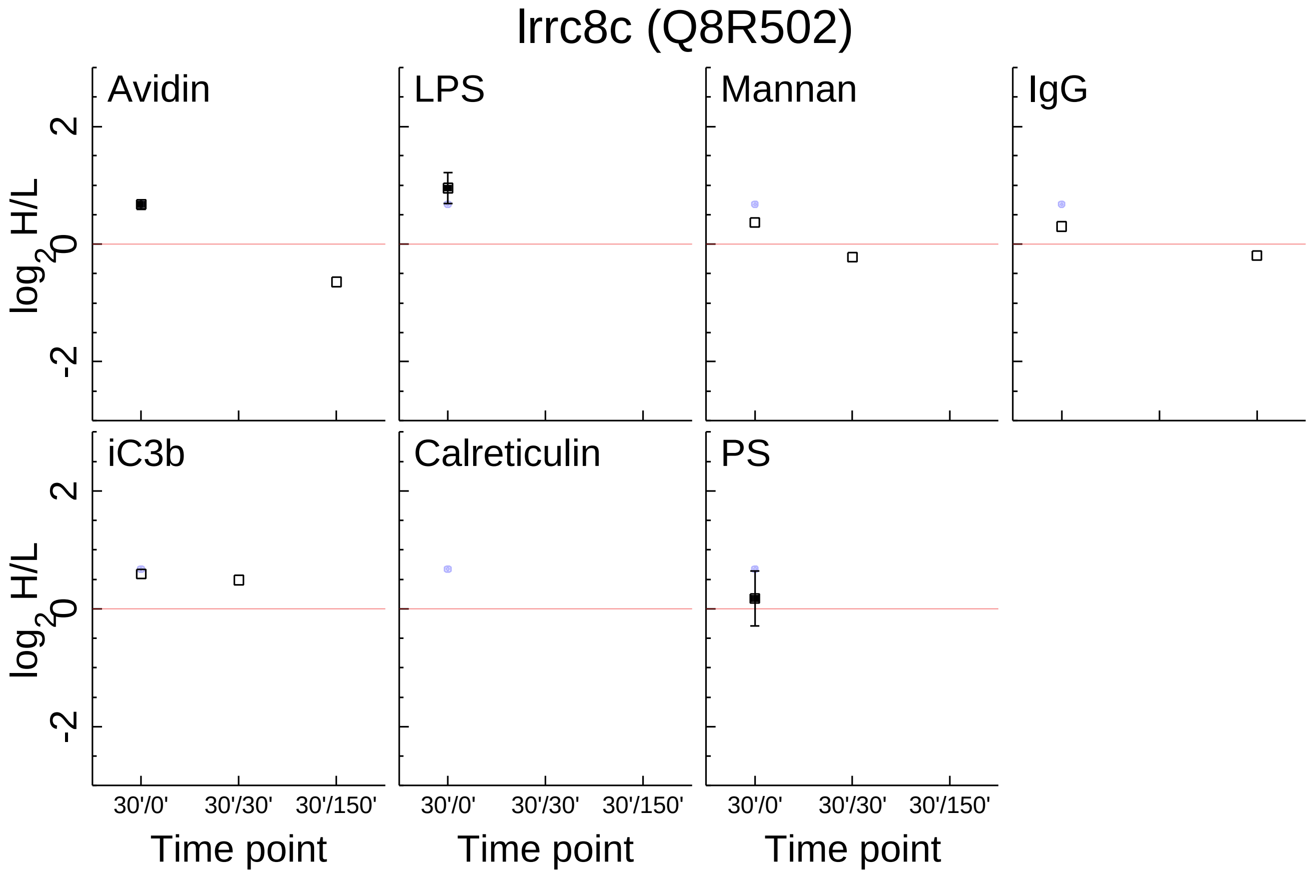
lrrc8c (Q8R502)
Avidin
LPS
Mannan
IgG
iC3b
Calreticulin
PS
log2 H/L
2
0
-2
30'/0'
30'/30'
30'/150'
Time point

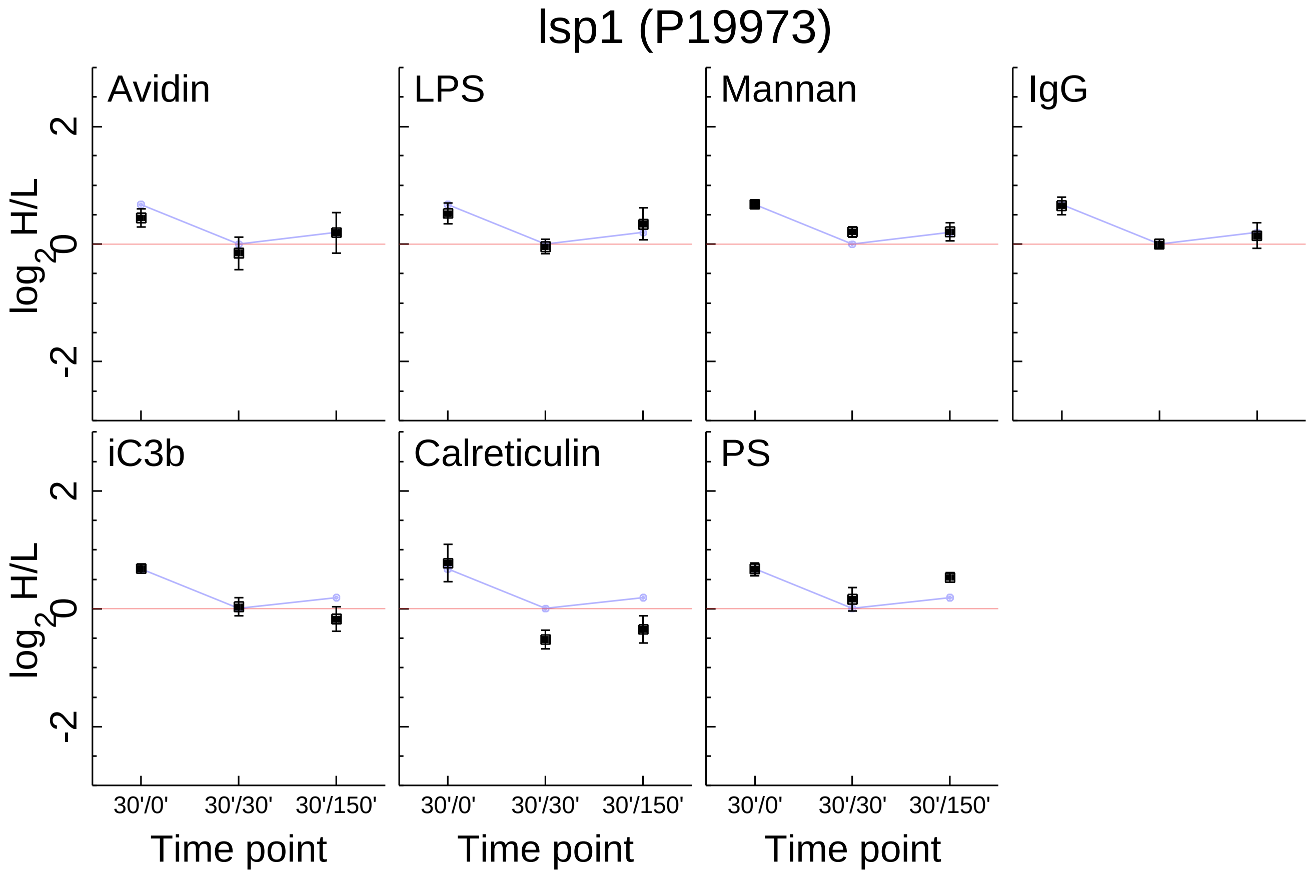
Isp1 (P19973)
Avidin
LPS
Mannan
IgG
iC3b
Calreticulin
PS
log2 H/L
2
0
-2
30'/0'
30'/30'
30'/150'
Time point

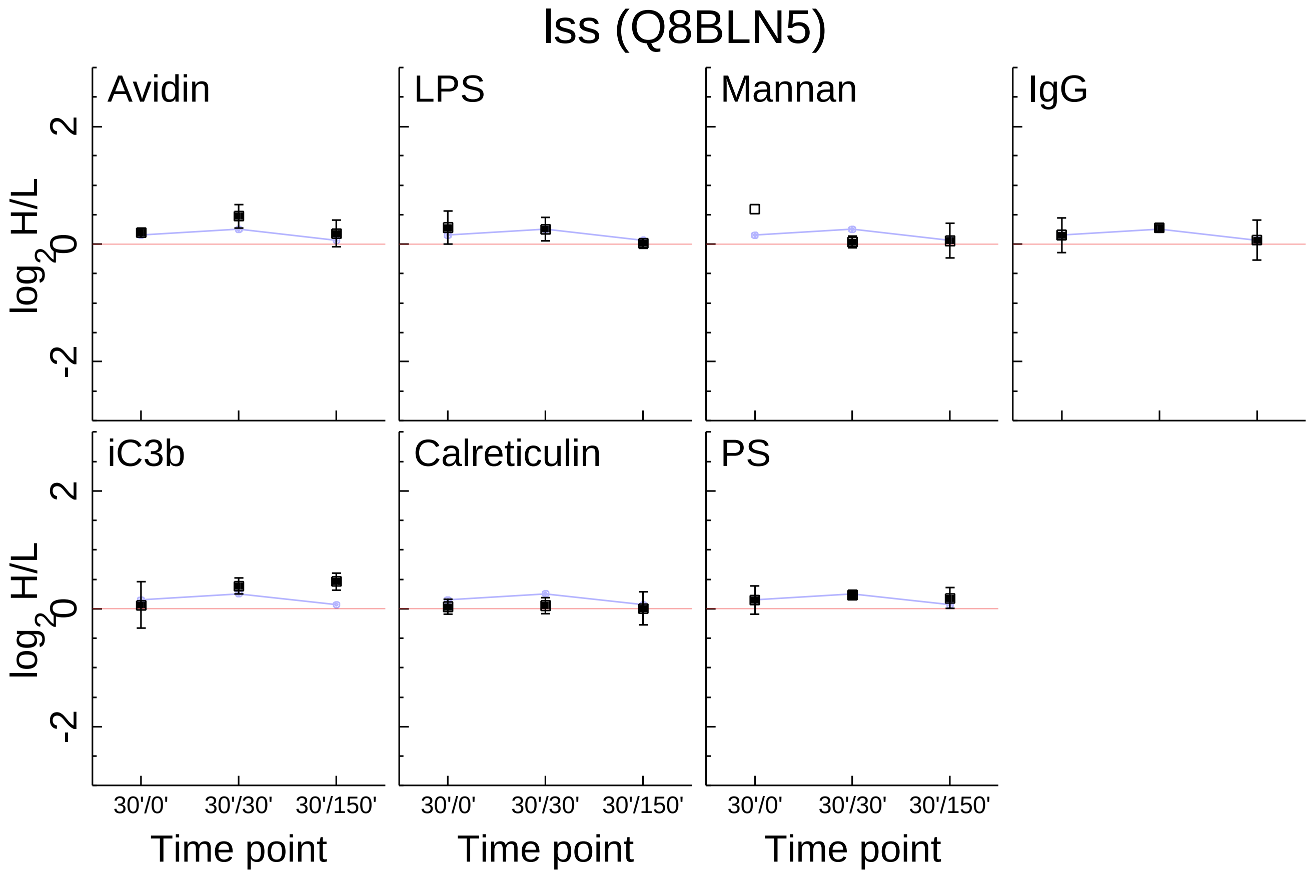
lss (Q8BLN5)
Avidin
LPS
Mannan
IgG
iC3b
Calreticulin
PS
log2 H/L
2
0
-2
30'/0'
30'/30'
30'/150'
Time point

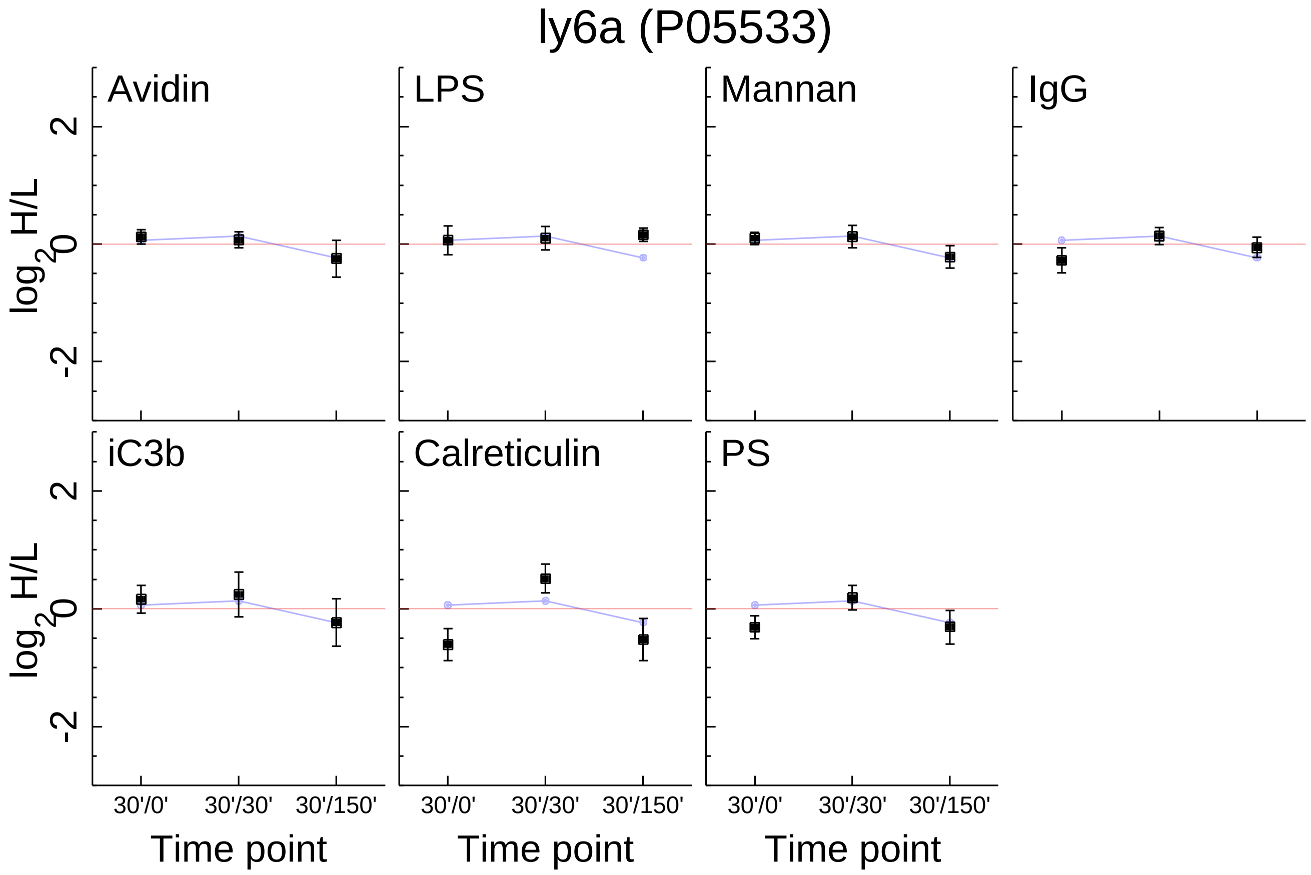
ly6a (P05533)
Avidin
LPS
Mannan
IgG
iC3b
Calreticulin
PS
log2 H/L
2
0
-2
30'/0'
30'/30'
30'/150'
Time point

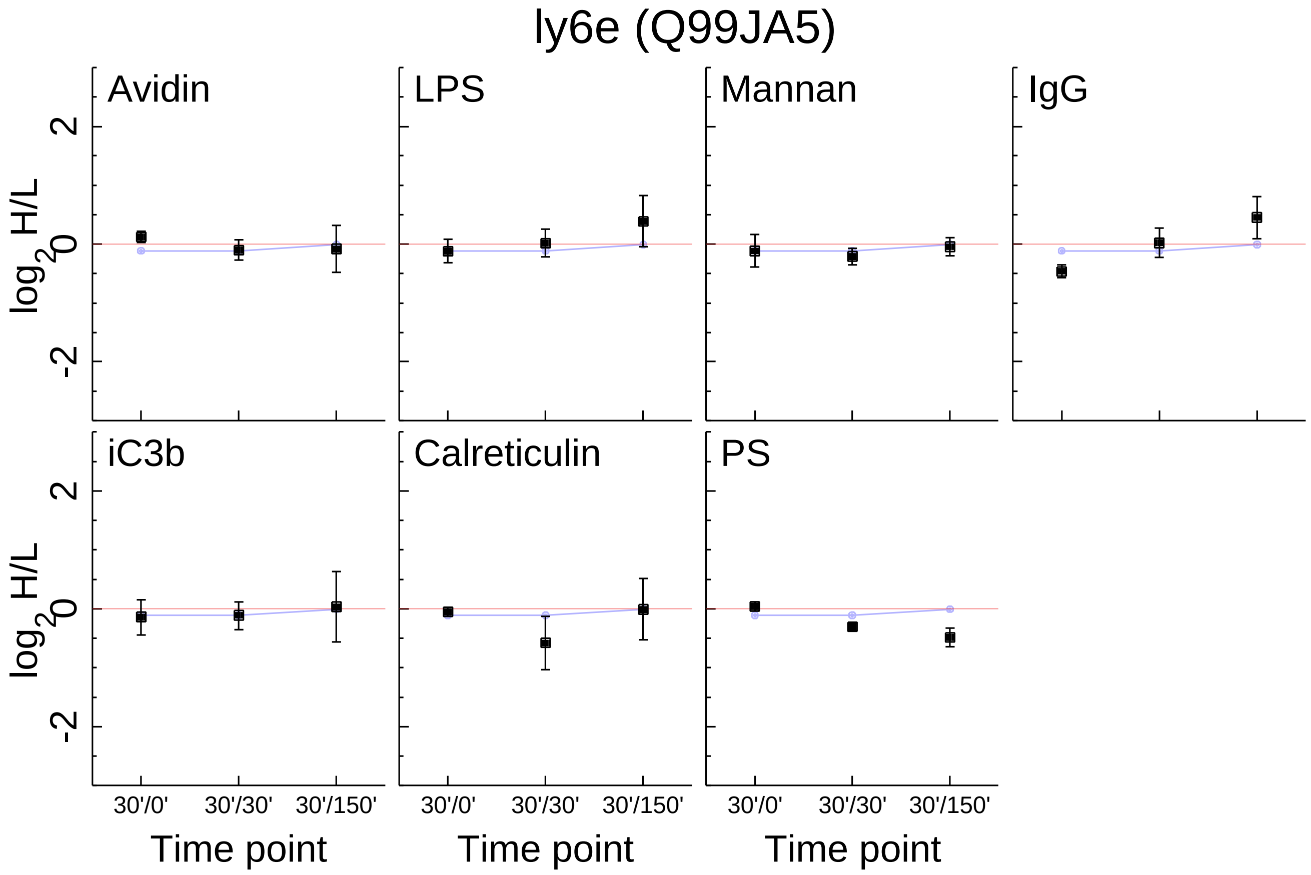

ly6e (Q99JA5)
Avidin
LPS
Mannan
IgG
iC3b
Calreticulin
PS
log2 H/L
2
0
-2
30'/0'
30'/30'
30'/150'
Time point

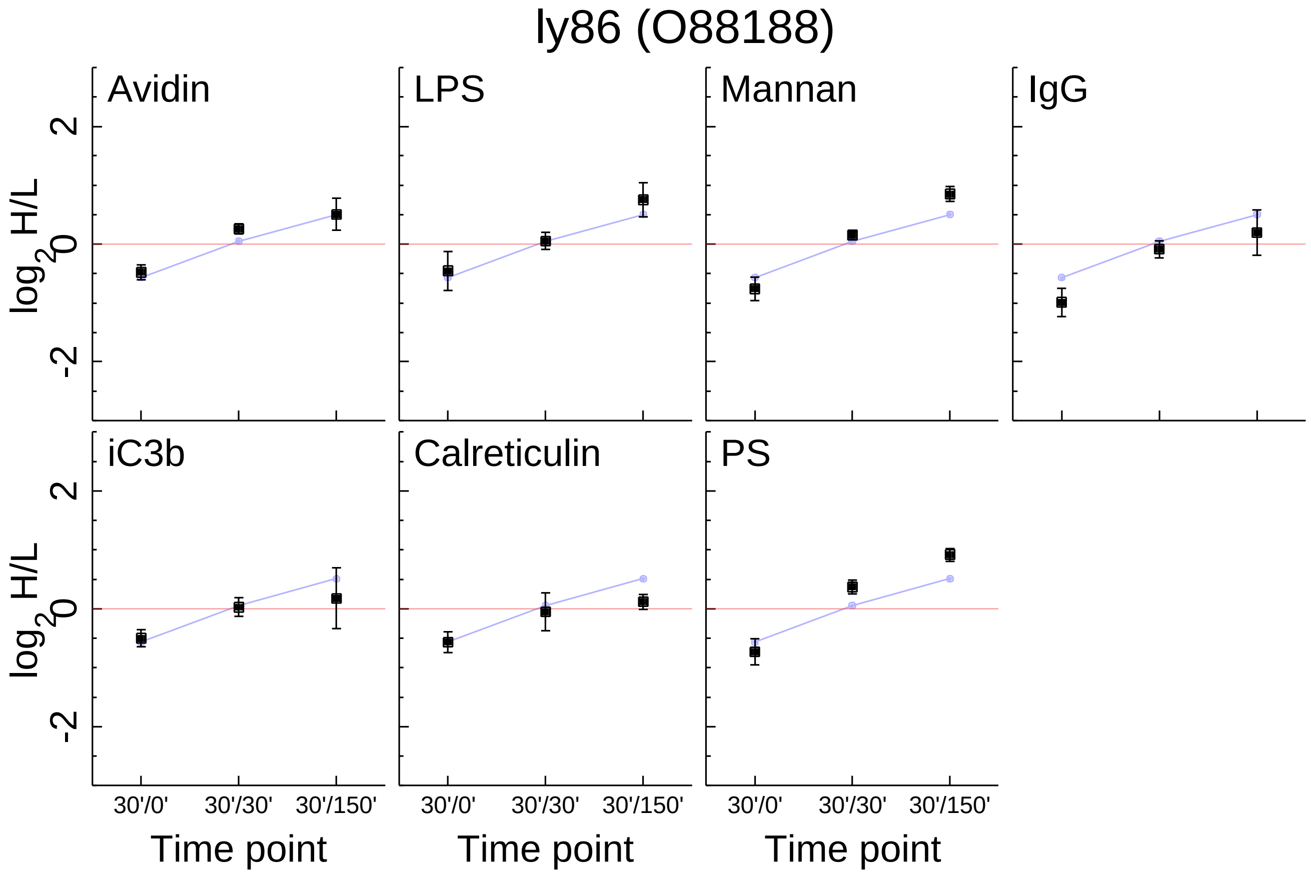
ly86 (O88188)
Avidin
LPS
Mannan
IgG
iC3b
Calreticulin
PS
log2 H/L
2
0
-2
30'/0'
30'/30'
30'/150'
Time point

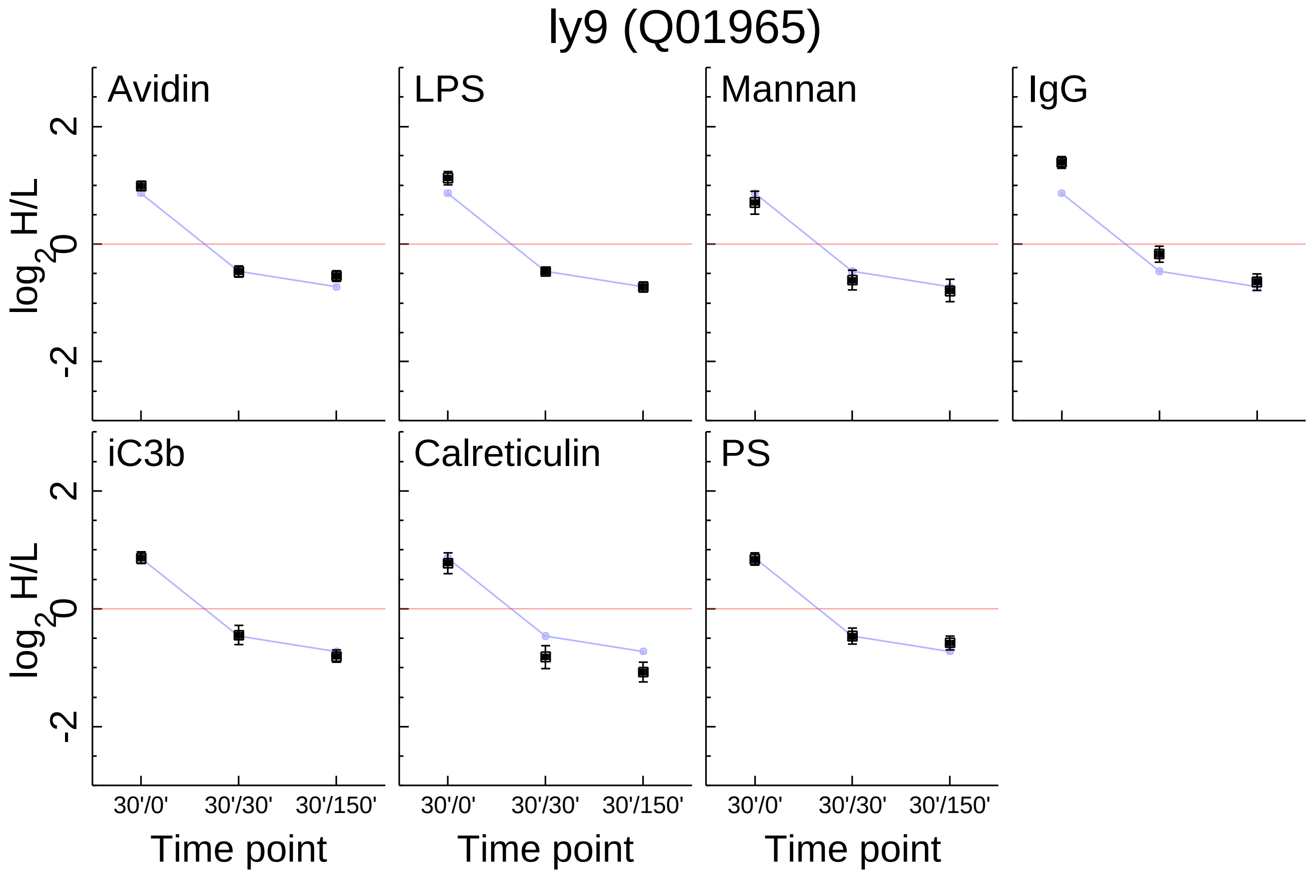
ly9 (Q01965)
Avidin
LPS
Mannan
IgG
iC3b
Calreticulin
PS
log2 H/L
2
0
-2
30'/0'
30'/30'
30'/150'
Time point

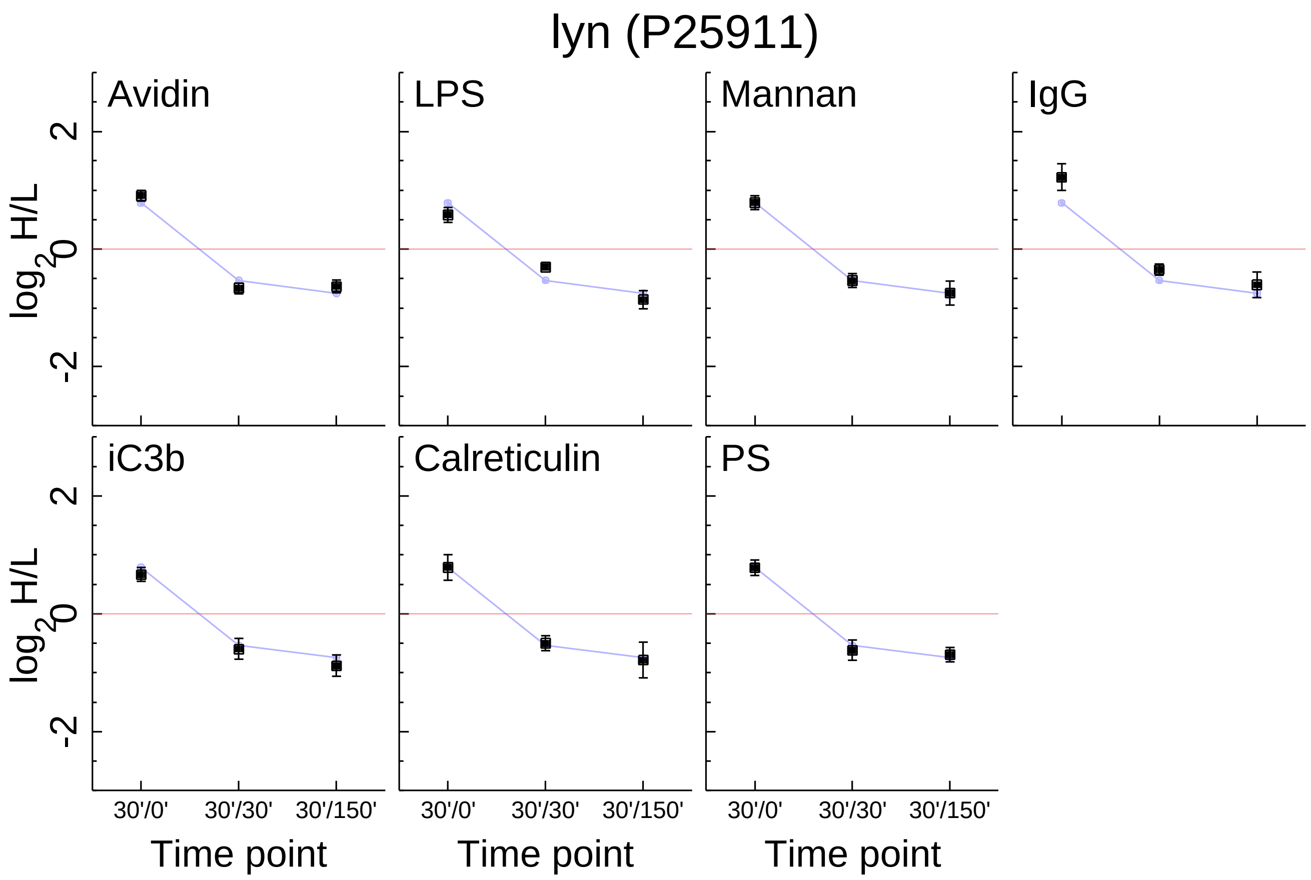
lyn (P25911)
Avidin
LPS
Mannan
IgG
iC3b
Calreticulin
PS
log2 H/L
2
0
-2
30'/0'
30'/30'
30'/150'
Time point

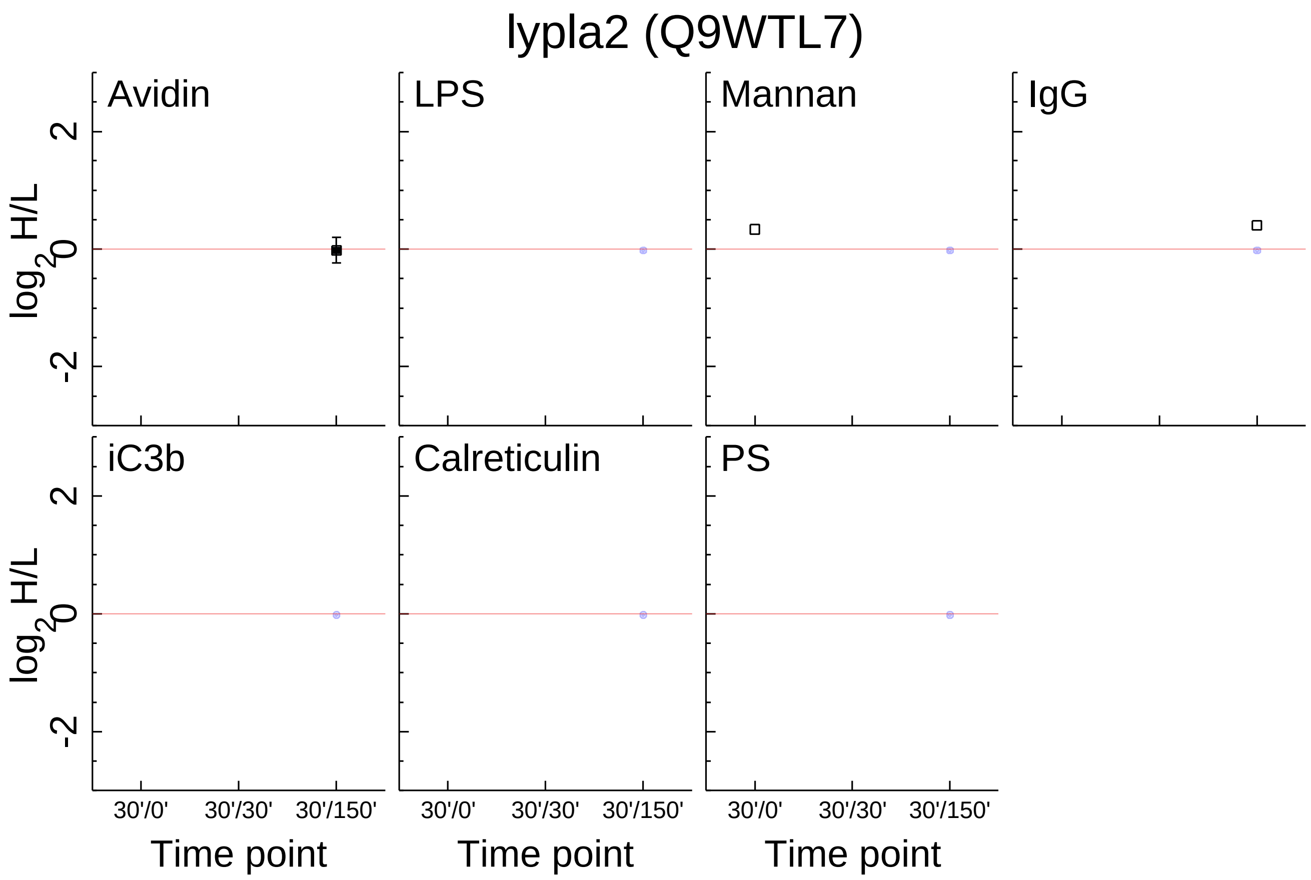
lypla2 (Q9WTL7)
Avidin
LPS
Mannan
IgG
iC3b
Calreticulin
PS
log2 H/L
2
0
-2
30'/0'
30'/30'
30'/150'
Time point

lyz1 (P17897)

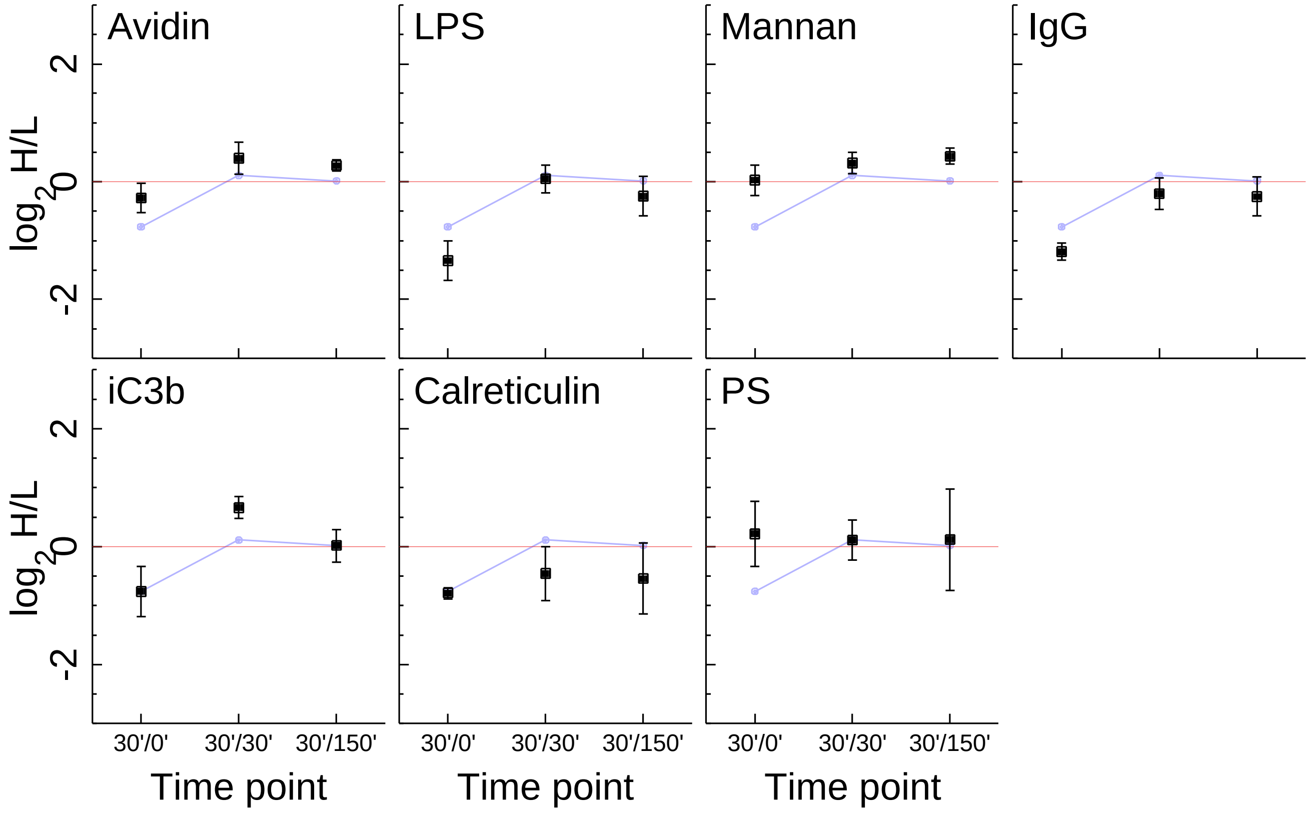

Avidin
LPS
Mannan
IgG
iC3b
Calreticulin
PS
log2 H/L
2
0
-2
30'/0'
30'/30'
30'/150'
Time point

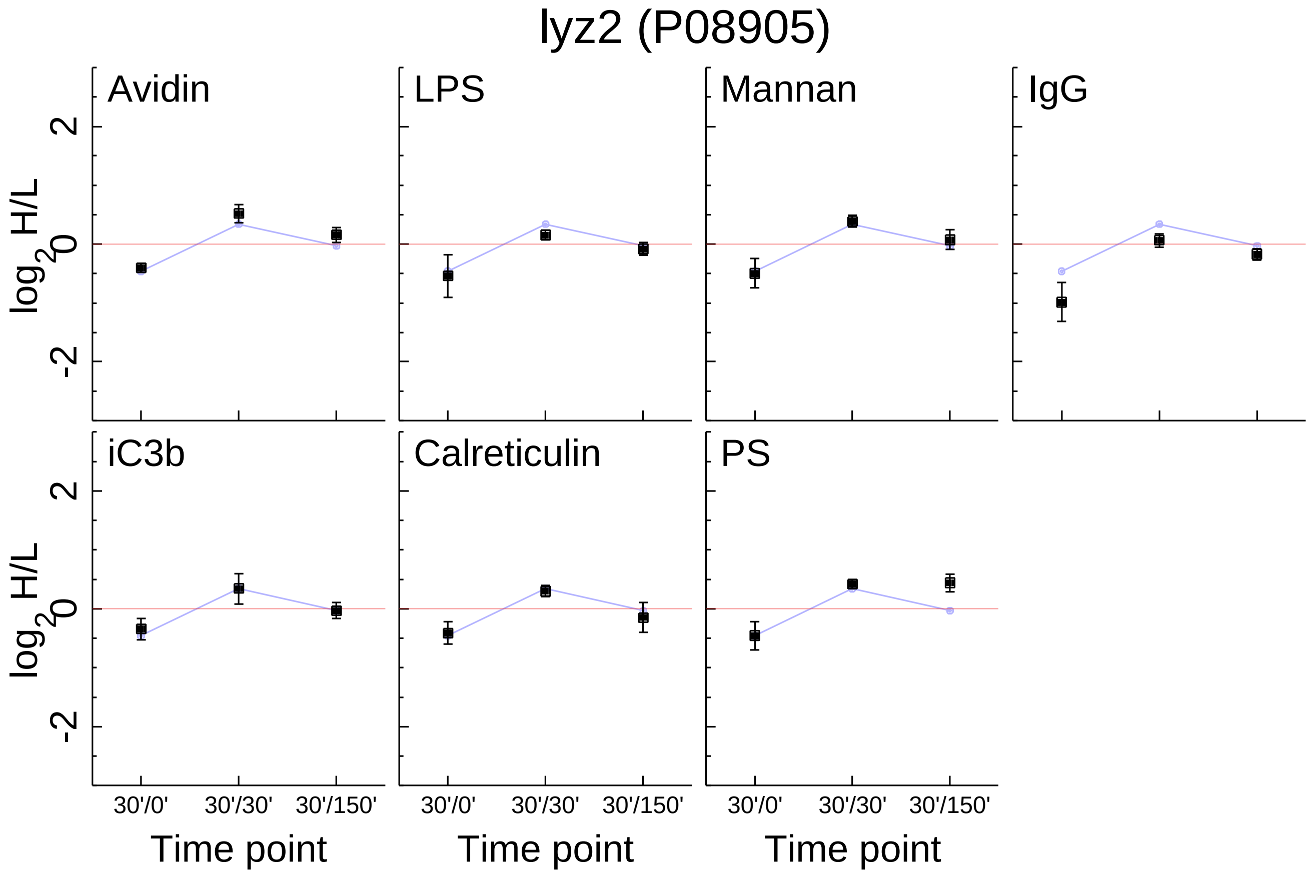

lyz2 (P08905)
Avidin
LPS
Mannan
IgG
iC3b
Calreticulin
PS
log2 H/L
2
0
-2
30'/0'
30'/30'
30'/150'
Time point

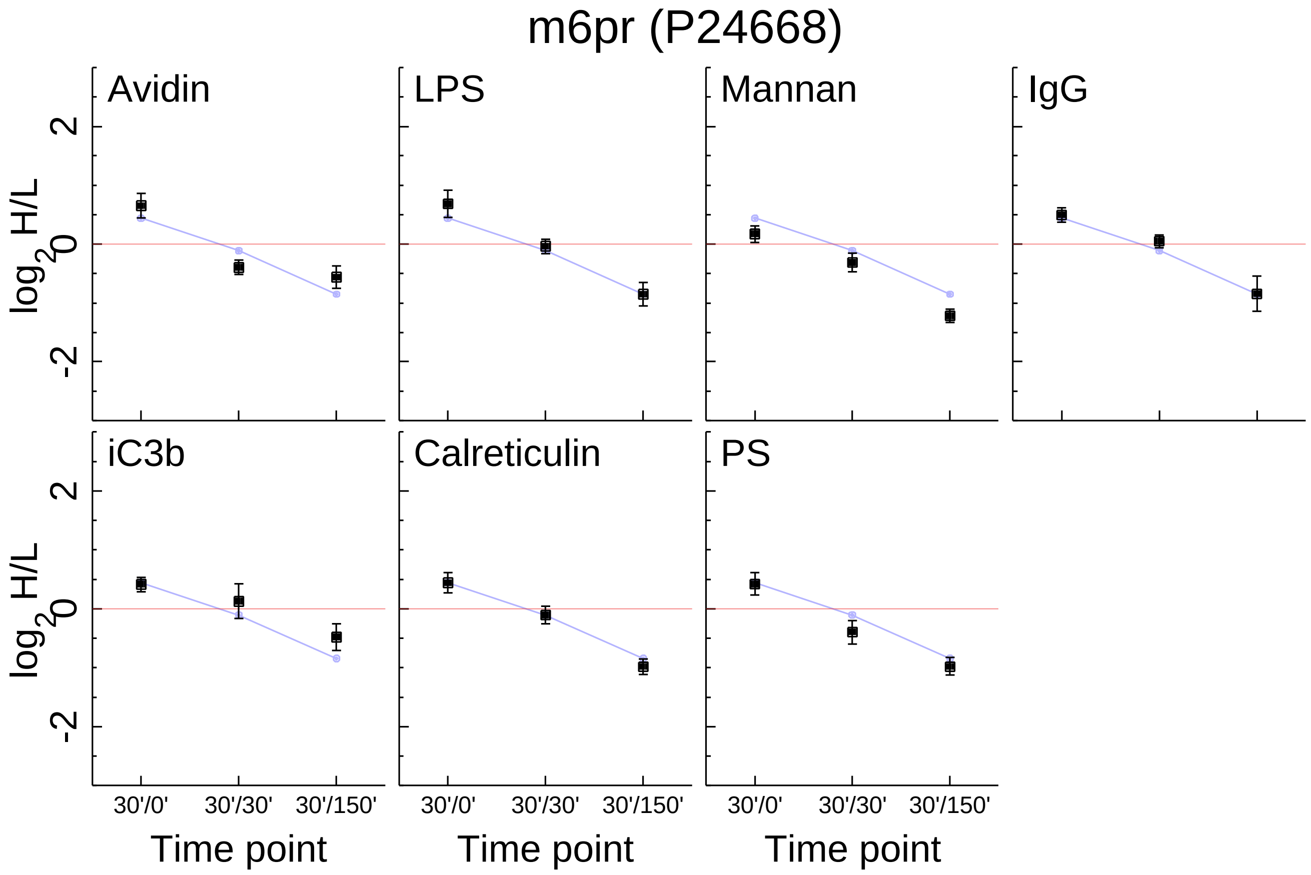

m6pr (P24668)
Avidin
LPS
Mannan
IgG
iC3b
Calreticulin
PS
log2 H/L
2
0
-2
30'/0'
30'/30'
30'/150'
Time point

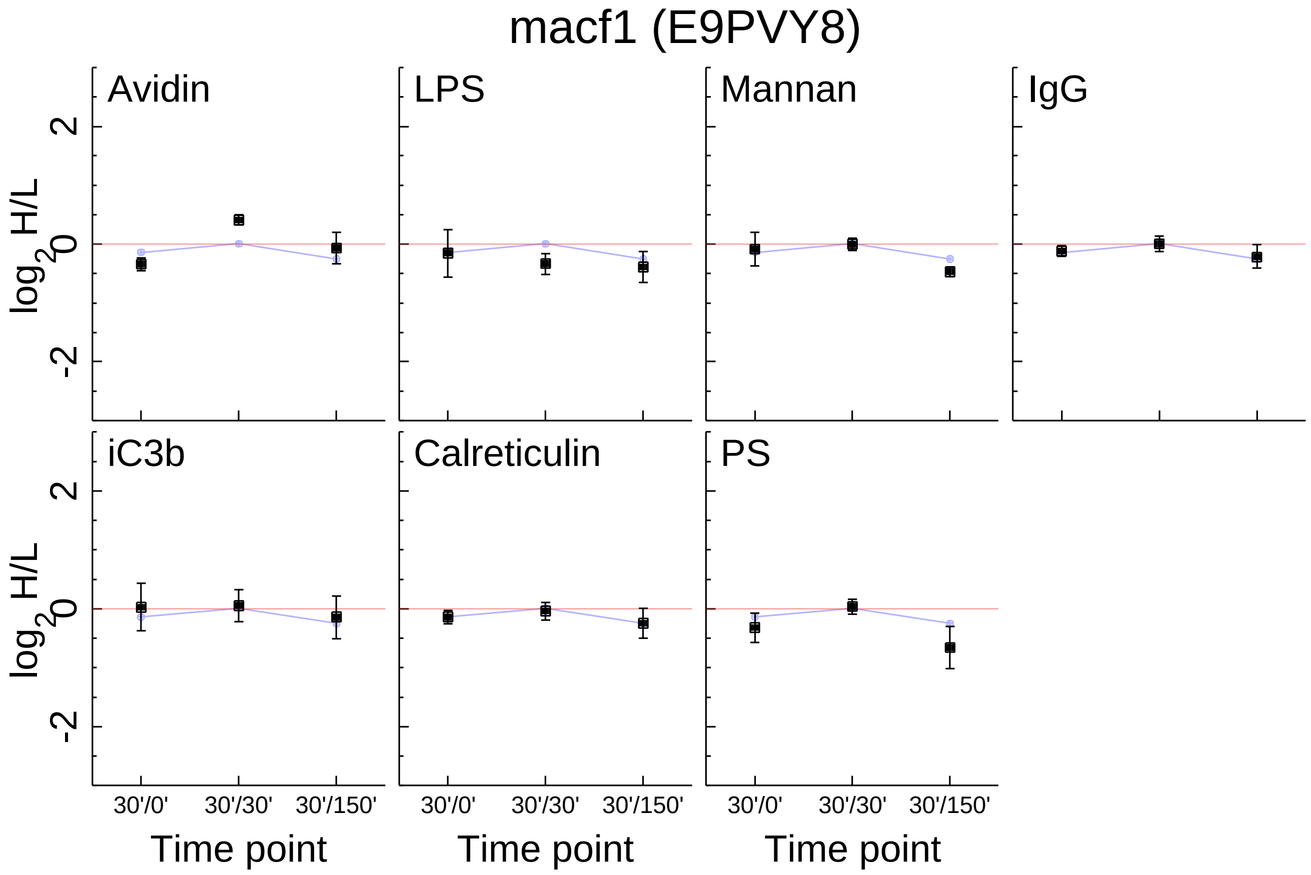
macf1 (E9PVY8)
Avidin
LPS
Mannan
IgG
iC3b
Calreticulin
PS
log2 H/L
2
0
-2
30'/0'
30'/30'
30'/150'
Time point

magt1 (A2ADH1)

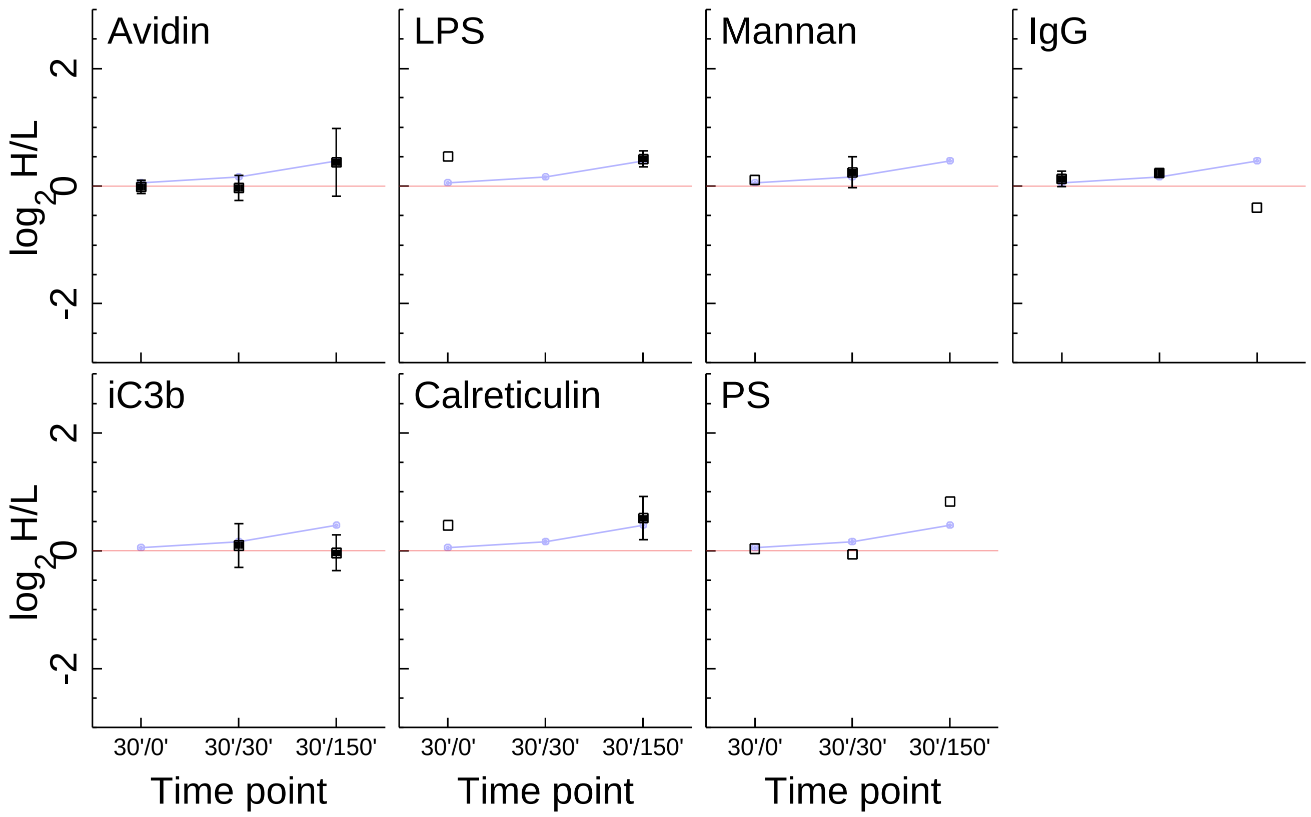

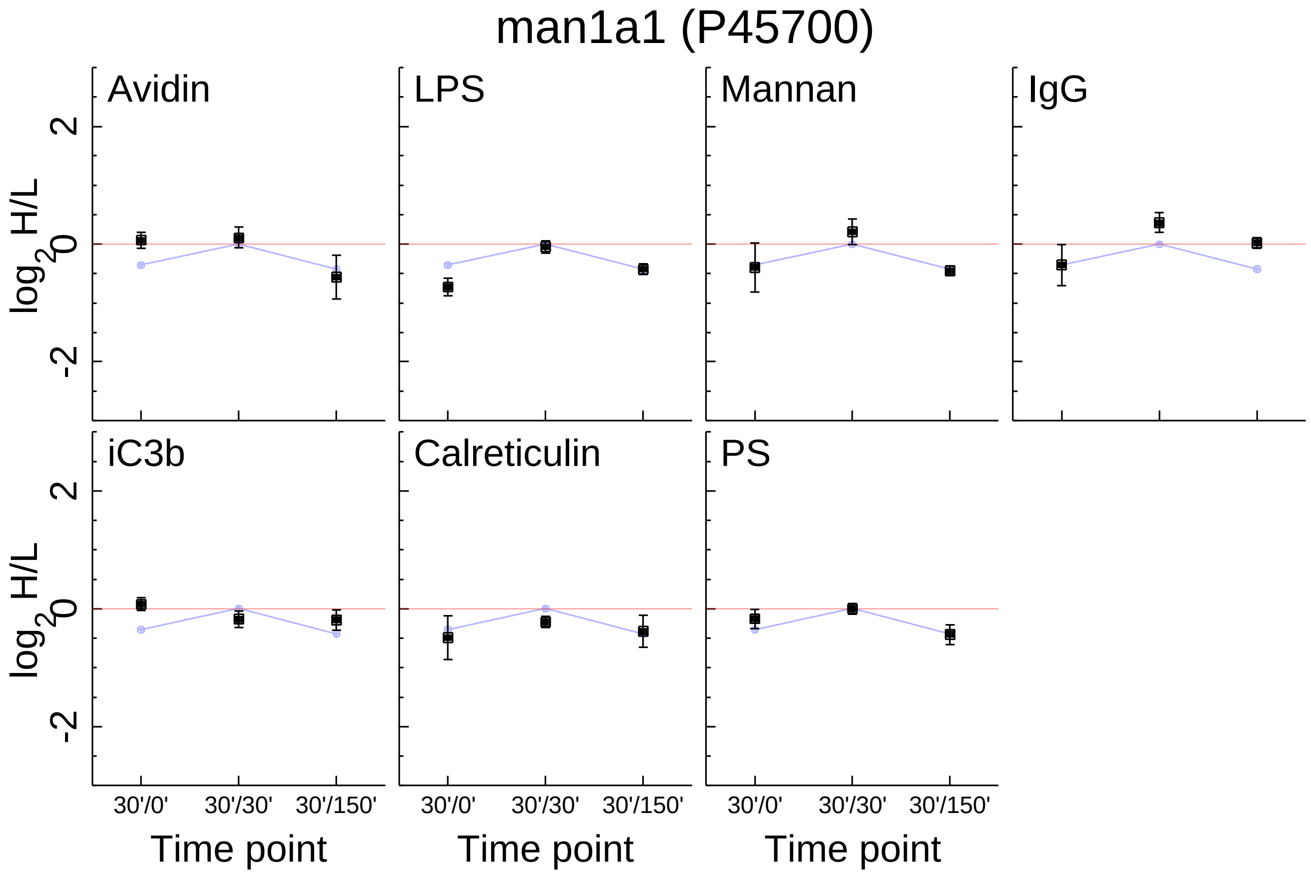
man1a1 (P45700)
Avidin
LPS
Mannan
IgG
iC3b
Calreticulin
PS
log2 H/L
2
0
-2
30'/0'
30'/30'
30'/150'
Time point

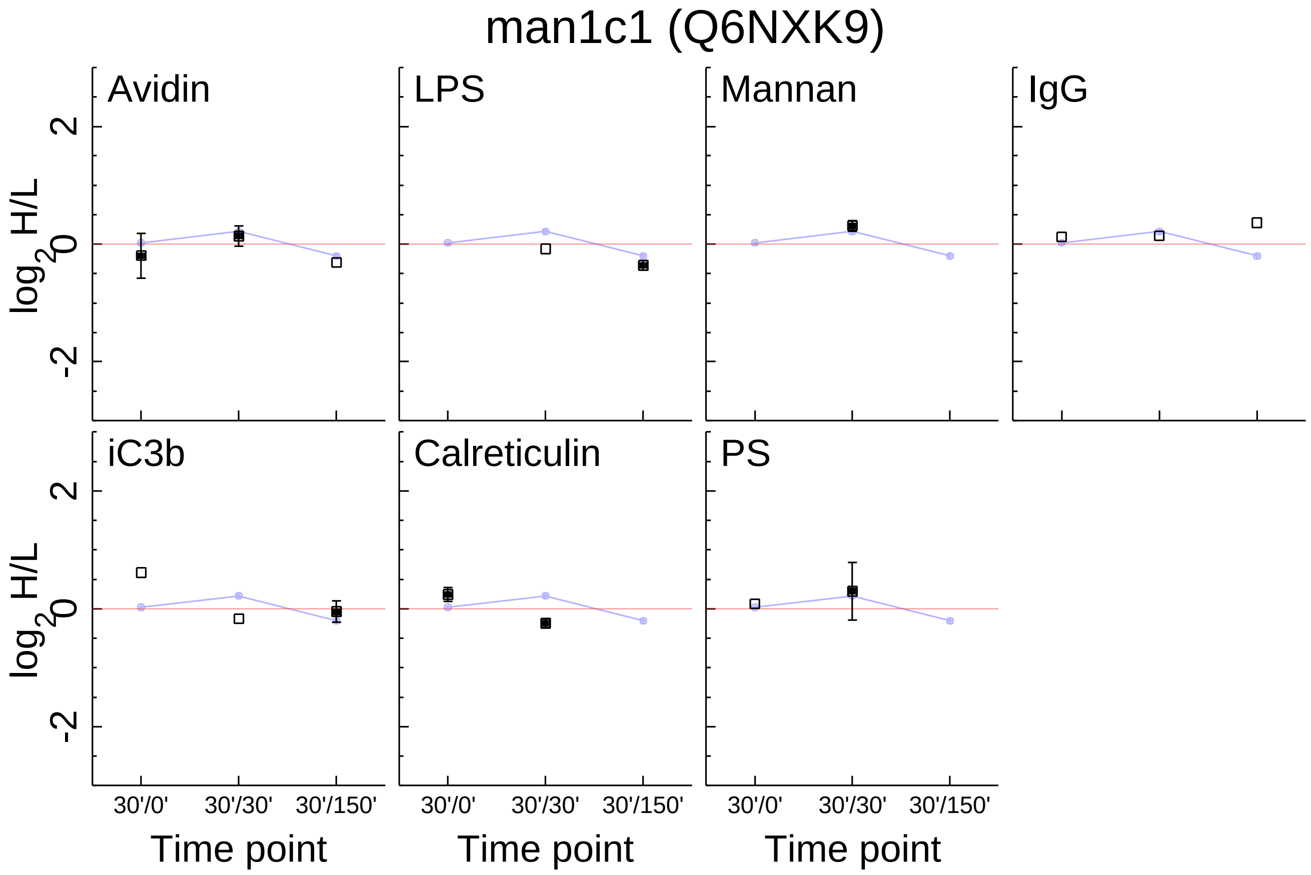

man1c1 (Q6NXK9)
Avidin
LPS
Mannan
IgG
iC3b
Calreticulin
PS
log2 H/L
2
0
-2
30'/0'
30'/30'
30'/150'
Time point

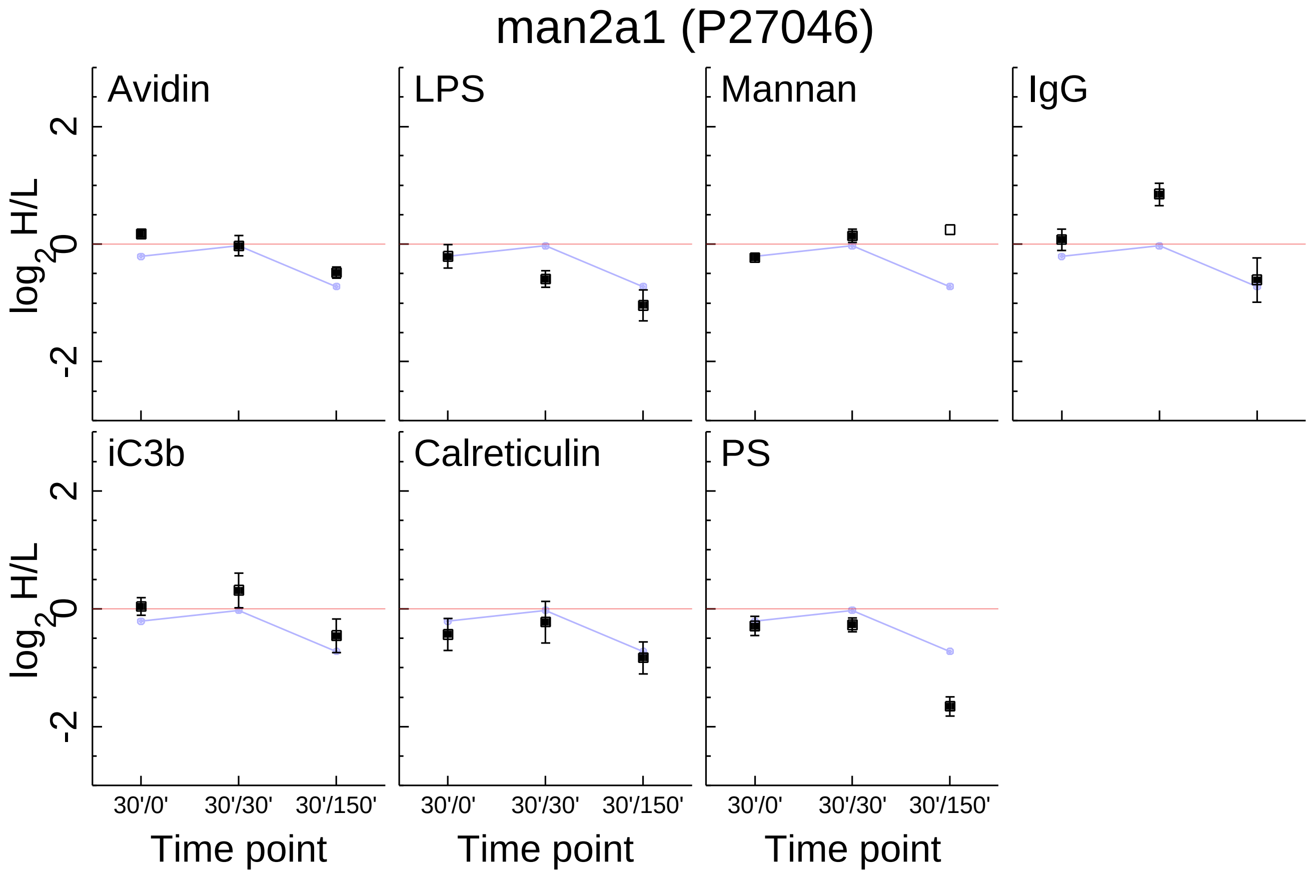

man2a1 (P27046)
Avidin
LPS
Mannan
IgG
iC3b
Calreticulin
PS
log2 H/L
2
0
-2
30'/0'
30'/30'
30'/150'
Time point

man2b1 (O09159)

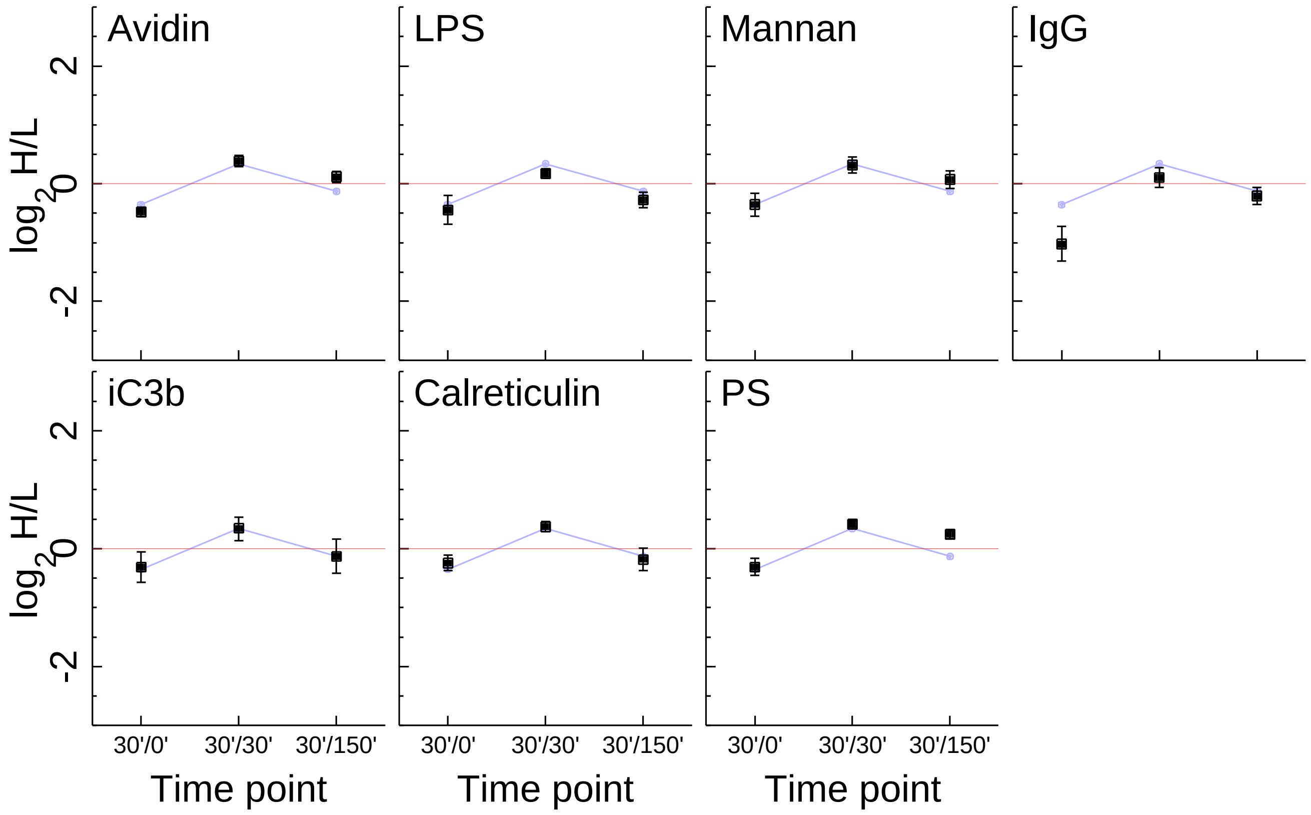

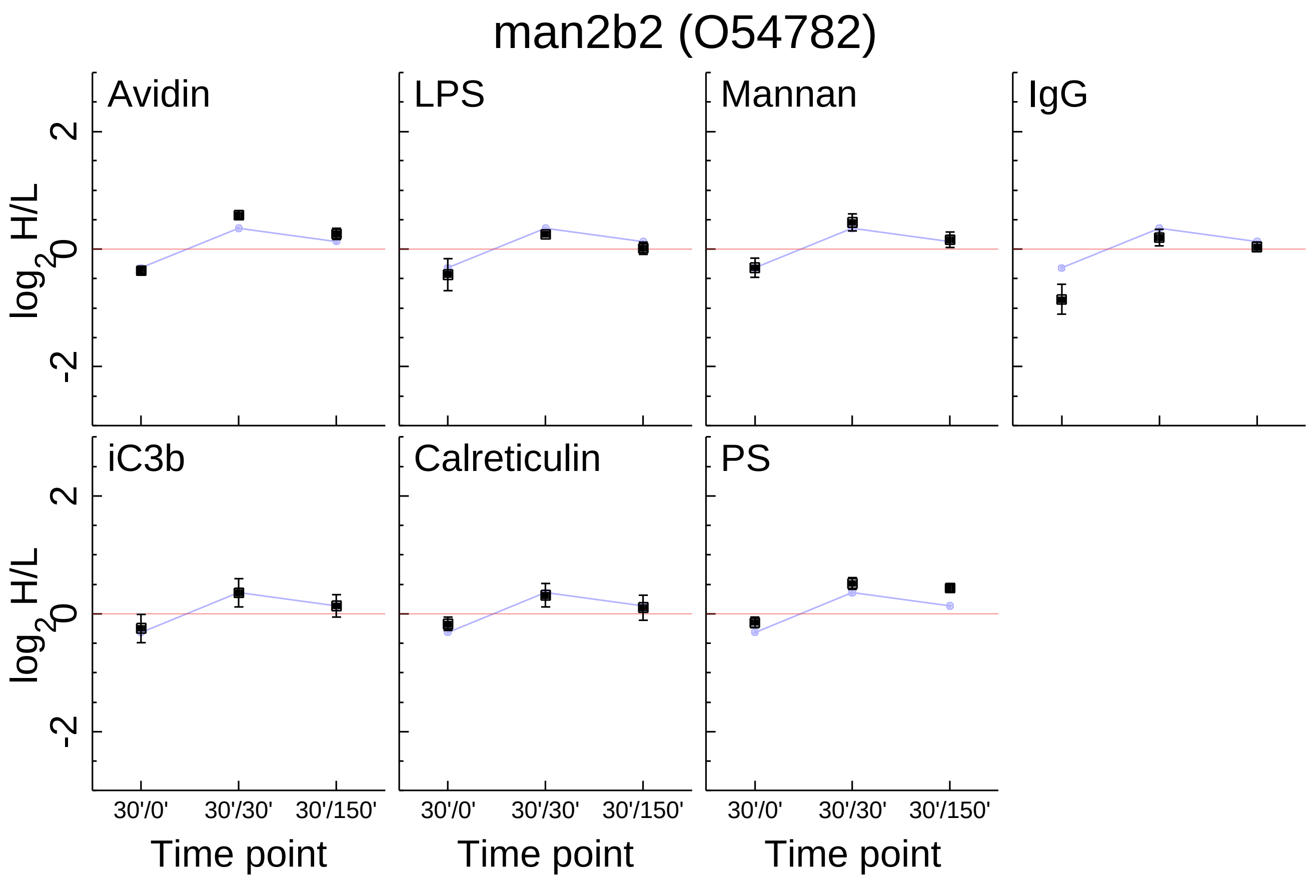

man2b2 (O54782)
Avidin
LPS
Mannan
IgG
iC3b
Calreticulin
PS
log2 H/L
2
0
-2
30'/0'
30'/30'
30'/150'
Time point

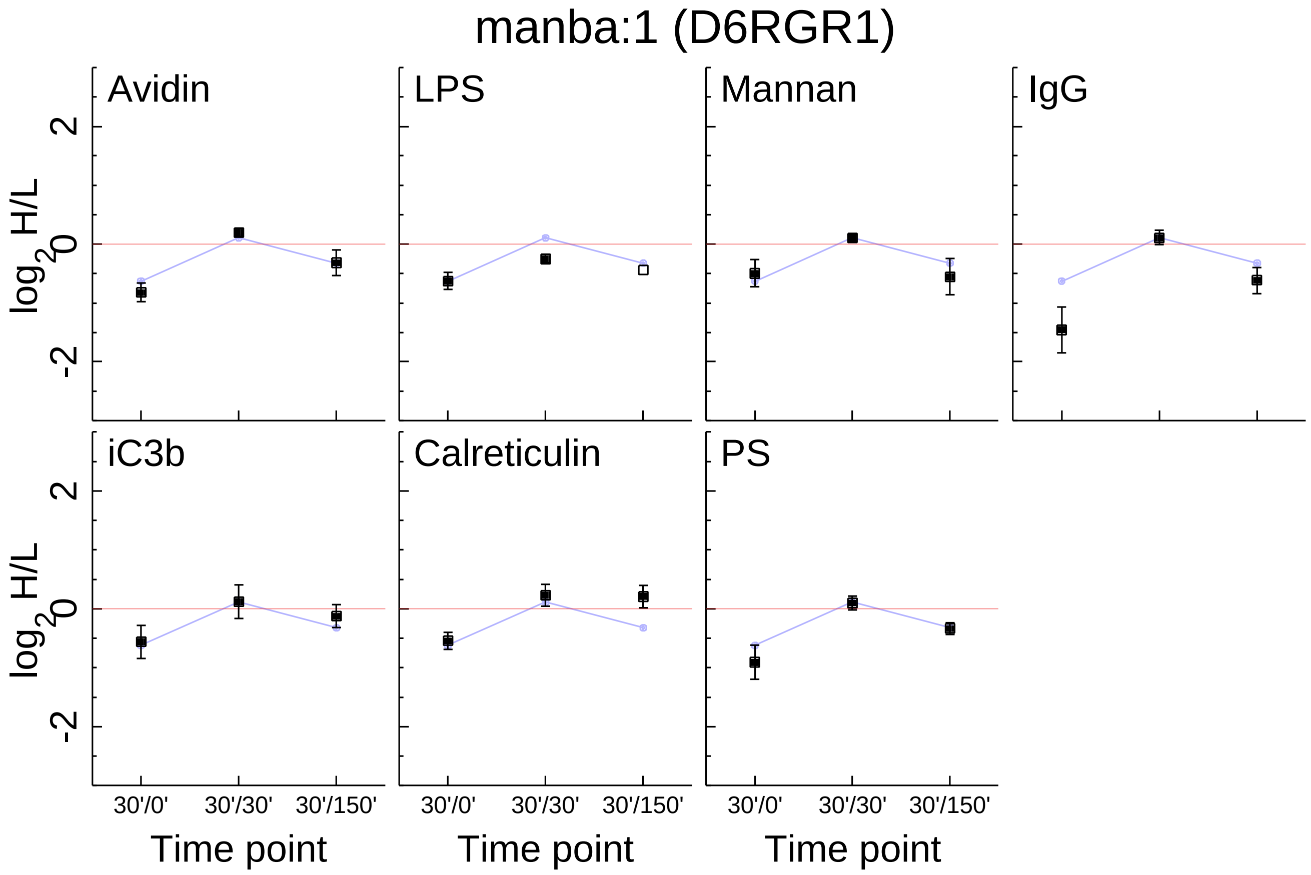
manba:1 (D6RGR1)
Avidin
LPS
Mannan
IgG
iC3b
Calreticulin
PS
log2 H/L
2
0
-2
30'/0'
30'/30'
30'/150'
Time point

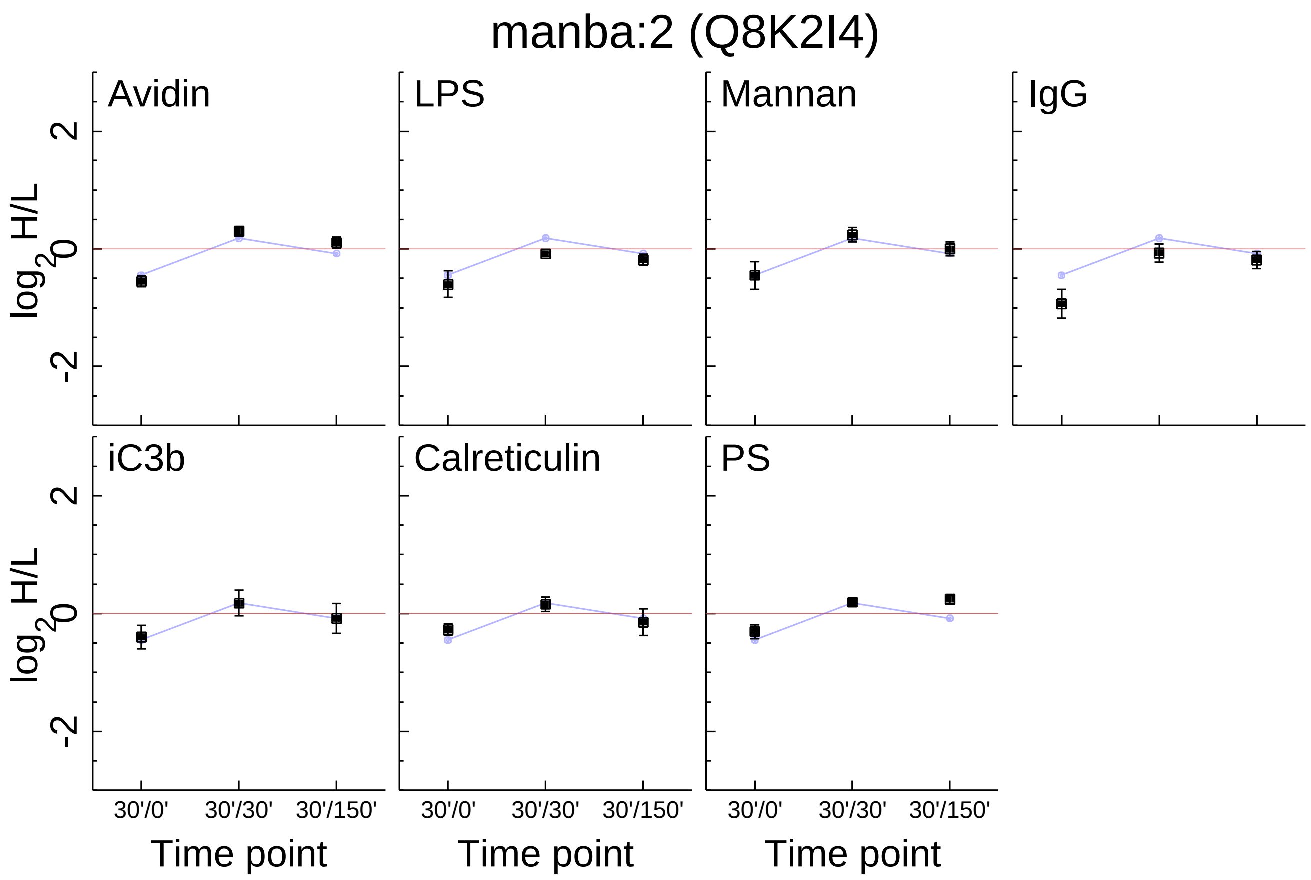

manba:2 (Q8K2I4)
Avidin
LPS
Mannan
IgG
iC3b
Calreticulin
PS
log2 H/L
2
0
-2
30'/0'
30'/30'
30'/150'
Time point

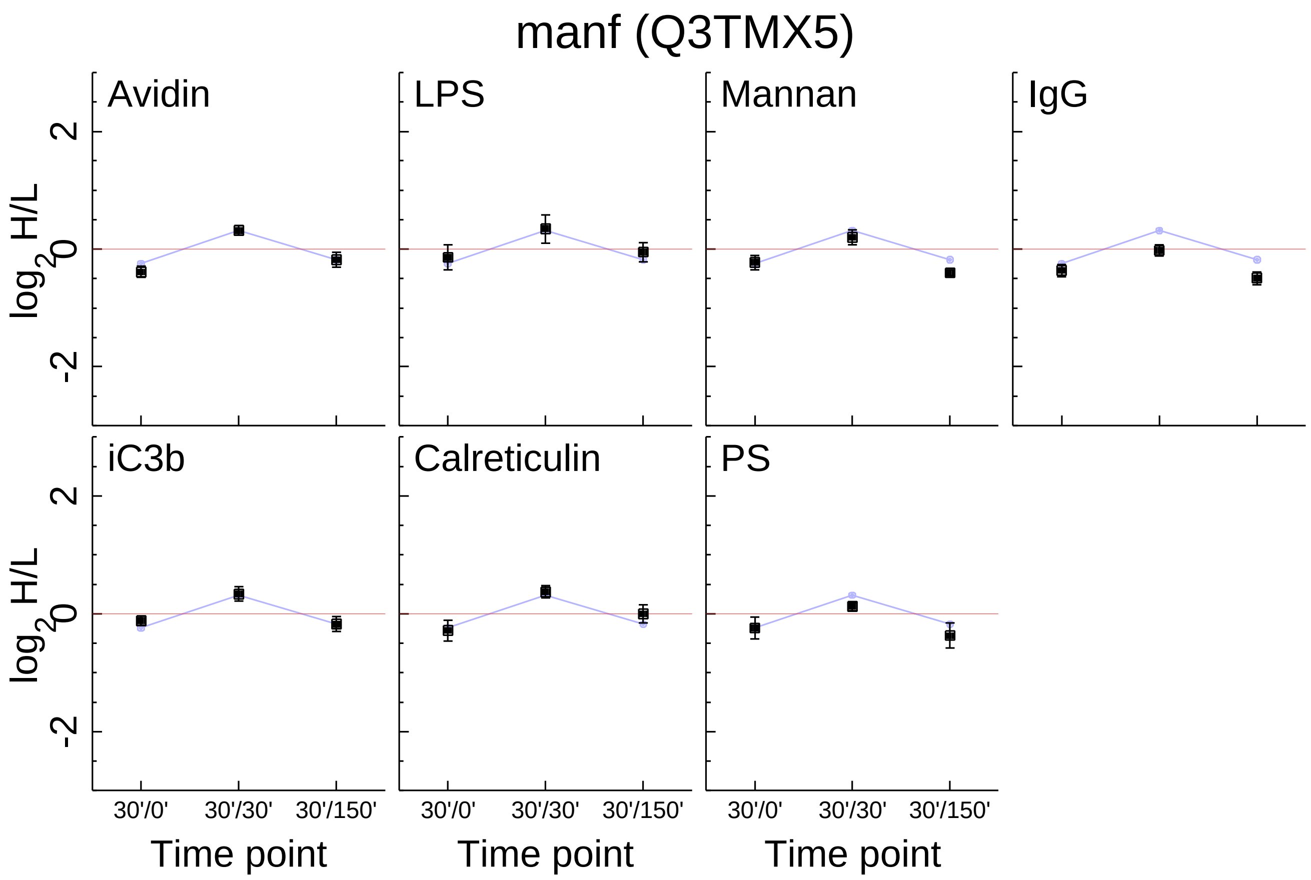
manf (Q3TMX5)
Avidin
LPS
Mannan
IgG
iC3b
Calreticulin
PS
log2 H/L
2
0
-2
30'/0'
30'/30'
30'/150'
Time point

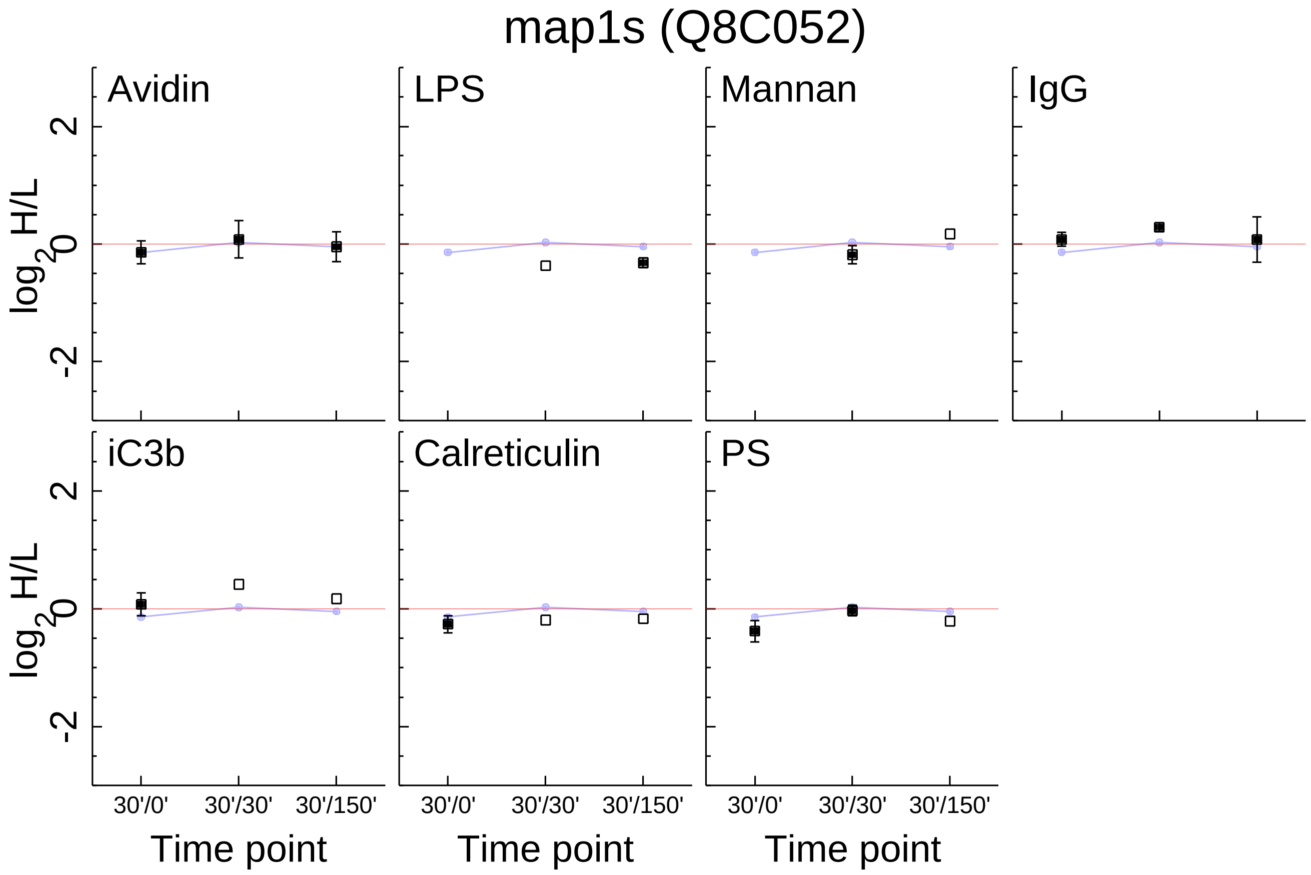

map1s (Q8C052)
Avidin
LPS
Mannan
IgG
iC3b
Calreticulin
PS
log2 H/L
2
0
-2
30'/0'
30'/30'
30'/150'
Time point

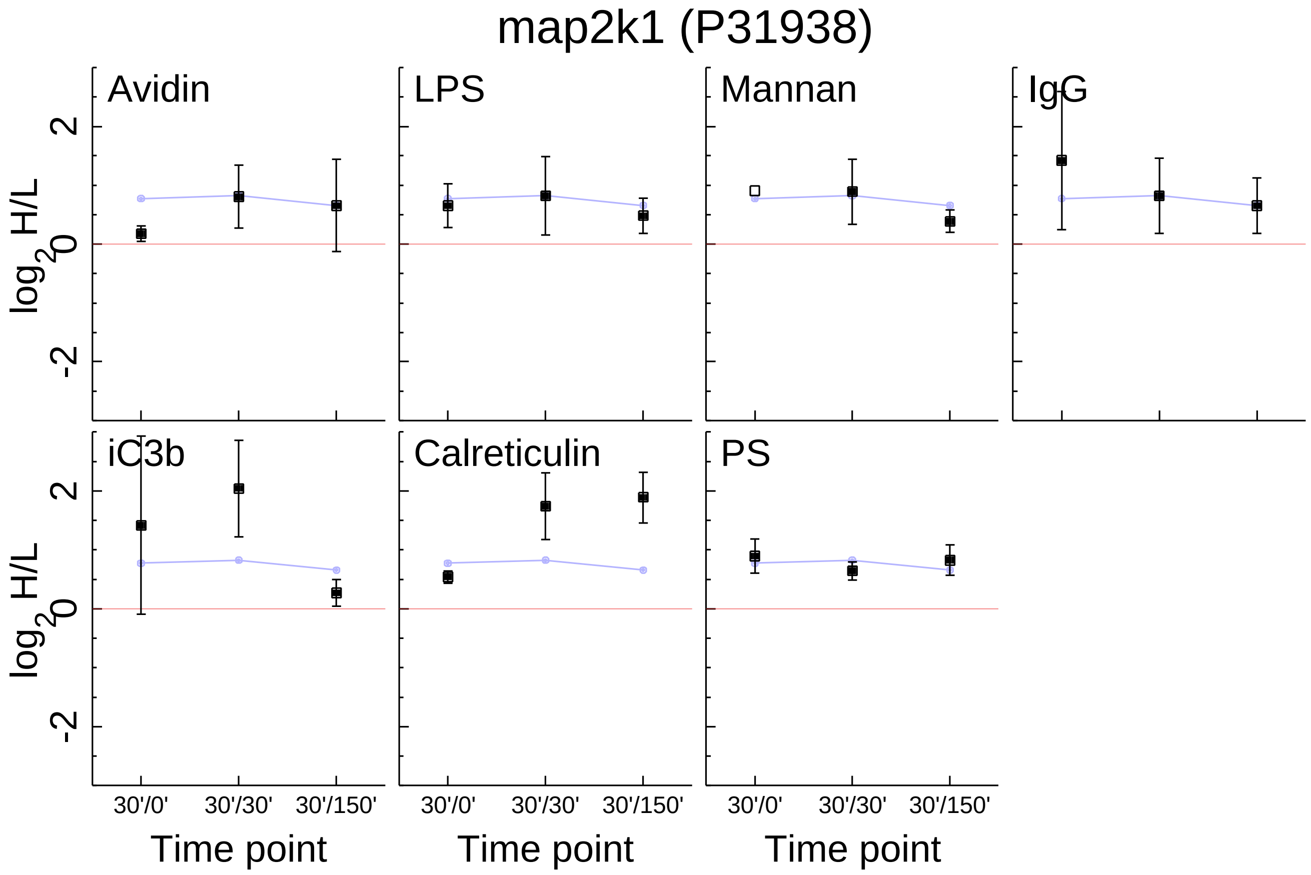

map2k1 (P31938)
Avidin
LPS
Mannan
IgG
iC3b
Calreticulin
PS
log2 H/L
2
0
-2
30'/0'
30'/30'
30'/150'
Time point

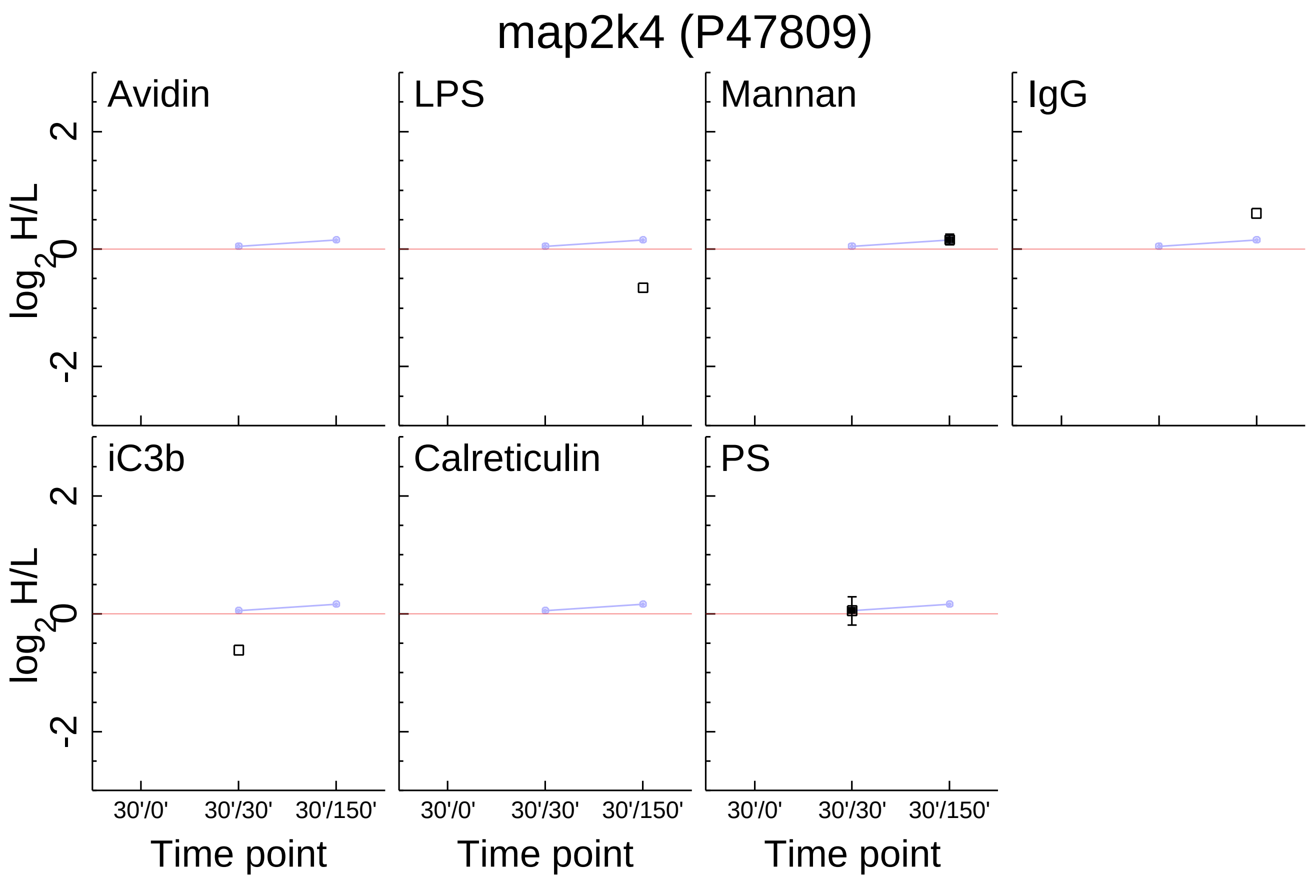
map2k4 (P47809)
Avidin
LPS
Mannan
IgG
iC3b
Calreticulin
PS
log2 H/L
2
0
-2
30'/0'
30'/30'
30'/150'
Time point

map3k11 (Q80XI6)

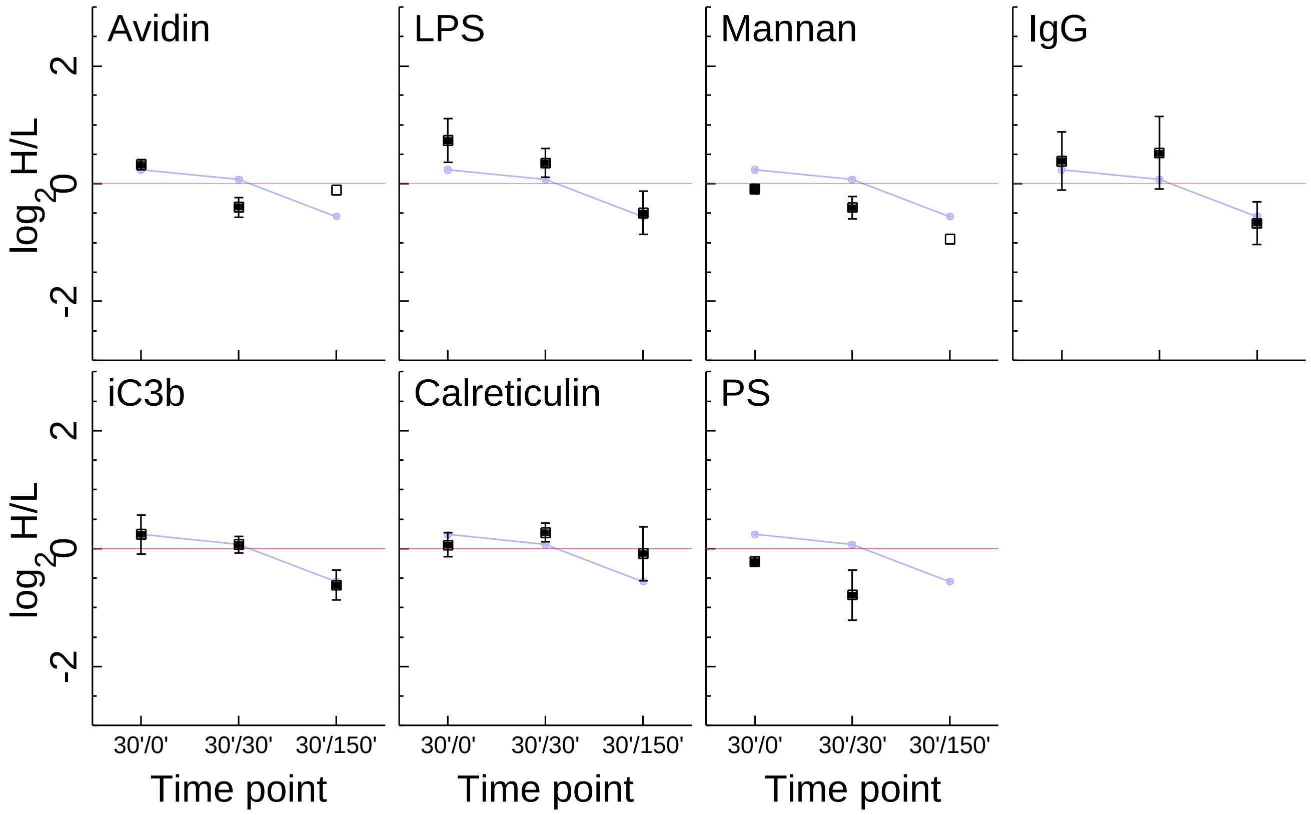

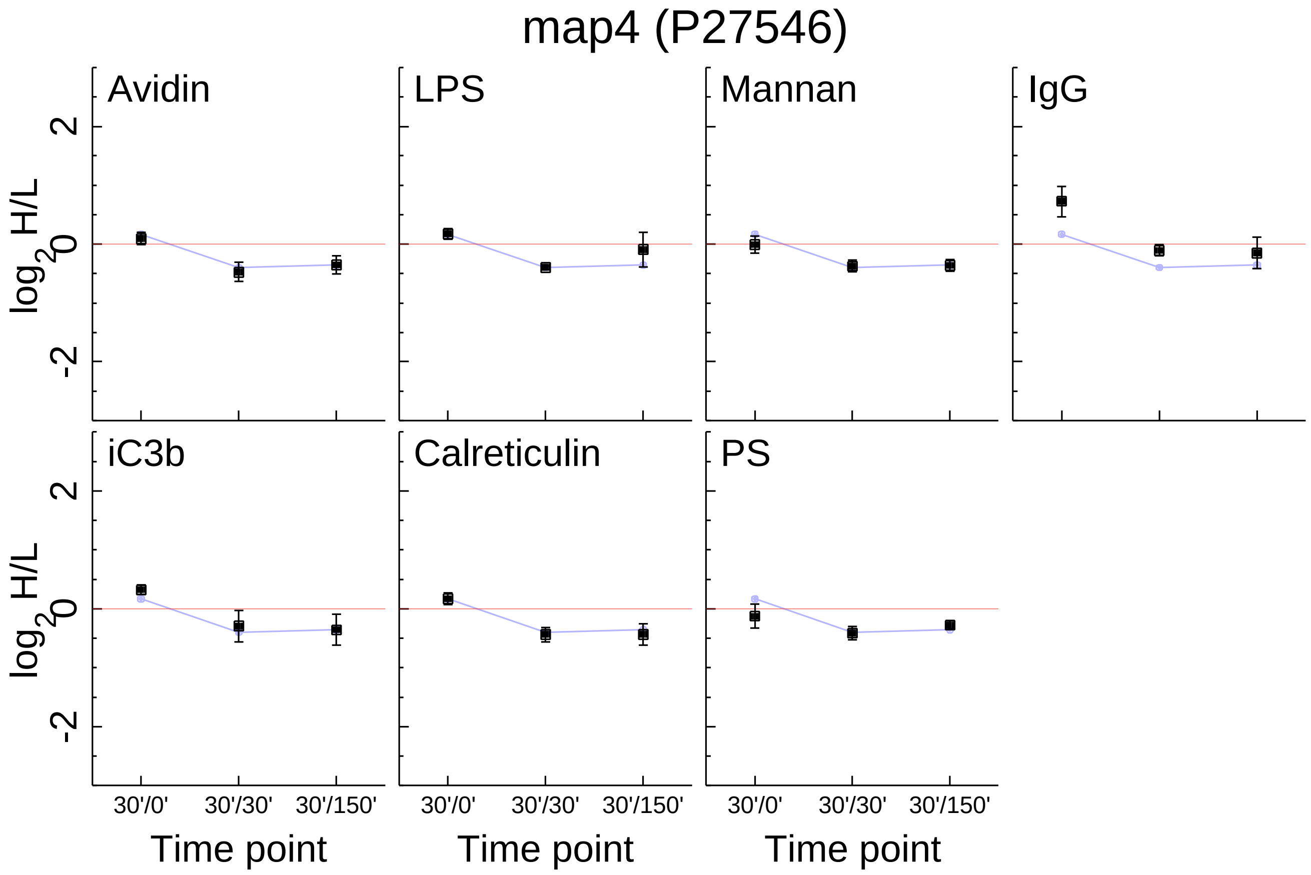
map4 (P27546)
Avidin
LPS
Mannan
IgG
iC3b
Calreticulin
PS
log2 H/L
2
0
-2
30'/0'
30'/30'
30'/150'
Time point

mapre1 (Q61166)

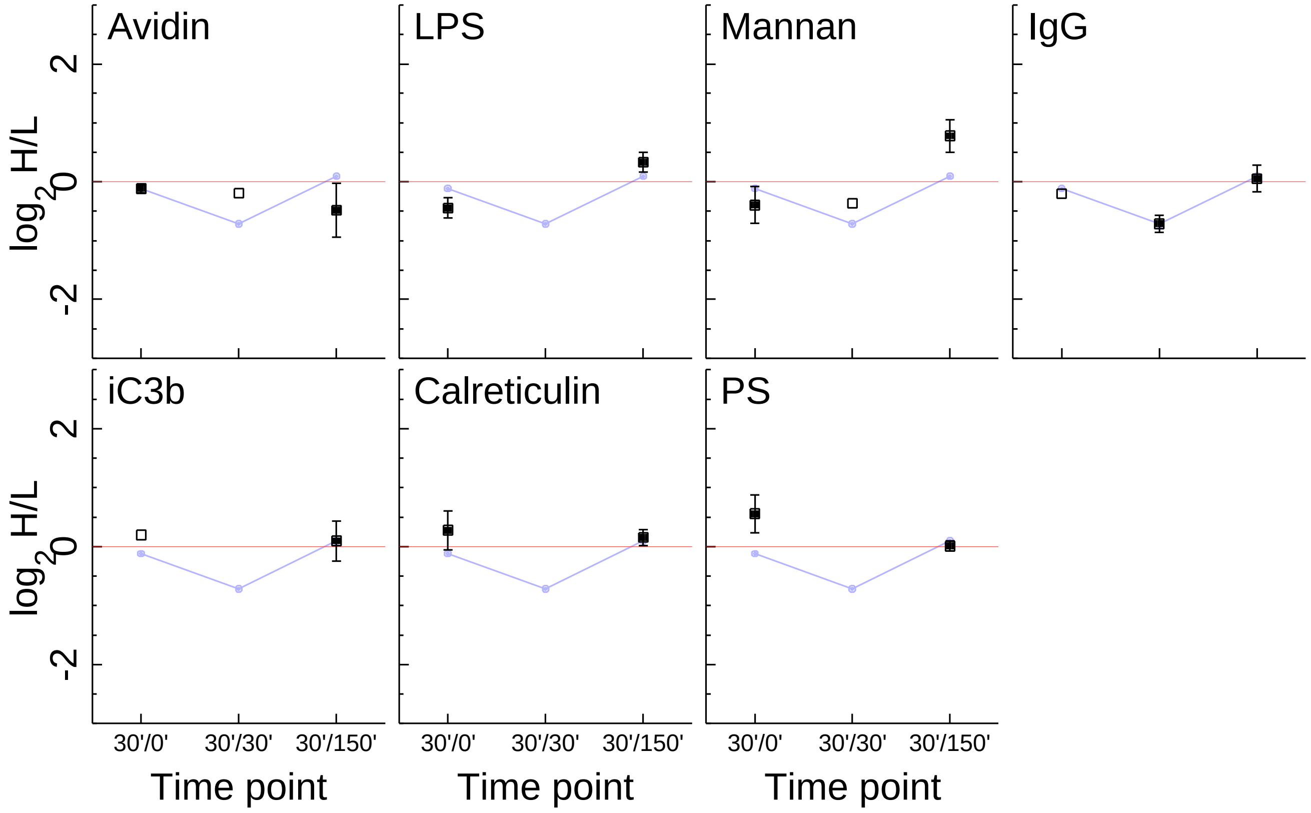

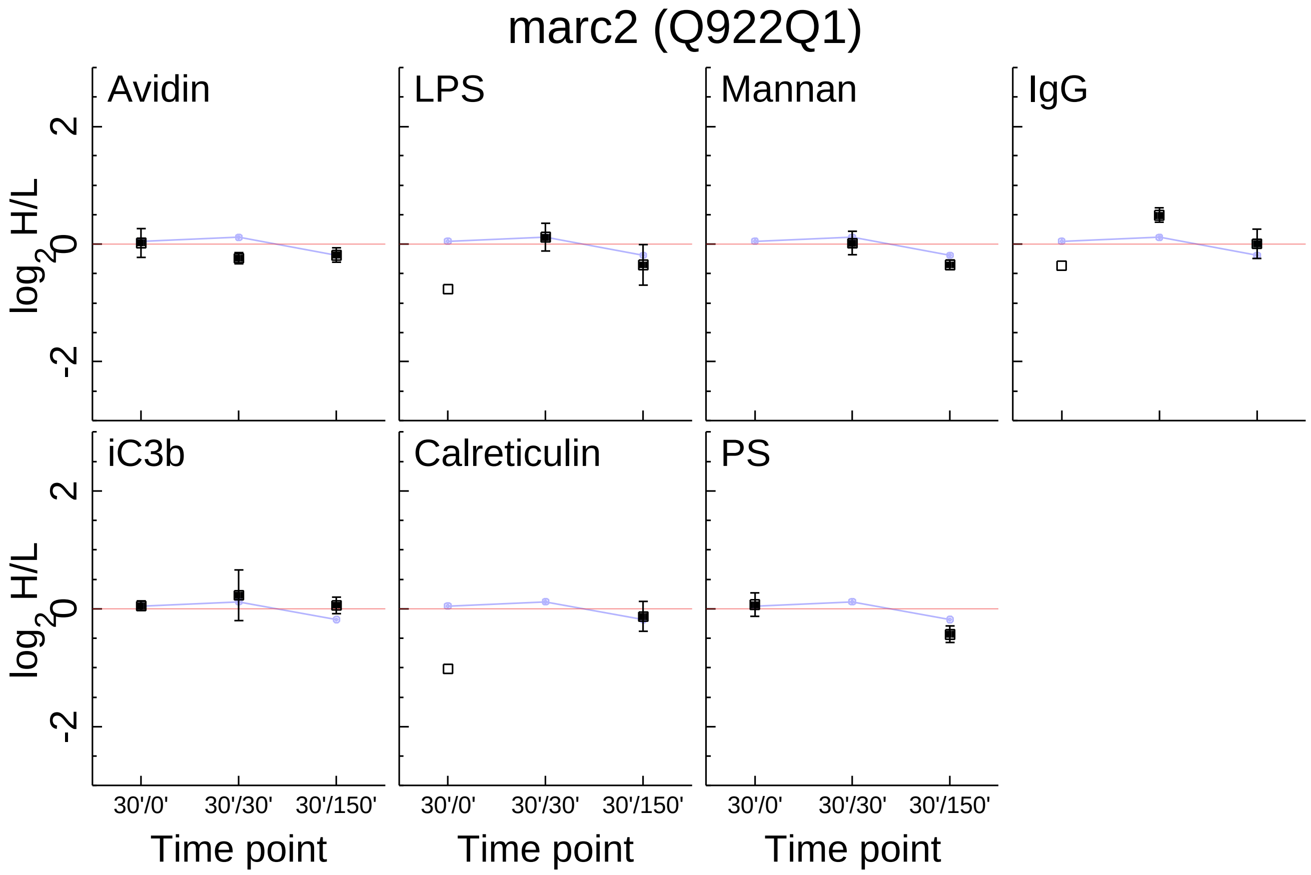
marc2 (Q922Q1)
Avidin
LPS
Mannan
IgG
iC3b
Calreticulin
PS
log2 H/L
2
0
-2
30'/0'
30'/30'
30'/150'
Time point

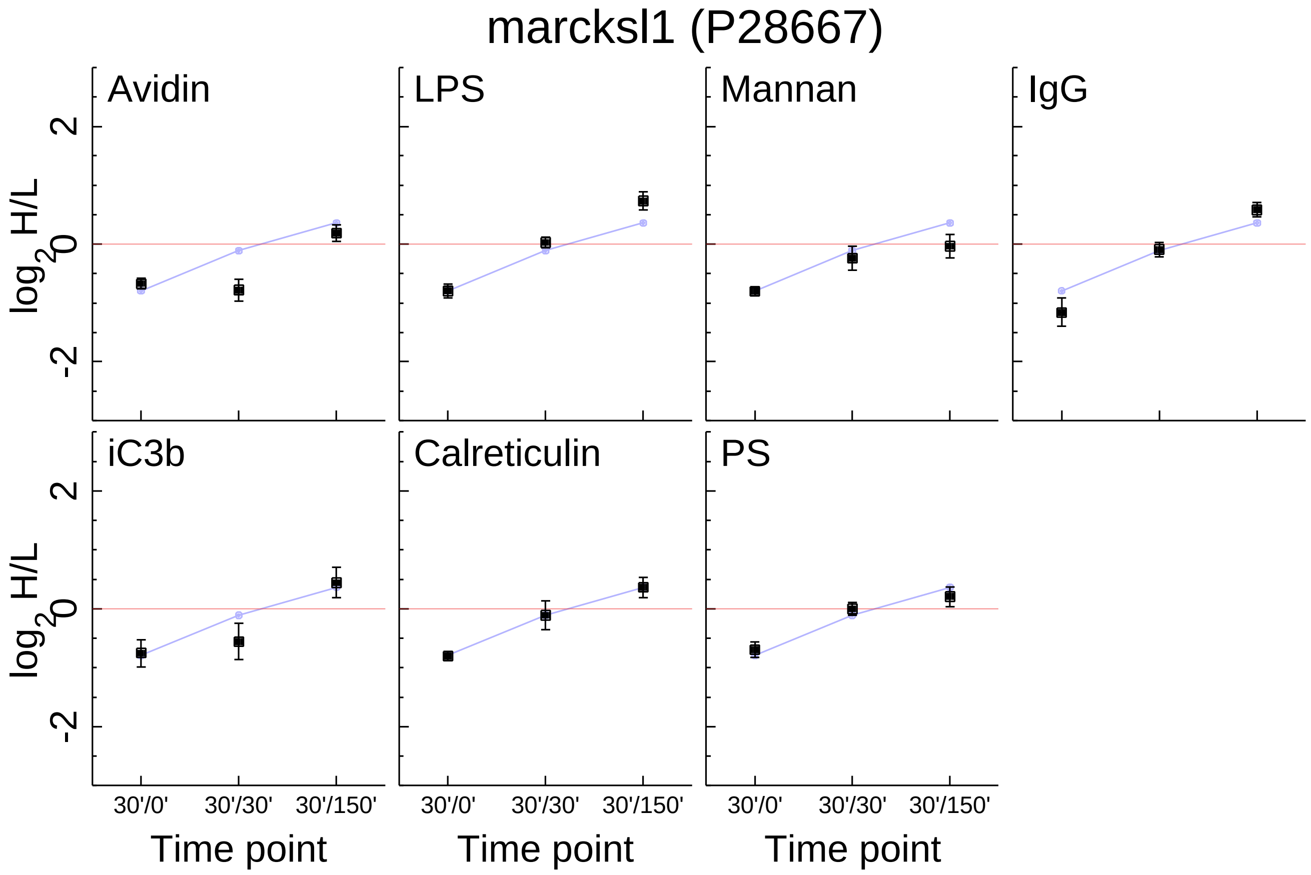
marcksl1 (P28667)
Avidin
LPS
Mannan
IgG
iC3b
Calreticulin
PS
log2 H/L
2
0
-2
30'/0'
30'/30'
30'/150'
Time point

marcks (P26645)

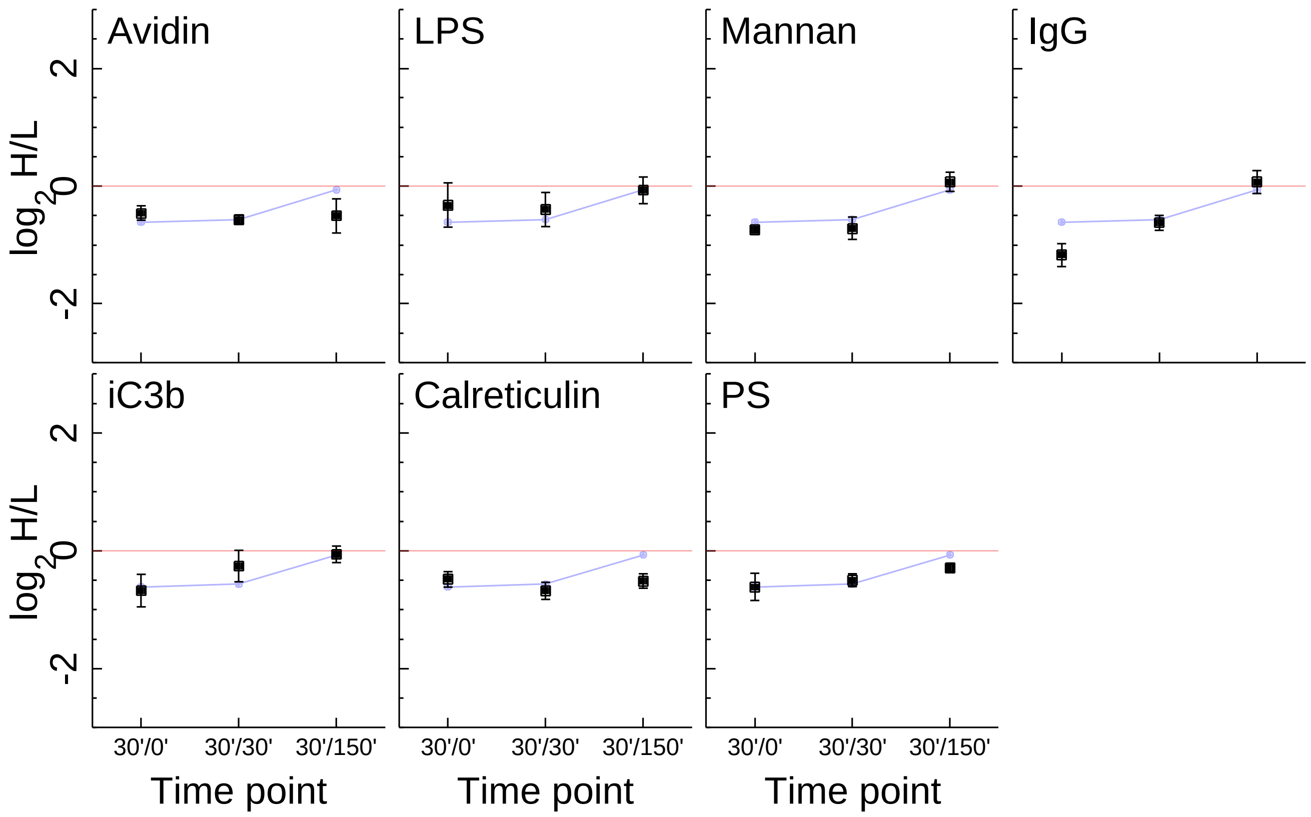

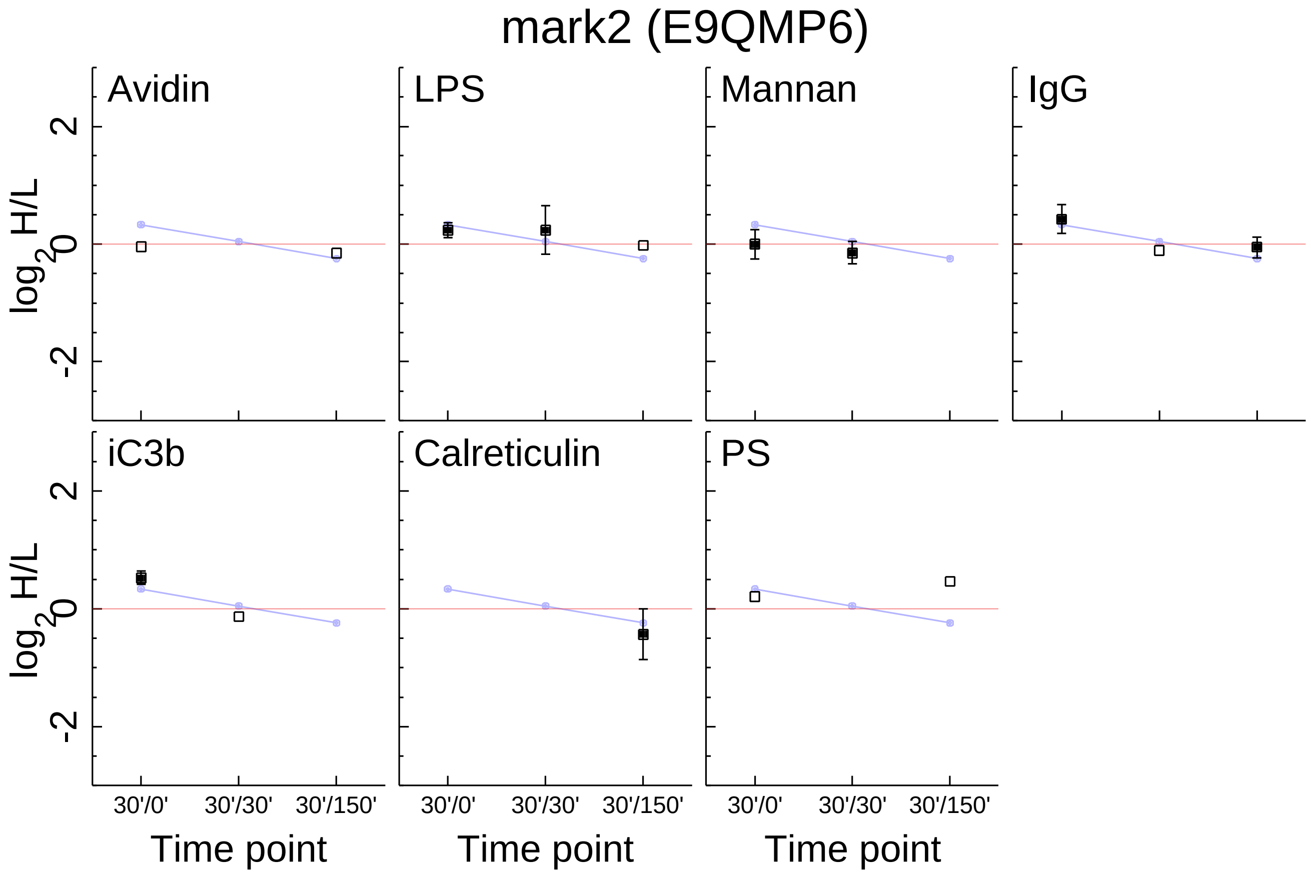

mark2 (E9QMP6)
Avidin
LPS
Mannan
IgG
iC3b
Calreticulin
PS
log2 H/L
2
0
-2
30'/0'
30'/30'
30'/150'
Time point

matr3 (Q8K310)

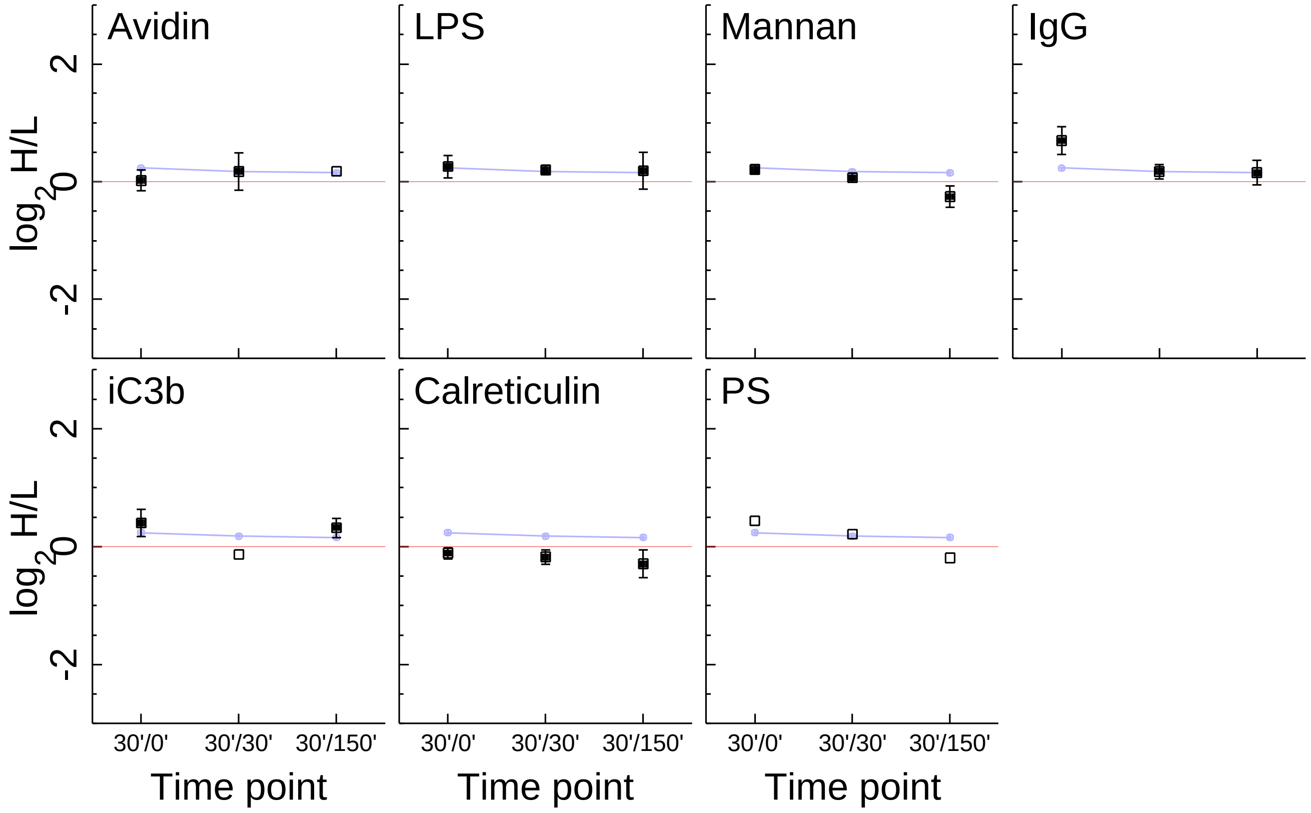

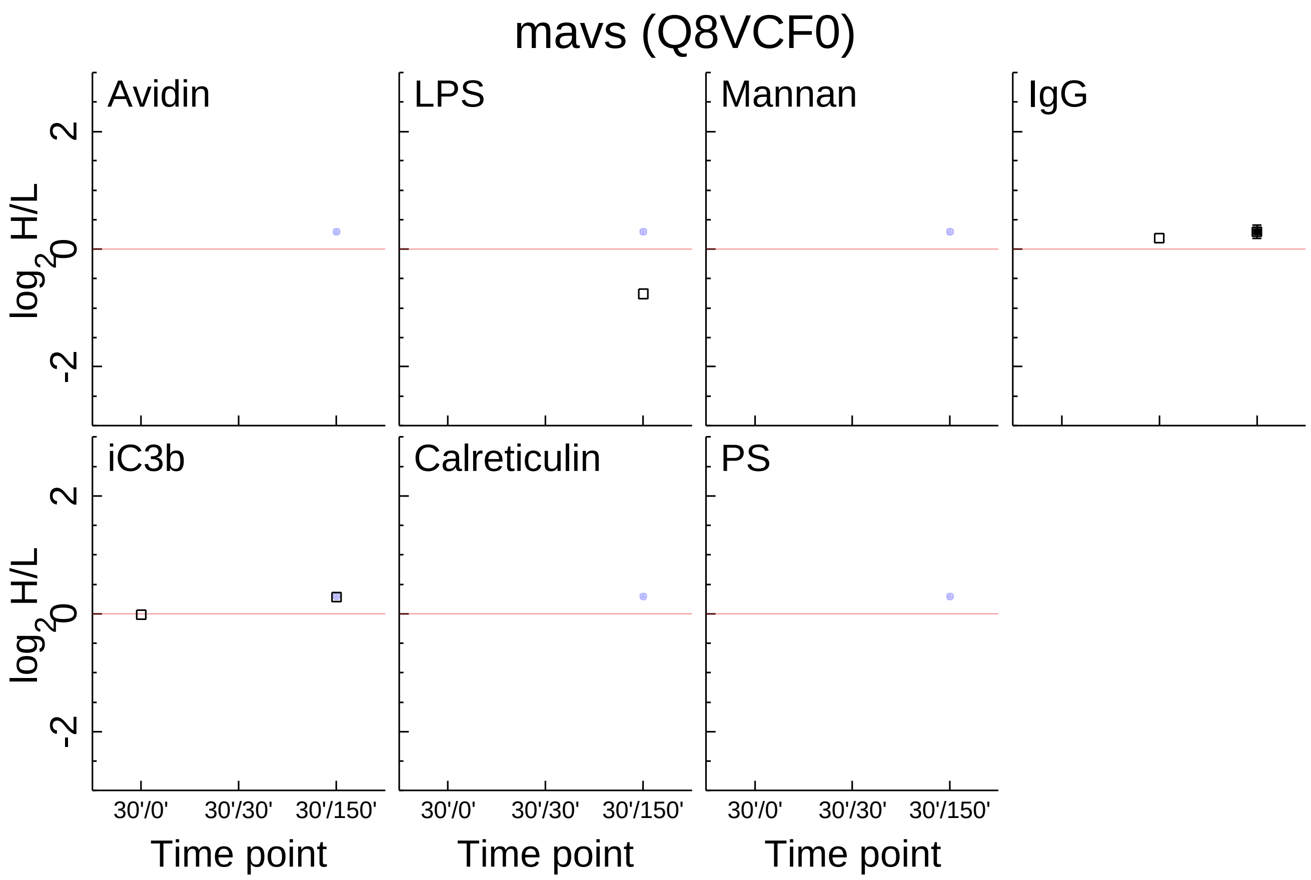
mavs (Q8VCF0)
Avidin
LPS
Mannan
IgG
iC3b
Calreticulin
PS
log2 H/L
2
0
-2
30'/0'
30'/30'
30'/150'
Time point

mboat7 (Q8CHK3)

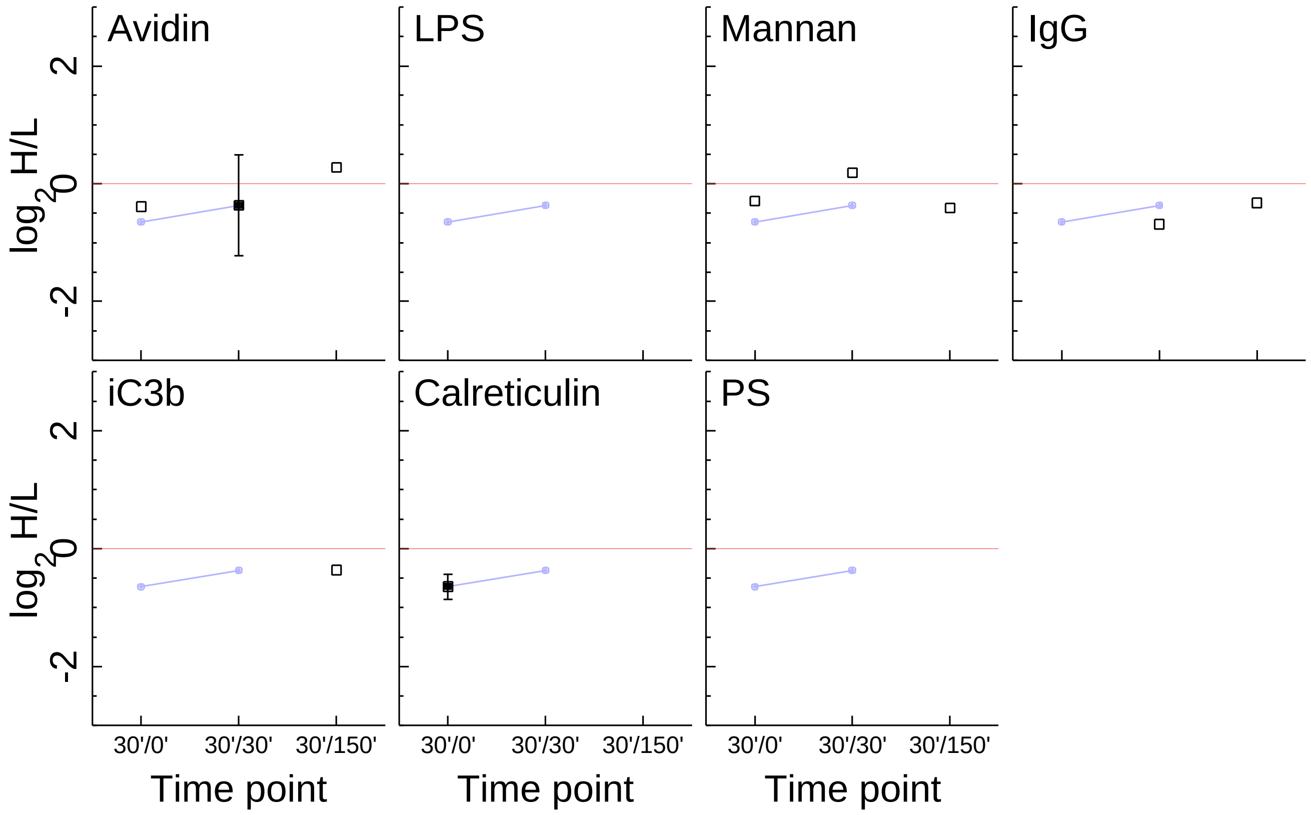

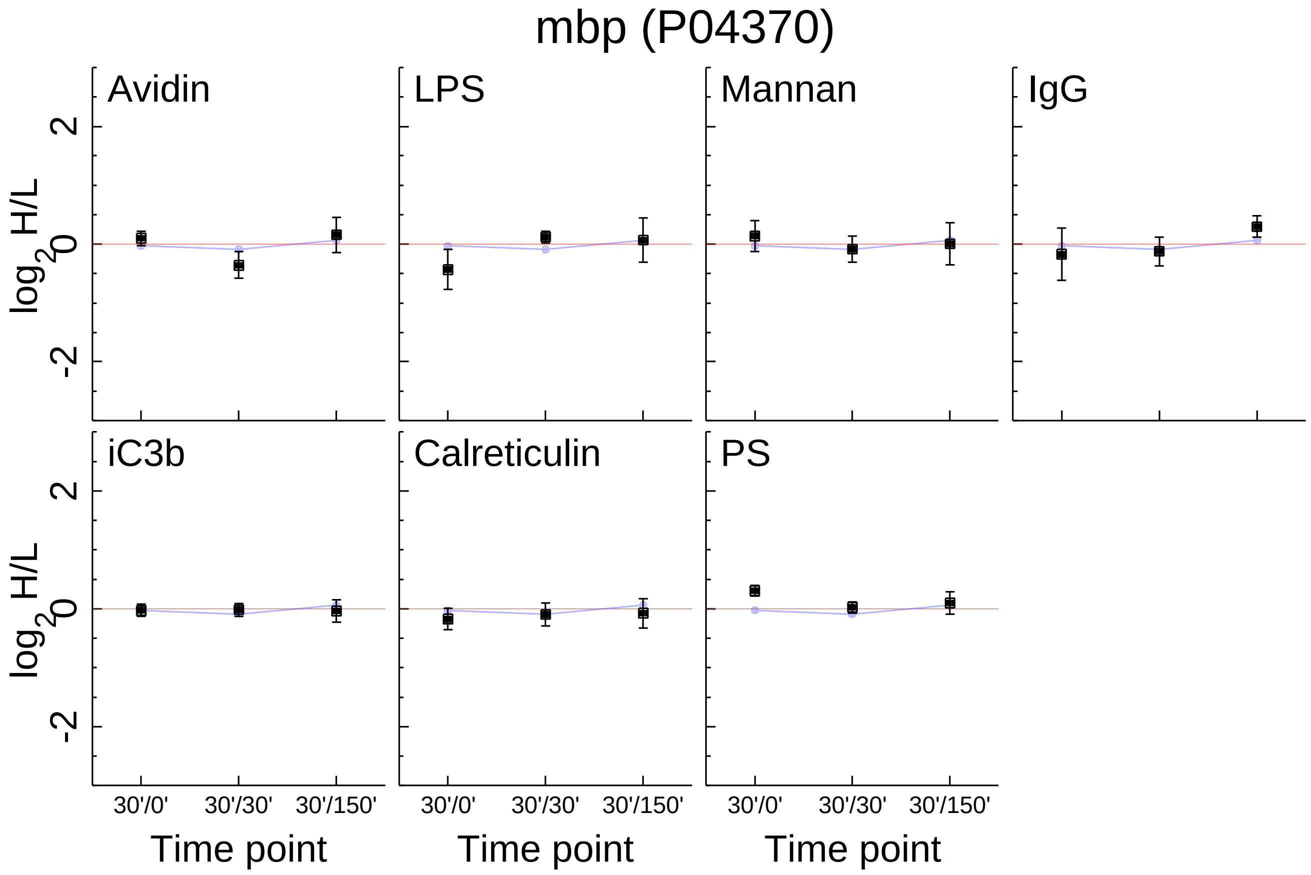

mbp (P04370)
Avidin
LPS
Mannan
IgG
iC3b
Calreticulin
PS
$\log_2$ H/L
2
0
-2
30'/0'
30'/30'
30'/150'
Time point

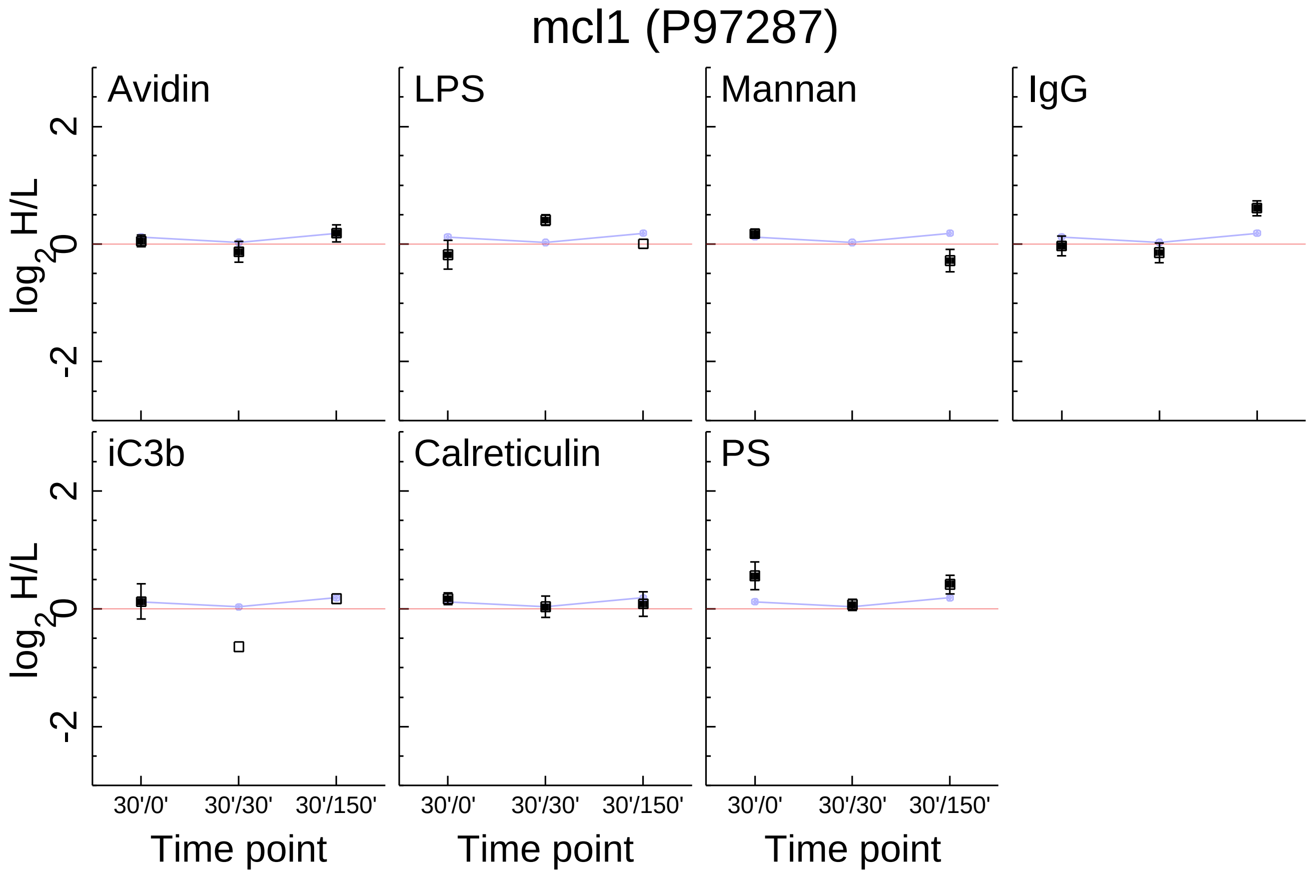
mcl1 (P97287)
Avidin
LPS
Mannan
IgG
iC3b
Calreticulin
PS
log2 H/L
2
0
-2
30'/0'
30'/30'
30'/150'
Time point

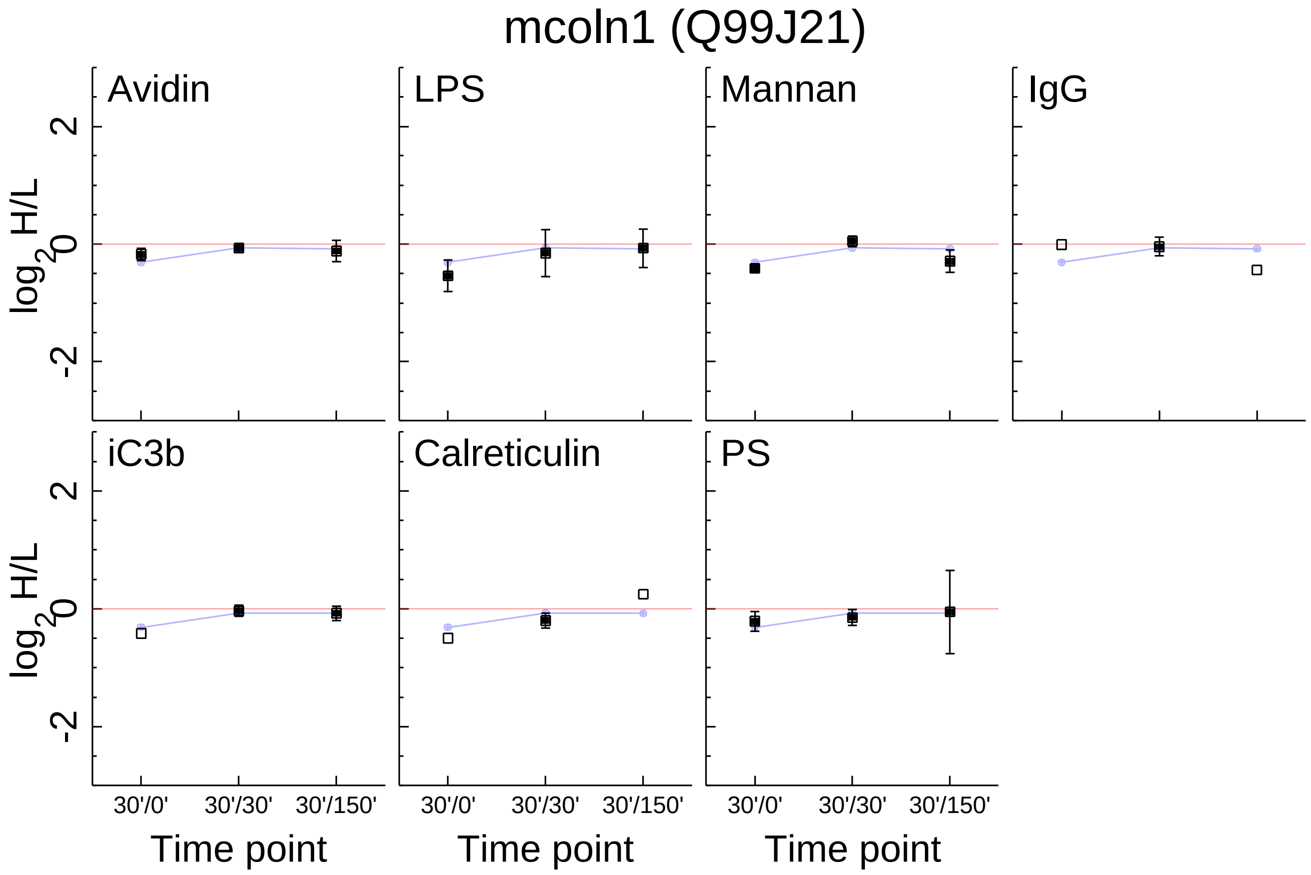

mcoln1 (Q99J21)
Avidin
LPS
Mannan
IgG
iC3b
Calreticulin
PS
log2 H/L
2
0
-2
30'/0'
30'/30'
30'/150'
Time point

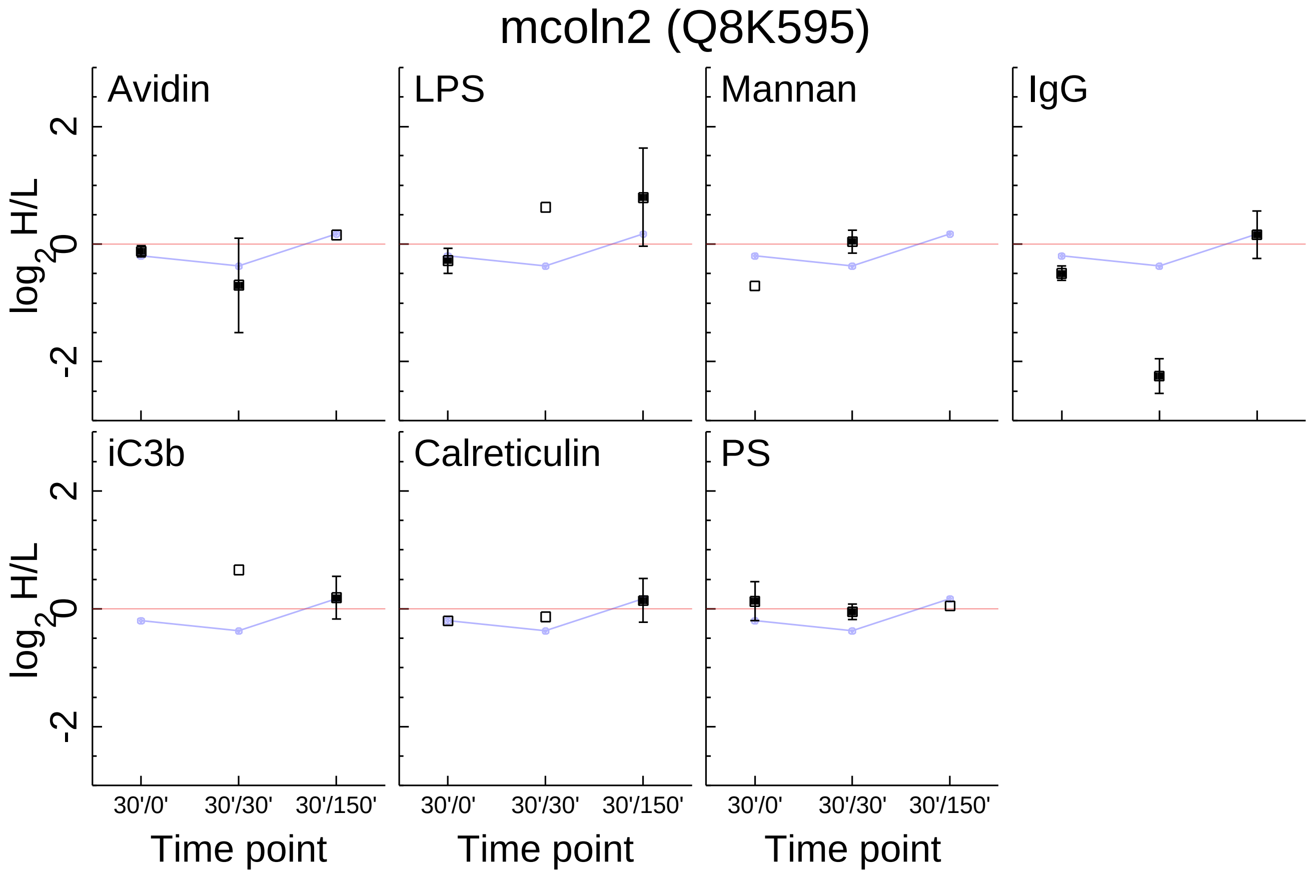

mcoln2 (Q8K595)
Avidin
LPS
Mannan
IgG
iC3b
Calreticulin
PS
log2 H/L
2
0
-2
30'/0'
30'/30'
30'/150'
Time point

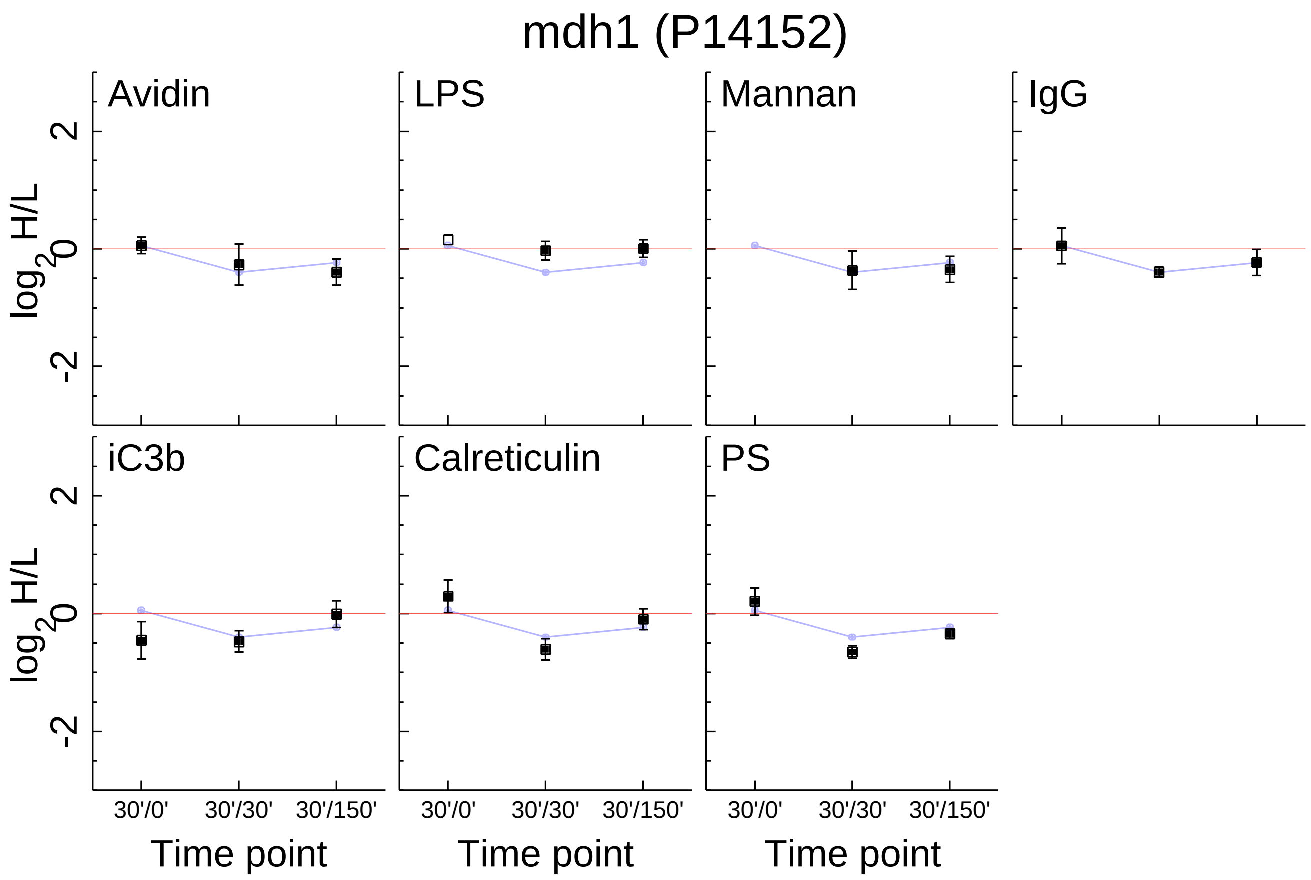

mdh1 (P14152)
Avidin
LPS
Mannan
IgG
iC3b
Calreticulin
PS
log2 H/L
2
0
-2
30'/0'
30'/30'
30'/150'
Time point

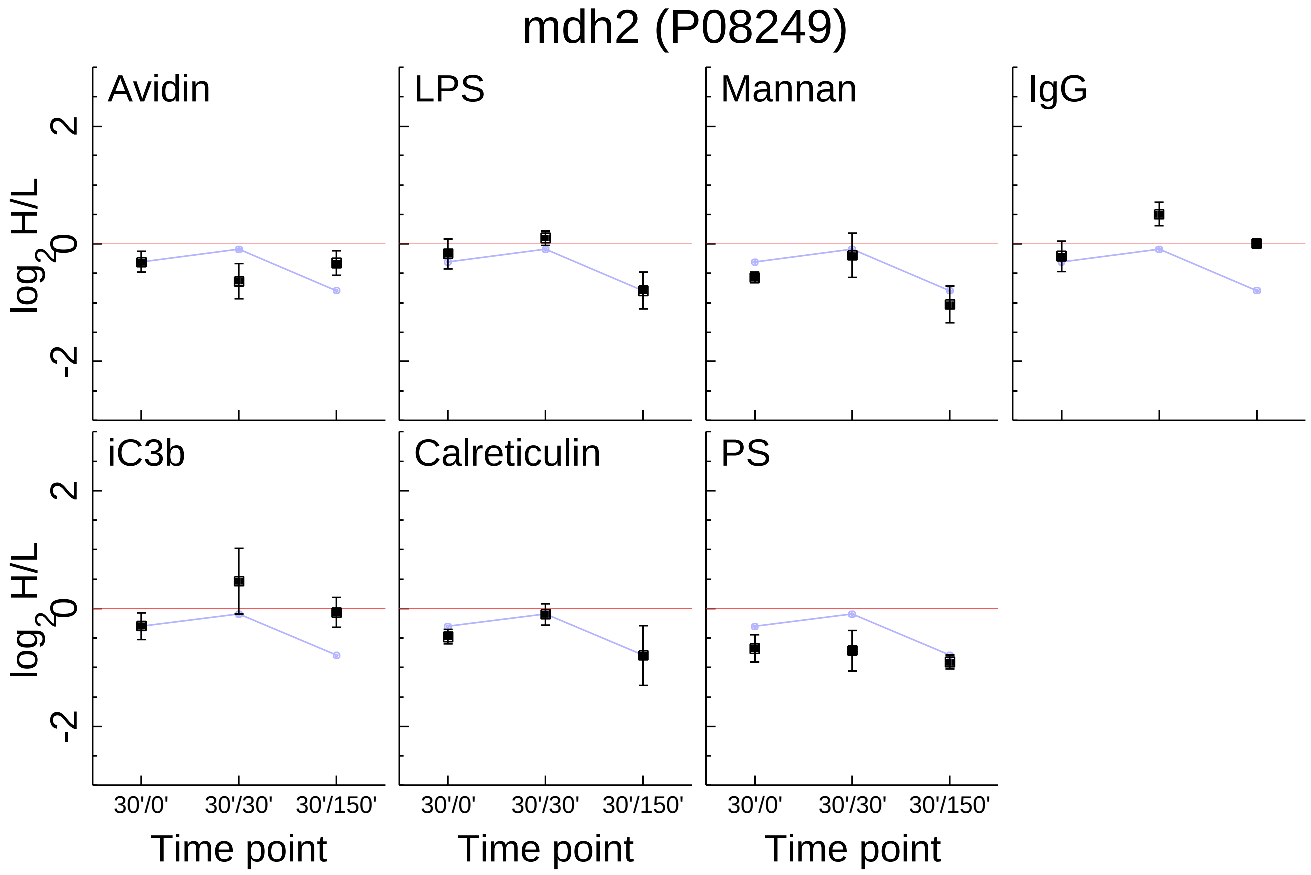

mdh2 (P08249)
Avidin
LPS
Mannan
IgG
iC3b
Calreticulin
PS
log2 H/L
2
0
-2
30'/0'
30'/30'
30'/150'
Time point

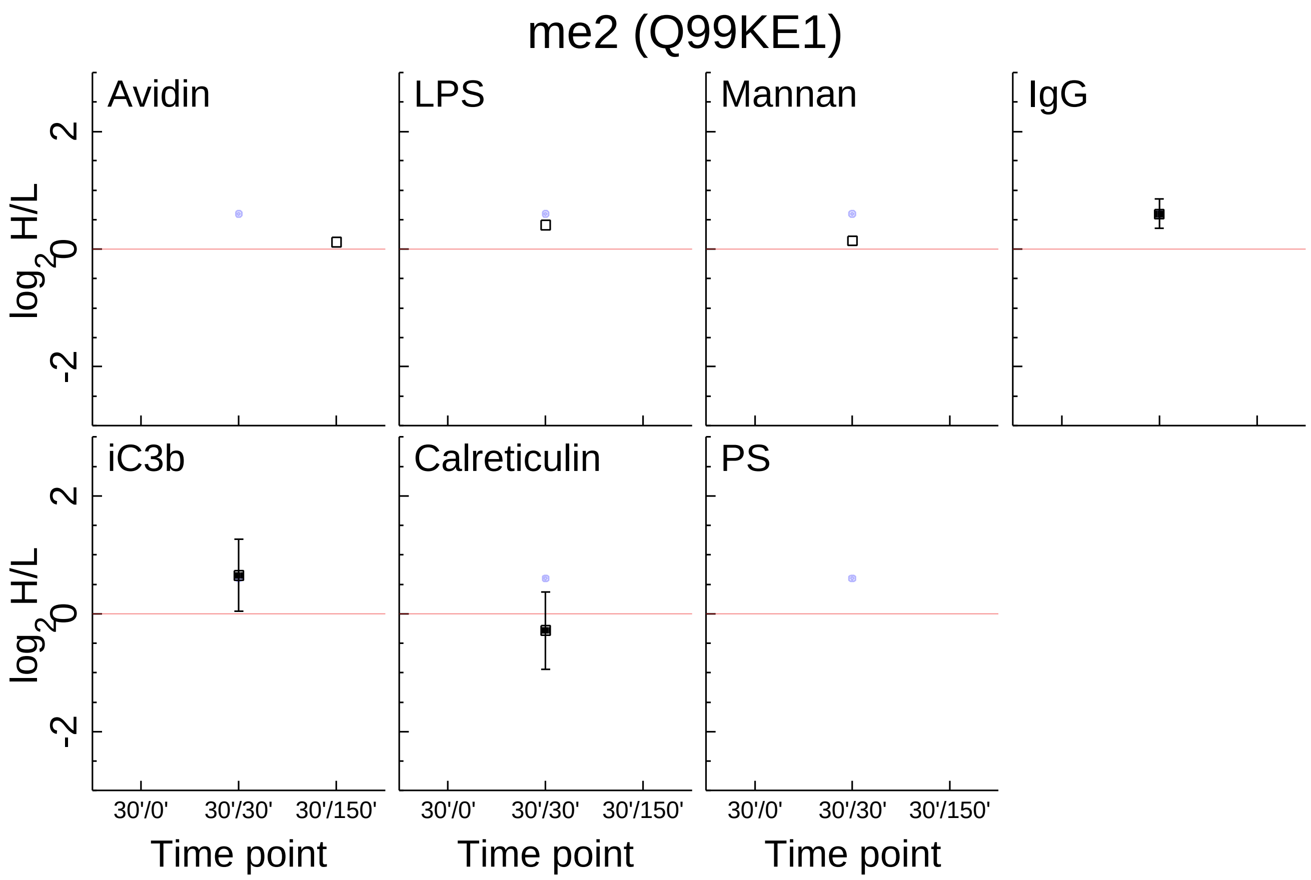
me2 (Q99KE1)
Avidin
LPS
Mannan
IgG
iC3b
Calreticulin
PS
log2 H/L
2
0
-2
30'/0'
30'/30'
30'/150'
Time point

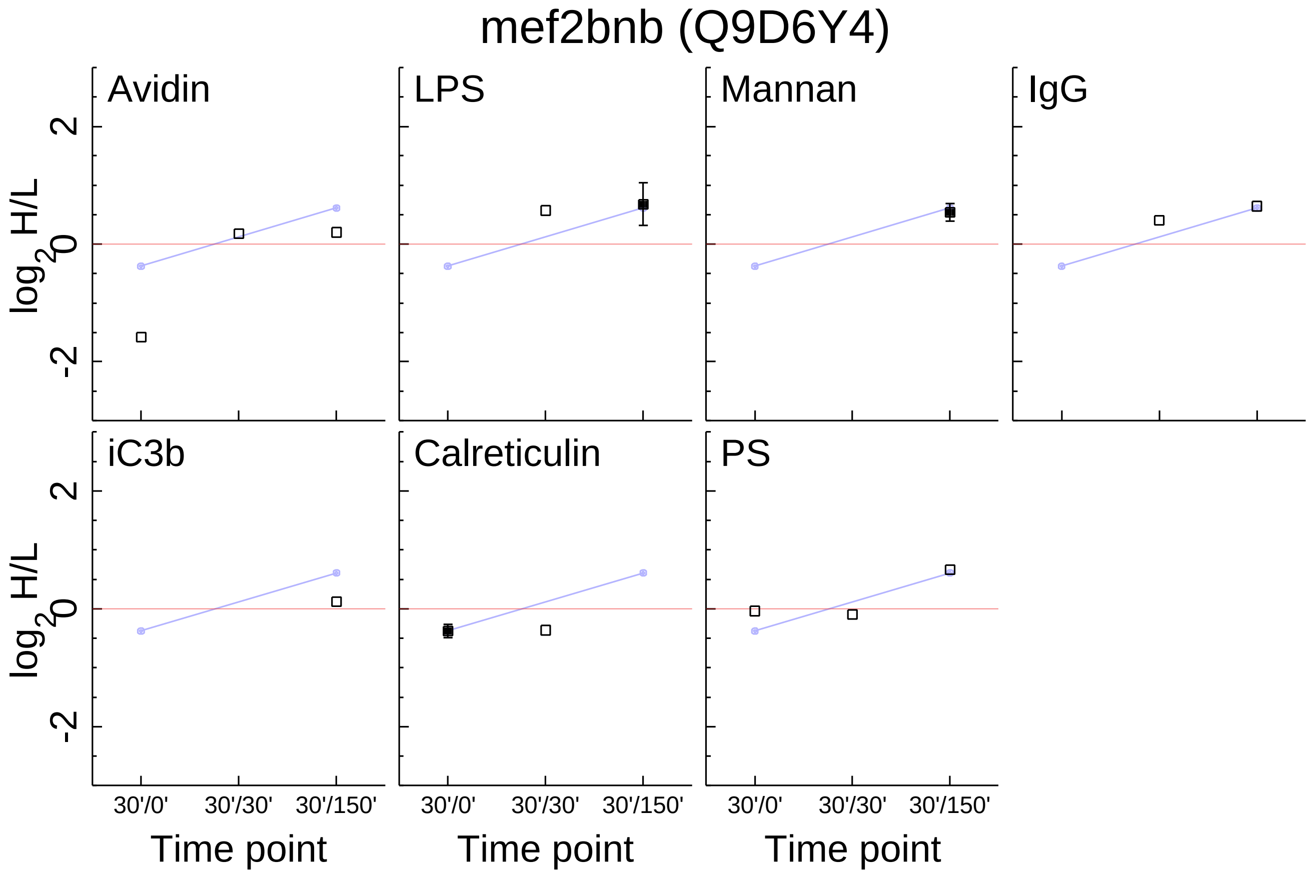
mef2bnb (Q9D6Y4)
Avidin
LPS
Mannan
IgG
iC3b
Calreticulin
PS
log2 H/L
2
0
-2
30'/0'
30'/30'
30'/150'
Time point

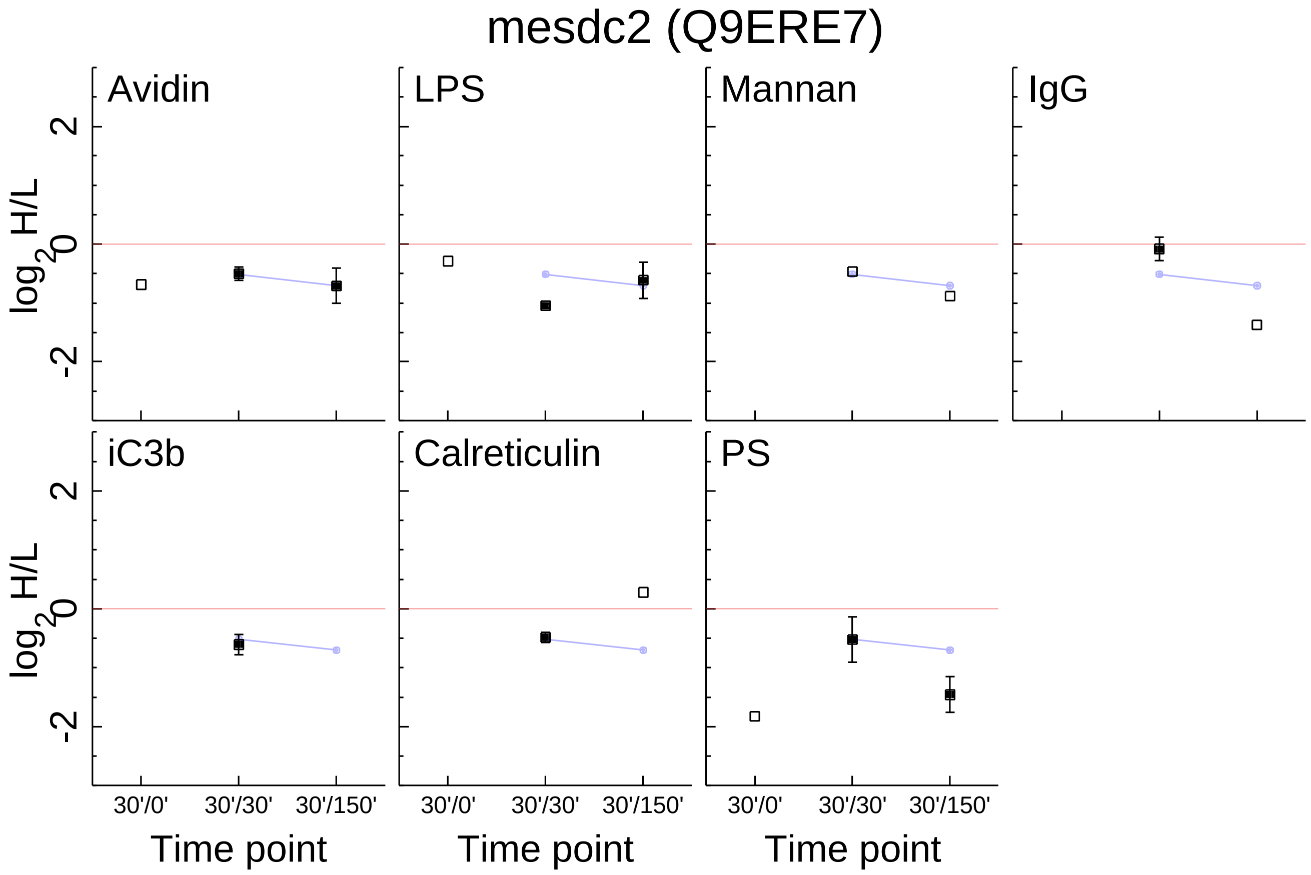
mesdc2 (Q9ERE7)
Avidin
LPS
Mannan
IgG
iC3b
Calreticulin
PS
log2 H/L
2
0
-2
30'/0'
30'/30'
30'/150'
Time point

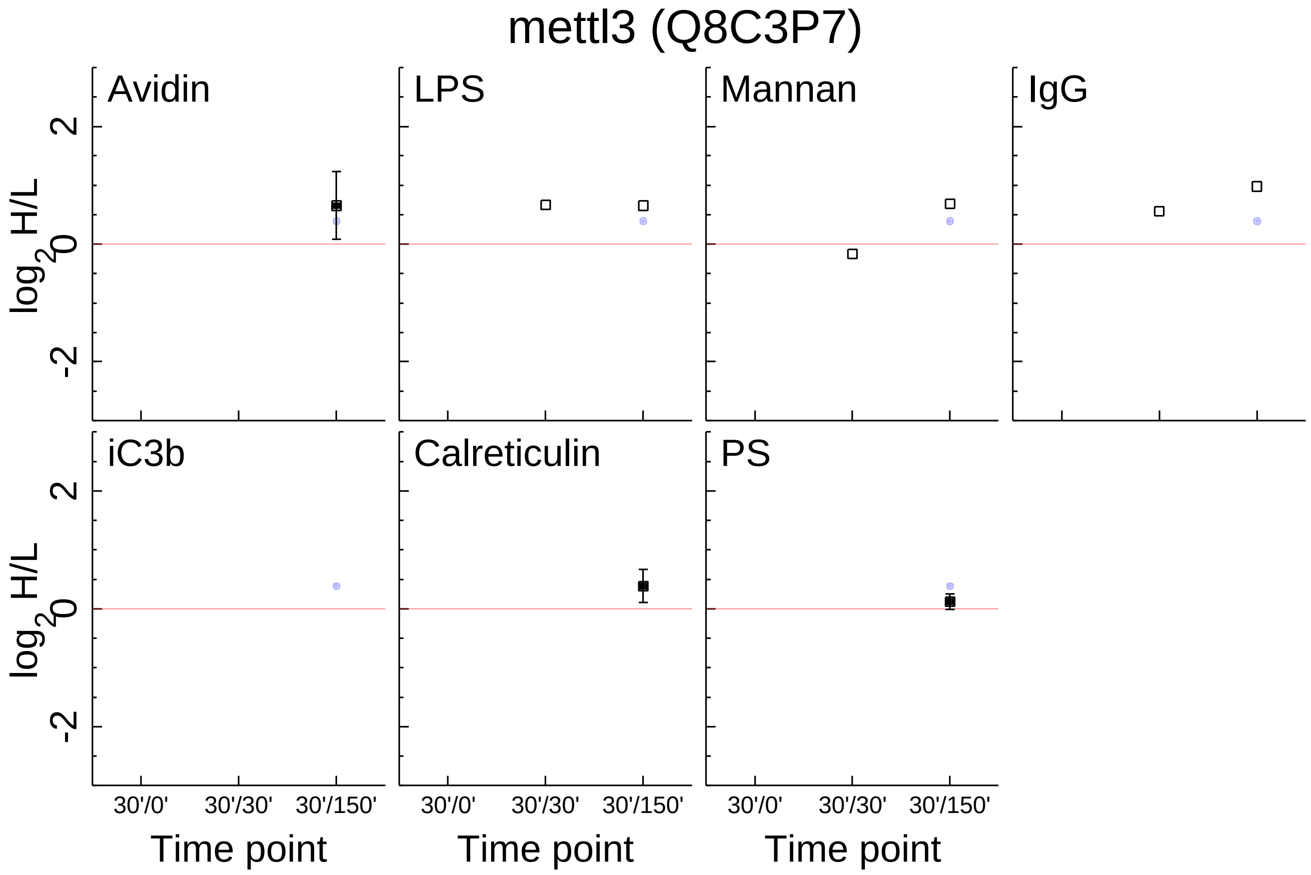
mettl3 (Q8C3P7)
Avidin
LPS
Mannan
IgG
iC3b
Calreticulin
PS
log2 H/L
2
0
-2
30'/0'
30'/30'
30'/150'
Time point

mettl7a1 (Q8C6B0)

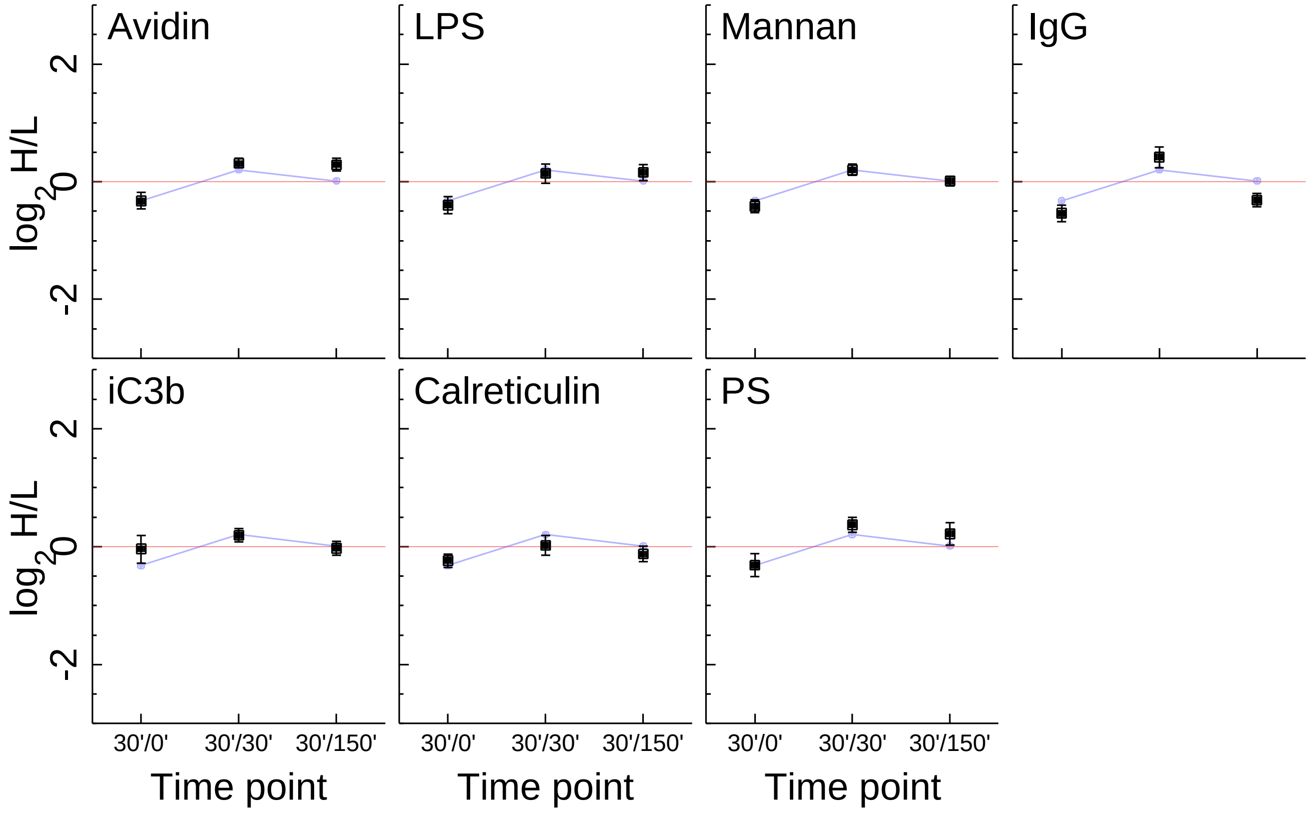

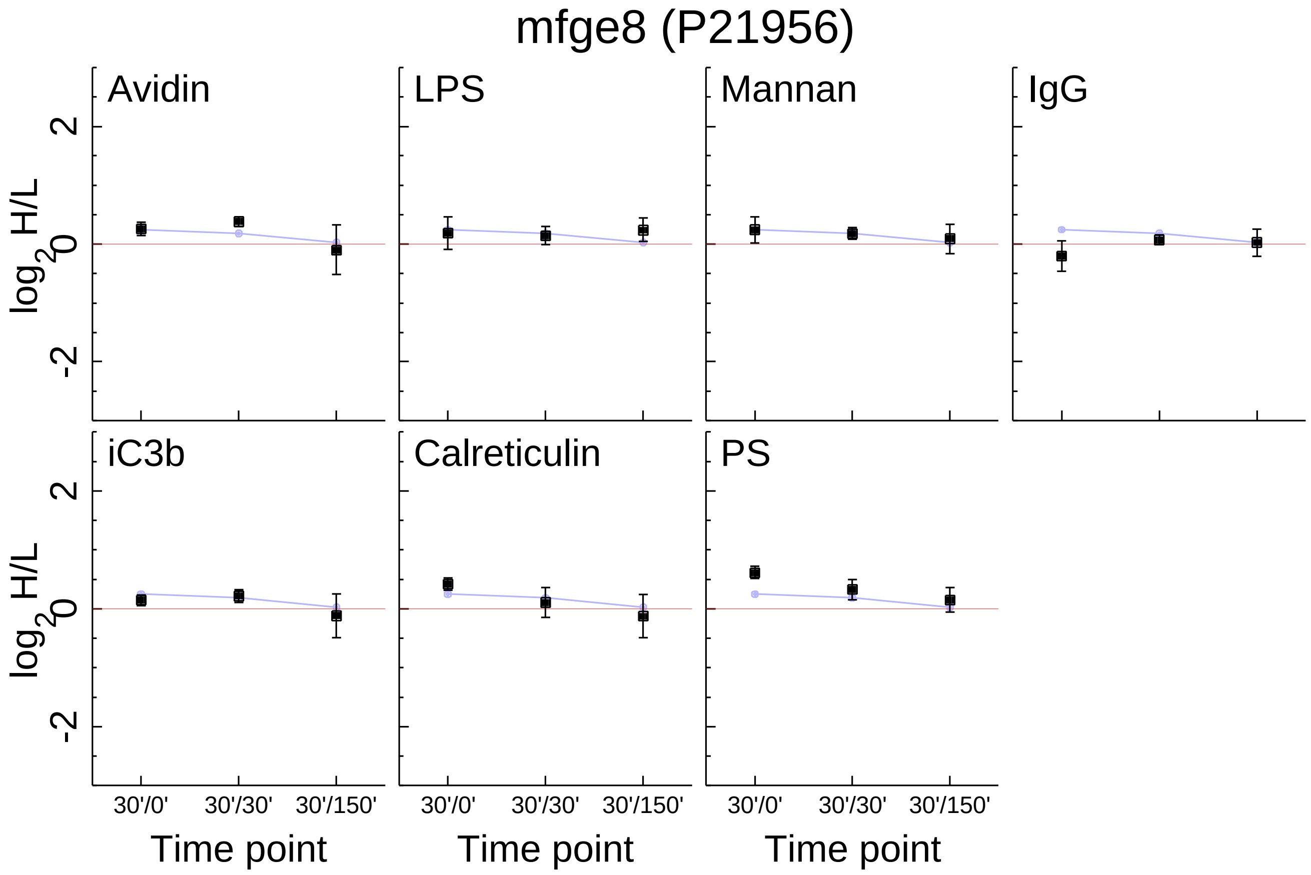

mfge8 (P21956)
Avidin
LPS
Mannan
IgG
iC3b
Calreticulin
PS
log2 H/L
2
0
-2
30'/0'
30'/30'
30'/150'
Time point

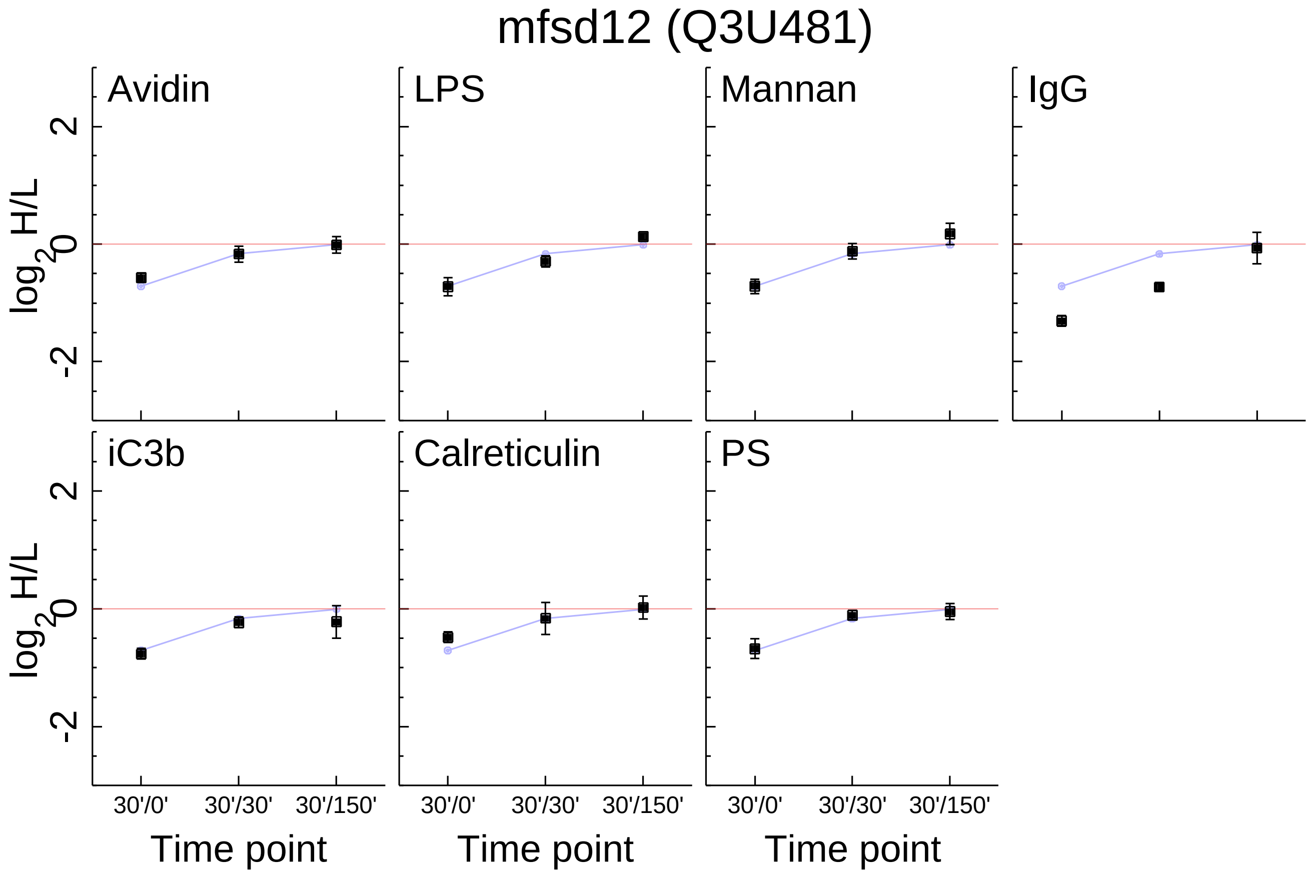
mfsd12 (Q3U481)
Avidin
LPS
Mannan
IgG
iC3b
Calreticulin
PS
log2 H/L
2
0
-2
30'/0'
30'/30'
30'/150'
Time point

mfsd1 (Q9DC37)

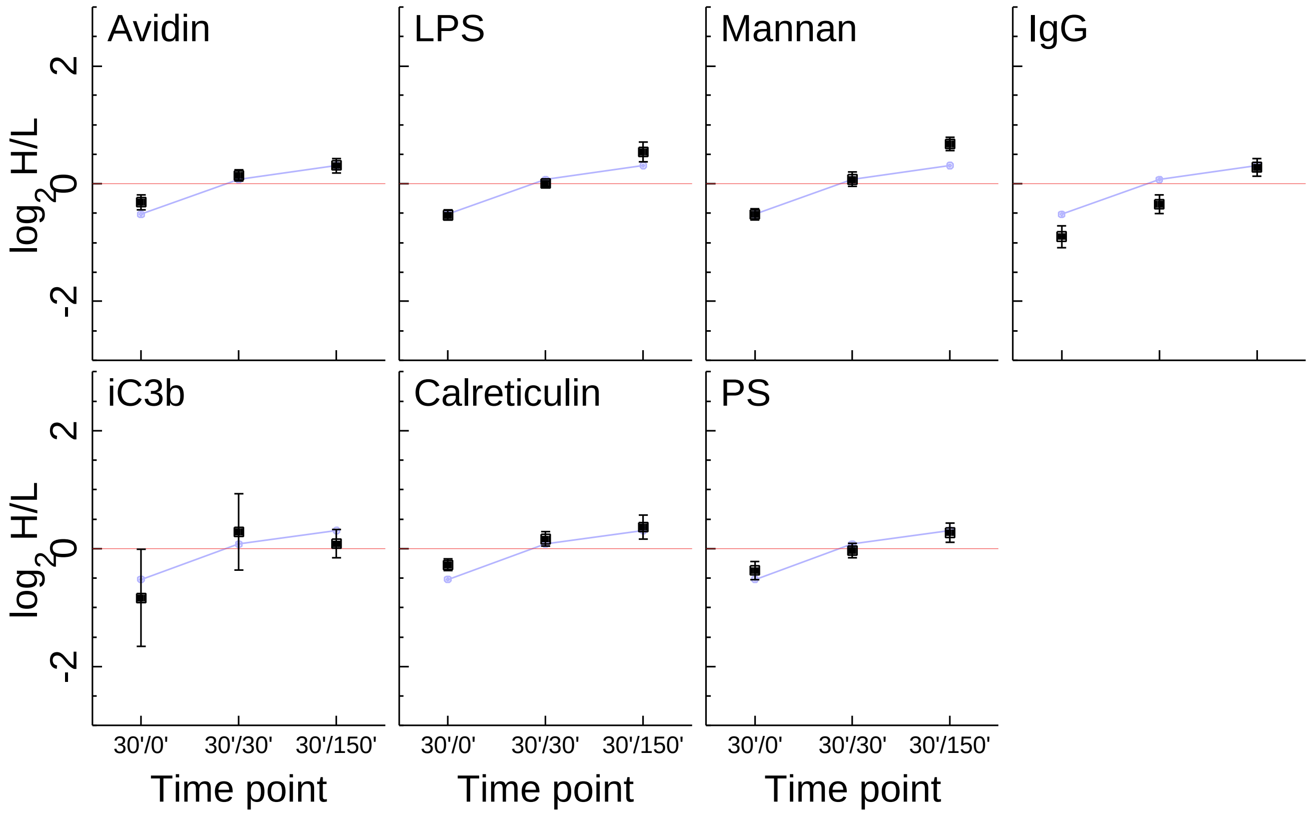

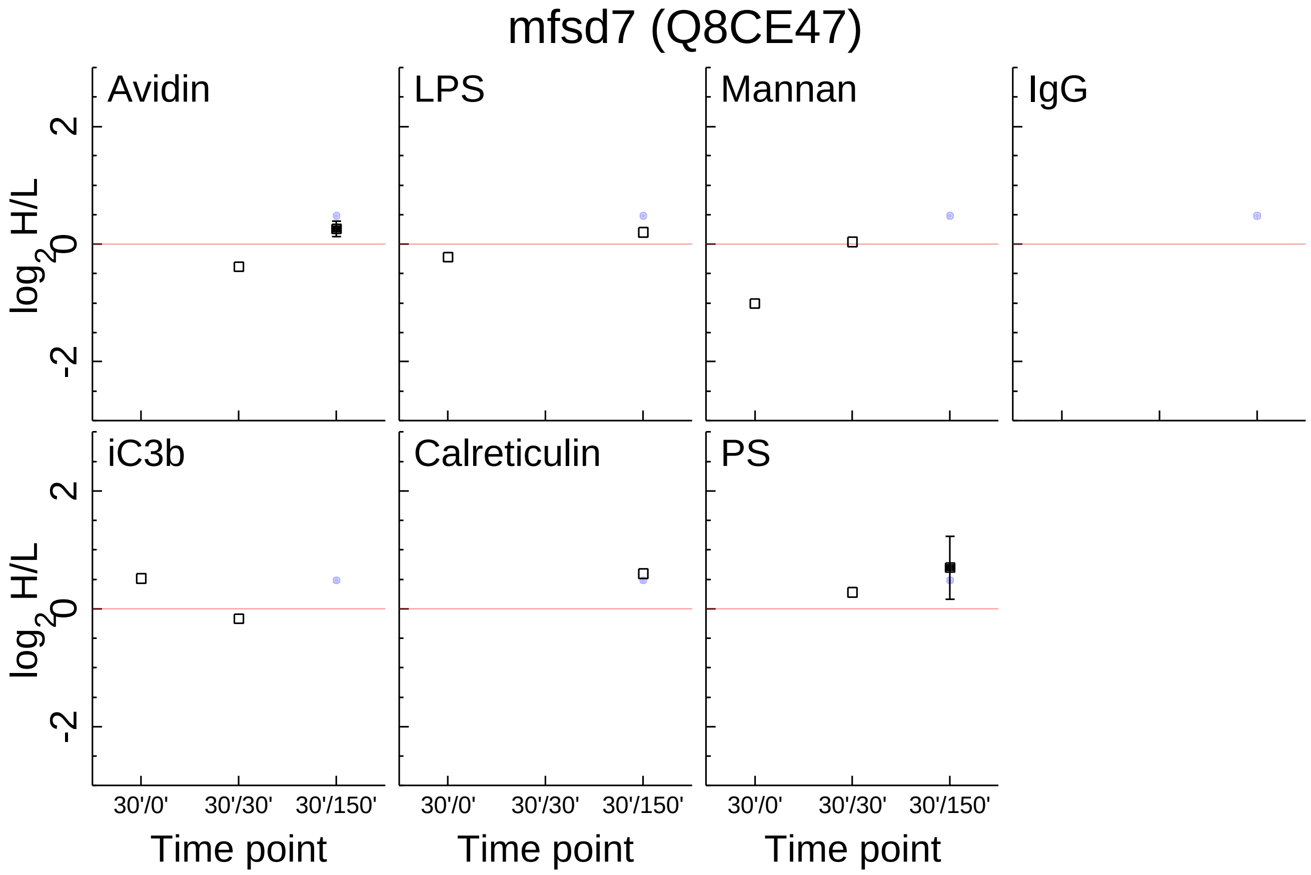

mfsd7 (Q8CE47)
Avidin
LPS
Mannan
IgG
iC3b
Calreticulin
PS
log2 H/L
2
0
-2
30'/0'
30'/30'
30'/150'
Time point

mfsd8 (Q8BH31)

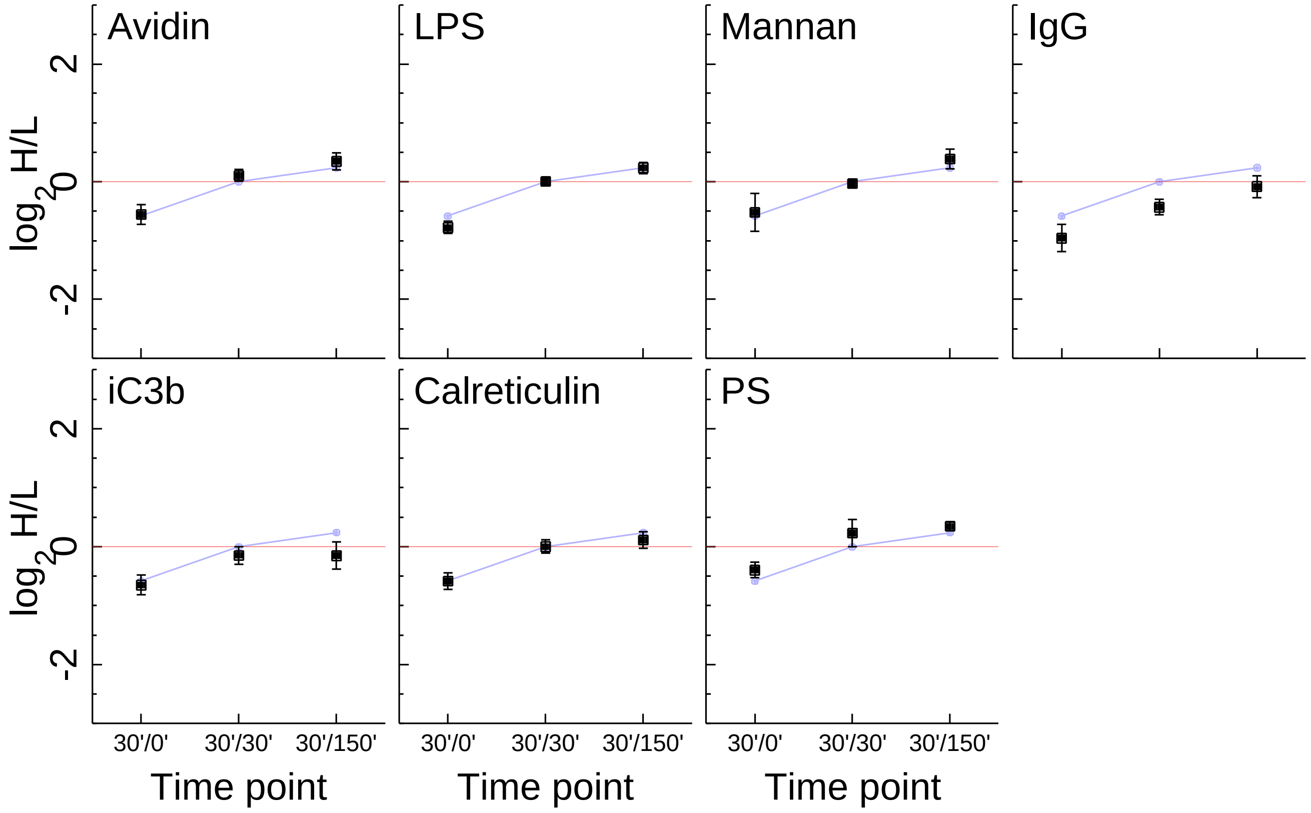

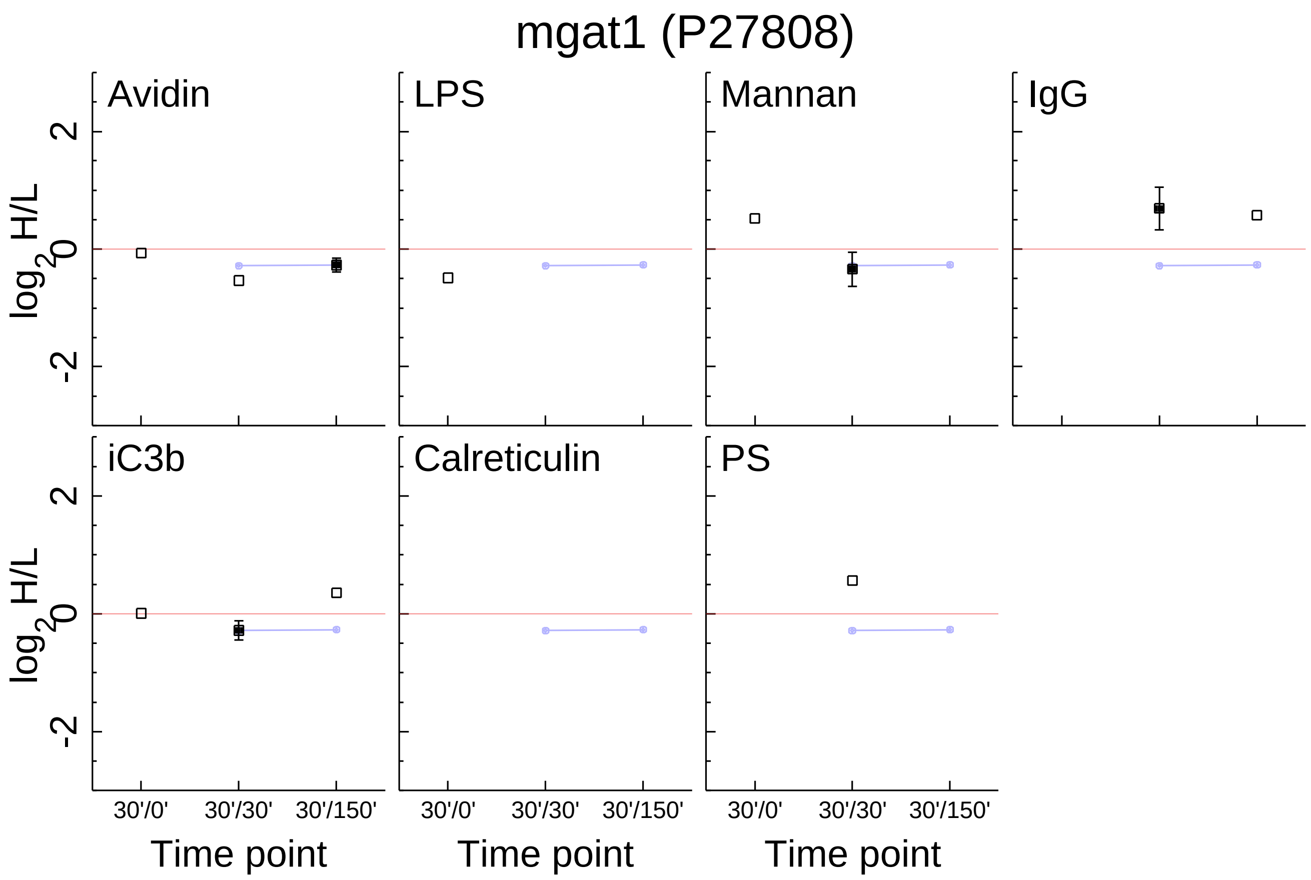
mgat1 (P27808)
Avidin
LPS
Mannan
IgG
iC3b
Calreticulin
PS
log2 H/L
2
0
-2
30'/0'
30'/30'
30'/150'
Time point

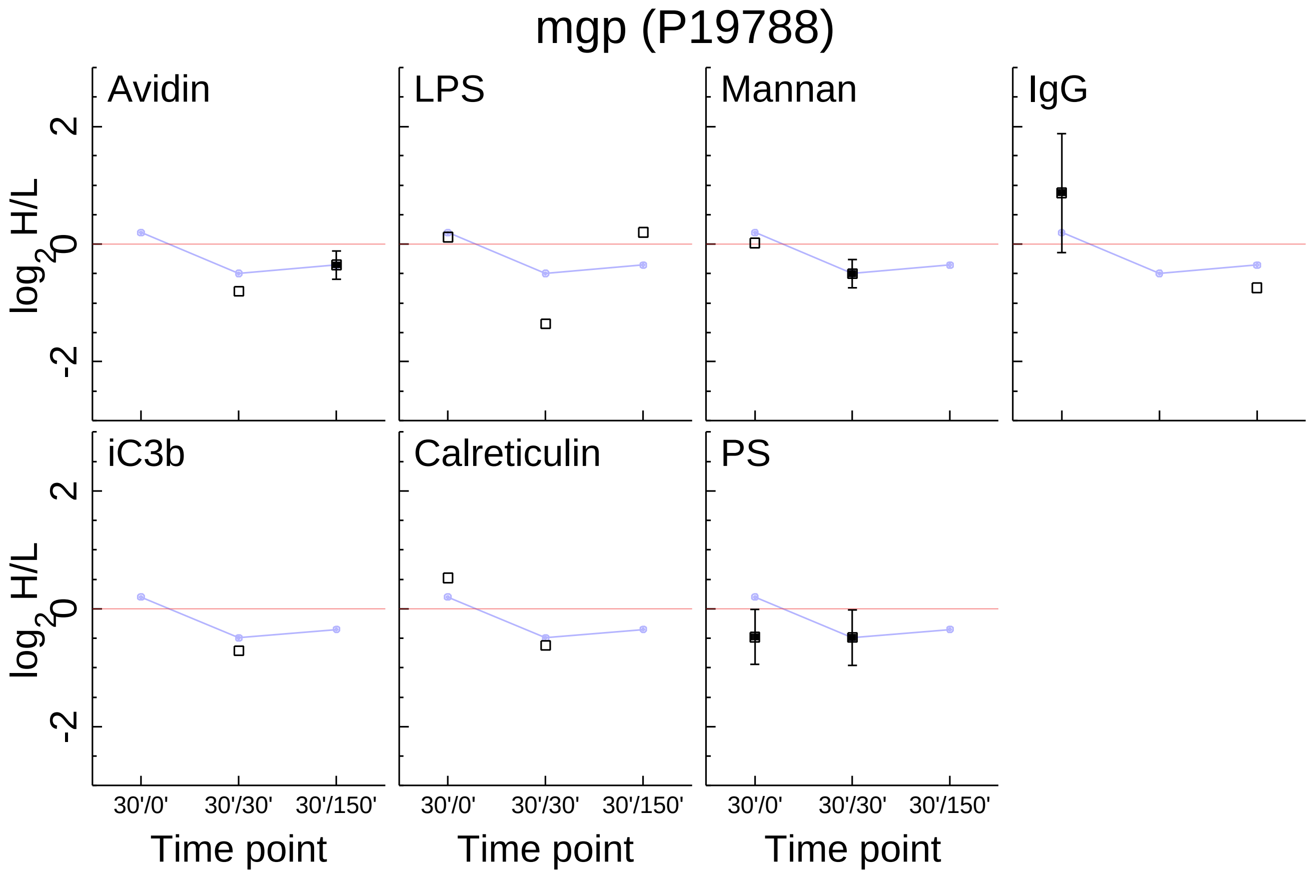
mgp (P19788)
Avidin
LPS
Mannan
IgG
iC3b
Calreticulin
PS
log2 H/L
2
0
-2
30'/0'
30'/30'
30'/150'
Time point

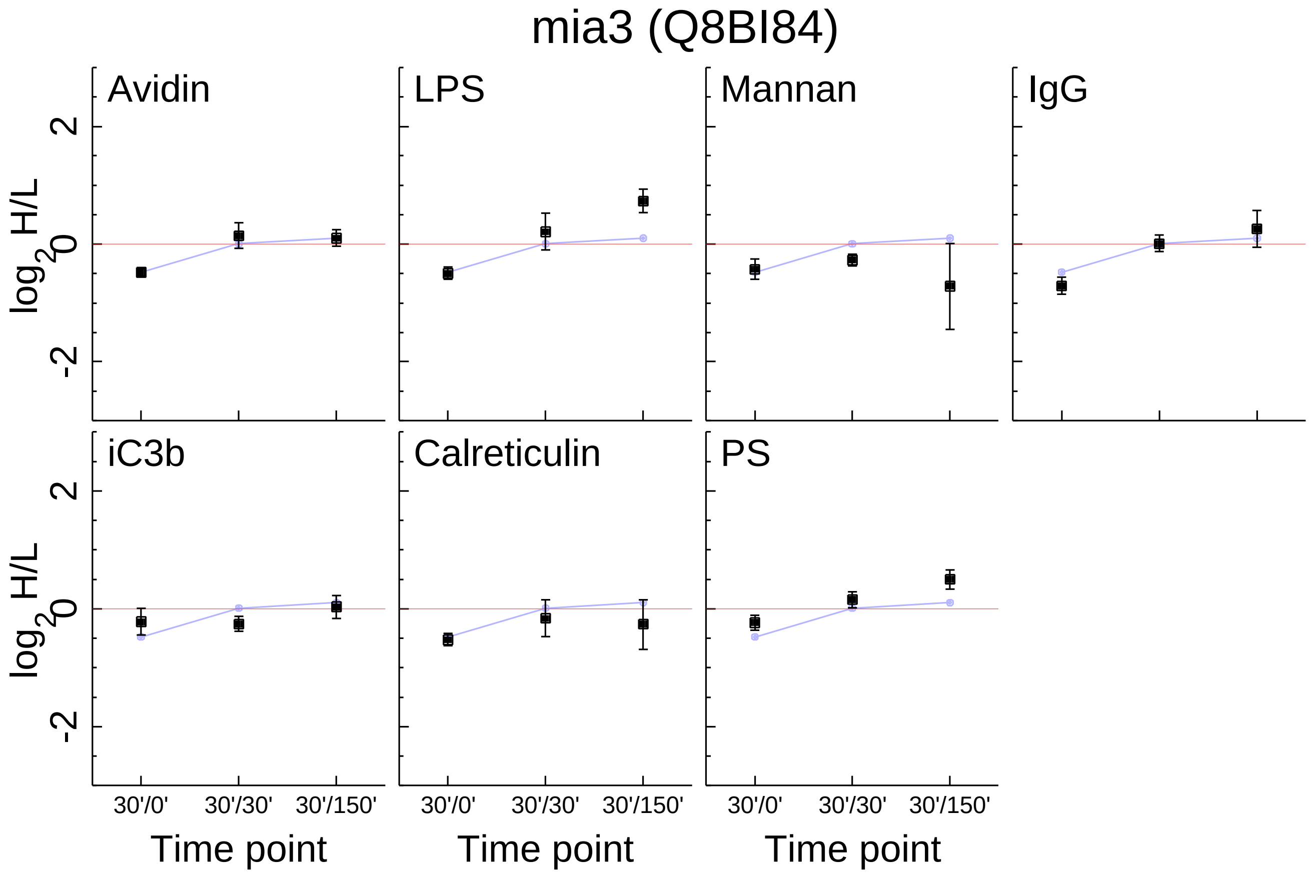
mia3 (Q8BI84)
Avidin
LPS
Mannan
IgG
iC3b
Calreticulin
PS
log2 H/L
2
0
-2
30'/0'
30'/30'
30'/150'
Time point

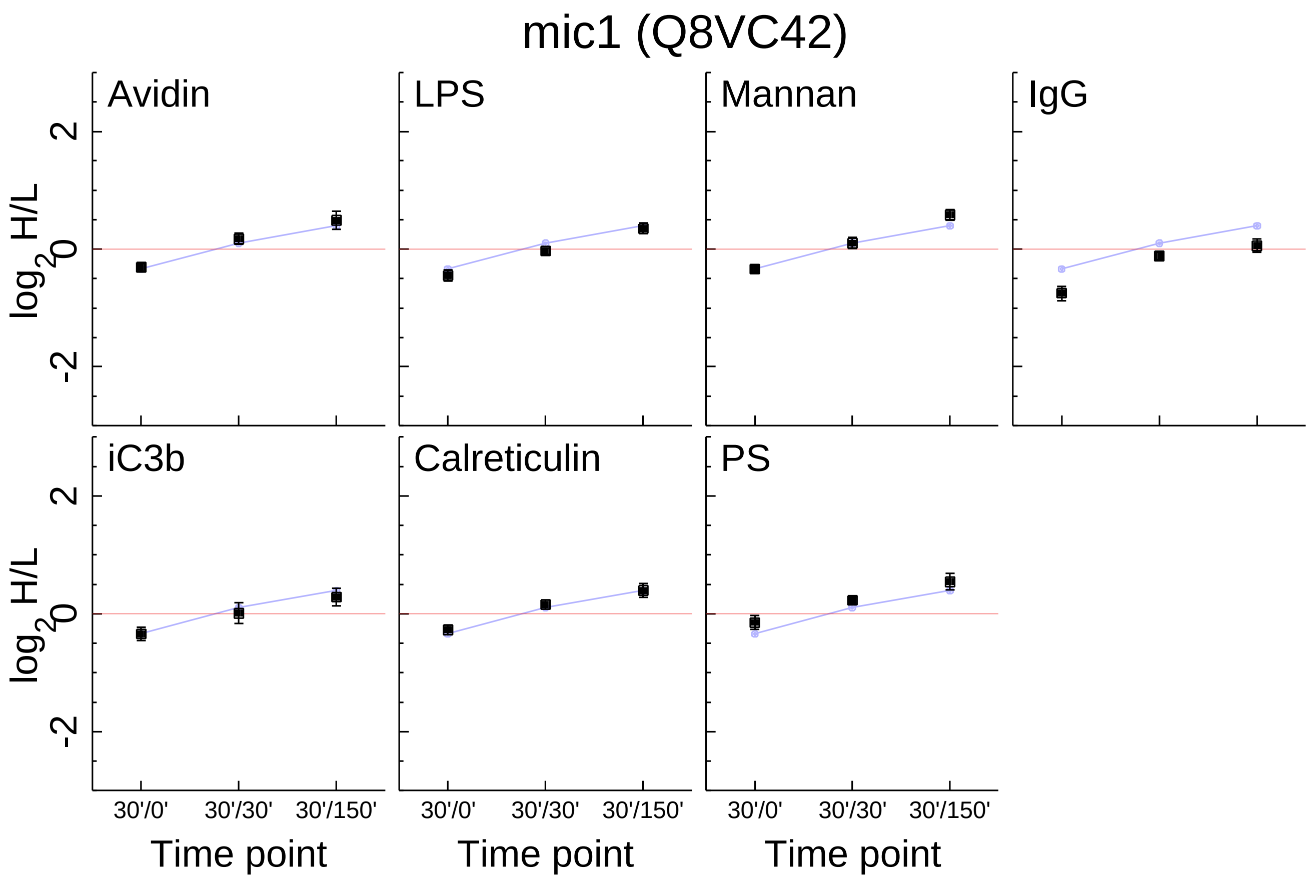

mic1 (Q8VC42)
Avidin
LPS
Mannan
IgG
iC3b
Calreticulin
PS
log2 H/L
2
0
-2
30'/0'
30'/30'
30'/150'
Time point

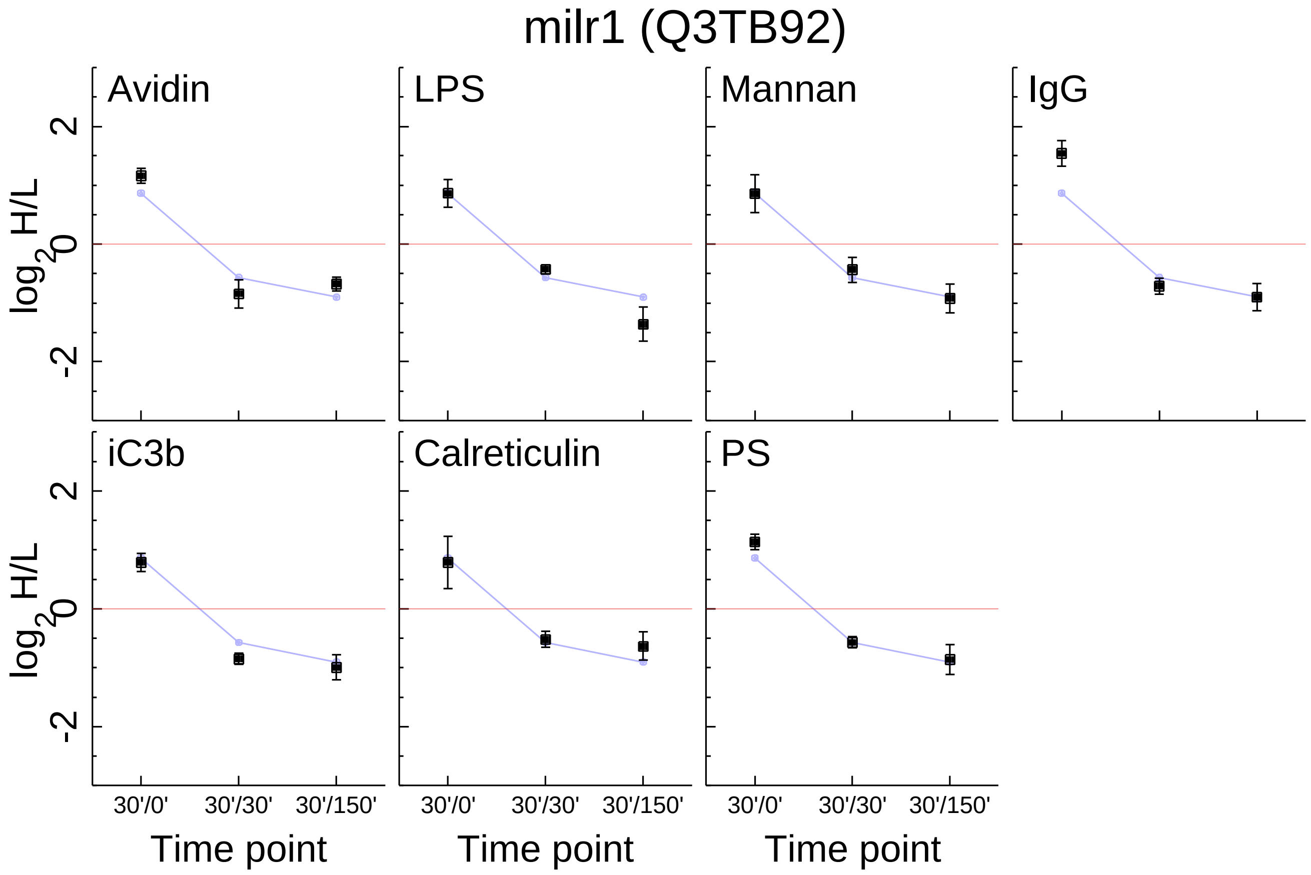
milr1 (Q3TB92)
Avidin
LPS
Mannan
IgG
iC3b
Calreticulin
PS
log2 H/L
2
0
-2
30'/0'
30'/30'
30'/150'
Time point

mitd1 (Q8VDV8)

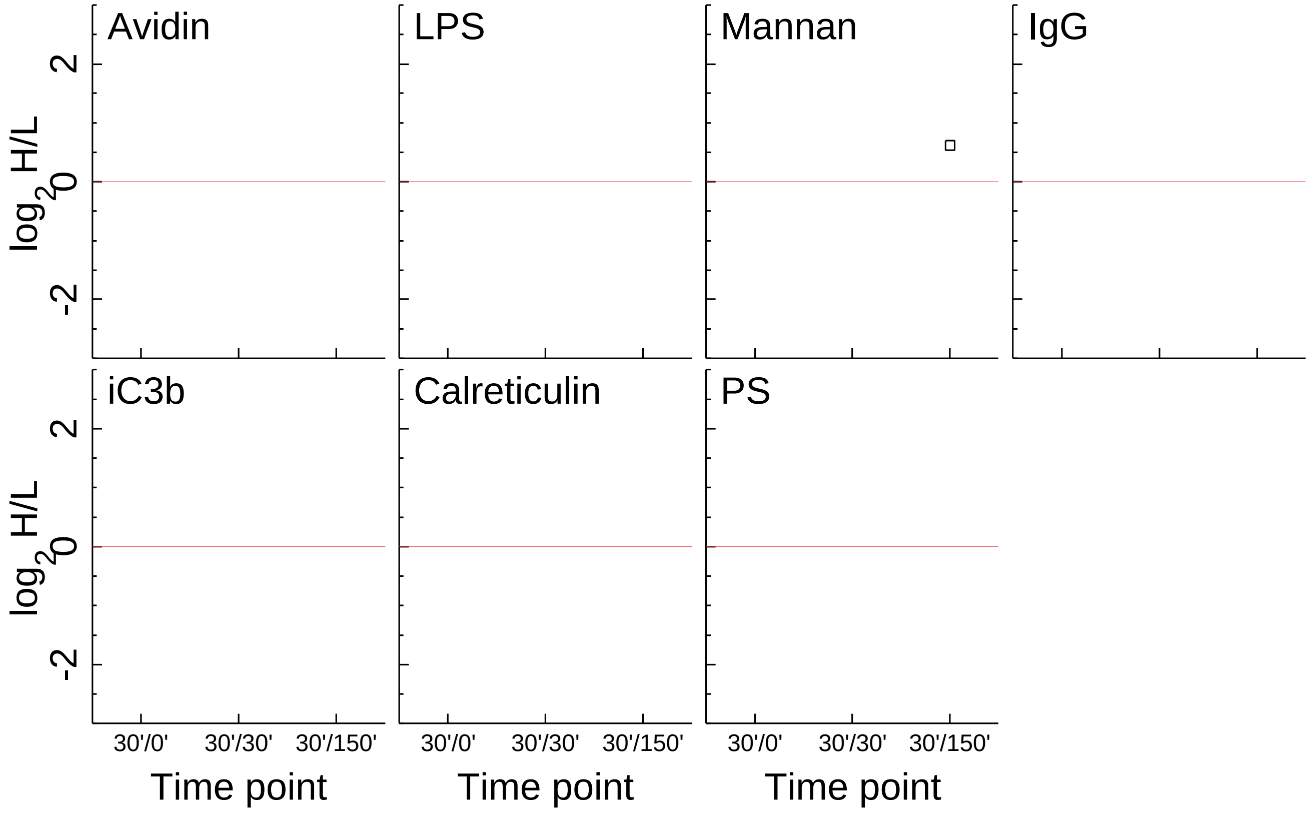

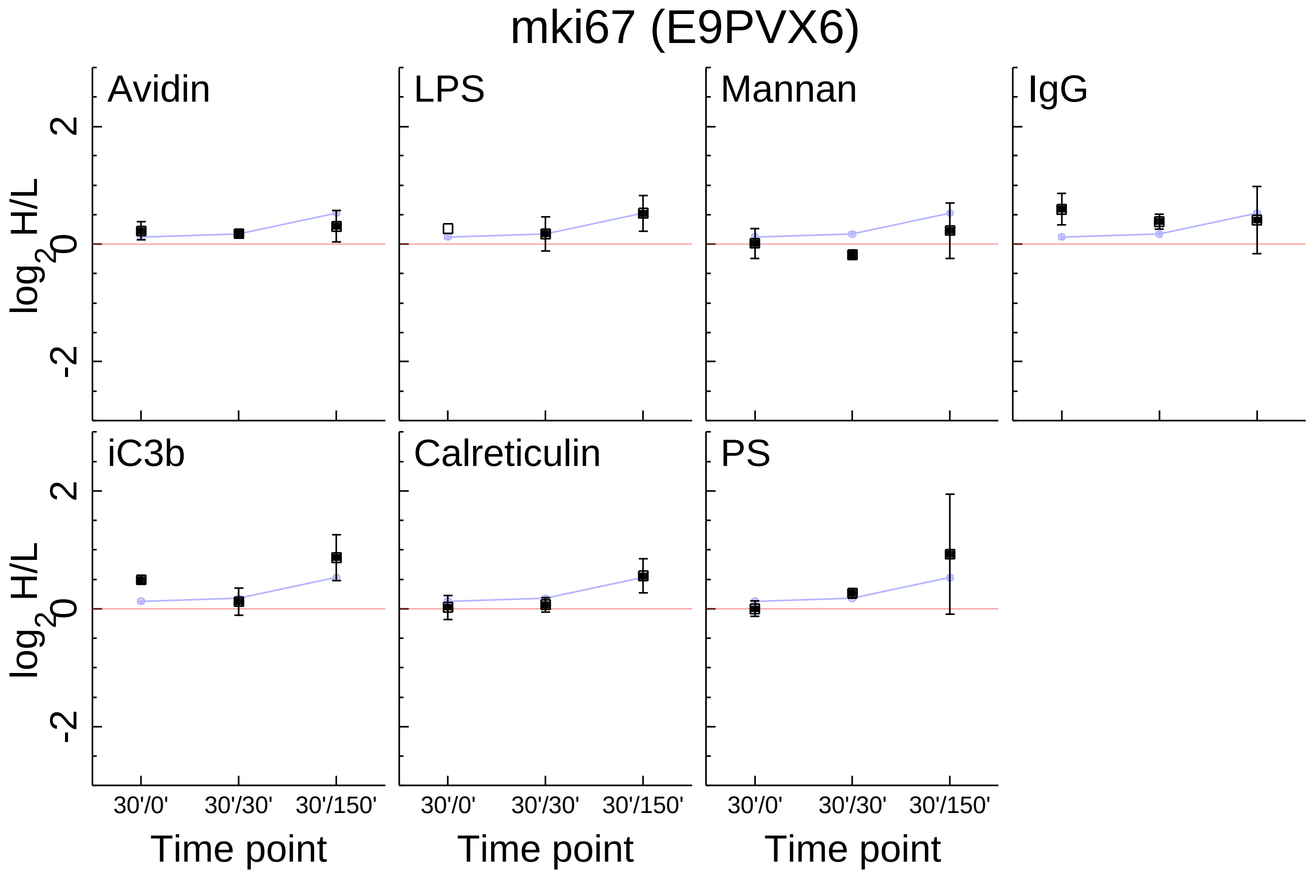
mki67 (E9PVX6)
Avidin
LPS
Mannan
IgG
iC3b
Calreticulin
PS
log2 H/L
2
0
-2
30'/0'
30'/30'
30'/150'
Time point

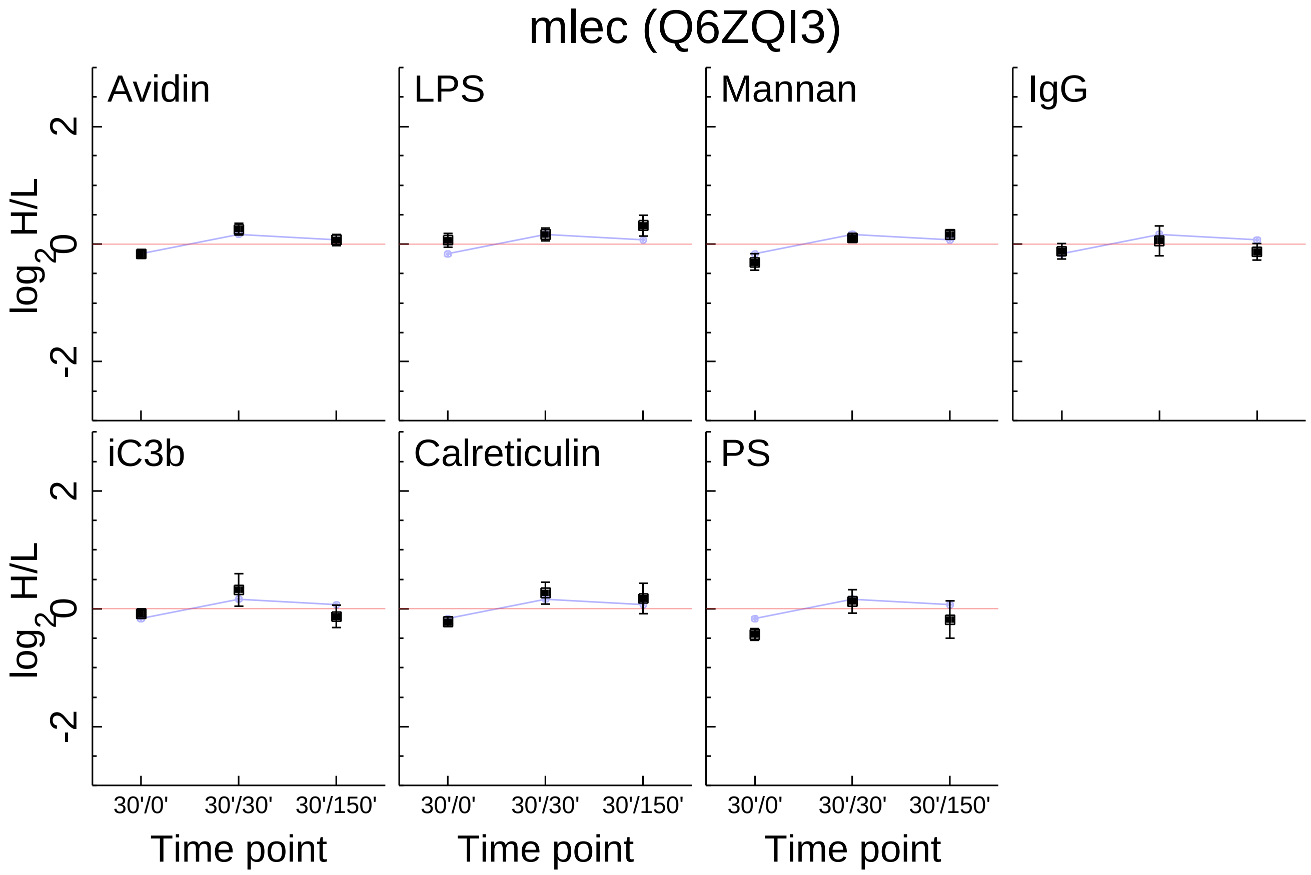
mlec (Q6ZQI3)
Avidin
LPS
Mannan
IgG
iC3b
Calreticulin
PS
log2 H/L
2
0
-2
30'/0'
30'/30'
30'/150'
Time point

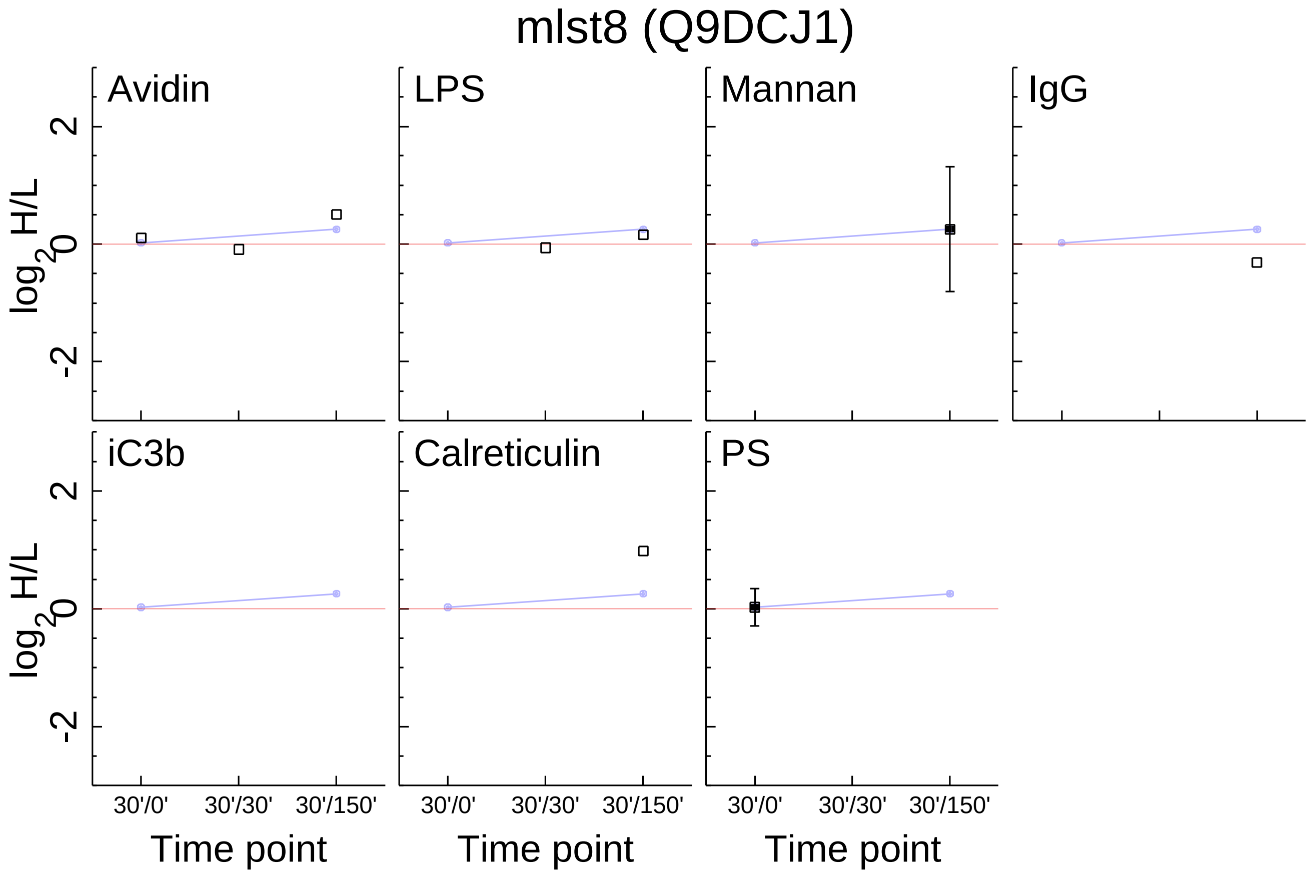

mlst8 (Q9DCJ1)
Avidin
LPS
Mannan
IgG
iC3b
Calreticulin
PS
log2 H/L
2
0
-2
30'/0'
30'/30'
30'/150'
Time point

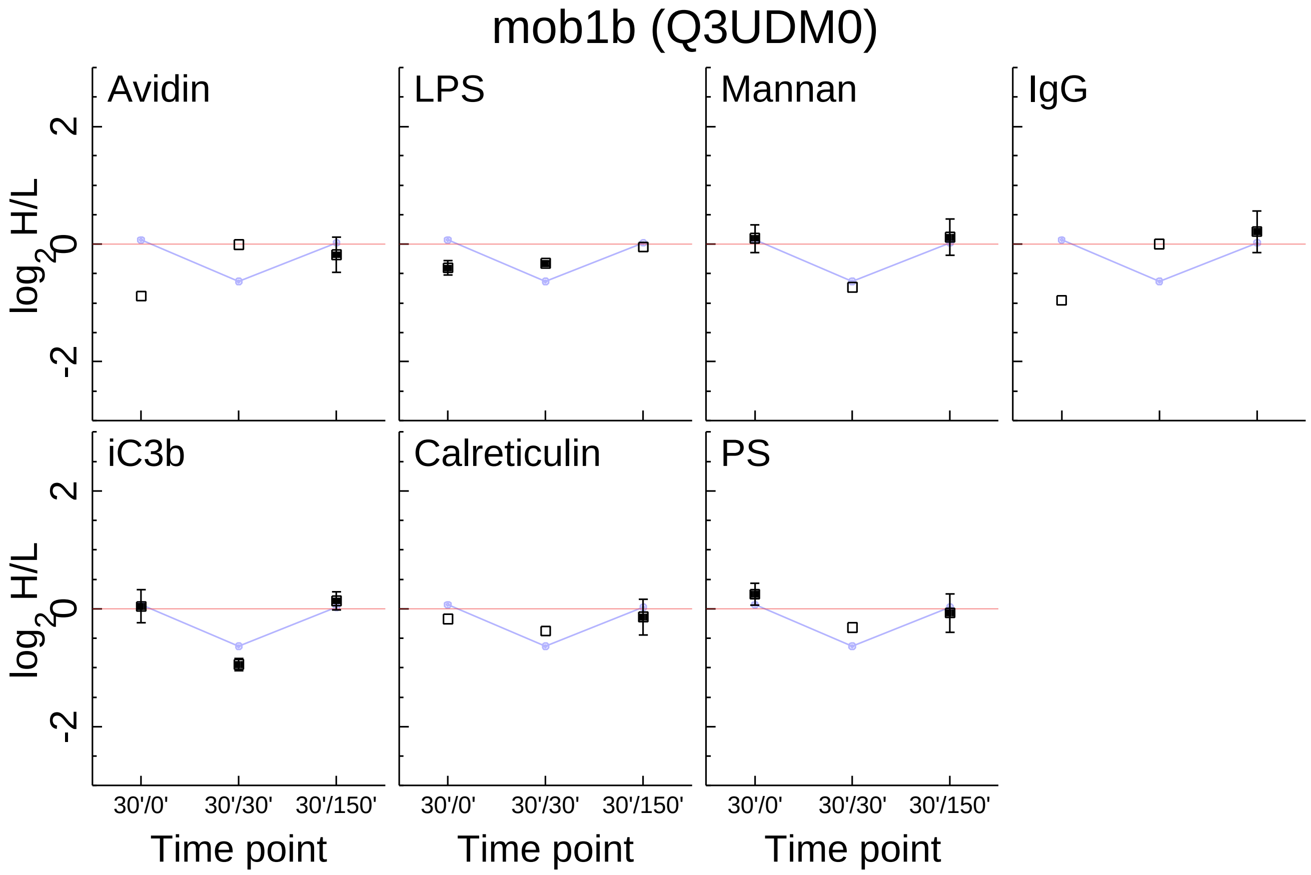
mob1b (Q3UDM0)
Avidin
LPS
Mannan
IgG
iC3b
Calreticulin
PS
log2 H/L
2
0
-2
30'/0'
30'/30'
30'/150'
Time point

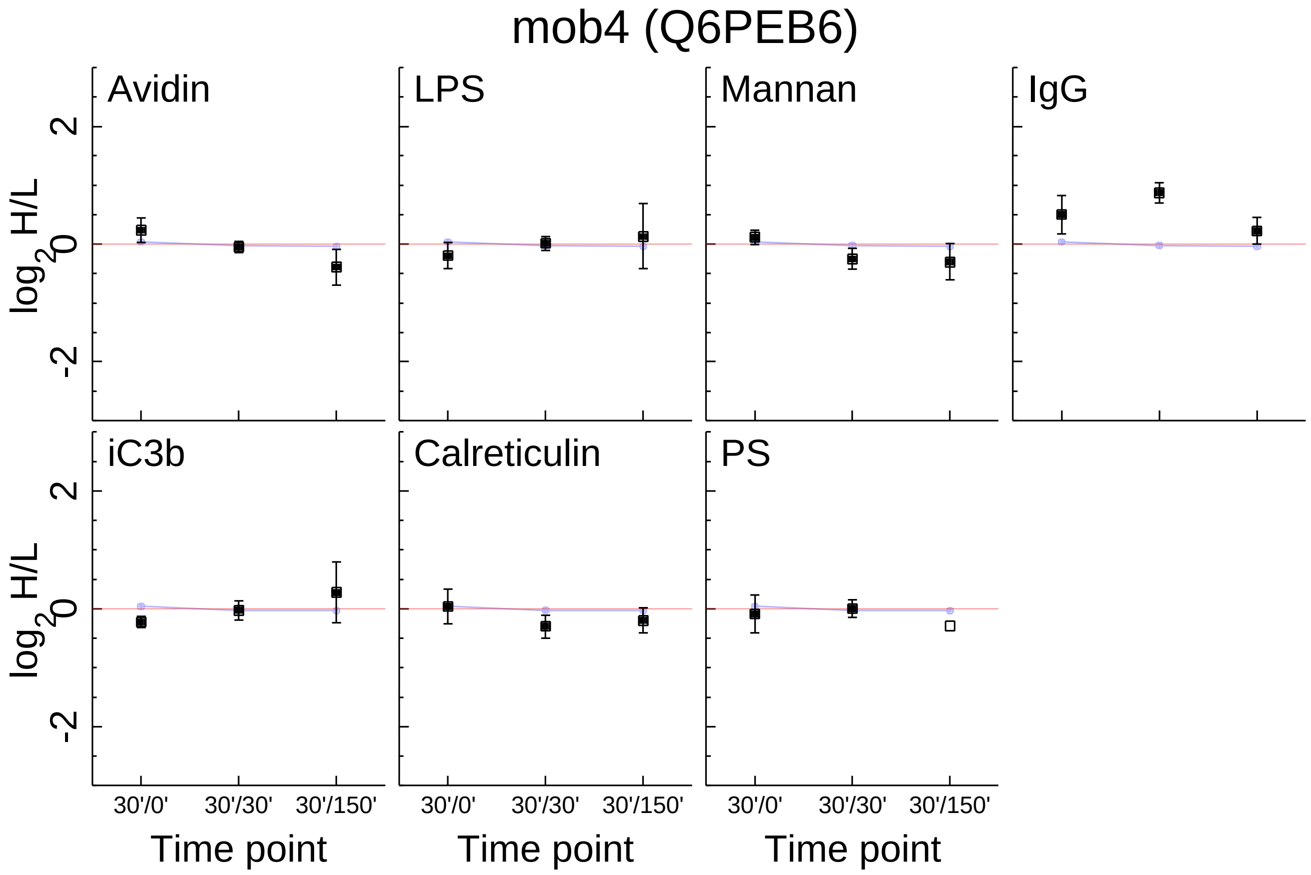

mob4 (Q6PEB6)
Avidin
LPS
Mannan
IgG
iC3b
Calreticulin
PS
2
0
-2
log2 H/L
30'/0'
30'/30'
30'/150'
Time point

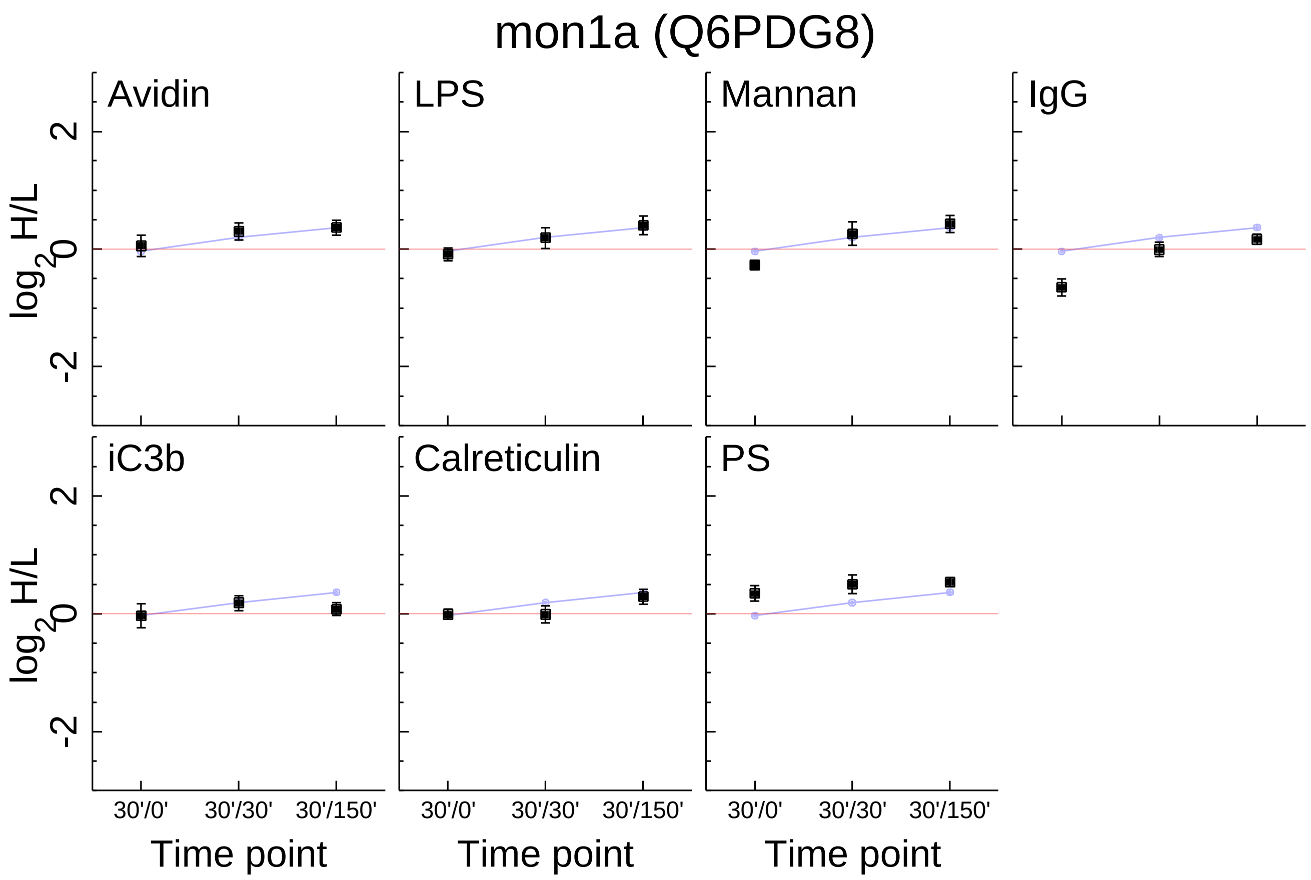
mon1a (Q6PDG8)
Avidin
LPS
Mannan
IgG
iC3b
Calreticulin
PS
log2 H/L
2
0
-2
30'/0'
30'/30'
30'/150'
Time point

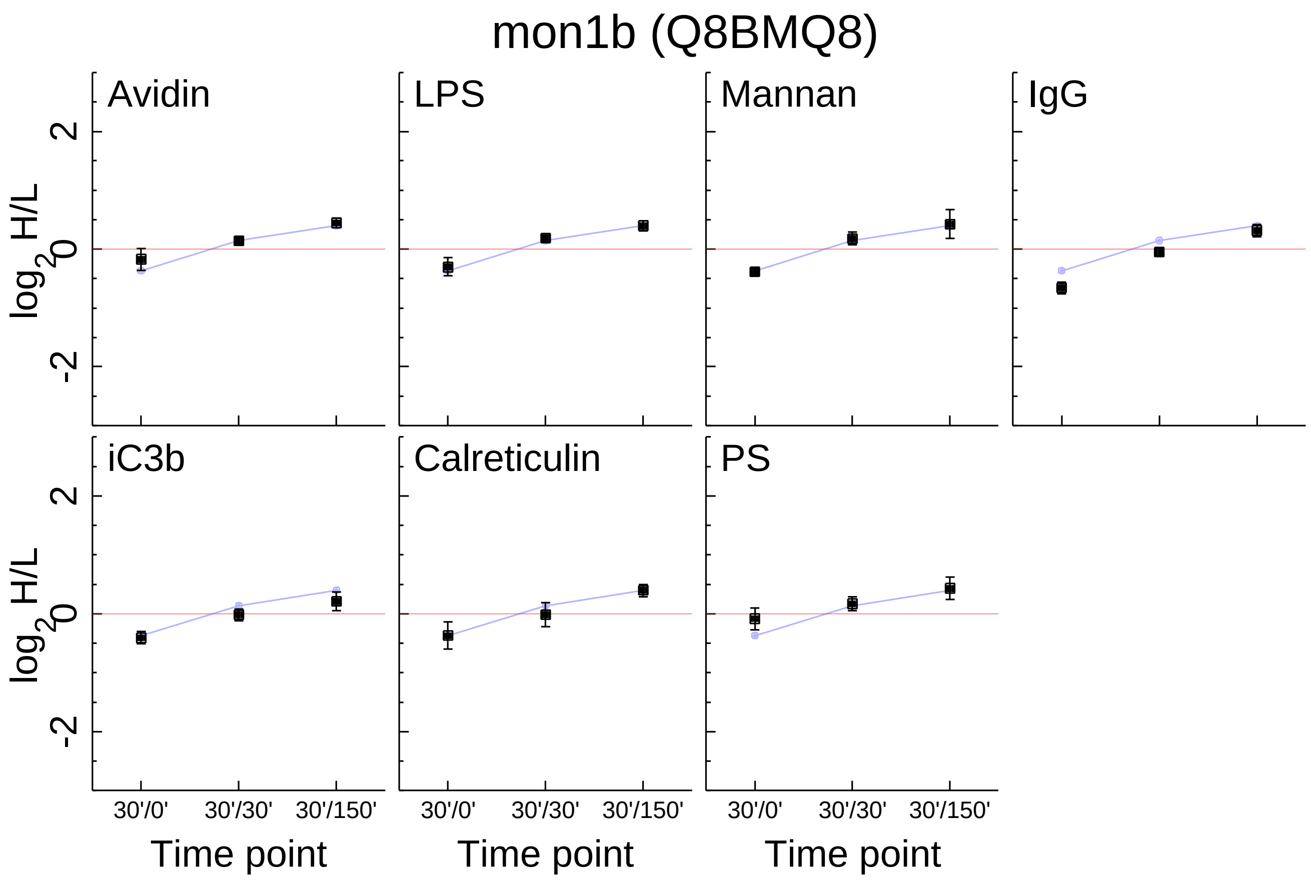
mon1b (Q8BMQ8)
Avidin
LPS
Mannan
IgG
iC3b
Calreticulin
PS
log2 H/L
2
0
-2
30'/0'
30'/30'
30'/150'
Time point

mpdu1 (Q8R0J2)

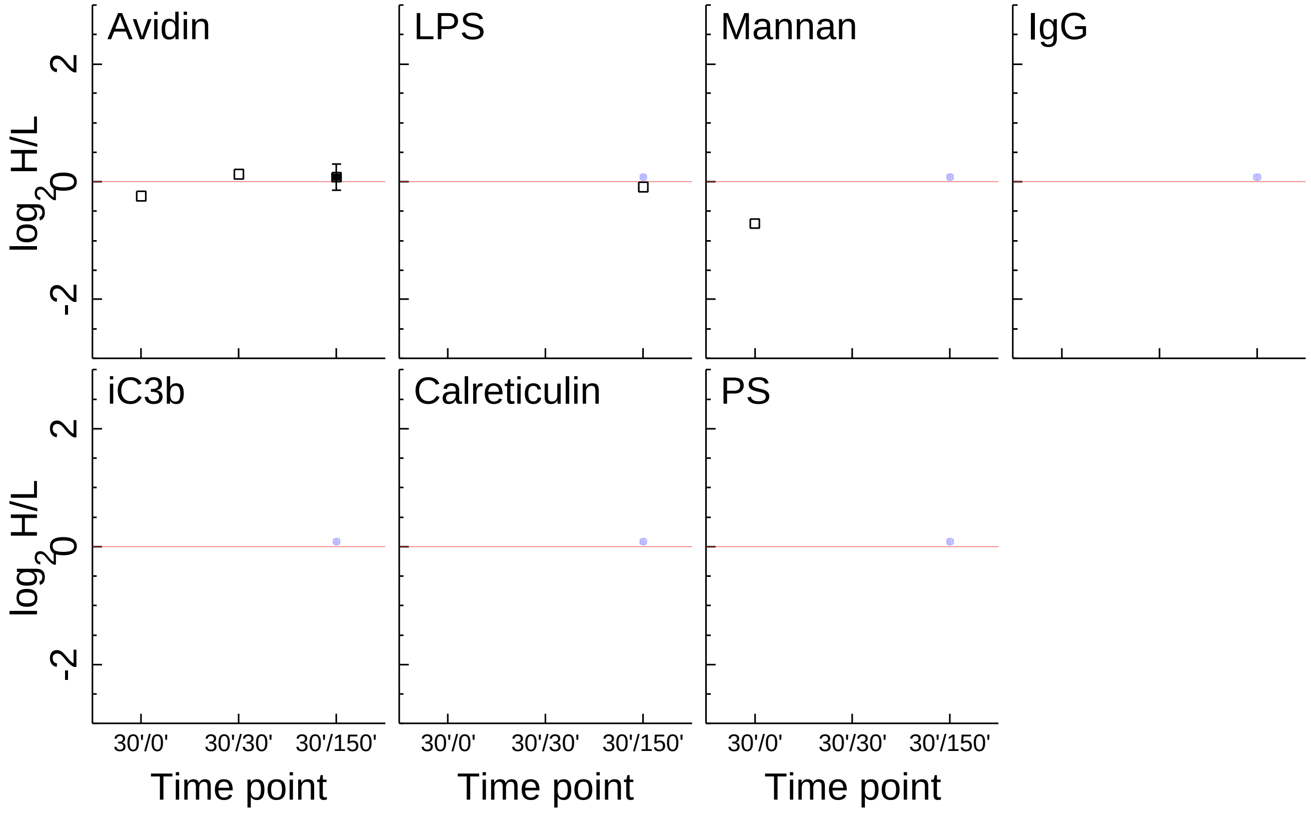

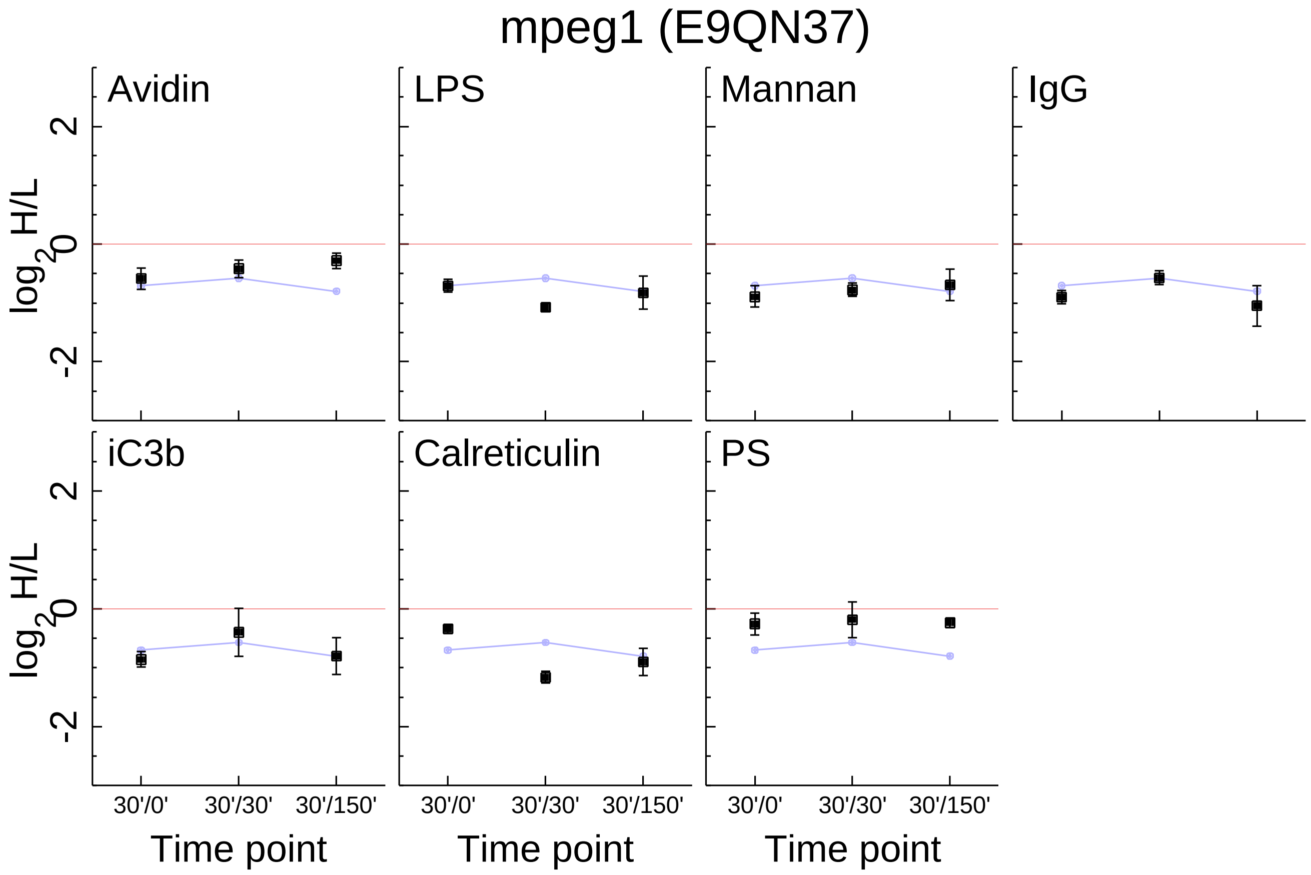

mpeg1 (E9QN37)
Avidin
LPS
Mannan
IgG
iC3b
Calreticulin
PS
log2 H/L
2
0
-2
30'/0'
30'/30'
30'/150'
Time point

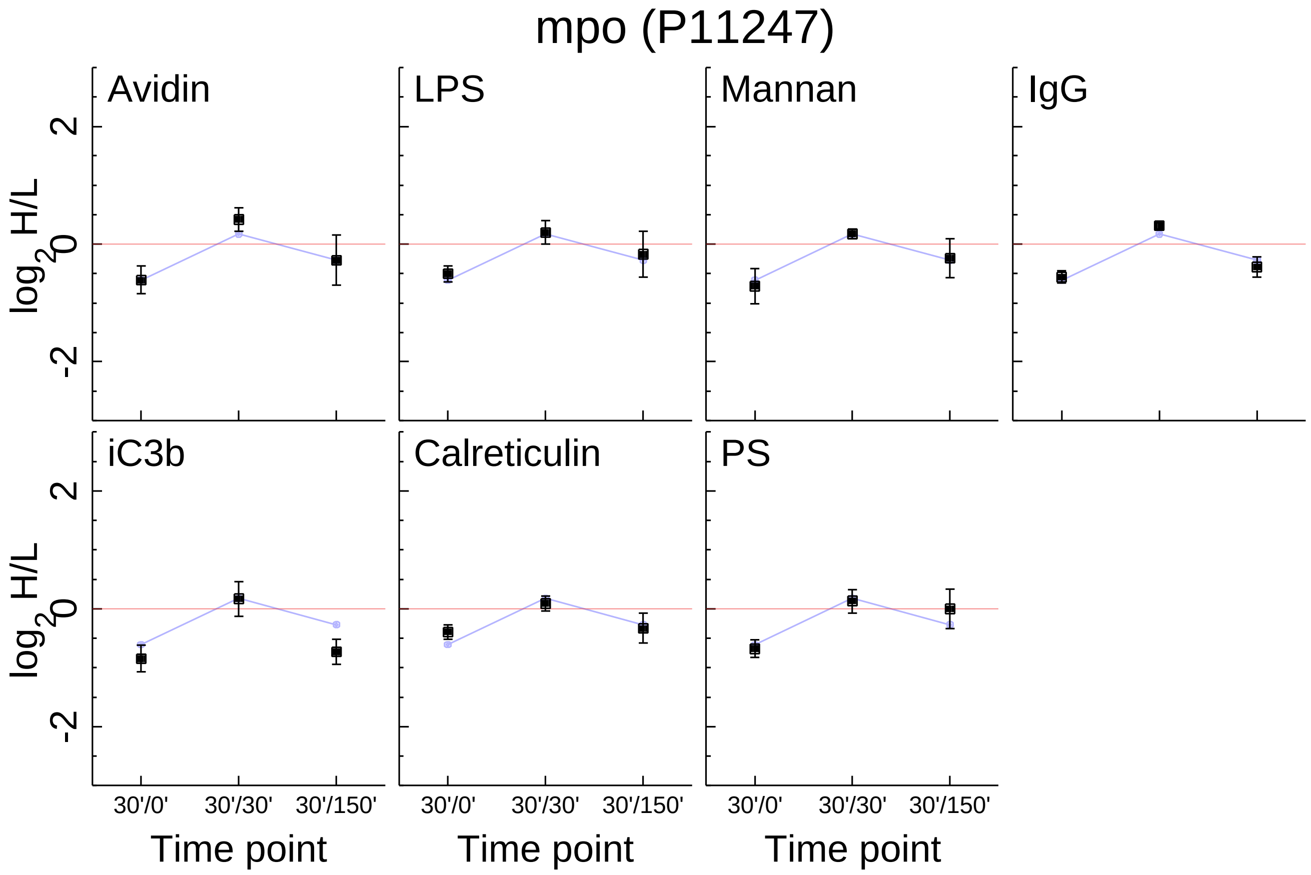
mpo (P11247)
Avidin
LPS
Mannan
IgG
iC3b
Calreticulin
PS
log2 H/L
2
0
-2
30'/0'
30'/30'
30'/150'
Time point

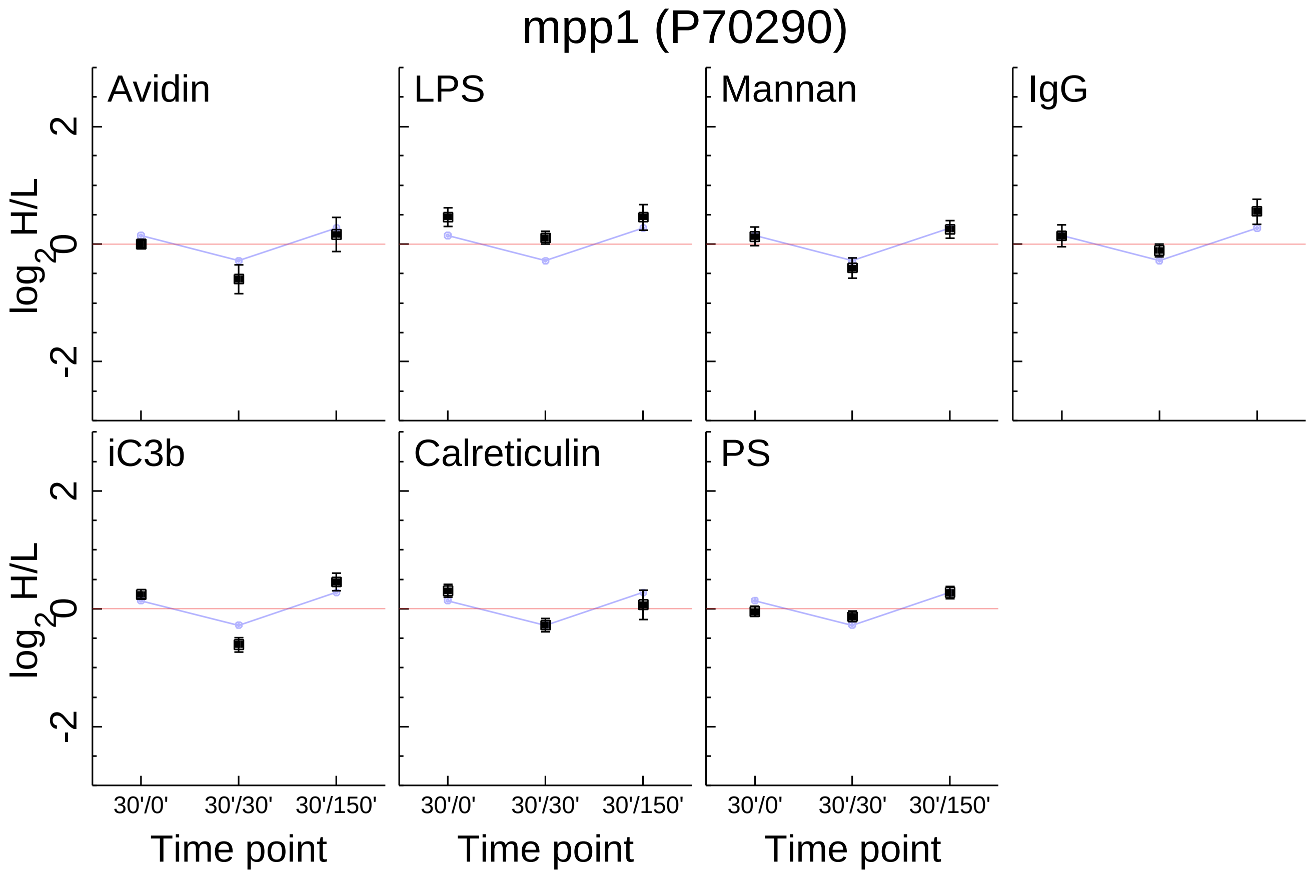
mpp1 (P70290)
Avidin
LPS
Mannan
IgG
iC3b
Calreticulin
PS
log2 H/L
2
0
-2
30'/0'
30'/30'
30'/150'
Time point

mrc1 (Q61830)

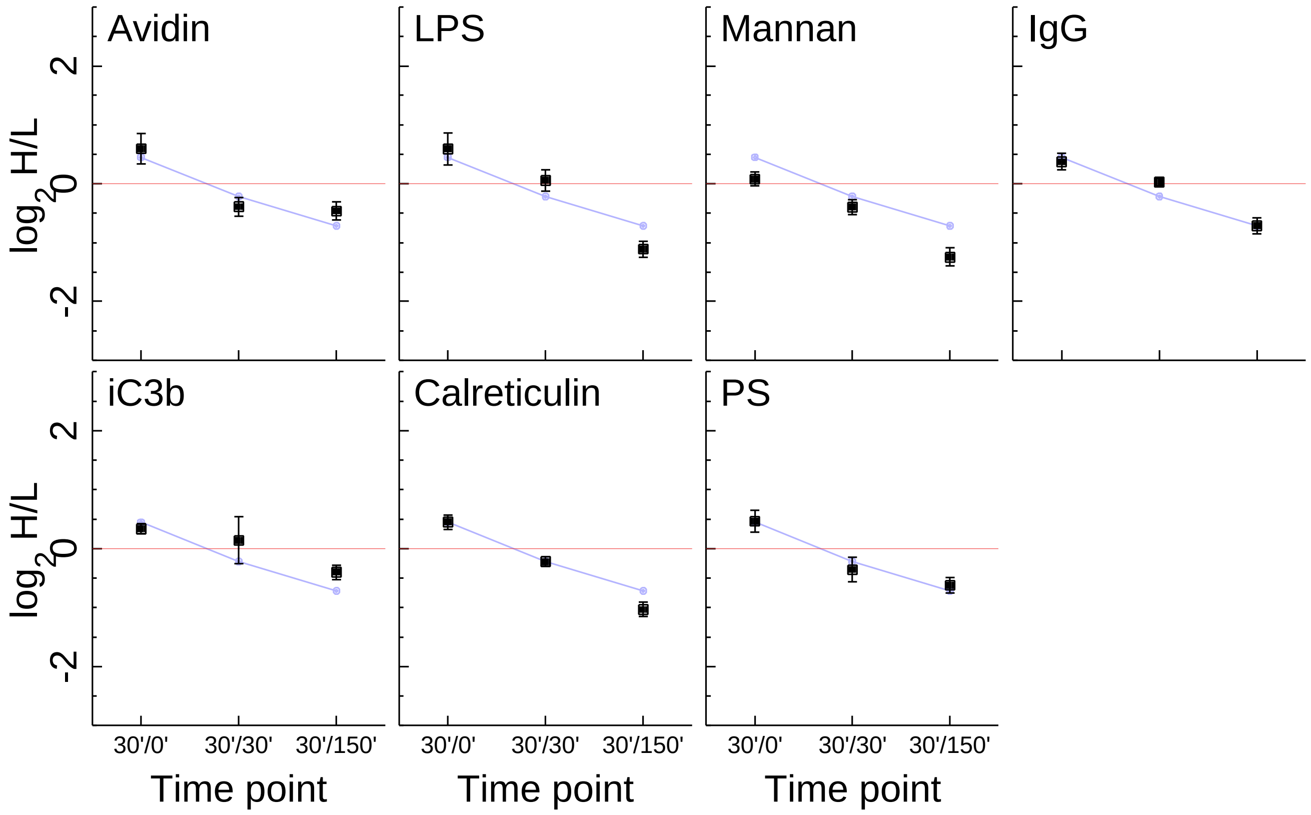

Avidin
LPS
Mannan
IgG
iC3b
Calreticulin
PS
log2 H/L
2
0
-2
30'/0'
30'/30'
30'/150'
Time point

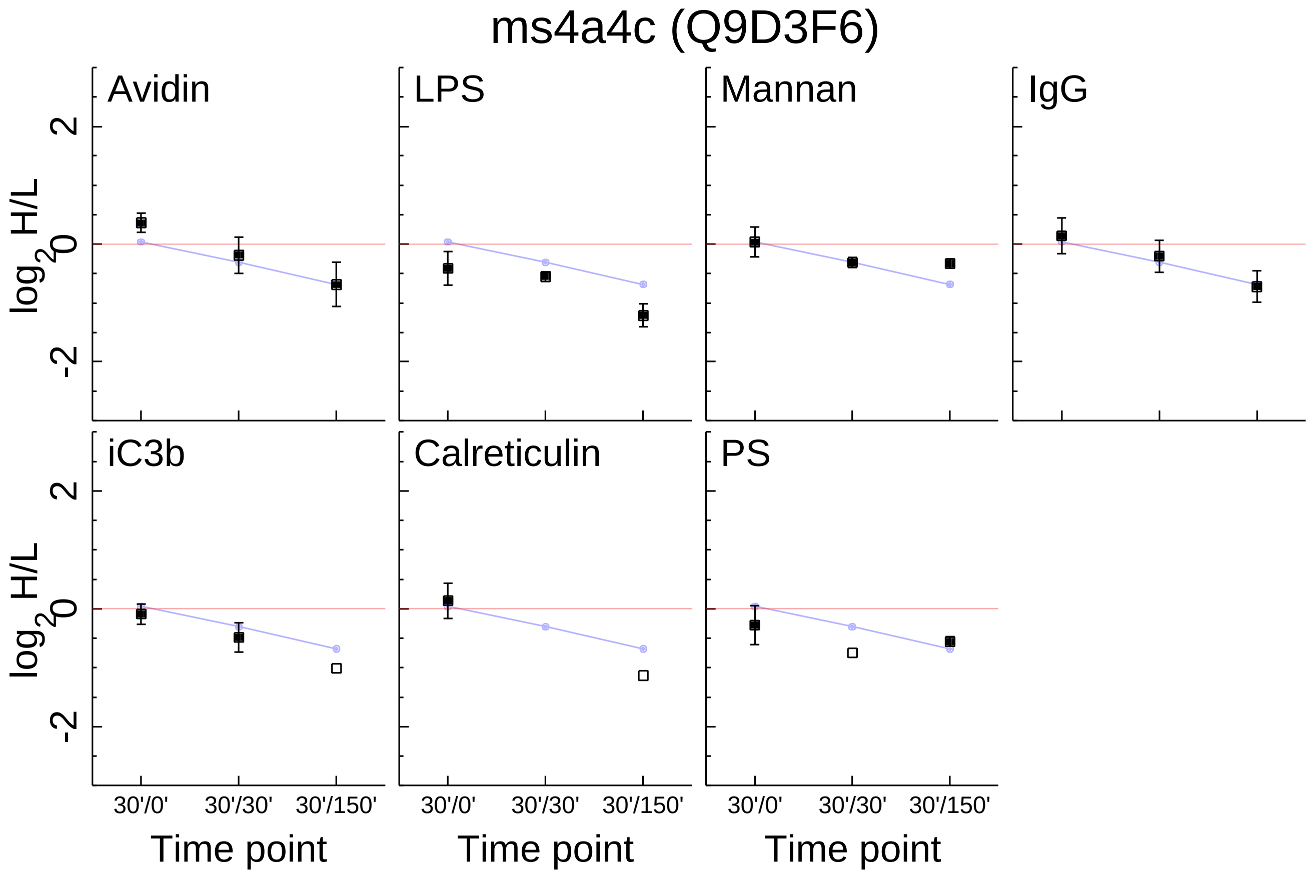

ms4a4c (Q9D3F6)
Avidin
LPS
Mannan
IgG
iC3b
Calreticulin
PS
log2 H/L
2
0
-2
30'/0'
30'/30'
30'/150'
Time point

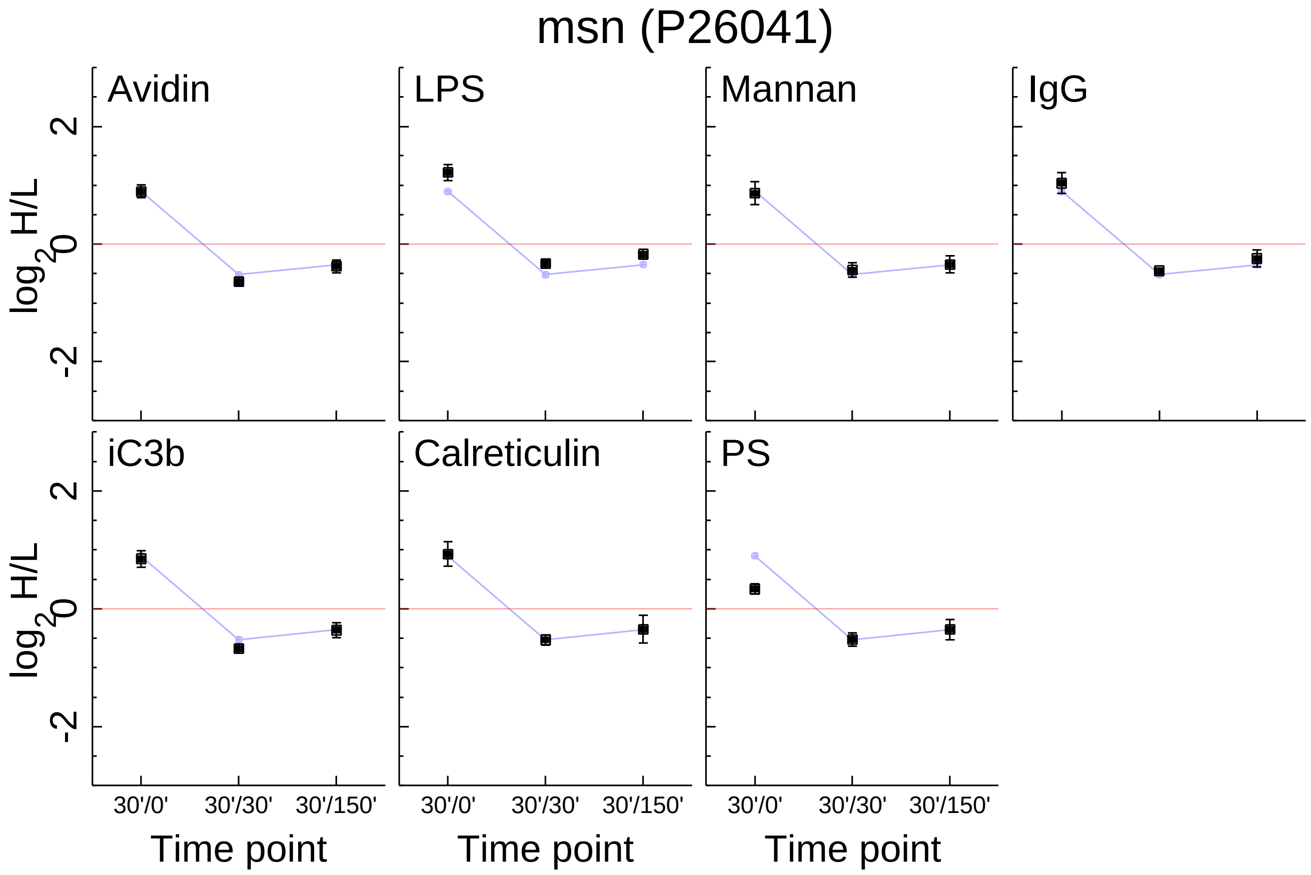

msn (P26041)
Avidin
LPS
Mannan
IgG
iC3b
Calreticulin
PS
log2 H/L
2
0
-2
30'/0'
30'/30'
30'/150'
Time point

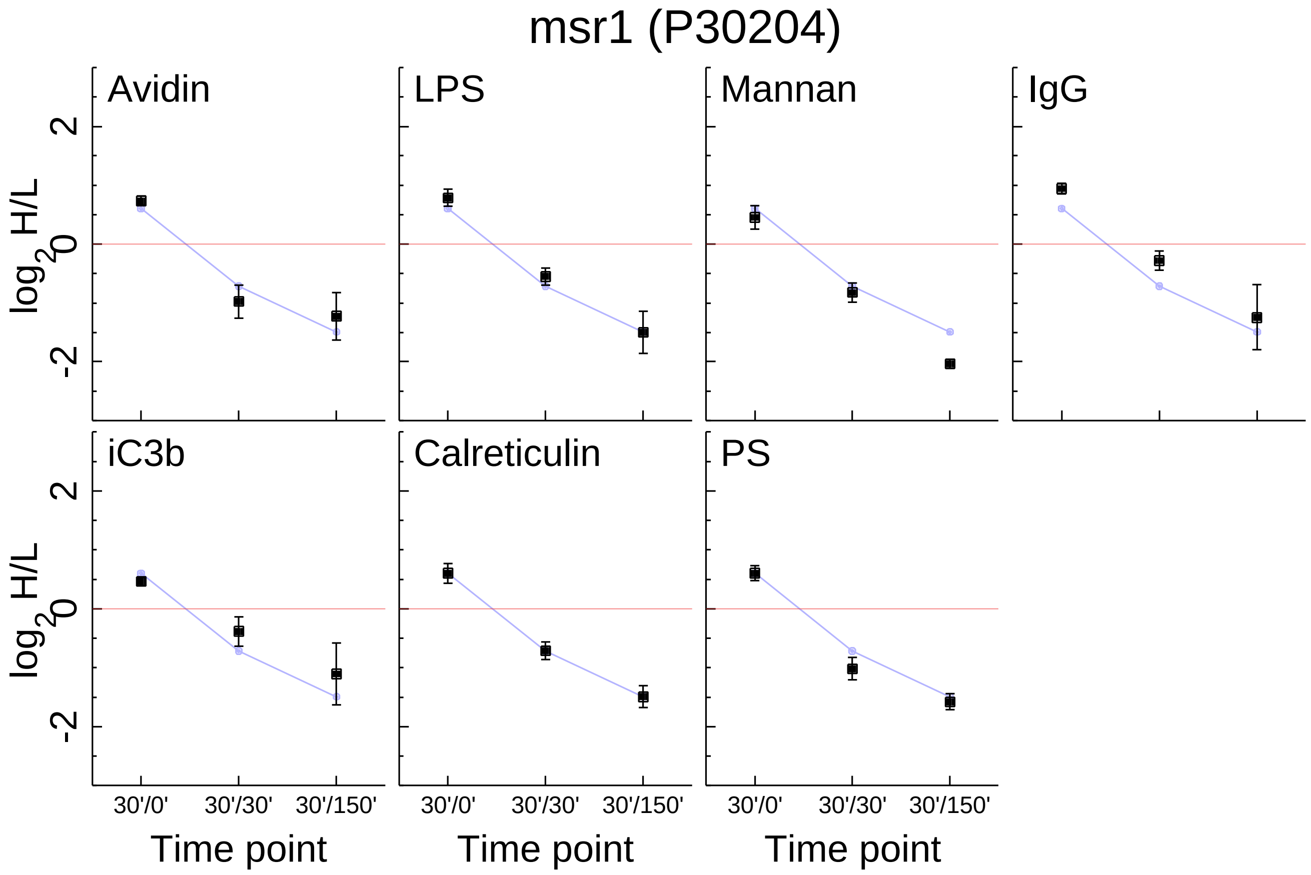

msr1 (P30204)
Avidin
LPS
Mannan
IgG
iC3b
Calreticulin
PS
log2 H/L
2
0
-2
30'/0'
30'/30'
30'/150'
Time point

msra (Q9D6Y7)

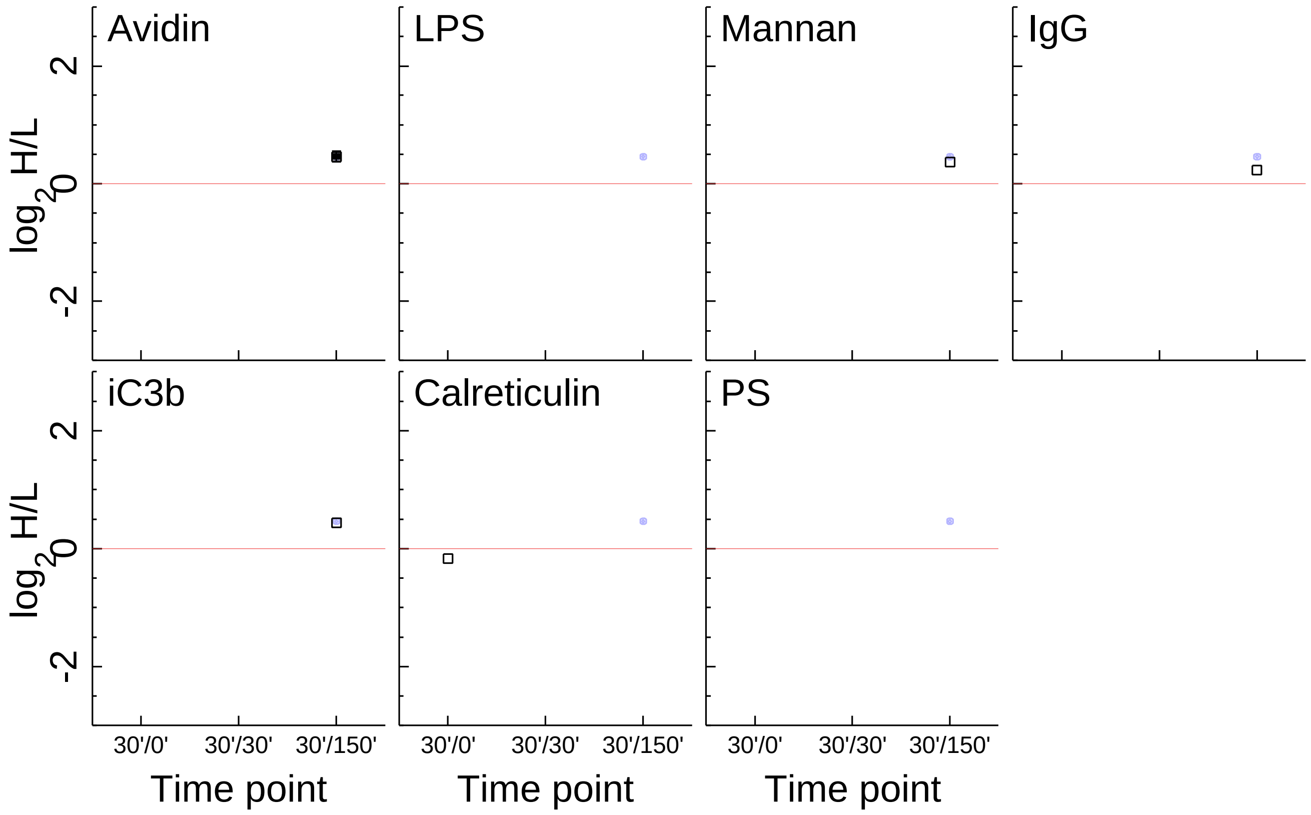

Avidin
LPS
Mannan
IgG
iC3b
Calreticulin
PS
log2 H/L
2
0
-2
30'/0'
30'/30'
30'/150'
Time point

mtatp8 (P03930)

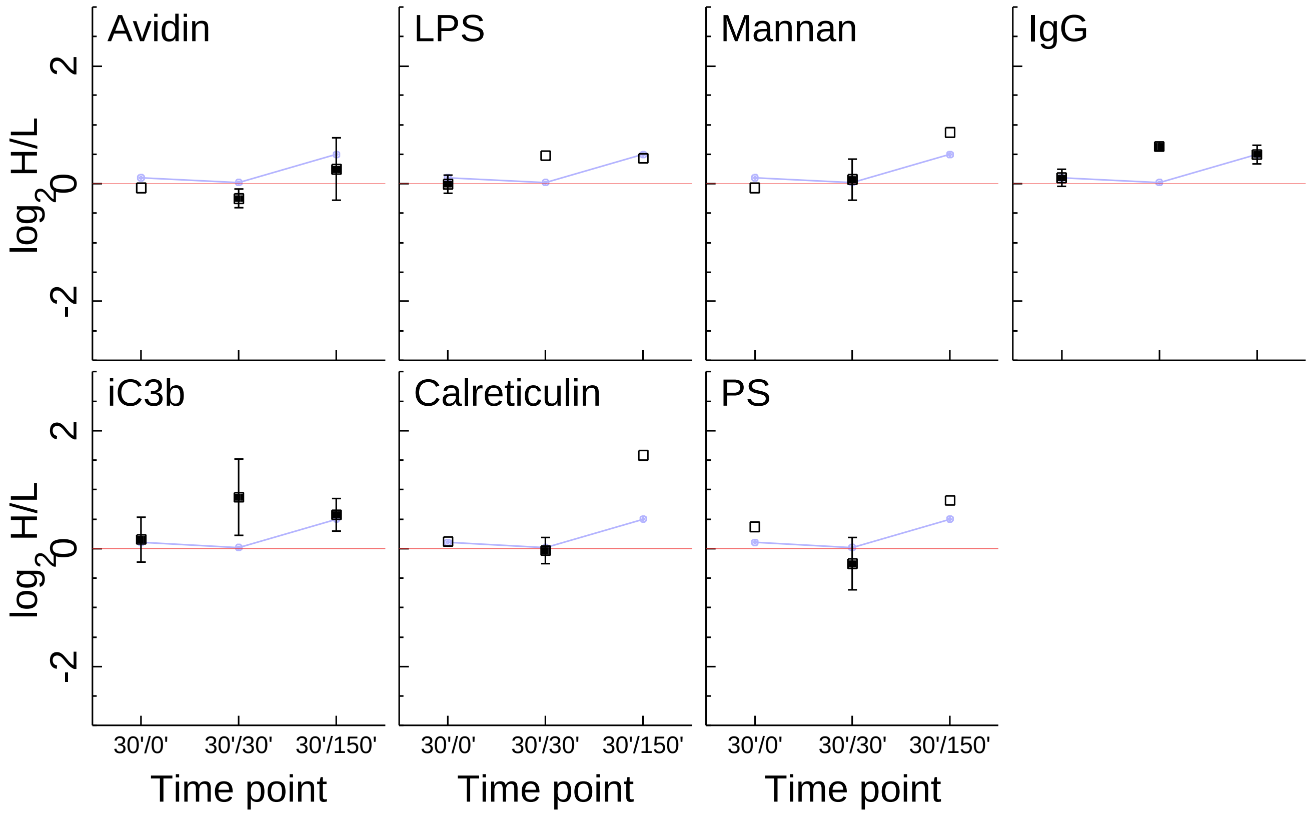

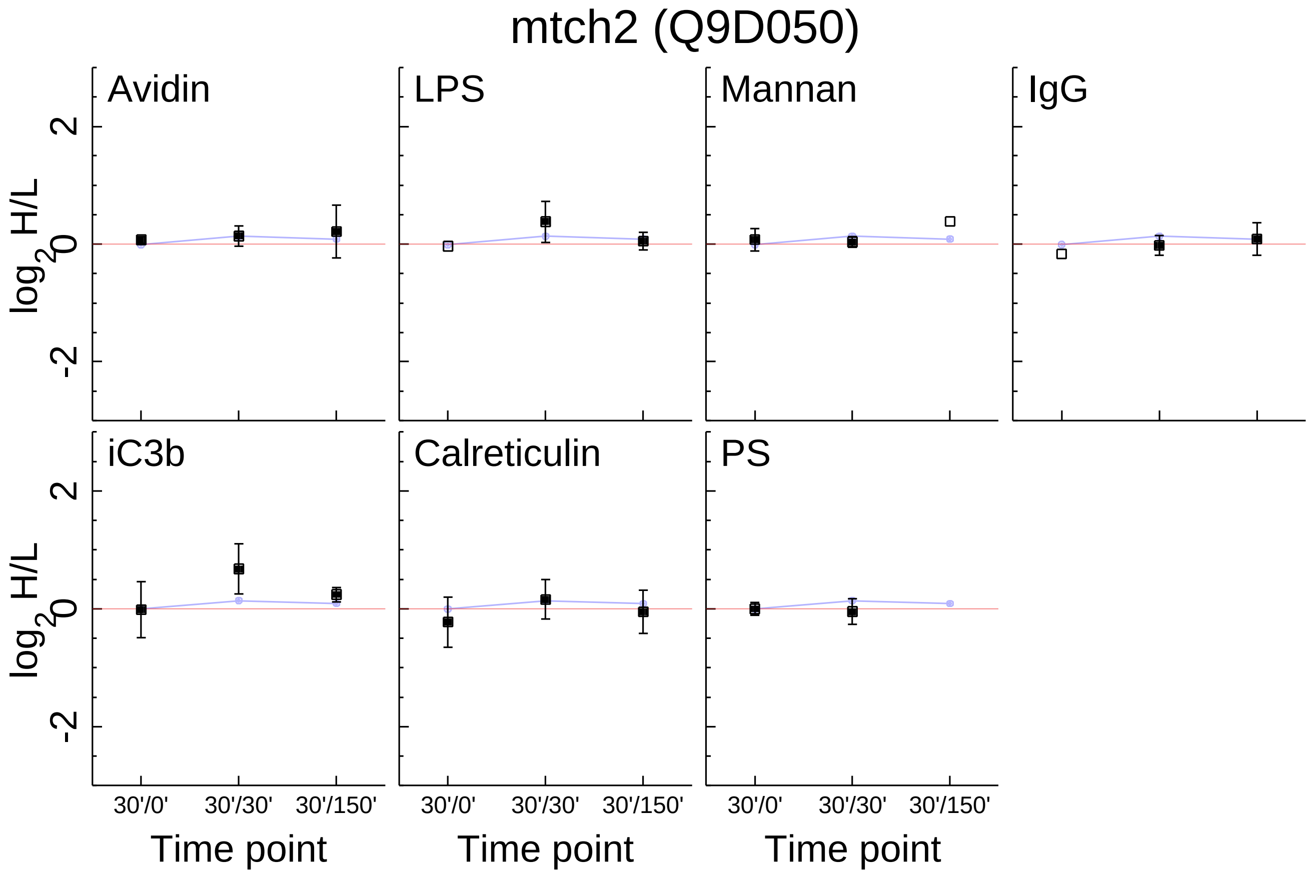
mtch2 (Q9D050)
Avidin
LPS
Mannan
IgG
iC3b
Calreticulin
PS
log2 H/L
2
0
-2
30'/0'
30'/30'
30'/150'
Time point

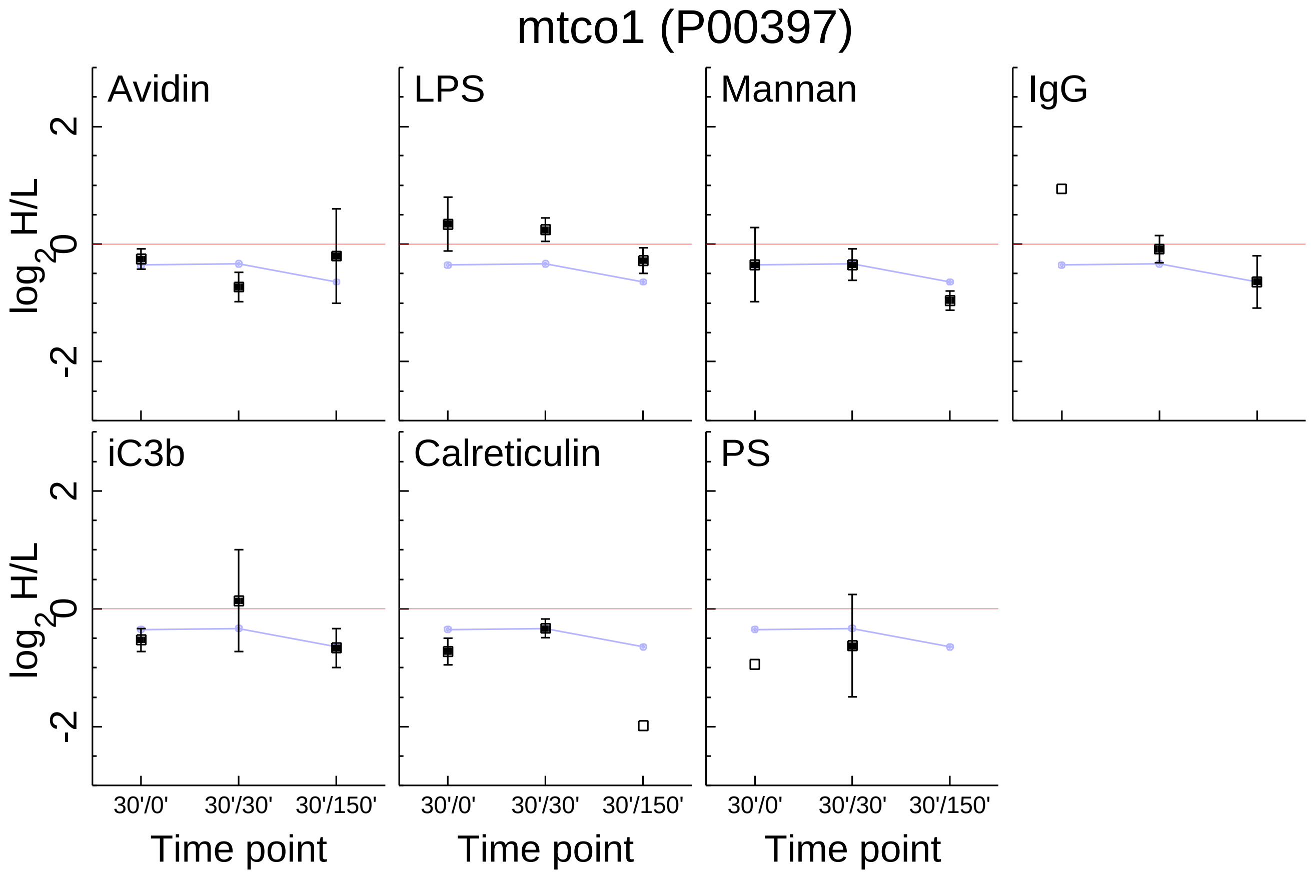
mtco1 (P00397)
Avidin
LPS
Mannan
IgG
iC3b
Calreticulin
PS
log2 H/L
2
0
-2
30'/0'
30'/30'
30'/150'
Time point

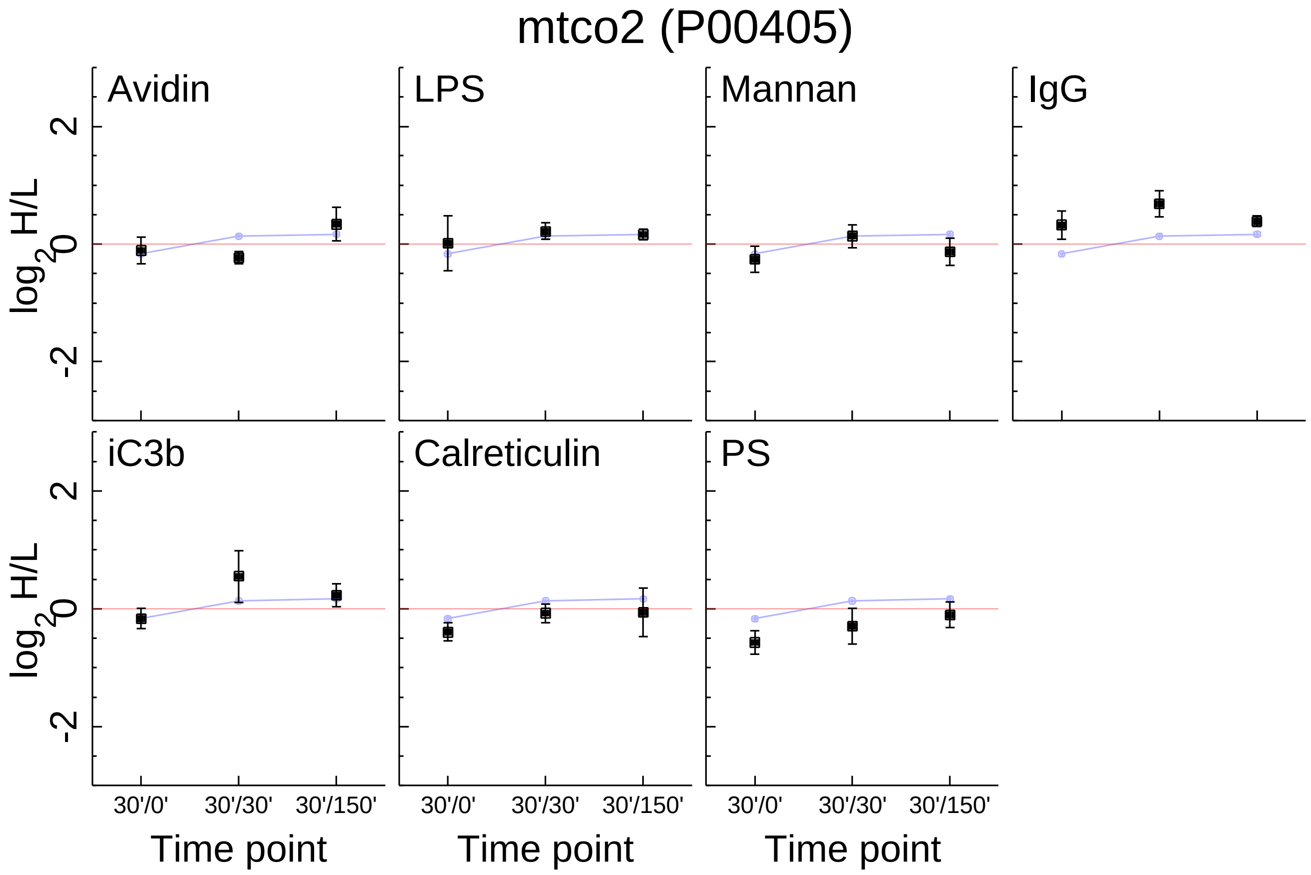

mtco2 (P00405)
Avidin
LPS
Mannan
IgG
iC3b
Calreticulin
PS
log2 H/L
2
0
-2
30'/0'
30'/30'
30'/150'
Time point

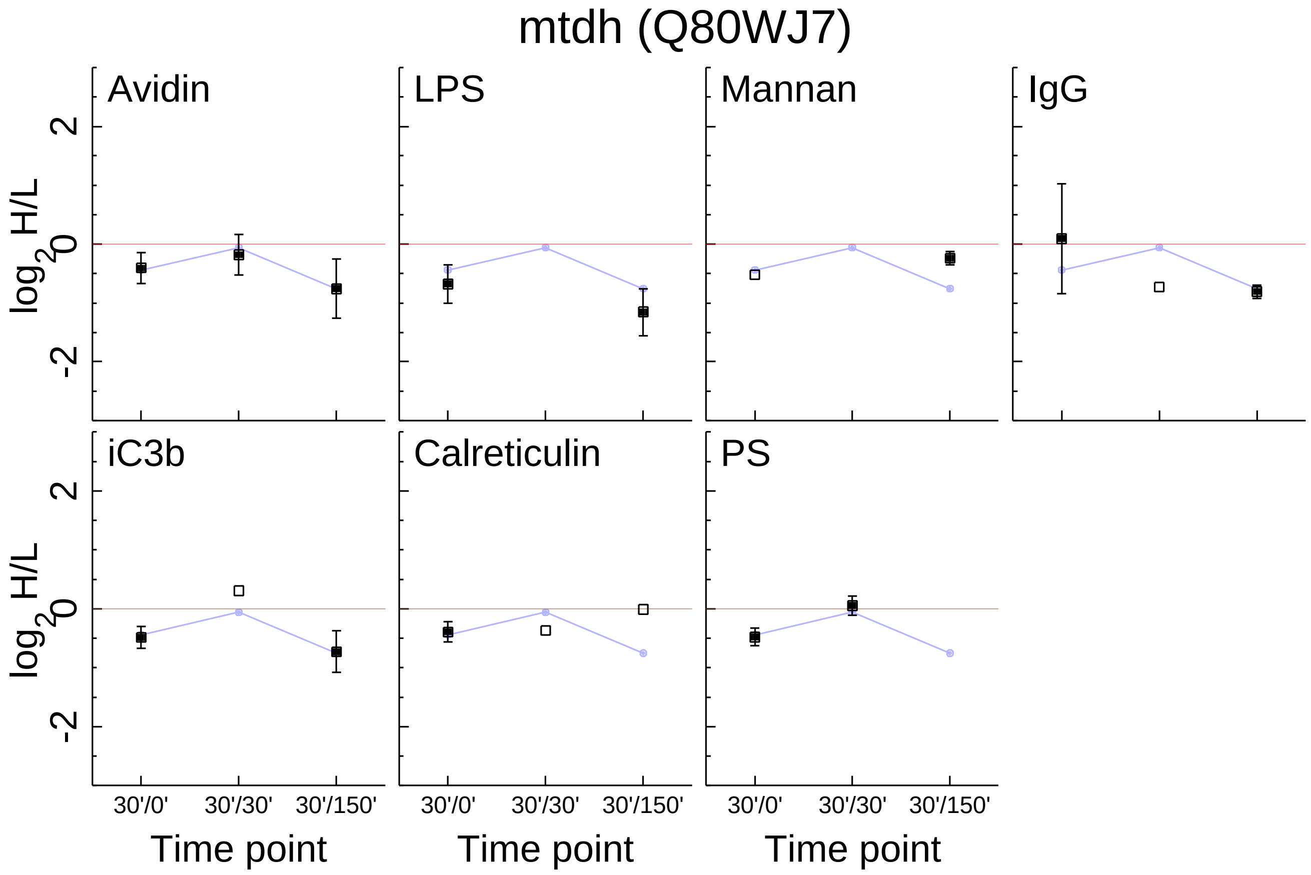
mtdh (Q80WJ7)
Avidin
LPS
Mannan
IgG
iC3b
Calreticulin
PS
log2 H/L
2
0
-2
30'/0'
30'/30'
30'/150'
Time point

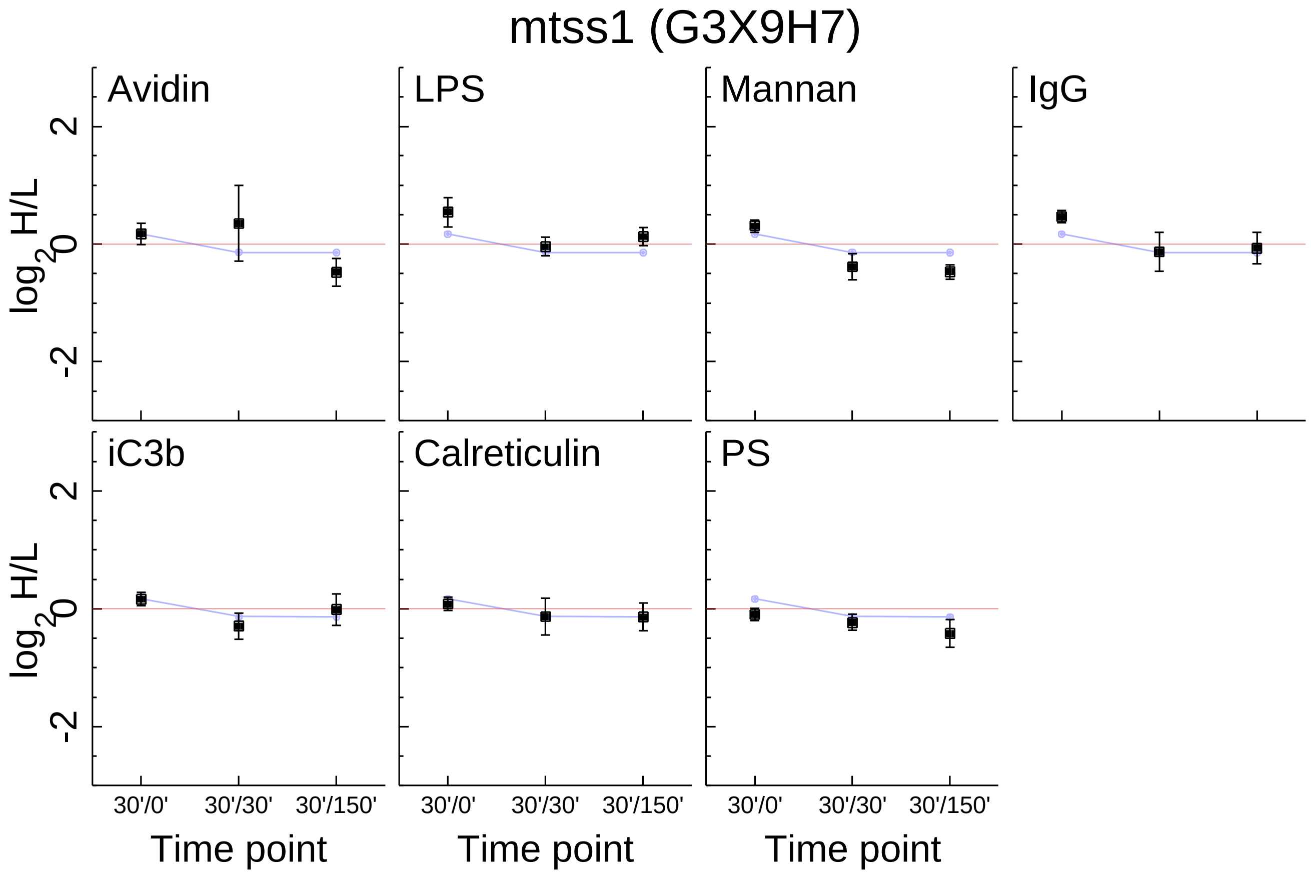
mtss1 (G3X9H7)
Avidin
LPS
Mannan
IgG
iC3b
Calreticulin
PS
log2 H/L
2
0
-2
30'/0'
30'/30'
30'/150'
Time point

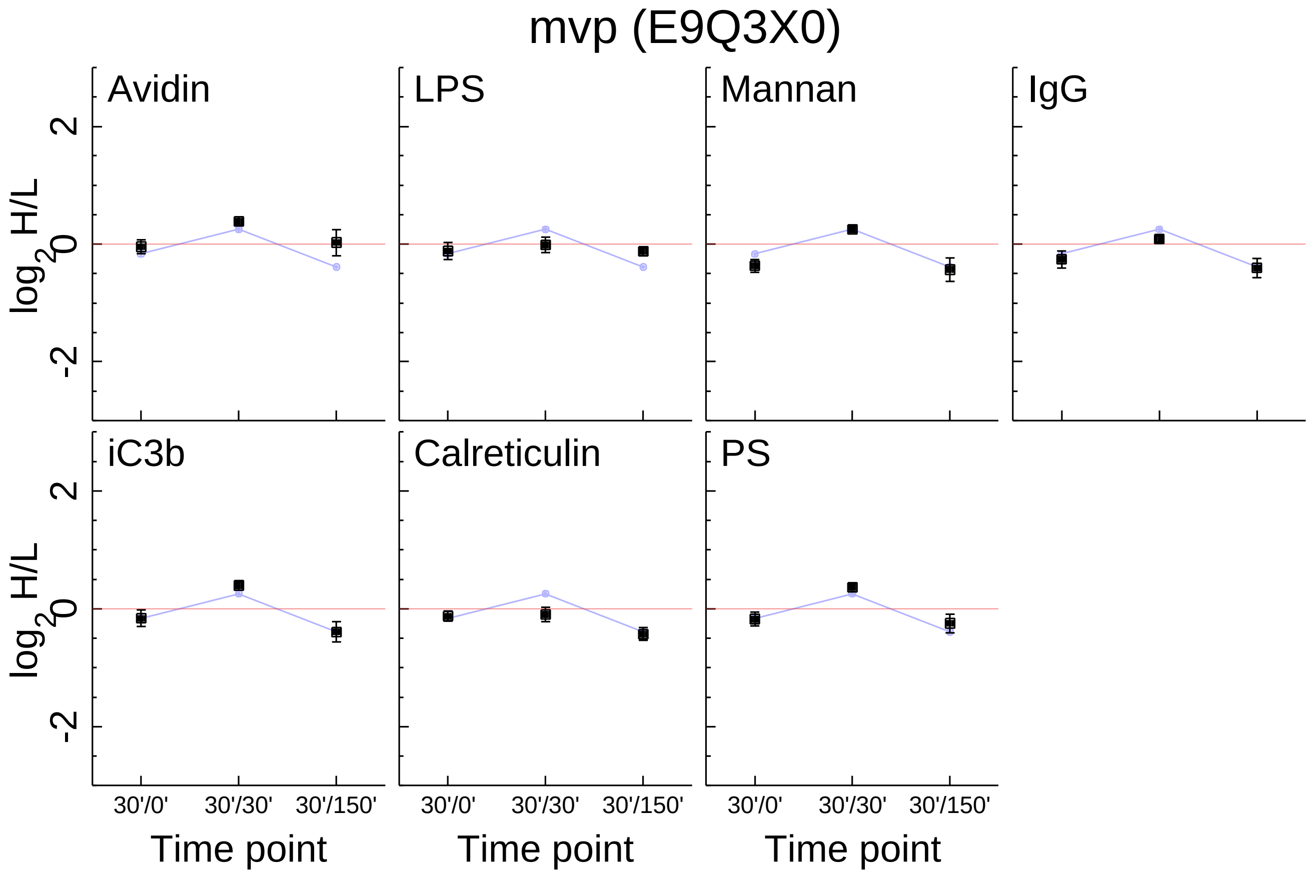
mvp (E9Q3X0)
Avidin
LPS
Mannan
IgG
iC3b
Calreticulin
PS
2
0
-2
log2 H/L
30'/0'
30'/30'
30'/150'
Time point

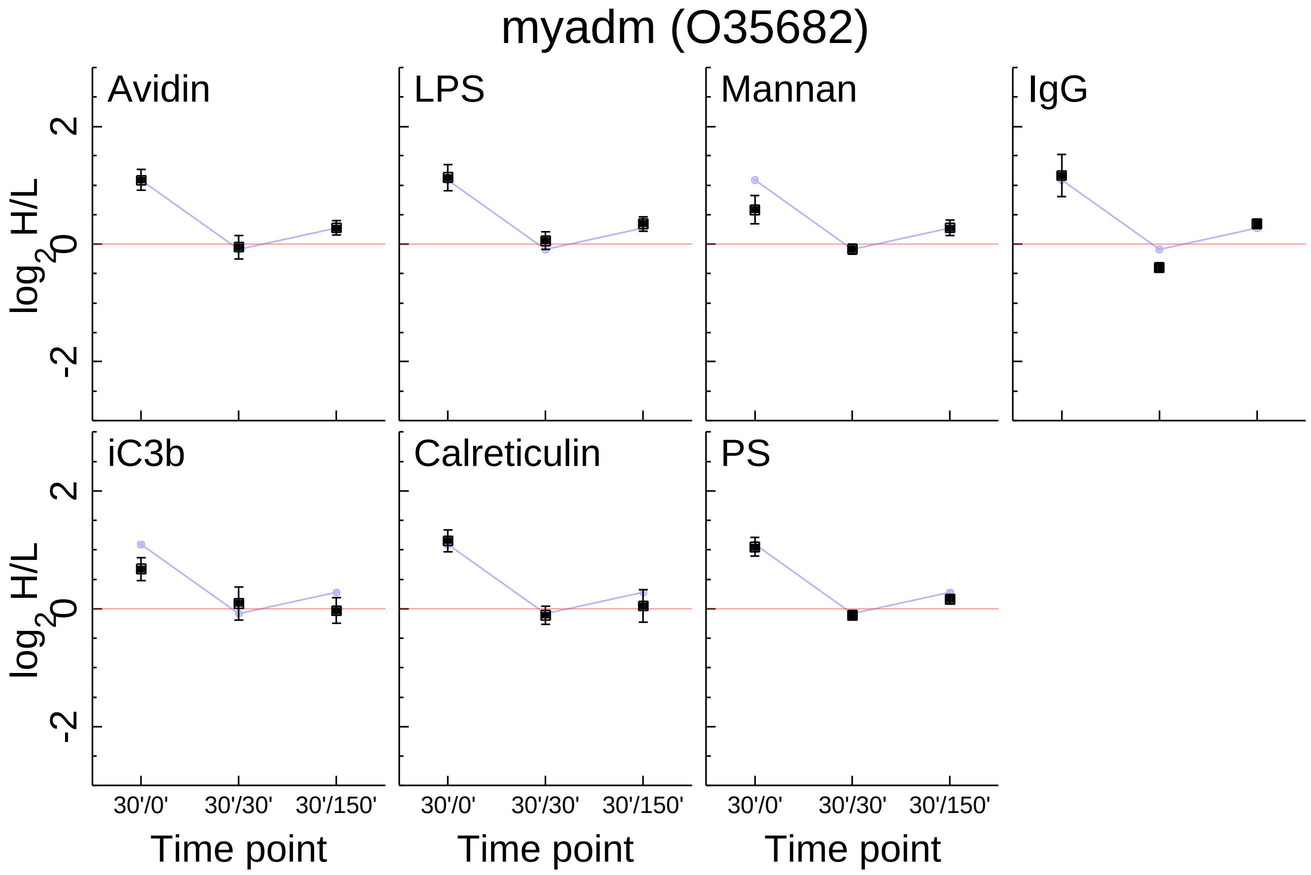
myadm (O35682)
Avidin
LPS
Mannan
IgG
iC3b
Calreticulin
PS
log2 H/L
2
0
-2
30'/0'
30'/30'
30'/150'
Time point

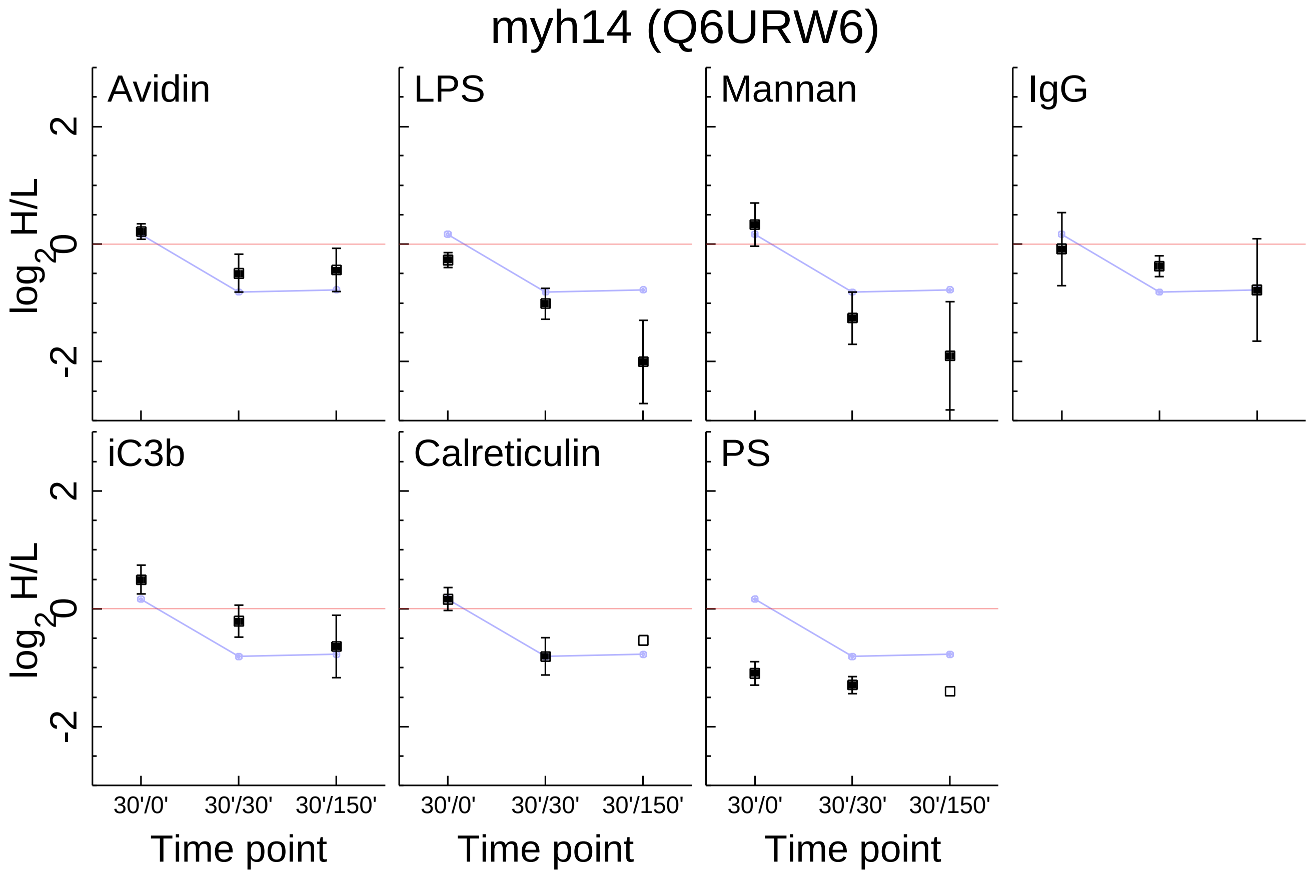

myh14 (Q6URW6)
Avidin
LPS
Mannan
IgG
iC3b
Calreticulin
PS
log2 H/L
2
0
-2
30'/0'
30'/30'
30'/150'
Time point

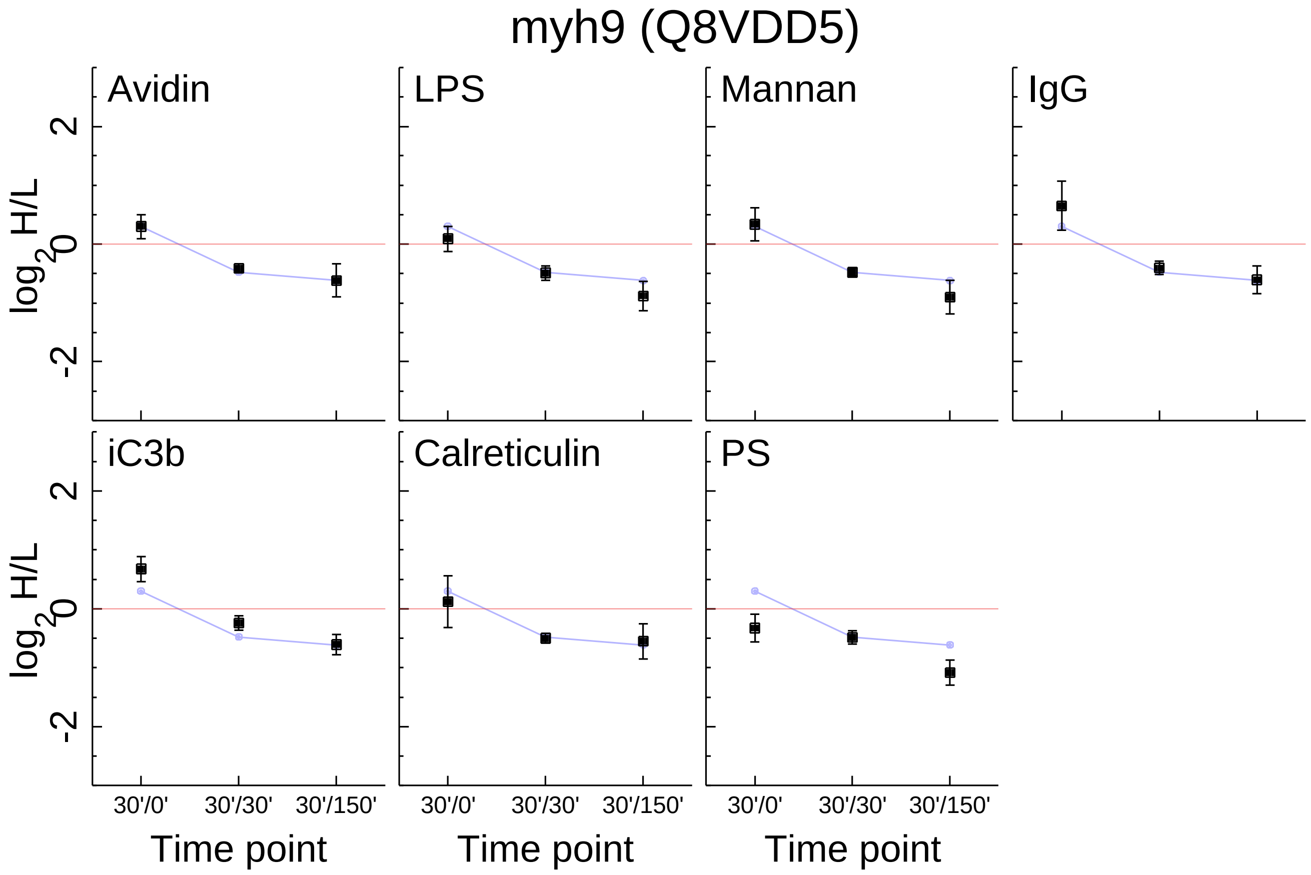

myh9 (Q8VDD5)
Avidin
LPS
Mannan
IgG
iC3b
Calreticulin
PS
log2 H/L
2
0
-2
30'/0'
30'/30'
30'/150'
Time point

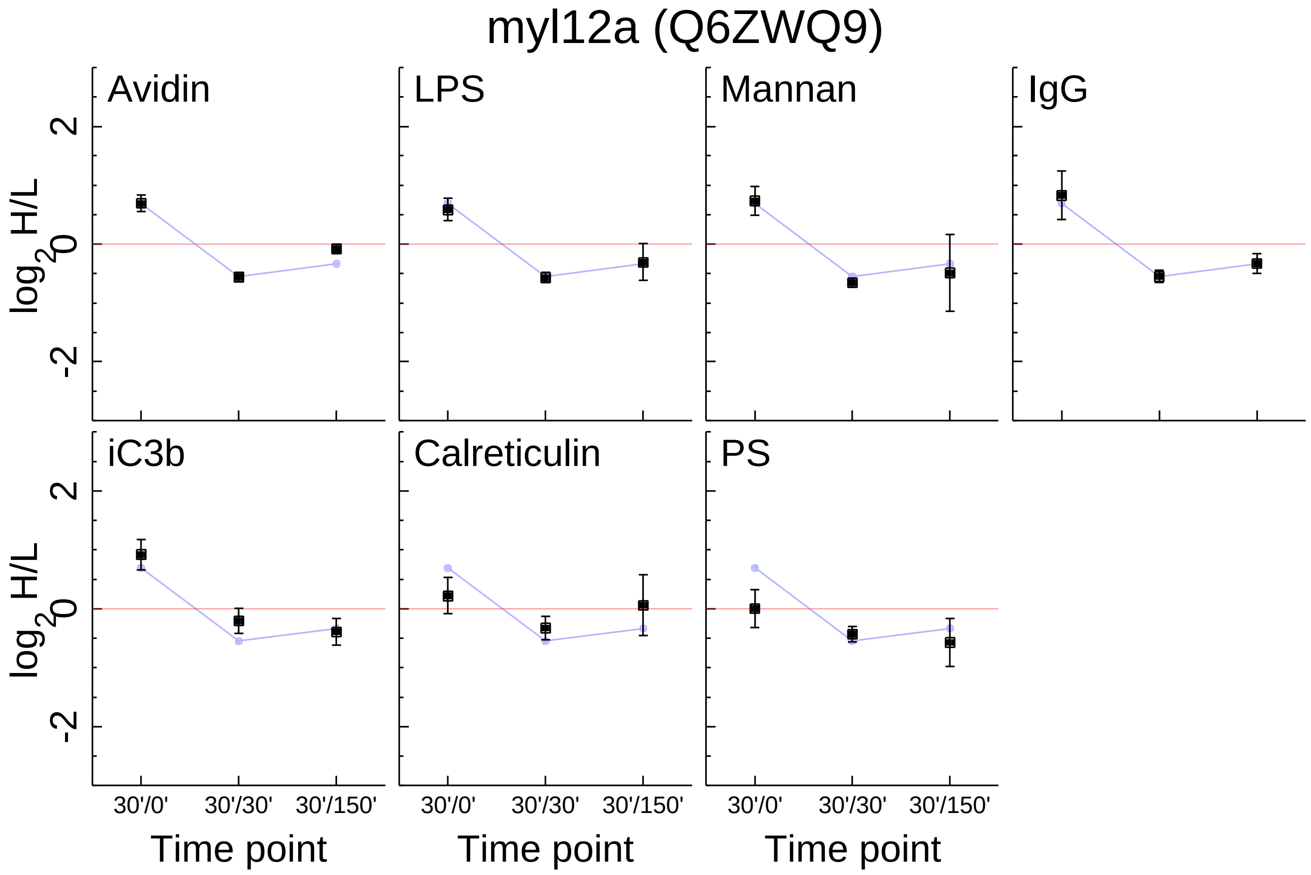

myl12a (Q6ZWQ9)
Avidin
LPS
Mannan
IgG
iC3b
Calreticulin
PS
2
0
-2
log2 H/L
30'/0'
30'/30'
30'/150'
Time point

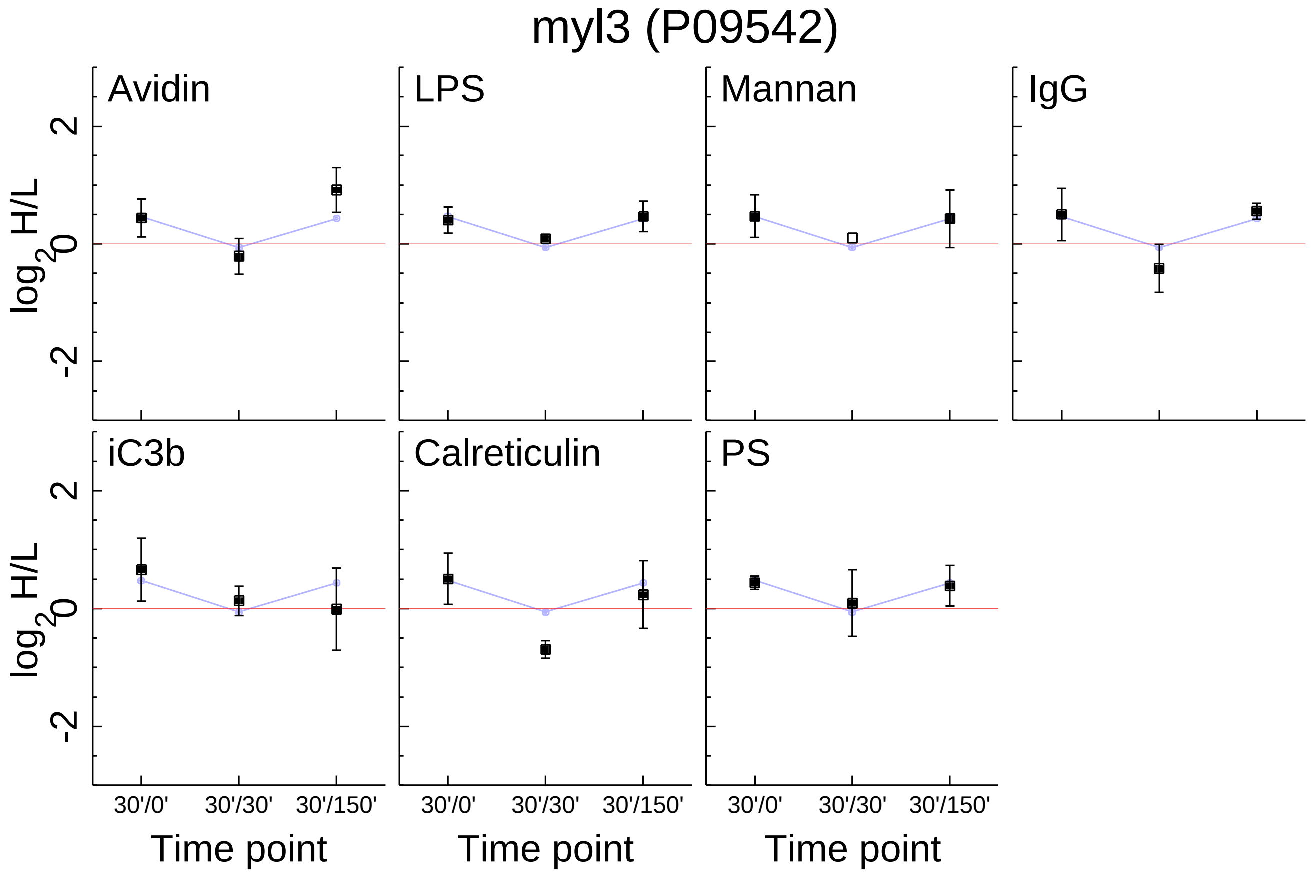
myl3 (P09542)
Avidin
LPS
Mannan
IgG
iC3b
Calreticulin
PS
log2 H/L
2
0
-2
30'/0'
30'/30'
30'/150'
Time point

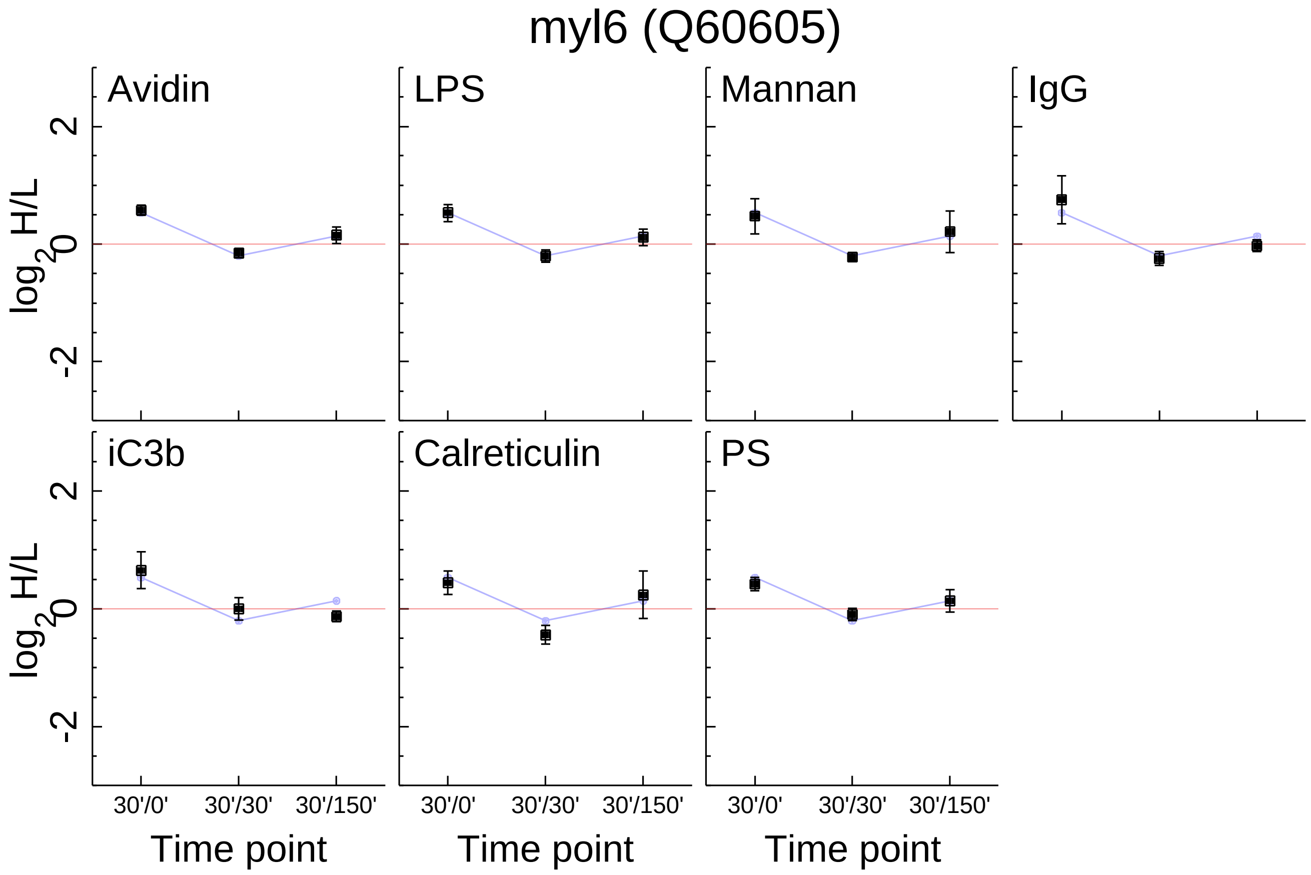

myl6 (Q60605)
Avidin
LPS
Mannan
IgG
iC3b
Calreticulin
PS
log2 H/L
2
0
-2
30'/0'
30'/30'
30'/150'
Time point

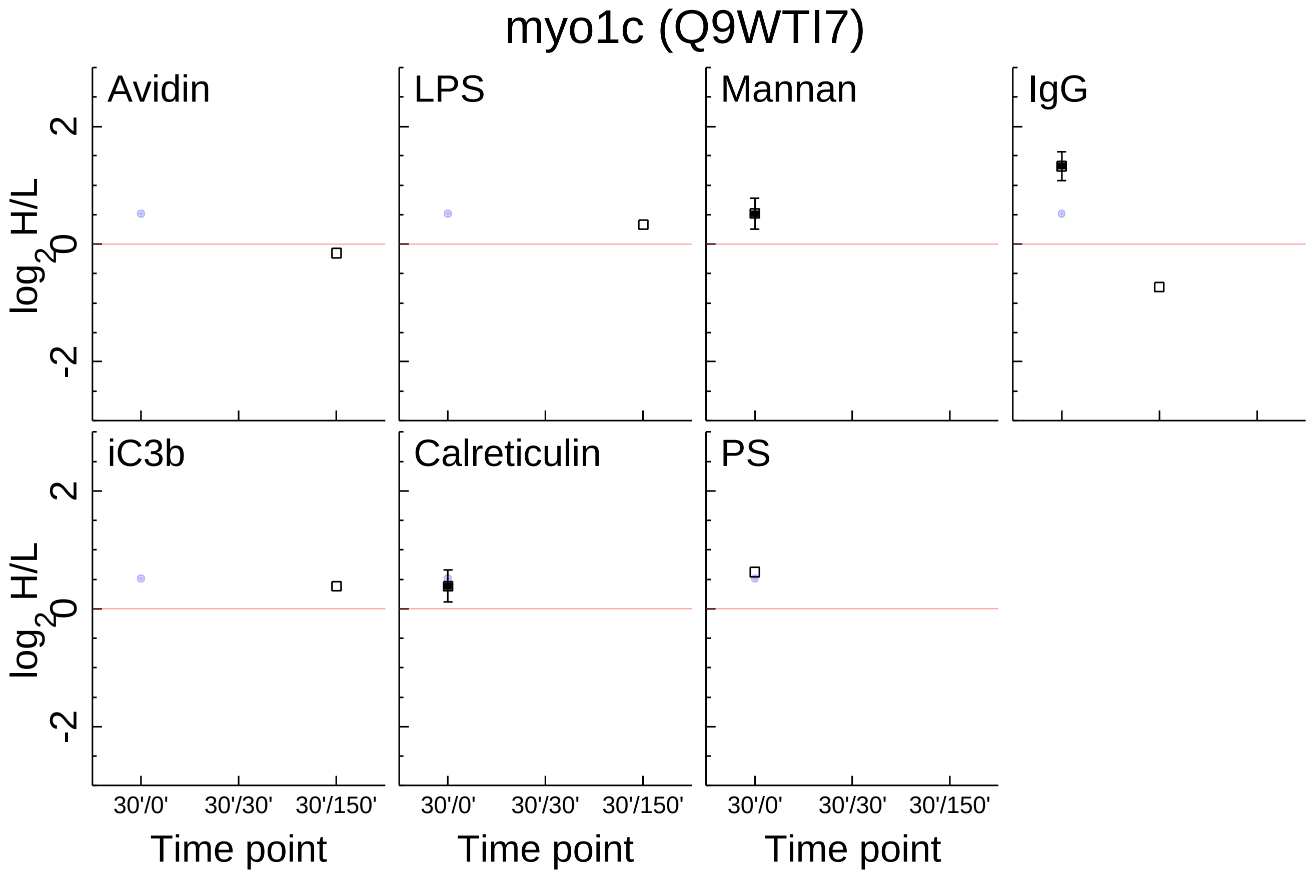
myo1c (Q9WTI7)
Avidin
LPS
Mannan
IgG
iC3b
Calreticulin
PS
log2 H/L
2
0
-2
30'/0'
30'/30'
30'/150'
Time point

myo1e (E9Q634)

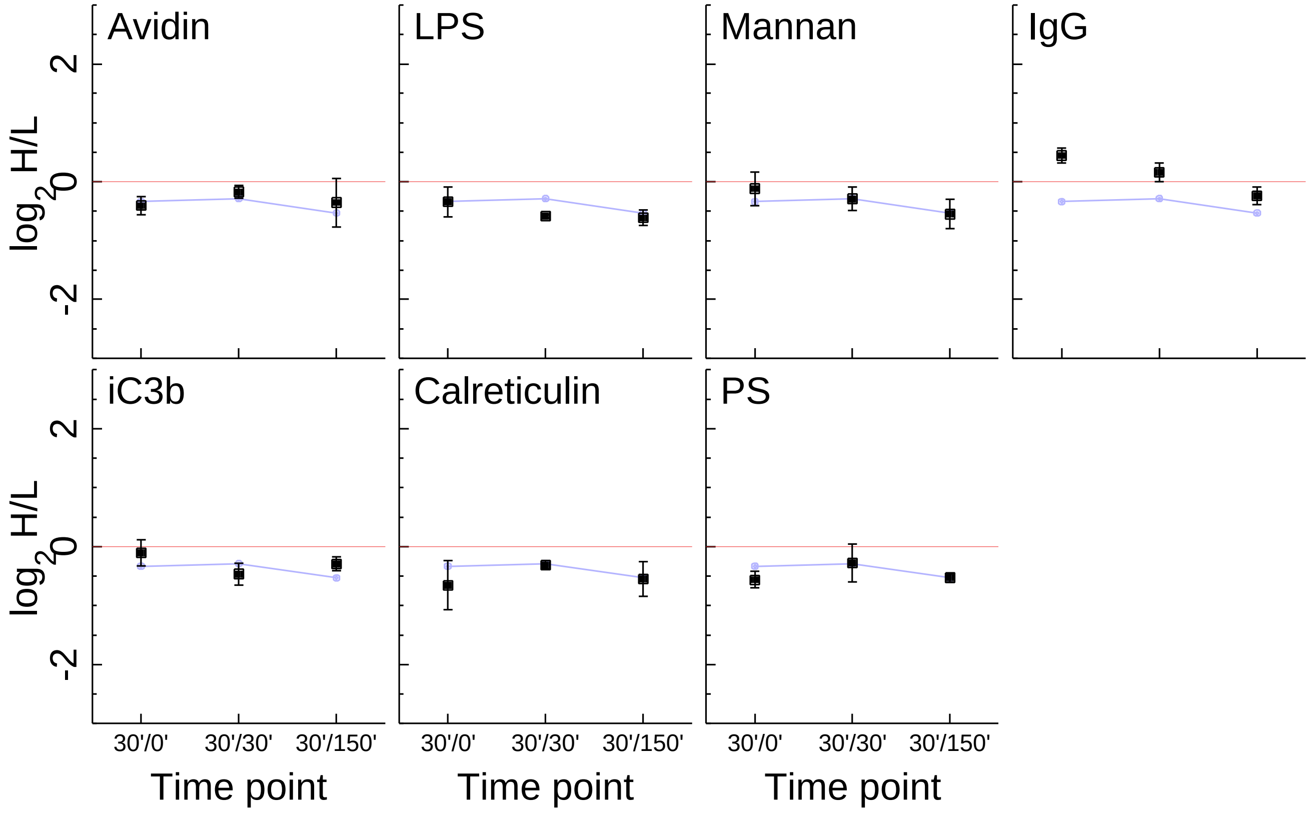

myo1f (Q8CG29)

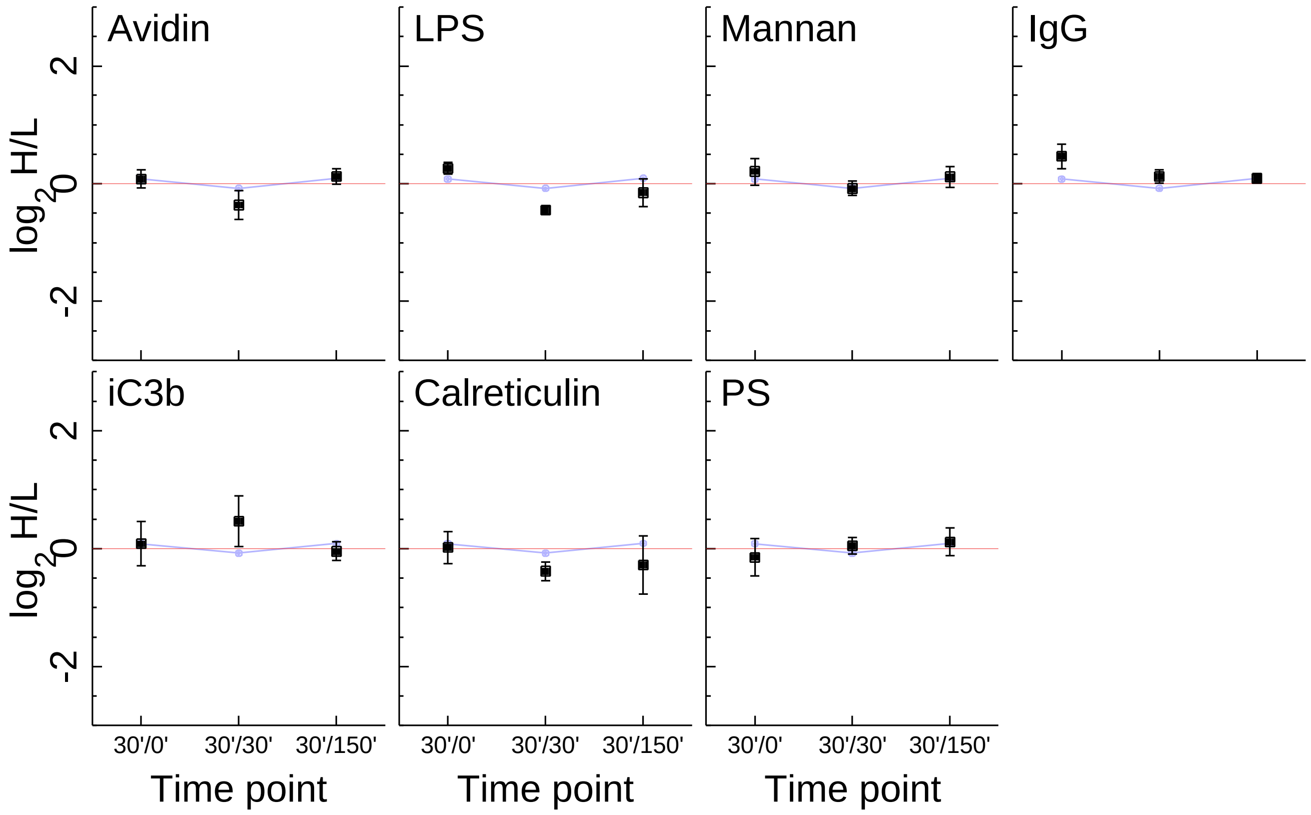

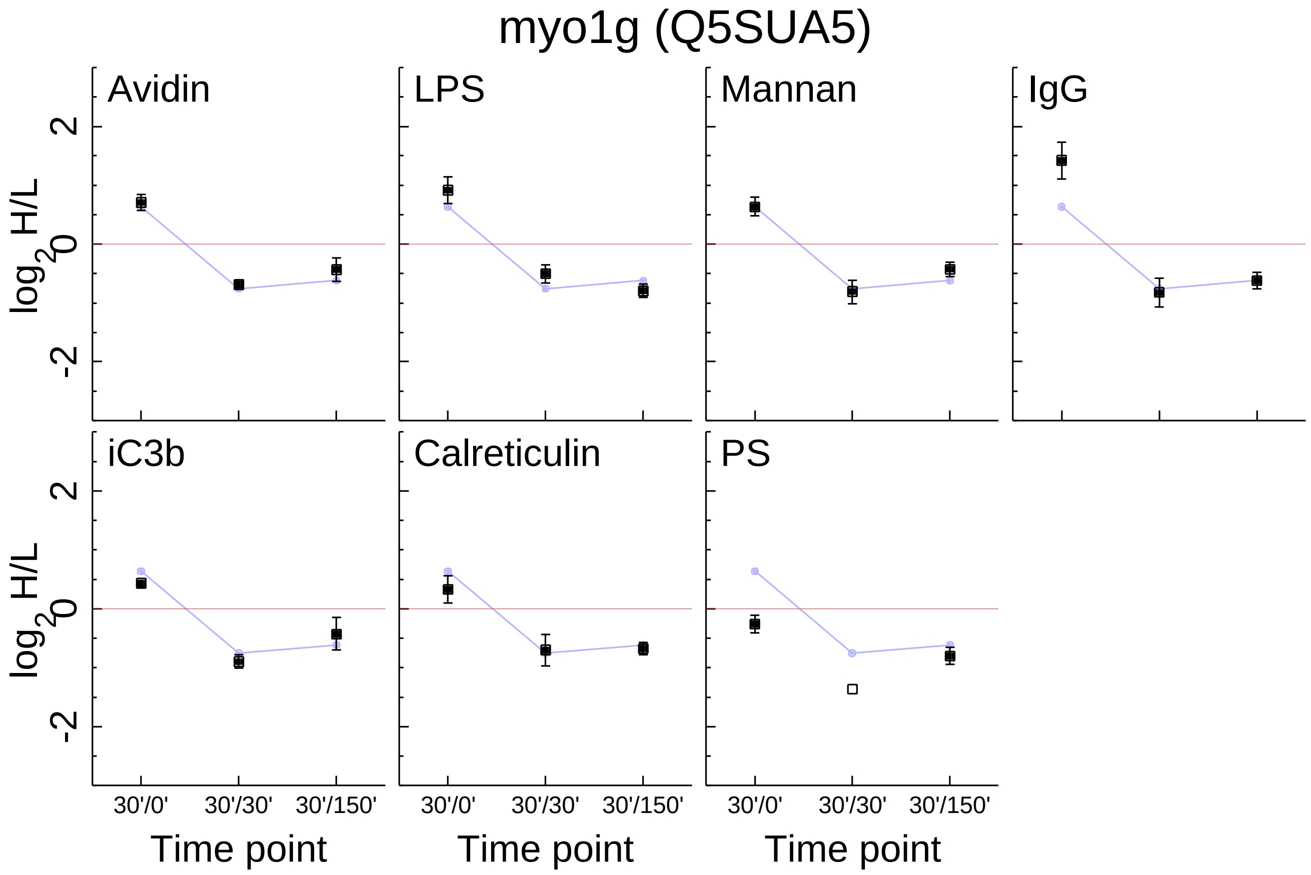
myo1g (Q5SUA5)
Avidin
LPS
Mannan
IgG
iC3b
Calreticulin
PS
log2 H/L
2
0
-2
30'/0'
30'/30'
30'/150'
Time point

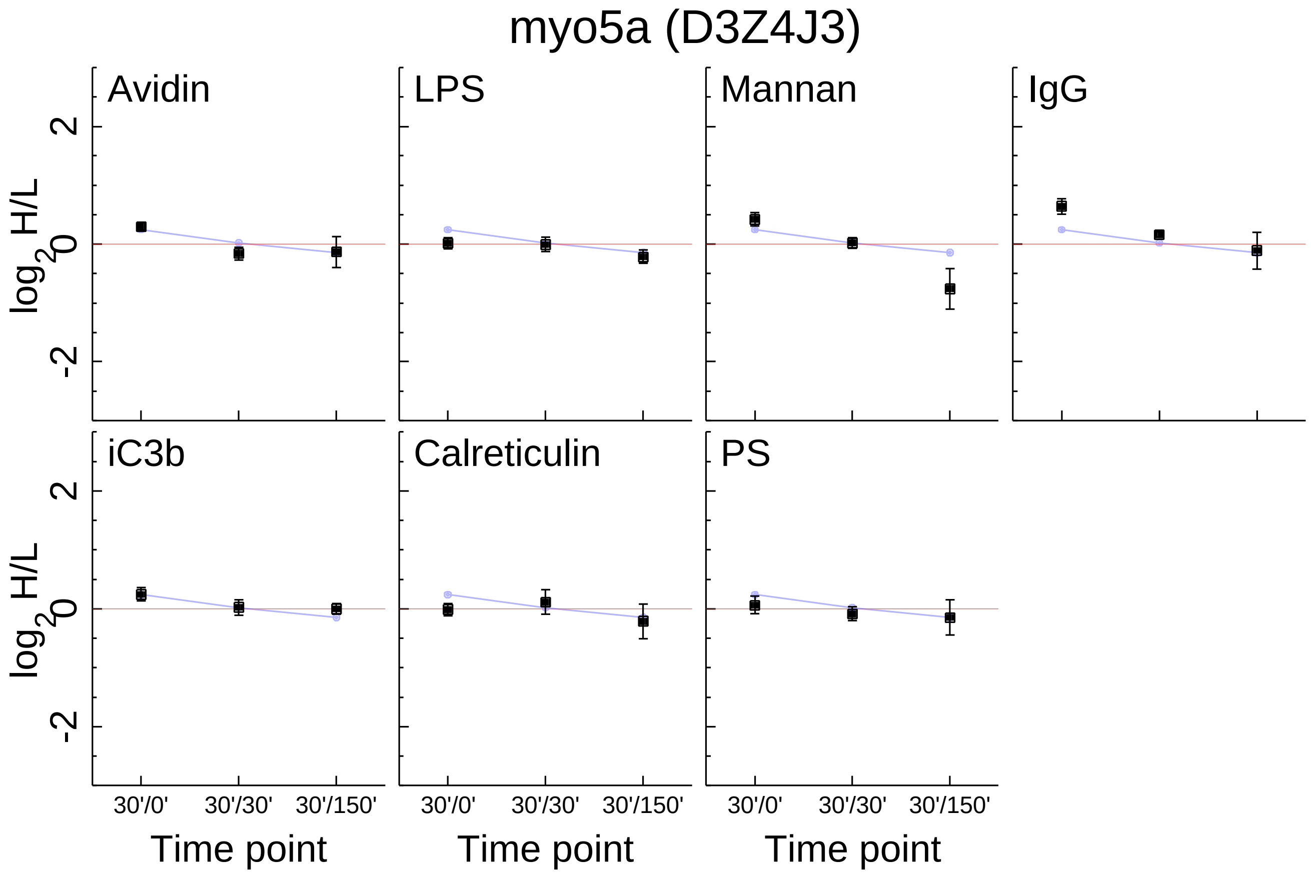
myo5a (D3Z4J3)
Avidin
LPS
Mannan
IgG
iC3b
Calreticulin
PS
$\log_2$ H/L
2
0
-2
30'/0'
30'/30'
30'/150'
Time point

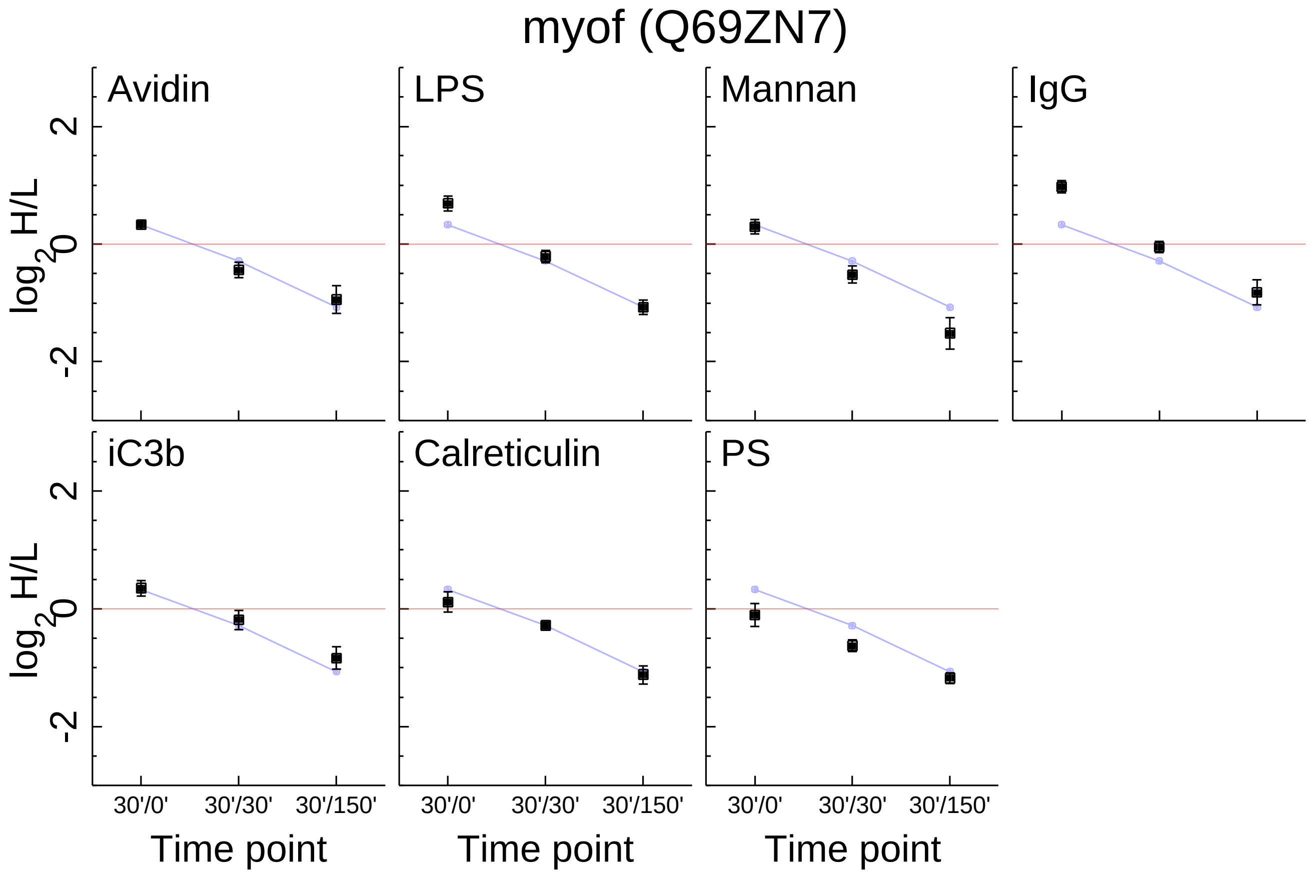

myof (Q69ZN7)
Avidin
LPS
Mannan
IgG
iC3b
Calreticulin
PS
log2 H/L
2
0
-2
30'/0'
30'/30'
30'/150'
Time point

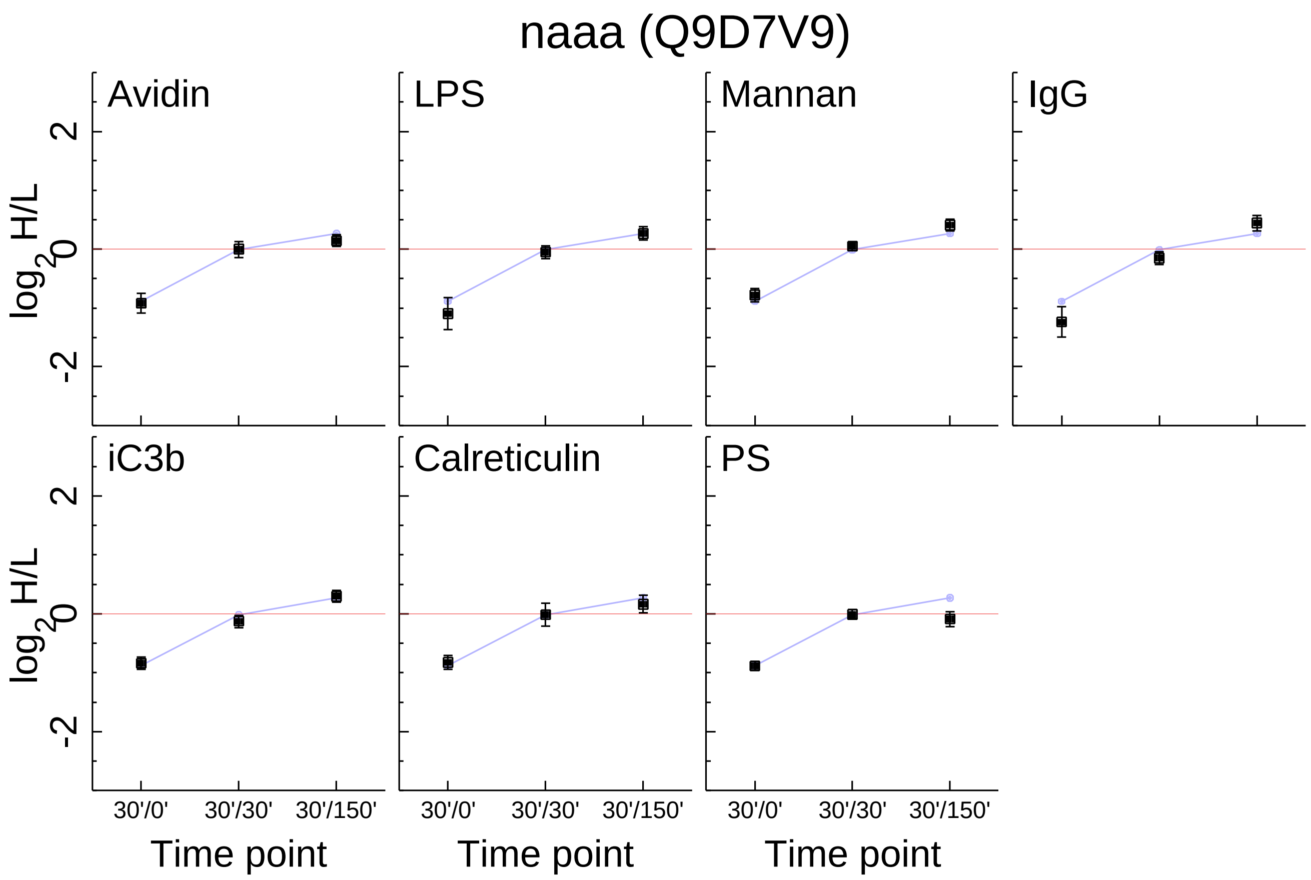
naaa (Q9D7V9)
Avidin
LPS
Mannan
IgG
iC3b
Calreticulin
PS
log2 H/L
2
0
-2
30'/0'
30'/30'
30'/150'
Time point

naca (P70670)

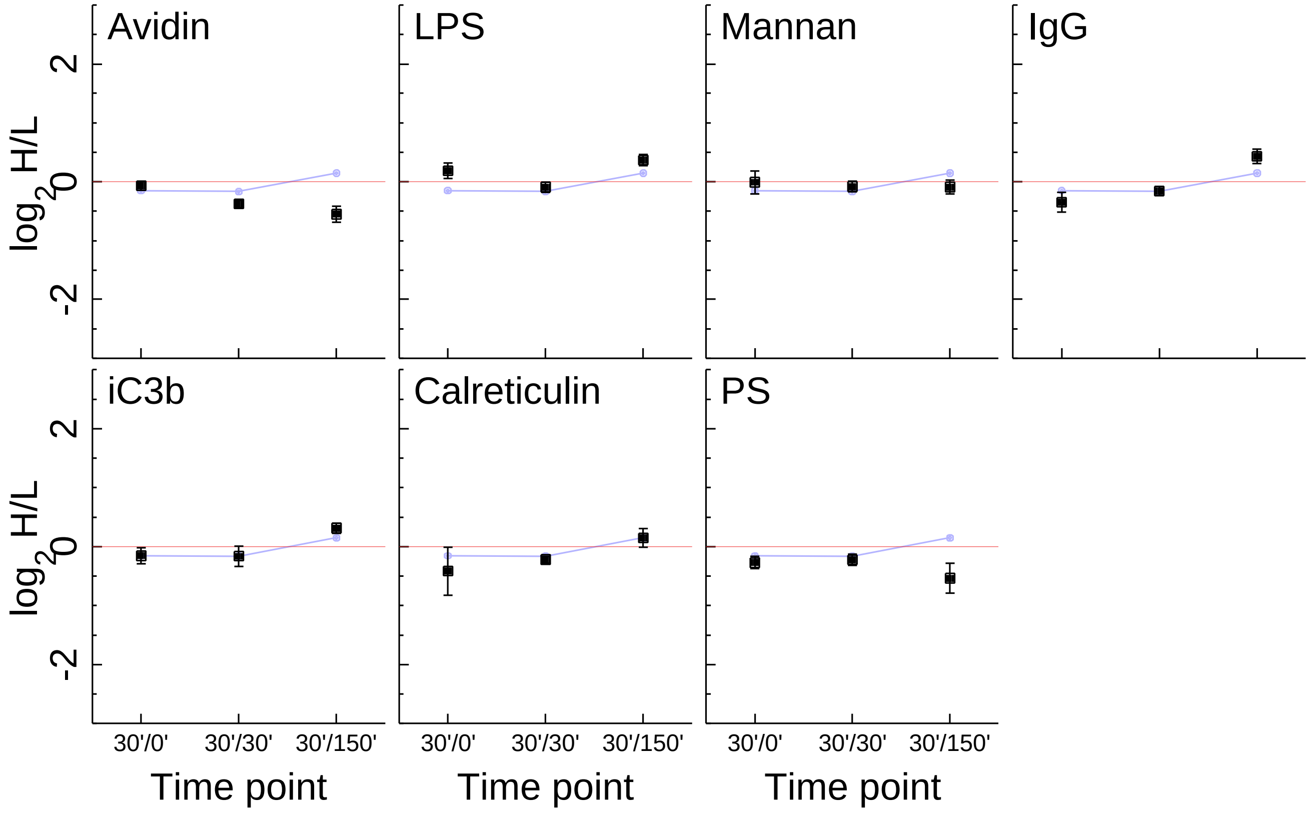
Avidin
LPS
Mannan
IgG
iC3b
Calreticulin
PS
log2 H/L
2
0
-2
30'/0'
30'/30'
30'/150'
Time point

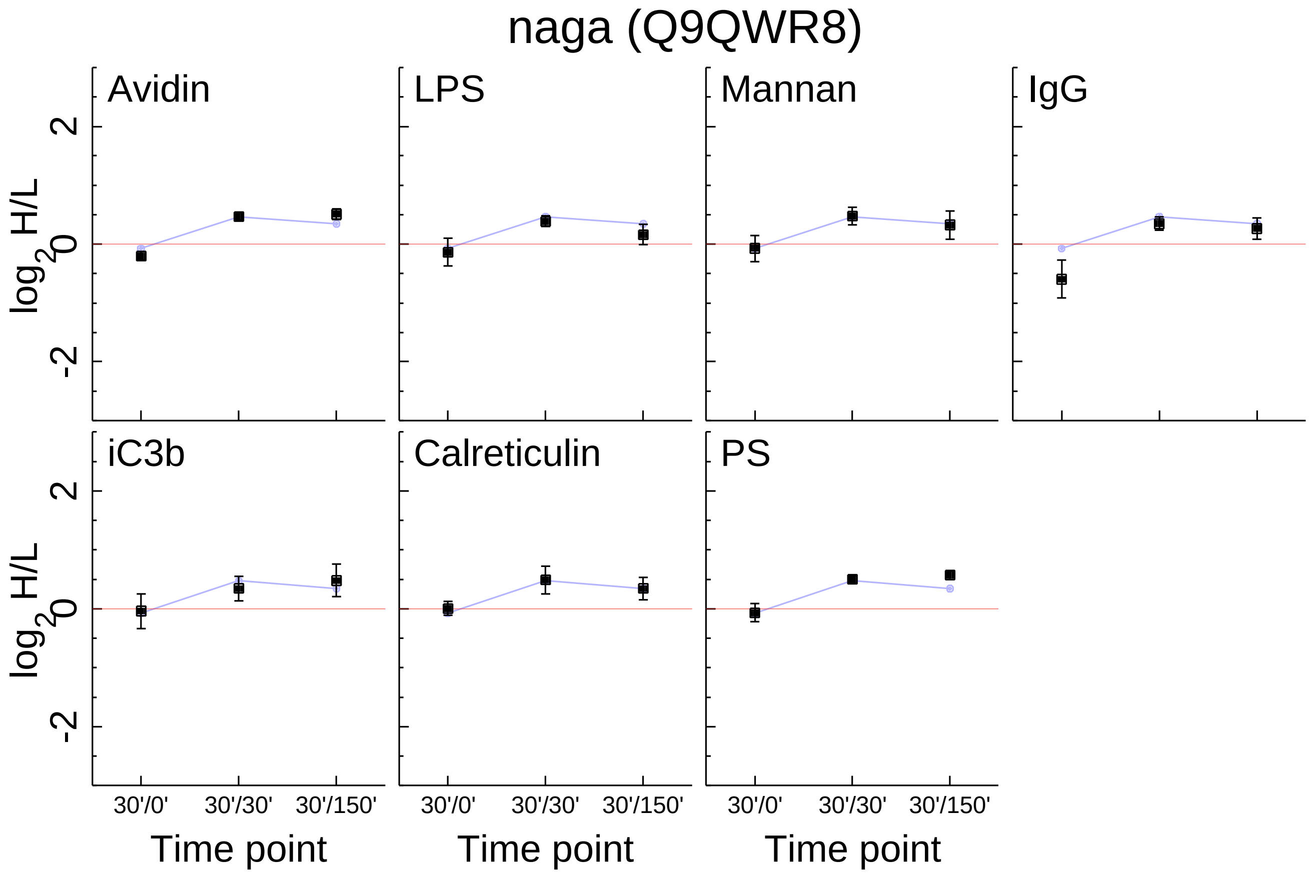
naga (Q9QWR8)
Avidin
LPS
Mannan
IgG
iC3b
Calreticulin
PS
2
0
-2
$\log_2$ H/L
30'/0'
30'/30'
30'/150'
Time point

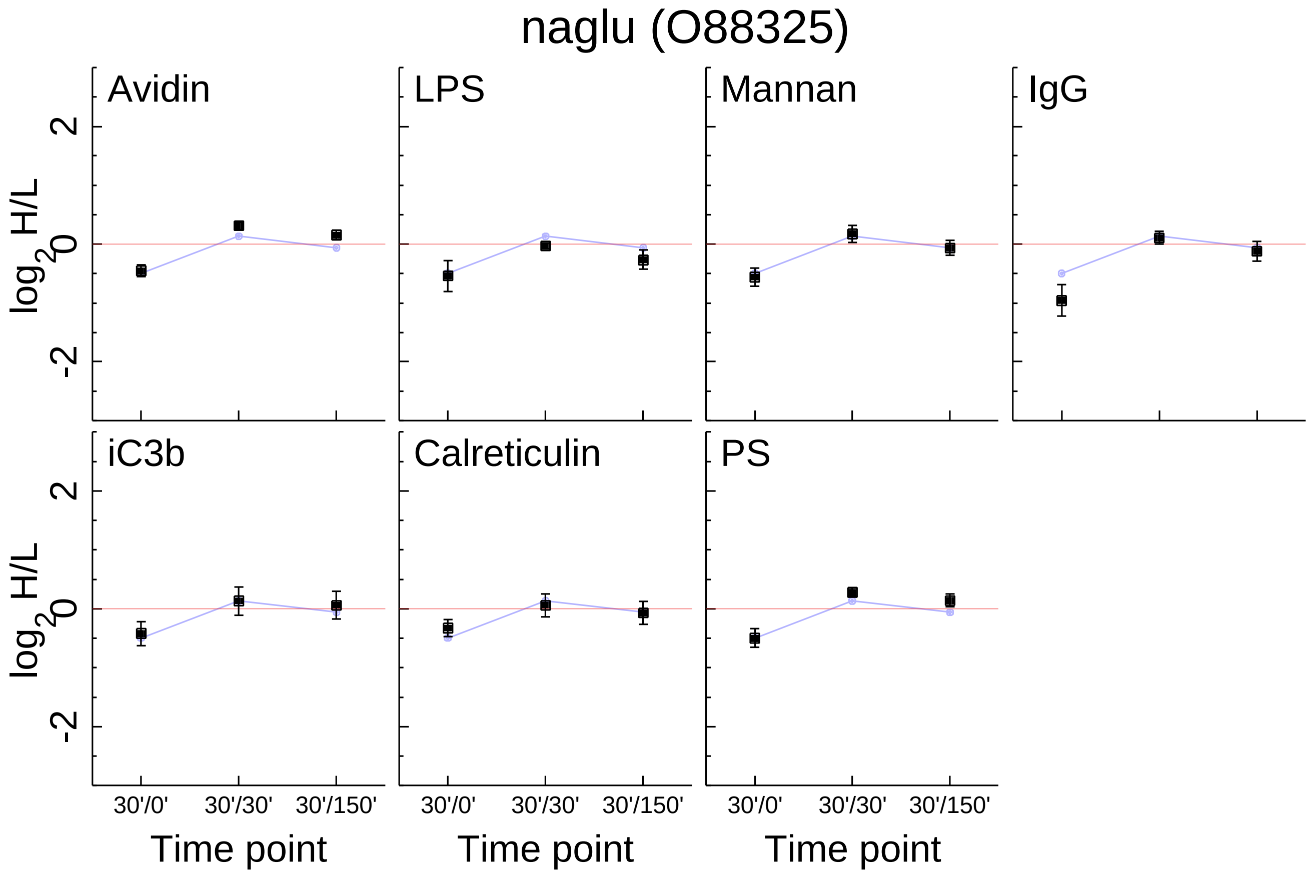
naglu (O88325)
Avidin
LPS
Mannan
IgG
iC3b
Calreticulin
PS
log2 H/L
2
0
-2
30'/0'
30'/30'
30'/150'
Time point

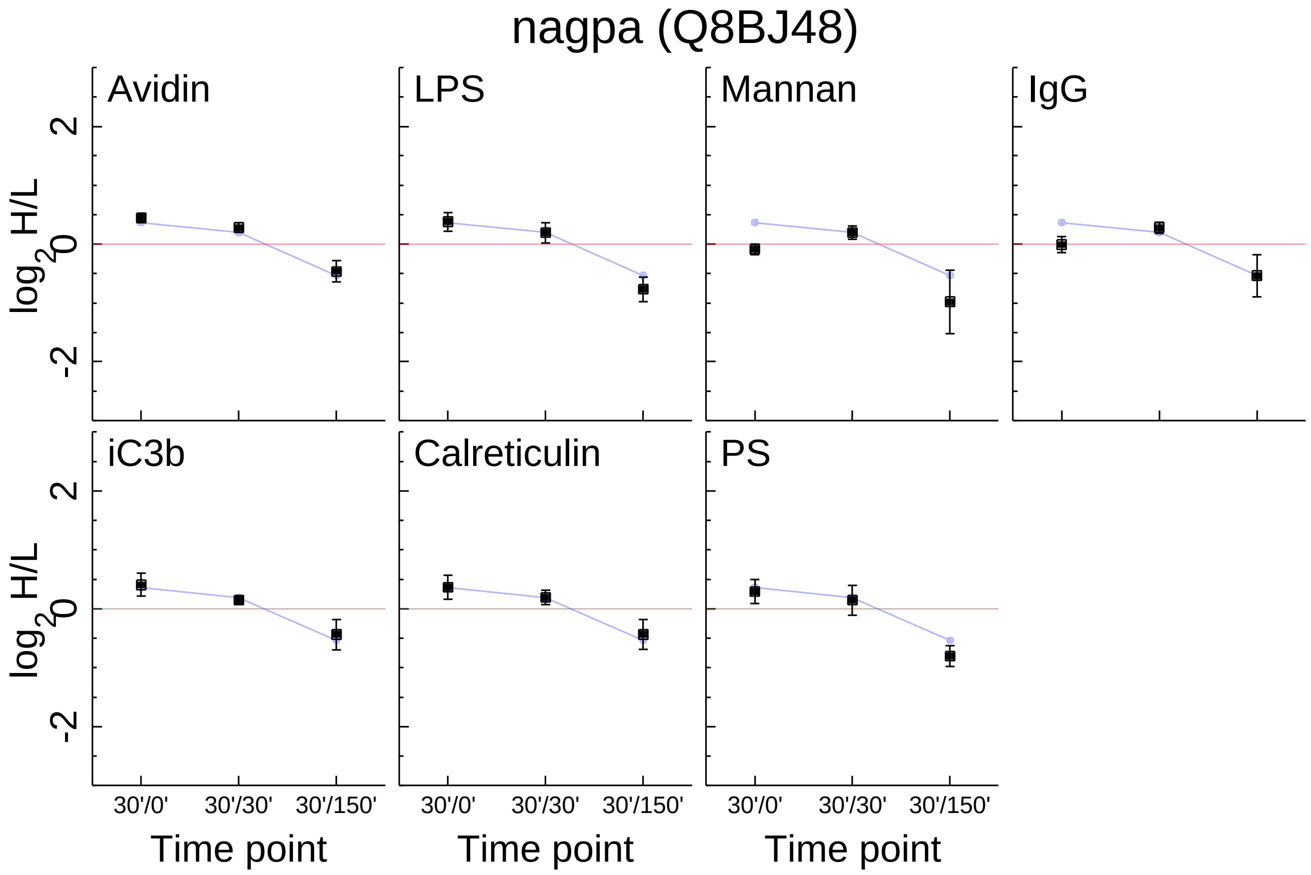

nagpa (Q8BJ48)
Avidin
LPS
Mannan
IgG
iC3b
Calreticulin
PS
log2 H/L
2
0
-2
30'/0'
30'/30'
30'/150'
Time point

nap1l1 (E9PW66)

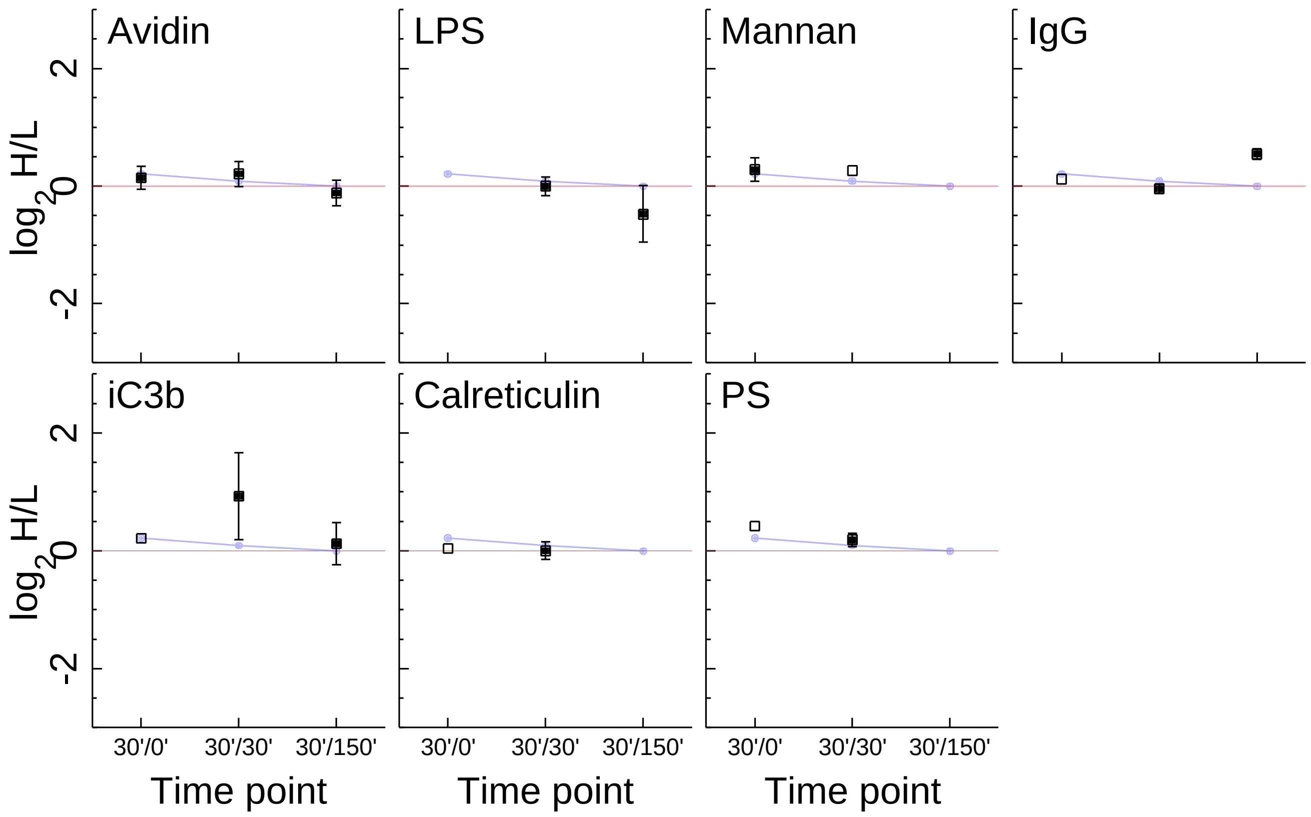

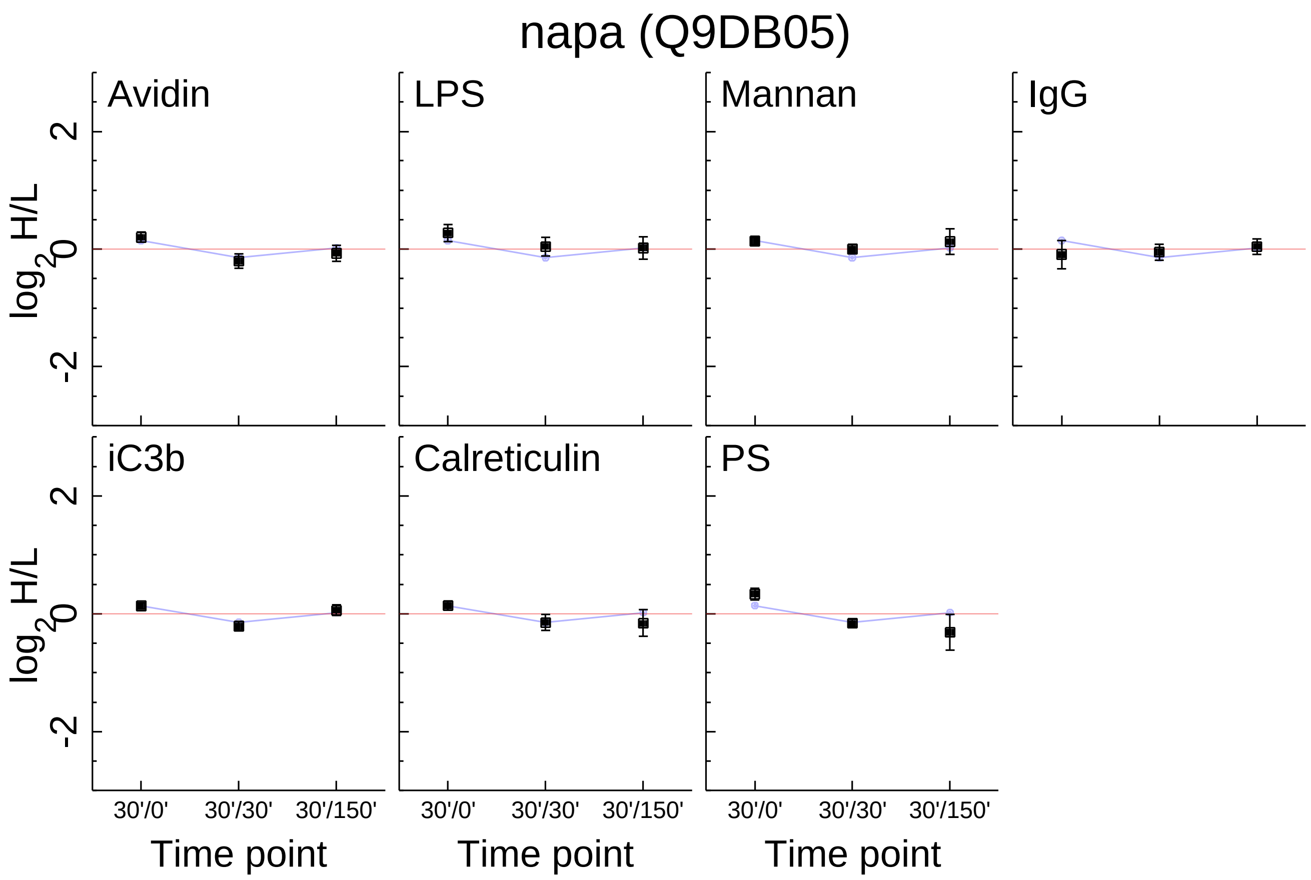
napa (Q9DB05)
Avidin
LPS
Mannan
IgG
iC3b
Calreticulin
PS
log2 H/L
2
0
-2
30'/0'
30'/30'
30'/150'
Time point

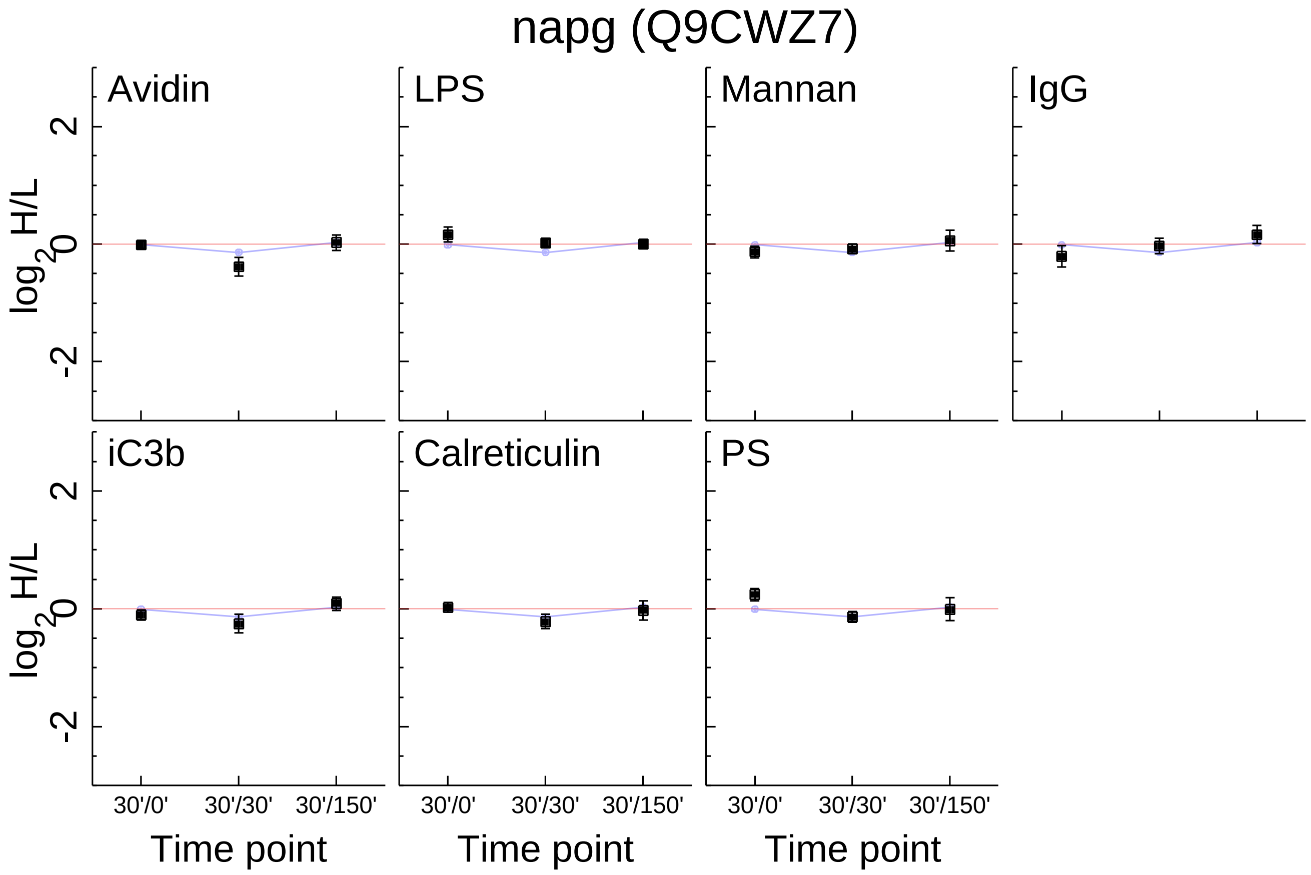
napg (Q9CWZ7)
Avidin
LPS
Mannan
IgG
iC3b
Calreticulin
PS
2
0
-2
log2 H/L
30'/0'
30'/30'
30'/150'
Time point

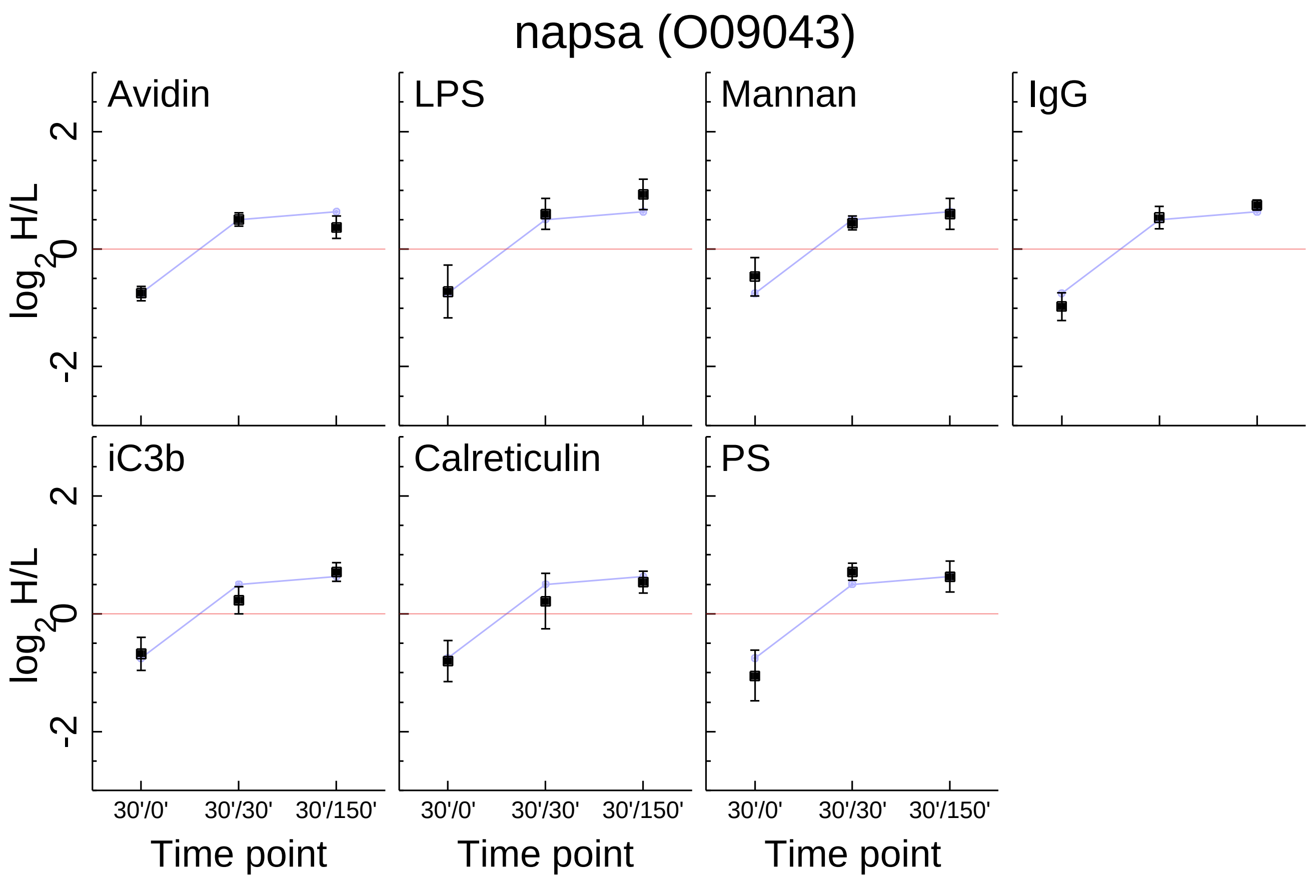
napsa (O09043)
Avidin
LPS
Mannan
IgG
iC3b
Calreticulin
PS
log2 H/L
2
0
-2
30'/0'
30'/30'
30'/150'
Time point

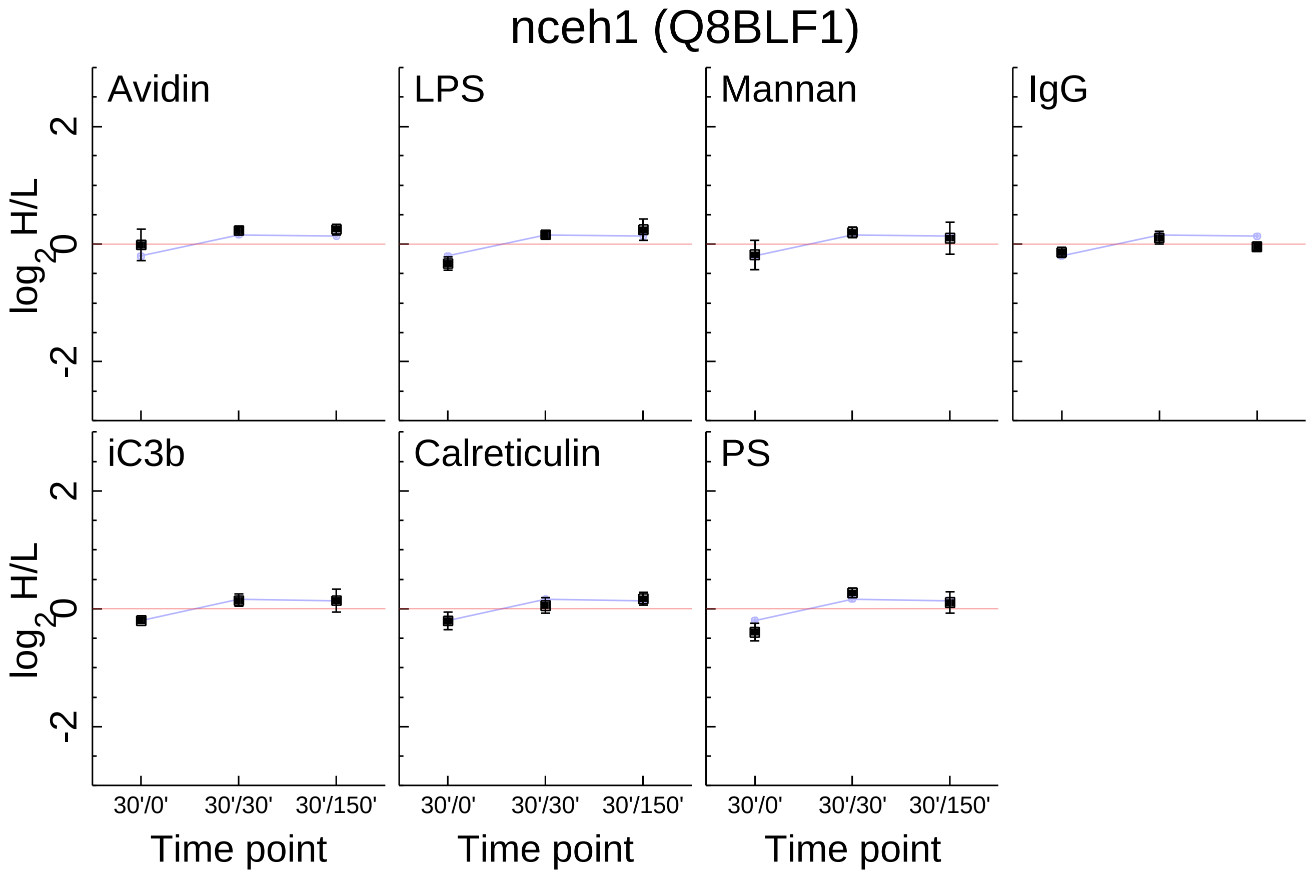
nceh1 (Q8BLF1)
Avidin
LPS
Mannan
IgG
iC3b
Calreticulin
PS
log2 H/L
2
0
-2
30'/0'
30'/30'
30'/150'
Time point

ncf1 (F8WH69)

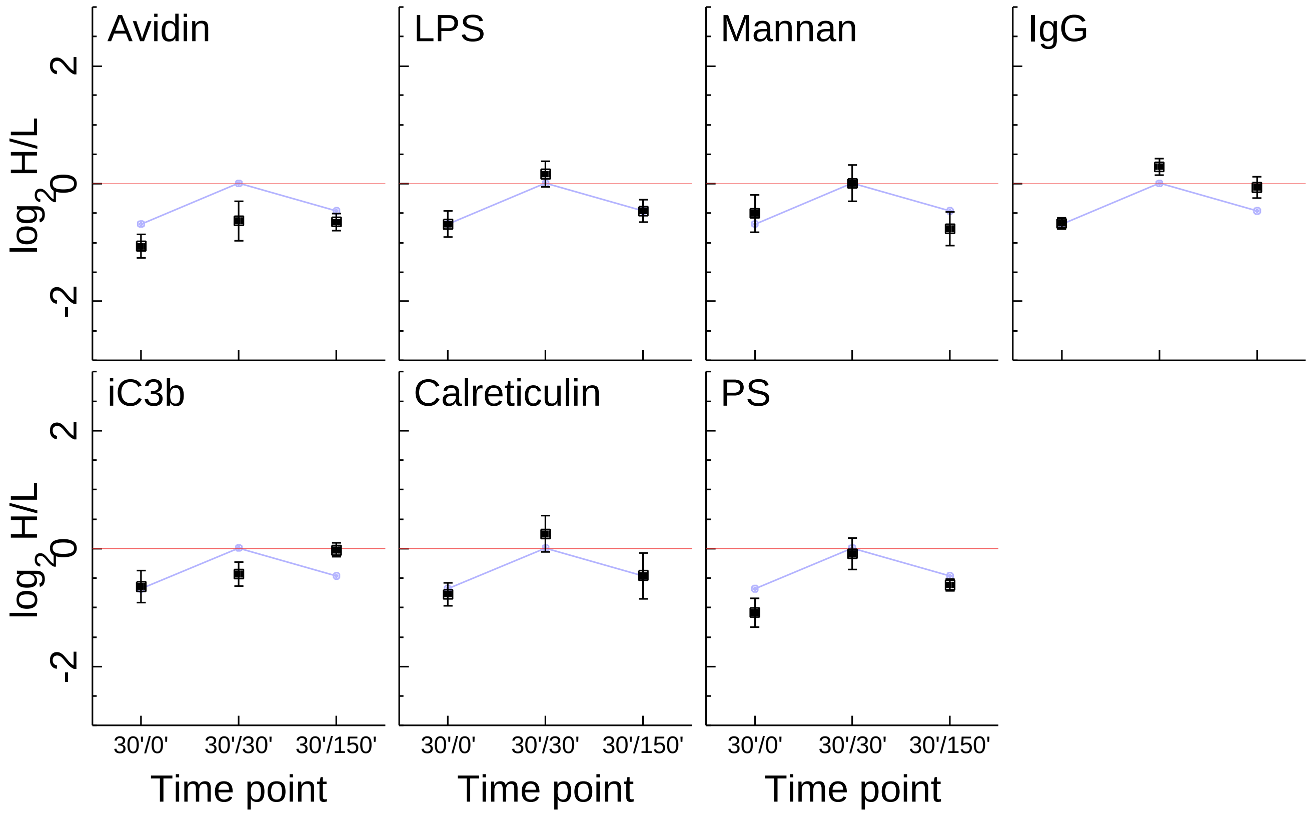

Avidin
LPS
Mannan
IgG
iC3b
Calreticulin
PS
log2 H/L
2
0
-2
30'/0'
30'/30'
30'/150'
Time point

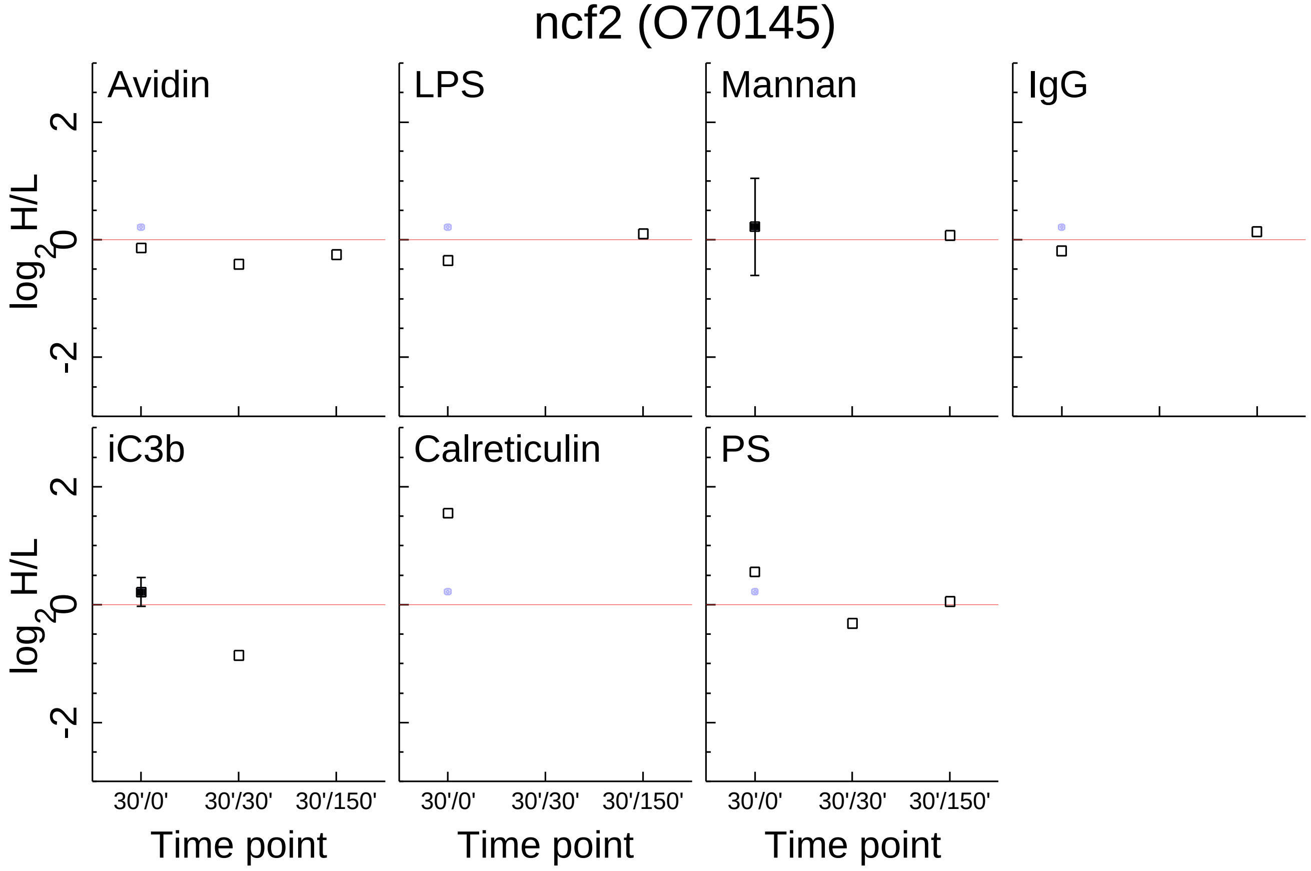

ncf2 (O70145)
Avidin
LPS
Mannan
IgG
iC3b
Calreticulin
PS
log2 H/L
2
0
-2
30'/0'
30'/30'
30'/150'
Time point

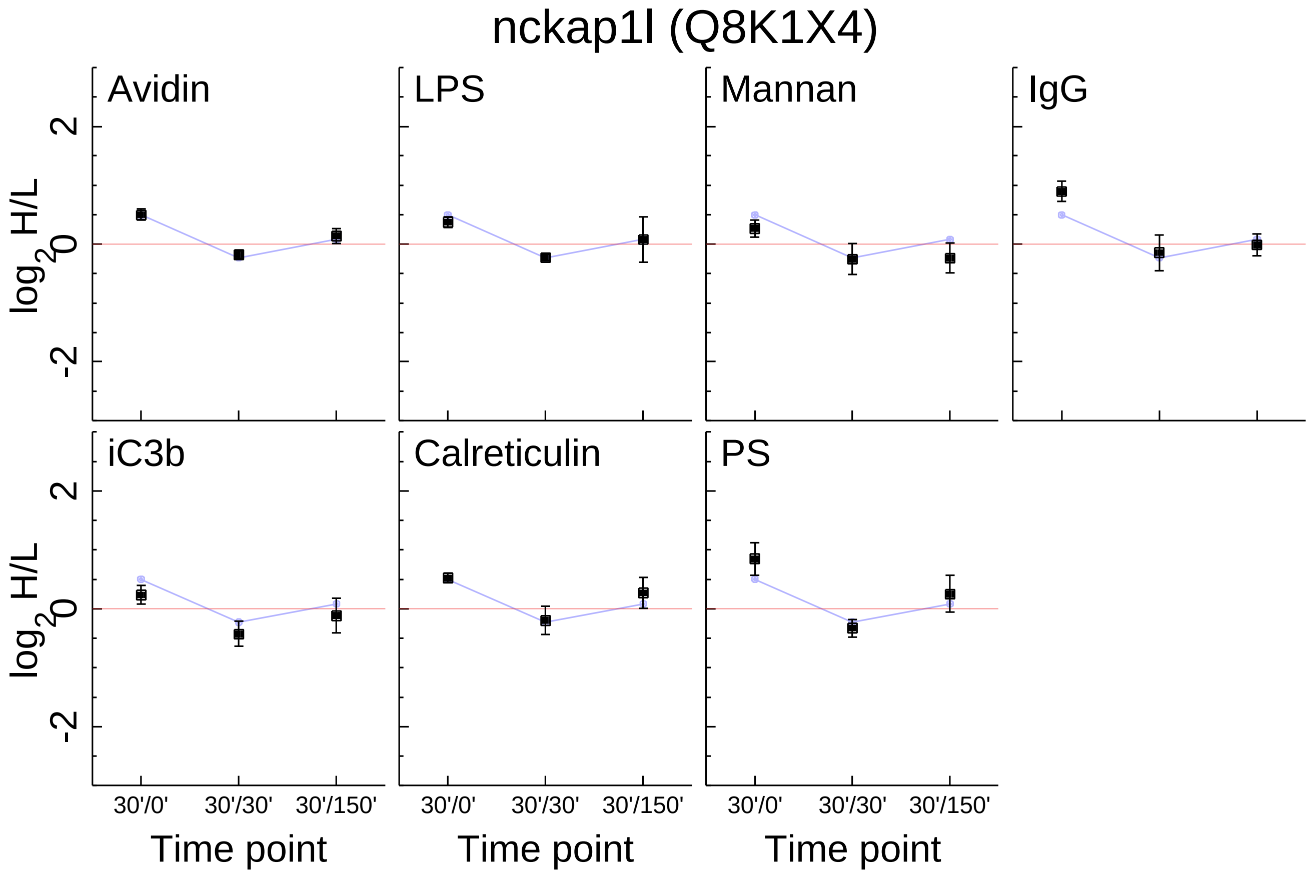
nckap1l (Q8K1X4)
Avidin
LPS
Mannan
IgG
iC3b
Calreticulin
PS
log2 H/L
2
0
-2
30'/0'
30'/30'
30'/150'
Time point

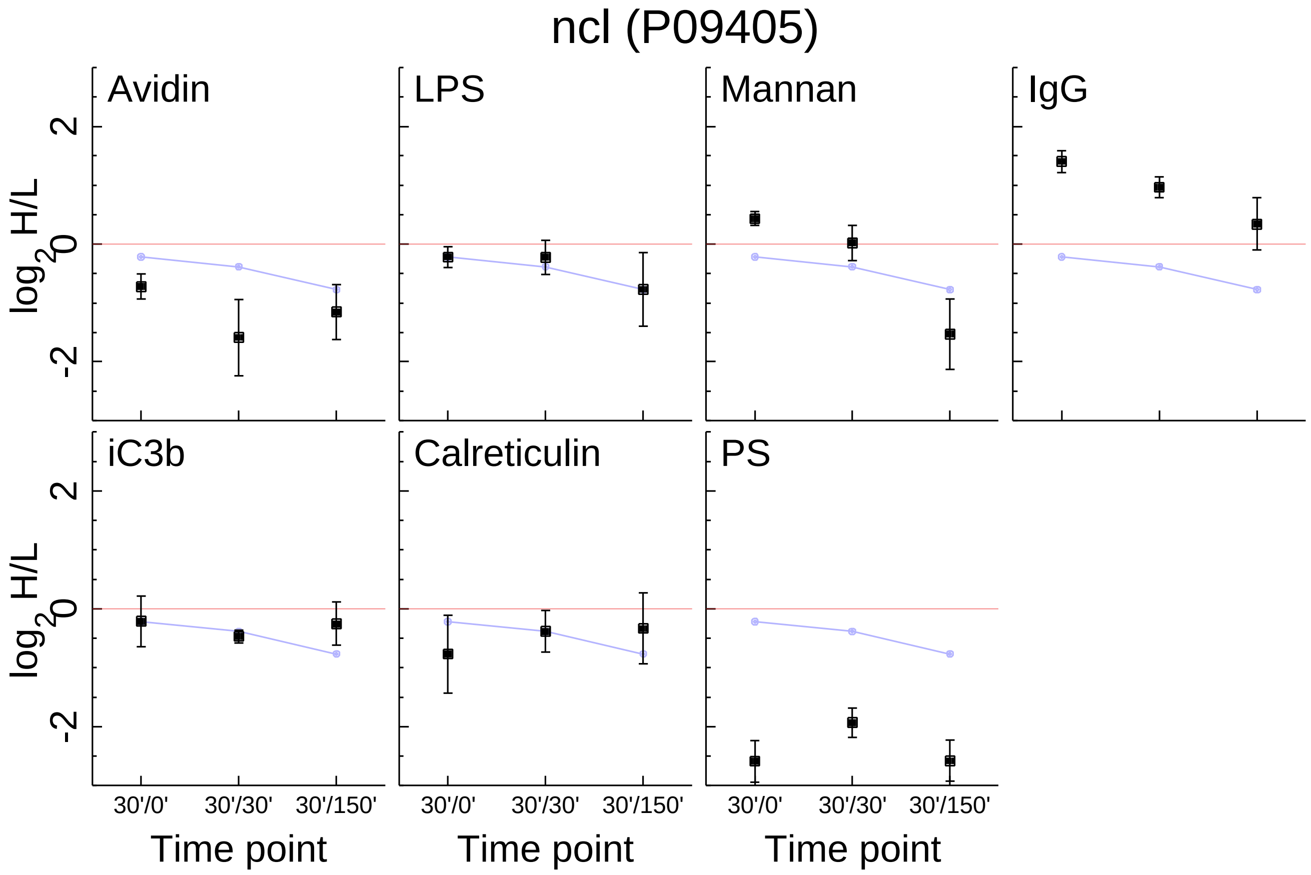

ncl (P09405)
Avidin
LPS
Mannan
IgG
iC3b
Calreticulin
PS
log2 H/L
2
0
-2
30'/0'
30'/30'
30'/150'
Time point

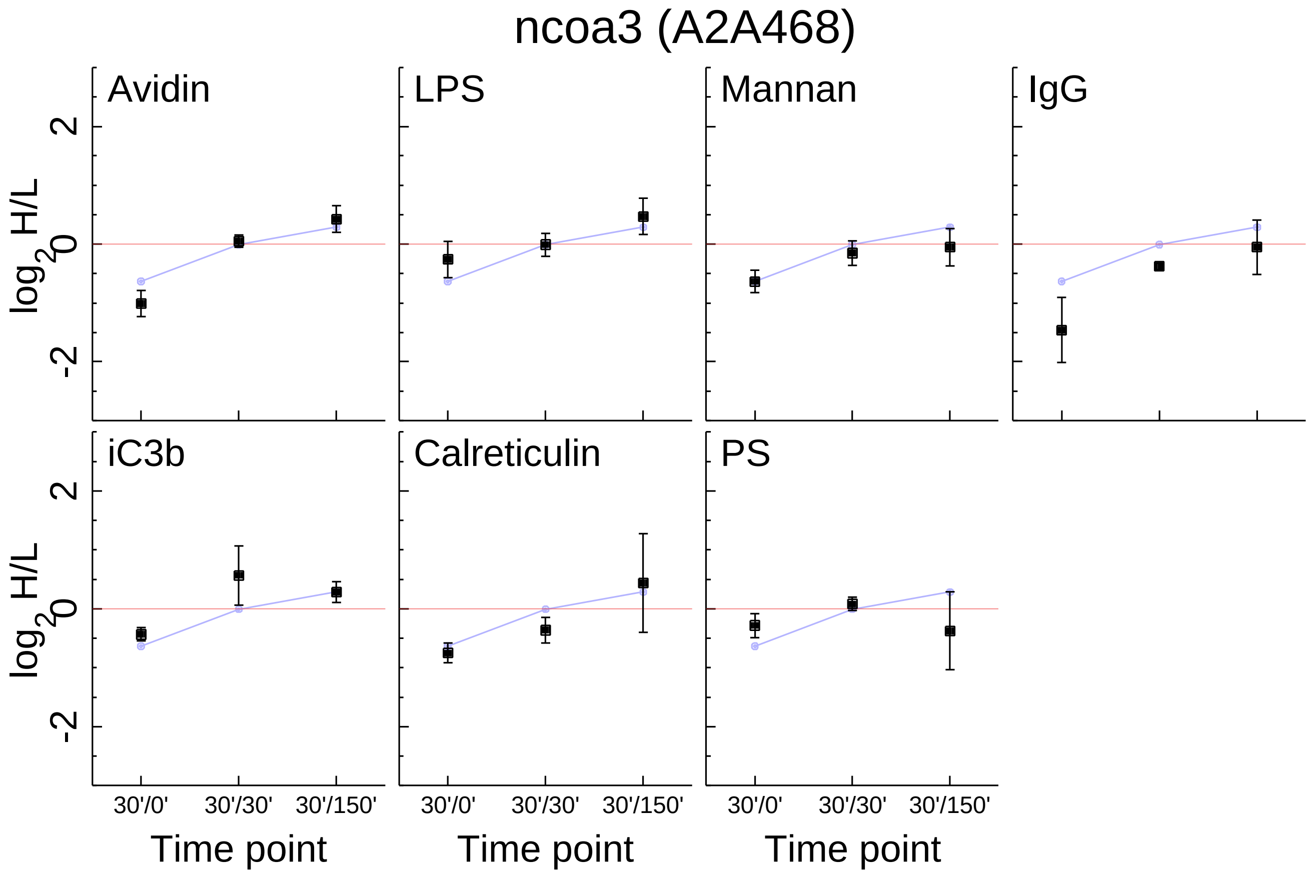

ncoa3 (A2A468)
Avidin
LPS
Mannan
IgG
iC3b
Calreticulin
PS
log2 H/L
2
0
-2
30'/0'
30'/30'
30'/150'
Time point

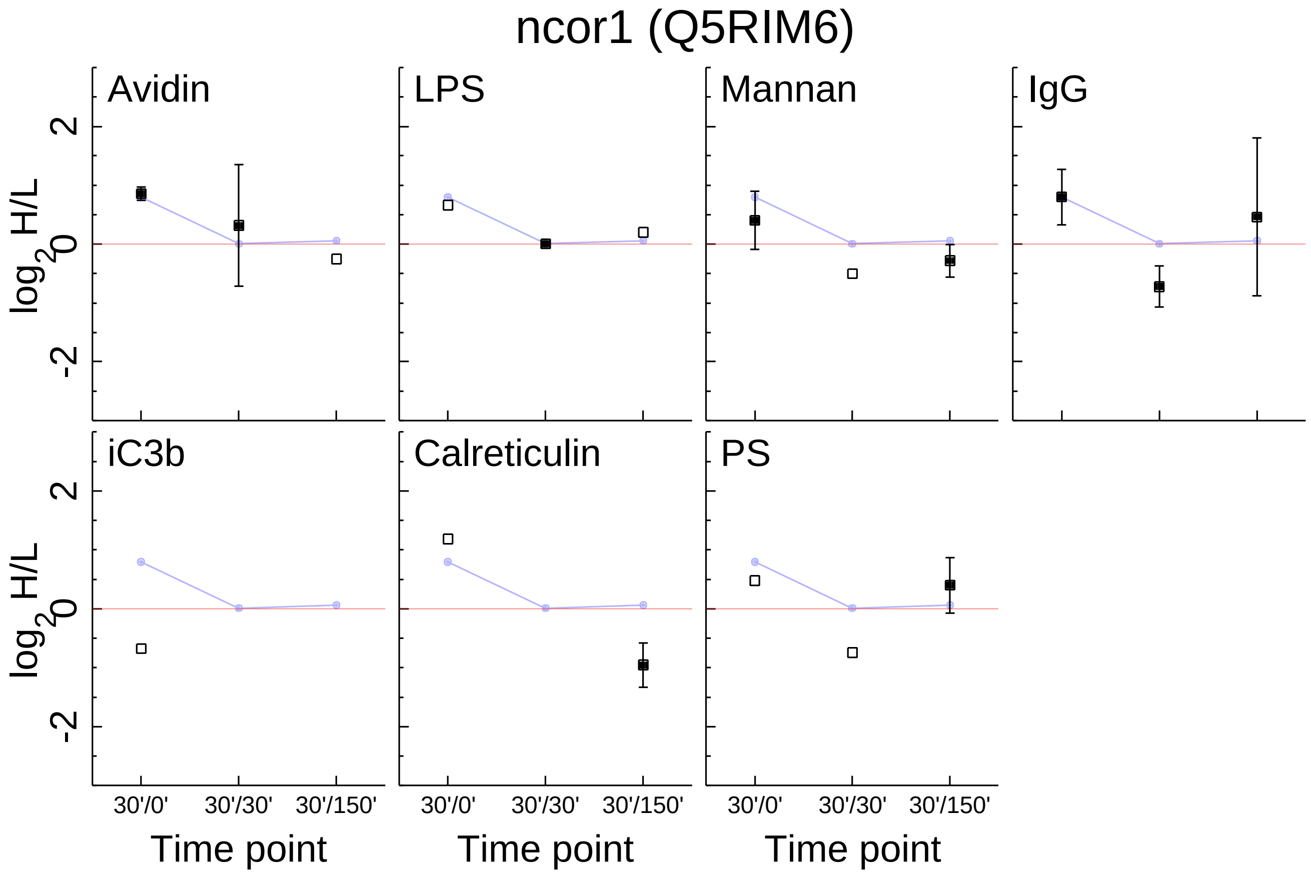

ncor1 (Q5RIM6)
Avidin
LPS
Mannan
IgG
iC3b
Calreticulin
PS
log2 H/L
2
0
-2
30'/0'
30'/30'
30'/150'
Time point

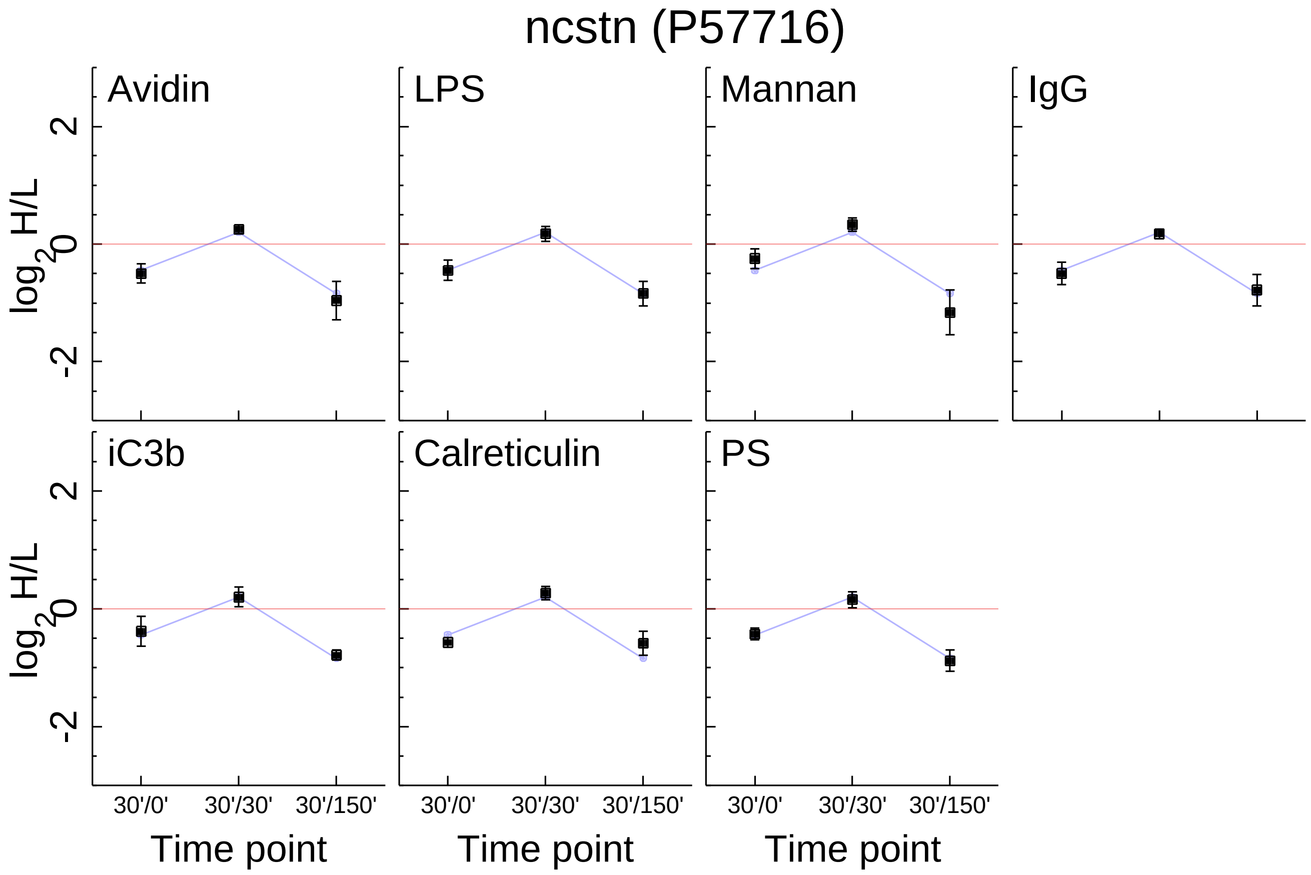
ncstn (P57716)
Avidin
LPS
Mannan
IgG
iC3b
Calreticulin
PS
log2 H/L
2
0
-2
30'/0'
30'/30'
30'/150'
Time point

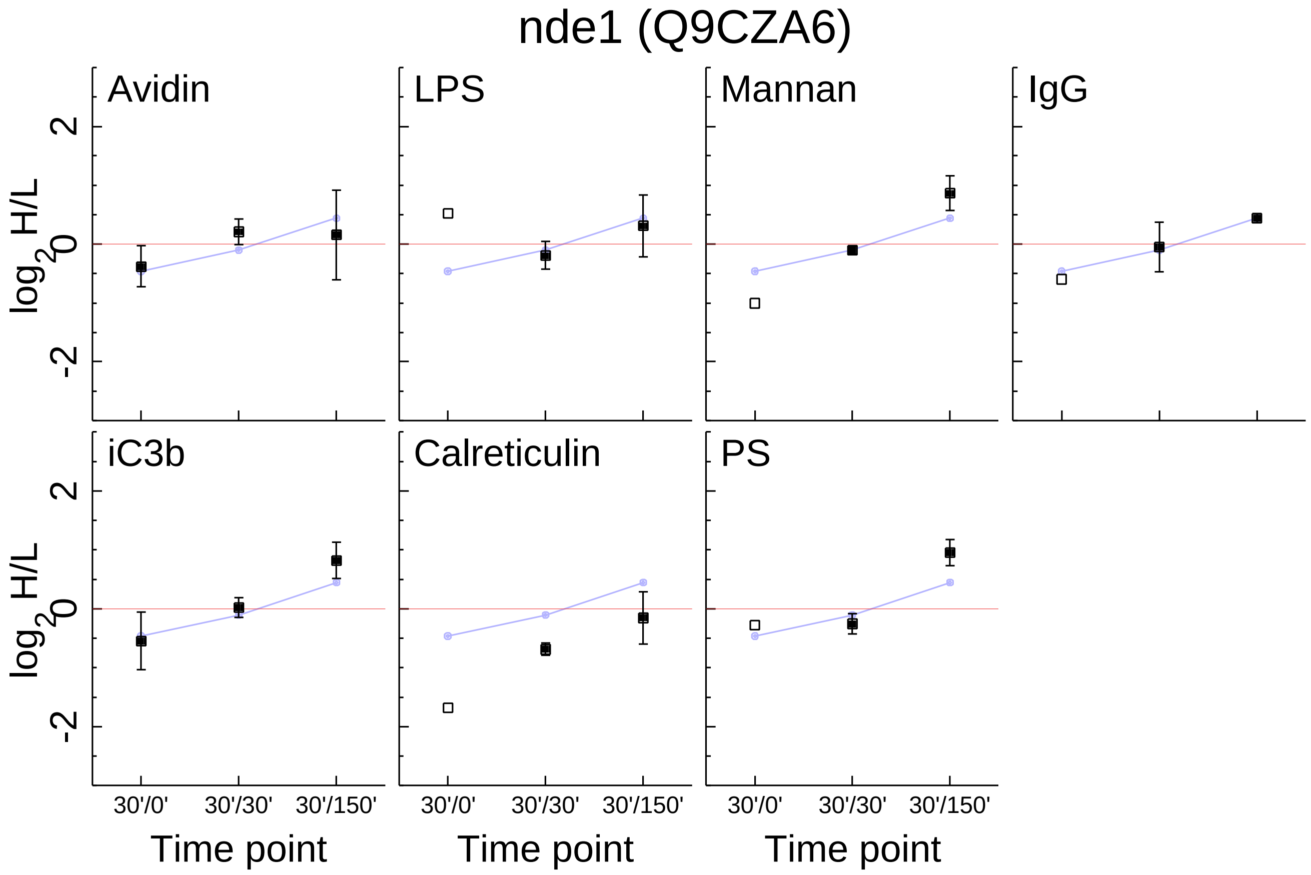

nde1 (Q9CZA6)
Avidin
LPS
Mannan
IgG
iC3b
Calreticulin
PS
log2 H/L
2
0
-2
30'/0'
30'/30'
30'/150'
Time point

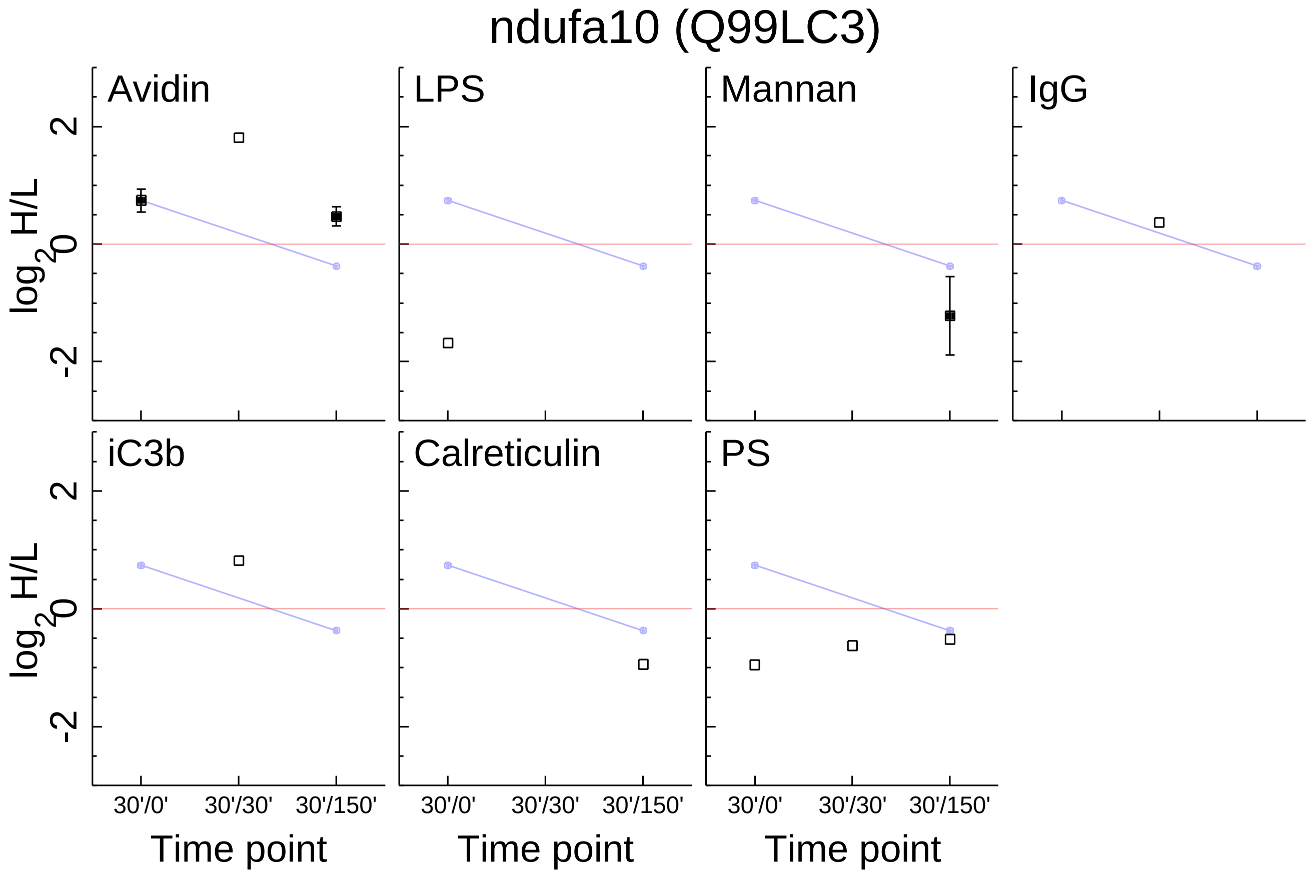

ndufa10 (Q99LC3)
Avidin
LPS
Mannan
IgG
iC3b
Calreticulin
PS
log2 H/L
2
0
-2
30'/0'
30'/30'
30'/150'
Time point

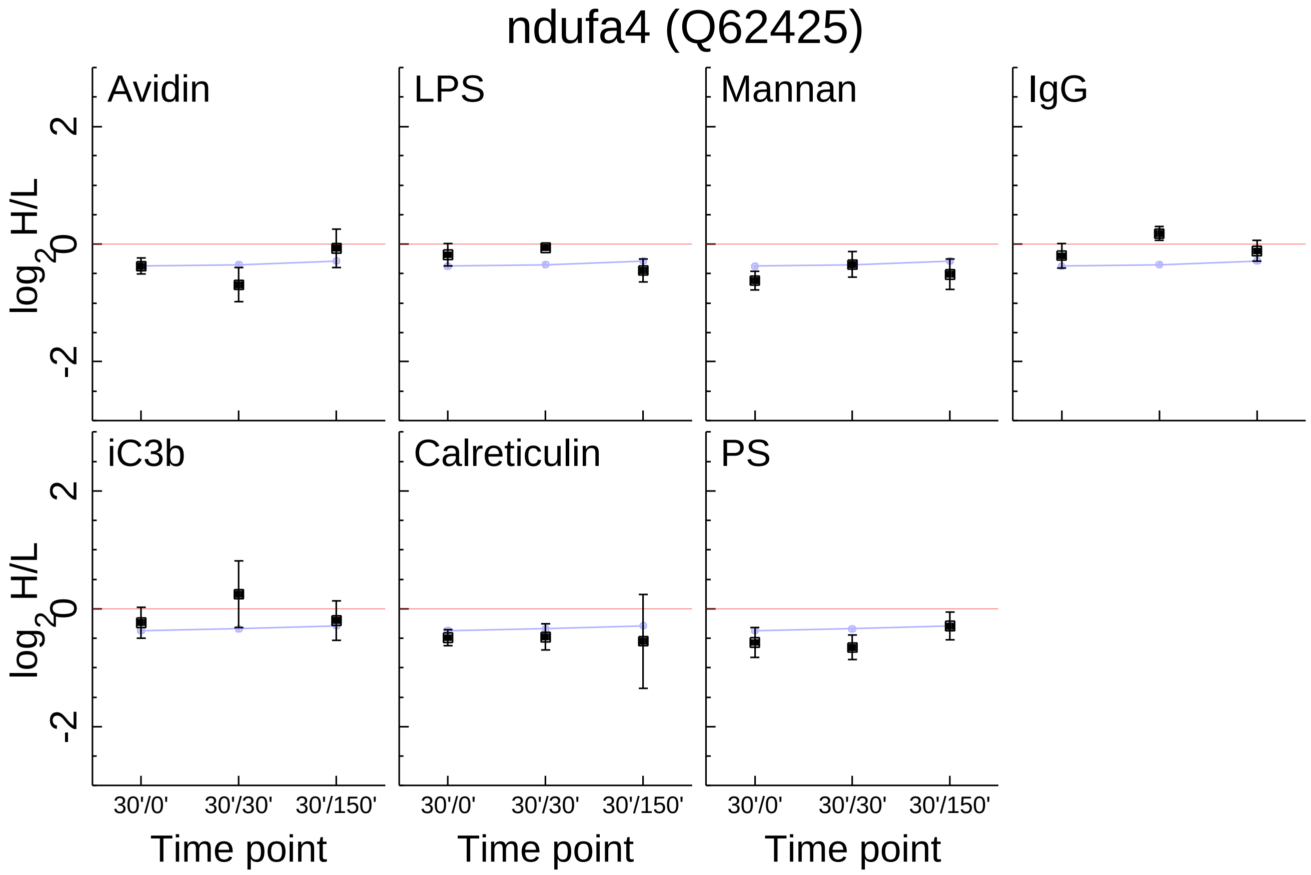
ndufa4 (Q62425)
Avidin
LPS
Mannan
IgG
iC3b
Calreticulin
PS
log2 H/L
2
0
-2
30'/0'
30'/30'
30'/150'
Time point

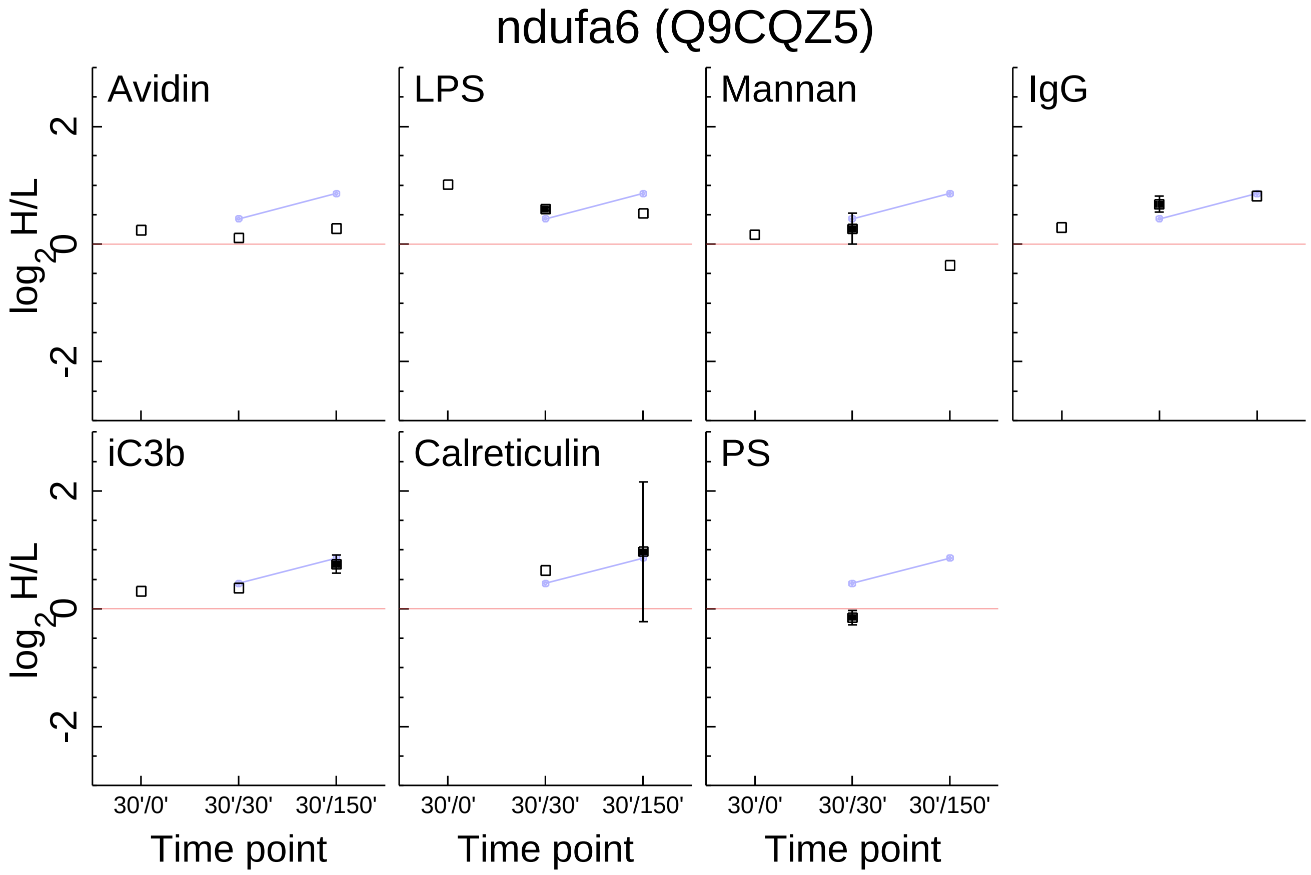

ndufa6 (Q9CQZ5)
Avidin
LPS
Mannan
IgG
iC3b
Calreticulin
PS
log2 H/L
2
0
-2
30'/0'
30'/30'
30'/150'
Time point

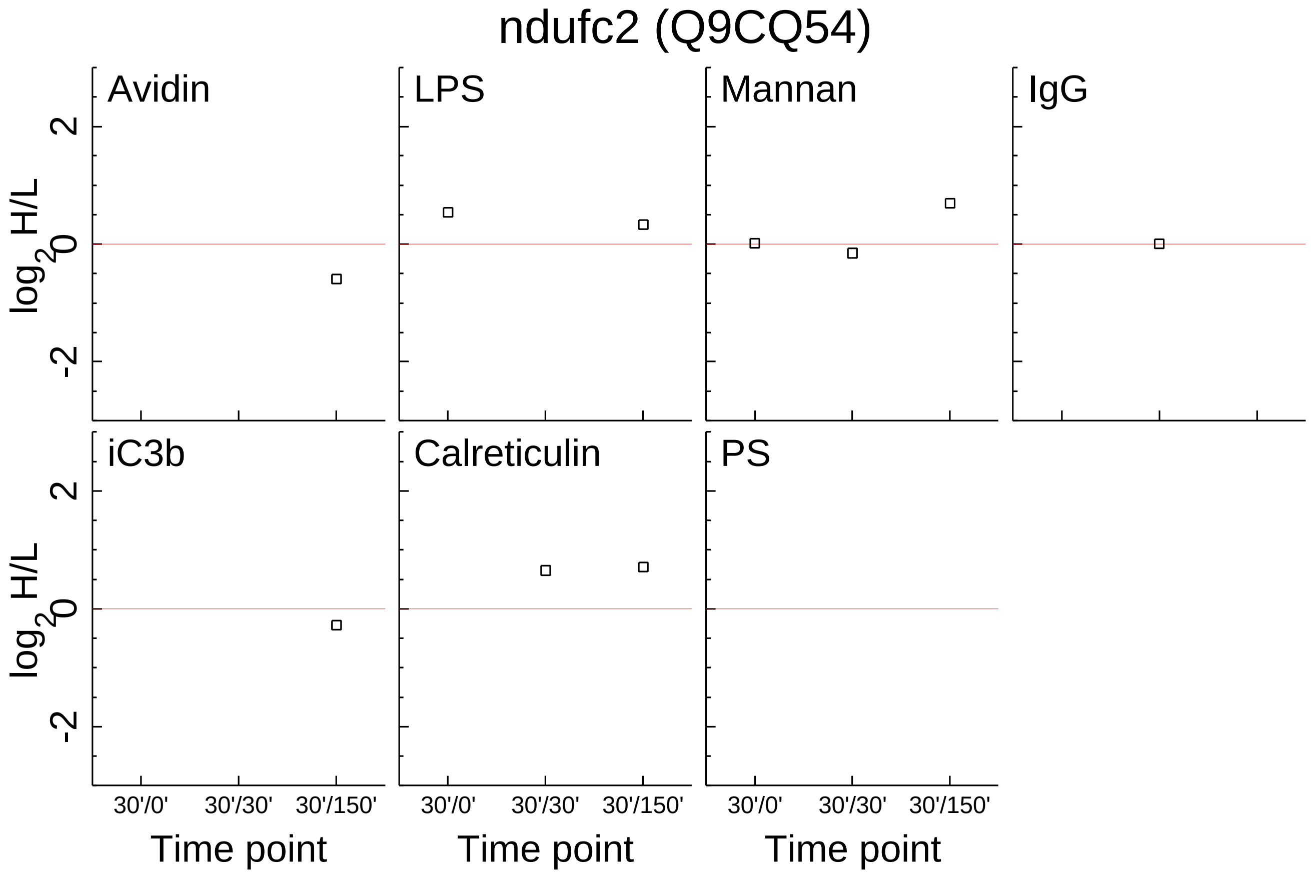
ndufc2 (Q9CQ54)
Avidin
LPS
Mannan
IgG
iC3b
Calreticulin
PS
$\log_2$ H/L
2
0
-2
30'/0'
30'/30'
30'/150'
Time point

ndufs1 (Q91VD9)

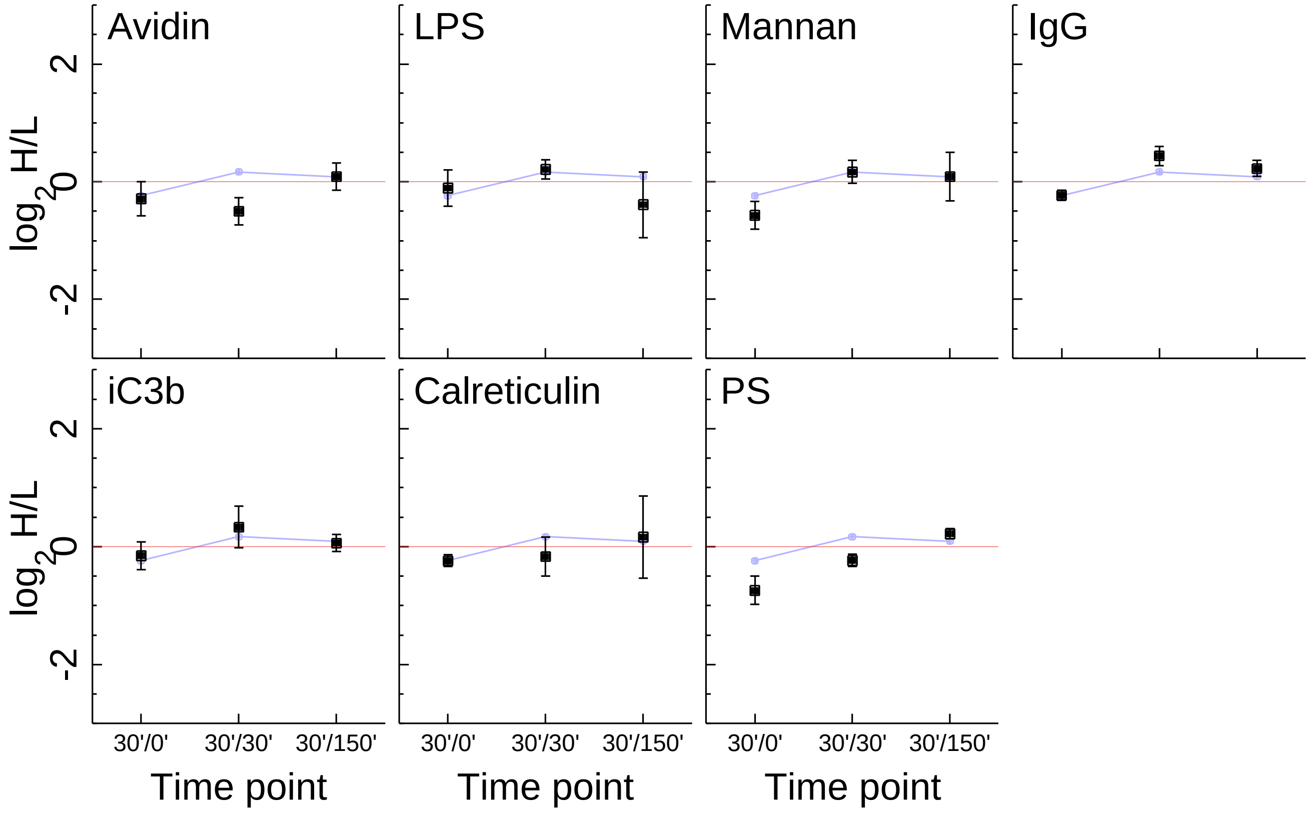

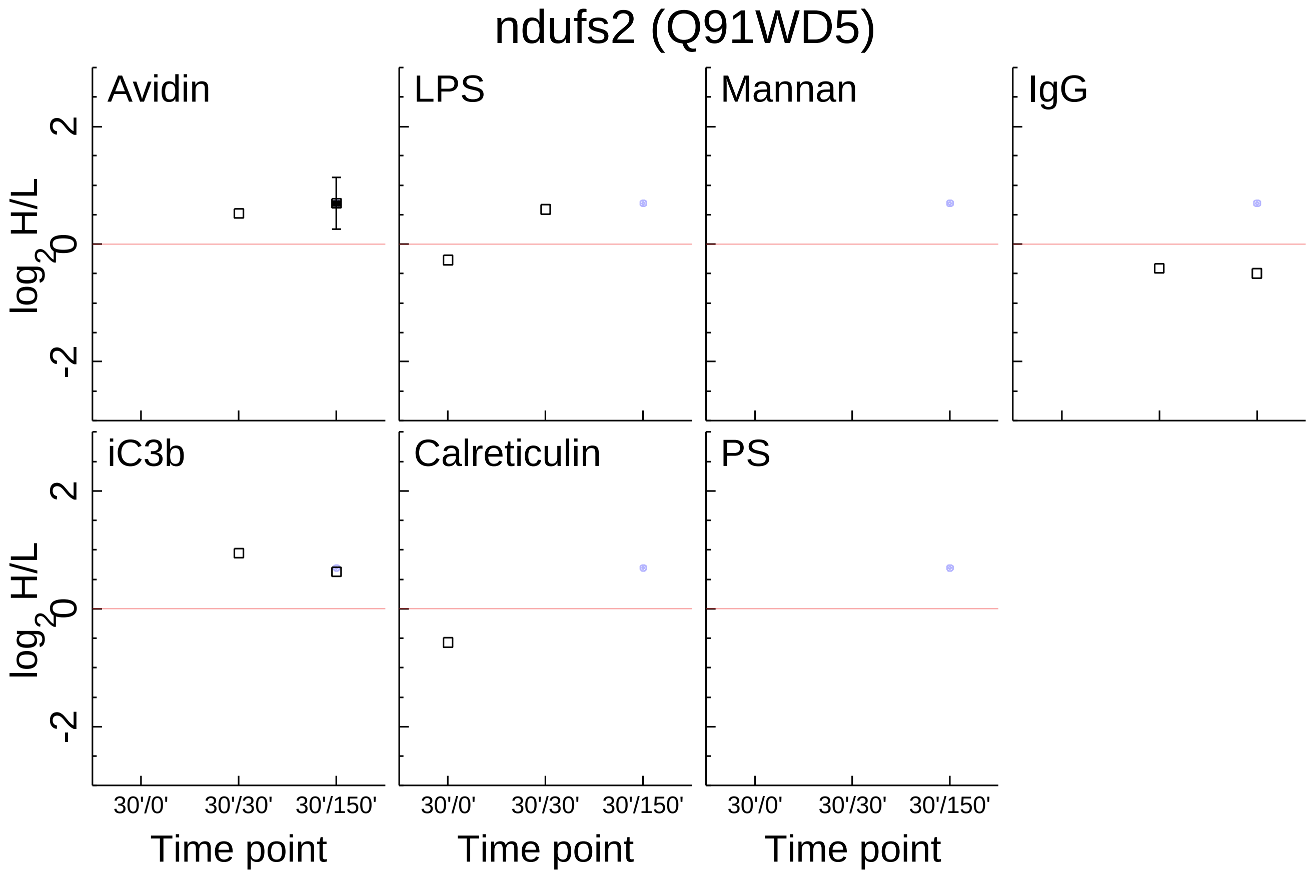

ndufs2 (Q91WD5)
Avidin
LPS
Mannan
IgG
iC3b
Calreticulin
PS
log2 H/L
2
0
-2
30'/0'
30'/30'
30'/150'
Time point

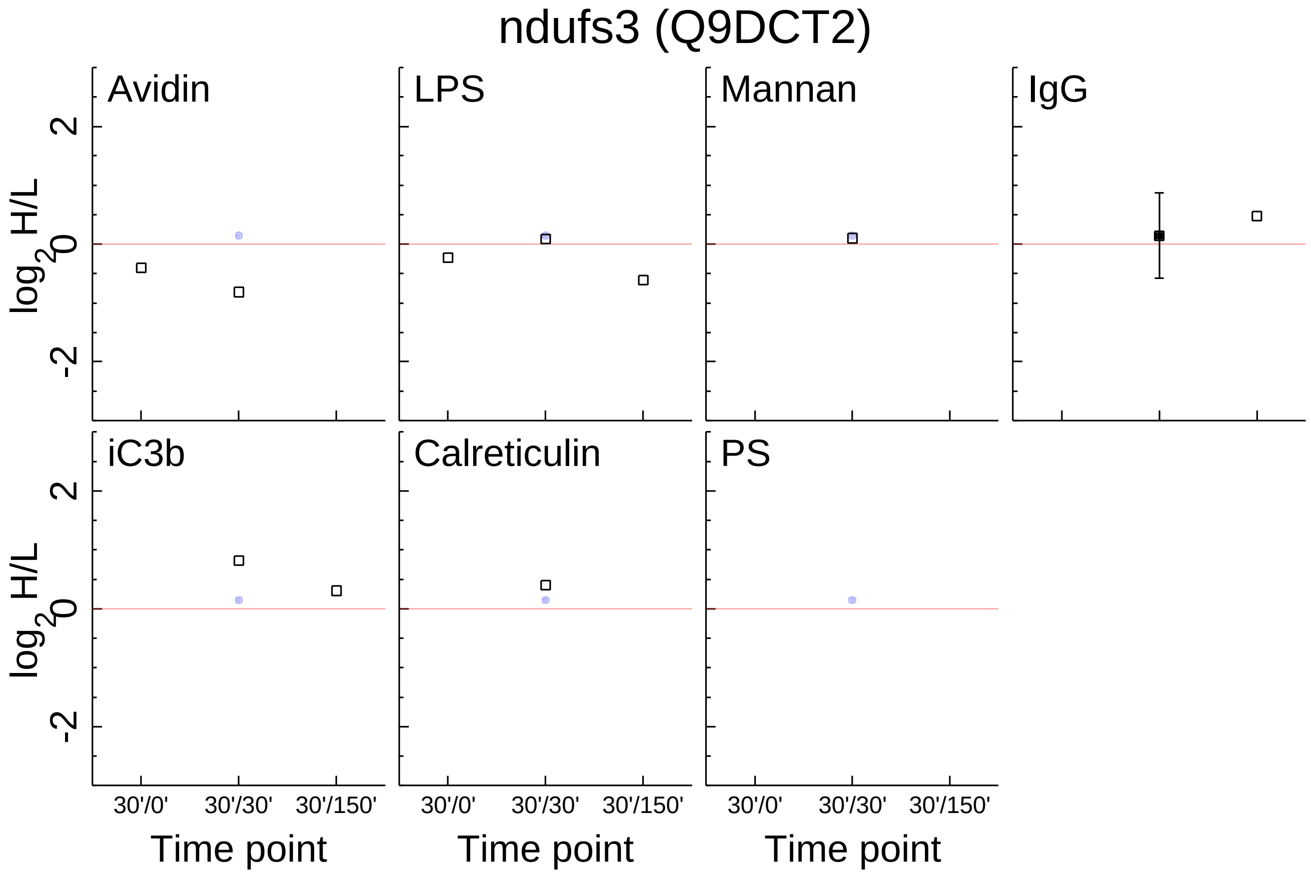

ndufs3 (Q9DCT2)
Avidin
LPS
Mannan
IgG
iC3b
Calreticulin
PS
log2 H/L
2
0
-2
30'/0'
30'/30'
30'/150'
Time point

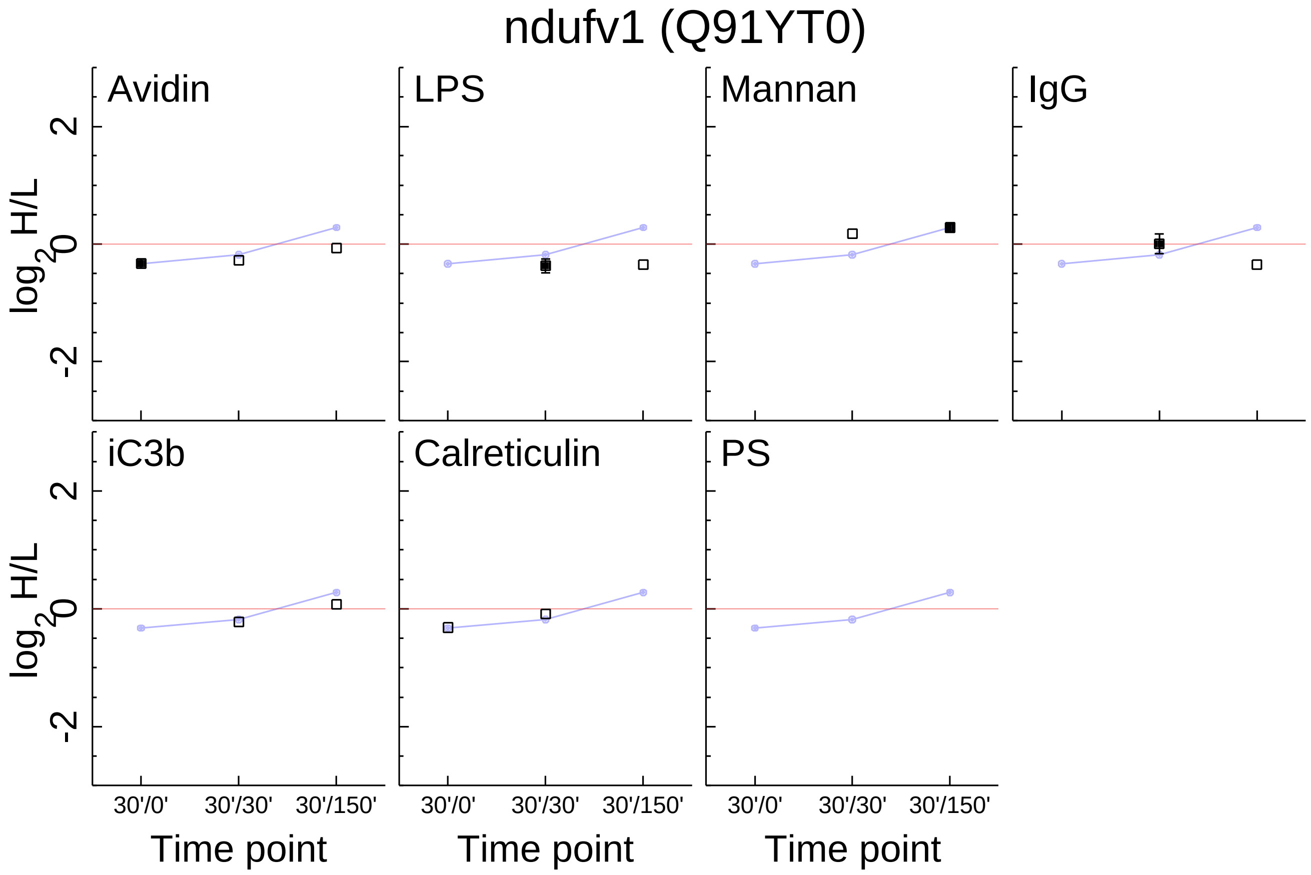

ndufv1 (Q91YT0)
Avidin
LPS
Mannan
IgG
iC3b
Calreticulin
PS
log2 H/L
2
0
-2
30'/0'
30'/30'
30'/150'
Time point

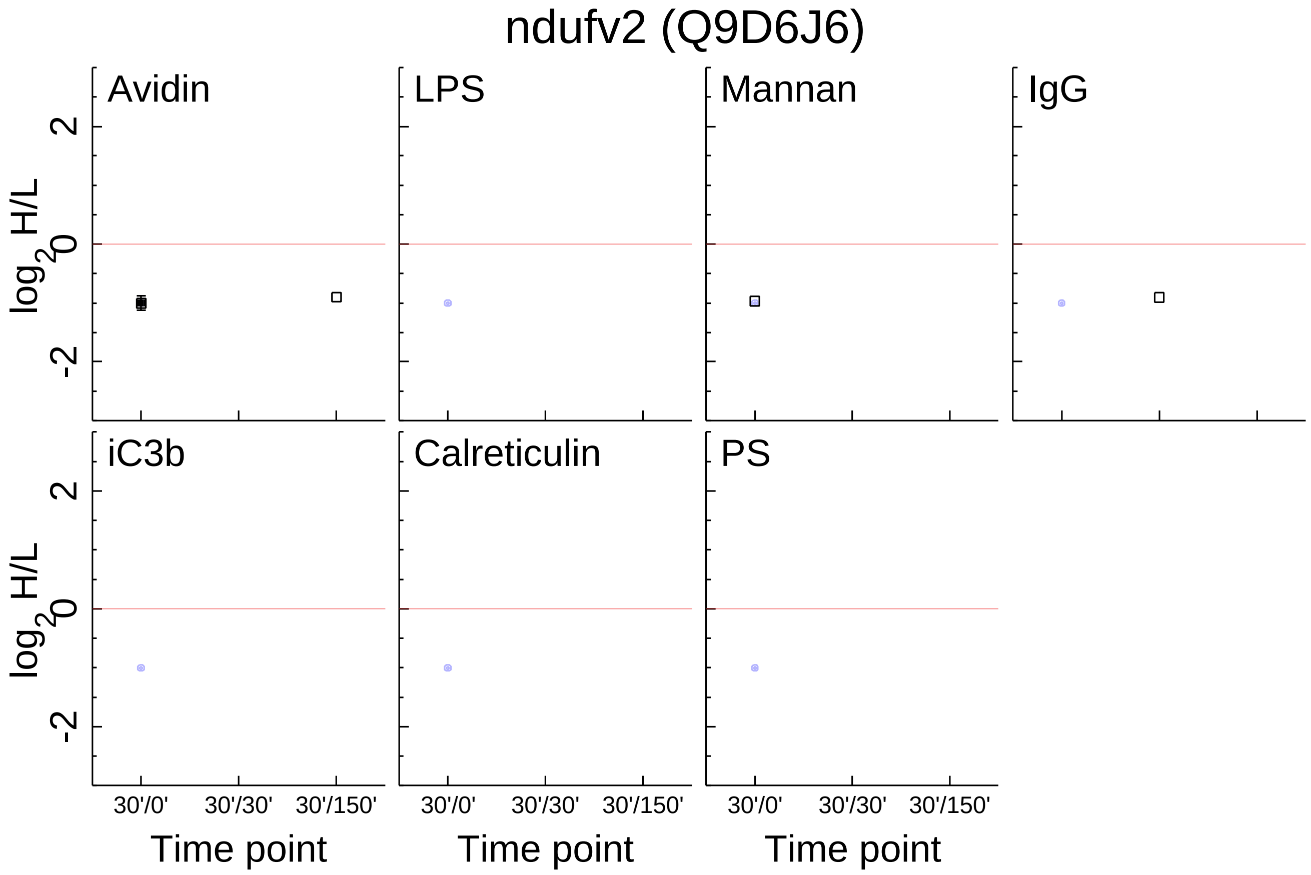
ndufv2 (Q9D6J6)
Avidin
LPS
Mannan
IgG
iC3b
Calreticulin
PS
log2 H/L
2
0
-2
30'/0'
30'/30'
30'/150'
Time point

neb (E9Q1W3)

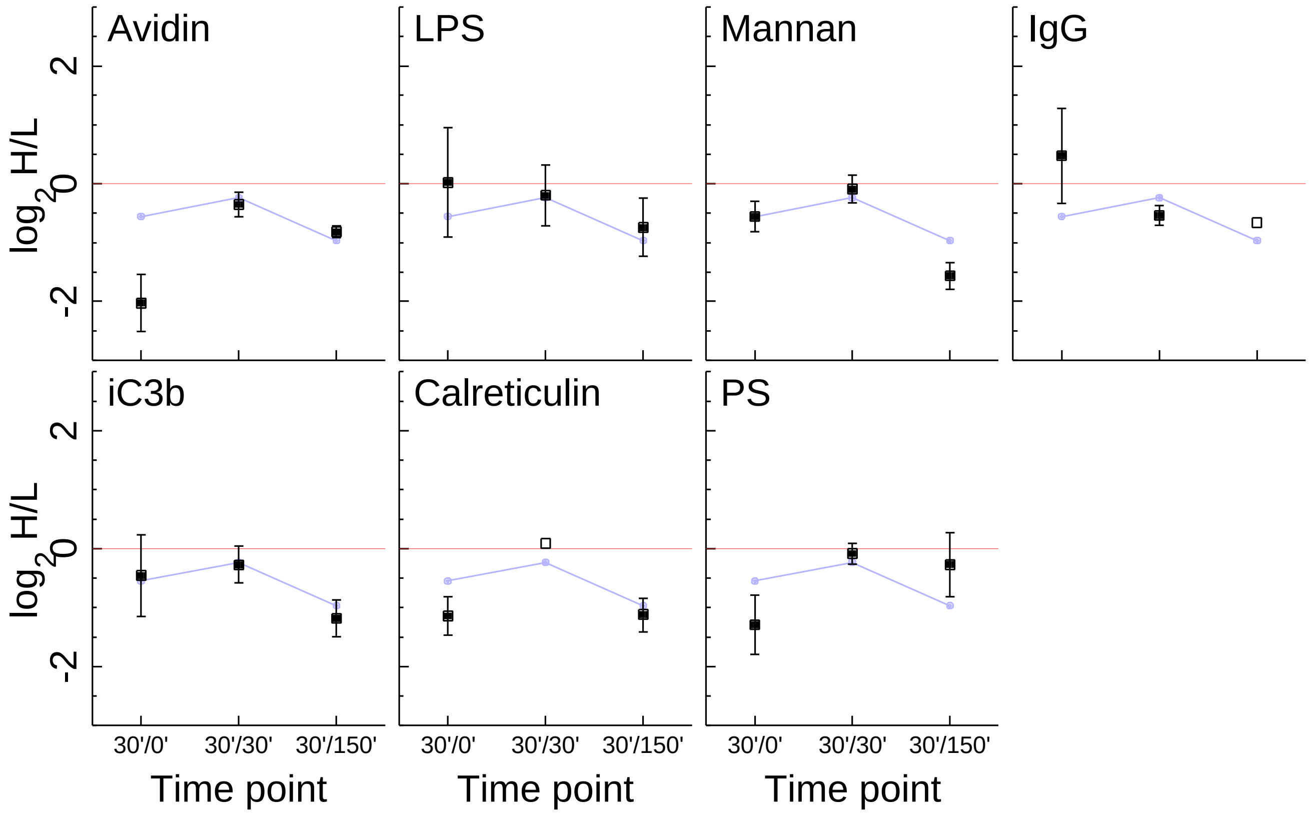

Avidin
LPS
Mannan
IgG
iC3b
Calreticulin
PS
log2 H/L
2
0
-2
30'/0'
30'/30'
30'/150'
Time point

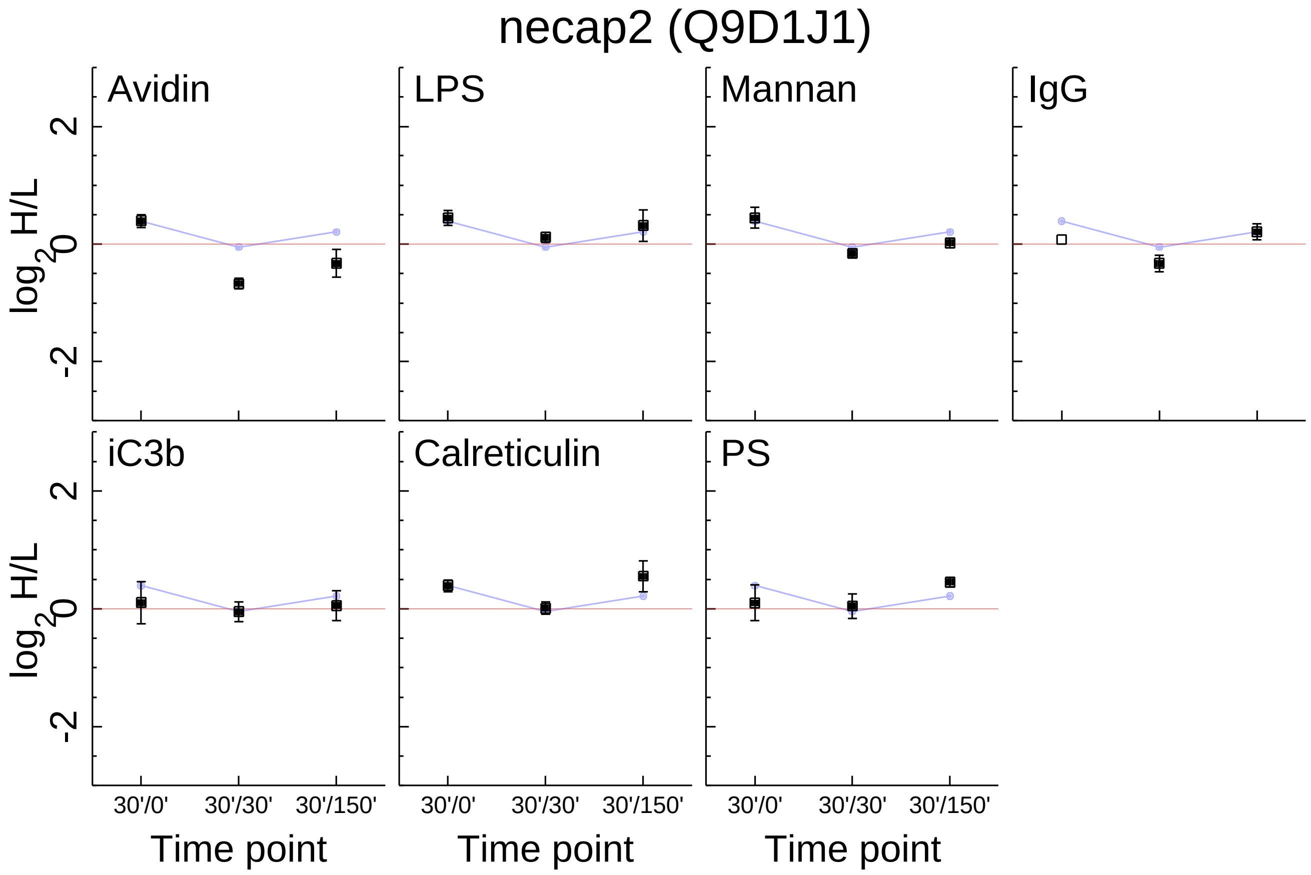
necap2 (Q9D1J1)
Avidin
LPS
Mannan
IgG
iC3b
Calreticulin
PS
log2 H/L
2
0
-2
30'/0'
30'/30'
30'/150'
Time point

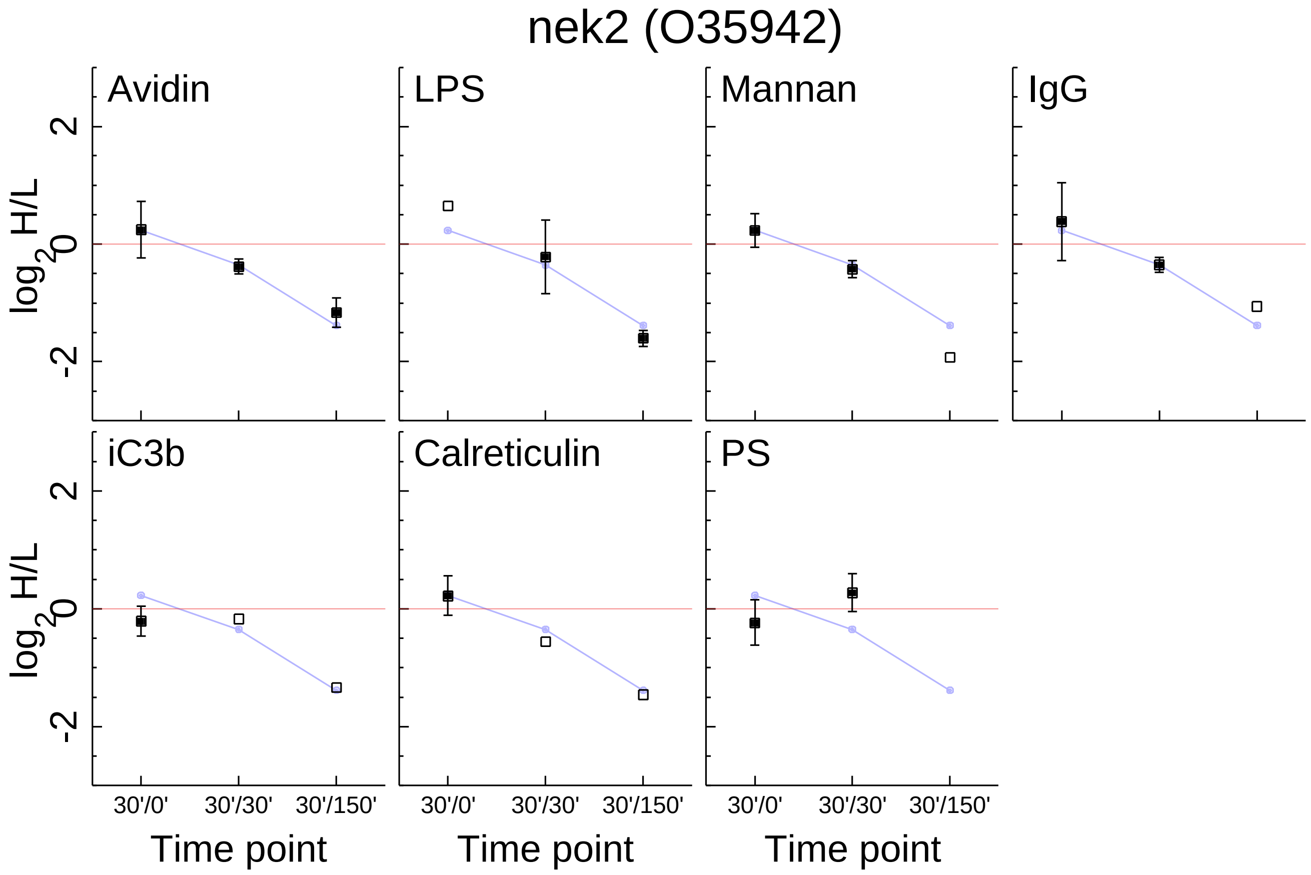
nek2 (O35942)
Avidin
LPS
Mannan
IgG
iC3b
Calreticulin
PS
log2 H/L
2
0
-2
30'/0'
30'/30'
30'/150'
Time point

neu1 (O35657)

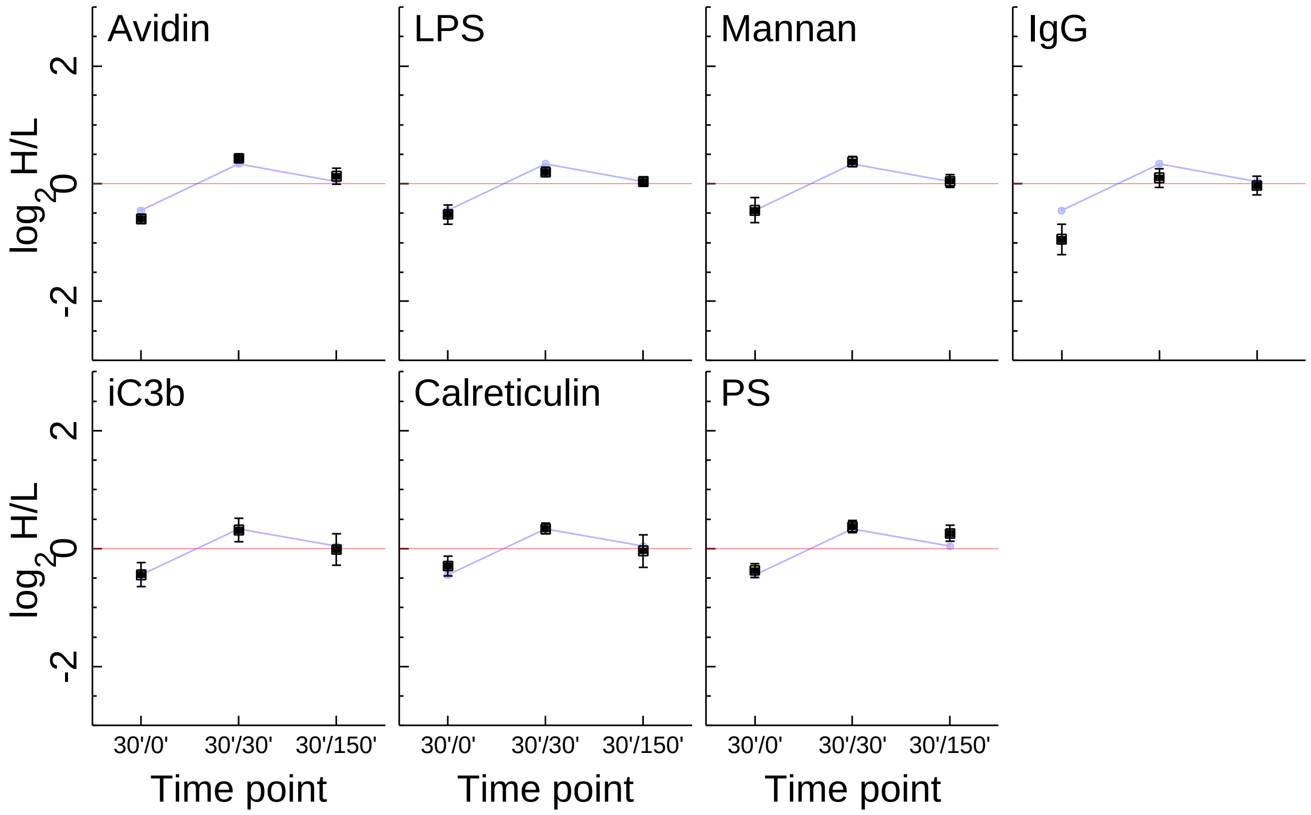
Avidin
LPS
Mannan
IgG
iC3b
Calreticulin
PS
log_2 H/L
log_2 H/L
2
0
-2
2
0
-2
30'/0'
30'/30'
30'/150'
30'/0'
30'/30'
30'/150'
30'/0'
30'/30'
30'/150'
Time point
Time point
Time point

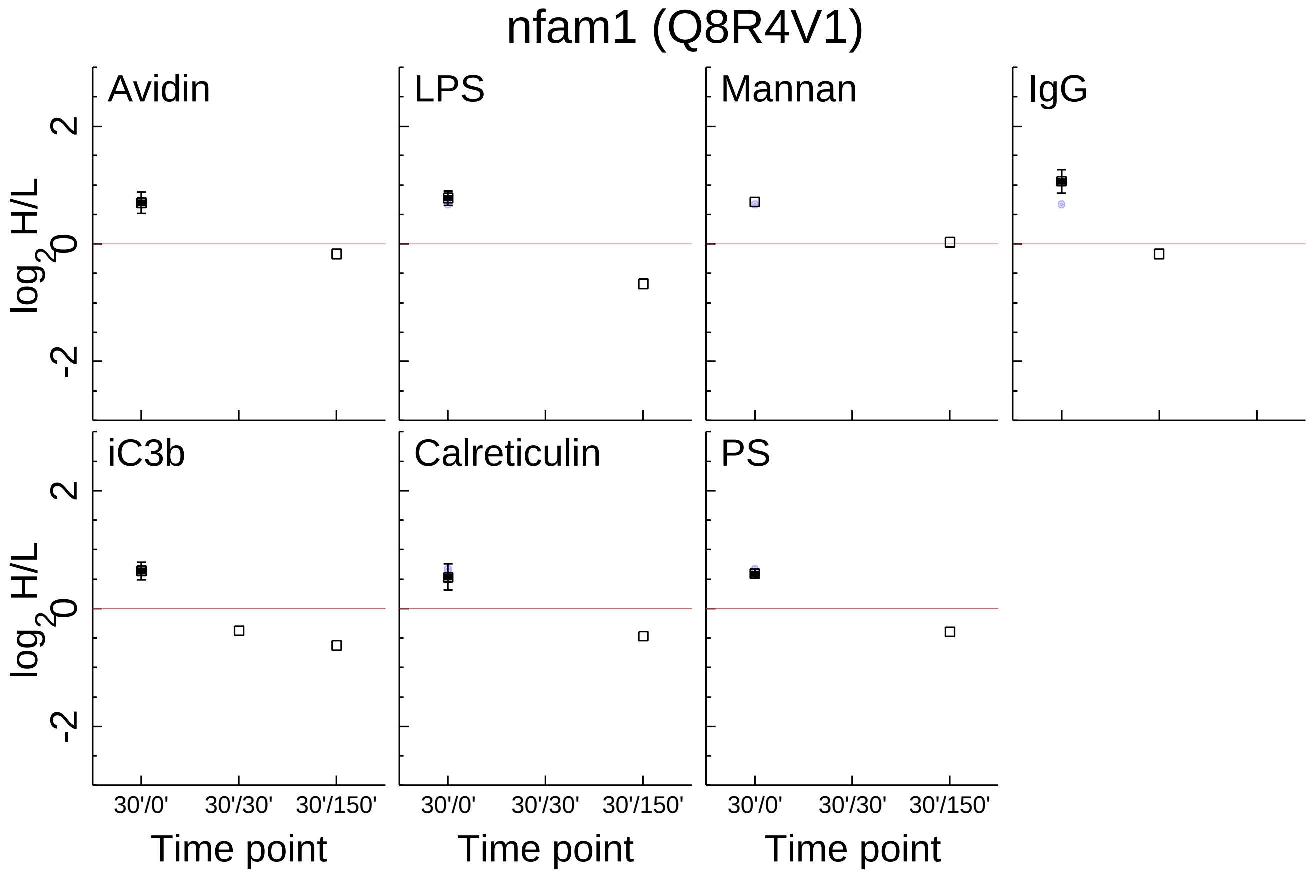
nfam1 (Q8R4V1)
Avidin
LPS
Mannan
IgG
iC3b
Calreticulin
PS
log2 H/L
2
0
-2
30'/0'
30'/30'
30'/150'
Time point

nfatc2ip (O09130)

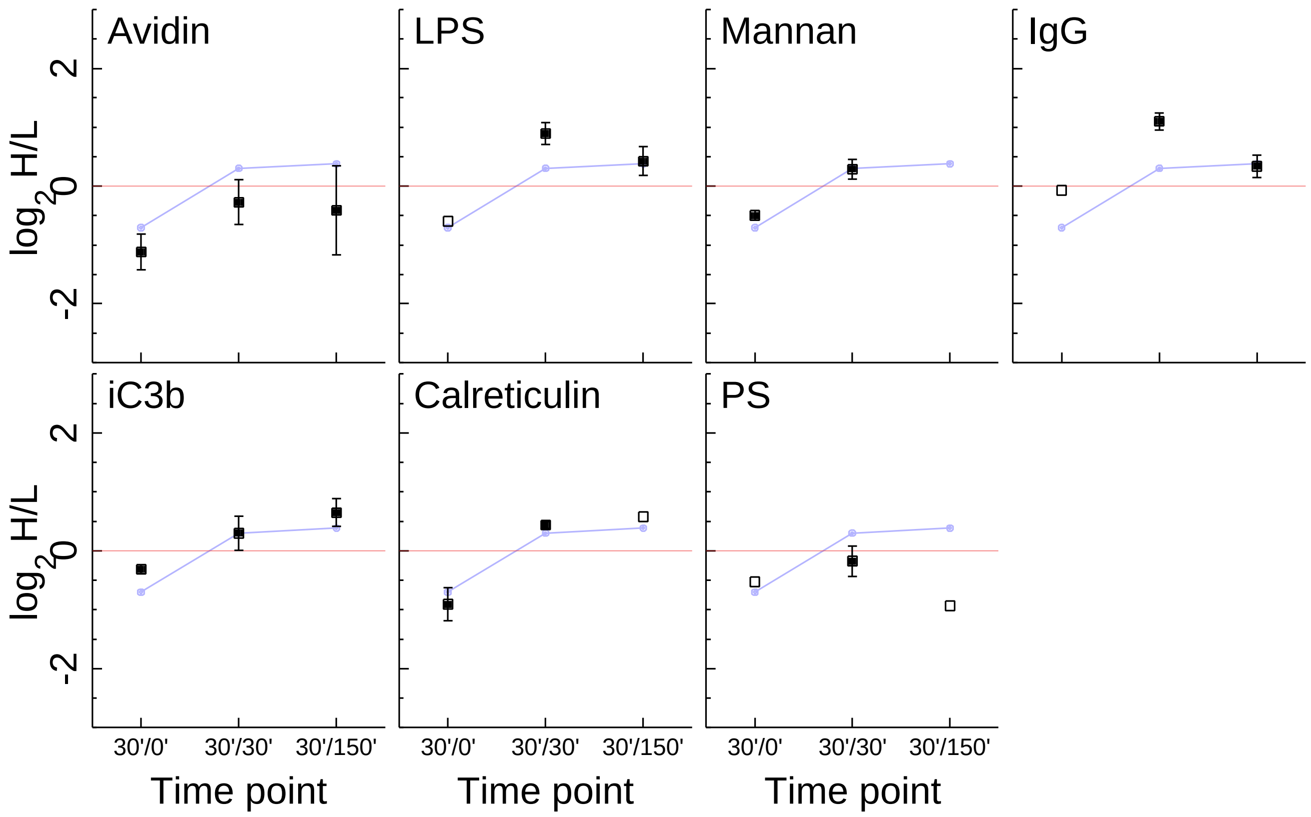

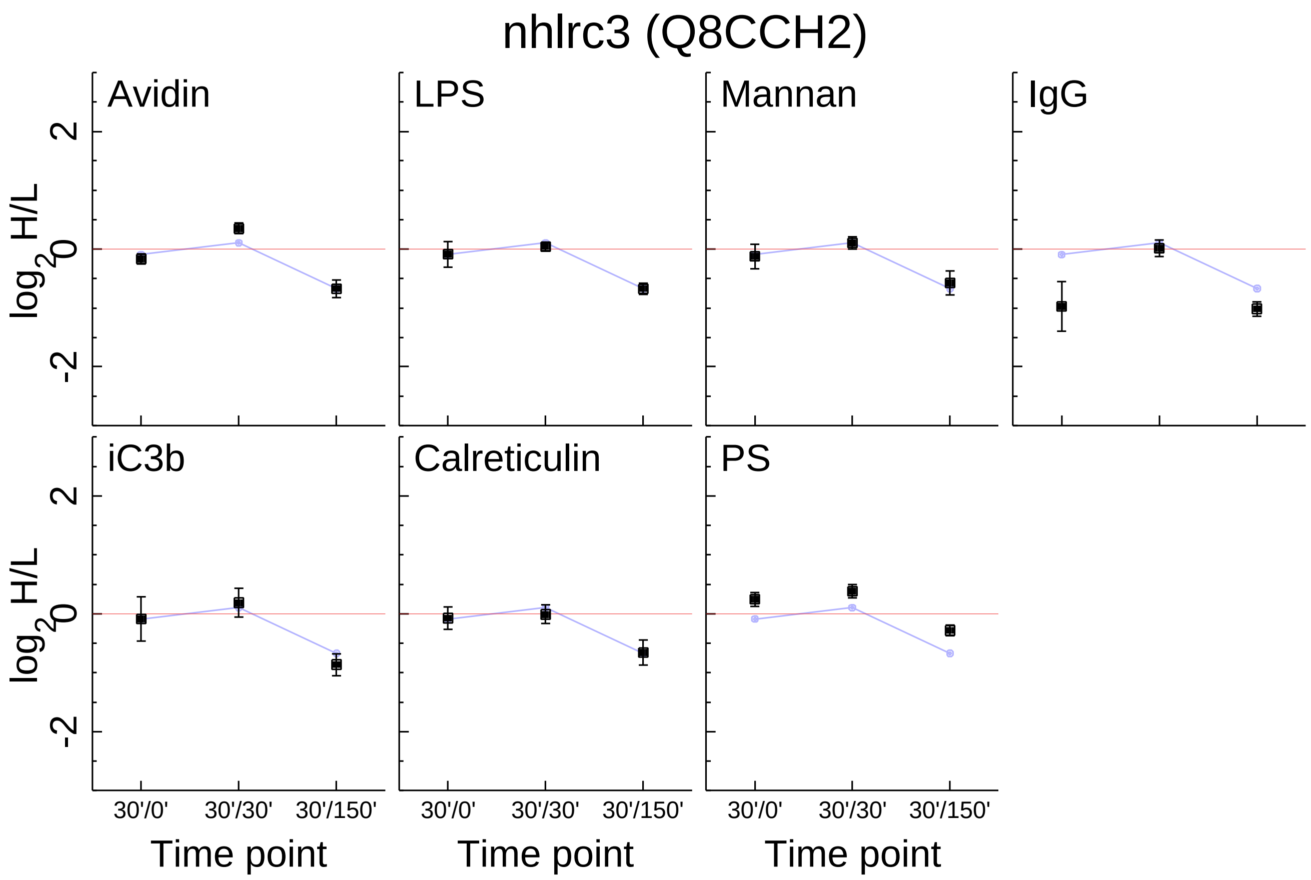
nhlrc3 (Q8CCH2)
Avidin
LPS
Mannan
IgG
iC3b
Calreticulin
PS
2
0
-2
log2 H/L
30'/0'
30'/30'
30'/150'
Time point

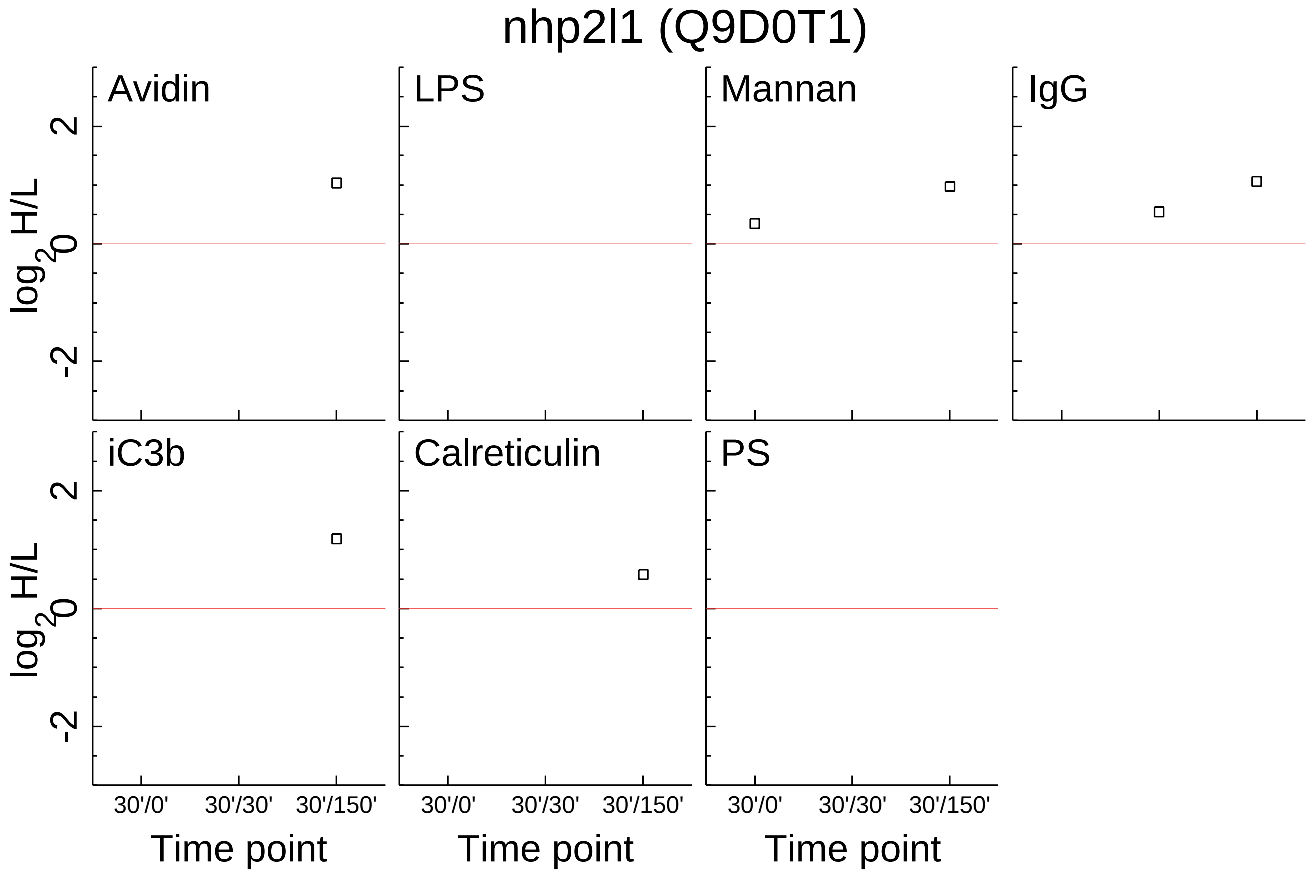
nhp2l1 (Q9D0T1)
Avidin
LPS
Mannan
IgG
iC3b
Calreticulin
PS
log2 H/L
2
0
-2
30'/0'
30'/30'
30'/150'
Time point

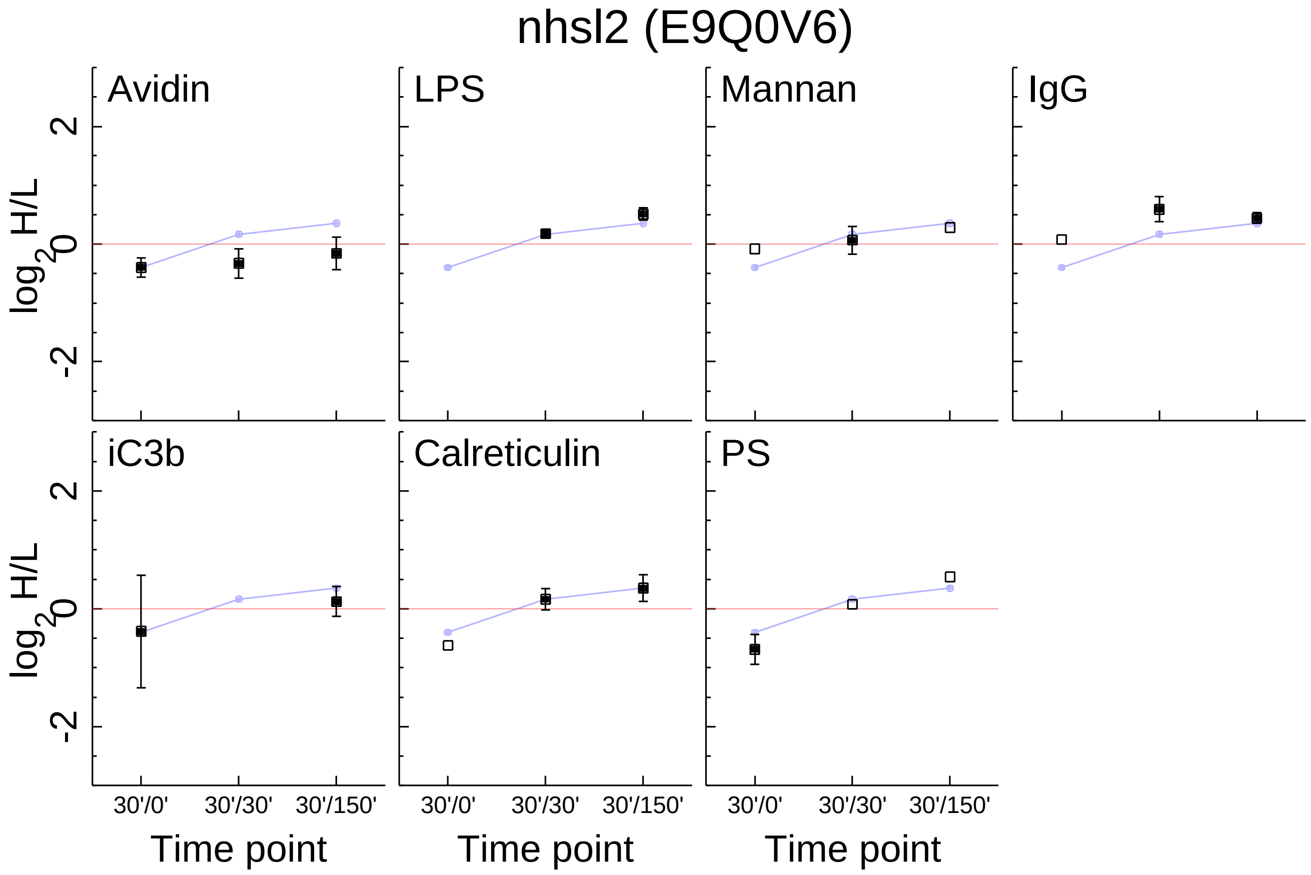
nhsl2 (E9Q0V6)
Avidin
LPS
Mannan
IgG
iC3b
Calreticulin
PS
log_2 H/L
2
0
-2
30'/0'
30'/30'
30'/150'
Time point

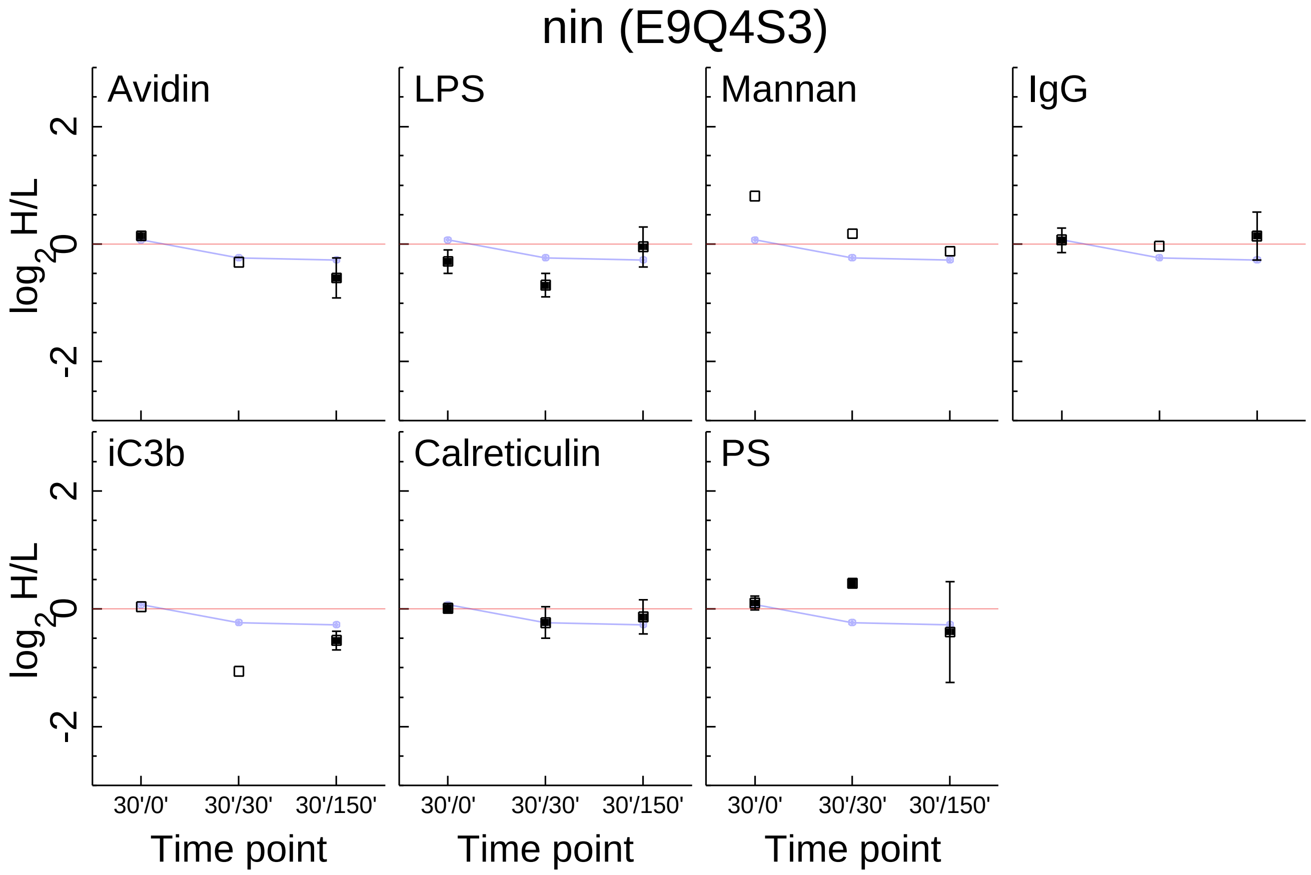
nin (E9Q4S3)
Avidin
LPS
Mannan
IgG
iC3b
Calreticulin
PS
log2 H/L
2
0
-2
30'/0'
30'/30'
30'/150'
Time point

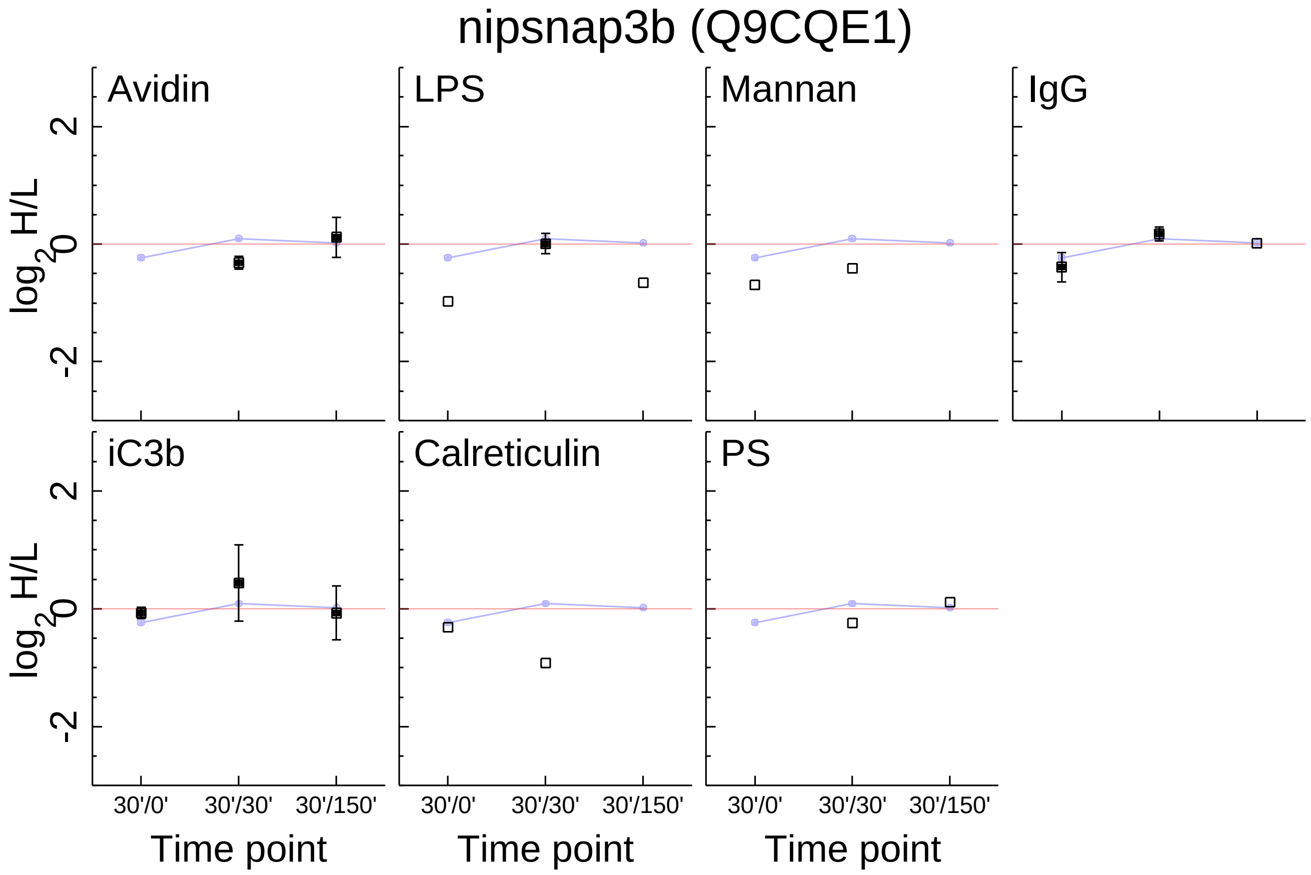
nipsnap3b (Q9CQE1)
Avidin
LPS
Mannan
IgG
iC3b
Calreticulin
PS
log2 H/L
2
0
-2
30'/0'
30'/30'
30'/150'
Time point

nisch (Q80TM9)

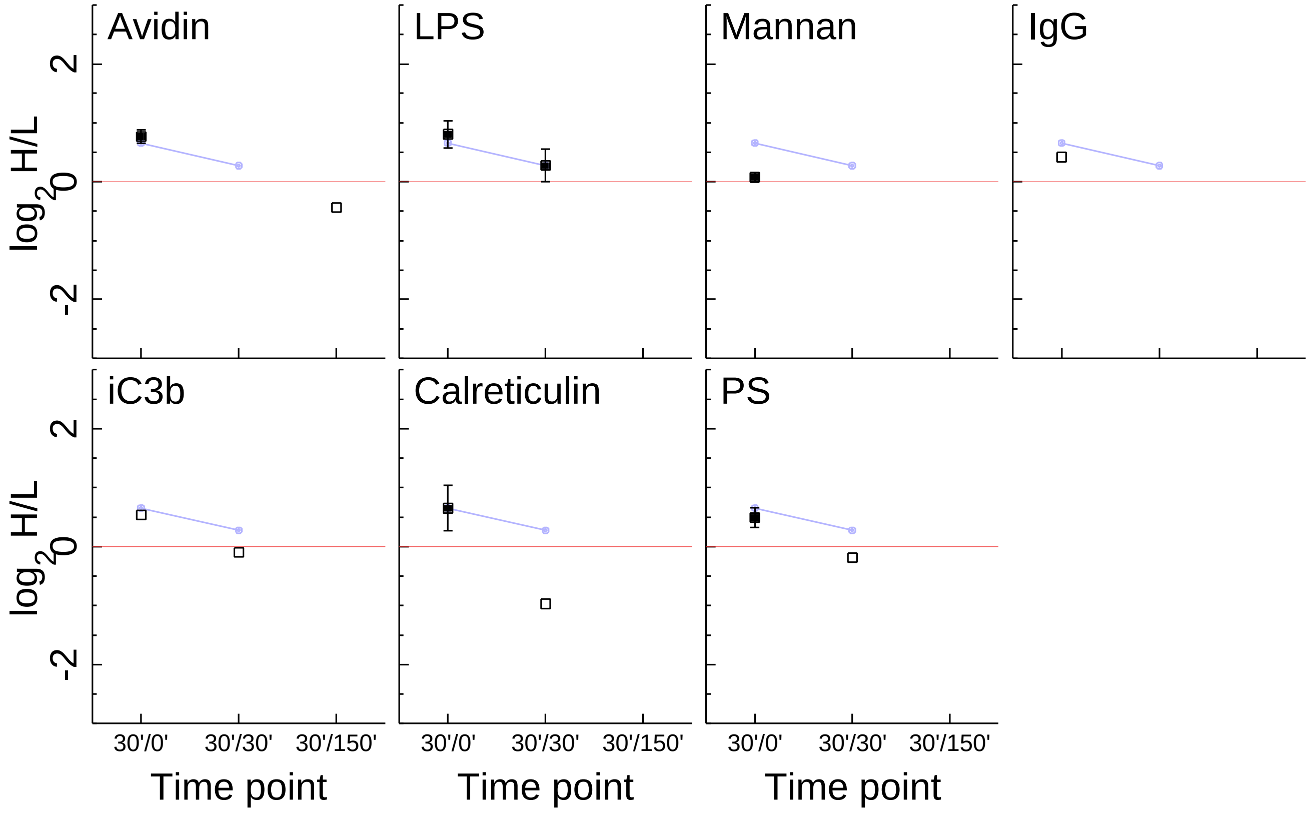

nmt1 (O70310)

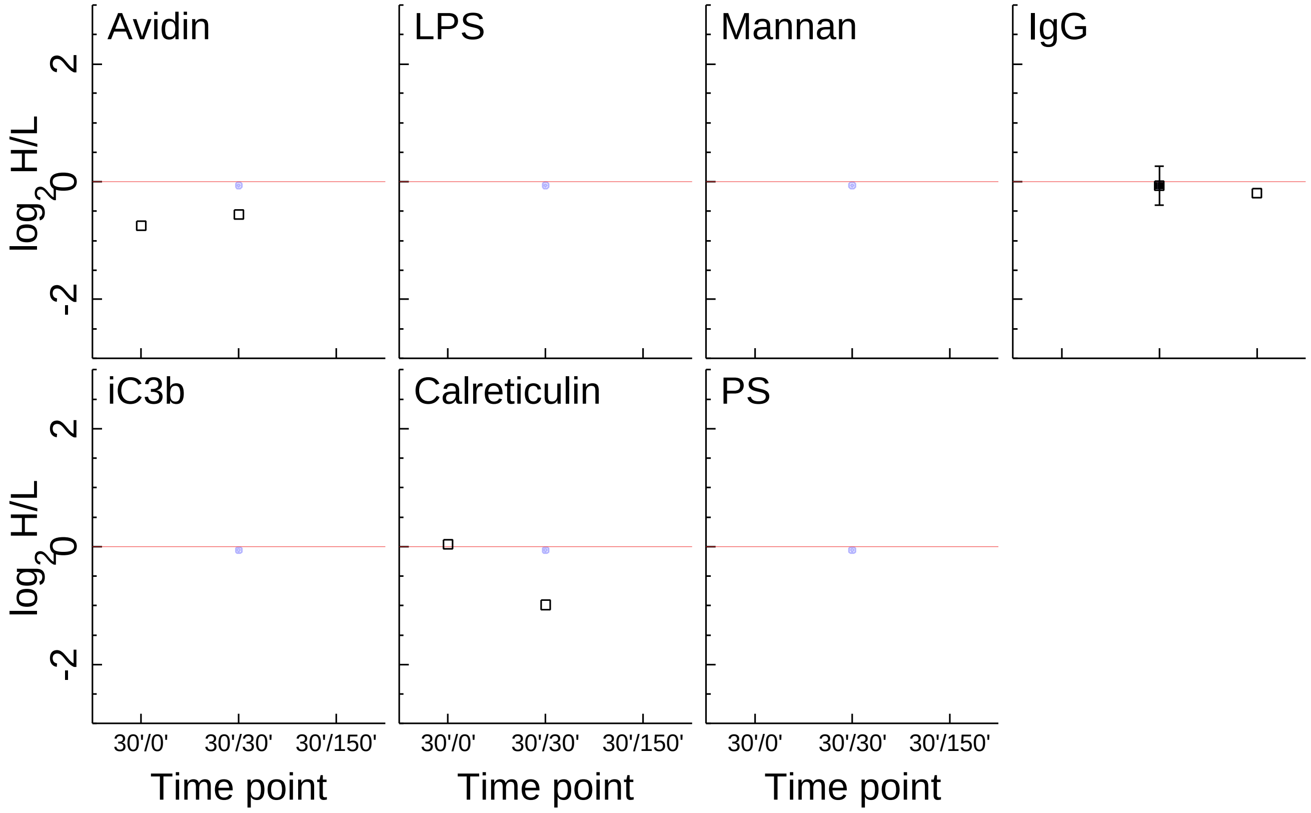

Avidin
LPS
Mannan
IgG
iC3b
Calreticulin
PS
log2 H/L
2
0
-2
30'/0'
30'/30'
30'/150'
Time point

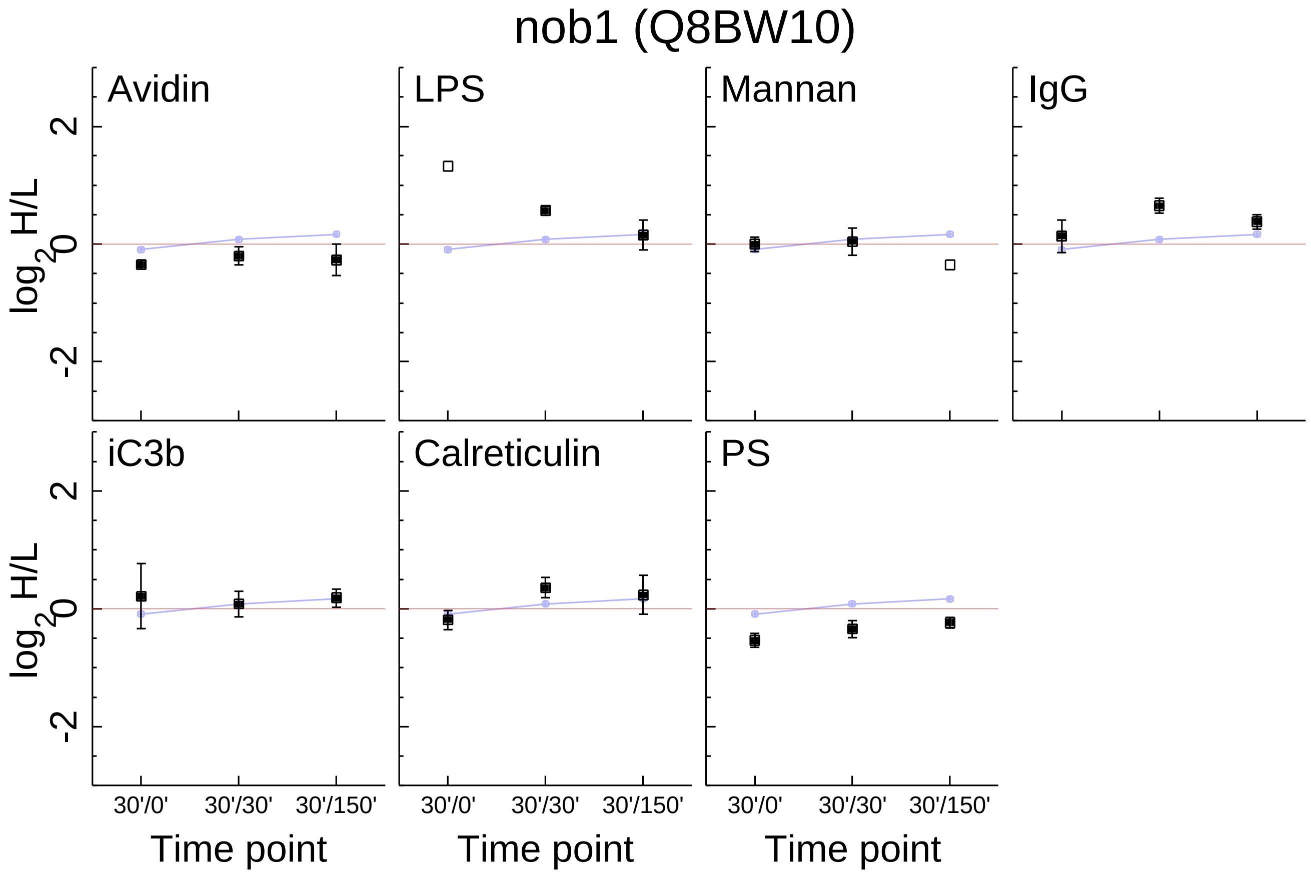
nob1 (Q8BW10)
Avidin
LPS
Mannan
IgG
iC3b
Calreticulin
PS
$\log_2$ H/L
2
0
-2
30'/0'
30'/30'
30'/150'
Time point

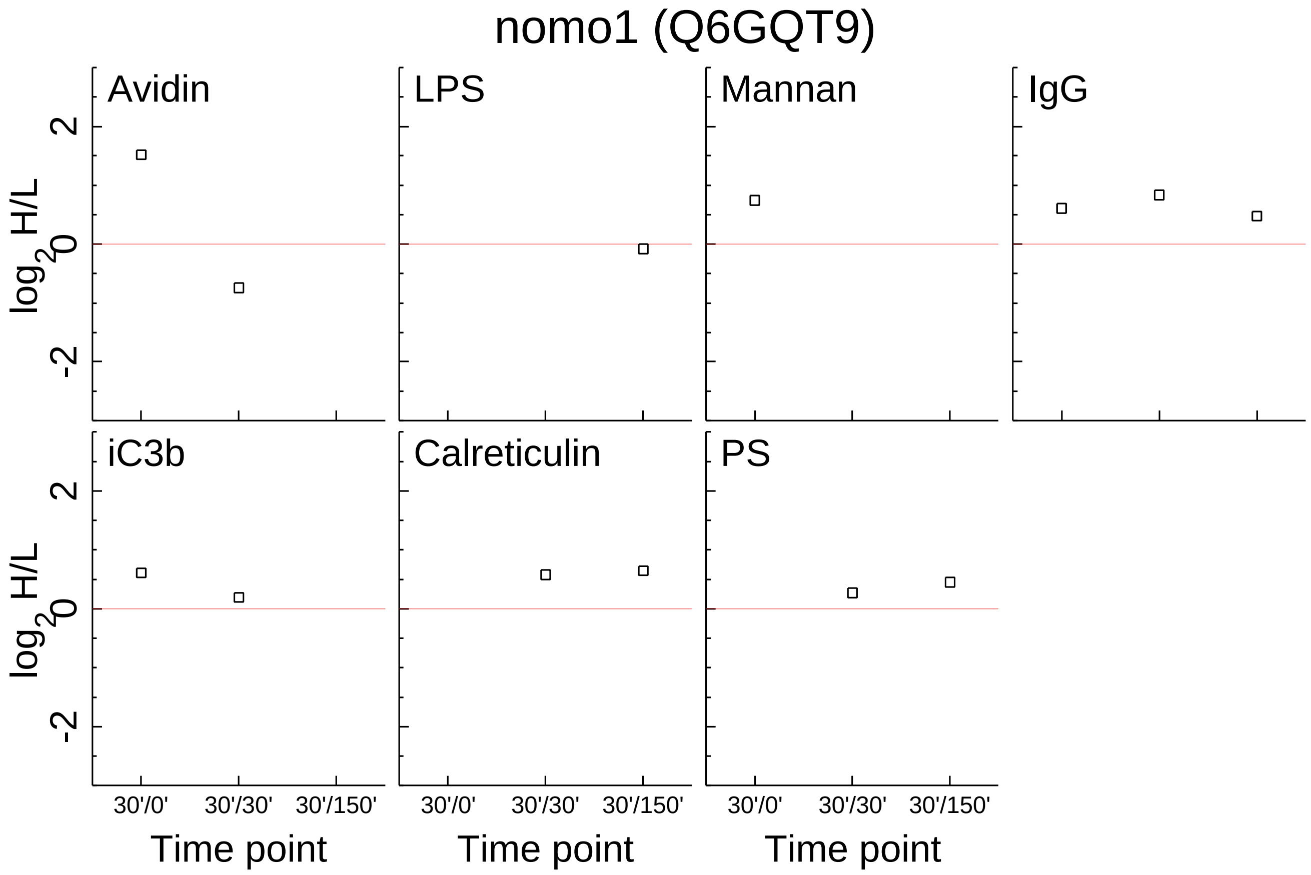

nomo1 (Q6GQT9)
Avidin
LPS
Mannan
IgG
iC3b
Calreticulin
PS
log2 H/L
2
0
-2
30'/0'
30'/30'
30'/150'
Time point

nono (Q99K48)

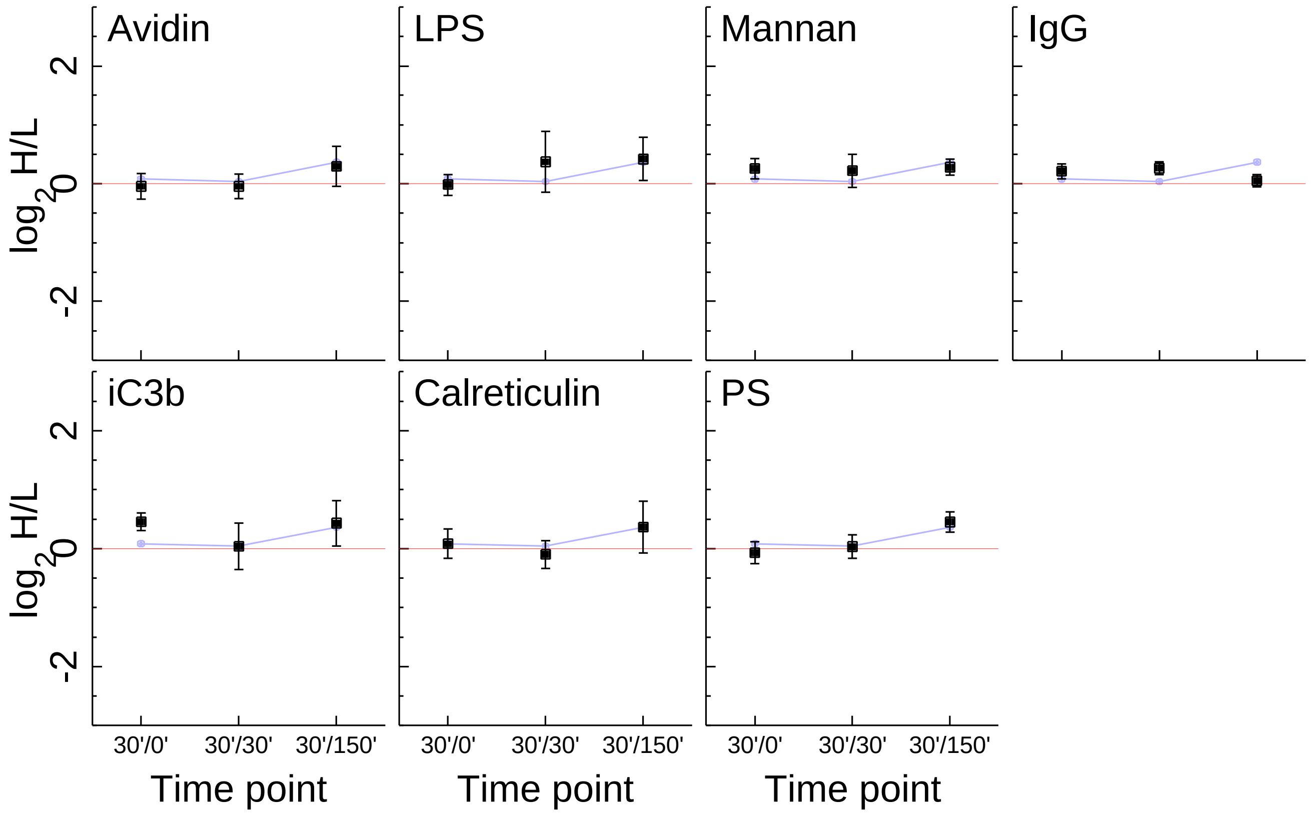

Avidin
LPS
Mannan
IgG
iC3b
Calreticulin
PS
log2 H/L
2
0
-2
30'/0'
30'/30'
30'/150'
Time point

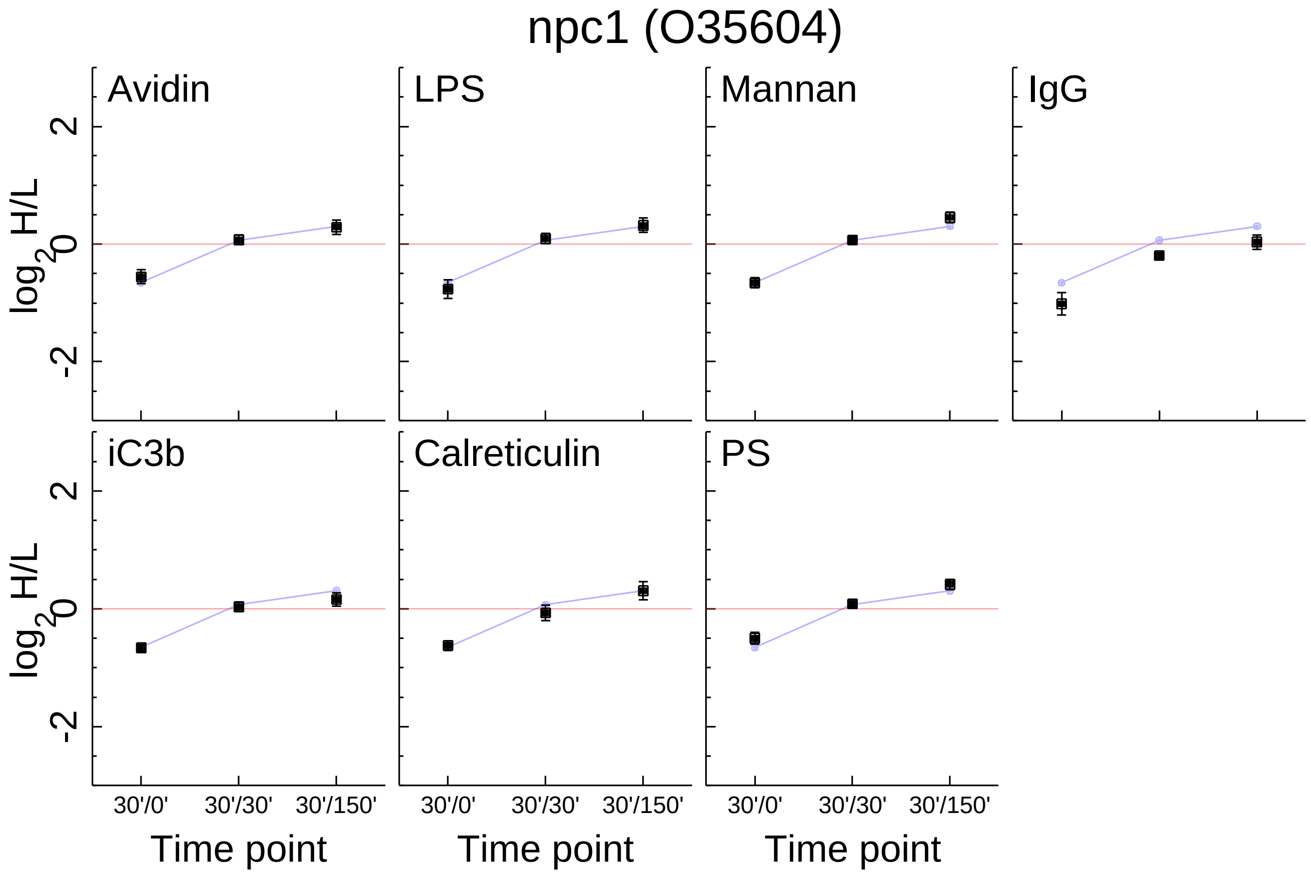
npc1 (O35604)
Avidin
LPS
Mannan
IgG
iC3b
Calreticulin
PS
log2 H/L
2
0
-2
30'/0'
30'/30'
30'/150'
Time point

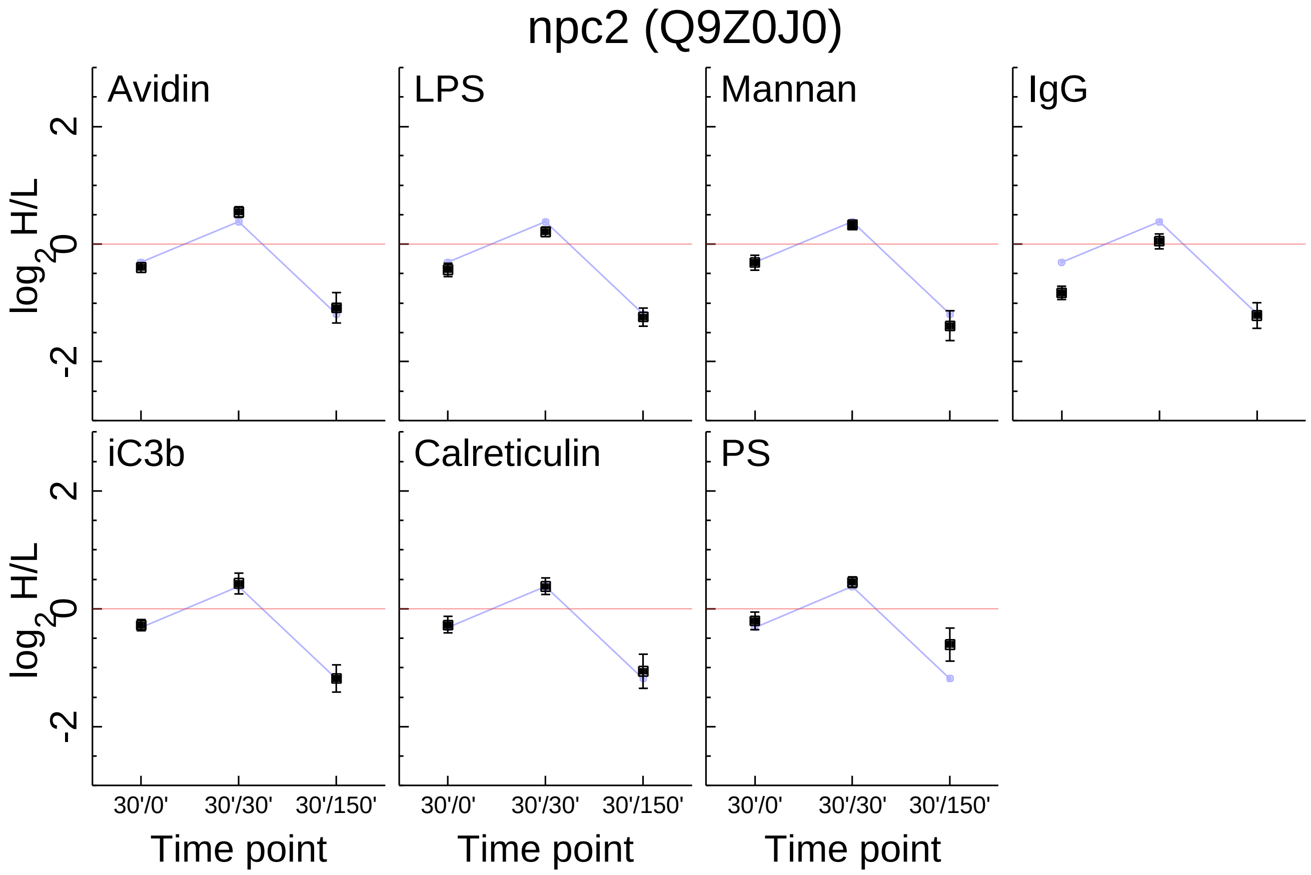
npc2 (Q9Z0J0)
Avidin
LPS
Mannan
IgG
iC3b
Calreticulin
PS
log2 H/L
2
0
-2
30'/0'
30'/30'
30'/150'
Time point

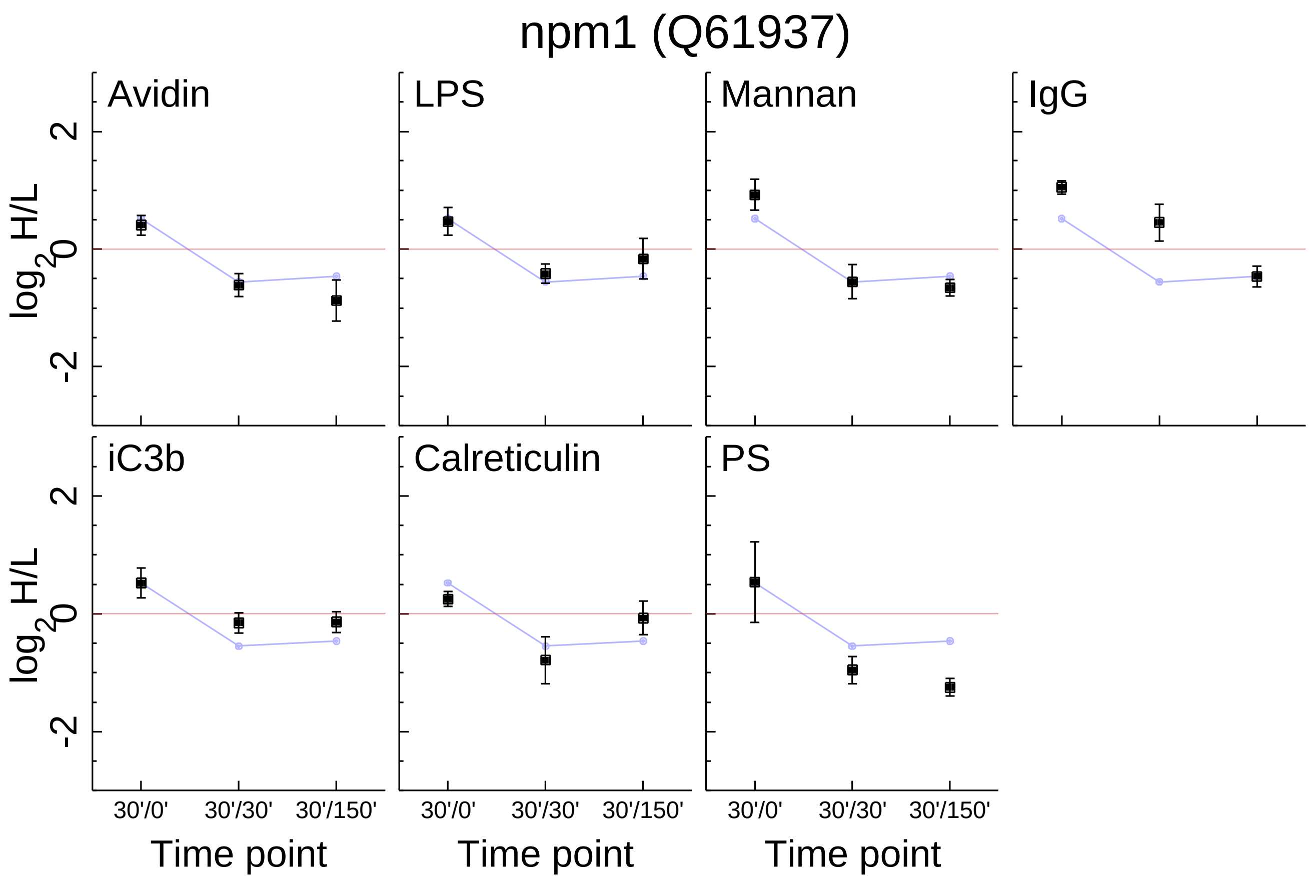
npm1 (Q61937)
Avidin
LPS
Mannan
IgG
iC3b
Calreticulin
PS
$\log_2$ H/L
2
0
-2
30'/0'
30'/30'
30'/150'
Time point

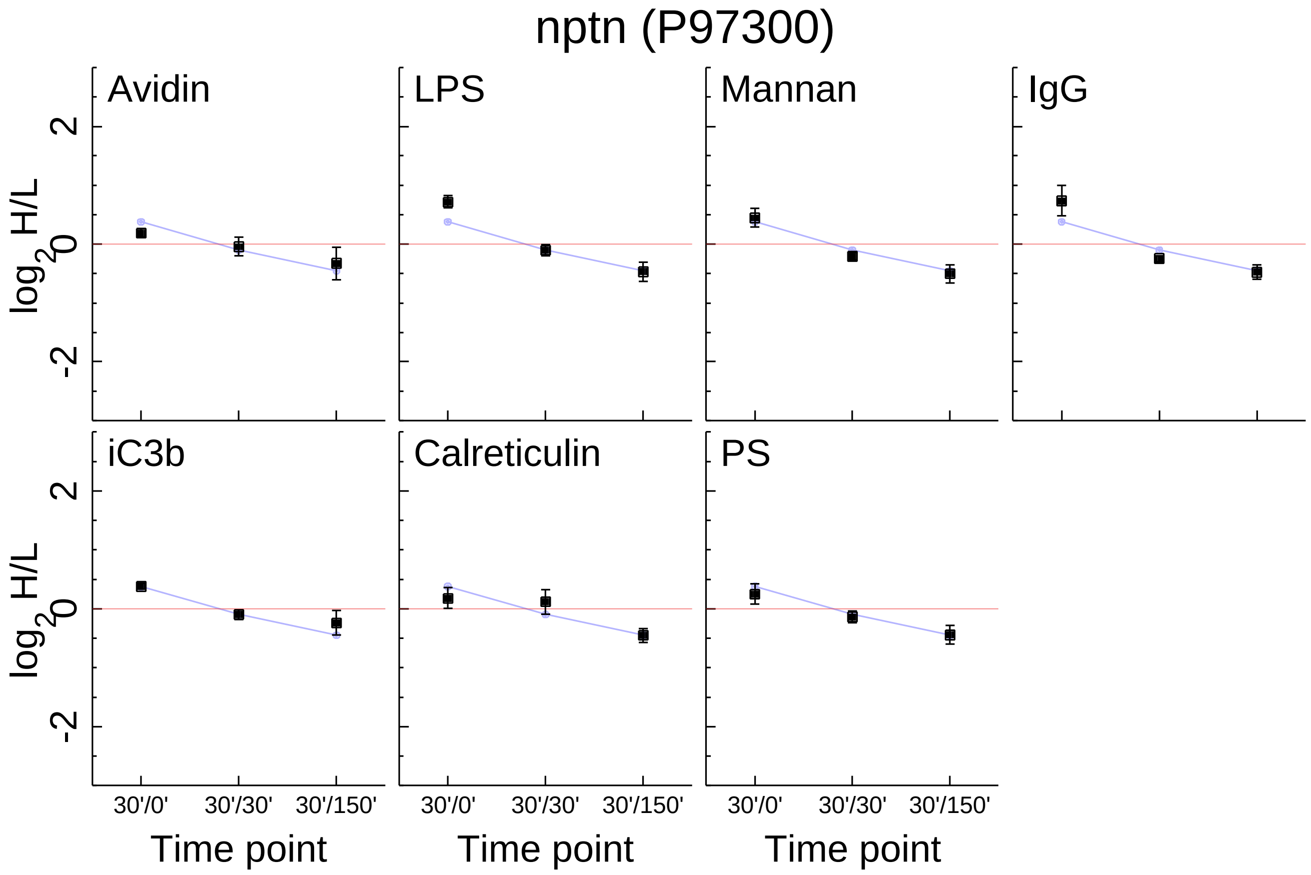

nptn (P97300)
Avidin
LPS
Mannan
IgG
iC3b
Calreticulin
PS
log2 H/L
2
0
-2
30'/0'
30'/30'
30'/150'
Time point

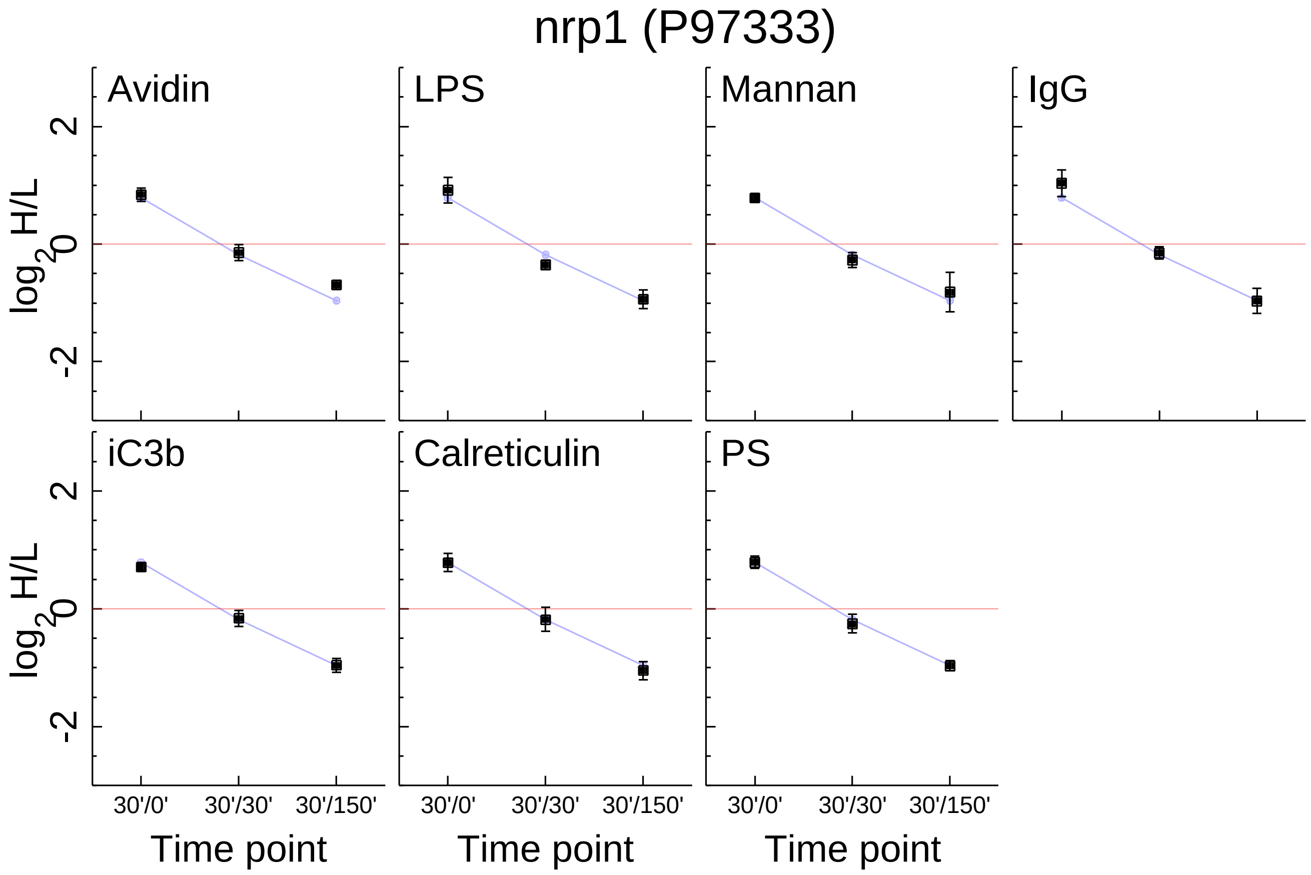
nrp1 (P97333)
Avidin
LPS
Mannan
IgG
iC3b
Calreticulin
PS
log2 H/L
2
0
-2
30'/0'
30'/30'
30'/150'
Time point

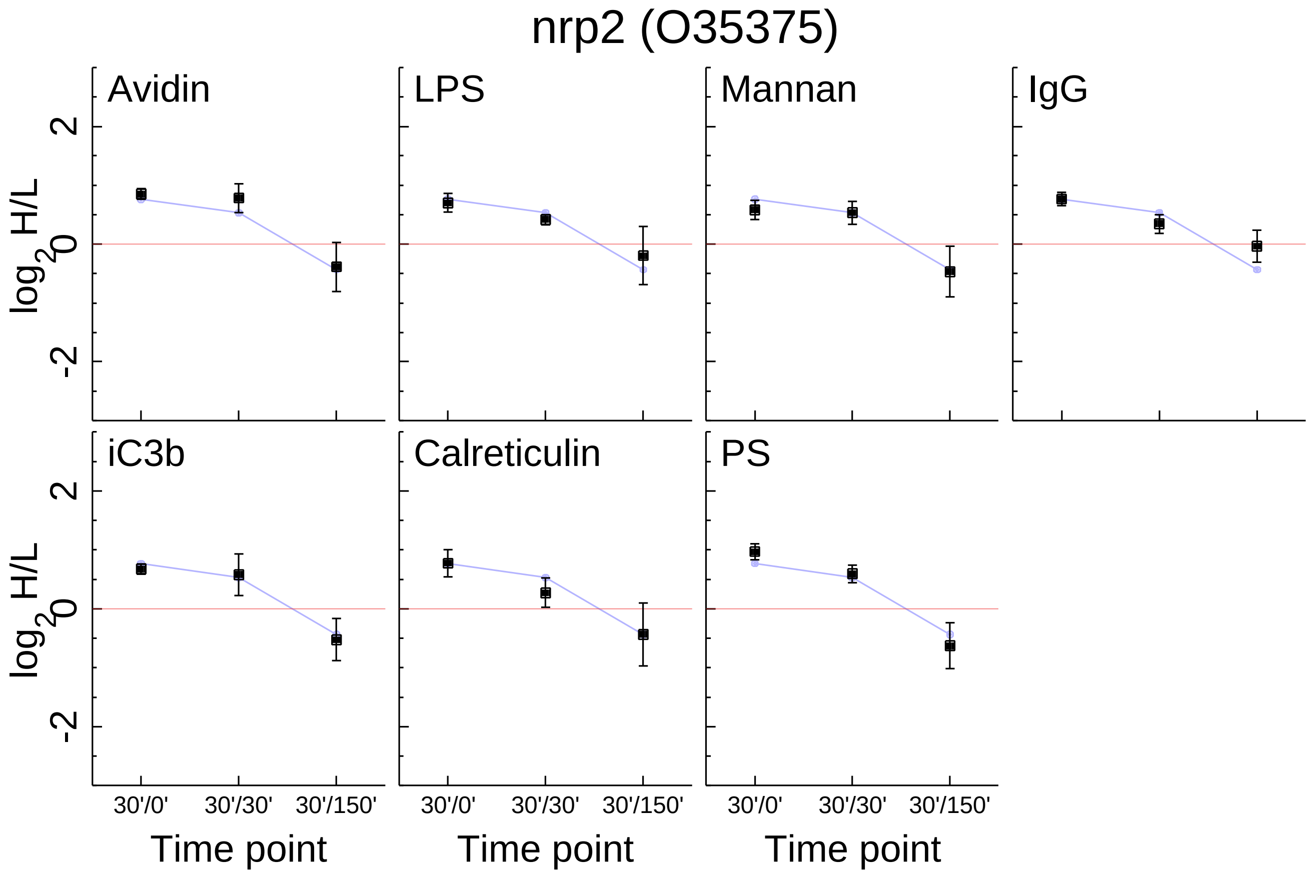

nrp2 (O35375)
Avidin
LPS
Mannan
IgG
iC3b
Calreticulin
PS
log2 H/L
2
0
-2
30'/0'
30'/30'
30'/150'
Time point

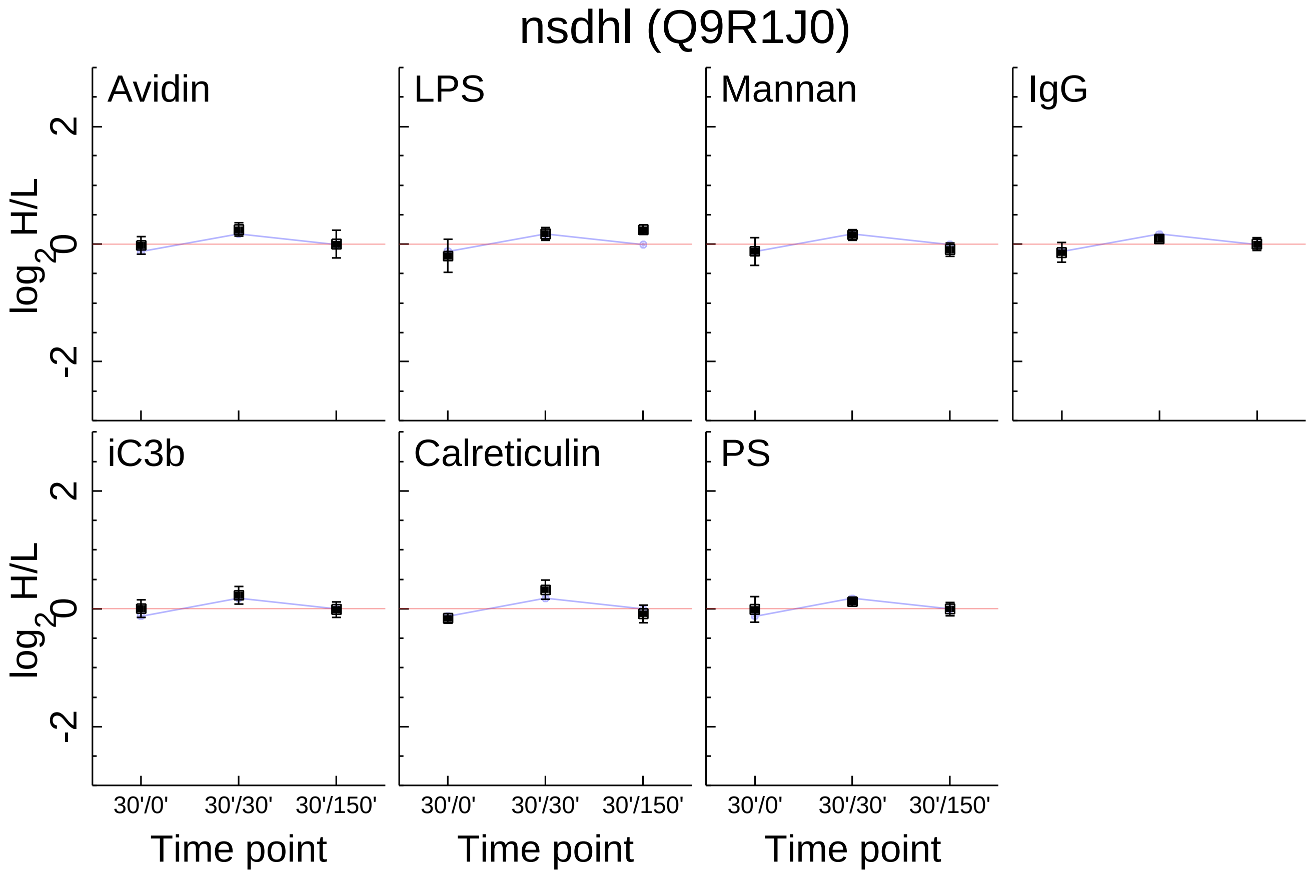
nsdhl (Q9R1J0)
Avidin
LPS
Mannan
IgG
iC3b
Calreticulin
PS
log2 H/L
2
0
-2
30'/0'
30'/30'
30'/150'
Time point

nsf (P46460)

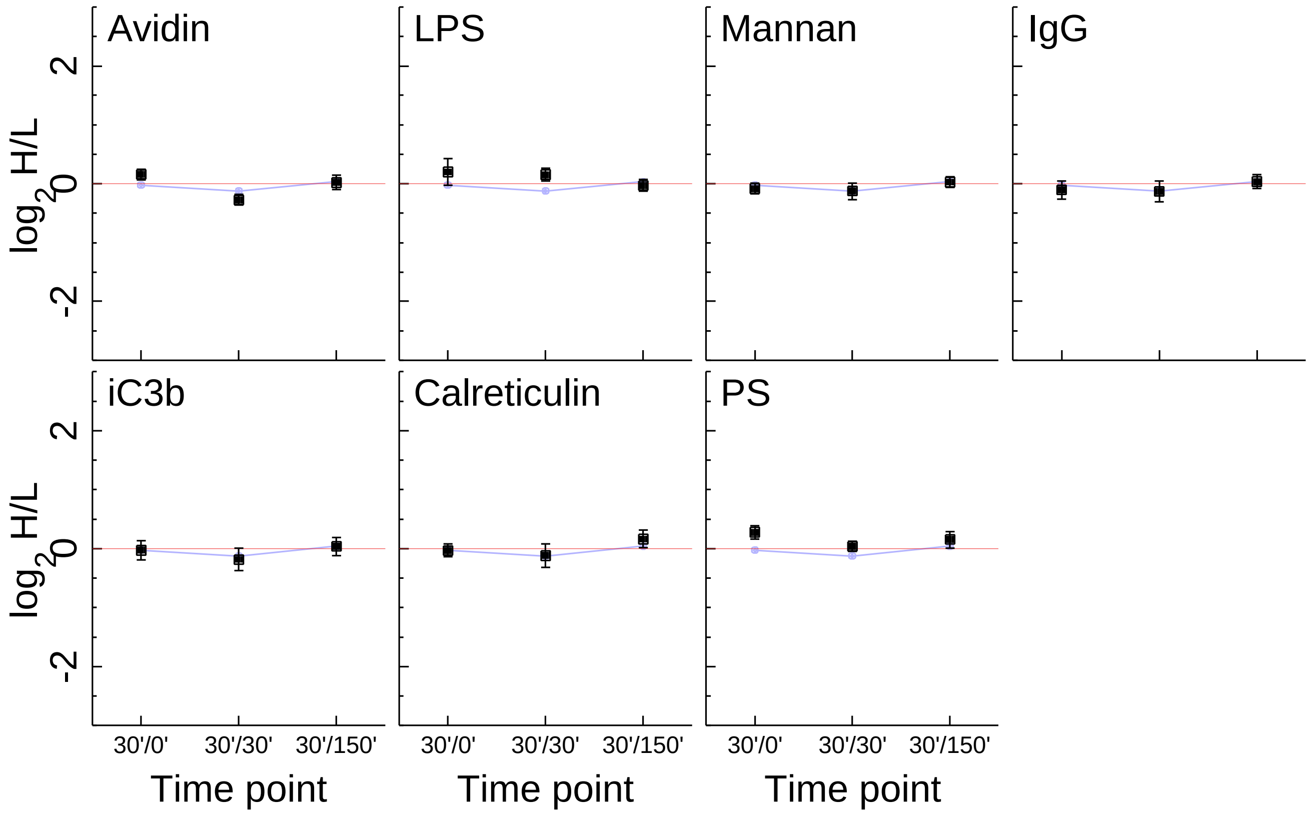

Avidin
LPS
Mannan
IgG
iC3b
Calreticulin
PS
log2 H/L
2
0
-2
30'/0'
30'/30'
30'/150'
Time point

nucb1 (Q02819)

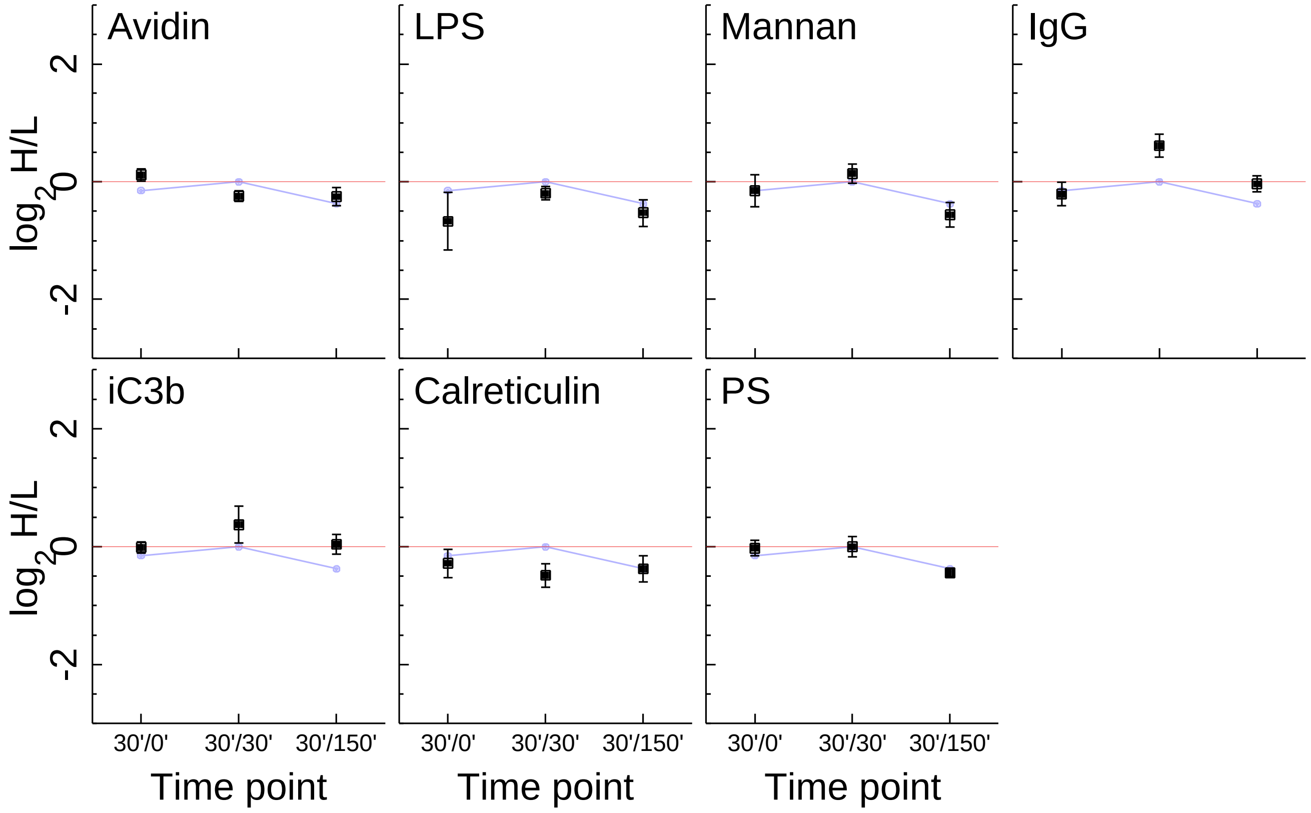

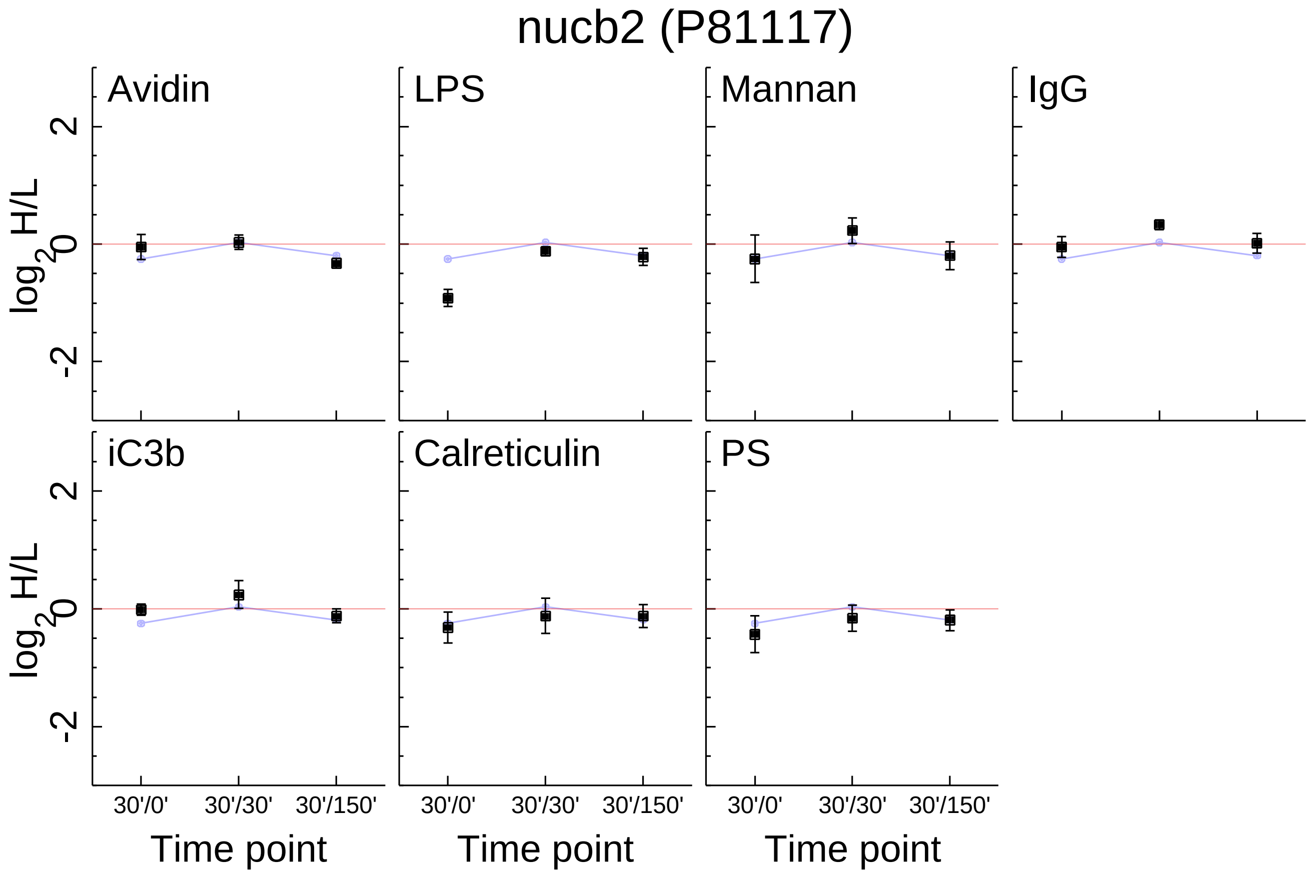

nucb2 (P81117)
Avidin
LPS
Mannan
IgG
iC3b
Calreticulin
PS
log2 H/L
2
0
-2
30'/0'
30'/30'
30'/150'
Time point

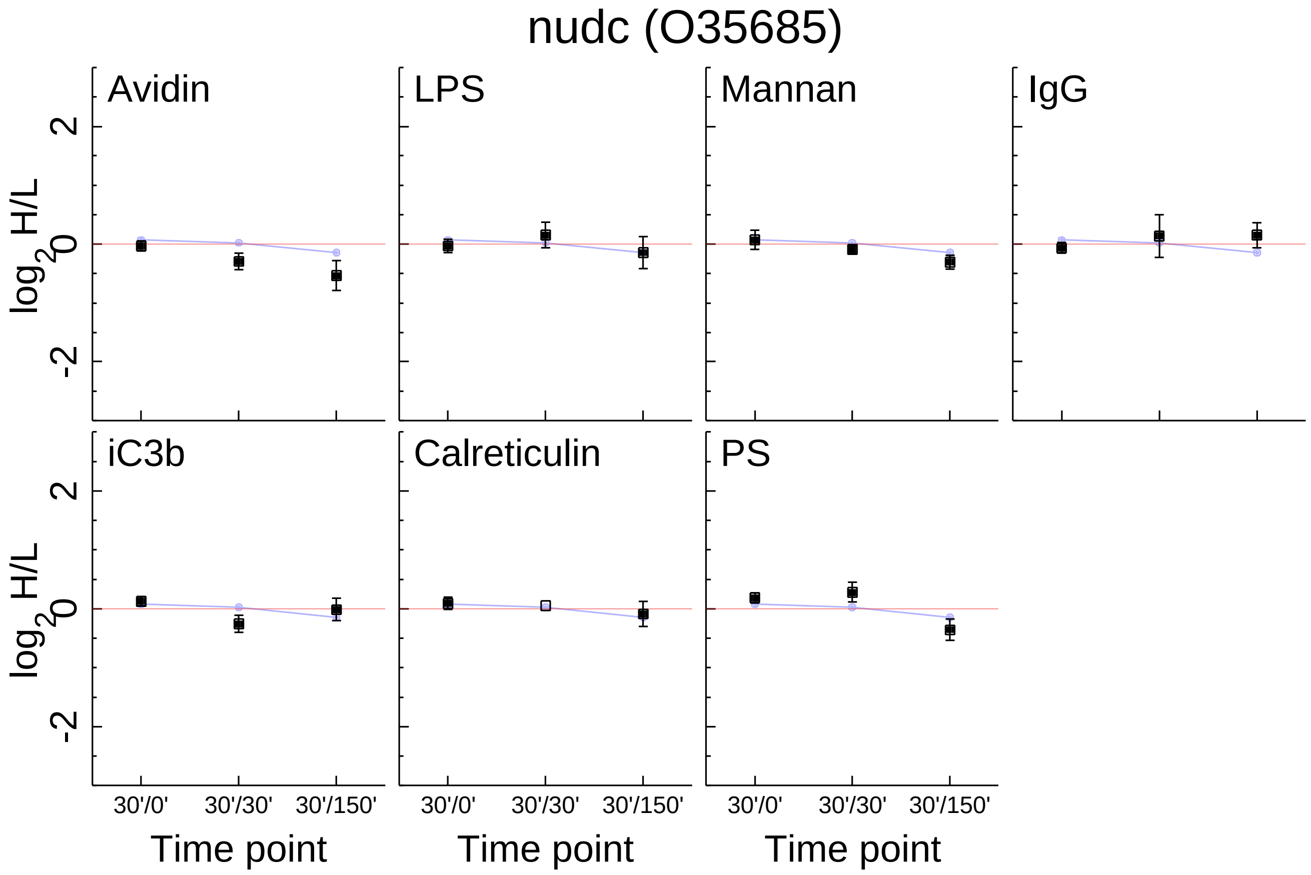
nudc (O35685)
Avidin
LPS
Mannan
IgG
iC3b
Calreticulin
PS
log2 H/L
2
0
-2
30'/0'
30'/30'
30'/150'
Time point

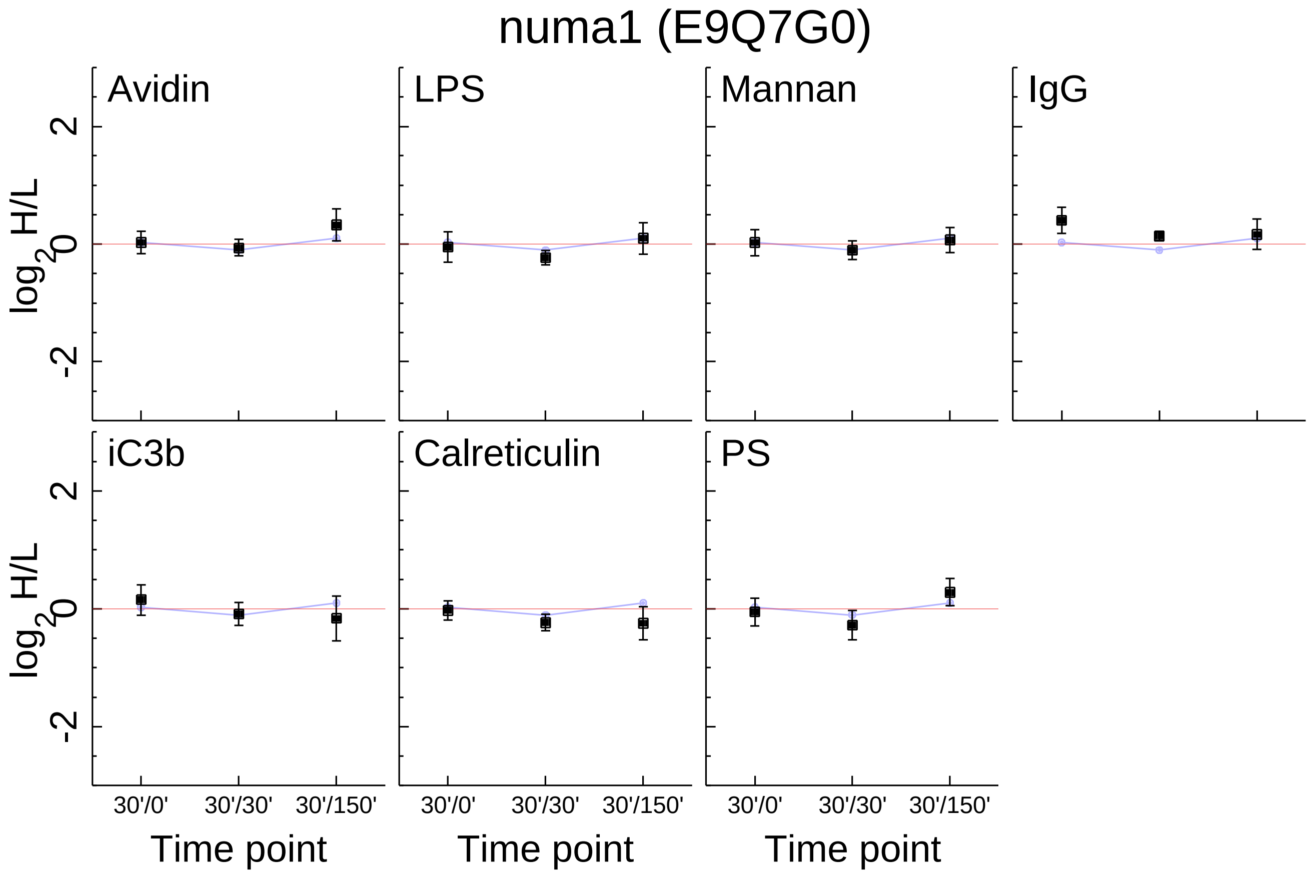

numa1 (E9Q7G0)
Avidin
LPS
Mannan
IgG
iC3b
Calreticulin
PS
log2 H/L
2
0
-2
30'/0'
30'/30'
30'/150'
Time point

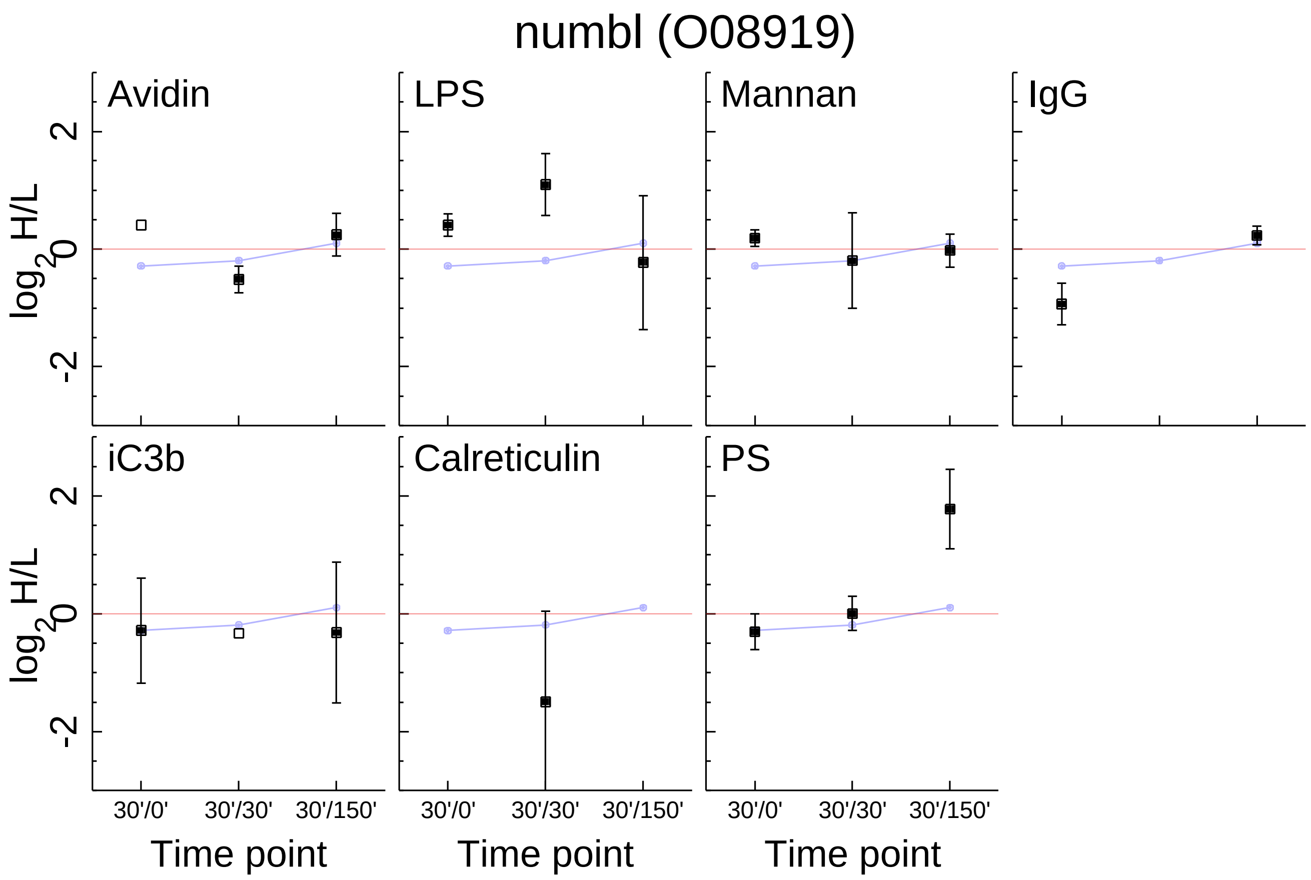
numbl (O08919)
Avidin
LPS
Mannan
IgG
iC3b
Calreticulin
PS
log2 H/L
2
0
-2
30'/0'
30'/30'
30'/150'
Time point

numb (Q9QZS3)

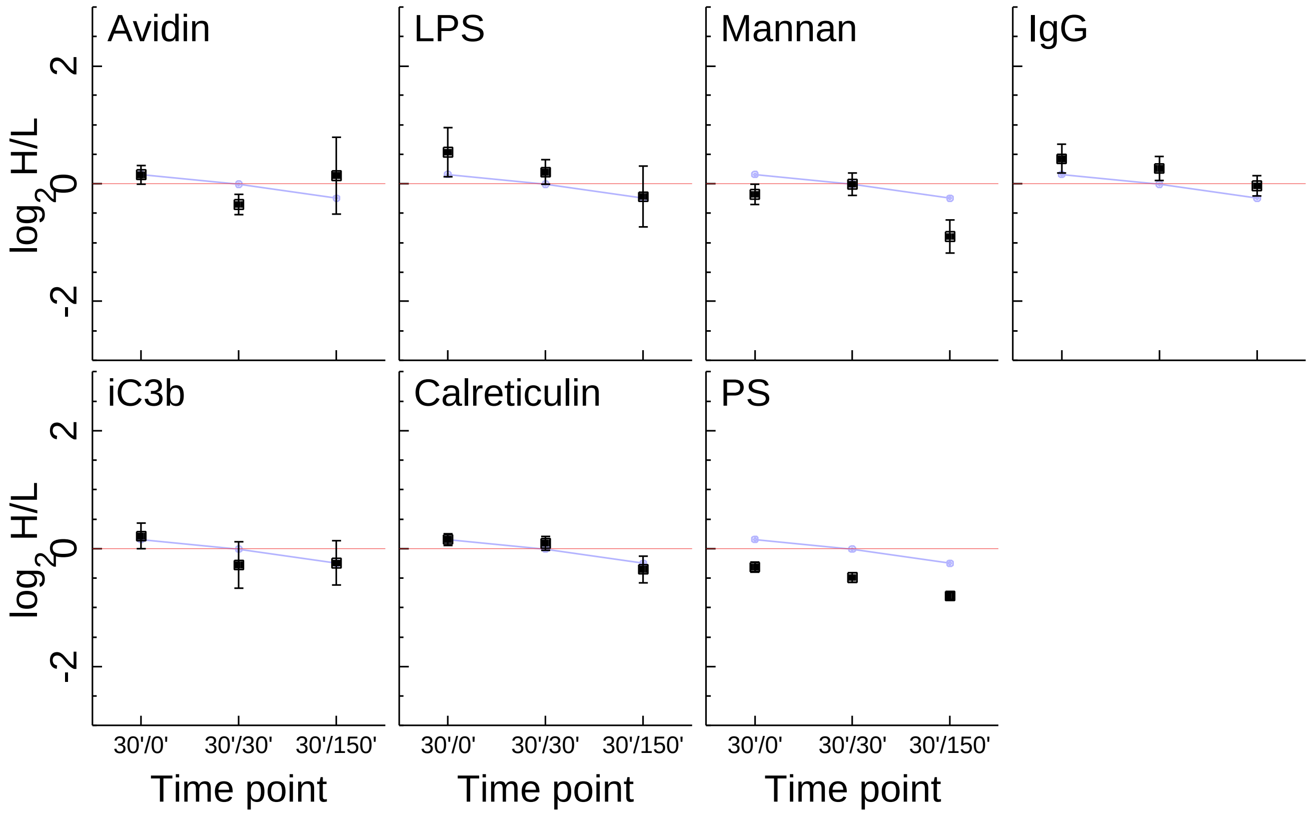

Avidin
LPS
Mannan
IgG
iC3b
Calreticulin
PS
log2 H/L
2
0
-2
30'/0'
30'/30'
30'/150'
Time point

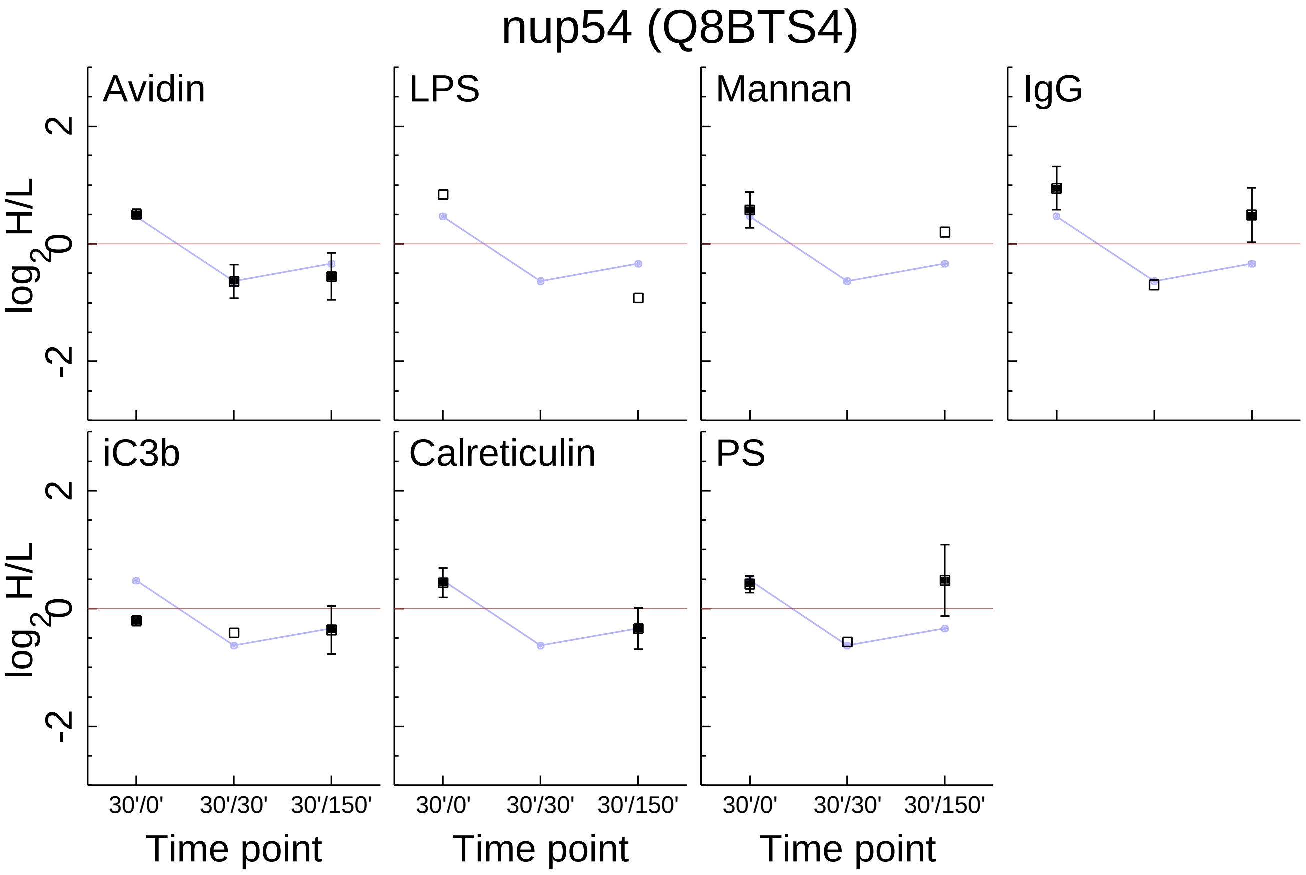
nup54 (Q8BTS4)
Avidin
LPS
Mannan
IgG
iC3b
Calreticulin
PS
log2 H/L
2
0
-2
30'/0'
30'/30'
30'/150'
Time point

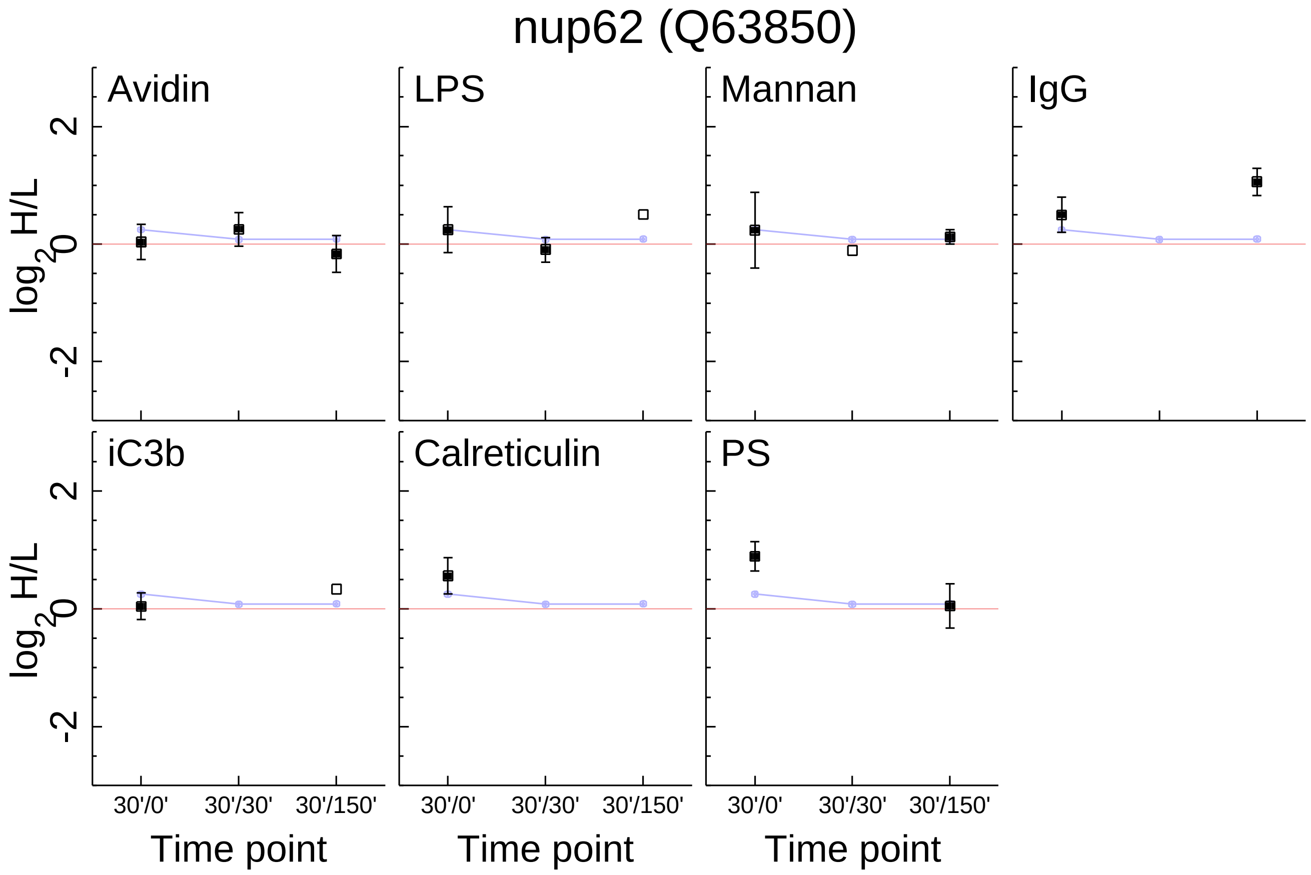
nup62 (Q63850)
Avidin
LPS
Mannan
IgG
iC3b
Calreticulin
PS
log2 H/L
2
0
-2
30'/0'
30'/30'
30'/150'
Time point

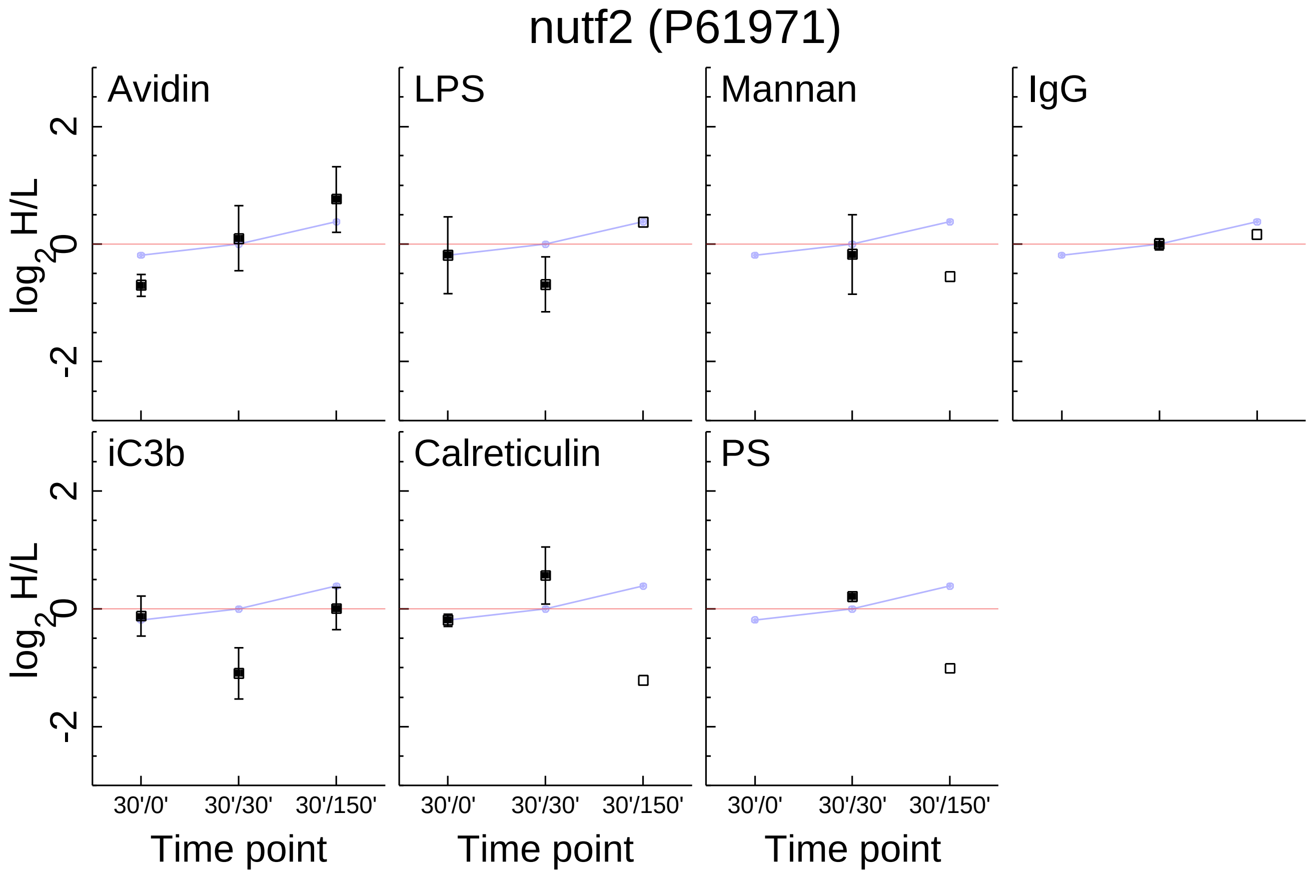

nutf2 (P61971)
Avidin
LPS
Mannan
IgG
iC3b
Calreticulin
PS
log2 H/L
2
0
-2
30'/0'
30'/30'
30'/150'
Time point

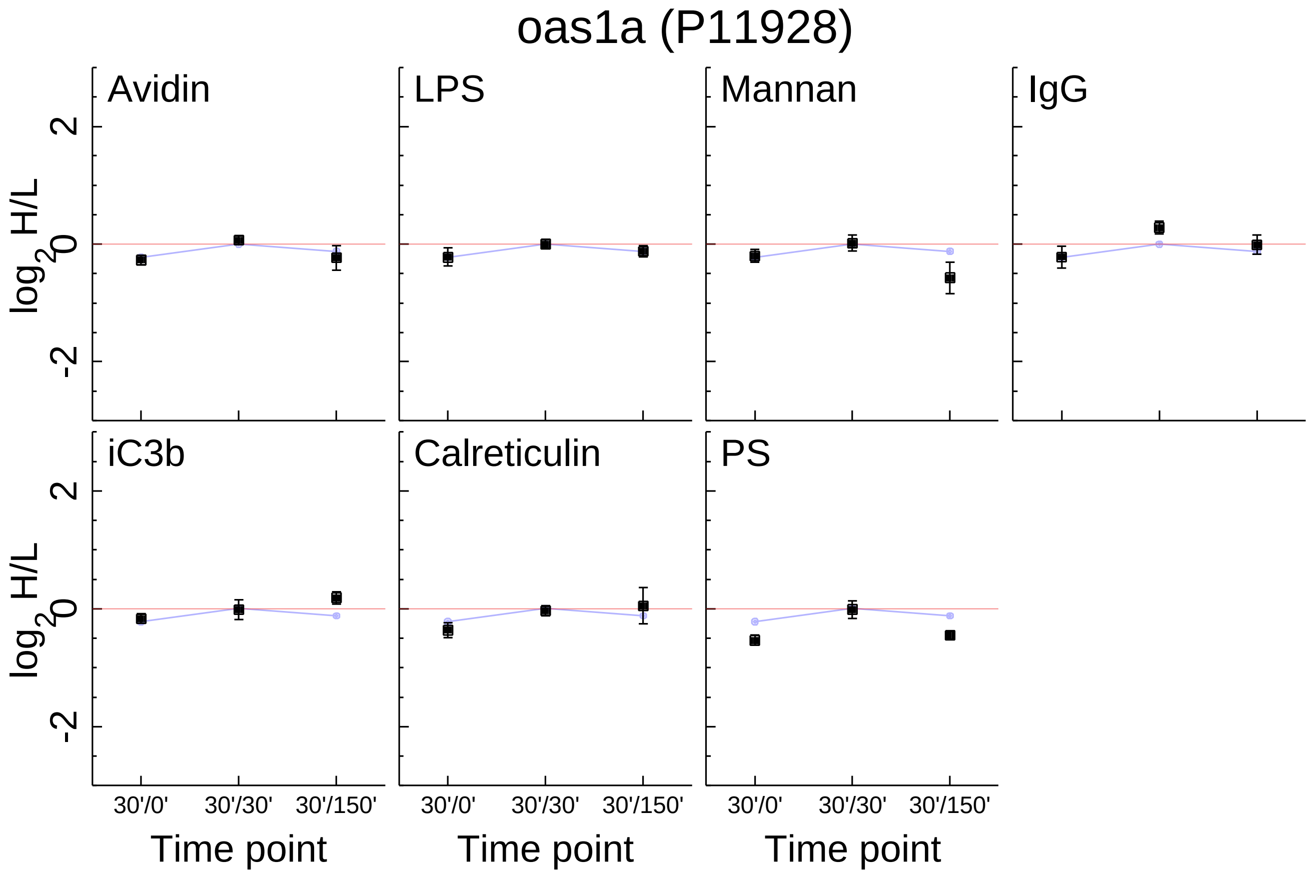

oas1a (P11928)
Avidin
LPS
Mannan
IgG
iC3b
Calreticulin
PS
log2 H/L
2
0
-2
30'/0'
30'/30'
30'/150'
Time point

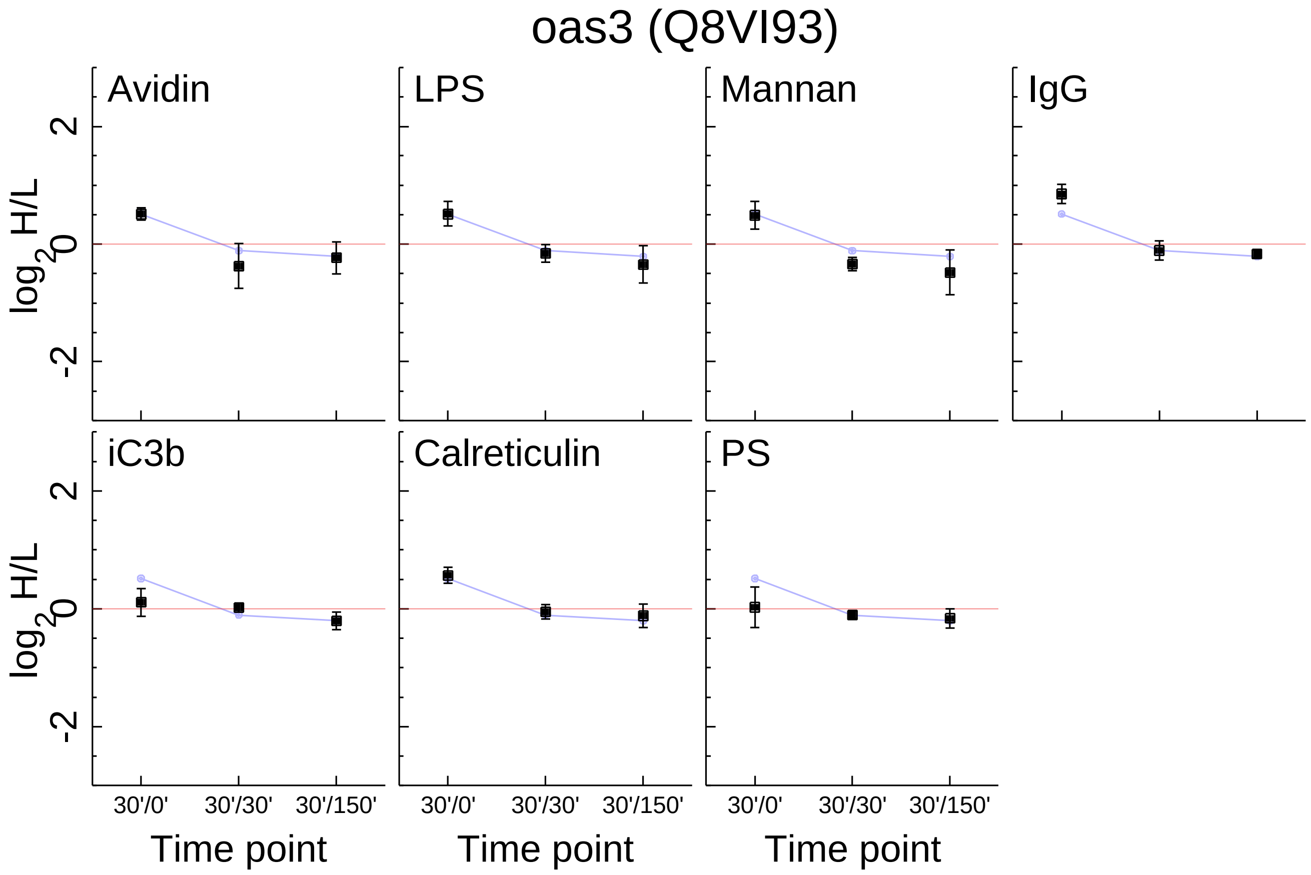
oas3 (Q8VI93)
Avidin
LPS
Mannan
IgG
iC3b
Calreticulin
PS
log2 H/L
2
0
-2
30'/0'
30'/30'
30'/150'
Time point

oat (P29758)

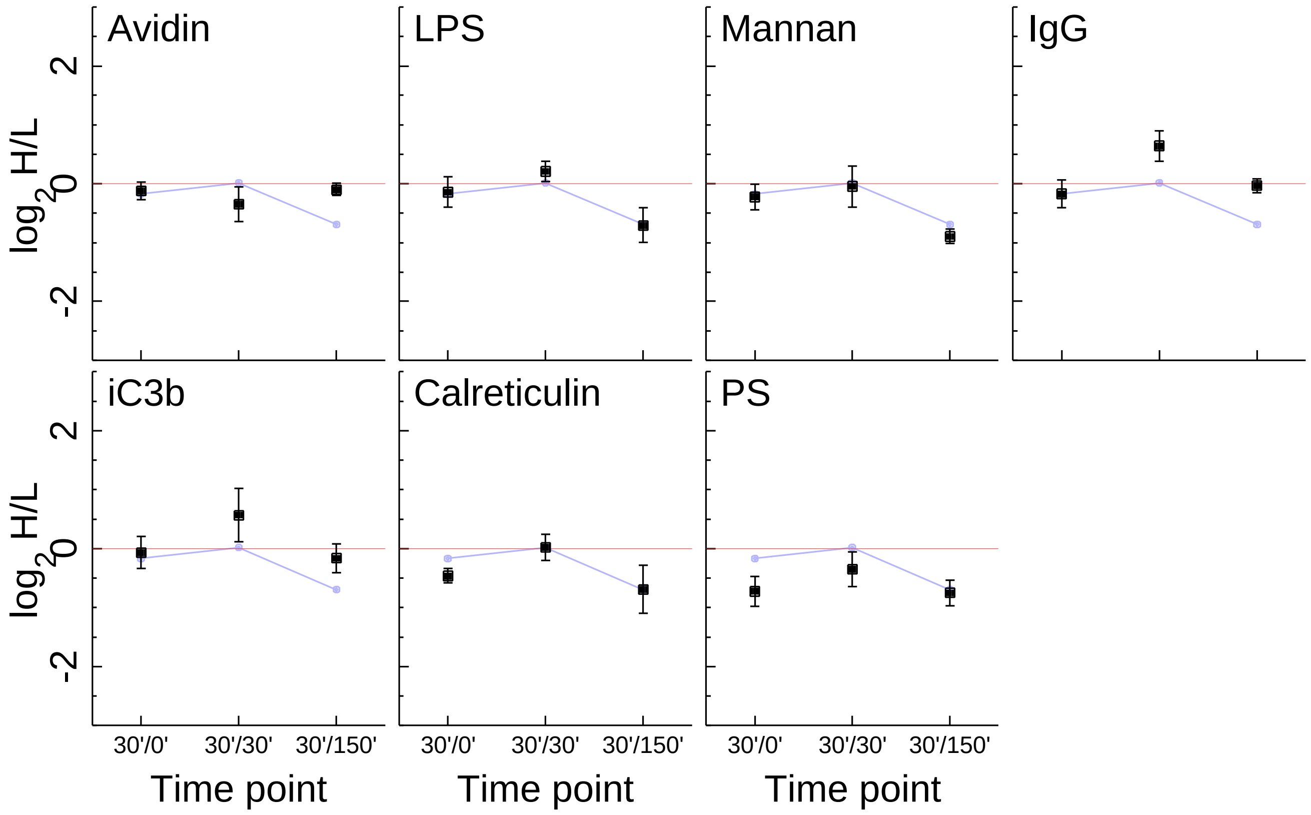
Avidin
LPS
Mannan
IgG
iC3b
Calreticulin
PS
2
0
-2
log2 H/L
30'/0'
30'/30'
30'/150'
Time point

ogdh (Q60597)

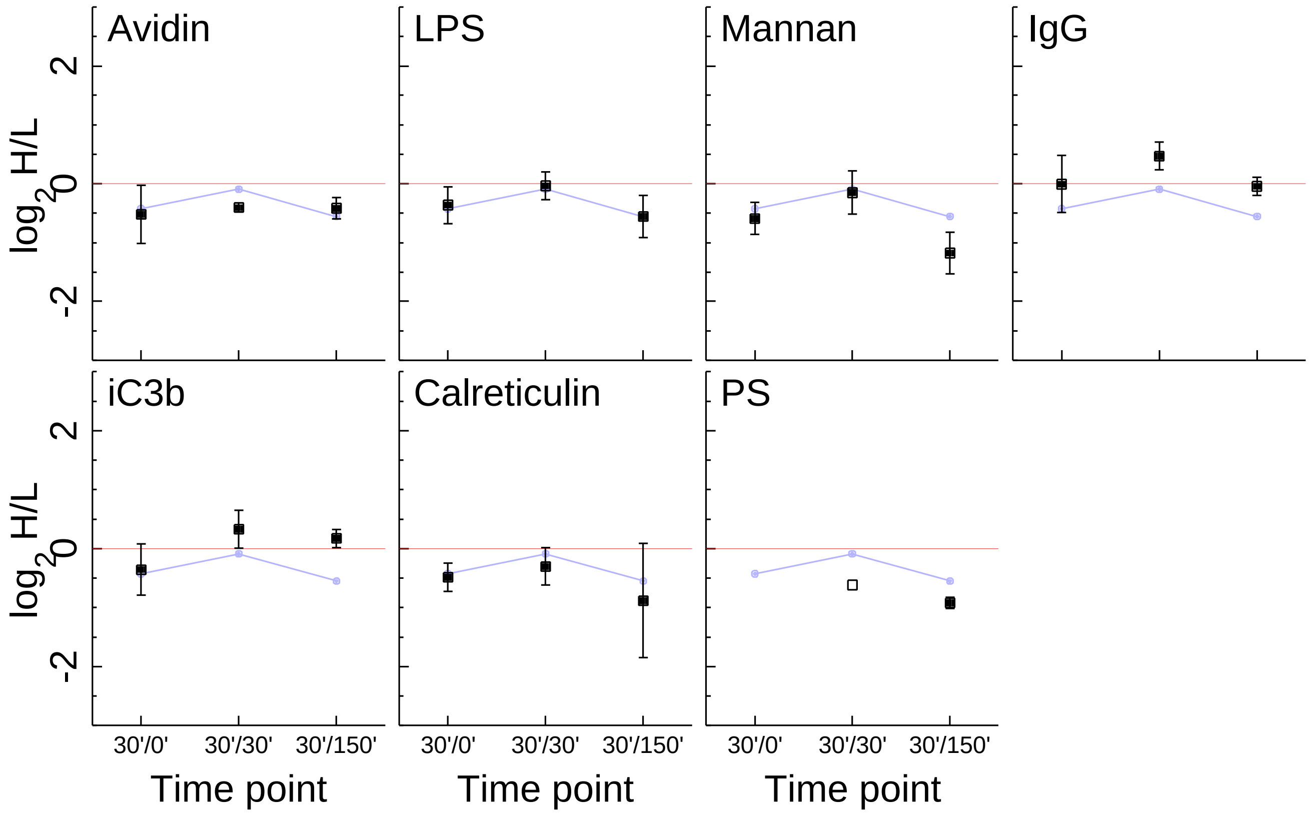

Avidin
LPS
Mannan
IgG
iC3b
Calreticulin
PS
log2 H/L
2
0
-2
30'/0'
30'/30'
30'/150'
Time point

orf3 (Q9EP72)

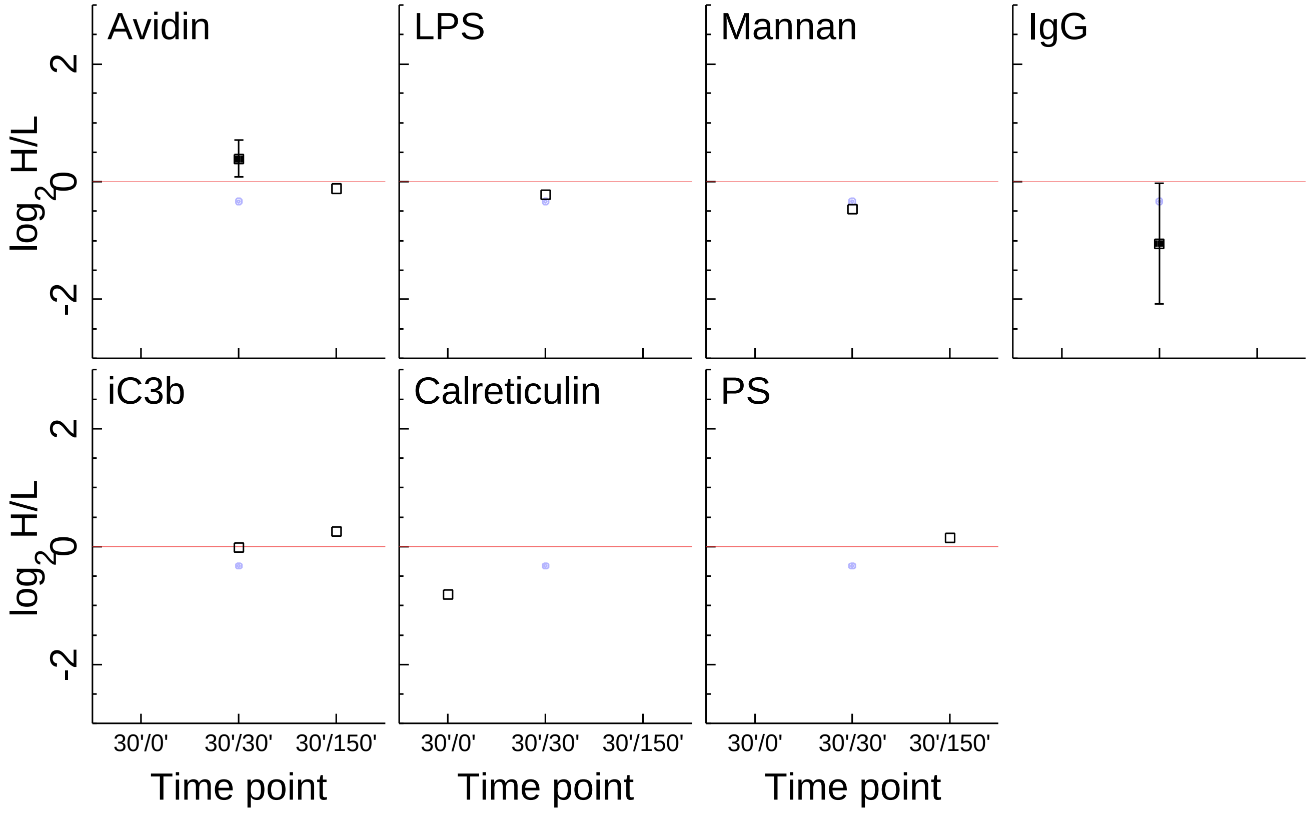
Avidin
LPS
Mannan
IgG
iC3b
Calreticulin
PS
log2 H/L
2
0
-2
30'/0'
30'/30'
30'/150'
Time point

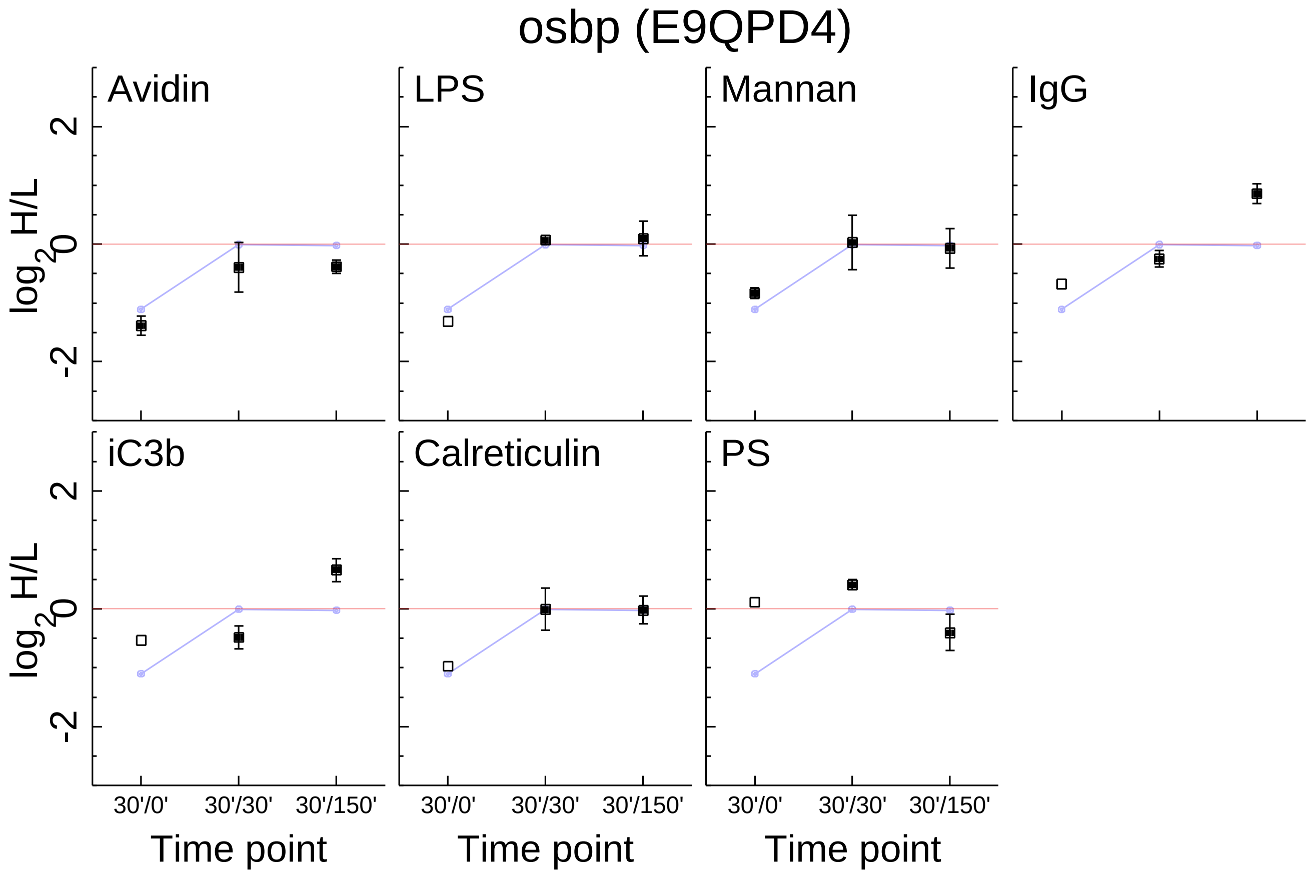
osbp (E9QPD4)
Avidin
LPS
Mannan
IgG
iC3b
Calreticulin
PS
log2 H/L
2
0
-2
30'/0'
30'/30'
30'/150'
Time point

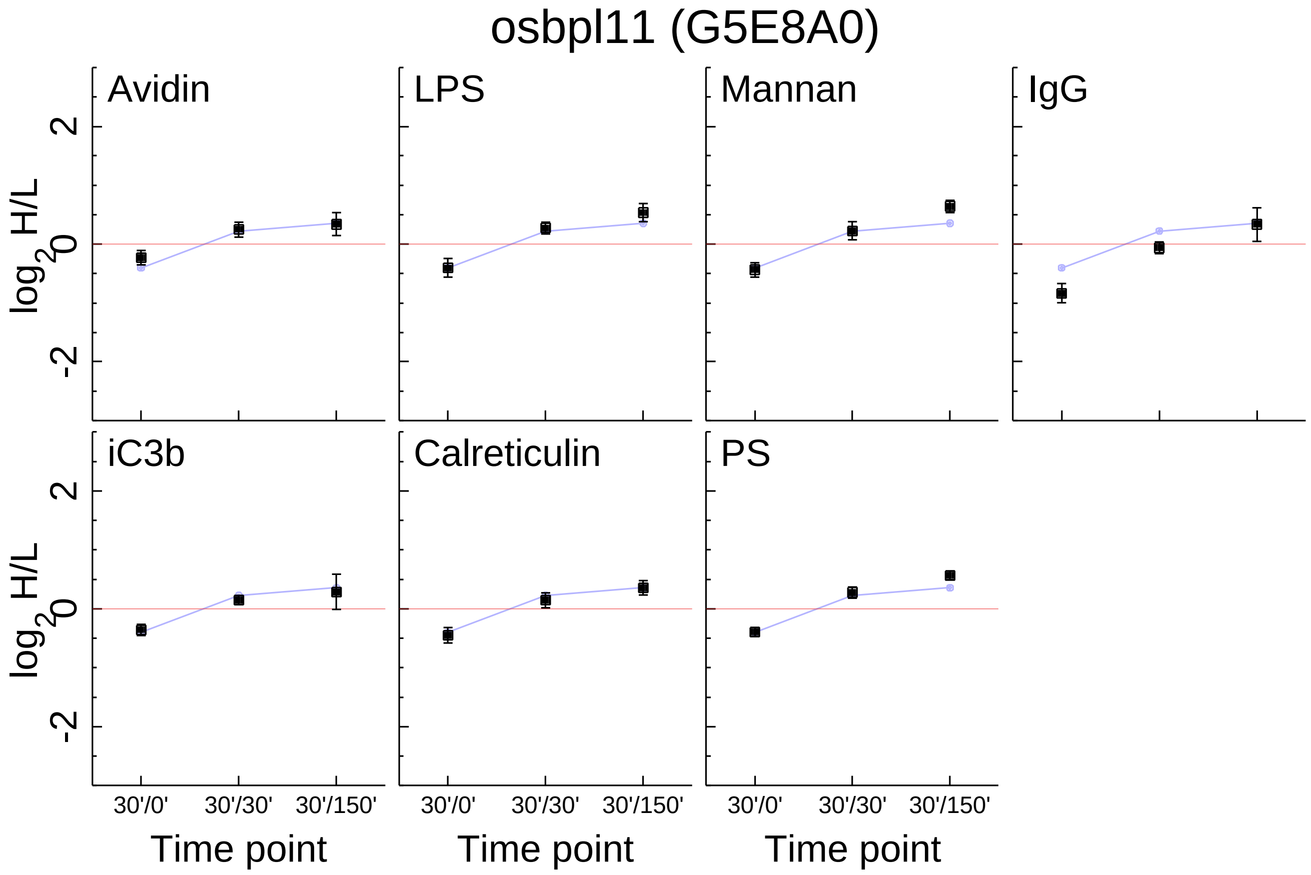
osbpl11 (G5E8A0)
Avidin
LPS
Mannan
IgG
iC3b
Calreticulin
PS
log2 H/L
2
0
-2
30'/0'
30'/30'
30'/150'
Time point

osbpl1a (Q91XL9)

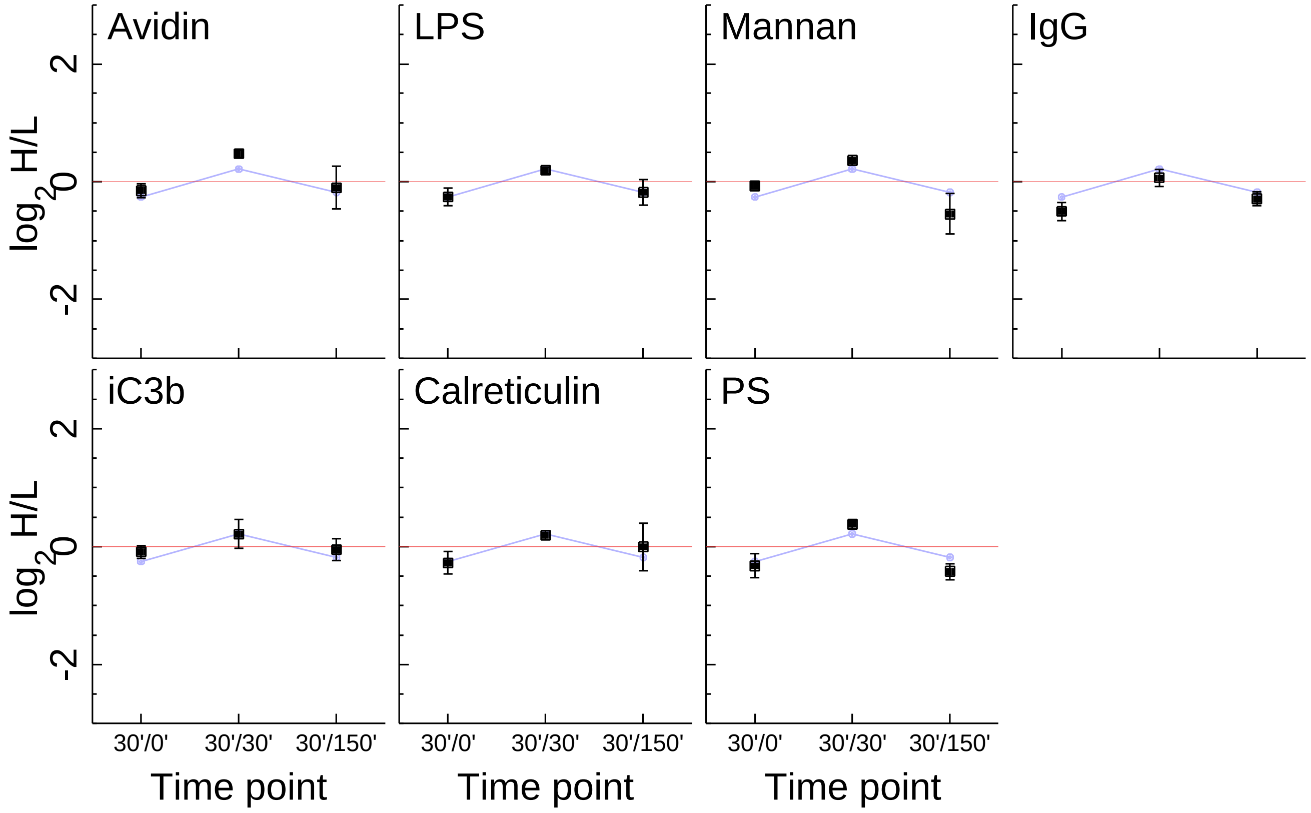

osbpl6 (Q8BXR9)

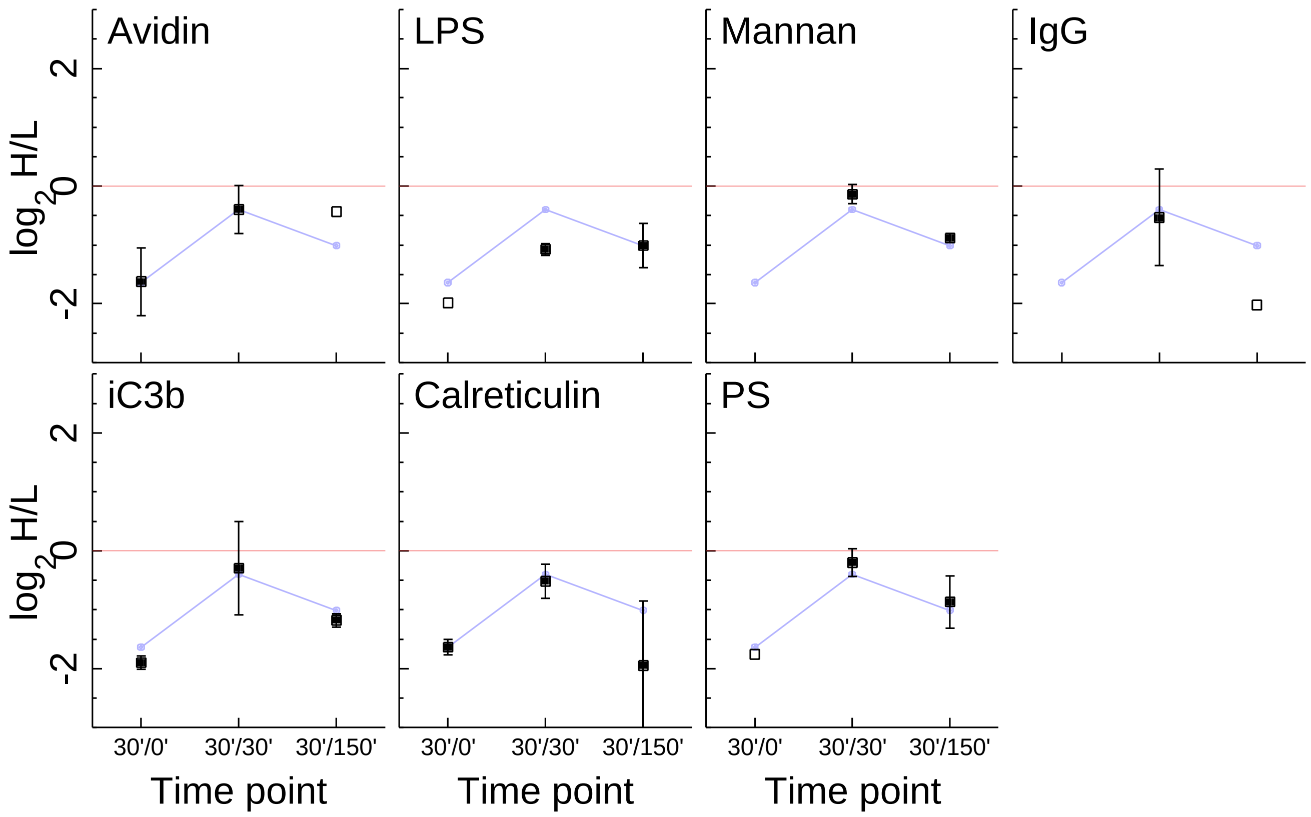

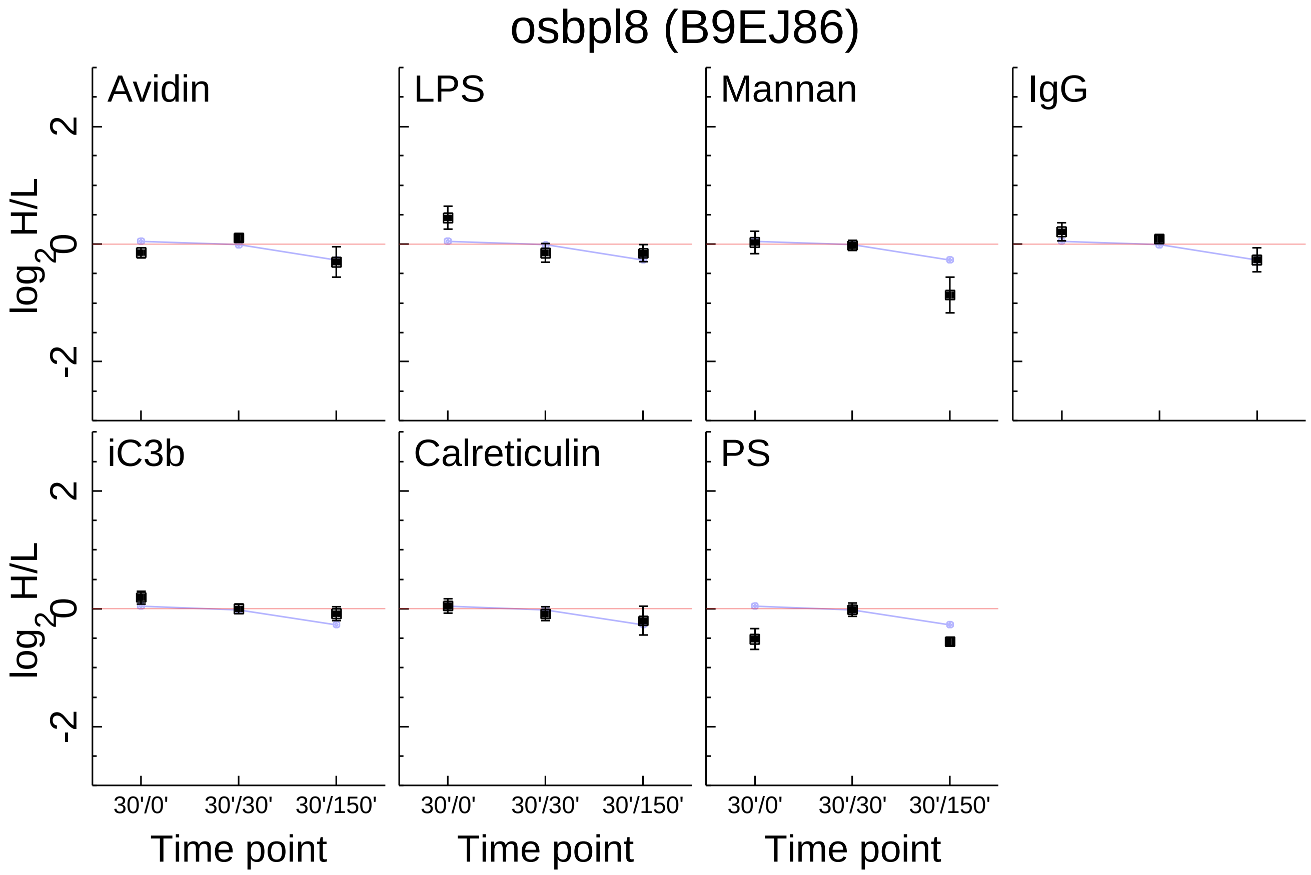

osbpl8 (B9EJ86)
Avidin
LPS
Mannan
IgG
iC3b
Calreticulin
PS
log2 H/L
2
0
-2
30'/0'
30'/30'
30'/150'
Time point

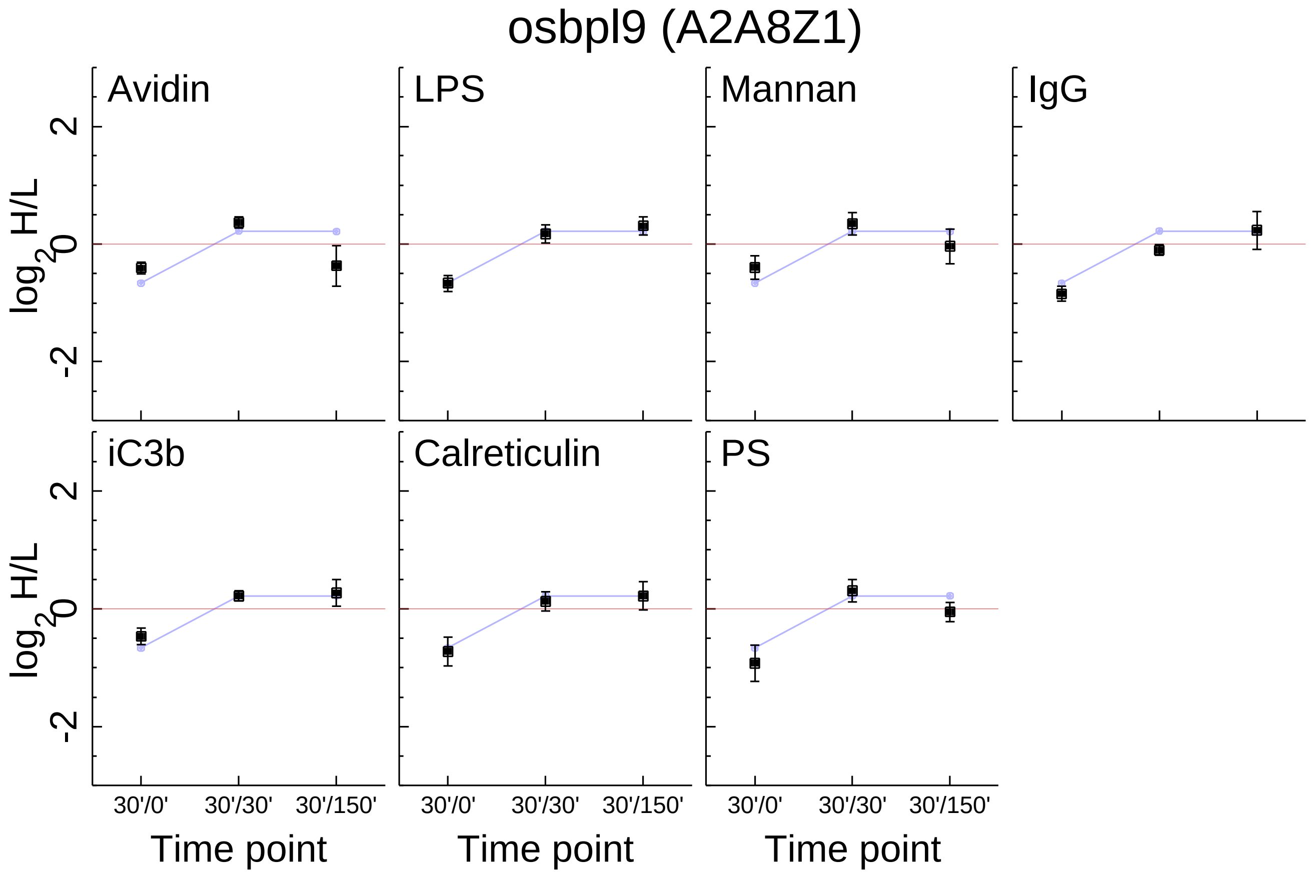
osbpl9 (A2A8Z1)
Avidin
LPS
Mannan
IgG
iC3b
Calreticulin
PS
log2 H/L
2
0
-2
30'/0'
30'/30'
30'/150'
Time point

ostf1 (Q62422)

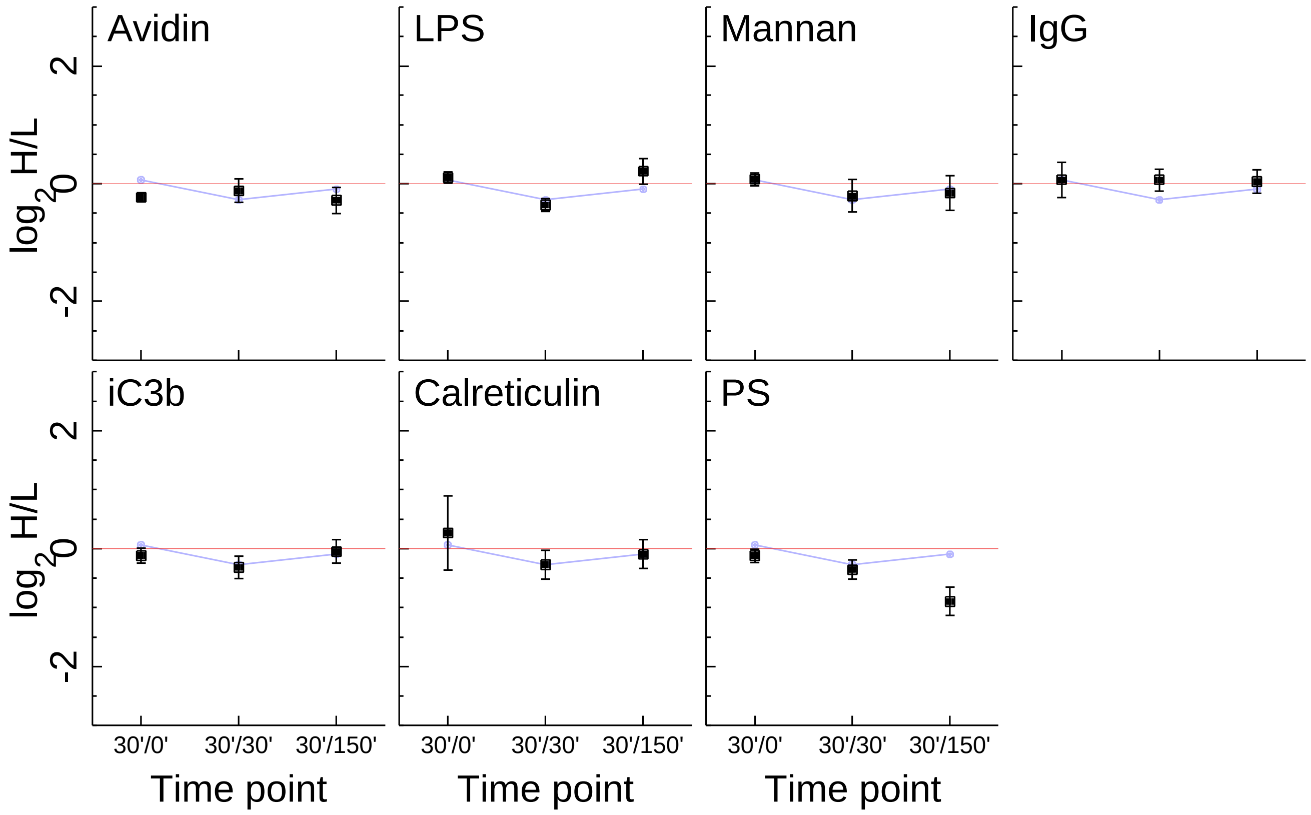

ostm1 (Q8BGT0)

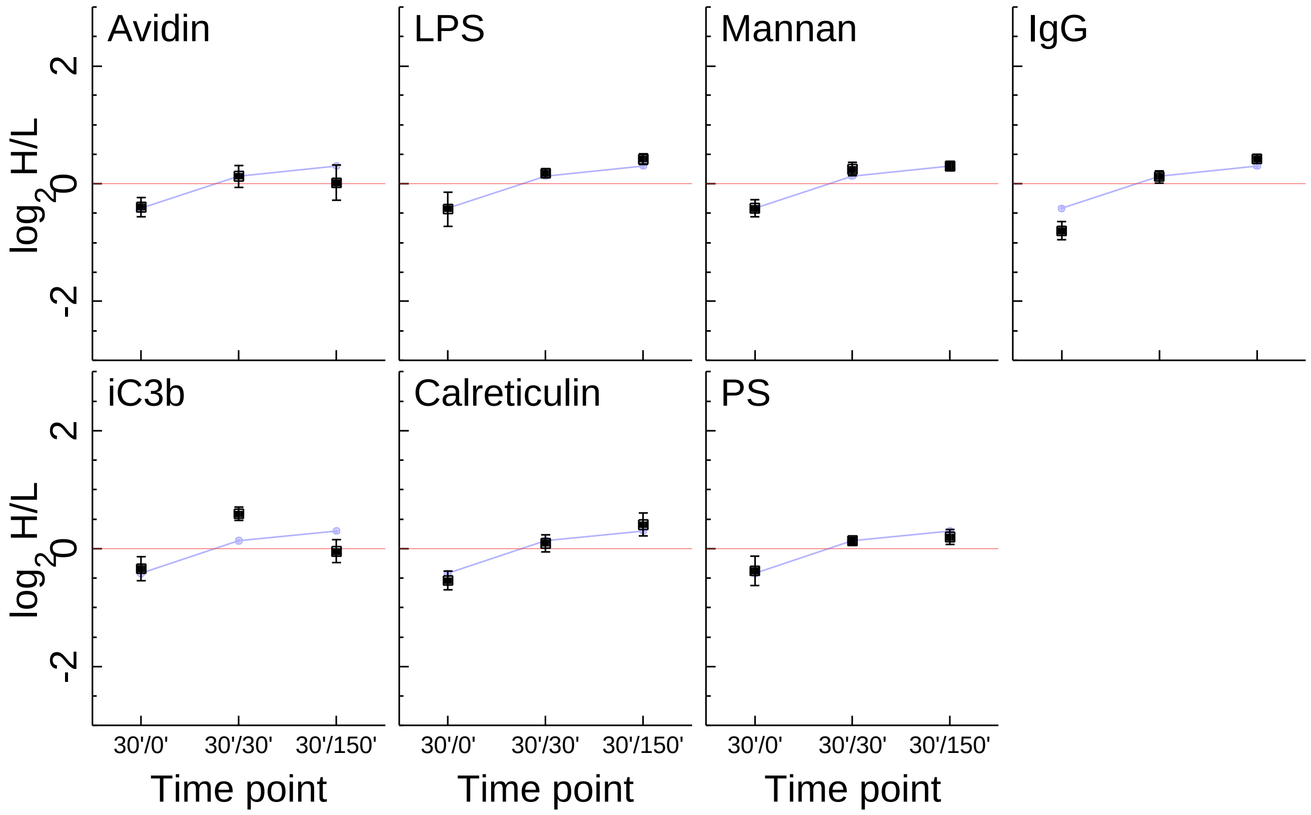

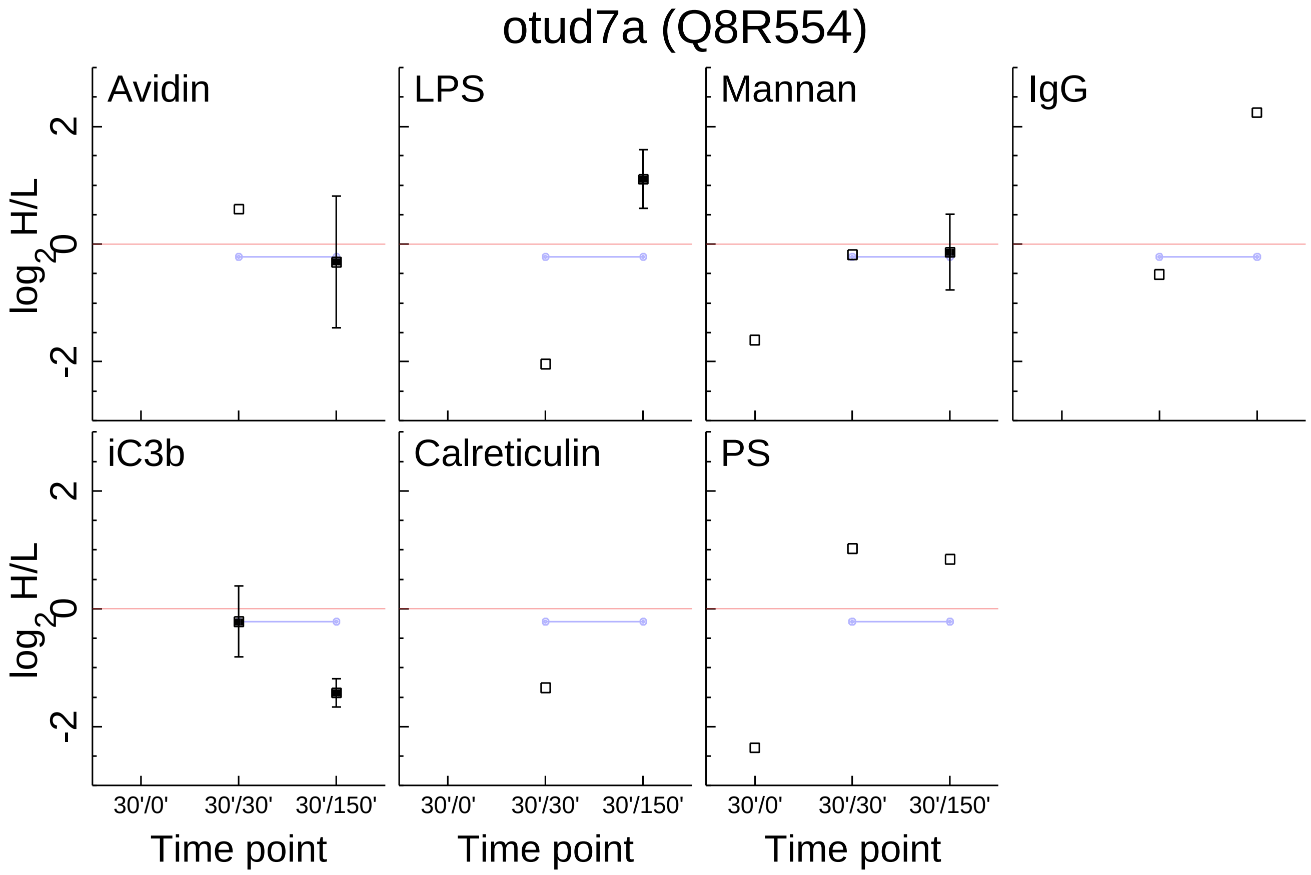
otud7a (Q8R554)
Avidin
LPS
Mannan
IgG
iC3b
Calreticulin
PS
log2 H/L
2
0
-2
30'/0'
30'/30'
30'/150'
Time point

oxct1 (Q9D0K2)

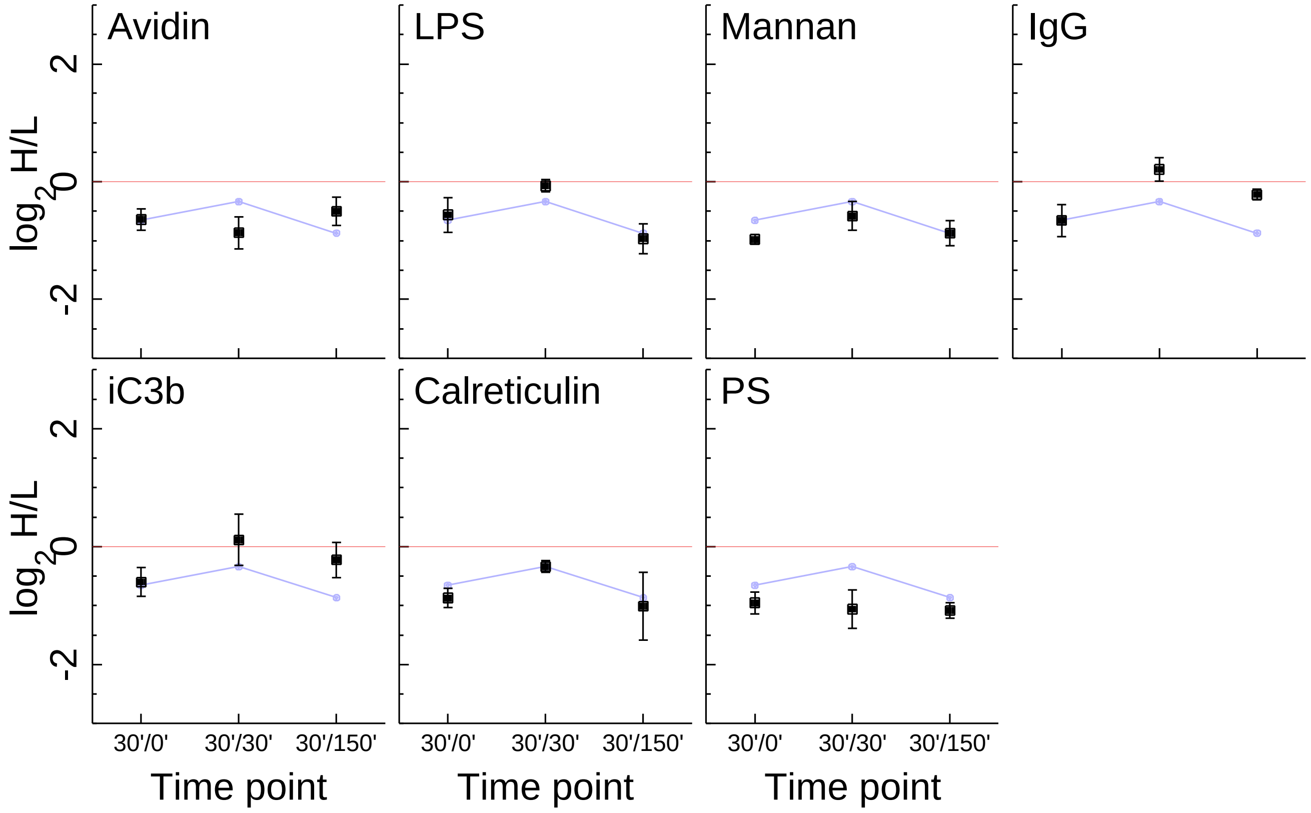

p10404 (P10404)

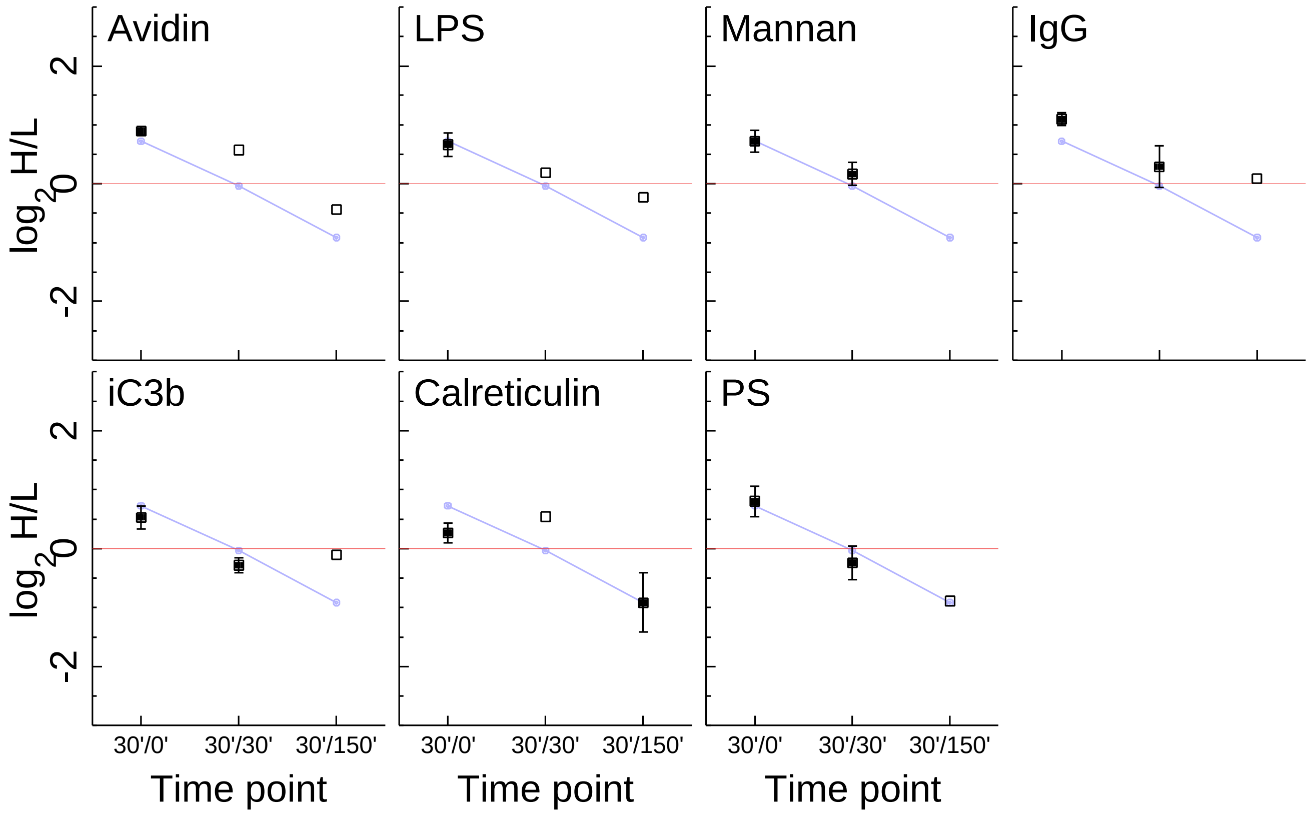

p17047-3 (P17047)

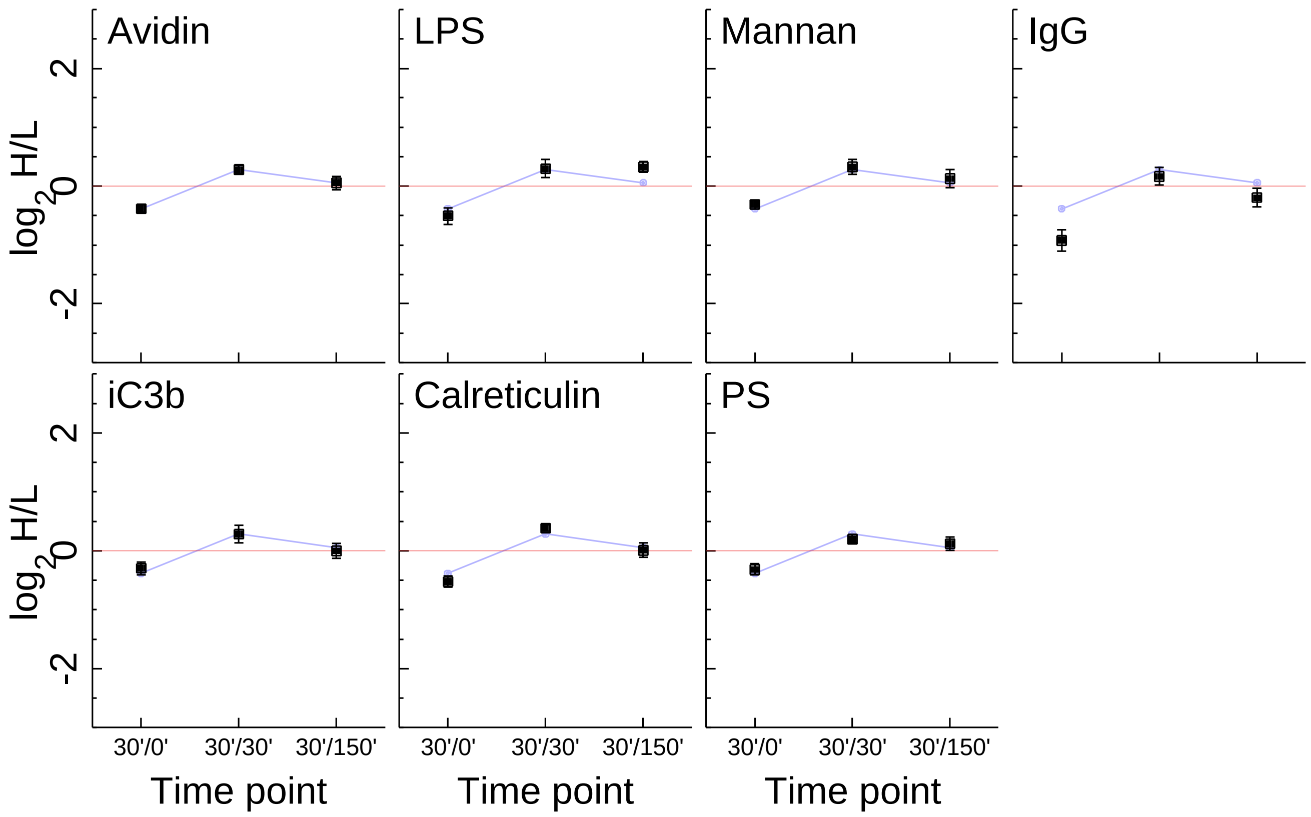

p2rx4 (Q9Z257)

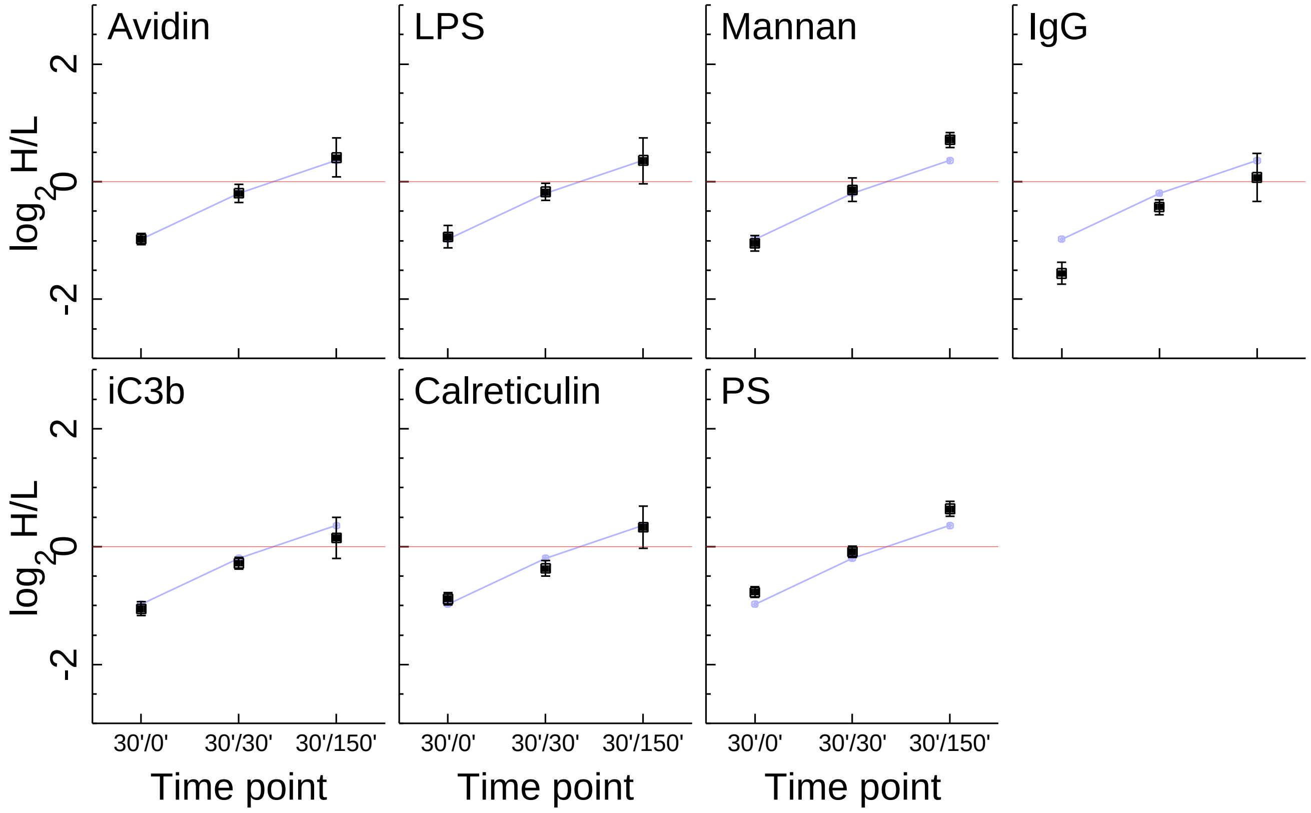

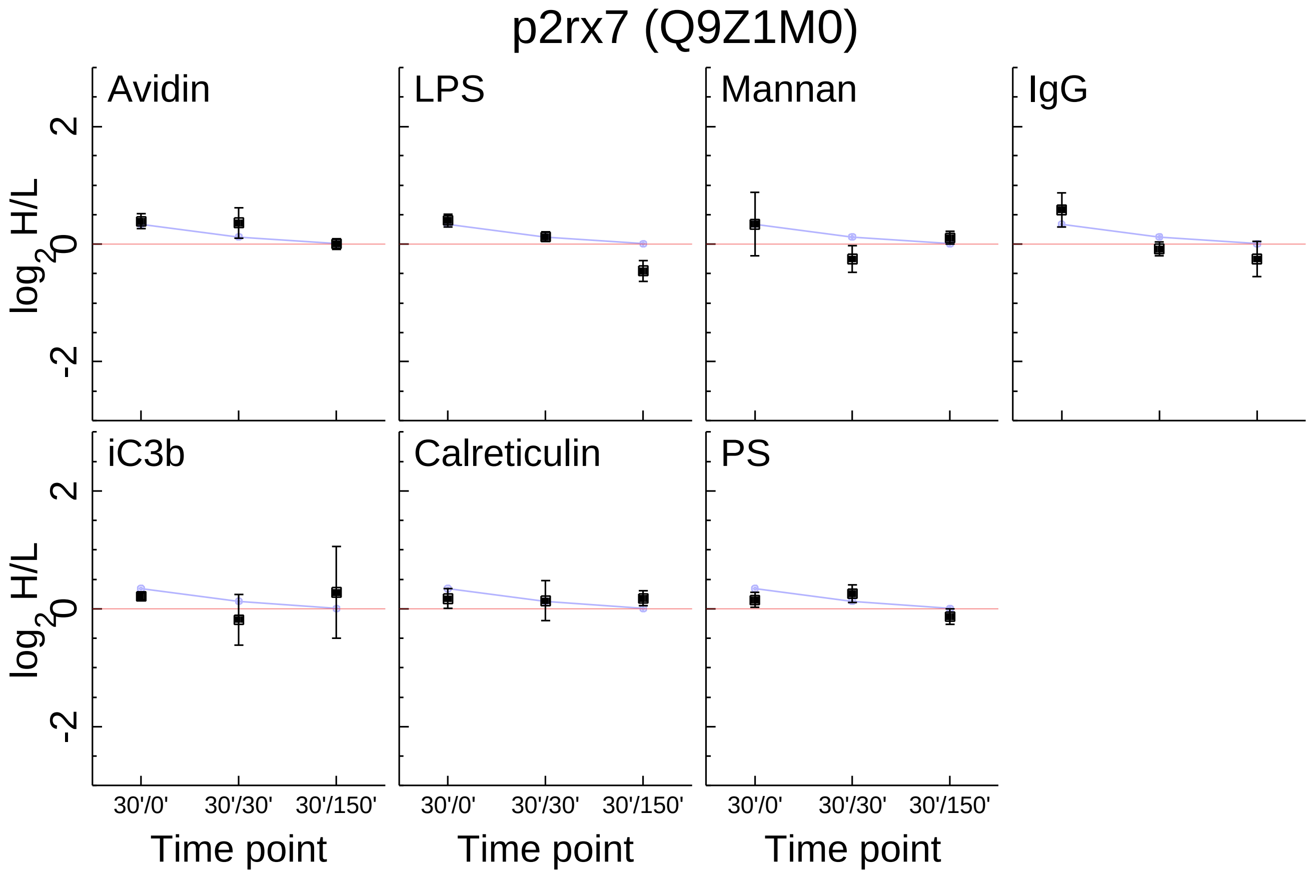

p2rx7 (Q9Z1M0)
Avidin
LPS
Mannan
IgG
iC3b
Calreticulin
PS
log2 H/L
2
0
-2
30'/0'
30'/30'
30'/150'
Time point

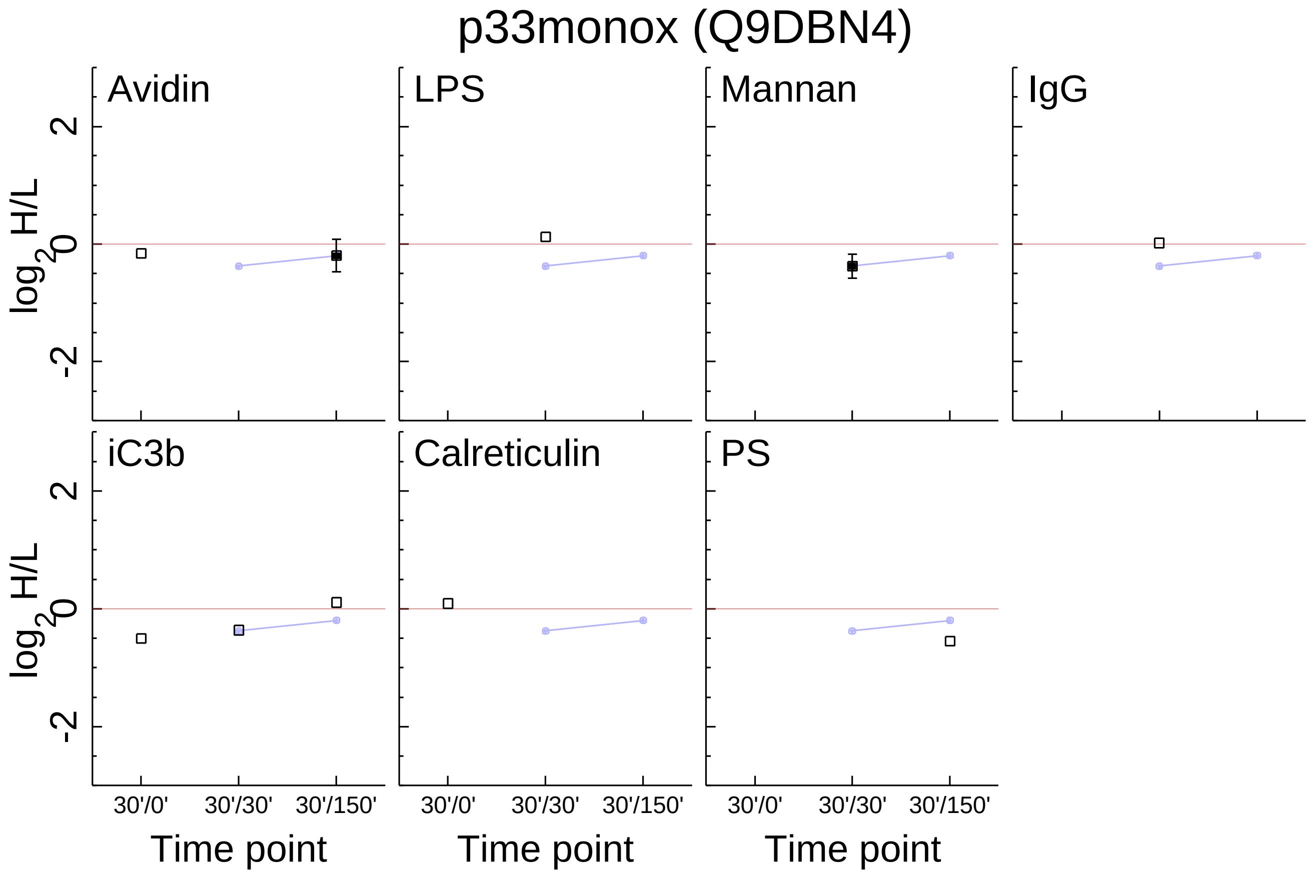

p33monox (Q9DBN4)
Avidin
LPS
Mannan
IgG
iC3b
Calreticulin
PS
2
0
-2
log2 H/L
30'/0'
30'/30'
30'/150'
Time point

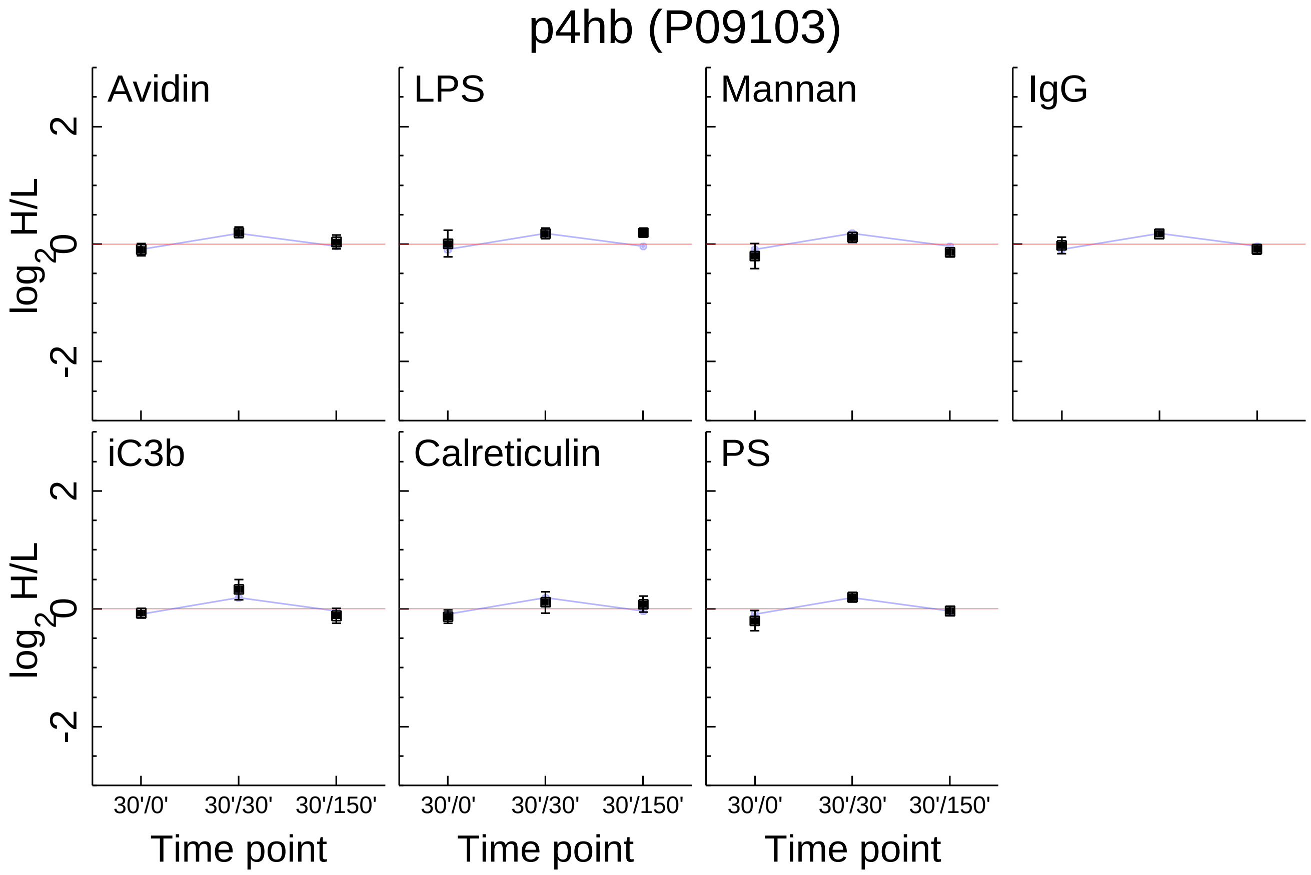
p4hb (P09103)
Avidin
LPS
Mannan
IgG
iC3b
Calreticulin
PS
log2 H/L
2
0
-2
30'/0'
30'/30'
30'/150'
Time point

pa2g4 (P50580)

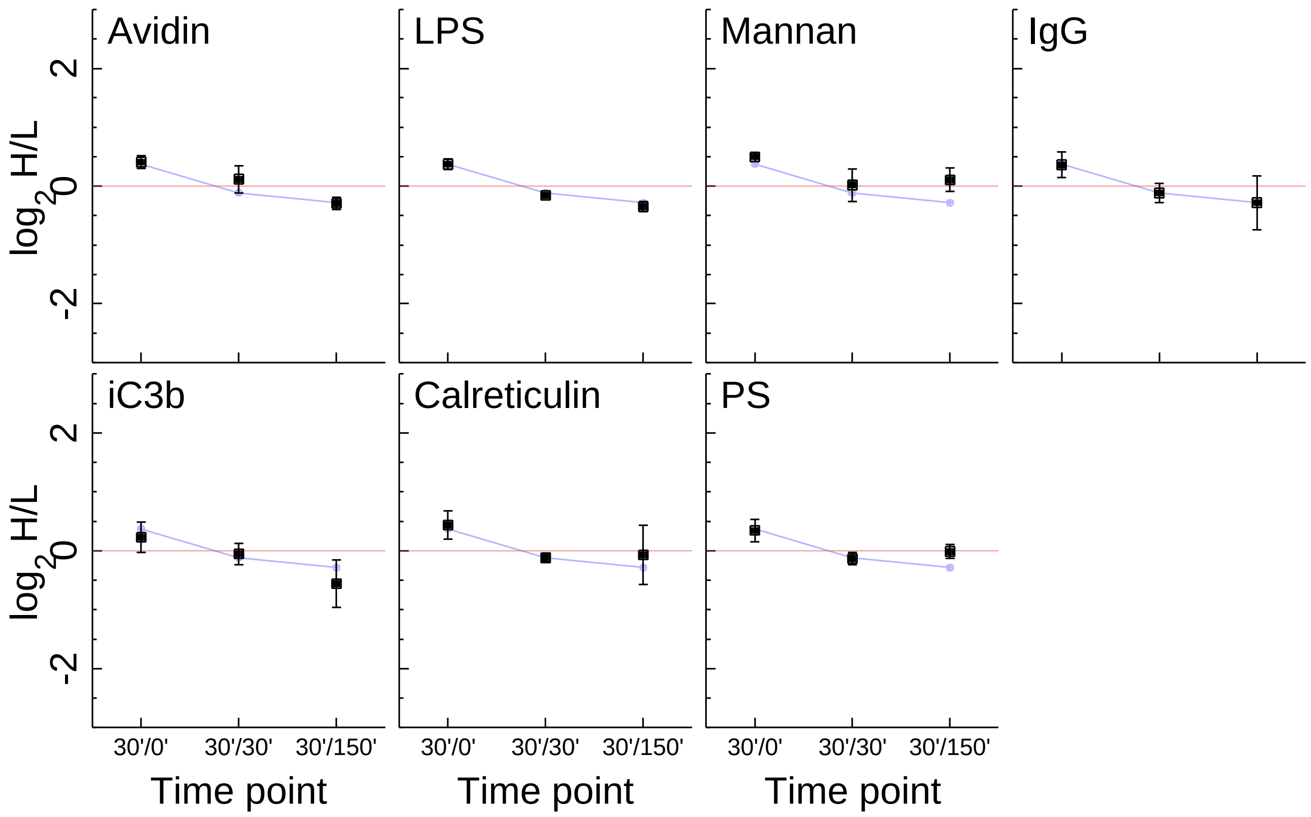

pabpc1 (P29341)

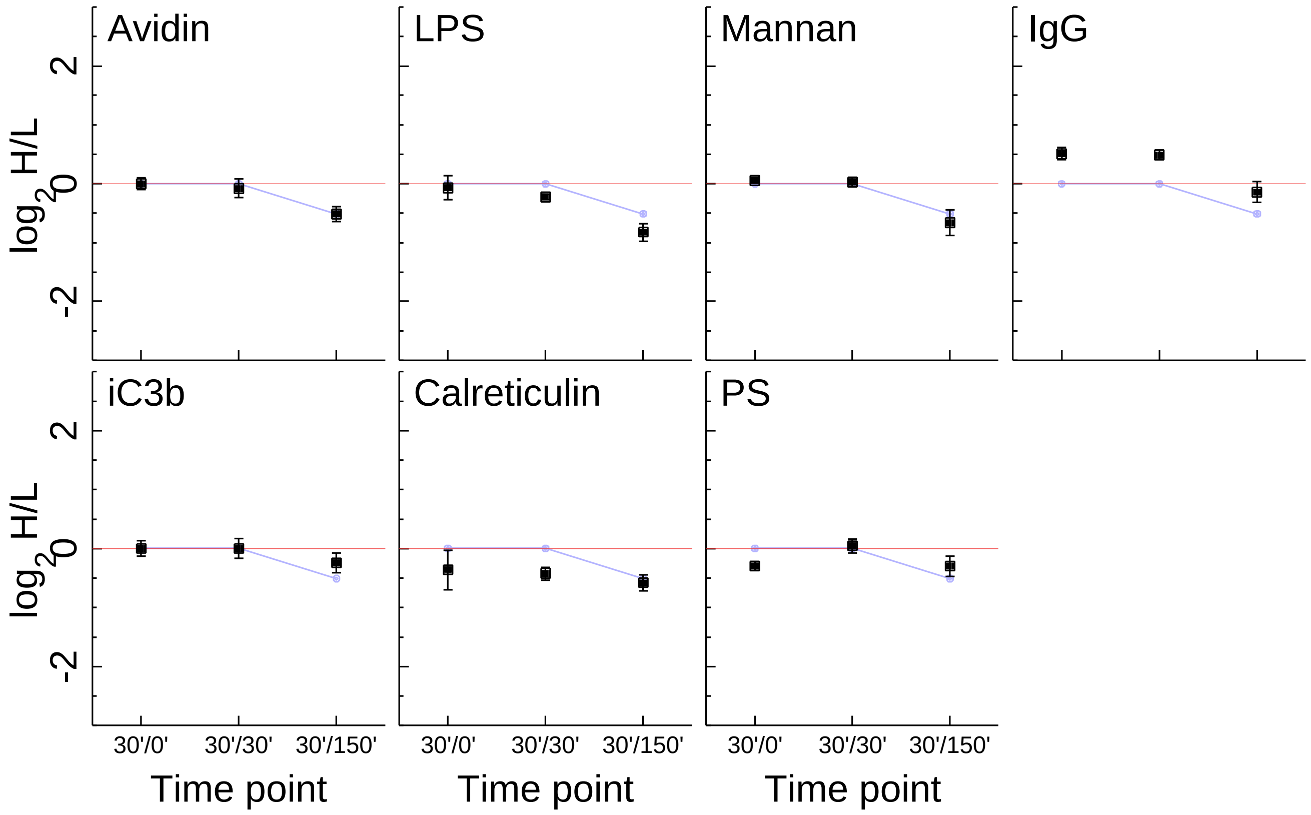

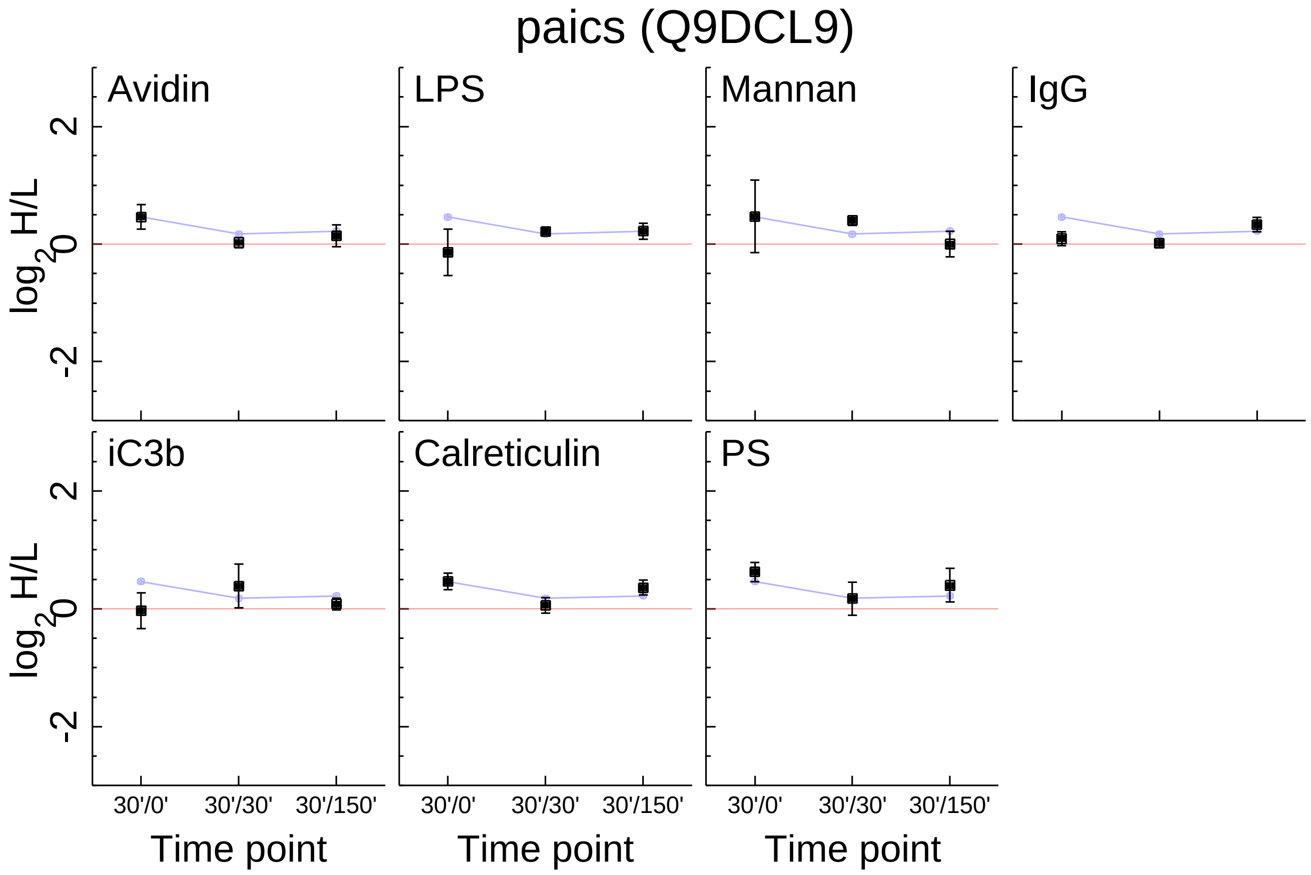

paics (Q9DCL9)
Avidin
LPS
Mannan
IgG
iC3b
Calreticulin
PS
log2 H/L
2
0
-2
30'/0'
30'/30'
30'/150'
Time point

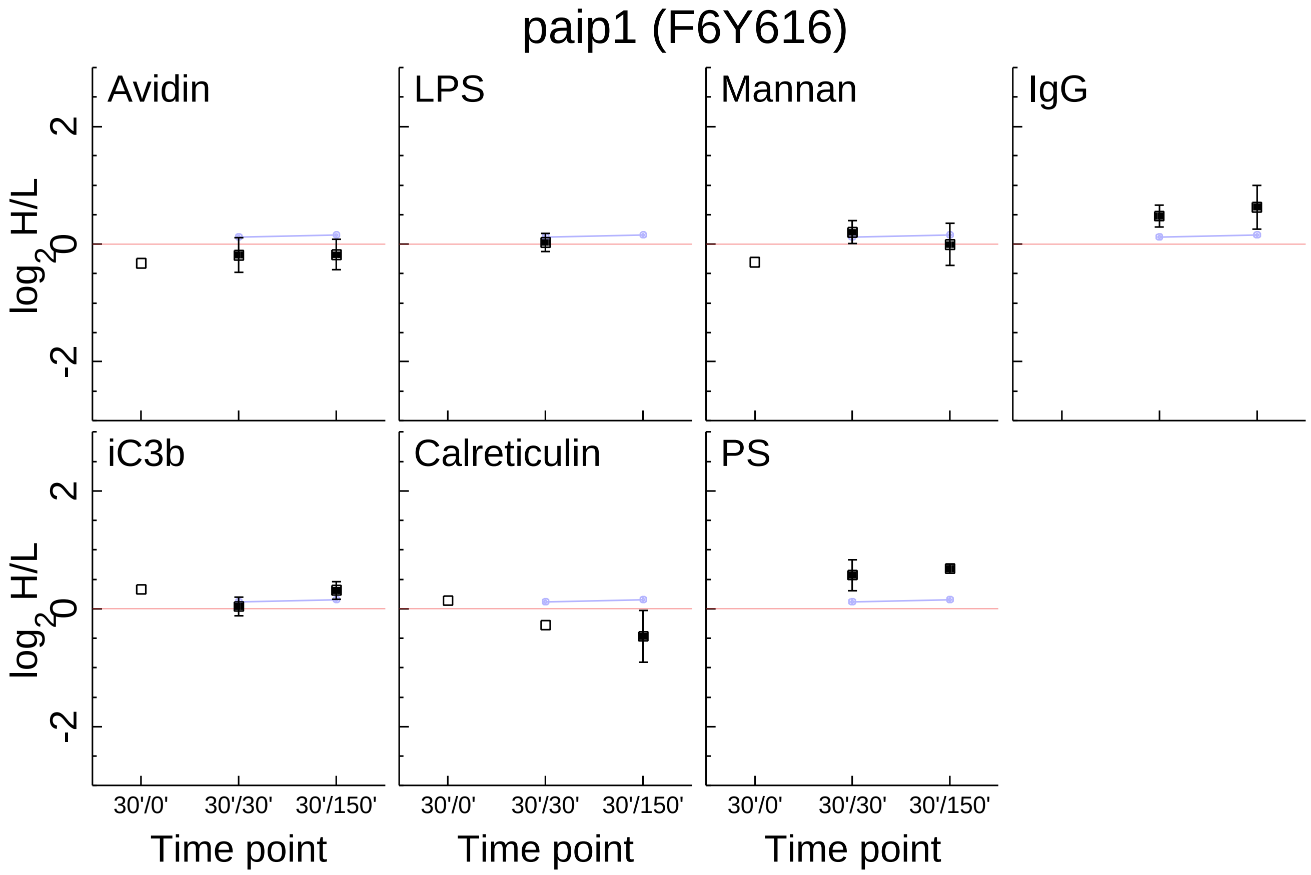
paip1 (F6Y616)
Avidin
LPS
Mannan
IgG
iC3b
Calreticulin
PS
log2 H/L
2
0
-2
30'/0'
30'/30'
30'/150'
Time point

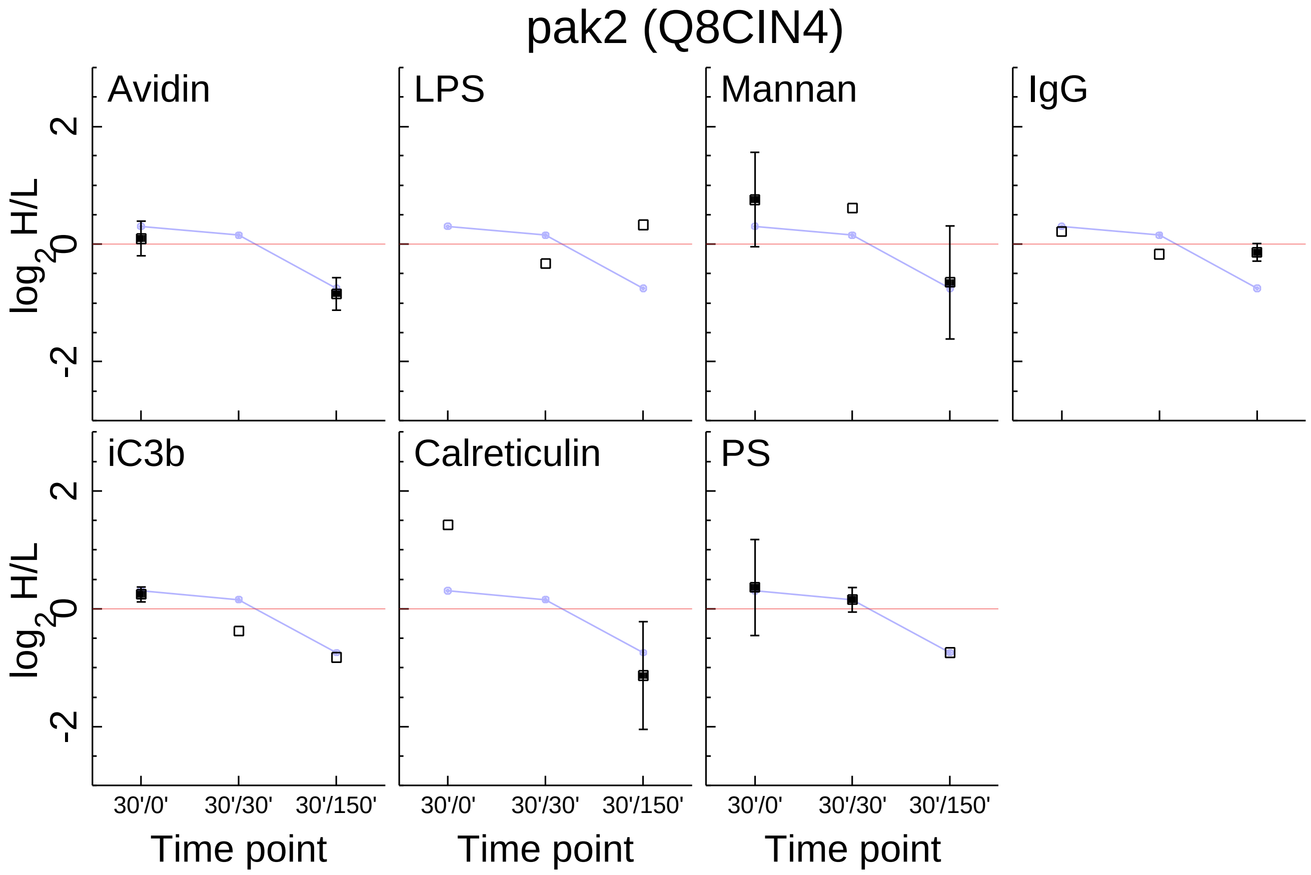

pak2 (Q8CIN4)
Avidin
LPS
Mannan
IgG
iC3b
Calreticulin
PS
log2 H/L
2
0
-2
30'/0'
30'/30'
30'/150'
Time point

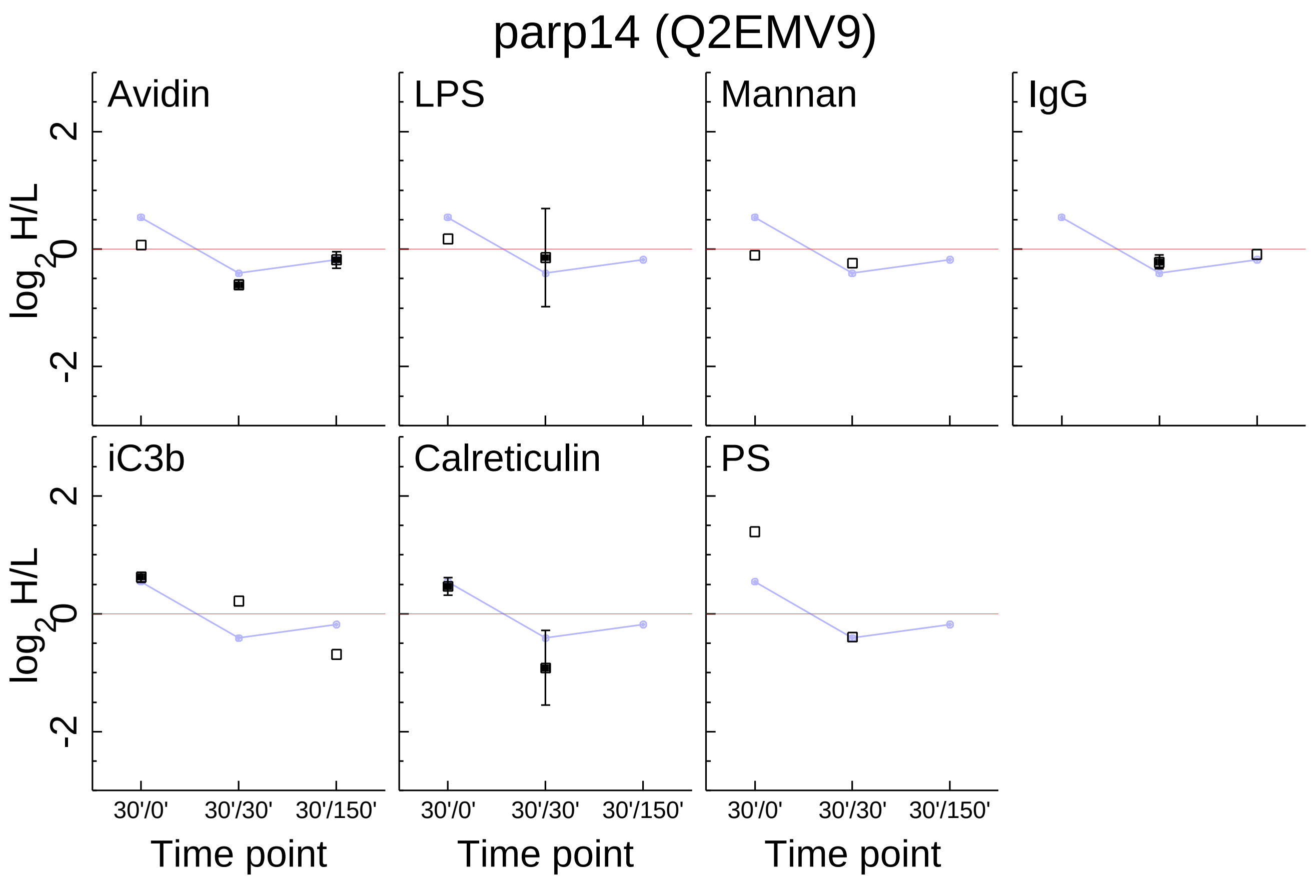

parp14 (Q2EMV9)
Avidin
LPS
Mannan
IgG
iC3b
Calreticulin
PS
log2 H/L
2
0
-2
30'/0'
30'/30'
30'/150'
Time point

parp1 (Q921K2)

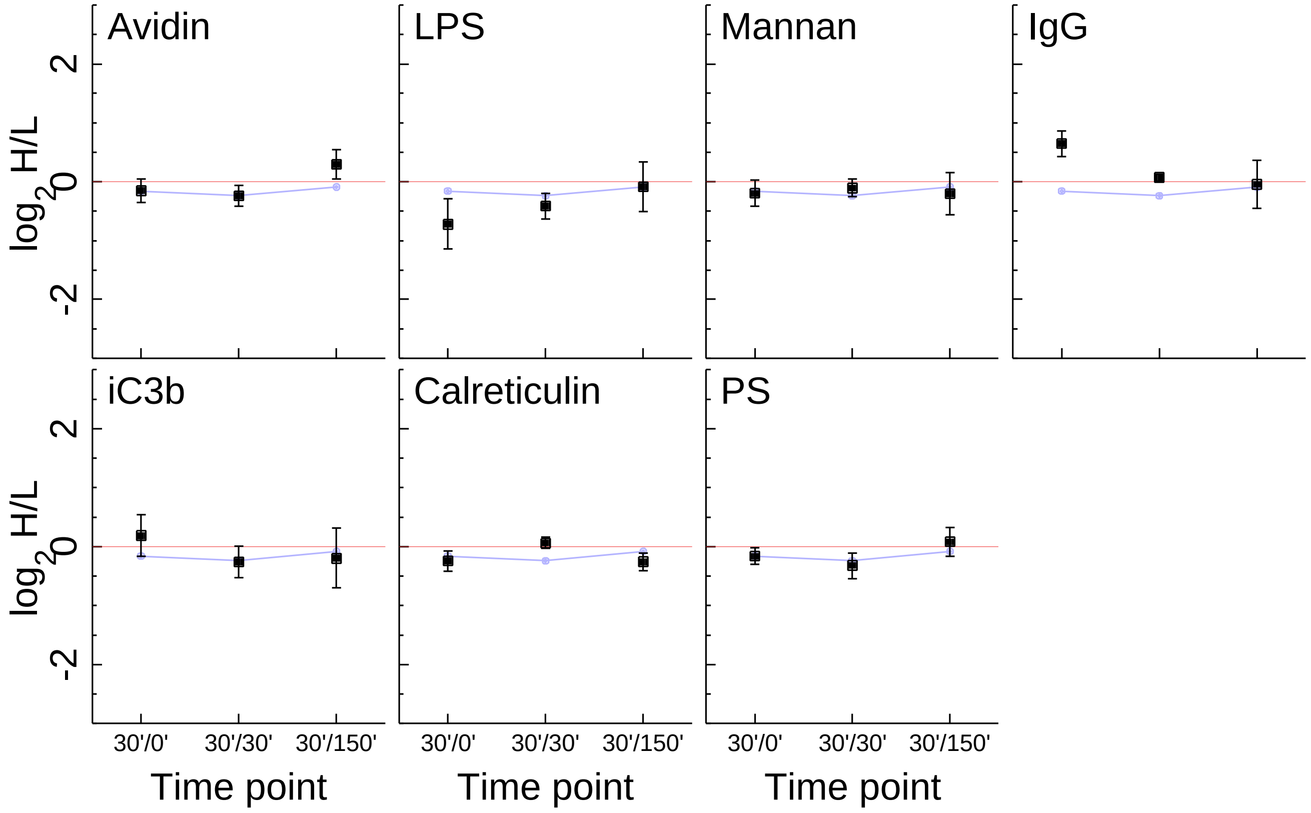

pcbp1 (P60335)

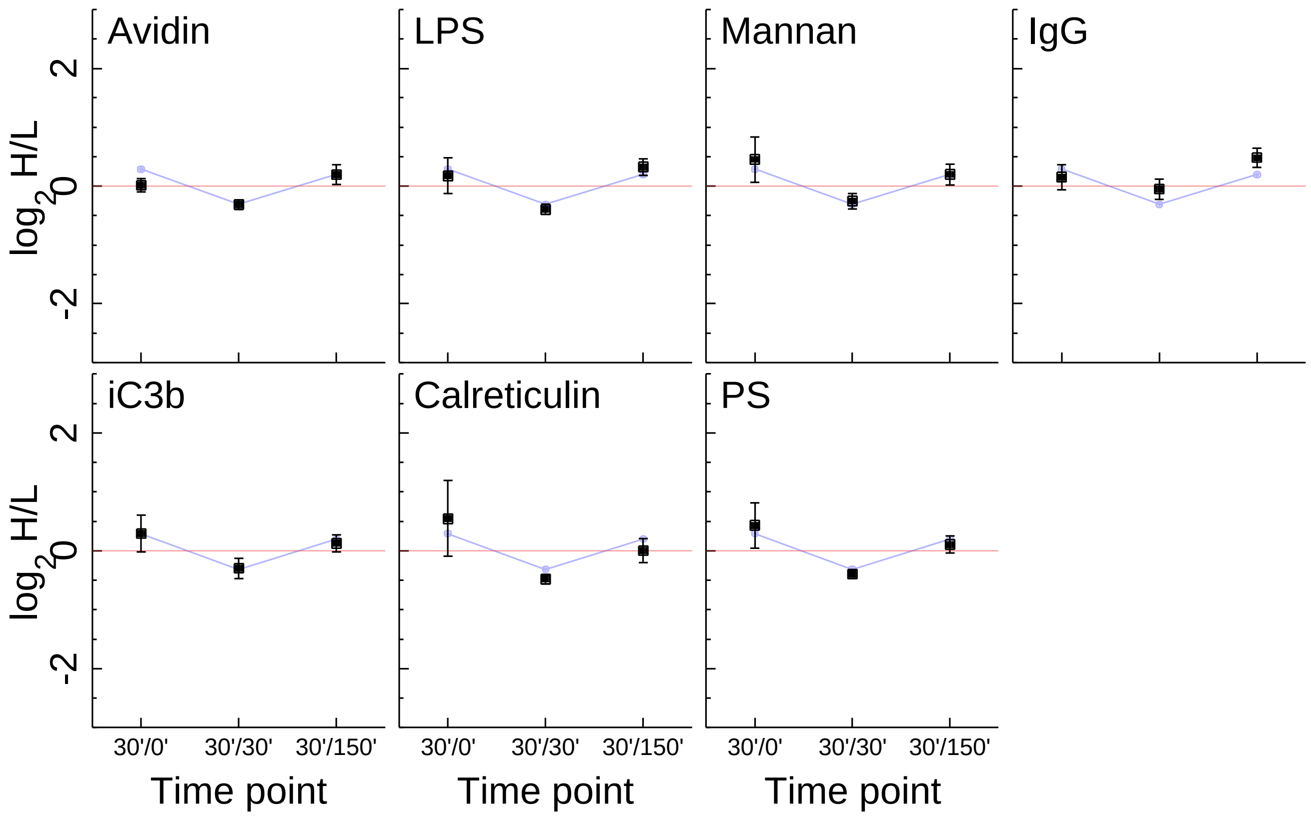

pcbp2 (Q61990)

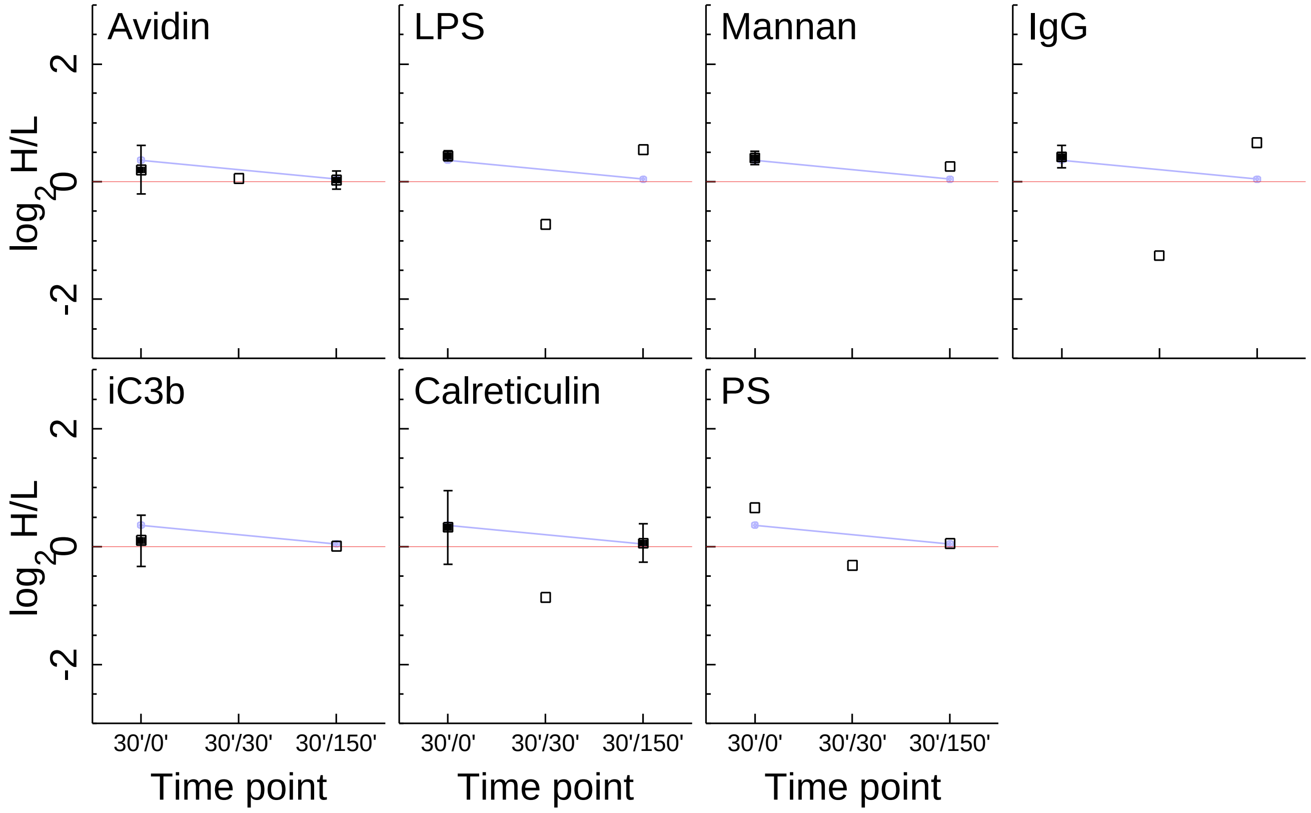

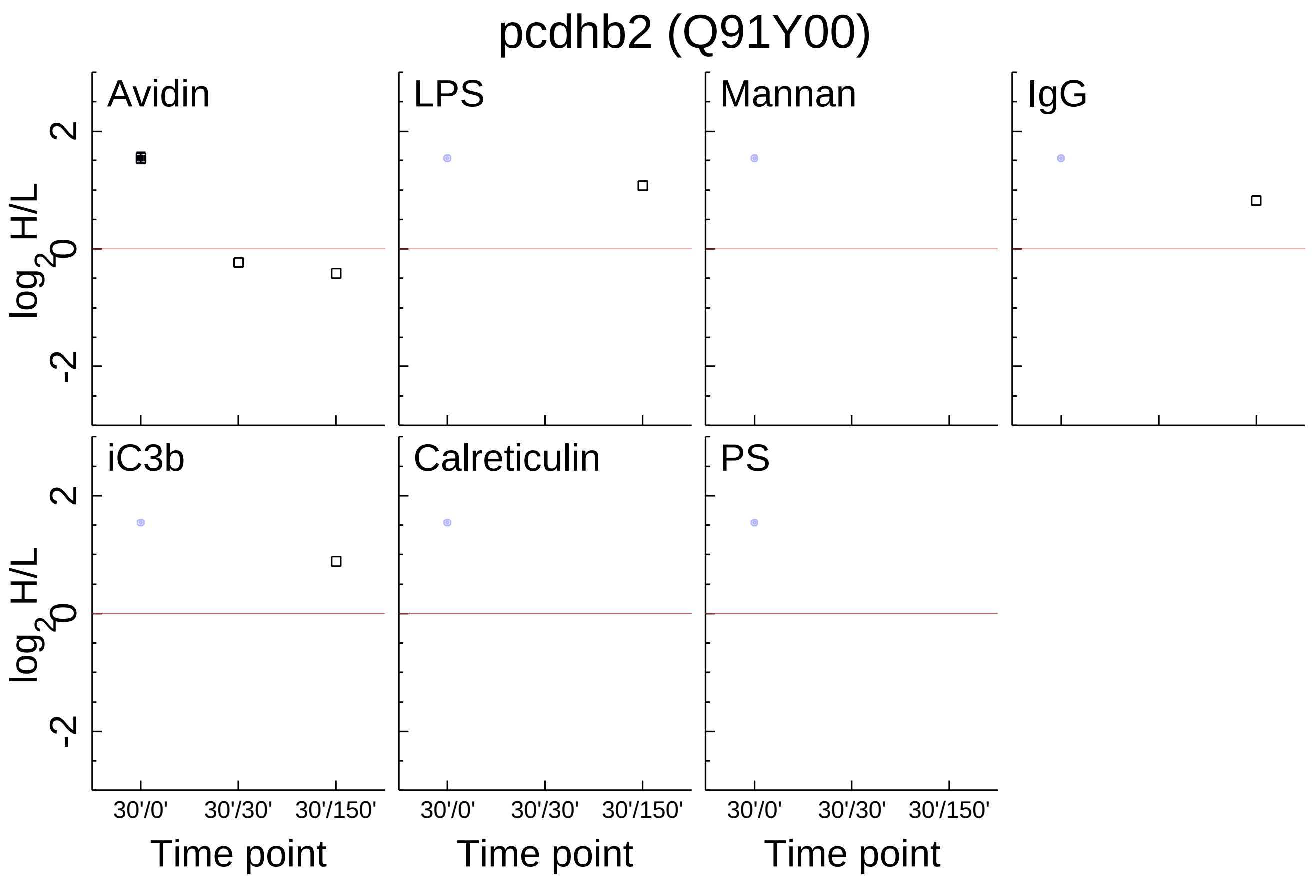

pcdhb2 (Q91Y00)
Avidin
LPS
Mannan
IgG
iC3b
Calreticulin
PS
log2 H/L
2
0
-2
30'/0'
30'/30'
30'/150'
Time point

pck2 (Q8BH04)

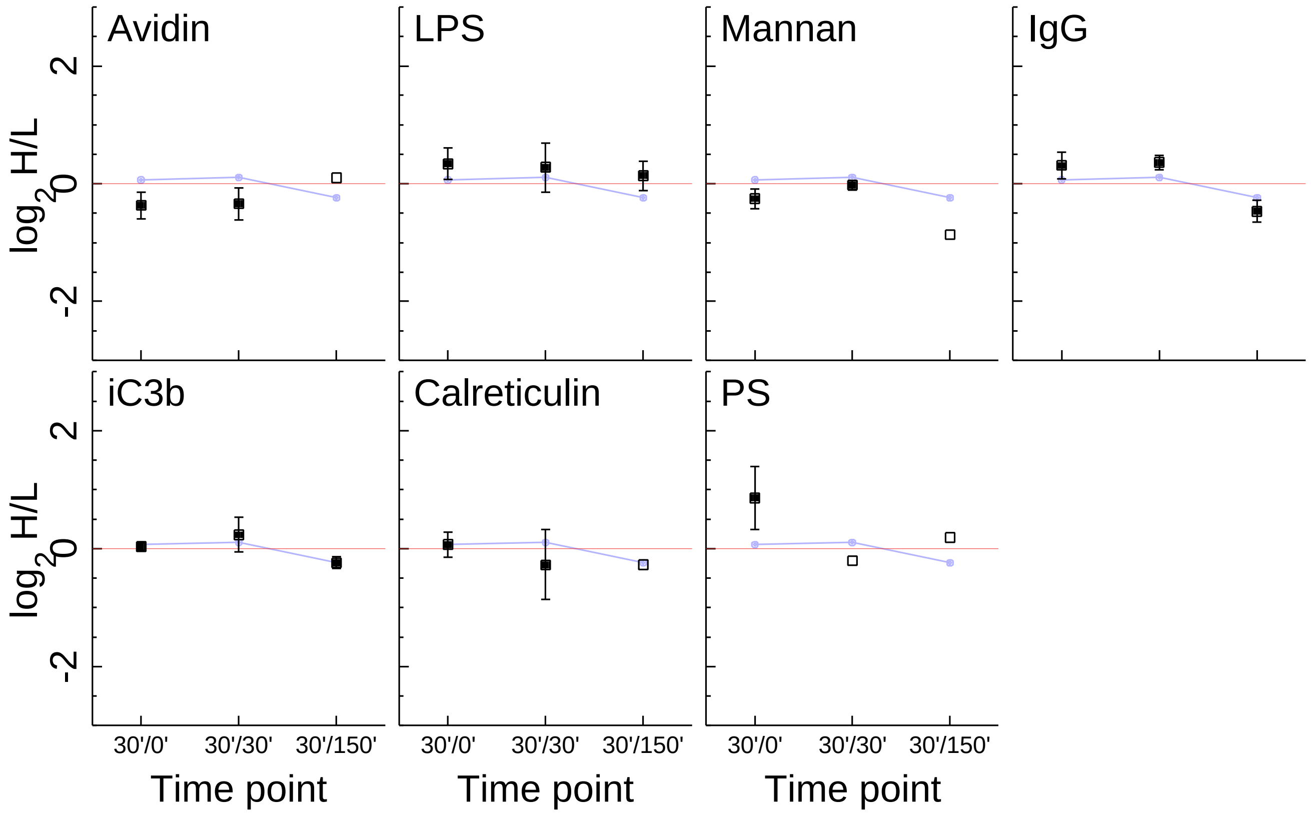

Avidin
LPS
Mannan
IgG
iC3b
Calreticulin
PS
log2 H/L
2
0
-2
30'/0'
30'/30'
30'/150'
Time point

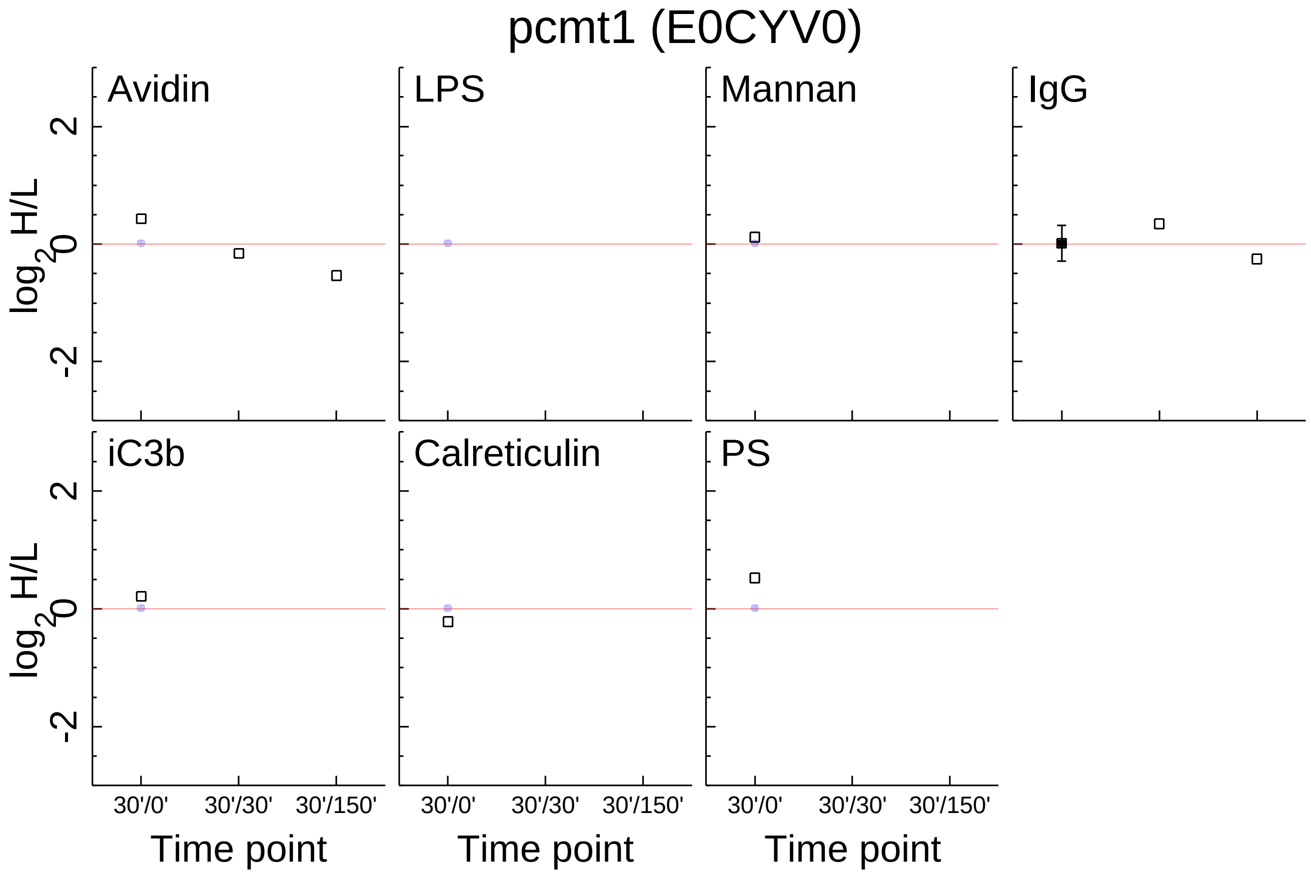
pcmt1 (E0CYV0)
Avidin
LPS
Mannan
IgG
iC3b
Calreticulin
PS
log2 H/L
2
0
-2
30'/0'
30'/30'
30'/150'
Time point

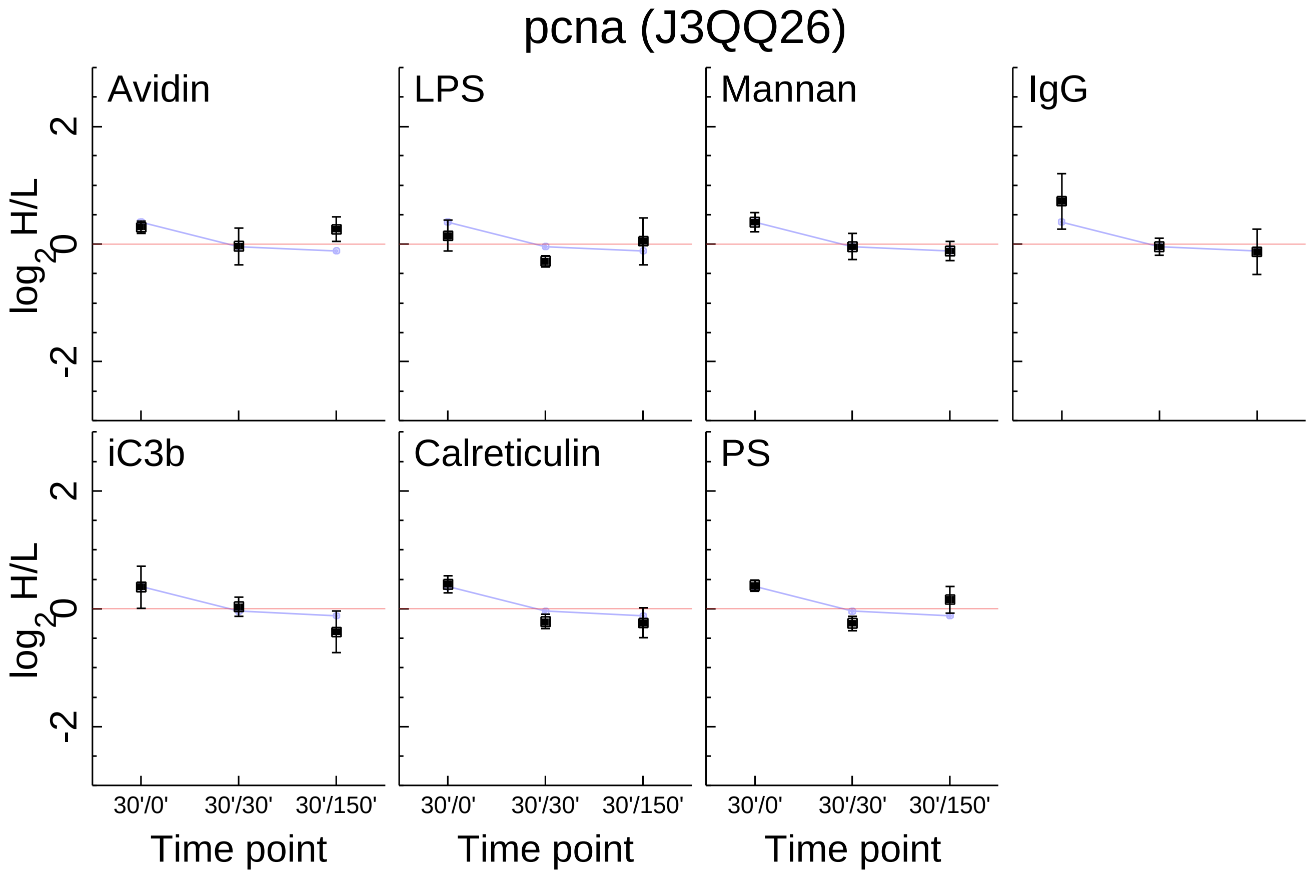

pcna (J3QQ26)
Avidin
LPS
Mannan
IgG
iC3b
Calreticulin
PS
log2 H/L
2
0
-2
30'/0'
30'/30'
30'/150'
Time point

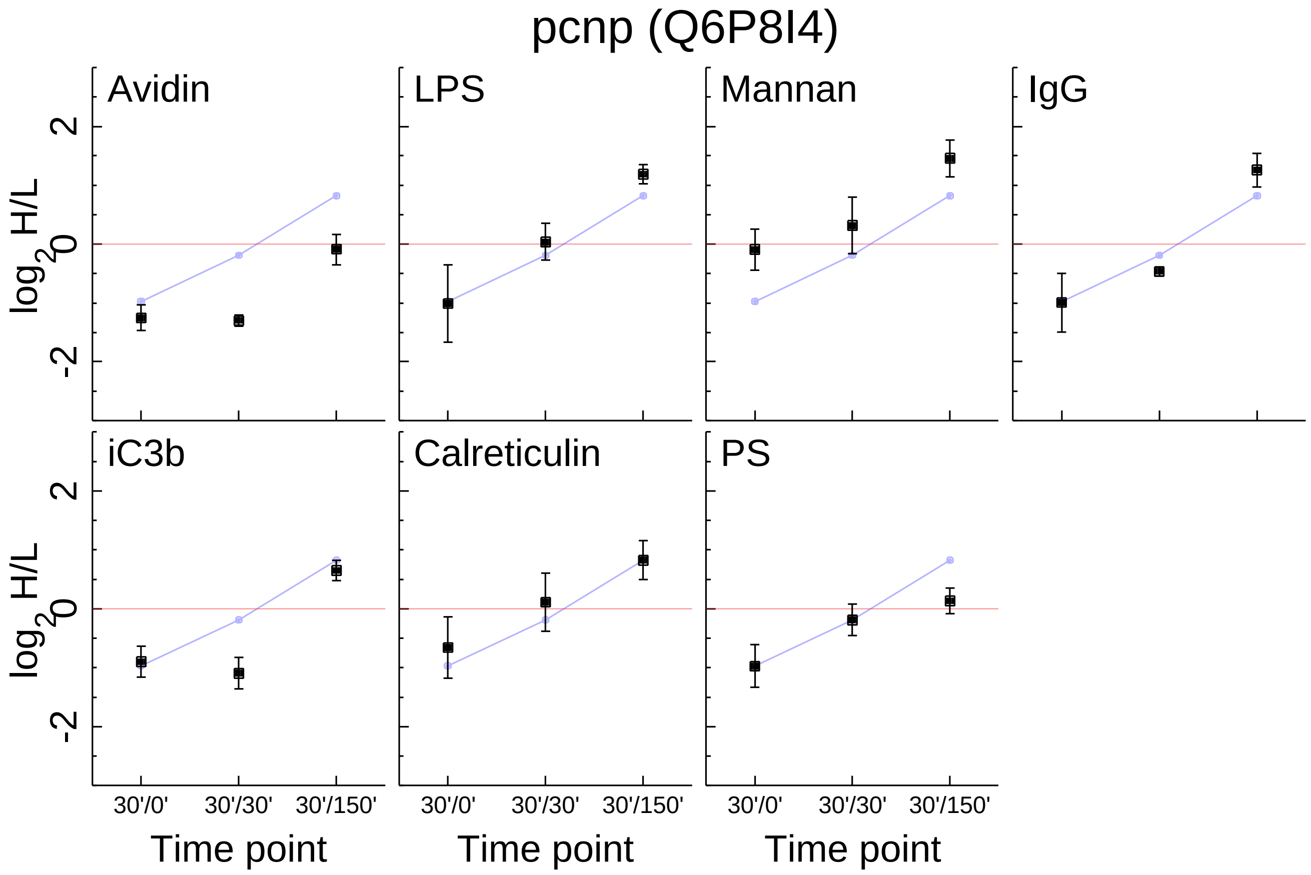
pcnp (Q6P8I4)
Avidin
LPS
Mannan
IgG
iC3b
Calreticulin
PS
log2 H/L
2
0
-2
30'/0'
30'/30'
30'/150'
Time point

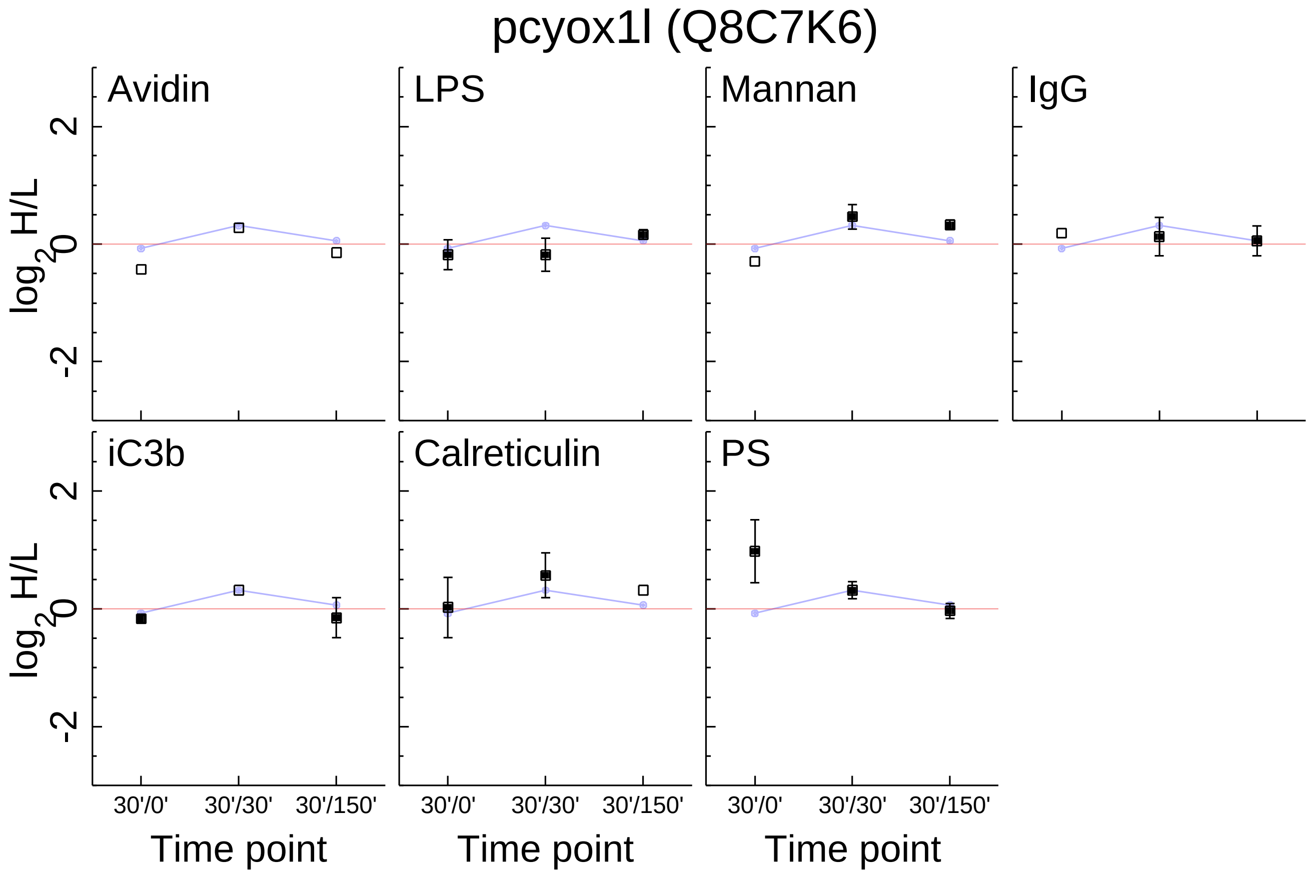
pcyox1l (Q8C7K6)
Avidin
LPS
Mannan
IgG
iC3b
Calreticulin
PS
log2 H/L
2
0
-2
30'/0'
30'/30'
30'/150'
Time point

pcyox1 (Q9CQF9)

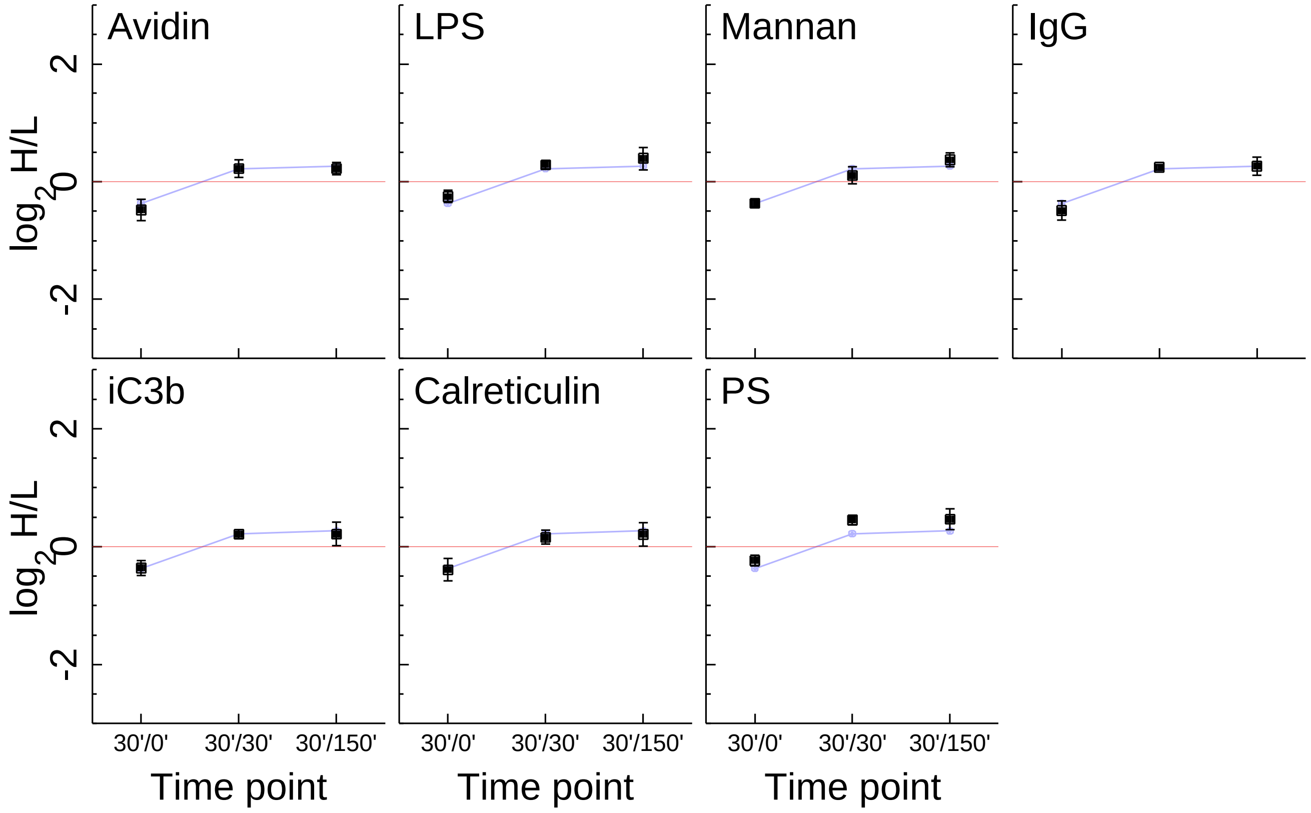

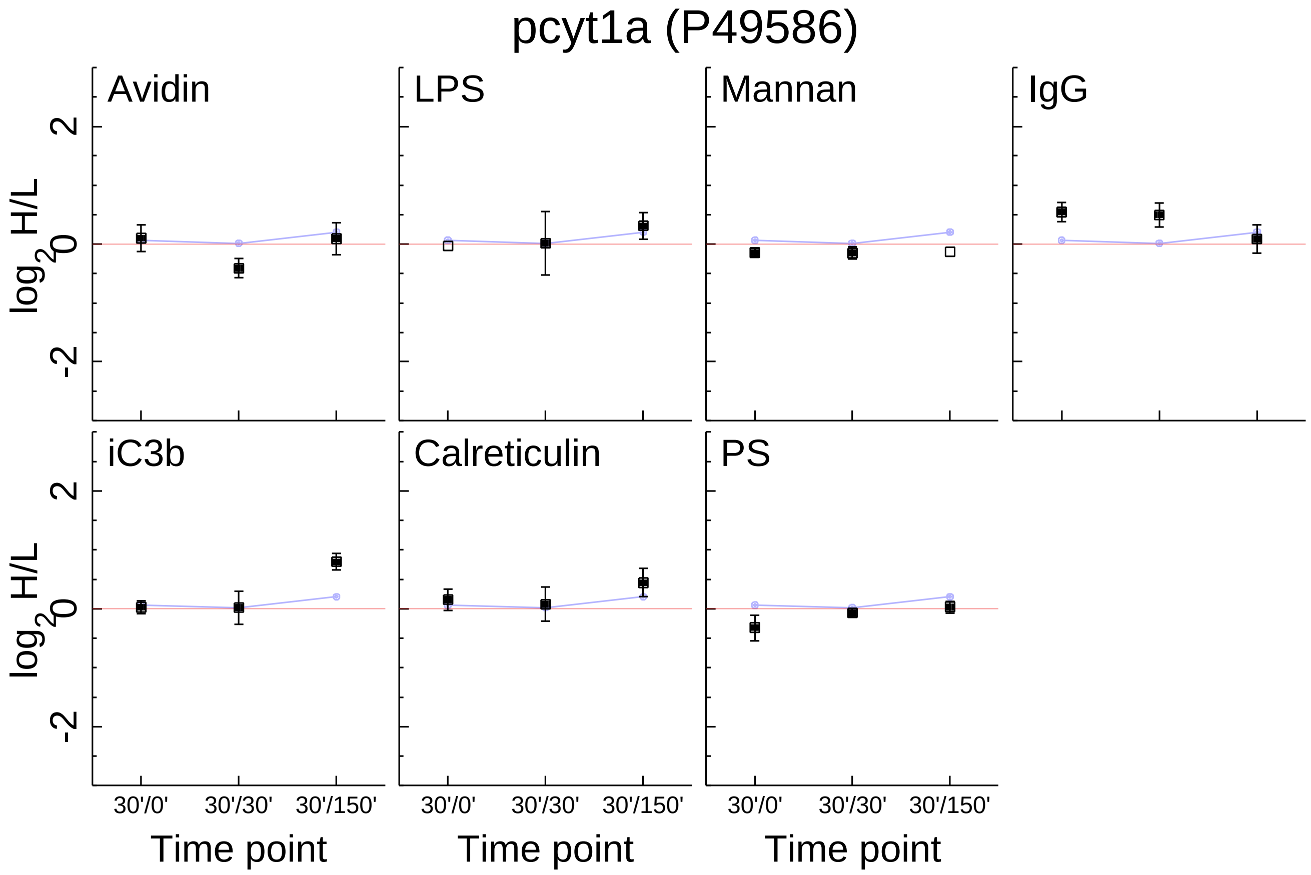
pcyt1a (P49586)
Avidin
LPS
Mannan
IgG
iC3b
Calreticulin
PS
log2 H/L
2
0
-2
30'/0'
30'/30'
30'/150'
Time point

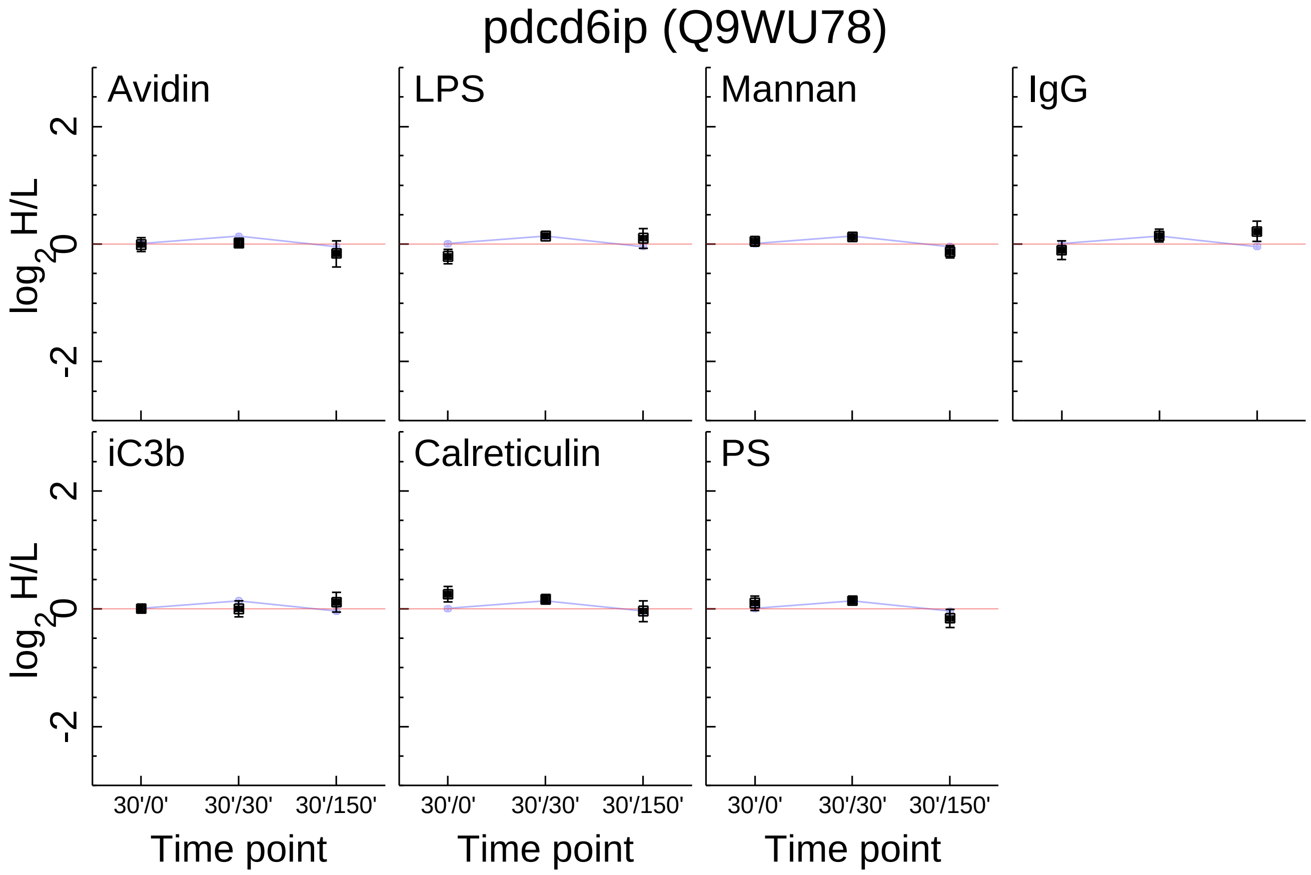
pdcd6ip (Q9WU78)
Avidin
LPS
Mannan
IgG
iC3b
Calreticulin
PS
log2 H/L
2
0
-2
30'/0'
30'/30'
30'/150'
Time point

pdcd6 (P12815)

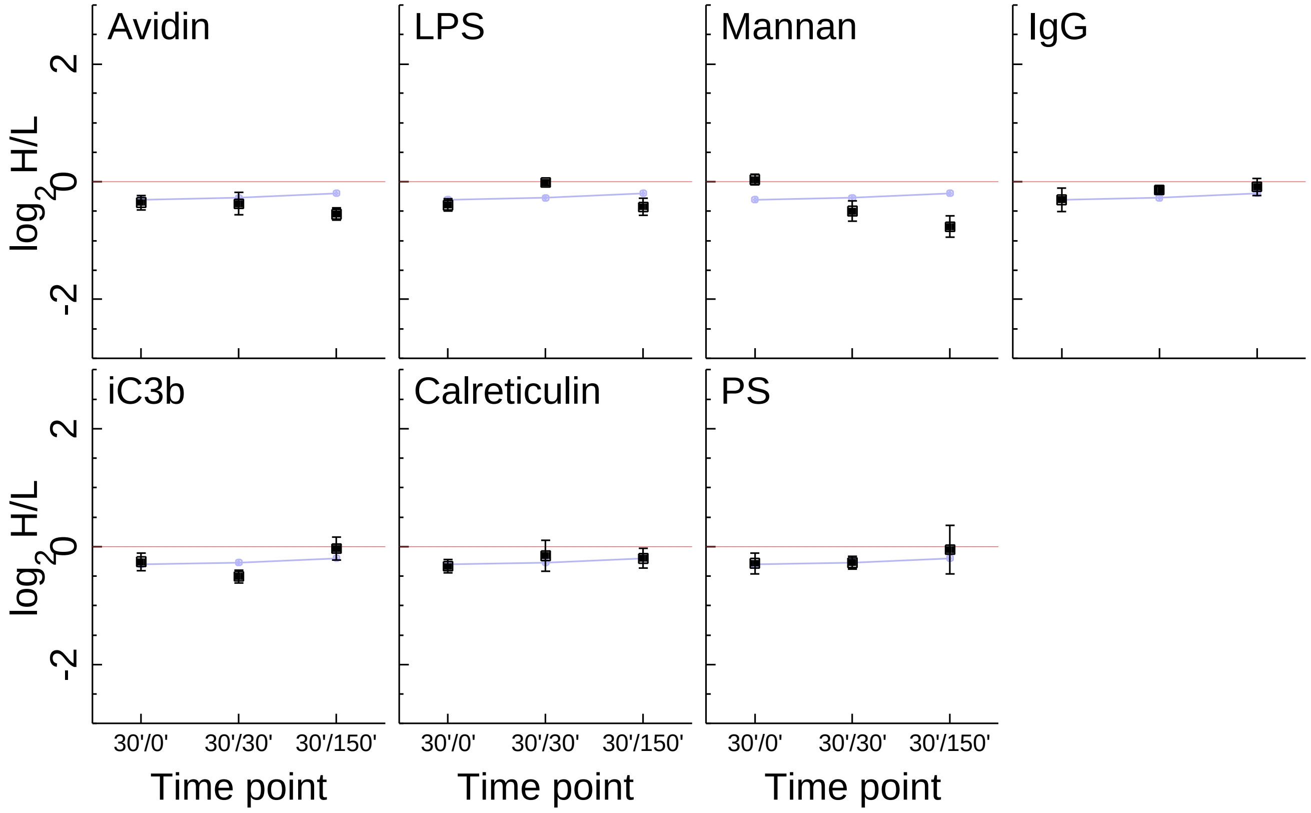

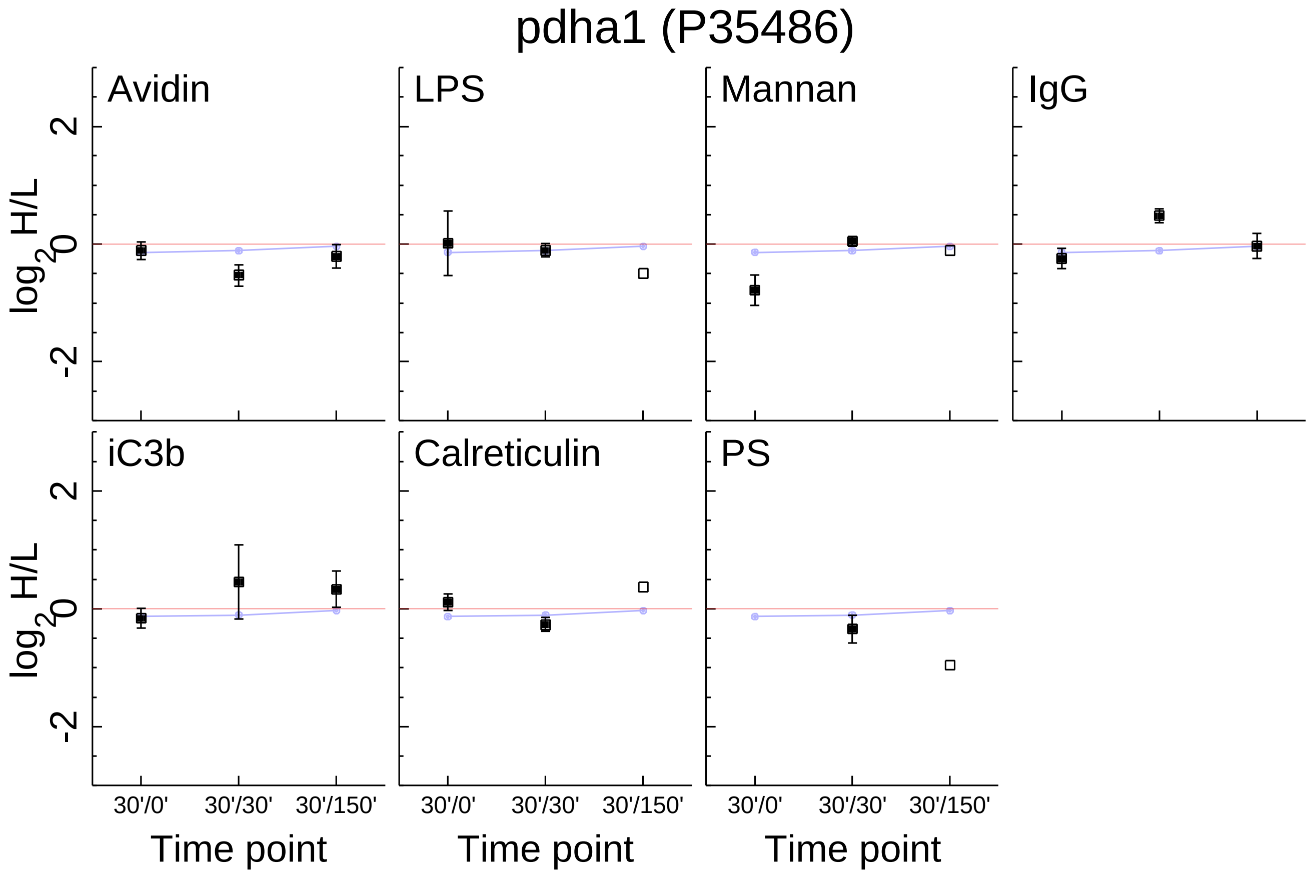
pdha1 (P35486)
Avidin
LPS
Mannan
IgG
iC3b
Calreticulin
PS
log2 H/L
2
0
-2
30'/0'
30'/30'
30'/150'
Time point

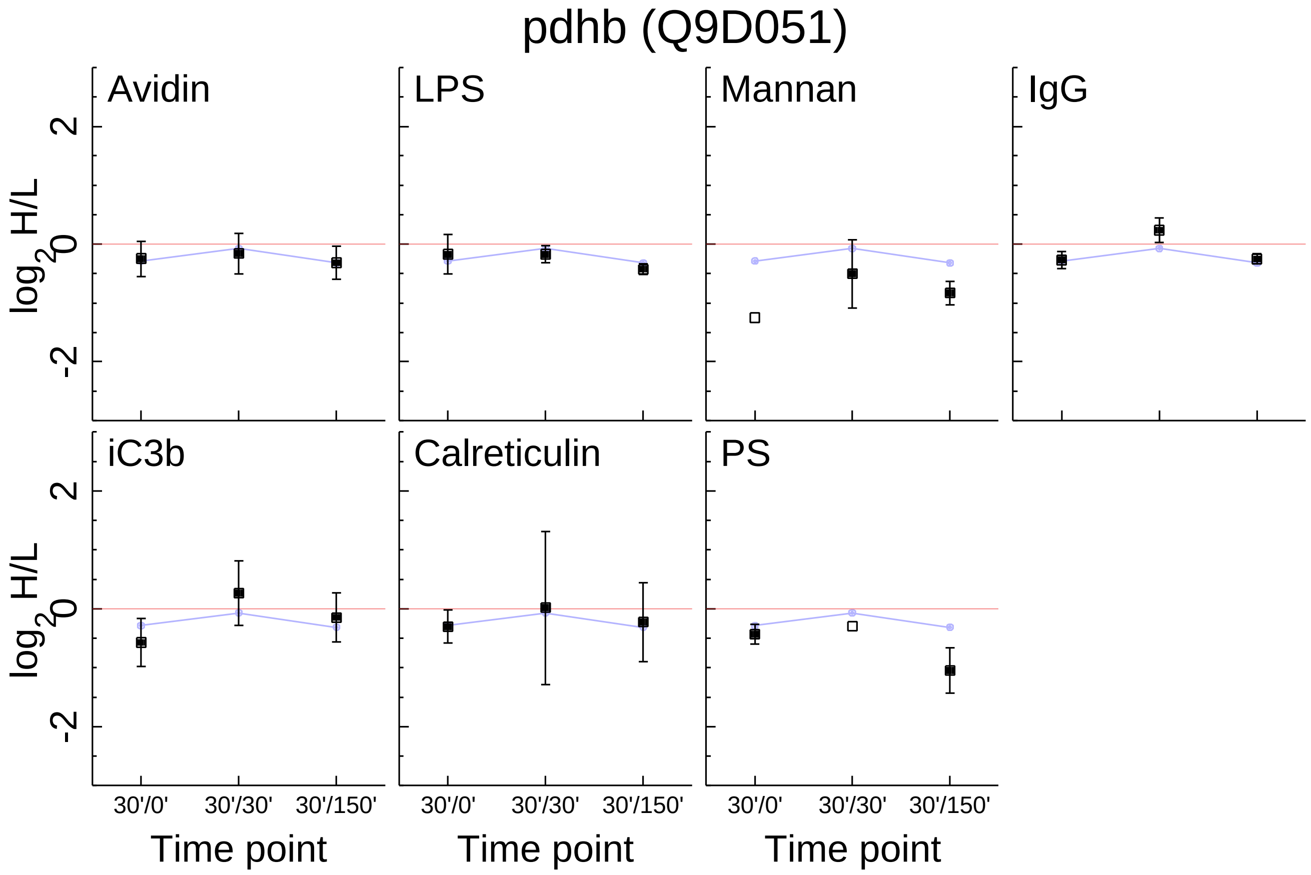
pdhb (Q9D051)
Avidin
LPS
Mannan
IgG
iC3b
Calreticulin
PS
log2 H/L
2
0
-2
30'/0'
30'/30'
30'/150'
Time point

pdia3 (P27773)

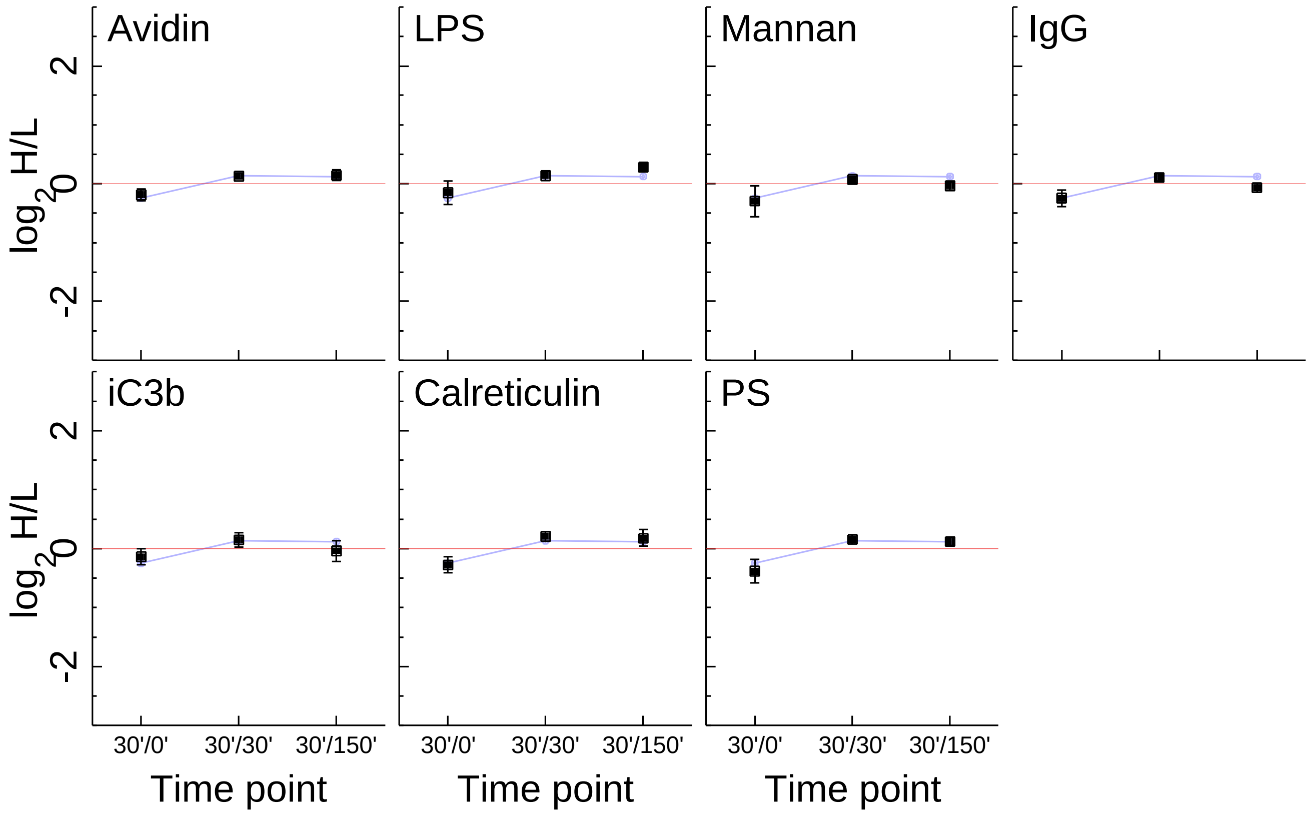

pdia4 (P08003)

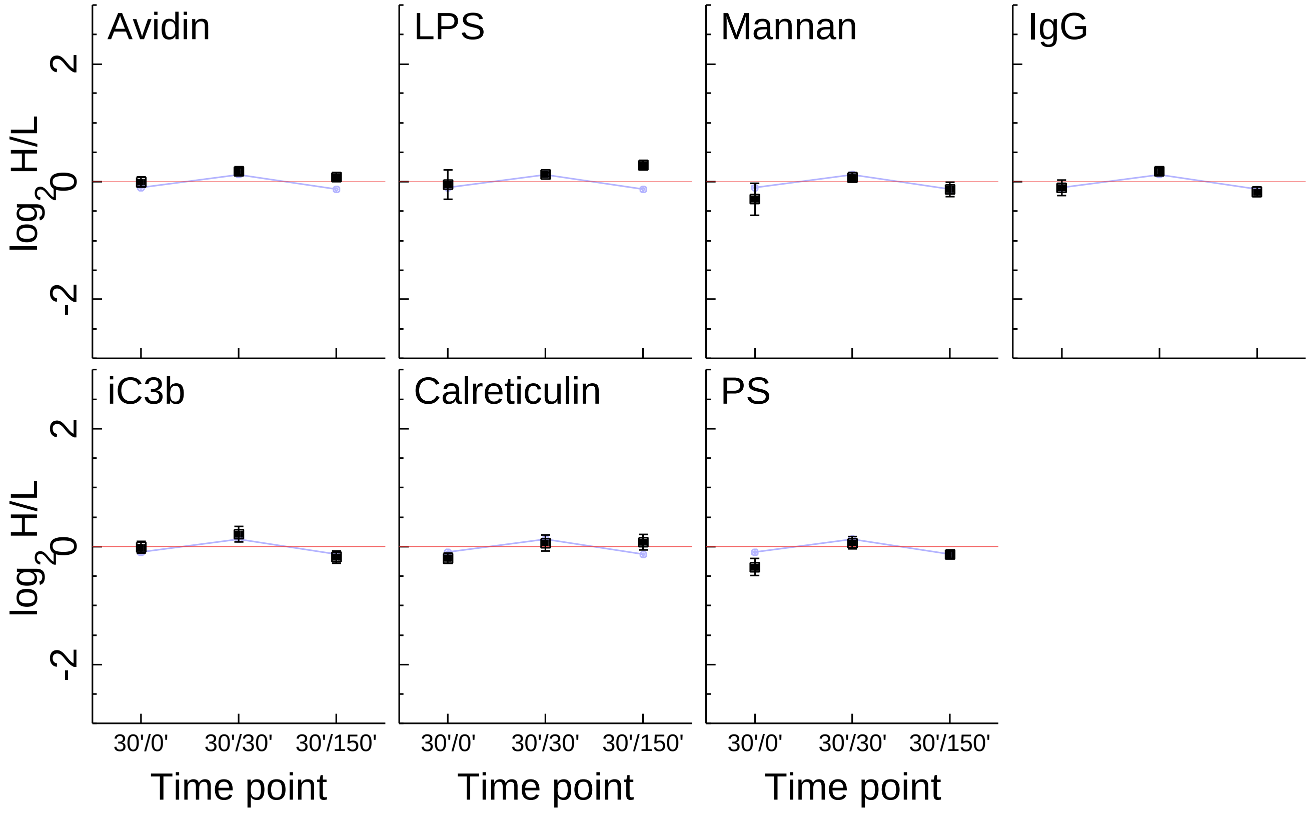

pdia6 (Q3TML0)

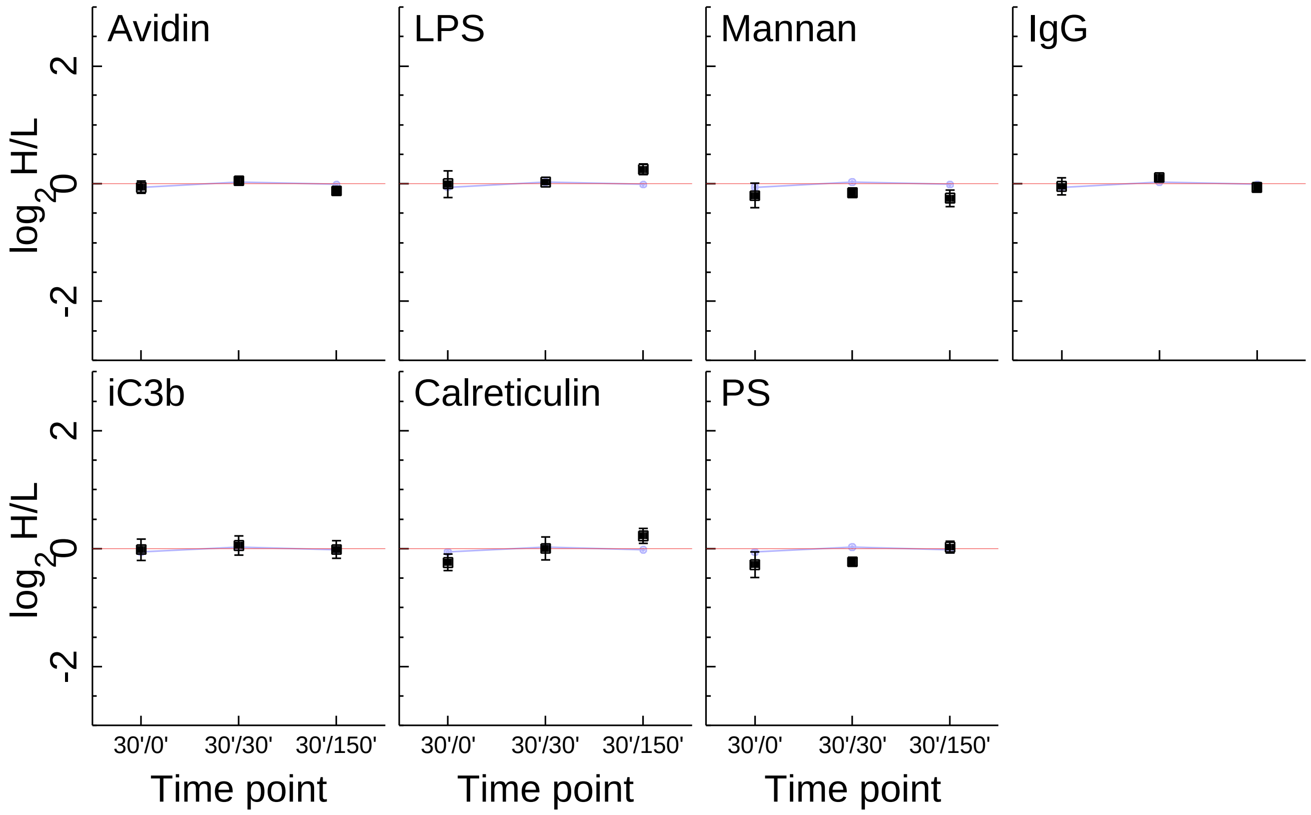

pdlim2 (Q8R1G6)

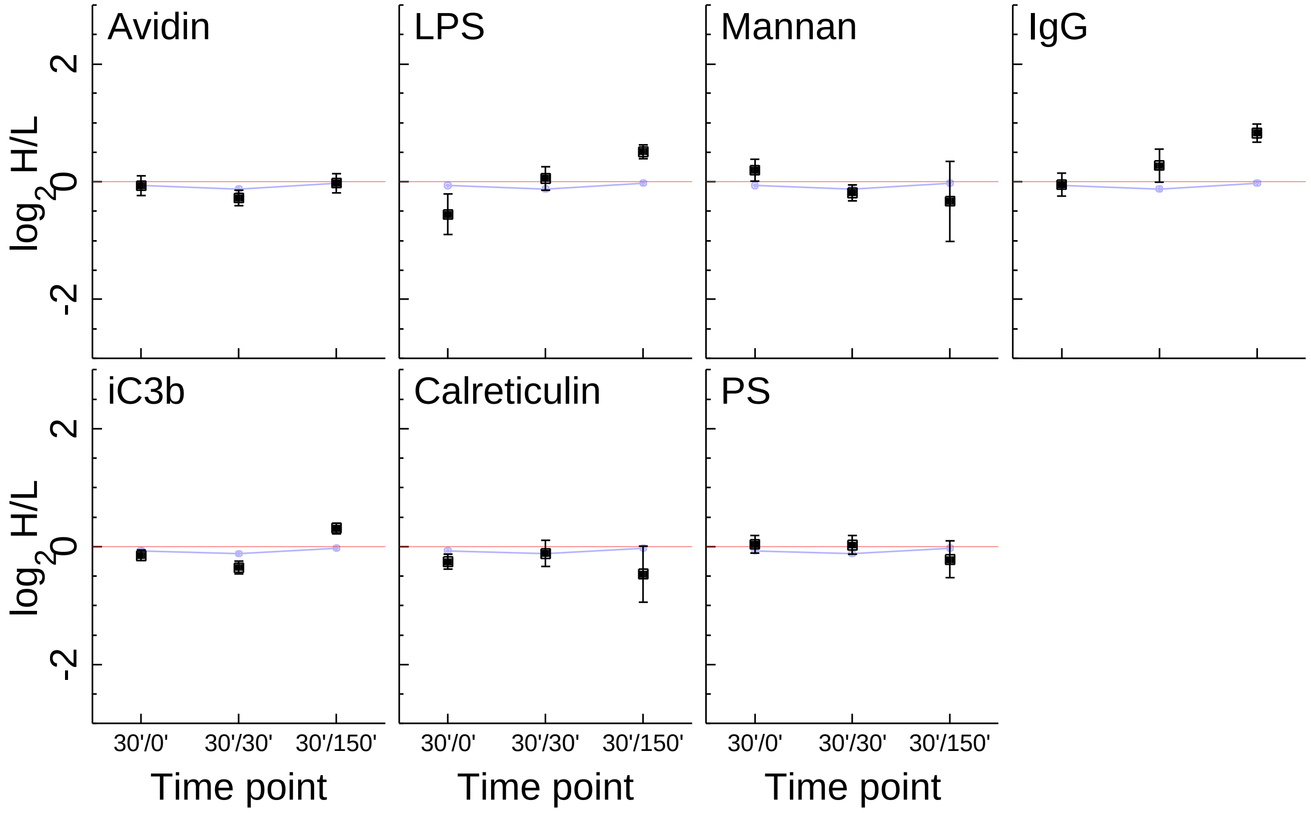

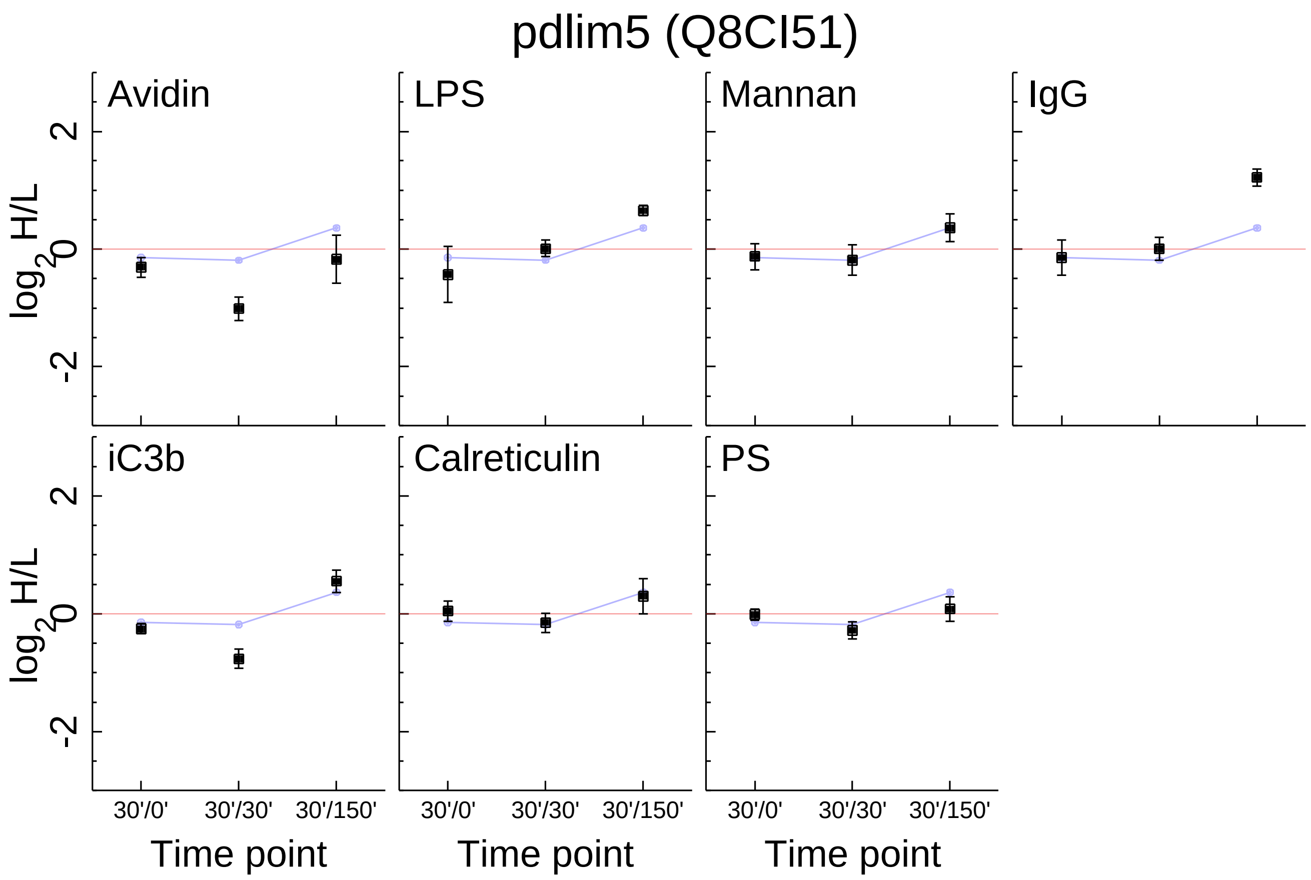
pdlim5 (Q8CI51)
Avidin
LPS
Mannan
IgG
iC3b
Calreticulin
PS
log2 H/L
2
0
-2
30'/0'
30'/30'
30'/150'
Time point

pdxk (Q8K183)

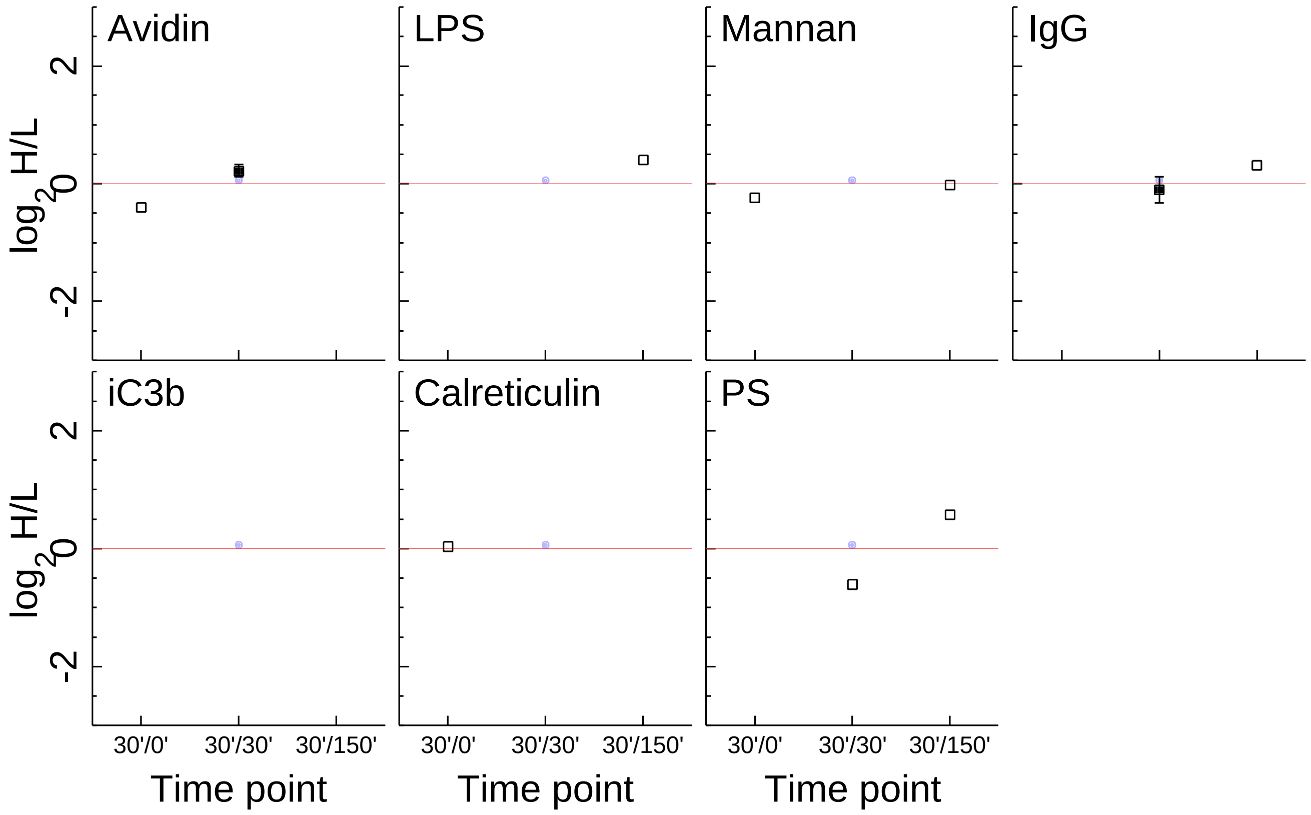
Avidin
LPS
Mannan
IgG
iC3b
Calreticulin
PS
2
0
-2
log2 H/L
30'/0'
30'/30'
30'/150'
Time point

pea15 (Q62048)

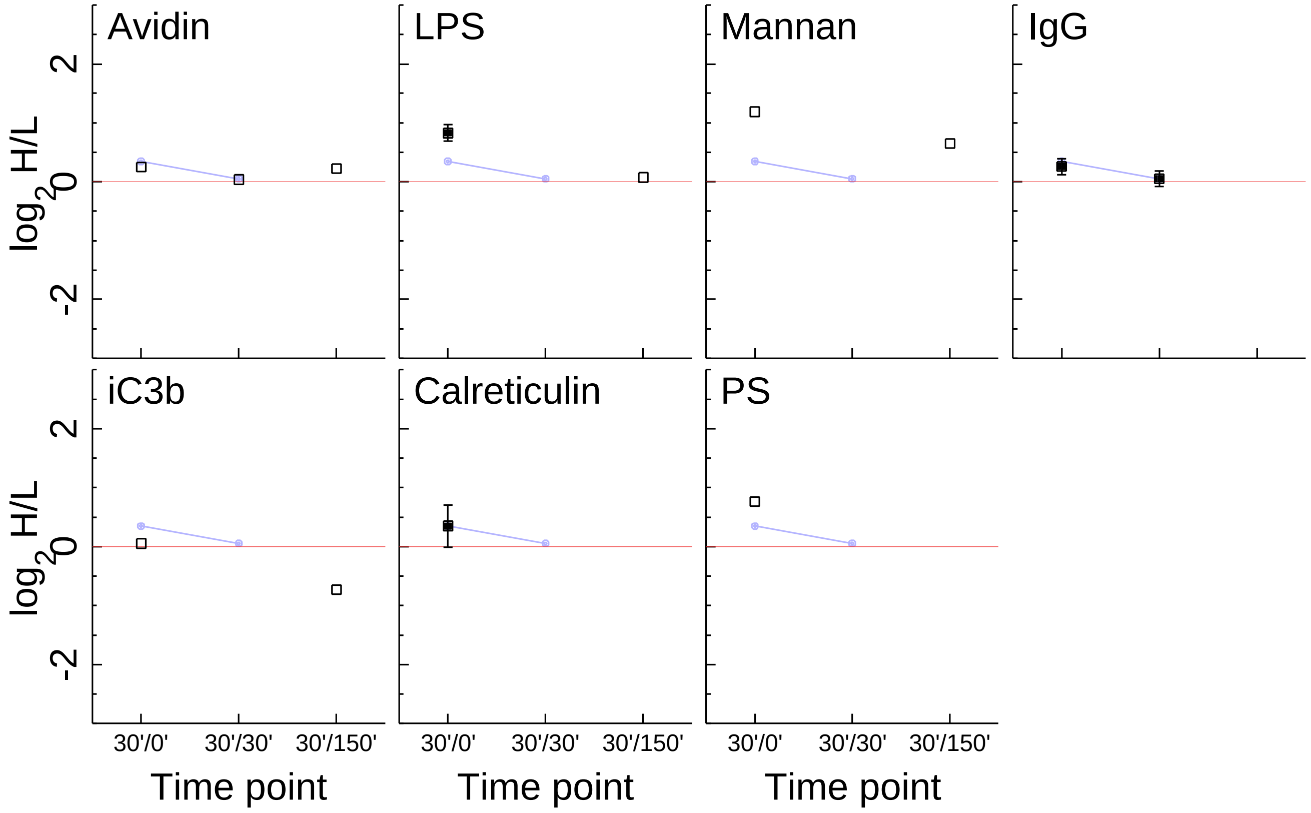

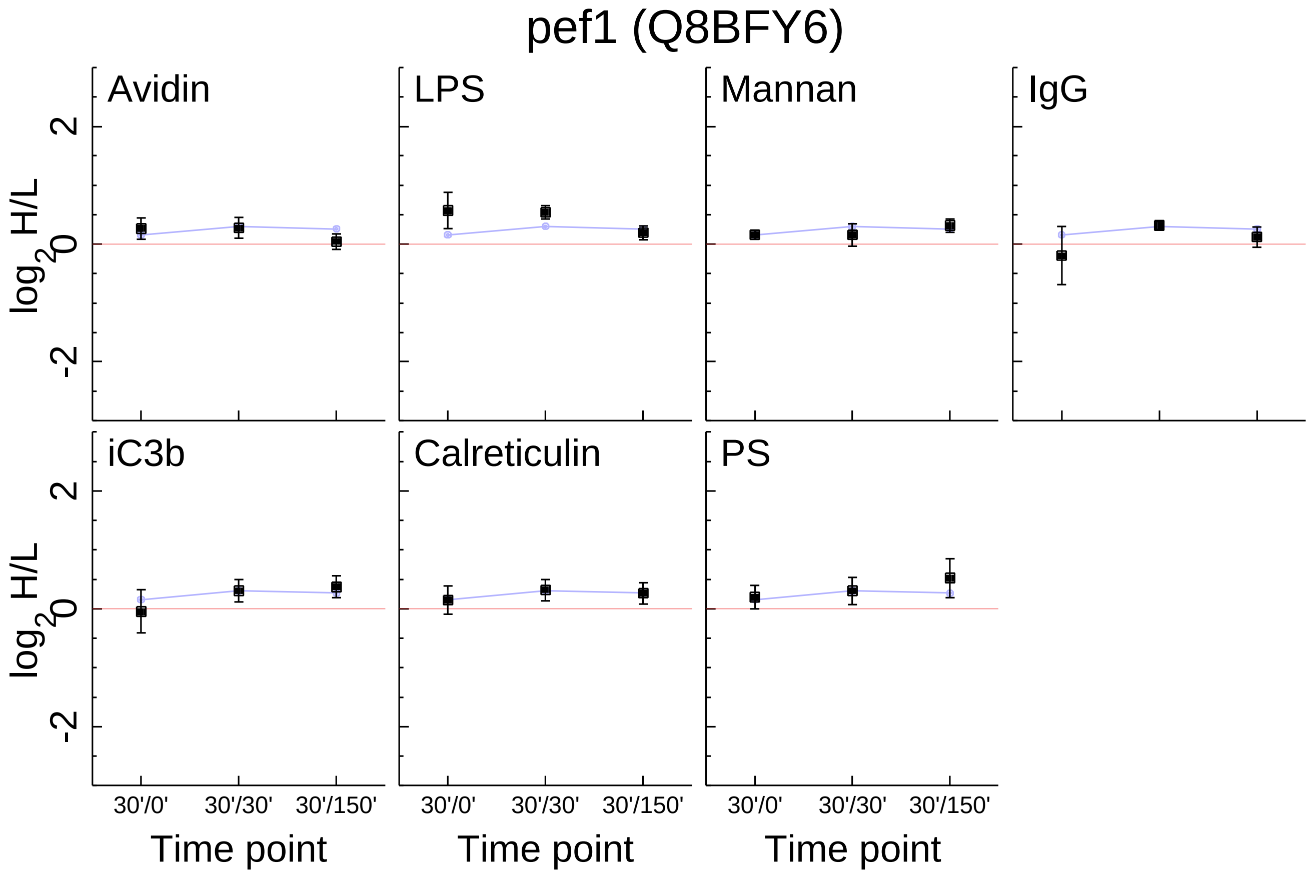
pef1 (Q8BFY6)
Avidin
LPS
Mannan
IgG
iC3b
Calreticulin
PS
log2 H/L
2
0
-2
30'/0'
30'/30'
30'/150'
Time point

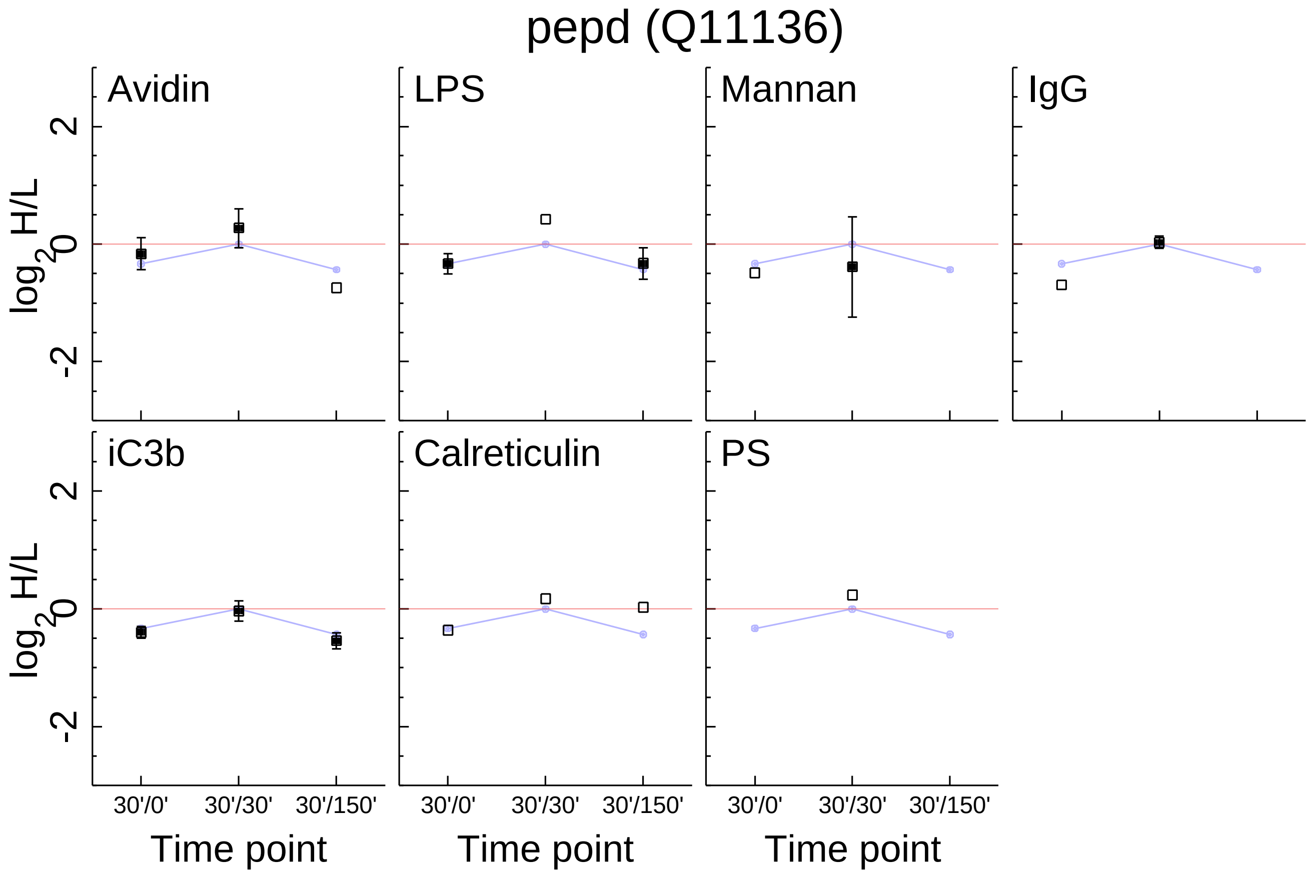
pepd (Q11136)
Avidin
LPS
Mannan
IgG
iC3b
Calreticulin
PS
log2 H/L
2
0
-2
30'/0'
30'/30'
30'/150'
Time point

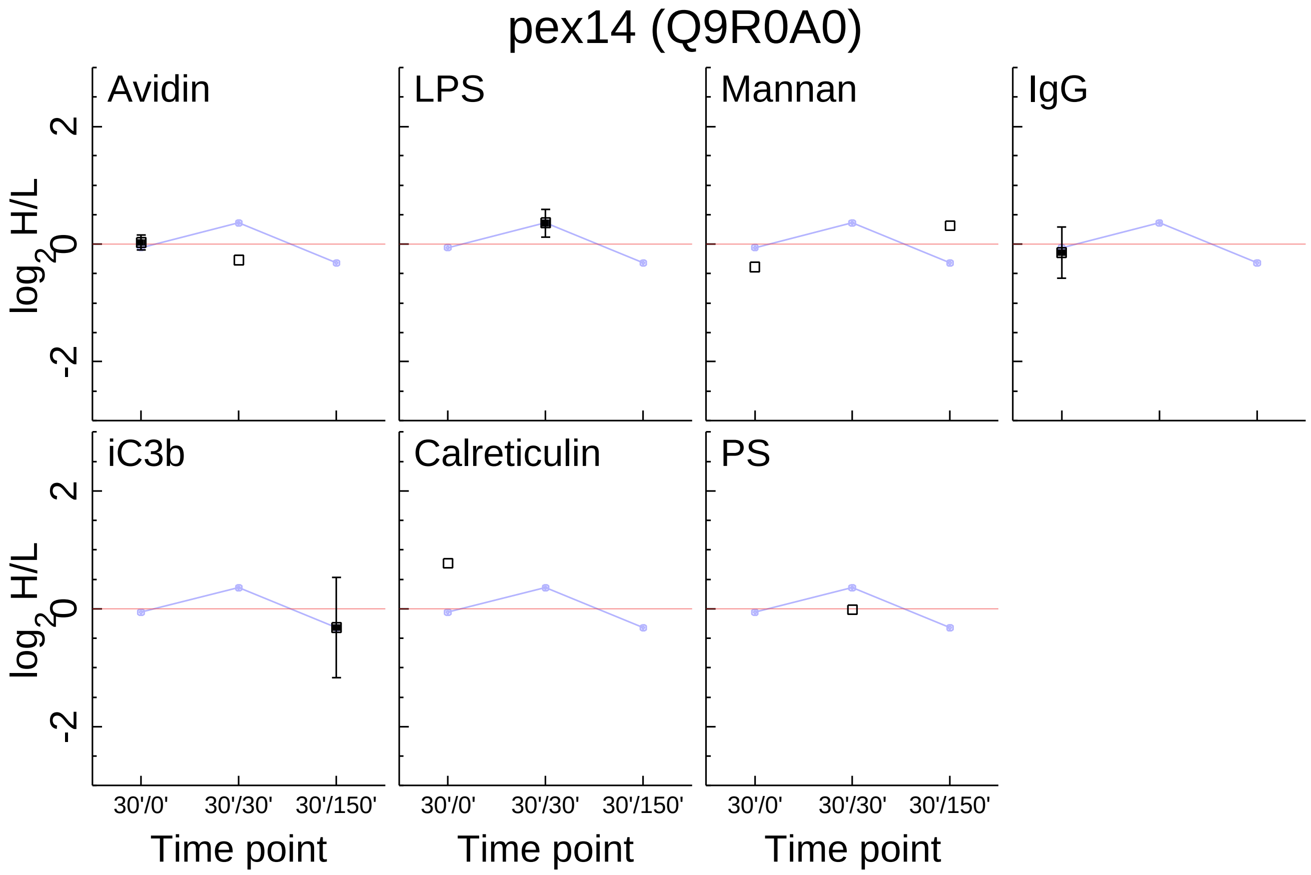
pex14 (Q9R0A0)
Avidin
LPS
Mannan
IgG
iC3b
Calreticulin
PS
log2 H/L
2
0
-2
30'/0'
30'/30'
30'/150'
Time point

pf4 (Q9Z126)

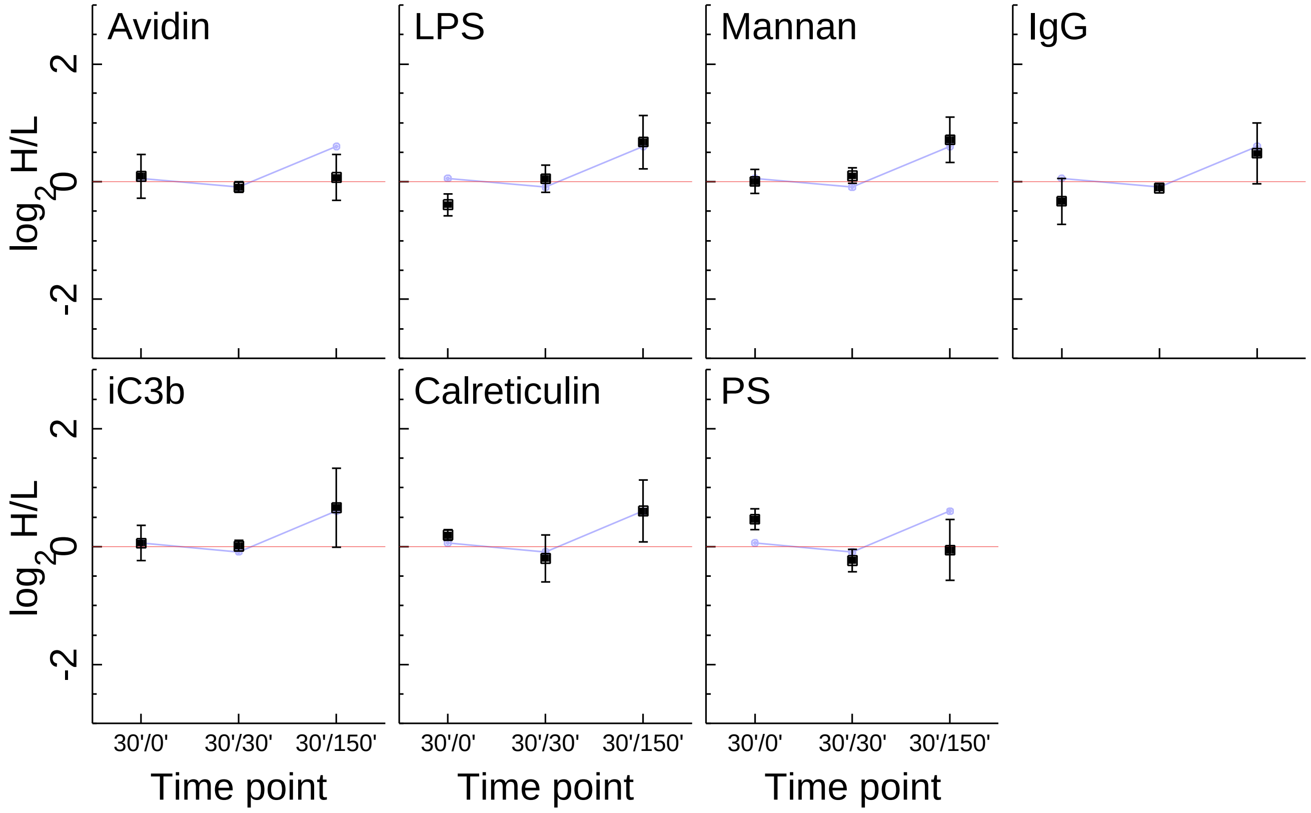

Avidin
LPS
Mannan
IgG
iC3b
Calreticulin
PS
log2 H/L
2
0
-2
30'/0'
30'/30'
30'/150'
Time point

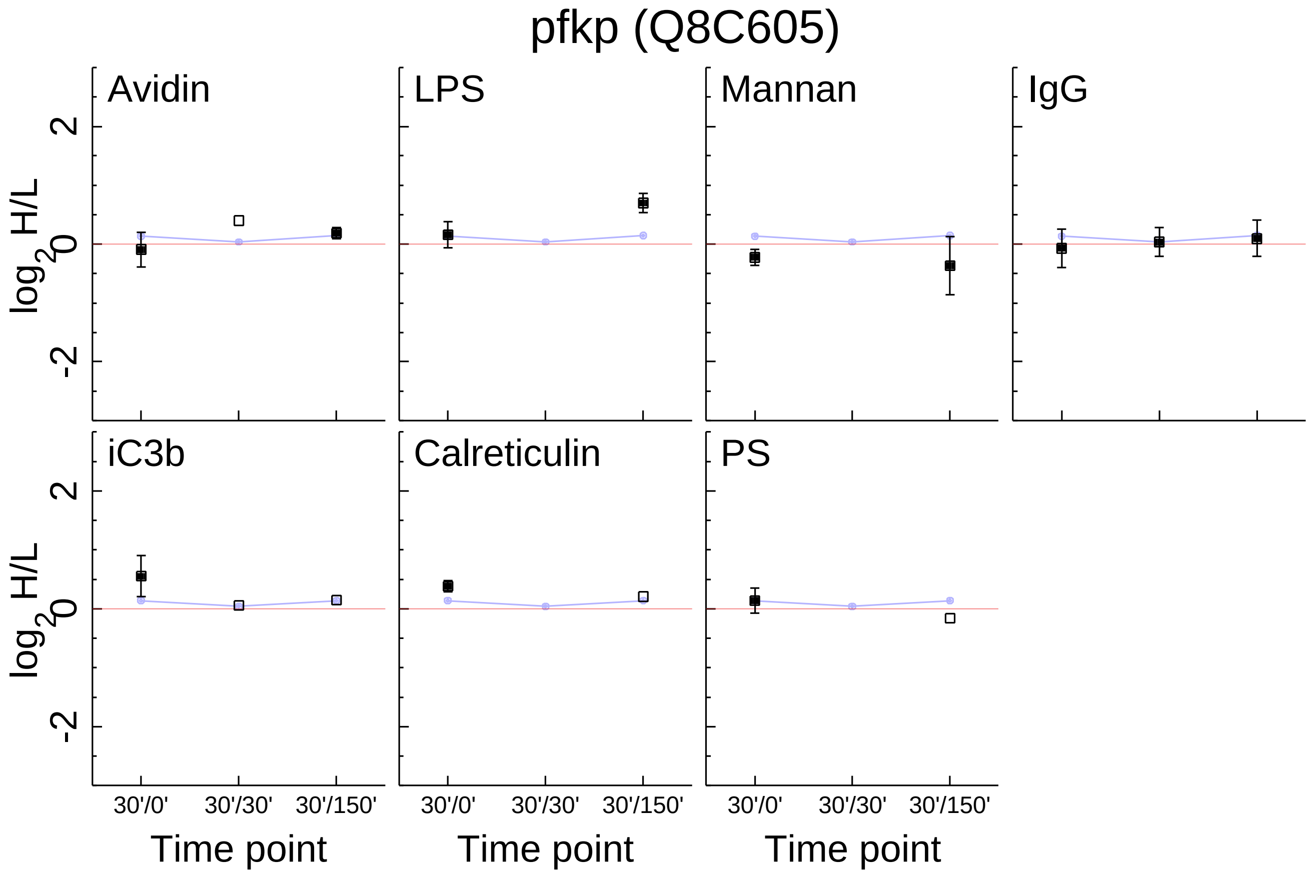

pfkp (Q8C605)
Avidin
LPS
Mannan
IgG
iC3b
Calreticulin
PS
log2 H/L
2
0
-2
30'/0'
30'/30'
30'/150'
Time point

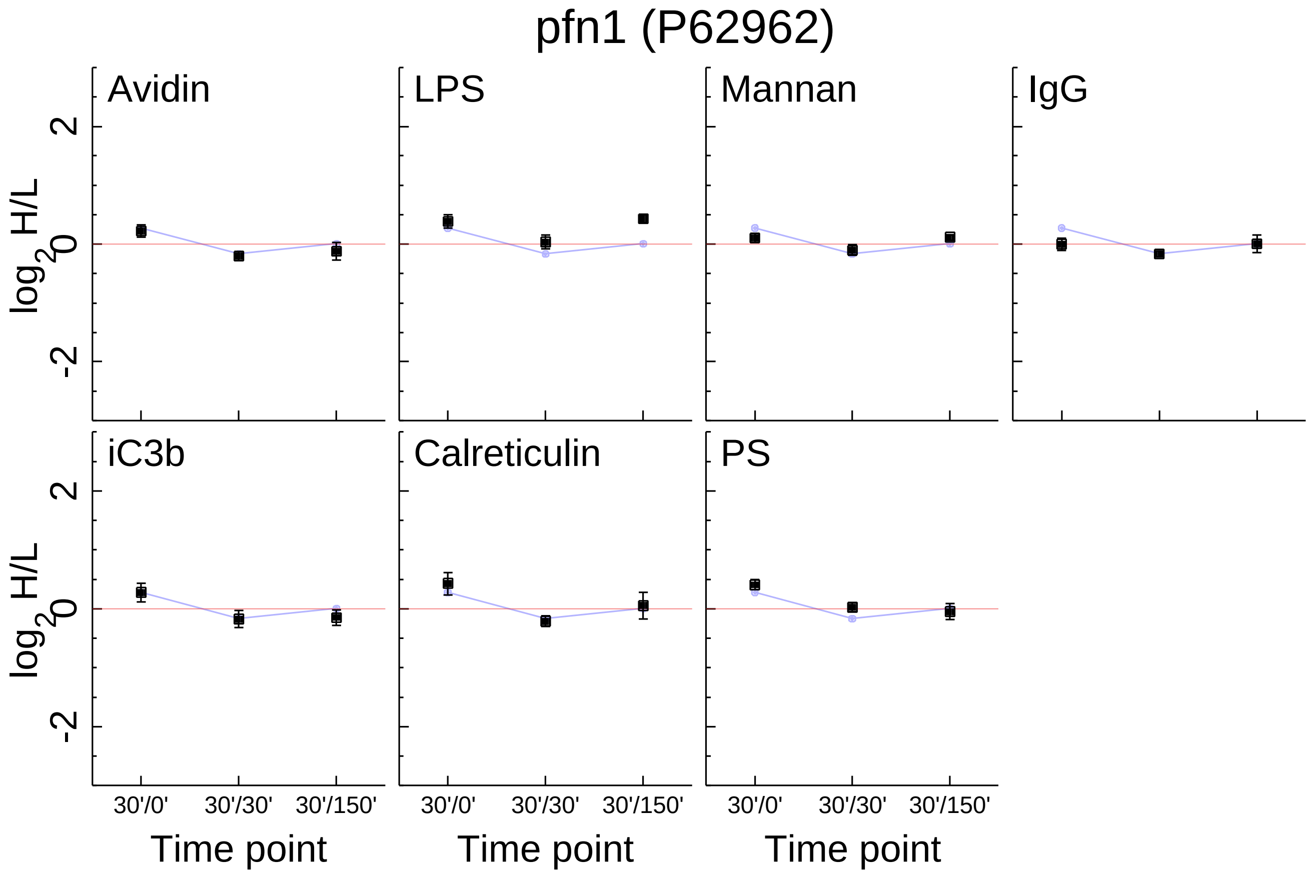

pfn1 (P62962)
Avidin
LPS
Mannan
IgG
iC3b
Calreticulin
PS
log2 H/L
2
0
-2
30'/0'
30'/30'
30'/150'
Time point

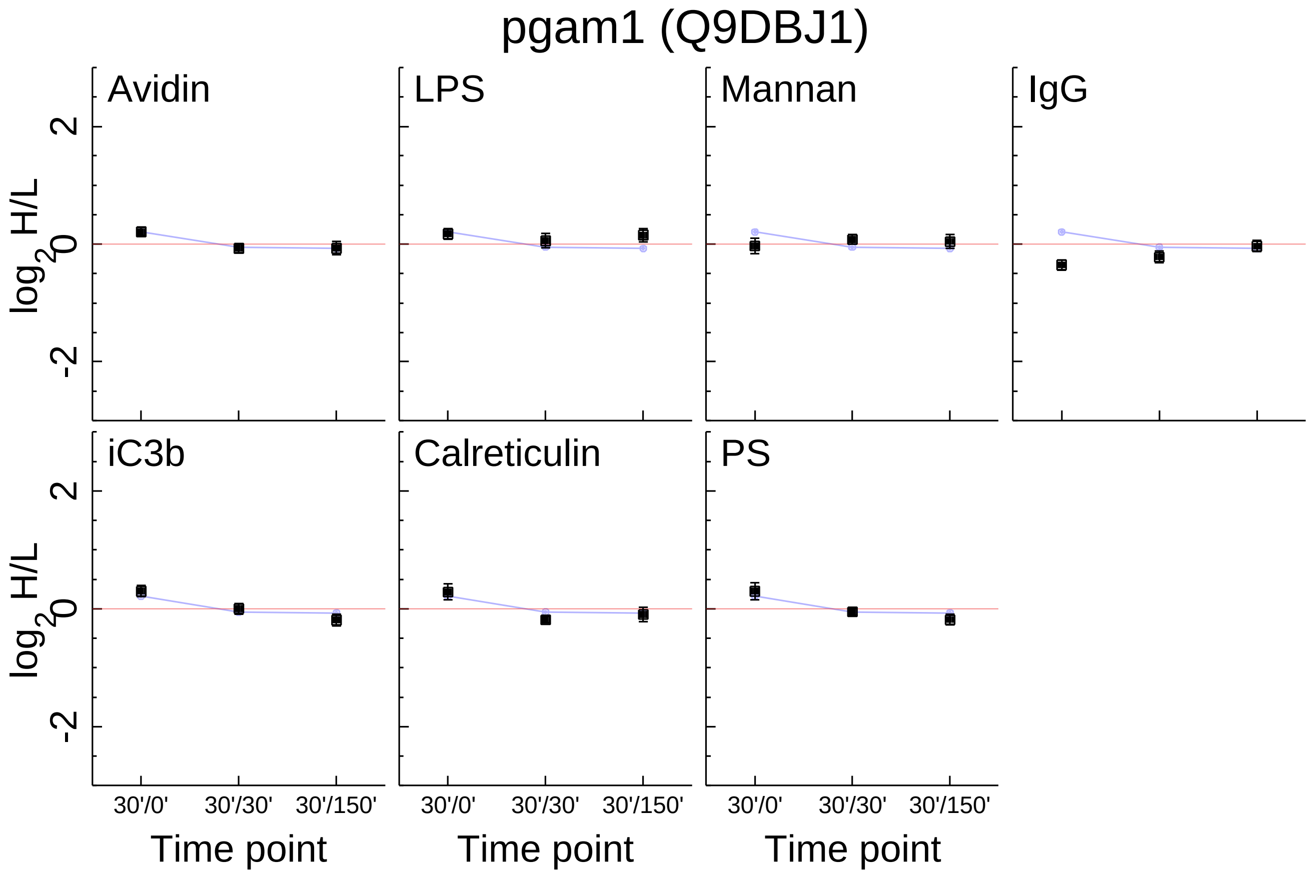
pgam1 (Q9DBJ1)
Avidin
LPS
Mannan
IgG
iC3b
Calreticulin
PS
log2 H/L
2
0
-2
30'/0'
30'/30'
30'/150'
Time point

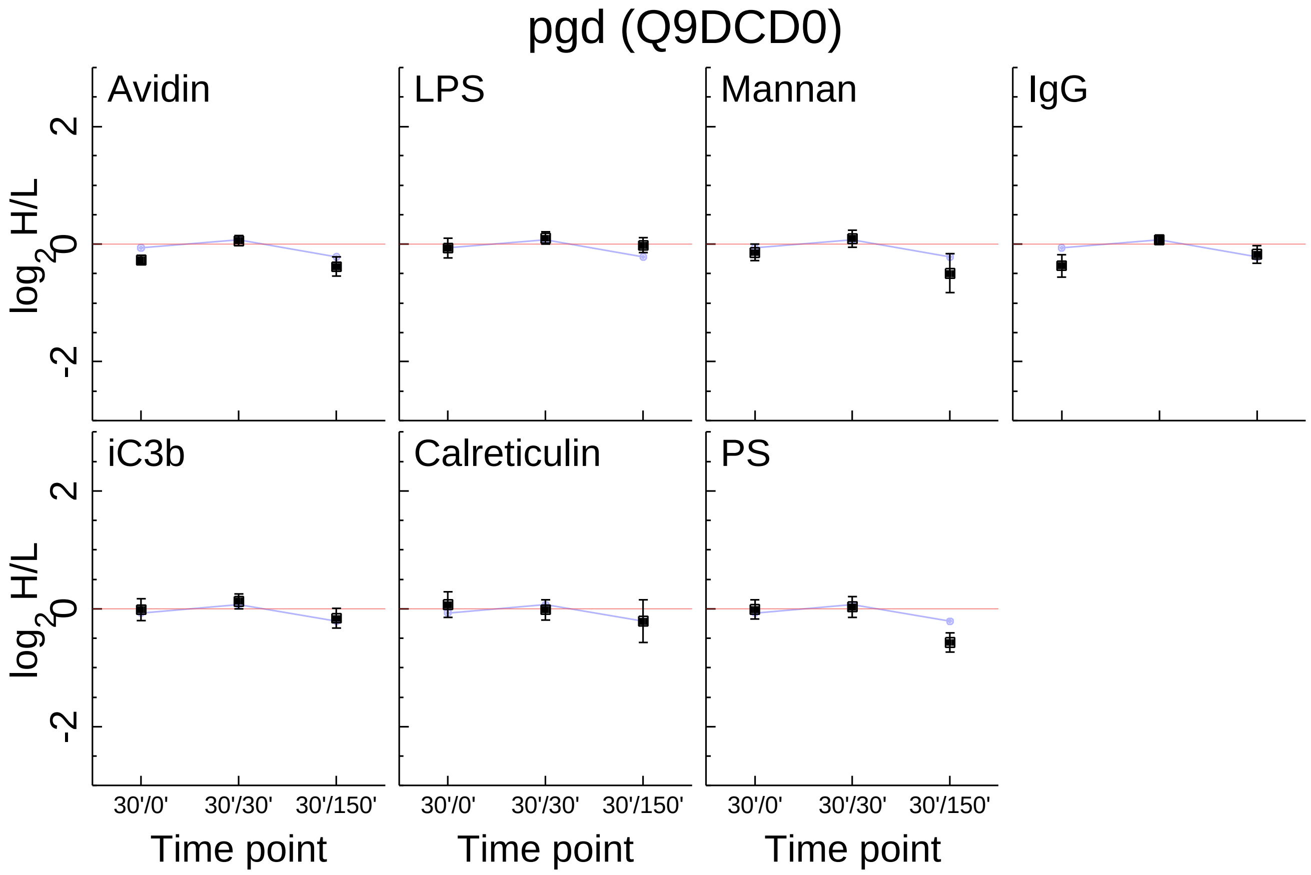
pgd (Q9DCD0)
Avidin
LPS
Mannan
IgG
iC3b
Calreticulin
PS
log2 H/L
2
0
-2
30'/0'
30'/30'
30'/150'
Time point

pgk1 (P09411)

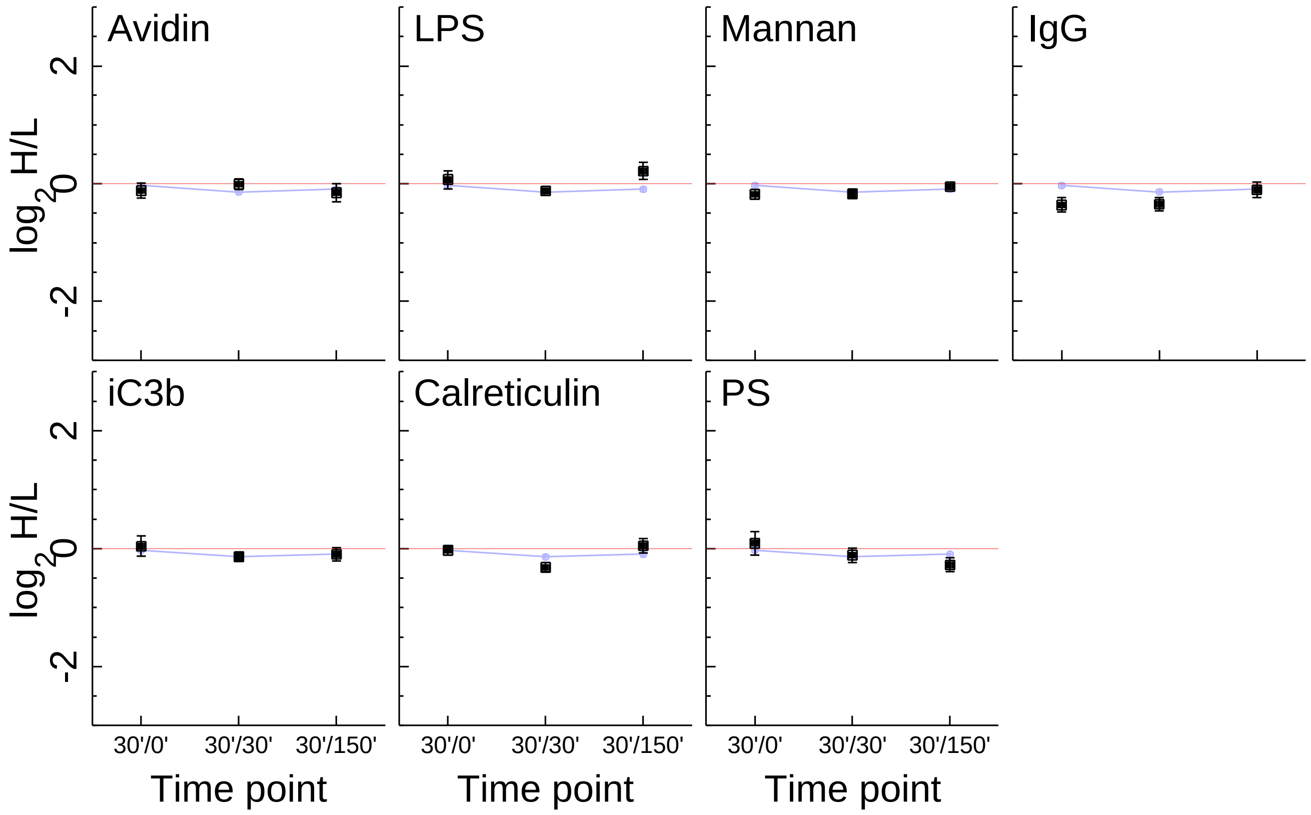

Avidin
LPS
Mannan
IgG
iC3b
Calreticulin
PS
log2 H/L
2
0
-2
30'/0'
30'/30'
30'/150'
Time point

pgrmc1 (O55022)

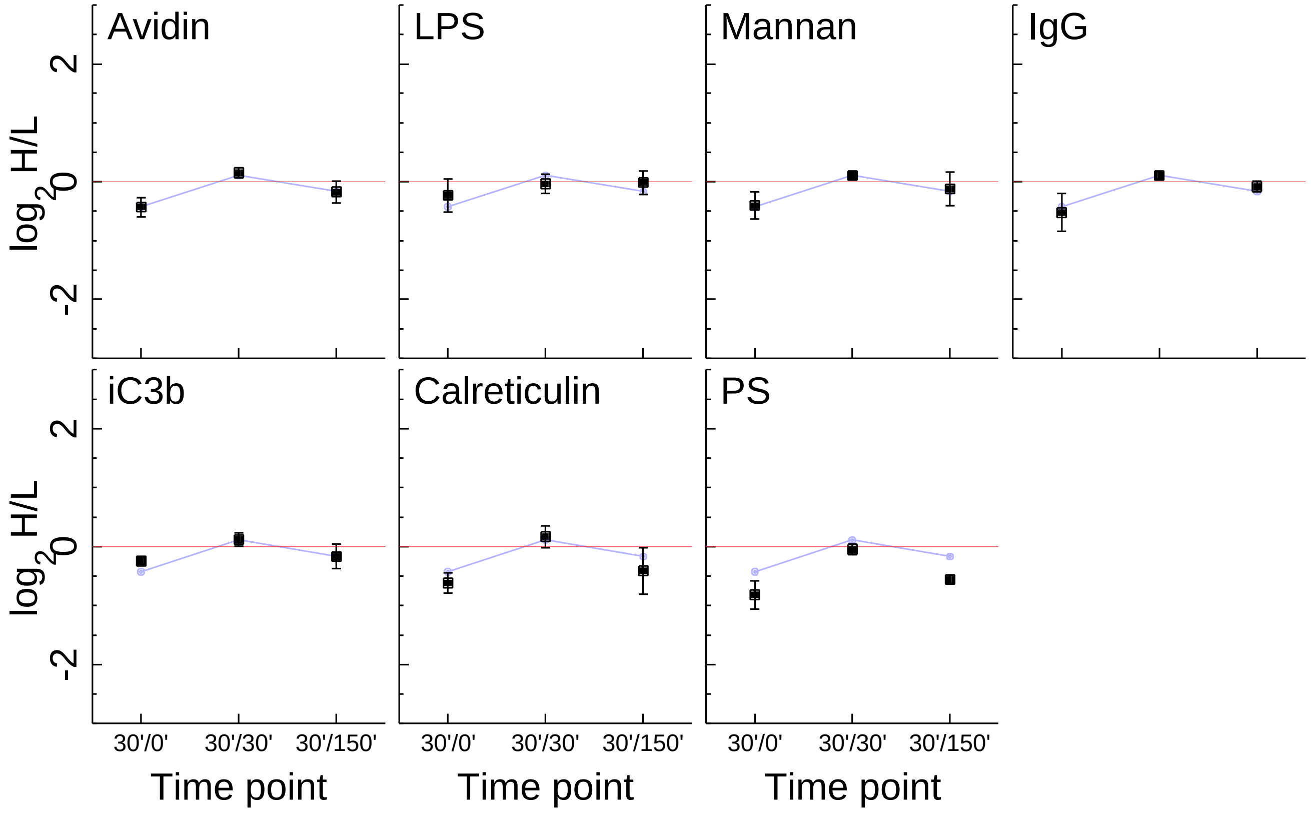

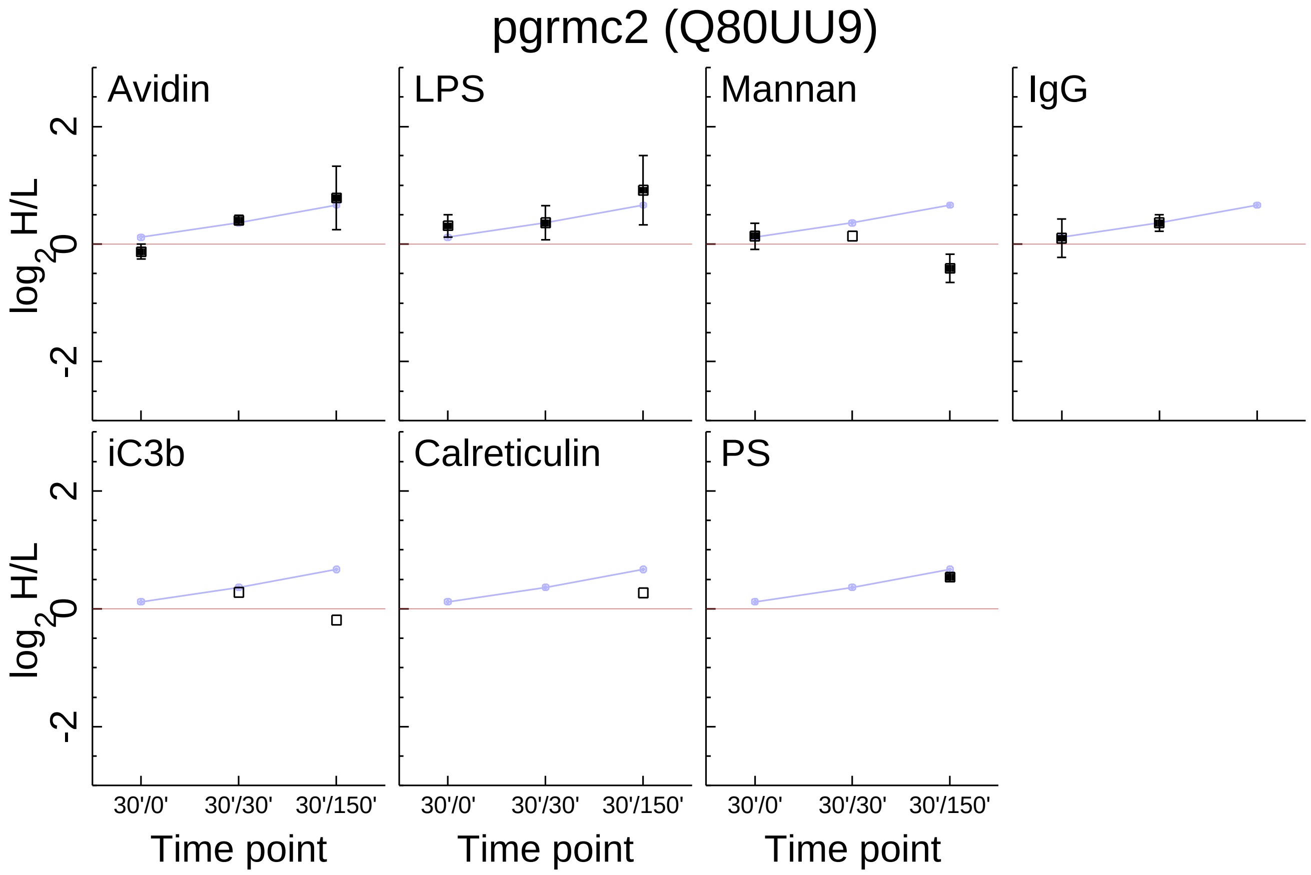
pgrmc2 (Q80UU9)
Avidin
LPS
Mannan
IgG
iC3b
Calreticulin
PS
log2 H/L
2
0
-2
30'/0'
30'/30'
30'/150'
Time point

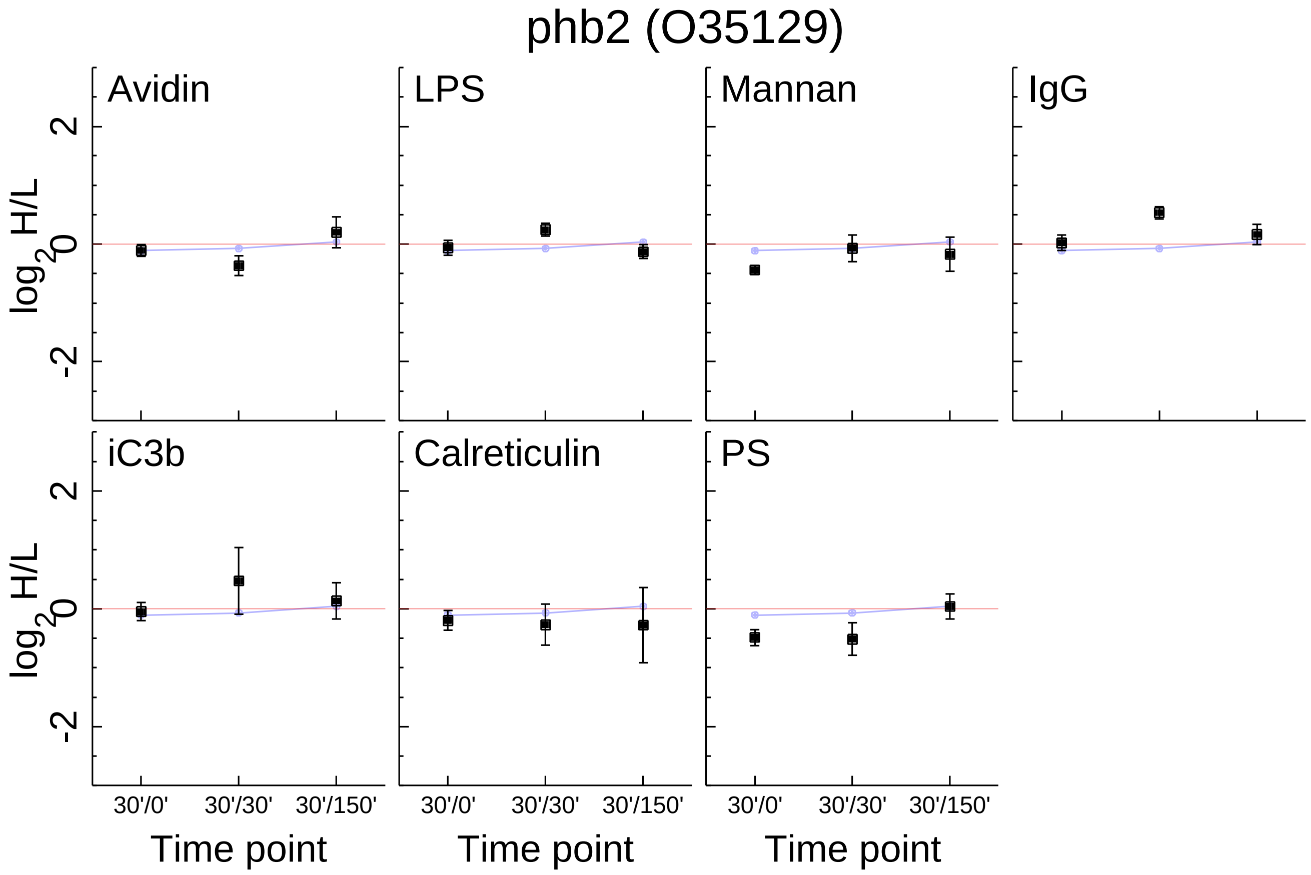

phb2 (O35129)
Avidin
LPS
Mannan
IgG
iC3b
Calreticulin
PS
log2 H/L
2
0
-2
30'/0'
30'/30'
30'/150'
Time point

phb (P67778)

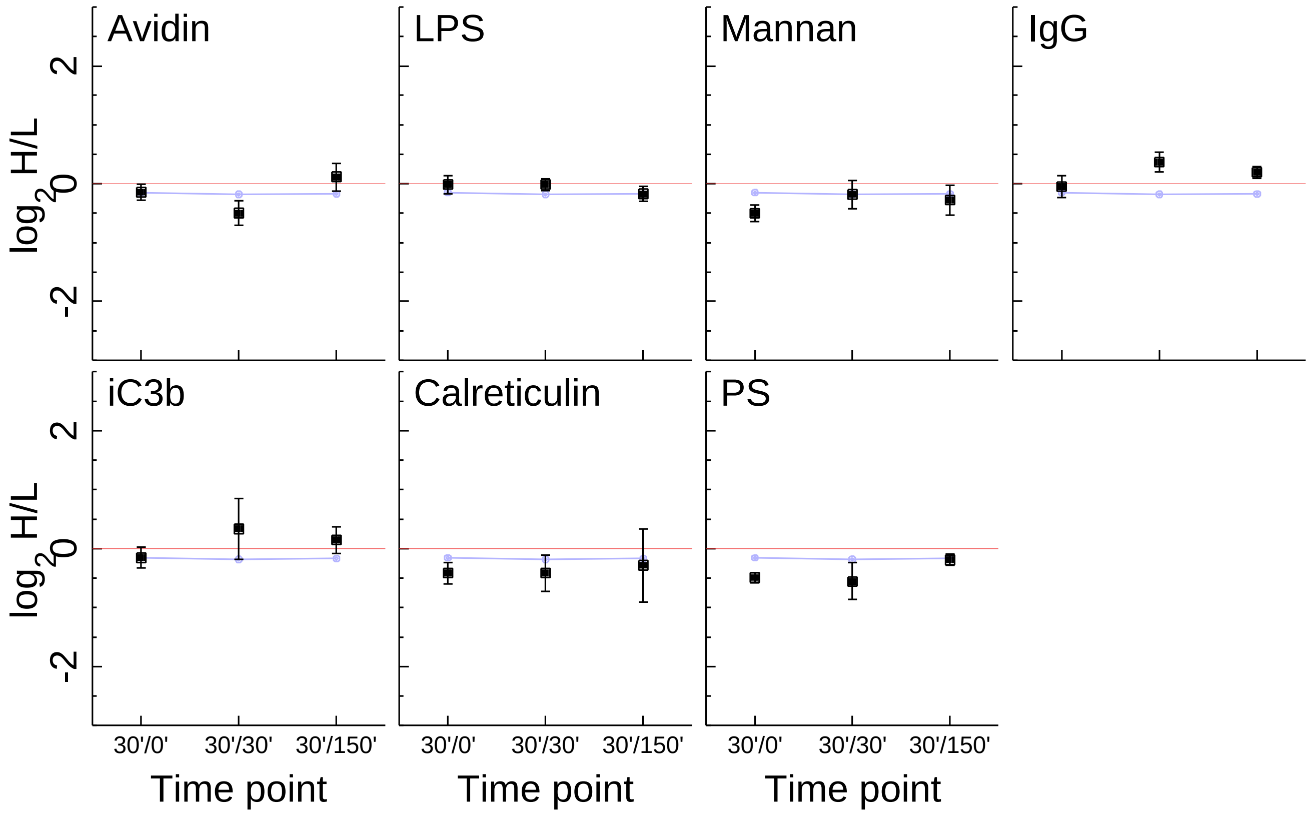

Avidin
LPS
Mannan
IgG
iC3b
Calreticulin
PS
log2 H/L
2
0
-2
30'/0'
30'/30'
30'/150'
Time point

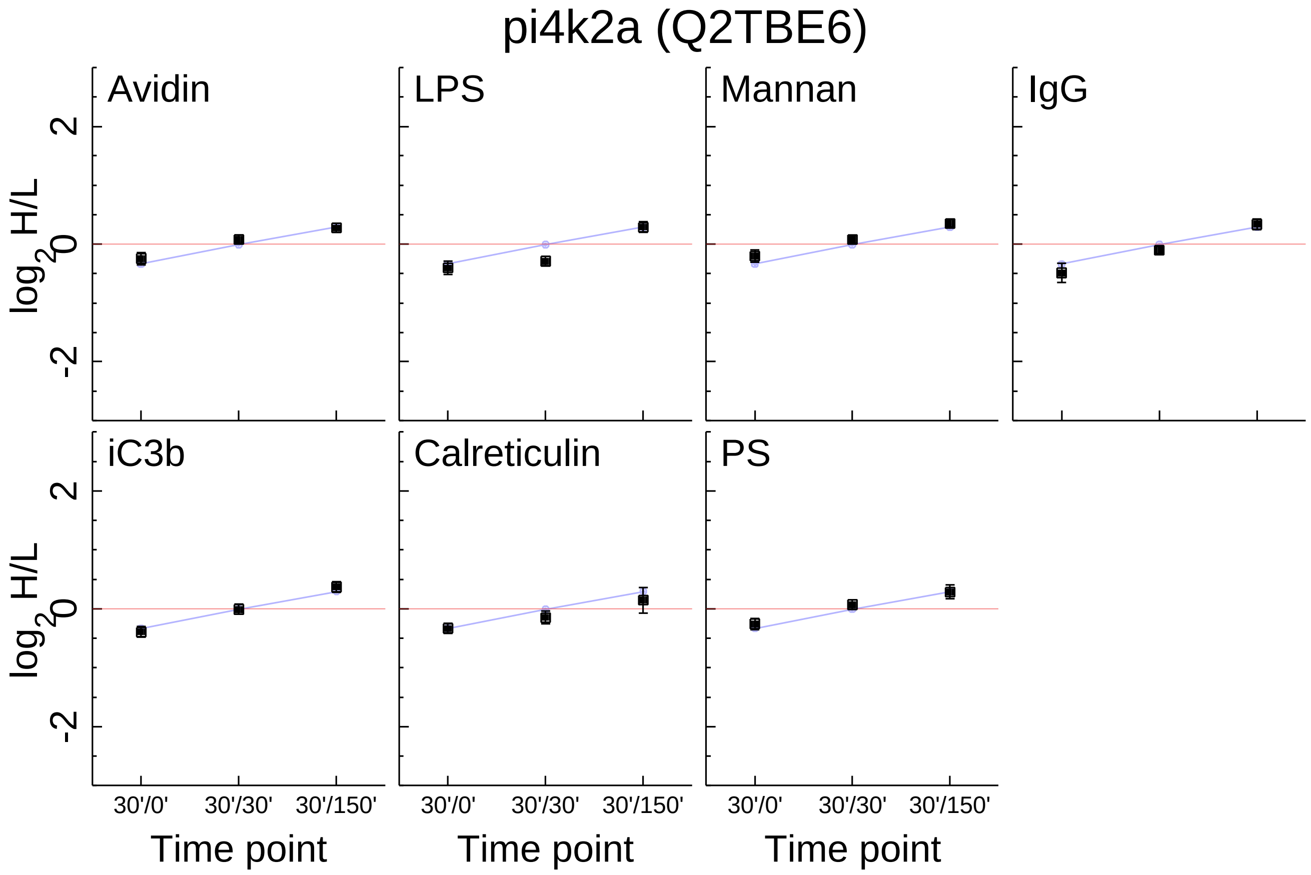
pi4k2a (Q2TBE6)
Avidin
LPS
Mannan
IgG
iC3b
Calreticulin
PS
log2 H/L
2
0
-2
30'/0'
30'/30'
30'/150'
Time point

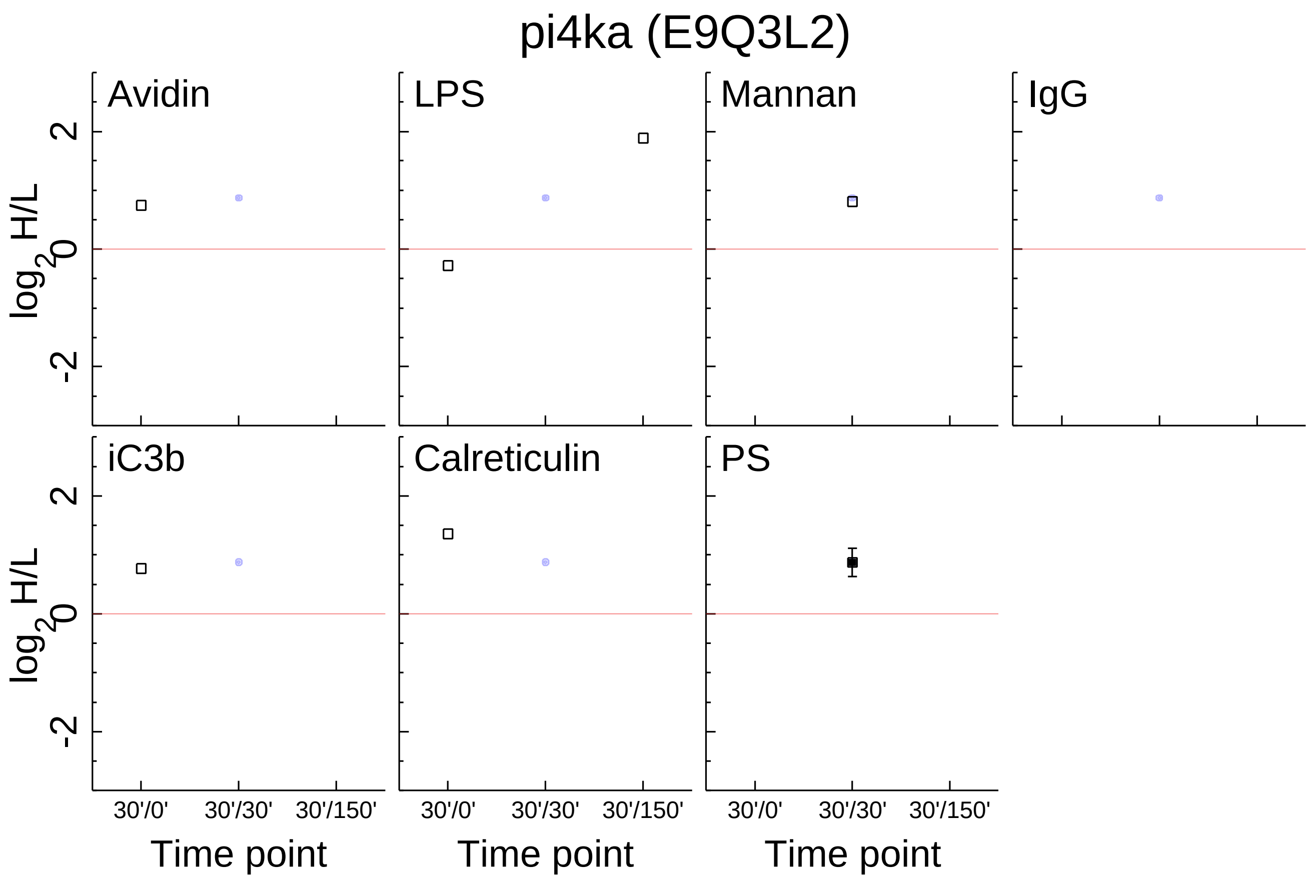
pi4ka (E9Q3L2)
Avidin
LPS
Mannan
IgG
iC3b
Calreticulin
PS
log2 H/L
2
0
-2
30'/0'
30'/30'
30'/150'
Time point

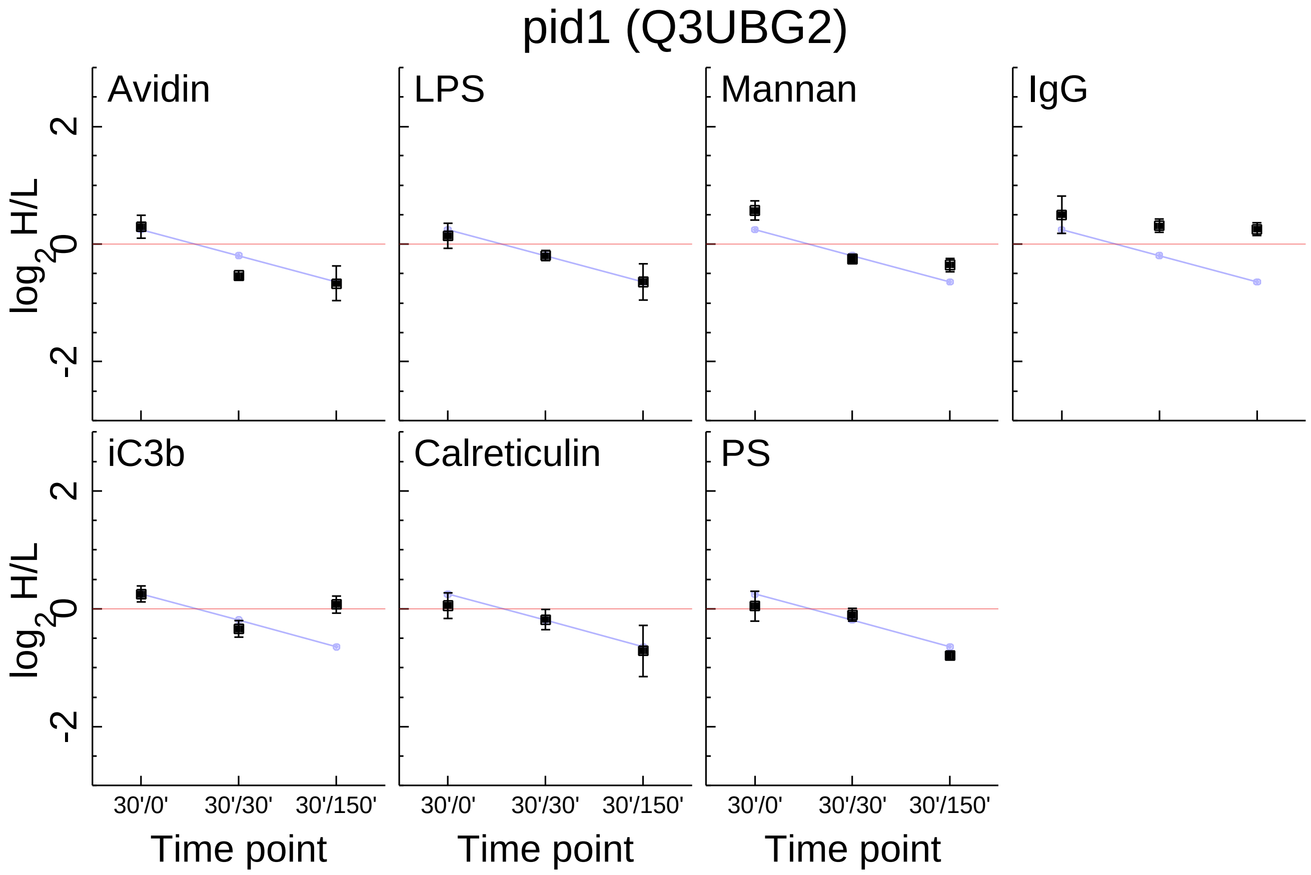
pid1 (Q3UBG2)
Avidin
LPS
Mannan
IgG
iC3b
Calreticulin
PS
log_2 H/L
2
0
-2
30'/0'
30'/30'
30'/150'
Time point

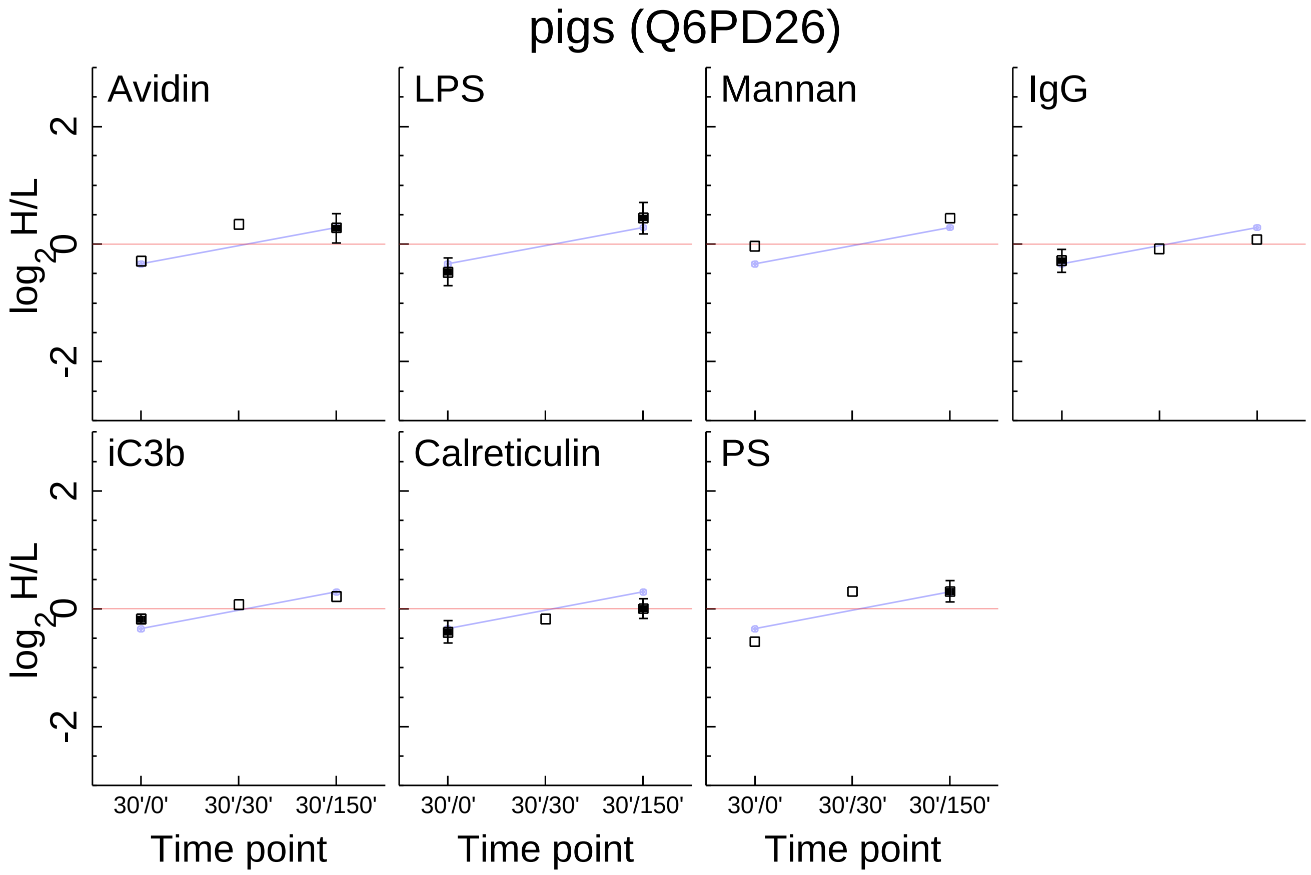
pigs (Q6PD26)
Avidin
LPS
Mannan
IgG
iC3b
Calreticulin
PS
log2 H/L
2
0
-2
30'/0'
30'/30'
30'/150'
Time point

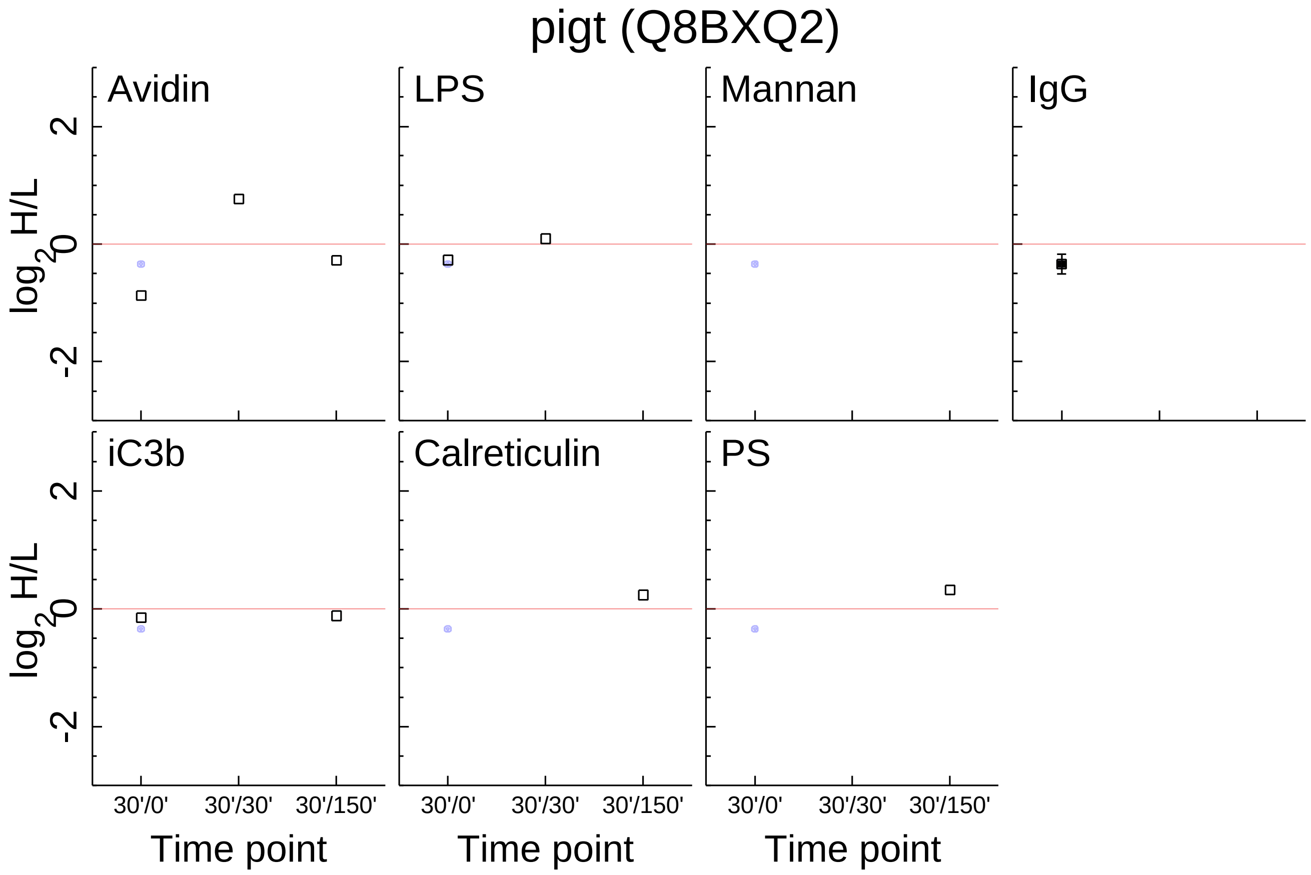

pigt (Q8BXQ2)
Avidin
LPS
Mannan
IgG
iC3b
Calreticulin
PS
log2 H/L
2
0
-2
30'/0'
30'/30'
30'/150'
Time point

pik3ap1 (Q9EQ32)

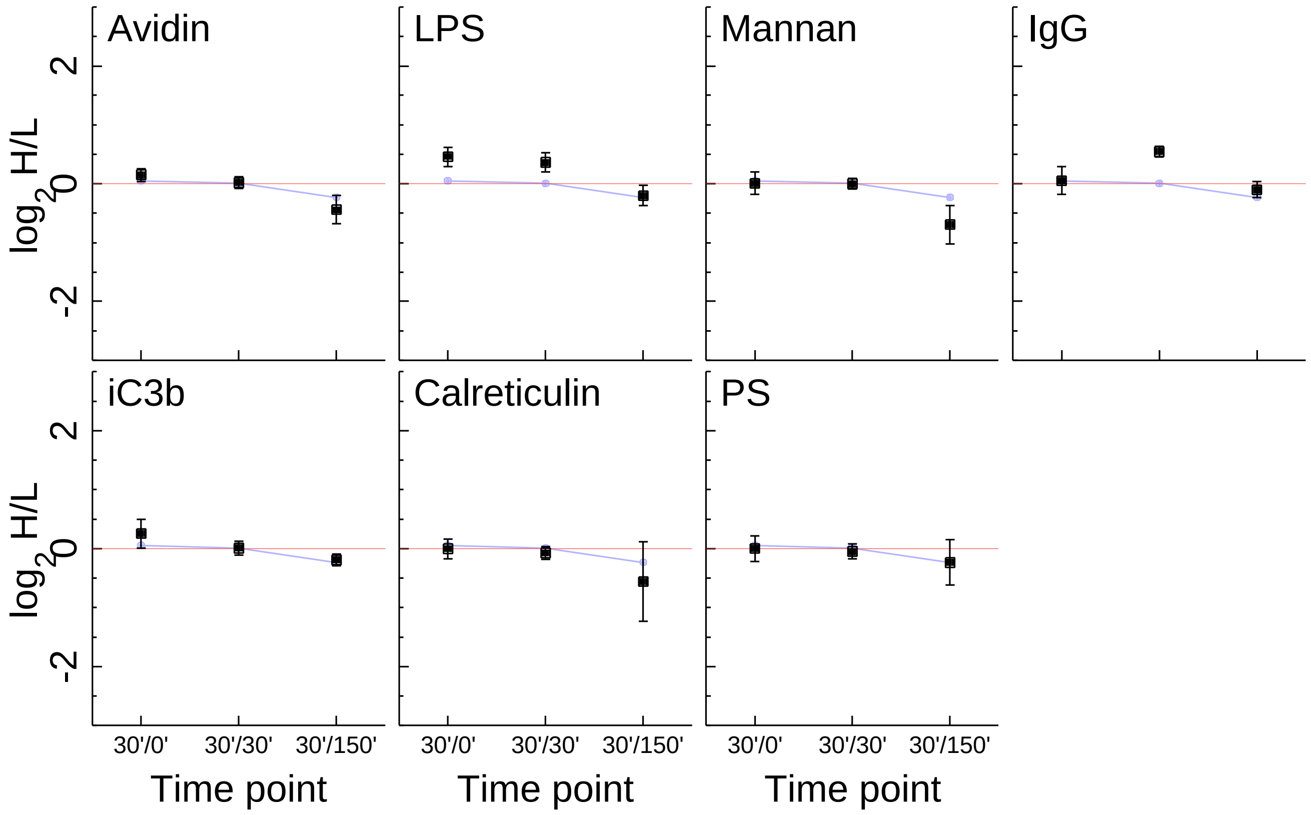

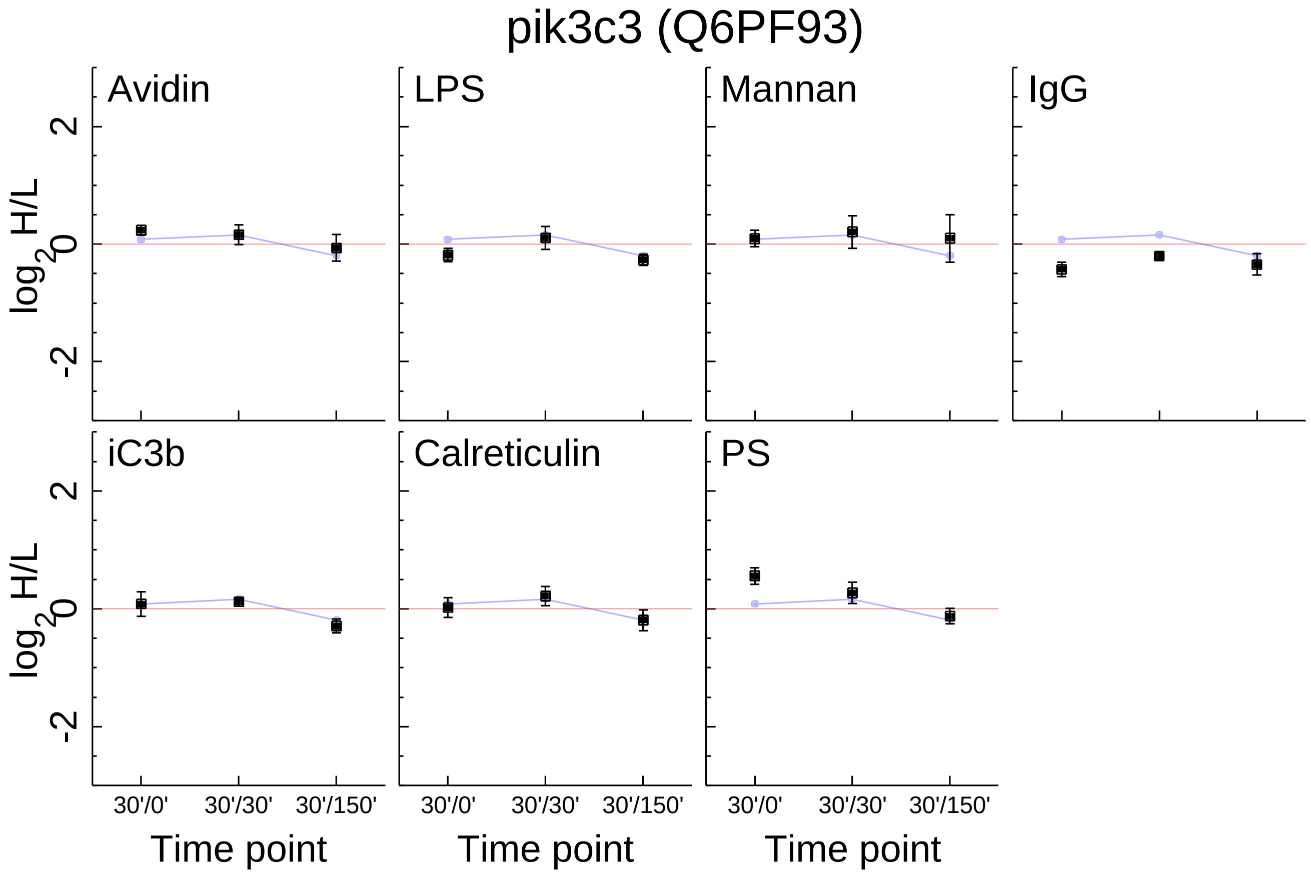

pik3c3 (Q6PF93)
Avidin
LPS
Mannan
IgG
iC3b
Calreticulin
PS
log2 H/L
2
0
-2
30'/0'
30'/30'
30'/150'
Time point

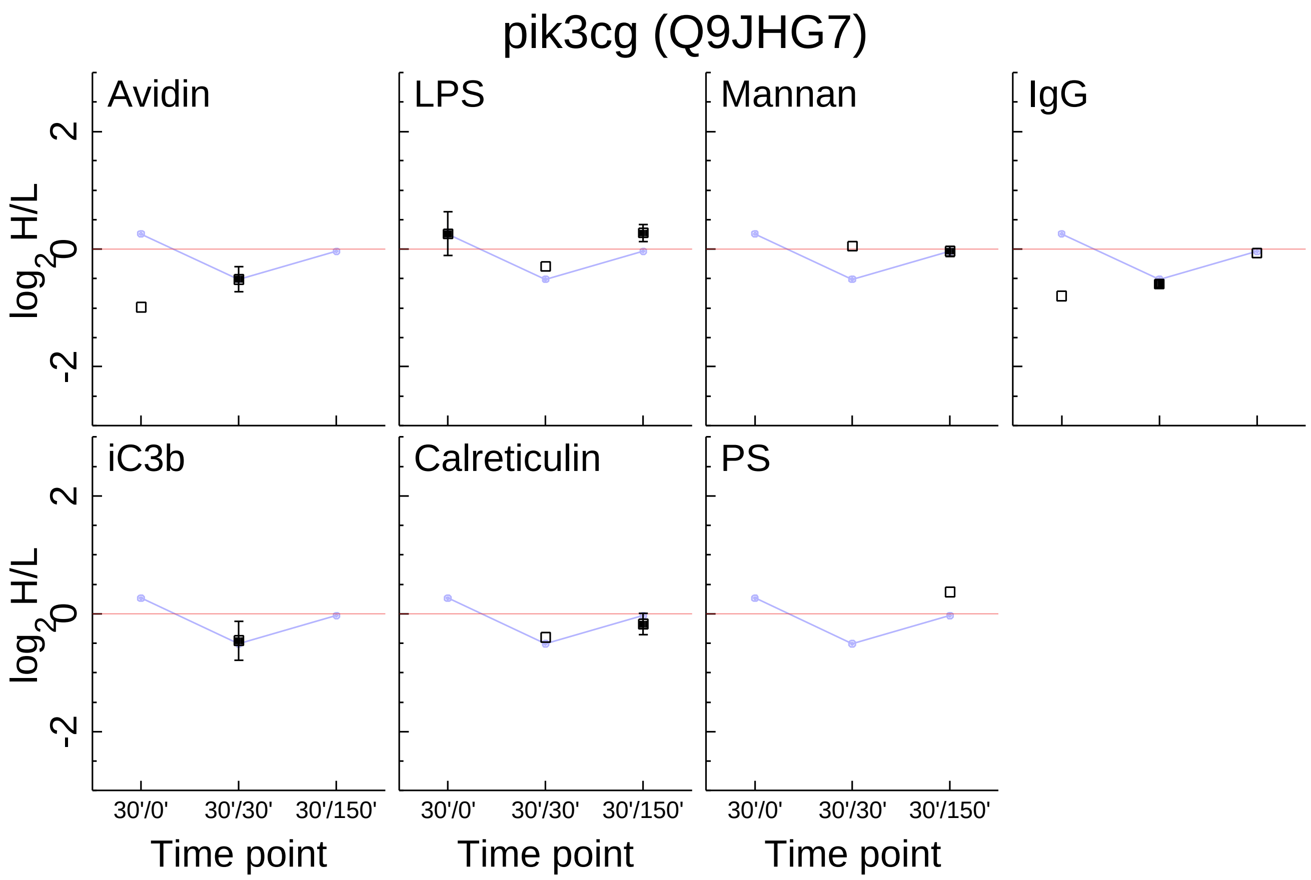

pik3cg (Q9JHG7)
Avidin
LPS
Mannan
IgG
iC3b
Calreticulin
PS
log2 H/L
2
0
-2
30'/0'
30'/30'
30'/150'
Time point

pik3r4 (Q8VD65)

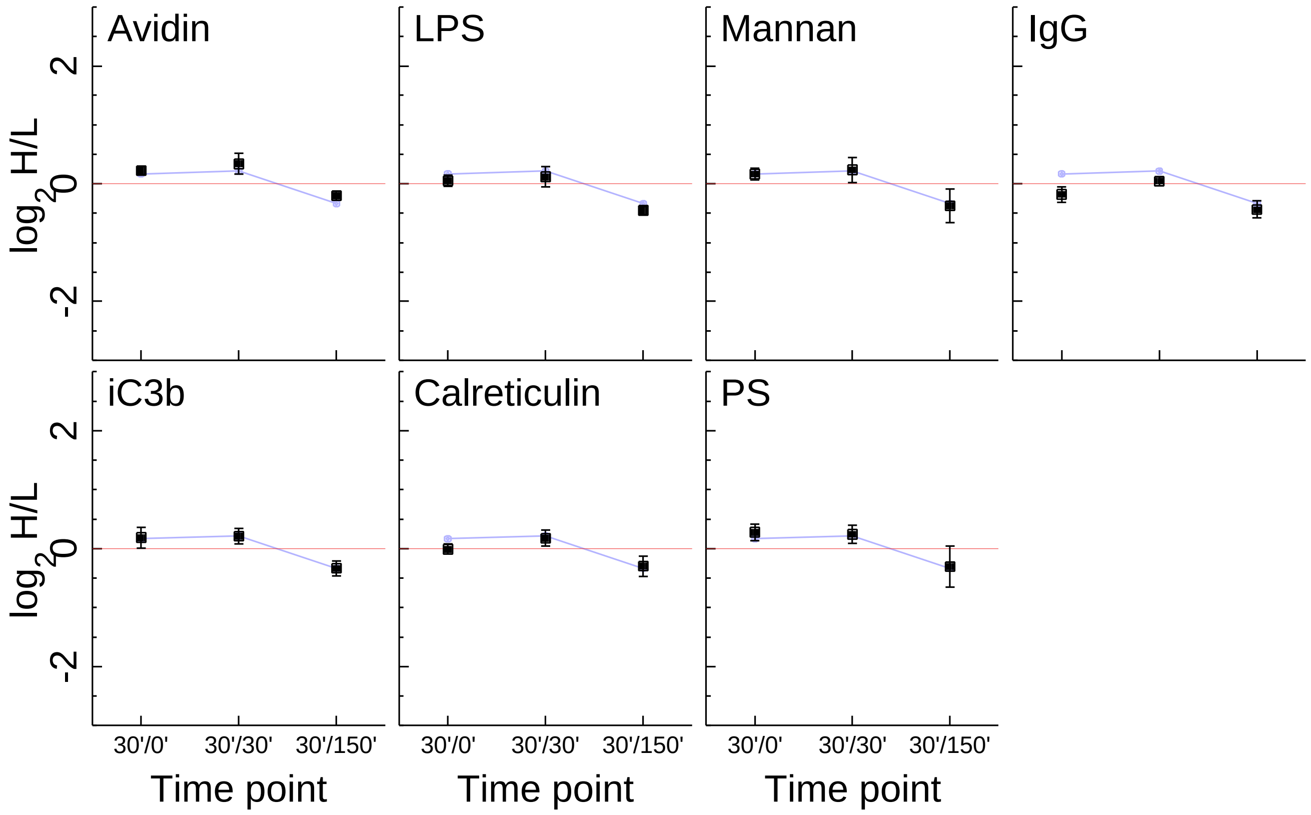

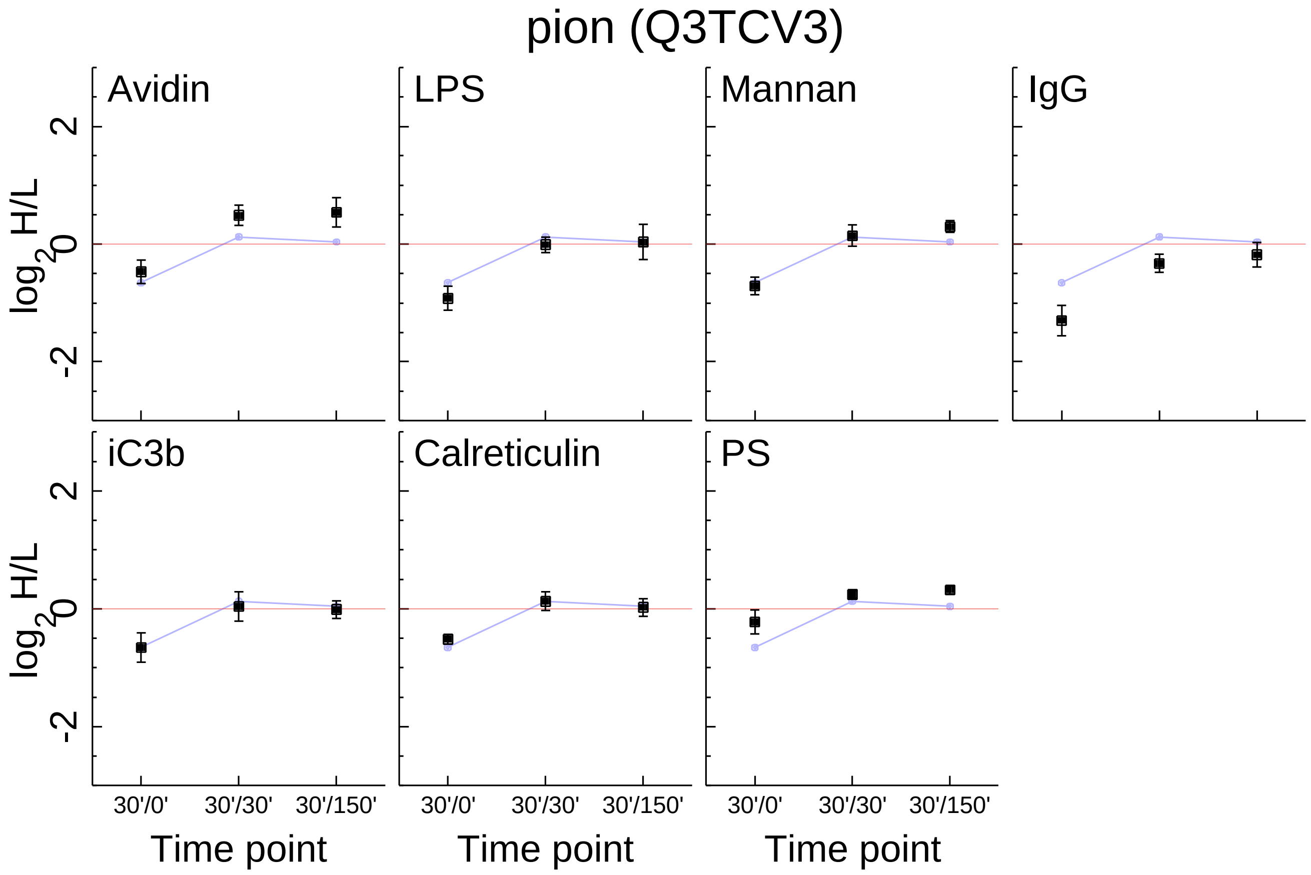

pion (Q3TCV3)
Avidin
LPS
Mannan
IgG
iC3b
Calreticulin
PS
log2 H/L
2
0
-2
30'/0'
30'/30'
30'/150'
Time point

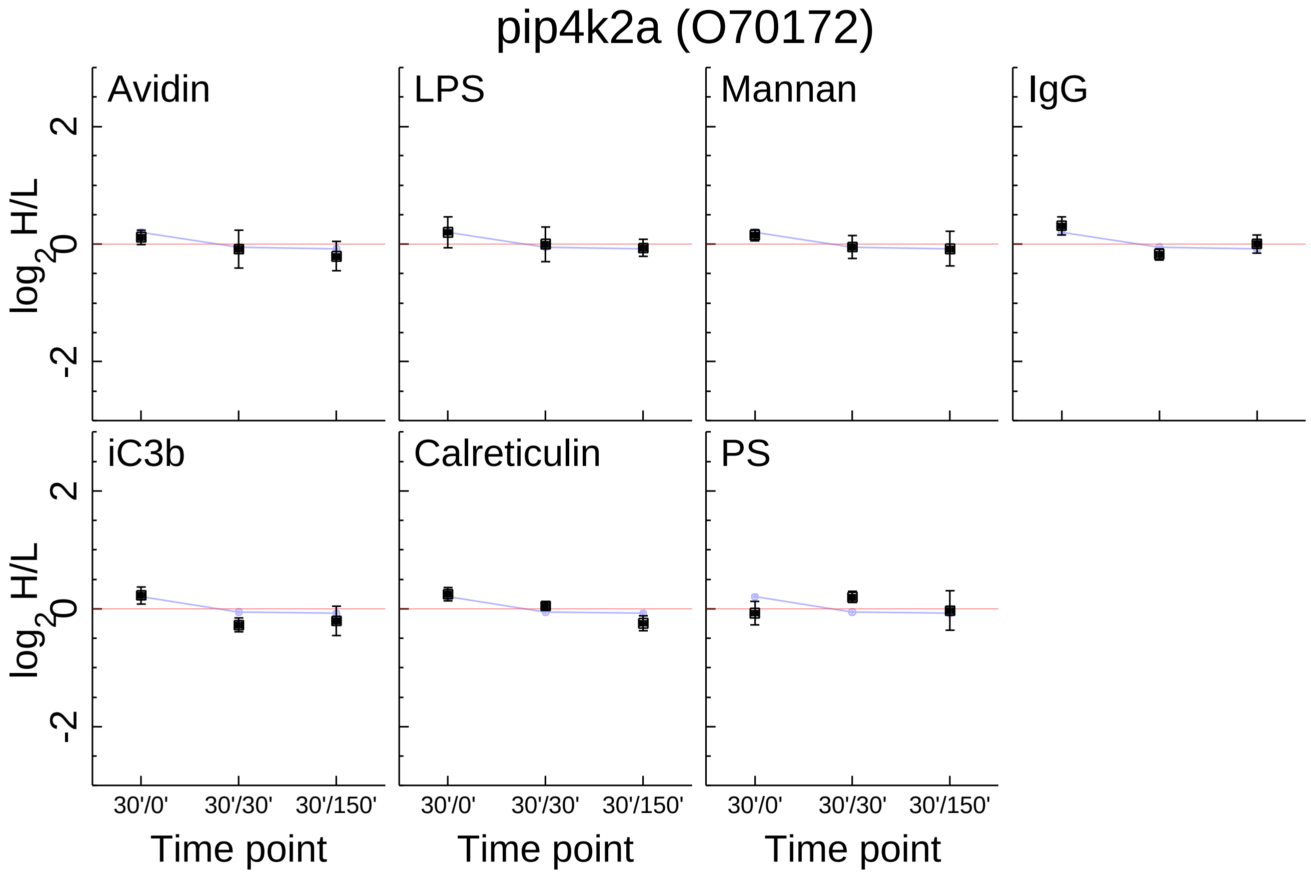
pip4k2a (O70172)
Avidin
LPS
Mannan
IgG
iC3b
Calreticulin
PS
log2 H/L
2
0
-2
30'/0'
30'/30'
30'/150'
Time point

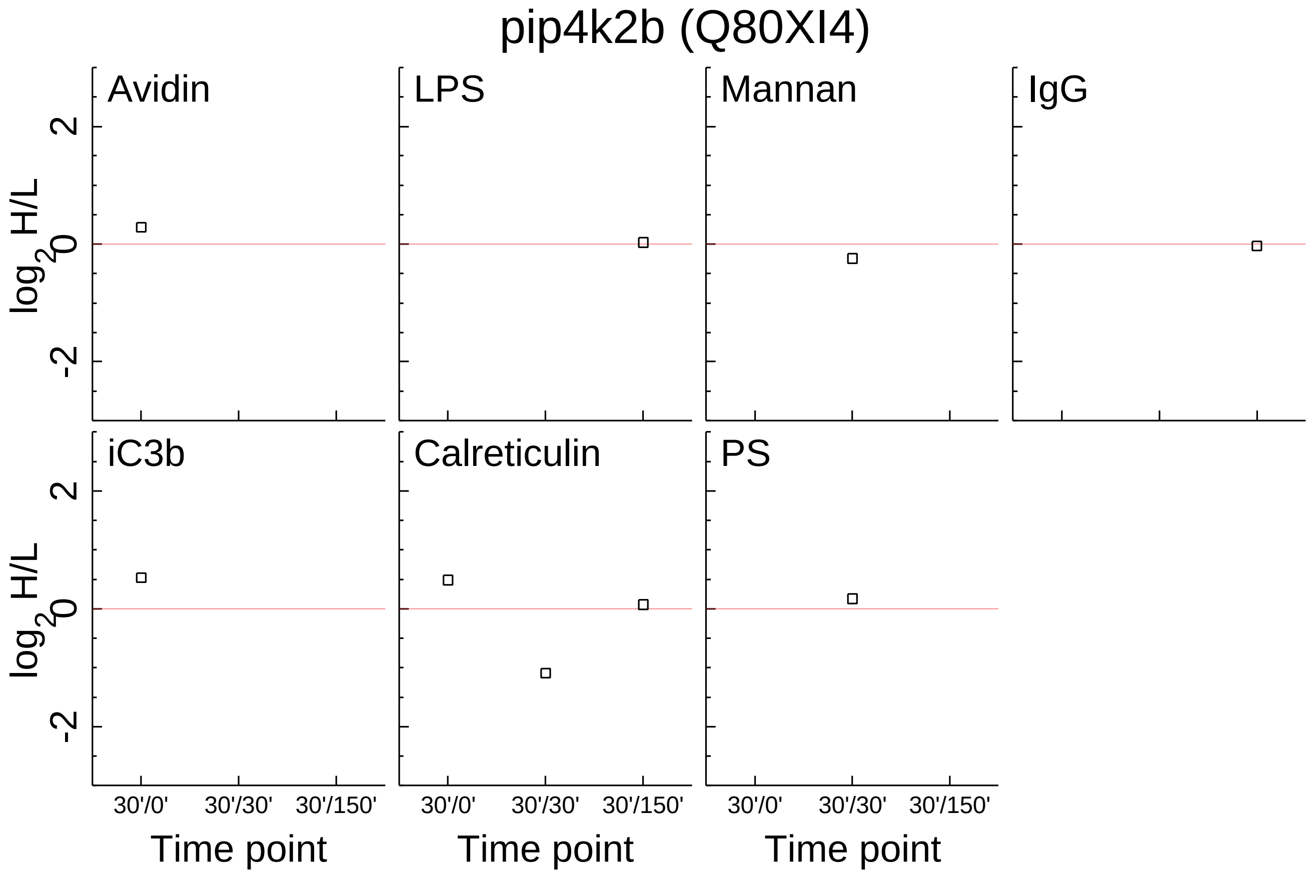
pip4k2b (Q80XI4)
Avidin
LPS
Mannan
IgG
iC3b
Calreticulin
PS
log2 H/L
2
0
-2
30'/0'
30'/30'
30'/150'
Time point

pitpna (P53810)

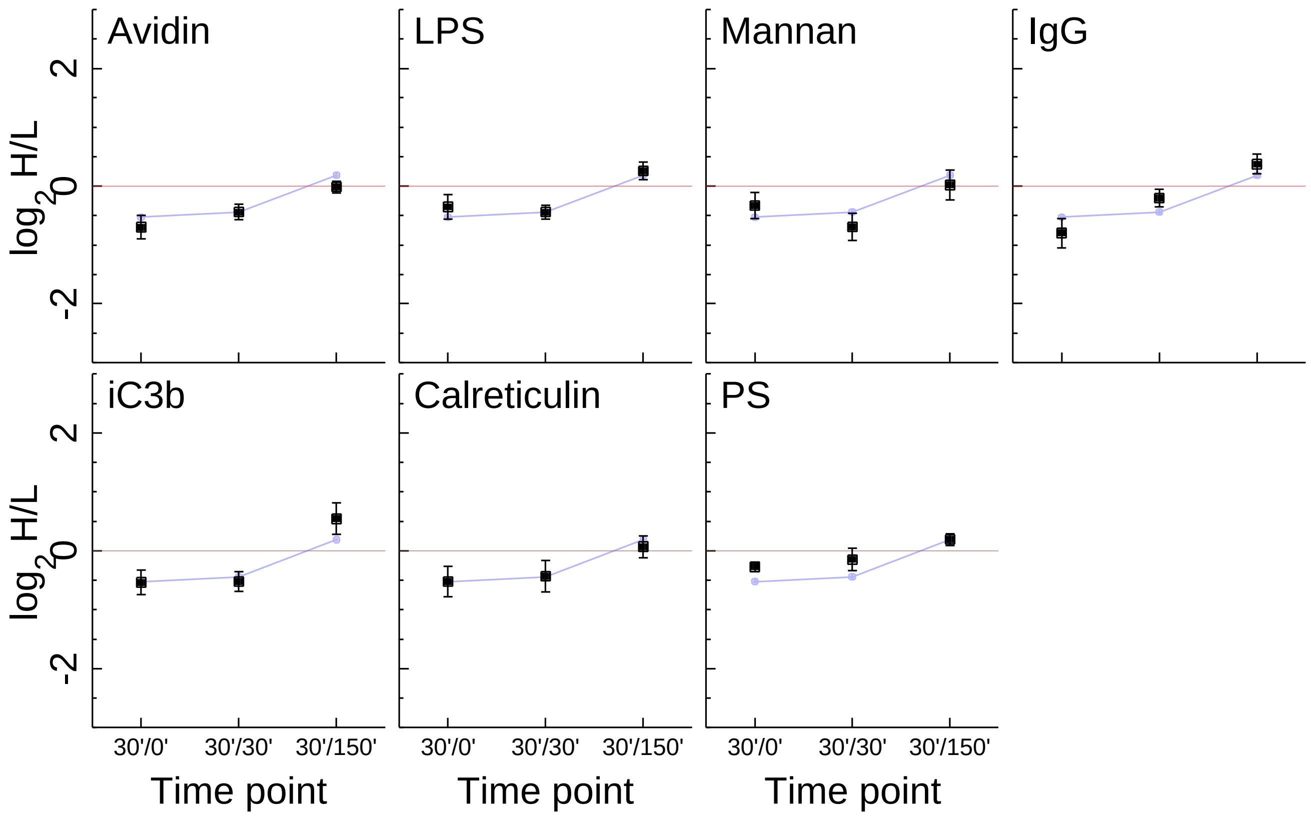

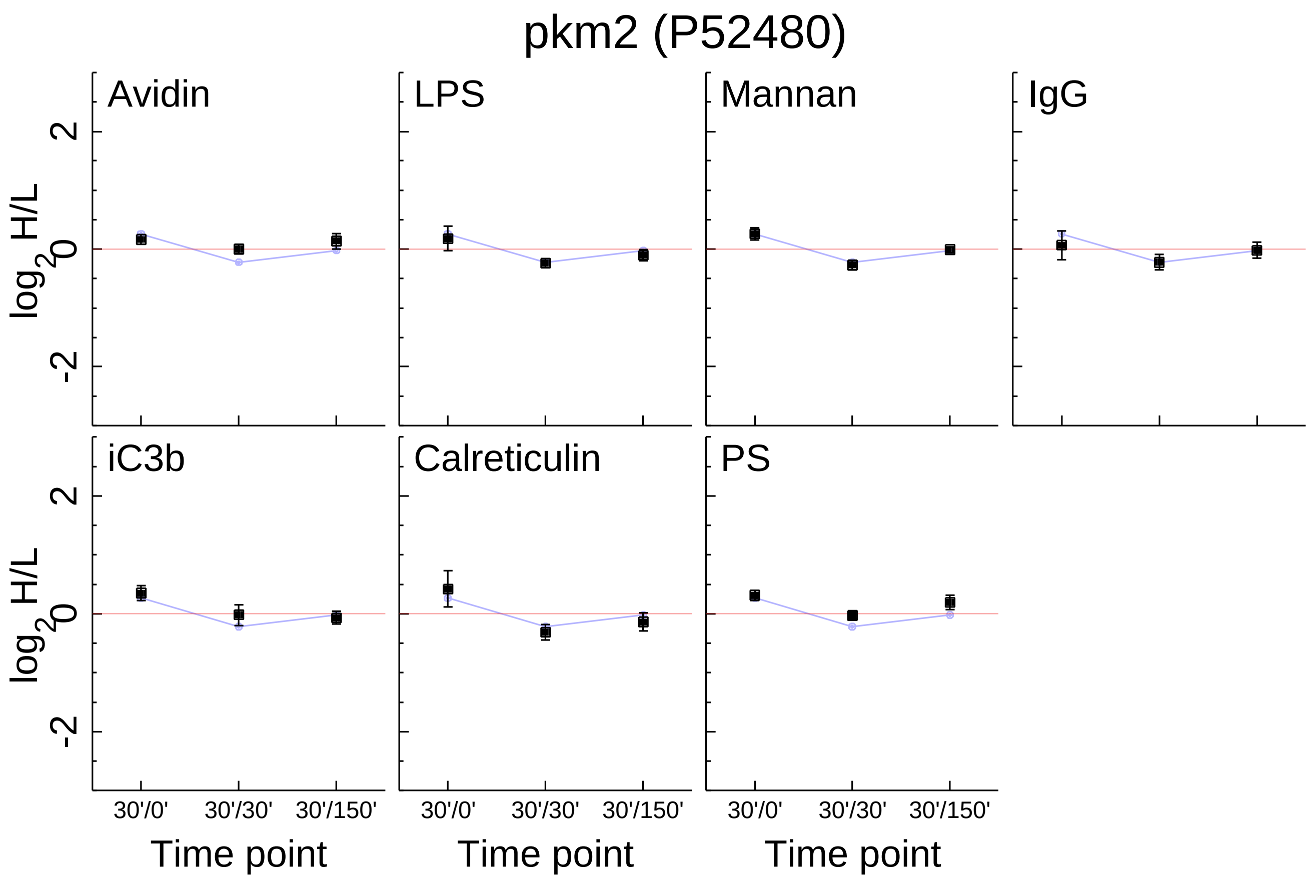

pkm2 (P52480)
Avidin
LPS
Mannan
IgG
iC3b
Calreticulin
PS
log2 H/L
2
0
-2
30'/0'
30'/30'
30'/150'
Time point

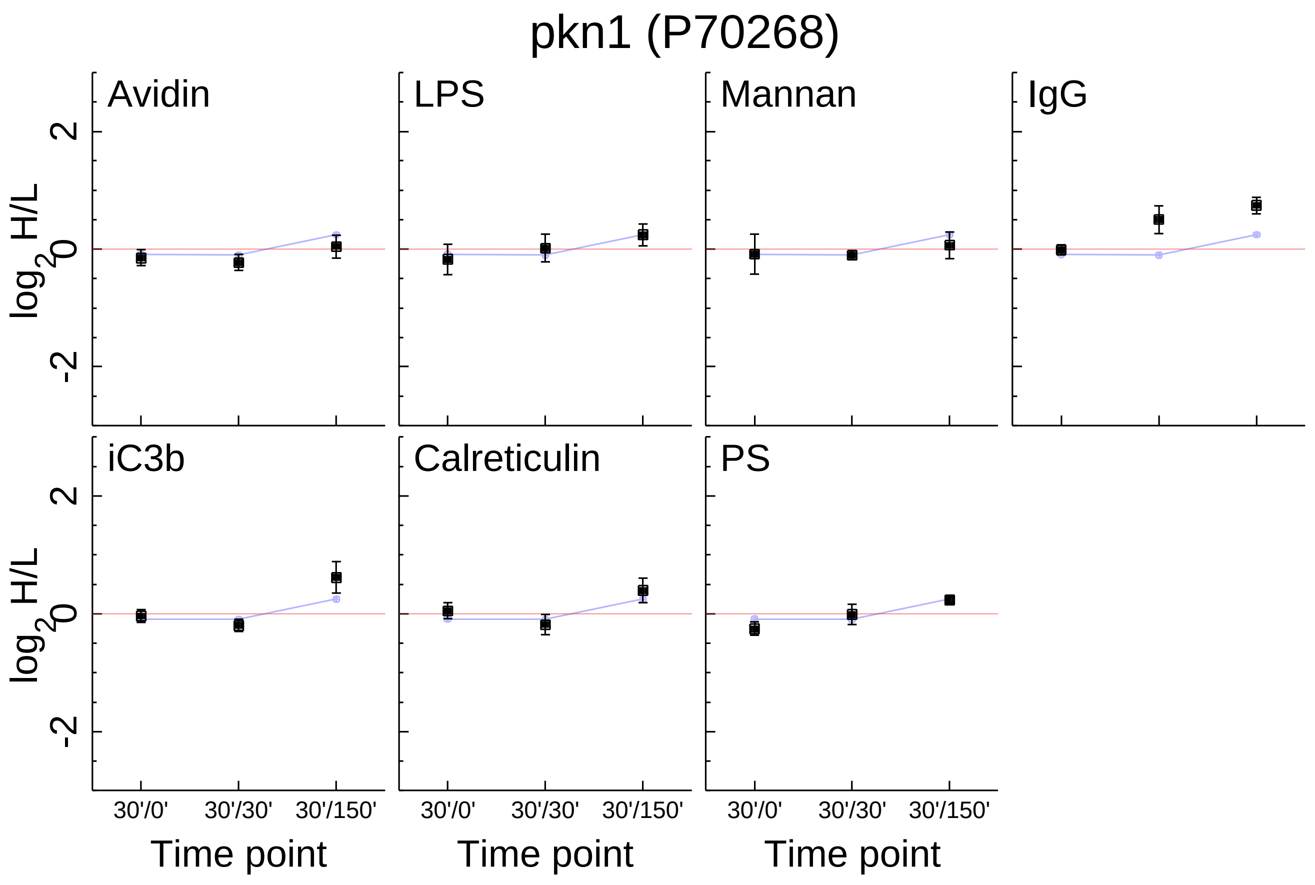

pkn1 (P70268)
Avidin
LPS
Mannan
IgG
iC3b
Calreticulin
PS
log2 H/L
2
0
-2
30'/0'
30'/30'
30'/150'
Time point

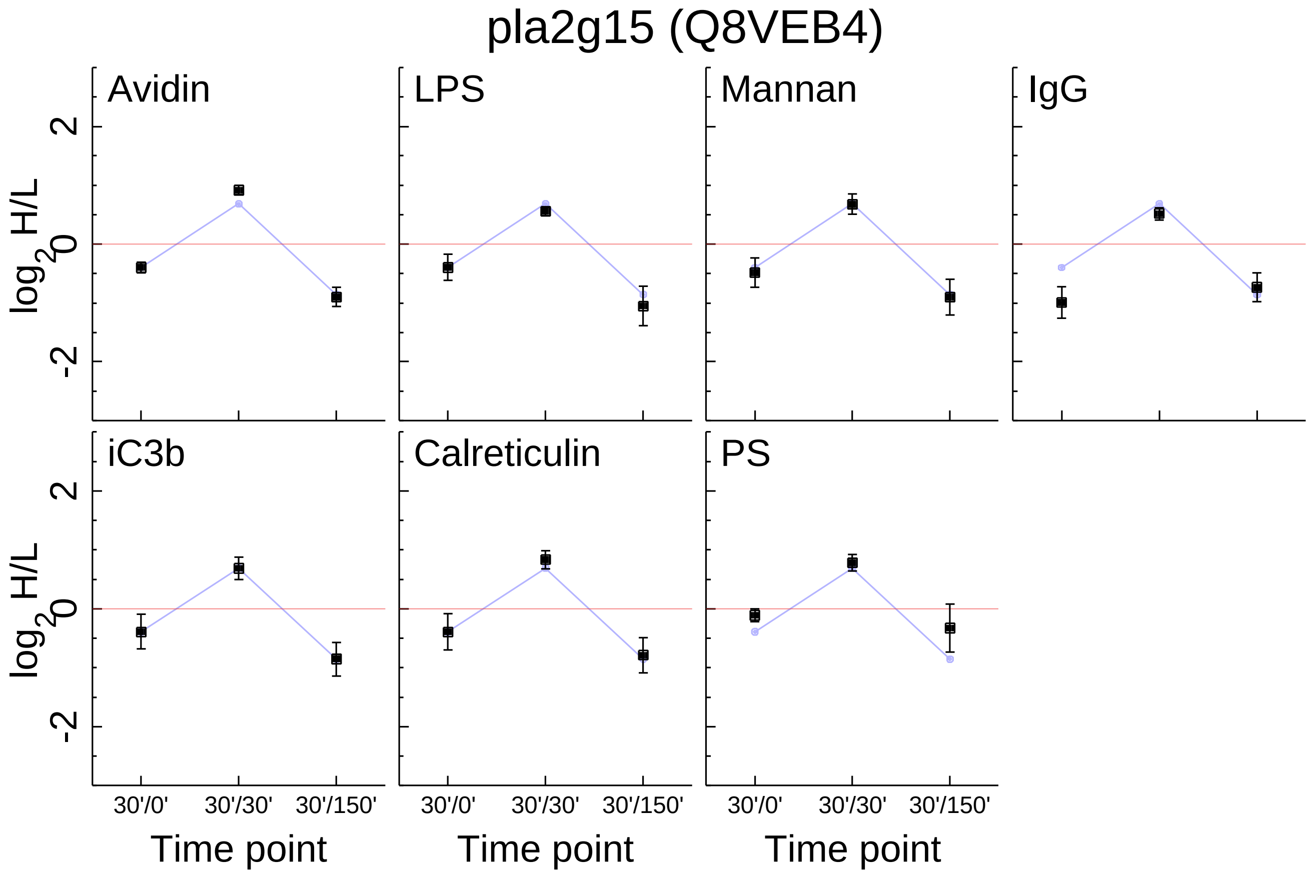
pla2g15 (Q8VEB4)
Avidin
LPS
Mannan
IgG
iC3b
Calreticulin
PS
log2 H/L
2
0
-2
30'/0'
30'/30'
30'/150'
Time point

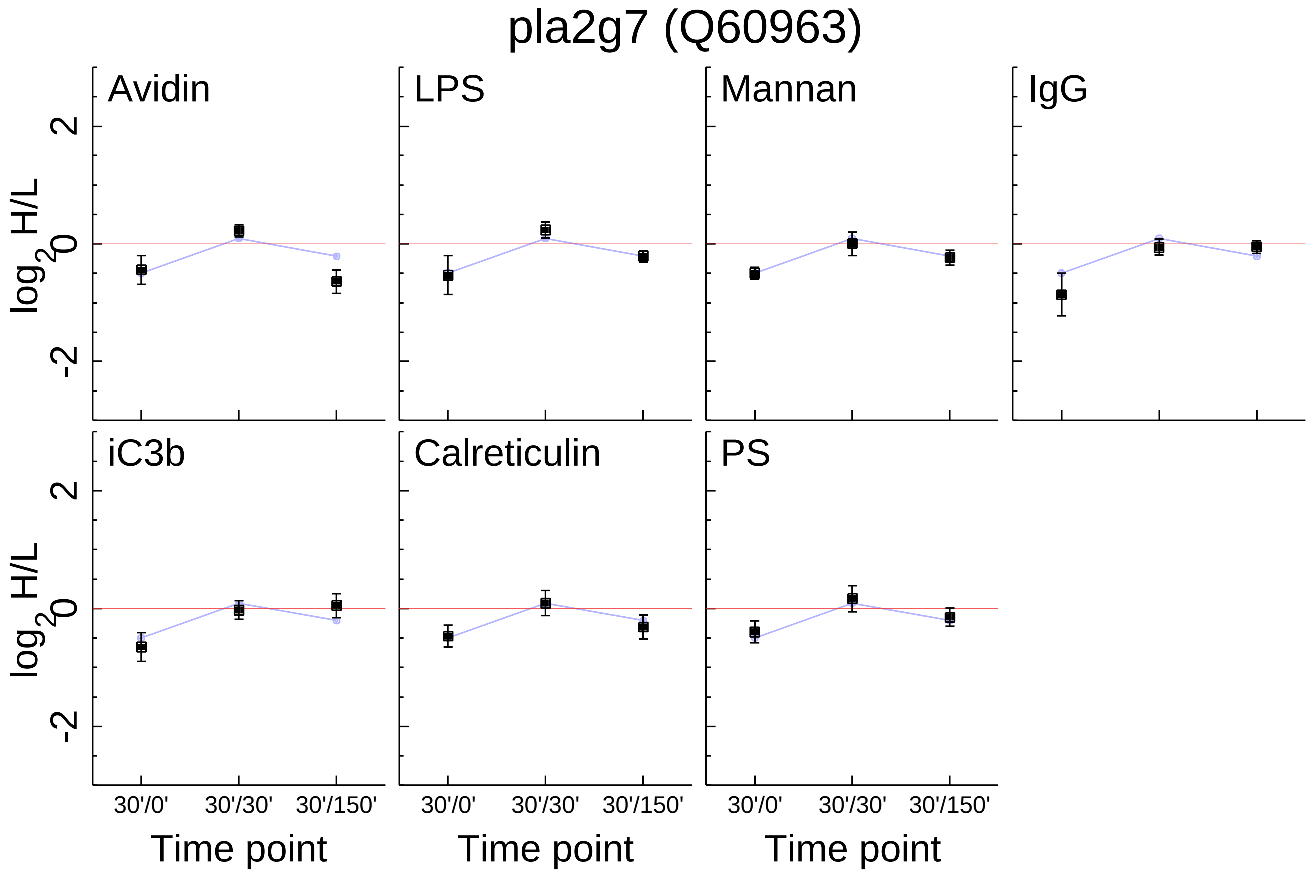
pla2g7 (Q60963)
Avidin
LPS
Mannan
IgG
iC3b
Calreticulin
PS
log2 H/L
2
0
-2
30'/0'
30'/30'
30'/150'
Time point

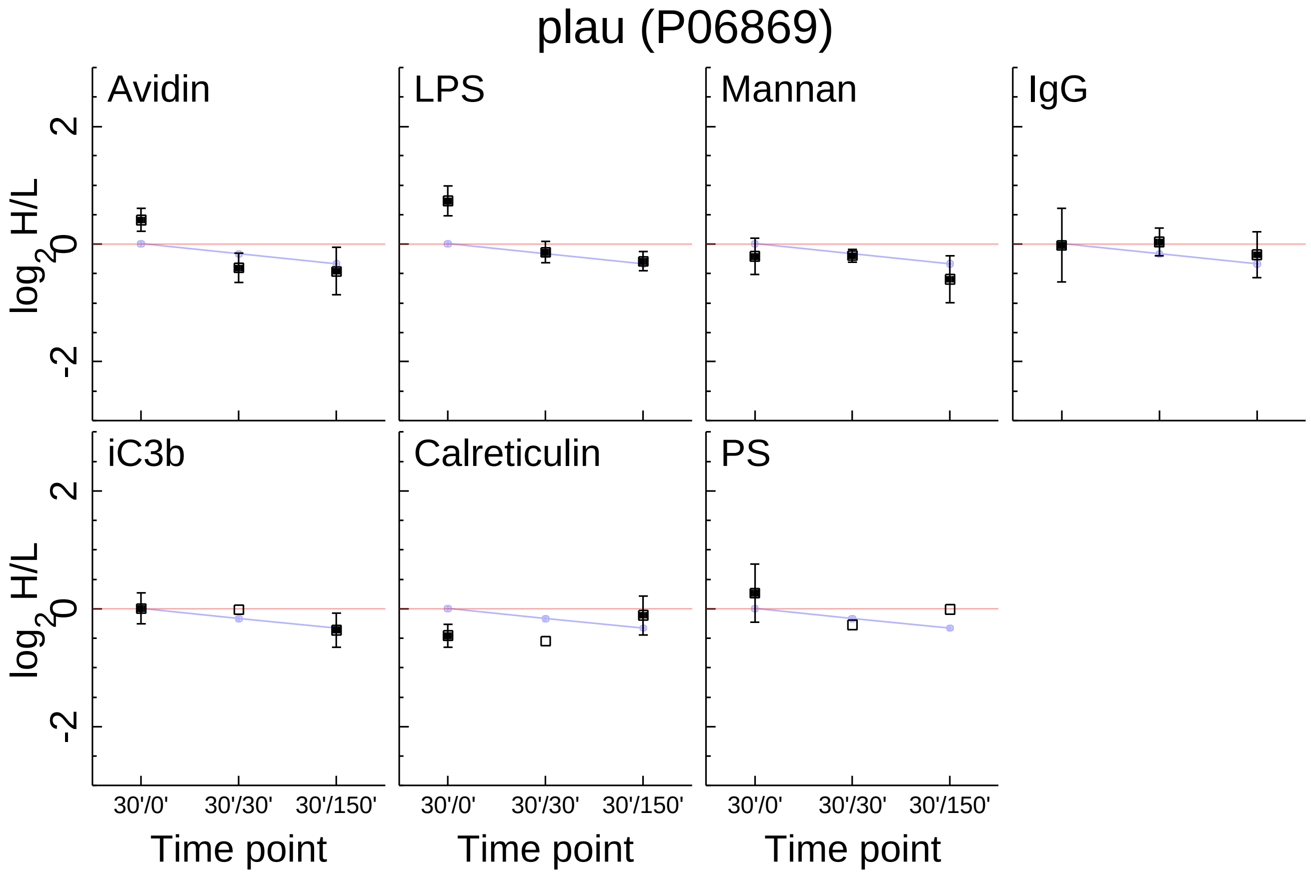
plau (P06869)
Avidin
LPS
Mannan
IgG
iC3b
Calreticulin
PS
log2 H/L
2
0
-2
30'/0'
30'/30'
30'/150'
Time point

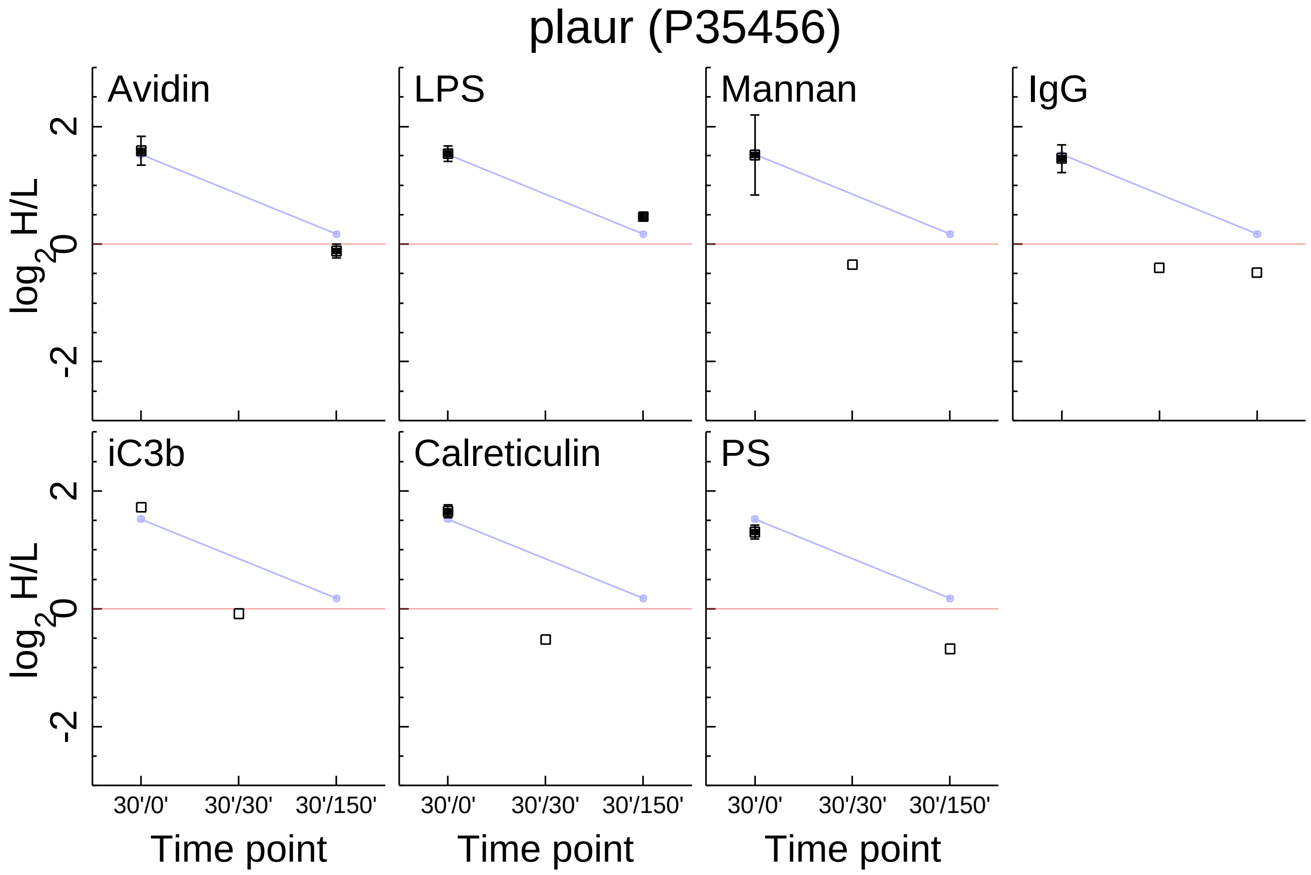
plaur (P35456)
Avidin
LPS
Mannan
IgG
iC3b
Calreticulin
PS
log2 H/L
2
0
-2
30'/0'
30'/30'
30'/150'
Time point

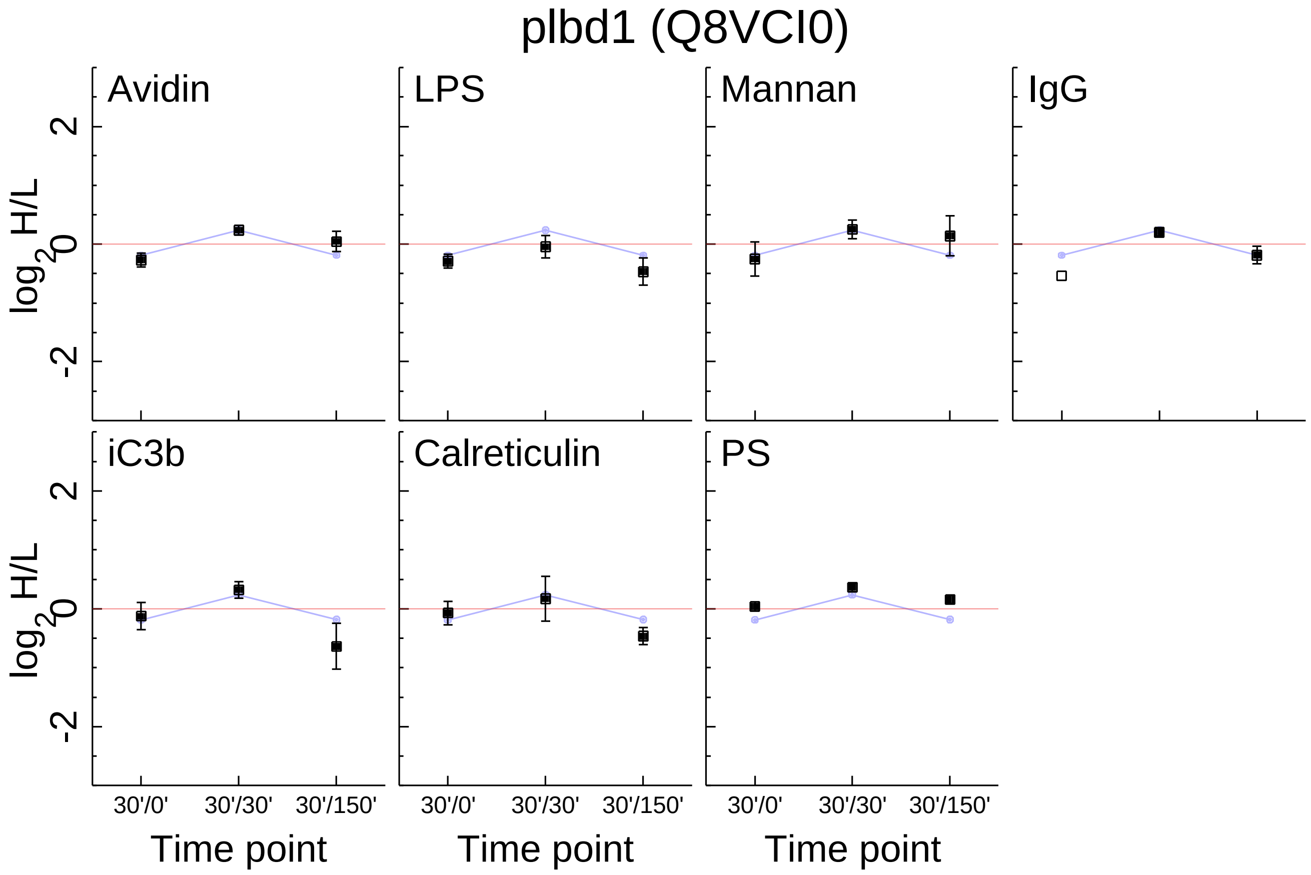
plbd1 (Q8VCI0)
Avidin
LPS
Mannan
IgG
iC3b
Calreticulin
PS
log2 H/L
2
0
-2
30'/0'
30'/30'
30'/150'
Time point

plbd2 (Q3TCN2)

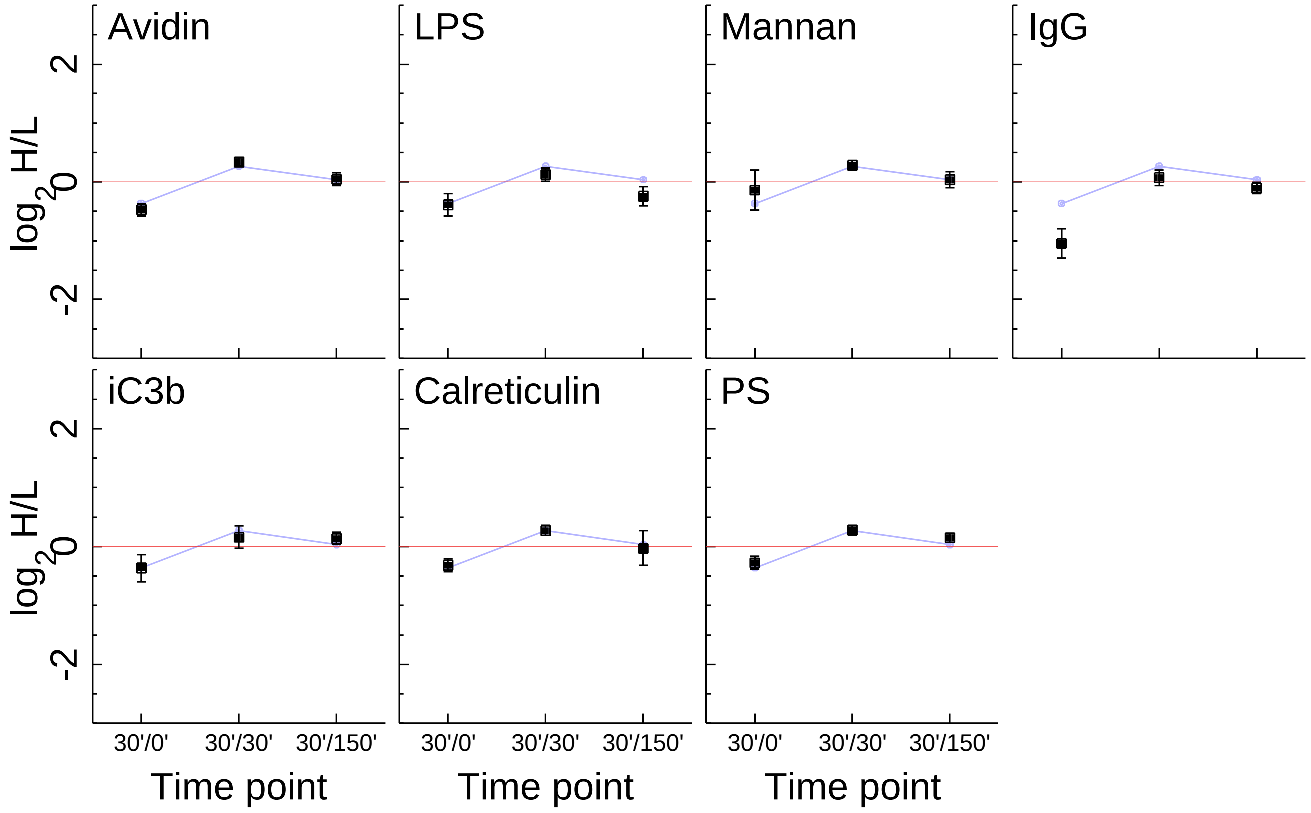

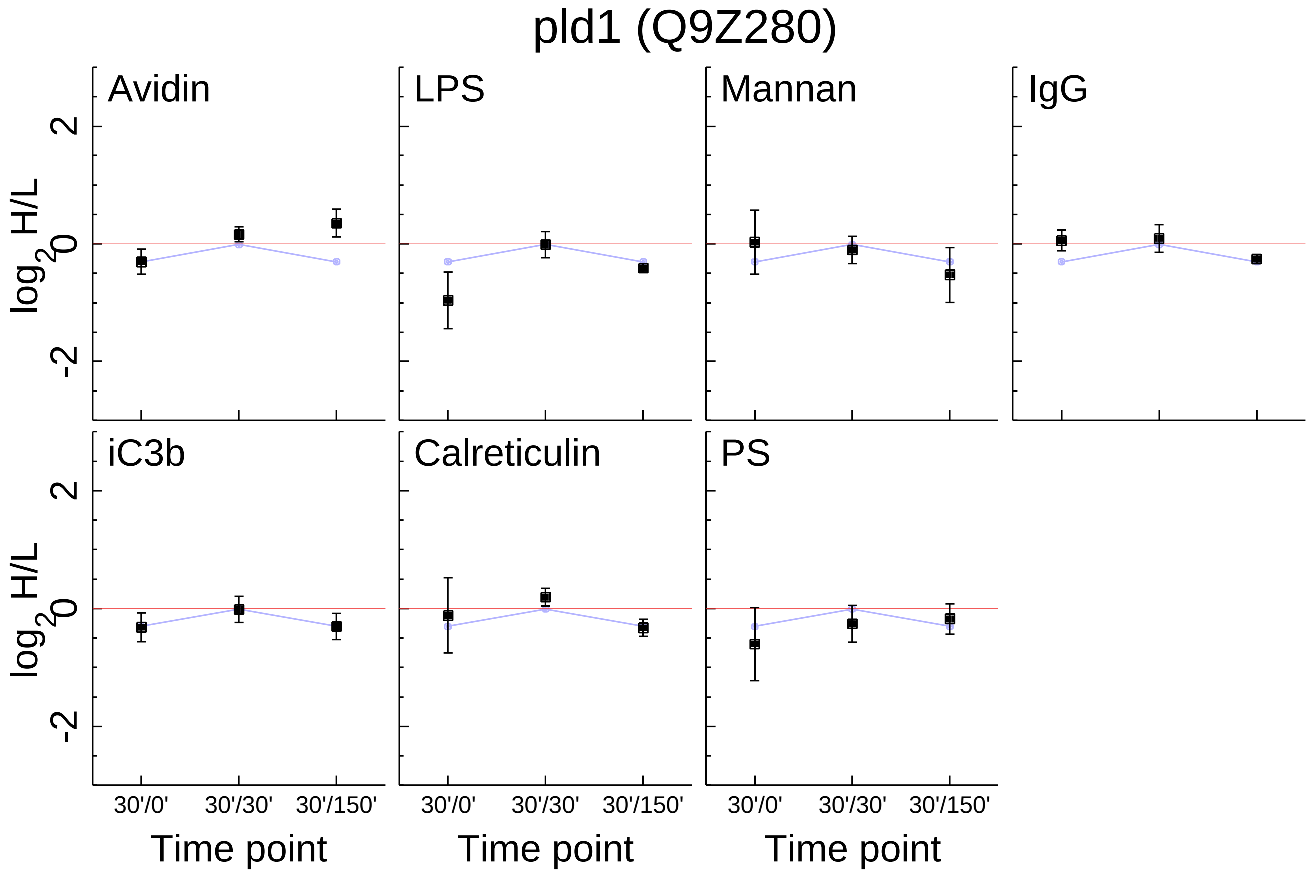
pld1 (Q9Z280)
Avidin
LPS
Mannan
IgG
iC3b
Calreticulin
PS
log2 H/L
2
0
-2
30'/0'
30'/30'
30'/150'
Time point

pld3 (O35405)

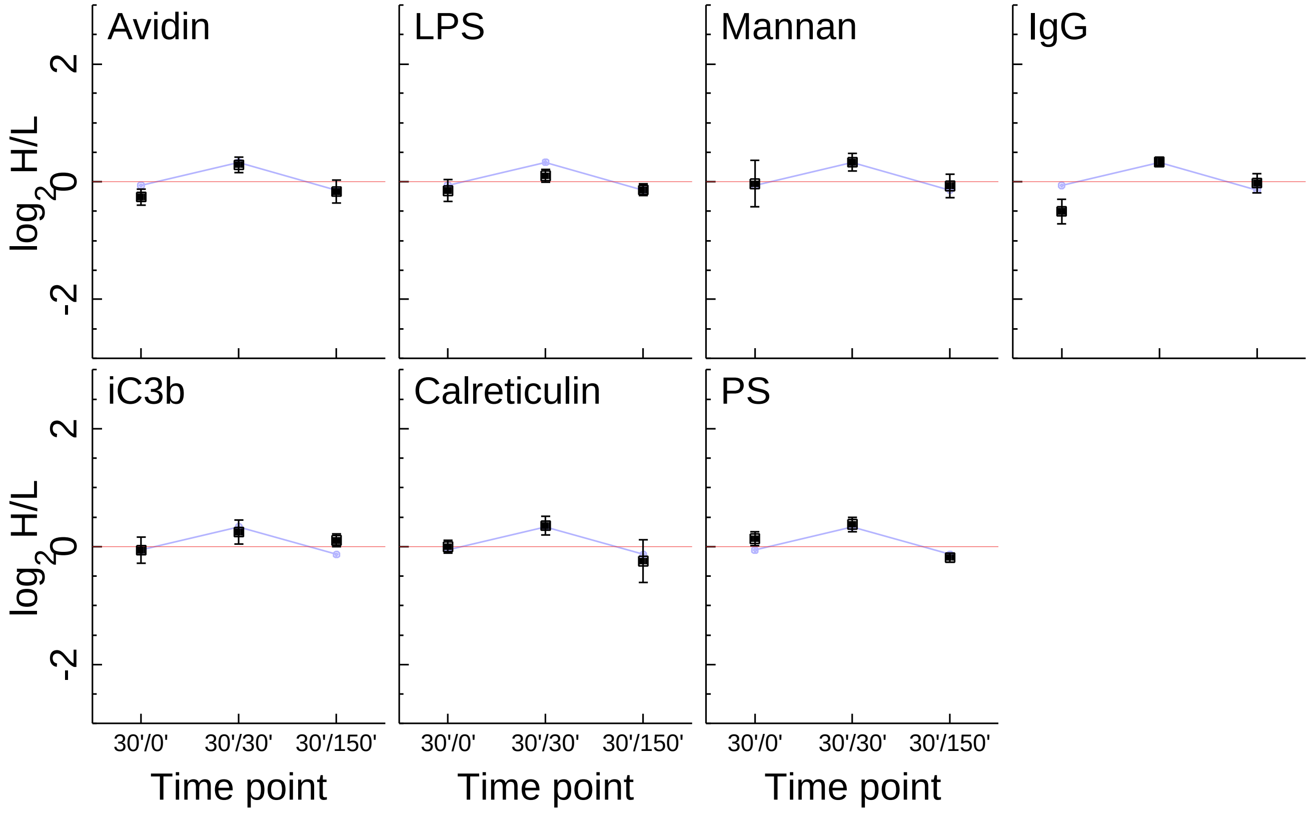

Avidin
LPS
Mannan
IgG
iC3b
Calreticulin
PS
log2 H/L
2
0
-2
30'/0'
30'/30'
30'/150'
Time point

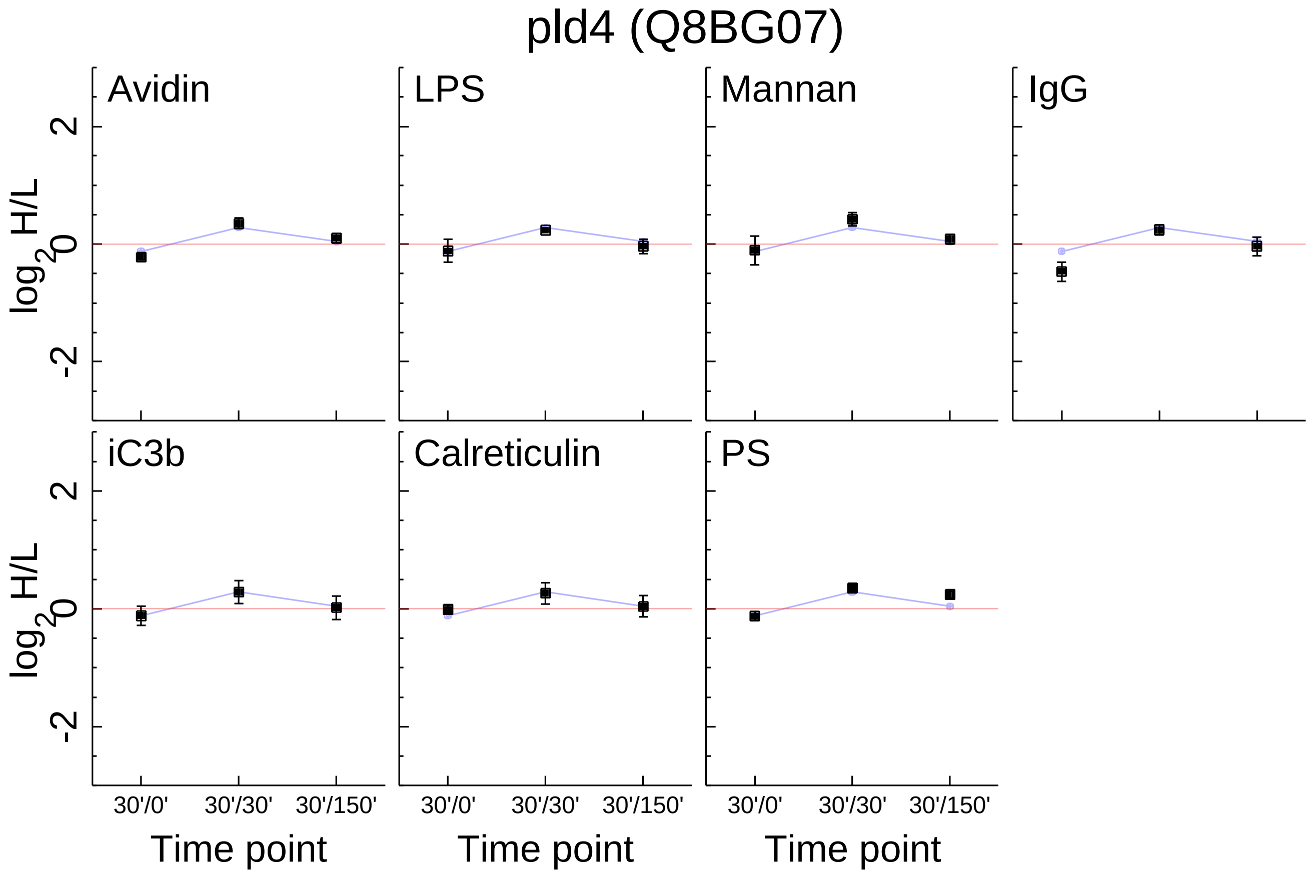
pld4 (Q8BG07)
Avidin
LPS
Mannan
IgG
iC3b
Calreticulin
PS
log2 H/L
2
0
-2
30'/0'
30'/30'
30'/150'
Time point

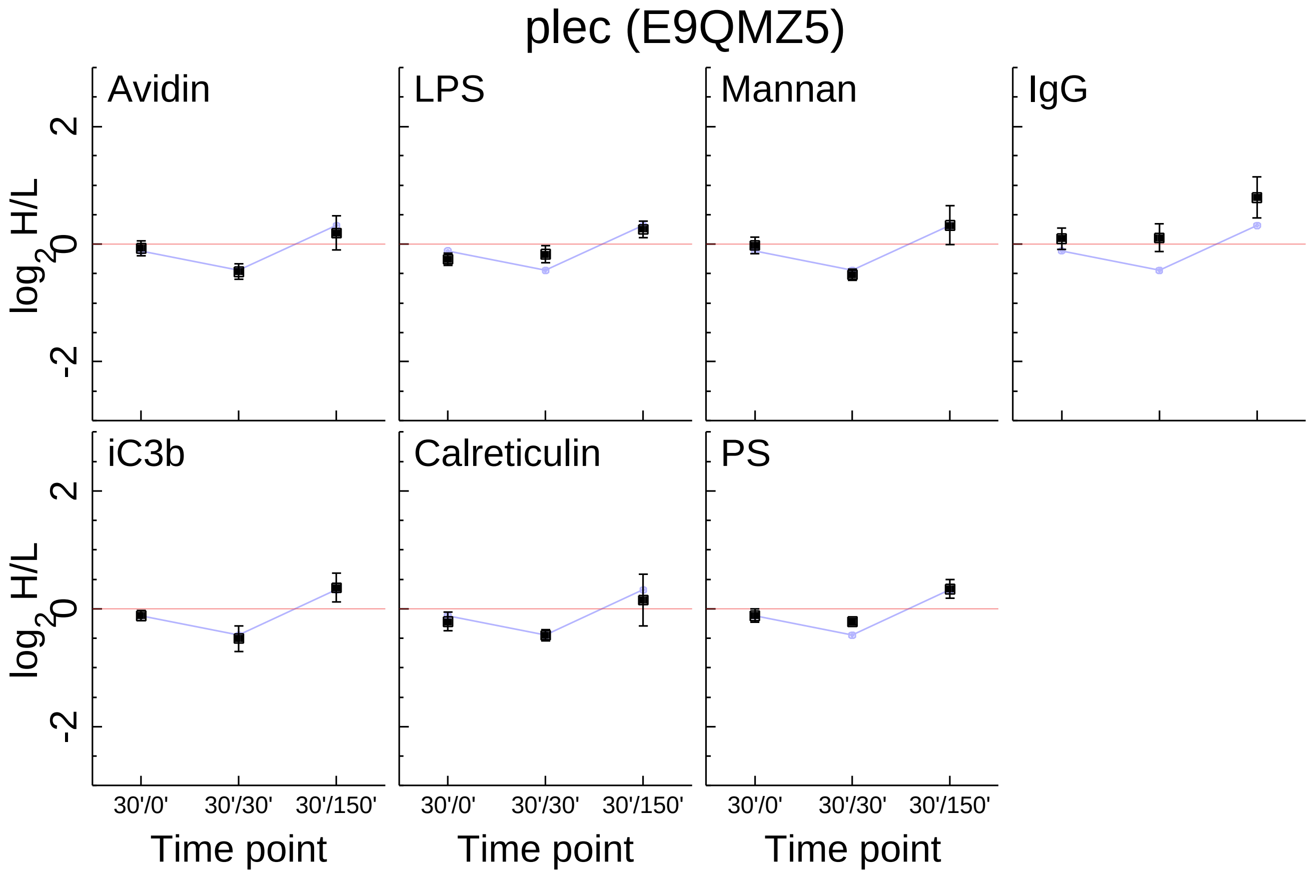

plec (E9QMZ5)
Avidin
LPS
Mannan
IgG
iC3b
Calreticulin
PS
log2 H/L
2
0
-2
30'/0'
30'/30'
30'/150'
Time point

plekha1 (Q8BUL6)

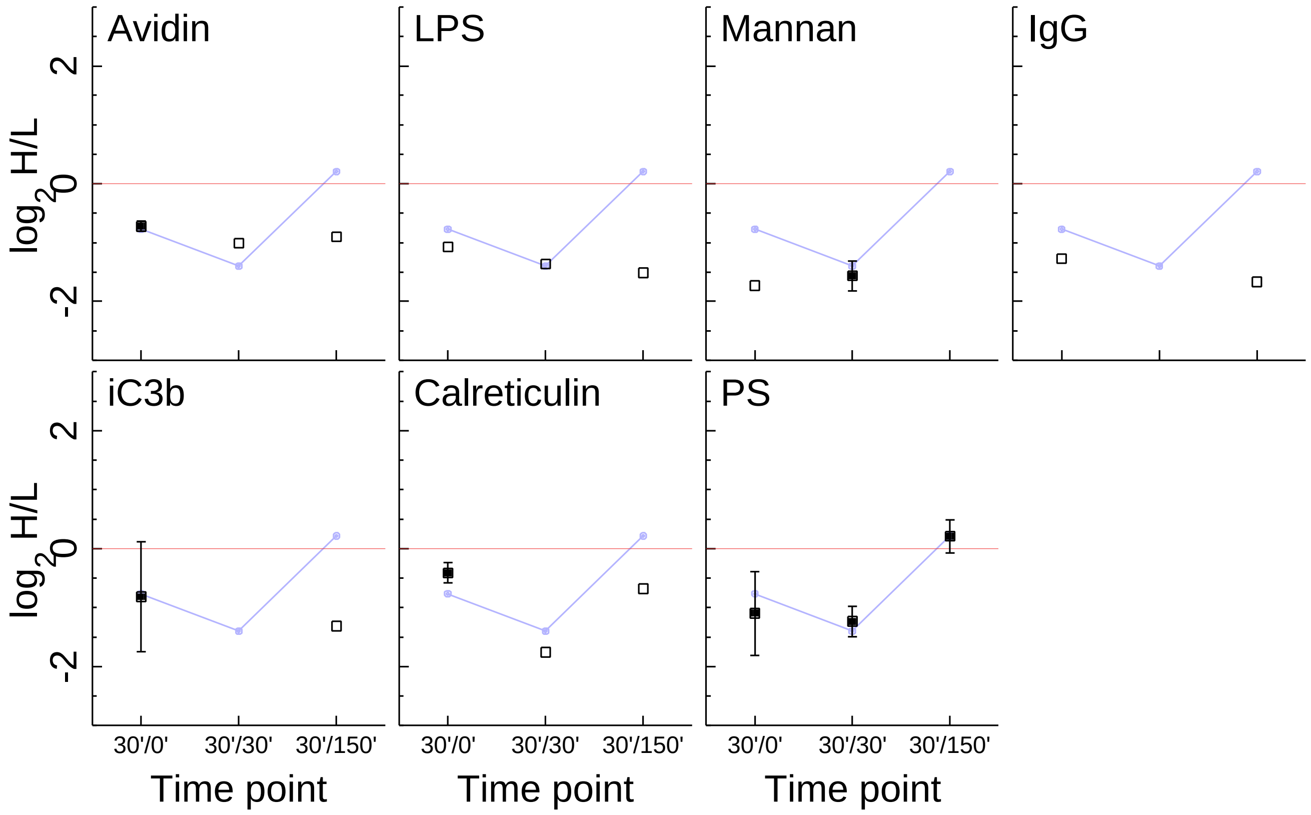

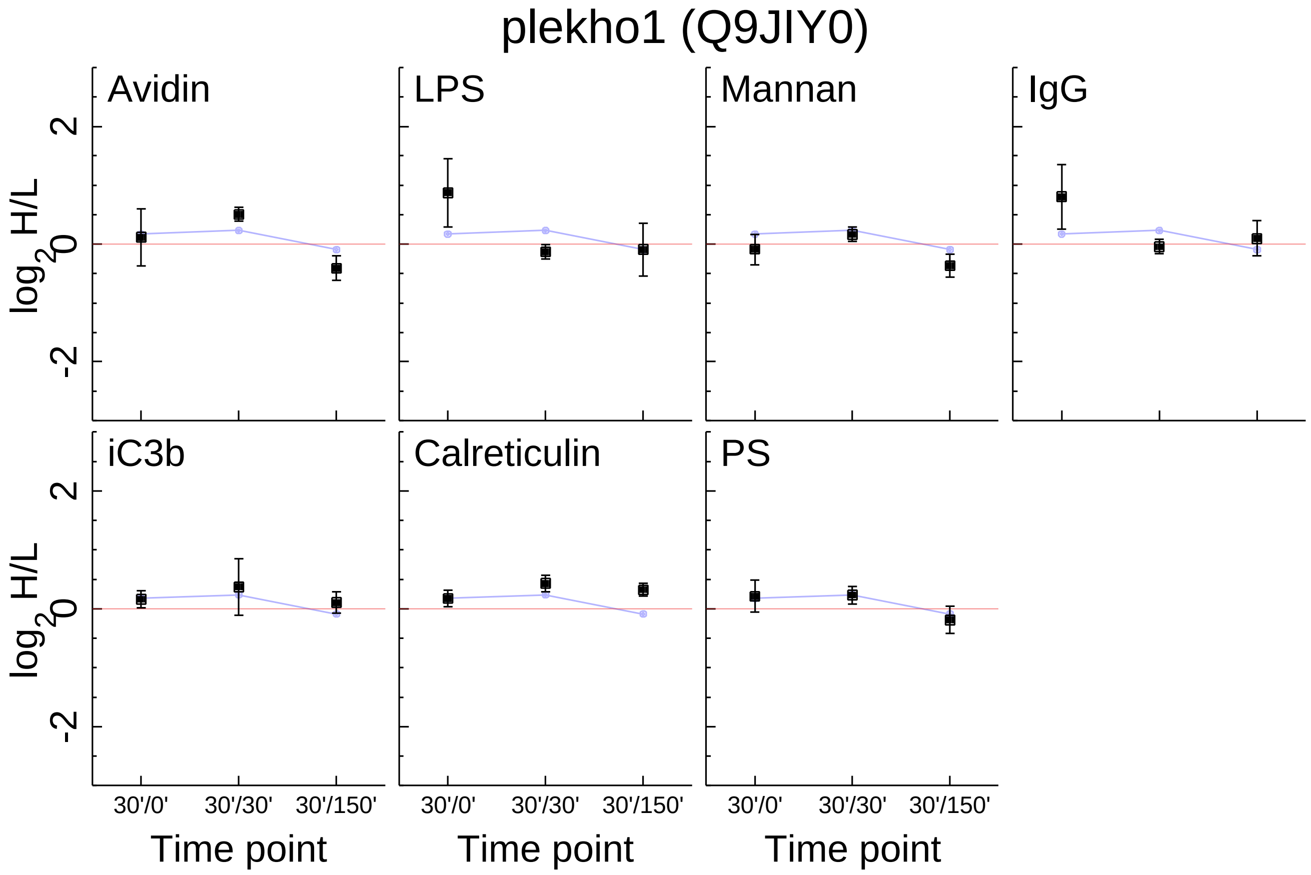

plekho1 (Q9JIY0)
Avidin
LPS
Mannan
IgG
iC3b
Calreticulin
PS
log2 H/L
2
0
-2
30'/0'
30'/30'
30'/150'
Time point

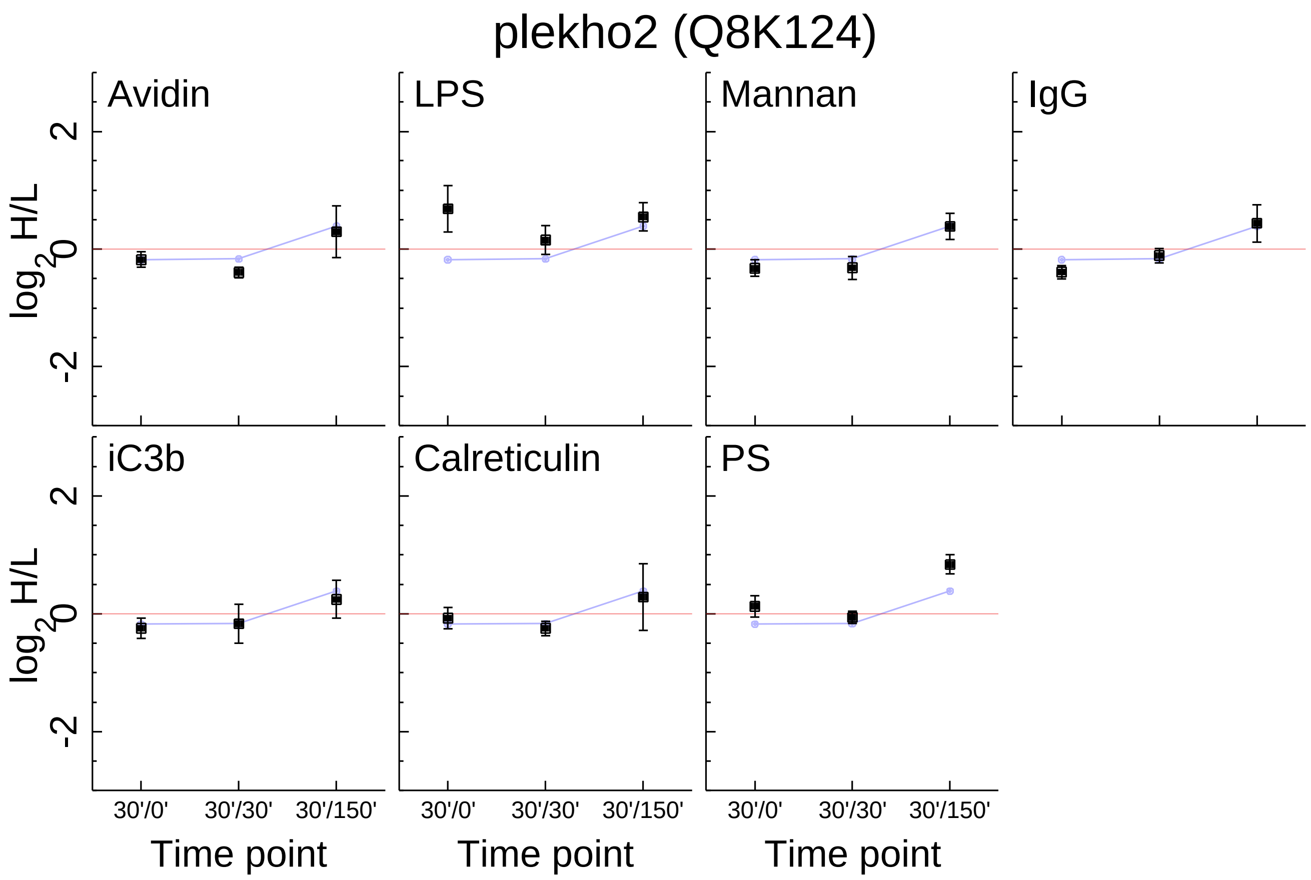
plekho2 (Q8K124)
Avidin
LPS
Mannan
IgG
iC3b
Calreticulin
PS
log2 H/L
2
0
-2
30'/0'
30'/30'
30'/150'
Time point

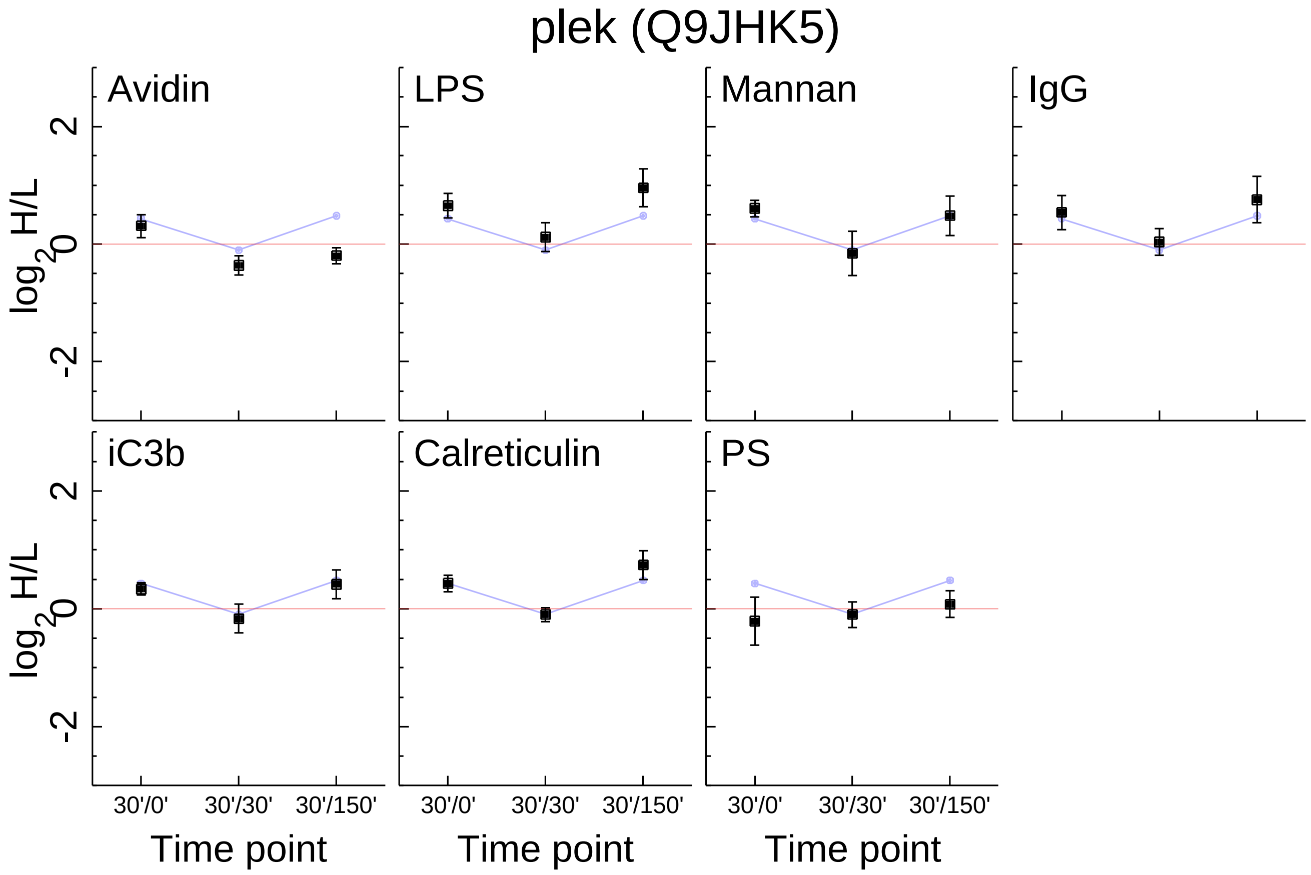
plek (Q9JHK5)
Avidin
LPS
Mannan
IgG
iC3b
Calreticulin
PS
log2 H/L
2
0
-2
30'/0'
30'/30'
30'/150'
Time point

plod1 (Q9R0E2)

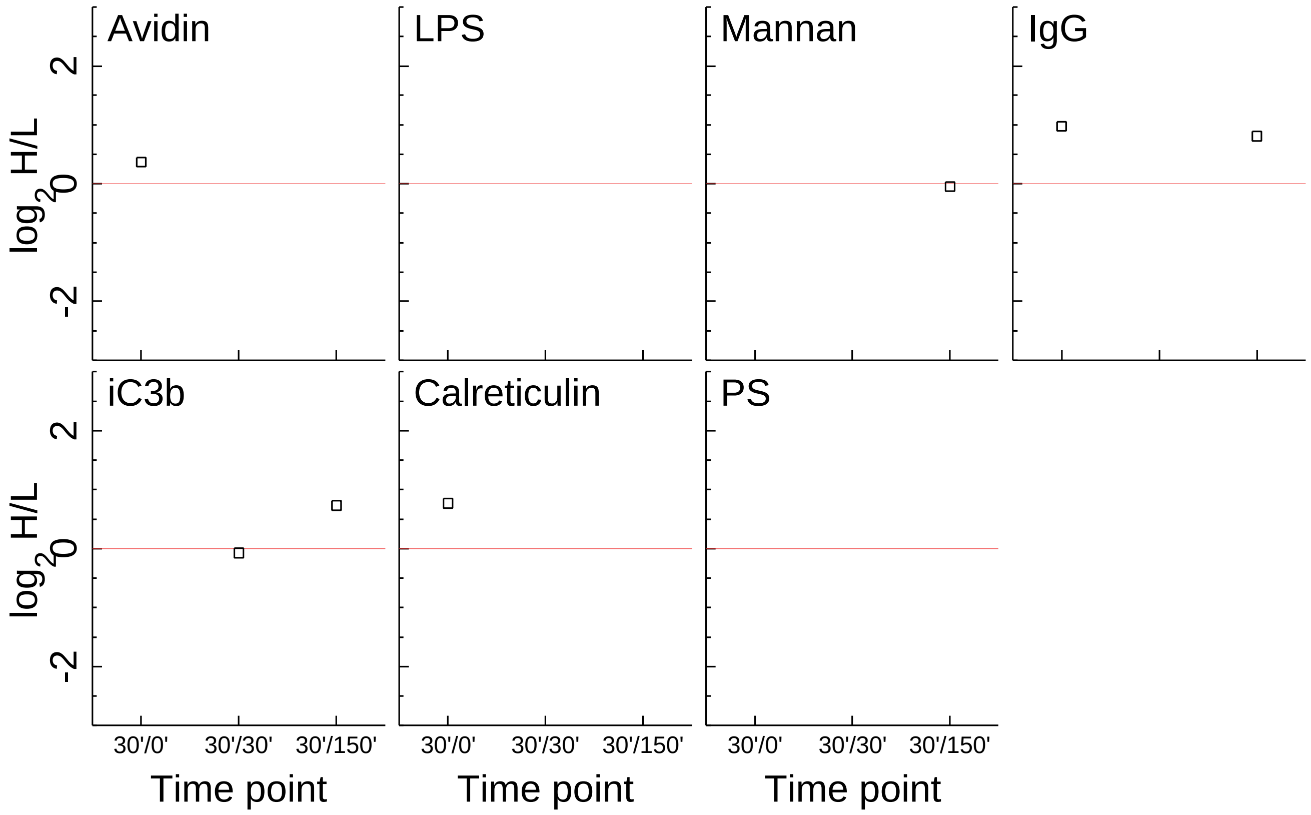

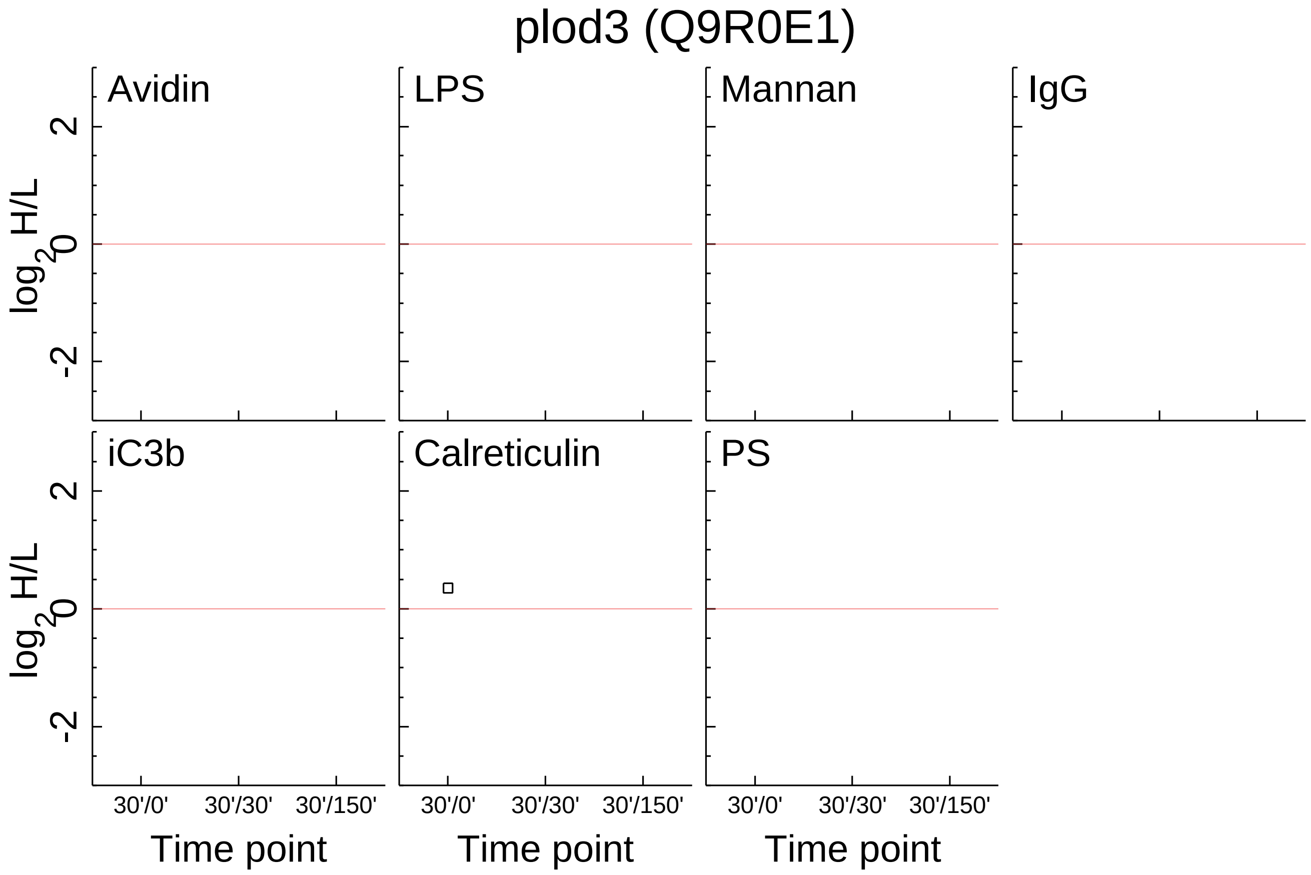
plod3 (Q9R0E1)
Avidin
LPS
Mannan
IgG
iC3b
Calreticulin
PS
log2 H/L
2
0
-2
30'/0'
30'/30'
30'/150'
Time point

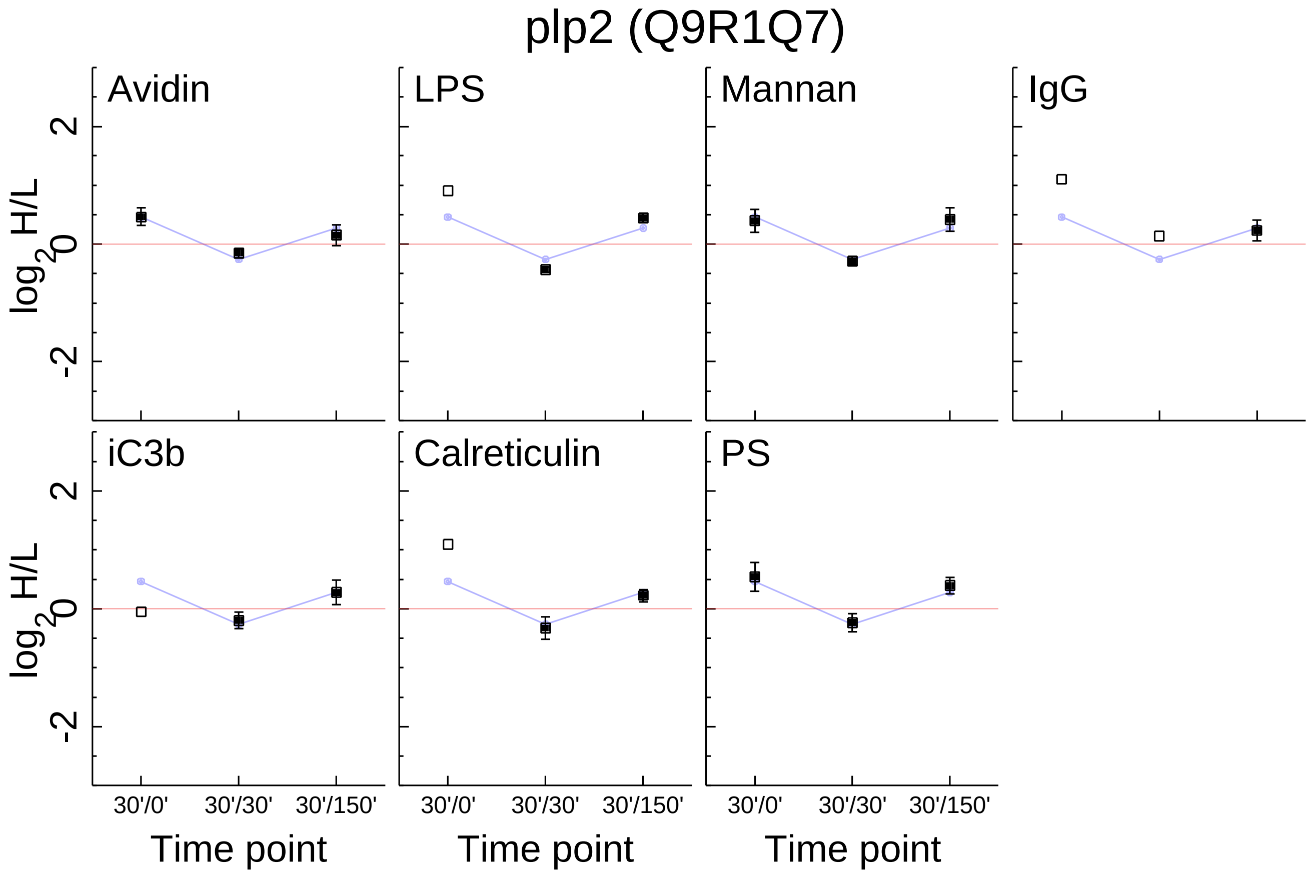
plp2 (Q9R1Q7)
Avidin
LPS
Mannan
IgG
iC3b
Calreticulin
PS
log2 H/L
2
0
-2
30'/0'
30'/30'
30'/150'
Time point

plscr3 (Q9JIZ9)

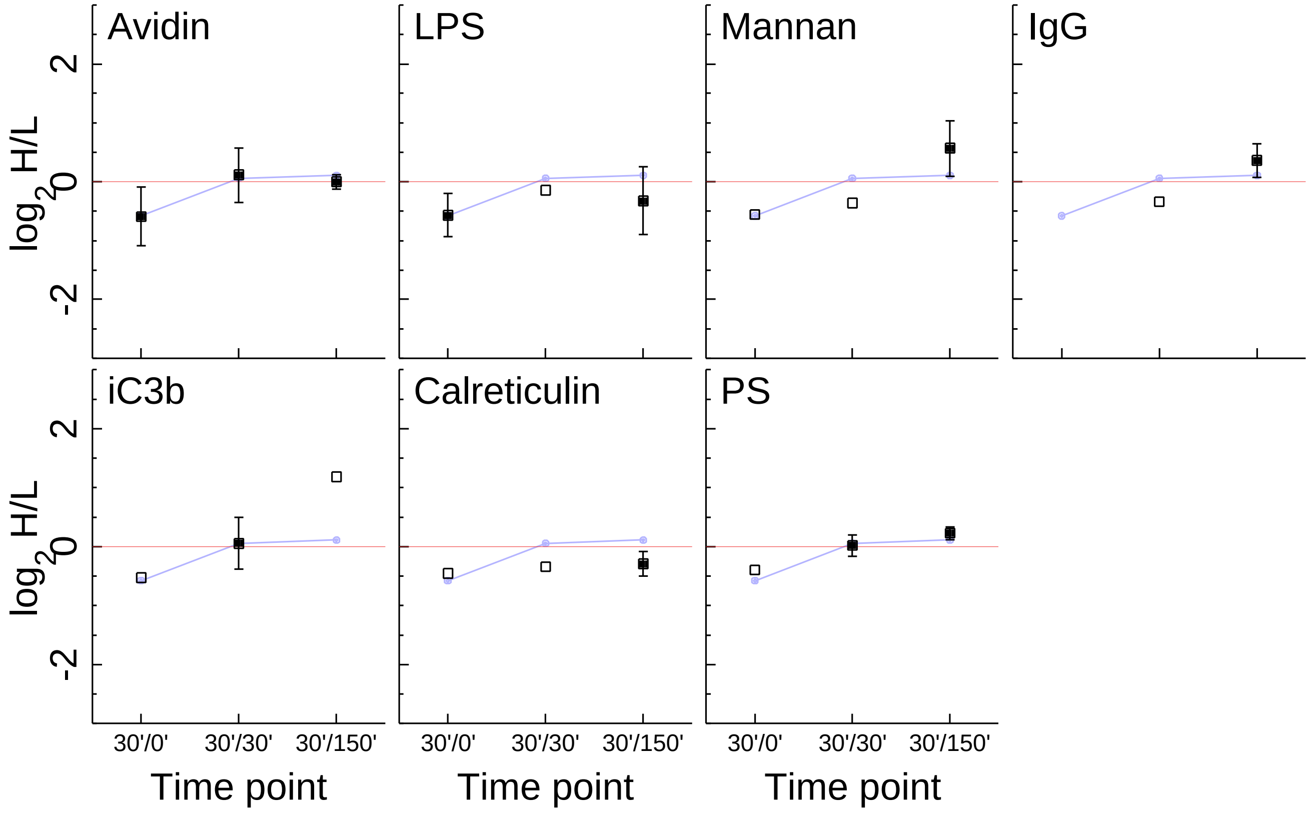

pltp (P55065)

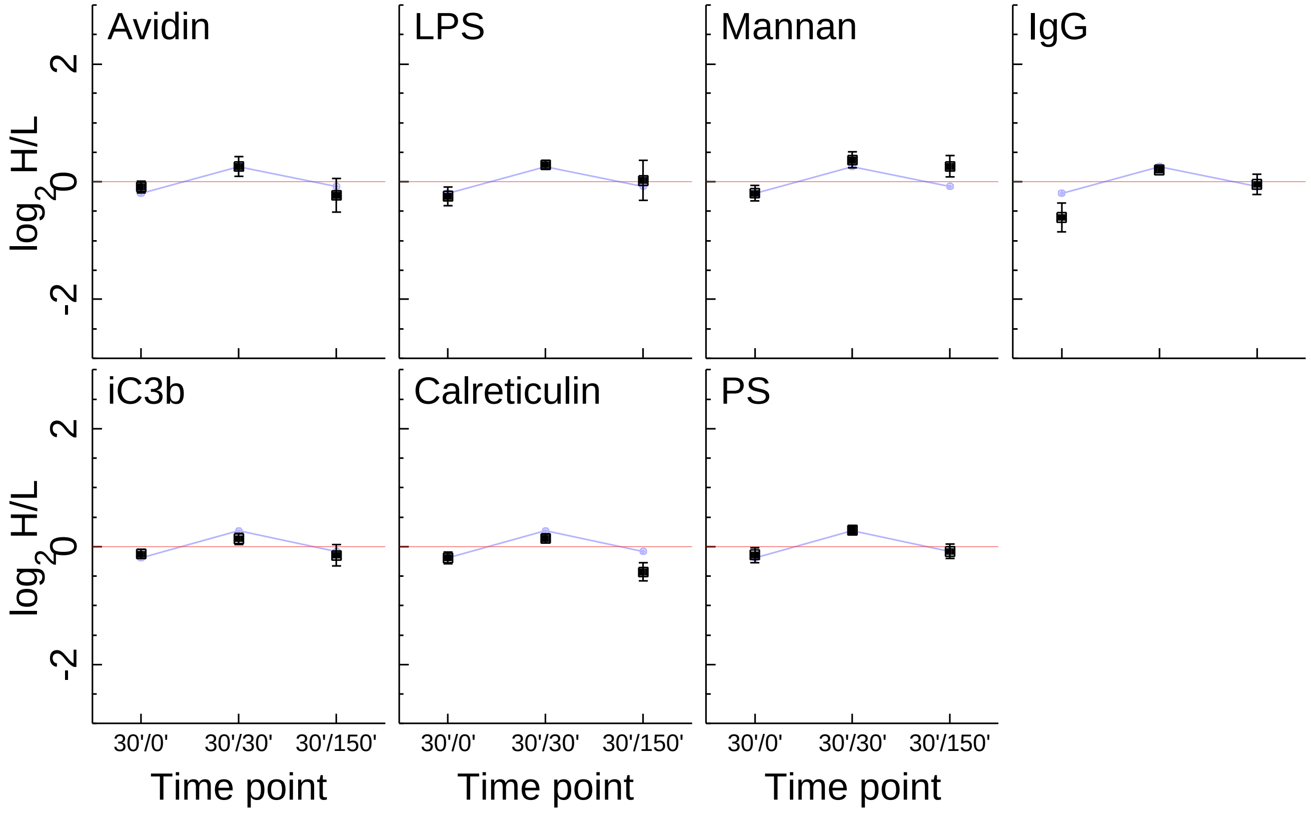
Avidin
LPS
Mannan
IgG
iC3b
Calreticulin
PS
2
0
-2
log_2 H/L
30'/0'
30'/30'
30'/150'
Time point

plxdc1 (Q91ZV7)

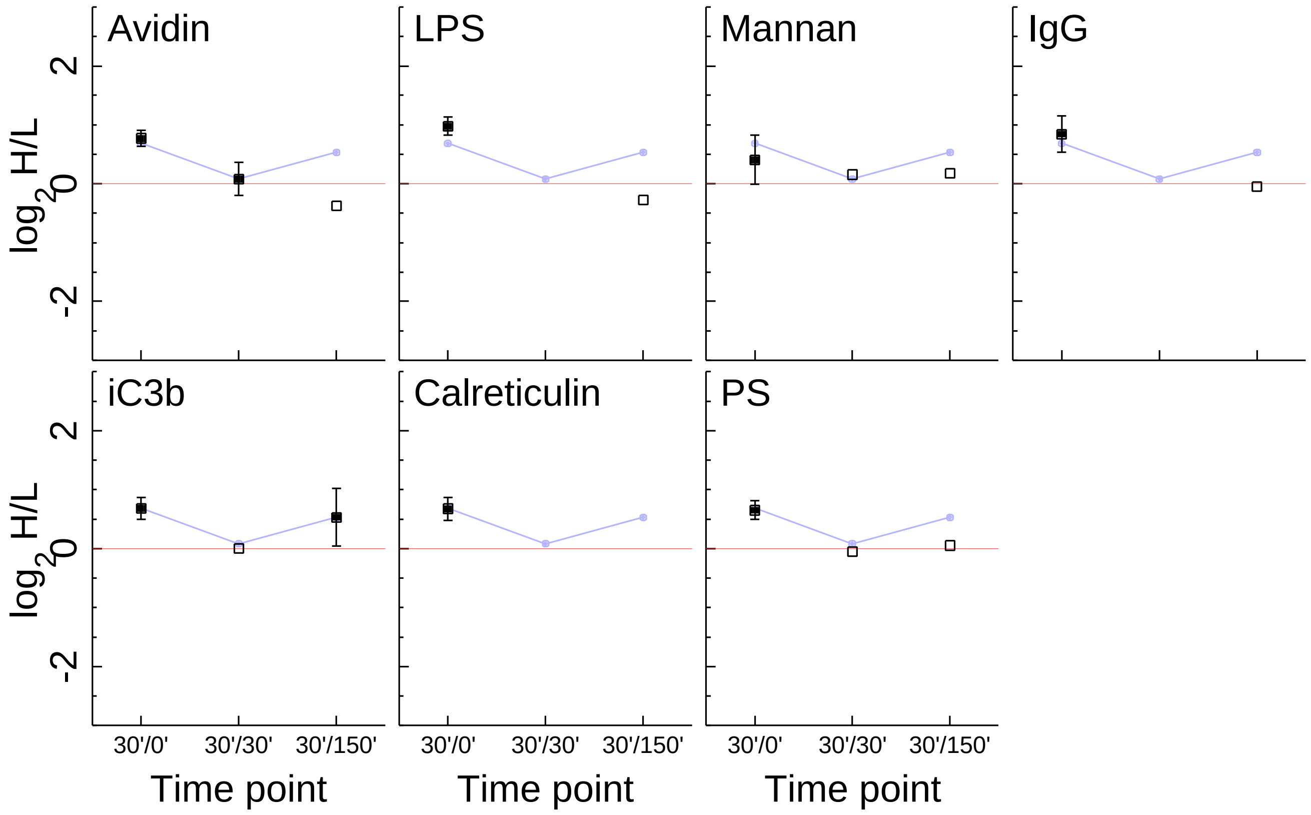

plxna1 (P70206)

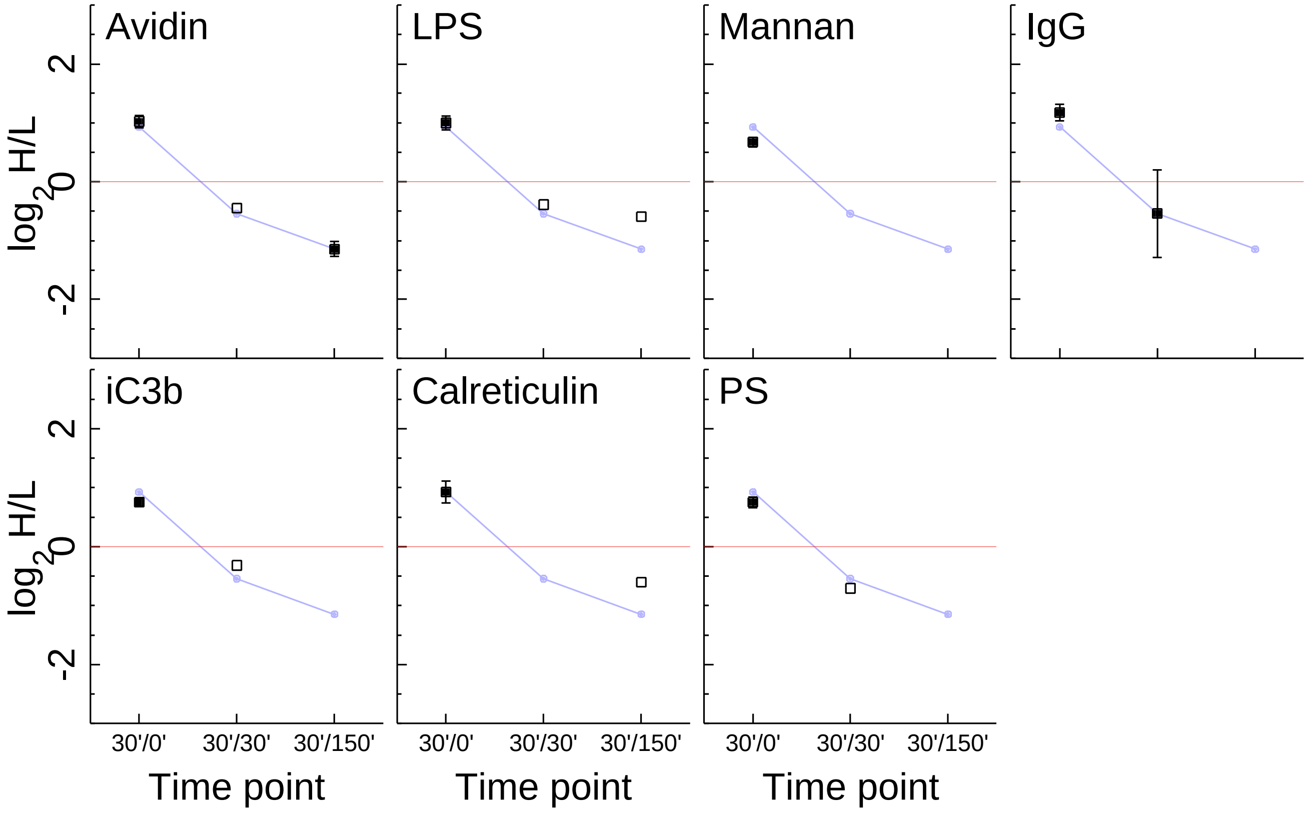

plxnb2 (B2RXS4)

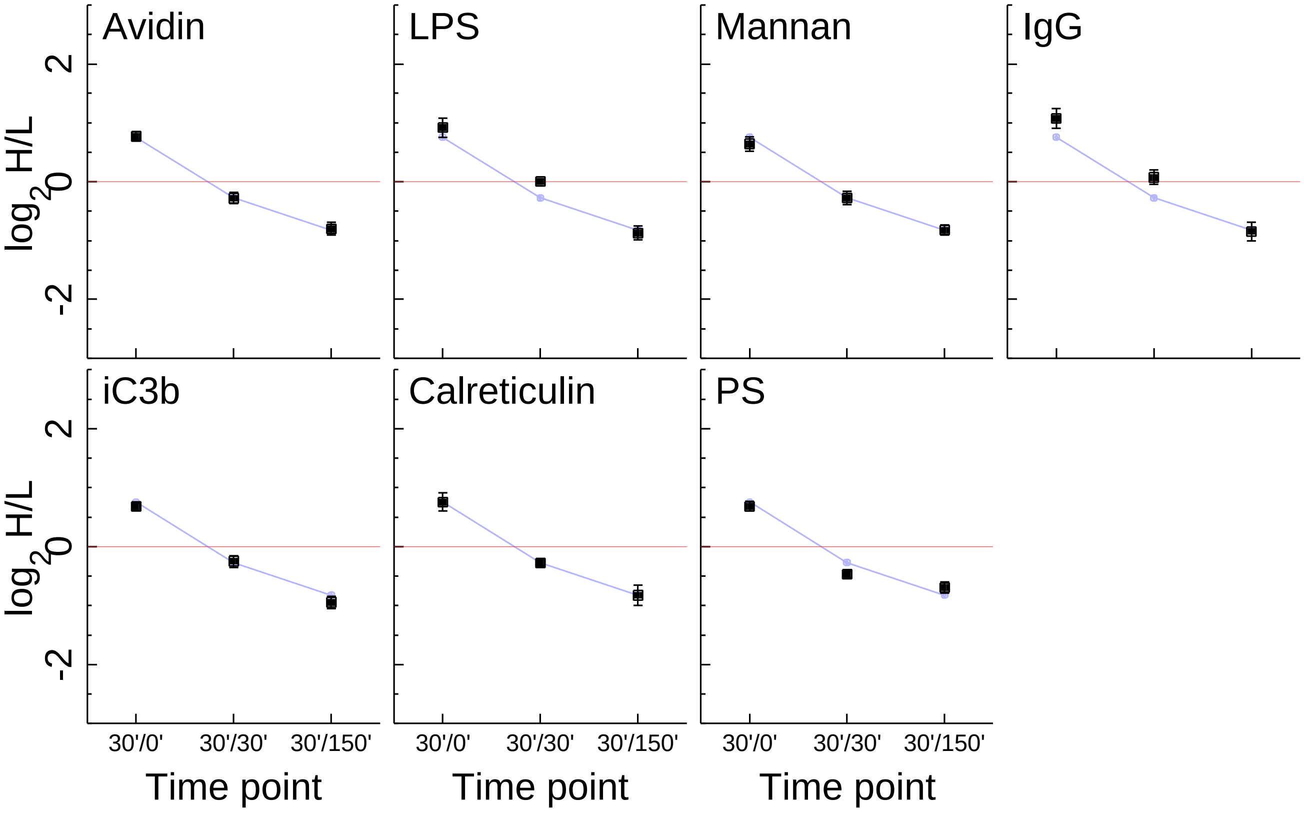

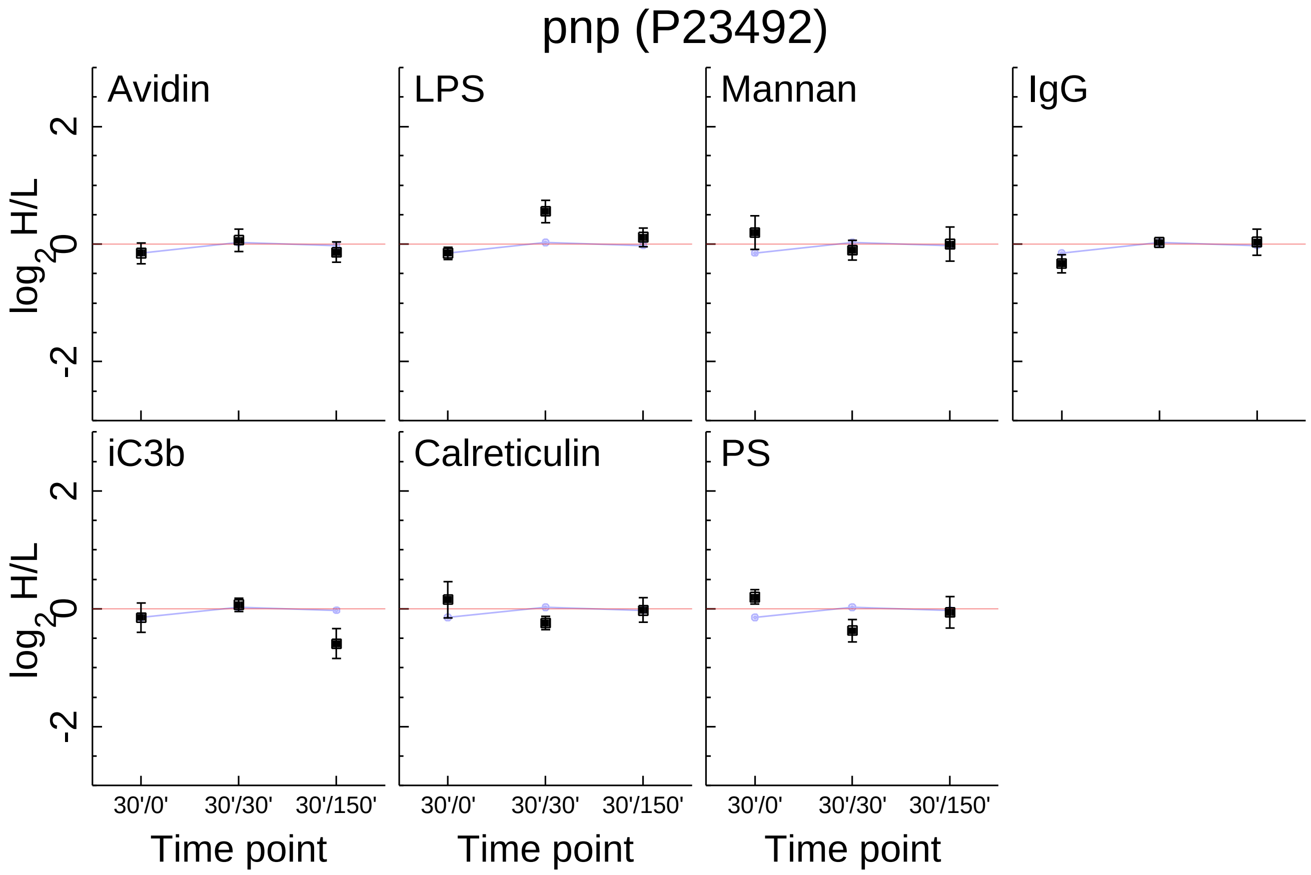
pnp (P23492)
Avidin
LPS
Mannan
IgG
iC3b
Calreticulin
PS
log2 H/L
2
0
-2
30'/0'
30'/30'
30'/150'
Time point

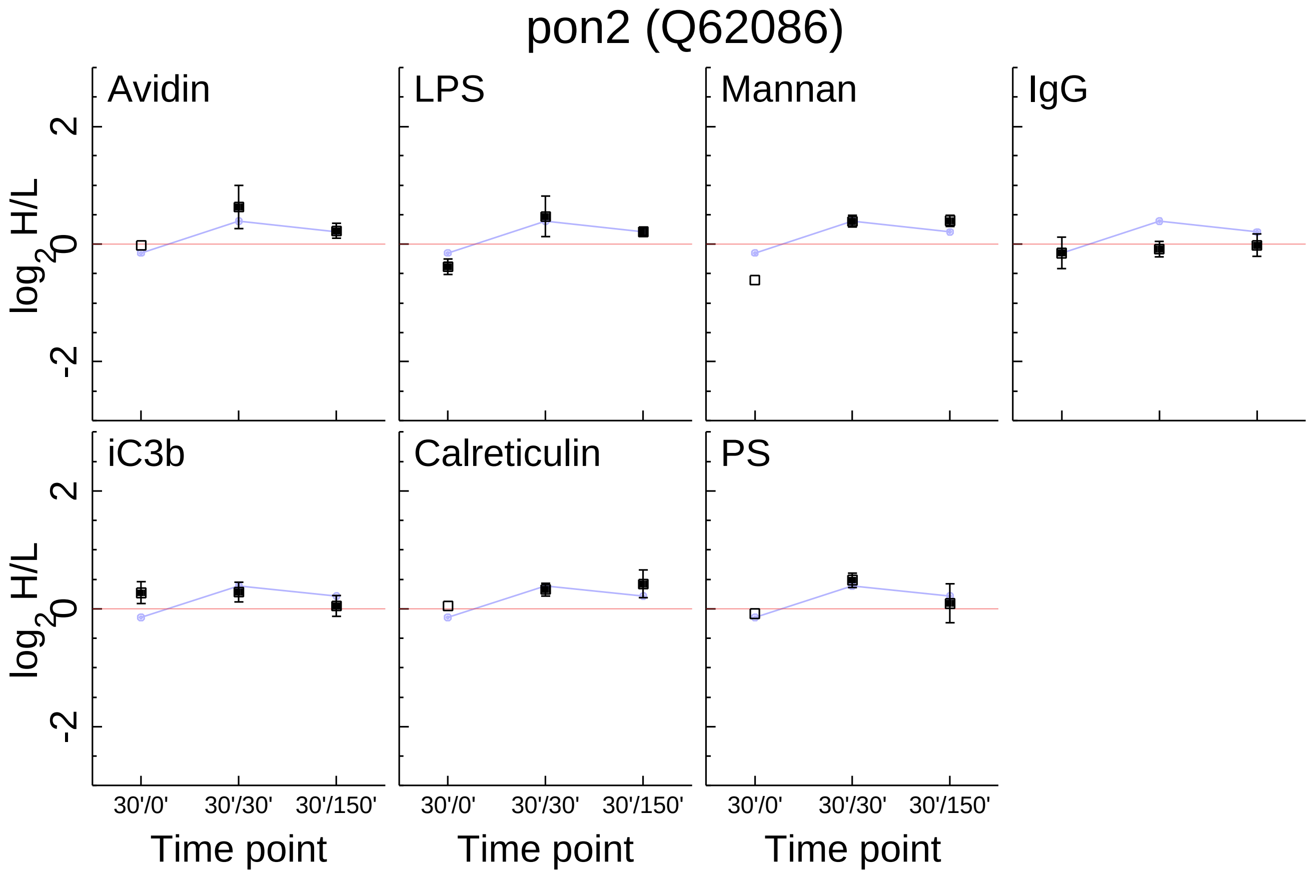

pon2 (Q62086)
Avidin
LPS
Mannan
IgG
iC3b
Calreticulin
PS
log2 H/L
2
0
-2
30'/0'
30'/30'
30'/150'
Time point

pon3 (Q62087)

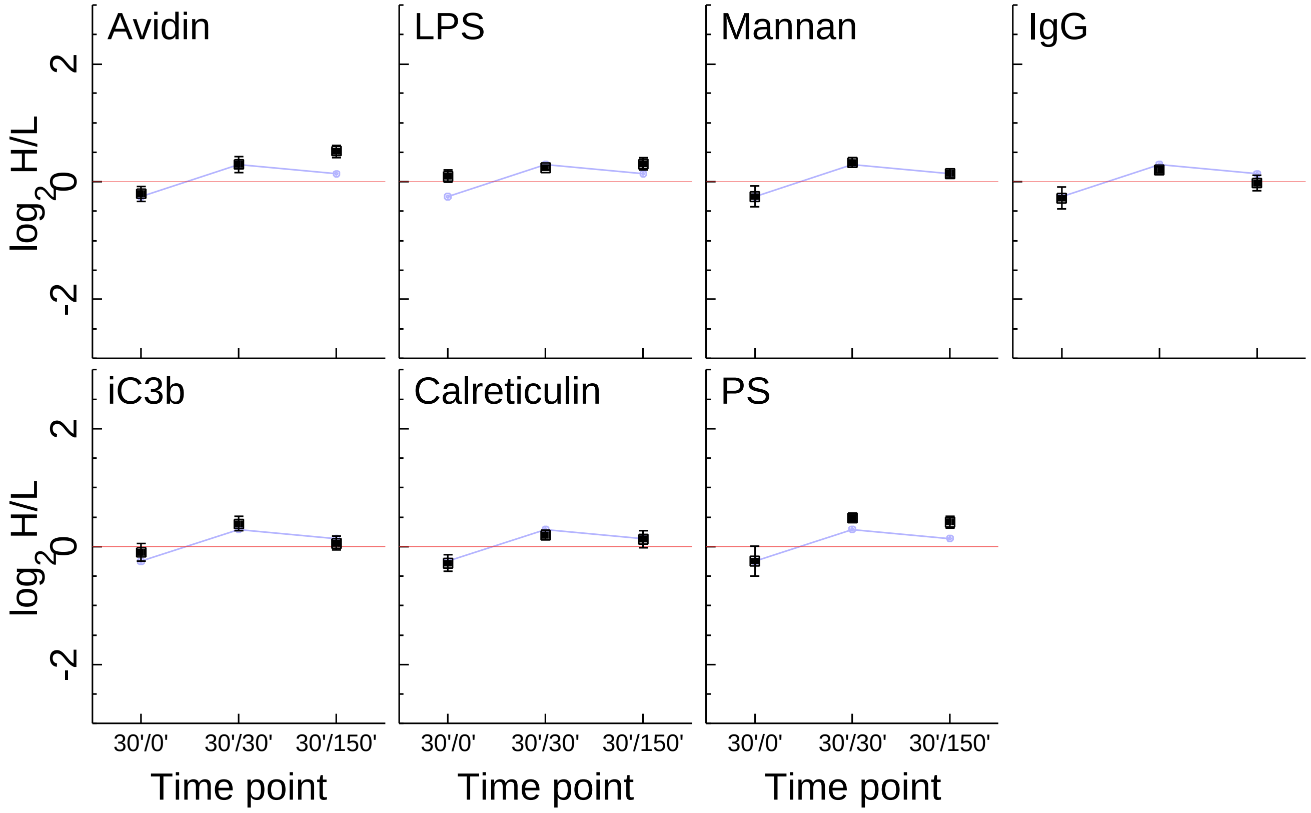

Avidin
LPS
Mannan
IgG
iC3b
Calreticulin
PS
log2 H/L
2
0
-2
30'/0'
30'/30'
30'/150'
Time point

por (P37040)

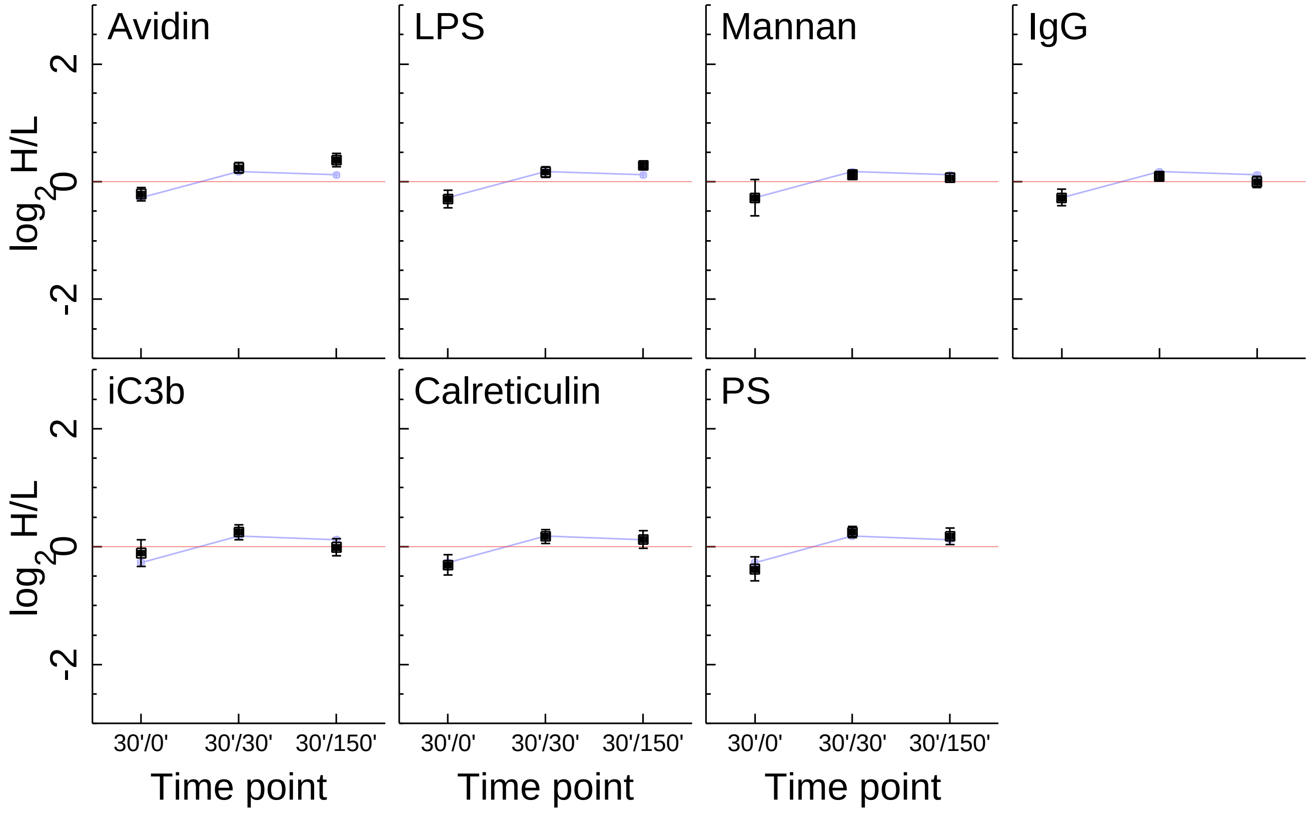

Avidin
LPS
Mannan
IgG
iC3b
Calreticulin
PS
log2 H/L
2
0
-2
30'/0'
30'/30'
30'/150'
Time point

ppia (P17742)

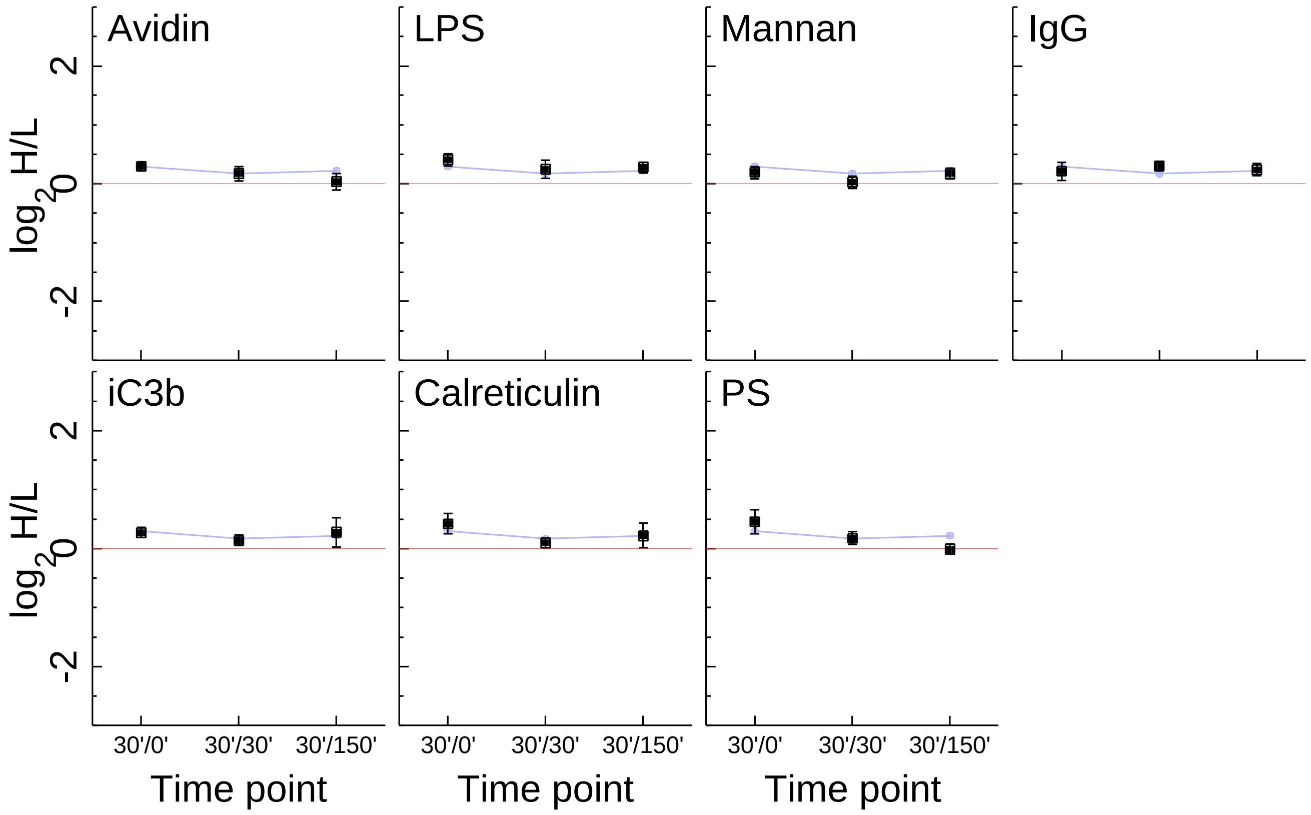

Avidin
LPS
Mannan
IgG
iC3b
Calreticulin
PS
log2 H/L
2
0
-2
30'/0'
30'/30'
30'/150'
Time point

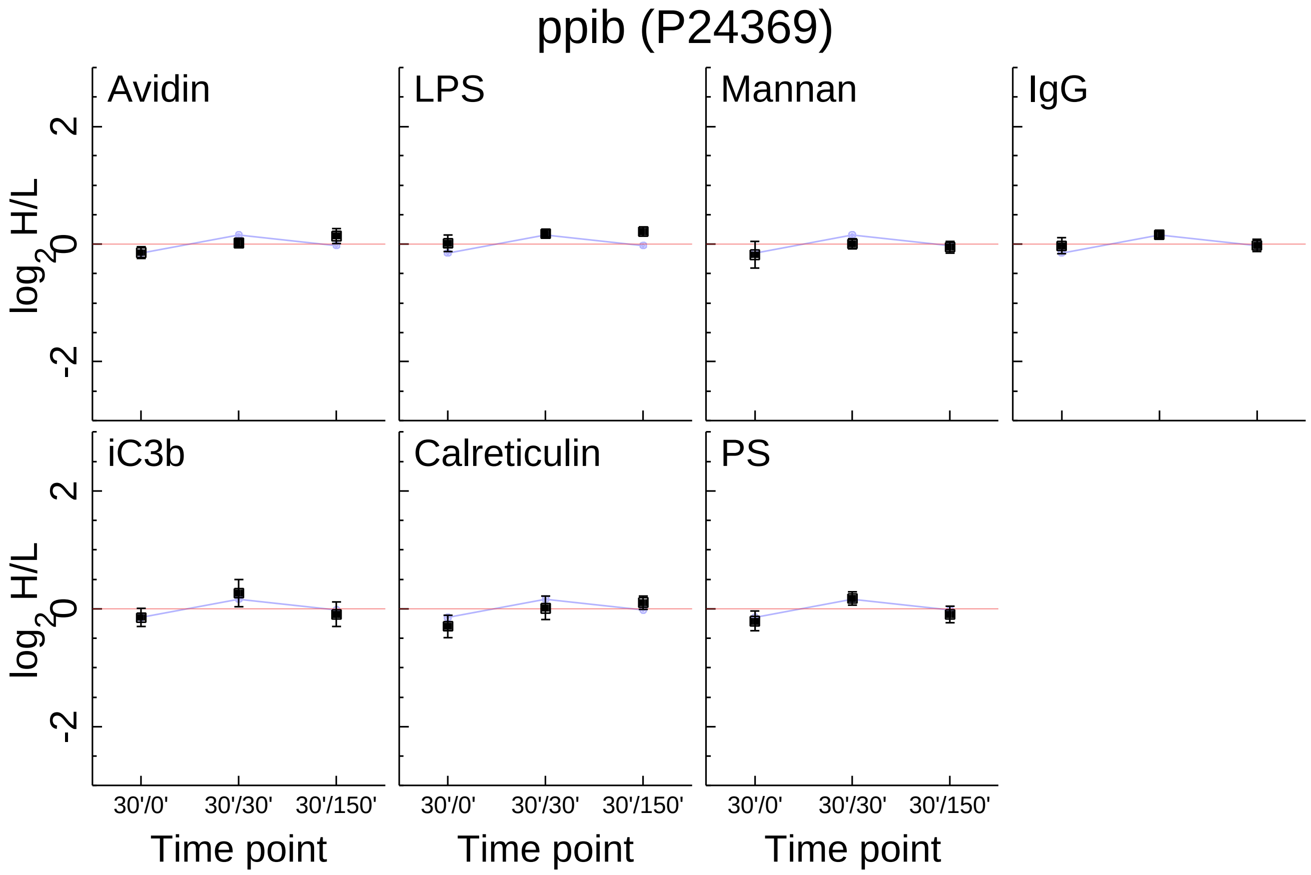
ppib (P24369)
Avidin
LPS
Mannan
IgG
iC3b
Calreticulin
PS
log2 H/L
2
0
-2
30'/0'
30'/30'
30'/150'
Time point

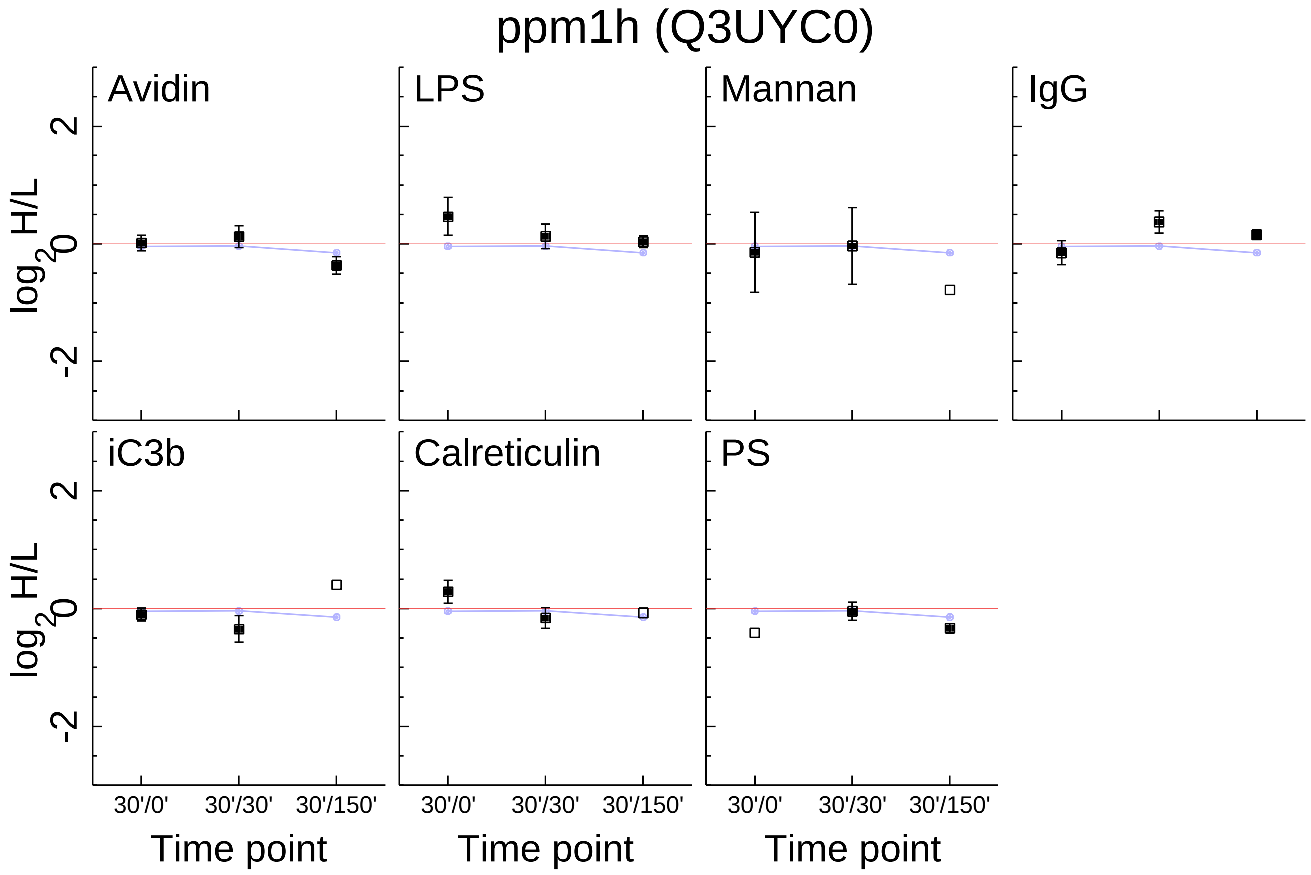
ppm1h (Q3UYC0)
Avidin
LPS
Mannan
IgG
iC3b
Calreticulin
PS
log2 H/L
2
0
-2
30'/0'
30'/30'
30'/150'
Time point

ppp1ca (P62137)

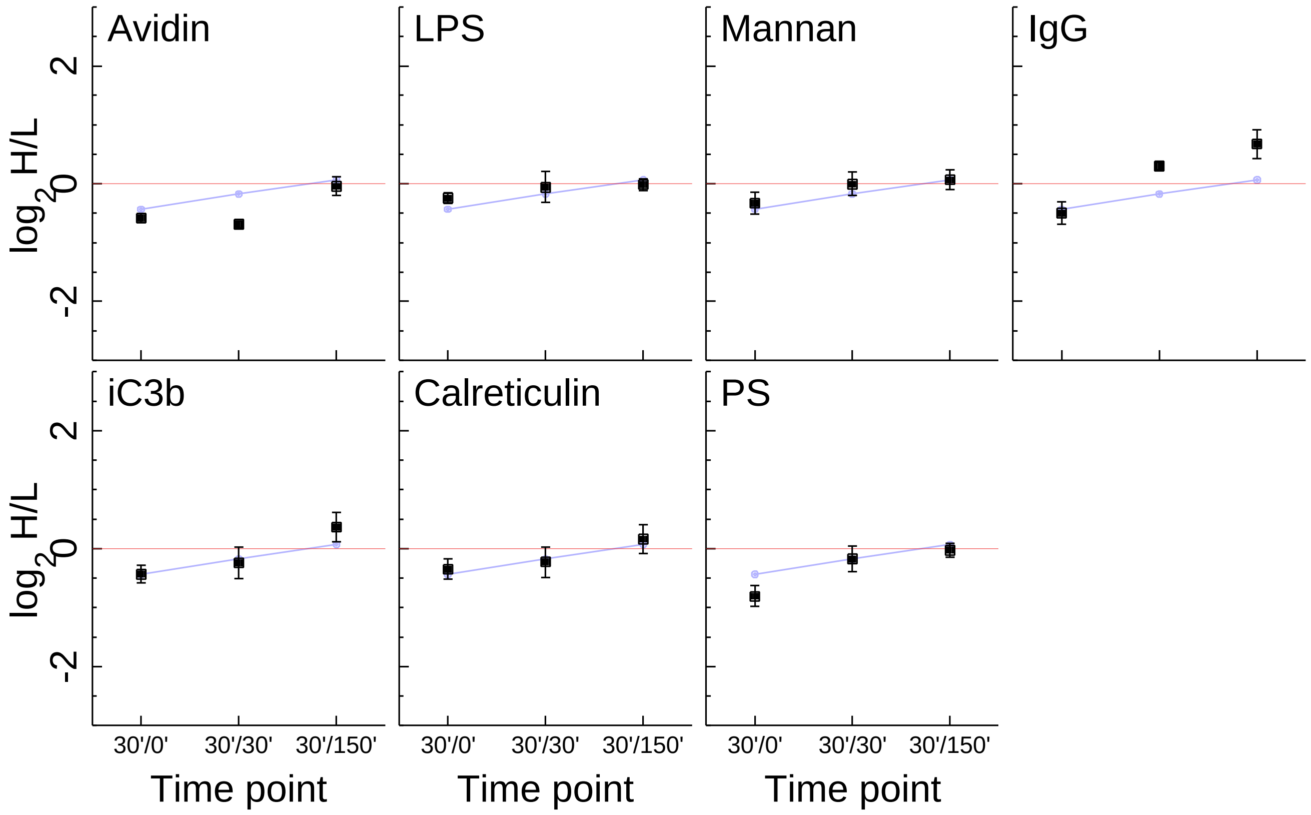

ppp1cb (P62141)

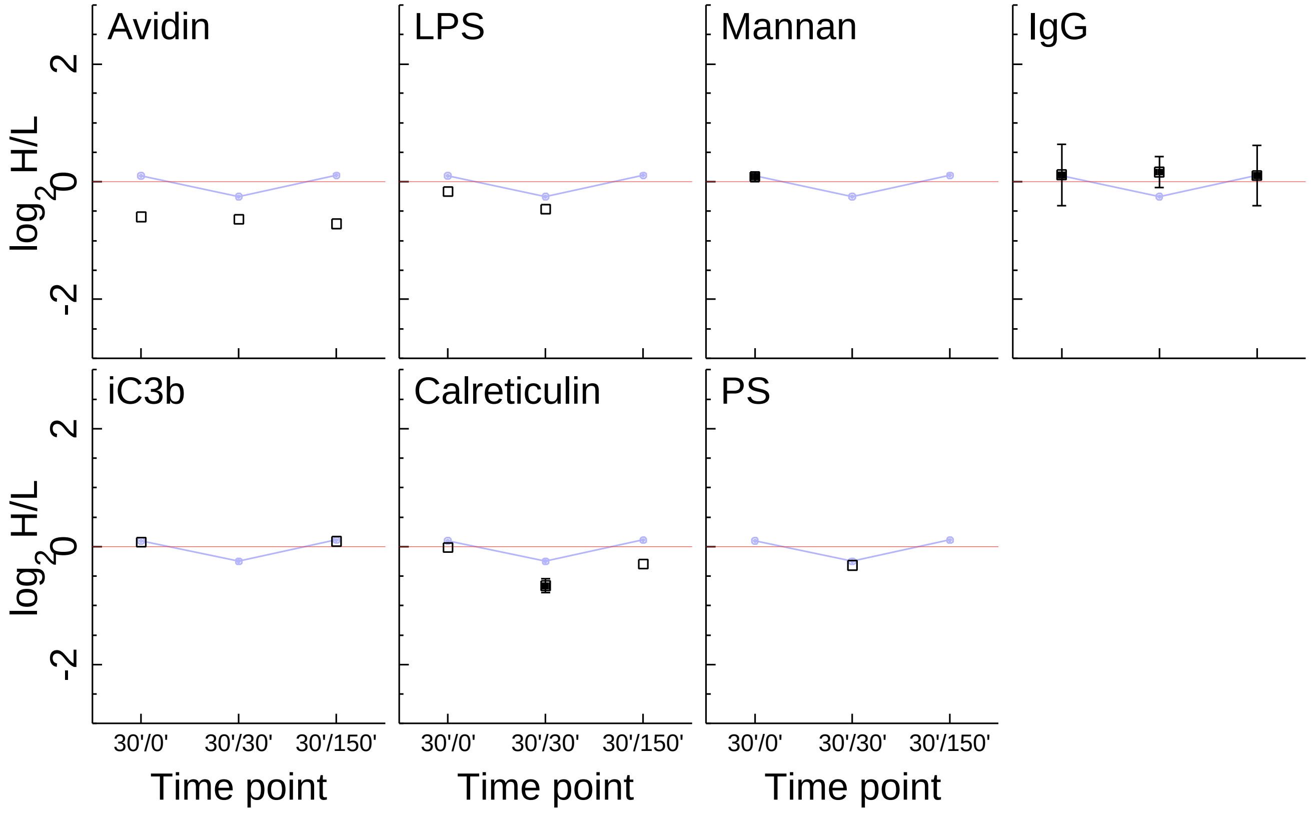

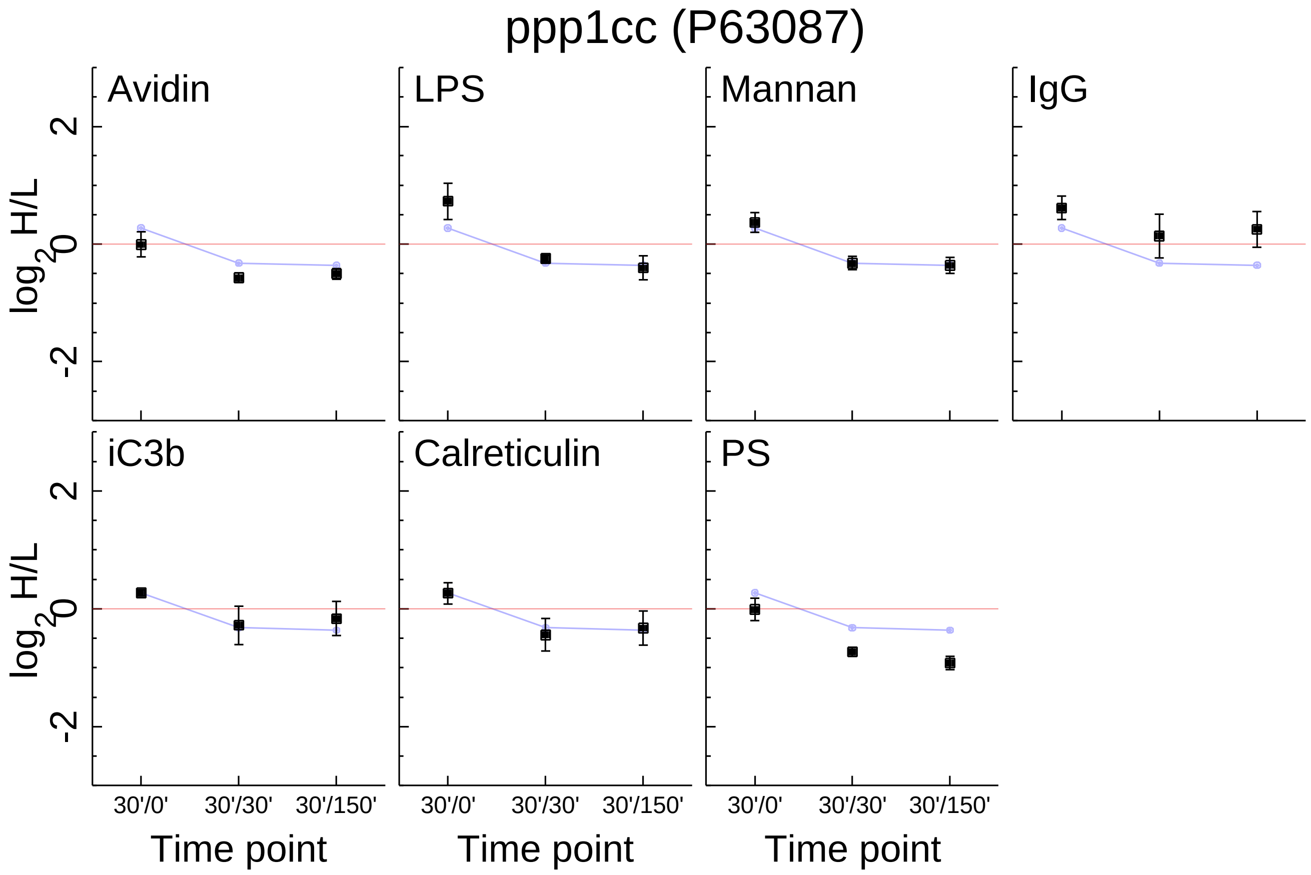
ppp1cc (P63087)
Avidin
LPS
Mannan
IgG
iC3b
Calreticulin
PS
log2 H/L
2
0
-2
30'/0'
30'/30'
30'/150'
Time point

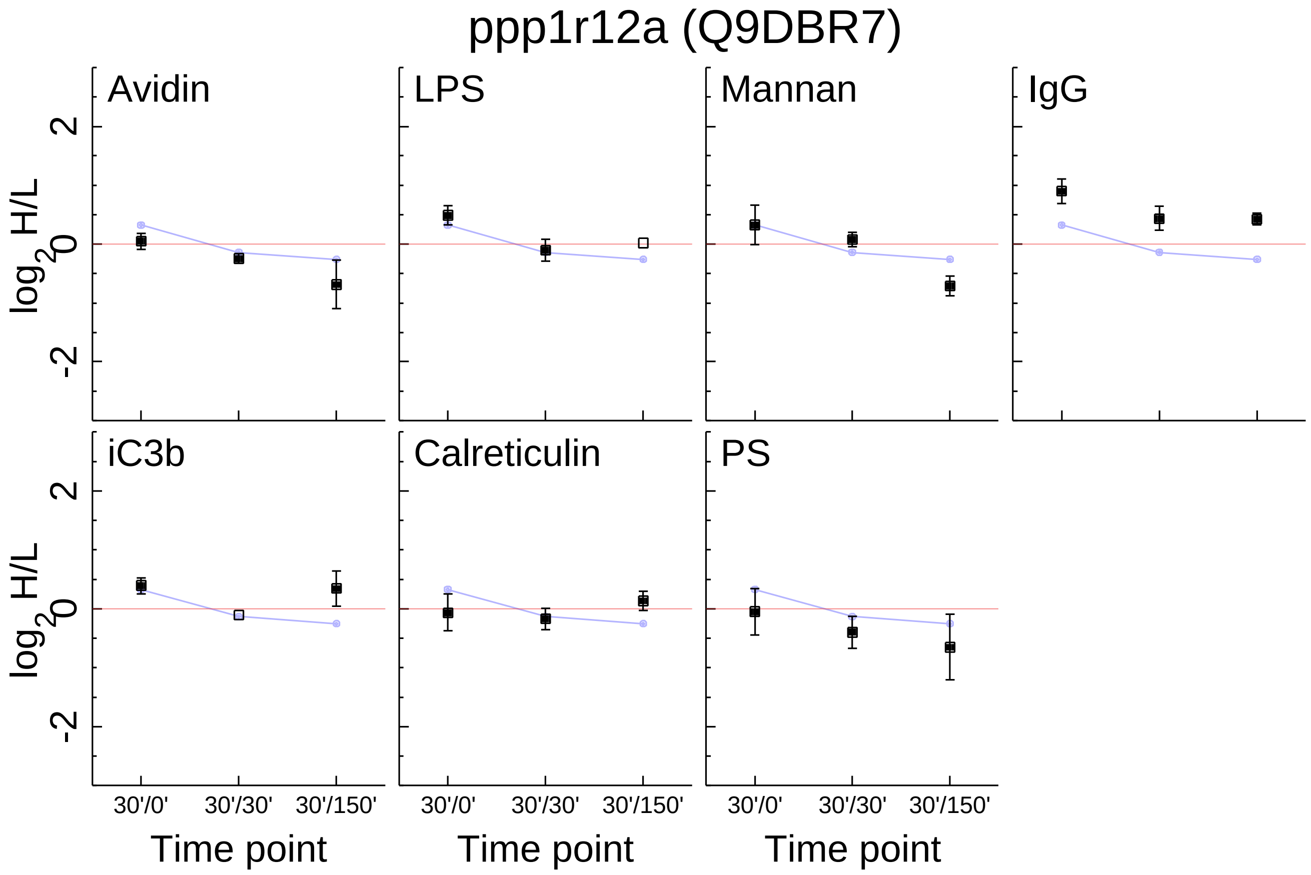
ppp1r12a (Q9DBR7)
Avidin
LPS
Mannan
IgG
iC3b
Calreticulin
PS
log2 H/L
2
0
-2
30'/0'
30'/30'
30'/150'
Time point

ppp1r12c (Q3UMT1)

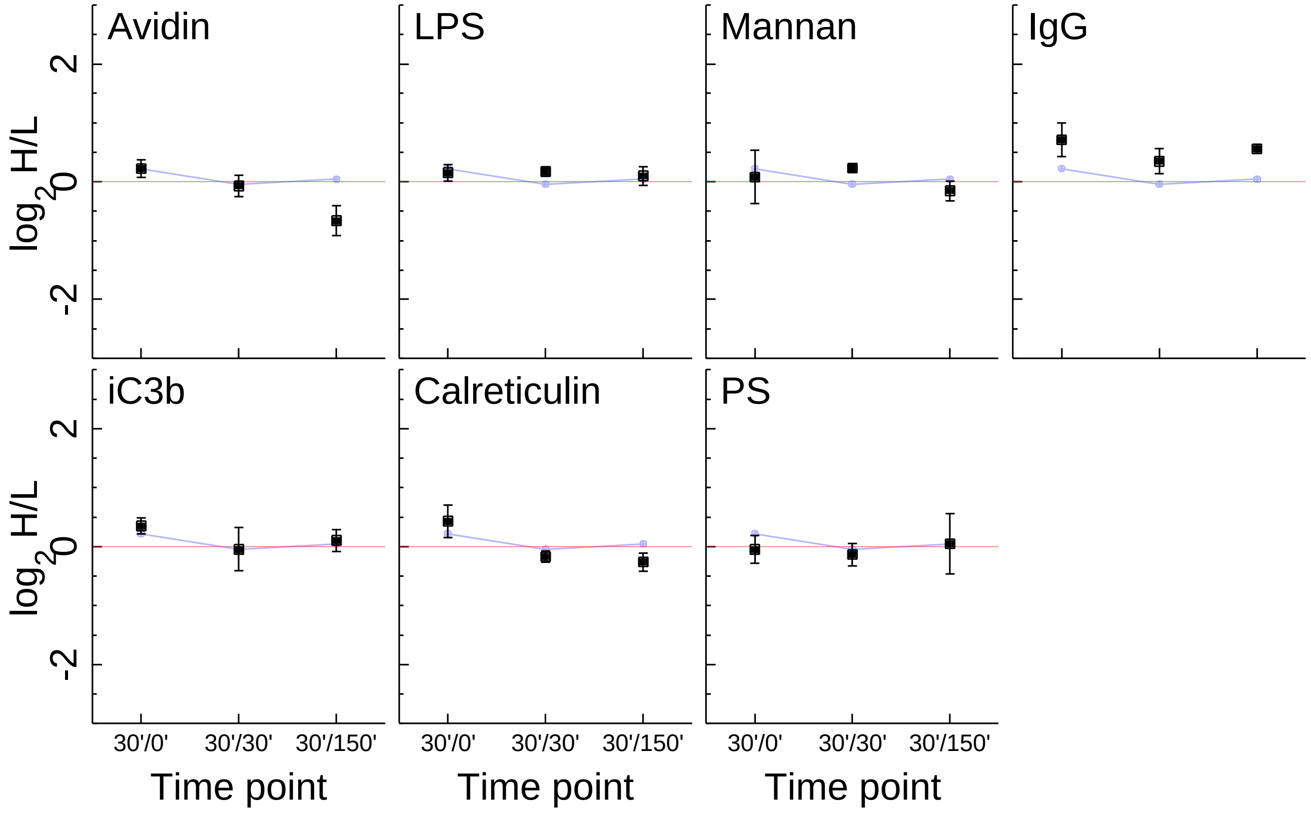

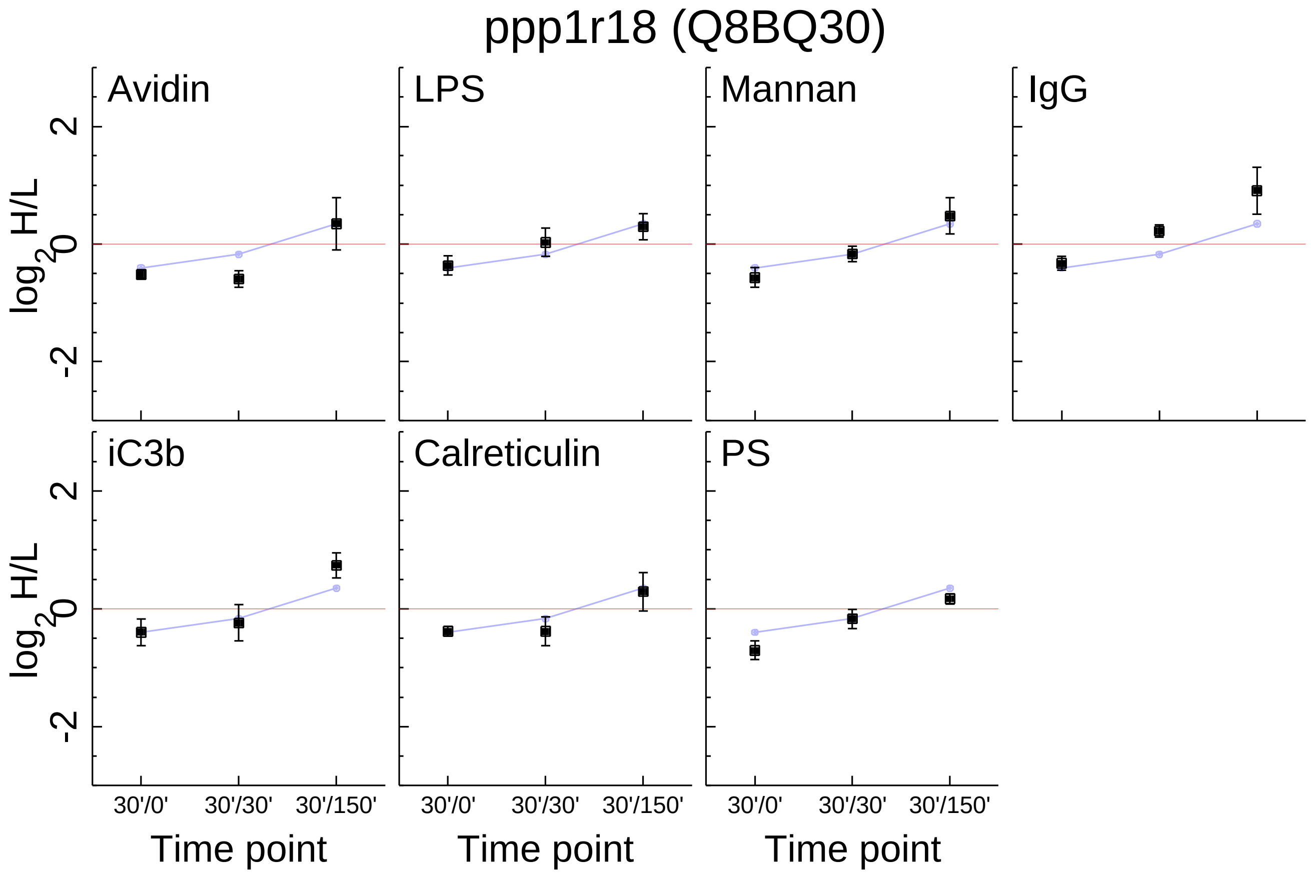

ppp1r18 (Q8BQ30)
Avidin
LPS
Mannan
IgG
iC3b
Calreticulin
PS
log2 H/L
2
0
-2
30'/0'
30'/30'
30'/150'
Time point

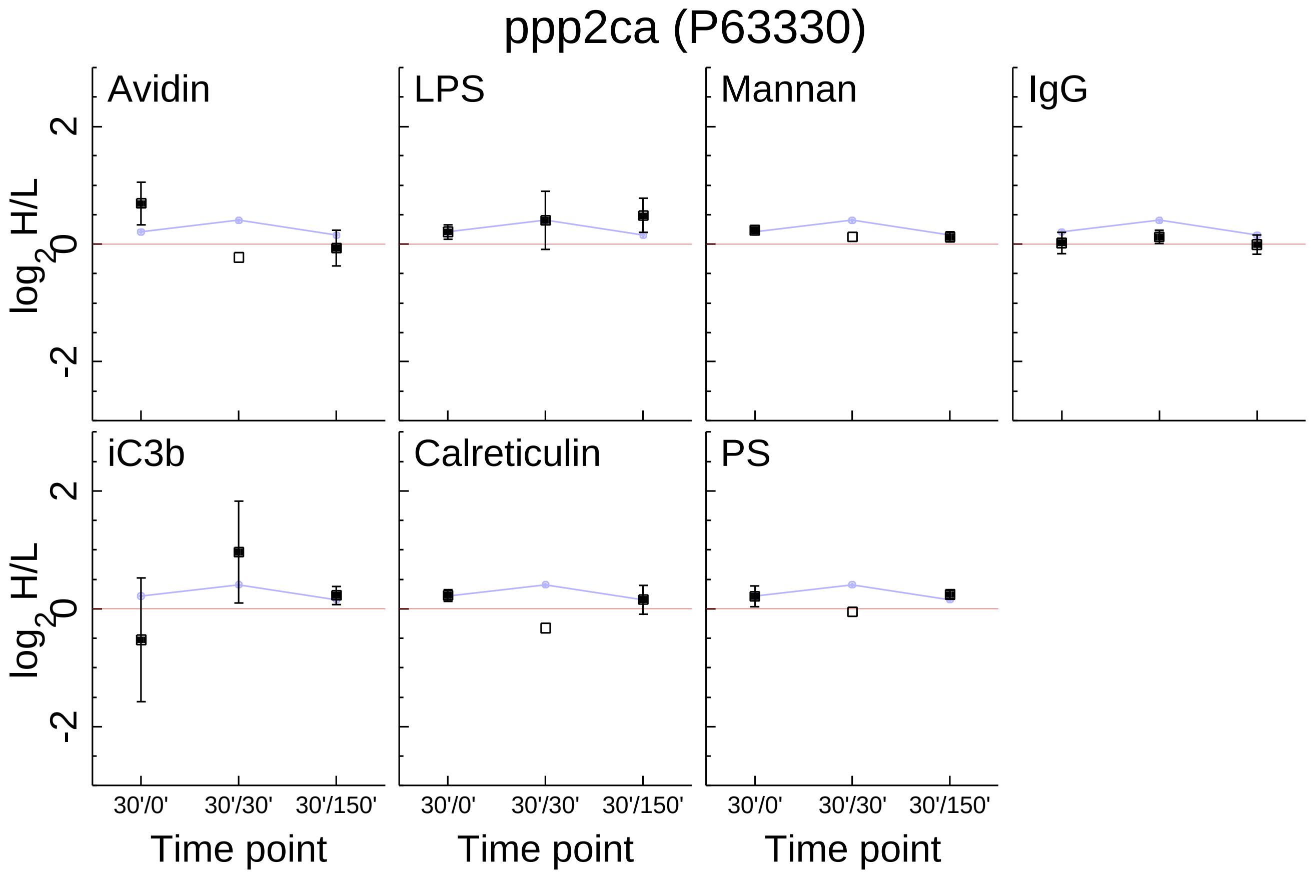
ppp2ca (P63330)
Avidin
LPS
Mannan
IgG
iC3b
Calreticulin
PS
log2 H/L
2
0
-2
30'/0'
30'/30'
30'/150'
Time point

ppp2r1a (Q76MZ3)

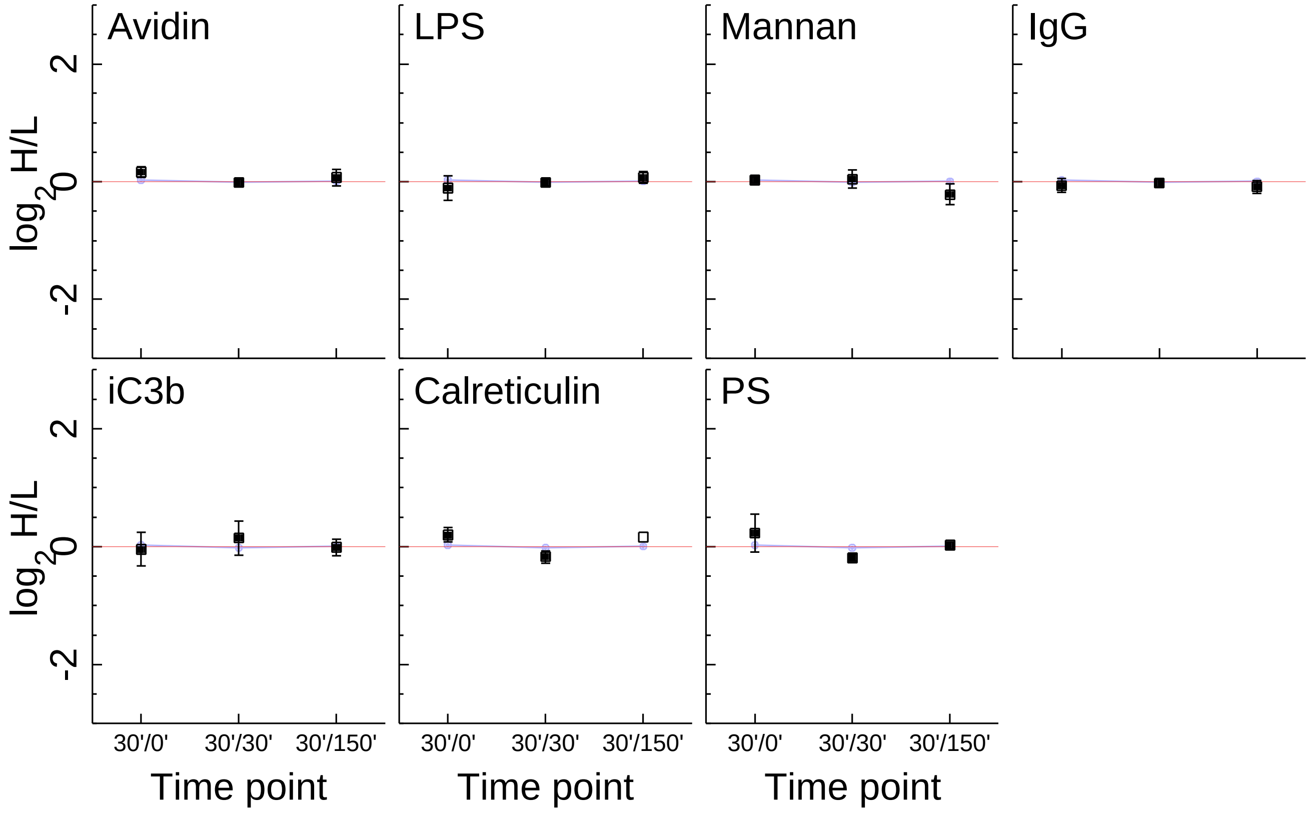

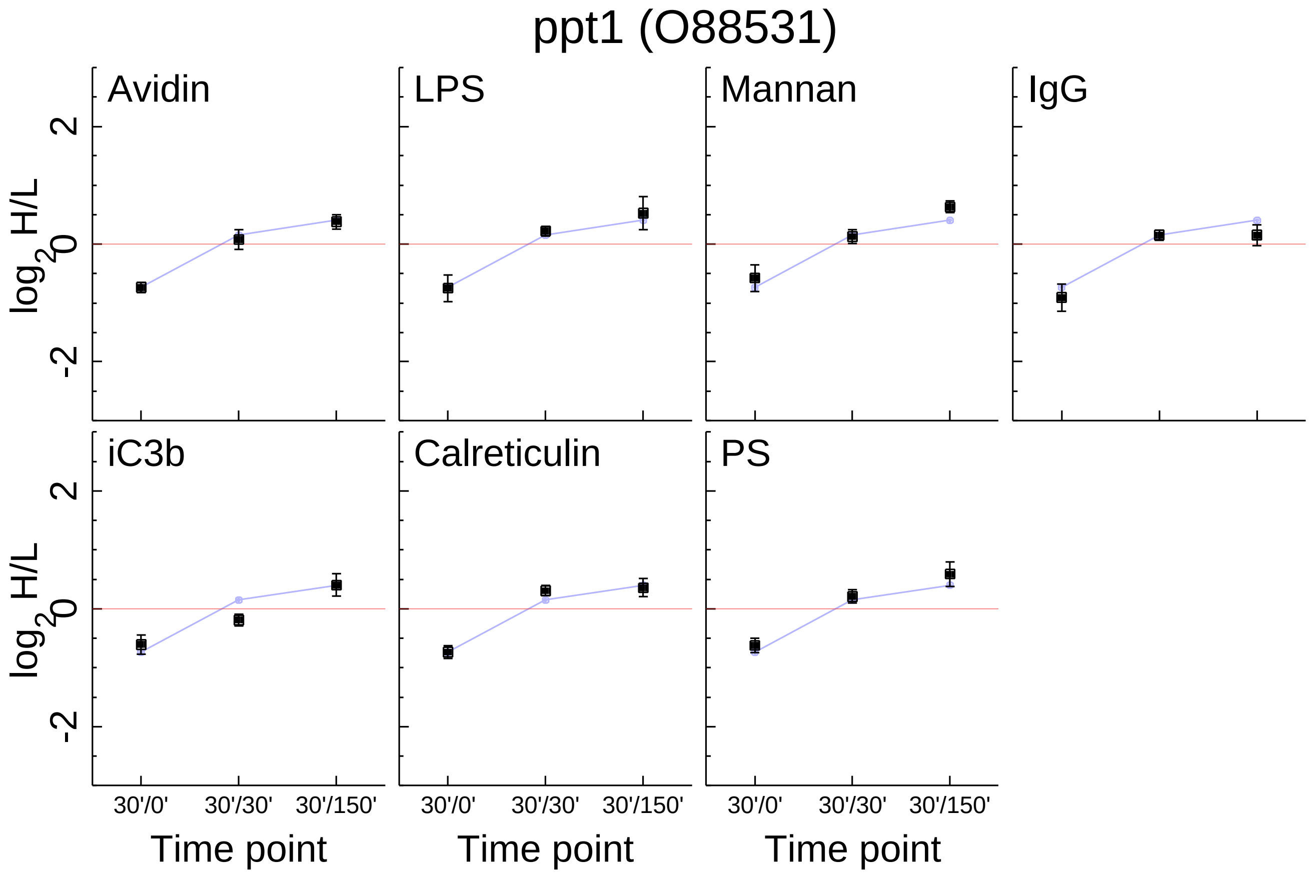
ppt1 (O88531)
Avidin
LPS
Mannan
IgG
iC3b
Calreticulin
PS
log2 H/L
2
0
-2
30'/0'
30'/30'
30'/150'
Time point

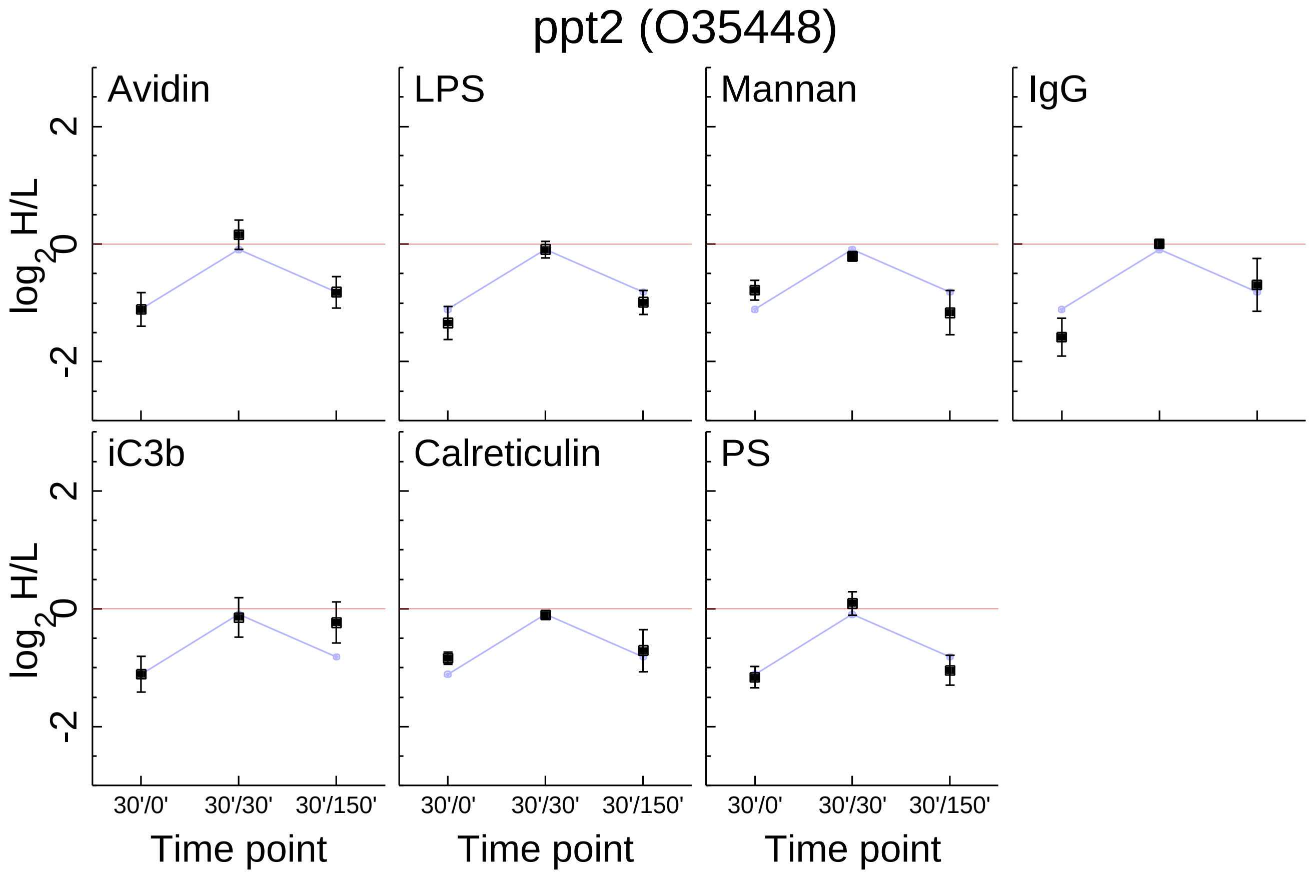
ppt2 (O35448)
Avidin
LPS
Mannan
IgG
iC3b
Calreticulin
PS
log2 H/L
2
0
-2
30'/0'
30'/30'
30'/150'
Time point

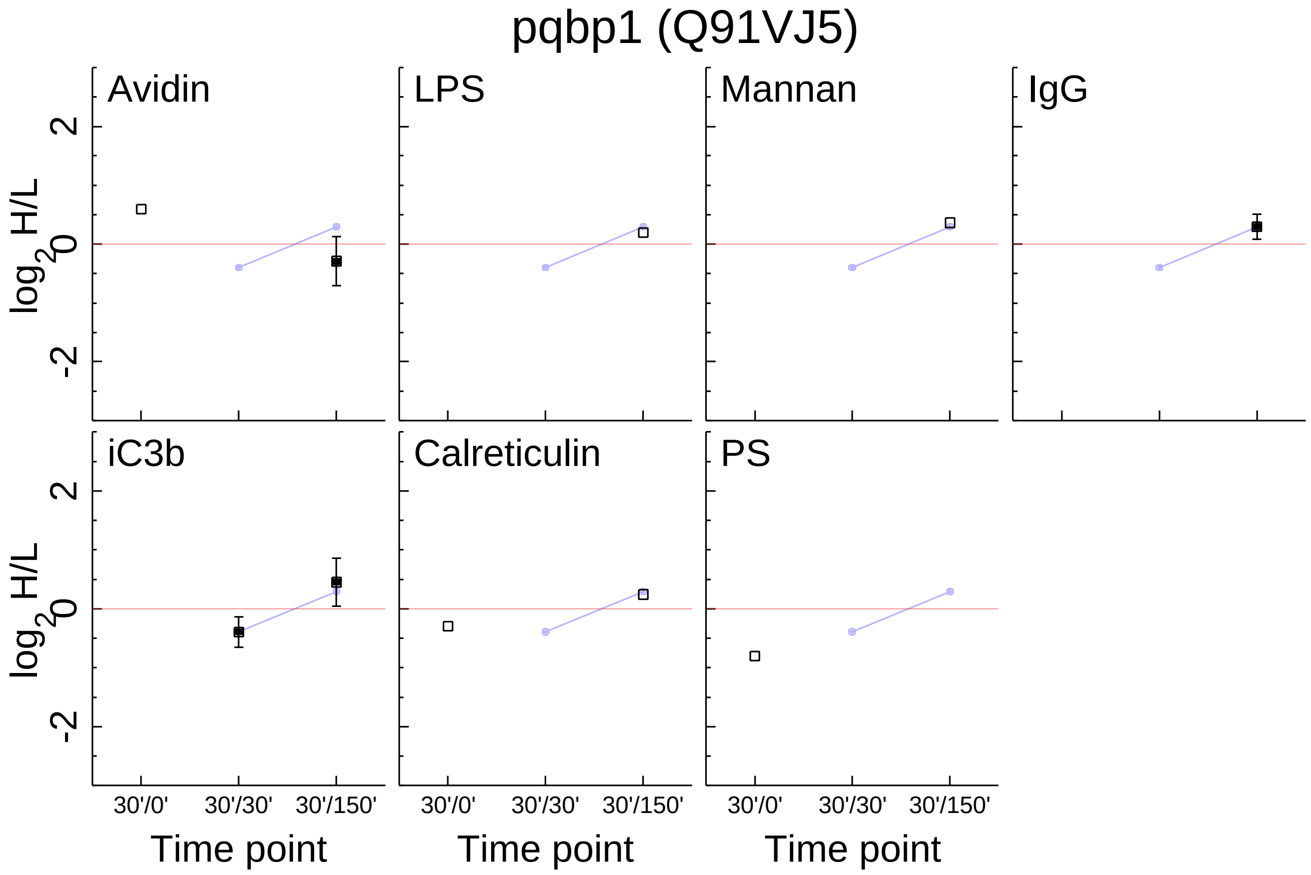
pqbp1 (Q91VJ5)
Avidin
LPS
Mannan
IgG
iC3b
Calreticulin
PS
log2 H/L
2
0
-2
30'/0'
30'/30'
30'/150'
Time point

pqlc2 (Q8C4N4)

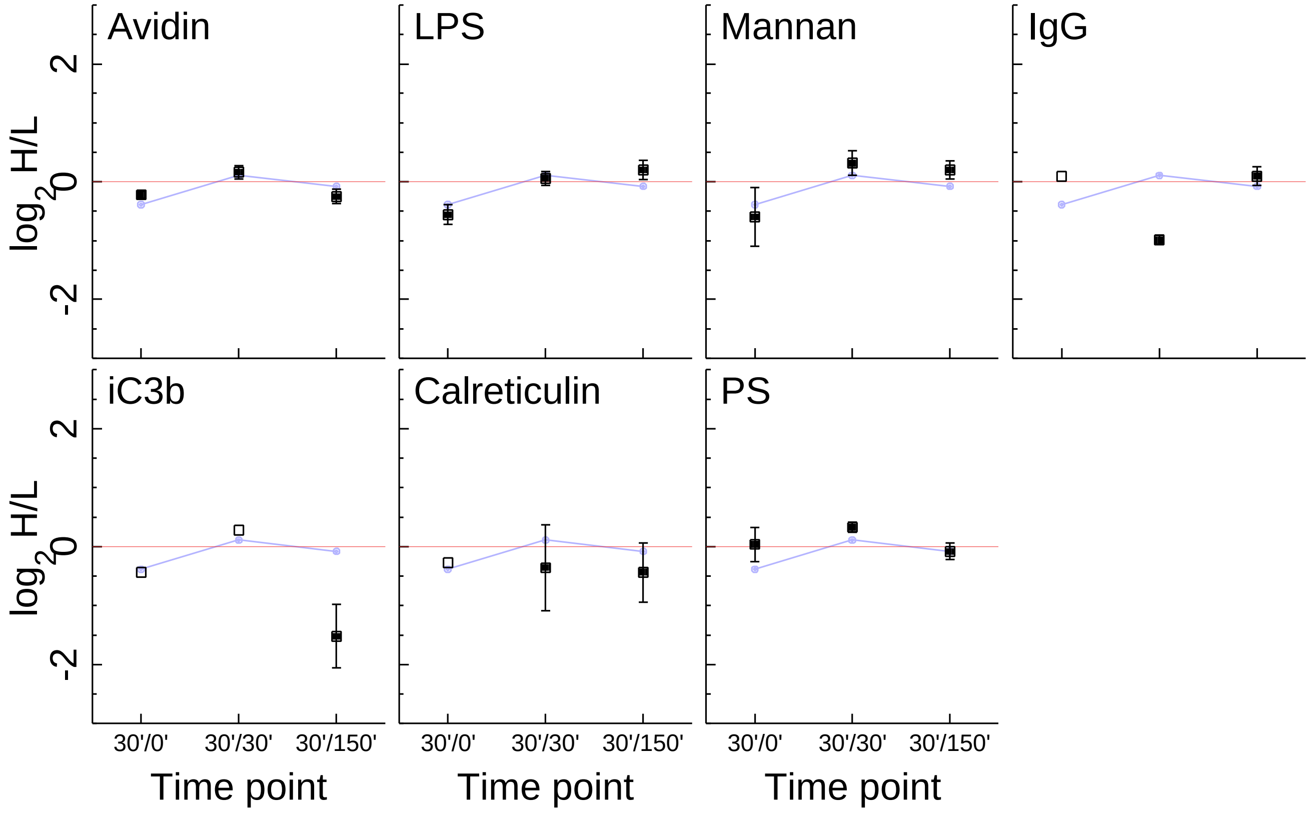

prcc (Q9EQC8)

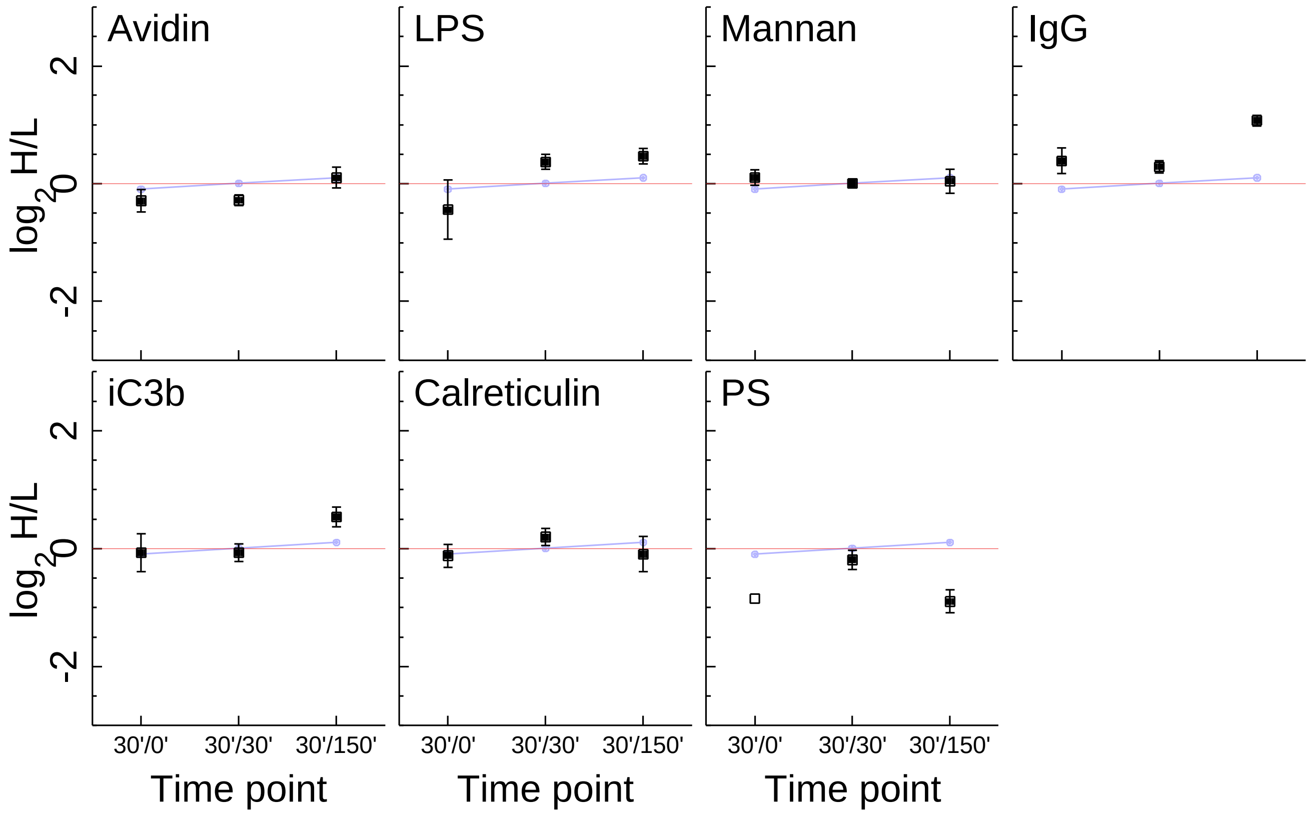

Avidin
LPS
Mannan
IgG
iC3b
Calreticulin
PS
log2 H/L
2
0
-2
30'/0'
30'/30'
30'/150'
Time point

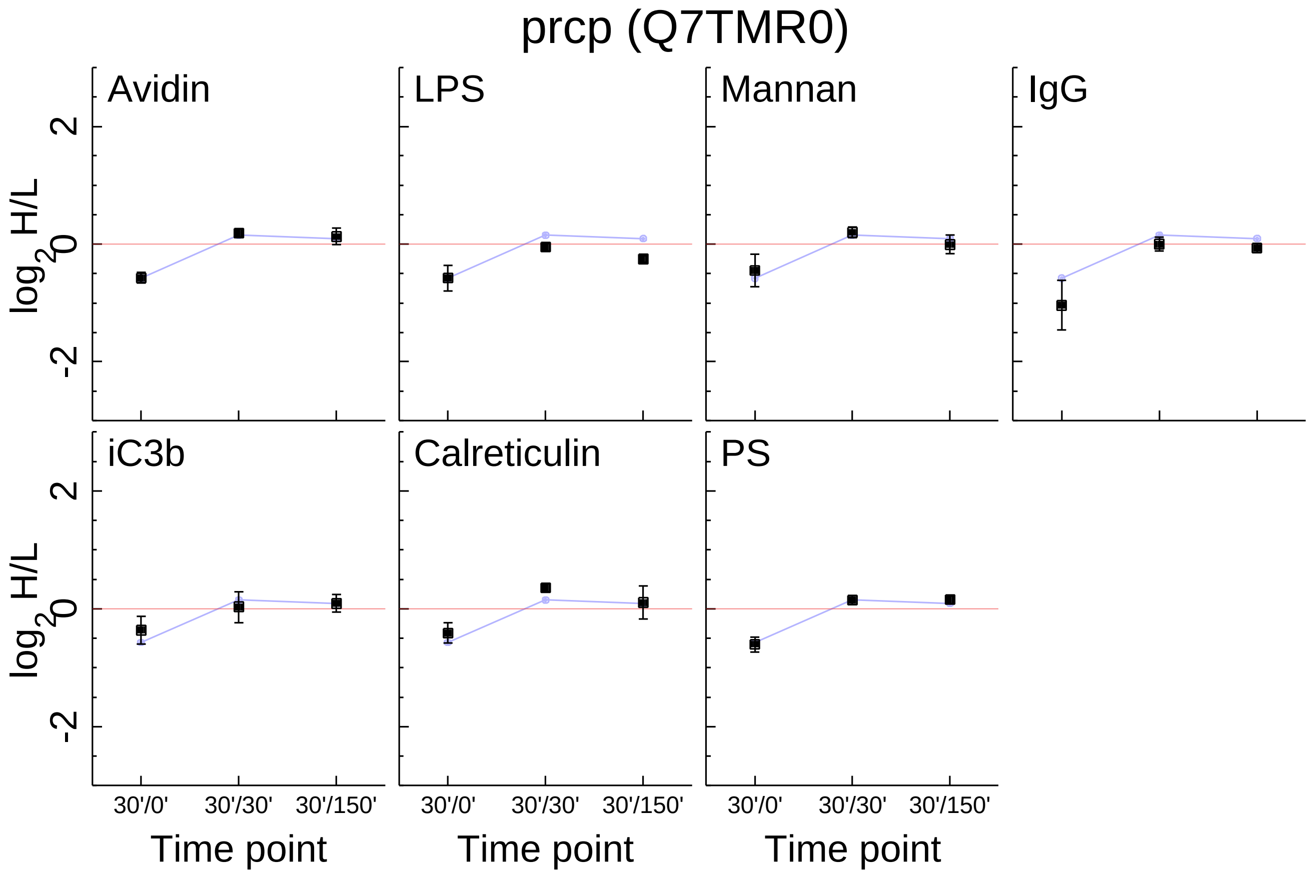
prcp (Q7TMR0)
Avidin
LPS
Mannan
IgG
iC3b
Calreticulin
PS
log2 H/L
2
0
-2
30'/0'
30'/30'
30'/150'
Time point

prdx1 (P35700)

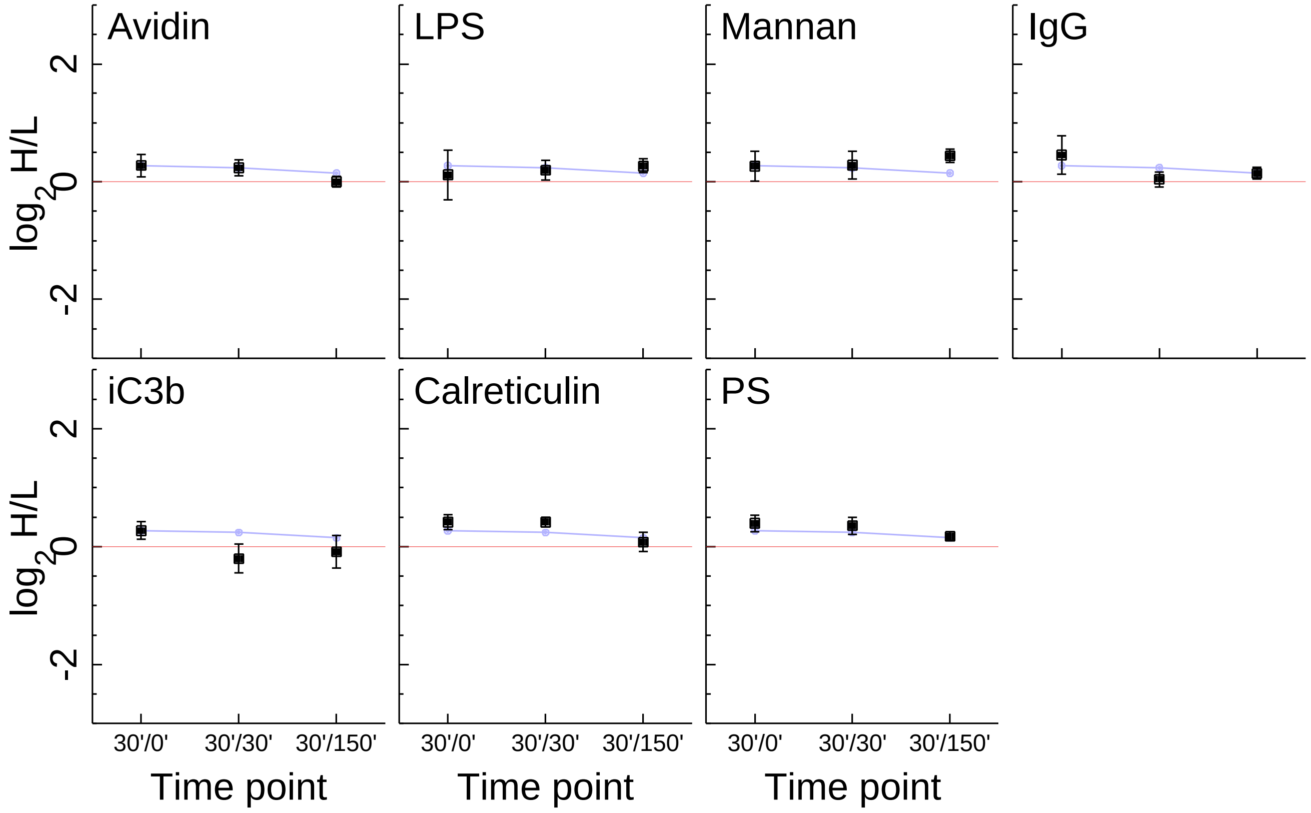

prdx2 (Q61171)

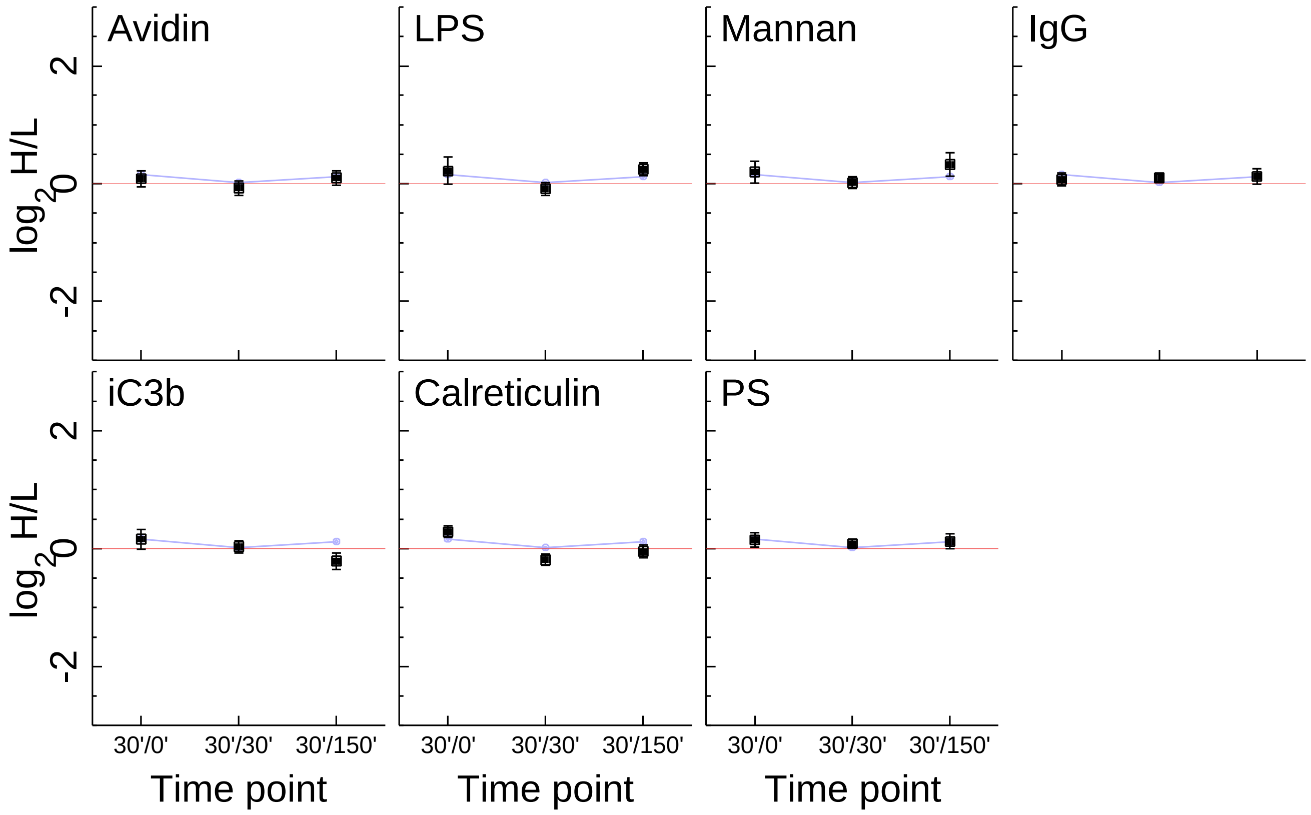

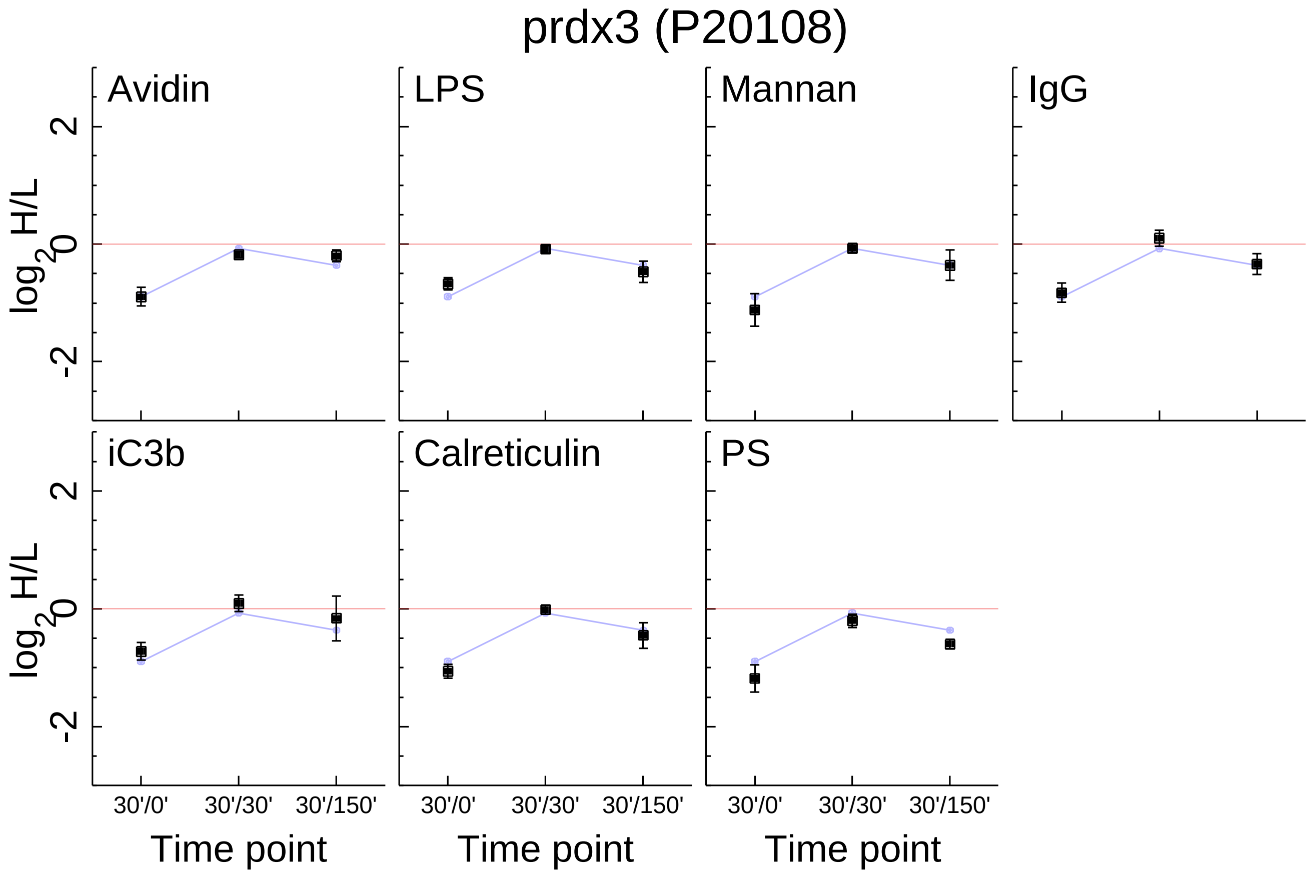

prdx3 (P20108)
Avidin
LPS
Mannan
IgG
iC3b
Calreticulin
PS
log2 H/L
2
0
-2
30'/0'
30'/30'
30'/150'
Time point

prdx4 (O08807)

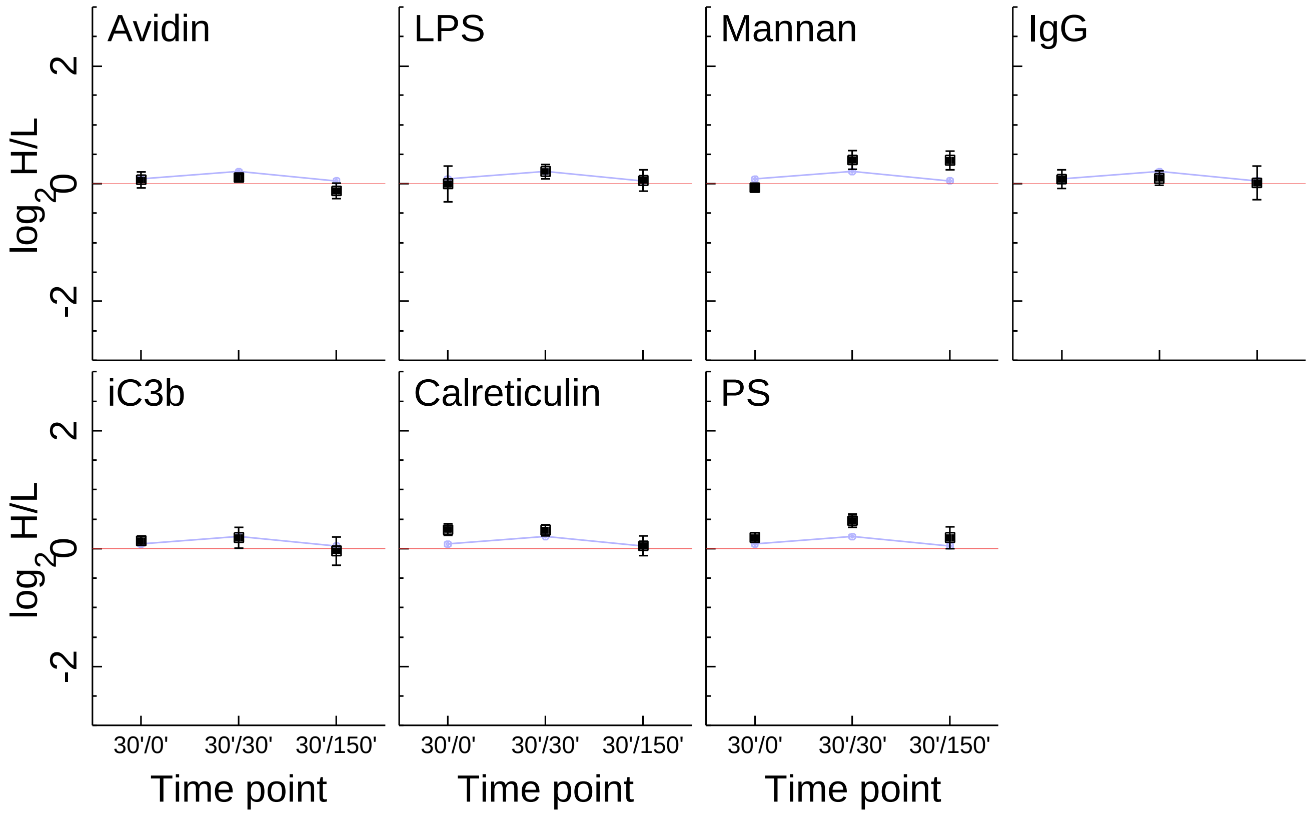

prdx5 (H3BJQ7)

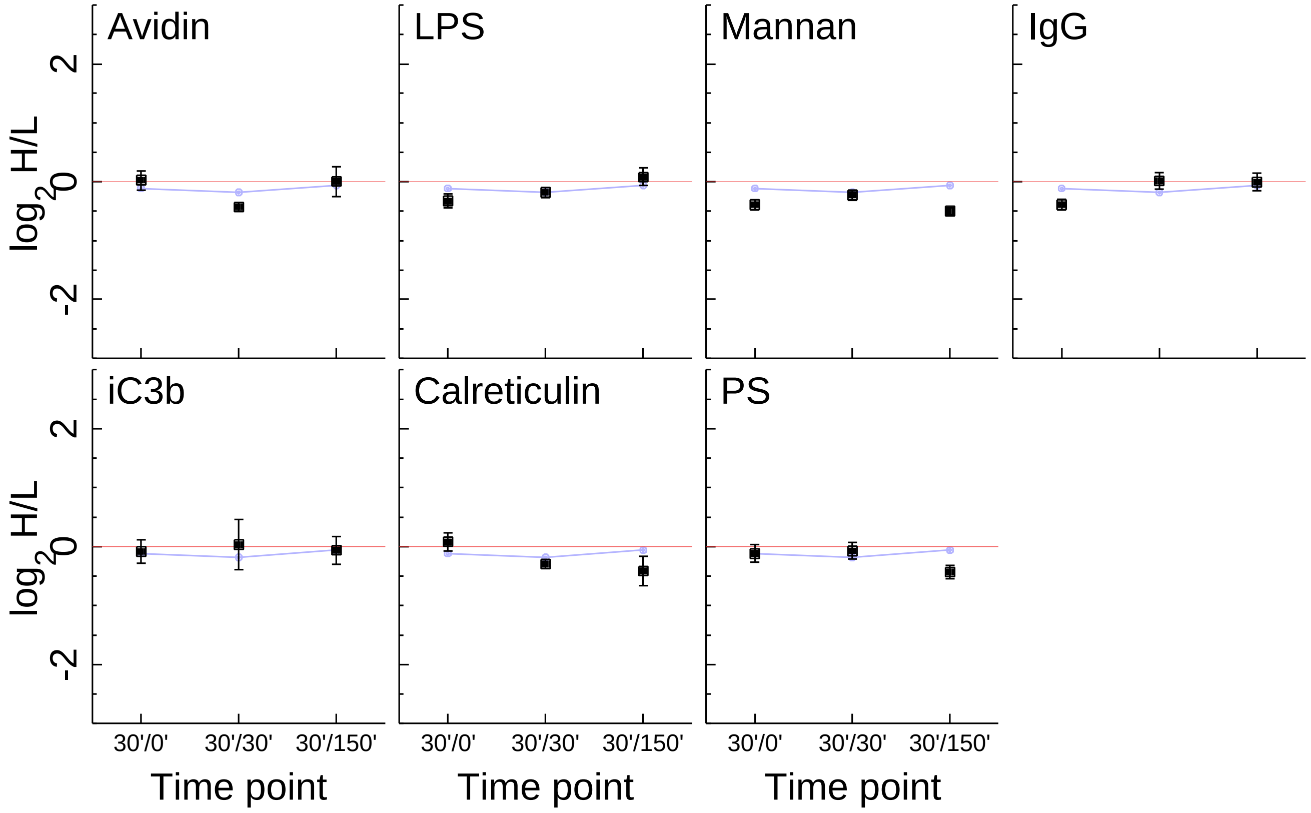

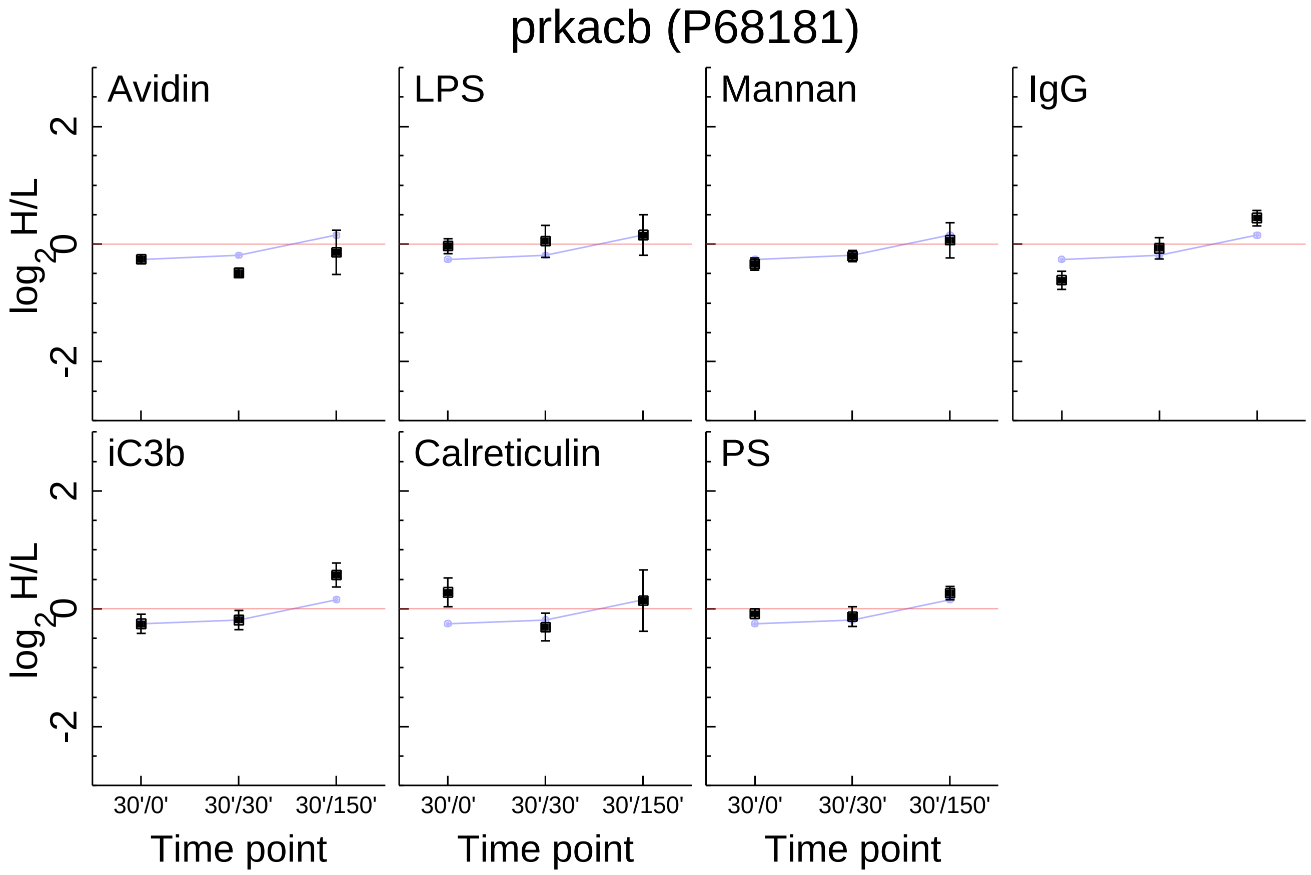
prkacb (P68181)
Avidin
LPS
Mannan
IgG
iC3b
Calreticulin
PS
log2 H/L
2
0
-2
30'/0'
30'/30'
30'/150'
Time point

prkar1a (Q9DBC7)

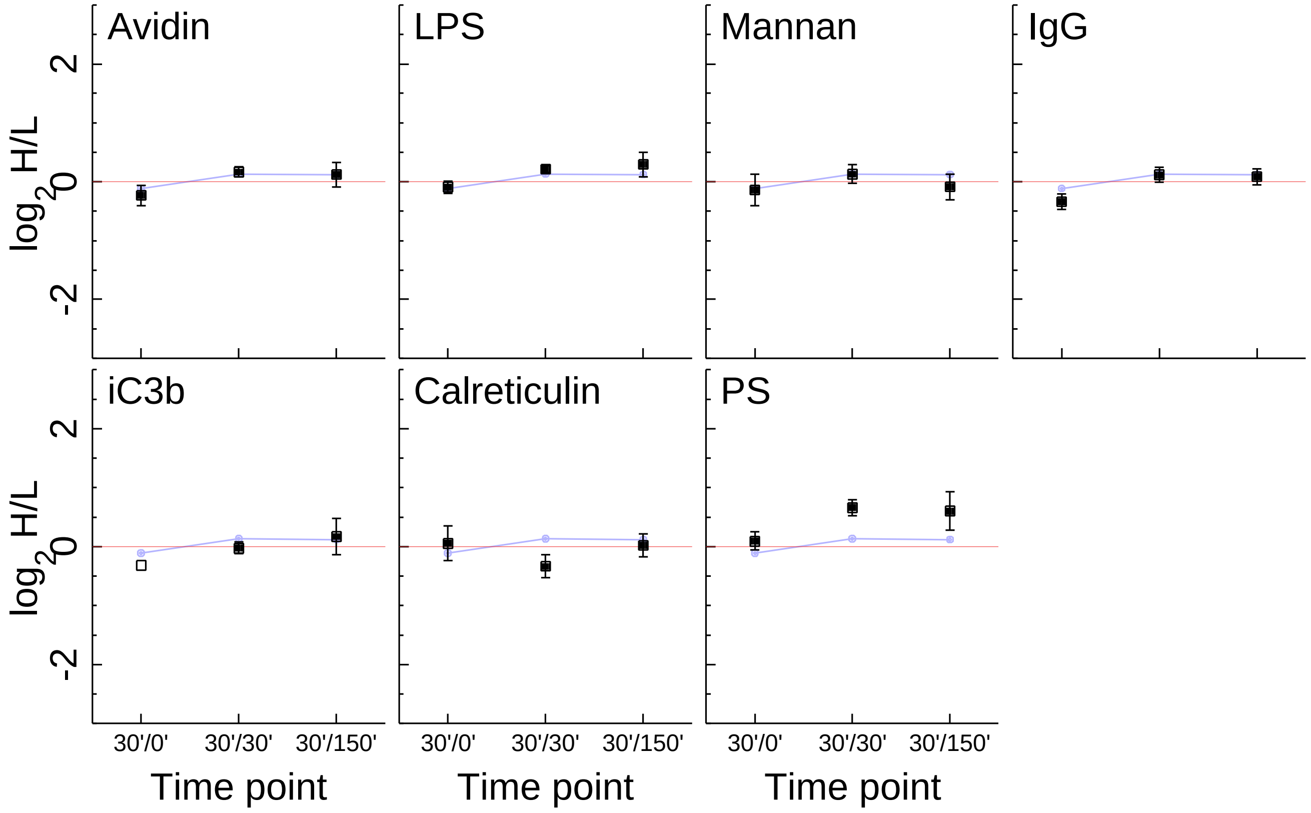

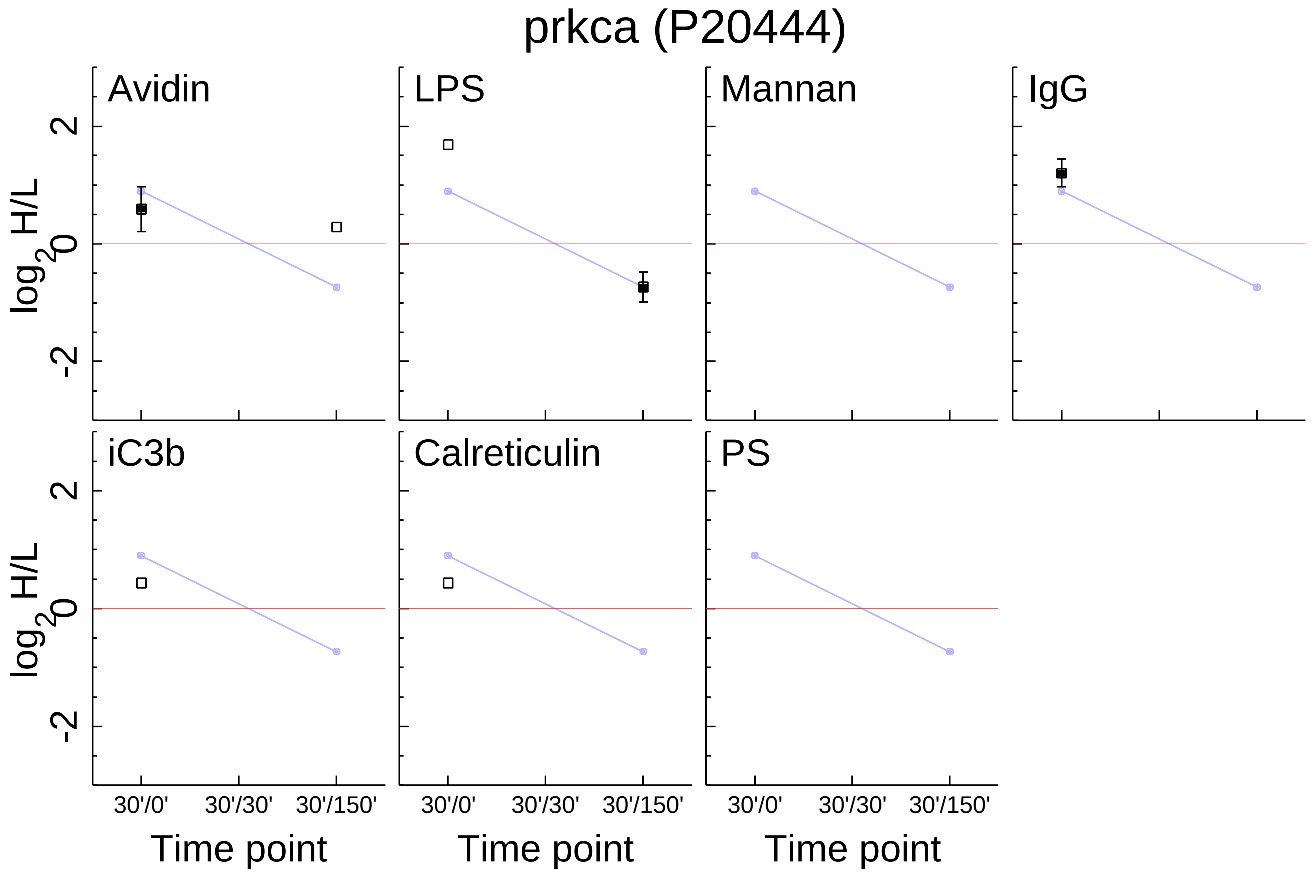
prkca (P20444)
Avidin
LPS
Mannan
IgG
iC3b
Calreticulin
PS
log2 H/L
2
0
-2
30'/0'
30'/30'
30'/150'
Time point

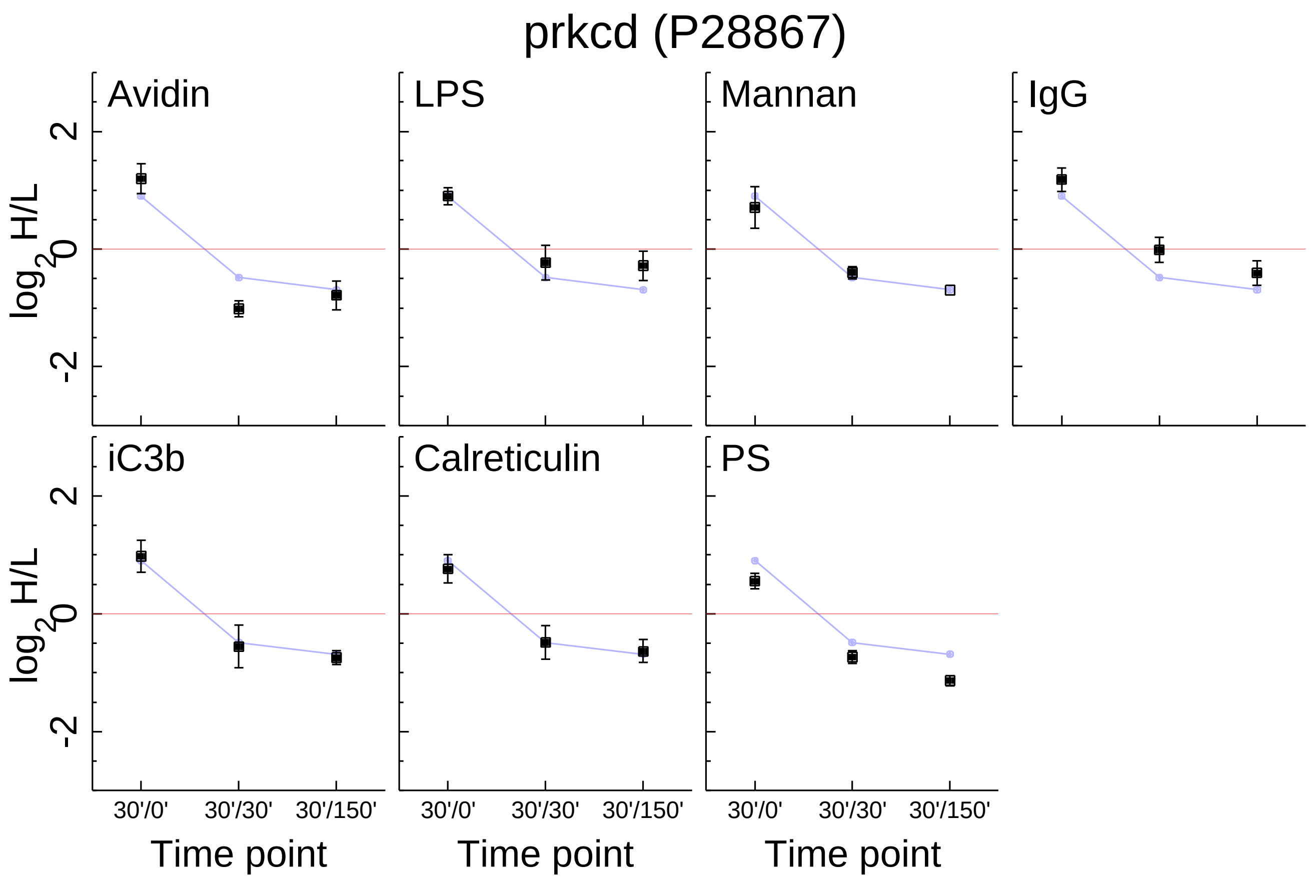
prkcd (P28867)
Avidin
LPS
Mannan
IgG
iC3b
Calreticulin
PS
log2 H/L
2
0
-2
30'/0'
30'/30'
30'/150'
Time point

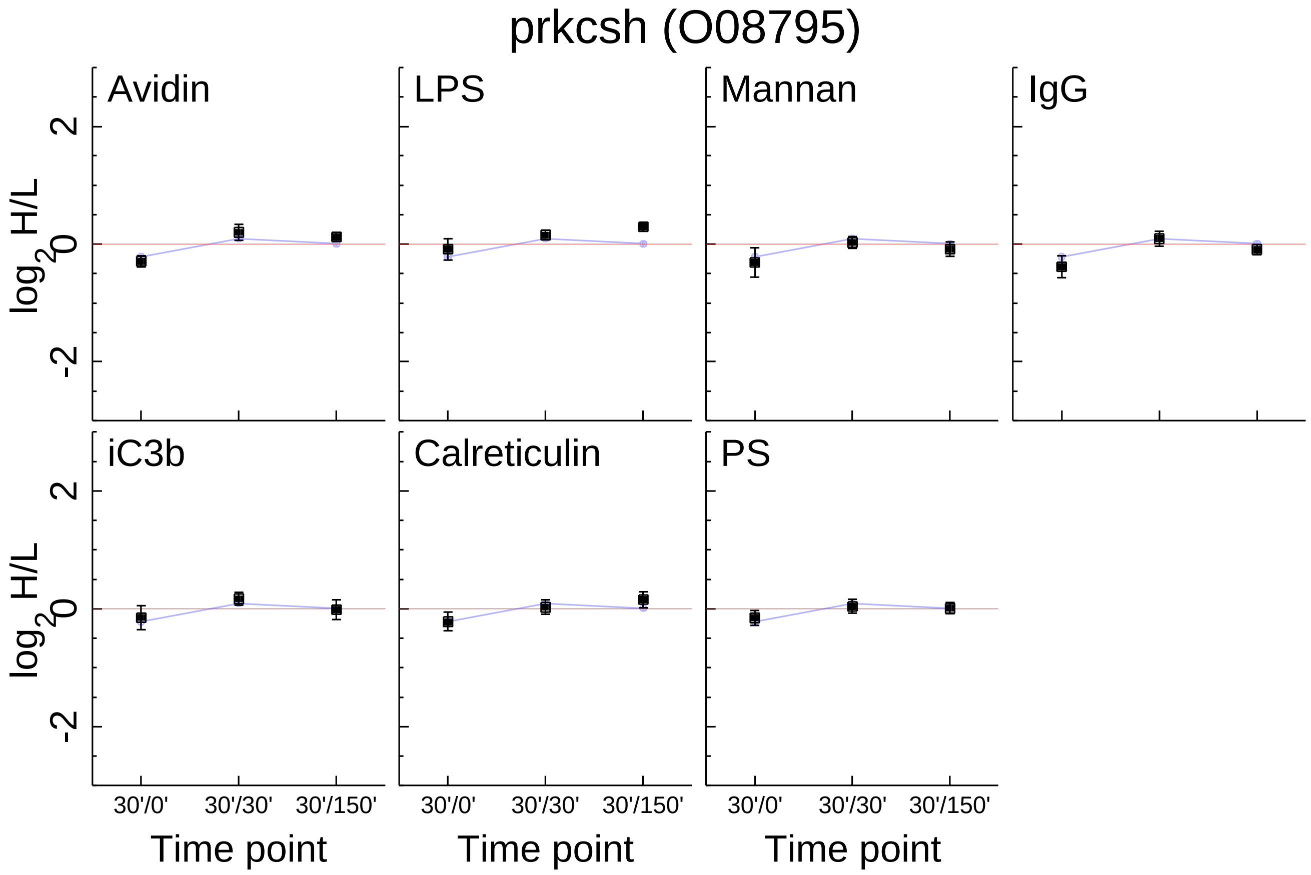
prkcsh (O08795)
Avidin
LPS
Mannan
IgG
iC3b
Calreticulin
PS
log2 H/L
2
0
-2
30'/0'
30'/30'
30'/150'
Time point

prpf19 (J3QNL8)

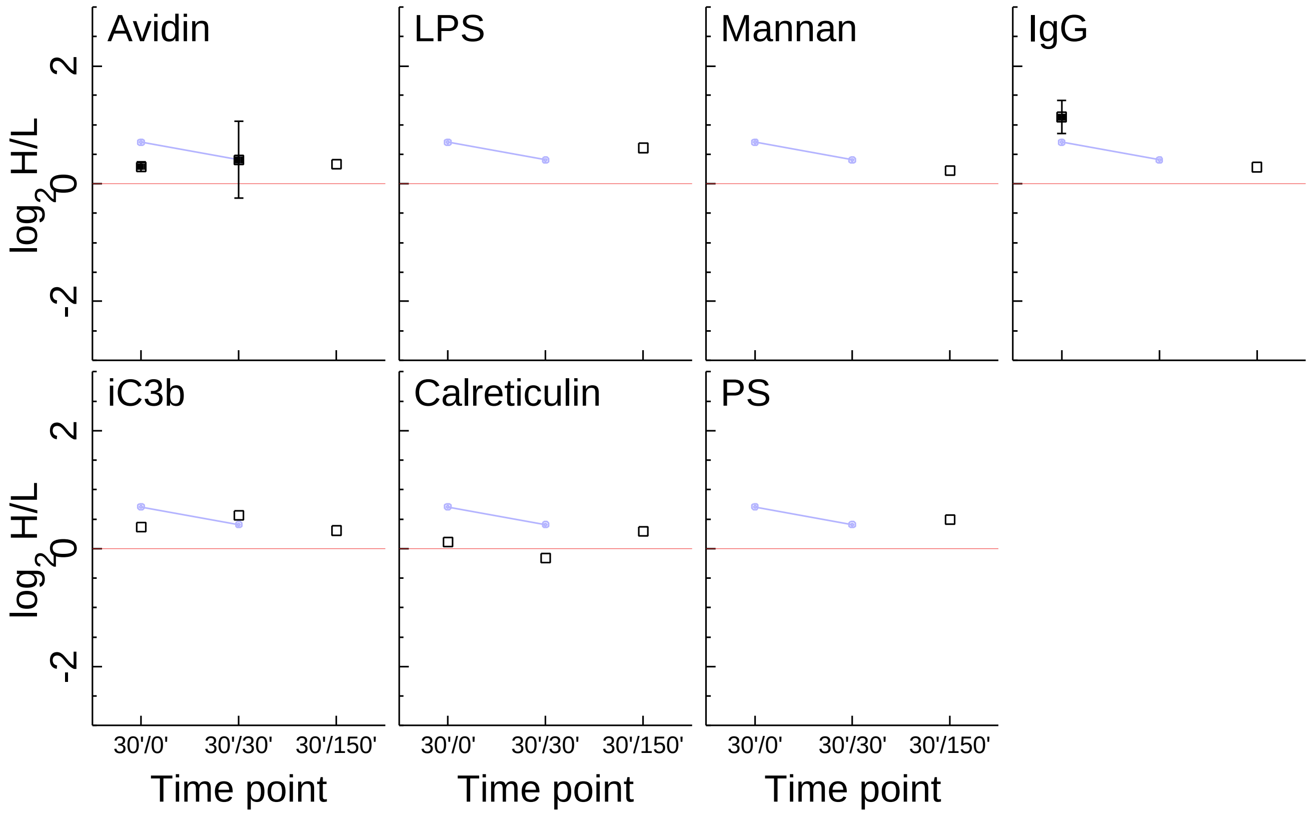

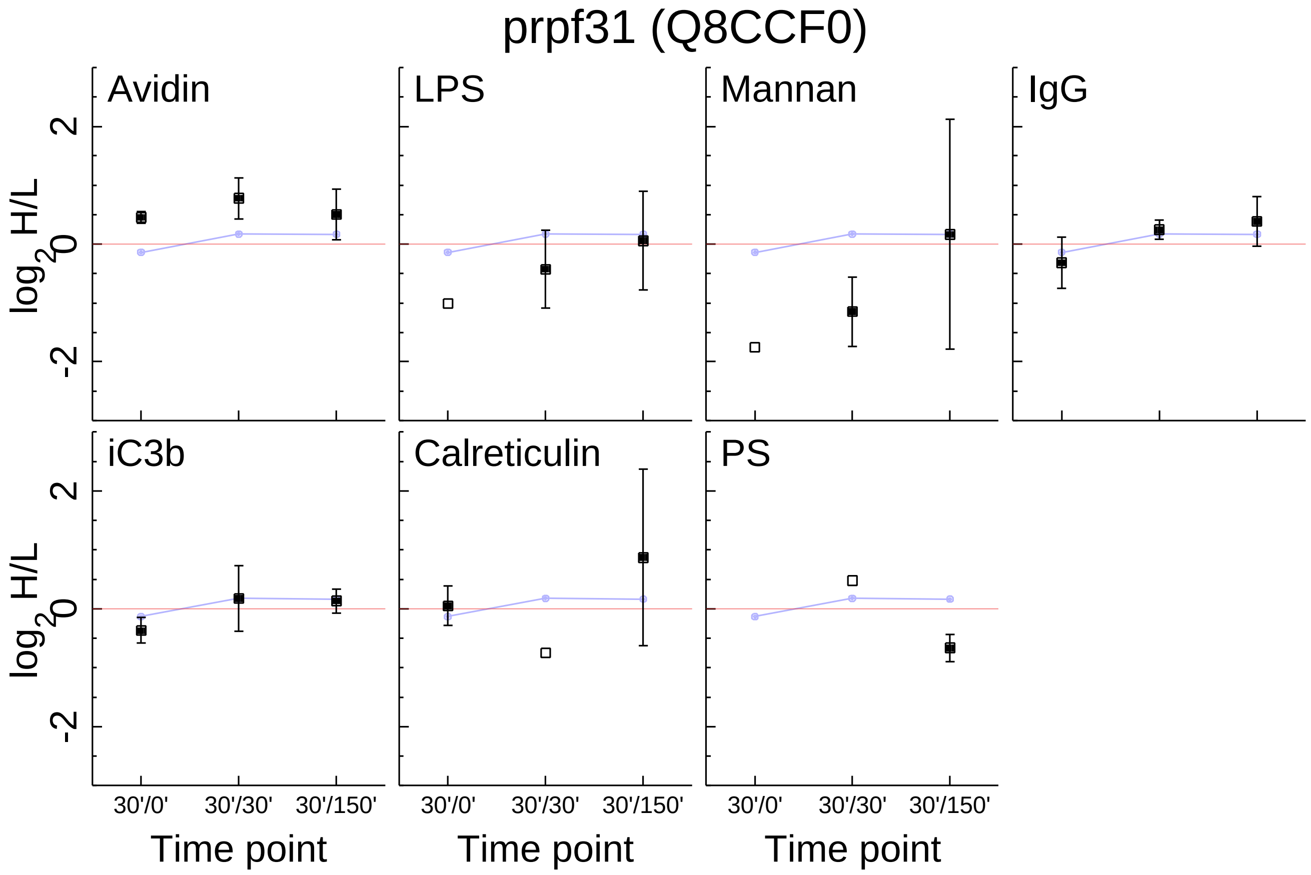

prpf31 (Q8CCF0)
Avidin
LPS
Mannan
IgG
iC3b
Calreticulin
PS
log2 H/L
2
0
-2
30'/0'
30'/30'
30'/150'
Time point

prpf40a (Q9R1C7)

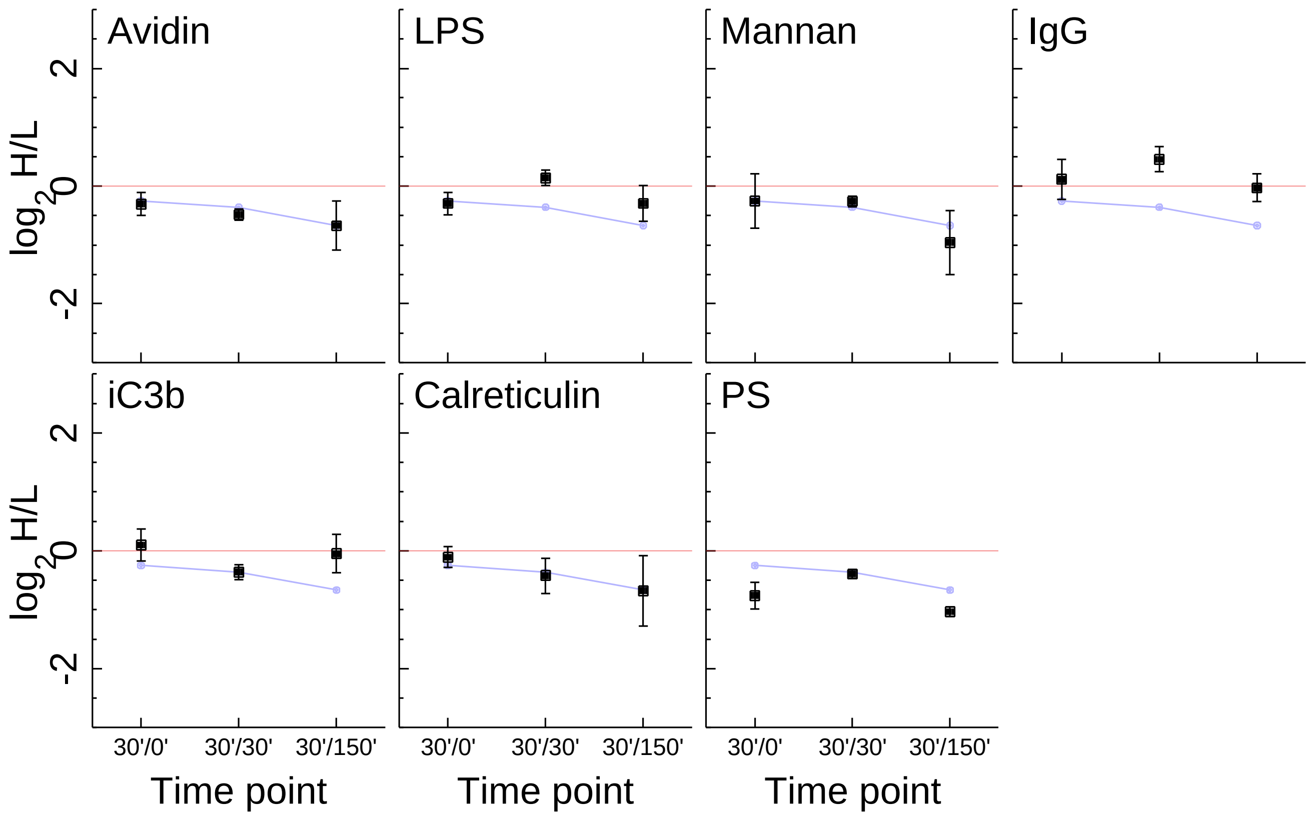

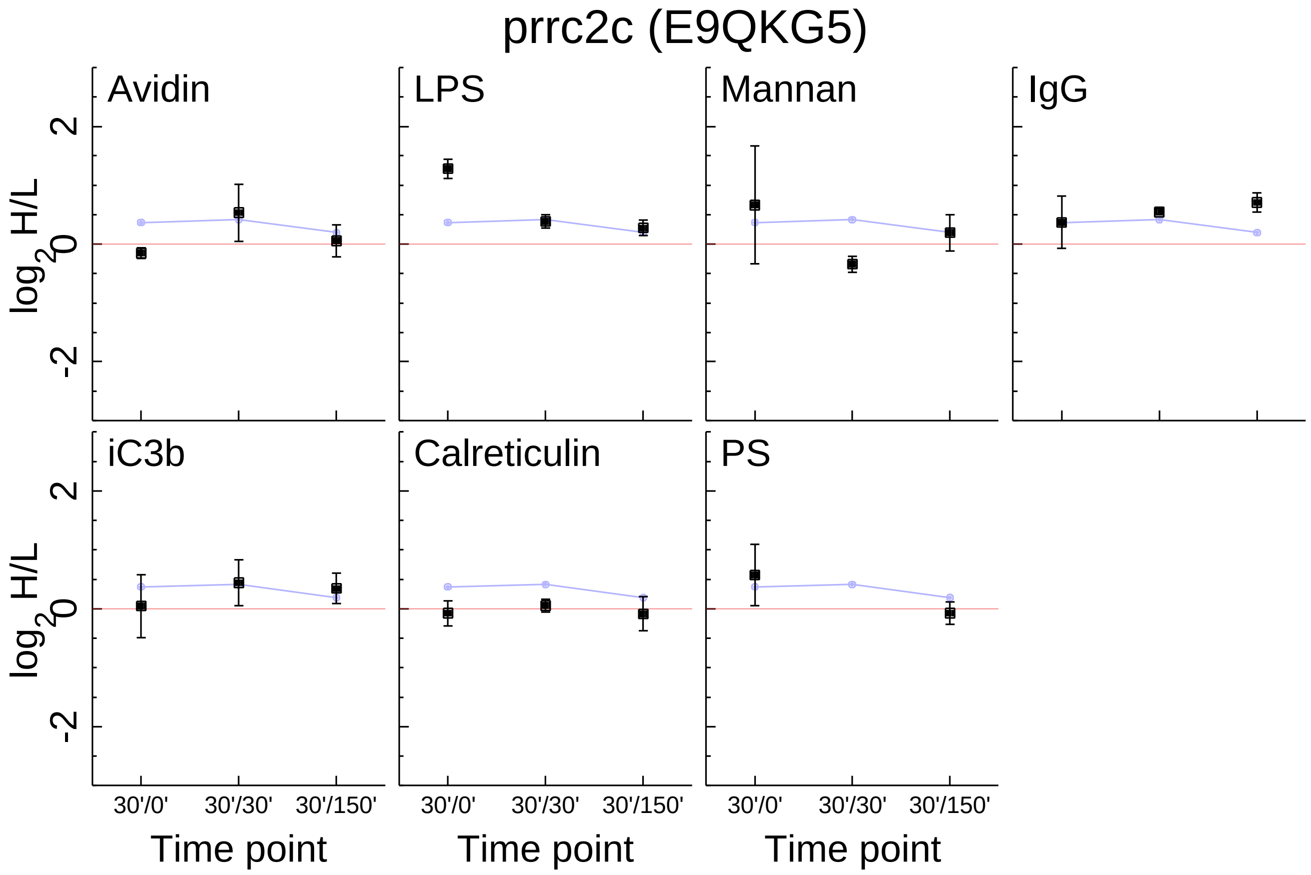

prrc2c (E9QKG5)
Avidin
LPS
Mannan
IgG
iC3b
Calreticulin
PS
log2 H/L
2
0
-2
30'/0'
30'/30'
30'/150'
Time point

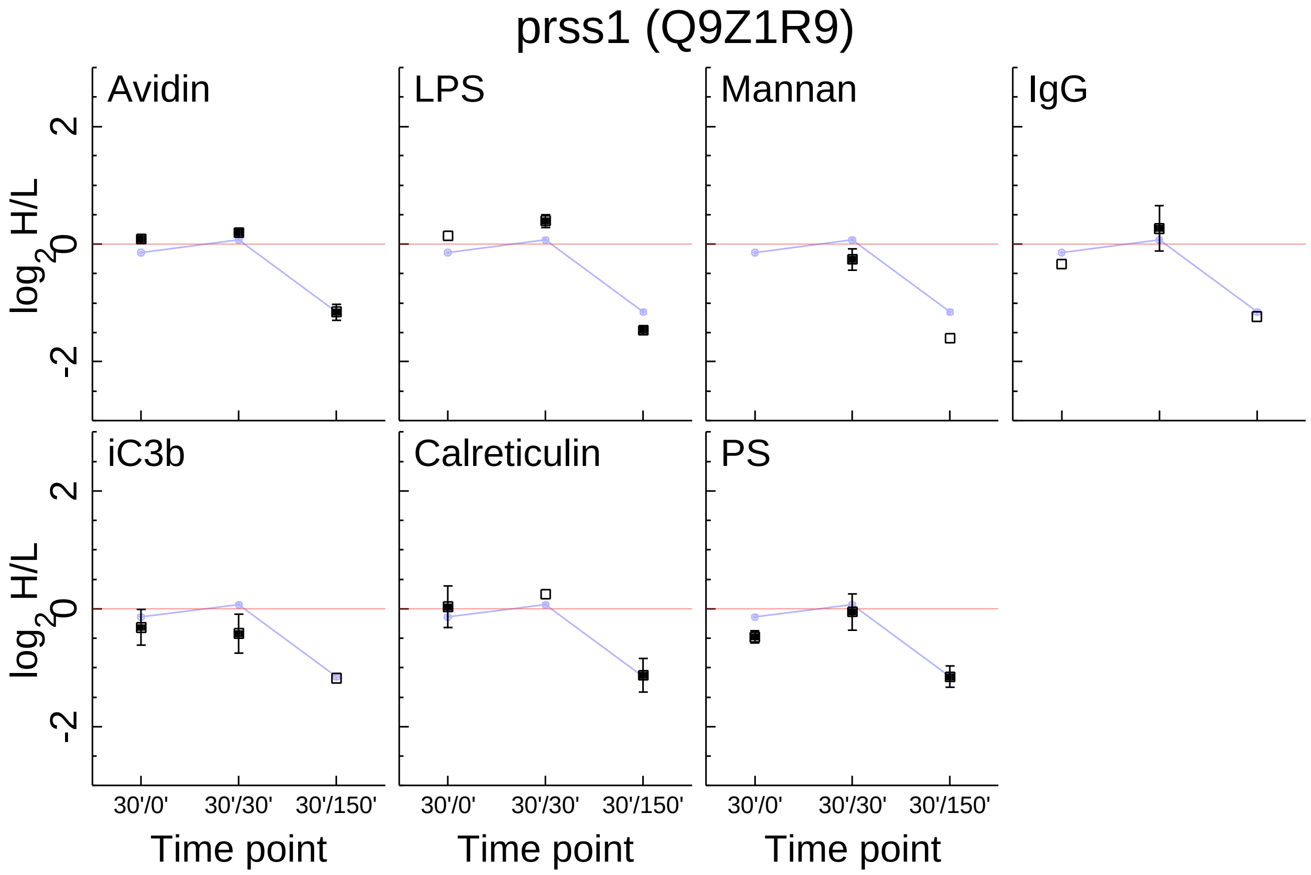
prss1 (Q9Z1R9)
Avidin
LPS
Mannan
IgG
iC3b
Calreticulin
PS
log_2 H/L
2
0
-2
30'/0'
30'/30'
30'/150'
Time point

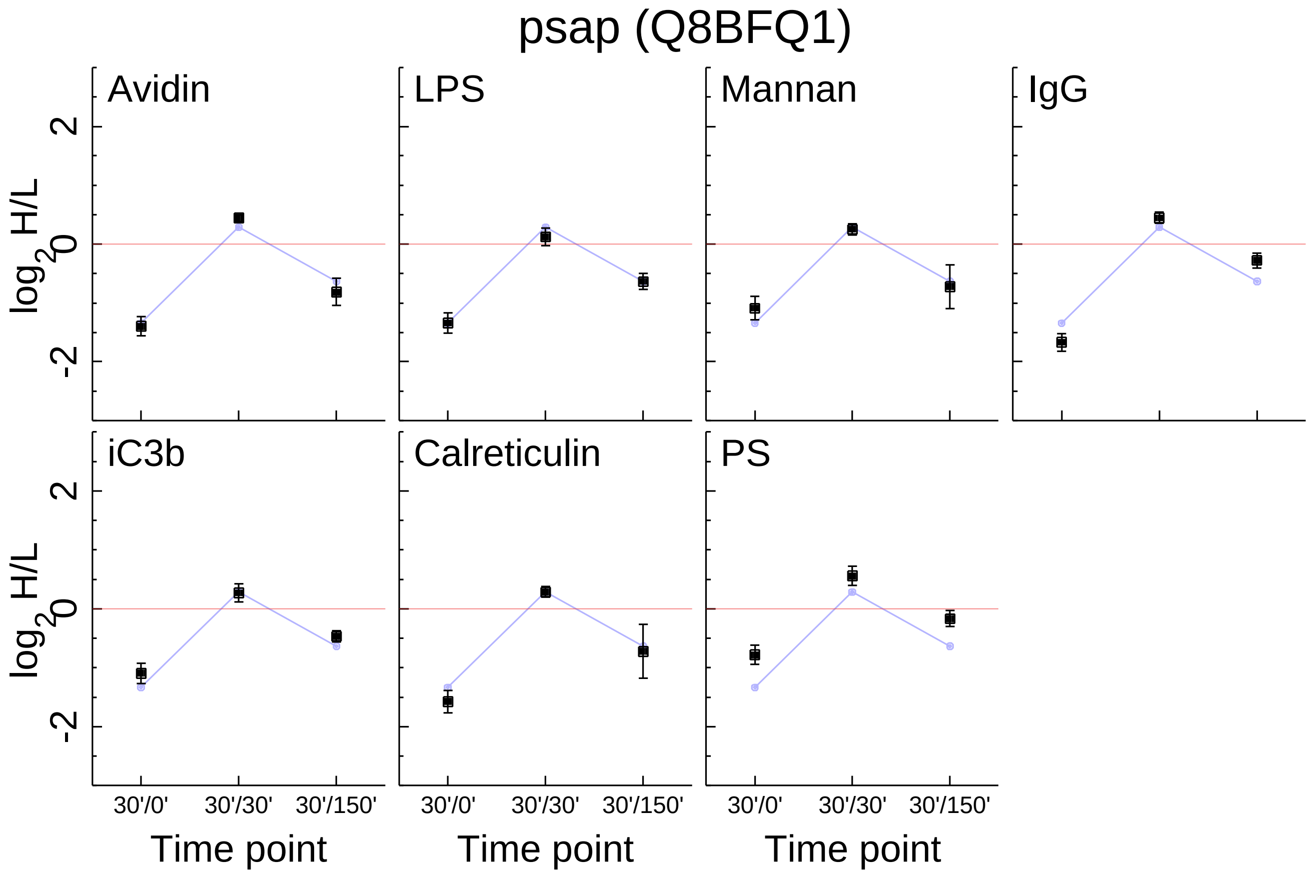
psap (Q8BFQ1)
Avidin
LPS
Mannan
IgG
iC3b
Calreticulin
PS
log2 H/L
2
0
-2
30'/0'
30'/30'
30'/150'
Time point

psen1 (P49769)

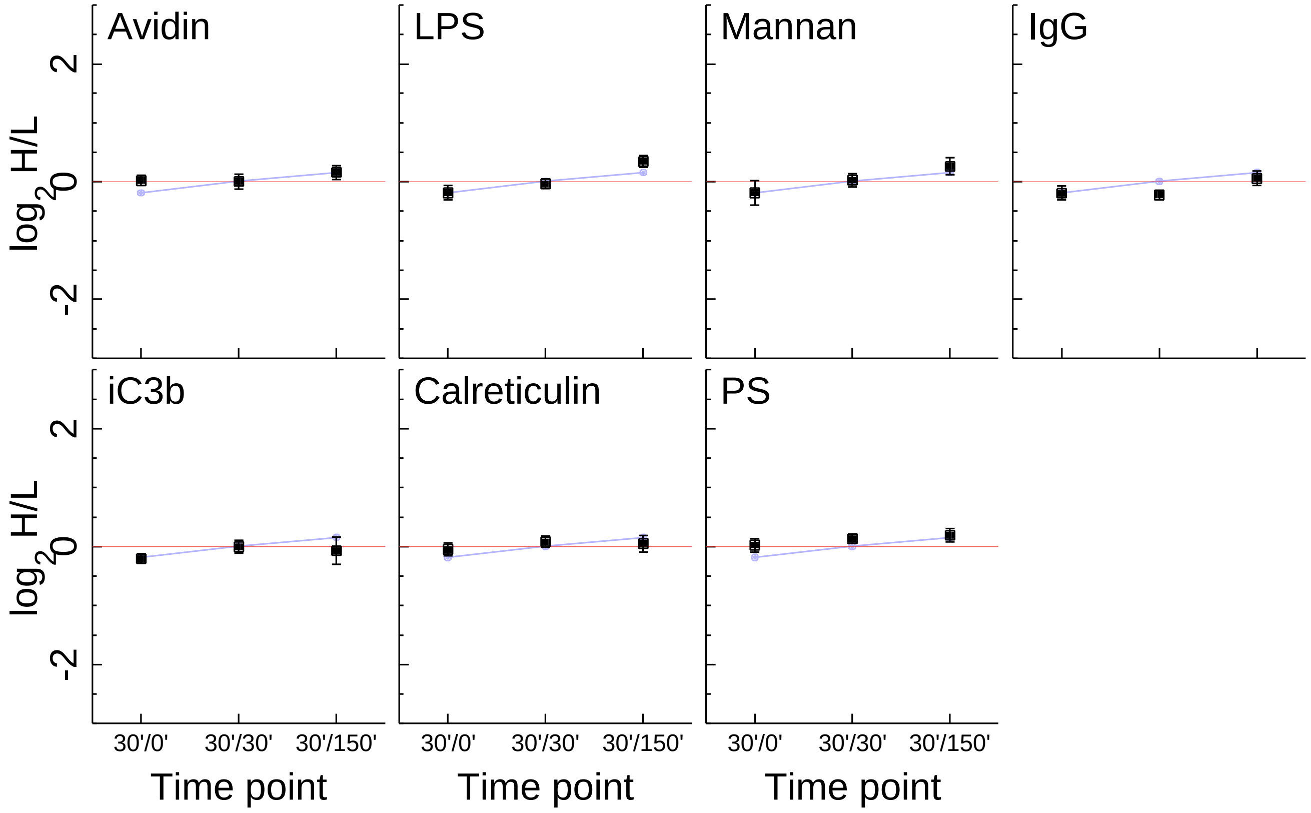

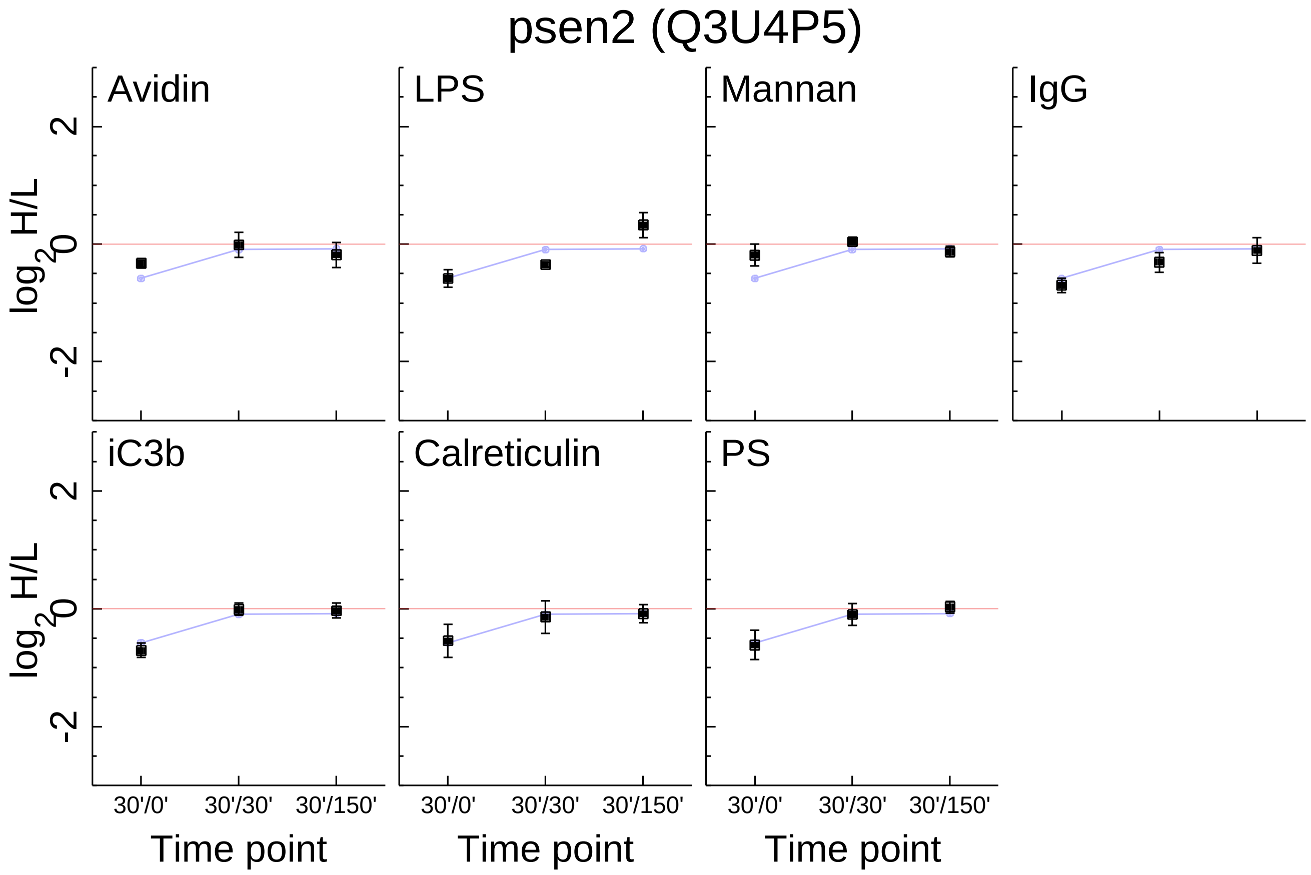
psen2 (Q3U4P5)
Avidin
LPS
Mannan
IgG
iC3b
Calreticulin
PS
log2 H/L
2
0
-2
30'/0'
30'/30'
30'/150'
Time point

psma1 (Q9R1P4)

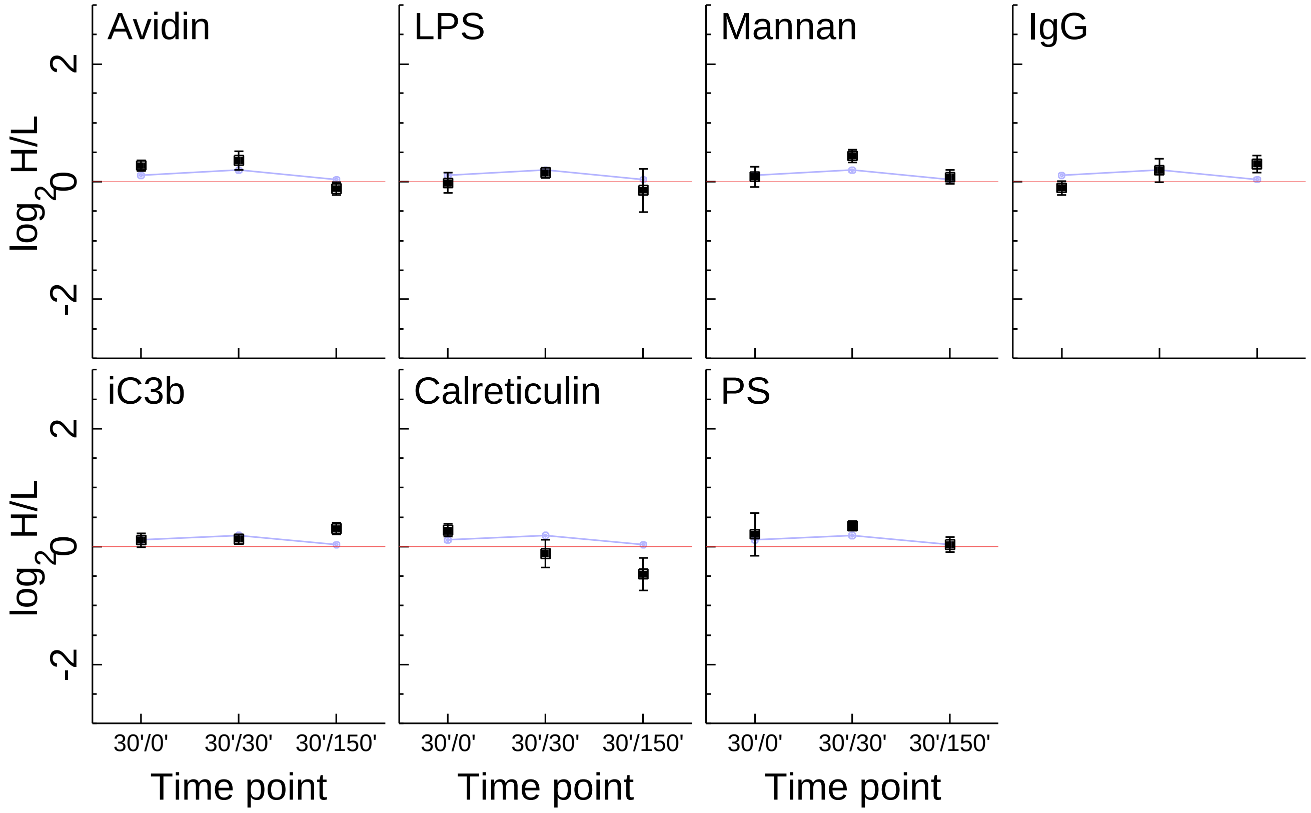

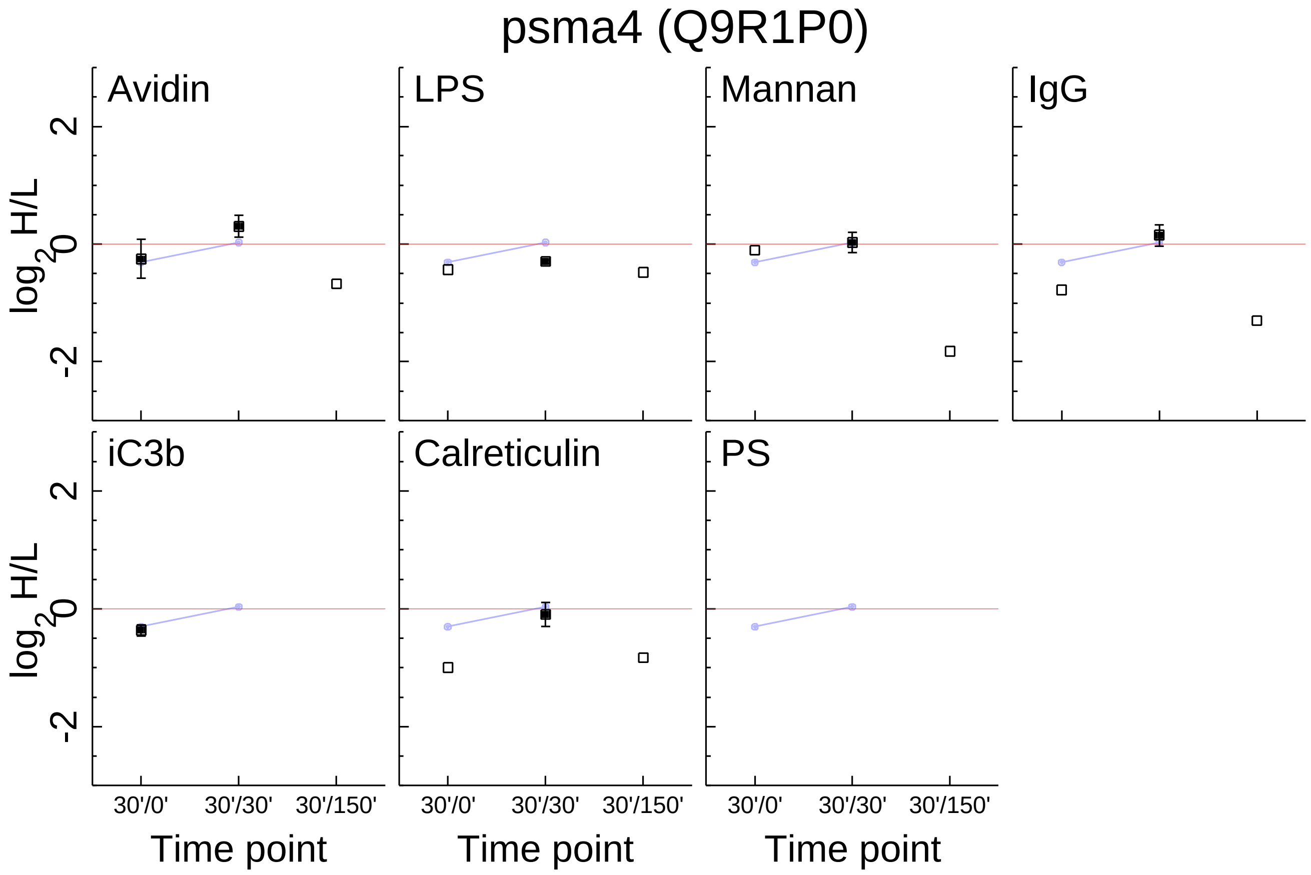
psma4 (Q9R1P0)
Avidin
LPS
Mannan
IgG
iC3b
Calreticulin
PS
log2 H/L
2
0
-2
30'/0'
30'/30'
30'/150'
Time point

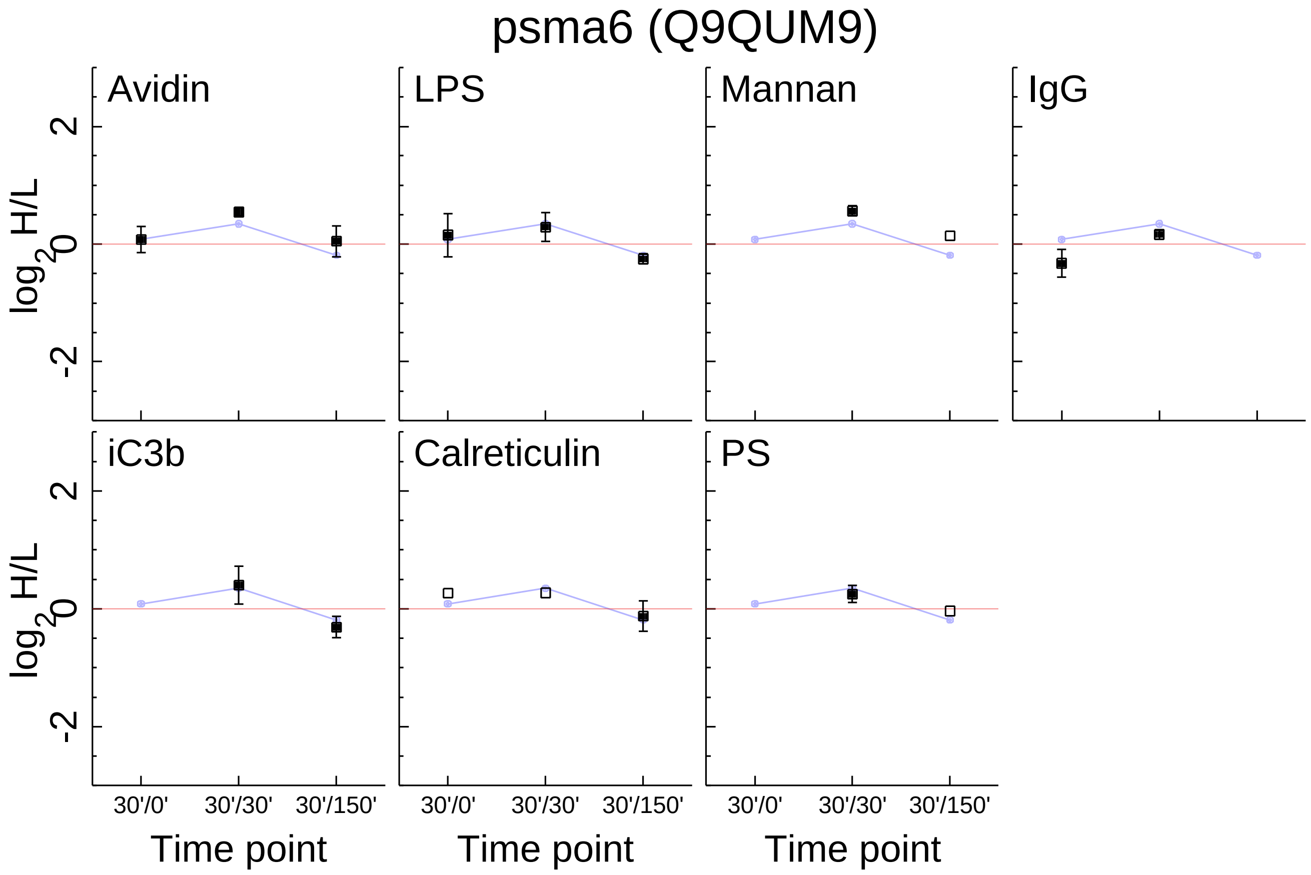
psma6 (Q9QUM9)
Avidin
LPS
Mannan
IgG
iC3b
Calreticulin
PS
log2 H/L
2
0
-2
30'/0'
30'/30'
30'/150'
Time point

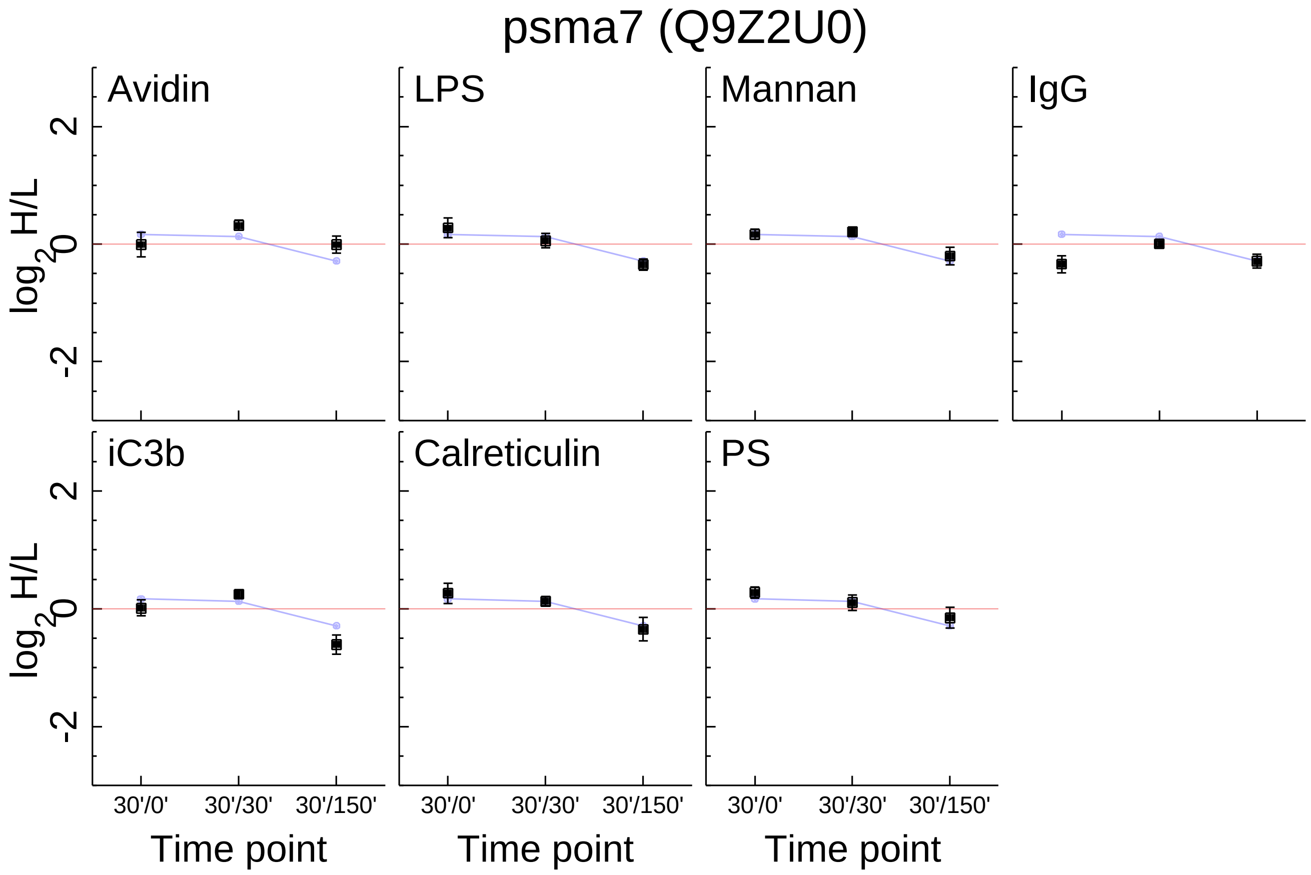
psma7 (Q9Z2U0)
Avidin
LPS
Mannan
IgG
iC3b
Calreticulin
PS
log2 H/L
2
0
-2
30'/0'
30'/30'
30'/150'
Time point

psmb10 (O35955)

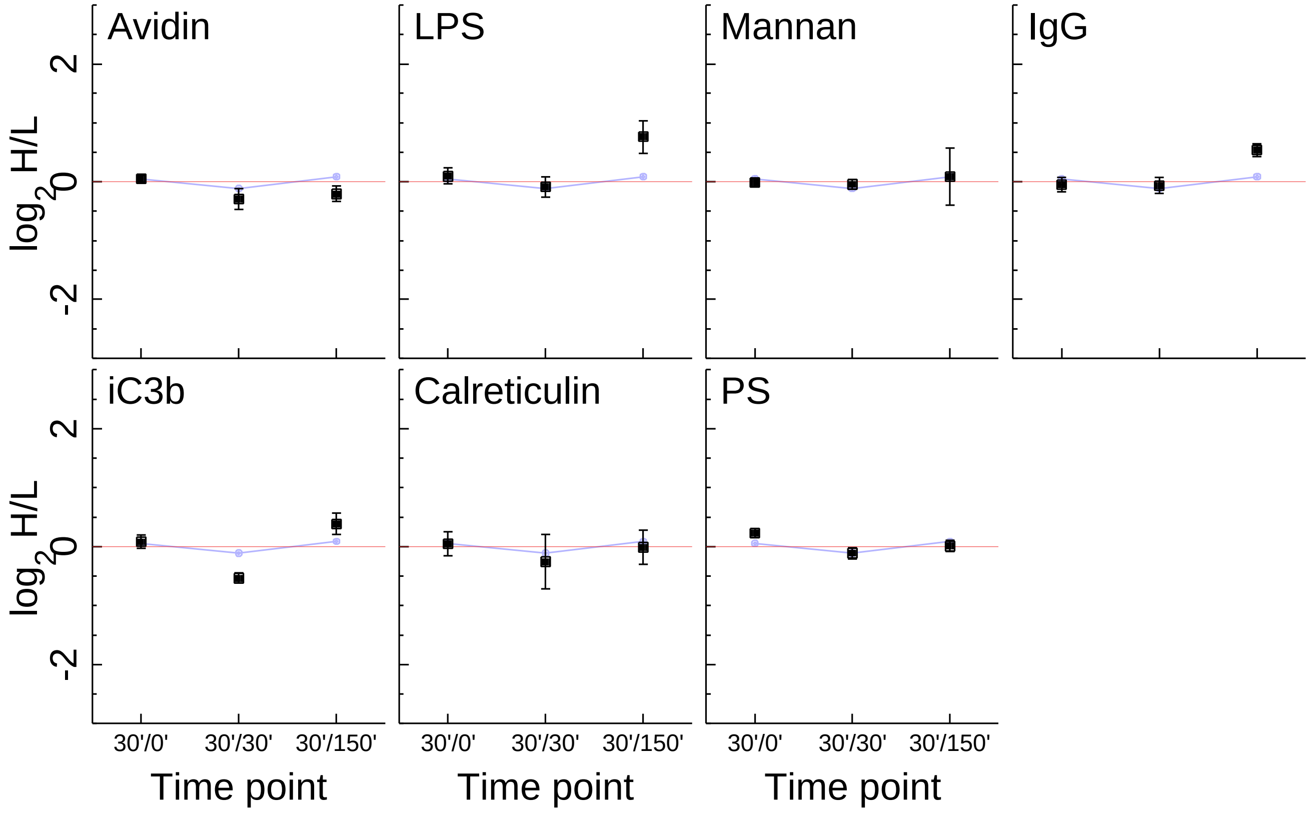

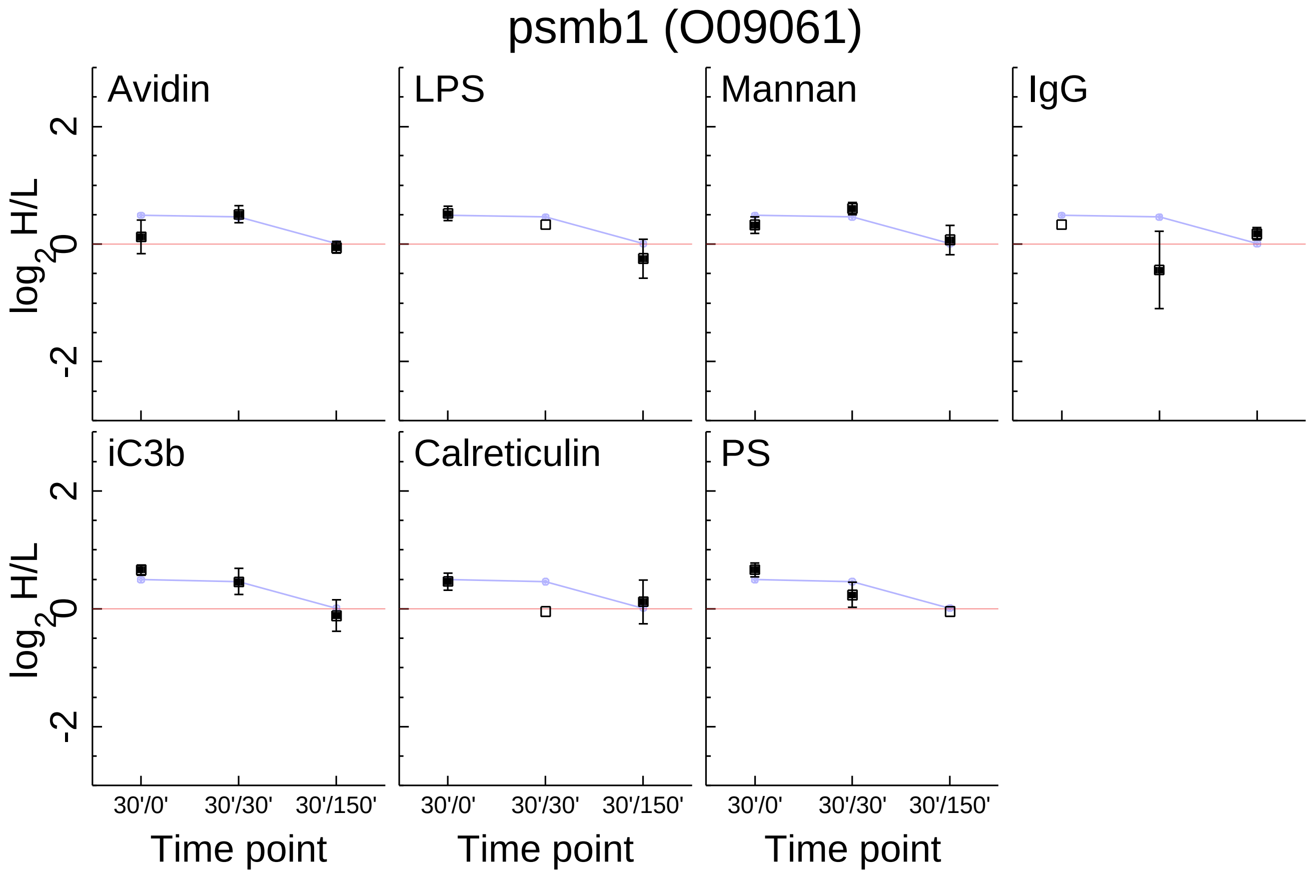
psmb1 (O09061)
Avidin
LPS
Mannan
IgG
iC3b
Calreticulin
PS
log2 H/L
2
0
-2
30'/0'
30'/30'
30'/150'
Time point

psmb3 (Q9R1P1)

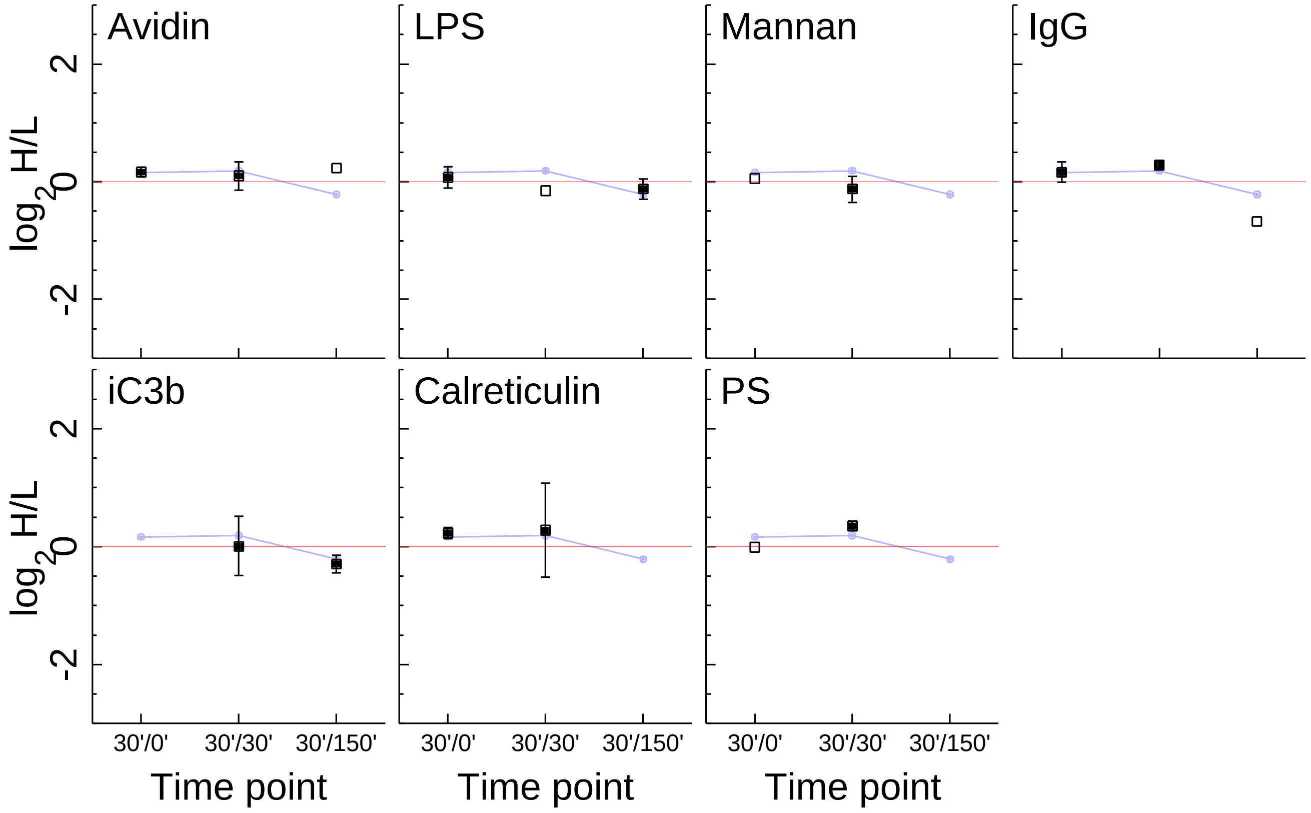

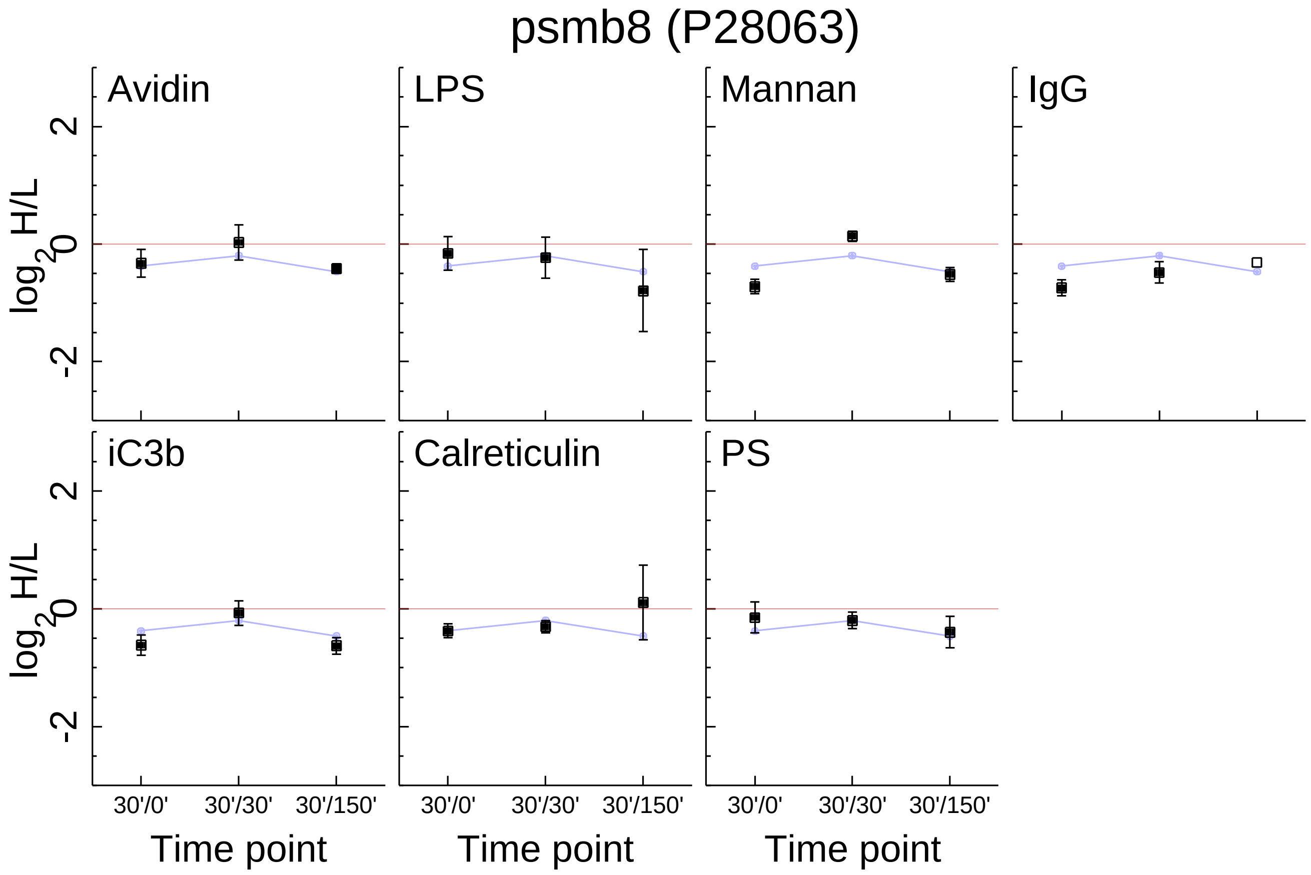
psmb8 (P28063)
Avidin
LPS
Mannan
IgG
iC3b
Calreticulin
PS
log2 H/L
2
0
-2
30'/0'
30'/30'
30'/150'
Time point

psme1 (P97371)

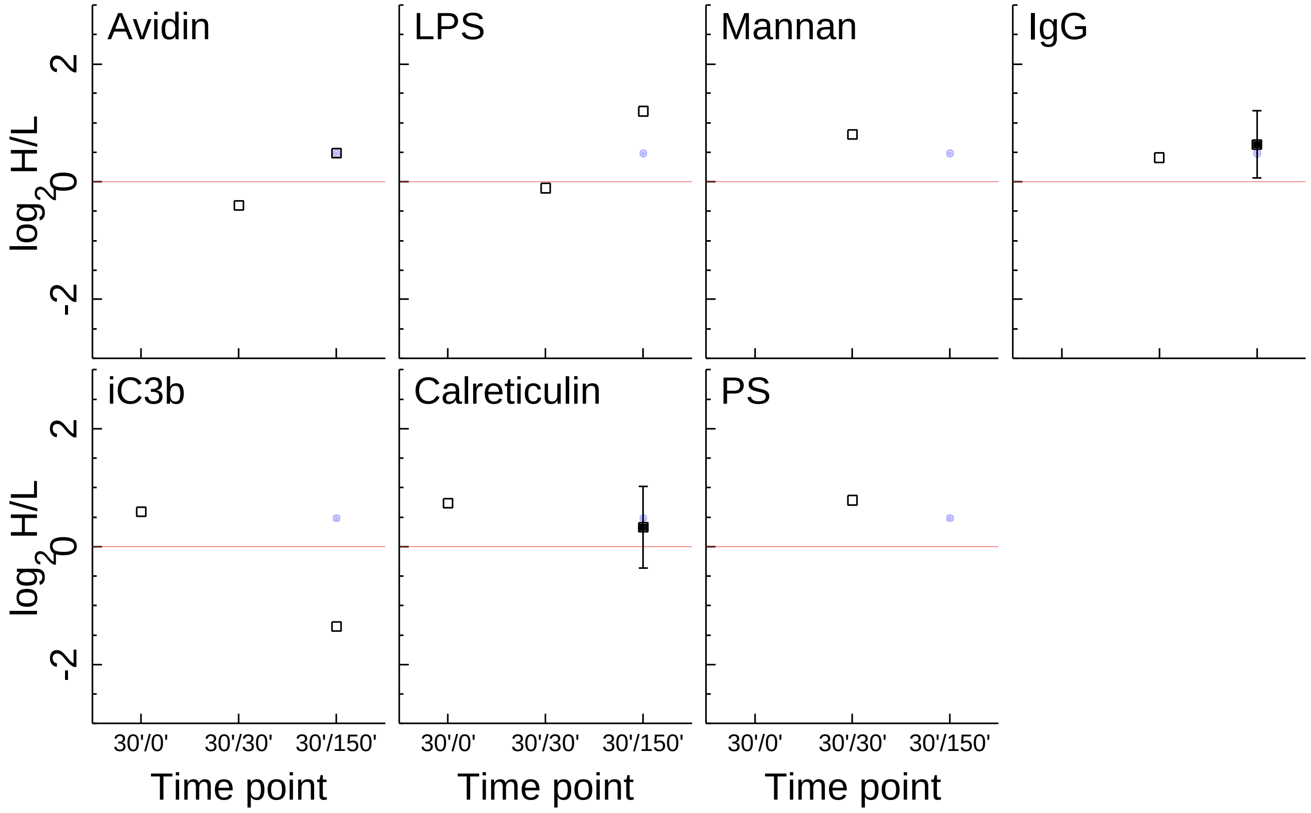

pstpip1 (P97814)

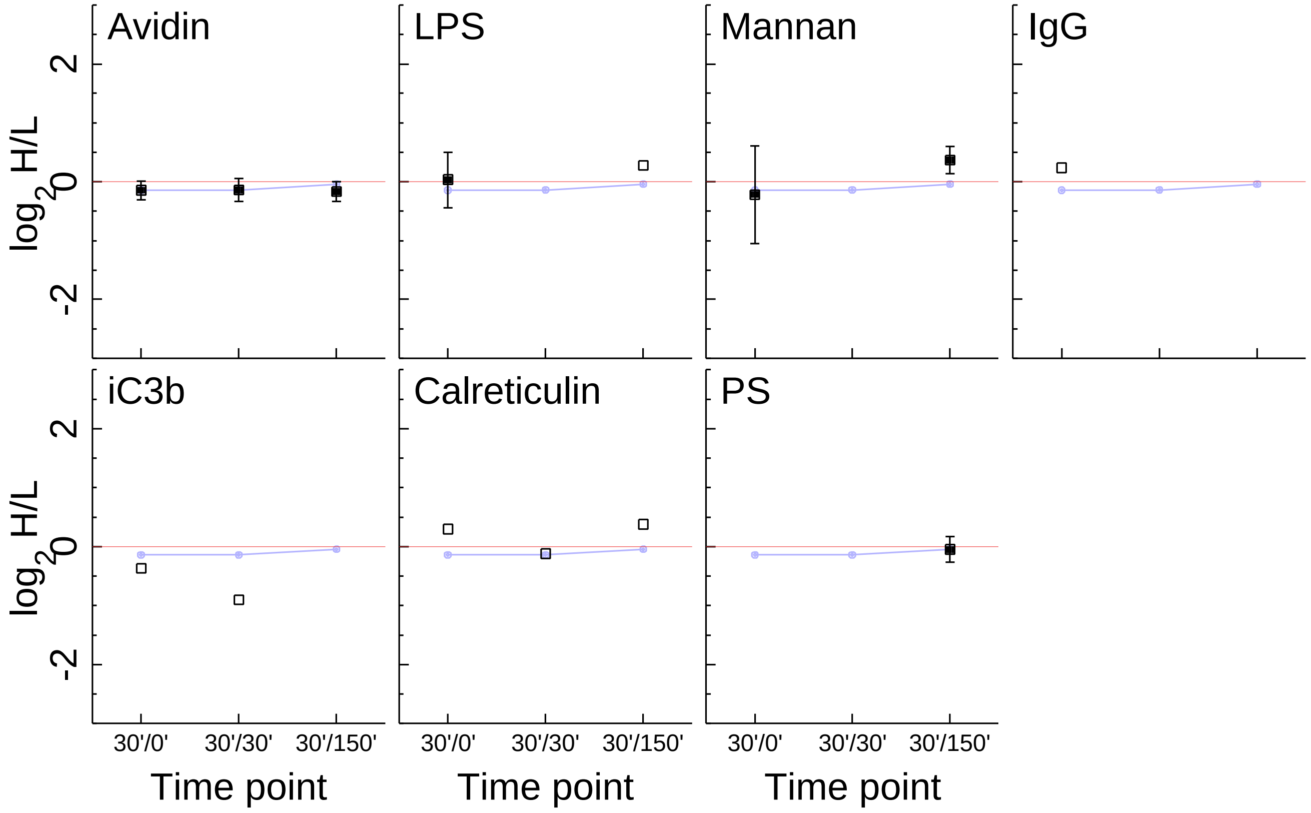

ptbp1 (Q922I7)

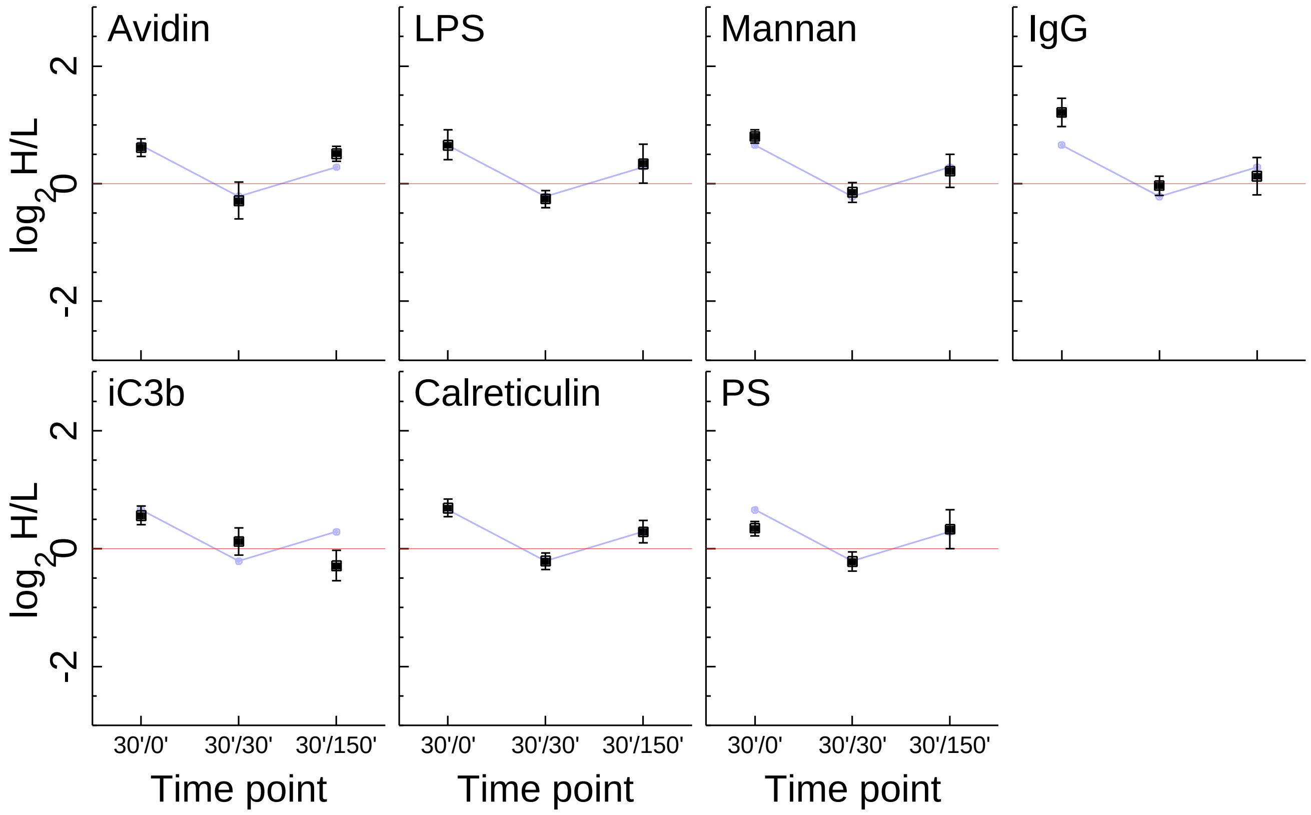

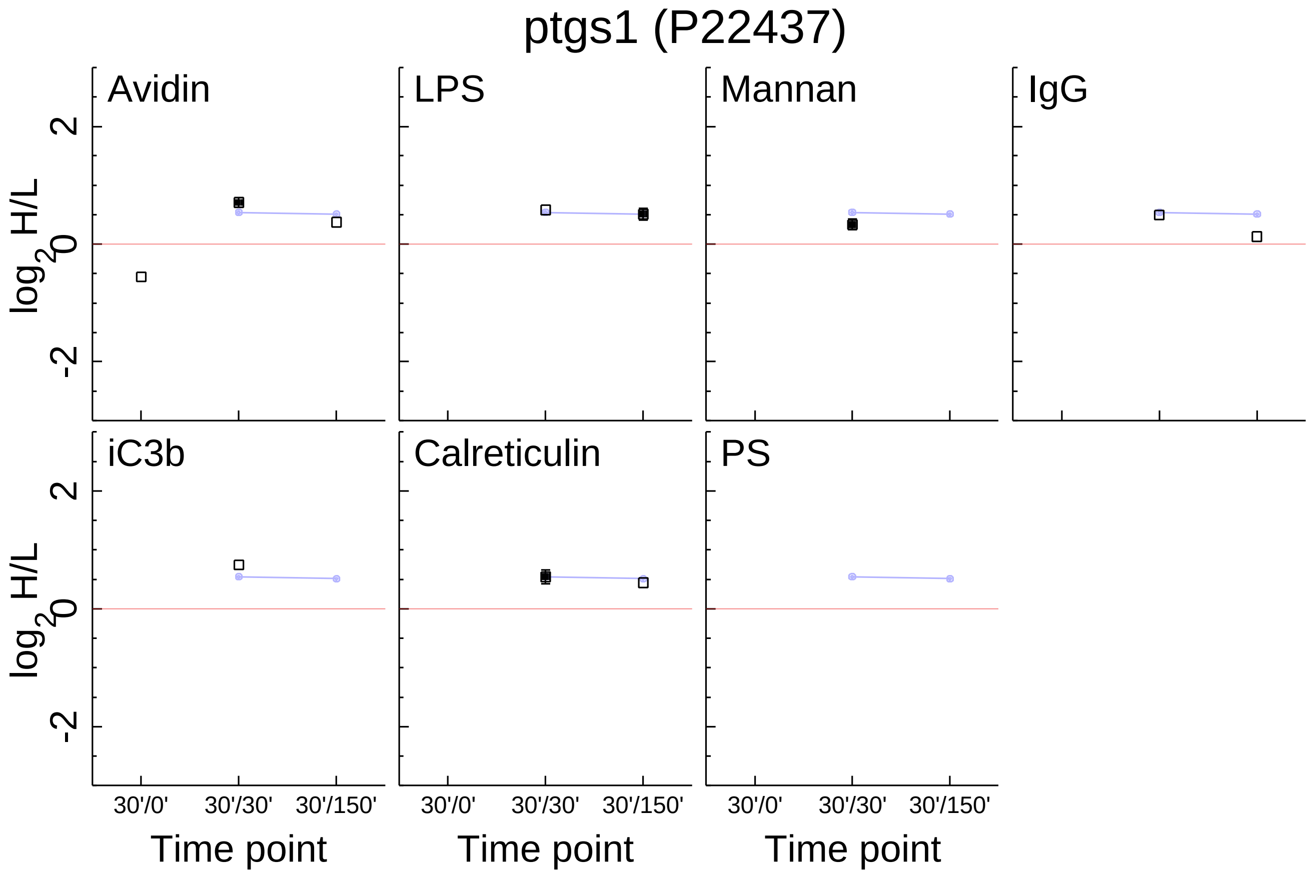

ptgs1 (P22437)
Avidin
LPS
Mannan
IgG
iC3b
Calreticulin
PS
log2 H/L
2
0
-2
30'/0'
30'/30'
30'/150'
Time point

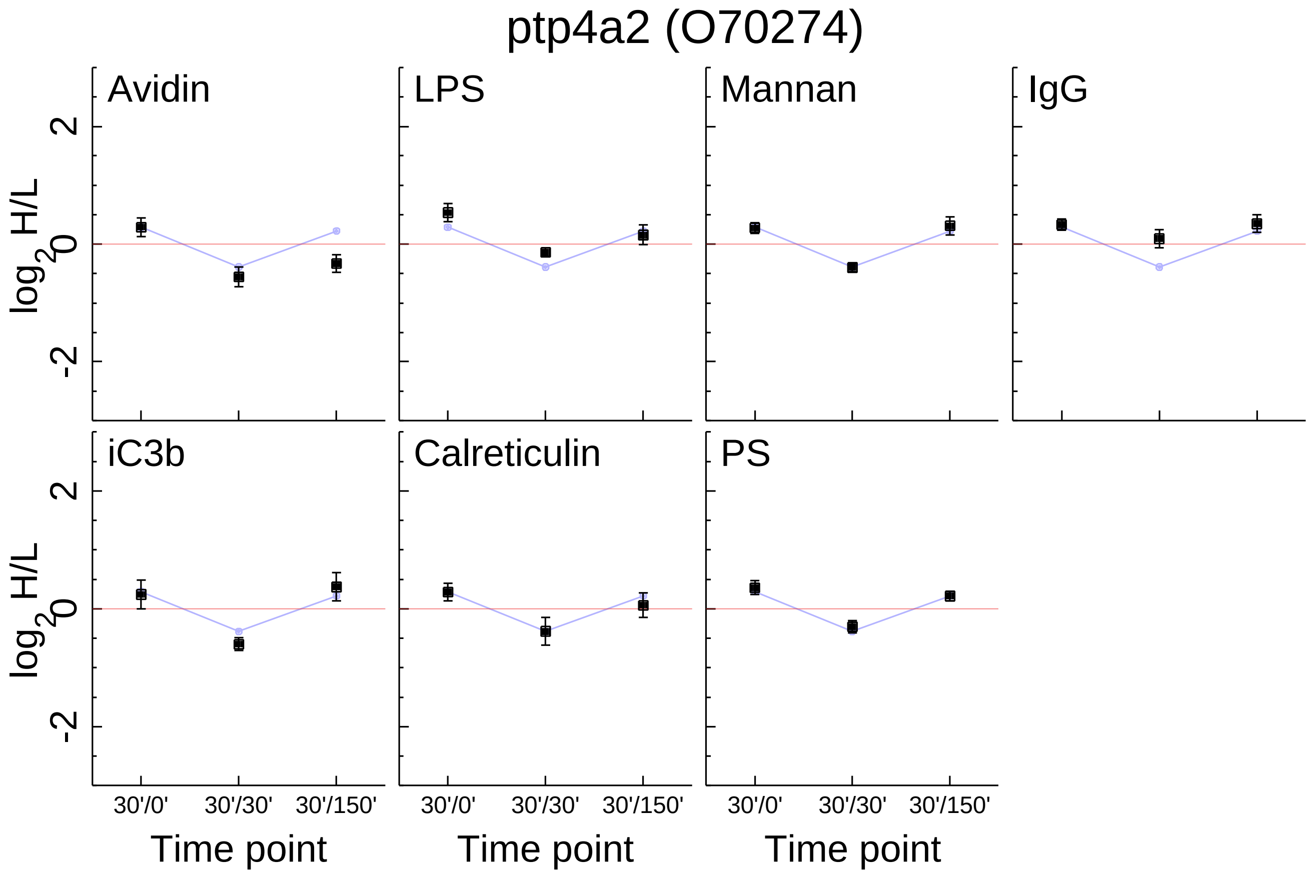
ptp4a2 (O70274)
Avidin
LPS
Mannan
IgG
iC3b
Calreticulin
PS
log2 H/L
2
0
-2
30'/0'
30'/30'
30'/150'
Time point

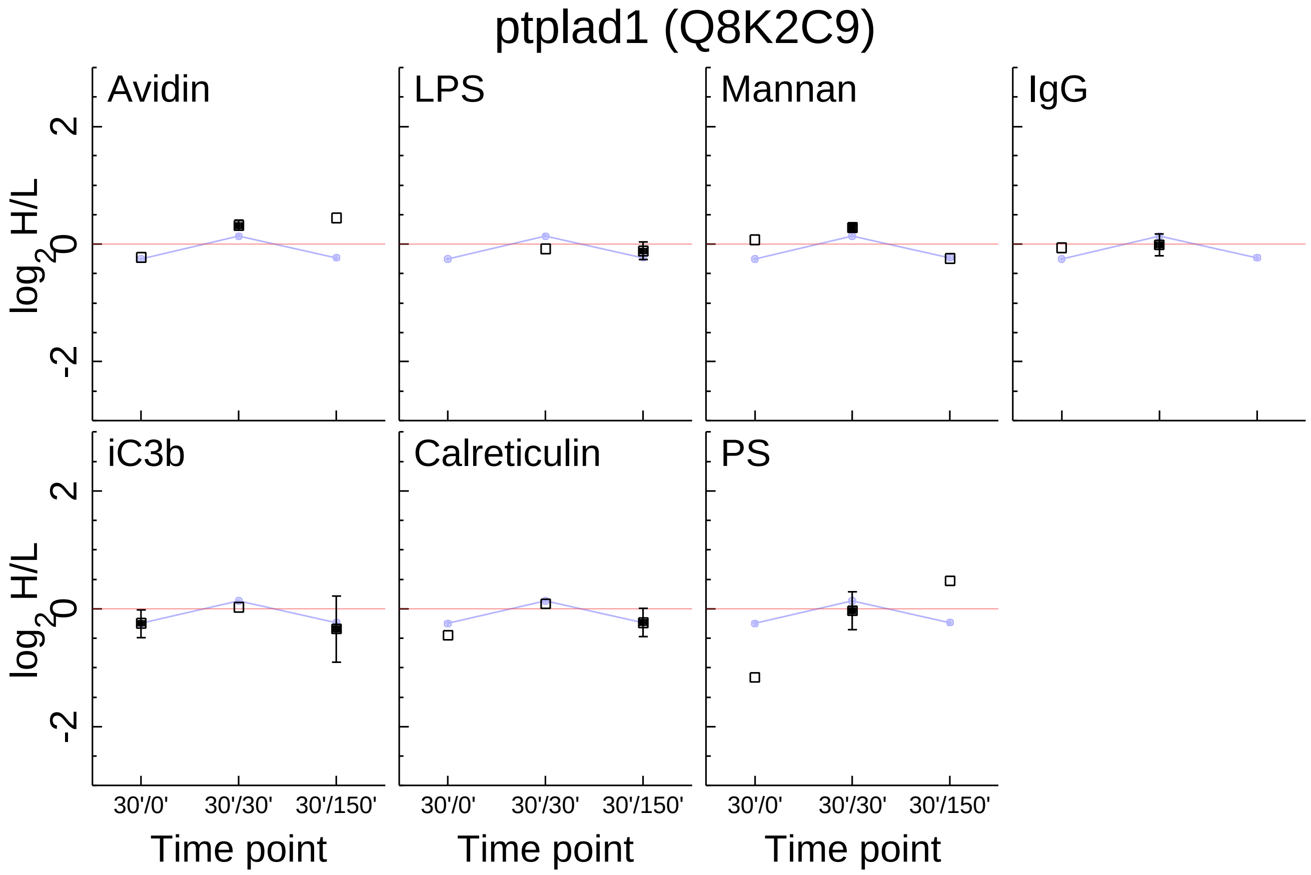
ptplad1 (Q8K2C9)
Avidin
LPS
Mannan
IgG
iC3b
Calreticulin
PS
log2 H/L
2
0
-2
30'/0'
30'/30'
30'/150'
Time point

ptpn1 (P35821)

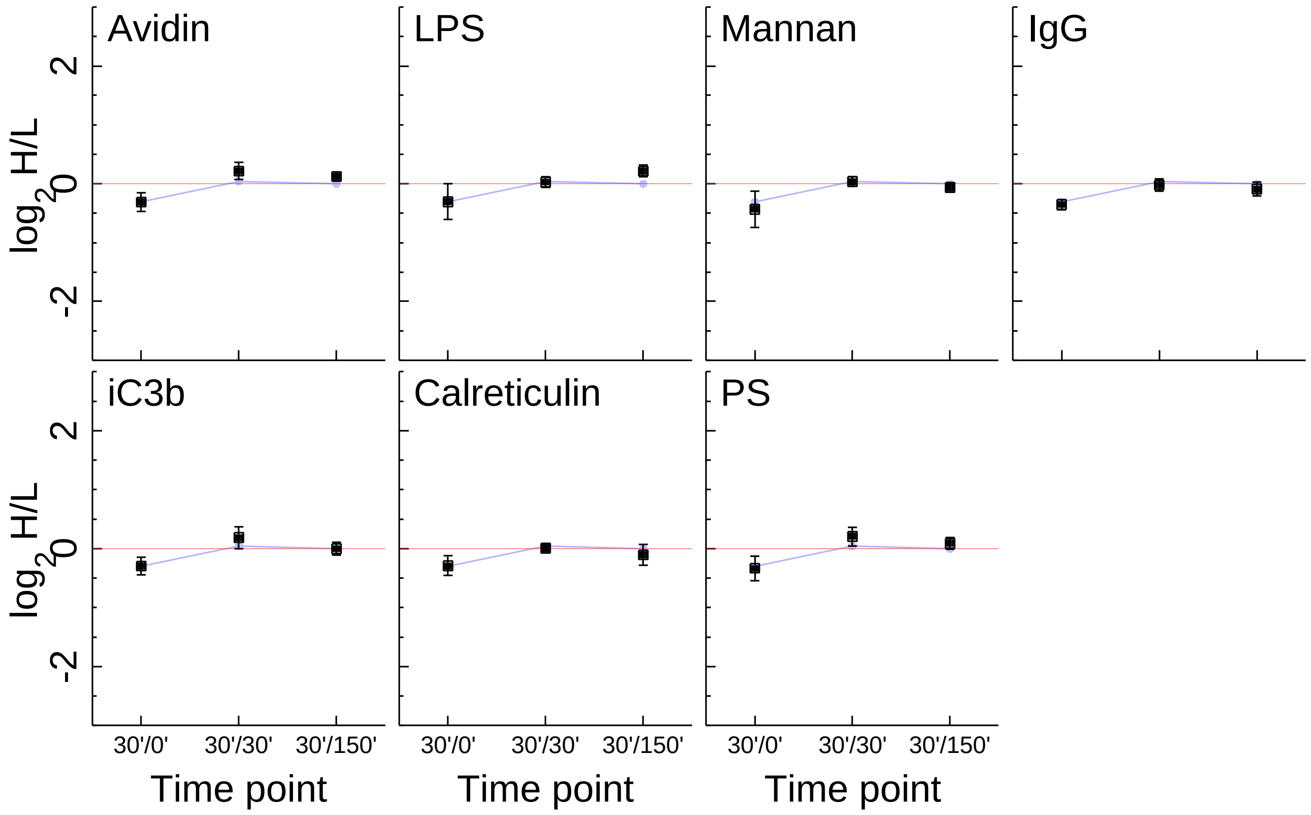

ptpn23 (Q6PB44)

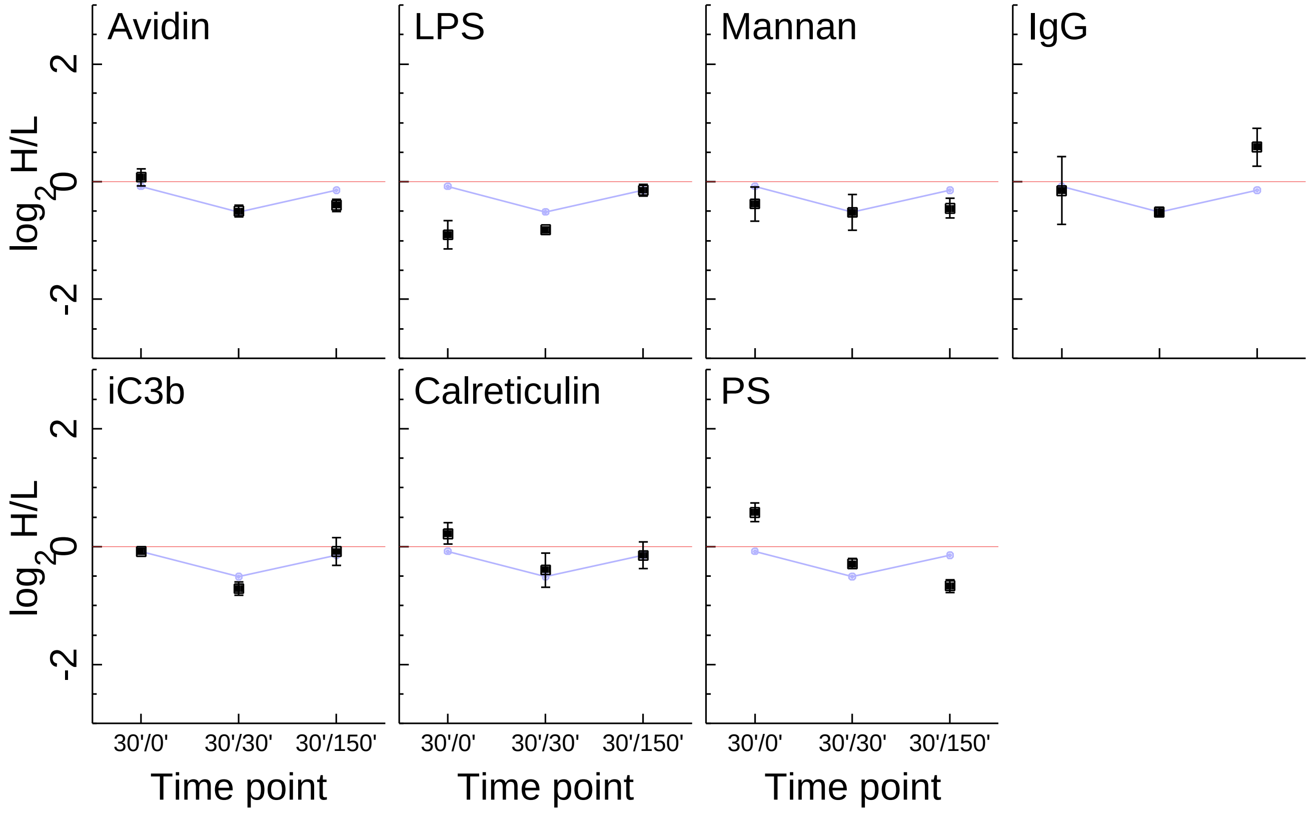

ptpn6 (P29351)

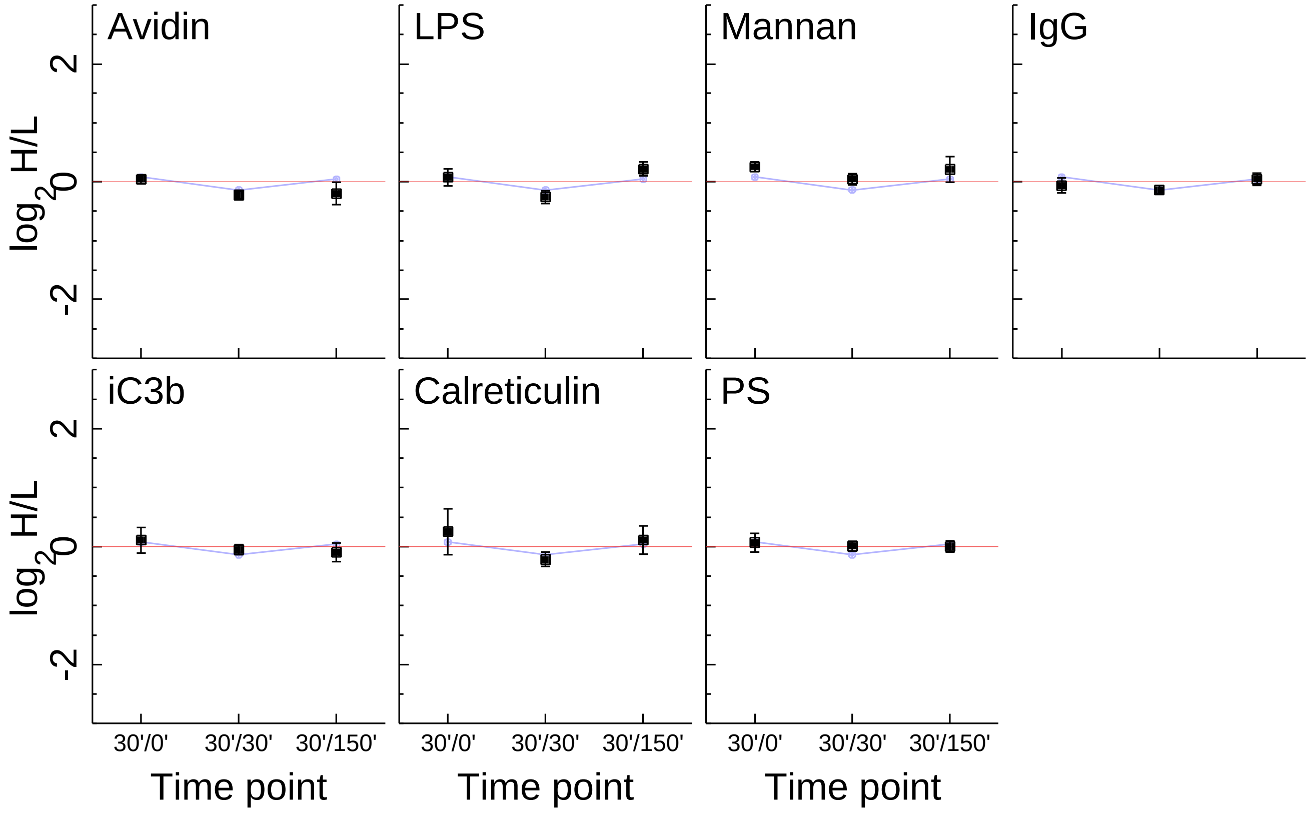

ptpn9 (O35239)

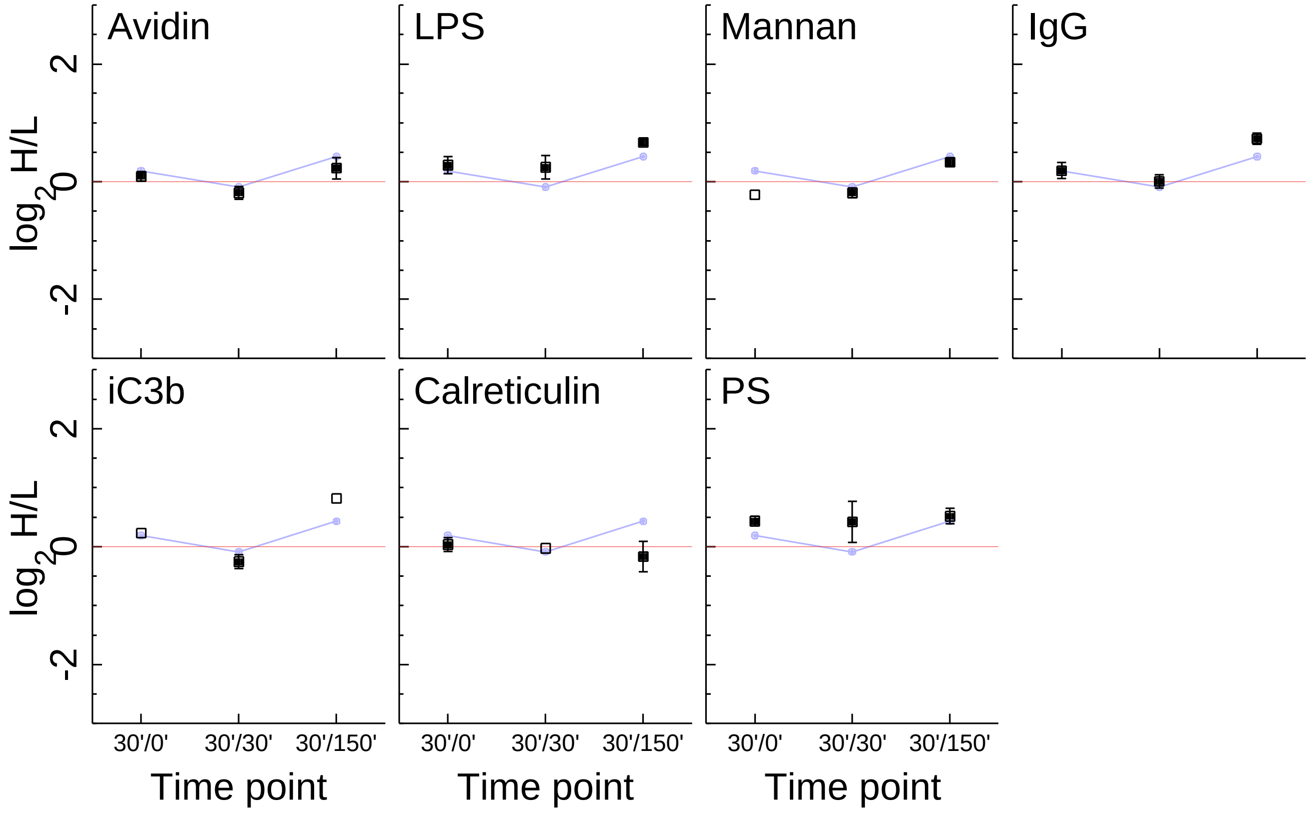

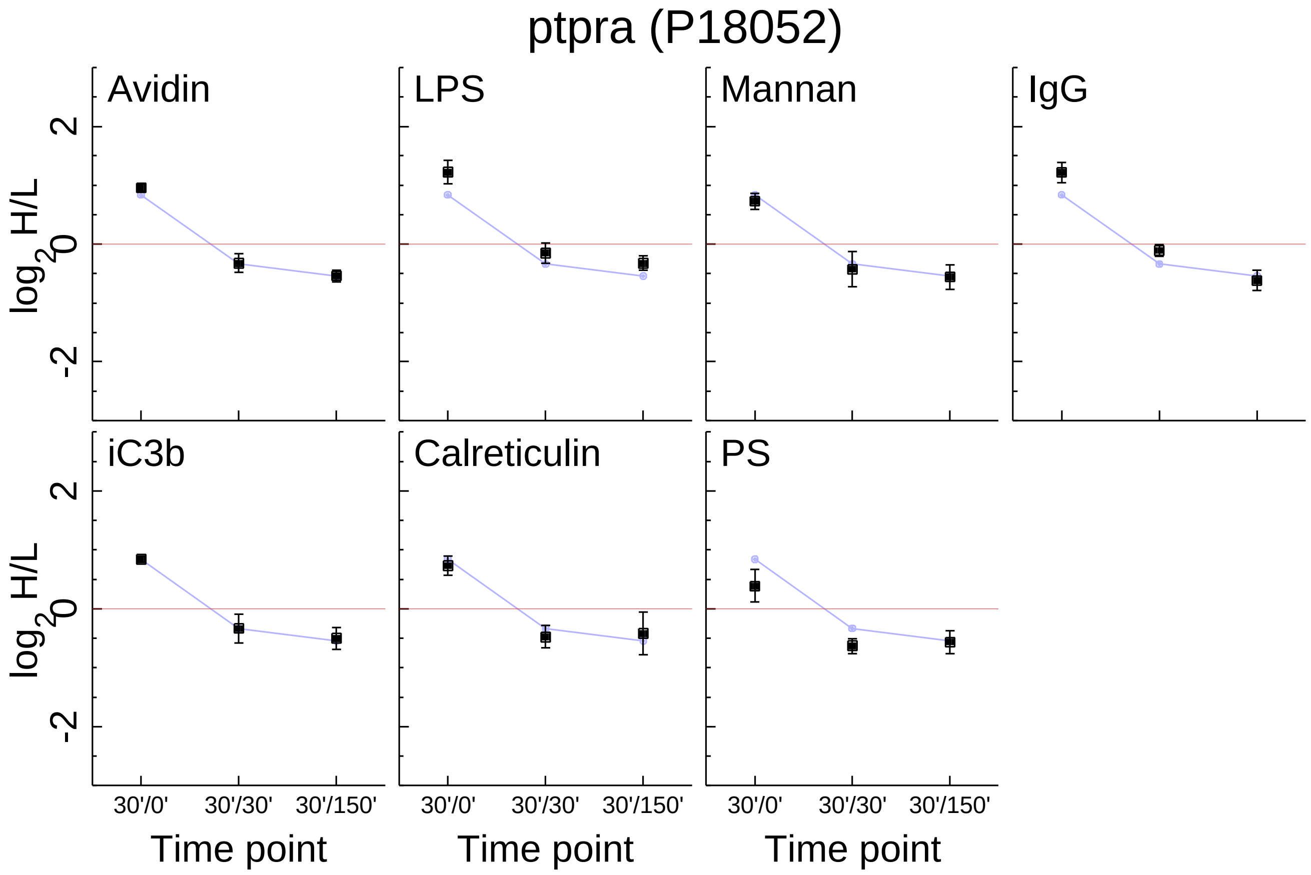
ptpra (P18052)
Avidin
LPS
Mannan
IgG
iC3b
Calreticulin
PS
log2 H/L
2
0
-2
30'/0'
30'/30'
30'/150'
Time point

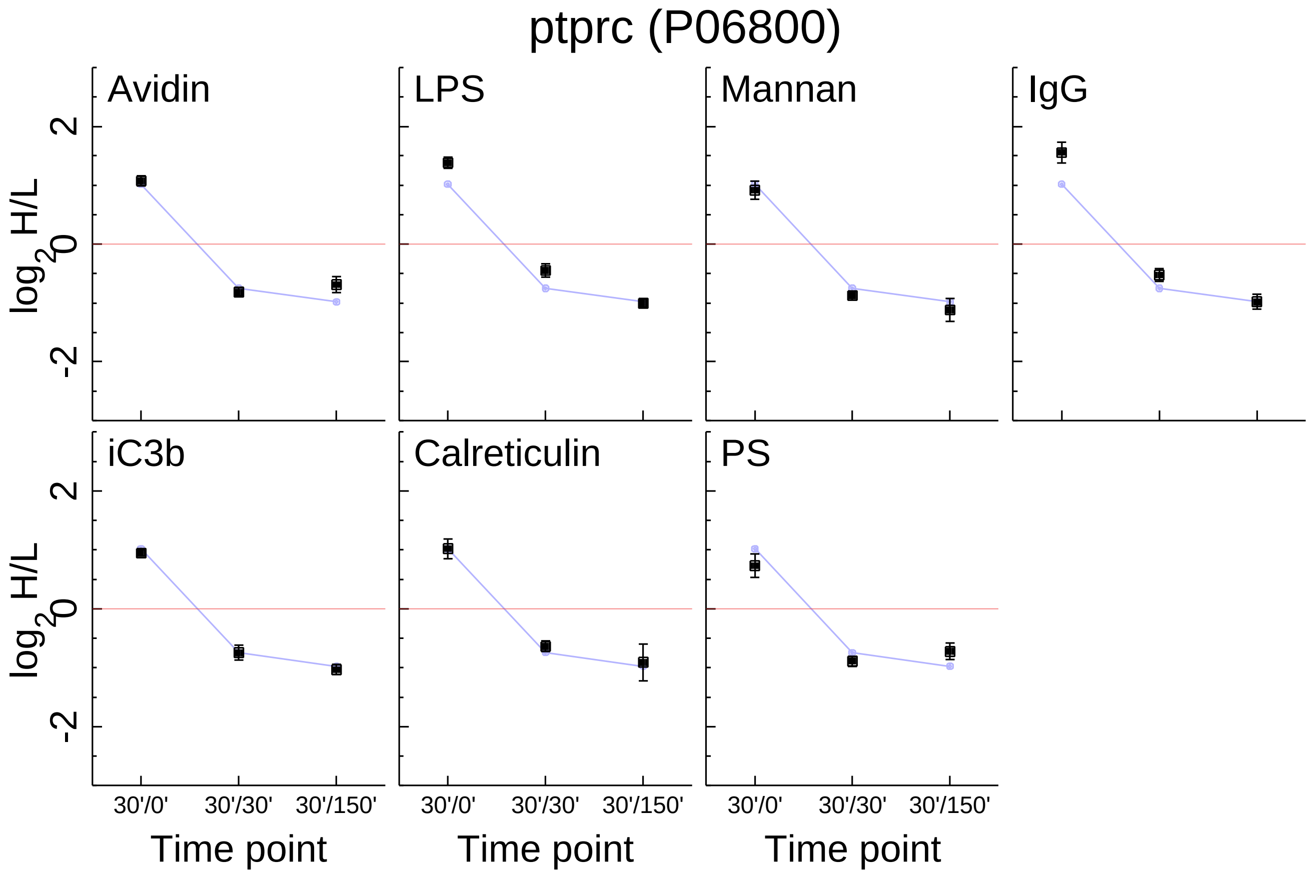

ptprc (P06800)
Avidin
LPS
Mannan
IgG
iC3b
Calreticulin
PS
log2 H/L
2
0
-2
30'/0'
30'/30'
30'/150'
Time point

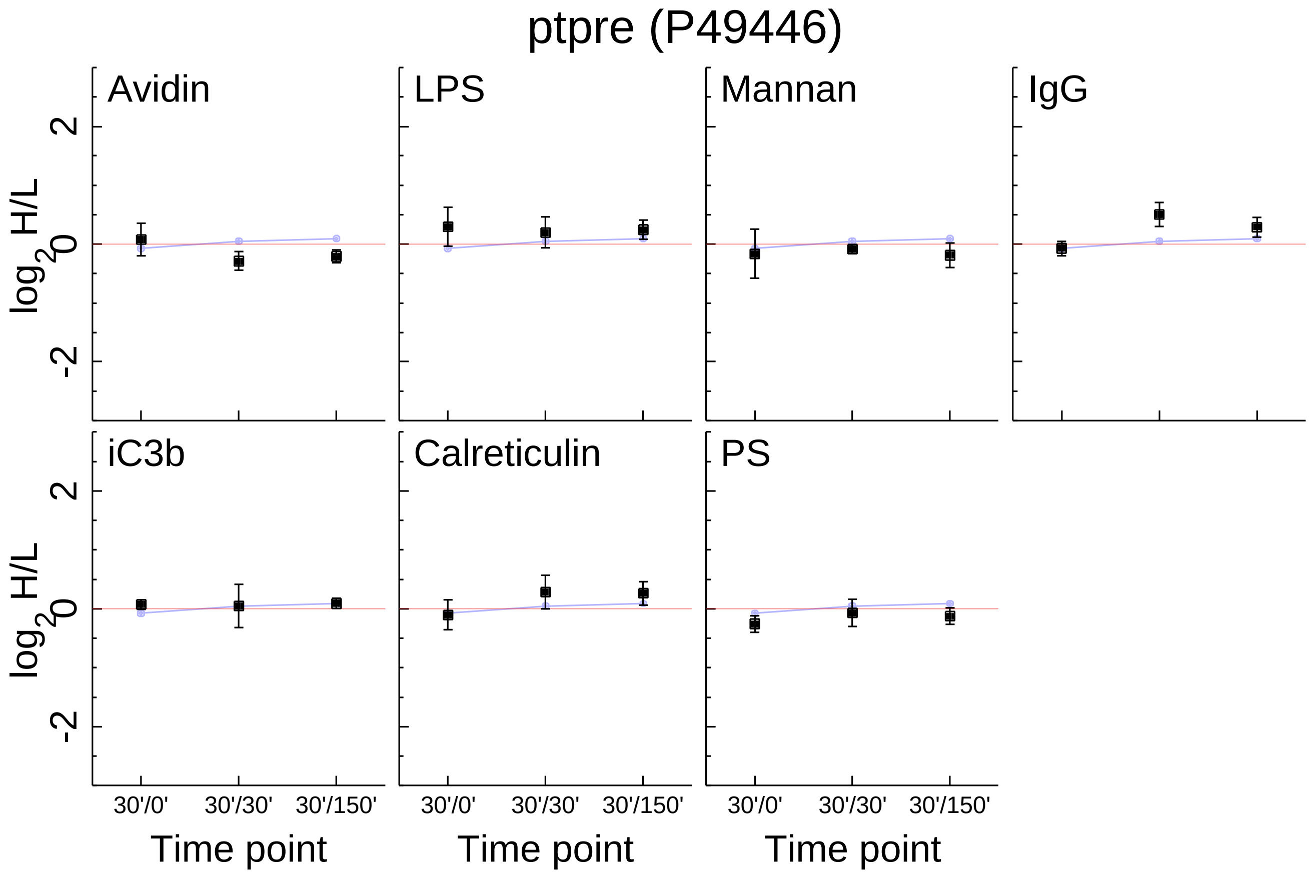
ptpre (P49446)
Avidin
LPS
Mannan
IgG
iC3b
Calreticulin
PS
log2 H/L
2
0
-2
30'/0'
30'/30'
30'/150'
Time point

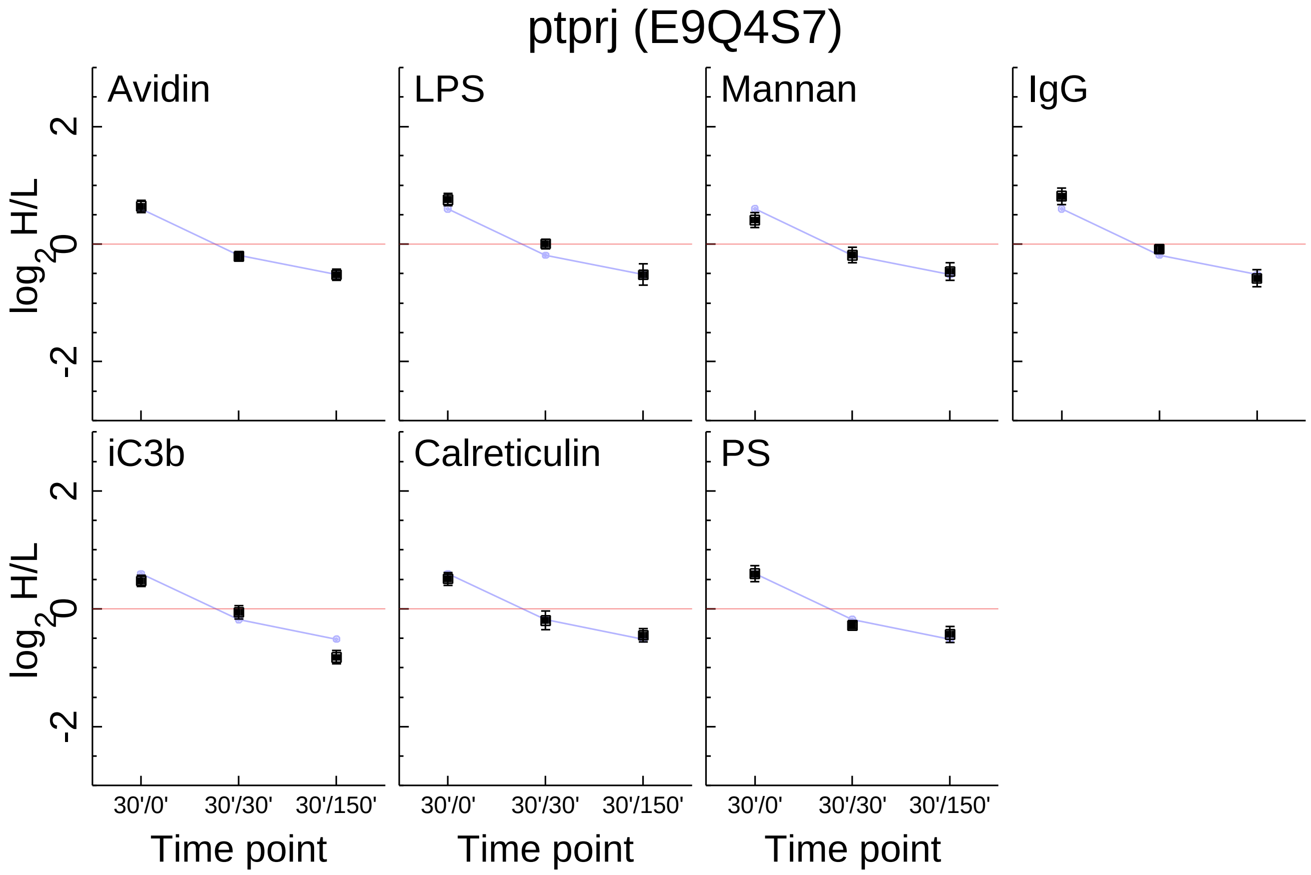
ptprj (E9Q4S7)
Avidin
LPS
Mannan
IgG
iC3b
Calreticulin
PS
log2 H/L
2
0
-2
30'/0'
30'/30'
30'/150'
Time point

ptpro (E9Q612)

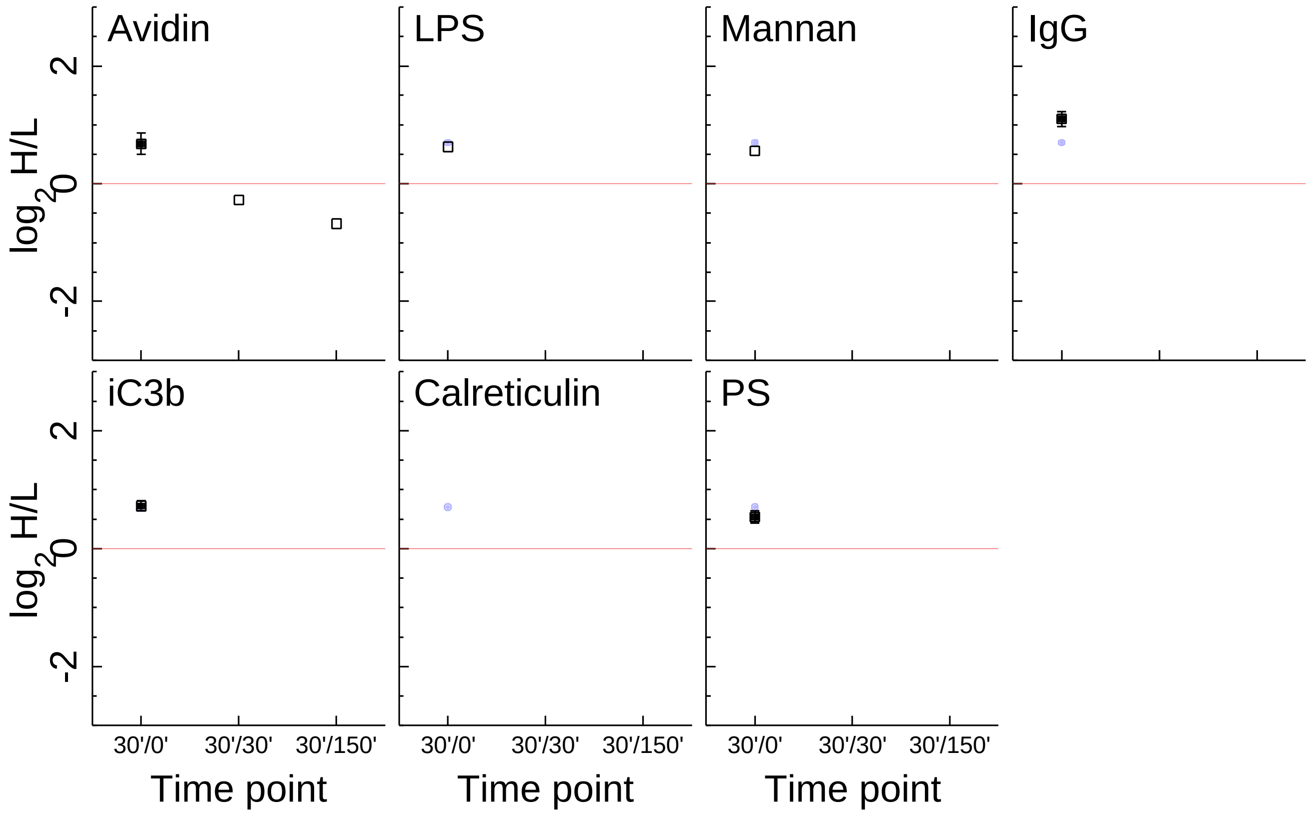

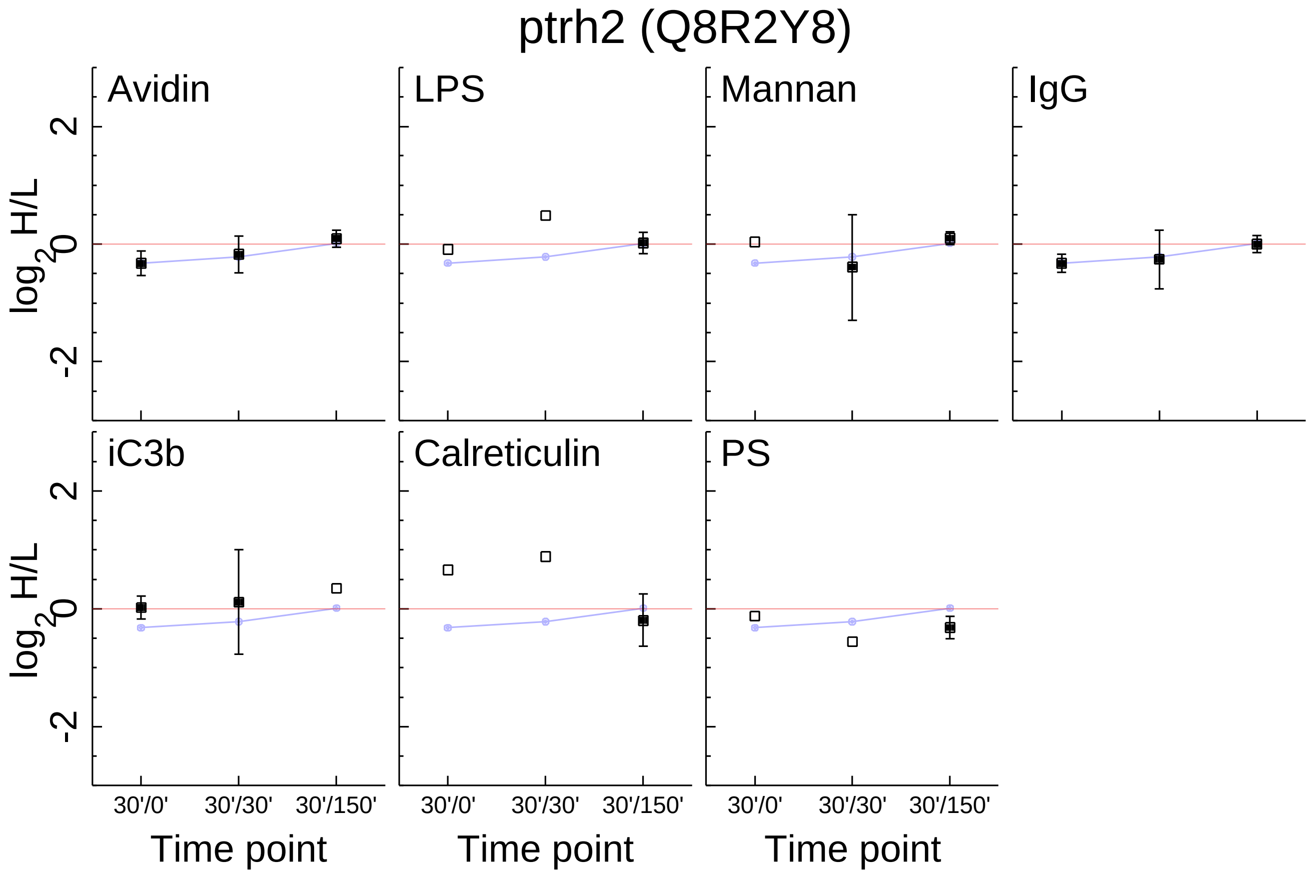
ptrh2 (Q8R2Y8)
Avidin
LPS
Mannan
IgG
iC3b
Calreticulin
PS
log2 H/L
2
0
-2
30'/0'
30'/30'
30'/150'
Time point

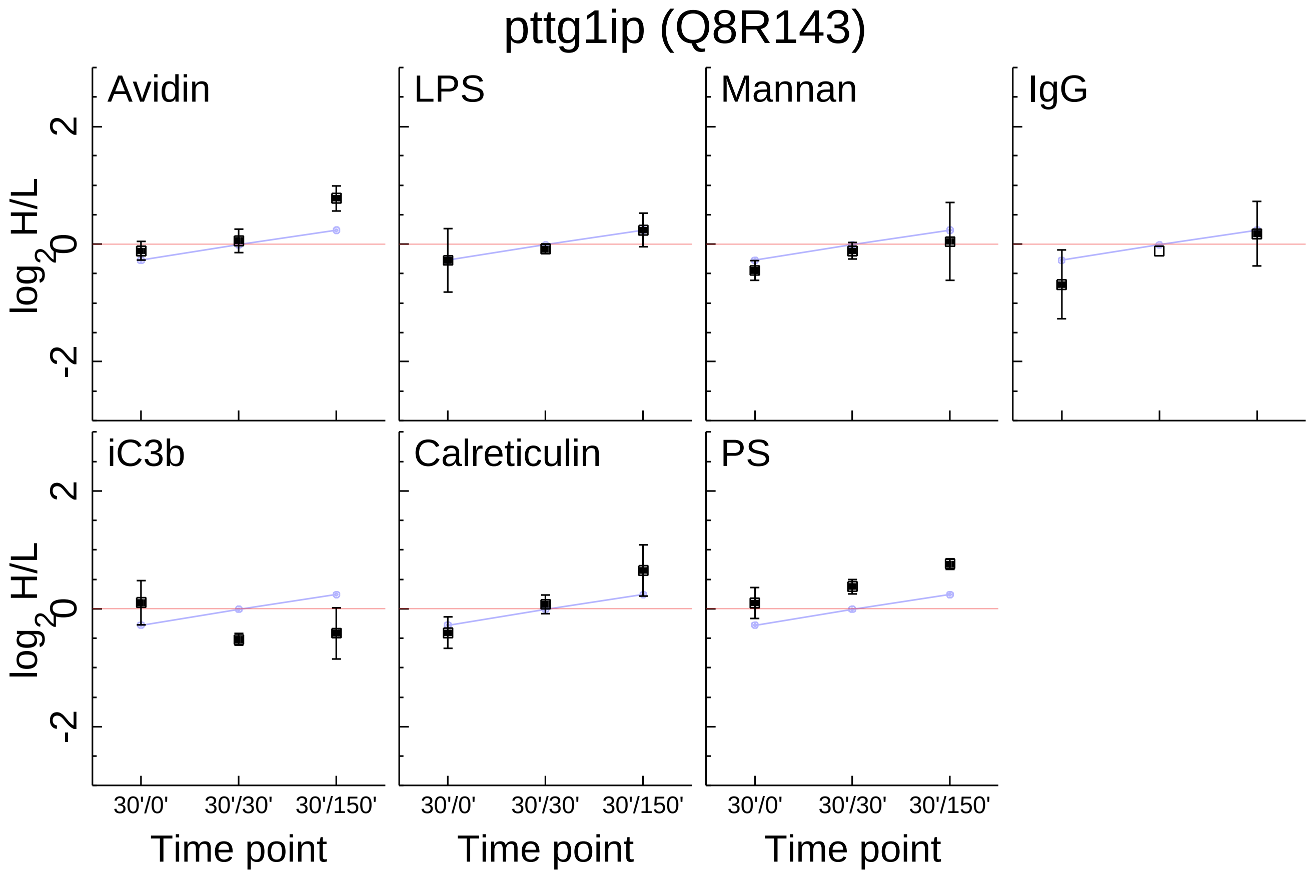

pttg1ip (Q8R143)
Avidin
LPS
Mannan
IgG
iC3b
Calreticulin
PS
$\log_2$ H/L
2
0
-2
30'/0'
30'/30'
30'/150'
Time point

pxk (Q8BX57)

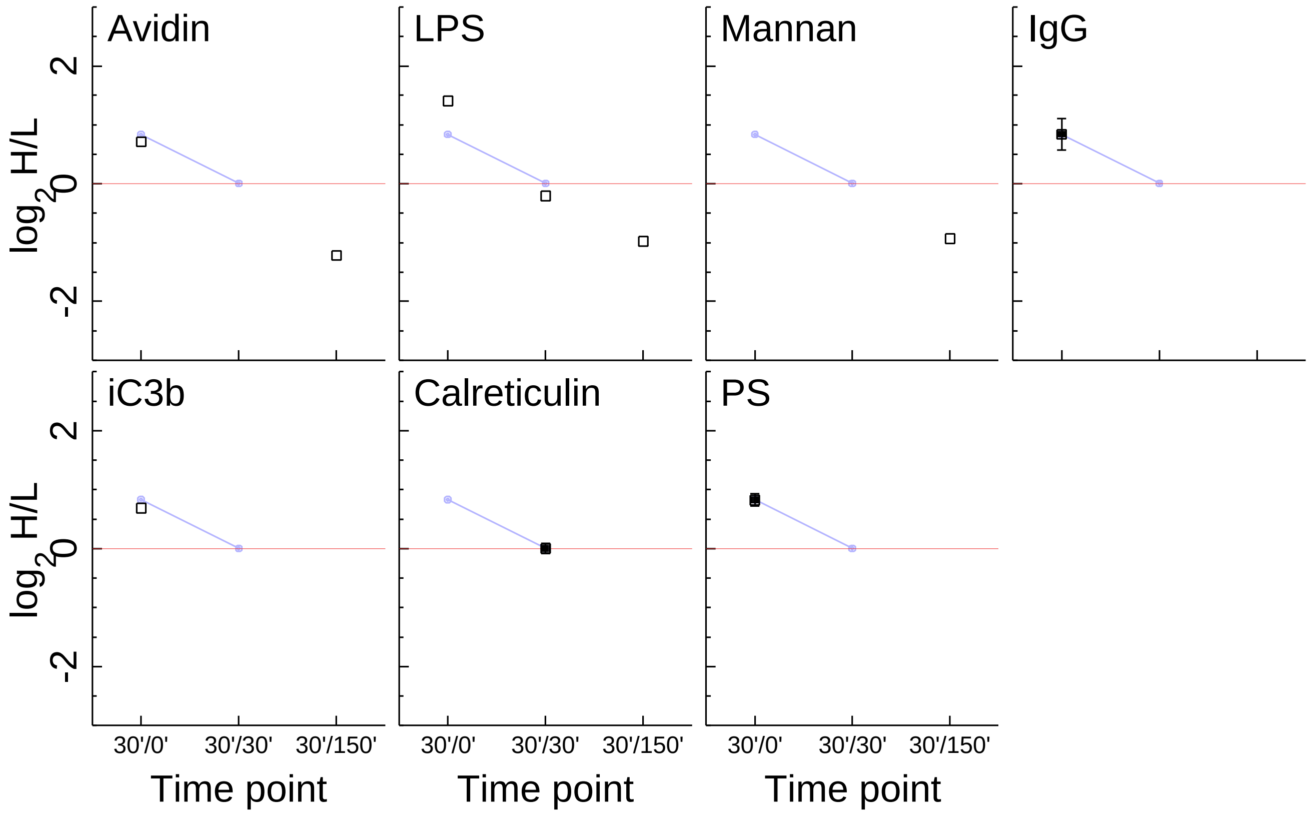
Avidin
LPS
Mannan
IgG
iC3b
Calreticulin
PS
log2 H/L
2
0
-2
30'/0'
30'/30'
30'/150'
Time point

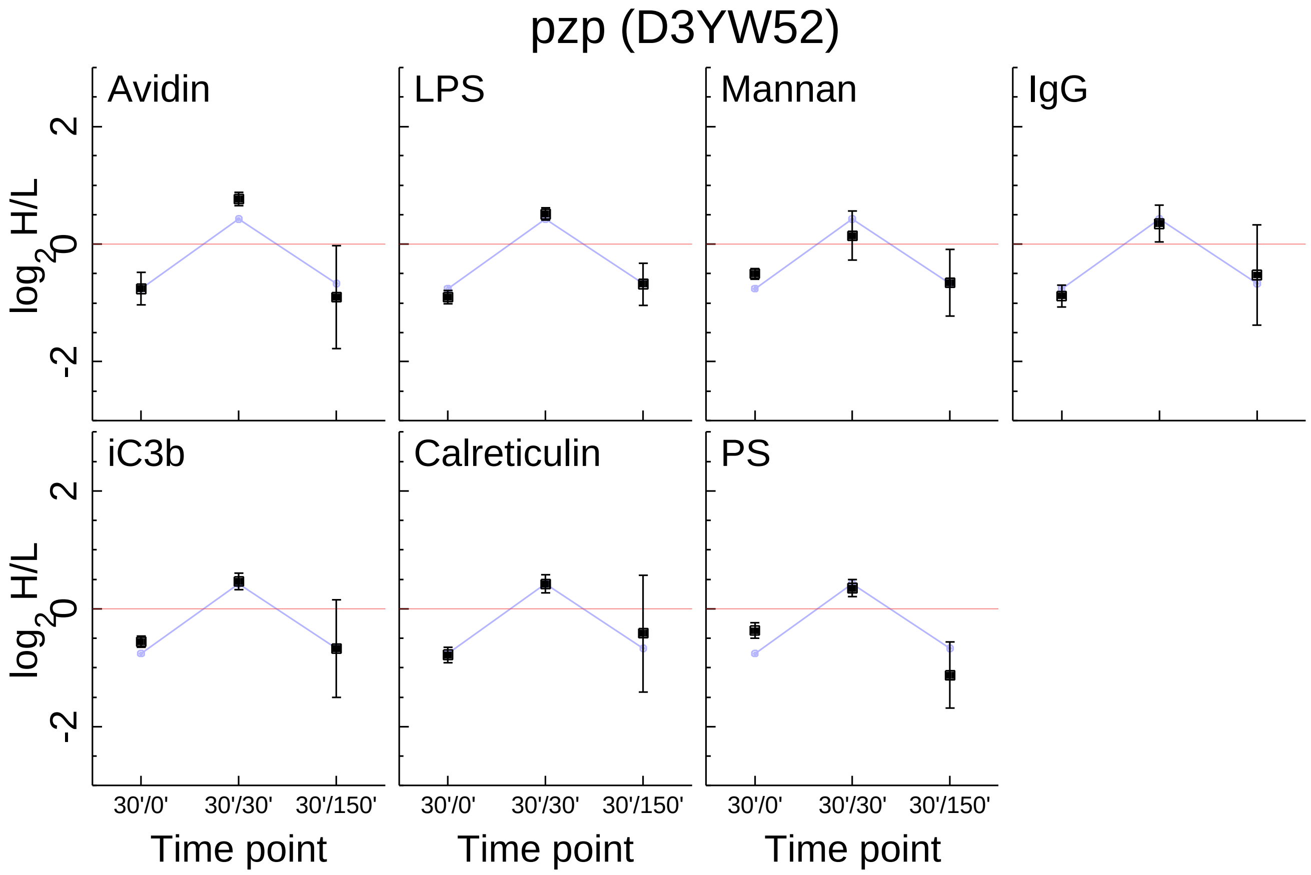
pzp (D3YW52)
Avidin
LPS
Mannan
IgG
iC3b
Calreticulin
PS
log2 H/L
2
0
-2
30'/0'
30'/30'
30'/150'
Time point

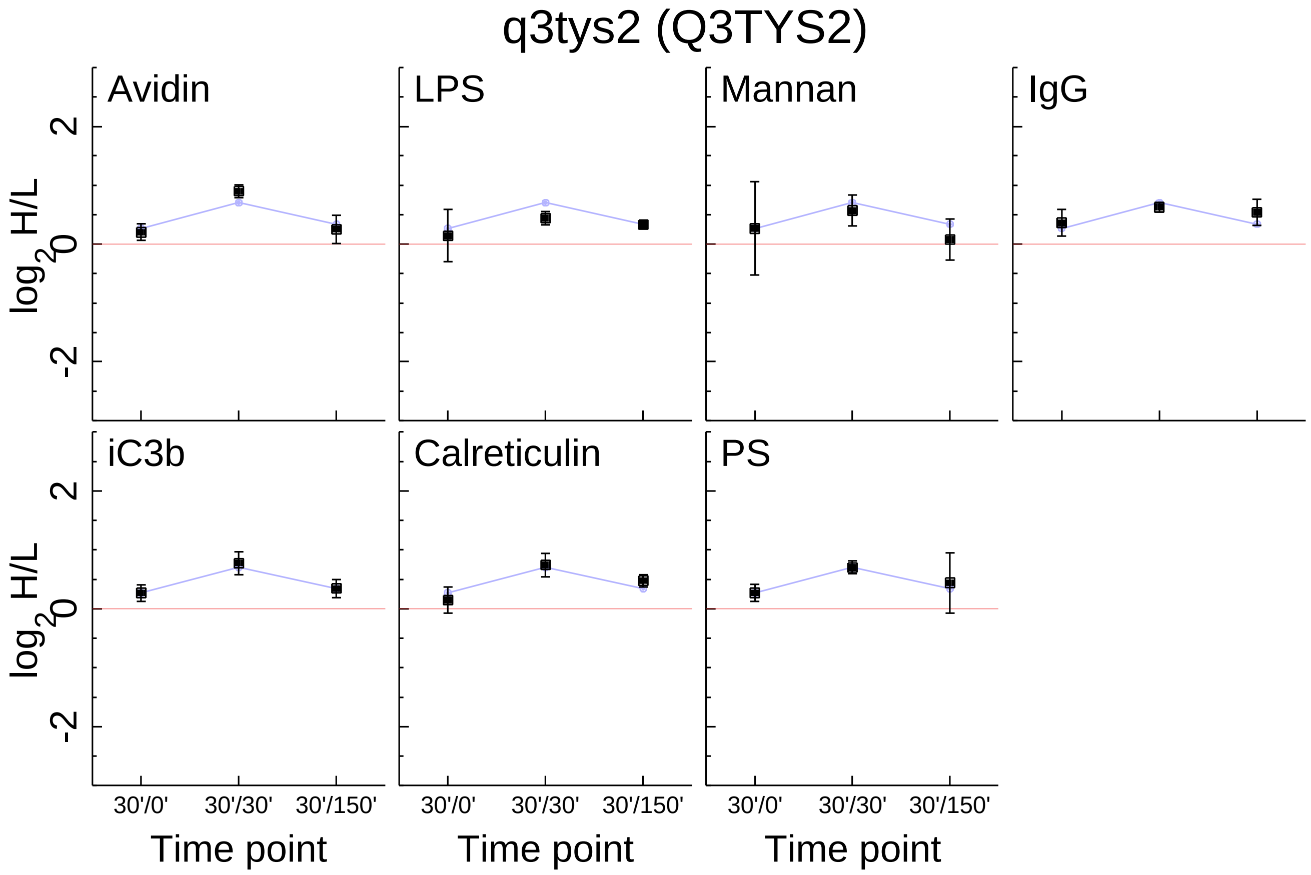
q3tys2 (Q3TYS2)
Avidin
LPS
Mannan
IgG
iC3b
Calreticulin
PS
log2 H/L
2
0
-2
30'/0'
30'/30'
30'/150'
Time point

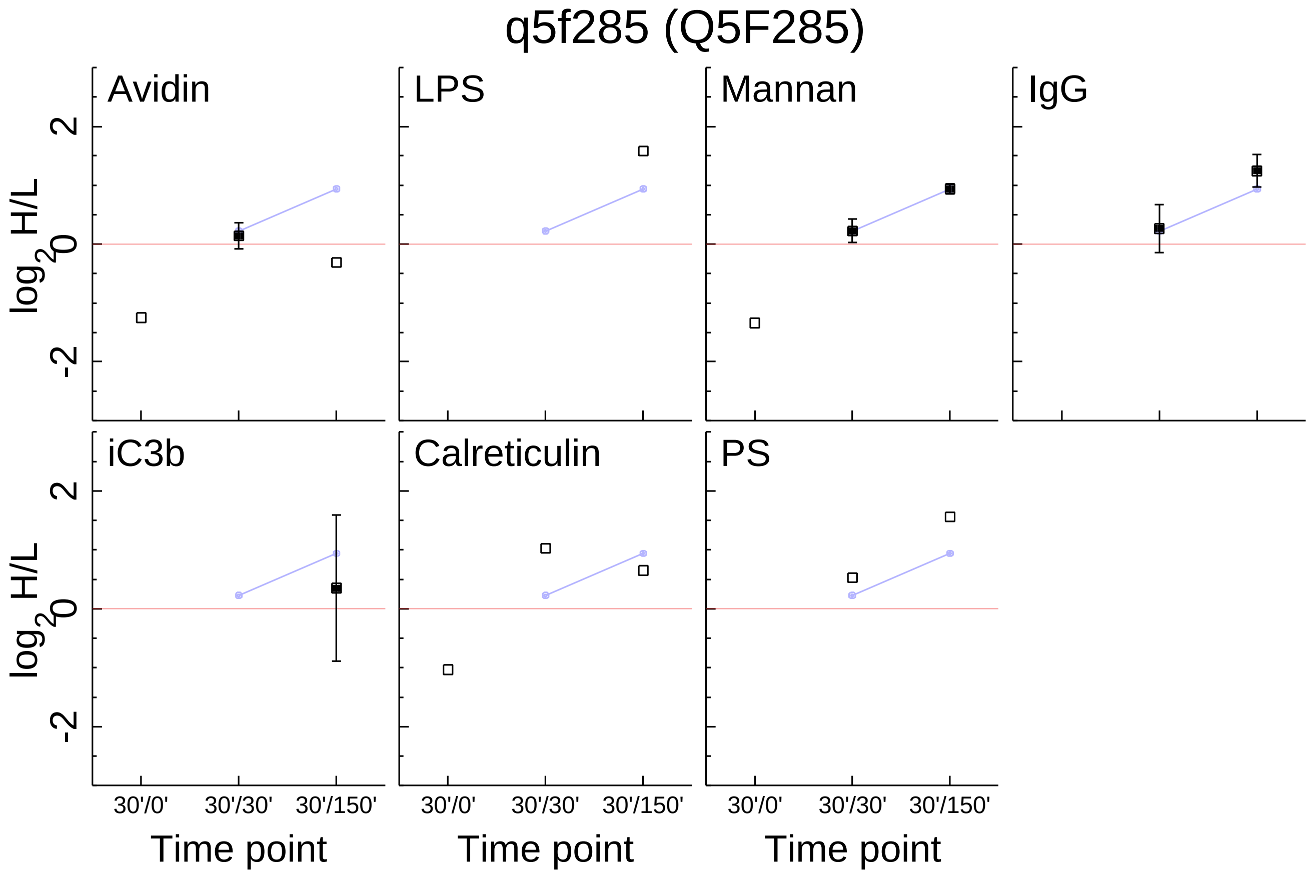
q5f285 (Q5F285)
Avidin
LPS
Mannan
IgG
iC3b
Calreticulin
PS
log2 H/L
2
0
-2
30'/0'
30'/30'
30'/150'
Time point

q6piu9 (Q6PIU9)

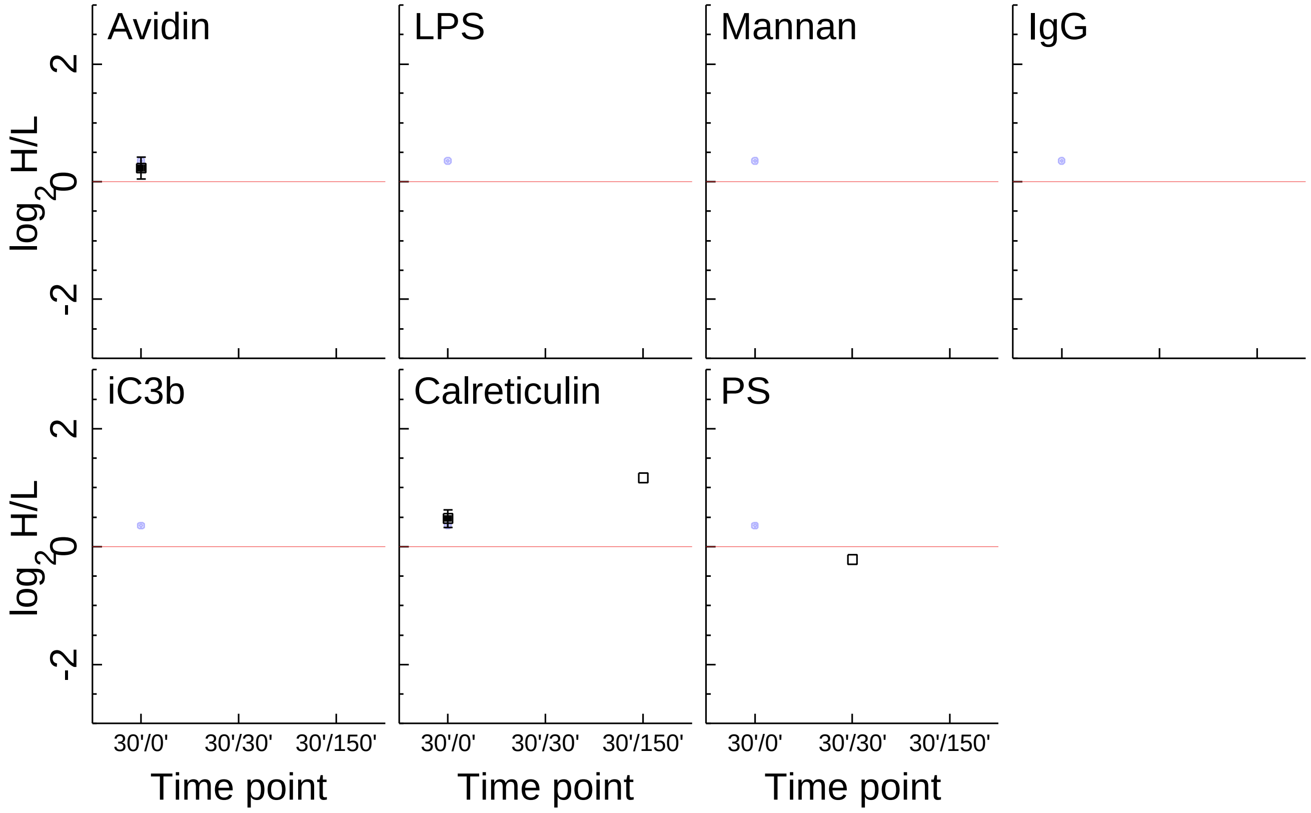

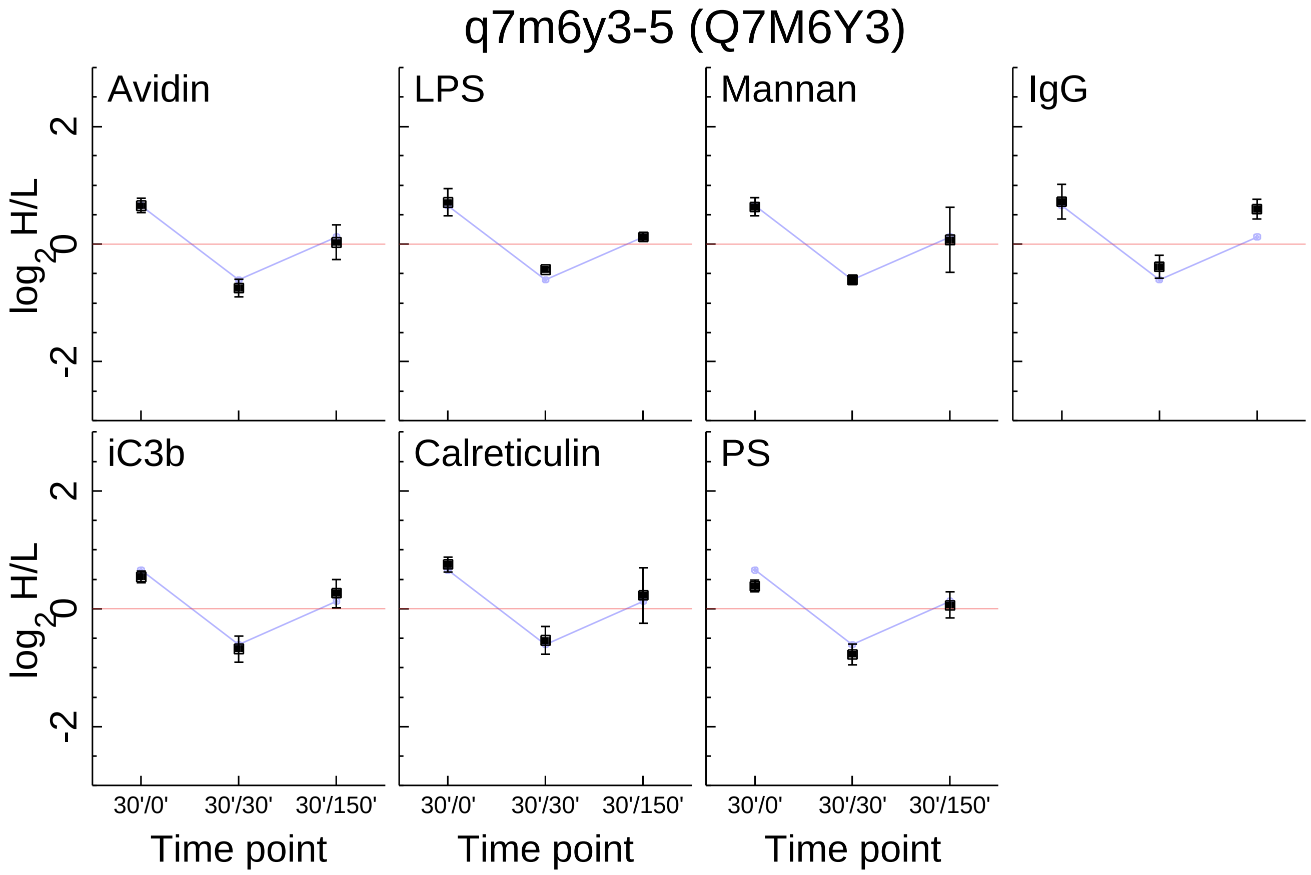
q7m6y3-5 (Q7M6Y3)
Avidin
LPS
Mannan
IgG
iC3b
Calreticulin
PS
log2 H/L
2
0
-2
30'/0'
30'/30'
30'/150'
Time point

q8c708 (Q8C708)

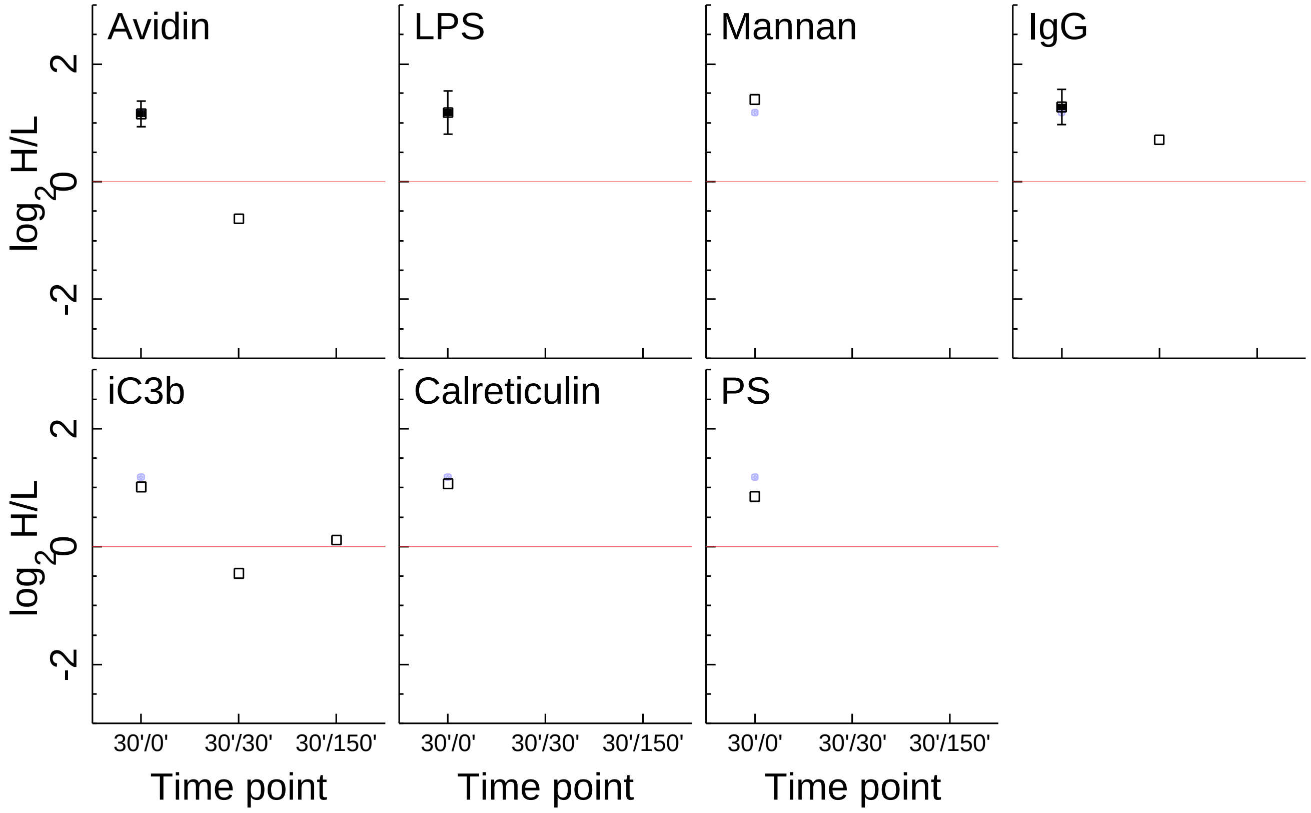

q8caq8-3 (Q8CAQ8)

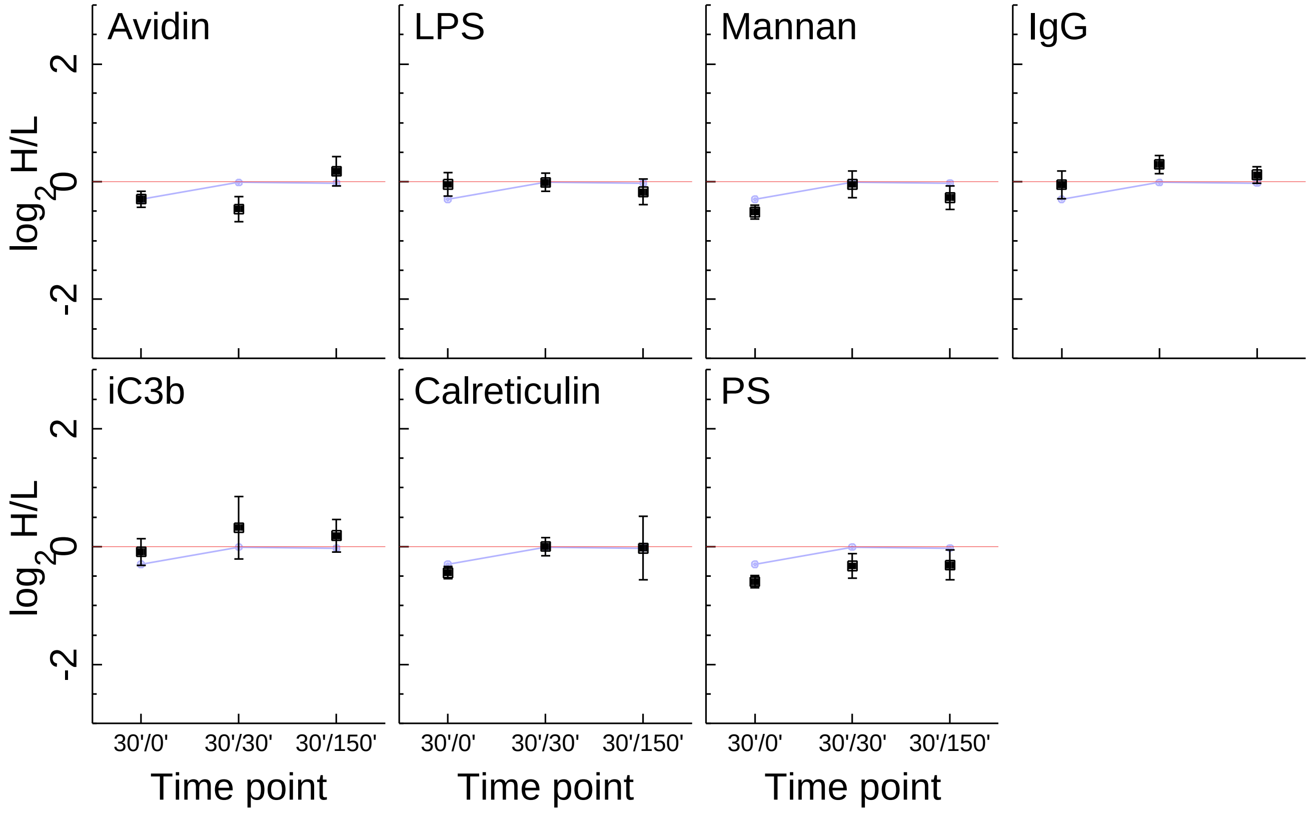

q8cf66 (Q8CF66)

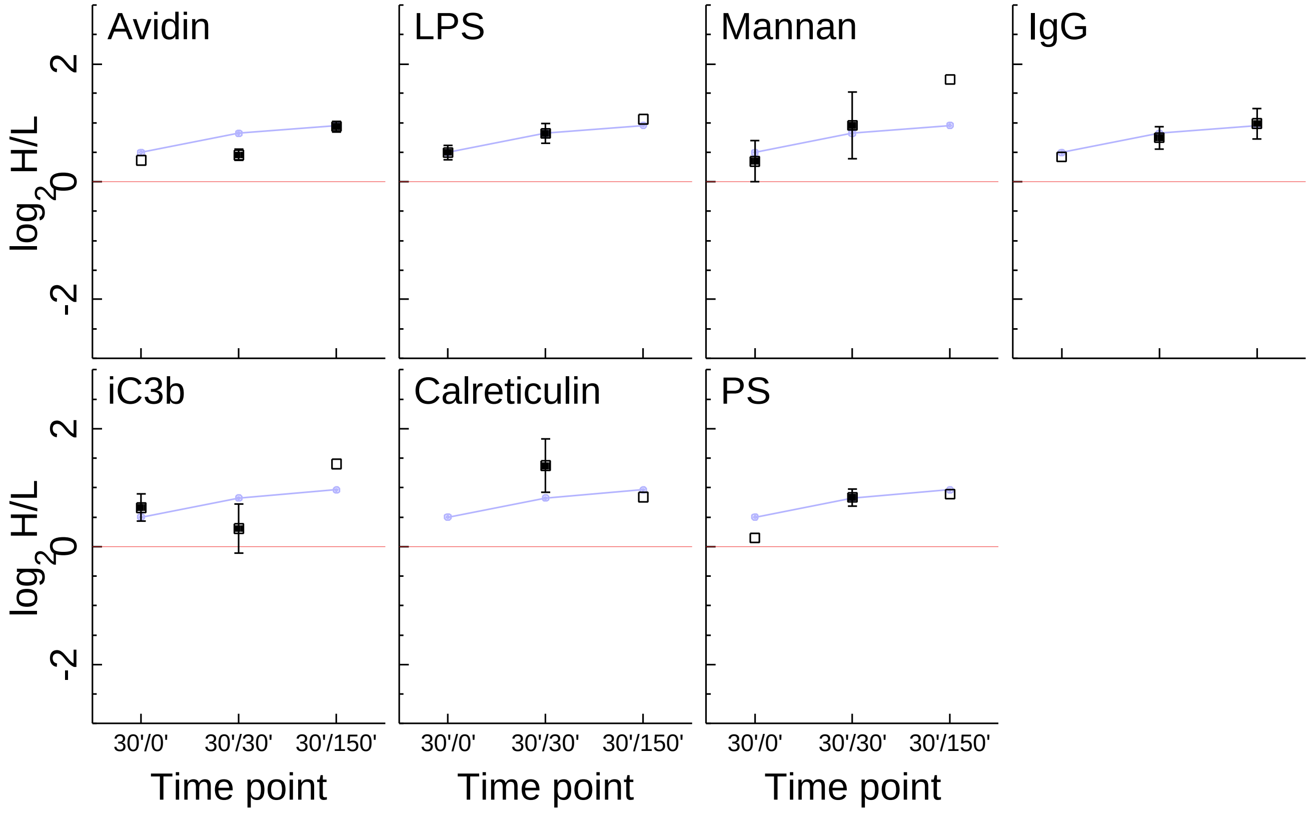

q8k039 (Q8K039)

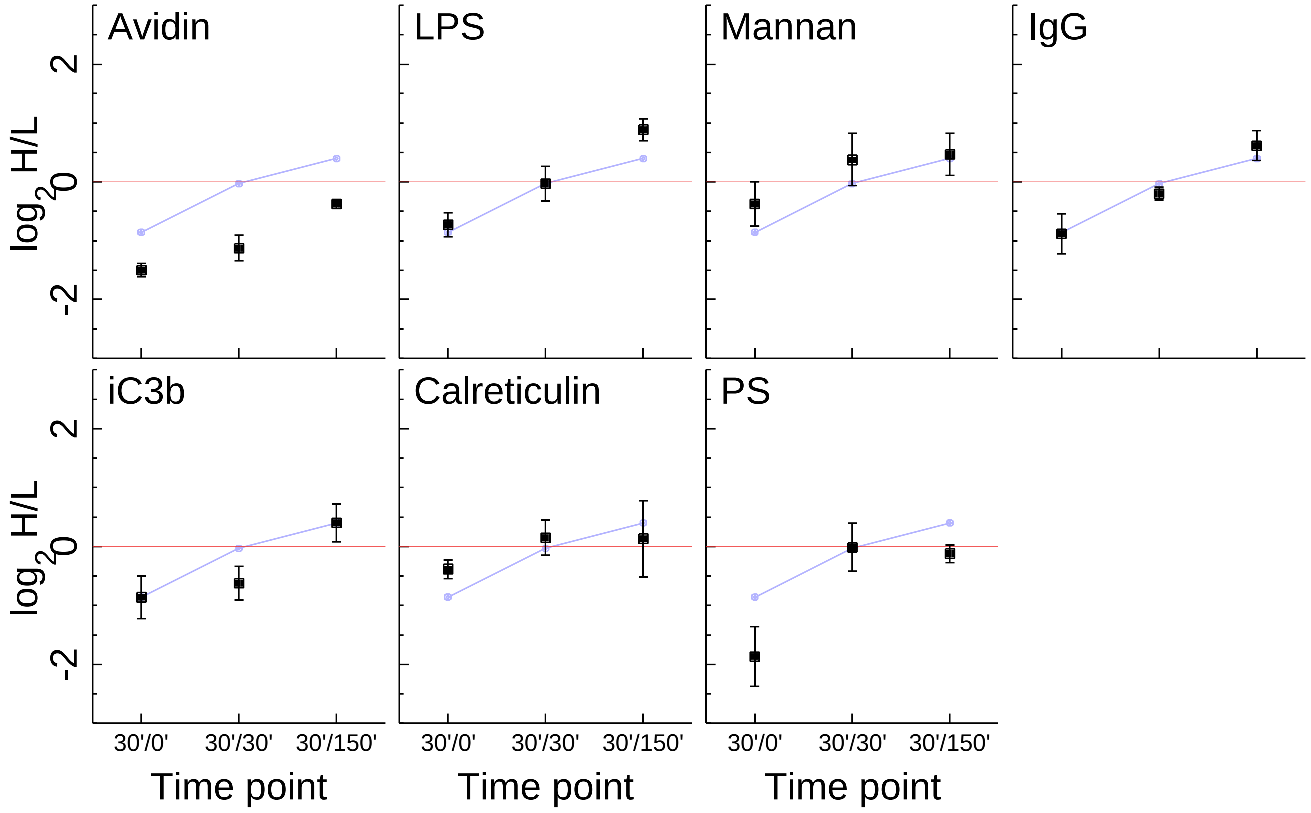

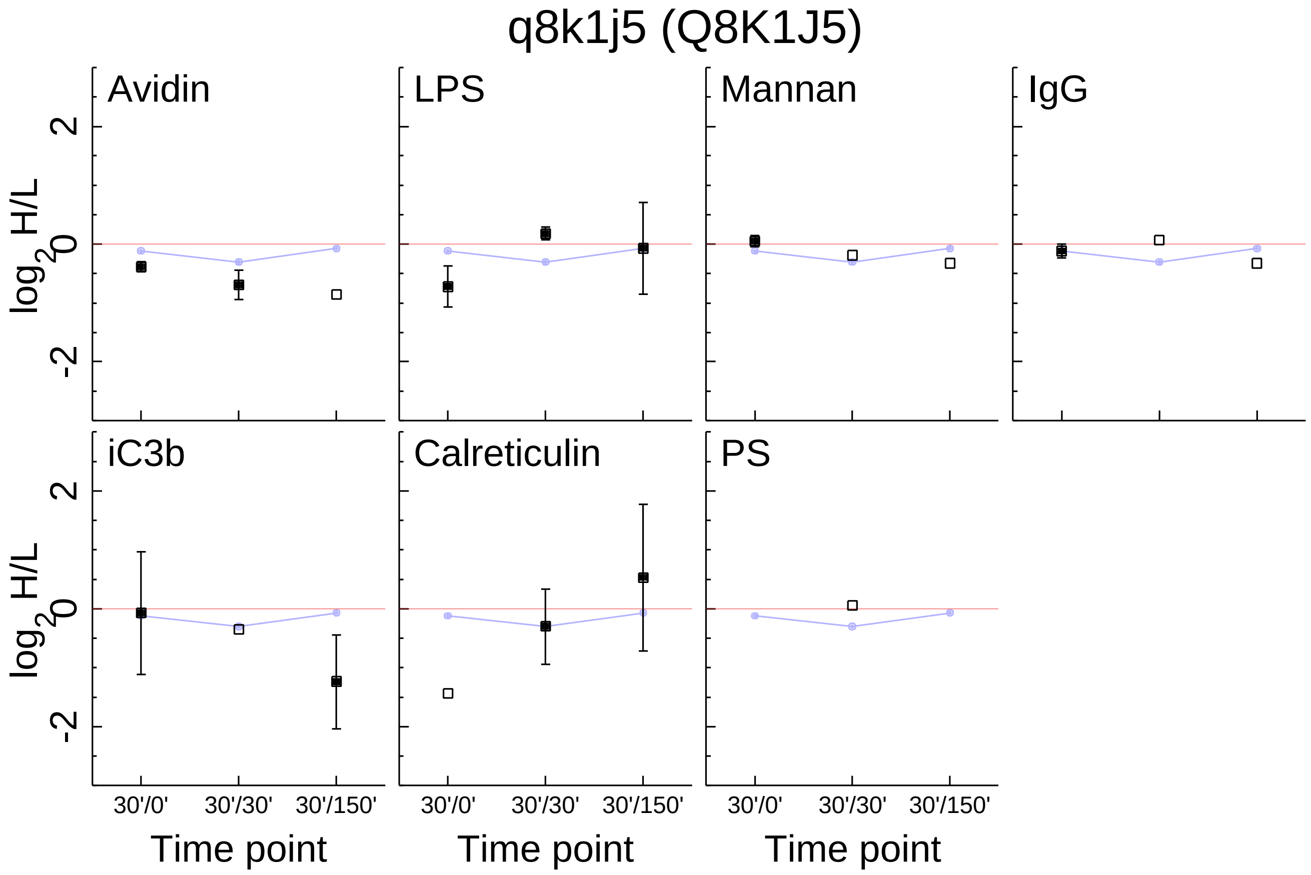
q8k1j5 (Q8K1J5)
Avidin
LPS
Mannan
IgG
iC3b
Calreticulin
PS
log2 H/L
2
0
-2
30'/0'
30'/30'
30'/150'
Time point

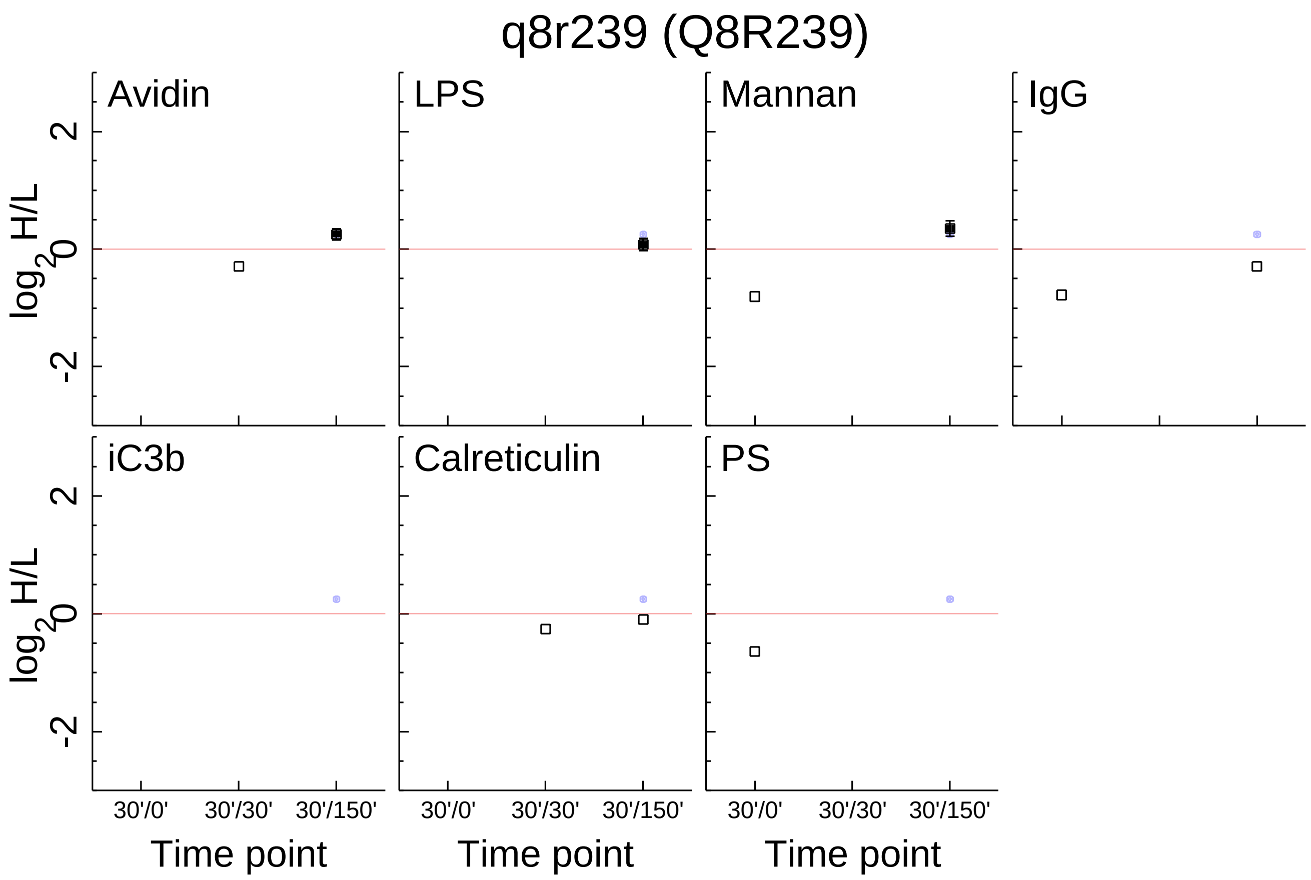
q8r239 (Q8R239)
Avidin
LPS
Mannan
IgG
iC3b
Calreticulin
PS
log2 H/L
2
0
-2
30'/0'
30'/30'
30'/150'
Time point

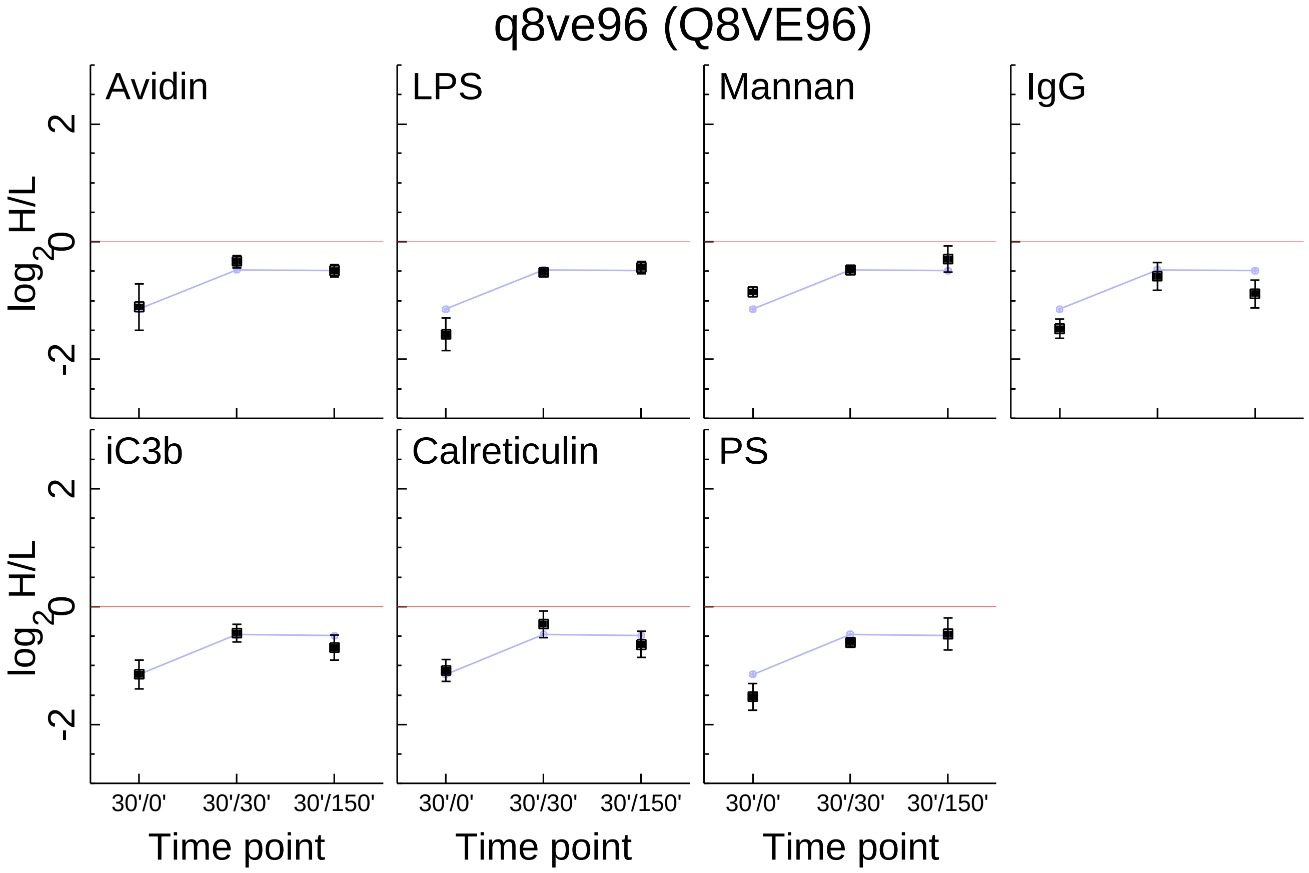
q8ve96 (Q8VE96)
Avidin
LPS
Mannan
IgG
iC3b
Calreticulin
PS
log2 H/L
2
0
-2
30'/0'
30'/30'
30'/150'
Time point

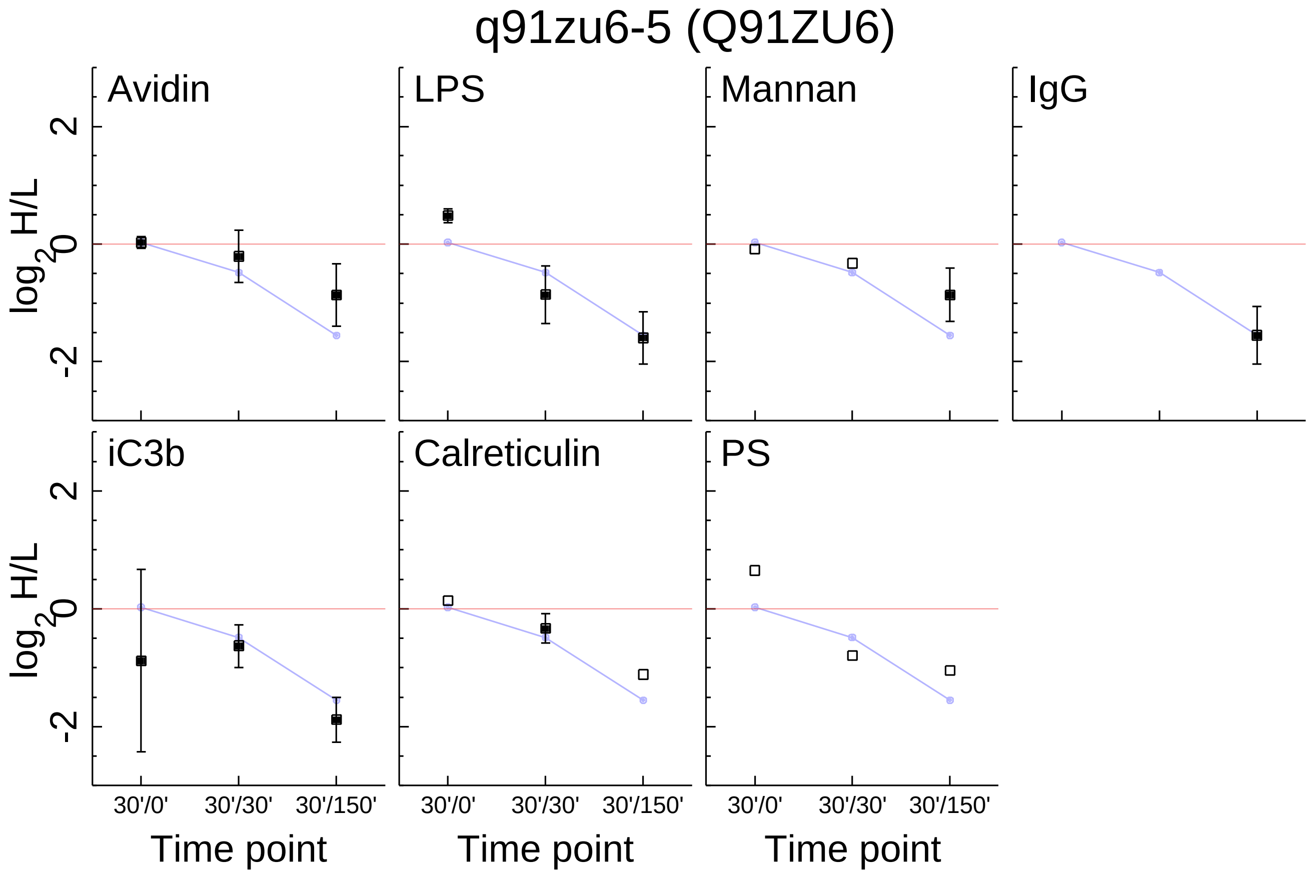

q91zu6-5 (Q91ZU6)
Avidin
LPS
Mannan
IgG
iC3b
Calreticulin
PS
log2 H/L
2
0
-2
30'/0'
30'/30'
30'/150'
Time point

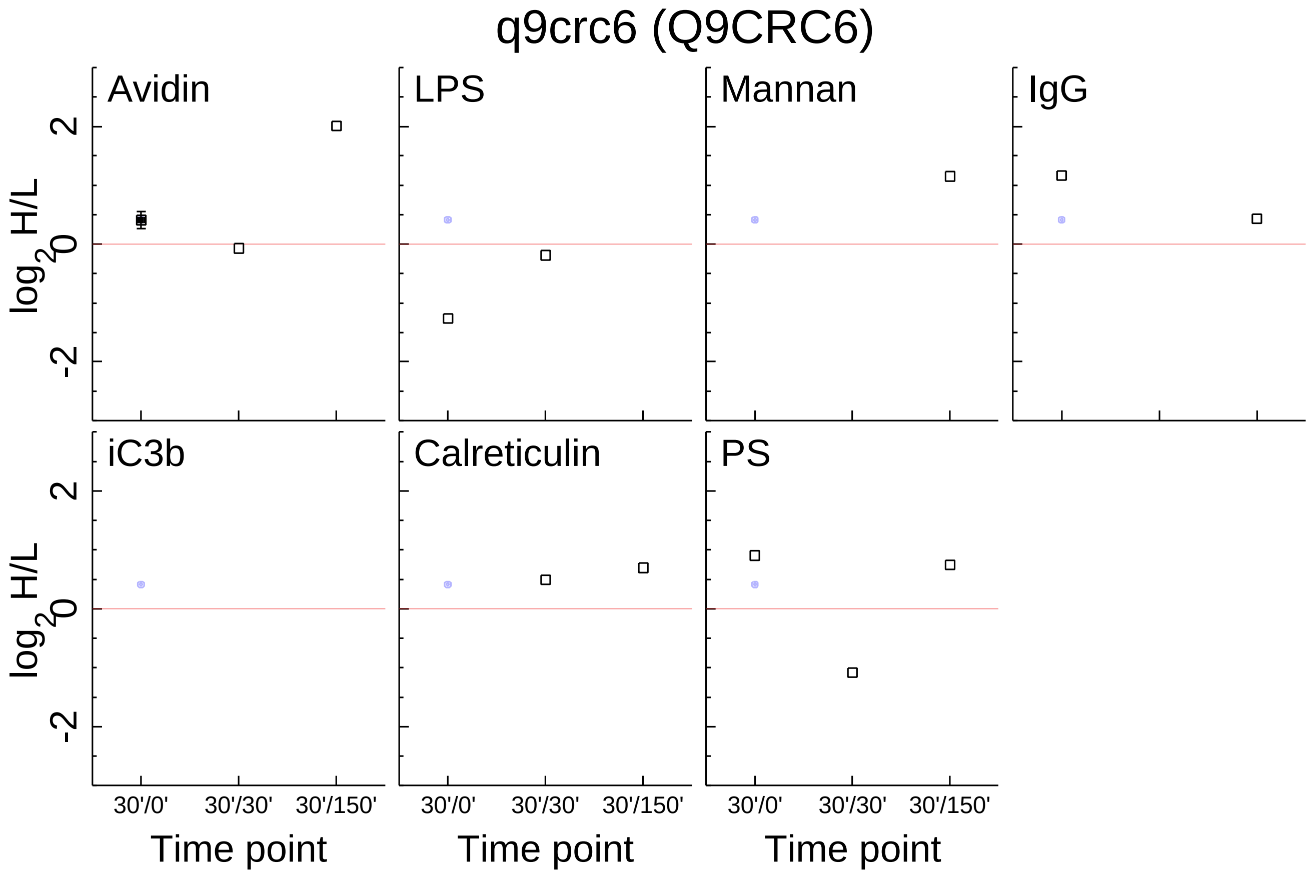
q9crc6 (Q9CRC6)
Avidin
LPS
Mannan
IgG
iC3b
Calreticulin
PS
log2 H/L
2
0
-2
30'/0'
30'/30'
30'/150'
Time point

q9d0b6 (Q9D0B6)

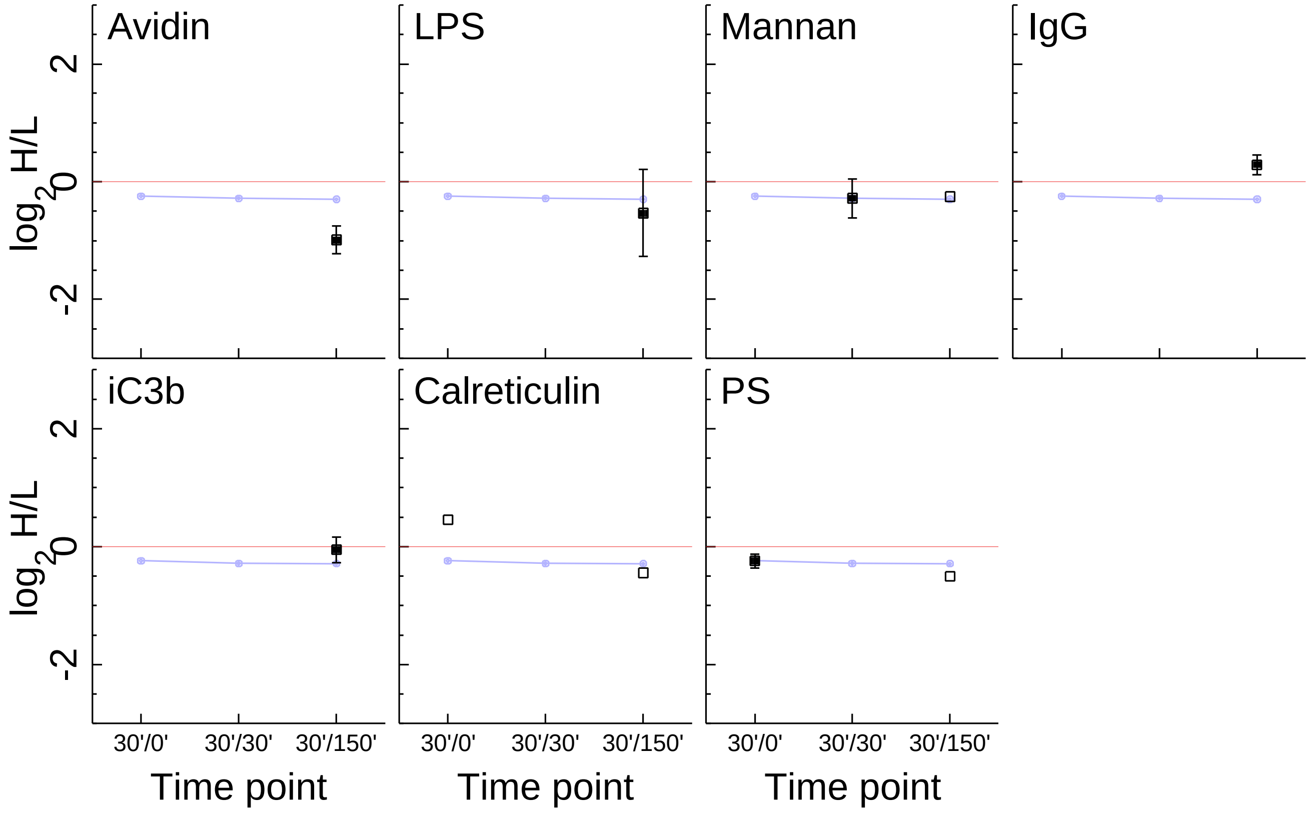

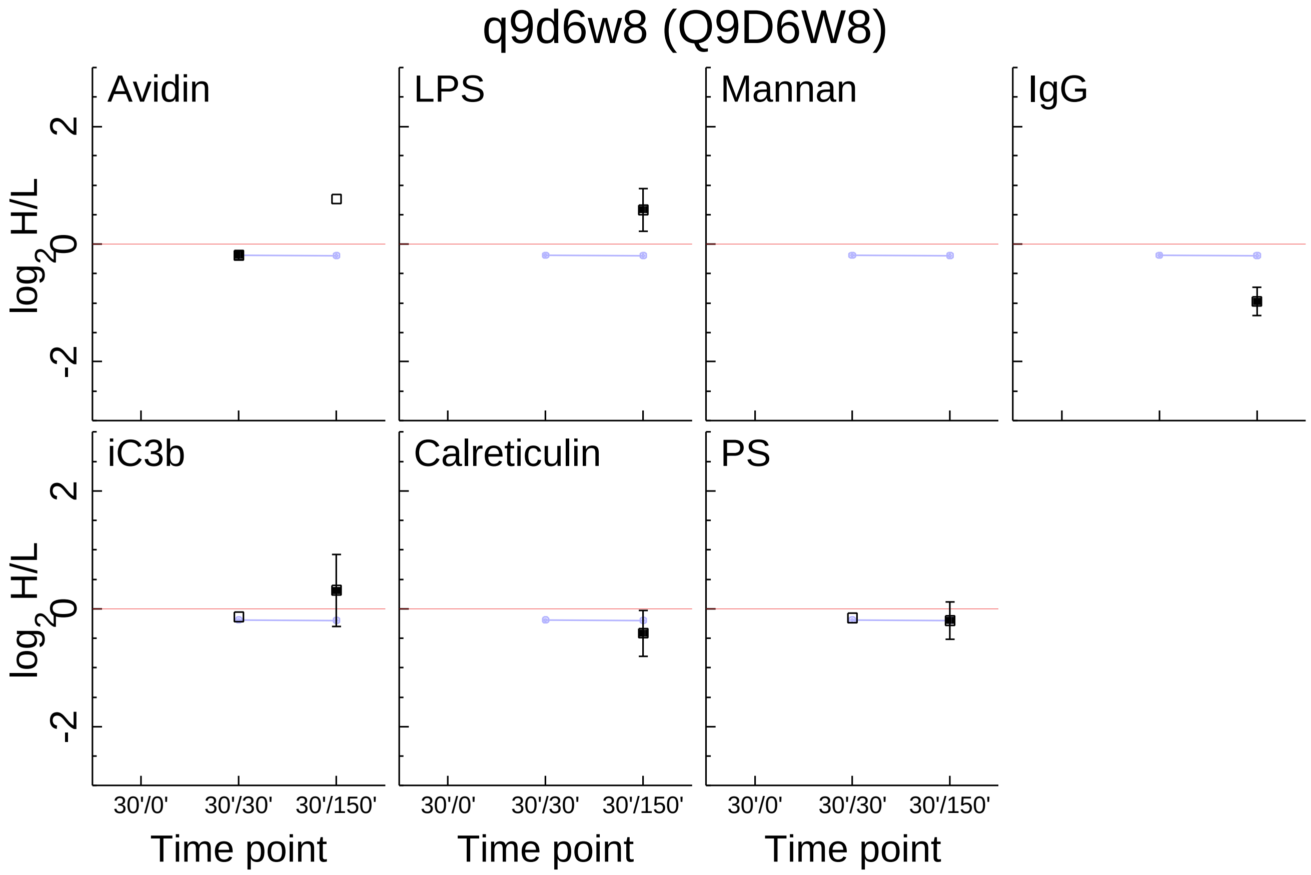
q9d6w8 (Q9D6W8)
Avidin
LPS
Mannan
IgG
iC3b
Calreticulin
PS
log2 H/L
2
0
-2
30'/0'
30'/30'
30'/150'
Time point

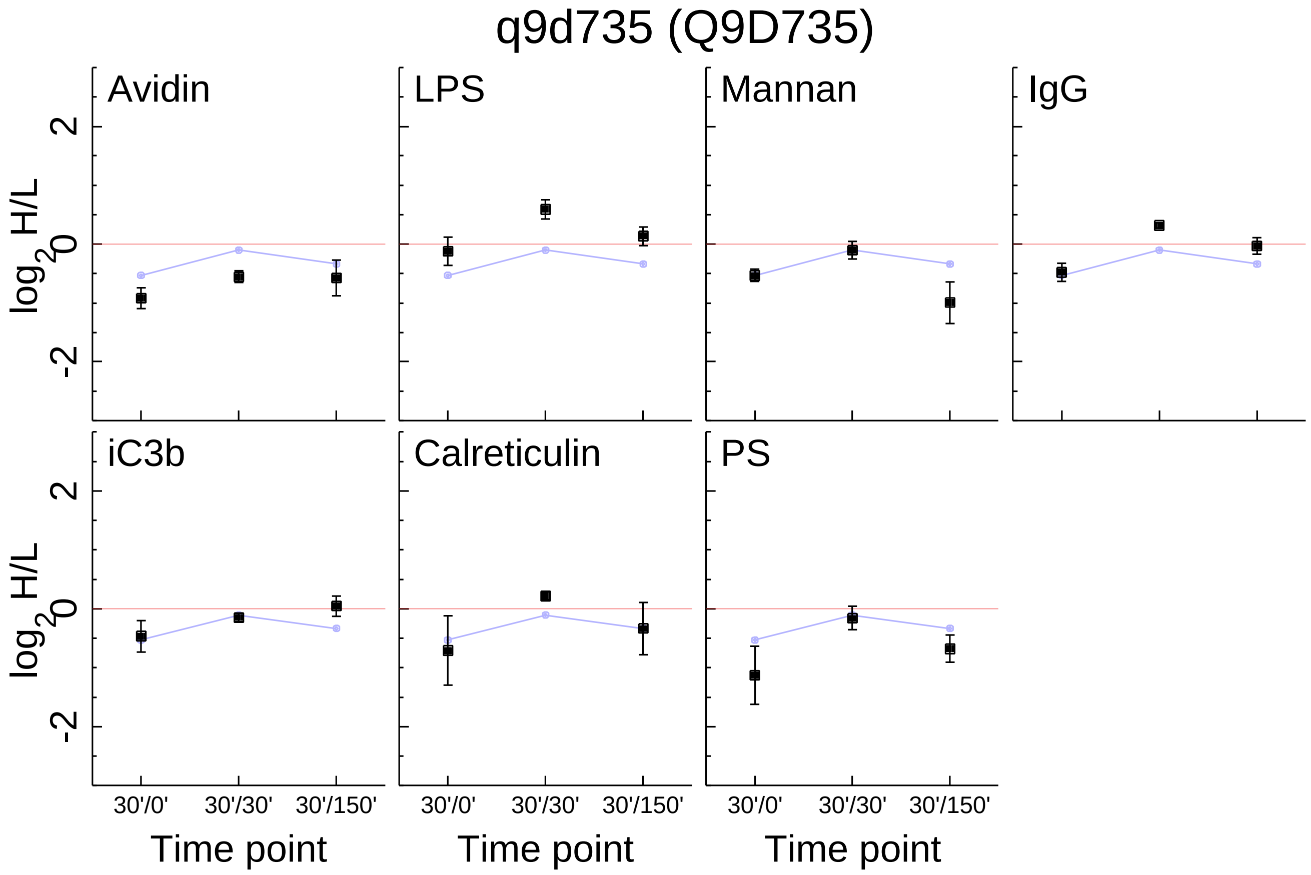
q9d735 (Q9D735)
Avidin
LPS
Mannan
IgG
iC3b
Calreticulin
PS
log_2 H/L
2
0
-2
30'/0'
30'/30'
30'/150'
Time point

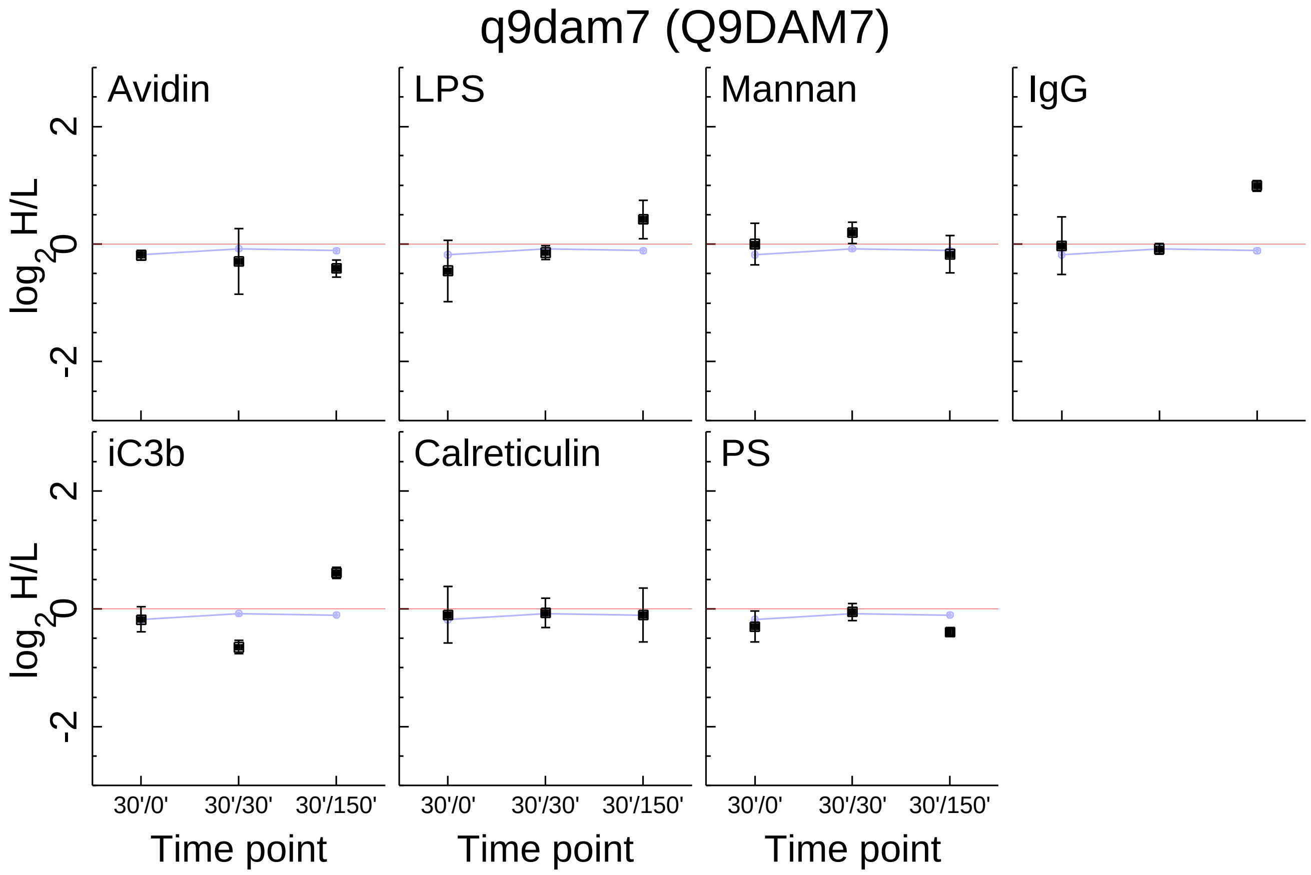

q9dam7 (Q9DAM7)
Avidin
LPS
Mannan
IgG
iC3b
Calreticulin
PS
log2 H/L
2
0
-2
30'/0'
30'/30'
30'/150'
Time point

q9es97-3 (Q9ES97)

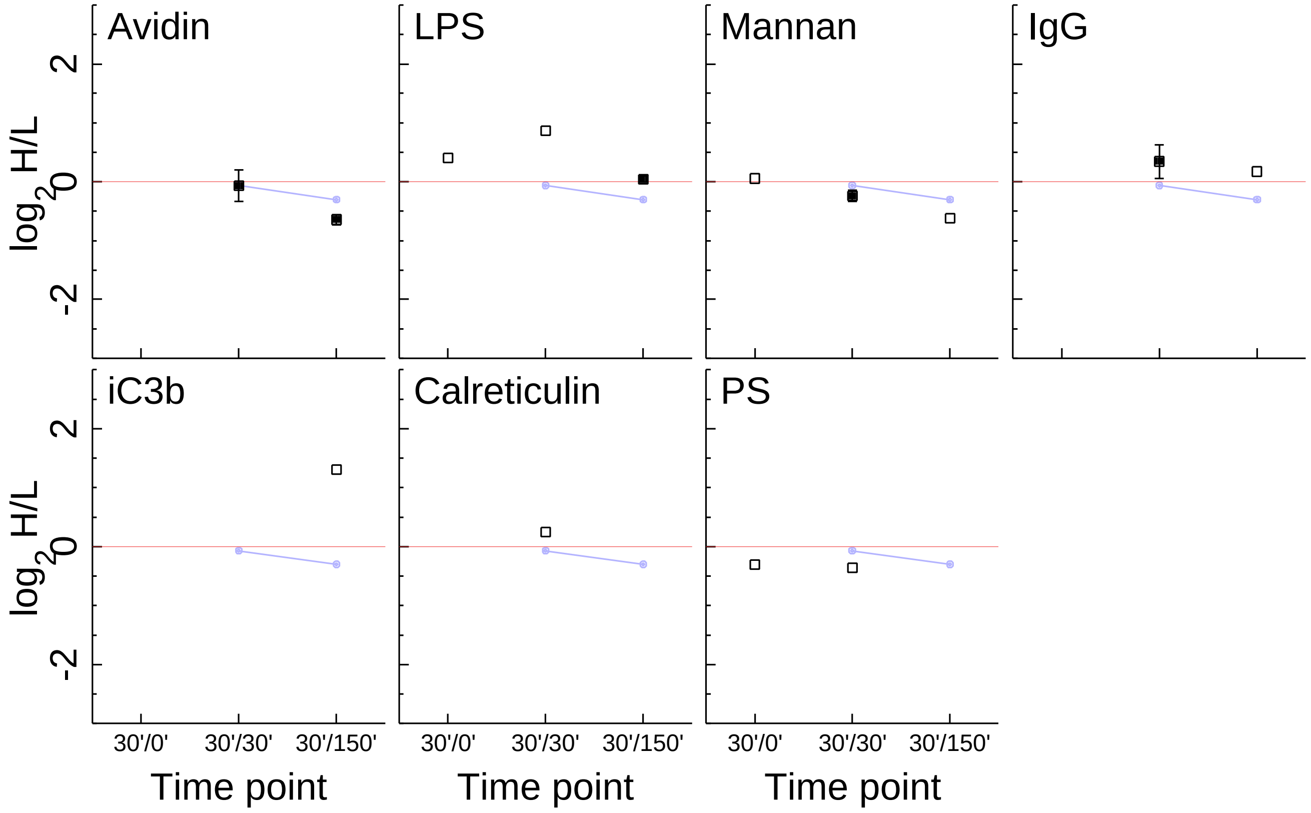

qki (Q9QYS9)

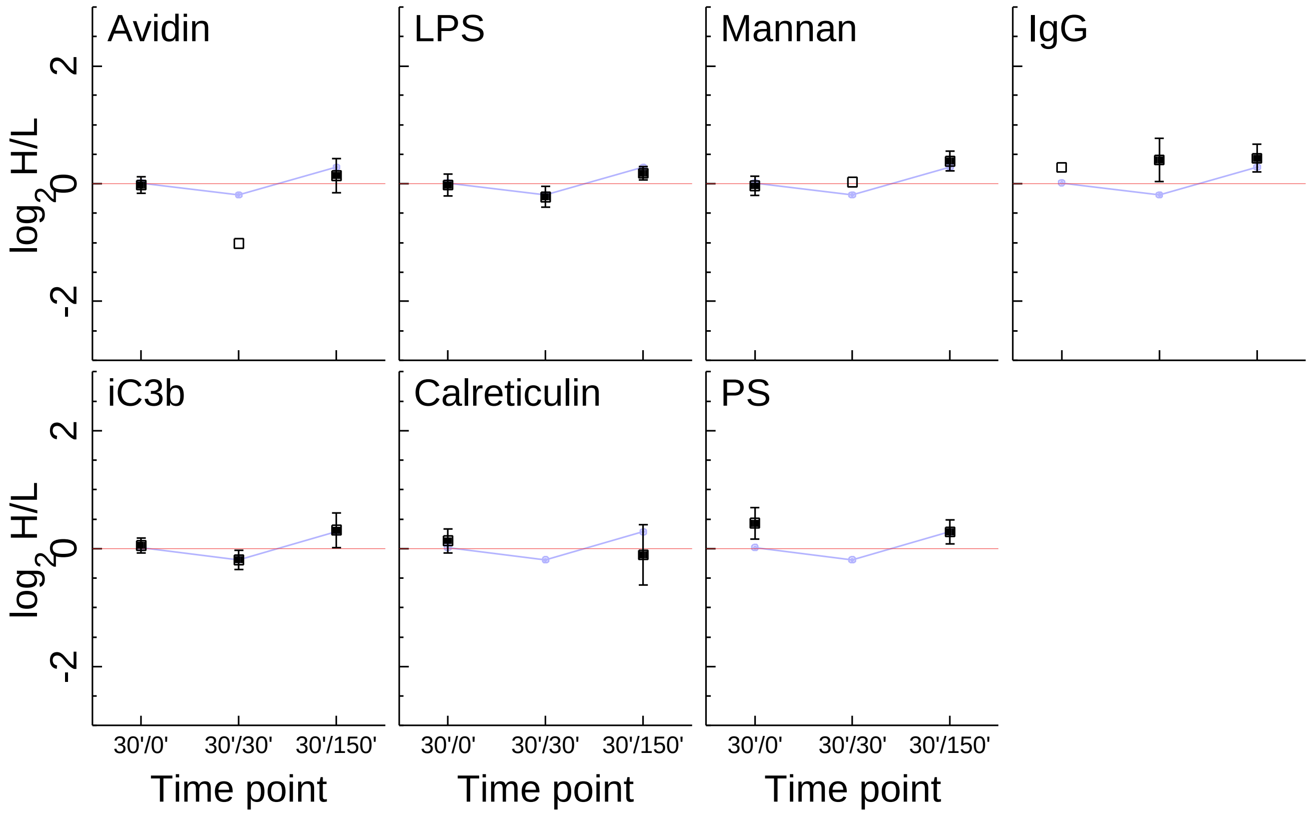
Avidin
LPS
Mannan
IgG
iC3b
Calreticulin
PS
log2 H/L
2
0
-2
30'/0'
30'/30'
30'/150'
Time point

rab10 (P61027)

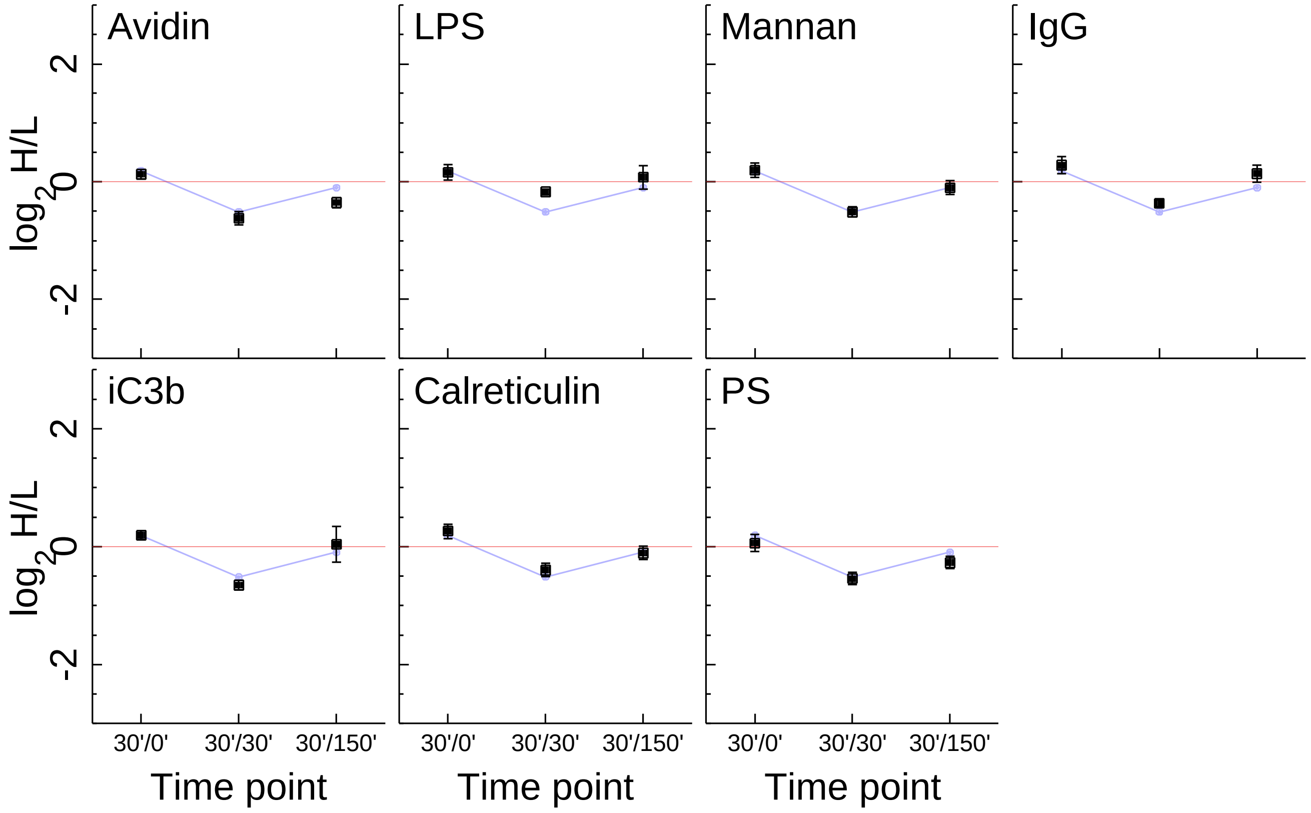

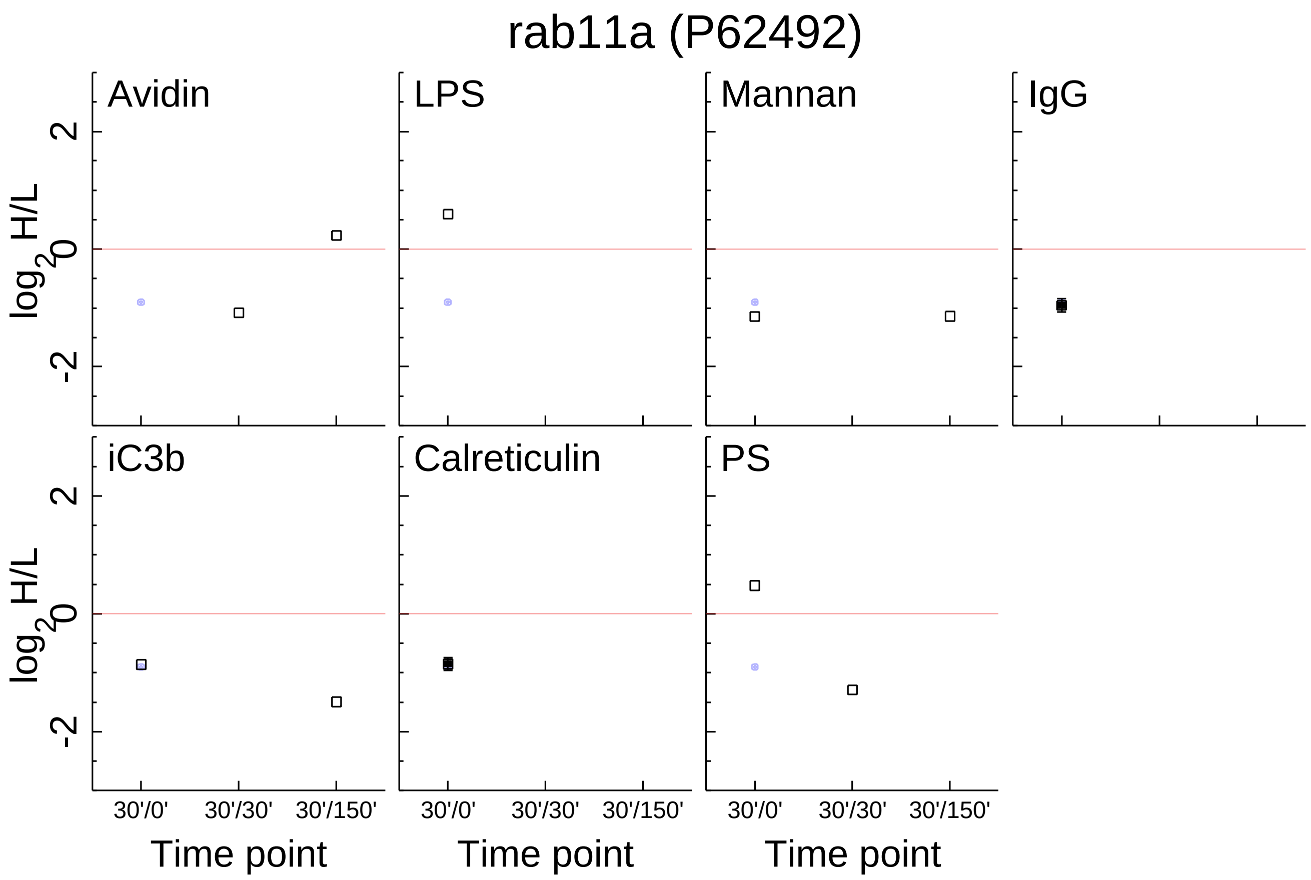
rab11a (P62492)
Avidin
LPS
Mannan
IgG
iC3b
Calreticulin
PS
log2 H/L
2
0
-2
30'/0'
30'/30'
30'/150'
Time point

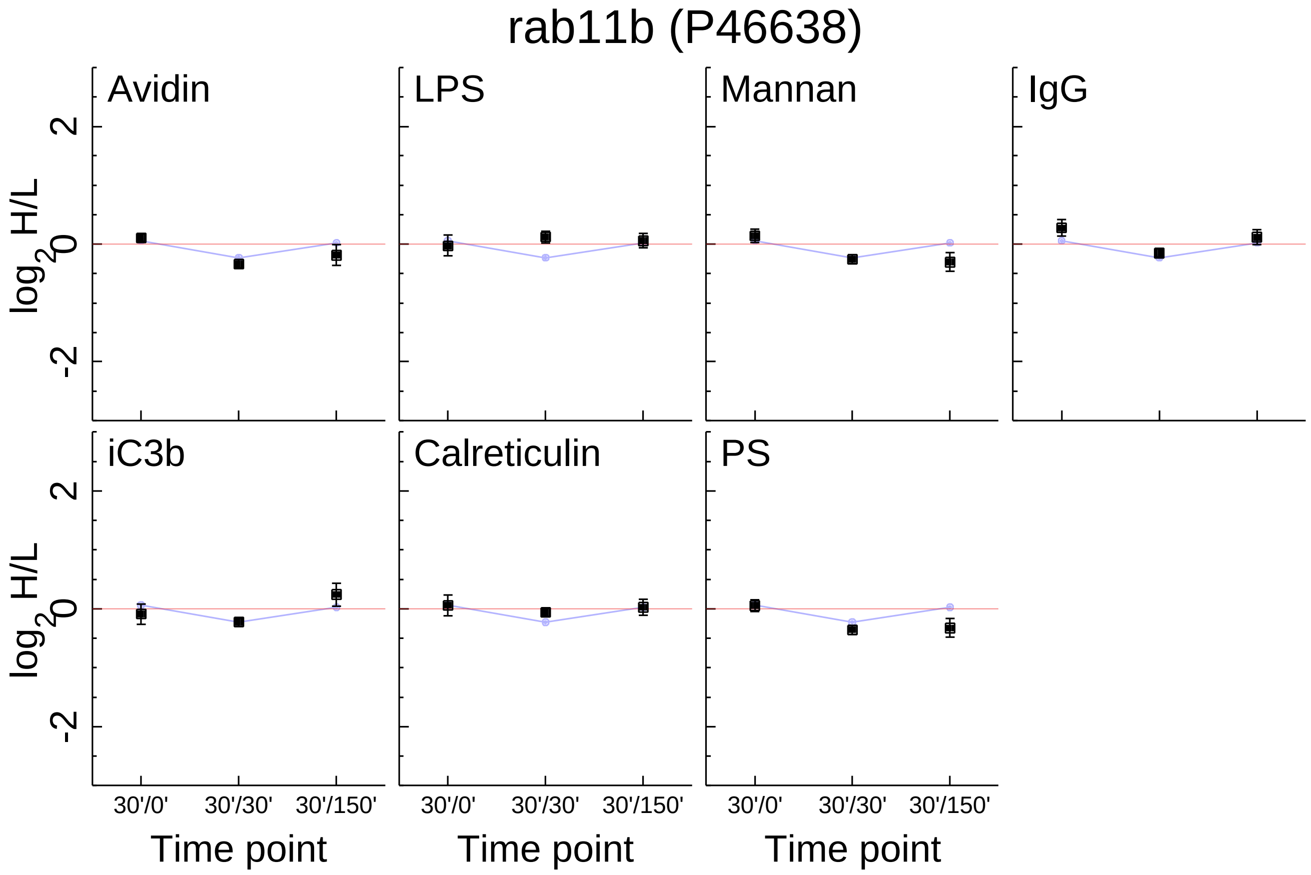

rab11b (P46638)
Avidin
LPS
Mannan
IgG
iC3b
Calreticulin
PS
log2 H/L
2
0
-2
30'/0'
30'/30'
30'/150'
Time point

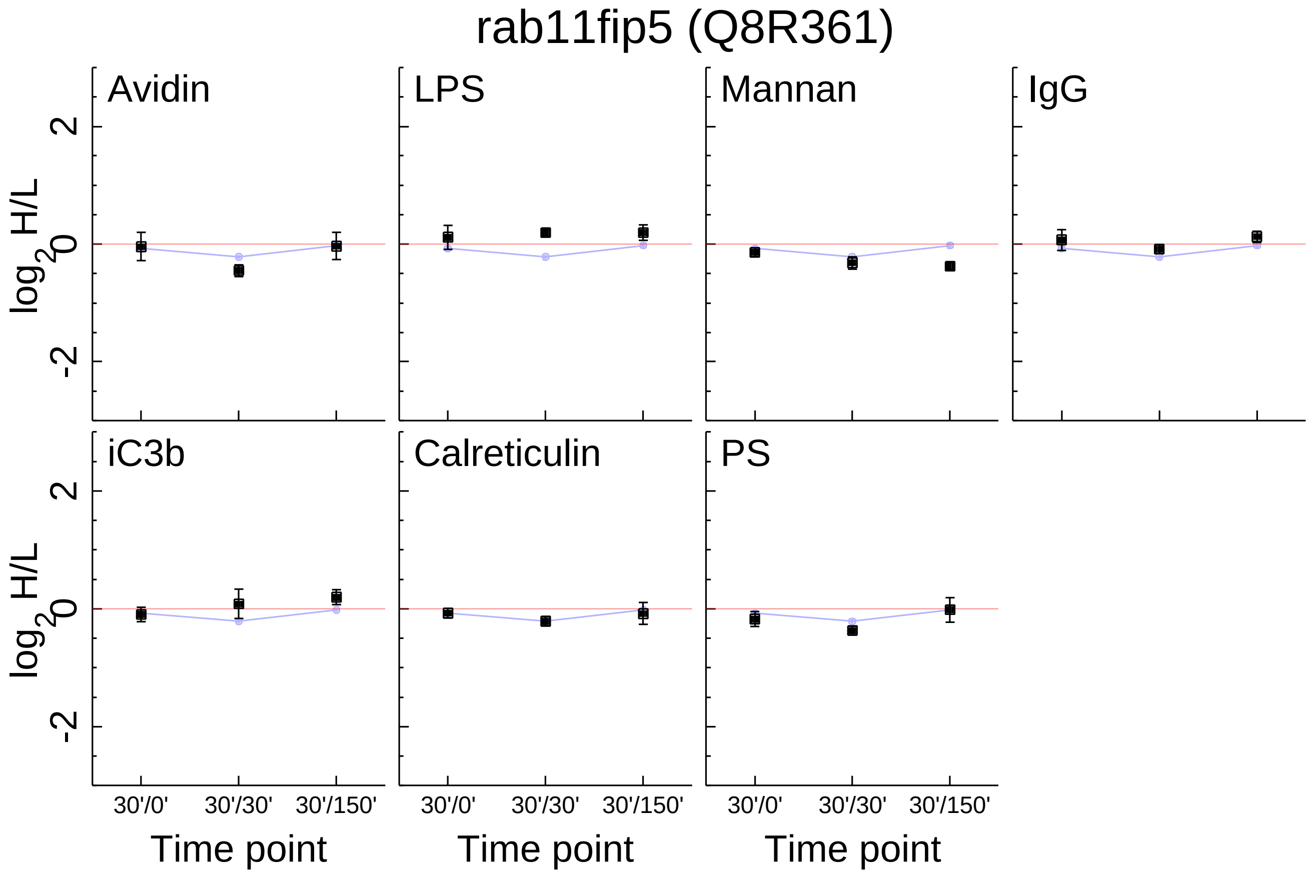

rab11fip5 (Q8R361)
Avidin
LPS
Mannan
IgG
iC3b
Calreticulin
PS
2
0
-2
log2 H/L
30'/0'
30'/30'
30'/150'
Time point

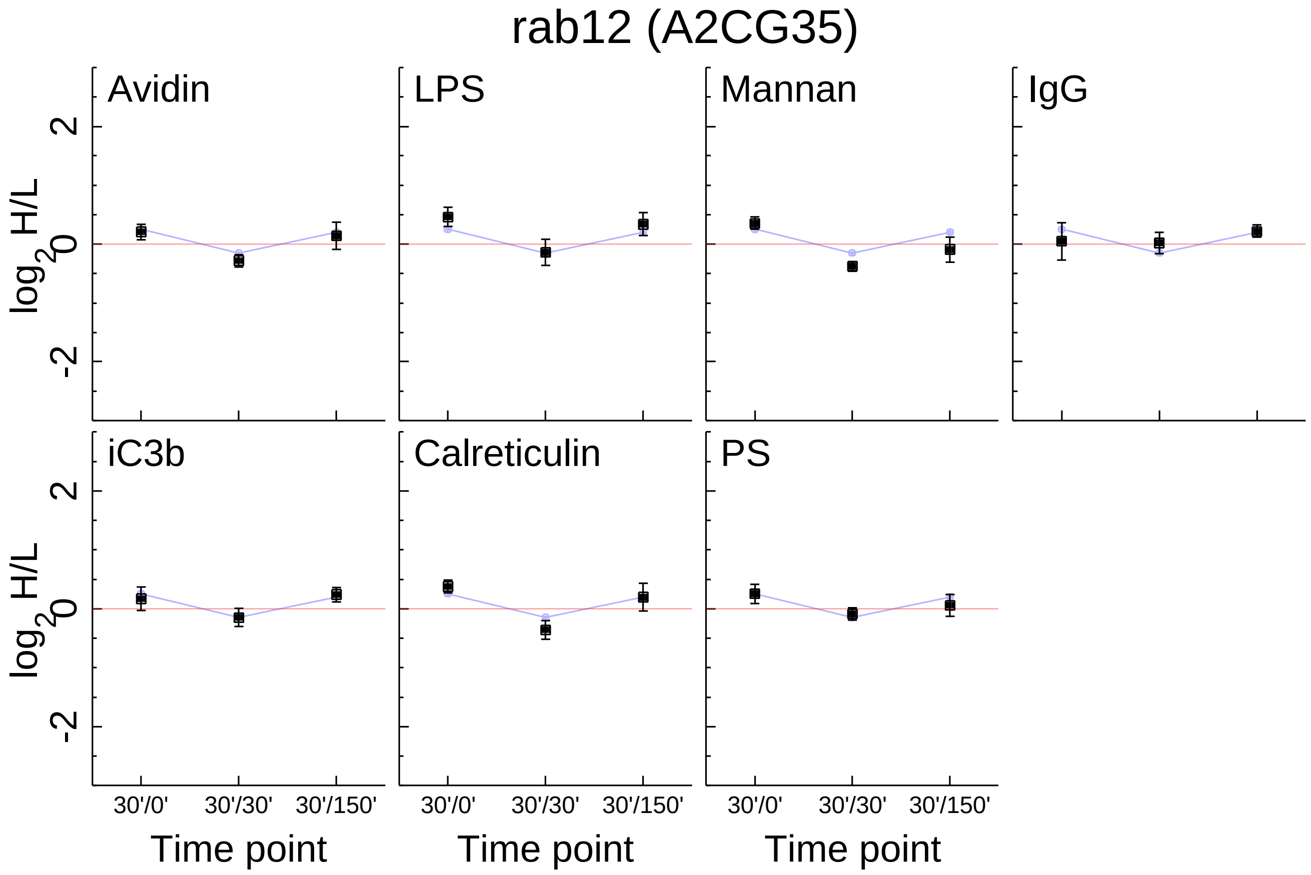
rab12 (A2CG35)
Avidin
LPS
Mannan
IgG
iC3b
Calreticulin
PS
log2 H/L
2
0
-2
30'/0'
30'/30'
30'/150'
Time point

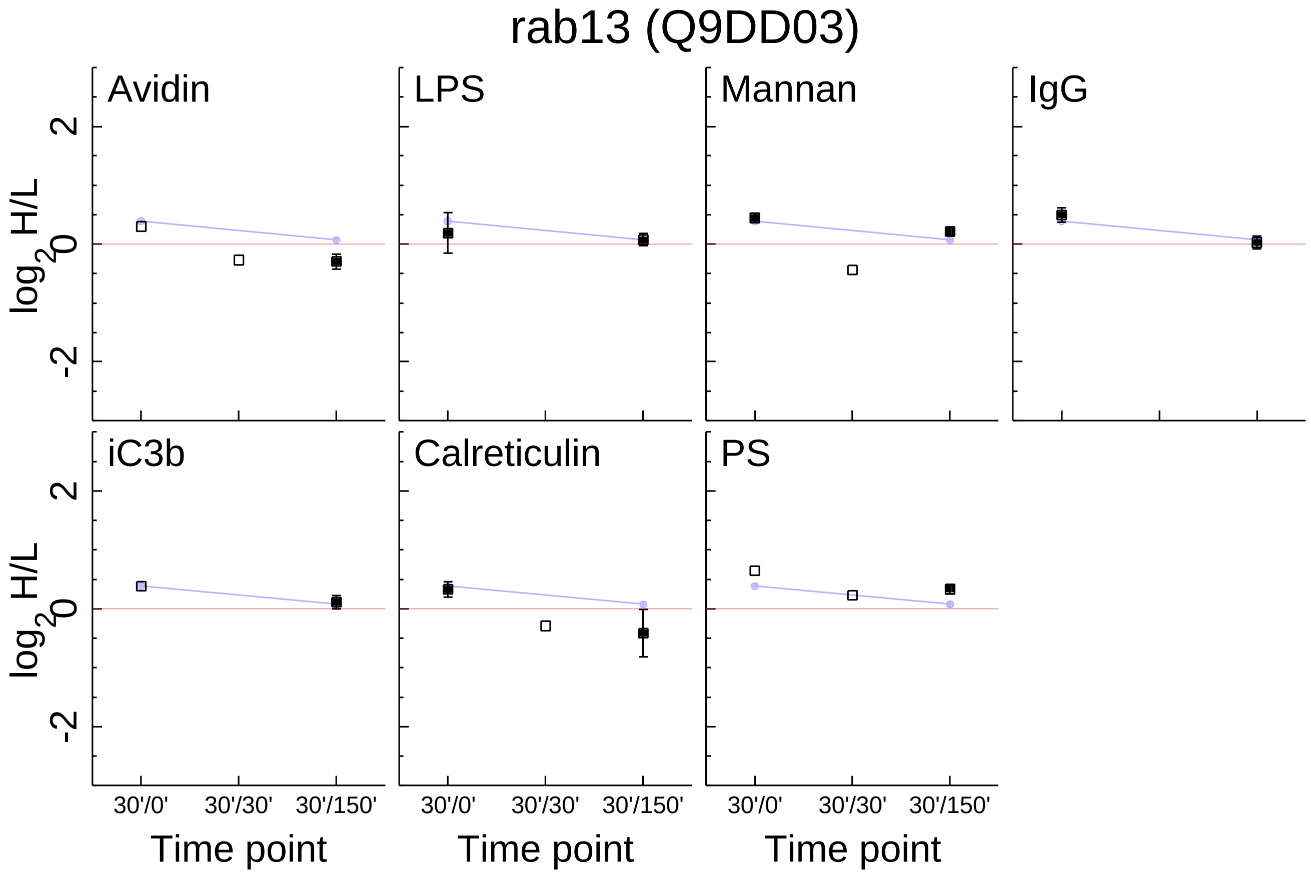

rab13 (Q9DD03)
Avidin
LPS
Mannan
IgG
iC3b
Calreticulin
PS
2
0
-2
log2 H/L
30'/0'
30'/30'
30'/150'
Time point

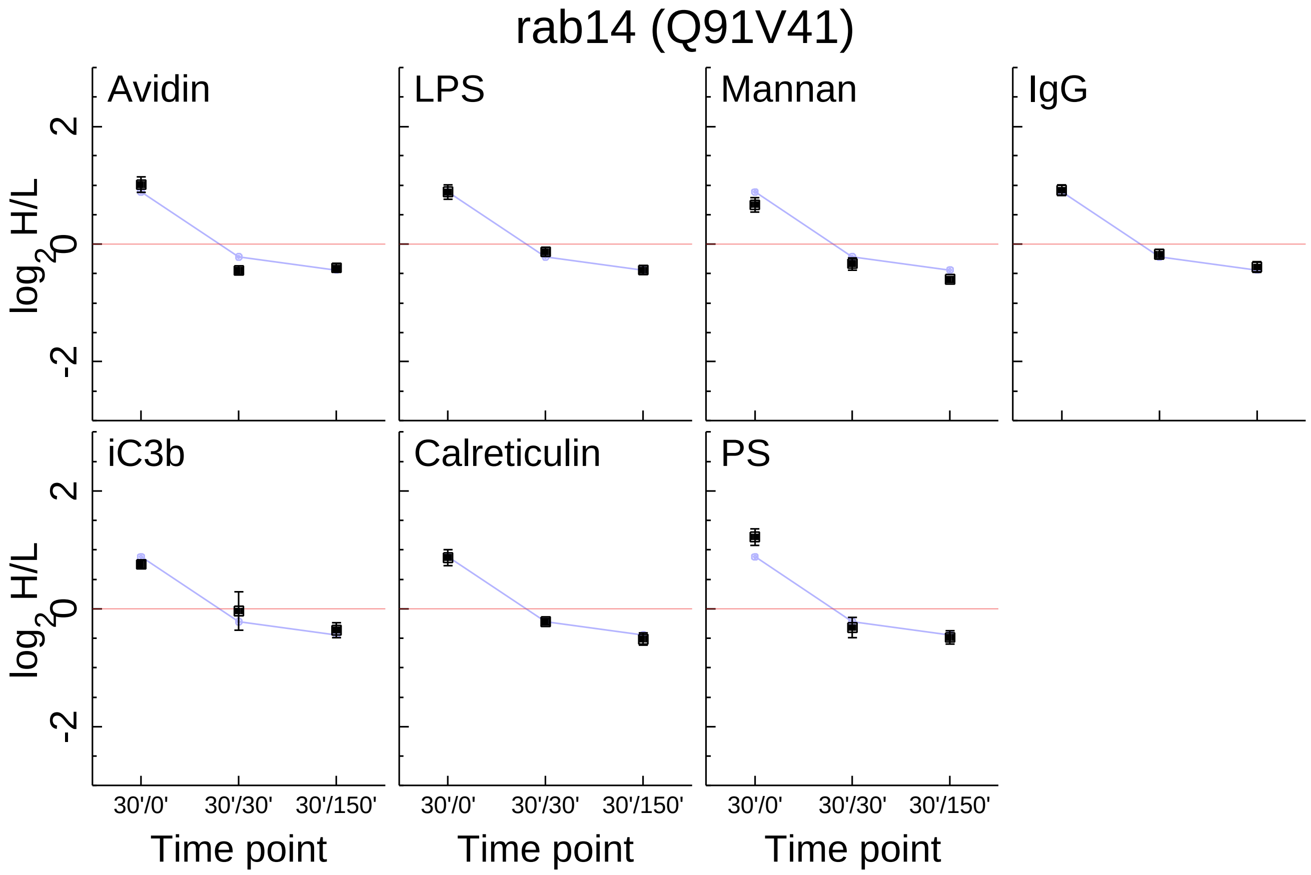

rab14 (Q91V41)
Avidin
LPS
Mannan
IgG
iC3b
Calreticulin
PS
log2 H/L
2
0
-2
30'/0'
30'/30'
30'/150'
Time point

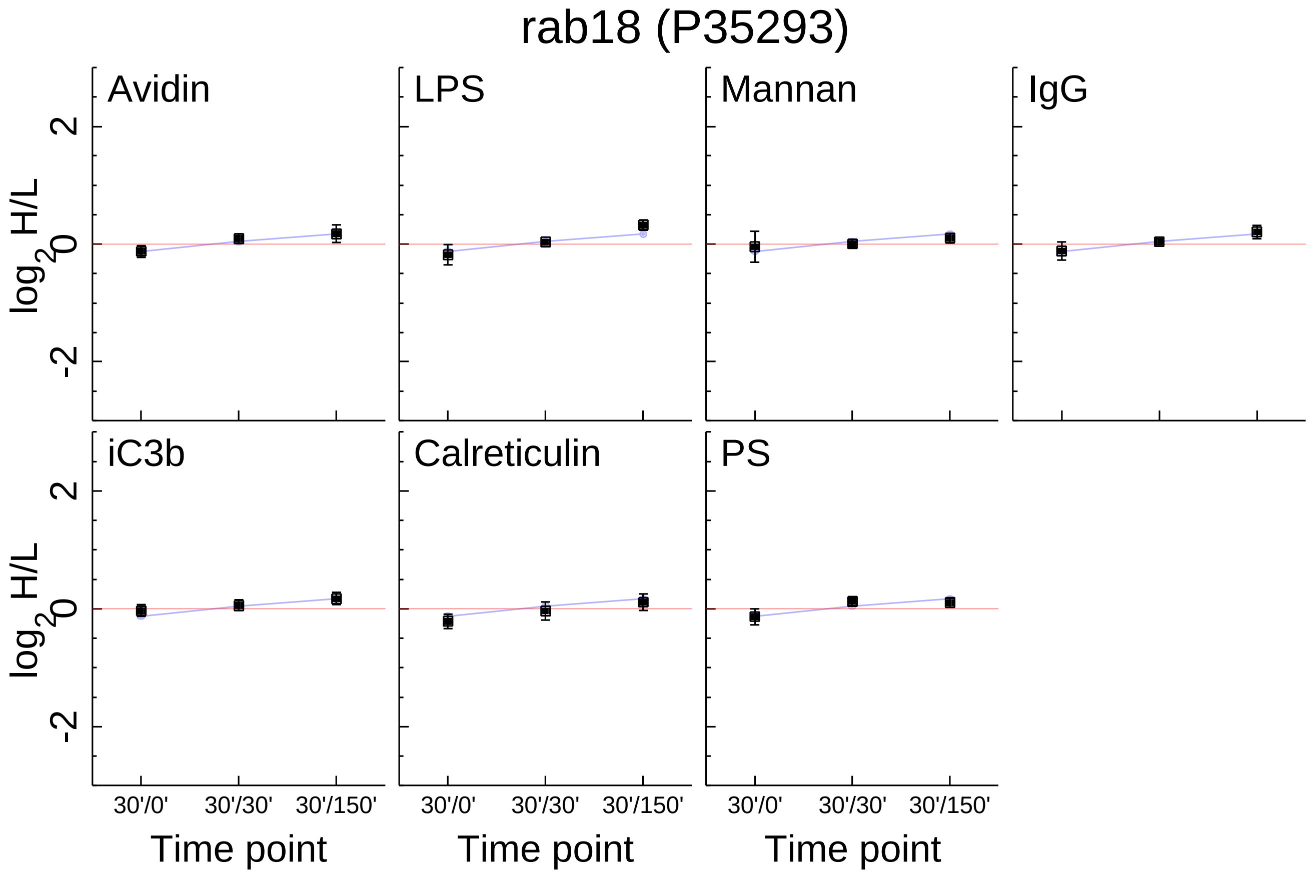

rab18 (P35293)
Avidin
LPS
Mannan
IgG
iC3b
Calreticulin
PS
log2 H/L
2
0
-2
30'/0'
30'/30'
30'/150'
Time point

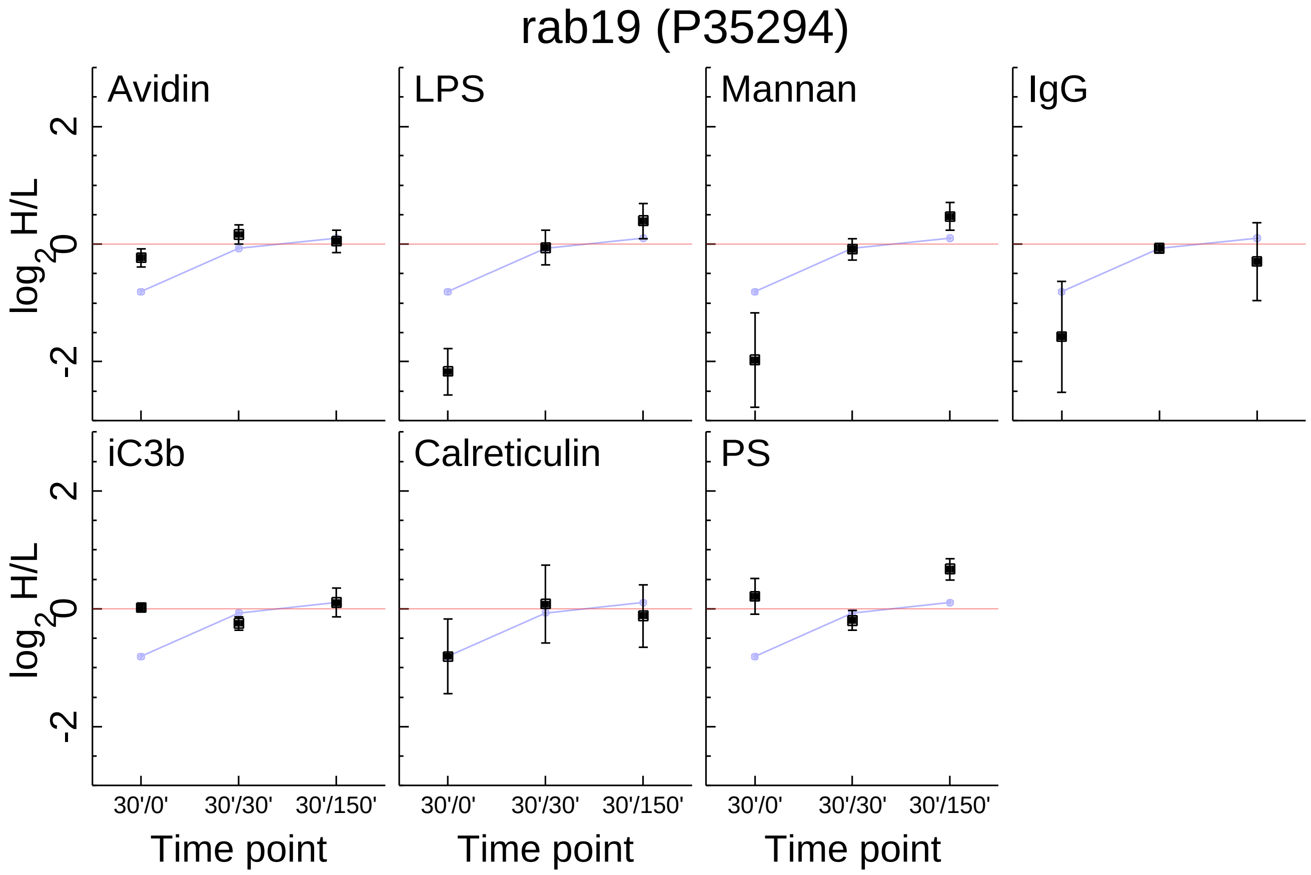

rab19 (P35294)
Avidin
LPS
Mannan
IgG
iC3b
Calreticulin
PS
log2 H/L
2
0
-2
30'/0'
30'/30'
30'/150'
Time point

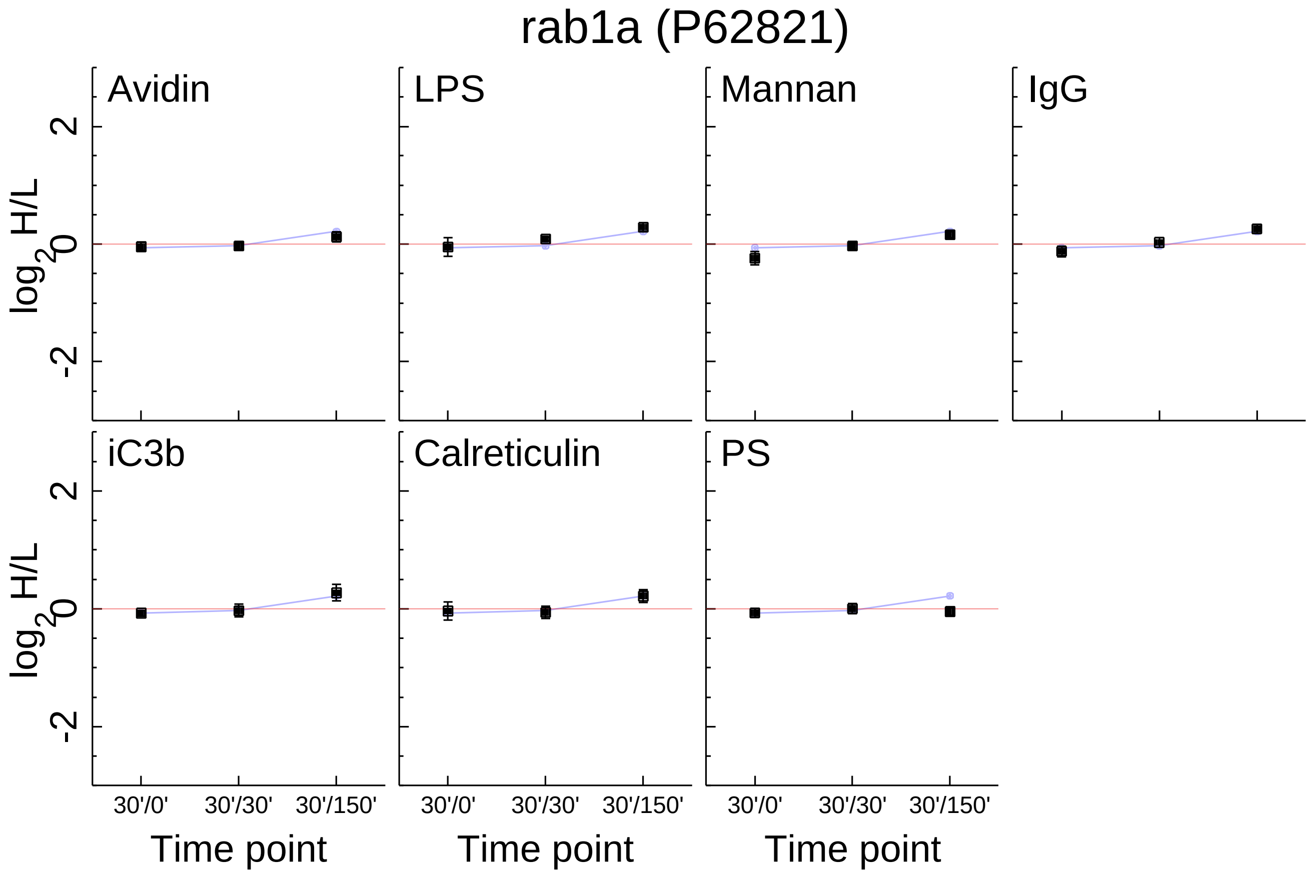

rab1a (P62821)
Avidin
LPS
Mannan
IgG
iC3b
Calreticulin
PS
log2 H/L
2
0
-2
30'/0'
30'/30'
30'/150'
Time point

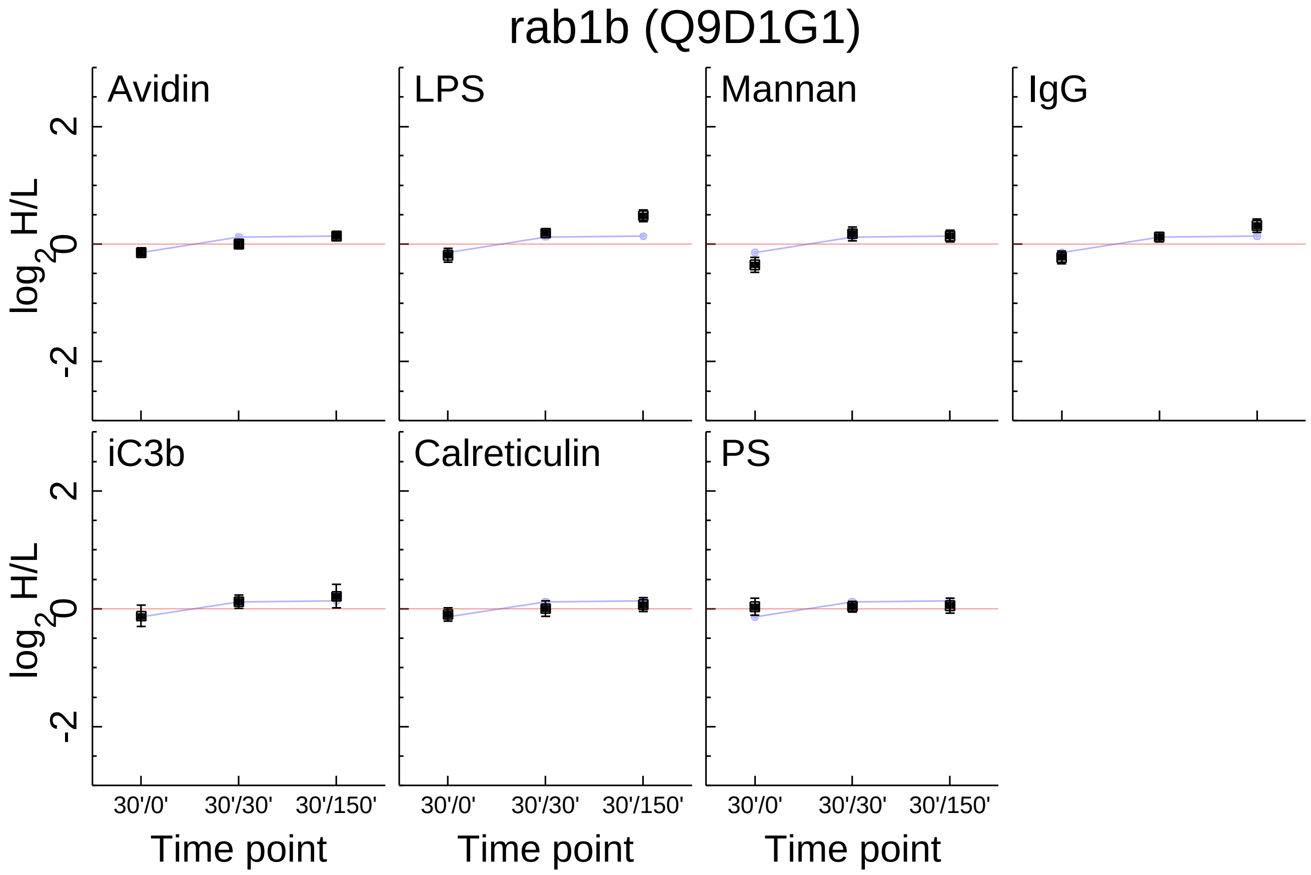

rab1b (Q9D1G1)
Avidin
LPS
Mannan
IgG
iC3b
Calreticulin
PS
log2 H/L
2
0
-2
30'/0'
30'/30'
30'/150'
Time point

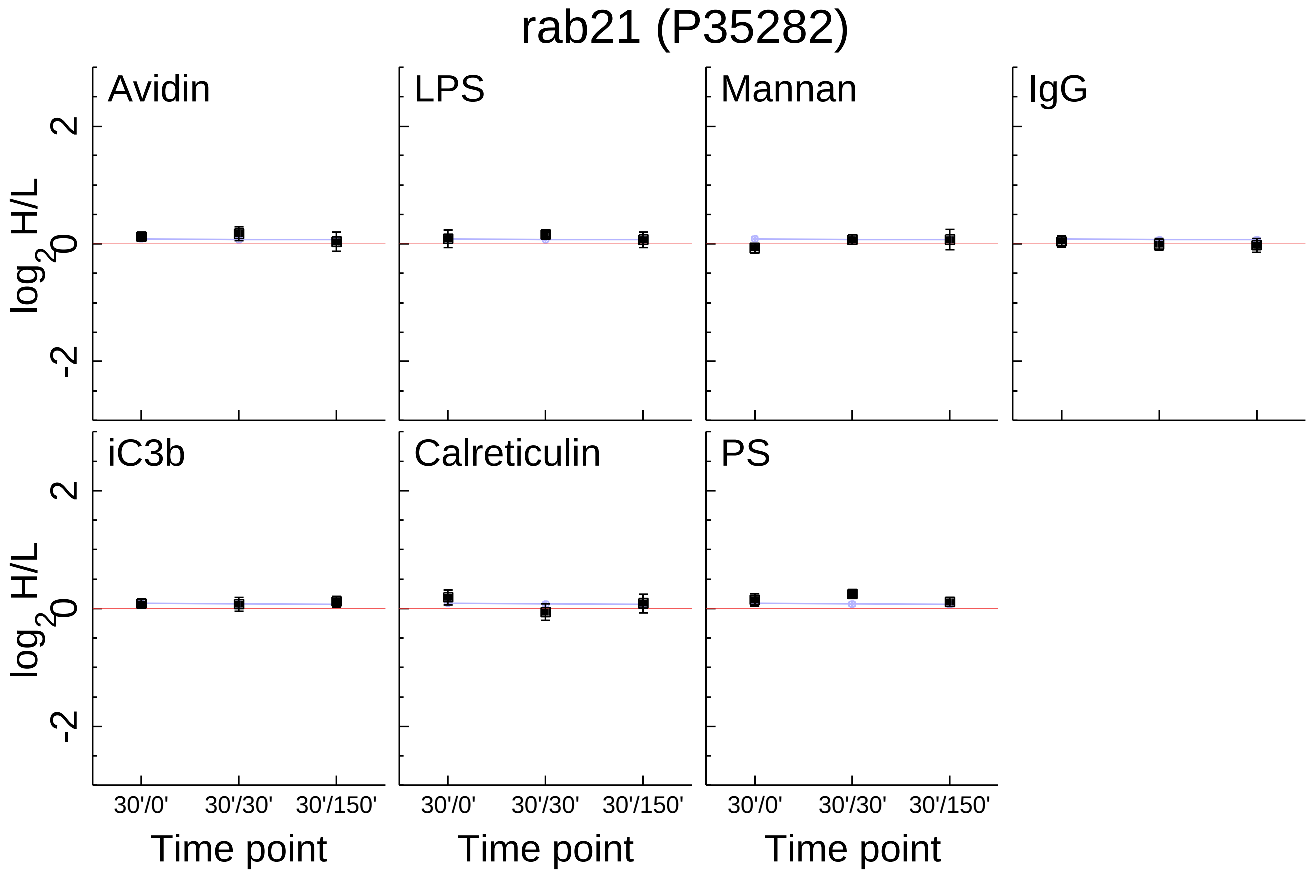
rab21 (P35282)
Avidin
LPS
Mannan
IgG
iC3b
Calreticulin
PS
log2 H/L
2
0
-2
30'/0'
30'/30'
30'/150'
Time point

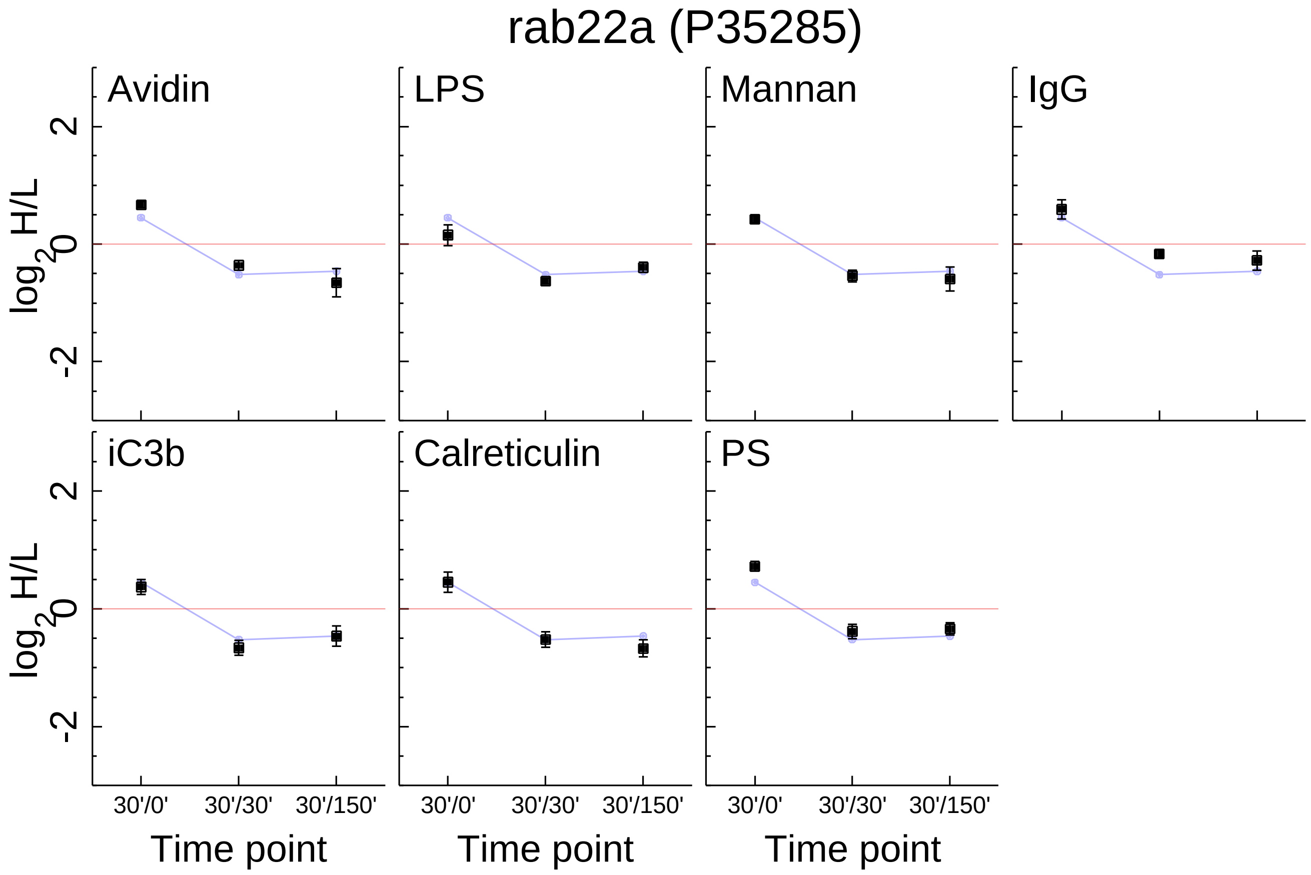

rab22a (P35285)
Avidin
LPS
Mannan
IgG
iC3b
Calreticulin
PS
log2 H/L
2
0
-2
30'/0'
30'/30'
30'/150'
Time point

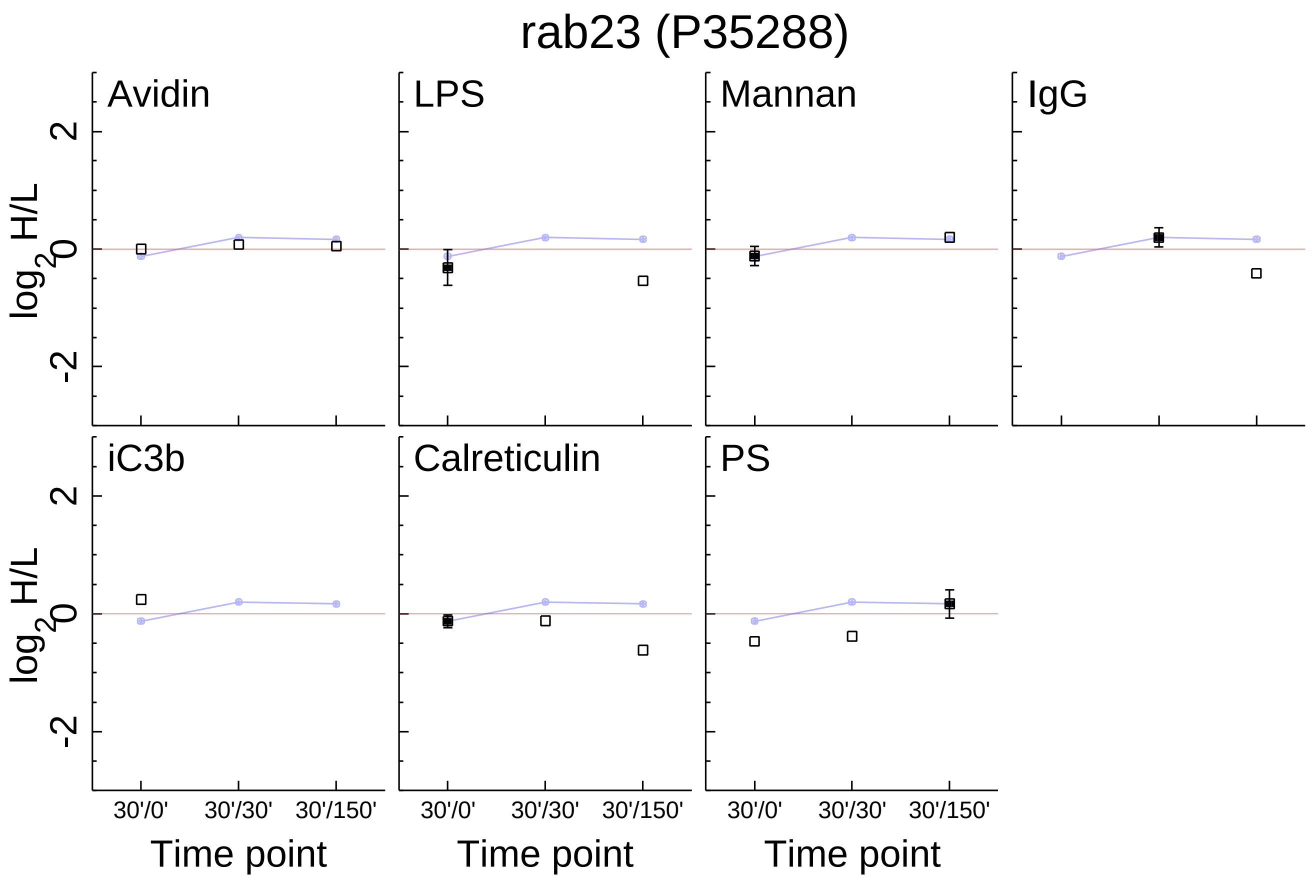
rab23 (P35288)
Avidin
LPS
Mannan
IgG
iC3b
Calreticulin
PS
log2 H/L
2
0
-2
30'/0'
30'/30'
30'/150'
Time point

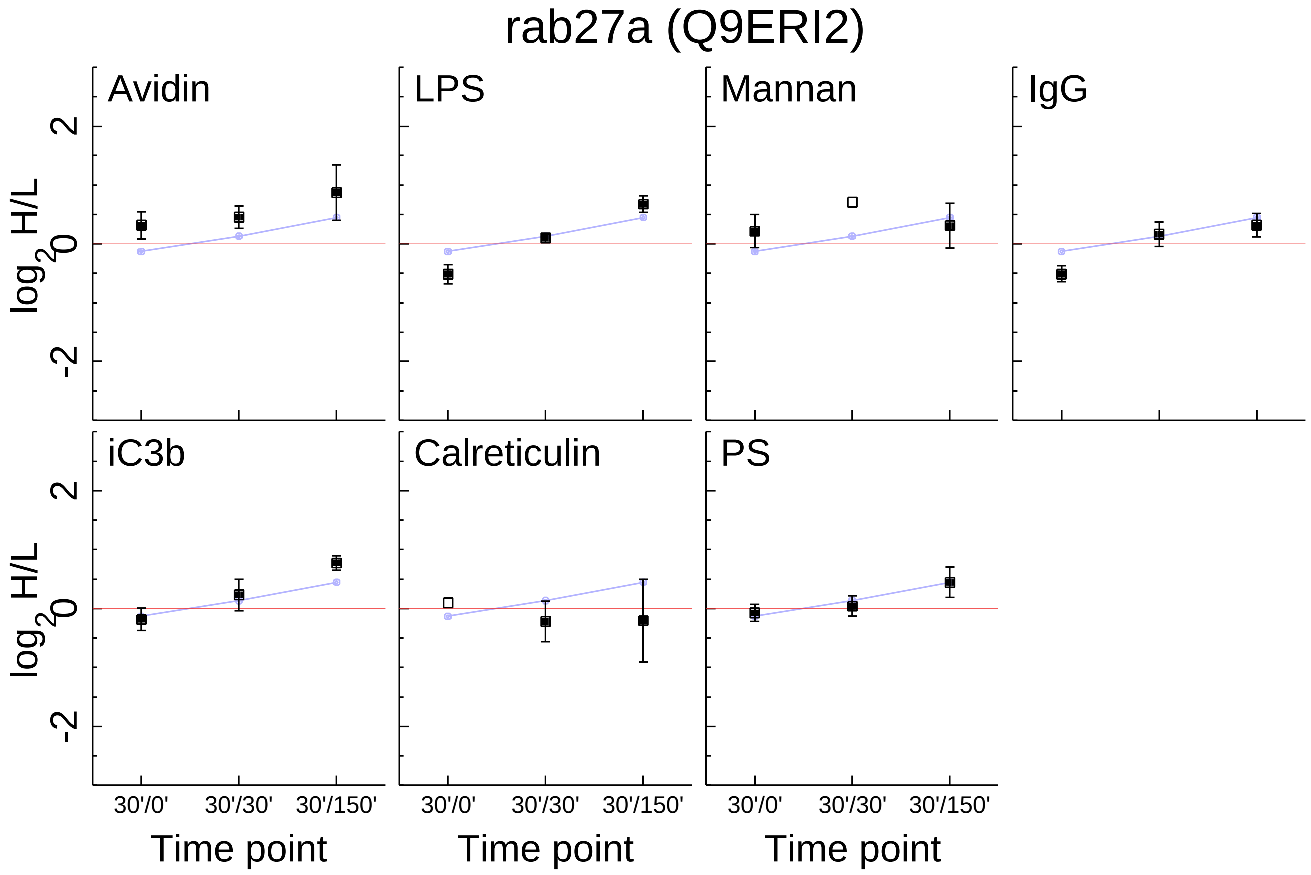

rab27a (Q9ERI2)
Avidin
LPS
Mannan
IgG
iC3b
Calreticulin
PS
log2 H/L
2
0
-2
30'/0'
30'/30'
30'/150'
Time point

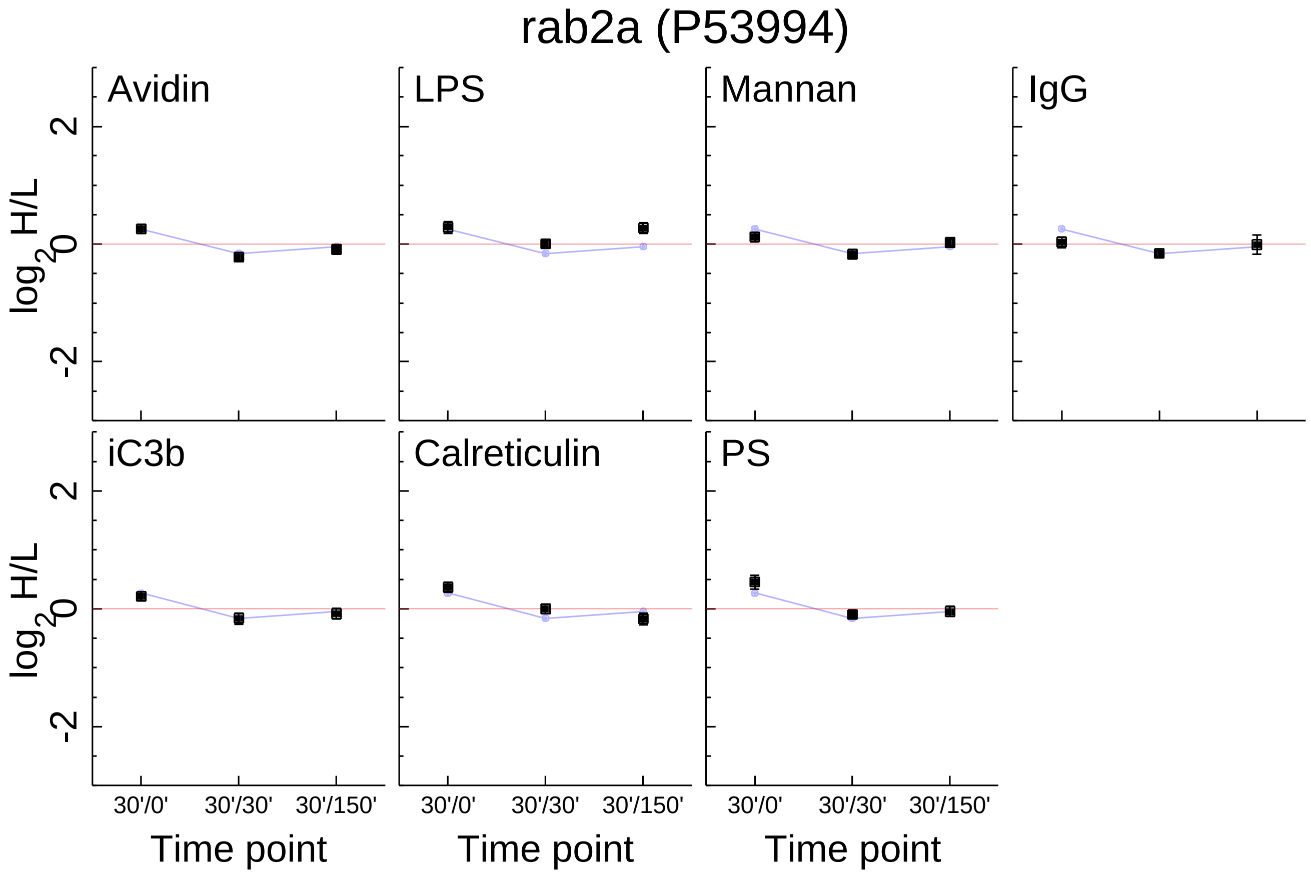

rab2a (P53994)
Avidin
LPS
Mannan
IgG
iC3b
Calreticulin
PS
log2 H/L
2
0
-2
30'/0'
30'/30'
30'/150'
Time point

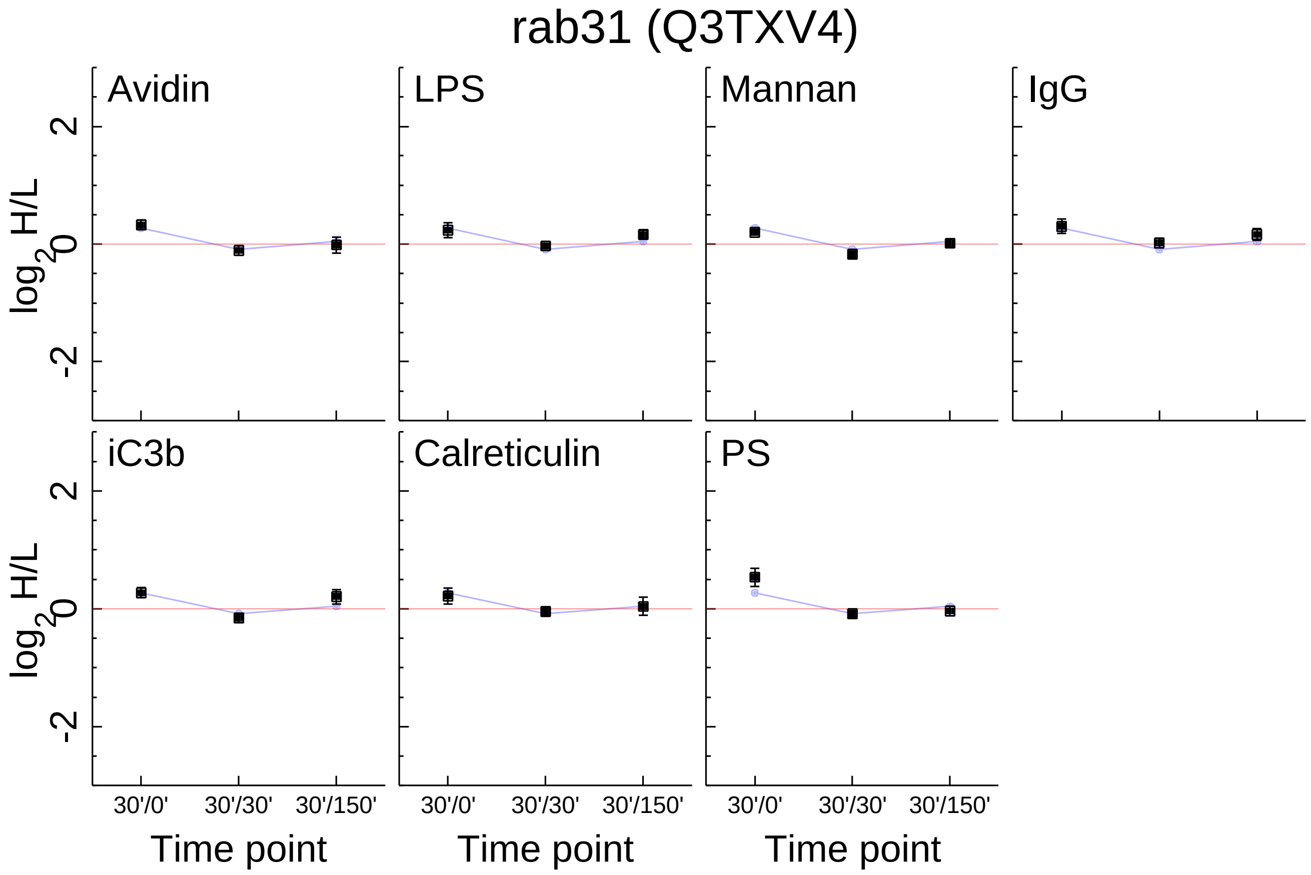
rab31 (Q3TXV4)
Avidin
LPS
Mannan
IgG
iC3b
Calreticulin
PS
log2 H/L
2
0
-2
30'/0'
30'/30'
30'/150'
Time point

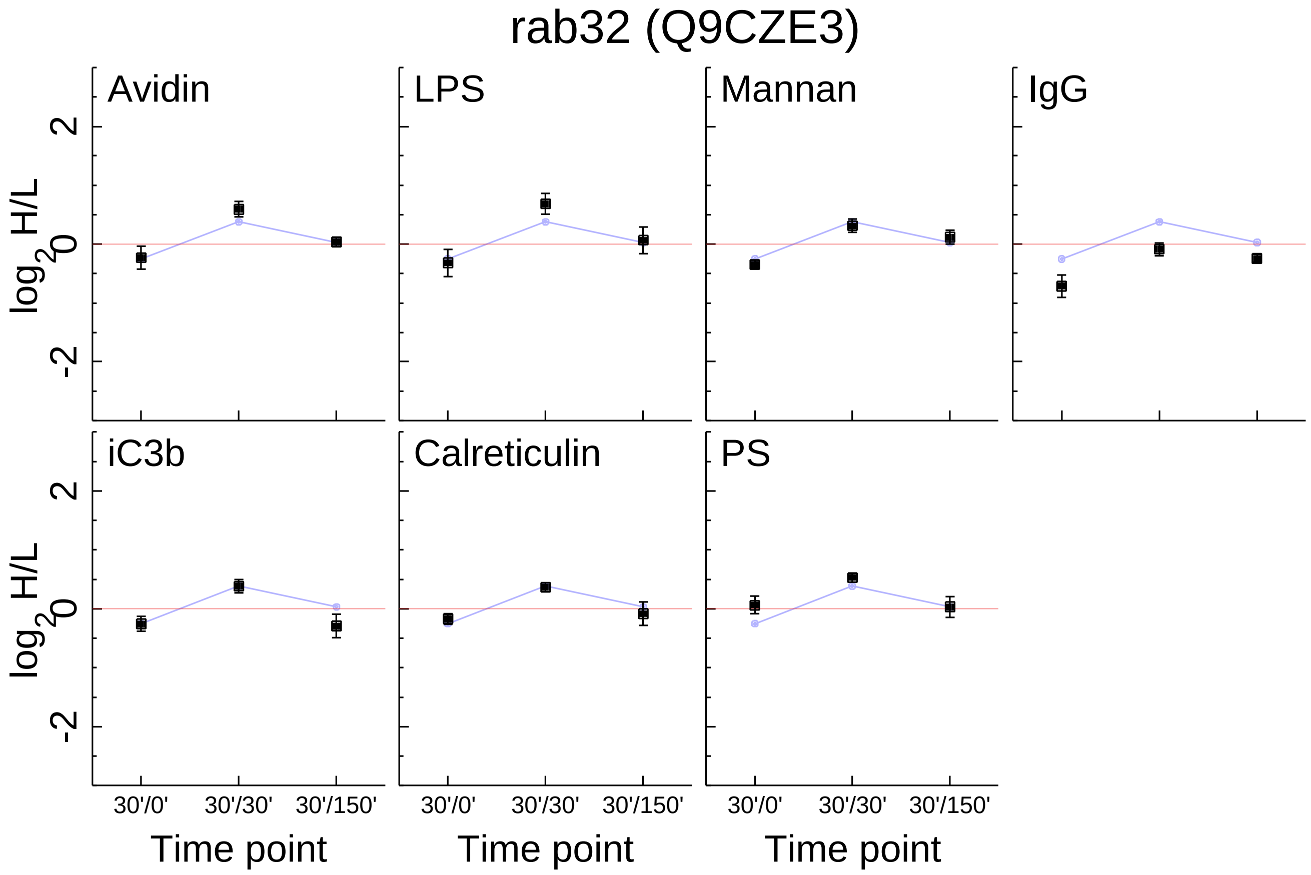

rab32 (Q9CZE3)
Avidin
LPS
Mannan
IgG
iC3b
Calreticulin
PS
log2 H/L
2
0
-2
30'/0'
30'/30'
30'/150'
Time point

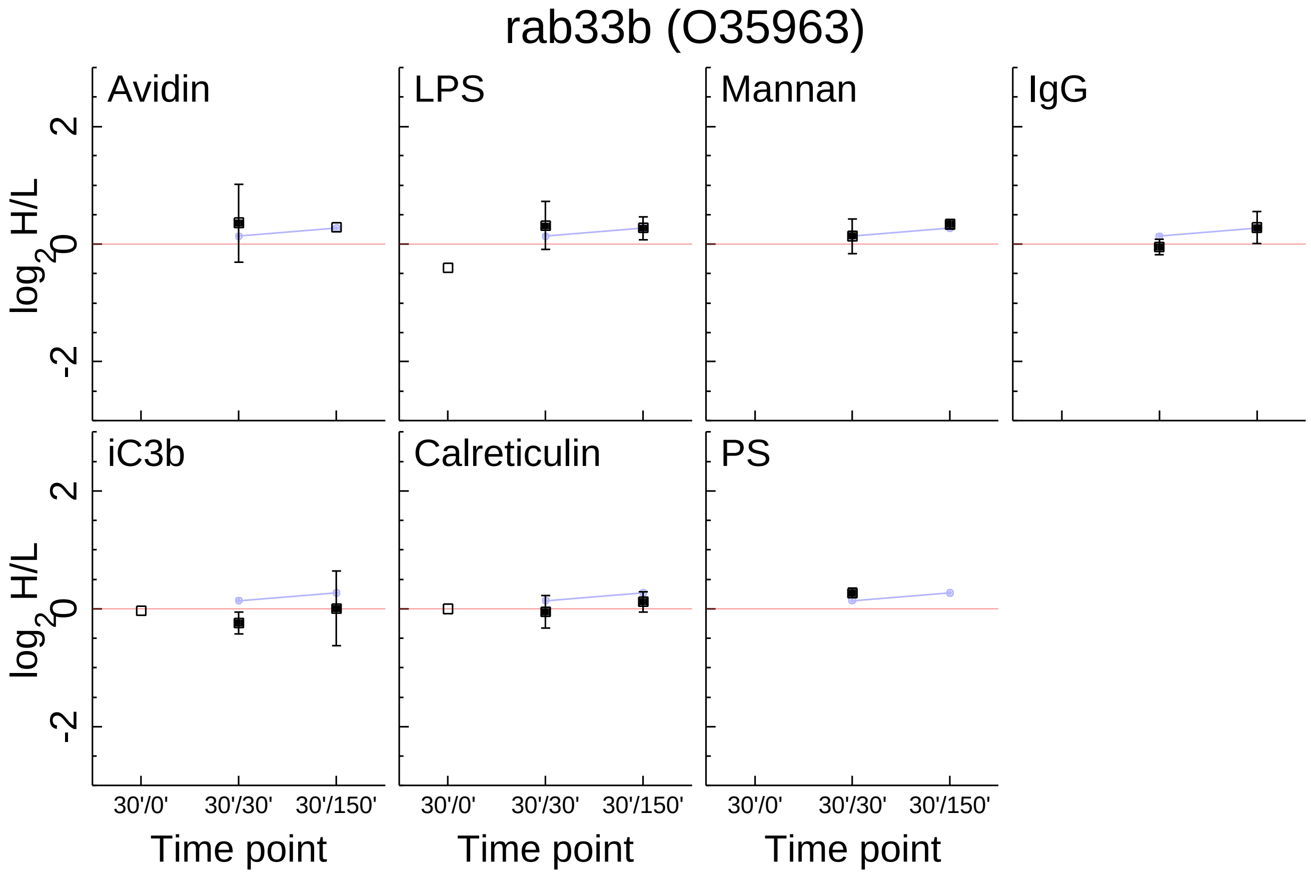
rab33b (O35963)
Avidin
LPS
Mannan
IgG
iC3b
Calreticulin
PS
log2 H/L
2
0
-2
30'/0'
30'/30'
30'/150'
Time point

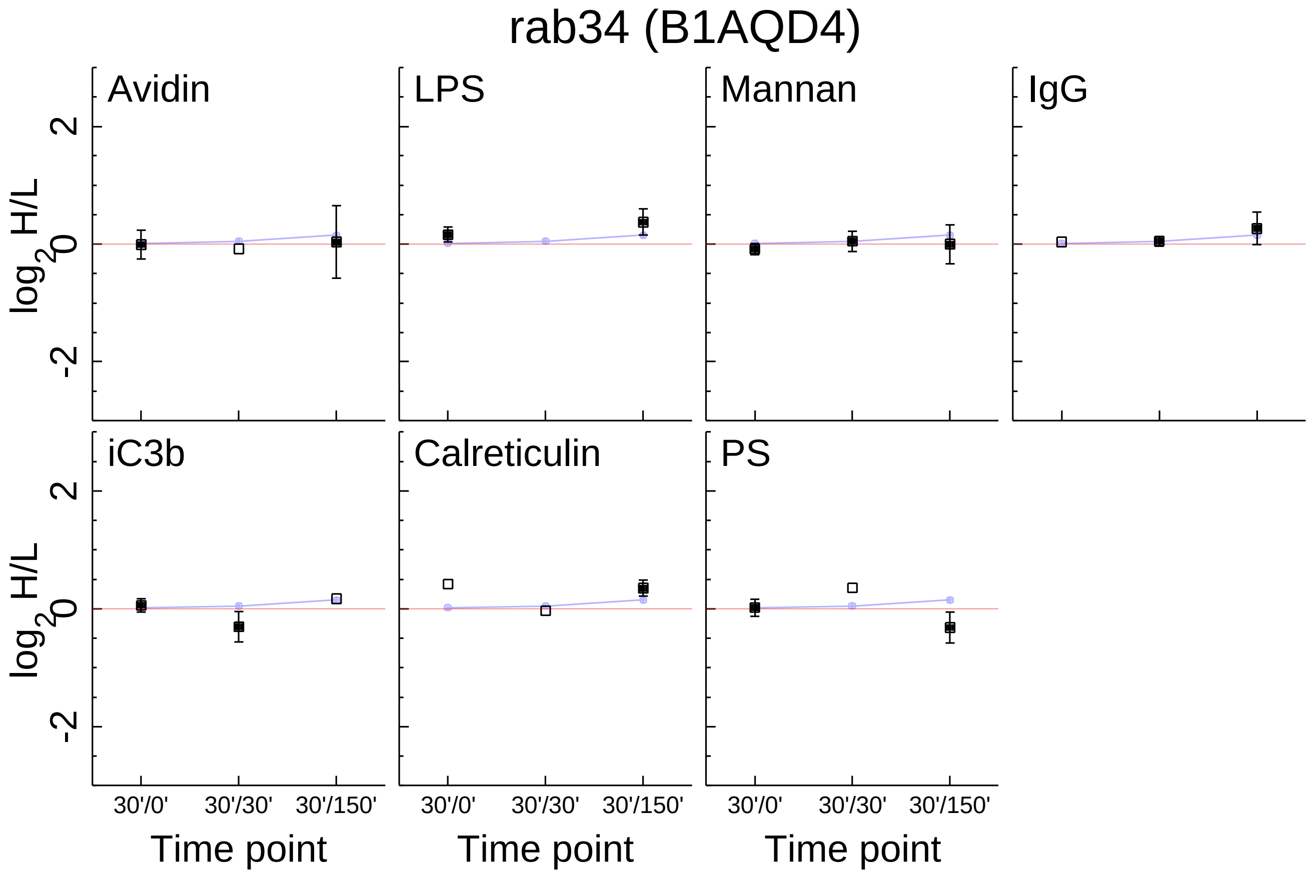

rab34 (B1AQD4)
Avidin
LPS
Mannan
IgG
iC3b
Calreticulin
PS
log2 H/L
2
0
-2
30'/0'
30'/30'
30'/150'
Time point

rab35 (Q6PHN9)

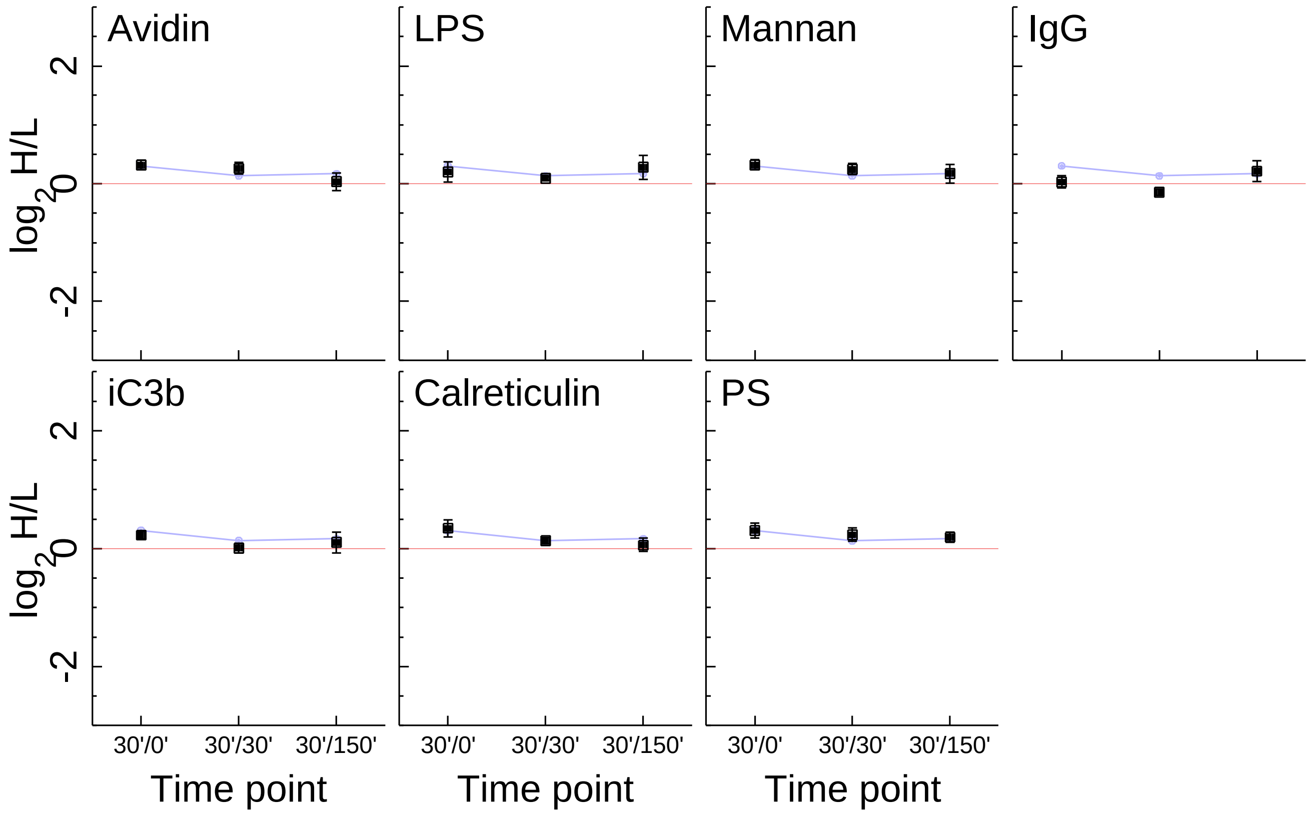

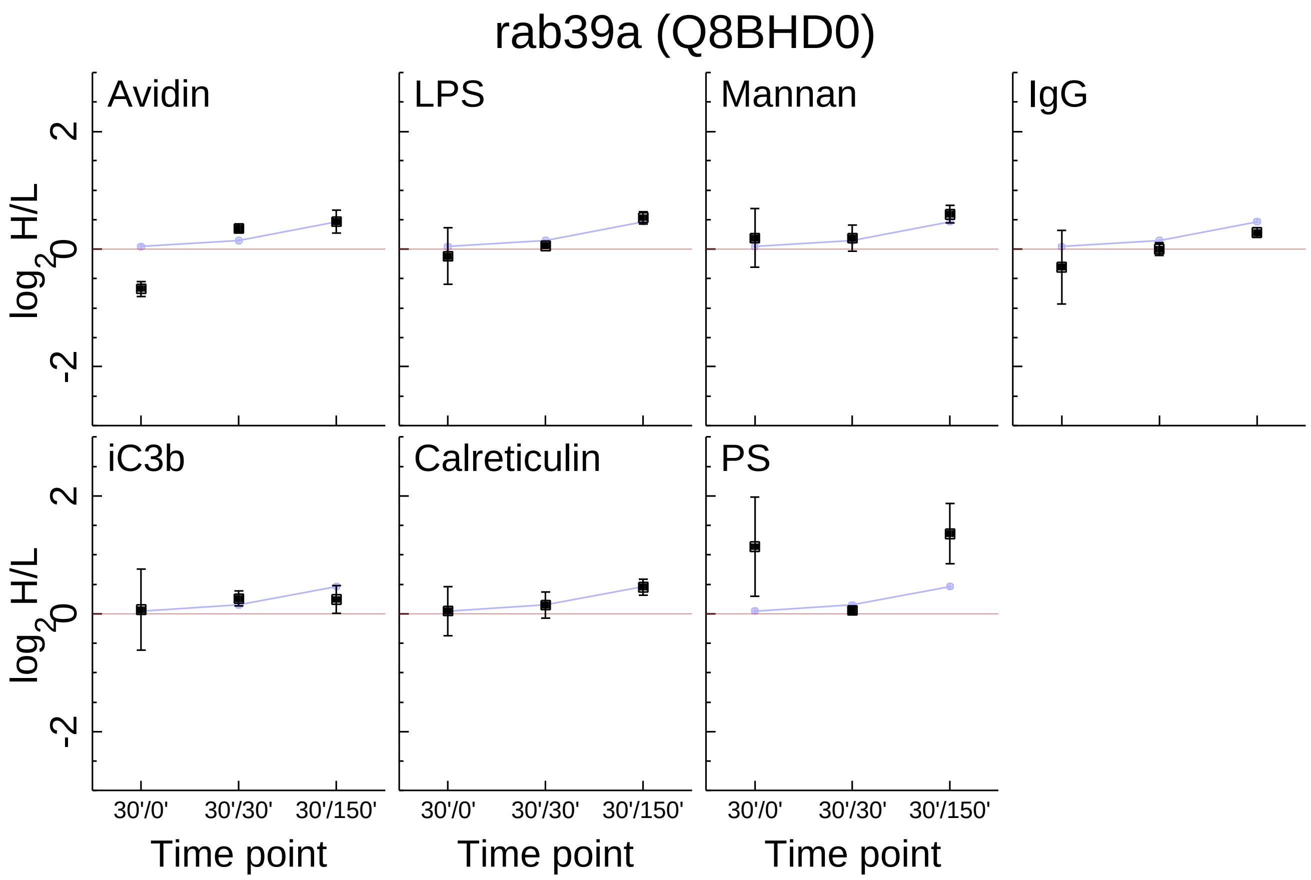
rab39a (Q8BHD0)
Avidin
LPS
Mannan
IgG
iC3b
Calreticulin
PS
2
0
-2
log2 H/L
30'/0'
30'/30'
30'/150'
Time point

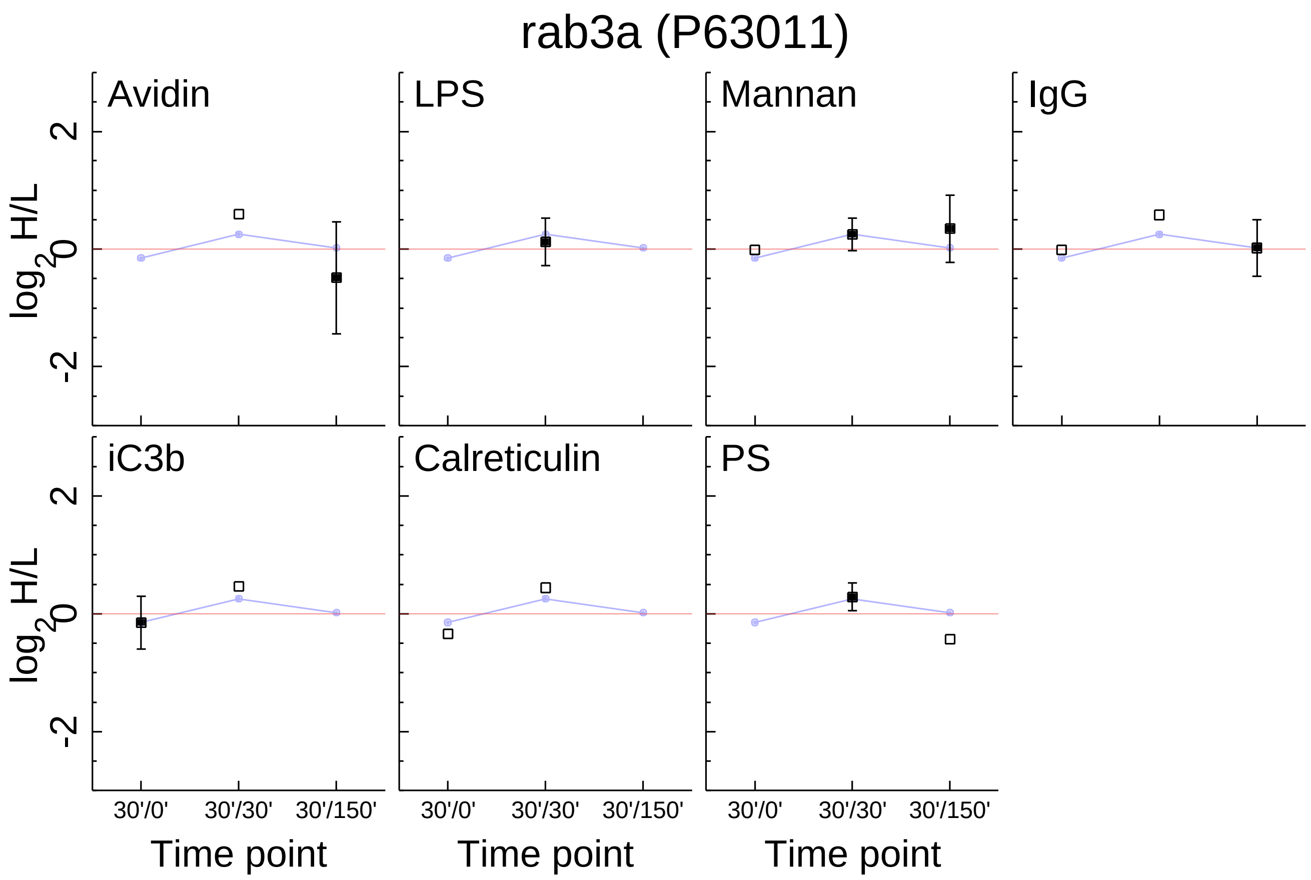

rab3a (P63011)
Avidin
LPS
Mannan
IgG
iC3b
Calreticulin
PS
log2 H/L
2
0
-2
30'/0'
30'/30'
30'/150'
Time point

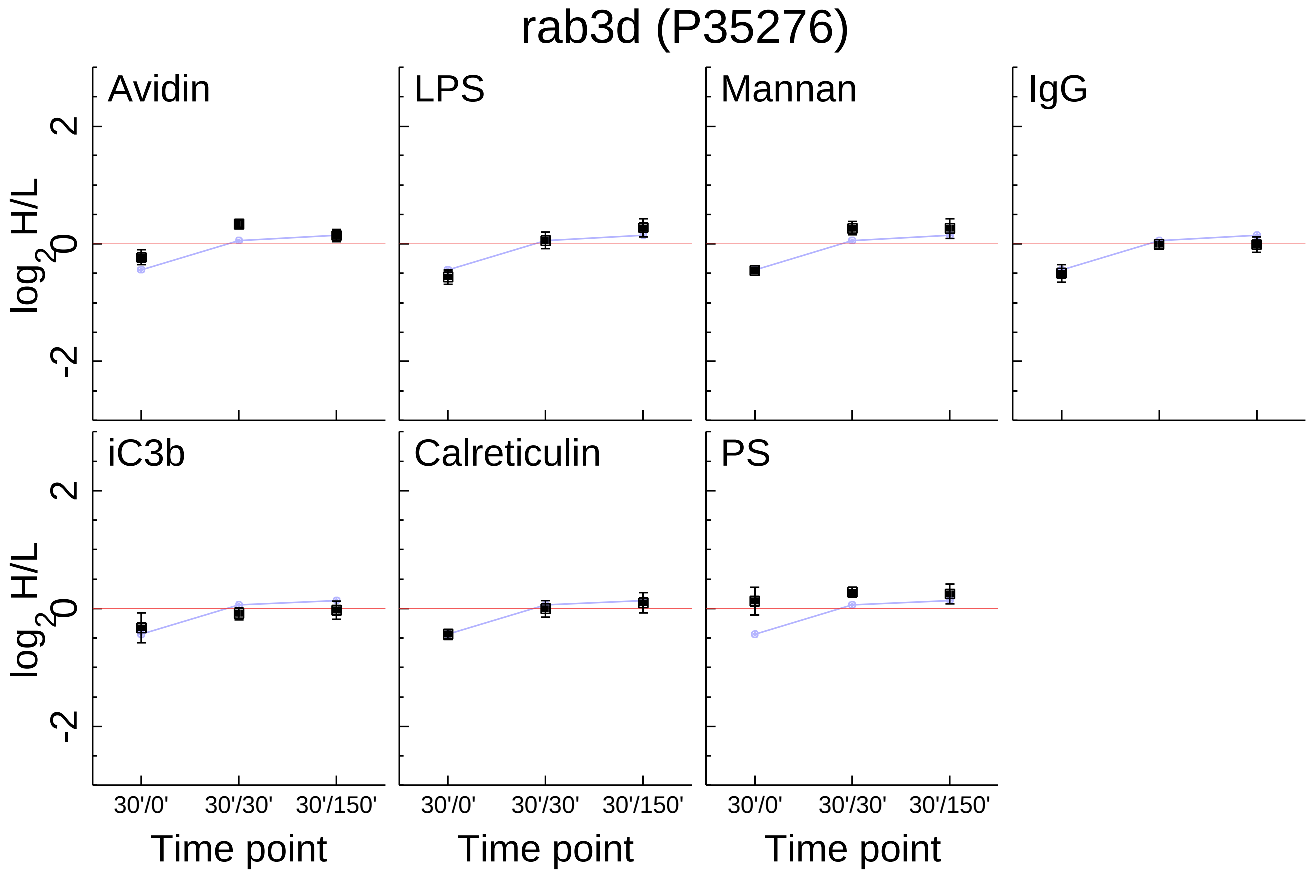

rab3d (P35276)
Avidin
LPS
Mannan
IgG
iC3b
Calreticulin
PS
log2 H/L
2
0
-2
30'/0'
30'/30'
30'/150'
Time point

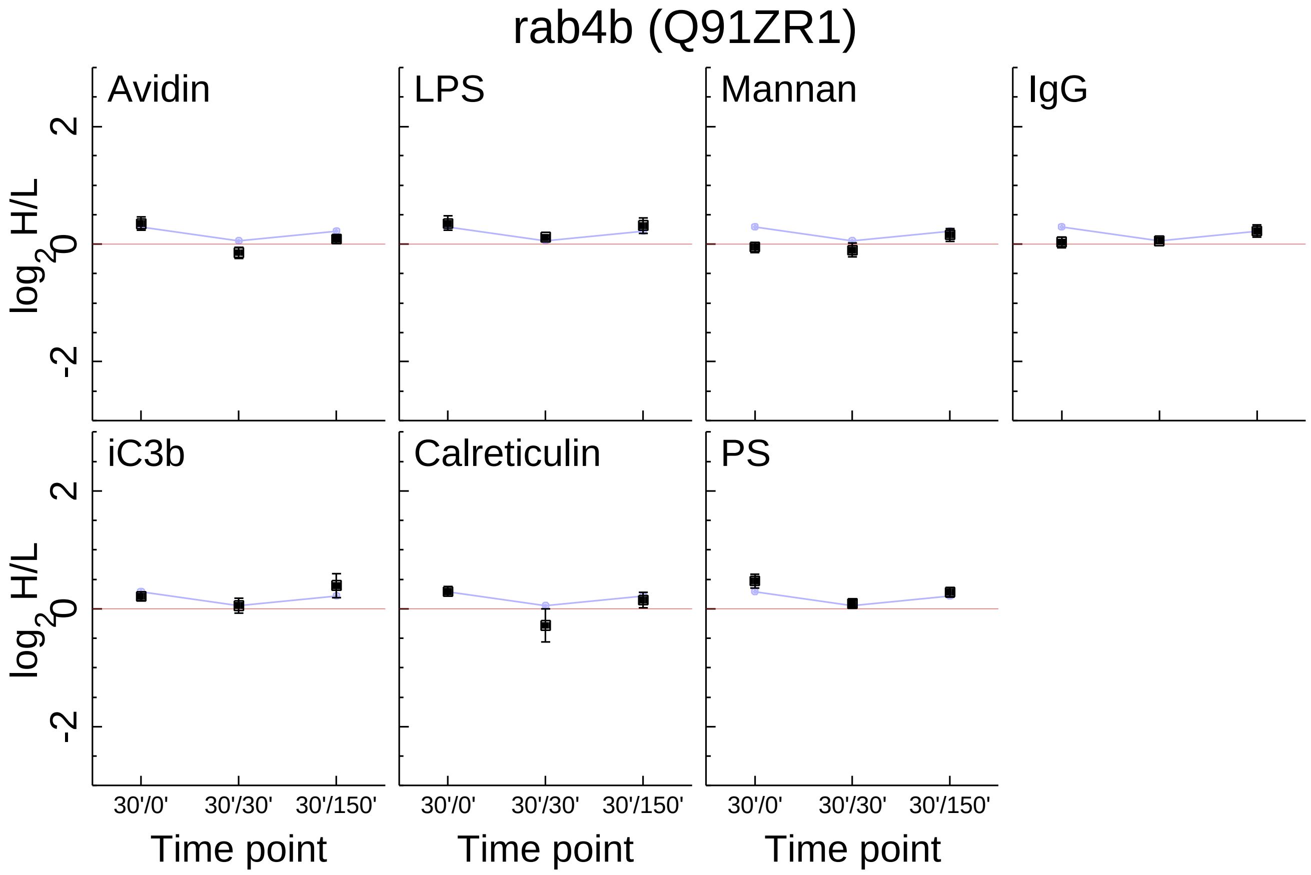
rab4b (Q91ZR1)
Avidin
LPS
Mannan
IgG
iC3b
Calreticulin
PS
log2 H/L
2
0
-2
30'/0'
30'/30'
30'/150'
Time point

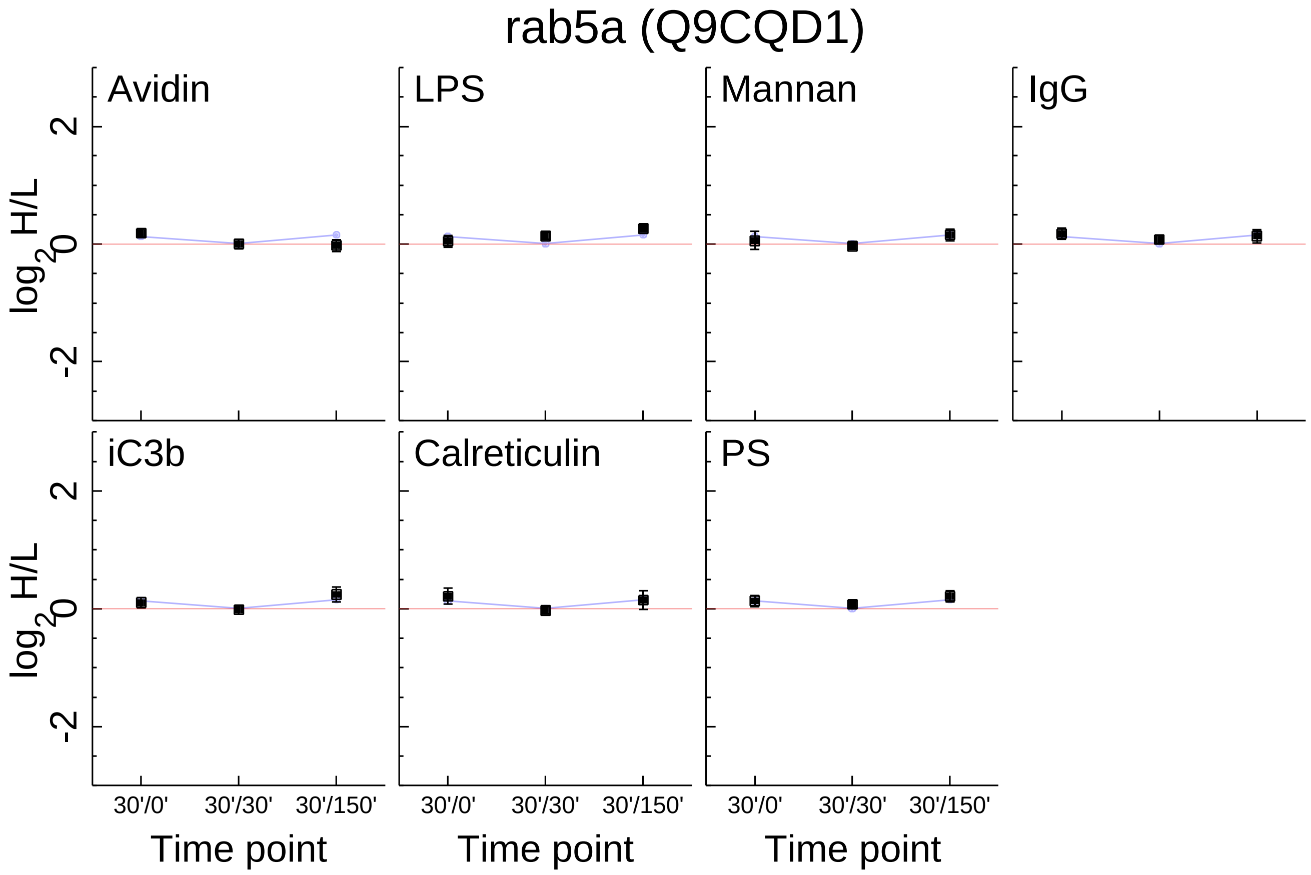
rab5a (Q9CQD1)
Avidin
LPS
Mannan
IgG
iC3b
Calreticulin
PS
log2 H/L
2
0
-2
30'/0'
30'/30'
30'/150'
Time point

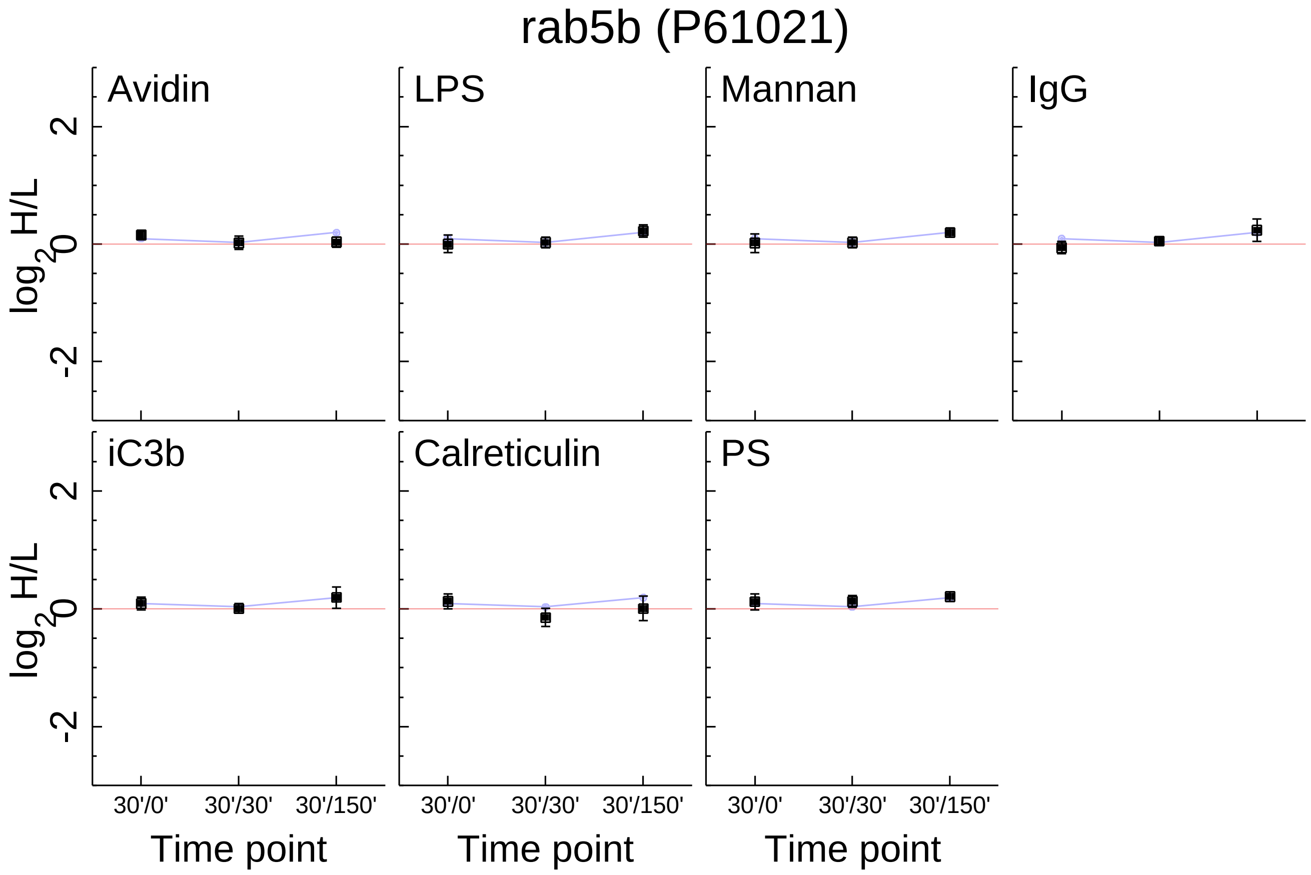

rab5b (P61021)
Avidin
LPS
Mannan
IgG
iC3b
Calreticulin
PS
log2 H/L
2
0
-2
30'/0'
30'/30'
30'/150'
Time point

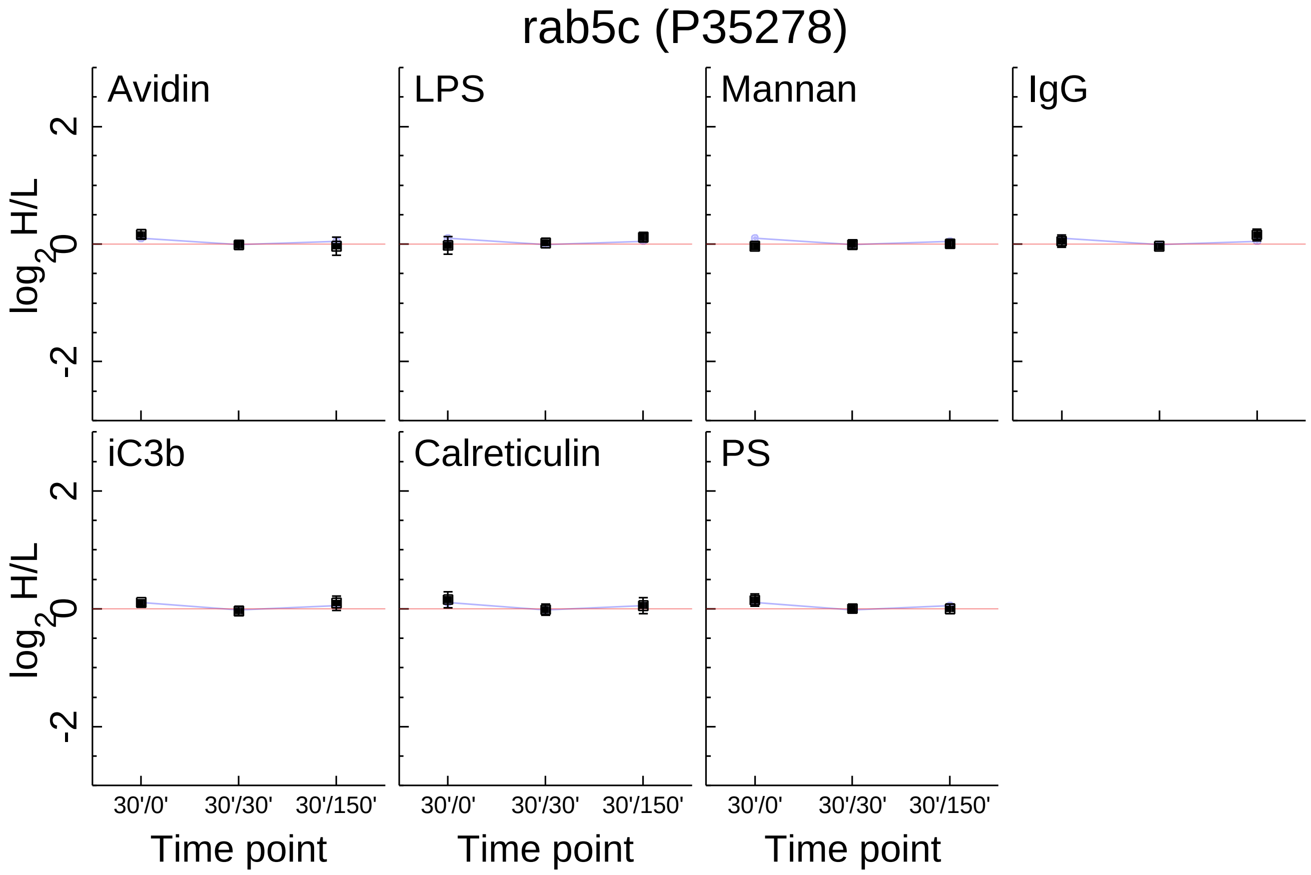

rab5c (P35278)
Avidin
LPS
Mannan
IgG
iC3b
Calreticulin
PS
log2 H/L
2
0
-2
30'/0'
30'/30'
30'/150'
Time point

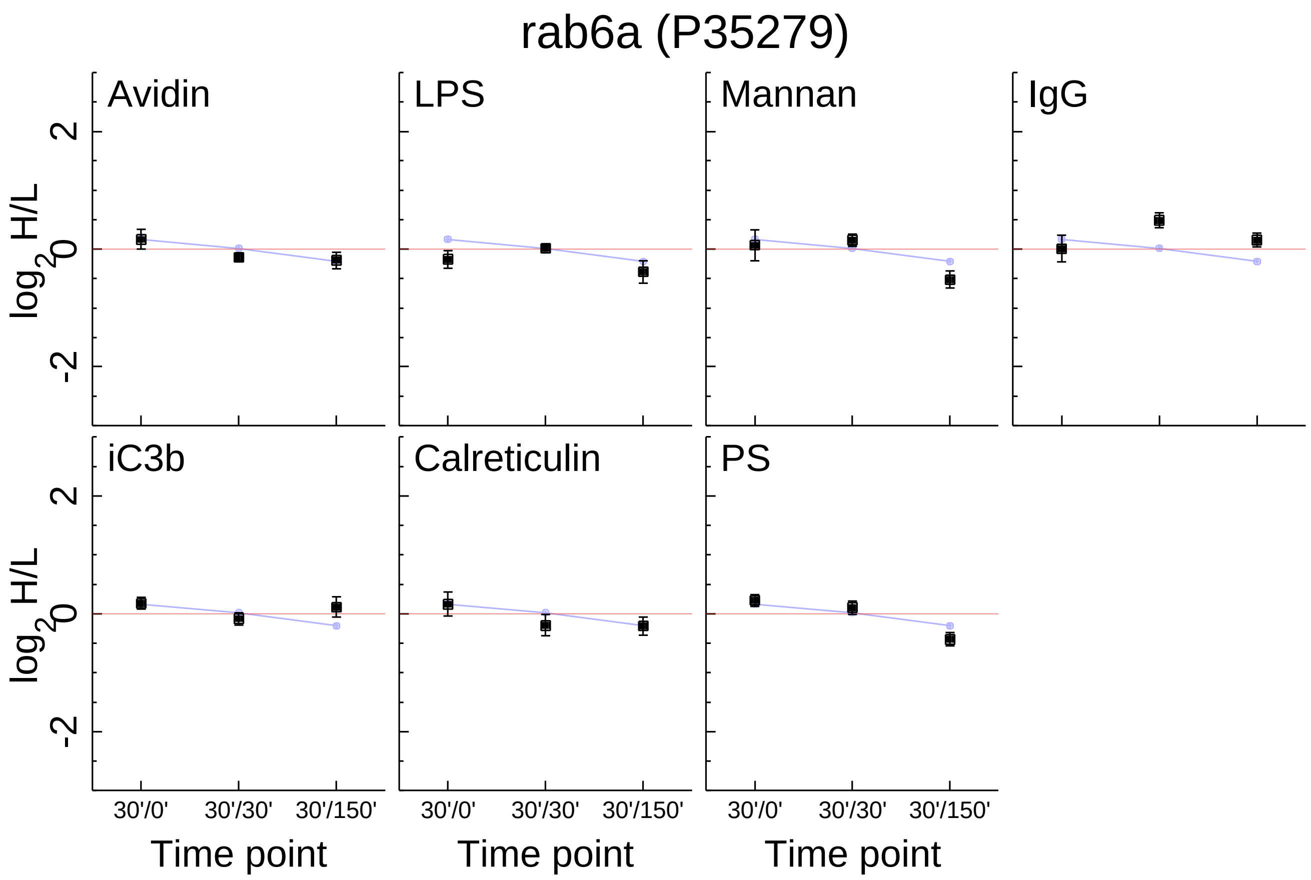
rab6a (P35279)
Avidin
LPS
Mannan
IgG
iC3b
Calreticulin
PS
log2 H/L
2
0
-2
30'/0'
30'/30'
30'/150'
Time point

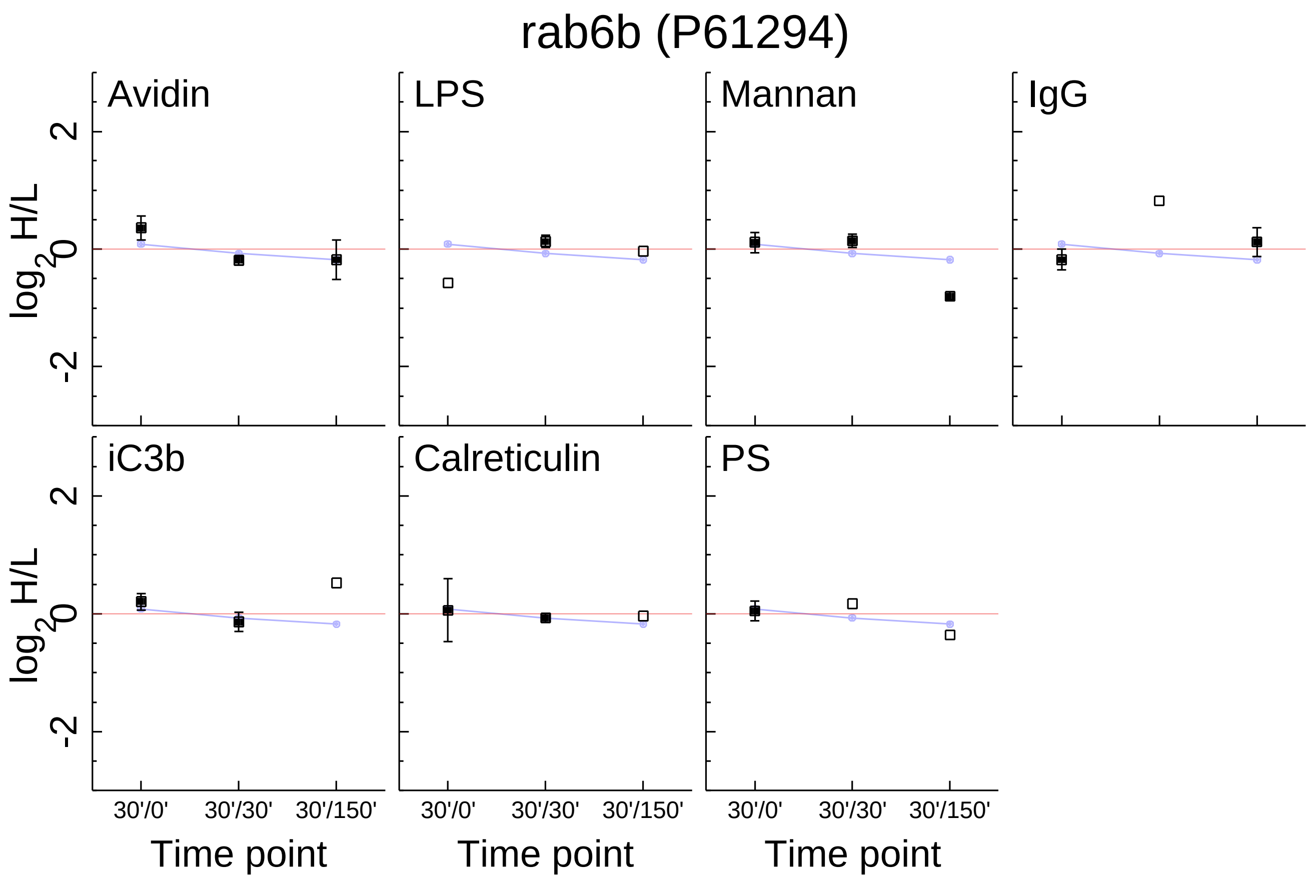
rab6b (P61294)
Avidin
LPS
Mannan
IgG
iC3b
Calreticulin
PS
log2 H/L
2
0
-2
30'/0'
30'/30'
30'/150'
Time point

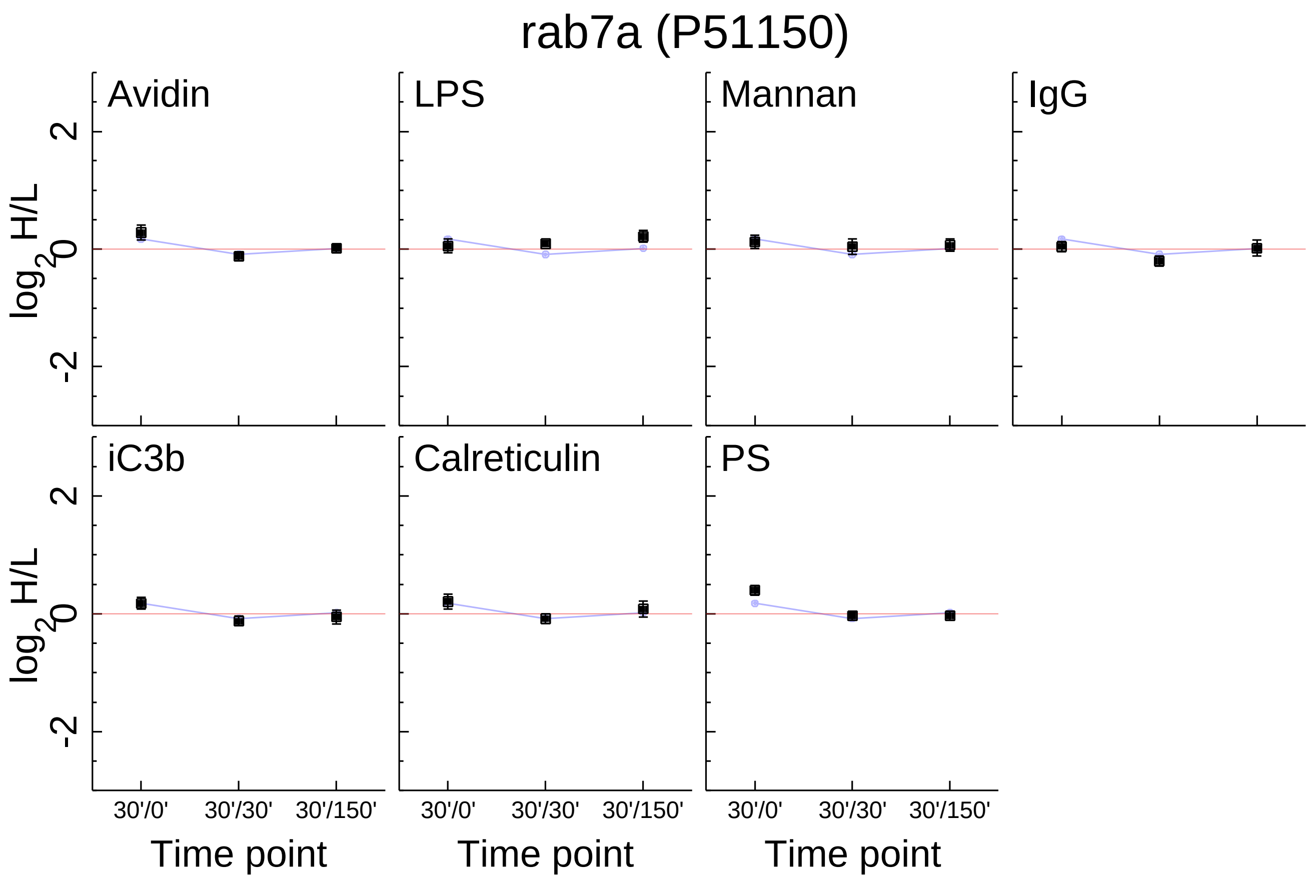
rab7a (P51150)
Avidin
LPS
Mannan
IgG
iC3b
Calreticulin
PS
log2 H/L
2
0
-2
30'/0'
30'/30'
30'/150'
Time point

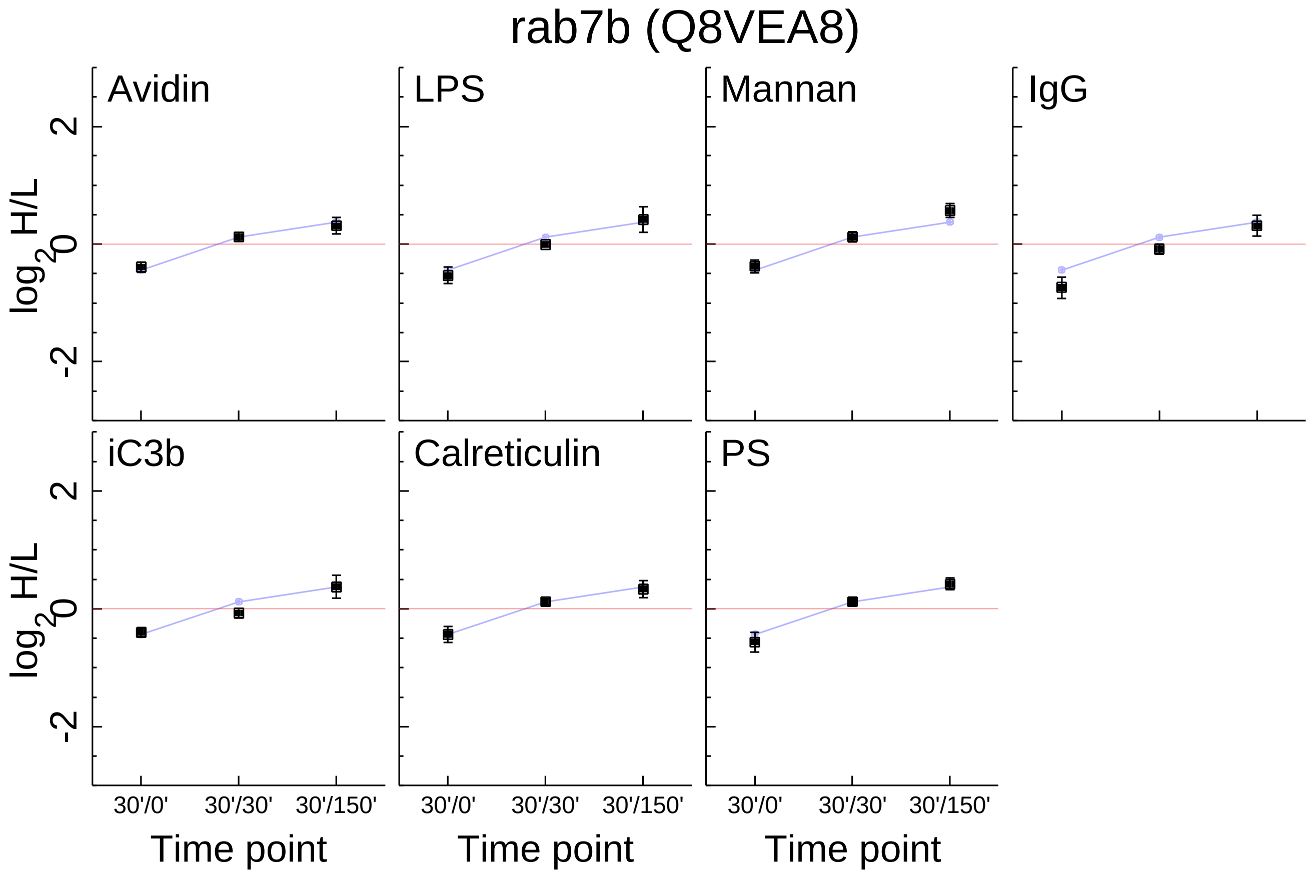
rab7b (Q8VEA8)
Avidin
LPS
Mannan
IgG
iC3b
Calreticulin
PS
log2 H/L
2
0
-2
30'/0'
30'/30'
30'/150'
Time point

rab7l1 (E9QLQ7)

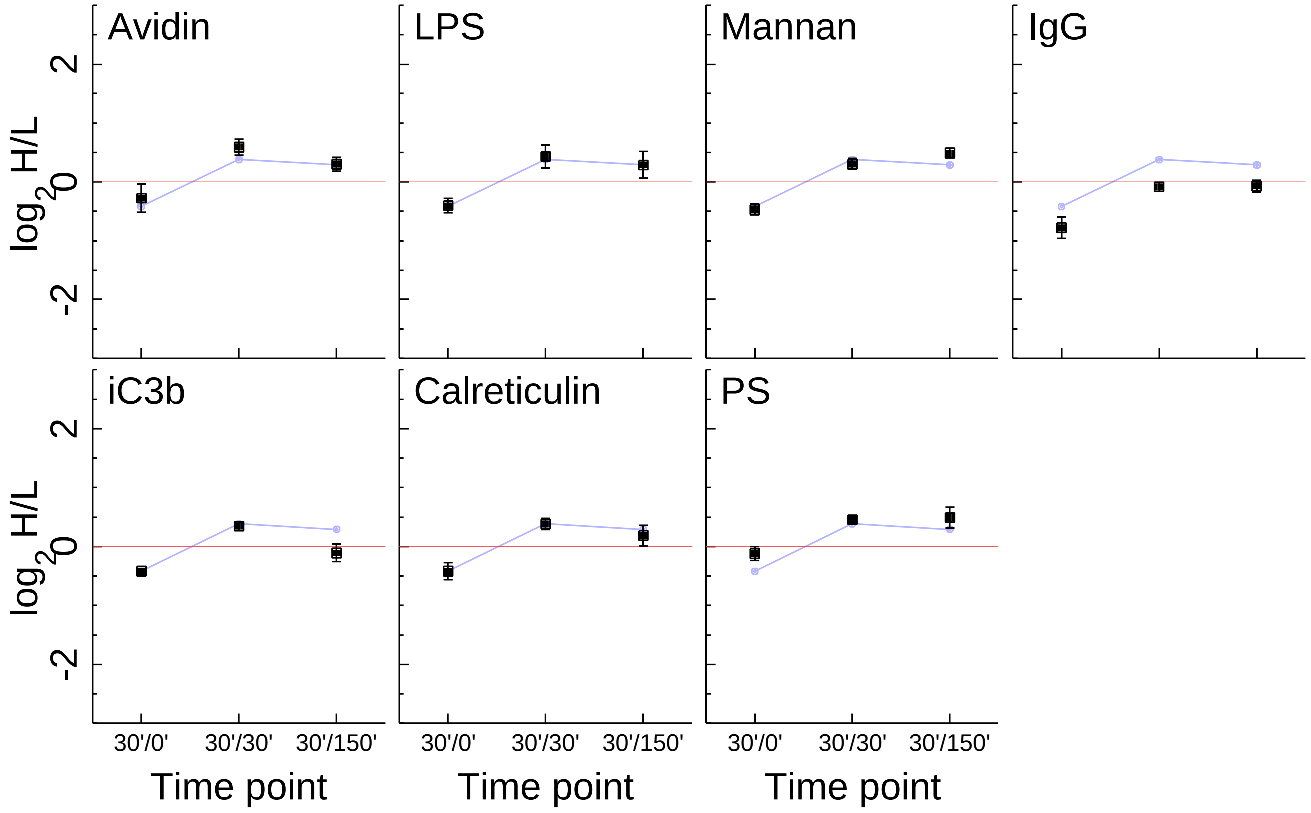

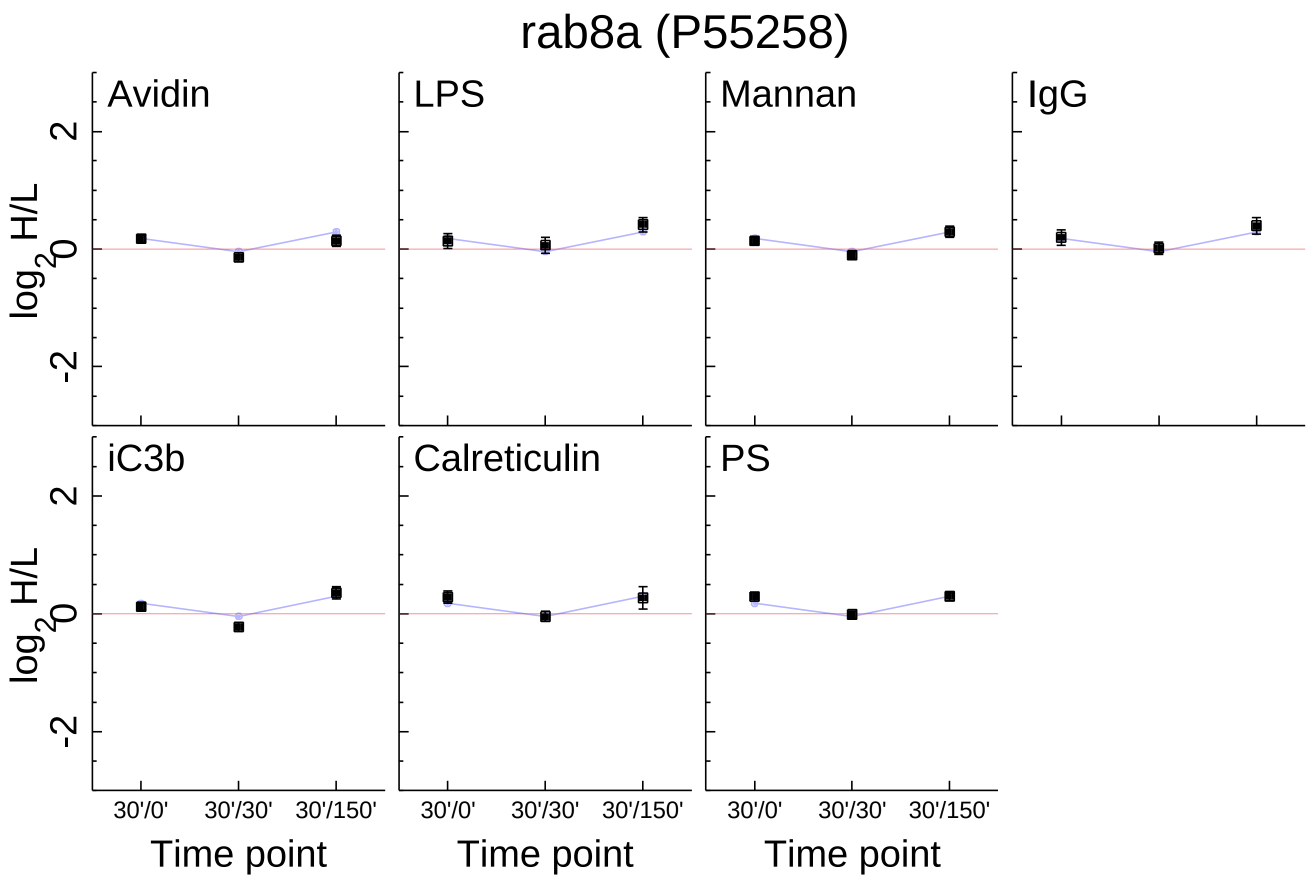
rab8a (P55258)
Avidin
LPS
Mannan
IgG
iC3b
Calreticulin
PS
log2 H/L
2
0
-2
30'/0'
30'/30'
30'/150'
Time point

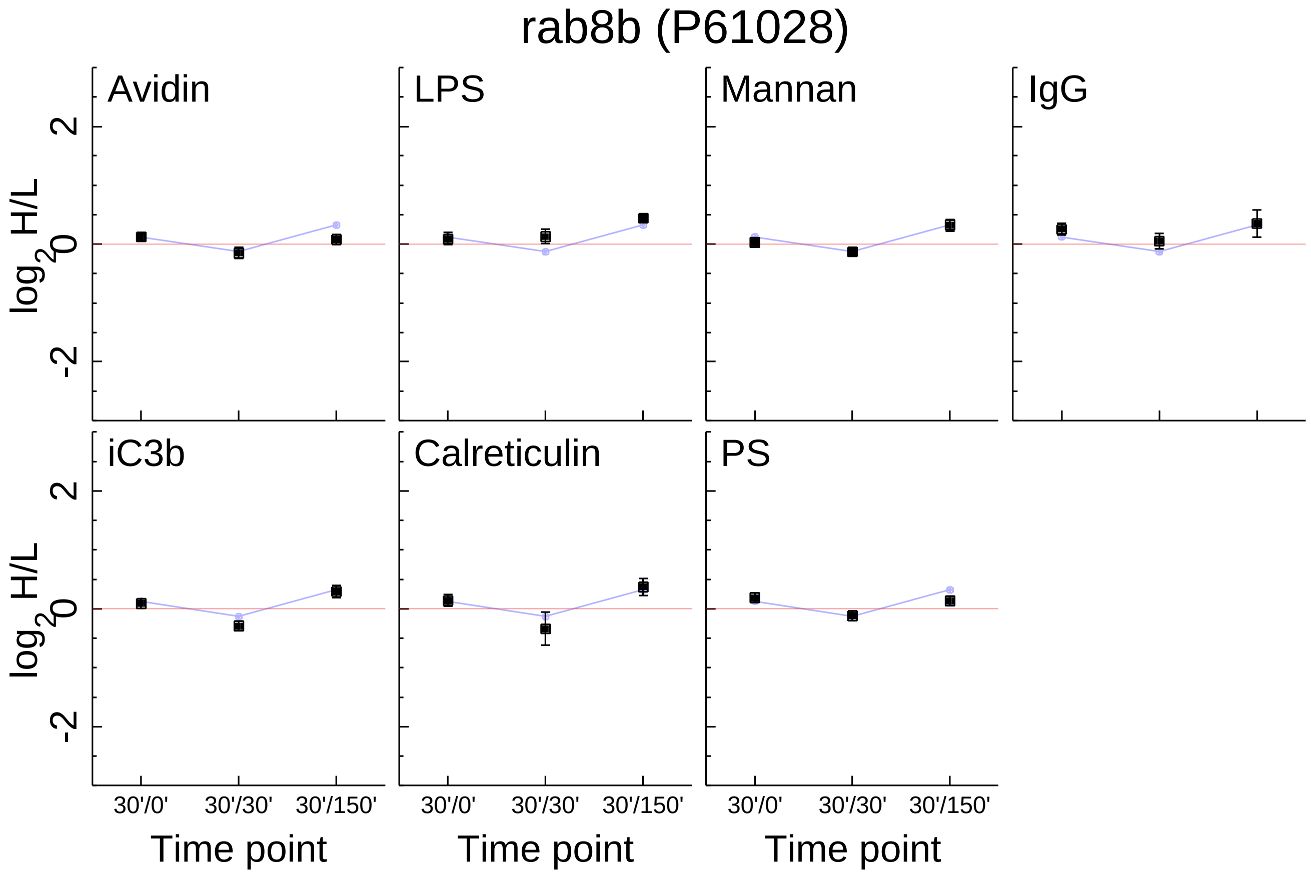
rab8b (P61028)
Avidin
LPS
Mannan
IgG
iC3b
Calreticulin
PS
log2 H/L
2
0
-2
30'/0'
30'/30'
30'/150'
Time point

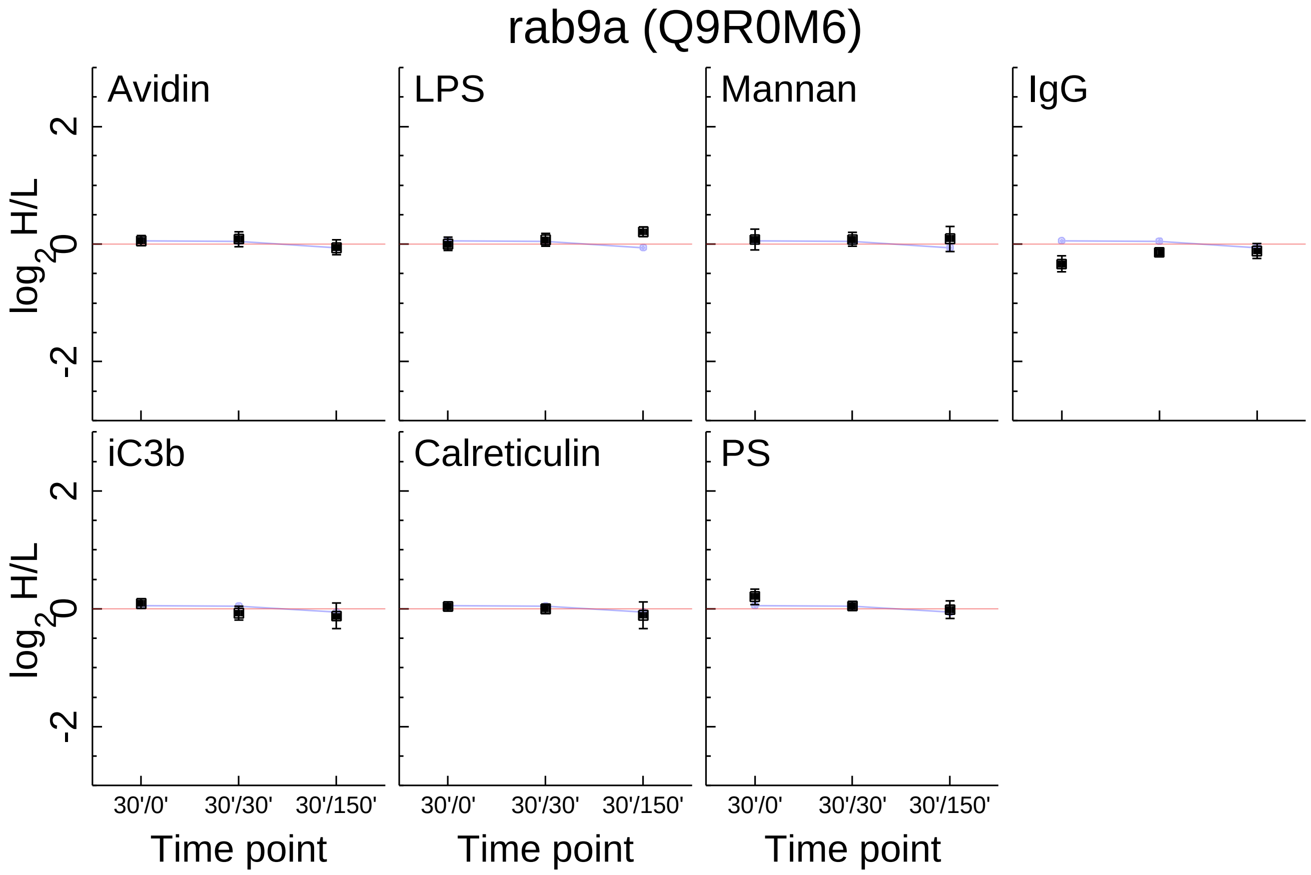

rab9a (Q9R0M6)
Avidin
LPS
Mannan
IgG
iC3b
Calreticulin
PS
log2 H/L
2
0
-2
30'/0'
30'/30'
30'/150'
Time point

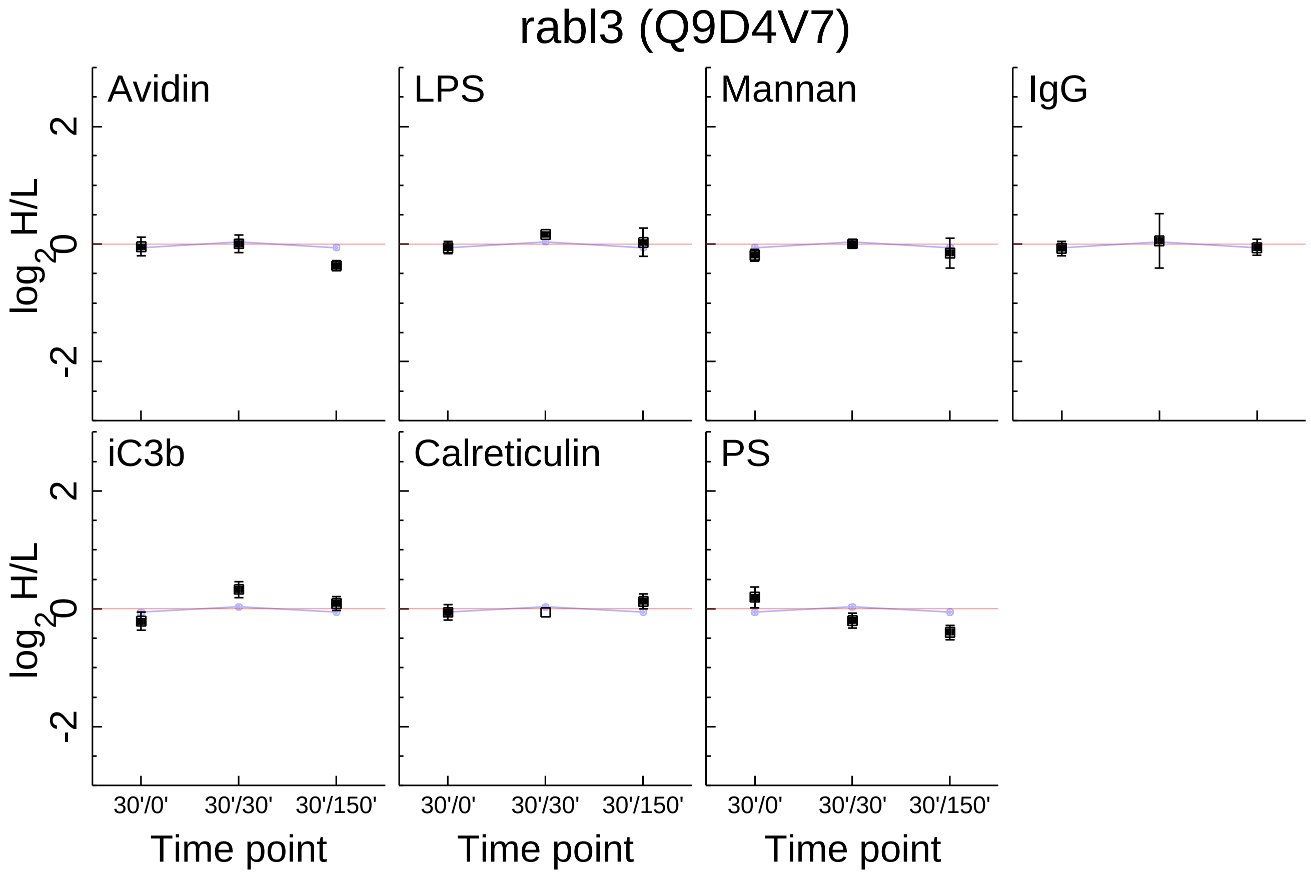
rabl3 (Q9D4V7)
Avidin
LPS
Mannan
IgG
iC3b
Calreticulin
PS
log2 H/L
2
0
-2
30'/0'
30'/30'
30'/150'
Time point

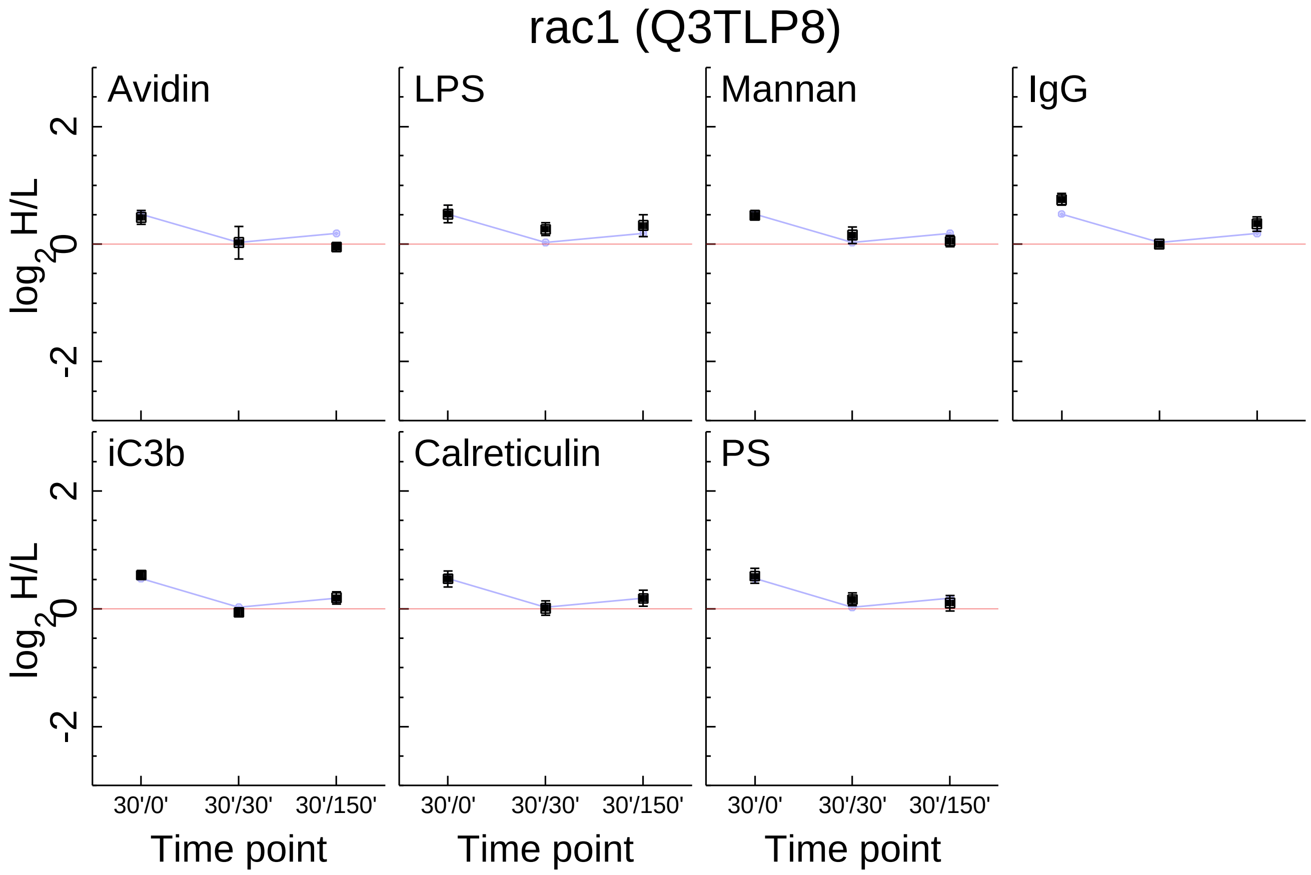
rac1 (Q3TLP8)
Avidin
LPS
Mannan
IgG
iC3b
Calreticulin
PS
log2 H/L
2
0
-2
30'/0'
30'/30'
30'/150'
Time point

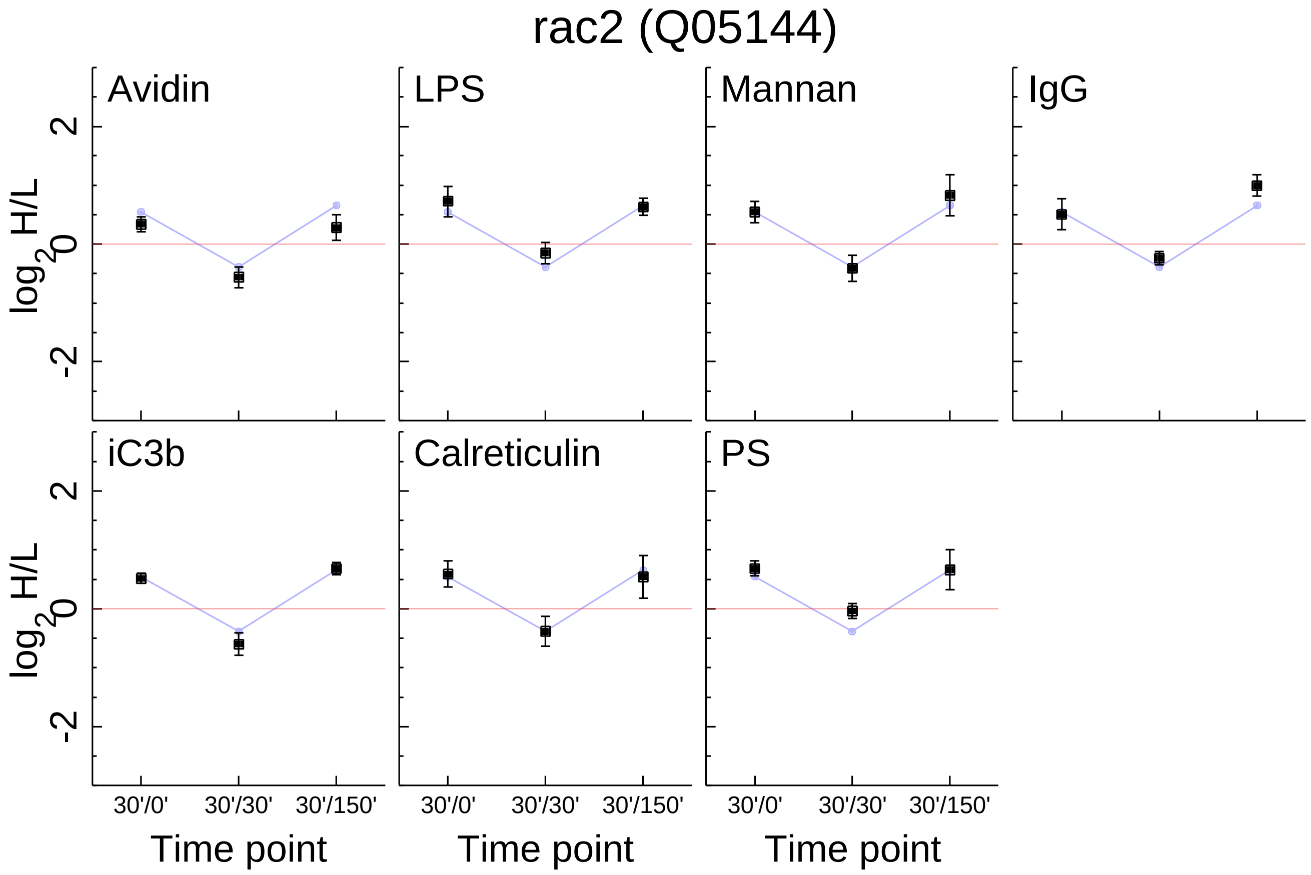

rac2 (Q05144)
Avidin
LPS
Mannan
IgG
iC3b
Calreticulin
PS
log2 H/L
2
0
-2
30'/0'
30'/30'
30'/150'
Time point

rala (P63321)

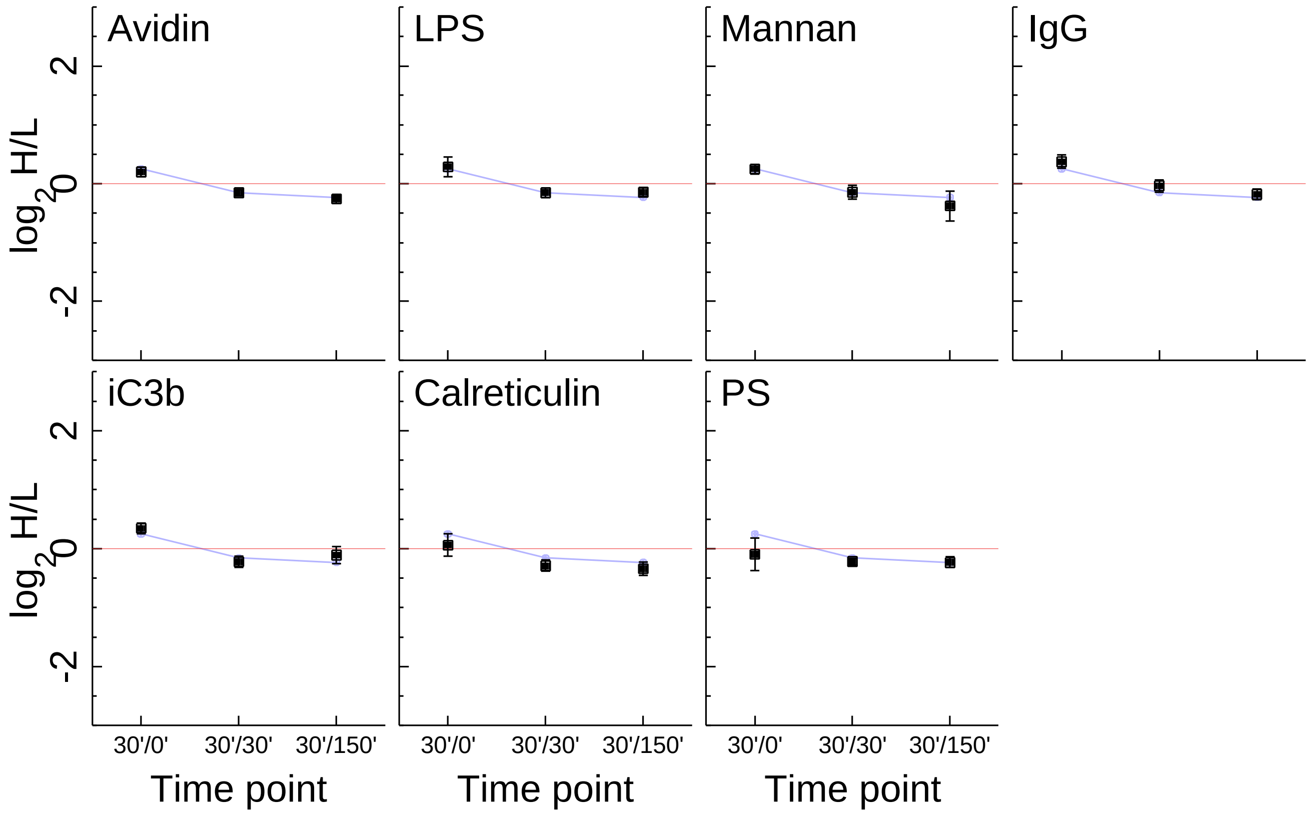

Avidin
LPS
Mannan
IgG
iC3b
Calreticulin
PS
log2 H/L
2
0
-2
30'/0'
30'/30'
30'/150'
Time point

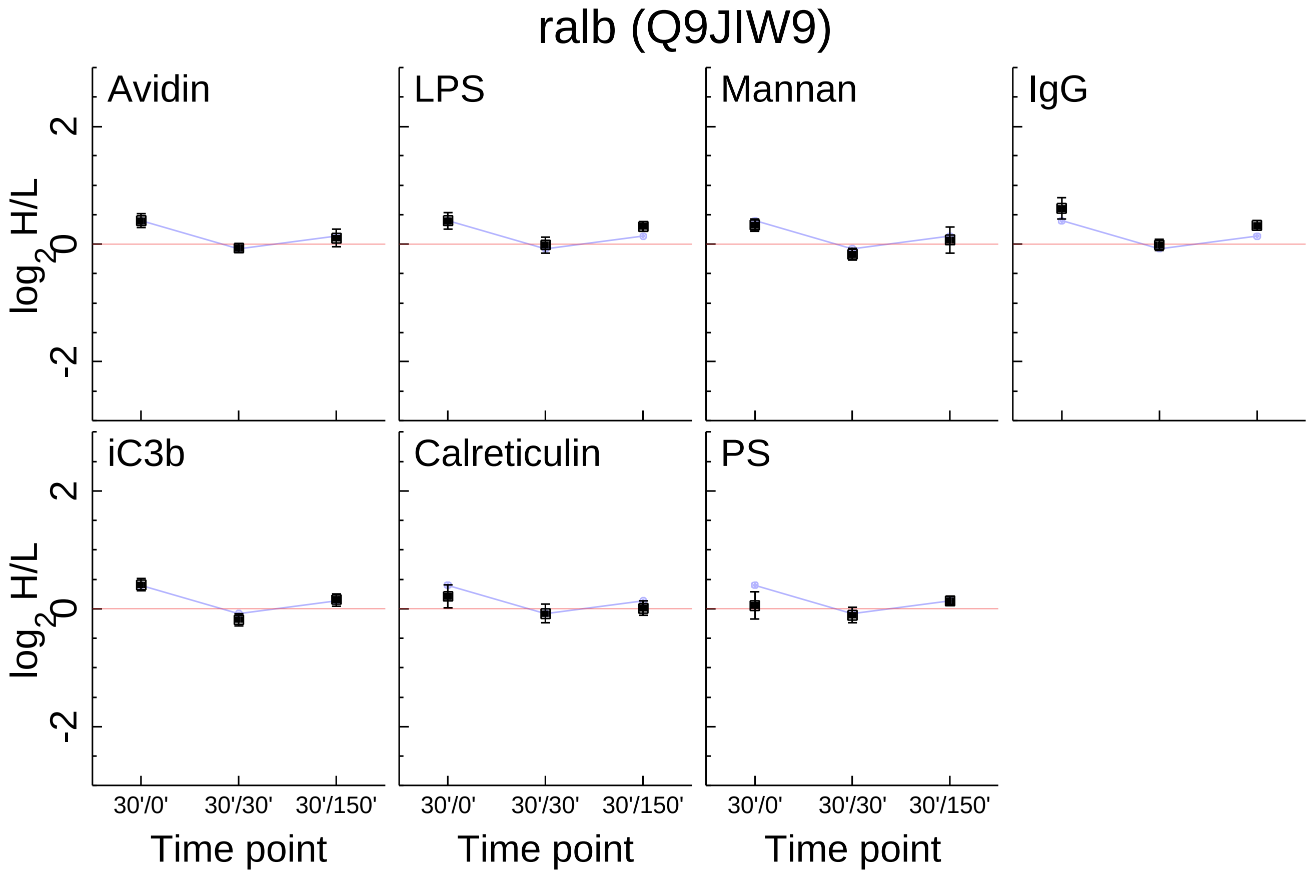
ralb (Q9JIW9)
Avidin
LPS
Mannan
IgG
iC3b
Calreticulin
PS
log2 H/L
2
0
-2
30'/0'
30'/30'
30'/150'
Time point

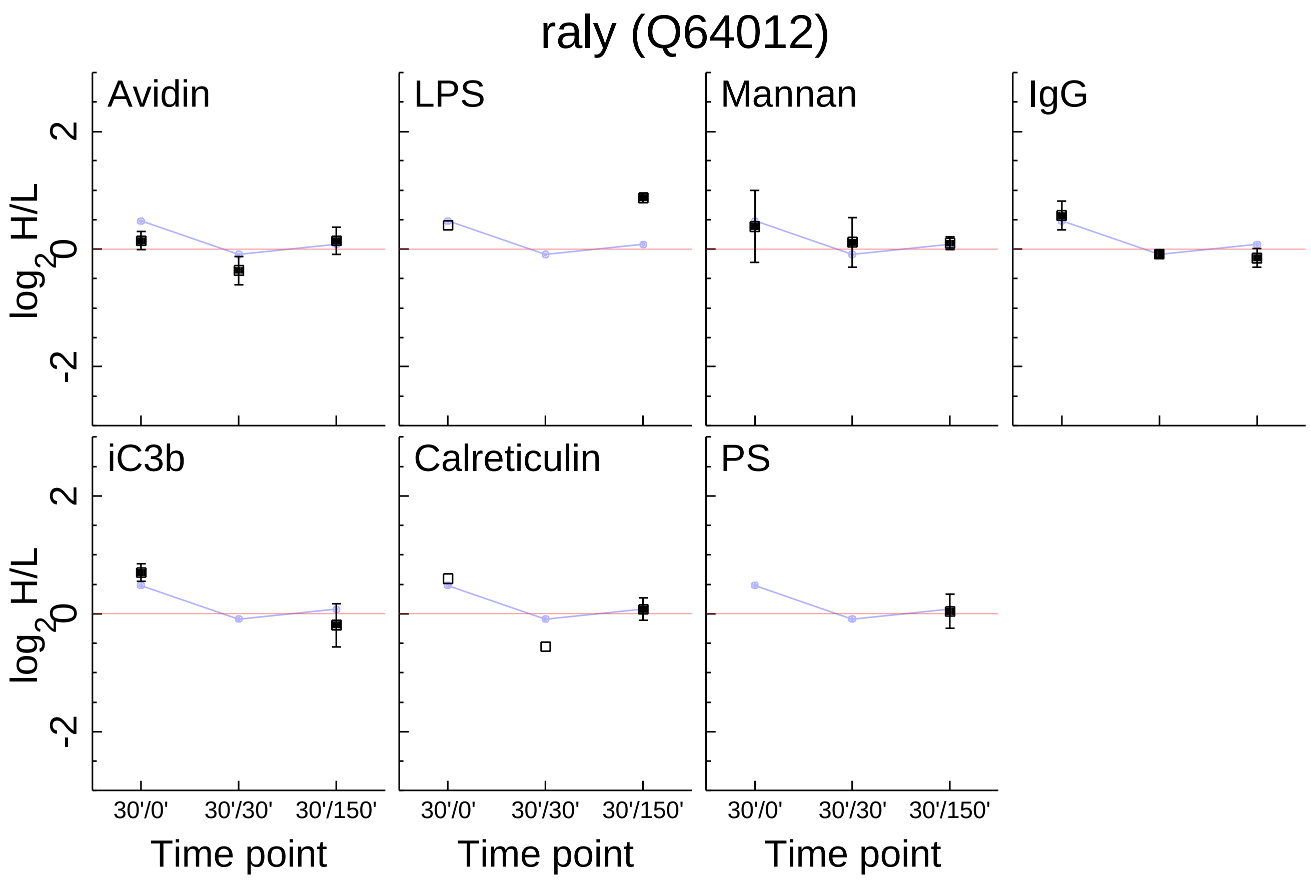

raly (Q64012)
Avidin
LPS
Mannan
IgG
iC3b
Calreticulin
PS
log2 H/L
2
0
-2
30'/0'
30'/30'
30'/150'
Time point

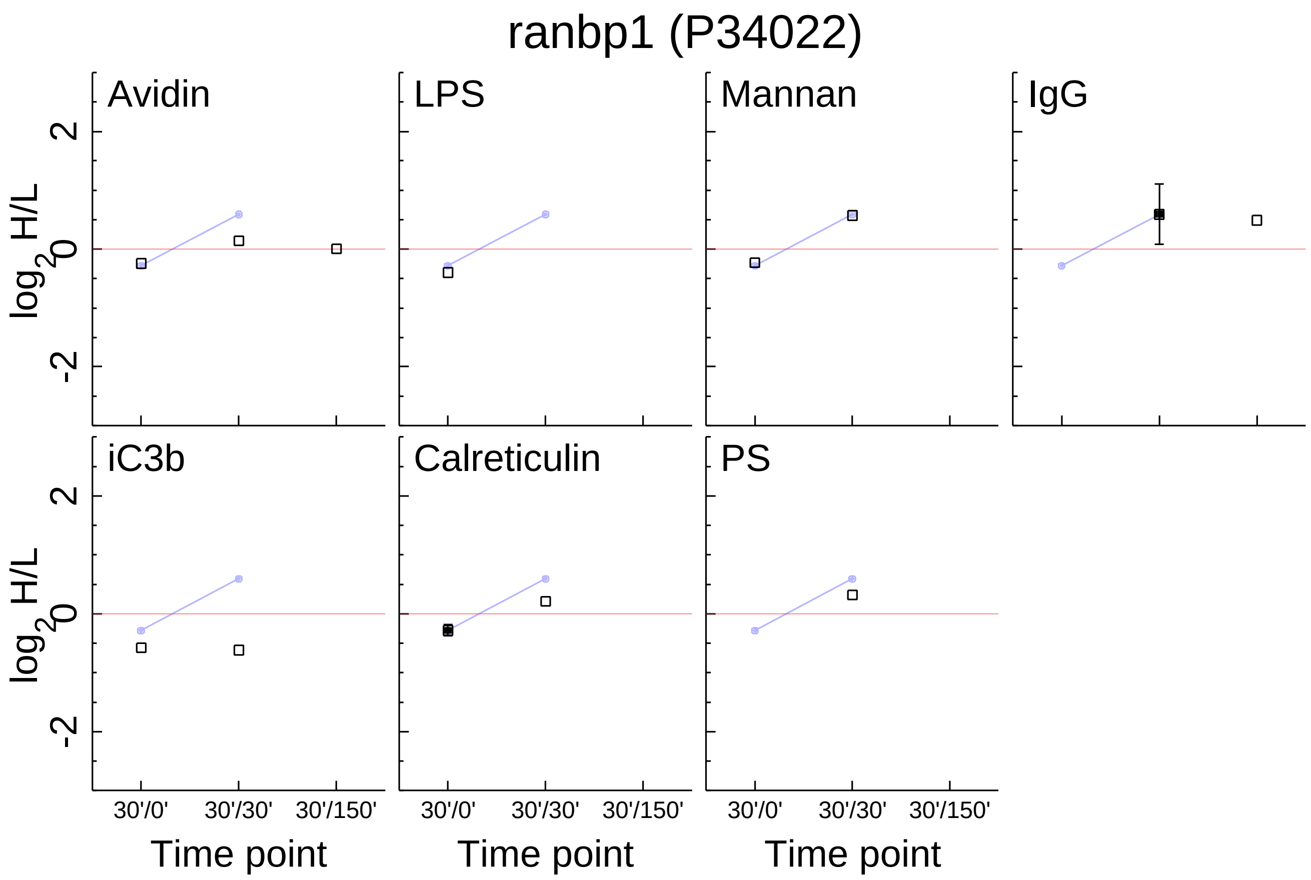
ranbp1 (P34022)
Avidin
LPS
Mannan
IgG
iC3b
Calreticulin
PS
log2 H/L
2
0
-2
30'/0'
30'/30'
30'/150'
Time point

ranbp2 (Q9ERU9)

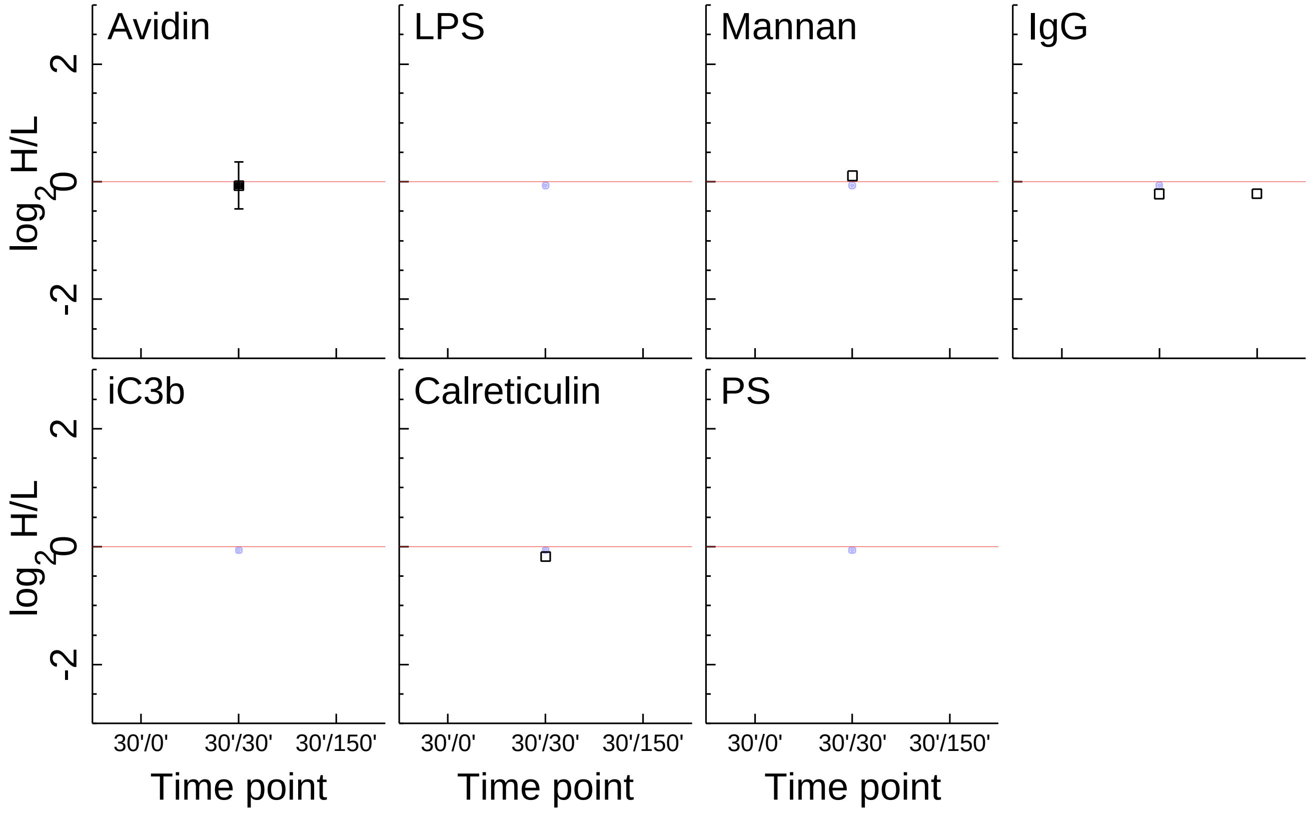

rangap1 (P46061)

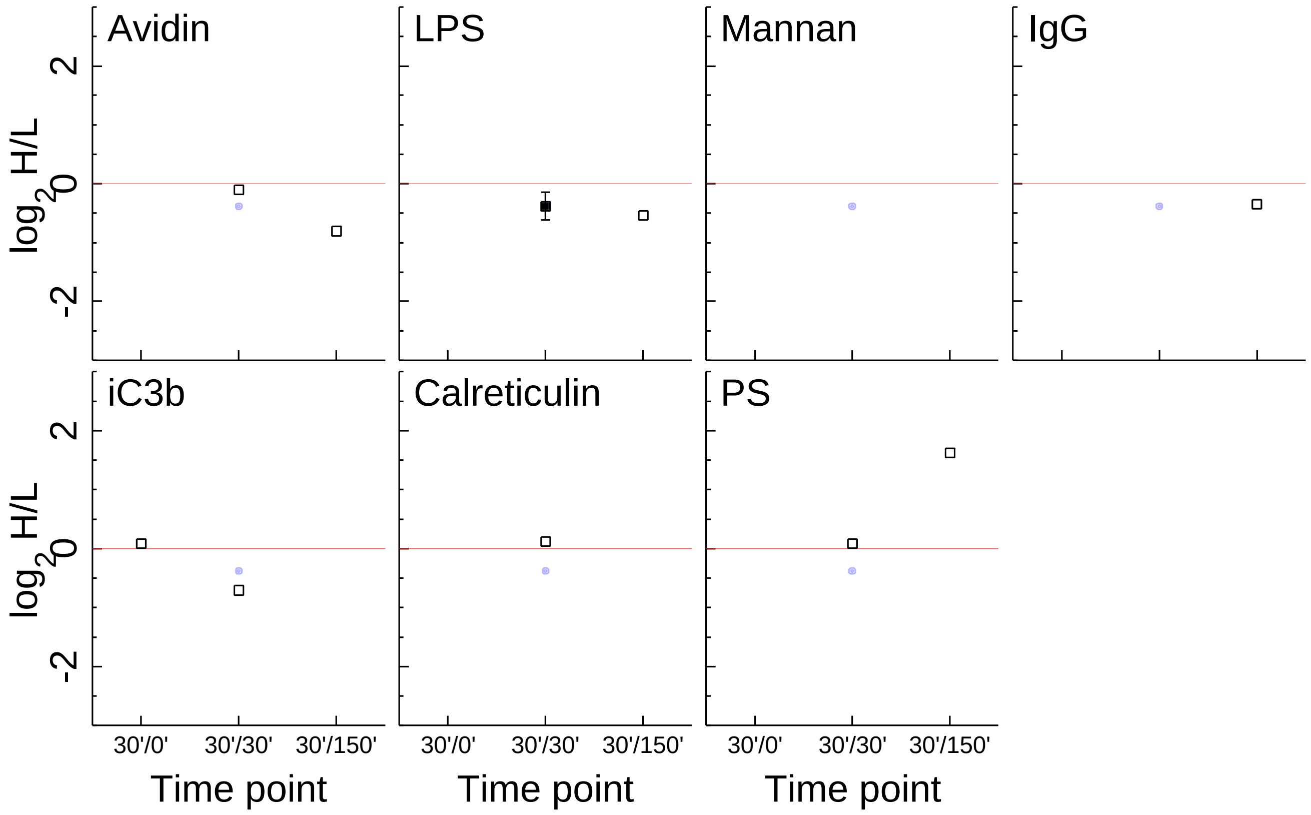

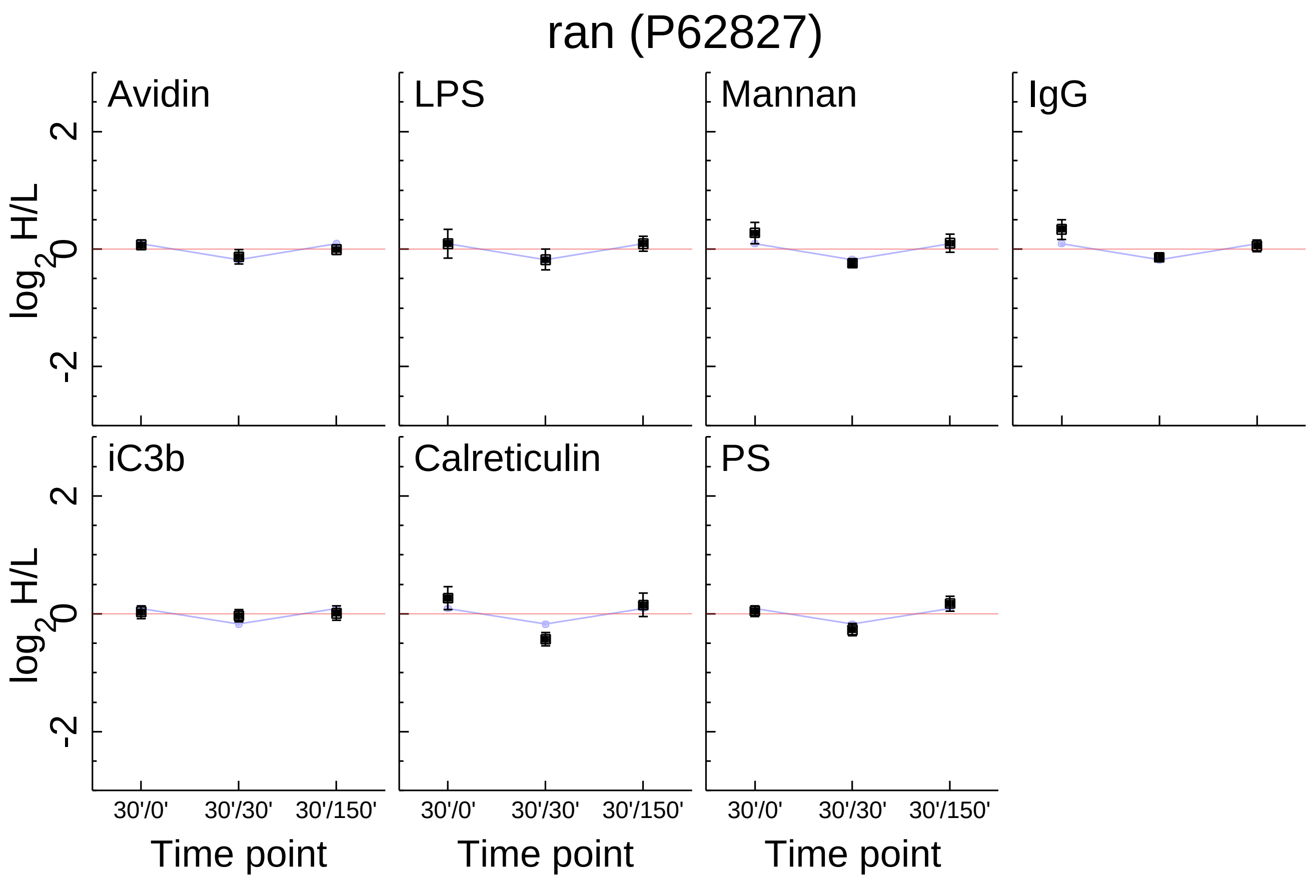
ran (P62827)
Avidin
LPS
Mannan
IgG
iC3b
Calreticulin
PS
log2 H/L
2
0
-2
30'/0'
30'/30'
30'/150'
Time point

rap1a (P62835)

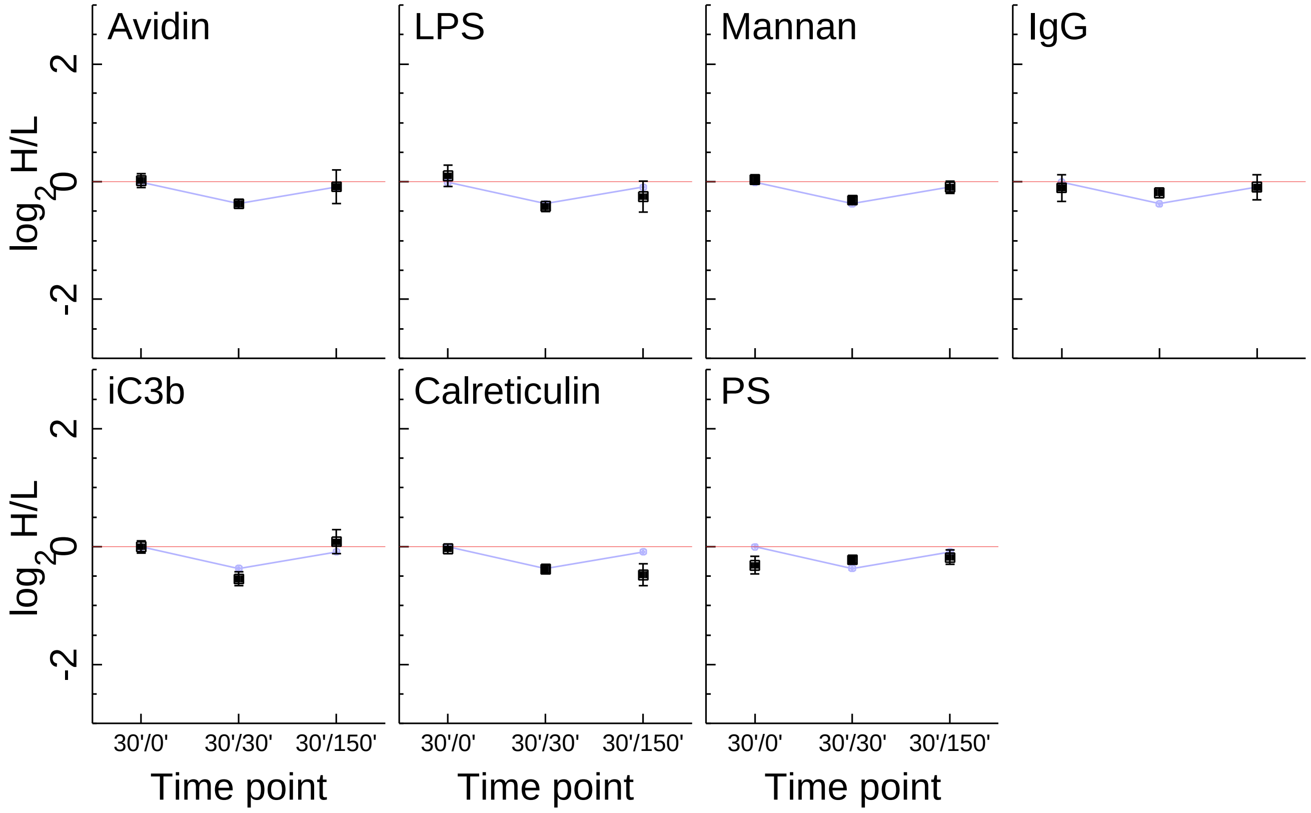

rap1b (Q99JI6)

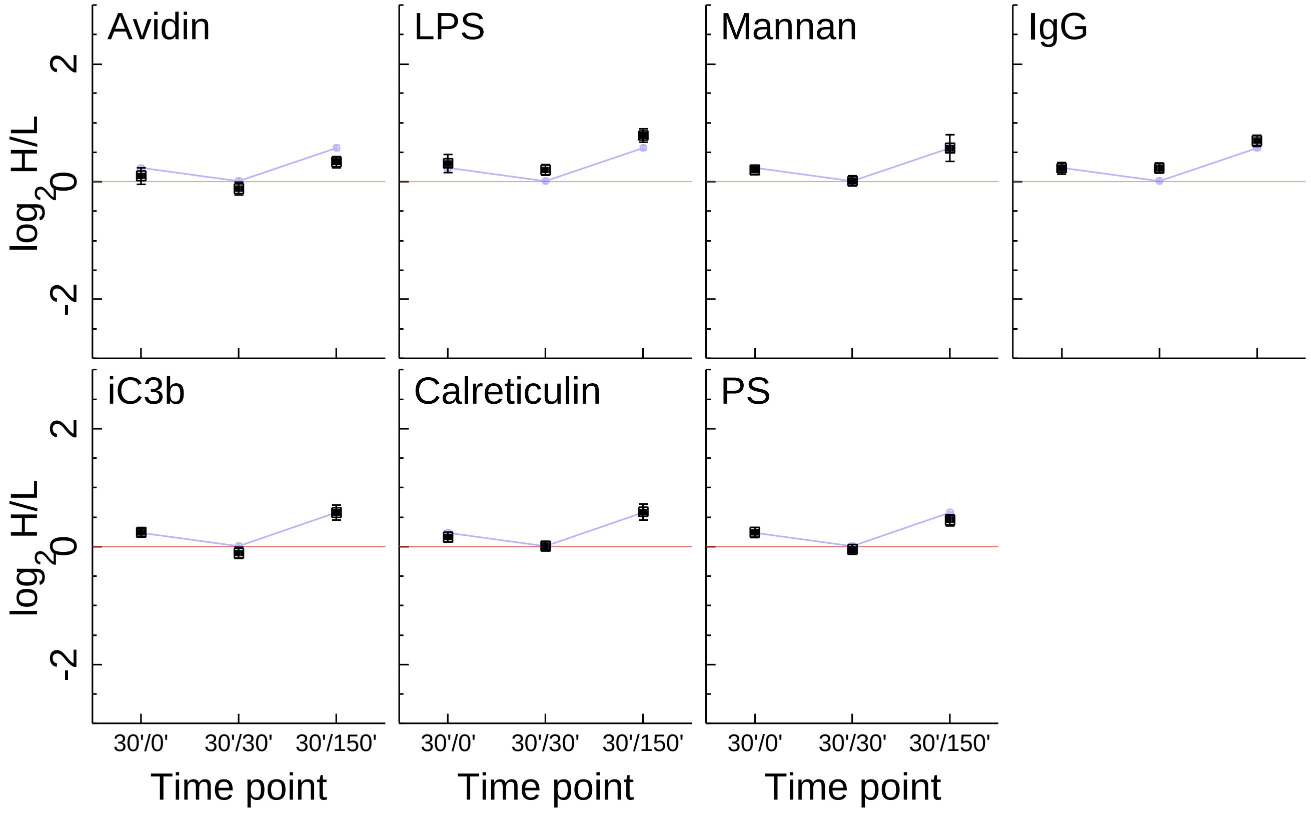

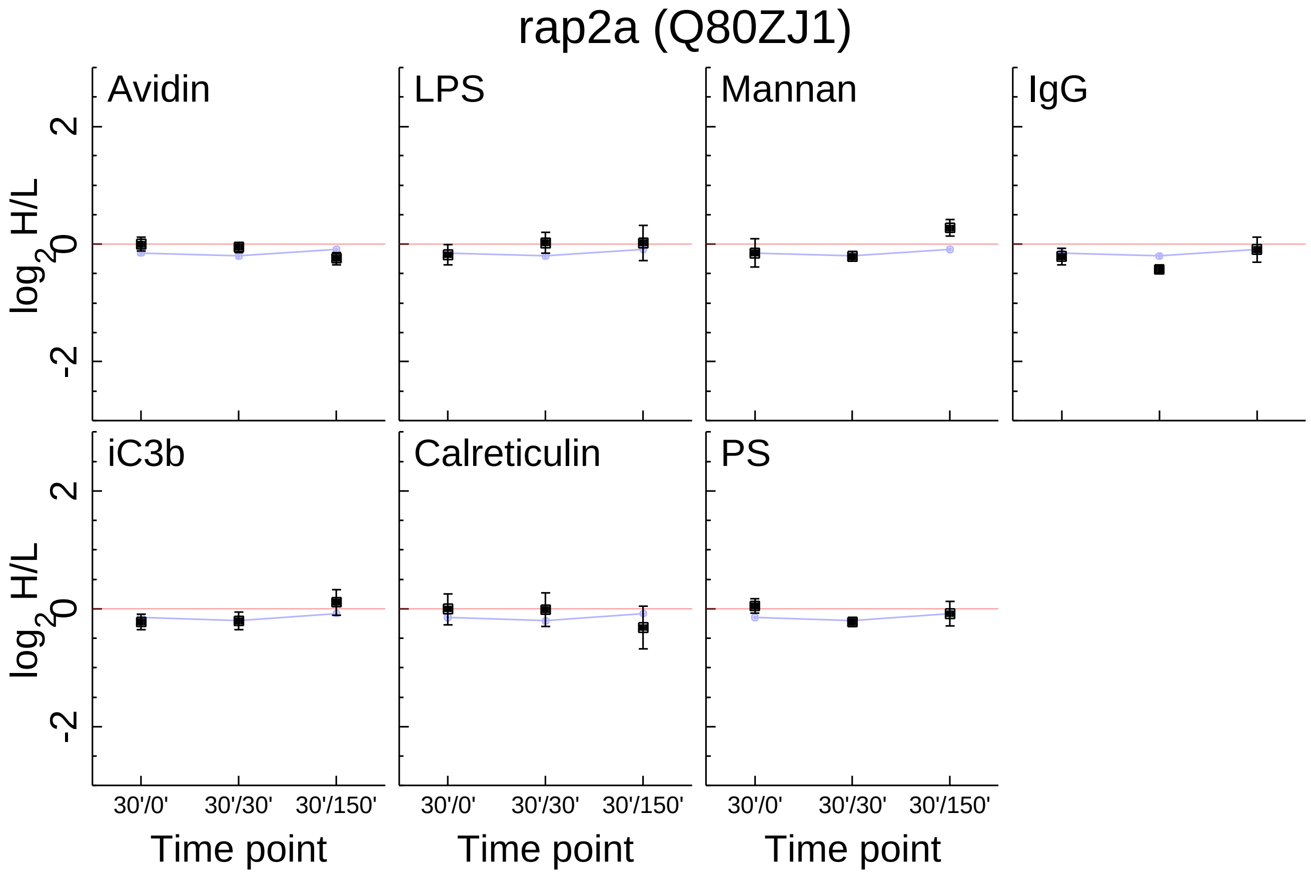
rap2a (Q80ZJ1)
Avidin
LPS
Mannan
IgG
iC3b
Calreticulin
PS
log2 H/L
2
0
-2
30'/0'
30'/30'
30'/150'
Time point

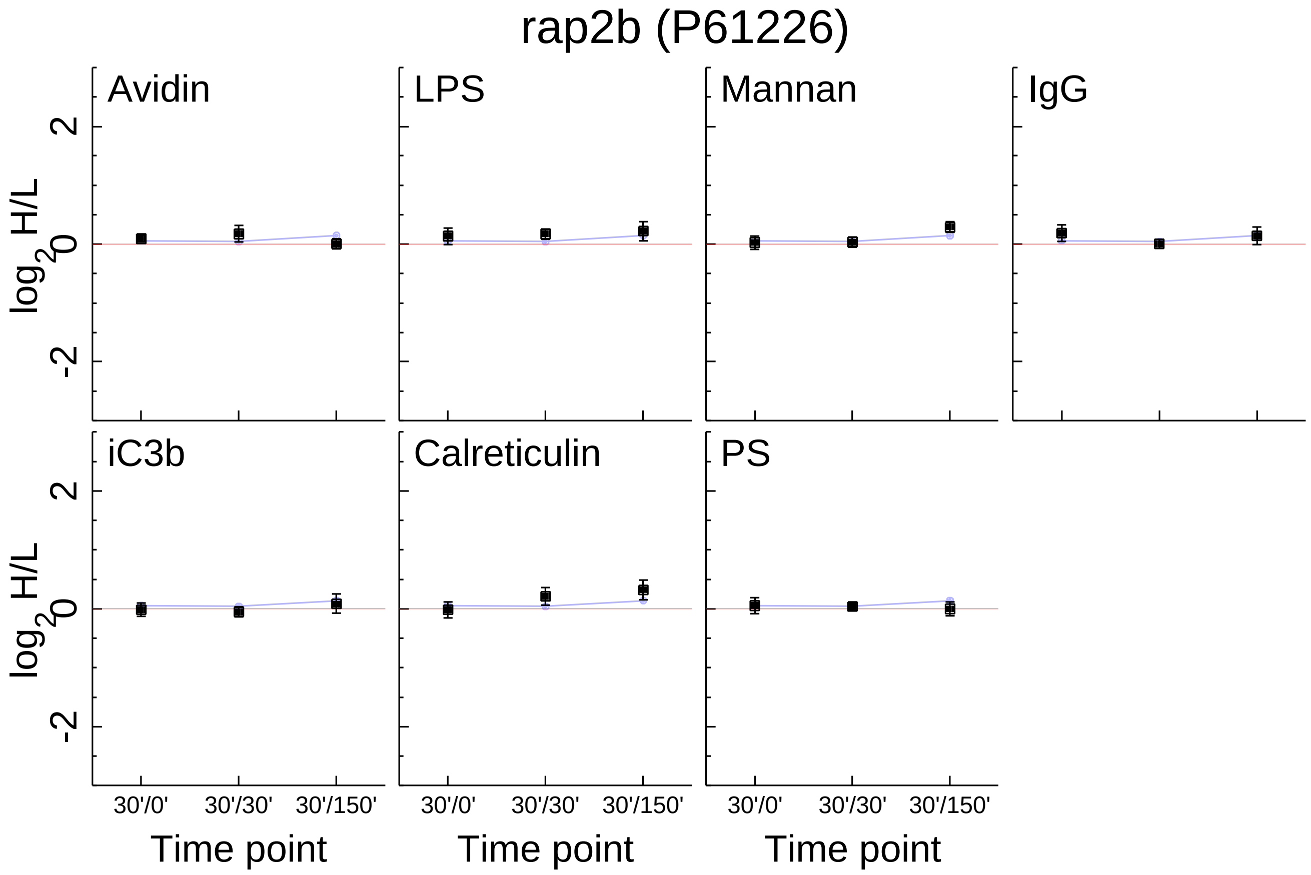

rap2b (P61226)
Avidin
LPS
Mannan
IgG
iC3b
Calreticulin
PS
log_2 H/L
2
0
-2
30'/0'
30'/30'
30'/150'
Time point

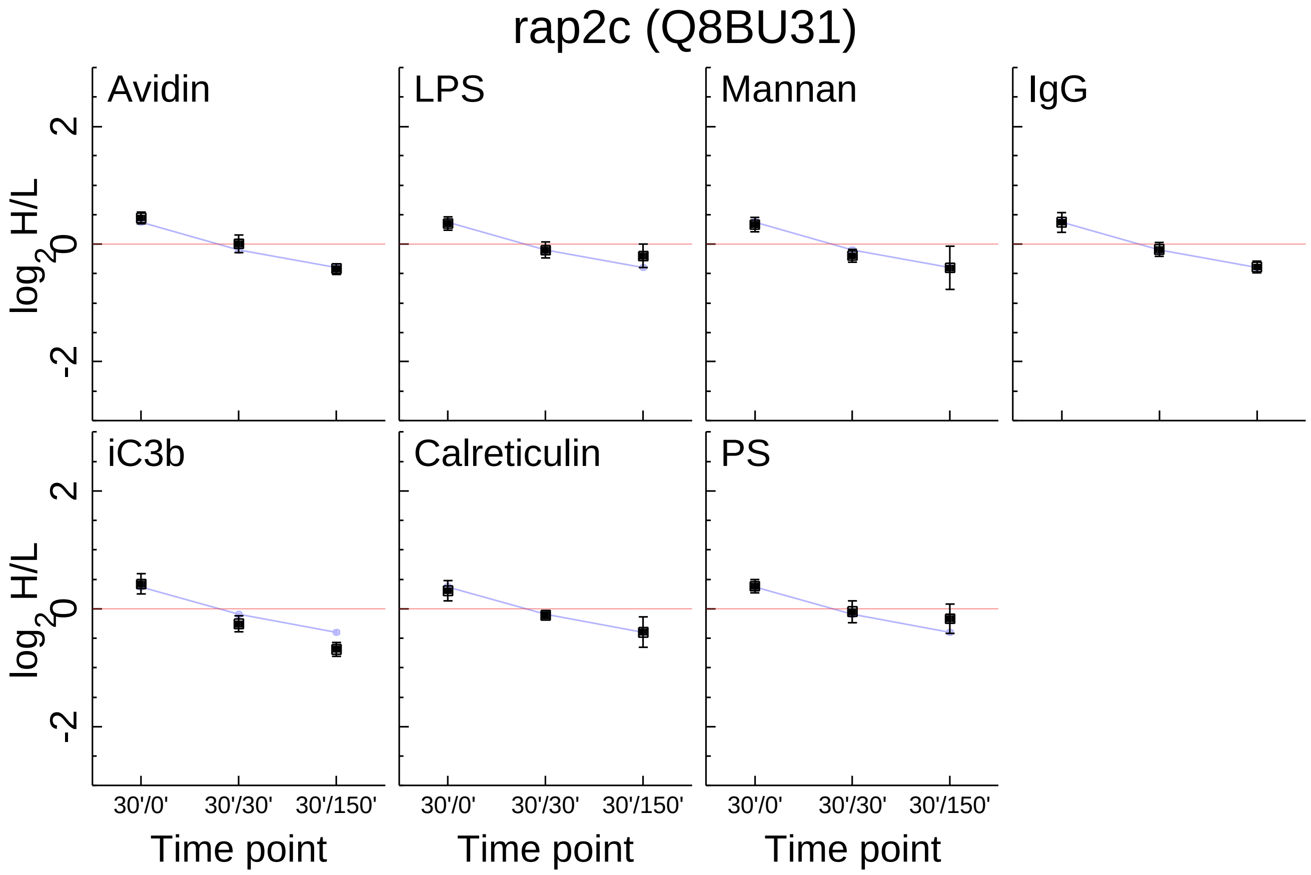

rap2c (Q8BU31)
Avidin
LPS
Mannan
IgG
iC3b
Calreticulin
PS
log2 H/L
2
0
-2
30'/0'
30'/30'
30'/150'
Time point

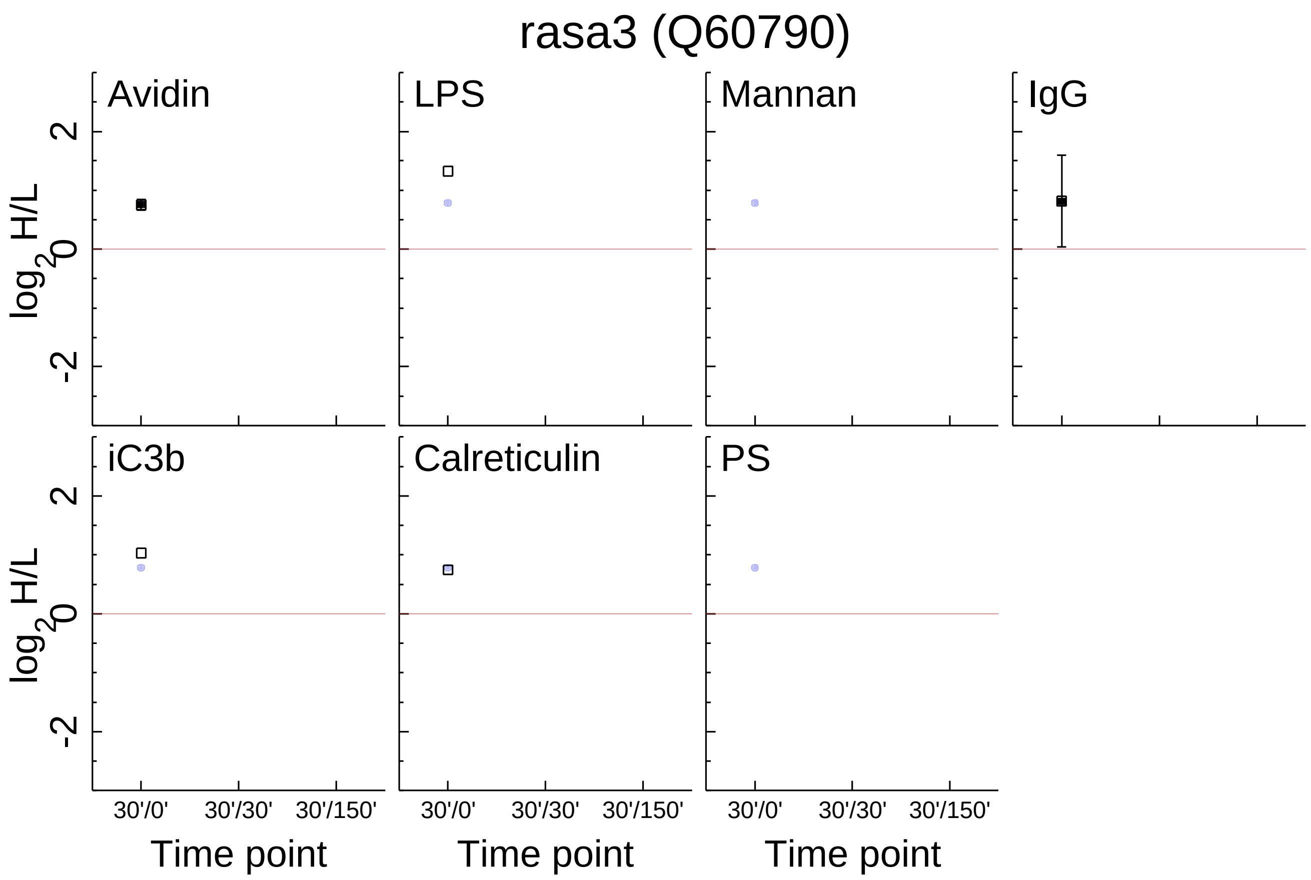
rasa3 (Q60790)
Avidin
LPS
Mannan
IgG
iC3b
Calreticulin
PS
log2 H/L
2
0
-2
30'/0'
30'/30'
30'/150'
Time point

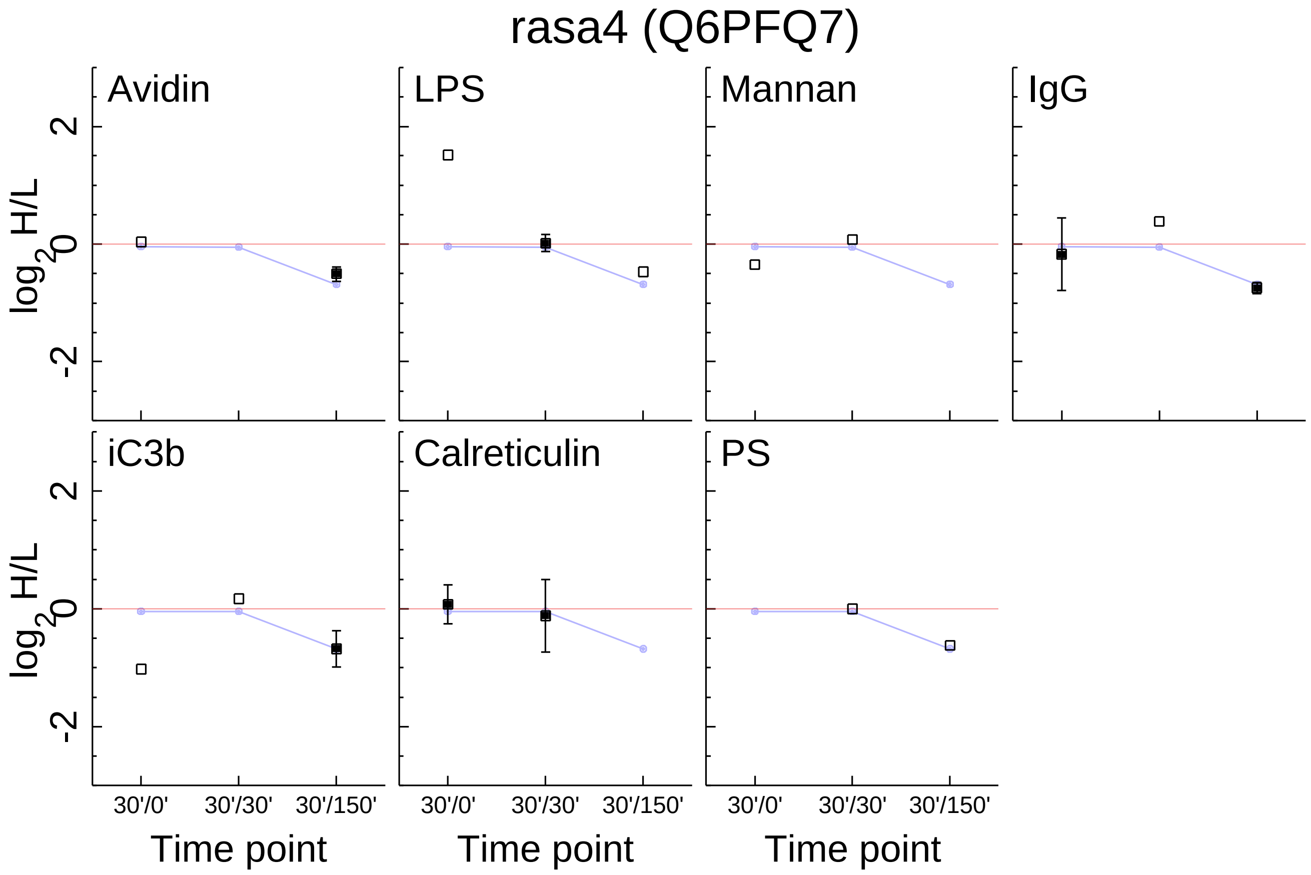

rasa4 (Q6PFQ7)
Avidin
LPS
Mannan
IgG
iC3b
Calreticulin
PS
log_2 H/L
2
0
-2
30'/0'
30'/30'
30'/150'
Time point

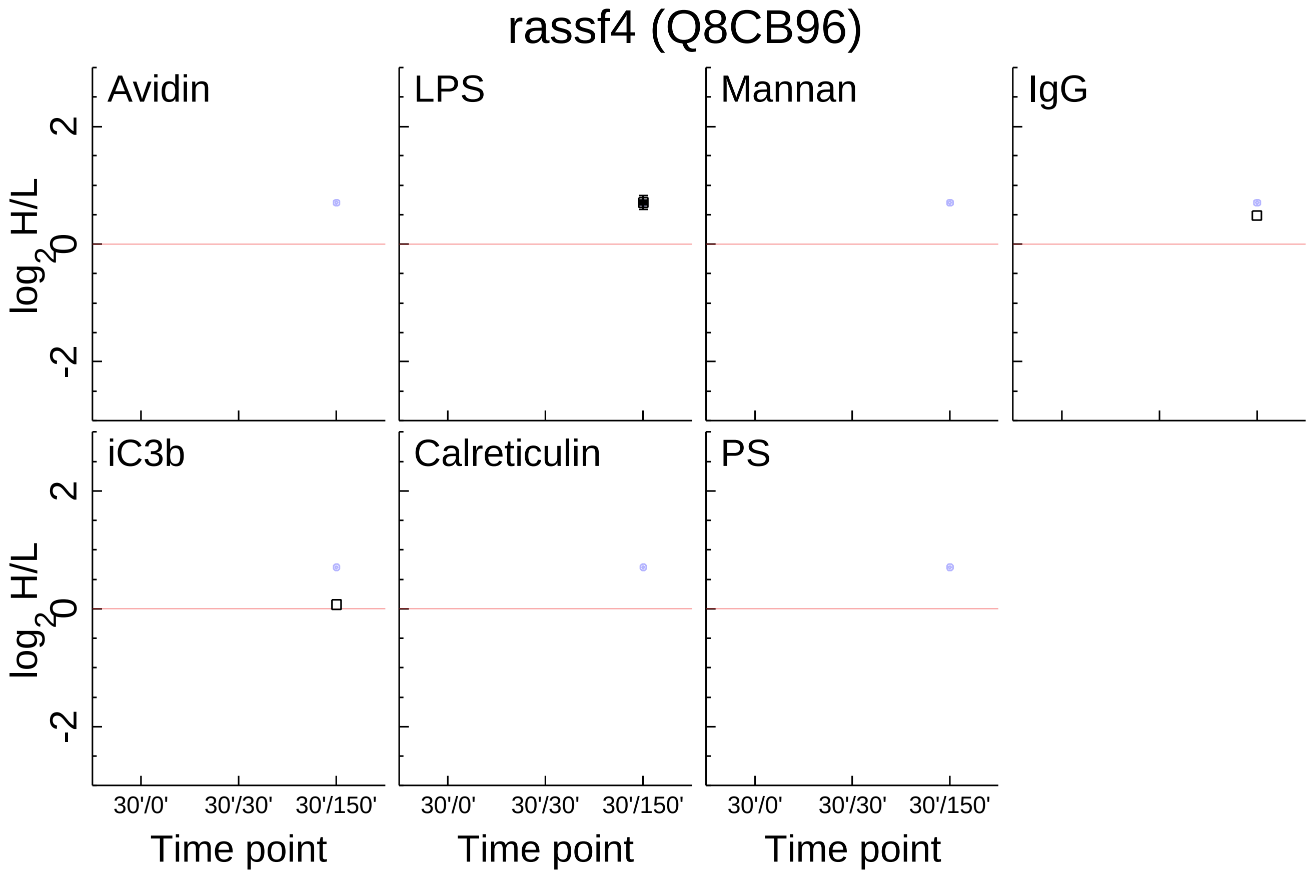

rassf4 (Q8CB96)
Avidin
LPS
Mannan
IgG
iC3b
Calreticulin
PS
log2 H/L
2
0
-2
30'/0'
30'/30'
30'/150'
Time point

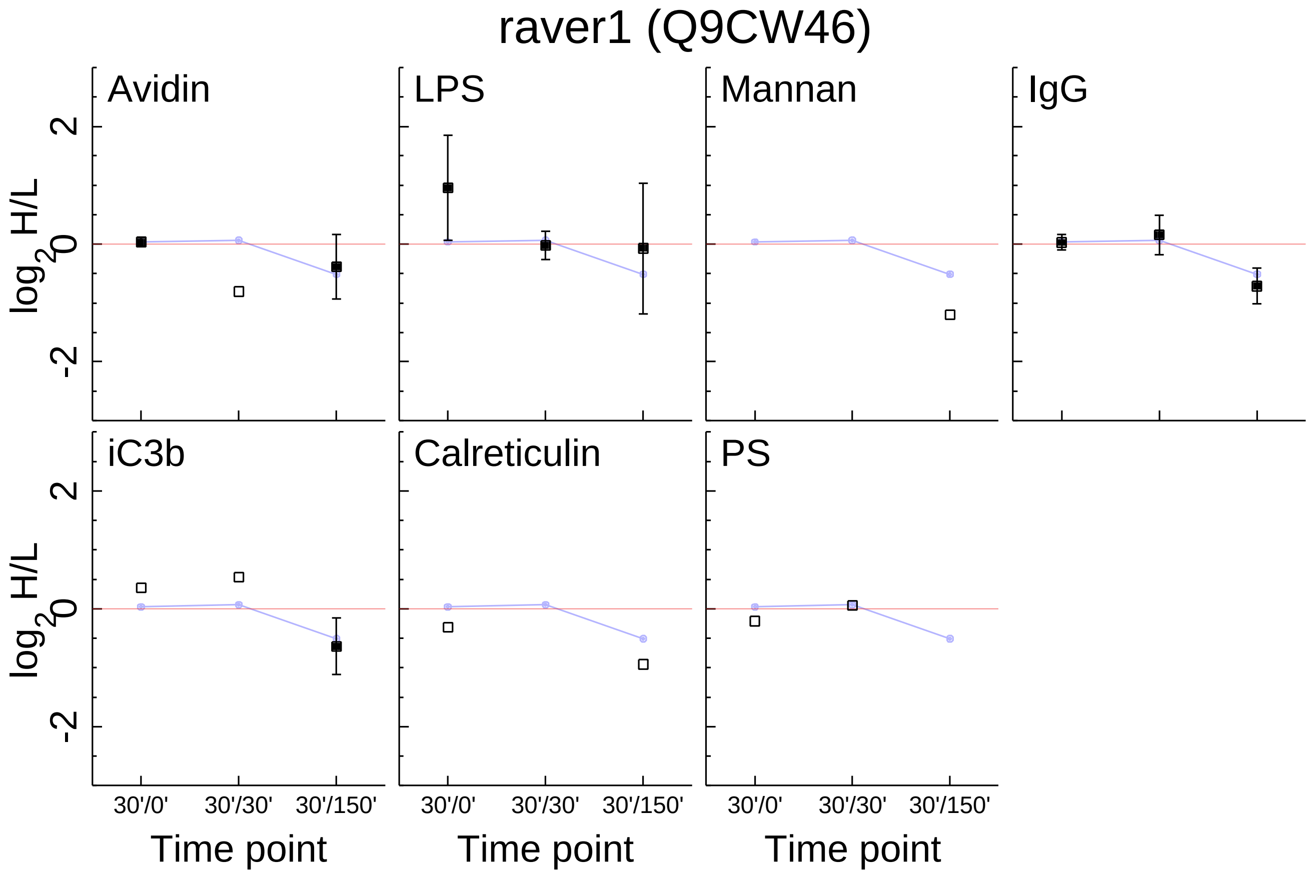

raver1 (Q9CW46)
Avidin
LPS
Mannan
IgG
iC3b
Calreticulin
PS
log2 H/L
2
0
-2
30'/0'
30'/30'
30'/150'
Time point

rbbp4 (Q60972)

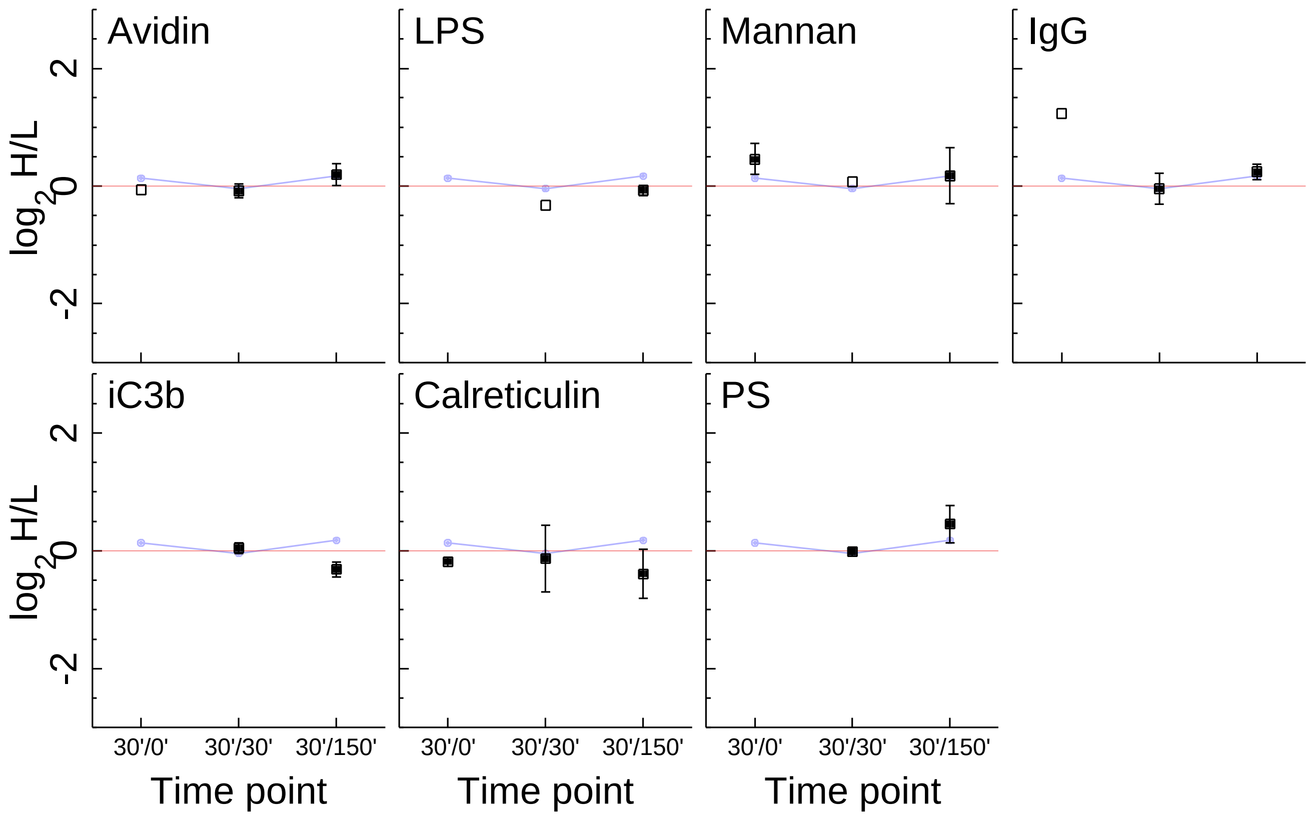

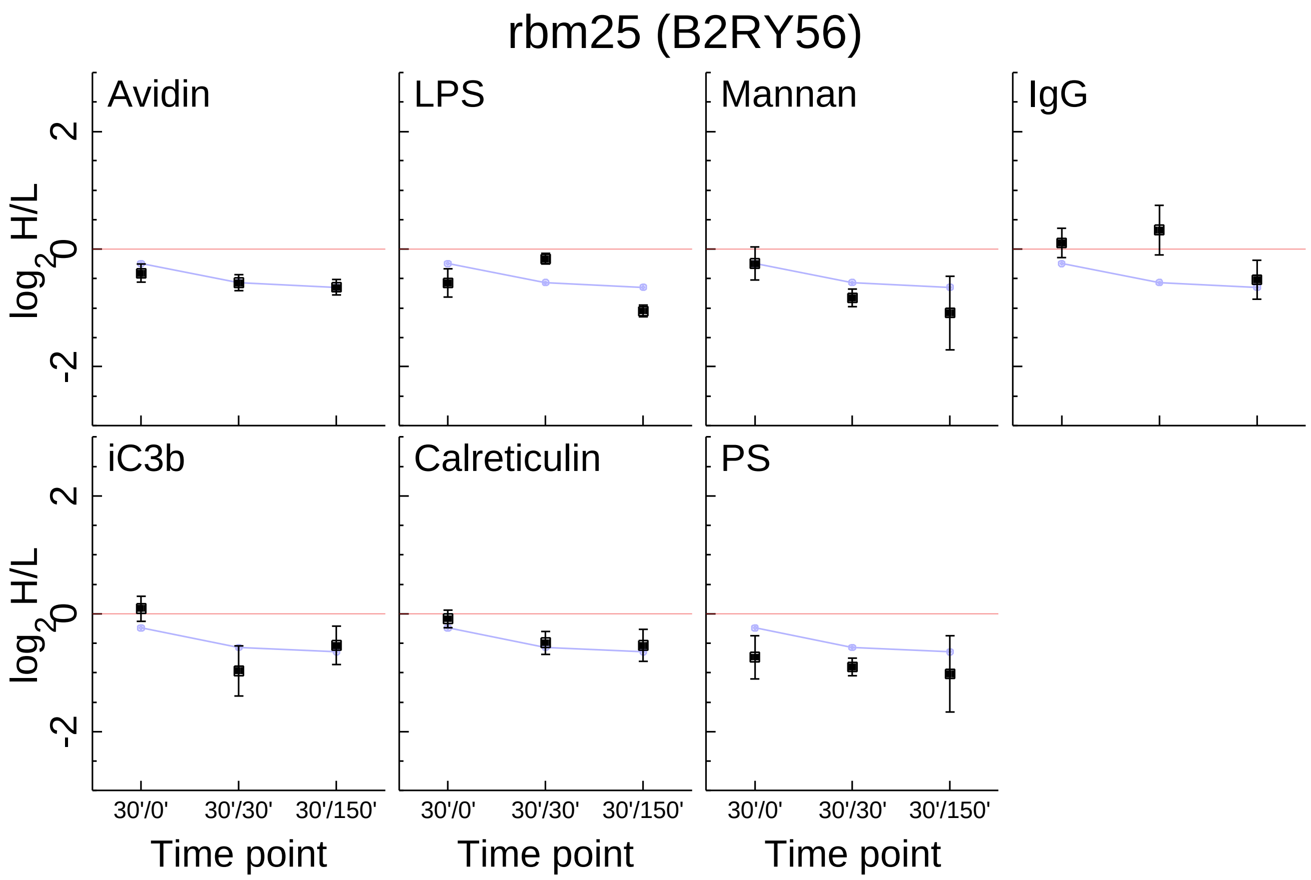
rbm25 (B2RY56)
Avidin
LPS
Mannan
IgG
iC3b
Calreticulin
PS
log2 H/L
2
0
-2
30'/0'
30'/30'
30'/150'
Time point

rbm3 (O89086)

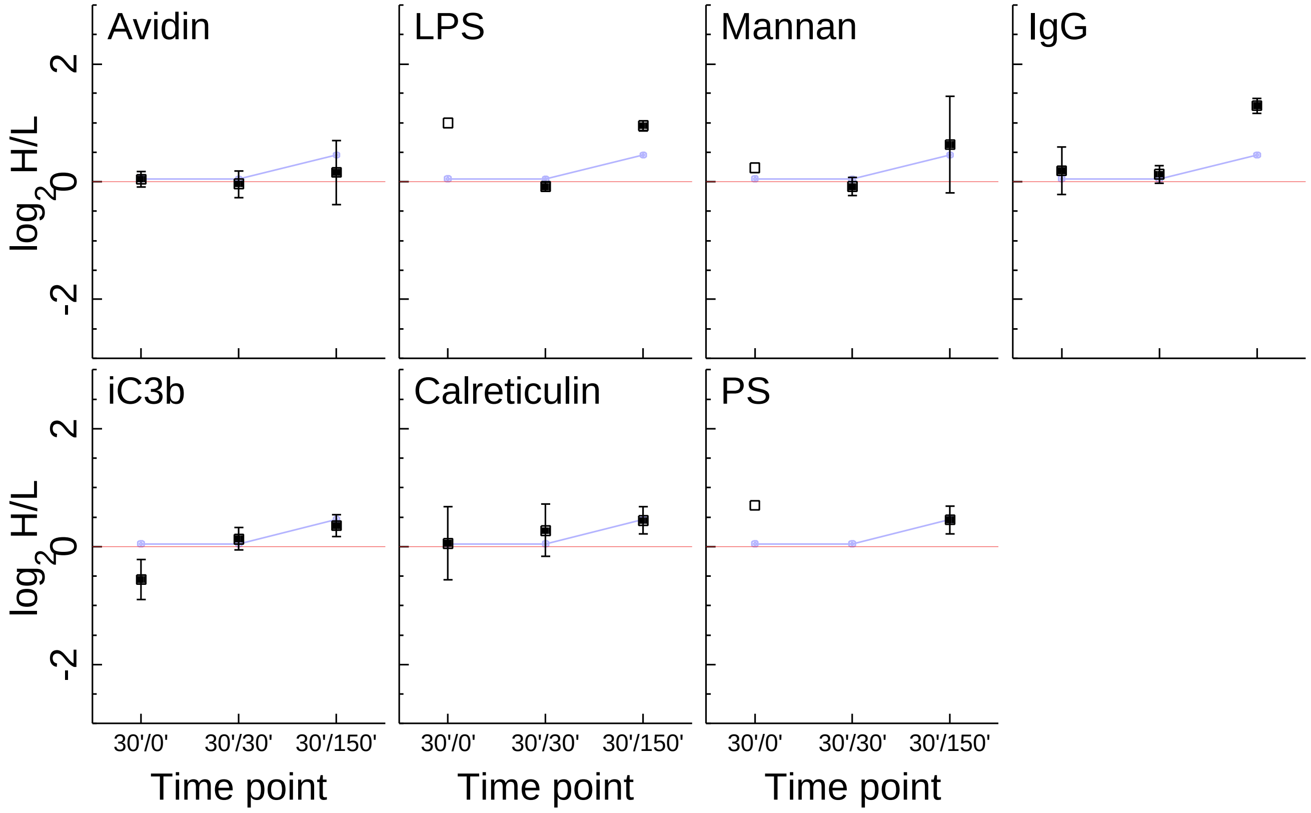

Avidin
LPS
Mannan
IgG
iC3b
Calreticulin
PS
log2 H/L
2
0
-2
30'/0'
30'/30'
30'/150'
Time point

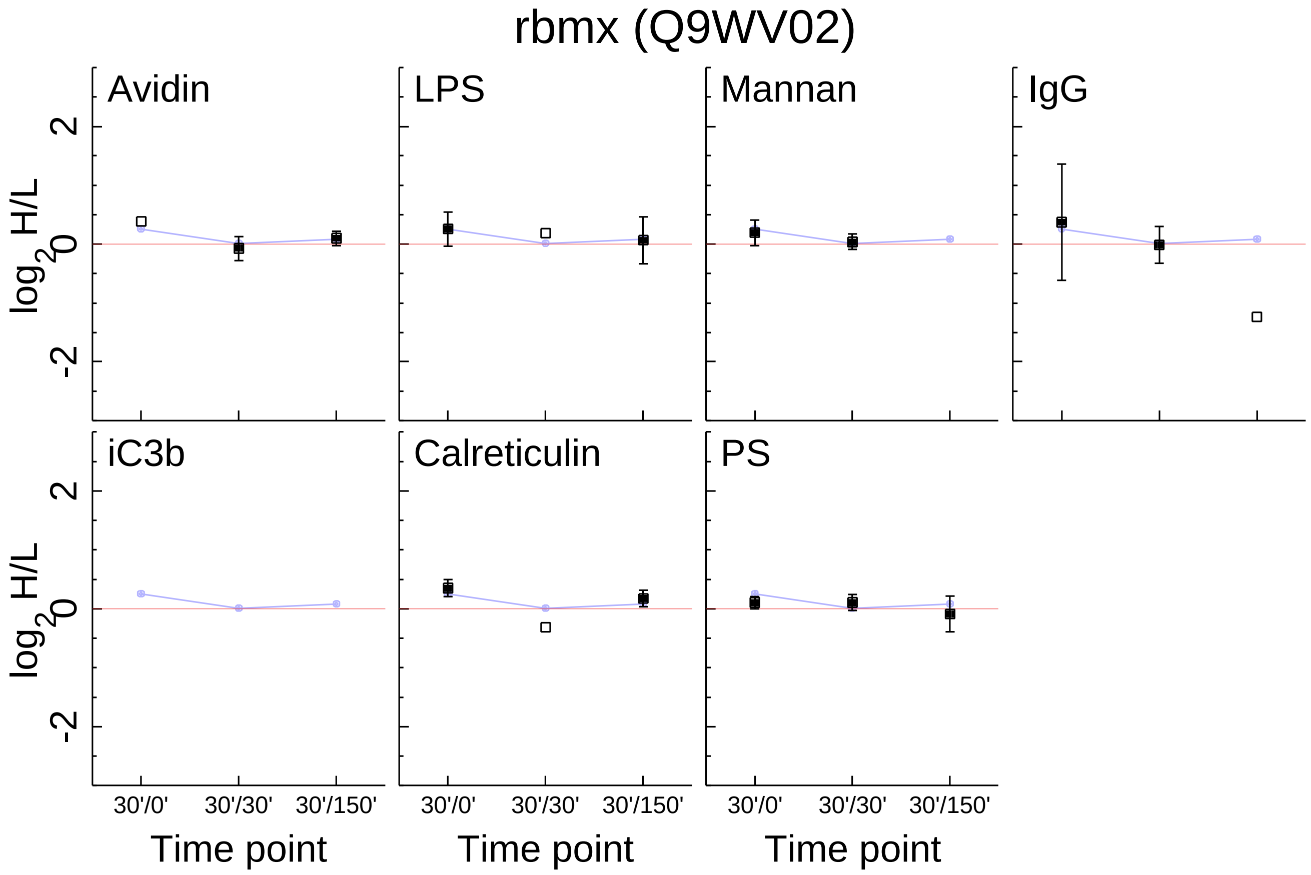
rbmx (Q9WV02)
Avidin
LPS
Mannan
IgG
iC3b
Calreticulin
PS
log2 H/L
2
0
-2
30'/0'
30'/30'
30'/150'
Time point

rbp4 (H7BWY6)

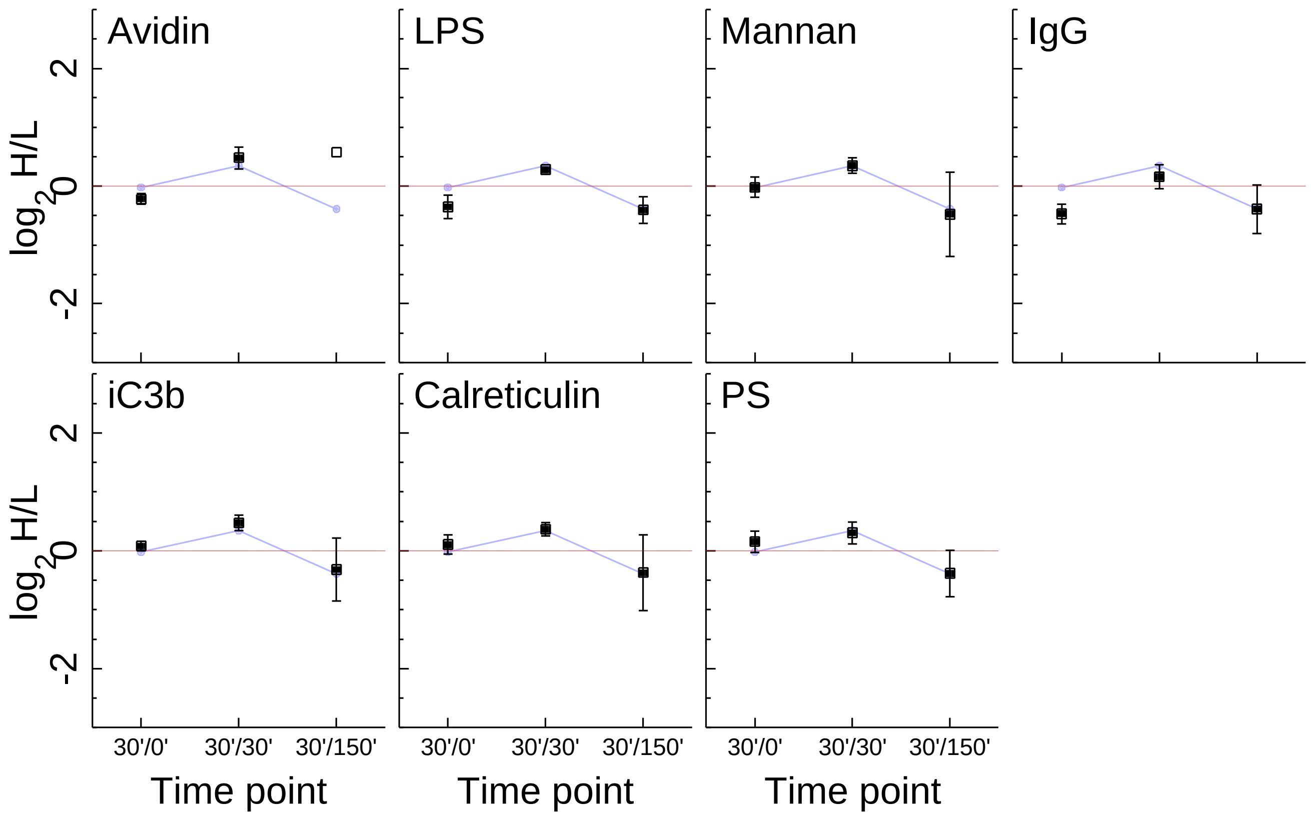

Avidin
LPS
Mannan
IgG
iC3b
Calreticulin
PS
log2 H/L
2
0
-2
30'/0'
30'/30'
30'/150'
Time point

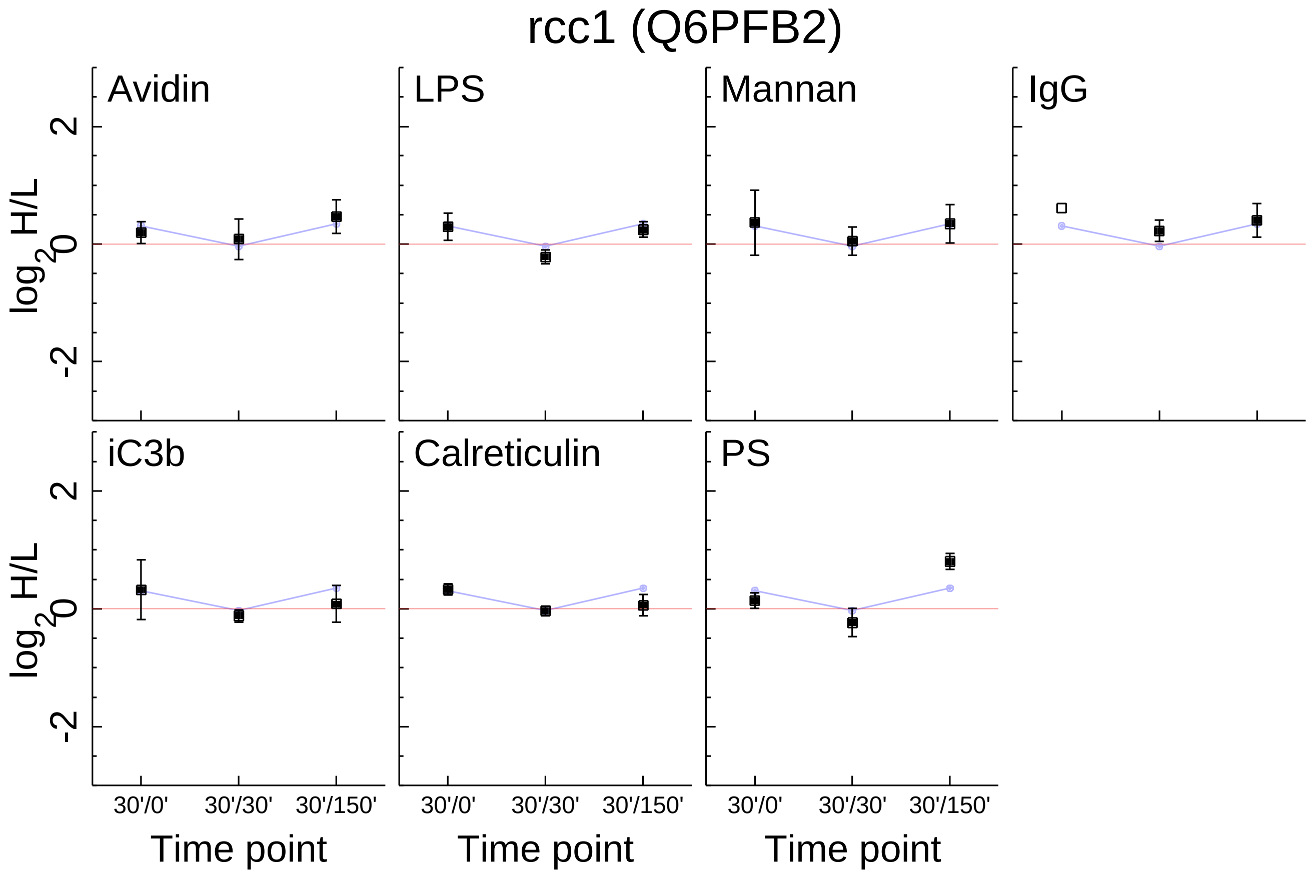

rcc1 (Q6PFB2)
Avidin
LPS
Mannan
IgG
iC3b
Calreticulin
PS
2
0
-2
log2 H/L
30'/0'
30'/30'
30'/150'
Time point

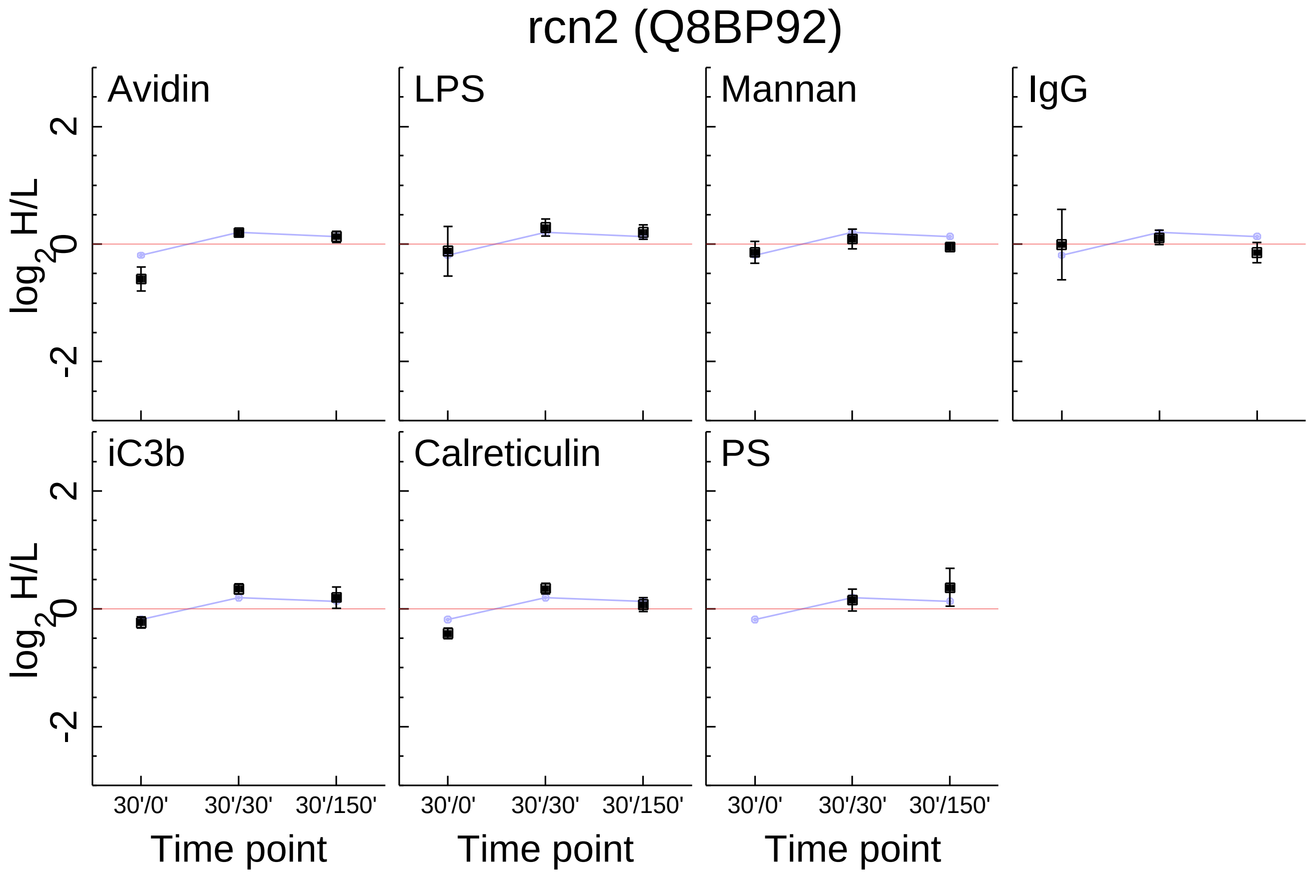

rcn2 (Q8BP92)
Avidin
LPS
Mannan
IgG
iC3b
Calreticulin
PS
log2 H/L
2
0
-2
30'/0'
30'/30'
30'/150'
Time point

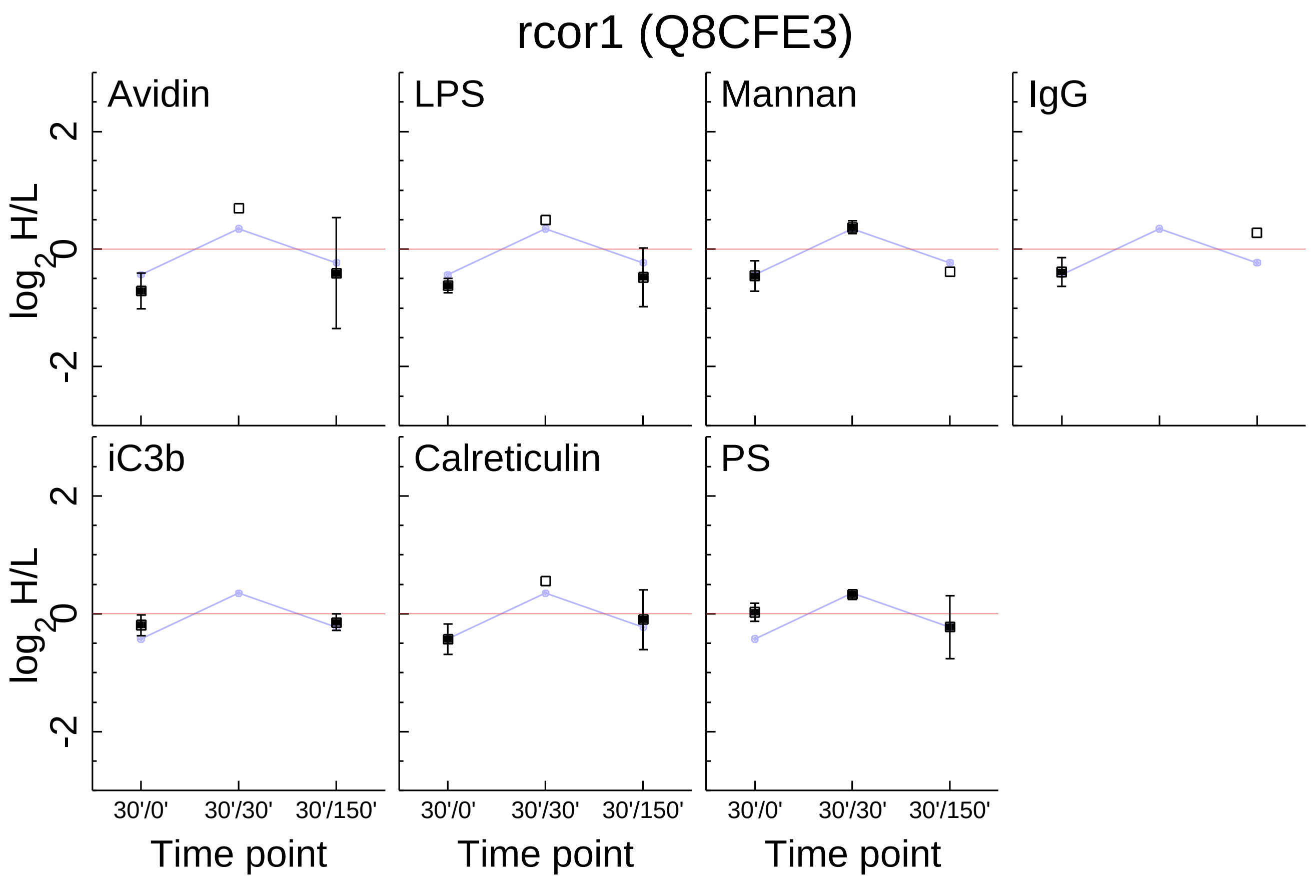

rcor1 (Q8CFE3)
Avidin
LPS
Mannan
IgG
iC3b
Calreticulin
PS
log2 H/L
2
0
-2
30'/0'
30'/30'
30'/150'
Time point

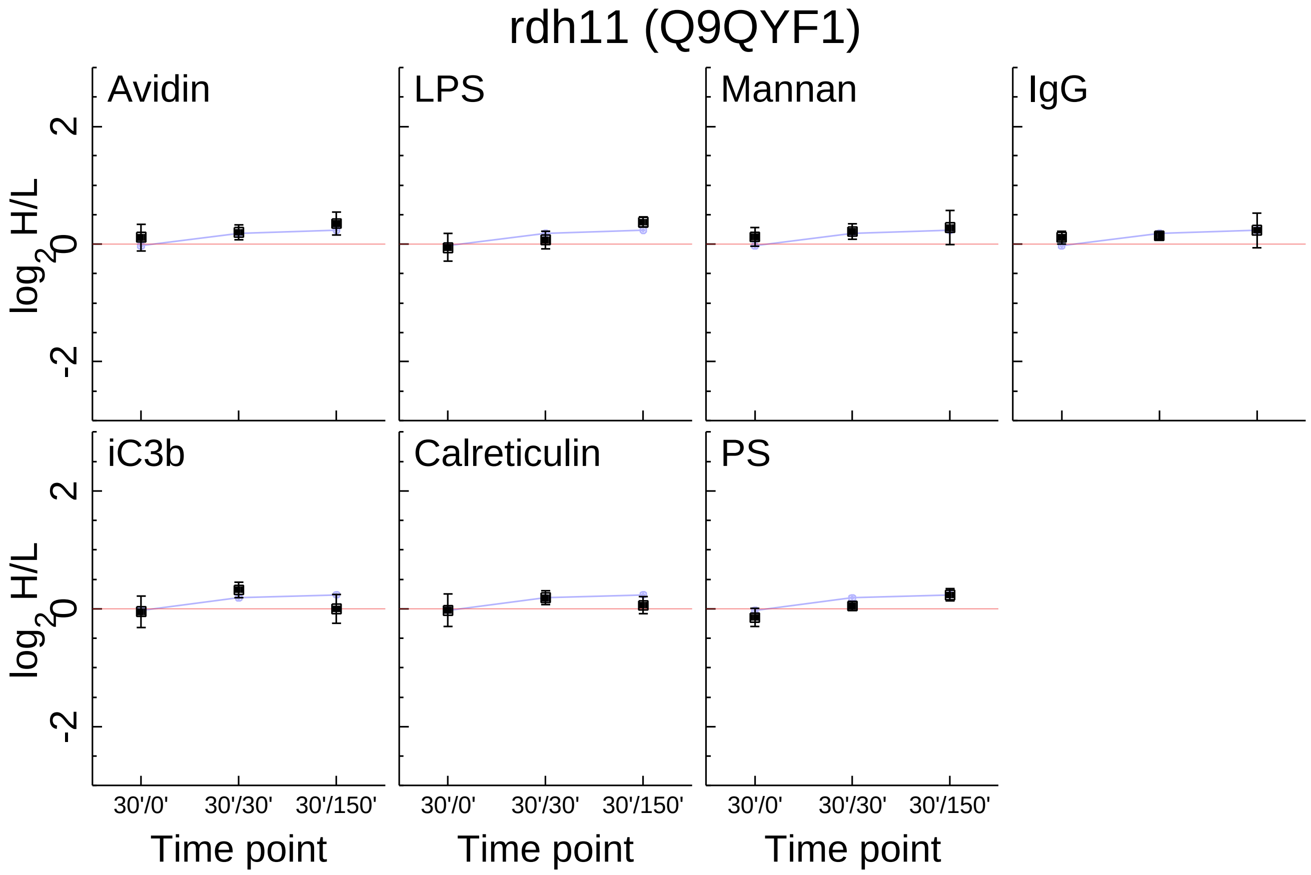
rdh11 (Q9QYF1)
Avidin
LPS
Mannan
IgG
iC3b
Calreticulin
PS
log2 H/L
2
0
-2
30'/0'
30'/30'
30'/150'
Time point

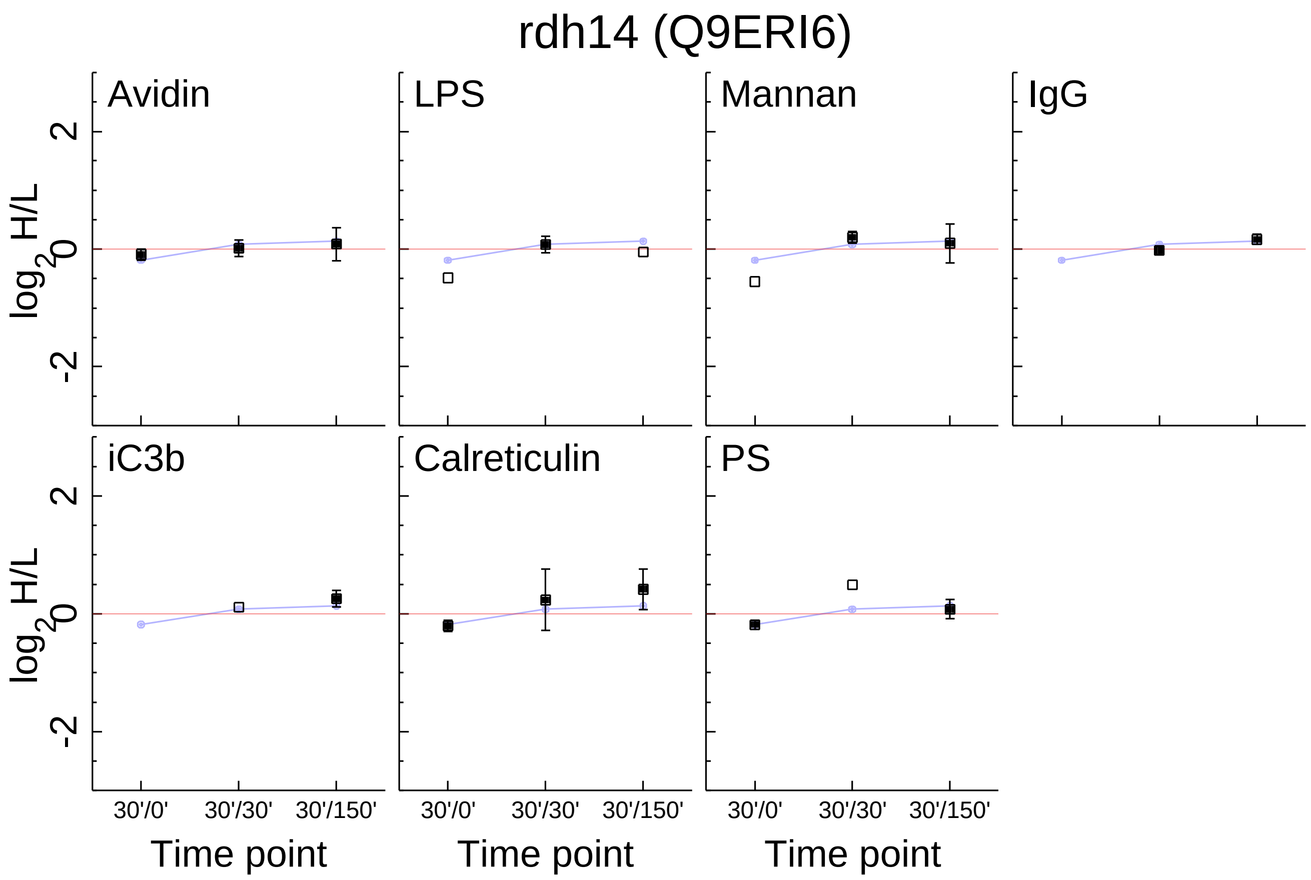
rdh14 (Q9ERI6)
Avidin
LPS
Mannan
IgG
iC3b
Calreticulin
PS
$\log_2$ H/L
2
0
-2
30'/0'
30'/30'
30'/150'
Time point

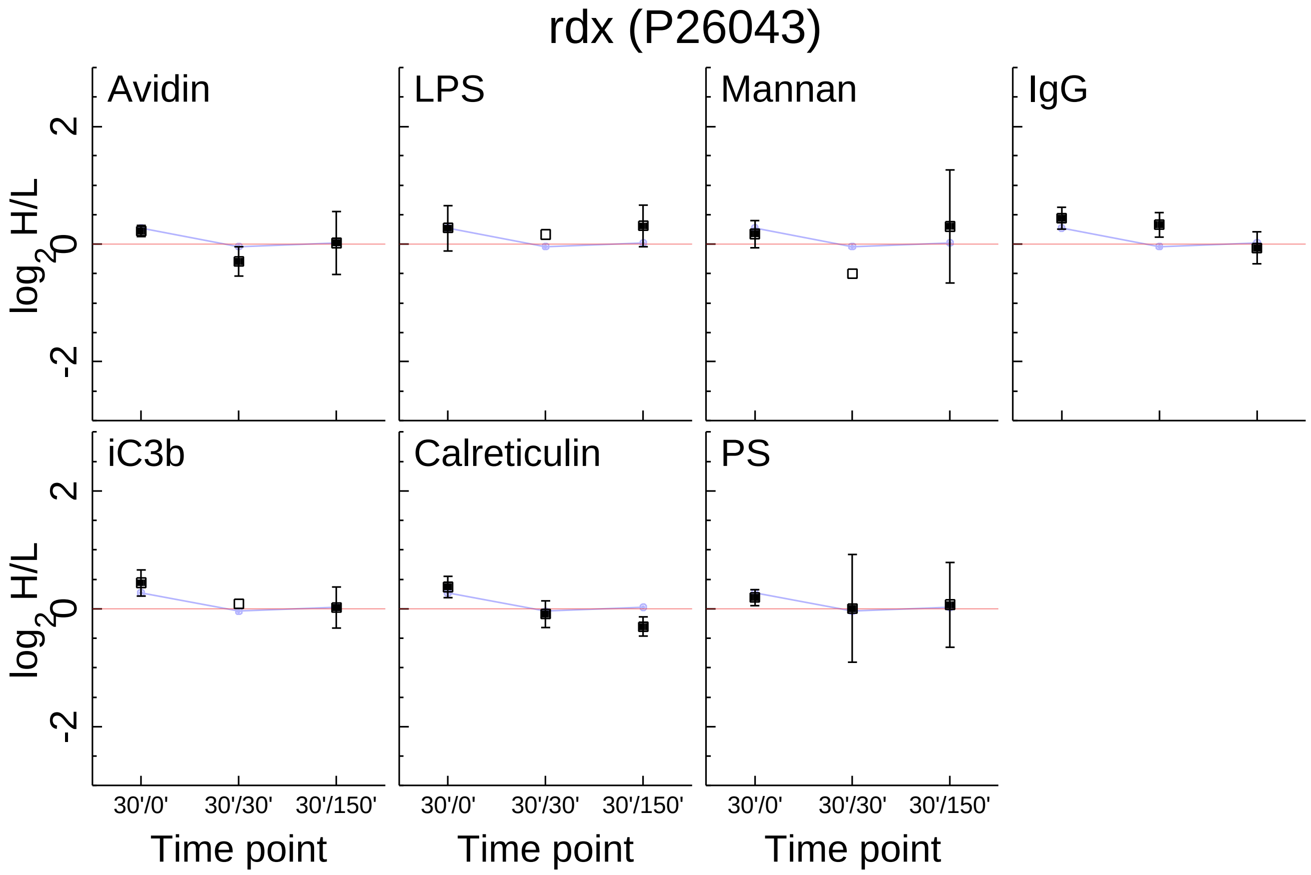
rdx (P26043)
Avidin
LPS
Mannan
IgG
iC3b
Calreticulin
PS
log2 H/L
2
0
-2
30'/0'
30'/30'
30'/150'
Time point

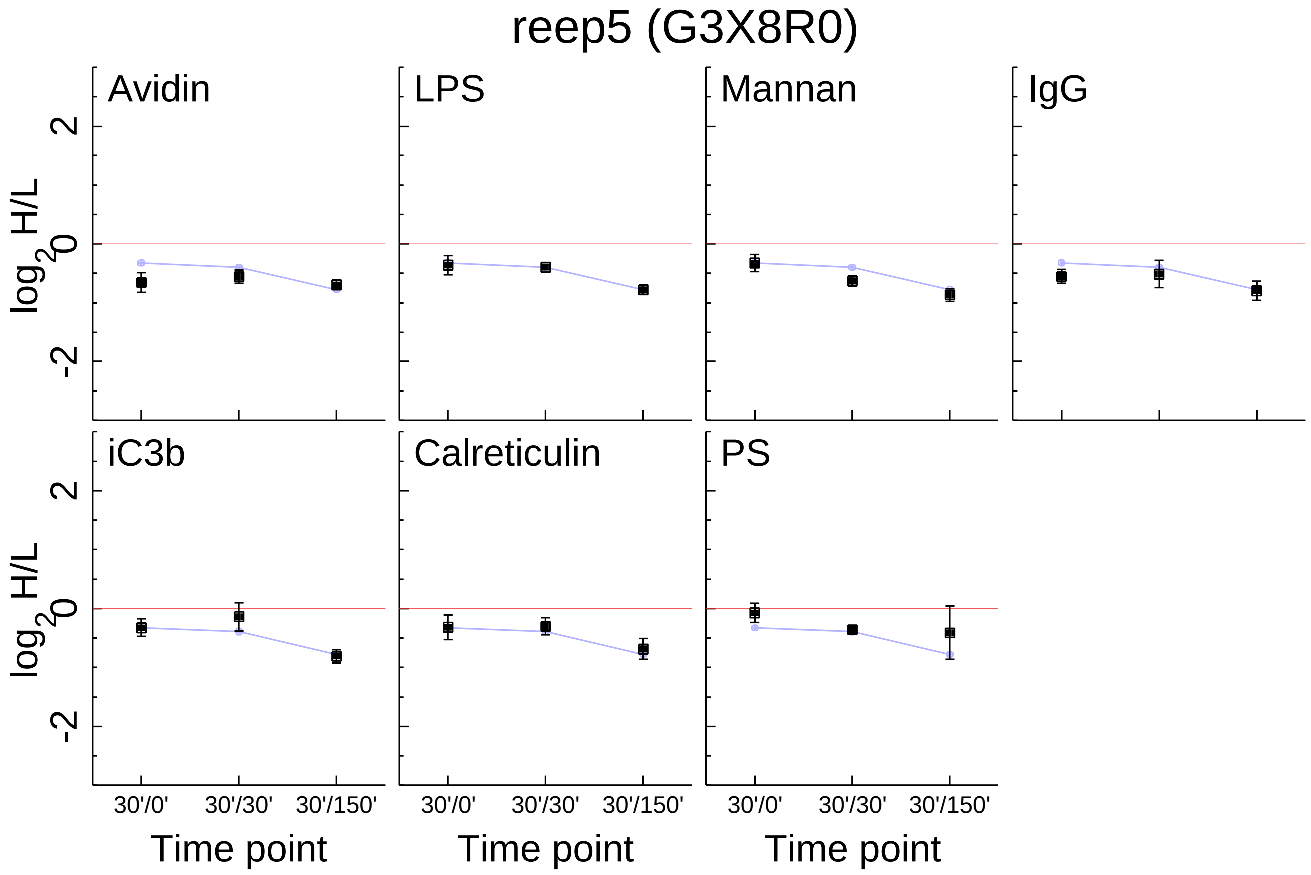
reep5 (G3X8R0)
Avidin
LPS
Mannan
IgG
iC3b
Calreticulin
PS
$\log_2$ H/L
2
0
-2
30'/0'
30'/30'
30'/150'
Time point

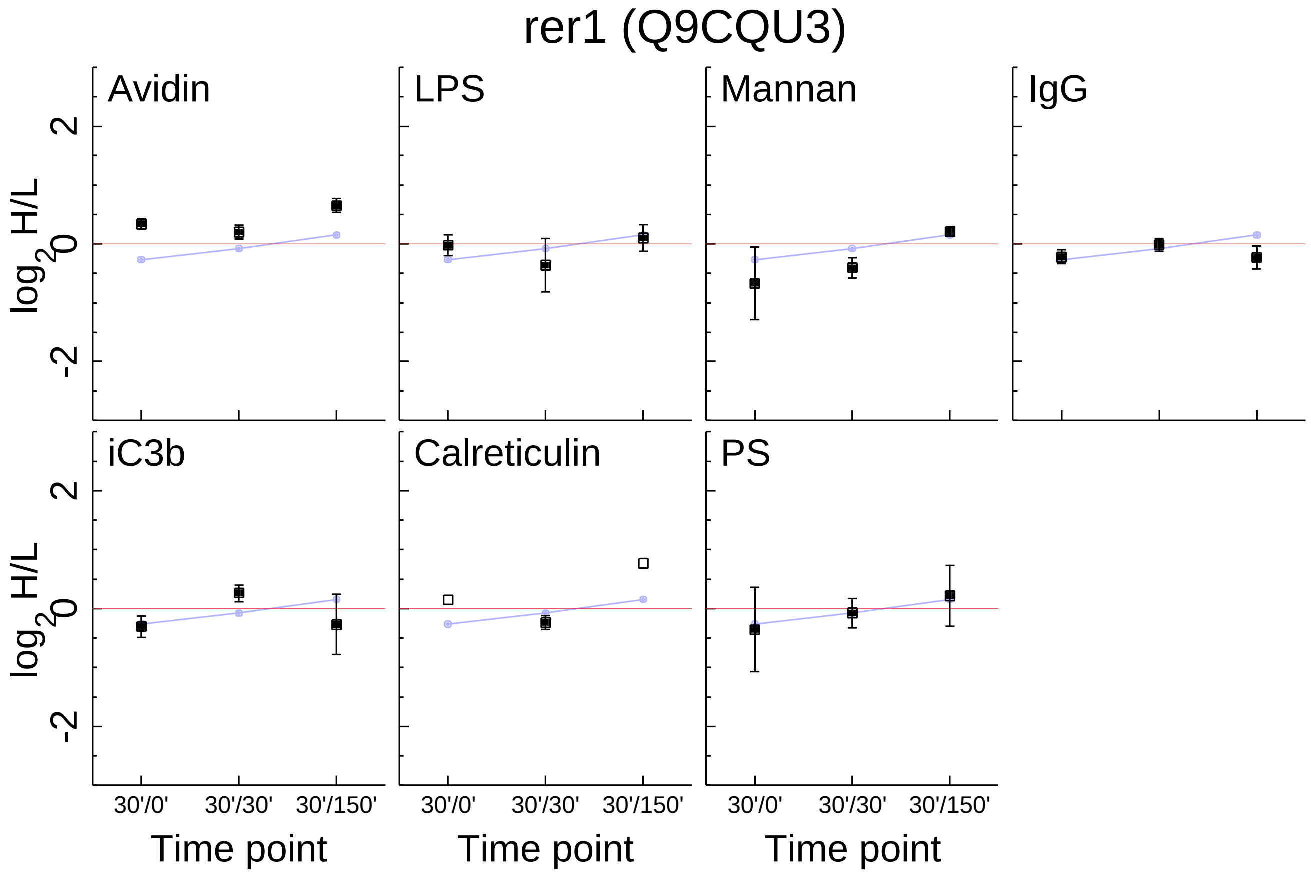
rer1 (Q9CQU3)
Avidin
LPS
Mannan
IgG
iC3b
Calreticulin
PS
log2 H/L
2
0
-2
30'/0'
30'/30'
30'/150'
Time point

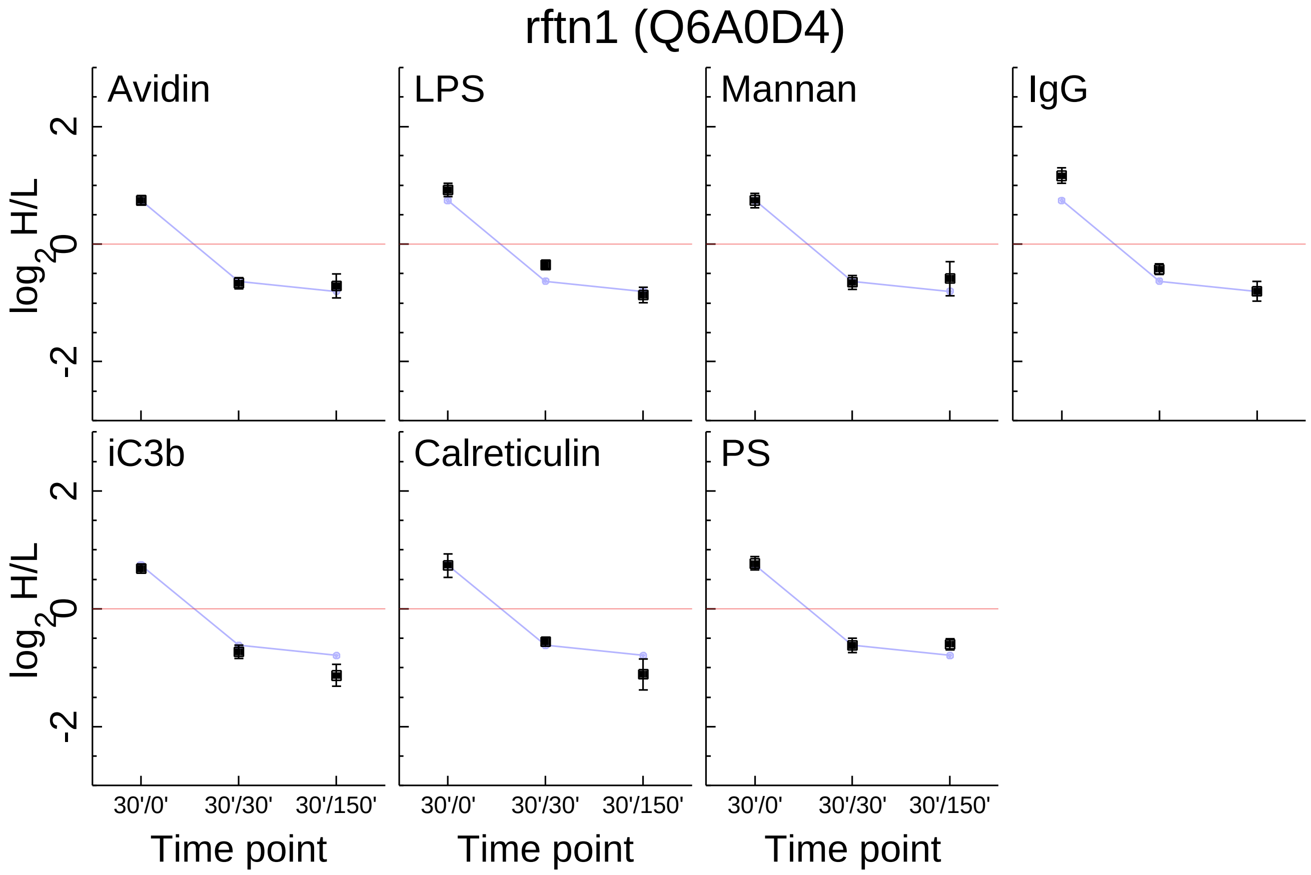
rftn1 (Q6A0D4)
Avidin
LPS
Mannan
IgG
iC3b
Calreticulin
PS
log2 H/L
2
0
-2
30'/0'
30'/30'
30'/150'
Time point

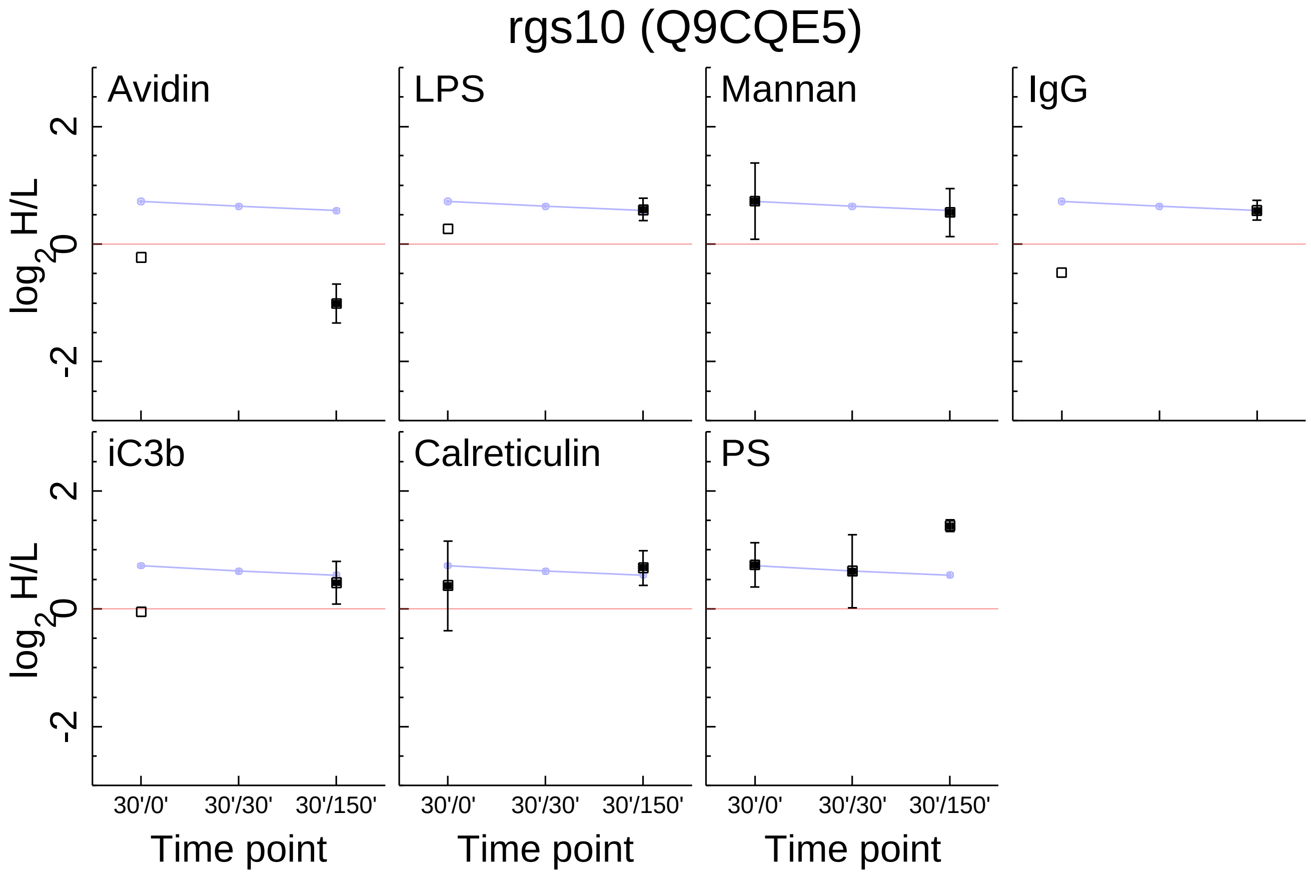
rgs10 (Q9CQE5)
Avidin
LPS
Mannan
IgG
iC3b
Calreticulin
PS
log2 H/L
2
0
-2
30'/0'
30'/30'
30'/150'
Time point

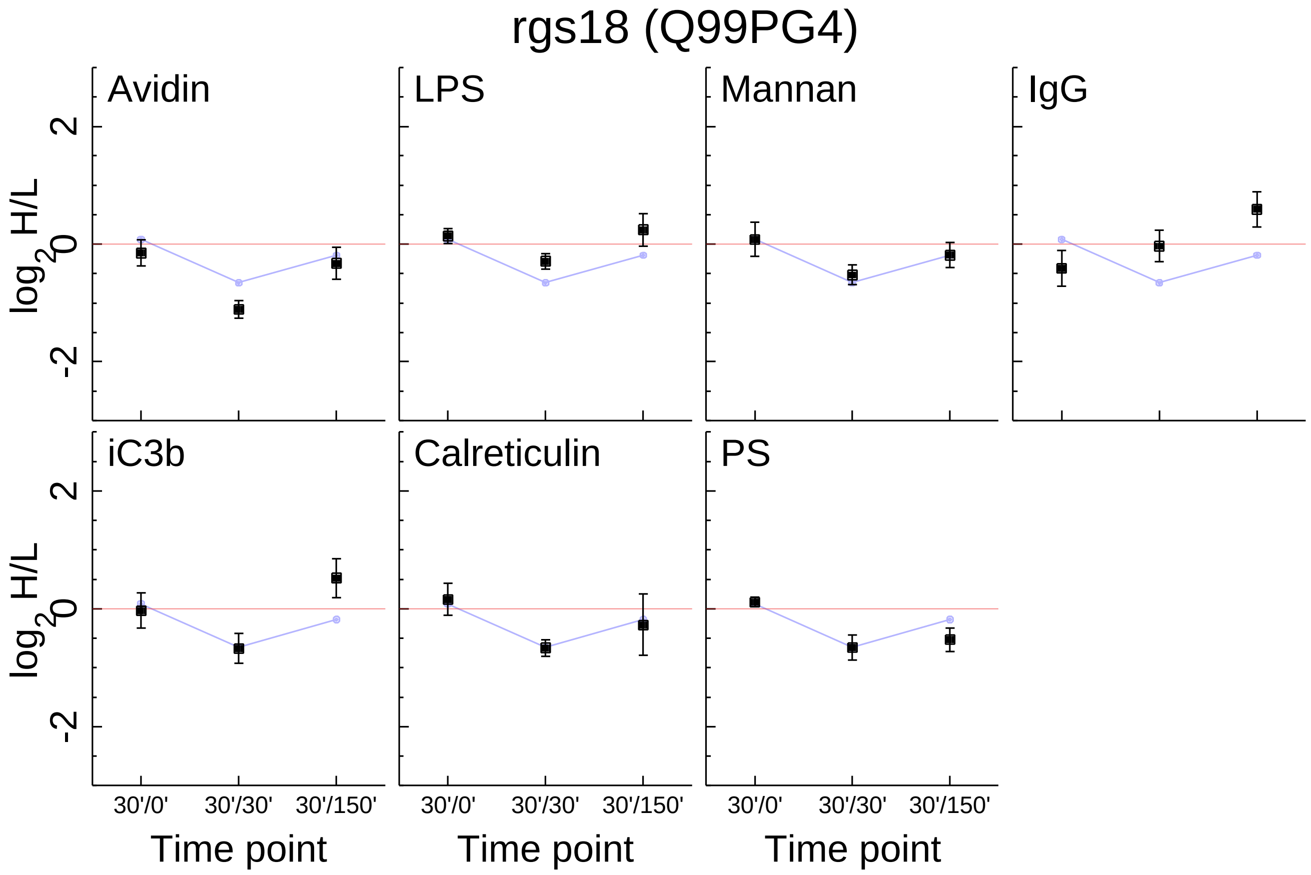

rgs18 (Q99PG4)
Avidin
LPS
Mannan
IgG
iC3b
Calreticulin
PS
log2 H/L
2
0
-2
30'/0'
30'/30'
30'/150'
Time point

rgs19 (B7ZCT1)

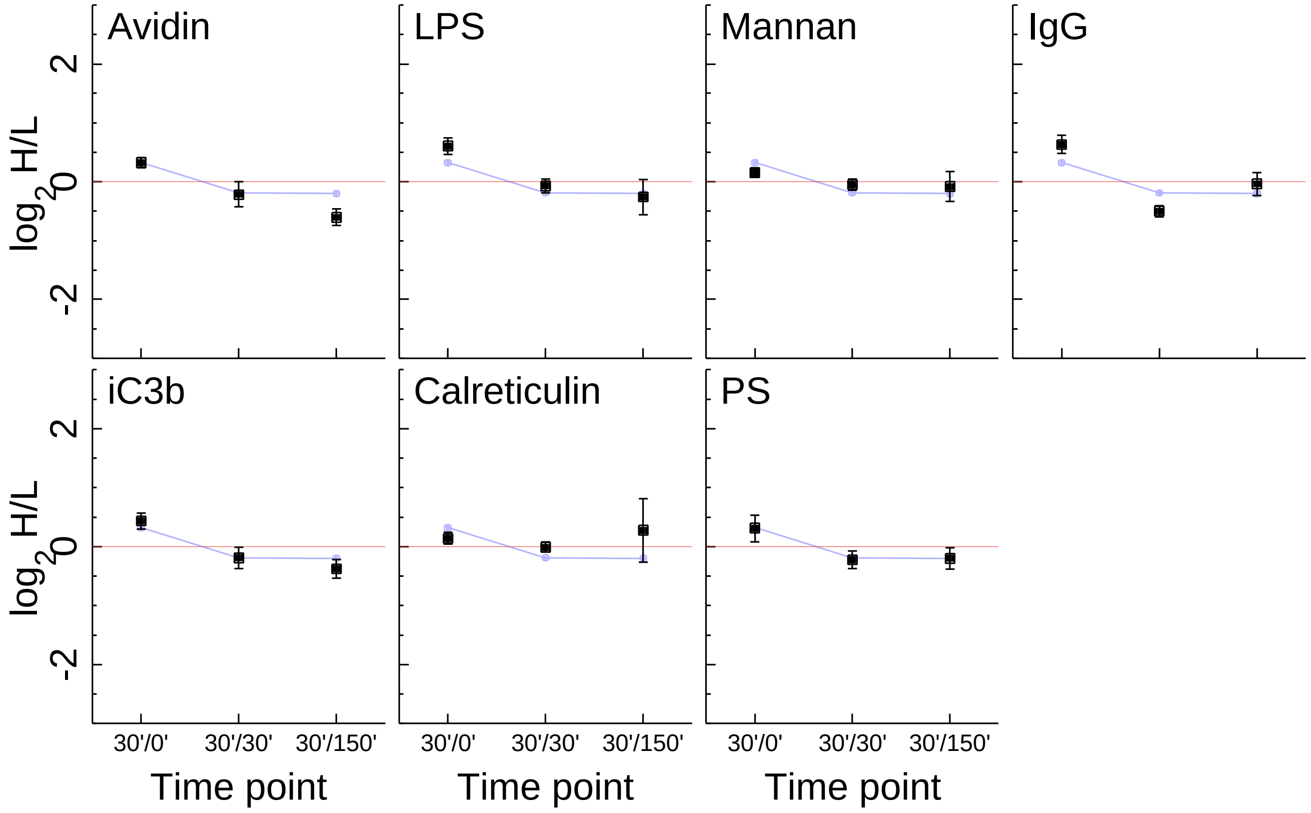

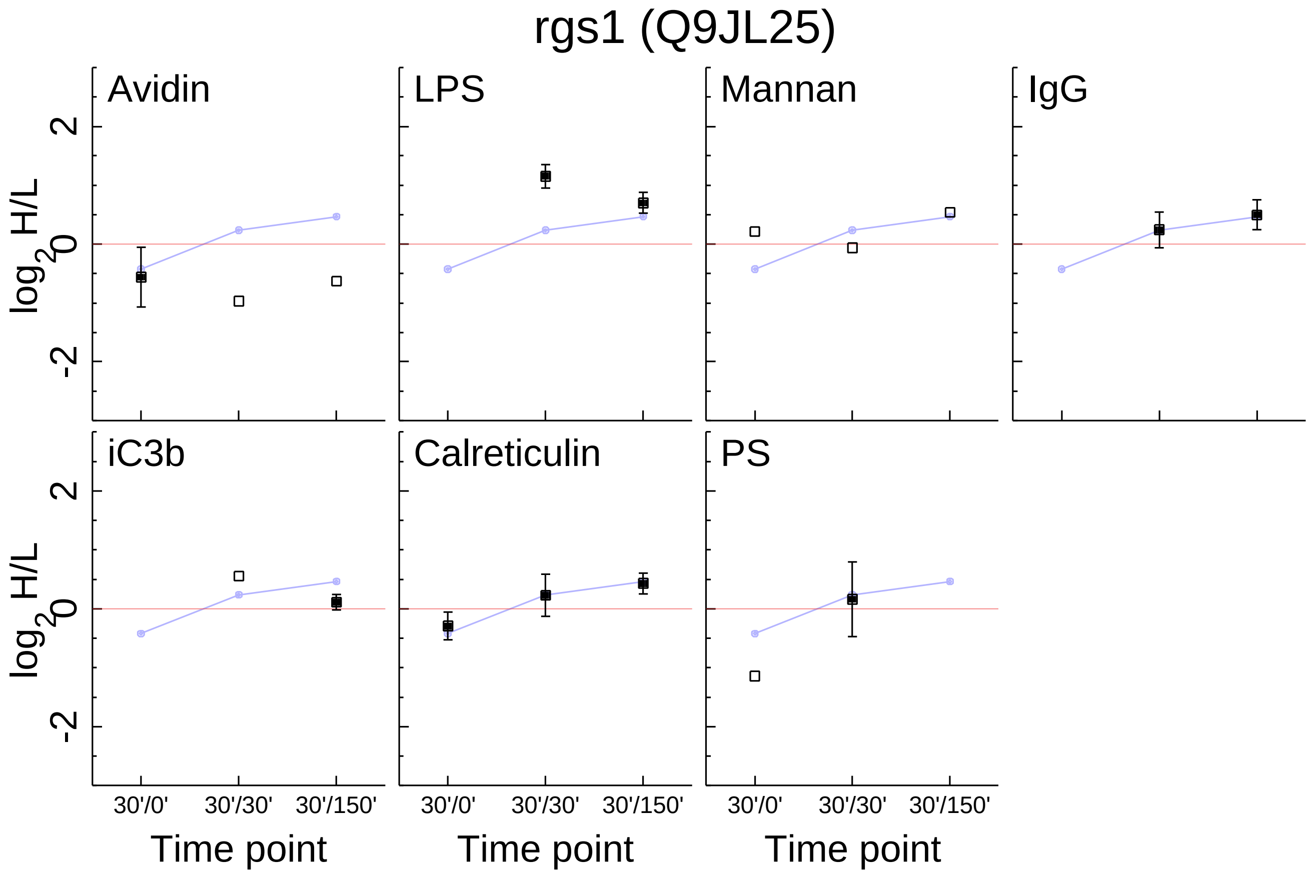
rgs1 (Q9JL25)
Avidin
LPS
Mannan
IgG
iC3b
Calreticulin
PS
log2 H/L
2
0
-2
30'/0'
30'/30'
30'/150'
Time point

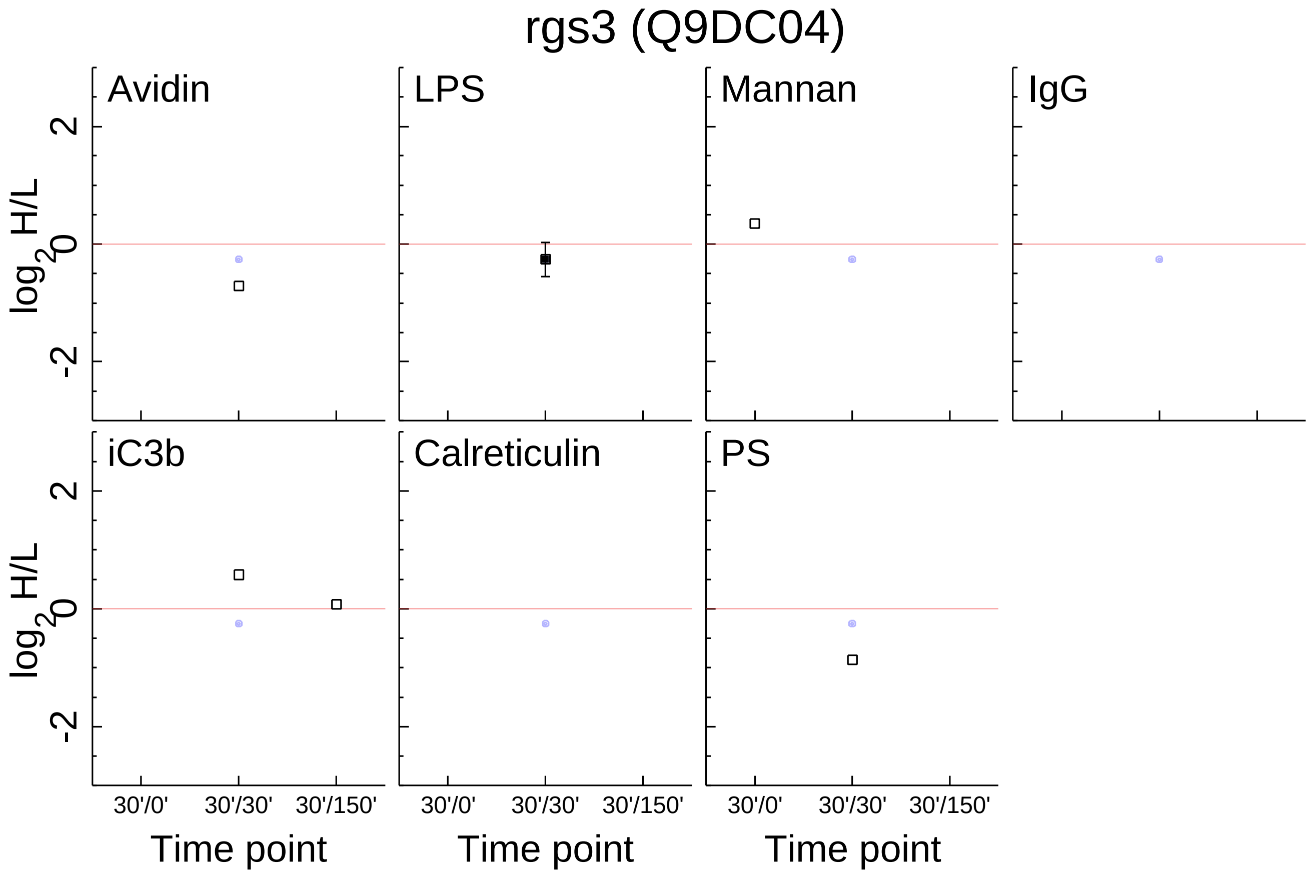
rgs3 (Q9DC04)
Avidin
LPS
Mannan
IgG
iC3b
Calreticulin
PS
log2 H/L
2
0
-2
30'/0'
30'/30'
30'/150'
Time point

rhbdf2 (Q80WQ6)

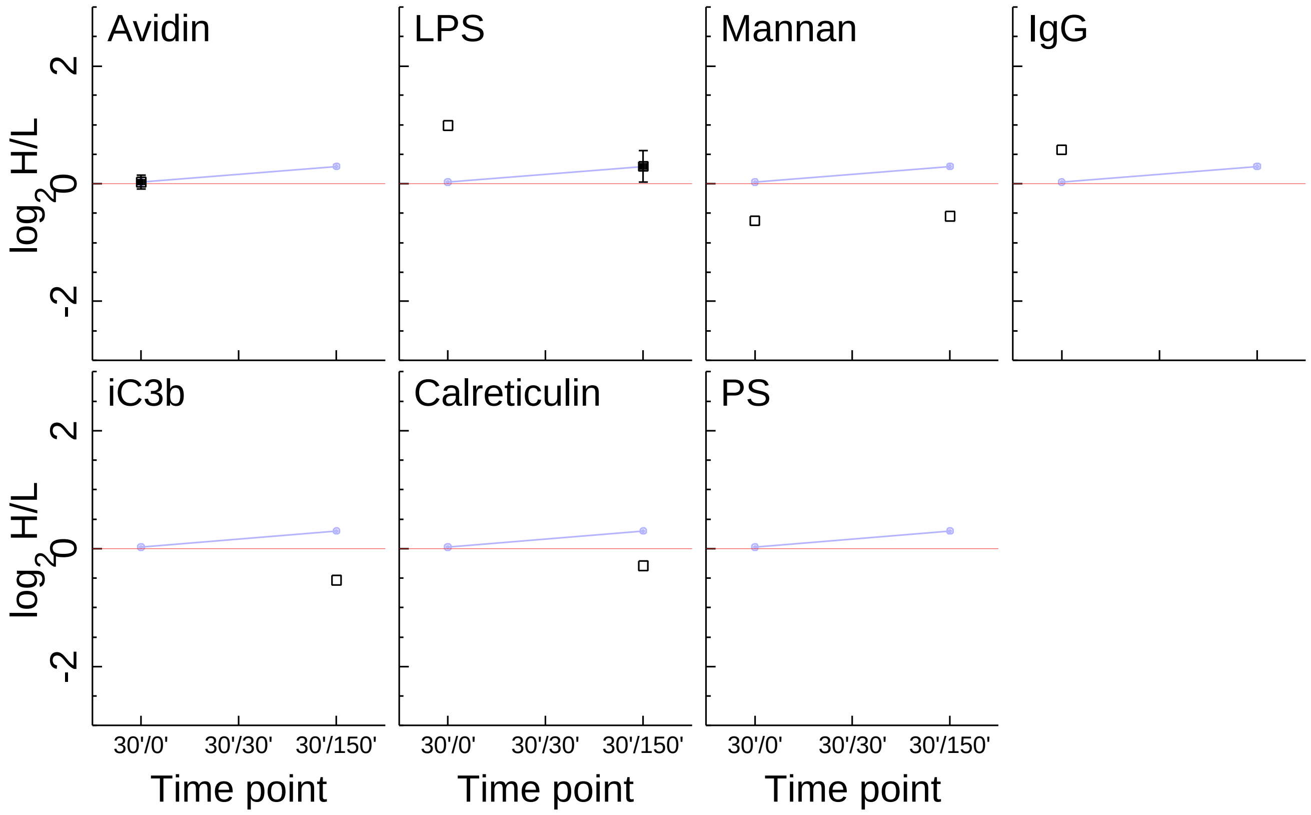

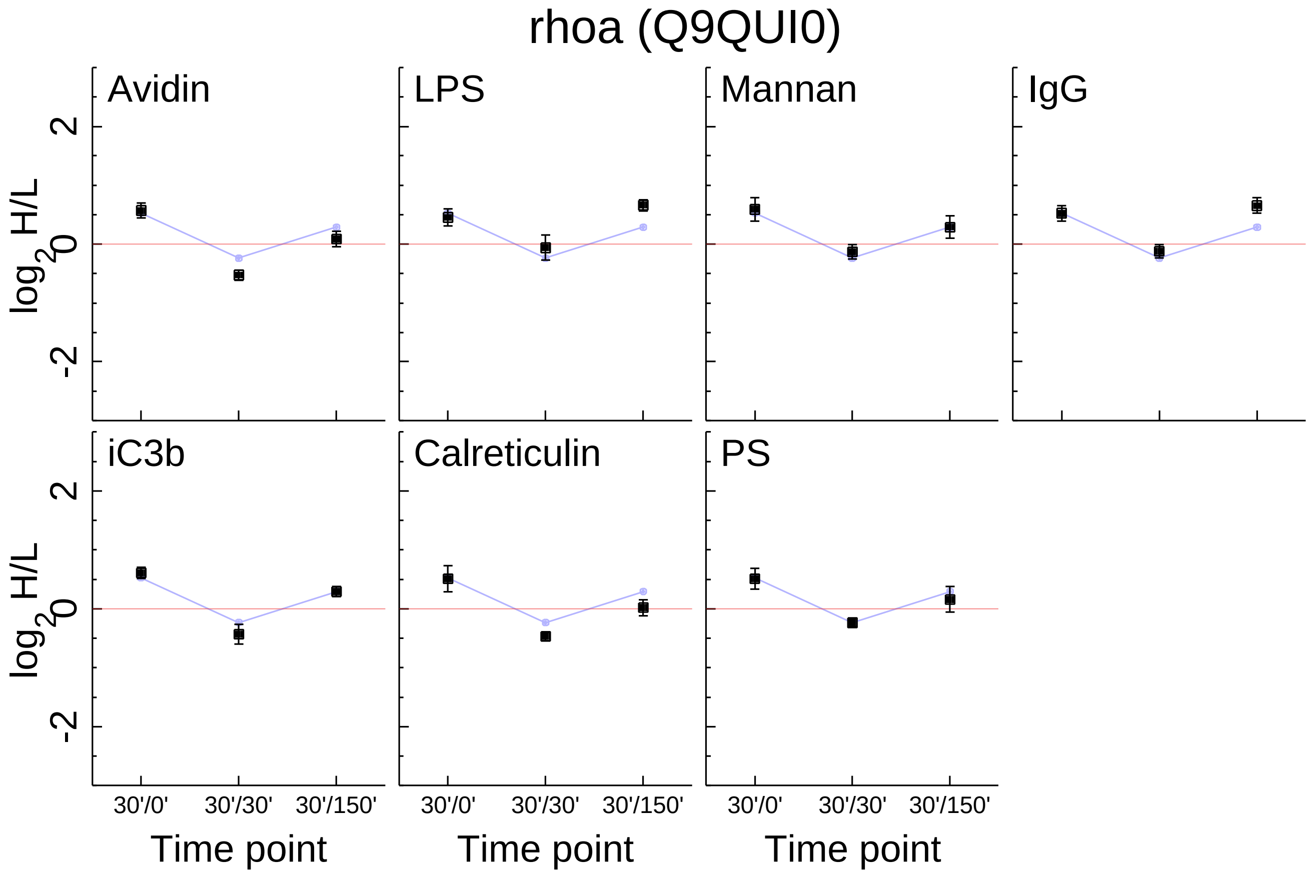
rhoa (Q9QUI0)
Avidin
LPS
Mannan
IgG
iC3b
Calreticulin
PS
log2 H/L
2
0
-2
30'/0'
30'/30'
30'/150'
Time point

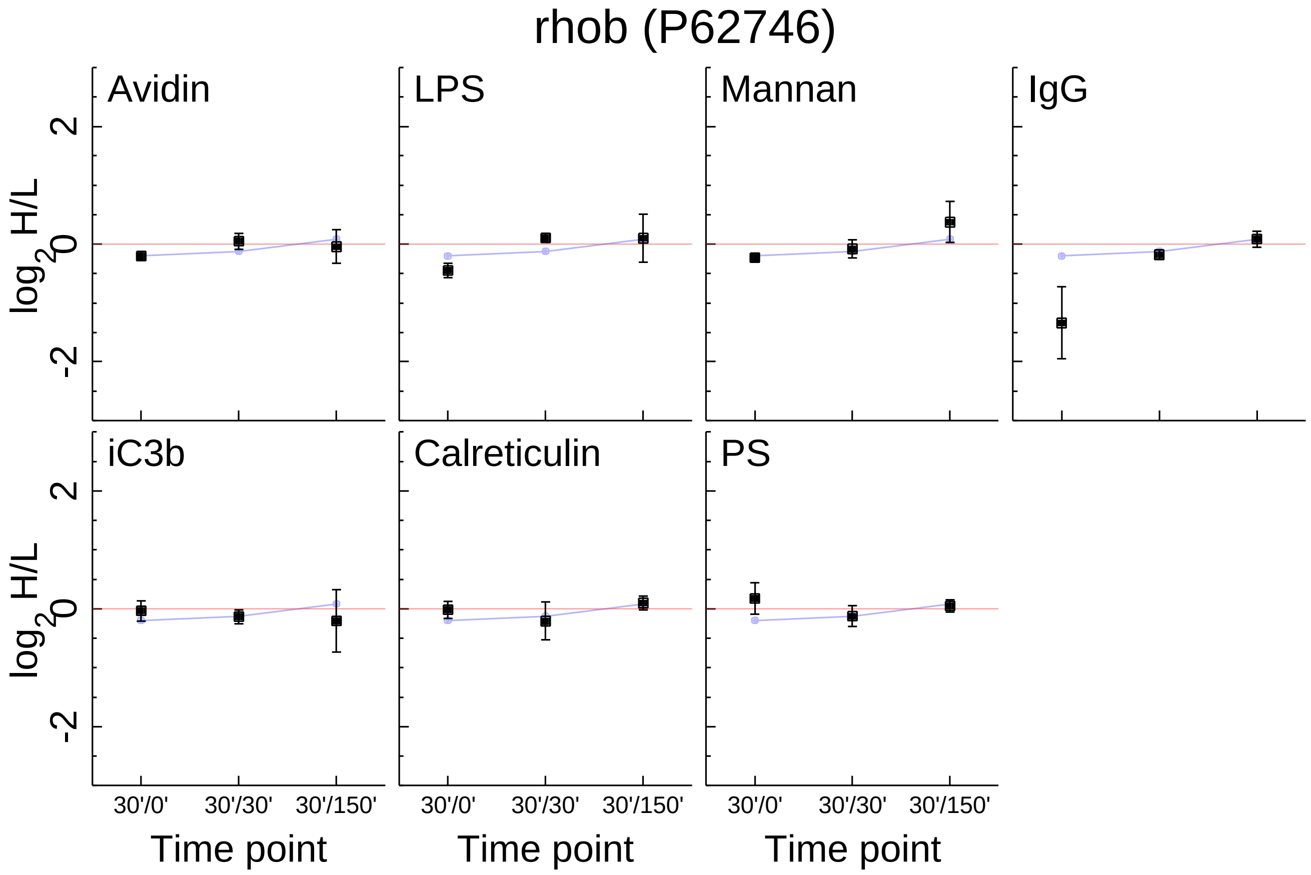

rhob (P62746)
Avidin
LPS
Mannan
IgG
iC3b
Calreticulin
PS
log2 H/L
2
0
-2
30'/0'
30'/30'
30'/150'
Time point

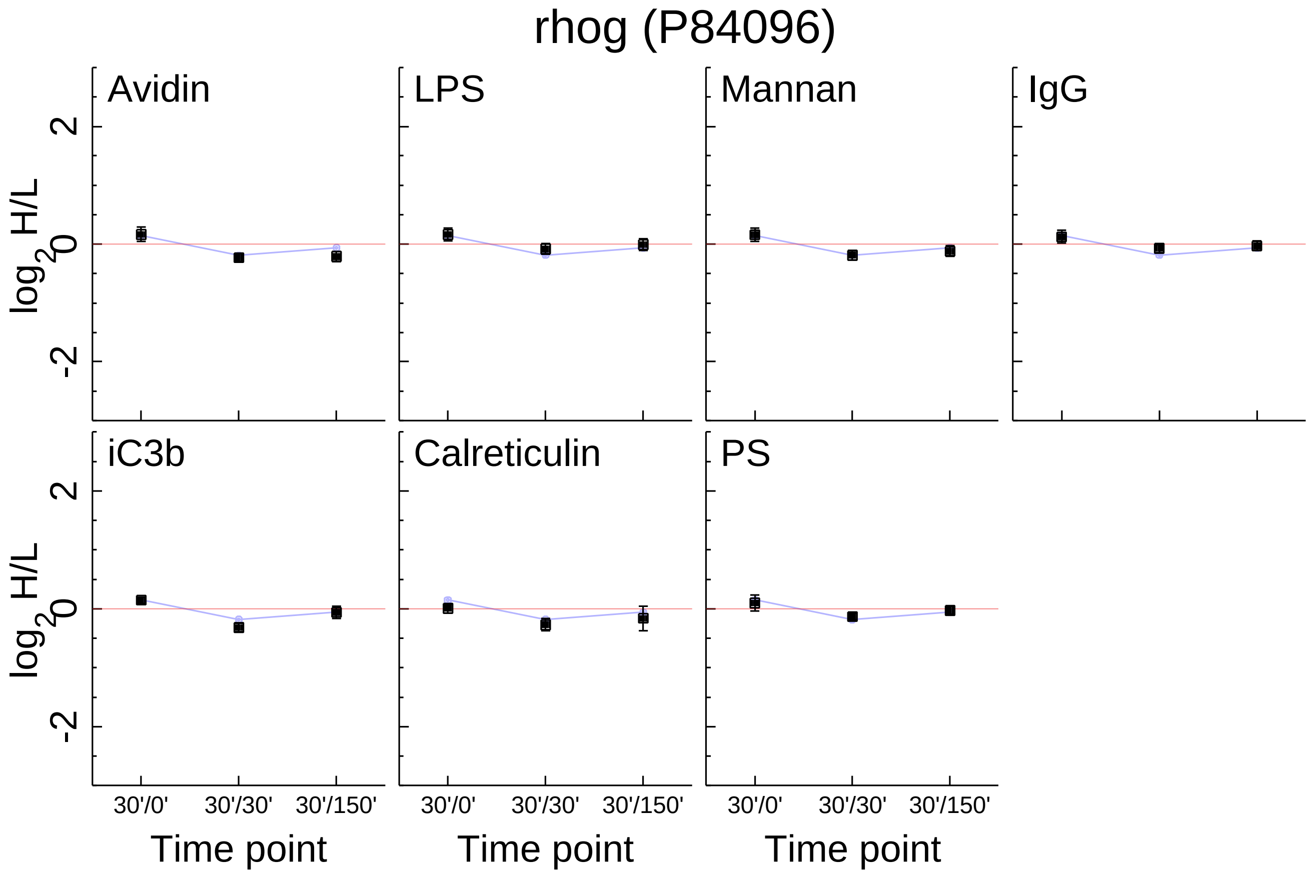

rhog (P84096)
Avidin
LPS
Mannan
IgG
iC3b
Calreticulin
PS
log2 H/L
2
0
-2
30'/0'
30'/30'
30'/150'
Time point

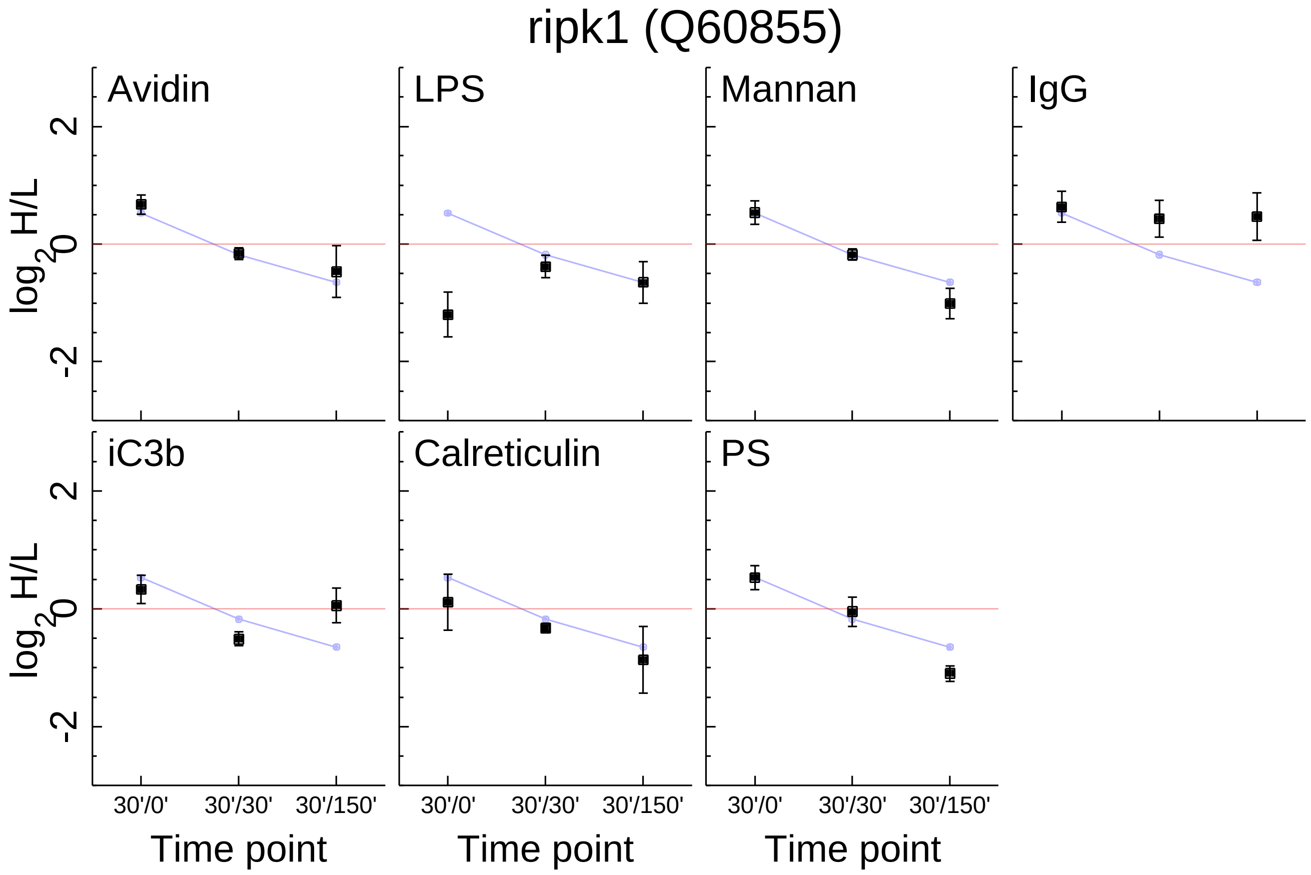

ripk1 (Q60855)
Avidin
LPS
Mannan
IgG
iC3b
Calreticulin
PS
log2 H/L
2
0
-2
30'/0'
30'/30'
30'/150'
Time point

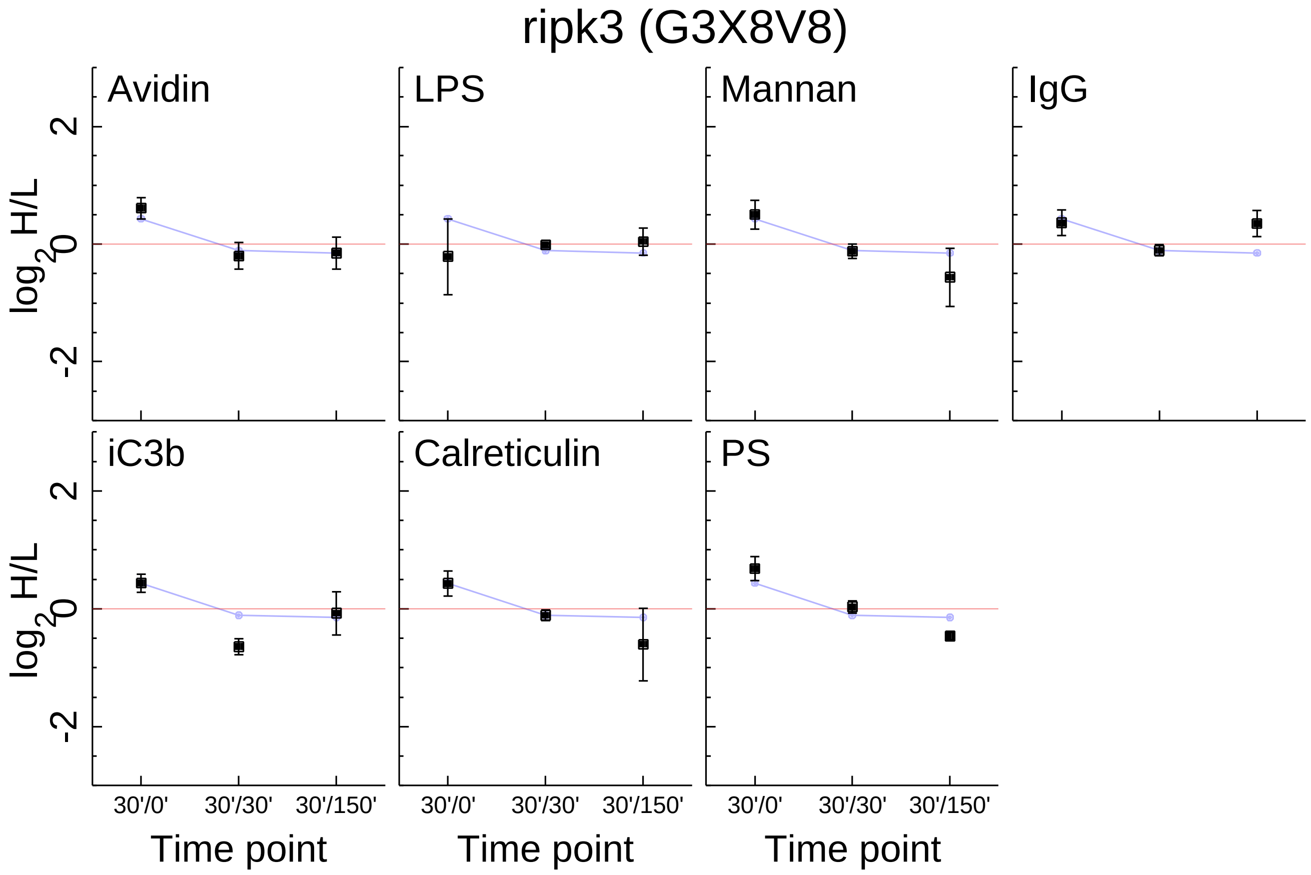

ripk3 (G3X8V8)
Avidin
LPS
Mannan
IgG
iC3b
Calreticulin
PS
log2 H/L
2
0
-2
30'/0'
30'/30'
30'/150'
Time point

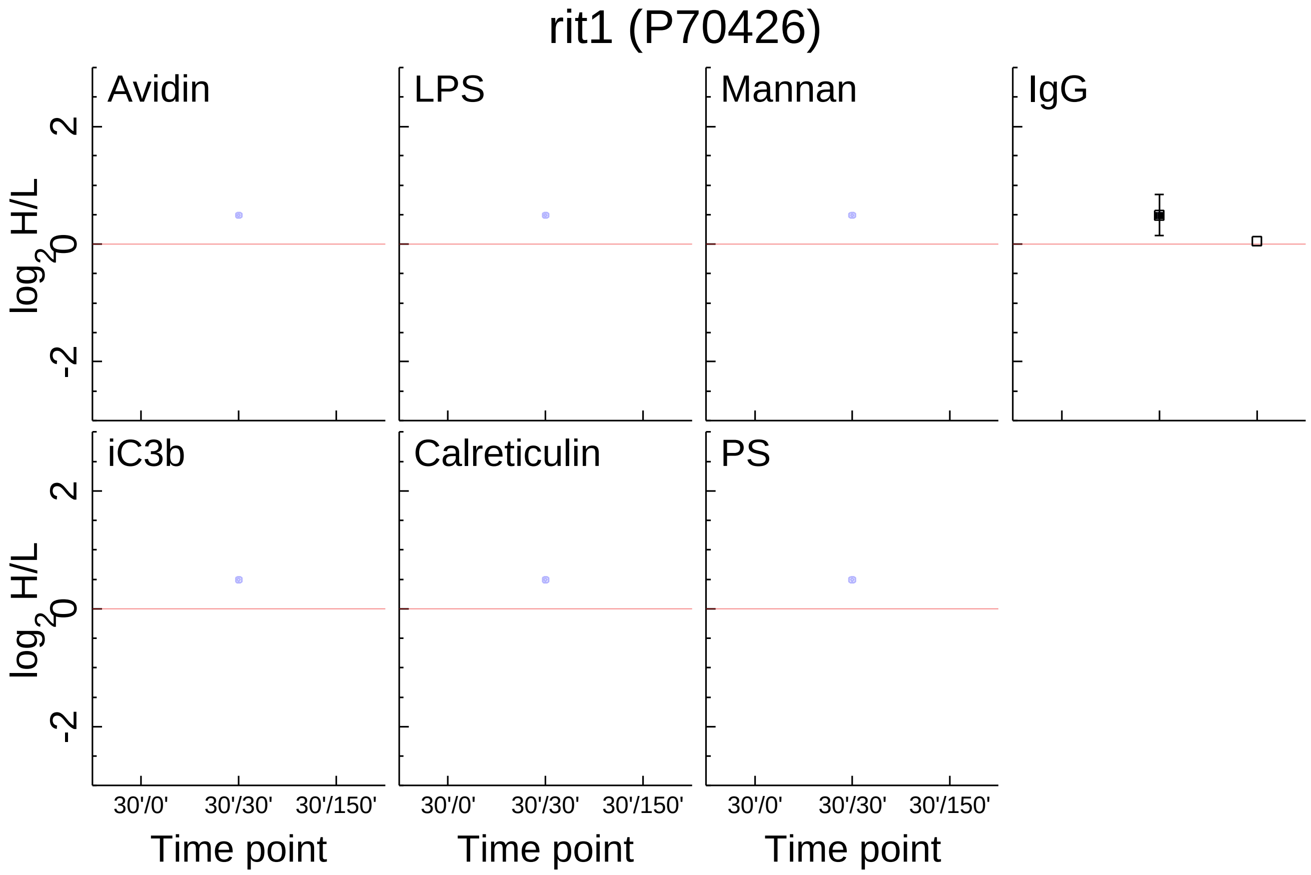
rit1 (P70426)
Avidin
LPS
Mannan
IgG
iC3b
Calreticulin
PS
log2 H/L
2
0
-2
30'/0'
30'/30'
30'/150'
Time point

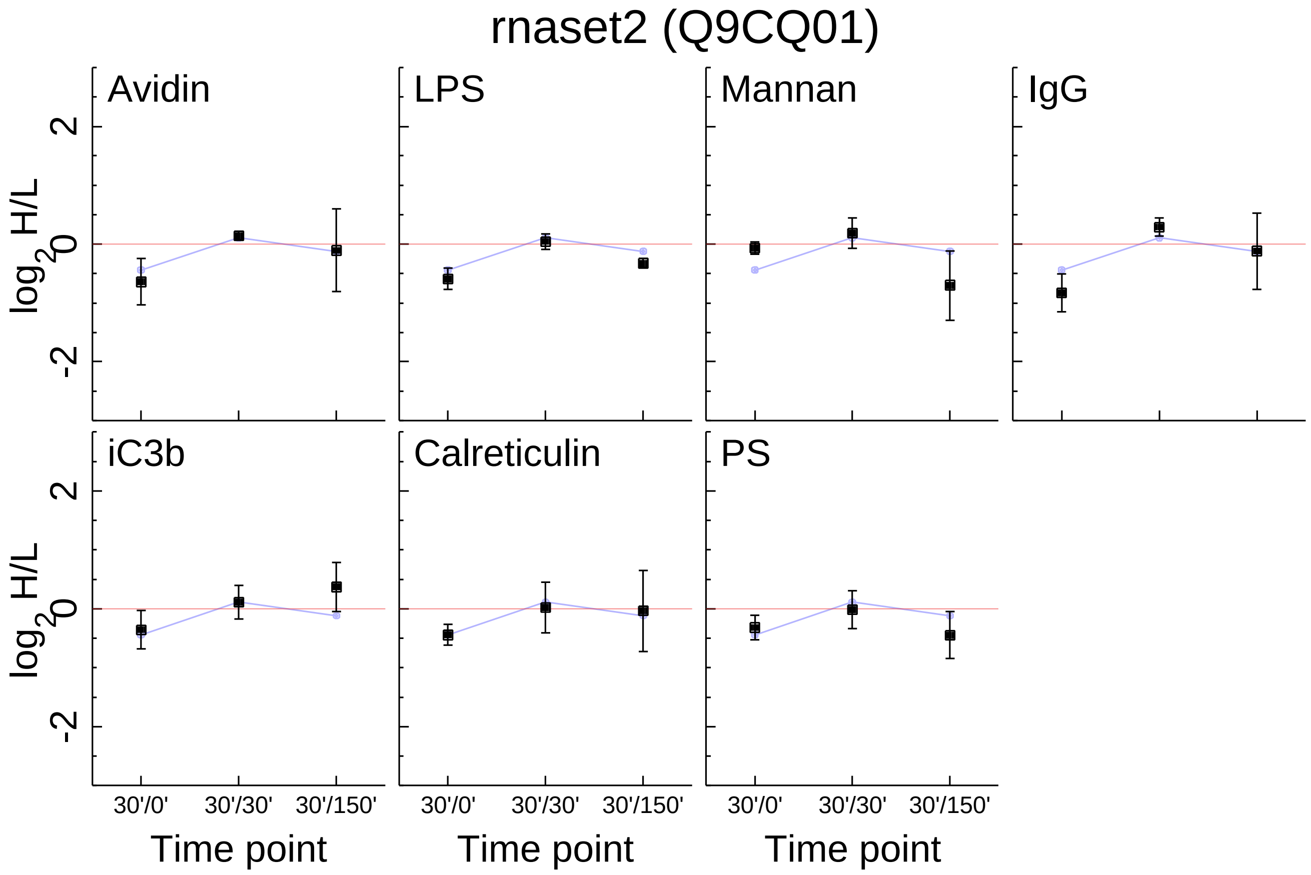

rnaset2 (Q9CQ01)
Avidin
LPS
Mannan
IgG
iC3b
Calreticulin
PS
log2 H/L
2
0
-2
30'/0'
30'/30'
30'/150'
Time point

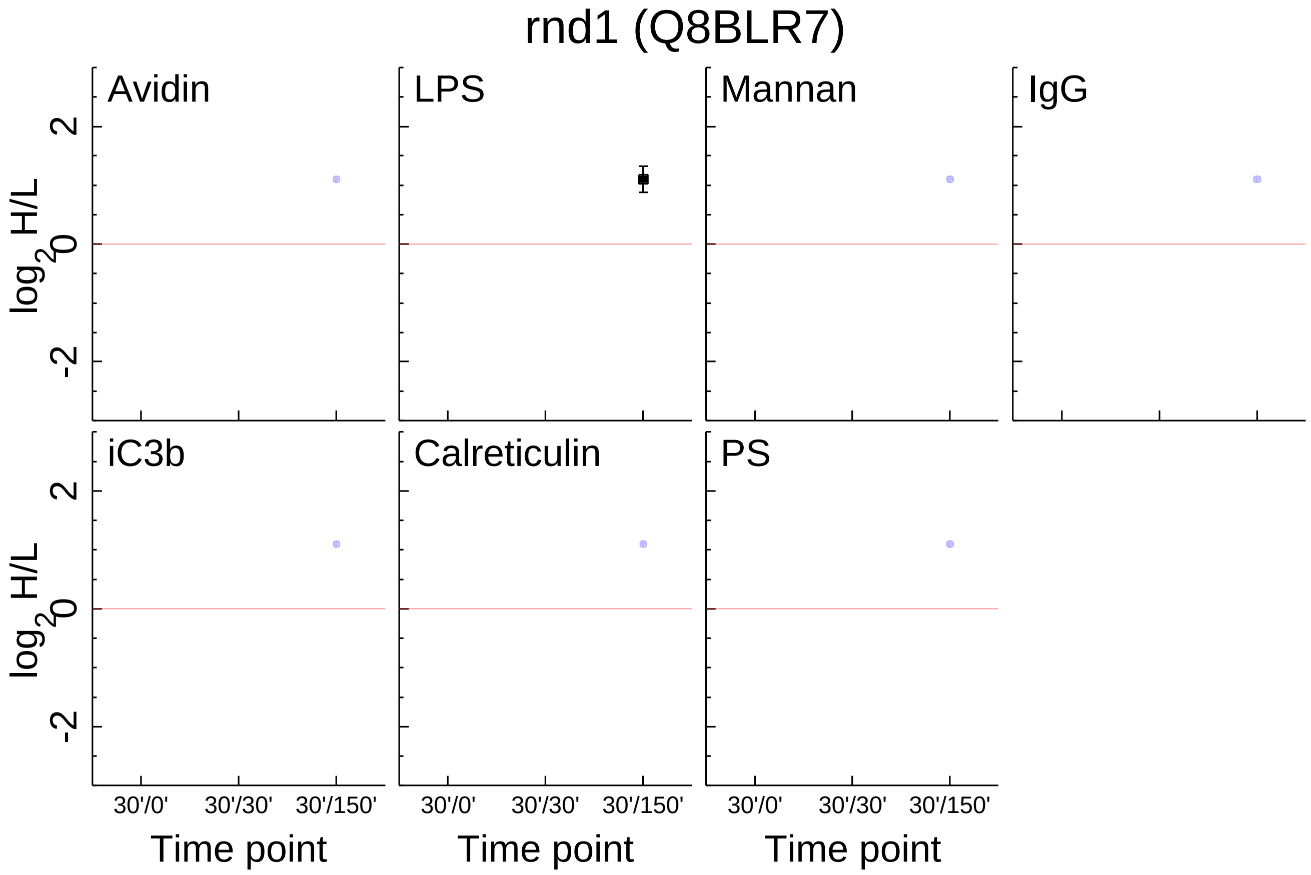

rnd1 (Q8BLR7)
Avidin
LPS
Mannan
IgG
iC3b
Calreticulin
PS
log2 H/L
2
0
-2
30'/0'
30'/30'
30'/150'
Time point

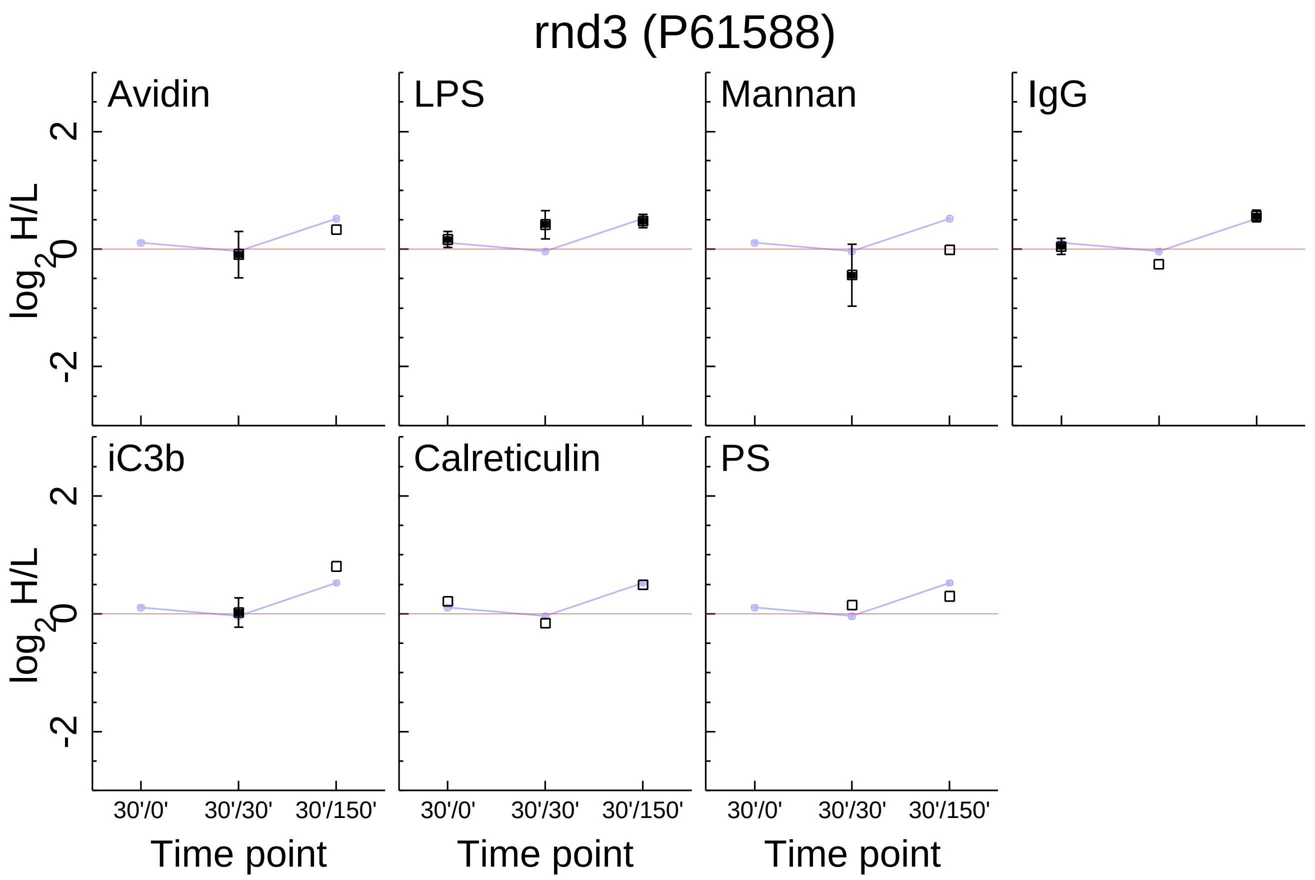

rnd3 (P61588)
Avidin
LPS
Mannan
IgG
iC3b
Calreticulin
PS
log2 H/L
2
0
-2
30'/0'
30'/30'
30'/150'
Time point

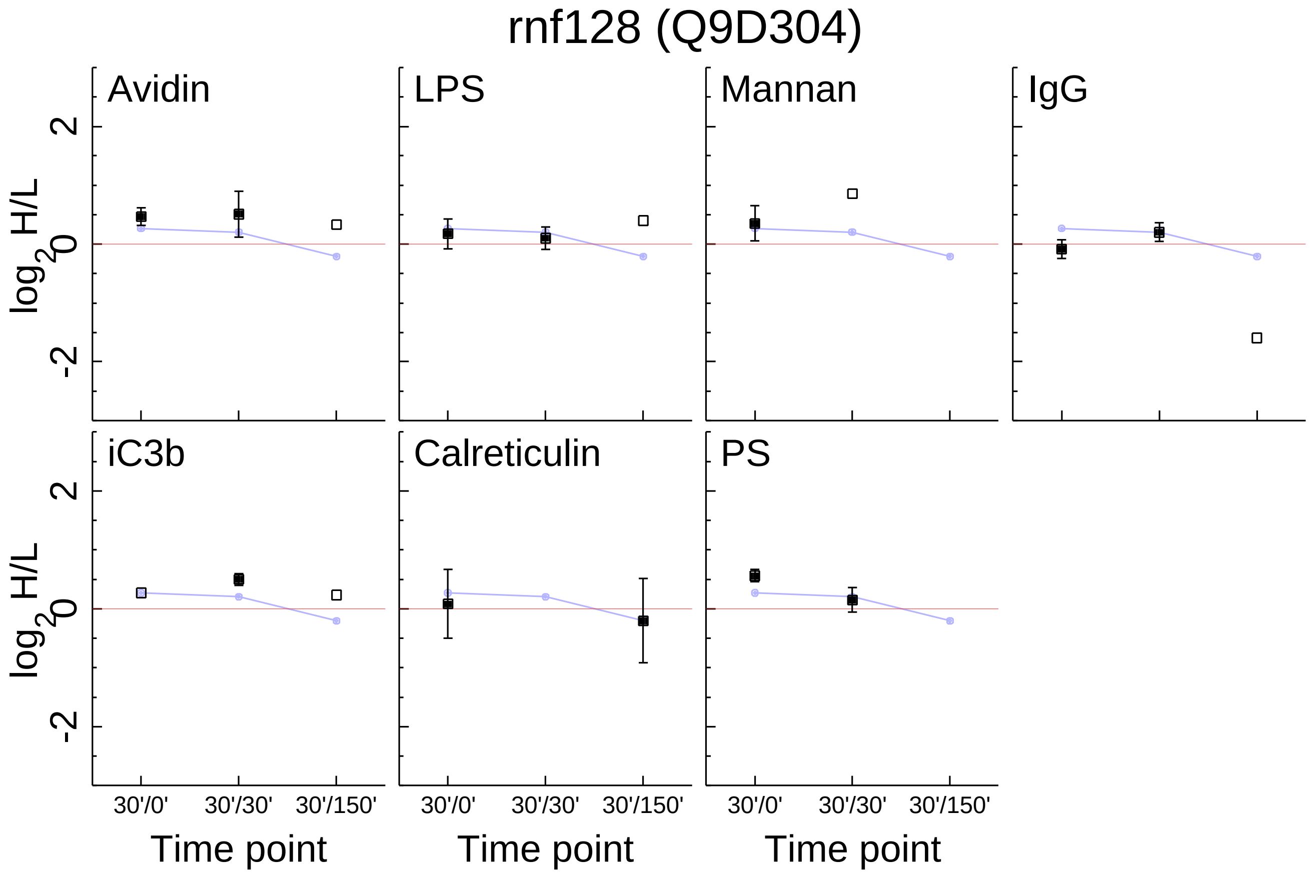

rnf128 (Q9D304)
Avidin
LPS
Mannan
IgG
iC3b
Calreticulin
PS
log2 H/L
2
0
-2
30'/0'
30'/30'
30'/150'
Time point

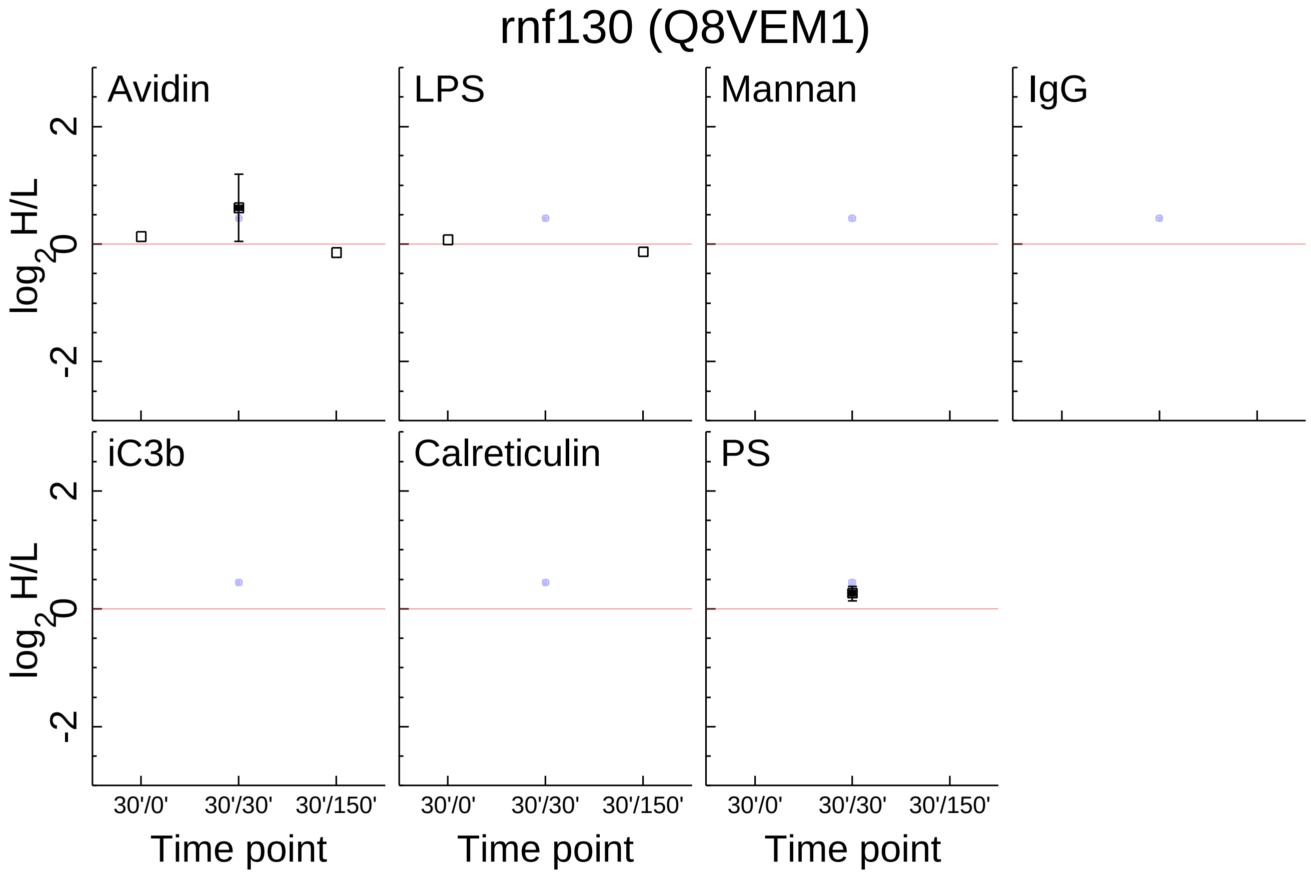

rnf130 (Q8VEM1)
Avidin
LPS
Mannan
IgG
iC3b
Calreticulin
PS
log2 H/L
2
0
-2
30'/0'
30'/30'
30'/150'
Time point

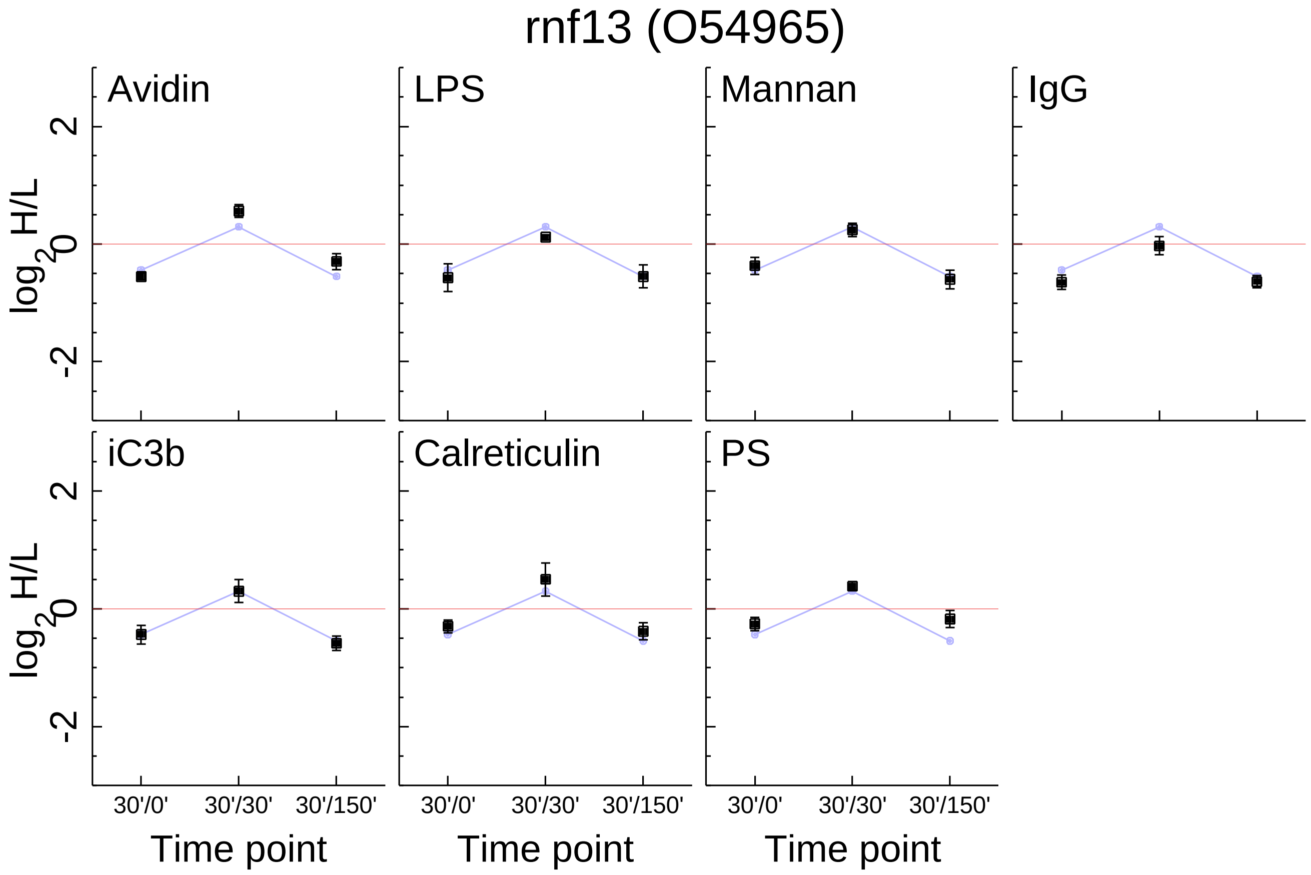

rnf13 (O54965)
Avidin
LPS
Mannan
IgG
iC3b
Calreticulin
PS
log2 H/L
2
0
-2
30'/0'
30'/30'
30'/150'
Time point

rnf213 (F7A6H4)

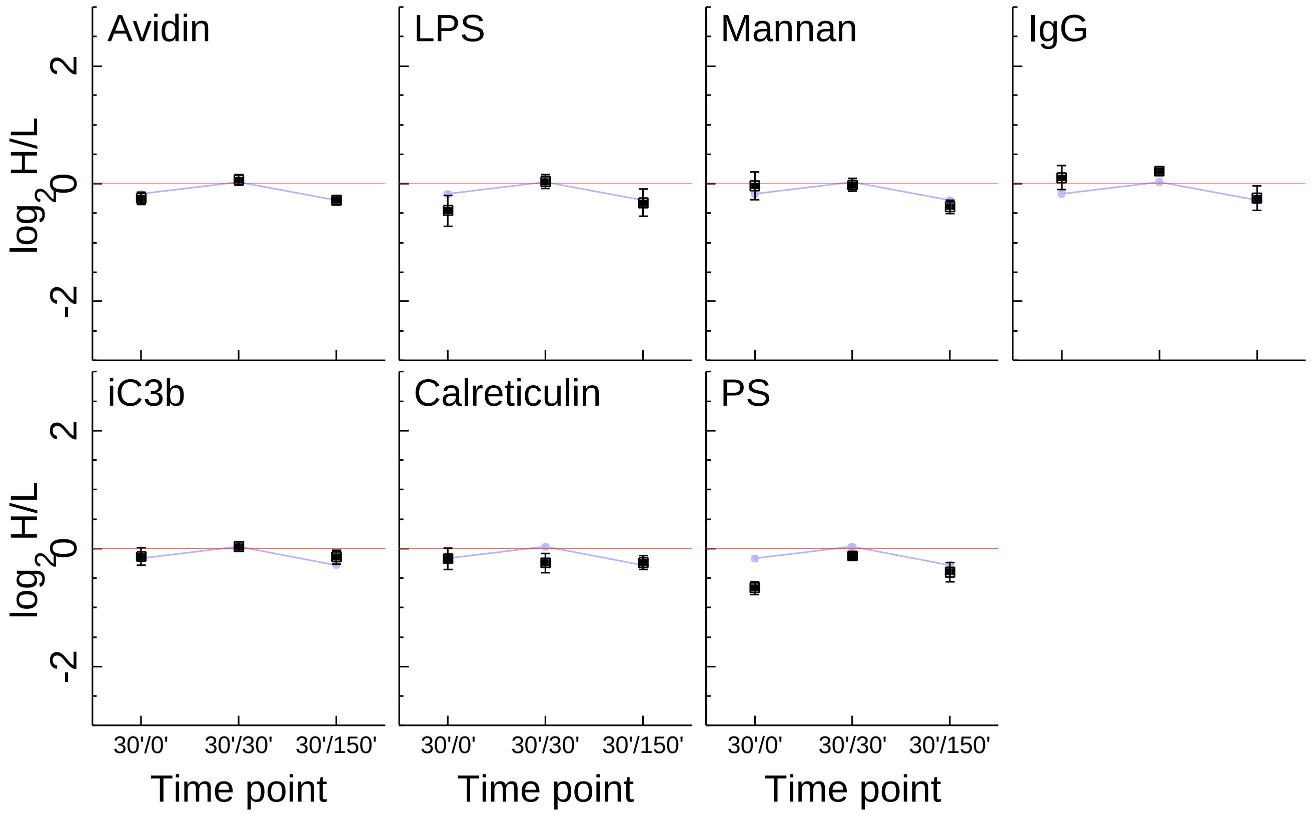

rnh1 (Q91VI7)

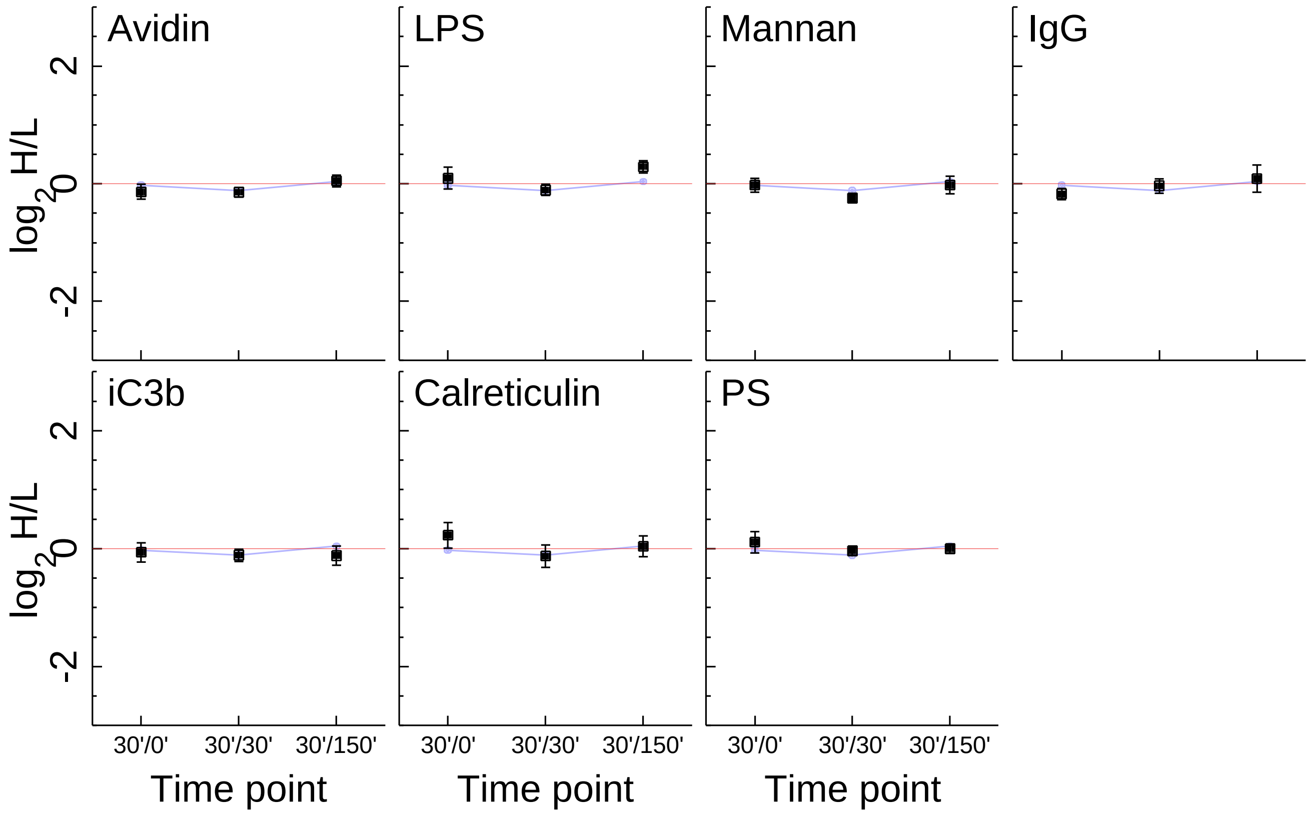

Avidin
LPS
Mannan
IgG
iC3b
Calreticulin
PS
log2 H/L
2
0
-2
30'/0'
30'/30'
30'/150'
Time point

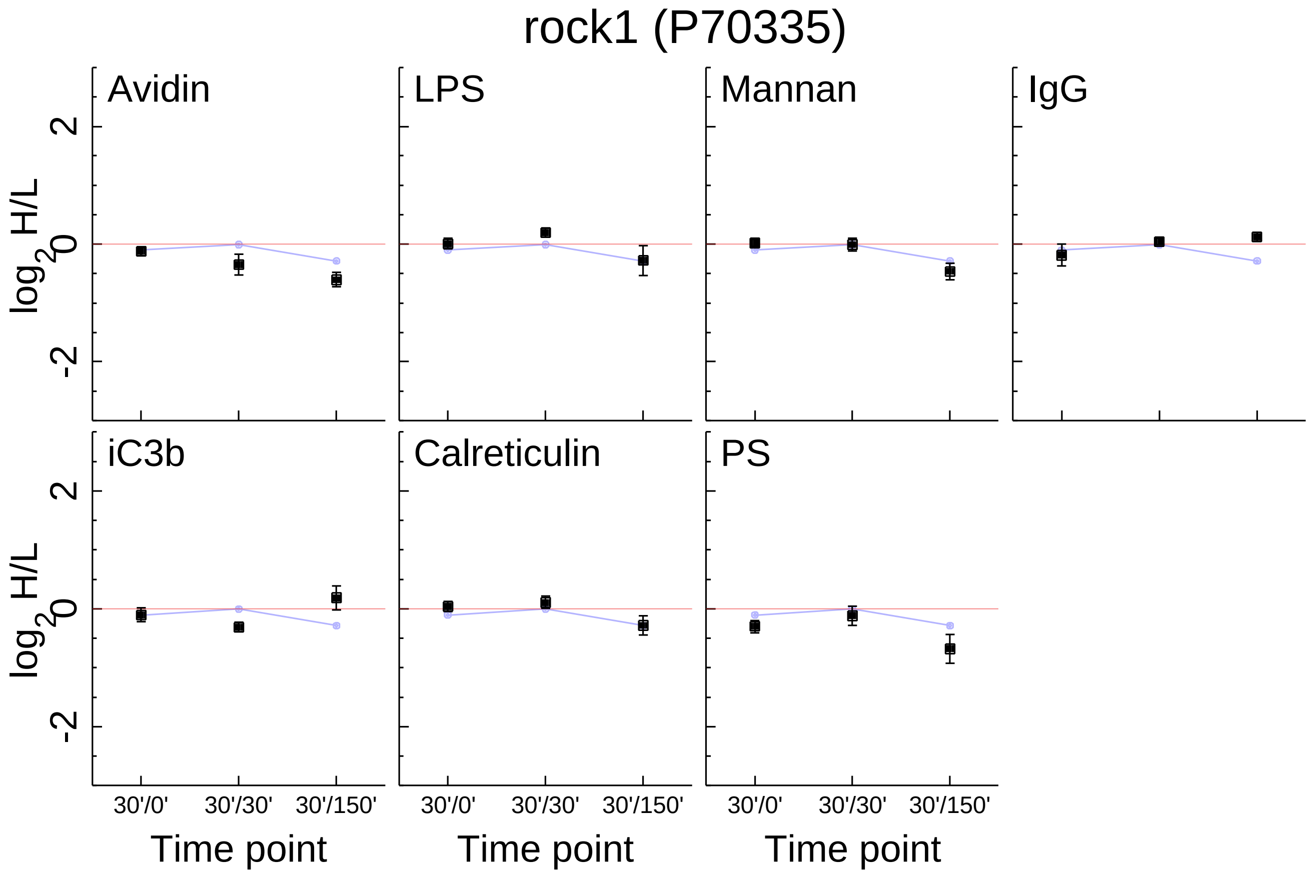
rock1 (P70335)
Avidin
LPS
Mannan
IgG
iC3b
Calreticulin
PS
log2 H/L
2
0
-2
30'/0'
30'/30'
30'/150'
Time point

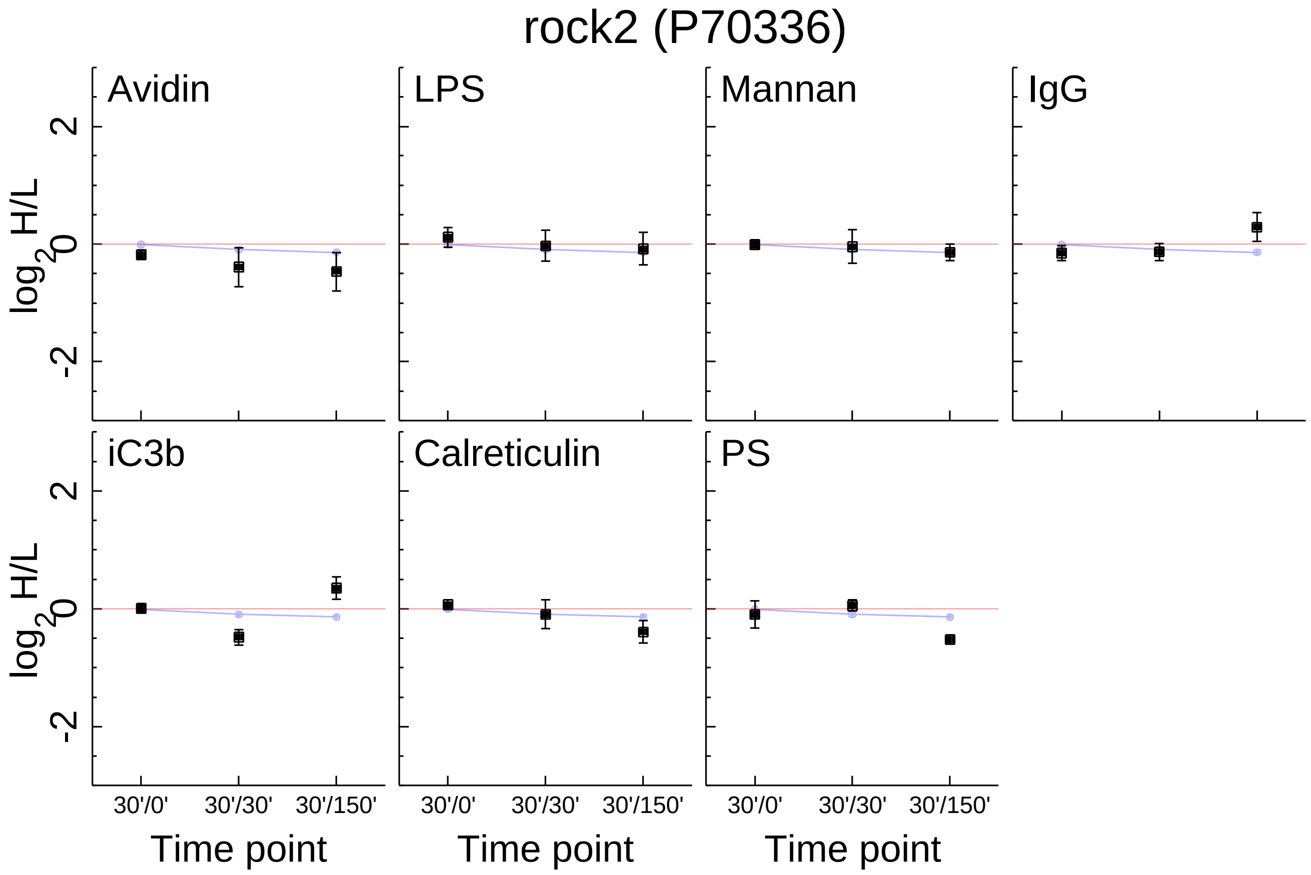

rock2 (P70336)
Avidin
LPS
Mannan
IgG
iC3b
Calreticulin
PS
log2 H/L
2
0
-2
30'/0'
30'/30'
30'/150'
Time point

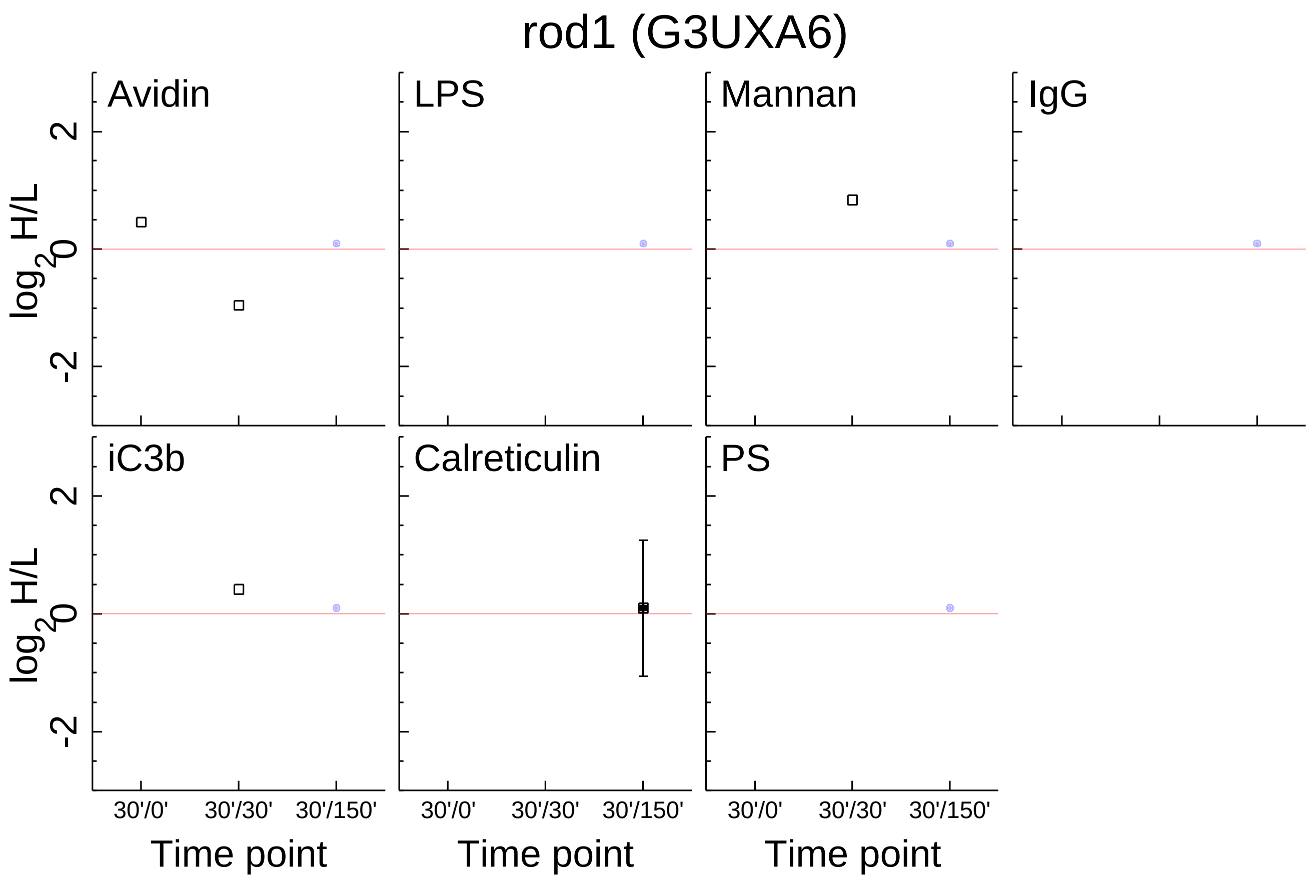

rod1 (G3UXA6)
Avidin
LPS
Mannan
IgG
iC3b
Calreticulin
PS
log2 H/L
2
0
-2
30'/0'
30'/30'
30'/150'
Time point

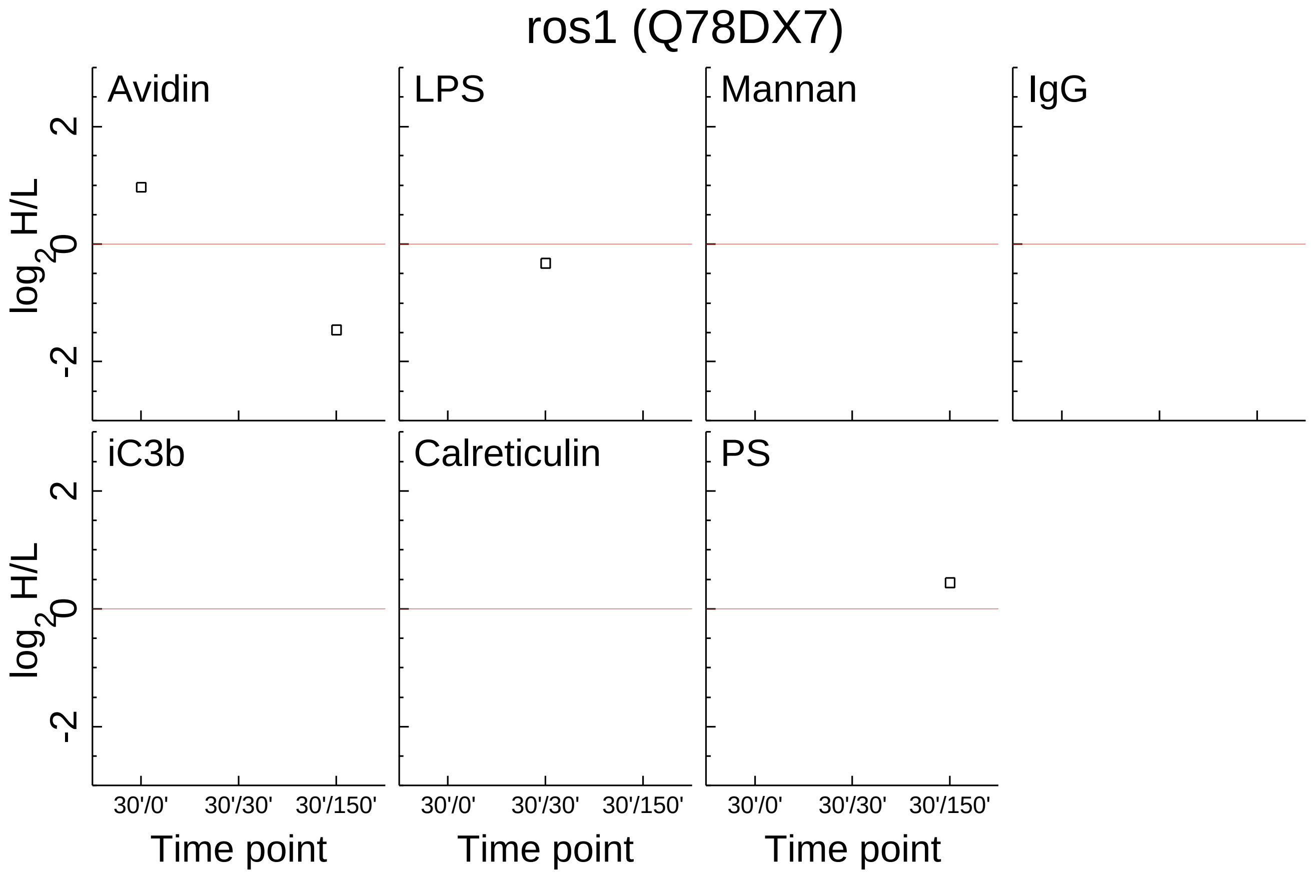

ros1 (Q78DX7)
Avidin
LPS
Mannan
IgG
iC3b
Calreticulin
PS
log2 H/L
2
0
-2
30'/0'
30'/30'
30'/150'
Time point

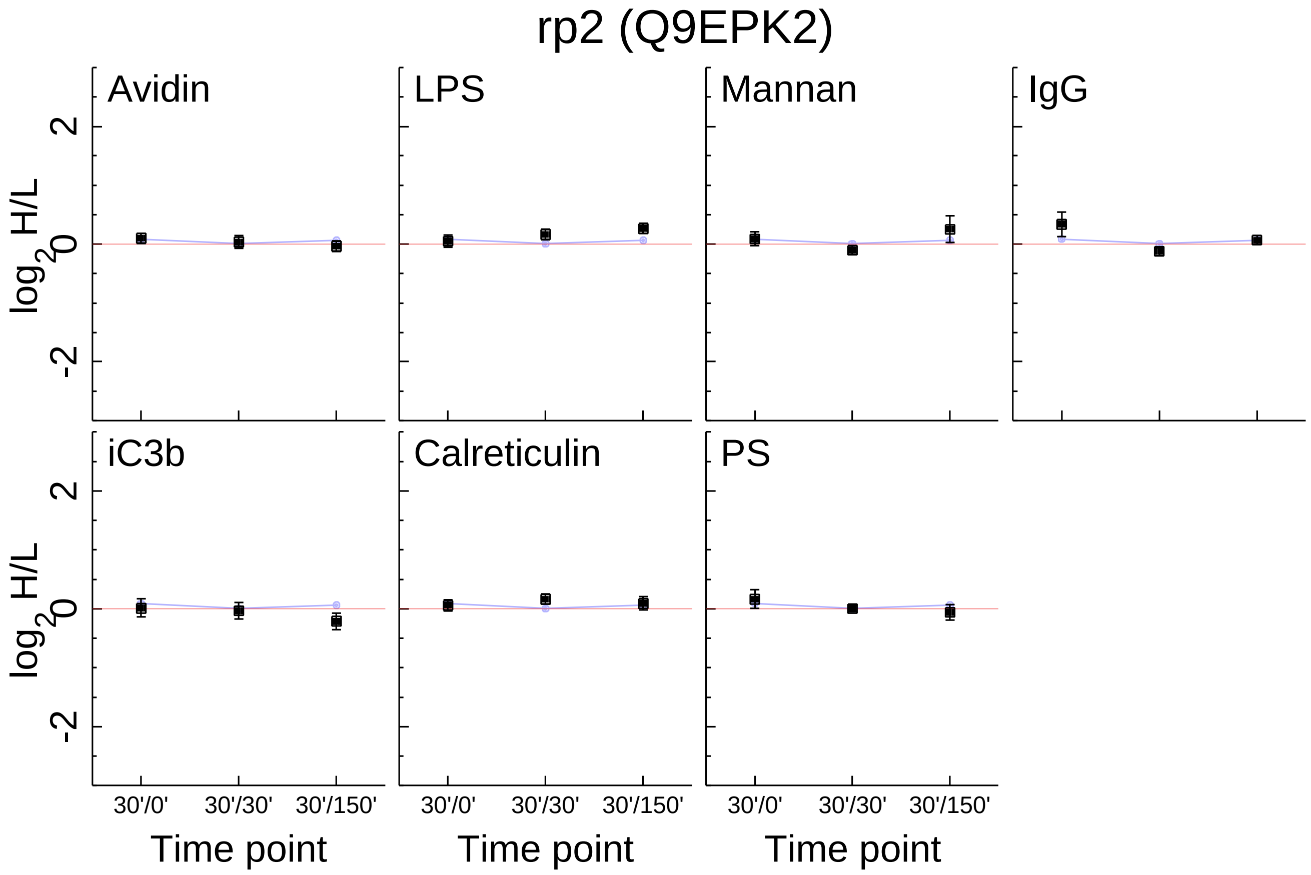
rp2 (Q9EPK2)
Avidin
LPS
Mannan
IgG
iC3b
Calreticulin
PS
log2 H/L
2
0
-2
30'/0'
30'/30'
30'/150'
Time point

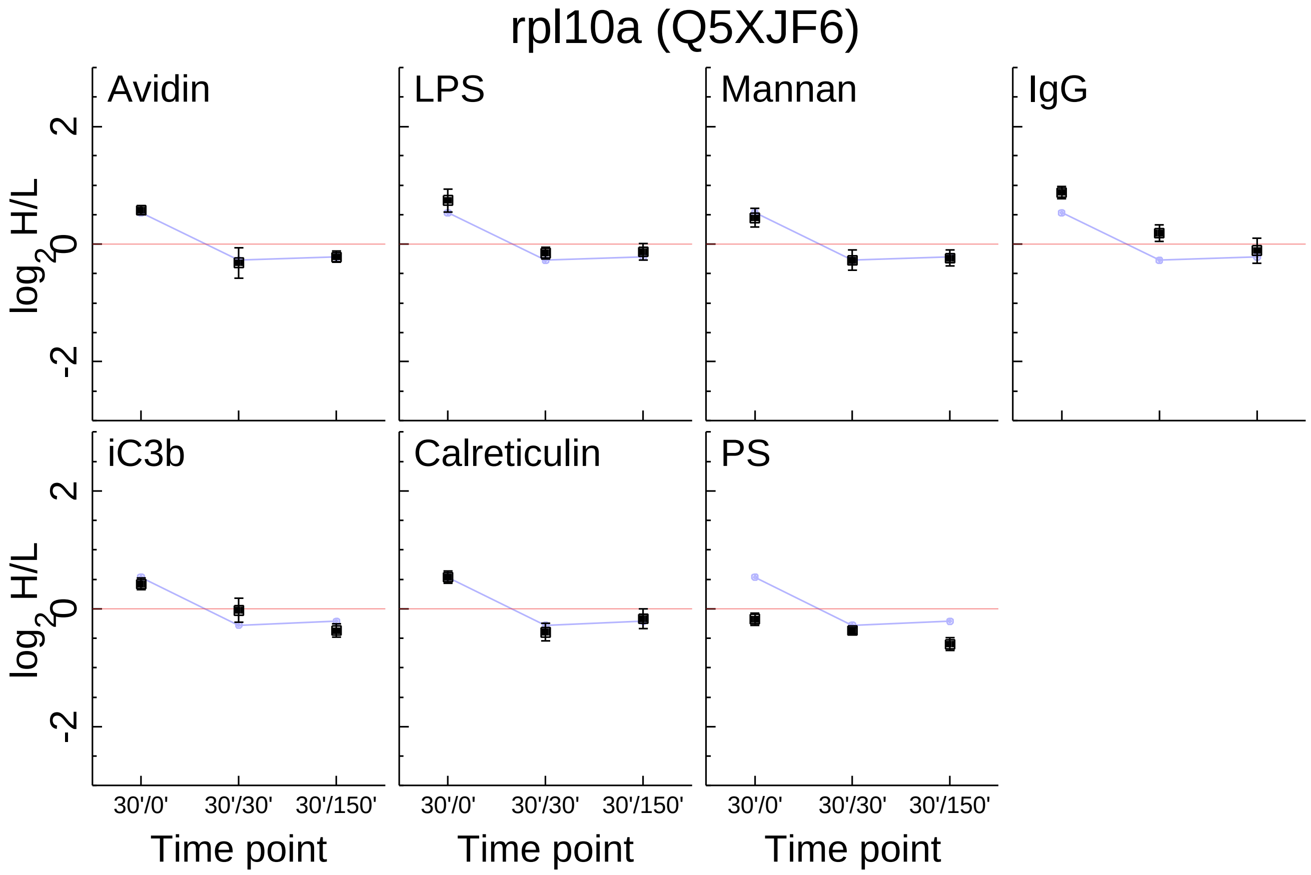

rpl10a (Q5XJF6)
Avidin
LPS
Mannan
IgG
iC3b
Calreticulin
PS
log2 H/L
2
0
-2
30'/0'
30'/30'
30'/150'
Time point

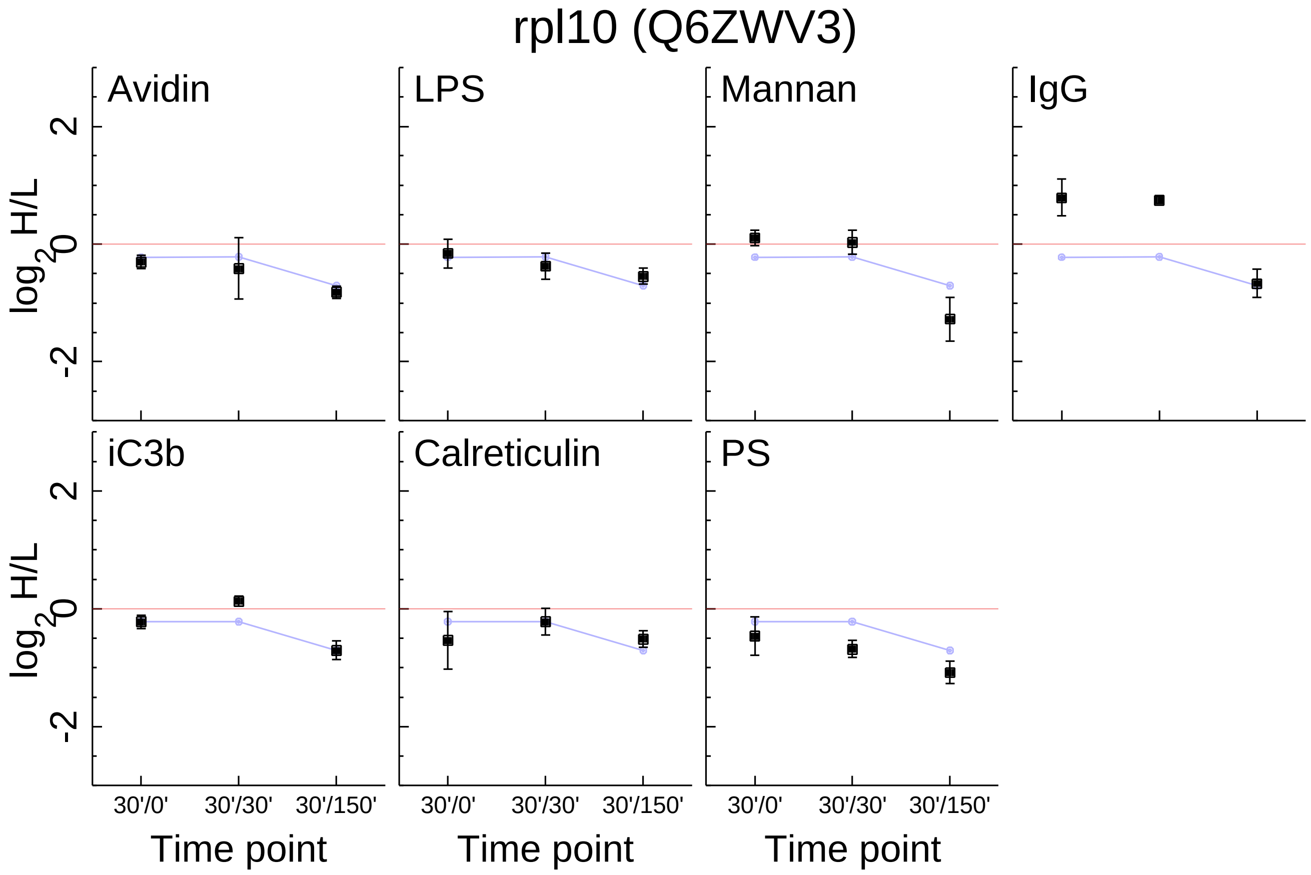
rpl10 (Q6ZWV3)
Avidin
LPS
Mannan
IgG
iC3b
Calreticulin
PS
log2 H/L
2
0
-2
30'/0'
30'/30'
30'/150'
Time point

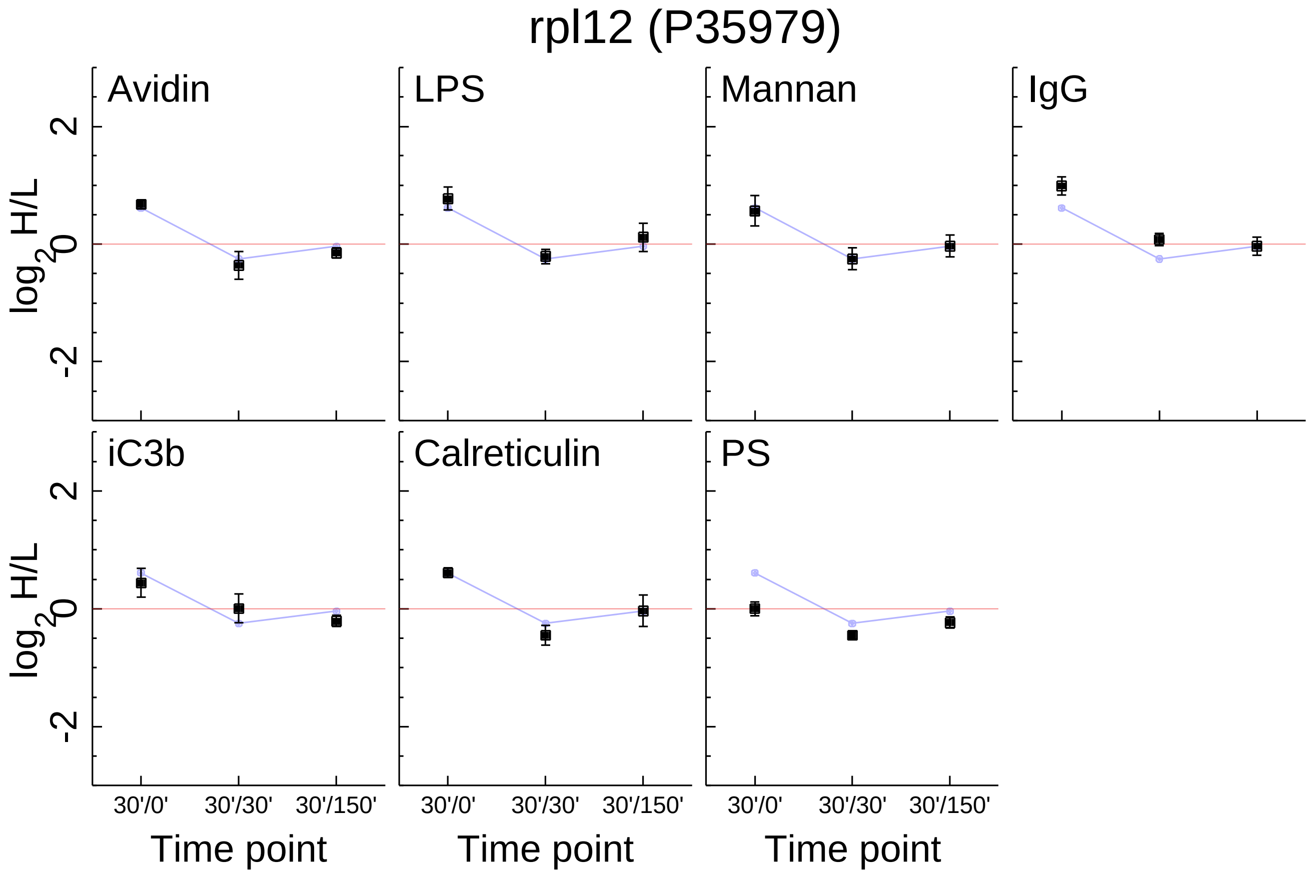

rpl12 (P35979)
Avidin
LPS
Mannan
IgG
iC3b
Calreticulin
PS
log2 H/L
2
0
-2
30'/0'
30'/30'
30'/150'
Time point

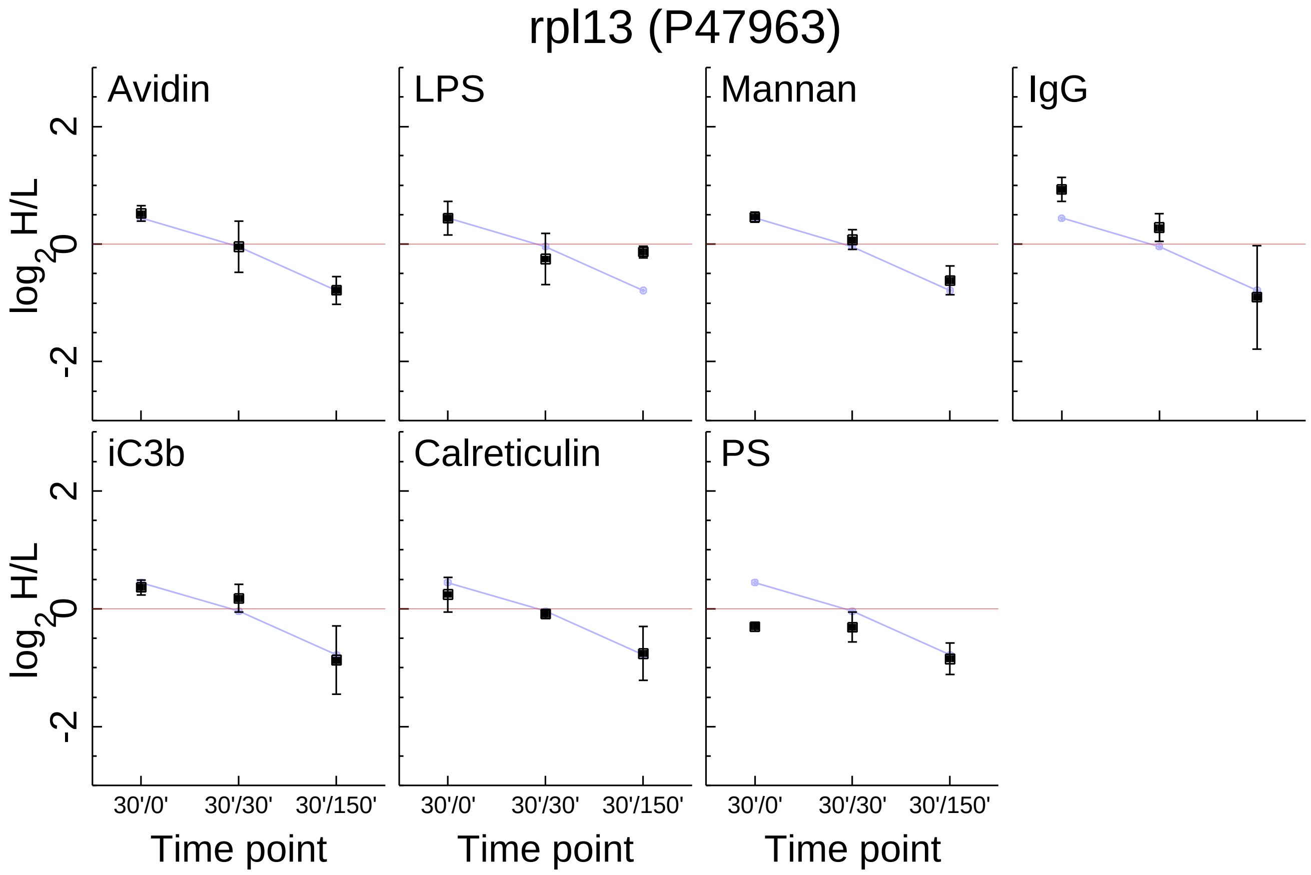
rpl13 (P47963)
Avidin
LPS
Mannan
IgG
iC3b
Calreticulin
PS
2
0
-2
log2 H/L
30'/0'
30'/30'
30'/150'
Time point

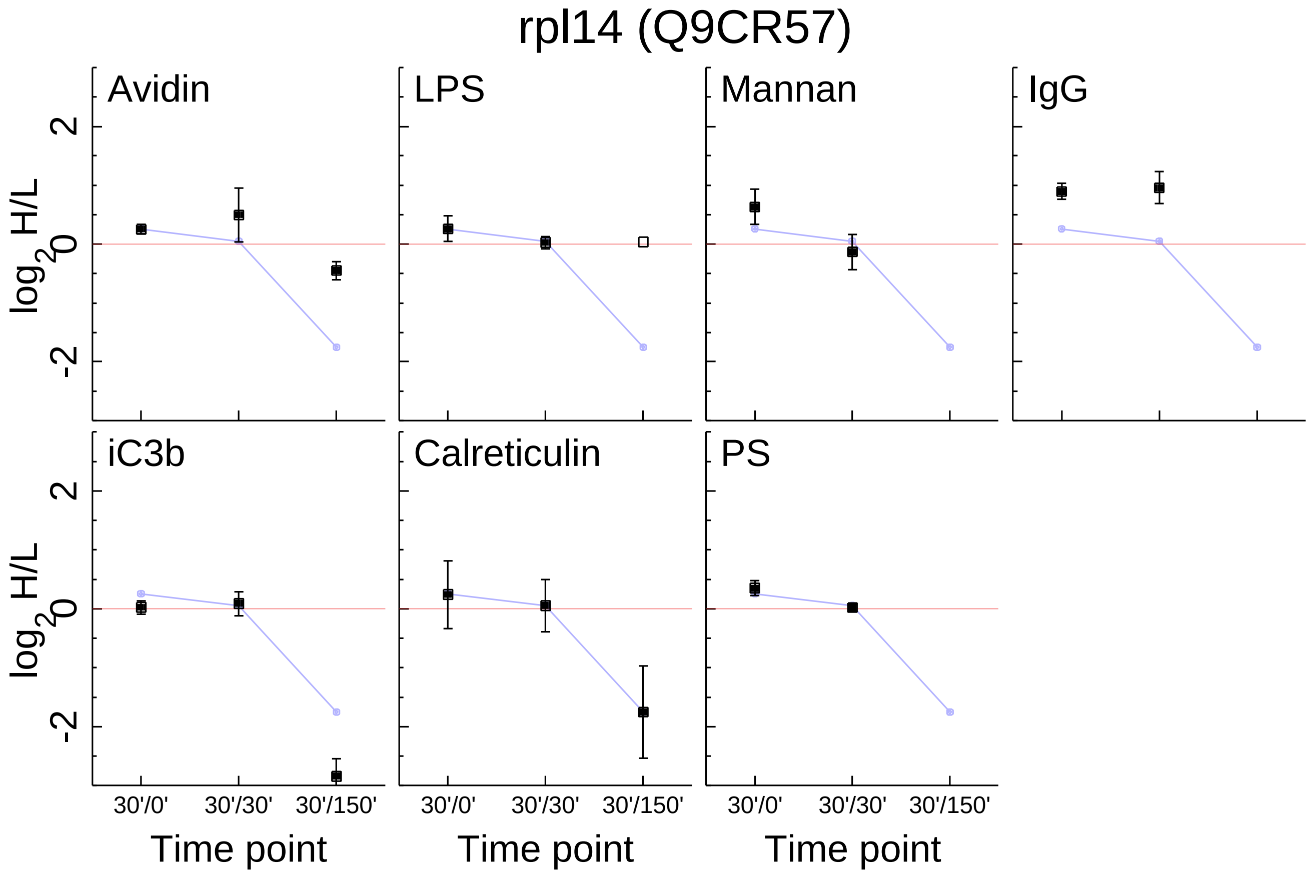
rpl14 (Q9CR57)
Avidin
LPS
Mannan
IgG
iC3b
Calreticulin
PS
log2 H/L
2
0
-2
30'/0'
30'/30'
30'/150'
Time point

rpl17 (Q6ZWZ7)

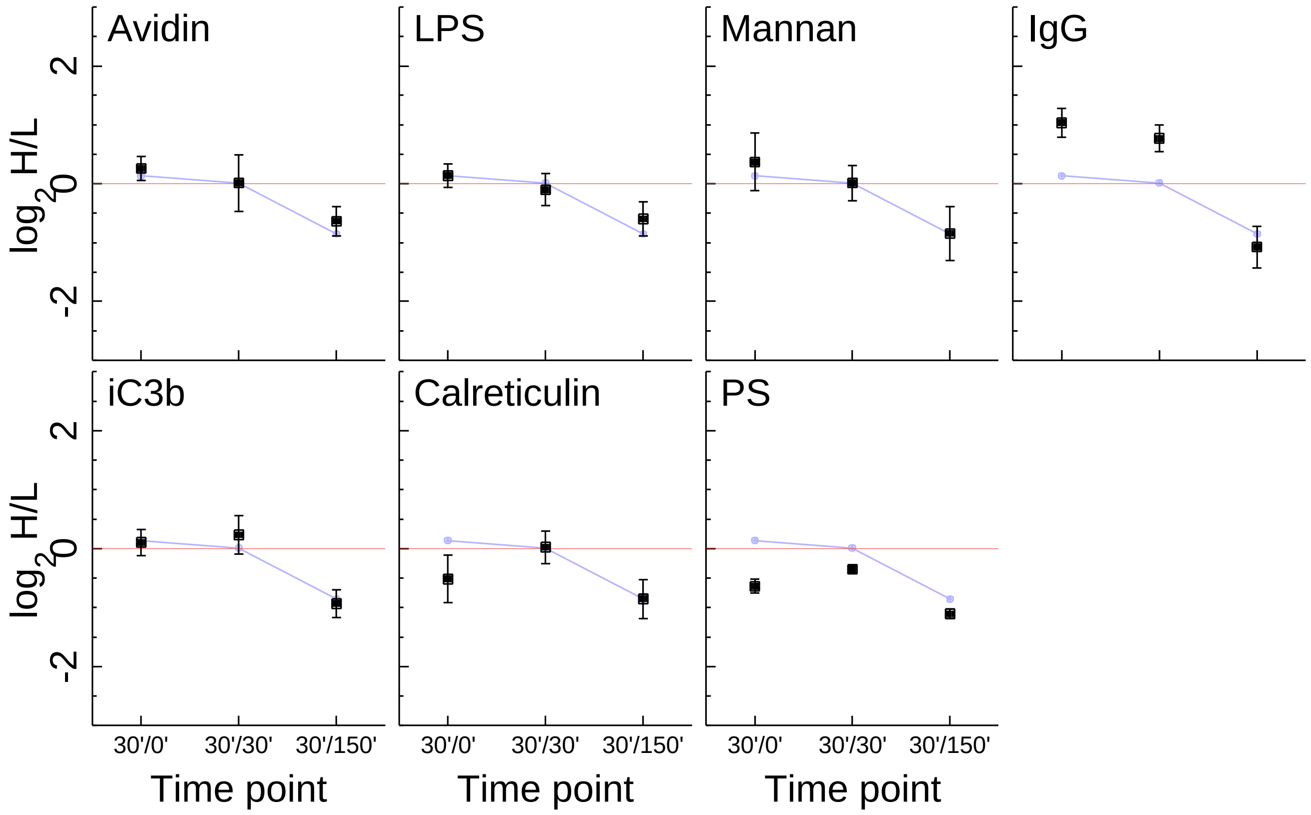

rpl21 (O09167)

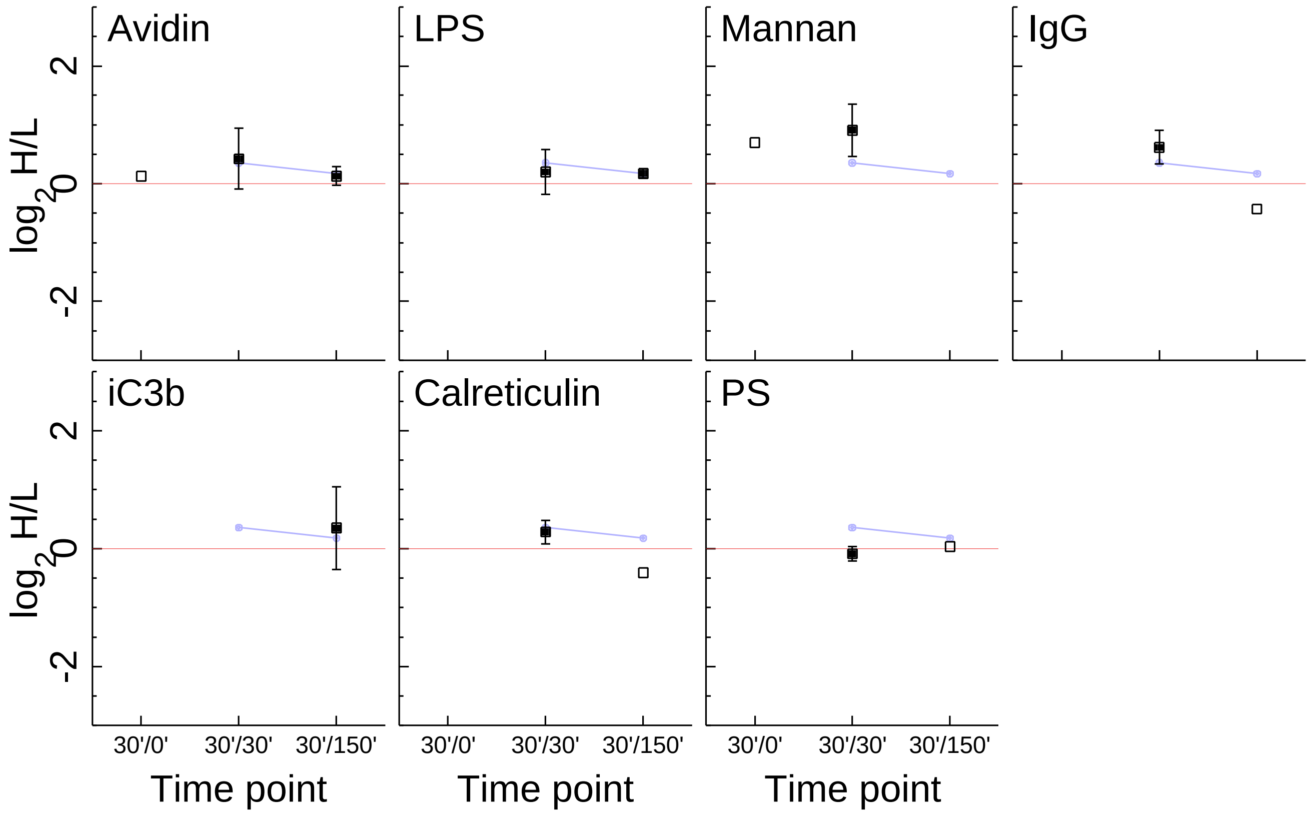

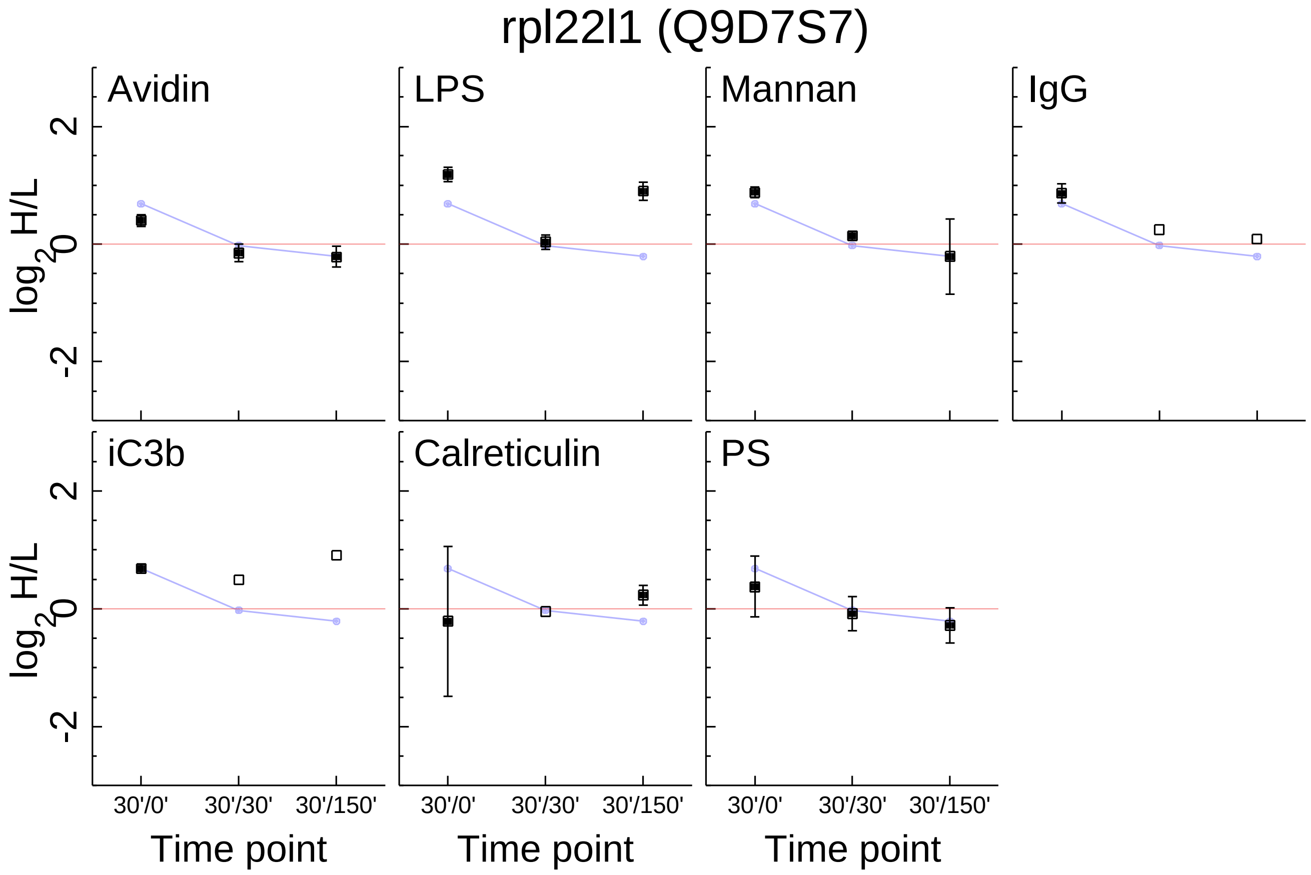

rpl22l1 (Q9D7S7)
Avidin
LPS
Mannan
IgG
iC3b
Calreticulin
PS
log2 H/L
2
0
-2
30'/0'
30'/30'
30'/150'
Time point

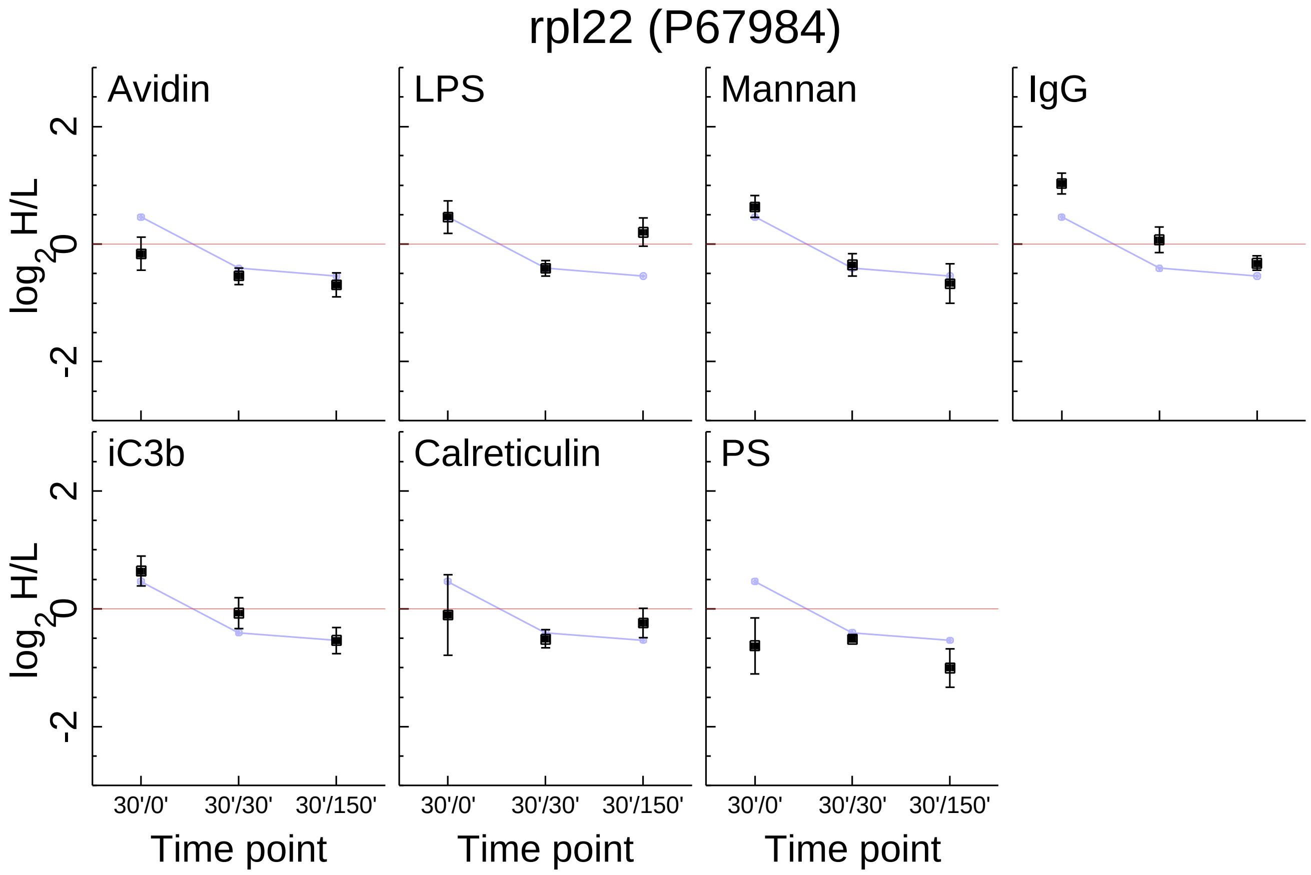

rpl22 (P67984)
Avidin
LPS
Mannan
IgG
iC3b
Calreticulin
PS
log2 H/L
2
0
-2
30'/0'
30'/30'
30'/150'
Time point

rpl23a-ps3 (D3YWP3)

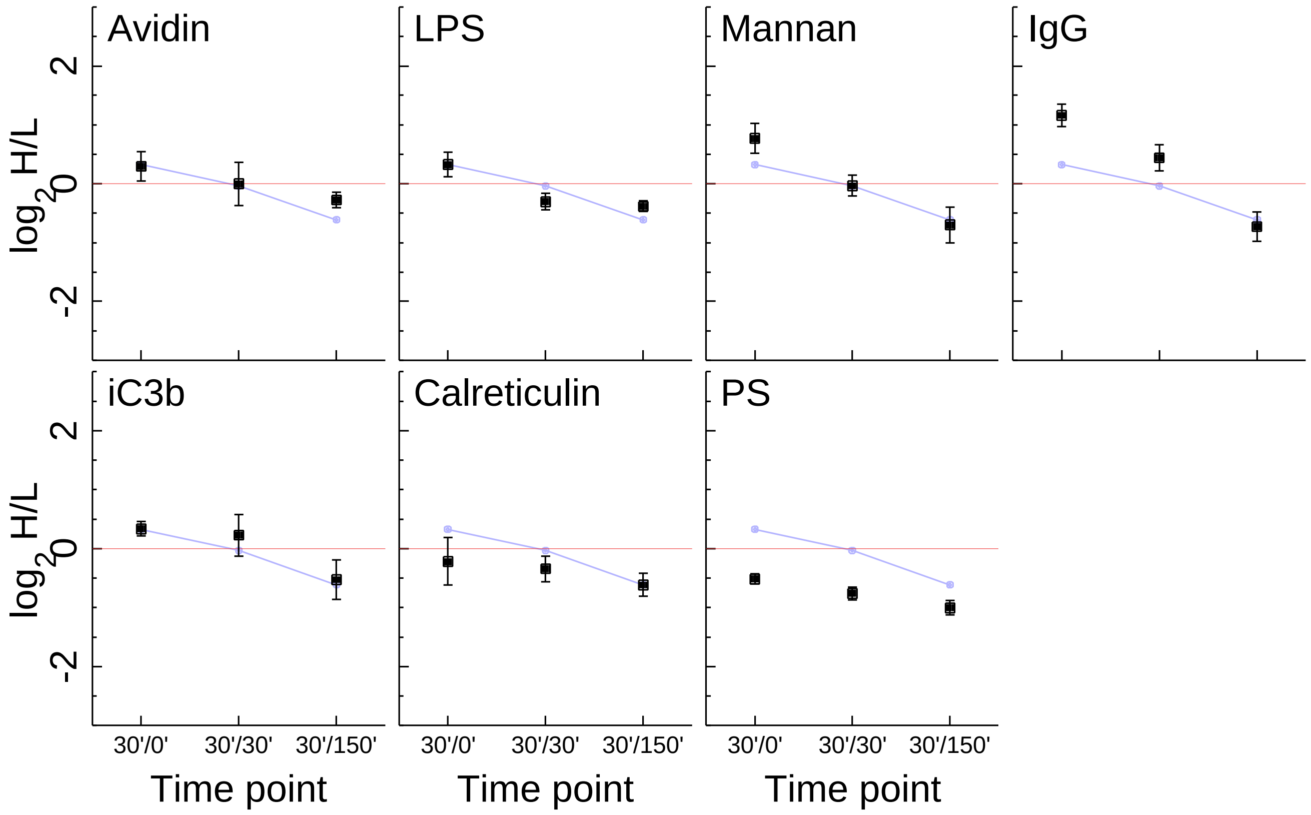

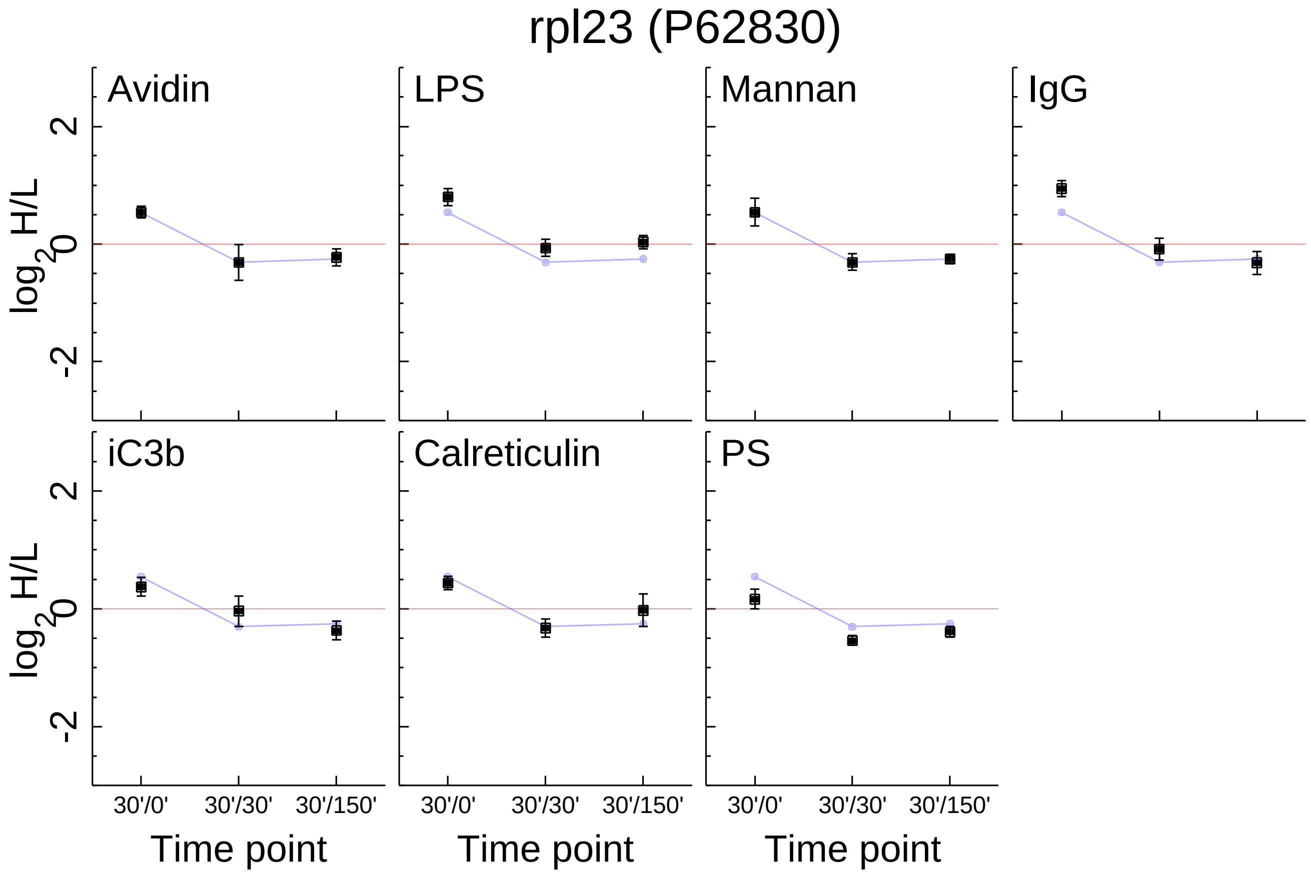

rpl23 (P62830)
Avidin
LPS
Mannan
IgG
iC3b
Calreticulin
PS
2
0
-2
log2 H/L
30'/0'
30'/30'
30'/150'
Time point

rpl24 (Q8BP67)

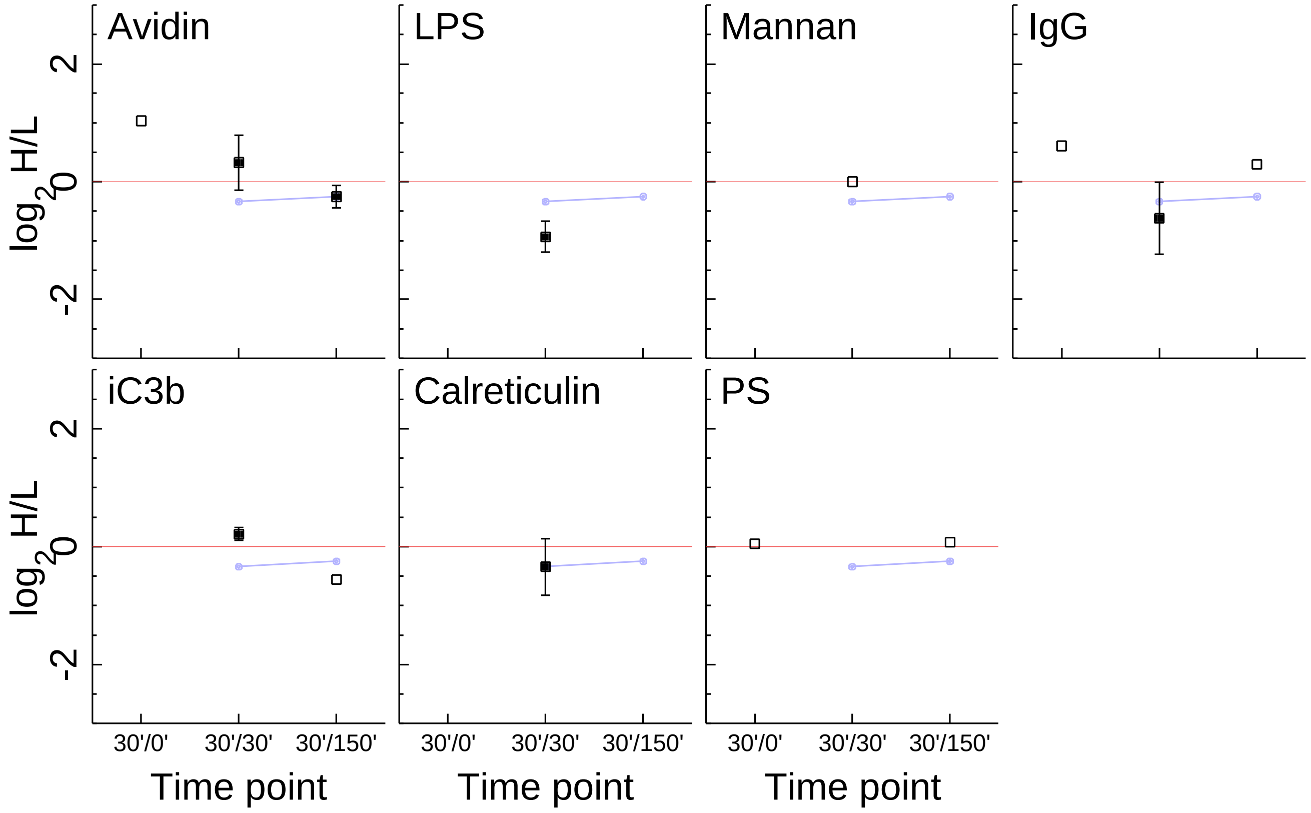

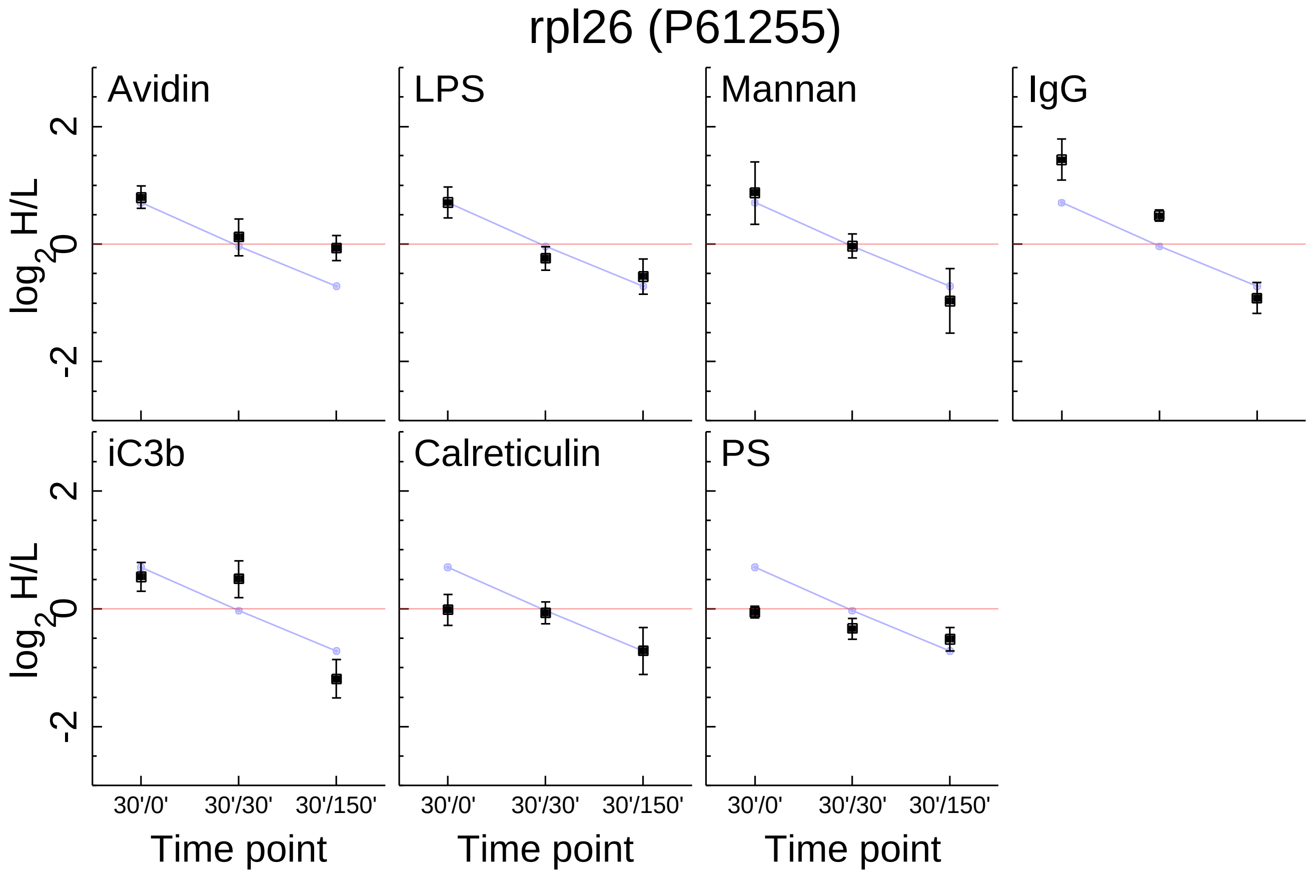
rpl26 (P61255)
Avidin
LPS
Mannan
IgG
iC3b
Calreticulin
PS
log2 H/L
2
0
-2
30'/0'
30'/30'
30'/150'
Time point

rpl27 (P61358)

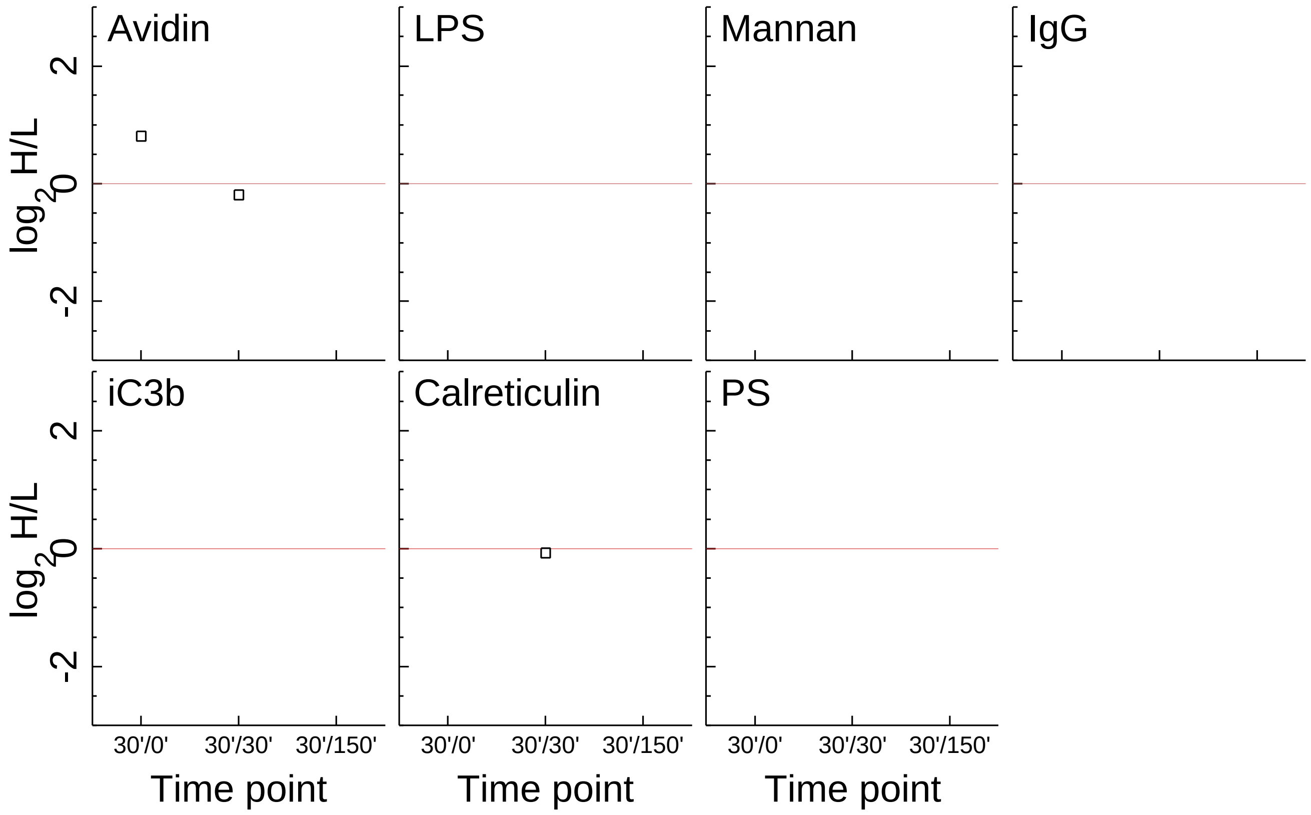

rpl28 (P41105)

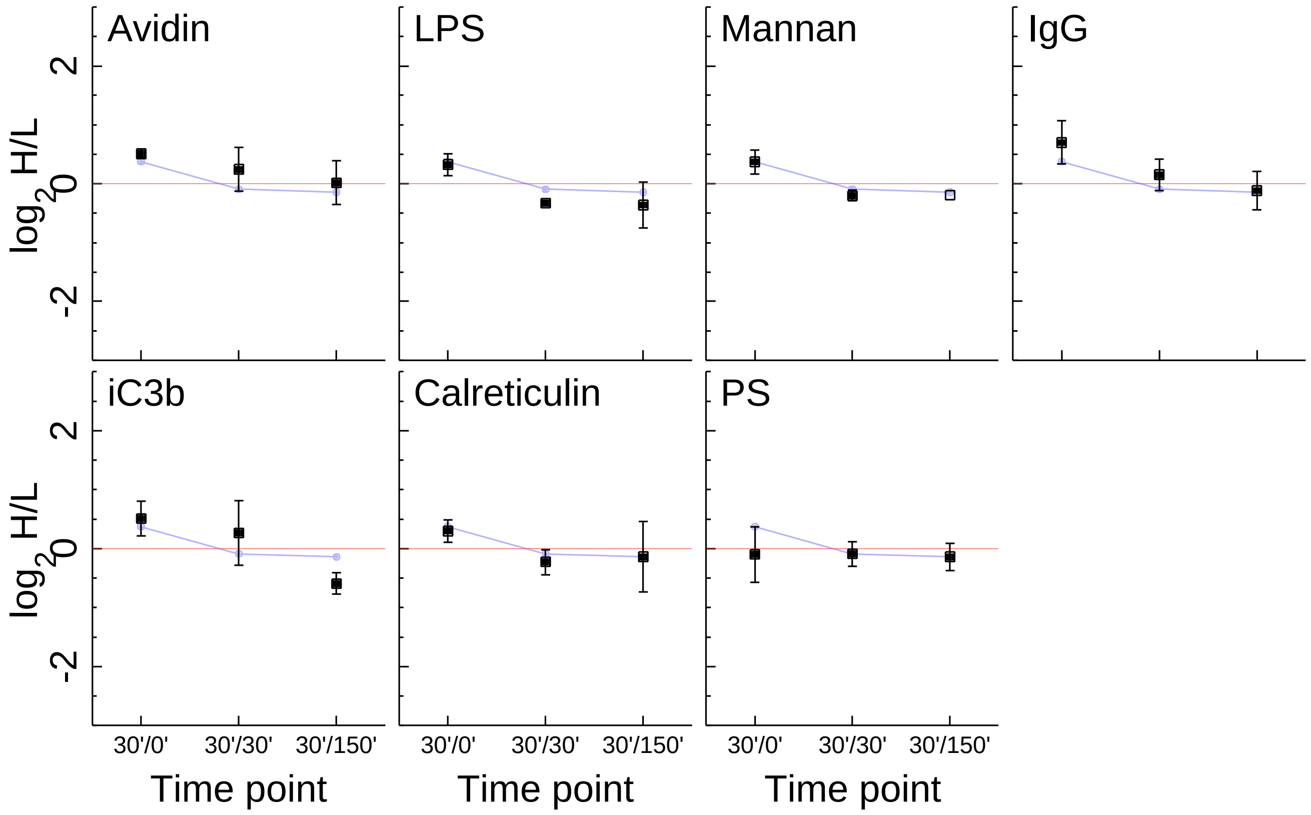

rpl30 (P62889)

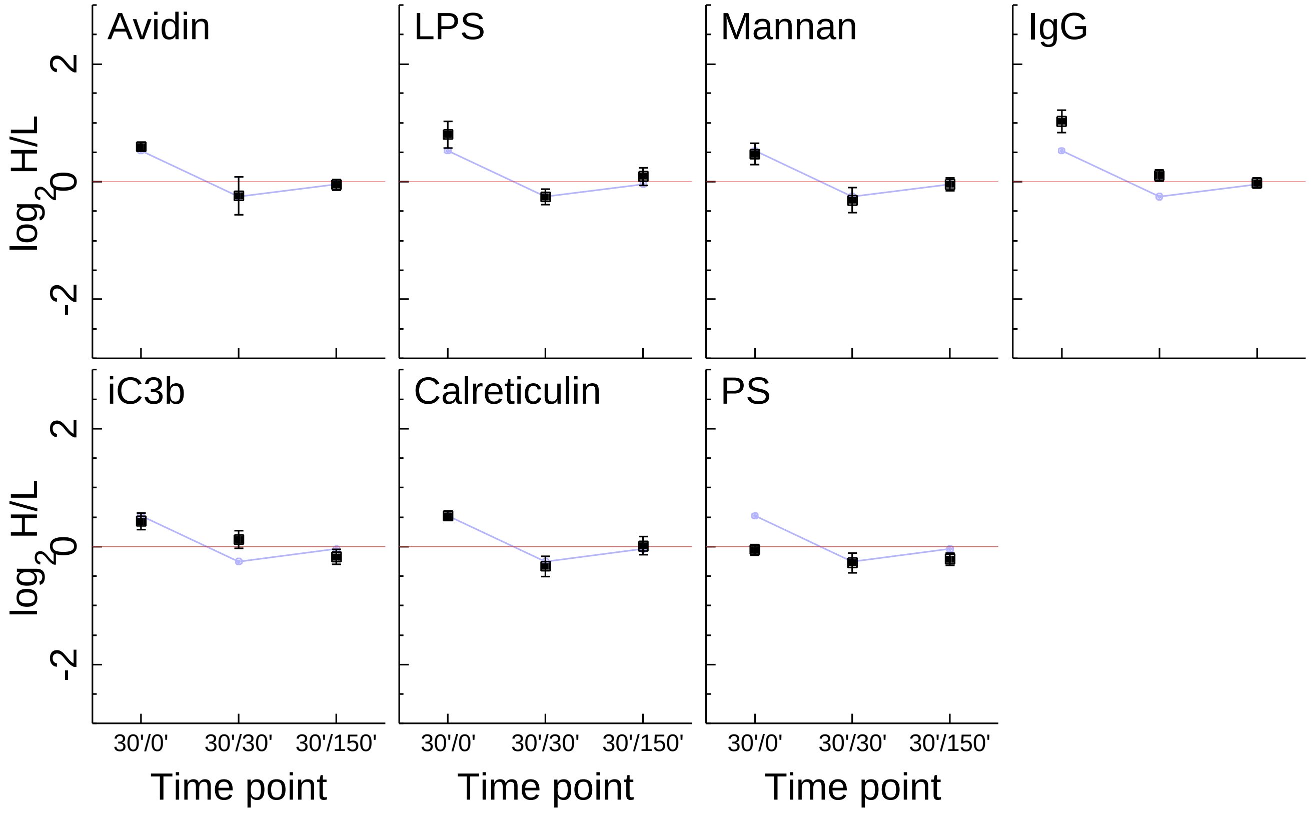

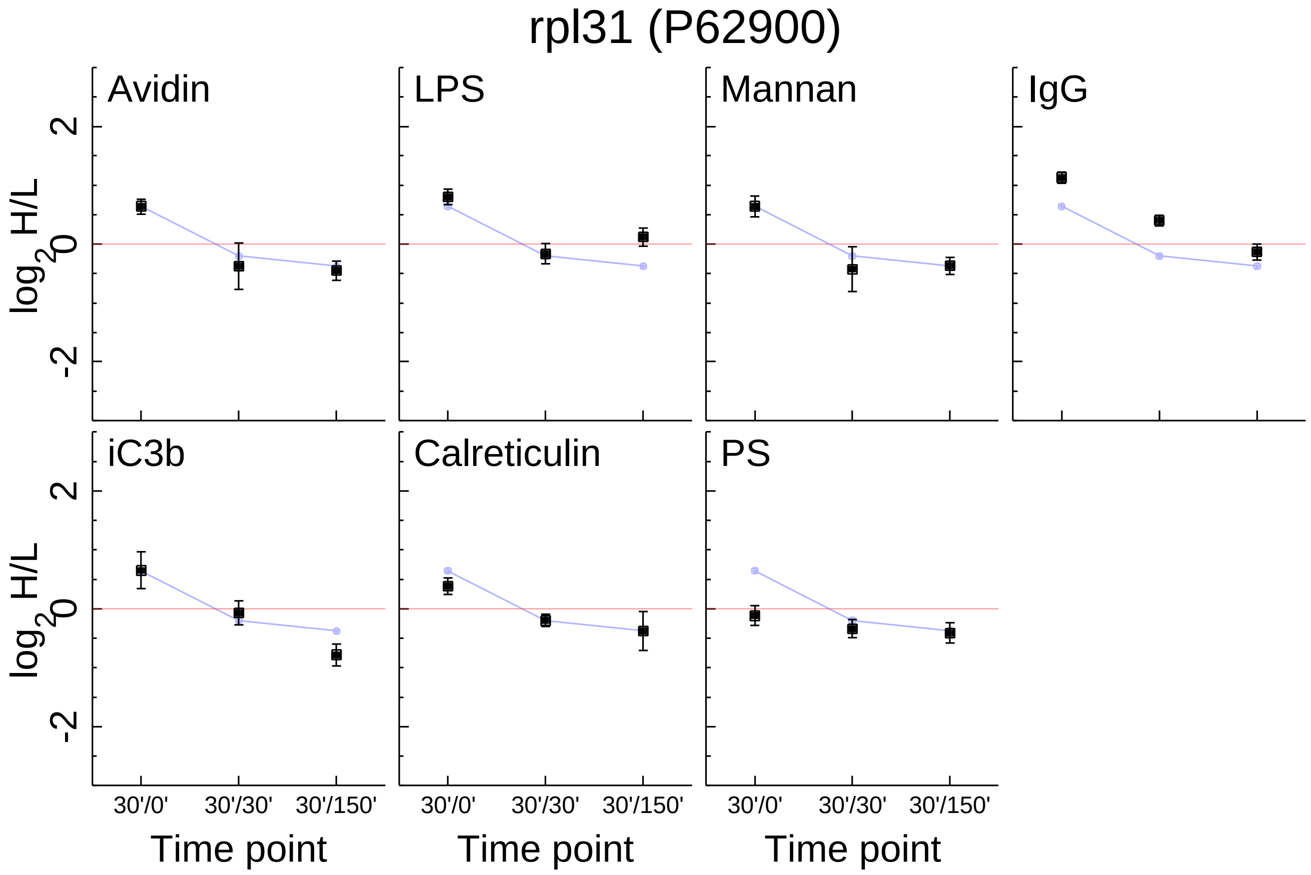

rpl31 (P62900)
Avidin
LPS
Mannan
IgG
iC3b
Calreticulin
PS
log2 H/L
2
0
-2
30'/0'
30'/30'
30'/150'
Time point

rpl34 (Q9D1R9)

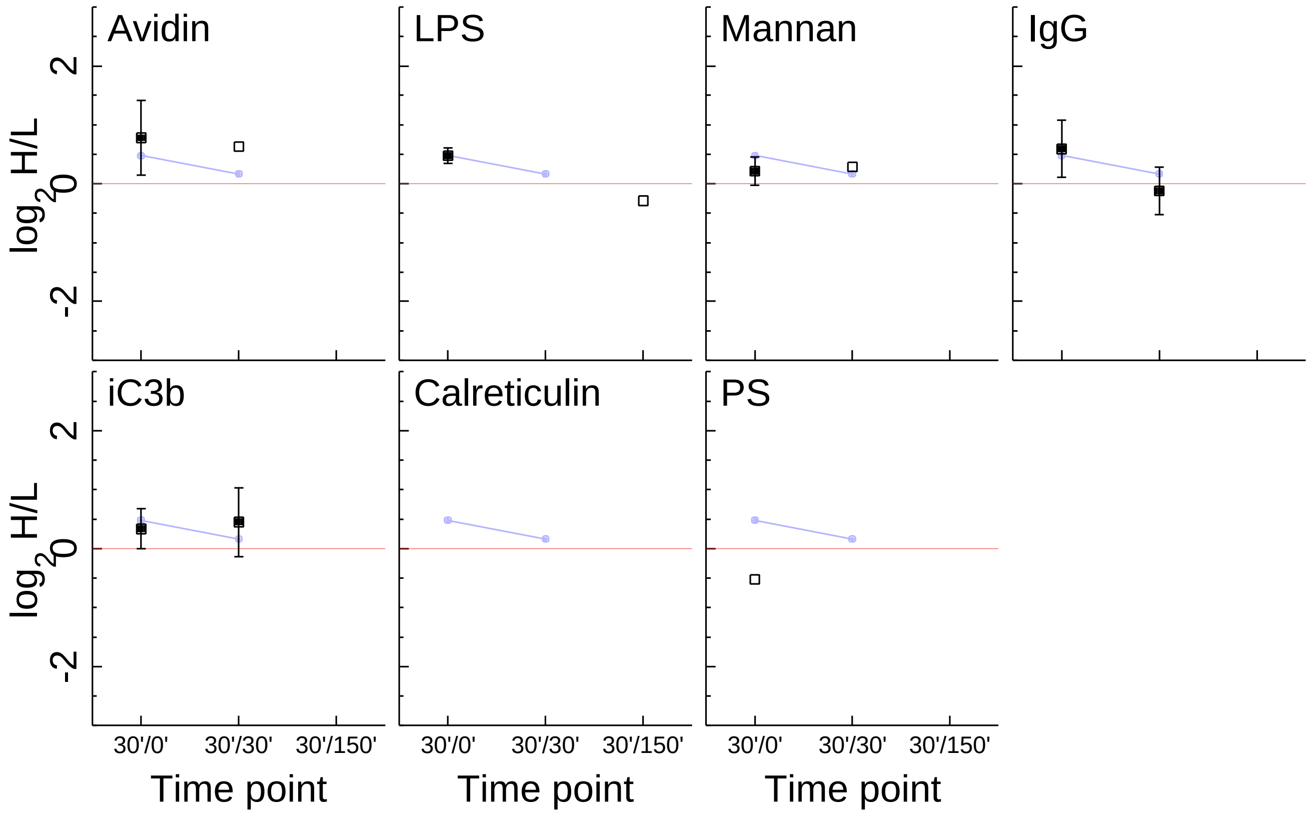

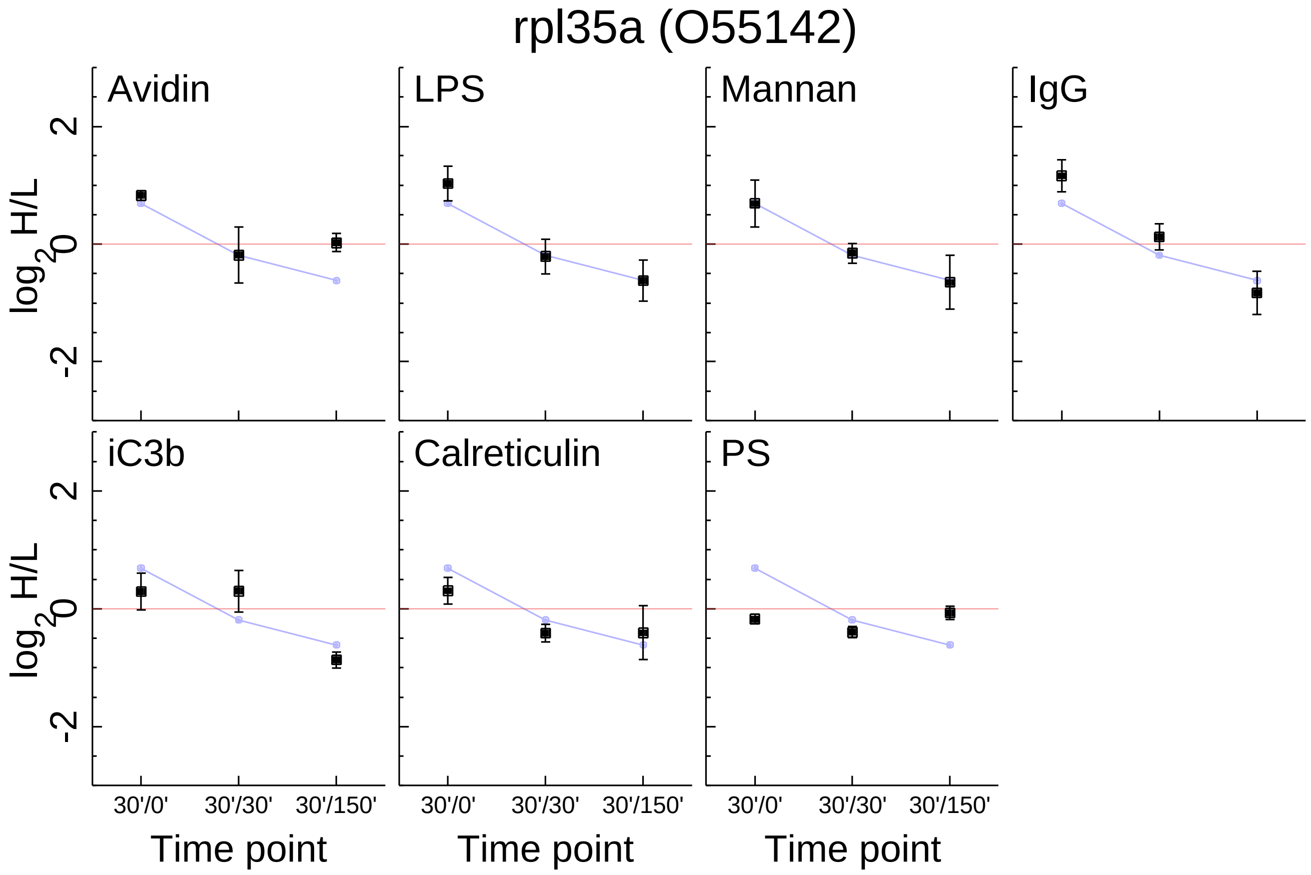
rpl35a (O55142)
Avidin
LPS
Mannan
IgG
iC3b
Calreticulin
PS
log2 H/L
2
0
-2
30'/0'
30'/30'
30'/150'
Time point

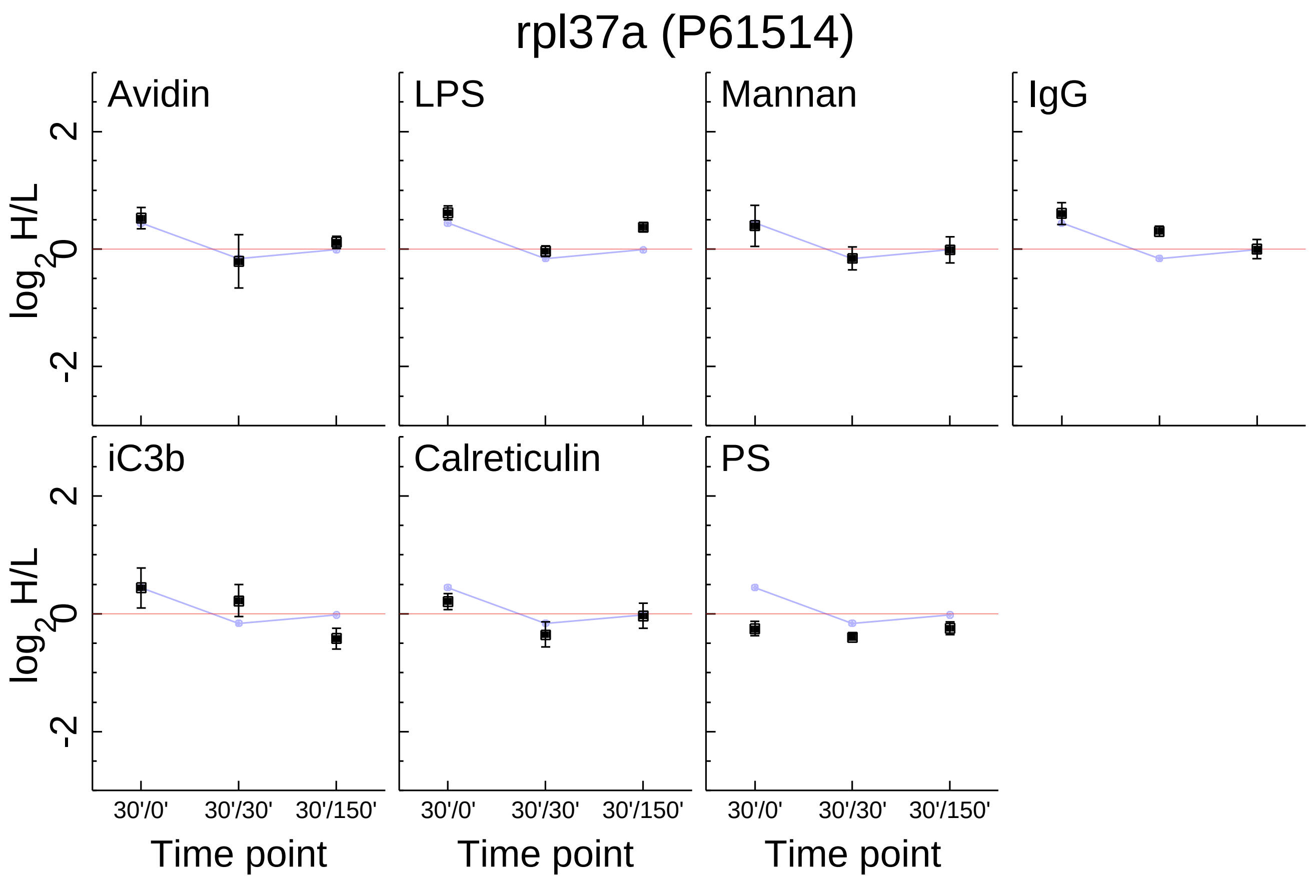

rpl37a (P61514)
Avidin
LPS
Mannan
IgG
iC3b
Calreticulin
PS
log2 H/L
2
0
-2
30'/0'
30'/30'
30'/150'
Time point

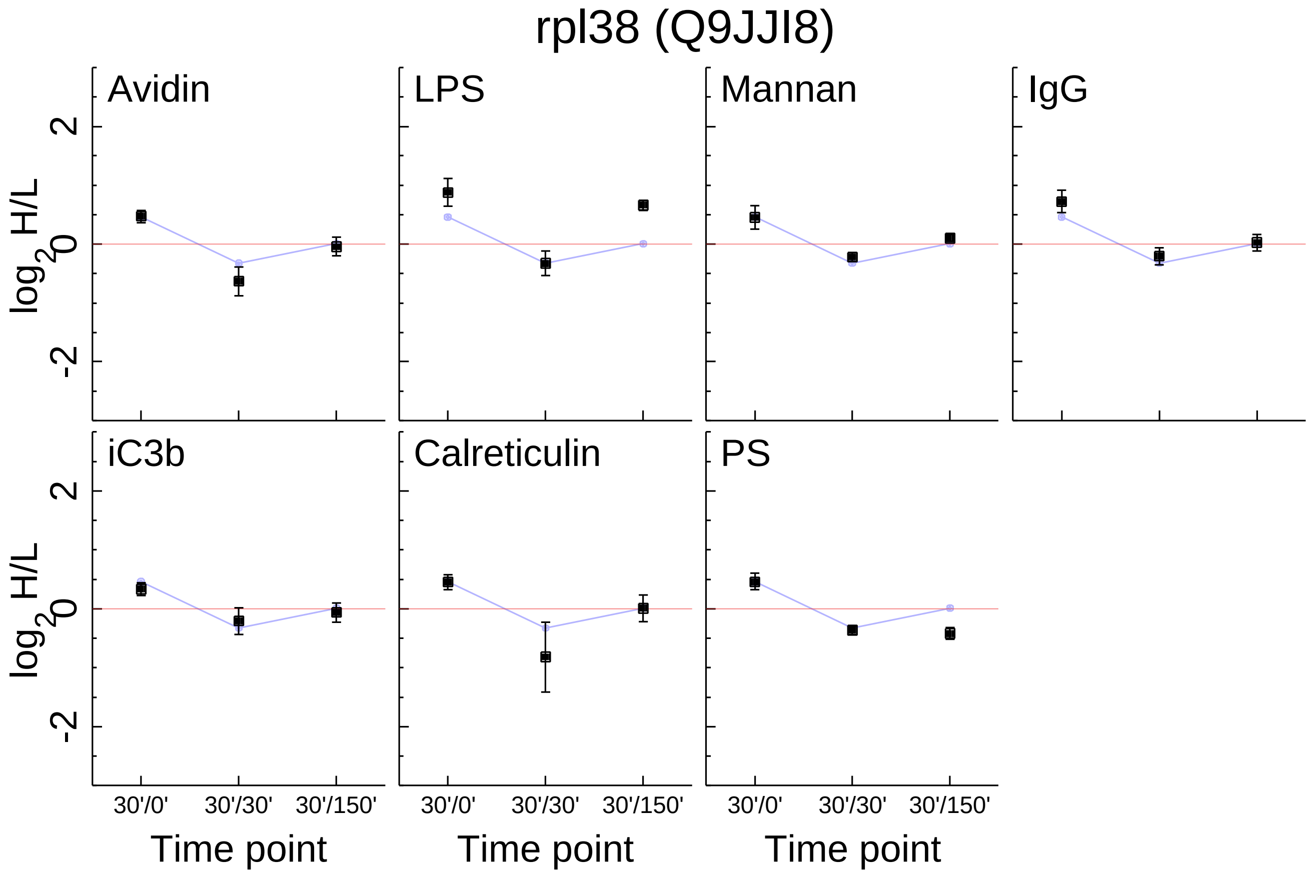
rpl38 (Q9JJI8)
Avidin
LPS
Mannan
IgG
iC3b
Calreticulin
PS
log2 H/L
2
0
-2
30'/0'
30'/30'
30'/150'
Time point

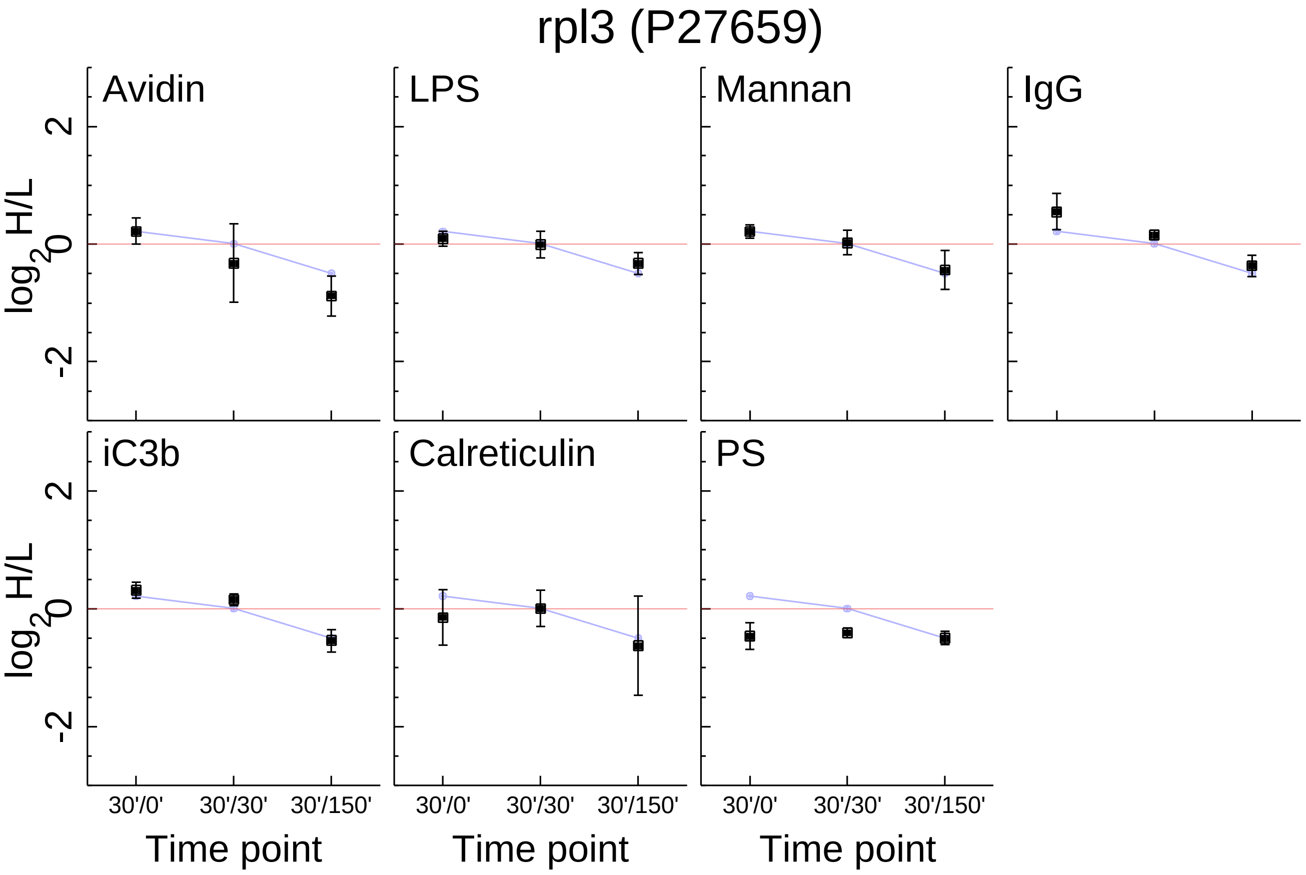

rpl3 (P27659)
Avidin
LPS
Mannan
IgG
iC3b
Calreticulin
PS
log2 H/L
2
0
-2
30'/0'
30'/30'
30'/150'
Time point

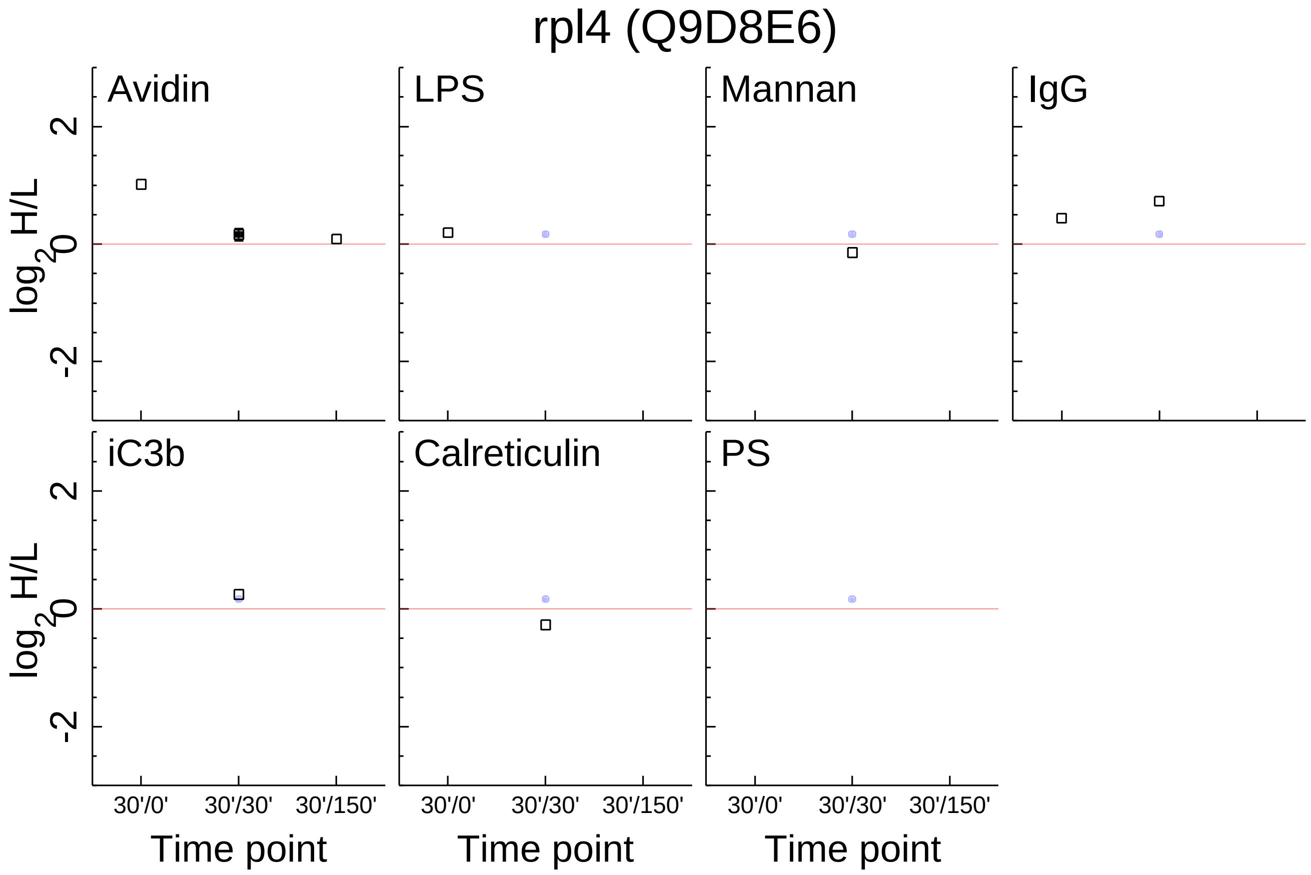
rpl4 (Q9D8E6)
Avidin
LPS
Mannan
IgG
iC3b
Calreticulin
PS
log2 H/L
2
0
-2
30'/0'
30'/30'
30'/150'
Time point

rpl5 (P47962)

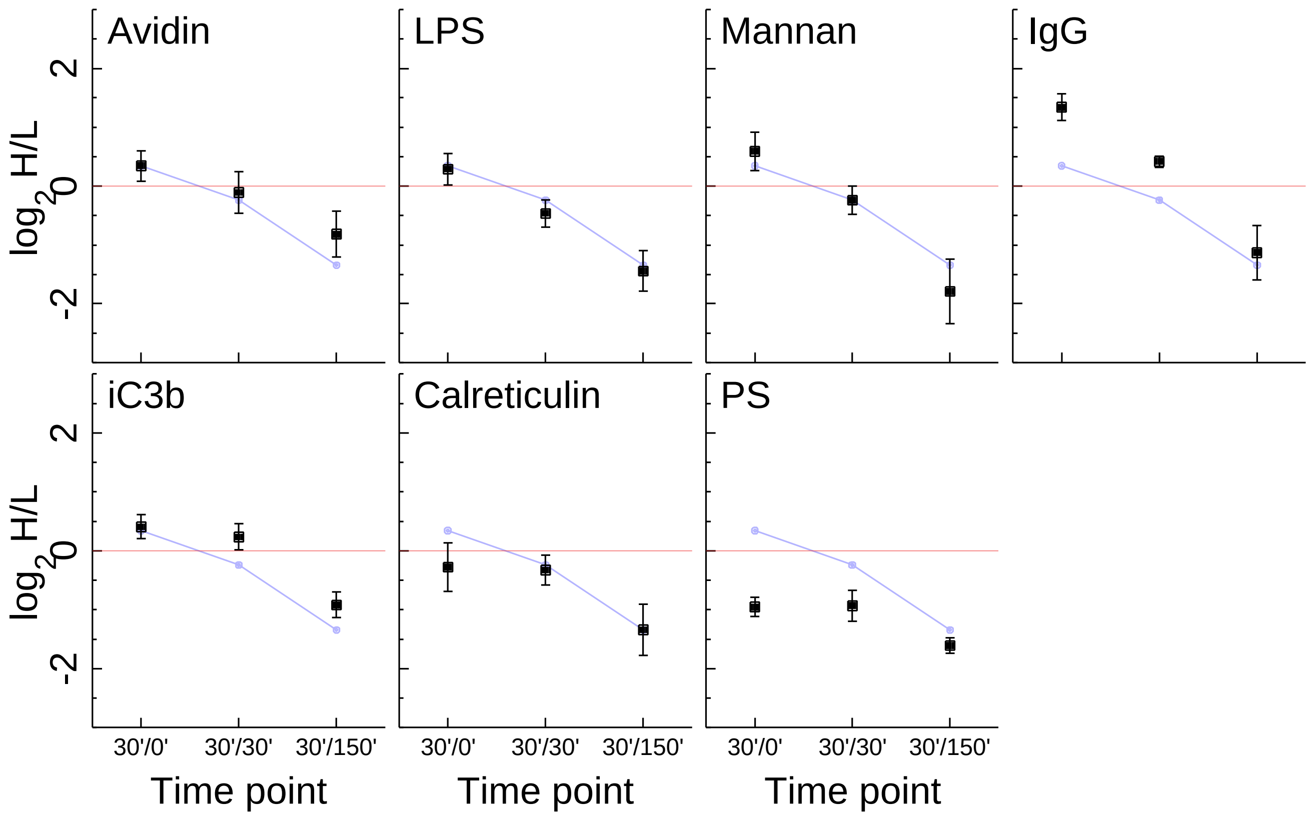

Avidin
LPS
Mannan
IgG
iC3b
Calreticulin
PS
log2 H/L
2
0
-2
30'/0'
30'/30'
30'/150'
Time point

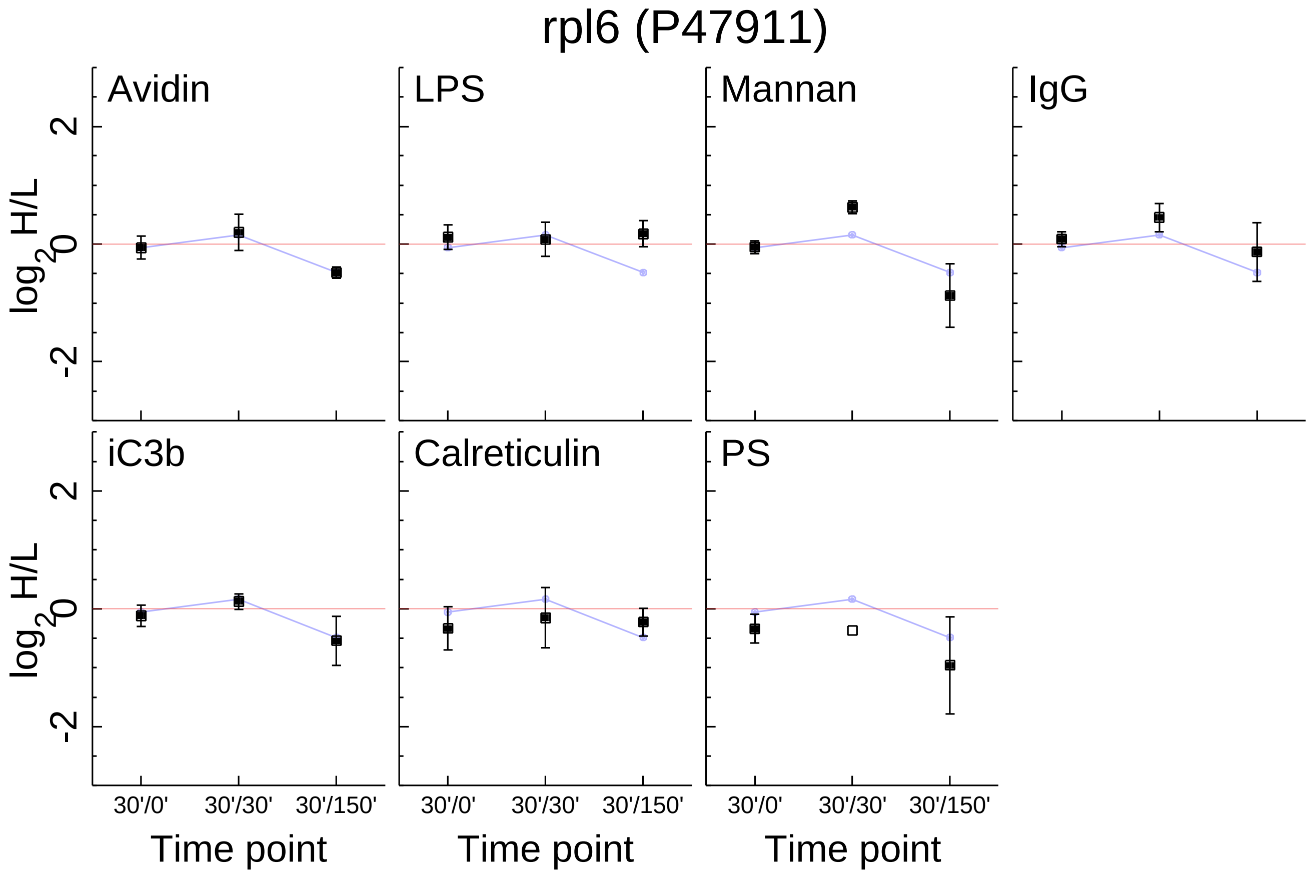
rpl6 (P47911)
Avidin
LPS
Mannan
IgG
iC3b
Calreticulin
PS
log2 H/L
2
0
-2
30'/0'
30'/30'
30'/150'
Time point

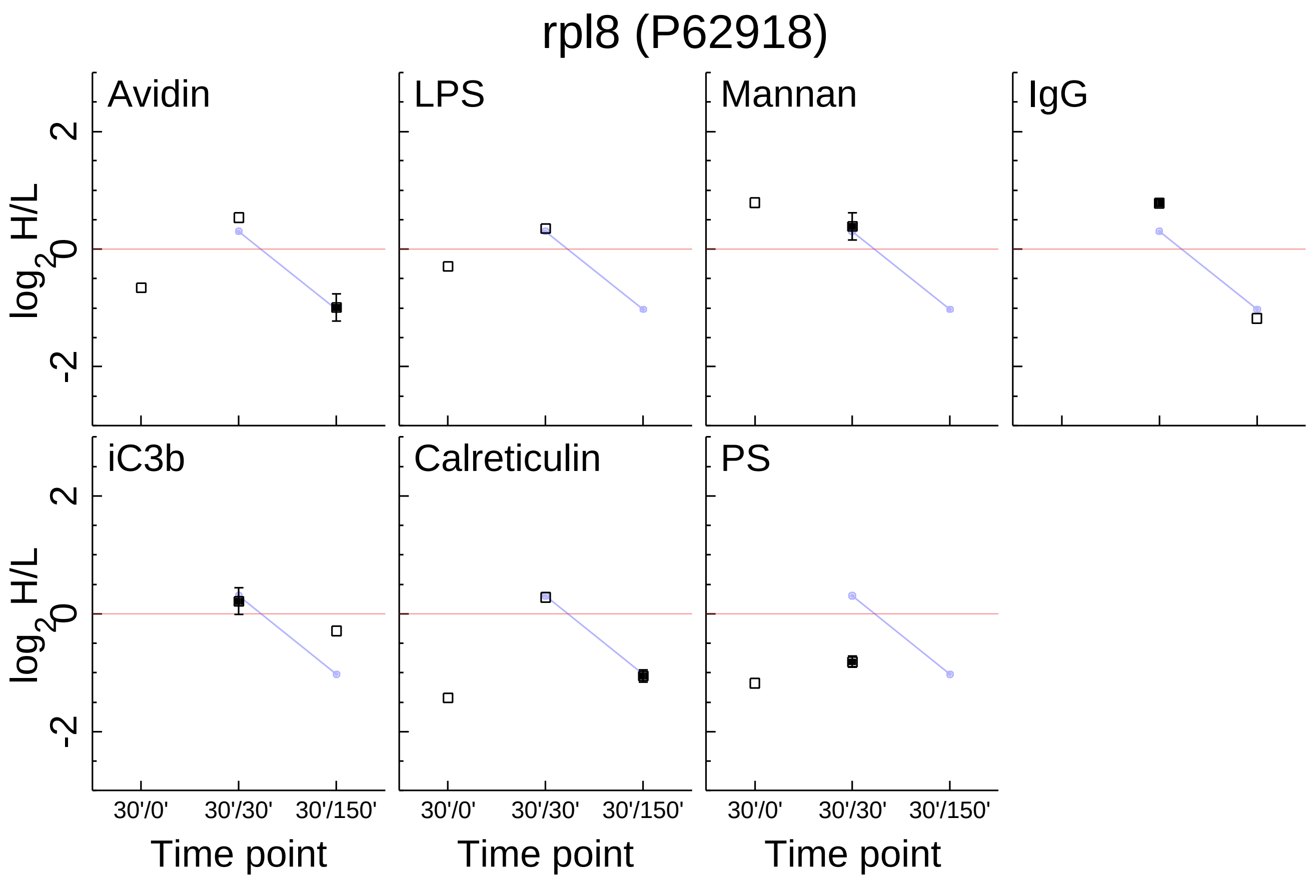
rpl8 (P62918)
Avidin
LPS
Mannan
IgG
iC3b
Calreticulin
PS
log2 H/L
2
0
-2
30'/0'
30'/30'
30'/150'
Time point

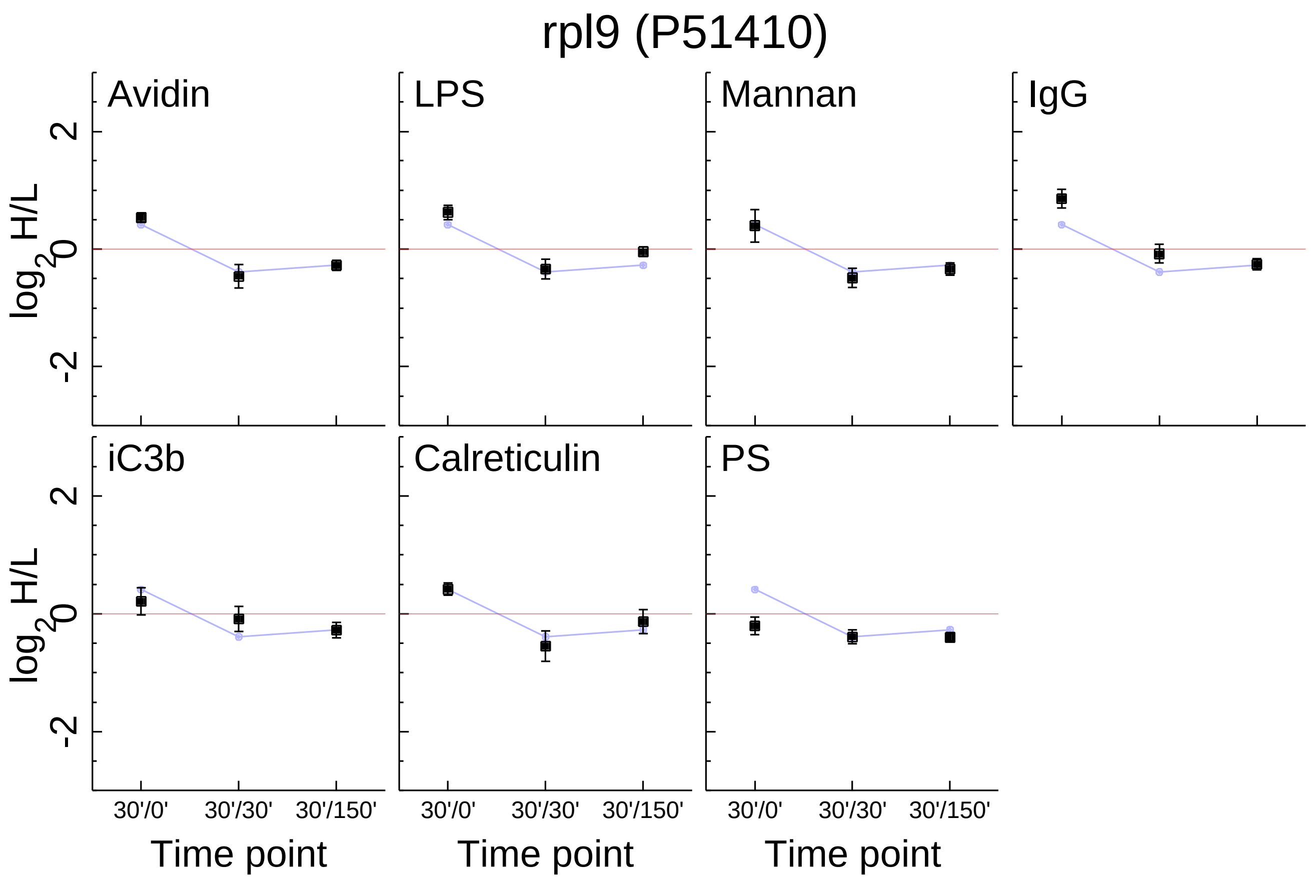
rpl9 (P51410)
Avidin
LPS
Mannan
IgG
iC3b
Calreticulin
PS
log2 H/L
2
0
-2
30'/0'
30'/30'
30'/150'
Time point

rplp0 (P14869)

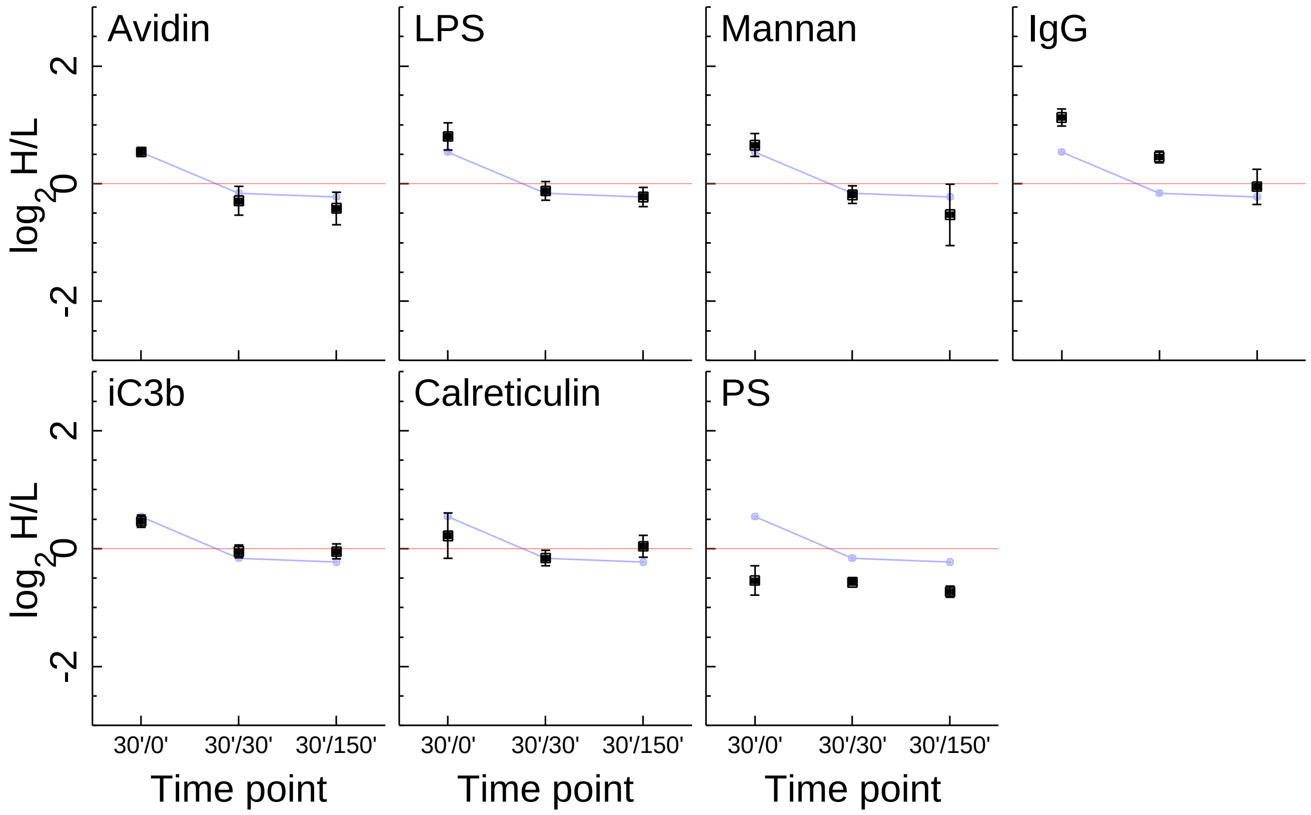

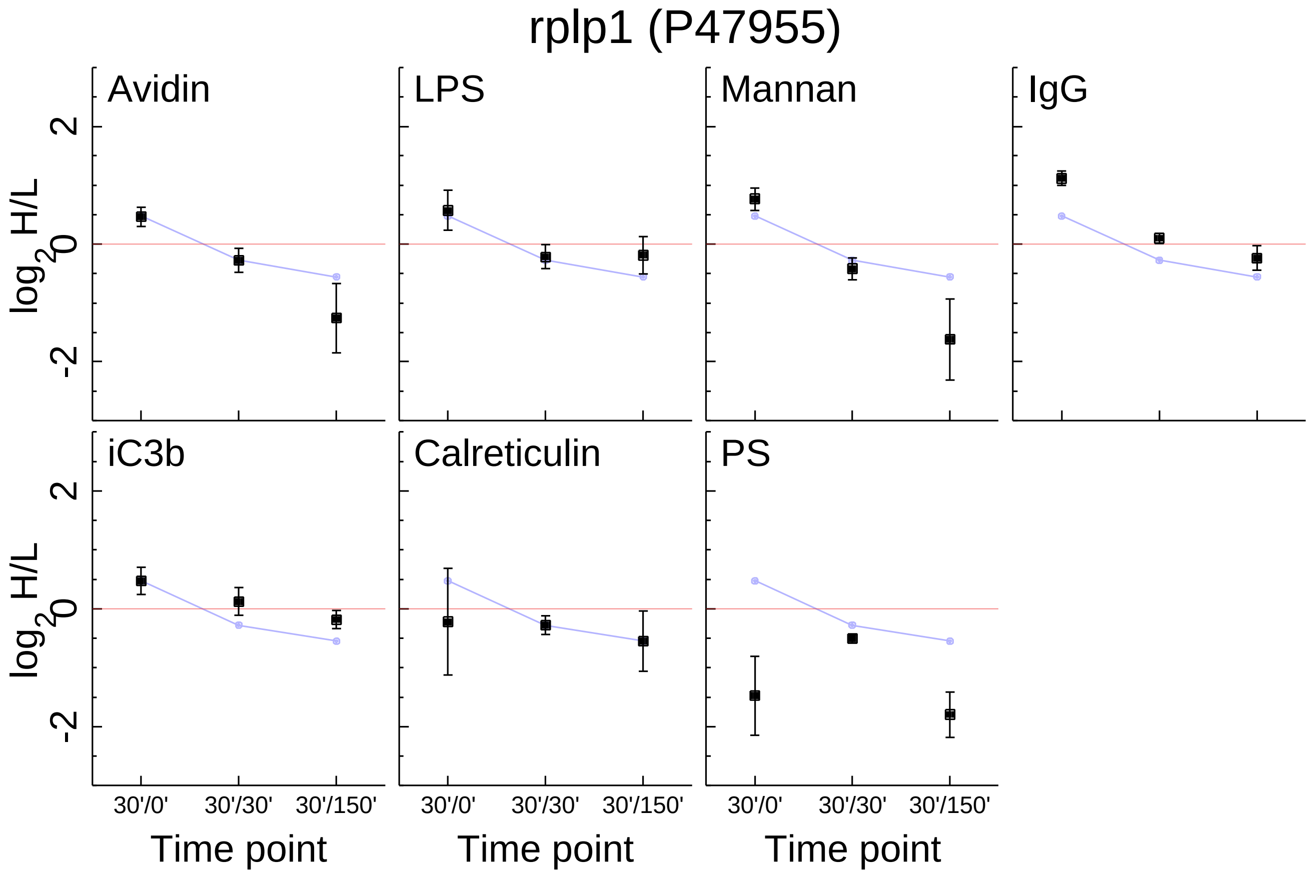

rplp1 (P47955)
Avidin
LPS
Mannan
IgG
iC3b
Calreticulin
PS
log2 H/L
2
0
-2
30'/0'
30'/30'
30'/150'
Time point

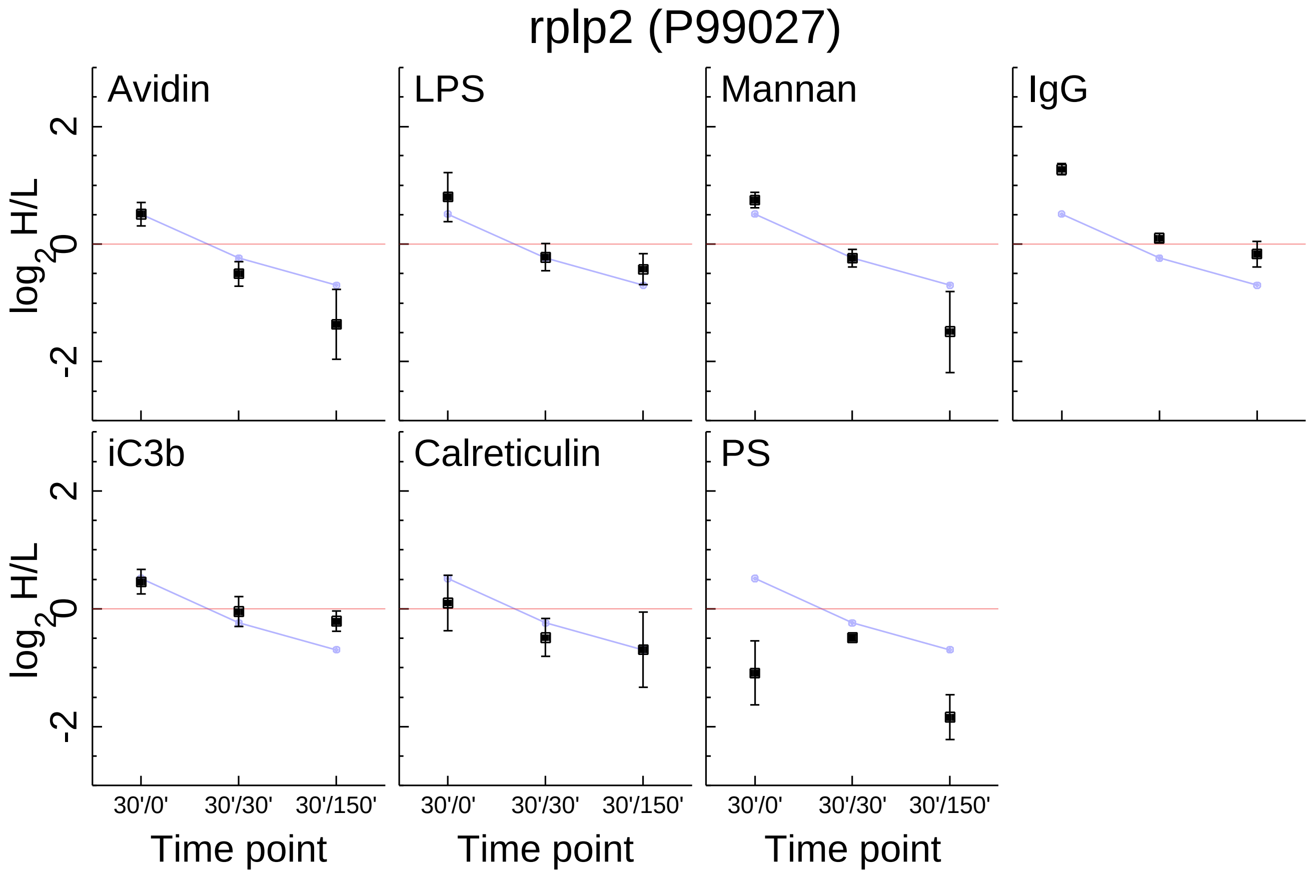
rplp2 (P99027)
Avidin
LPS
Mannan
IgG
iC3b
Calreticulin
PS
log2 H/L
2
0
-2
30'/0'
30'/30'
30'/150'
Time point

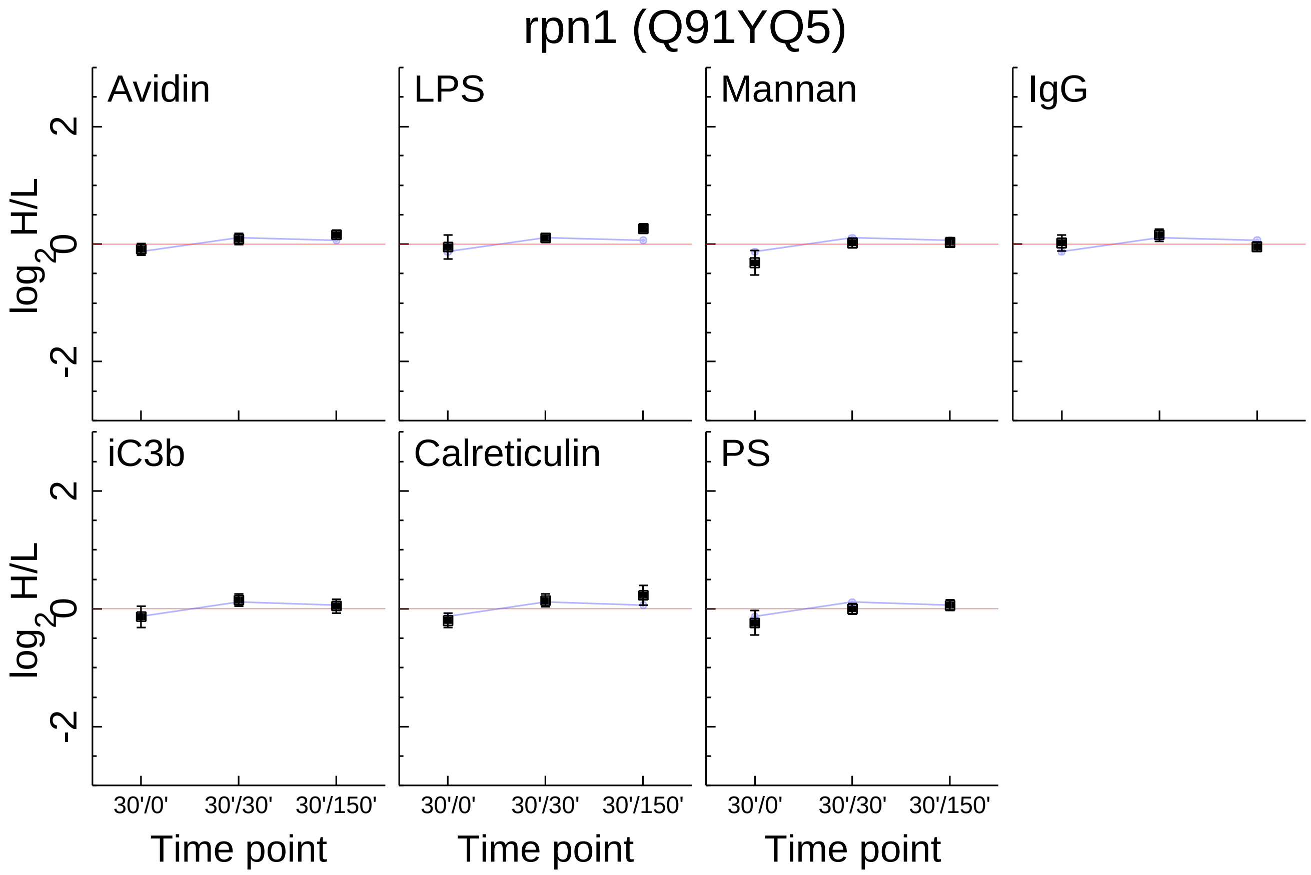

rpn1 (Q91YQ5)
Avidin
LPS
Mannan
IgG
iC3b
Calreticulin
PS
log2 H/L
2
0
-2
30'/0'
30'/30'
30'/150'
Time point

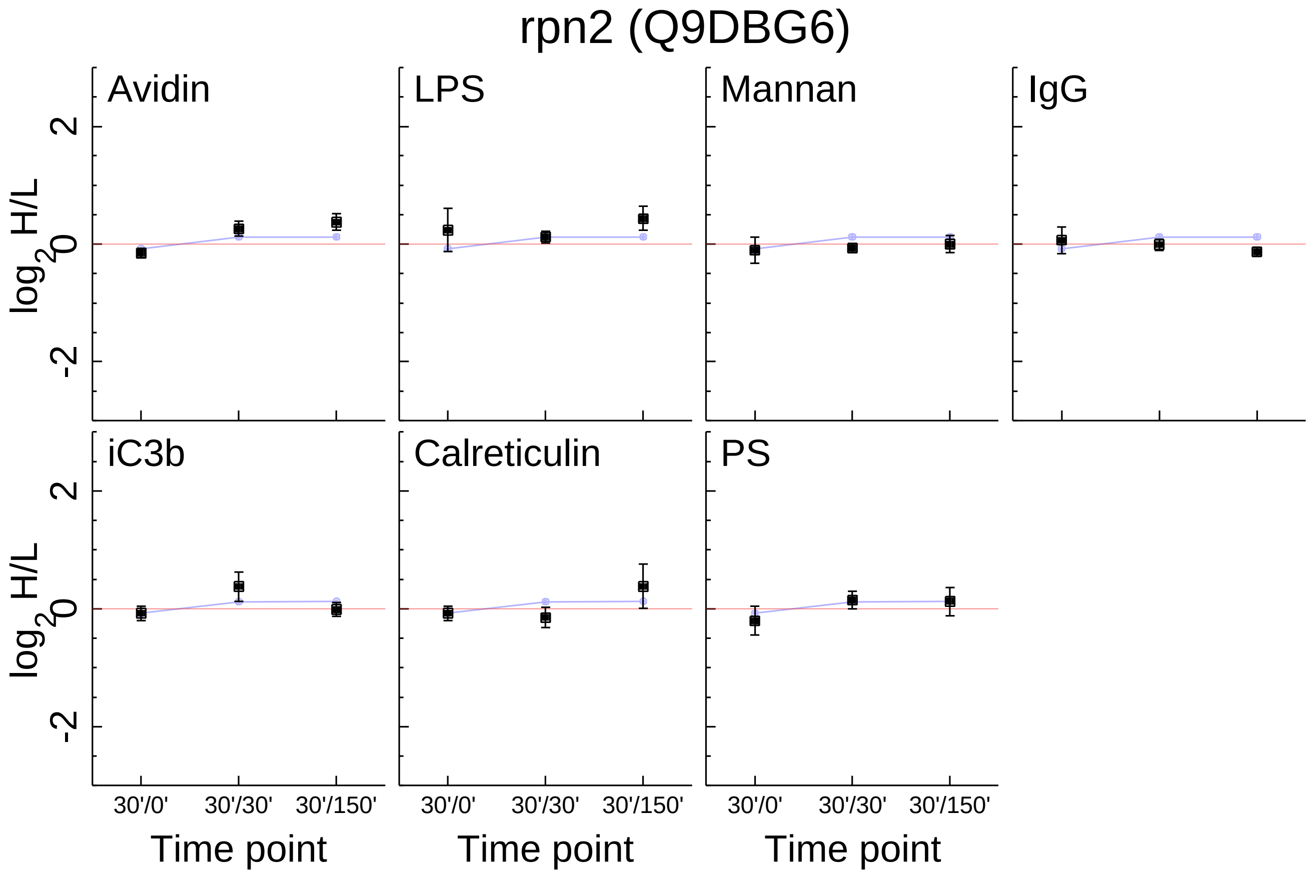
rpn2 (Q9DBG6)
Avidin
LPS
Mannan
IgG
iC3b
Calreticulin
PS
log2 H/L
2
0
-2
30'/0'
30'/30'
30'/150'
Time point

rprd2 (Q6NXI6)

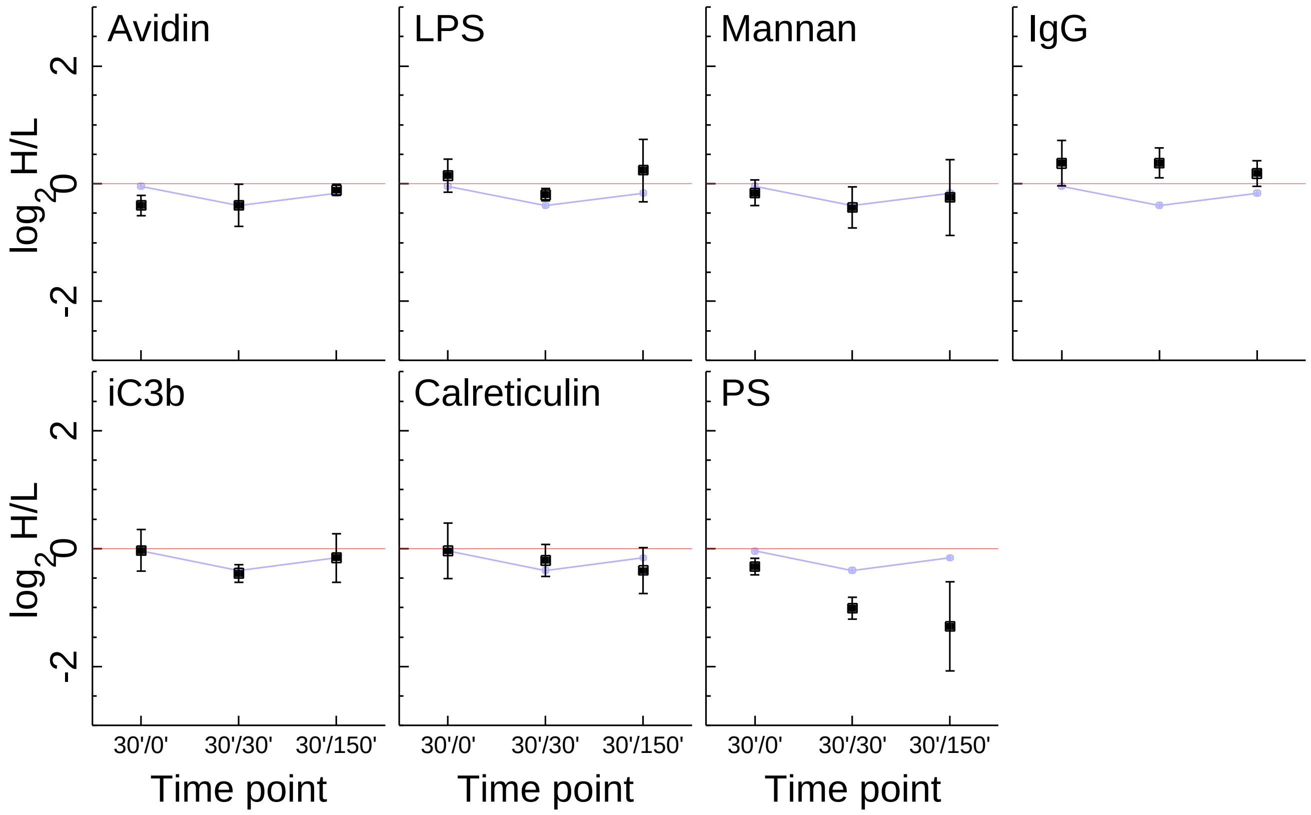

rps10 (P63325)

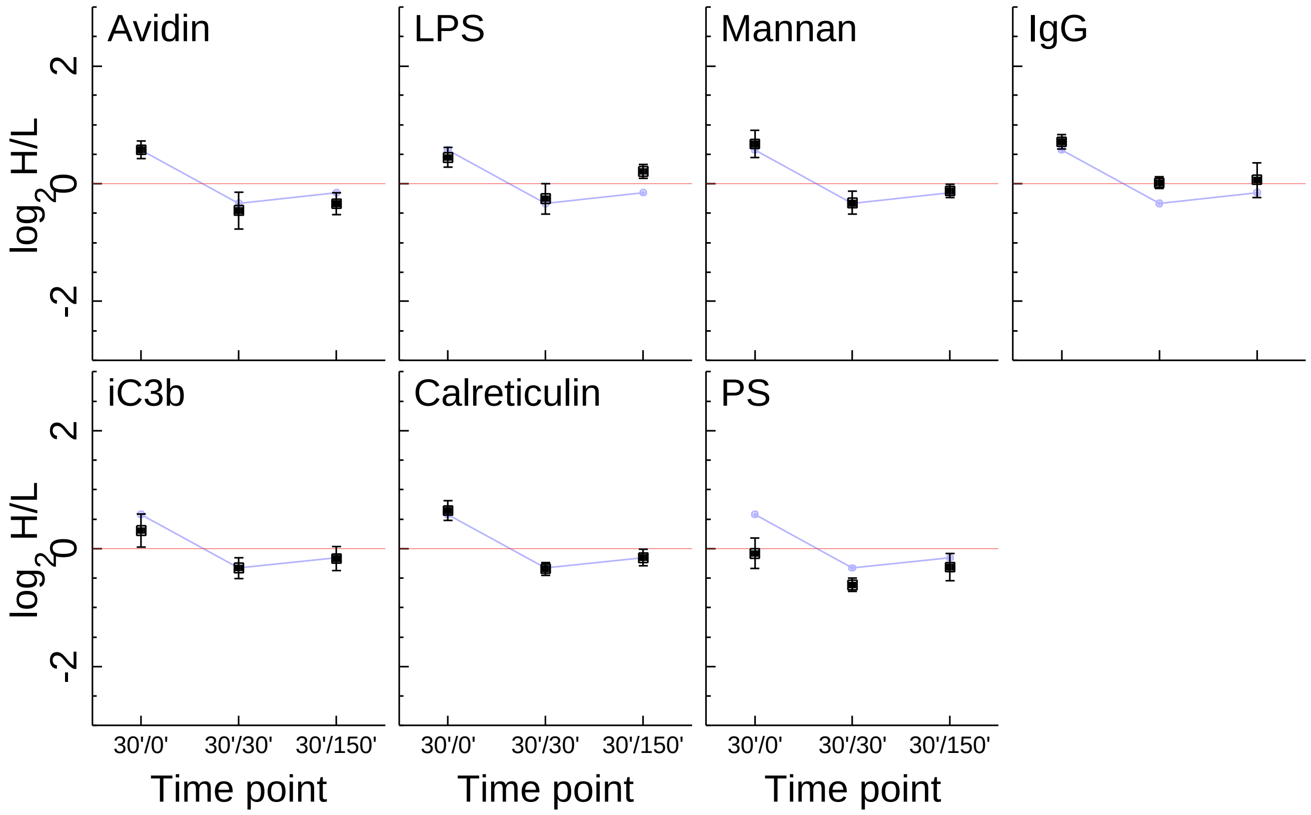

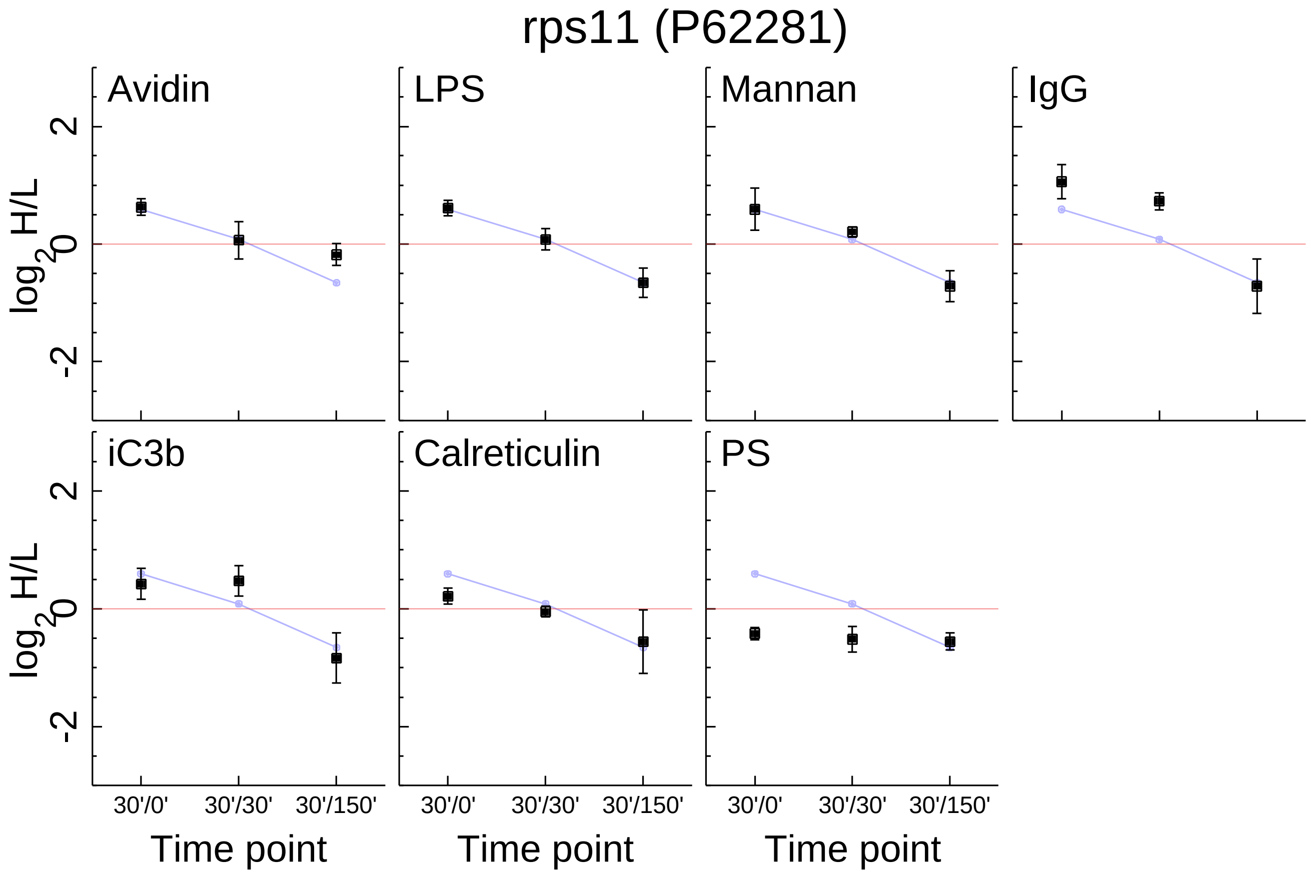

rps11 (P62281)
Avidin
LPS
Mannan
IgG
iC3b
Calreticulin
PS
log2 H/L
2
0
-2
30'/0'
30'/30'
30'/150'
Time point

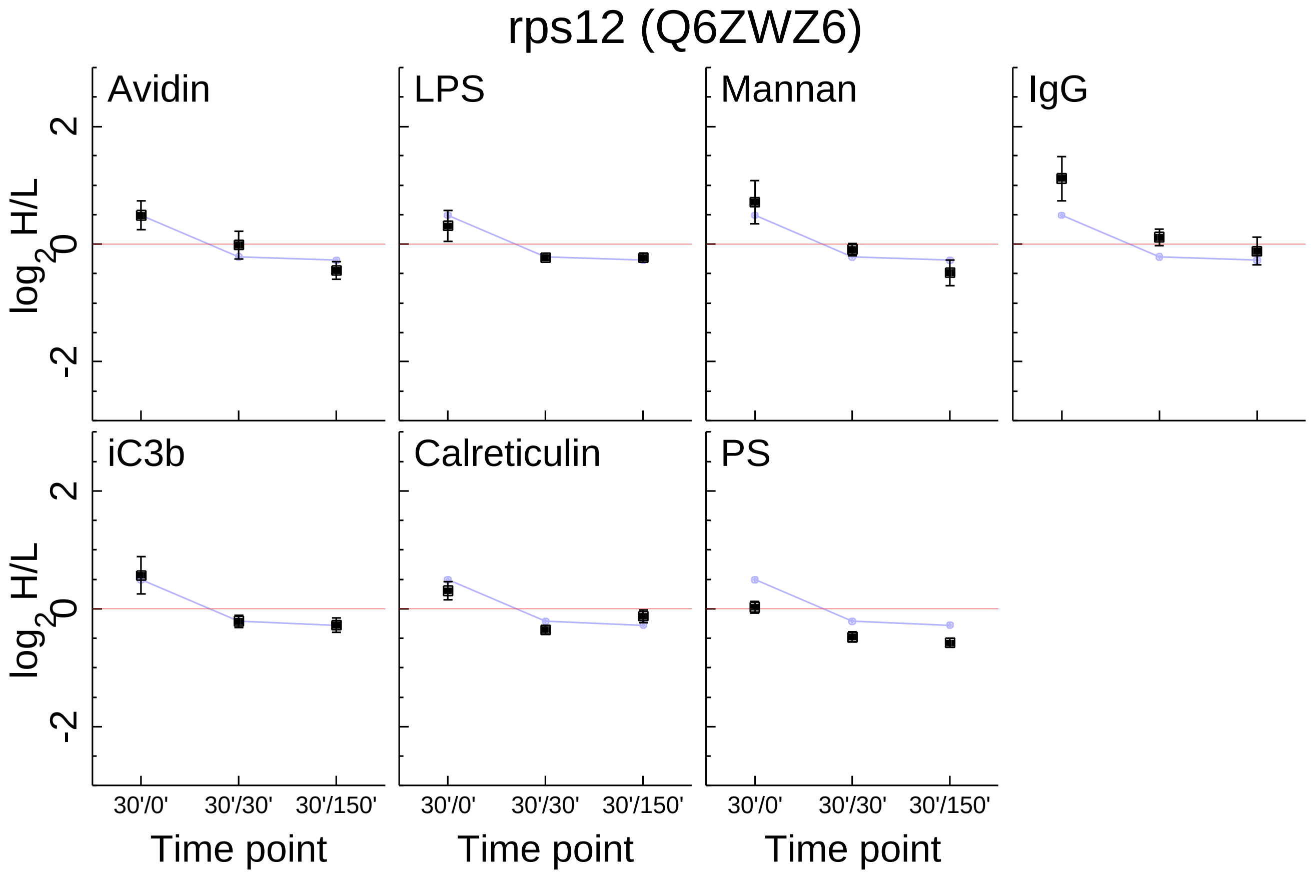

rps12 (Q6ZWZ6)
Avidin
LPS
Mannan
IgG
iC3b
Calreticulin
PS
log2 H/L
2
0
-2
30'/0'
30'/30'
30'/150'
Time point

rps13 (P62301)

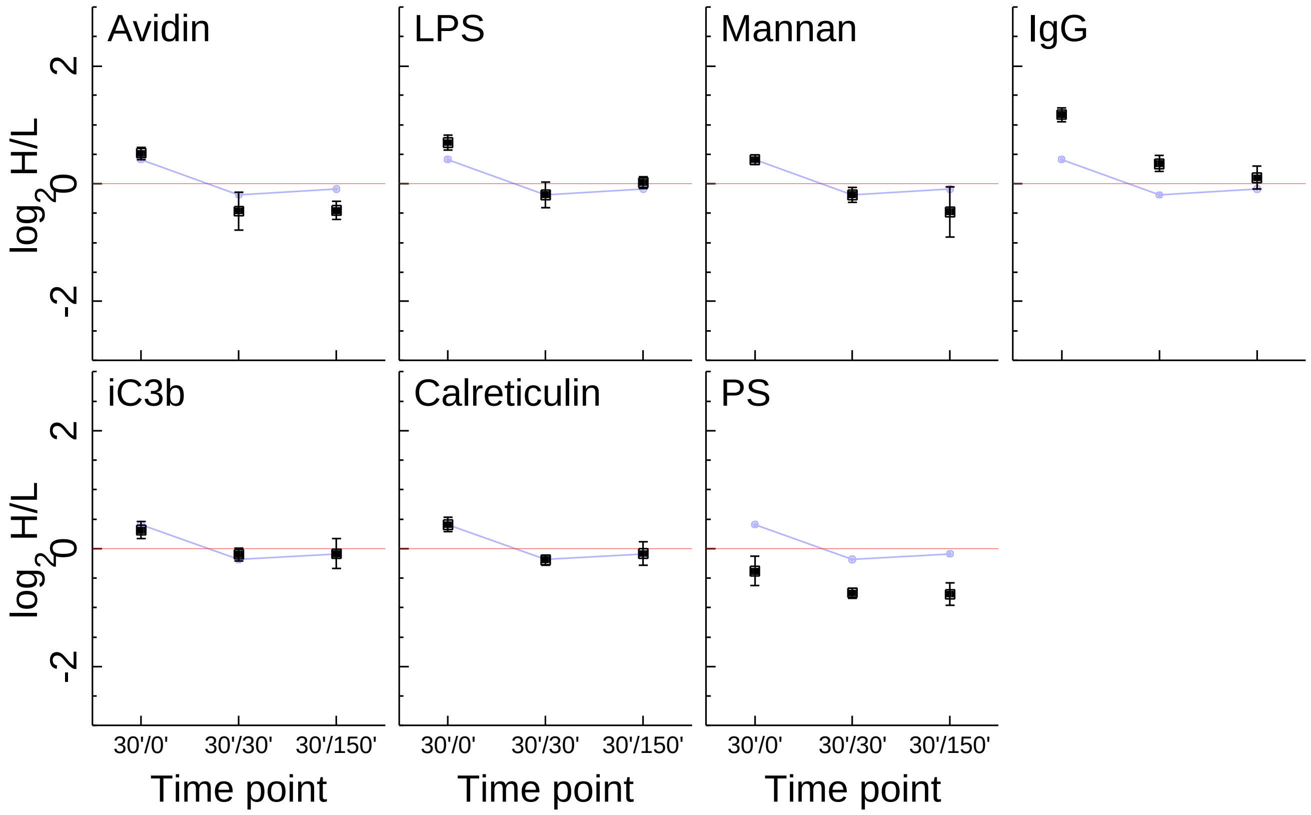

rps14 (P62264)

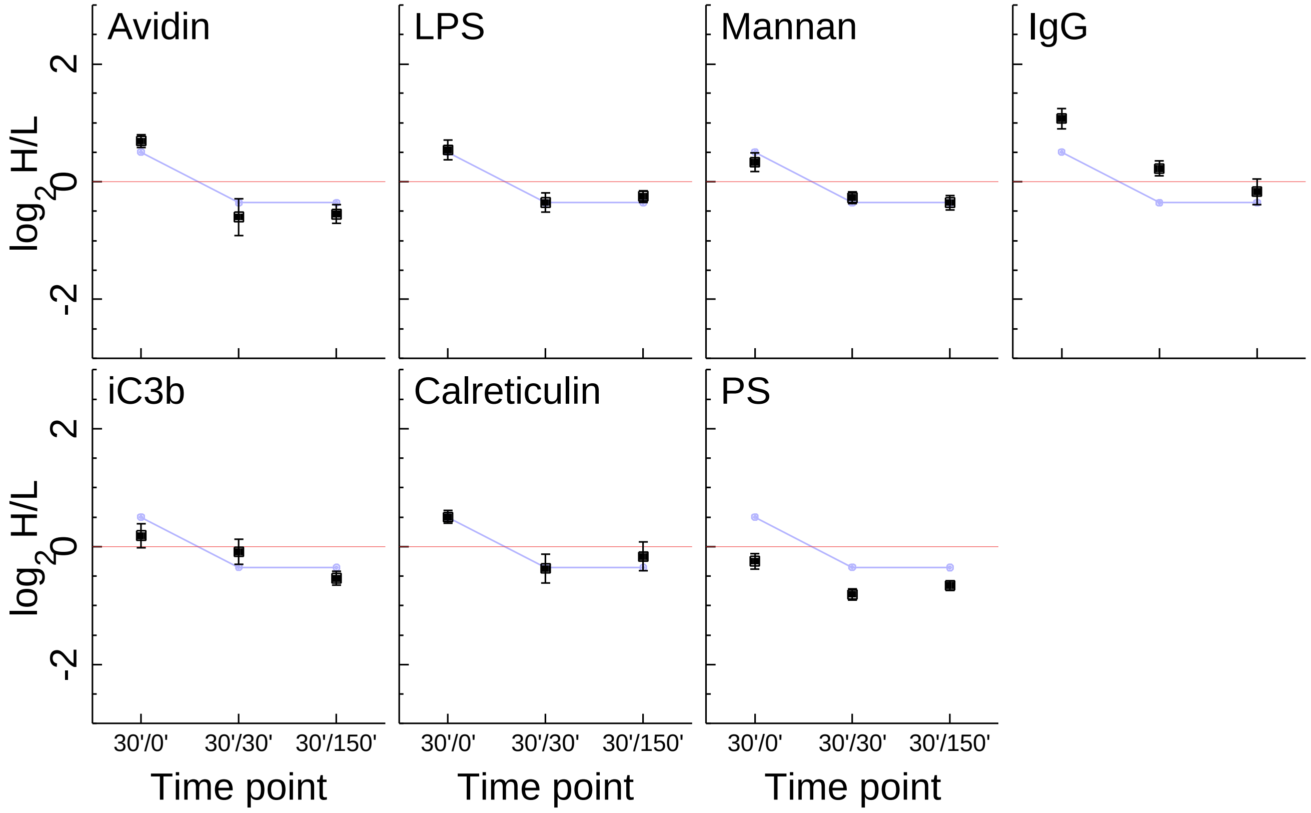

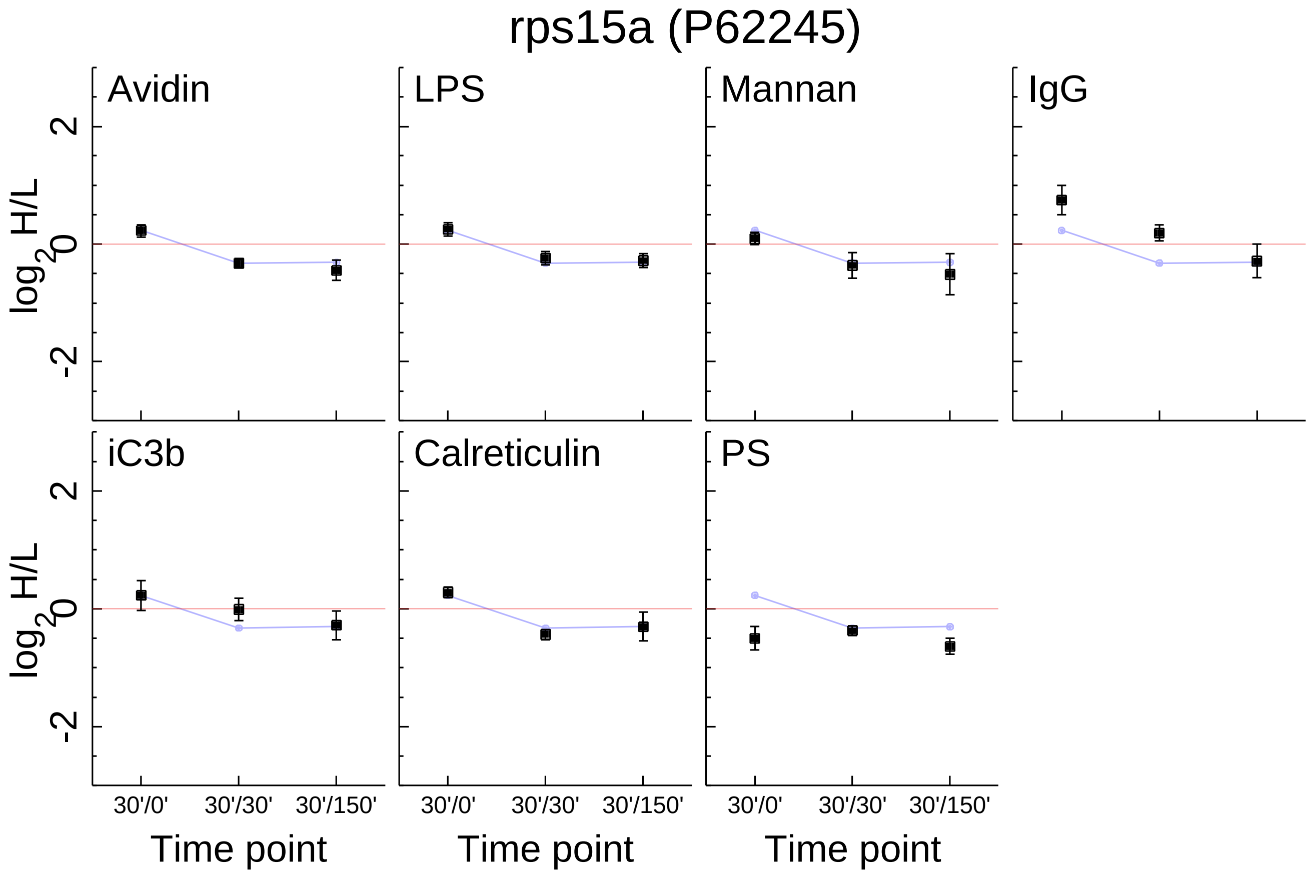
rps15a (P62245)
Avidin
LPS
Mannan
IgG
iC3b
Calreticulin
PS
log2 H/L
2
0
-2
30'/0'
30'/30'
30'/150'
Time point

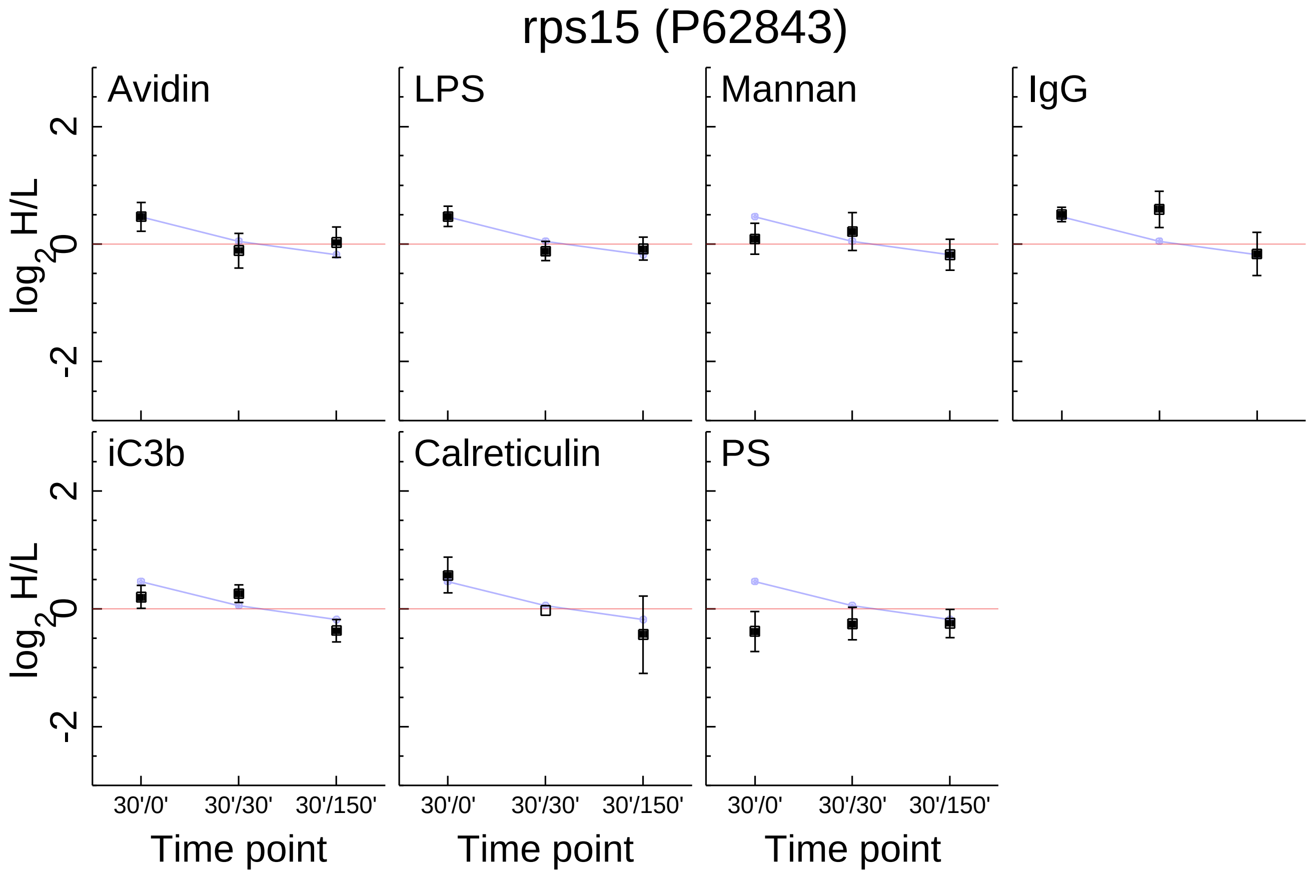
rps15 (P62843)
Avidin
LPS
Mannan
IgG
iC3b
Calreticulin
PS
log2 H/L
2
0
-2
30'/0'
30'/30'
30'/150'
Time point

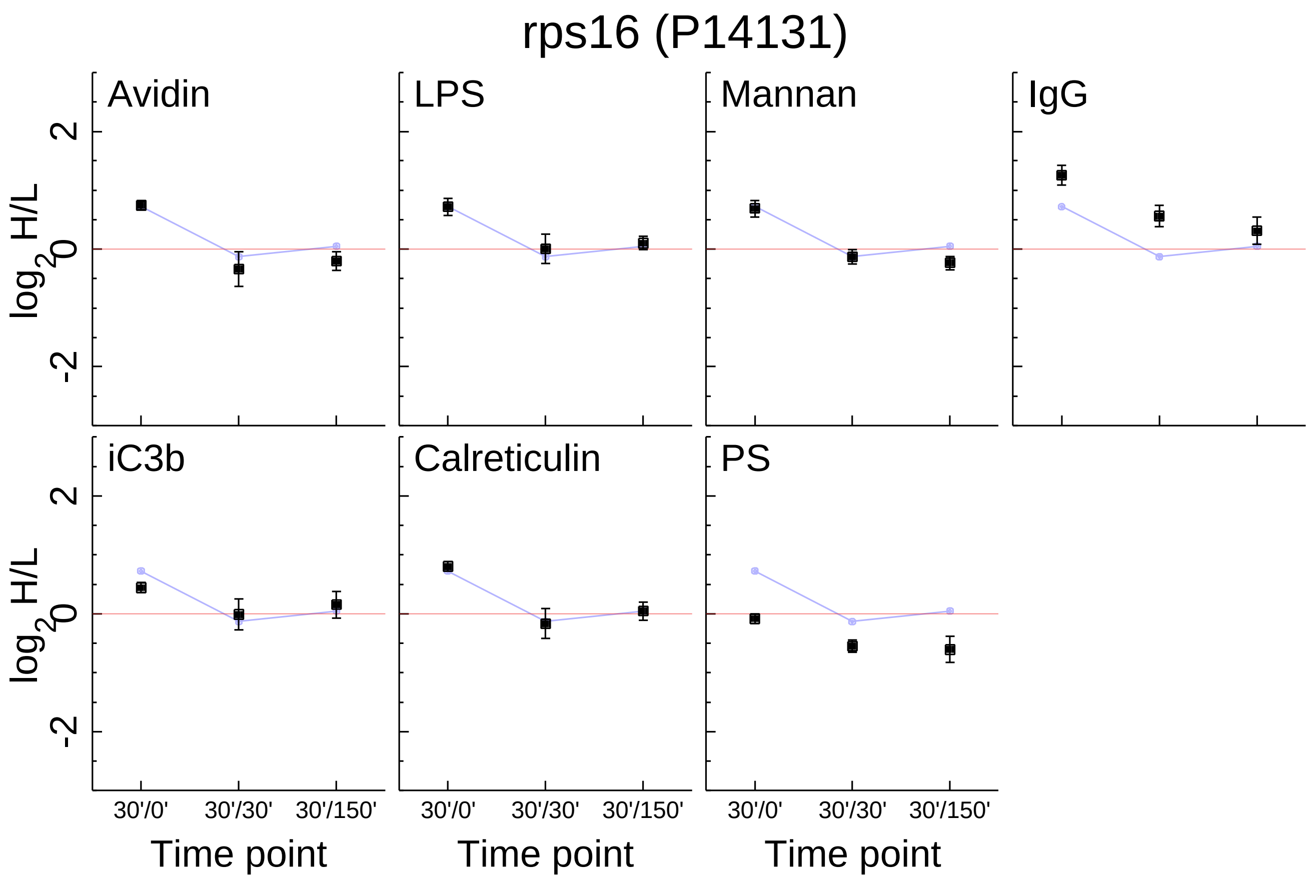

rps16 (P14131)
Avidin
LPS
Mannan
IgG
iC3b
Calreticulin
PS
log2 H/L
2
0
-2
30'/0'
30'/30'
30'/150'
Time point

rps17 (P63276)

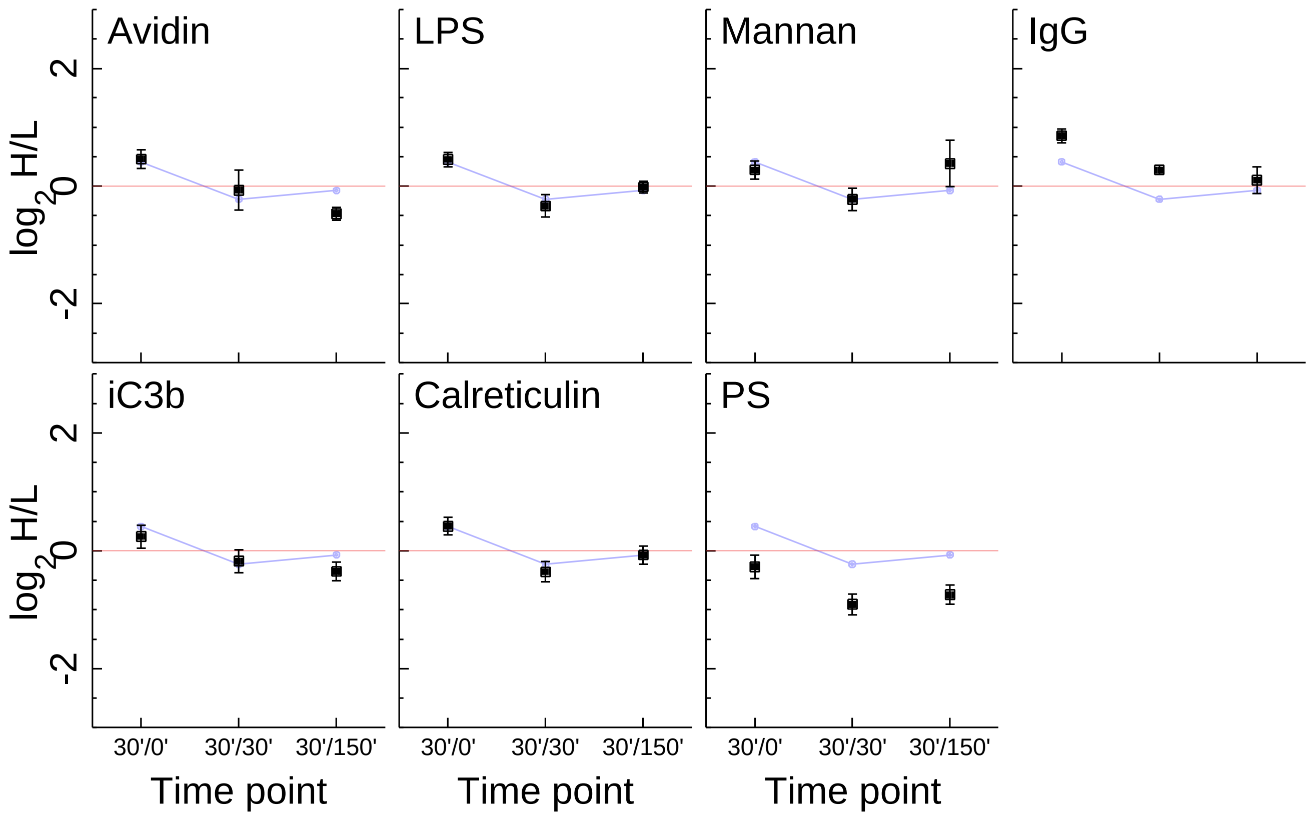

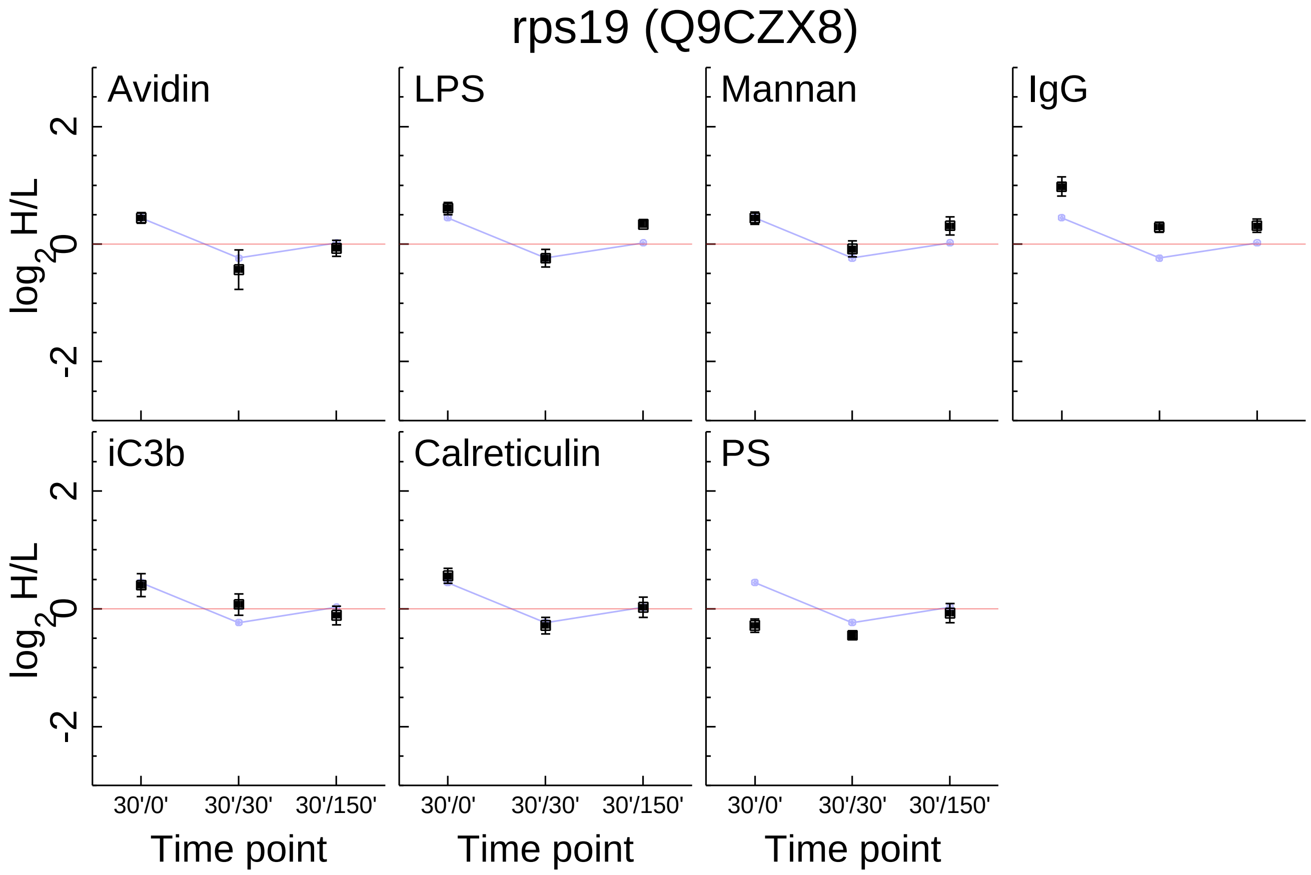
rps19 (Q9CZX8)
Avidin
LPS
Mannan
IgG
iC3b
Calreticulin
PS
log2 H/L
2
0
-2
30'/0'
30'/30'
30'/150'
Time point

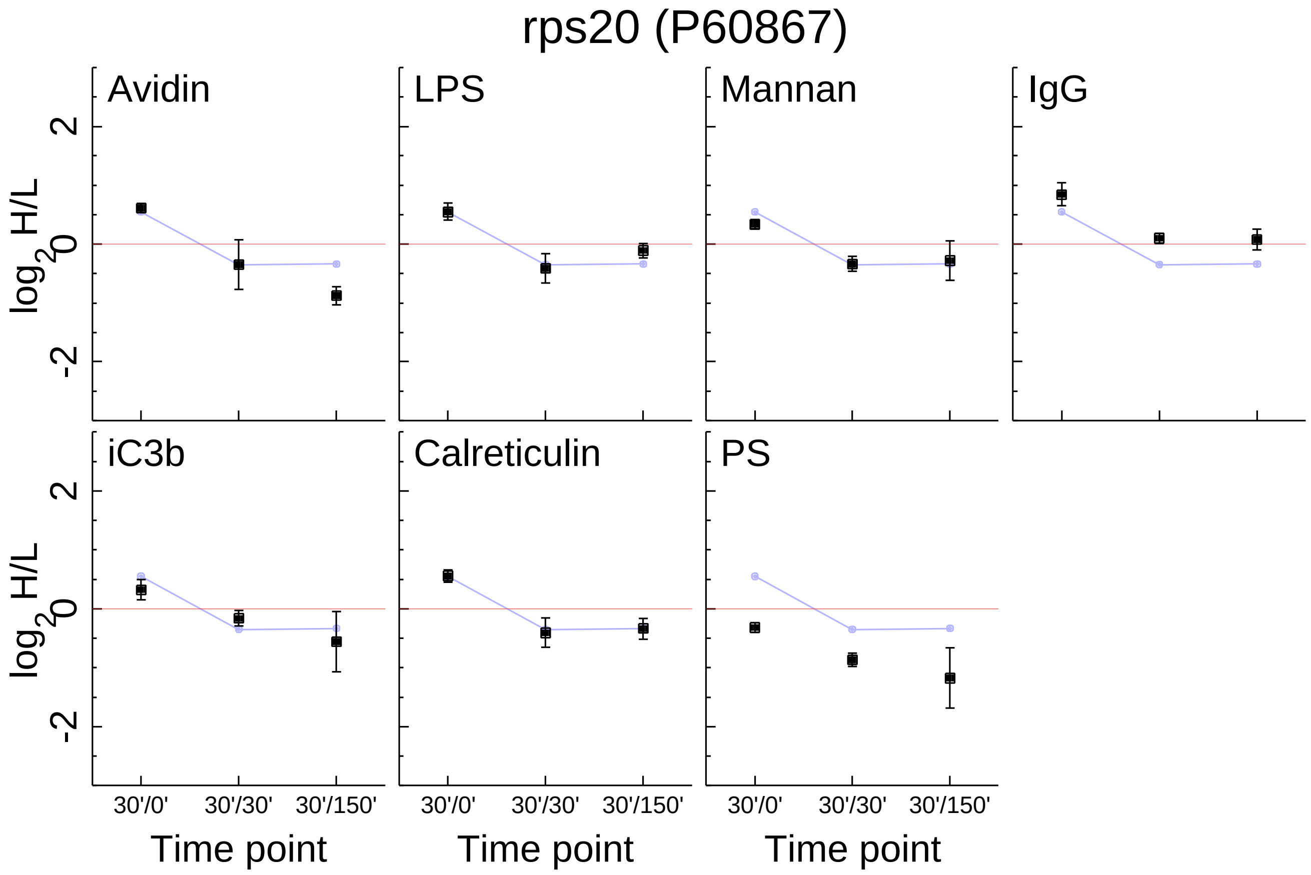

rps20 (P60867)
Avidin
LPS
Mannan
IgG
iC3b
Calreticulin
PS
log2 H/L
2
0
-2
30'/0'
30'/30'
30'/150'
Time point

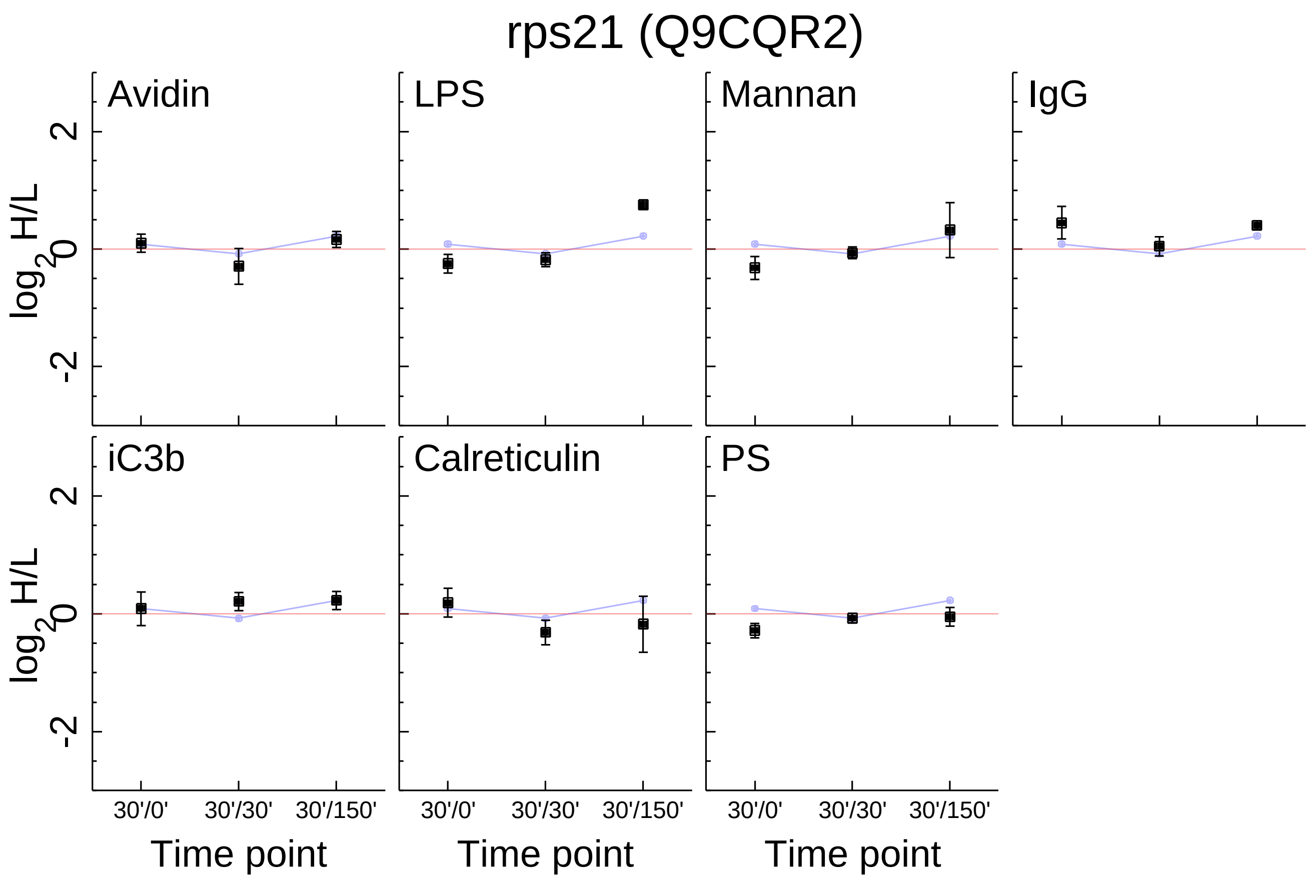
rps21 (Q9CQR2)
Avidin
LPS
Mannan
IgG
iC3b
Calreticulin
PS
log2 H/L
2
0
-2
30'/0'
30'/30'
30'/150'
Time point

rps23 (P62267)

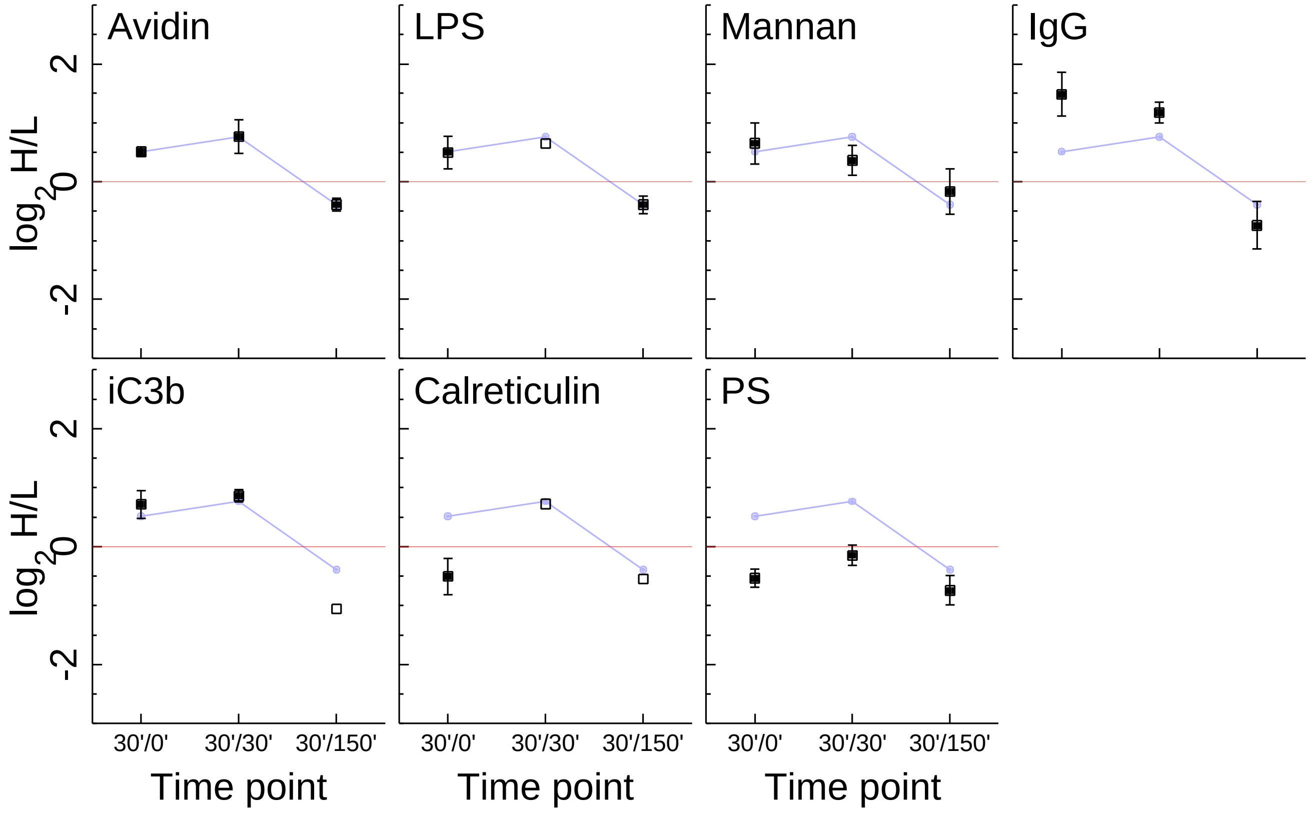

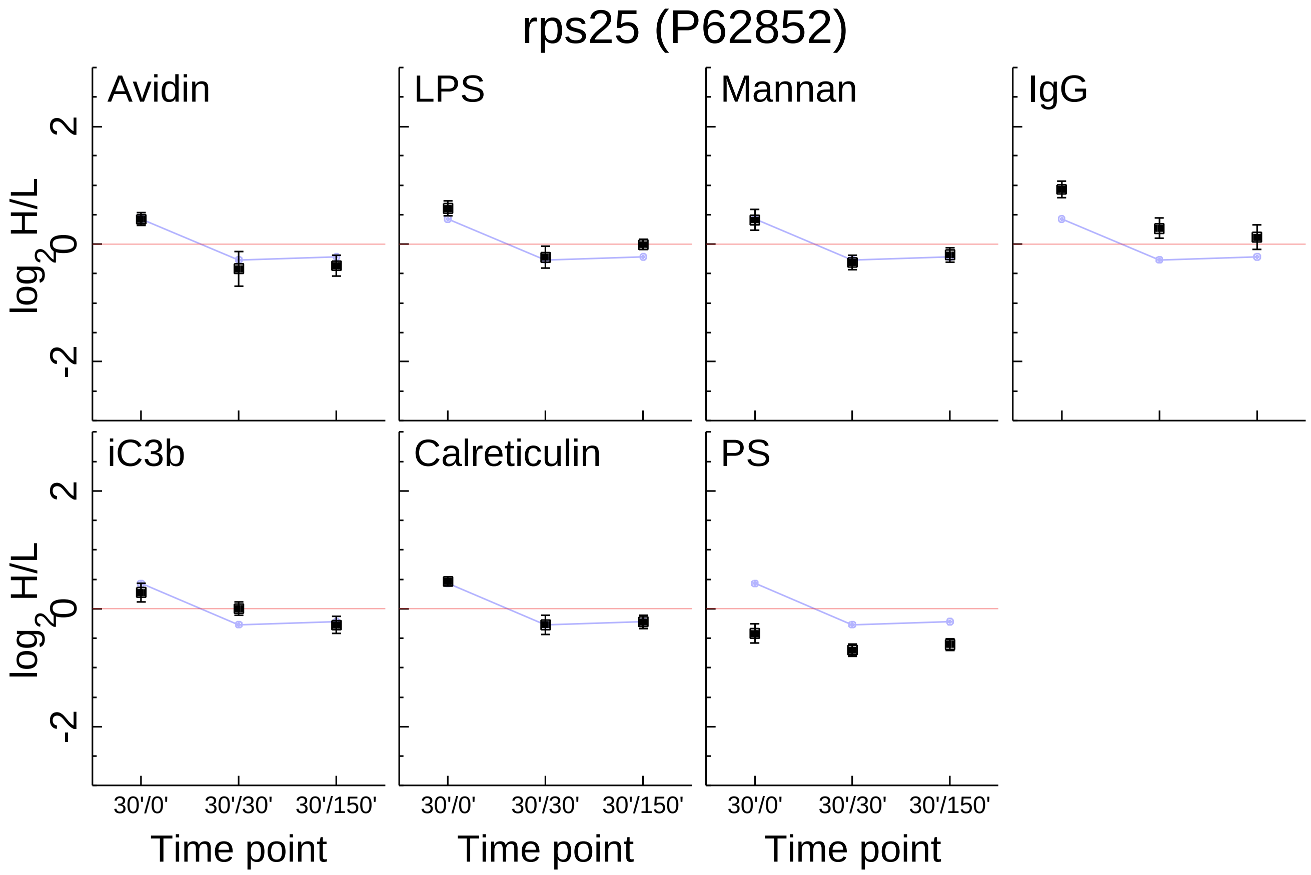

rps25 (P62852)
Avidin
LPS
Mannan
IgG
iC3b
Calreticulin
PS
log2 H/L
2
0
-2
30'/0'
30'/30'
30'/150'
Time point

rps26 (P62855)

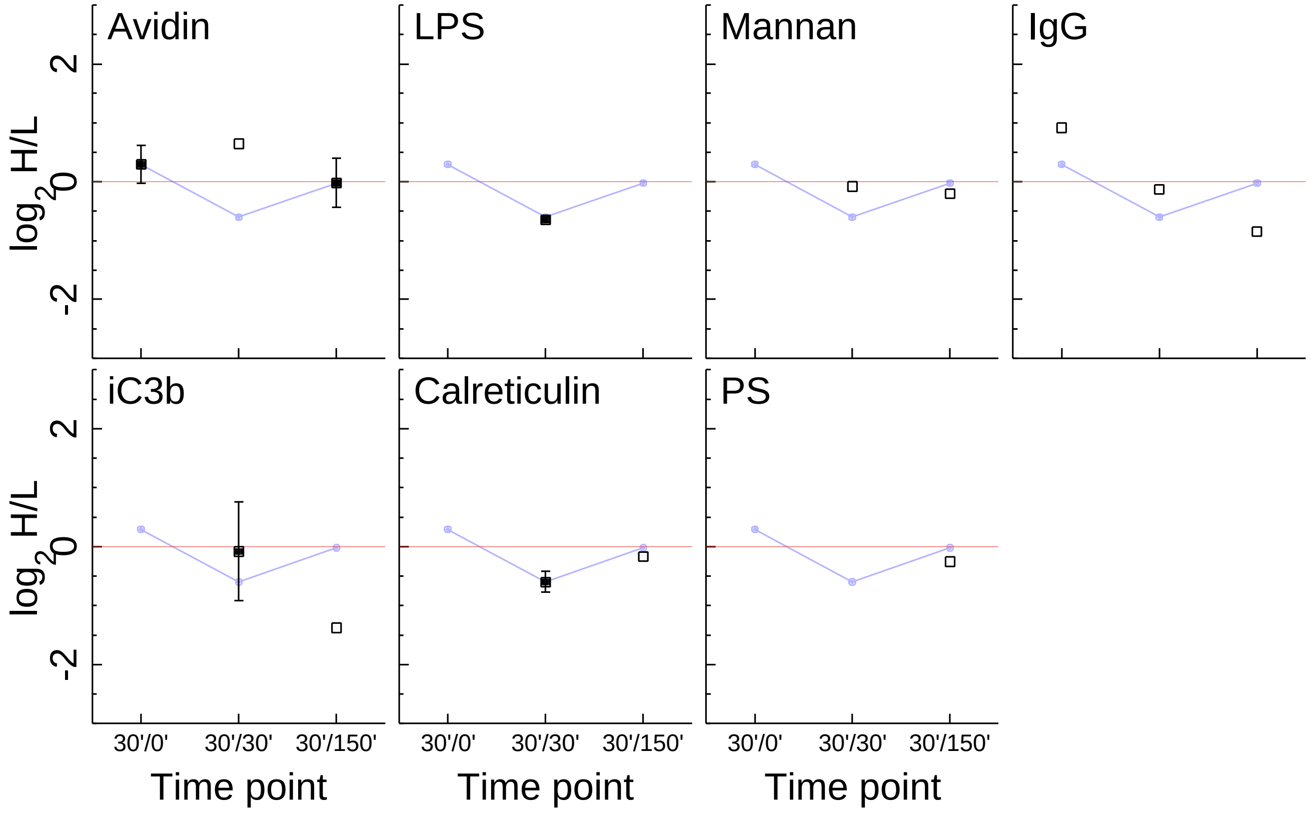

rps27a (P62983)

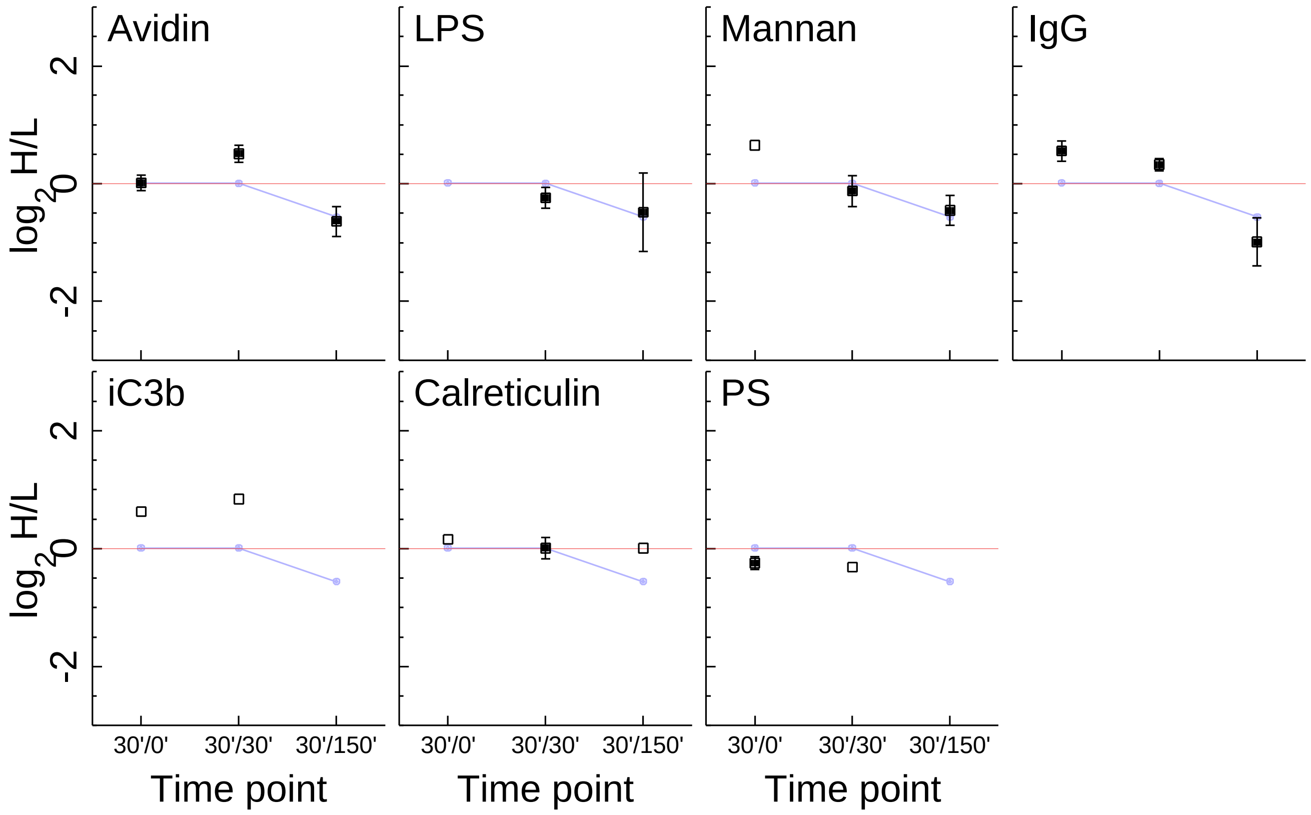

rps27l (Q6ZWY3)

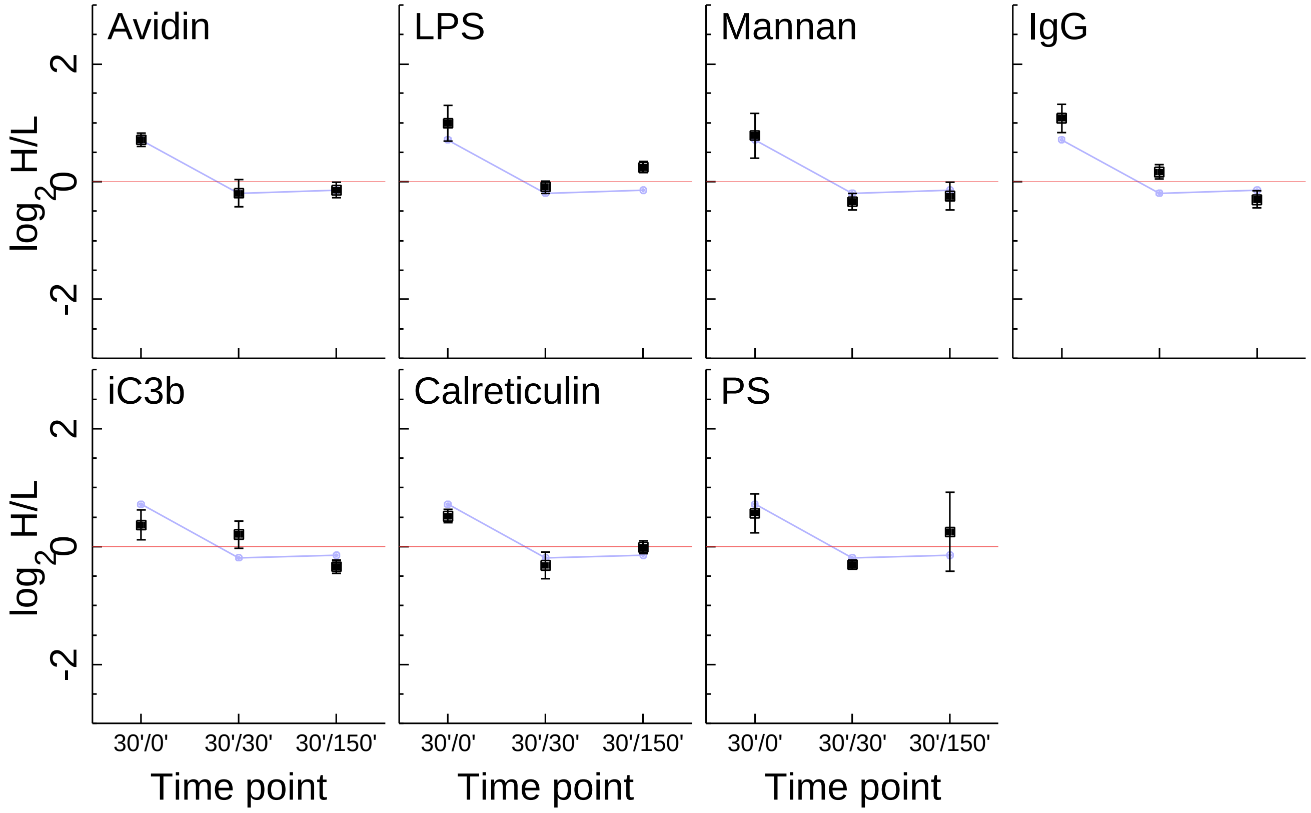

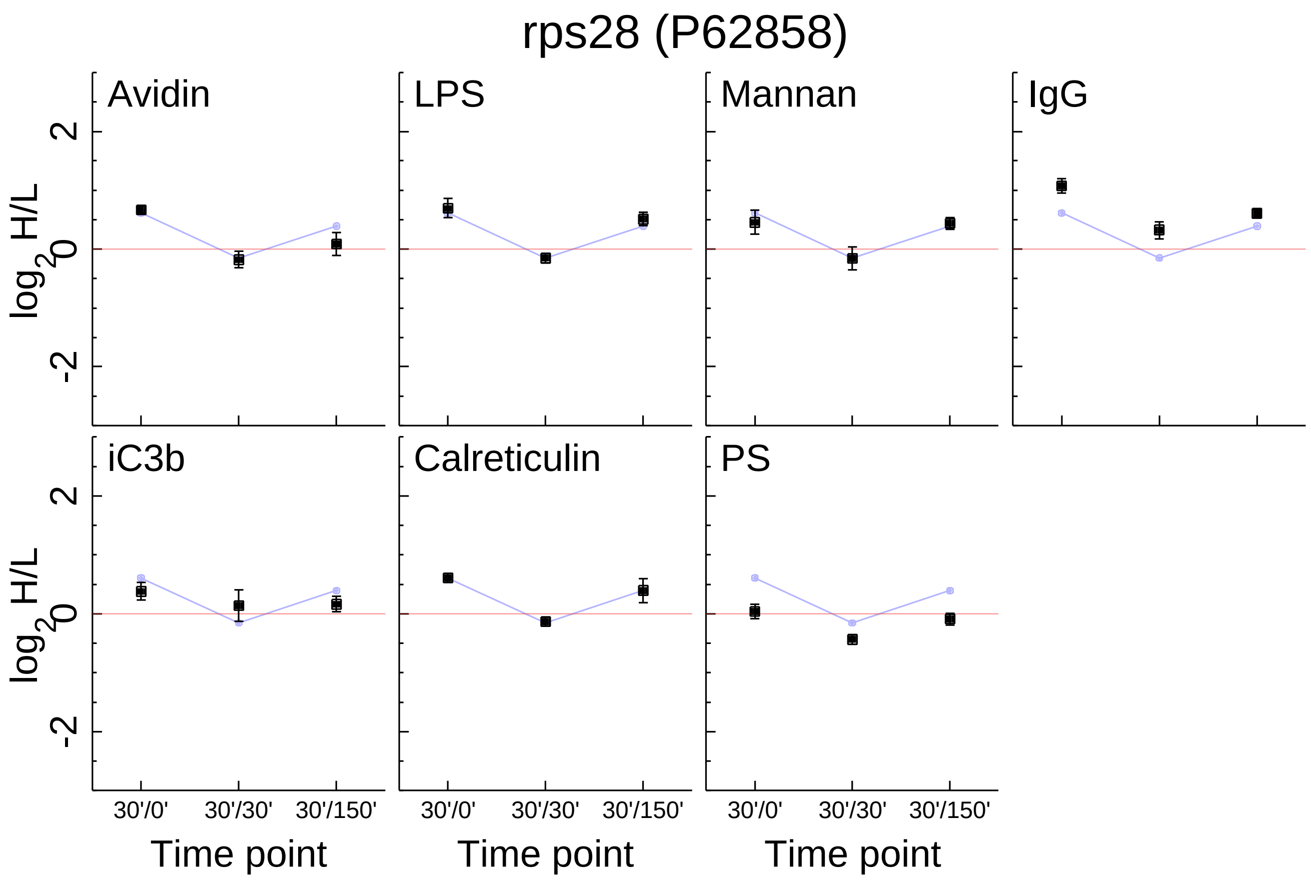
rps28 (P62858)
Avidin
LPS
Mannan
IgG
iC3b
Calreticulin
PS
log2 H/L
2
0
-2
30'/0'
30'/30'
30'/150'
Time point

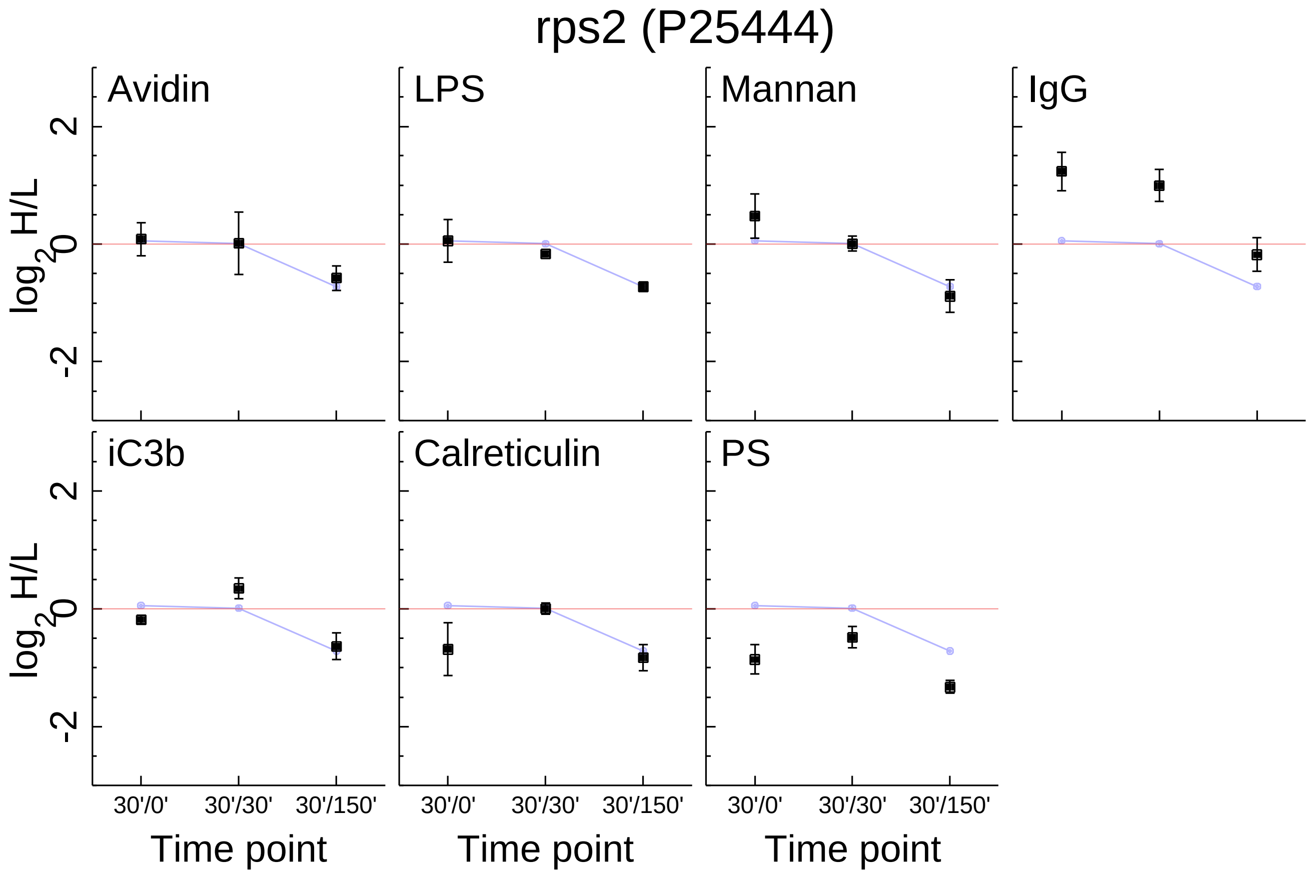
rps2 (P25444)
Avidin
LPS
Mannan
IgG
iC3b
Calreticulin
PS
log2 H/L
2
0
-2
30'/0'
30'/30'
30'/150'
Time point

rps3 (P62908)

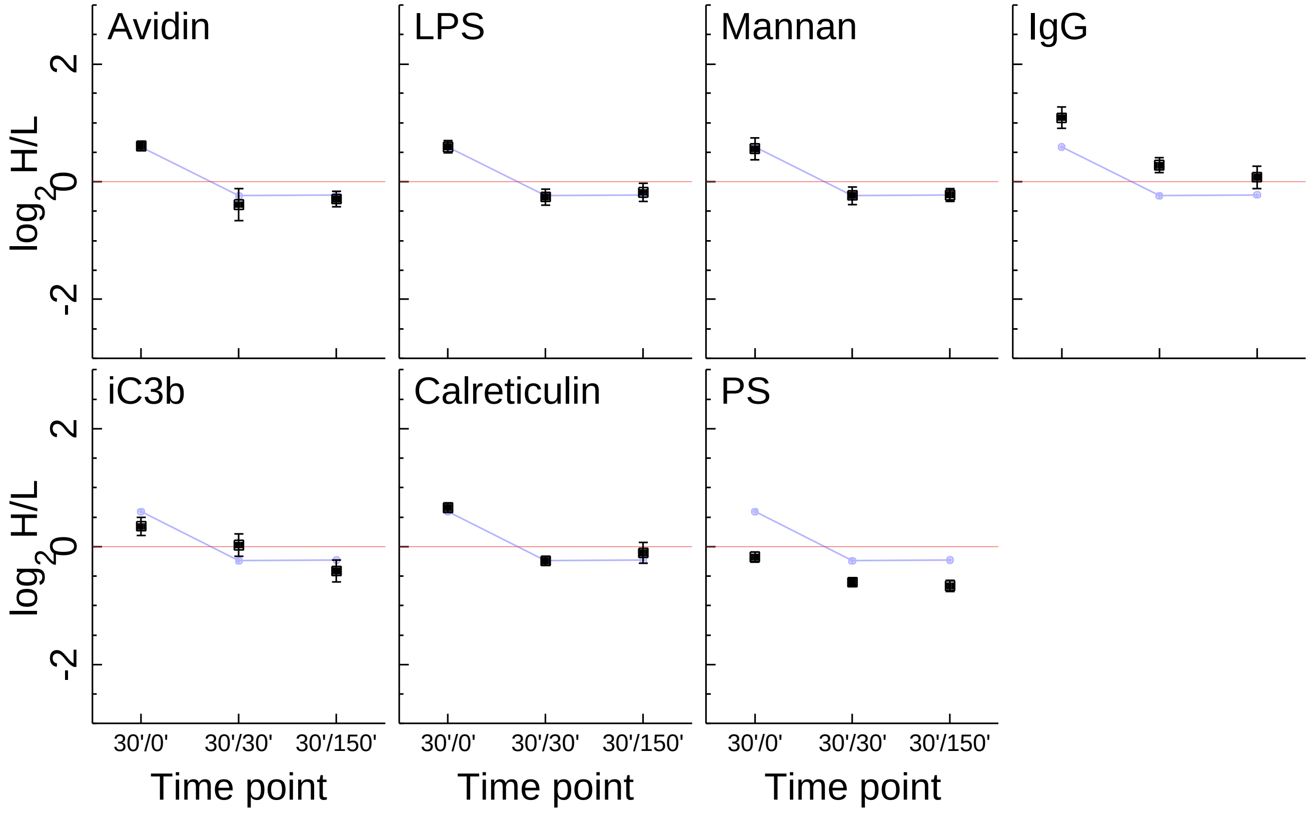
Avidin
LPS
Mannan
IgG
iC3b
Calreticulin
PS
log2 H/L
2
0
-2
30'/0'
30'/30'
30'/150'
Time point

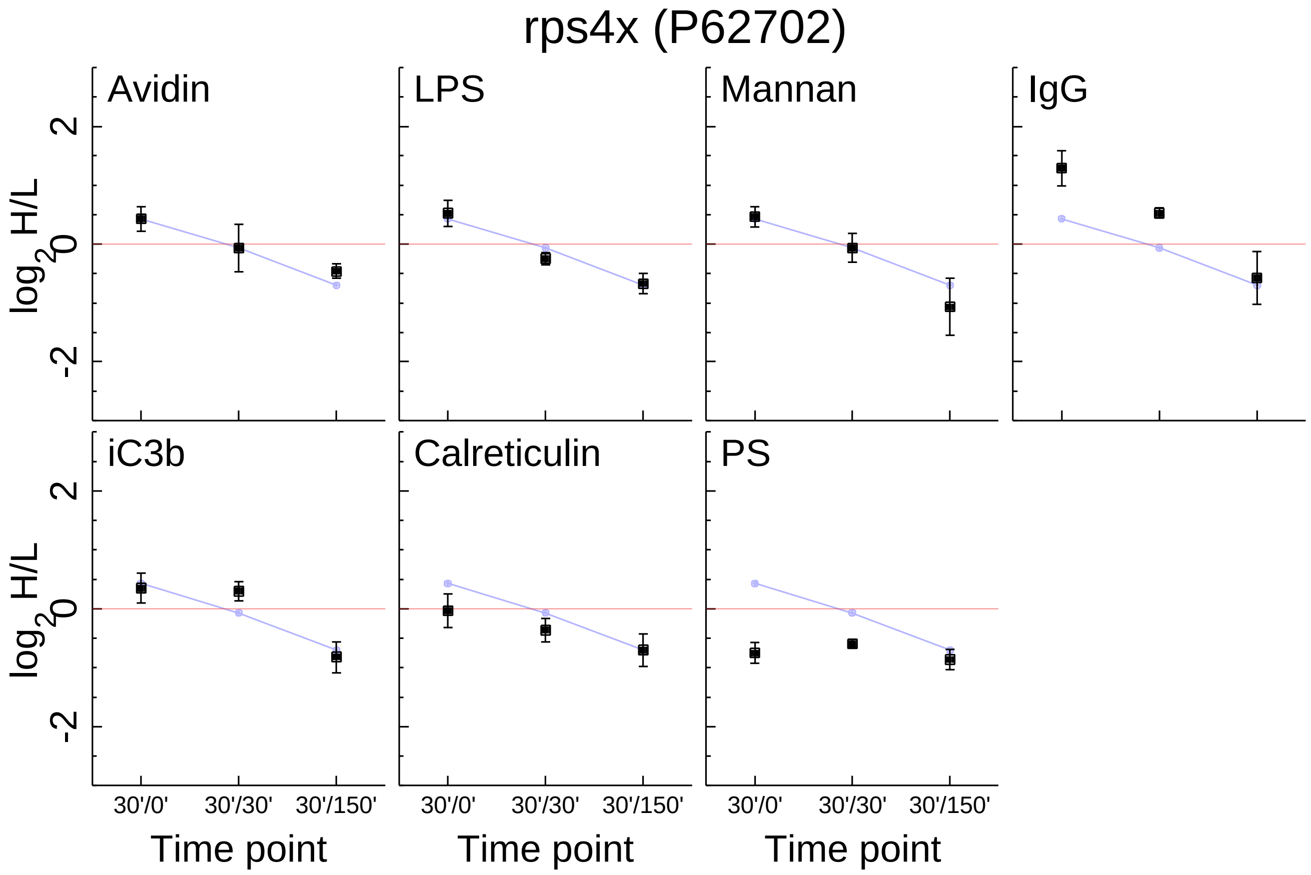
rps4x (P62702)
Avidin
LPS
Mannan
IgG
iC3b
Calreticulin
PS
log2 H/L
2
0
-2
30'/0'
30'/30'
30'/150'
Time point

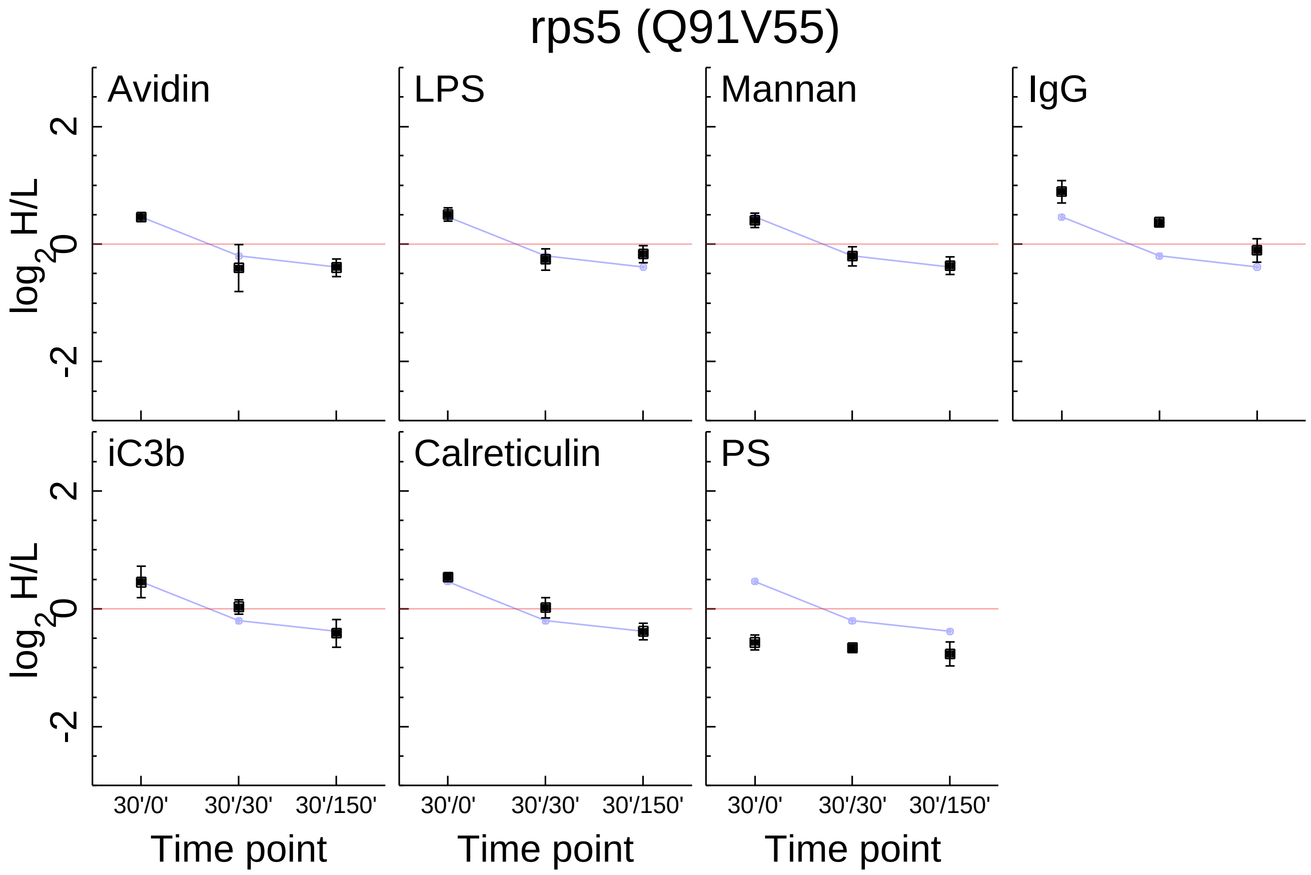

rps5 (Q91V55)
Avidin
LPS
Mannan
IgG
iC3b
Calreticulin
PS
log2 H/L
2
0
-2
30'/0'
30'/30'
30'/150'
Time point

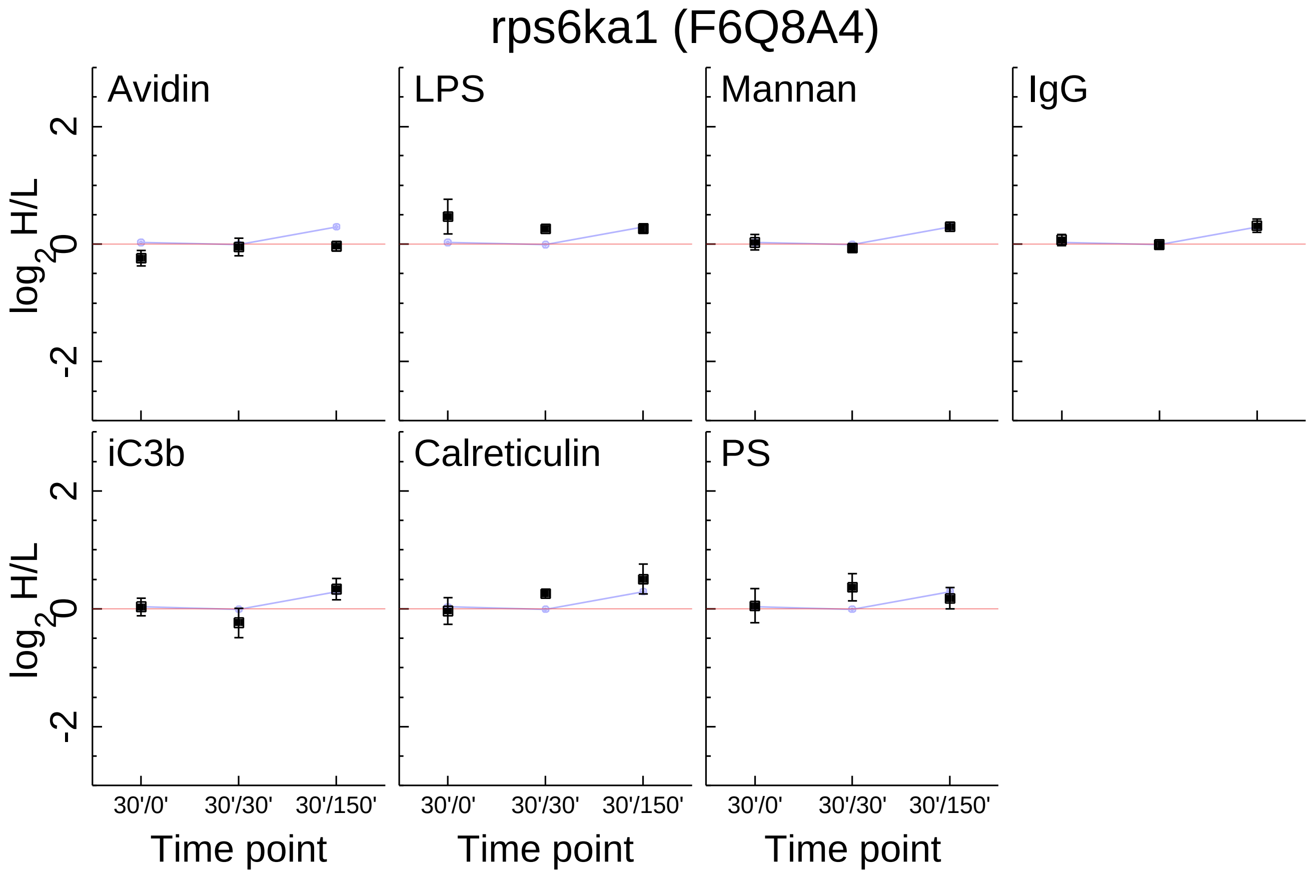
rps6ka1 (F6Q8A4)
Avidin
LPS
Mannan
IgG
iC3b
Calreticulin
PS
log2 H/L
2
0
-2
30'/0'
30'/30'
30'/150'
Time point

rps6 (P62754)

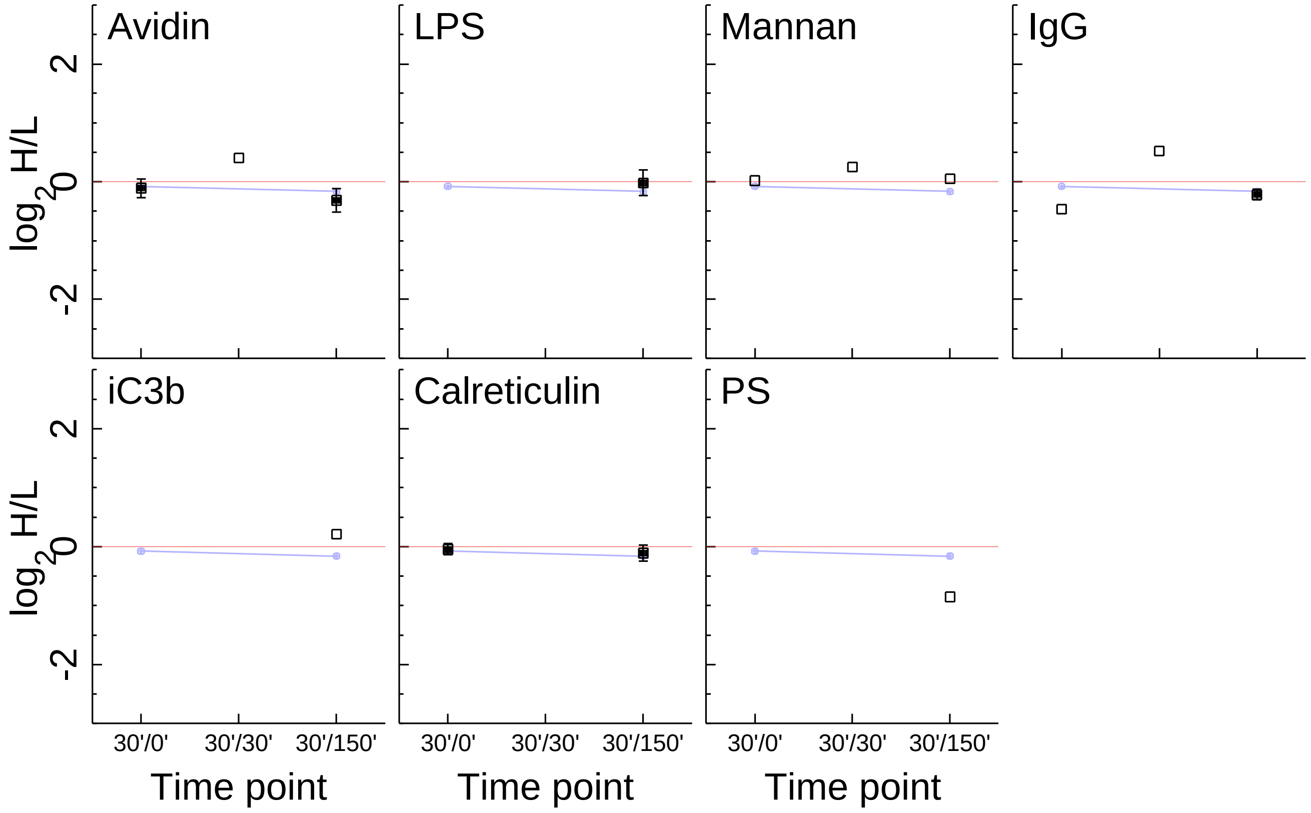
Avidin
LPS
Mannan
IgG
iC3b
Calreticulin
PS
2
0
-2
$\log_2$ H/L
30'/0'
30'/30'
30'/150'
Time point

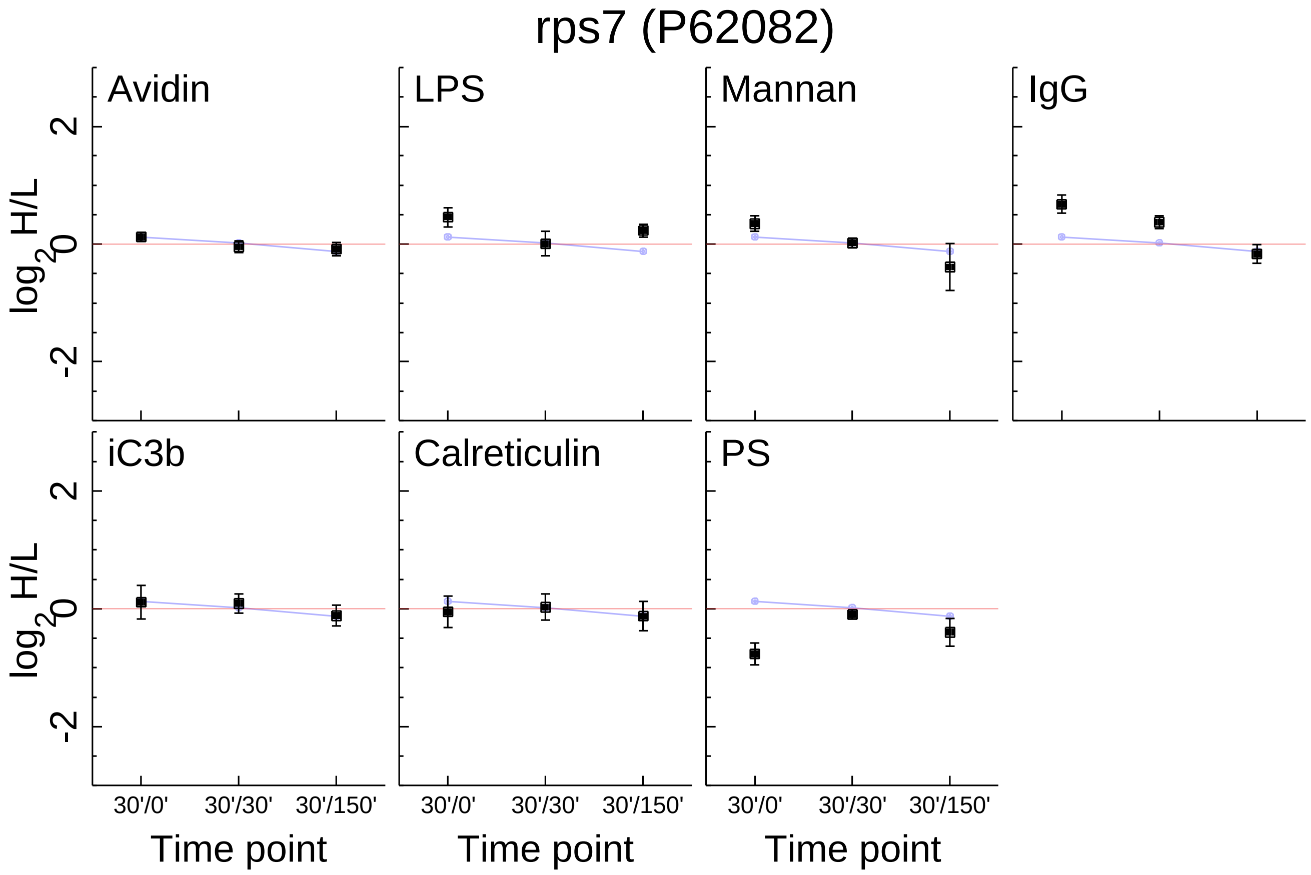

rps7 (P62082)
Avidin
LPS
Mannan
IgG
iC3b
Calreticulin
PS
log2 H/L
2
0
-2
30'/0'
30'/30'
30'/150'
Time point

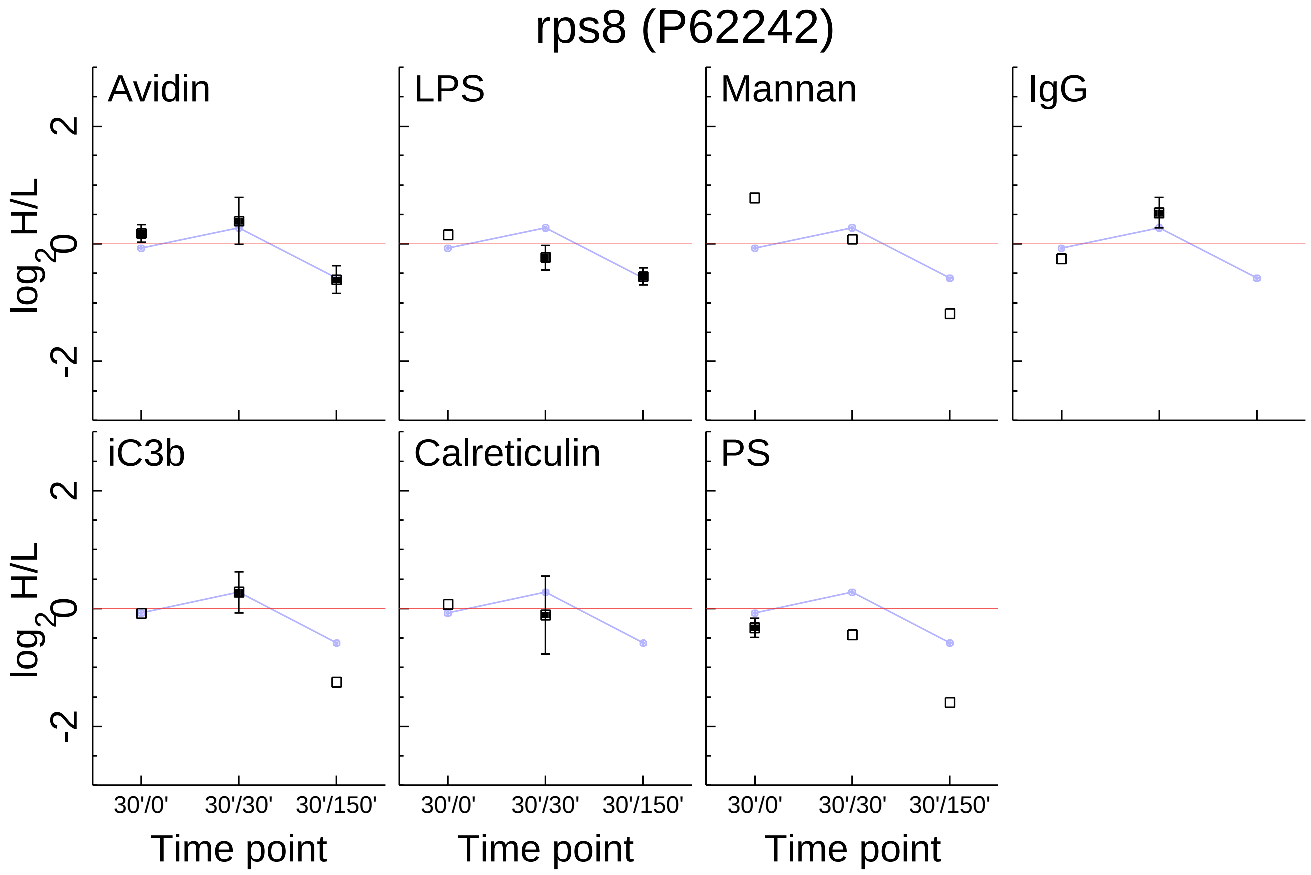

rps8 (P62242)
Avidin
LPS
Mannan
IgG
iC3b
Calreticulin
PS
log2 H/L
2
0
-2
30'/0'
30'/30'
30'/150'
Time point

rps9 (Q6ZWN5)

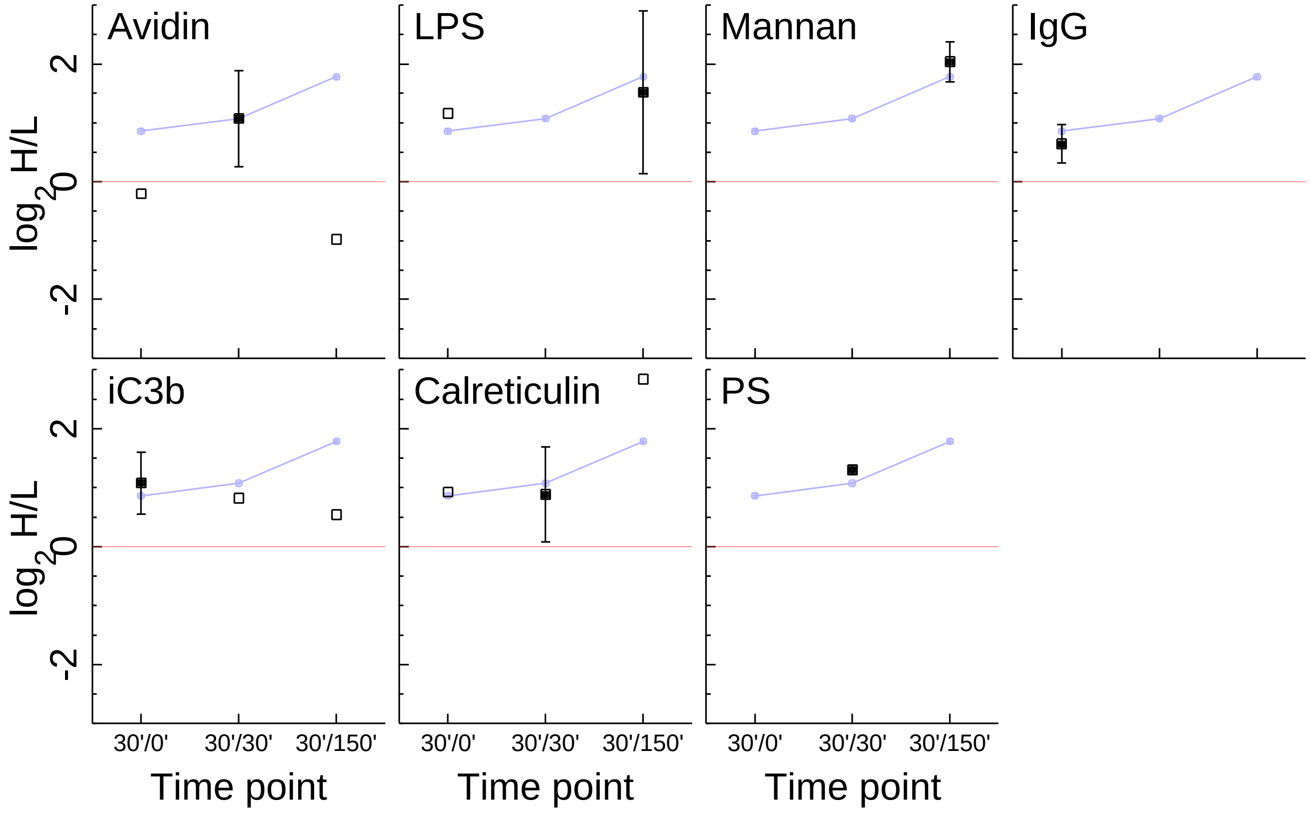

Avidin
LPS
Mannan
IgG
iC3b
Calreticulin
PS
log2 H/L
2
0
-2
30'/0'
30'/30'
30'/150'
Time point

rpsa-ps10 (D3YTT7)

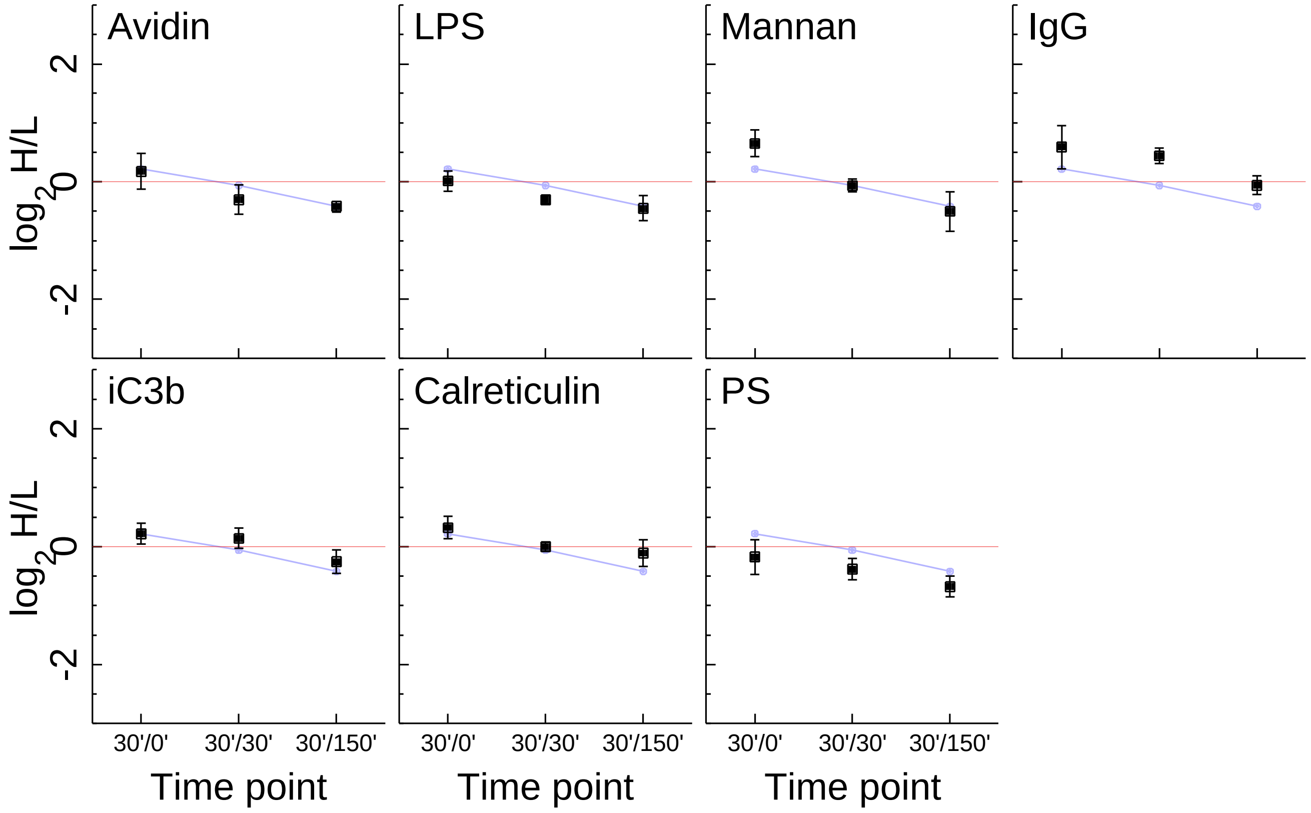

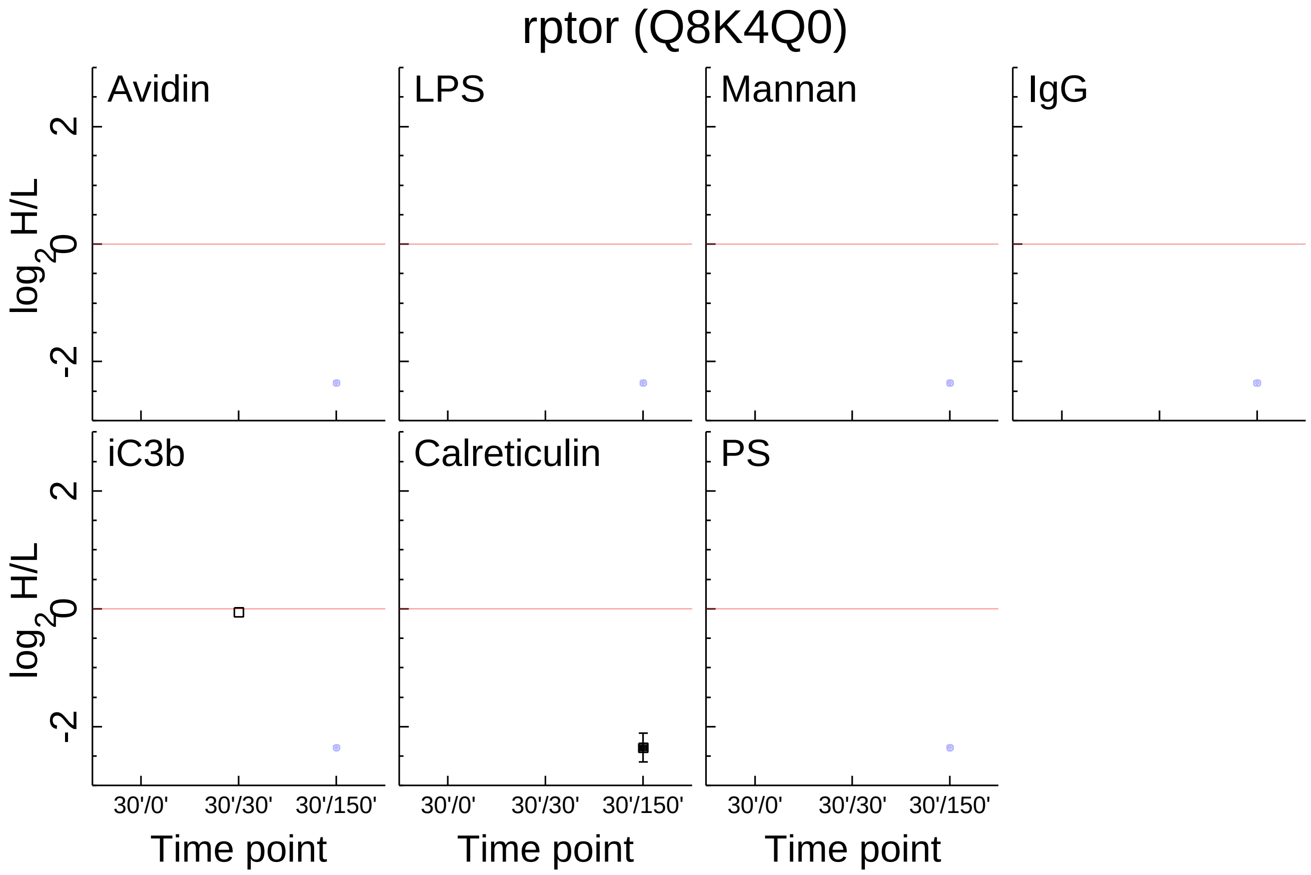

rptor (Q8K4Q0)
Avidin
LPS
Mannan
IgG
iC3b
Calreticulin
PS
2
0
-2
$\log_2$ H/L
30'/0'
30'/30'
30'/150'
Time point

rraga (Q80X95)

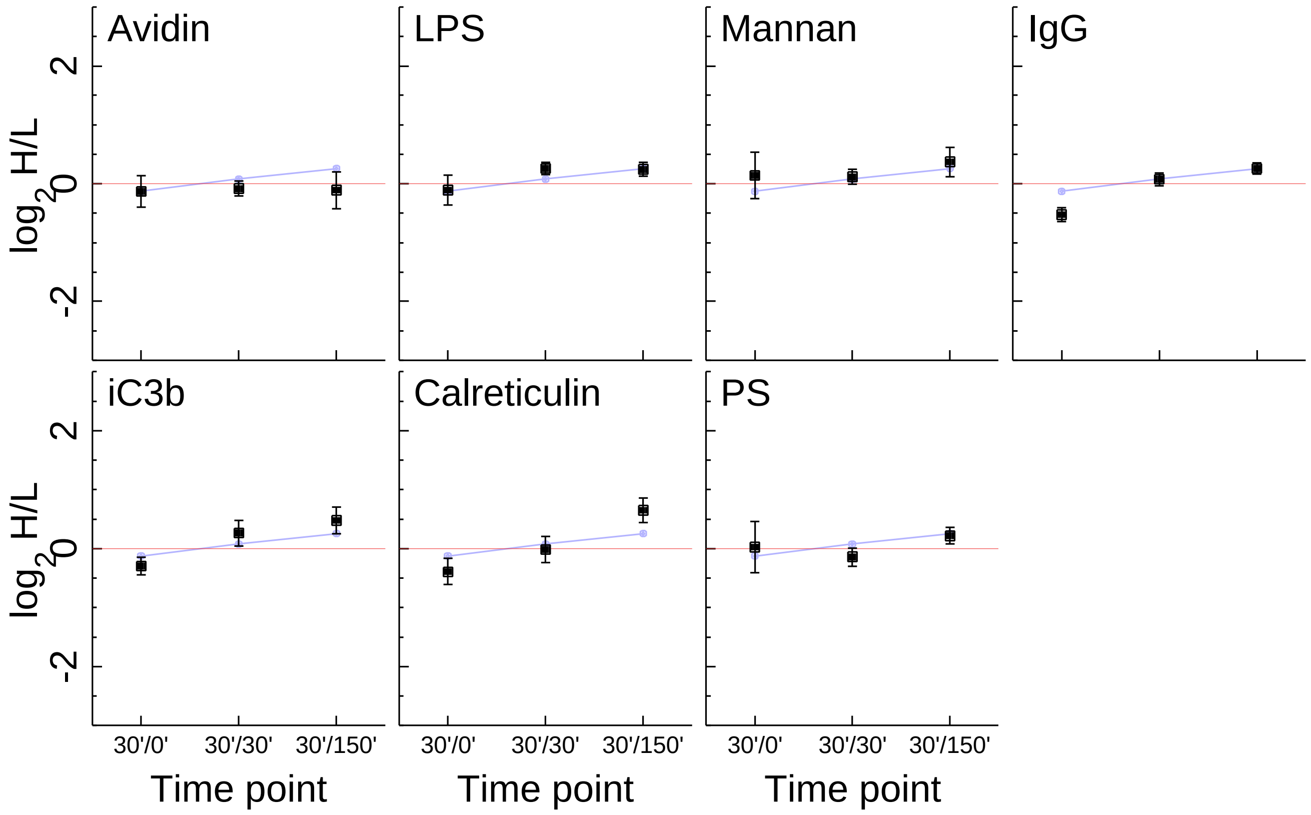

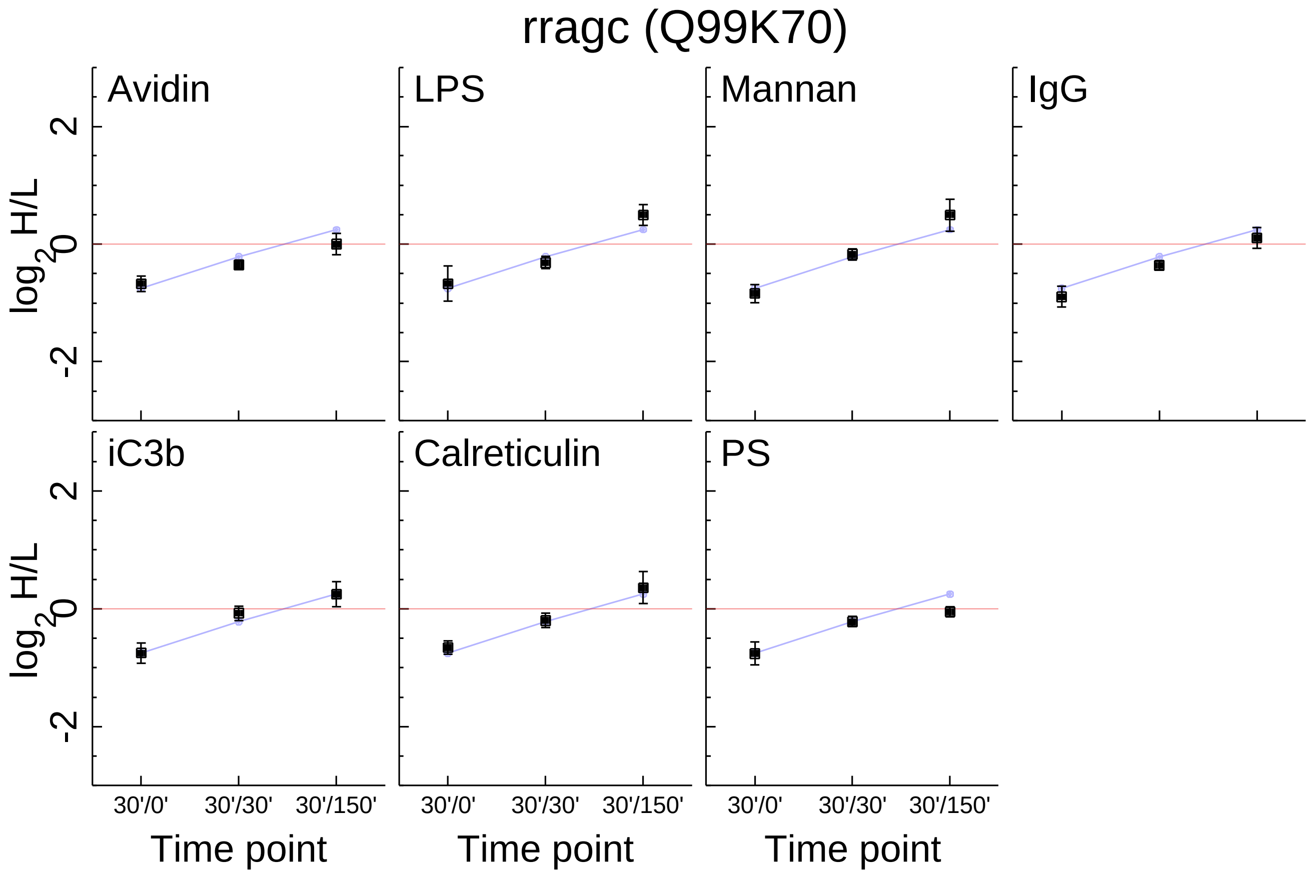
rragc (Q99K70)
Avidin
LPS
Mannan
IgG
iC3b
Calreticulin
PS
log2 H/L
2
0
-2
30'/0'
30'/30'
30'/150'
Time point

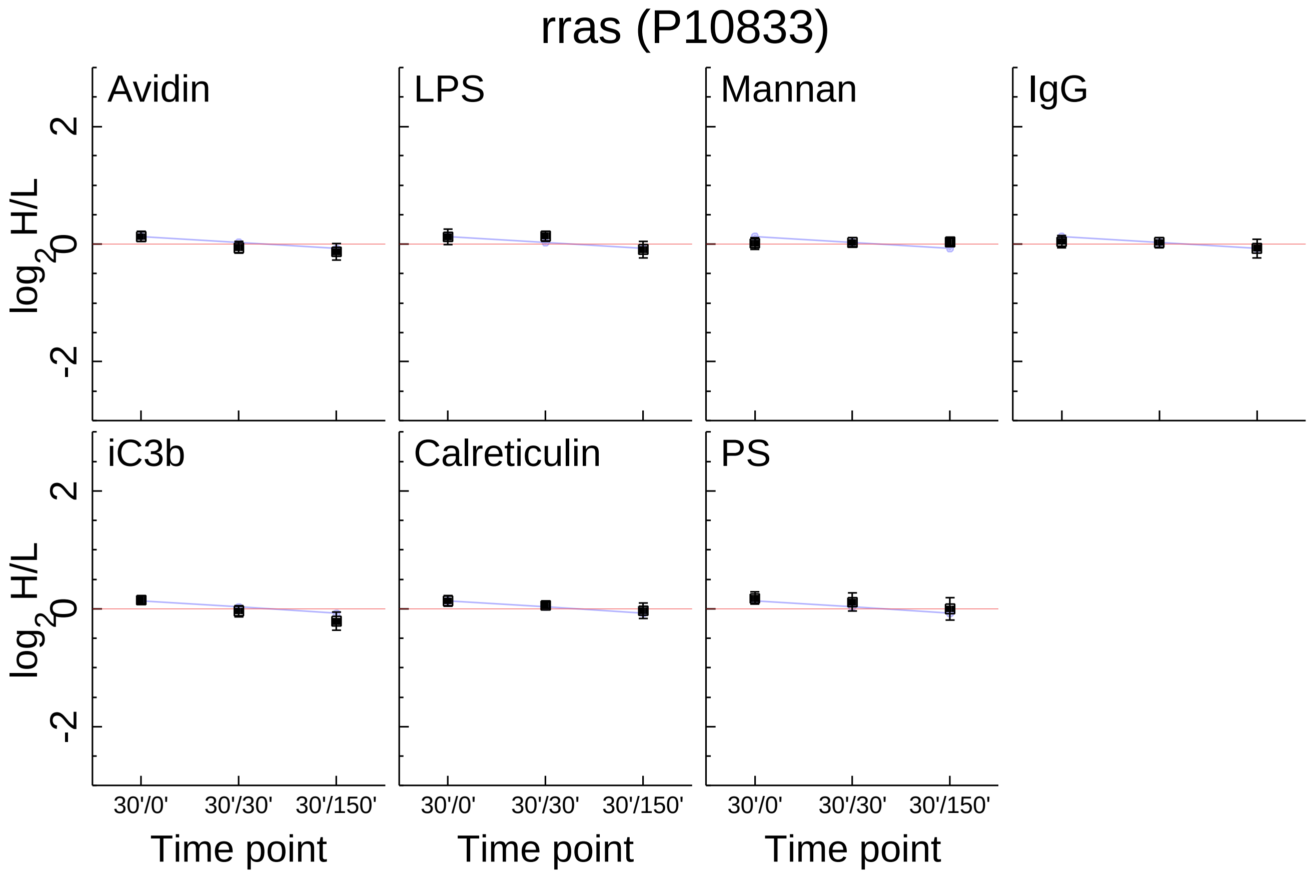
rras (P10833)
Avidin
LPS
Mannan
IgG
iC3b
Calreticulin
PS
log2 H/L
2
0
-2
30'/0'
30'/30'
30'/150'
Time point

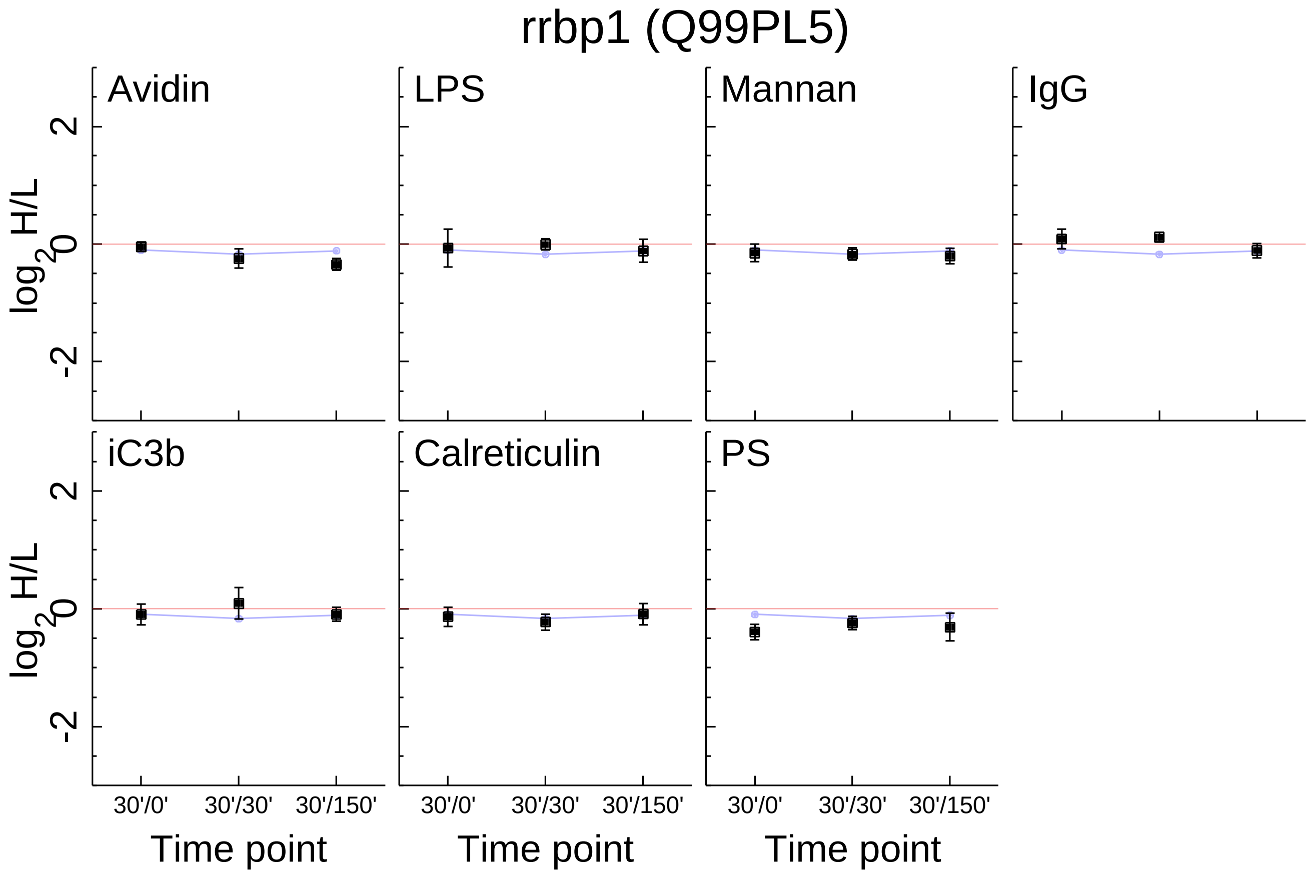

rrbp1 (Q99PL5)
Avidin
LPS
Mannan
IgG
iC3b
Calreticulin
PS
2
0
-2
log2 H/L
30'/0'
30'/30'
30'/150'
Time point

rsad2 (Q8CBB9)

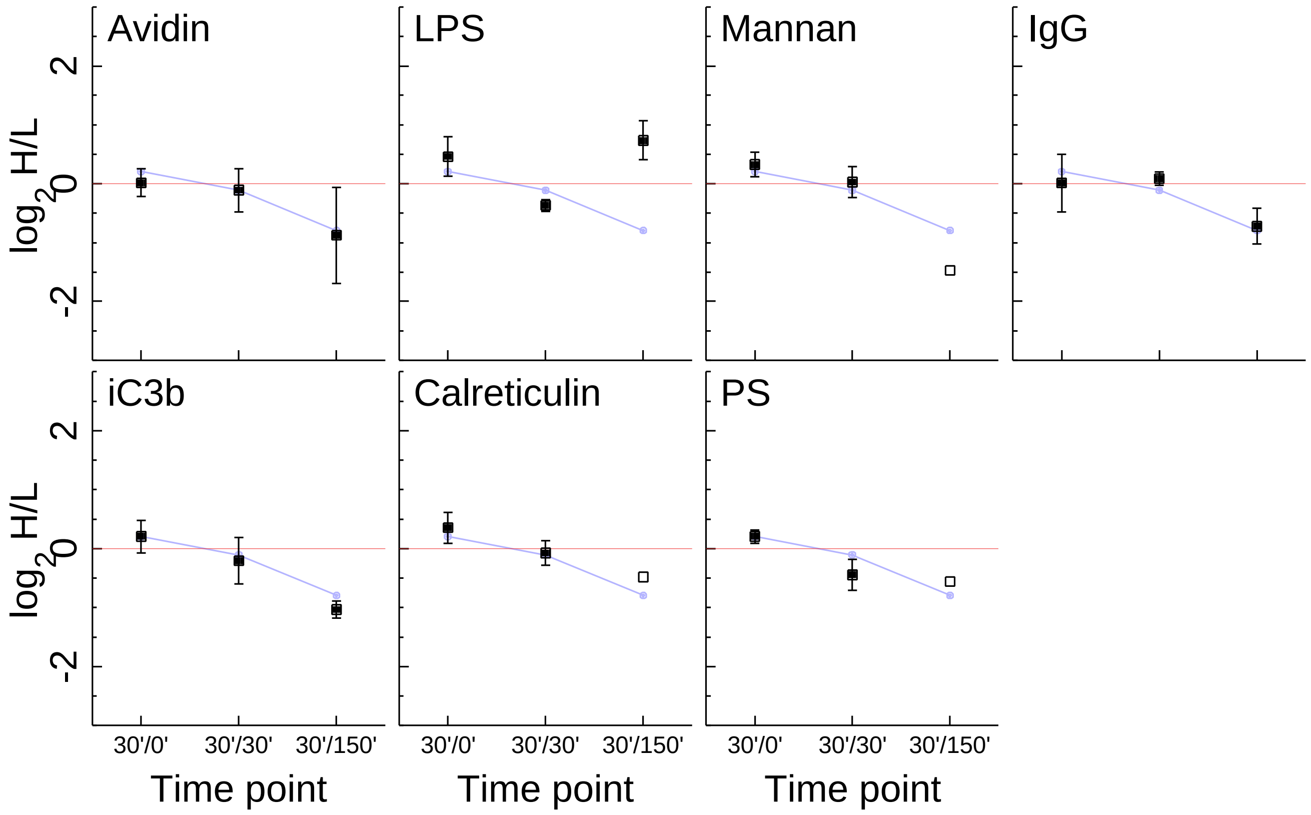

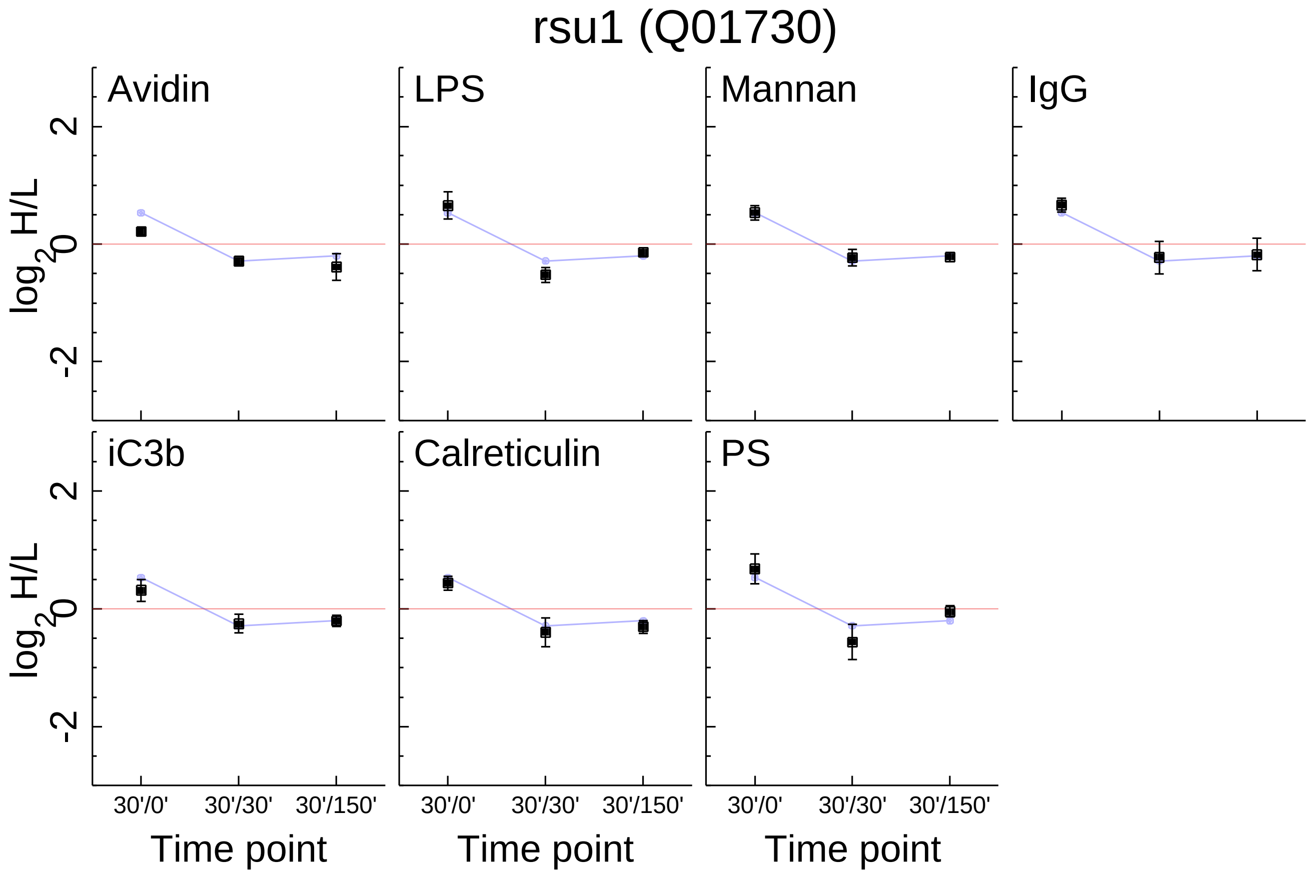

rsu1 (Q01730)
Avidin
LPS
Mannan
IgG
iC3b
Calreticulin
PS
log2 H/L
2
0
-2
30'/0'
30'/30'
30'/150'
Time point

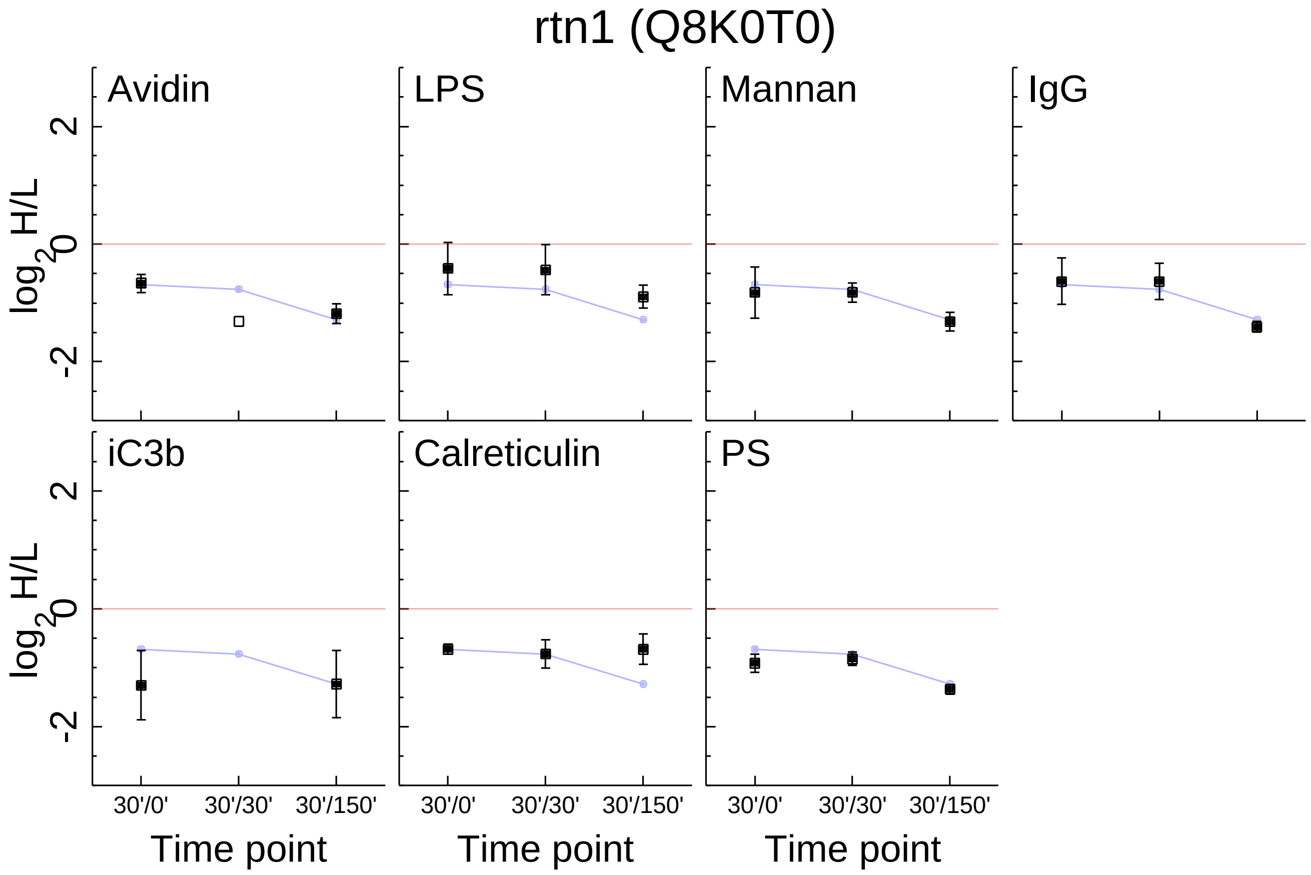

rtn1 (Q8K0T0)
Avidin
LPS
Mannan
IgG
iC3b
Calreticulin
PS
log2 H/L
2
0
-2
30'/0'
30'/30'
30'/150'
Time point

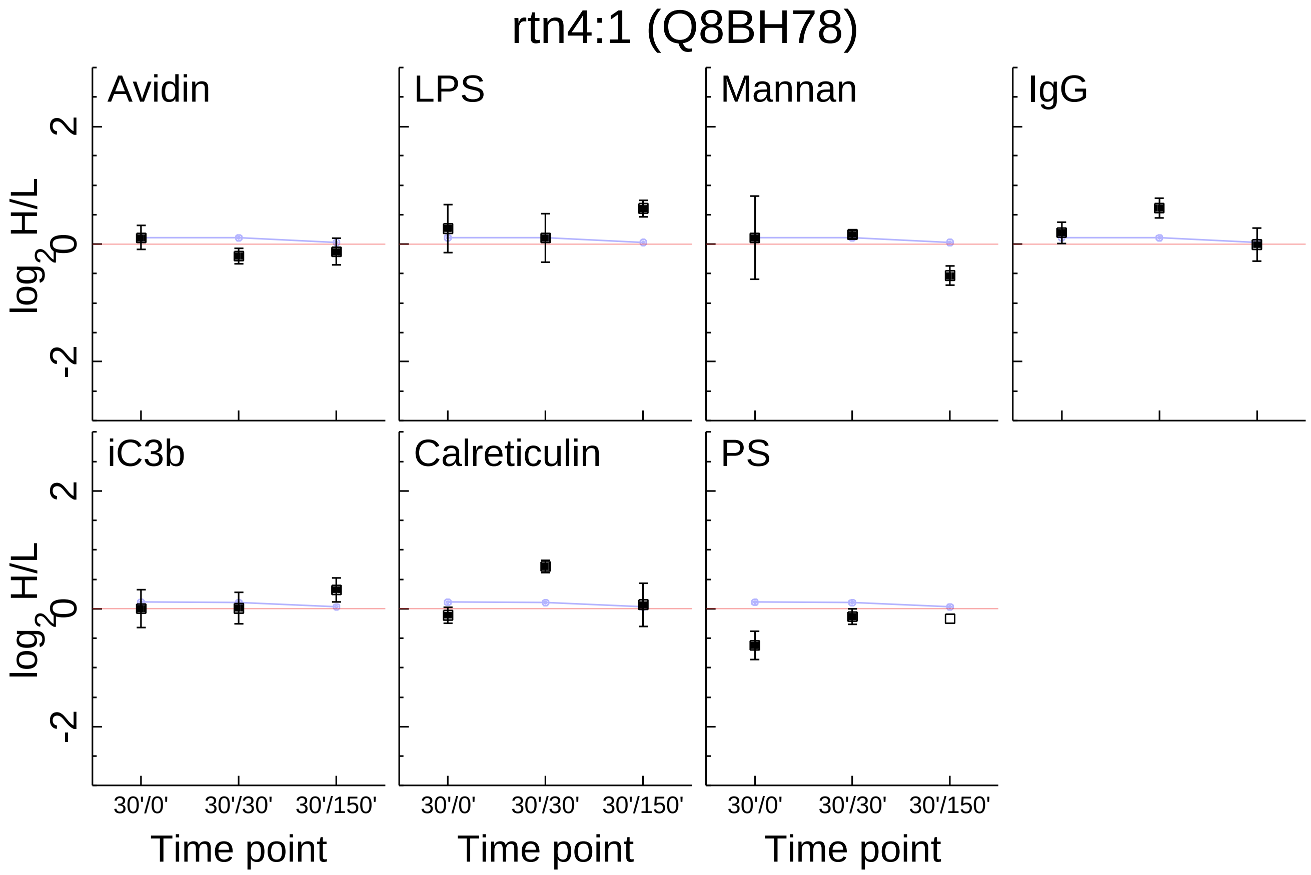
rtn4:1 (Q8BH78)
Avidin
LPS
Mannan
IgG
iC3b
Calreticulin
PS
log2 H/L
2
0
-2
30'/0'
30'/30'
30'/150'
Time point

rtn4:2 (Q99P72)

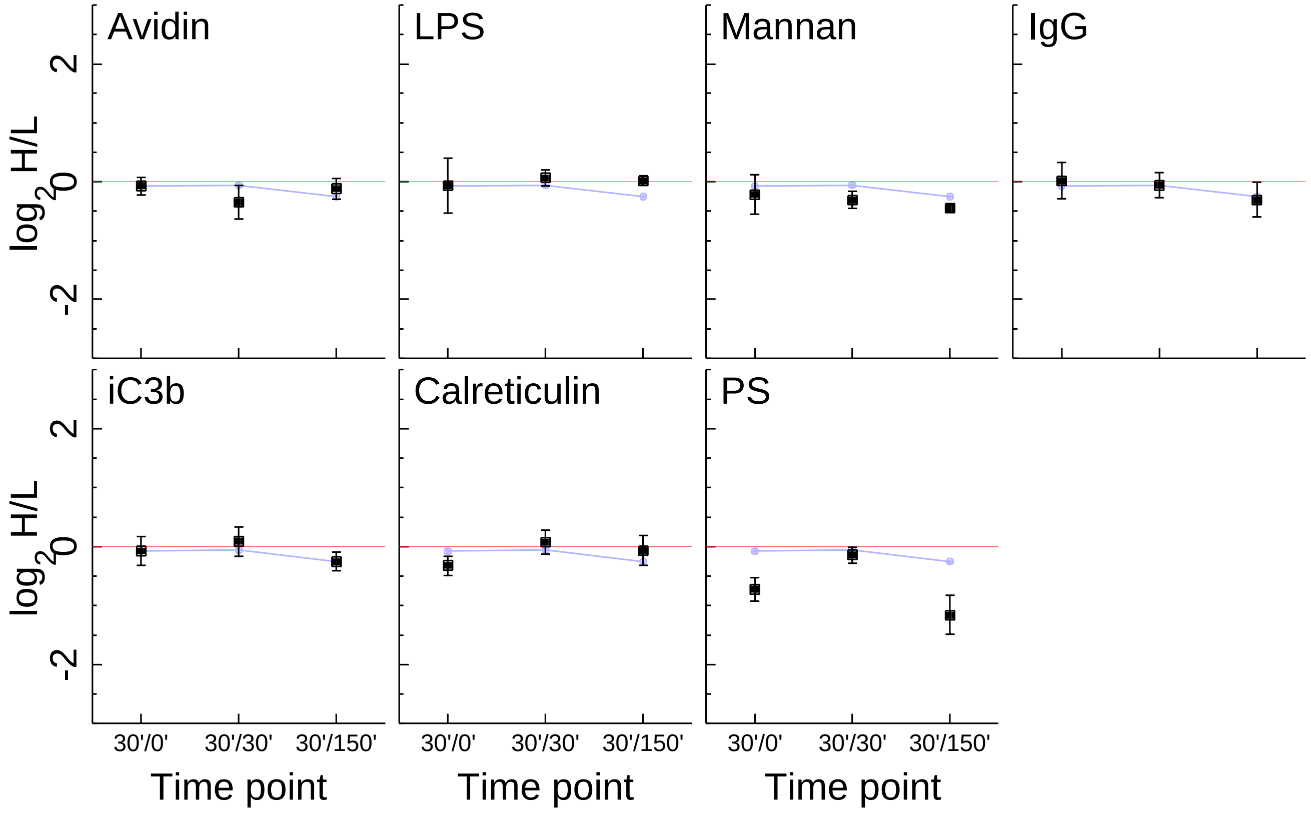

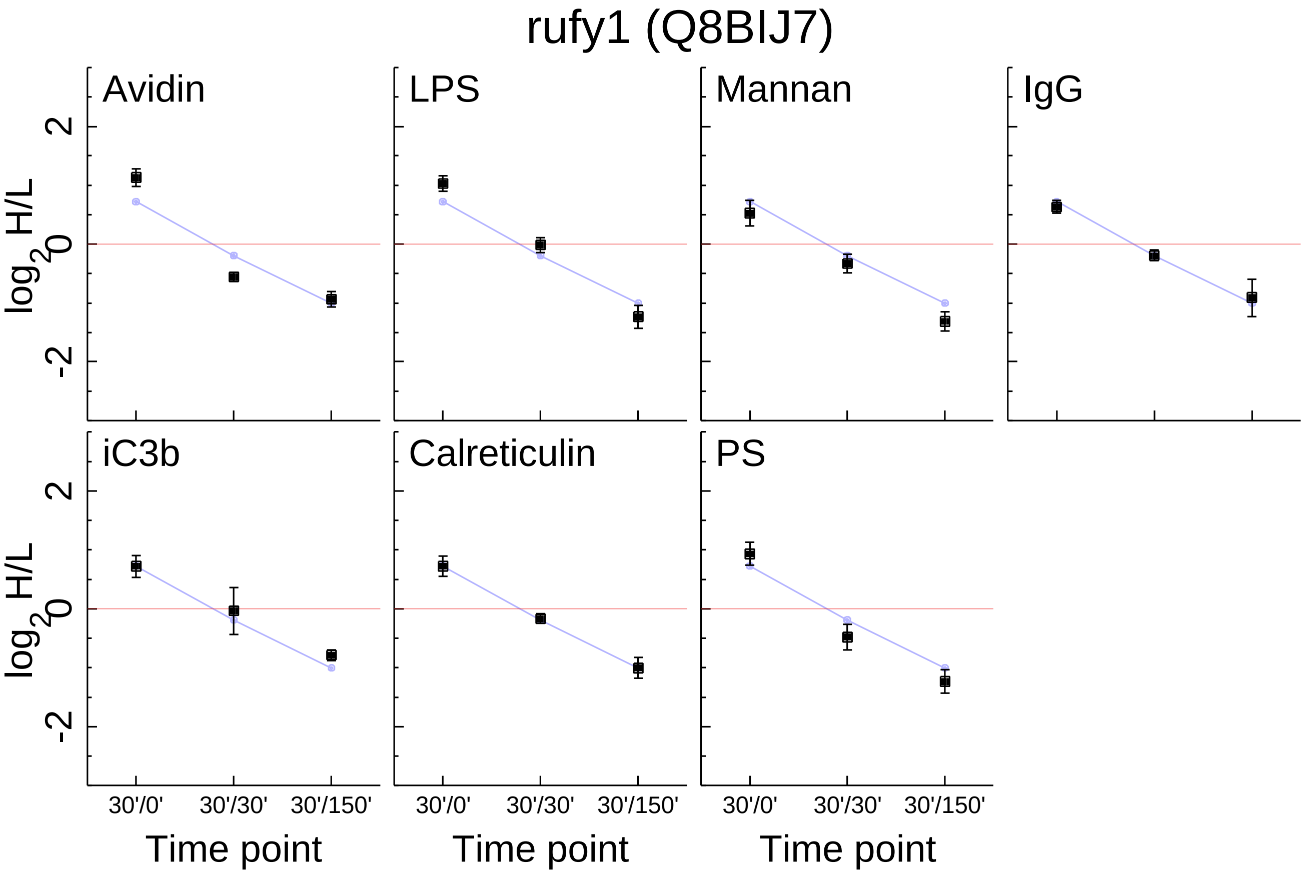
rufy1 (Q8BIJ7)
Avidin
LPS
Mannan
IgG
iC3b
Calreticulin
PS
log2 H/L
2
0
-2
30'/0'
30'/30'
30'/150'
Time point

rufy2 (Q8R4C2)

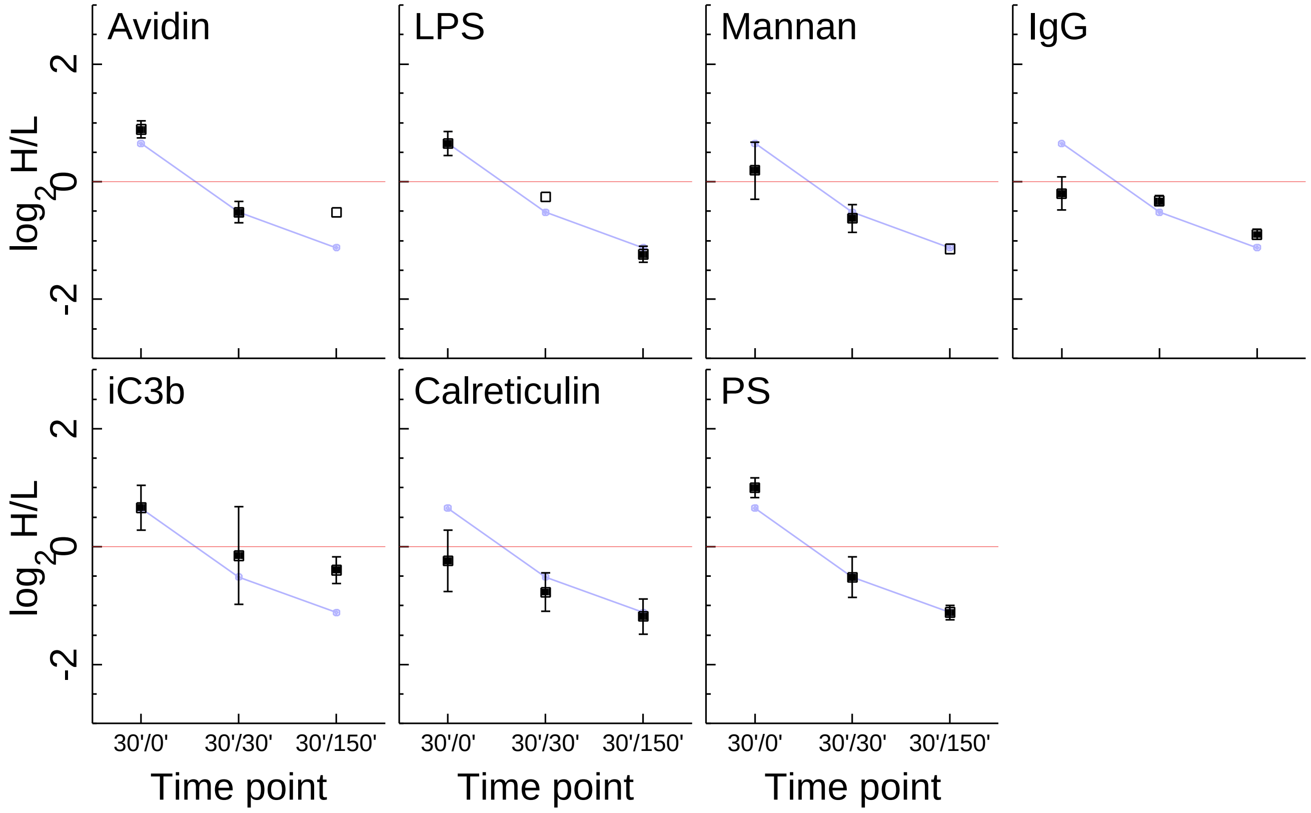

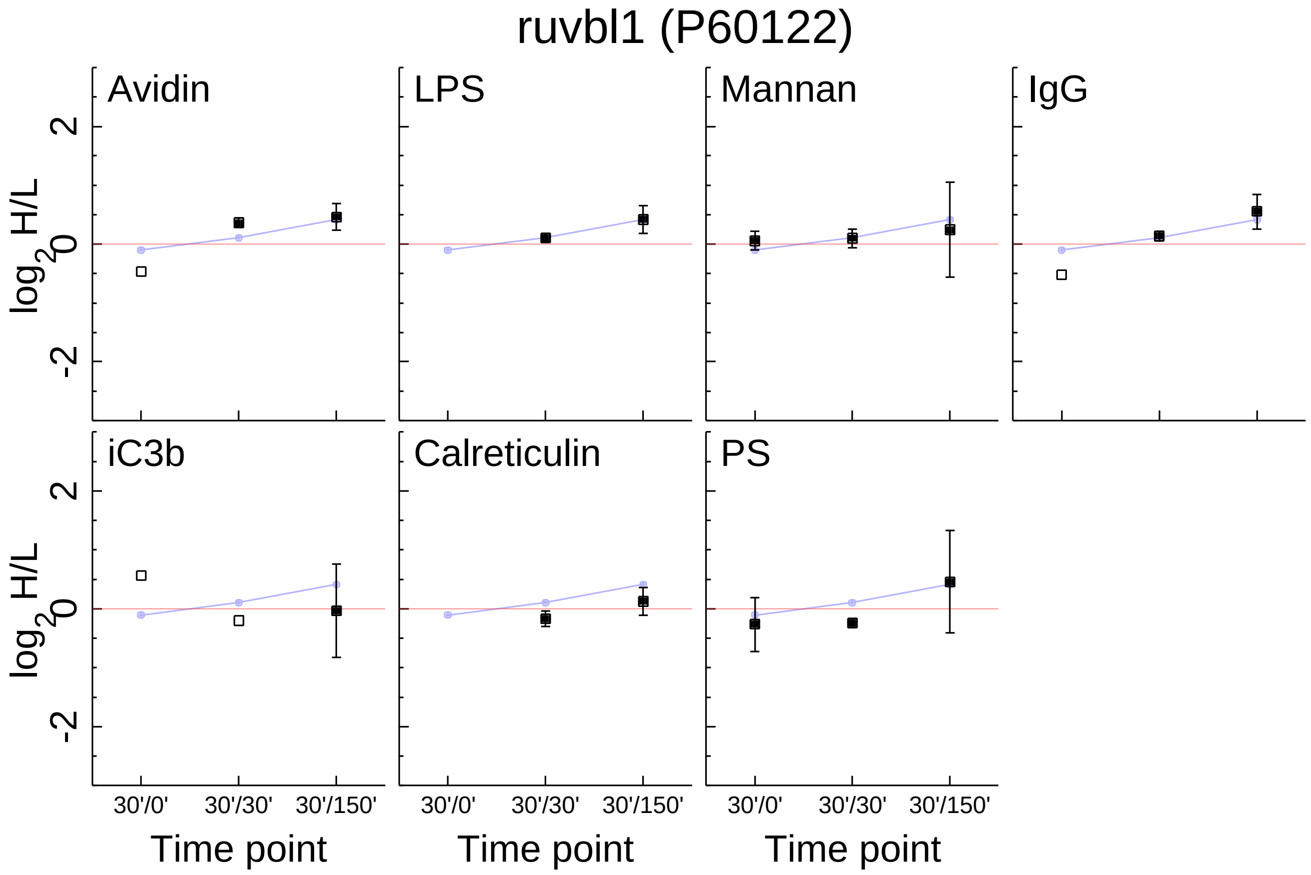

ruvbl1 (P60122)
Avidin
LPS
Mannan
IgG
iC3b
Calreticulin
PS
log2 H/L
2
0
-2
30'/0'
30'/30'
30'/150'
Time point

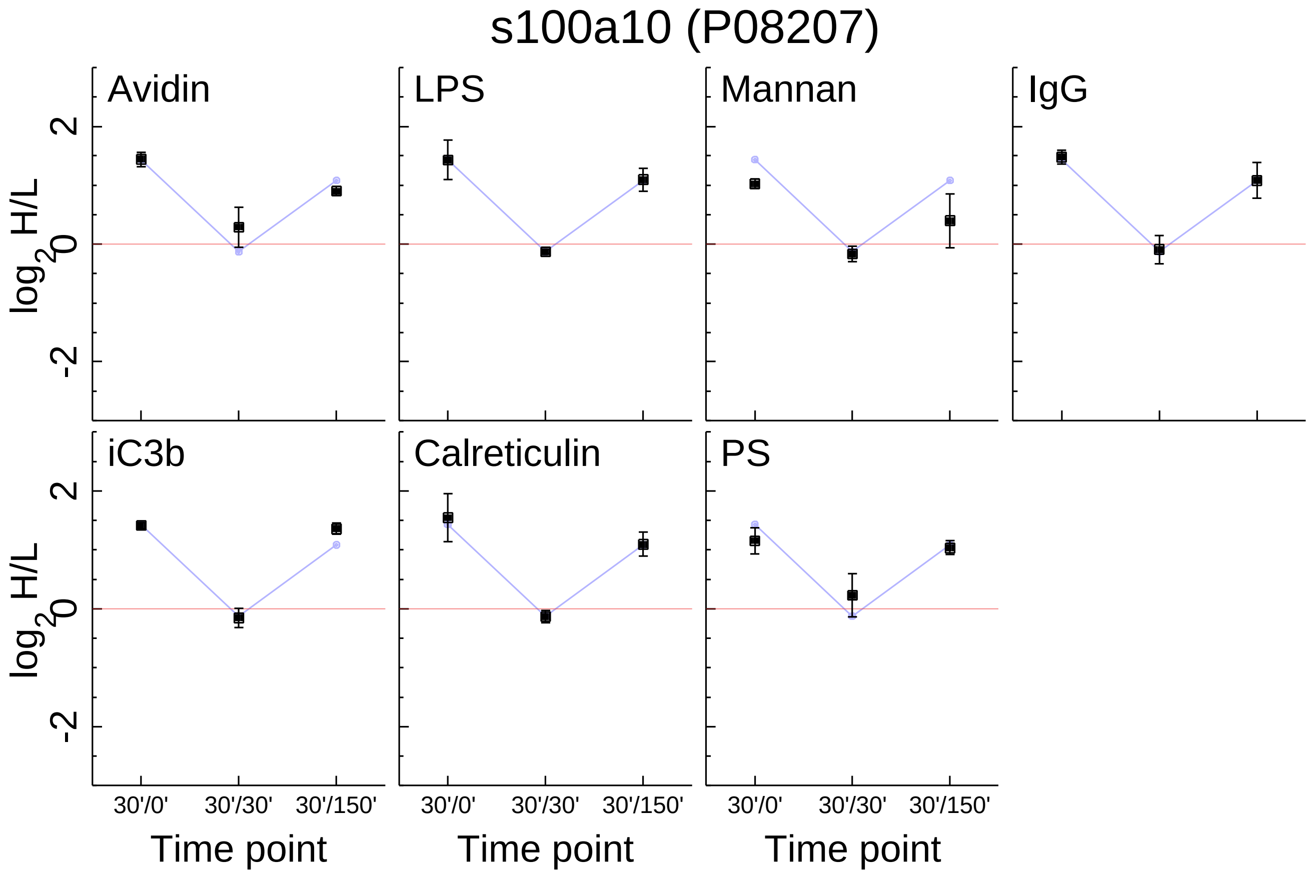
s100a10 (P08207)
Avidin
LPS
Mannan
IgG
iC3b
Calreticulin
PS
log2 H/L
2
0
-2
30'/0'
30'/30'
30'/150'
Time point

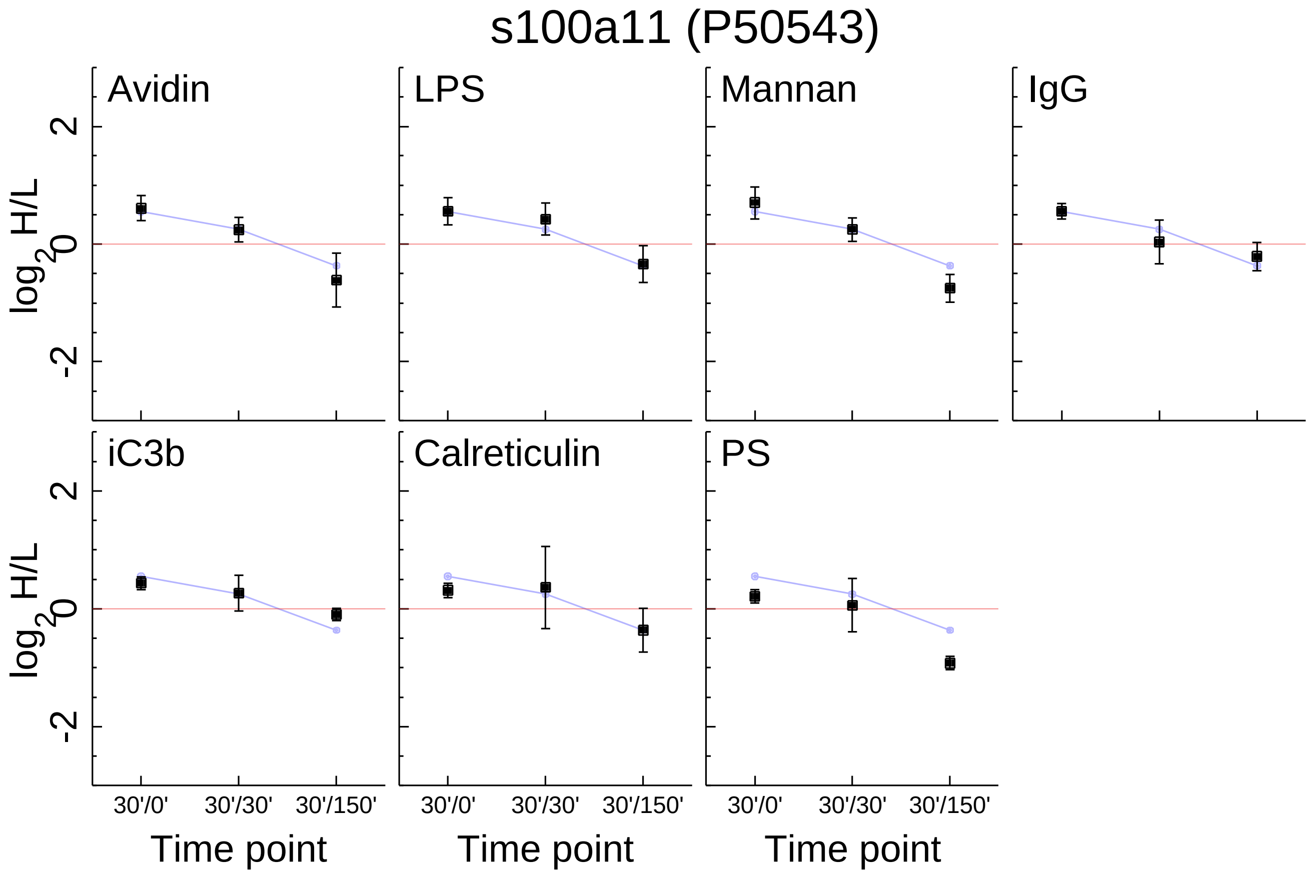
s100a11 (P50543)
Avidin
LPS
Mannan
IgG
iC3b
Calreticulin
PS
log2 H/L
2
0
-2
30'/0'
30'/30'
30'/150'
Time point

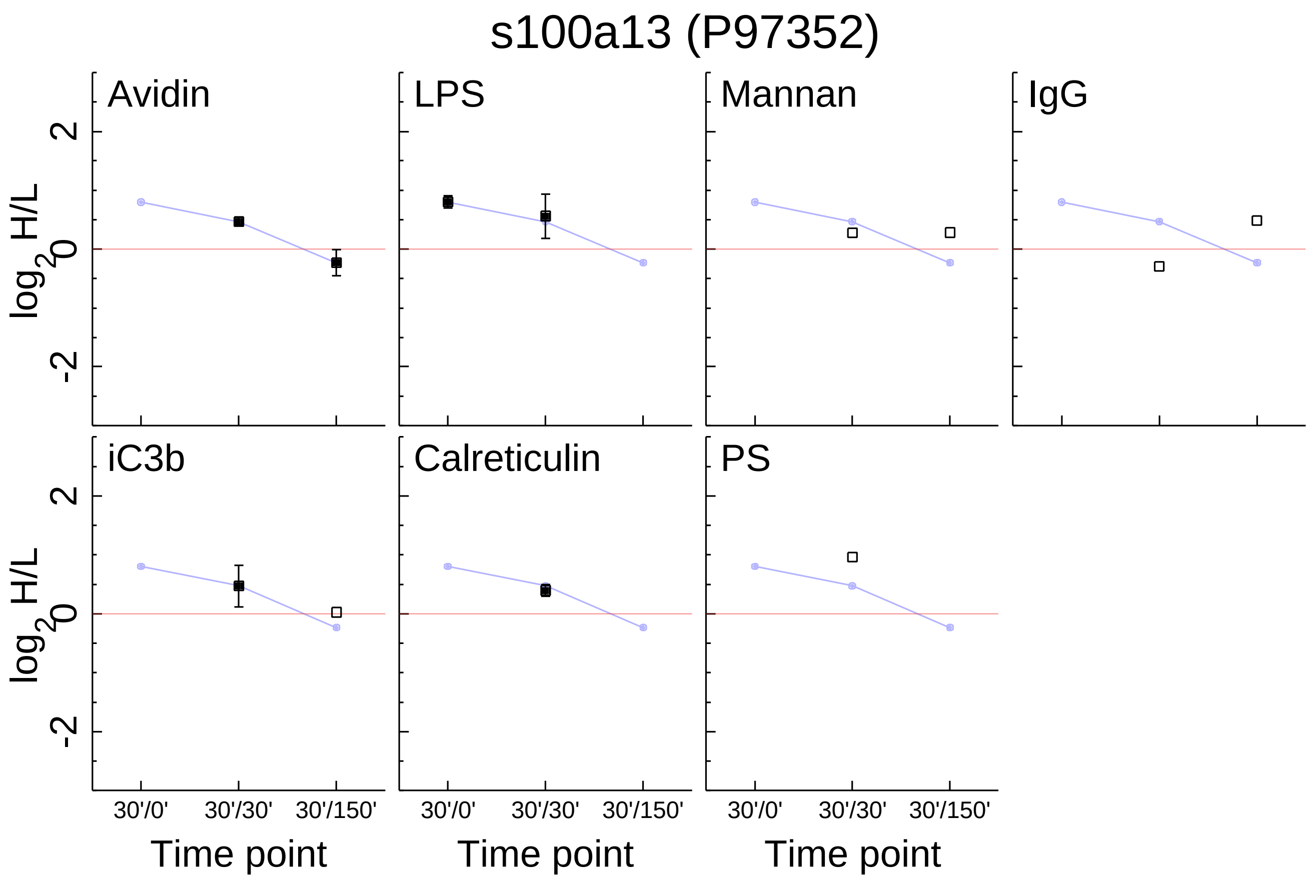
s100a13 (P97352)
Avidin
LPS
Mannan
IgG
iC3b
Calreticulin
PS
log2 H/L
2
0
-2
30'/0'
30'/30'
30'/150'
Time point

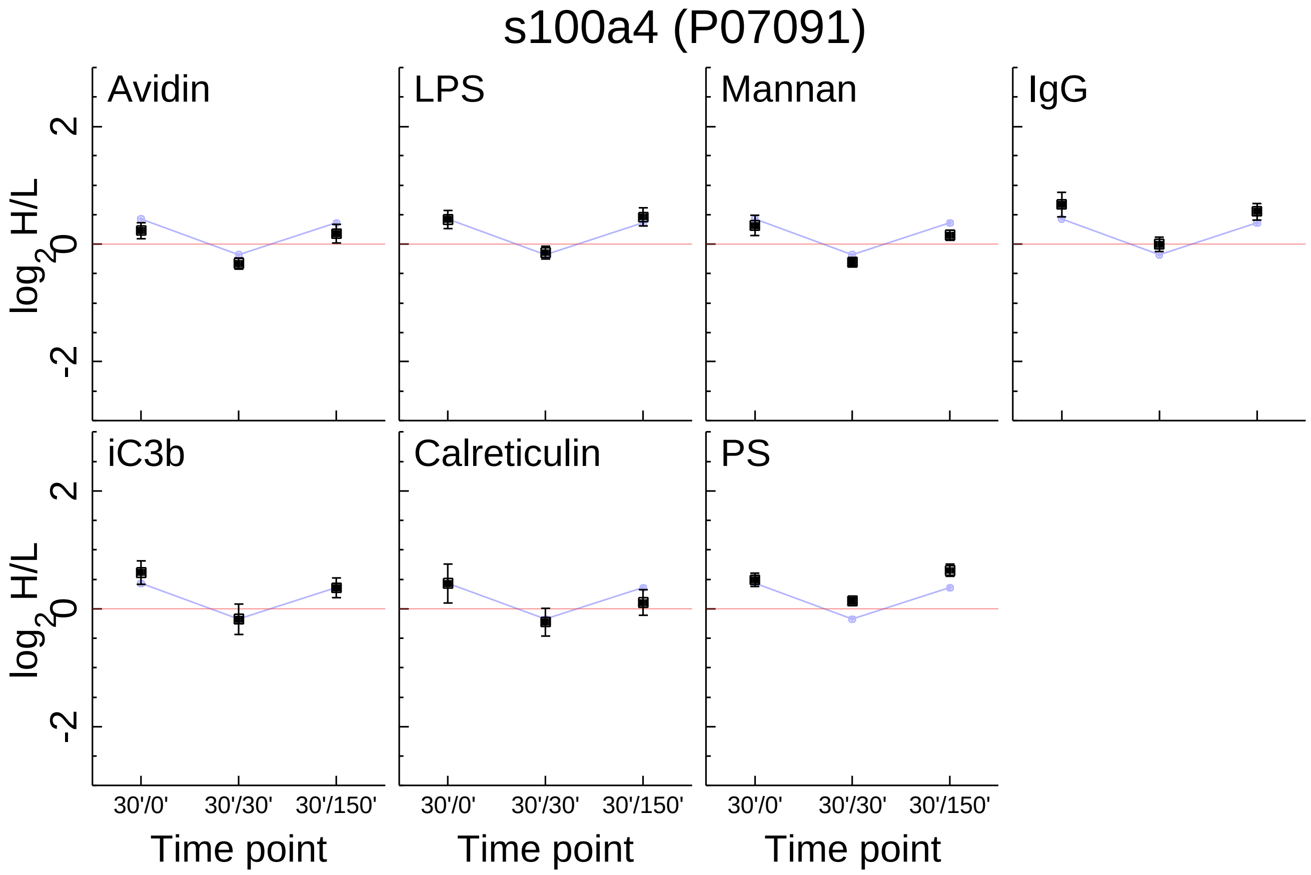
s100a4 (P07091)
Avidin
LPS
Mannan
IgG
iC3b
Calreticulin
PS
log2 H/L
2
0
-2
30'/0'
30'/30'
30'/150'
Time point

s100a6 (P14069)

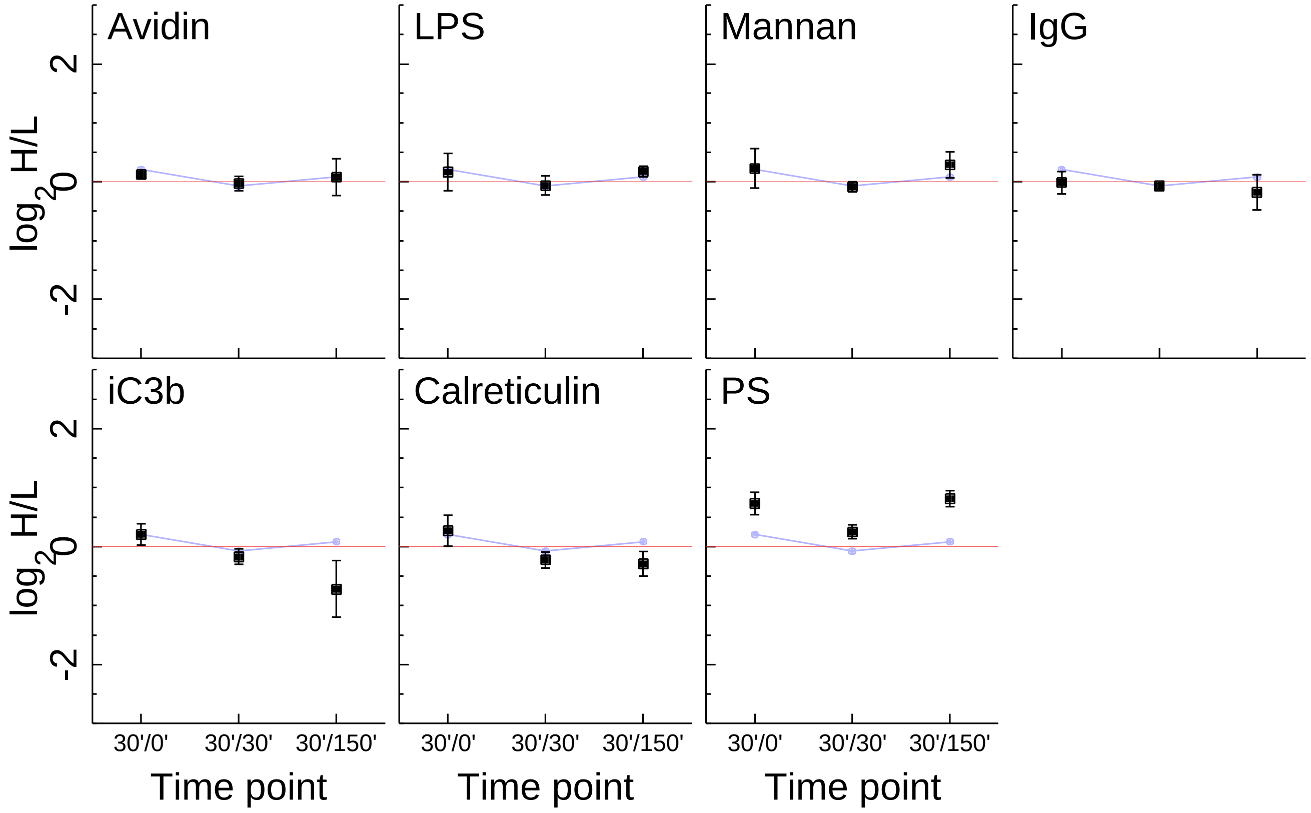

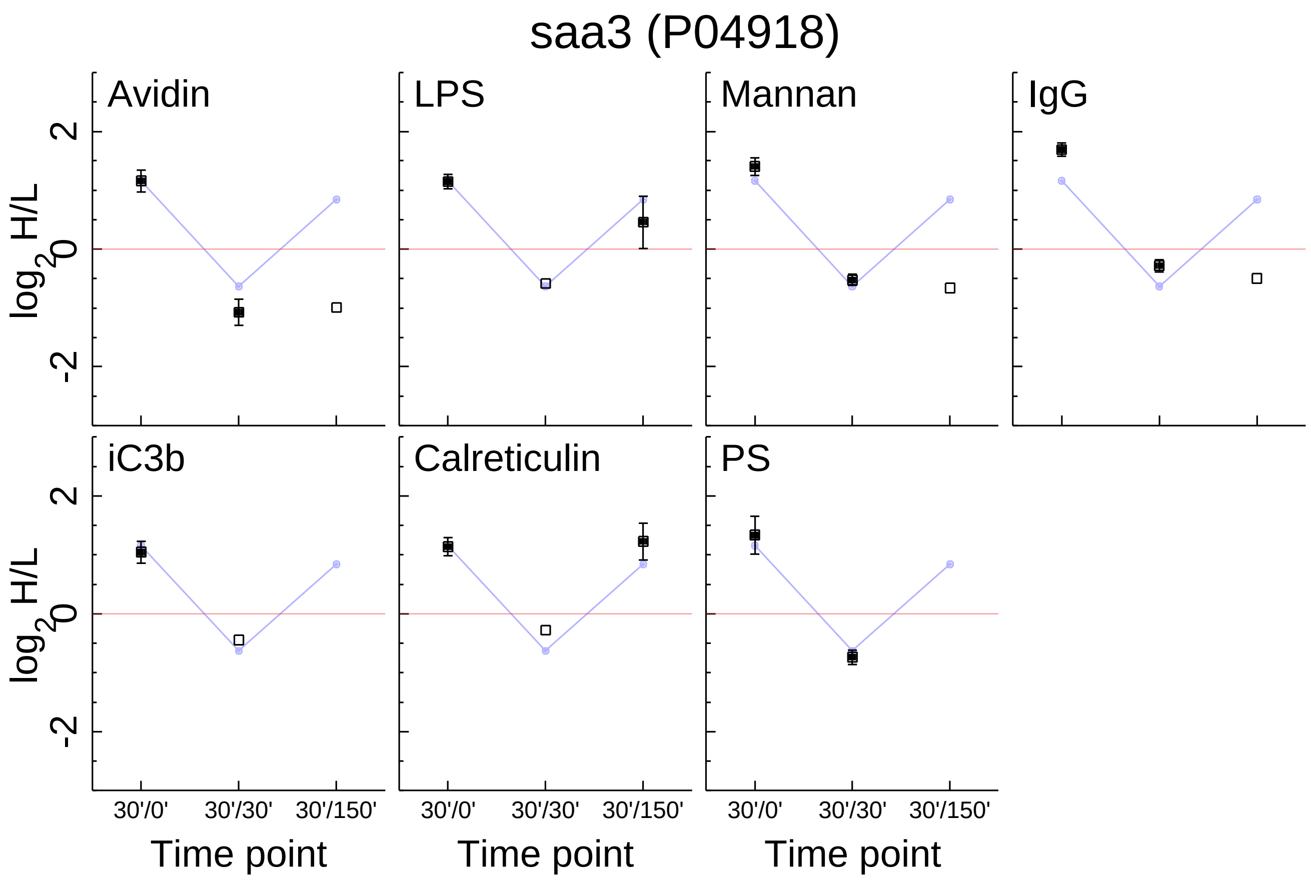
saa3 (P04918)
Avidin
LPS
Mannan
IgG
iC3b
Calreticulin
PS
log2 H/L
2
0
-2
30'/0'
30'/30'
30'/150'
Time point

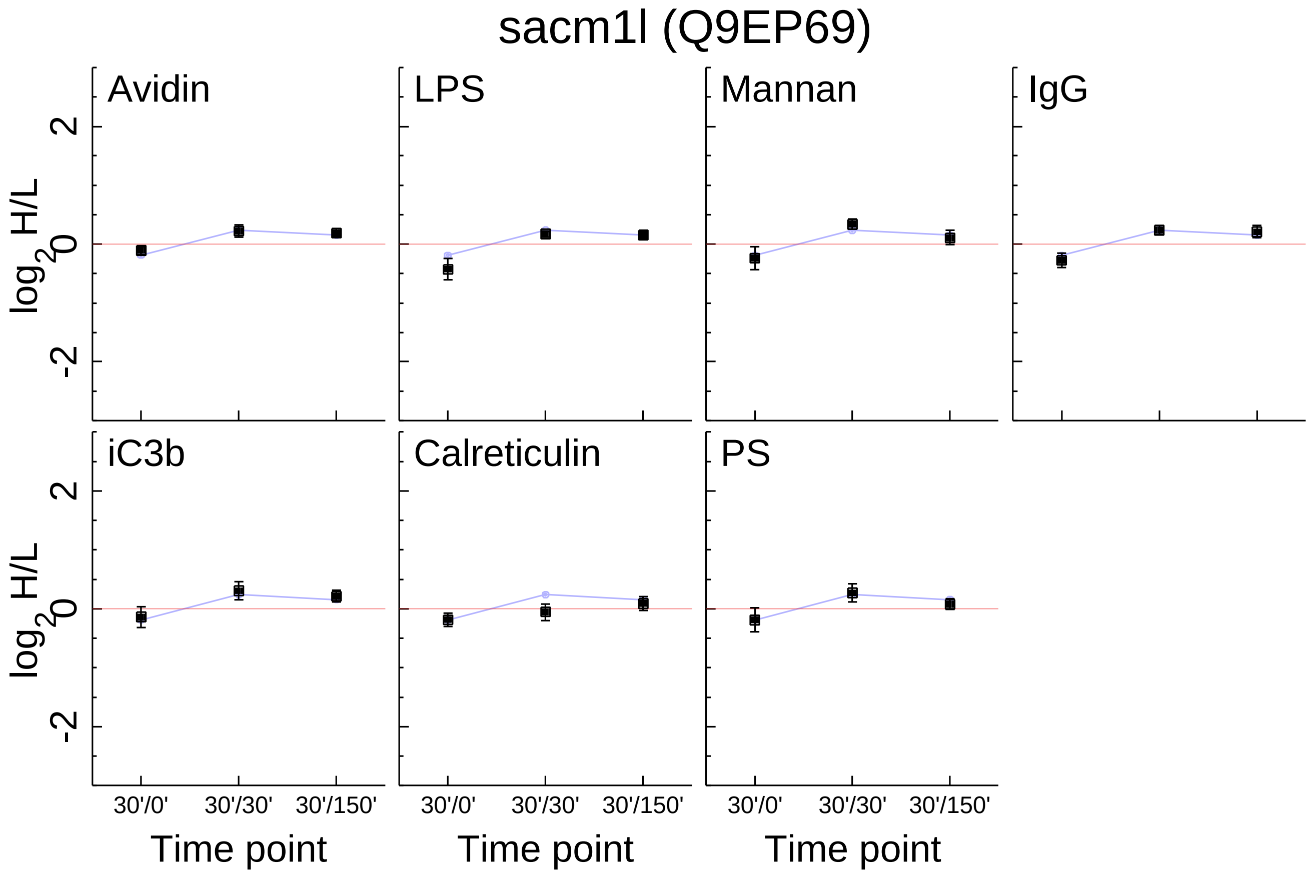

sacm1l (Q9EP69)
Avidin
LPS
Mannan
IgG
iC3b
Calreticulin
PS
log2 H/L
2
0
-2
30'/0'
30'/30'
30'/150'
Time point

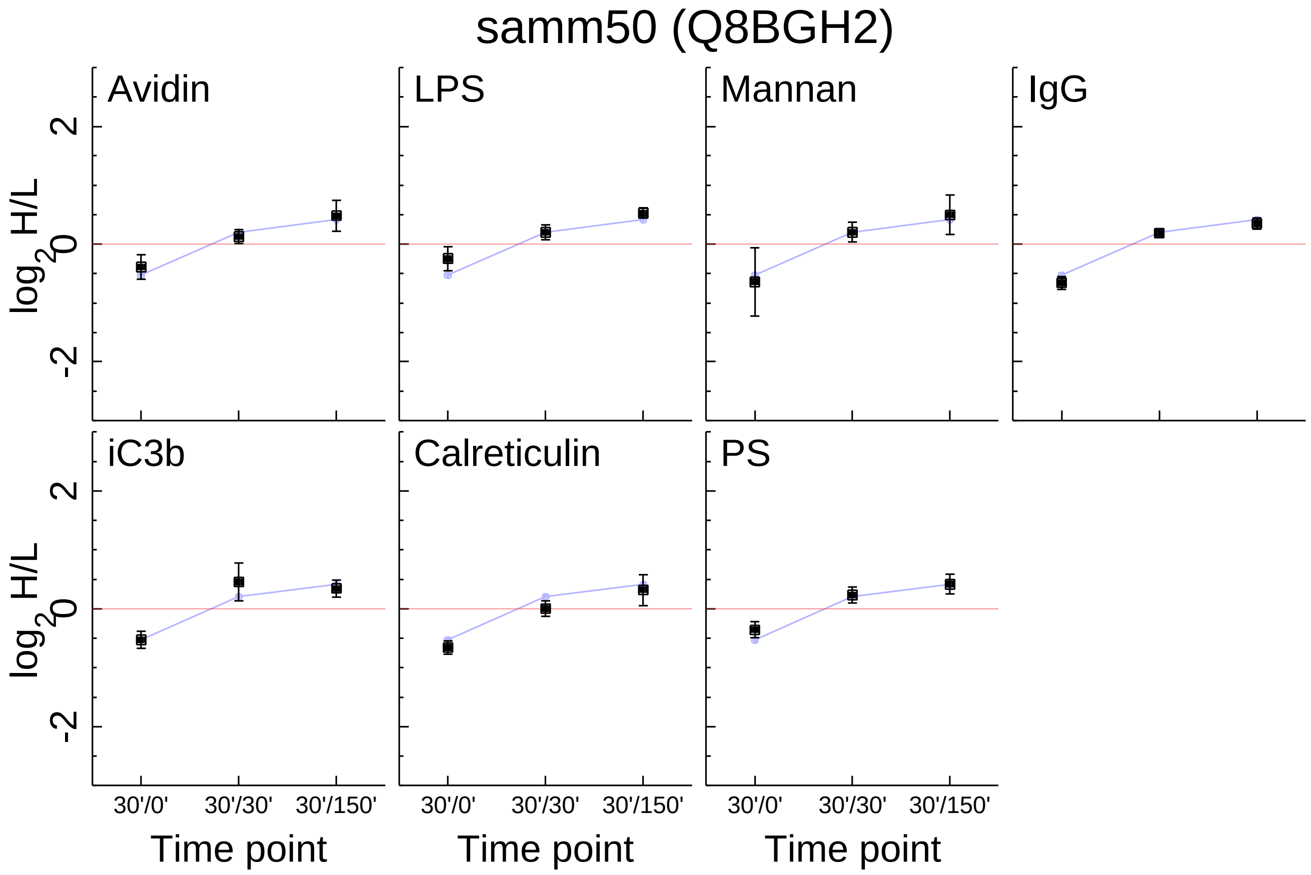
samm50 (Q8BGH2)
Avidin
LPS
Mannan
IgG
iC3b
Calreticulin
PS
log2 H/L
2
0
-2
30'/0'
30'/30'
30'/150'
Time point

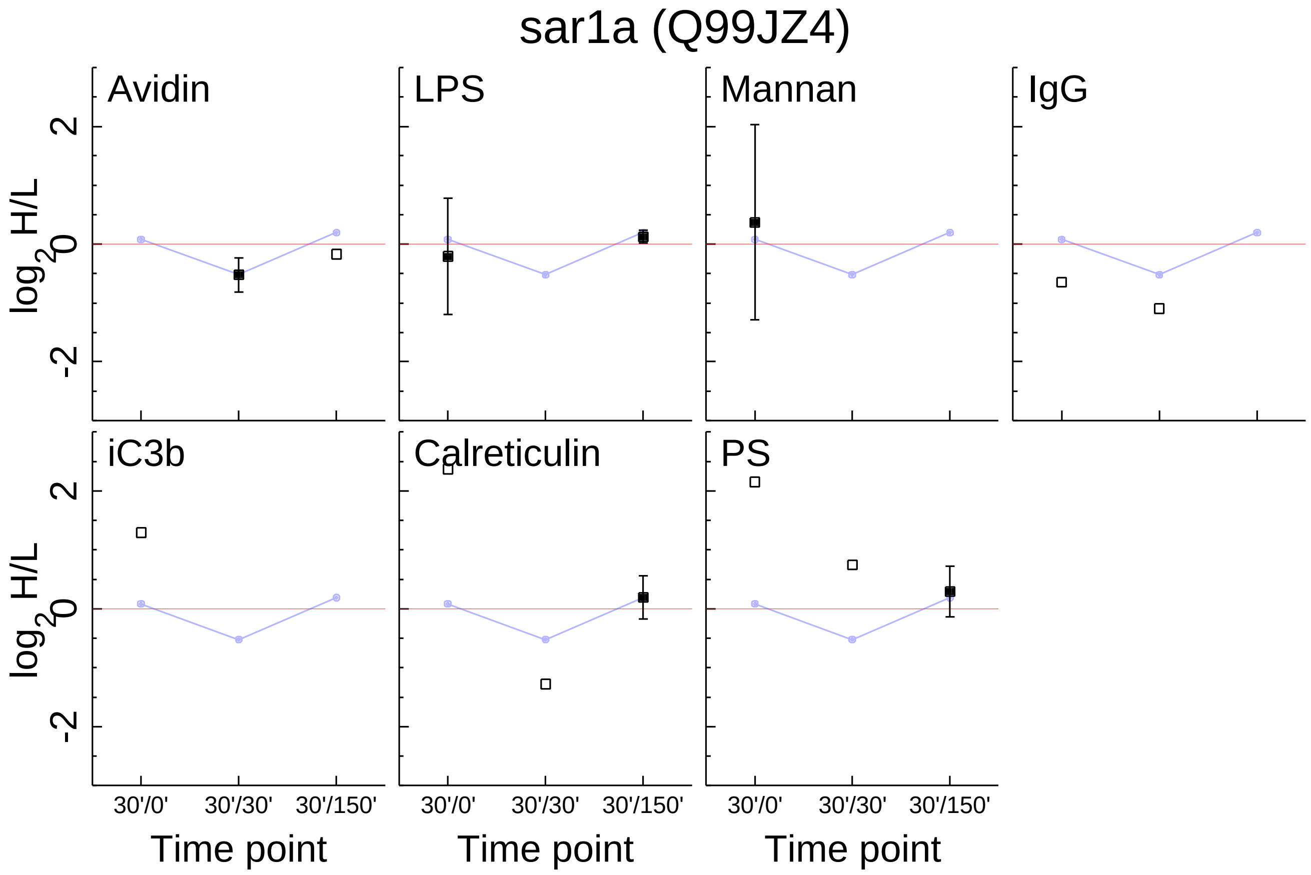
sar1a (Q99JZ4)
Avidin
LPS
Mannan
IgG
iC3b
Calreticulin
PS
log2 H/L
2
0
-2
30'/0'
30'/30'
30'/150'
Time point

sarnp (Q9D1J3)

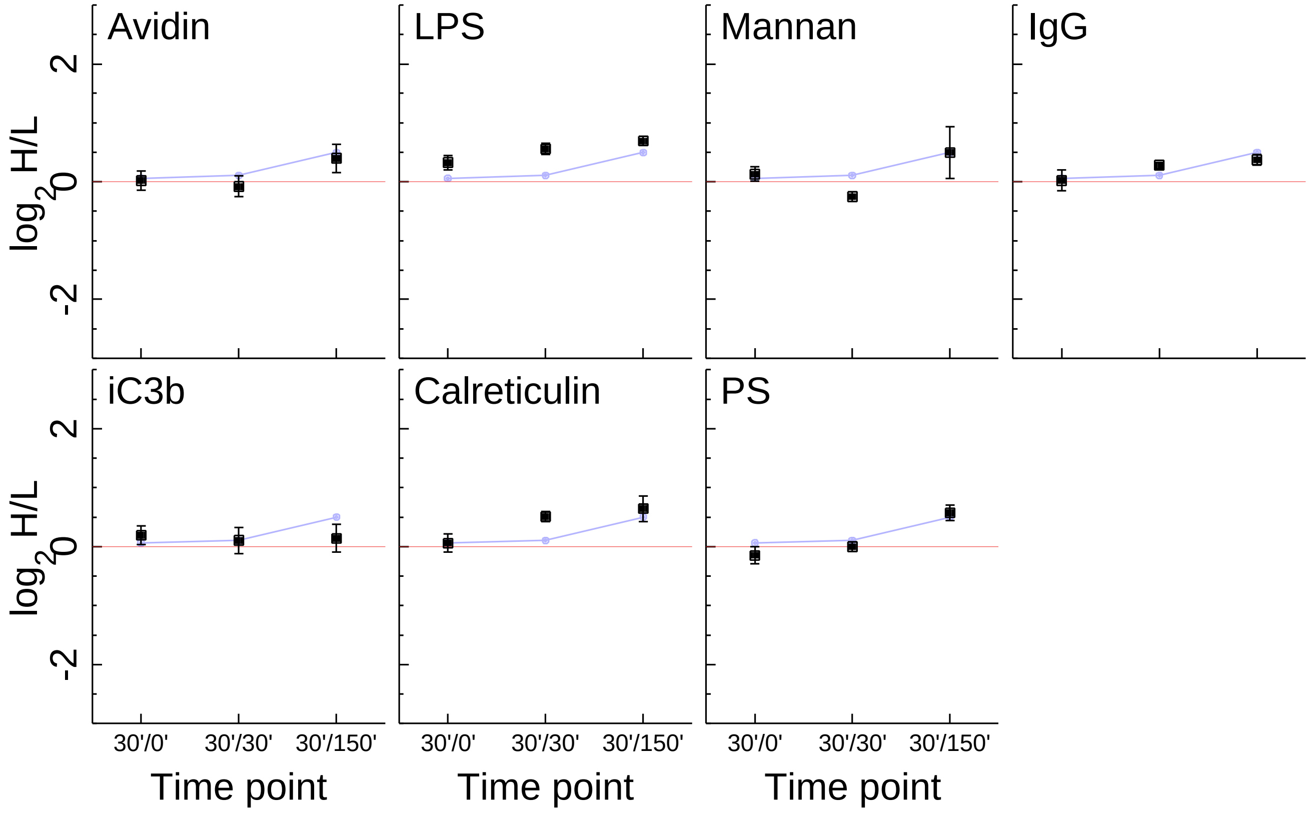

sash3 (Q8K352)

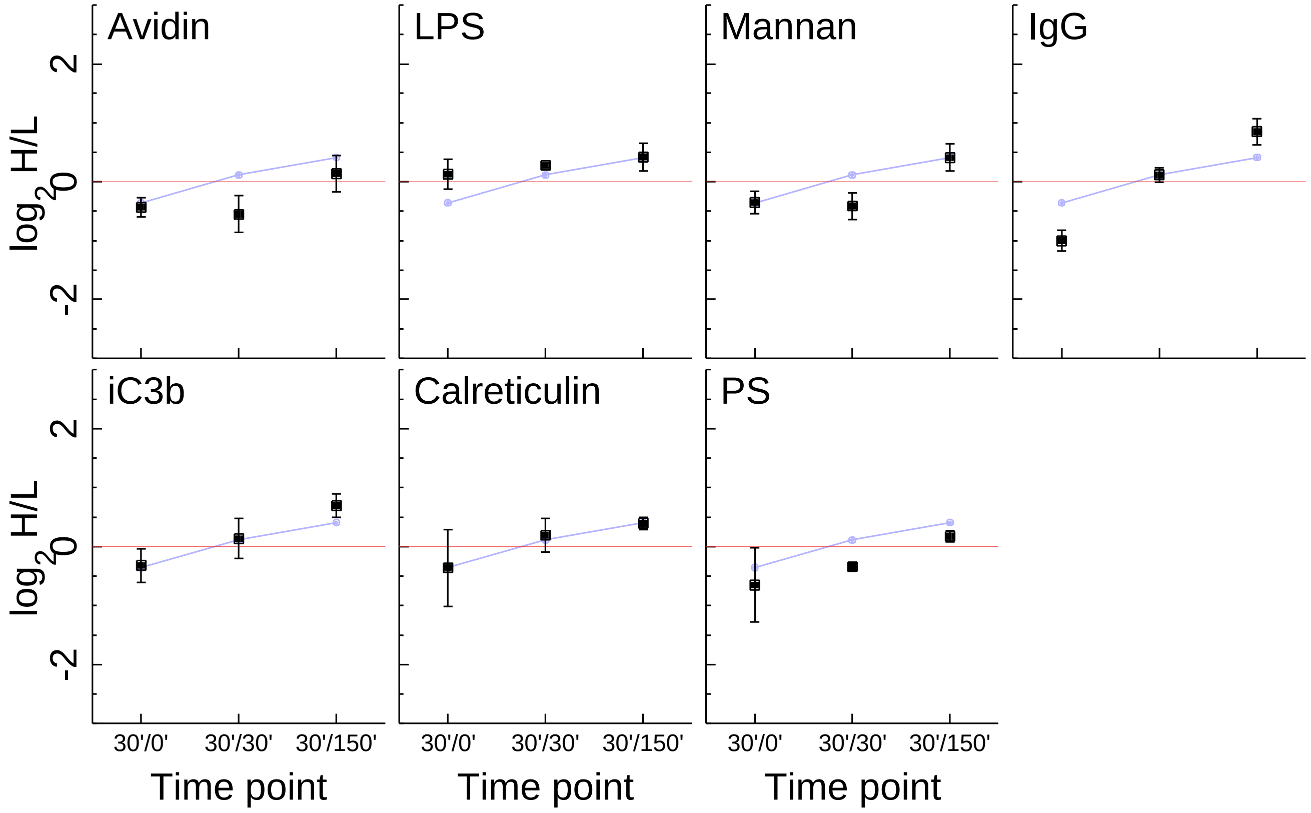

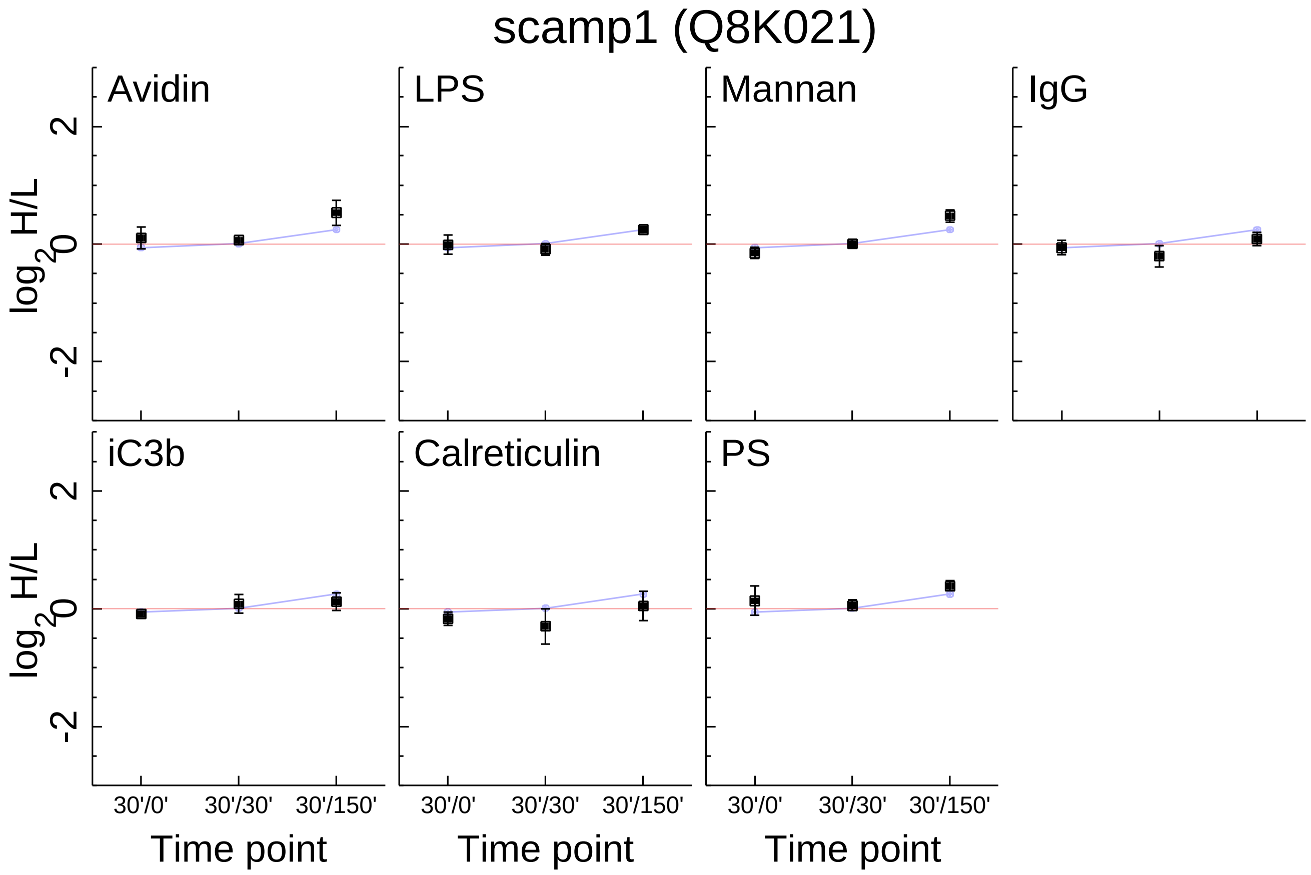

scamp1 (Q8K021)
Avidin
LPS
Mannan
IgG
iC3b
Calreticulin
PS
log2 H/L
2
0
-2
30'/0'
30'/30'
30'/150'
Time point

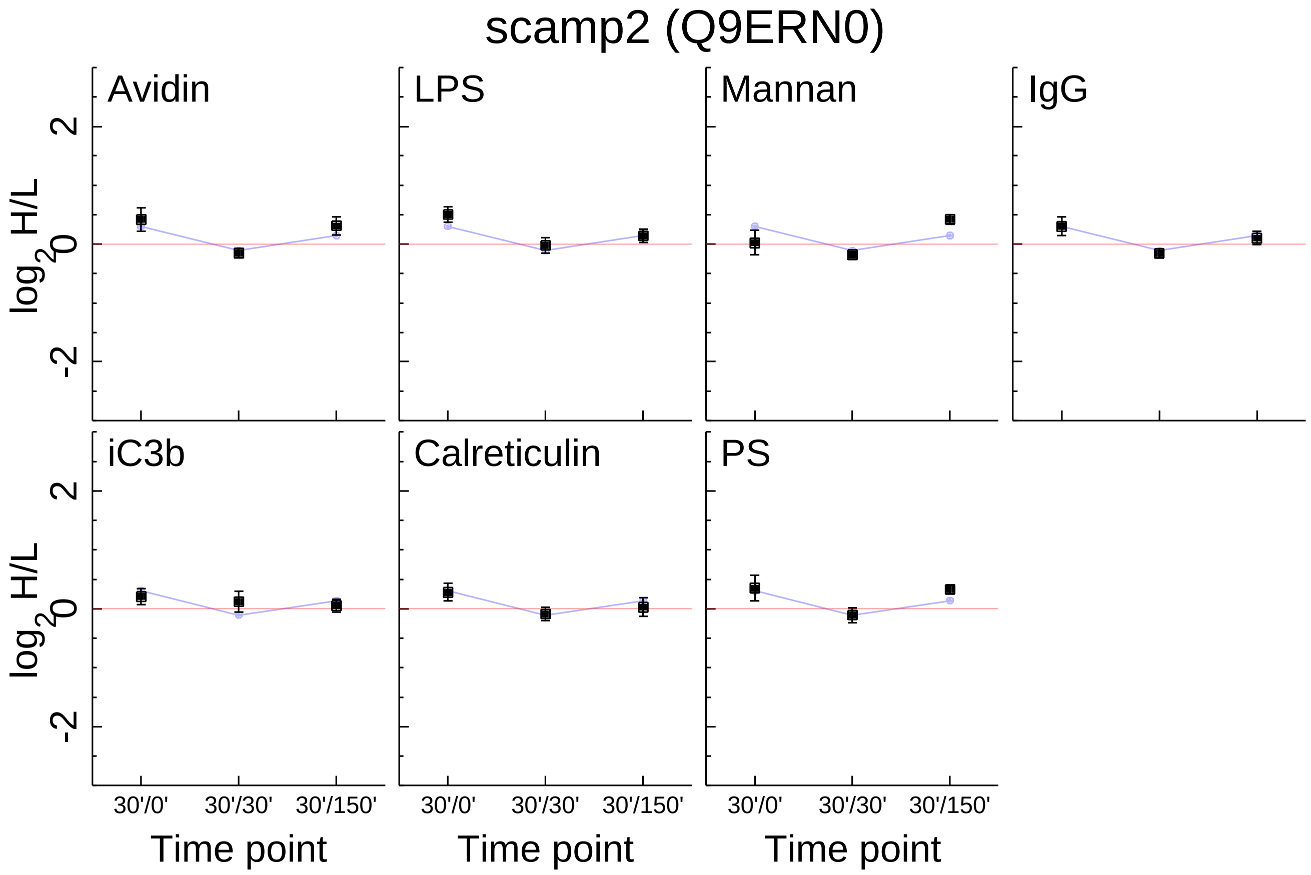
scamp2 (Q9ERN0)
Avidin
LPS
Mannan
IgG
iC3b
Calreticulin
PS
$\log_2$ H/L
2
0
-2
30'/0'
30'/30'
30'/150'
Time point

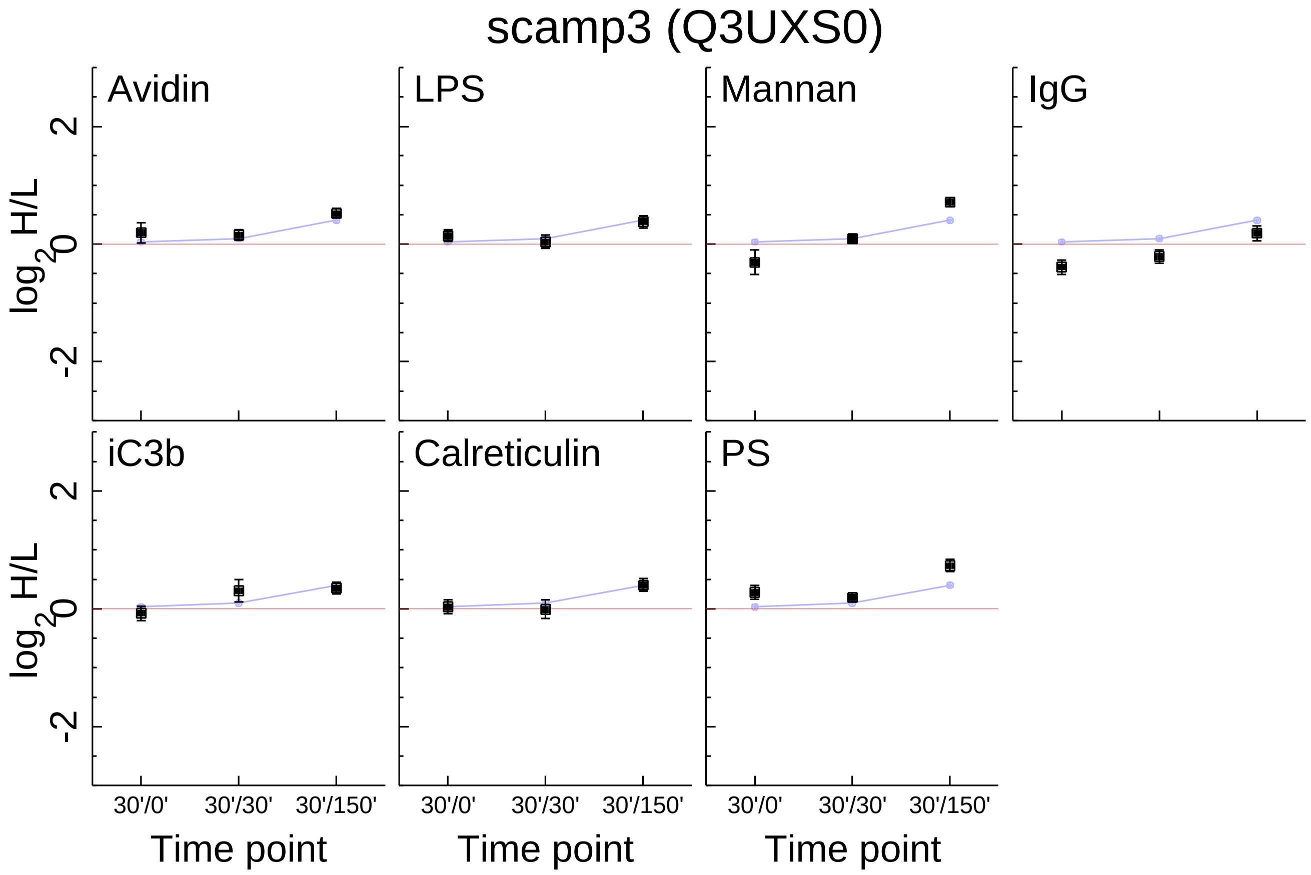
scamp3 (Q3UXS0)
Avidin
LPS
Mannan
IgG
iC3b
Calreticulin
PS
log2 H/L
2
0
-2
30'/0'
30'/30'
30'/150'
Time point

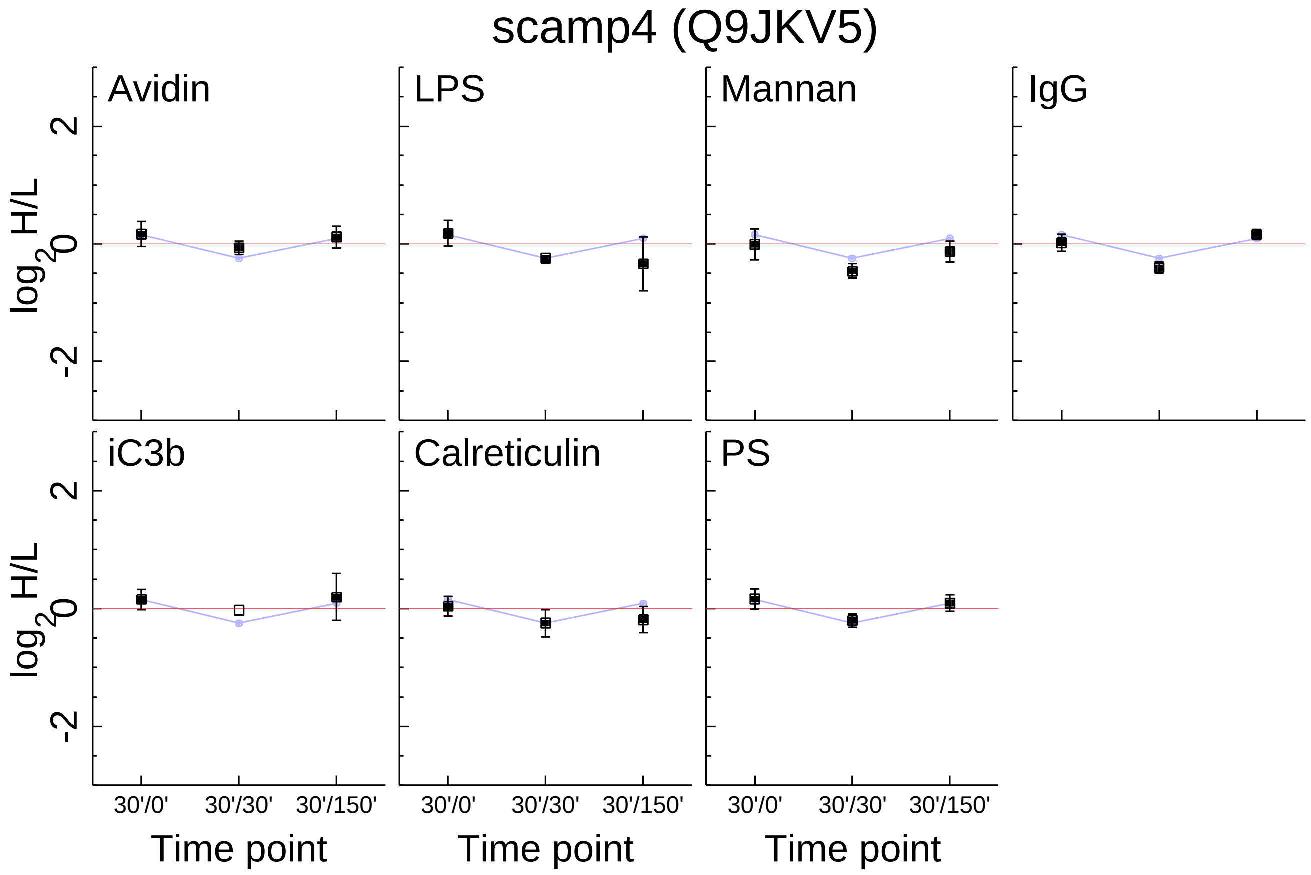
scamp4 (Q9JKV5)
Avidin
LPS
Mannan
IgG
iC3b
Calreticulin
PS
log2 H/L
2
0
-2
30'/0'
30'/30'
30'/150'
Time point

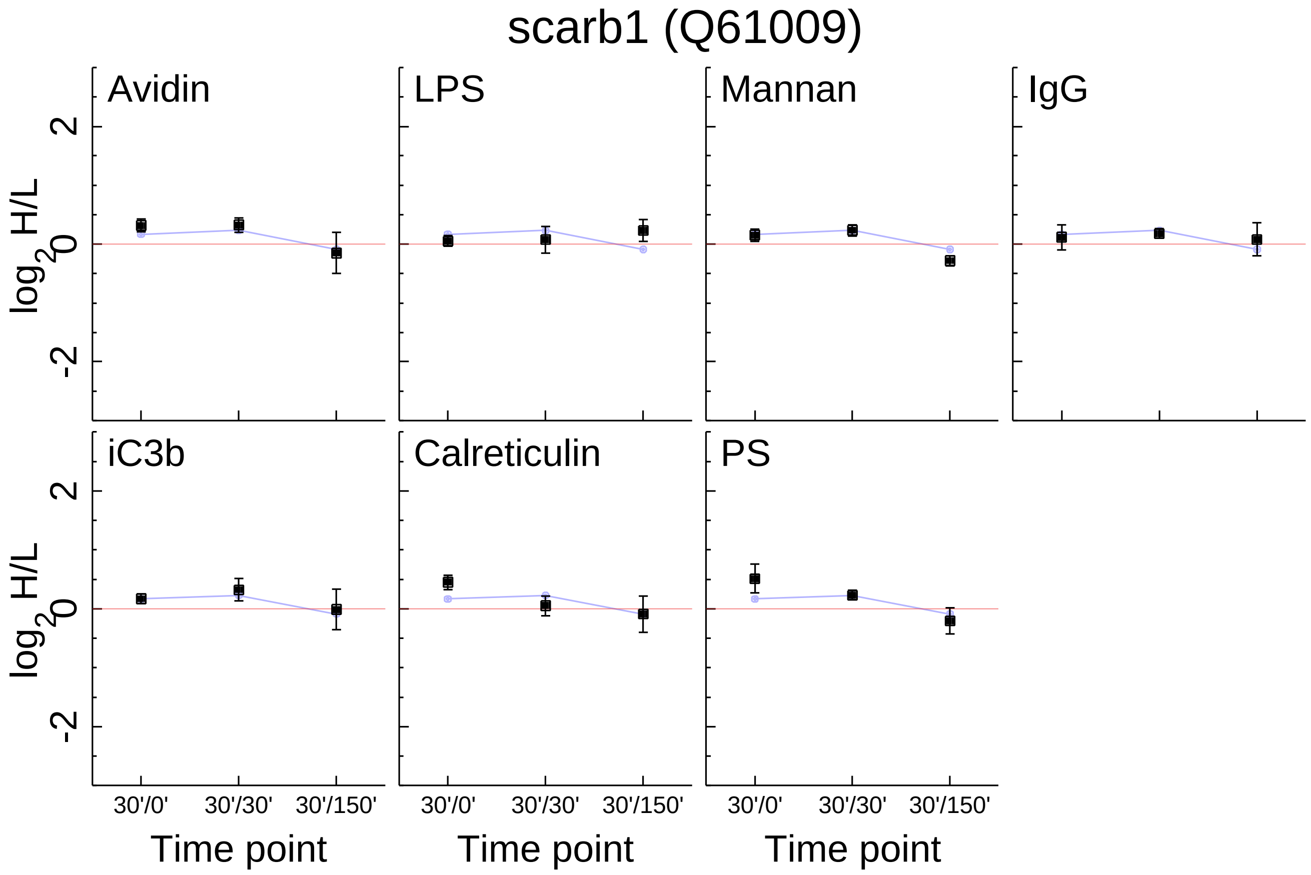

scarb1 (Q61009)
Avidin
LPS
Mannan
IgG
iC3b
Calreticulin
PS
log2 H/L
2
0
-2
30'/0'
30'/30'
30'/150'
Time point

scarb2 (O35114)

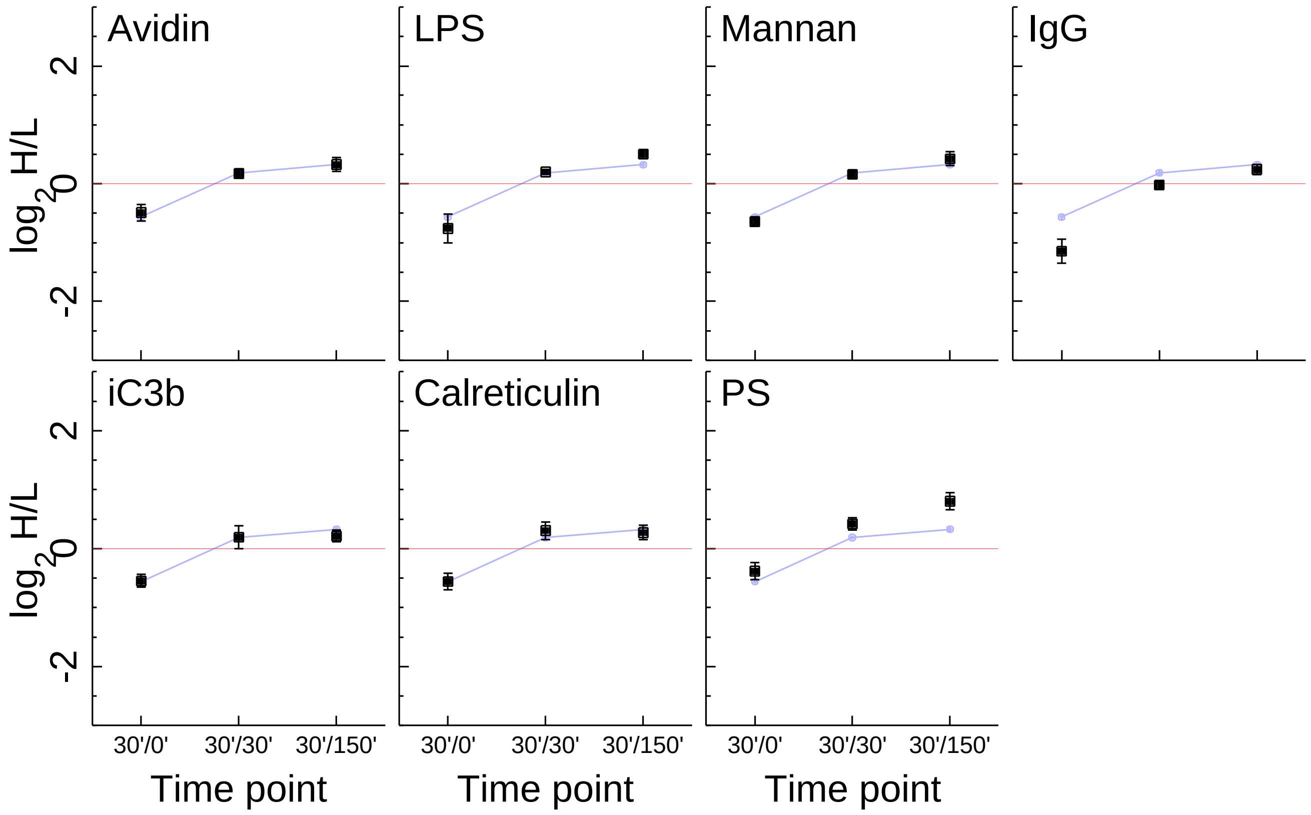

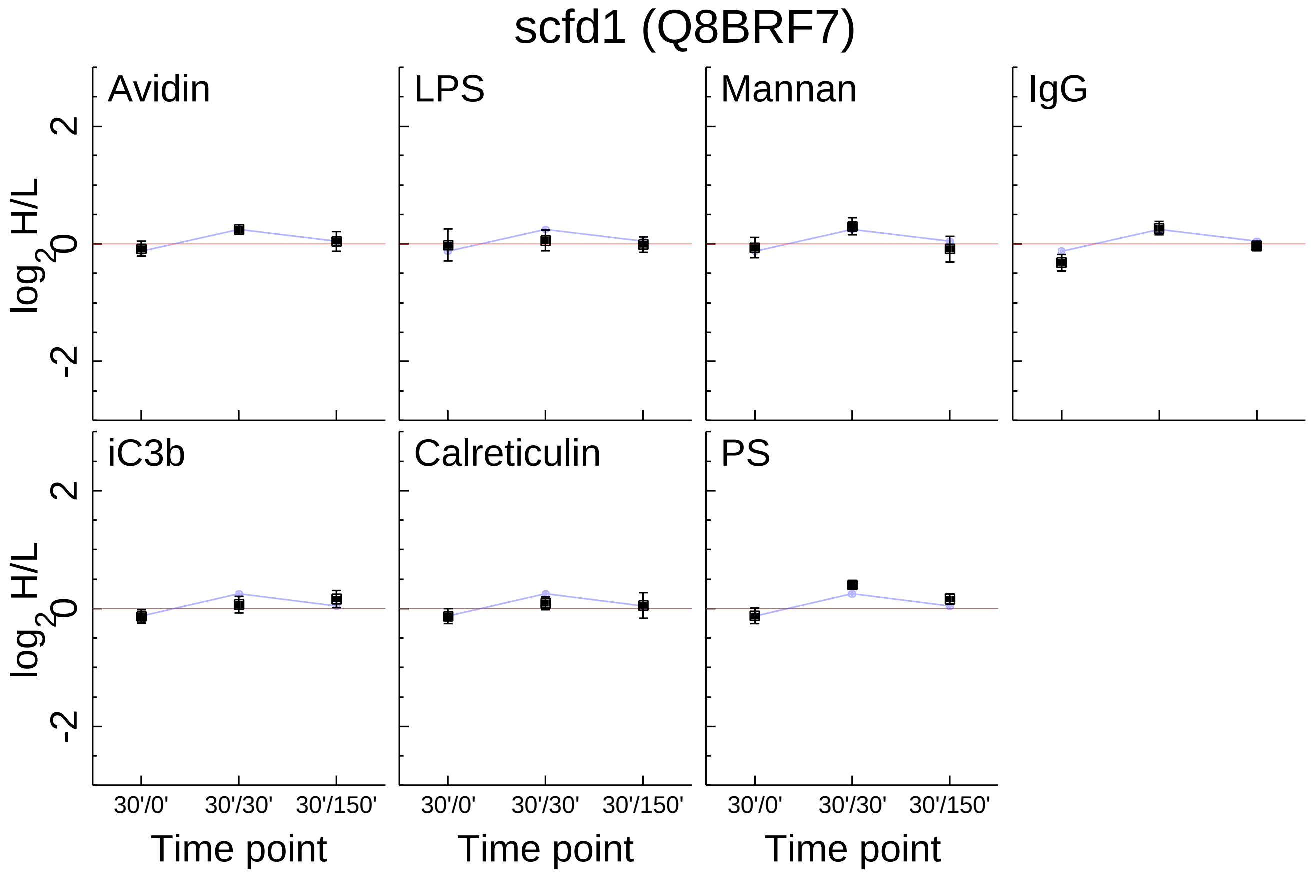
scfd1 (Q8BRF7)
Avidin
LPS
Mannan
IgG
iC3b
Calreticulin
PS
log2 H/L
2
0
-2
30'/0'
30'/30'
30'/150'
Time point

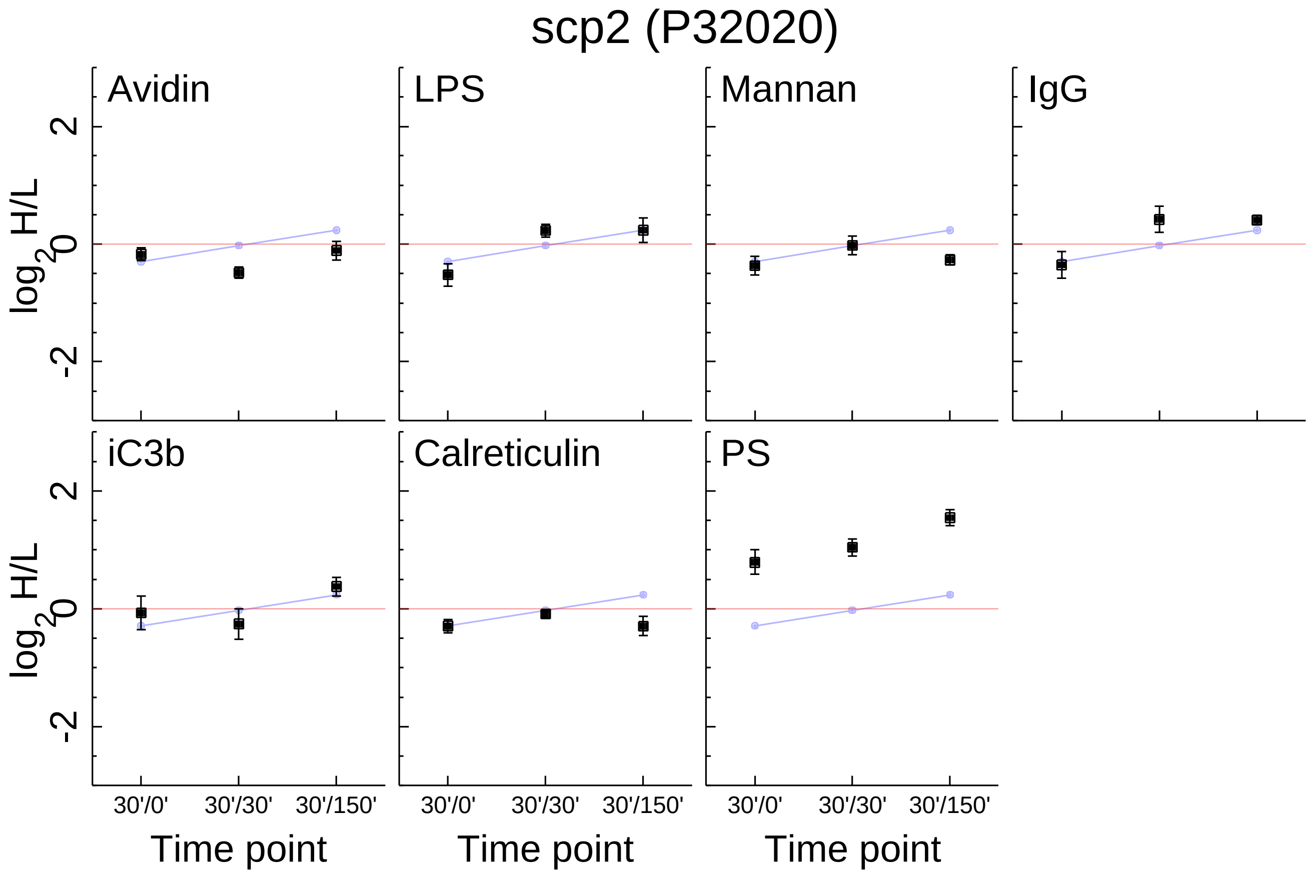

scp2 (P32020)
Avidin
LPS
Mannan
IgG
iC3b
Calreticulin
PS
log_2 H/L
2
0
-2
30'/0'
30'/30'
30'/150'
Time point

scpep1 (Q920A5)

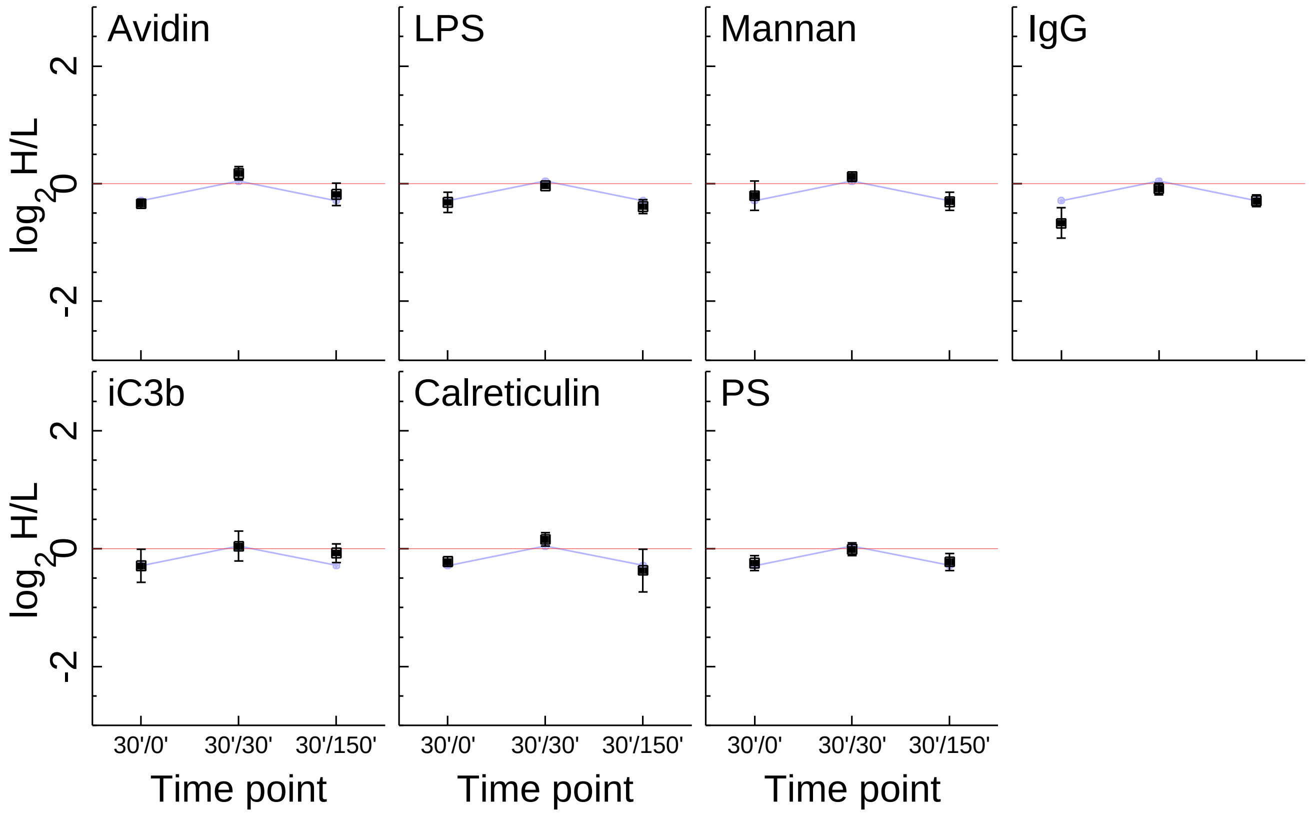

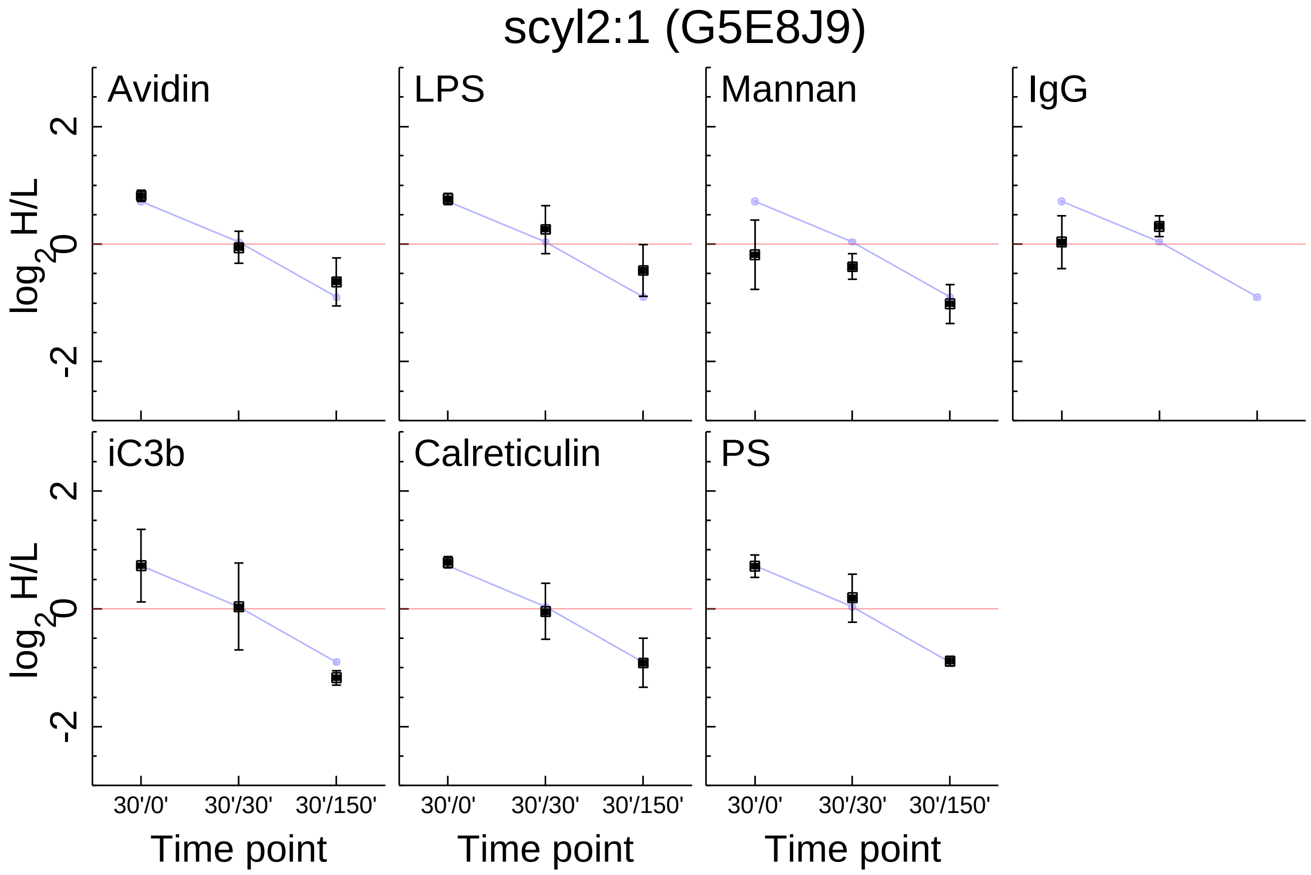

scyl2:1 (G5E8J9)
Avidin
LPS
Mannan
IgG
iC3b
Calreticulin
PS
log2 H/L
2
0
-2
30'/0'
30'/30'
30'/150'
Time point

scyl2:2 (Q8CFE4)

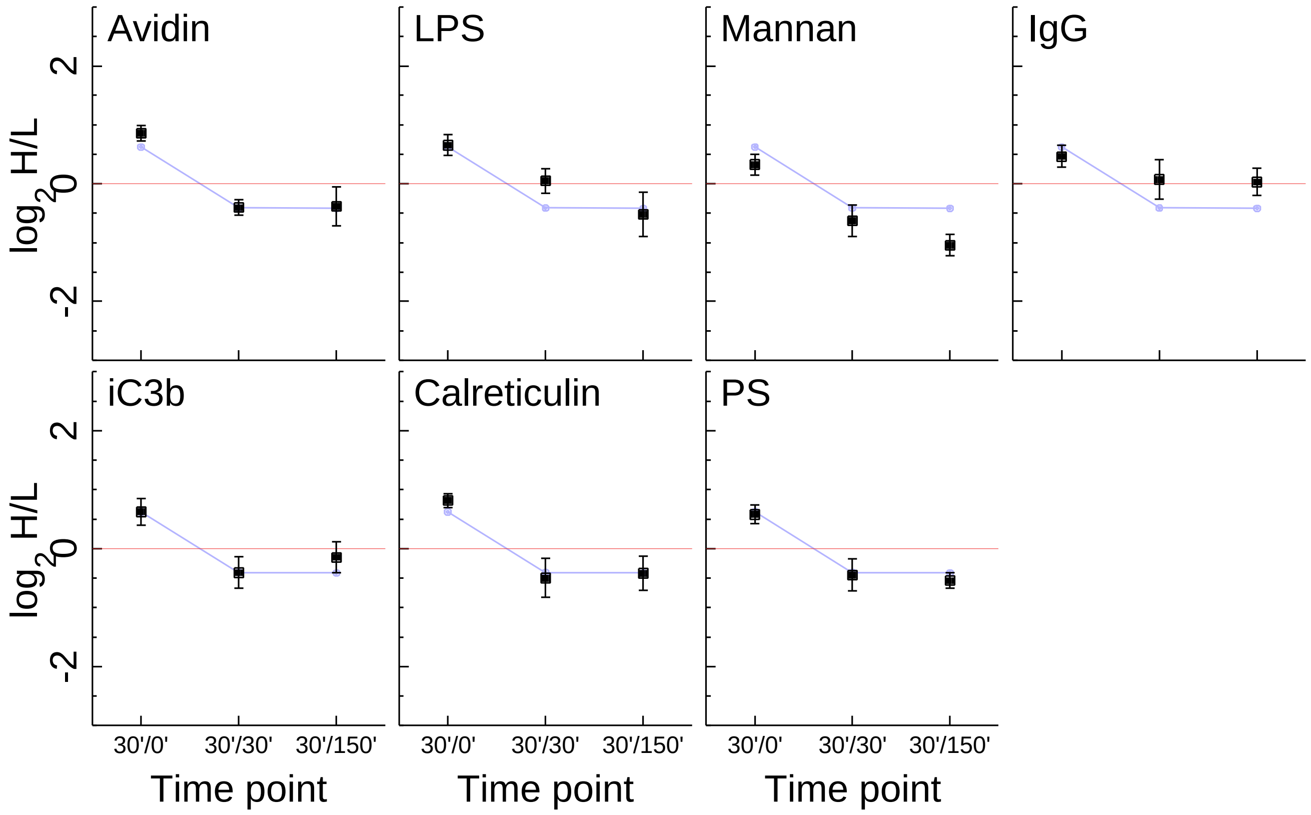

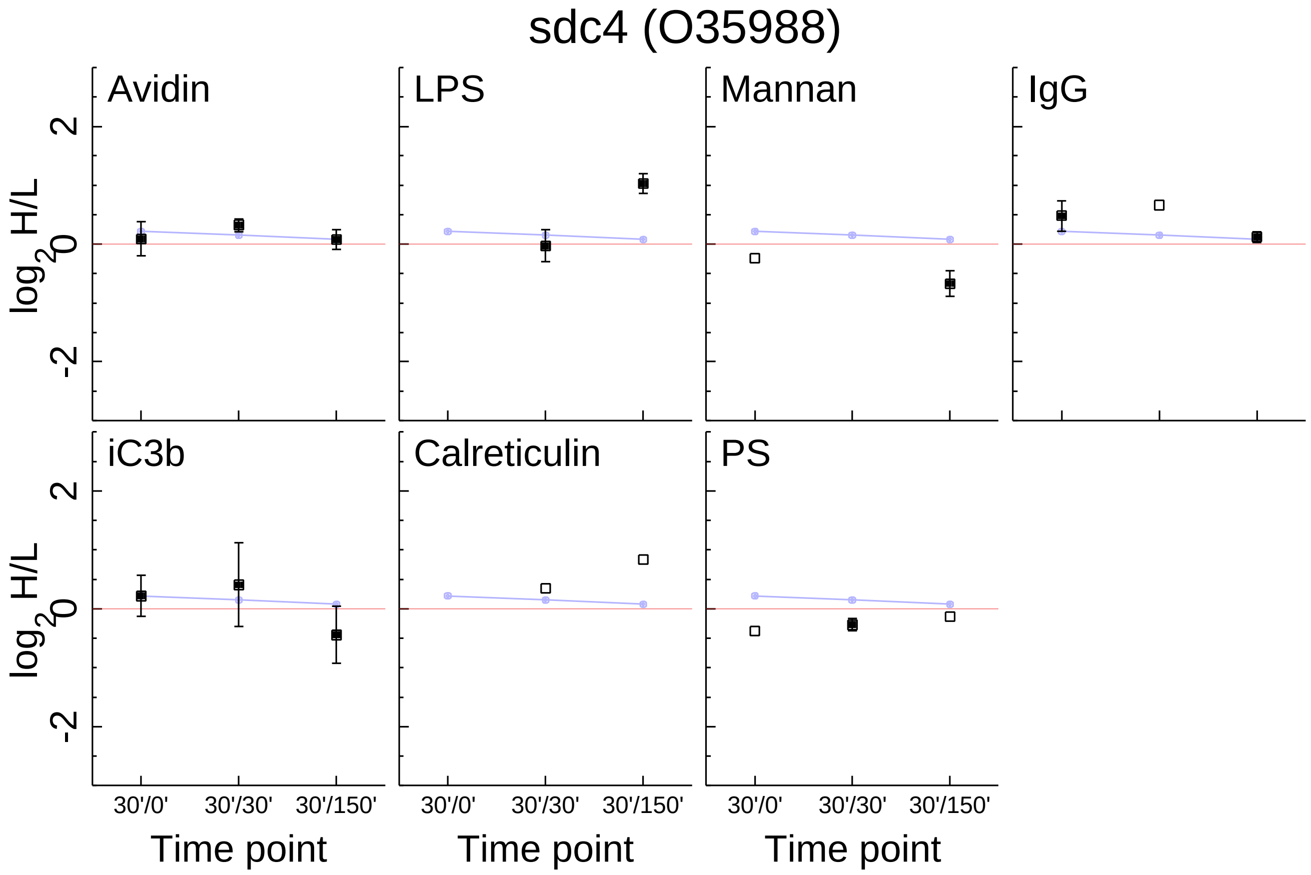

sdc4 (O35988)
Avidin
LPS
Mannan
IgG
iC3b
Calreticulin
PS
log2 H/L
2
0
-2
30'/0'
30'/30'
30'/150'
Time point

sdcbp (O08992)

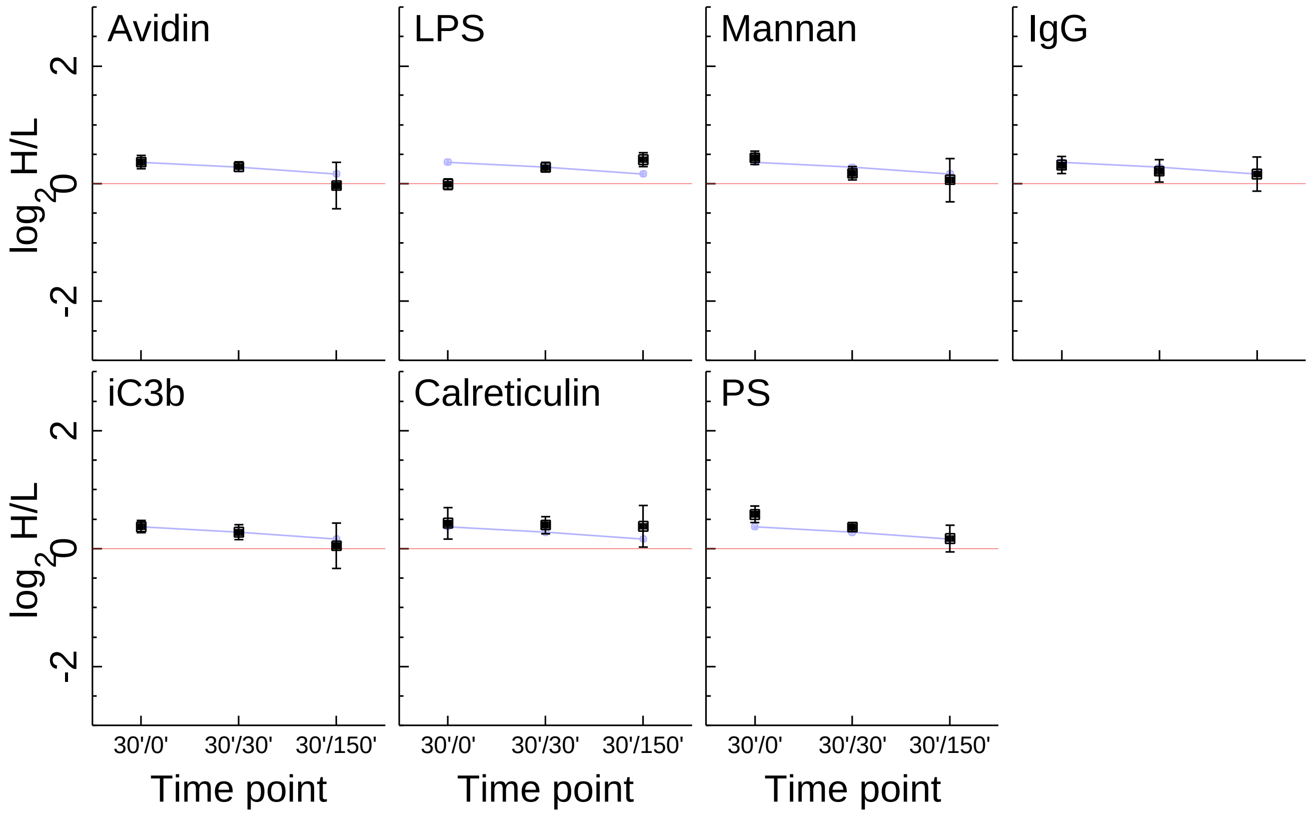

sdf2l1 (Q9ESP1)

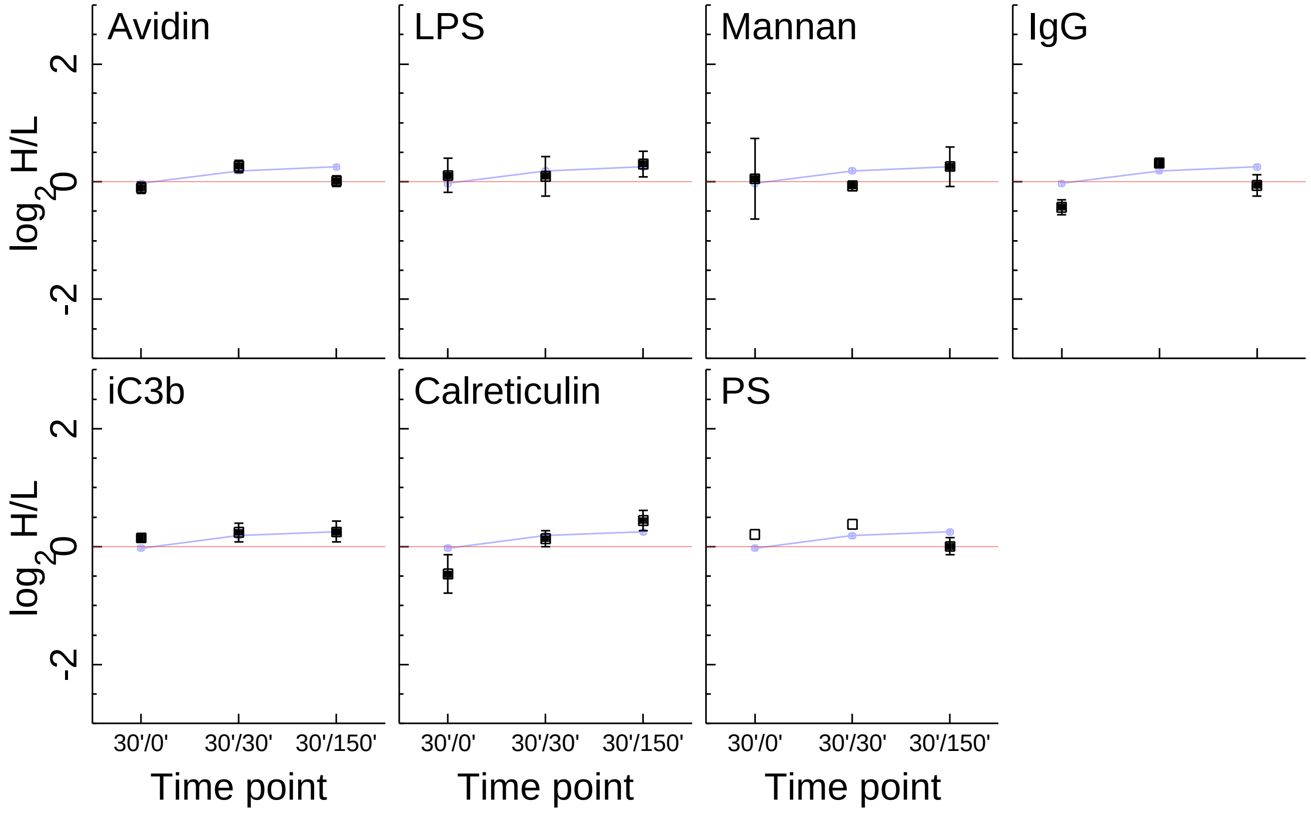

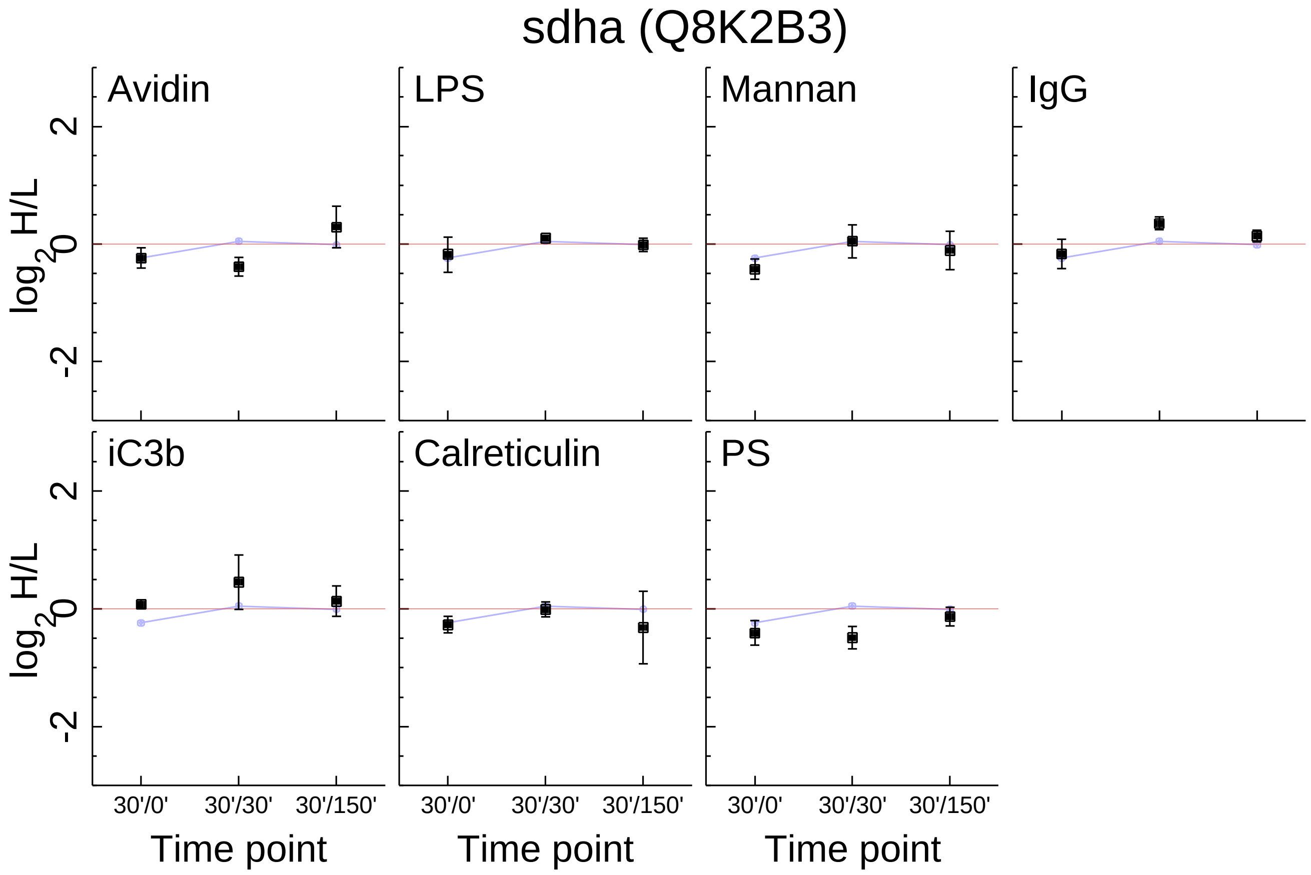
sdha (Q8K2B3)
Avidin
LPS
Mannan
IgG
iC3b
Calreticulin
PS
log2 H/L
2
0
-2
30'/0'
30'/30'
30'/150'
Time point

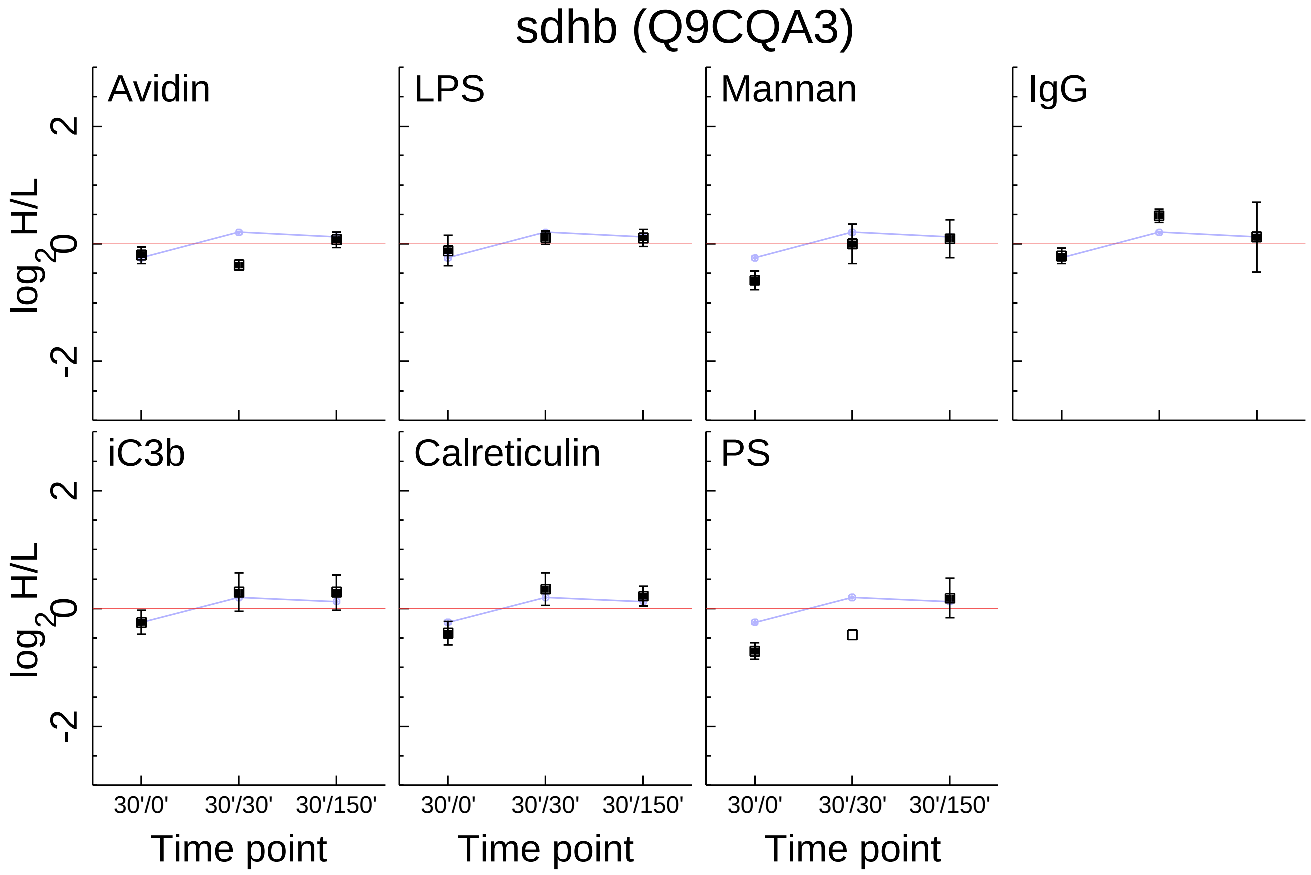

sdhb (Q9CQA3)
Avidin
LPS
Mannan
IgG
iC3b
Calreticulin
PS
log2 H/L
2
0
-2
30'/0'
30'/30'
30'/150'
Time point

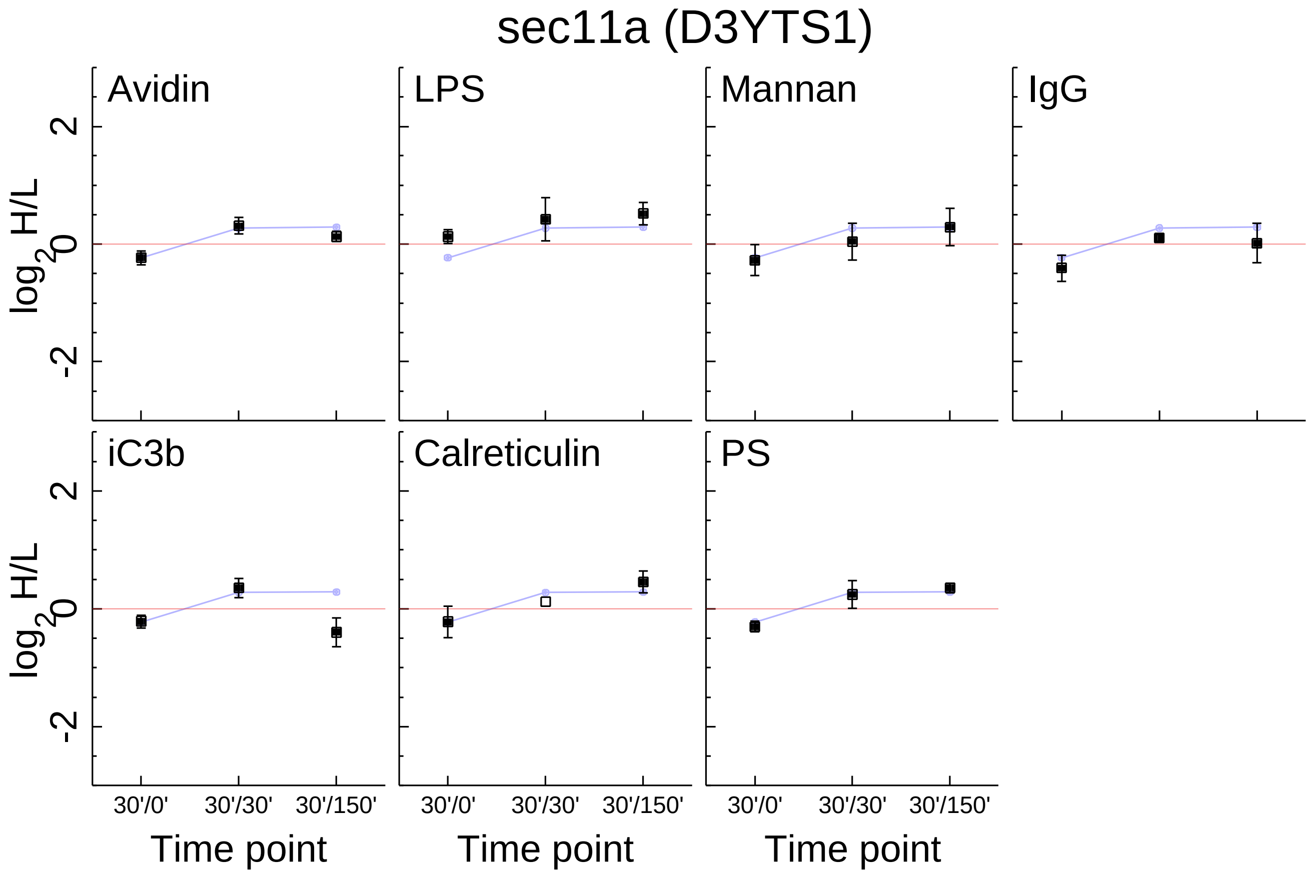

sec11a (D3YTS1)
Avidin
LPS
Mannan
IgG
iC3b
Calreticulin
PS
log2 H/L
2
0
-2
30'/0'
30'/30'
30'/150'
Time point

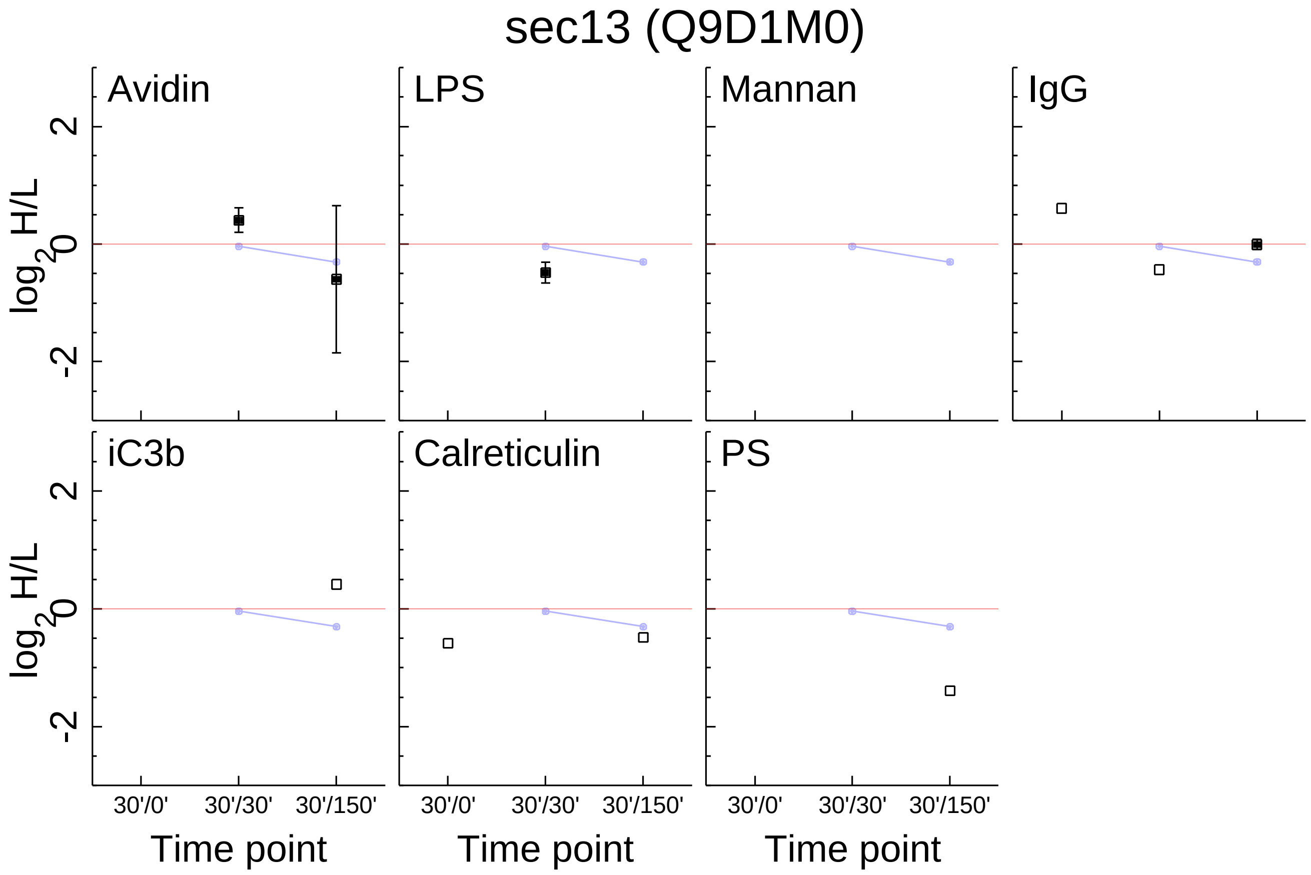

sec13 (Q9D1M0)
Avidin
LPS
Mannan
IgG
iC3b
Calreticulin
PS
log2 H/L
2
0
-2
30'/0'
30'/30'
30'/150'
Time point

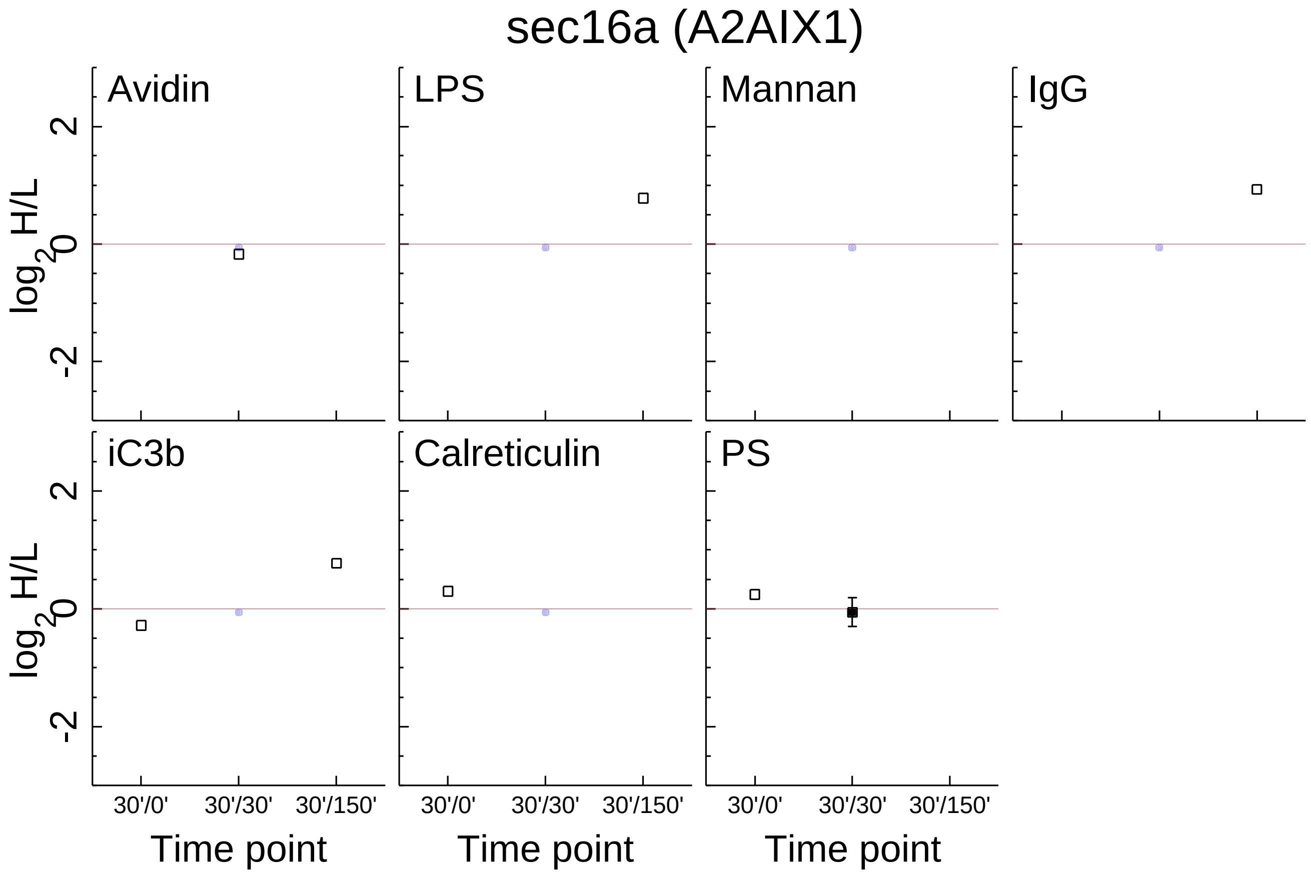

sec16a (A2AIX1)
Avidin
LPS
Mannan
IgG
iC3b
Calreticulin
PS
log2 H/L
2
0
-2
30'/0'
30'/30'
30'/150'
Time point

sec22b (O08547)

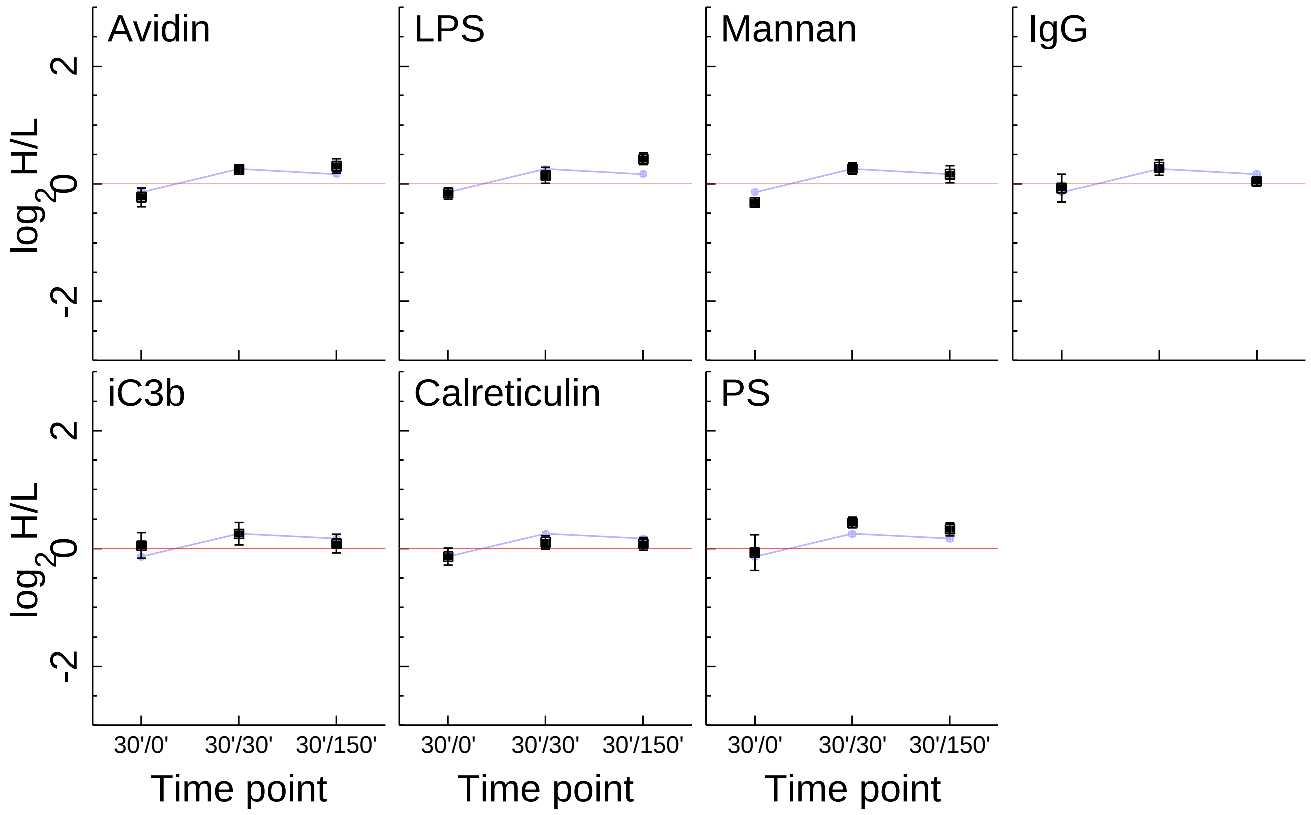

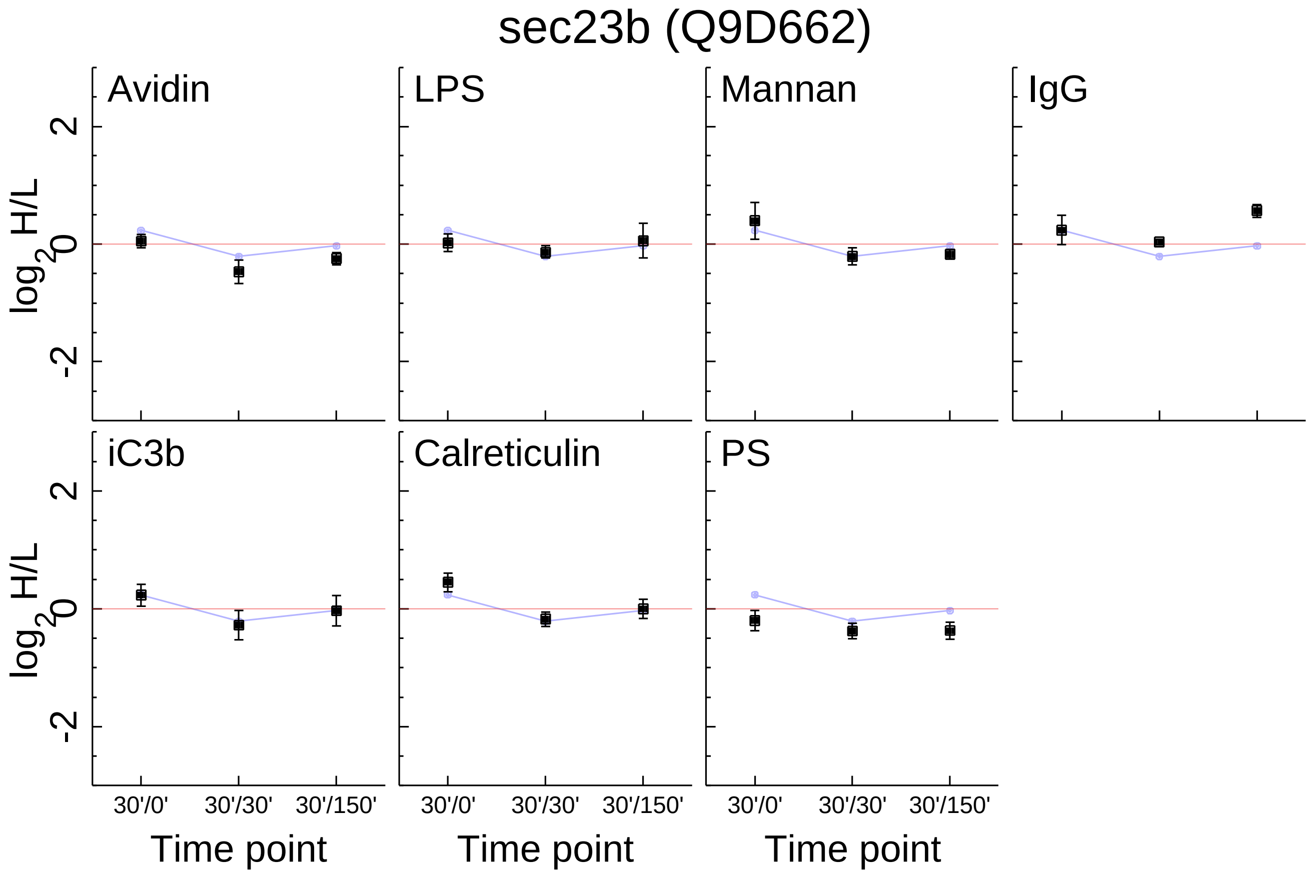

sec23b (Q9D662)
Avidin
LPS
Mannan
IgG
iC3b
Calreticulin
PS
log2 H/L
2
0
-2
30'/0'
30'/30'
30'/150'
Time point

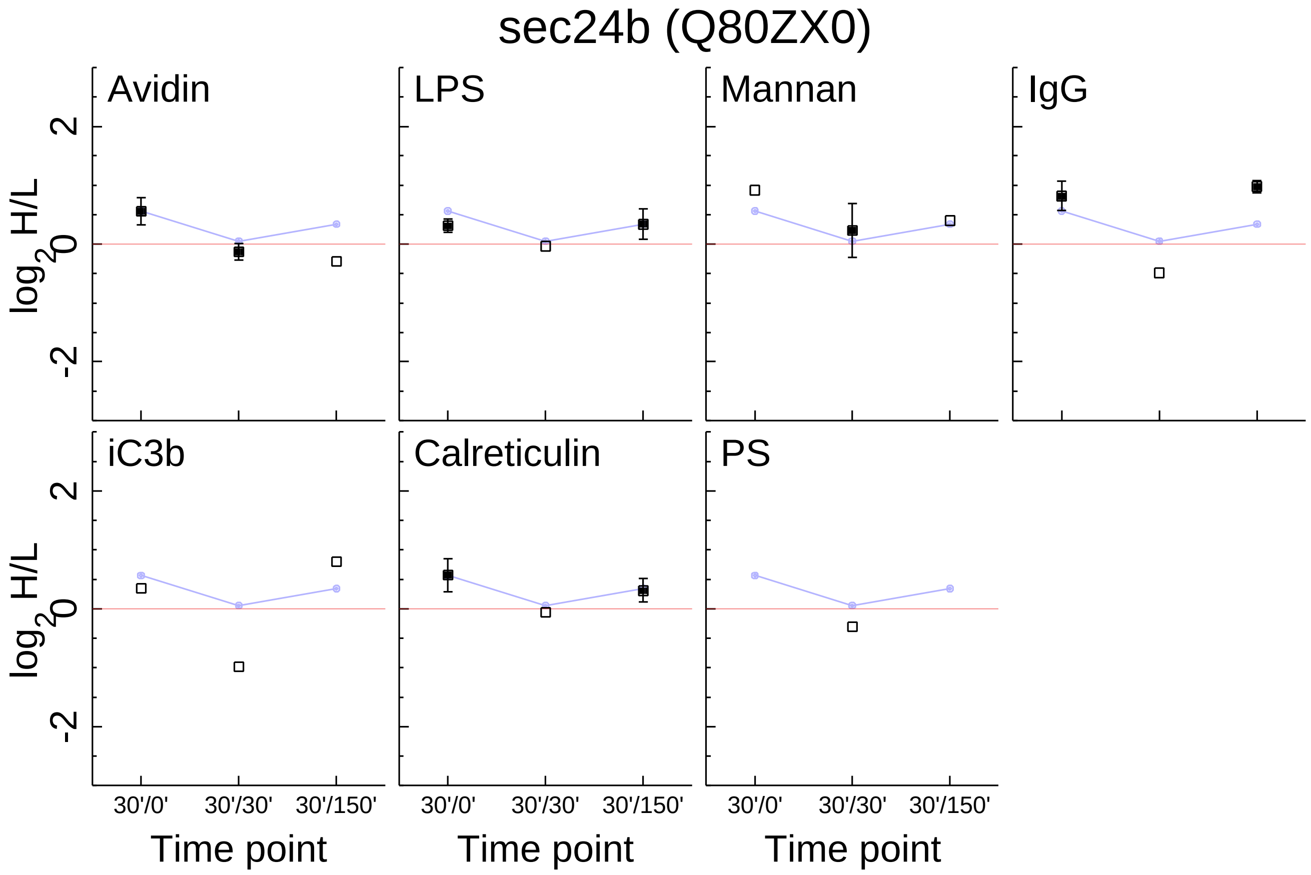

sec24b (Q80ZX0)
Avidin
LPS
Mannan
IgG
iC3b
Calreticulin
PS
log2 H/L
2
0
-2
30'/0'
30'/30'
30'/150'
Time point

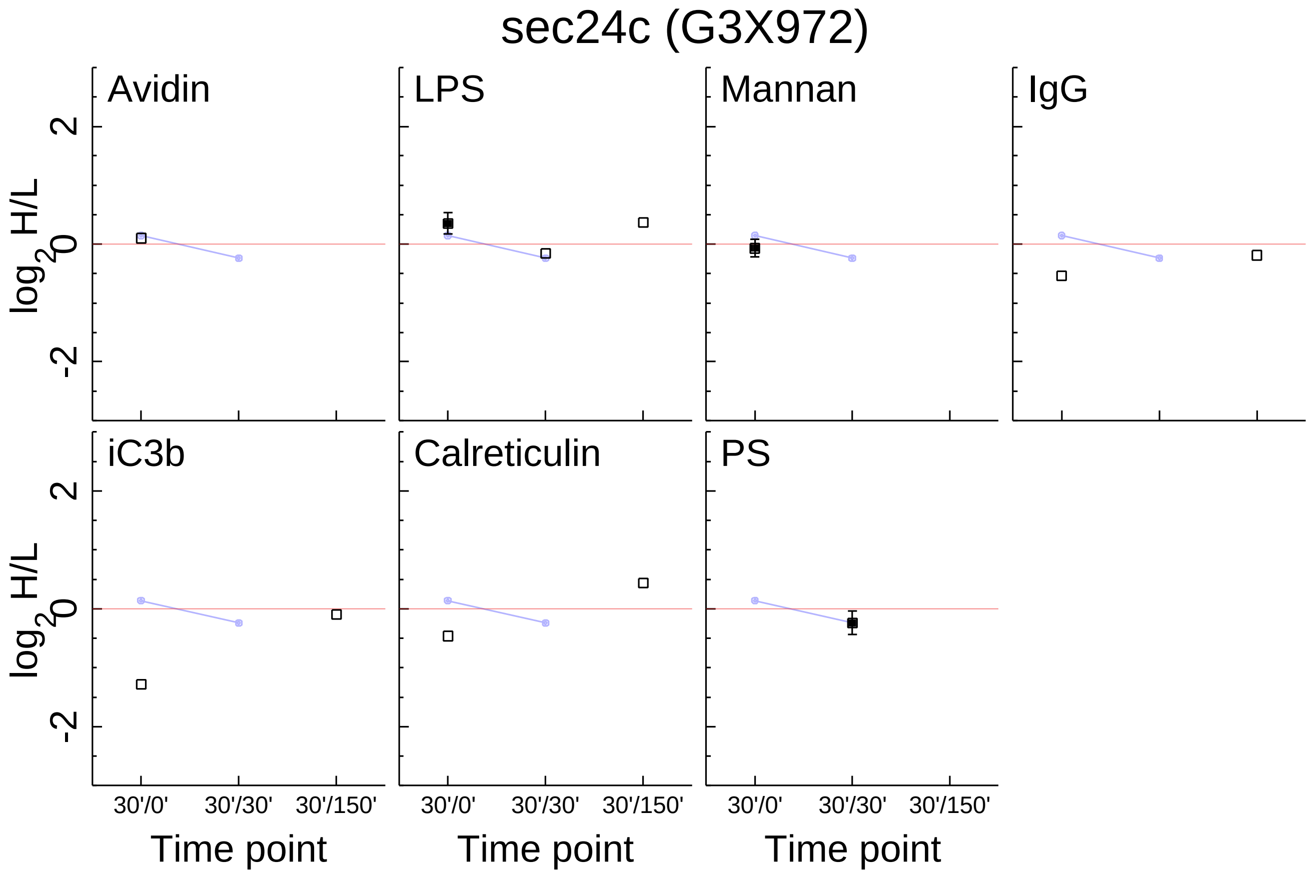

sec24c (G3X972)
Avidin
LPS
Mannan
IgG
iC3b
Calreticulin
PS
log2 H/L
2
0
-2
30'/0'
30'/30'
30'/150'
Time point

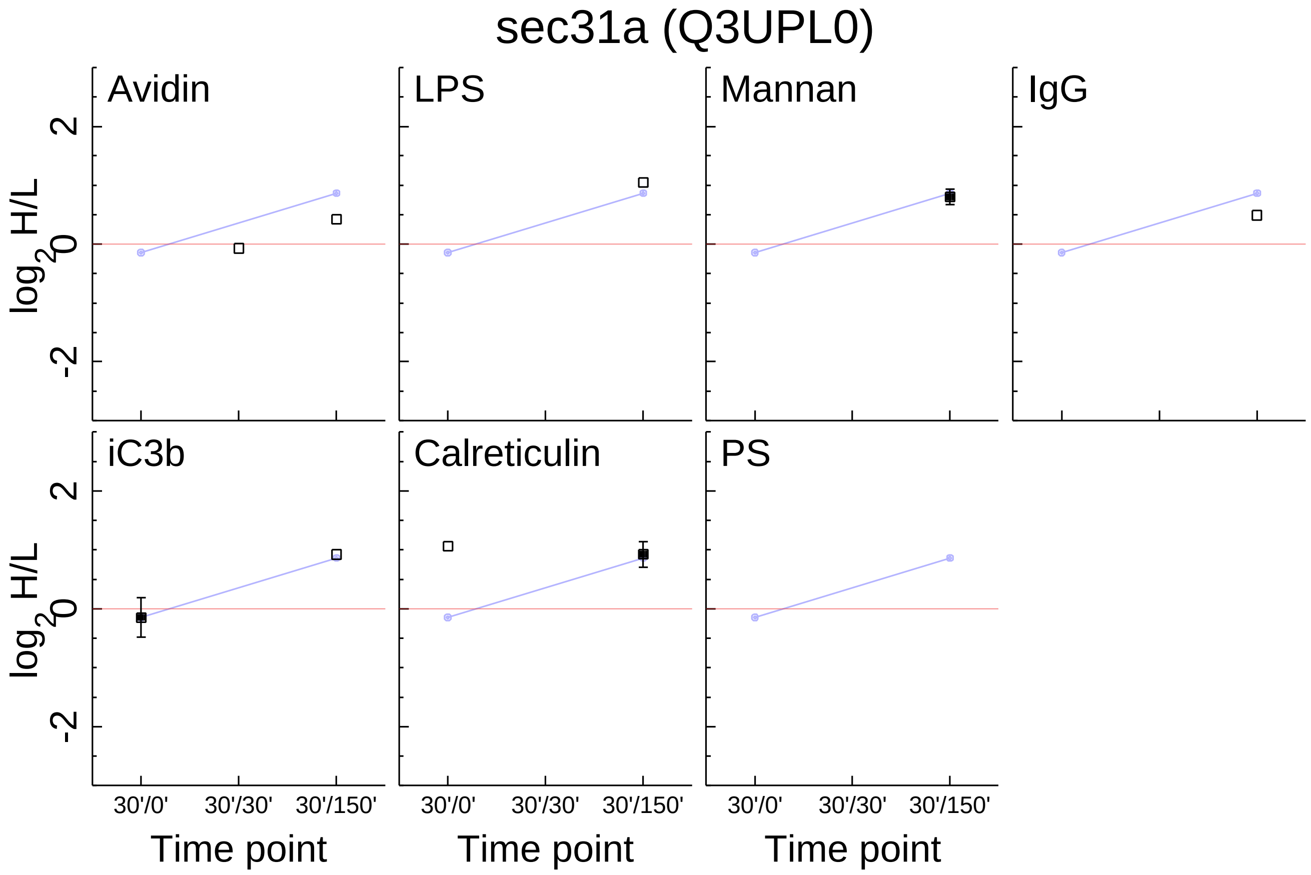
sec31a (Q3UPL0)
Avidin
LPS
Mannan
IgG
iC3b
Calreticulin
PS
log2 H/L
2
0
-2
30'/0'
30'/30'
30'/150'
Time point

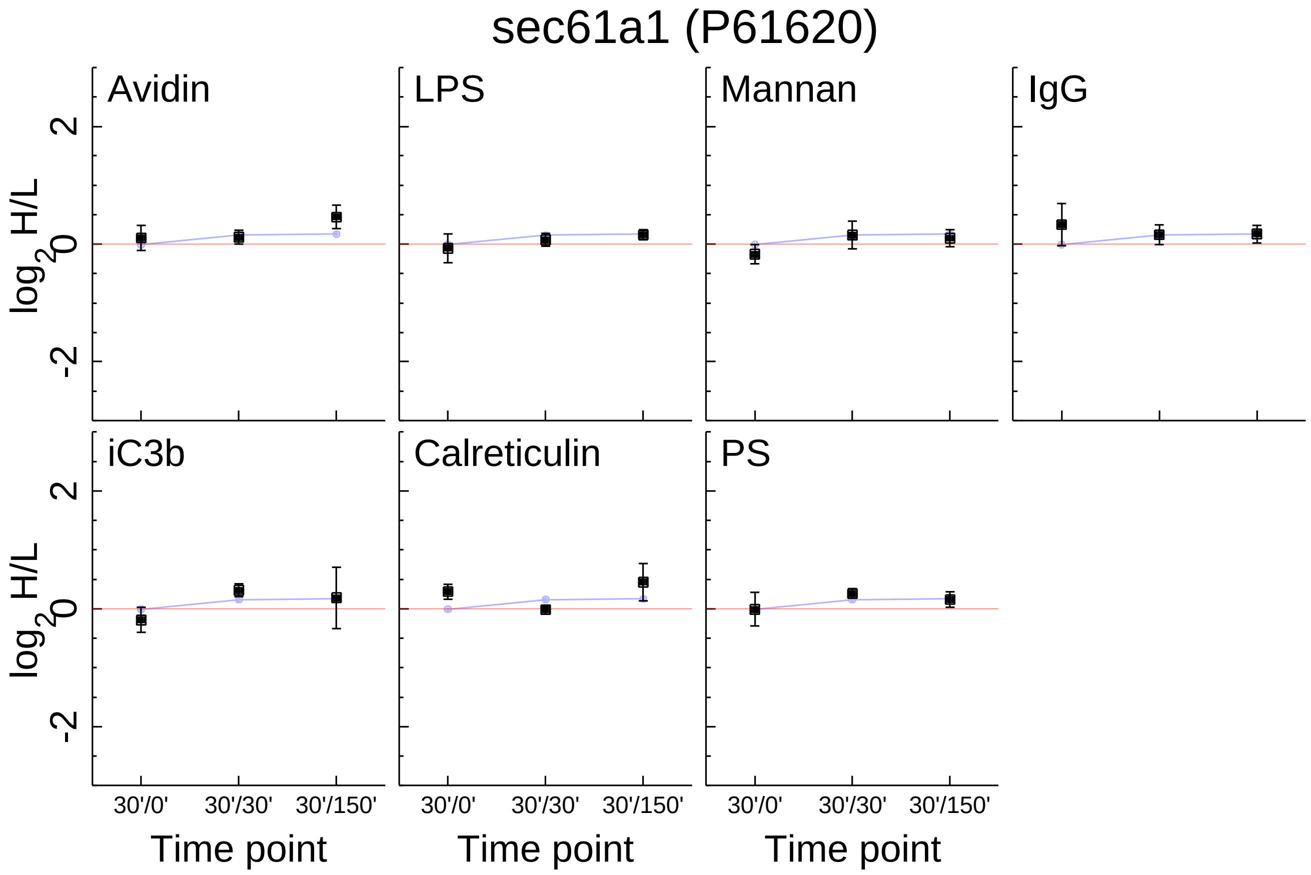
sec61a1 (P61620)
Avidin
LPS
Mannan
IgG
iC3b
Calreticulin
PS
log2 H/L
2
0
-2
30'/0'
30'/30'
30'/150'
Time point

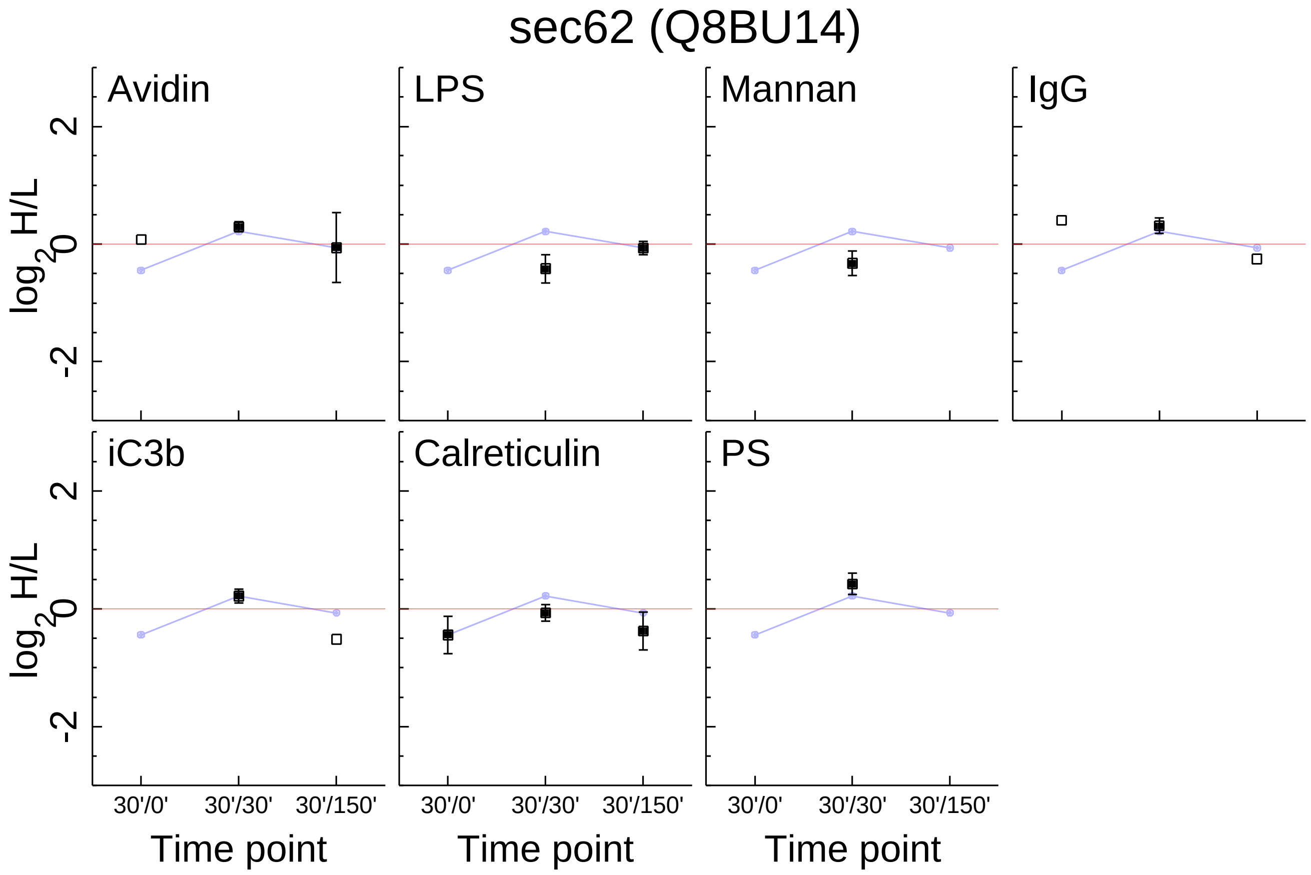
sec62 (Q8BU14)
Avidin
LPS
Mannan
IgG
iC3b
Calreticulin
PS
log2 H/L
2
0
-2
30'/0'
30'/30'
30'/150'
Time point

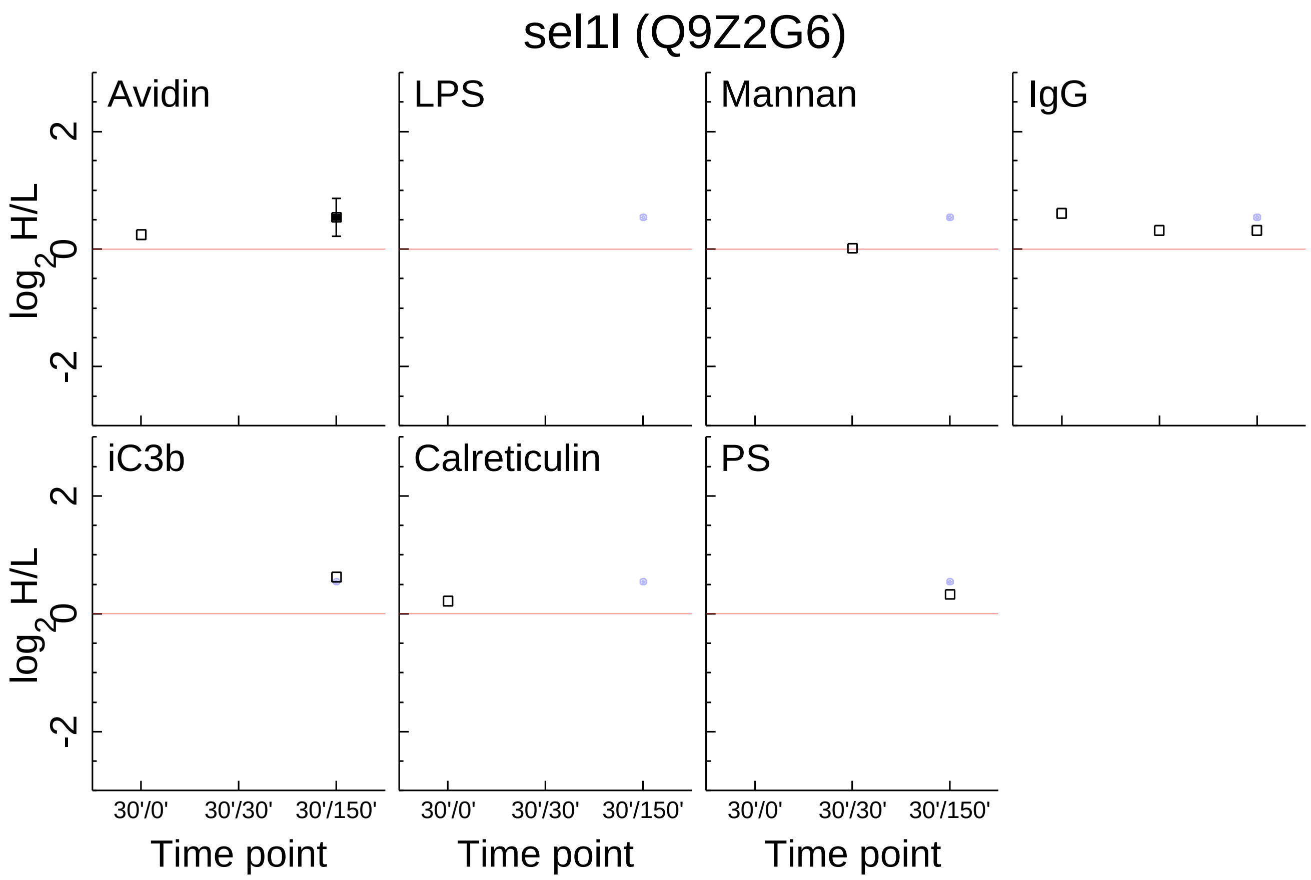

sel1l (Q9Z2G6)
Avidin
LPS
Mannan
IgG
iC3b
Calreticulin
PS
log2 H/L
2
0
-2
30'/0'
30'/30'
30'/150'
Time point

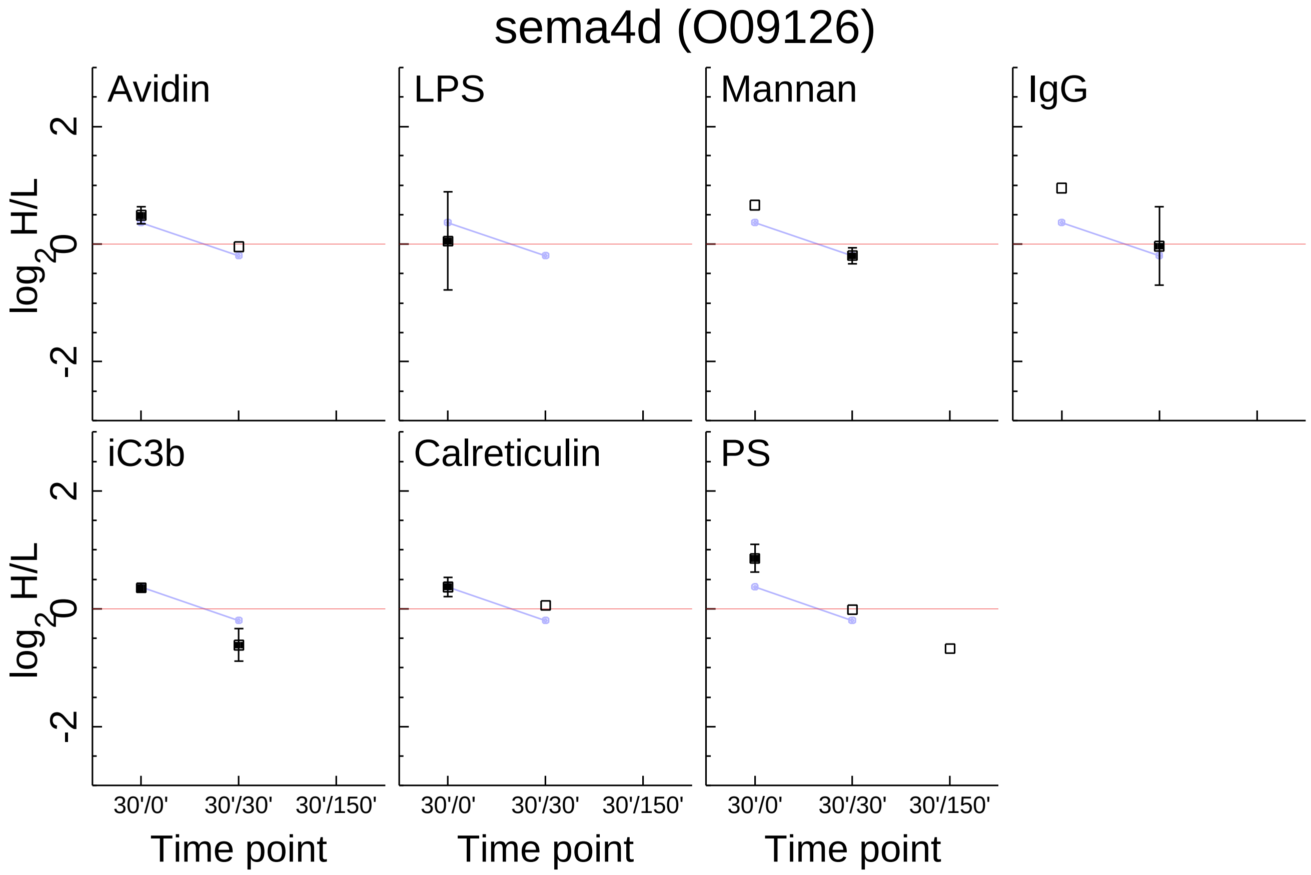

sema4d (O09126)
Avidin
LPS
Mannan
IgG
iC3b
Calreticulin
PS
log2 H/L
2
0
-2
30'/0'
30'/30'
30'/150'
Time point

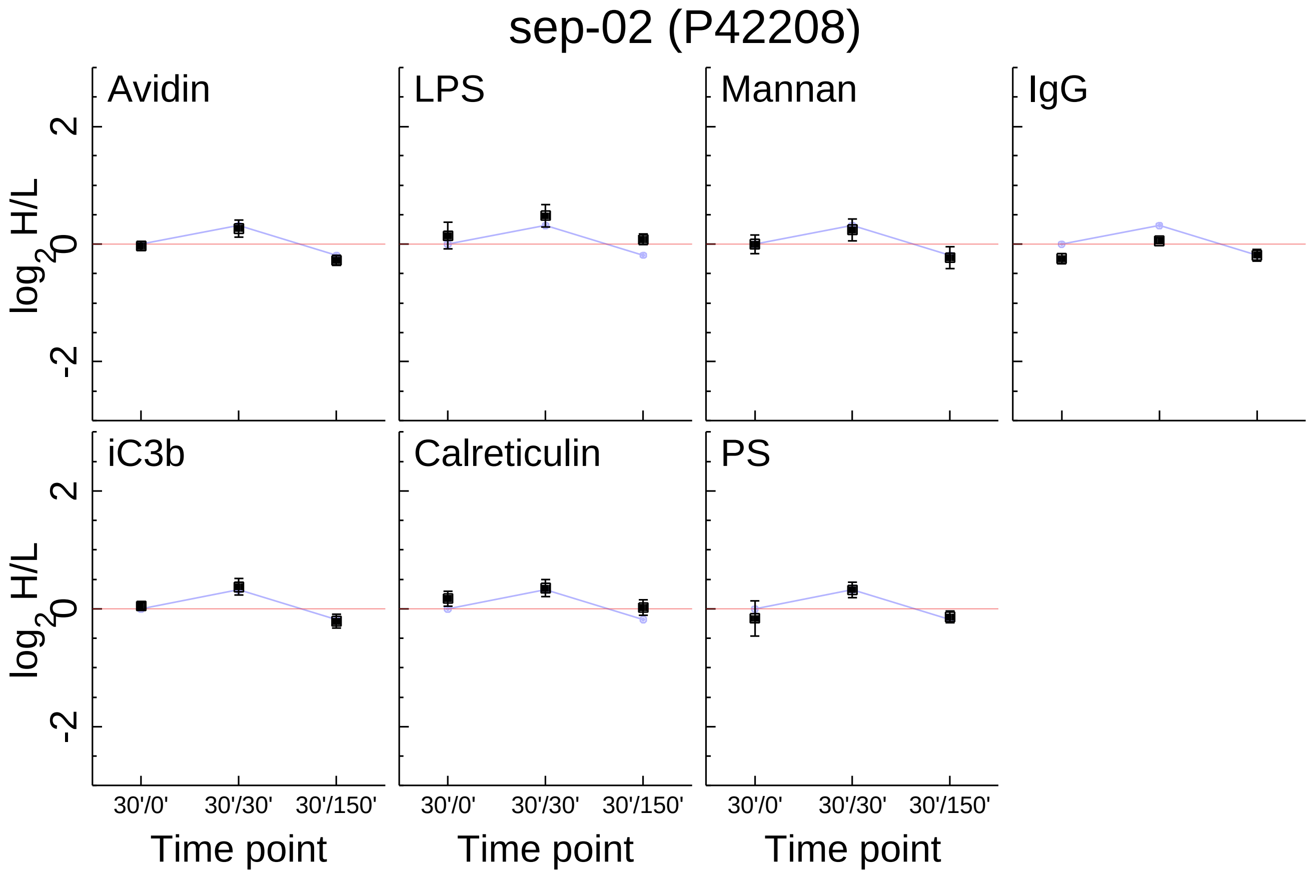
sep-02 (P42208)
Avidin
LPS
Mannan
IgG
iC3b
Calreticulin
PS
log2 H/L
2
0
-2
30'/0'
30'/30'
30'/150'
Time point

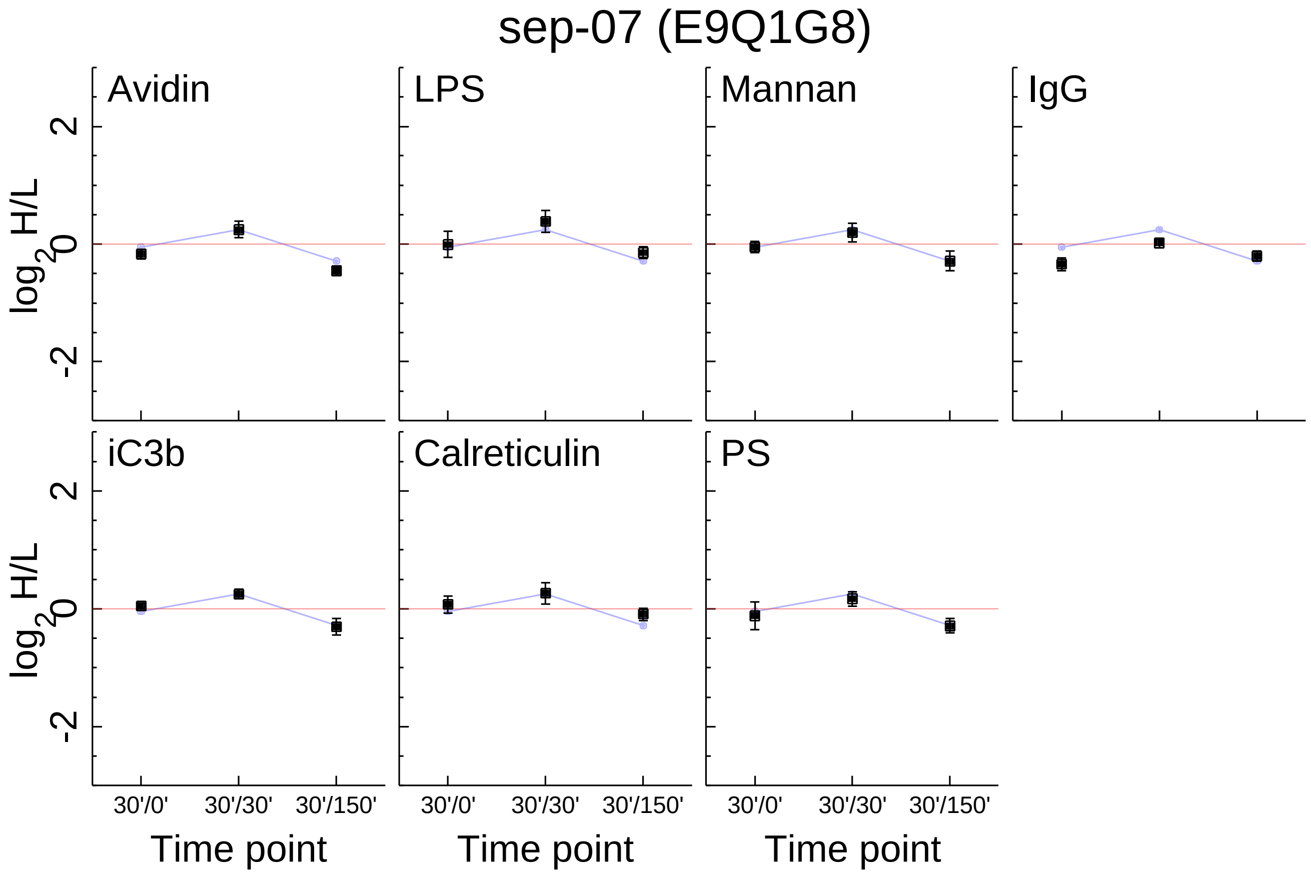

sep-07 (E9Q1G8)
Avidin
LPS
Mannan
IgG
iC3b
Calreticulin
PS
2
0
-2
log_2 H/L
30'/0'
30'/30'
30'/150'
Time point

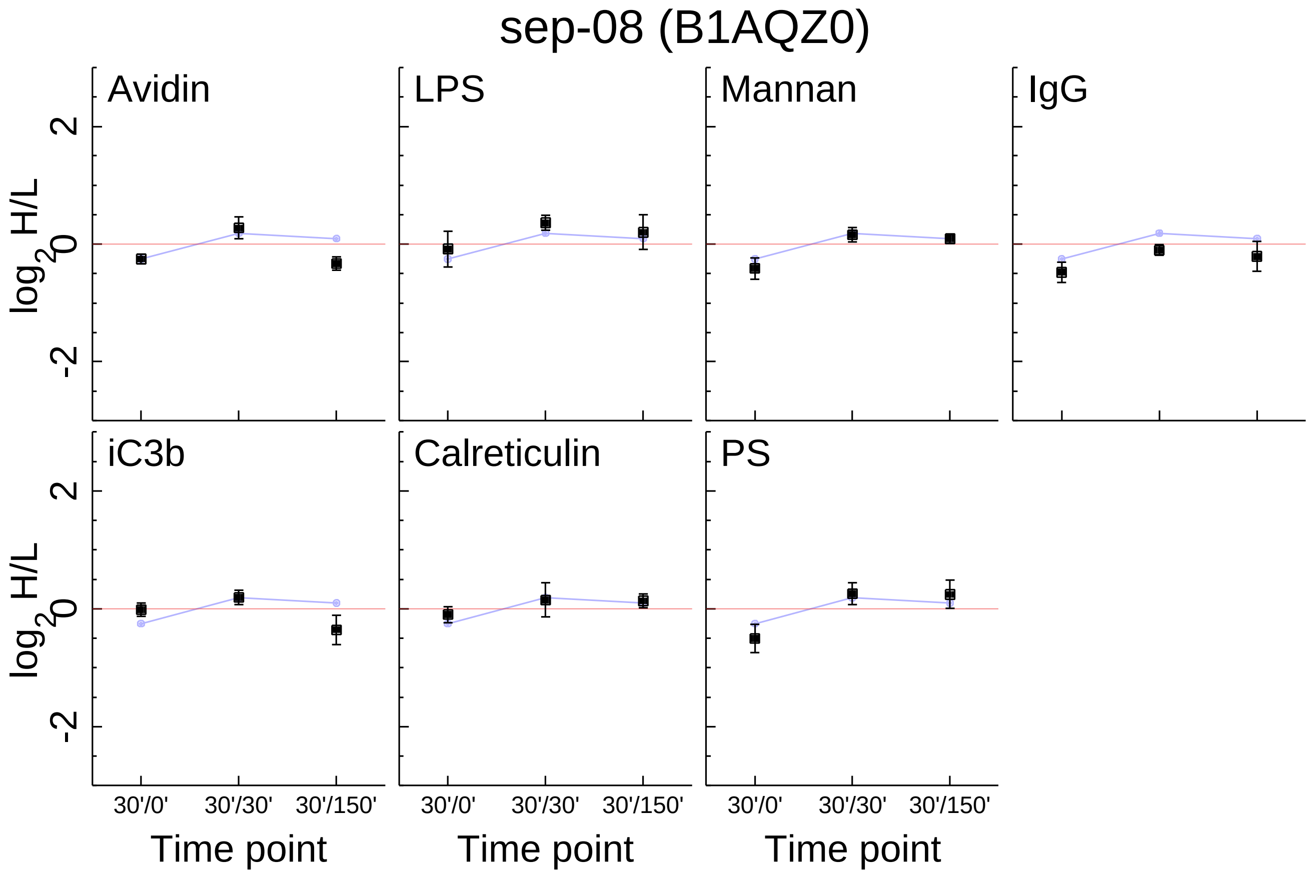

sep-08 (B1AQZ0)
Avidin
LPS
Mannan
IgG
iC3b
Calreticulin
PS
log2 H/L
2
0
-2
30'/0'
30'/30'
30'/150'
Time point

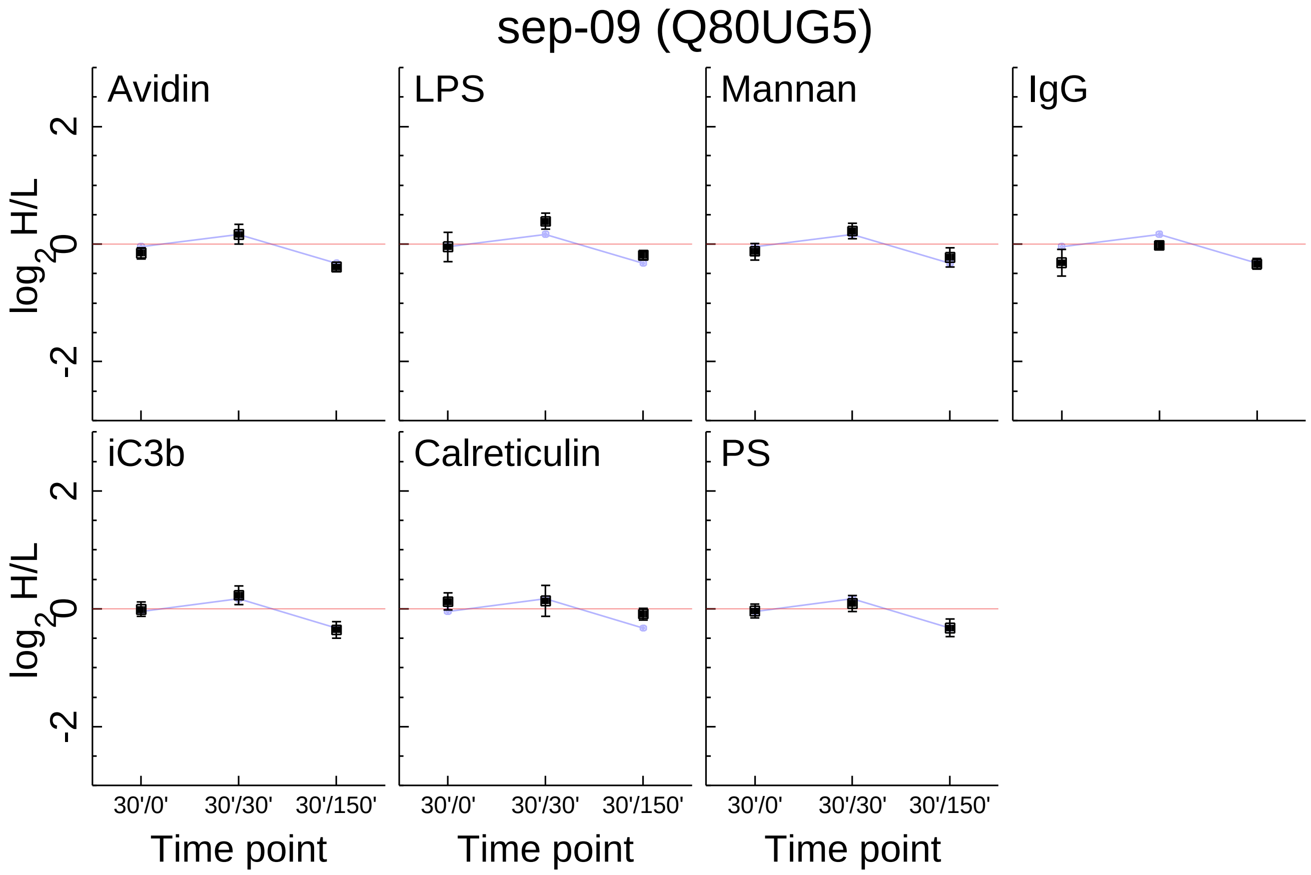
sep-09 (Q80UG5)
Avidin
LPS
Mannan
IgG
iC3b
Calreticulin
PS
2
0
-2
log2 H/L
30'/0'
30'/30'
30'/150'
Time point

sep-11 (Q8C1B7)

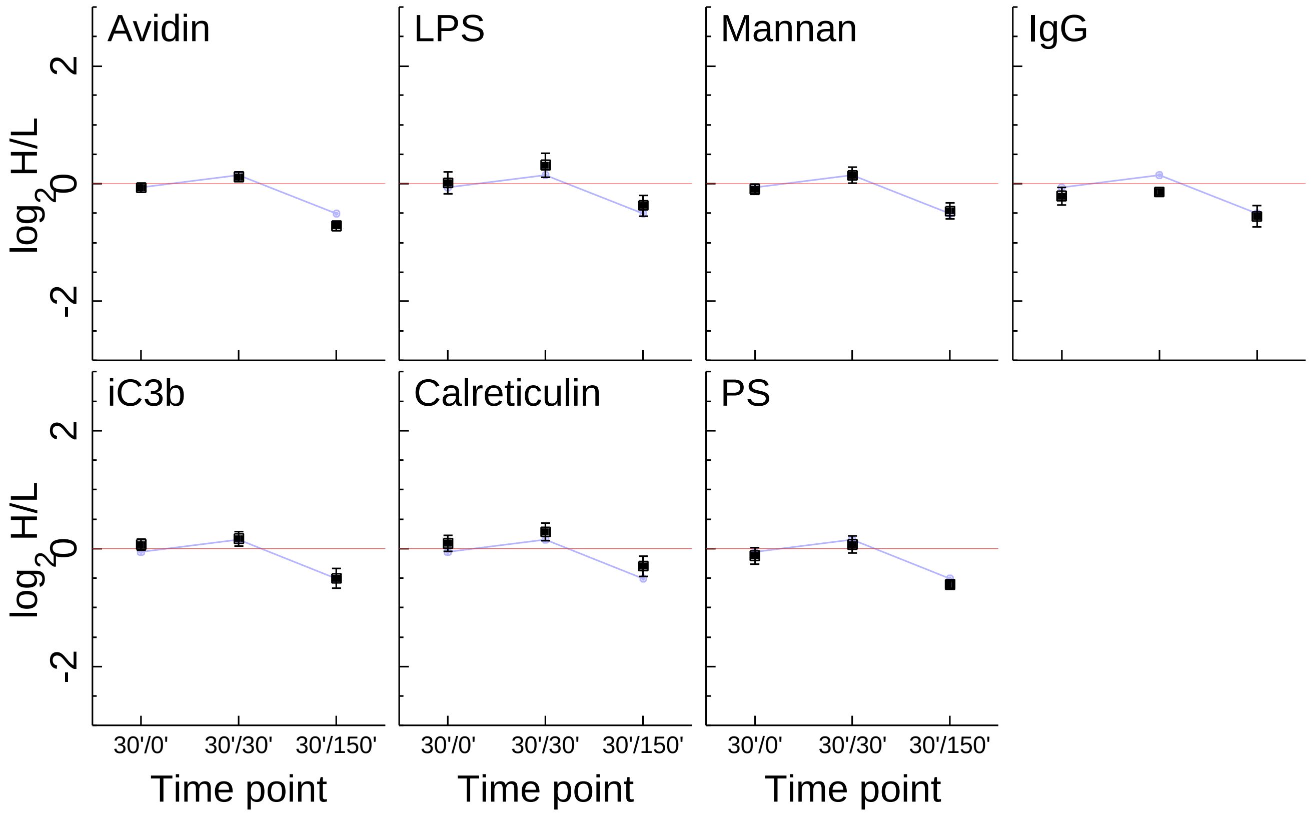

sep-15 (Q9ERR7)

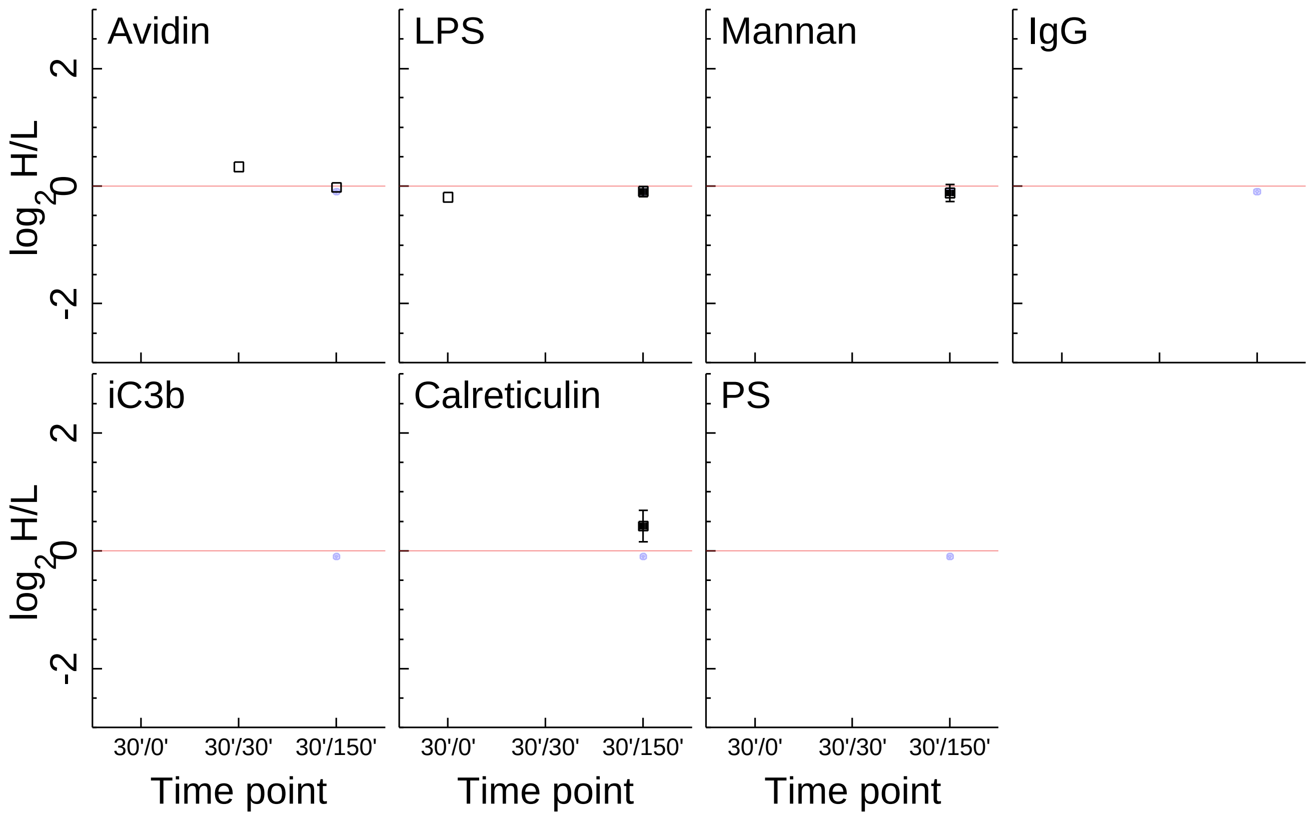

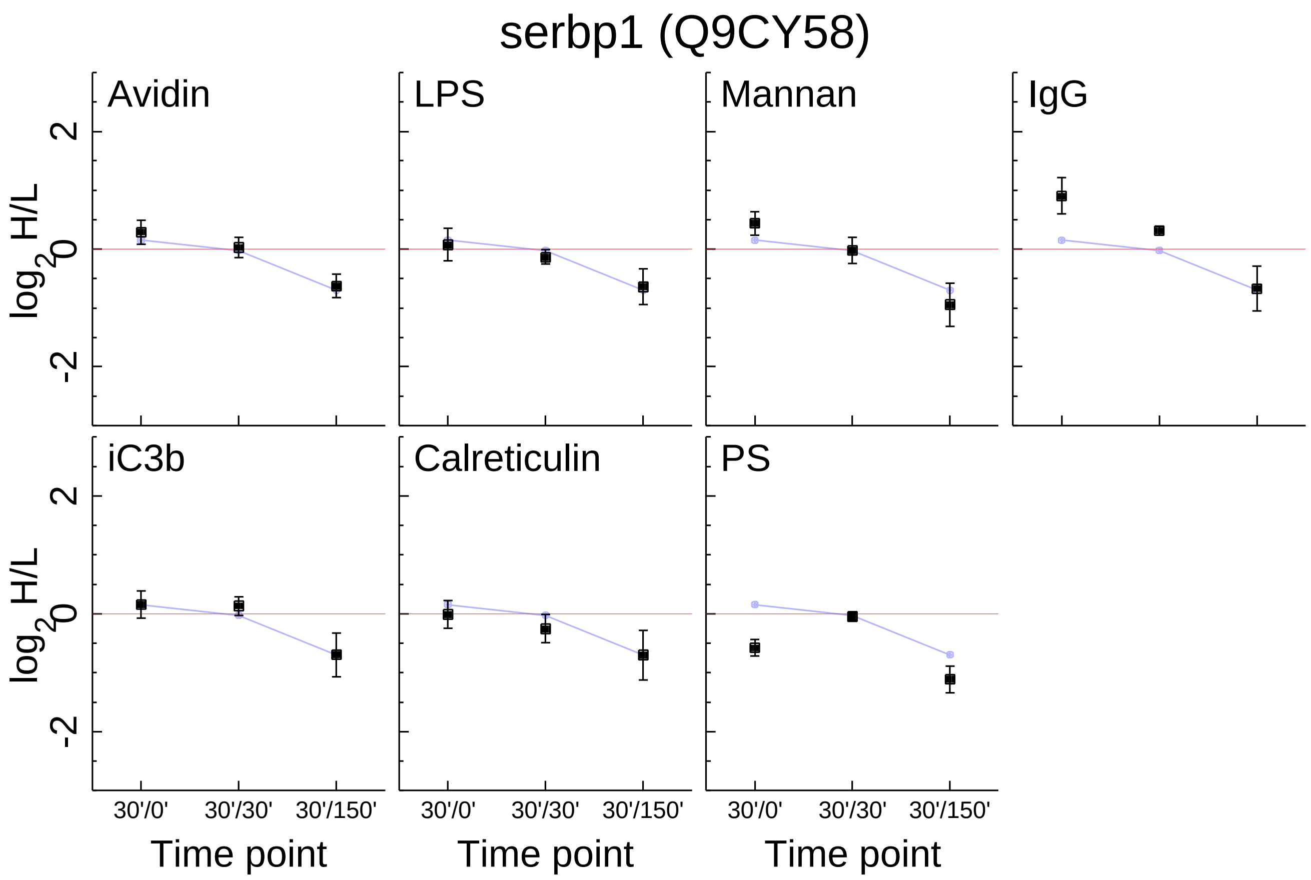
serbp1 (Q9CY58)
Avidin
LPS
Mannan
IgG
iC3b
Calreticulin
PS
log2 H/L
2
0
-2
30'/0'
30'/30'
30'/150'
Time point

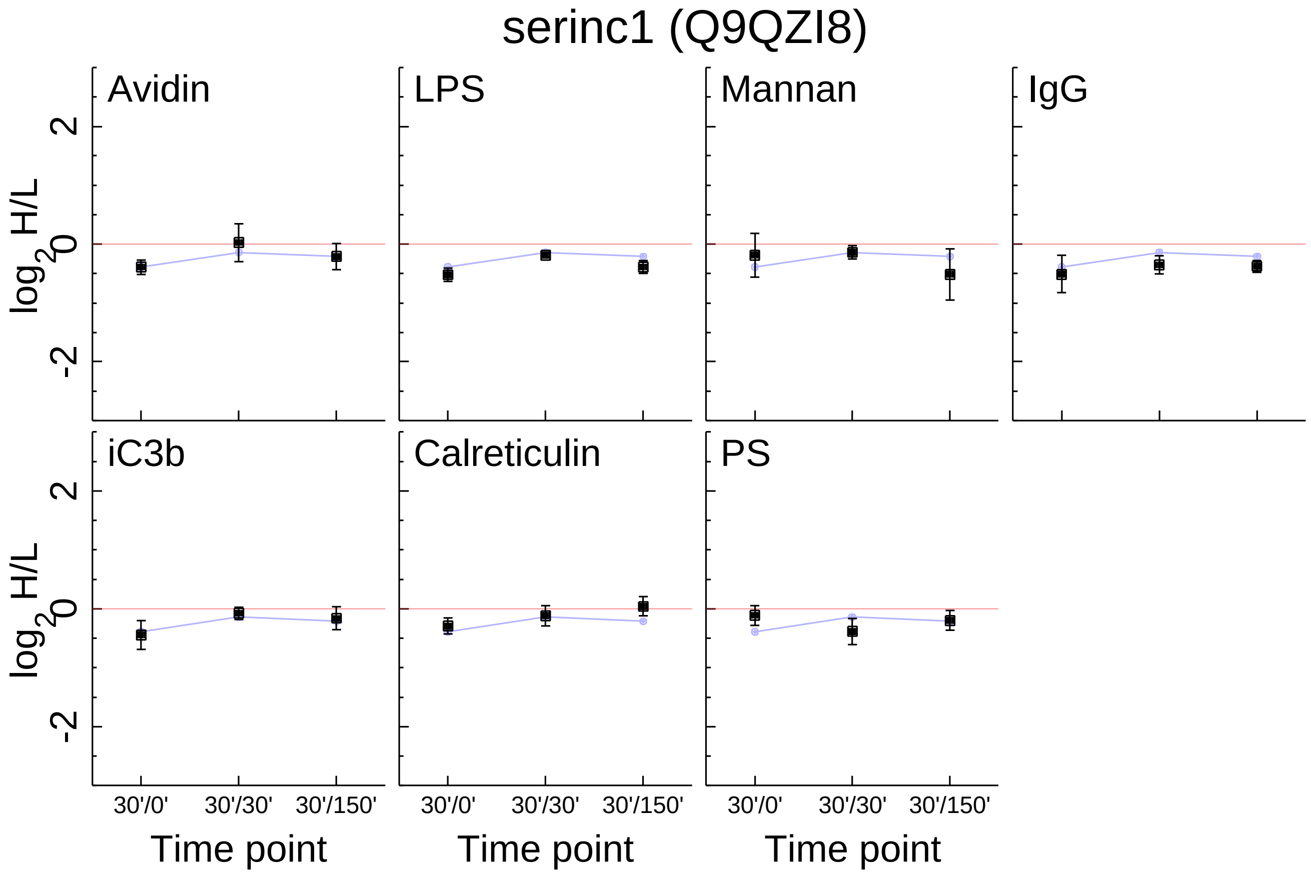

serinc1 (Q9QZI8)
Avidin
LPS
Mannan
IgG
iC3b
Calreticulin
PS
log2 H/L
2
0
-2
30'/0'
30'/30'
30'/150'
Time point

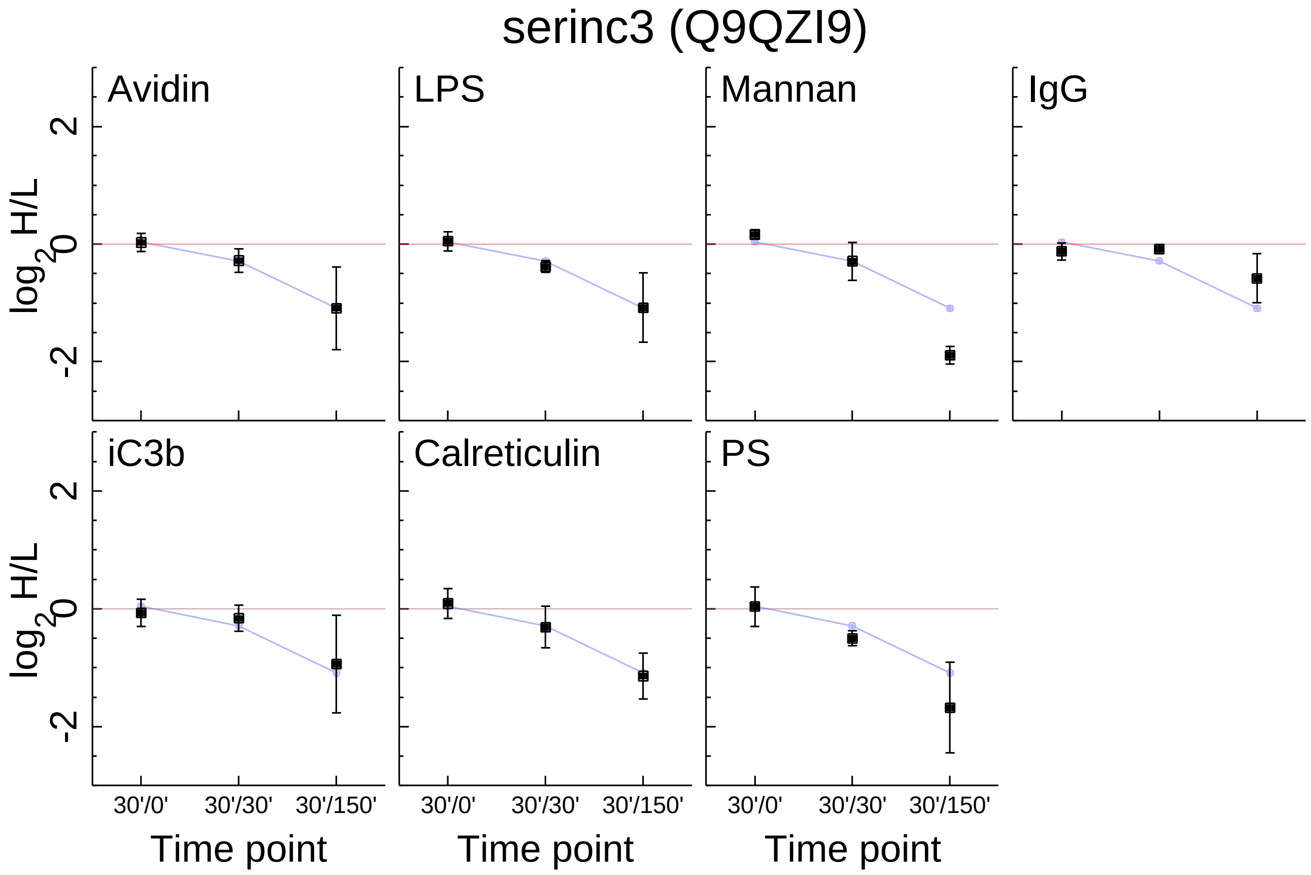
serinc3 (Q9QZI9)
Avidin
LPS
Mannan
IgG
iC3b
Calreticulin
PS
log2 H/L
2
0
-2
30'/0'
30'/30'
30'/150'
Time point

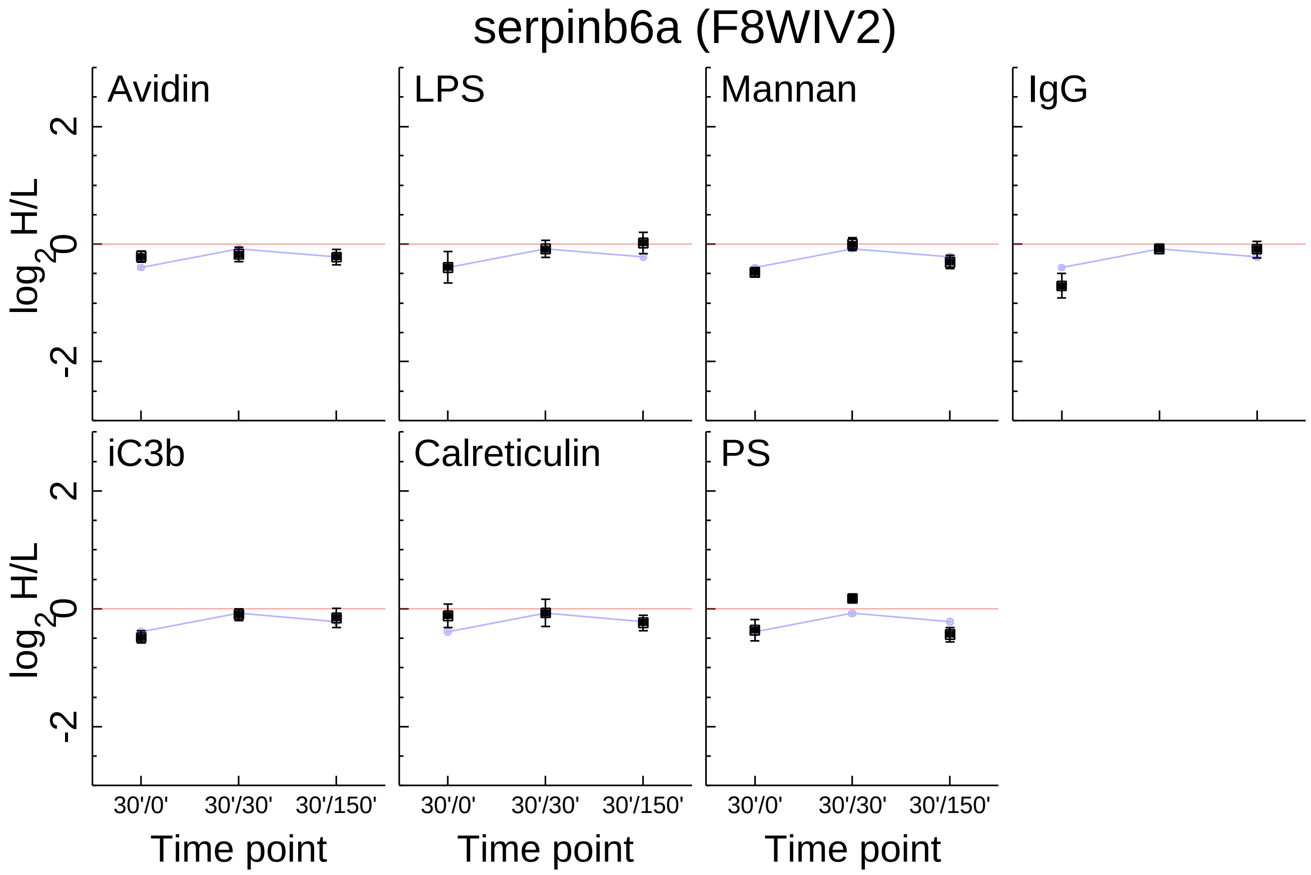
serpinb6a (F8WIV2)
Avidin
LPS
Mannan
IgG
iC3b
Calreticulin
PS
log2 H/L
2
0
-2
30'/0'
30'/30'
30'/150'
Time point

serpinb6d (Q3UWK8)

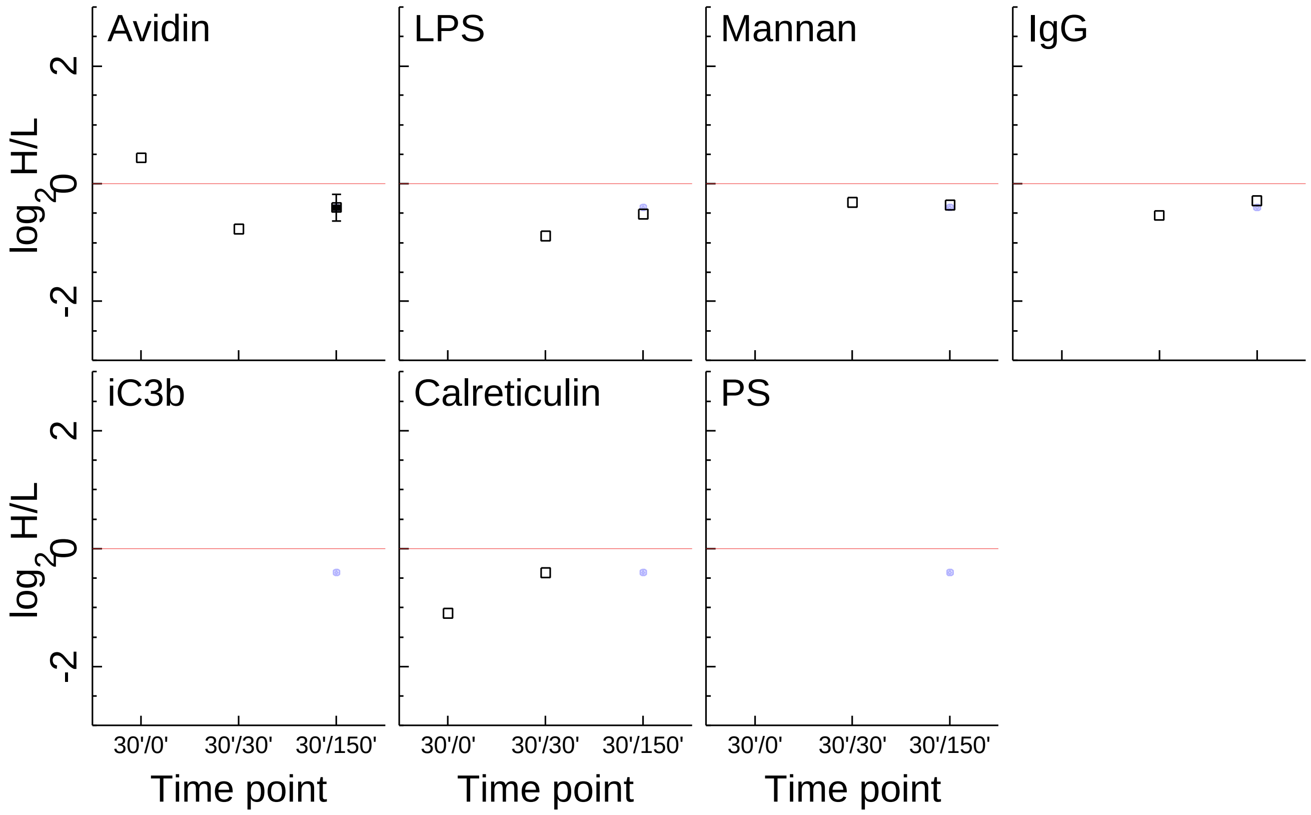

serpinc1 (P32261)

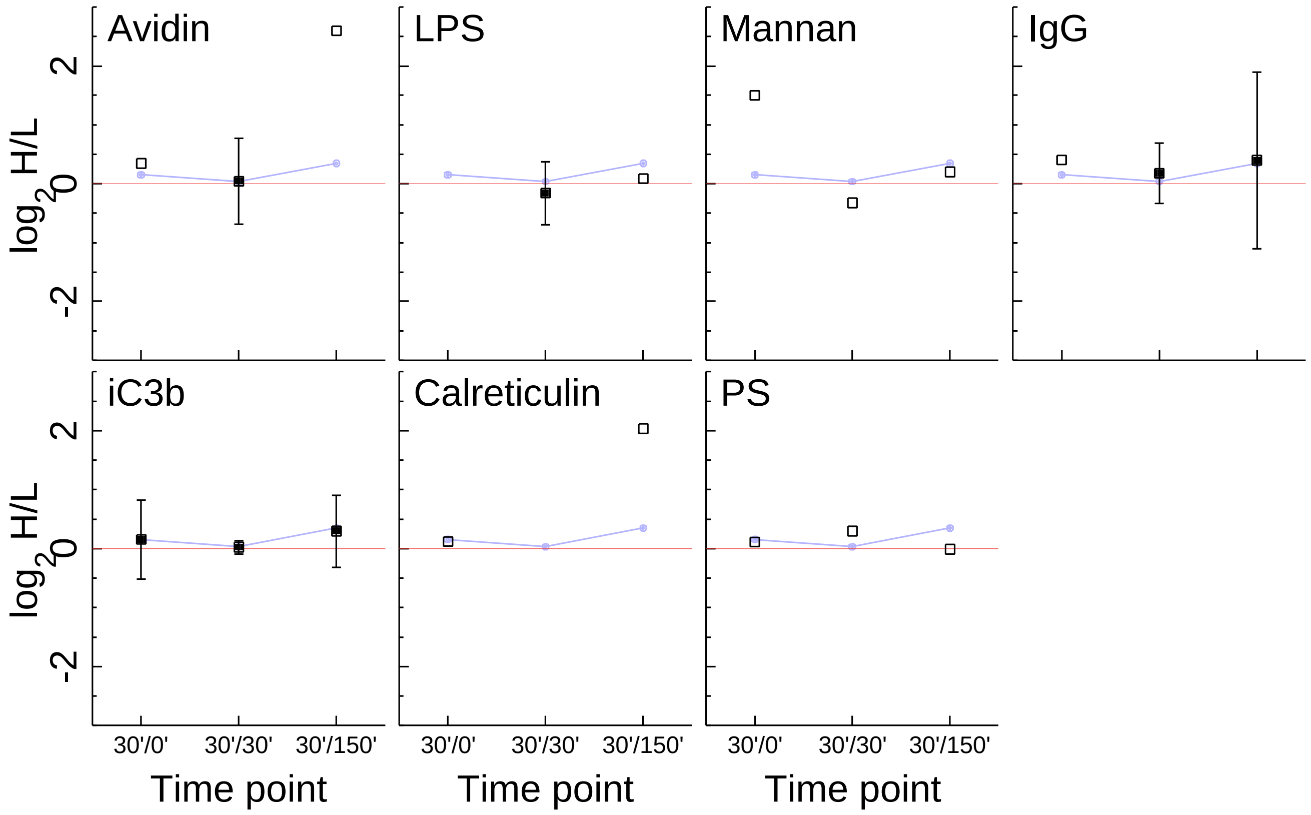

sf1 (E9Q4Q2)

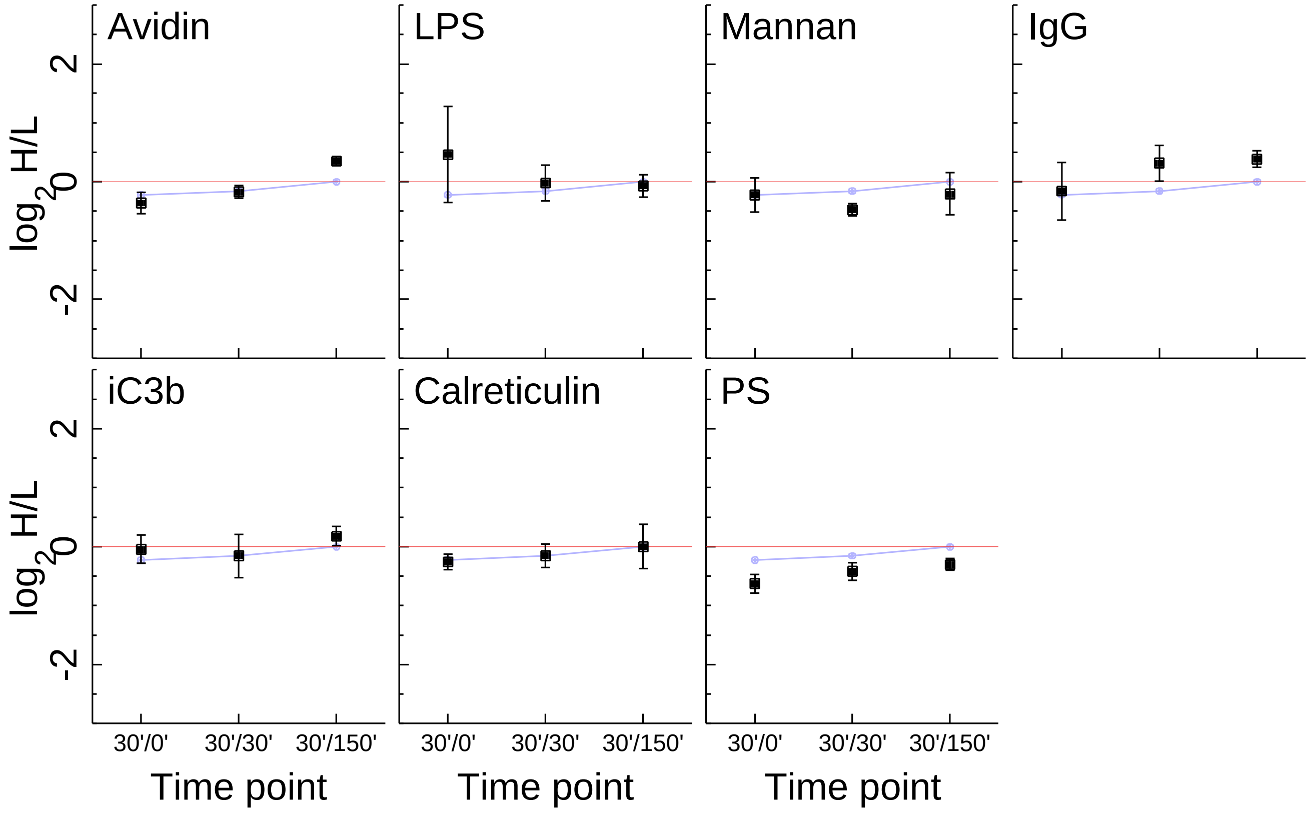

Avidin
LPS
Mannan
IgG
iC3b
Calreticulin
PS
log2 H/L
2
0
-2
30'/0'
30'/30'
30'/150'
Time point

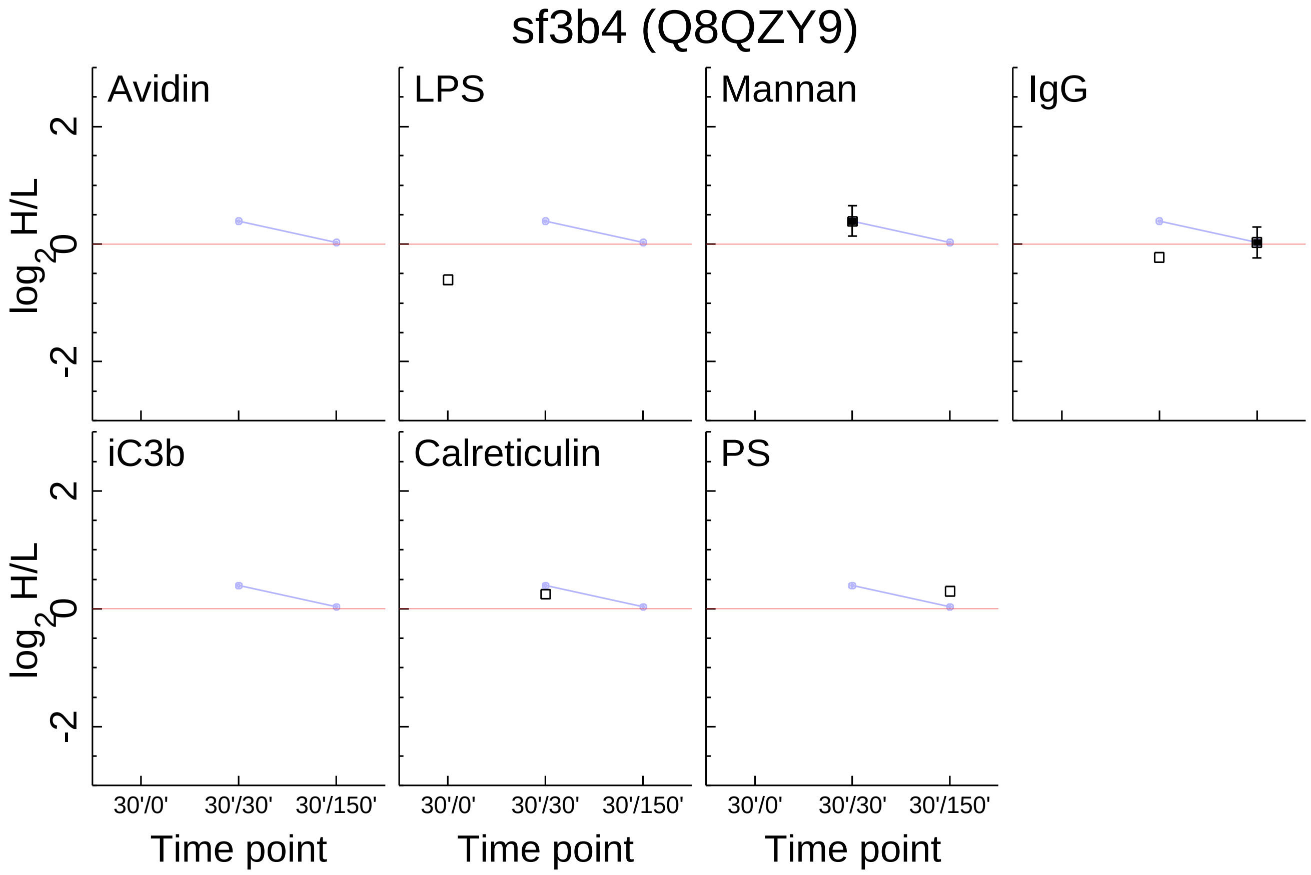
sf3b4 (Q8QZY9)
Avidin
LPS
Mannan
IgG
iC3b
Calreticulin
PS
log2 H/L
2
0
-2
30'/0'
30'/30'
30'/150'
Time point

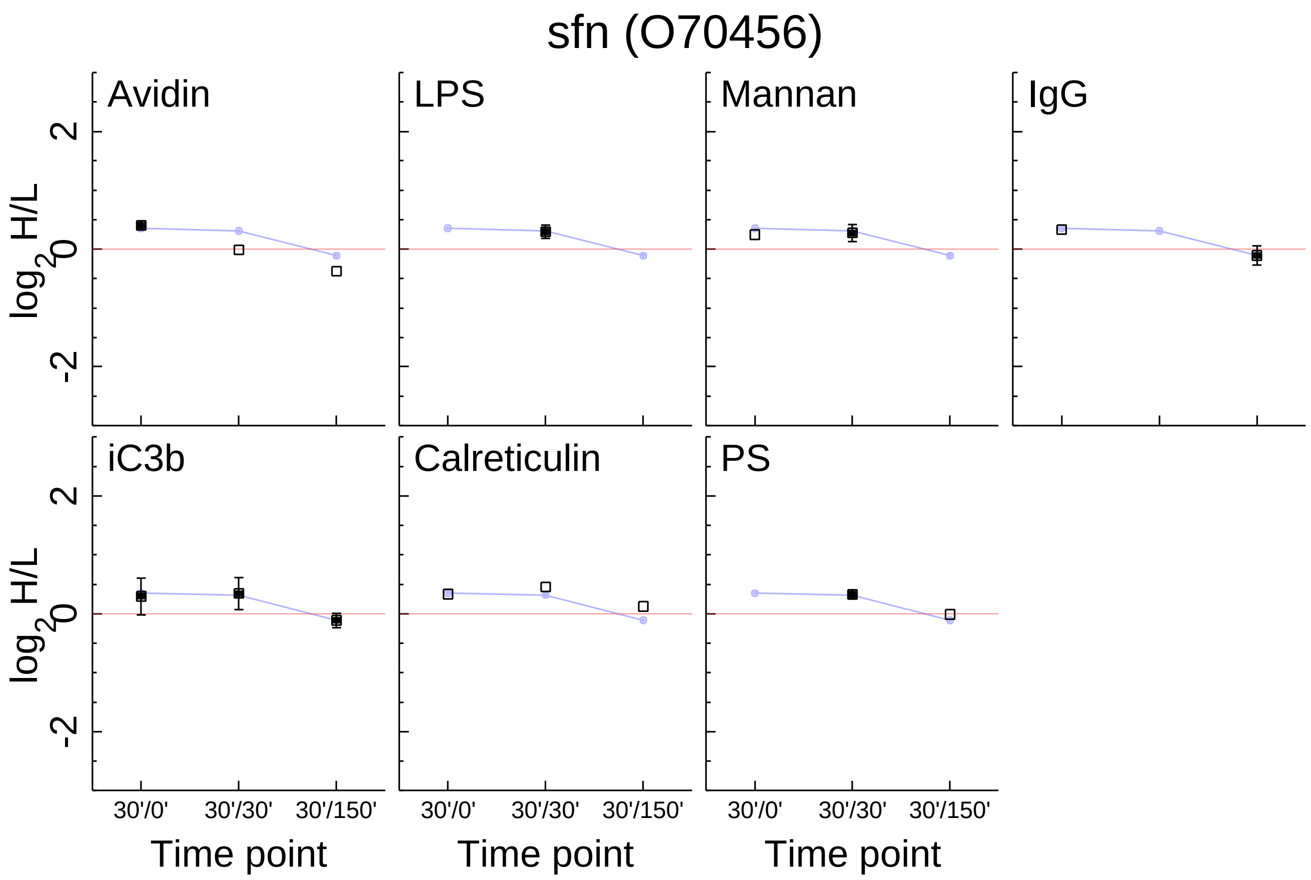

sfn (O70456)
Avidin
LPS
Mannan
IgG
iC3b
Calreticulin
PS
log2 H/L
2
0
-2
30'/0'
30'/30'
30'/150'
Time point

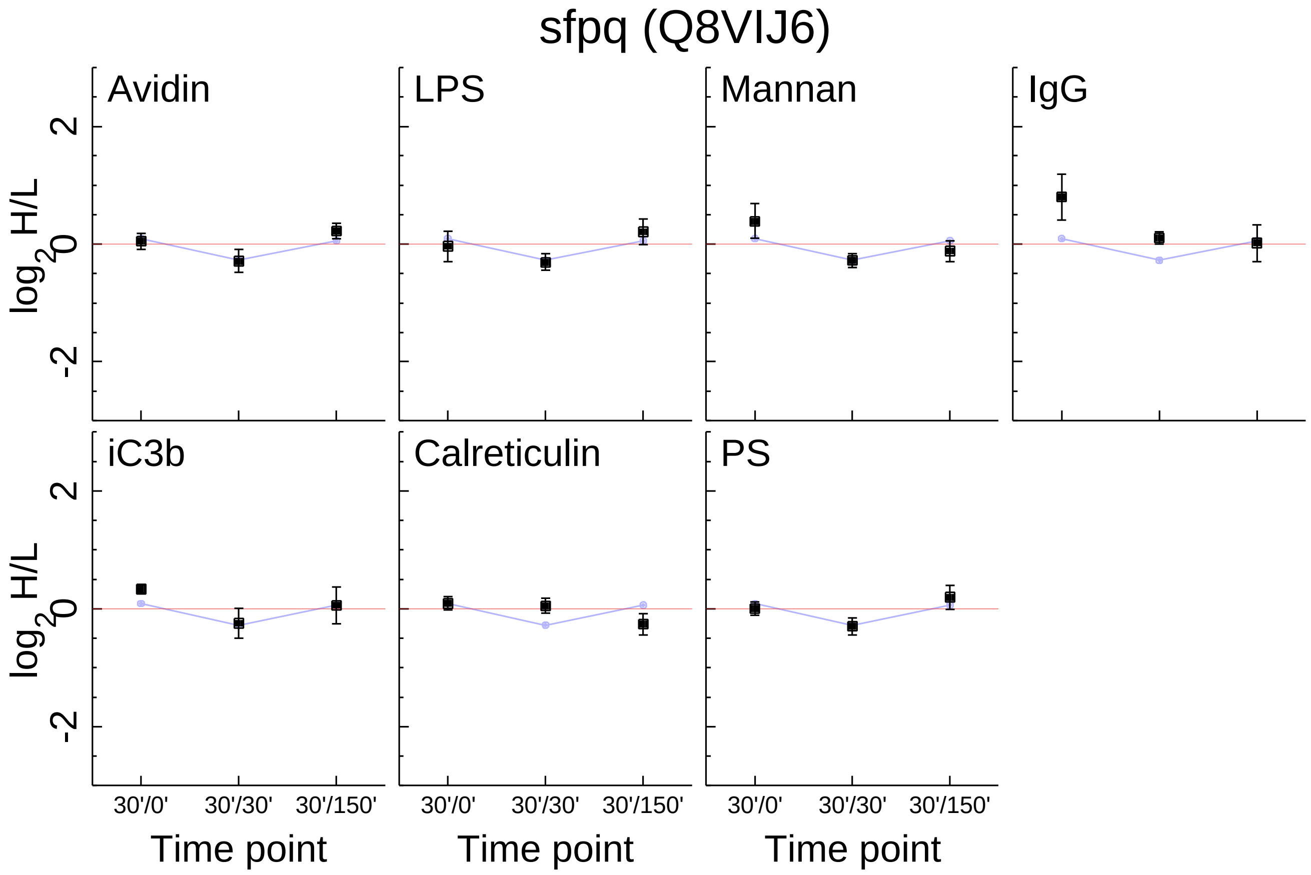
sfpq (Q8VIJ6)
Avidin
LPS
Mannan
IgG
iC3b
Calreticulin
PS
2
0
-2
log2 H/L
30'/0'
30'/30'
30'/150'
Time point

sft2d2 (Q8VD57)

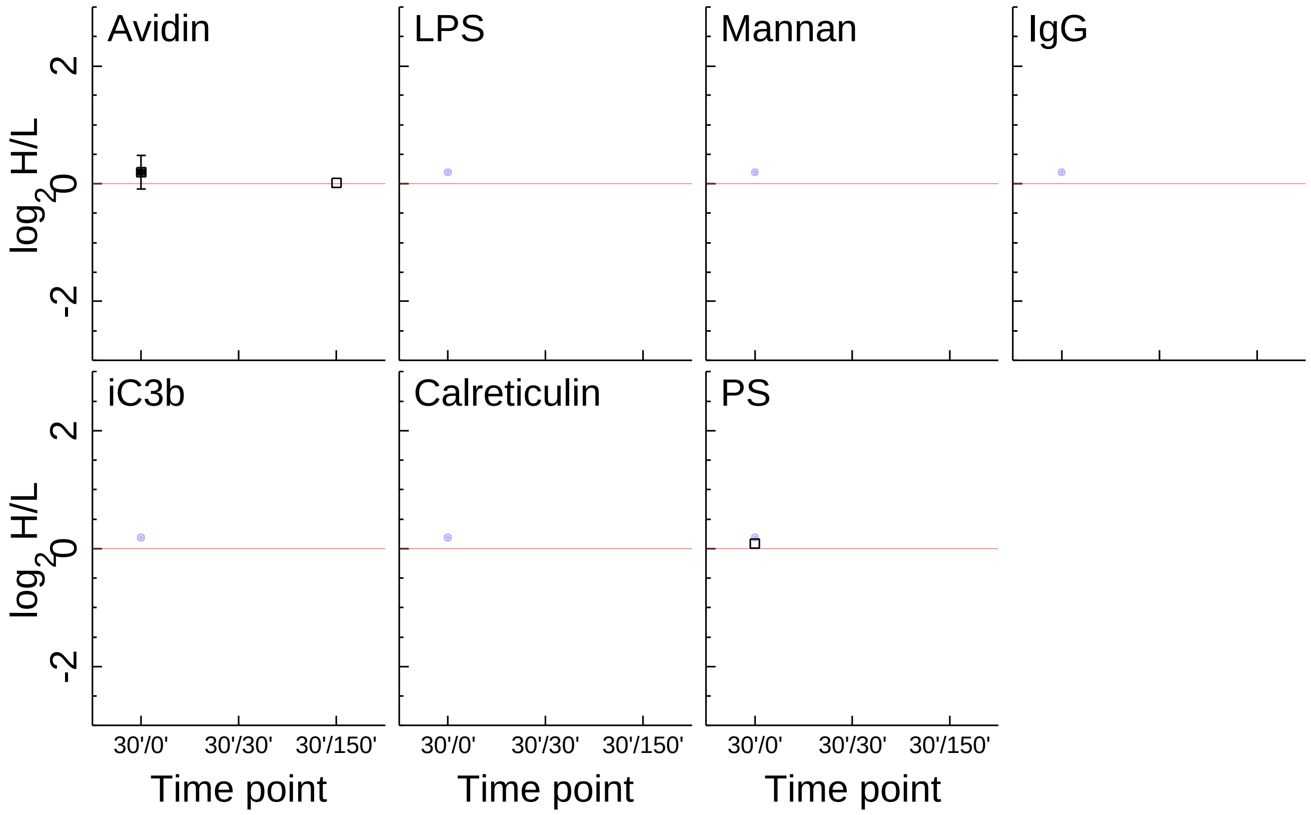

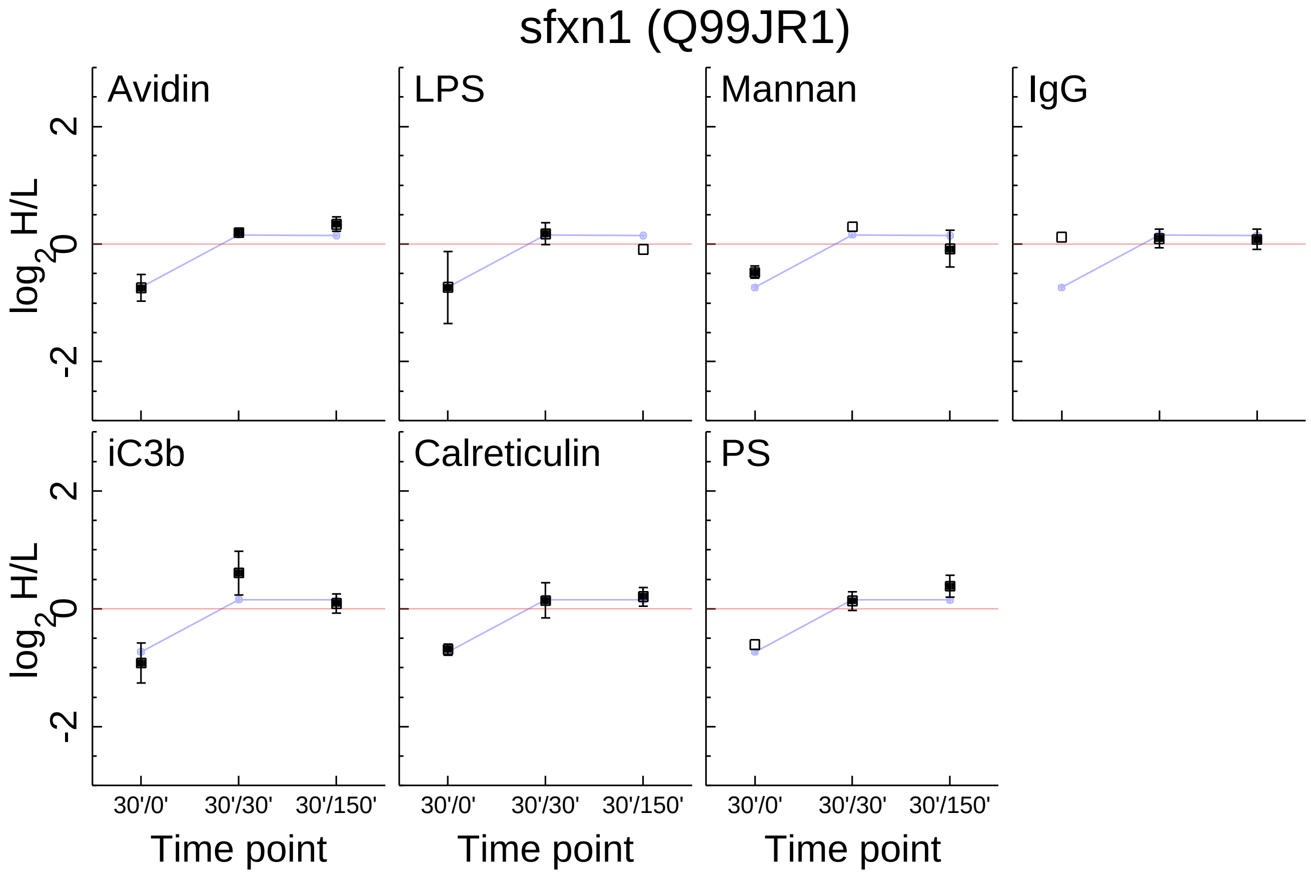
sfxn1 (Q99JR1)
Avidin
LPS
Mannan
IgG
iC3b
Calreticulin
PS
log2 H/L
2
0
-2
30'/0'
30'/30'
30'/150'
Time point

sfxn3 (Q91V61)

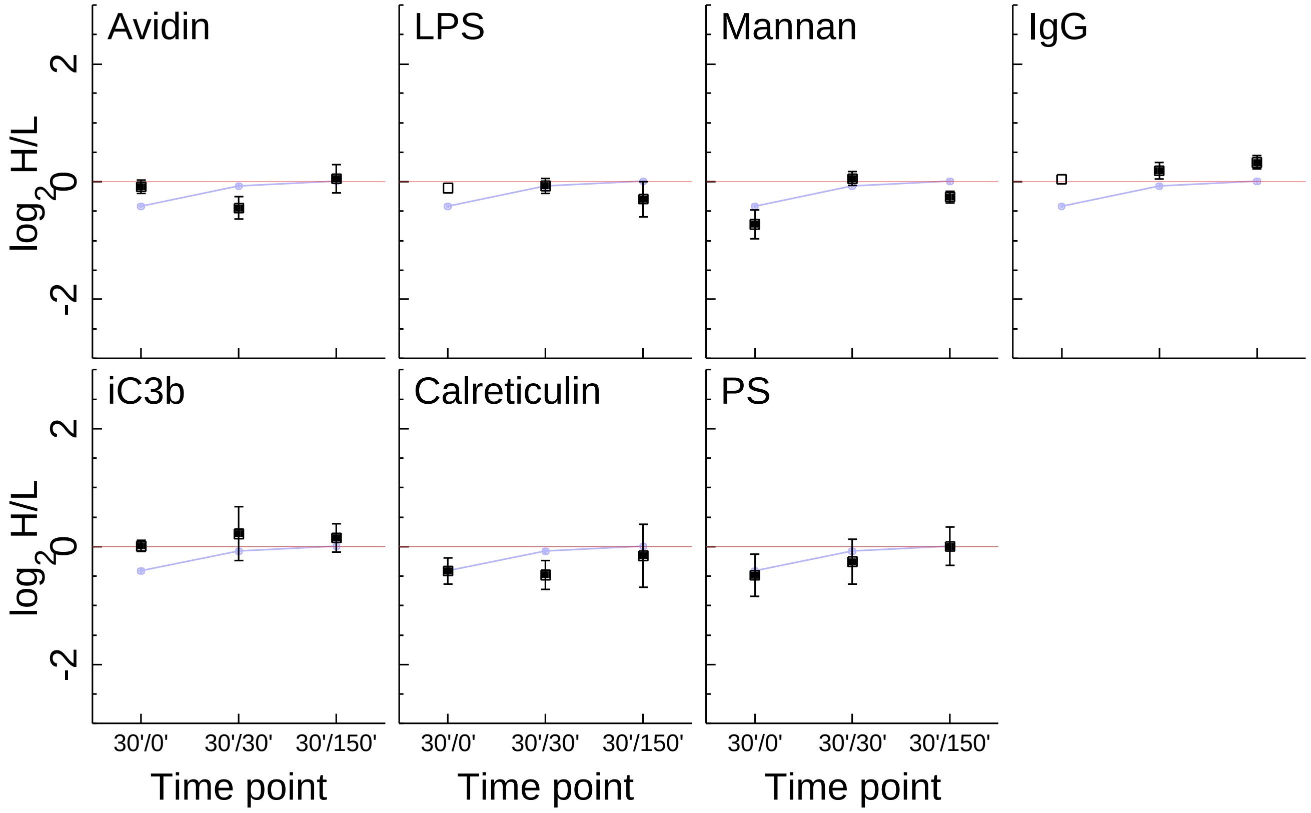

sgpl1 (Q8R0X7)

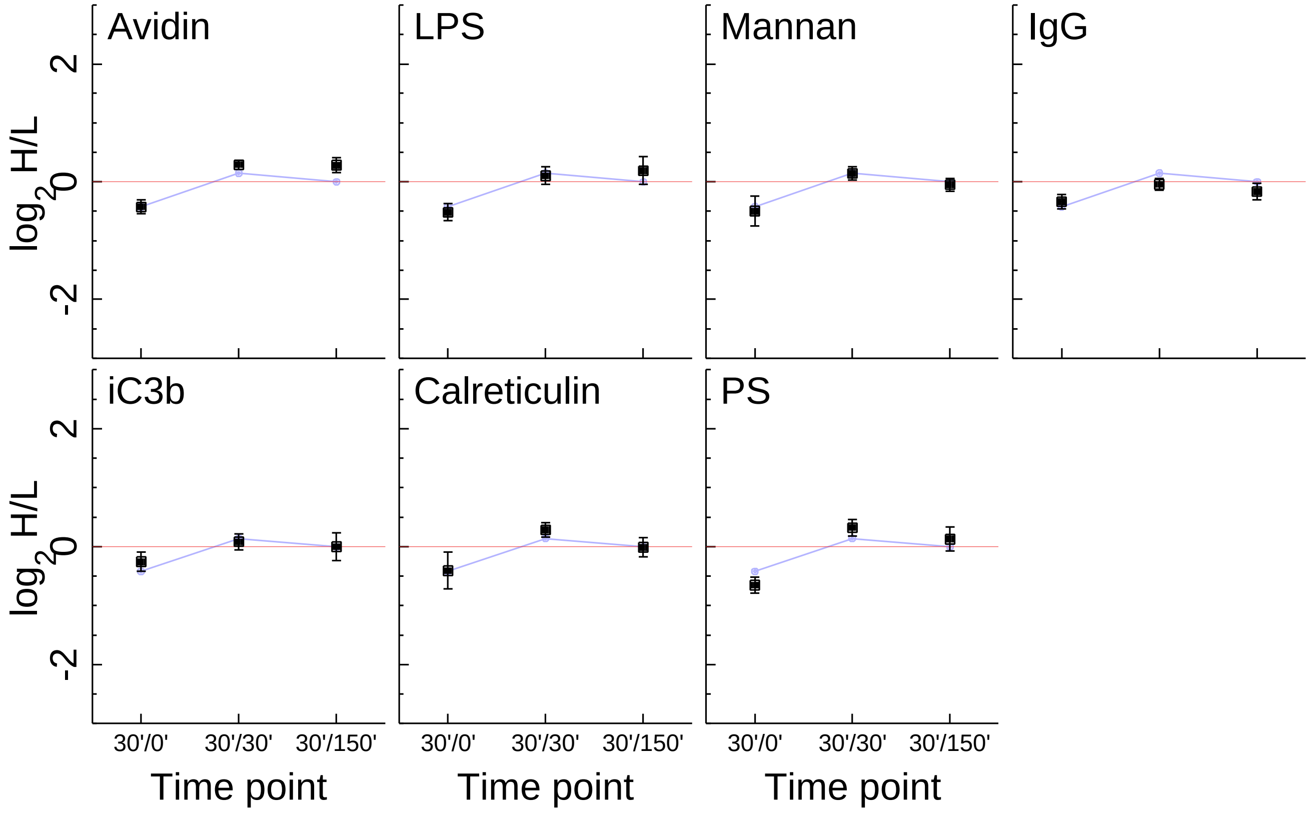

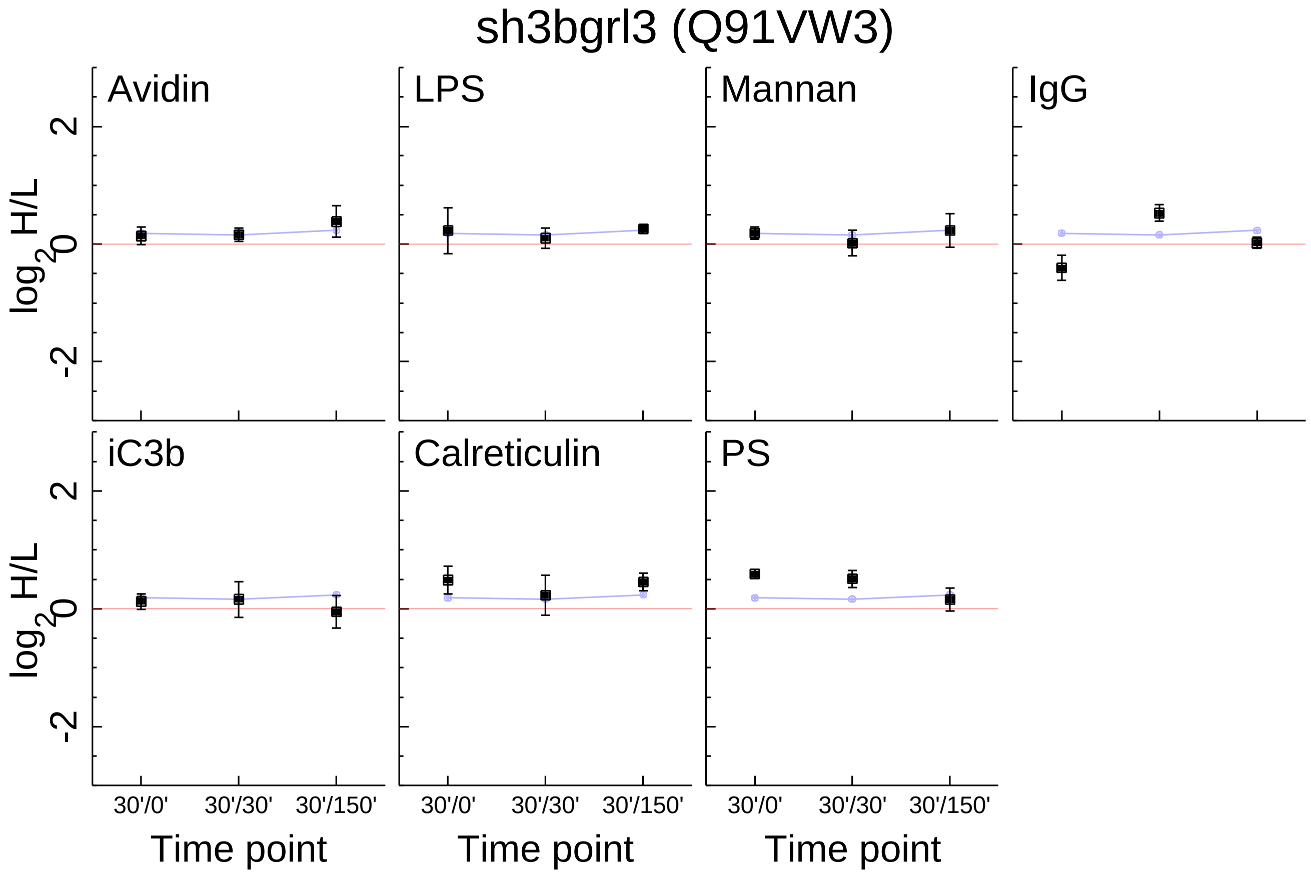
sh3bgrl3 (Q91VW3)
Avidin
LPS
Mannan
IgG
iC3b
Calreticulin
PS
log2 H/L
2
0
-2
30'/0'
30'/30'
30'/150'
Time point

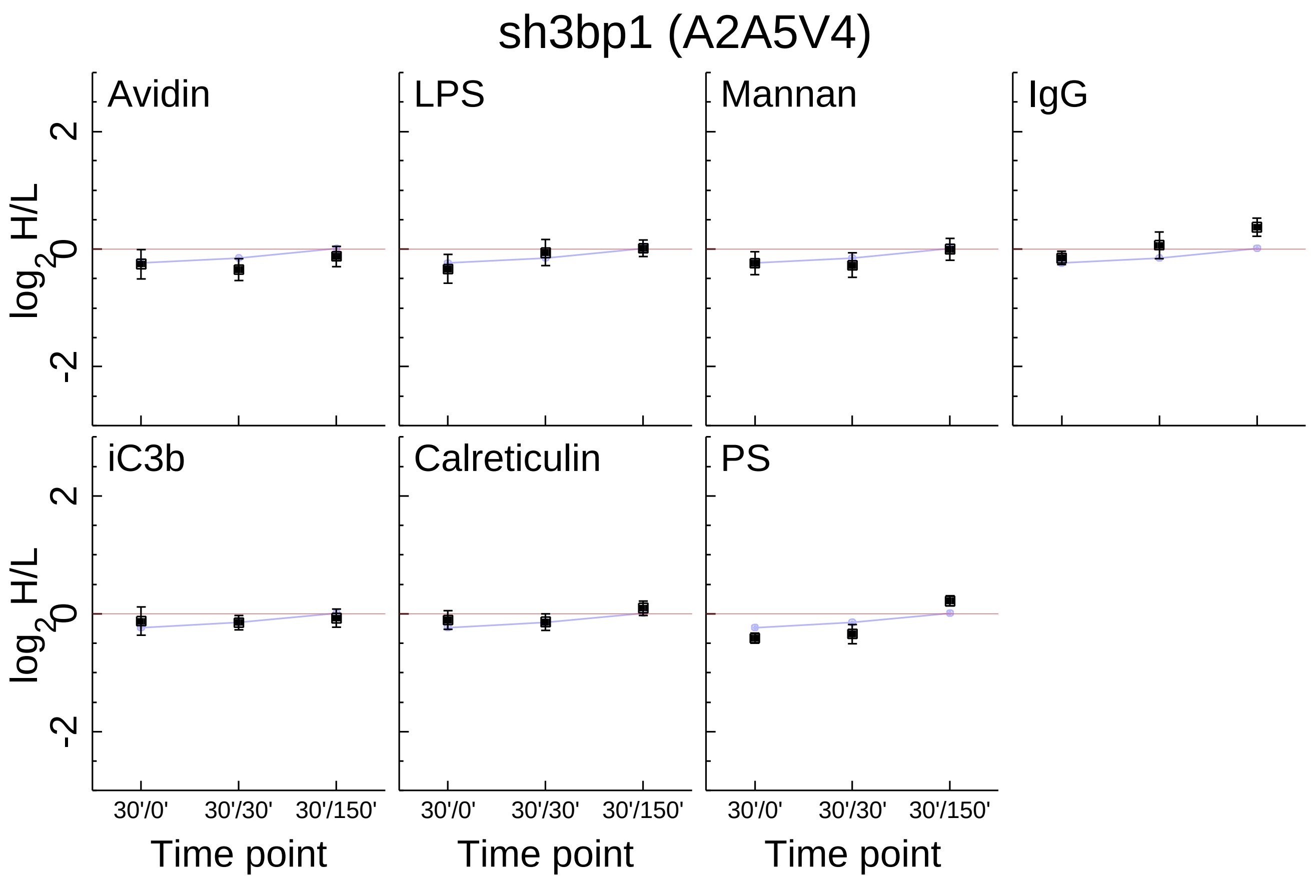
sh3bp1 (A2A5V4)
Avidin
LPS
Mannan
IgG
iC3b
Calreticulin
PS
log2 H/L
2
0
-2
30'/0'
30'/30'
30'/150'
Time point

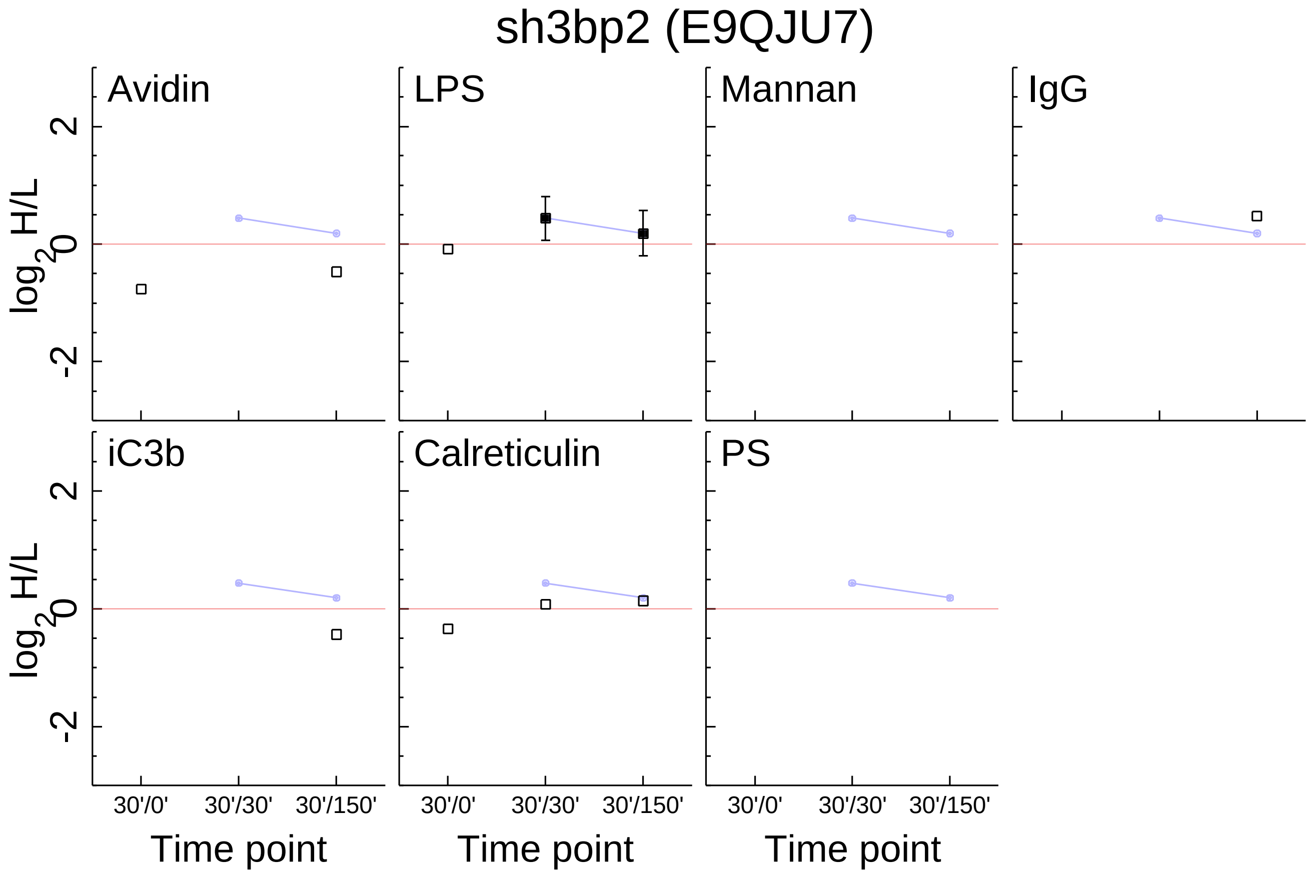

sh3bp2 (E9QJU7)
Avidin
LPS
Mannan
IgG
iC3b
Calreticulin
PS
log2 H/L
2
0
-2
30'/0'
30'/30'
30'/150'
Time point

sh3gl1 (Q62419)

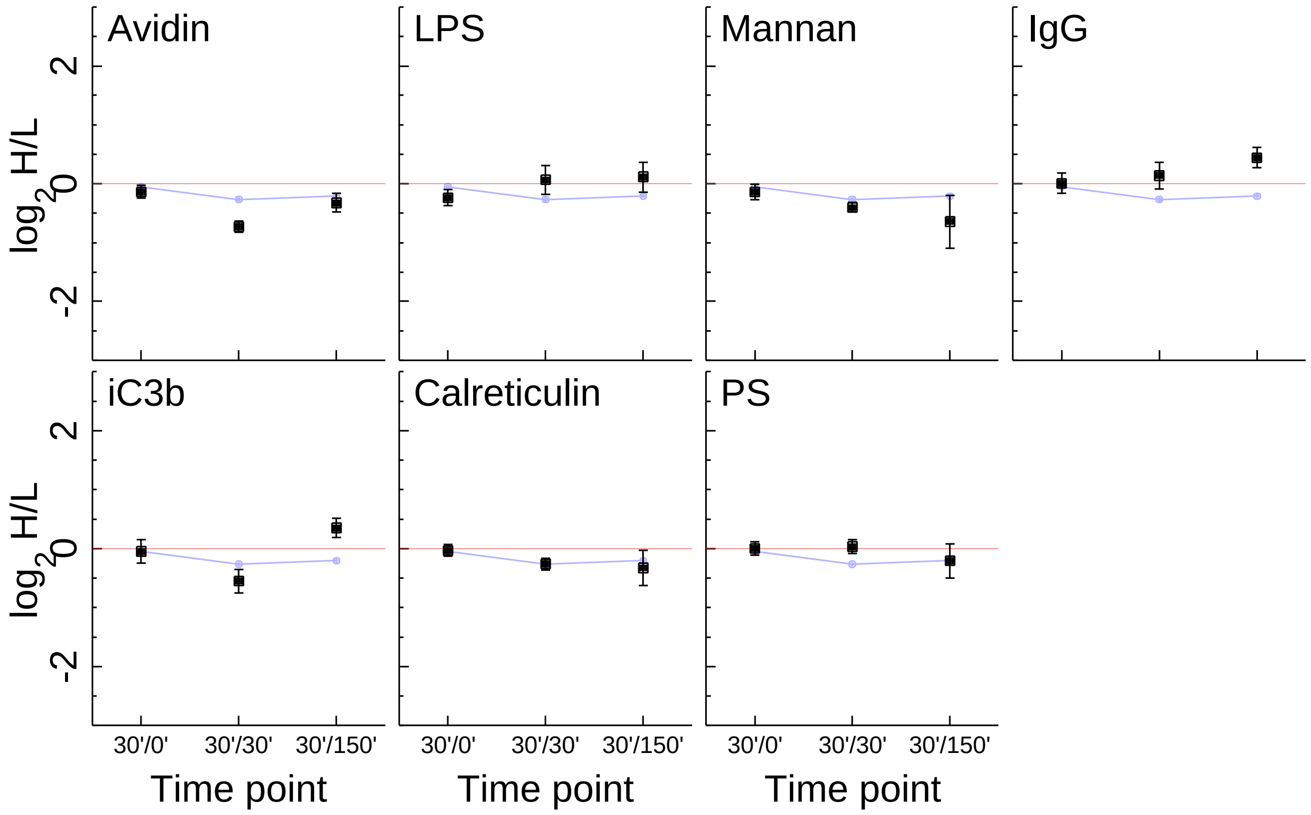

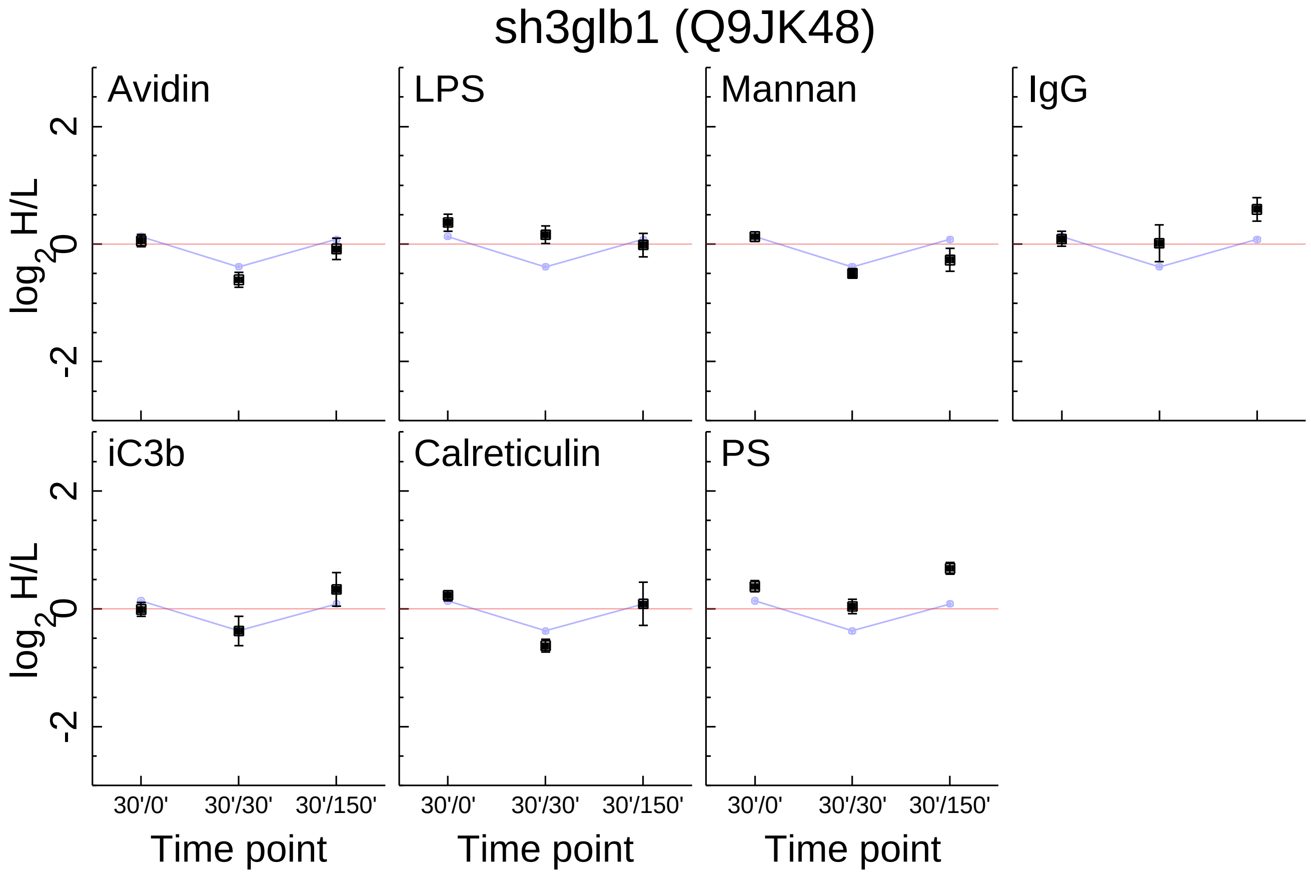

sh3glb1 (Q9JK48)
Avidin
LPS
Mannan
IgG
iC3b
Calreticulin
PS
log2 H/L
2
0
-2
30'/0'
30'/30'
30'/150'
Time point

sh3glb2 (A2AWI7)

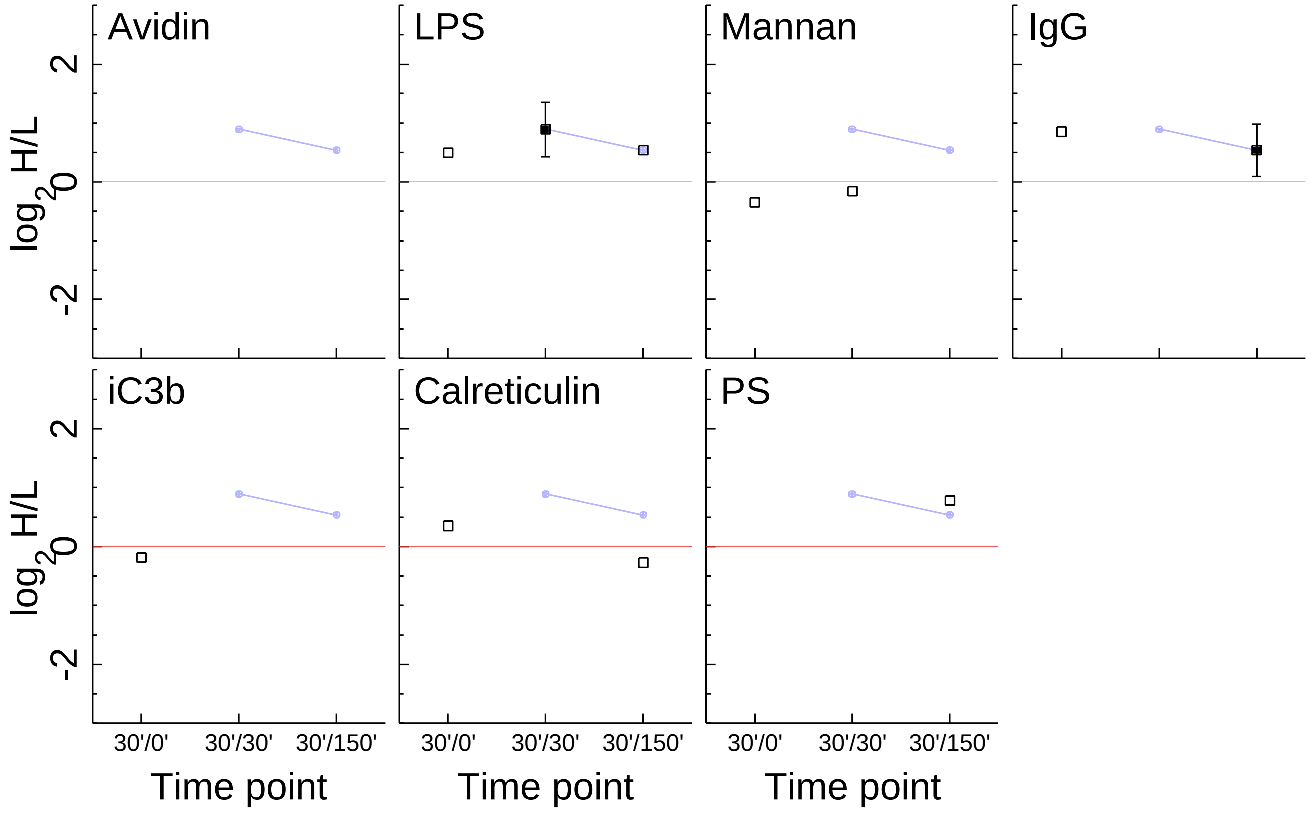

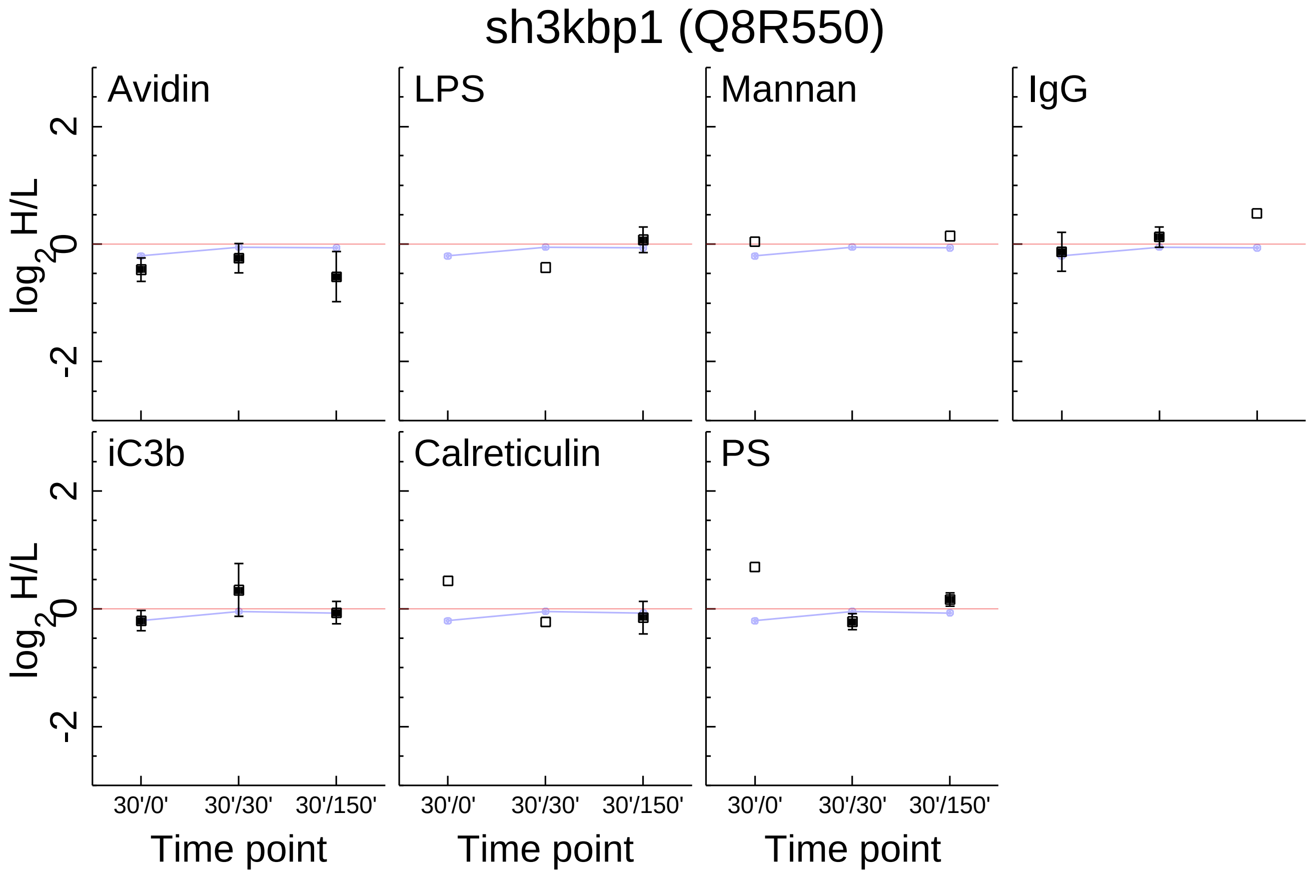

sh3kbp1 (Q8R550)
Avidin
LPS
Mannan
IgG
iC3b
Calreticulin
PS
log2 H/L
2
0
-2
30'/0'
30'/30'
30'/150'
Time point

sh3pxd2b (A2AAY5)

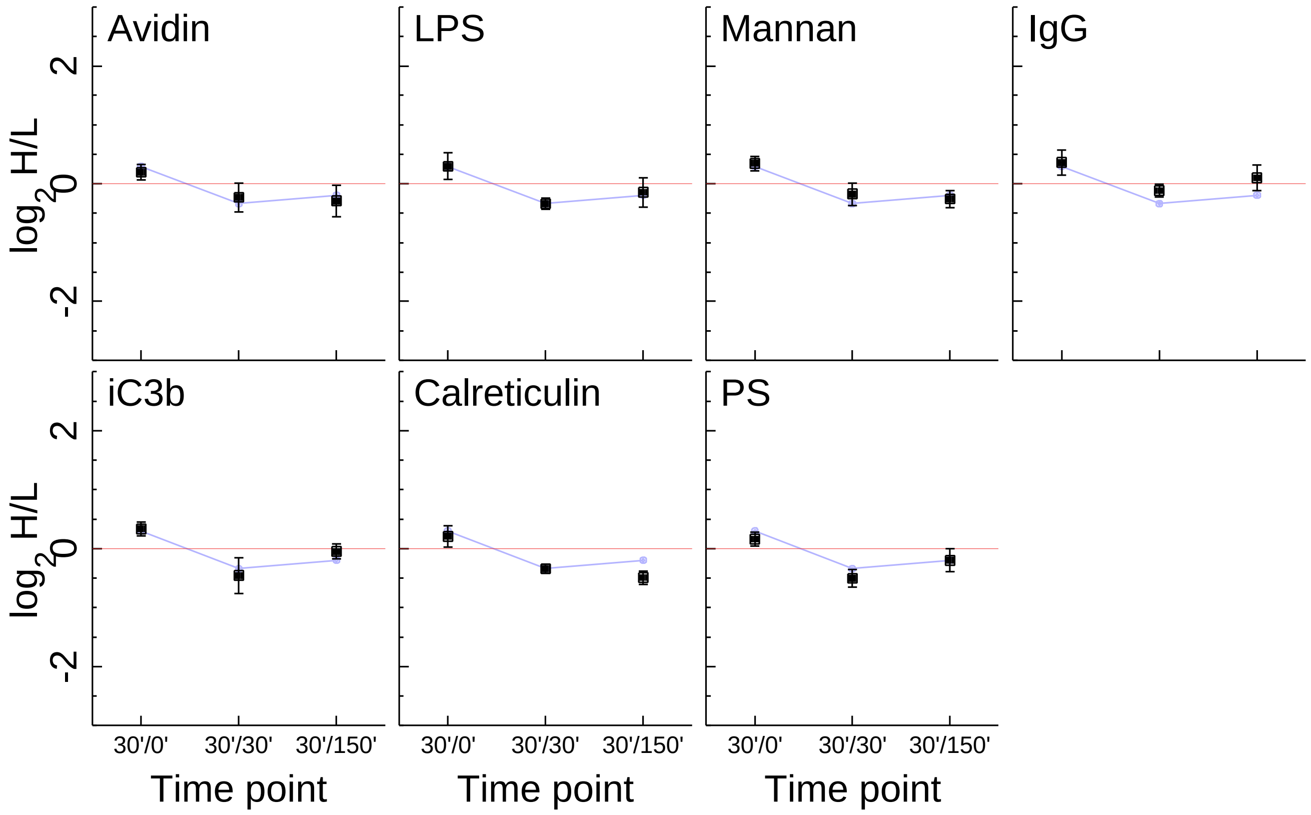

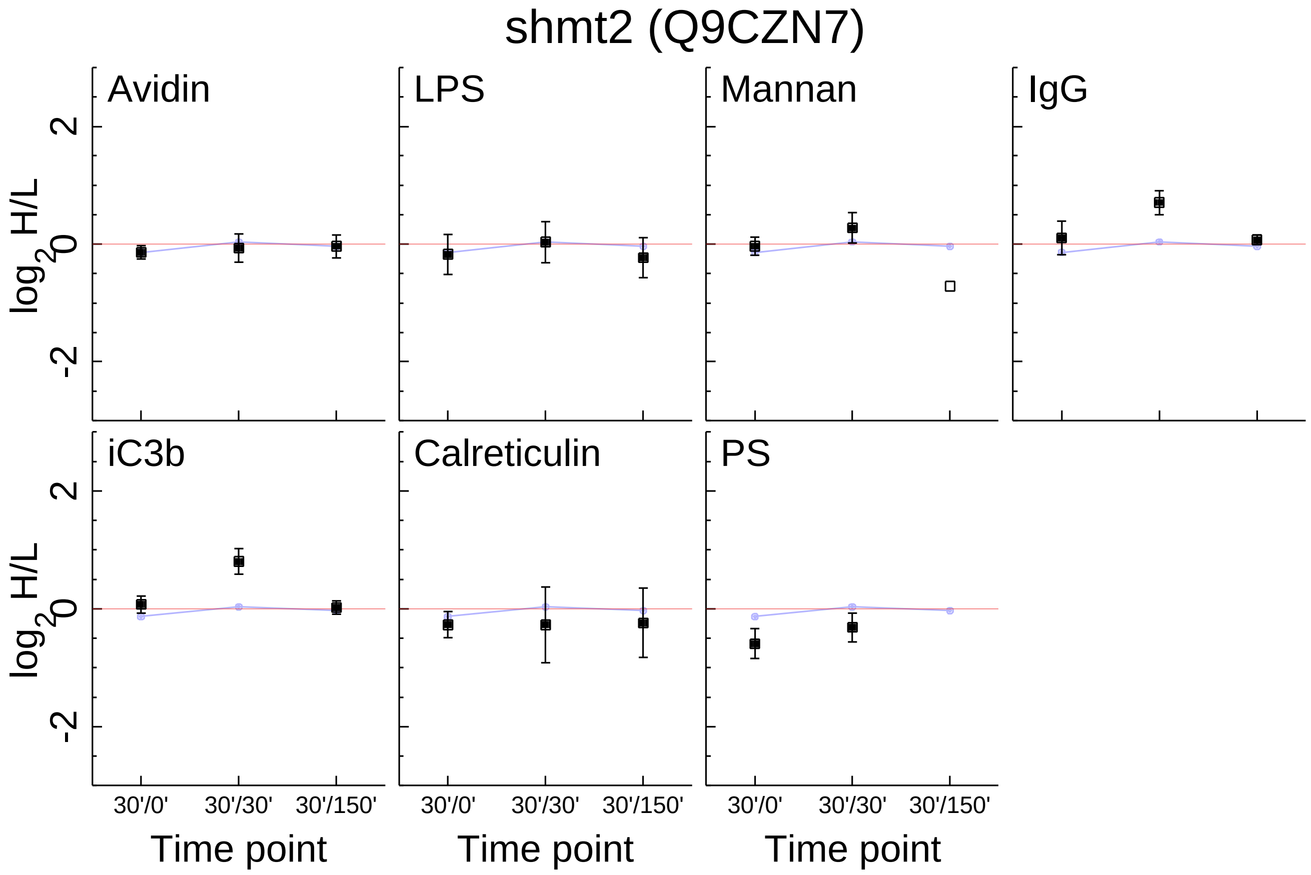
shmt2 (Q9CZN7)
Avidin
LPS
Mannan
IgG
iC3b
Calreticulin
PS
log2 H/L
2
0
-2
30'/0'
30'/30'
30'/150'
Time point

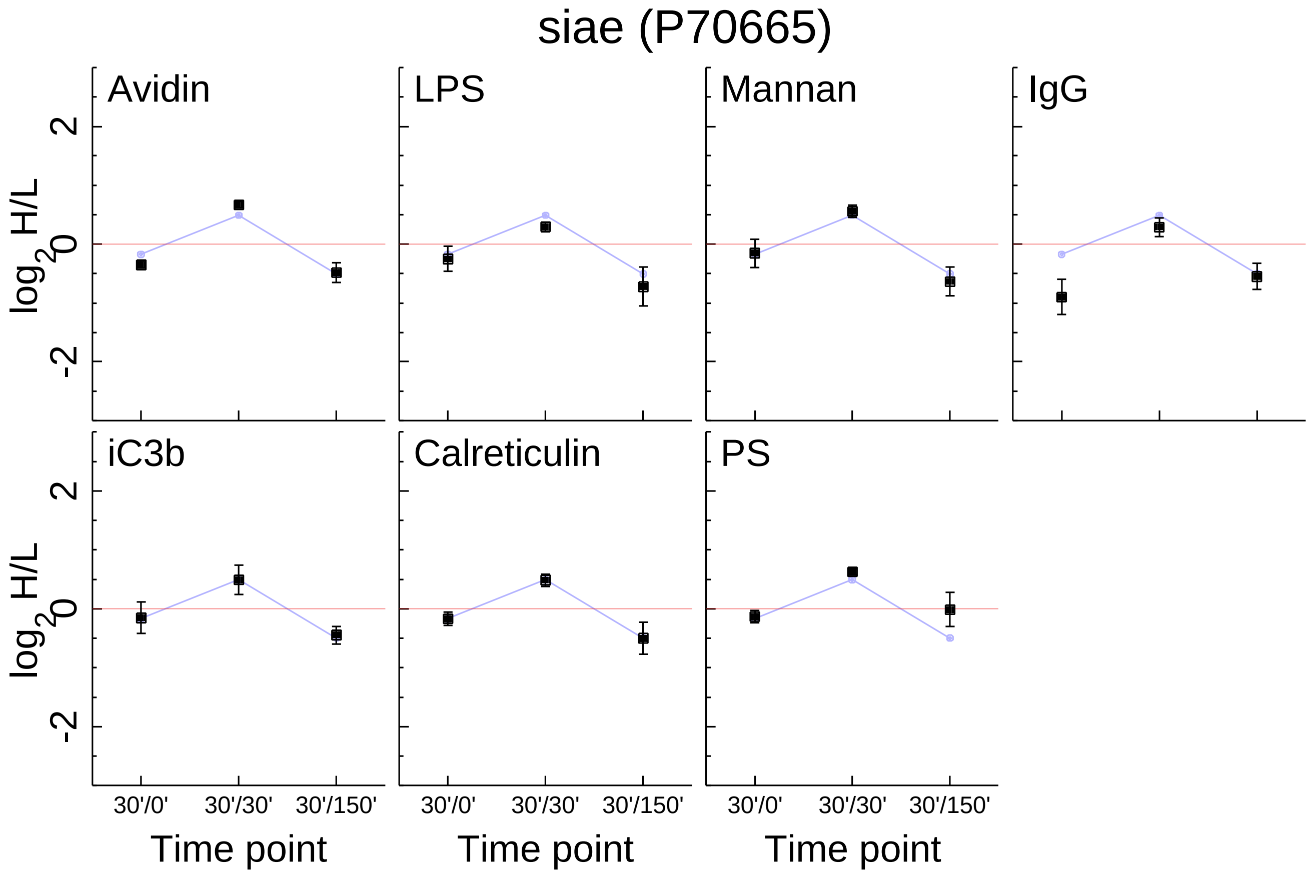
siae (P70665)
Avidin
LPS
Mannan
IgG
iC3b
Calreticulin
PS
log2 H/L
2
0
-2
30'/0'
30'/30'
30'/150'
Time point

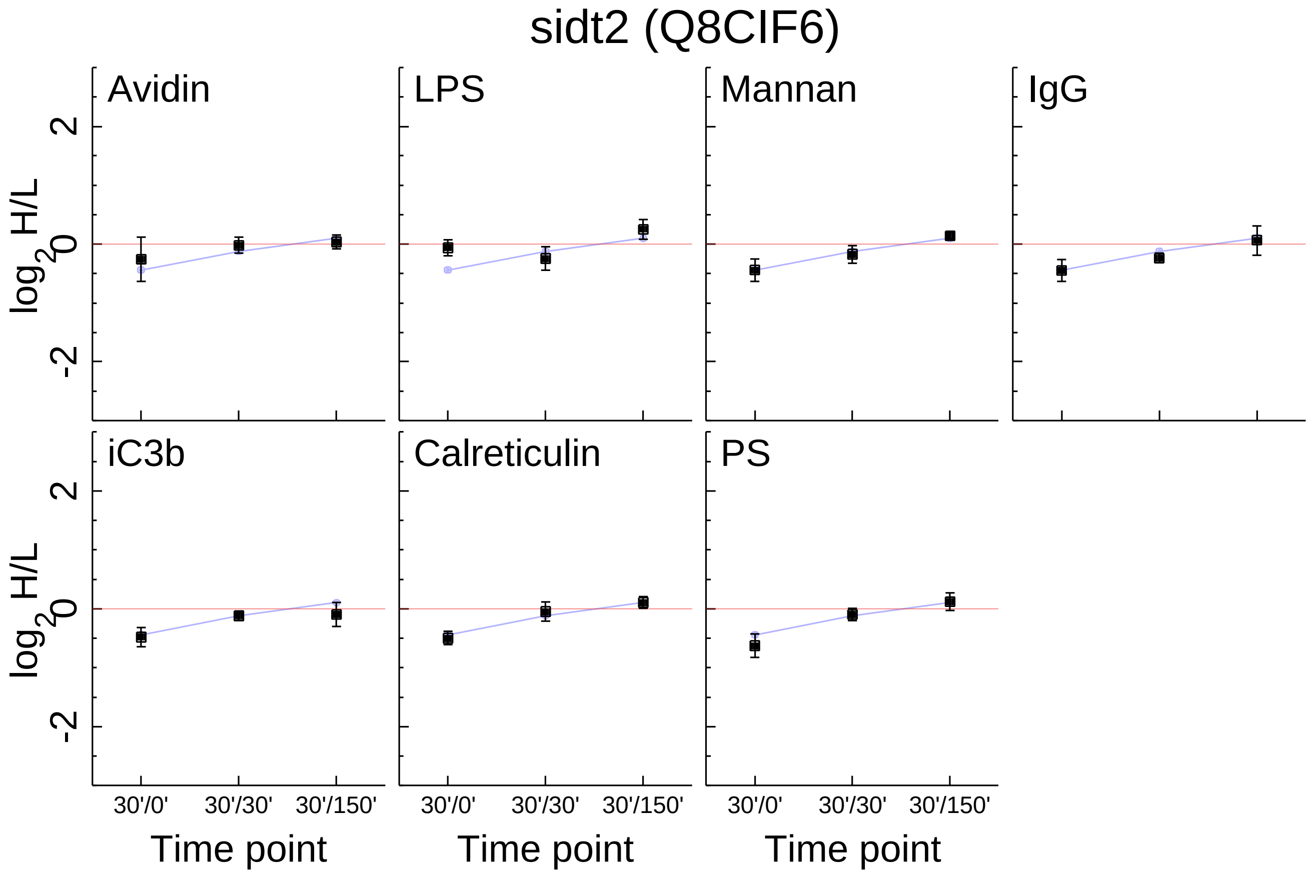
sidt2 (Q8CIF6)
Avidin
LPS
Mannan
IgG
iC3b
Calreticulin
PS
log2 H/L
2
0
-2
30'/0'
30'/30'
30'/150'
Time point

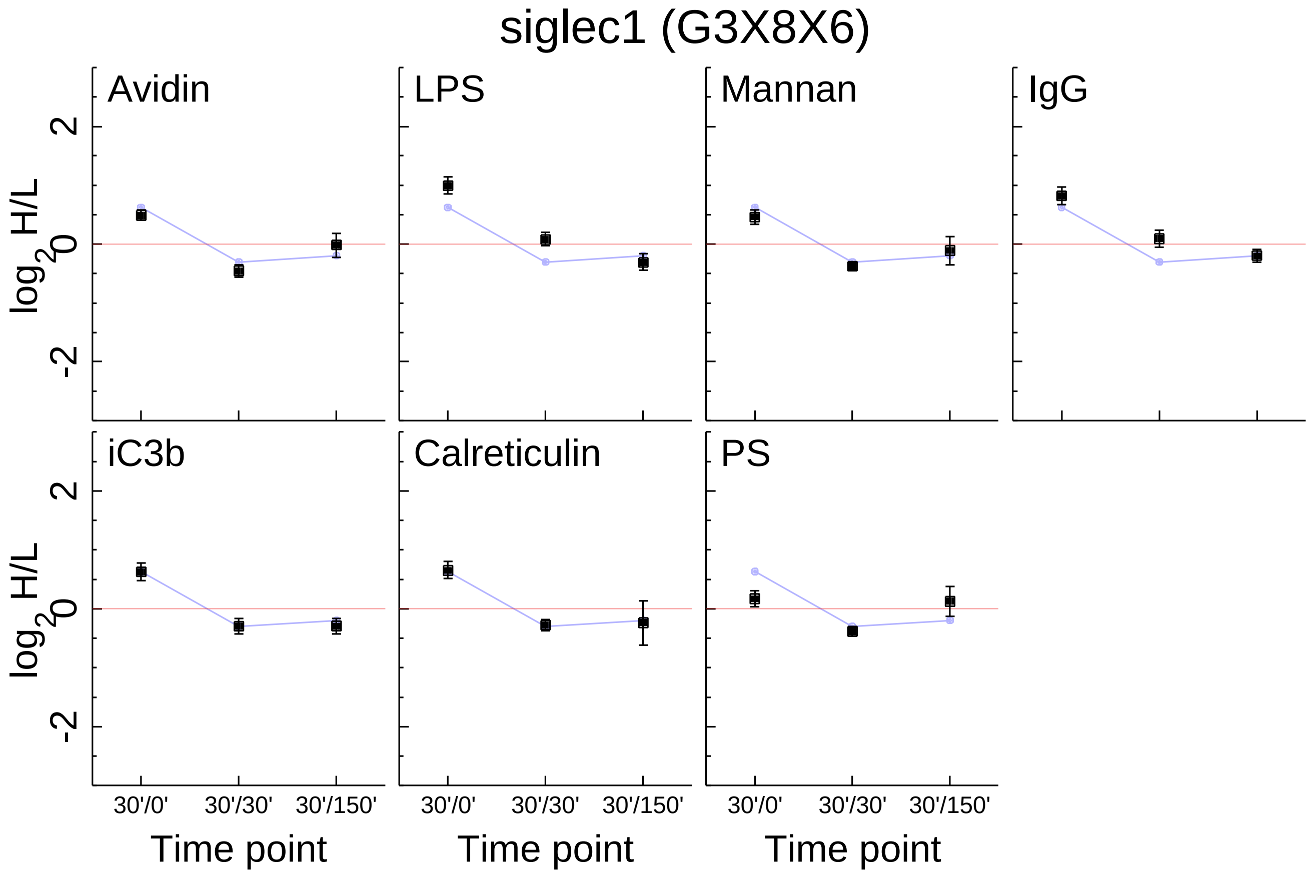

siglec1 (G3X8X6)
Avidin
LPS
Mannan
IgG
iC3b
Calreticulin
PS
log2 H/L
2
0
-2
30'/0'
30'/30'
30'/150'
Time point

sigmar1 (O55242)

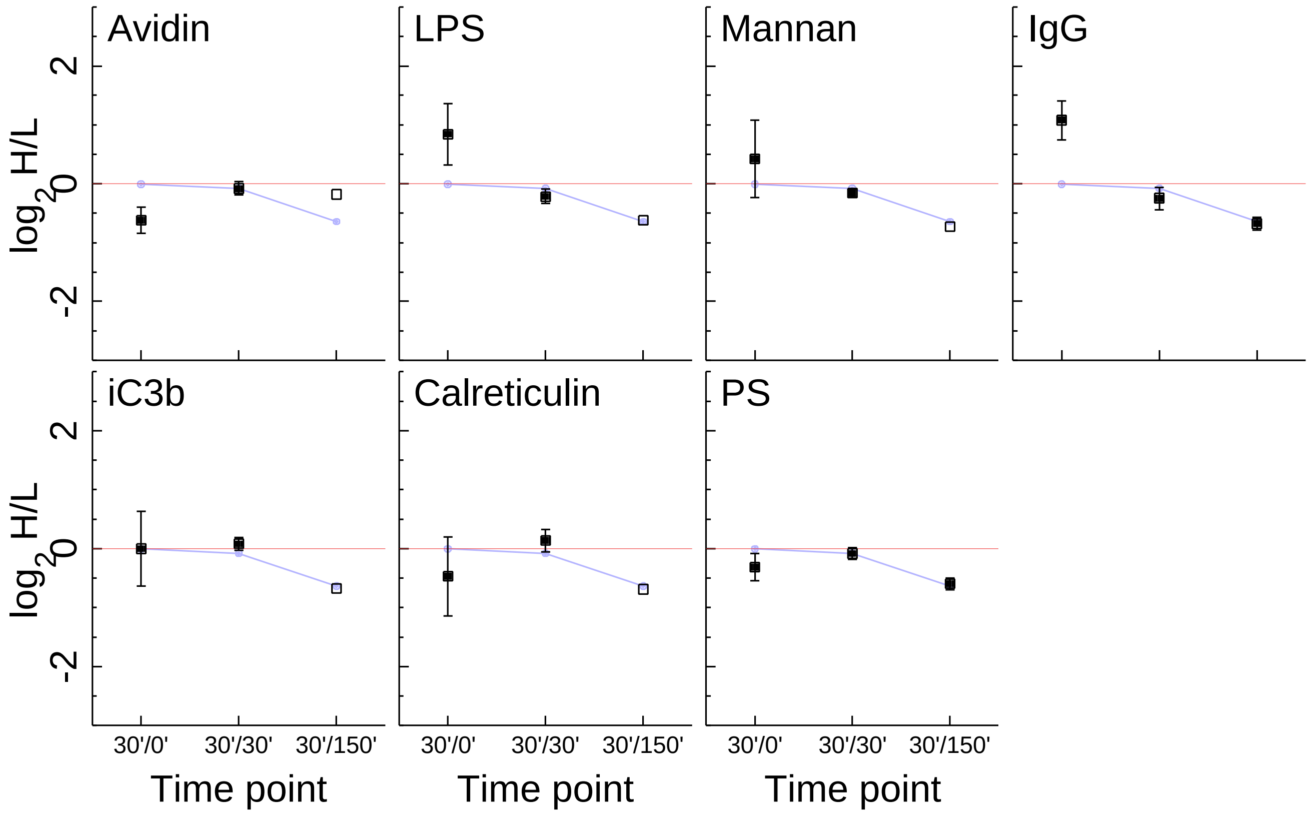

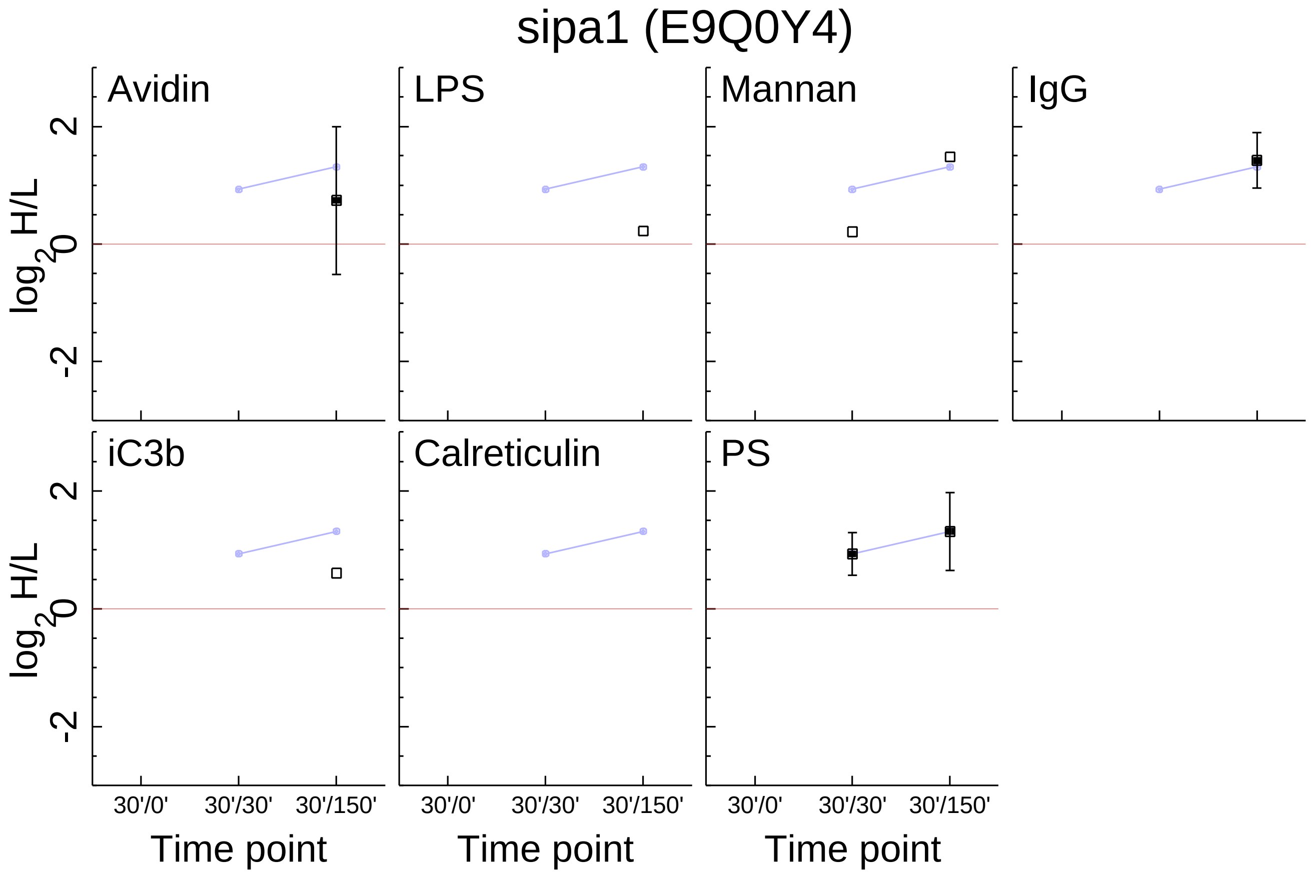
sipa1 (E9Q0Y4)
Avidin
LPS
Mannan
IgG
iC3b
Calreticulin
PS
log2 H/L
2
0
-2
30'/0'
30'/30'
30'/150'
Time point

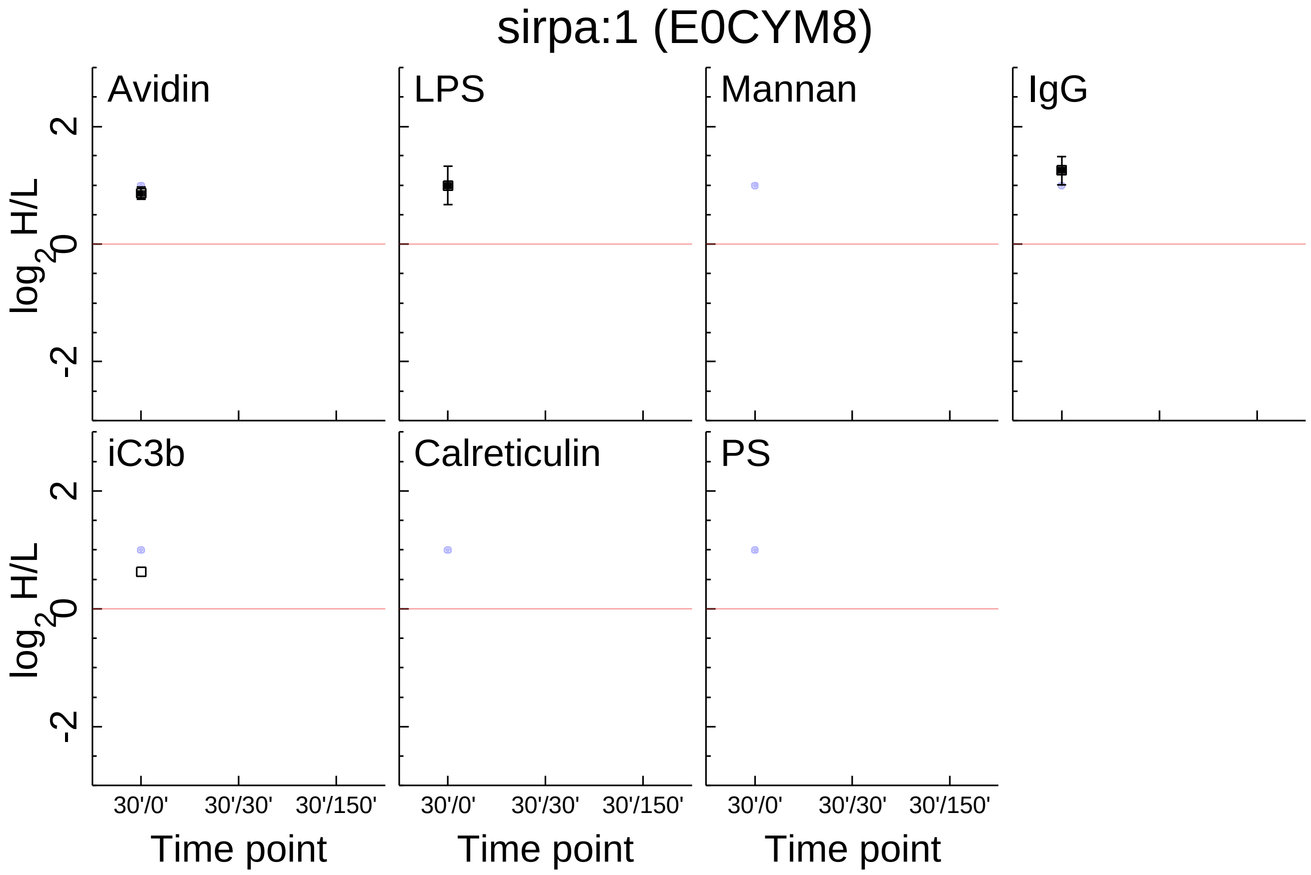
sirpa:1 (E0CYM8)
Avidin
LPS
Mannan
IgG
iC3b
Calreticulin
PS
log2 H/L
2
0
-2
30'/0'
30'/30'
30'/150'
Time point

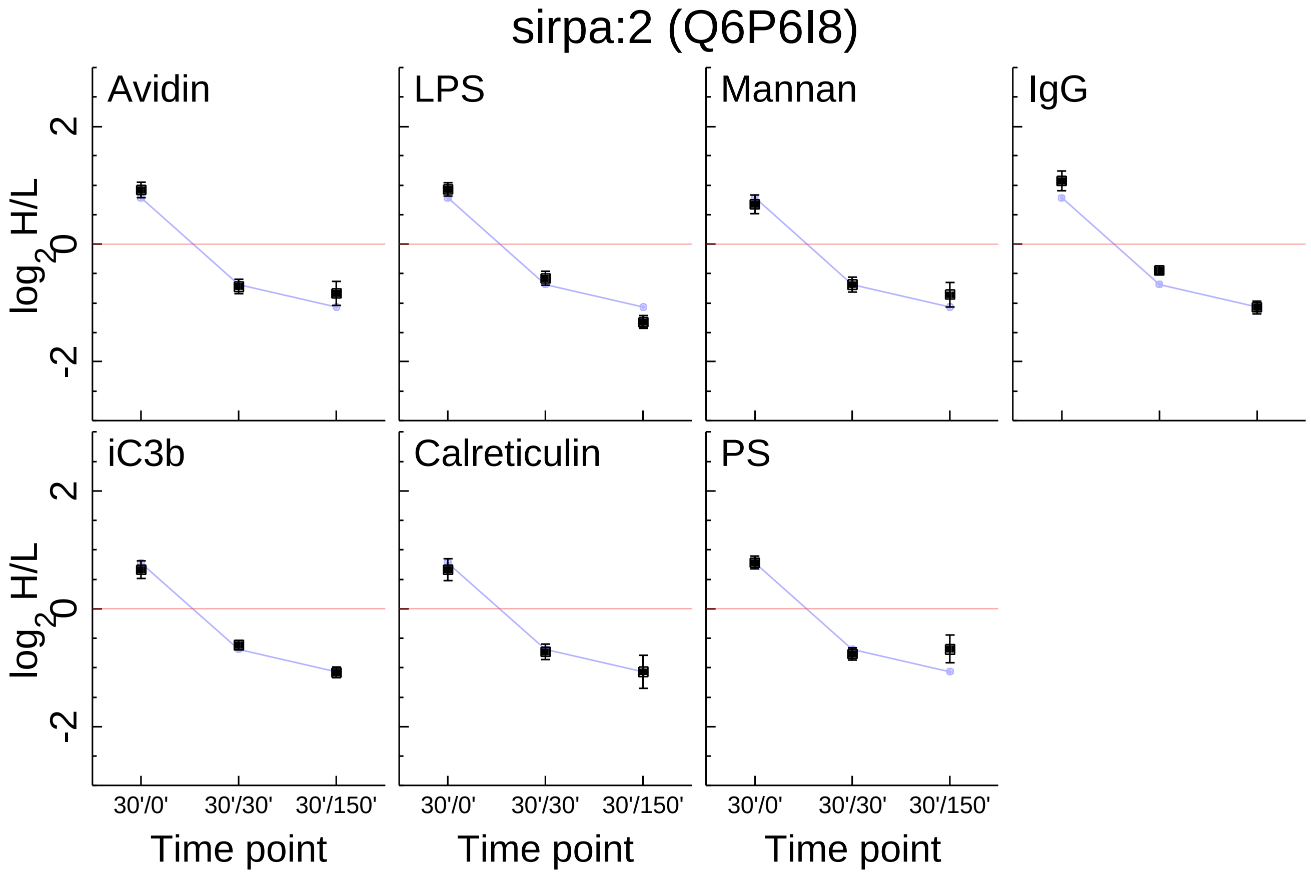
sirpa:2 (Q6P6I8)
Avidin
LPS
Mannan
IgG
iC3b
Calreticulin
PS
log2 H/L
2
0
-2
30'/0'
30'/30'
30'/150'
Time point

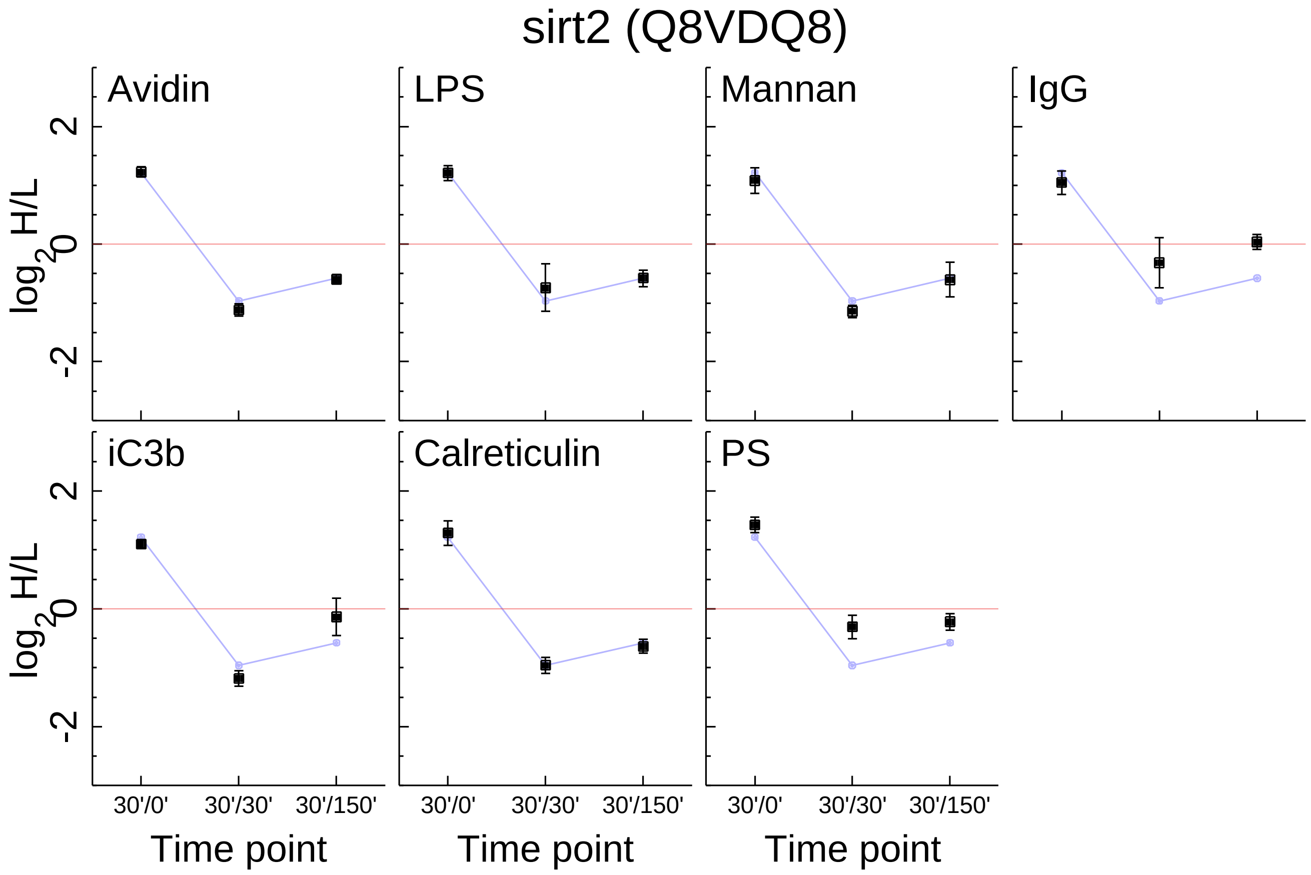
sirt2 (Q8VDQ8)
Avidin
LPS
Mannan
IgG
iC3b
Calreticulin
PS
log_2 H/L
2
0
-2
30'/0'
30'/30'
30'/150'
Time point

skap2 (Q3UND0)

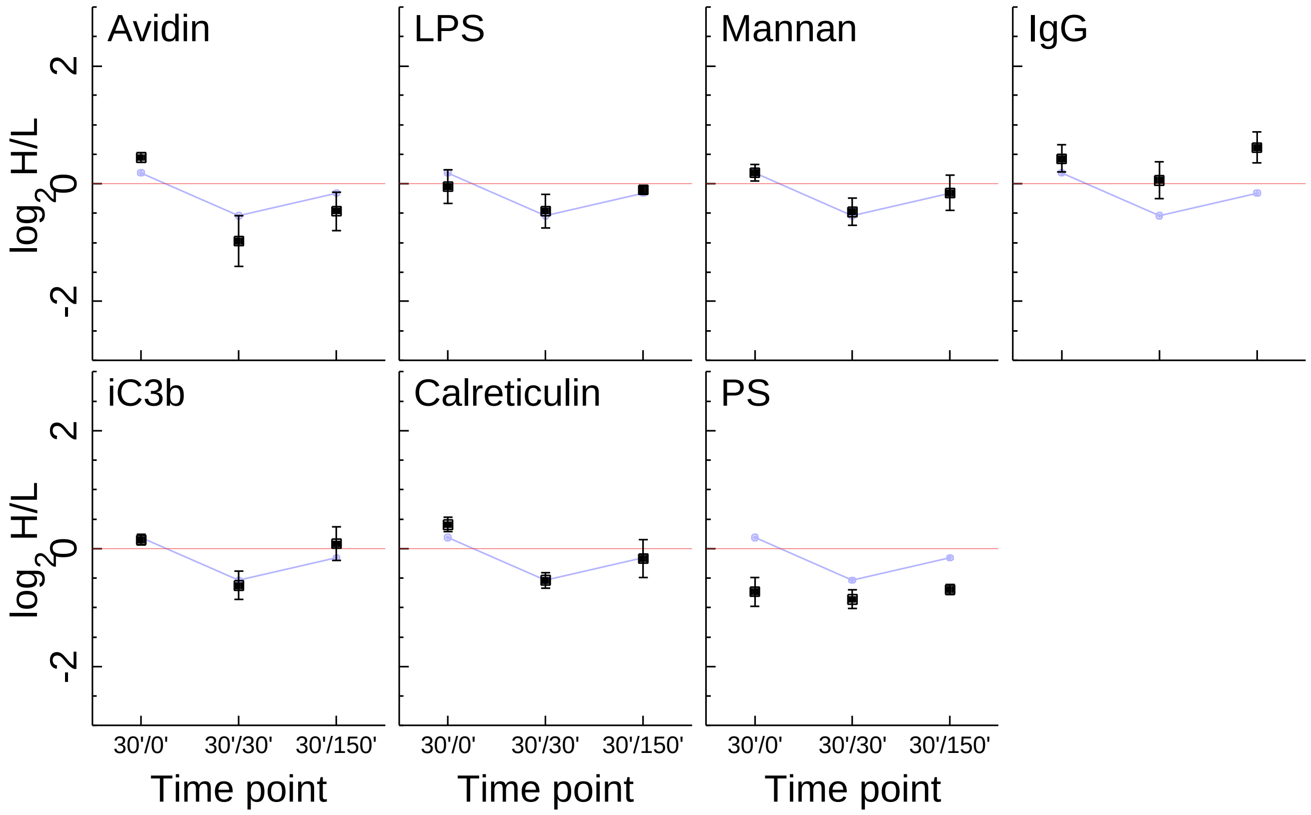

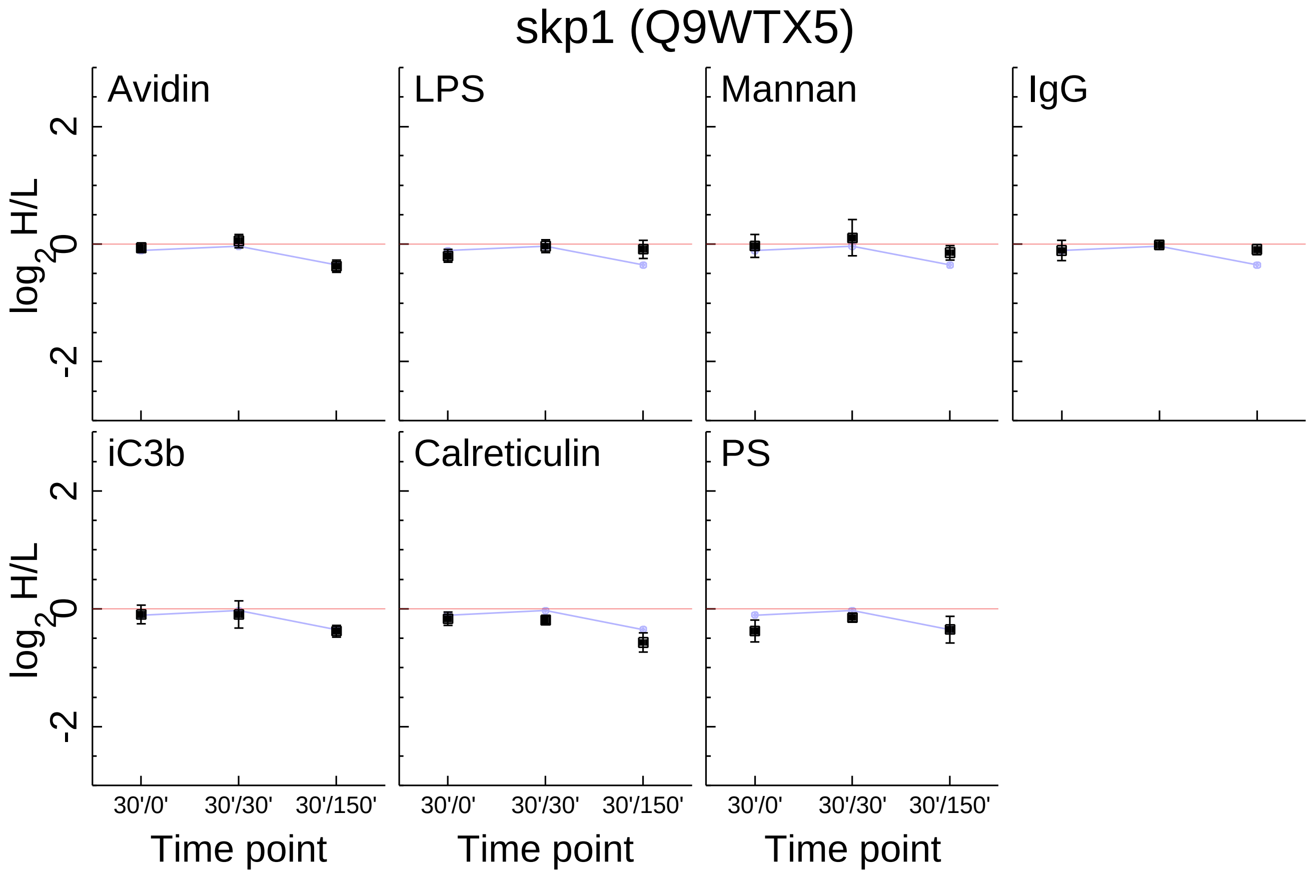

skp1 (Q9WTX5)
Avidin
LPS
Mannan
IgG
iC3b
Calreticulin
PS
log2 H/L
2
0
-2
30'/0'
30'/30'
30'/150'
Time point

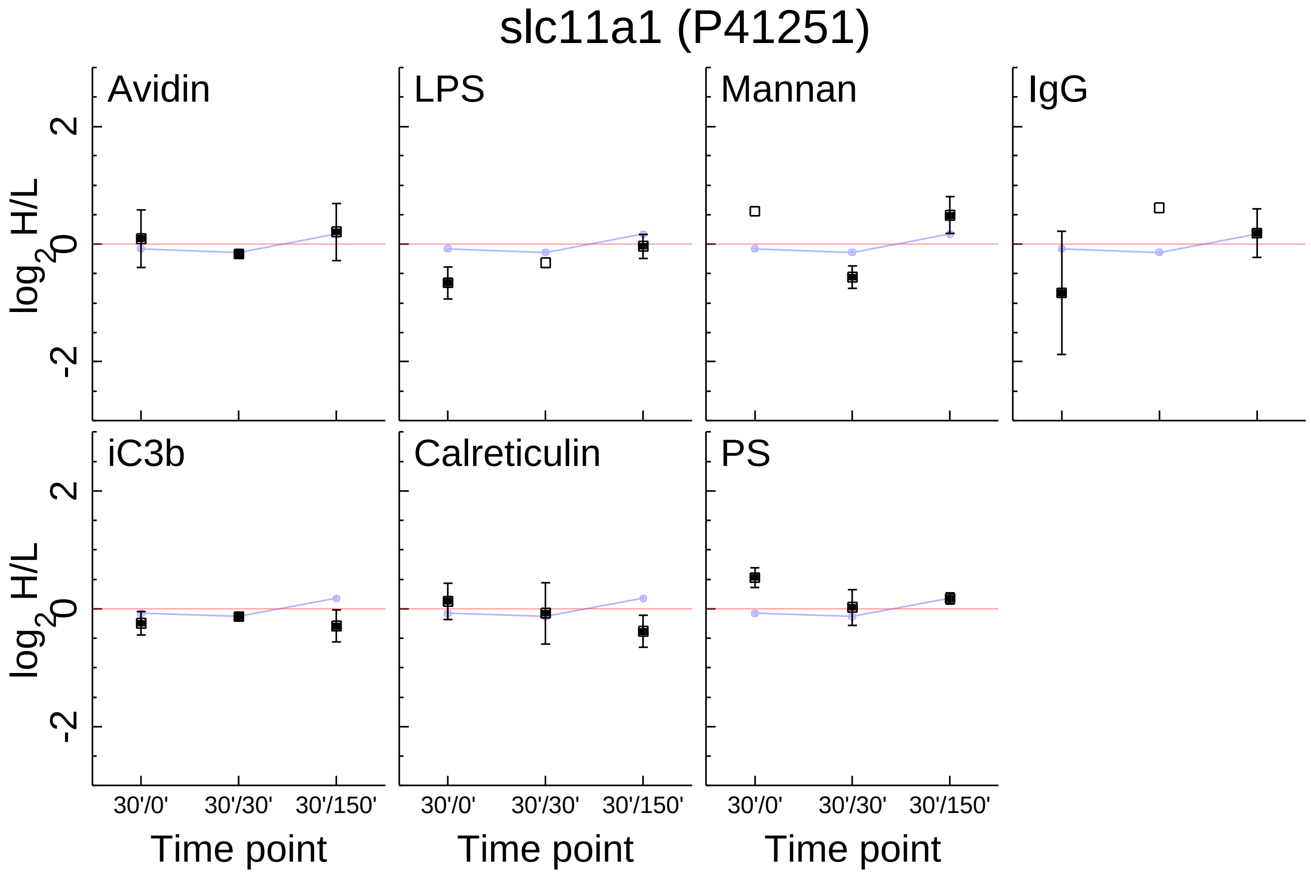
slc11a1 (P41251)
Avidin
LPS
Mannan
IgG
iC3b
Calreticulin
PS
log2 H/L
2
0
-2
30'/0'
30'/30'
30'/150'
Time point

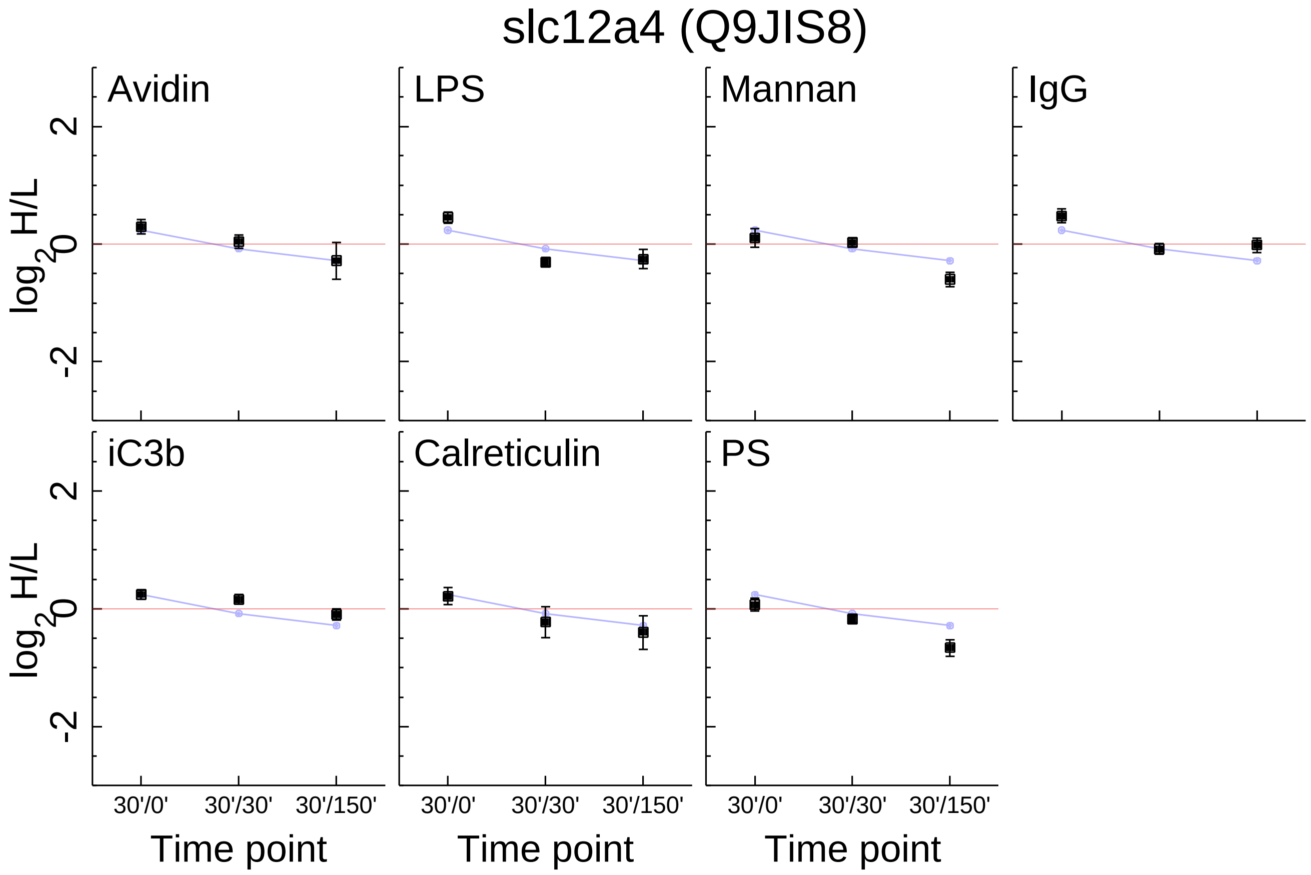
slc12a4 (Q9JIS8)
Avidin
LPS
Mannan
IgG
iC3b
Calreticulin
PS
log2 H/L
2
0
-2
30'/0'
30'/30'
30'/150'
Time point

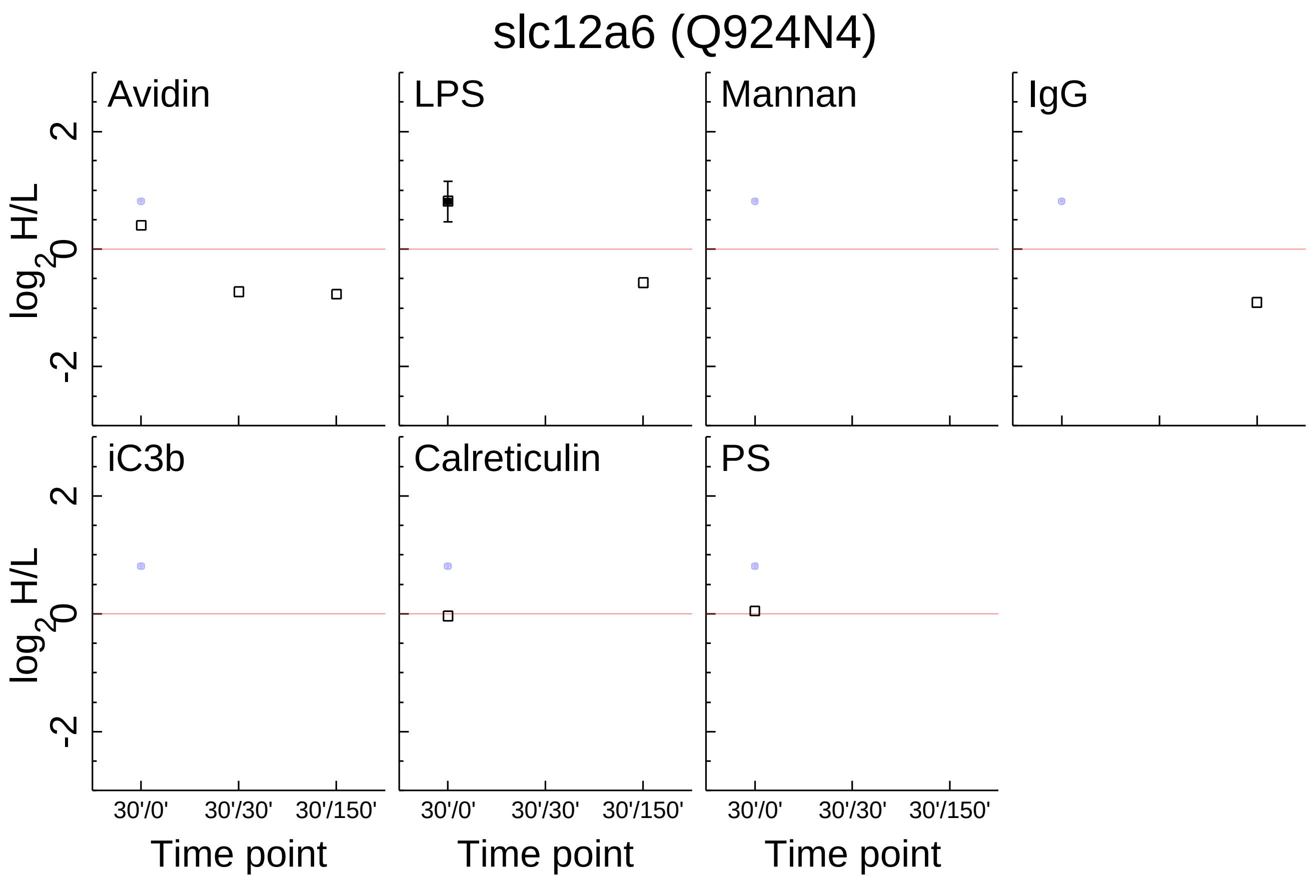

slc12a6 (Q924N4)
Avidin
LPS
Mannan
IgG
iC3b
Calreticulin
PS
log2 H/L
2
0
-2
30'/0'
30'/30'
30'/150'
Time point

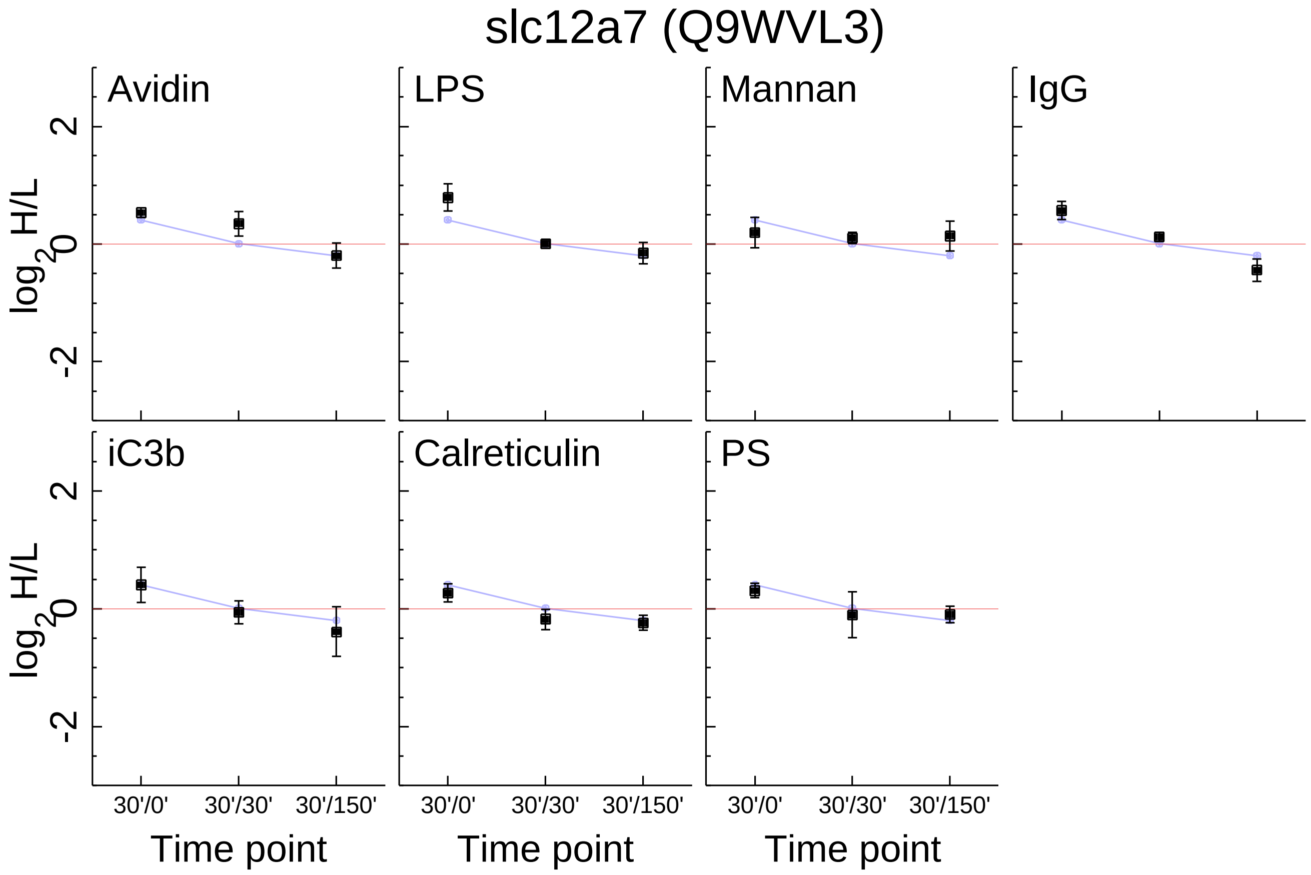
slc12a7 (Q9WVL3)
Avidin
LPS
Mannan
IgG
iC3b
Calreticulin
PS
log2 H/L
2
0
-2
30'/0'
30'/30'
30'/150'
Time point

slc12a9 (Q99MR3)

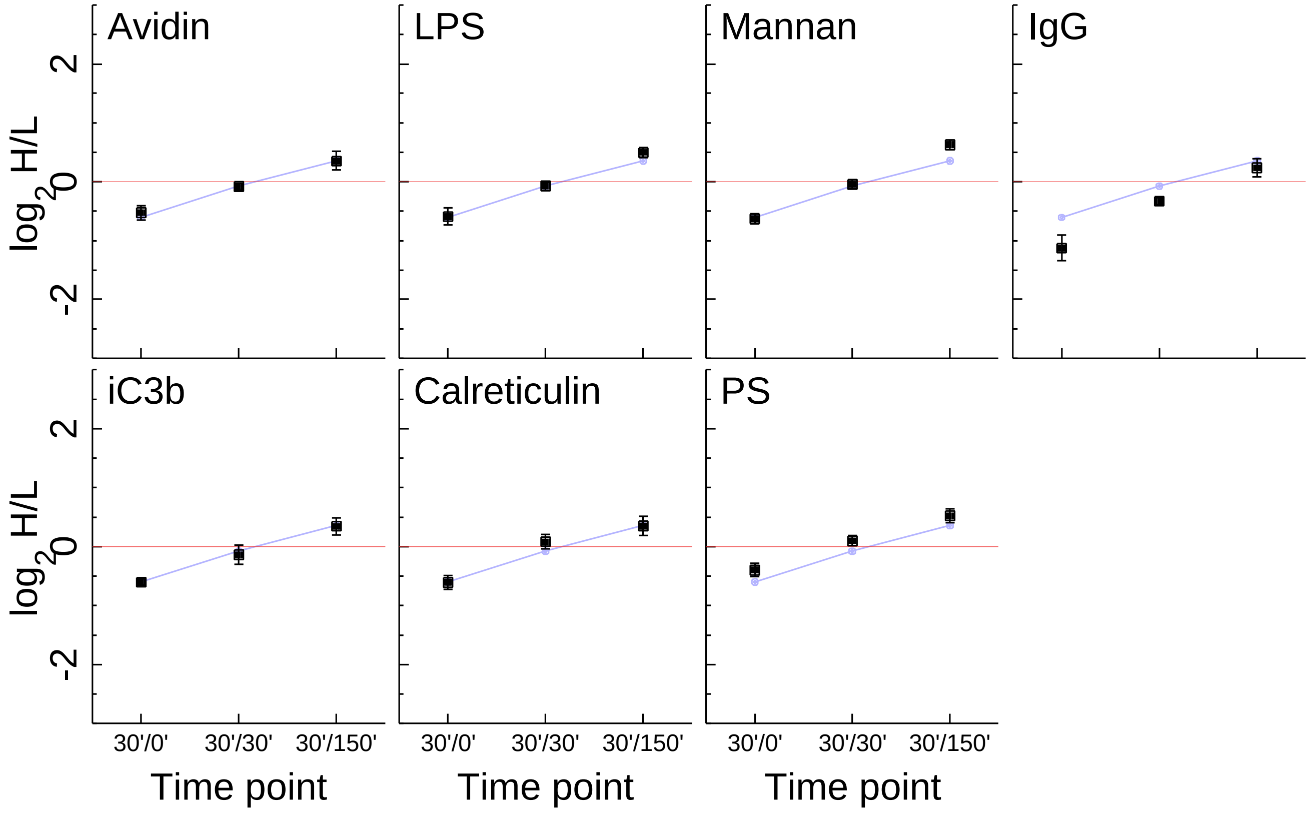

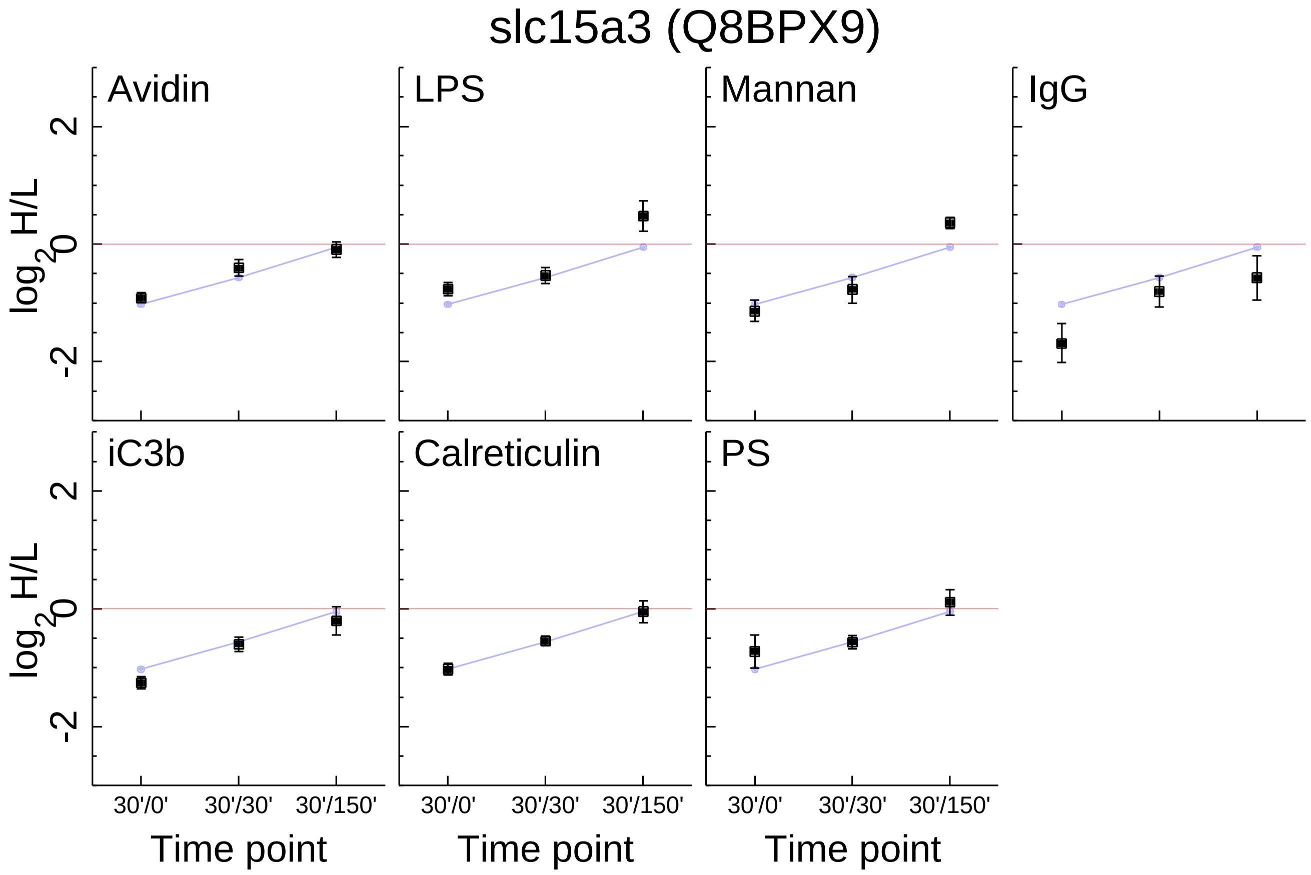
slc15a3 (Q8BPX9)
Avidin
LPS
Mannan
IgG
iC3b
Calreticulin
PS
log2 H/L
2
0
-2
30'/0'
30'/30'
30'/150'
Time point

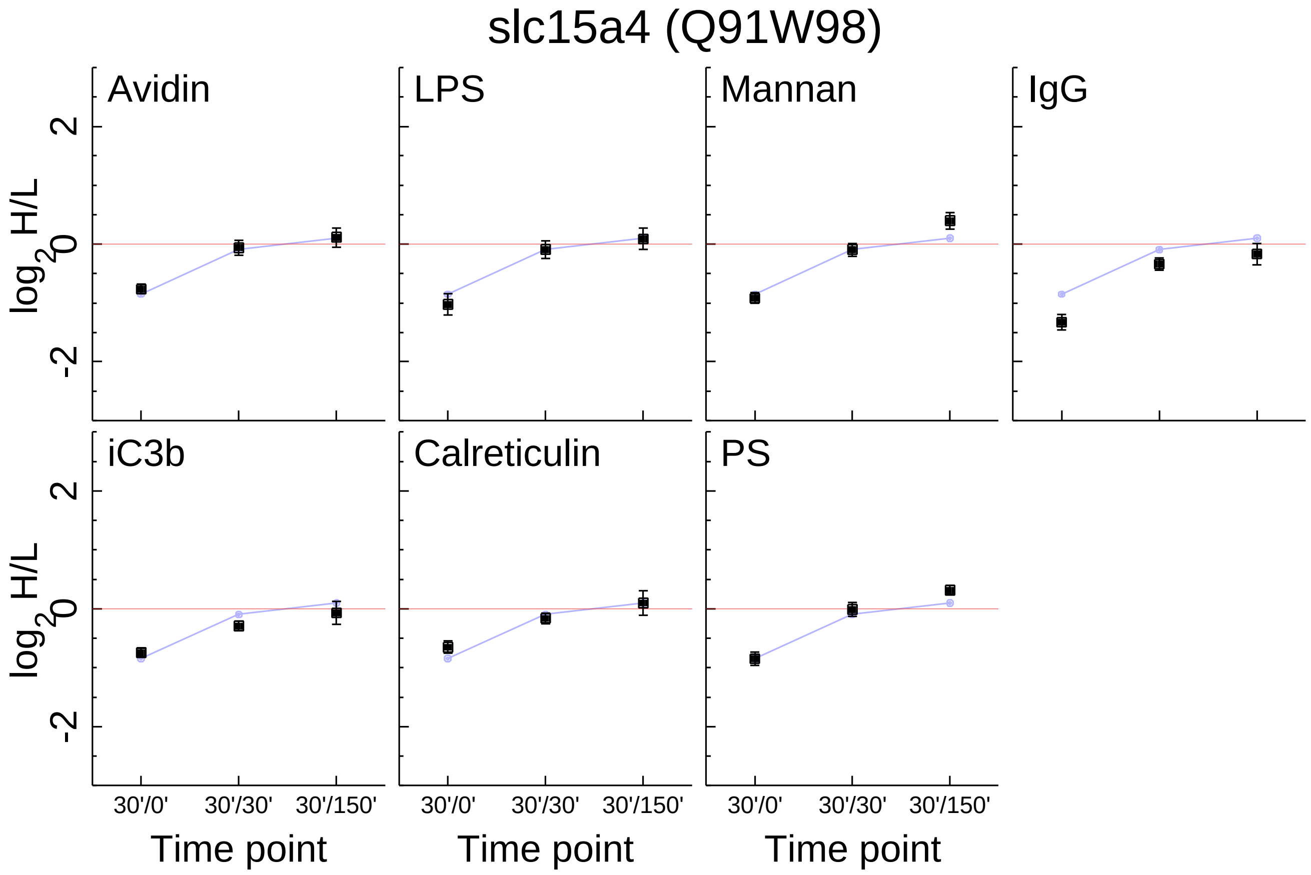

slc15a4 (Q91W98)
Avidin
LPS
Mannan
IgG
iC3b
Calreticulin
PS
log2 H/L
2
0
-2
30'/0'
30'/30'
30'/150'
Time point

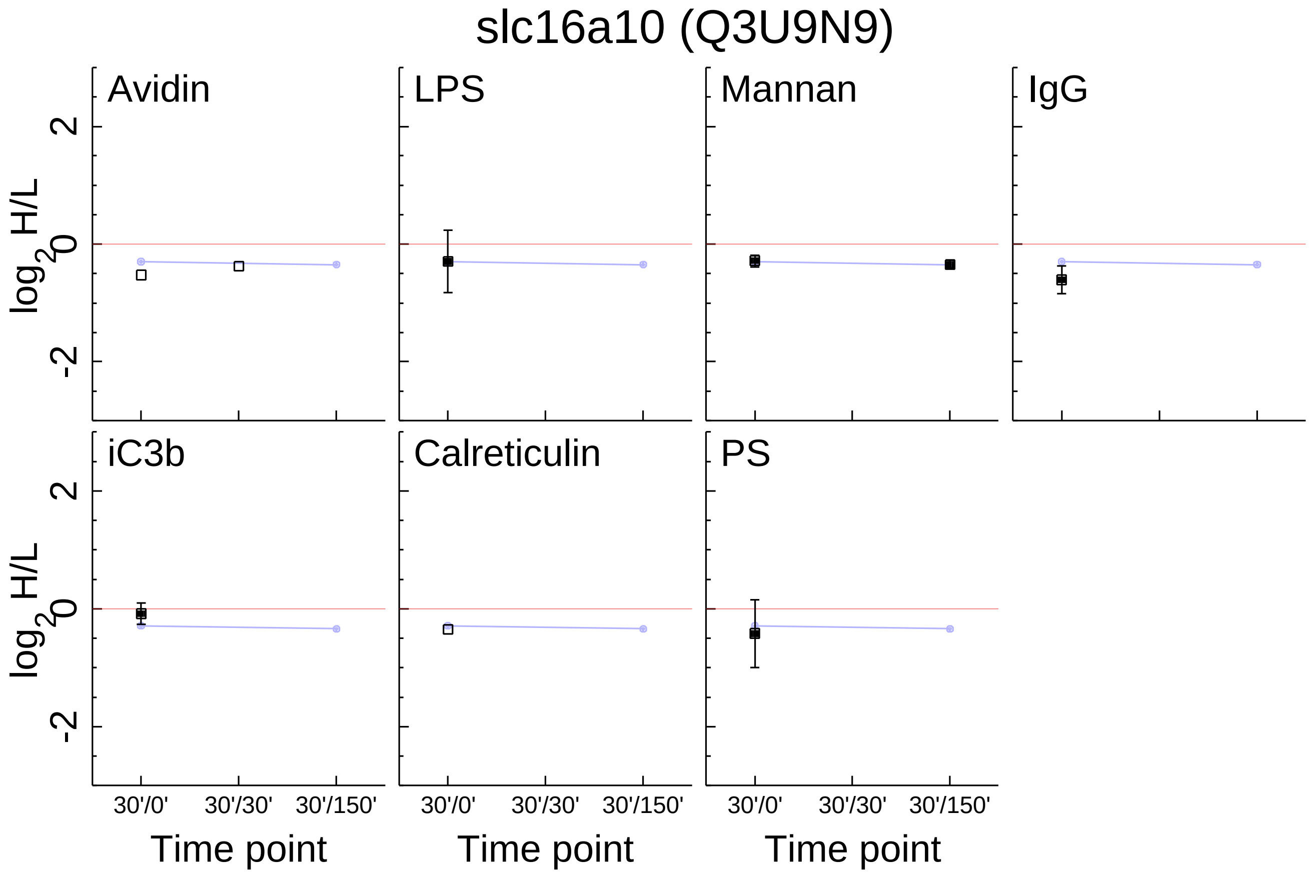

slc16a10 (Q3U9N9)
Avidin
LPS
Mannan
IgG
iC3b
Calreticulin
PS
log2 H/L
2
0
-2
30'/0'
30'/30'
30'/150'
Time point

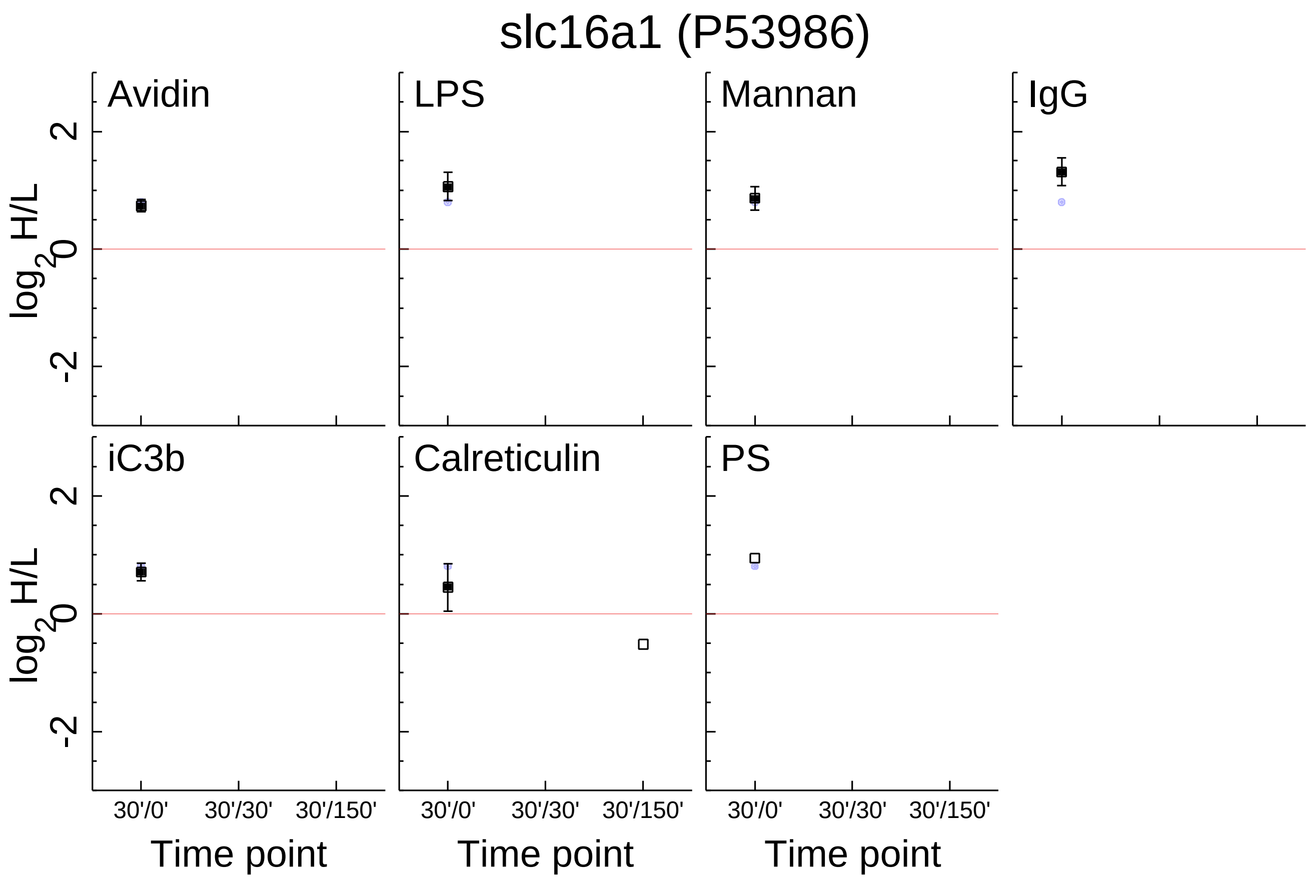
slc16a1 (P53986)
Avidin
LPS
Mannan
IgG
iC3b
Calreticulin
PS
log2 H/L
2
0
-2
30'/0'
30'/30'
30'/150'
Time point

slc16a3 (P57787)

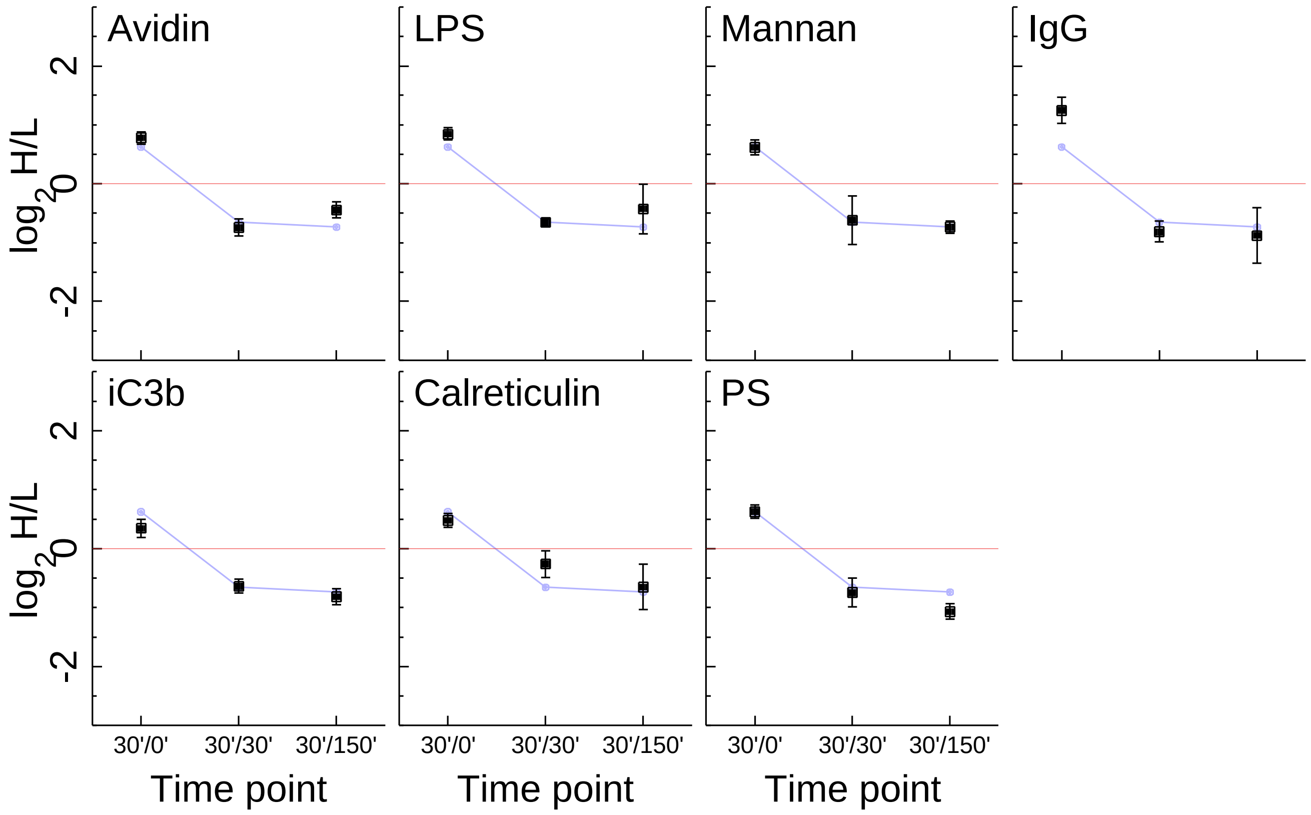

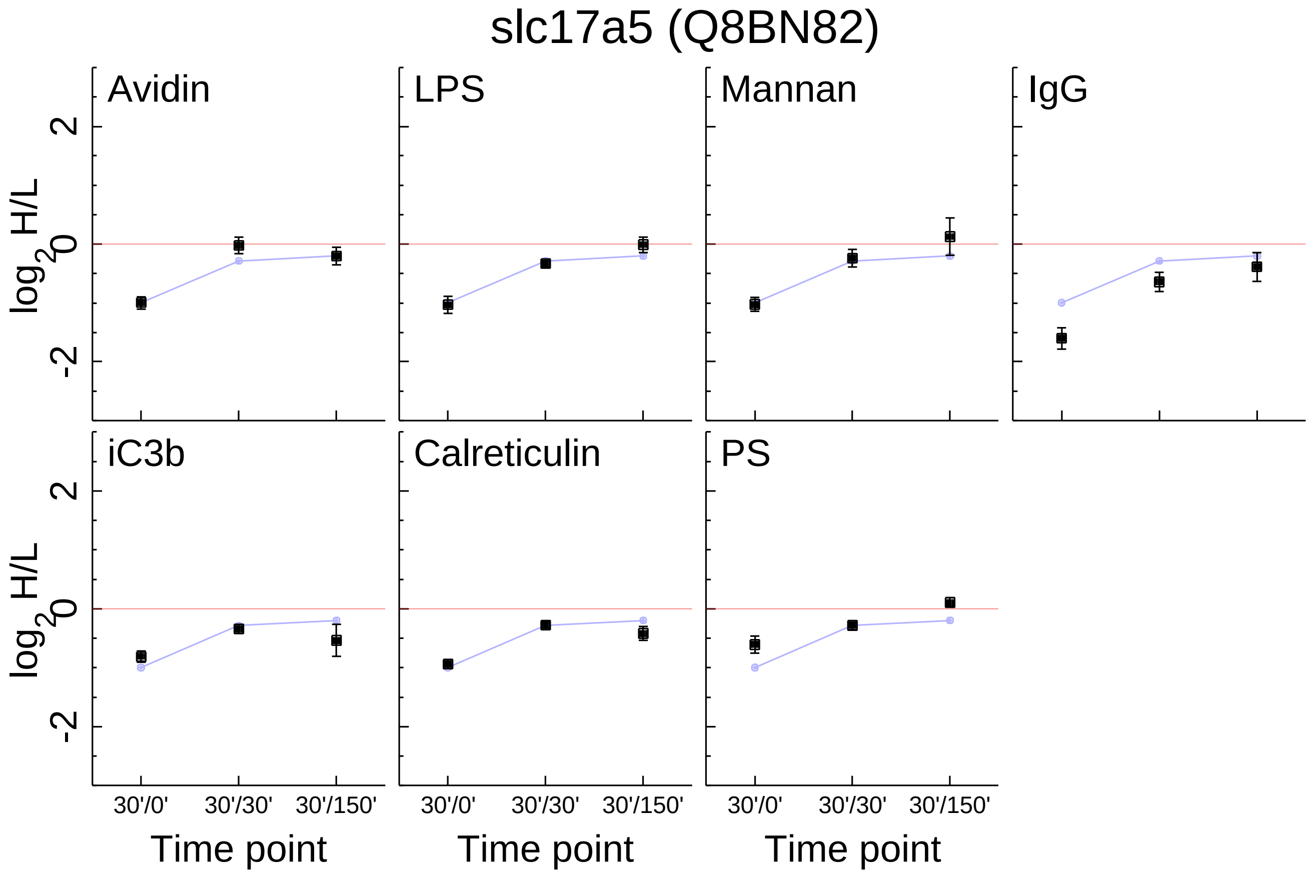

slc17a5 (Q8BN82)
Avidin
LPS
Mannan
IgG
iC3b
Calreticulin
PS
log2 H/L
2
0
-2
30'/0'
30'/30'
30'/150'
Time point

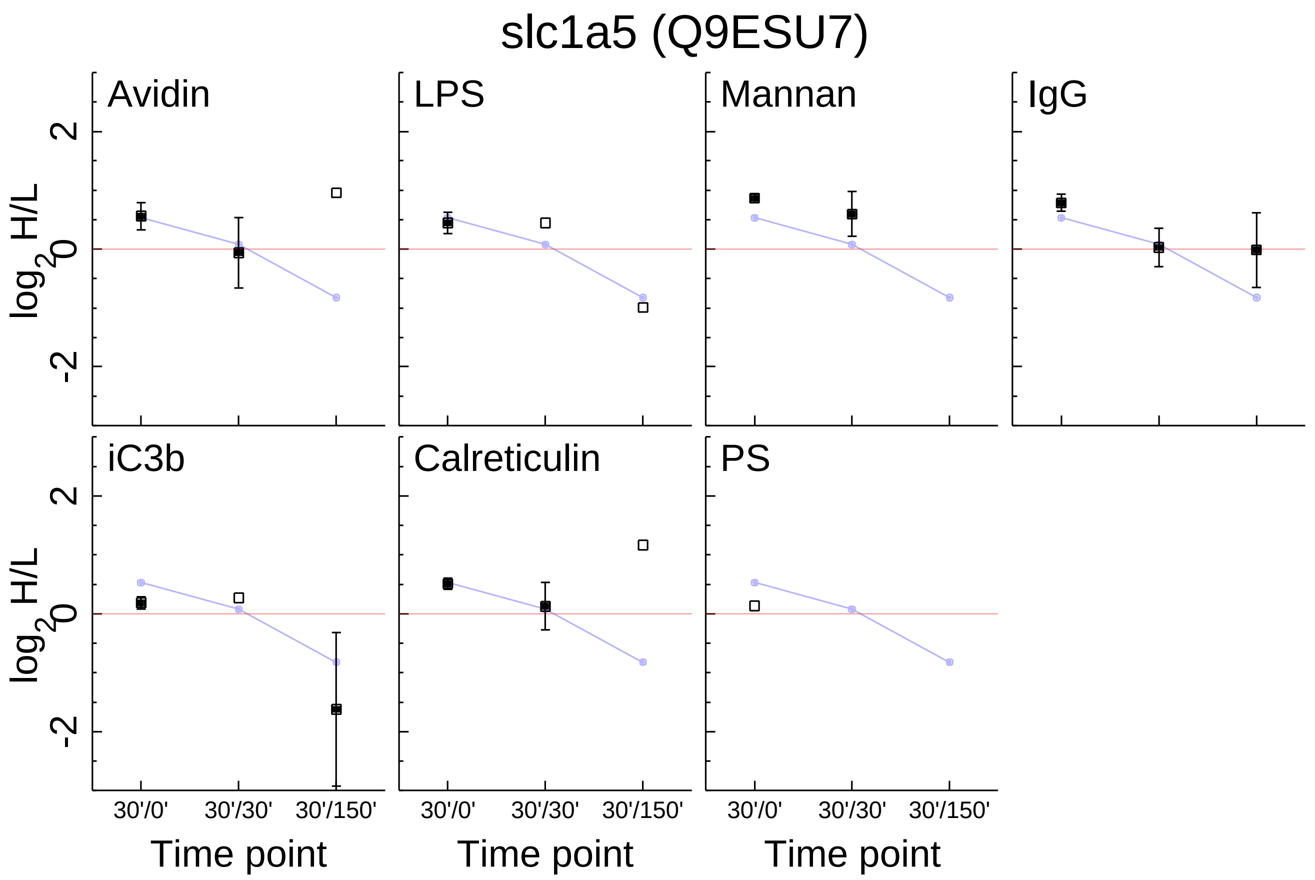

slc1a5 (Q9ESU7)
Avidin
LPS
Mannan
IgG
iC3b
Calreticulin
PS
$\log_2$ H/L
2
0
-2
30'/0'
30'/30'
30'/150'
Time point

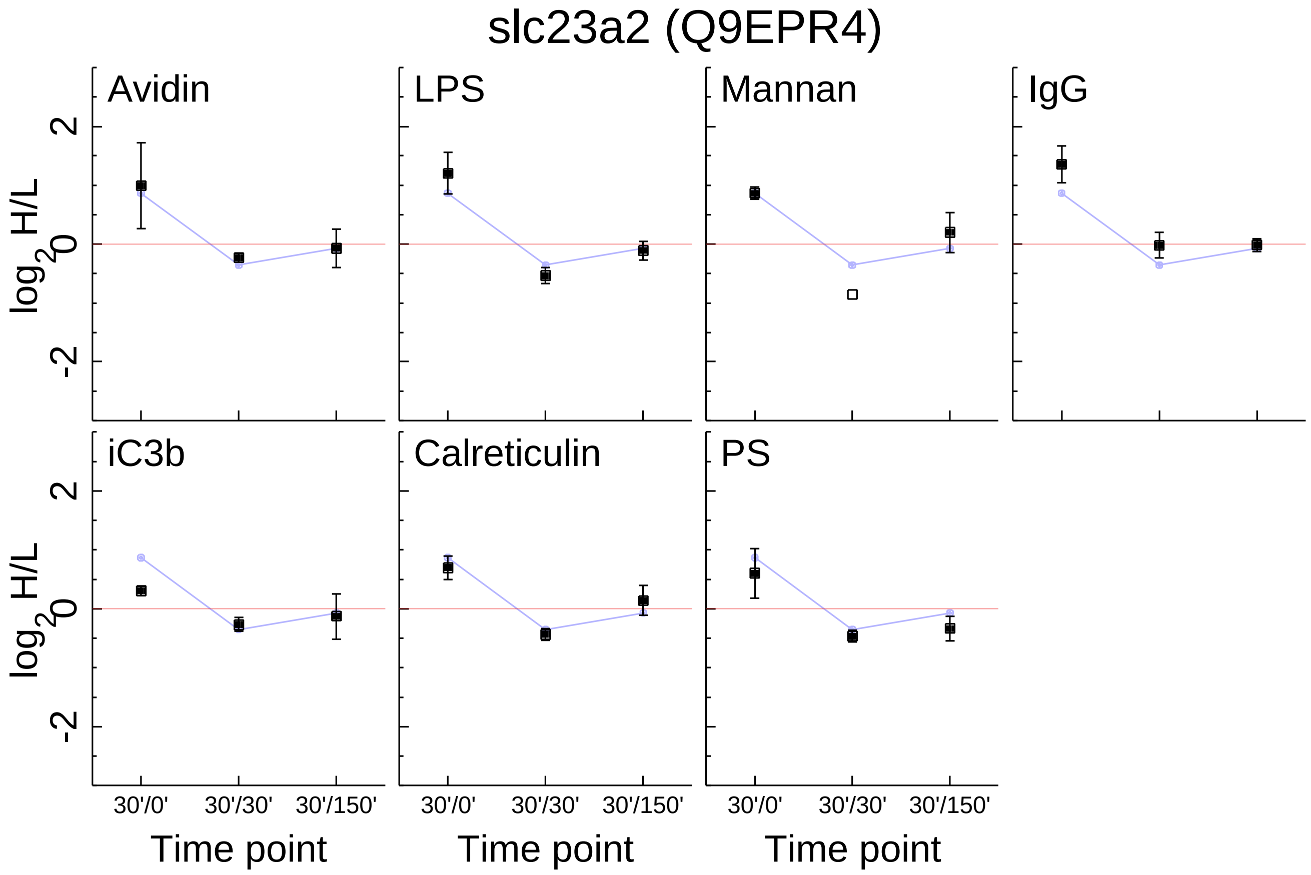
slc23a2 (Q9EPR4)
Avidin
LPS
Mannan
IgG
iC3b
Calreticulin
PS
log2 H/L
2
0
-2
30'/0'
30'/30'
30'/150'
Time point

slc25a10 (Q9QZD8)

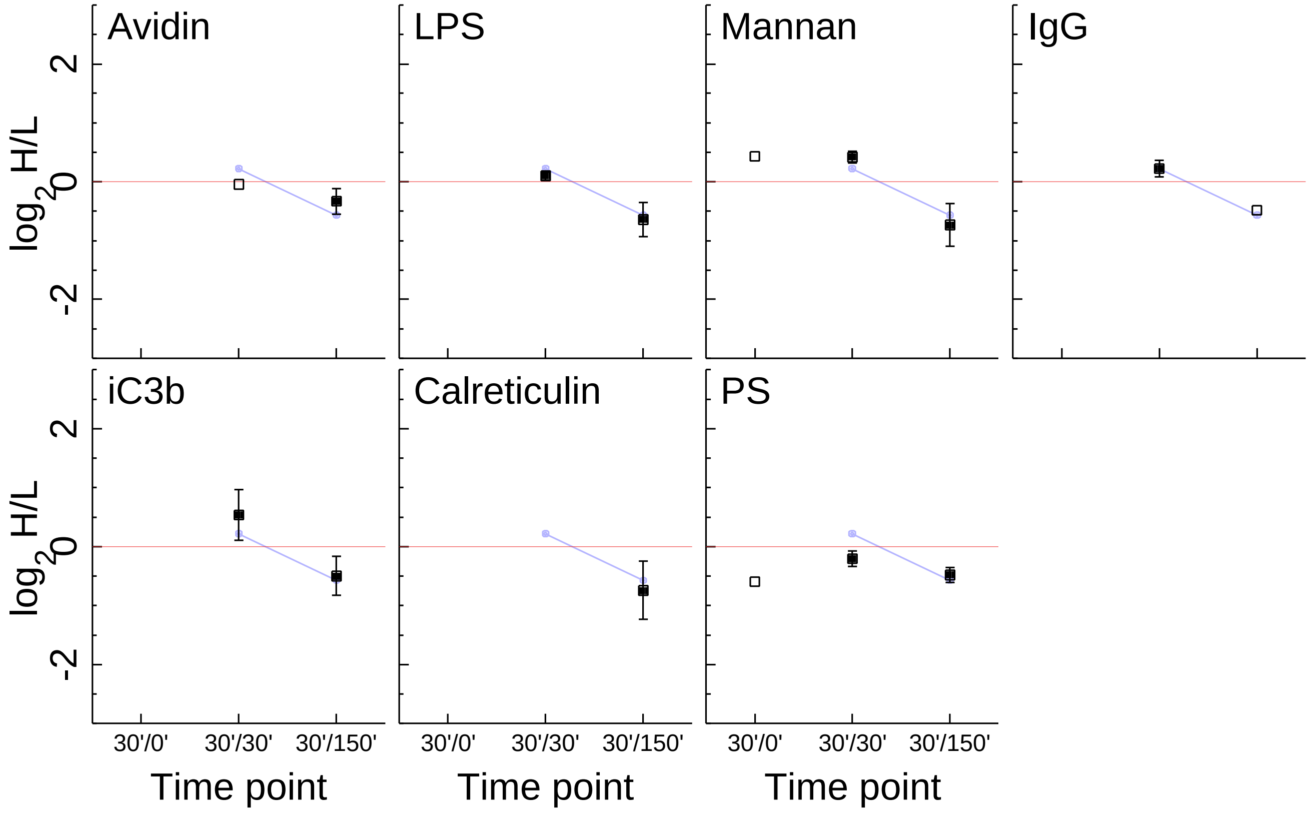

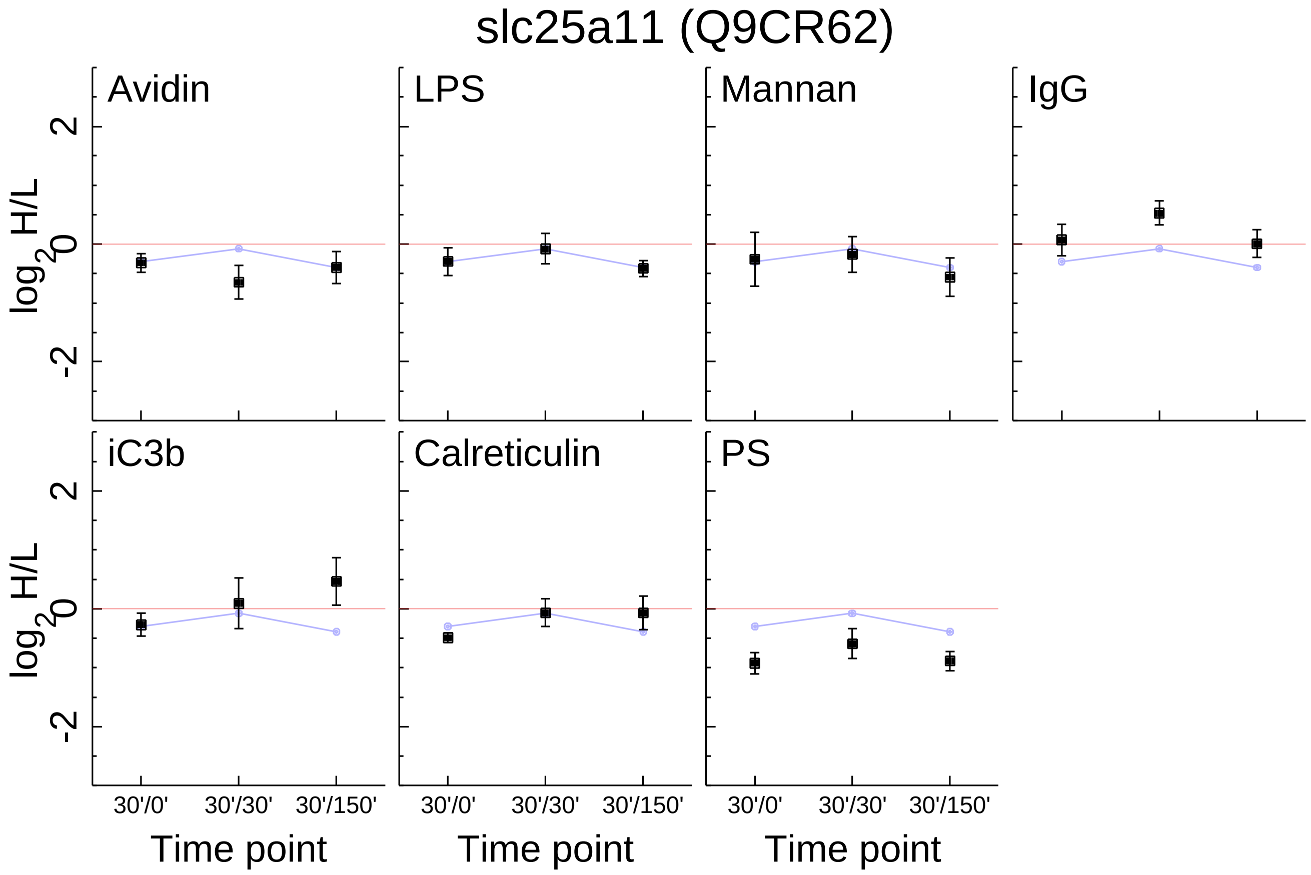
slc25a11 (Q9CR62)
Avidin
LPS
Mannan
IgG
iC3b
Calreticulin
PS
log2 H/L
2
0
-2
30'/0'
30'/30'
30'/150'
Time point

slc25a12 (Q8BH59)

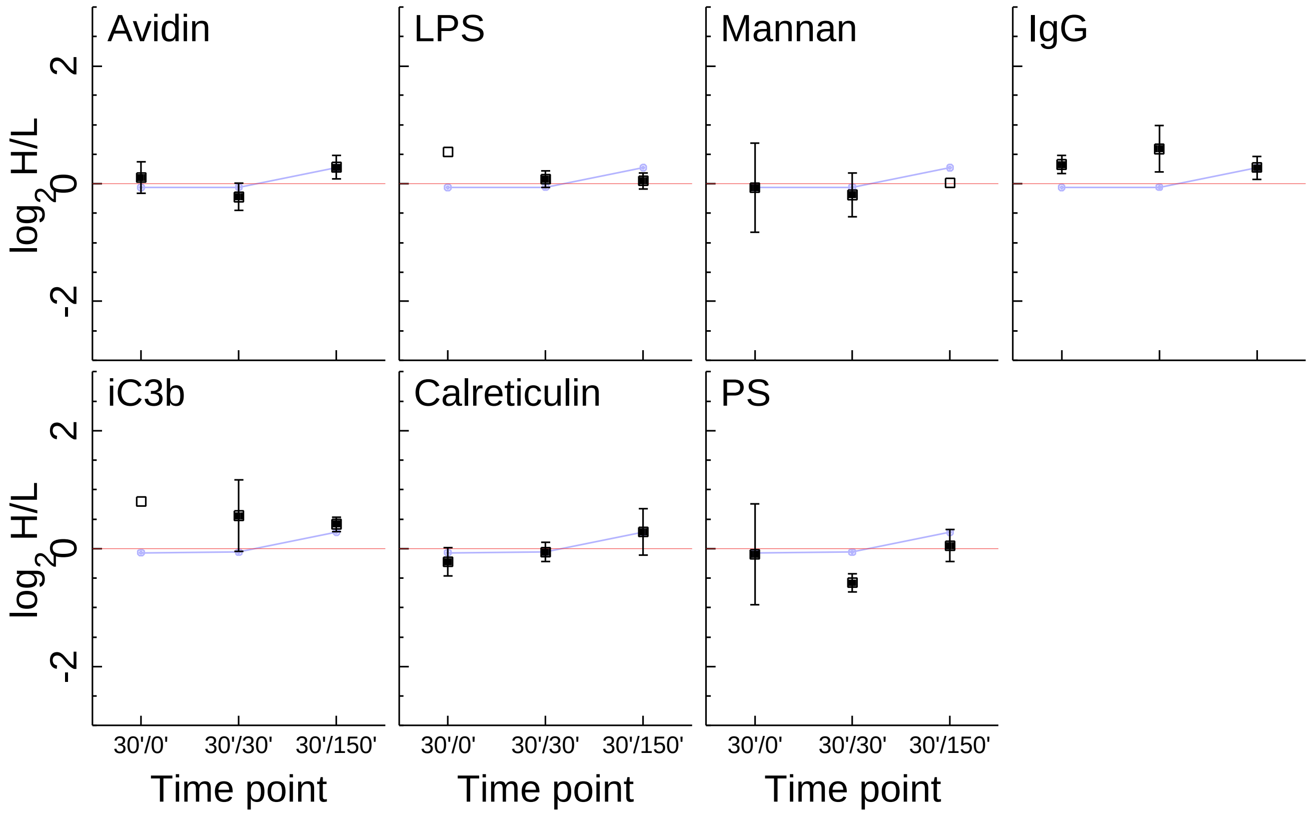

slc25a13 (Q9QXX4)

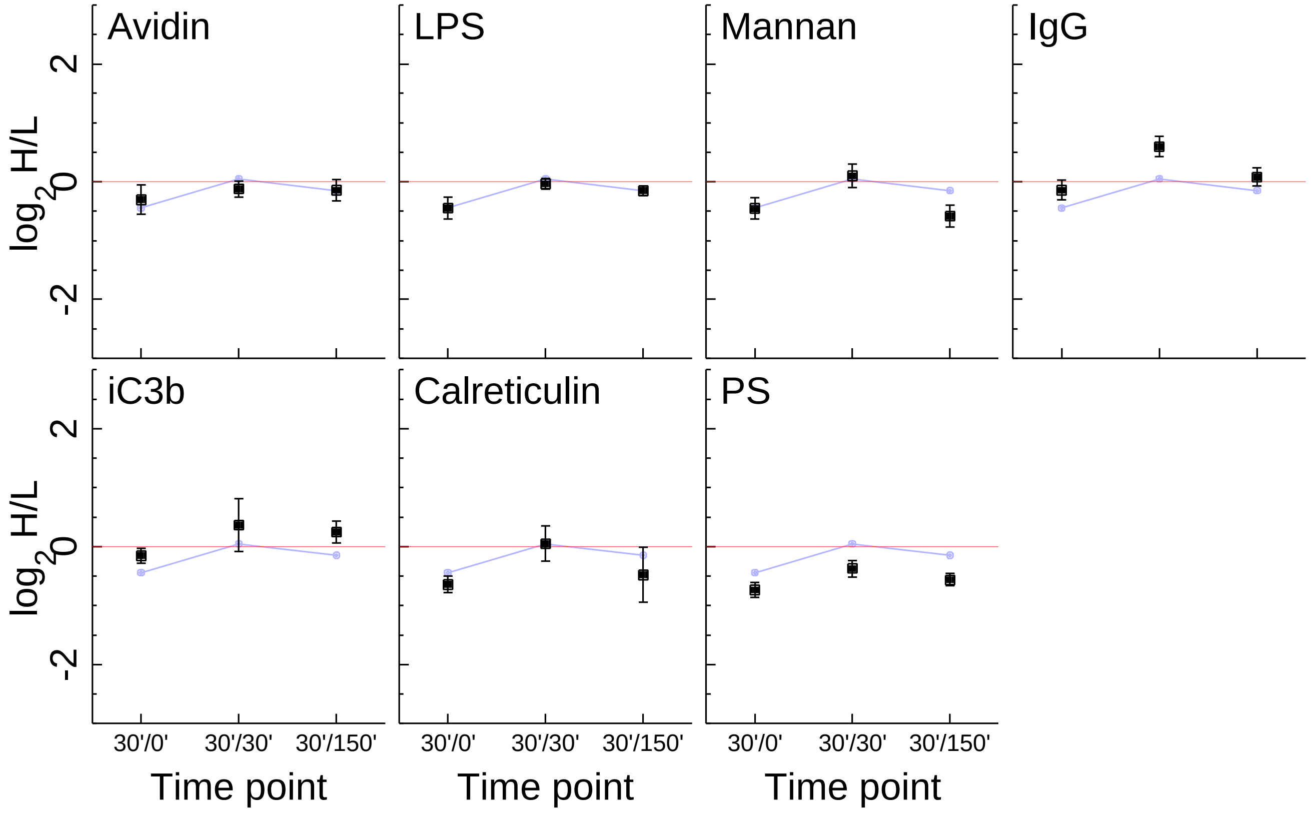

slc25a1 (Q8JZU2)

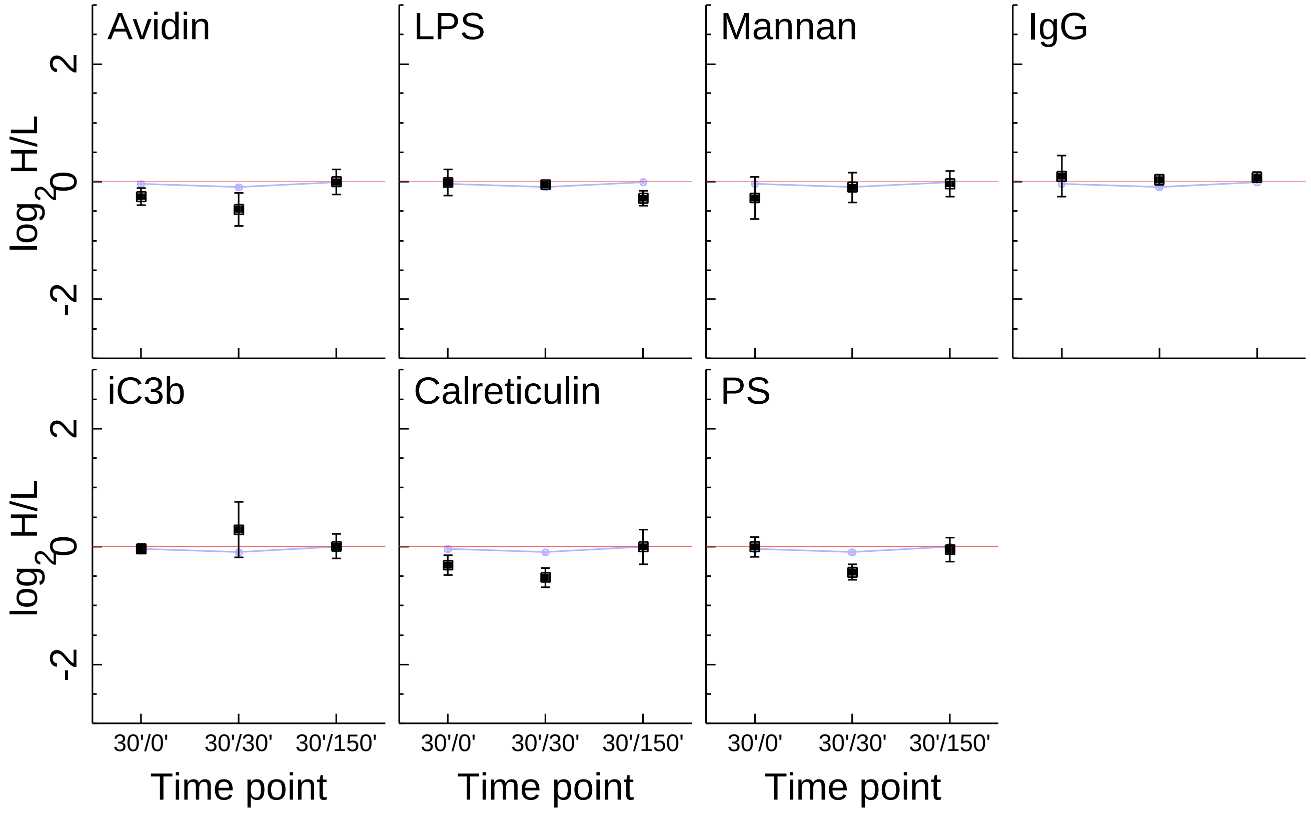

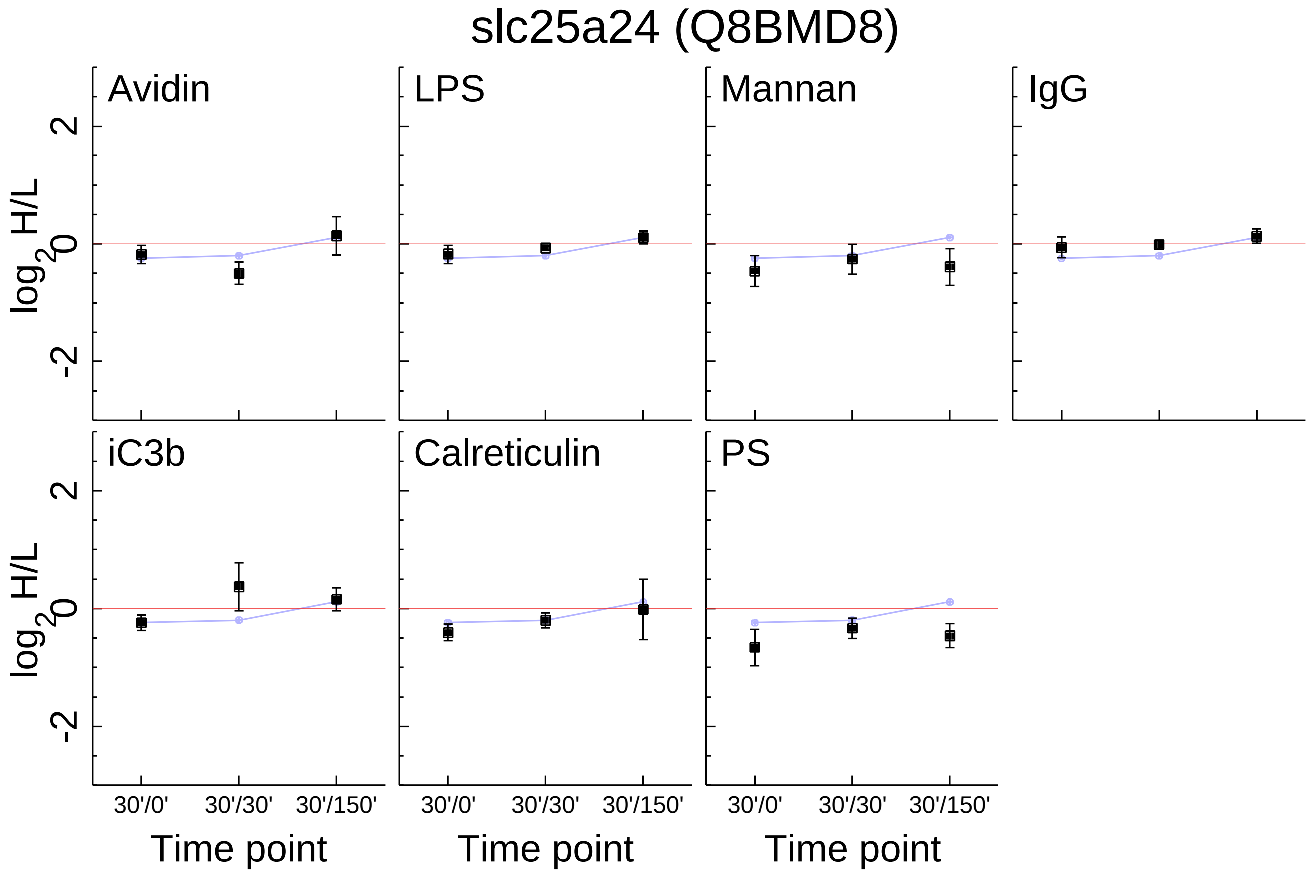
slc25a24 (Q8BMD8)
Avidin
LPS
Mannan
IgG
iC3b
Calreticulin
PS
log2 H/L
2
0
-2
30'/0'
30'/30'
30'/150'
Time point

slc25a3 (Q8VEM8)

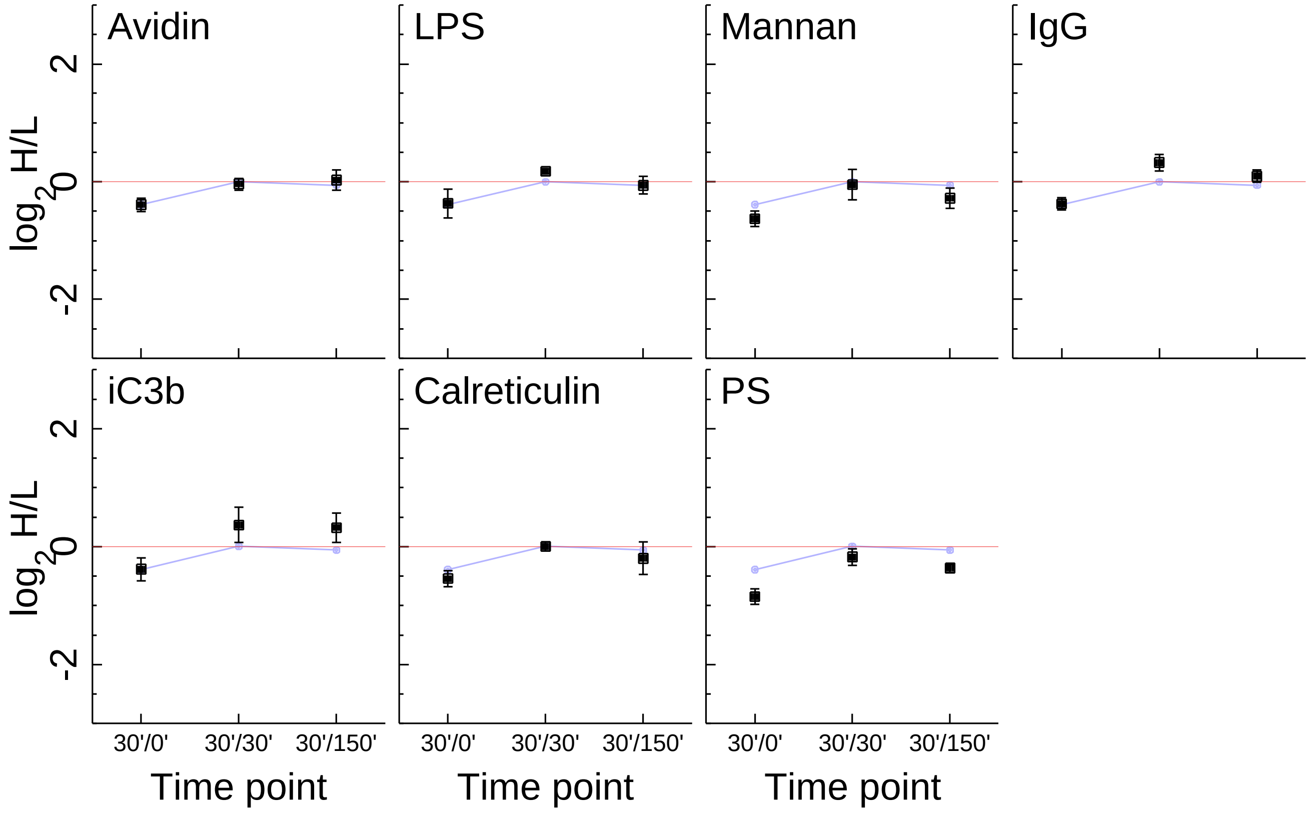

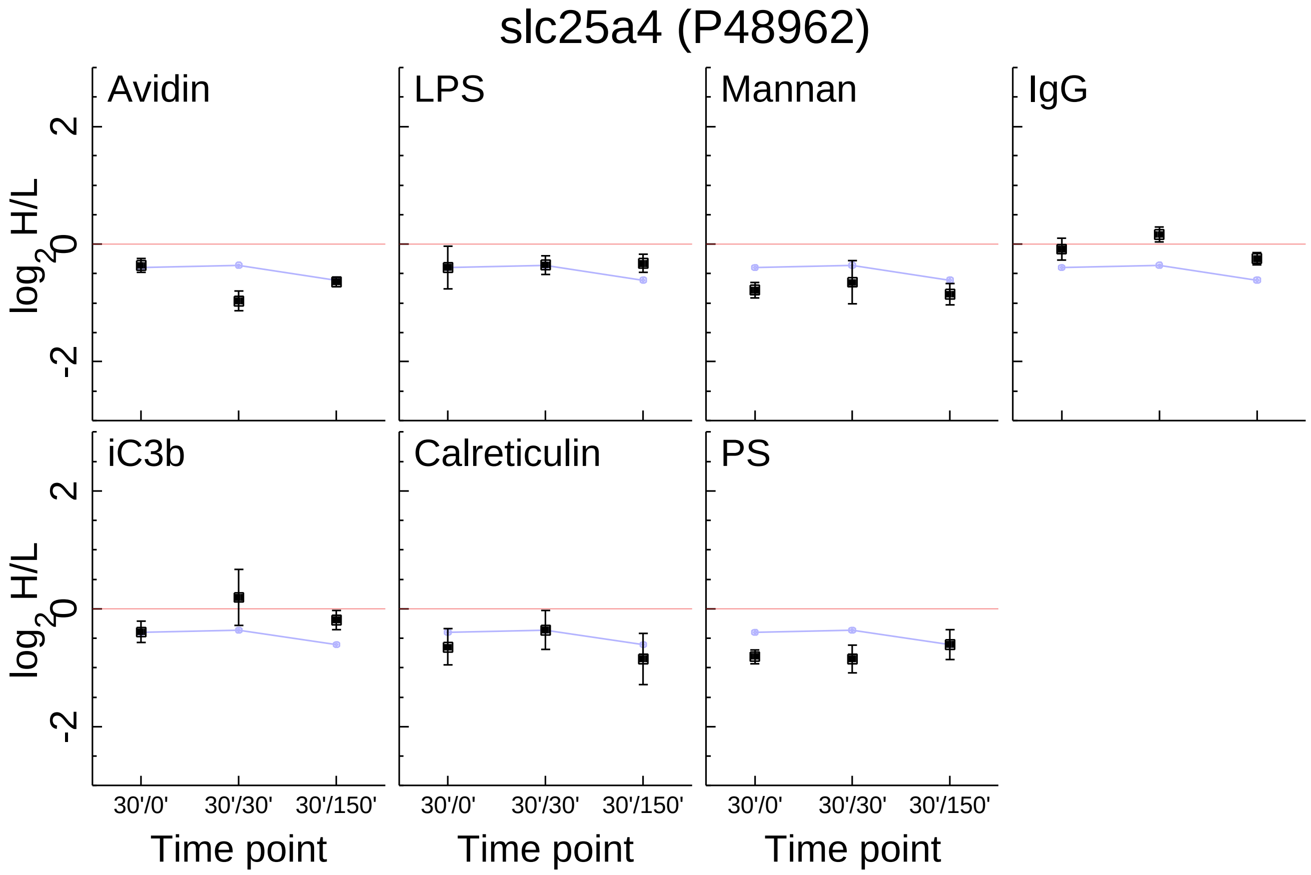

slc25a4 (P48962)
Avidin
LPS
Mannan
IgG
iC3b
Calreticulin
PS
log2 H/L
2
0
-2
30'/0'
30'/30'
30'/150'
Time point

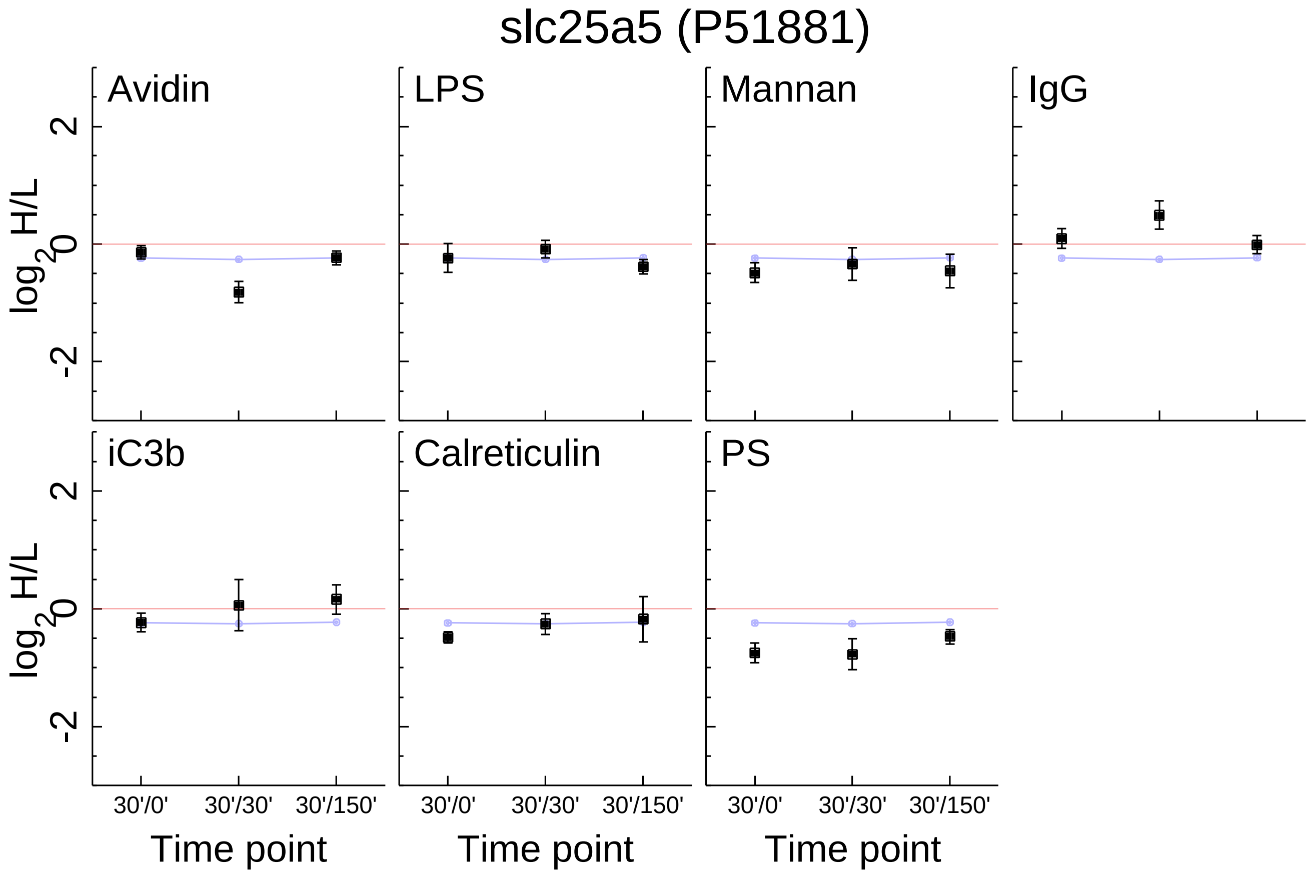
slc25a5 (P51881)
Avidin
LPS
Mannan
IgG
iC3b
Calreticulin
PS
log2 H/L
2
0
-2
30'/0'
30'/30'
30'/150'
Time point

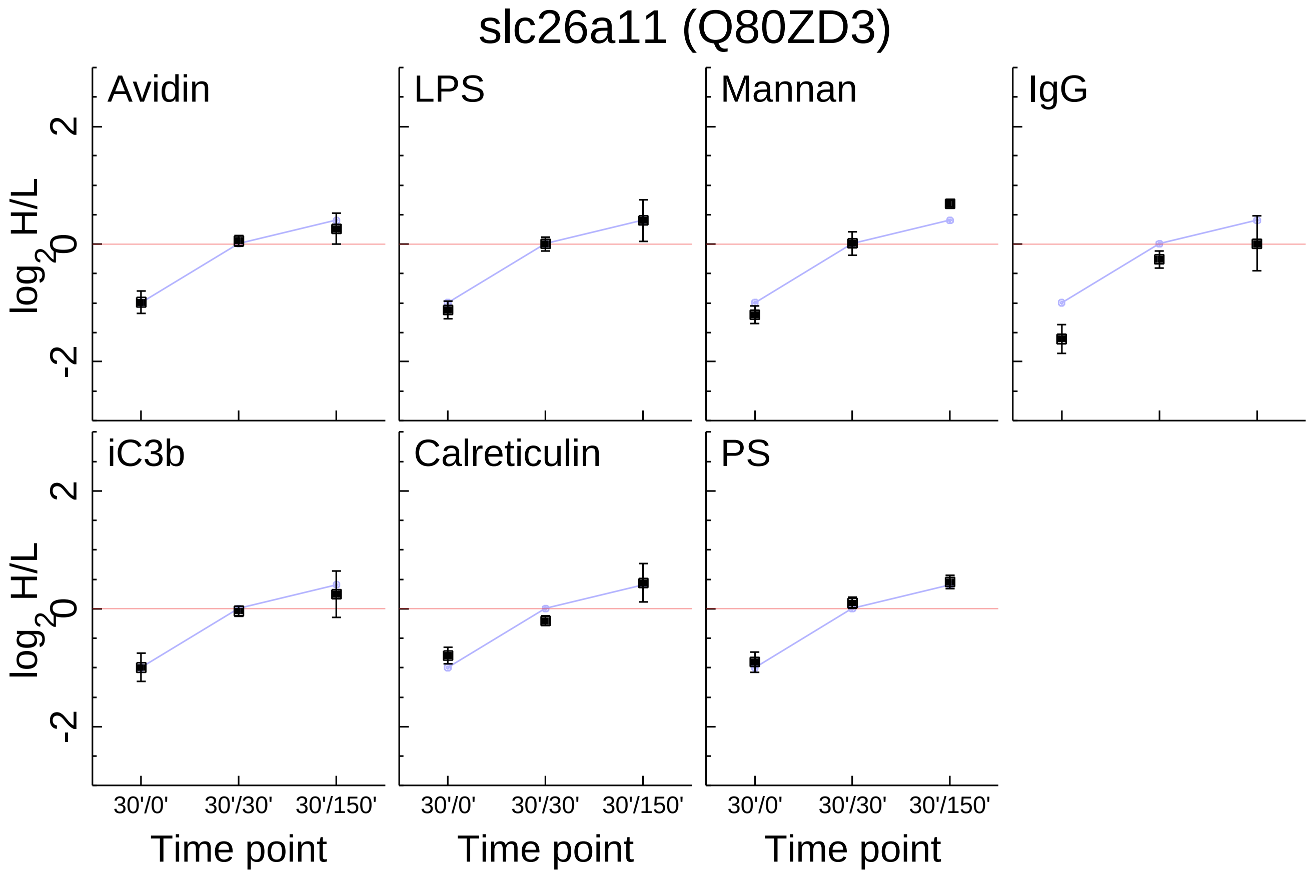

slc26a11 (Q80ZD3)
Avidin
LPS
Mannan
IgG
iC3b
Calreticulin
PS
log2 H/L
2
0
-2
30'/0'
30'/30'
30'/150'
Time point

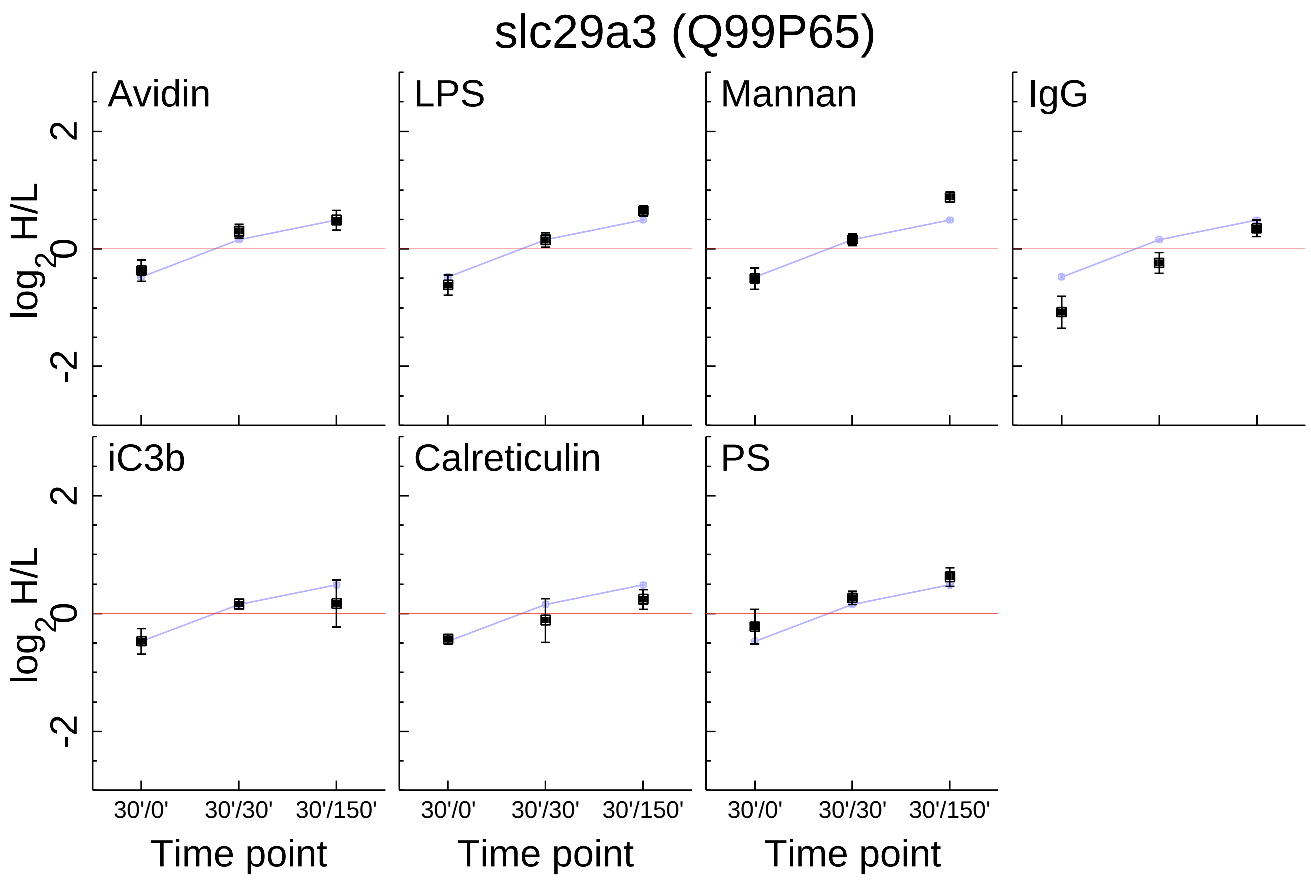

slc29a3 (Q99P65)
Avidin
LPS
Mannan
IgG
iC3b
Calreticulin
PS
log2 H/L
2
0
-2
30'/0'
30'/30'
30'/150'
Time point

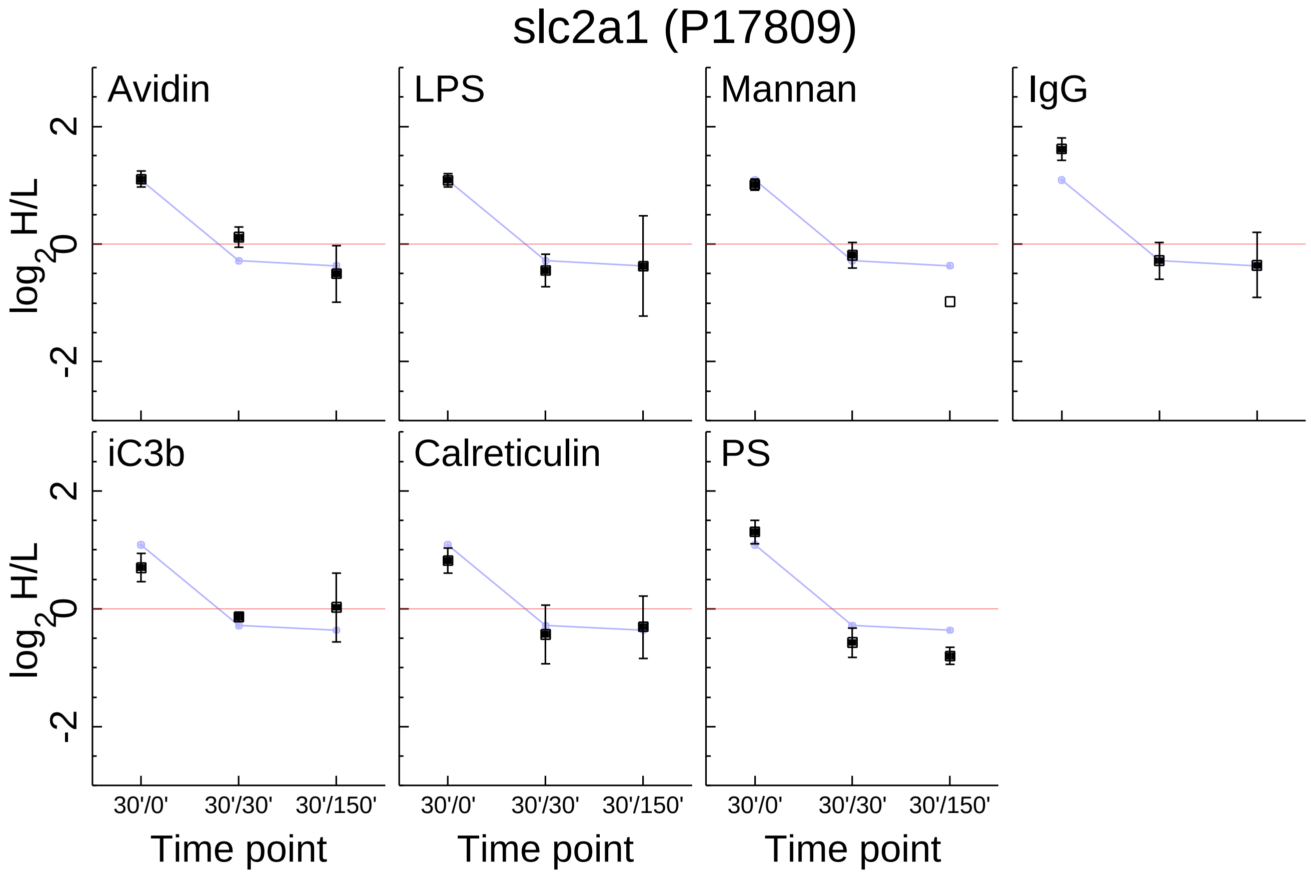
slc2a1 (P17809)
Avidin
LPS
Mannan
IgG
iC3b
Calreticulin
PS
log2 H/L
2
0
-2
30'/0'
30'/30'
30'/150'
Time point

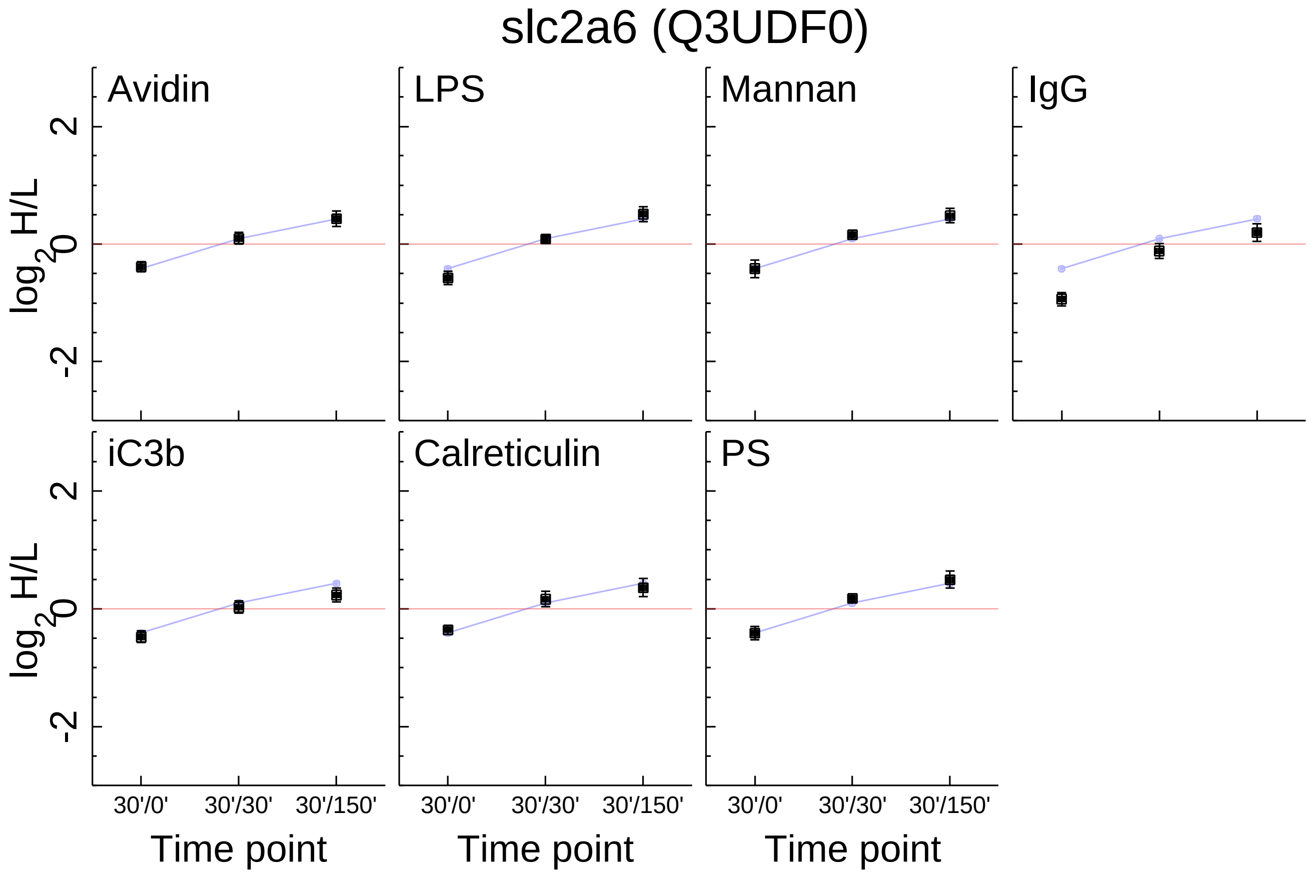
slc2a6 (Q3UDF0)
Avidin
LPS
Mannan
IgG
iC3b
Calreticulin
PS
log2 H/L
2
0
-2
30'/0'
30'/30'
30'/150'
Time point

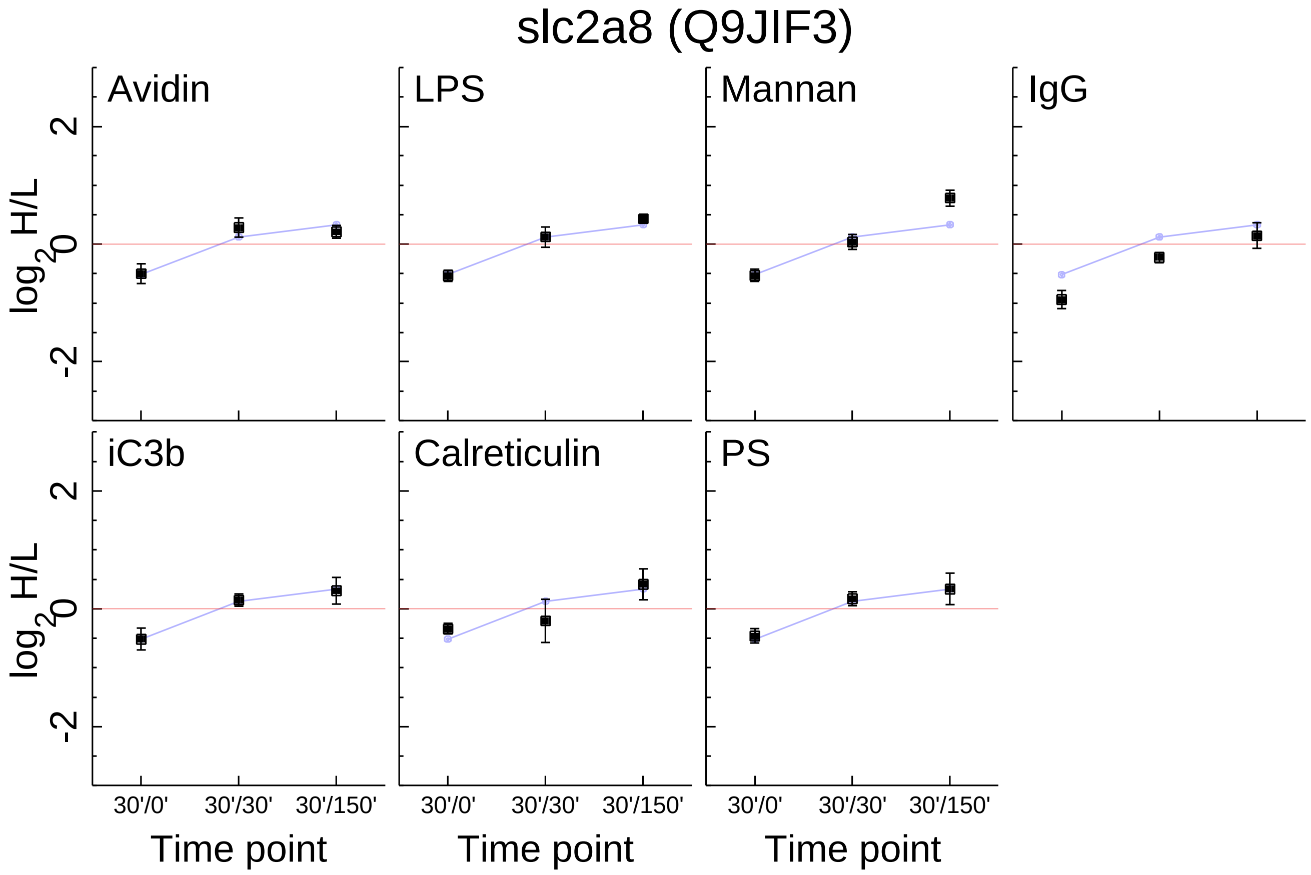
slc2a8 (Q9JIF3)
Avidin
LPS
Mannan
IgG
iC3b
Calreticulin
PS
log2 H/L
2
0
-2
30'/0'
30'/30'
30'/150'
Time point

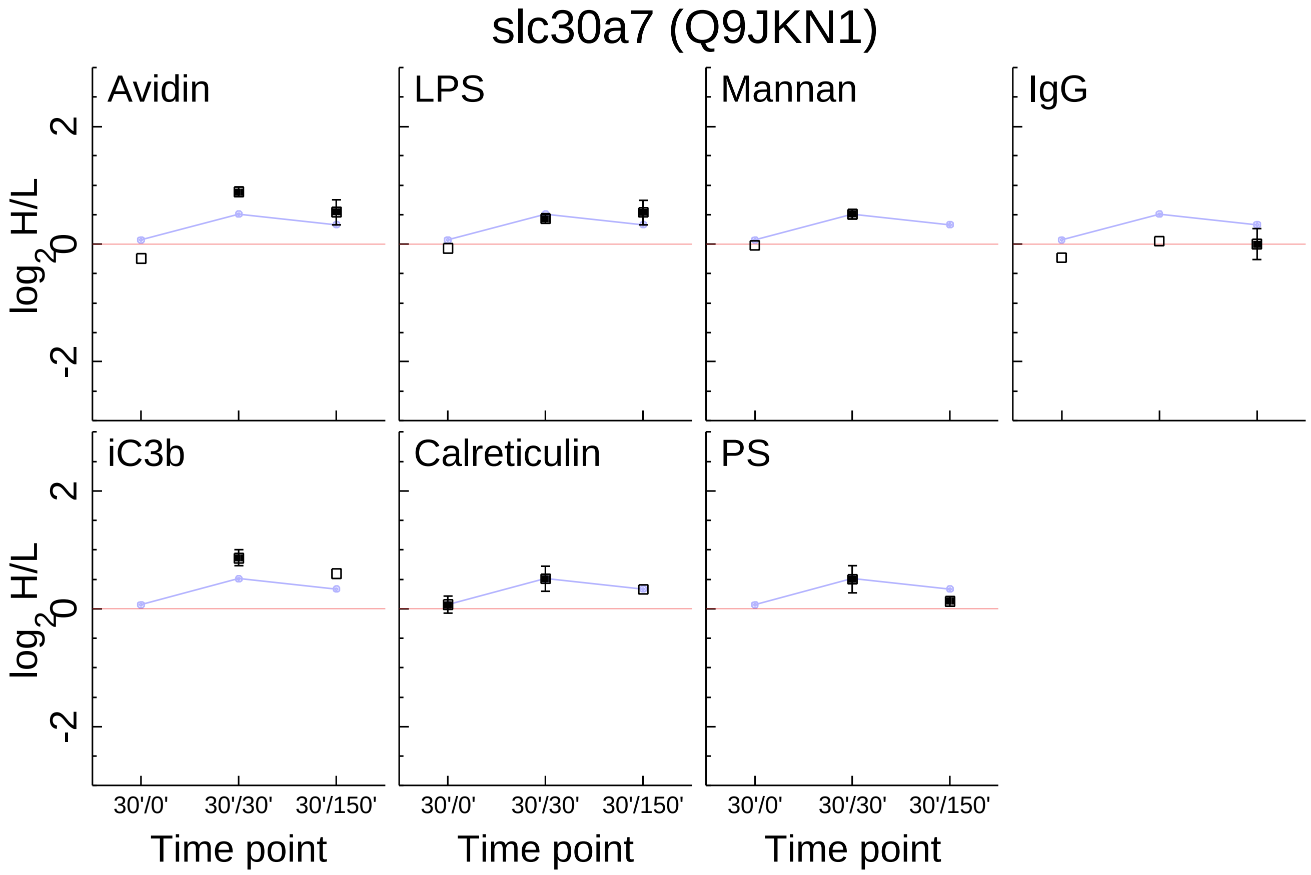
slc30a7 (Q9JKN1)
Avidin
LPS
Mannan
IgG
iC3b
Calreticulin
PS
log2 H/L
2
0
-2
30'/0'
30'/30'
30'/150'
Time point

slc31a1 (Q8K211)

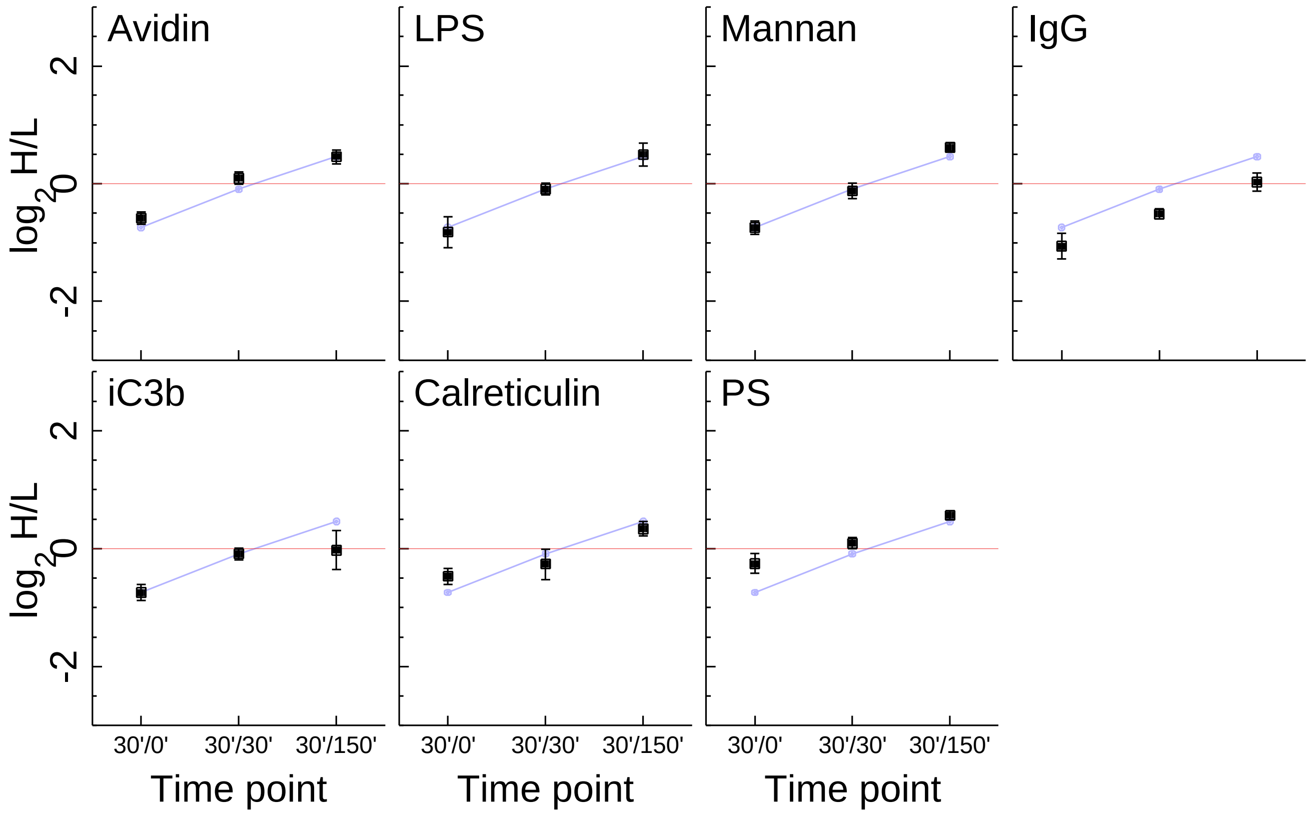

slc31a2 (Q9CPU9)

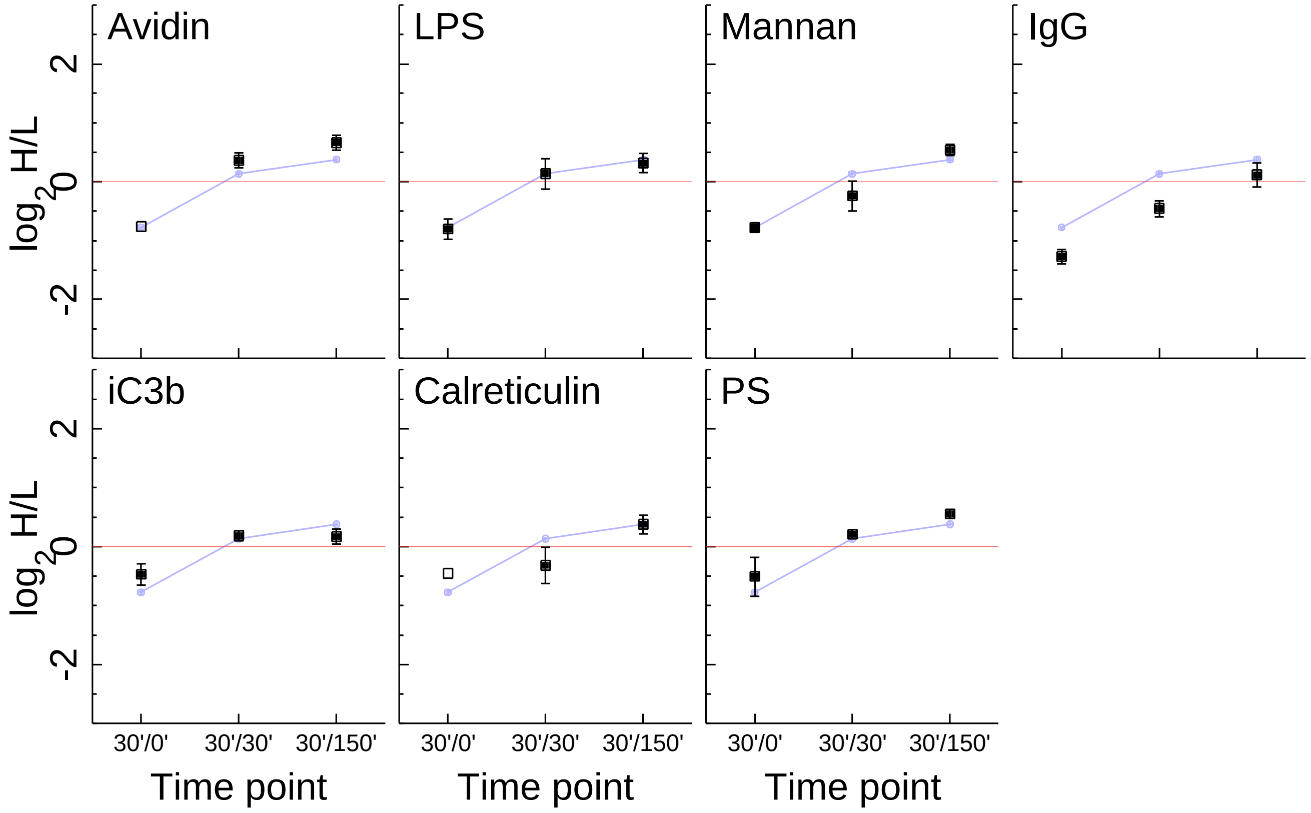

slc36a1 (Q8K4D3)

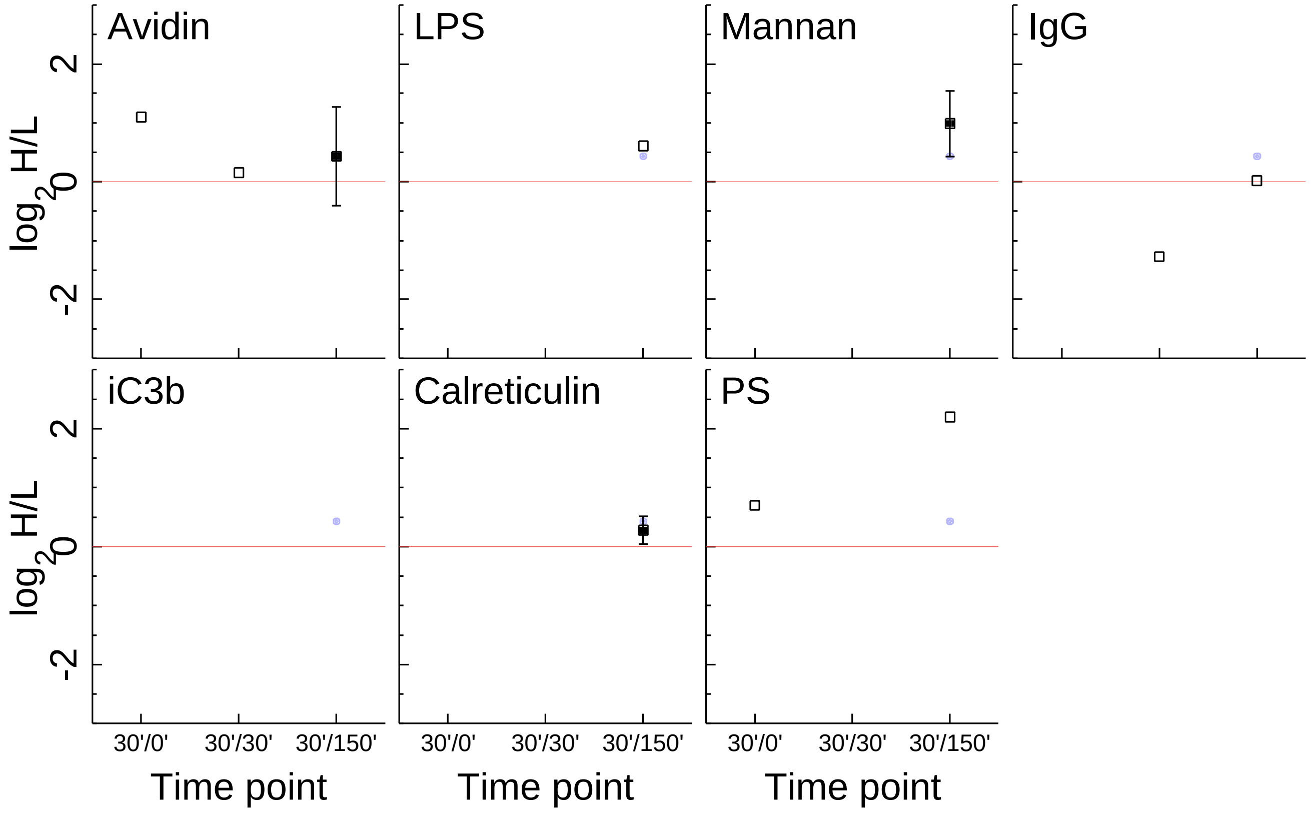

slc37a2 (Q9WU81)

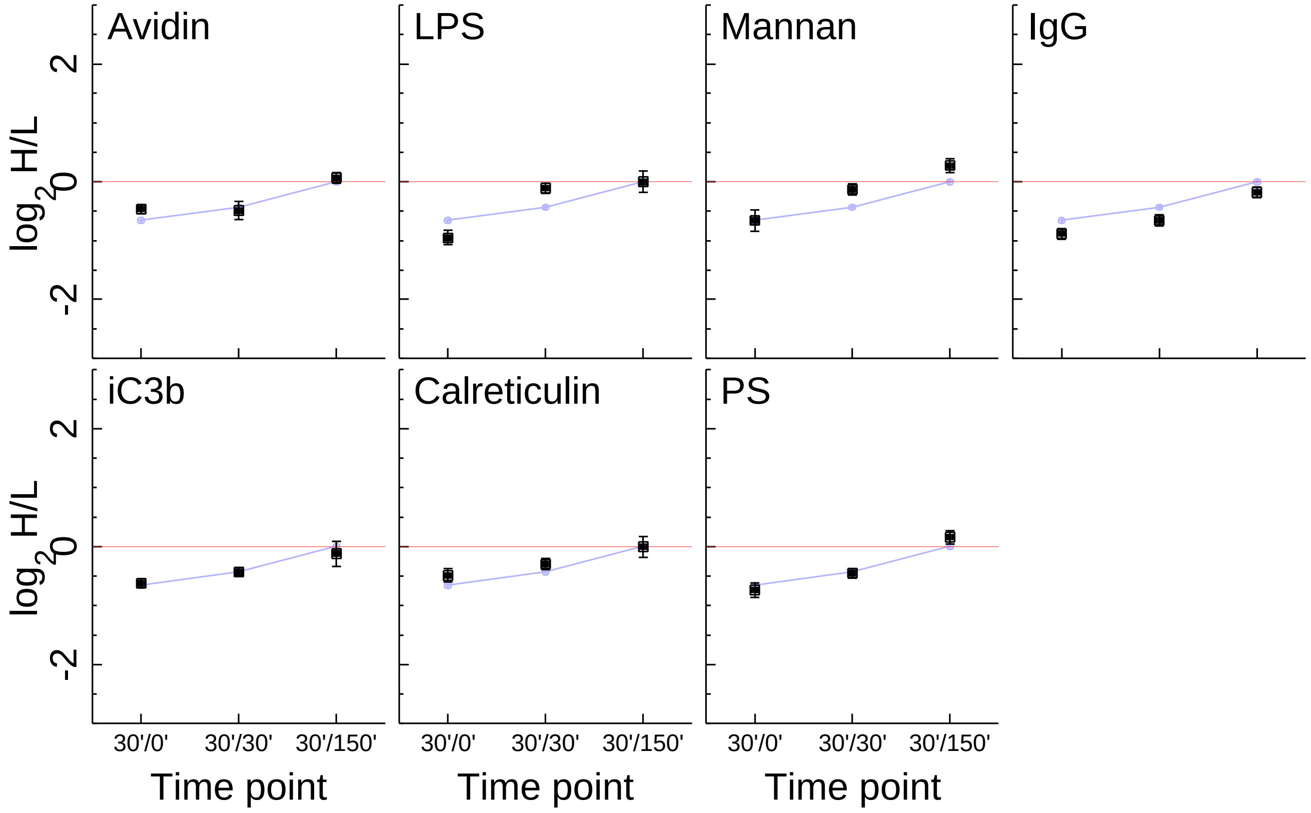

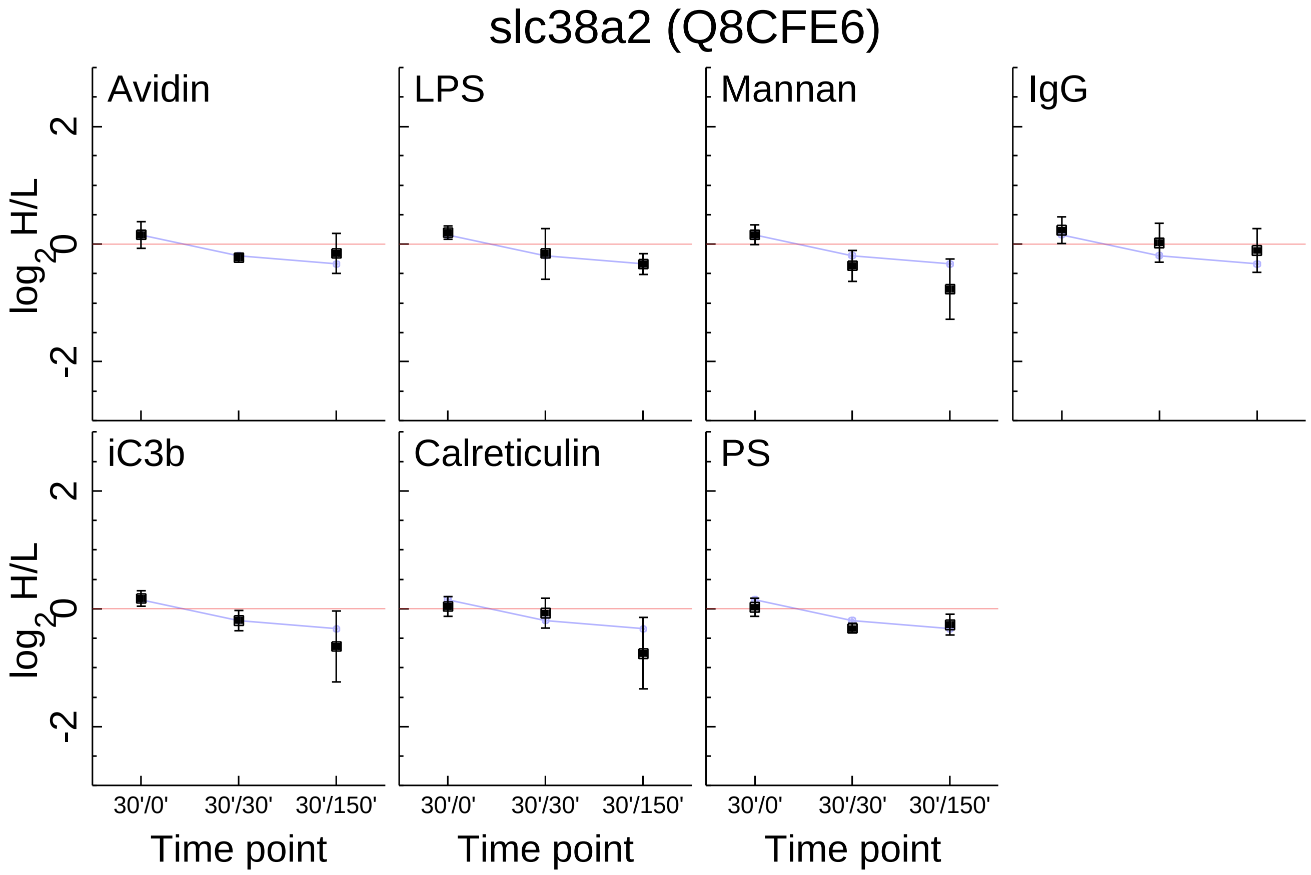

slc38a2 (Q8CFE6)
Avidin
LPS
Mannan
IgG
iC3b
Calreticulin
PS
log2 H/L
2
0
-2
30'/0'
30'/30'
30'/150'
Time point

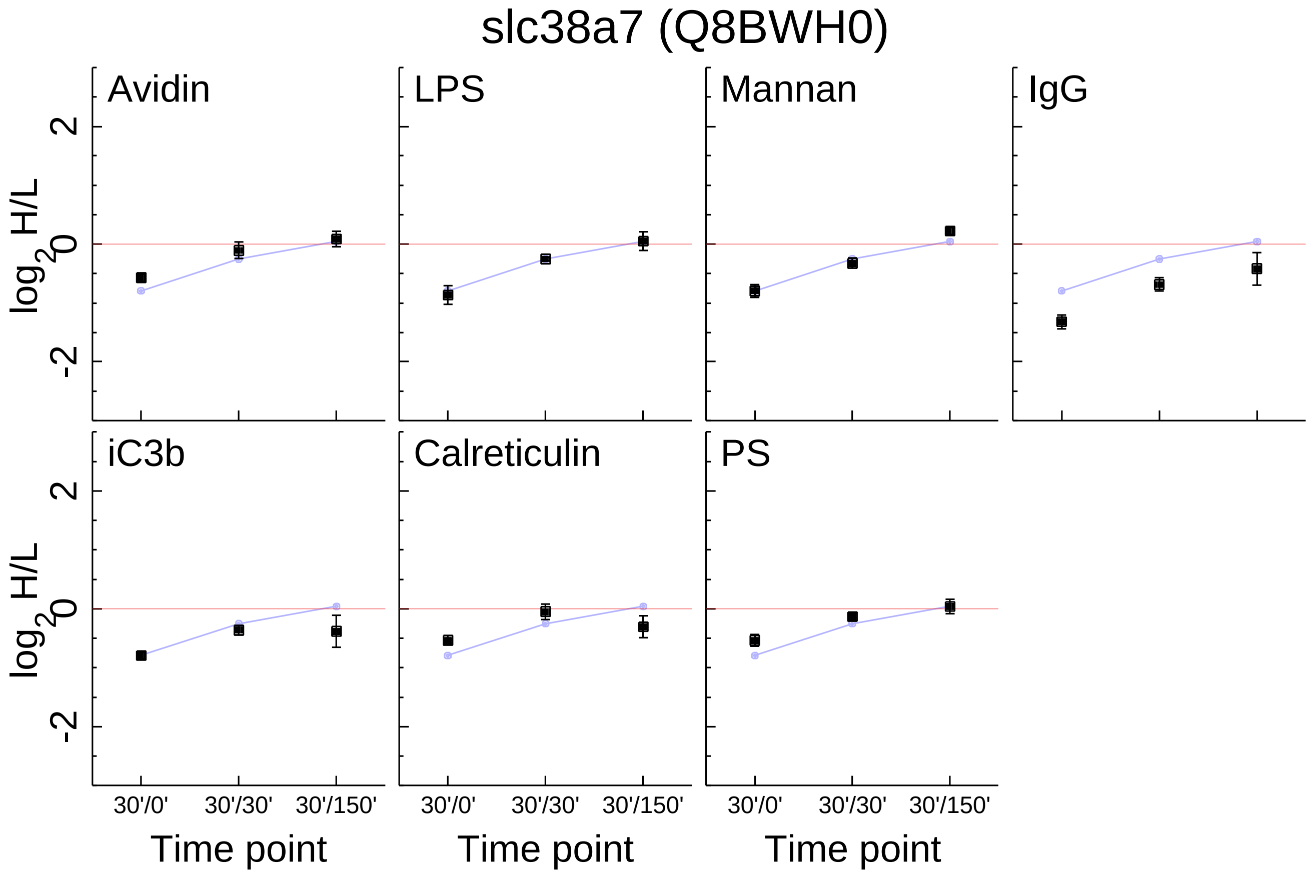
slc38a7 (Q8BWH0)
Avidin
LPS
Mannan
IgG
iC3b
Calreticulin
PS
log2 H/L
2
0
-2
30'/0'
30'/30'
30'/150'
Time point

slc38a9 (Q8BGD6)

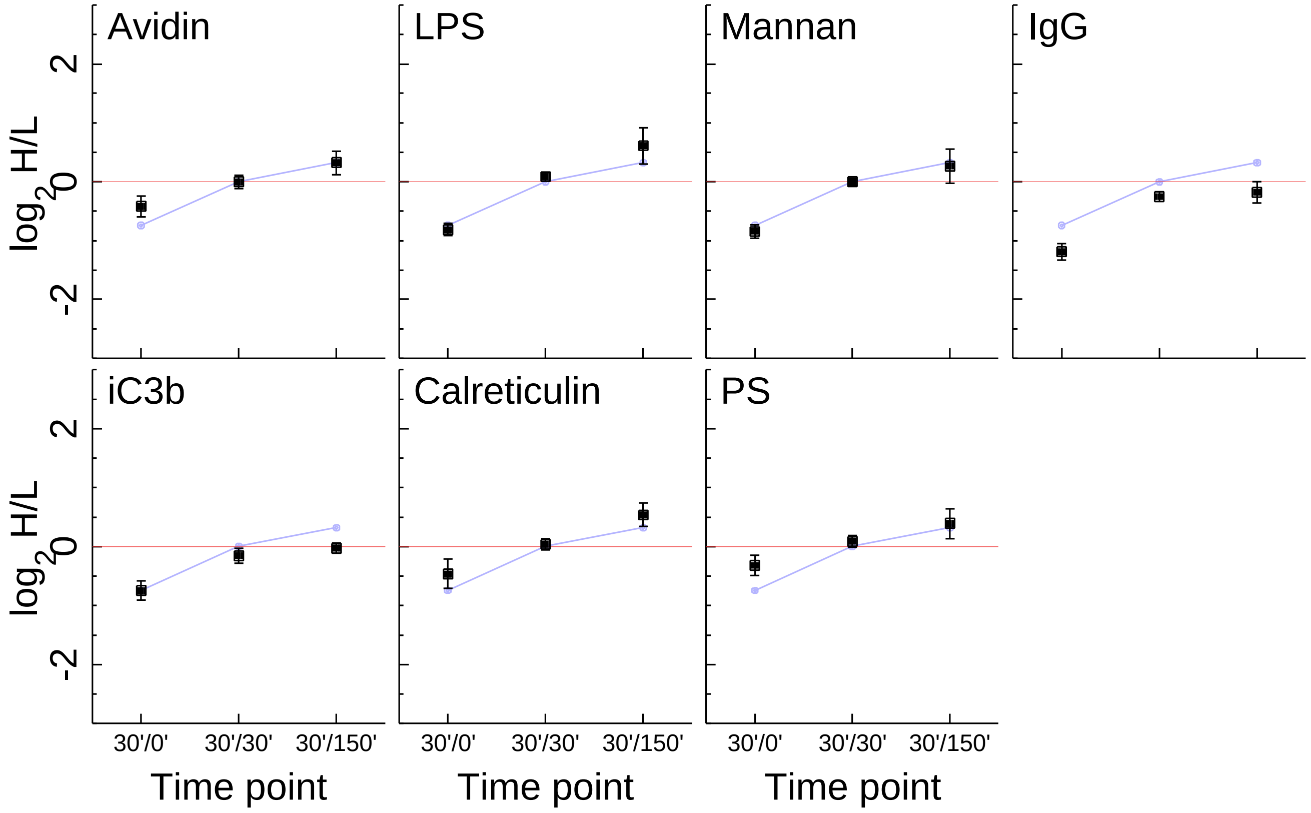

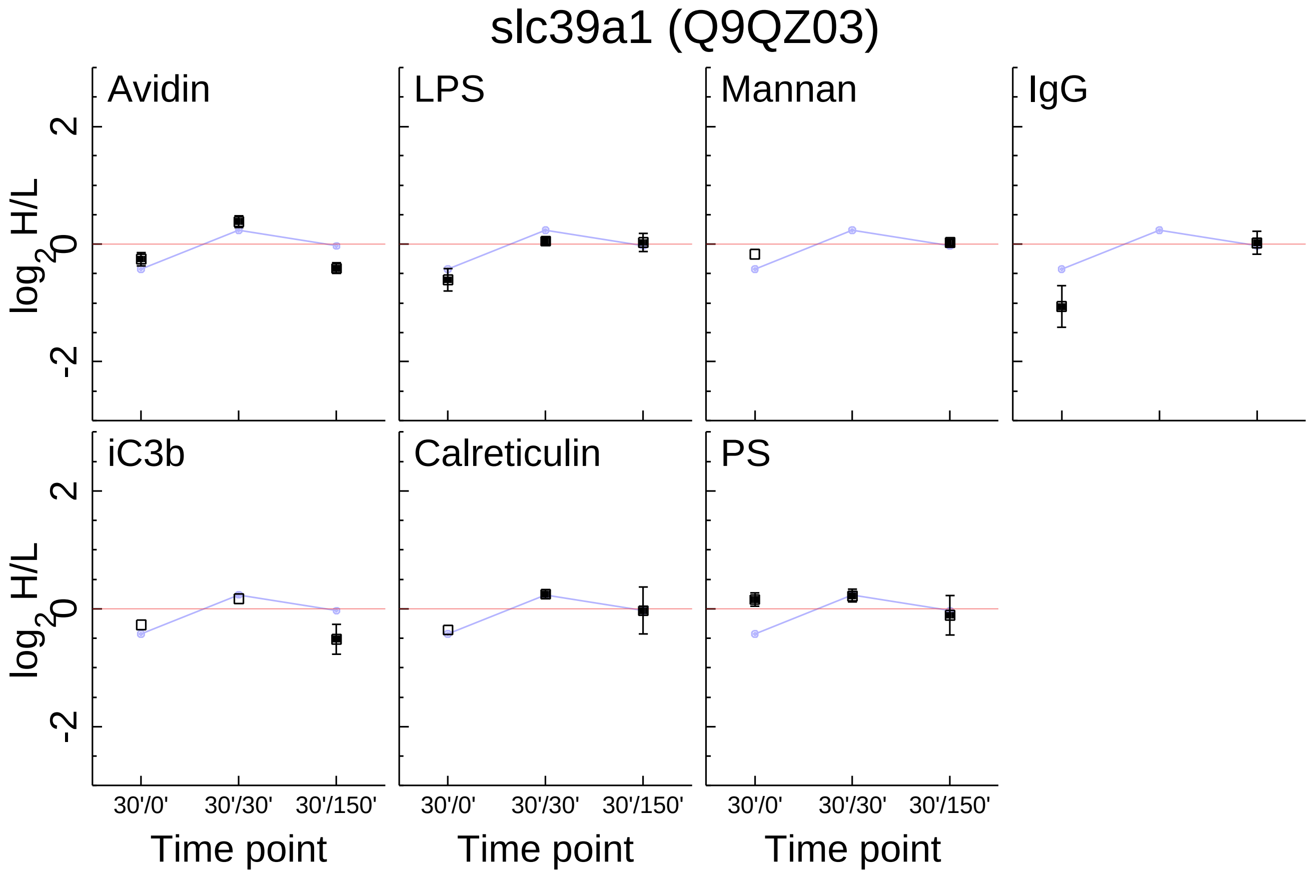

slc39a1 (Q9QZ03)
Avidin
LPS
Mannan
IgG
iC3b
Calreticulin
PS
log2 H/L
2
0
-2
30'/0'
30'/30'
30'/150'
Time point

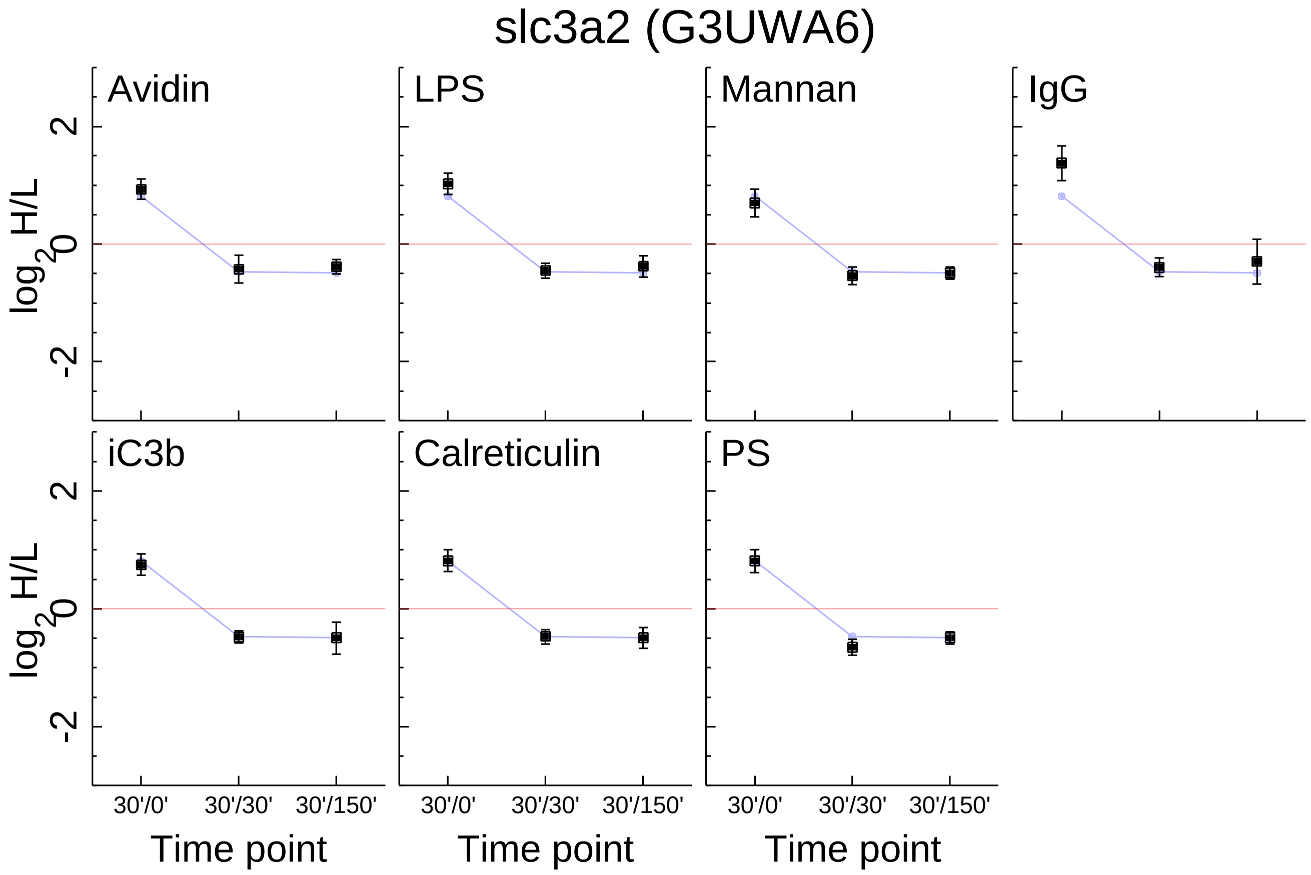
slc3a2 (G3UWA6)
Avidin
LPS
Mannan
IgG
iC3b
Calreticulin
PS
log2 H/L
2
0
-2
30'/0'
30'/30'
30'/150'
Time point

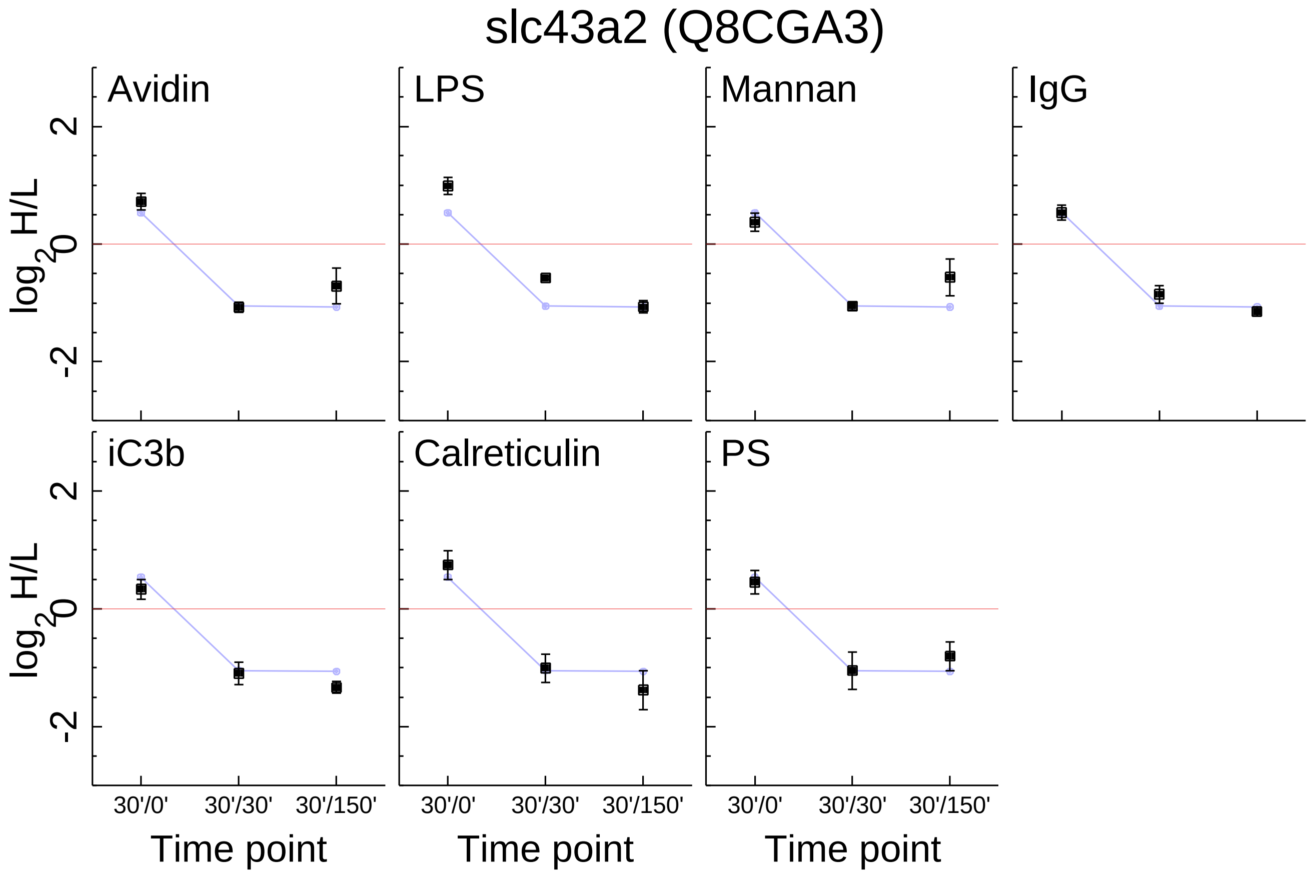
slc43a2 (Q8CGA3)
Avidin
LPS
Mannan
IgG
iC3b
Calreticulin
PS
log2 H/L
2
0
-2
30'/0'
30'/30'
30'/150'
Time point

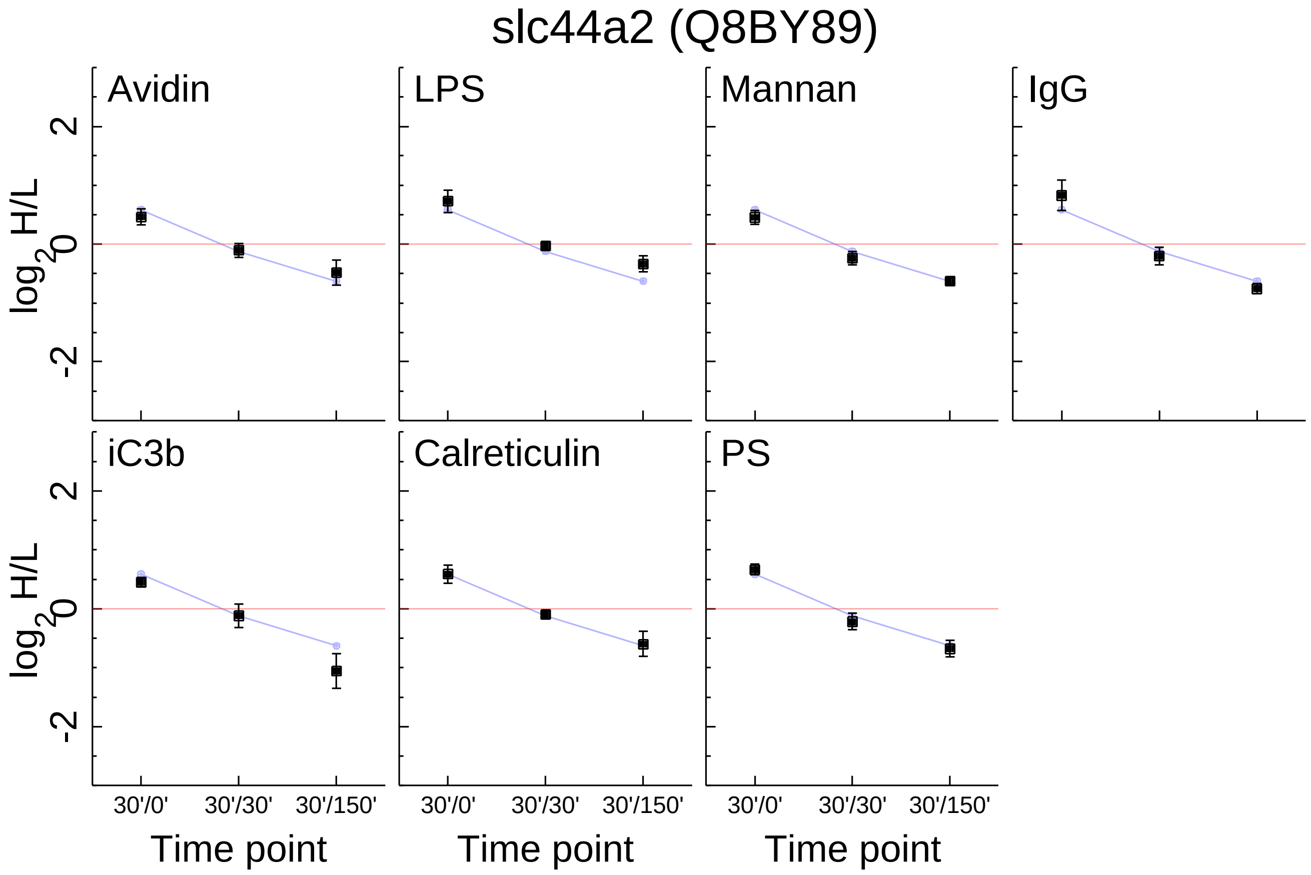
slc44a2 (Q8BY89)
Avidin
LPS
Mannan
IgG
iC3b
Calreticulin
PS
log2 H/L
2
0
-2
30'/0'
30'/30'
30'/150'
Time point

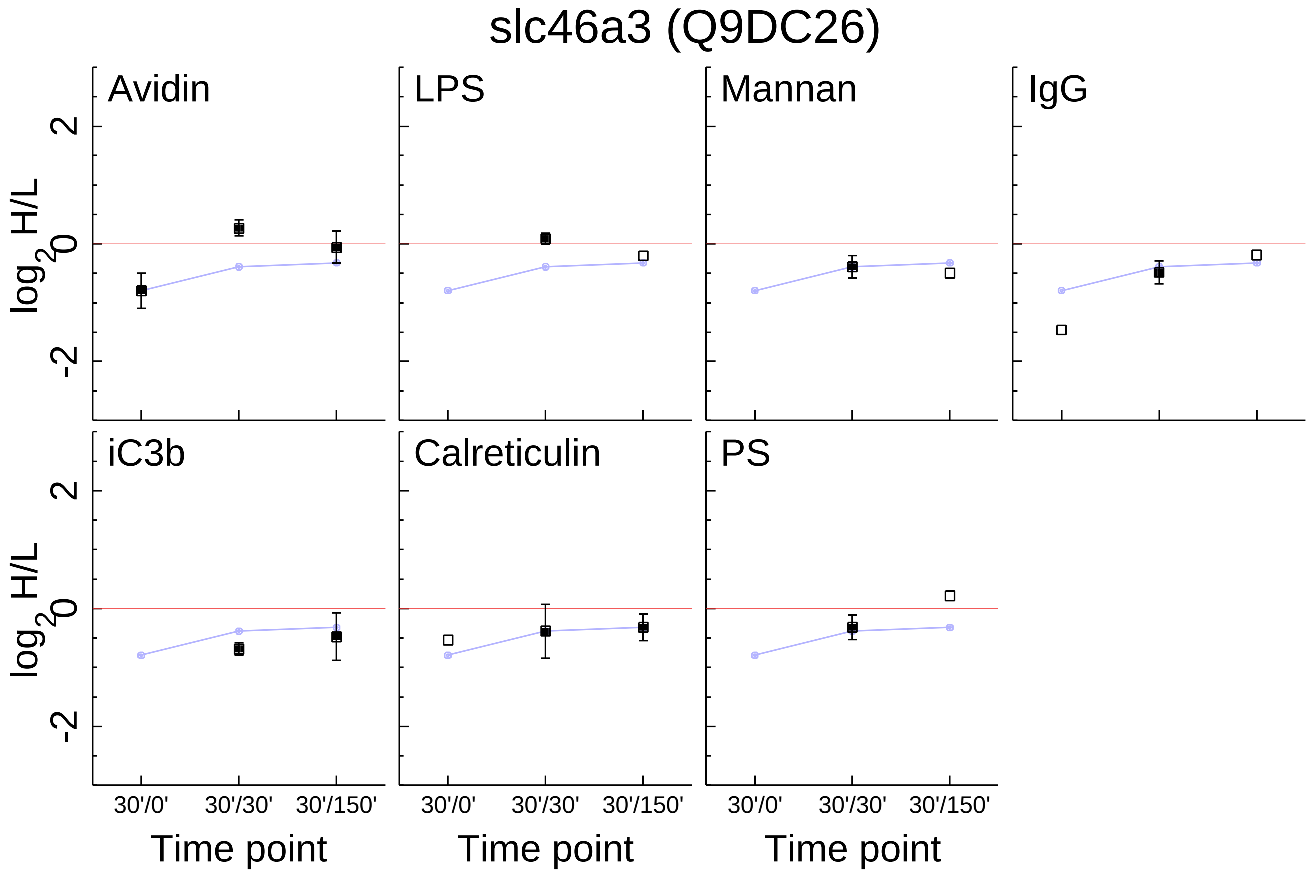

slc46a3 (Q9DC26)
Avidin
LPS
Mannan
IgG
iC3b
Calreticulin
PS
log2 H/L
2
0
-2
30'/0'
30'/30'
30'/150'
Time point

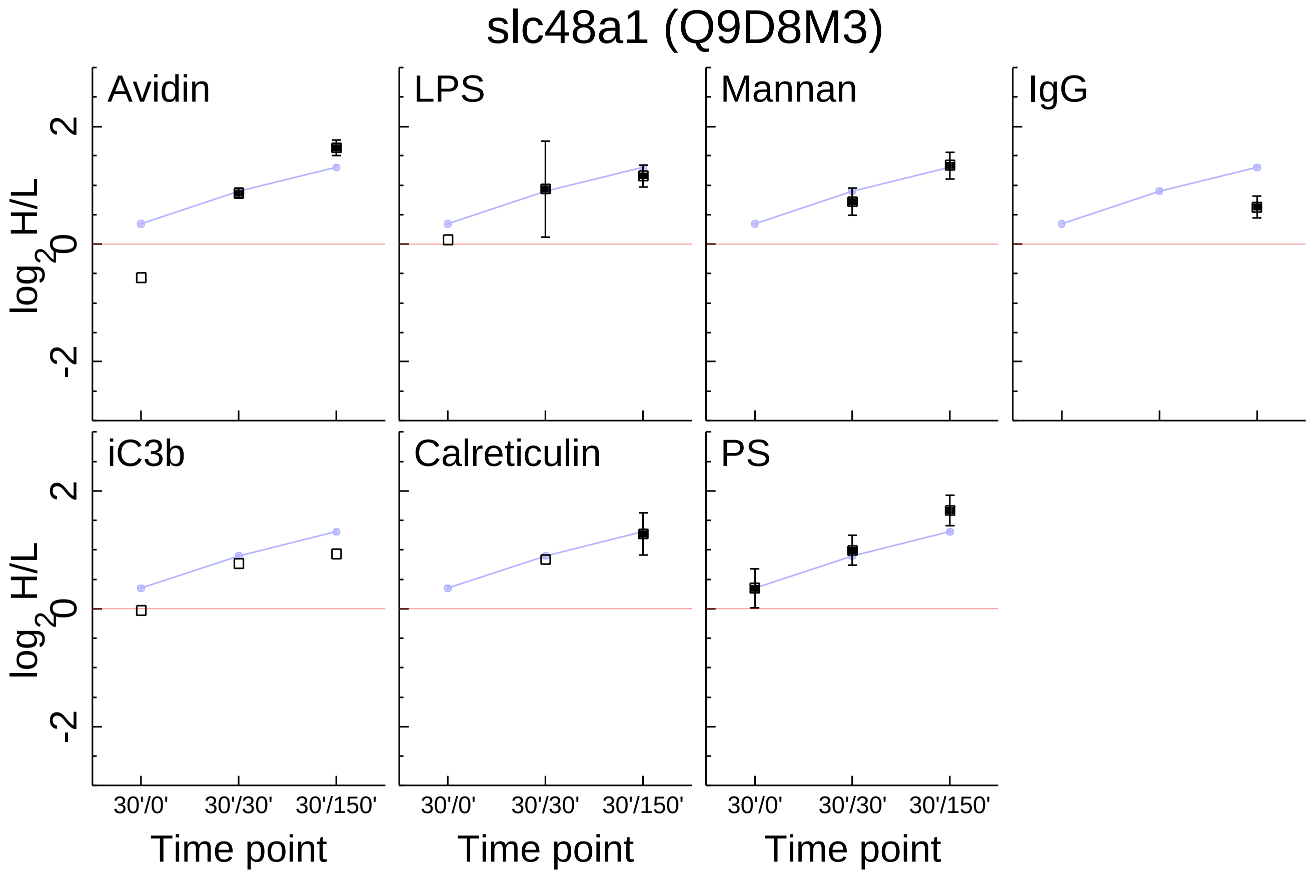
slc48a1 (Q9D8M3)
Avidin
LPS
Mannan
IgG
iC3b
Calreticulin
PS
log2 H/L
2
0
-2
30'/0'
30'/30'
30'/150'
Time point

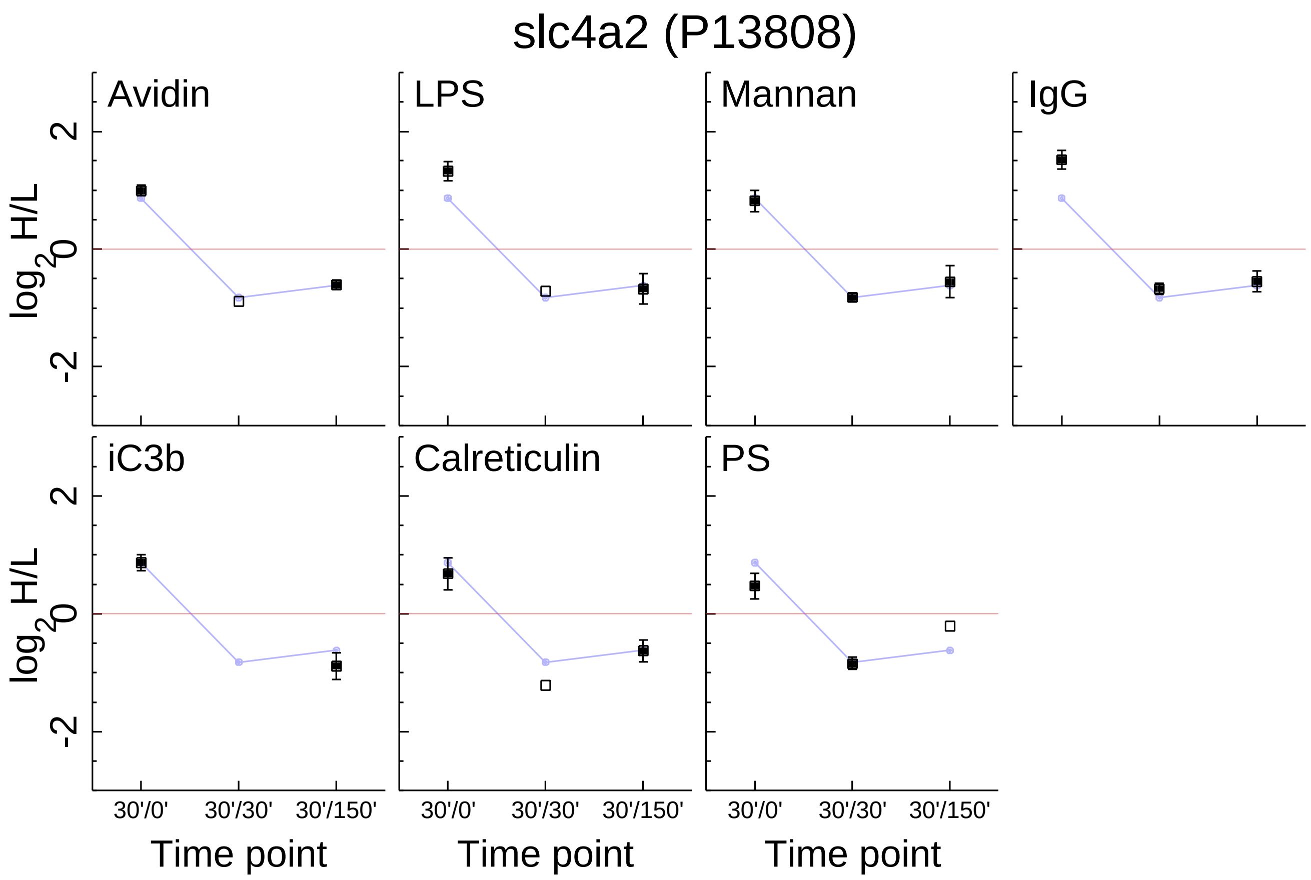
slc4a2 (P13808)
Avidin
LPS
Mannan
IgG
iC3b
Calreticulin
PS
log2 H/L
2
0
-2
30'/0'
30'/30'
30'/150'
Time point

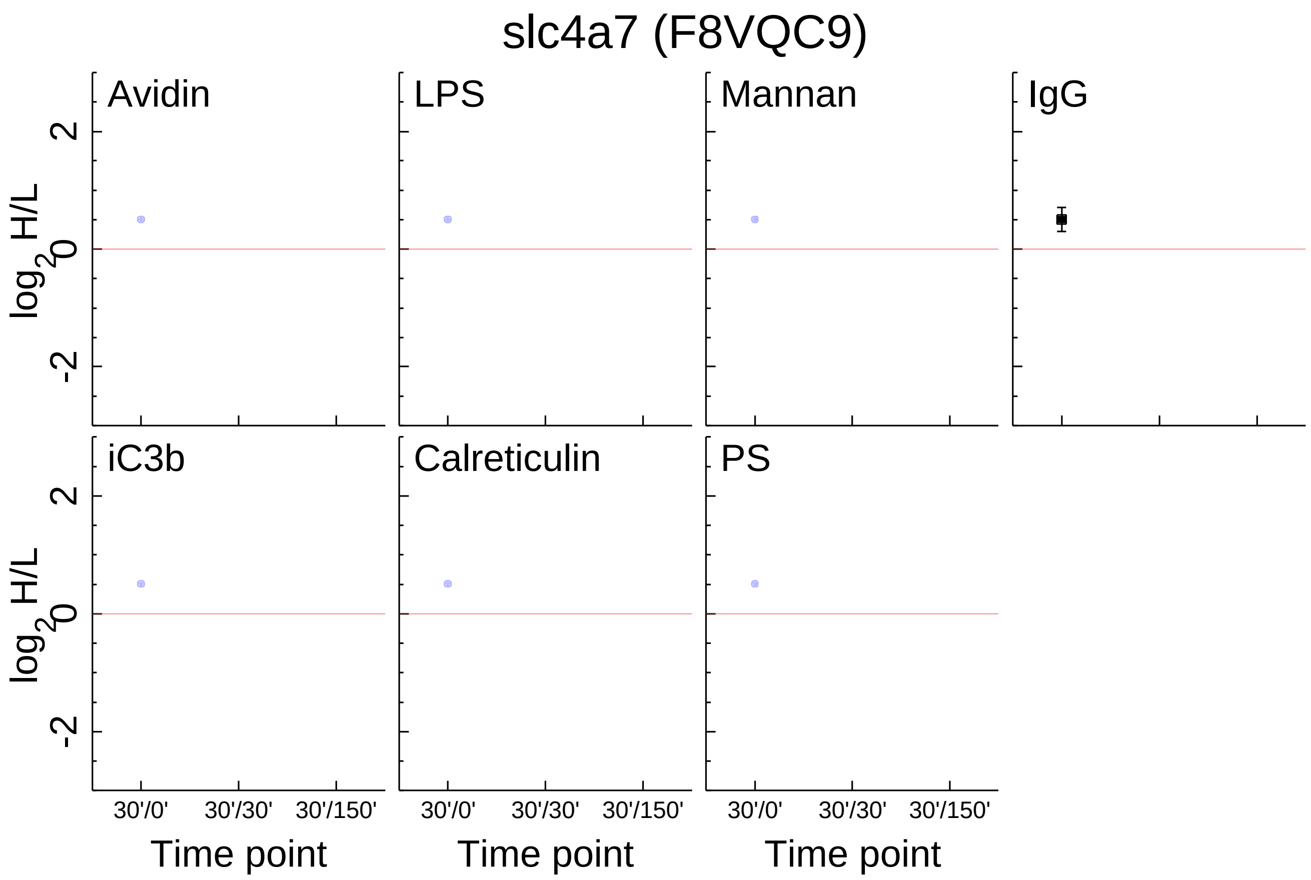

slc4a7 (F8VQC9)
Avidin
LPS
Mannan
IgG
iC3b
Calreticulin
PS
log2 H/L
2
0
-2
30'/0'
30'/30'
30'/150'
Time point

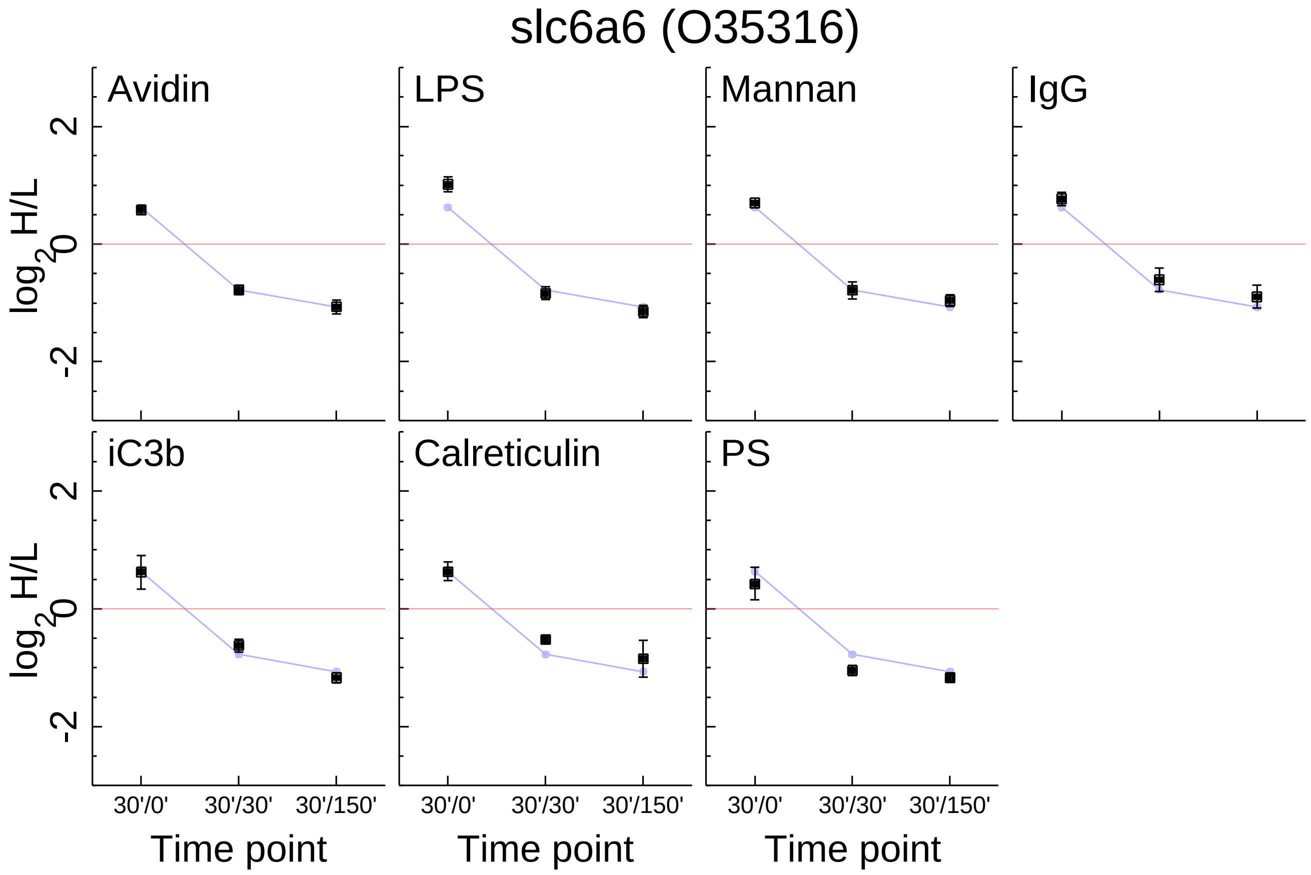
slc6a6 (O35316)
Avidin
LPS
Mannan
IgG
iC3b
Calreticulin
PS
log2 H/L
2
0
-2
30'/0'
30'/30'
30'/150'
Time point

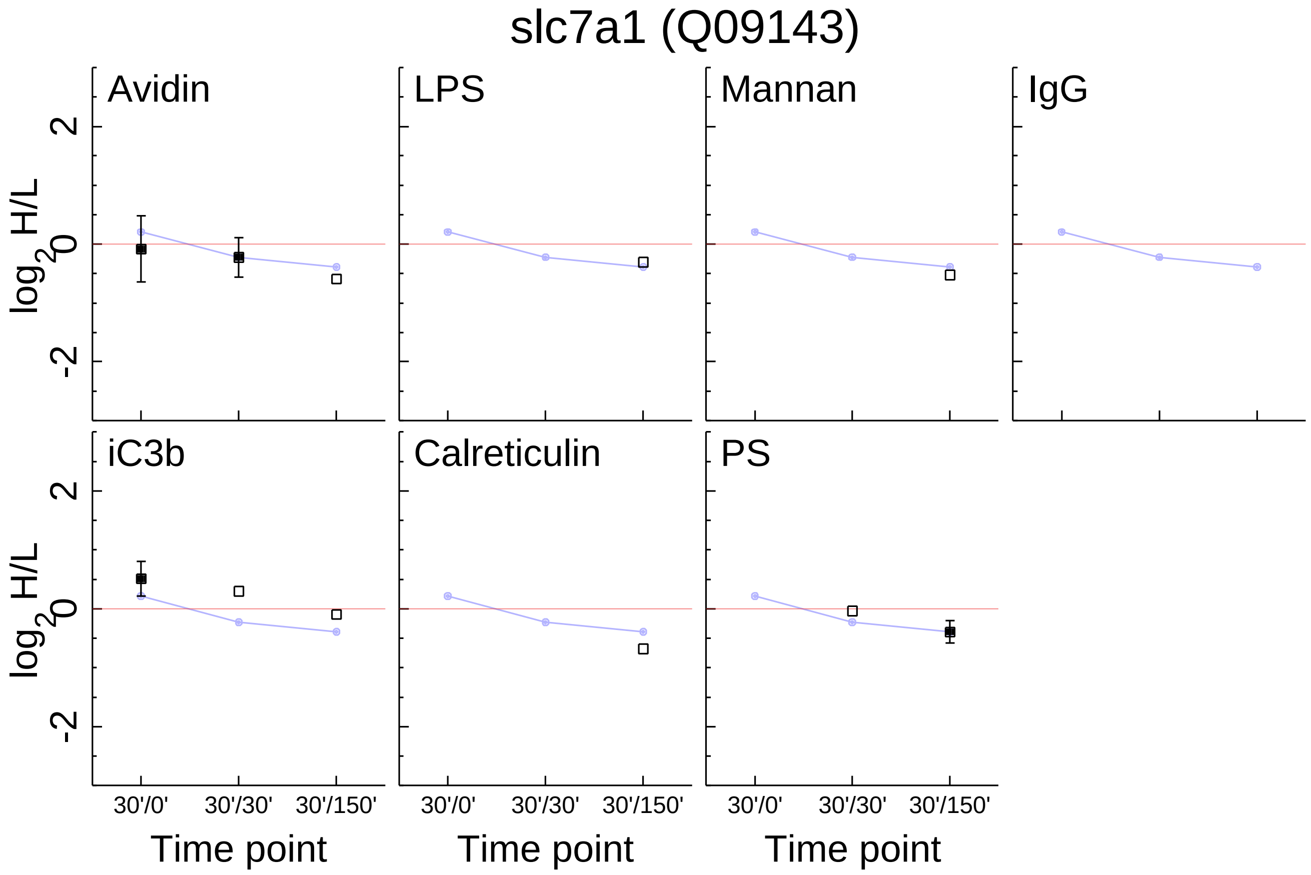
slc7a1 (Q09143)
Avidin
LPS
Mannan
IgG
iC3b
Calreticulin
PS
log2 H/L
2
0
-2
30'/0'
30'/30'
30'/150'
Time point

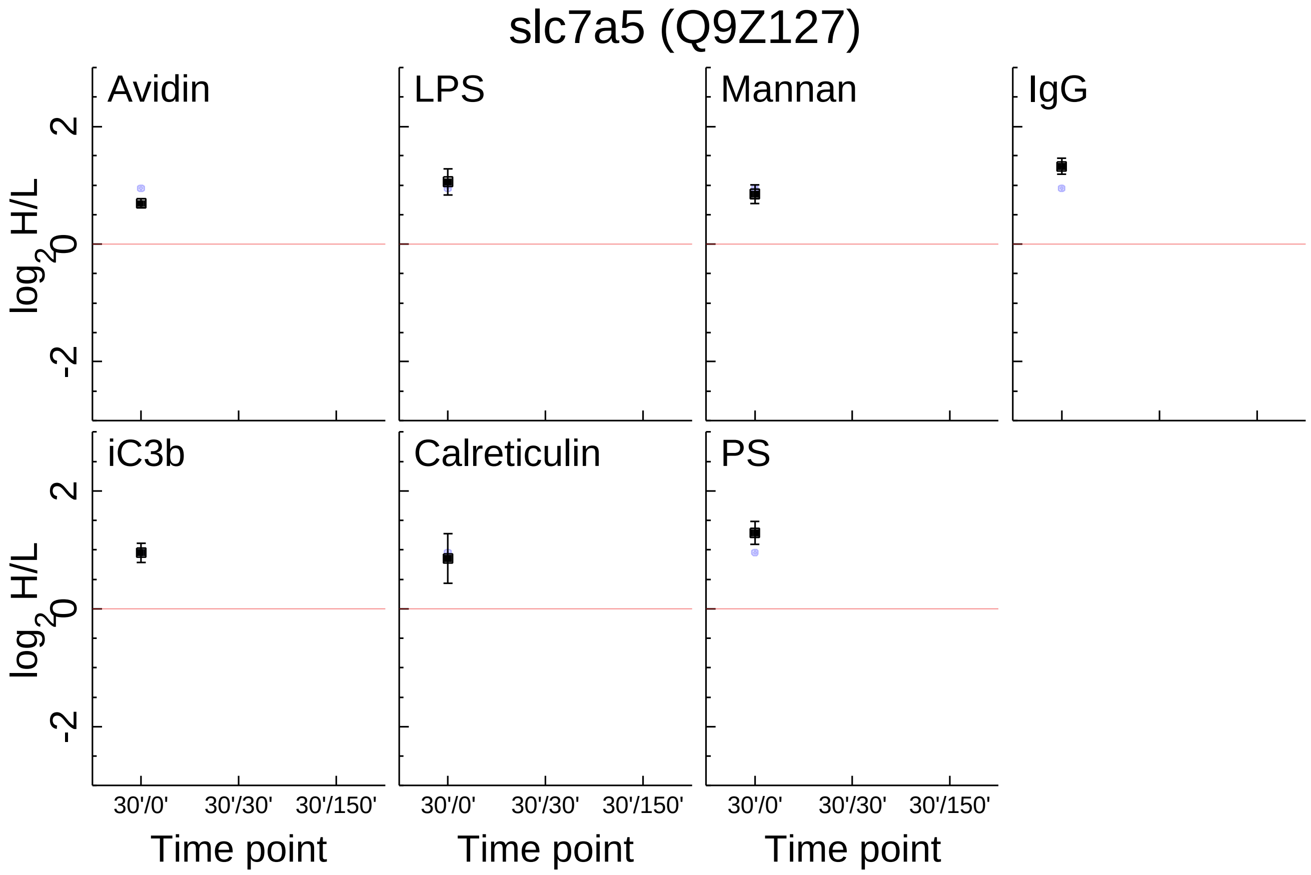
slc7a5 (Q9Z127)
Avidin
LPS
Mannan
IgG
iC3b
Calreticulin
PS
2
0
-2
log2 H/L
30'/0'
30'/30'
30'/150'
Time point

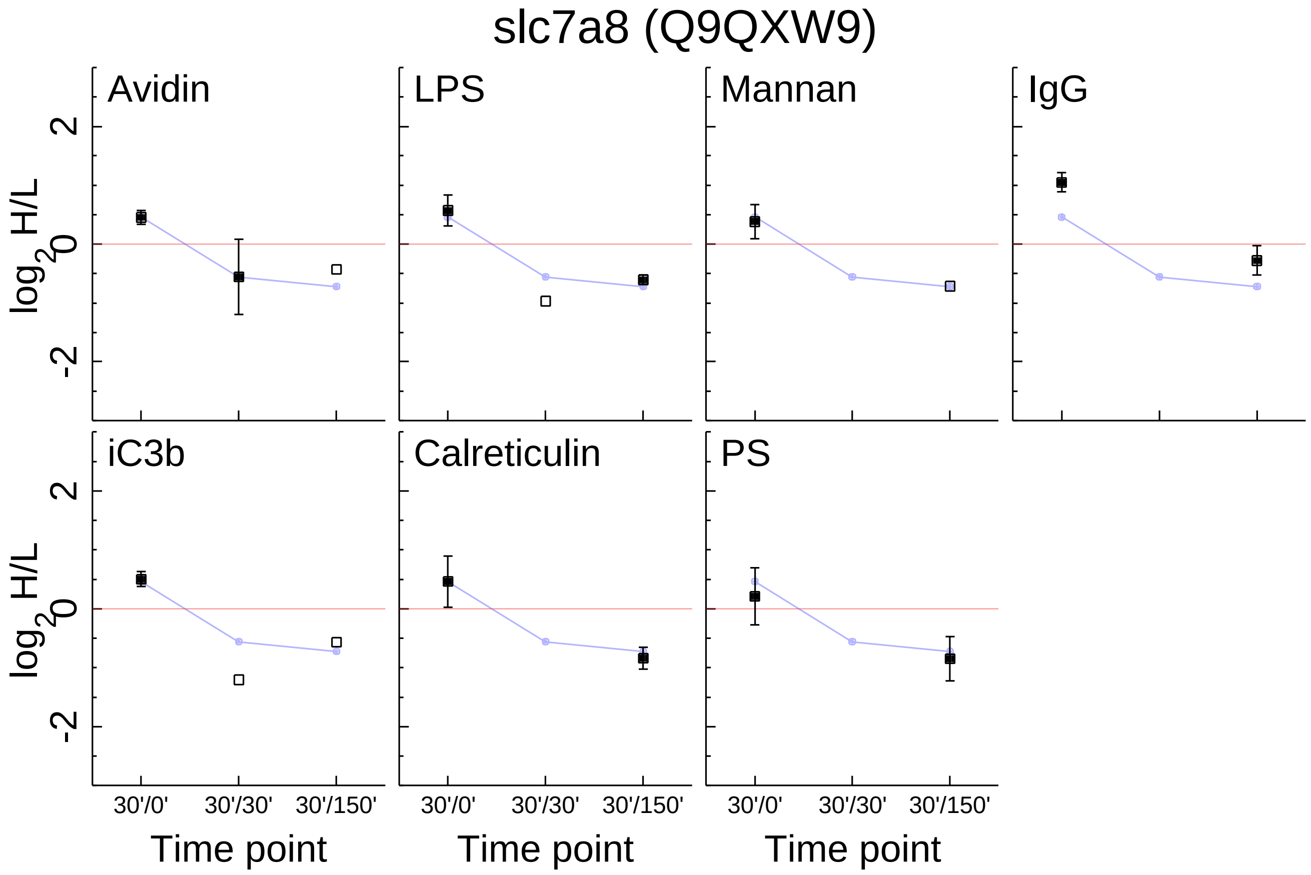

slc7a8 (Q9QXW9)
Avidin
LPS
Mannan
IgG
iC3b
Calreticulin
PS
log2 H/L
2
0
-2
30'/0'
30'/30'
30'/150'
Time point

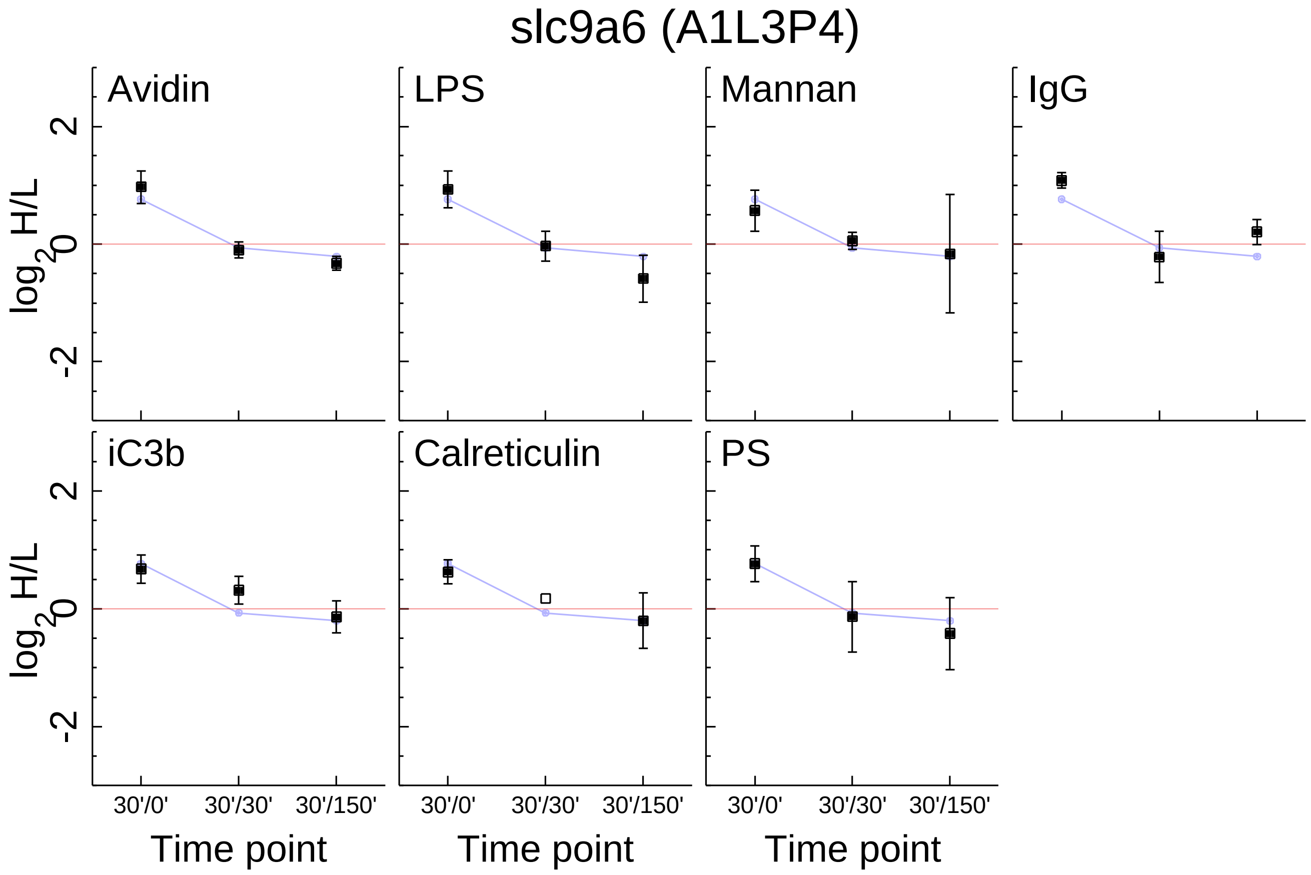

slc9a6 (A1L3P4)
Avidin
LPS
Mannan
IgG
iC3b
Calreticulin
PS
log2 H/L
2
0
-2
30'/0'
30'/30'
30'/150'
Time point

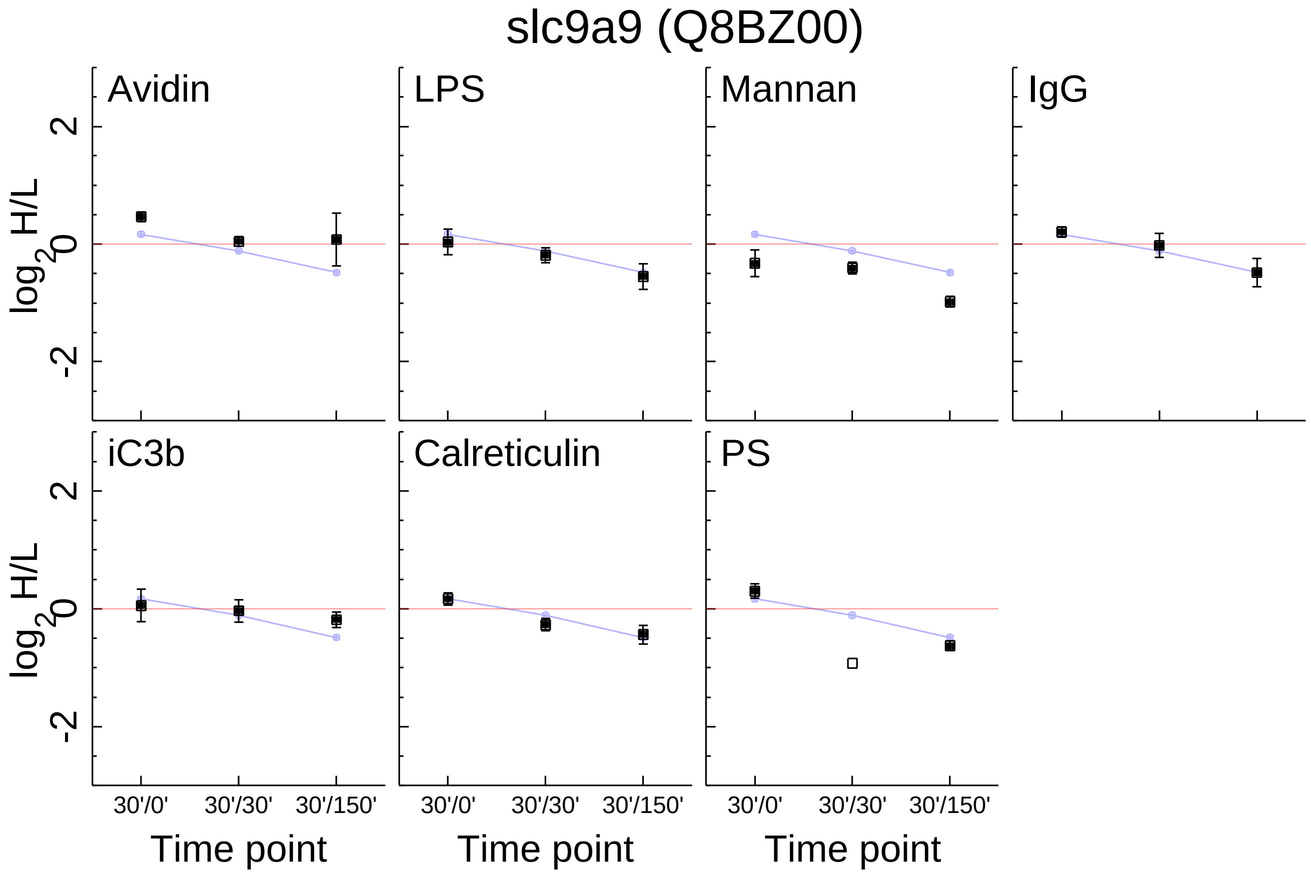

slc9a9 (Q8BZ00)
Avidin
LPS
Mannan
IgG
iC3b
Calreticulin
PS
log2 H/L
2
0
-2
30'/0'
30'/30'
30'/150'
Time point

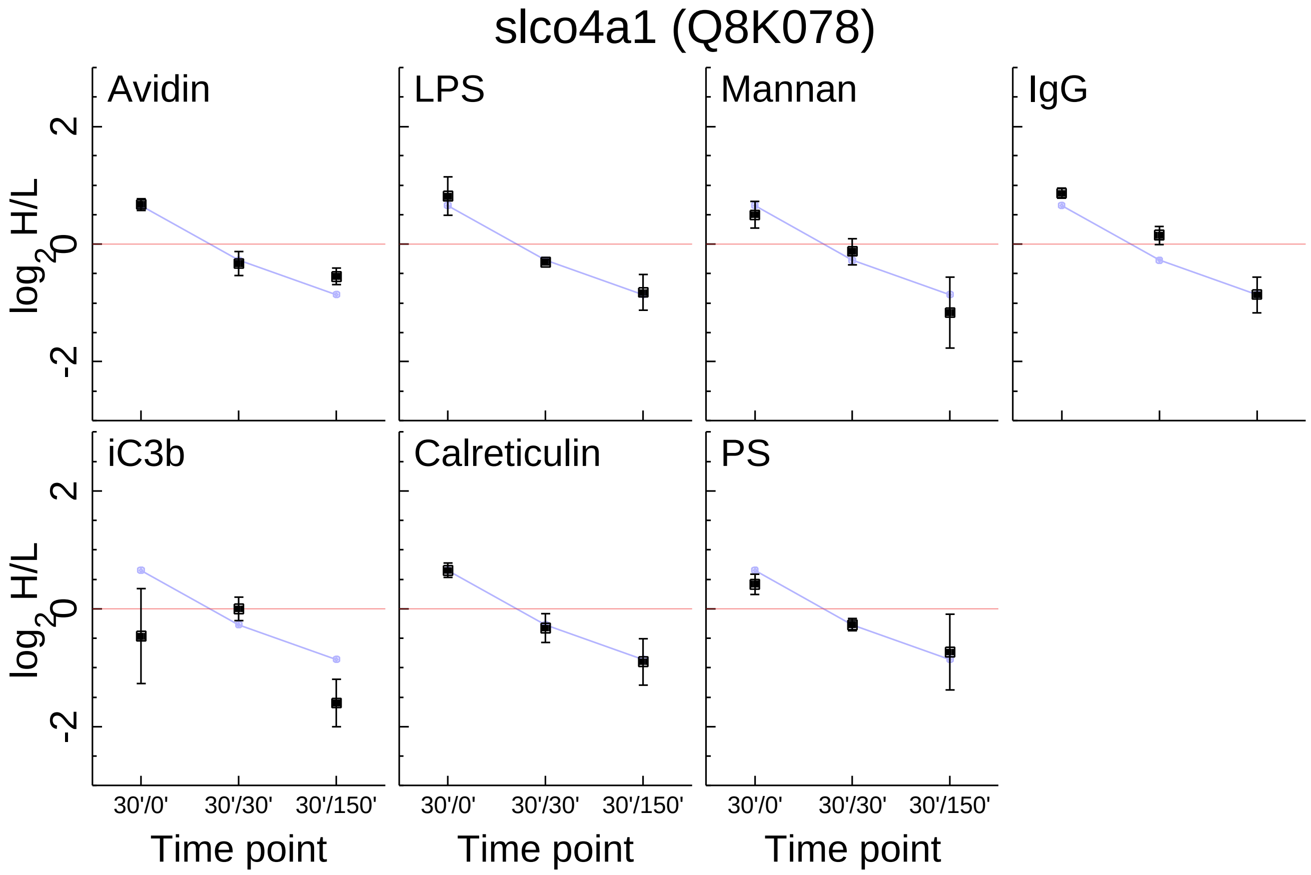

slco4a1 (Q8K078)
Avidin
LPS
Mannan
IgG
iC3b
Calreticulin
PS
$\log_2$ H/L
2
0
-2
30'/0'
30'/30'
30'/150'
Time point

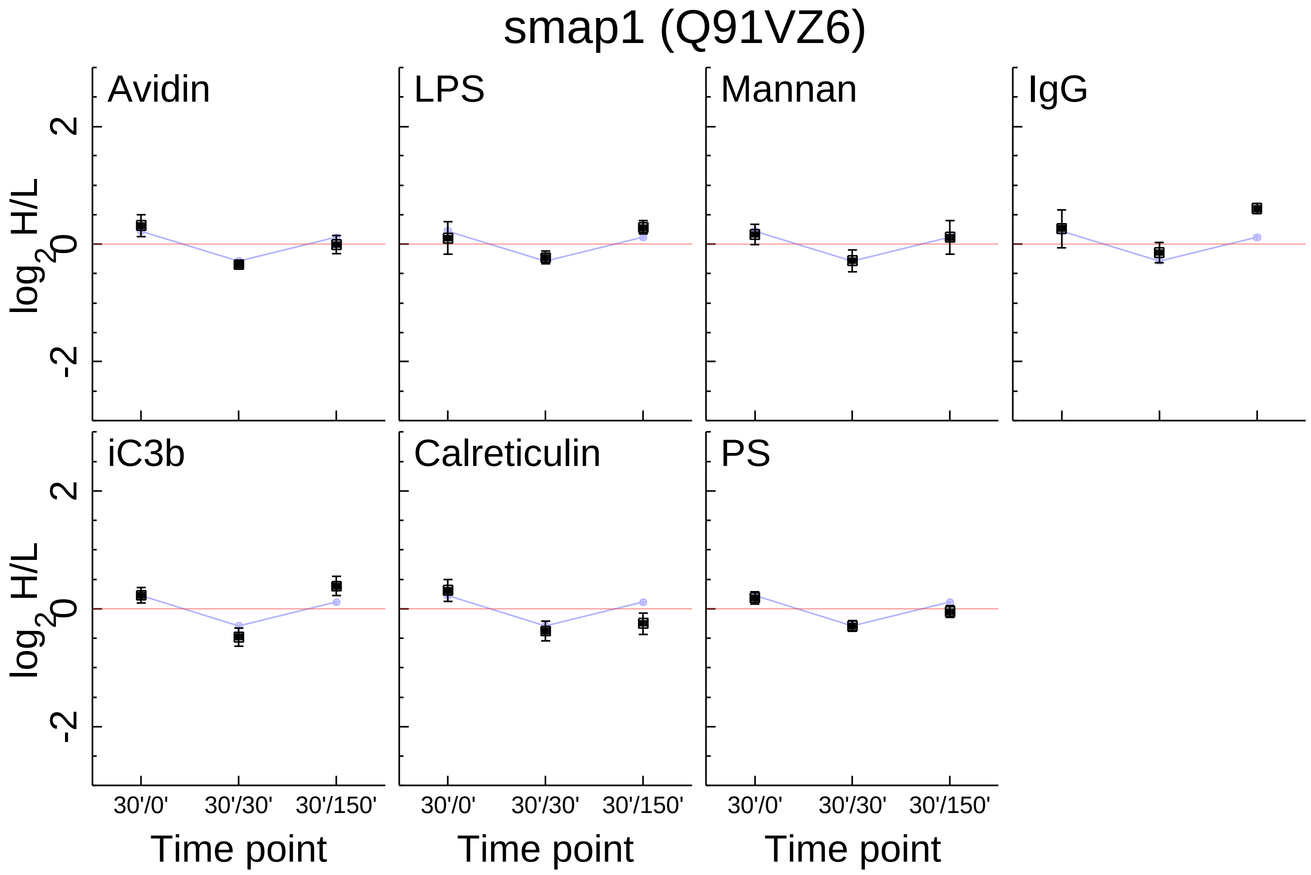
smap1 (Q91VZ6)
Avidin
LPS
Mannan
IgG
iC3b
Calreticulin
PS
log2 H/L
2
0
-2
30'/0'
30'/30'
30'/150'
Time point

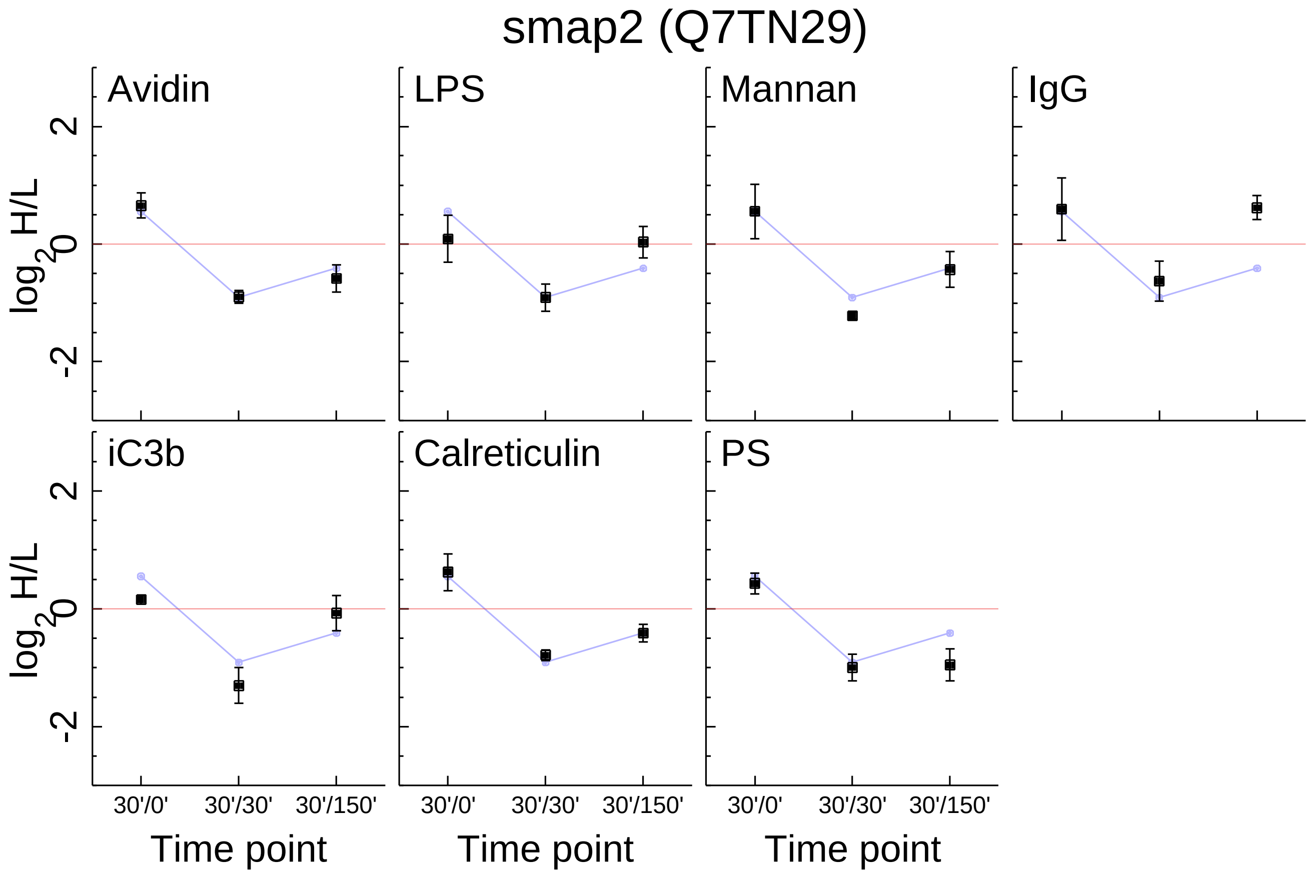

smap2 (Q7TN29)
Avidin
LPS
Mannan
IgG
iC3b
Calreticulin
PS
log2 H/L
2
0
-2
30'/0'
30'/30'
30'/150'
Time point

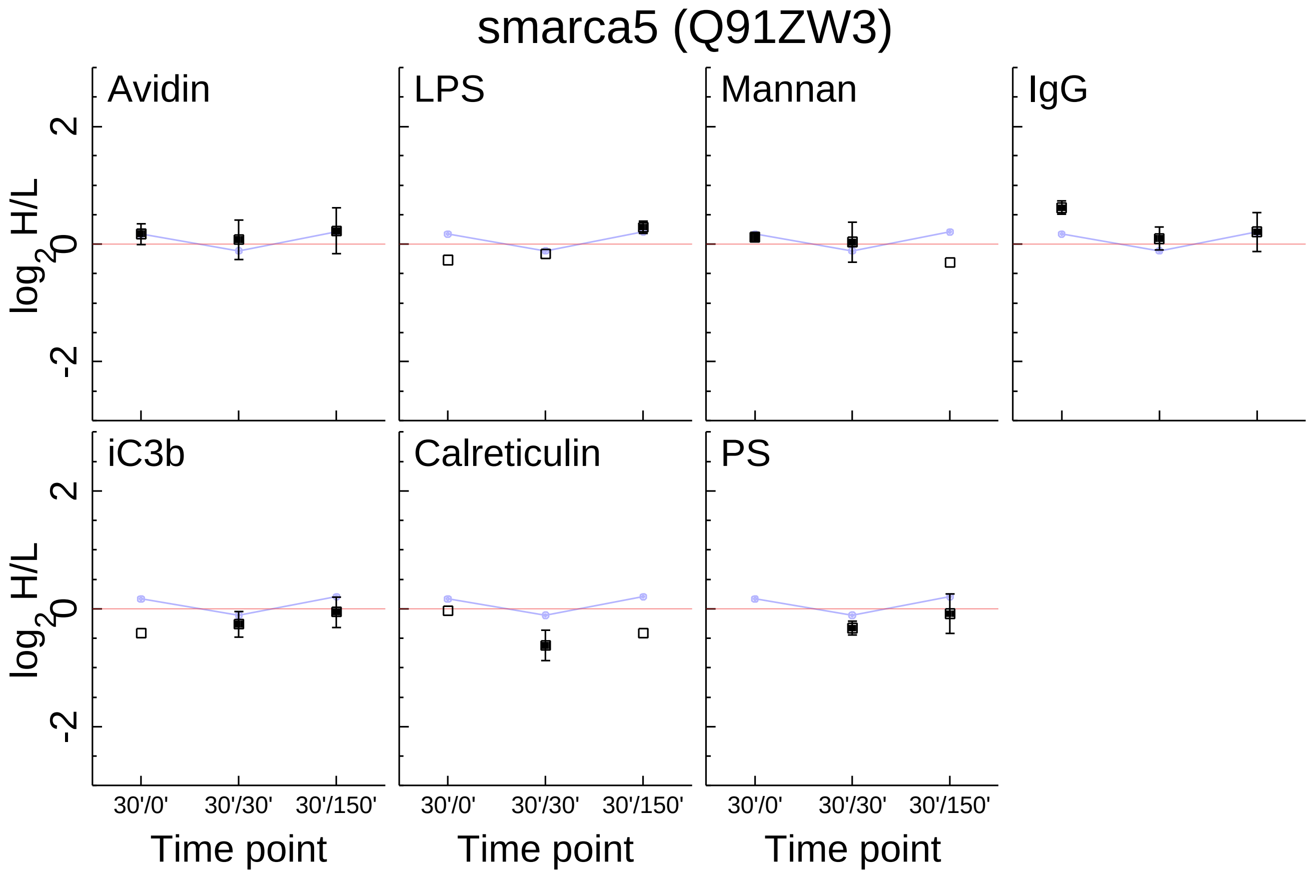
smarca5 (Q91ZW3)
Avidin
LPS
Mannan
IgG
iC3b
Calreticulin
PS
log2 H/L
2
0
-2
30'/0'
30'/30'
30'/150'
Time point

smarcc2 (Q6PDG5)

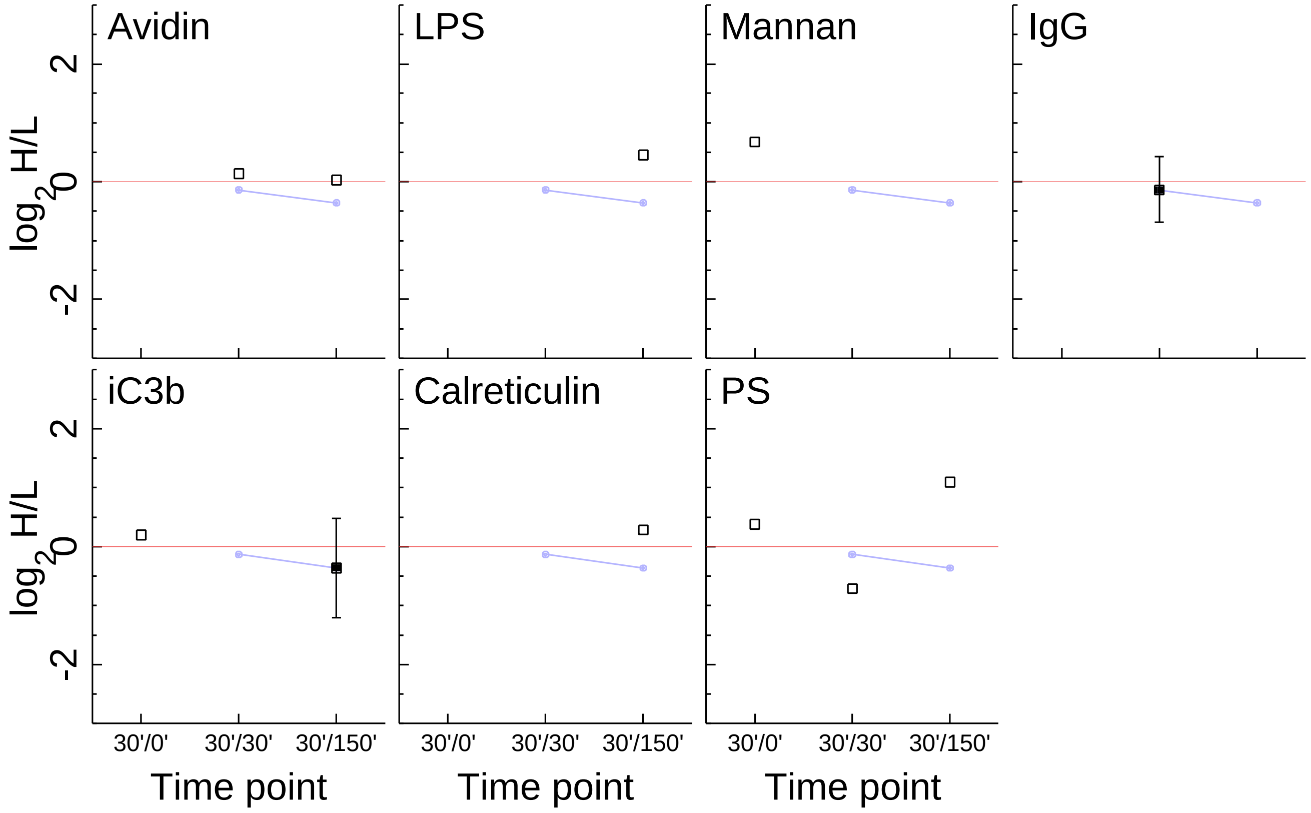

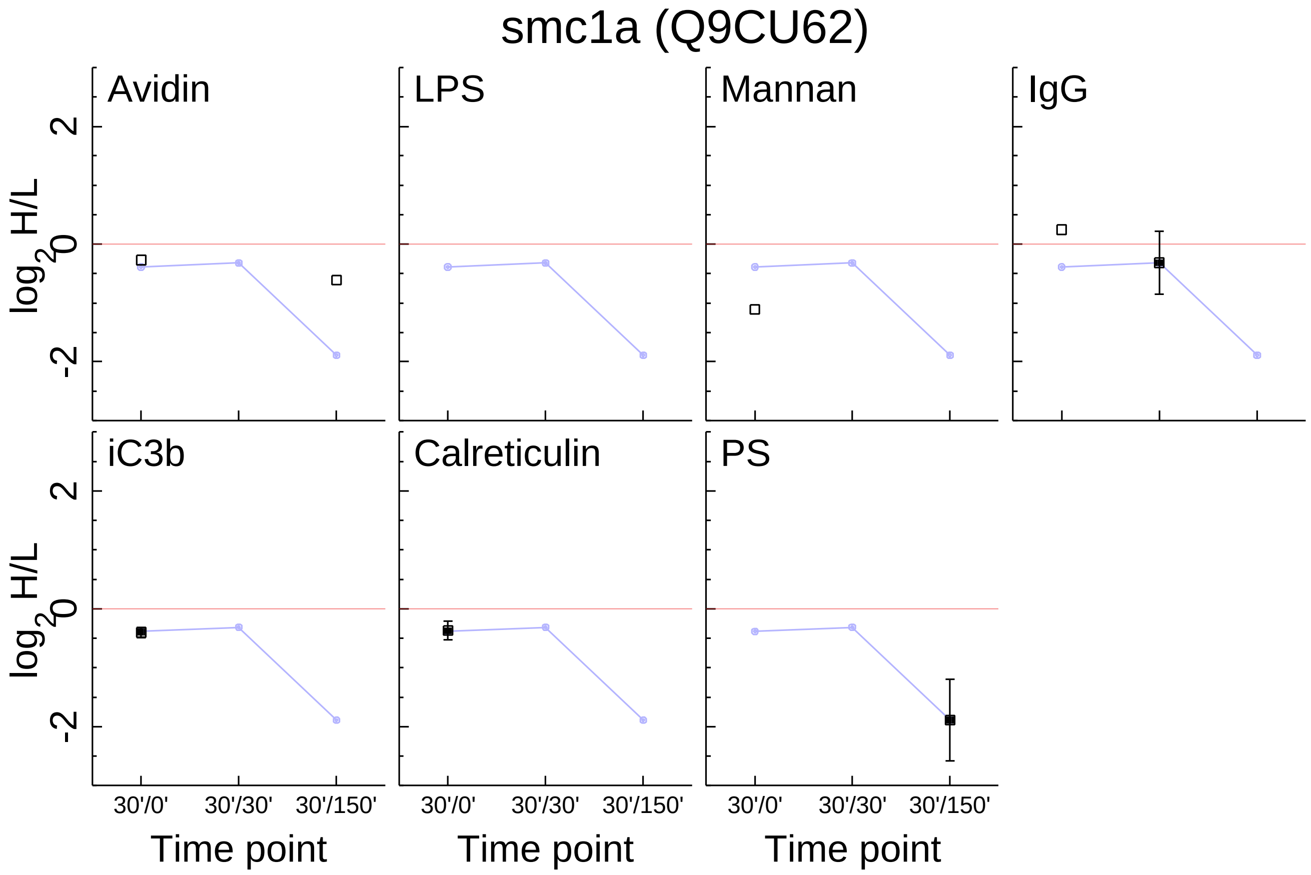

smc1a (Q9CU62)
Avidin
LPS
Mannan
IgG
iC3b
Calreticulin
PS
log2 H/L
2
0
-2
30'/0'
30'/30'
30'/150'
Time point

smc2 (Q8CG48)

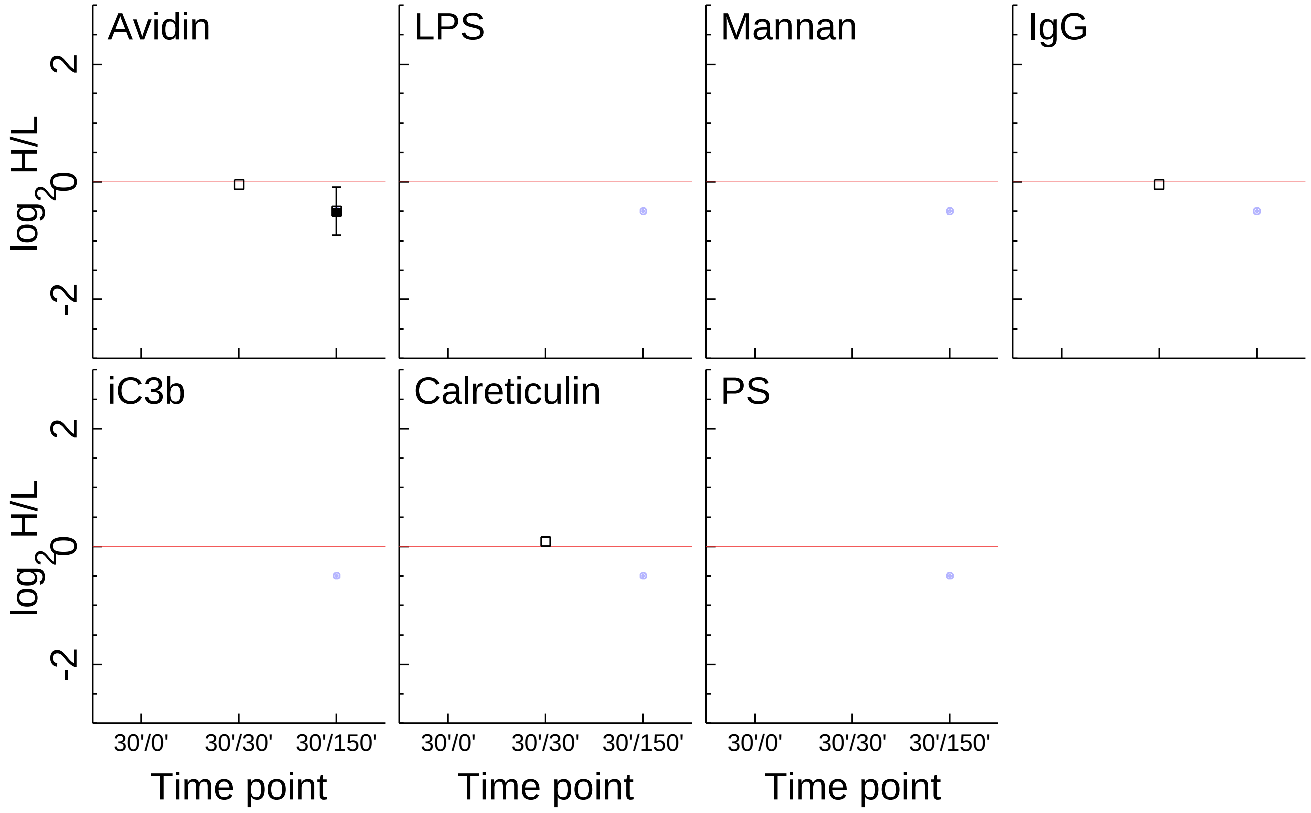

Avidin
LPS
Mannan
IgG
iC3b
Calreticulin
PS
log2 H/L
2
0
-2
30'/0'
30'/30'
30'/150'
Time point

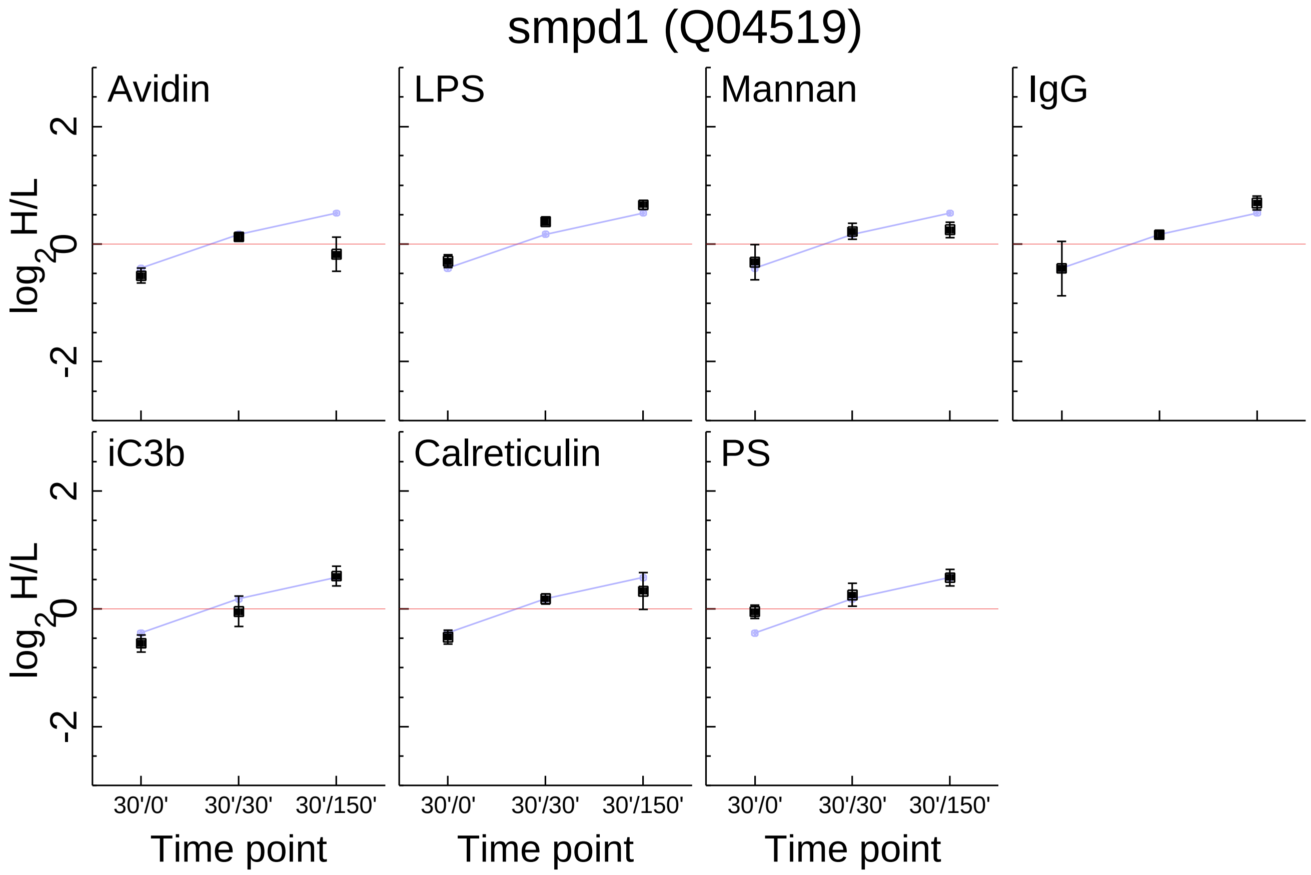
smpd1 (Q04519)
Avidin
LPS
Mannan
IgG
iC3b
Calreticulin
PS
2
0
-2
log2 H/L
30'/0'
30'/30'
30'/150'
Time point

smpdl3a (P70158)

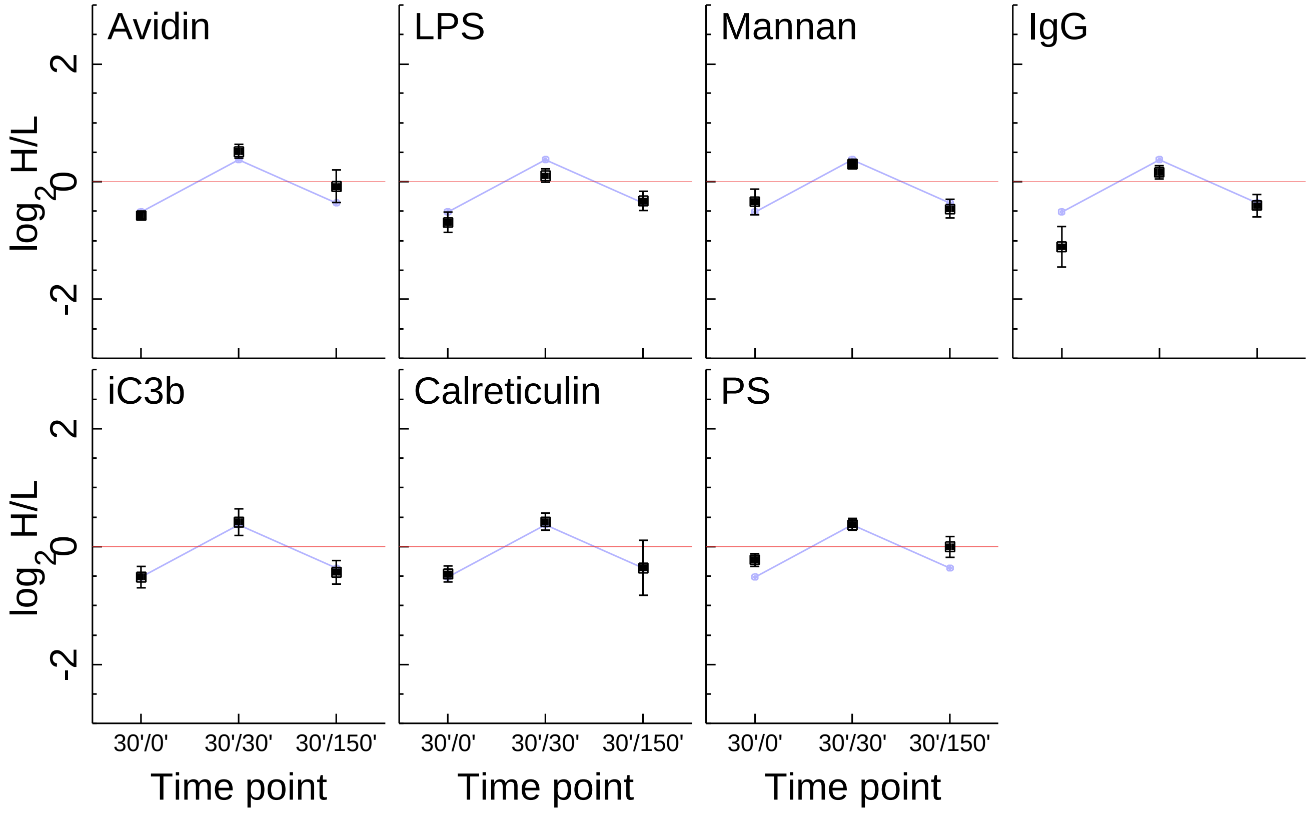

smpdl3b (P58242)

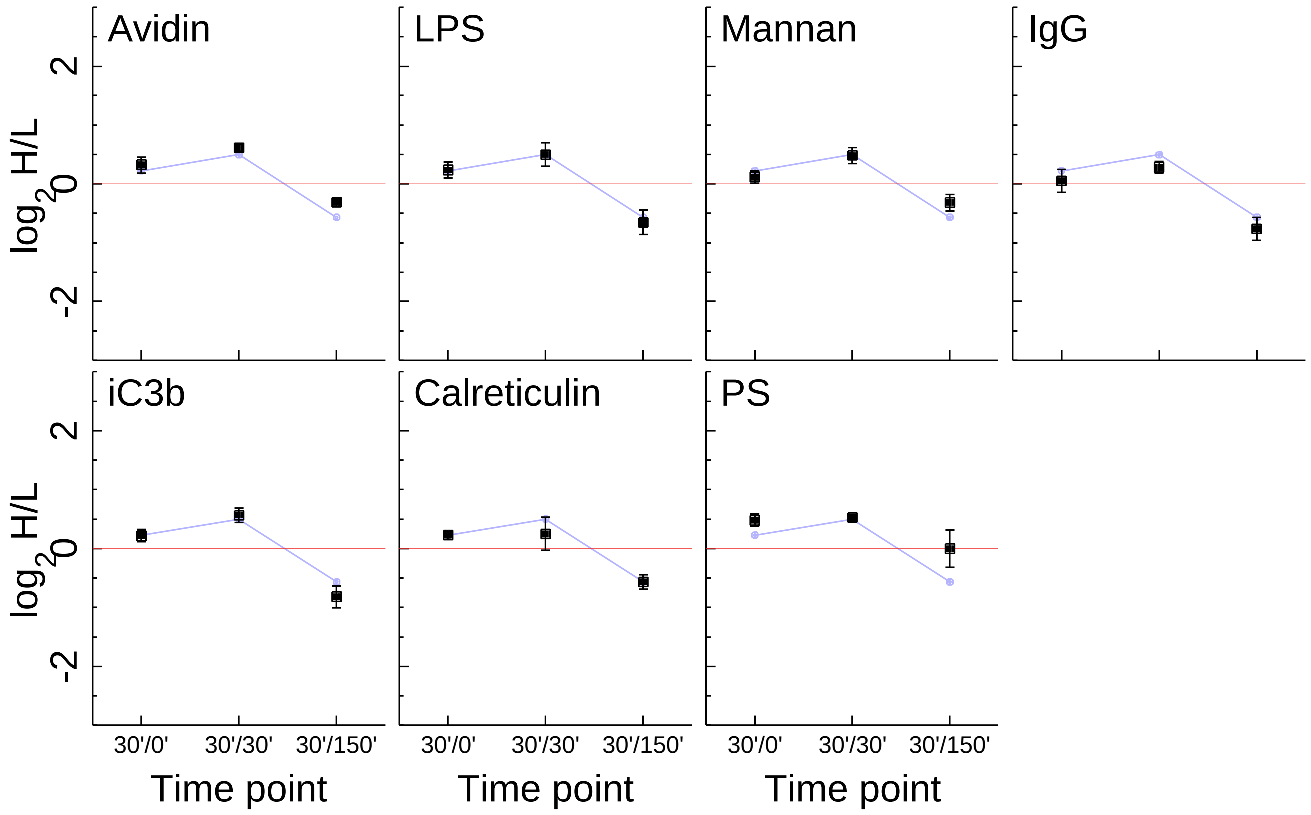

snap23 (Q9D3L3)

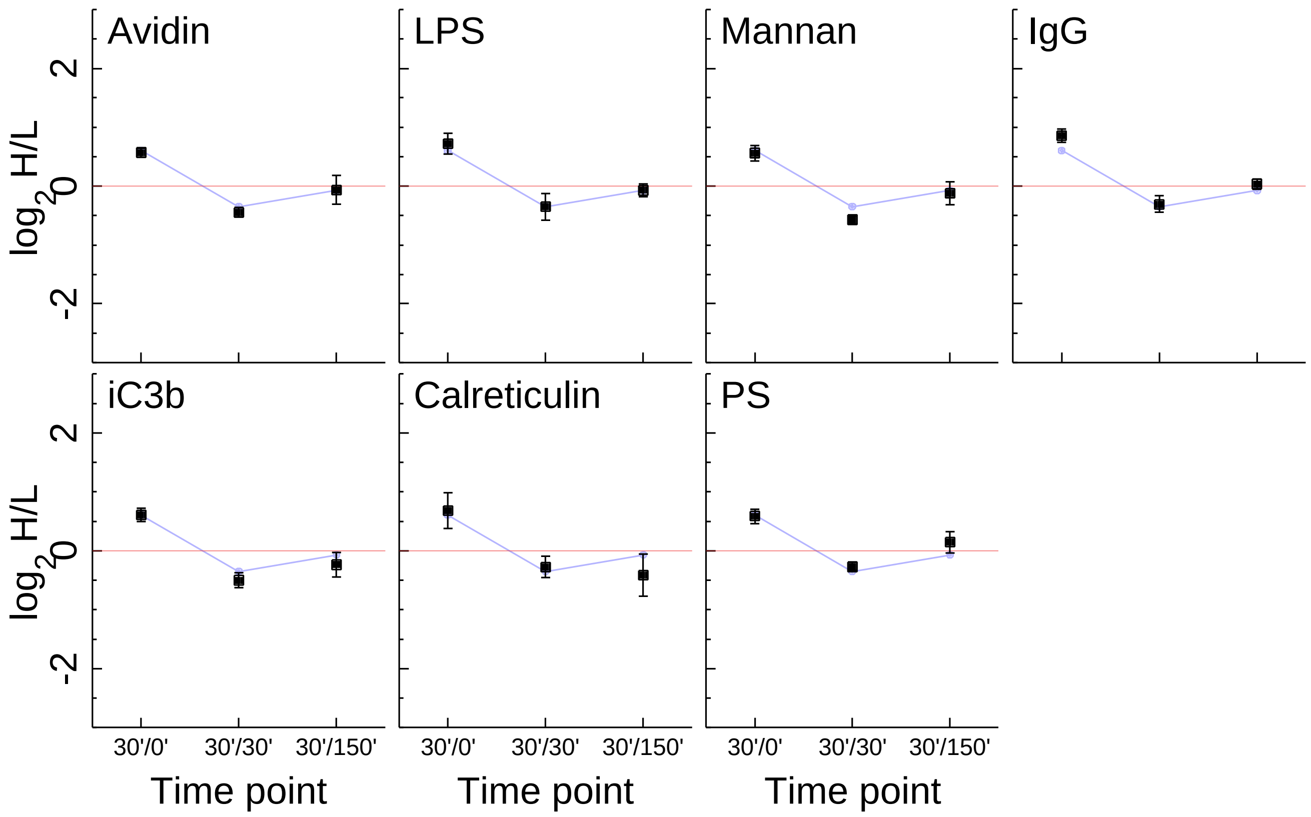

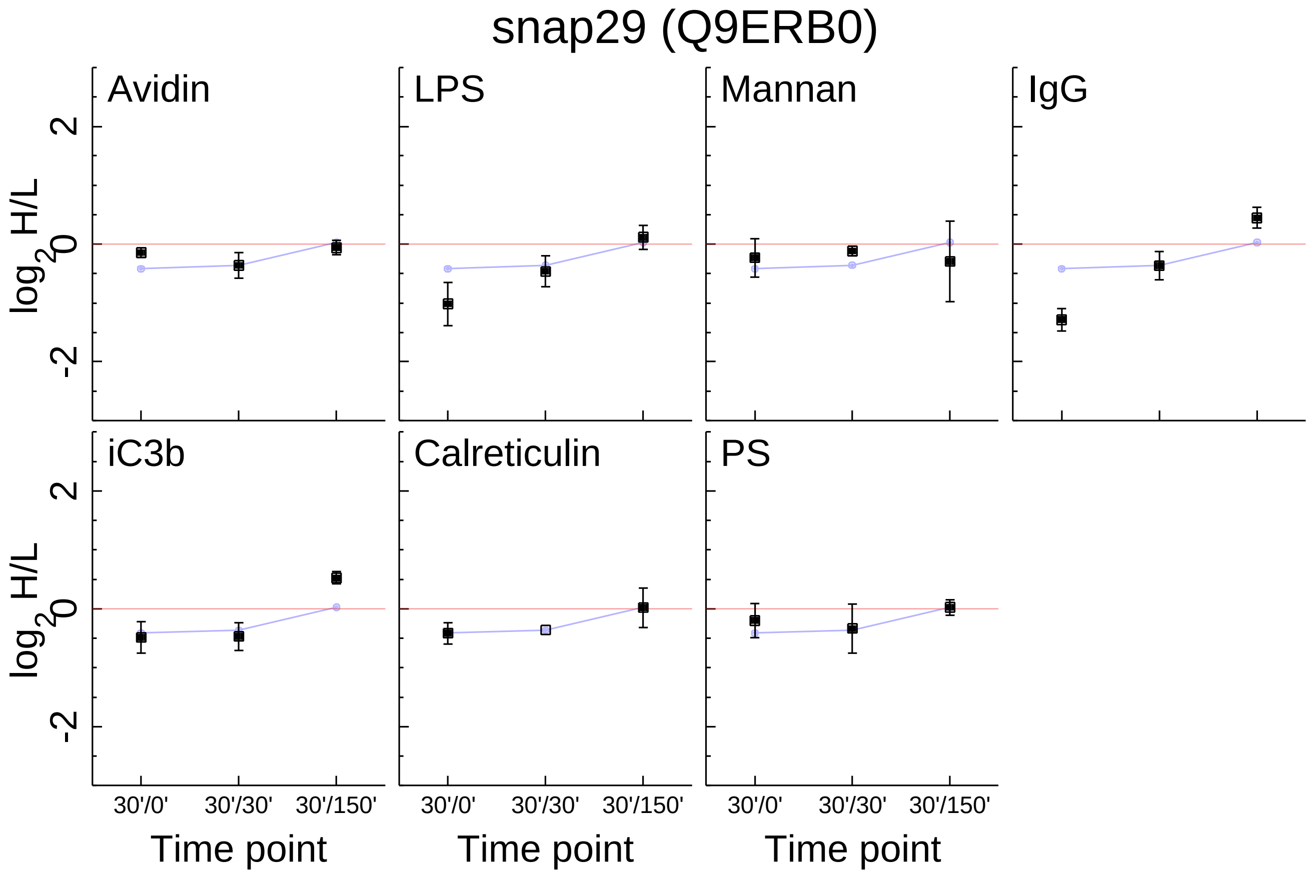

snap29 (Q9ERB0)
Avidin
LPS
Mannan
IgG
iC3b
Calreticulin
PS
log2 H/L
2
0
-2
30'/0'
30'/30'
30'/150'
Time point

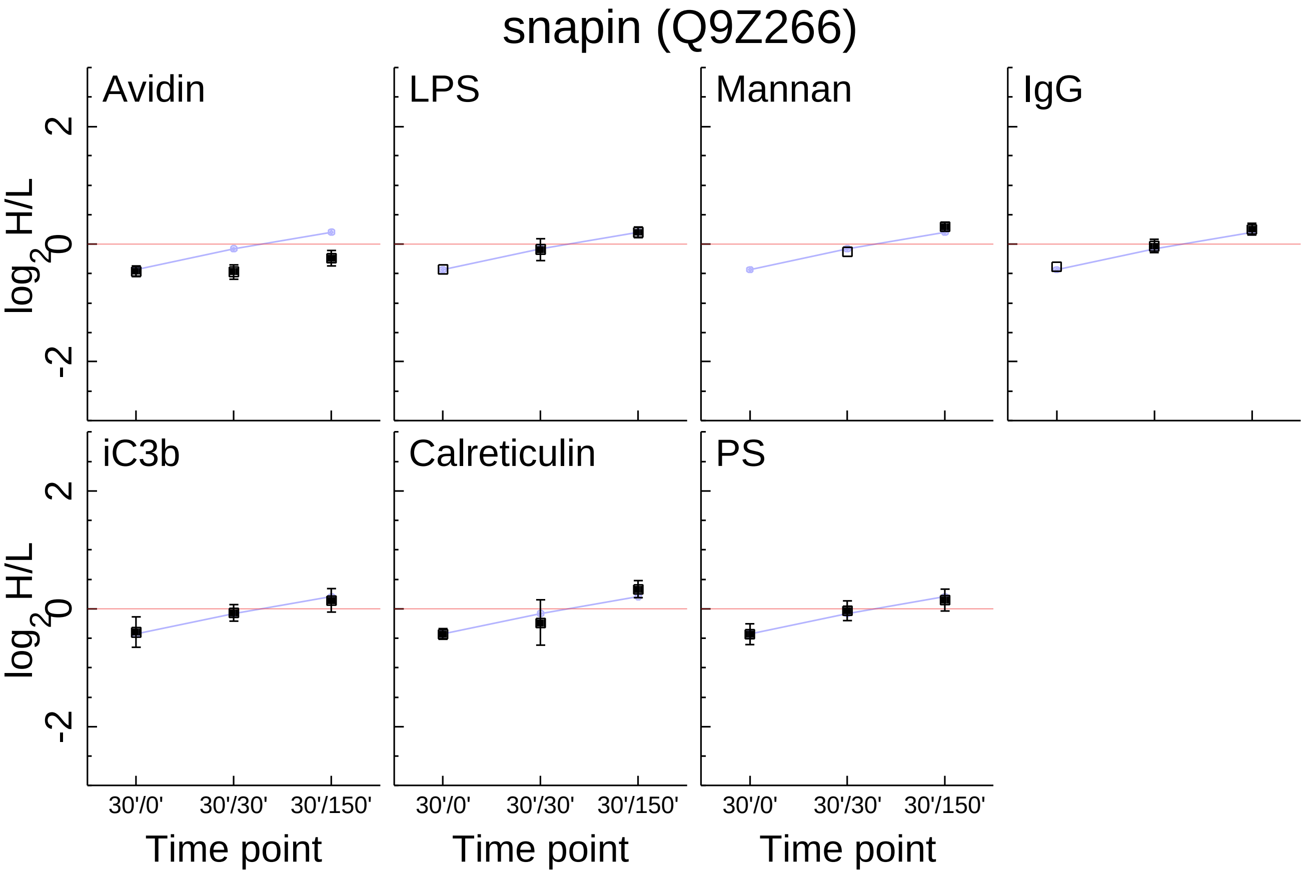

snapin (Q9Z266)
Avidin
LPS
Mannan
IgG
iC3b
Calreticulin
PS
log2 H/L
2
0
-2
30'/0'
30'/30'
30'/150'
Time point

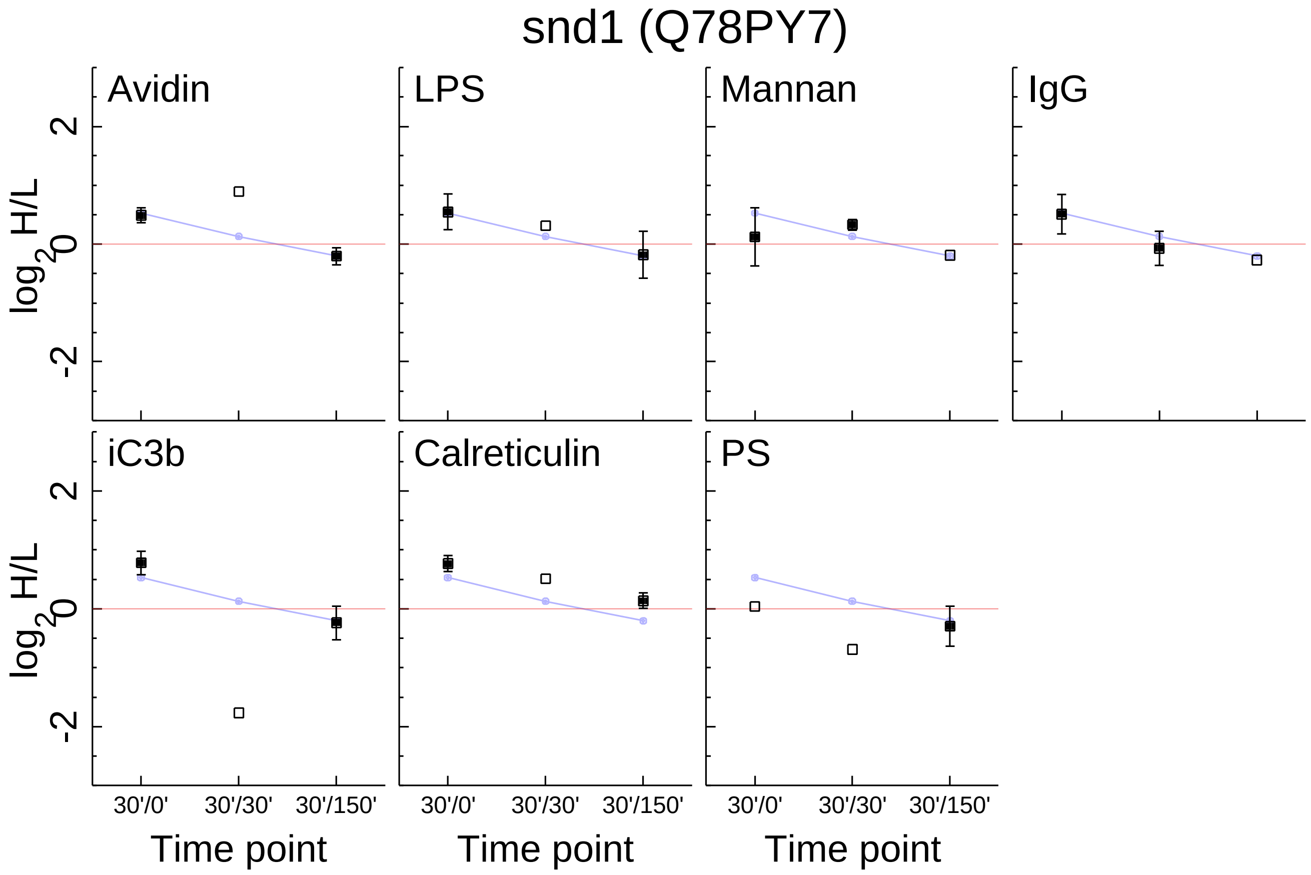
snd1 (Q78PY7)
Avidin
LPS
Mannan
IgG
iC3b
Calreticulin
PS
2
0
-2
log2 H/L
30'/0'
30'/30'
30'/150'
Time point

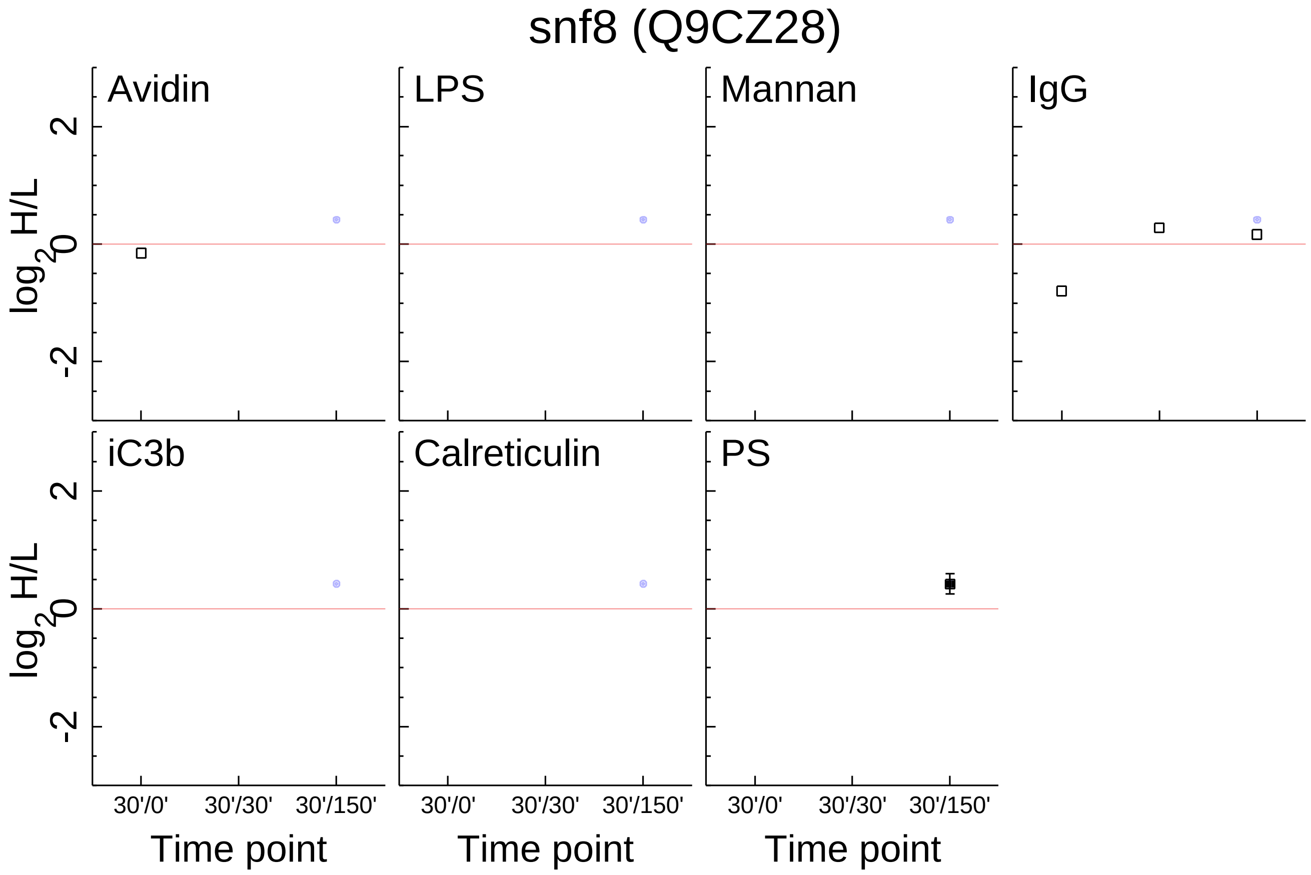

snf8 (Q9CZ28)
Avidin
LPS
Mannan
IgG
iC3b
Calreticulin
PS
log2 H/L
2
0
-2
30'/0'
30'/30'
30'/150'
Time point

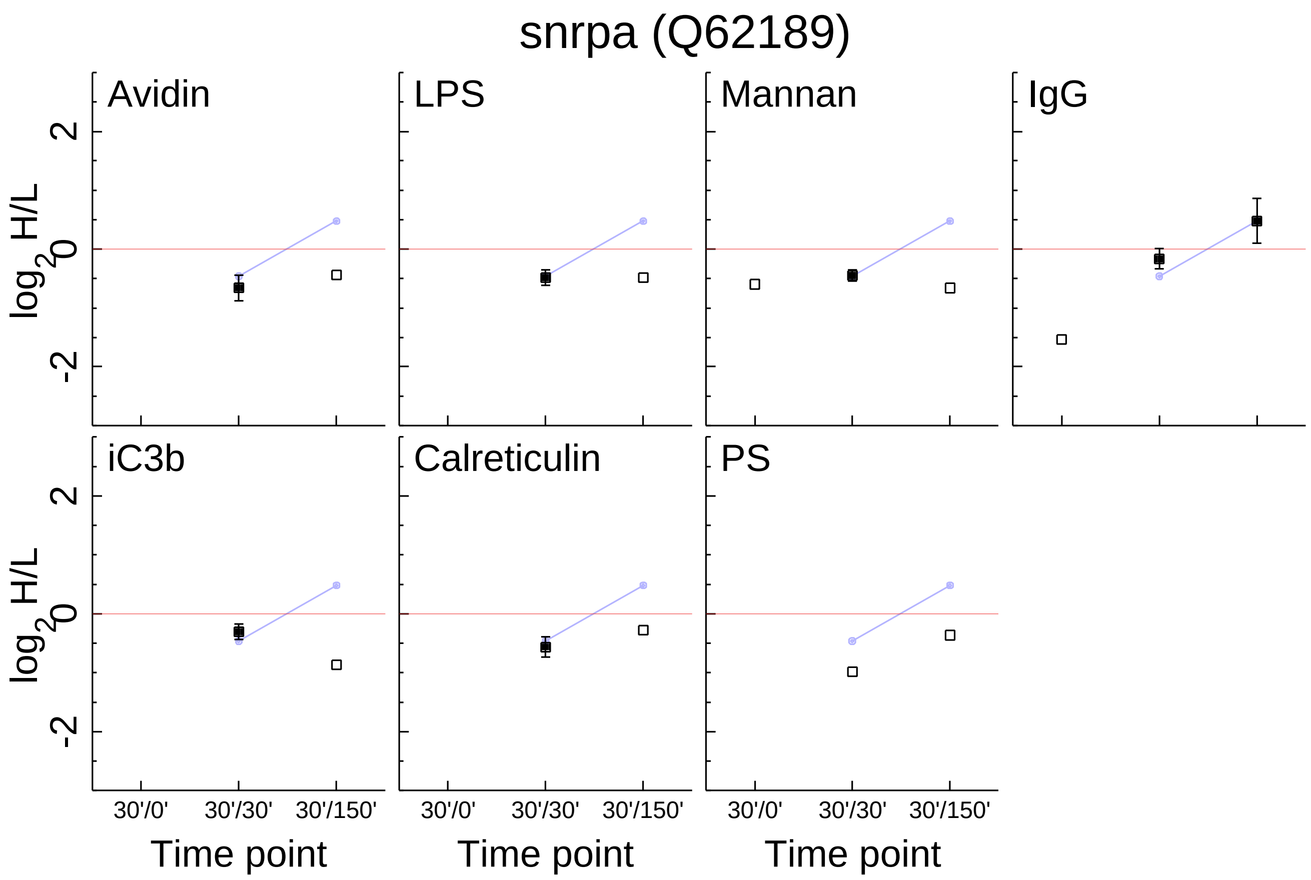
snrpa (Q62189)
Avidin
LPS
Mannan
IgG
iC3b
Calreticulin
PS
log2 H/L
2
0
-2
30'/0'
30'/30'
30'/150'
Time point

snrpc (Q62241)

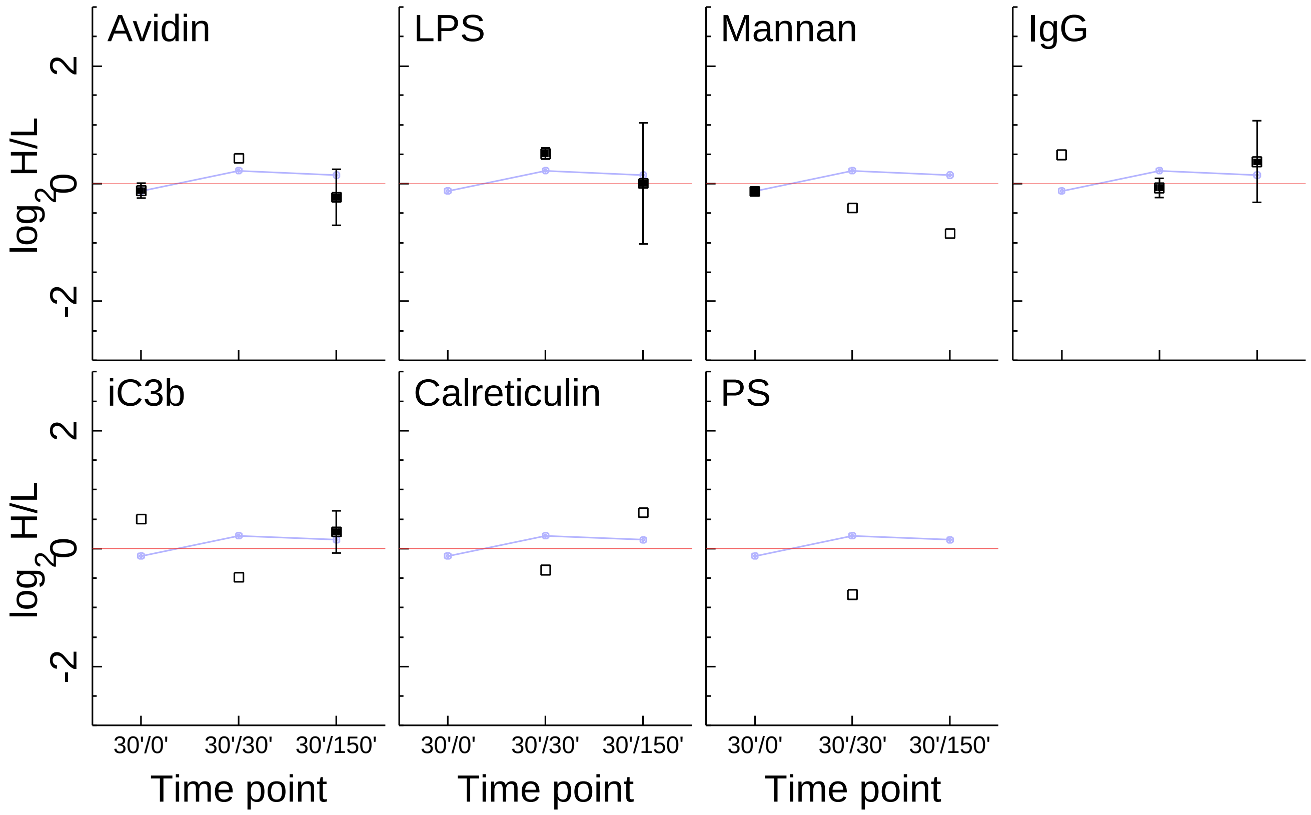

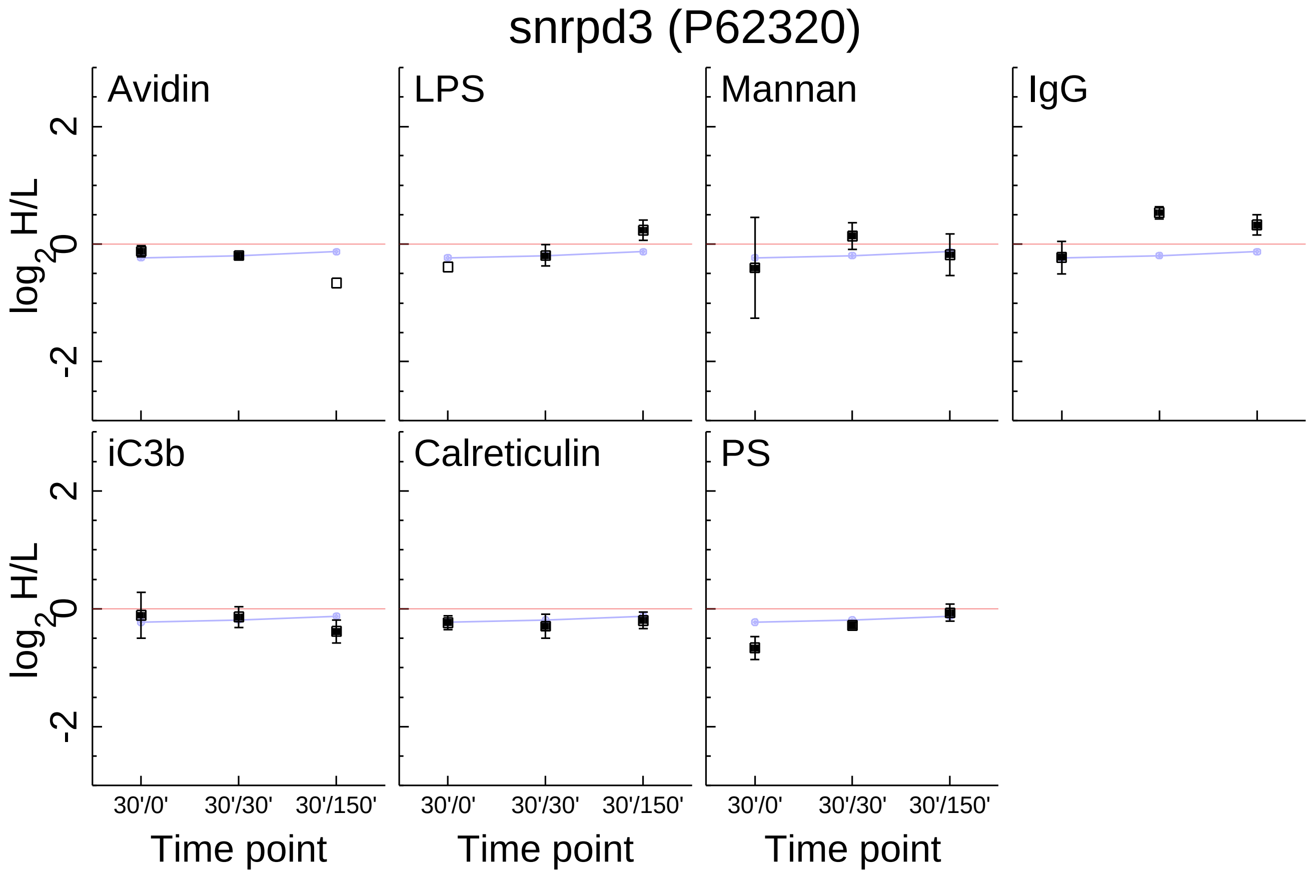

snrpd3 (P62320)
Avidin
LPS
Mannan
IgG
iC3b
Calreticulin
PS
log2 H/L
2
0
-2
30'/0'
30'/30'
30'/150'
Time point

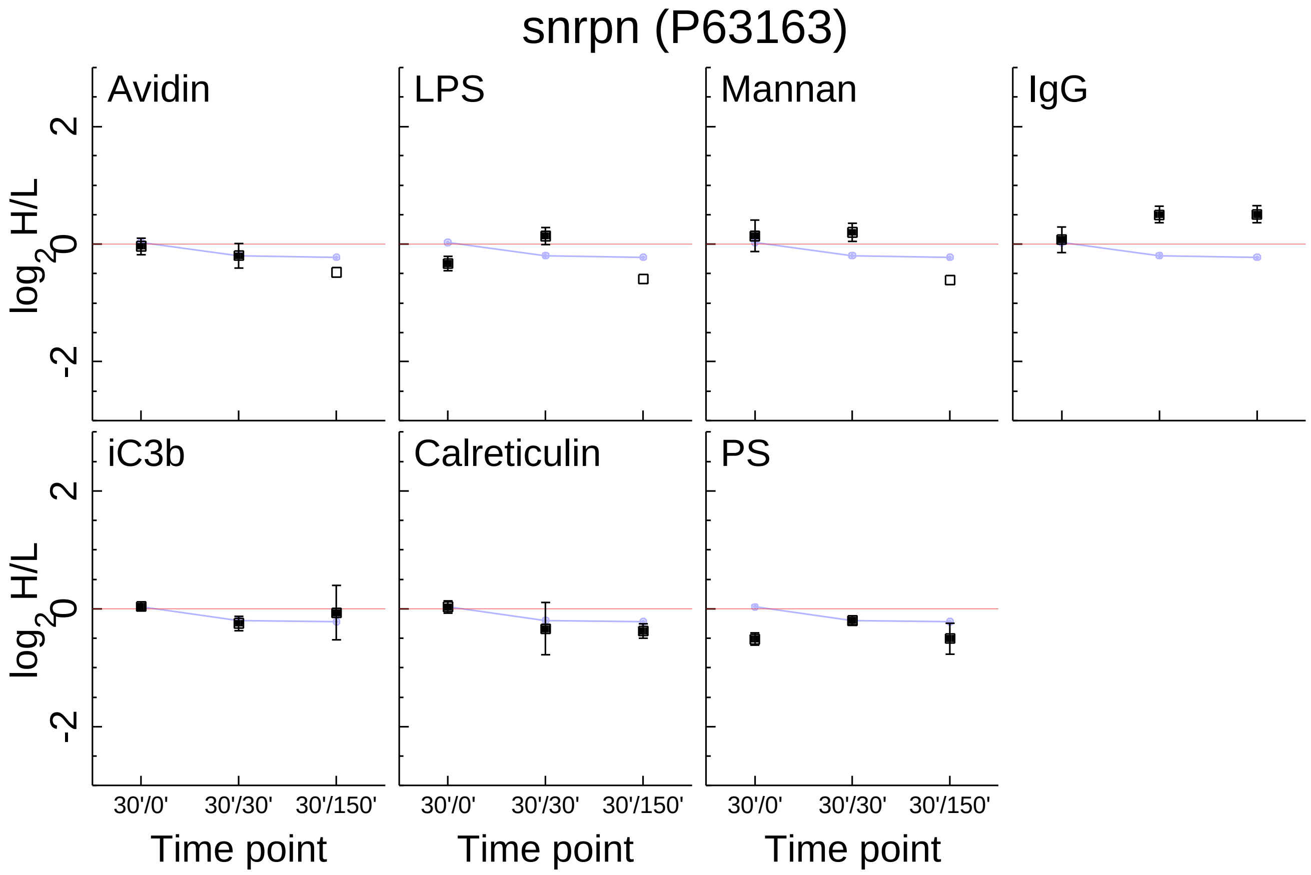

snrpn (P63163)
Avidin
LPS
Mannan
IgG
iC3b
Calreticulin
PS
log2 H/L
2
0
-2
30'/0'
30'/30'
30'/150'
Time point

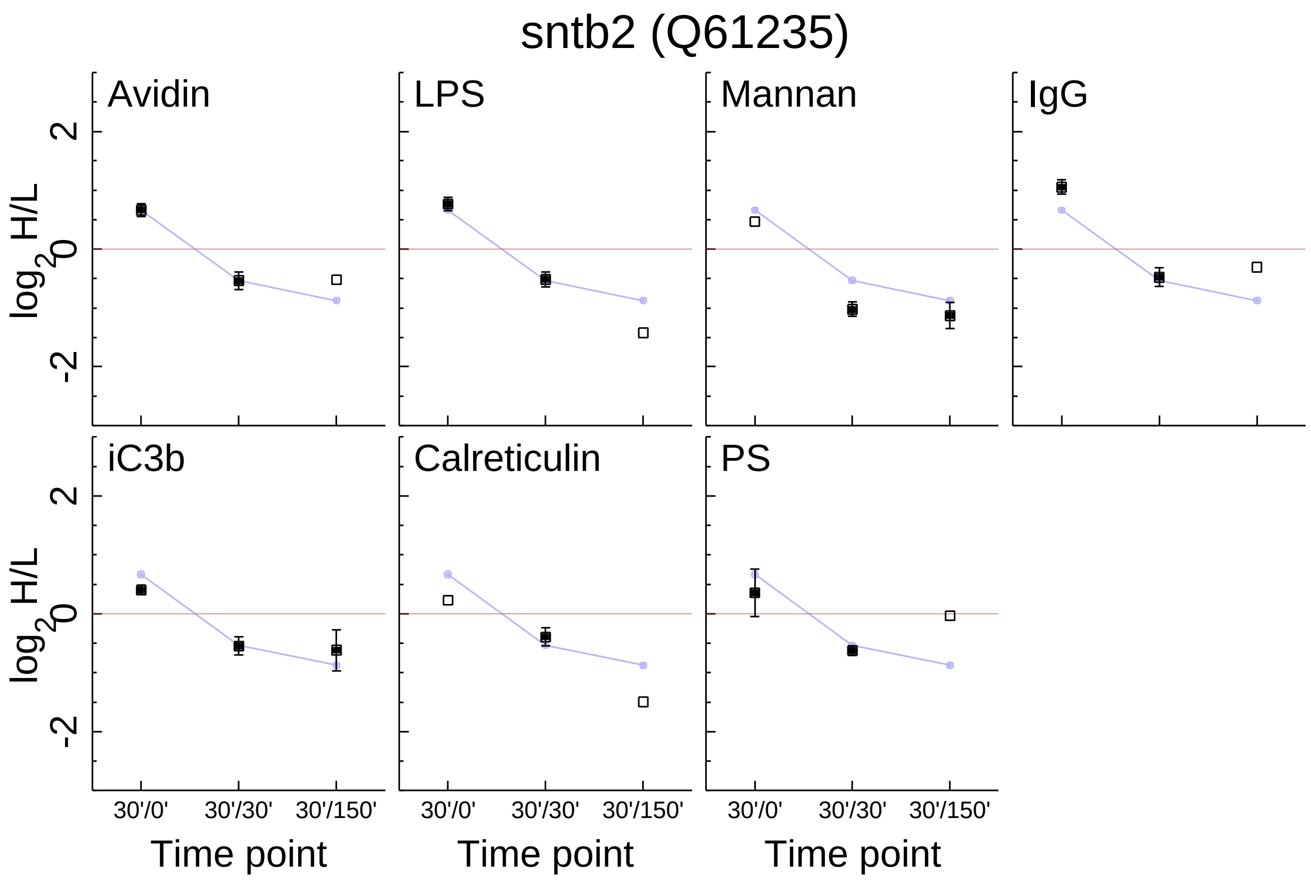

sntb2 (Q61235)
Avidin
LPS
Mannan
IgG
iC3b
Calreticulin
PS
log2 H/L
2
0
-2
30'/0'
30'/30'
30'/150'
Time point

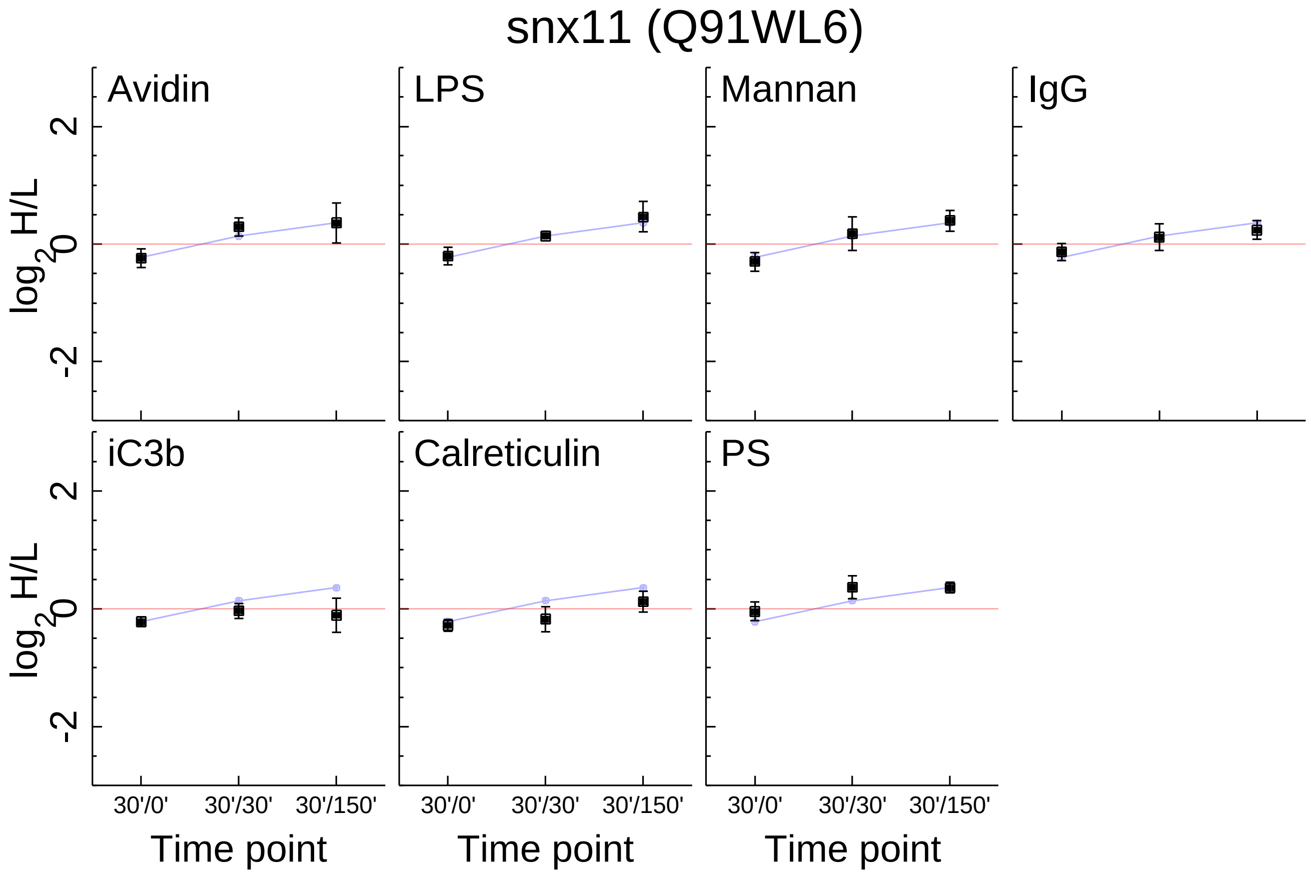
snx11 (Q91WL6)
Avidin
LPS
Mannan
IgG
iC3b
Calreticulin
PS
log2 H/L
2
0
-2
30'/0'
30'/30'
30'/150'
Time point

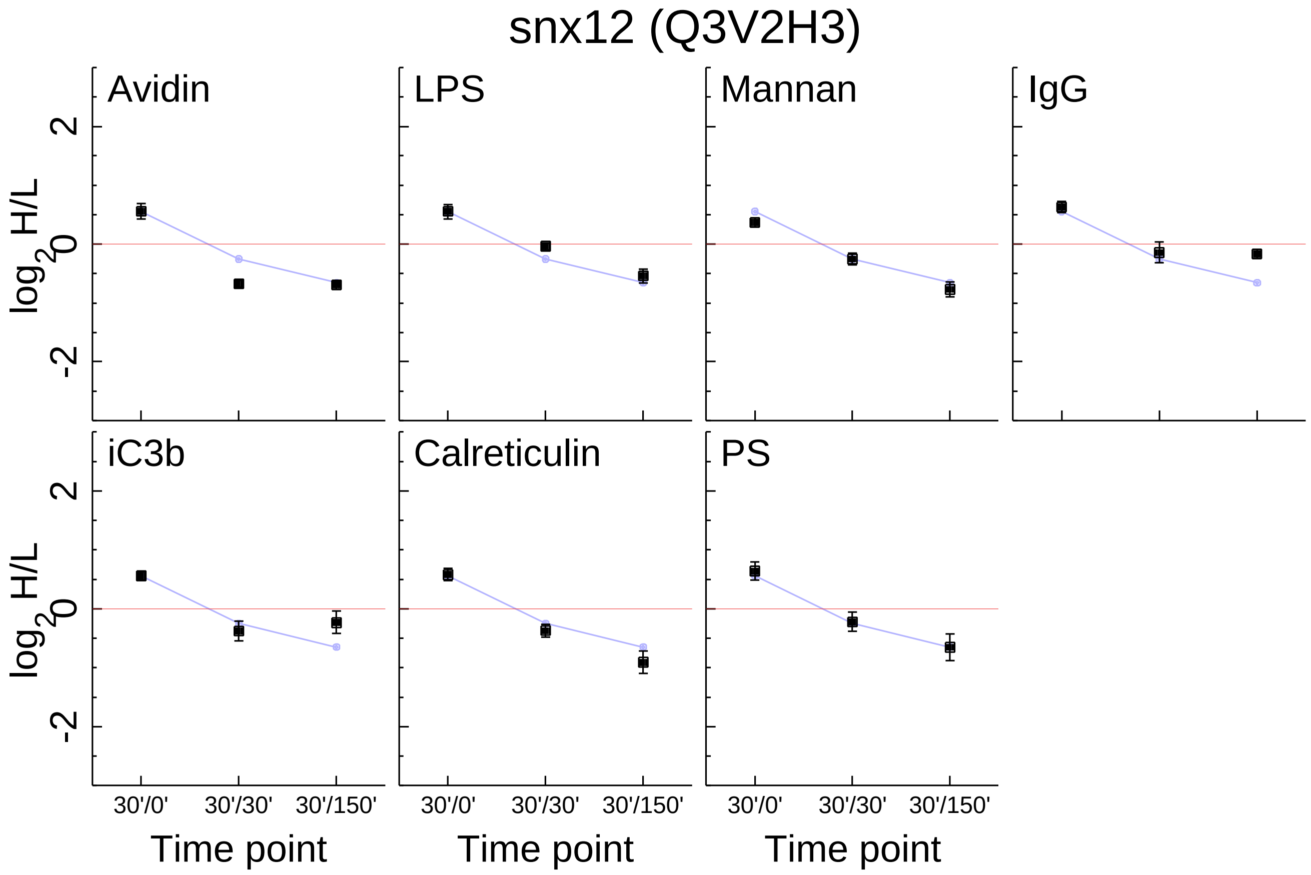

snx12 (Q3V2H3)
Avidin
LPS
Mannan
IgG
iC3b
Calreticulin
PS
log2 H/L
2
0
-2
30'/0'
30'/30'
30'/150'
Time point

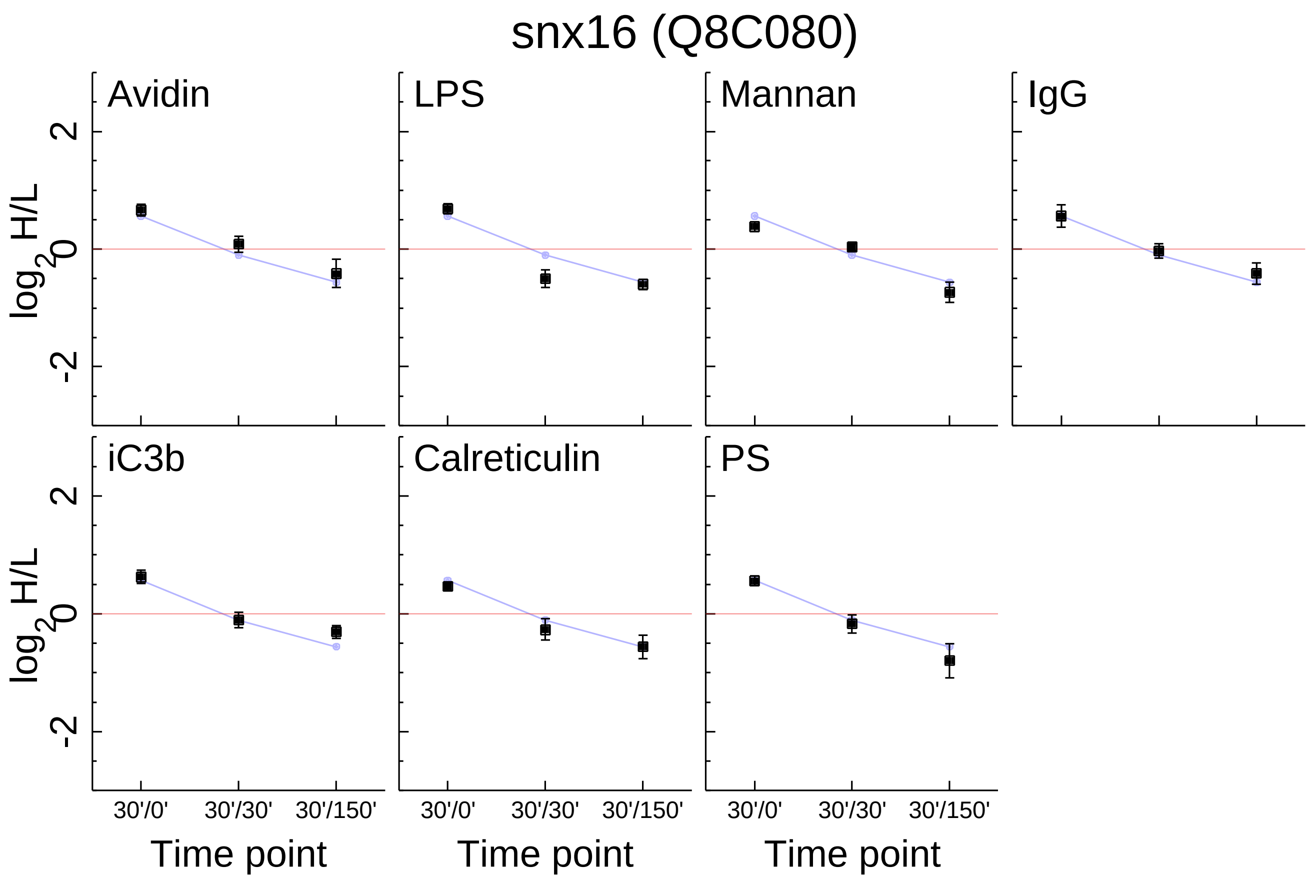
snx16 (Q8C080)
Avidin
LPS
Mannan
IgG
iC3b
Calreticulin
PS
log2 H/L
2
0
-2
30'/0'
30'/30'
30'/150'
Time point

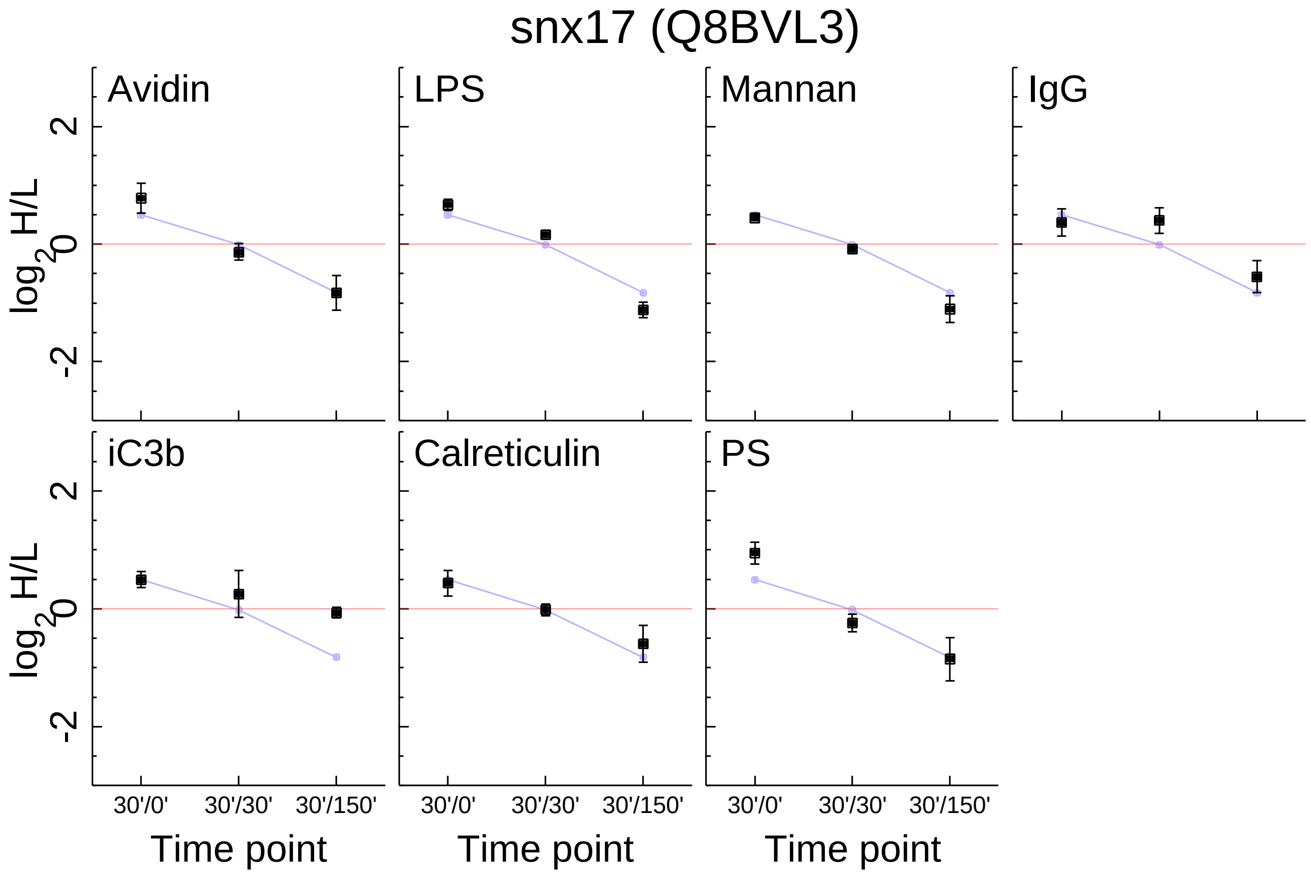
snx17 (Q8BVL3)
Avidin
LPS
Mannan
IgG
iC3b
Calreticulin
PS
log2 H/L
2
0
-2
30'/0'
30'/30'
30'/150'
Time point

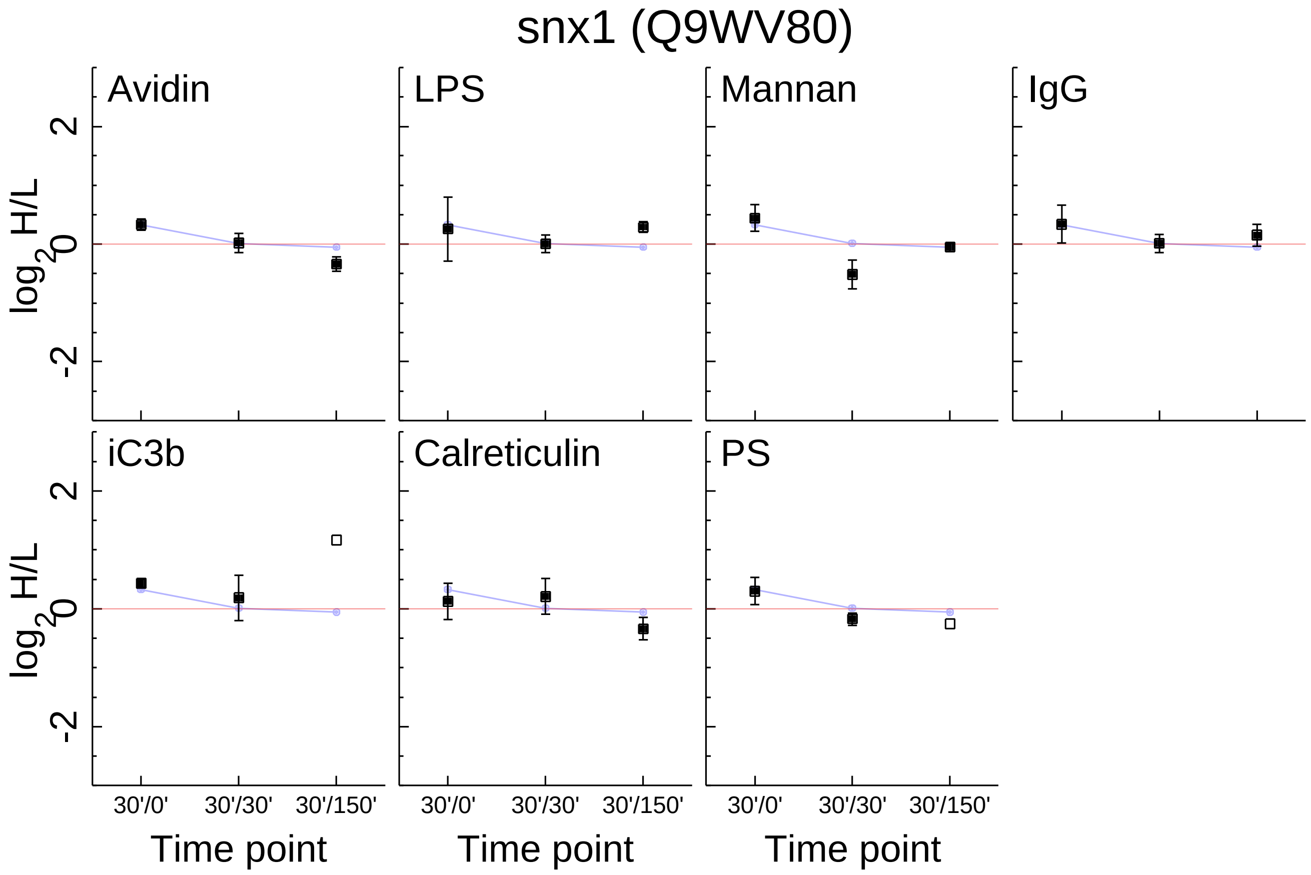

snx1 (Q9WV80)
Avidin
LPS
Mannan
IgG
iC3b
Calreticulin
PS
log2 H/L
2
0
-2
30'/0'
30'/30'
30'/150'
Time point

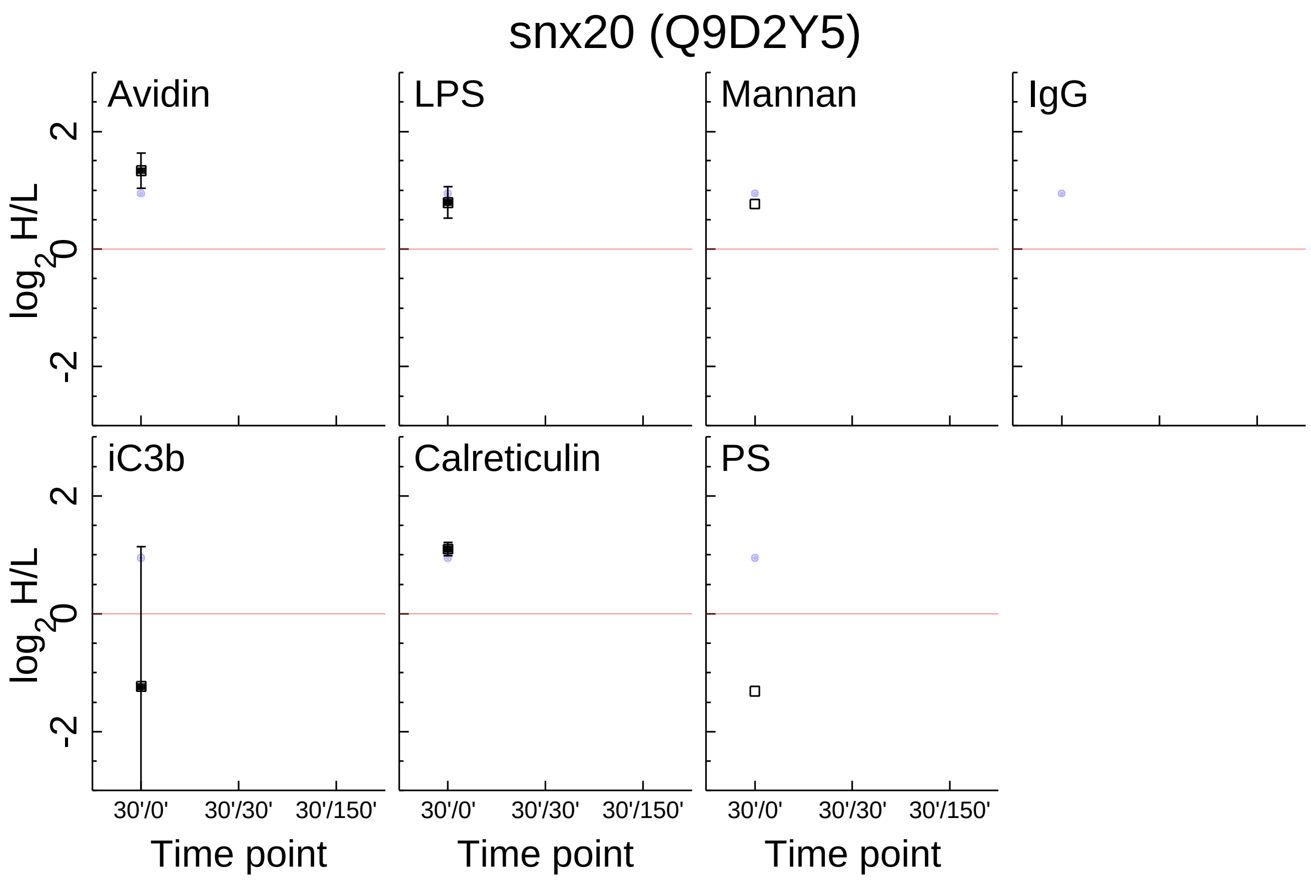
snx20 (Q9D2Y5)
Avidin
LPS
Mannan
IgG
iC3b
Calreticulin
PS
log2 H/L
2
0
-2
30'/0'
30'/30'
30'/150'
Time point

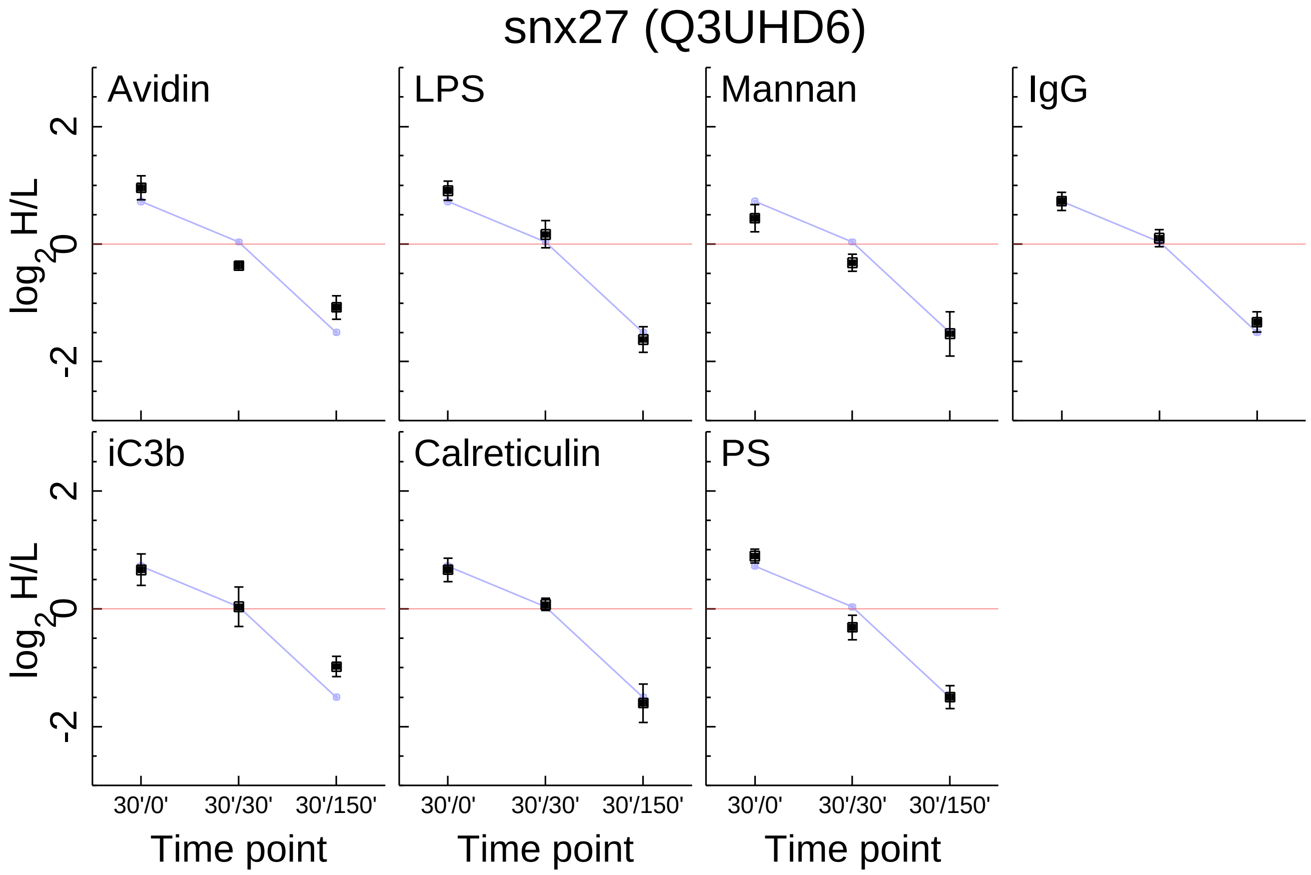
snx27 (Q3UHD6)
Avidin
LPS
Mannan
IgG
iC3b
Calreticulin
PS
log2 H/L
2
0
-2
30'/0'
30'/30'
30'/150'
Time point

snx29 (Q9D3S3)

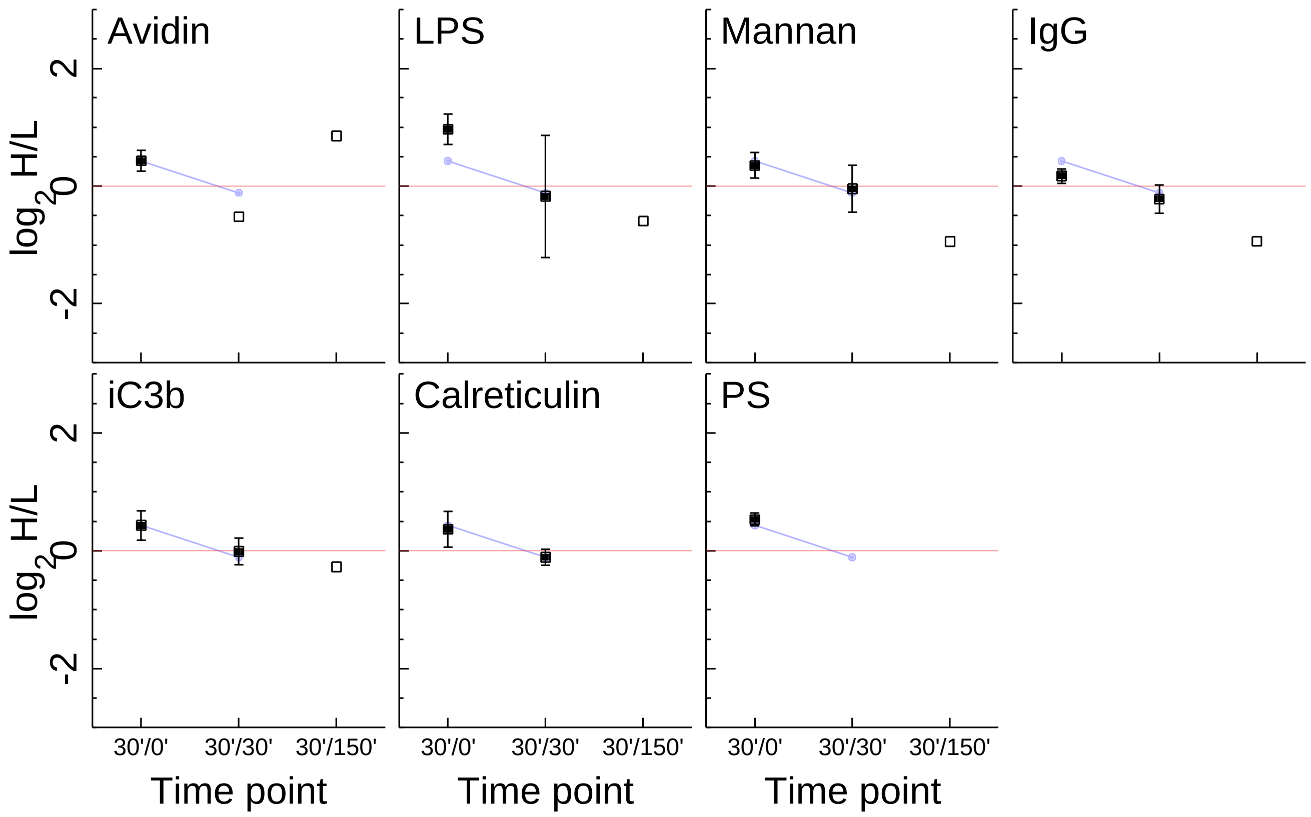

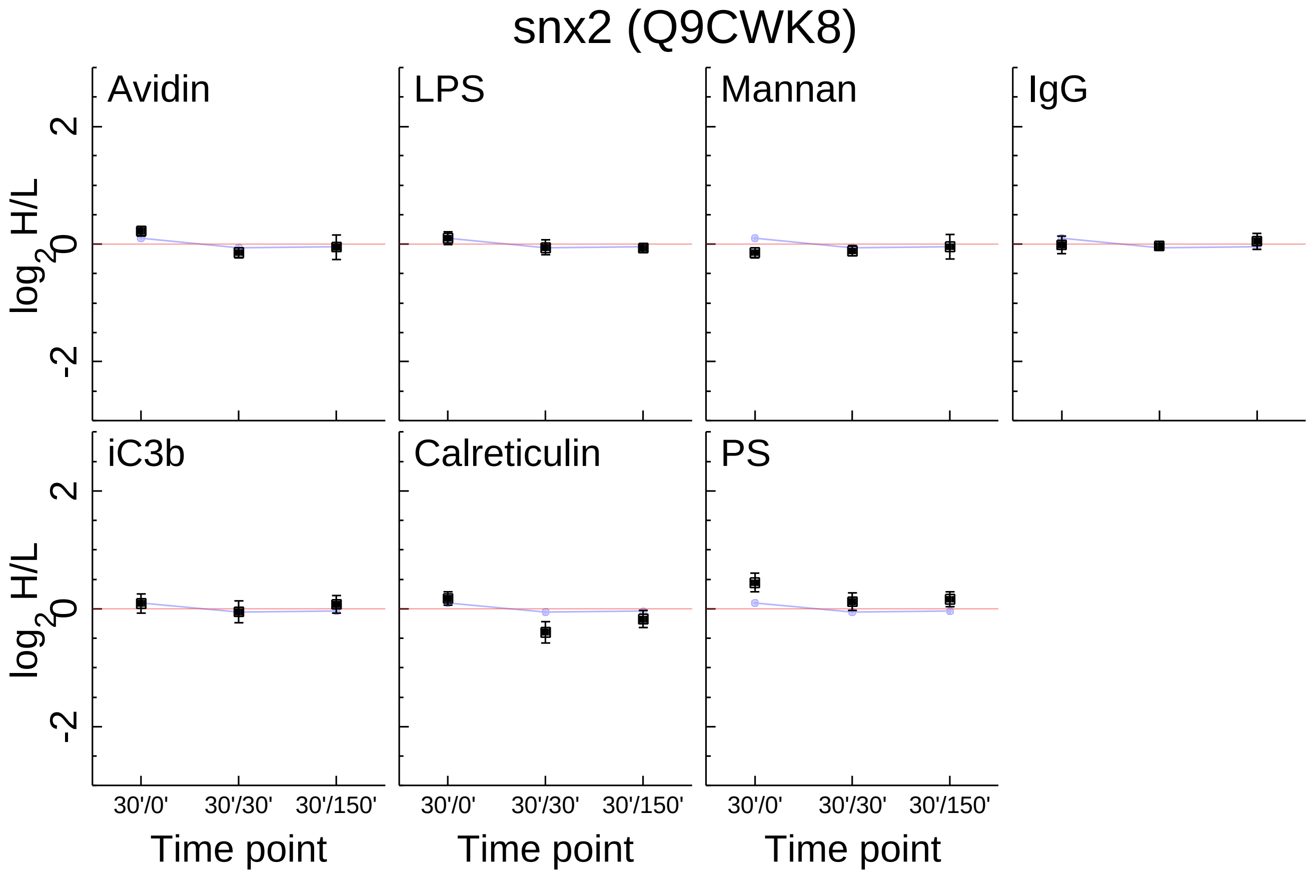

snx2 (Q9CWK8)
Avidin
LPS
Mannan
IgG
iC3b
Calreticulin
PS
log2 H/L
2
0
-2
30'/0'
30'/30'
30'/150'
Time point

snx30 (Q8CE50)

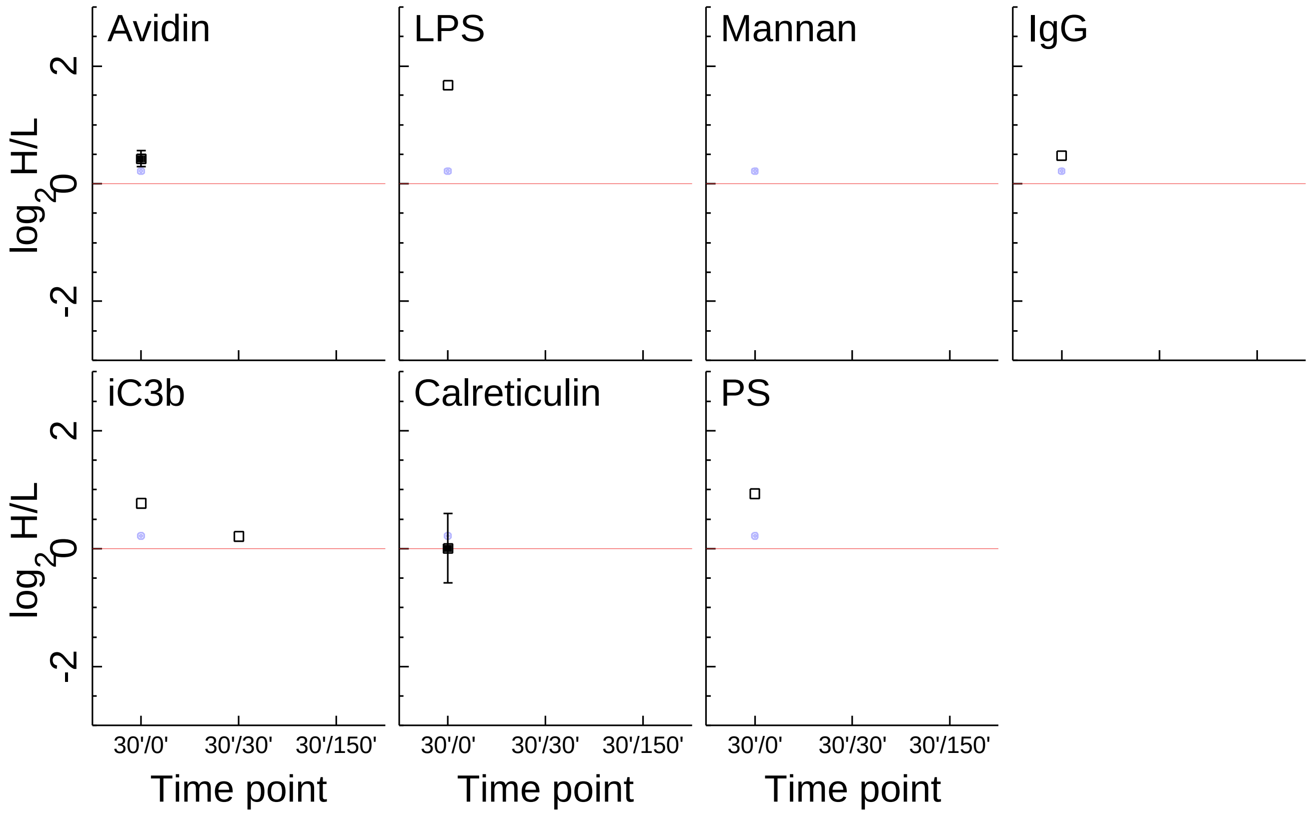

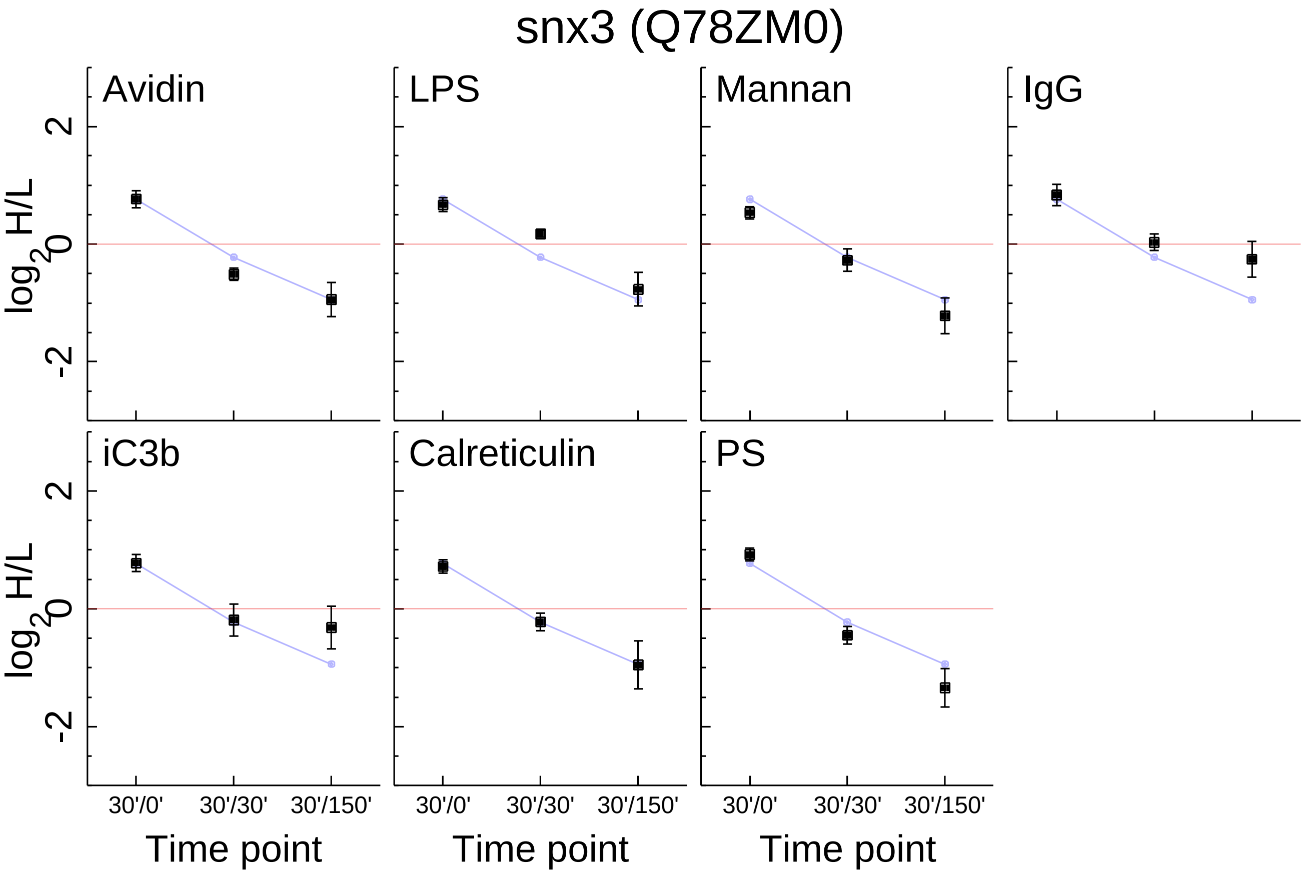

snx3 (Q78ZM0)
Avidin
LPS
Mannan
IgG
iC3b
Calreticulin
PS
log_2 H/L
2
0
-2
30'/0'
30'/30'
30'/150'
Time point

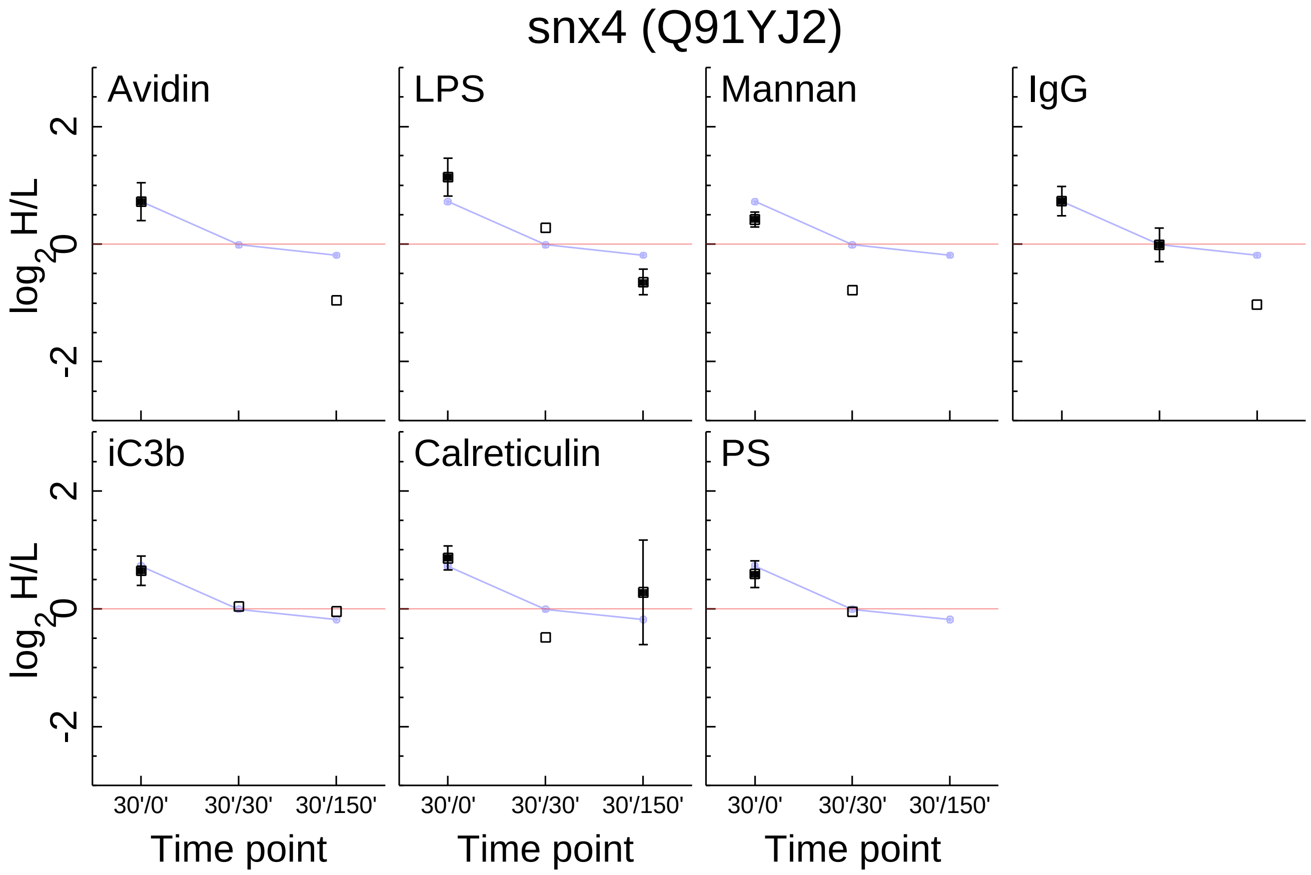
snx4 (Q91YJ2)
Avidin
LPS
Mannan
IgG
iC3b
Calreticulin
PS
log2 H/L
2
0
-2
30'/0'
30'/30'
30'/150'
Time point

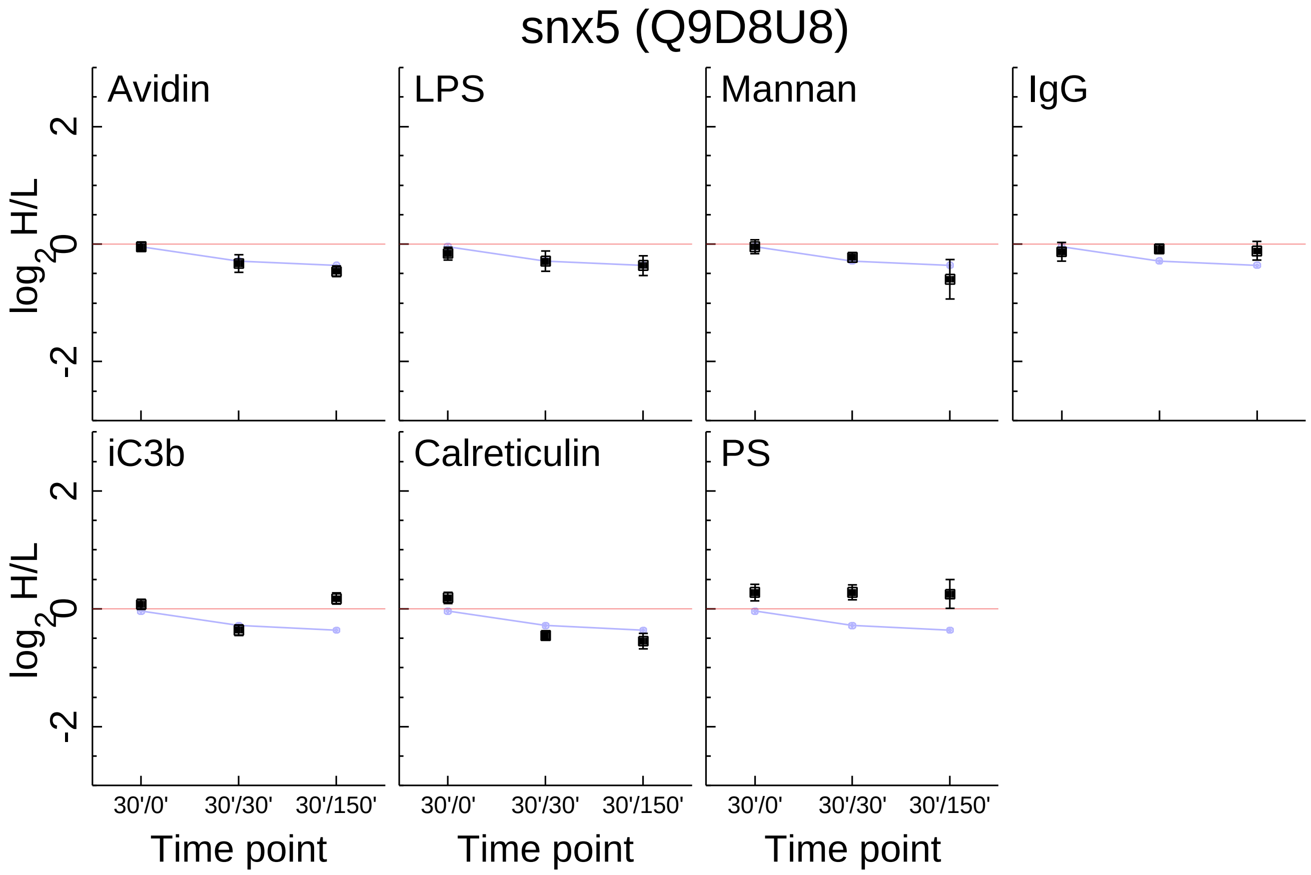

snx5 (Q9D8U8)
Avidin
LPS
Mannan
IgG
iC3b
Calreticulin
PS
log2 H/L
2
0
-2
30'/0'
30'/30'
30'/150'
Time point

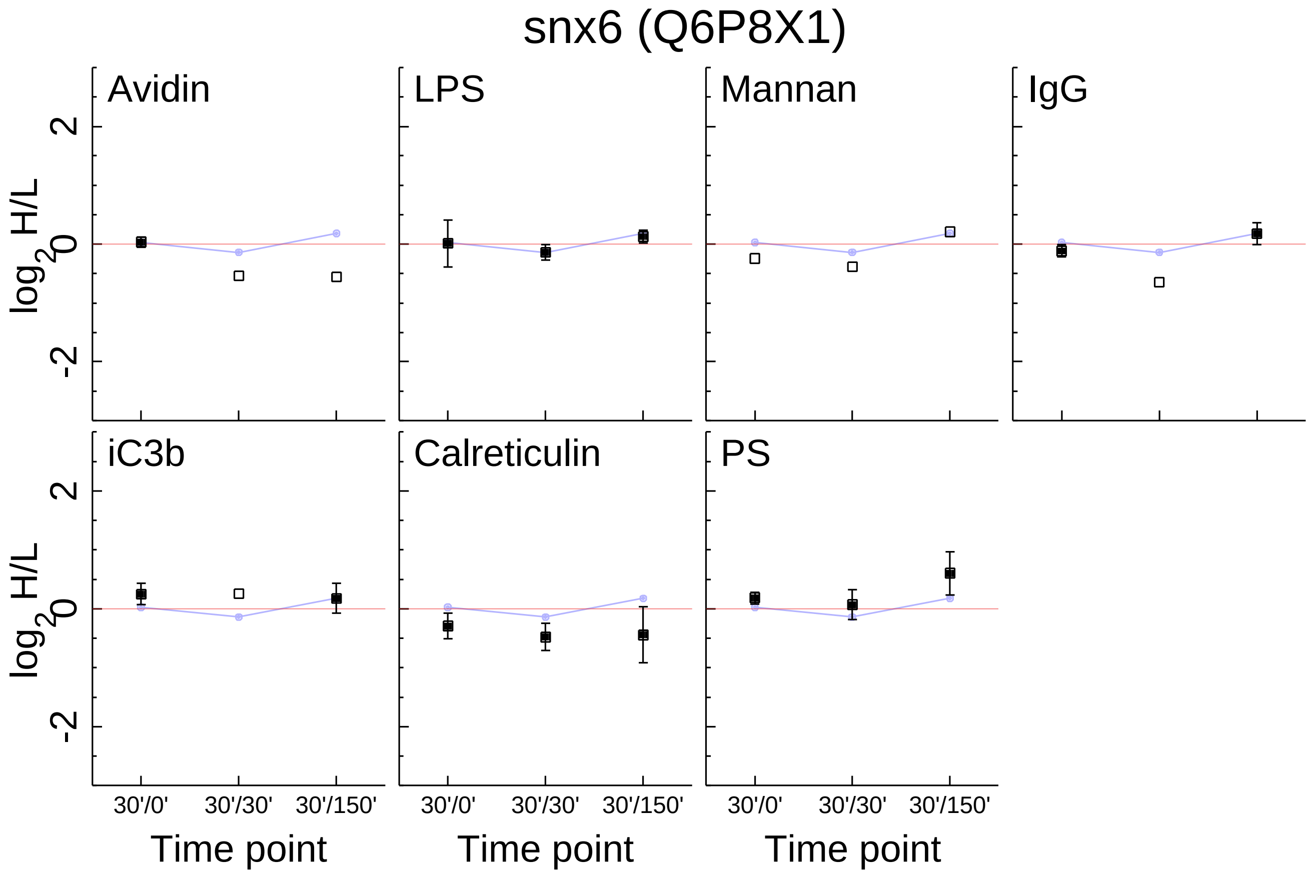
snx6 (Q6P8X1)
Avidin
LPS
Mannan
IgG
iC3b
Calreticulin
PS
log2 H/L
2
0
-2
30'/0'
30'/30'
30'/150'
Time point

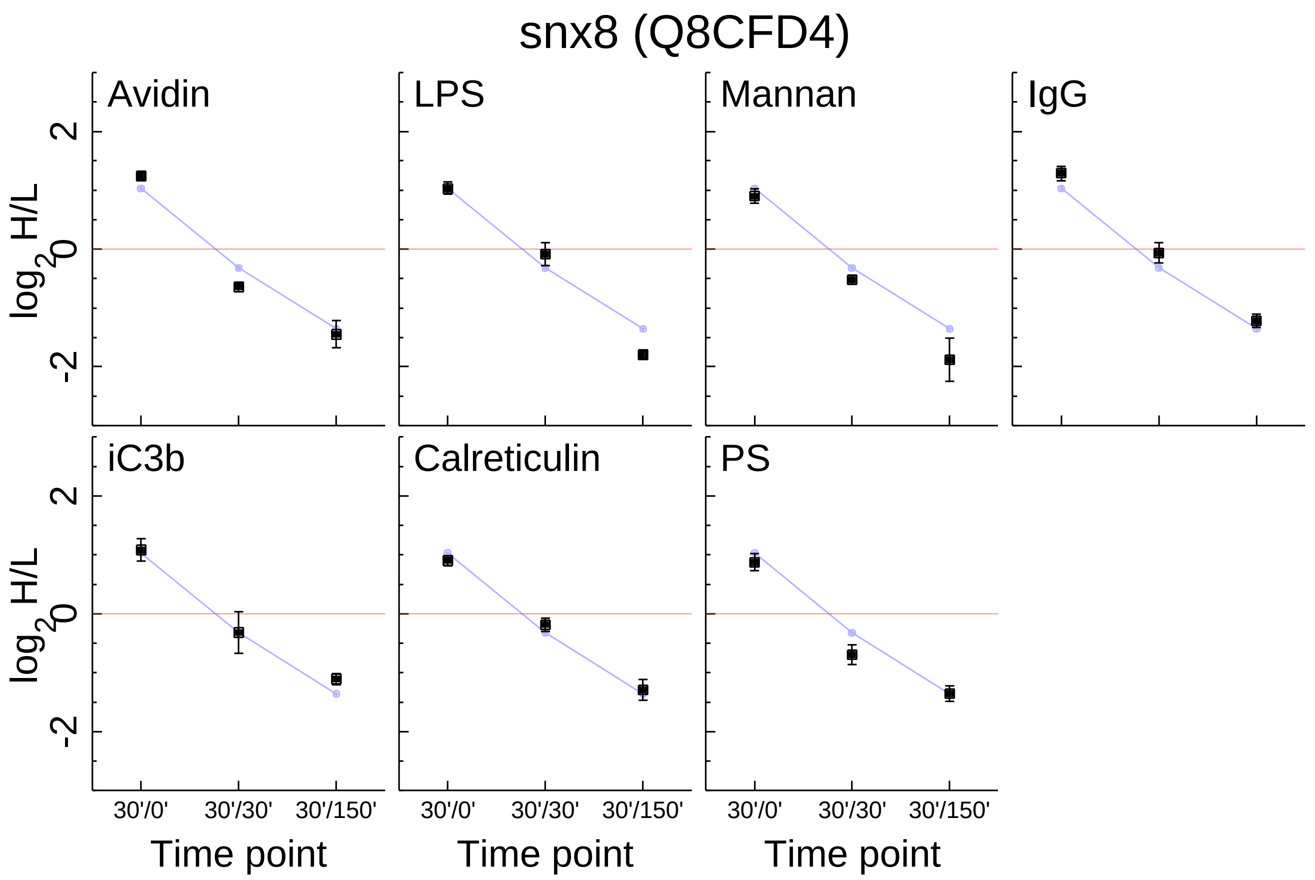
snx8 (Q8CFD4)
Avidin
LPS
Mannan
IgG
iC3b
Calreticulin
PS
log2 H/L
2
0
-2
30'/0'
30'/30'
30'/150'
Time point

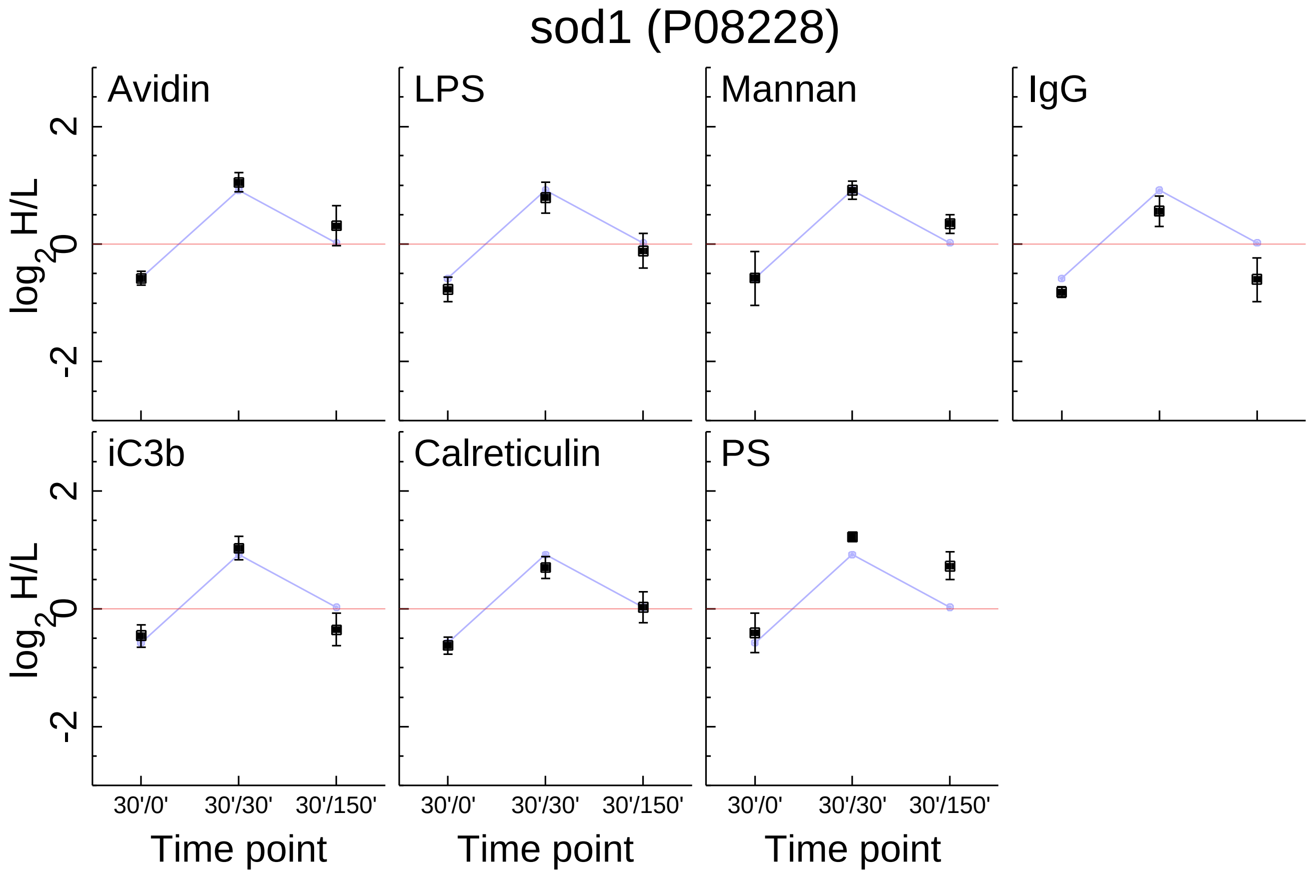

sod1 (P08228)
Avidin
LPS
Mannan
IgG
iC3b
Calreticulin
PS
log2 H/L
2
0
-2
30'/0'
30'/30'
30'/150'
Time point

sod2 (P09671)

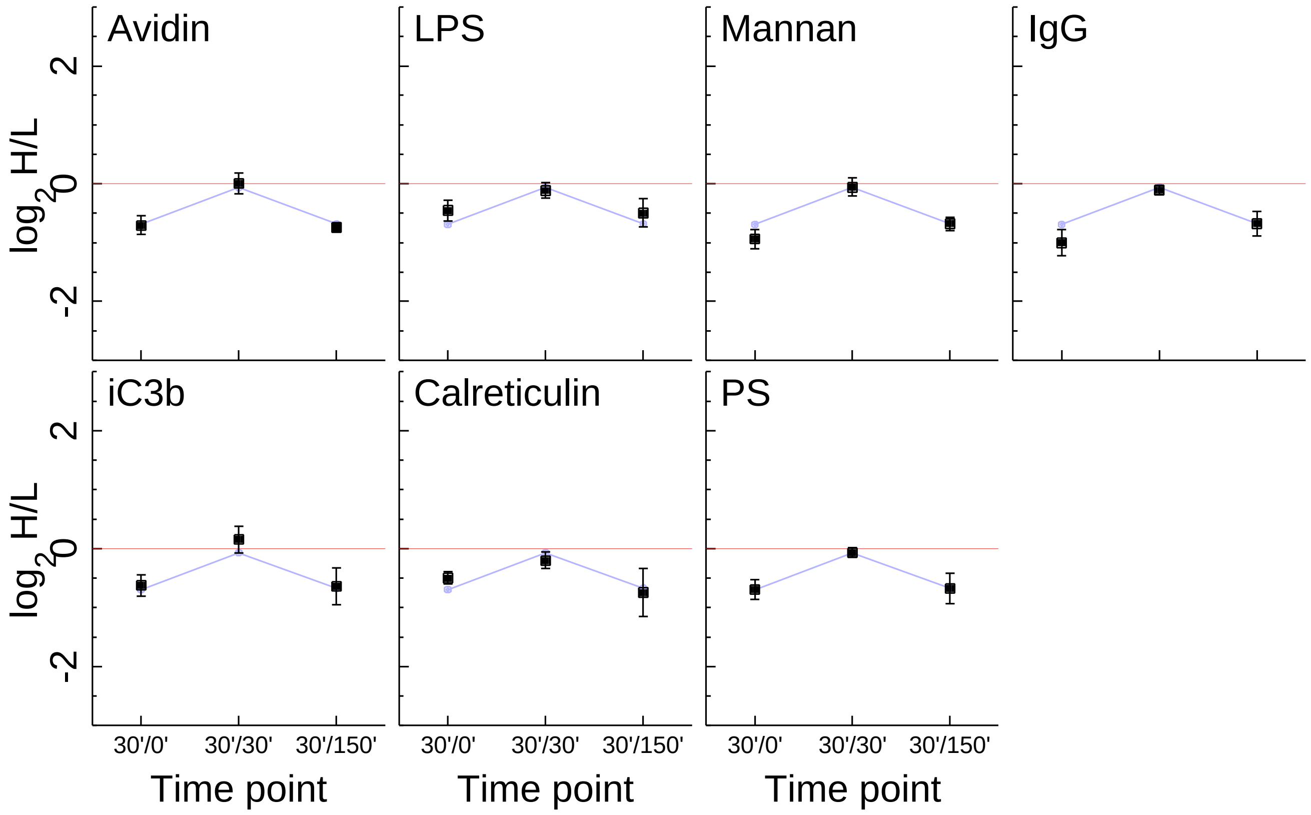

Avidin
LPS
Mannan
IgG
iC3b
Calreticulin
PS
log2 H/L
2
0
-2
30'/0'
30'/30'
30'/150'
Time point

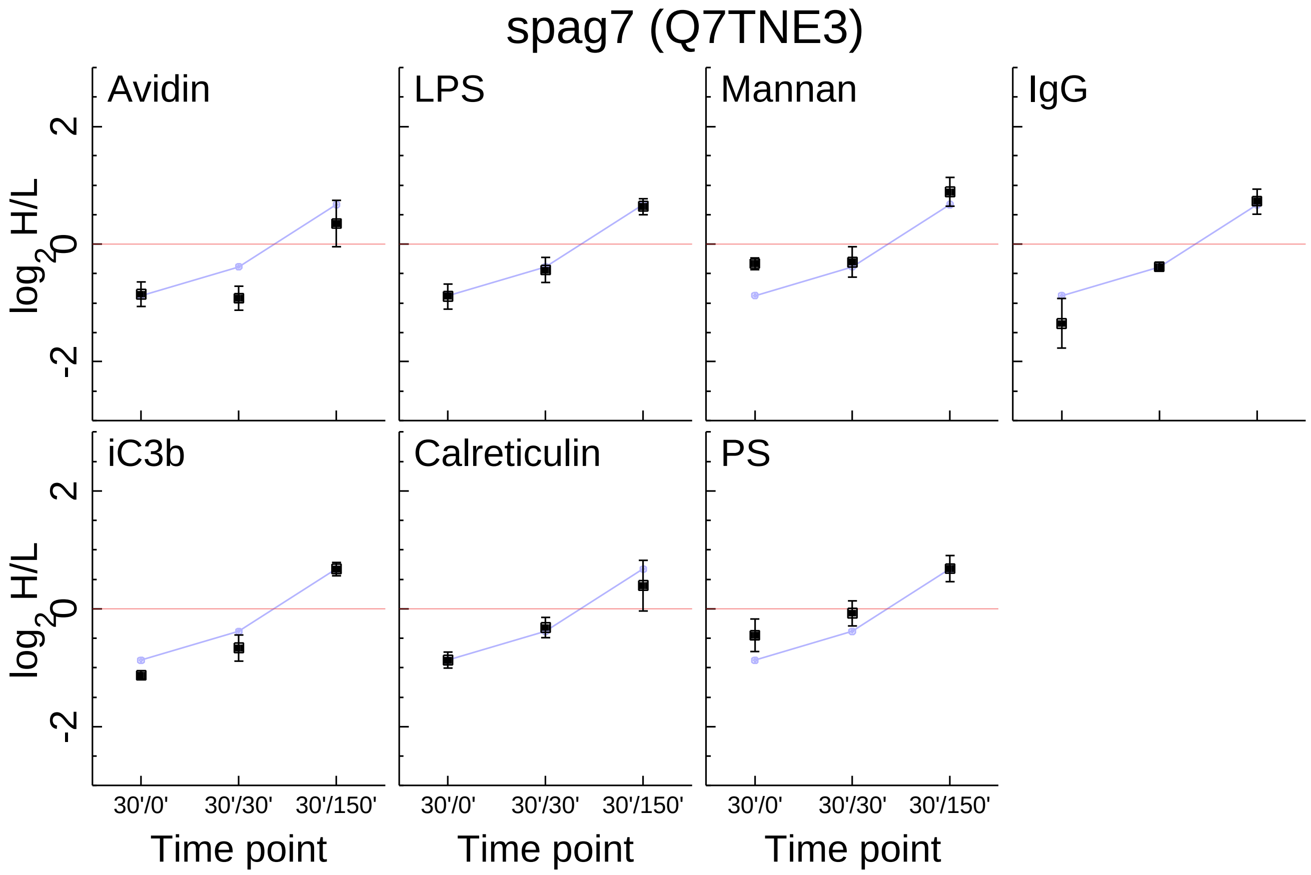
spag7 (Q7TNE3)
Avidin
LPS
Mannan
IgG
iC3b
Calreticulin
PS
log2 H/L
2
0
-2
30'/0'
30'/30'
30'/150'
Time point

spcs2 (Q9CYN2)

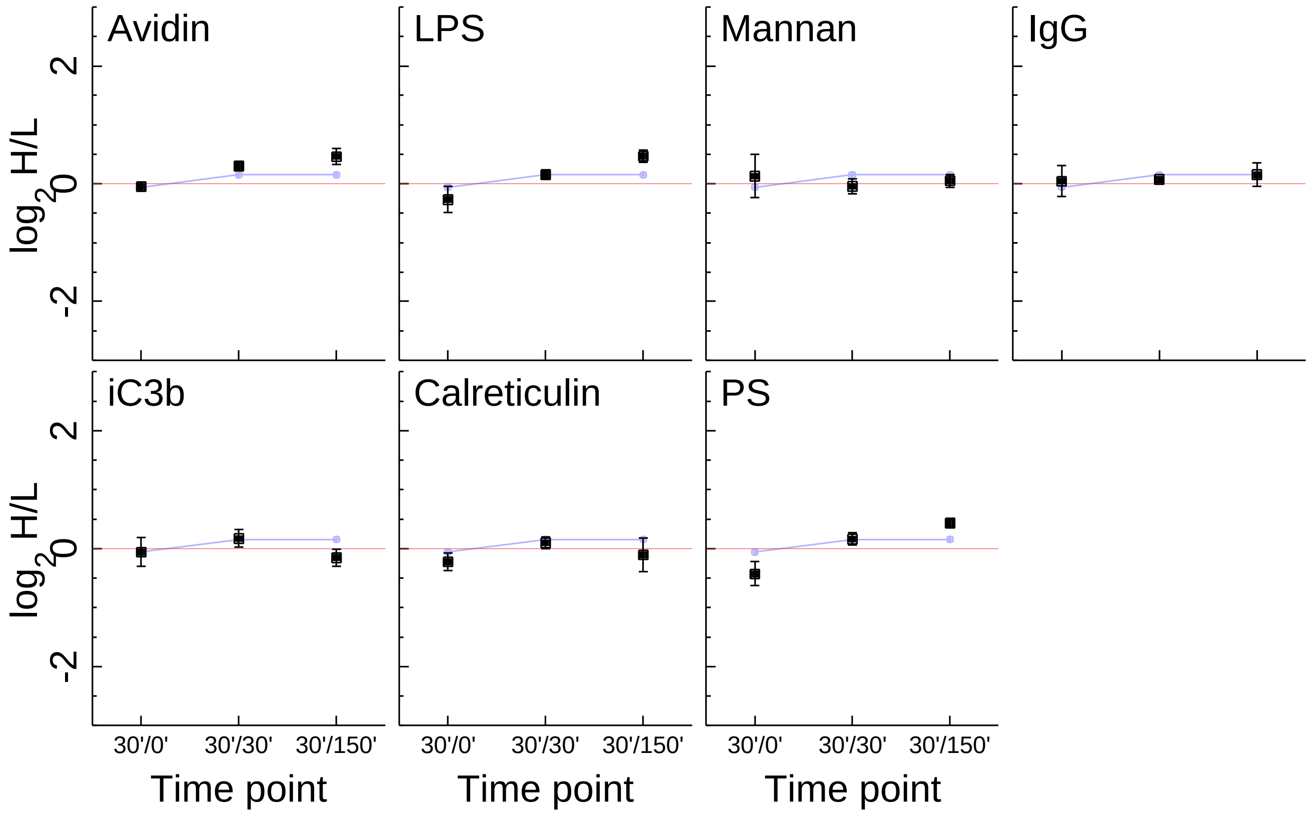

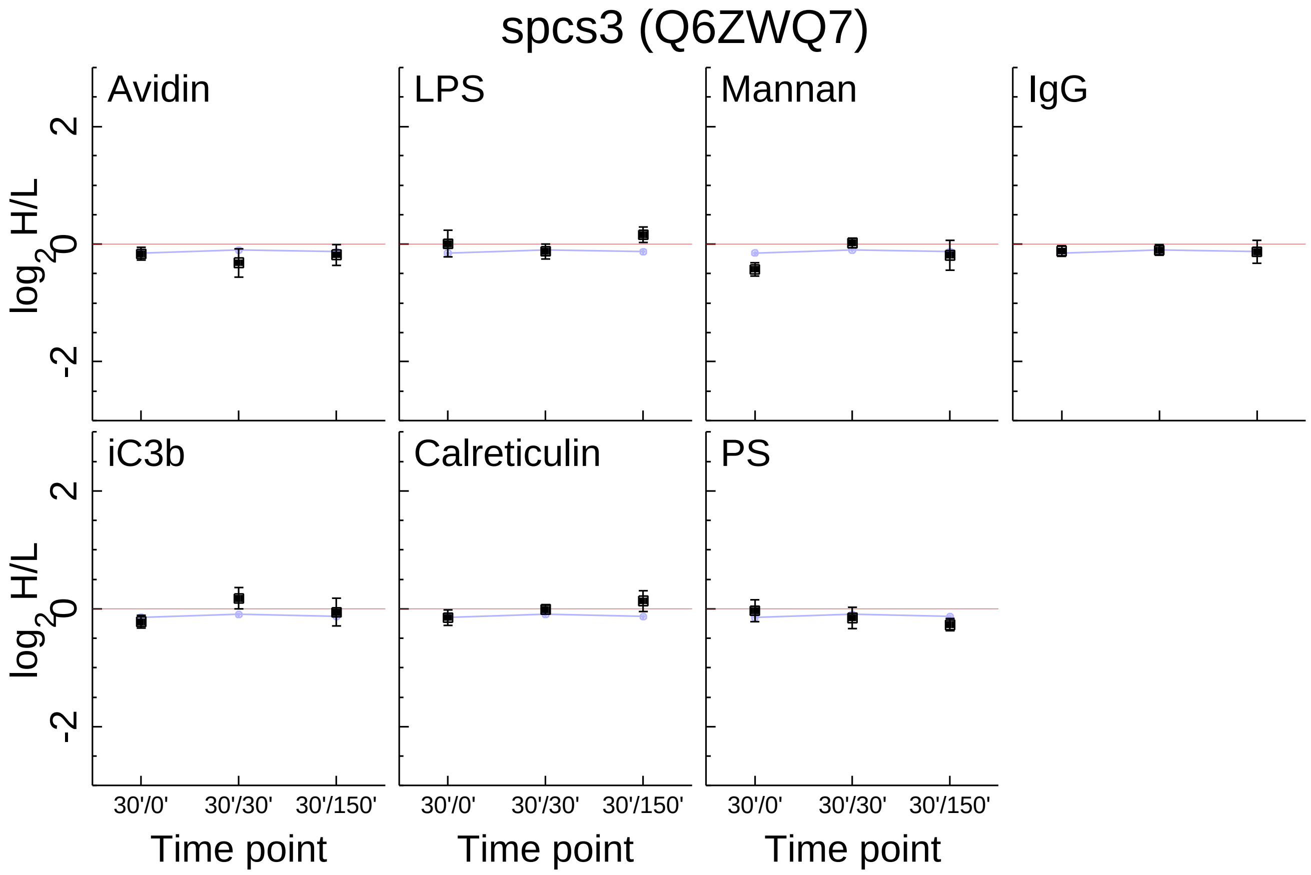
spcs3 (Q6ZWQ7)
Avidin
LPS
Mannan
IgG
iC3b
Calreticulin
PS
log2 H/L
2
0
-2
30'/0'
30'/30'
30'/150'
Time point

spe39 (Q8BGQ1)

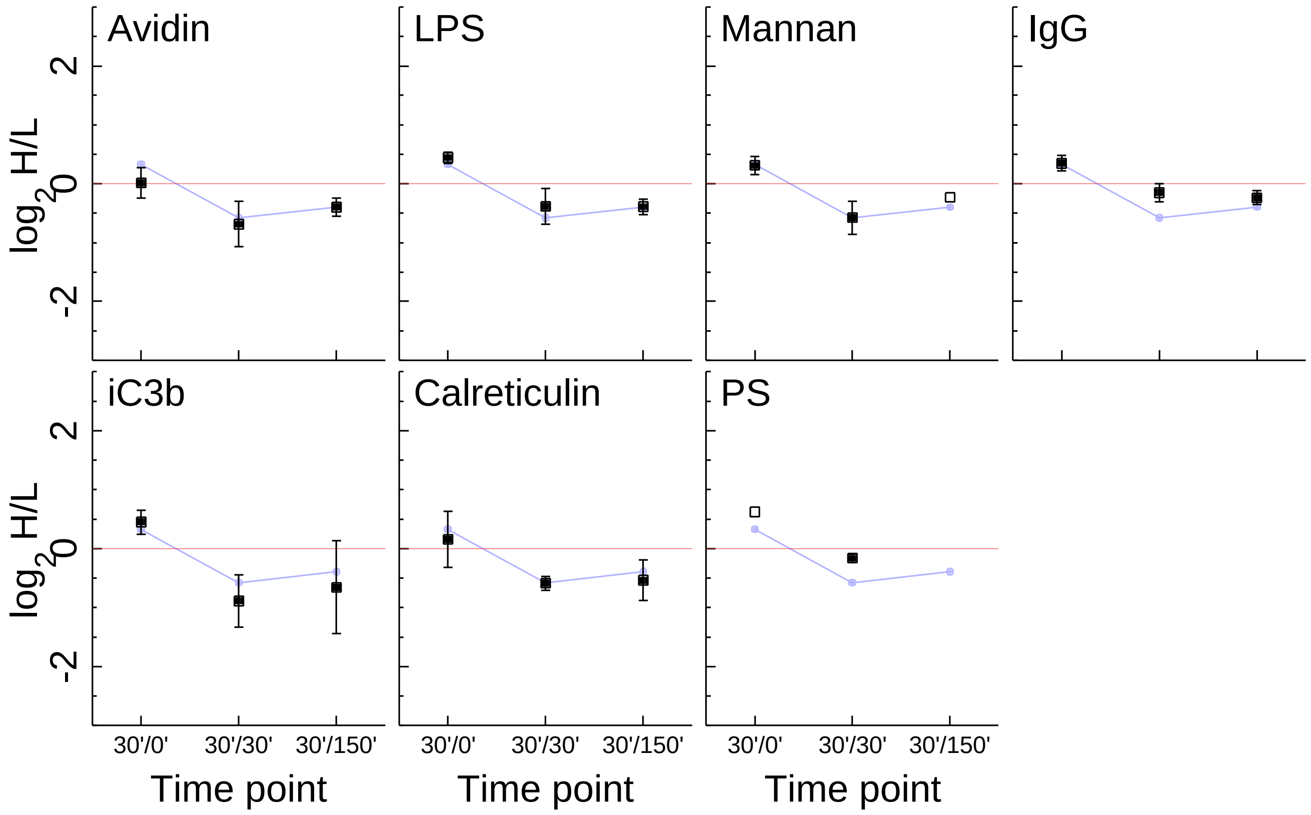

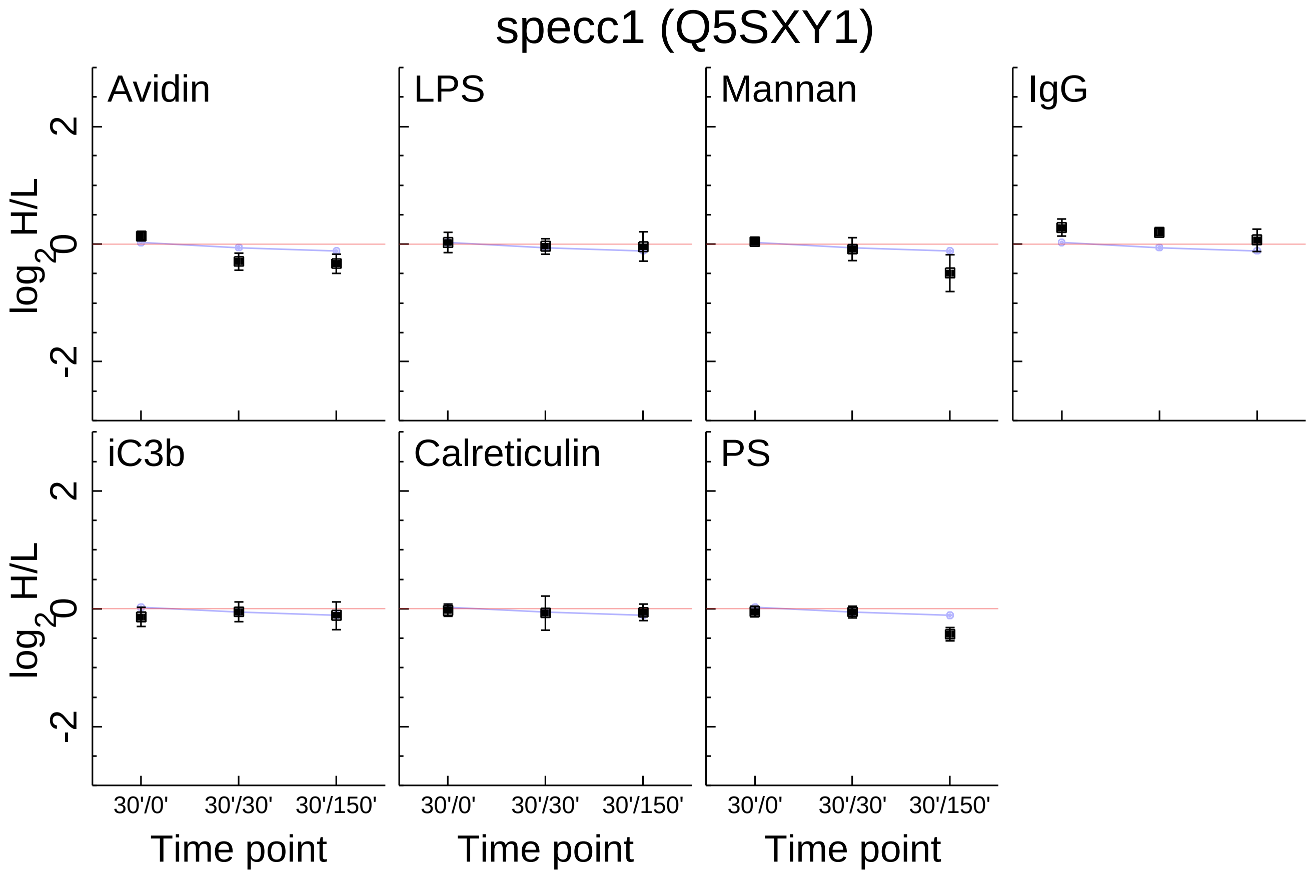
specc1 (Q5SXY1)
Avidin
LPS
Mannan
IgG
iC3b
Calreticulin
PS
log2 H/L
2
0
-2
30'/0'
30'/30'
30'/150'
Time point

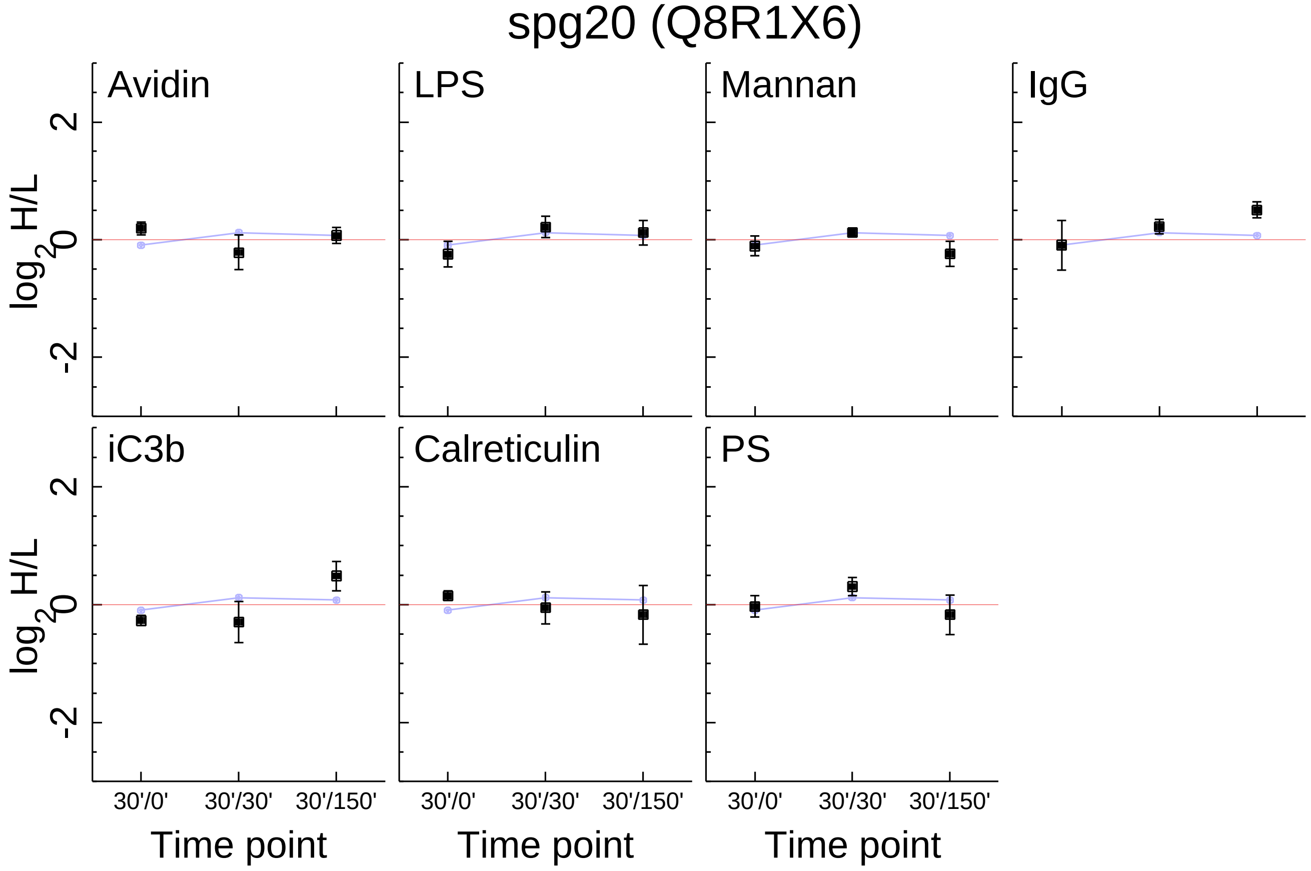

spg20 (Q8R1X6)
Avidin
LPS
Mannan
IgG
iC3b
Calreticulin
PS
log2 H/L
2
0
-2
30'/0'
30'/30'
30'/150'
Time point

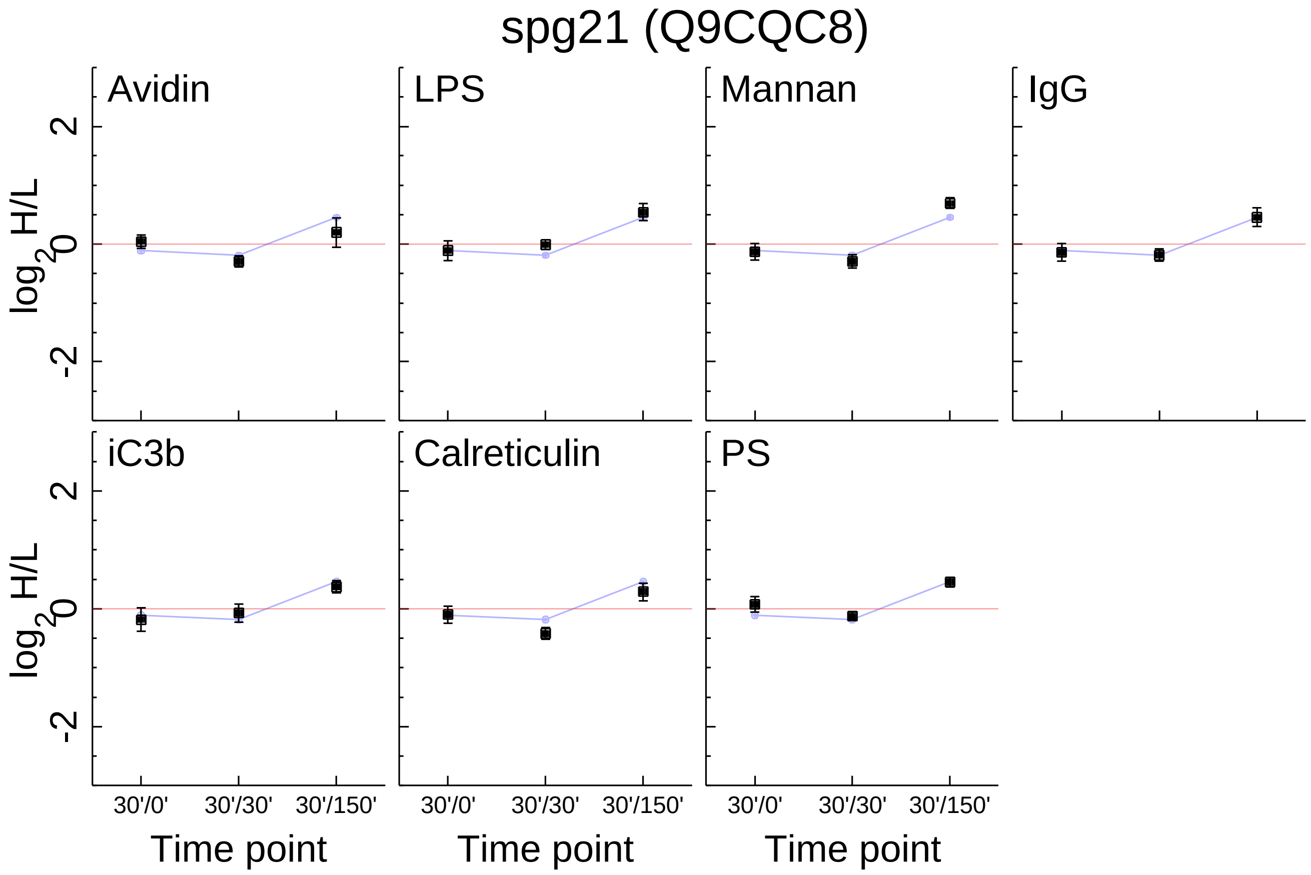
spg21 (Q9CQC8)
Avidin
LPS
Mannan
IgG
iC3b
Calreticulin
PS
log2 H/L
2
0
-2
30'/0'
30'/30'
30'/150'
Time point

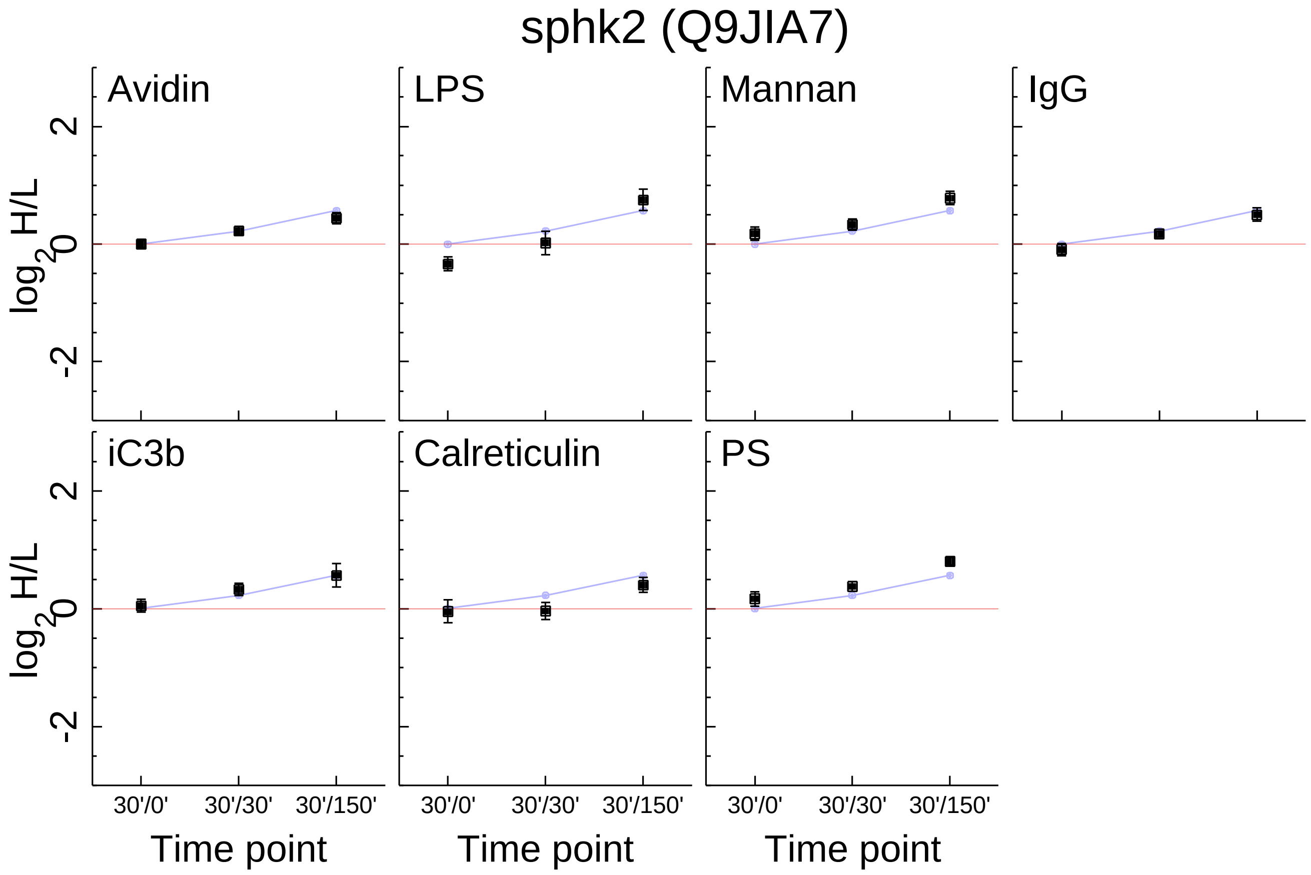
sphk2 (Q9JIA7)
Avidin
LPS
Mannan
IgG
iC3b
Calreticulin
PS
log2 H/L
2
0
-2
30'/0'
30'/30'
30'/150'
Time point

spn (P15702)

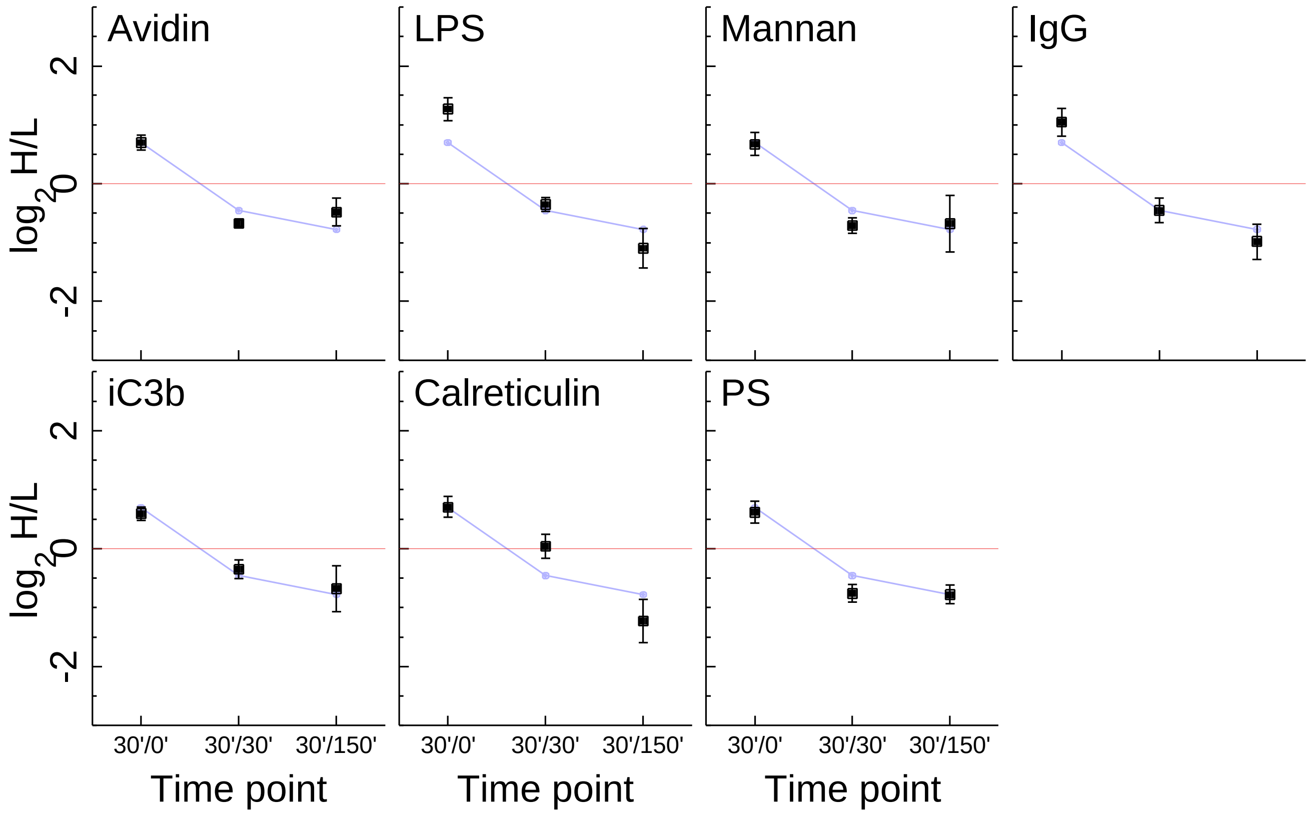

Avidin
LPS
Mannan
IgG
iC3b
Calreticulin
PS
log2 H/L
2
0
-2
30'/0'
30'/30'
30'/150'
Time point

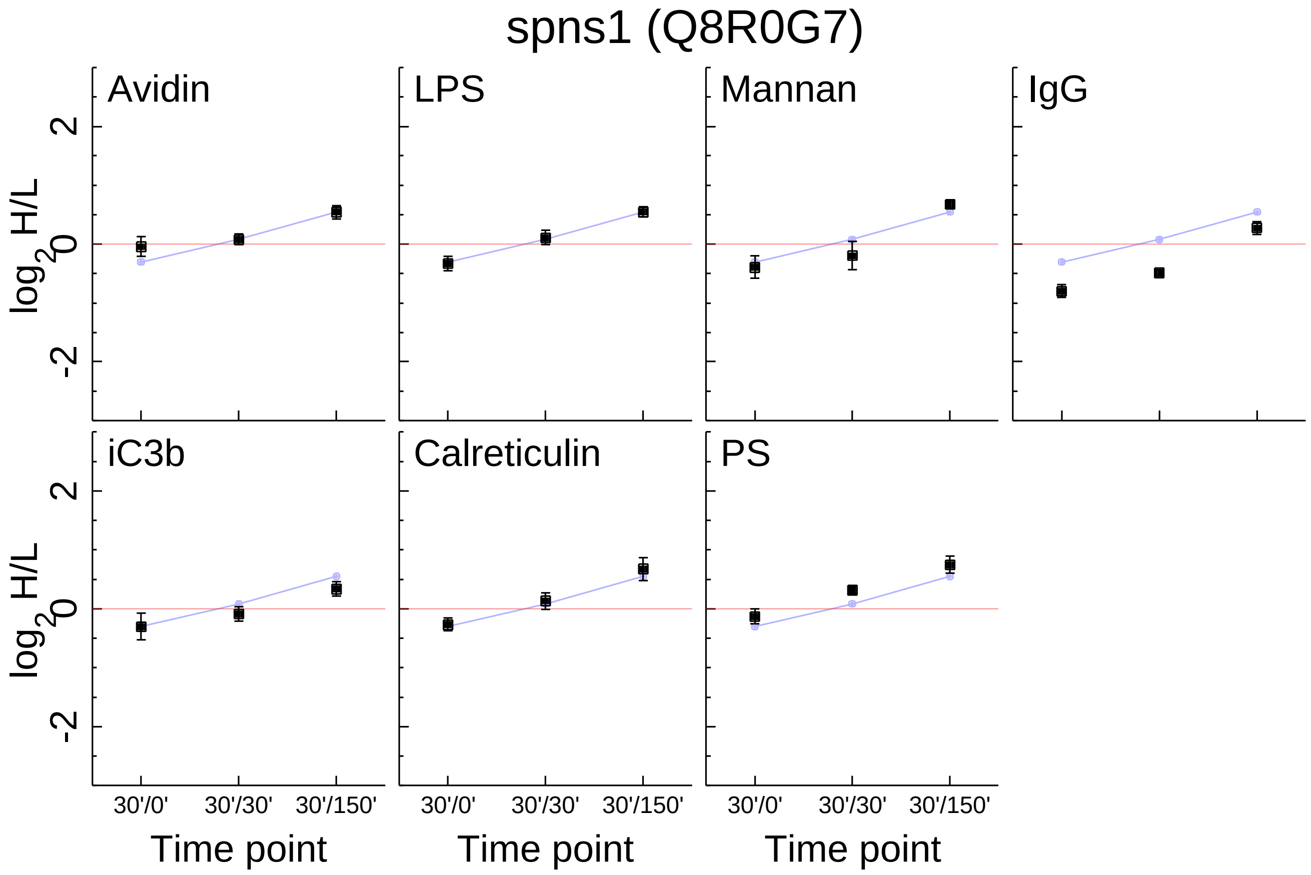
spns1 (Q8R0G7)
Avidin
LPS
Mannan
IgG
iC3b
Calreticulin
PS
log2 H/L
2
0
-2
30'/0'
30'/30'
30'/150'
Time point

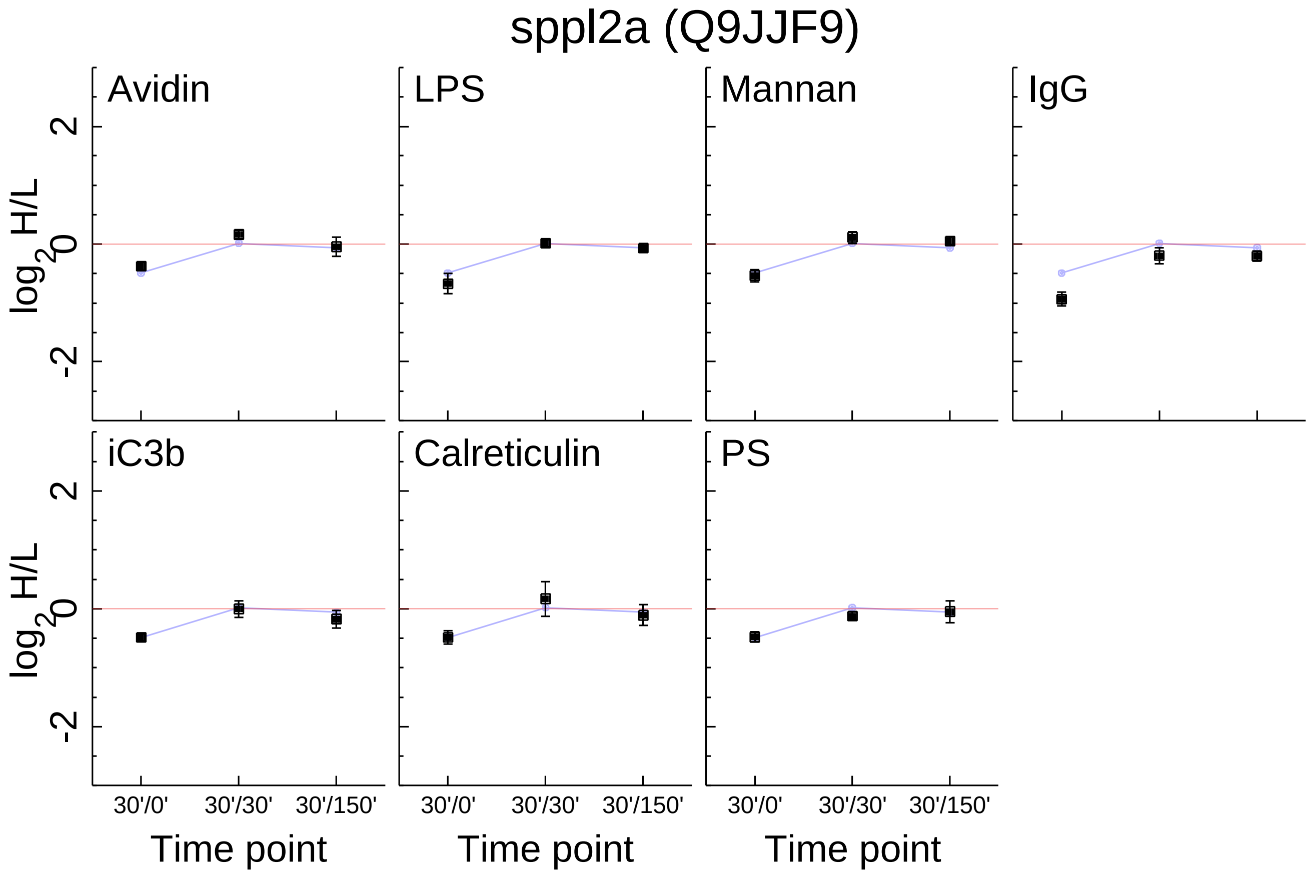

sppl2a (Q9JJF9)
Avidin
LPS
Mannan
IgG
iC3b
Calreticulin
PS
log2 H/L
2
0
-2
30'/0'
30'/30'
30'/150'
Time point

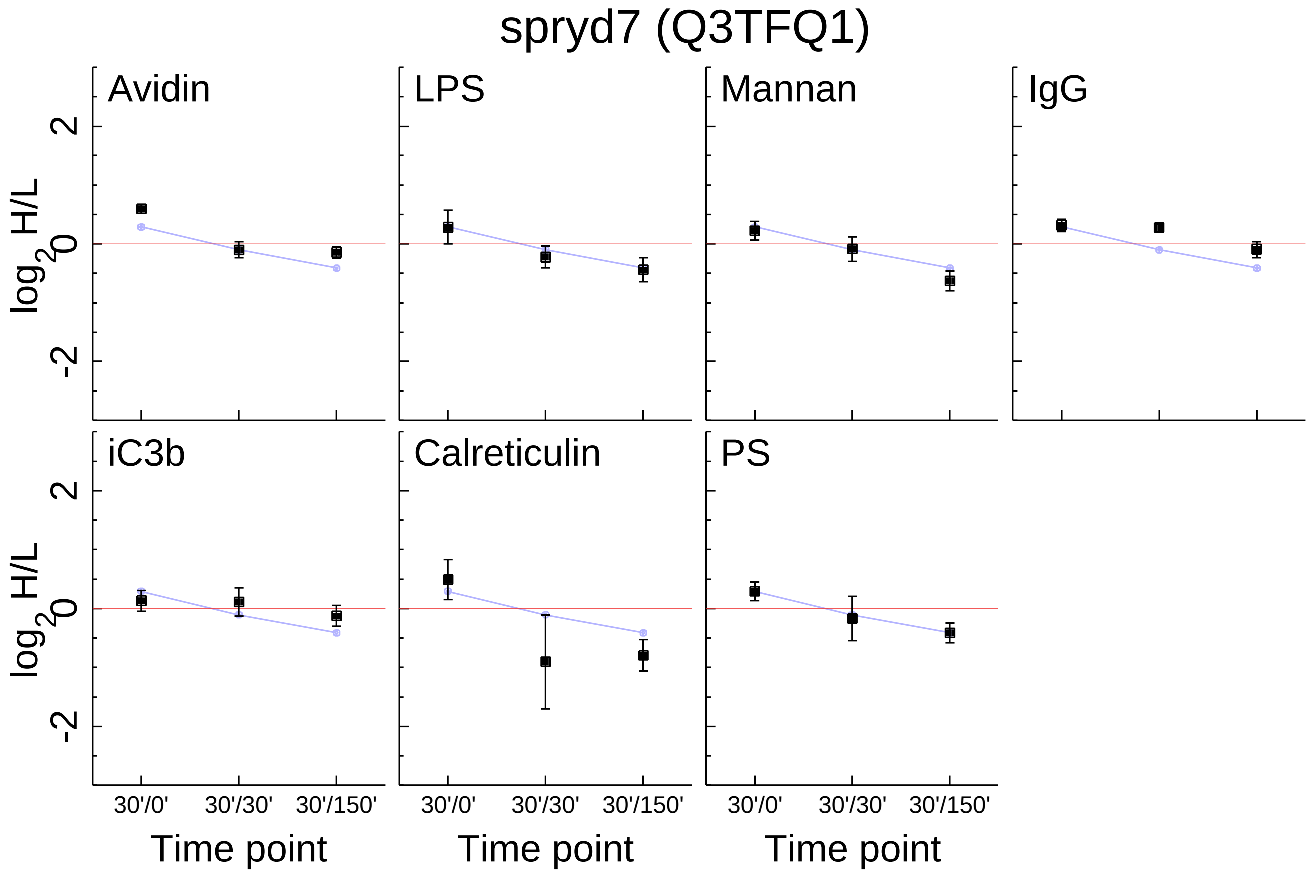
spryd7 (Q3TFQ1)
Avidin
LPS
Mannan
IgG
iC3b
Calreticulin
PS
log2 H/L
2
0
-2
30'/0'
30'/30'
30'/150'
Time point

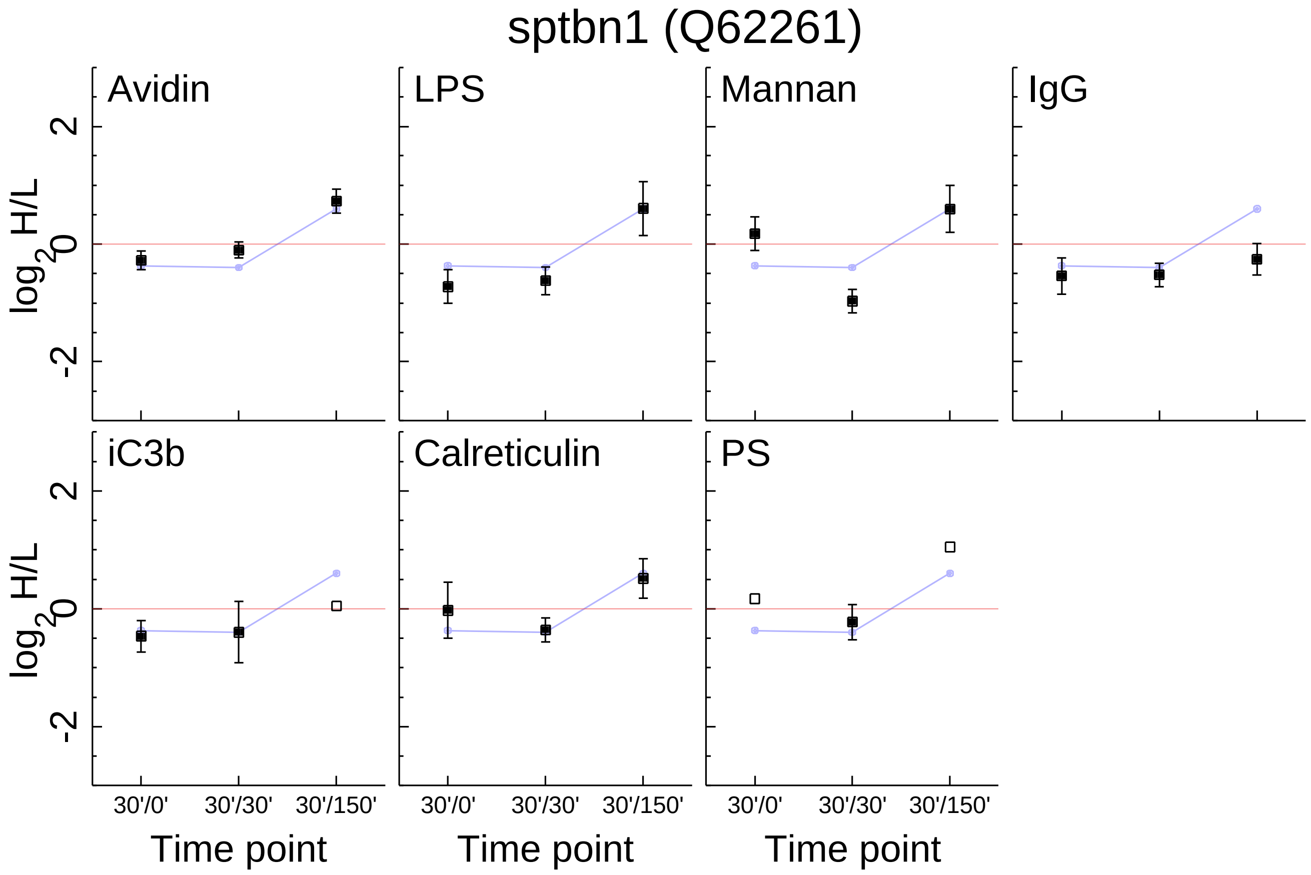

sptbn1 (Q62261)
Avidin
LPS
Mannan
IgG
iC3b
Calreticulin
PS
log2 H/L
2
0
-2
30'/0'
30'/30'
30'/150'
Time point

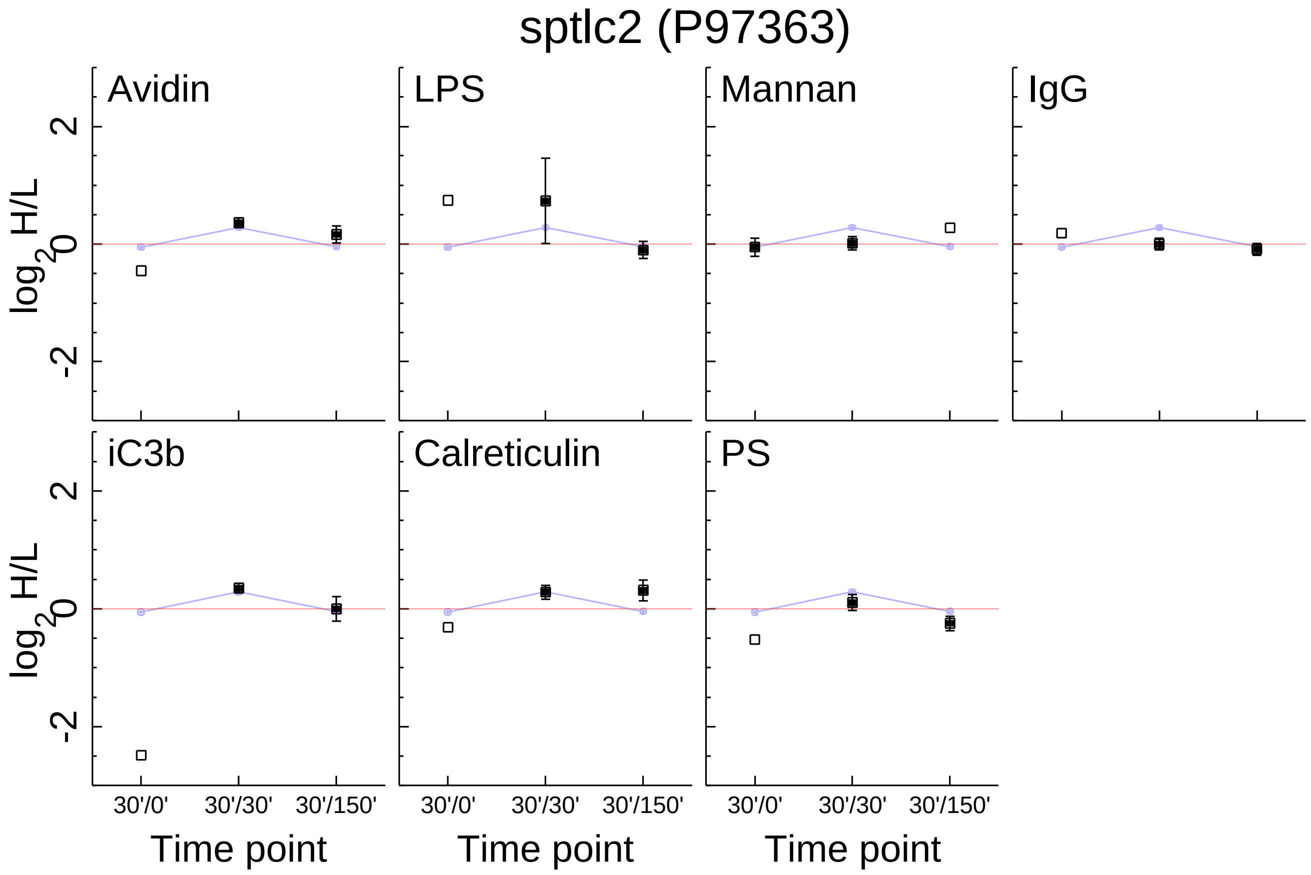

sptlc2 (P97363)
Avidin
LPS
Mannan
IgG
iC3b
Calreticulin
PS
log2 H/L
2
0
-2
30'/0'
30'/30'
30'/150'
Time point

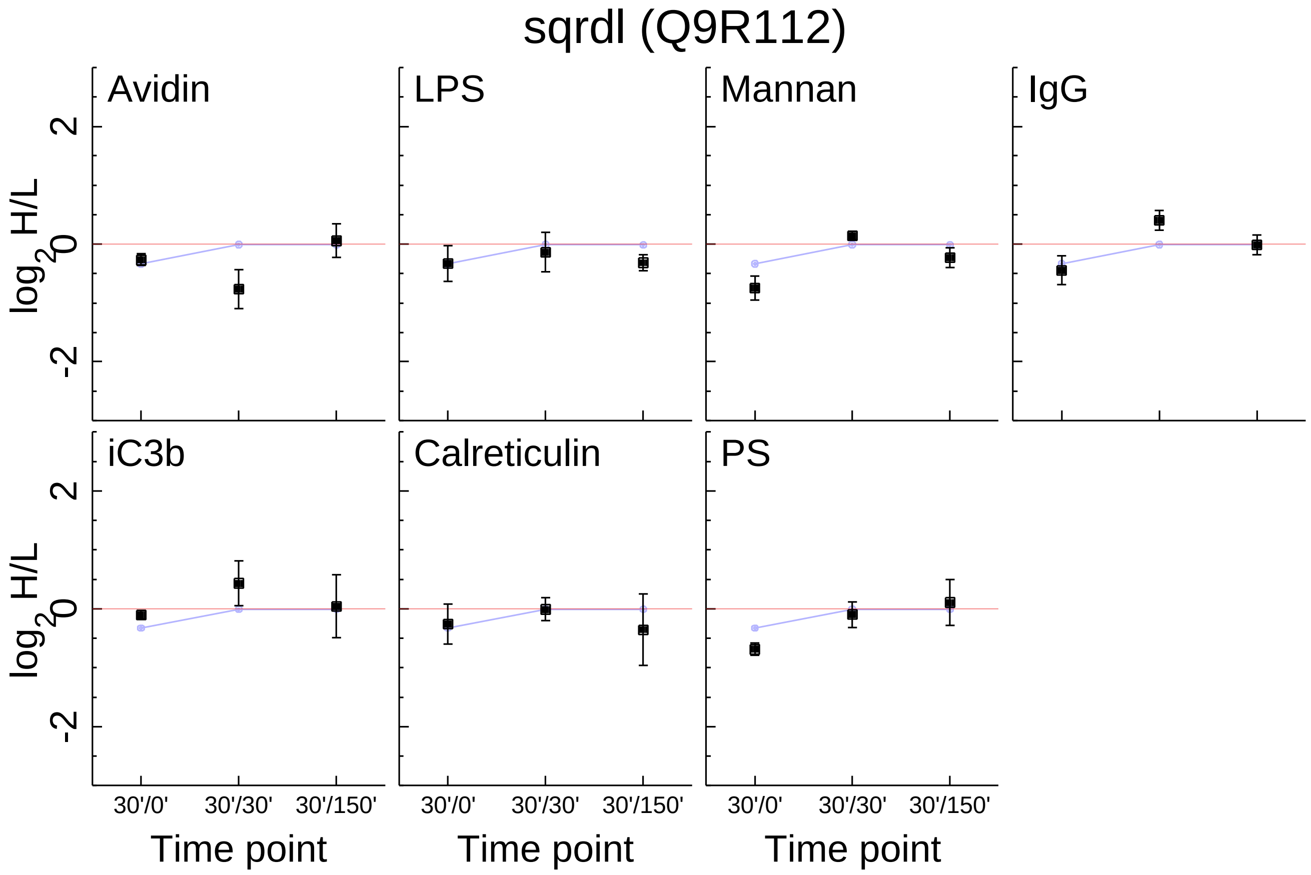

sqrdl (Q9R112)
Avidin
LPS
Mannan
IgG
iC3b
Calreticulin
PS
log2 H/L
2
0
-2
30'/0'
30'/30'
30'/150'
Time point

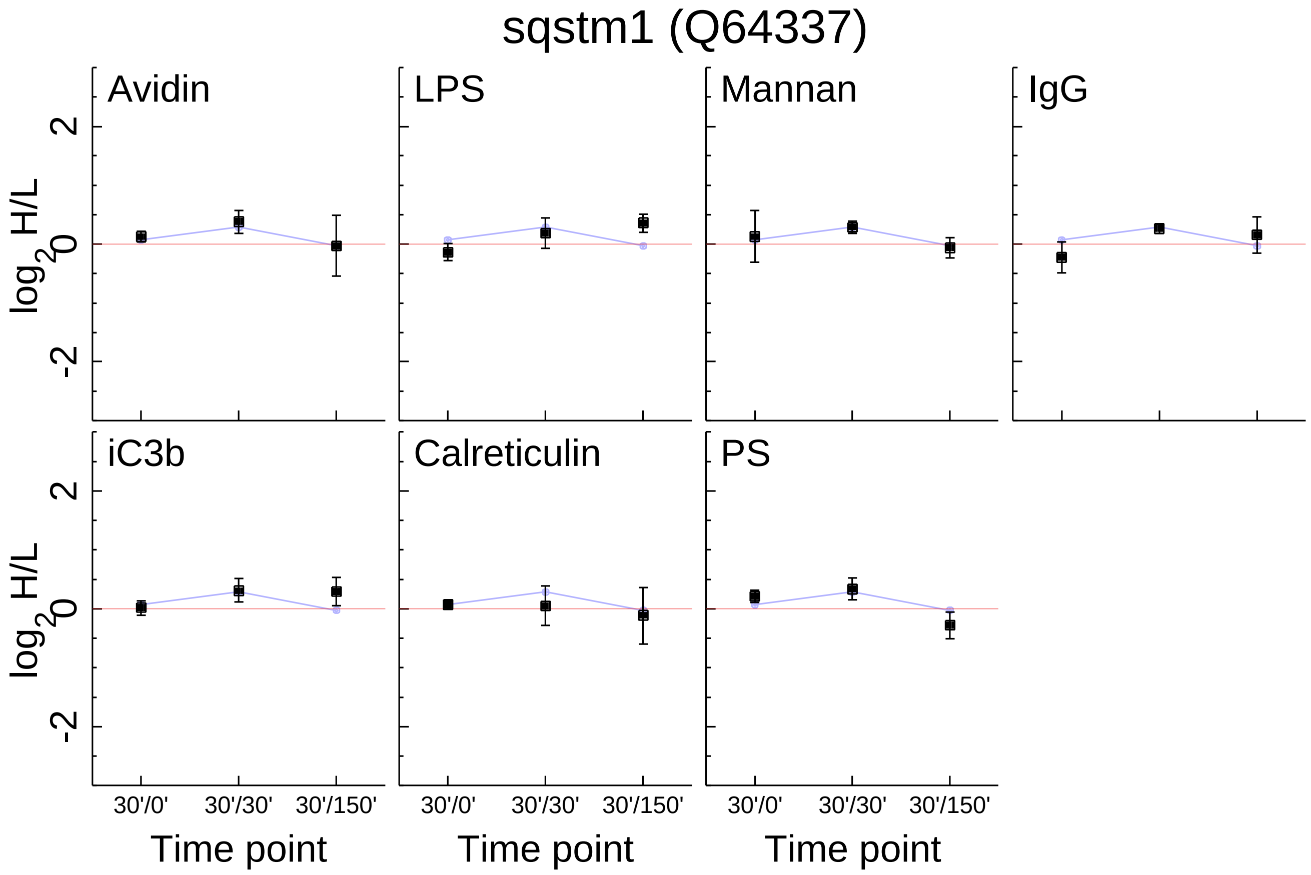
sqstm1 (Q64337)
Avidin
LPS
Mannan
IgG
iC3b
Calreticulin
PS
log2 H/L
2
0
-2
30'/0'
30'/30'
30'/150'
Time point

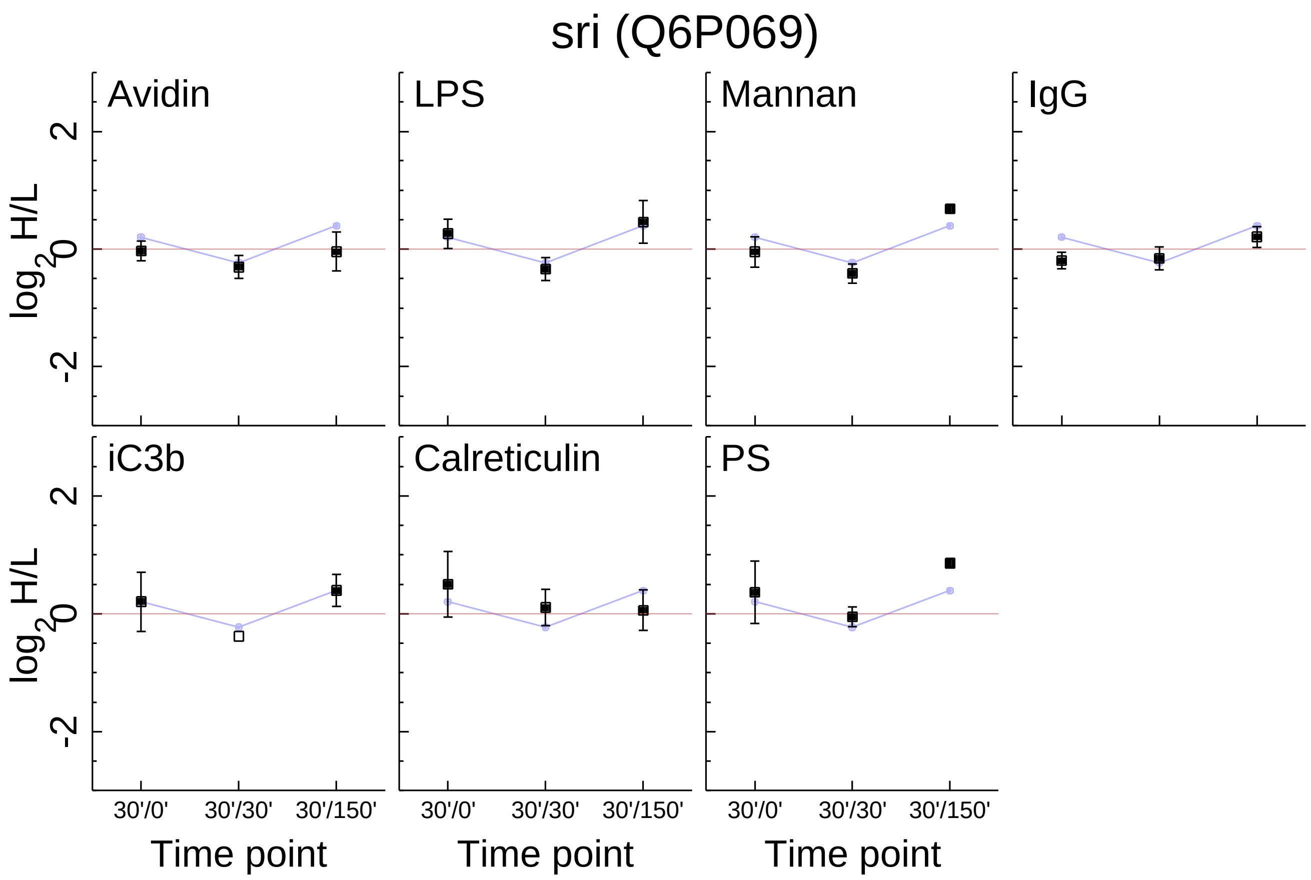
sri (Q6P069)
Avidin
LPS
Mannan
IgG
iC3b
Calreticulin
PS
2
0
-2
log2 H/L
30'/0'
30'/30'
30'/150'
Time point

srp14 (P16254)

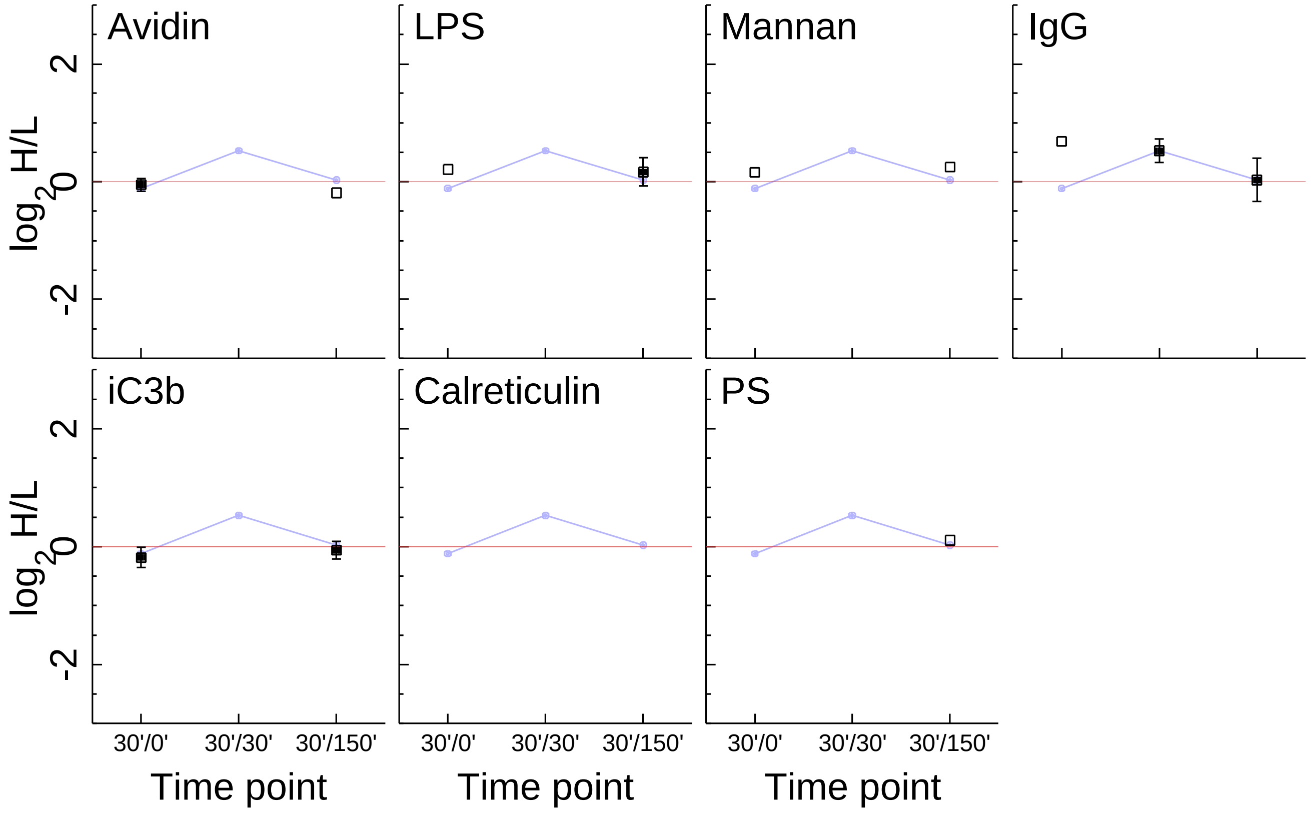

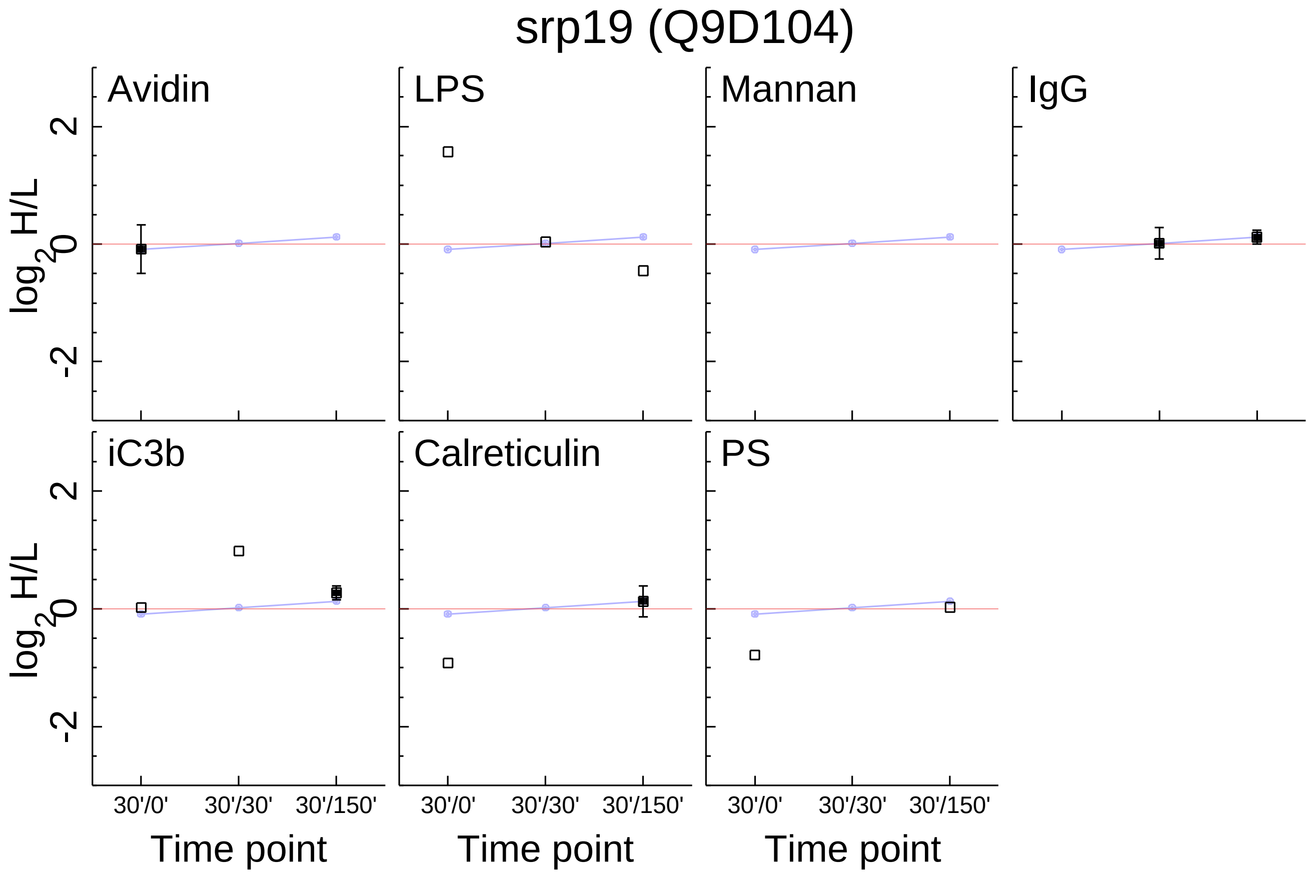
srp19 (Q9D104)
Avidin
LPS
Mannan
IgG
iC3b
Calreticulin
PS
log2 H/L
2
0
-2
30'/0'
30'/30'
30'/150'
Time point

srp54 (P14576)

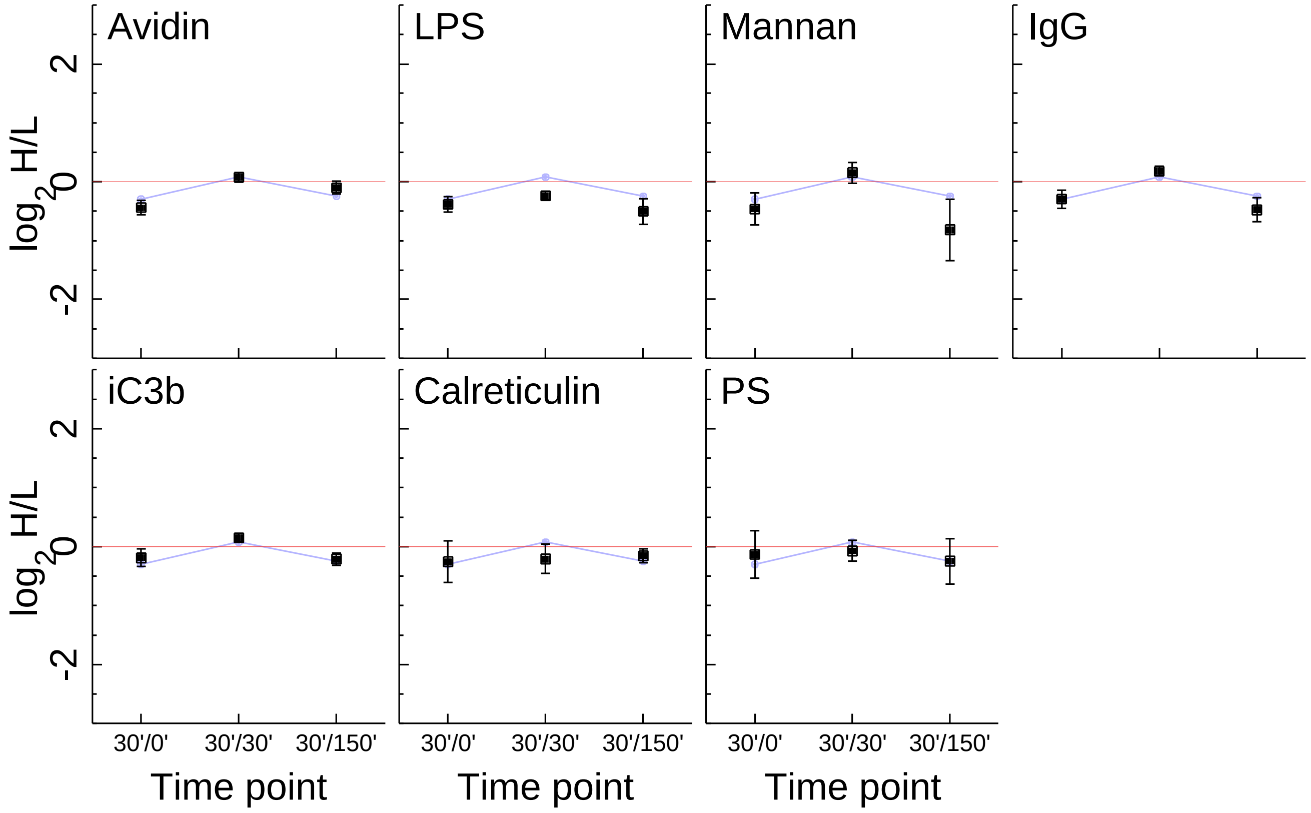

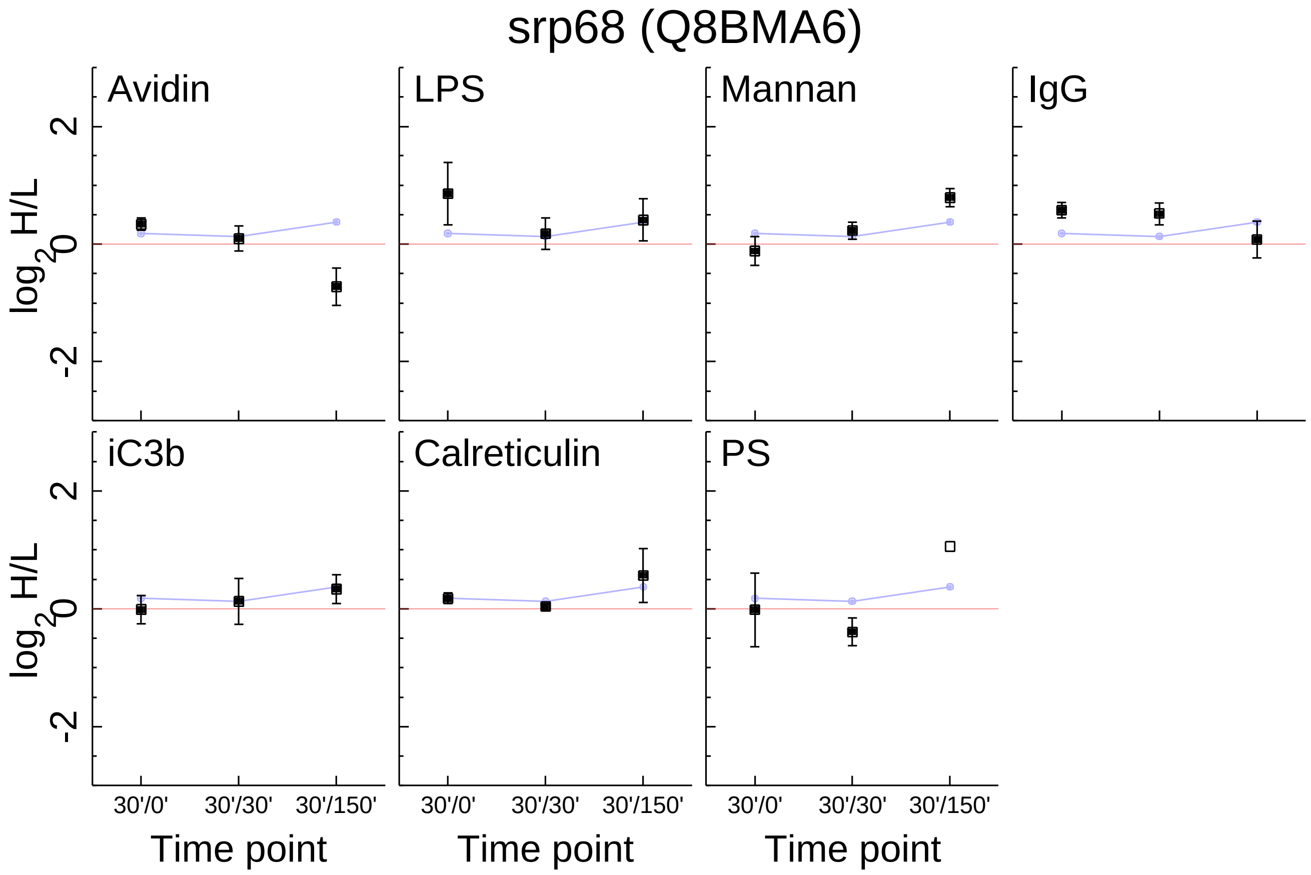

srp68 (Q8BMA6)
Avidin
LPS
Mannan
IgG
iC3b
Calreticulin
PS
2
0
-2
log2 H/L
30'/0'
30'/30'
30'/150'
Time point

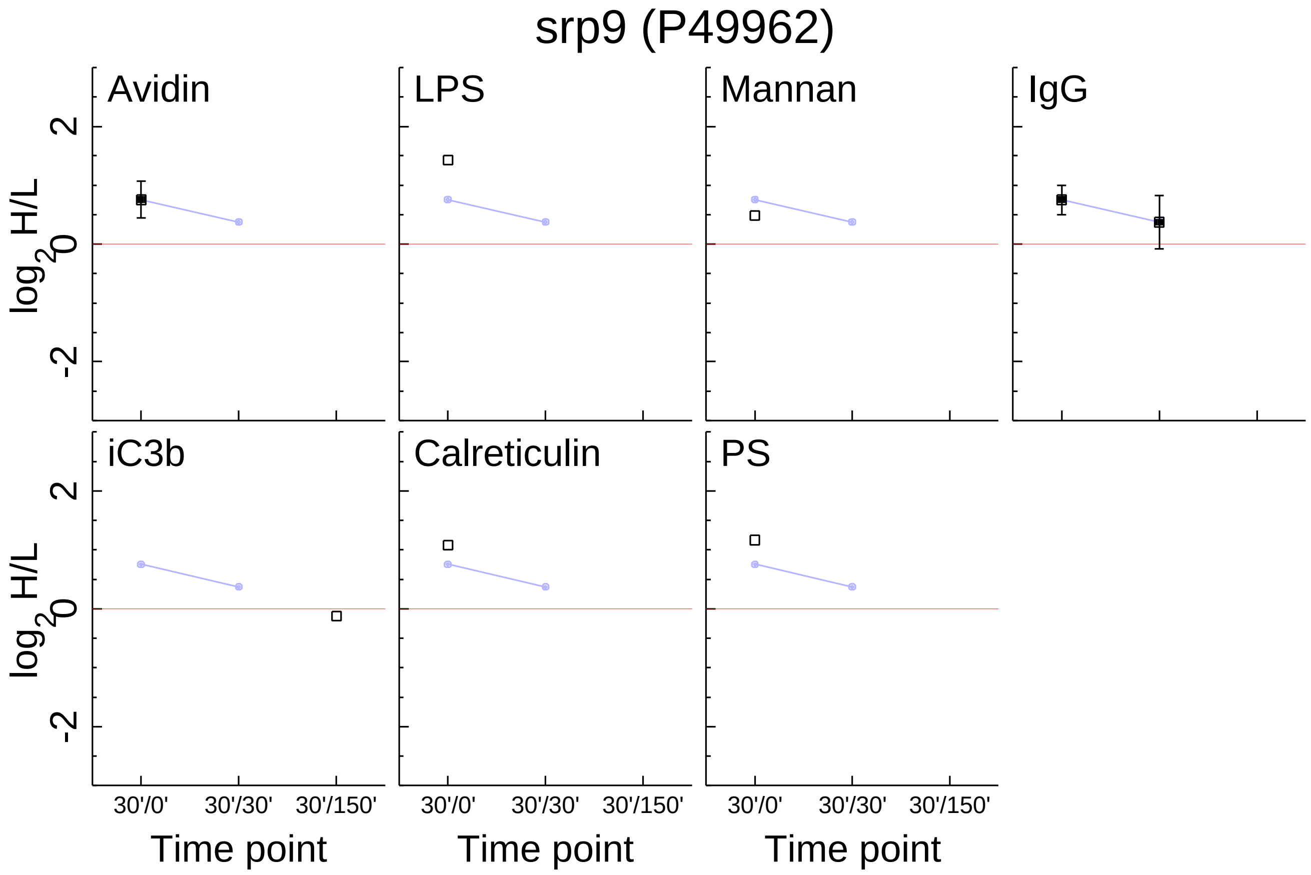
srp9 (P49962)
Avidin
LPS
Mannan
IgG
iC3b
Calreticulin
PS
2
0
-2
$\log_2$ H/L
30'/0'
30'/30'
30'/150'
Time point

srsf3 (P84104)

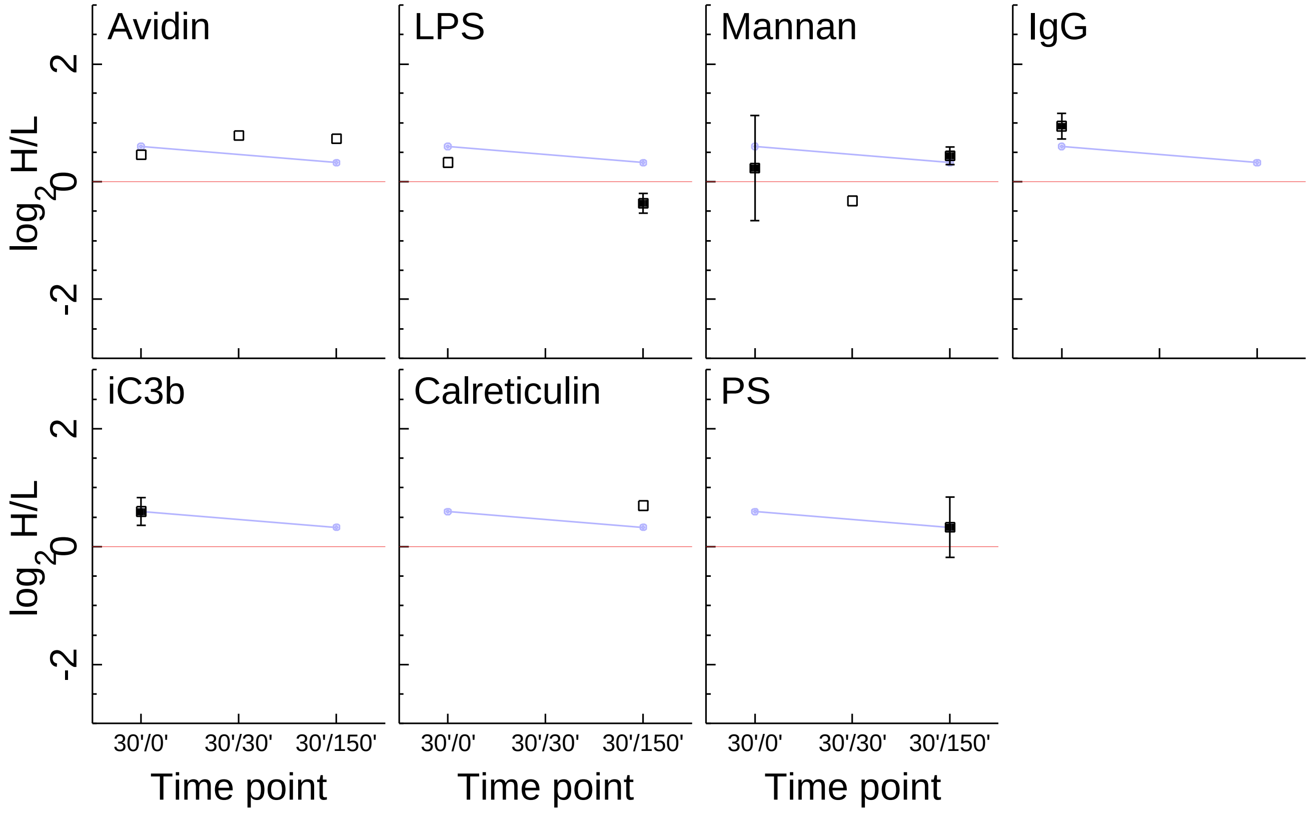

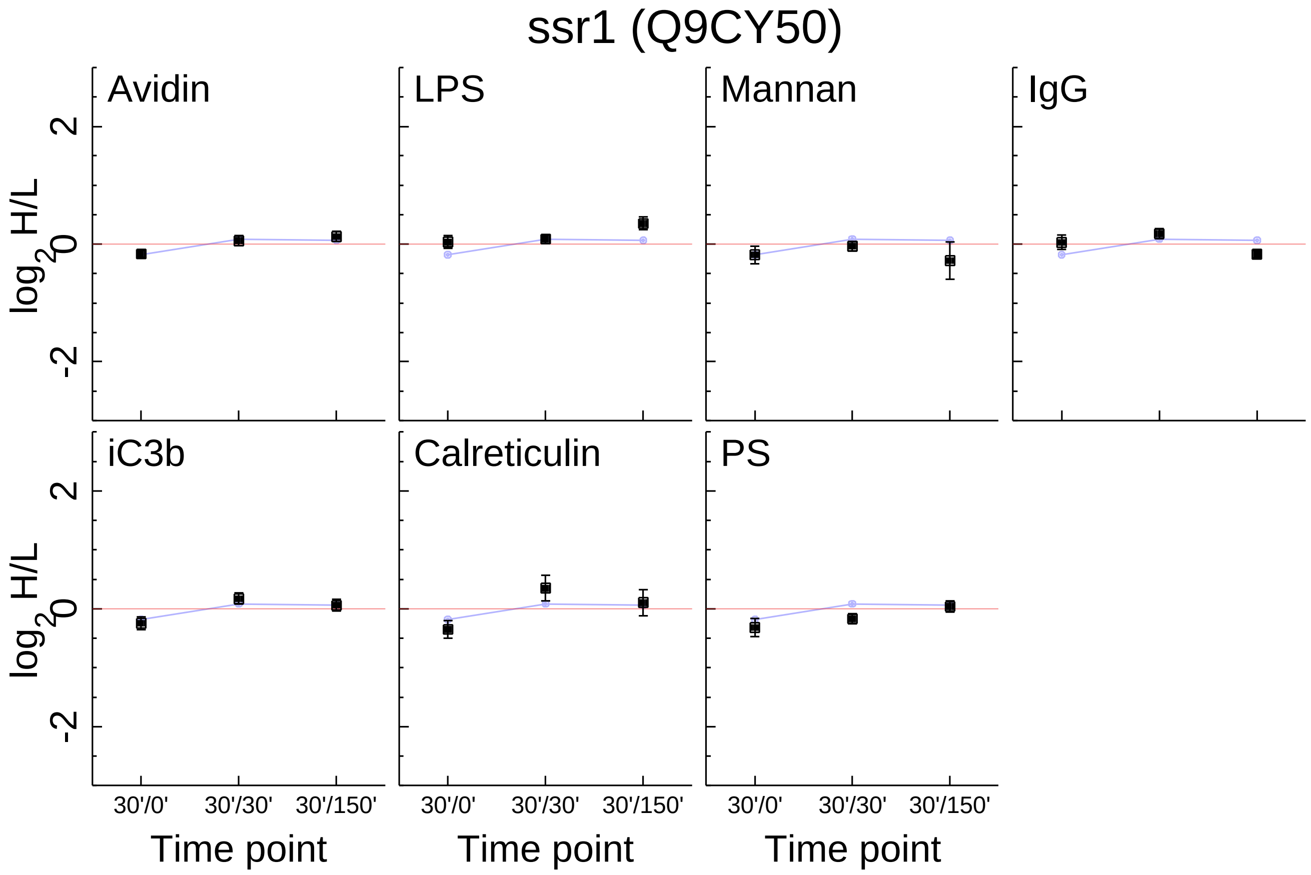

ssr1 (Q9CY50)
Avidin
LPS
Mannan
IgG
iC3b
Calreticulin
PS
log2 H/L
2
0
-2
30'/0'
30'/30'
30'/150'
Time point

ssr3 (Q9DCF9)

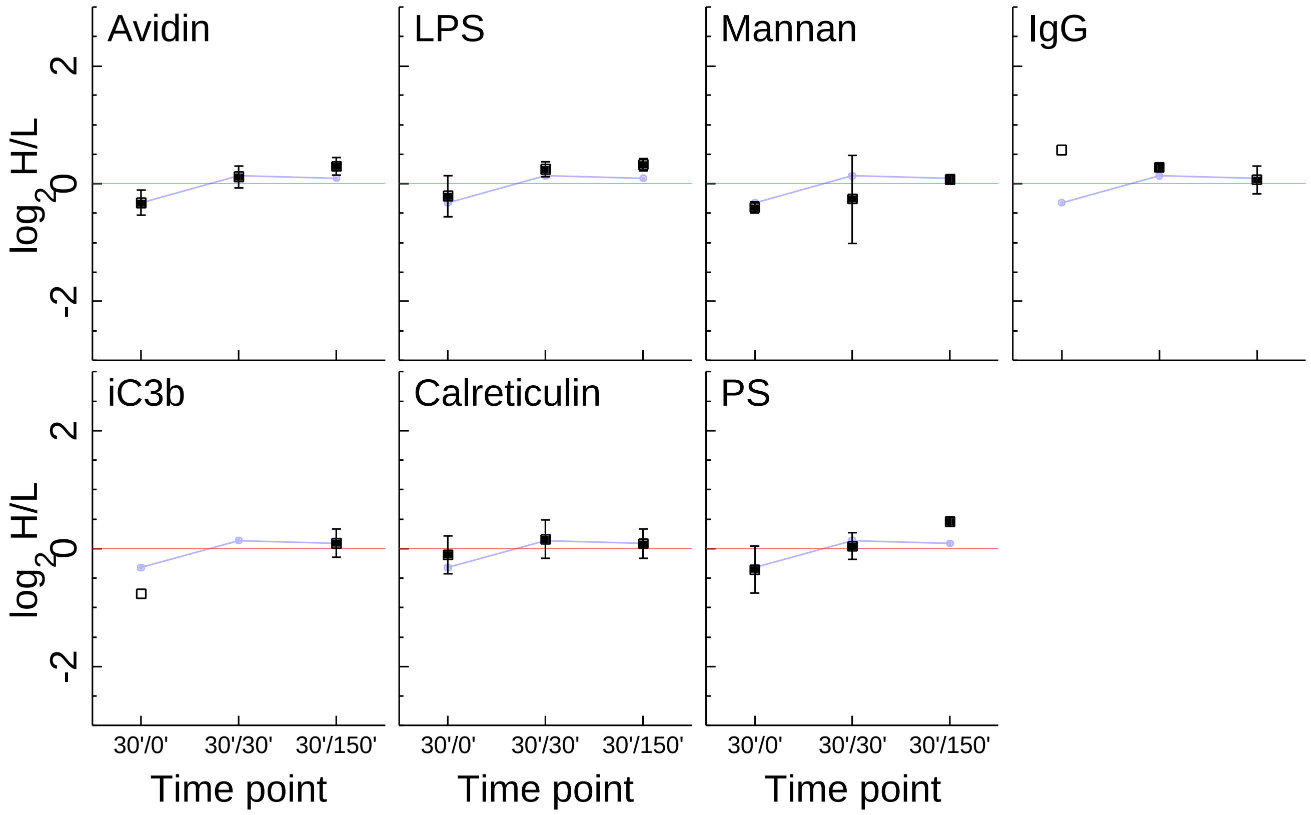

Avidin
LPS
Mannan
IgG
iC3b
Calreticulin
PS
log2 H/L
2
0
-2
30'/0'
30'/30'
30'/150'
Time point

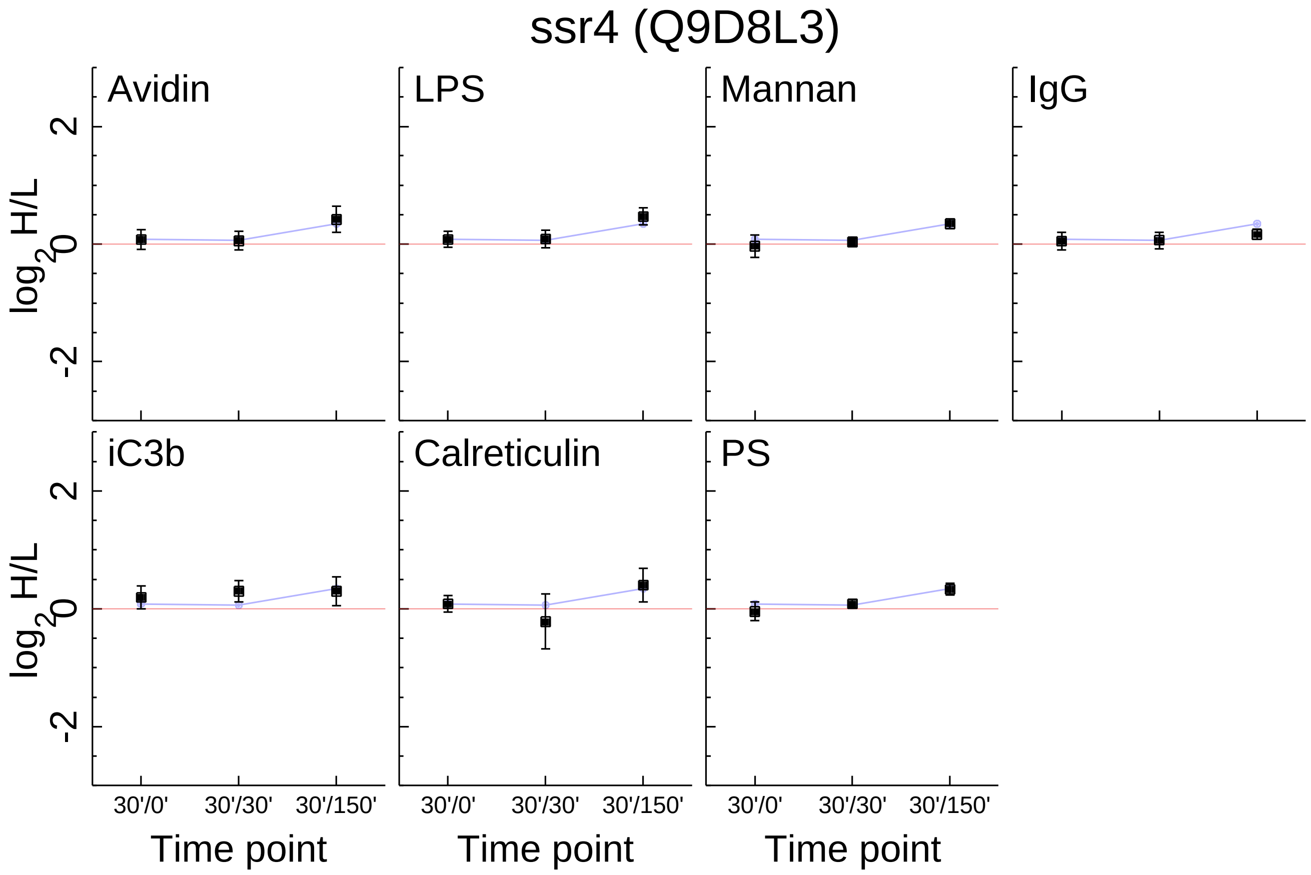
ssr4 (Q9D8L3)
Avidin
LPS
Mannan
IgG
iC3b
Calreticulin
PS
log2 H/L
2
0
-2
30'/0'
30'/30'
30'/150'
Time point

ssrp1 (Q08943)

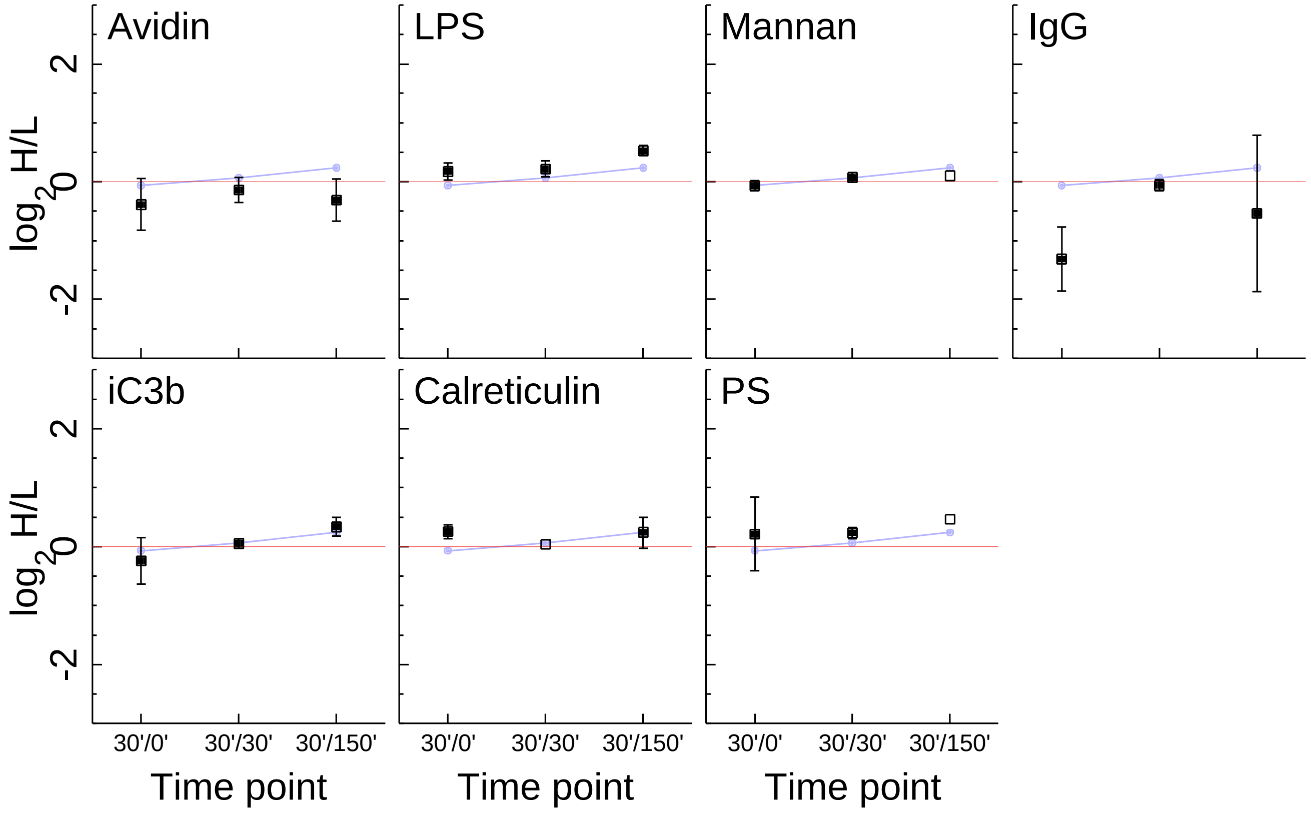

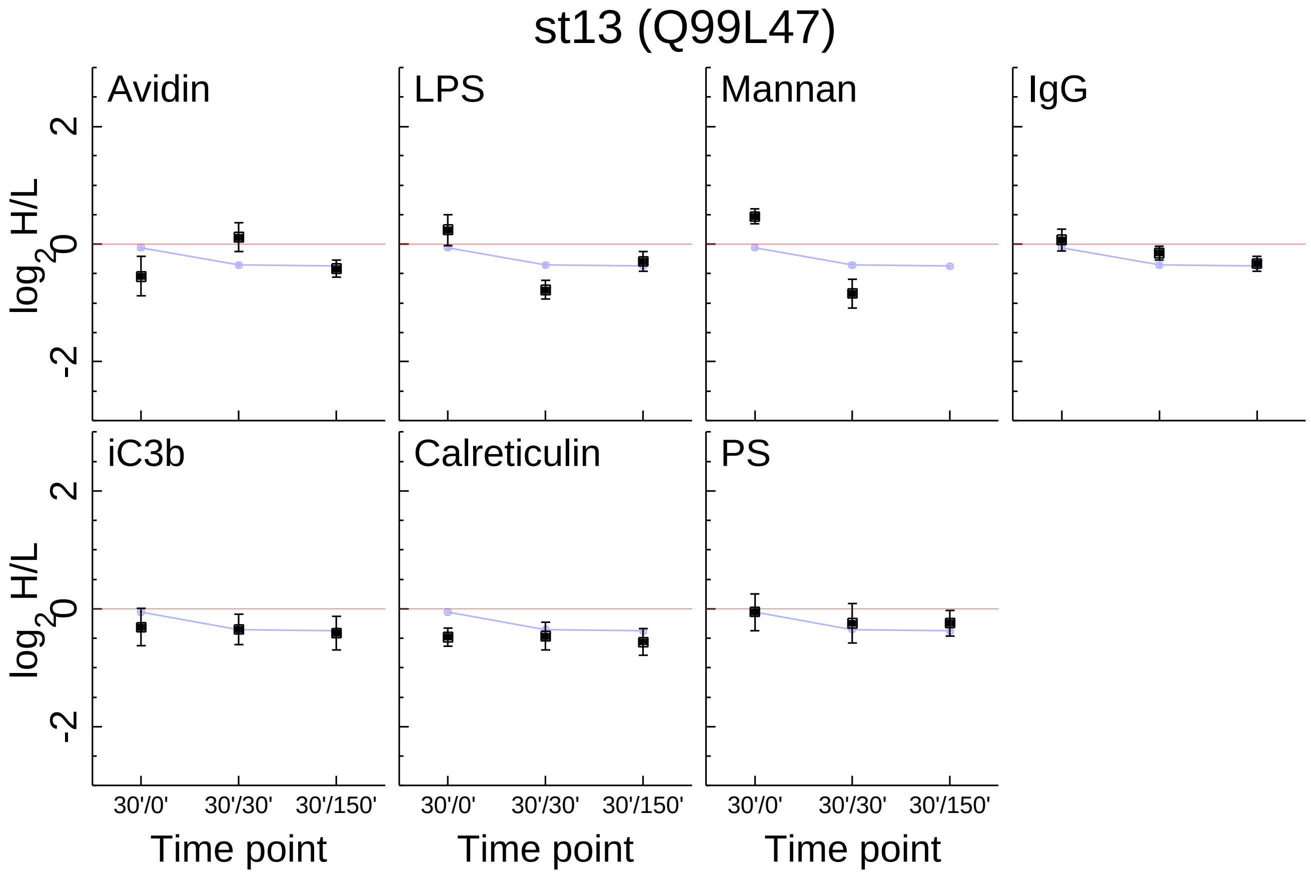

st13 (Q99L47)
Avidin
LPS
Mannan
IgG
iC3b
Calreticulin
PS
2
0
-2
log2 H/L
30'/0'
30'/30'
30'/150'
Time point

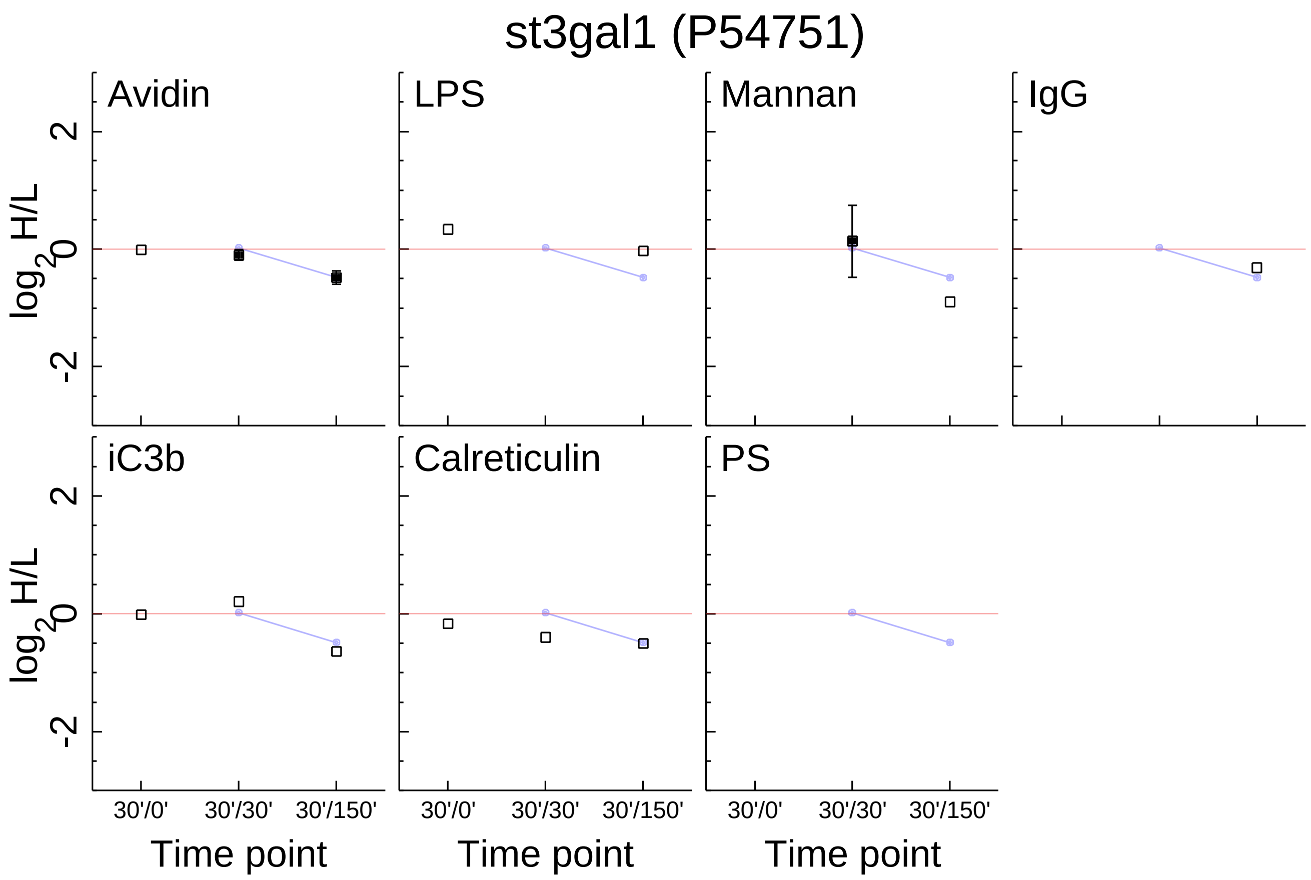
st3gal1 (P54751)
Avidin
LPS
Mannan
IgG
iC3b
Calreticulin
PS
log2 H/L
2
0
-2
30'/0'
30'/30'
30'/150'
Time point

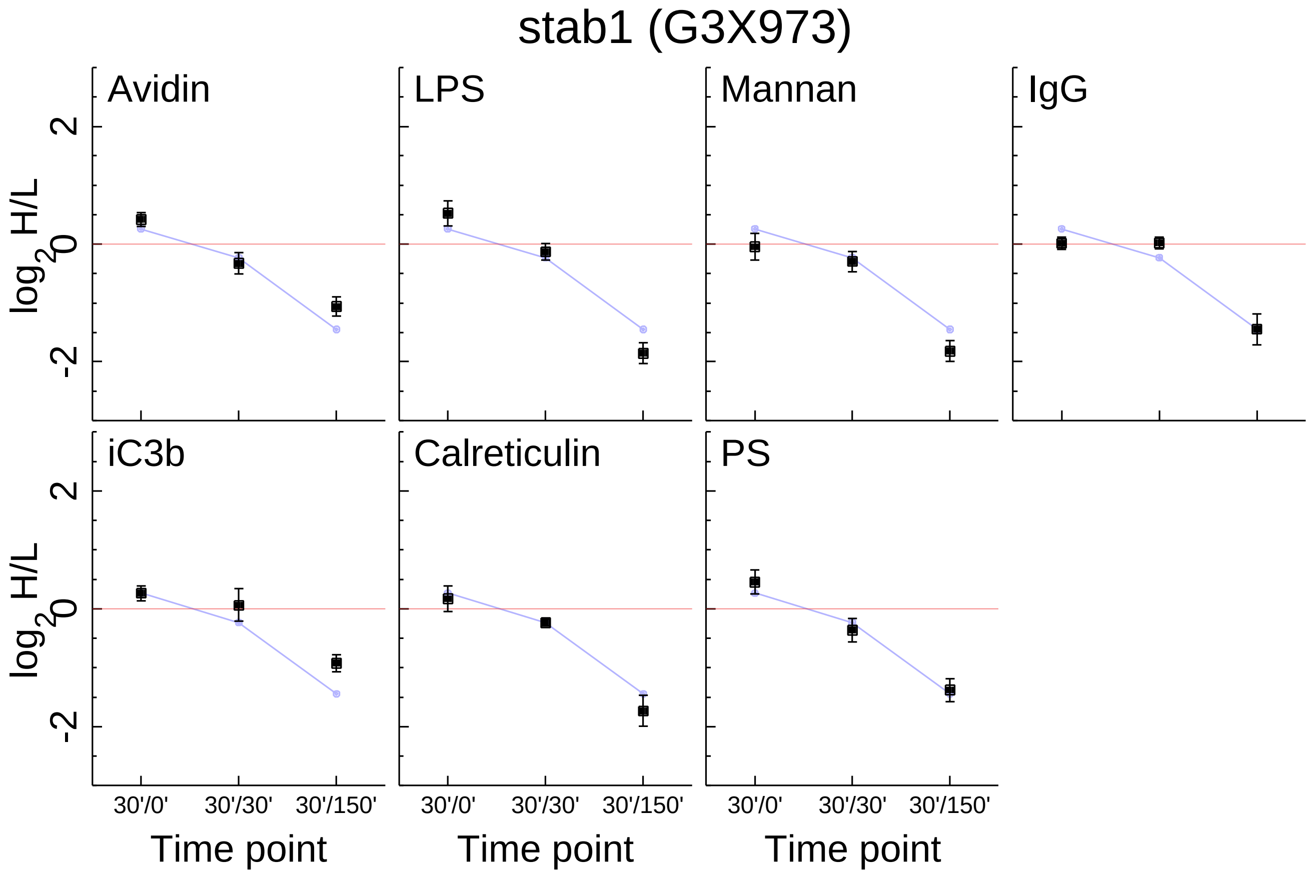
stab1 (G3X973)
Avidin
LPS
Mannan
IgG
iC3b
Calreticulin
PS
log2 H/L
2
0
-2
30'/0'
30'/30'
30'/150'
Time point

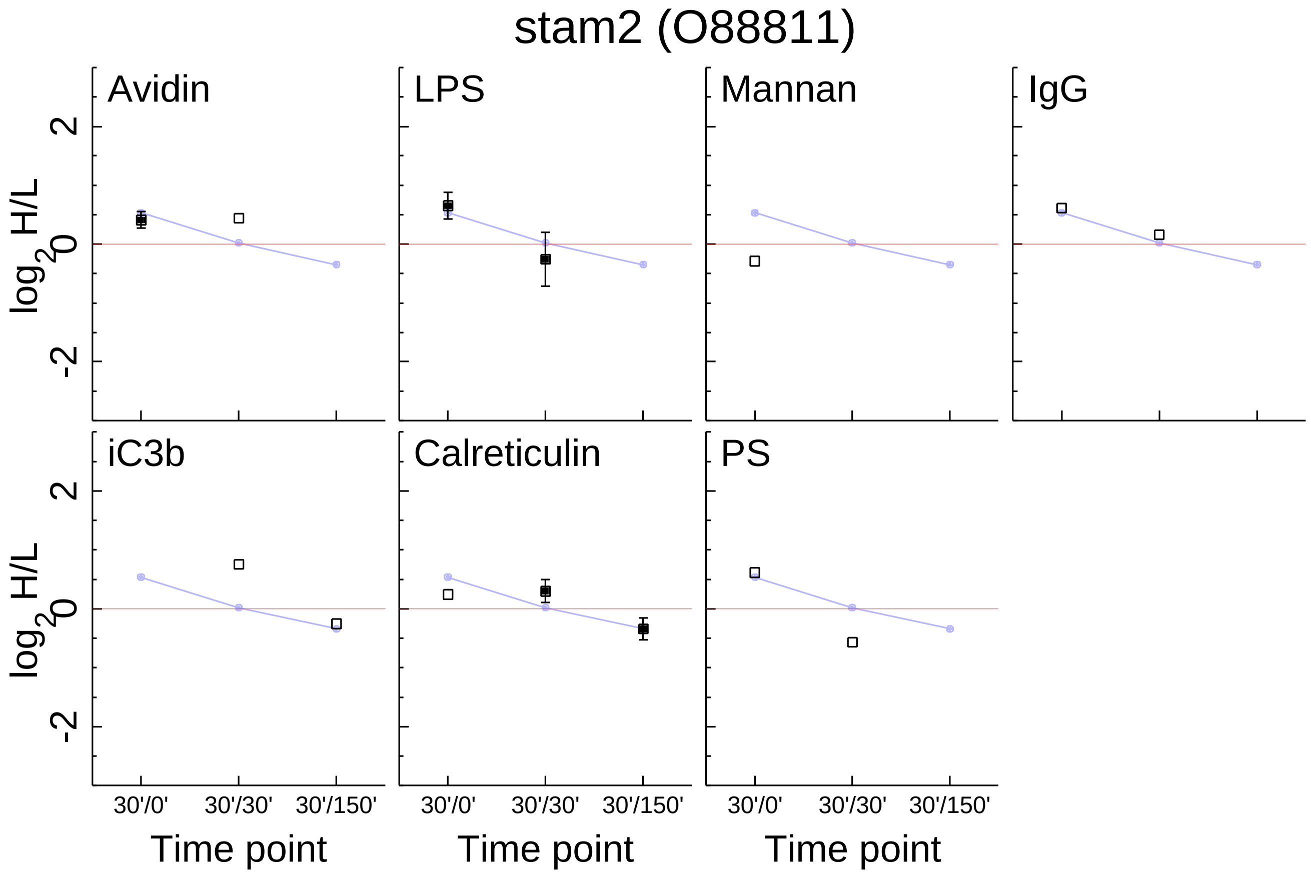
stam2 (O88811)
Avidin
LPS
Mannan
IgG
iC3b
Calreticulin
PS
log2 H/L
2
0
-2
30'/0'
30'/30'
30'/150'
Time point

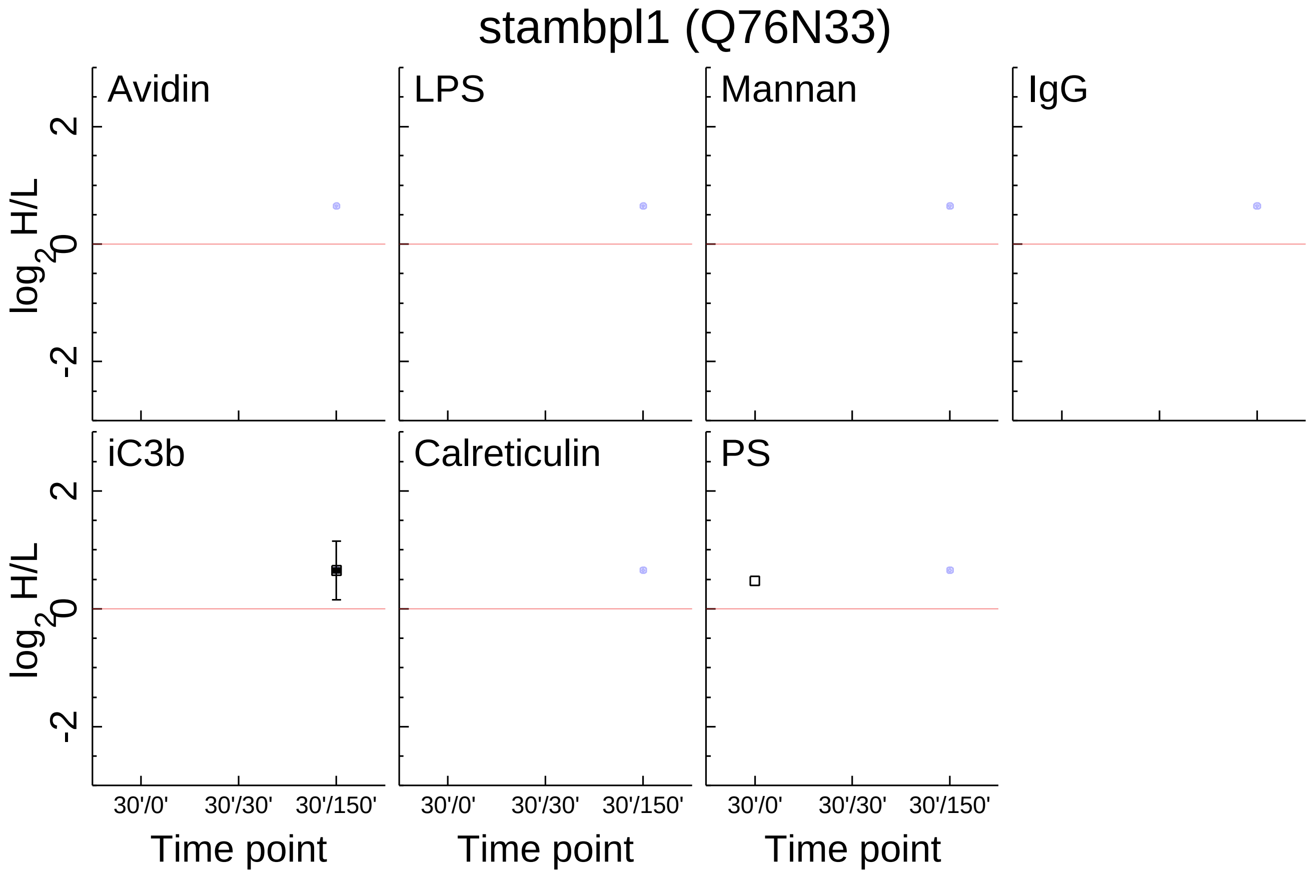
stambpl1 (Q76N33)
Avidin
LPS
Mannan
IgG
iC3b
Calreticulin
PS
log2 H/L
2
0
-2
30'/0'
30'/30'
30'/150'
Time point

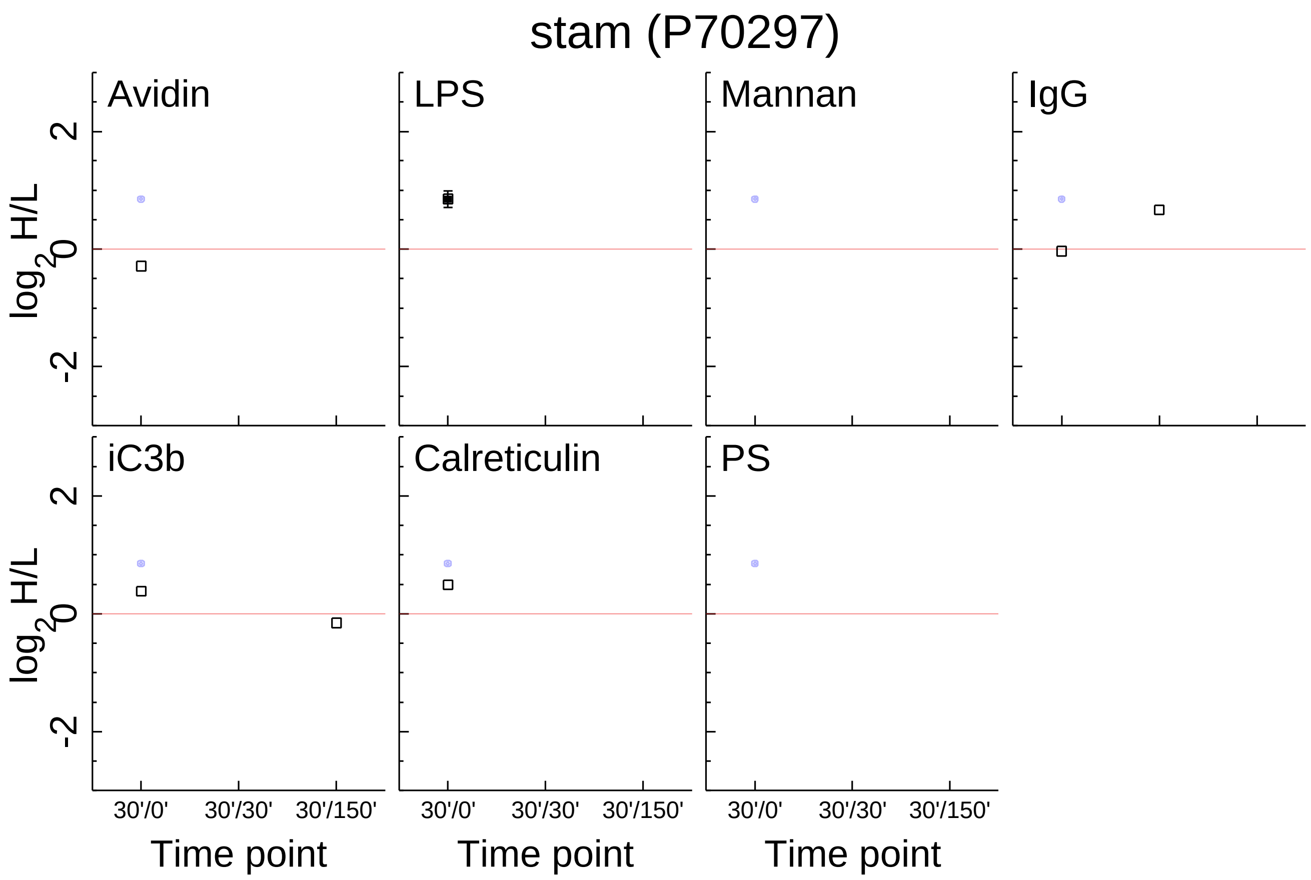
stam (P70297)
Avidin
LPS
Mannan
IgG
iC3b
Calreticulin
PS
log2 H/L
2
0
-2
30'/0'
30'/30'
30'/150'
Time point

stard3nl (Q9DCI3)

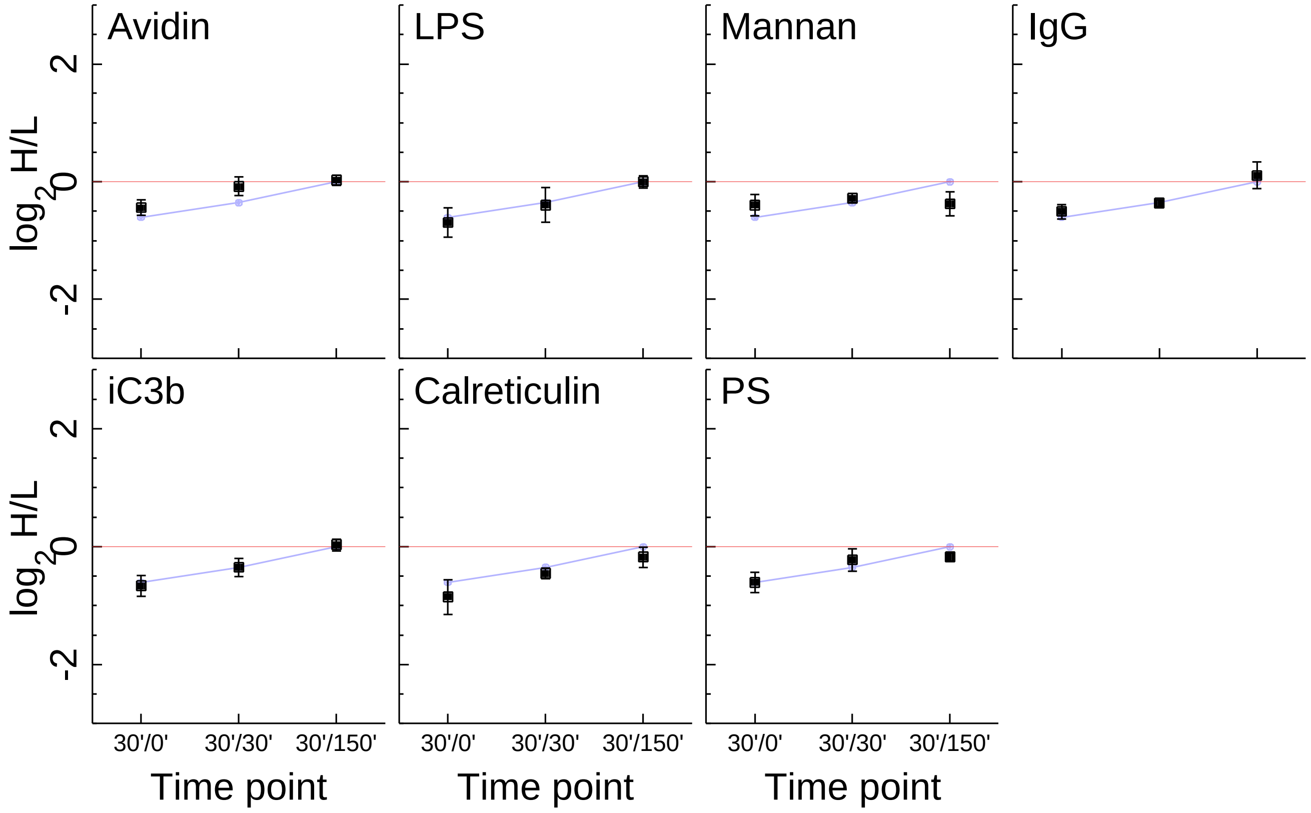

stard3 (Q61542)

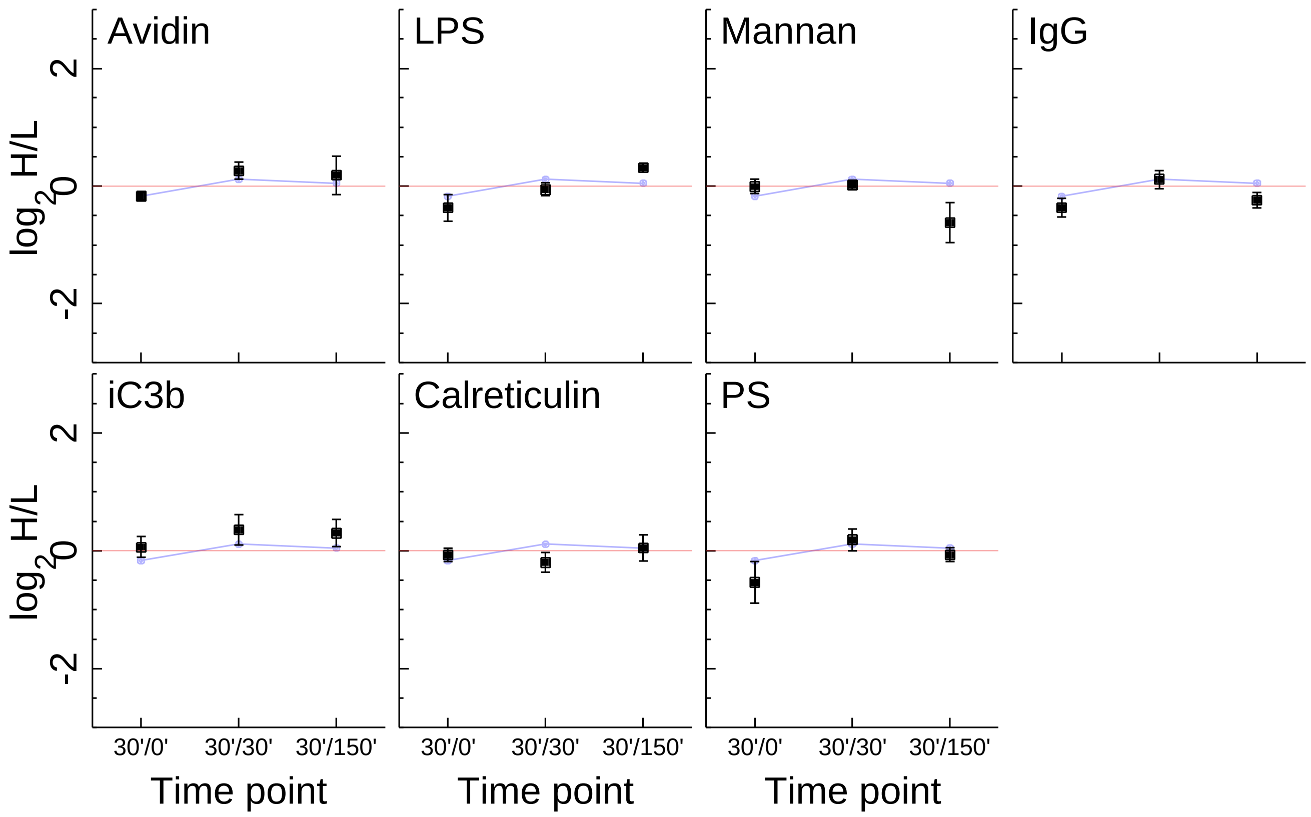

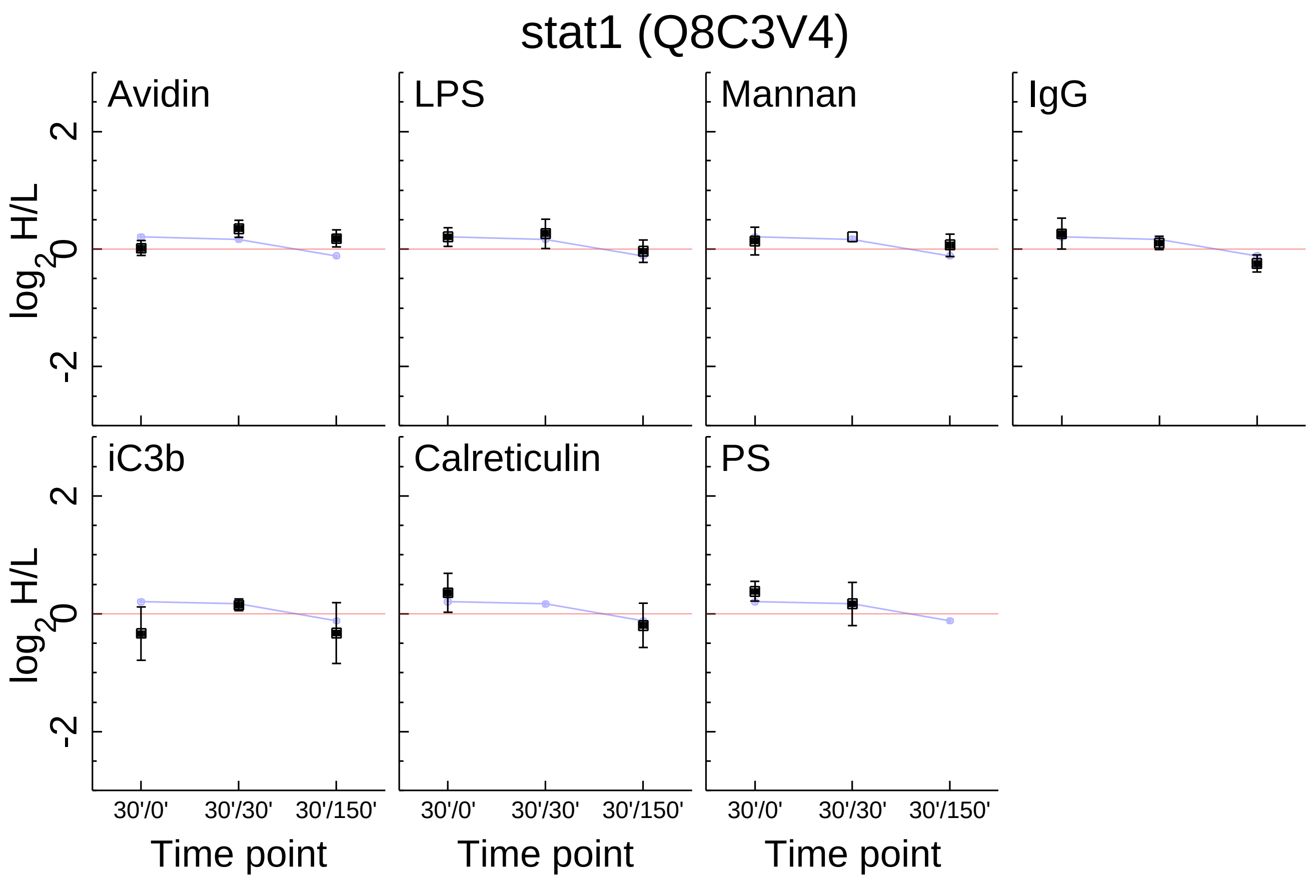
stat1 (Q8C3V4)
Avidin
LPS
Mannan
IgG
iC3b
Calreticulin
PS
log2 H/L
2
0
-2
30'/0'
30'/30'
30'/150'
Time point

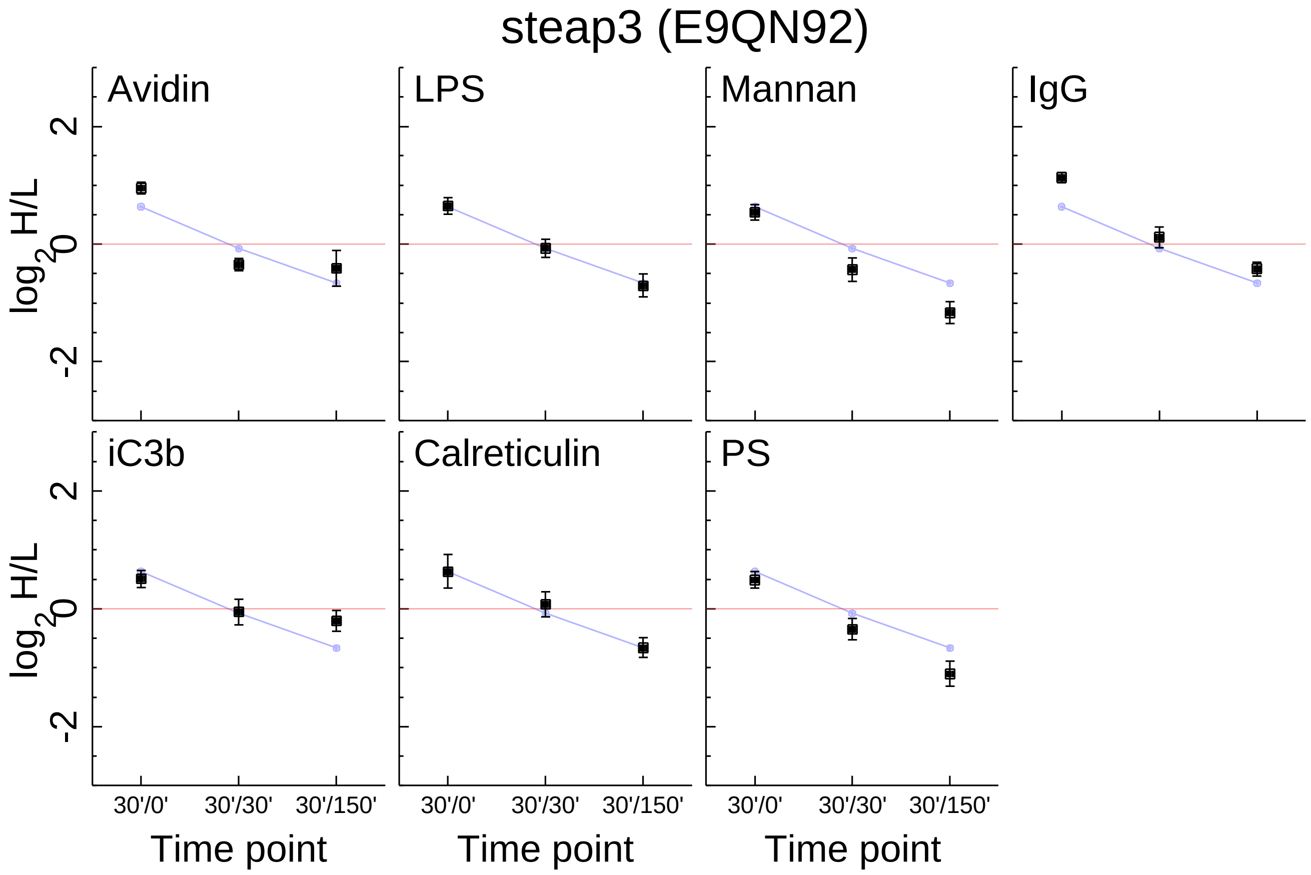

steap3 (E9QN92)
Avidin
LPS
Mannan
IgG
iC3b
Calreticulin
PS
log2 H/L
2
0
-2
30'/0'
30'/30'
30'/150'
Time point

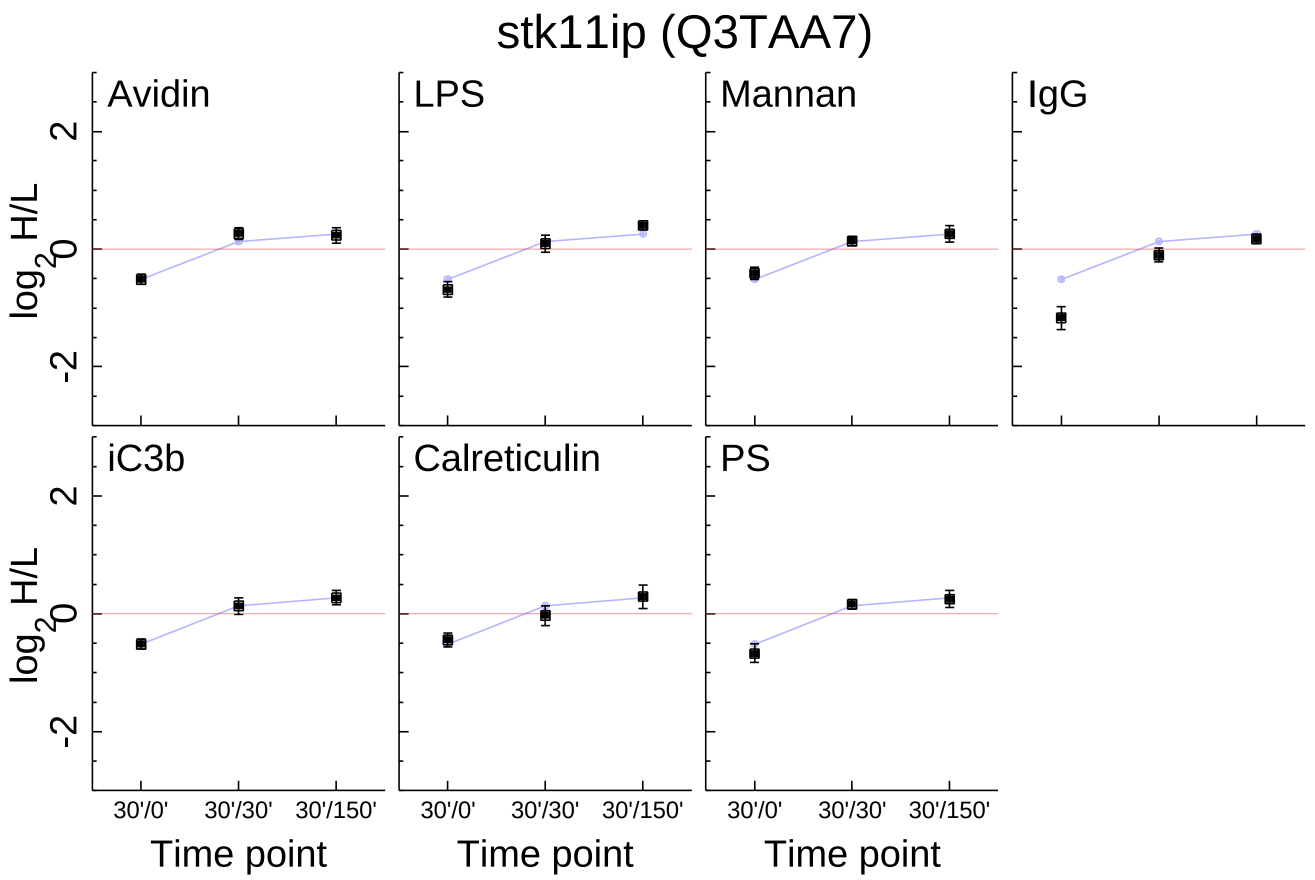
stk11ip (Q3TAA7)
Avidin
LPS
Mannan
IgG
iC3b
Calreticulin
PS
log2 H/L
2
0
-2
30'/0'
30'/30'
30'/150'
Time point

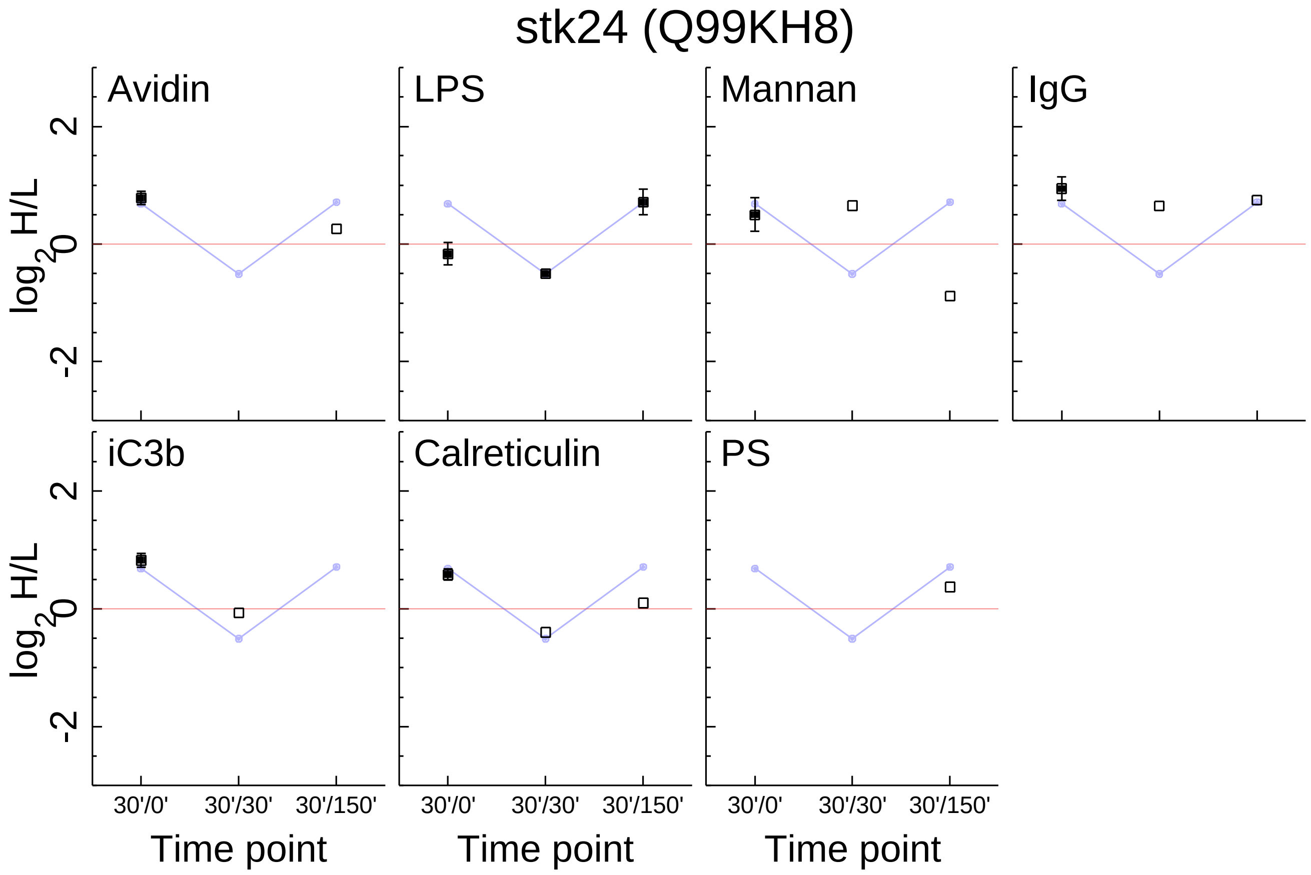
stk24 (Q99KH8)
Avidin
LPS
Mannan
IgG
iC3b
Calreticulin
PS
log2 H/L
2
0
-2
30'/0'
30'/30'
30'/150'
Time point

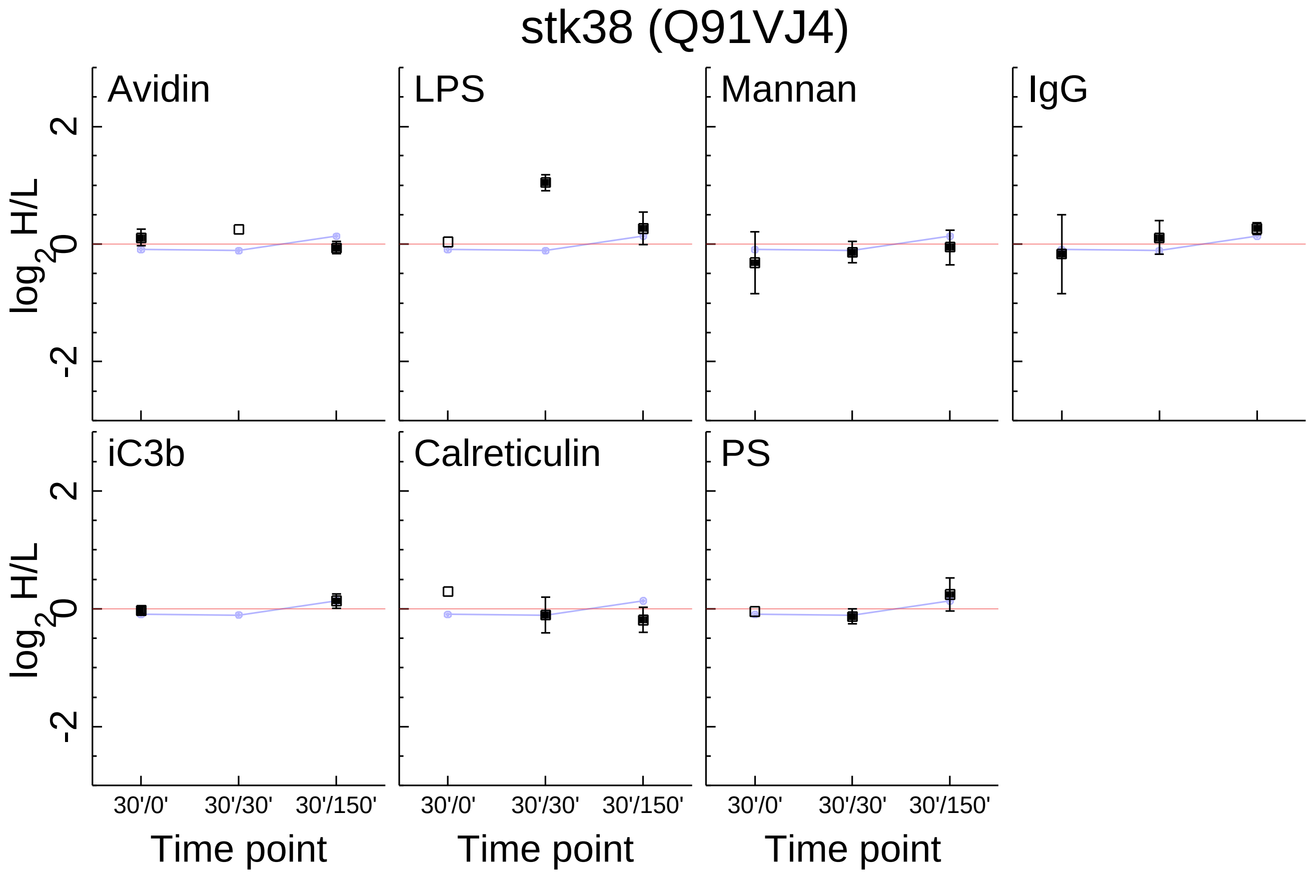
stk38 (Q91VJ4)
Avidin
LPS
Mannan
IgG
iC3b
Calreticulin
PS
log2 H/L
2
0
-2
30'/0'
30'/30'
30'/150'
Time point

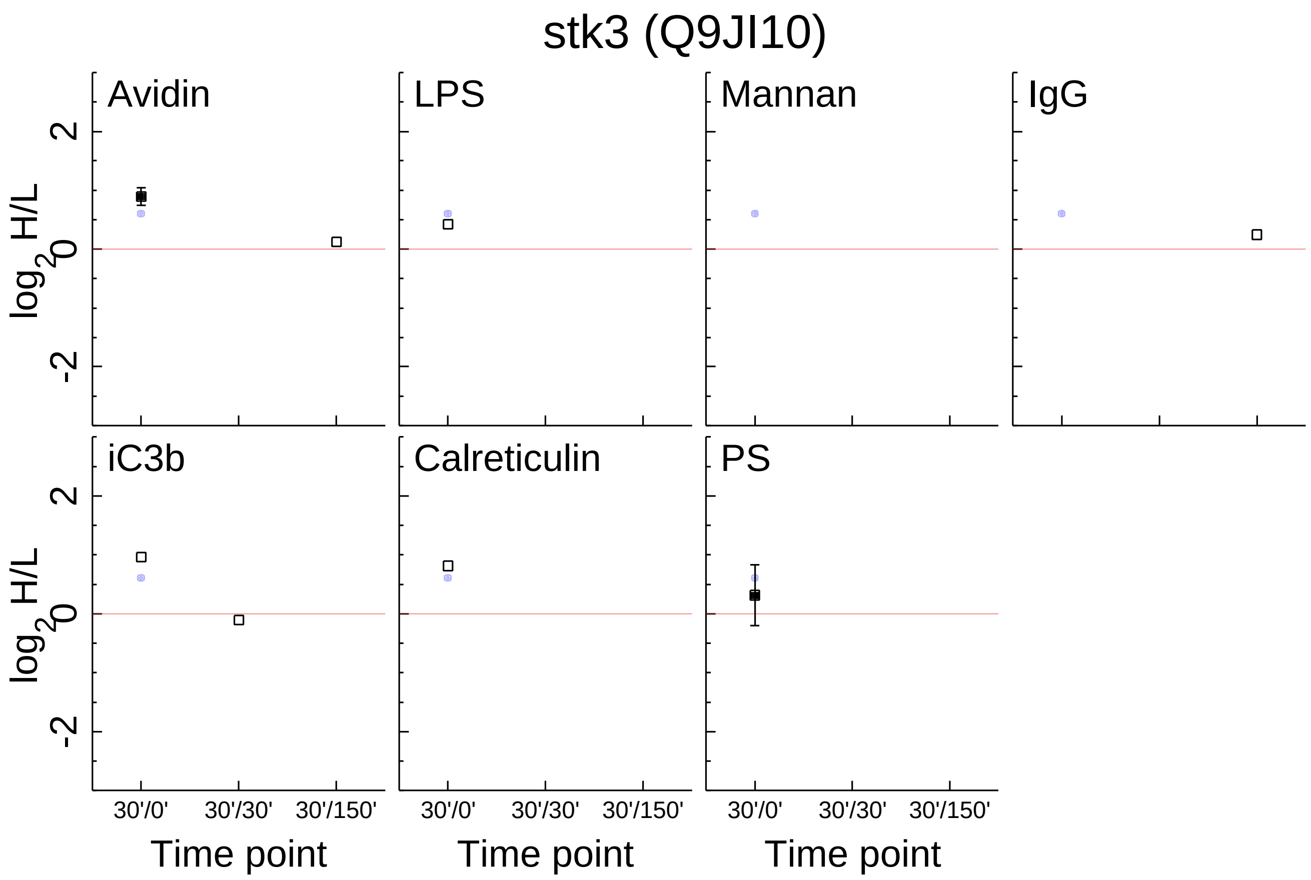

stk3 (Q9JI10)
Avidin
LPS
Mannan
IgG
iC3b
Calreticulin
PS
log2 H/L
2
0
-2
30'/0'
30'/30'
30'/150'
Time point

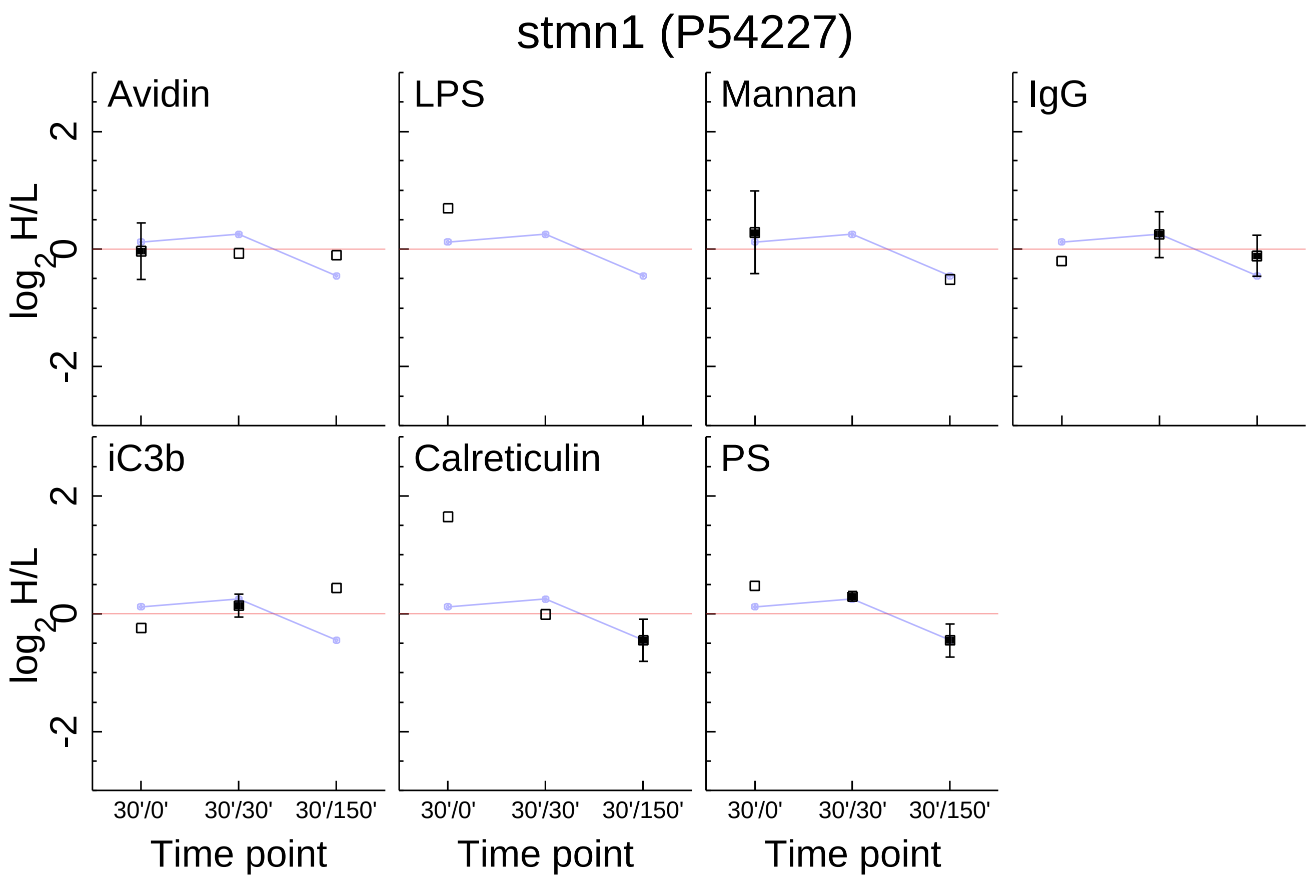

stmn1 (P54227)
Avidin
LPS
Mannan
IgG
iC3b
Calreticulin
PS
2
0
-2
log2 H/L
30'/0'
30'/30'
30'/150'
Time point

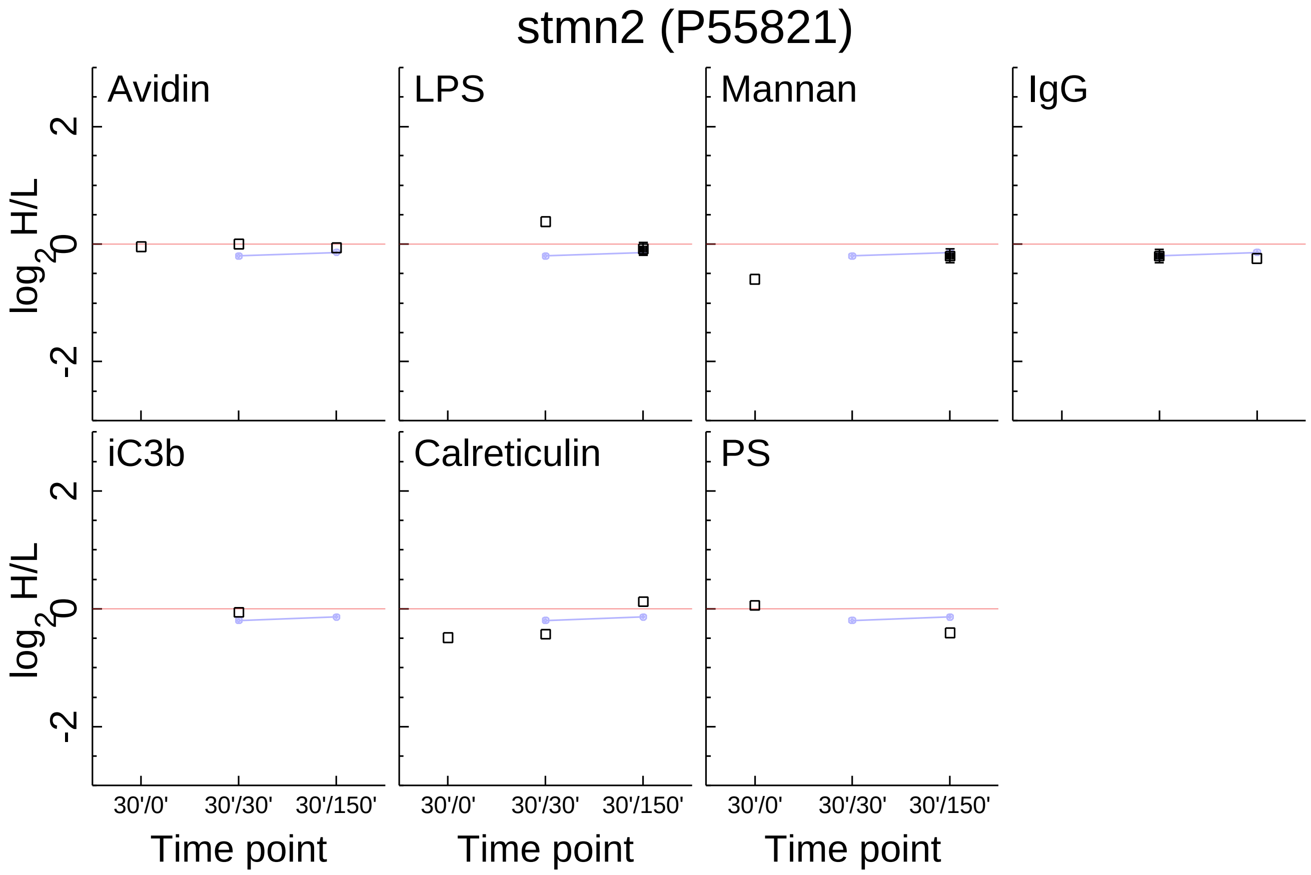
stmn2 (P55821)
Avidin
LPS
Mannan
IgG
iC3b
Calreticulin
PS
log2 H/L
2
0
-2
30'/0'
30'/30'
30'/150'
Time point

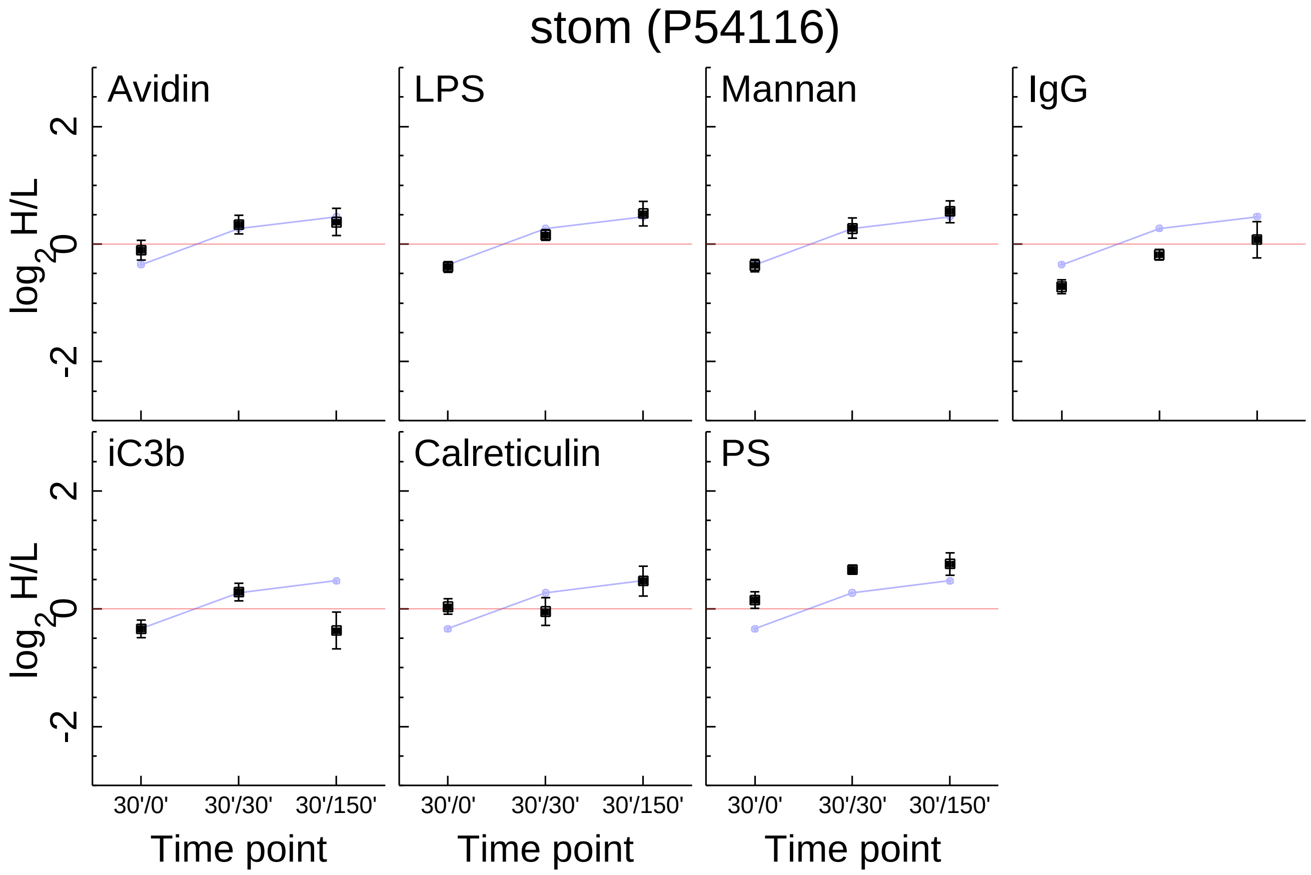
stom (P54116)
Avidin
LPS
Mannan
IgG
iC3b
Calreticulin
PS
log2 H/L
2
0
-2
30'/0'
30'/30'
30'/150'
Time point

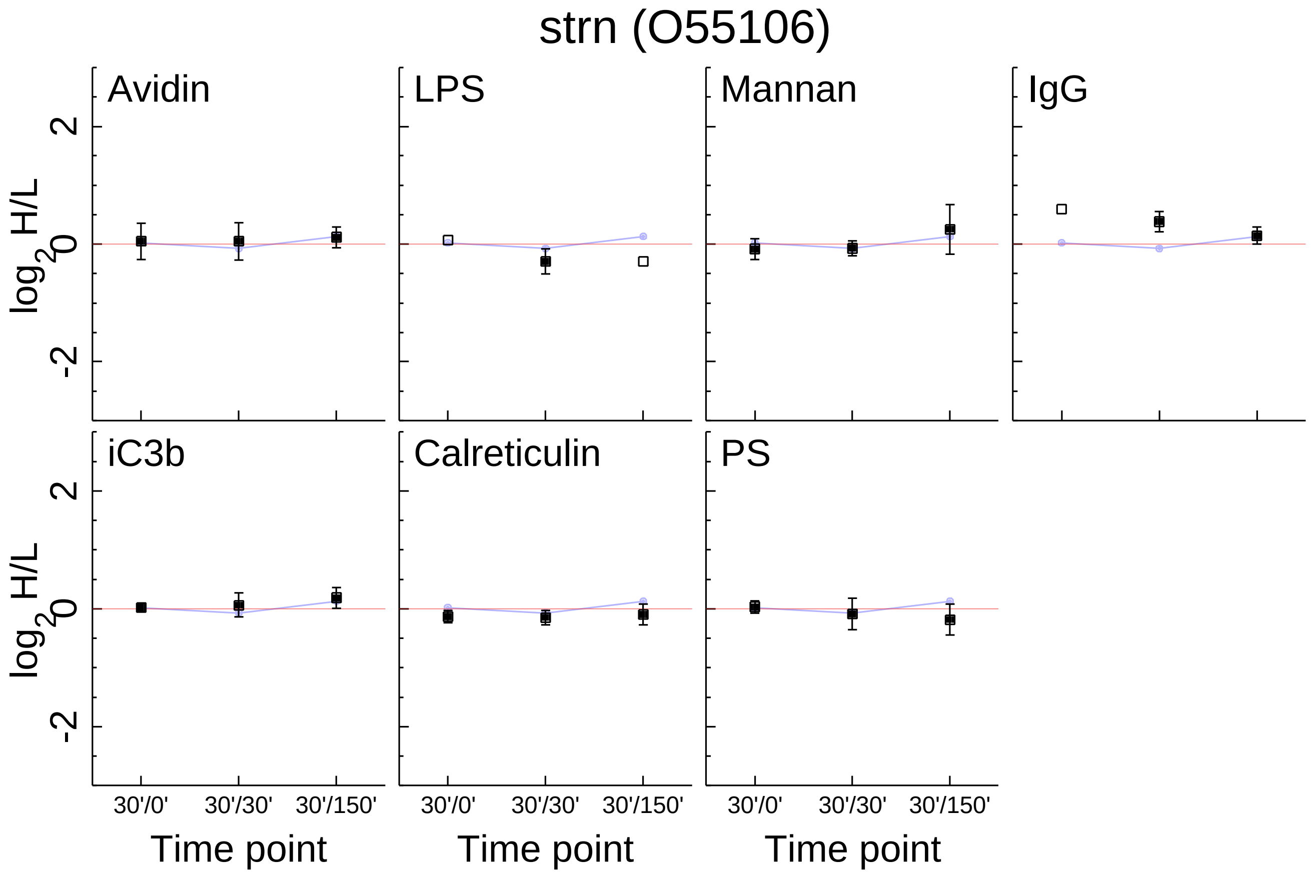
strn (O55106)
Avidin
LPS
Mannan
IgG
iC3b
Calreticulin
PS
log2 H/L
2
0
-2
30'/0'
30'/30'
30'/150'
Time point

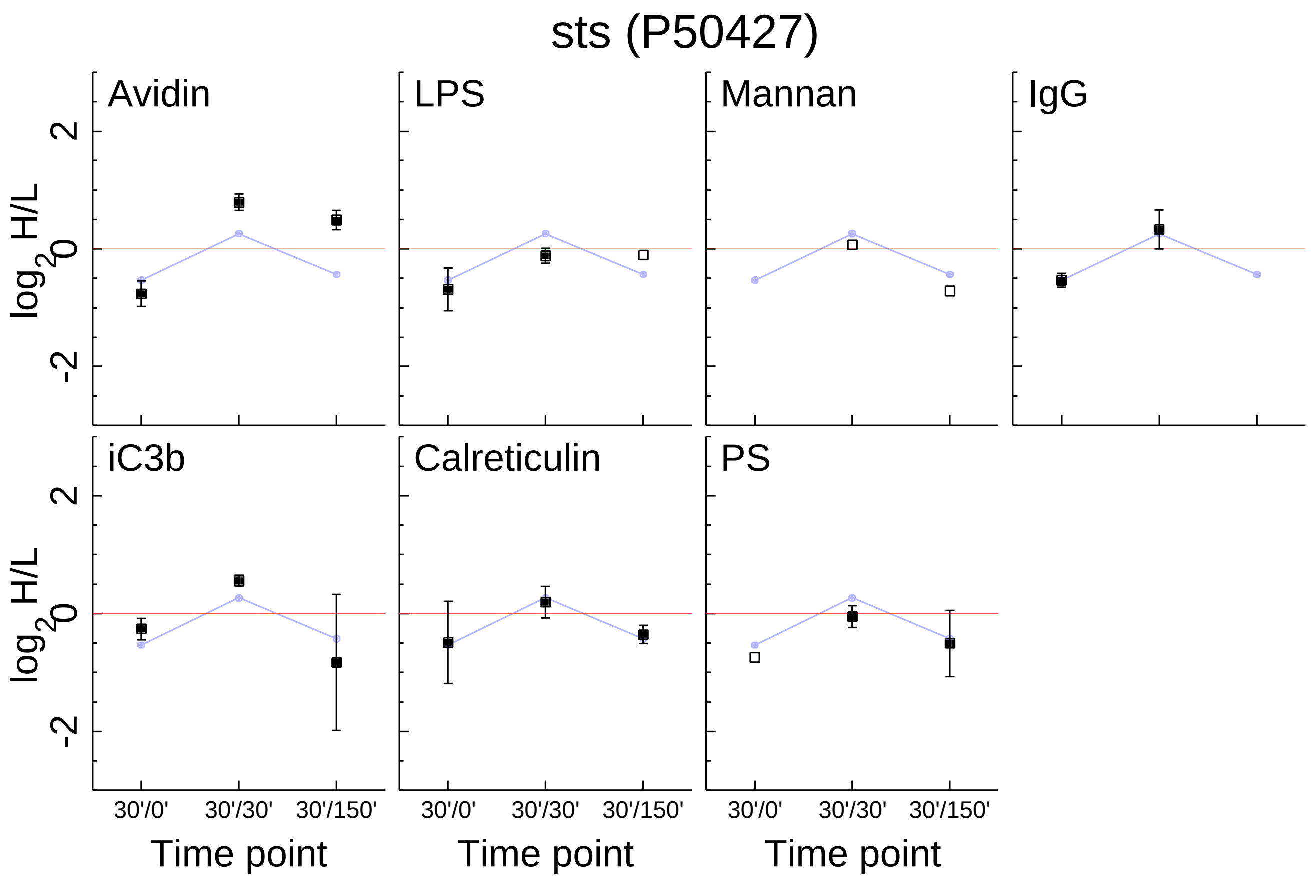

sts (P50427)
Avidin
LPS
Mannan
IgG
iC3b
Calreticulin
PS
log2 H/L
2
0
-2
30'/0'
30'/30'
30'/150'
Time point

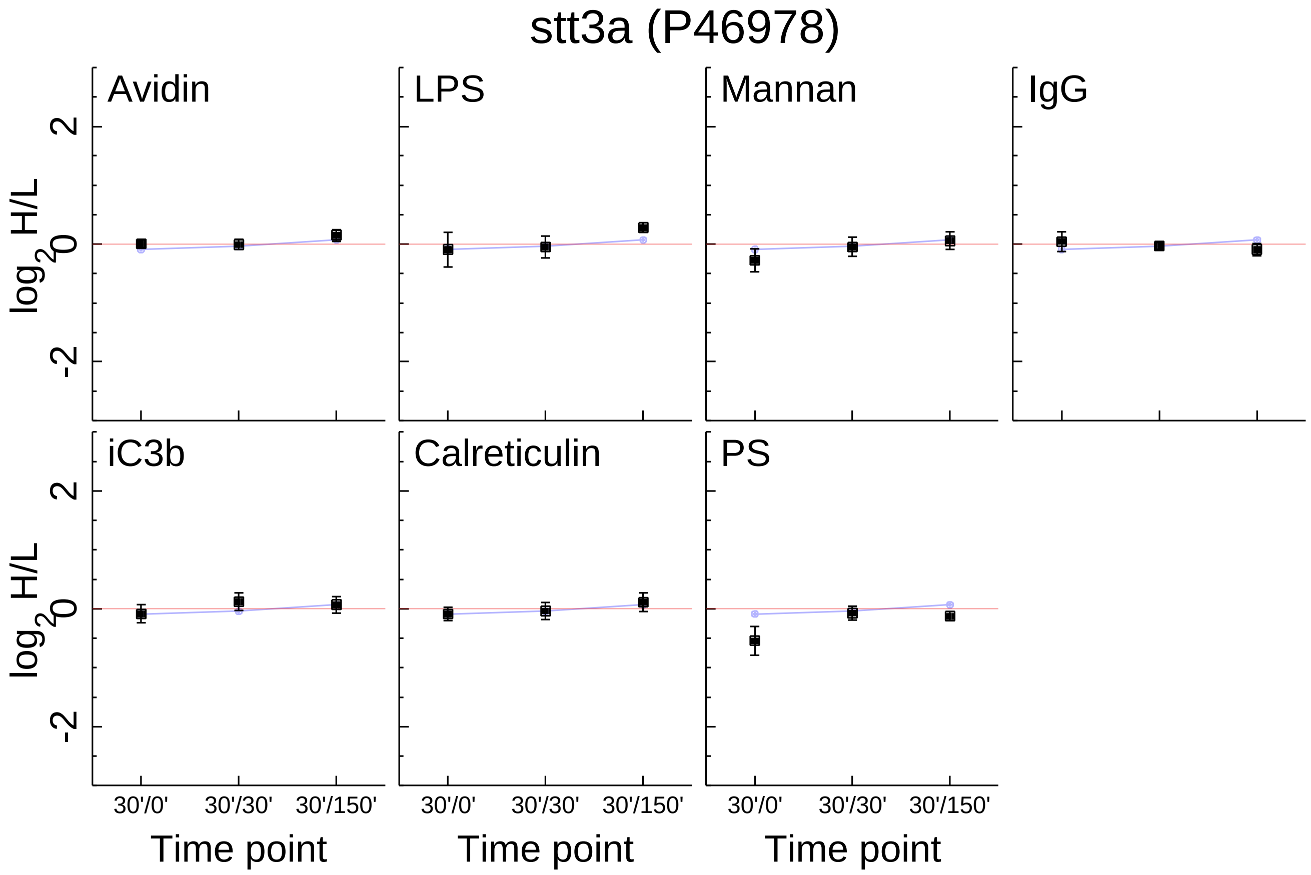
stt3a (P46978)
Avidin
LPS
Mannan
IgG
iC3b
Calreticulin
PS
log2 H/L
2
0
-2
30'/0'
30'/30'
30'/150'
Time point

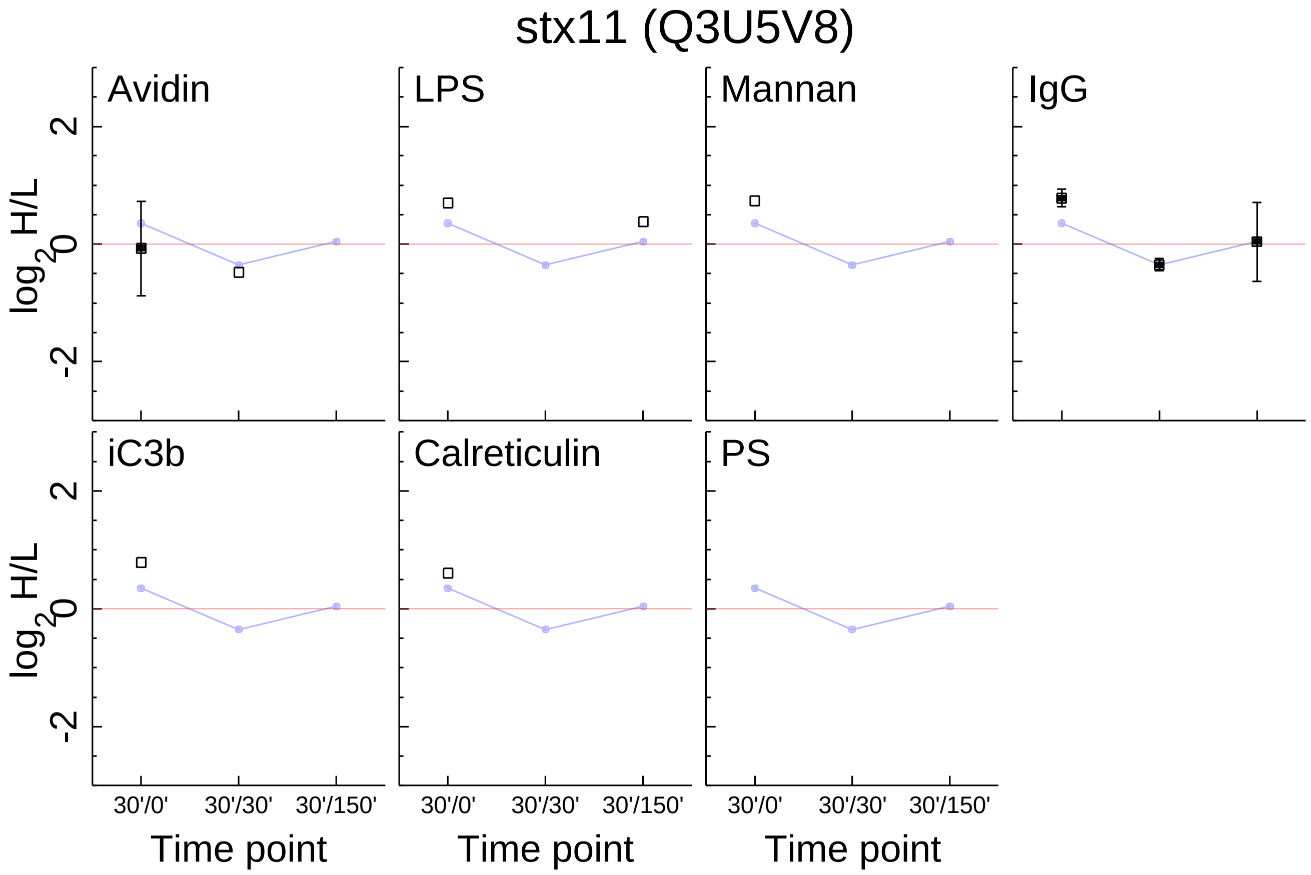
stx11 (Q3U5V8)
Avidin
LPS
Mannan
IgG
iC3b
Calreticulin
PS
log2 H/L
2
0
-2
30'/0'
30'/30'
30'/150'
Time point

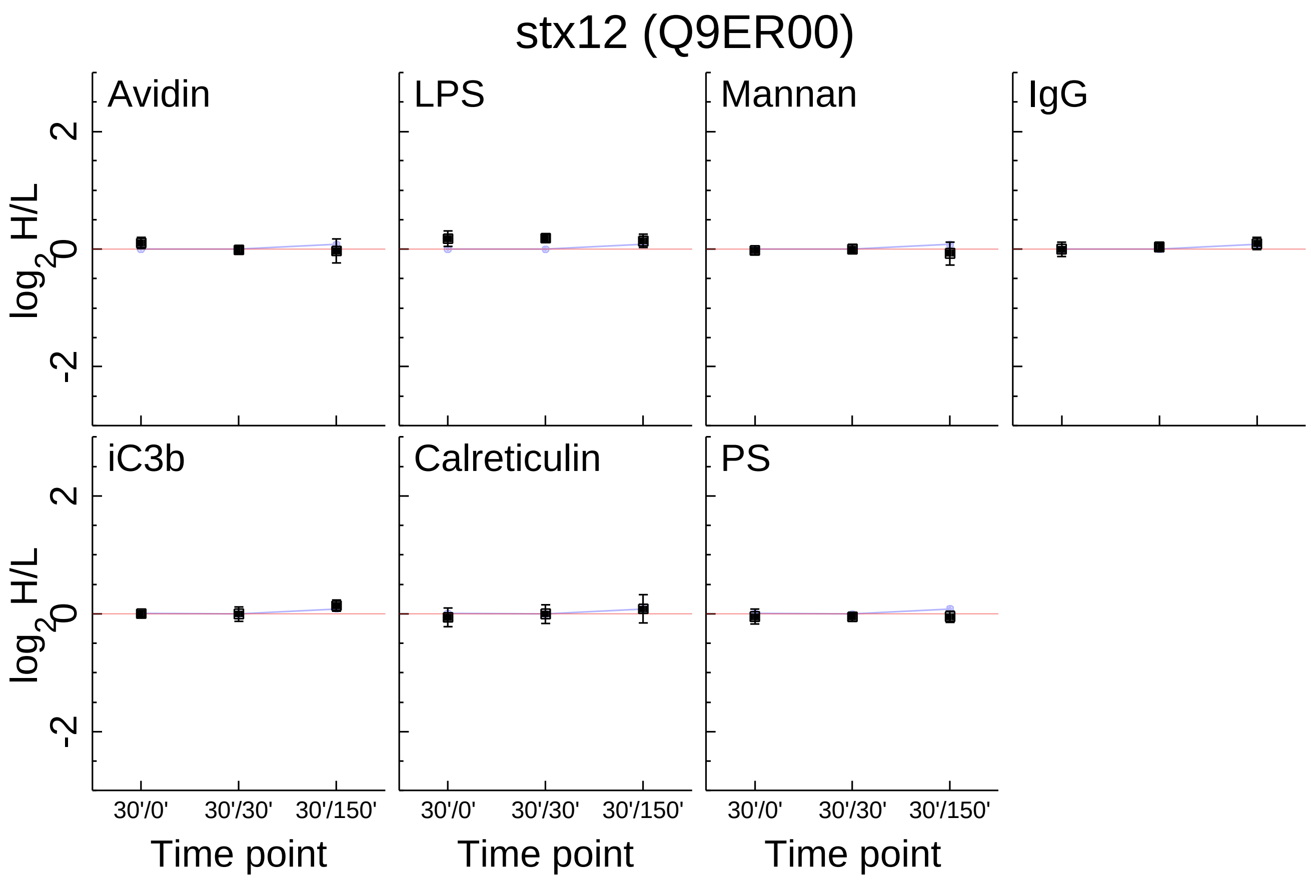

stx12 (Q9ER00)
Avidin
LPS
Mannan
IgG
iC3b
Calreticulin
PS
log2 H/L
2
0
-2
30'/0'
30'/30'
30'/150'
Time point

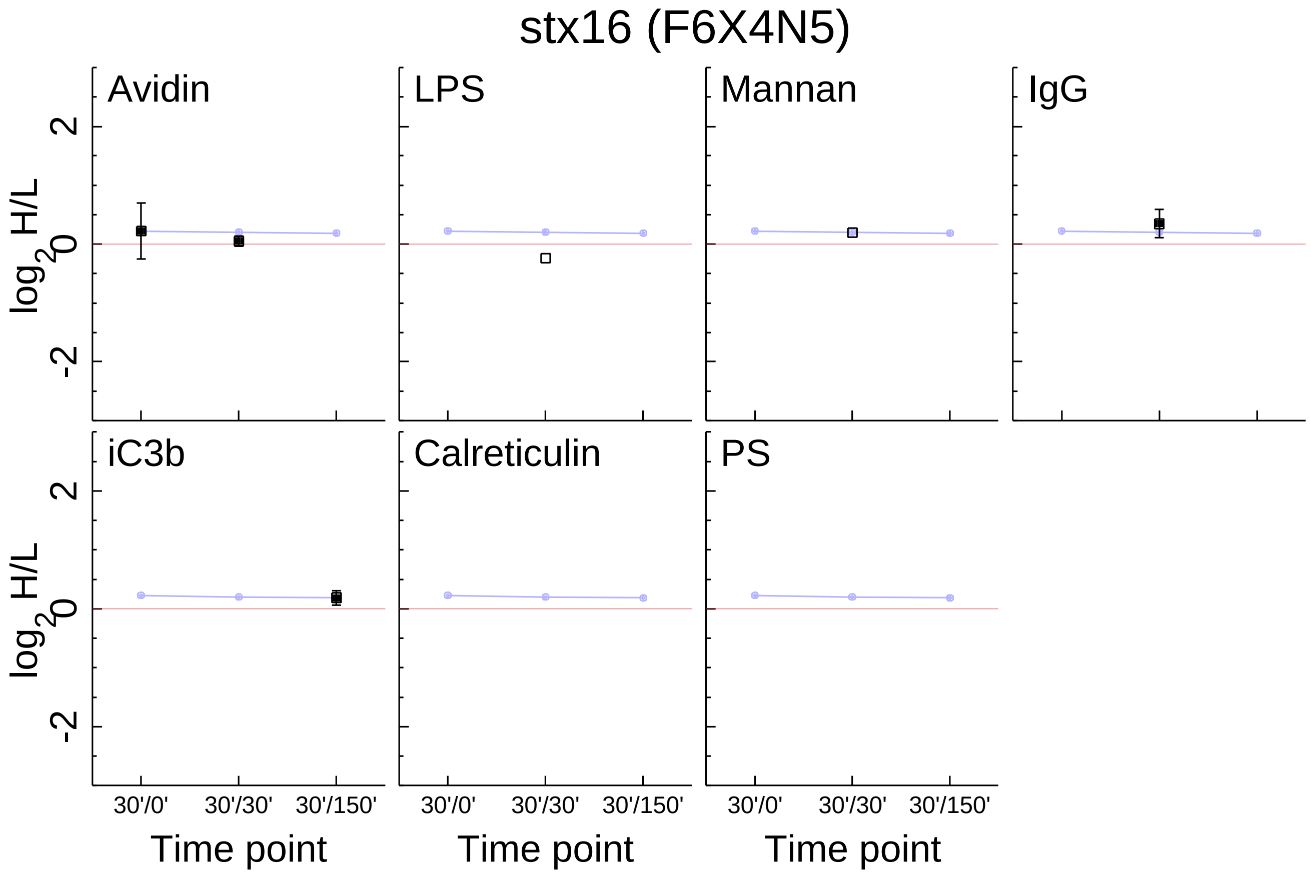
stx16 (F6X4N5)
Avidin
LPS
Mannan
IgG
iC3b
Calreticulin
PS
log2 H/L
2
0
-2
30'/0'
30'/30'
30'/150'
Time point

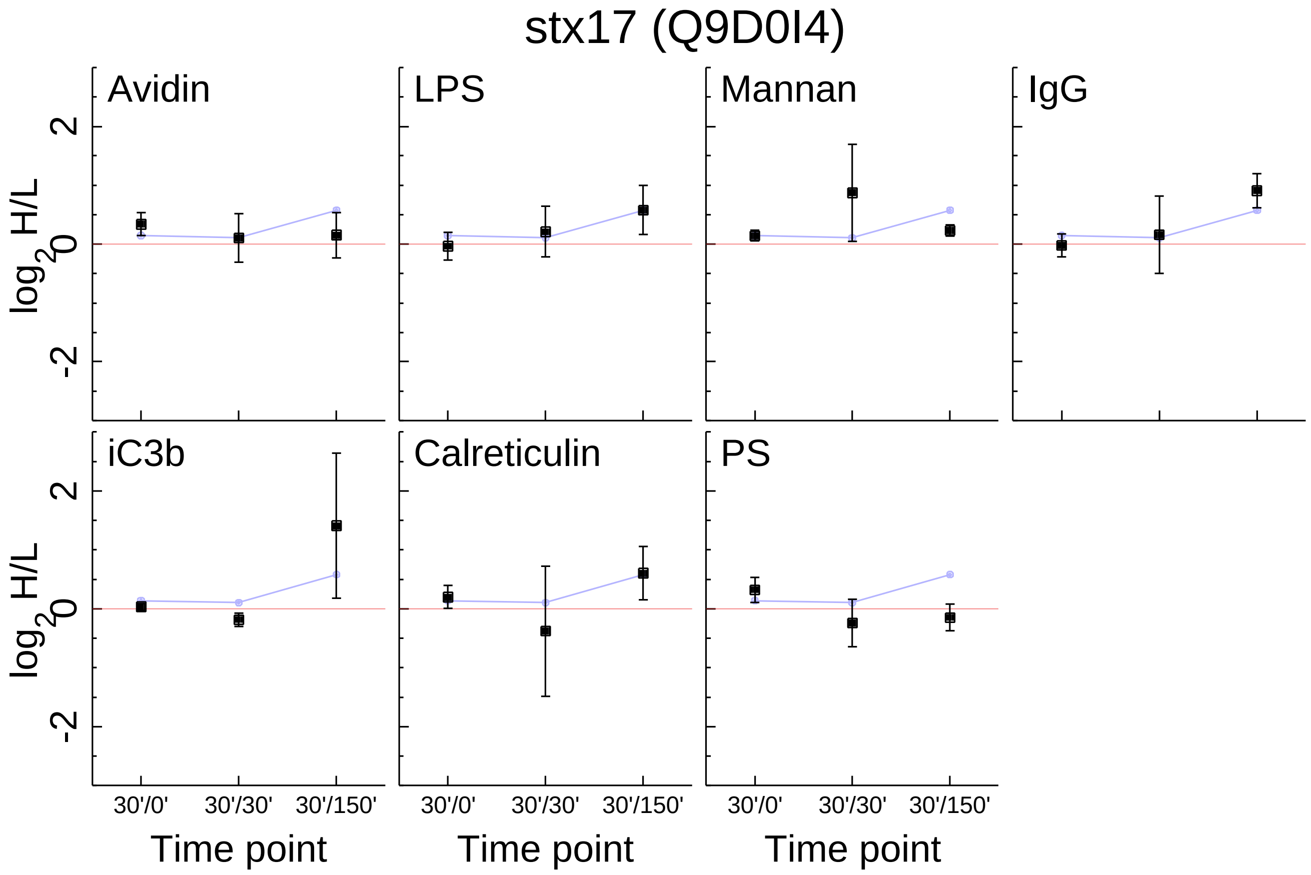

stx17 (Q9D0I4)
Avidin
LPS
Mannan
IgG
iC3b
Calreticulin
PS
$\log_2$ H/L
2
0
-2
30'/0'
30'/30'
30'/150'
Time point

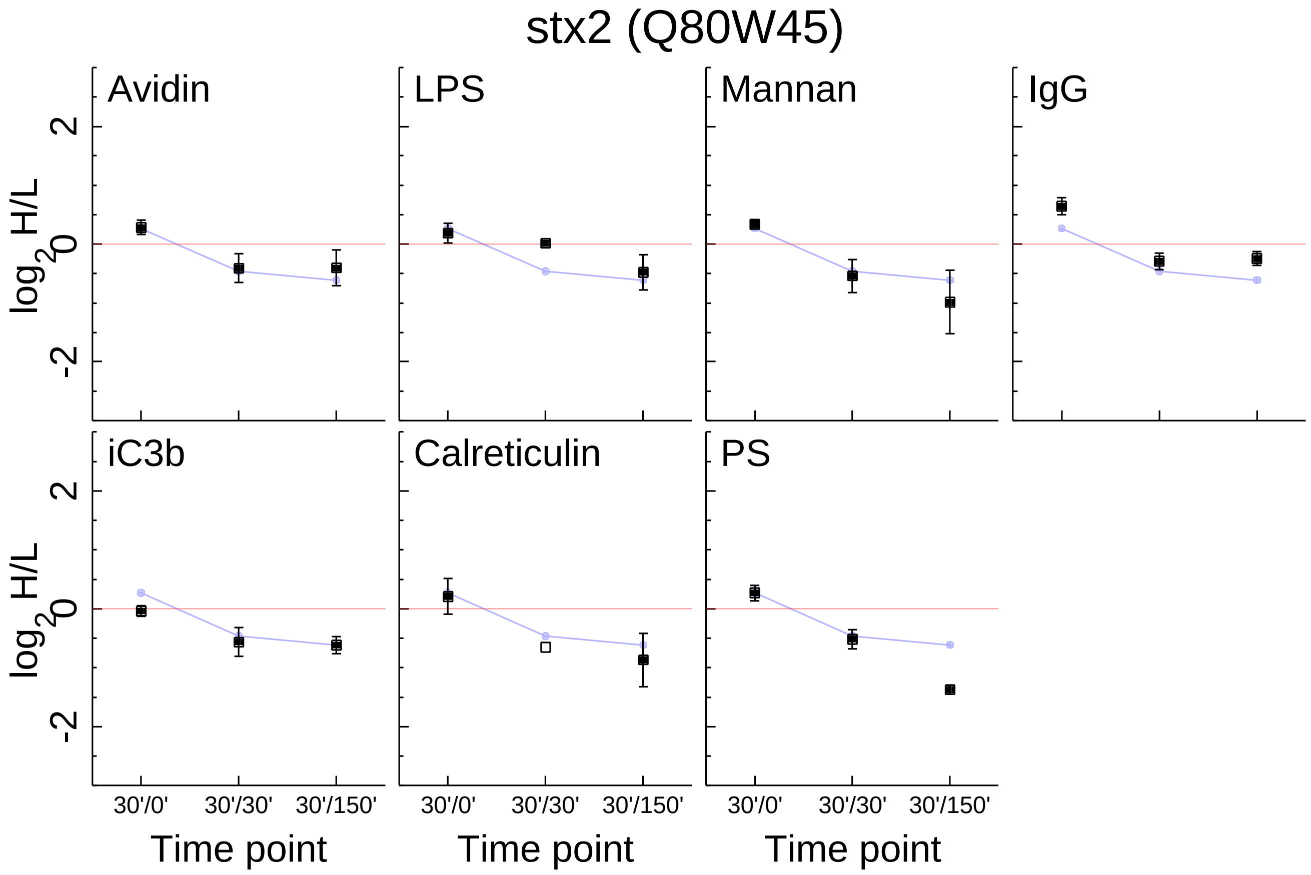
stx2 (Q80W45)
Avidin
LPS
Mannan
IgG
iC3b
Calreticulin
PS
log2 H/L
2
0
-2
30'/0'
30'/30'
30'/150'
Time point

stx3 (Q64704)

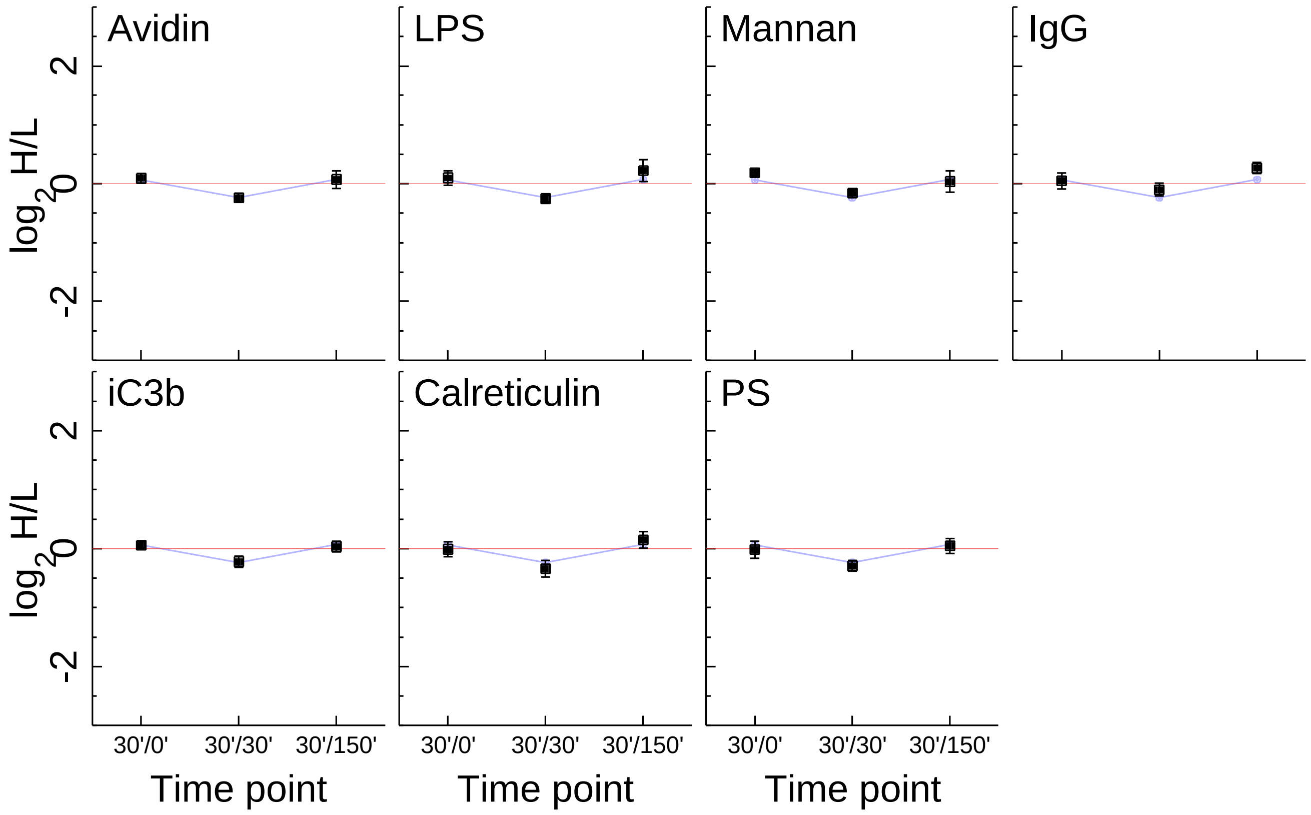

Avidin
LPS
Mannan
IgG
iC3b
Calreticulin
PS
log2 H/L
2
0
-2
30'/0'
30'/30'
30'/150'
Time point

stx4 (P70452)

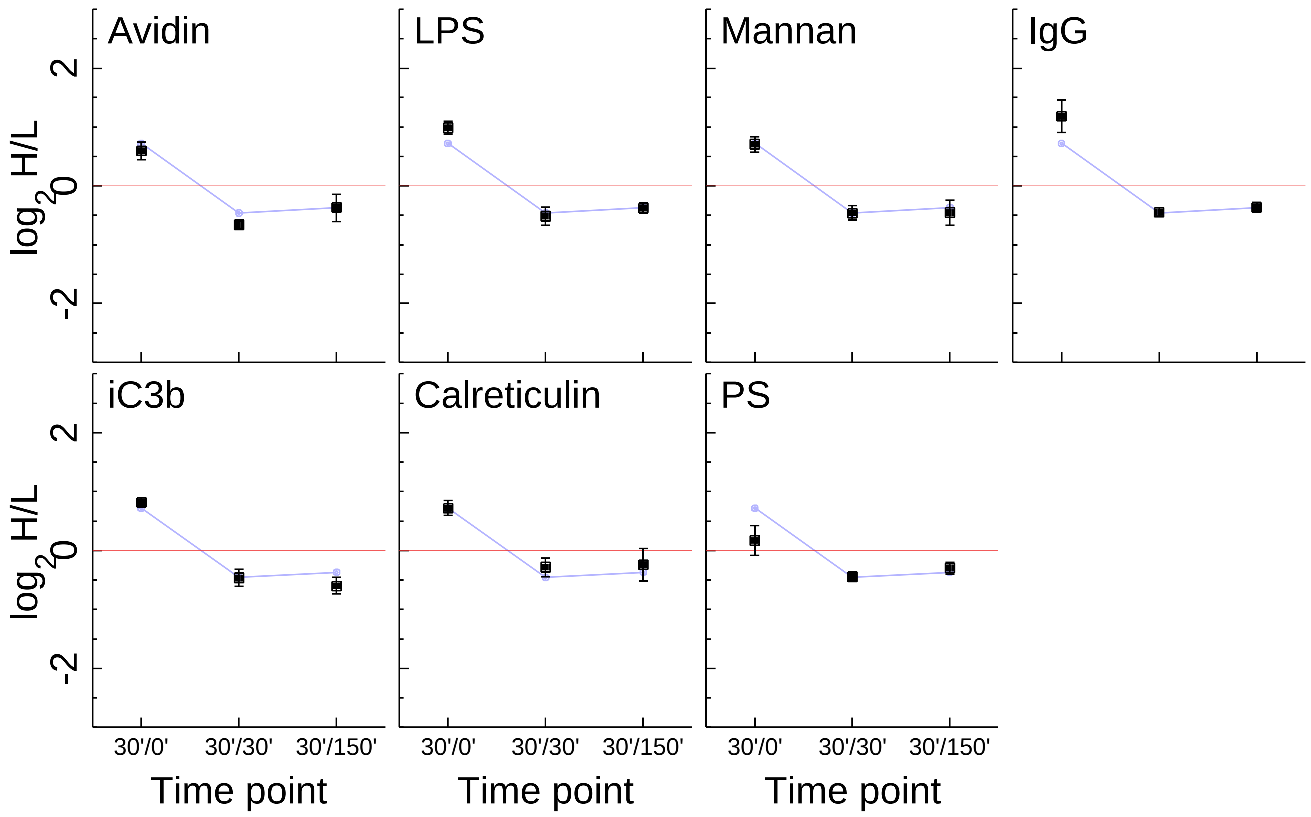
Avidin
LPS
Mannan
IgG
iC3b
Calreticulin
PS
log2 H/L
2
0
-2
30'/0'
30'/30'
30'/150'
Time point

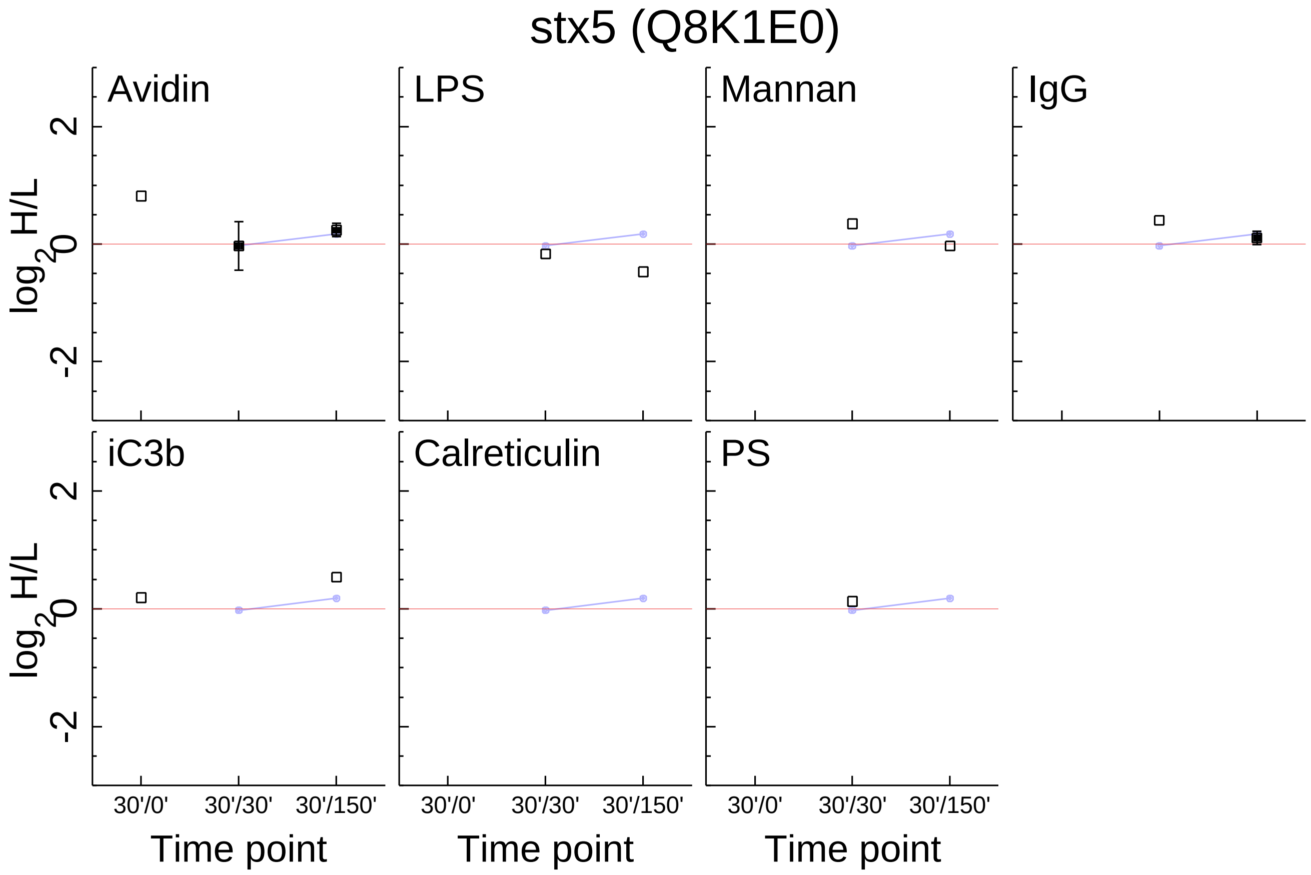

stx5 (Q8K1E0)
Avidin
LPS
Mannan
IgG
iC3b
Calreticulin
PS
log2 H/L
2
0
-2
30'/0'
30'/30'
30'/150'
Time point

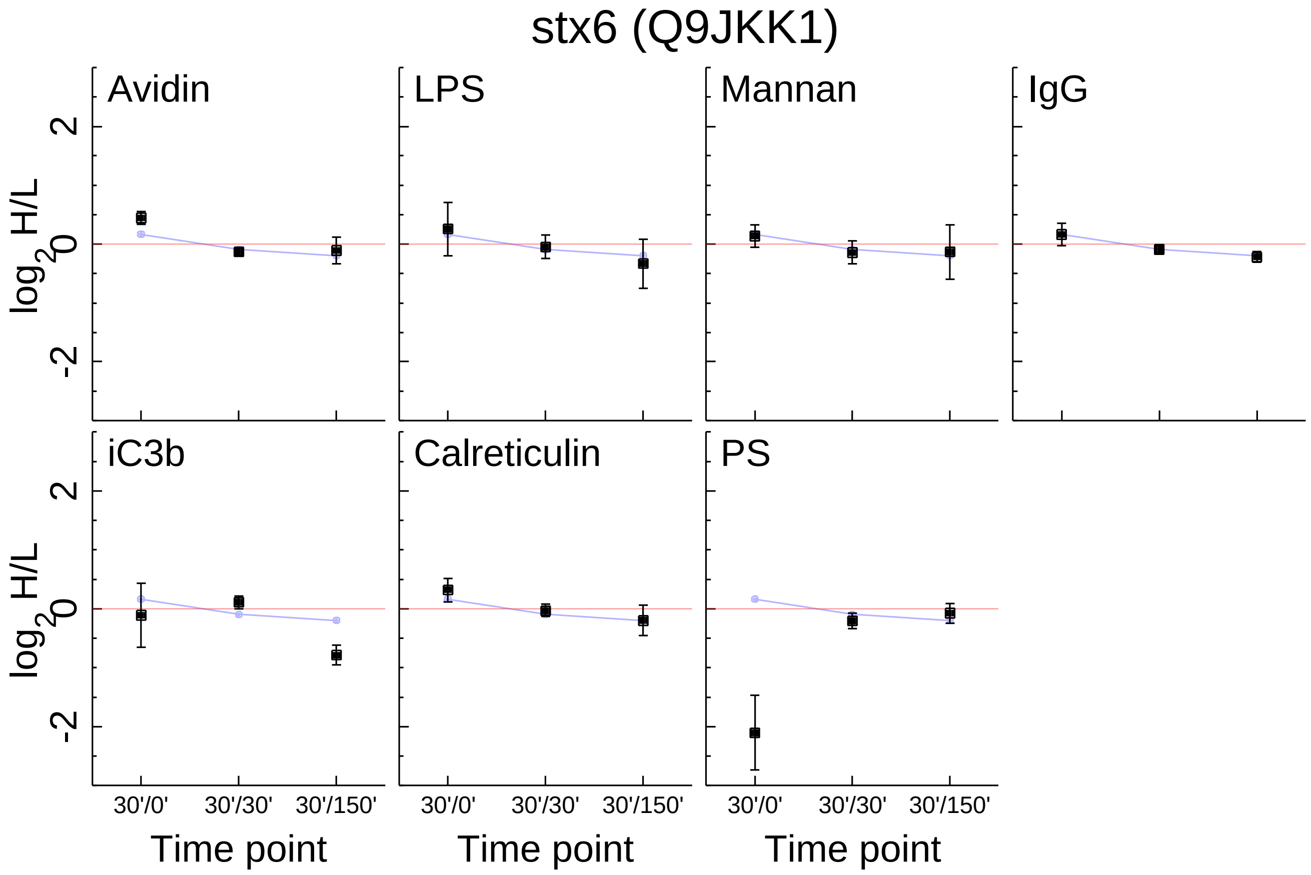

stx6 (Q9JKK1)
Avidin
LPS
Mannan
IgG
iC3b
Calreticulin
PS
2
0
-2
log2 H/L
30'/0'
30'/30'
30'/150'
Time point

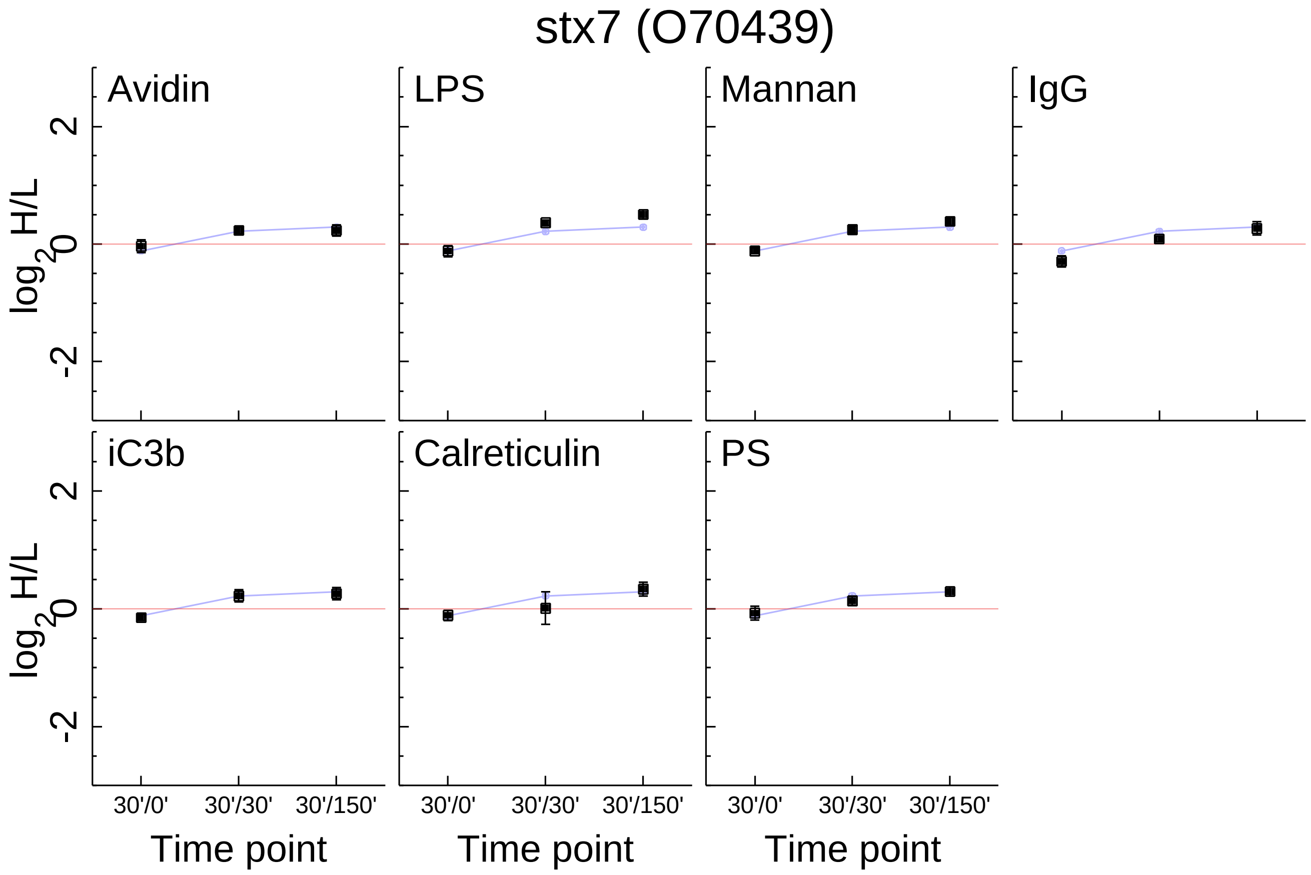
stx7 (O70439)
Avidin
LPS
Mannan
IgG
iC3b
Calreticulin
PS
log2 H/L
2
0
-2
30'/0'
30'/30'
30'/150'
Time point

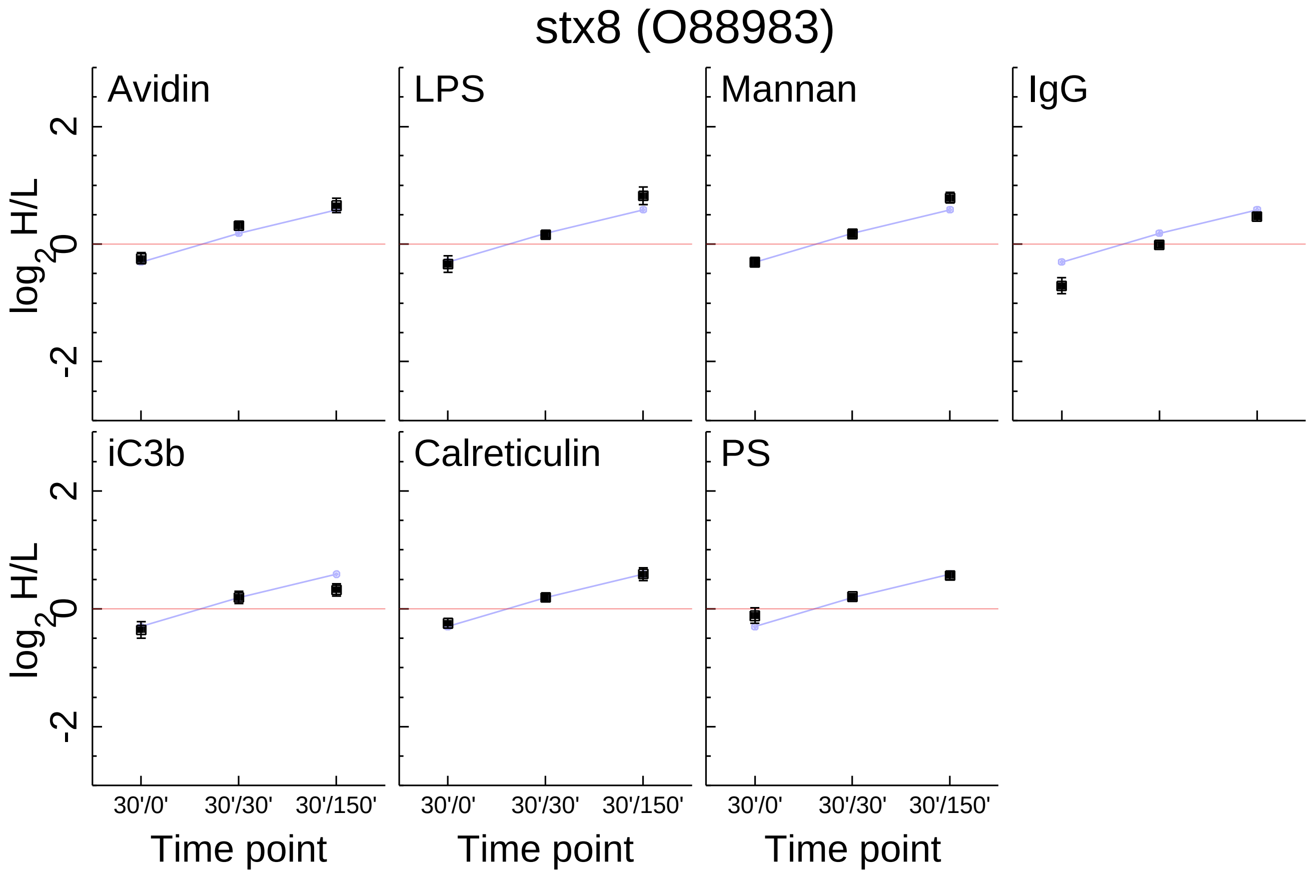

stx8 (O88983)
Avidin
LPS
Mannan
IgG
iC3b
Calreticulin
PS
log2 H/L
2
0
-2
30'/0'
30'/30'
30'/150'
Time point

stxbp1 (O08599)

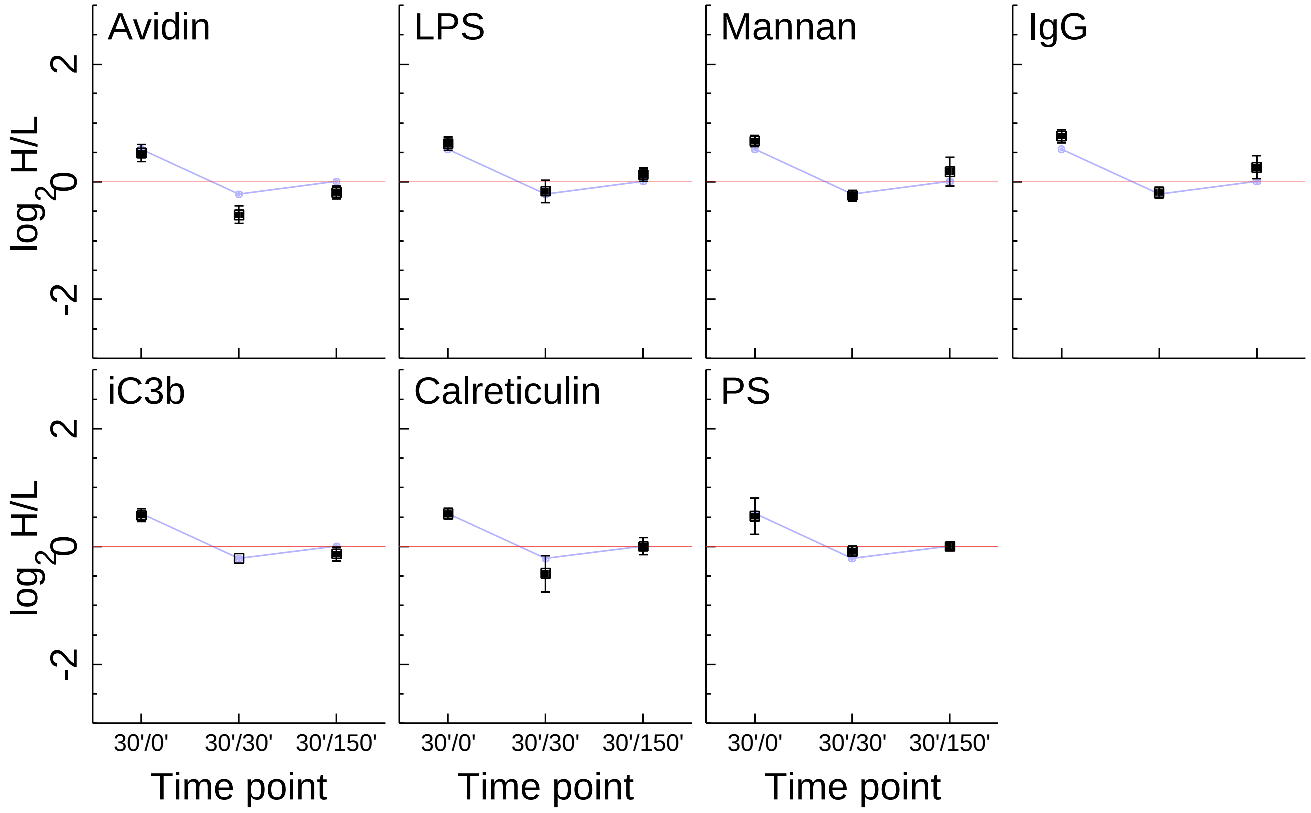

stxbp2 (Q64324)

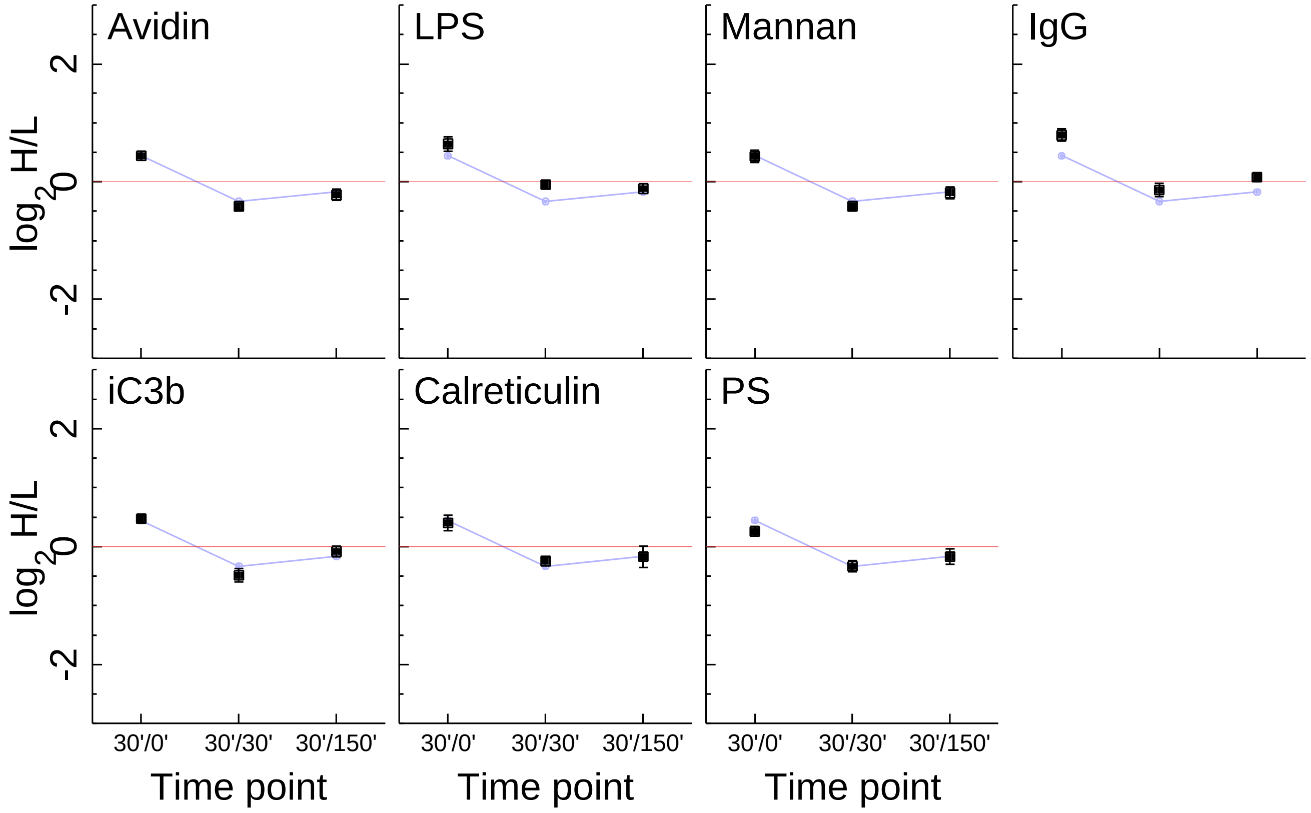

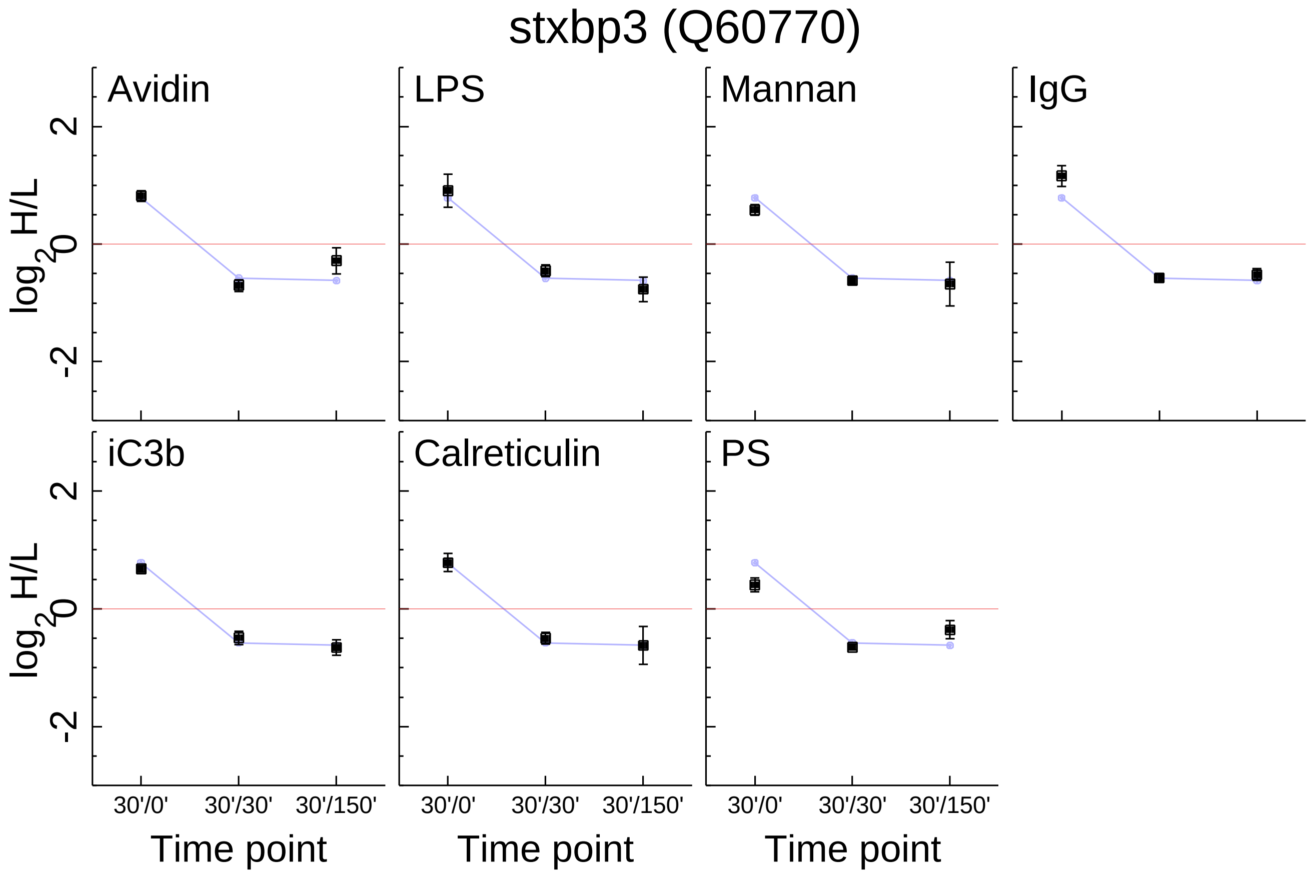

stxbp3 (Q60770)
Avidin
LPS
Mannan
IgG
iC3b
Calreticulin
PS
log2 H/L
2
0
-2
30'/0'
30'/30'
30'/150'
Time point

stxbp5 (Q8K400)

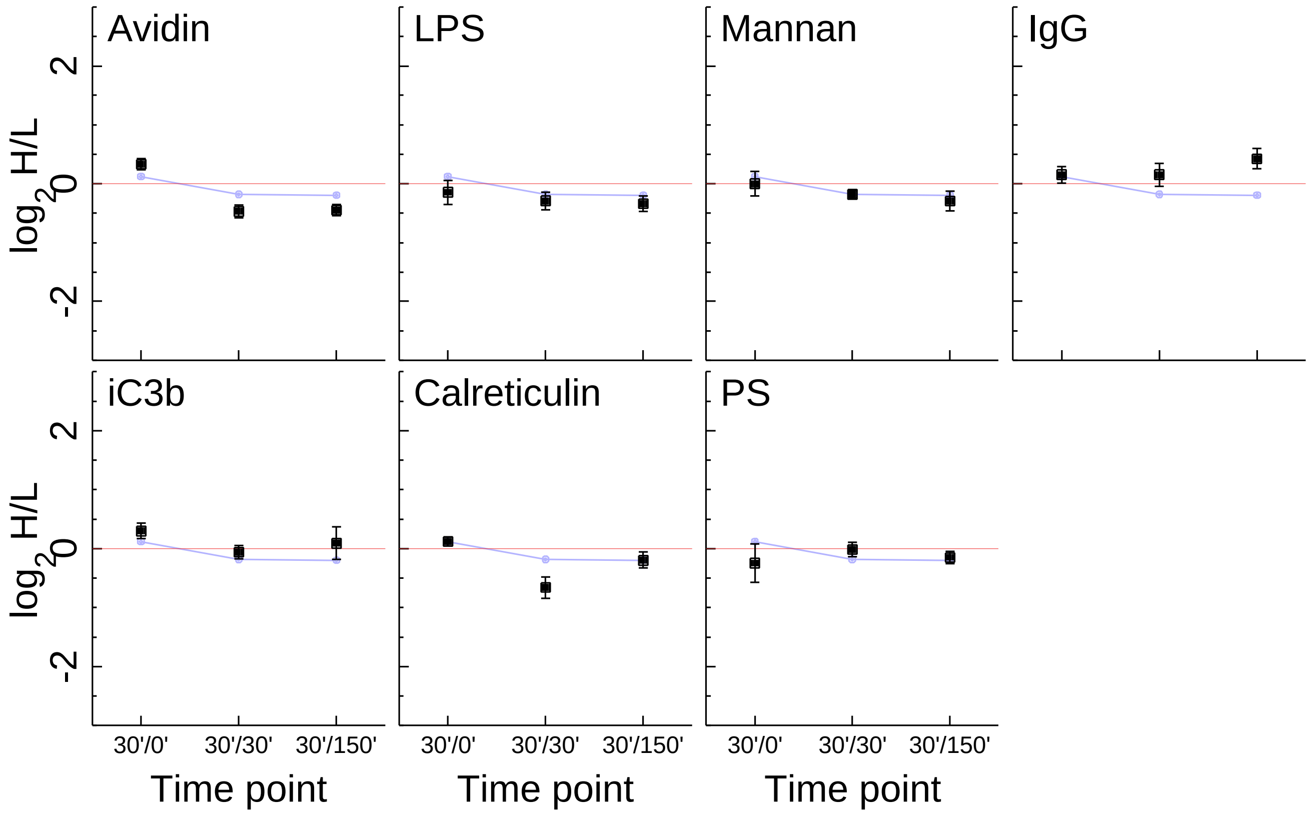

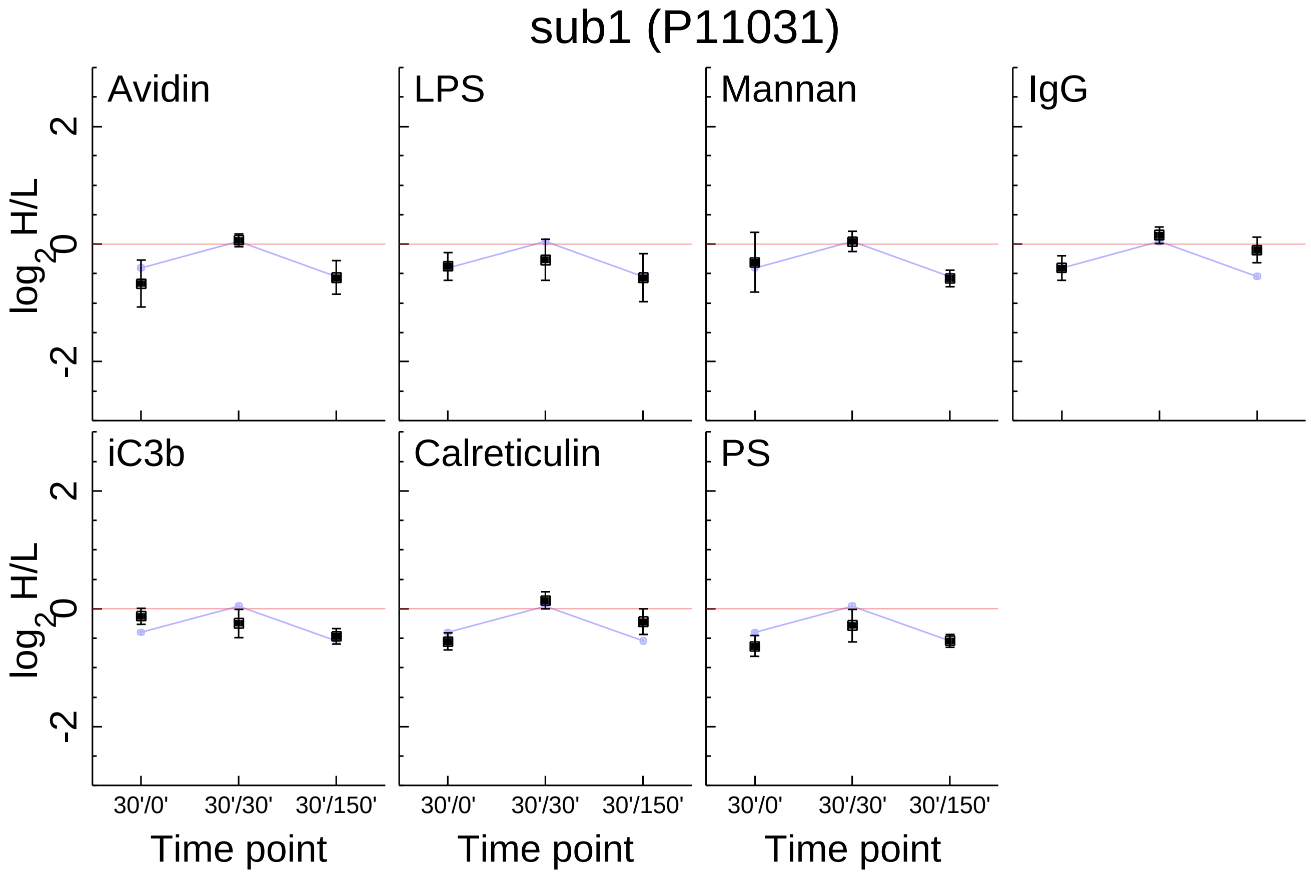

sub1 (P11031)
Avidin
LPS
Mannan
IgG
iC3b
Calreticulin
PS
log2 H/L
2
0
-2
30'/0'
30'/30'
30'/150'
Time point

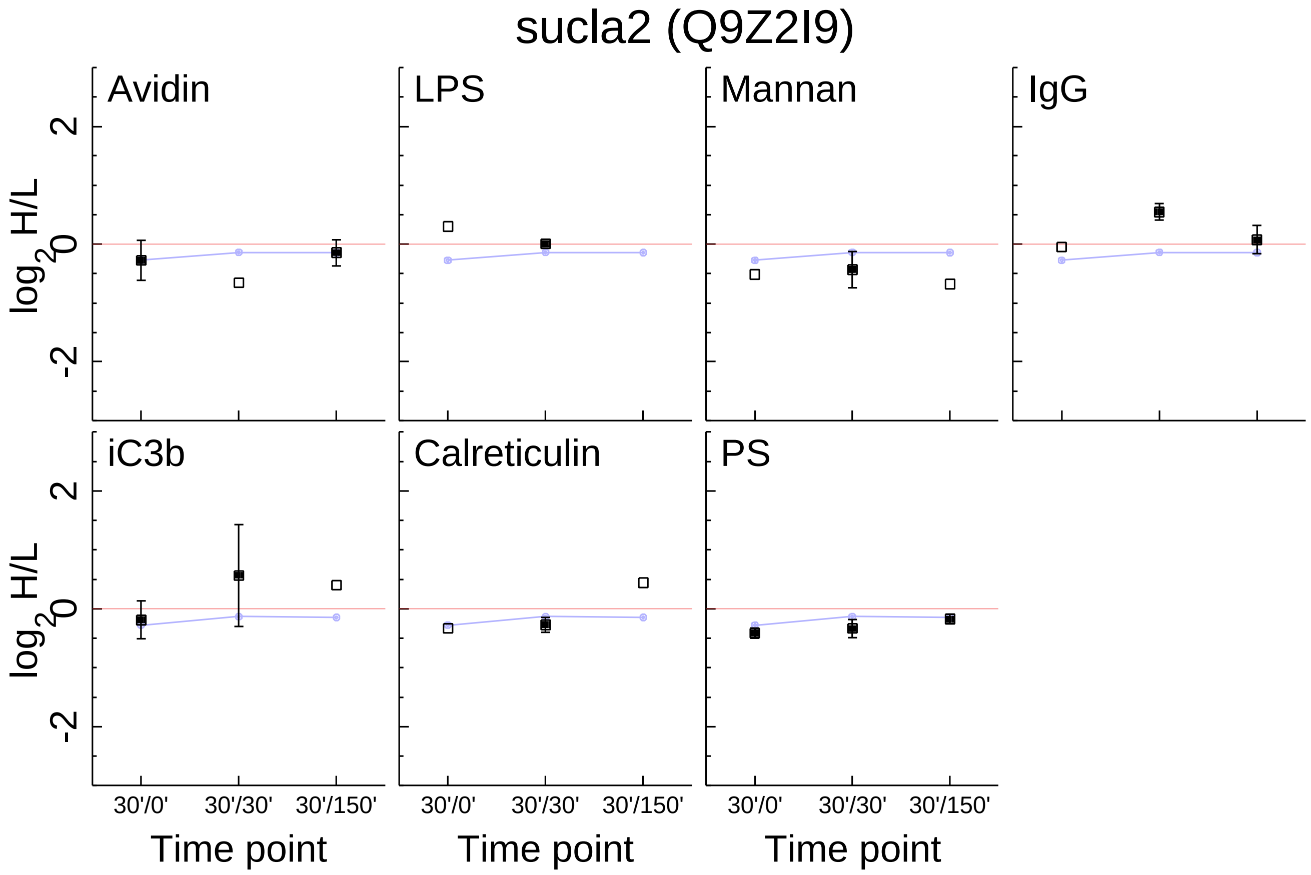
sucla2 (Q9Z2I9)
Avidin
LPS
Mannan
IgG
iC3b
Calreticulin
PS
log2 H/L
2
0
-2
30'/0'
30'/30'
30'/150'
Time point

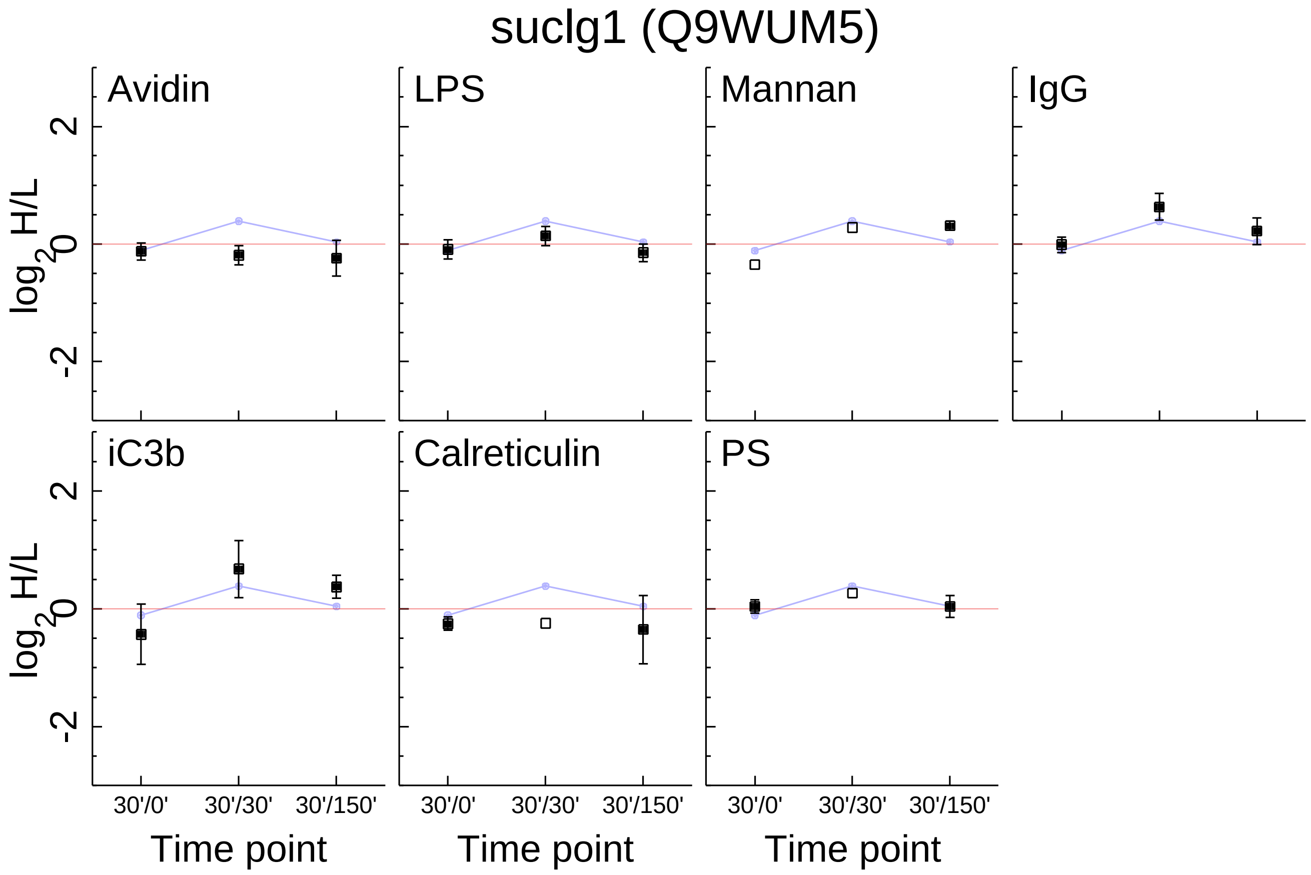
suclg1 (Q9WUM5)
Avidin
LPS
Mannan
IgG
iC3b
Calreticulin
PS
log2 H/L
2
0
-2
30'/0'
30'/30'
30'/150'
Time point

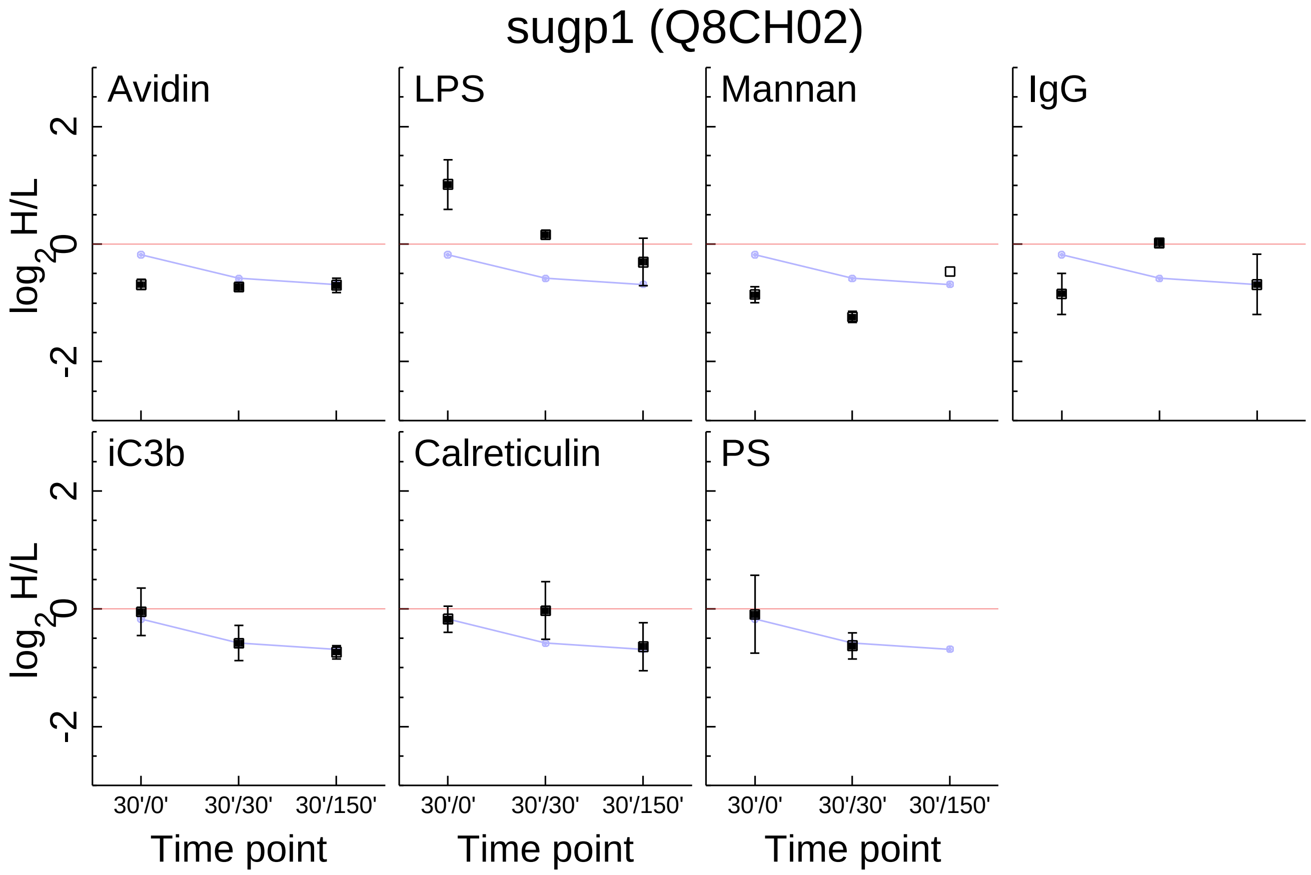
sugp1 (Q8CH02)
Avidin
LPS
Mannan
IgG
iC3b
Calreticulin
PS
log2 H/L
2
0
-2
30'/0'
30'/30'
30'/150'
Time point

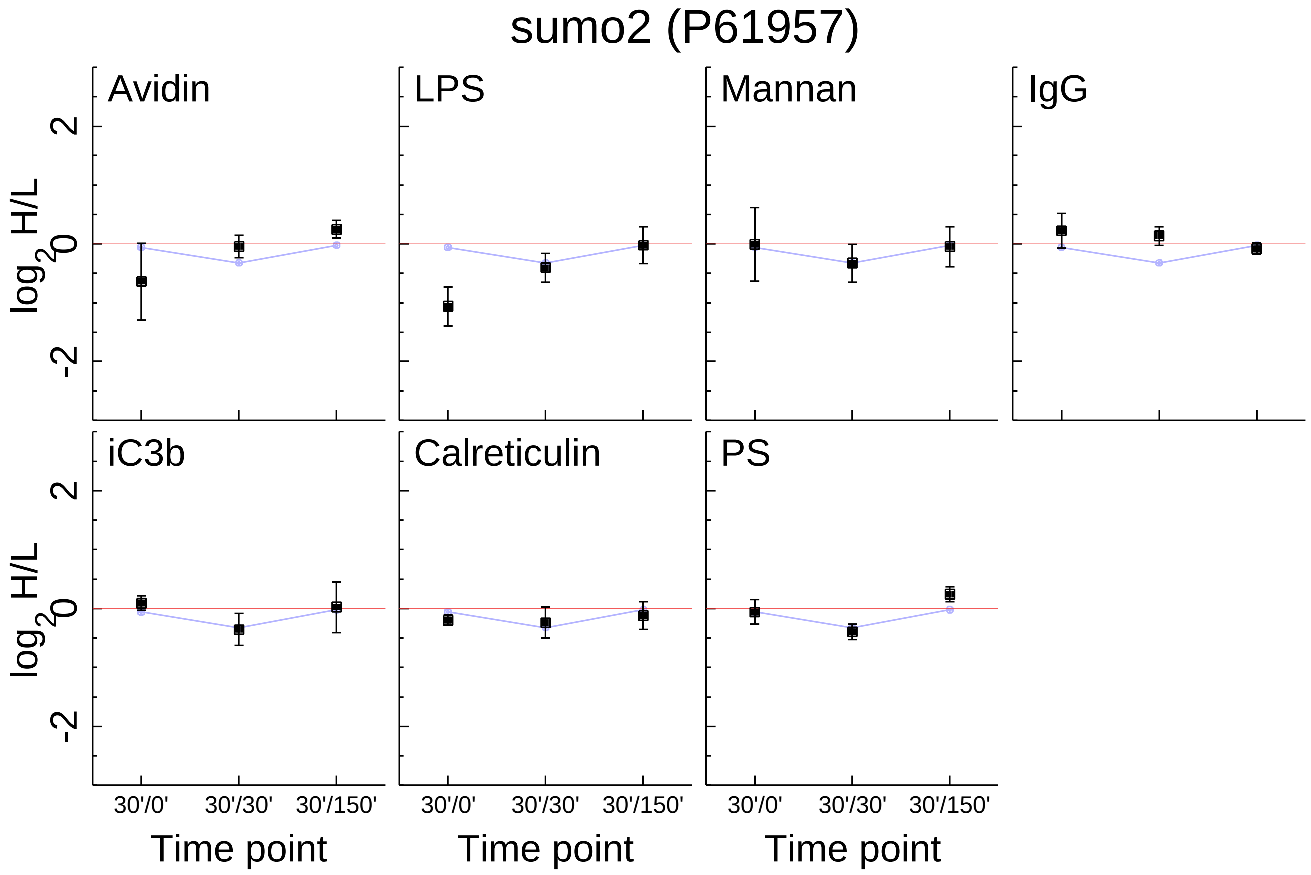

sumo2 (P61957)
Avidin
LPS
Mannan
IgG
iC3b
Calreticulin
PS
log2 H/L
2
0
-2
30'/0'
30'/30'
30'/150'
Time point

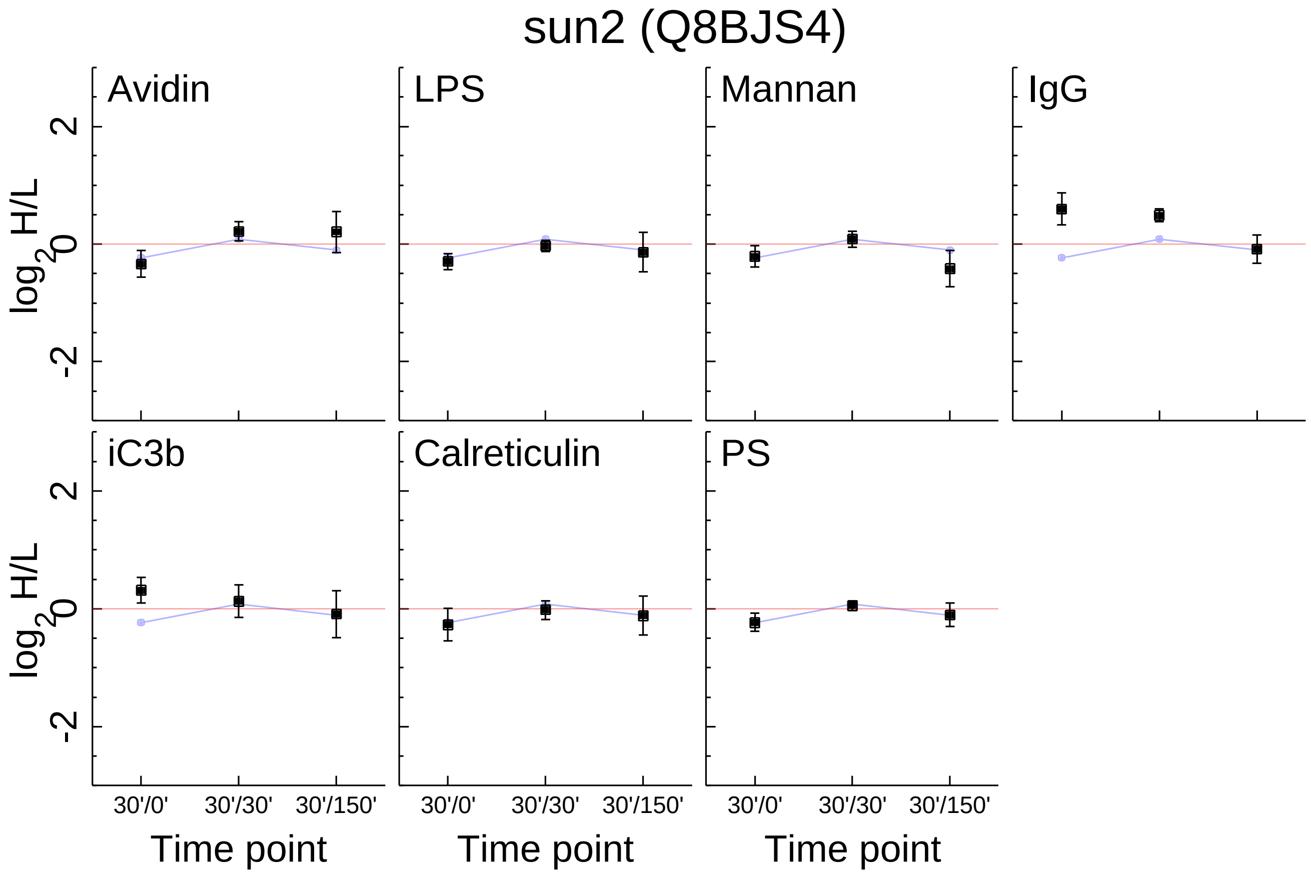
sun2 (Q8BJS4)
Avidin
LPS
Mannan
IgG
iC3b
Calreticulin
PS
log2 H/L
2
0
-2
30'/0'
30'/30'
30'/150'
Time point

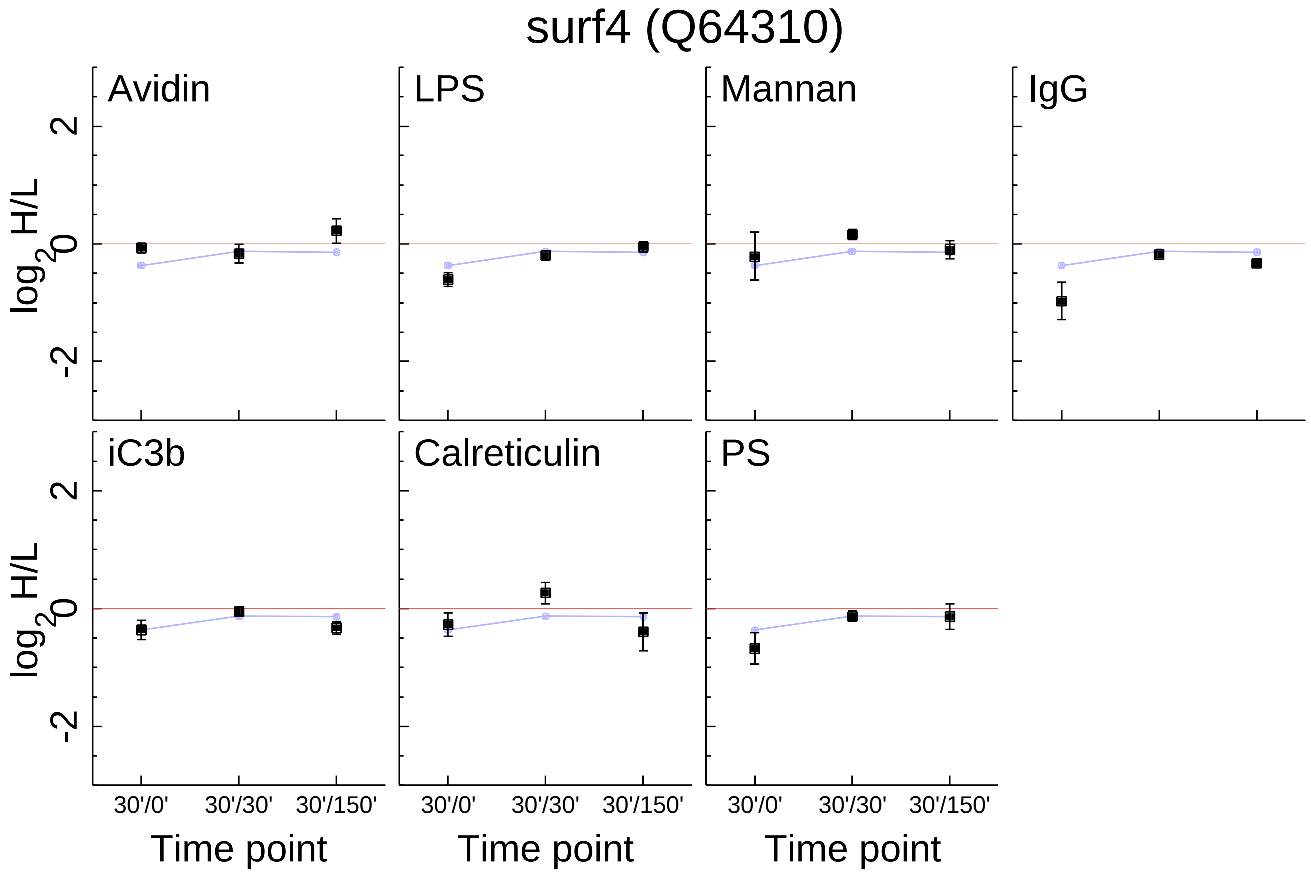
surf4 (Q64310)
Avidin
LPS
Mannan
IgG
iC3b
Calreticulin
PS
log2 H/L
2
0
-2
30'/0'
30'/30'
30'/150'
Time point

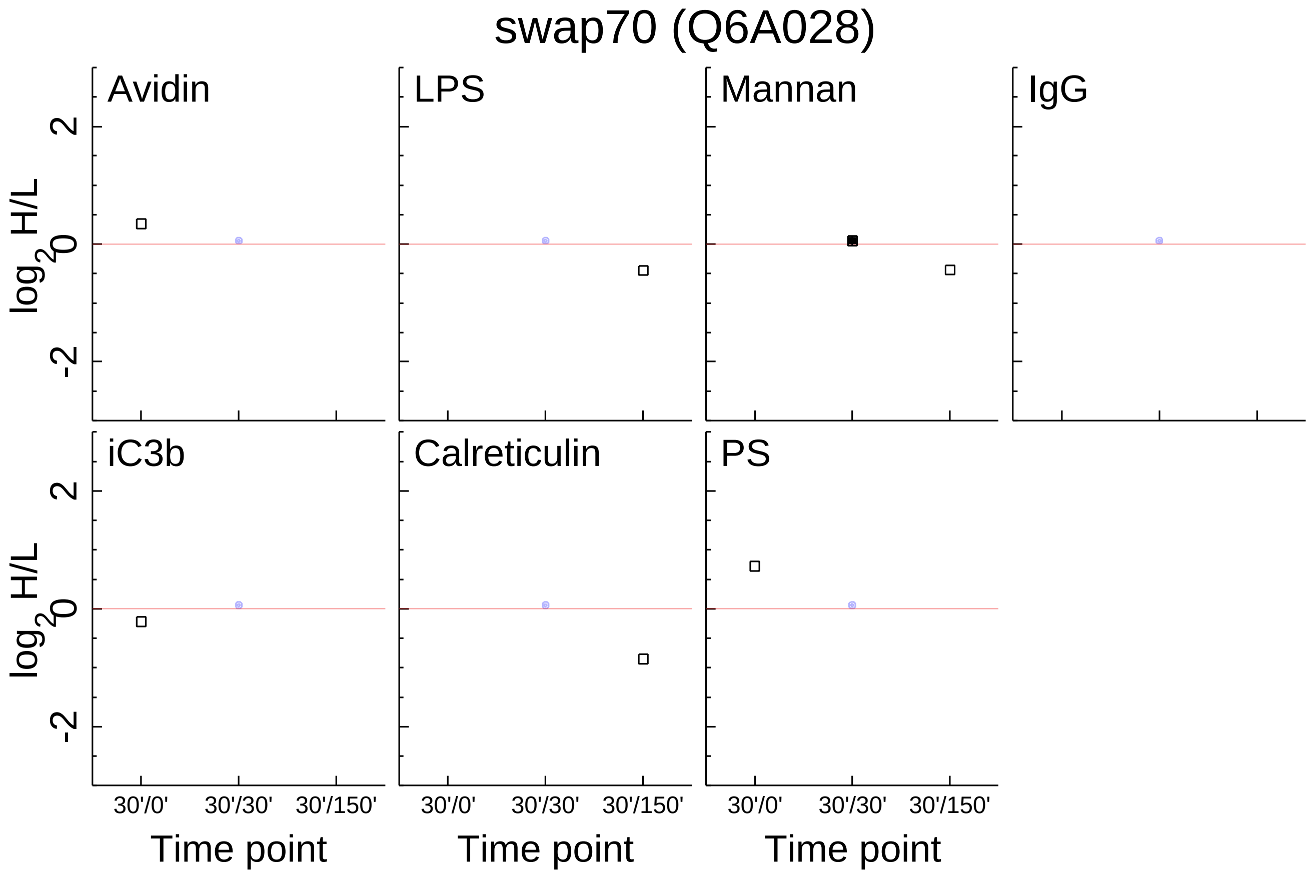

swap70 (Q6A028)
Avidin
LPS
Mannan
IgG
iC3b
Calreticulin
PS
log2 H/L
2
0
-2
30'/0'
30'/30'
30'/150'
Time point

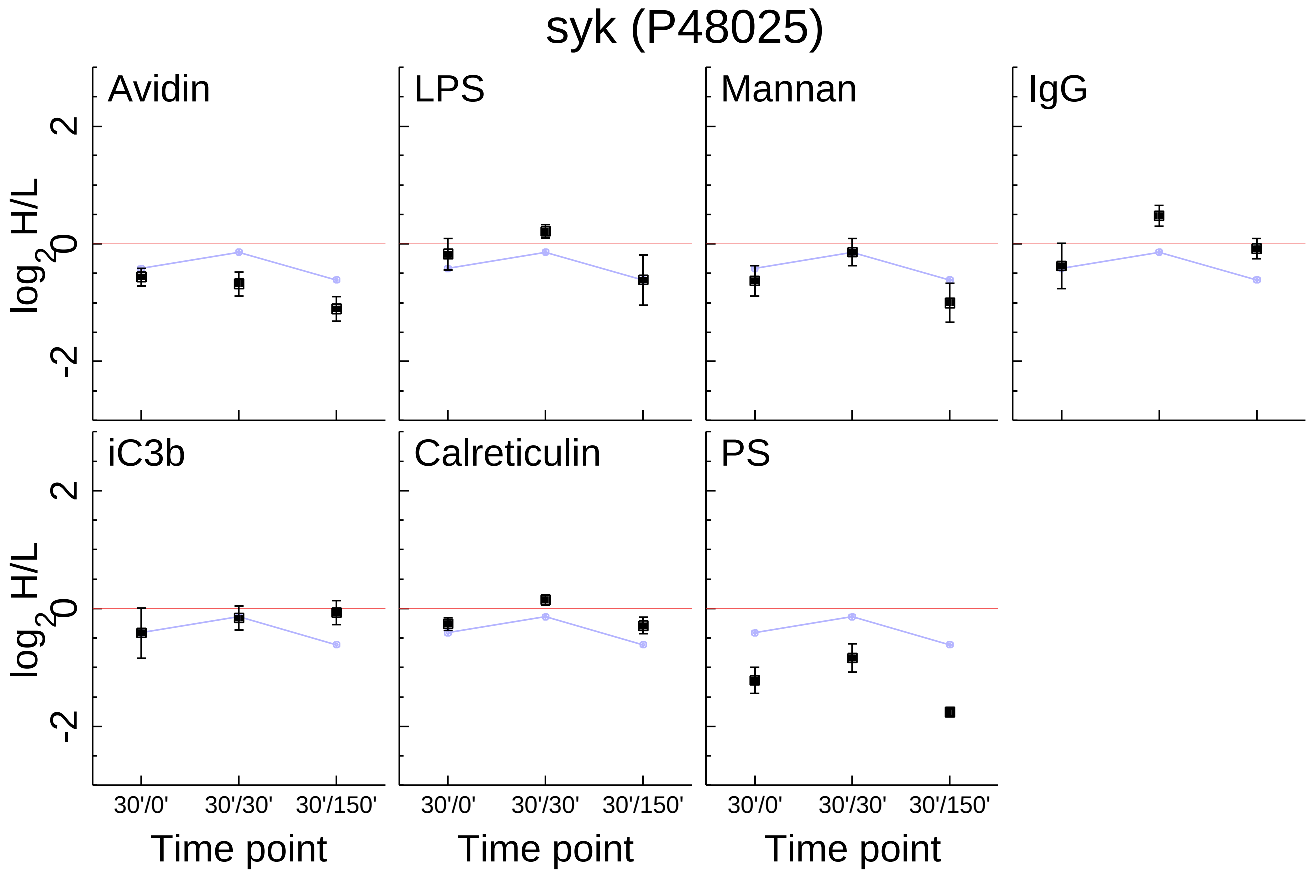

syk (P48025)
Avidin
LPS
Mannan
IgG
iC3b
Calreticulin
PS
log2 H/L
2
0
-2
30'/0'
30'/30'
30'/150'
Time point

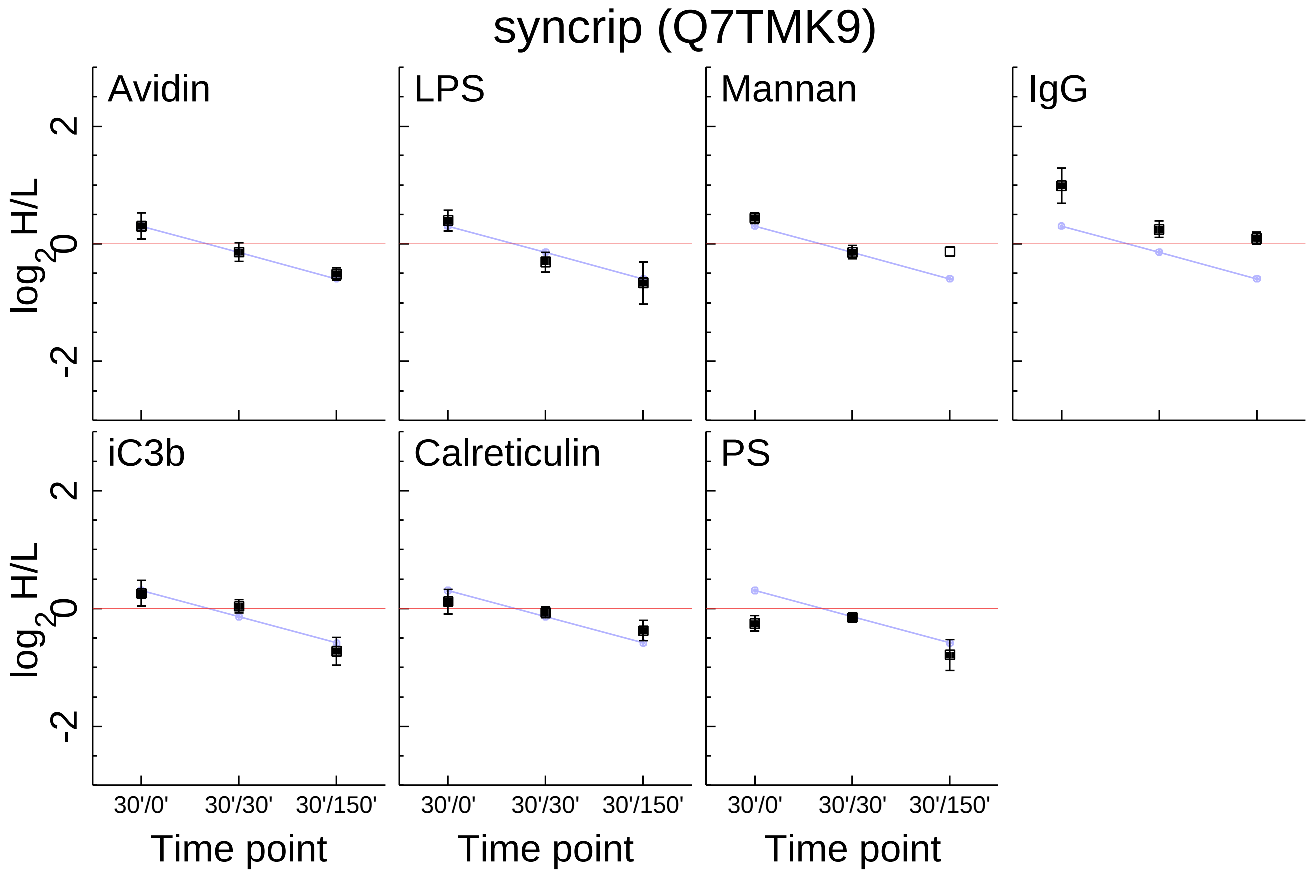

syncrip (Q7TMK9)
Avidin
LPS
Mannan
IgG
iC3b
Calreticulin
PS
log2 H/L
2
0
-2
30'/0'
30'/30'
30'/150'
Time point

syngr1 (O55100)

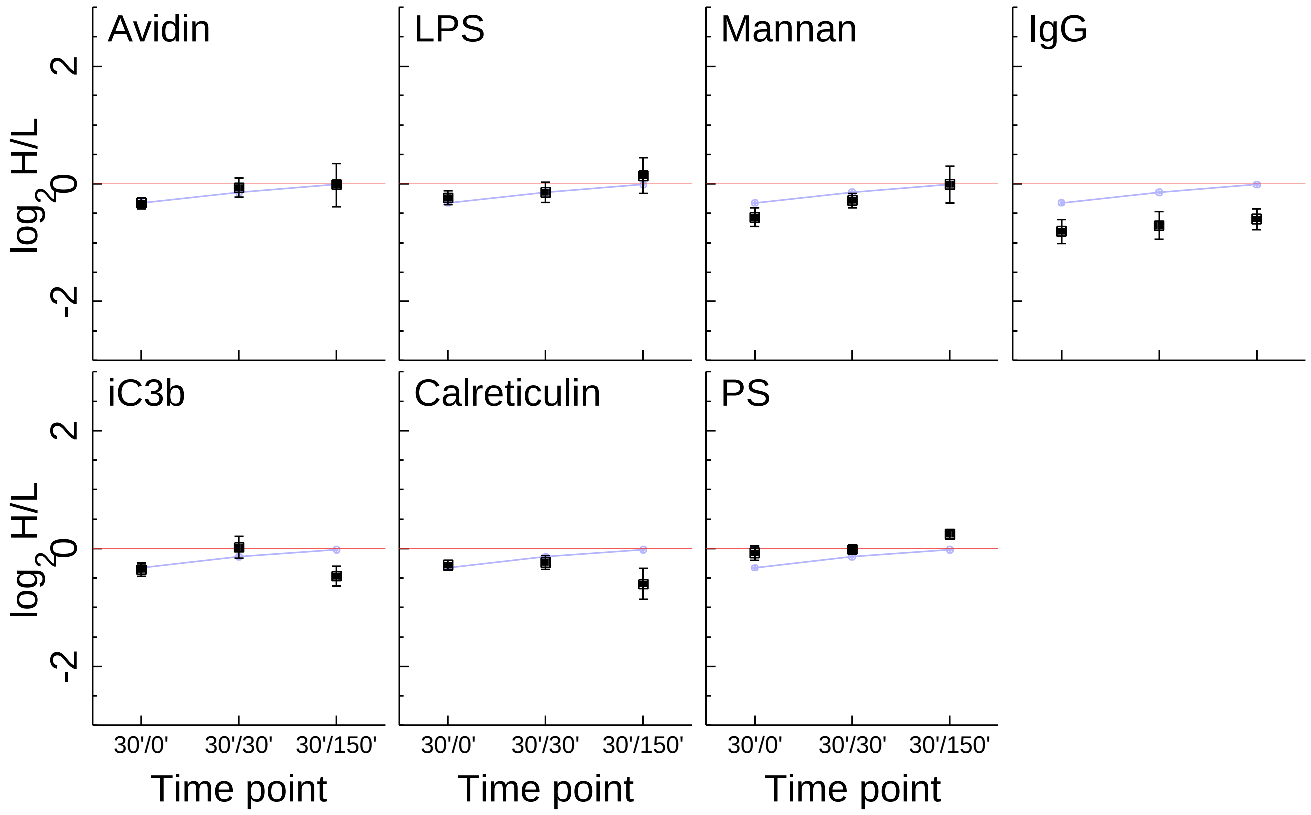

syngr2 (O55101)

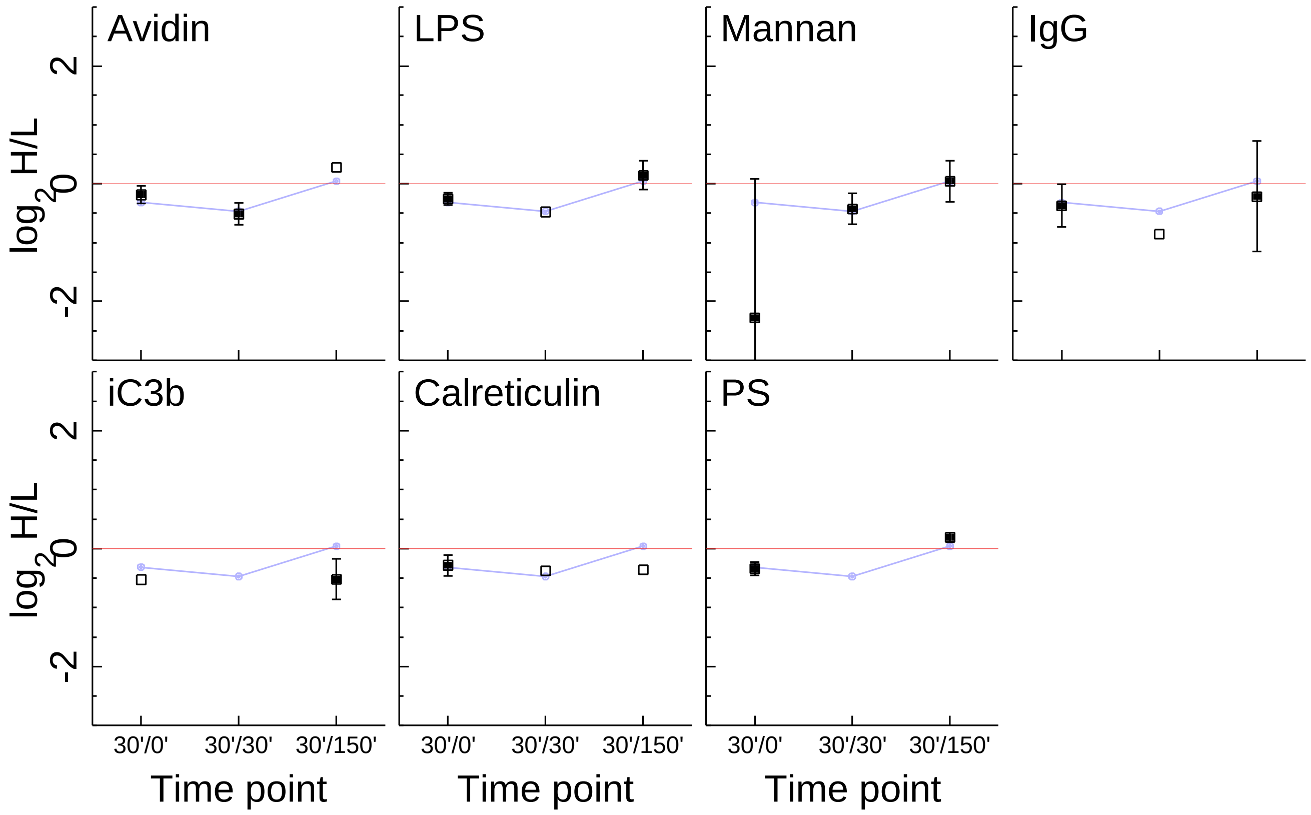

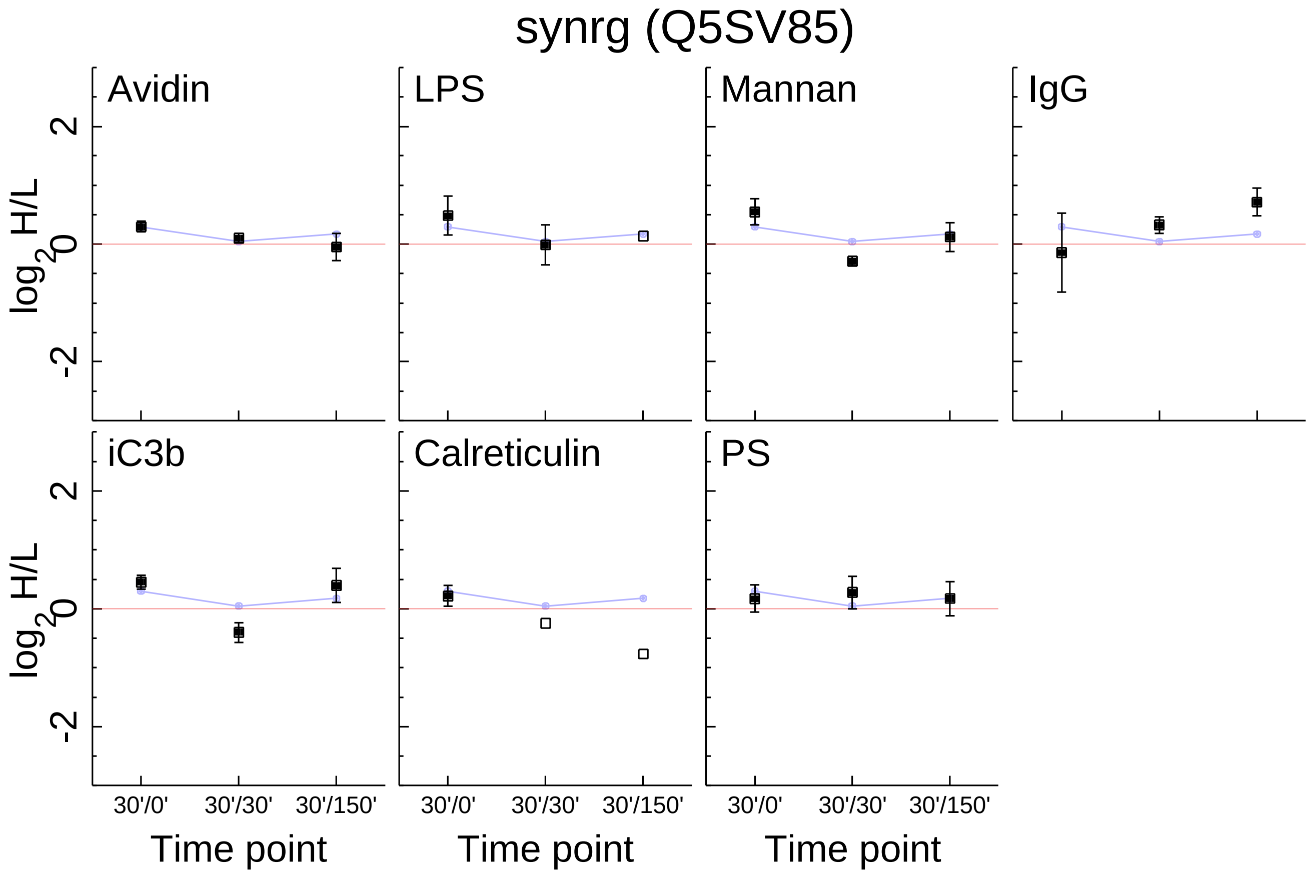
synrg (Q5SV85)
Avidin
LPS
Mannan
IgG
iC3b
Calreticulin
PS
log2 H/L
2
0
-2
30'/0'
30'/30'
30'/150'
Time point

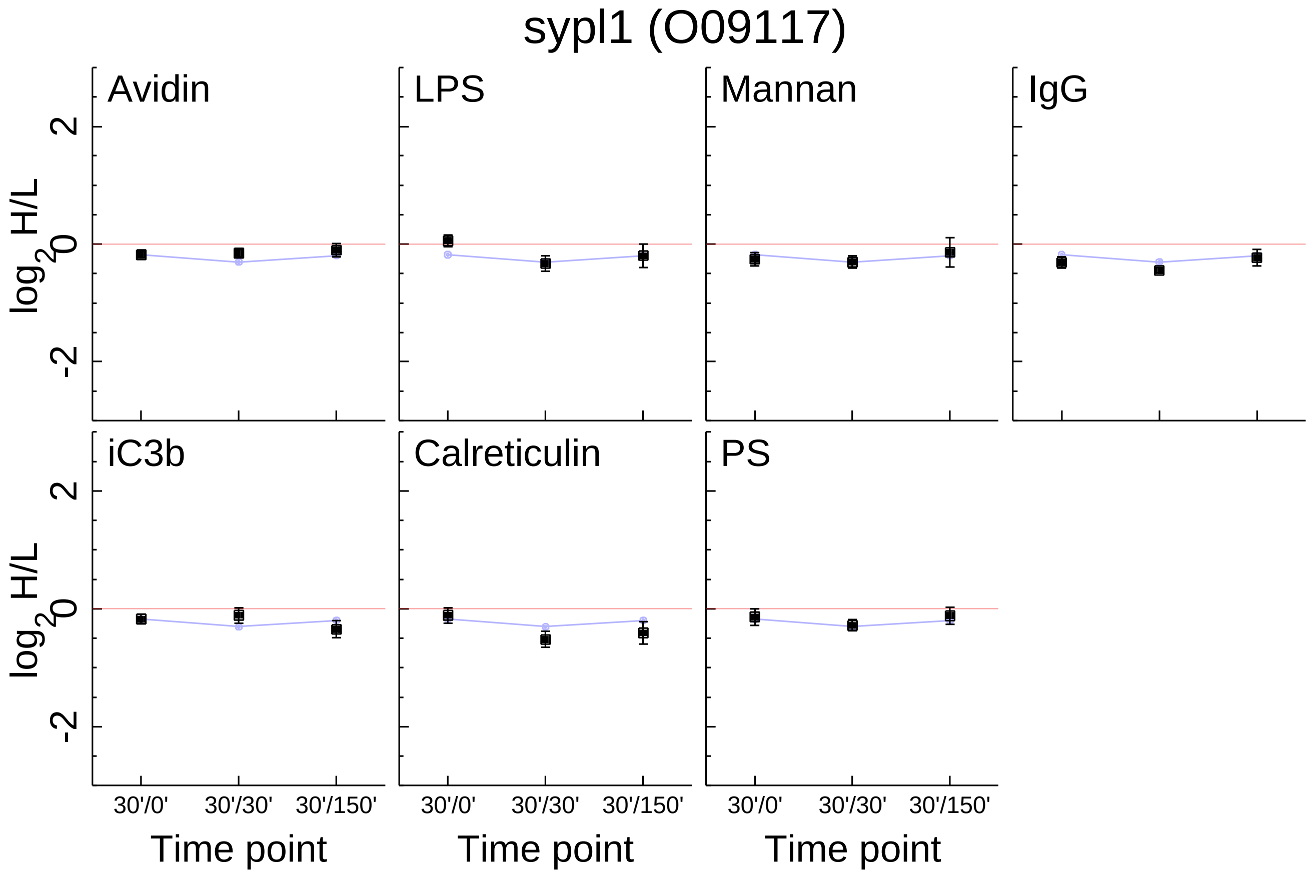
sypl1 (O09117)
Avidin
LPS
Mannan
IgG
iC3b
Calreticulin
PS
log2 H/L
2
0
-2
30'/0'
30'/30'
30'/150'
Time point

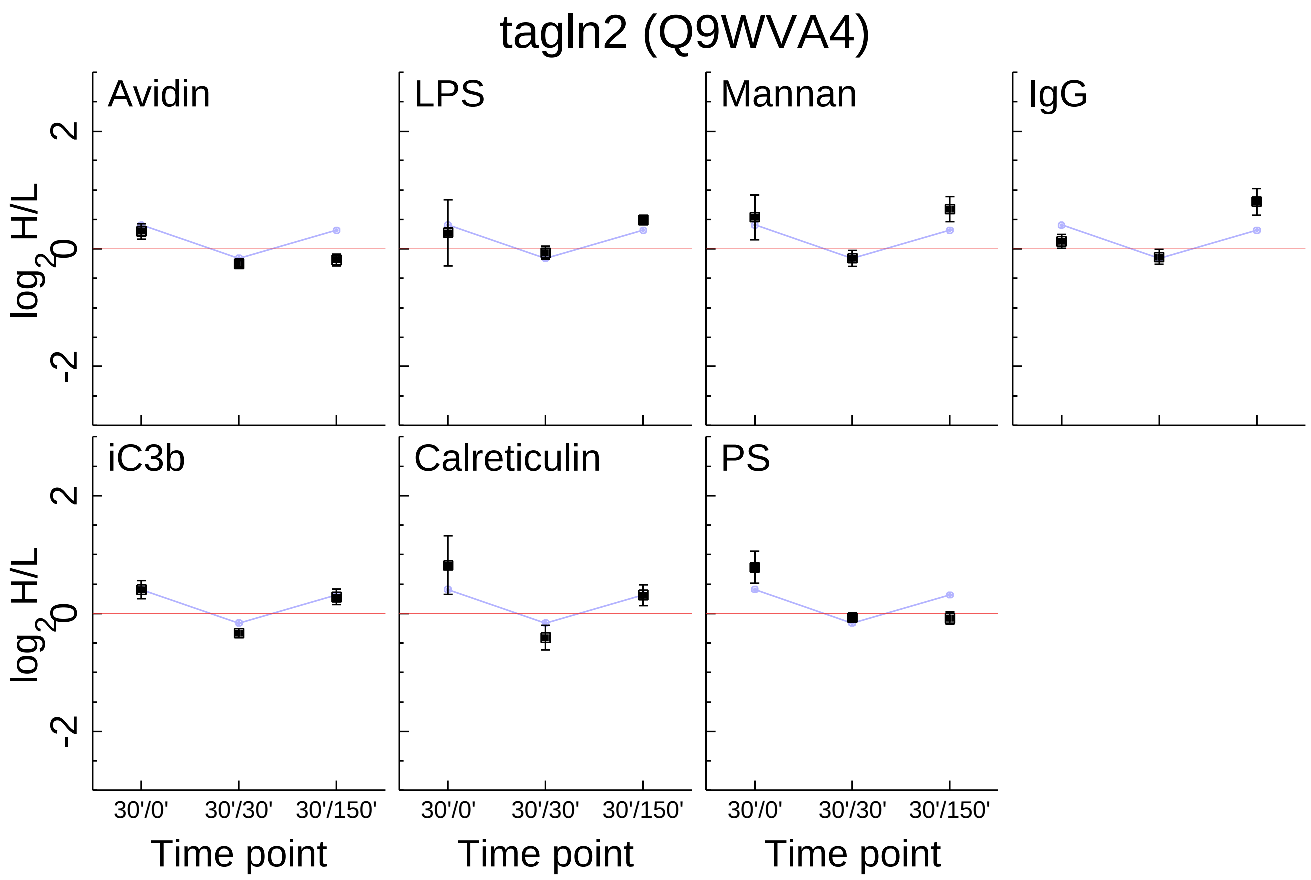

tagln2 (Q9WVA4)
Avidin
LPS
Mannan
IgG
iC3b
Calreticulin
PS
log2 H/L
2
0
-2
30'/0'
30'/30'
30'/150'
Time point

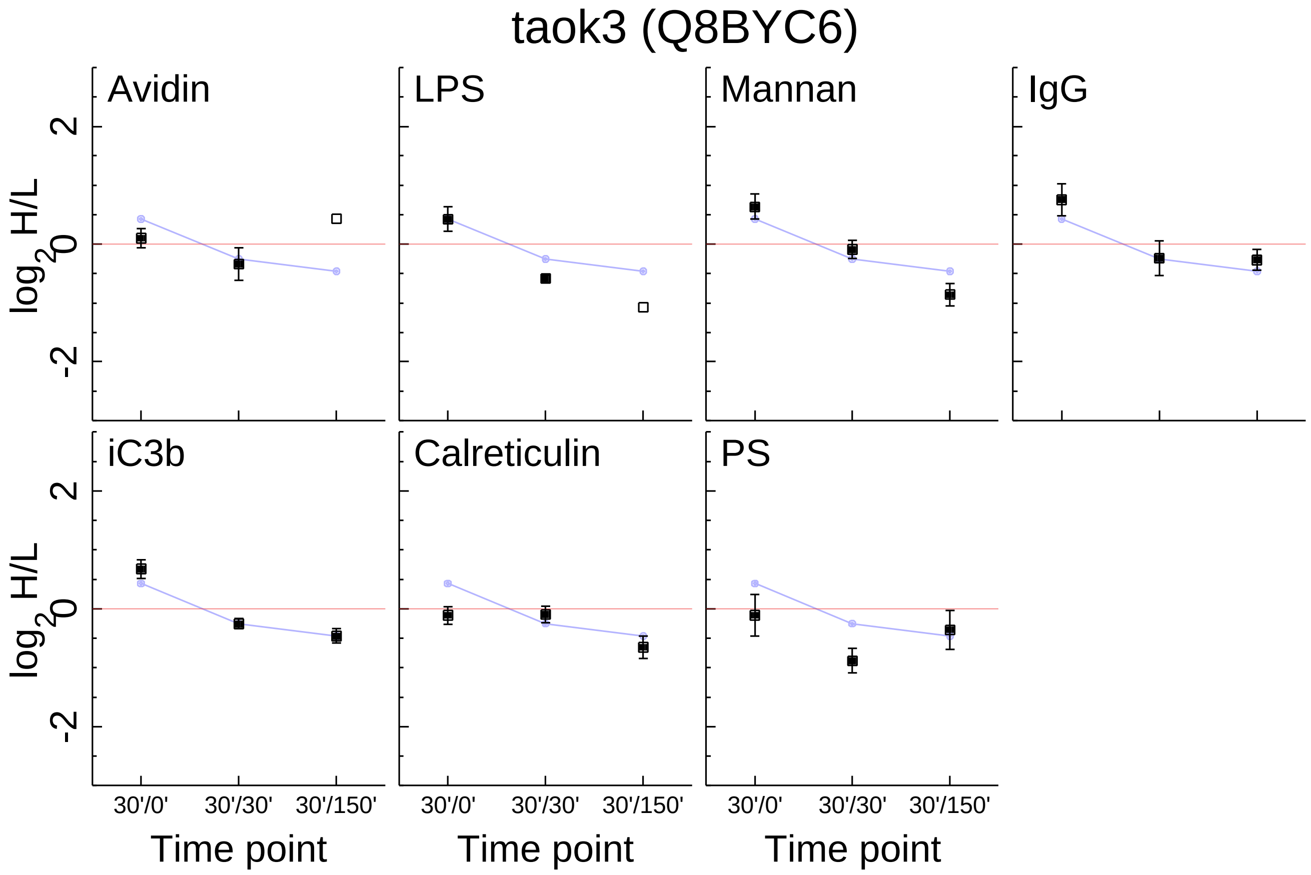
taok3 (Q8BYC6)
Avidin
LPS
Mannan
IgG
iC3b
Calreticulin
PS
log2 H/L
2
0
-2
30'/0'
30'/30'
30'/150'
Time point

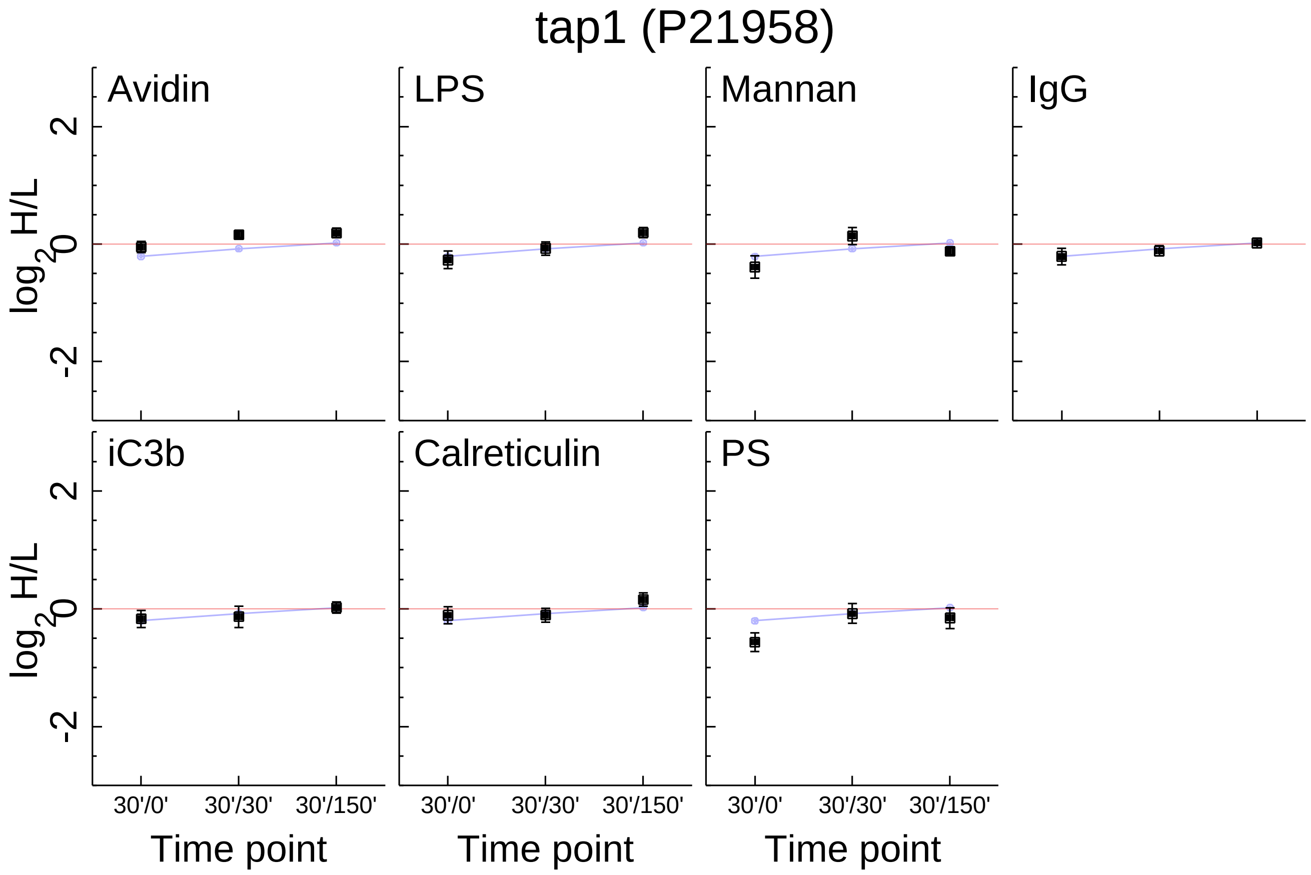
tap1 (P21958)
Avidin
LPS
Mannan
IgG
iC3b
Calreticulin
PS
log2 H/L
2
0
-2
30'/0'
30'/30'
30'/150'
Time point

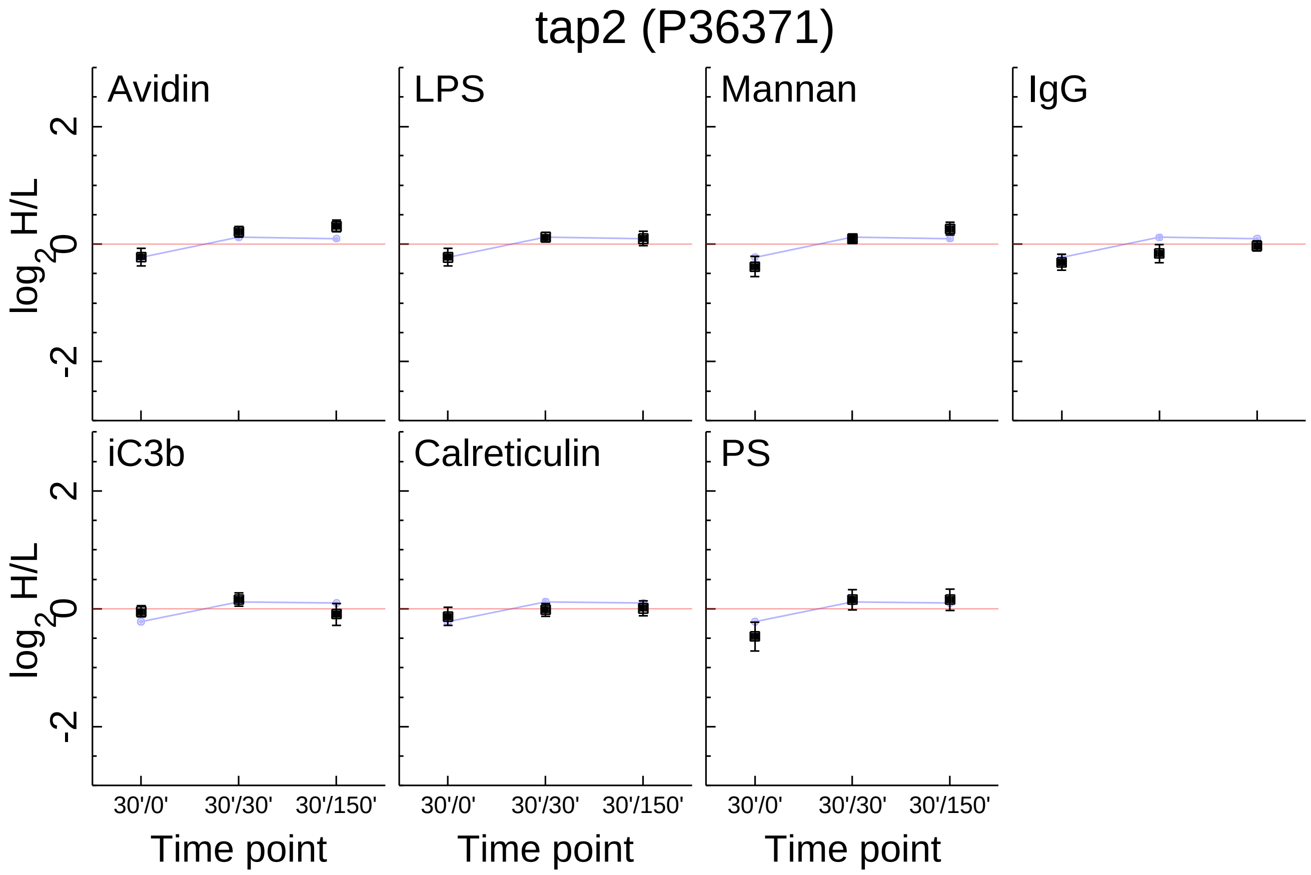
tap2 (P36371)
Avidin
LPS
Mannan
IgG
iC3b
Calreticulin
PS
log2 H/L
2
0
-2
30'/0'
30'/30'
30'/150'
Time point

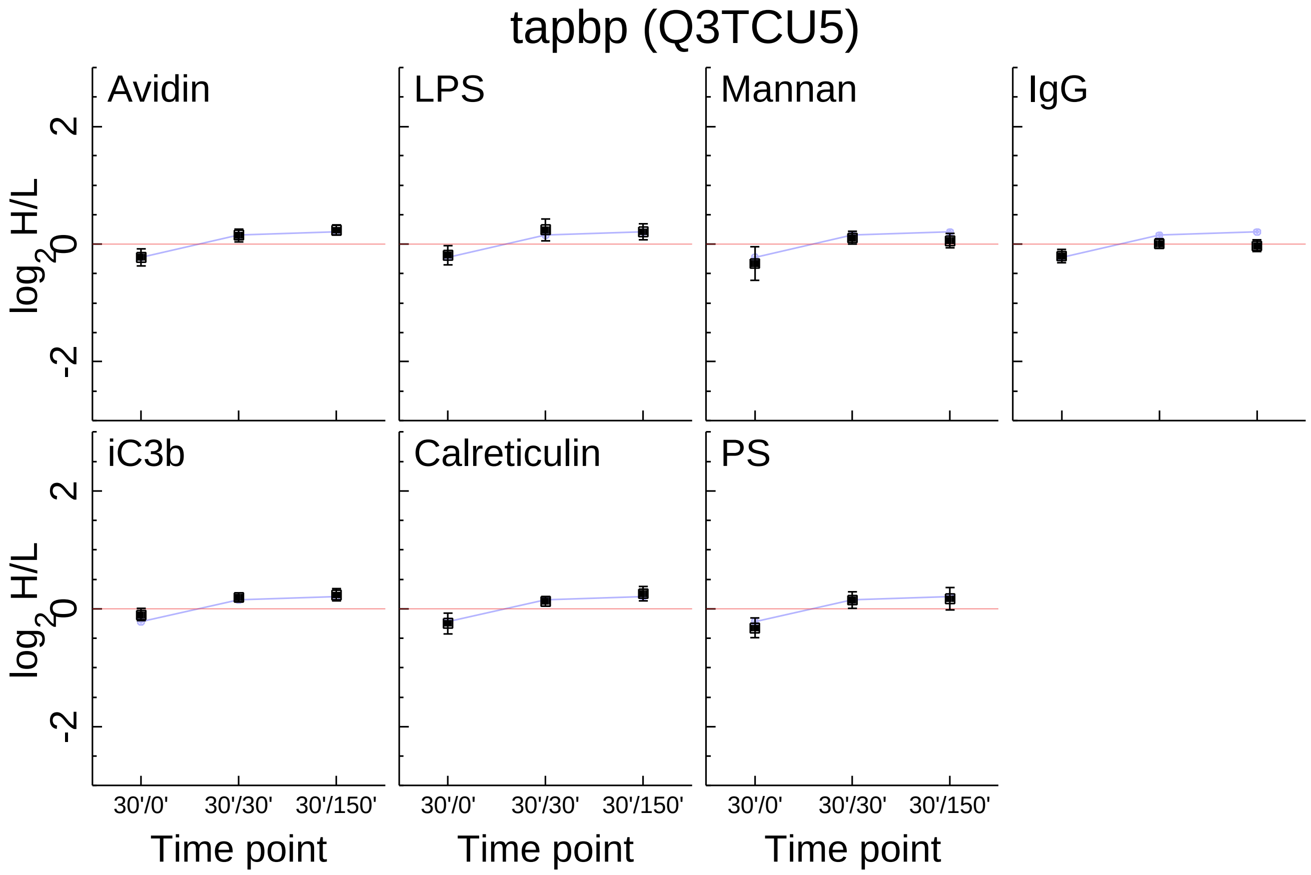
tapbp (Q3TCU5)
Avidin
LPS
Mannan
IgG
iC3b
Calreticulin
PS
log2 H/L
2
0
-2
30'/0'
30'/30'
30'/150'
Time point

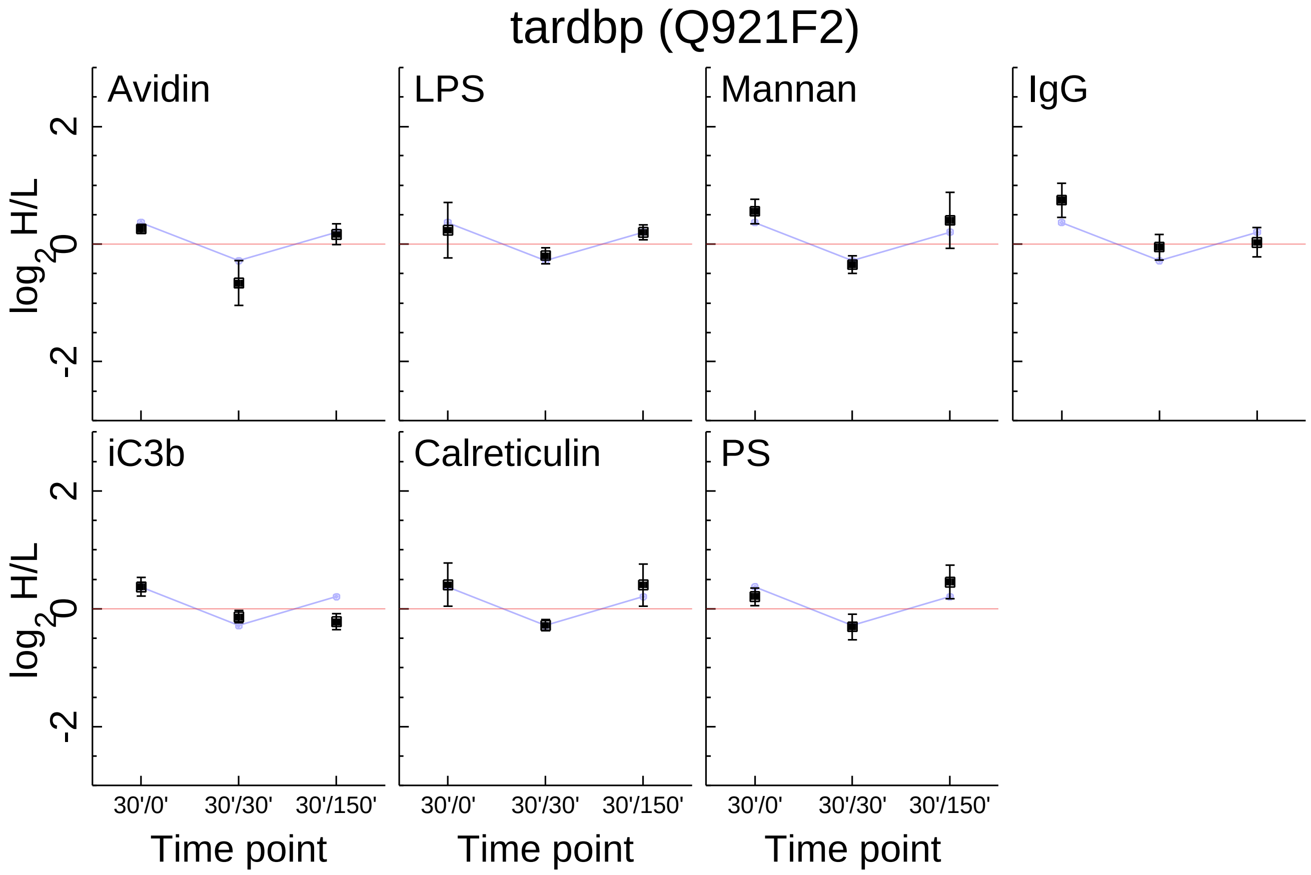

tardbp (Q921F2)
Avidin
LPS
Mannan
IgG
iC3b
Calreticulin
PS
log2 H/L
2
0
-2
30'/0'
30'/30'
30'/150'
Time point

tbc1d22a (Q8R5A6)

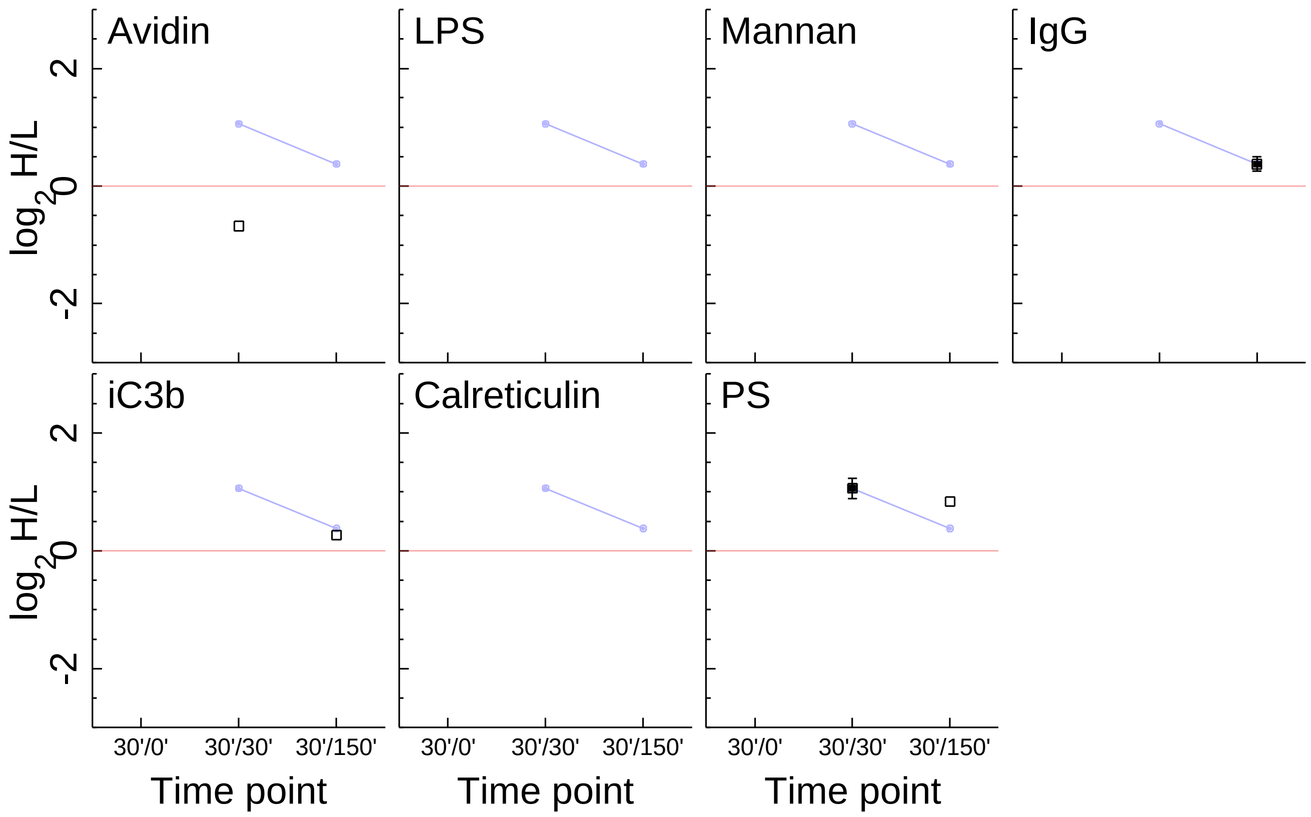

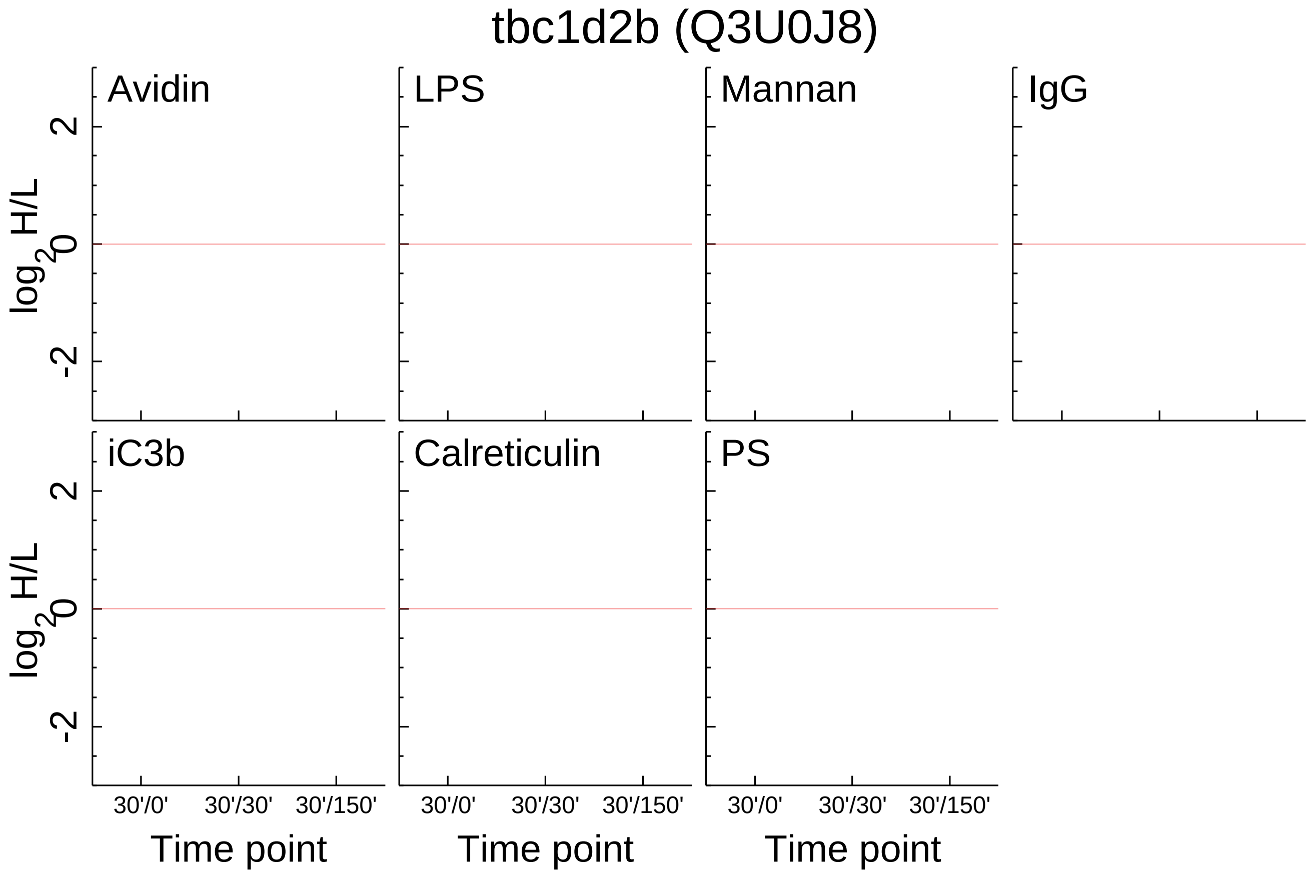
tbc1d2b (Q3U0J8)
Avidin
LPS
Mannan
IgG
iC3b
Calreticulin
PS
log2 H/L
2
0
-2
30'/0'
30'/30'
30'/150'
Time point

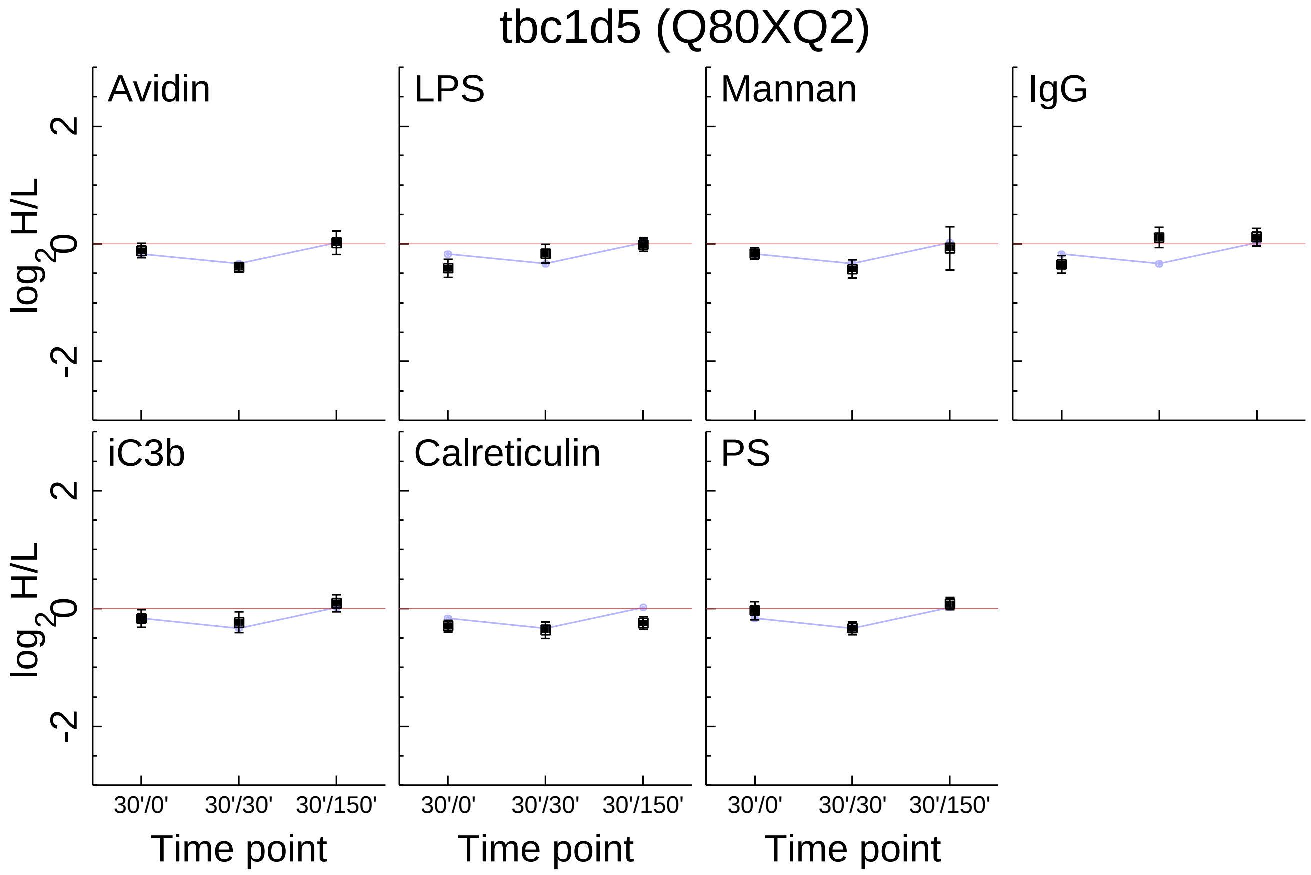

tbc1d5 (Q80XQ2)
Avidin
LPS
Mannan
IgG
iC3b
Calreticulin
PS
log2 H/L
2
0
-2
30'/0'
30'/30'
30'/150'
Time point

tbl1x (Q9QXE7)

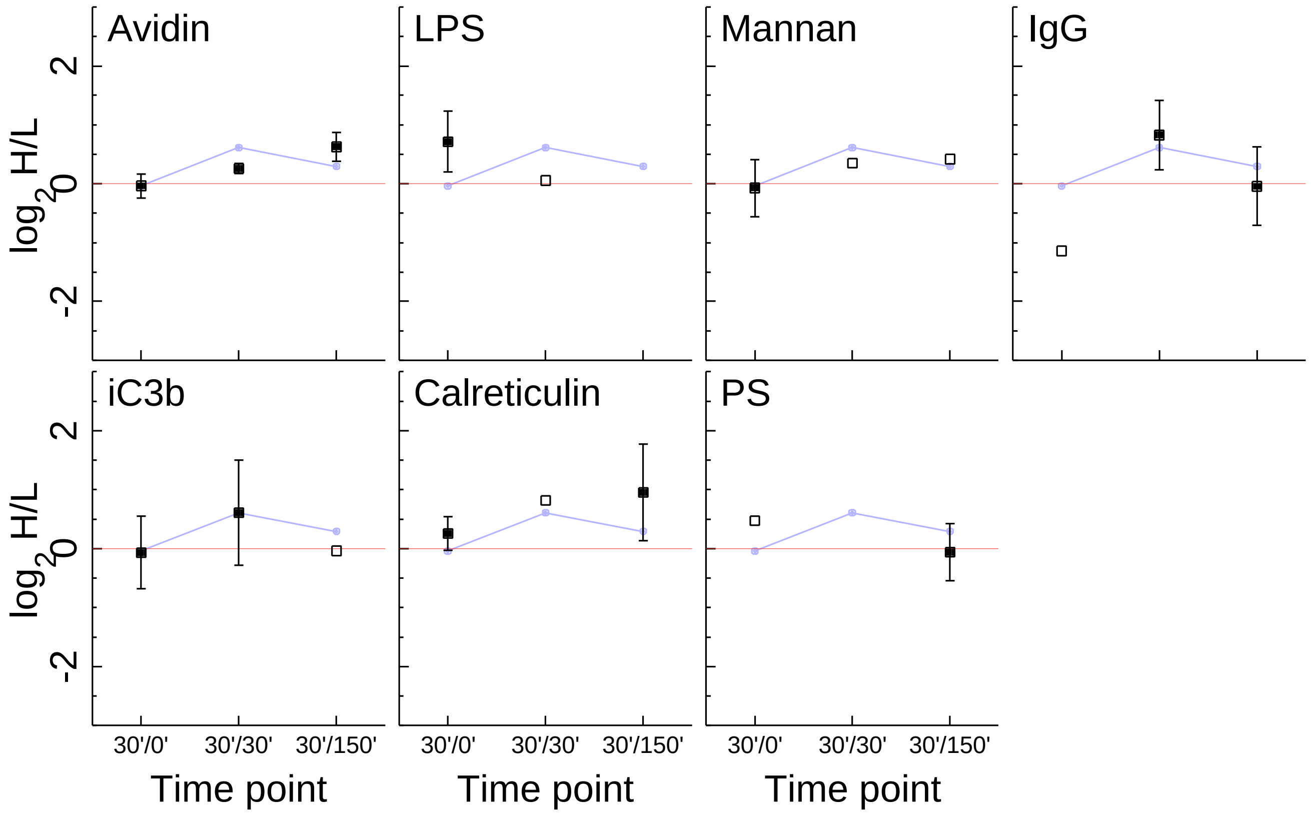

tbxas1 (P36423)

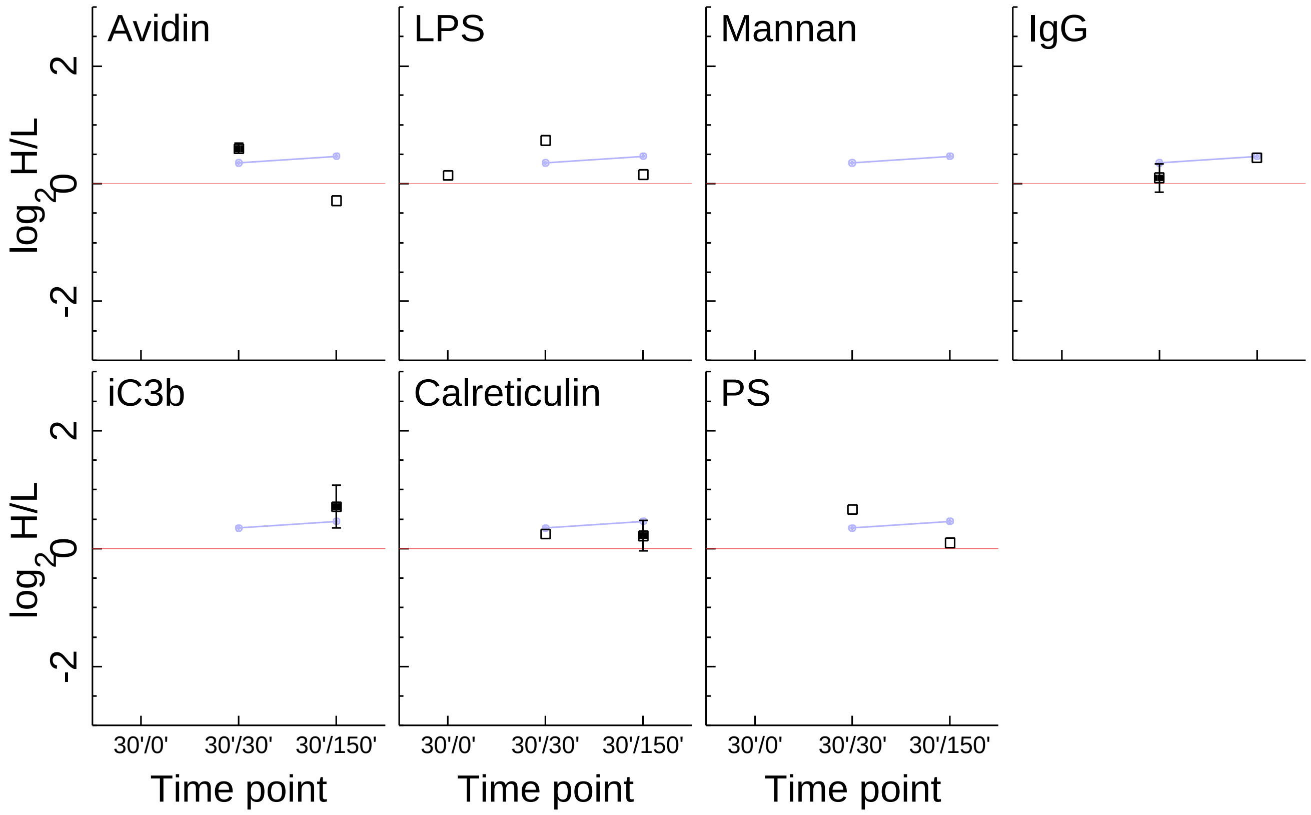

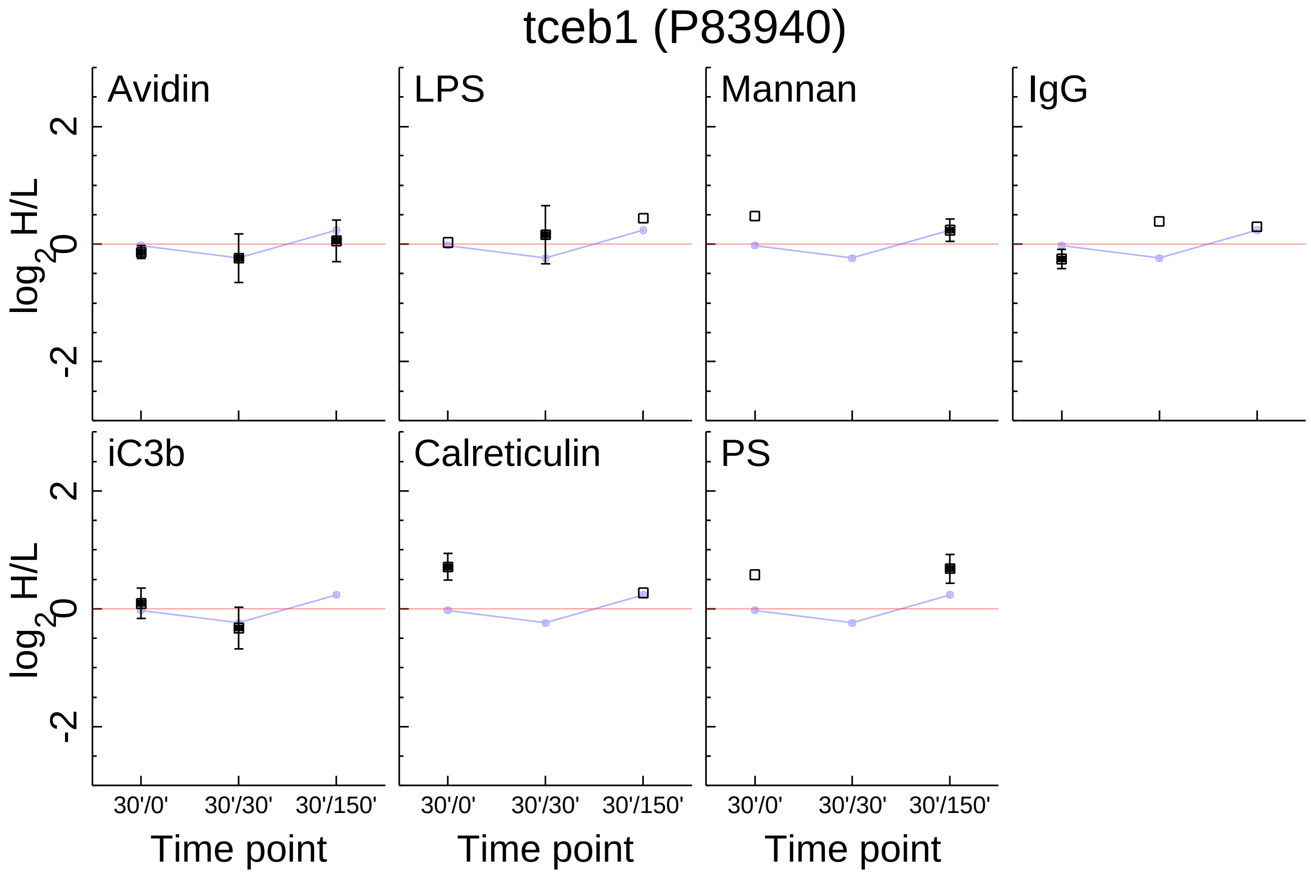
tceb1 (P83940)
Avidin
LPS
Mannan
IgG
iC3b
Calreticulin
PS
log2 H/L
2
0
-2
30'/0'
30'/30'
30'/150'
Time point

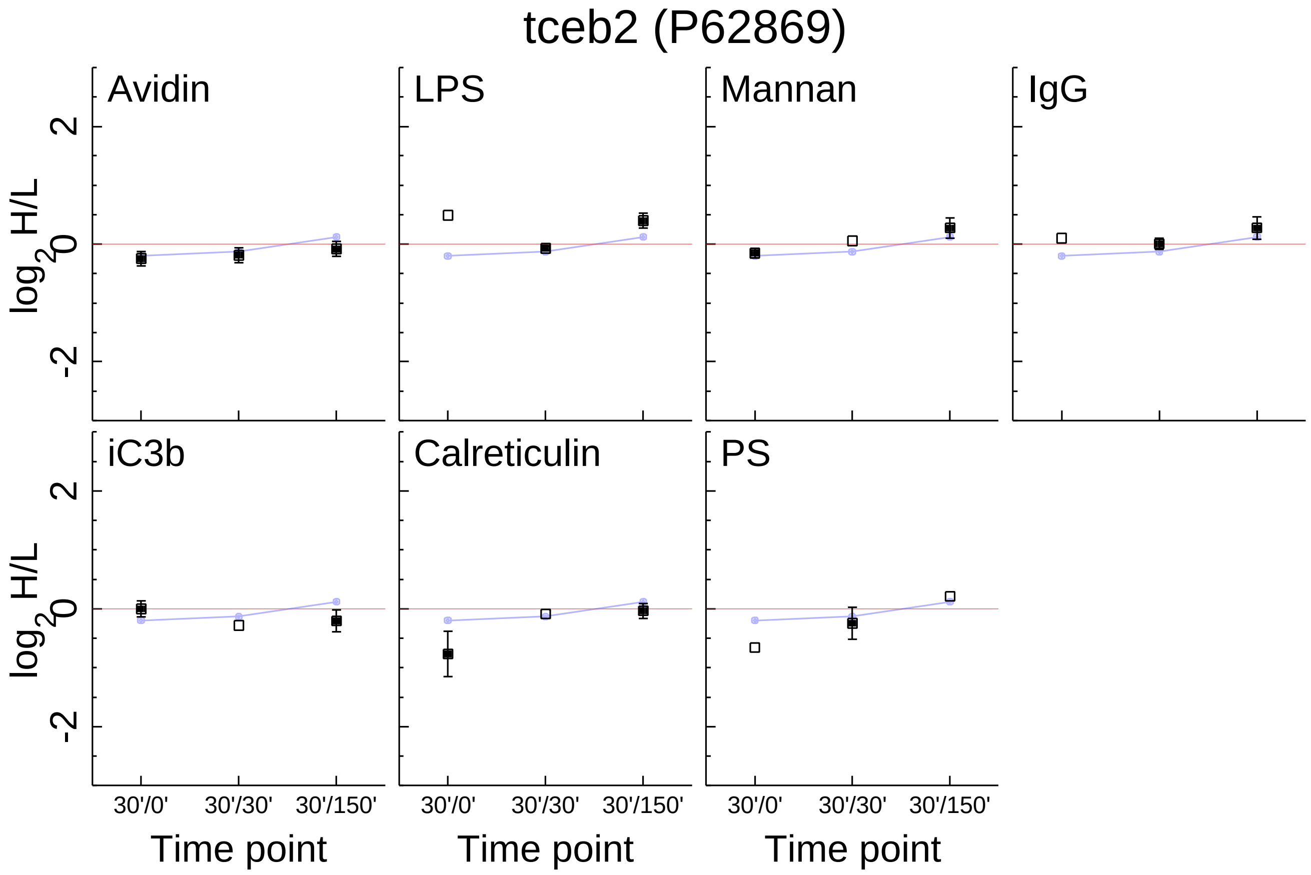
tceb2 (P62869)
Avidin
LPS
Mannan
IgG
iC3b
Calreticulin
PS
log2 H/L
2
0
-2
30'/0'
30'/30'
30'/150'
Time point

tcirg1 (Q9JHF5)

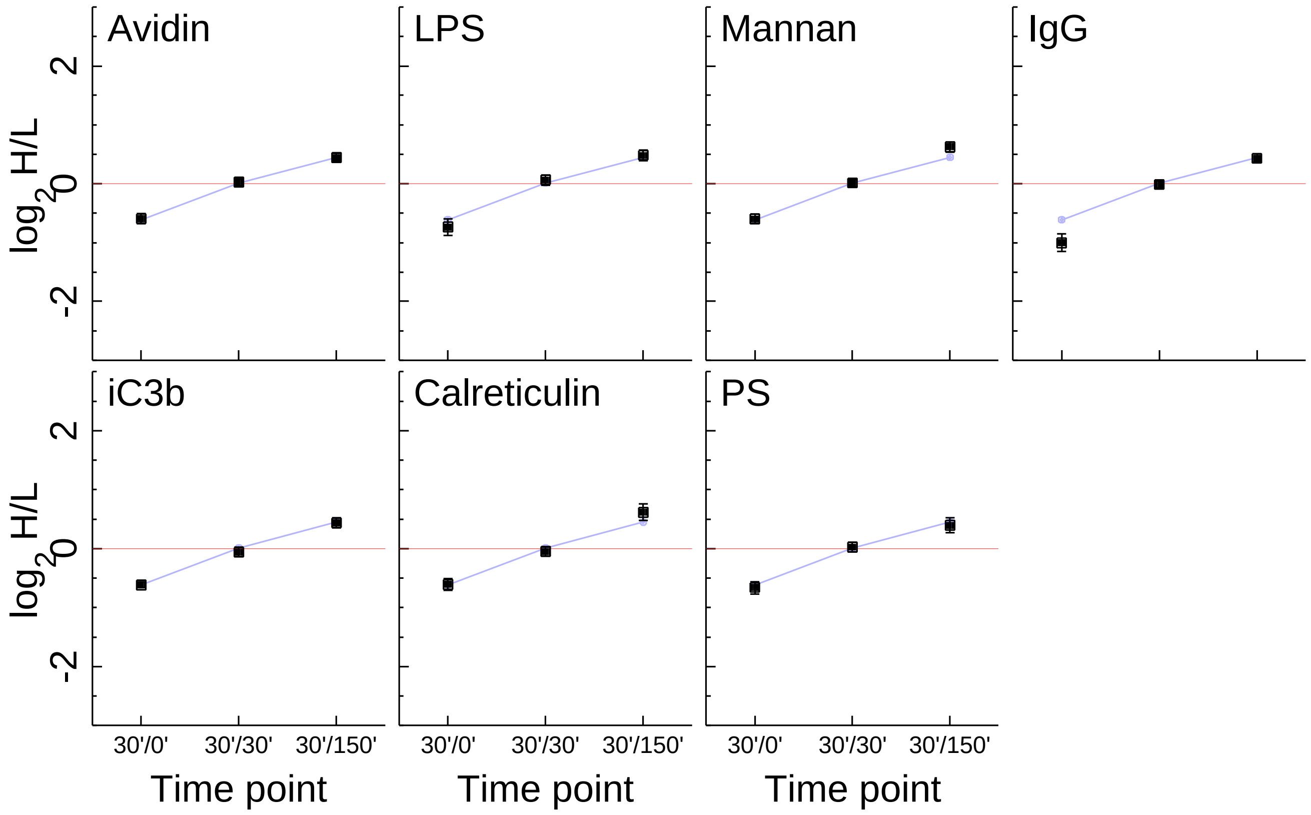

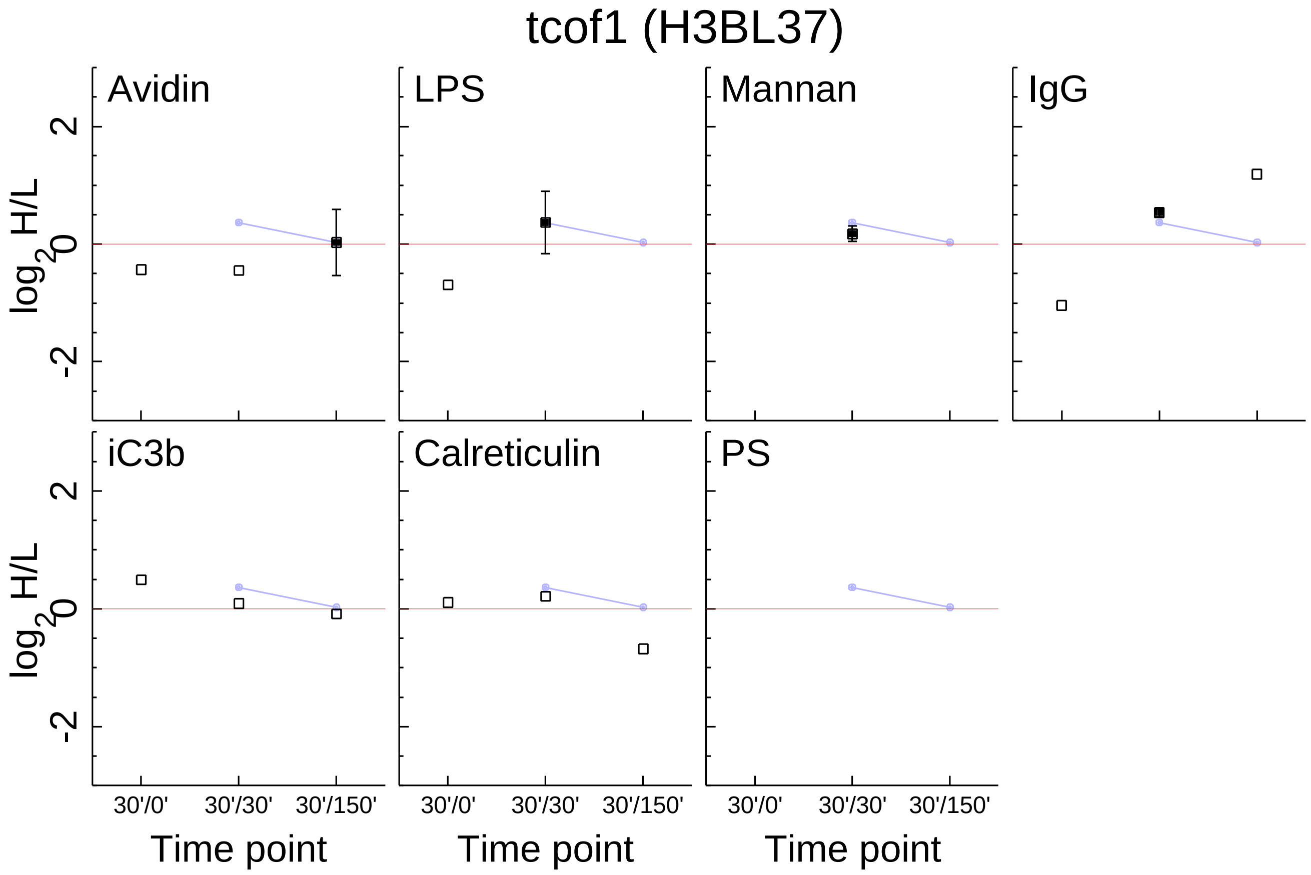

tcof1 (H3BL37)
Avidin
LPS
Mannan
IgG
iC3b
Calreticulin
PS
log2 H/L
2
0
-2
30'/0'
30'/30'
30'/150'
Time point

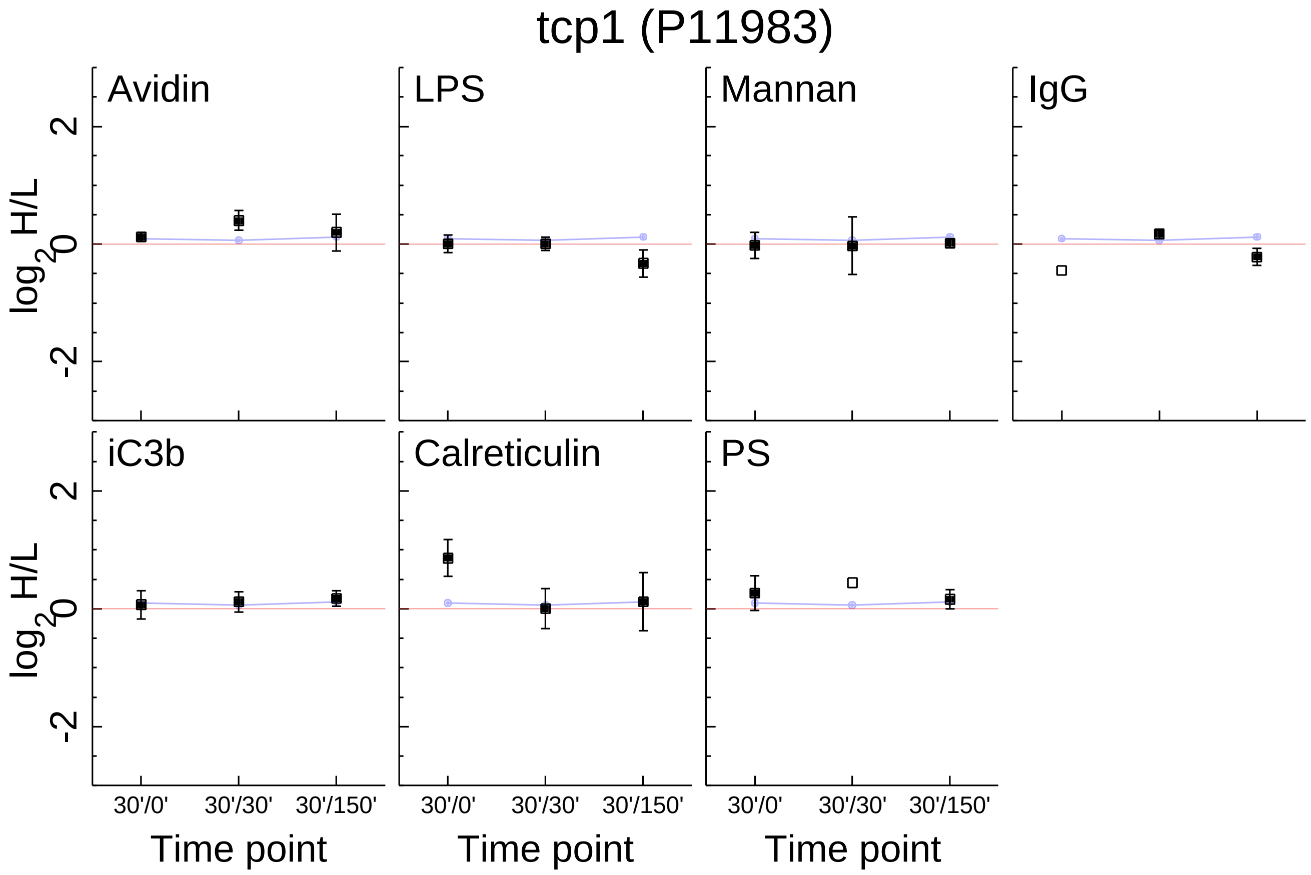

tcp1 (P11983)
Avidin
LPS
Mannan
IgG
iC3b
Calreticulin
PS
log2 H/L
2
0
-2
30'/0'
30'/30'
30'/150'
Time point

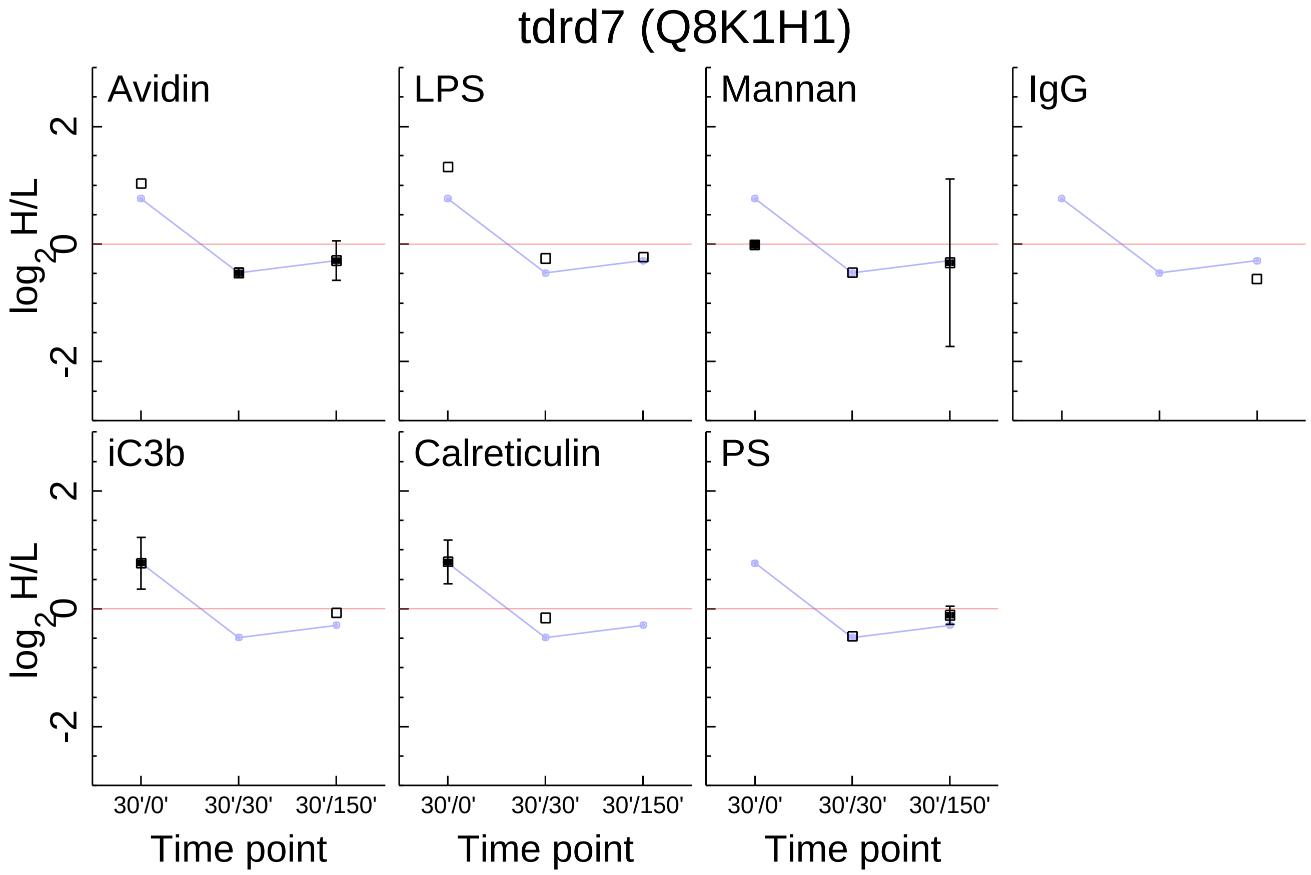

tdrd7 (Q8K1H1)
Avidin
LPS
Mannan
IgG
iC3b
Calreticulin
PS
log2 H/L
2
0
-2
30'/0'
30'/30'
30'/150'
Time point

tecpr1 (Q80VP0)

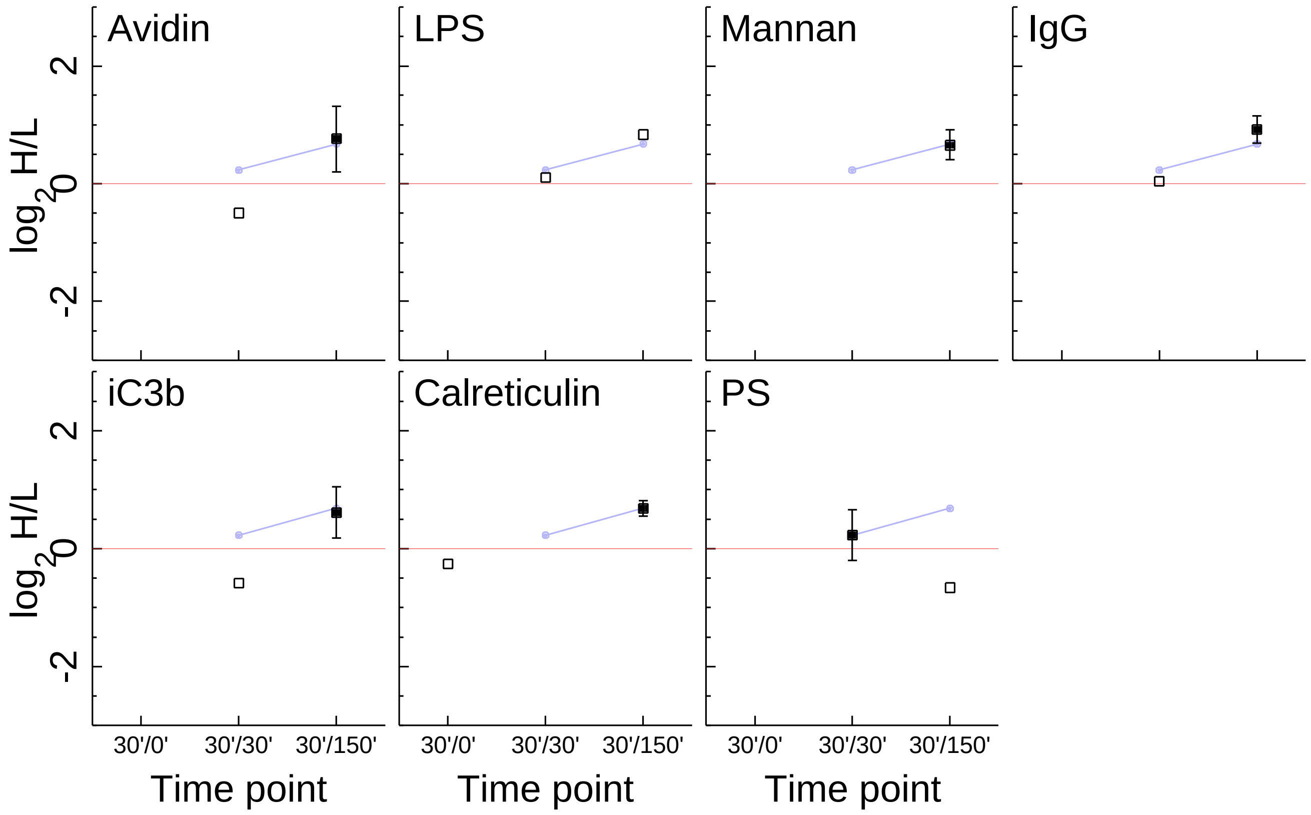

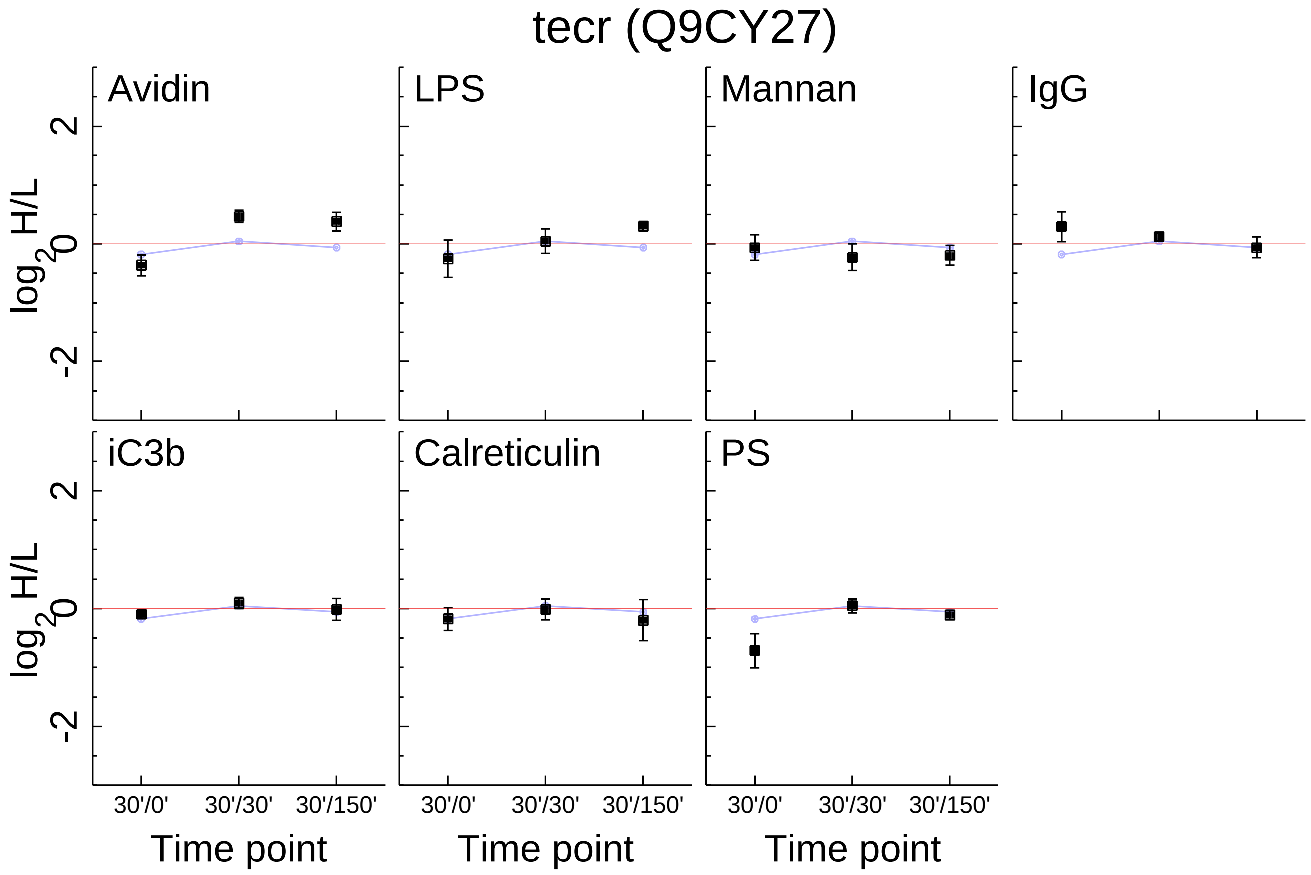

tecr (Q9CY27)
Avidin
LPS
Mannan
IgG
iC3b
Calreticulin
PS
log2 H/L
2
0
-2
30'/0'
30'/30'
30'/150'
Time point

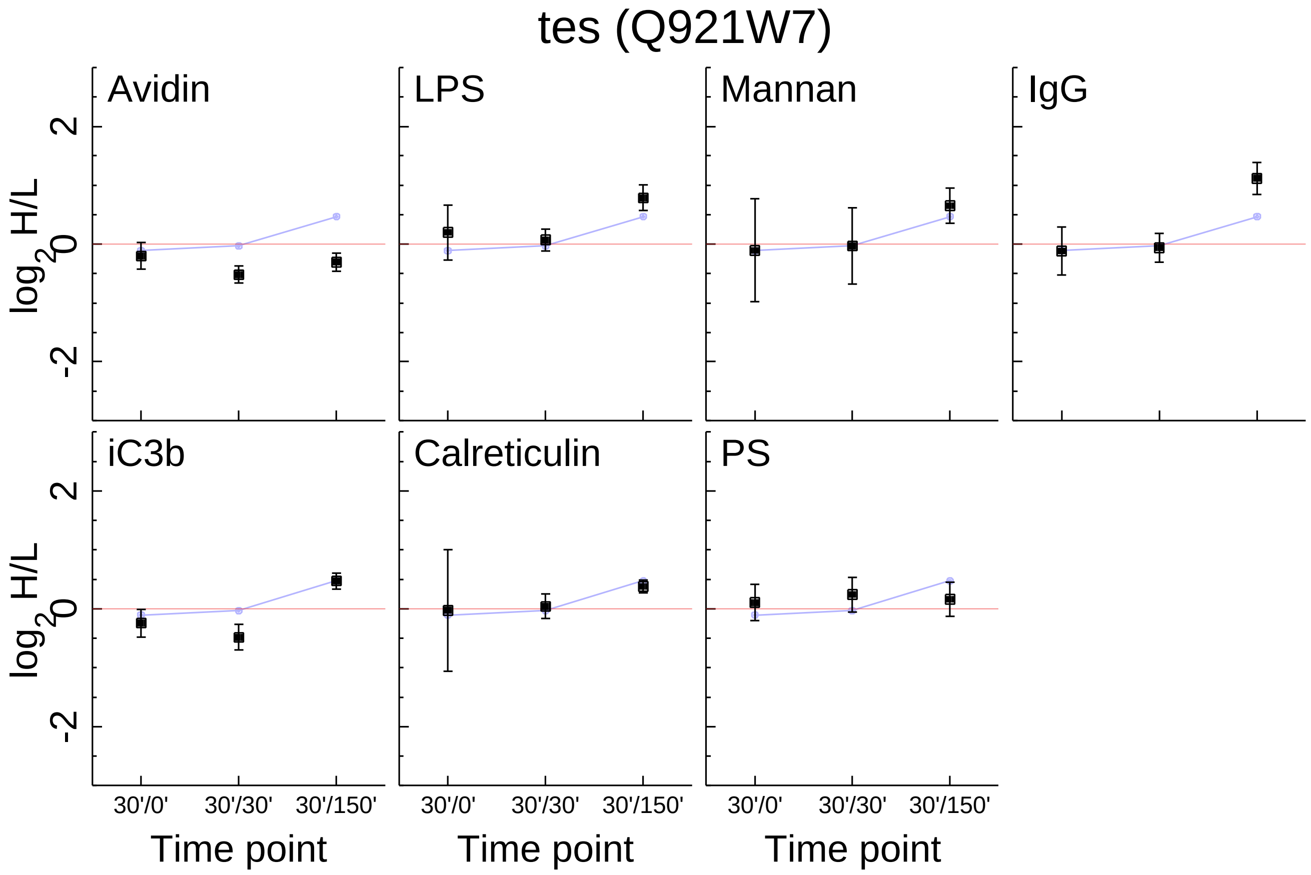

tes (Q921W7)
Avidin
LPS
Mannan
IgG
iC3b
Calreticulin
PS
log2 H/L
2
0
-2
30'/0'
30'/30'
30'/150'
Time point

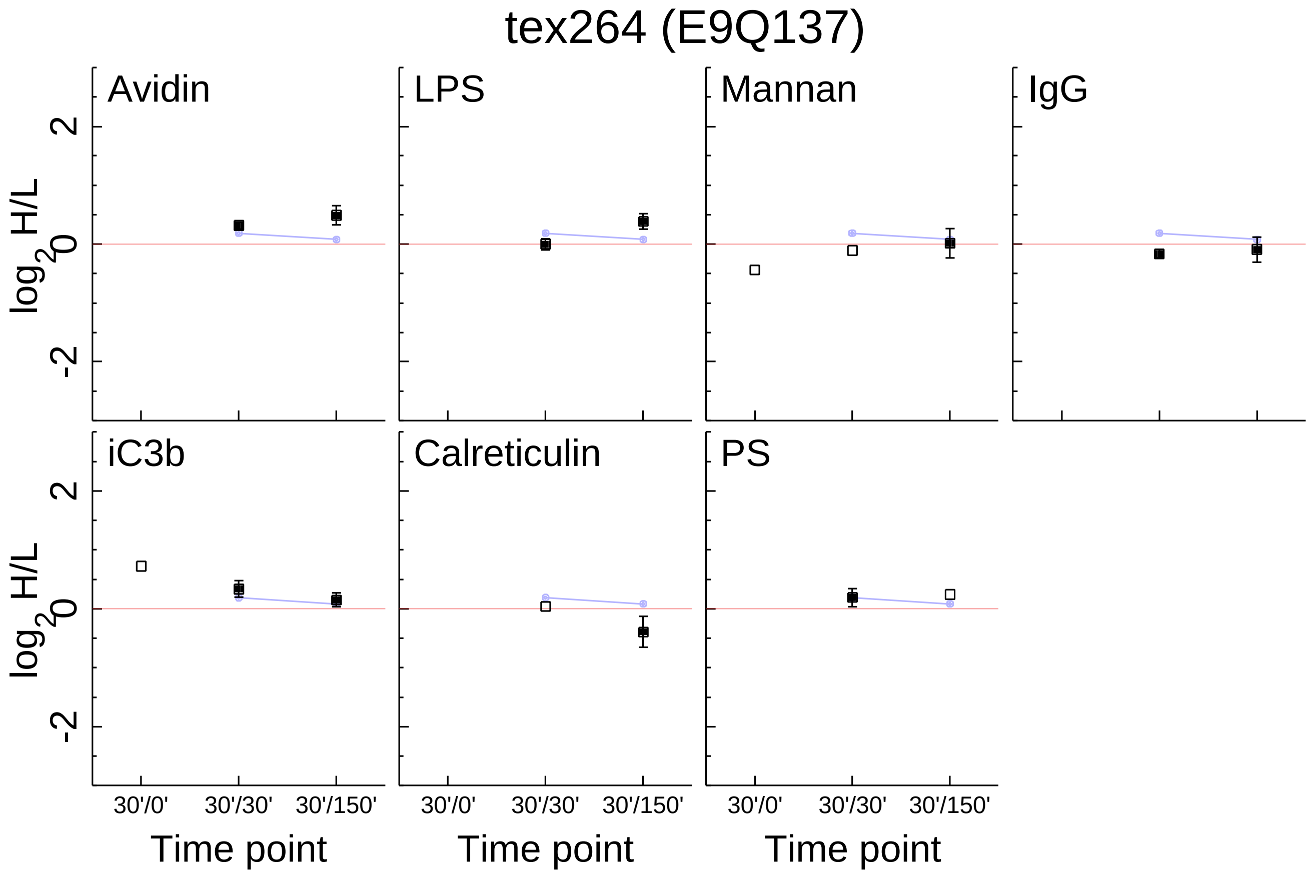

tex264 (E9Q137)
Avidin
LPS
Mannan
IgG
iC3b
Calreticulin
PS
log2 H/L
2
0
-2
30'/0'
30'/30'
30'/150'
Time point

tfe3 (A2AEW1)

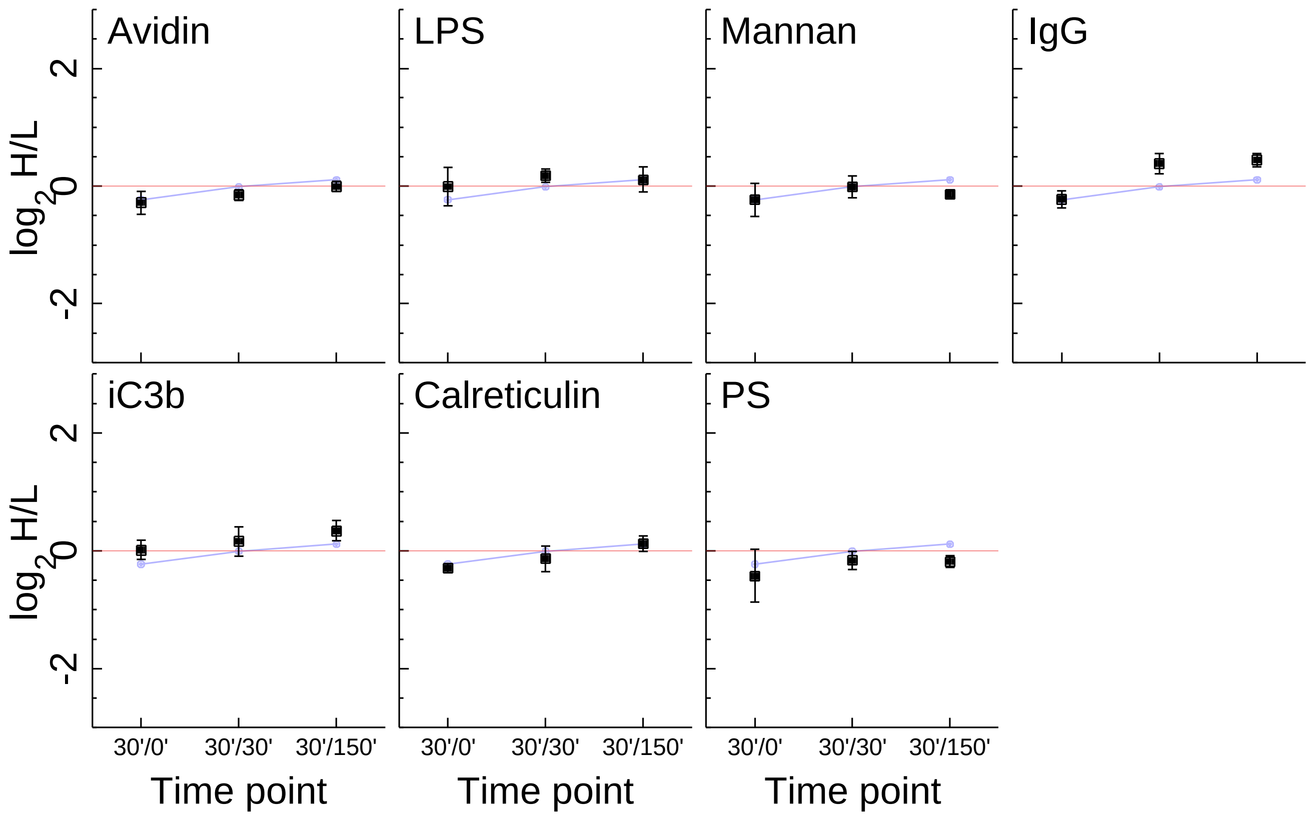

Avidin
LPS
Mannan
IgG
iC3b
Calreticulin
PS
2
0
-2
log2 H/L
30'/0'
30'/30'
30'/150'
Time point

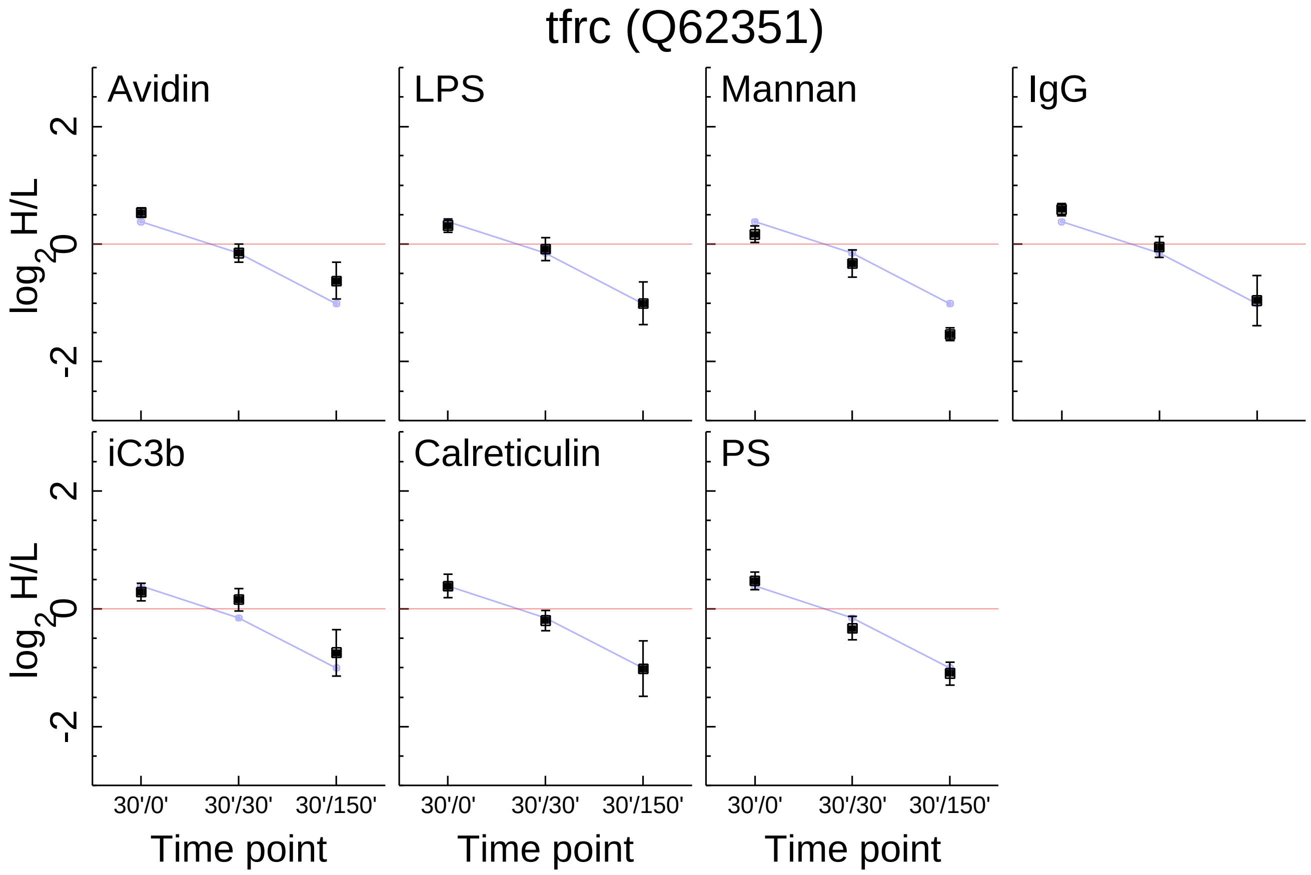

tfrc (Q62351)
Avidin
LPS
Mannan
IgG
iC3b
Calreticulin
PS
log2 H/L
2
0
-2
30'/0'
30'/30'
30'/150'
Time point

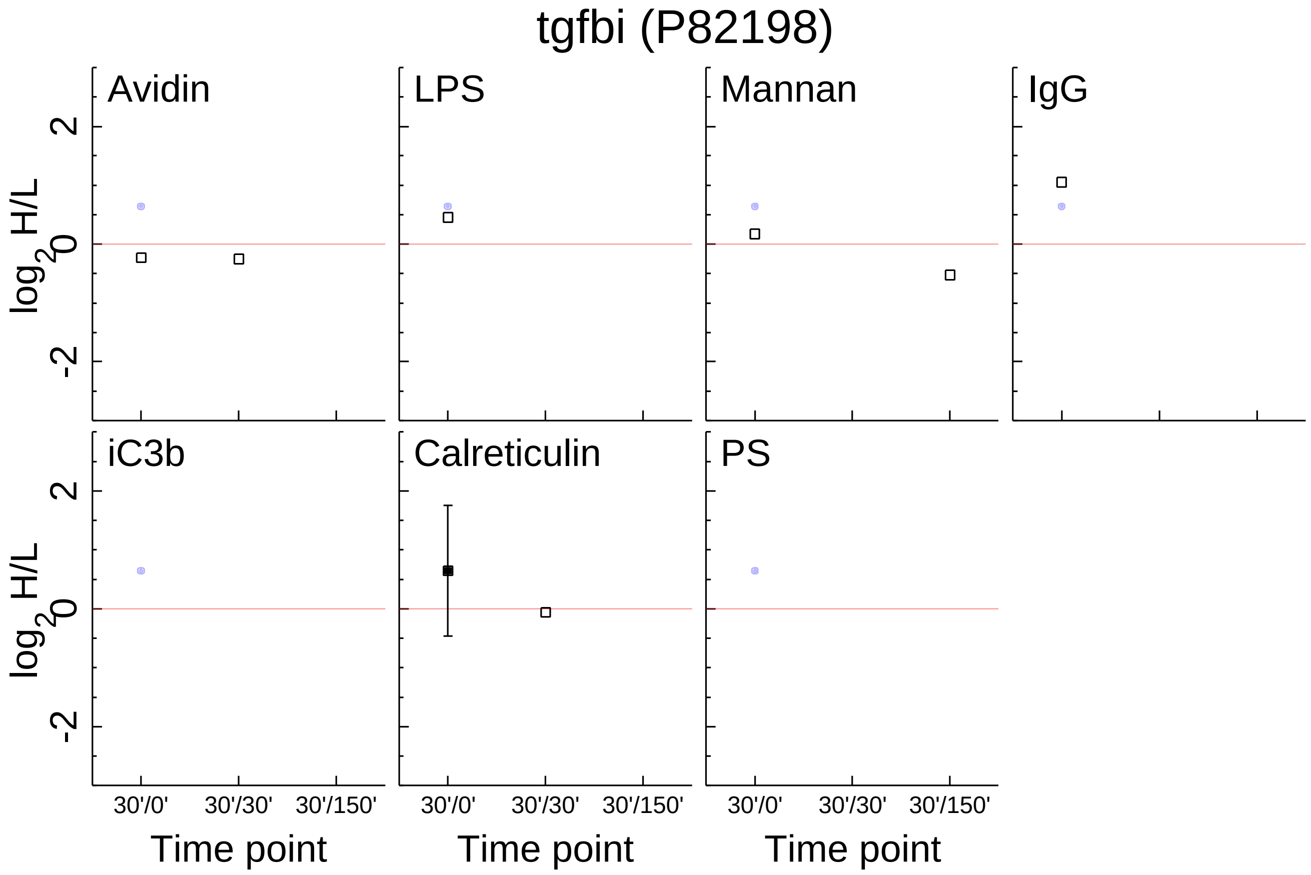
tgfbi (P82198)
Avidin
LPS
Mannan
IgG
iC3b
Calreticulin
PS
log2 H/L
2
0
-2
30'/0'
30'/30'
30'/150'
Time point

tgm2 (P21981)

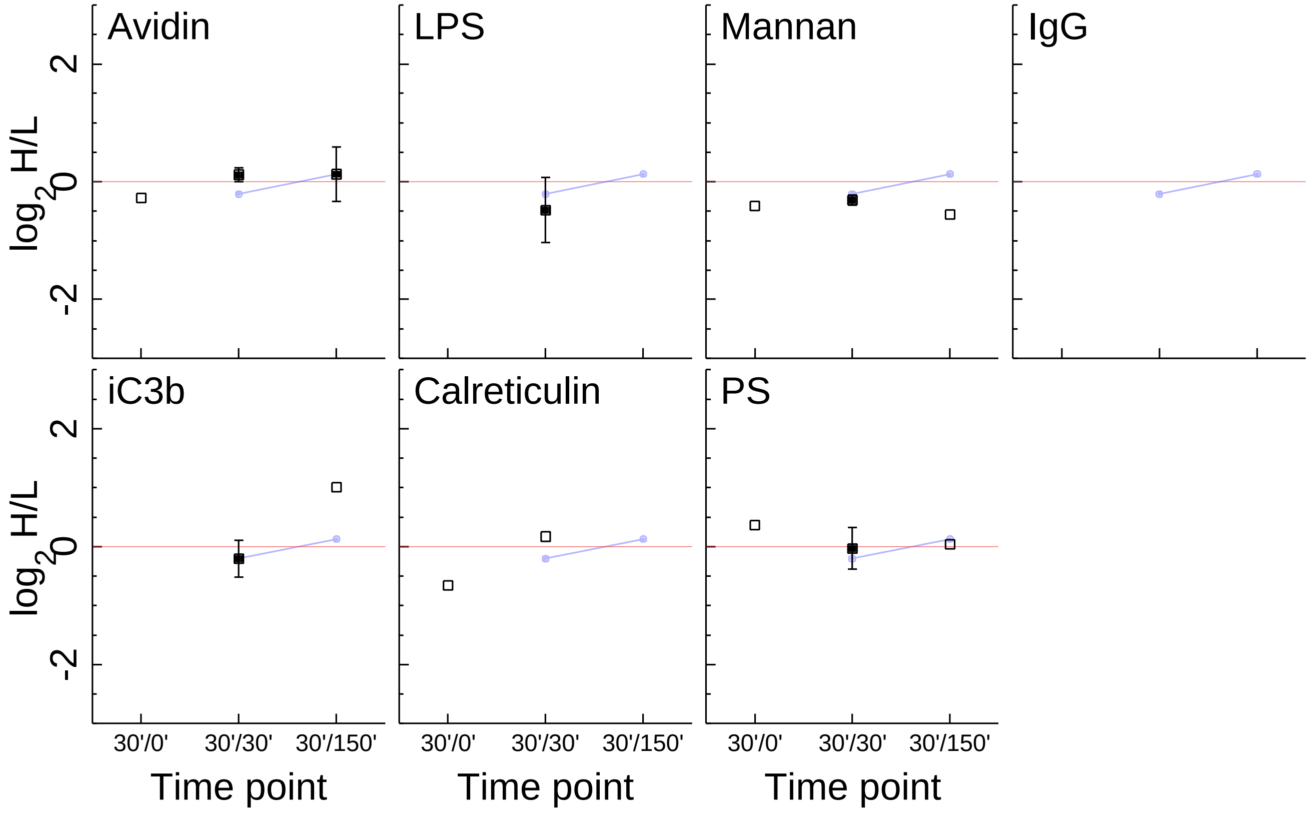

Avidin
LPS
Mannan
IgG
iC3b
Calreticulin
PS
log2 H/L
2
0
-2
30'/0'
30'/30'
30'/150'
Time point

themis2 (Q91YX0)

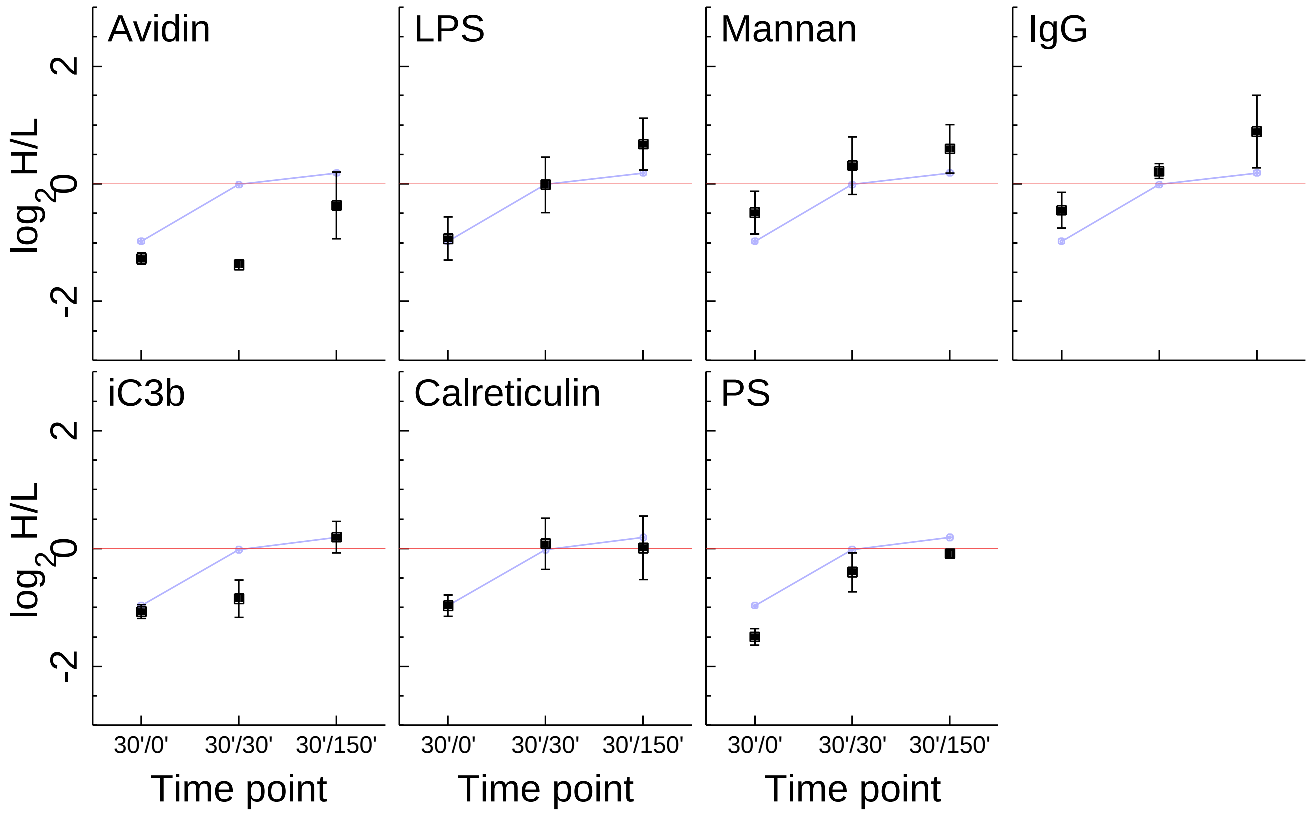

thrap3 (Q569Z6)

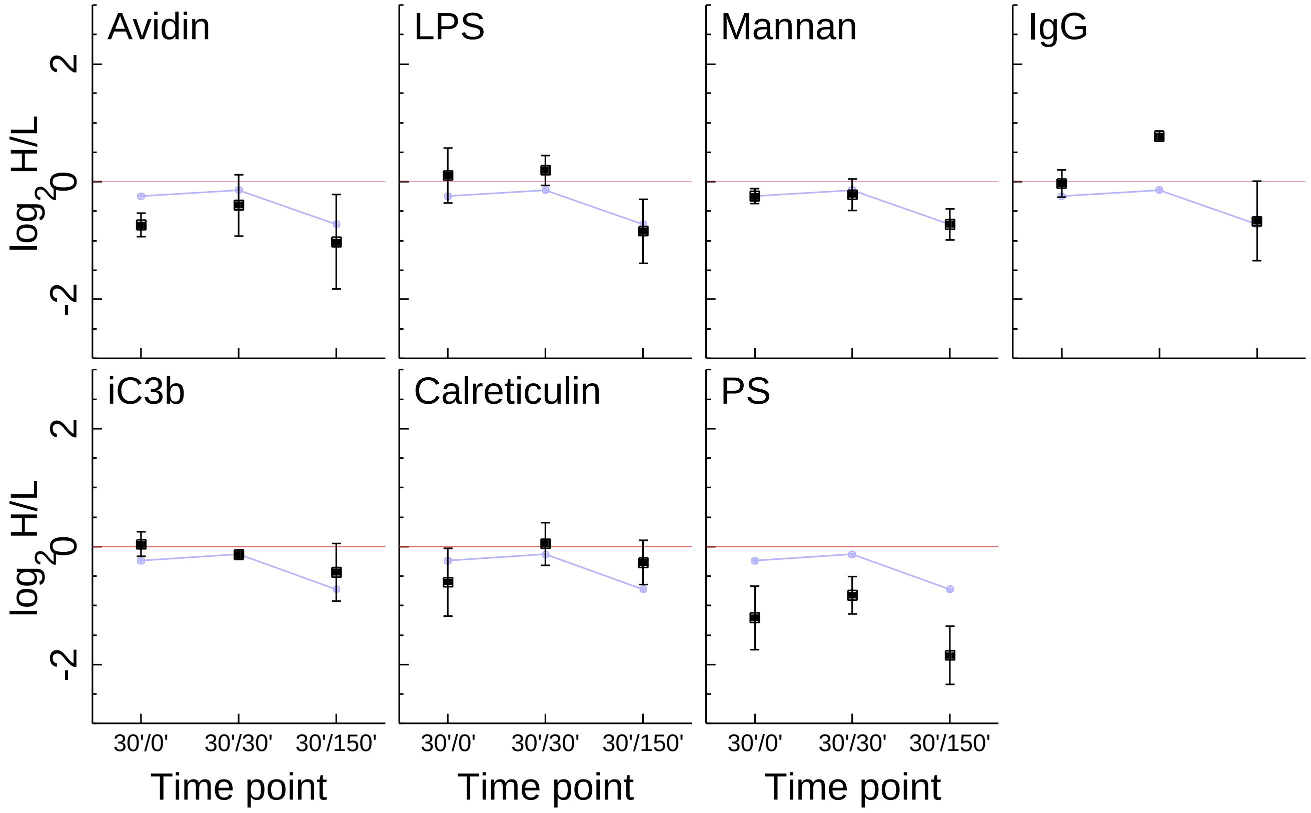

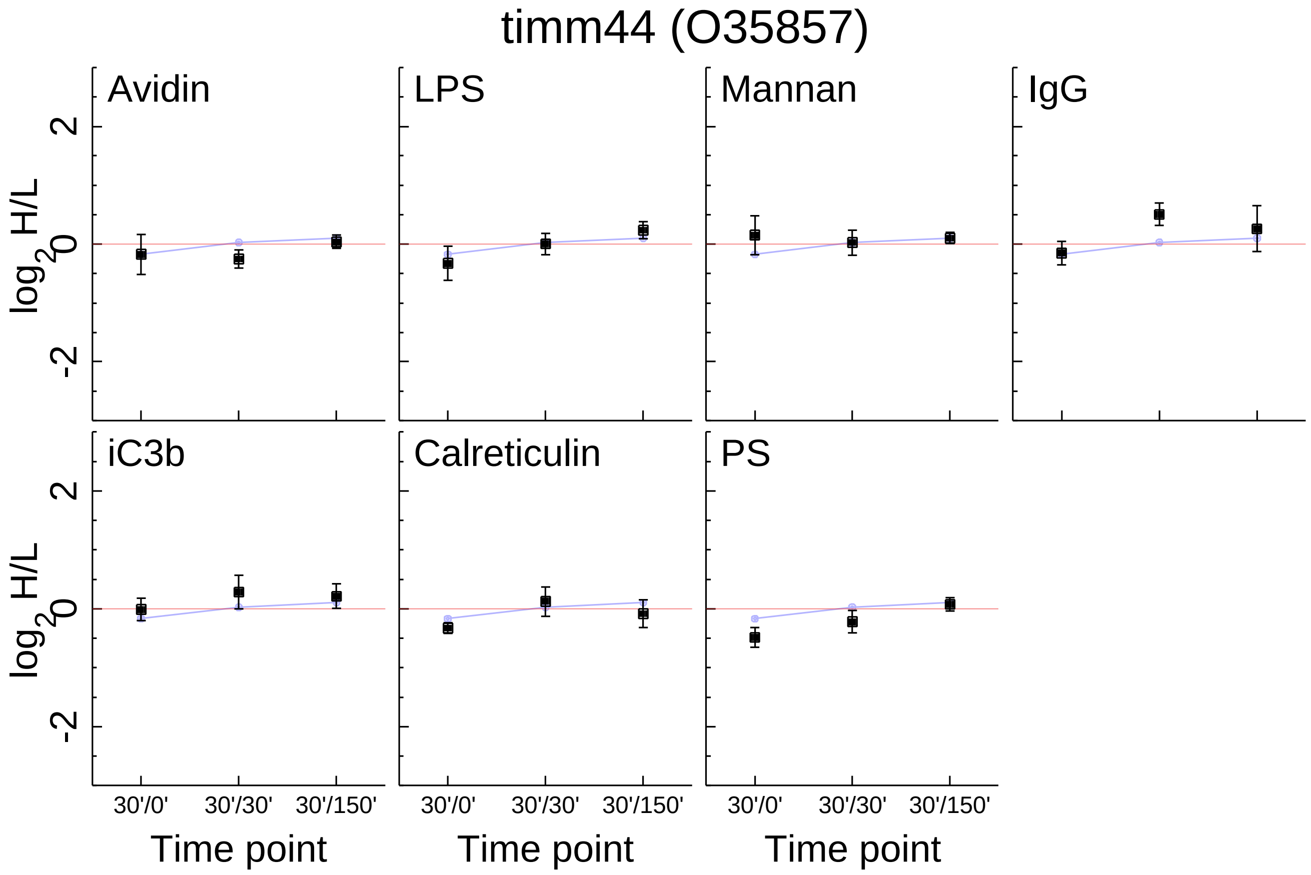
timm44 (O35857)
Avidin
LPS
Mannan
IgG
iC3b
Calreticulin
PS
log2 H/L
2
0
-2
30'/0'
30'/30'
30'/150'
Time point

timm50 (Q9D880)

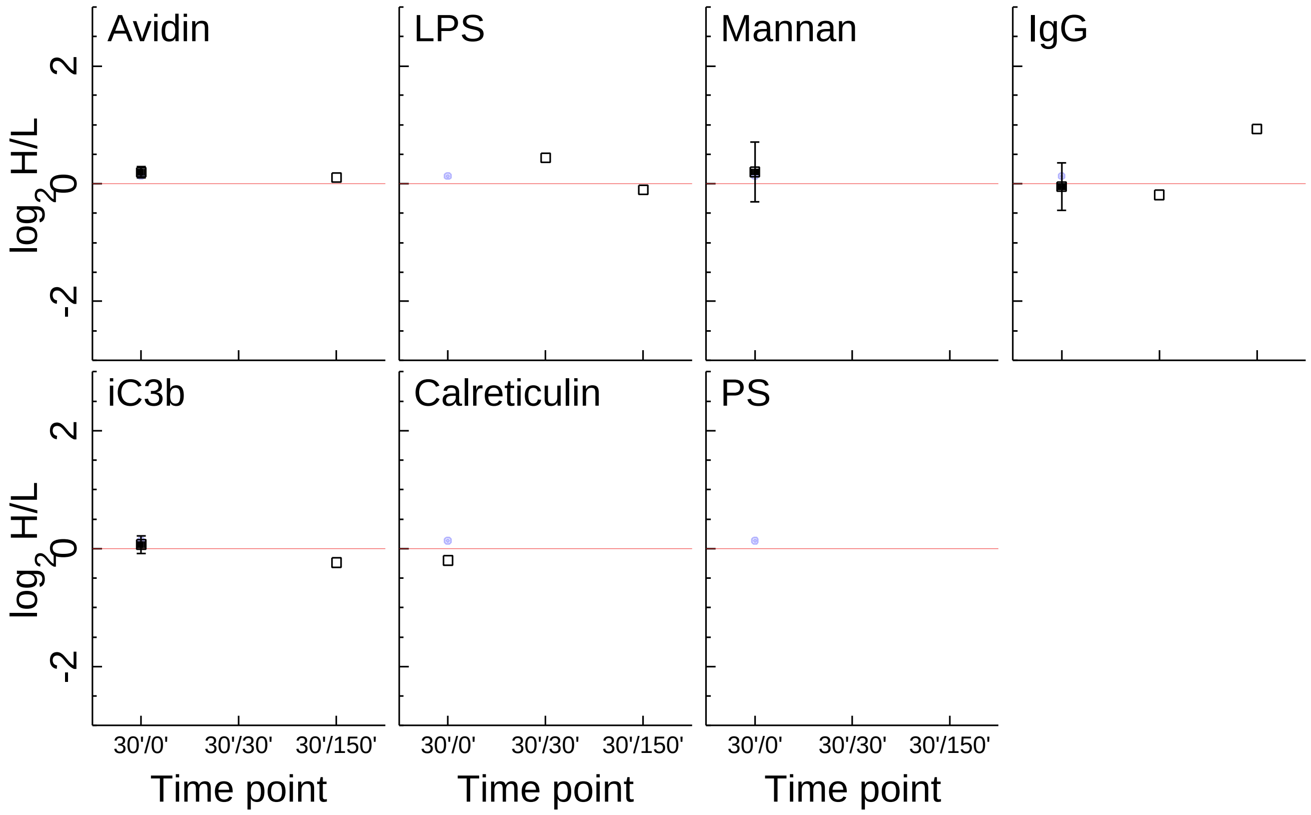

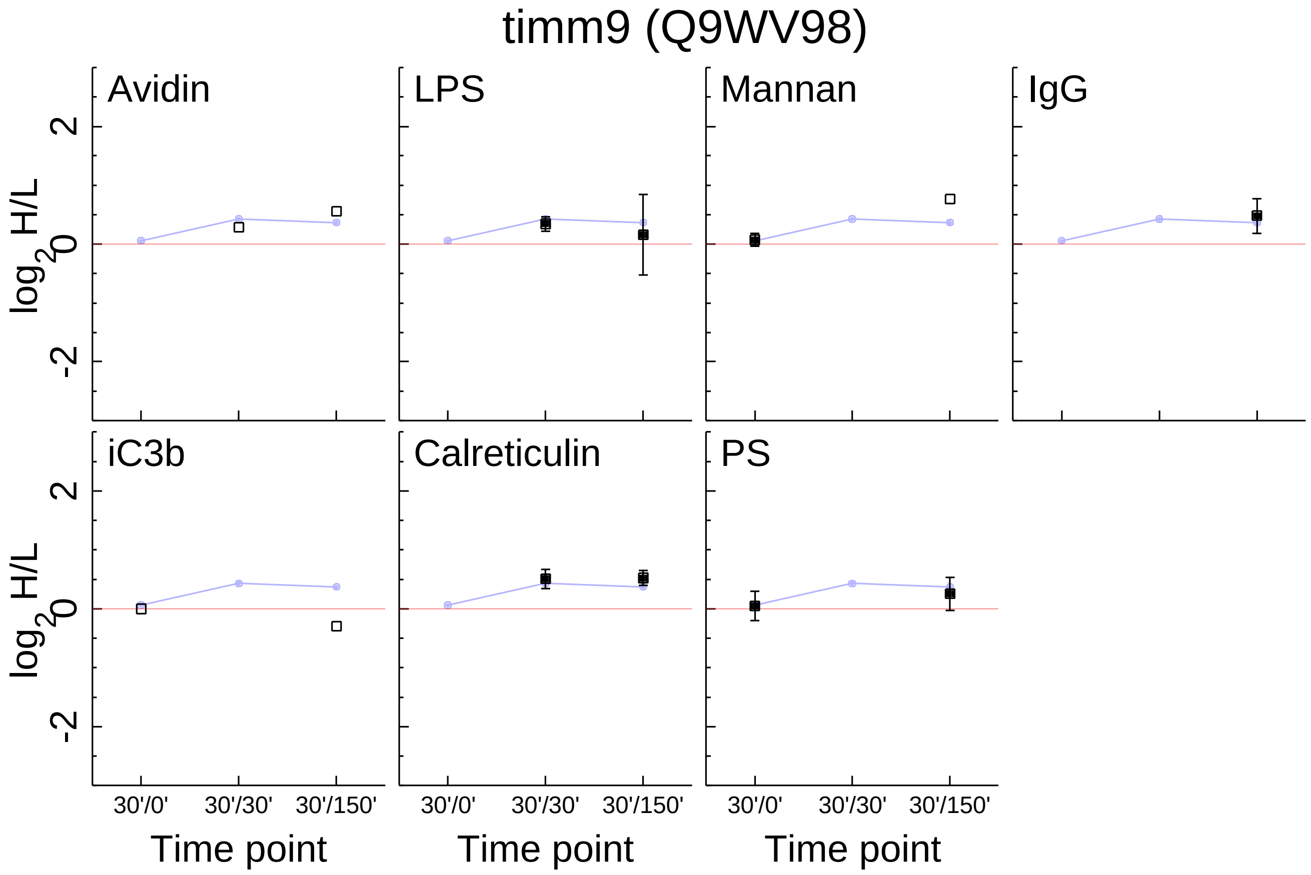
timm9 (Q9WV98)
Avidin
LPS
Mannan
IgG
iC3b
Calreticulin
PS
log2 H/L
2
0
-2
30'/0'
30'/30'
30'/150'
Time point

tkt (P40142)

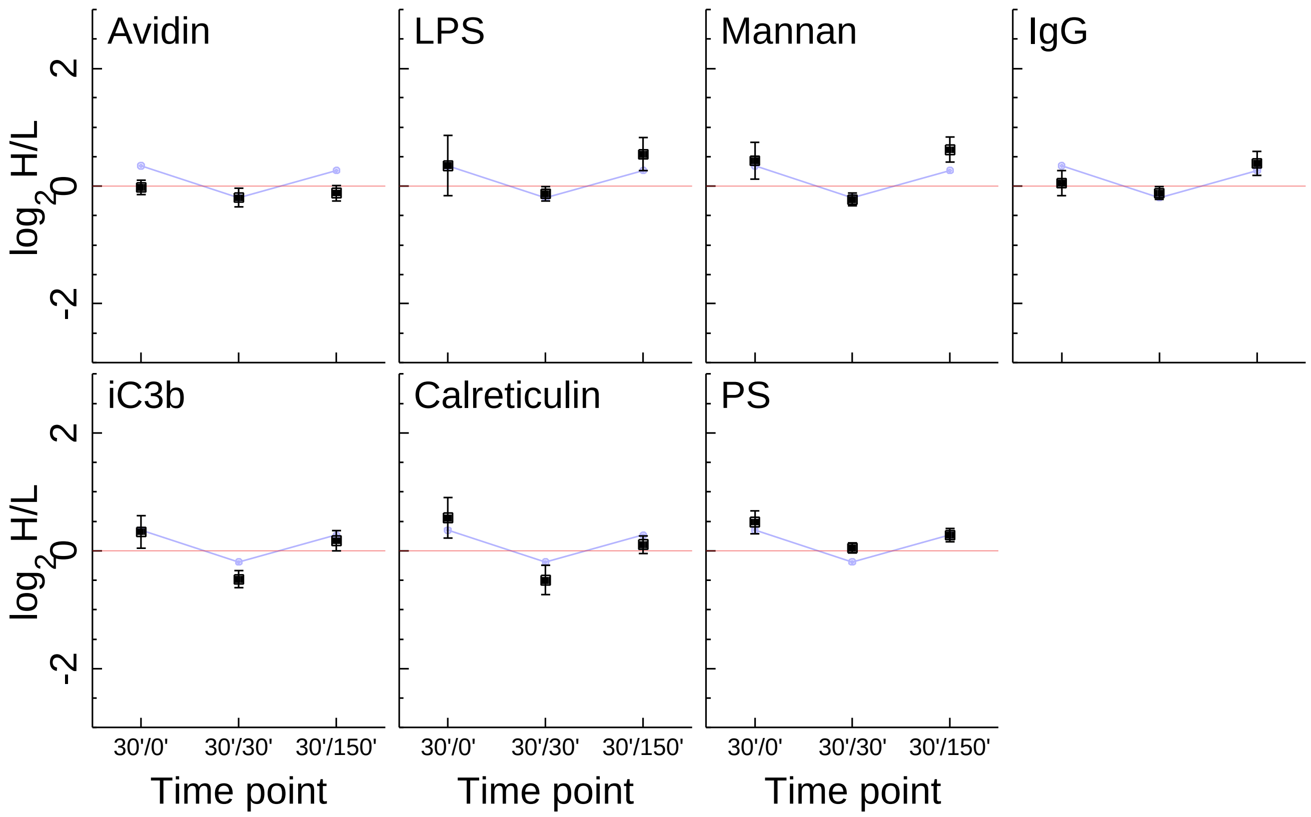

Avidin
LPS
Mannan
IgG
iC3b
Calreticulin
PS
log2 H/L
2
0
-2
30'/0'
30'/30'
30'/150'
Time point

tln1 (P26039)

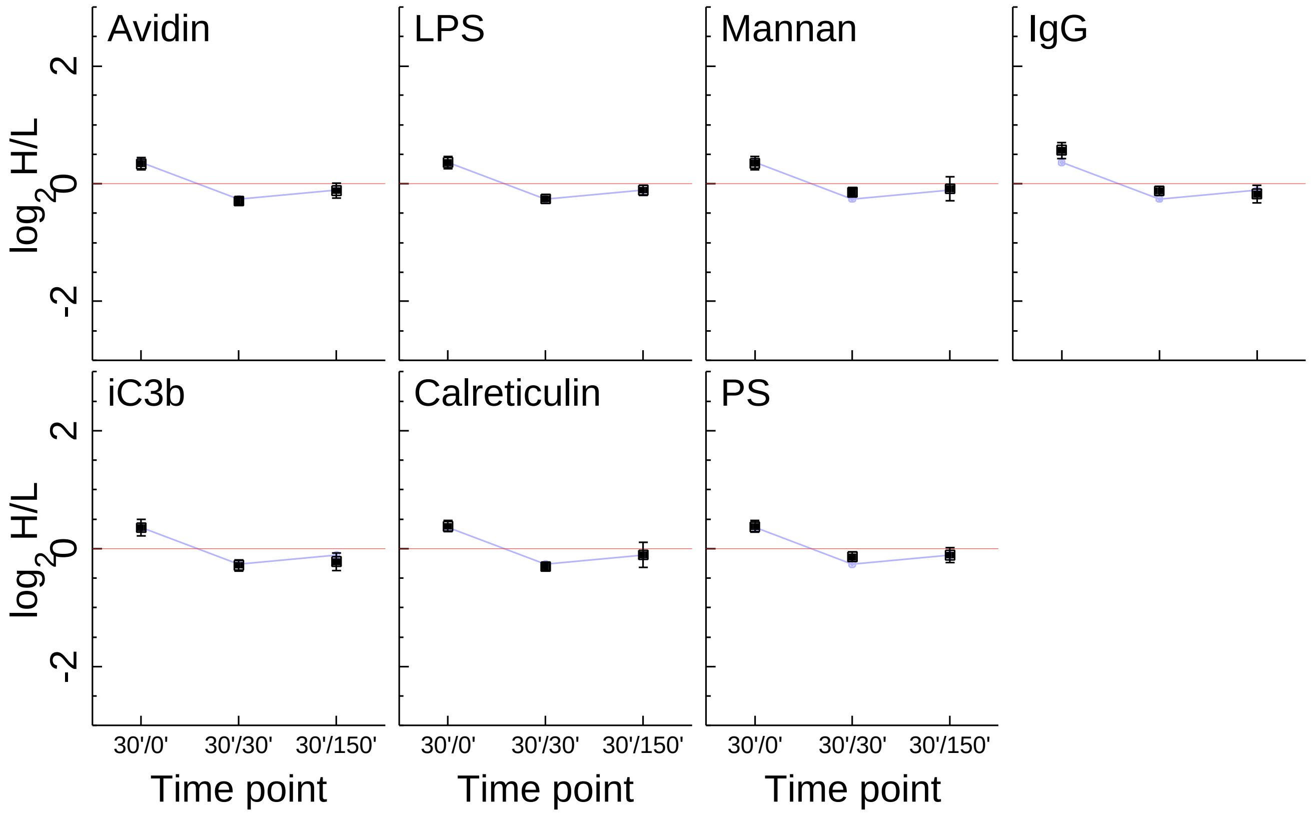

Avidin
LPS
Mannan
IgG
iC3b
Calreticulin
PS
log2 H/L
2
0
-2
30'/0'
30'/30'
30'/150'
Time point

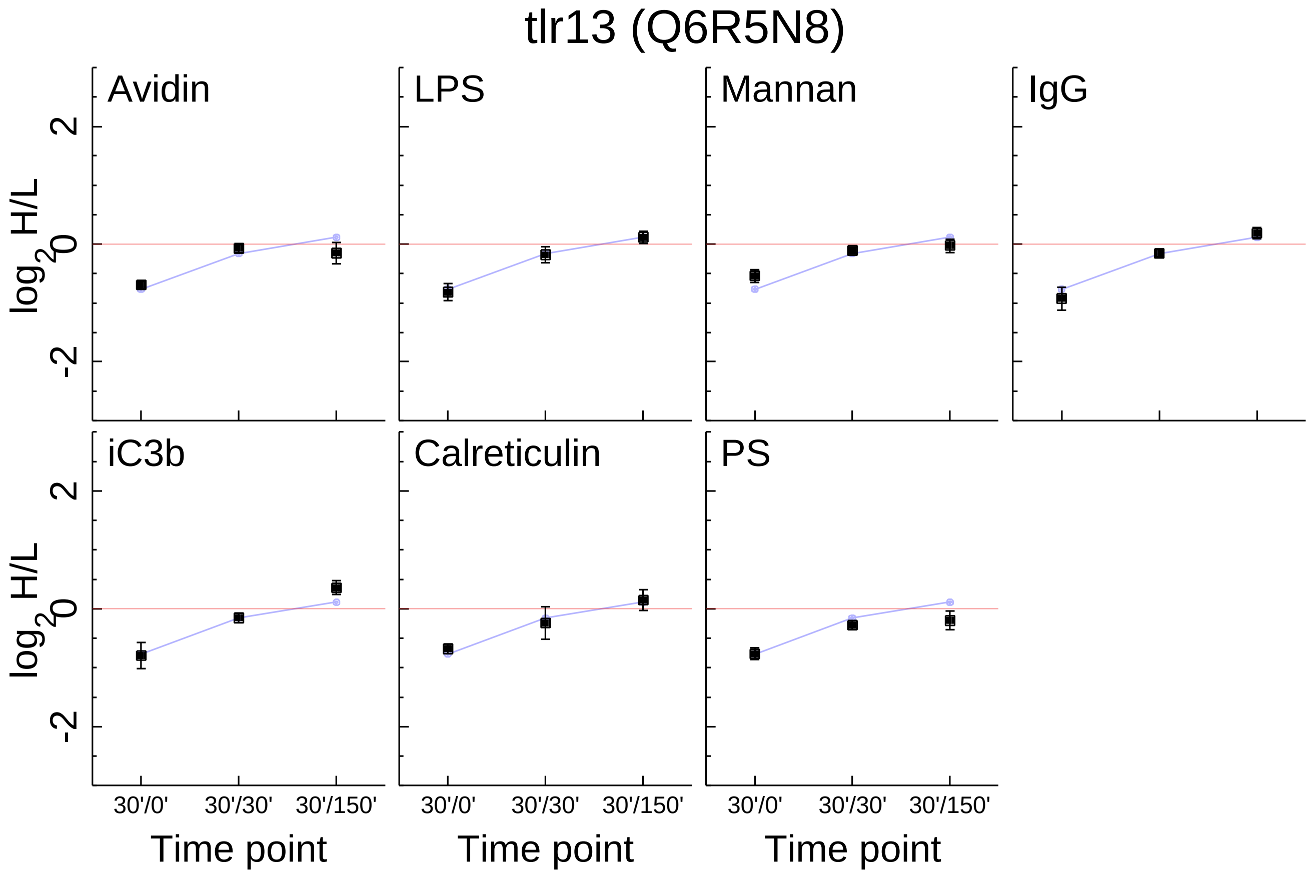
tlr13 (Q6R5N8)
Avidin
LPS
Mannan
IgG
iC3b
Calreticulin
PS
log2 H/L
2
0
-2
30'/0'
30'/30'
30'/150'
Time point

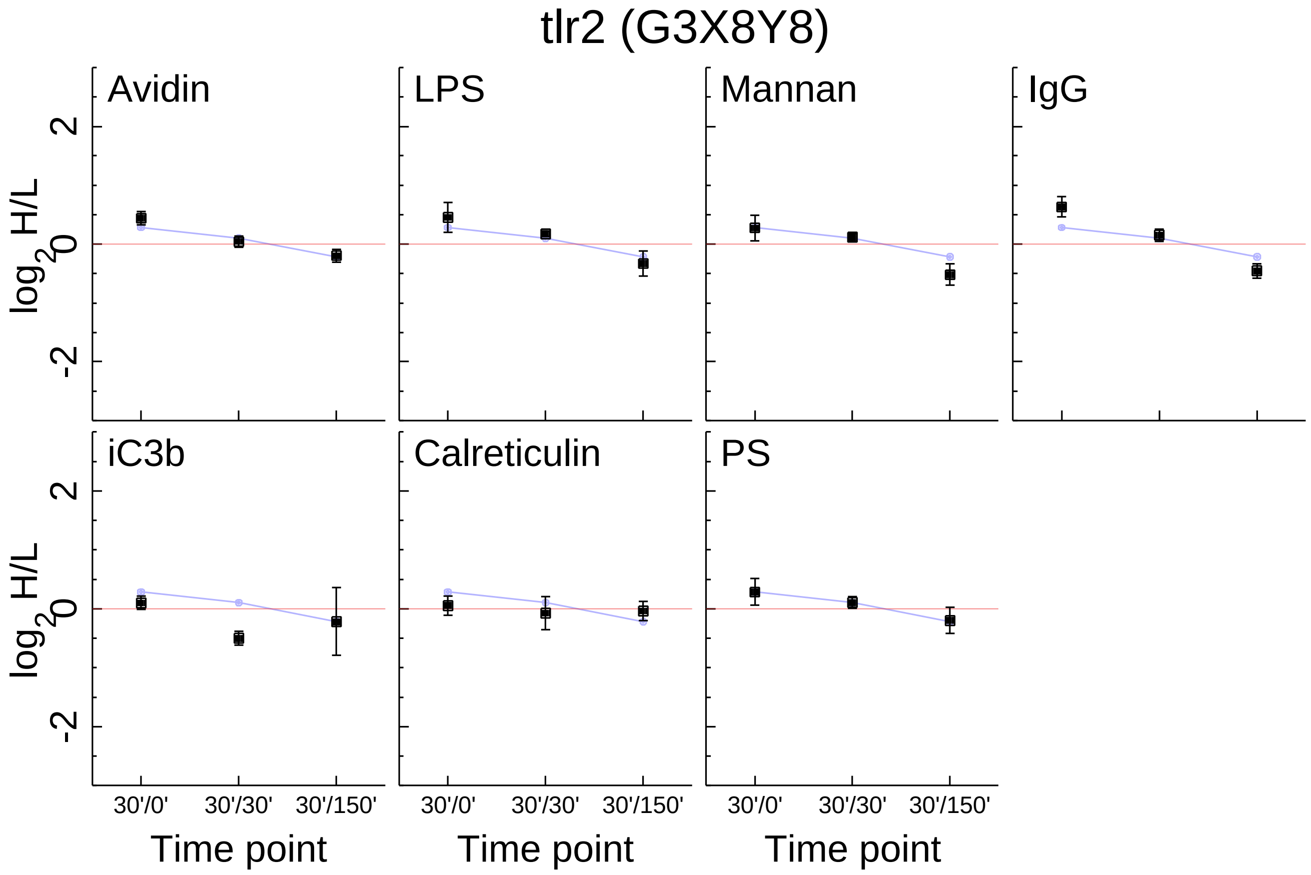

tlr2 (G3X8Y8)
Avidin
LPS
Mannan
IgG
iC3b
Calreticulin
PS
log2 H/L
2
0
-2
30'/0'
30'/30'
30'/150'
Time point

tlr3 (Q99MB1)

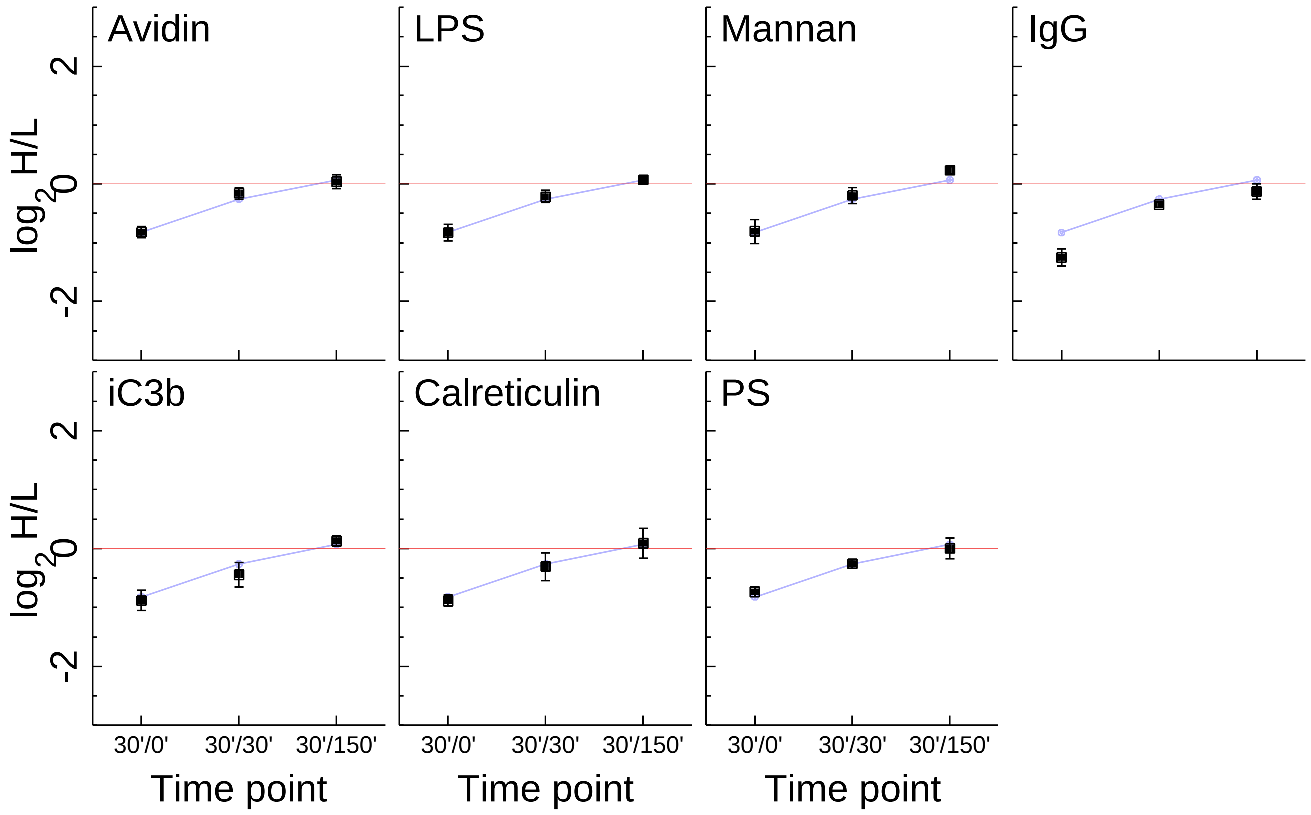
Avidin
LPS
Mannan
IgG
iC3b
Calreticulin
PS
log2 H/L
2
0
-2
30'/0'
30'/30'
30'/150'
Time point

tlr7 (P58681)

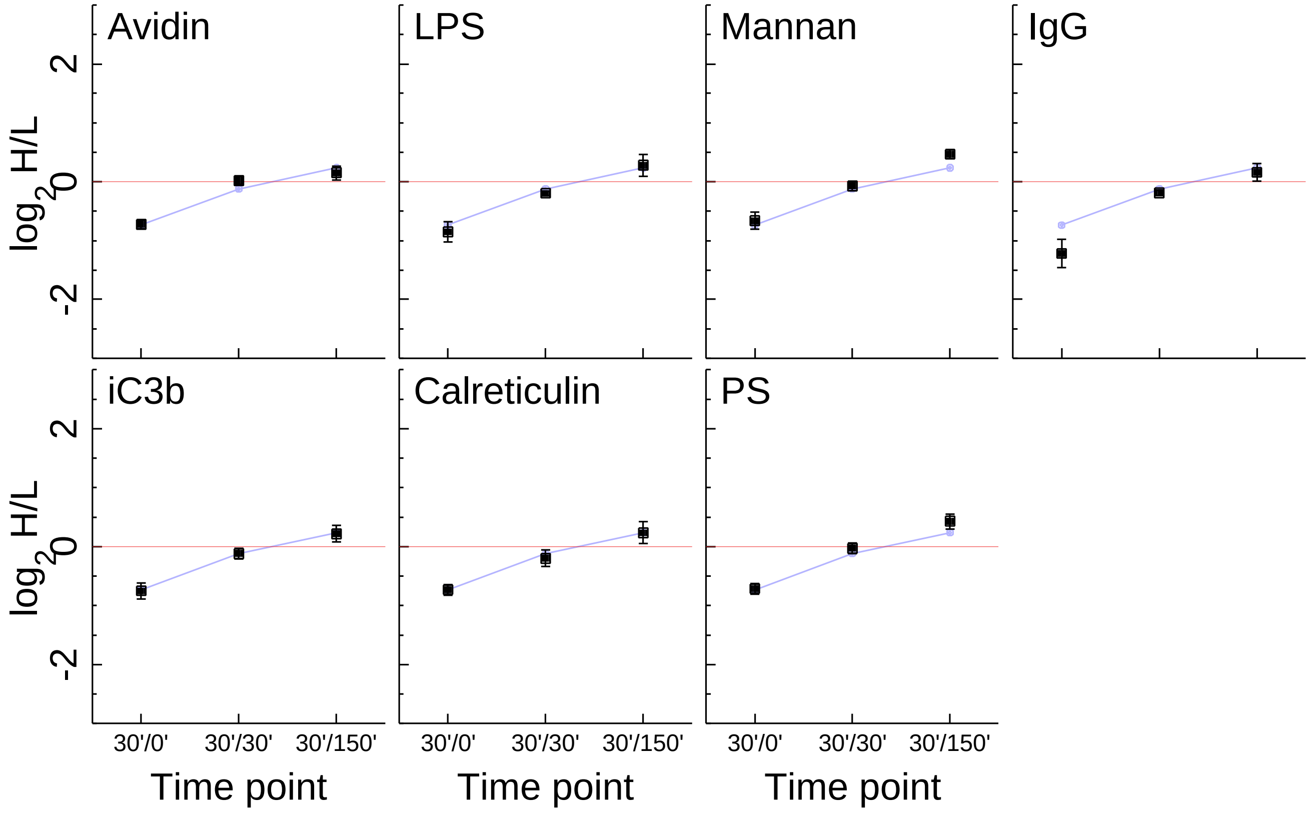
Avidin
LPS
Mannan
IgG
iC3b
Calreticulin
PS
log2 H/L
2
0
-2
30'/0'
30'/30'
30'/150'
Time point

tlr8 (P58682)

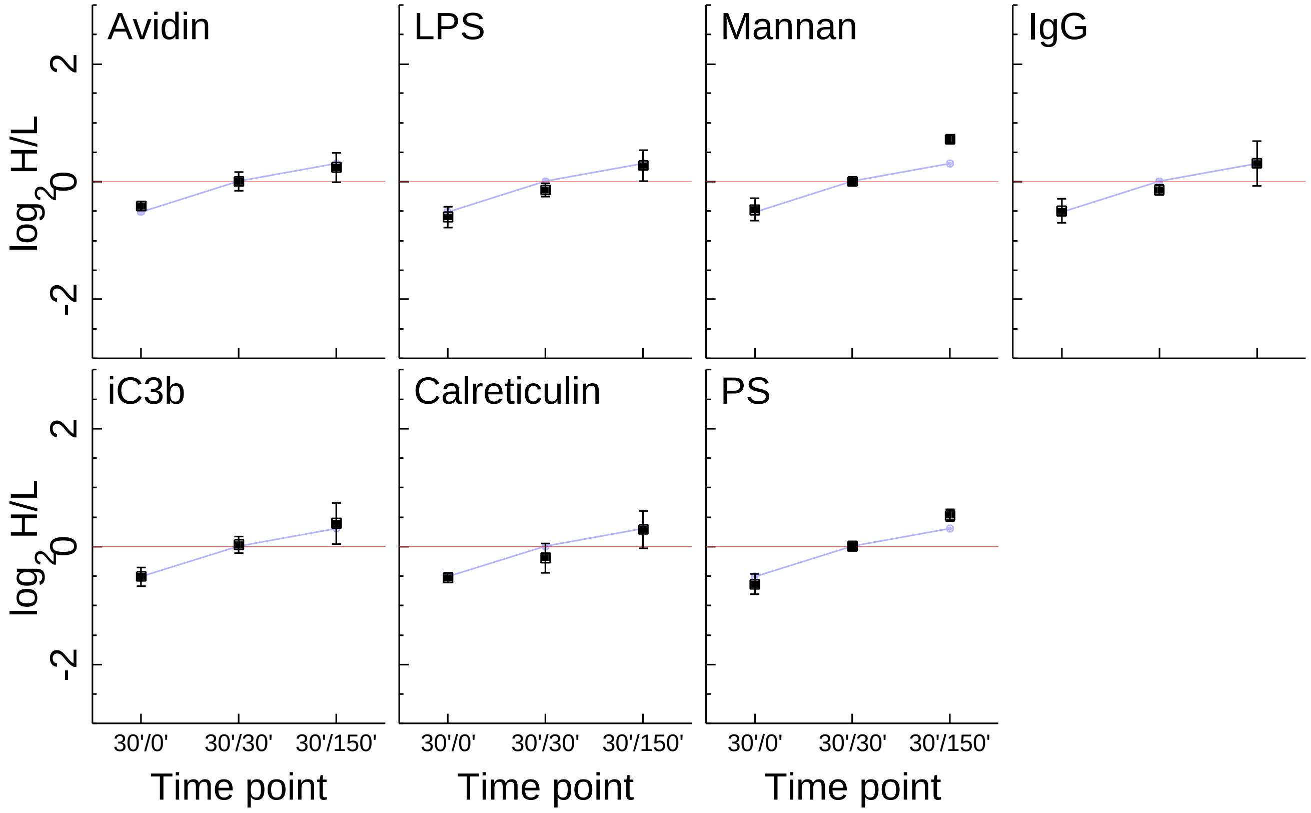
Avidin
LPS
Mannan
IgG
iC3b
Calreticulin
PS
log2 H/L
2
0
-2
30'/0'
30'/30'
30'/150'
Time point

tlr9 (Q9EQU3)

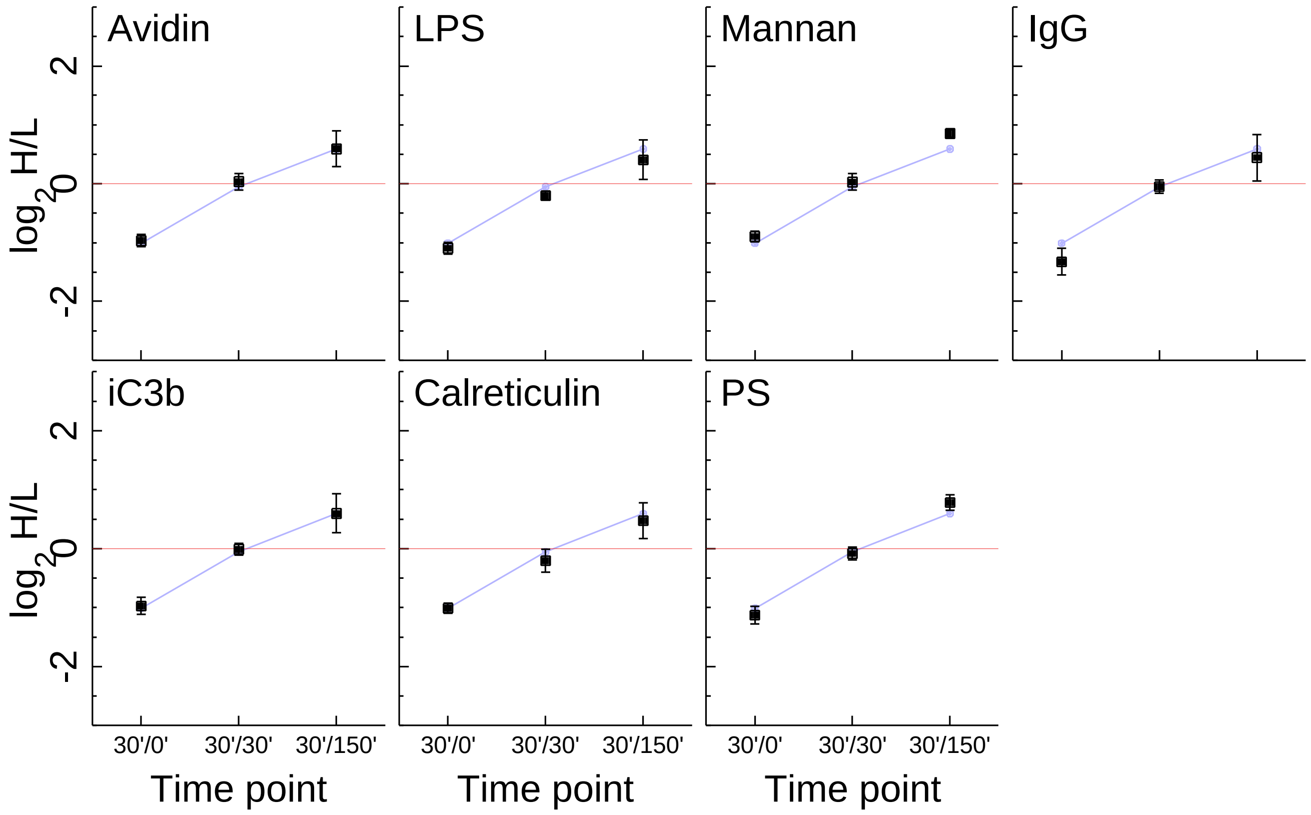

Avidin
LPS
Mannan
IgG
iC3b
Calreticulin
PS
2
0
-2
log2 H/L
30'/0'
30'/30'
30'/150'
Time point

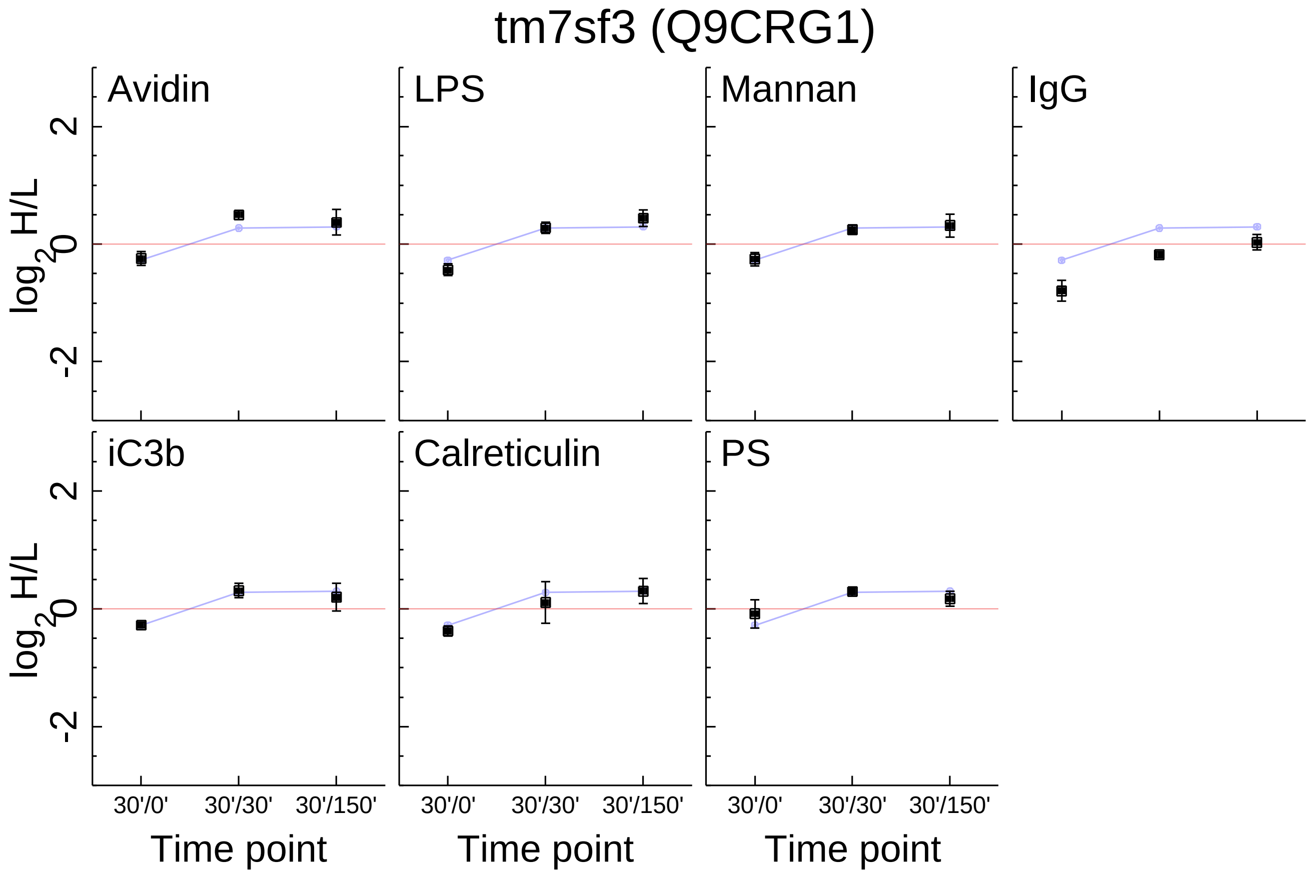
tm7sf3 (Q9CRG1)
Avidin
LPS
Mannan
IgG
iC3b
Calreticulin
PS
log2 H/L
2
0
-2
30'/0'
30'/30'
30'/150'
Time point

tm9sf1 (Q9DBU0)

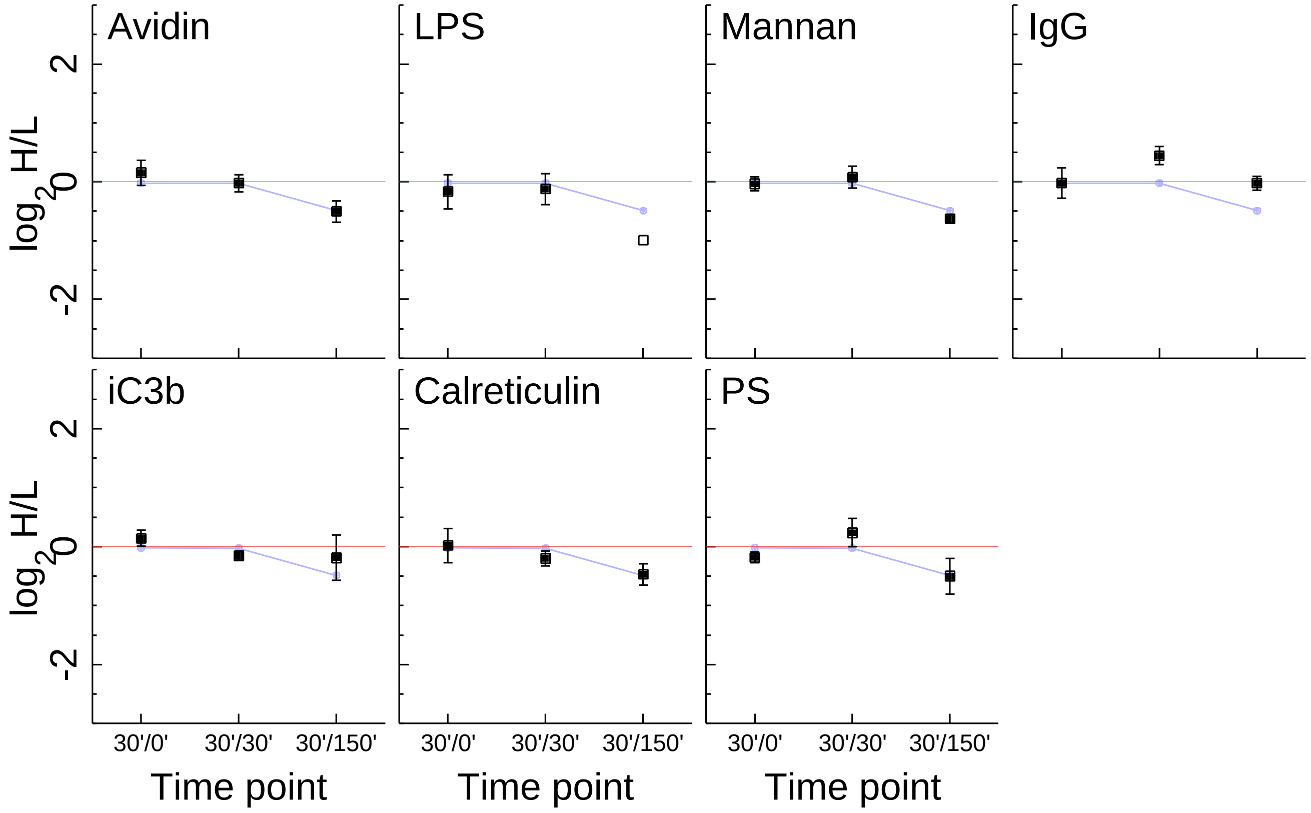

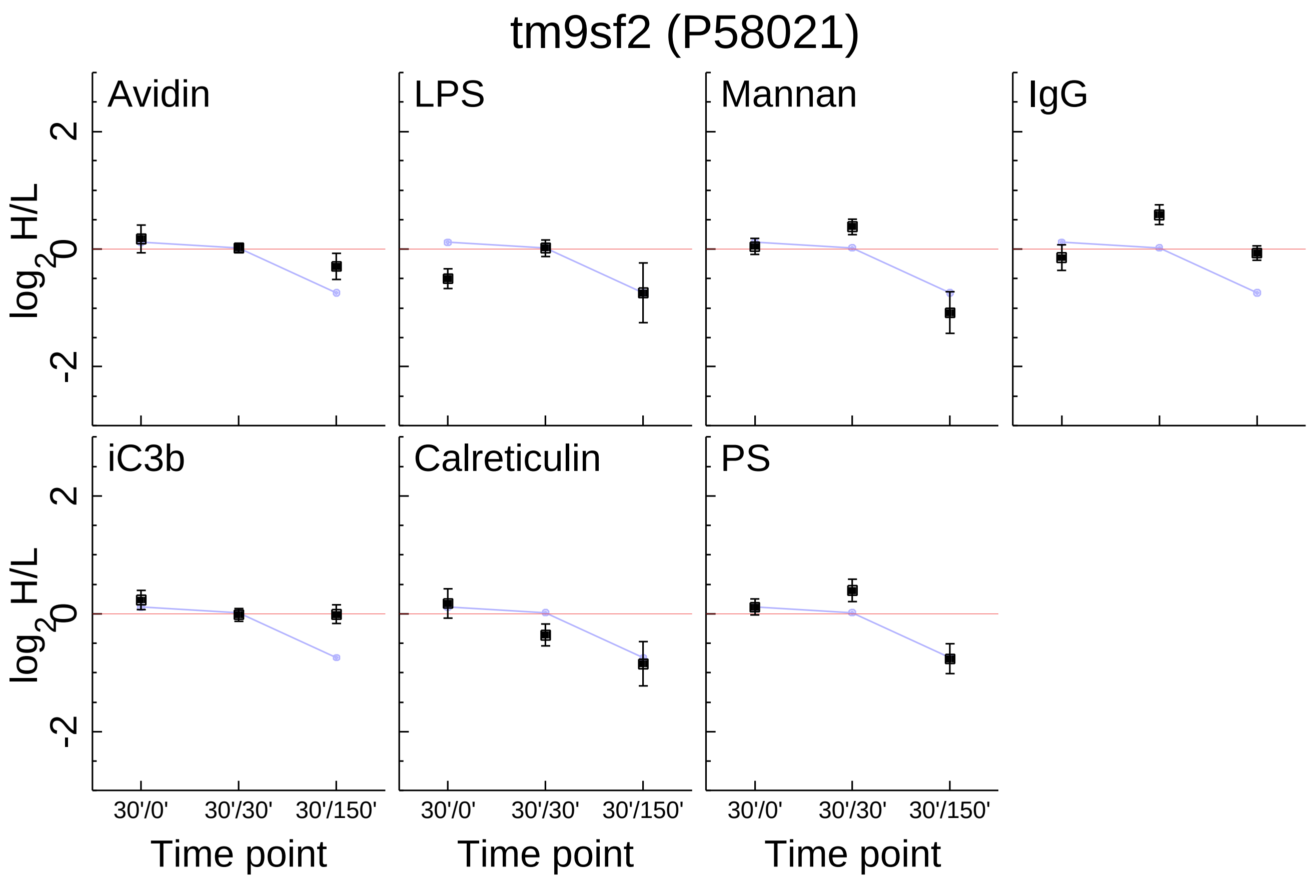
tm9sf2 (P58021)
Avidin
LPS
Mannan
IgG
iC3b
Calreticulin
PS
log2 H/L
2
0
-2
30'/0'
30'/30'
30'/150'
Time point

tm9sf3 (Q9ET30)

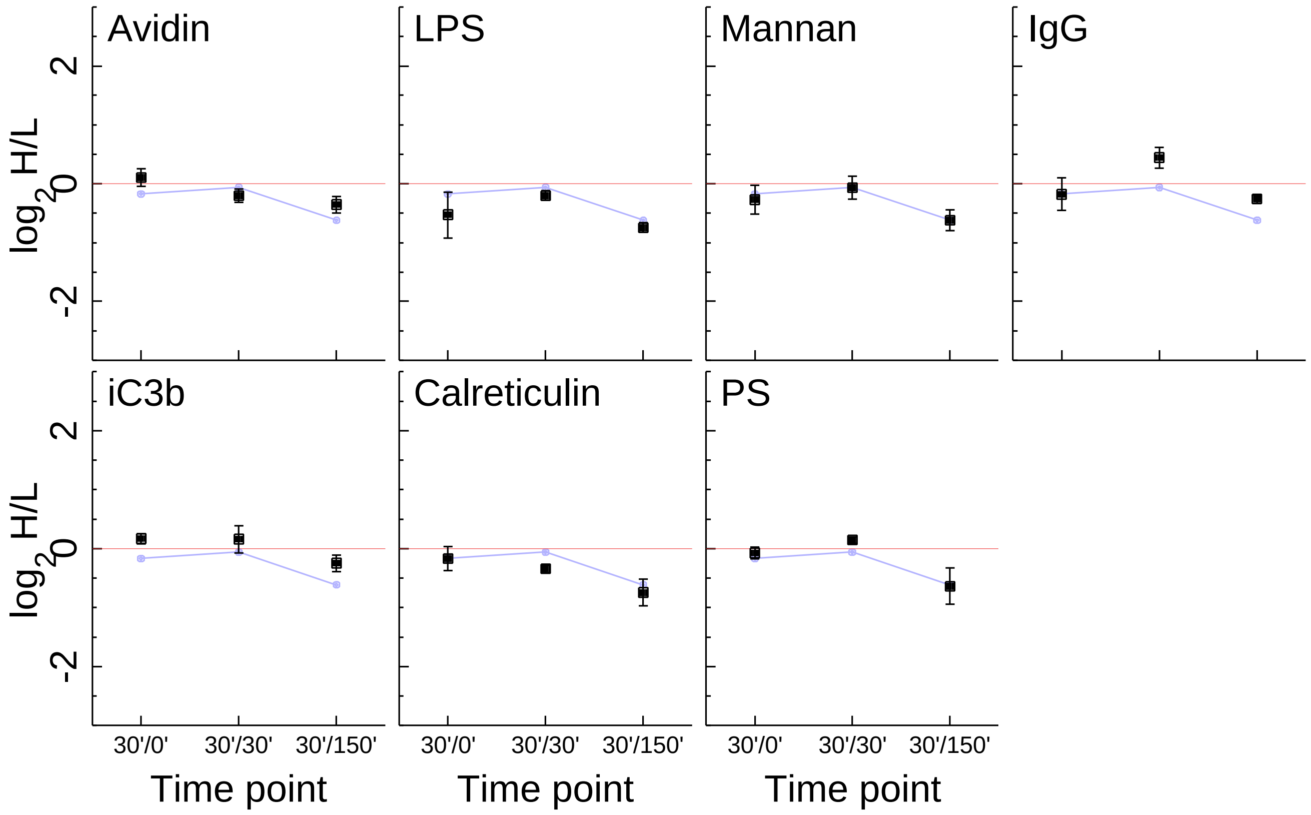

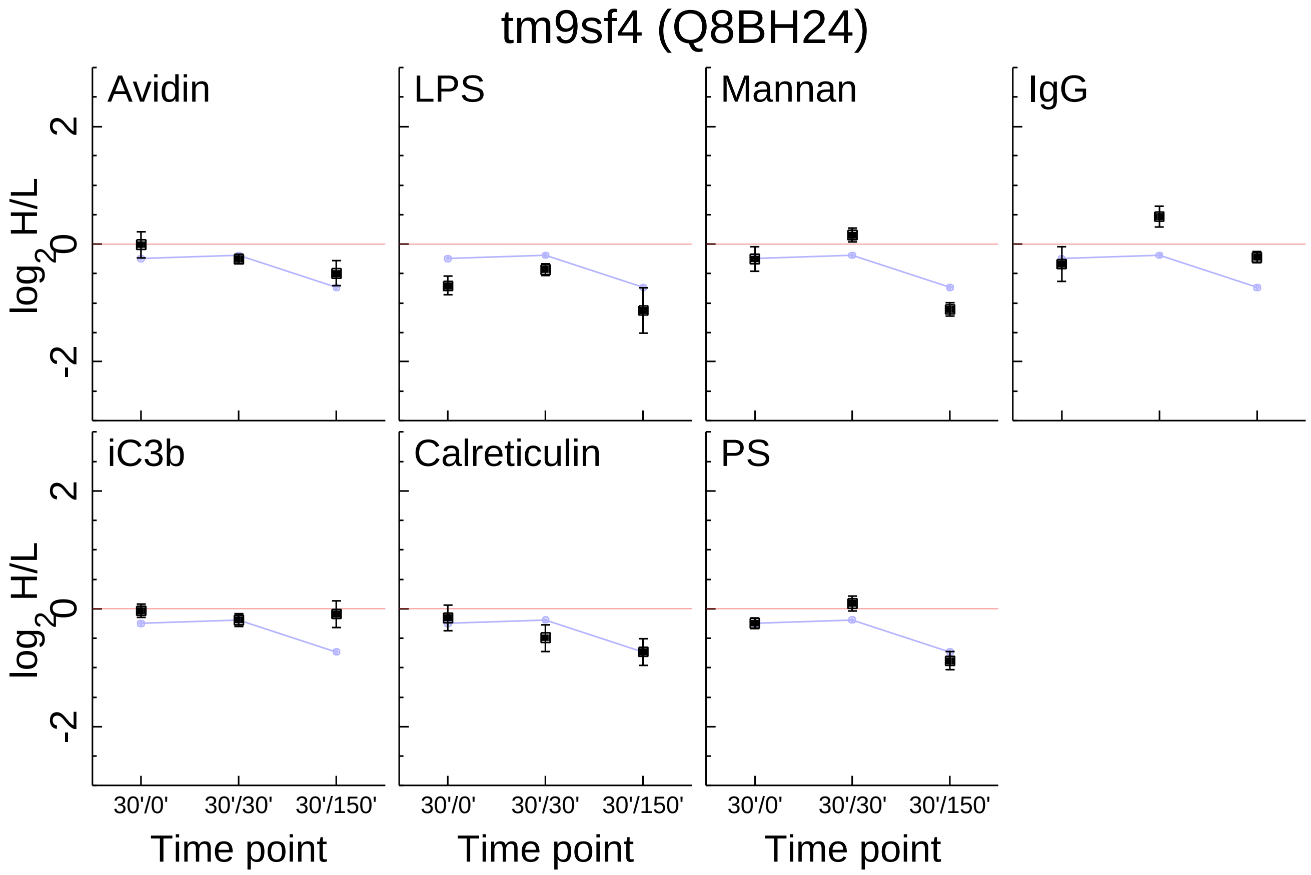

tm9sf4 (Q8BH24)
Avidin
LPS
Mannan
IgG
iC3b
Calreticulin
PS
log2 H/L
2
0
-2
30'/0'
30'/30'
30'/150'
Time point

tmco1 (Q921L3)

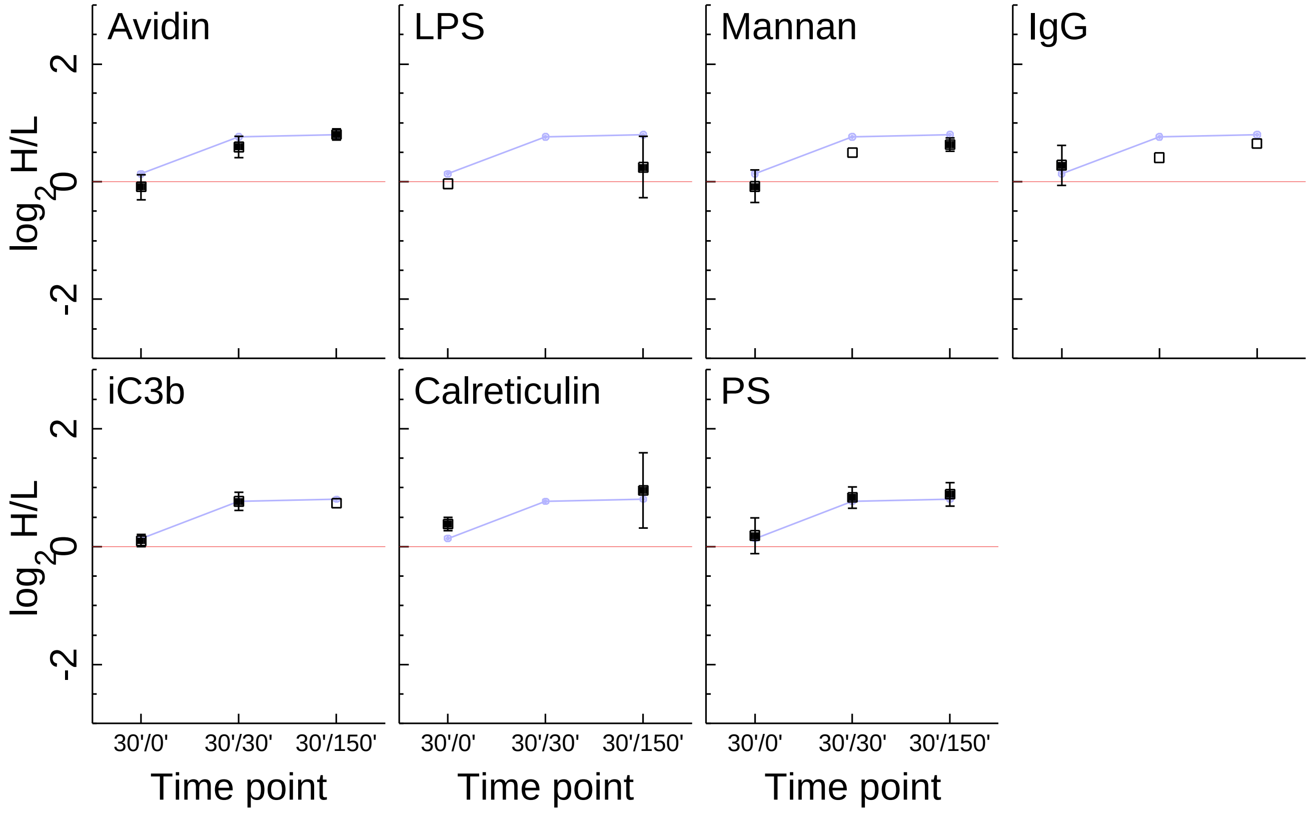

tmed10 (Q9D1D4)

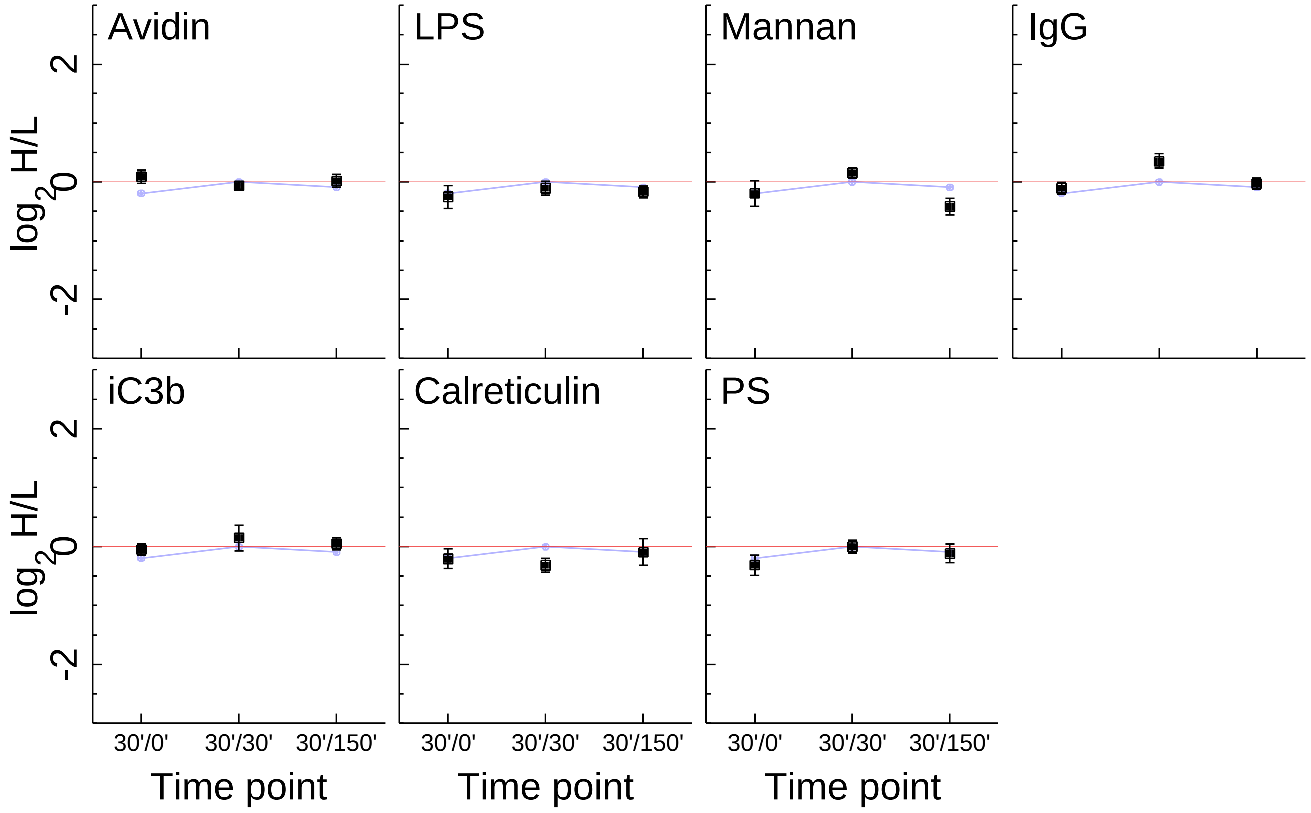

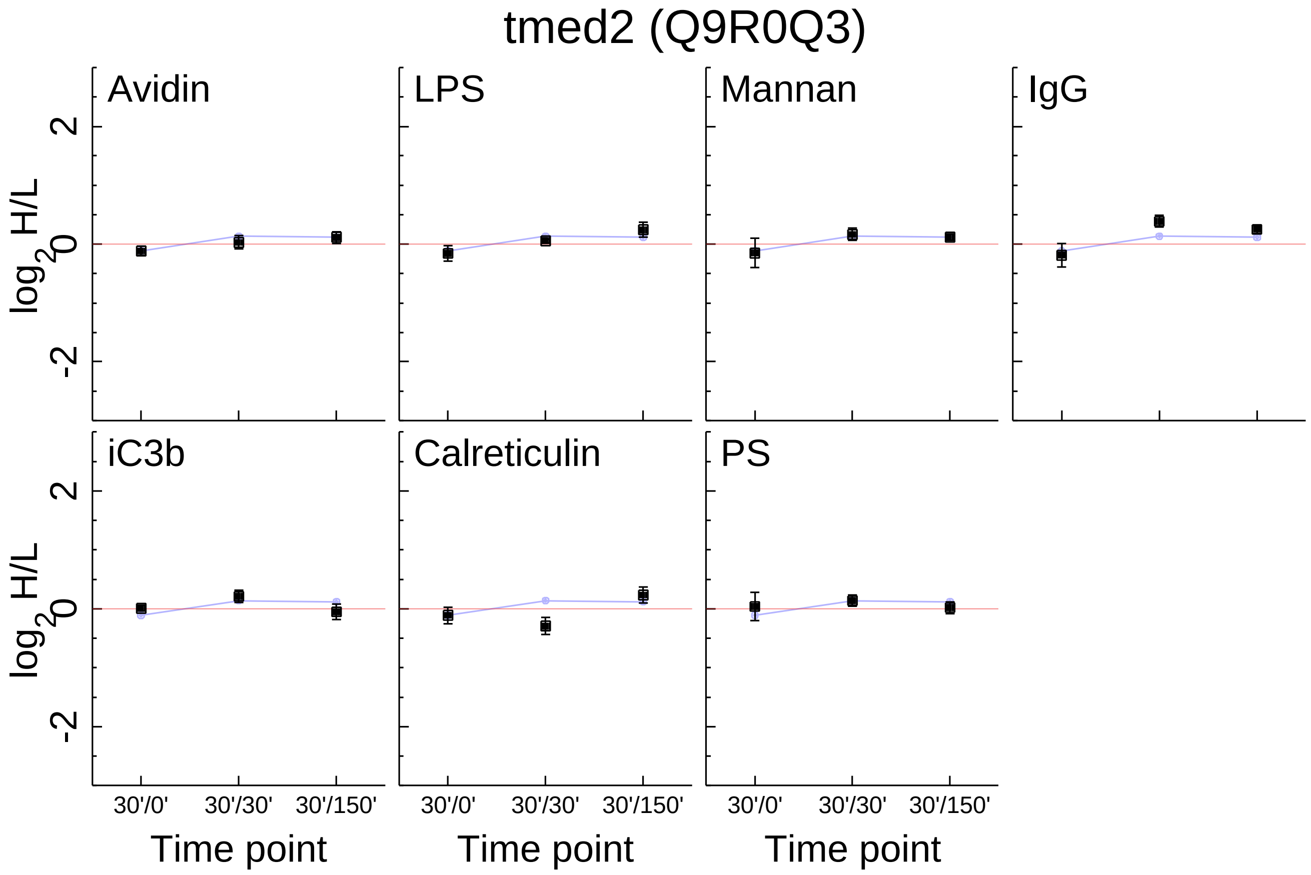
tmed2 (Q9R0Q3)
Avidin
LPS
Mannan
IgG
iC3b
Calreticulin
PS
2
0
-2
log2 H/L
30'/0'
30'/30'
30'/150'
Time point

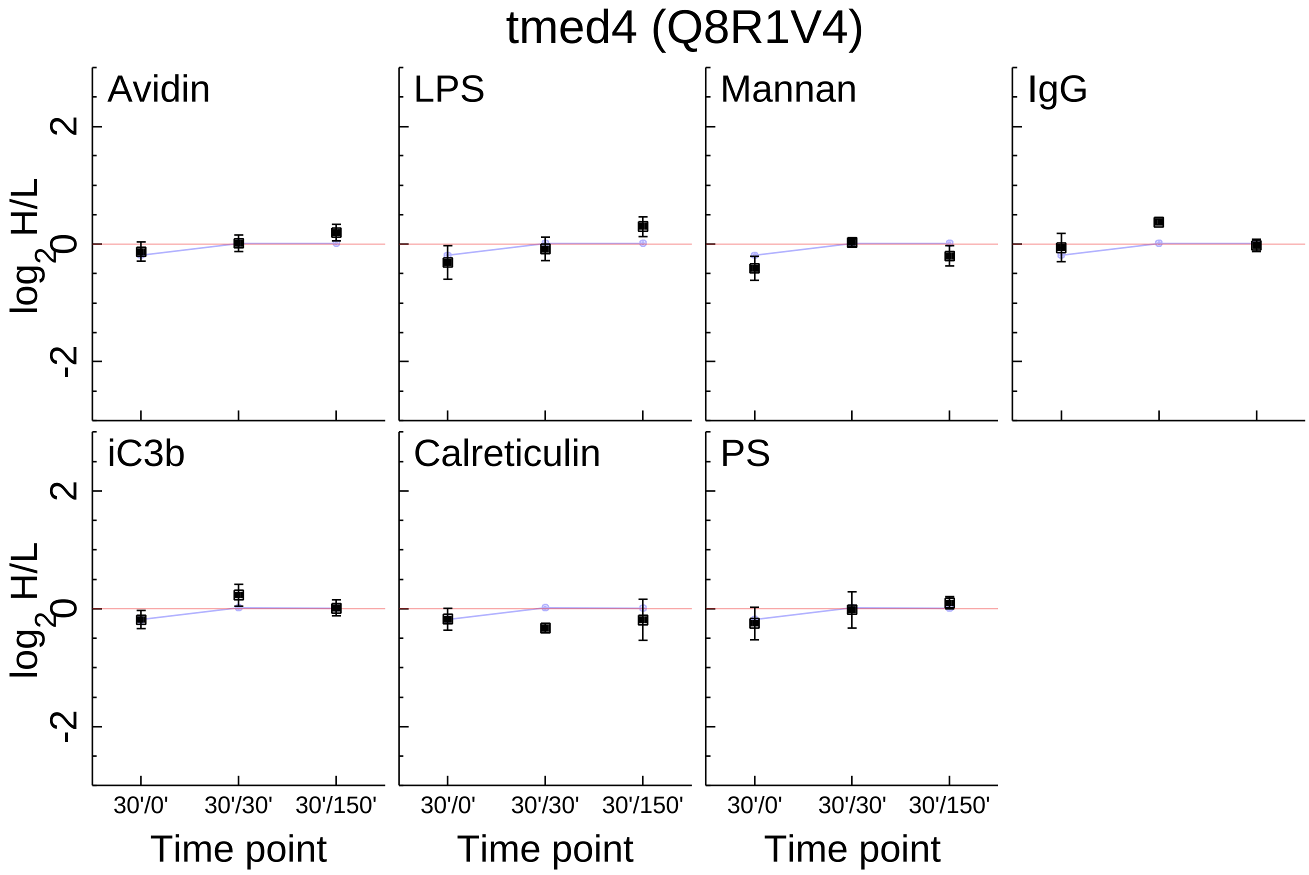

tmed4 (Q8R1V4)
Avidin
LPS
Mannan
IgG
iC3b
Calreticulin
PS
log2 H/L
2
0
-2
30'/0'
30'/30'
30'/150'
Time point

tmed5 (Q9CXE7)

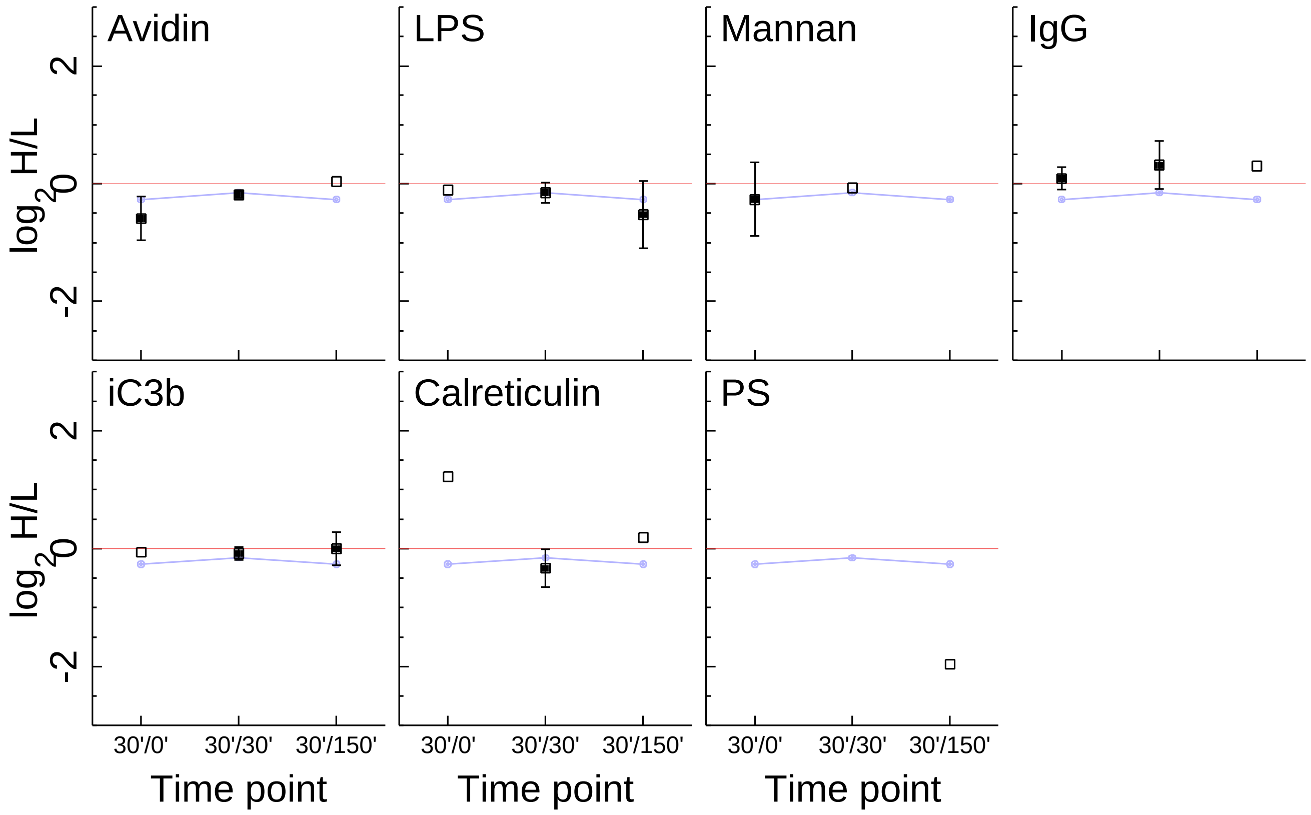

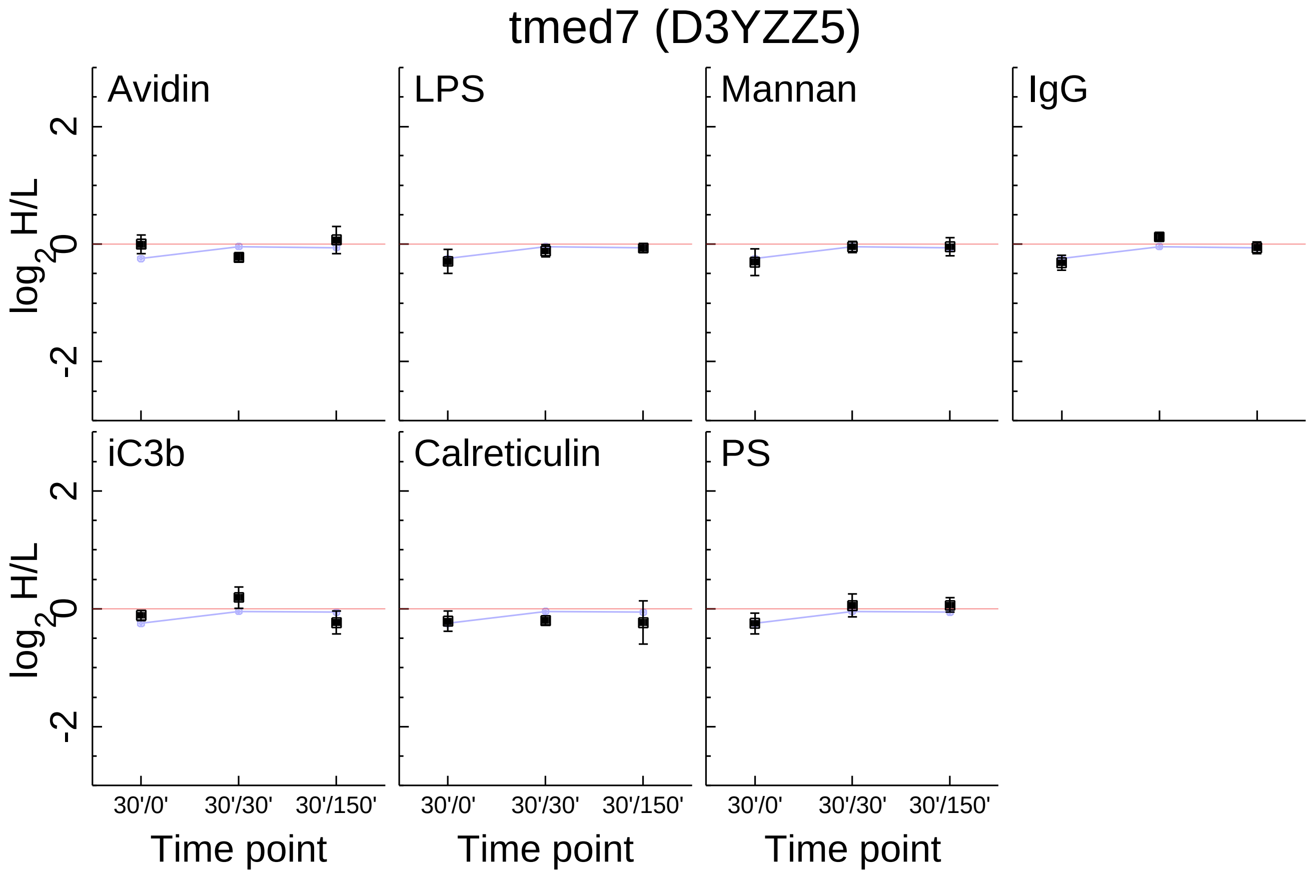
tmed7 (D3YZZ5)
Avidin
LPS
Mannan
IgG
iC3b
Calreticulin
PS
log2 H/L
2
0
-2
30'/0'
30'/30'
30'/150'
Time point

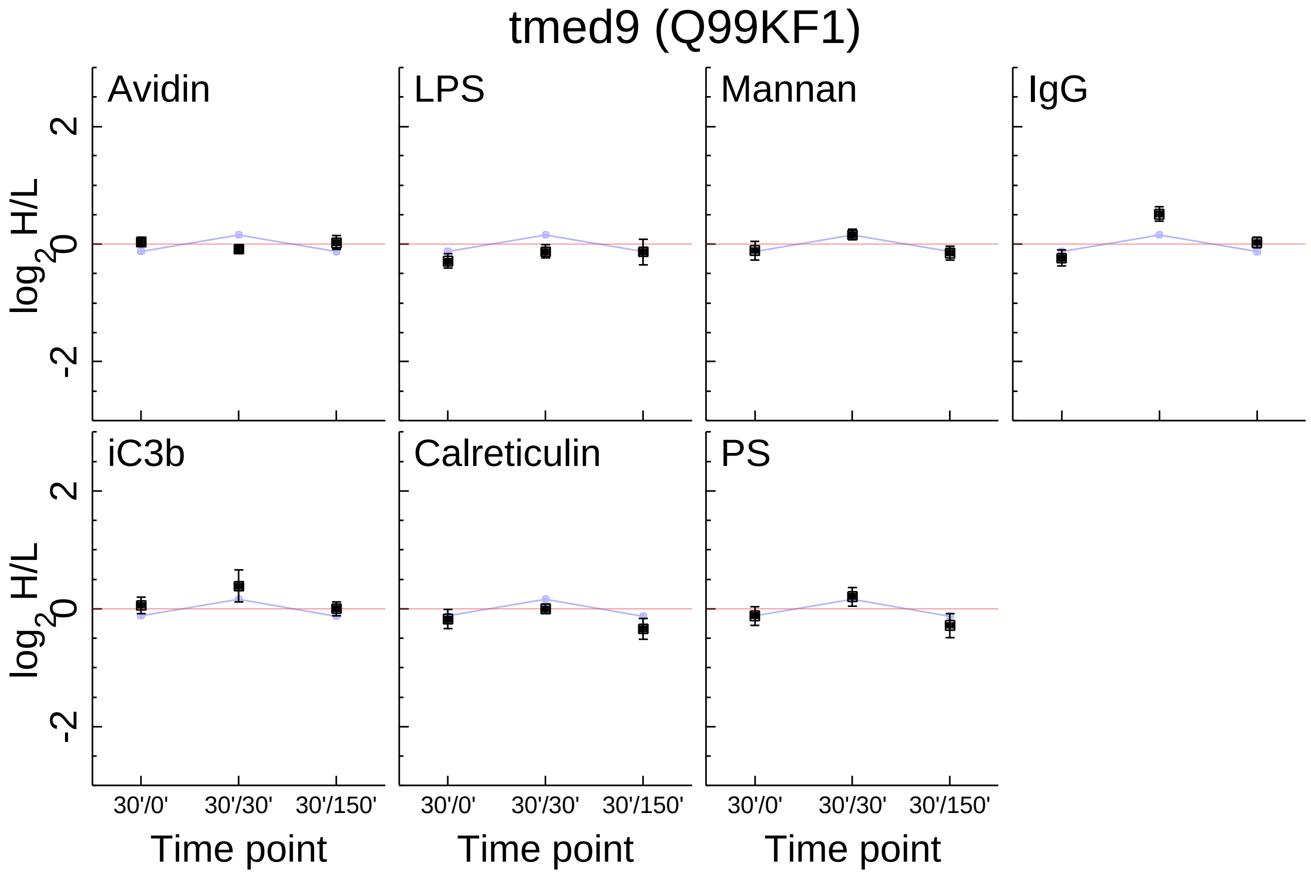

tmed9 (Q99KF1)
Avidin
LPS
Mannan
IgG
iC3b
Calreticulin
PS
log2 H/L
2
0
-2
30'/0'
30'/30'
30'/150'
Time point

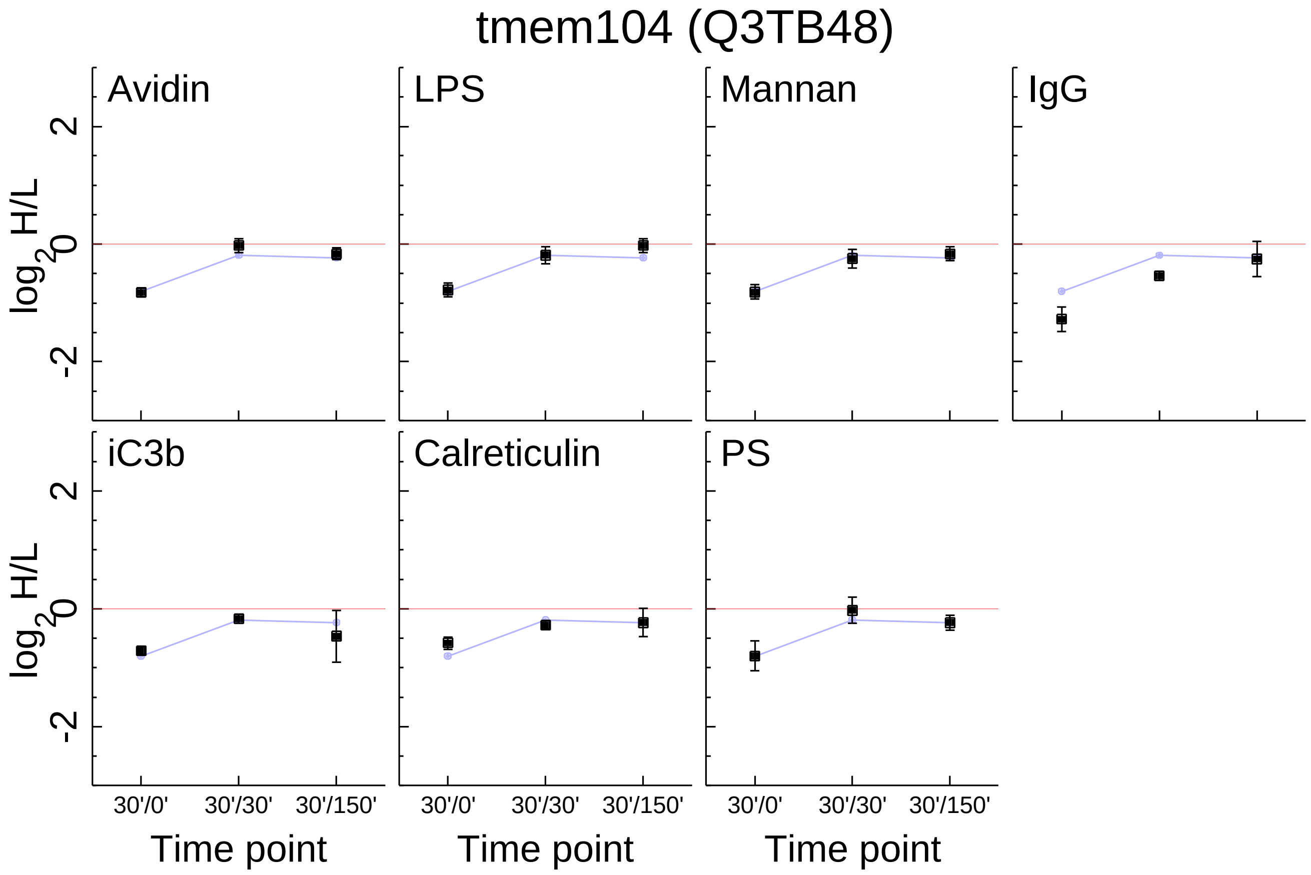
tmem104 (Q3TB48)
Avidin
LPS
Mannan
IgG
iC3b
Calreticulin
PS
log2 H/L
2
0
-2
30'/0'
30'/30'
30'/150'
Time point

tmem106a (Q8VC04)

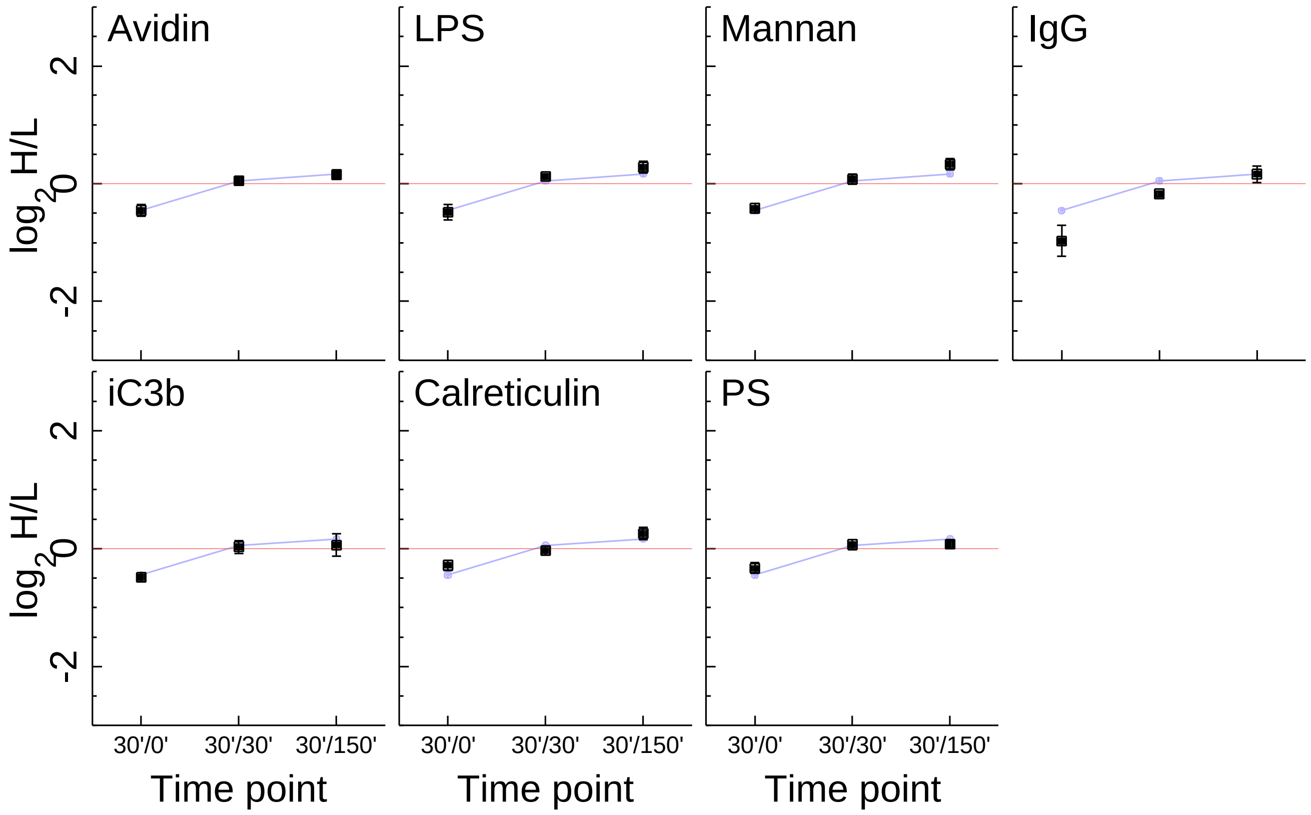

tmem106b (Q80X71)

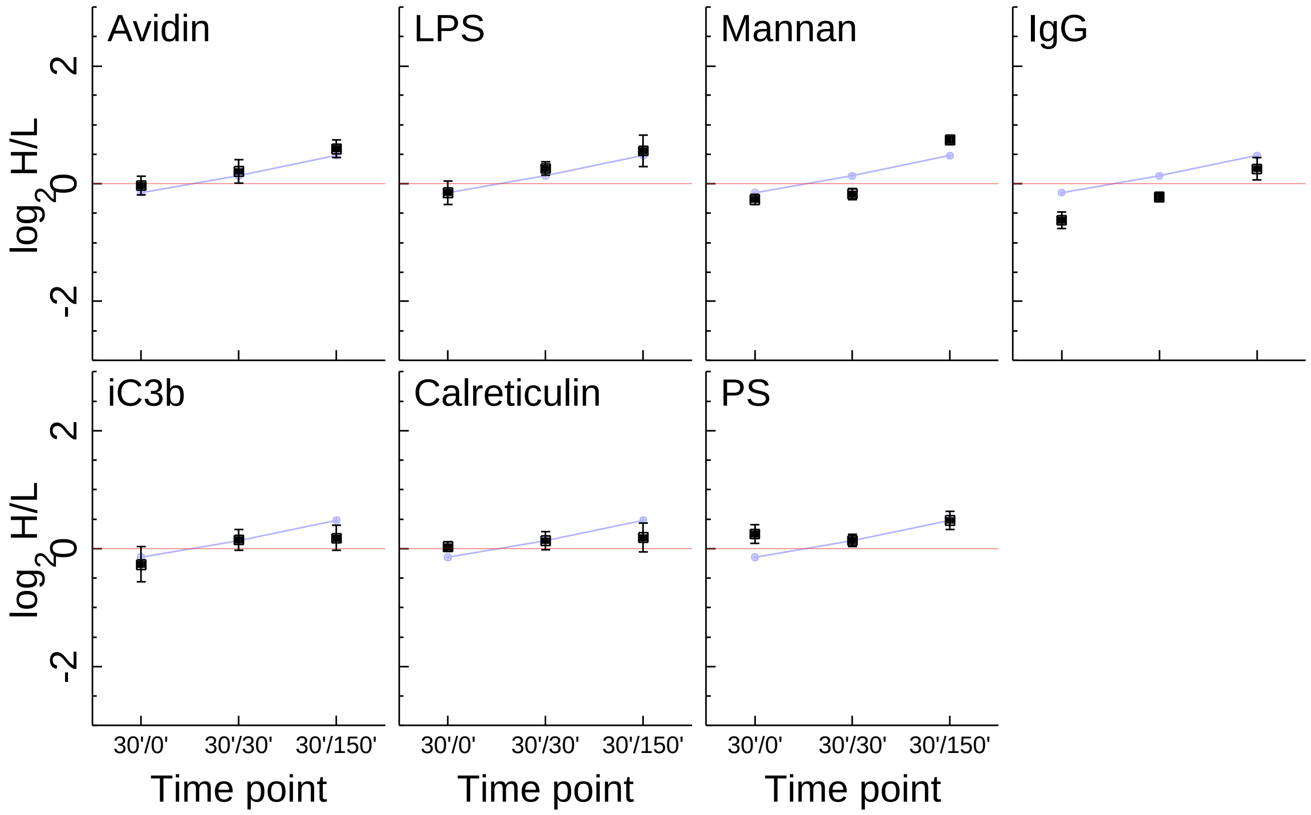

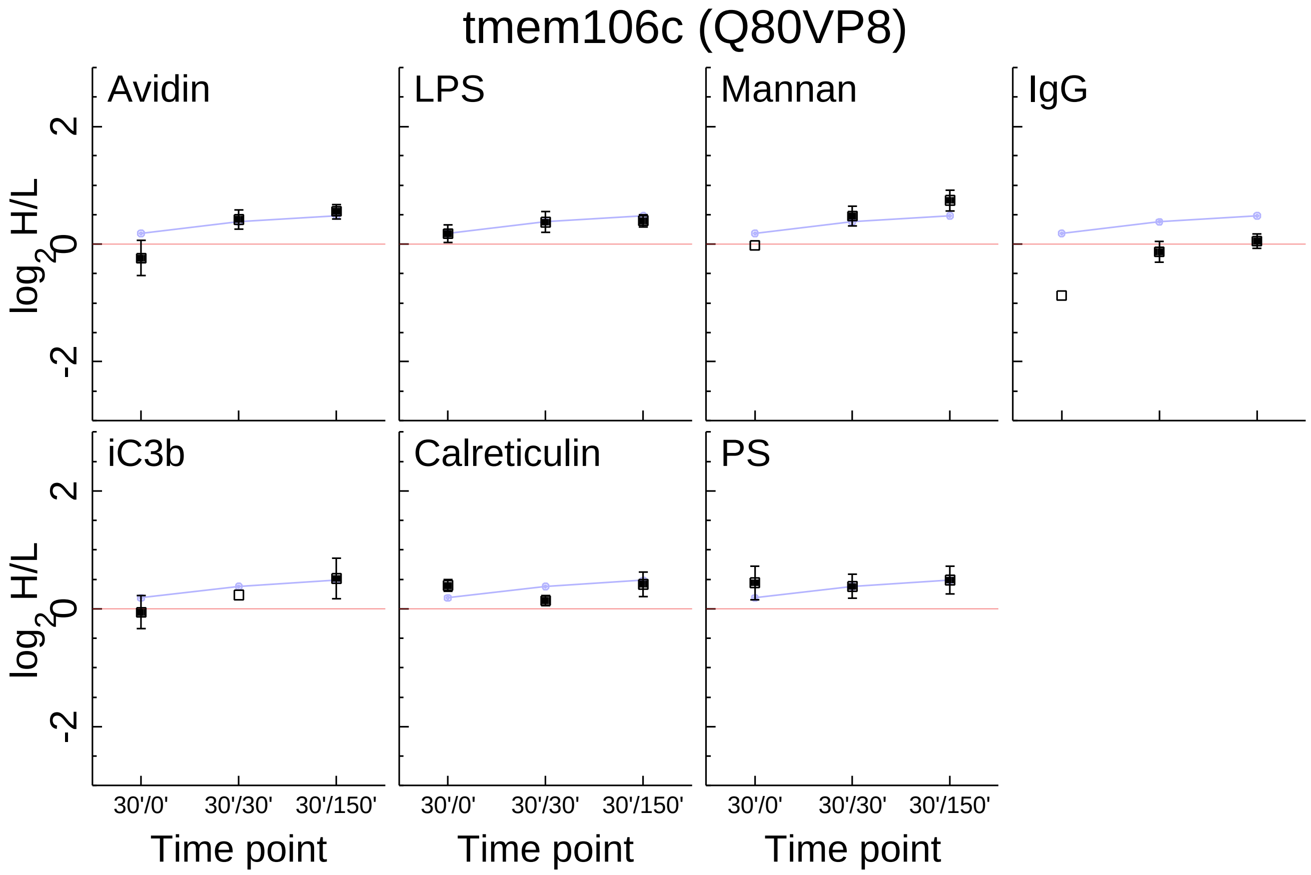
tmem106c (Q80VP8)
Avidin
LPS
Mannan
IgG
iC3b
Calreticulin
PS
log2 H/L
2
0
-2
30'/0'
30'/30'
30'/150'
Time point

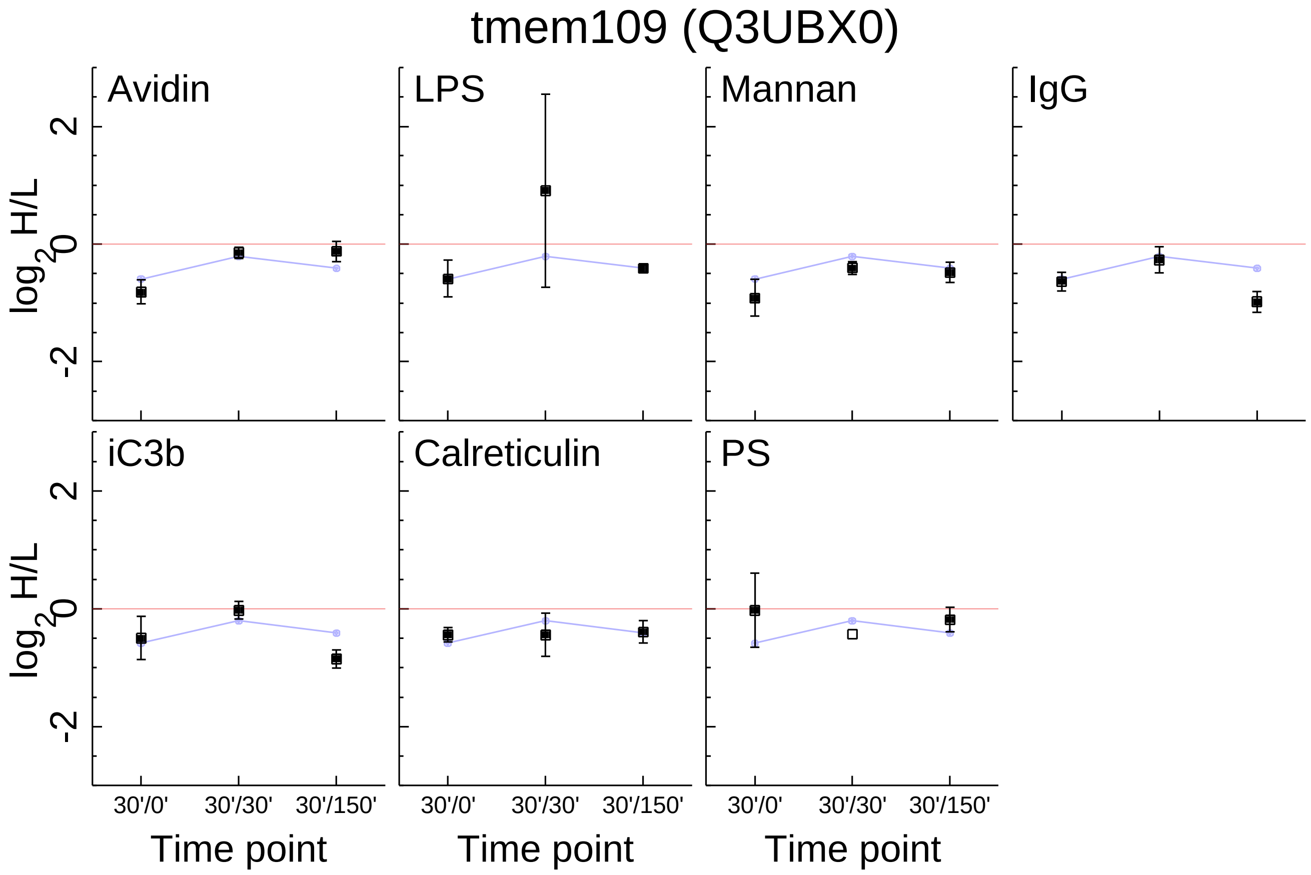

tmem109 (Q3UBX0)
Avidin
LPS
Mannan
IgG
iC3b
Calreticulin
PS
log2 H/L
2
0
-2
30'/0'
30'/30'
30'/150'
Time point

tmem120a (Q8C1E7)

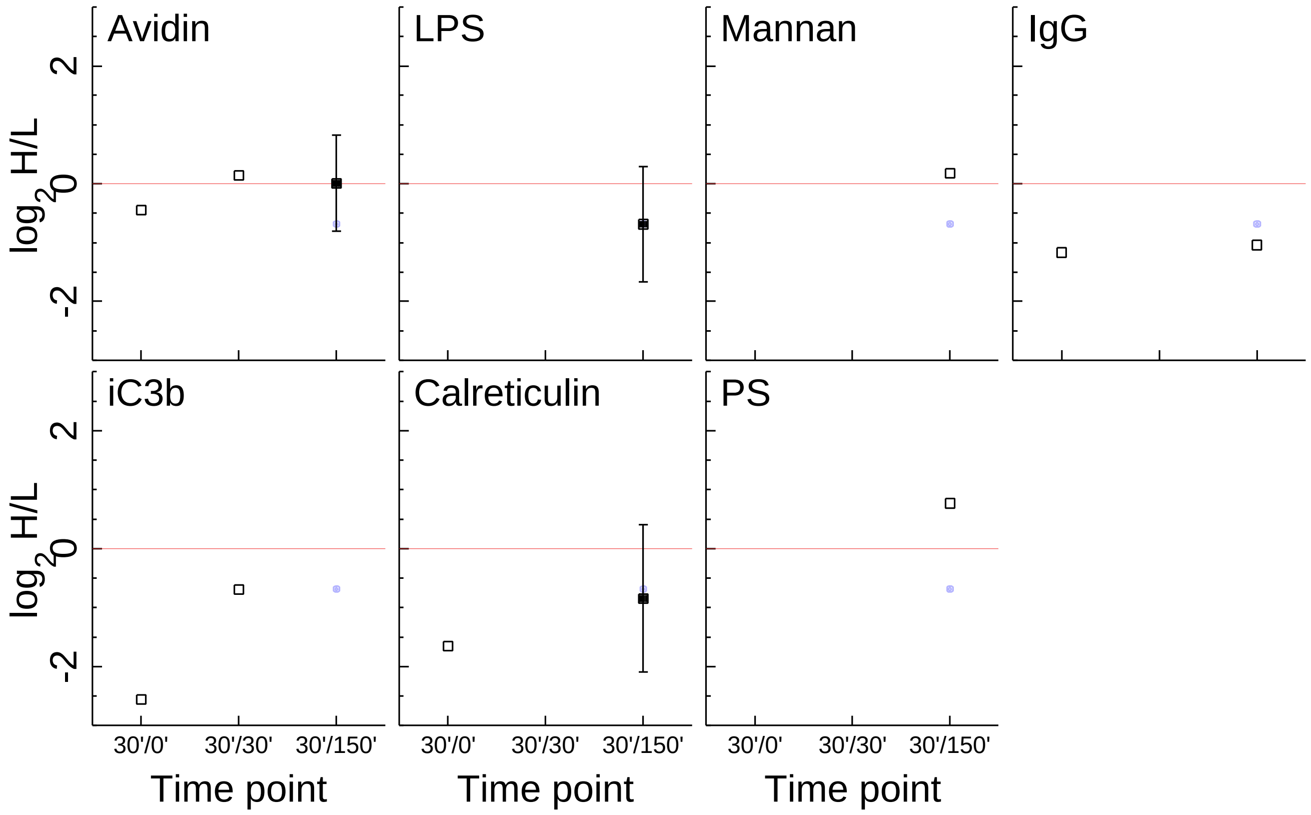

tmem144 (Q8VEH0)

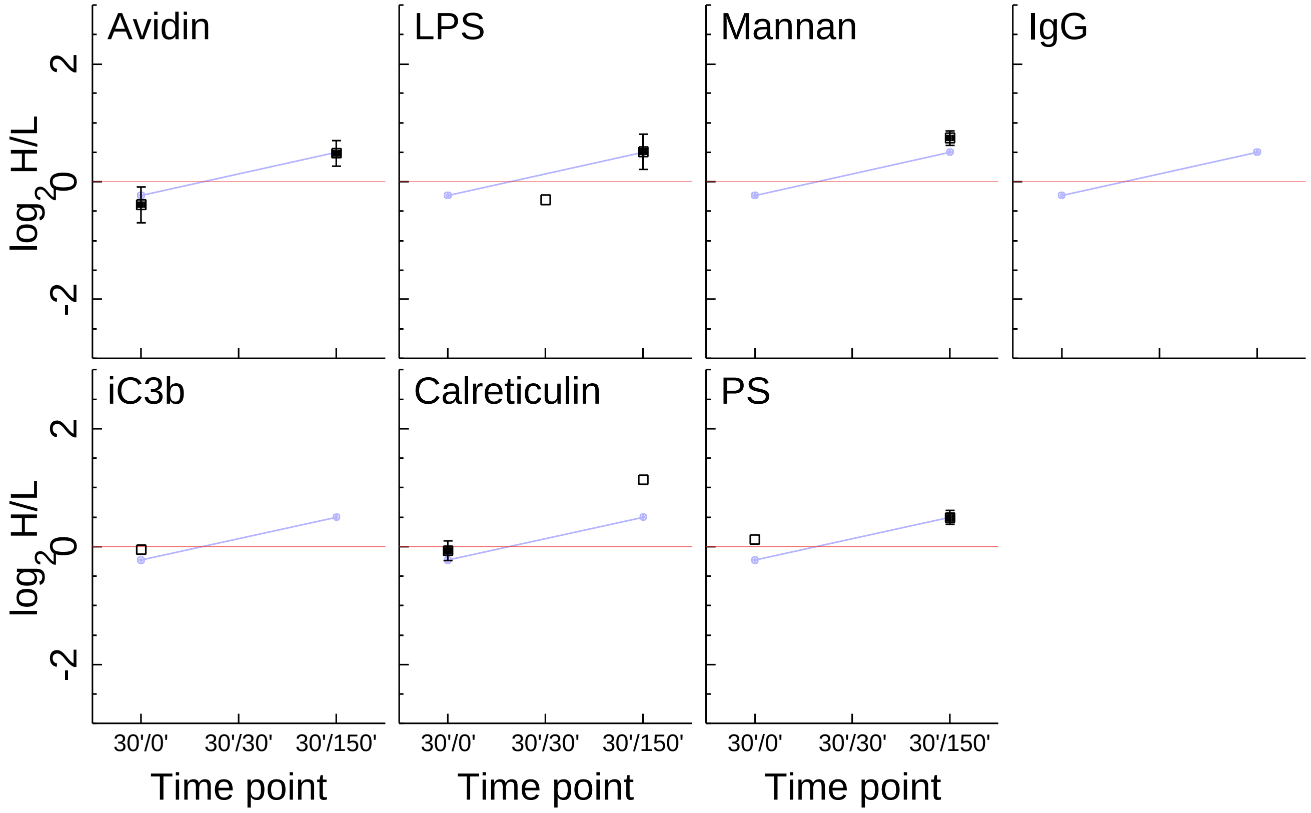

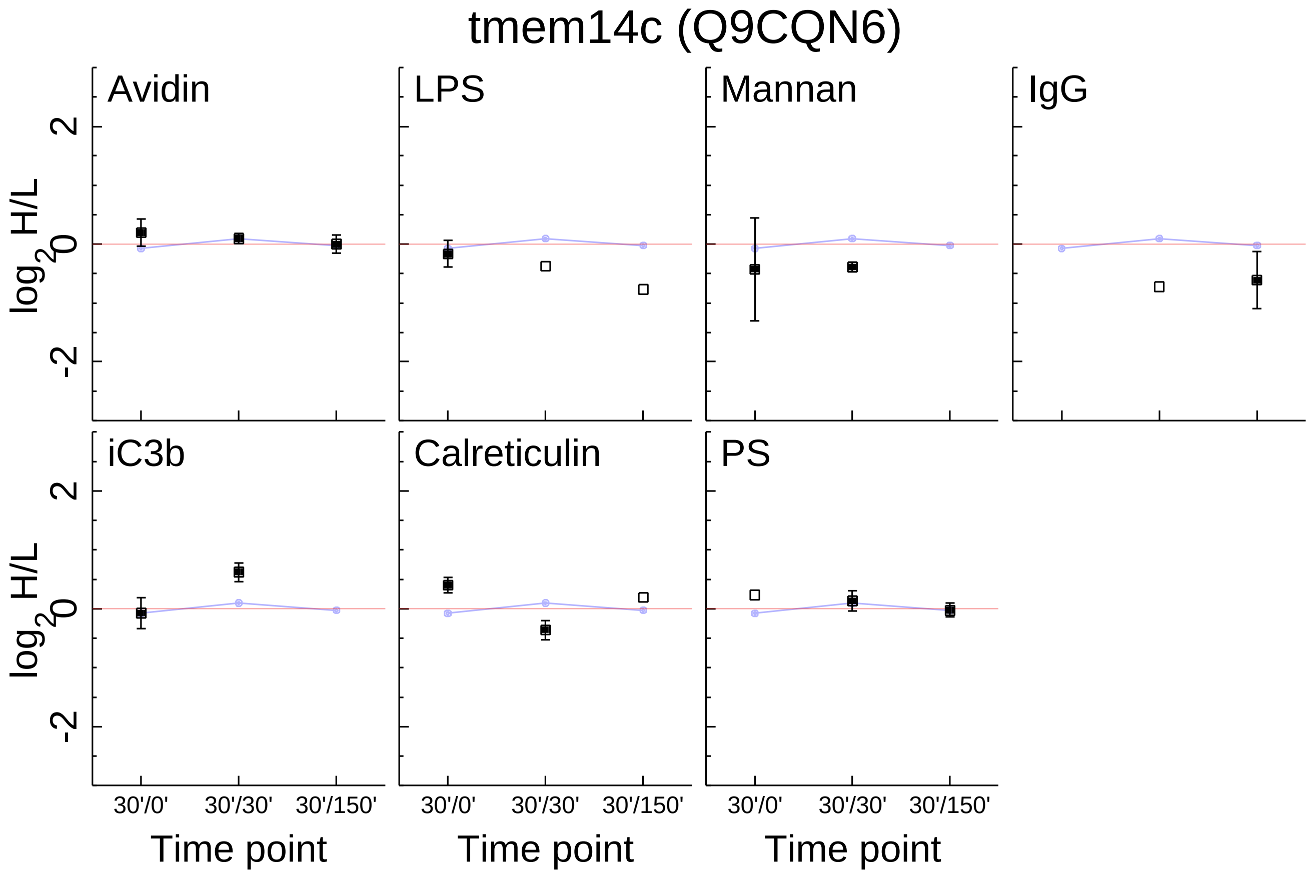
tmem14c (Q9CQN6)
Avidin
LPS
Mannan
IgG
iC3b
Calreticulin
PS
log2 H/L
2
0
-2
30'/0'
30'/30'
30'/150'
Time point

tmem165 (P52875)

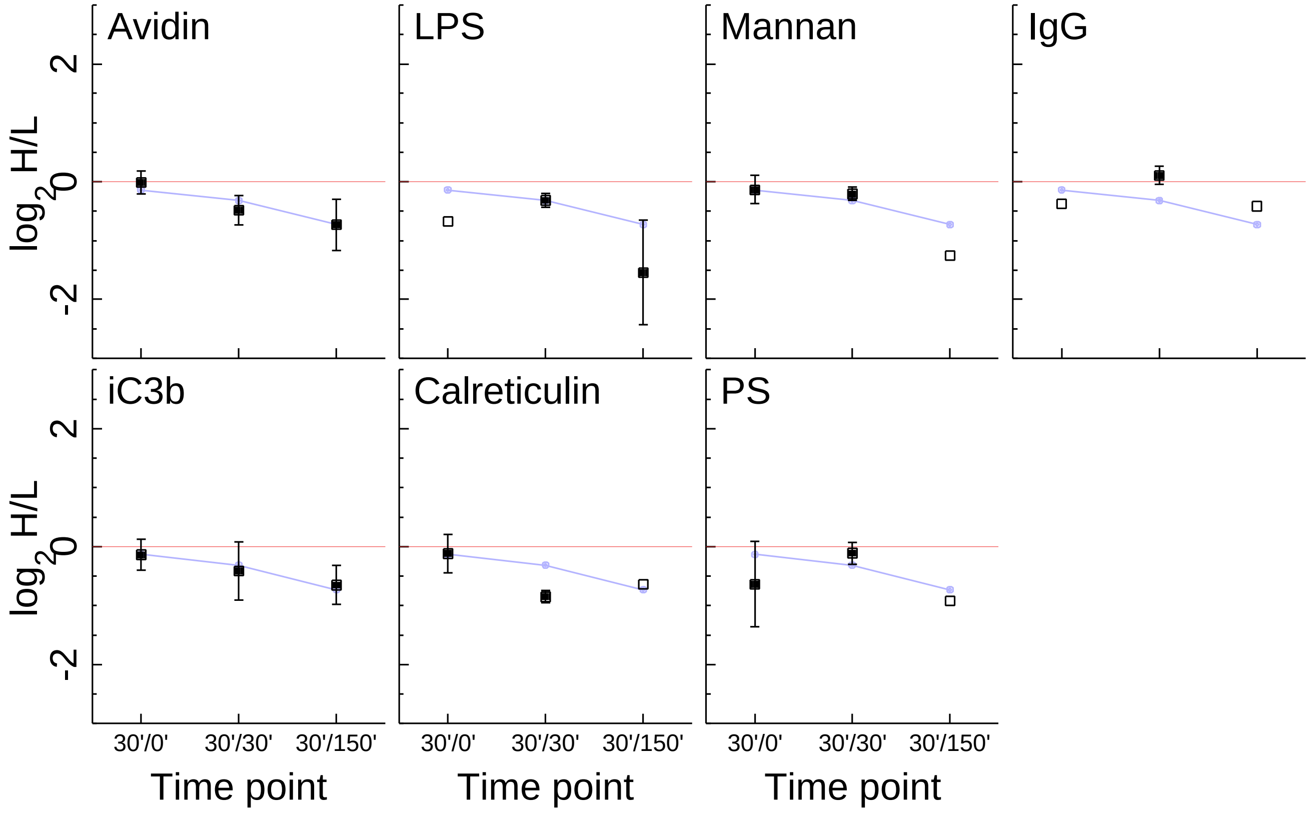

tmem173 (Q3TBT3)

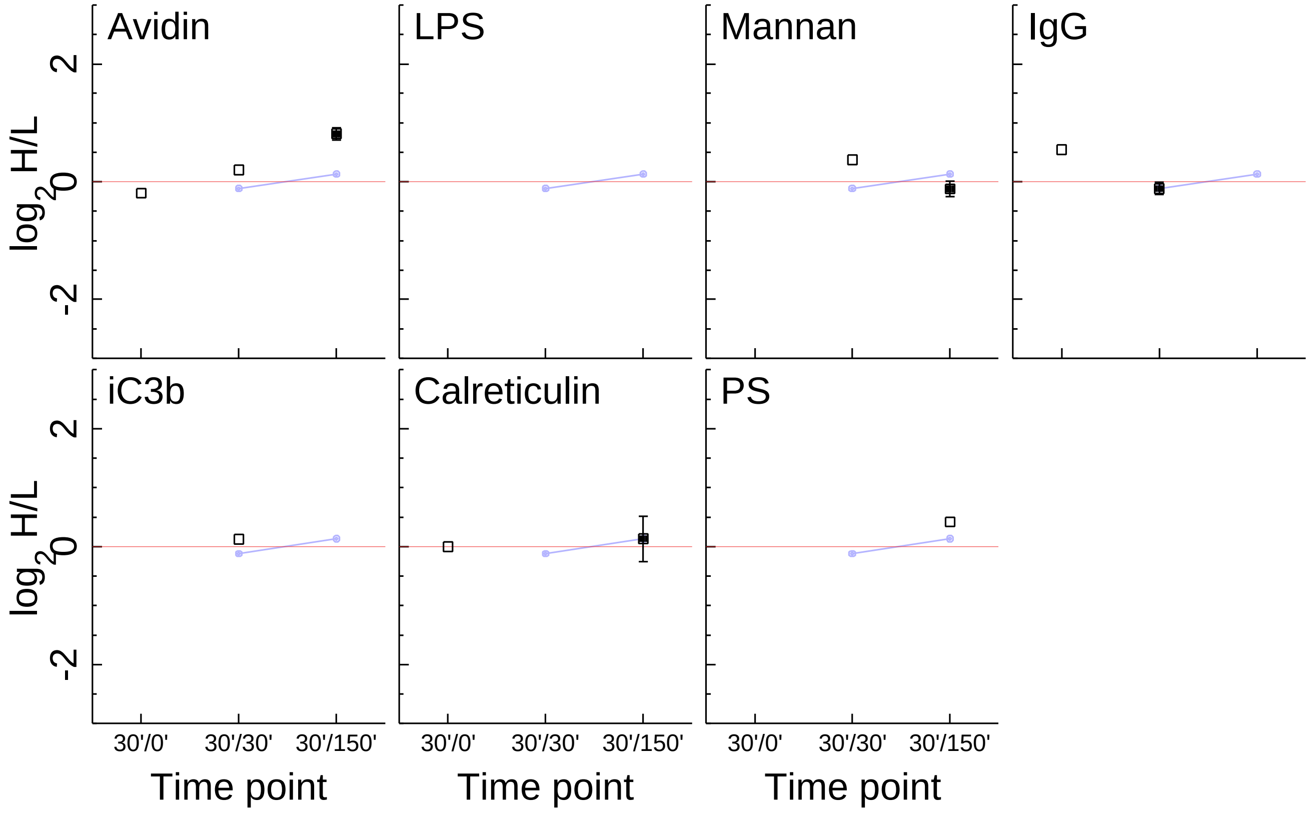

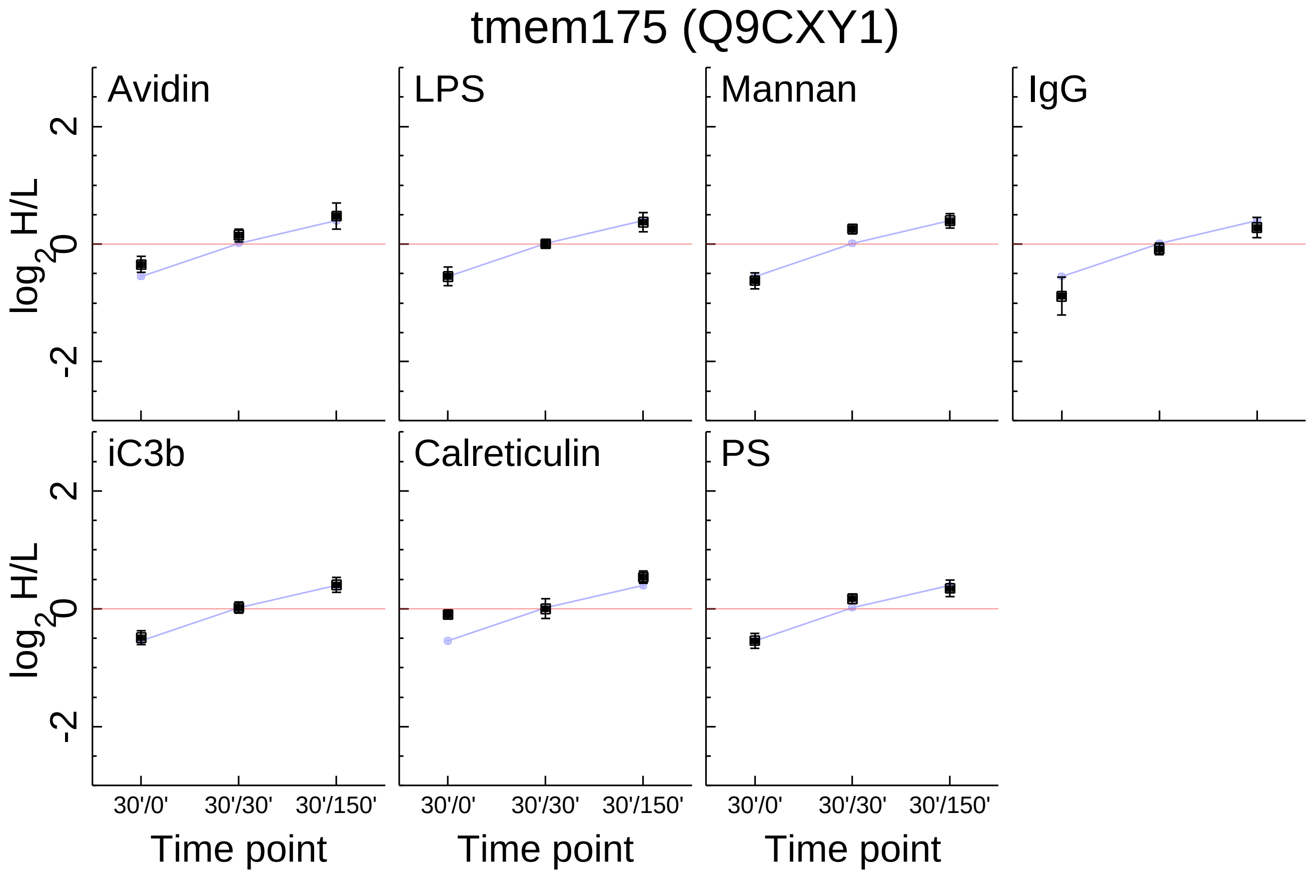
tmem175 (Q9CXY1)
Avidin
LPS
Mannan
IgG
iC3b
Calreticulin
PS
log2 H/L
2
0
-2
30'/0'
30'/30'
30'/150'
Time point

tmem176b (Q9R1Q6)

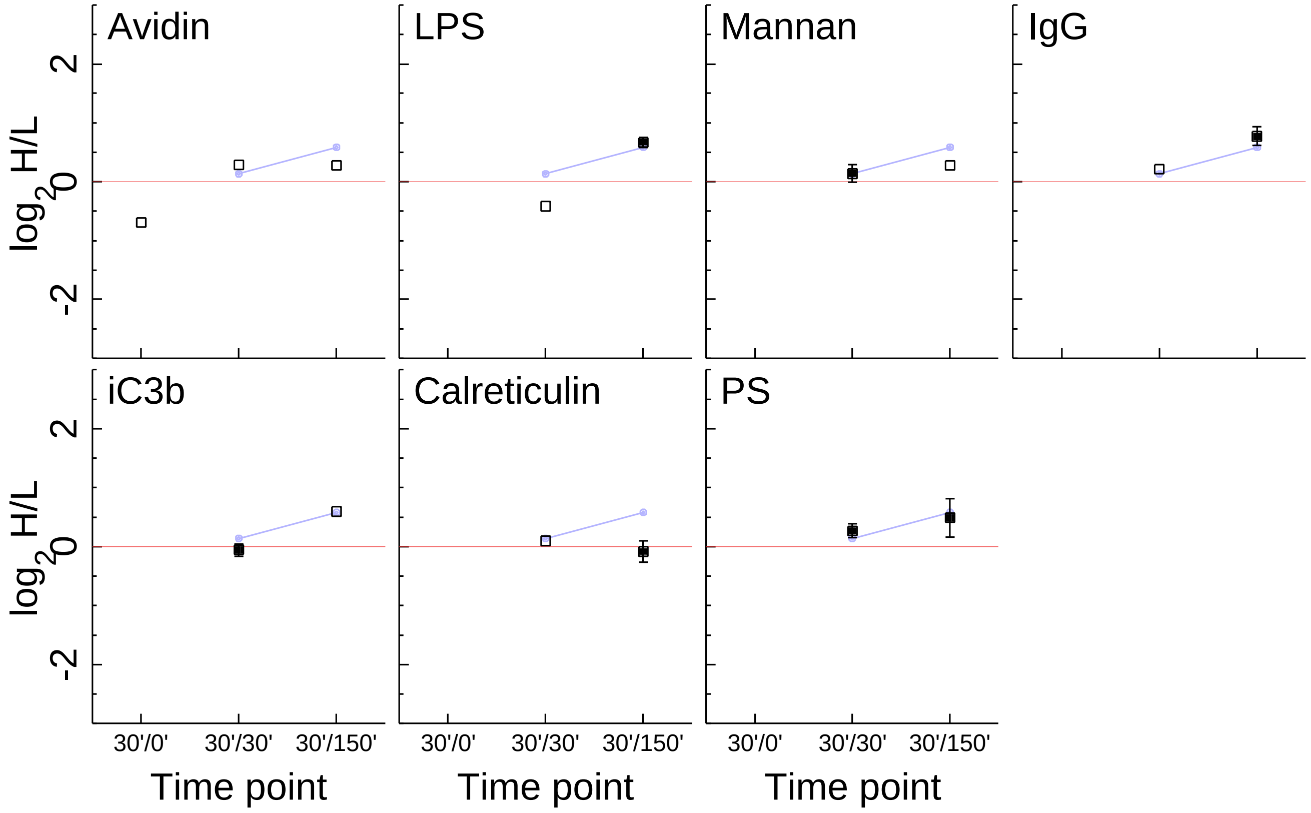

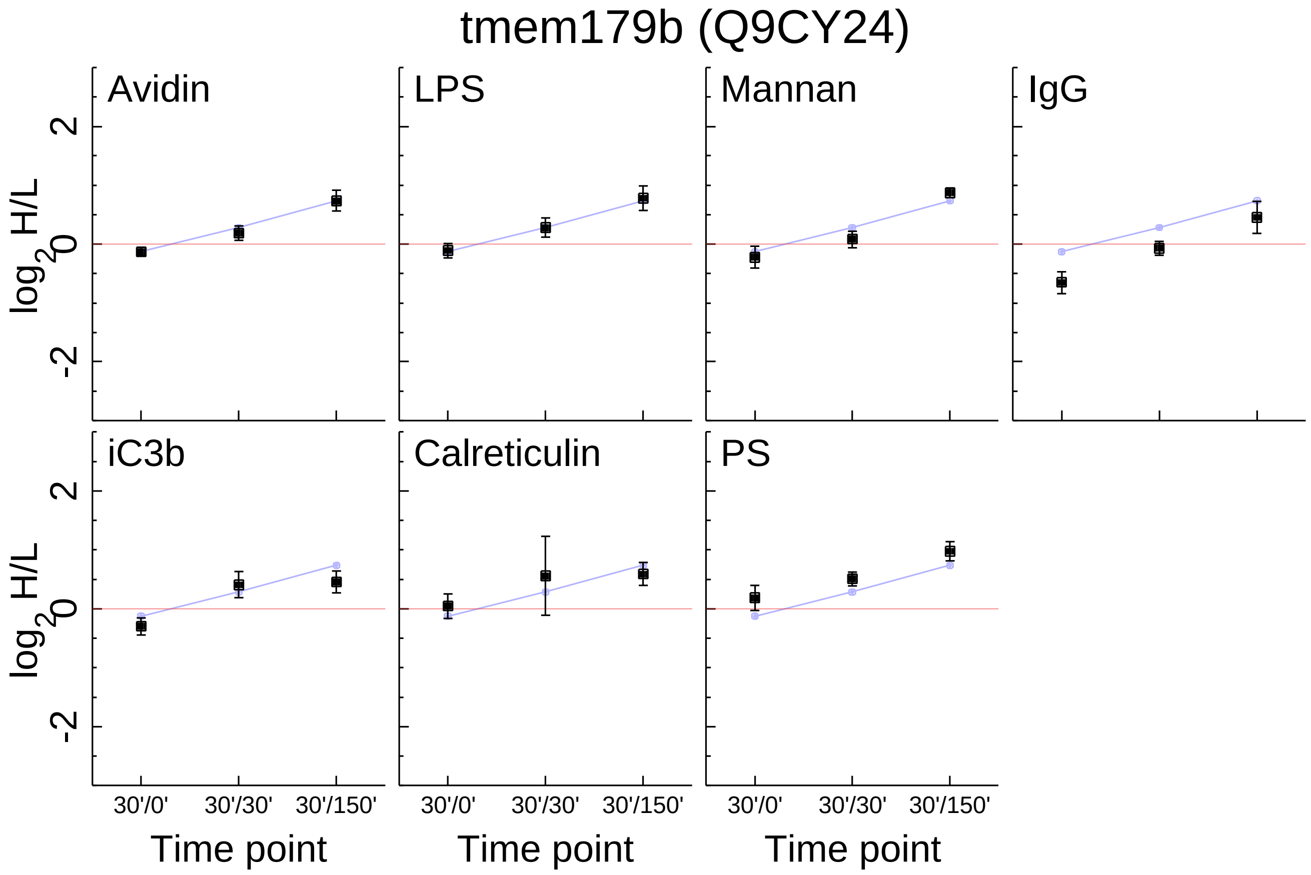
tmem179b (Q9CY24)
Avidin
LPS
Mannan
IgG
iC3b
Calreticulin
PS
log2 H/L
2
0
-2
30'/0'
30'/30'
30'/150'
Time point

tmem192 (Q9CXT7)

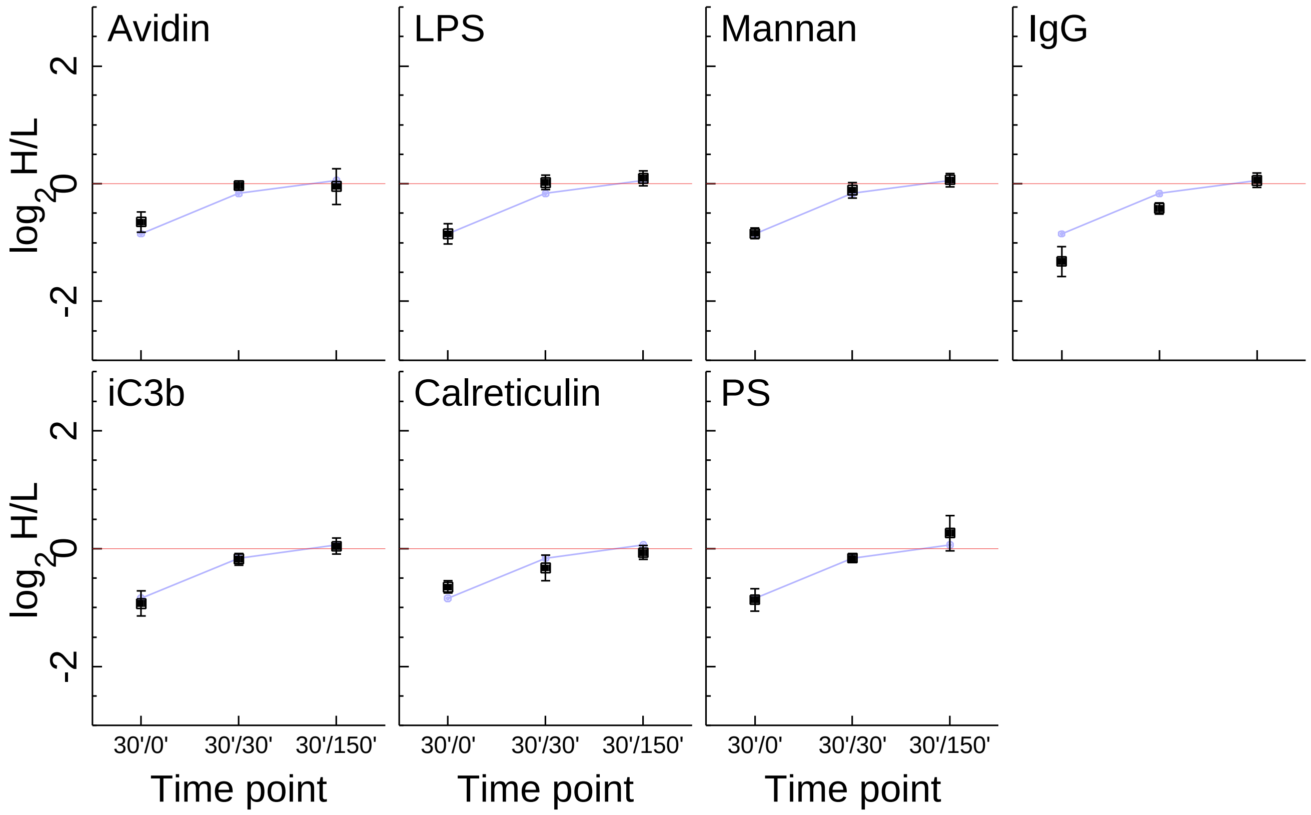

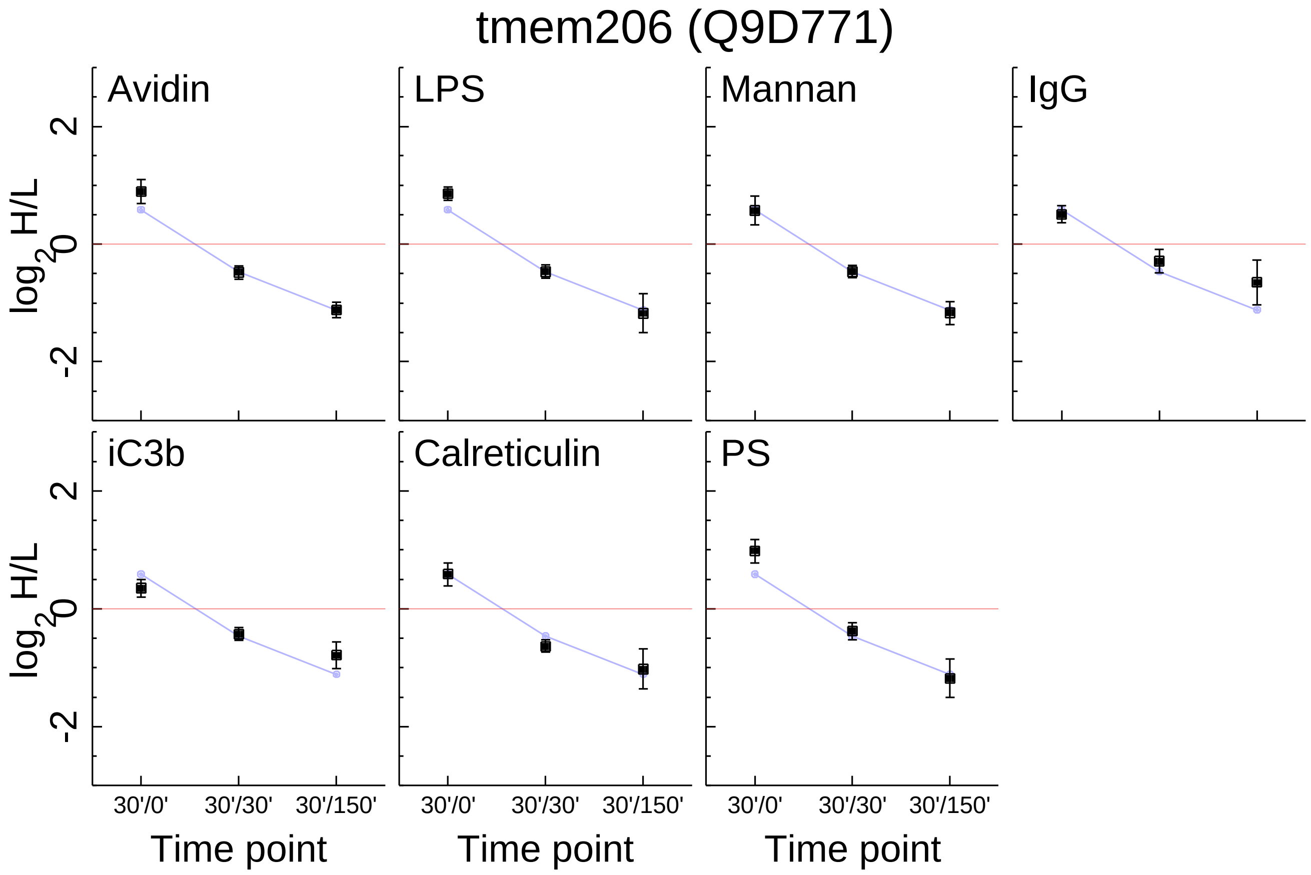
tmem206 (Q9D771)
Avidin
LPS
Mannan
IgG
iC3b
Calreticulin
PS
log2 H/L
2
0
-2
30'/0'
30'/30'
30'/150'
Time point

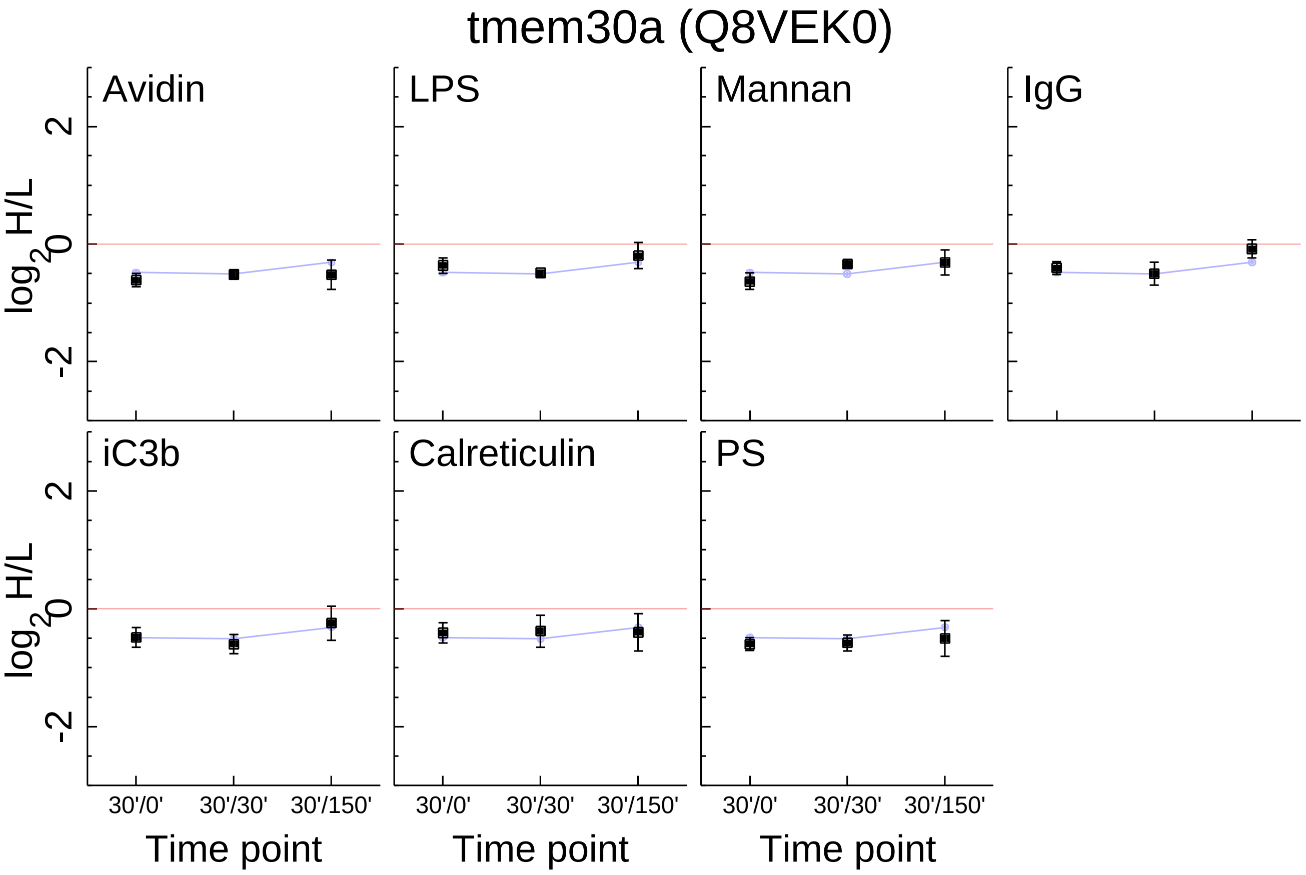
tmem30a (Q8VEK0)
Avidin
LPS
Mannan
IgG
iC3b
Calreticulin
PS
log2 H/L
2
0
-2
30'/0'
30'/30'
30'/150'
Time point

tmem43 (Q9DBS1)

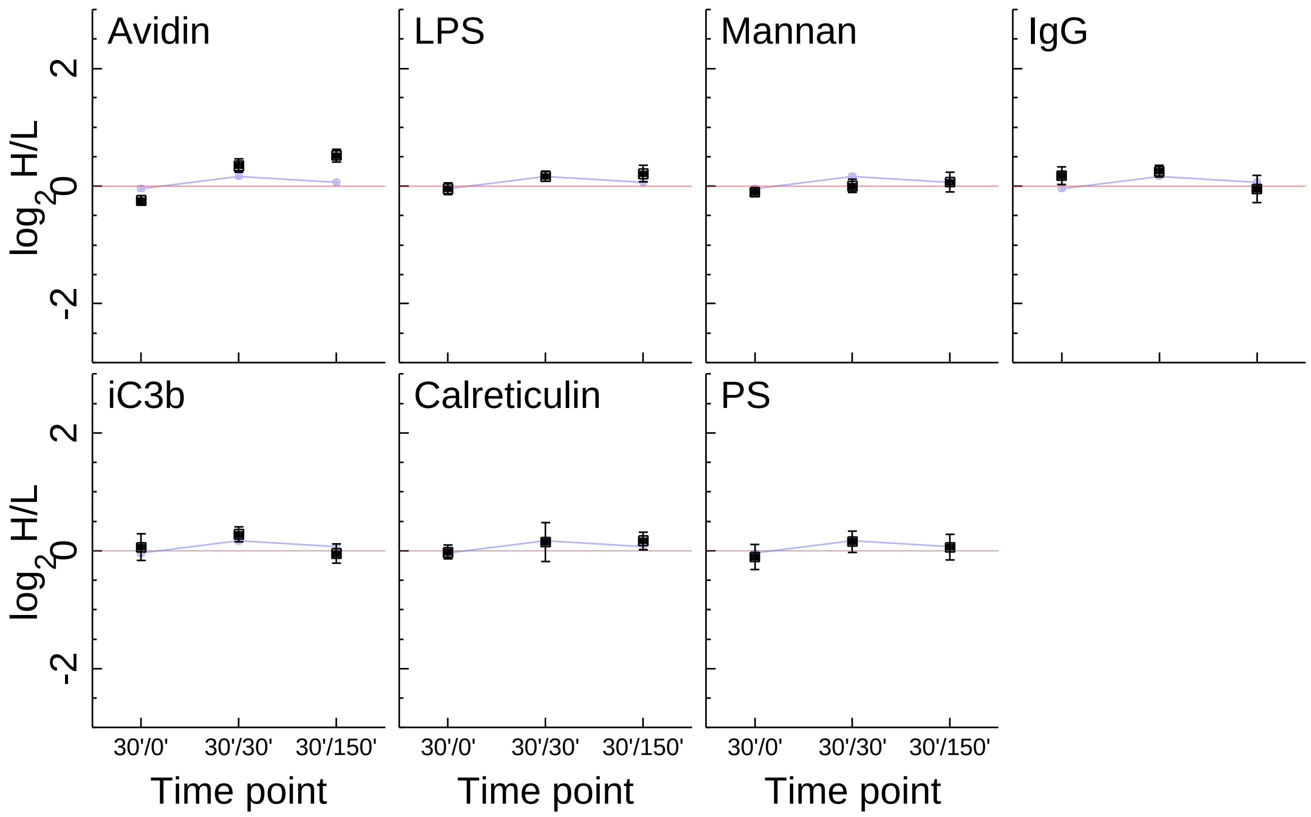

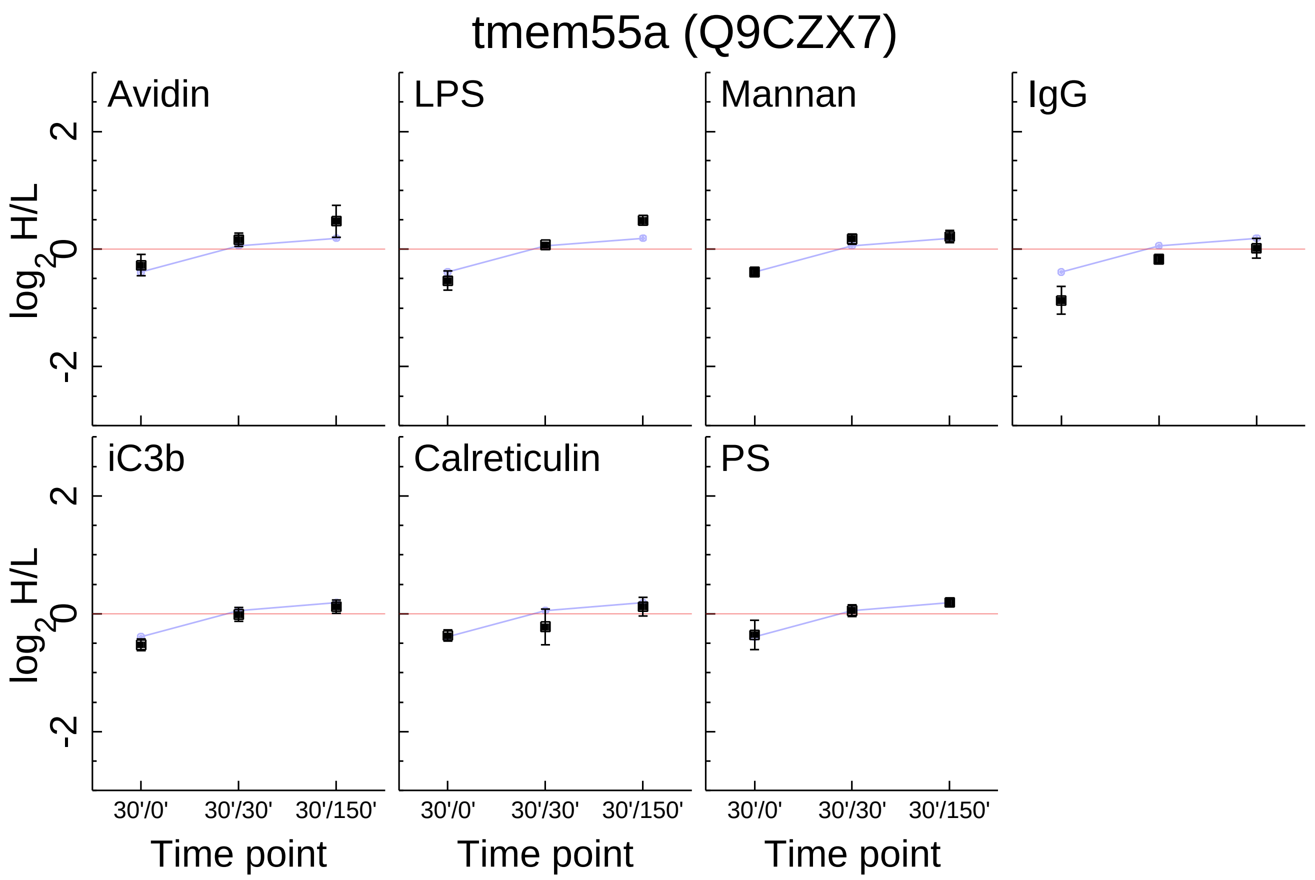

tmem55a (Q9CZX7)
Avidin
LPS
Mannan
IgG
iC3b
Calreticulin
PS
log2 H/L
2
0
-2
30'/0'
30'/30'
30'/150'
Time point

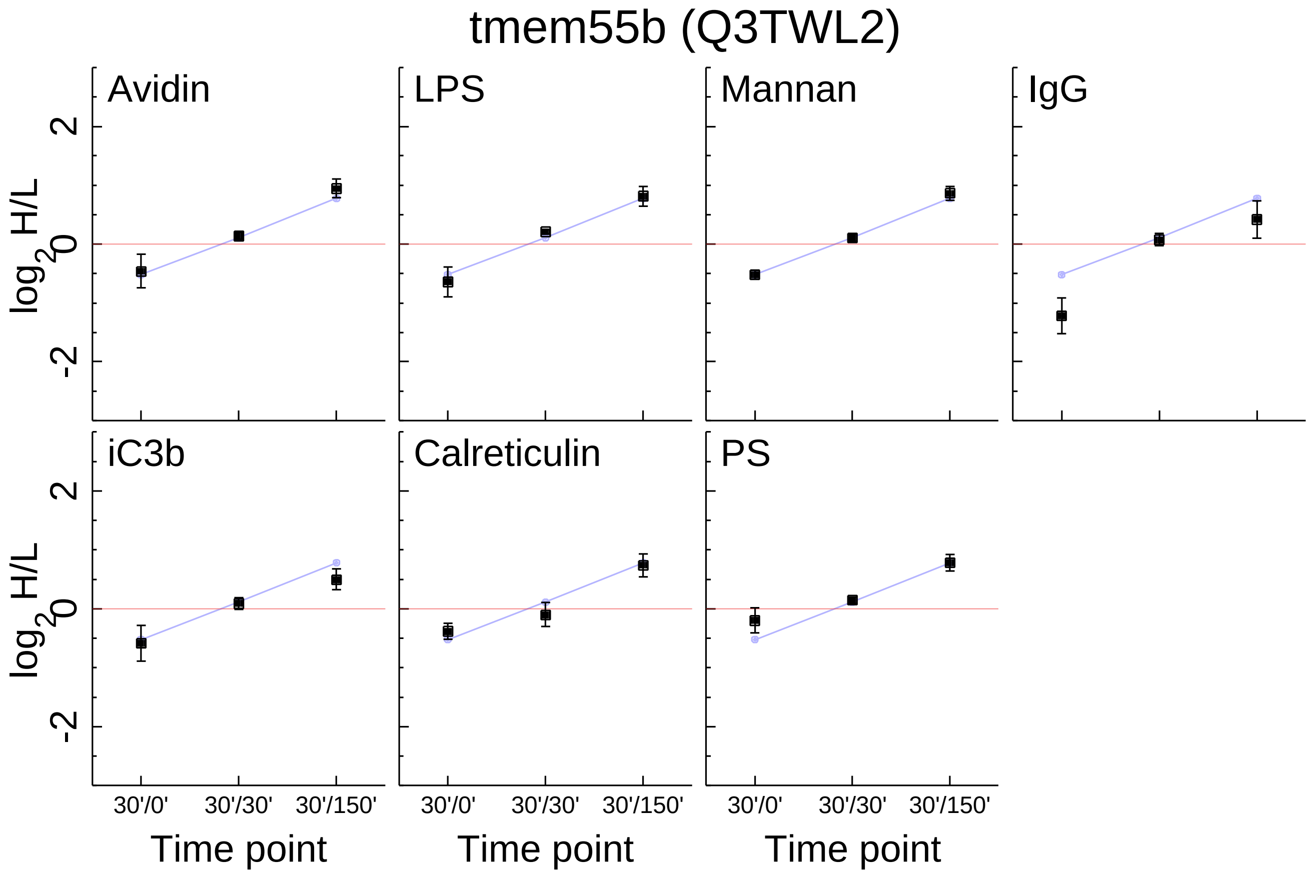
tmem55b (Q3TWL2)
Avidin
LPS
Mannan
IgG
iC3b
Calreticulin
PS
log2 H/L
2
0
-2
30'/0'
30'/30'
30'/150'
Time point

tmem63a (Q91YT8)

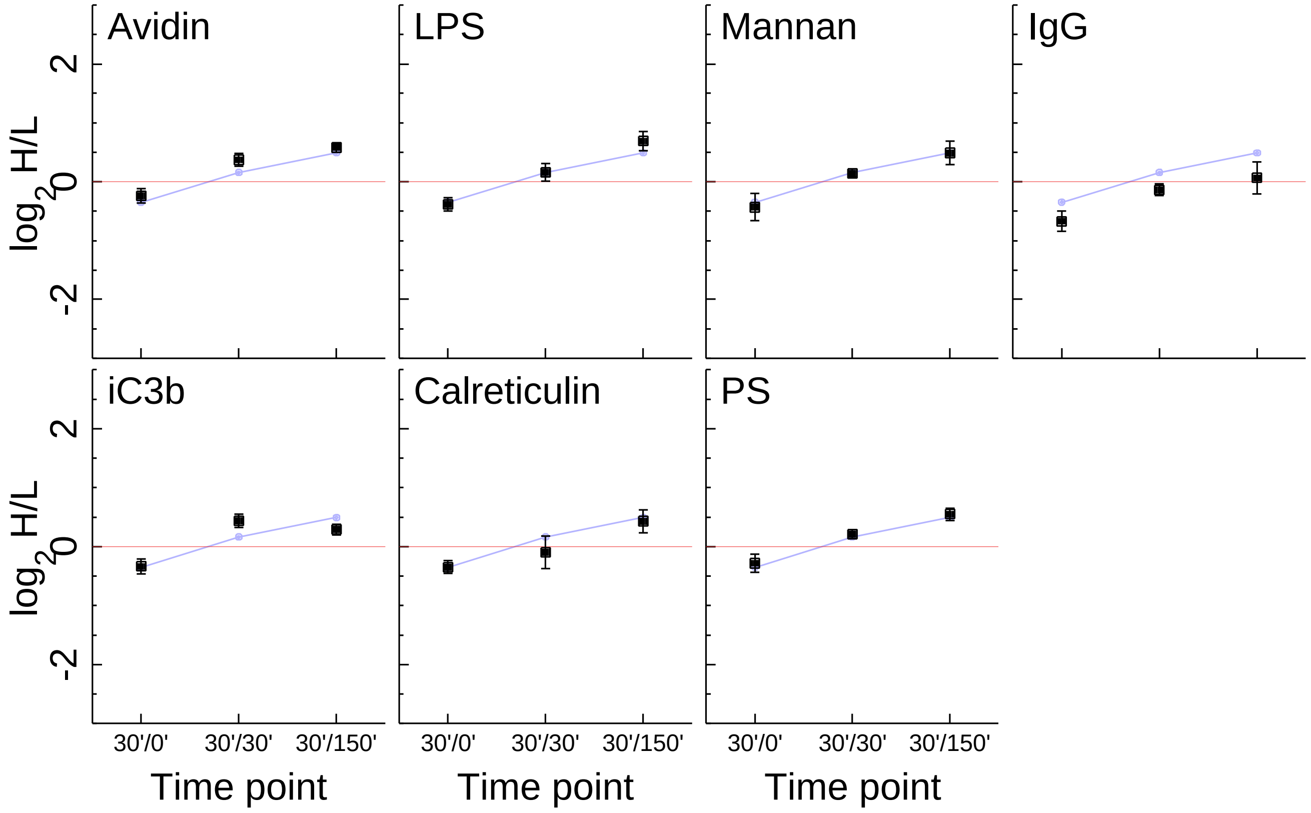

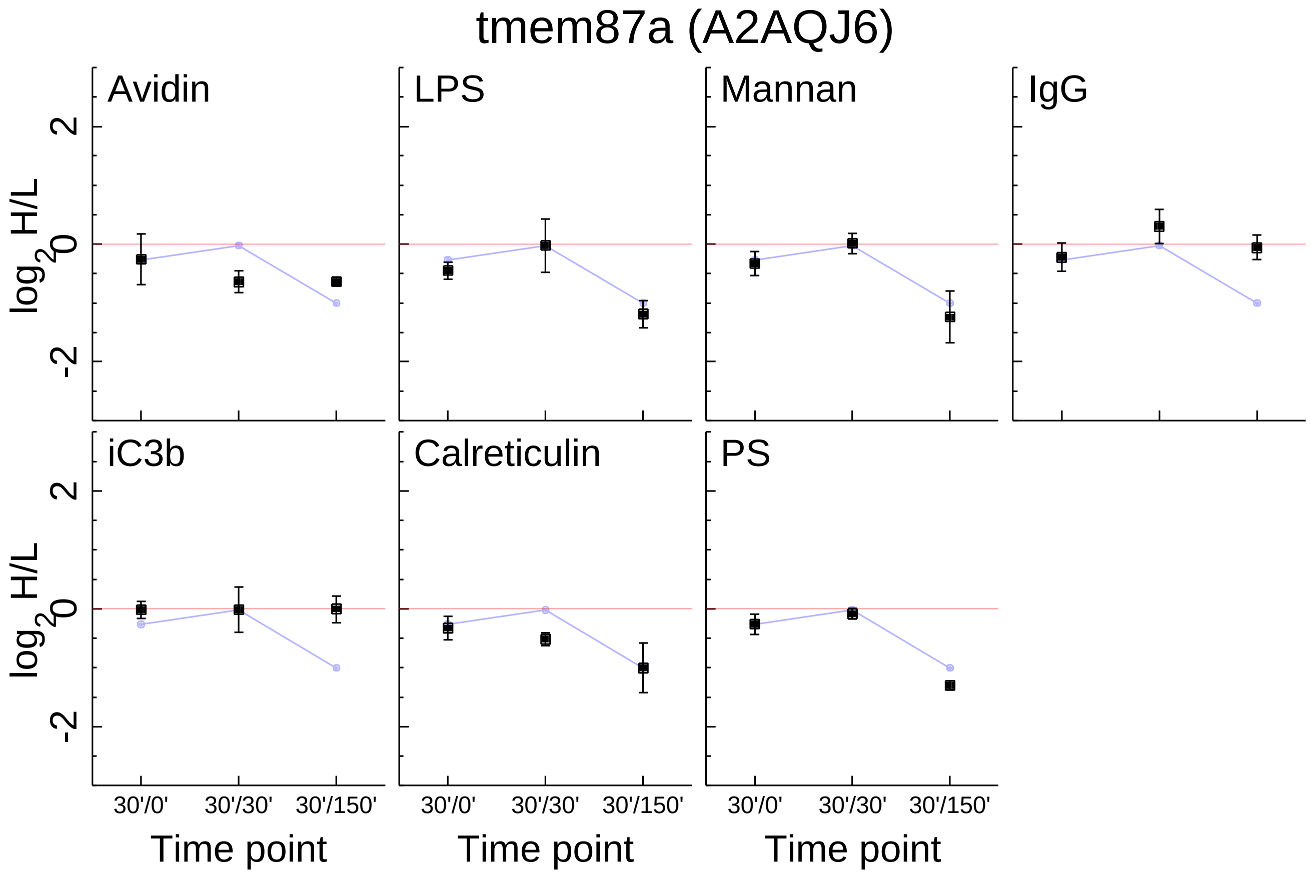
tmem87a (A2AQJ6)
Avidin
LPS
Mannan
IgG
iC3b
Calreticulin
PS
$\log_2$ H/L
2
0
-2
30'/0'
30'/30'
30'/150'
Time point

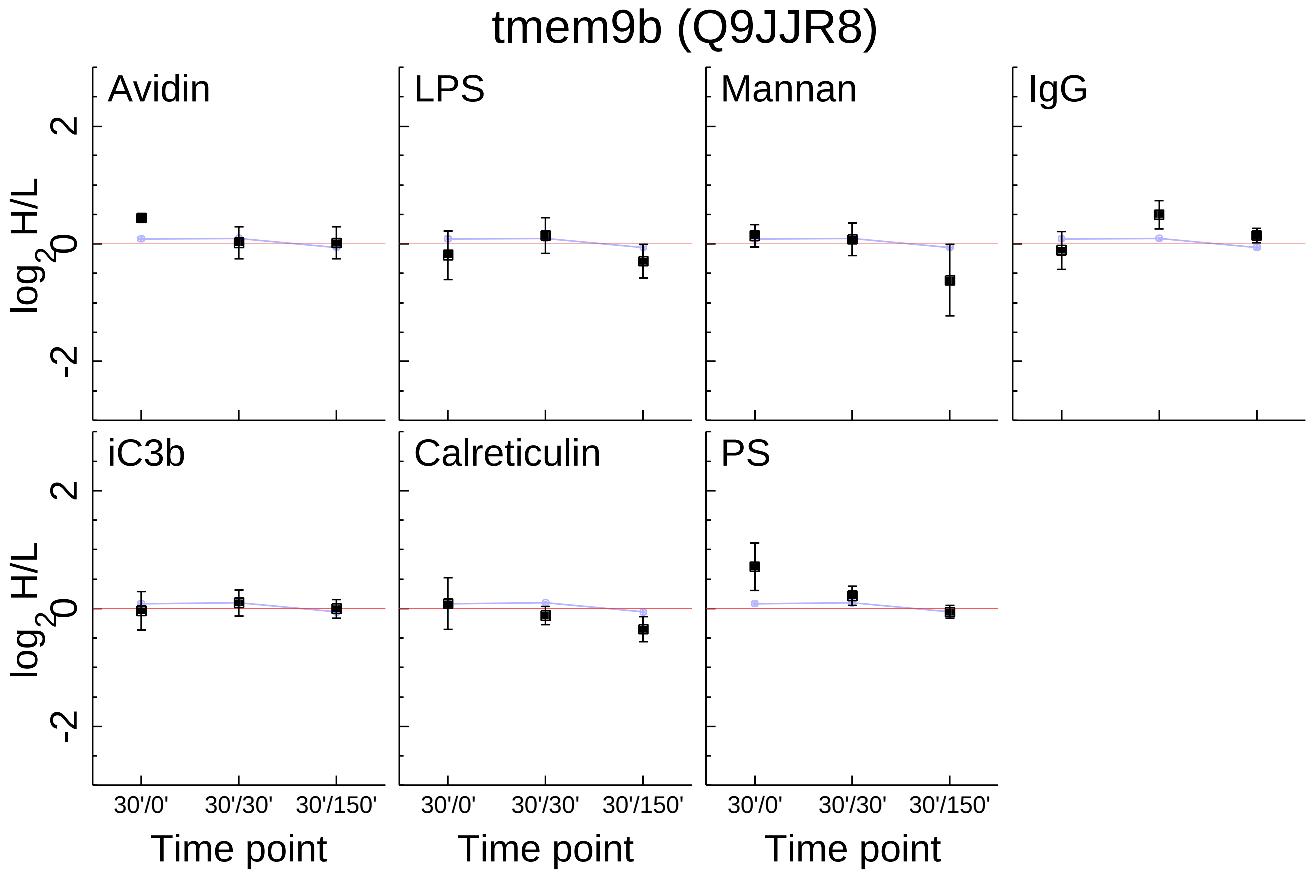
tmem9b (Q9JJR8)
Avidin
LPS
Mannan
IgG
iC3b
Calreticulin
PS
log2 H/L
2
0
-2
30'/0'
30'/30'
30'/150'
Time point

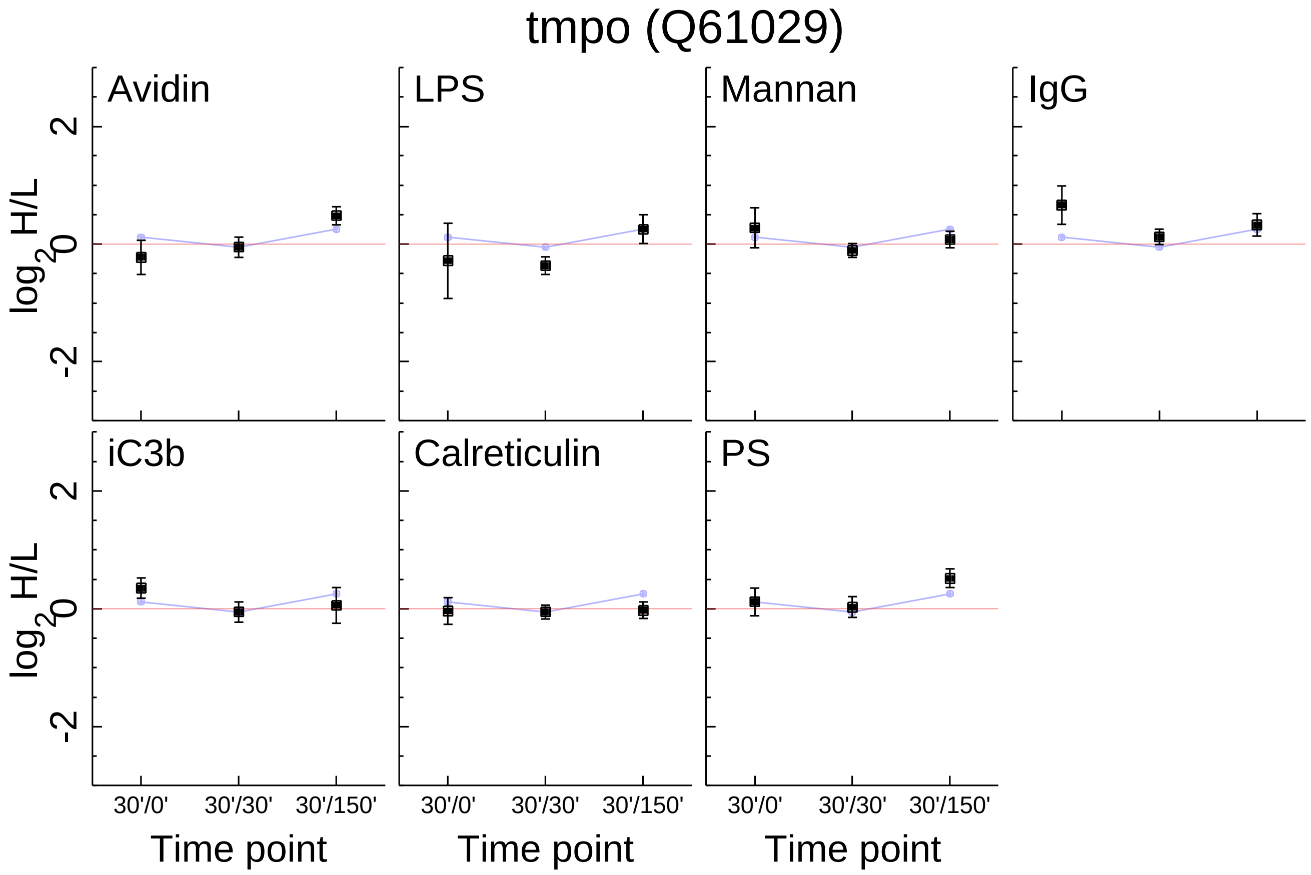

tmpo (Q61029)
Avidin
LPS
Mannan
IgG
iC3b
Calreticulin
PS
log2 H/L
2
0
-2
30'/0'
30'/30'
30'/150'
Time point

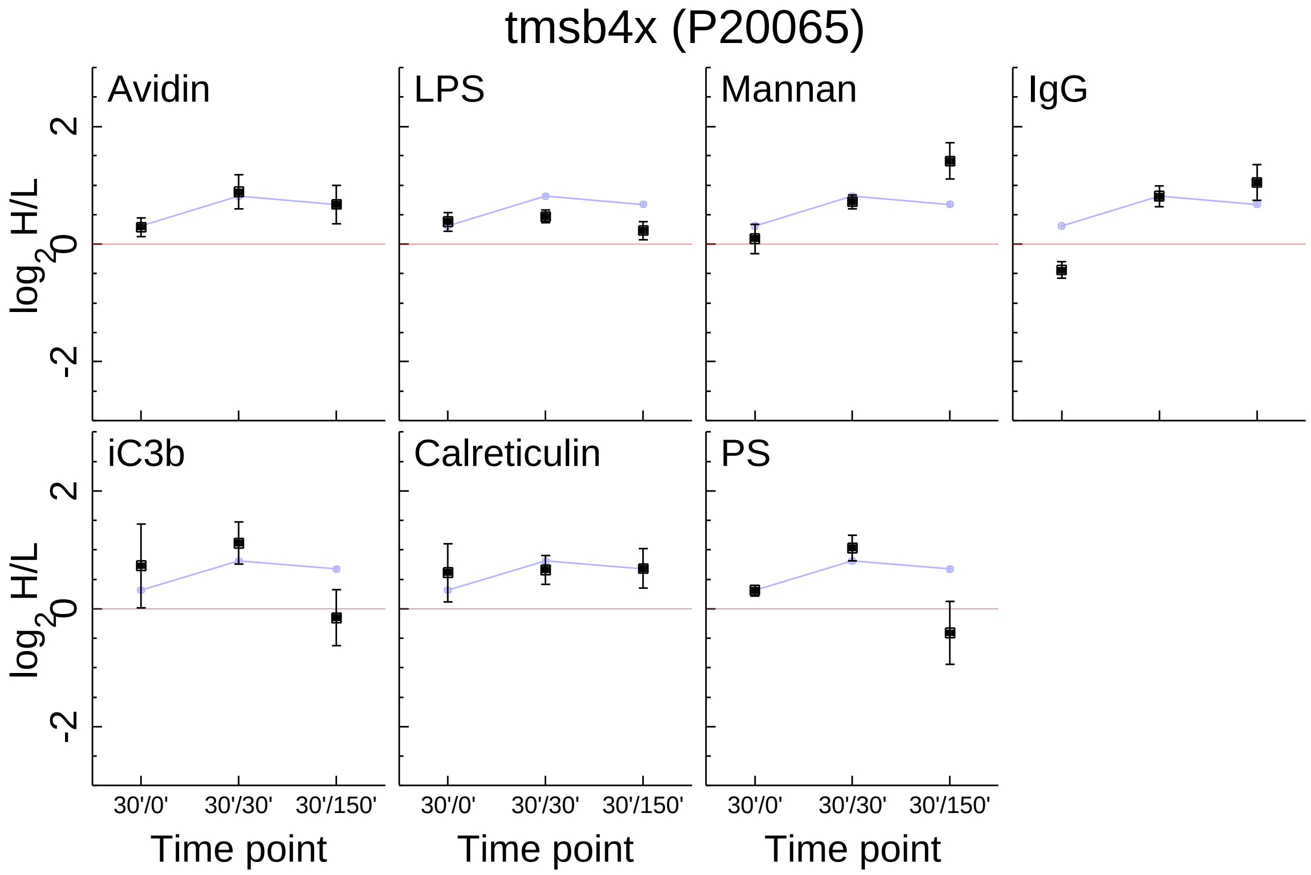
tmsb4x (P20065)
Avidin
LPS
Mannan
IgG
iC3b
Calreticulin
PS
log2 H/L
2
0
-2
30'/0'
30'/30'
30'/150'
Time point

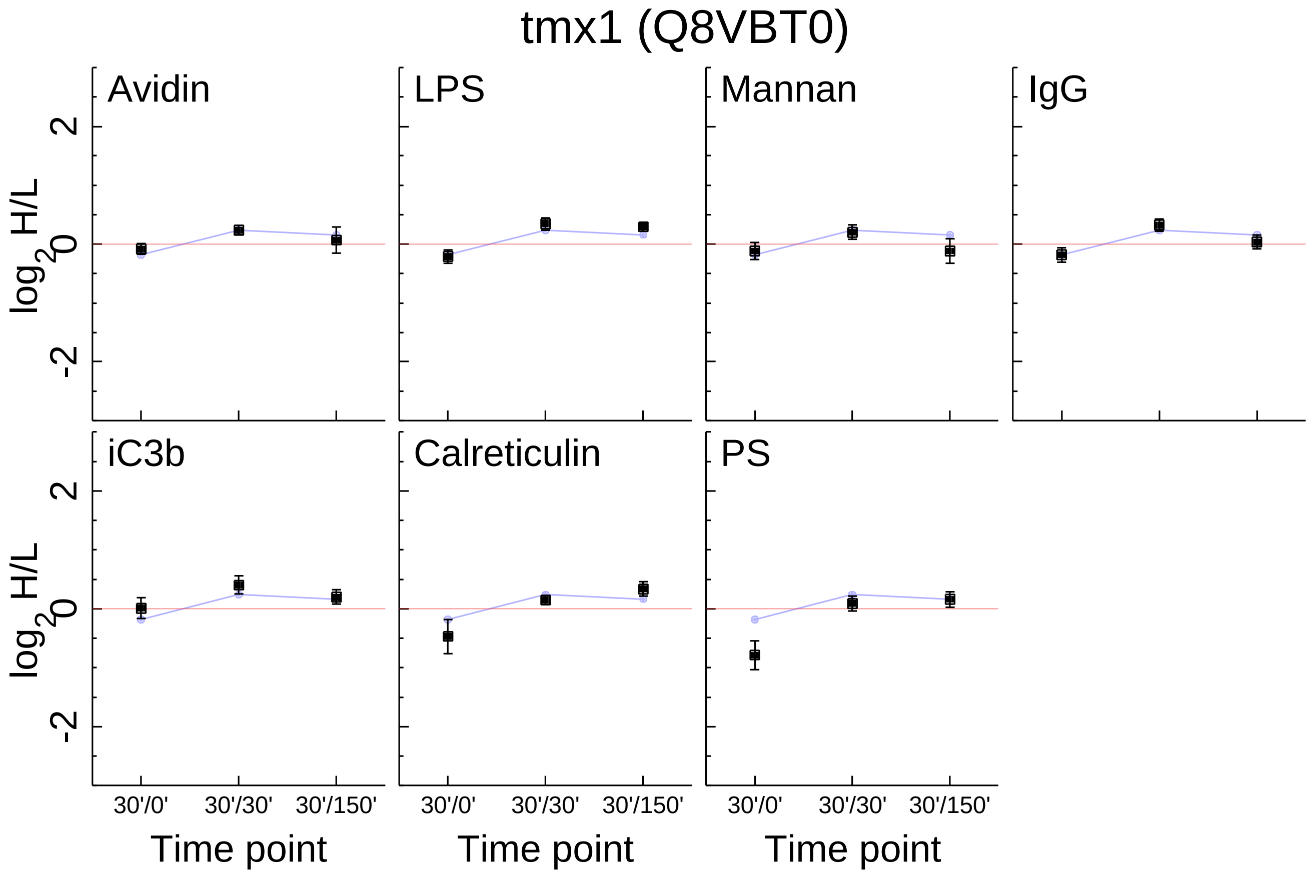
tmx1 (Q8VBT0)
Avidin
LPS
Mannan
IgG
iC3b
Calreticulin
PS
log2 H/L
2
0
-2
30'/0'
30'/30'
30'/150'
Time point

tmx2 (Q9D710)

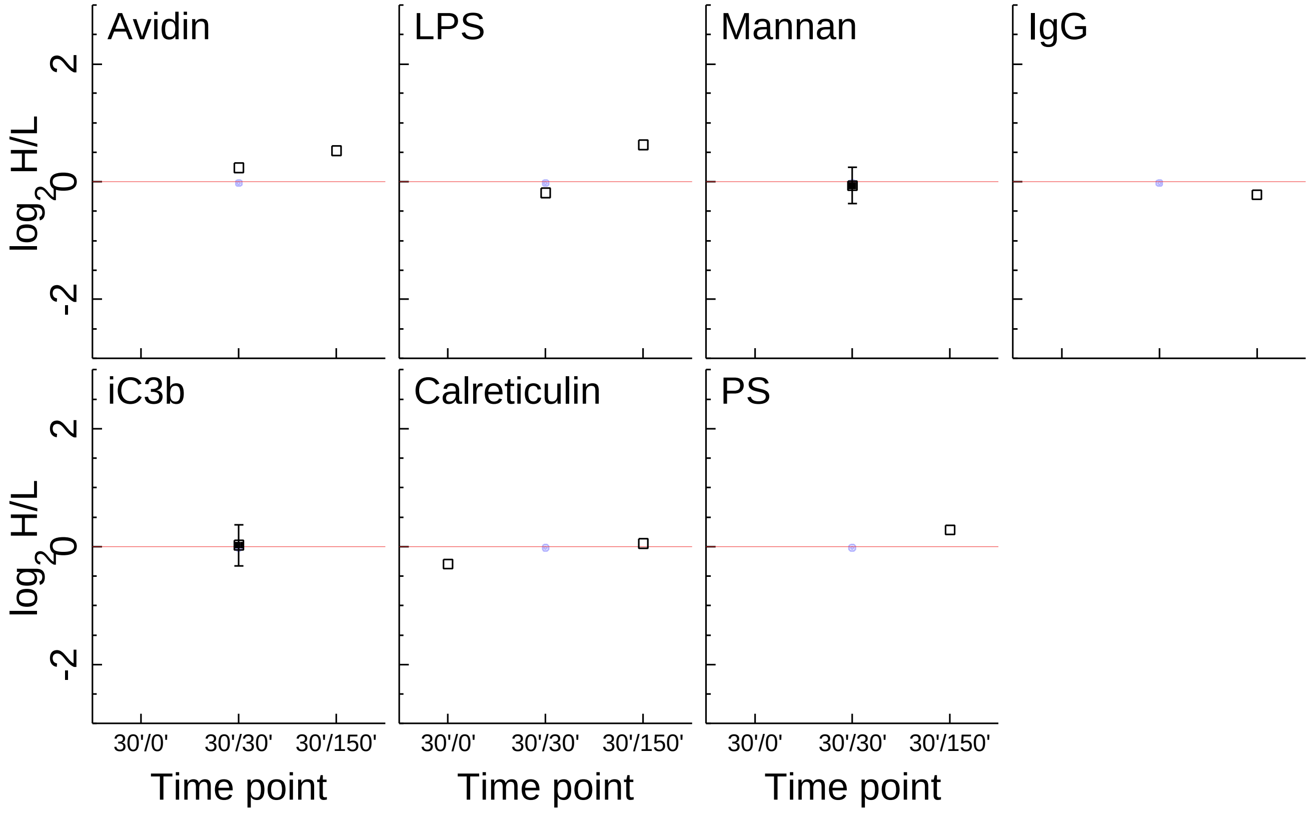
Avidin
LPS
Mannan
IgG
iC3b
Calreticulin
PS
log2 H/L
2
0
-2
30'/0'
30'/30'
30'/150'
Time point

tmx3 (Q8BXZ1)

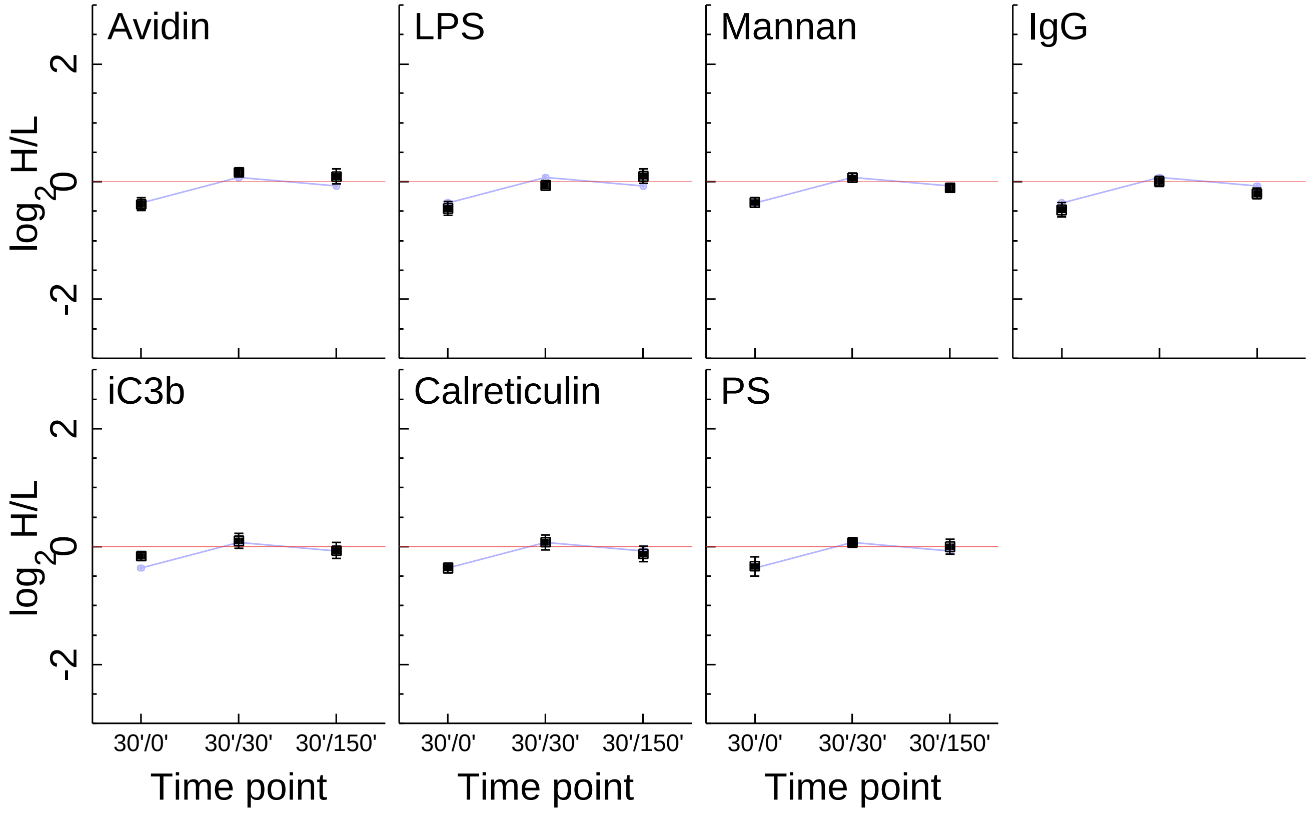

Avidin
LPS
Mannan
IgG
iC3b
Calreticulin
PS
log2 H/L
2
0
-2
30'/0'
30'/30'
30'/150'
Time point

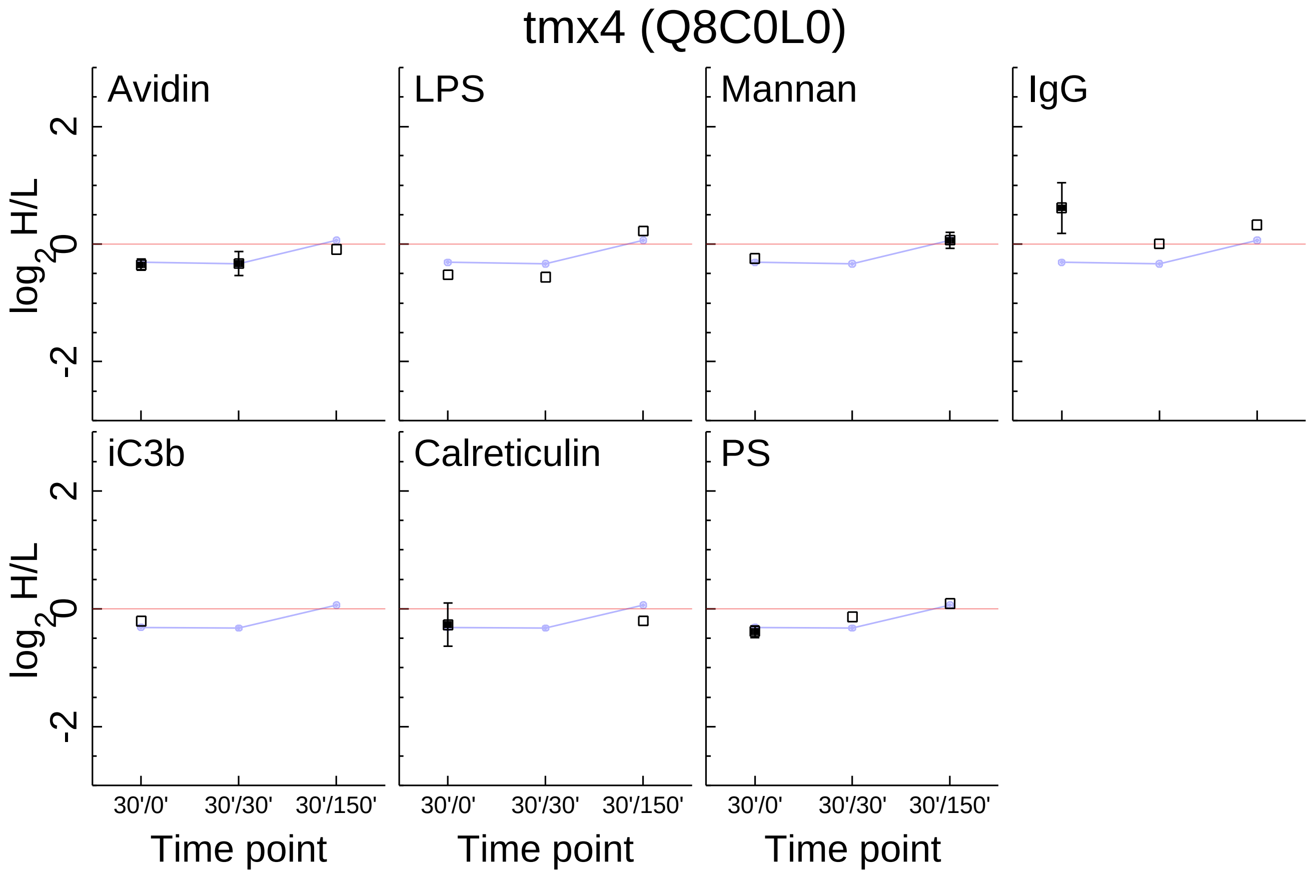
tmx4 (Q8C0L0)
Avidin
LPS
Mannan
IgG
iC3b
Calreticulin
PS
log2 H/L
2
0
-2
30'/0'
30'/30'
30'/150'
Time point

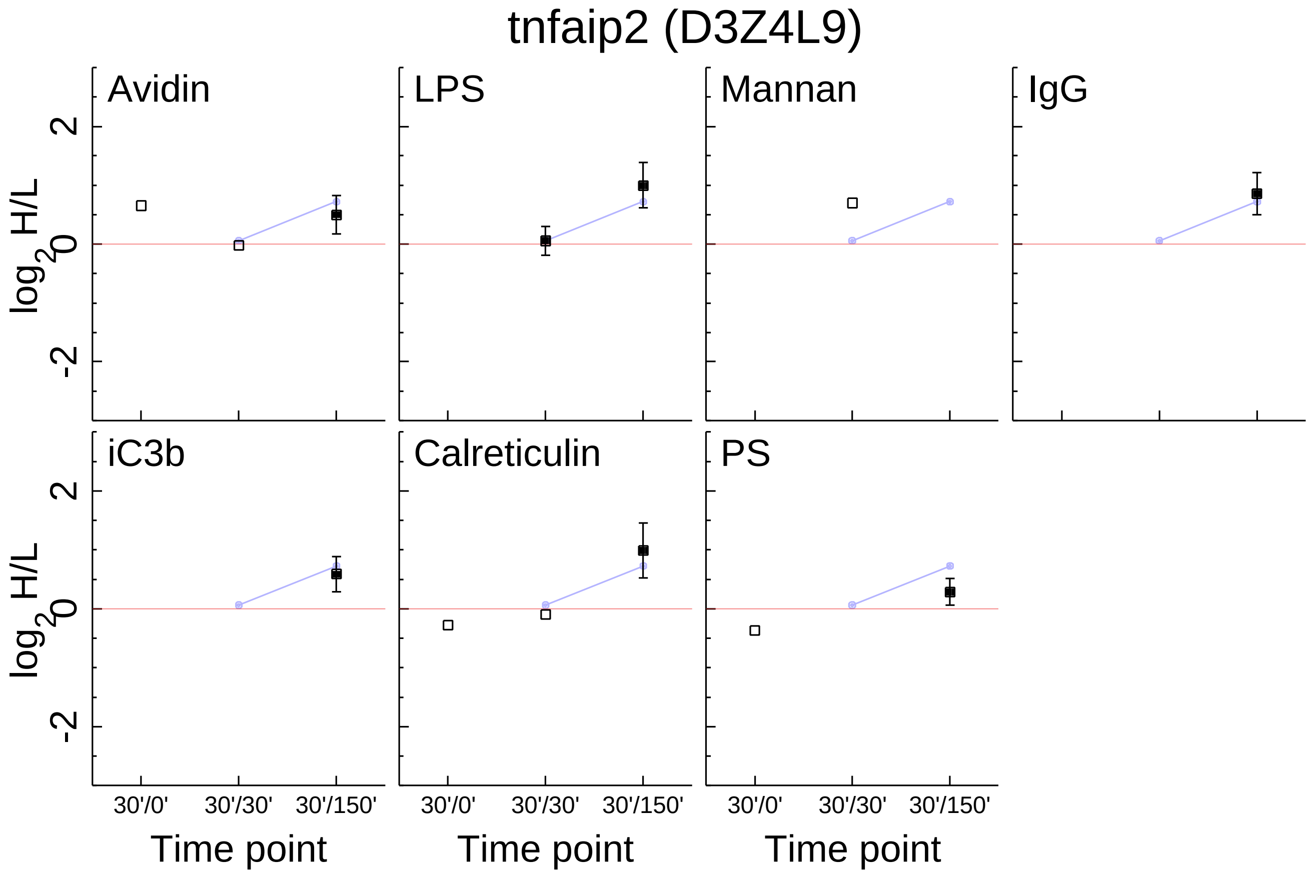
tnfaip2 (D3Z4L9)
Avidin
LPS
Mannan
IgG
iC3b
Calreticulin
PS
log2 H/L
2
0
-2
30'/0'
30'/30'
30'/150'
Time point

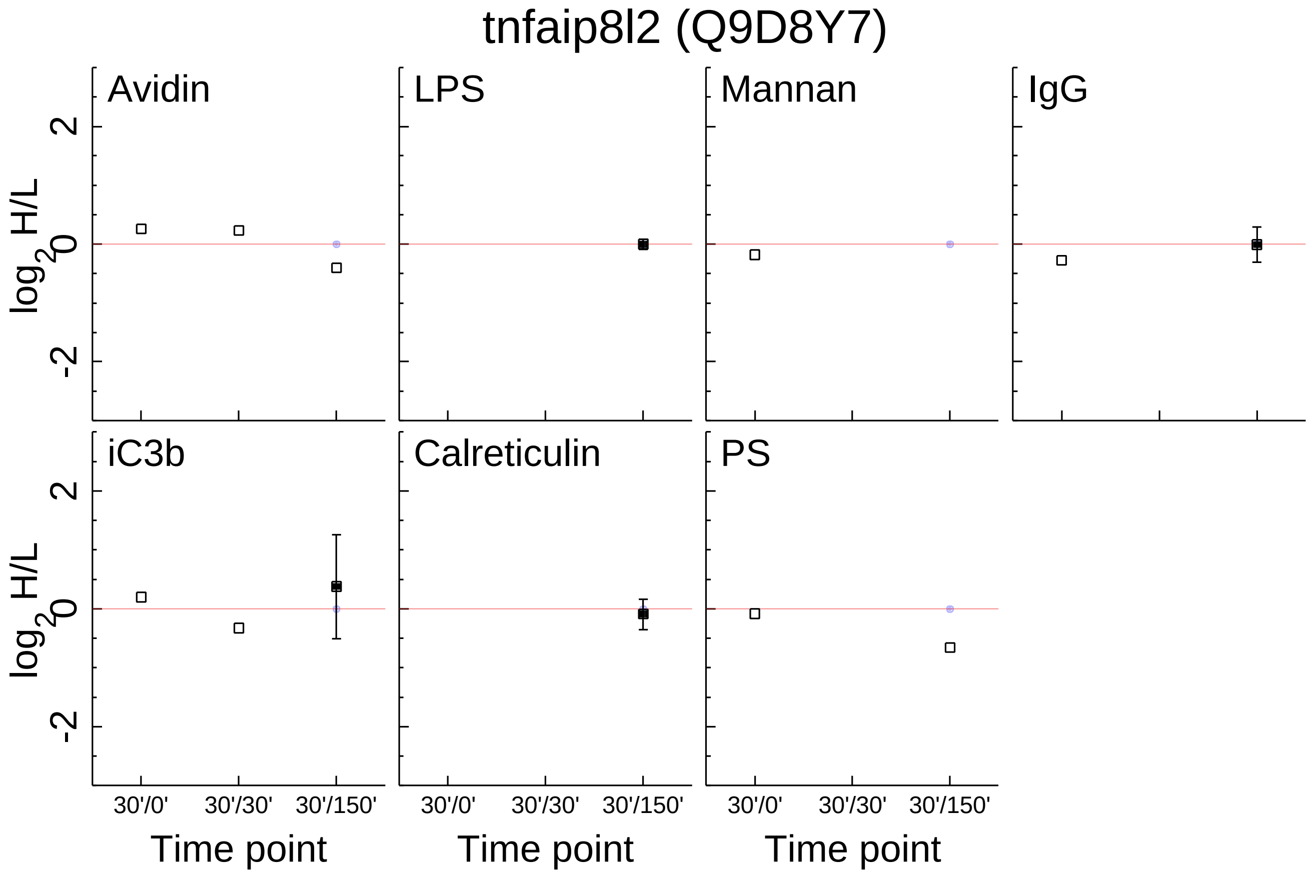

tnfaip8l2 (Q9D8Y7)
Avidin
LPS
Mannan
IgG
iC3b
Calreticulin
PS
log2 H/L
2
0
-2
30'/0'
30'/30'
30'/150'
Time point

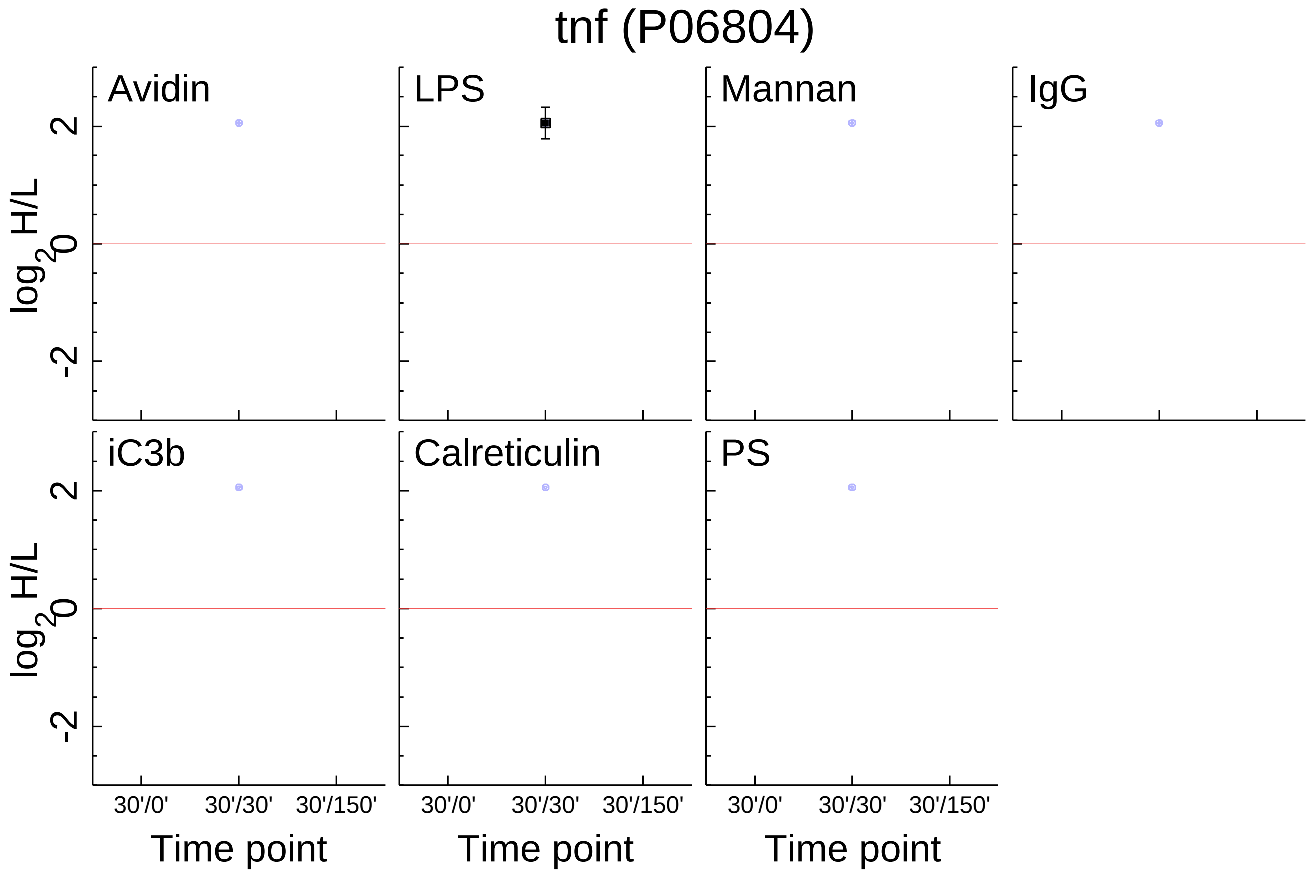
tnf (P06804)
Avidin
LPS
Mannan
IgG
iC3b
Calreticulin
PS
log2 H/L
2
0
-2
30'/0'
30'/30'
30'/150'
Time point

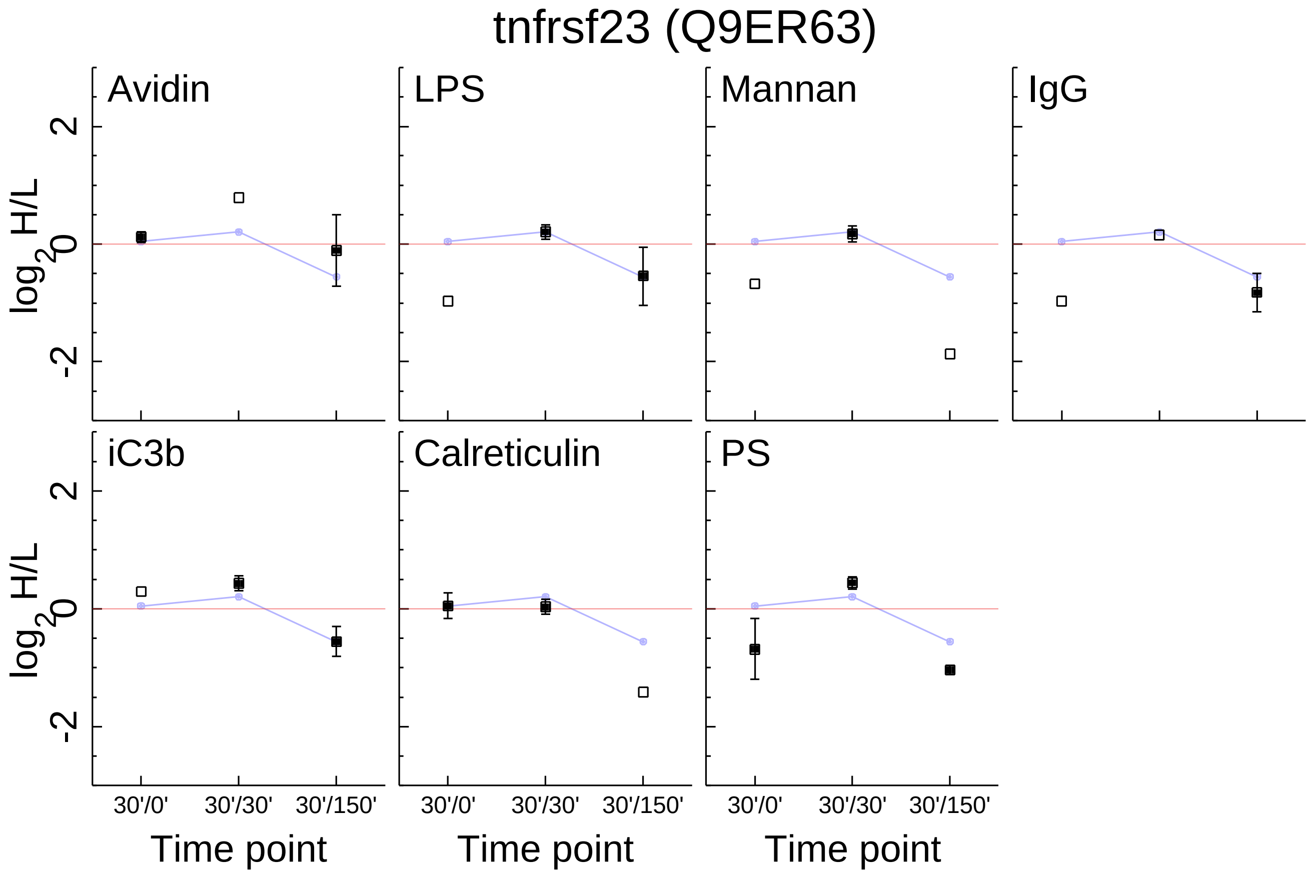
tnfrsf23 (Q9ER63)
Avidin
LPS
Mannan
IgG
iC3b
Calreticulin
PS
log2 H/L
2
0
-2
30'/0'
30'/30'
30'/150'
Time point

tns1 (E9Q0S6)

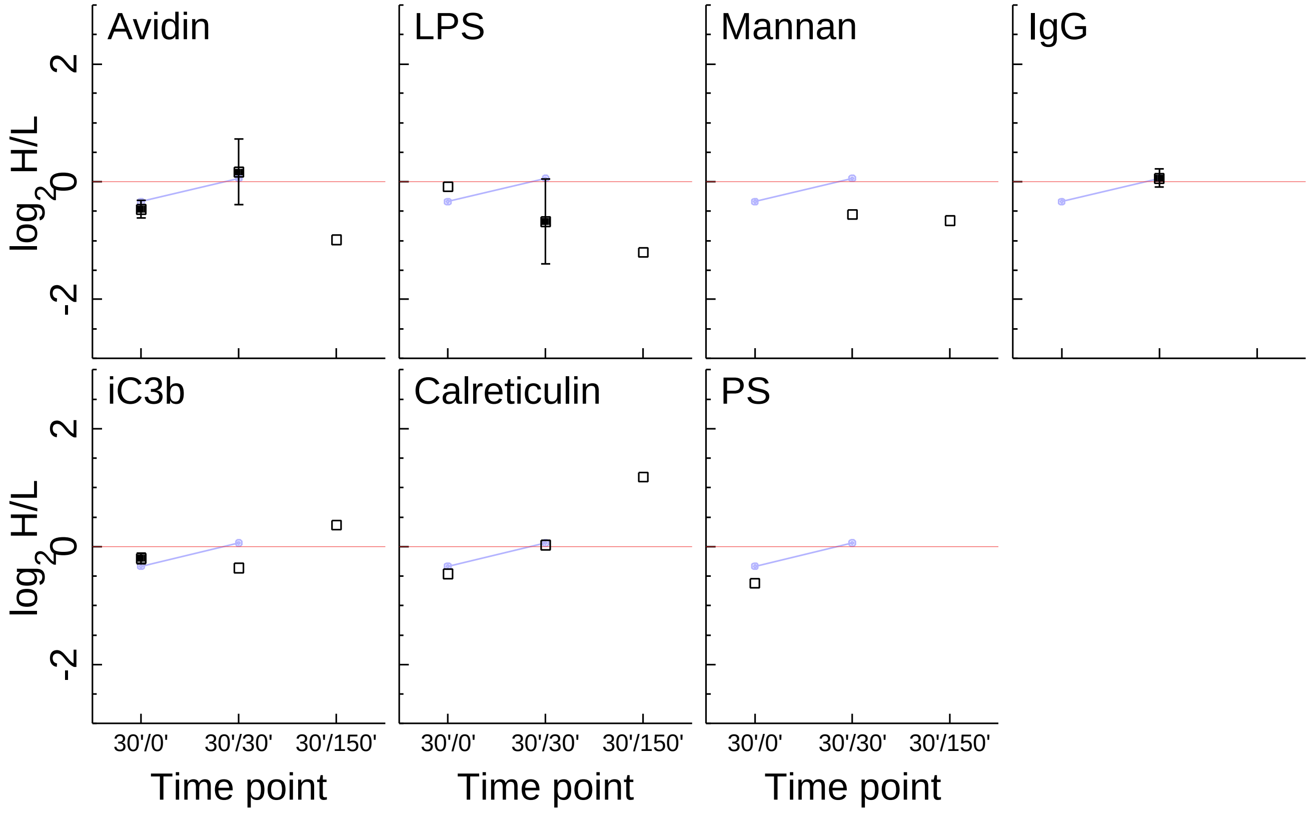

Avidin
LPS
Mannan
IgG
iC3b
Calreticulin
PS
log2 H/L
2
0
-2
30'/0'
30'/30'
30'/150'
Time point

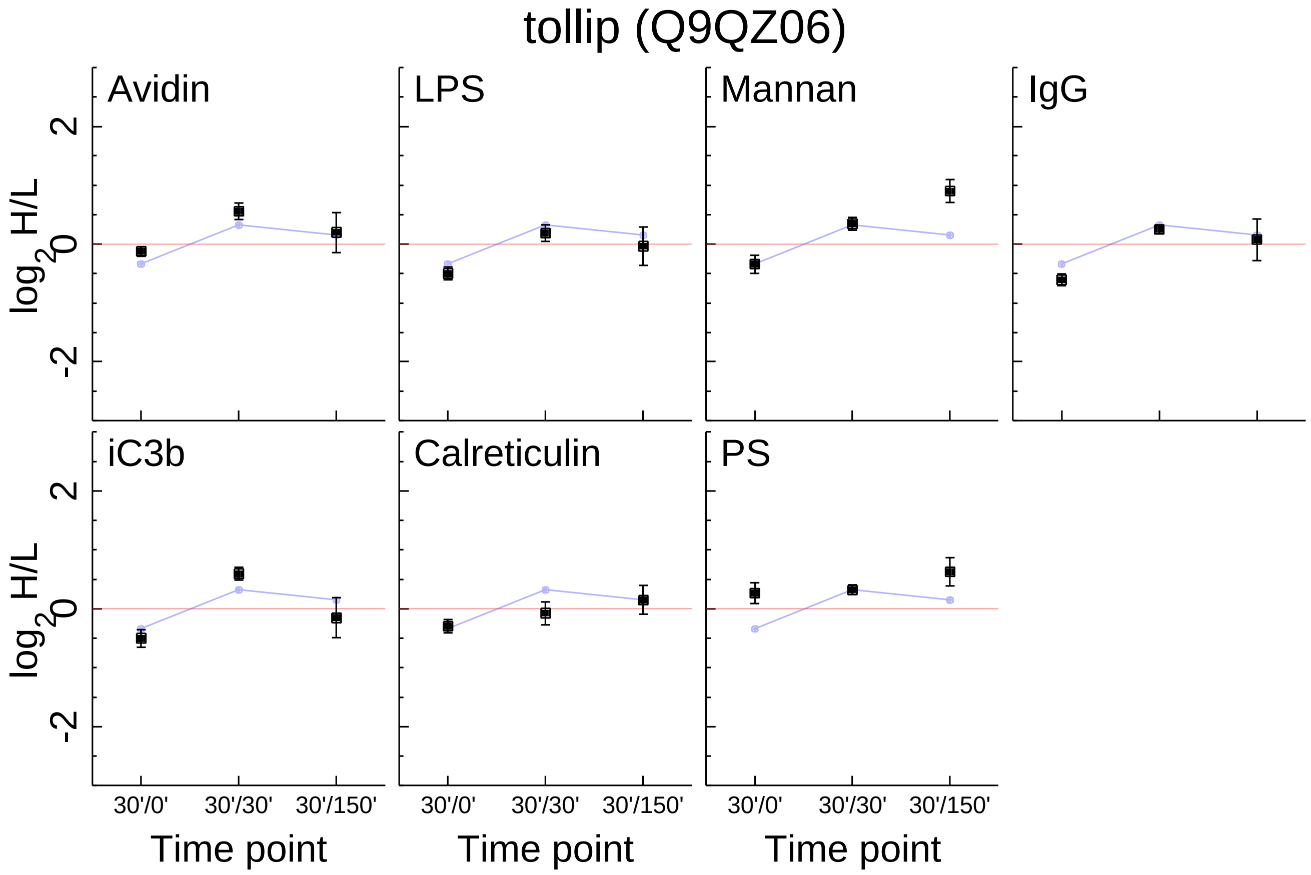
tollip (Q9QZ06)
Avidin
LPS
Mannan
IgG
iC3b
Calreticulin
PS
log2 H/L
2
0
-2
30'/0'
30'/30'
30'/150'
Time point

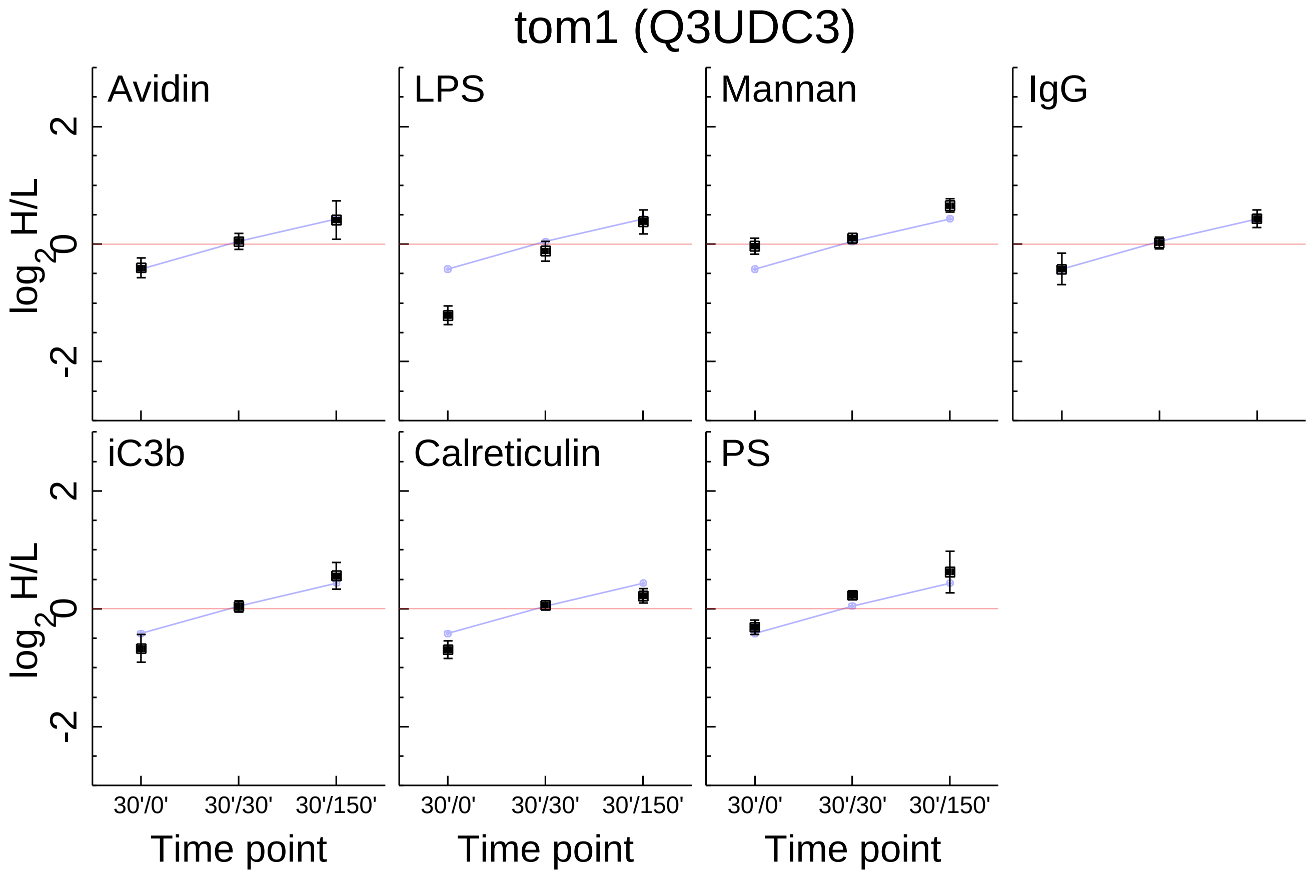

tom1 (Q3UDC3)
Avidin
LPS
Mannan
IgG
iC3b
Calreticulin
PS
log2 H/L
2
0
-2
30'/0'
30'/30'
30'/150'
Time point

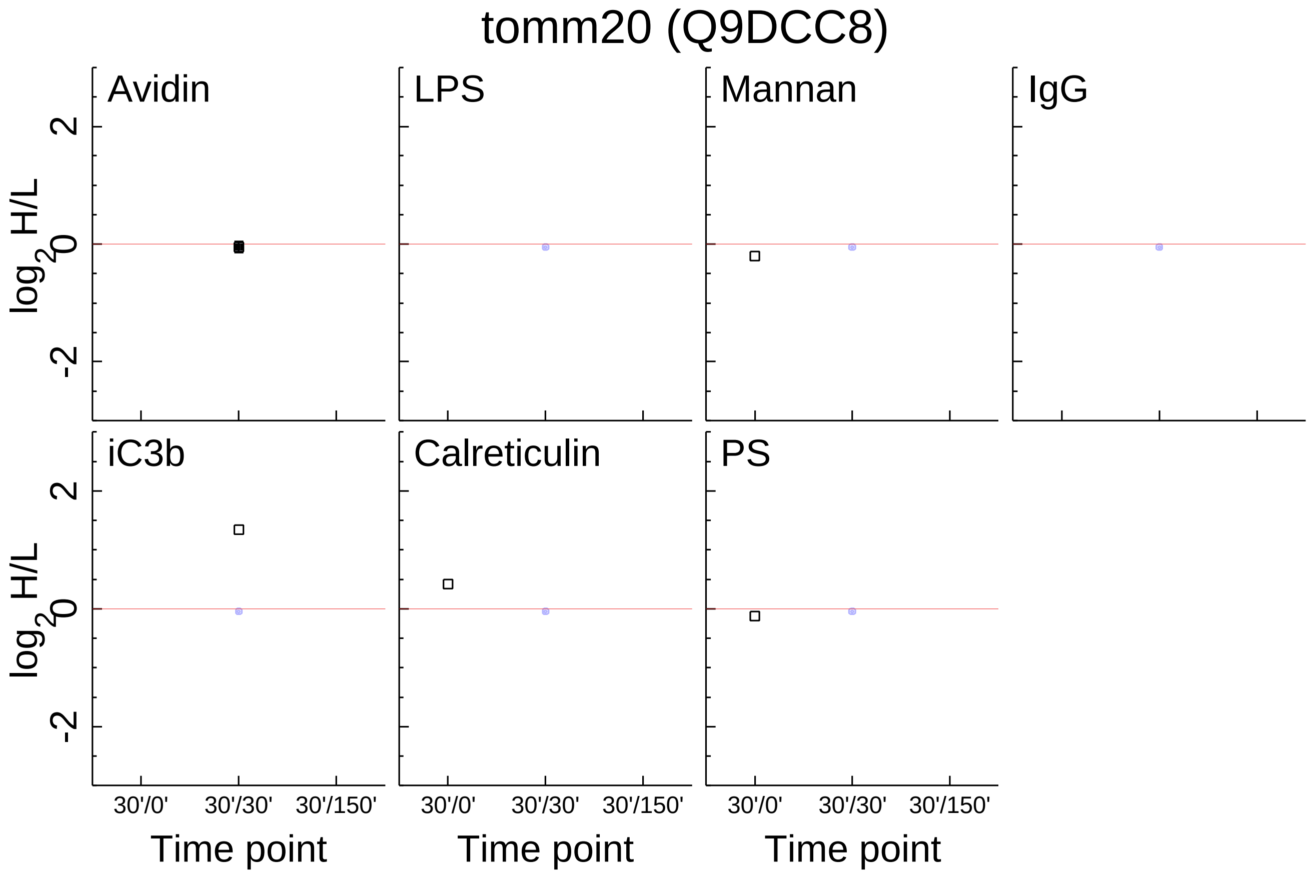
tomm20 (Q9DCC8)
Avidin
LPS
Mannan
IgG
iC3b
Calreticulin
PS
log2 H/L
2
0
-2
30'/0'
30'/30'
30'/150'
Time point

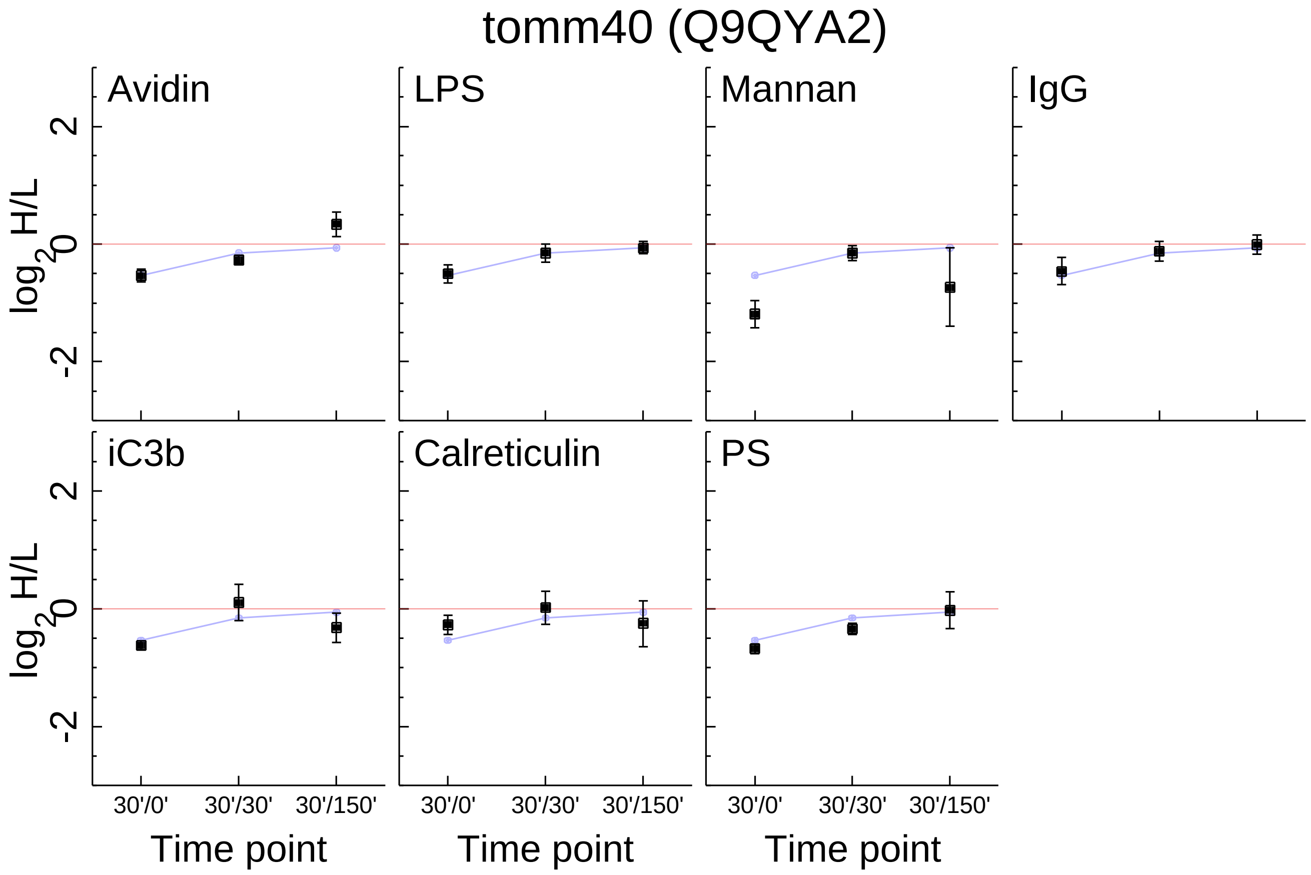
tomm40 (Q9QYA2)
Avidin
LPS
Mannan
IgG
iC3b
Calreticulin
PS
log2 H/L
2
0
-2
30'/0'
30'/30'
30'/150'
Time point

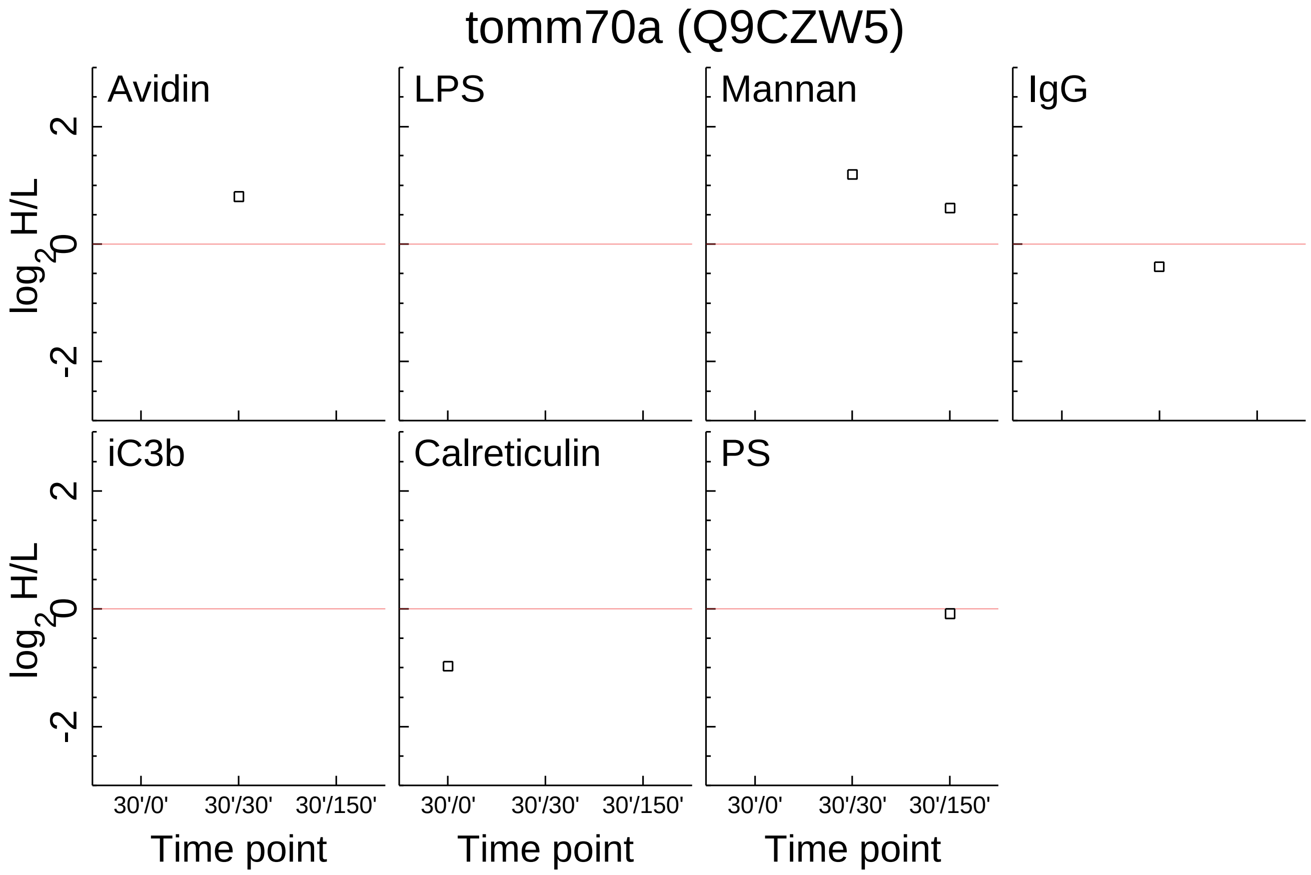

tomm70a (Q9CZW5)
Avidin
LPS
Mannan
IgG
iC3b
Calreticulin
PS
log2 H/L
2
0
-2
30'/0'
30'/30'
30'/150'
Time point

top2a (Q01320)

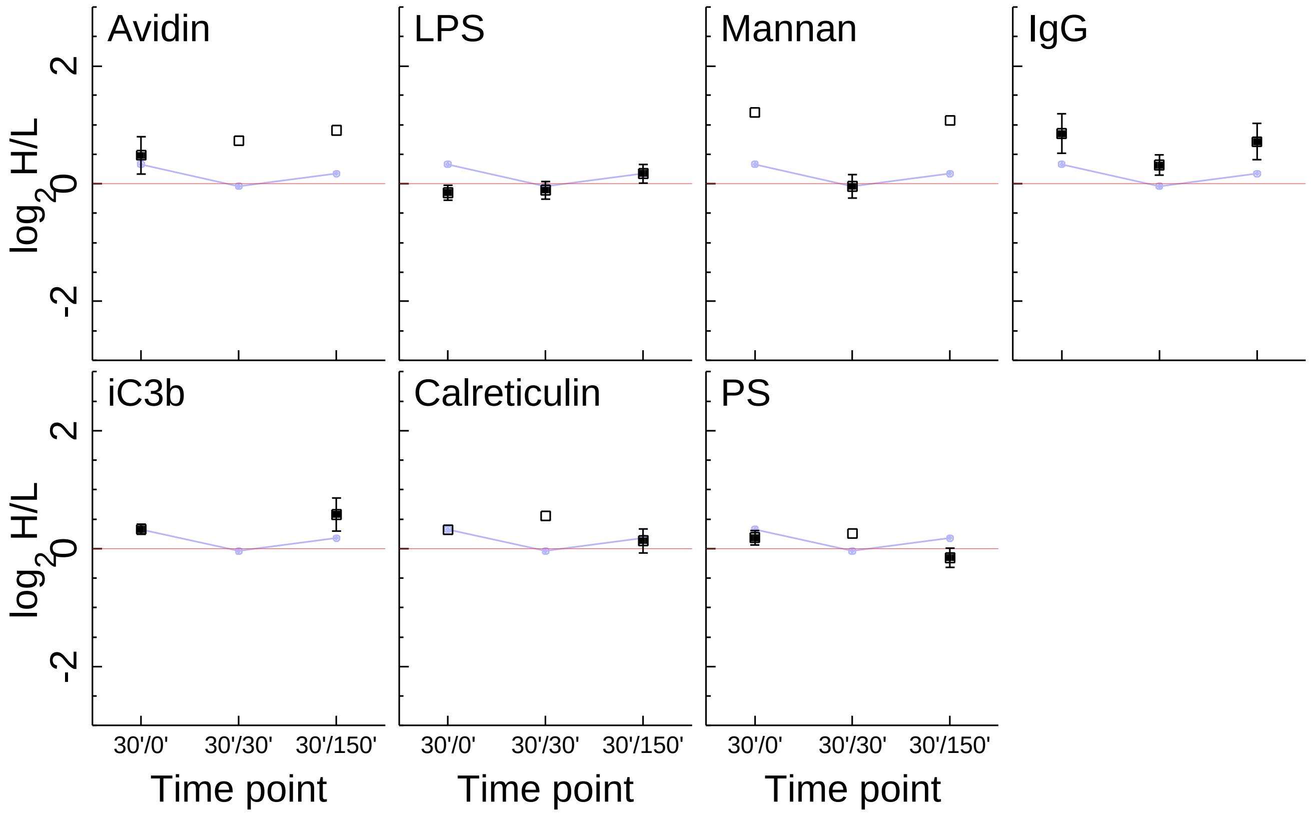

tor1aip1 (Q921T2)

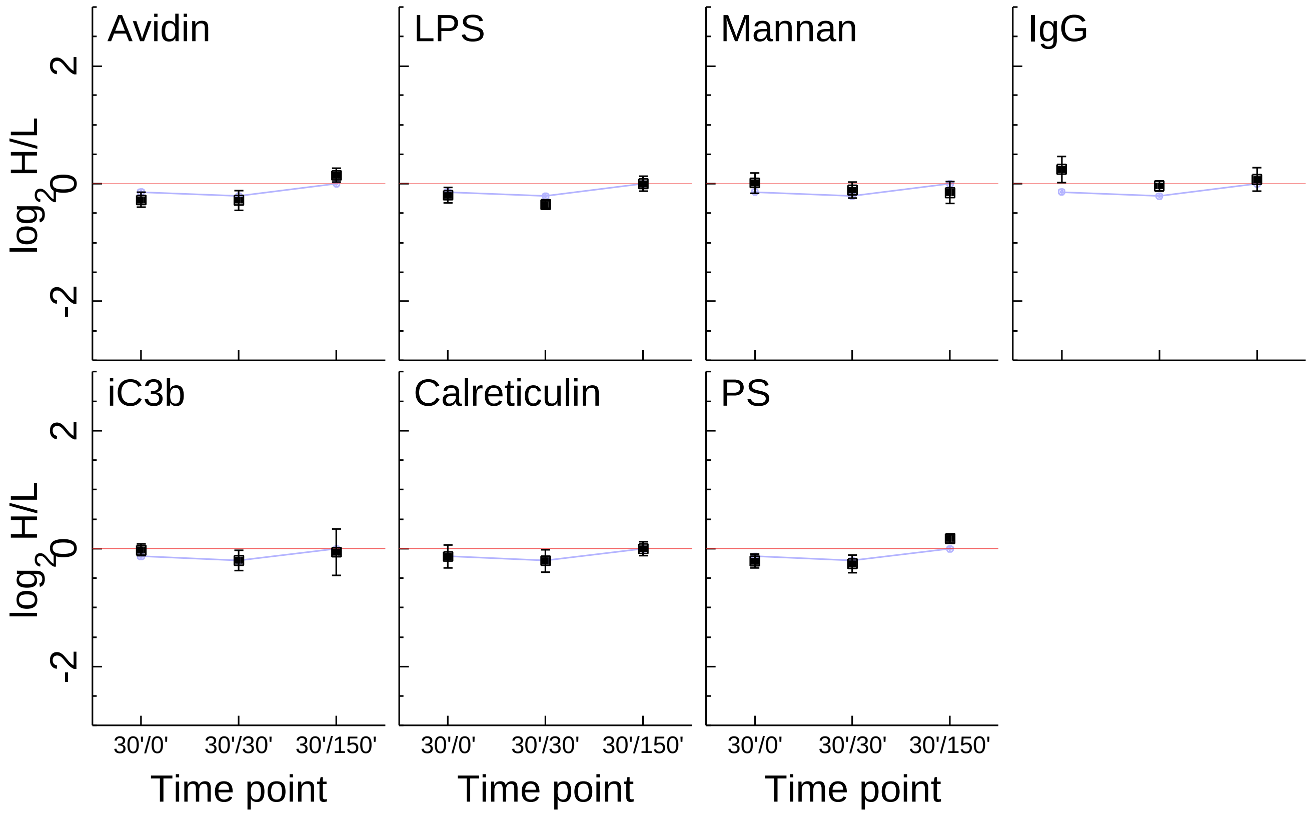

tor1aip2 (Q8BYU6)

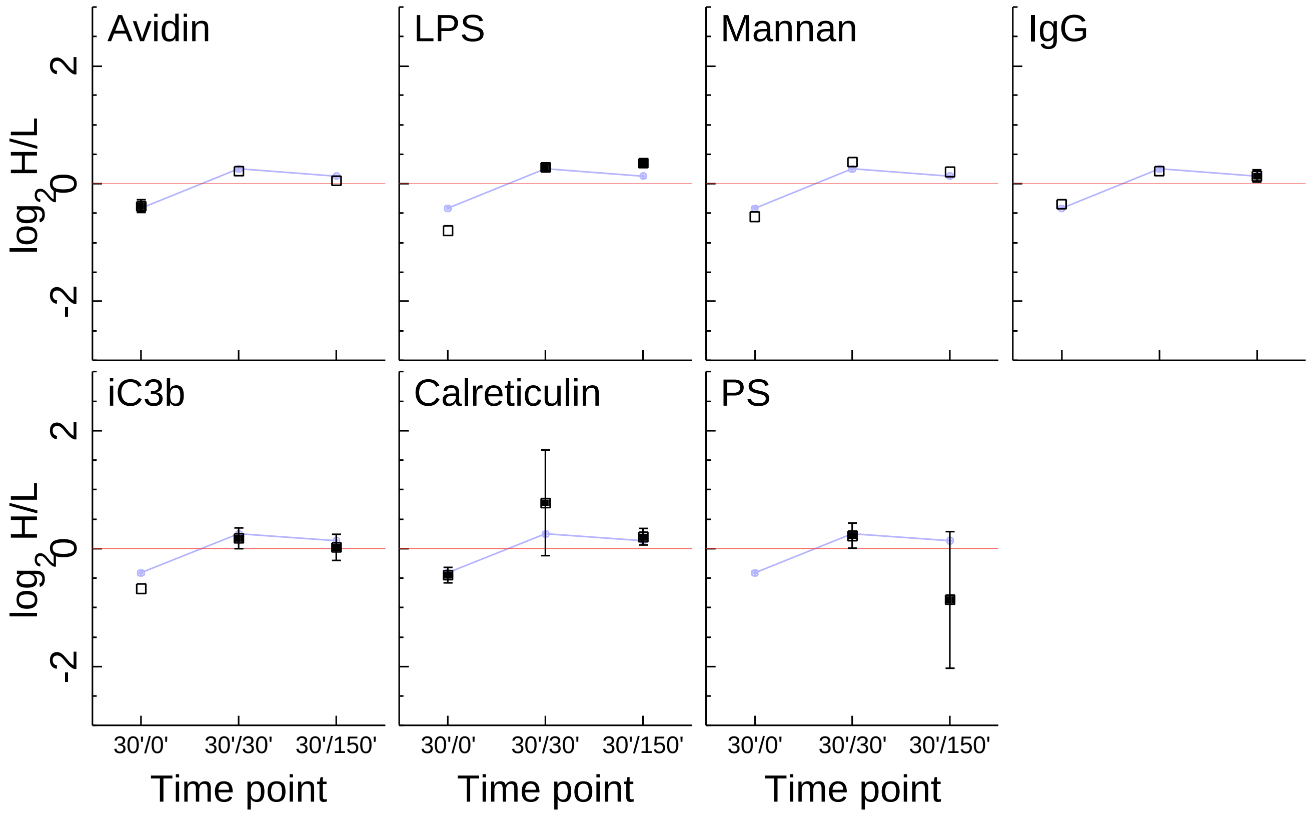

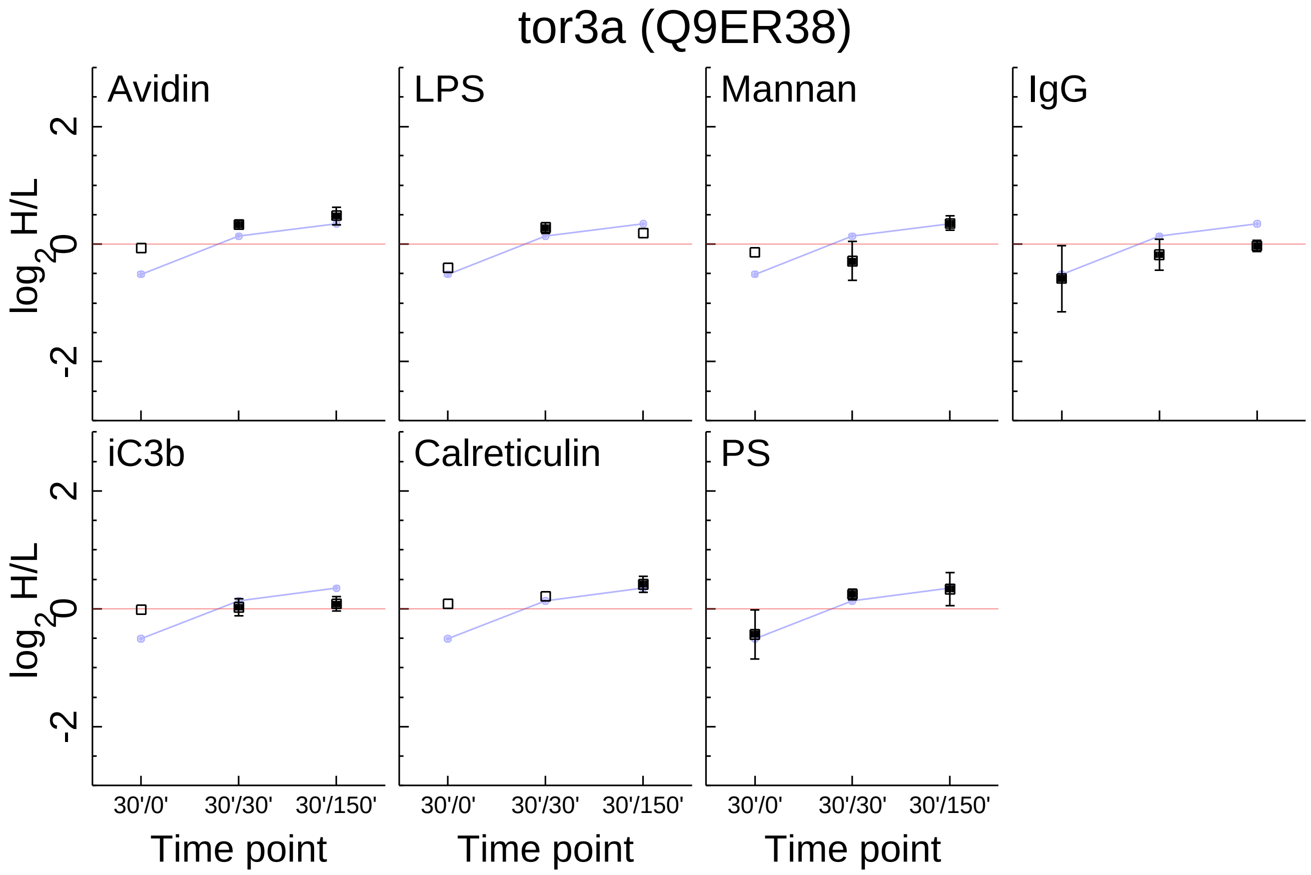

tor3a (Q9ER38)
Avidin
LPS
Mannan
IgG
iC3b
Calreticulin
PS
log2 H/L
2
0
-2
30'/0'
30'/30'
30'/150'
Time point

tpcn1 (Q9EQJ0)

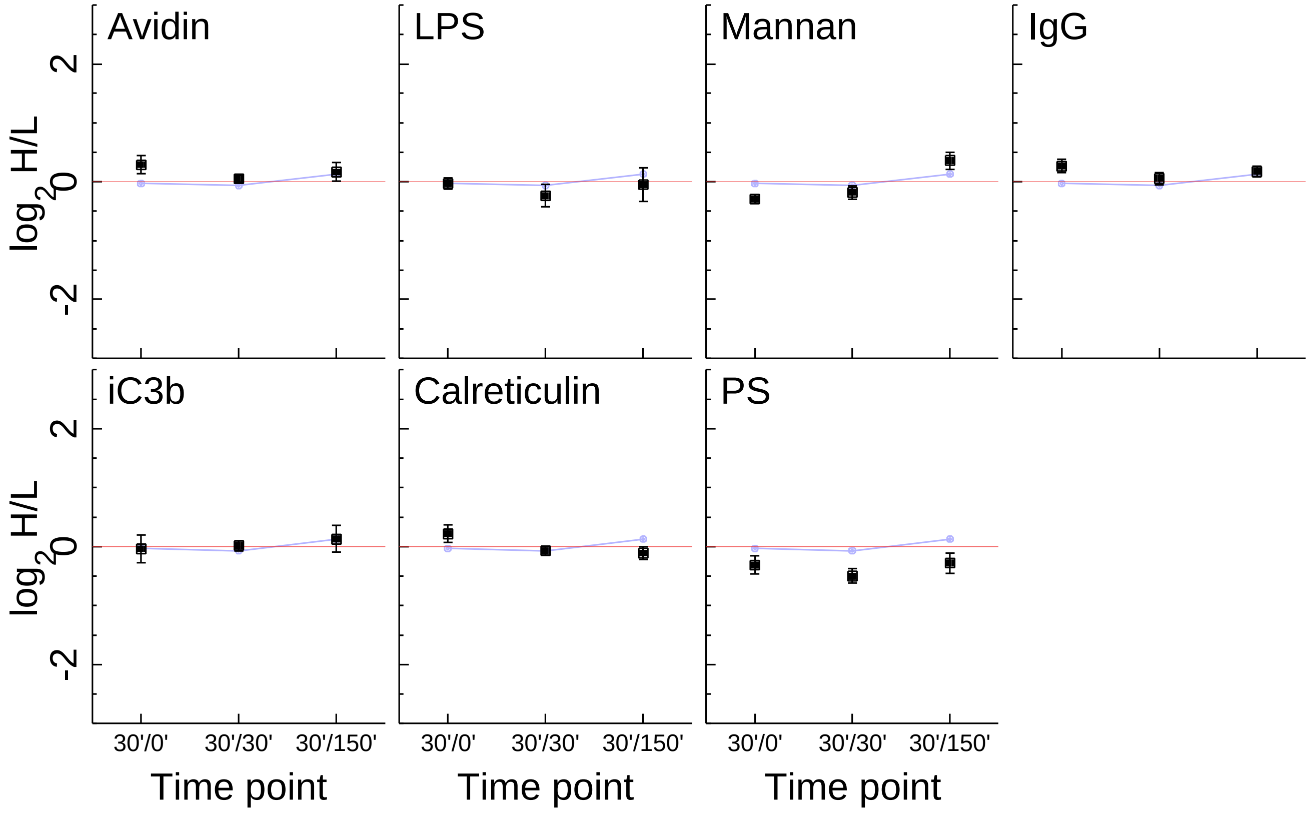

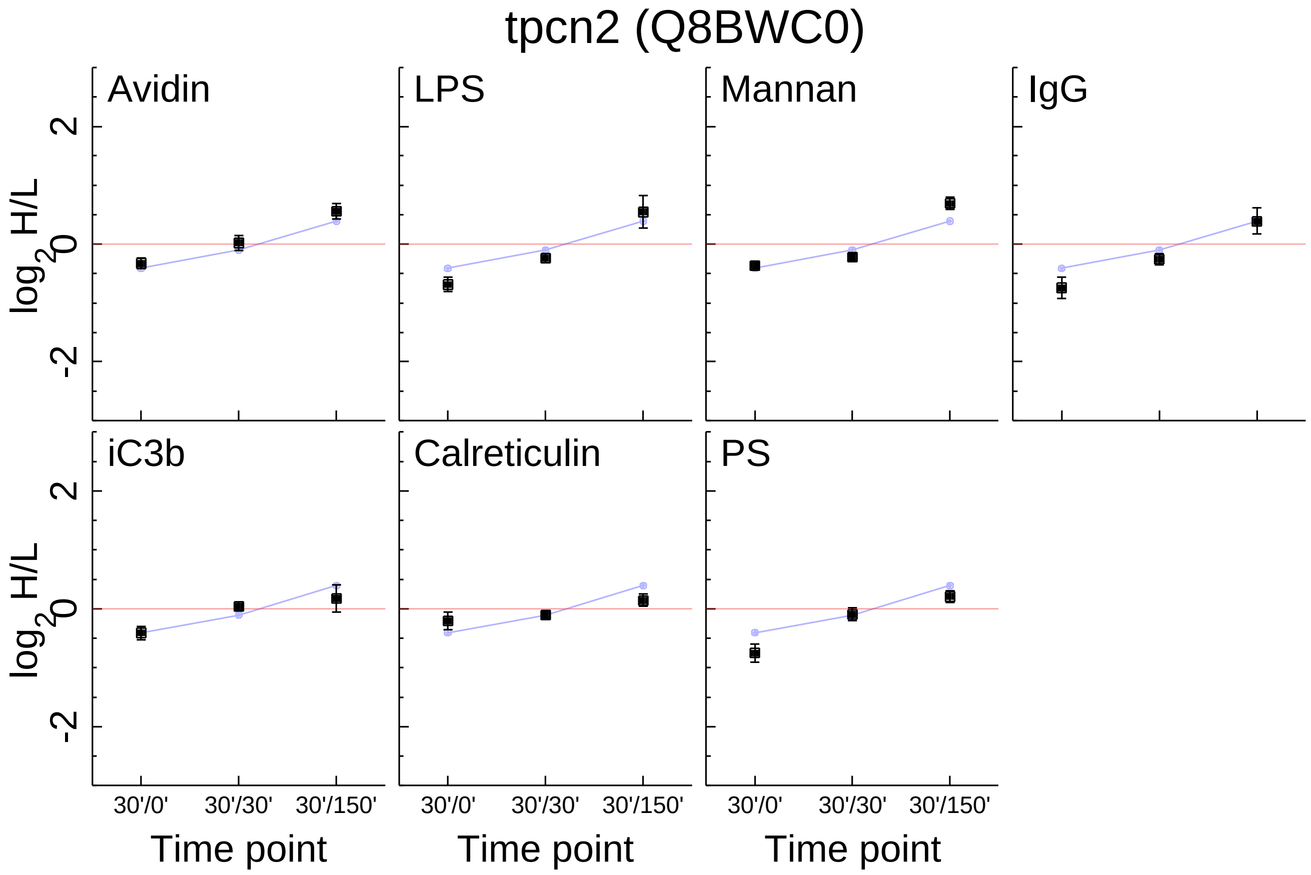

tpcn2 (Q8BWC0)
Avidin
LPS
Mannan
IgG
iC3b
Calreticulin
PS
2
0
-2
log2 H/L
30'/0'
30'/30'
30'/150'
Time point

tpd52 (F8WHQ1)

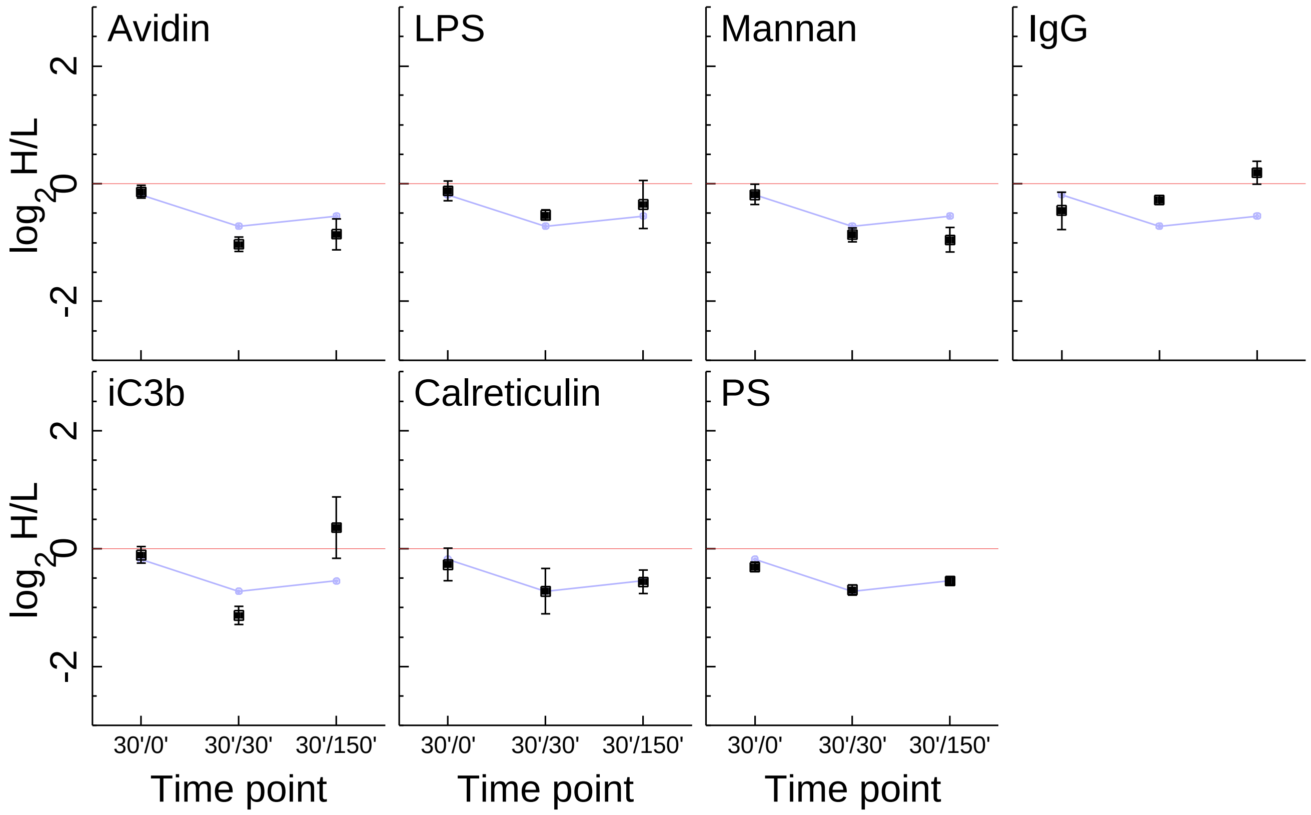

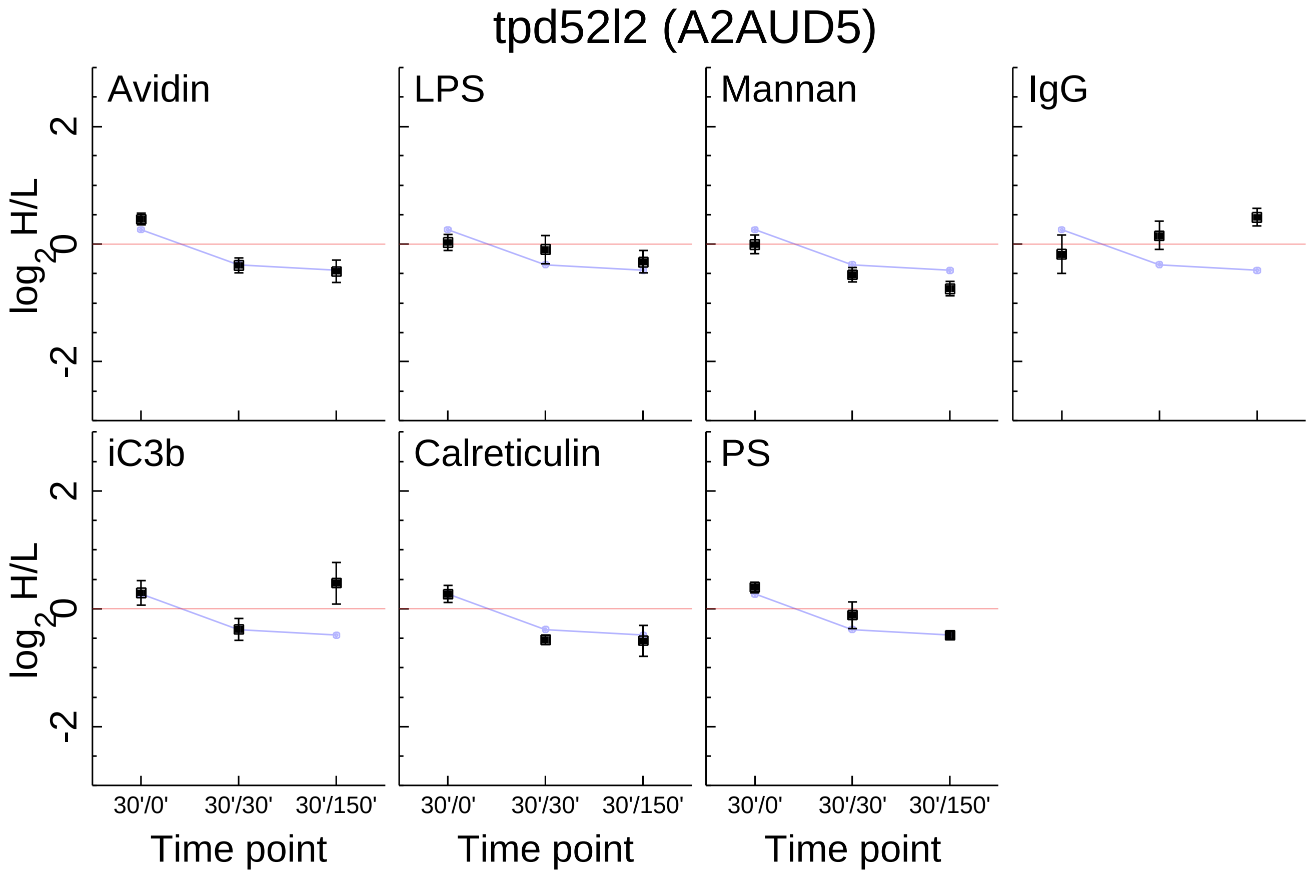

tpd52l2 (A2AUD5)
Avidin
LPS
Mannan
IgG
iC3b
Calreticulin
PS
log2 H/L
2
0
-2
30'/0'
30'/30'
30'/150'
Time point

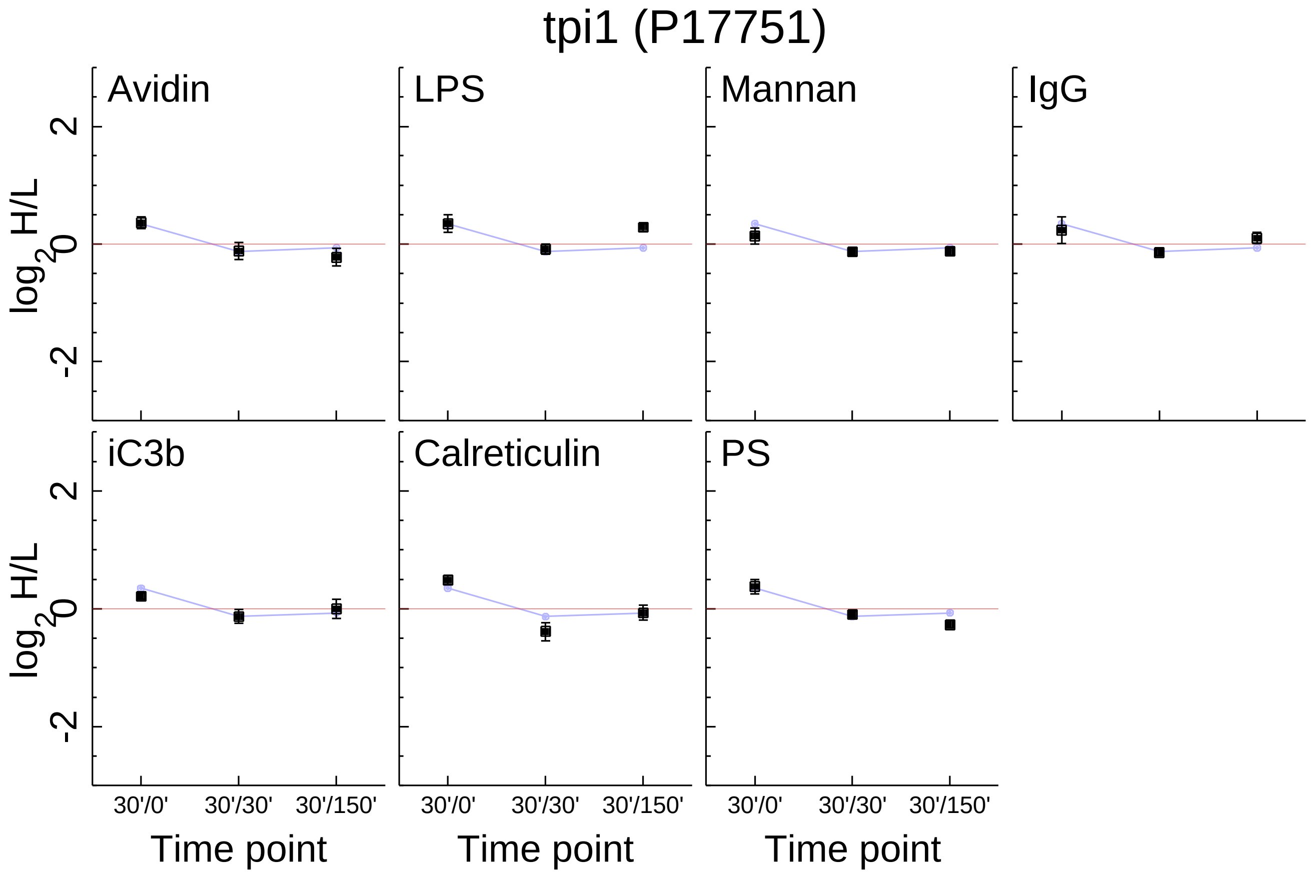
tpi1 (P17751)
Avidin
LPS
Mannan
IgG
iC3b
Calreticulin
PS
log2 H/L
2
0
-2
30'/0'
30'/30'
30'/150'
Time point

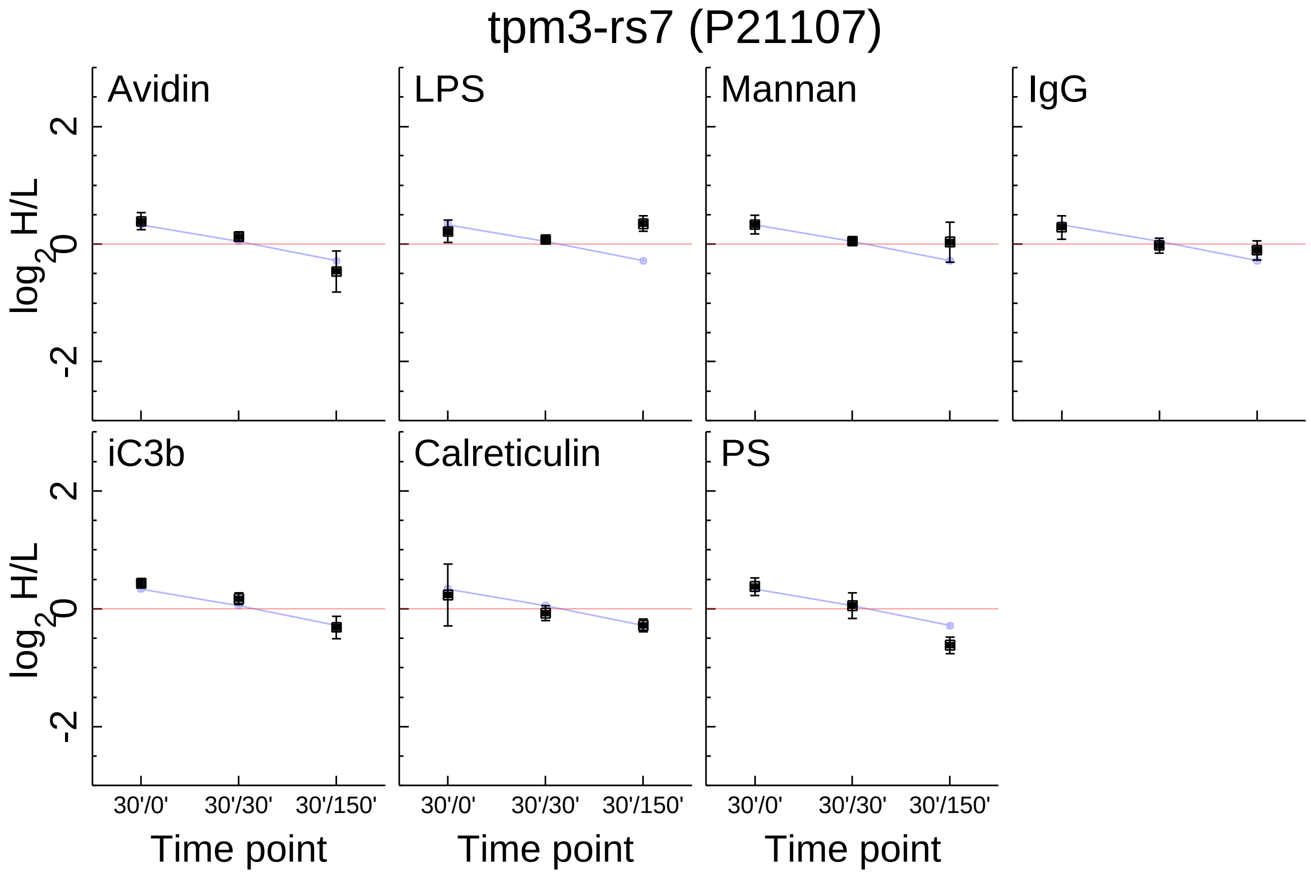
tpm3-rs7 (P21107)
Avidin
LPS
Mannan
IgG
iC3b
Calreticulin
PS
log2 H/L
2
0
-2
30'/0'
30'/30'
30'/150'
Time point

tpm4 (Q6IRU2)

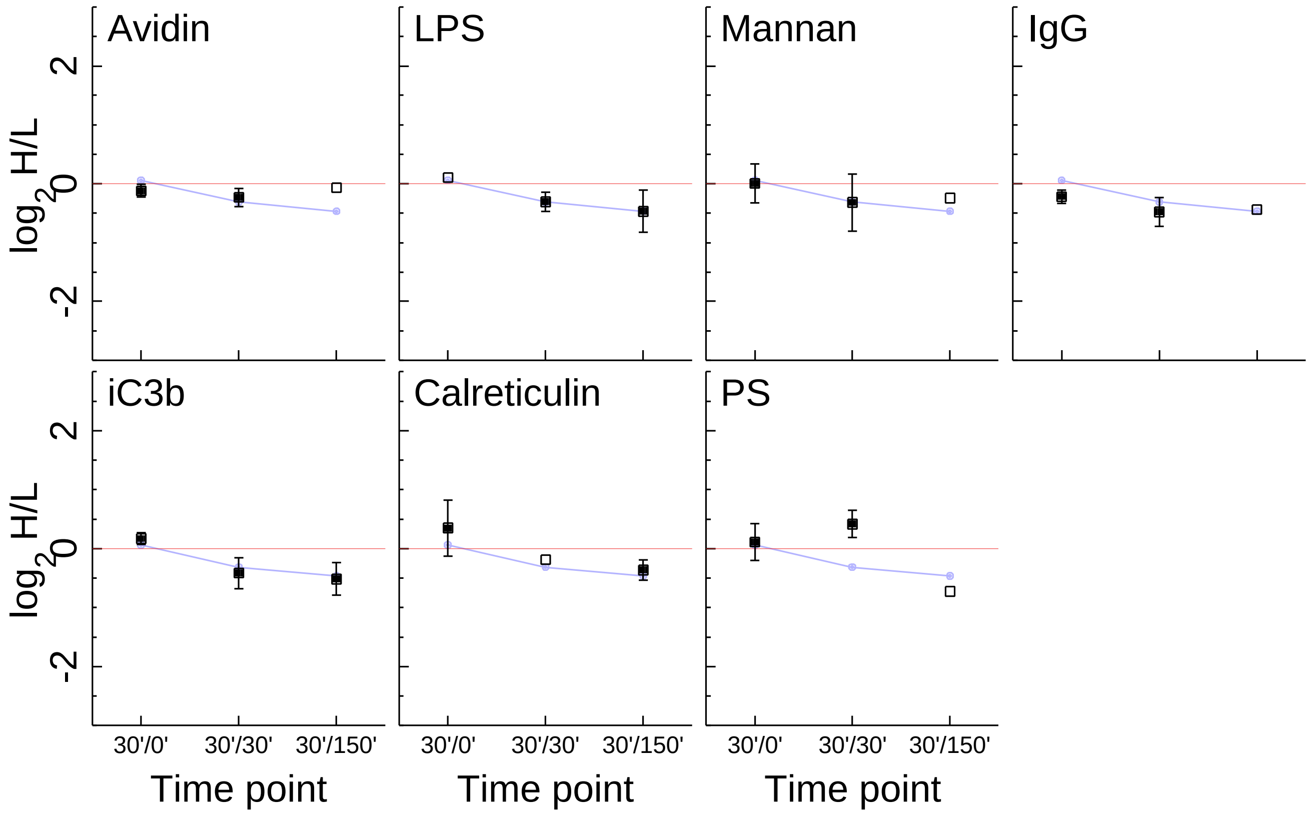
Avidin
LPS
Mannan
IgG
iC3b
Calreticulin
PS
log2 H/L
2
0
-2
30'/0'
30'/30'
30'/150'
Time point

tpp1 (O89023)

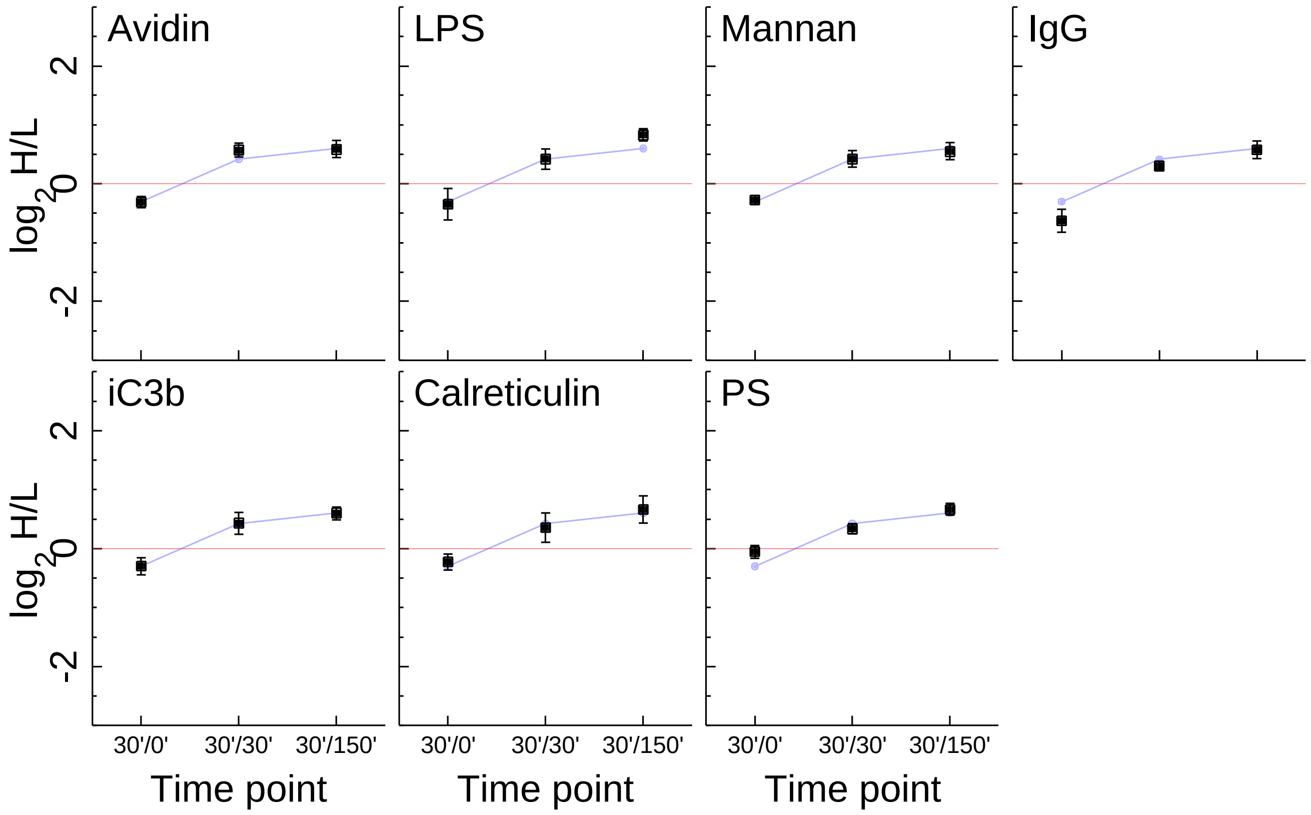
Avidin
LPS
Mannan
IgG
iC3b
Calreticulin
PS
log2 H/L
2
0
-2
30'/0'
30'/30'
30'/150'
Time point

tpt1 (P63028)

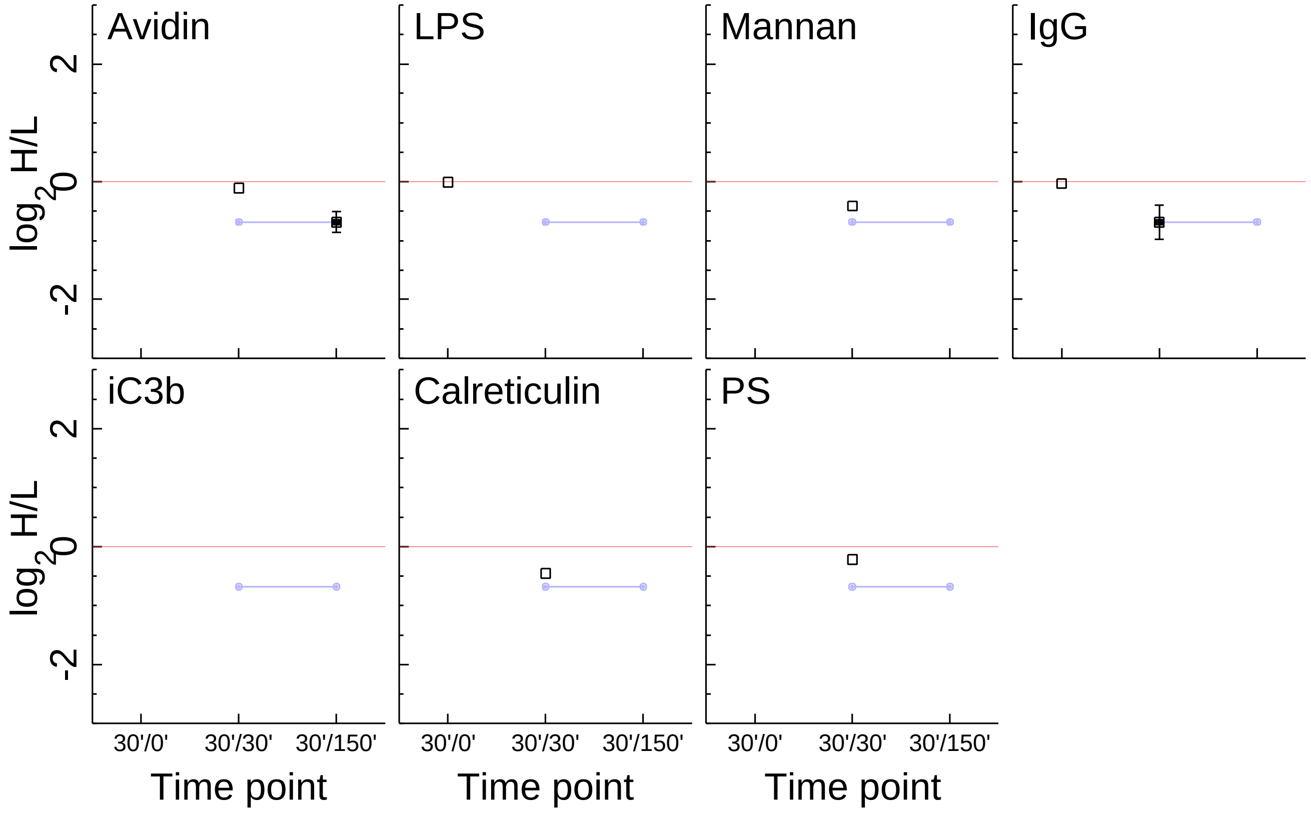

Avidin
LPS
Mannan
IgG
iC3b
Calreticulin
PS
log2 H/L
2
0
-2
30'/0'
30'/30'
30'/150'
Time point

trafd1 (Q3UDK1)

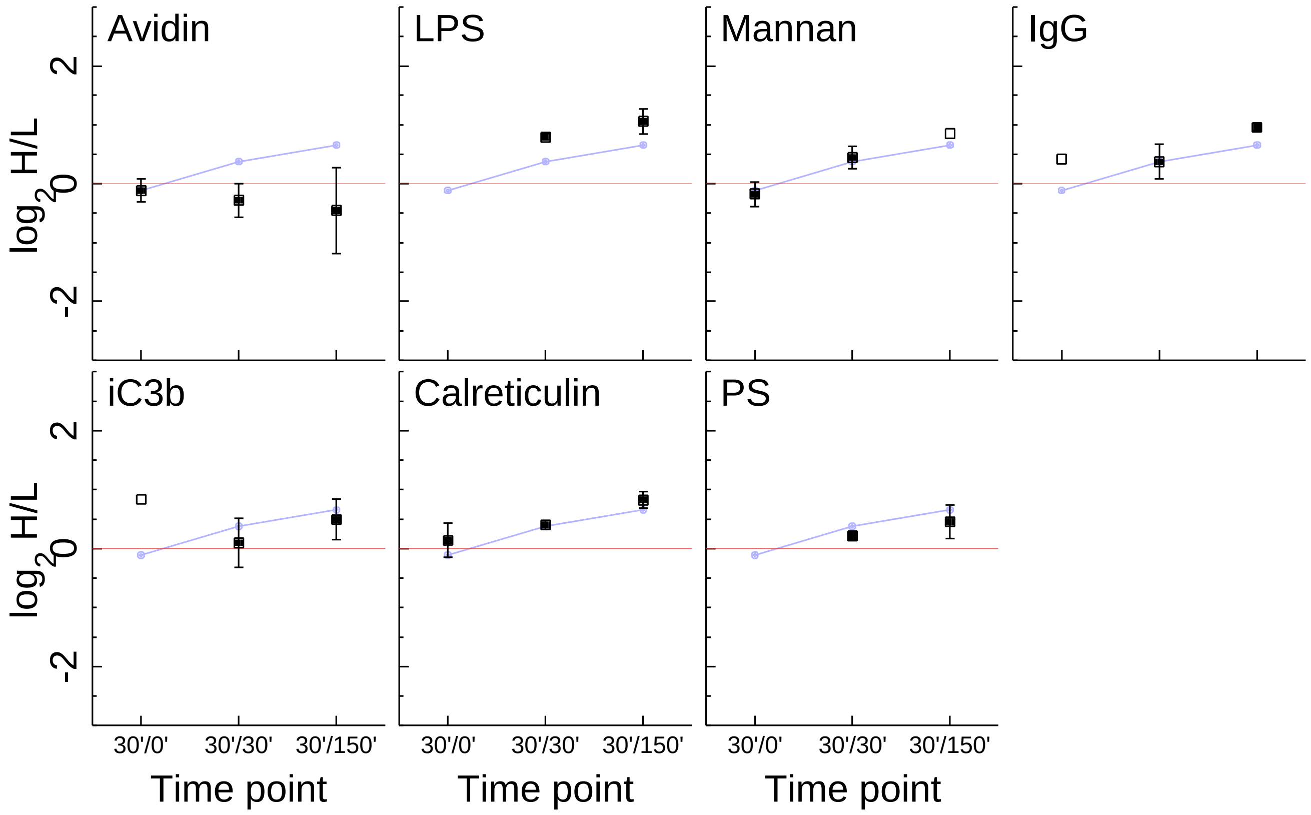

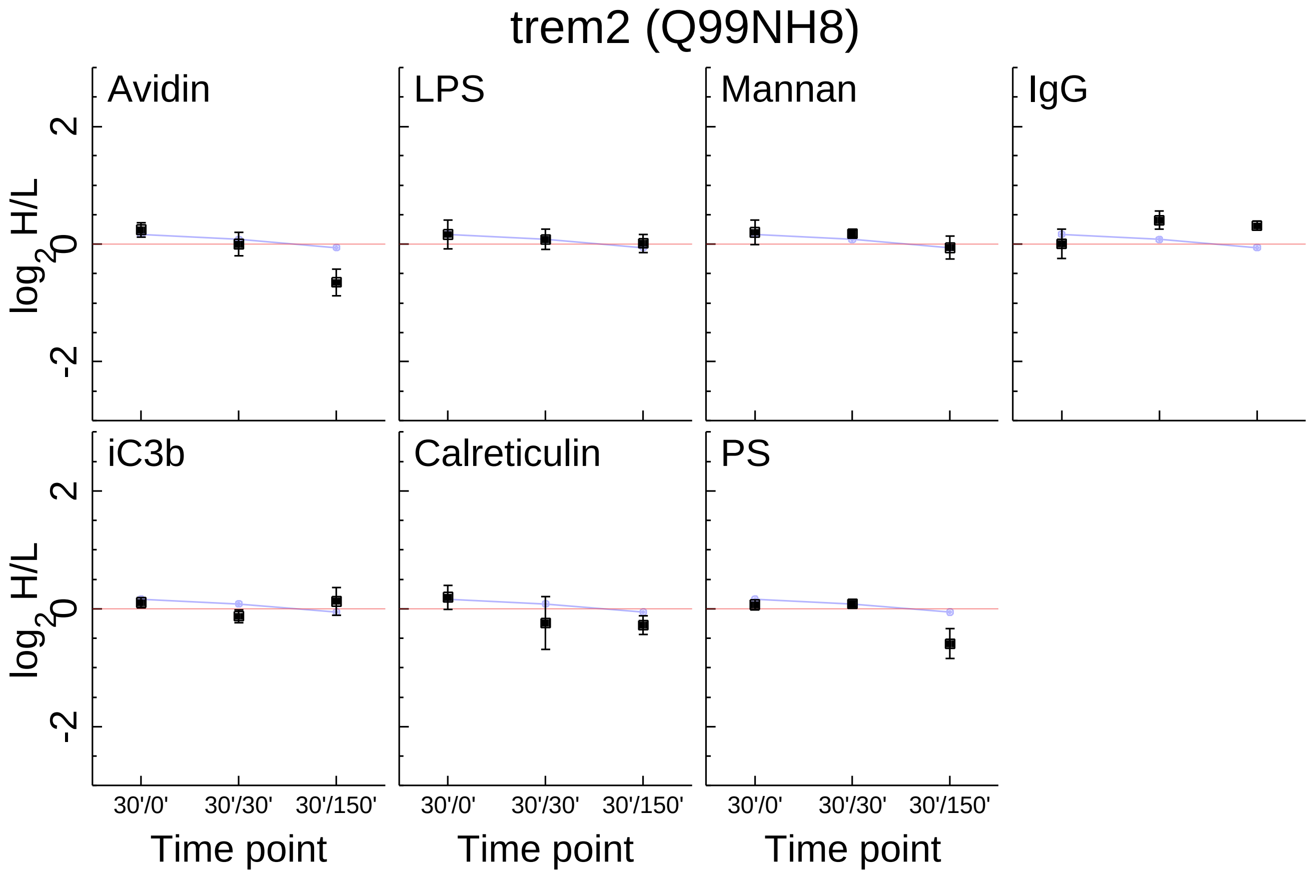
trem2 (Q99NH8)
Avidin
LPS
Mannan
IgG
iC3b
Calreticulin
PS
log2 H/L
2
0
-2
30'/0'
30'/30'
30'/150'
Time point

trex1 (Q91XB0)

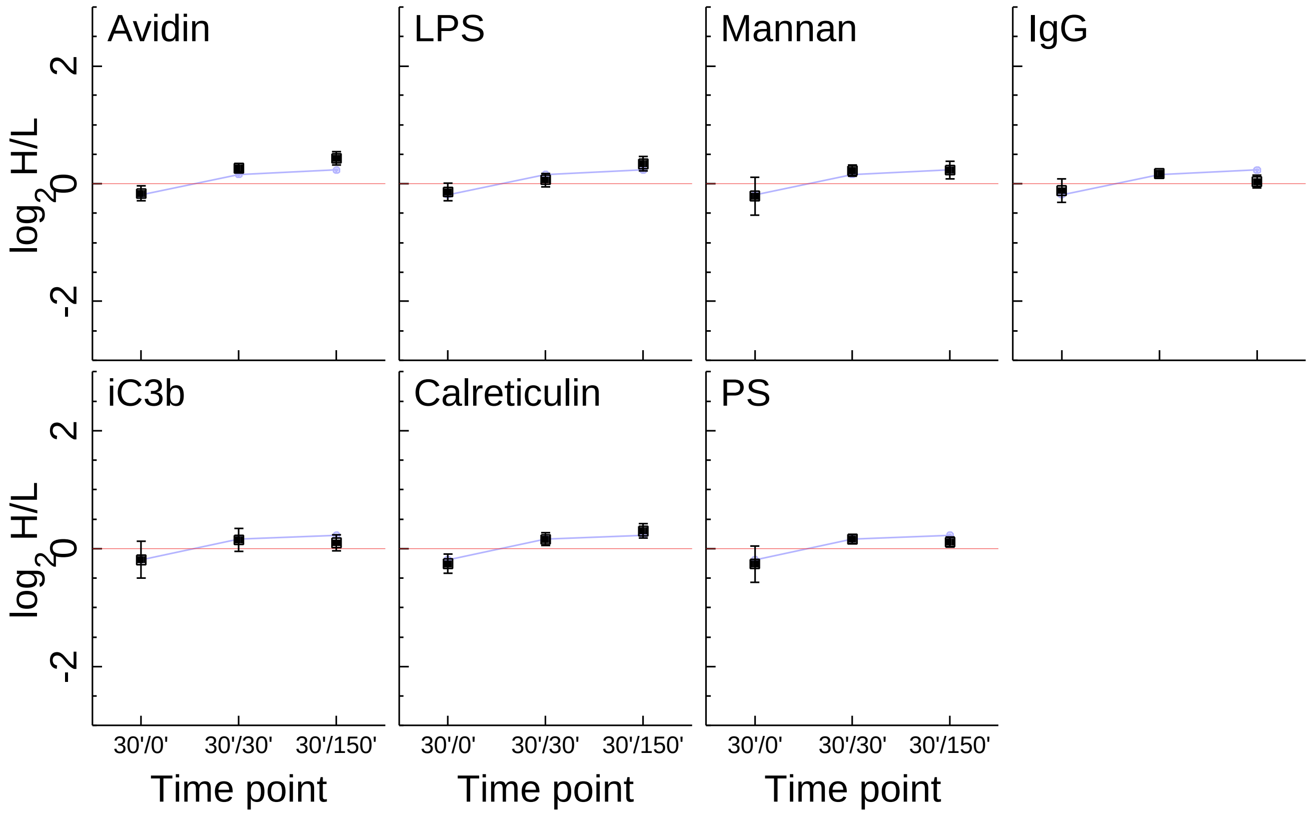

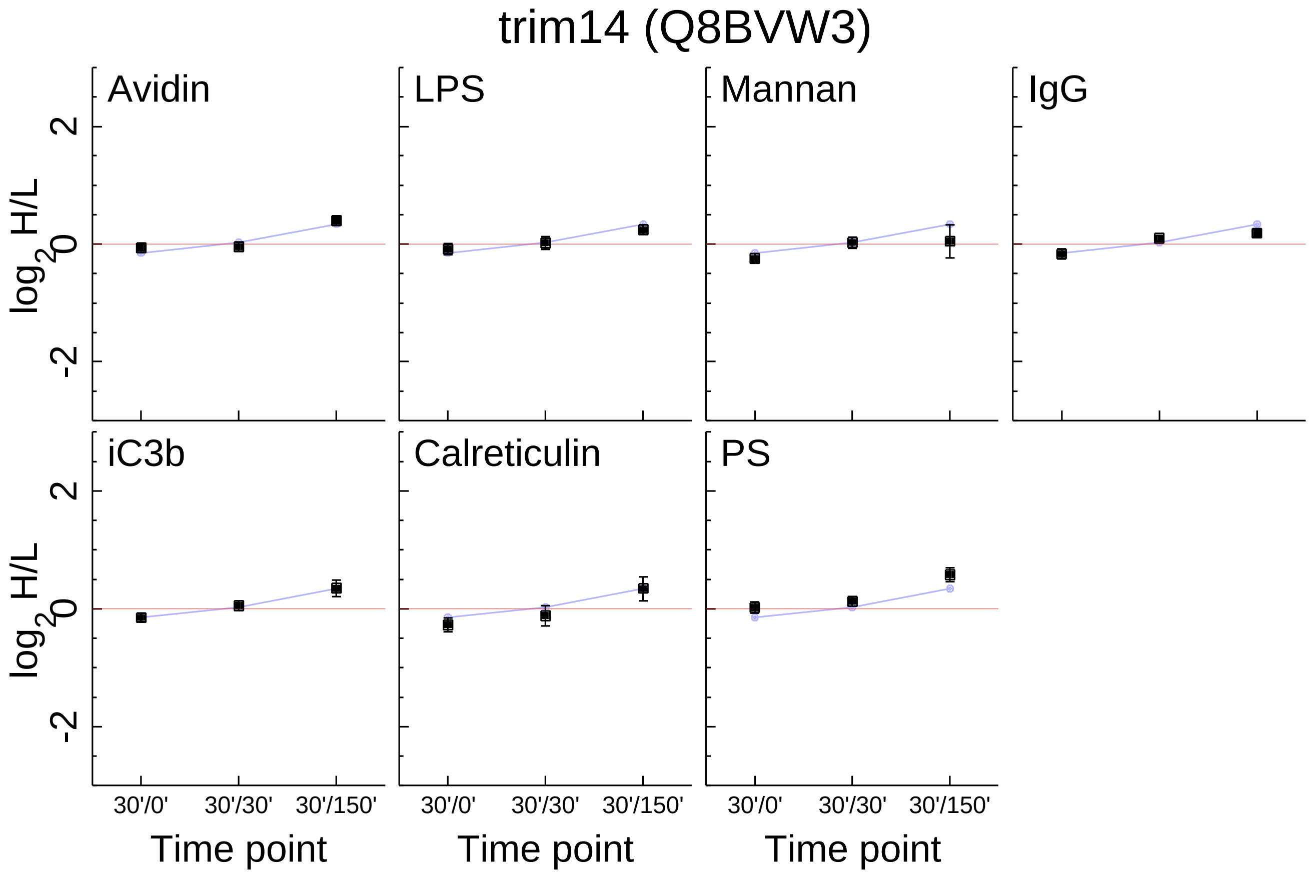
trim14 (Q8BVW3)
Avidin
LPS
Mannan
IgG
iC3b
Calreticulin
PS
log2 H/L
2
0
-2
30'/0'
30'/30'
30'/150'
Time point

trim21 (Q3U7K7)

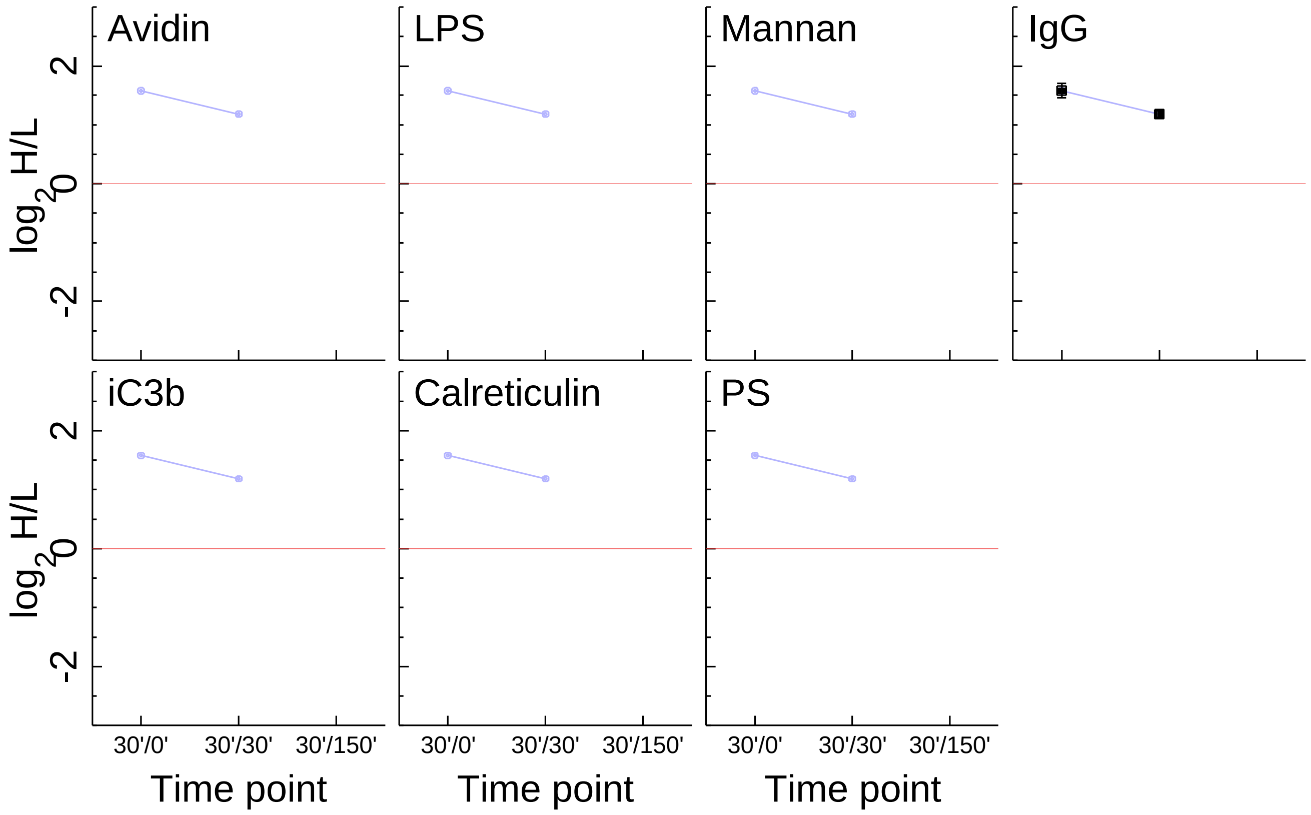

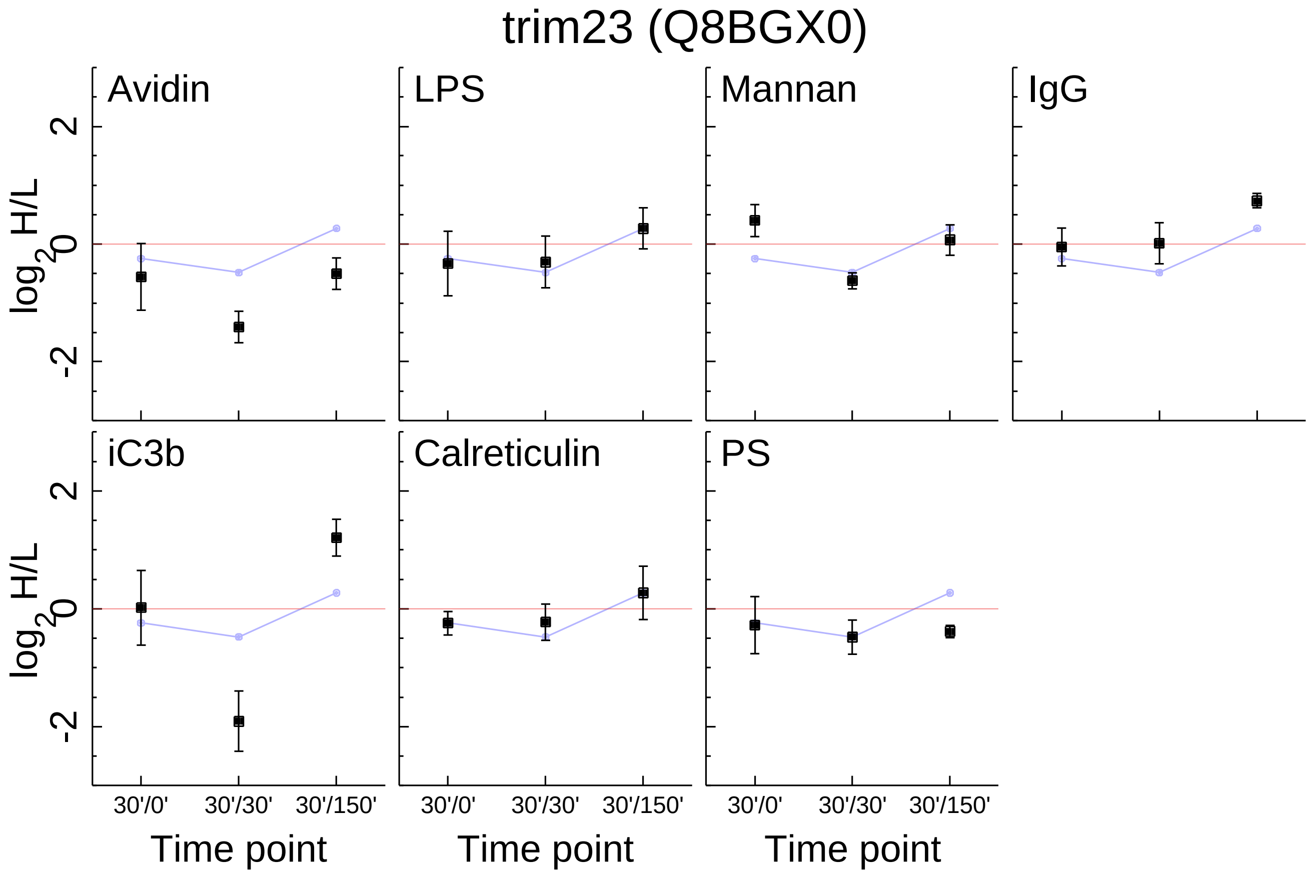

trim23 (Q8BGX0)
Avidin
LPS
Mannan
IgG
iC3b
Calreticulin
PS
log2 H/L
2
0
-2
30'/0'
30'/30'
30'/150'
Time point

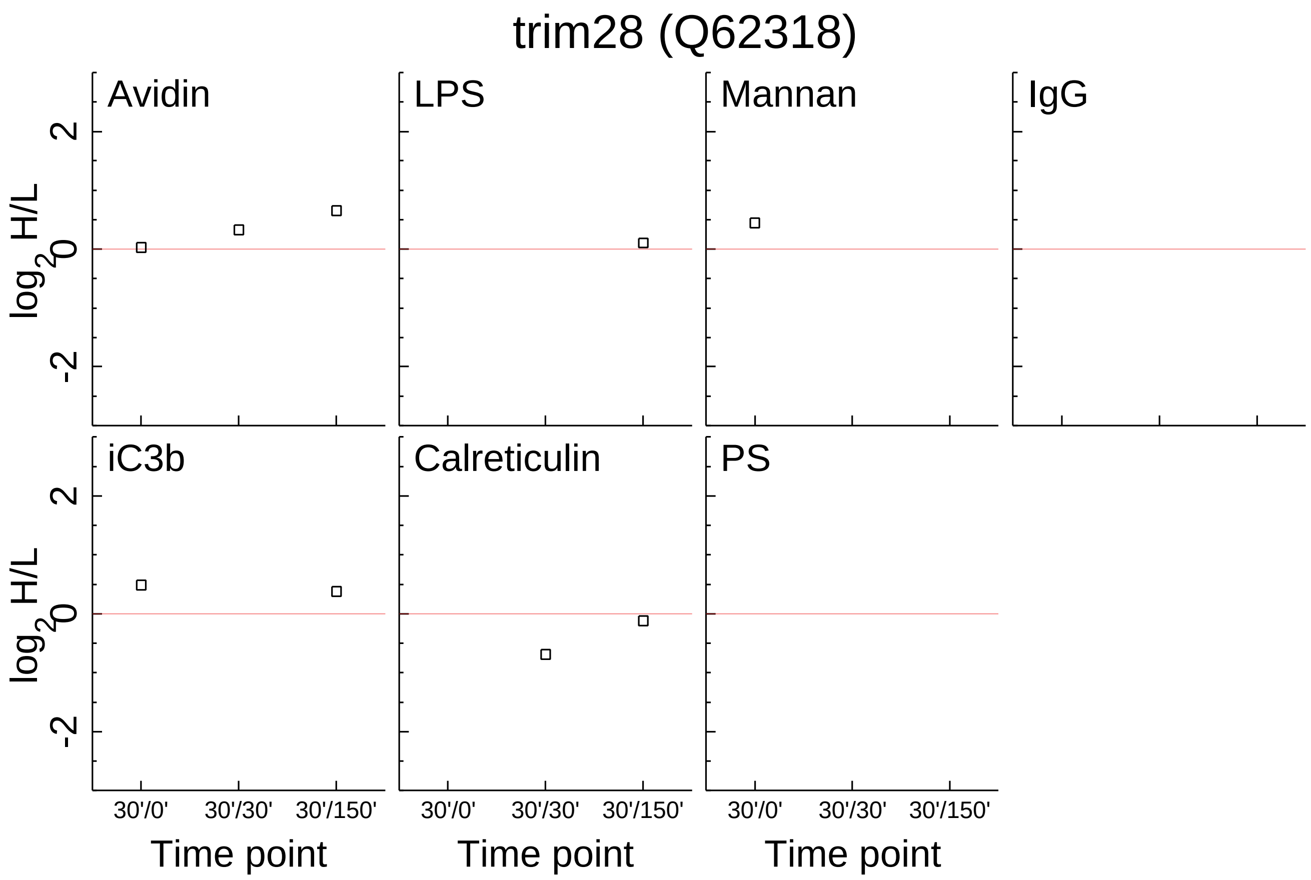
trim28 (Q62318)
Avidin
LPS
Mannan
IgG
iC3b
Calreticulin
PS
log2 H/L
2
0
-2
30'/0'
30'/30'
30'/150'
Time point

trpv2 (Q9WTR1)

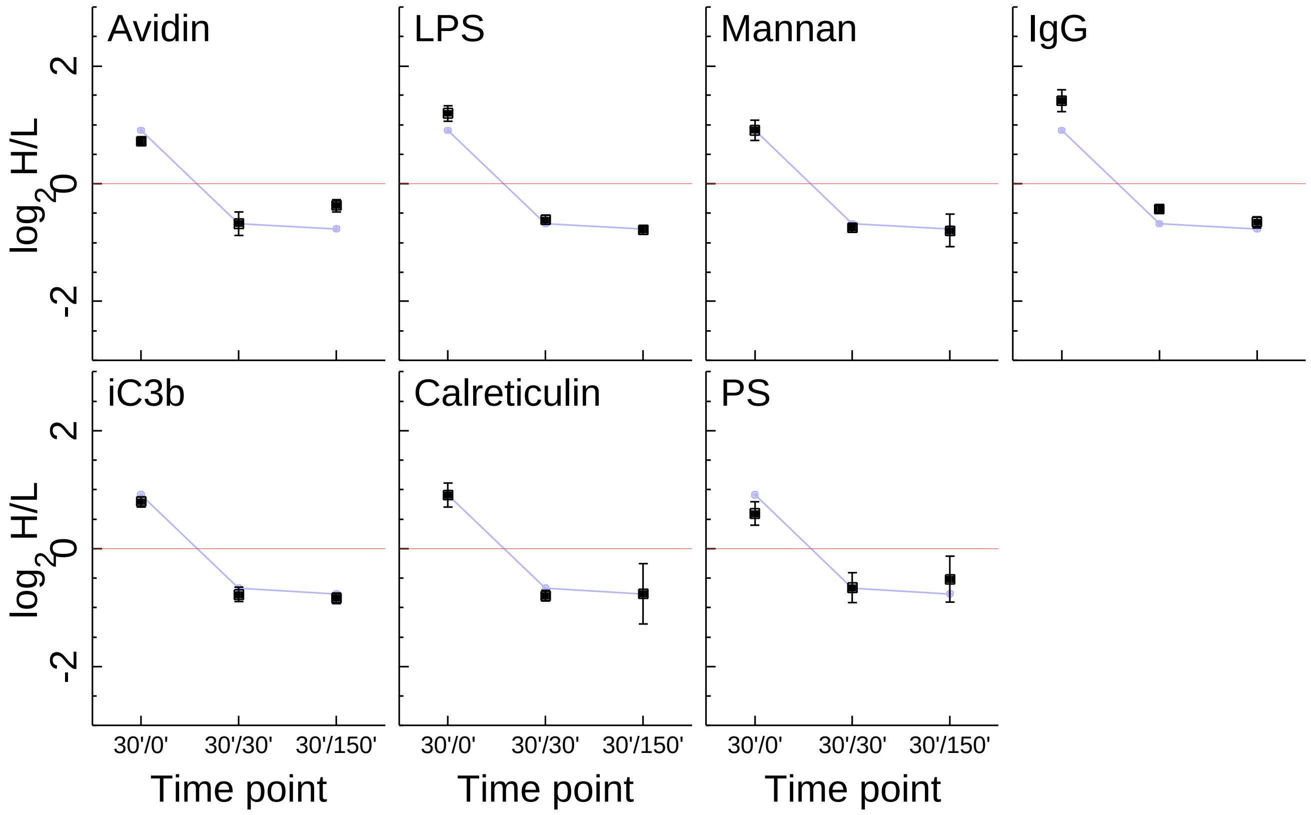

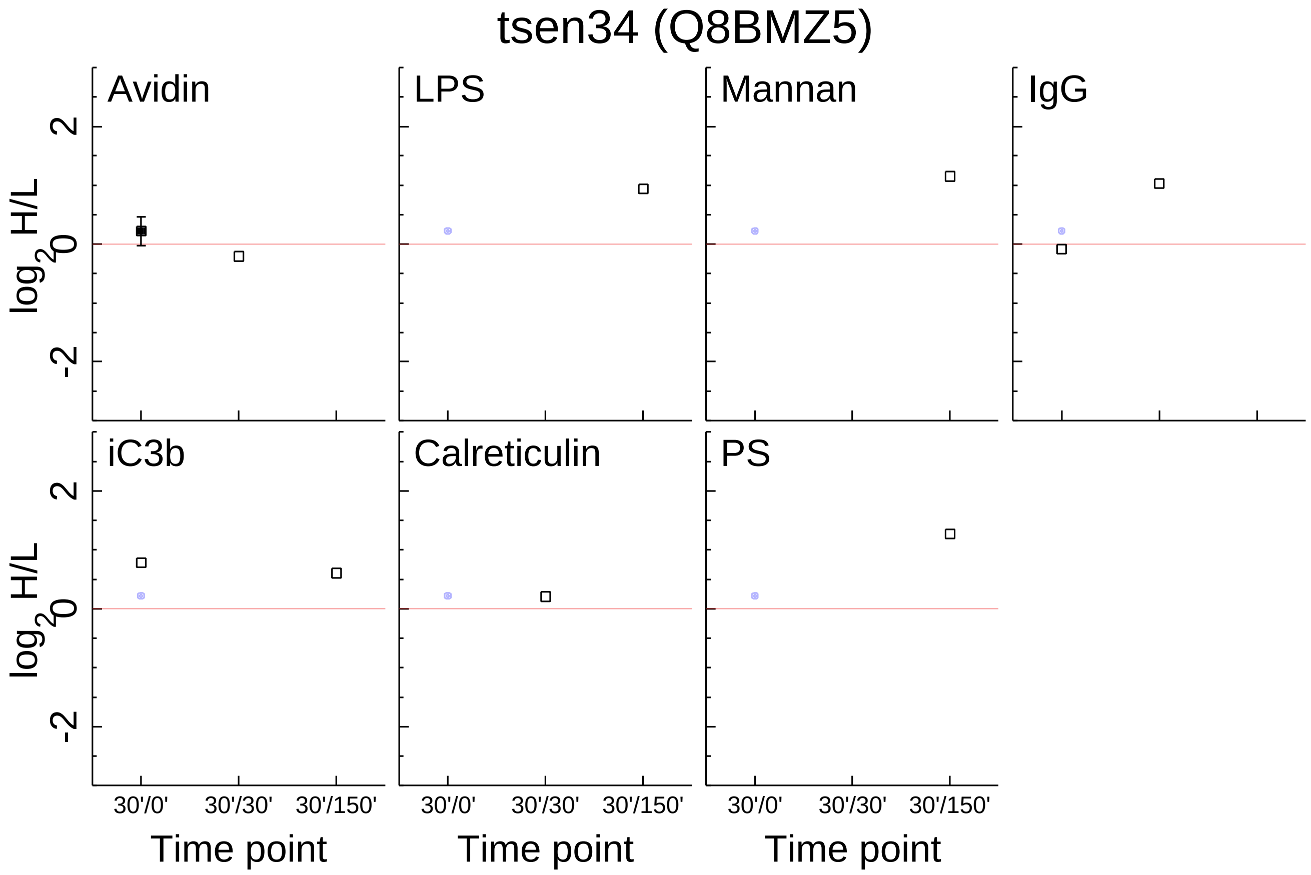

tsen34 (Q8BMZ5)
Avidin
LPS
Mannan
IgG
iC3b
Calreticulin
PS
log2 H/L
2
0
-2
30'/0'
30'/30'
30'/150'
Time point

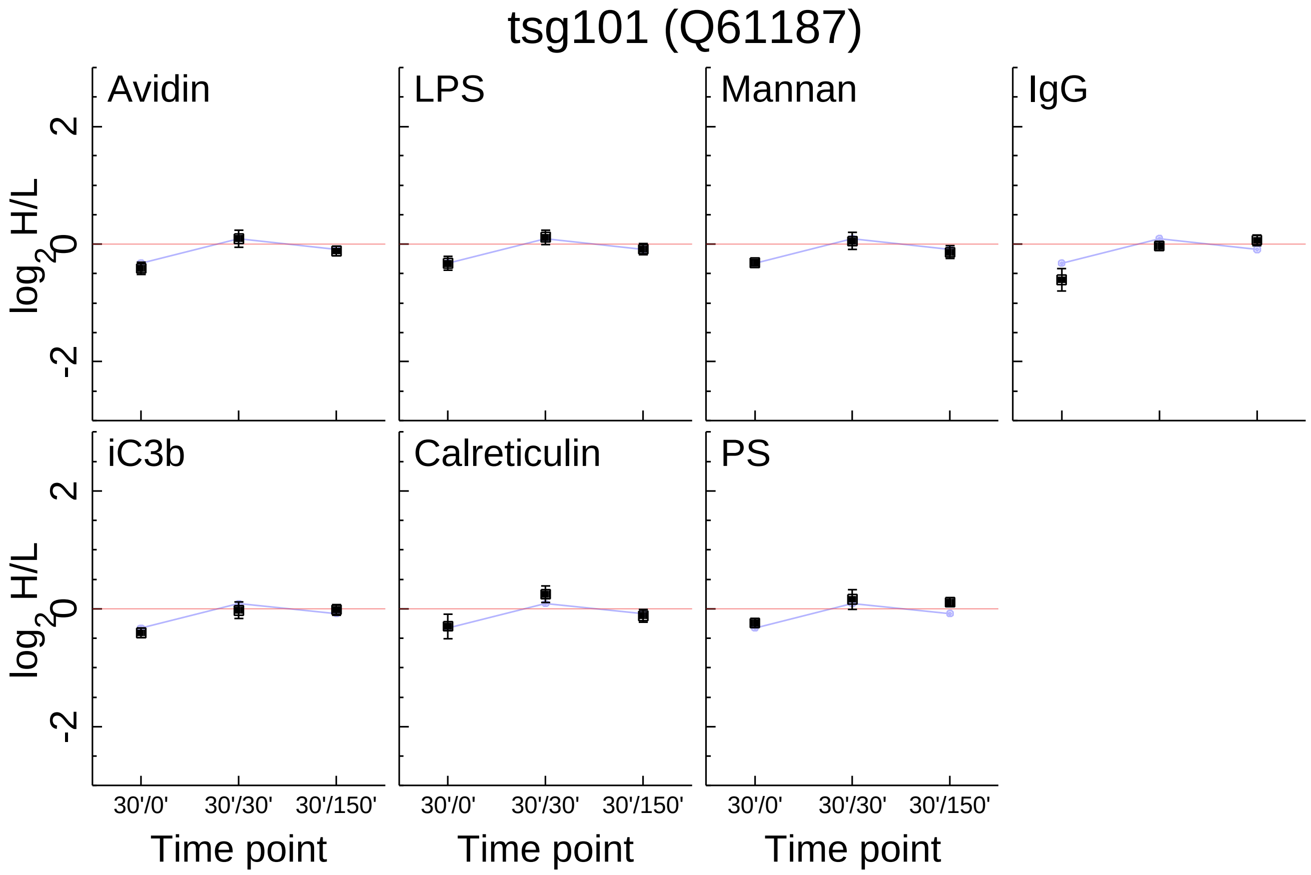

tsg101 (Q61187)
Avidin
LPS
Mannan
IgG
iC3b
Calreticulin
PS
2
0
-2
log2 H/L
30'/0'
30'/30'
30'/150'
Time point

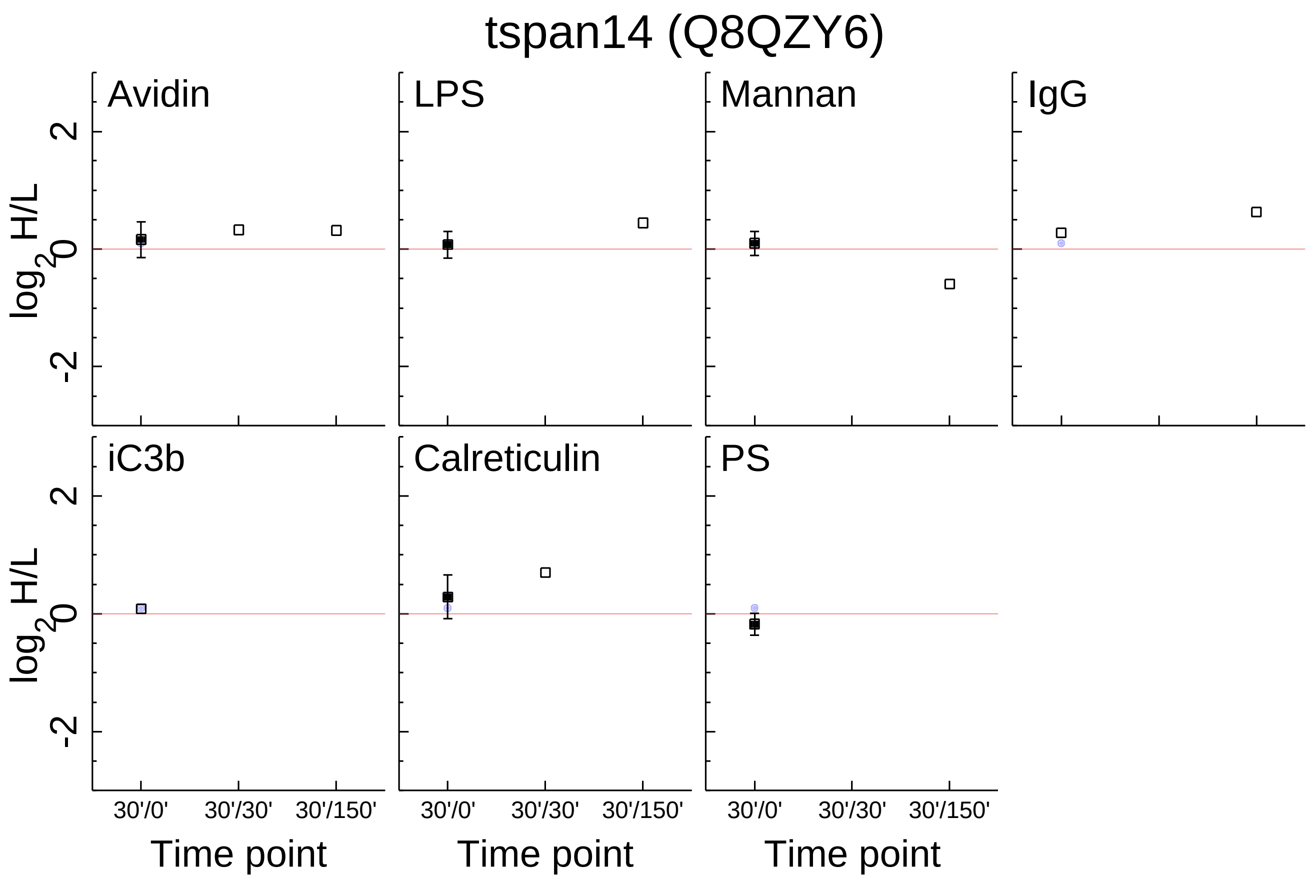

tspan14 (Q8QZY6)
Avidin
LPS
Mannan
IgG
iC3b
Calreticulin
PS
log2 H/L
2
0
-2
30'/0'
30'/30'
30'/150'
Time point

tspan31 (Q9CQ88)

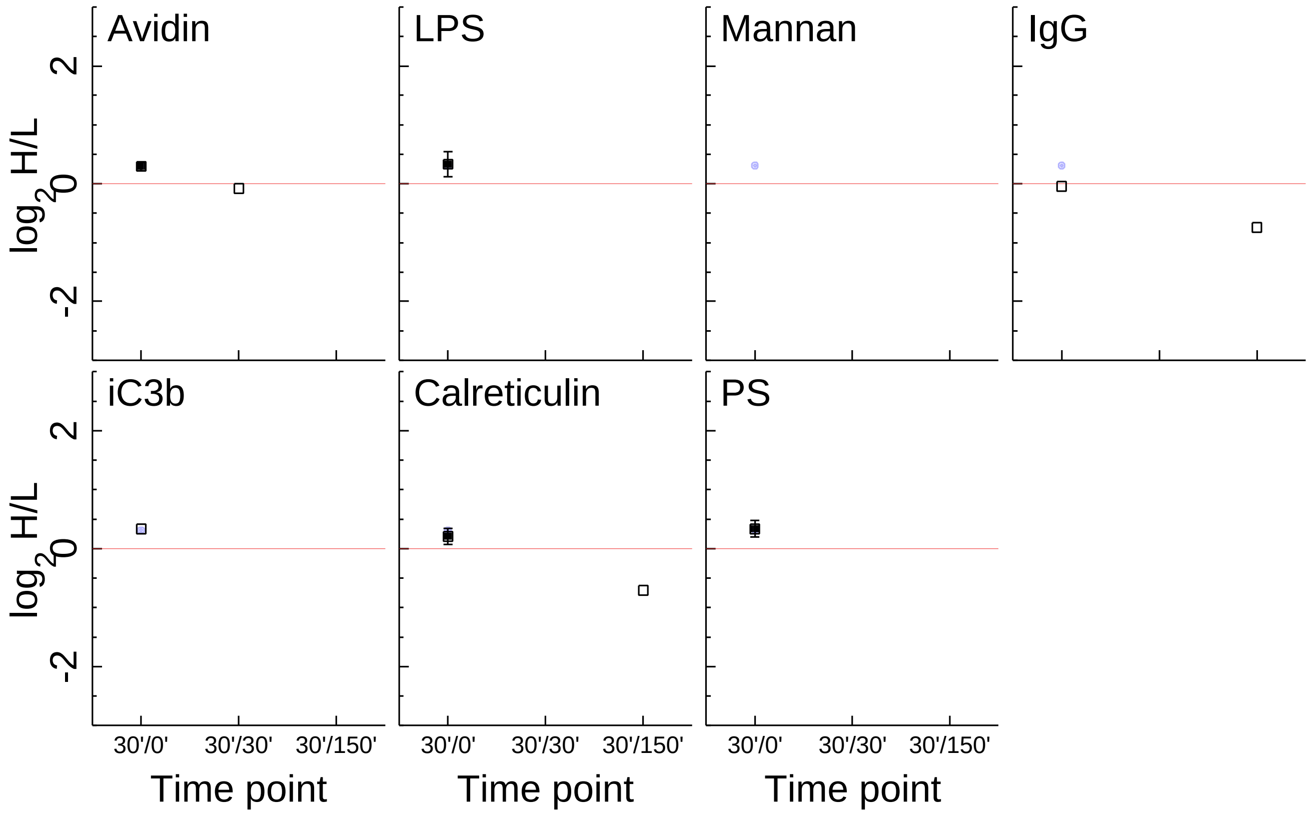

ttc35 (Q9CRD2)

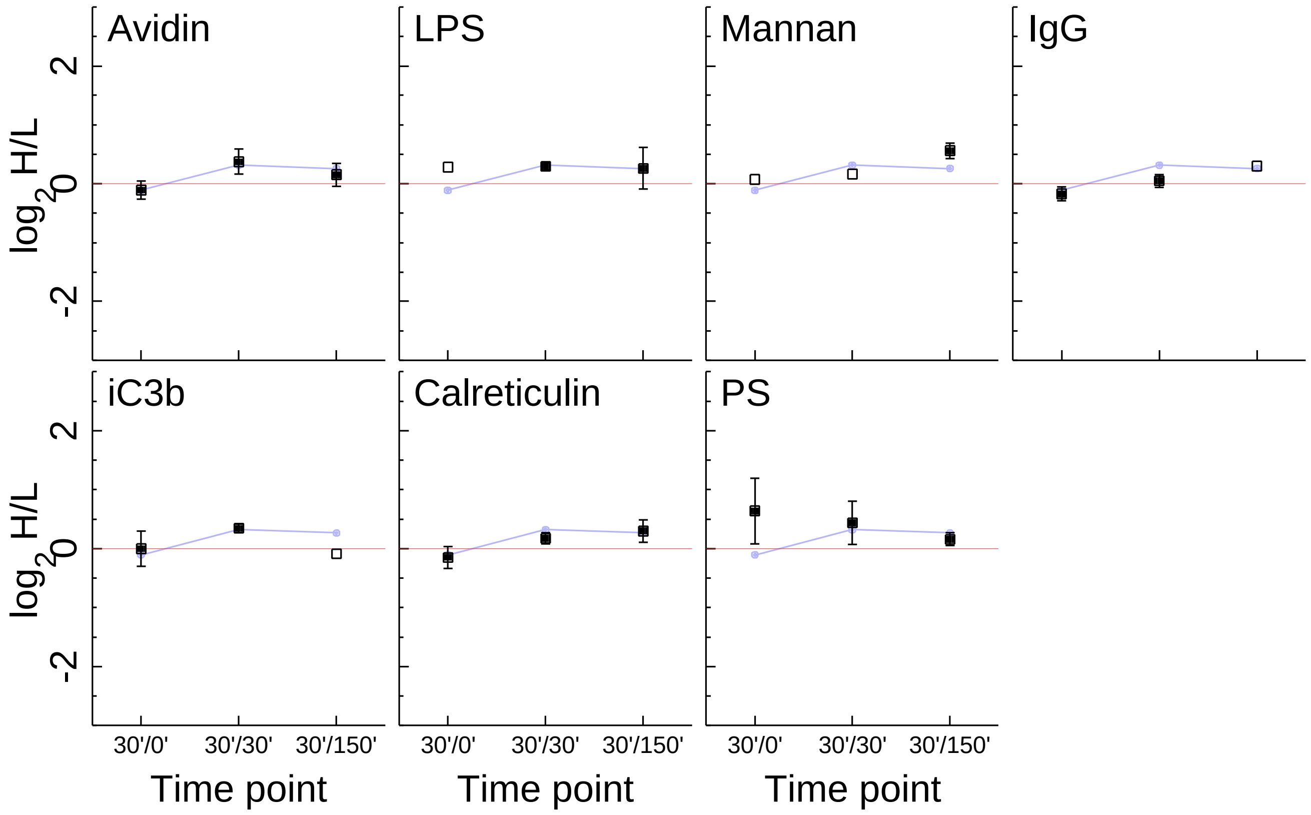

ttk (P35761)

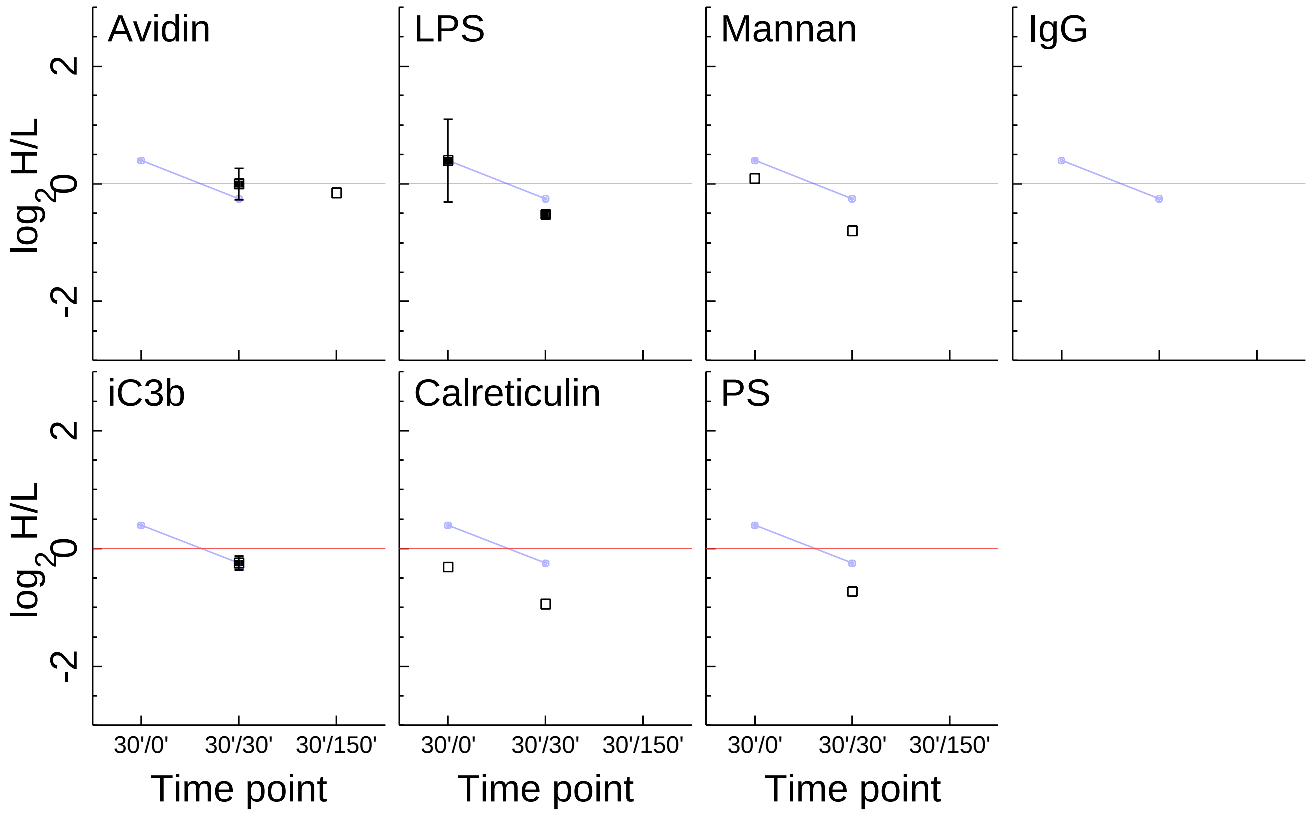
Avidin
LPS
Mannan
IgG
iC3b
Calreticulin
PS
log2 H/L
2
0
-2
30'/0'
30'/30'
30'/150'
Time point

ttn (A2ASS6)

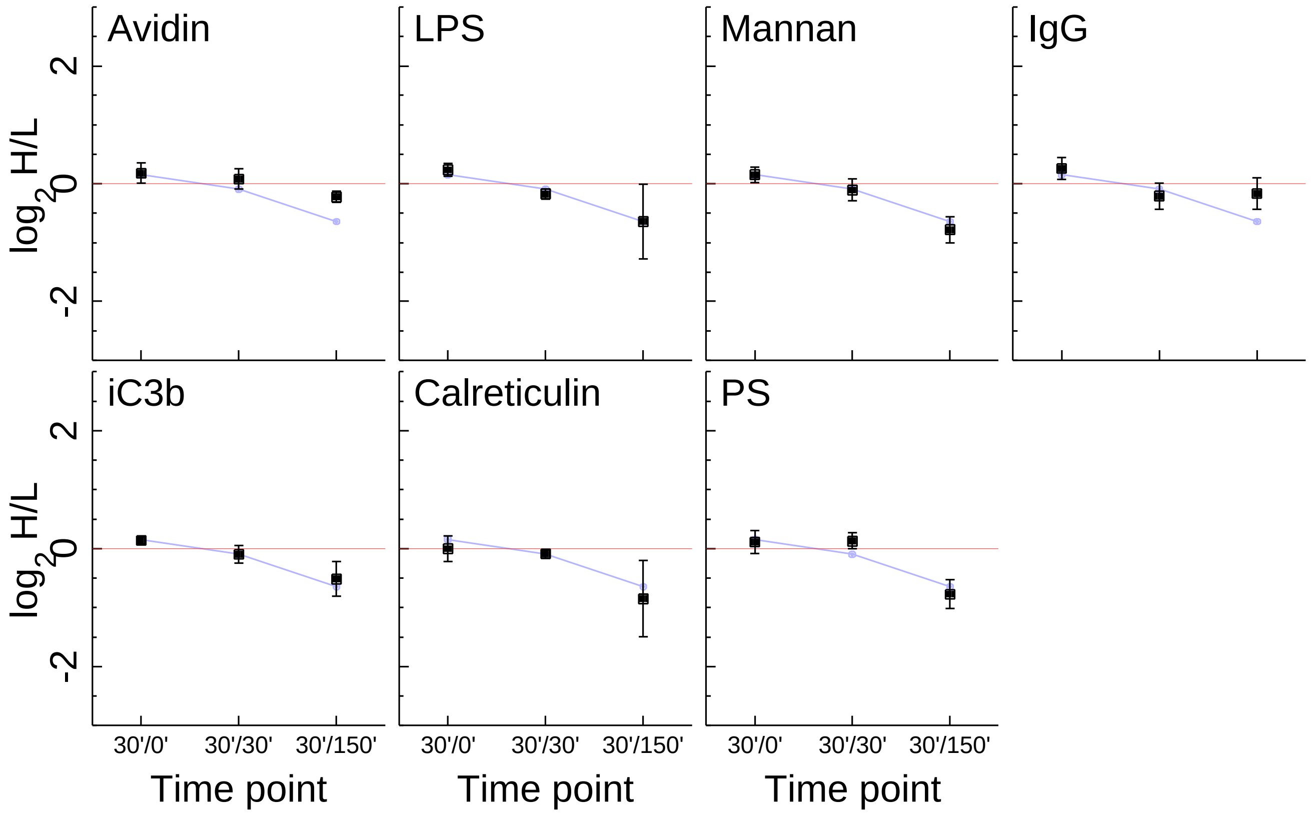

Avidin
LPS
Mannan
IgG
iC3b
Calreticulin
PS
2
0
-2
$\log_2$ H/L
30'/0'
30'/30'
30'/150'
Time point

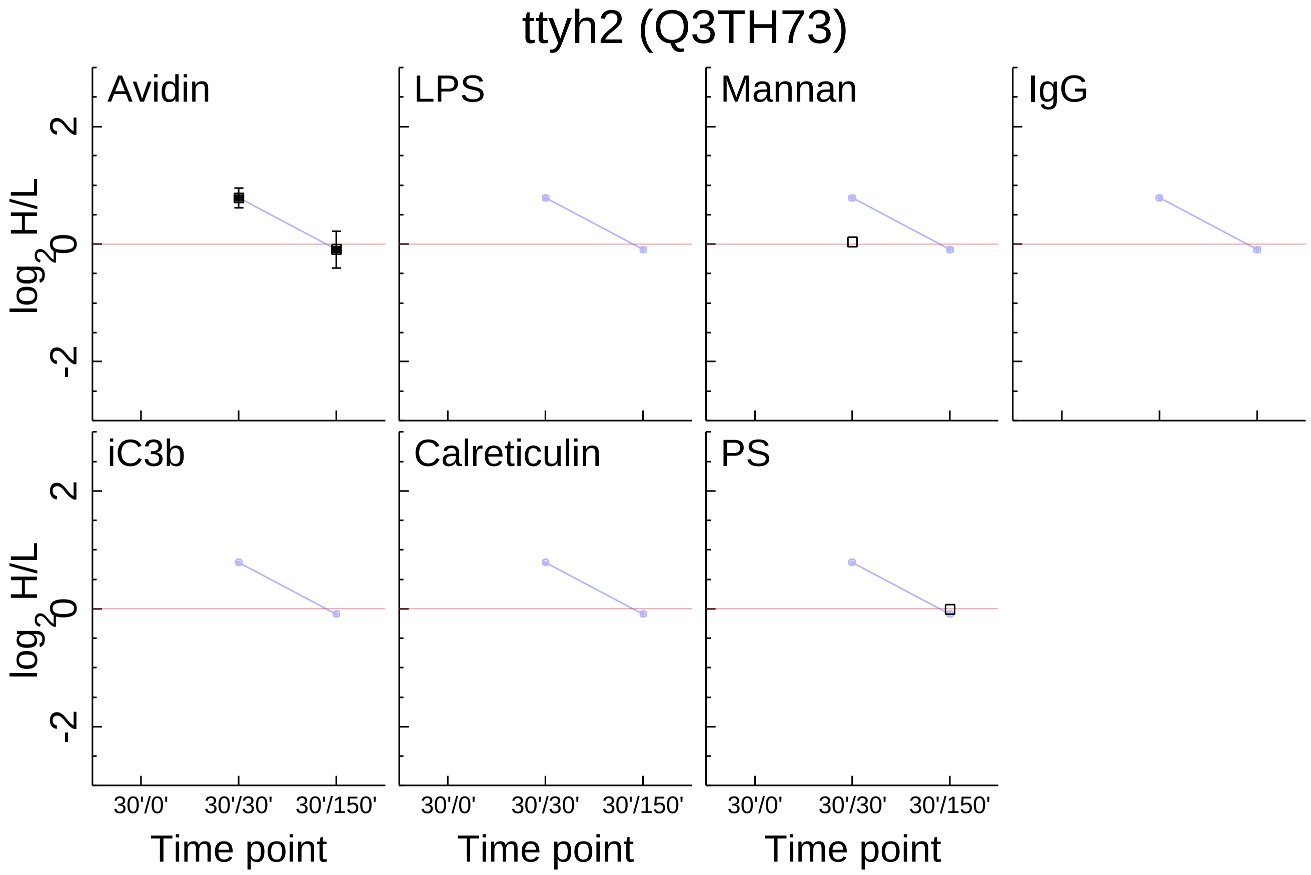
ttyh2 (Q3TH73)
Avidin
LPS
Mannan
IgG
iC3b
Calreticulin
PS
log2 H/L
2
0
-2
30'/0'
30'/30'
30'/150'
Time point

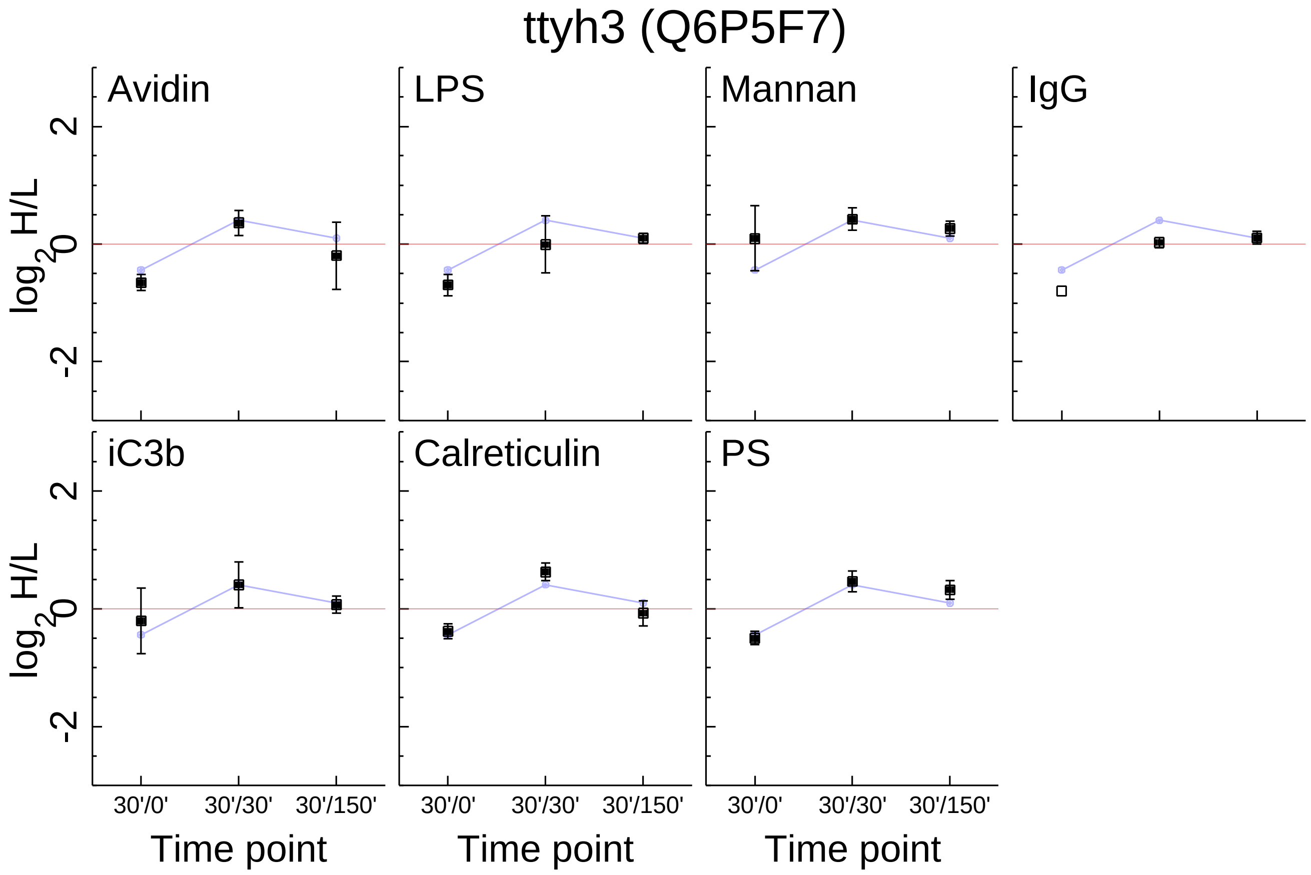
ttyh3 (Q6P5F7)
Avidin
LPS
Mannan
IgG
iC3b
Calreticulin
PS
log2 H/L
2
0
-2
30'/0'
30'/30'
30'/150'
Time point

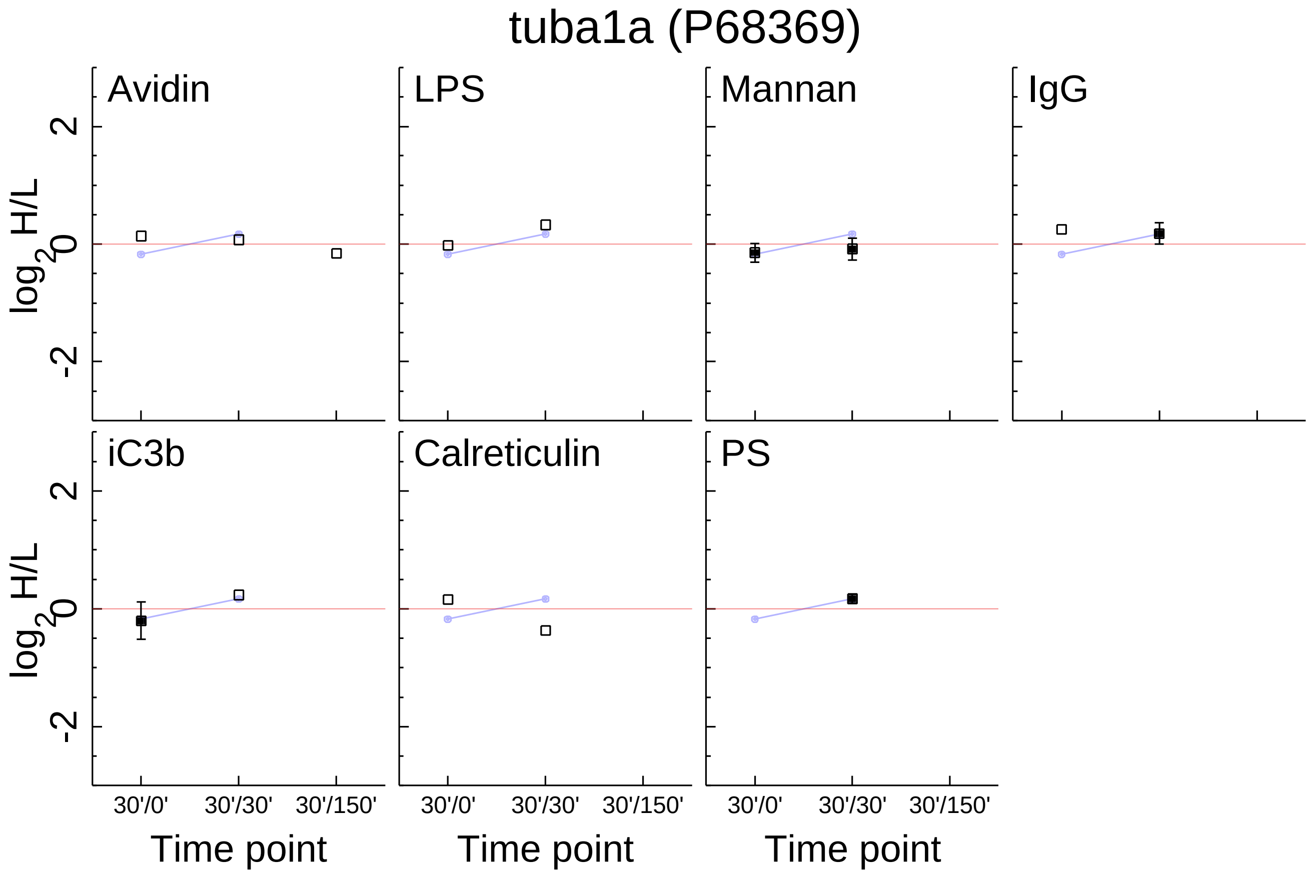

tuba1a (P68369)
Avidin
LPS
Mannan
IgG
iC3b
Calreticulin
PS
log2 H/L
2
0
-2
30'/0'
30'/30'
30'/150'
Time point

tuba1b (P05213)

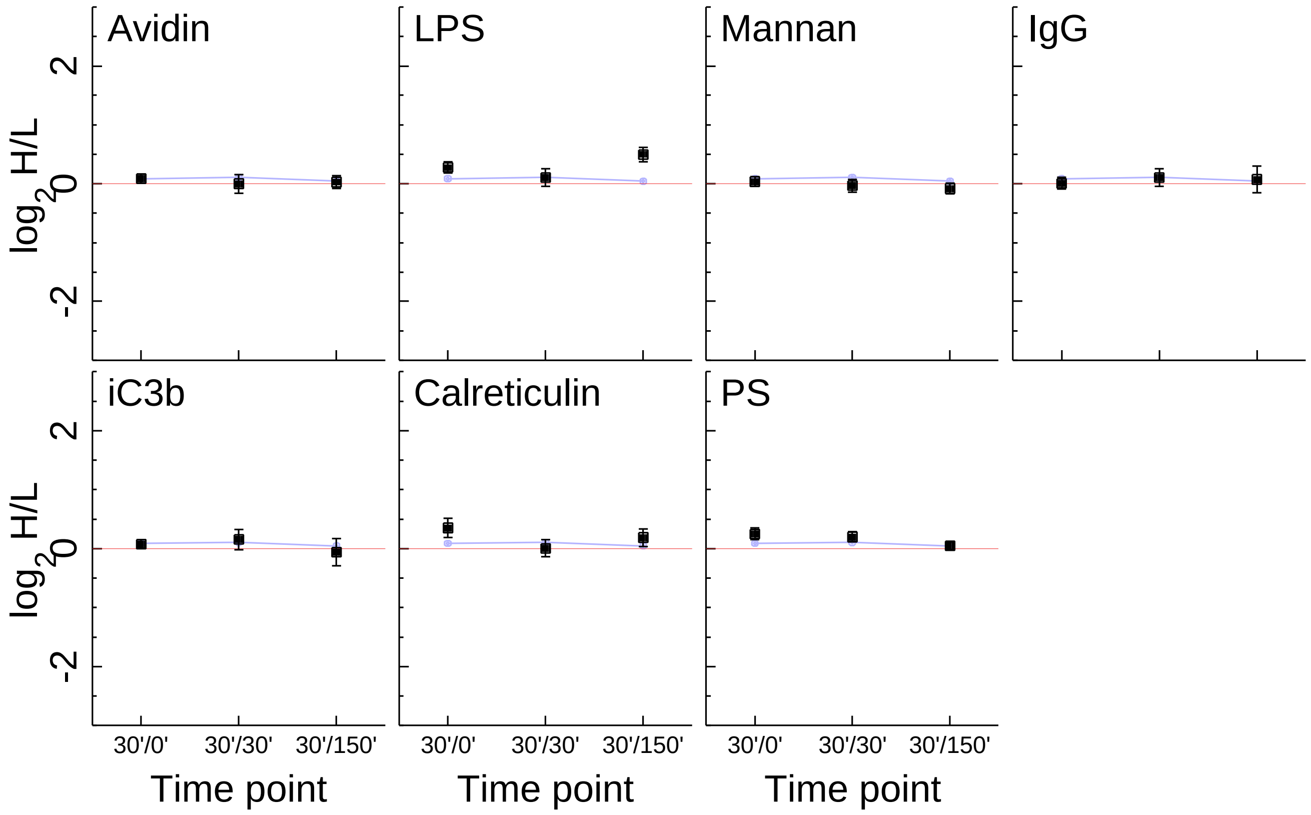

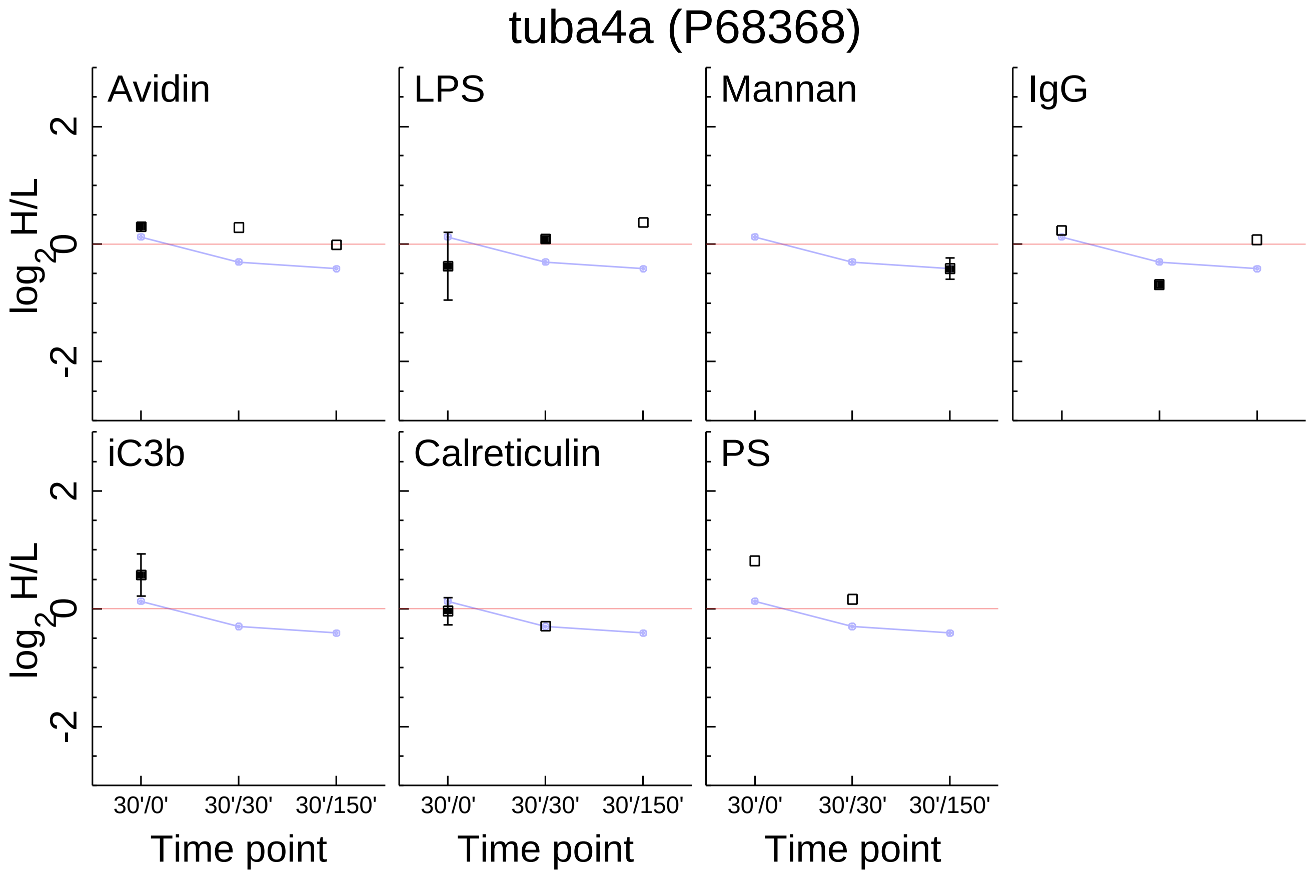

tuba4a (P68368)
Avidin
LPS
Mannan
IgG
iC3b
Calreticulin
PS
log2 H/L
2
0
-2
30'/0'
30'/30'
30'/150'
Time point

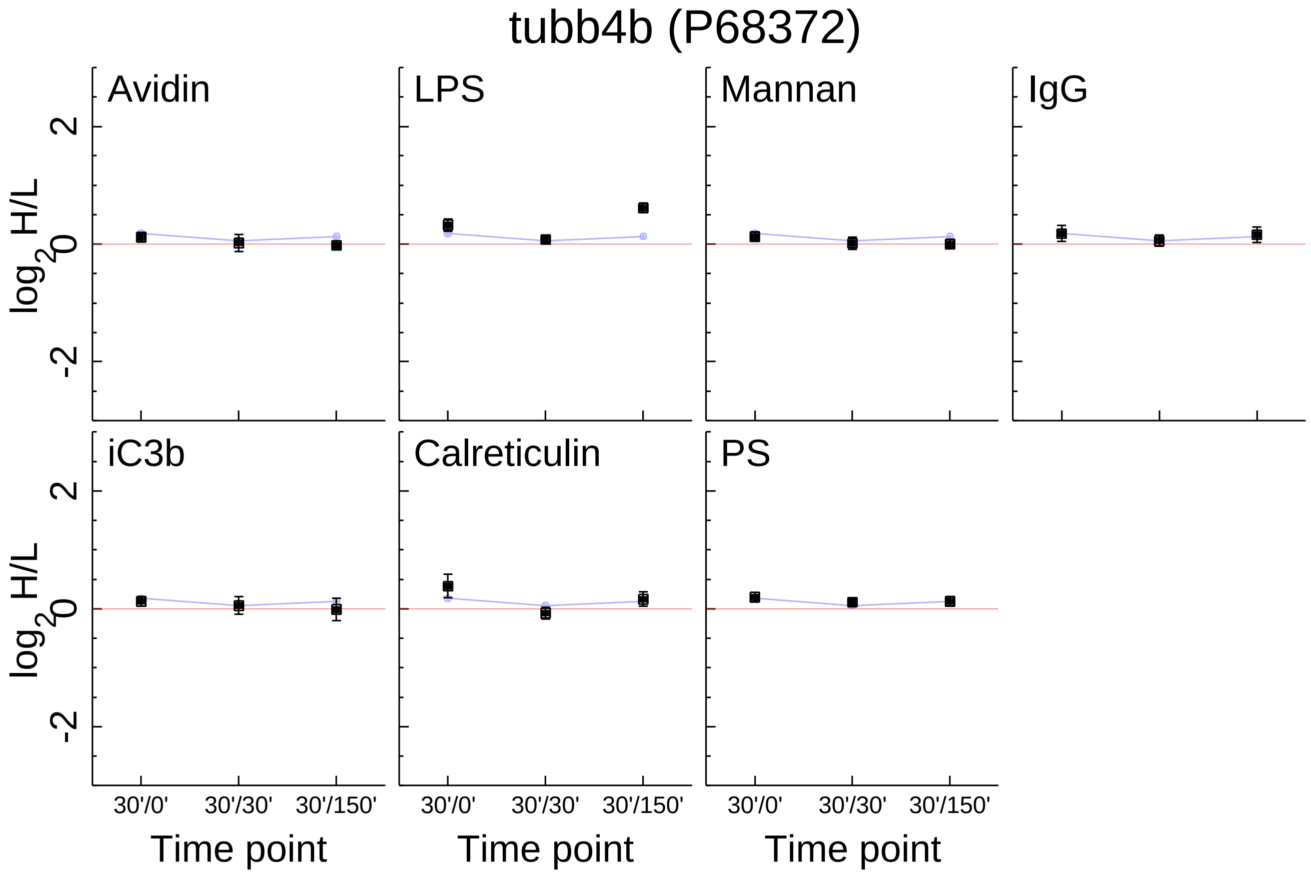

tubb4b (P68372)
Avidin
LPS
Mannan
IgG
iC3b
Calreticulin
PS
log2 H/L
2
0
-2
30'/0'
30'/30'
30'/150'
Time point

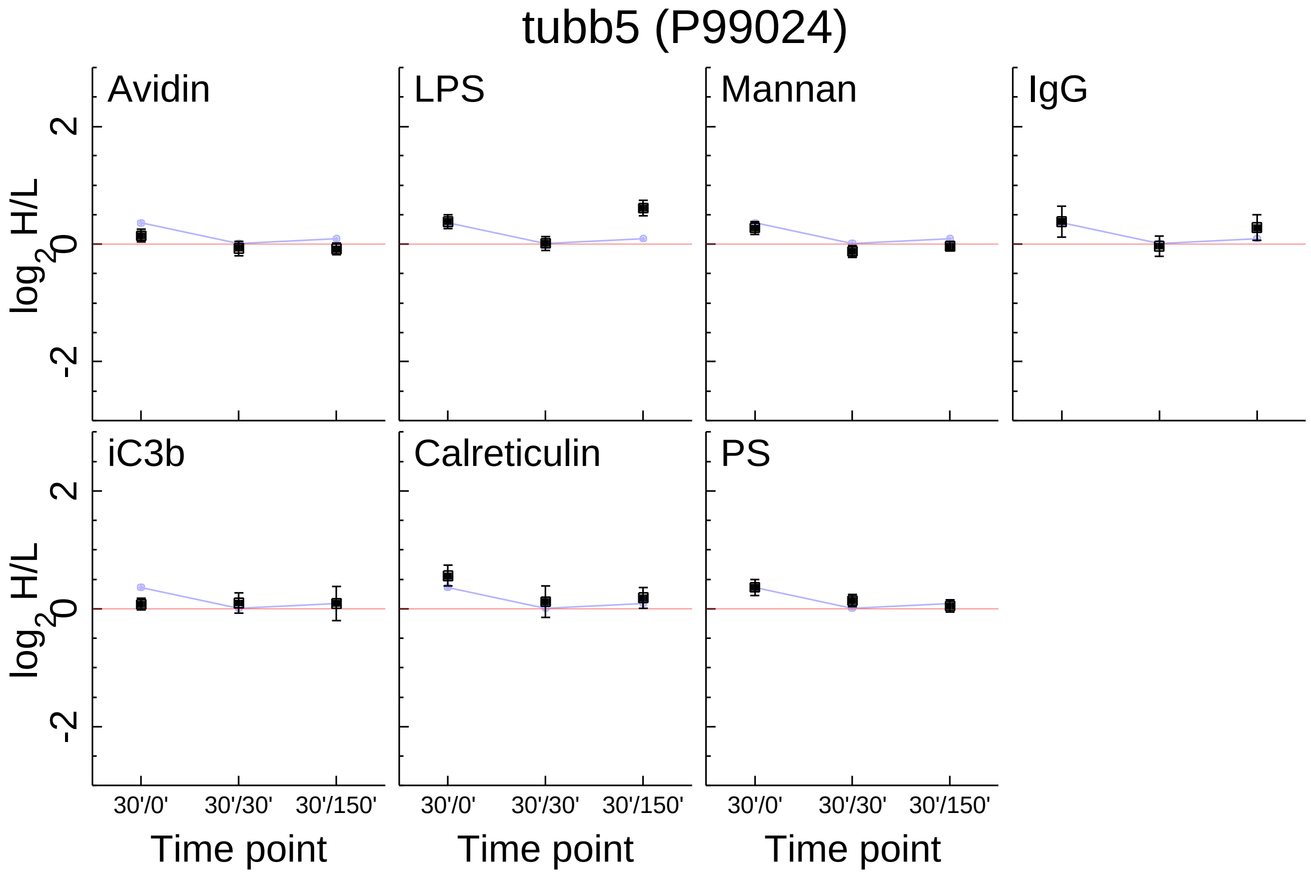

tubb5 (P99024)
Avidin
LPS
Mannan
IgG
iC3b
Calreticulin
PS
log2 H/L
2
0
-2
30'/0'
30'/30'
30'/150'
Time point

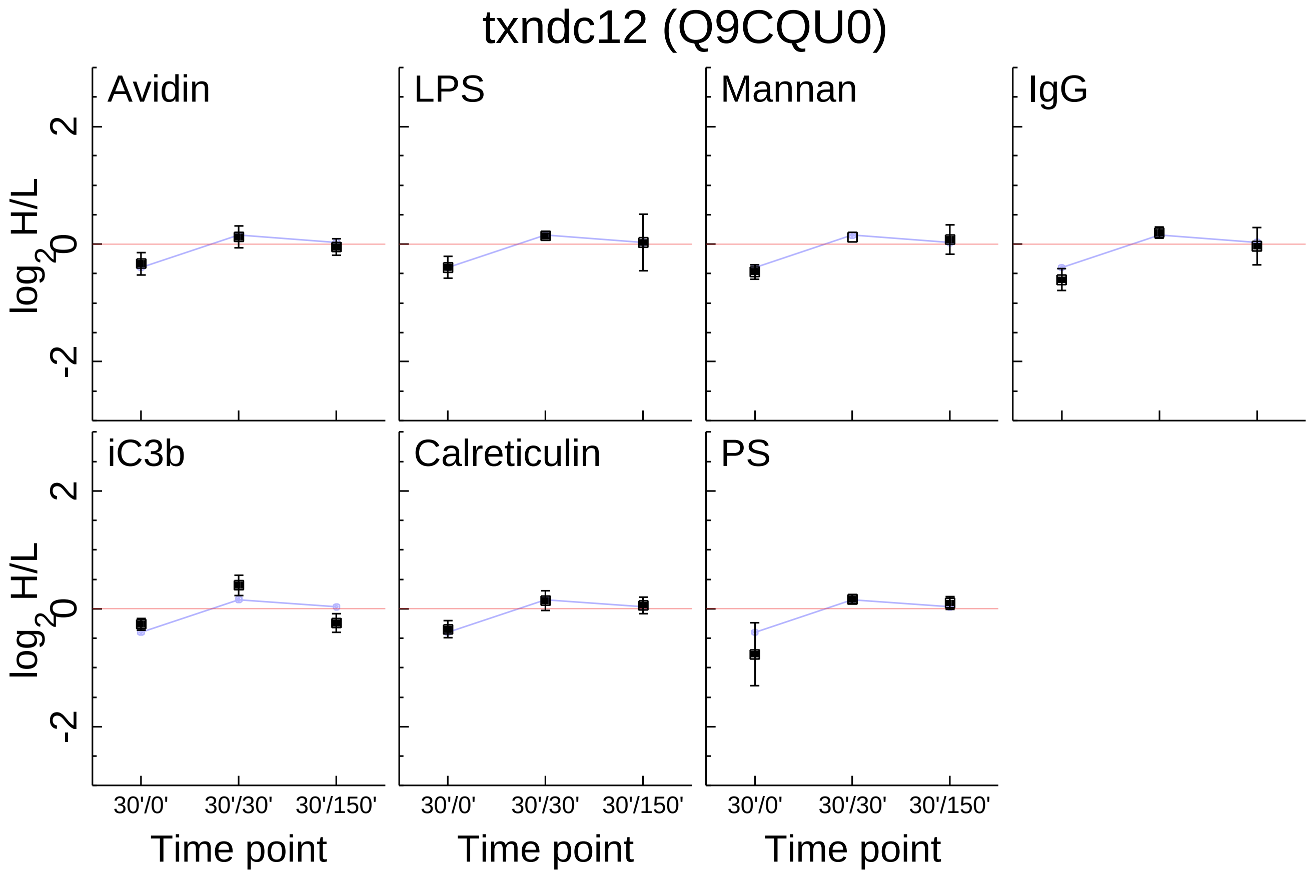
txndc12 (Q9CQU0)
Avidin
LPS
Mannan
IgG
iC3b
Calreticulin
PS
log2 H/L
2
0
-2
30'/0'
30'/30'
30'/150'
Time point

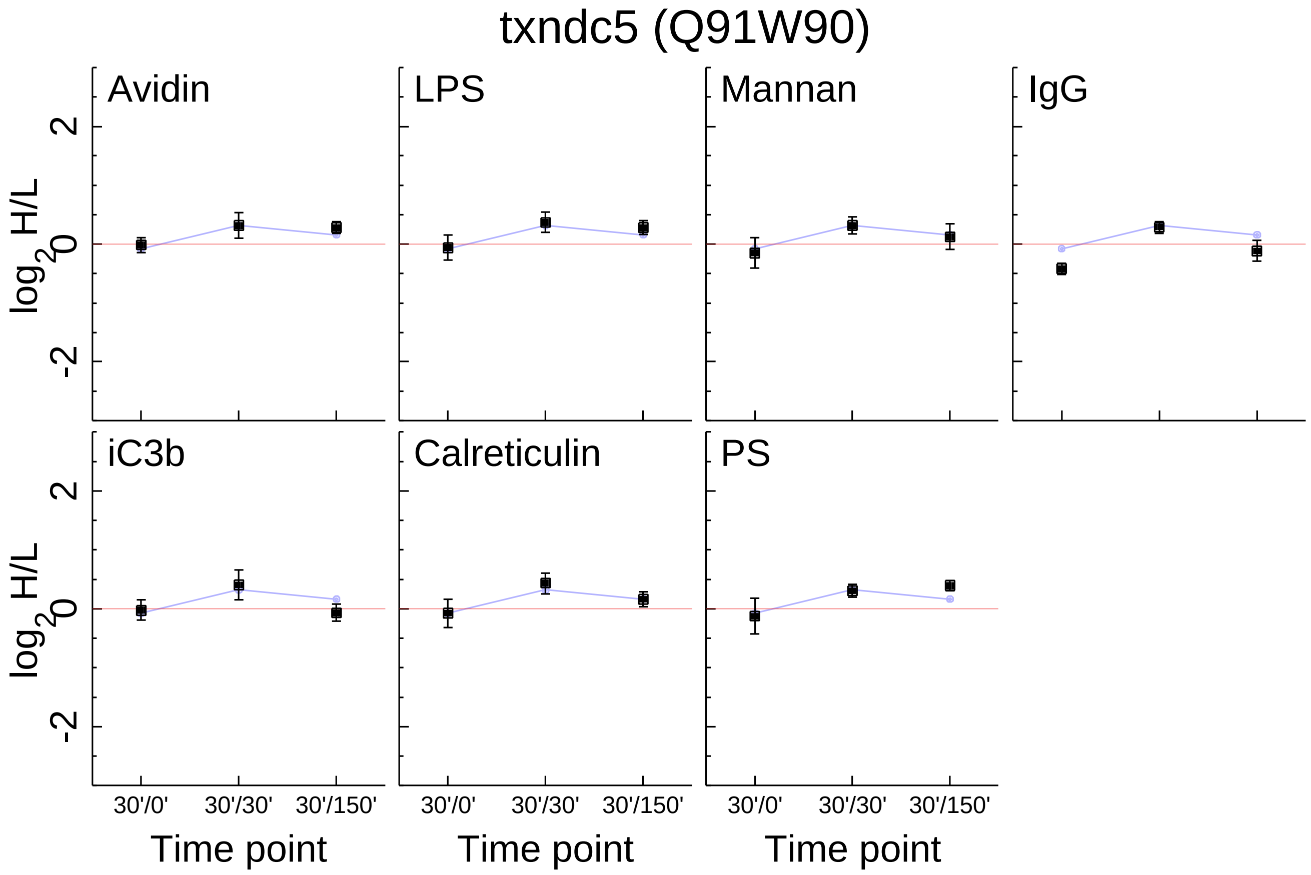

txndc5 (Q91W90)
Avidin
LPS
Mannan
IgG
iC3b
Calreticulin
PS
log2 H/L
2
0
-2
30'/0'
30'/30'
30'/150'
Time point

txn (P10639)

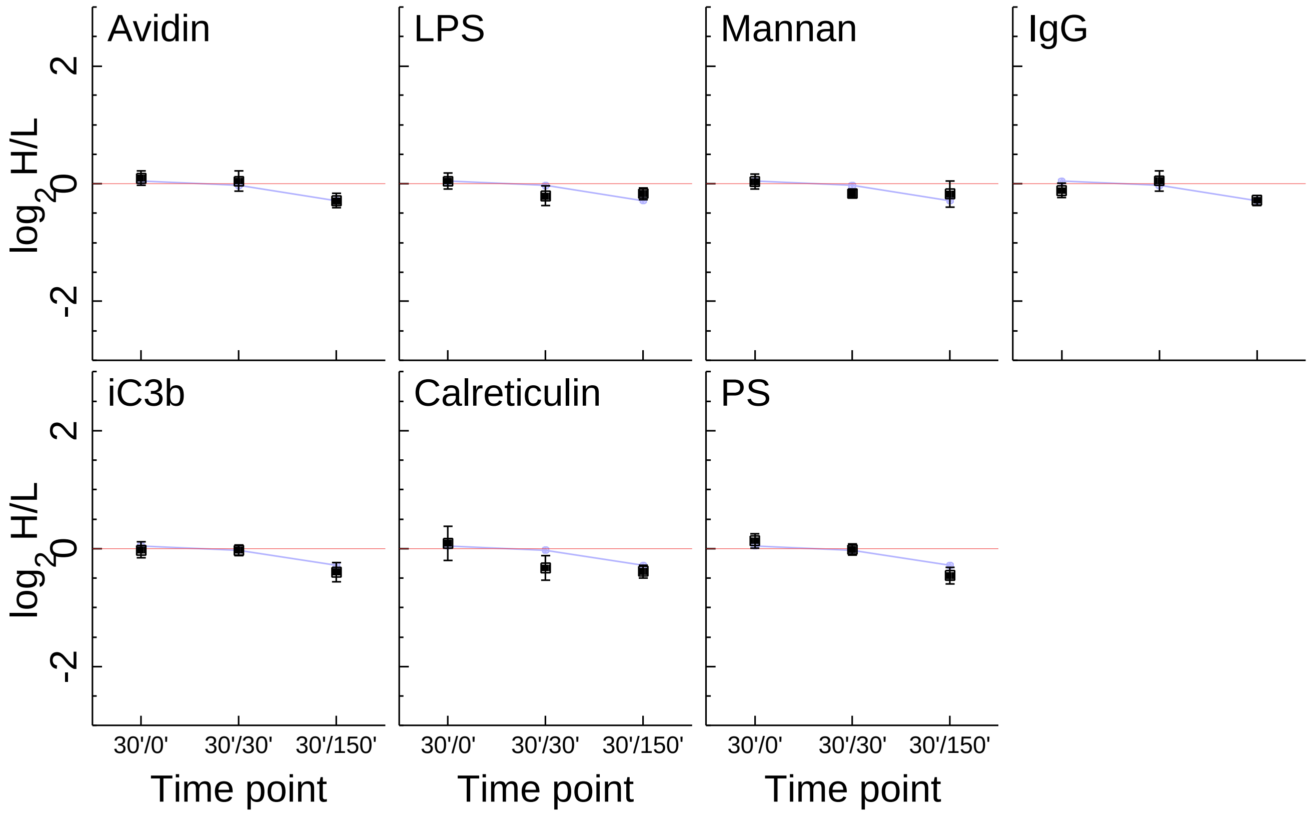

Avidin
LPS
Mannan
IgG
iC3b
Calreticulin
PS
log2 H/L
2
0
-2
30'/0'
30'/30'
30'/150'
Time point

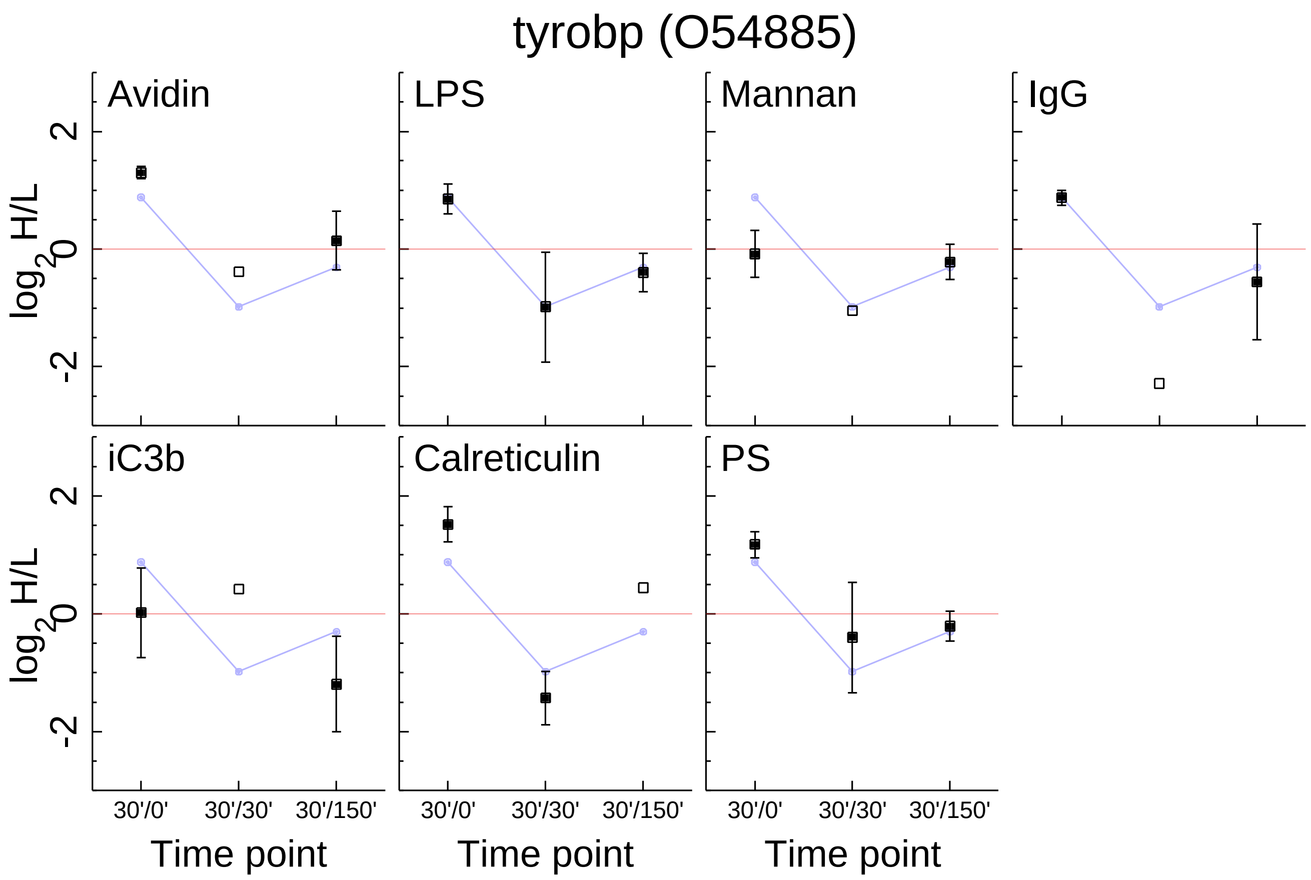
tyrobp (O54885)
Avidin
LPS
Mannan
IgG
iC3b
Calreticulin
PS
log2 H/L
2
0
-2
30'/0'
30'/30'
30'/150'
Time point

uba1 (Q02053)

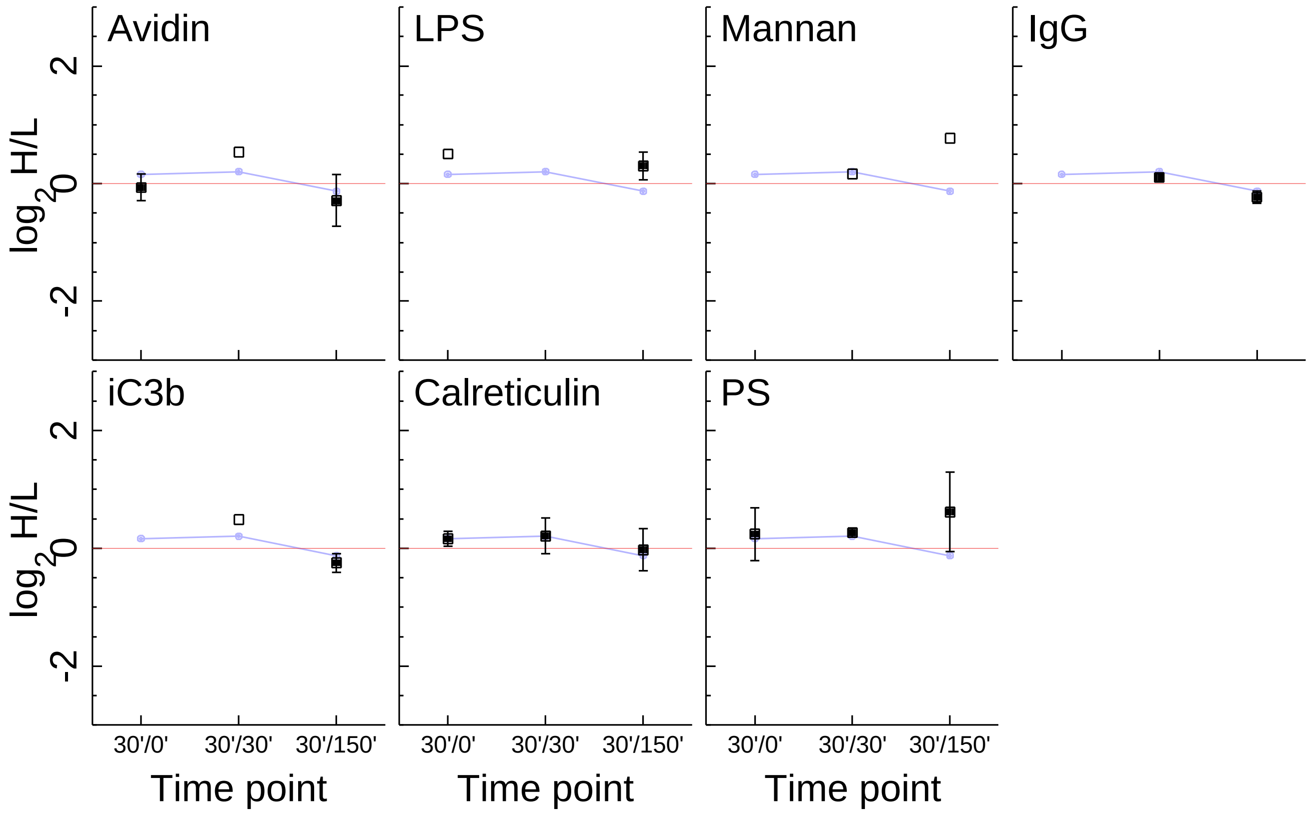

Avidin
LPS
Mannan
IgG
iC3b
Calreticulin
PS
log2 H/L
2
0
-2
30'/0'
30'/30'
30'/150'
Time point

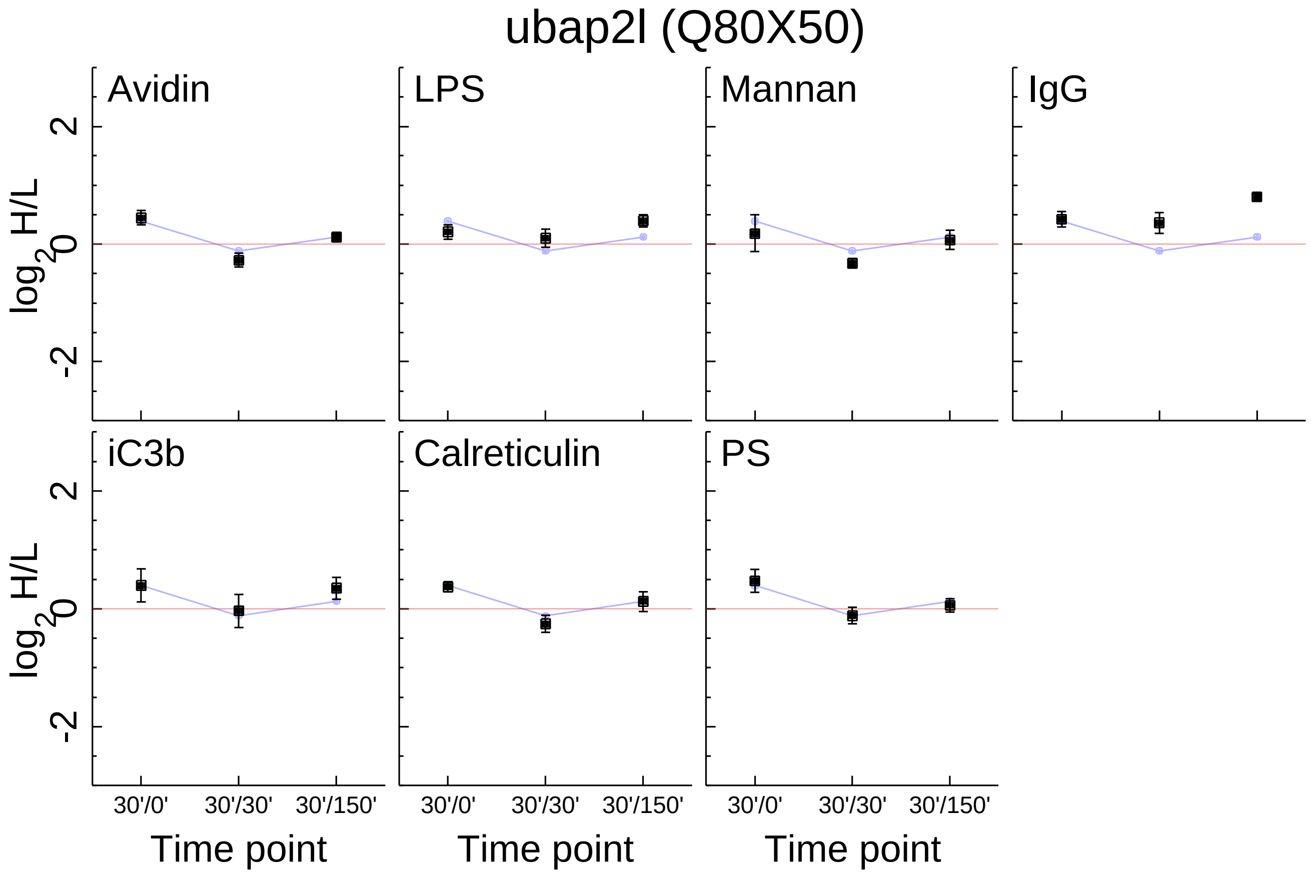
ubap2l (Q80X50)
Avidin
LPS
Mannan
IgG
iC3b
Calreticulin
PS
log2 H/L
2
0
-2
30'/0'
30'/30'
30'/150'
Time point

ubap2 (Q91VX2)

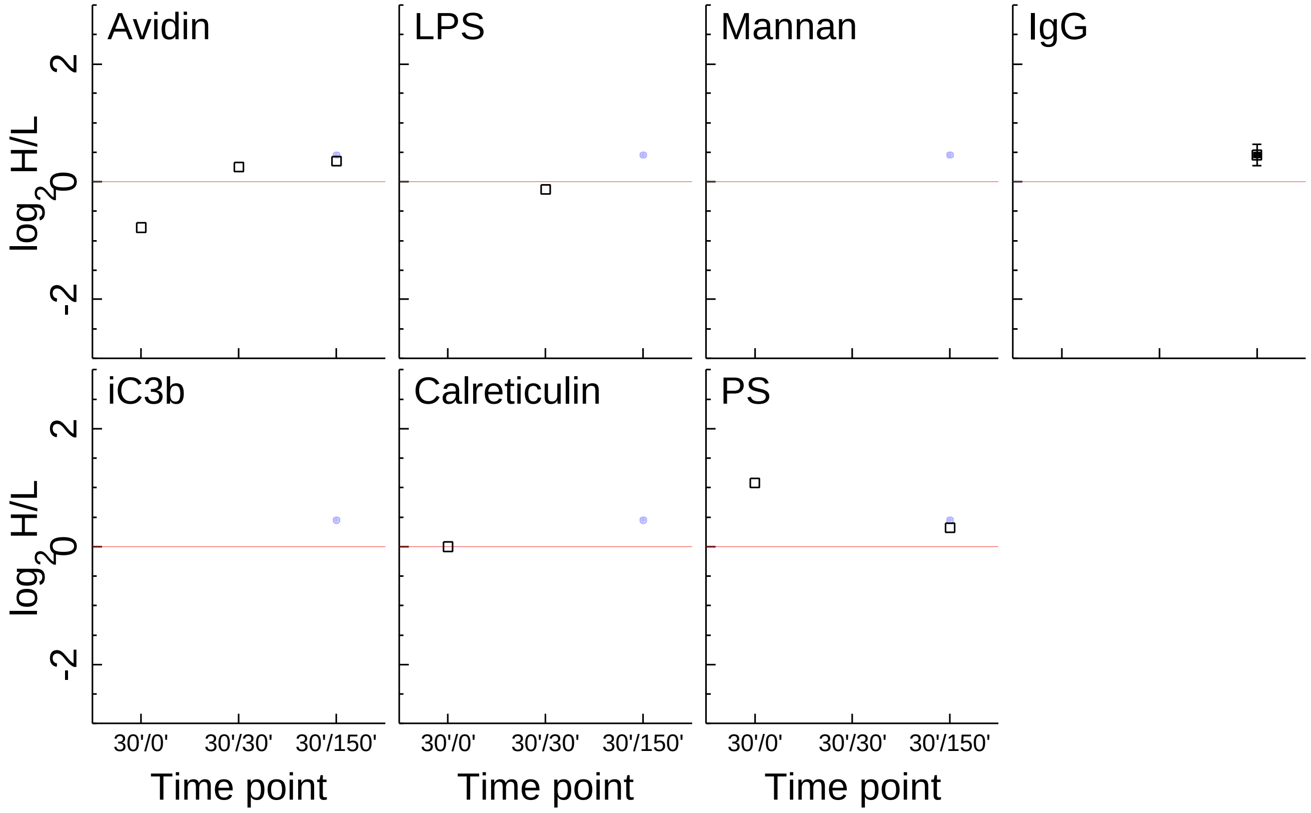

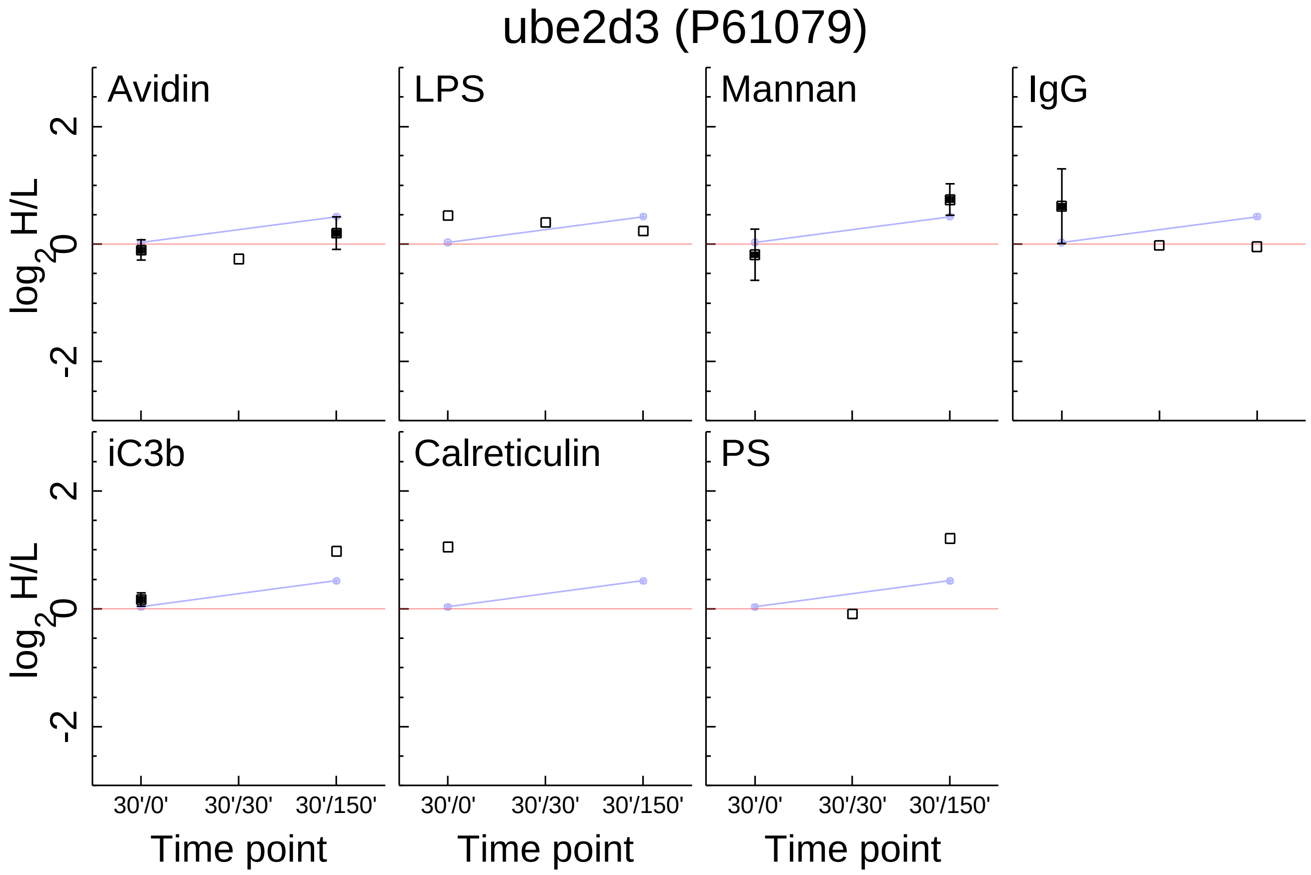
ube2d3 (P61079)
Avidin
LPS
Mannan
IgG
iC3b
Calreticulin
PS
log2 H/L
2
0
-2
30'/0'
30'/30'
30'/150'
Time point

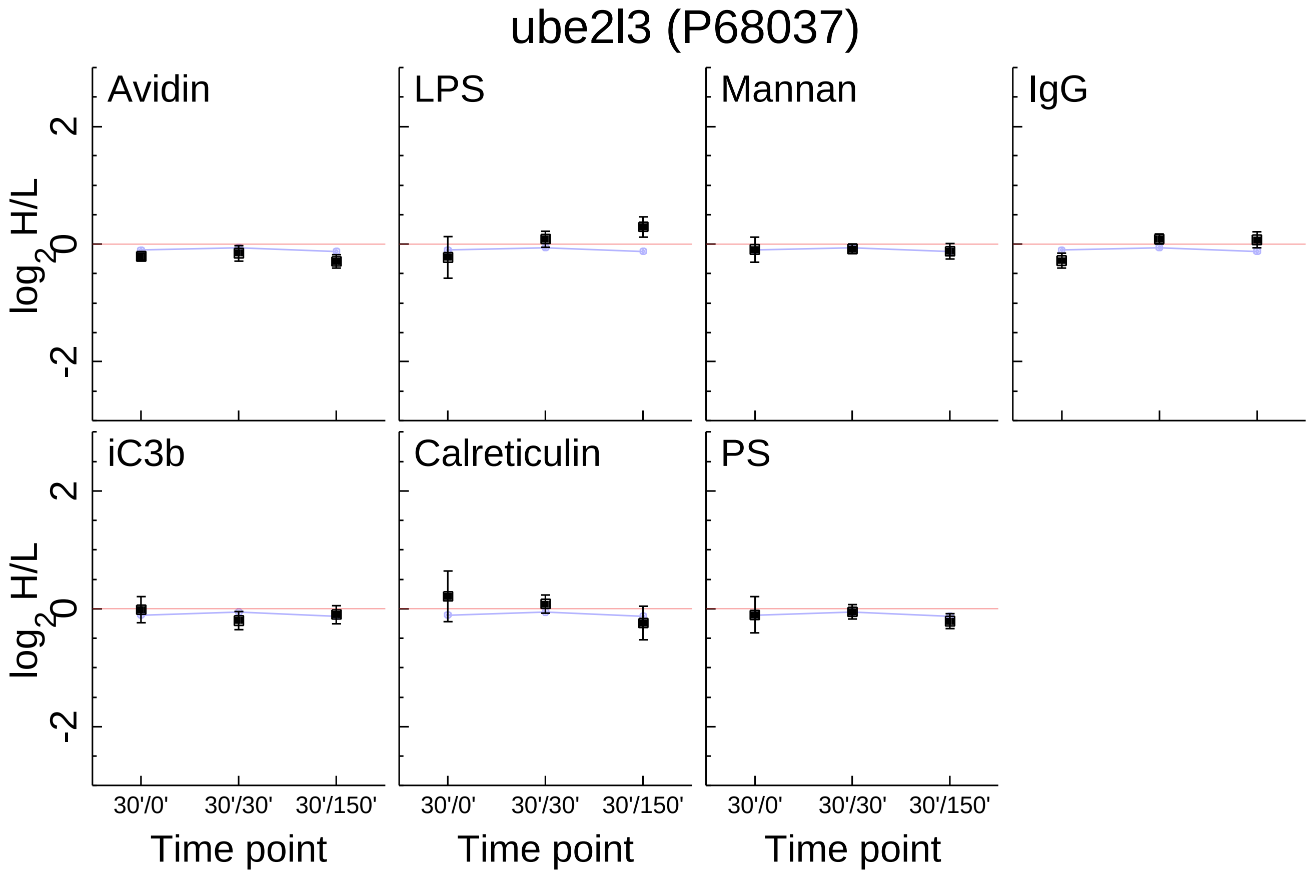

ube2l3 (P68037)
Avidin
LPS
Mannan
IgG
iC3b
Calreticulin
PS
log2 H/L
2
0
-2
30'/0'
30'/30'
30'/150'
Time point

ube2n (P61089)

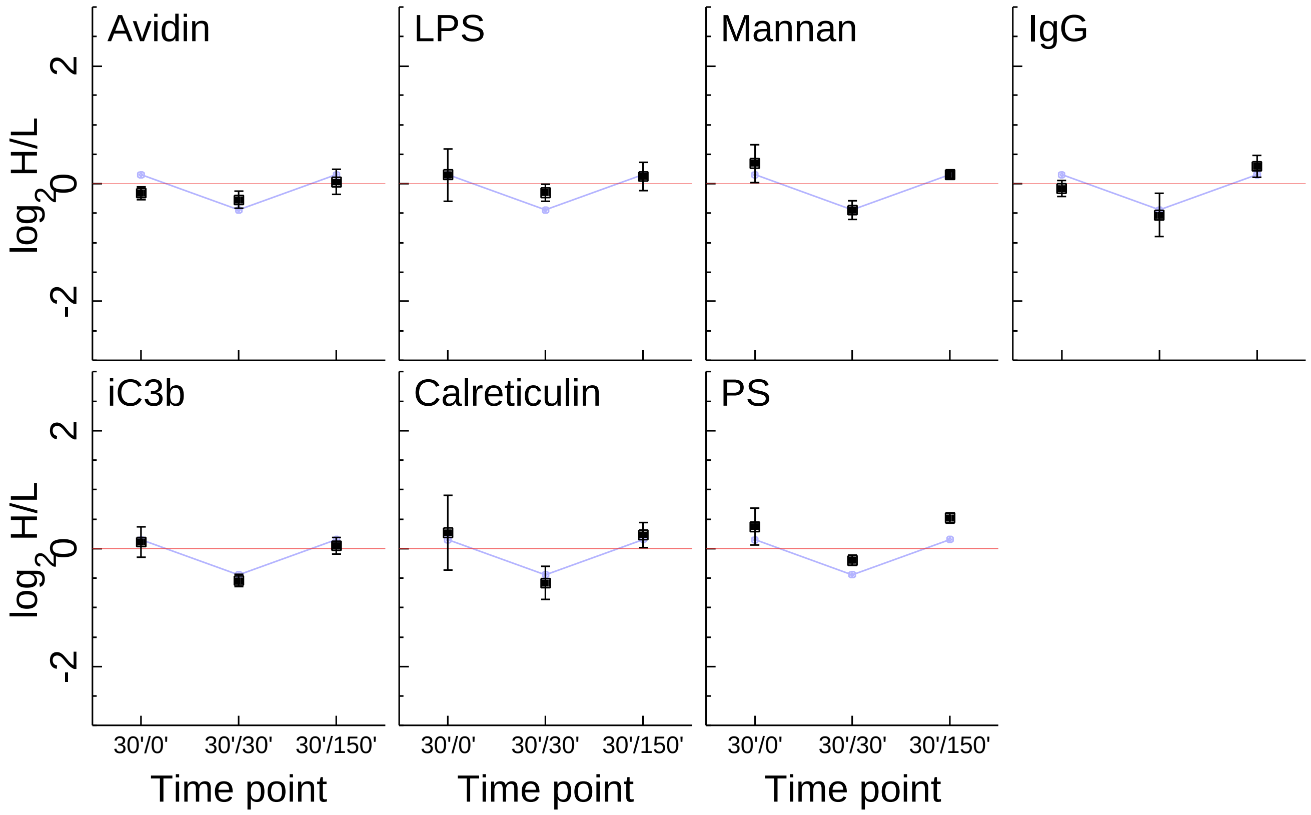

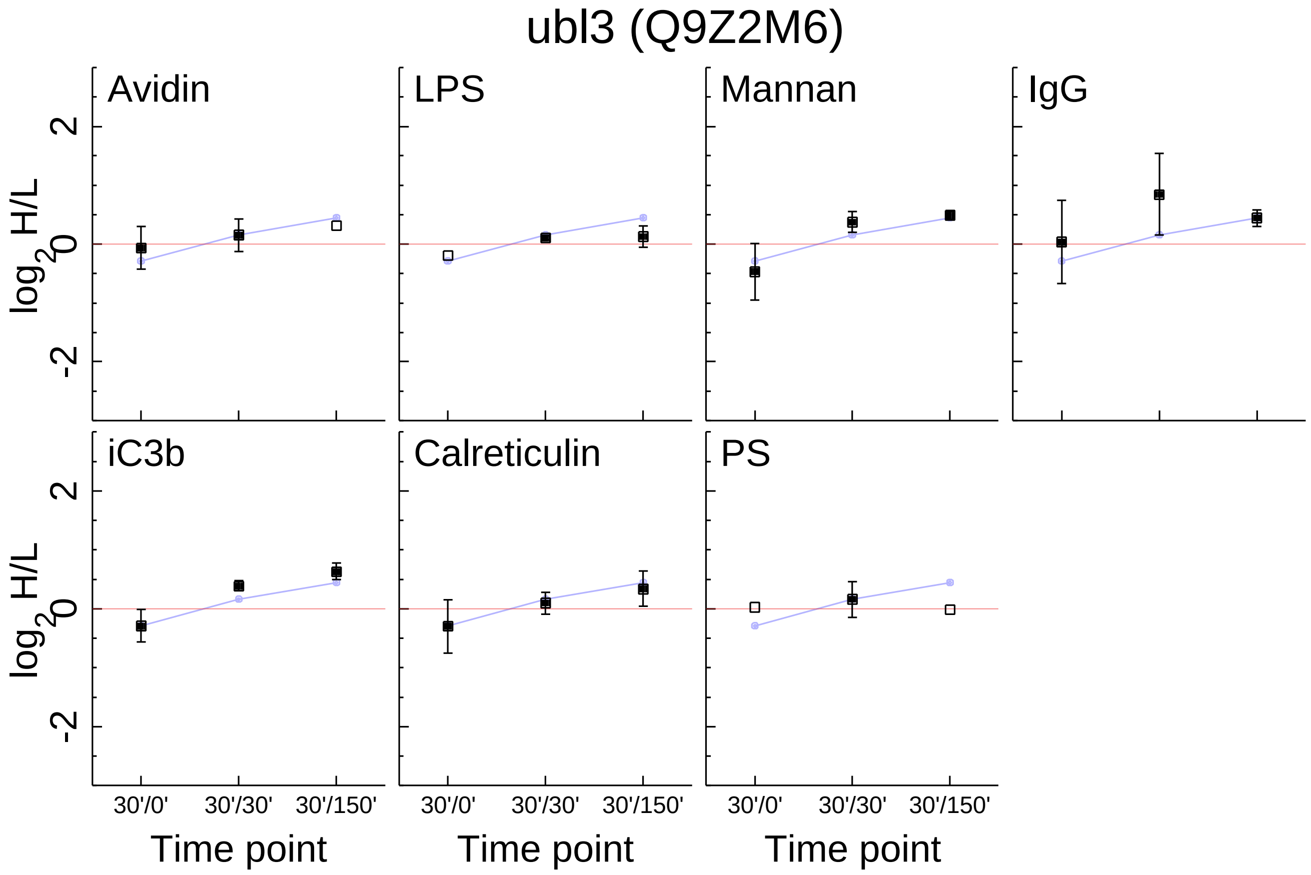

ubl3 (Q9Z2M6)
Avidin
LPS
Mannan
IgG
iC3b
Calreticulin
PS
log2 H/L
2
0
-2
30'/0'
30'/30'
30'/150'
Time point

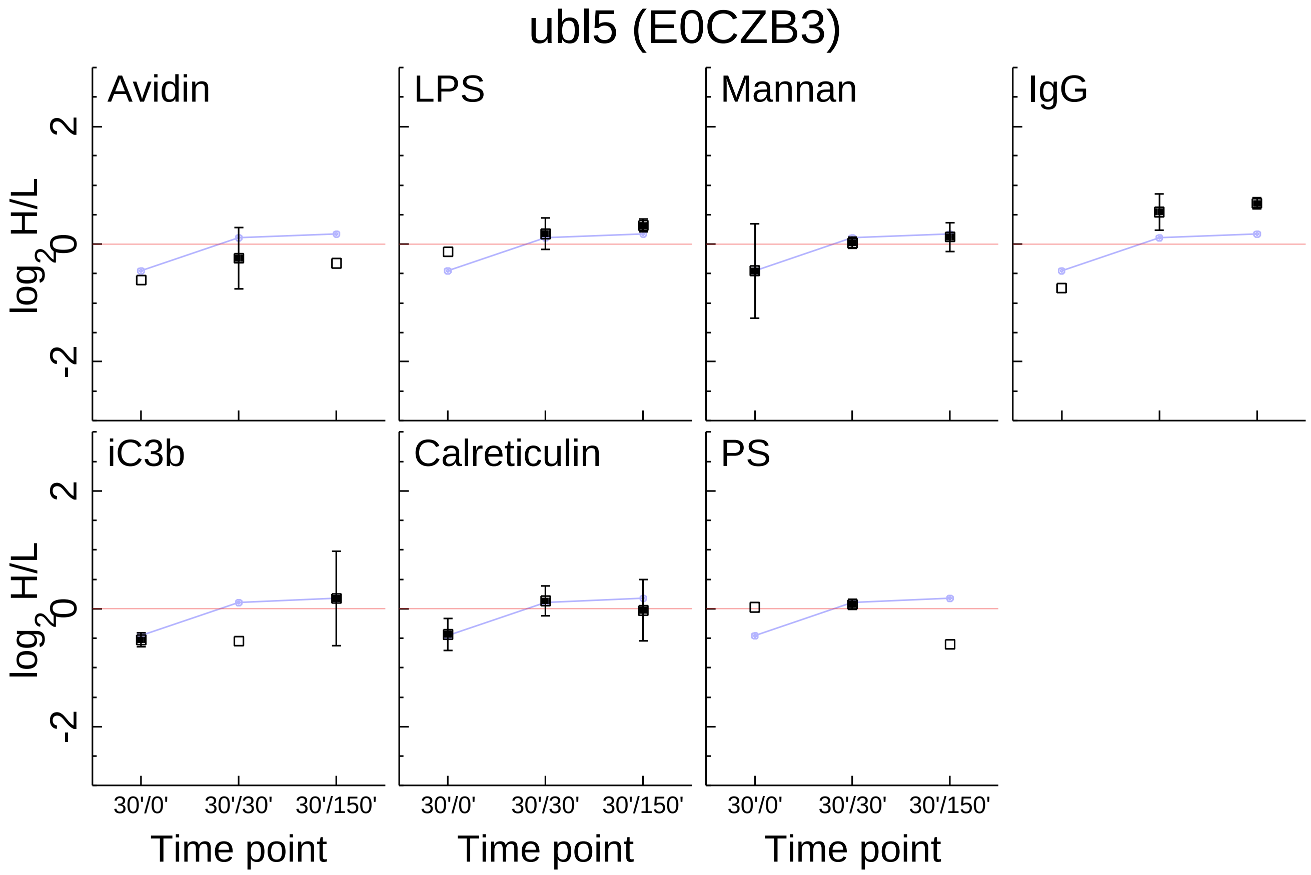

ubl5 (E0CZB3)
Avidin
LPS
Mannan
IgG
iC3b
Calreticulin
PS
log2 H/L
2
0
-2
30'/0'
30'/30'
30'/150'
Time point

ubtf (P25976)

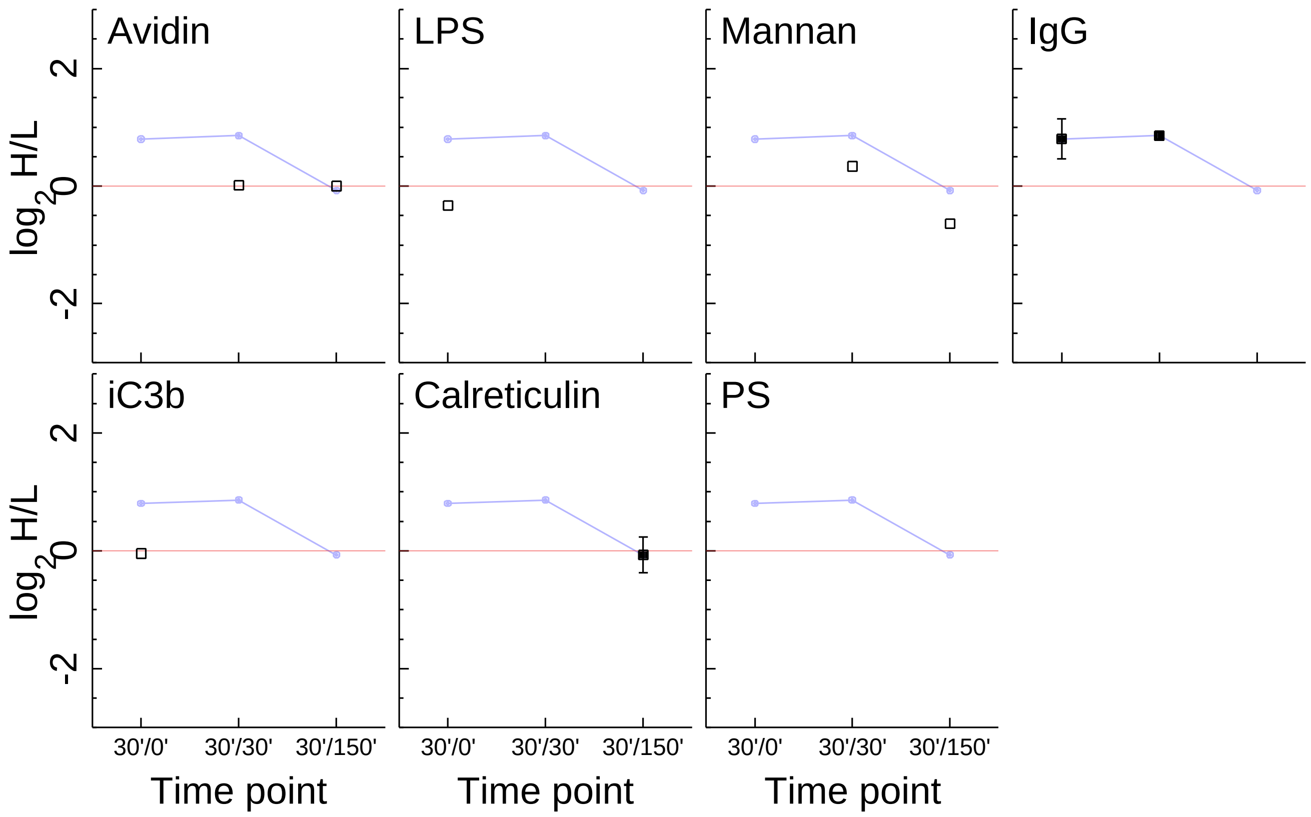

Avidin
LPS
Mannan
IgG
iC3b
Calreticulin
PS
2
0
-2
log2 H/L
30'/0'
30'/30'
30'/150'
Time point

ubxn4 (Q8VCH8)

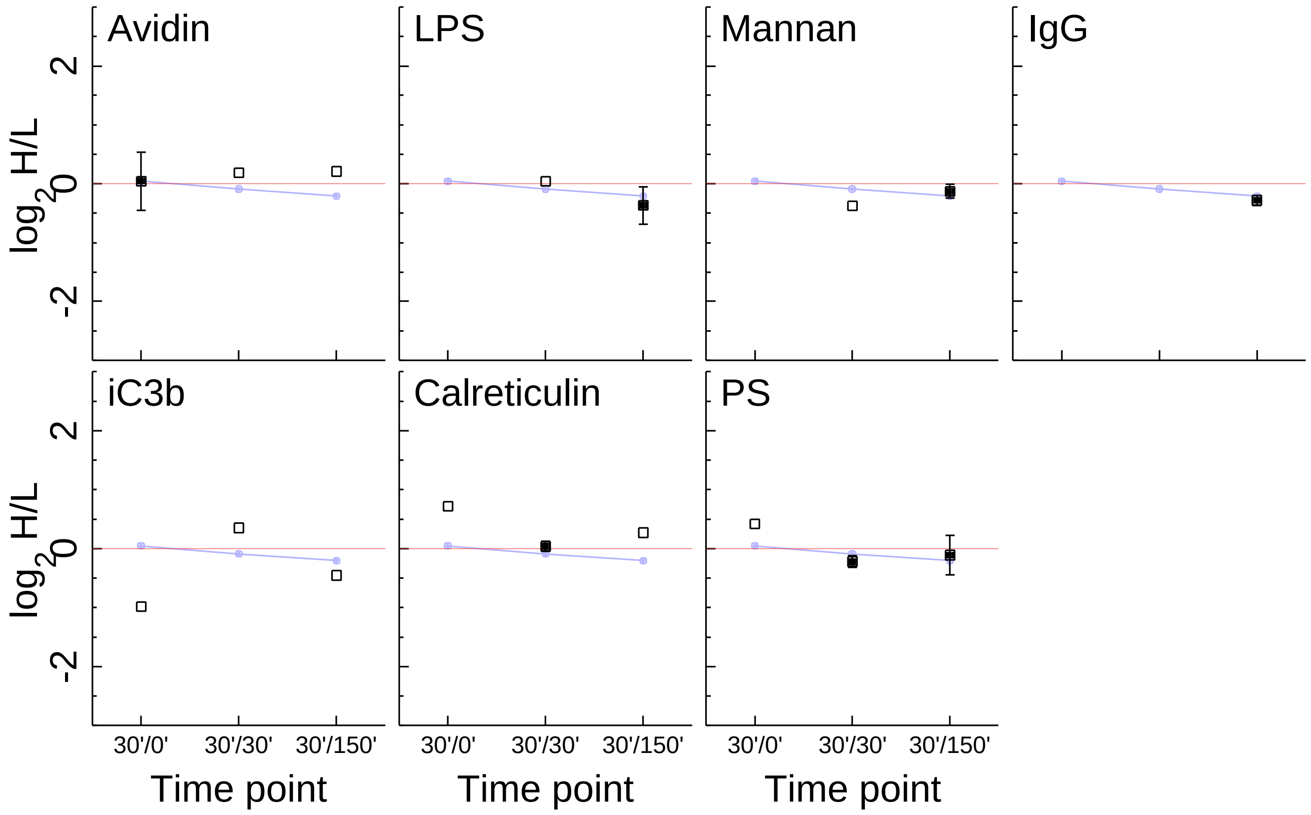

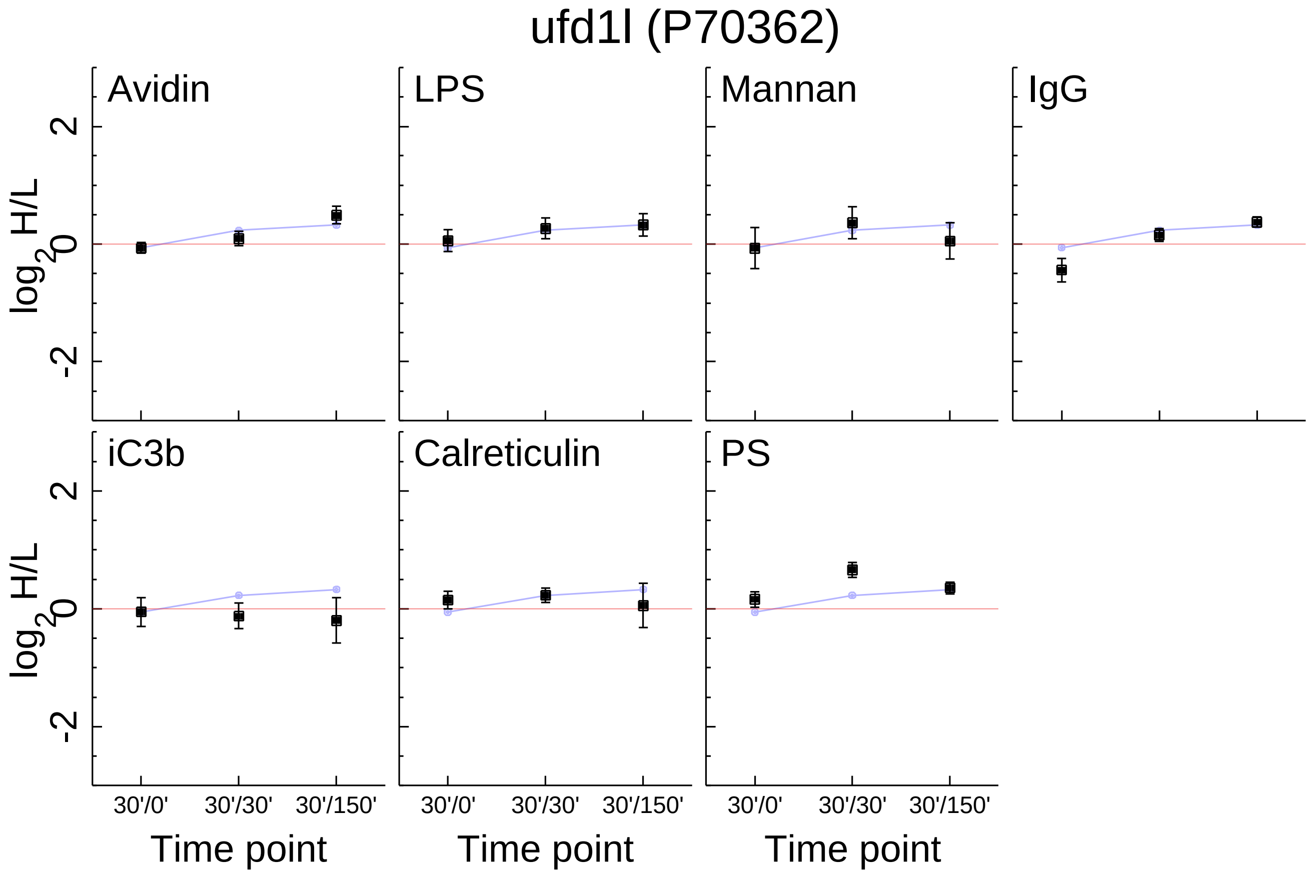

ufd1l (P70362)
Avidin
LPS
Mannan
IgG
iC3b
Calreticulin
PS
log2 H/L
2
0
-2
30'/0'
30'/30'
30'/150'
Time point

ufm1 (F6XUP5)

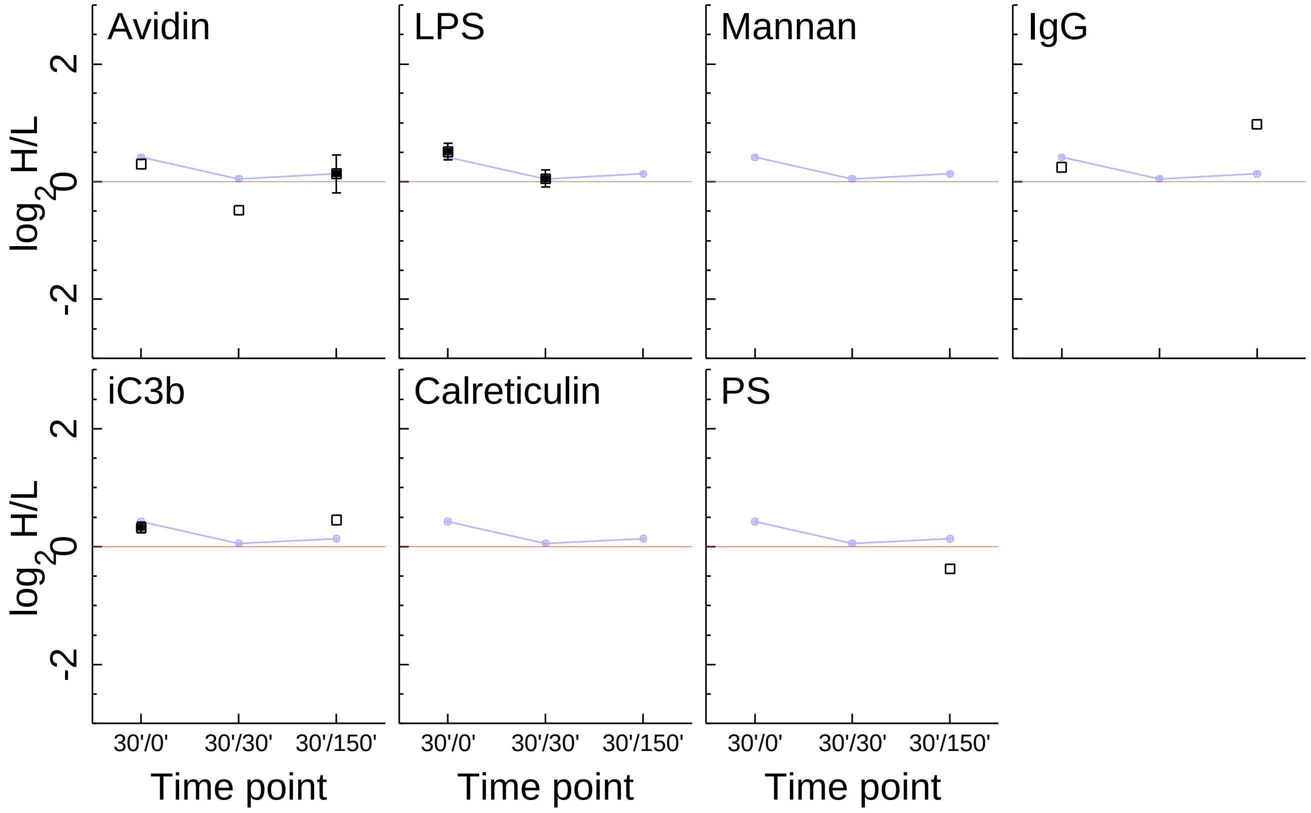

Avidin
LPS
Mannan
IgG
iC3b
Calreticulin
PS
log2 H/L
2
0
-2
30'/0'
30'/30'
30'/150'
Time point

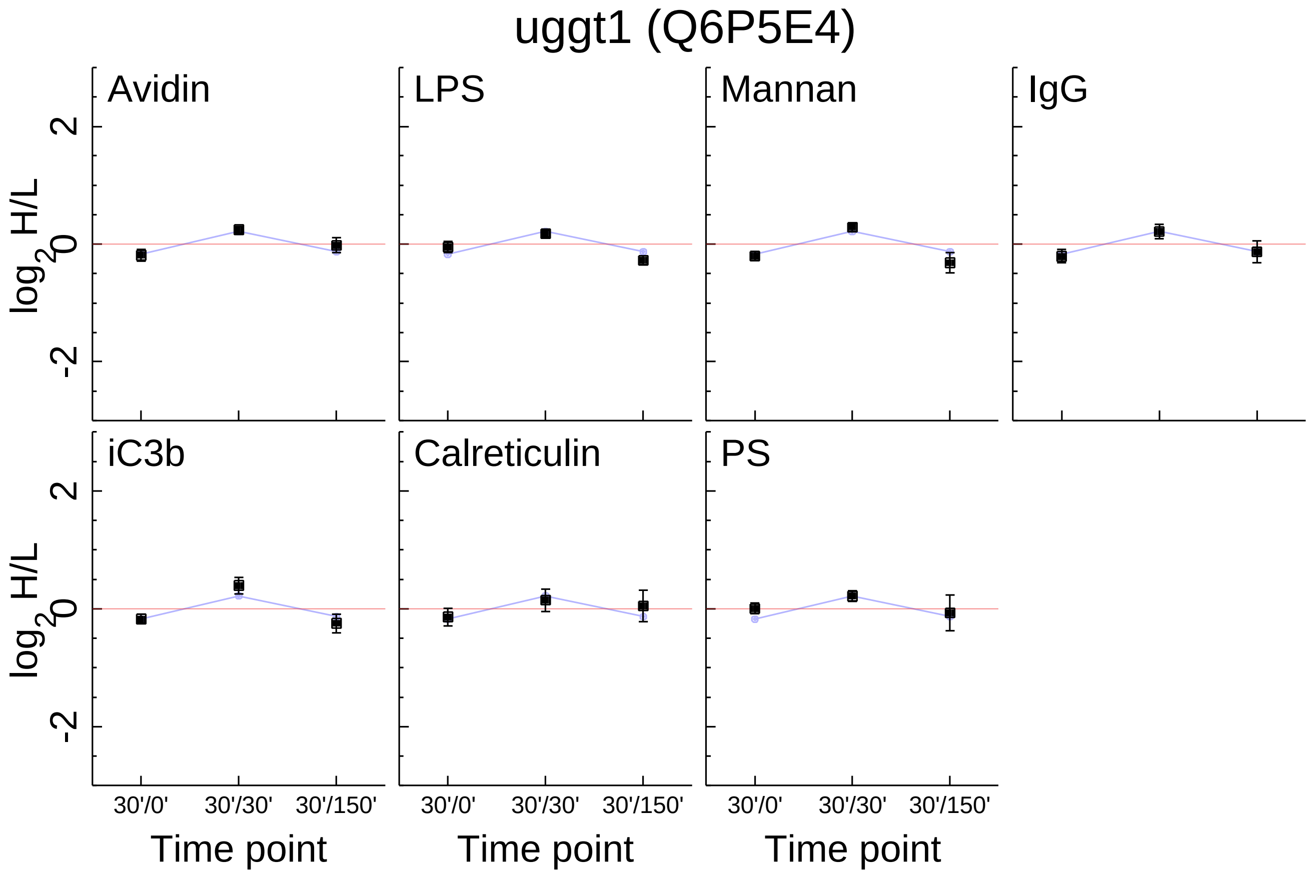

uggt1 (Q6P5E4)
Avidin
LPS
Mannan
IgG
iC3b
Calreticulin
PS
2
0
-2
log2 H/L
30'/0'
30'/30'
30'/150'
Time point

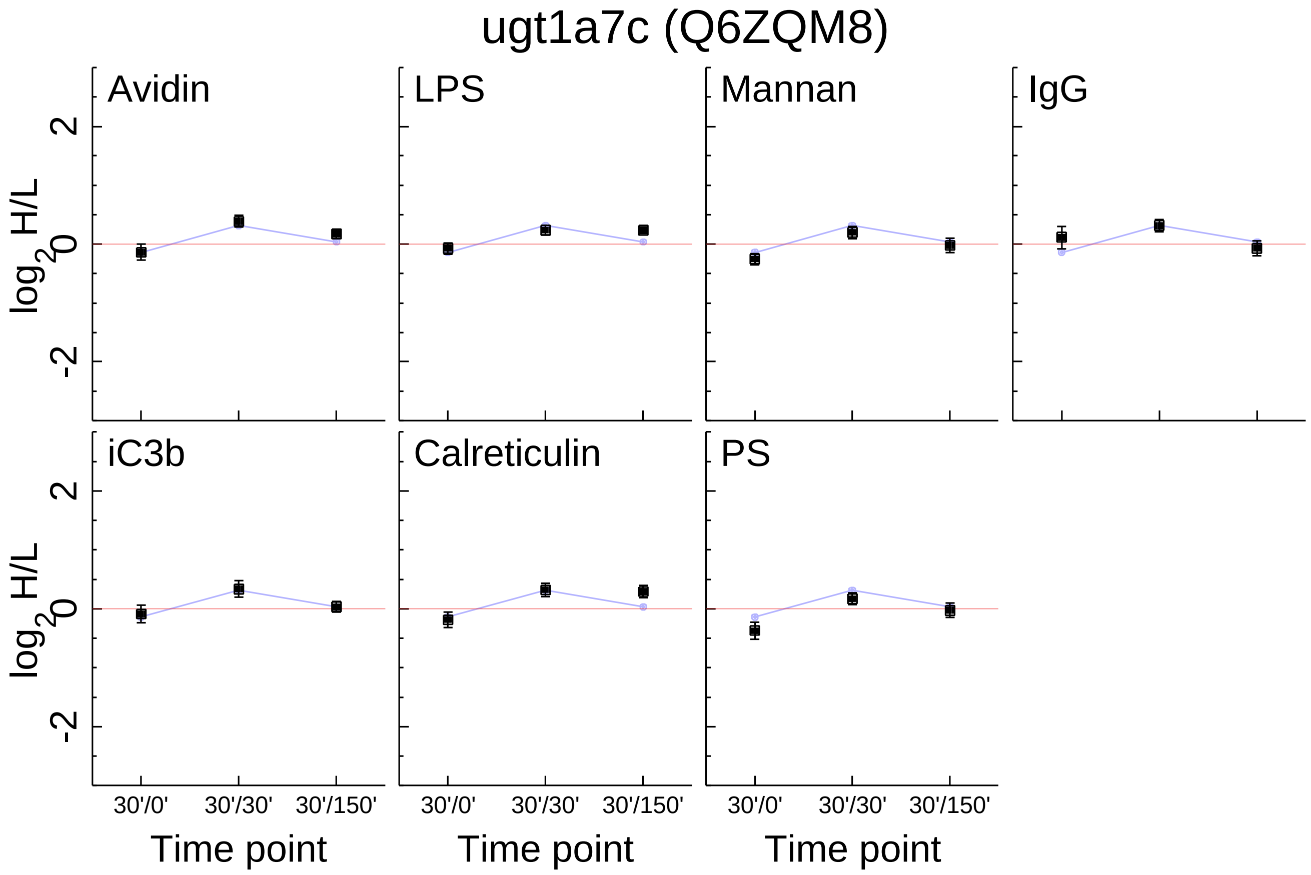
ugt1a7c (Q6ZQM8)
Avidin
LPS
Mannan
IgG
iC3b
Calreticulin
PS
log2 H/L
2
0
-2
30'/0'
30'/30'
30'/150'
Time point

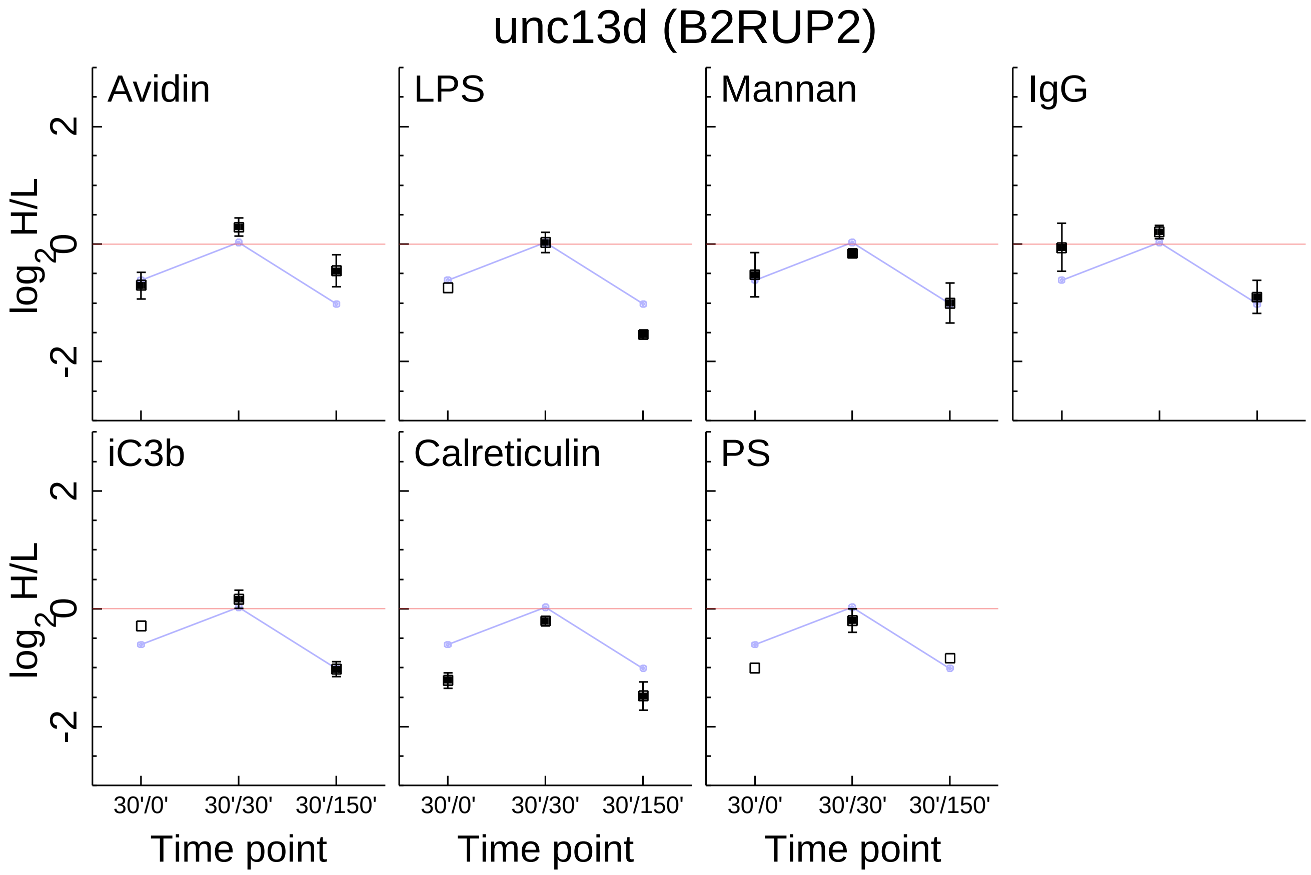
unc13d (B2RUP2)
Avidin
LPS
Mannan
IgG
iC3b
Calreticulin
PS
log2 H/L
2
0
-2
30'/0'
30'/30'
30'/150'
Time point

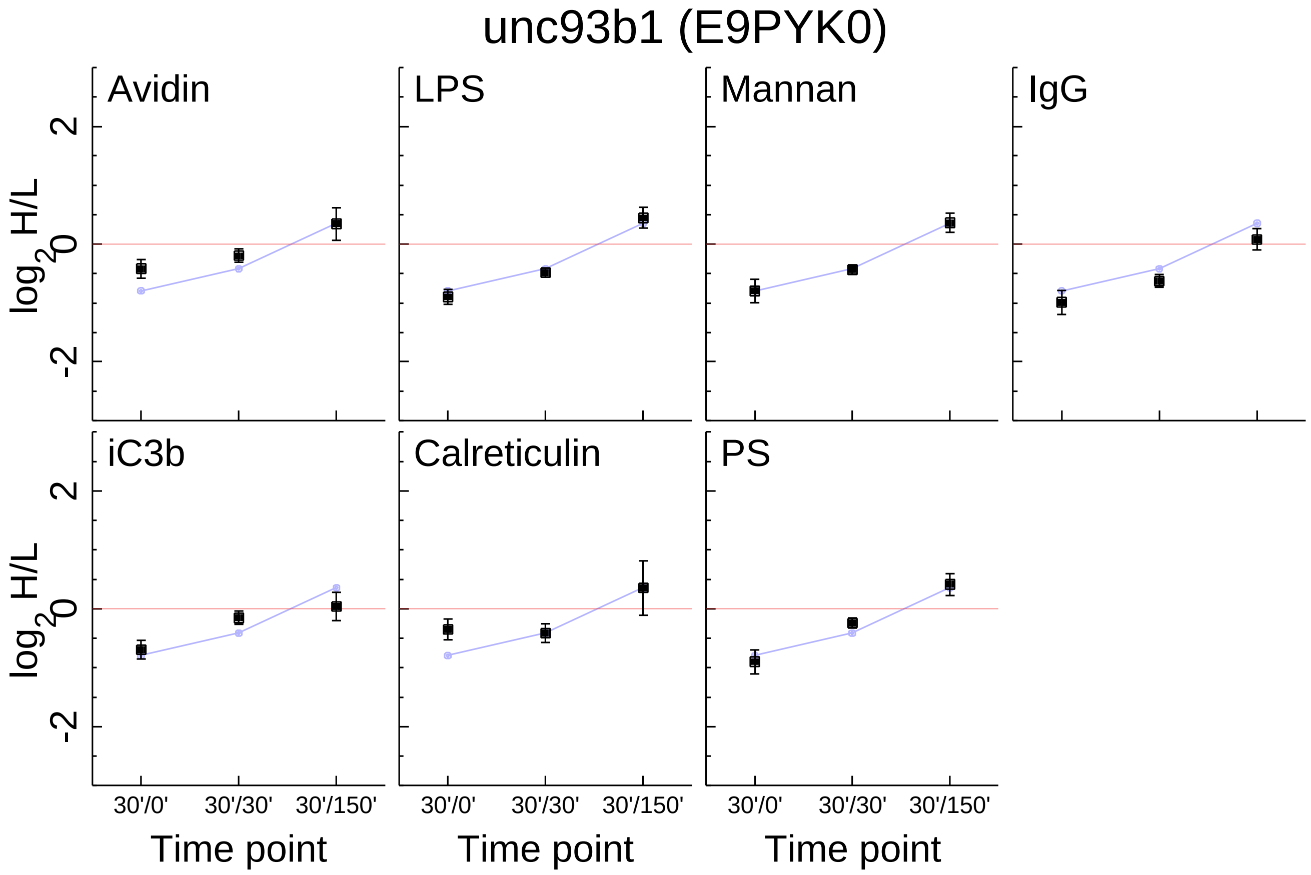
unc93b1 (E9PYK0)
Avidin
LPS
Mannan
IgG
iC3b
Calreticulin
PS
log2 H/L
2
0
-2
30'/0'
30'/30'
30'/150'
Time point

upf1 (Q9EPU0)

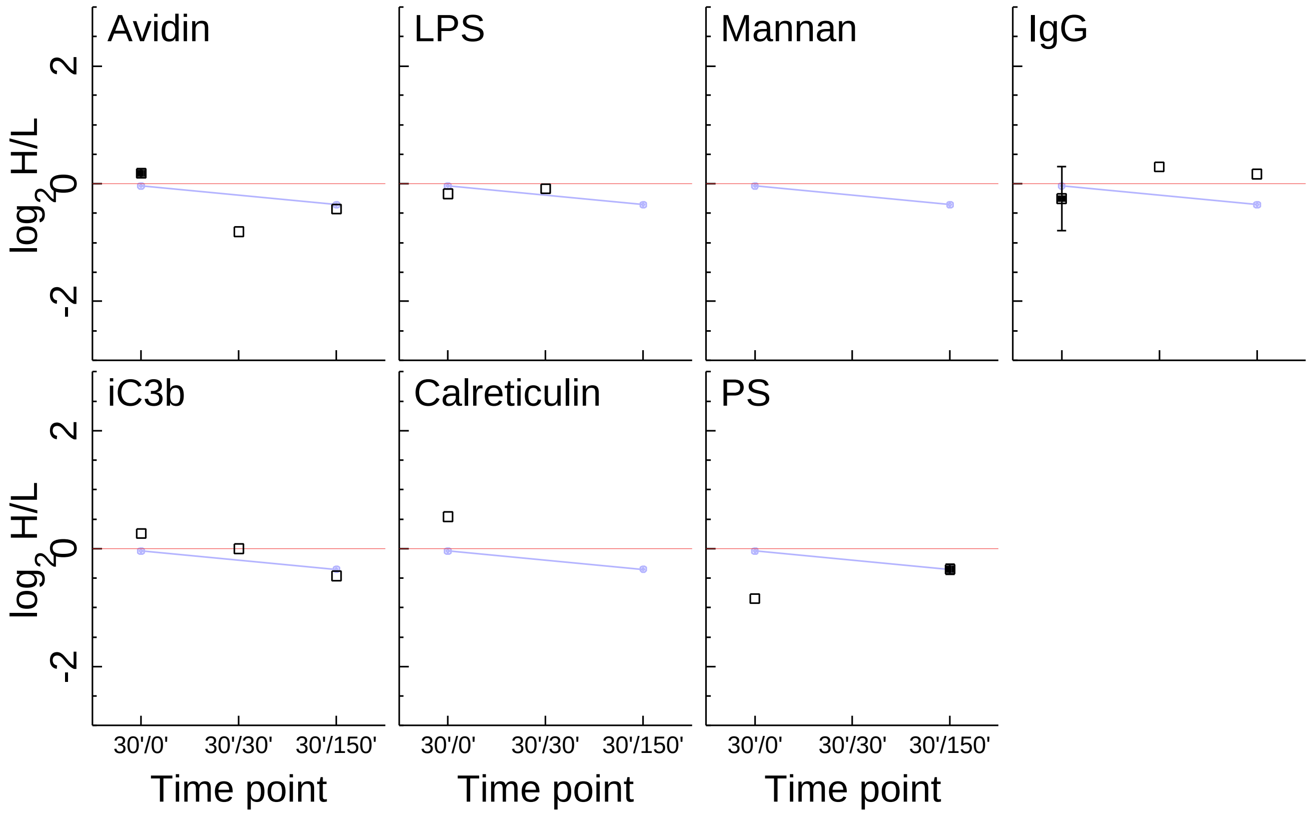

Avidin
LPS
Mannan
IgG
iC3b
Calreticulin
PS
log2 H/L
2
0
-2
30'/0'
30'/30'
30'/150'
Time point

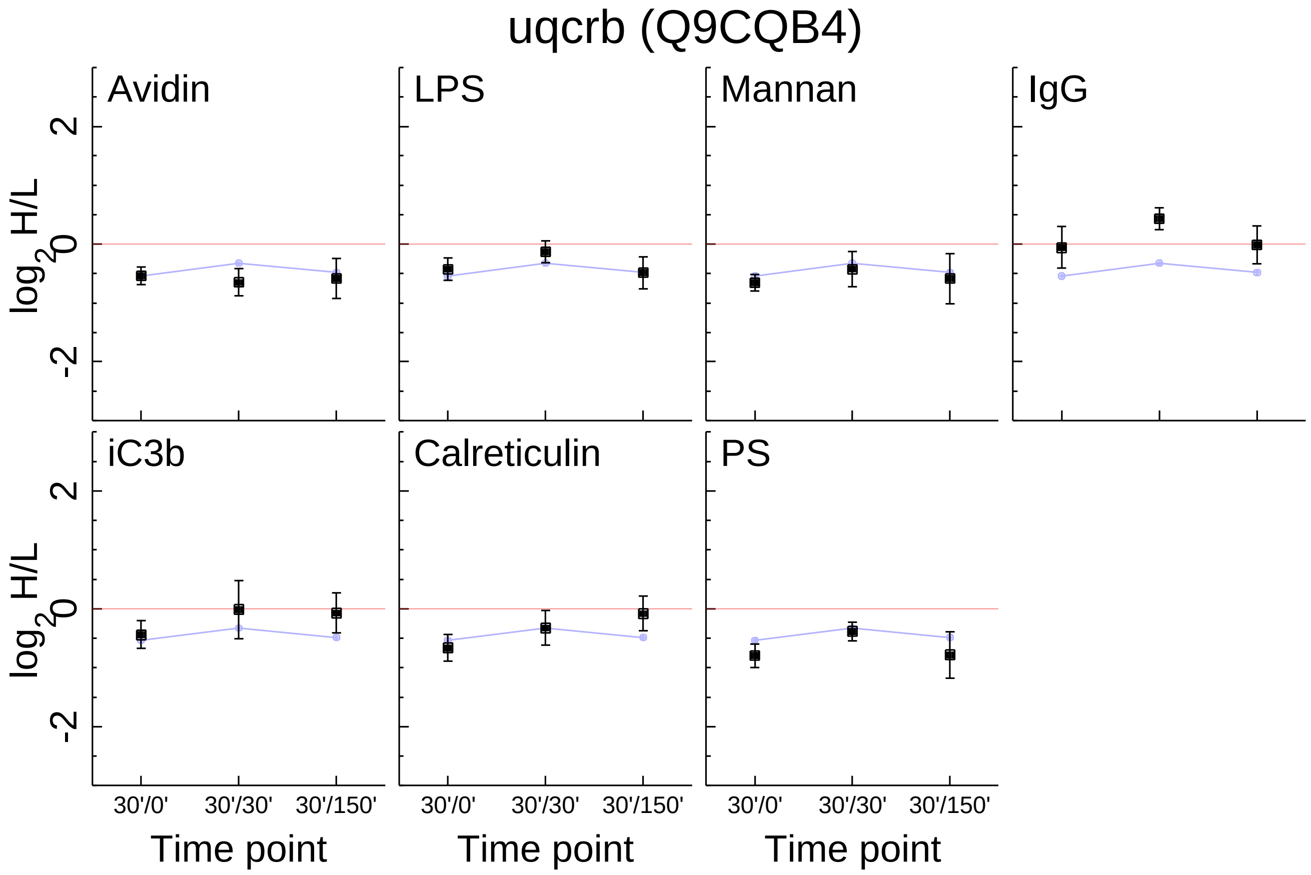
uqcrb (Q9CQB4)
Avidin
LPS
Mannan
IgG
iC3b
Calreticulin
PS
log2 H/L
2
0
-2
30'/0'
30'/30'
30'/150'
Time point

uqcrc1 (Q9CZ13)

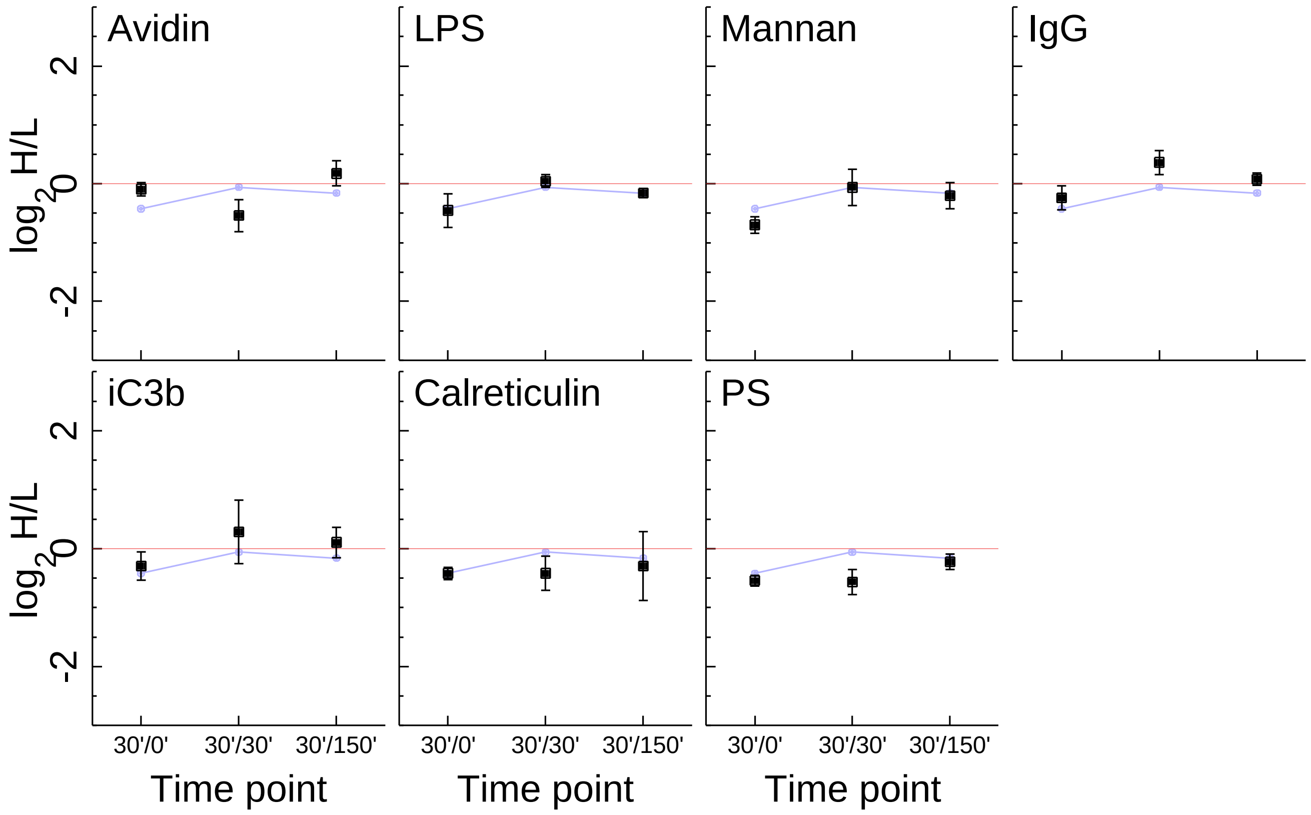

uqcrc2 (Q9DB77)

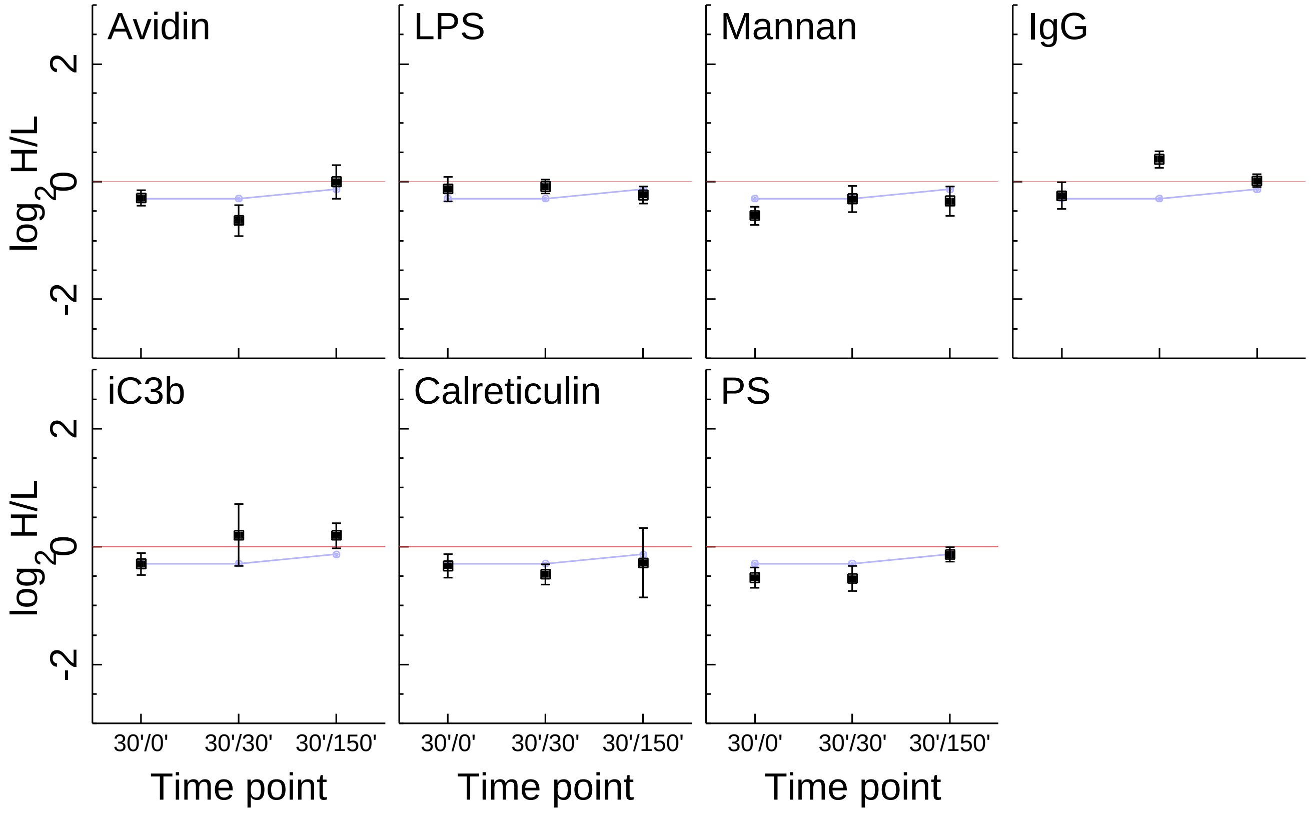

uqcrfs1 (Q9CR68)

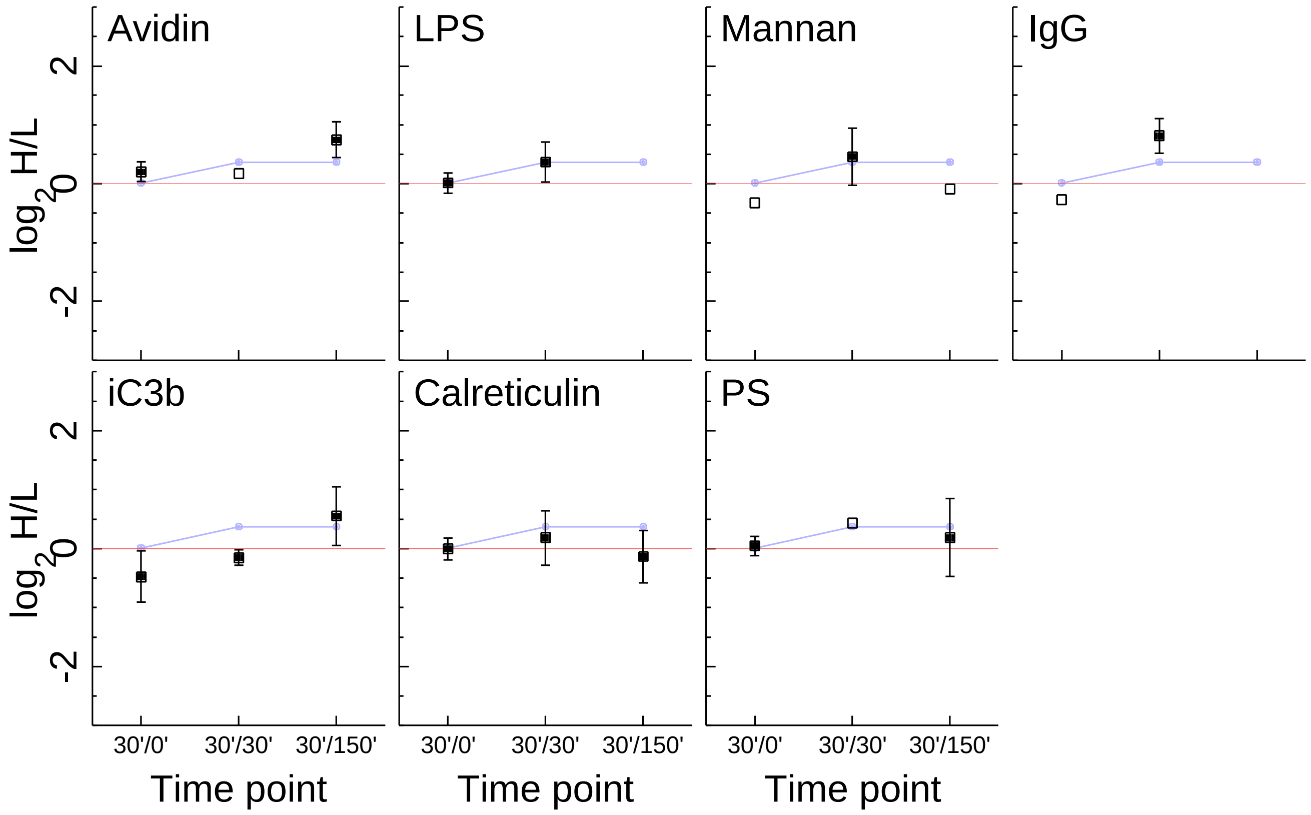

uqcrh (P99028)

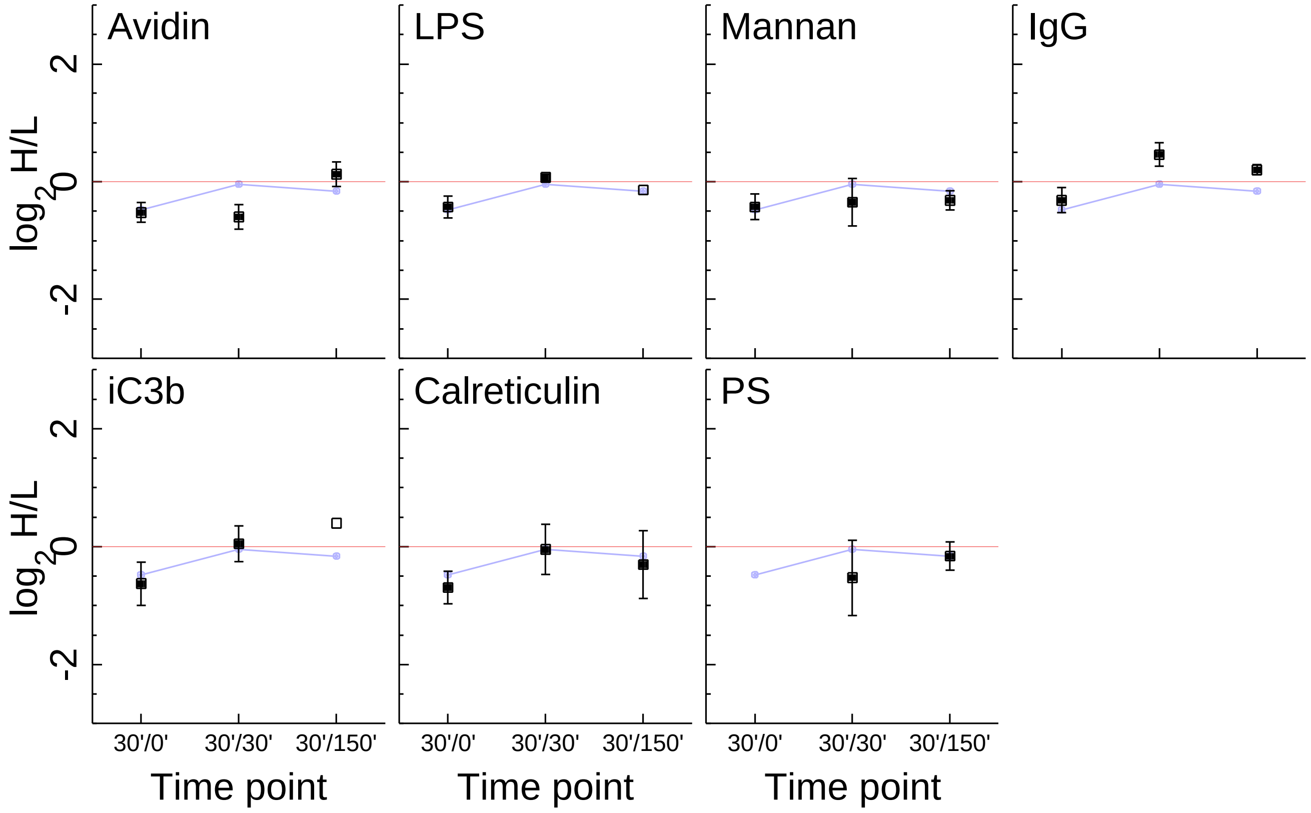

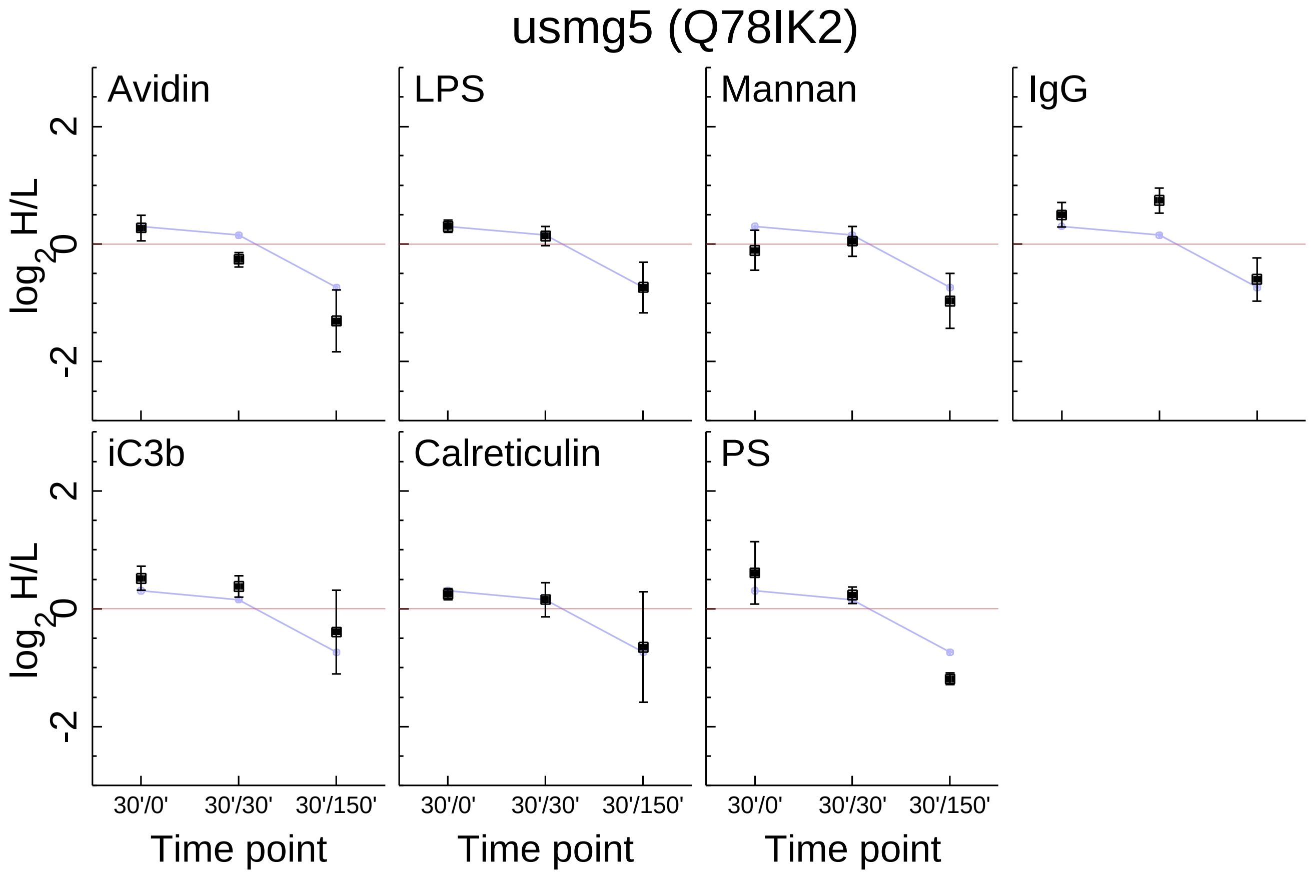
usmg5 (Q78IK2)
Avidin
LPS
Mannan
IgG
iC3b
Calreticulin
PS
log2 H/L
2
0
-2
30'/0'
30'/30'
30'/150'
Time point

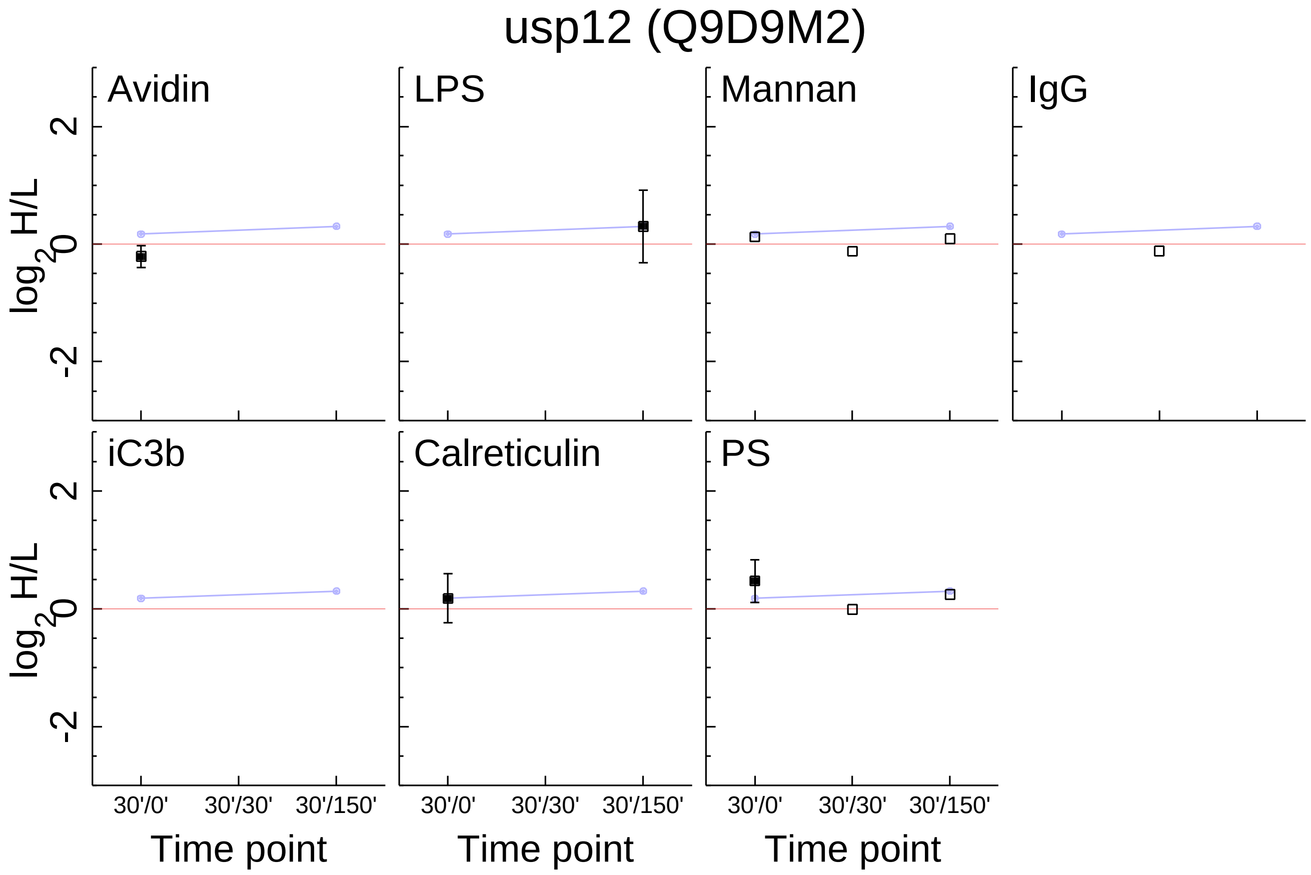
usp12 (Q9D9M2)
Avidin
LPS
Mannan
IgG
iC3b
Calreticulin
PS
2
0
-2
log2 H/L
30'/0'
30'/30'
30'/150'
Time point

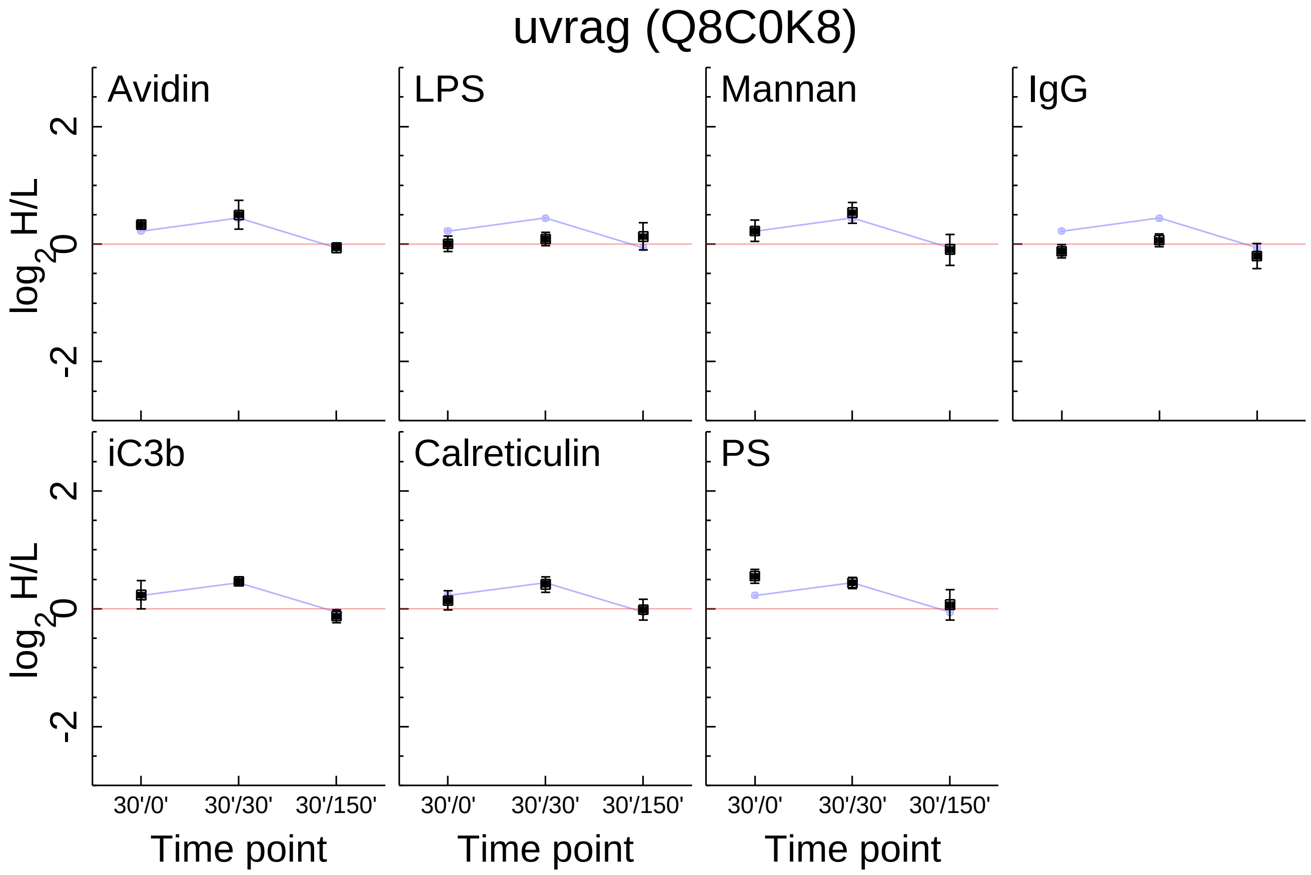

uvrag (Q8C0K8)
Avidin
LPS
Mannan
IgG
iC3b
Calreticulin
PS
2
0
-2
log2 H/L
30'/0'
30'/30'
30'/150'
Time point

vac14 (Q80WQ2)

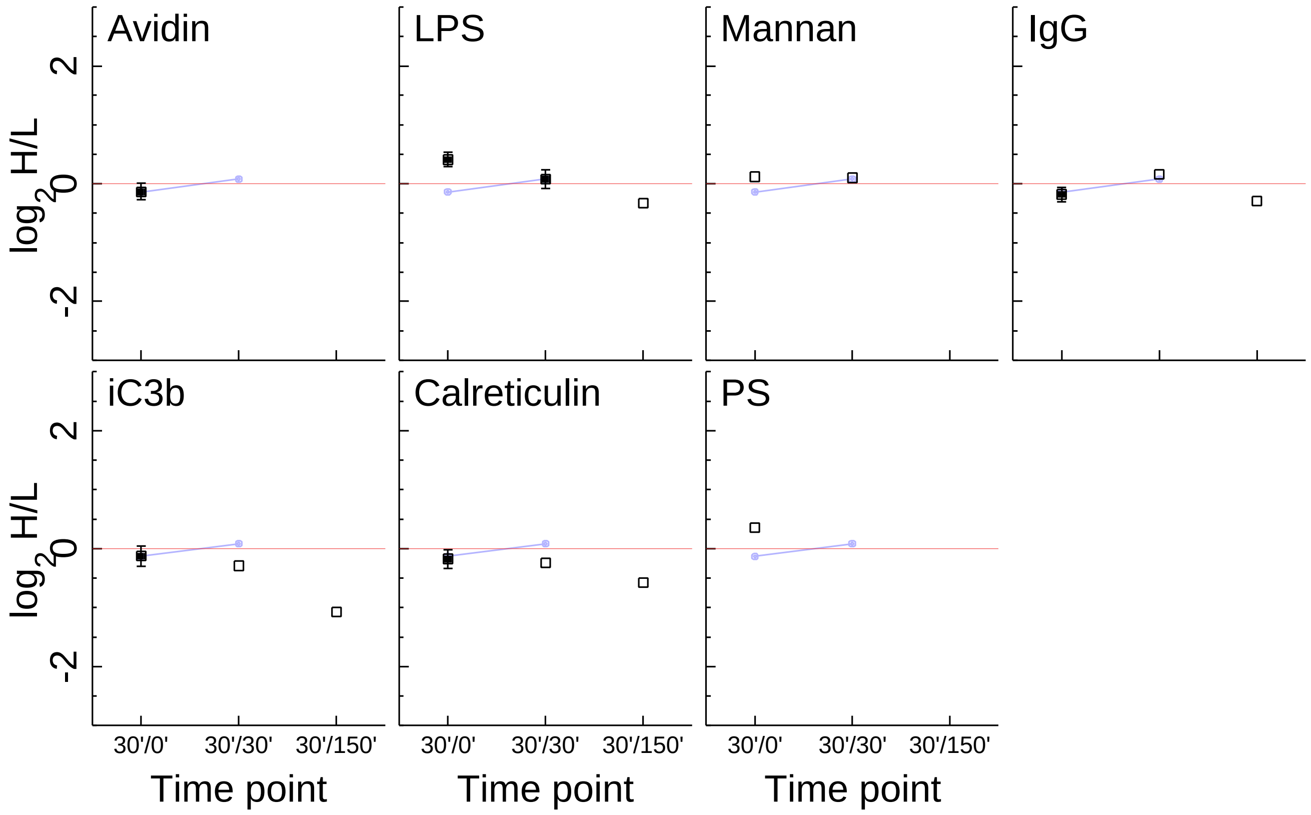

vamp3 (P63024)

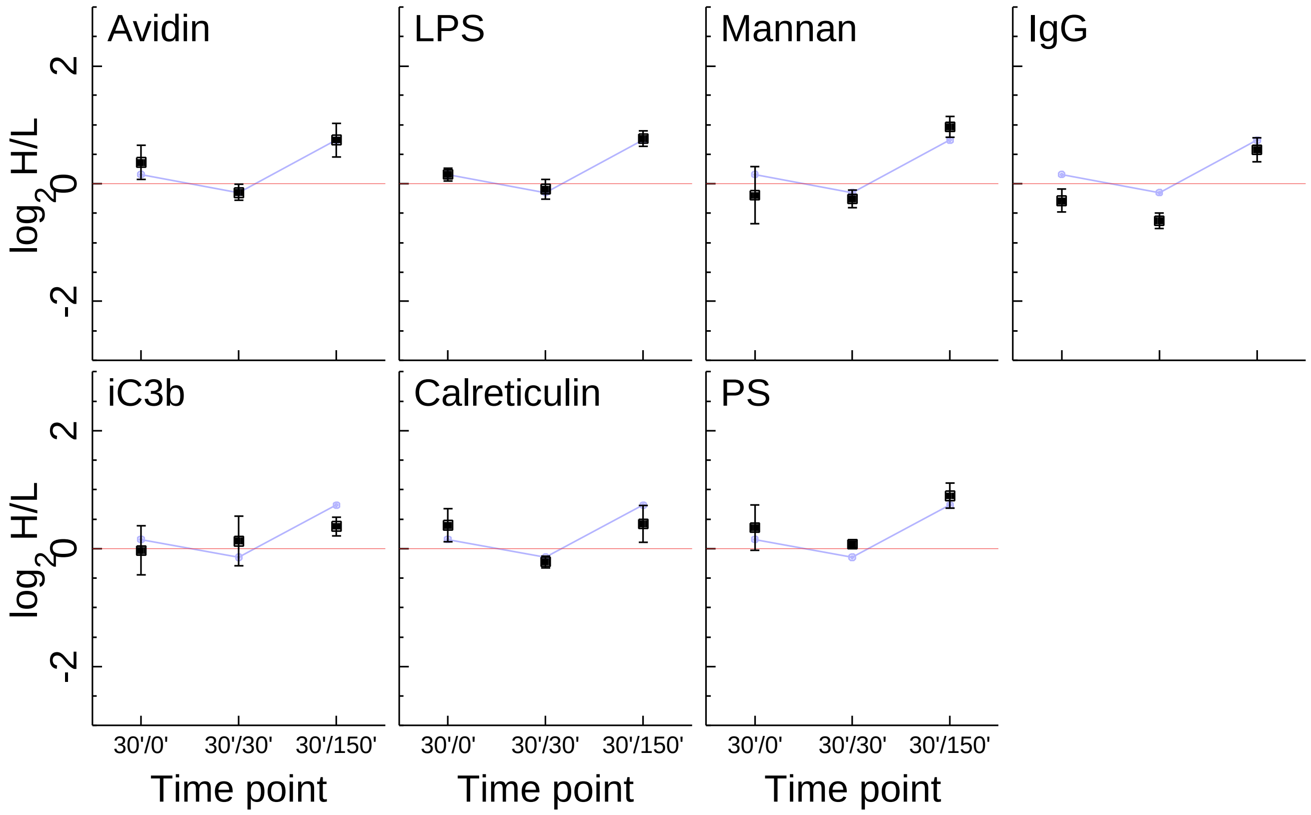

vamp4 (O70480)

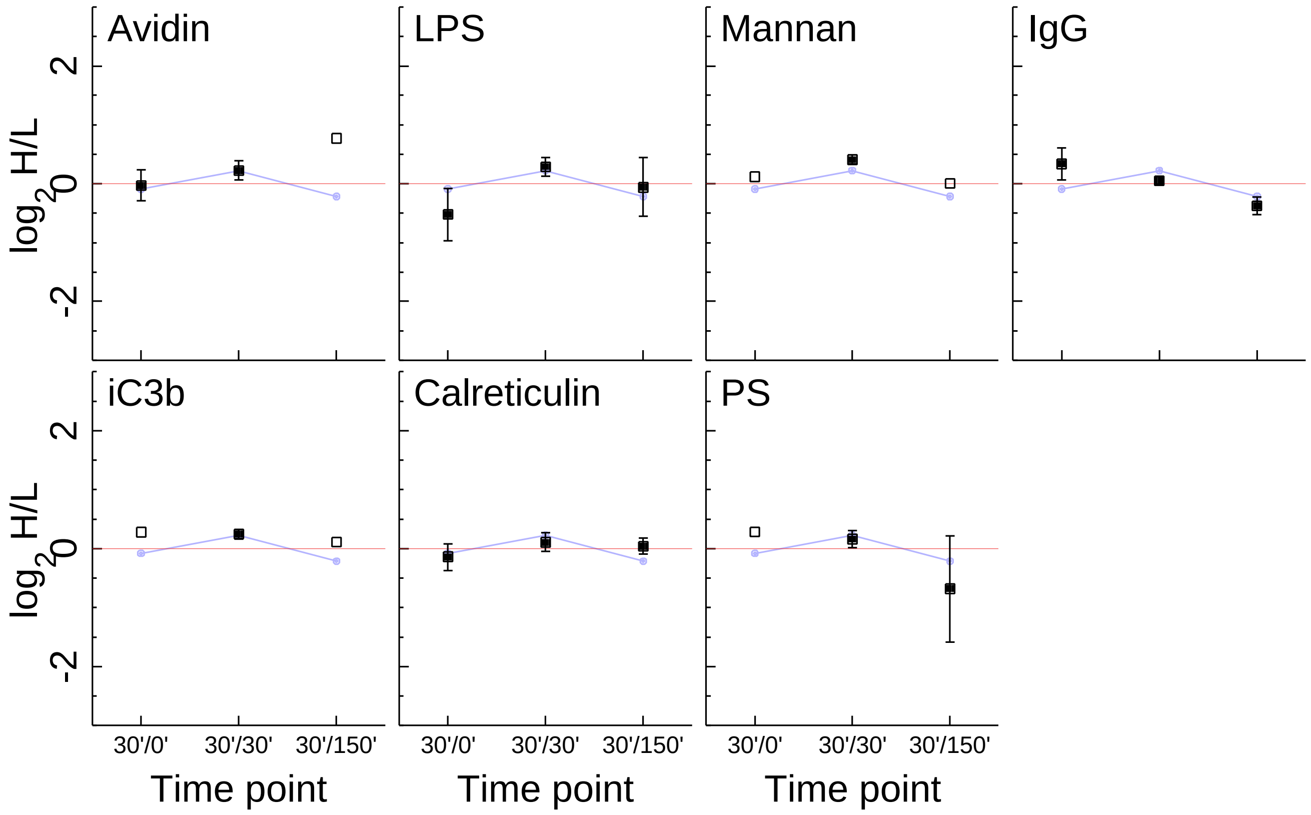

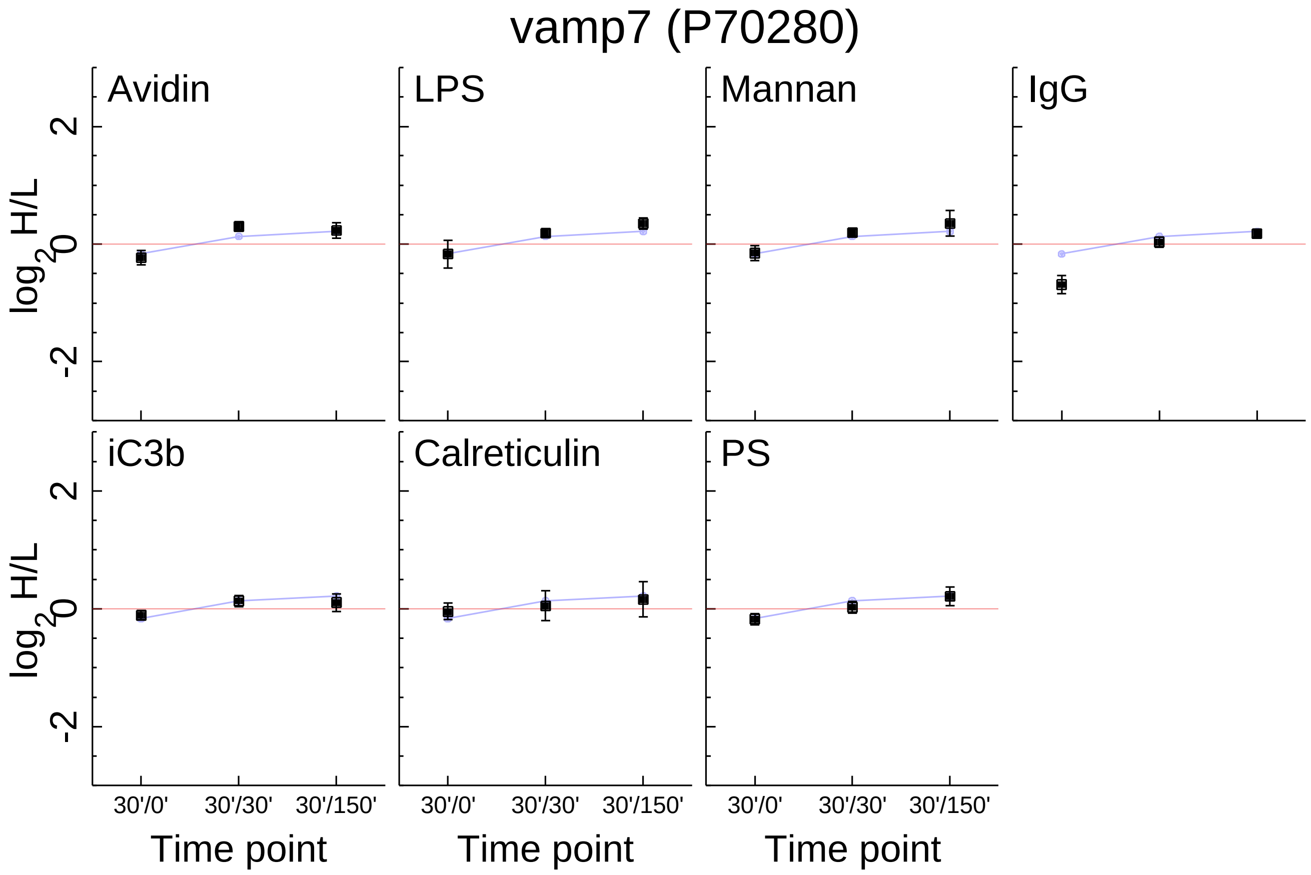
vamp7 (P70280)
Avidin
LPS
Mannan
IgG
iC3b
Calreticulin
PS
log2 H/L
2
0
-2
30'/0'
30'/30'
30'/150'
Time point

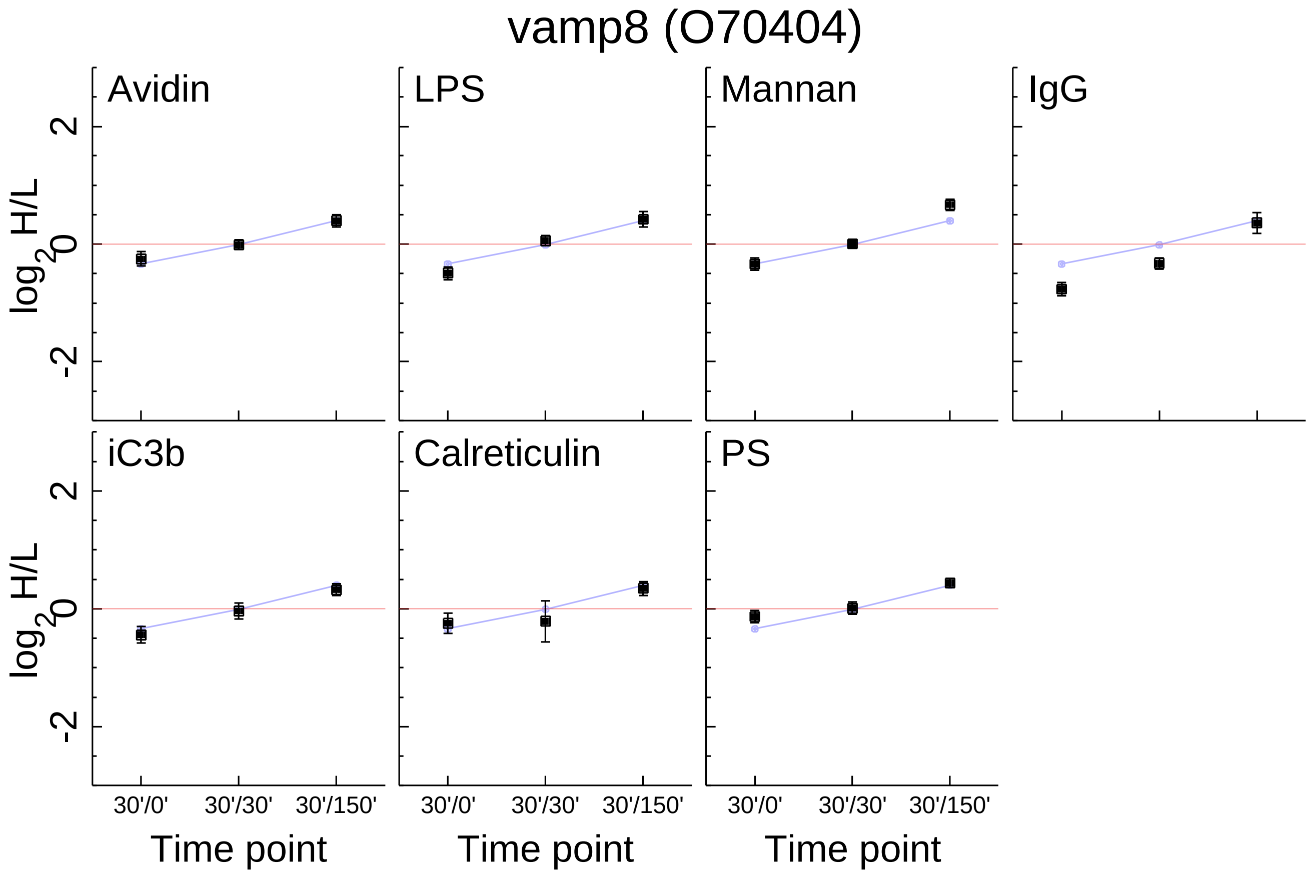
vamp8 (O70404)
Avidin
LPS
Mannan
IgG
iC3b
Calreticulin
PS
log2 H/L
2
0
-2
30'/0'
30'/30'
30'/150'
Time point

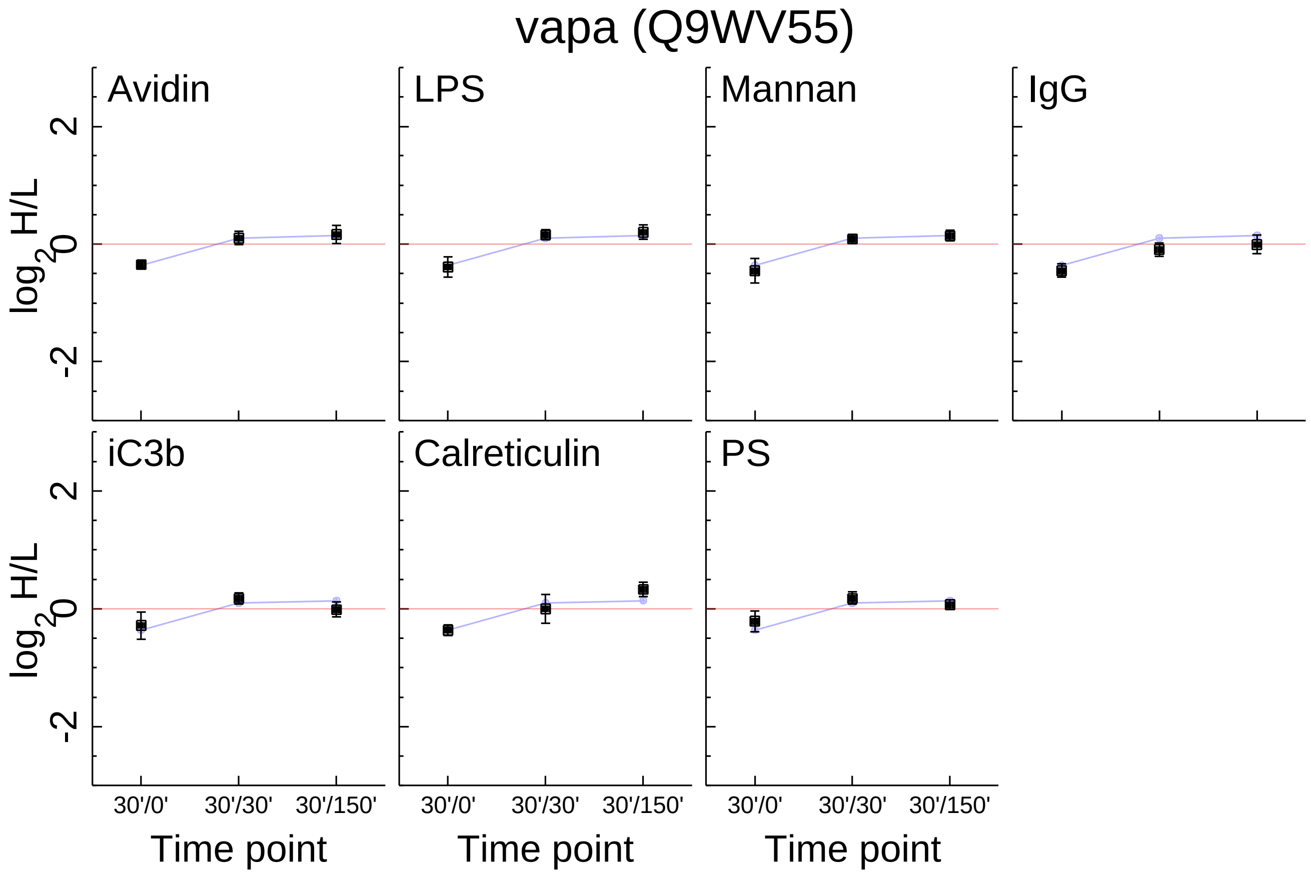
vapa (Q9WV55)
Avidin
LPS
Mannan
IgG
iC3b
Calreticulin
PS
log2 H/L
2
0
-2
30'/0'
30'/30'
30'/150'
Time point

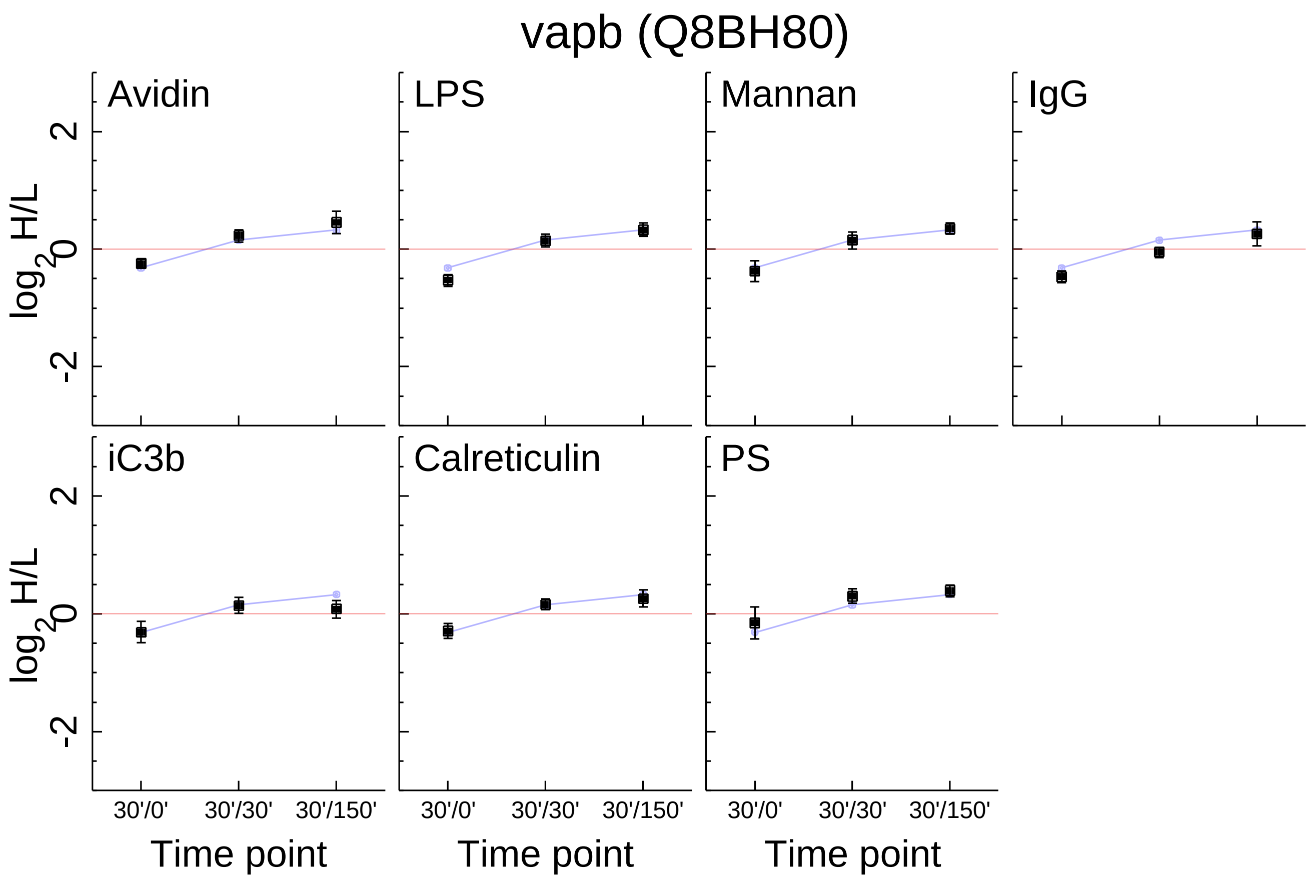

vapb (Q8BH80)
Avidin
LPS
Mannan
IgG
iC3b
Calreticulin
PS
log2 H/L
2
0
-2
30'/0'
30'/30'
30'/150'
Time point

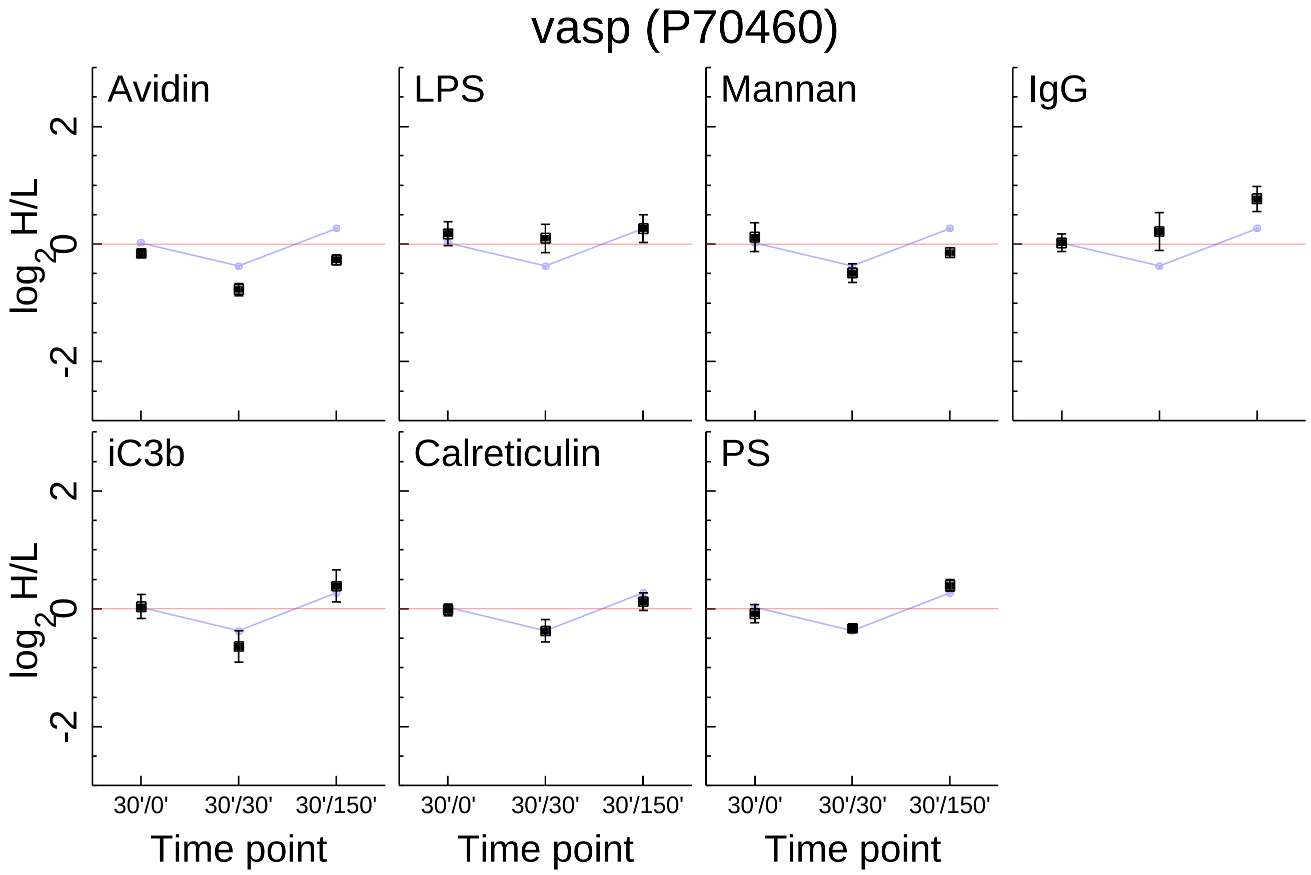

vasp (P70460)
Avidin
LPS
Mannan
IgG
iC3b
Calreticulin
PS
log2 H/L
2
0
-2
30'/0'
30'/30'
30'/150'
Time point

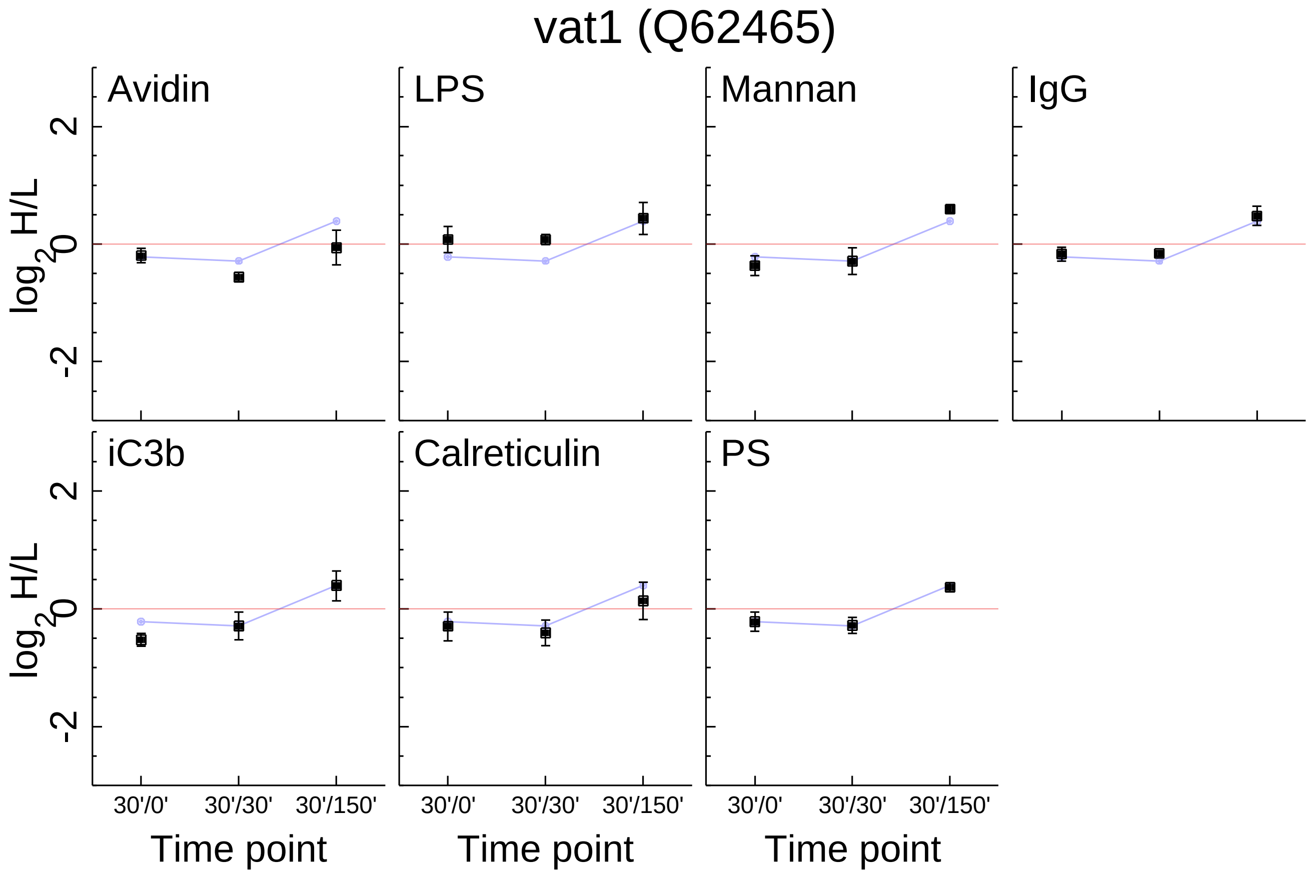
vat1 (Q62465)
Avidin
LPS
Mannan
IgG
iC3b
Calreticulin
PS
log2 H/L
2
0
-2
30'/0'
30'/30'
30'/150'
Time point

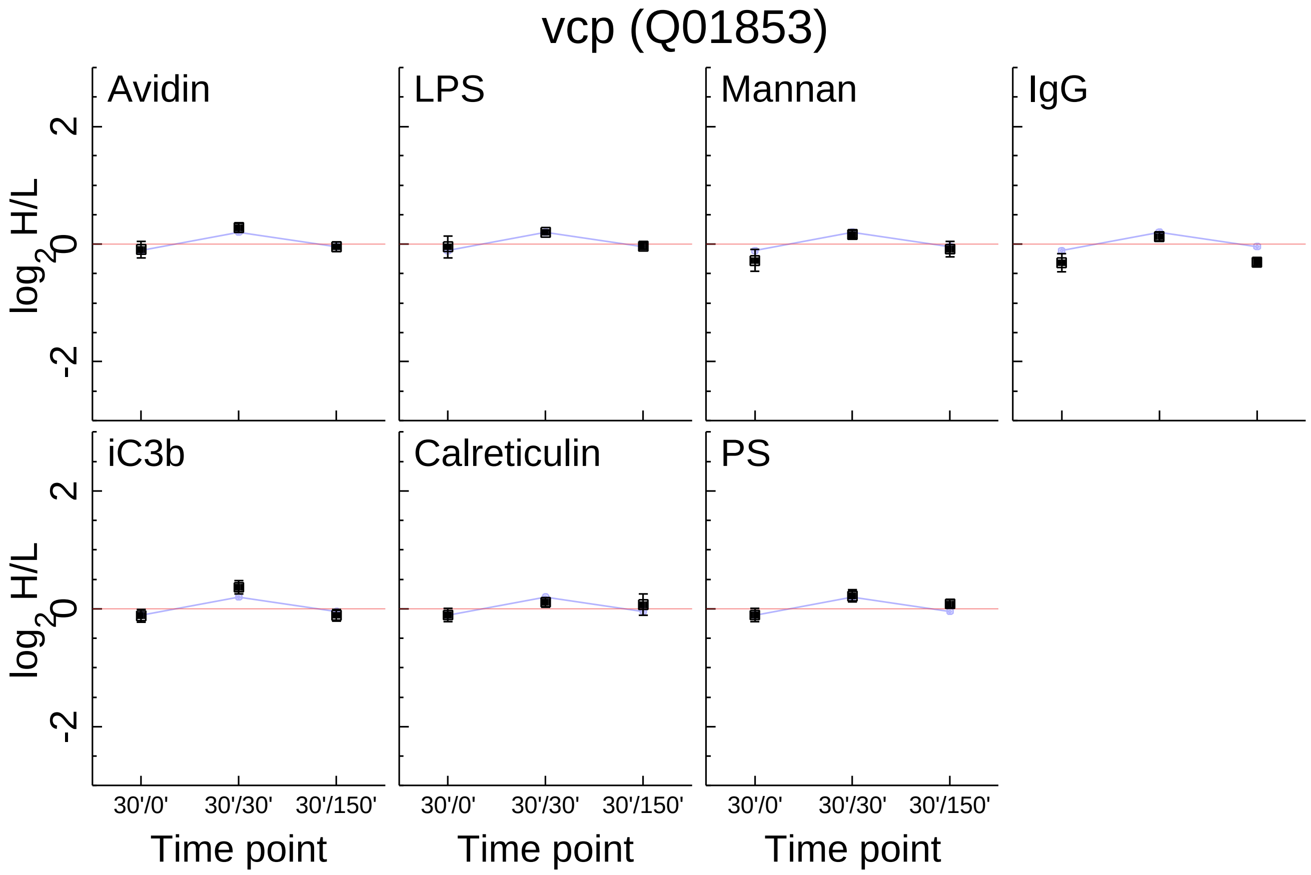
vcp (Q01853)
Avidin
LPS
Mannan
IgG
iC3b
Calreticulin
PS
log2 H/L
2
0
-2
30'/0'
30'/30'
30'/150'
Time point

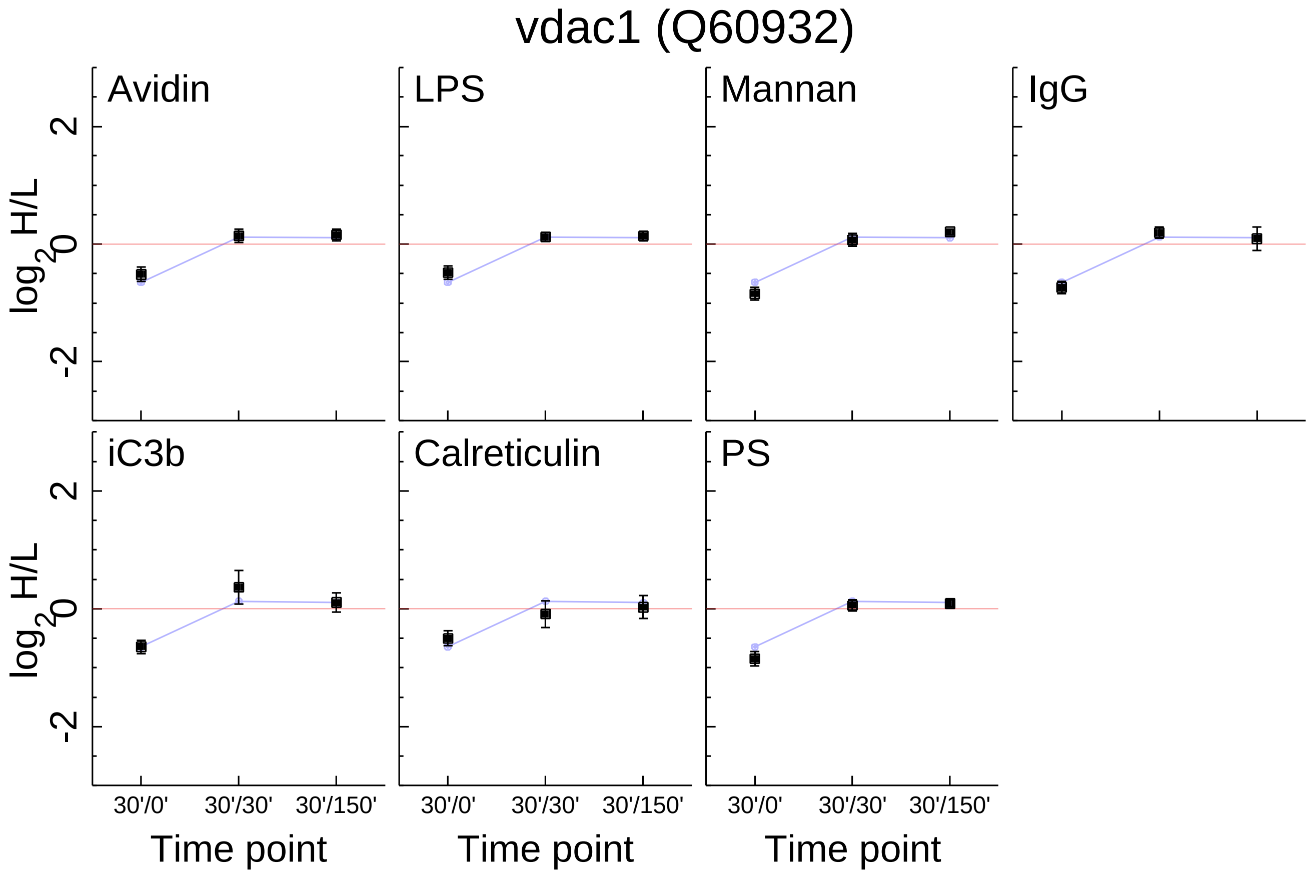

vdac1 (Q60932)
Avidin
LPS
Mannan
IgG
iC3b
Calreticulin
PS
log2 H/L
2
0
-2
30'/0'
30'/30'
30'/150'
Time point

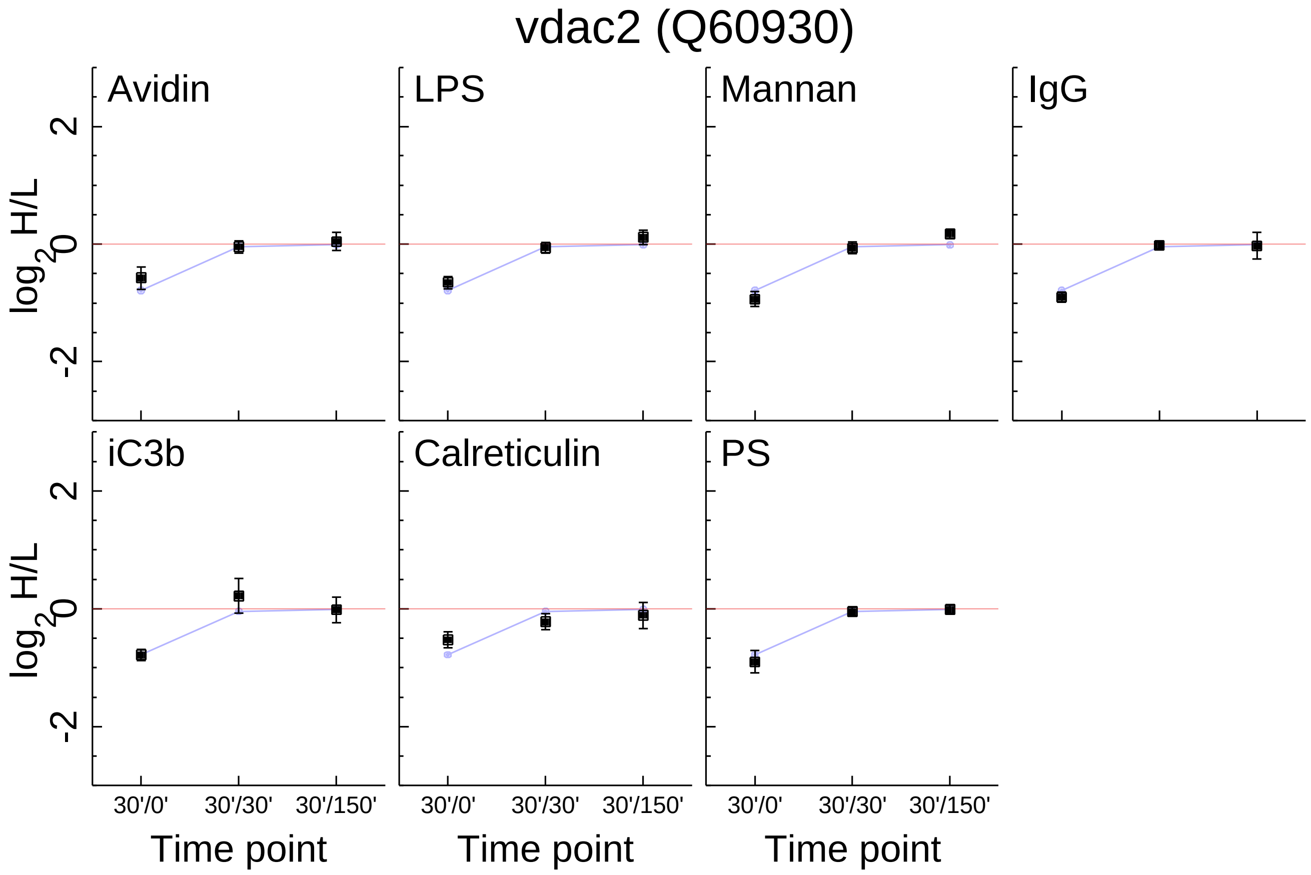
vdac2 (Q60930)
Avidin
LPS
Mannan
IgG
iC3b
Calreticulin
PS
log2 H/L
2
0
-2
30'/0'
30'/30'
30'/150'
Time point

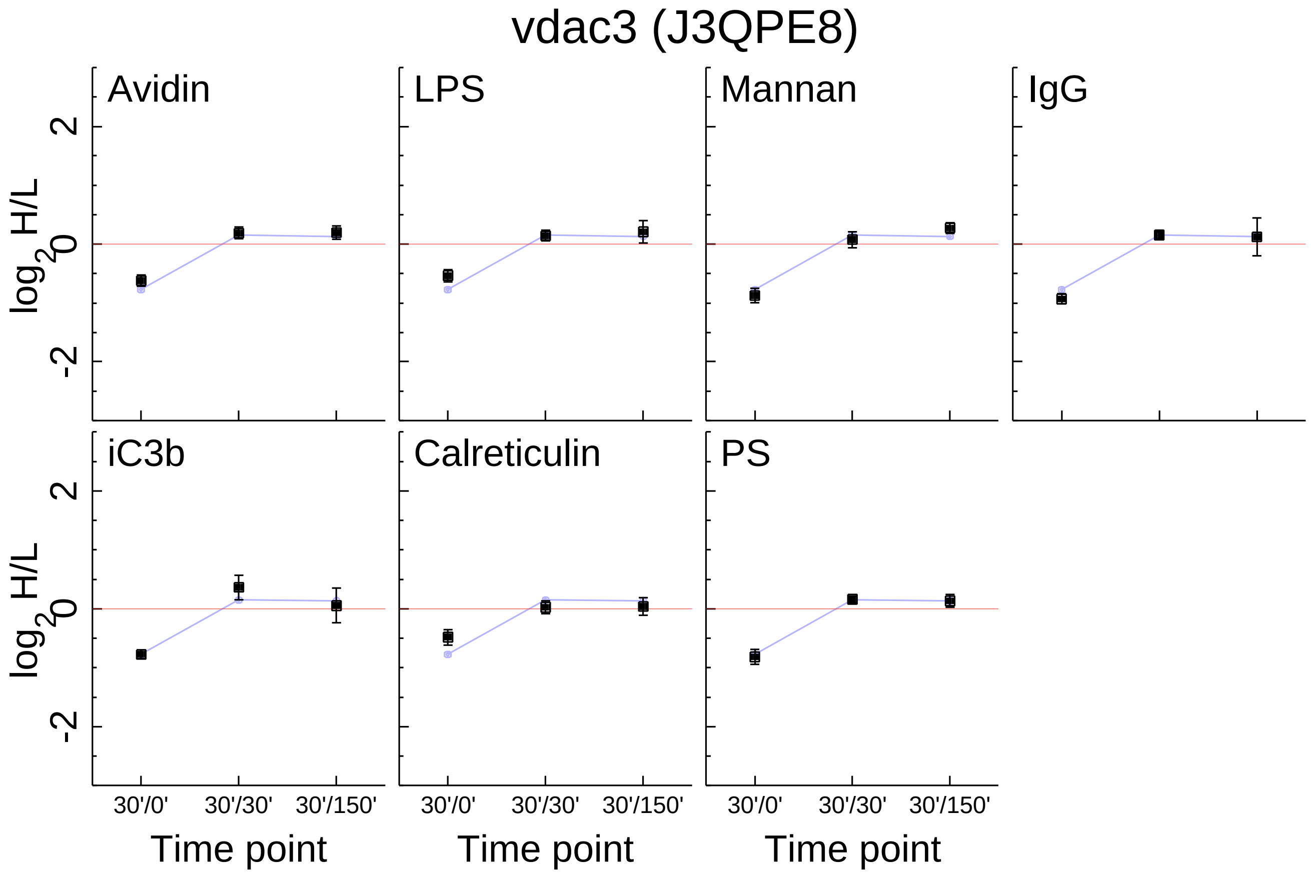
vdac3 (J3QPE8)
Avidin
LPS
Mannan
IgG
iC3b
Calreticulin
PS
log2 H/L
2
0
-2
30'/0'
30'/30'
30'/150'
Time point

vim (P20152)

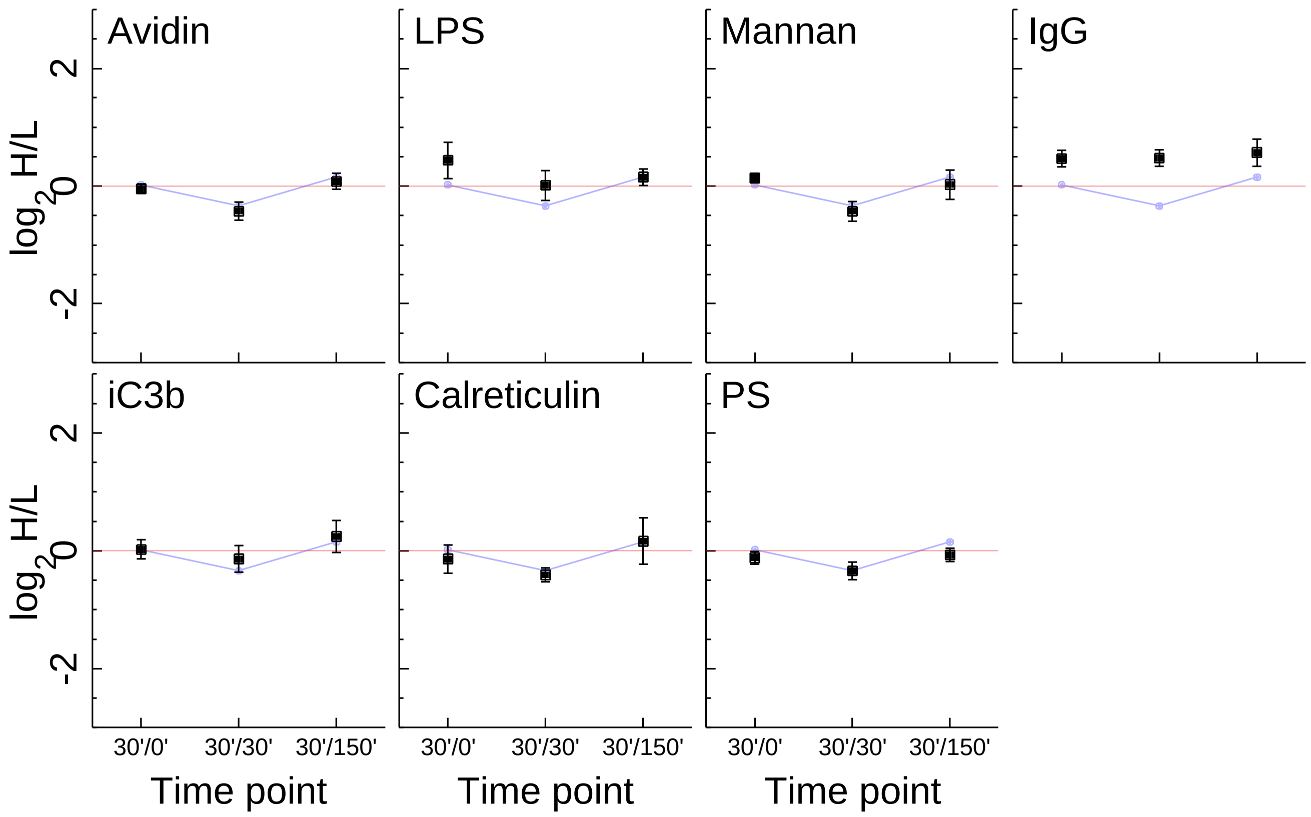
Avidin
LPS
Mannan
IgG
iC3b
Calreticulin
PS
log2 H/L
2
0
-2
30'/0'
30'/30'
30'/150'
Time point

vmp1 (Q99KU0)

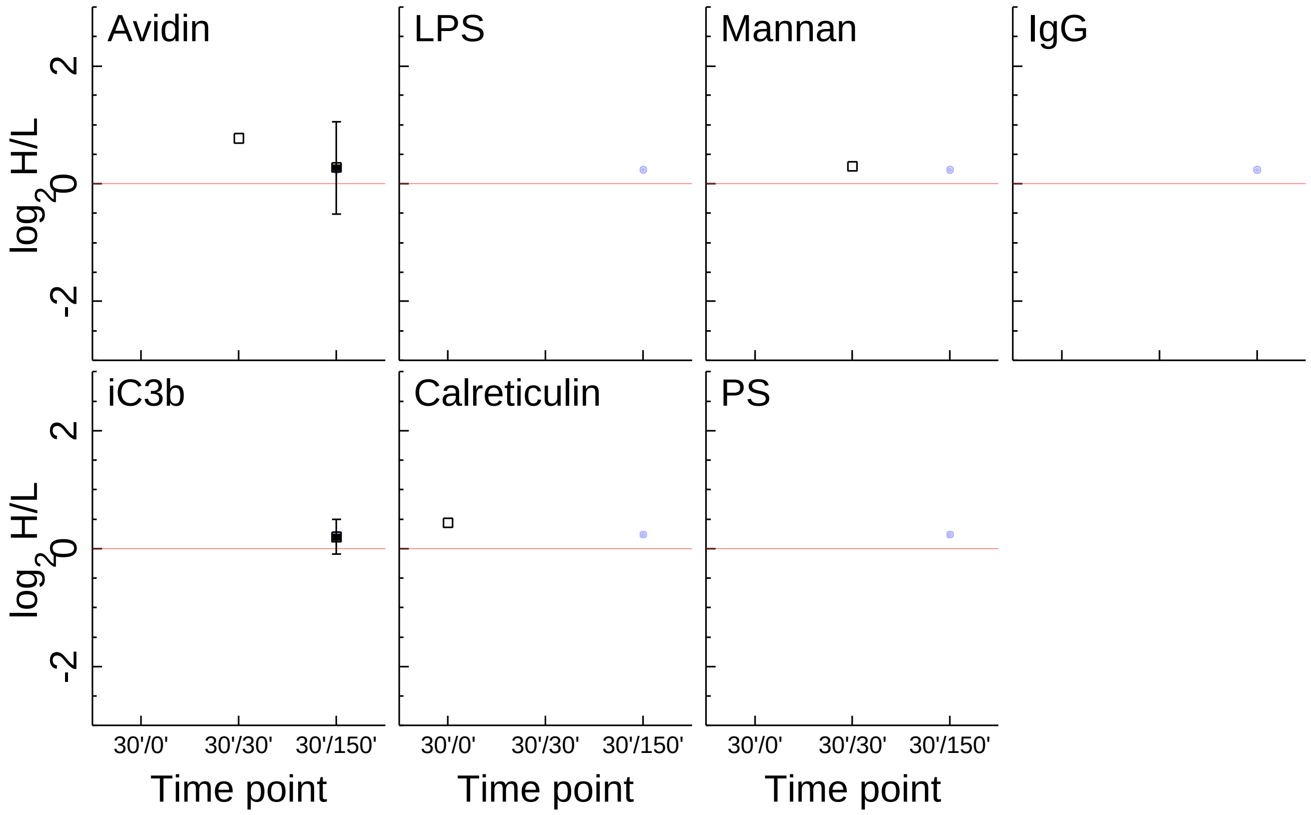
Avidin
LPS
Mannan
IgG
iC3b
Calreticulin
PS
$\log_2$ H/L
2
0
-2
30'/0'
30'/30'
30'/150'
Time point

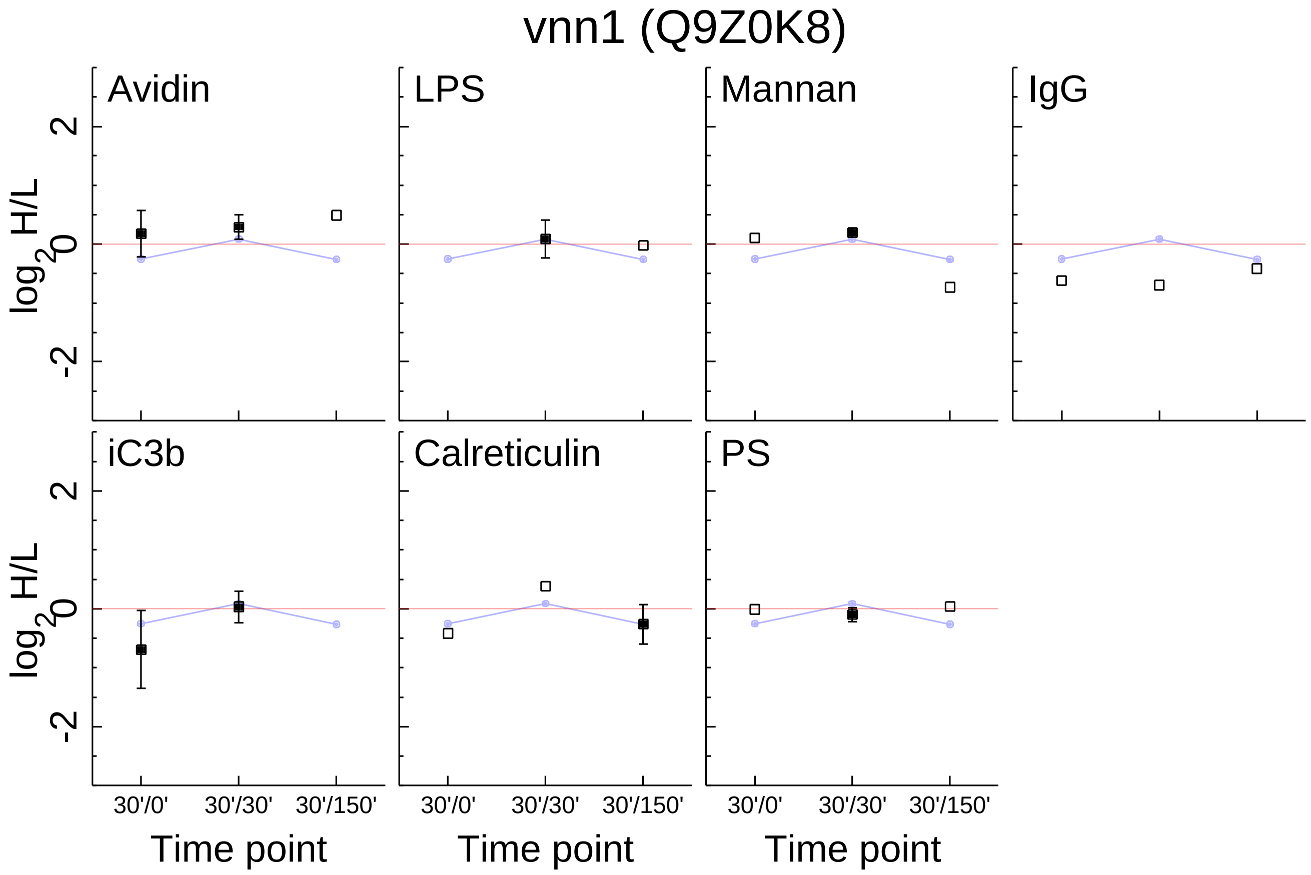

vnn1 (Q9Z0K8)
Avidin
LPS
Mannan
IgG
iC3b
Calreticulin
PS
log2 H/L
2
0
-2
30'/0'
30'/30'
30'/150'
Time point

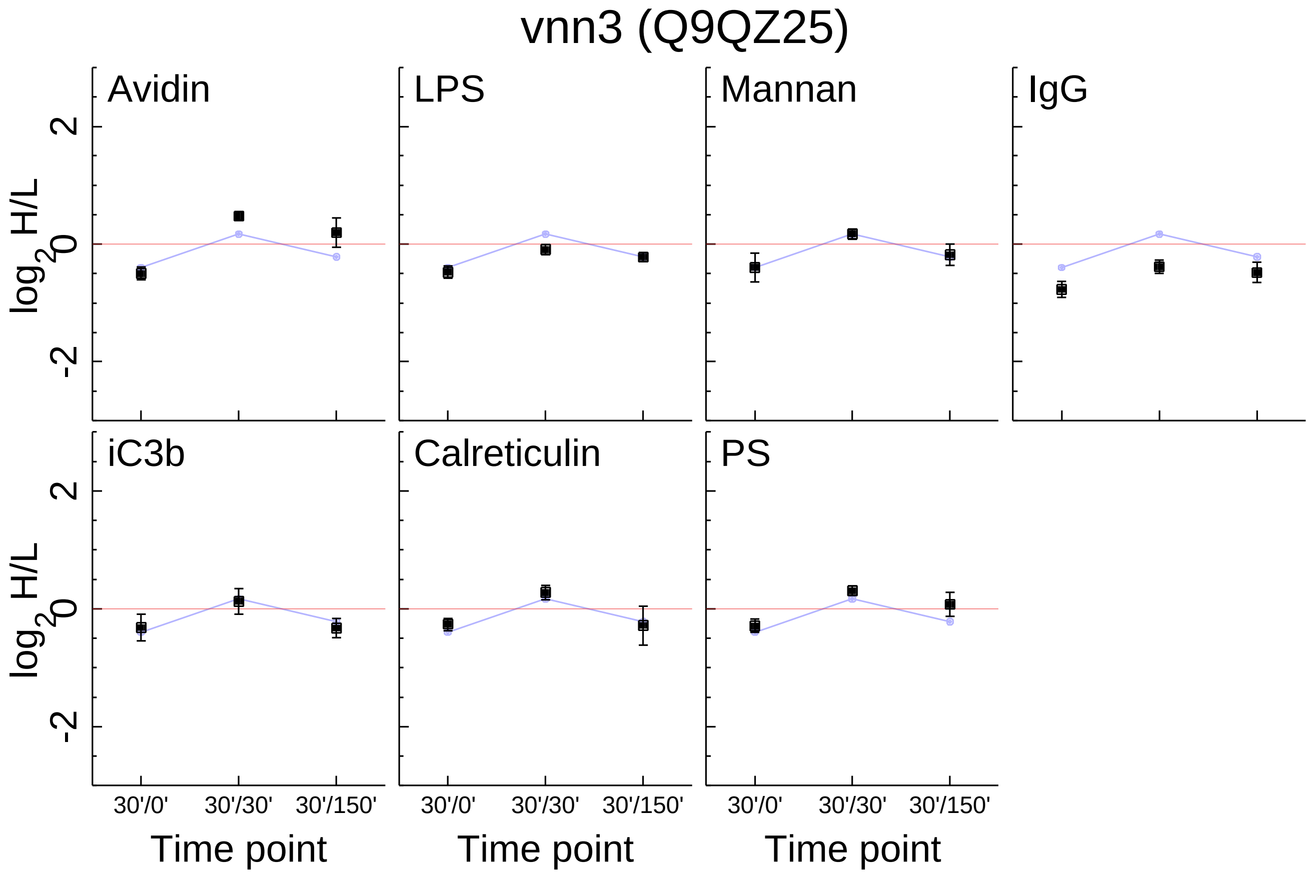
vnn3 (Q9QZ25)
Avidin
LPS
Mannan
IgG
iC3b
Calreticulin
PS
log_2 H/L
2
0
-2
30'/0'
30'/30'
30'/150'
Time point

vps11 (Q91W86)

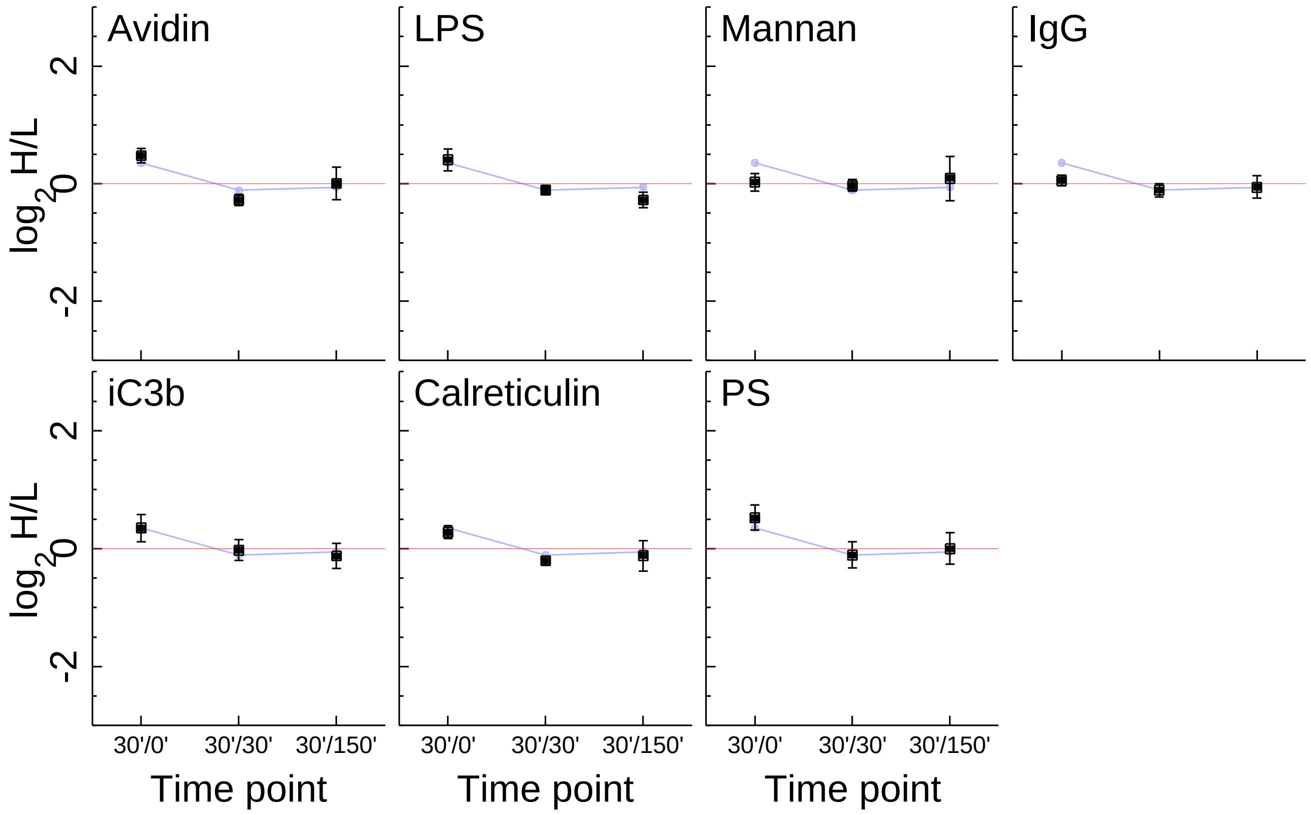

vps13c (Q8BX70)

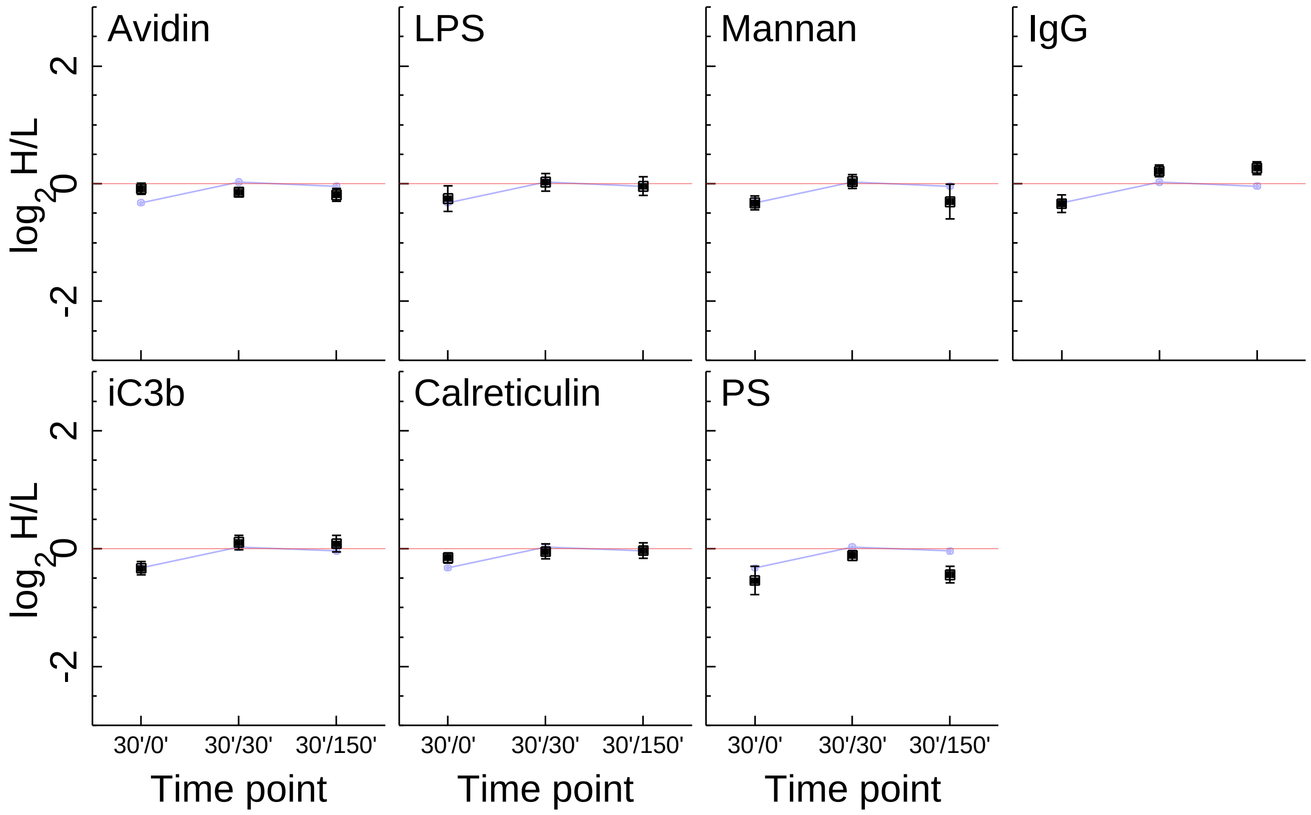

vps13d (B1ART2)

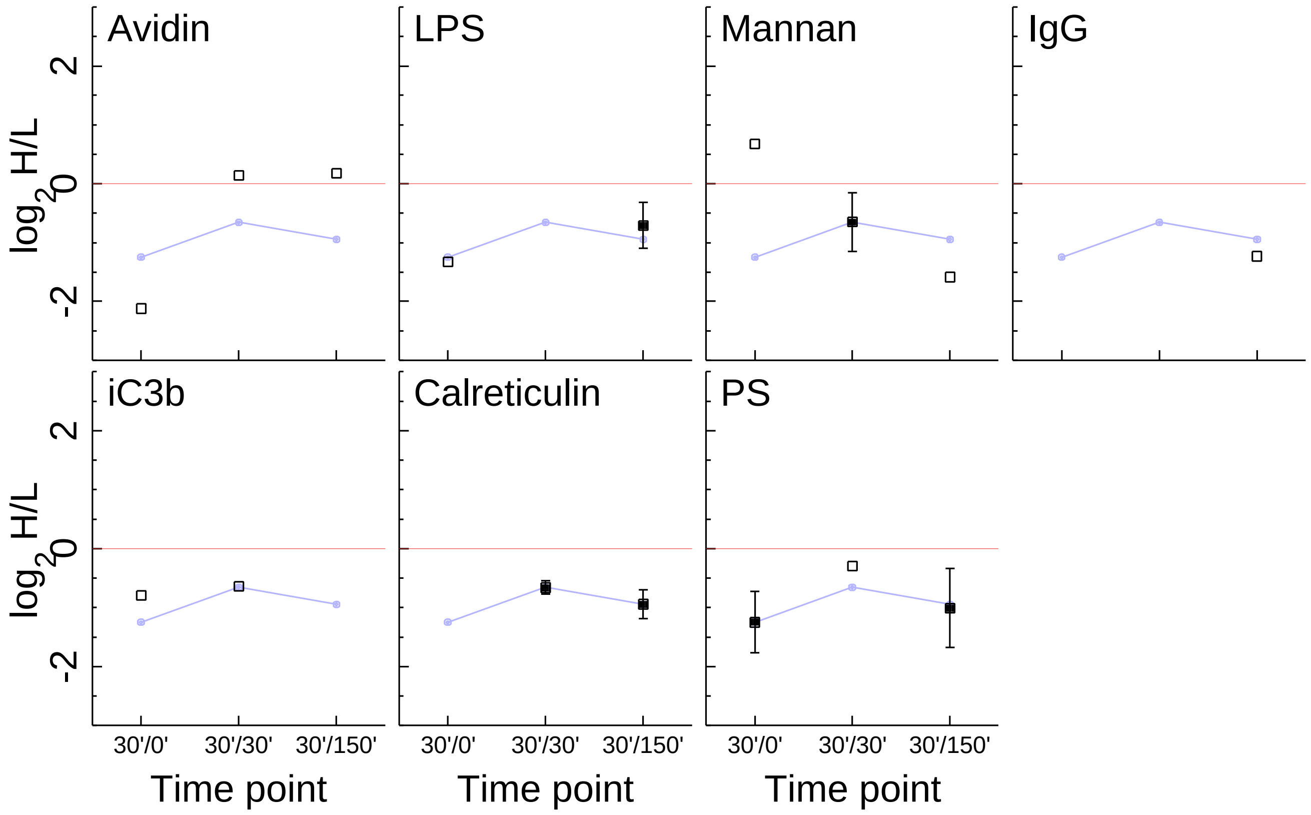

vps16 (G3X8X7)

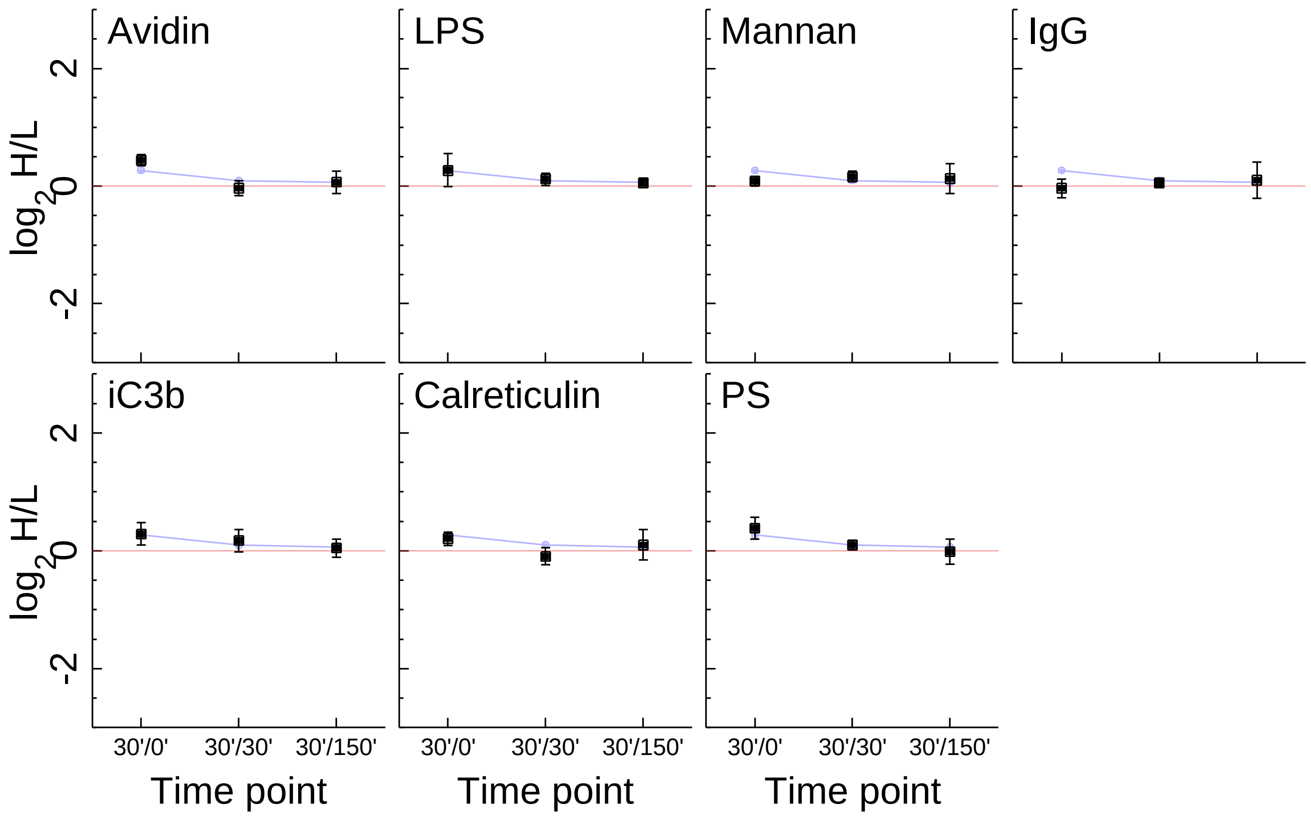

vps18 (Q8R307)

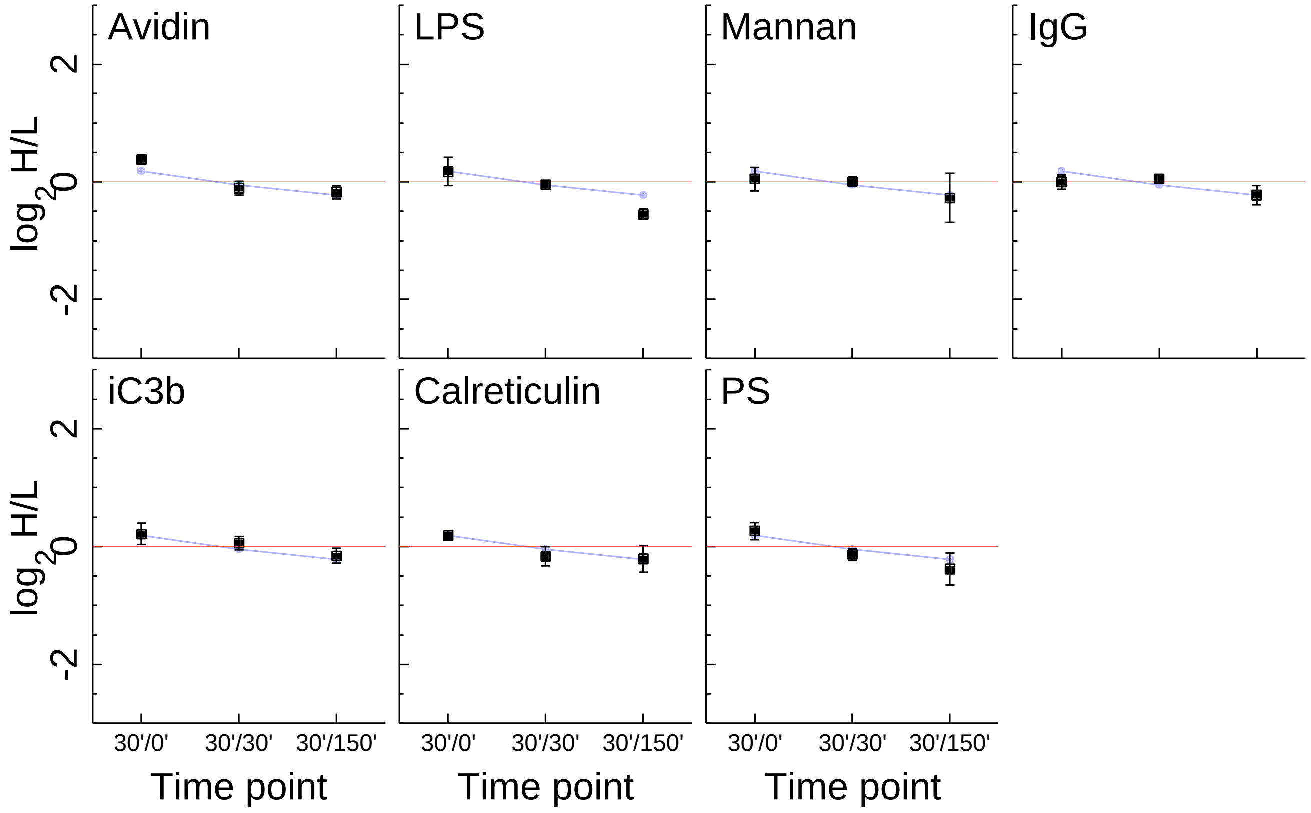

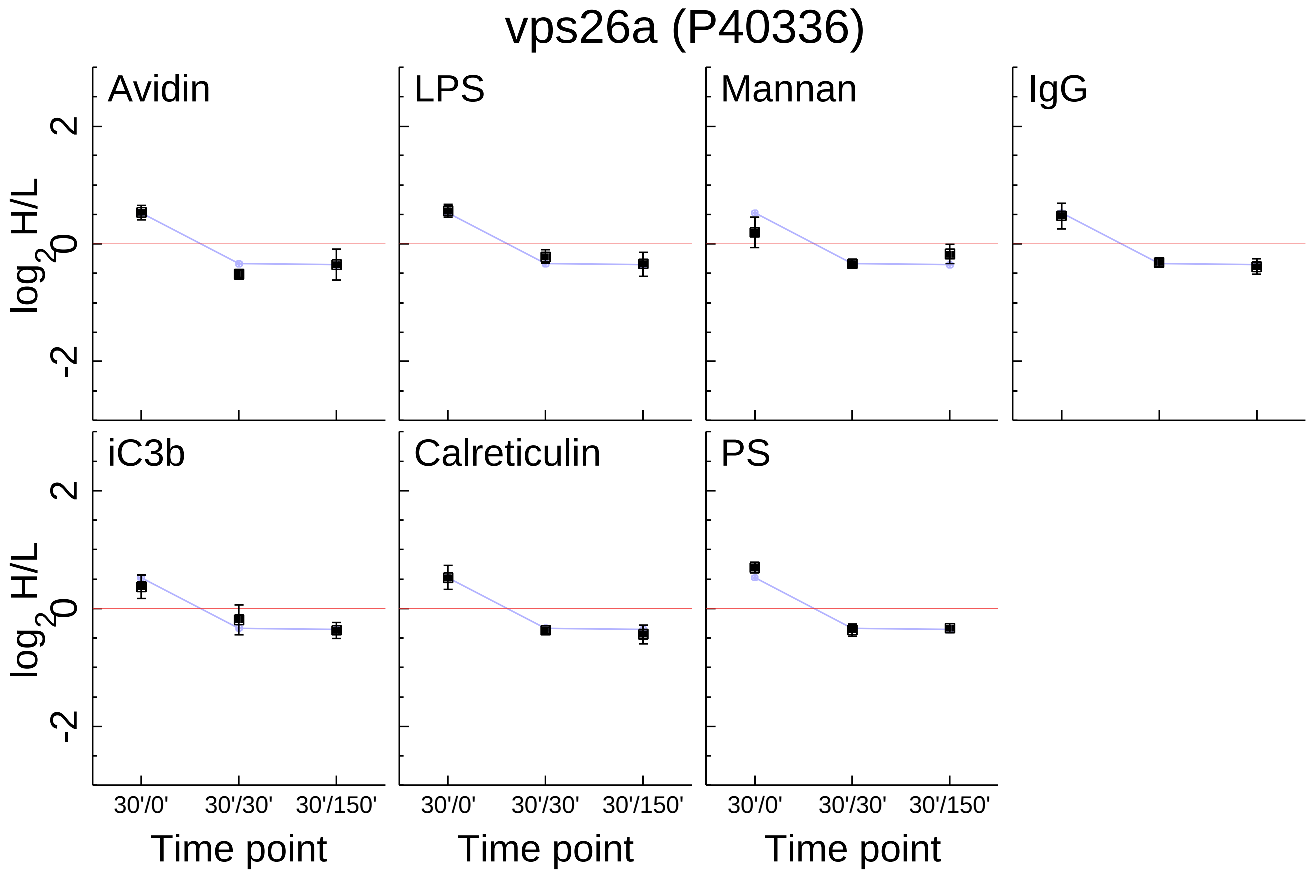
vps26a (P40336)
Avidin
LPS
Mannan
IgG
iC3b
Calreticulin
PS
log2 H/L
2
0
-2
30'/0'
30'/30'
30'/150'
Time point

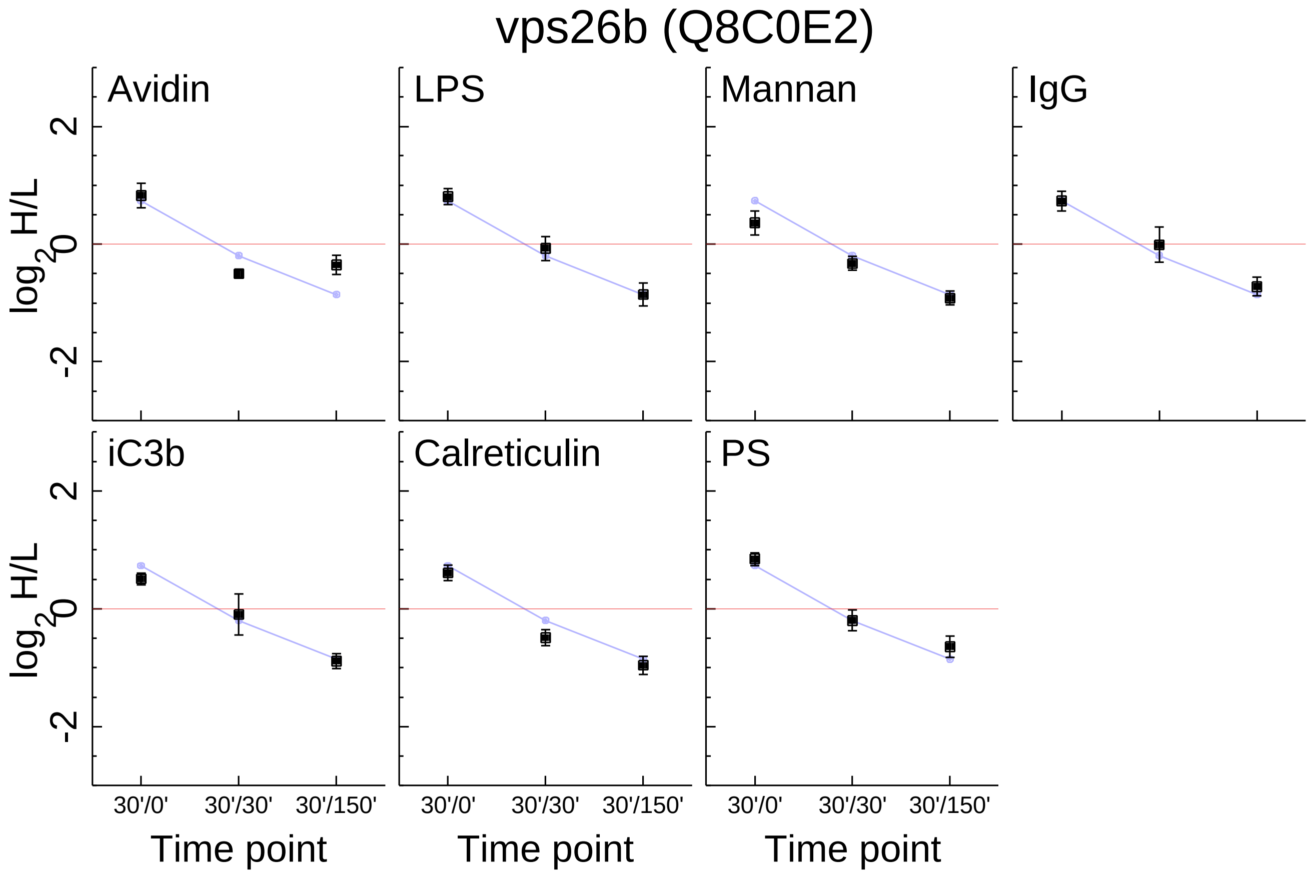
vps26b (Q8C0E2)
Avidin
LPS
Mannan
IgG
iC3b
Calreticulin
PS
2
0
-2
log2 H/L
30'/0'
30'/30'
30'/150'
Time point

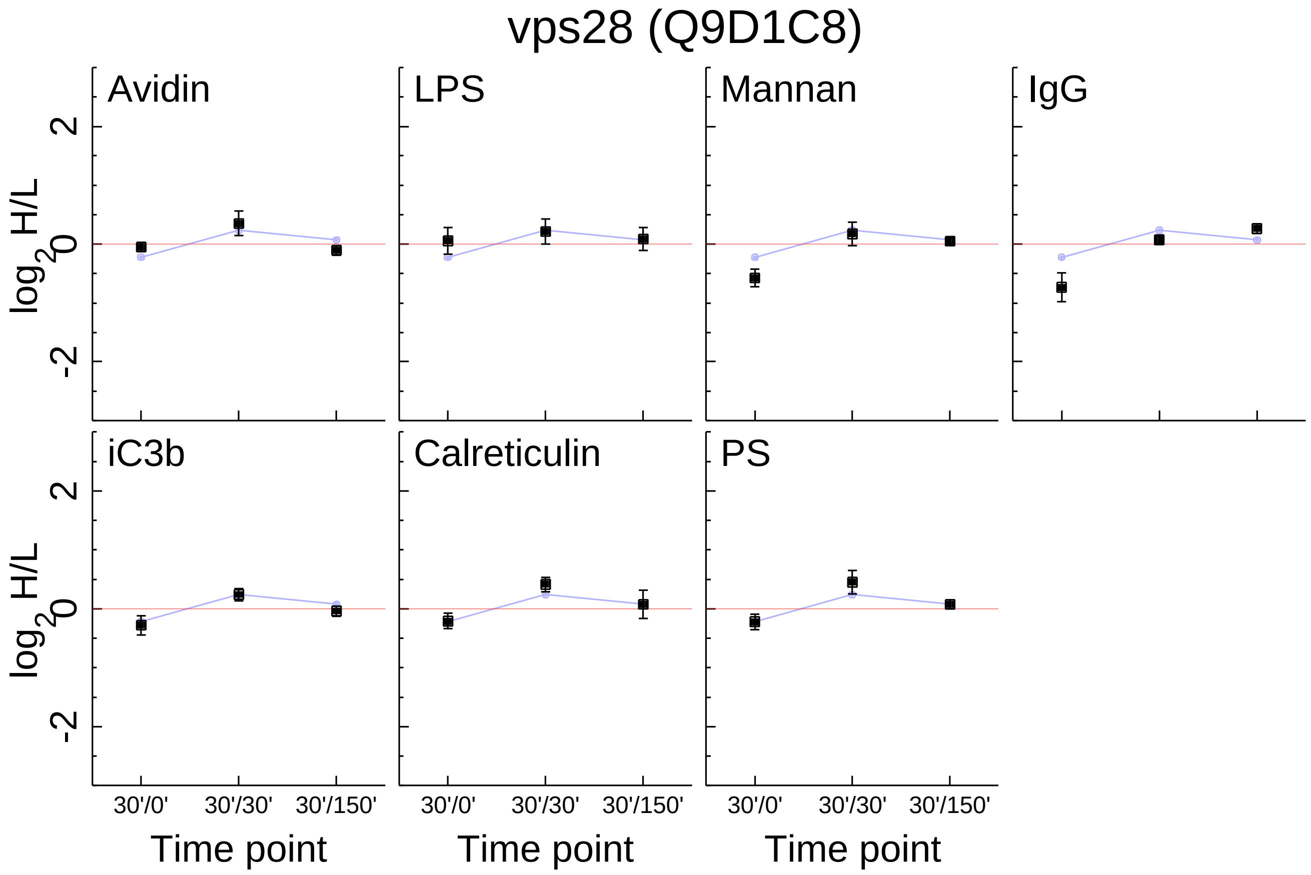

vps28 (Q9D1C8)
Avidin
LPS
Mannan
IgG
iC3b
Calreticulin
PS
log2 H/L
2
0
-2
30'/0'
30'/30'
30'/150'
Time point

vps29 (Q9QZ88)

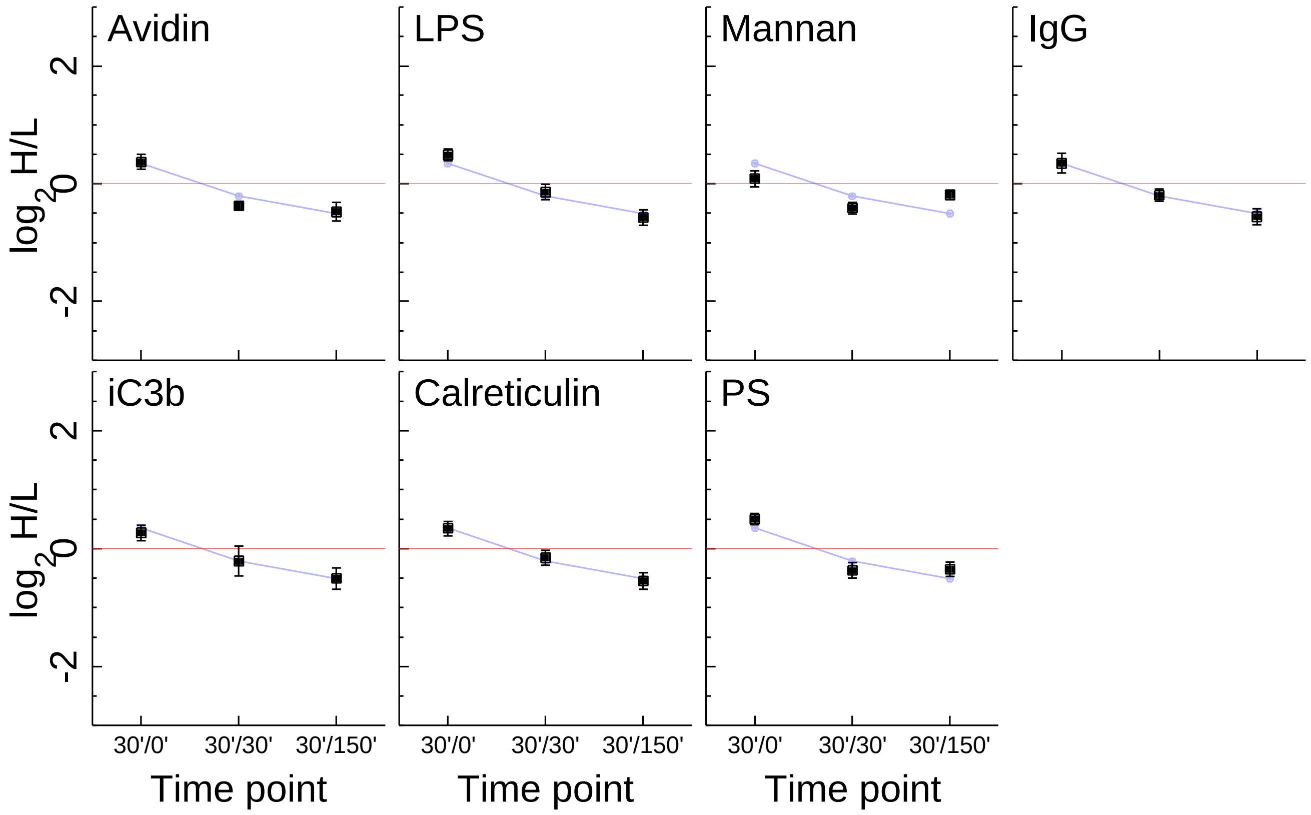

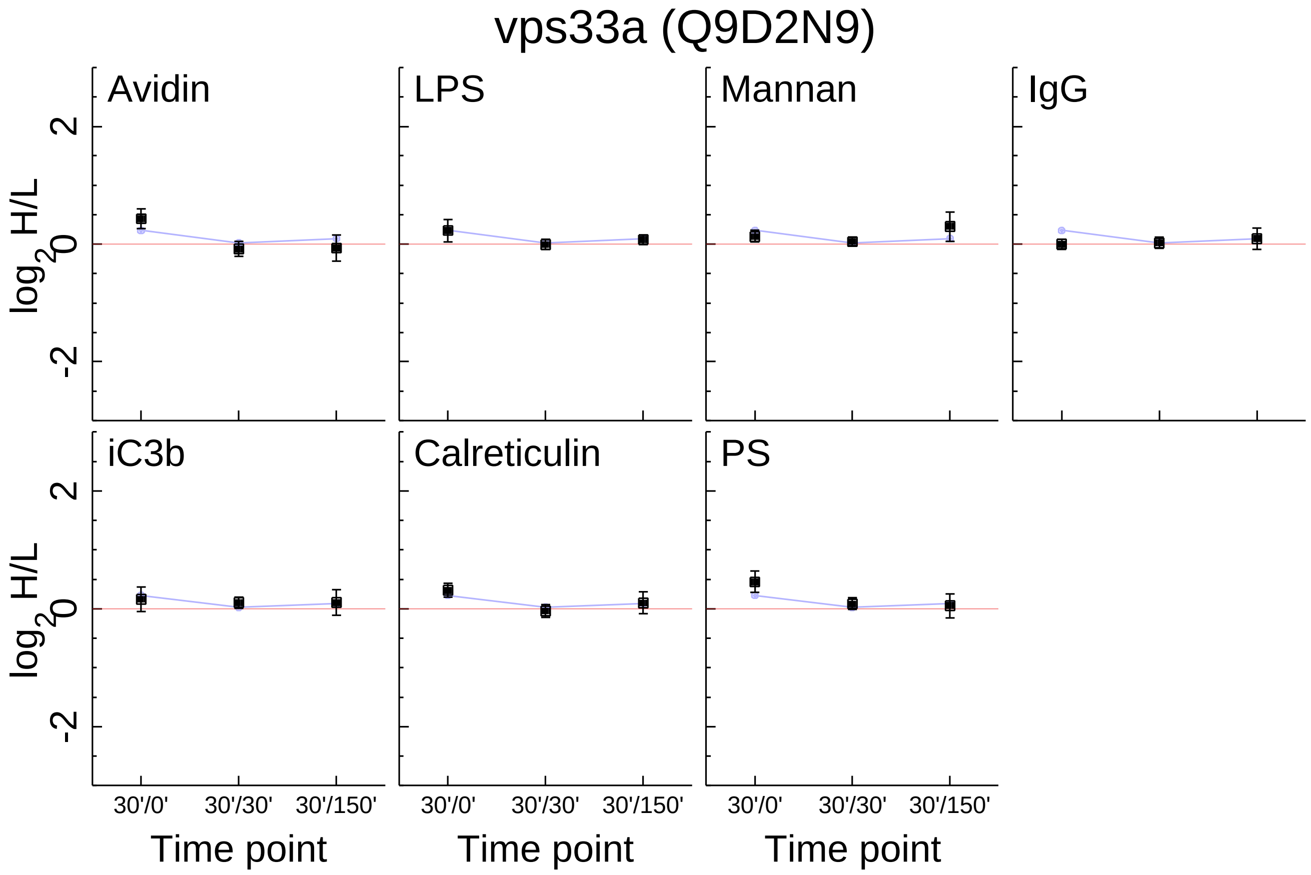

vps33a (Q9D2N9)
Avidin
LPS
Mannan
IgG
iC3b
Calreticulin
PS
log2 H/L
2
0
-2
30'/0'
30'/30'
30'/150'
Time point

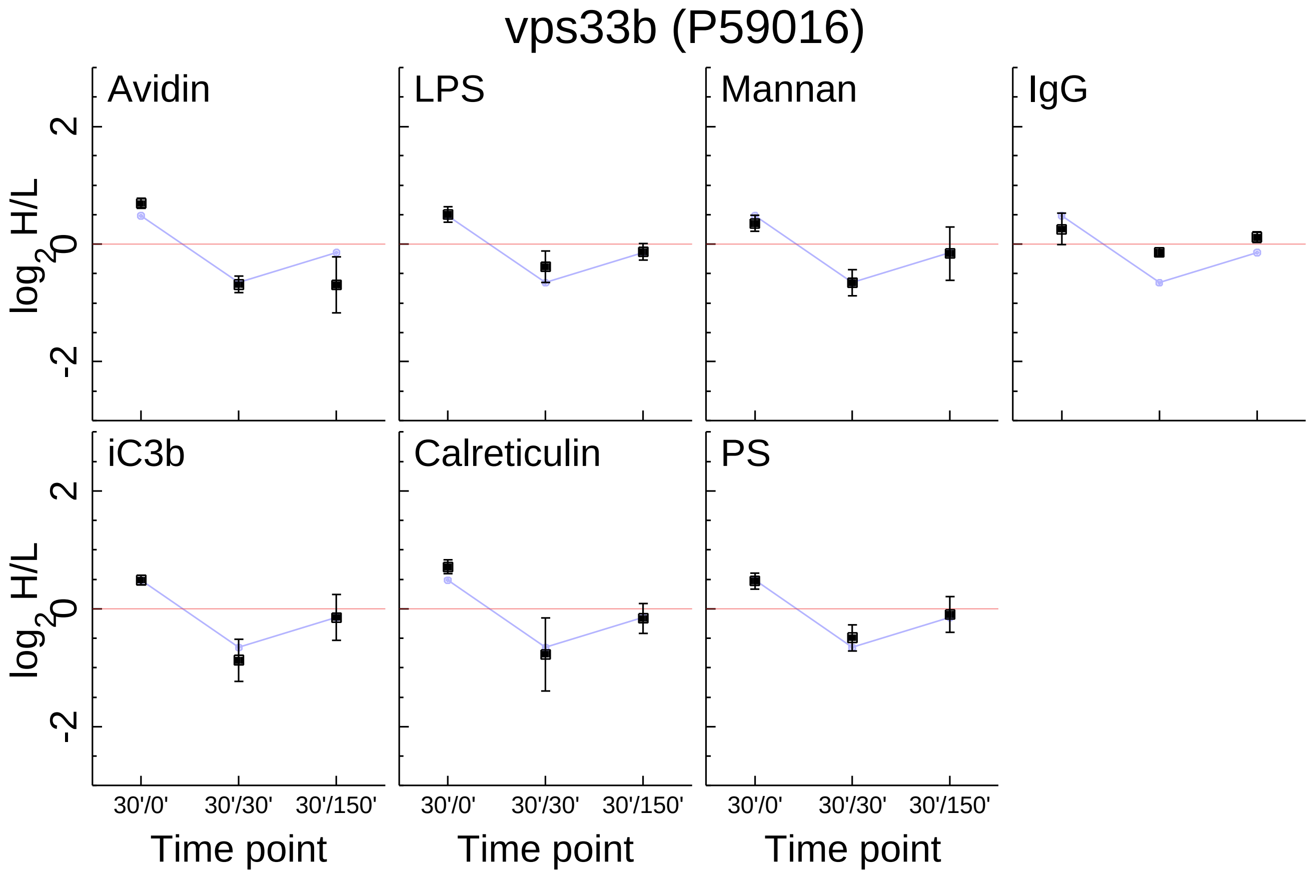
vps33b (P59016)
Avidin
LPS
Mannan
IgG
iC3b
Calreticulin
PS
log2 H/L
2
0
-2
30'/0'
30'/30'
30'/150'
Time point

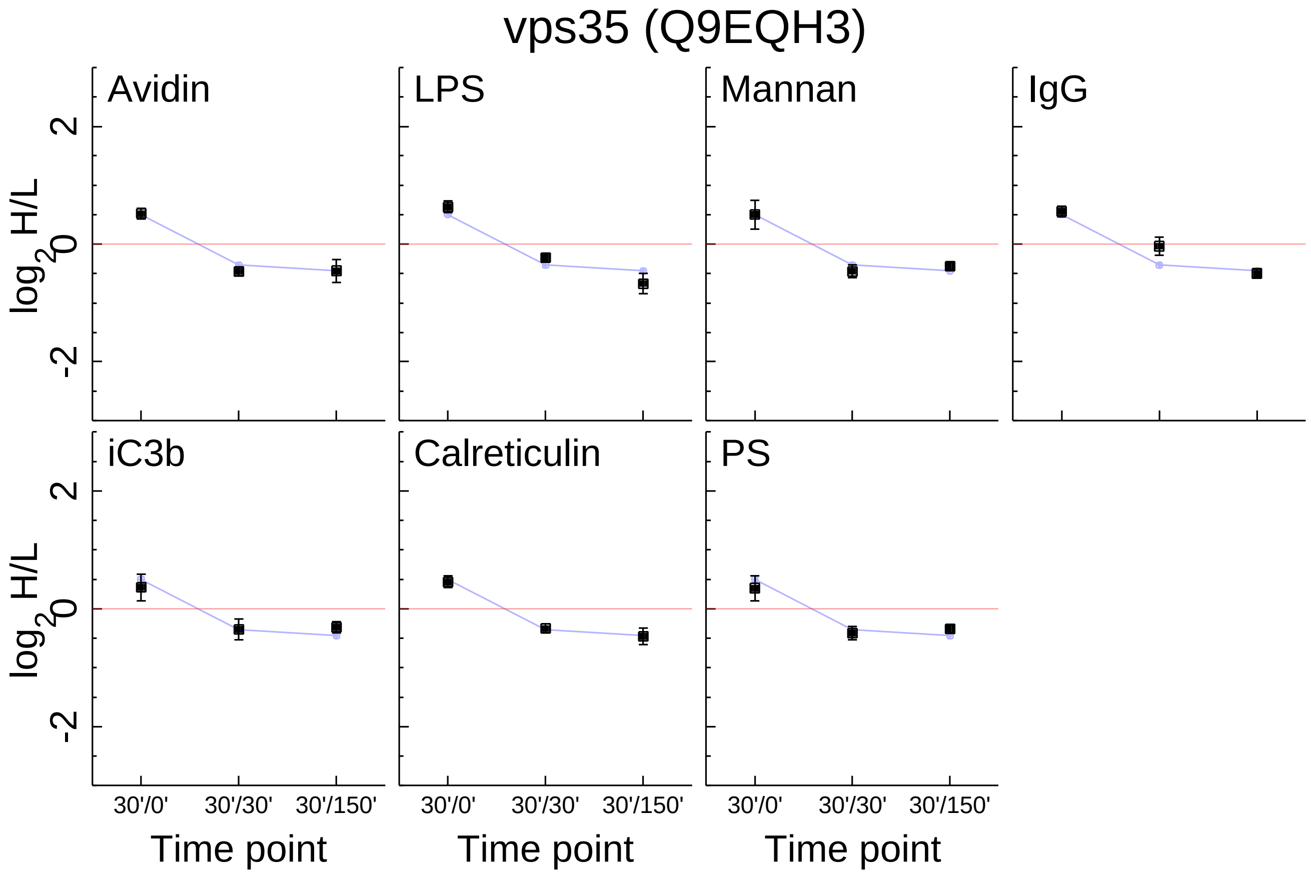

vps35 (Q9EQH3)
Avidin
LPS
Mannan
IgG
iC3b
Calreticulin
PS
log2 H/L
2
0
-2
30'/0'
30'/30'
30'/150'
Time point

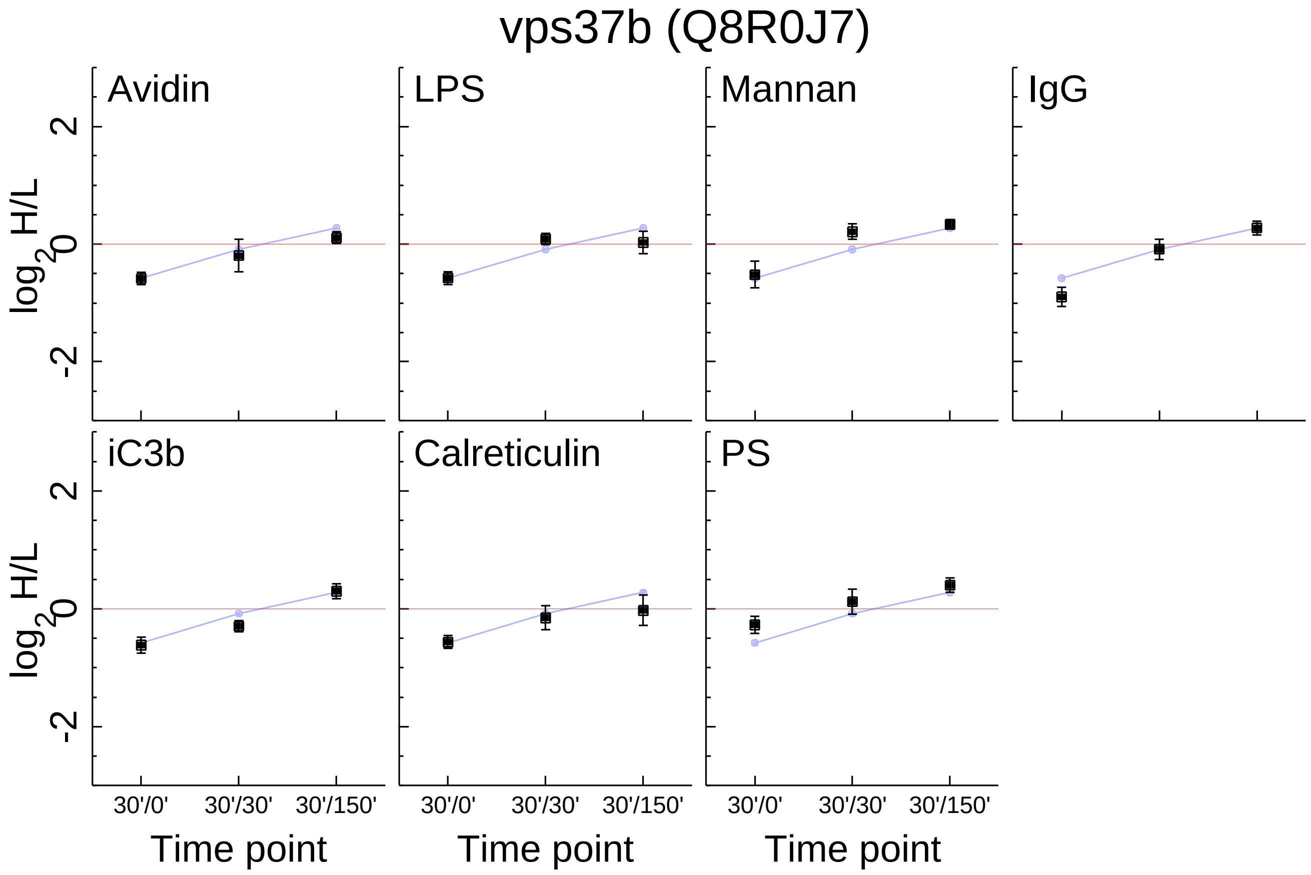
vps37b (Q8R0J7)
Avidin
LPS
Mannan
IgG
iC3b
Calreticulin
PS
log2 H/L
2
0
-2
30'/0'
30'/30'
30'/150'
Time point

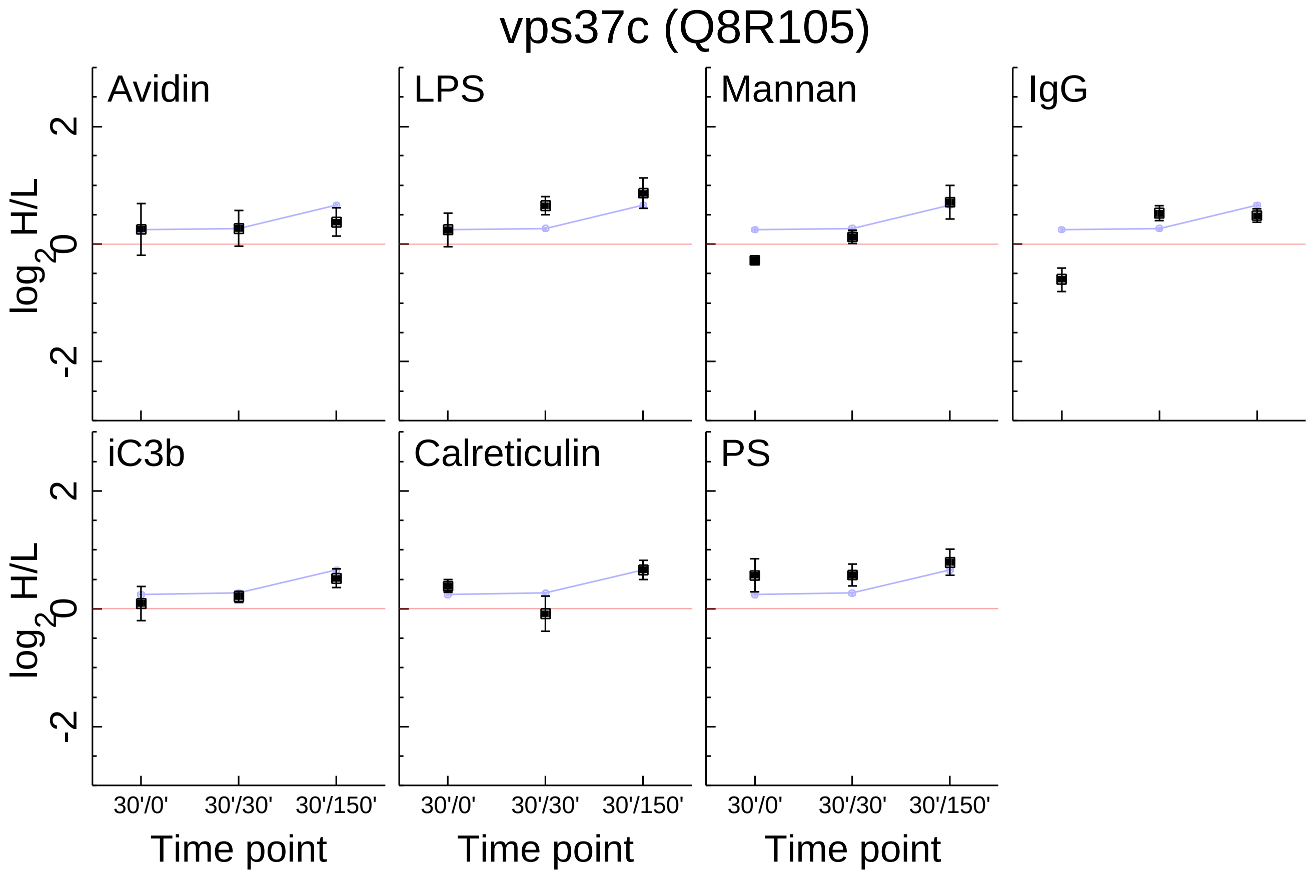

vps37c (Q8R105)
Avidin
LPS
Mannan
IgG
iC3b
Calreticulin
PS
log2 H/L
2
0
-2
30'/0'
30'/30'
30'/150'
Time point

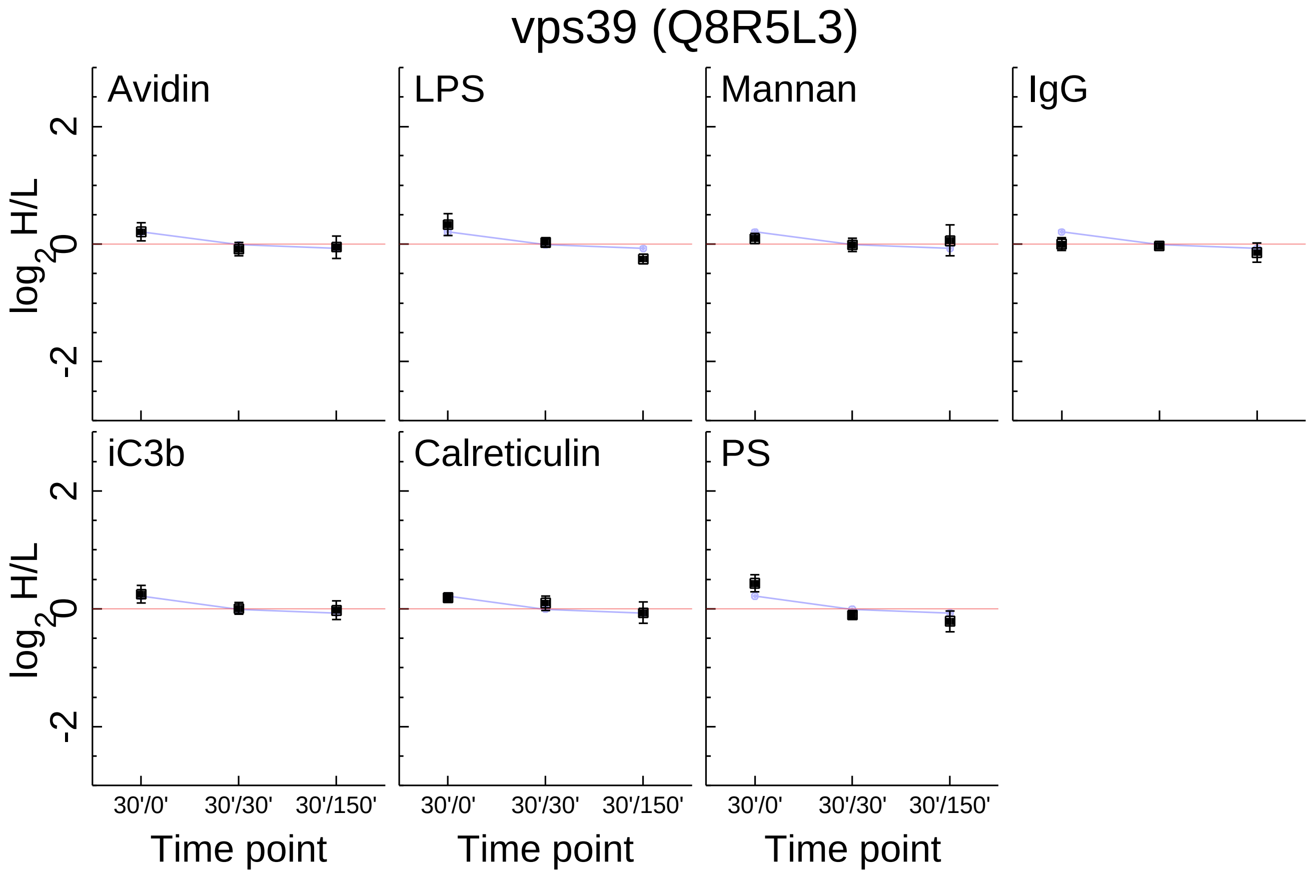
vps39 (Q8R5L3)
Avidin
LPS
Mannan
IgG
iC3b
Calreticulin
PS
log2 H/L
2
0
-2
30'/0'
30'/30'
30'/150'
Time point

vps41 (Q5KU39)

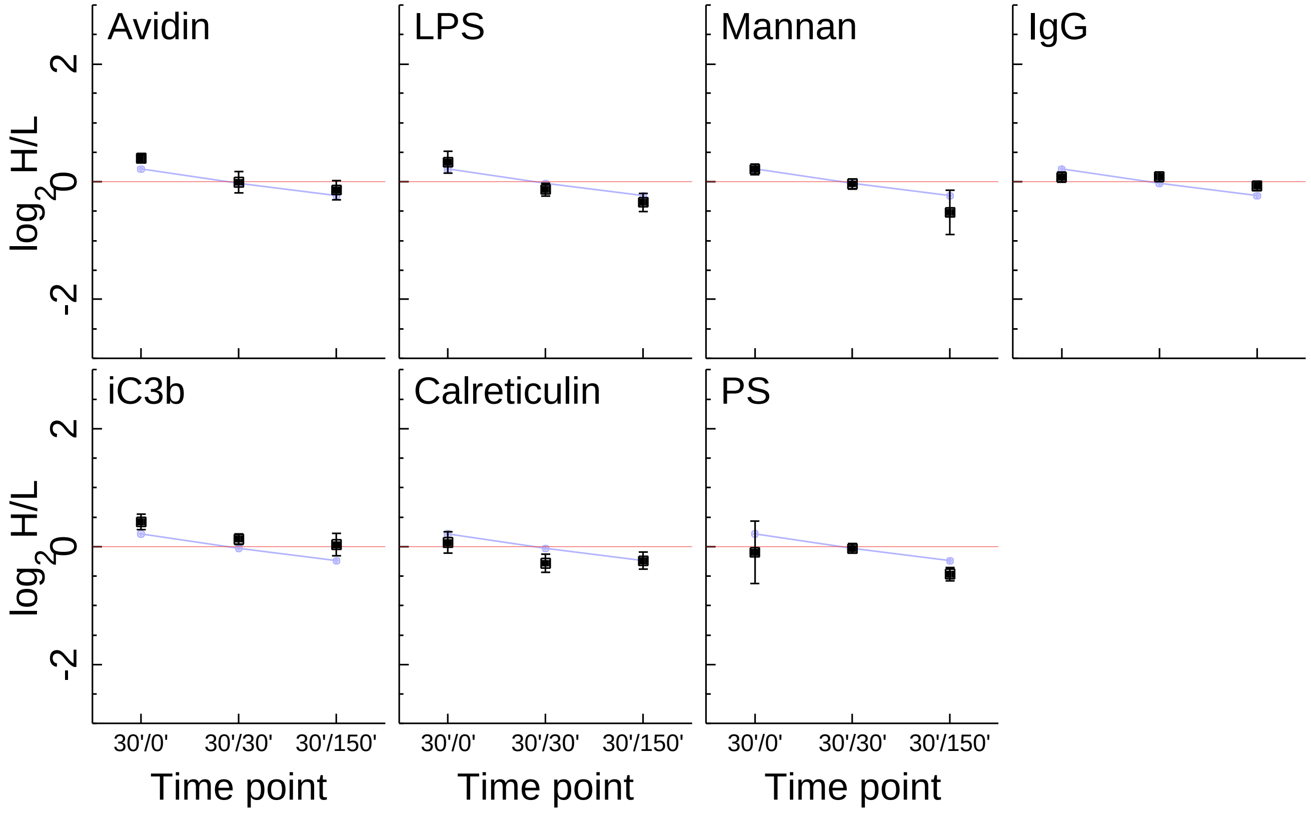

vps45 (P97390)

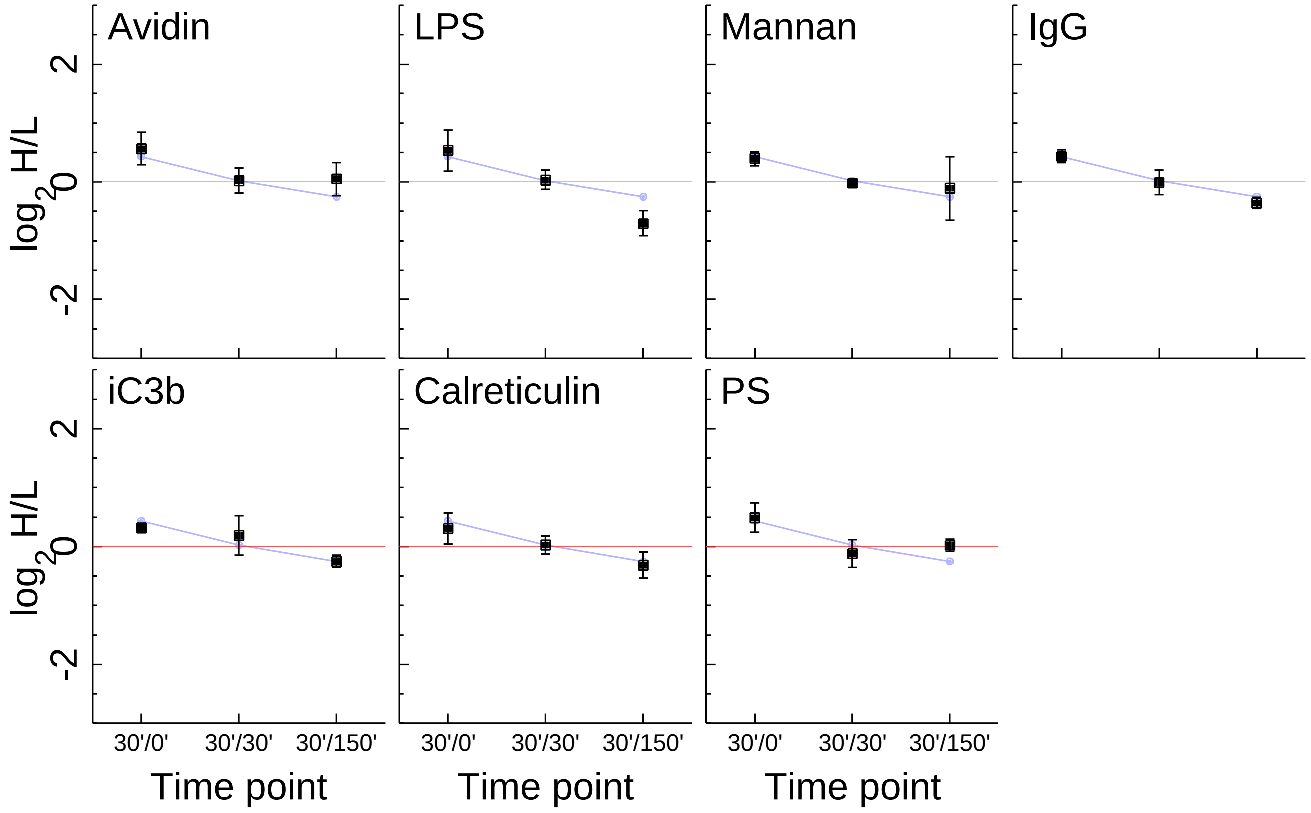

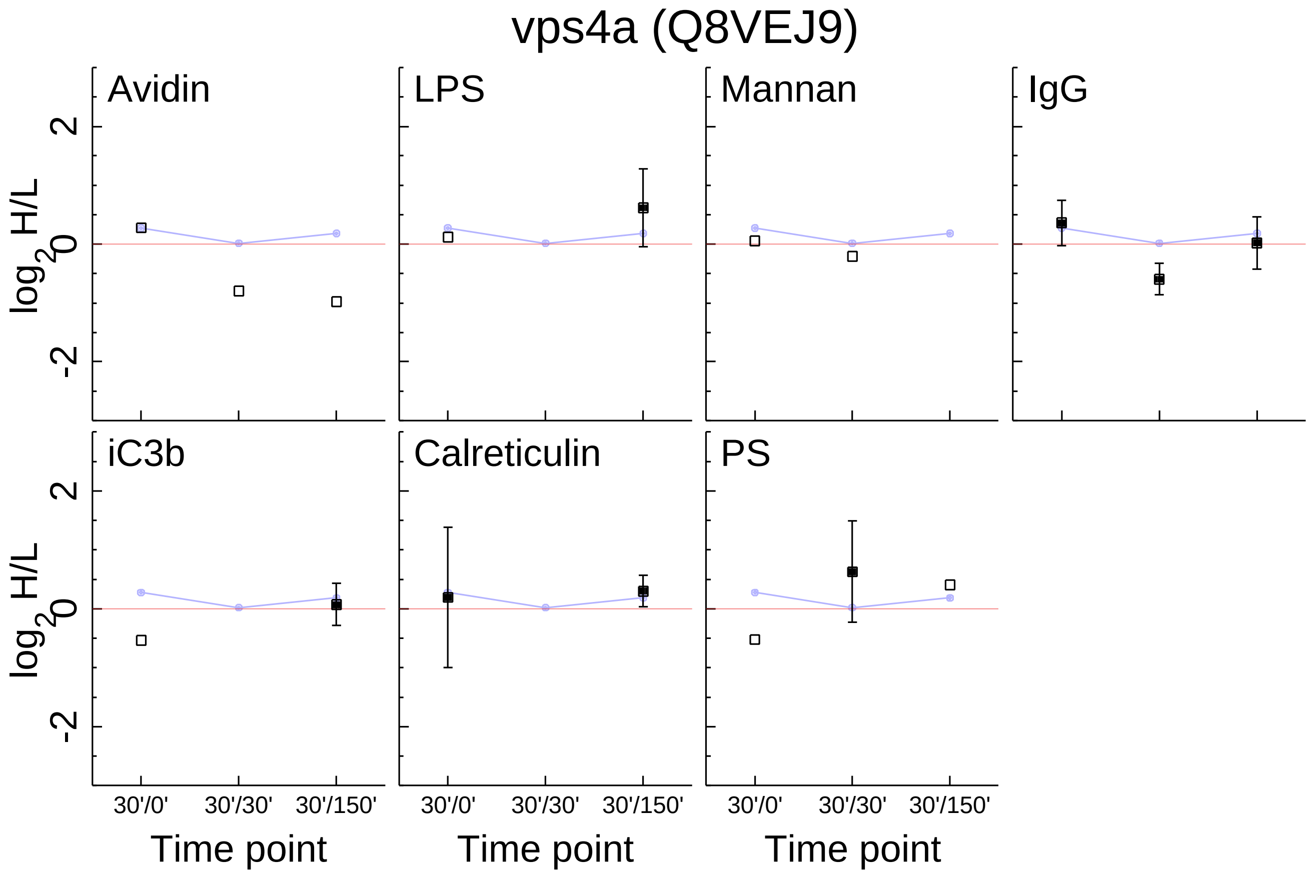
vps4a (Q8VEJ9)
Avidin
LPS
Mannan
IgG
iC3b
Calreticulin
PS
log2 H/L
2
0
-2
30'/0'
30'/30'
30'/150'
Time point

vps4b (P46467)

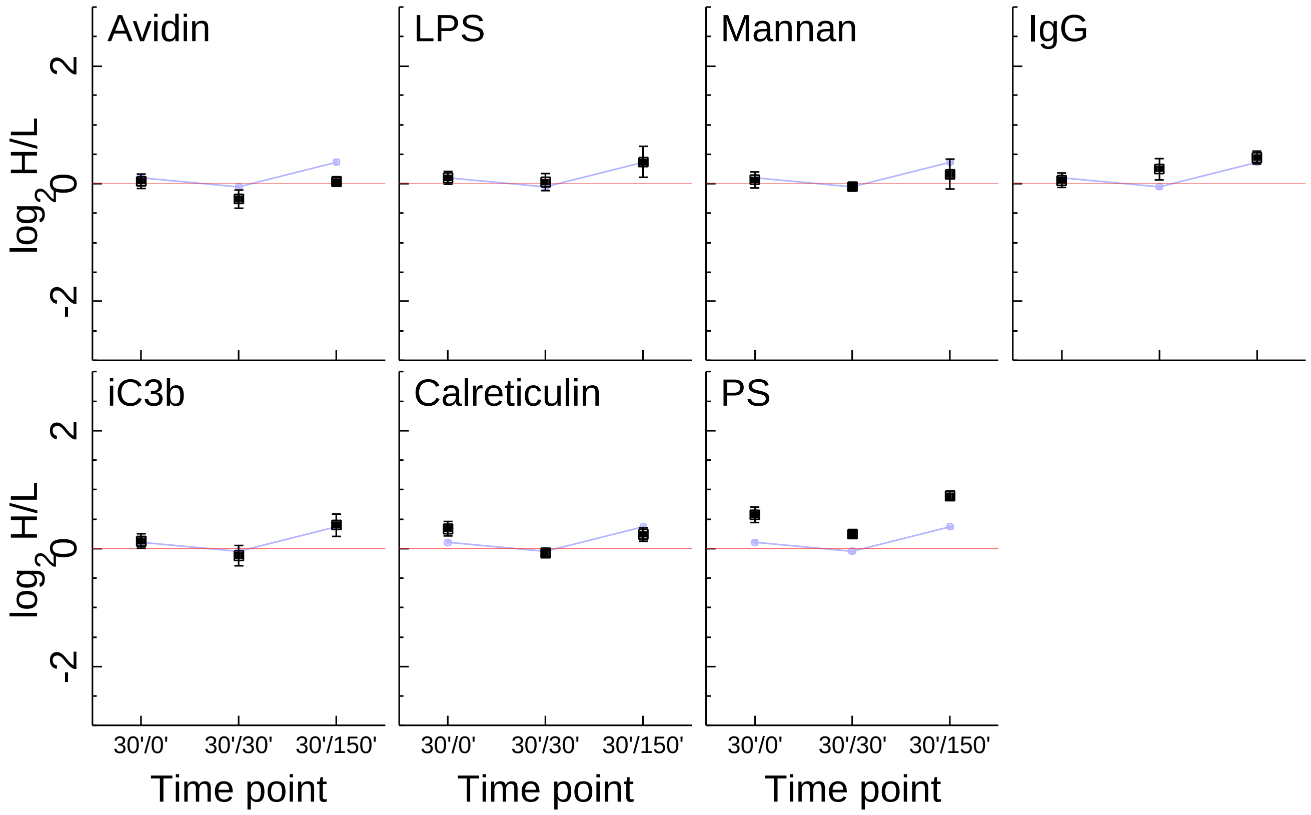

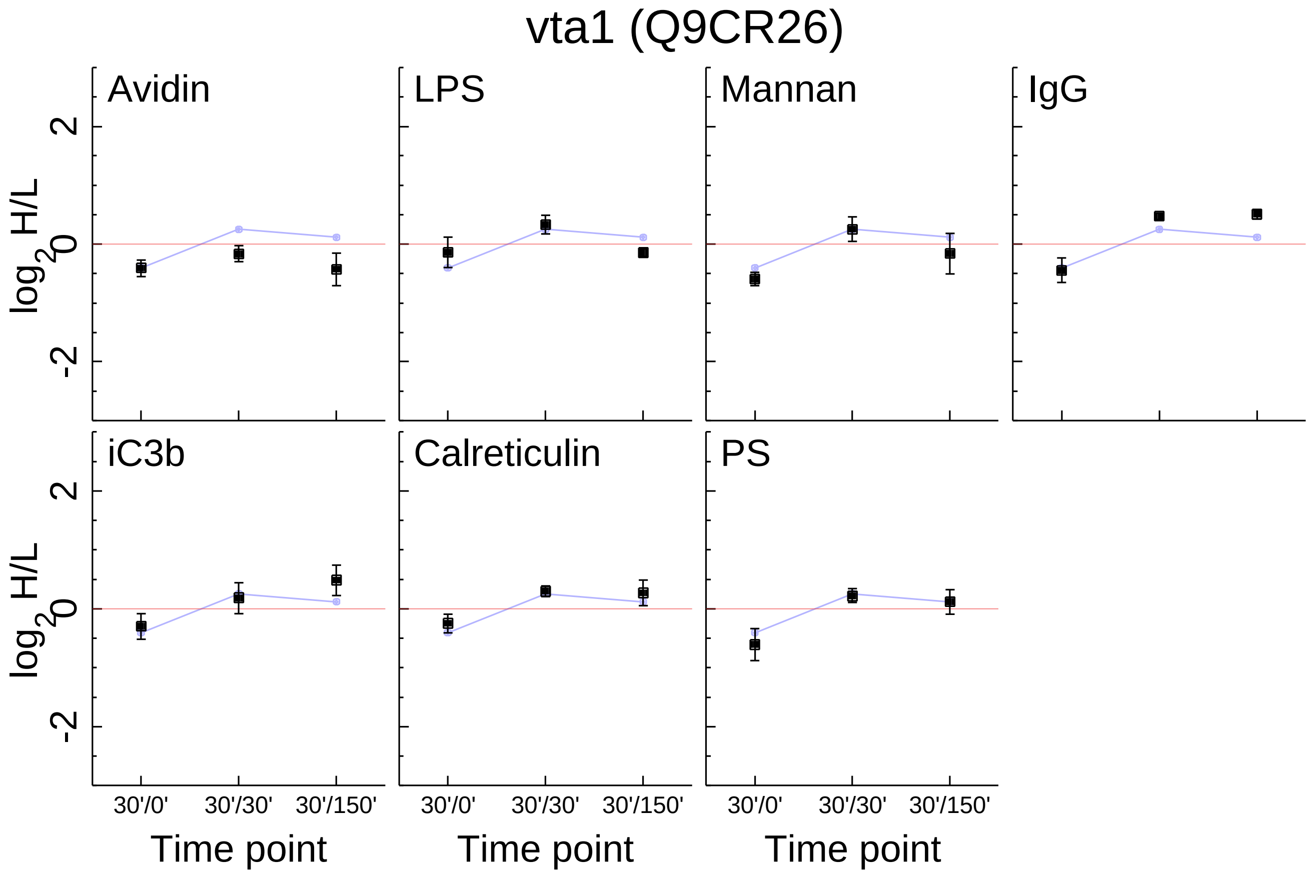
vta1 (Q9CR26)
Avidin
LPS
Mannan
IgG
iC3b
Calreticulin
PS
log2 H/L
2
0
-2
30'/0'
30'/30'
30'/150'
Time point

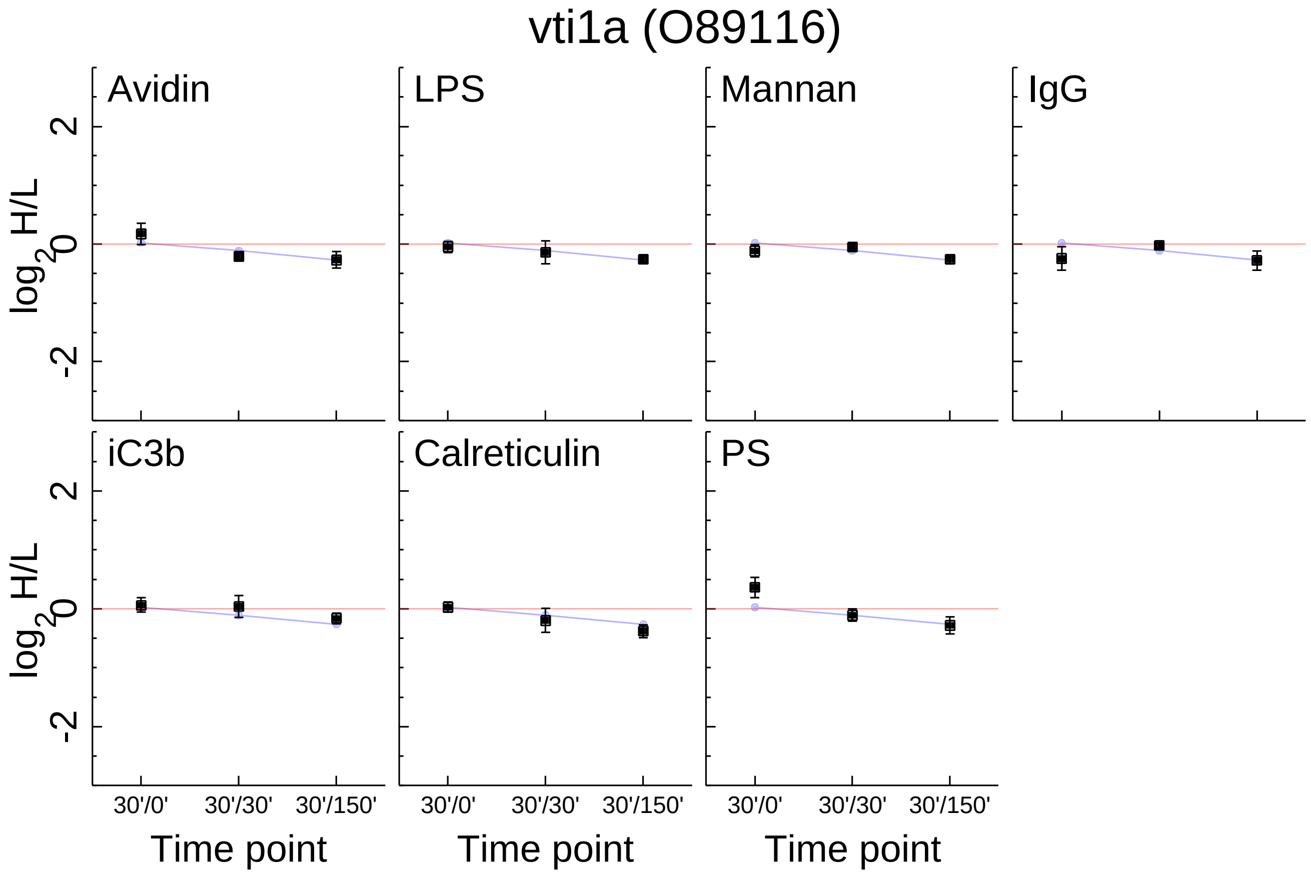

vti1a (O89116)
Avidin
LPS
Mannan
IgG
iC3b
Calreticulin
PS
log2 H/L
2
0
-2
30'/0'
30'/30'
30'/150'
Time point

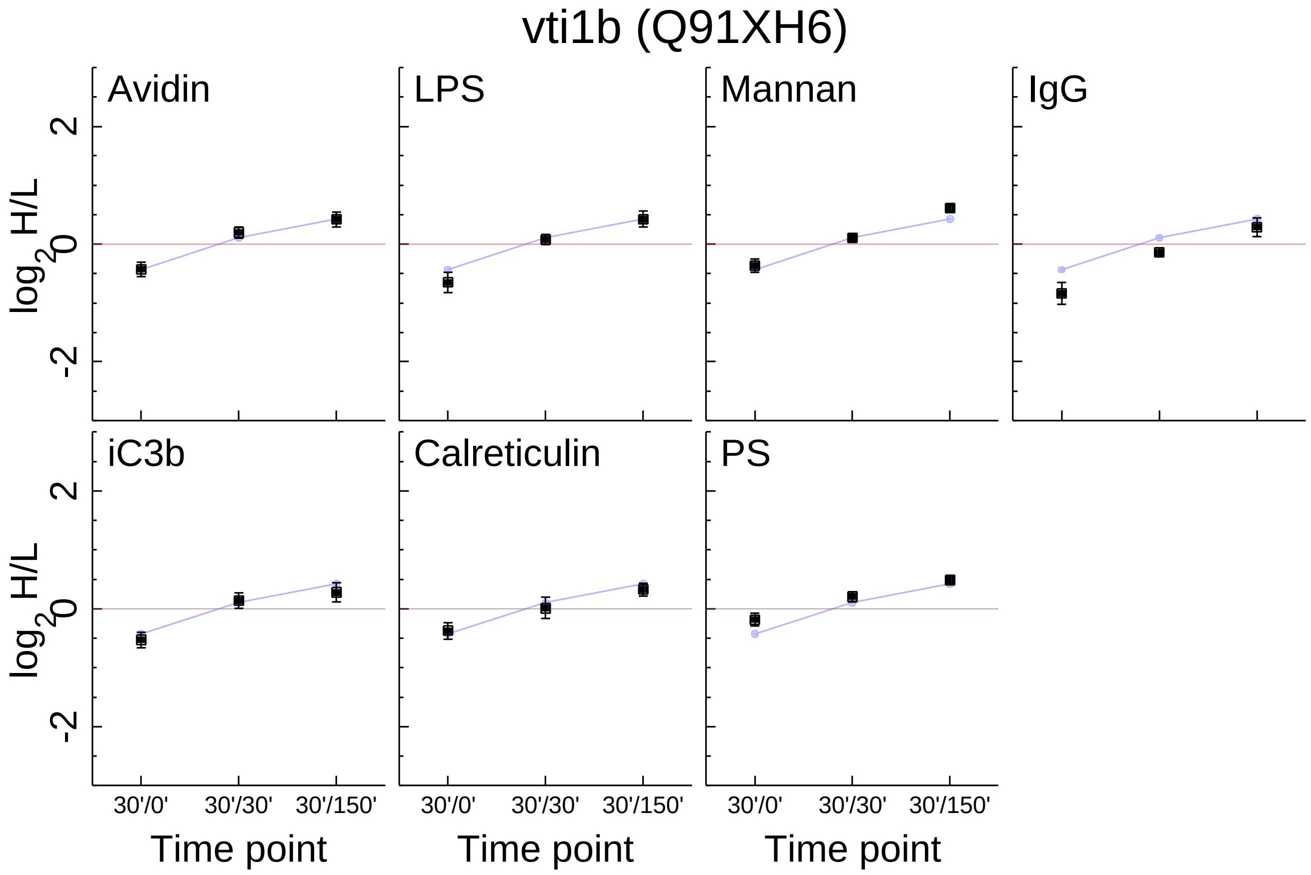

vti1b (Q91XH6)
Avidin
LPS
Mannan
IgG
iC3b
Calreticulin
PS
log2 H/L
2
0
-2
30'/0'
30'/30'
30'/150'
Time point

vwa5a (Q99KC8)

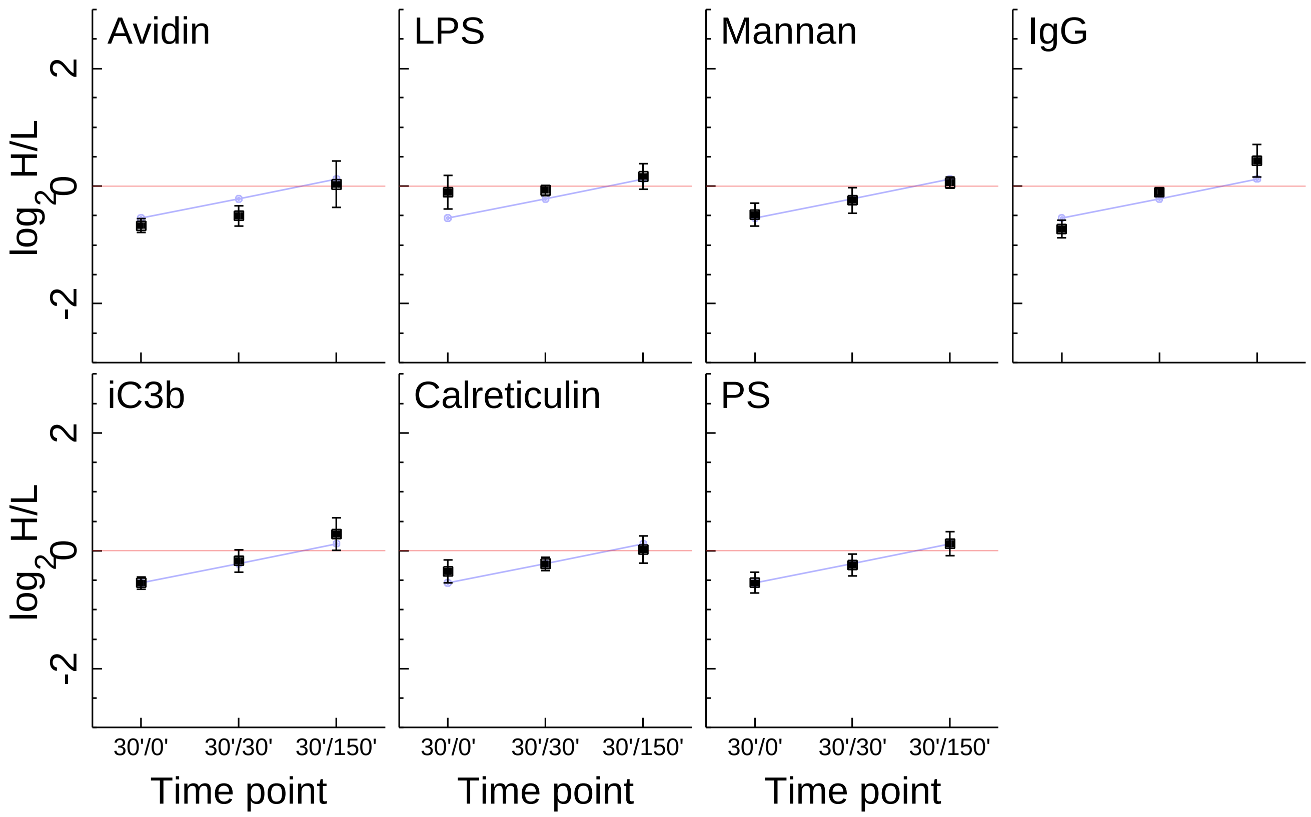

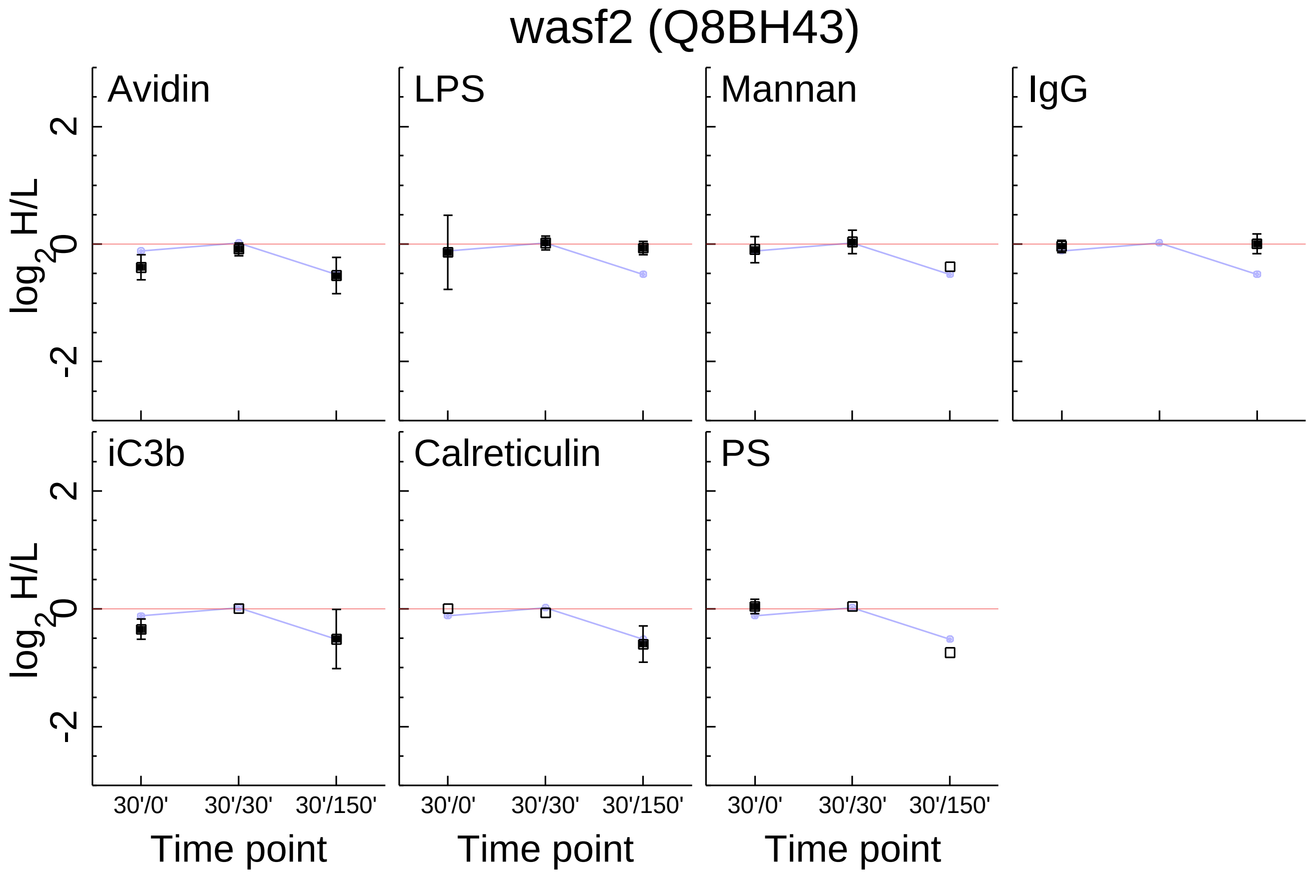

wasf2 (Q8BH43)
Avidin
LPS
Mannan
IgG
iC3b
Calreticulin
PS
log2 H/L
2
0
-2
30'/0'
30'/30'
30'/150'
Time point

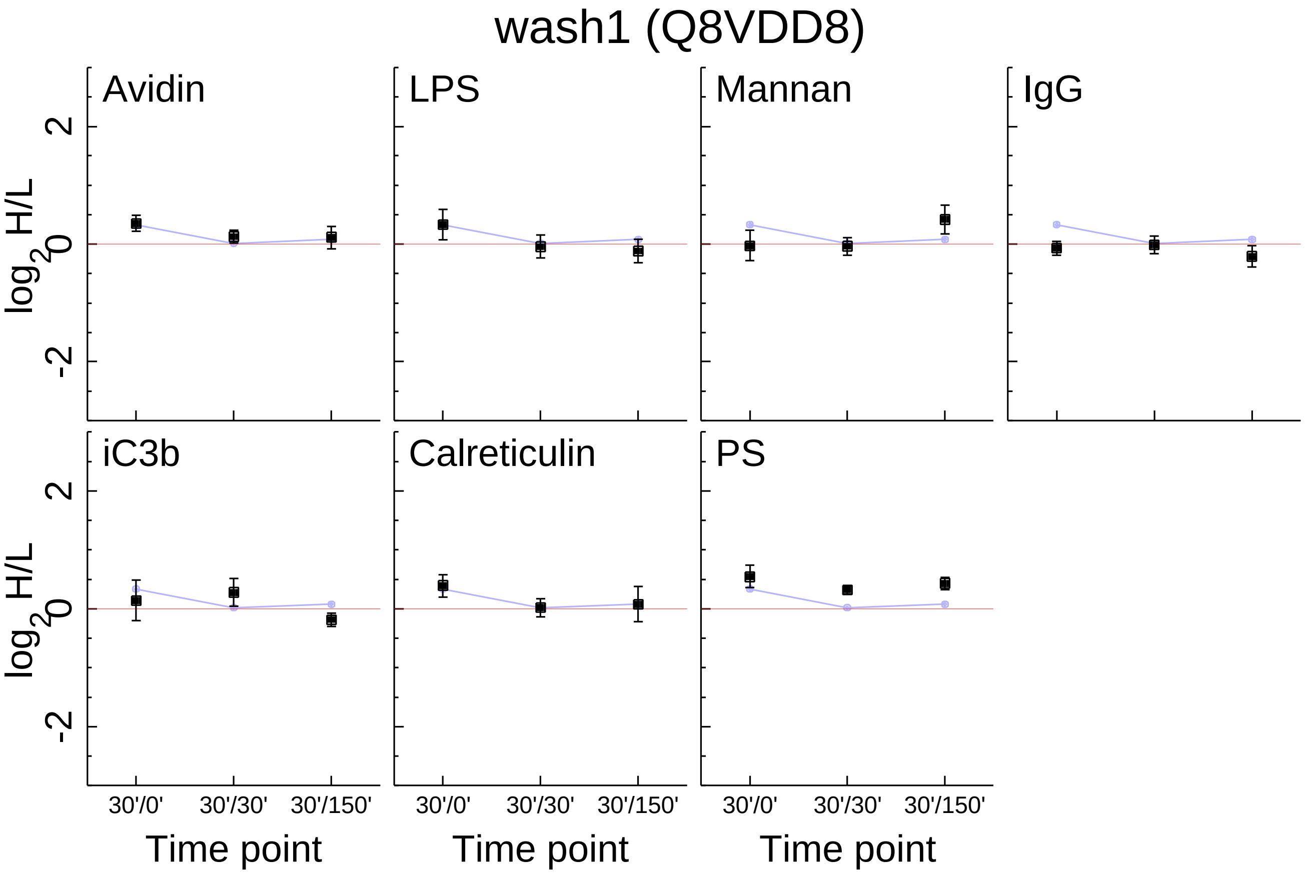
wash1 (Q8VDD8)
Avidin
LPS
Mannan
IgG
iC3b
Calreticulin
PS
log2 H/L
2
0
-2
30'/0'
30'/30'
30'/150'
Time point

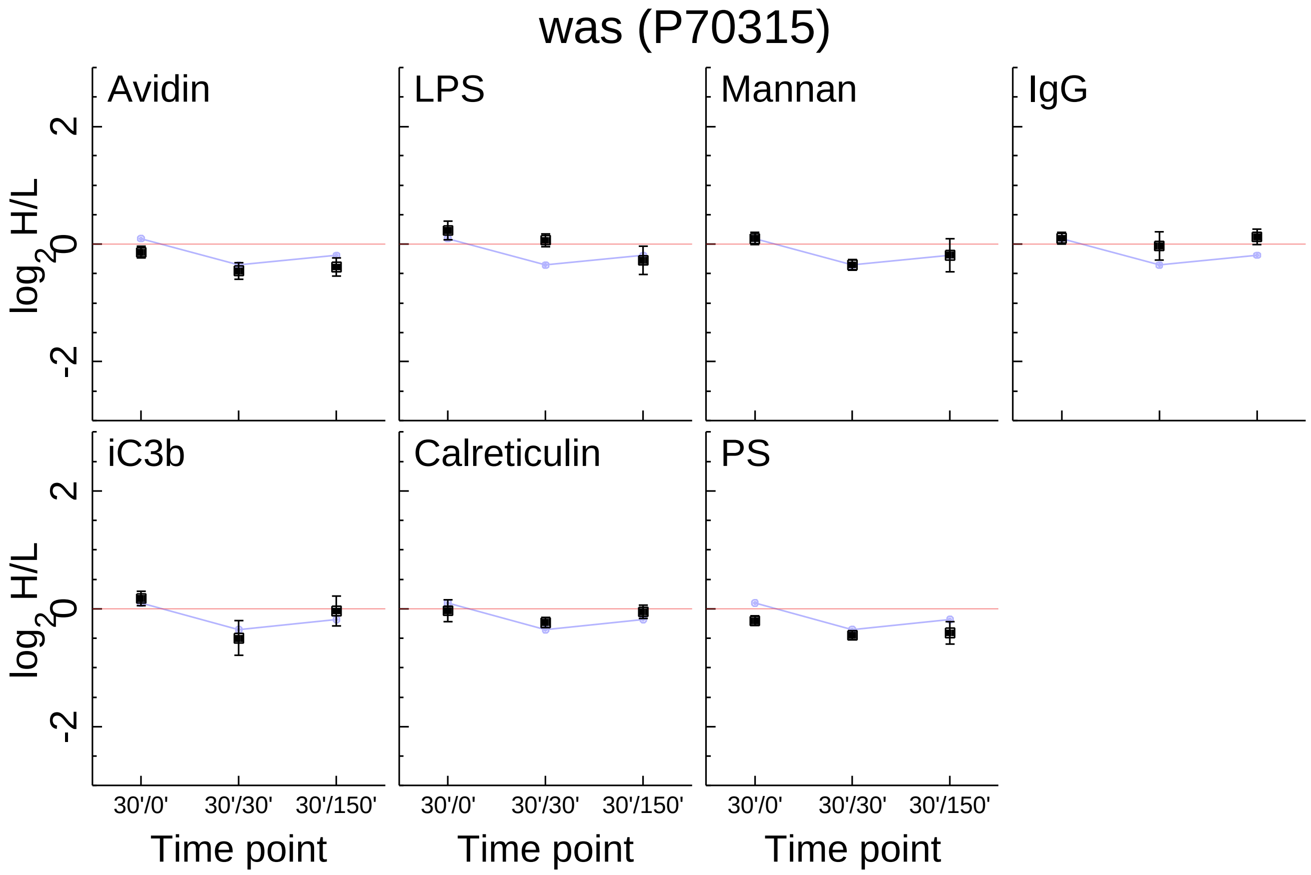
was (P70315)
Avidin
LPS
Mannan
IgG
iC3b
Calreticulin
PS
log2 H/L
2
0
-2
30'/0'
30'/30'
30'/150'
Time point

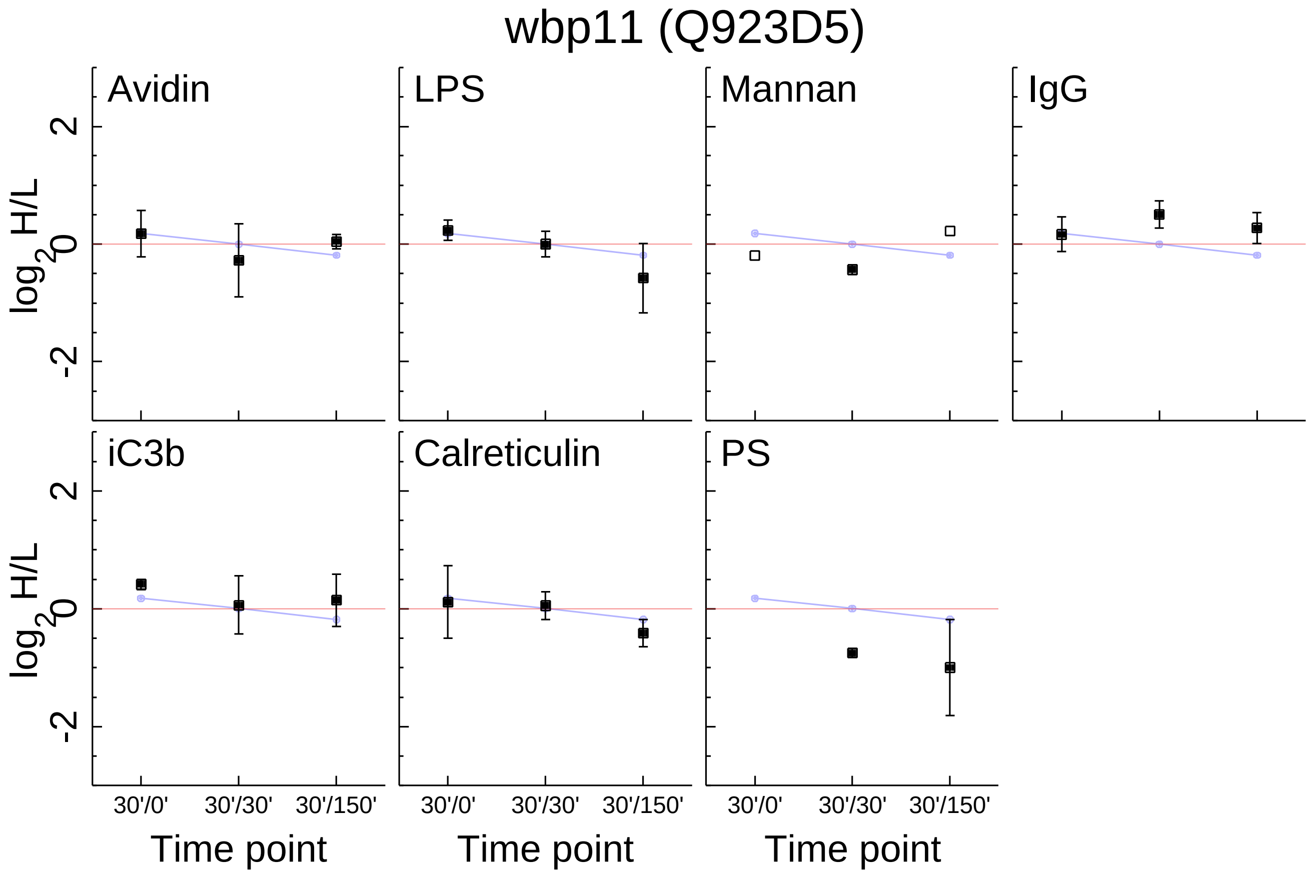

wbp11 (Q923D5)
Avidin
LPS
Mannan
IgG
iC3b
Calreticulin
PS
log2 H/L
2
0
-2
30'/0'
30'/30'
30'/150'
Time point

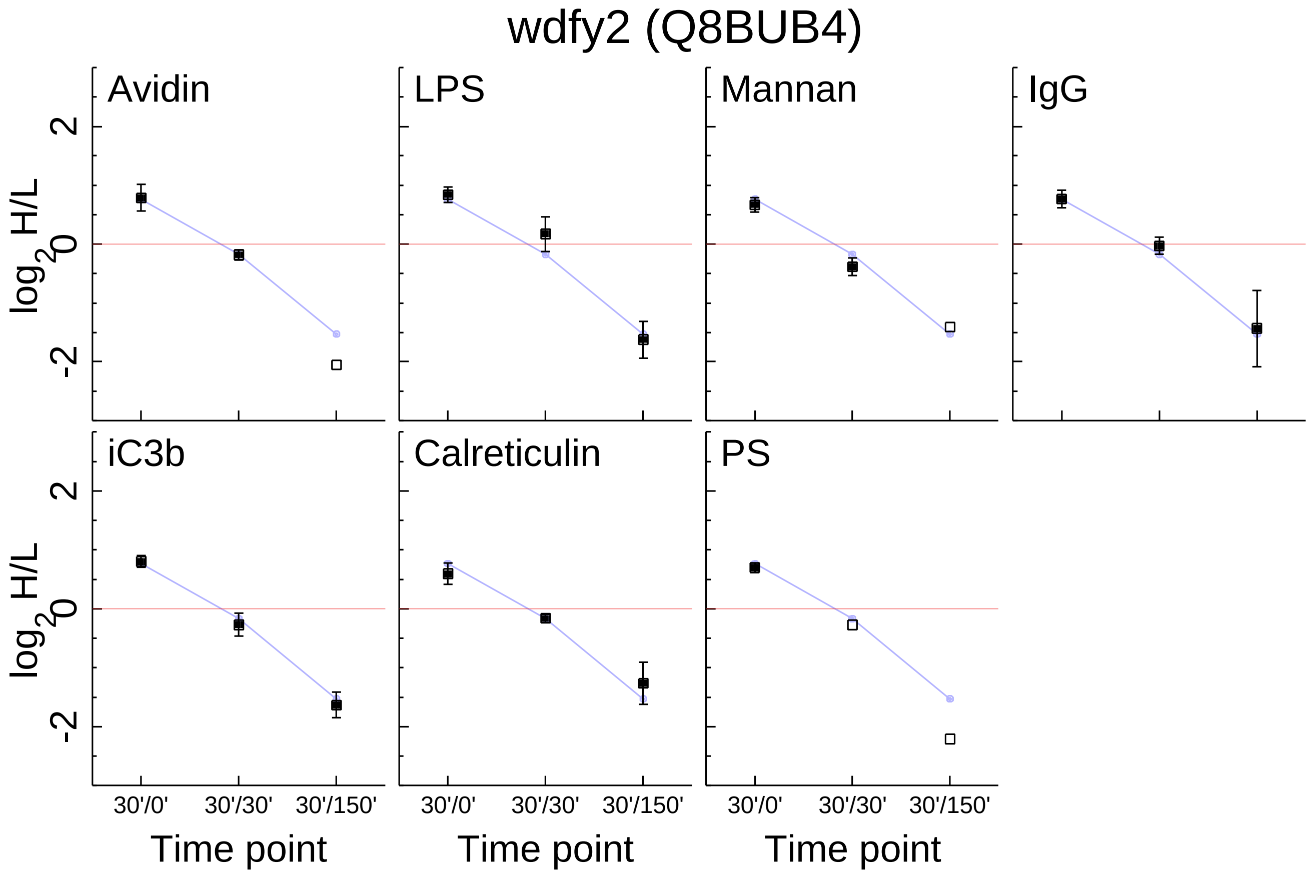
wdfy2 (Q8BUB4)
Avidin
LPS
Mannan
IgG
iC3b
Calreticulin
PS
log2 H/L
2
0
-2
30'/0'
30'/30'
30'/150'
Time point

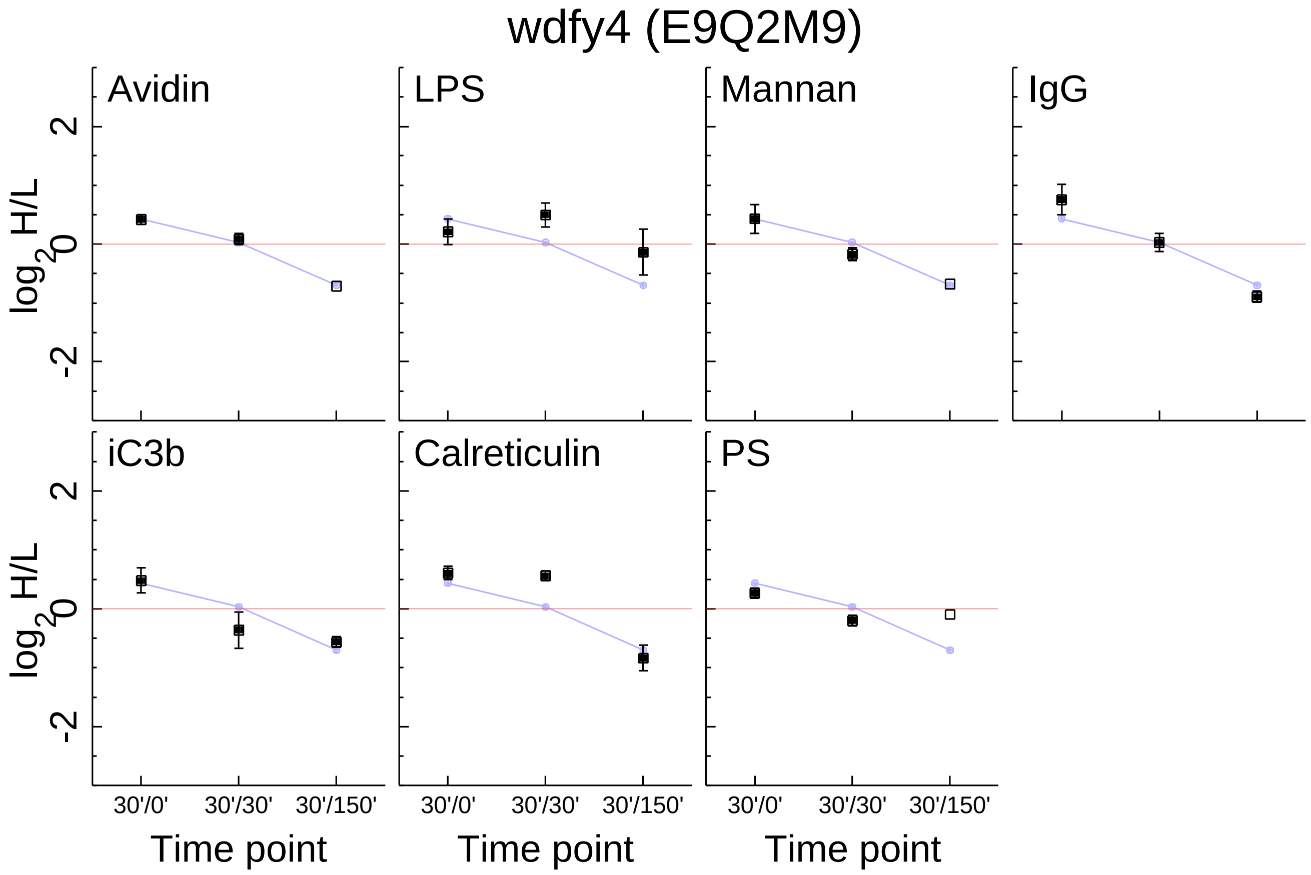
wdfy4 (E9Q2M9)
Avidin
LPS
Mannan
IgG
iC3b
Calreticulin
PS
log2 H/L
2
0
-2
30'/0'
30'/30'
30'/150'
Time point

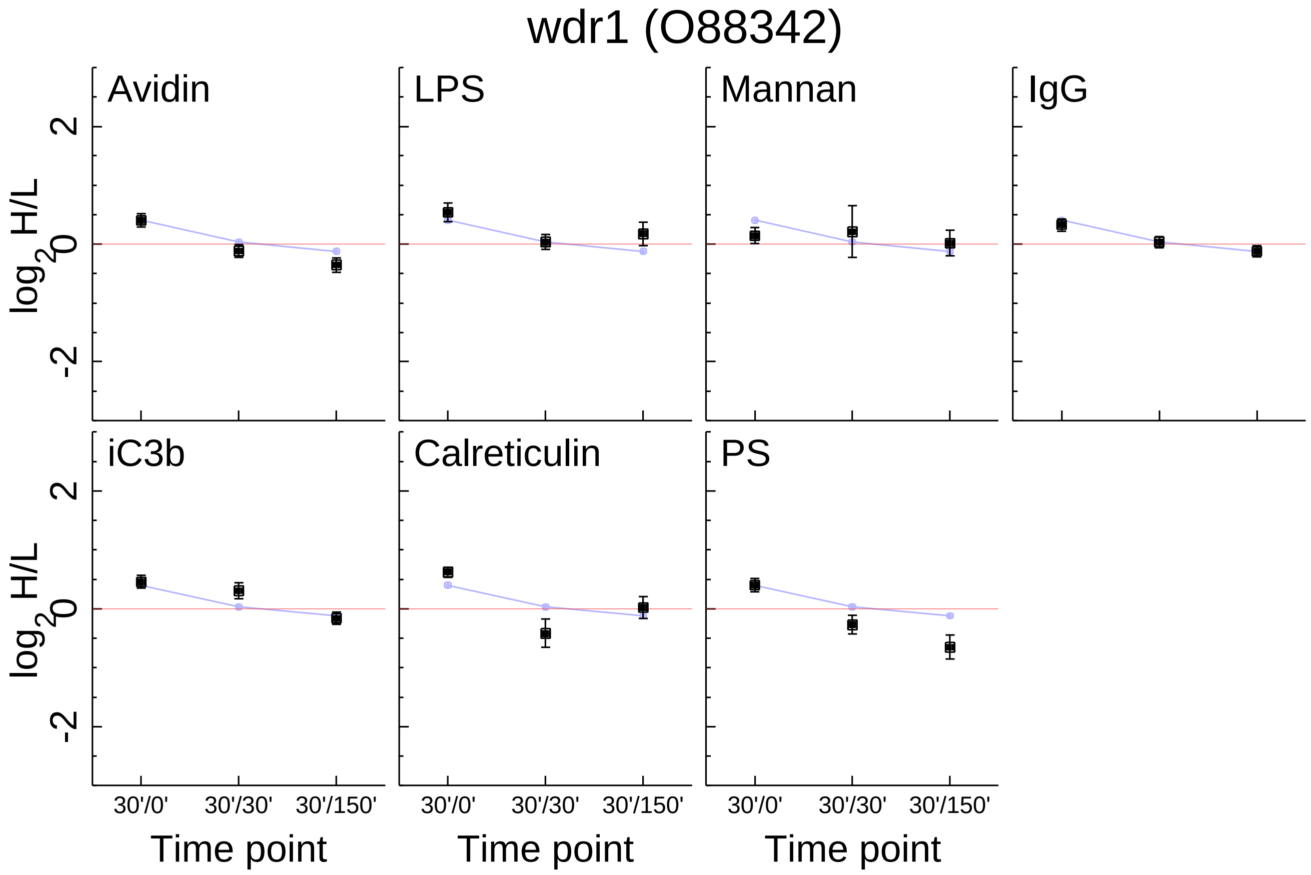

wdr1 (O88342)
Avidin
LPS
Mannan
IgG
iC3b
Calreticulin
PS
log2 H/L
2
0
-2
30'/0'
30'/30'
30'/150'
Time point

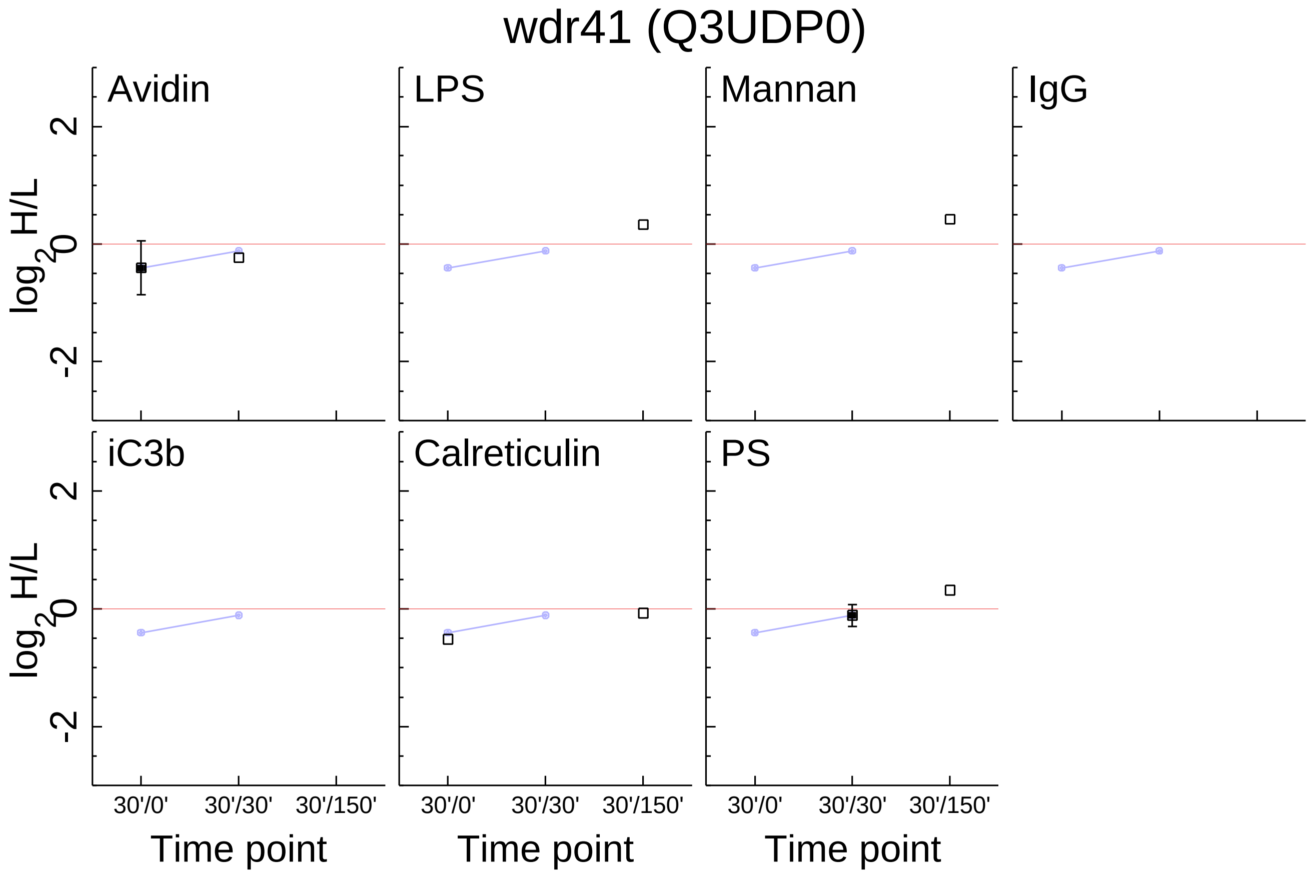

wdr41 (Q3UDP0)
Avidin
LPS
Mannan
IgG
iC3b
Calreticulin
PS
log2 H/L
2
0
-2
30'/0'
30'/30'
30'/150'
Time point

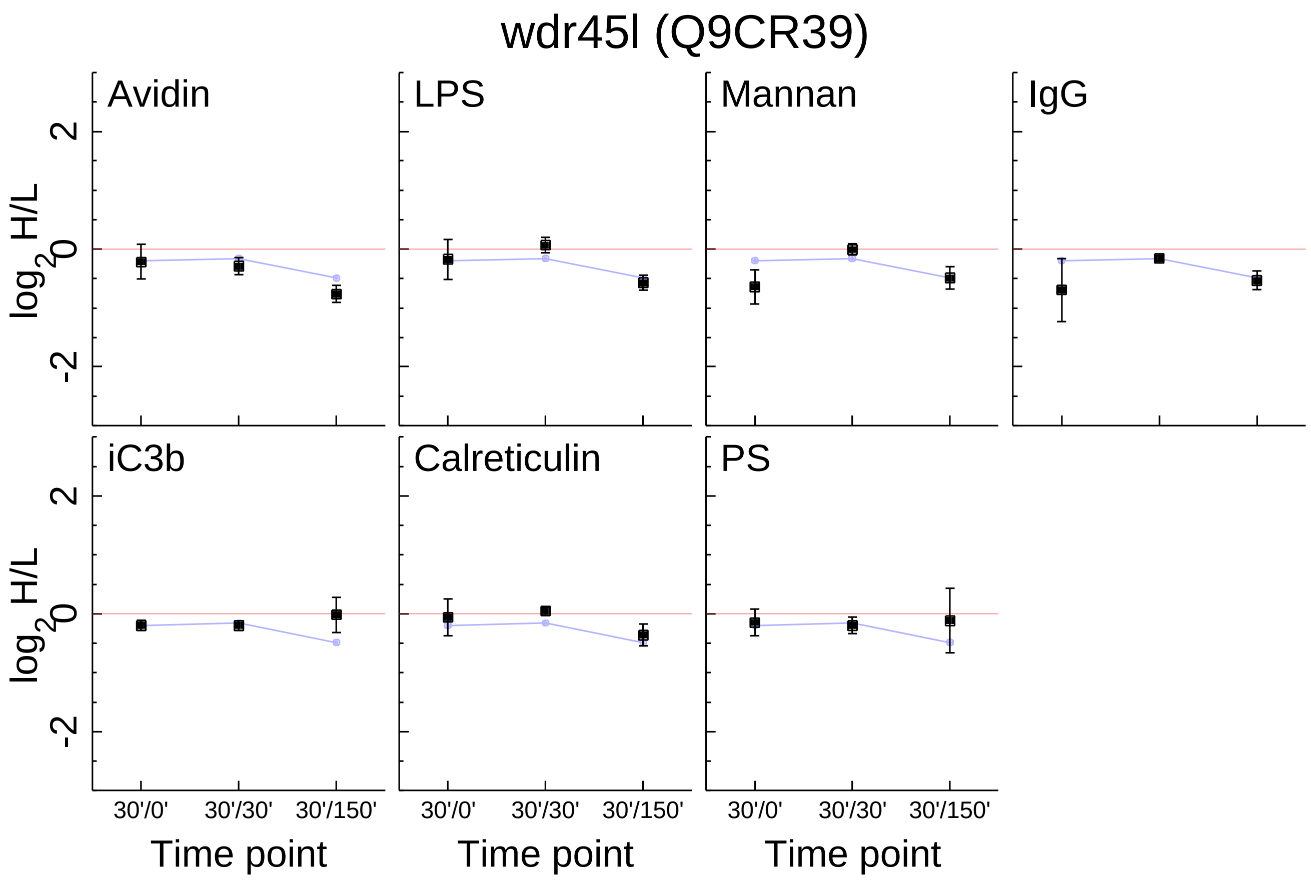
wdr45l (Q9CR39)
Avidin
LPS
Mannan
IgG
iC3b
Calreticulin
PS
log2 H/L
2
0
-2
30'/0'
30'/30'
30'/150'
Time point

wdr5 (P61965)

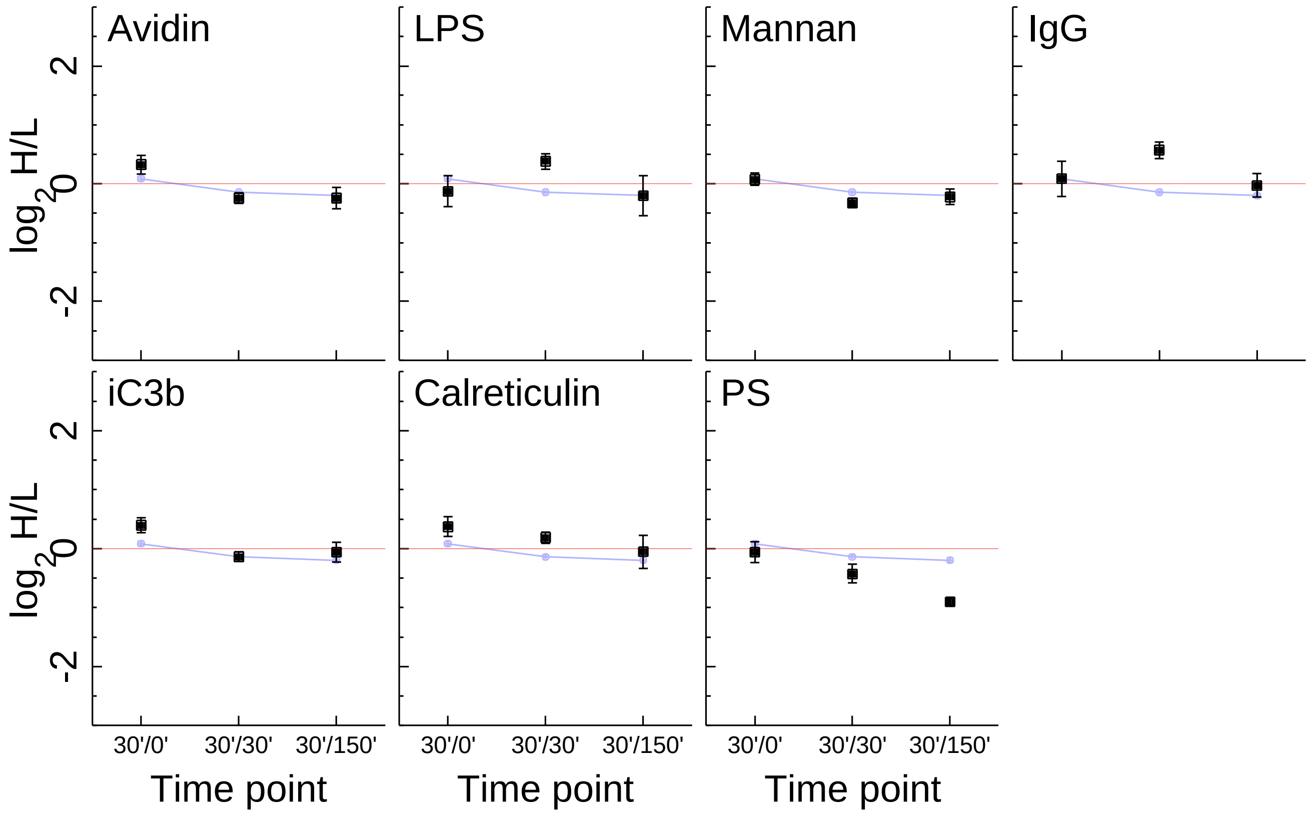
Avidin
LPS
Mannan
IgG
iC3b
Calreticulin
PS
log2 H/L
2
0
-2
30'/0'
30'/30'
30'/150'
Time point

wdr70 (G3X934)

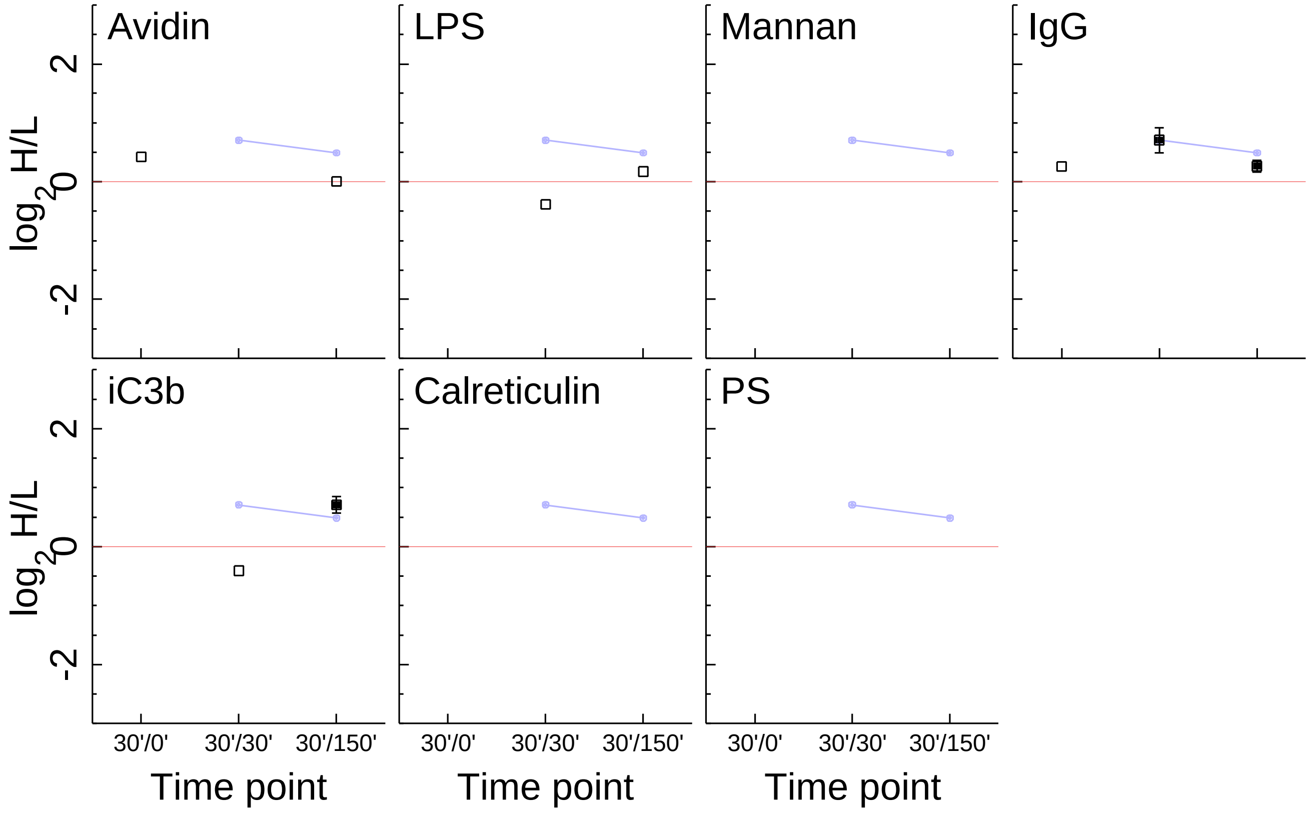

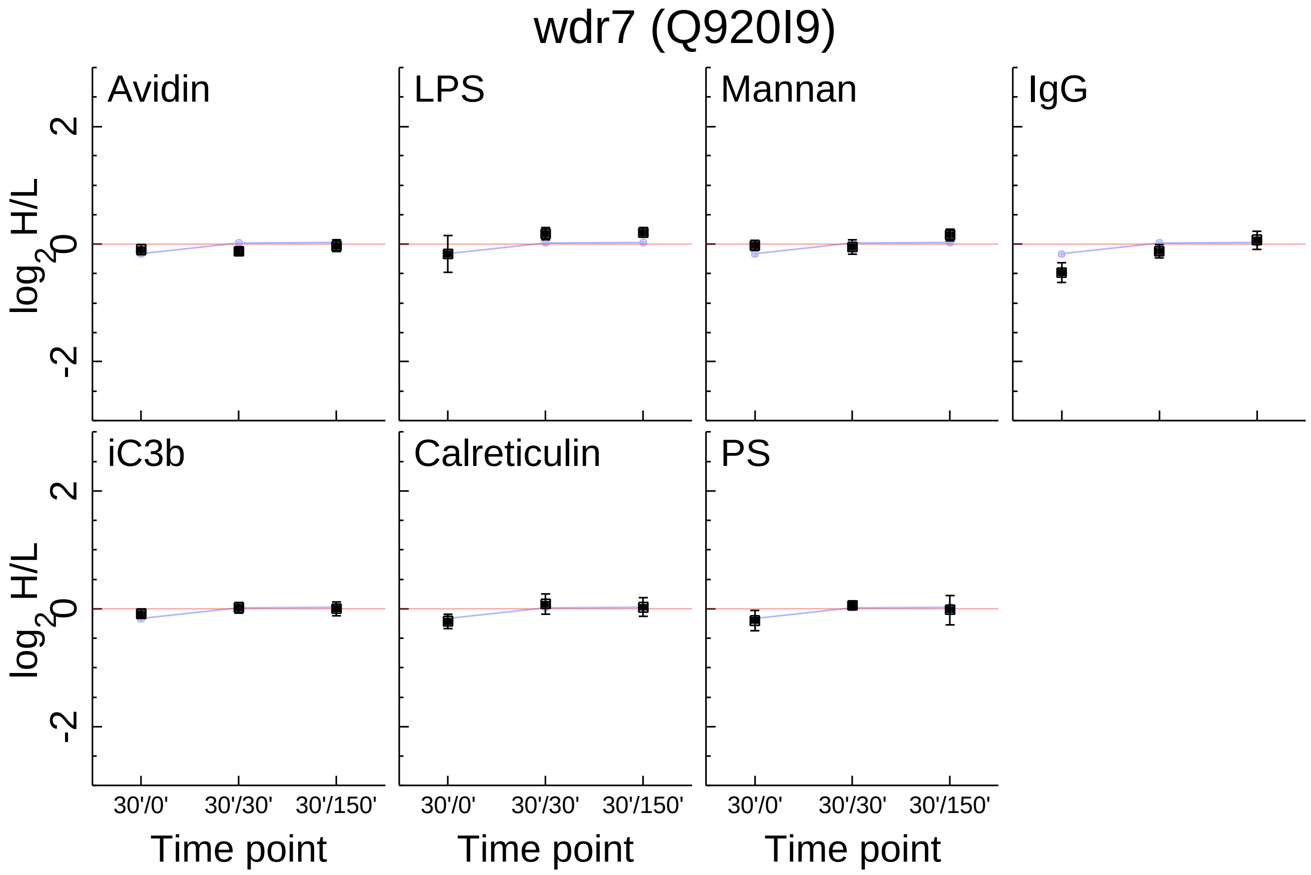

wdr7 (Q920I9)
Avidin
LPS
Mannan
IgG
iC3b
Calreticulin
PS
log2 H/L
2
0
-2
30'/0'
30'/30'
30'/150'
Time point

wdr81 (Q5ND34)

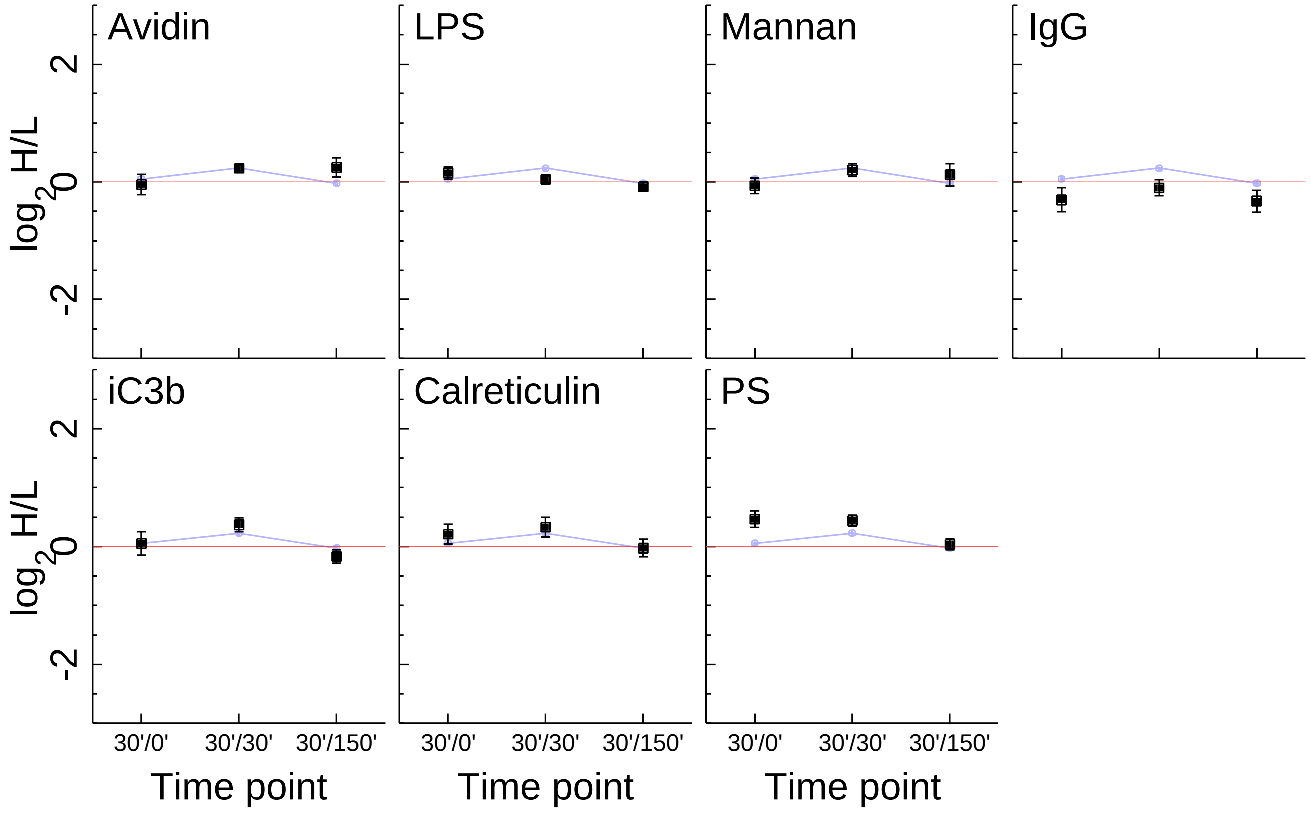

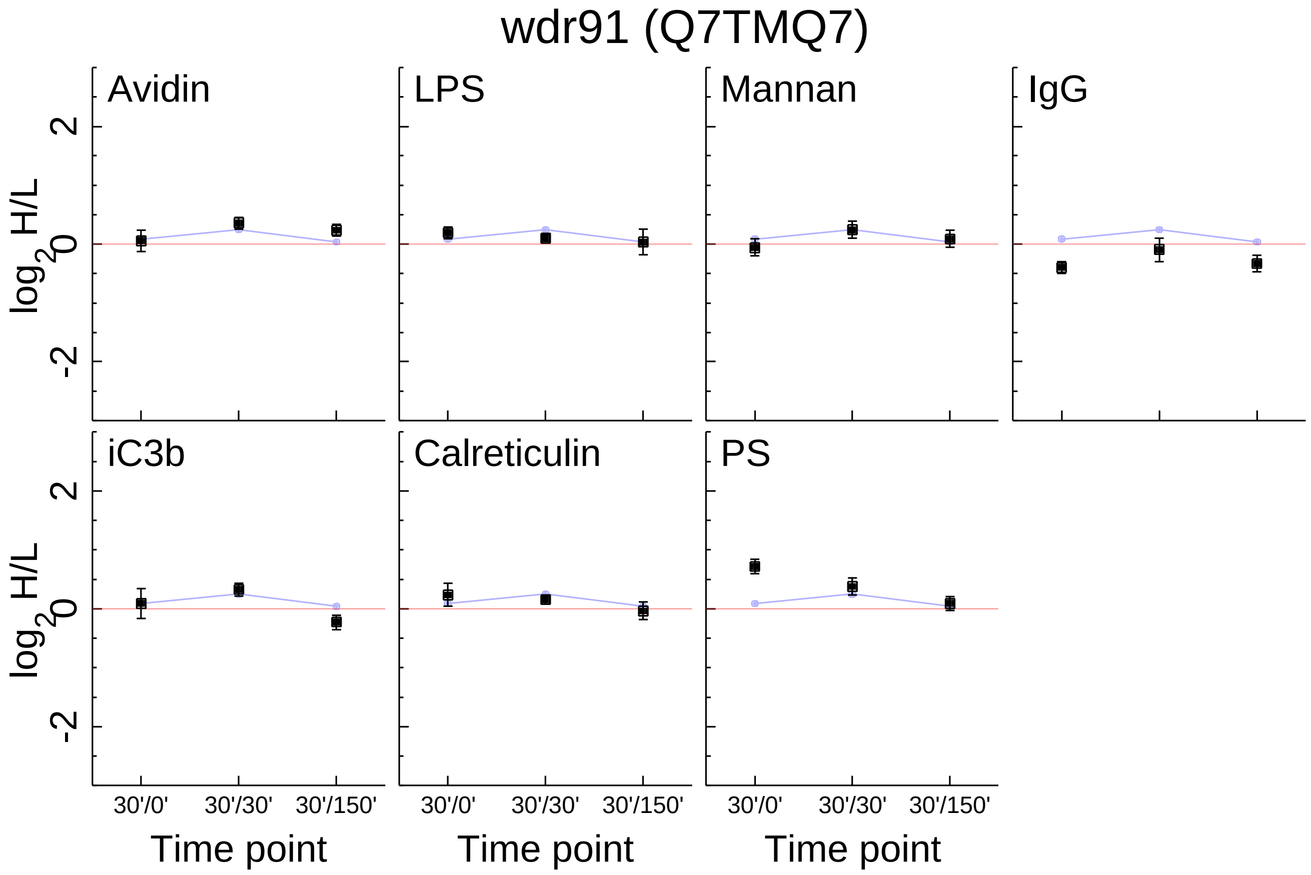

wdr91 (Q7TMQ7)
Avidin
LPS
Mannan
IgG
iC3b
Calreticulin
PS
log2 H/L
2
0
-2
30'/0'
30'/30'
30'/150'
Time point

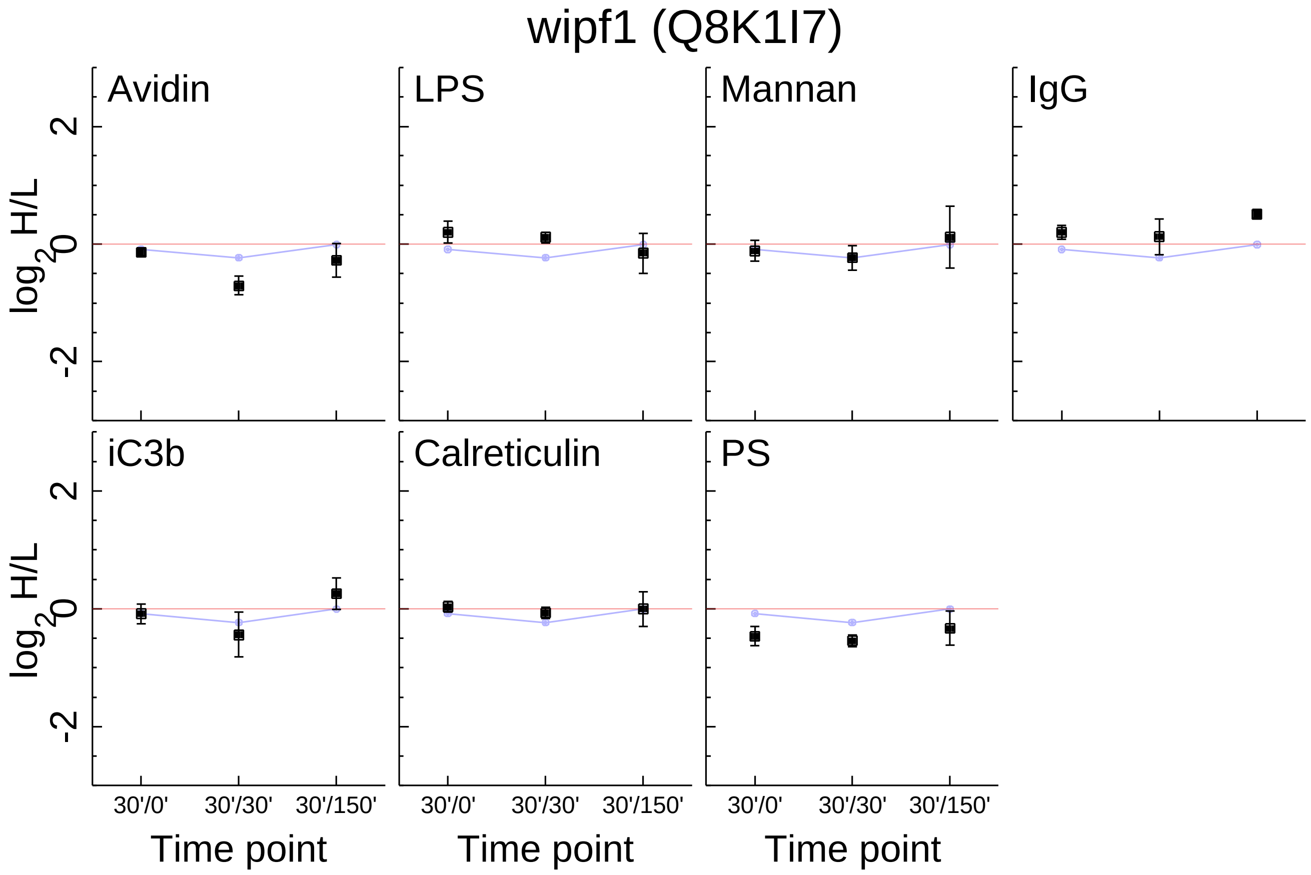

wipf1 (Q8K1I7)
Avidin
LPS
Mannan
IgG
iC3b
Calreticulin
PS
log2 H/L
2
0
-2
30'/0'
30'/30'
30'/150'
Time point

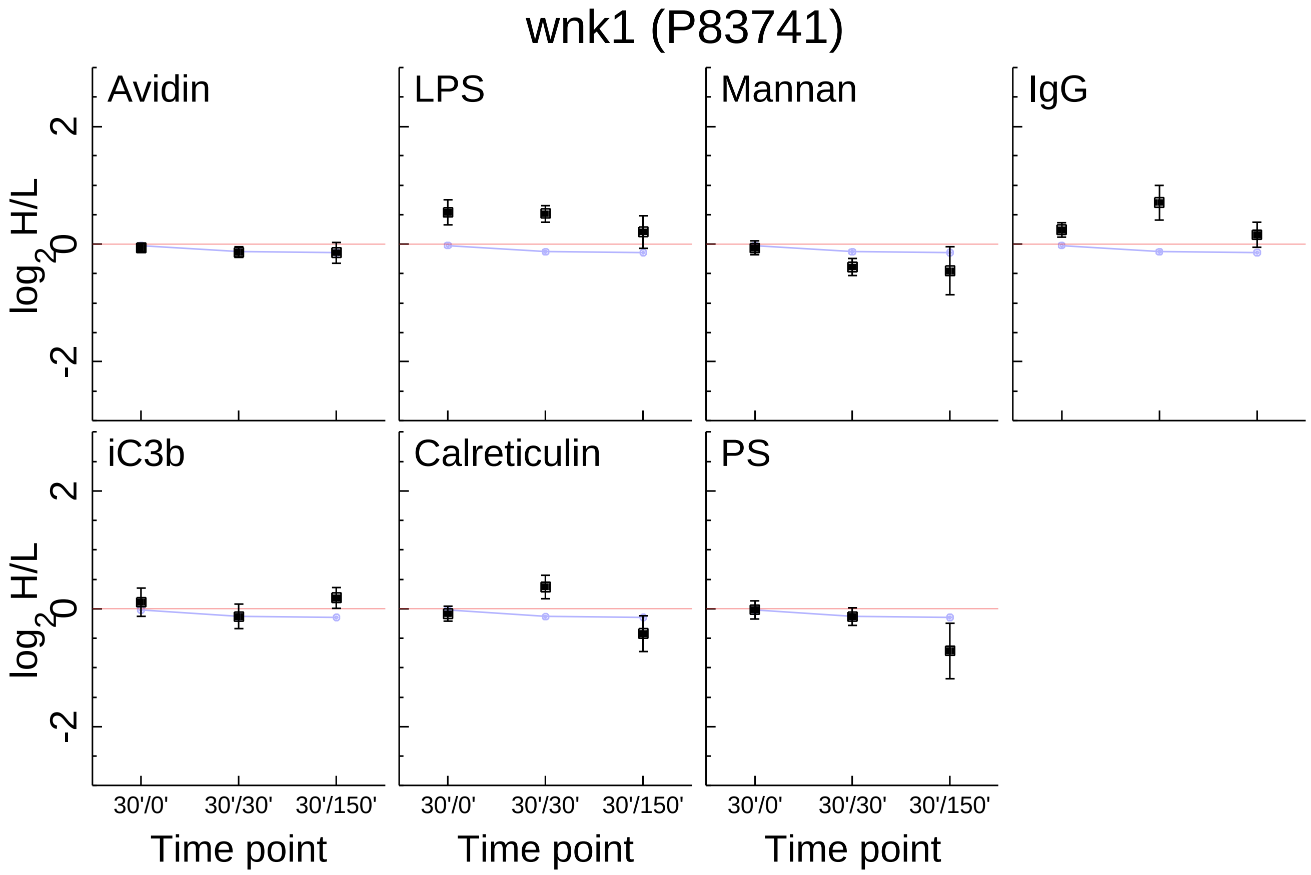

wnk1 (P83741)
Avidin
LPS
Mannan
IgG
iC3b
Calreticulin
PS
log2 H/L
2
0
-2
30'/0'
30'/30'
30'/150'
Time point

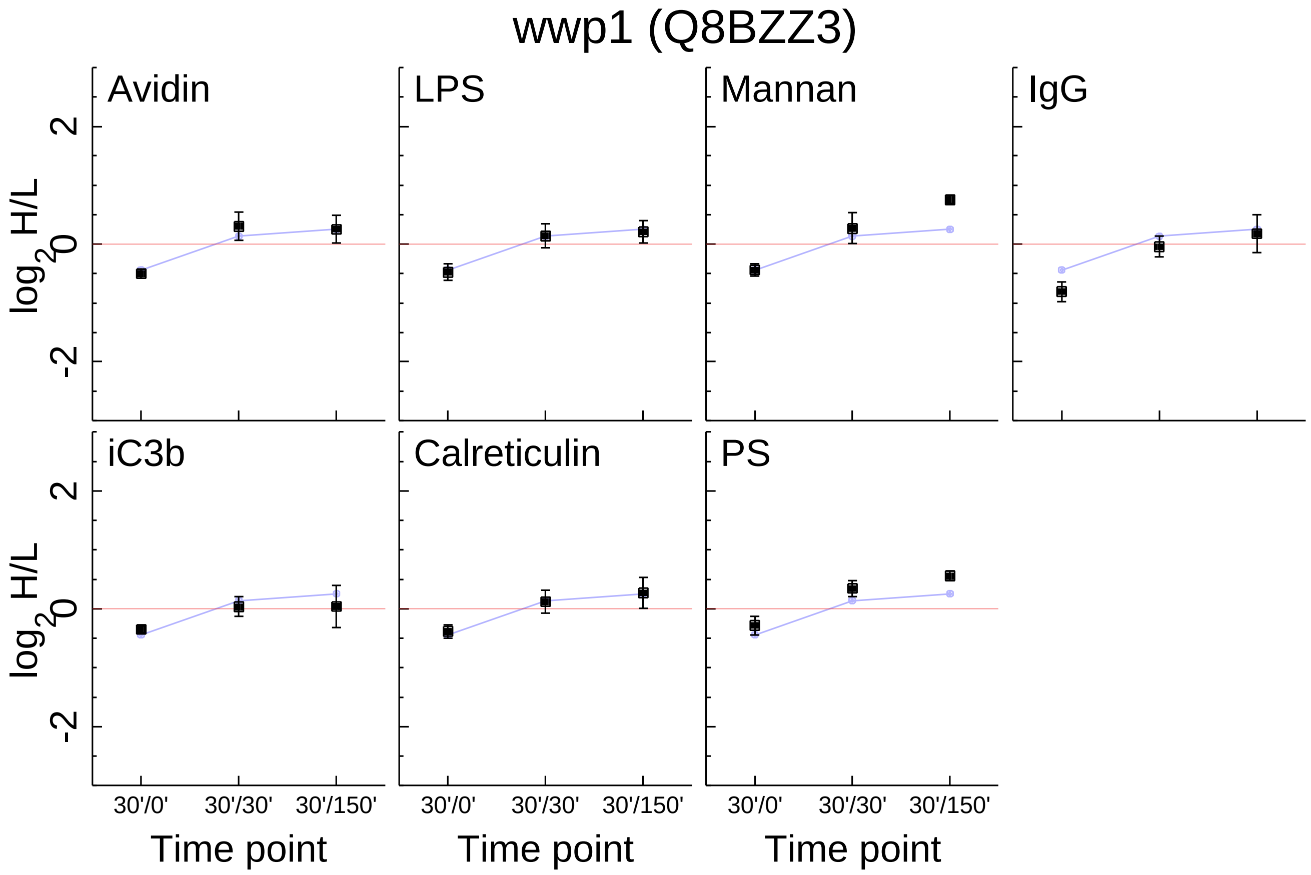

wwp1 (Q8BZZ3)
Avidin
LPS
Mannan
IgG
iC3b
Calreticulin
PS
log2 H/L
2
0
-2
30'/0'
30'/30'
30'/150'
Time point

wwp2 (Q9DBH0)

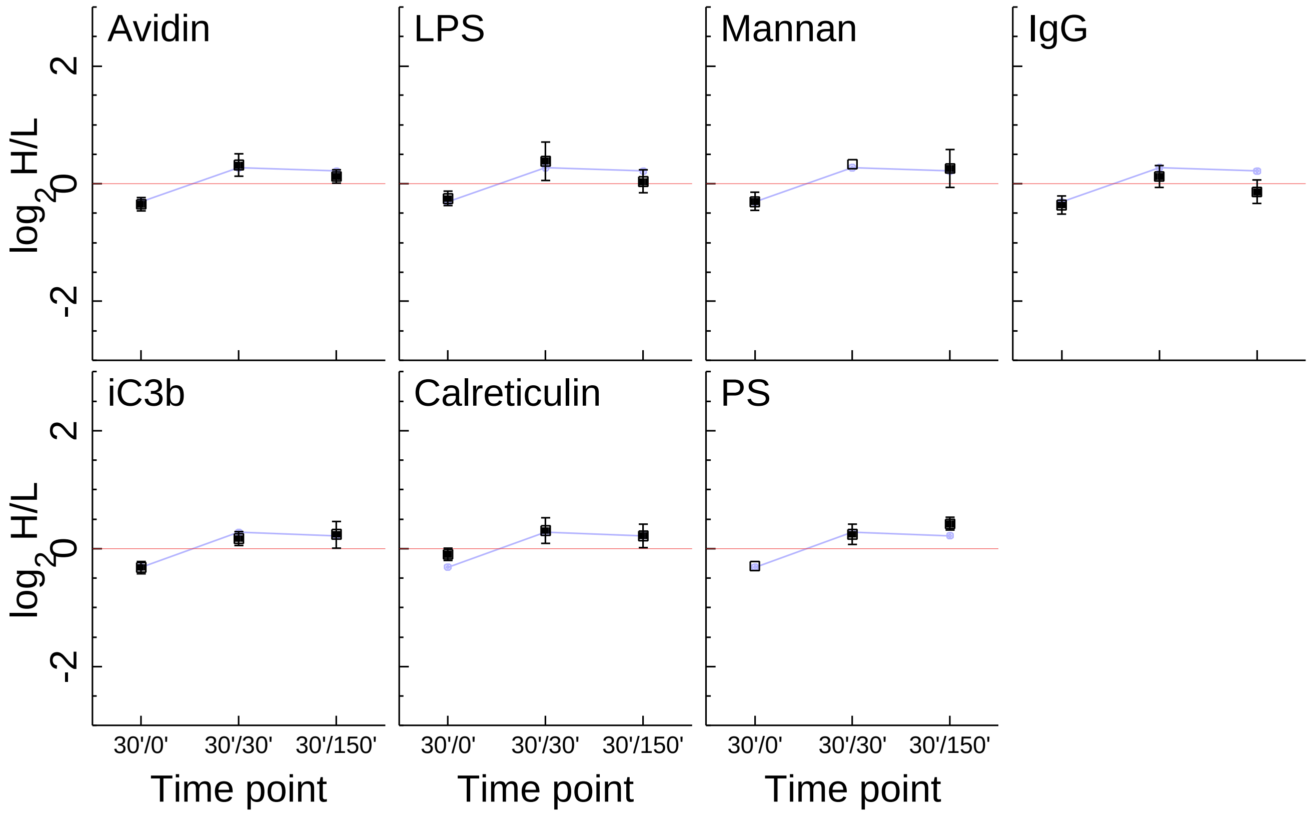

Avidin
LPS
Mannan
IgG
iC3b
Calreticulin
PS
log2 H/L
2
0
-2
30'/0'
30'/30'
30'/150'
Time point

xaf1 (Q5NBU8)

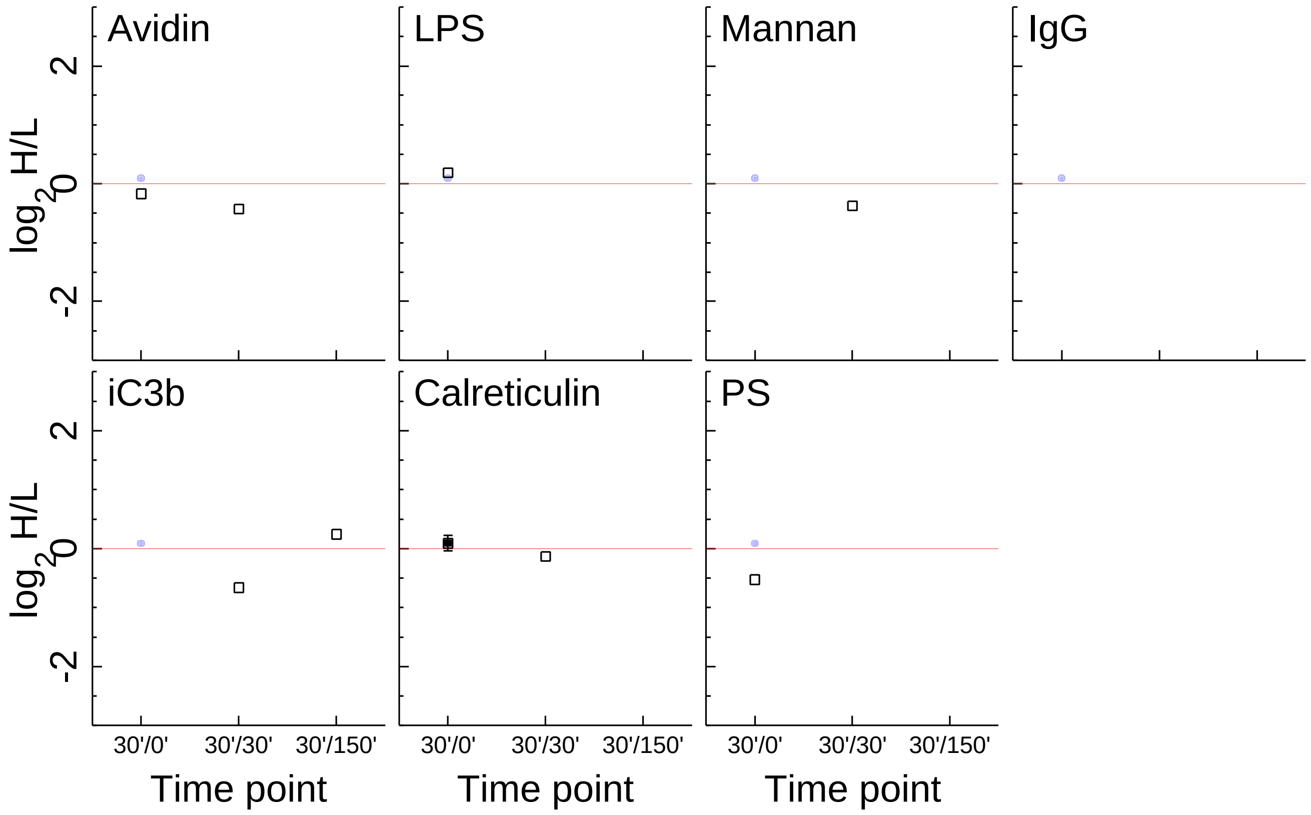

Avidin
LPS
Mannan
IgG
iC3b
Calreticulin
PS
log2 H/L
2
0
-2
30'/0'
30'/30'
30'/150'
Time point

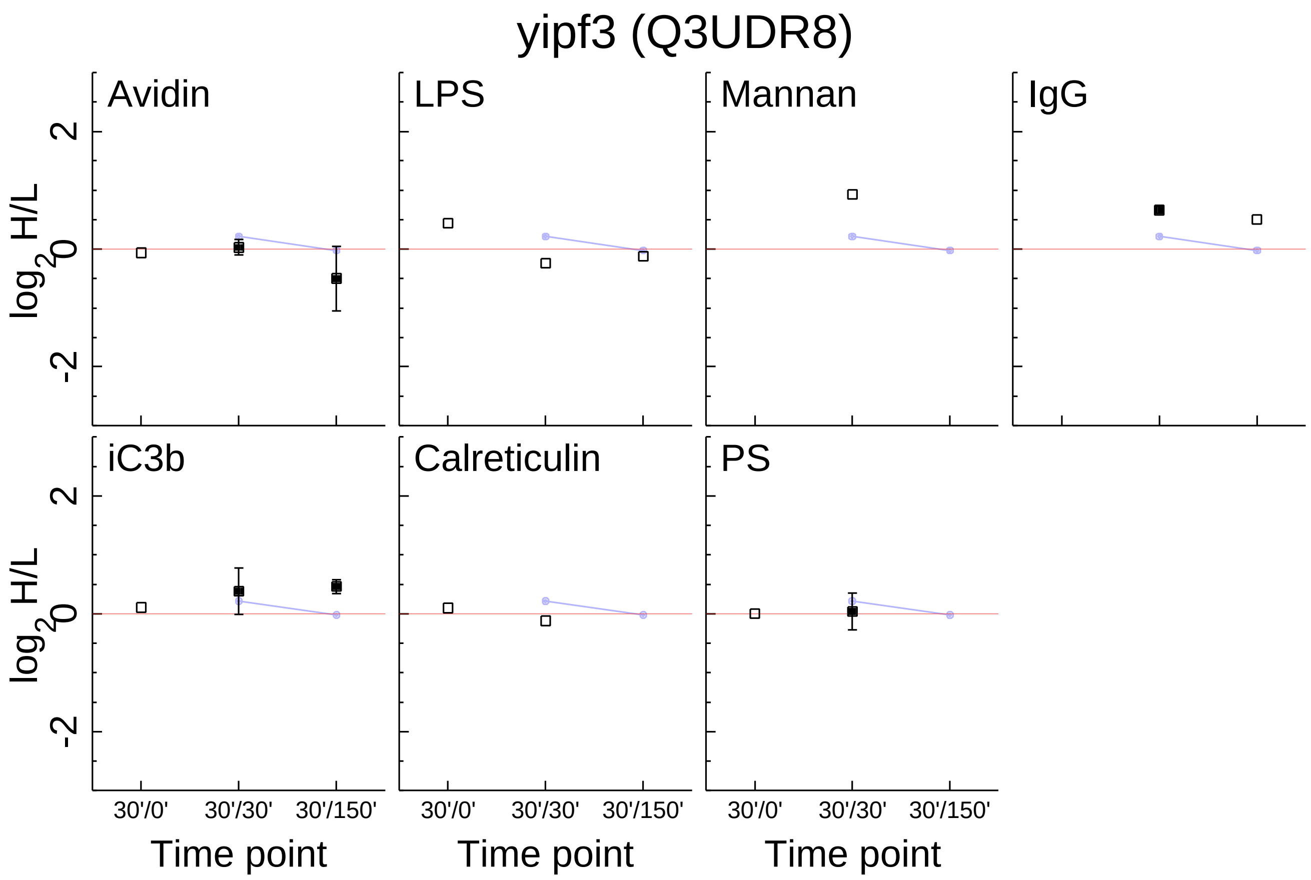

yipf3 (Q3UDR8)
Avidin
LPS
Mannan
IgG
iC3b
Calreticulin
PS
log2 H/L
2
0
-2
30'/0'
30'/30'
30'/150'
Time point

ykt6 (Q9CQW1)

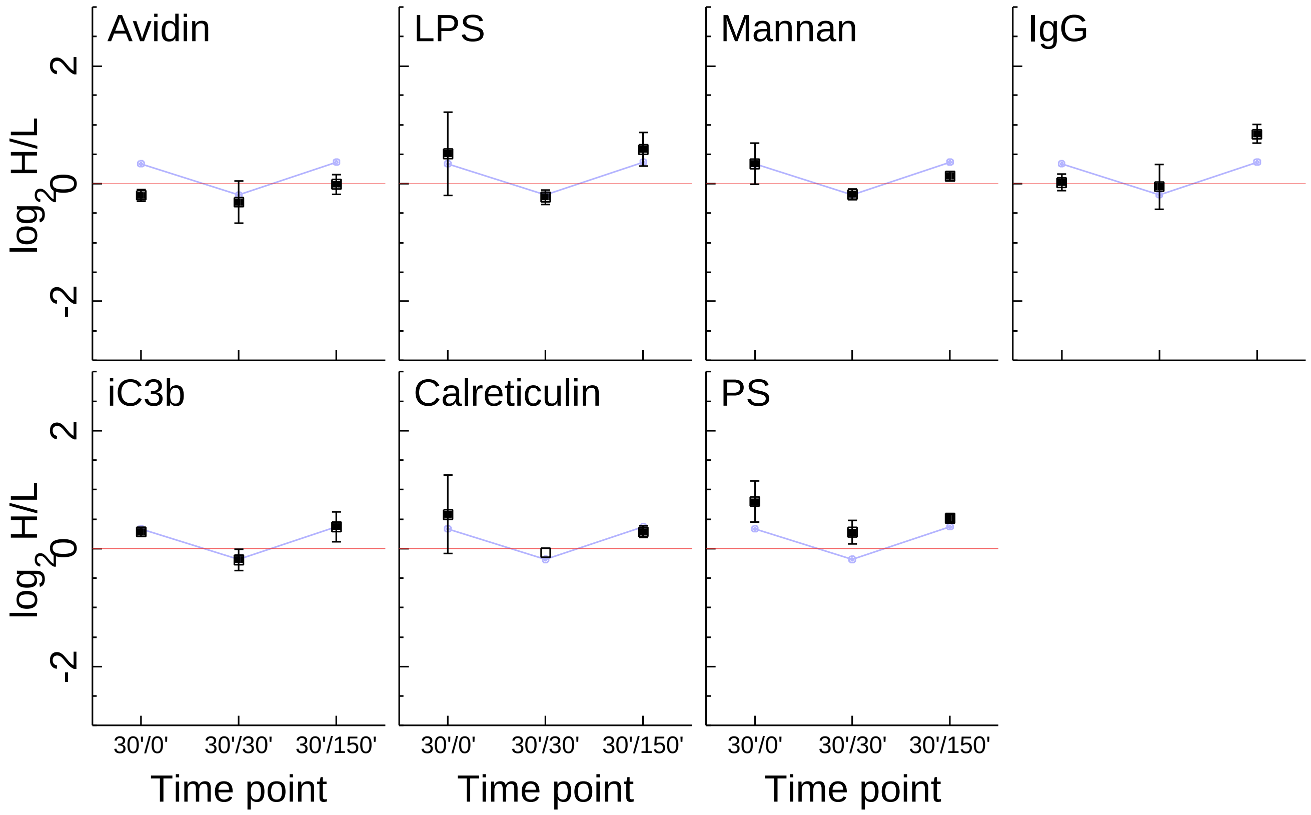

Avidin
LPS
Mannan
IgG
iC3b
Calreticulin
PS
log2 H/L
2
0
-2
30'/0'
30'/30'
30'/150'
Time point

ythdf3 (Q8BYK6)

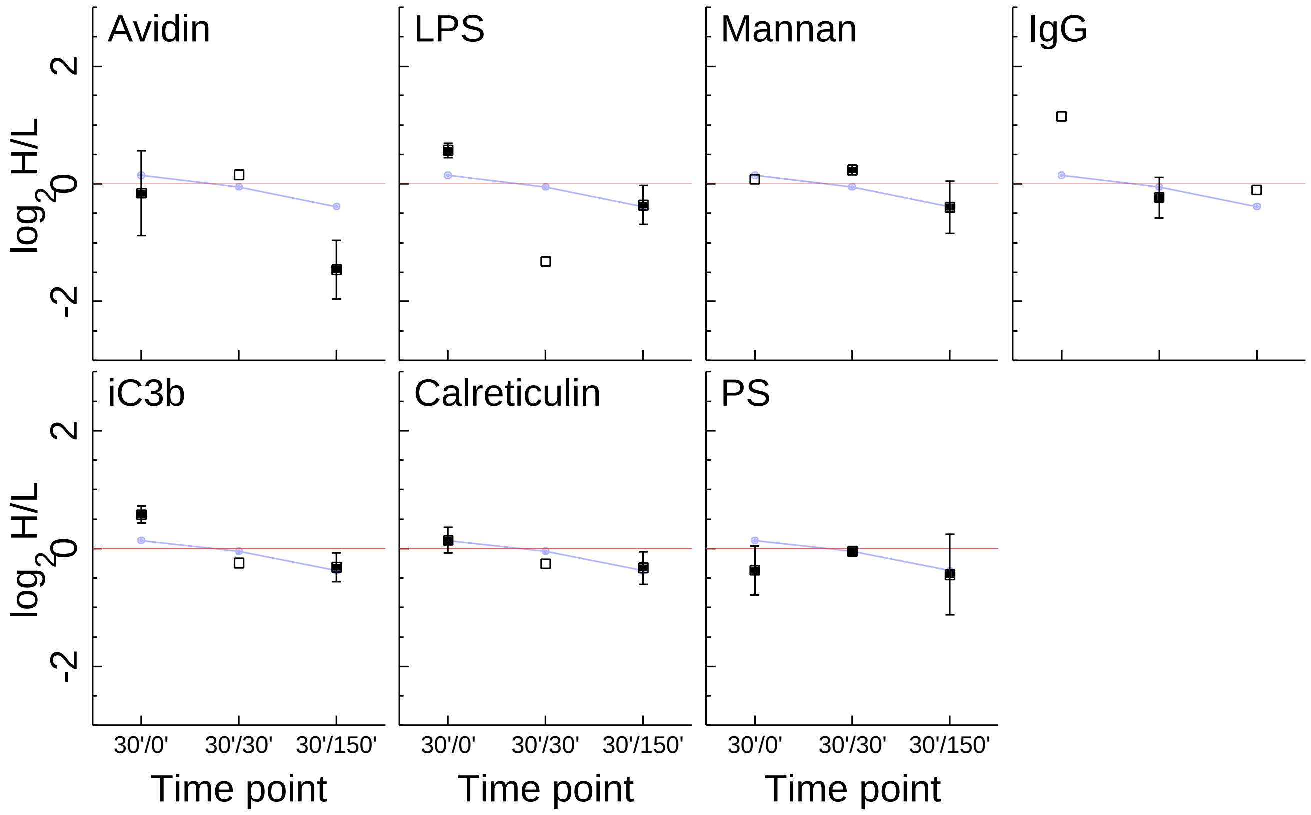

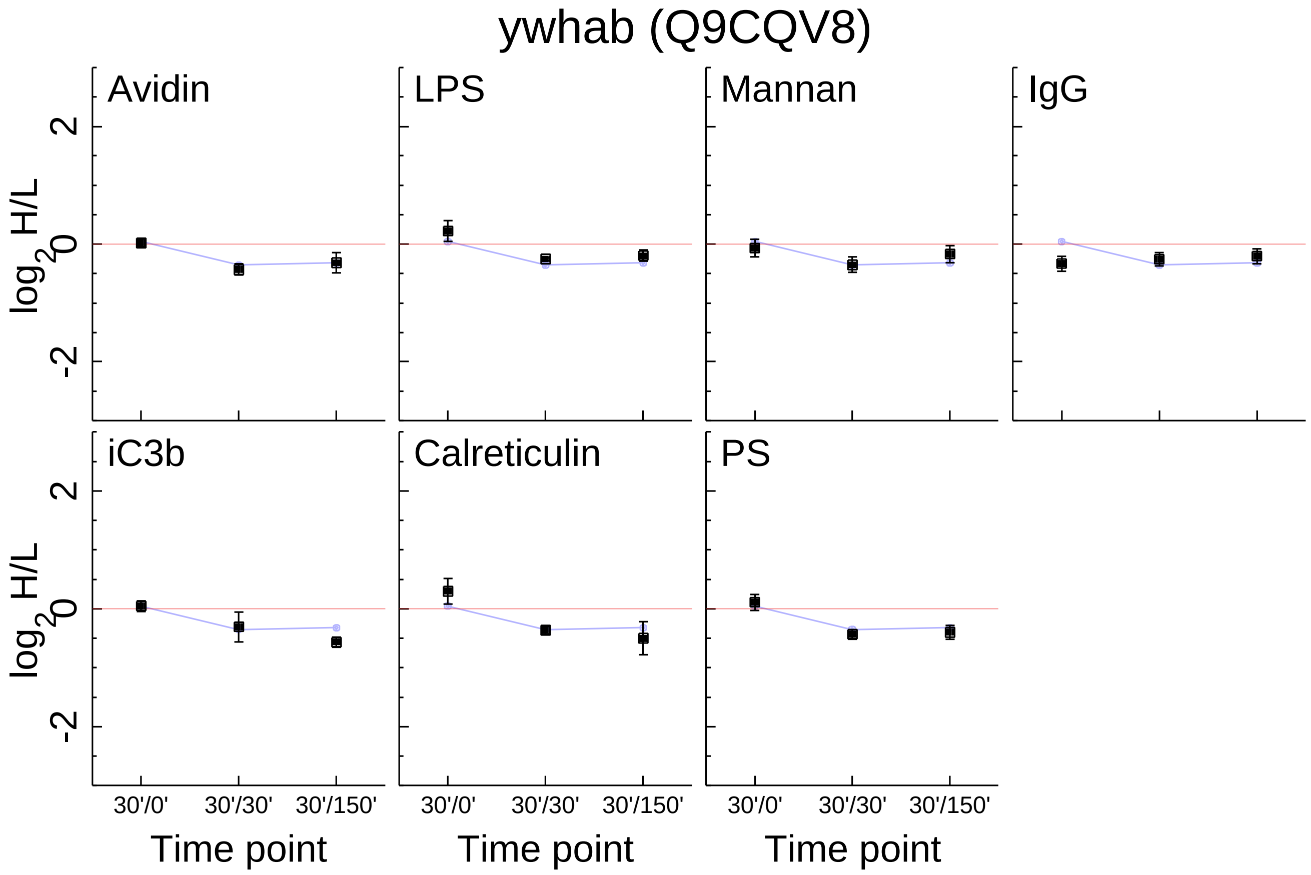
ywhab (Q9CQV8)
Avidin
LPS
Mannan
IgG
iC3b
Calreticulin
PS
log2 H/L
2
0
-2
30'/0'
30'/30'
30'/150'
Time point

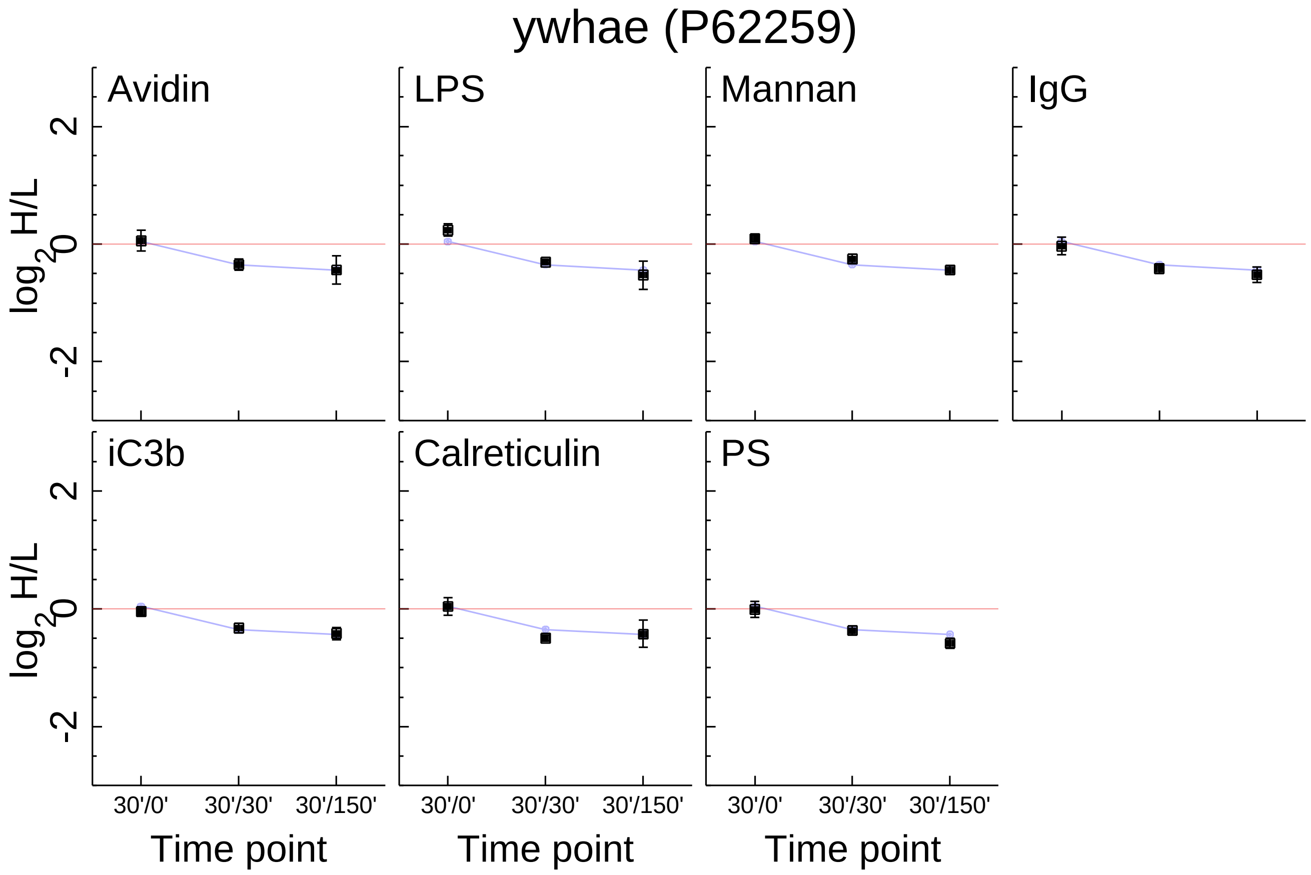
ywhae (P62259)
Avidin
LPS
Mannan
IgG
iC3b
Calreticulin
PS
log2 H/L
2
0
-2
30'/0'
30'/30'
30'/150'
Time point

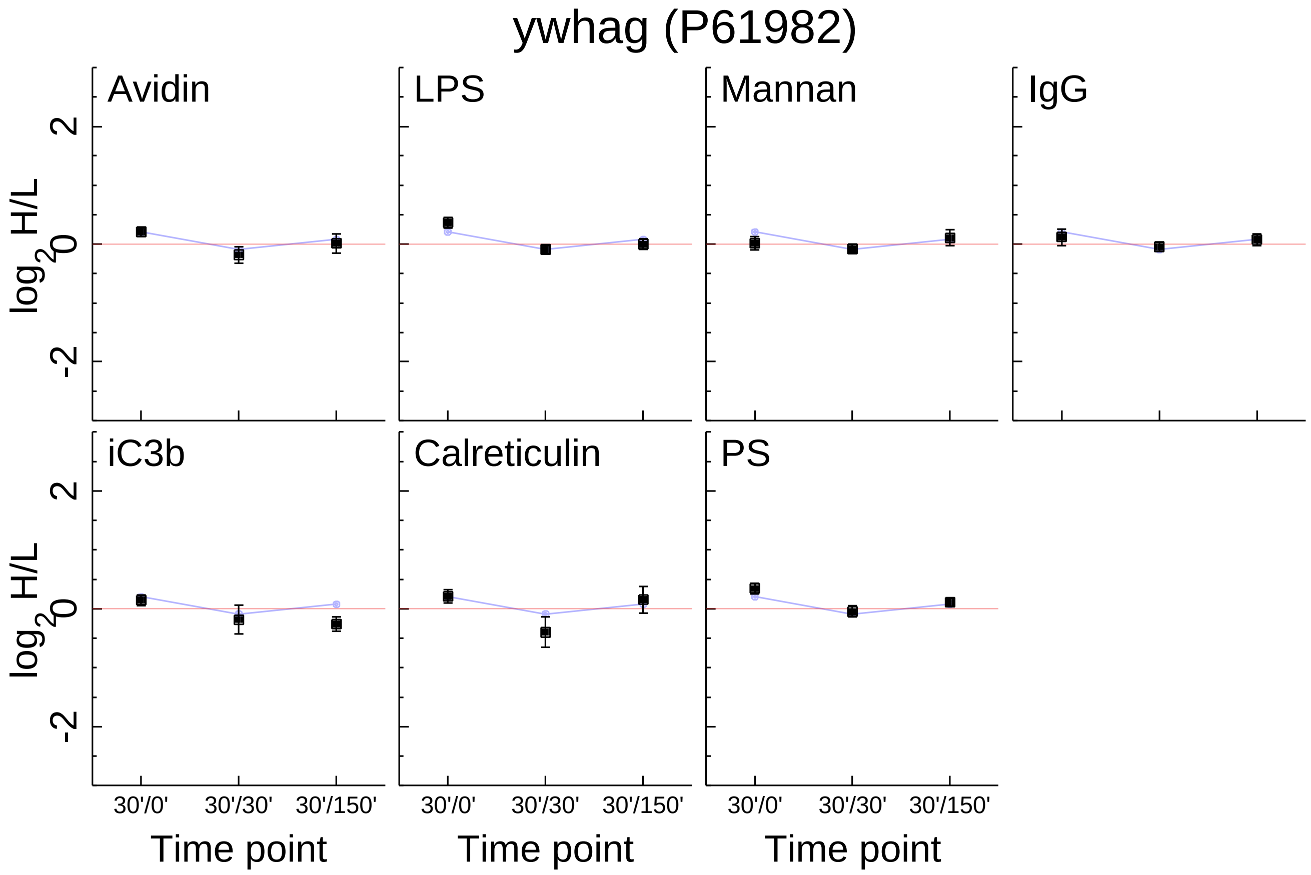

ywhag (P61982)
Avidin
LPS
Mannan
IgG
iC3b
Calreticulin
PS
log2 H/L
2
0
-2
30'/0'
30'/30'
30'/150'
Time point

ywhah (P68510)

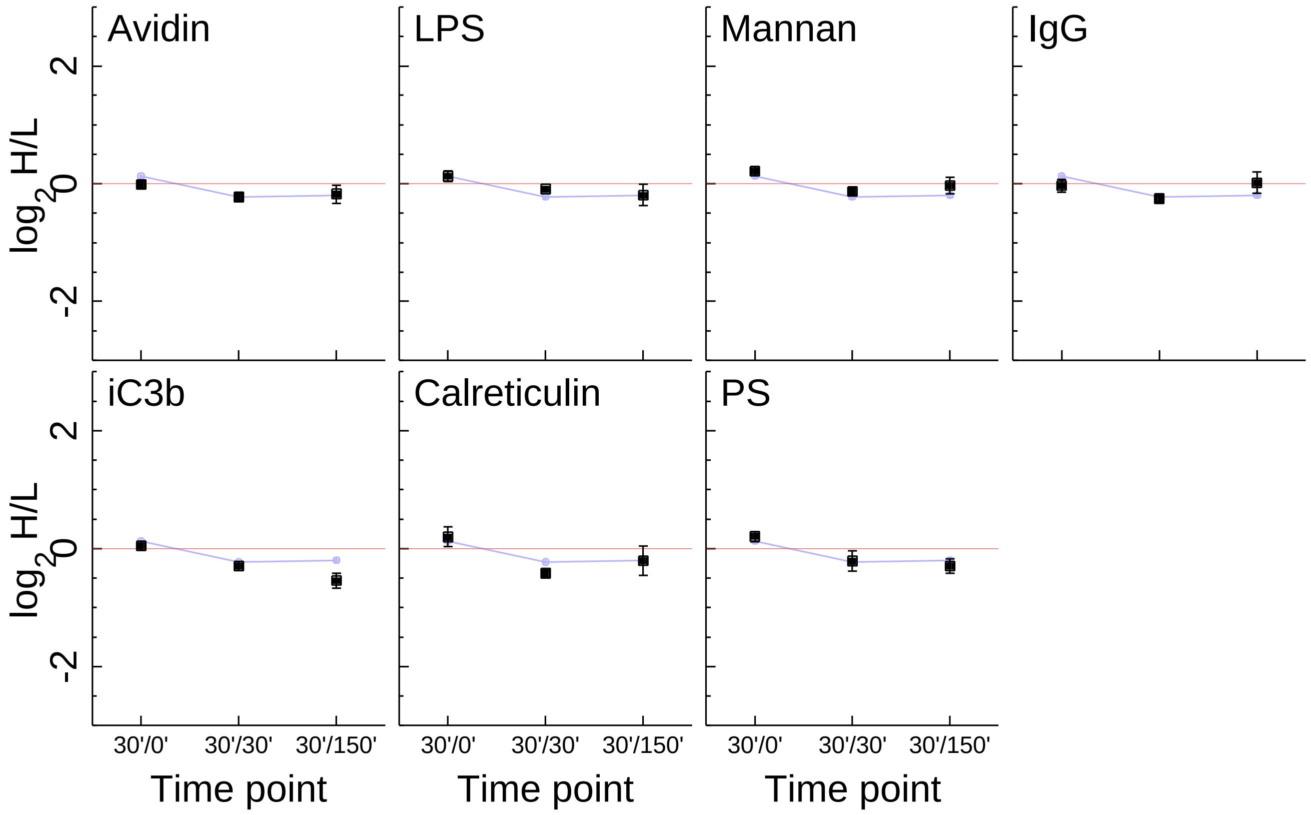

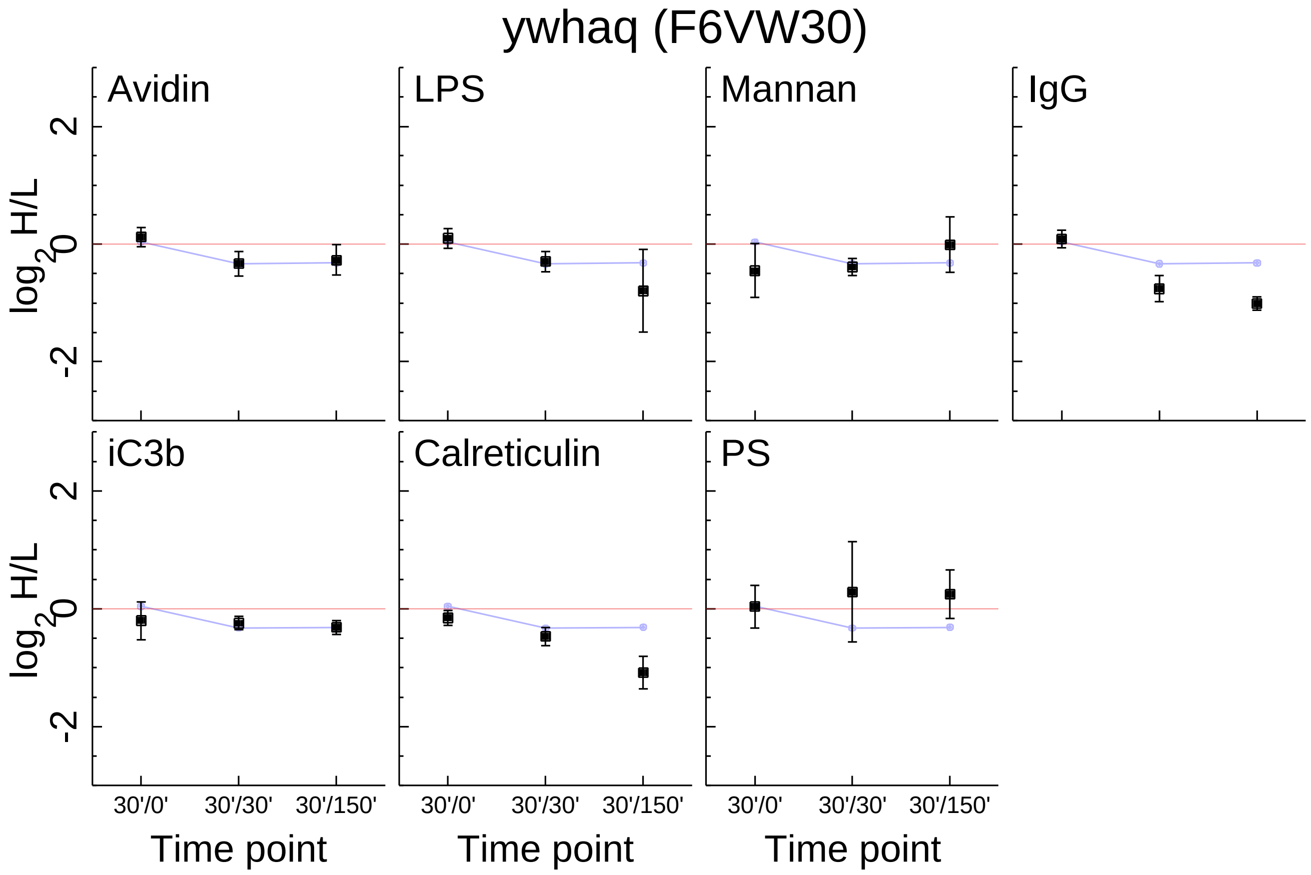
ywhaq (F6VW30)
Avidin
LPS
Mannan
IgG
iC3b
Calreticulin
PS
log2 H/L
2
0
-2
30'/0'
30'/30'
30'/150'
Time point

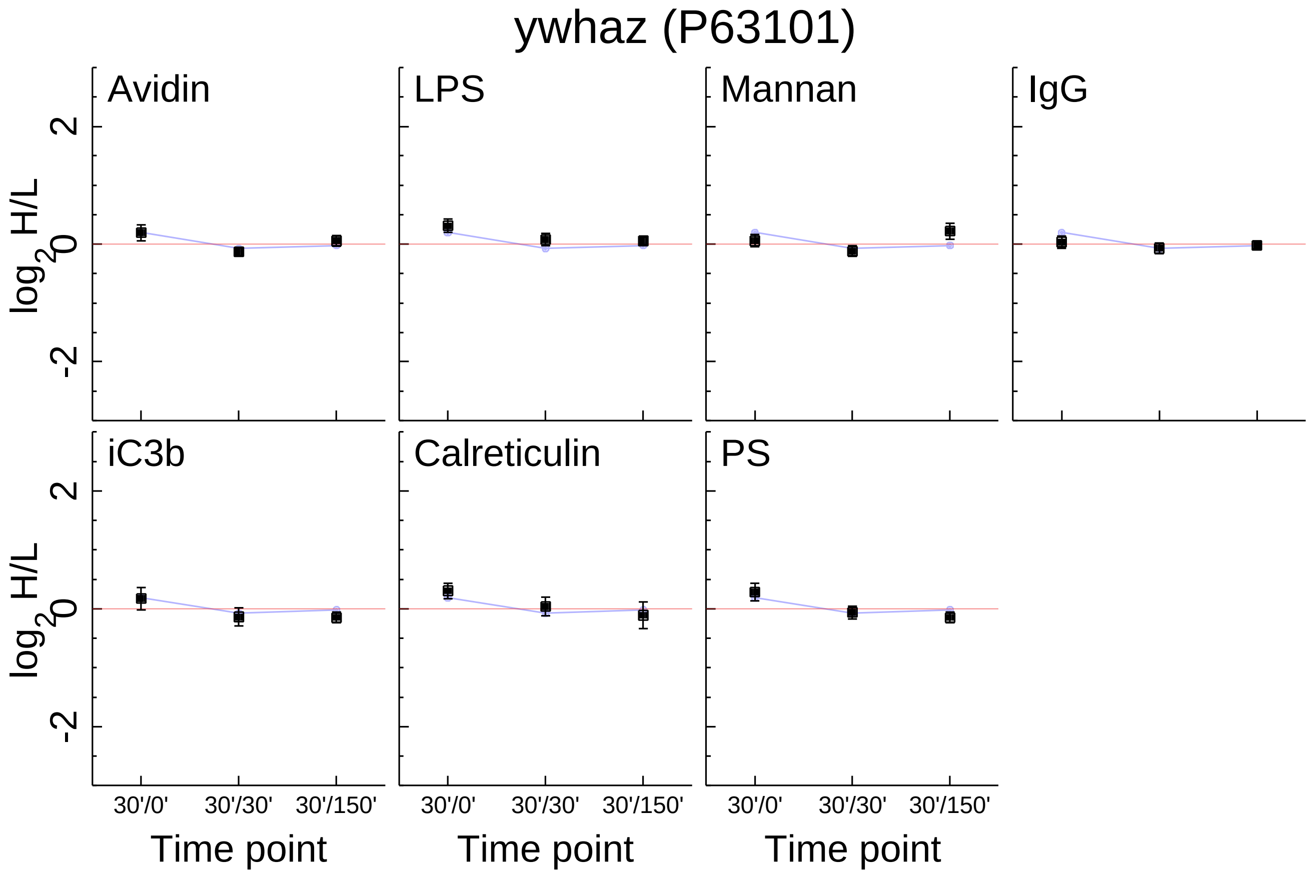
ywhaz (P63101)
Avidin
LPS
Mannan
IgG
iC3b
Calreticulin
PS
log2 H/L
2
0
-2
30'/0'
30'/30'
30'/150'
Time point

zadh2 (Q8BGC4)

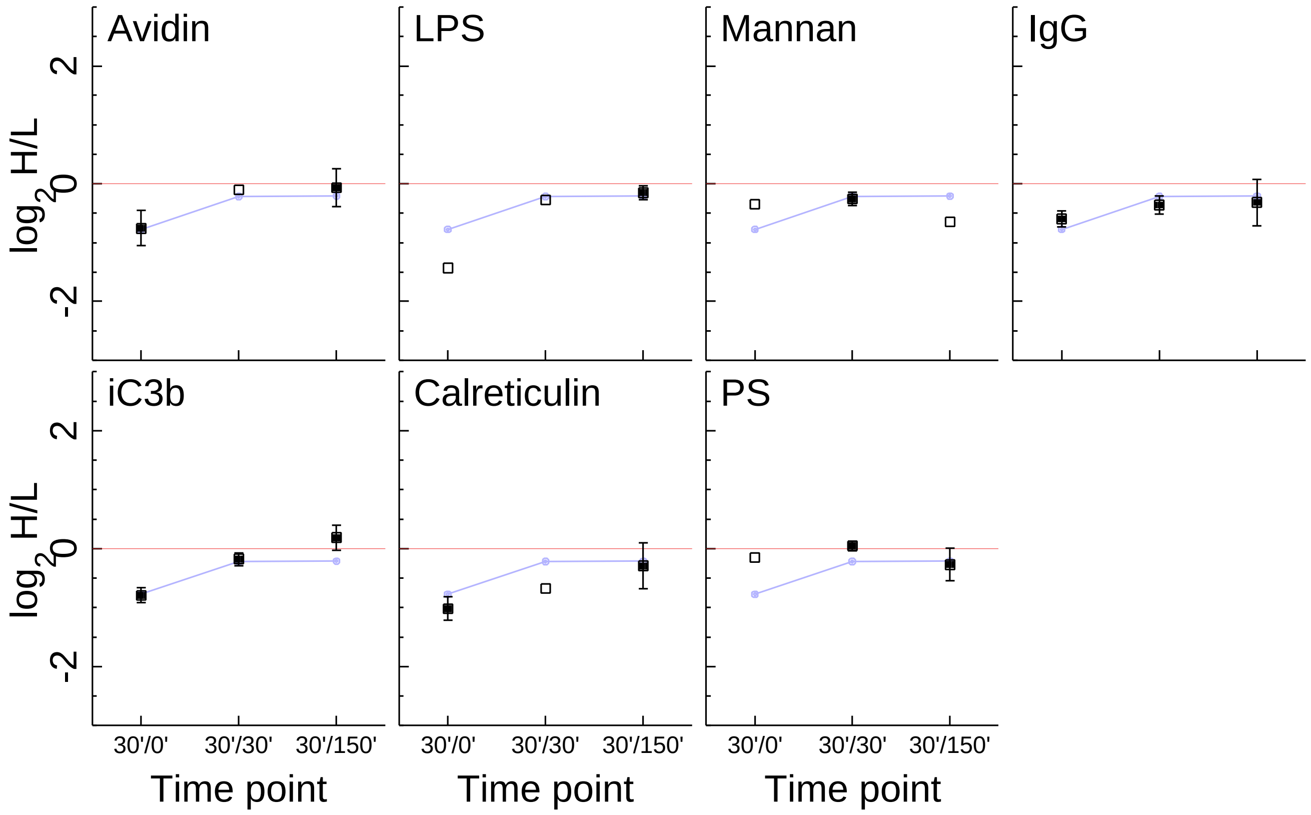

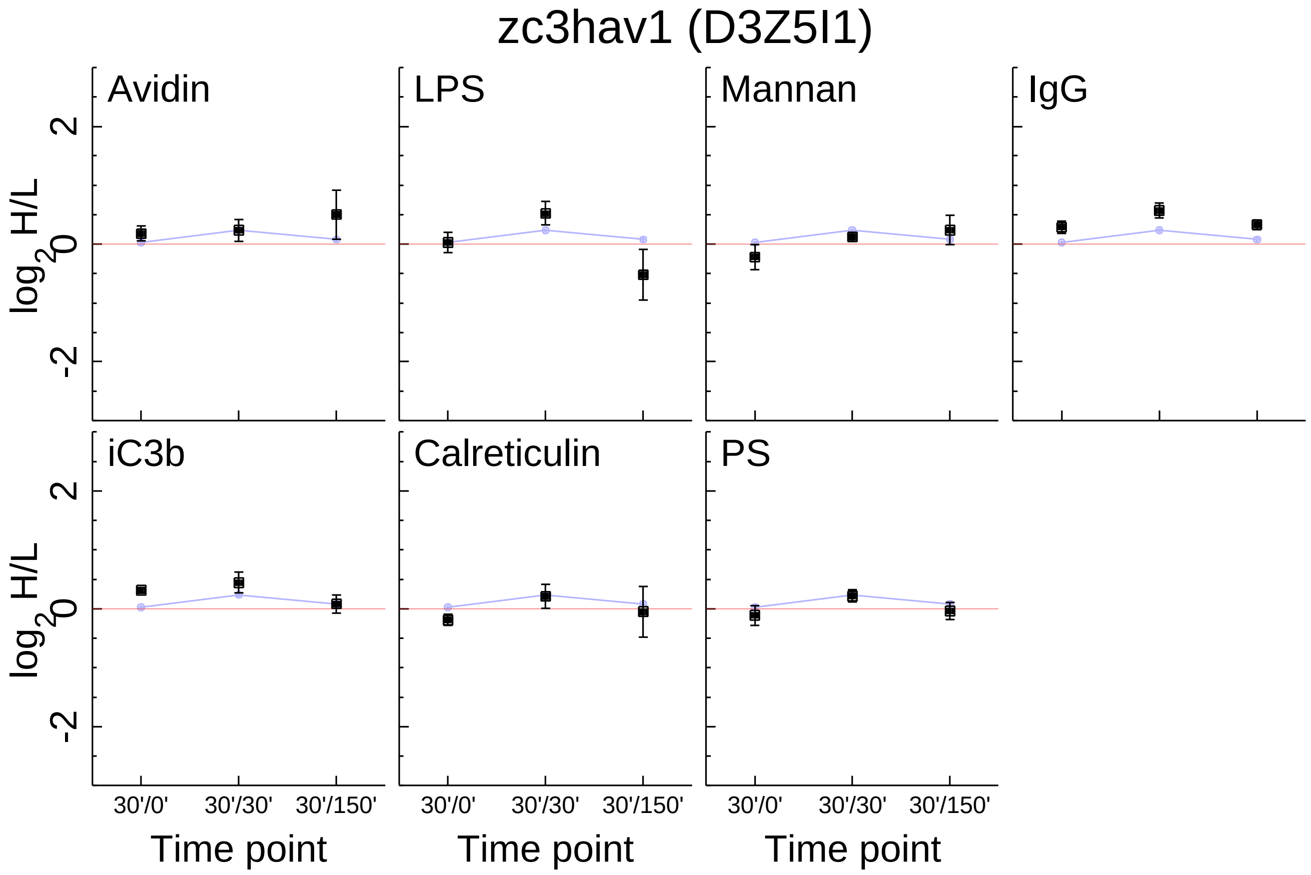

zc3hav1 (D3Z5I1)
Avidin
LPS
Mannan
IgG
iC3b
Calreticulin
PS
log2 H/L
2
0
-2
30'/0'
30'/30'
30'/150'
Time point

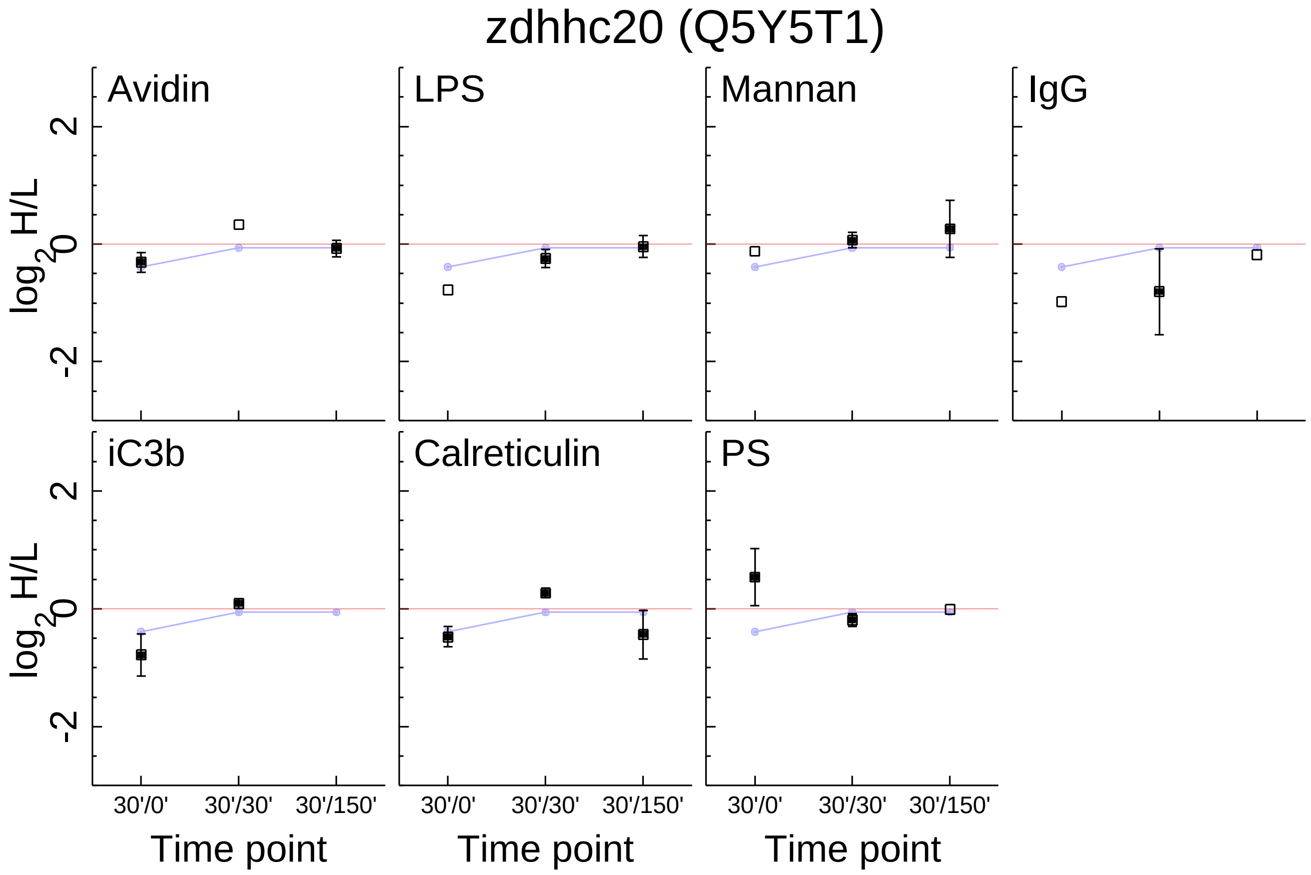

zdhhc20 (Q5Y5T1)
Avidin
LPS
Mannan
IgG
iC3b
Calreticulin
PS
log2 H/L
2
0
-2
30'/0'
30'/30'
30'/150'
Time point

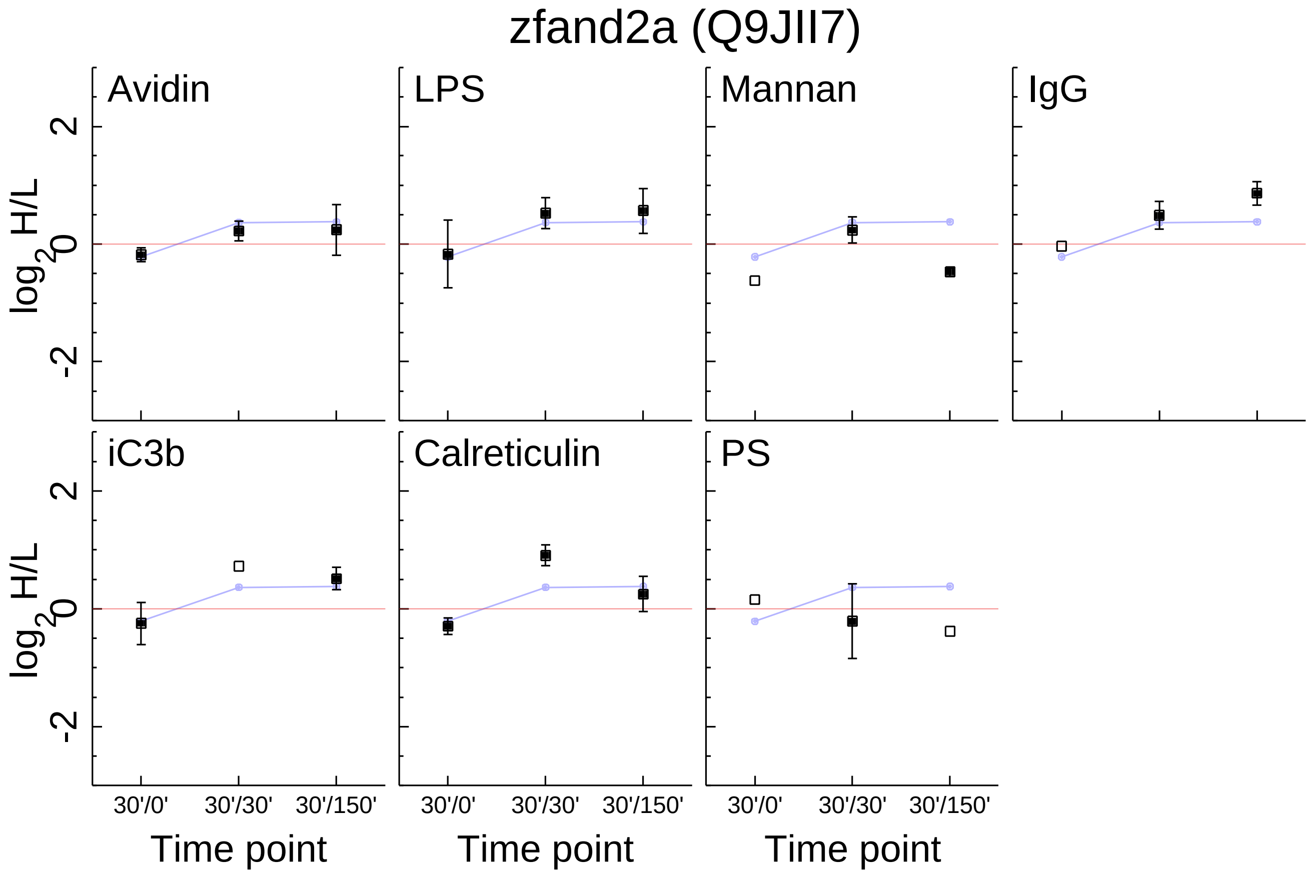

zfand2a (Q9JII7)
Avidin
LPS
Mannan
IgG
iC3b
Calreticulin
PS
log2 H/L
2
0
-2
30'/0'
30'/30'
30'/150'
Time point

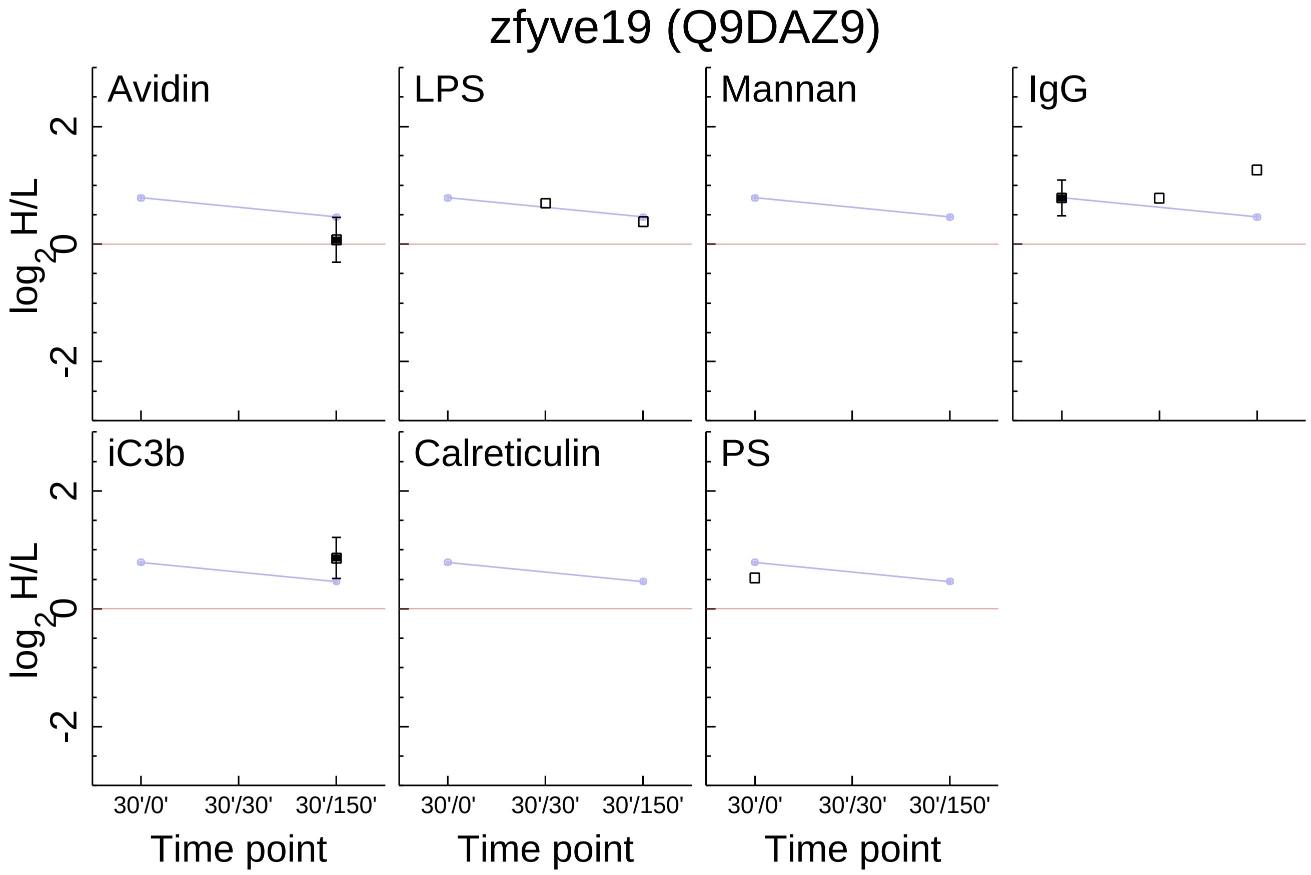
zfyve19 (Q9DAZ9)
Avidin
LPS
Mannan
IgG
iC3b
Calreticulin
PS
log2 H/L
2
0
-2
30'/0'
30'/30'
30'/150'
Time point

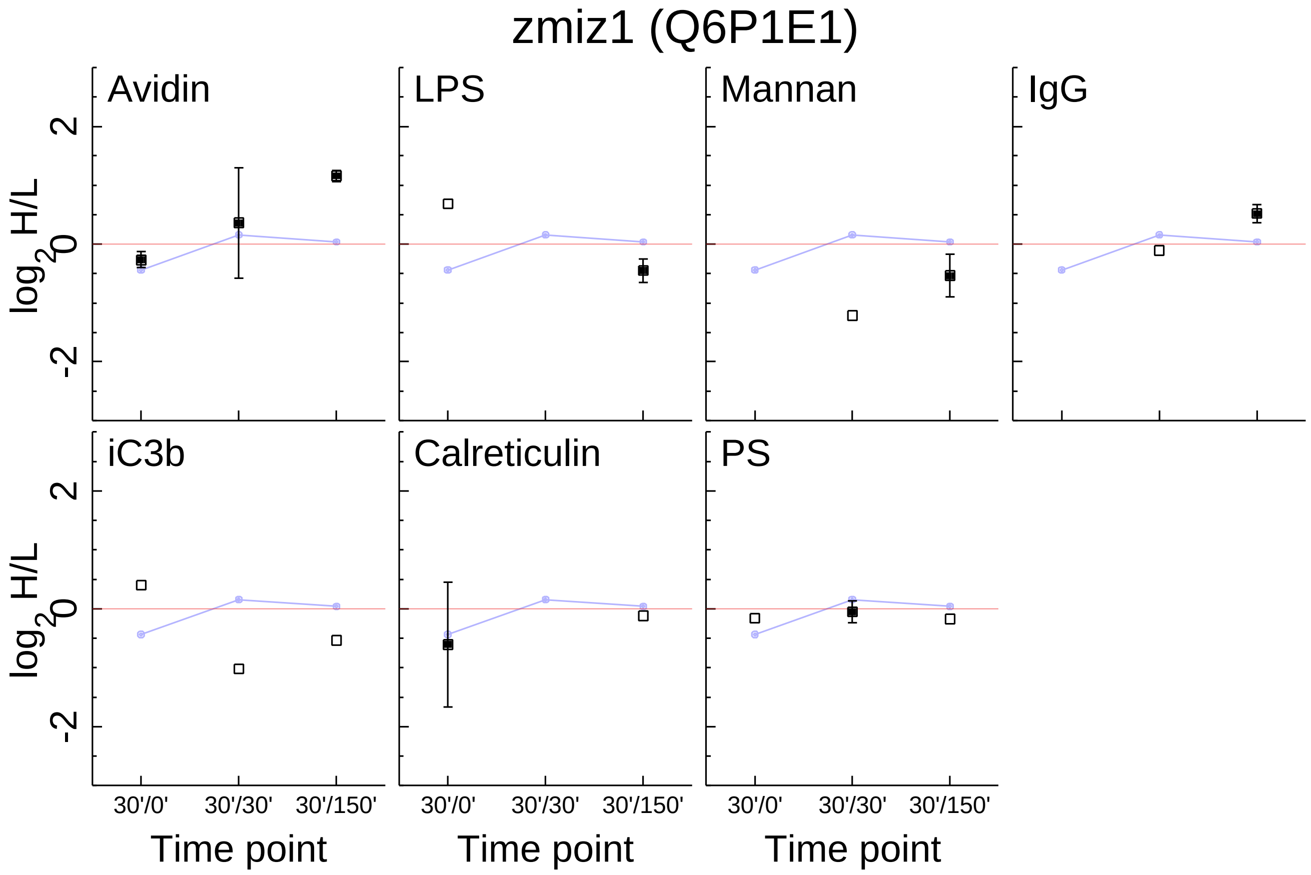

zmiz1 (Q6P1E1)
Avidin
LPS
Mannan
IgG
iC3b
Calreticulin
PS
log2 H/L
2
0
-2
30'/0'
30'/30'
30'/150'
Time point

zmpste24 (Q80W54)

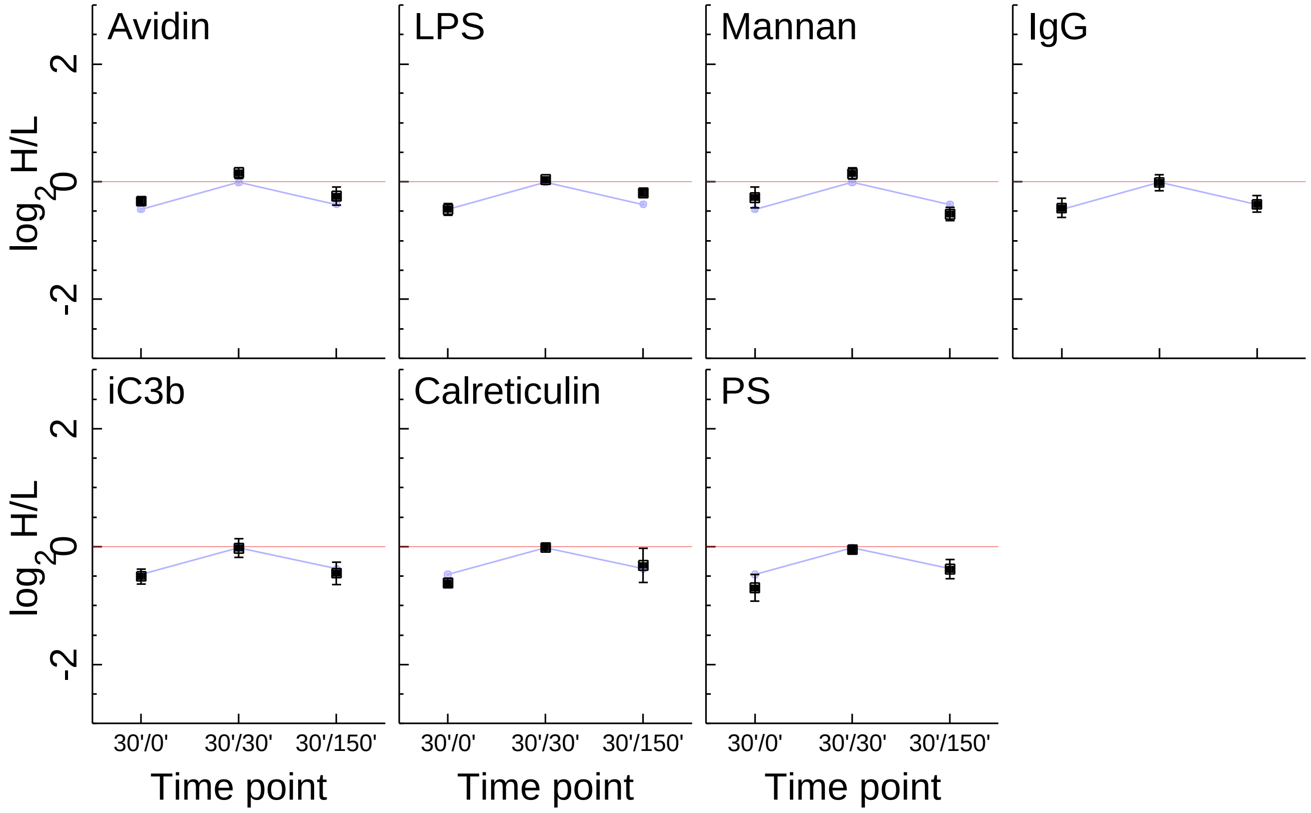

znf703 (P0CL69)

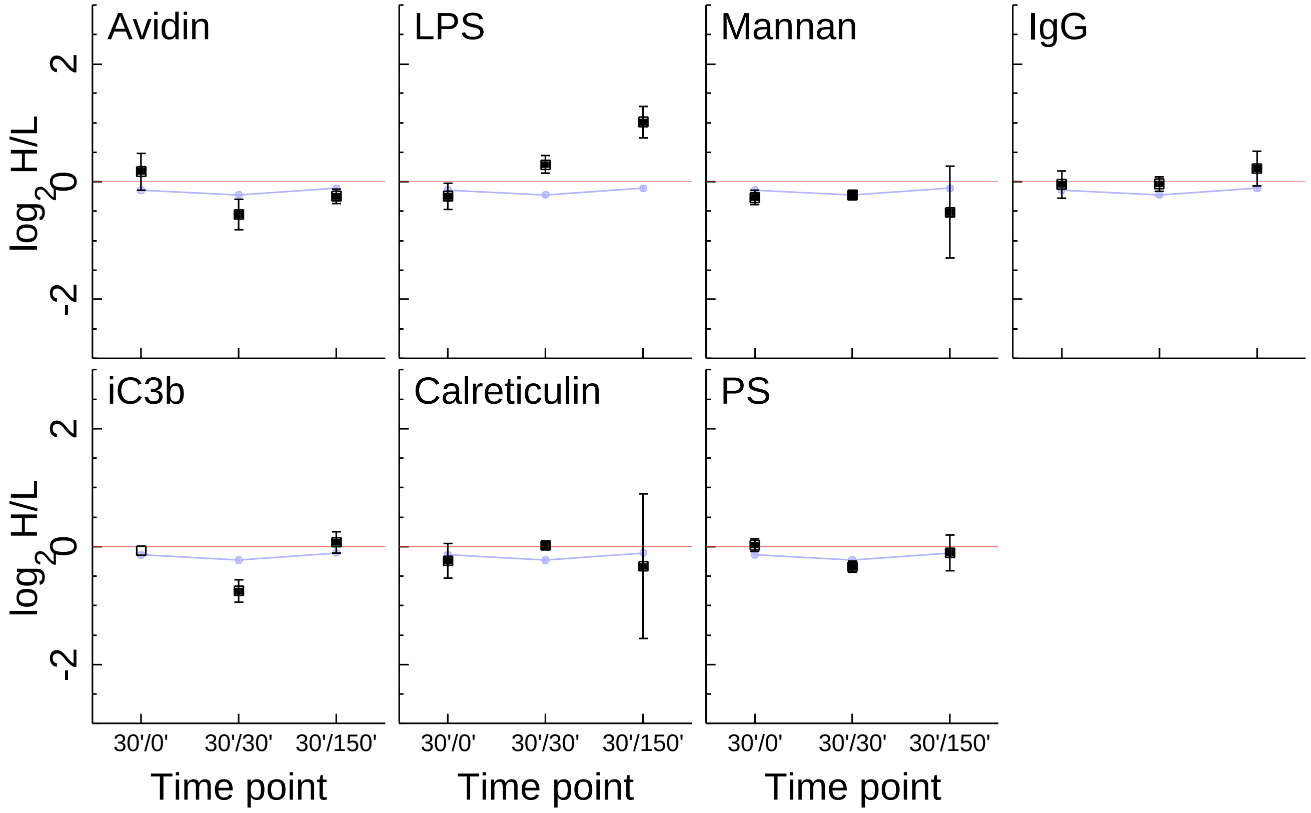

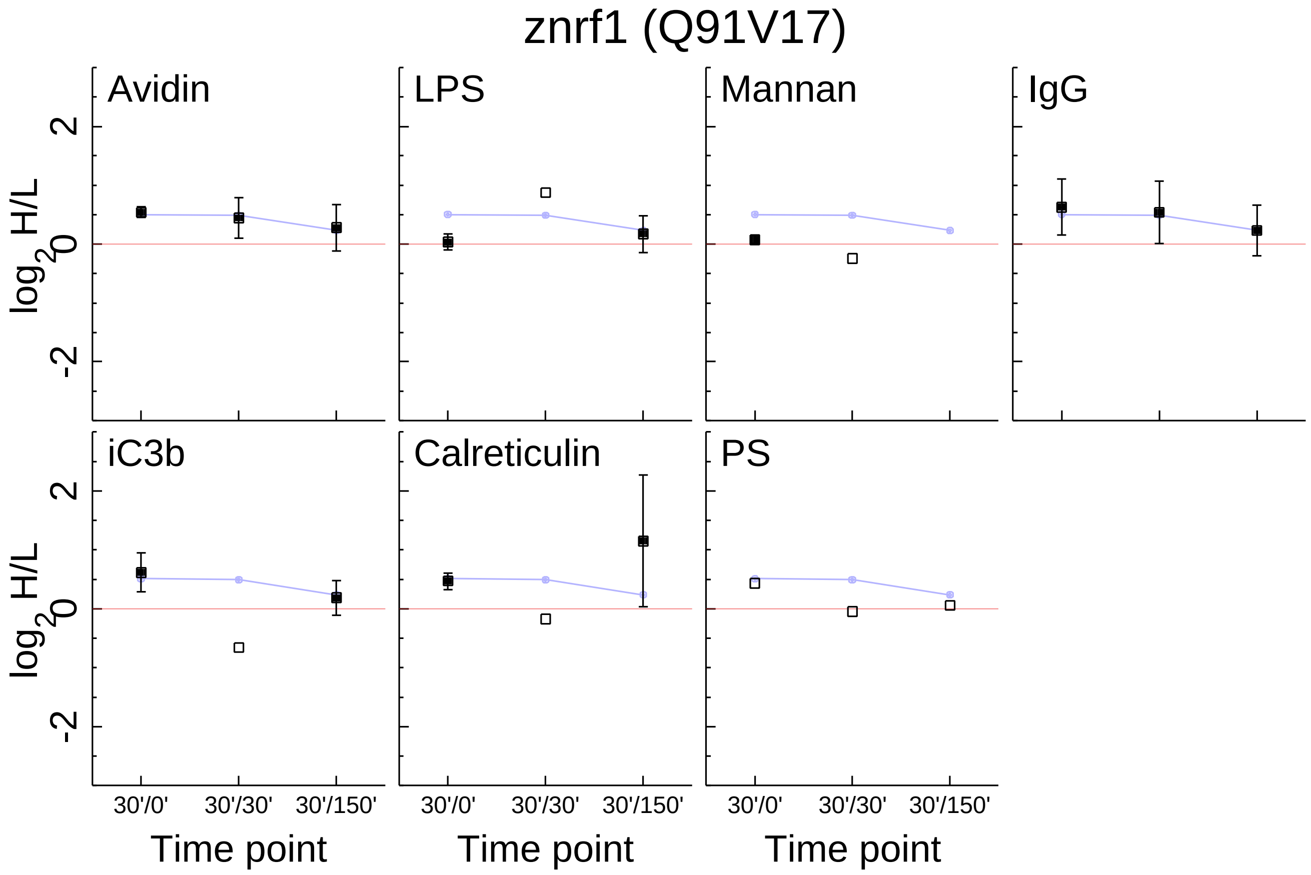
znrf1 (Q91V17)
Avidin
LPS
Mannan
IgG
iC3b
Calreticulin
PS
log2 H/L
2
0
-2
30'/0'
30'/30'
30'/150'
Time point

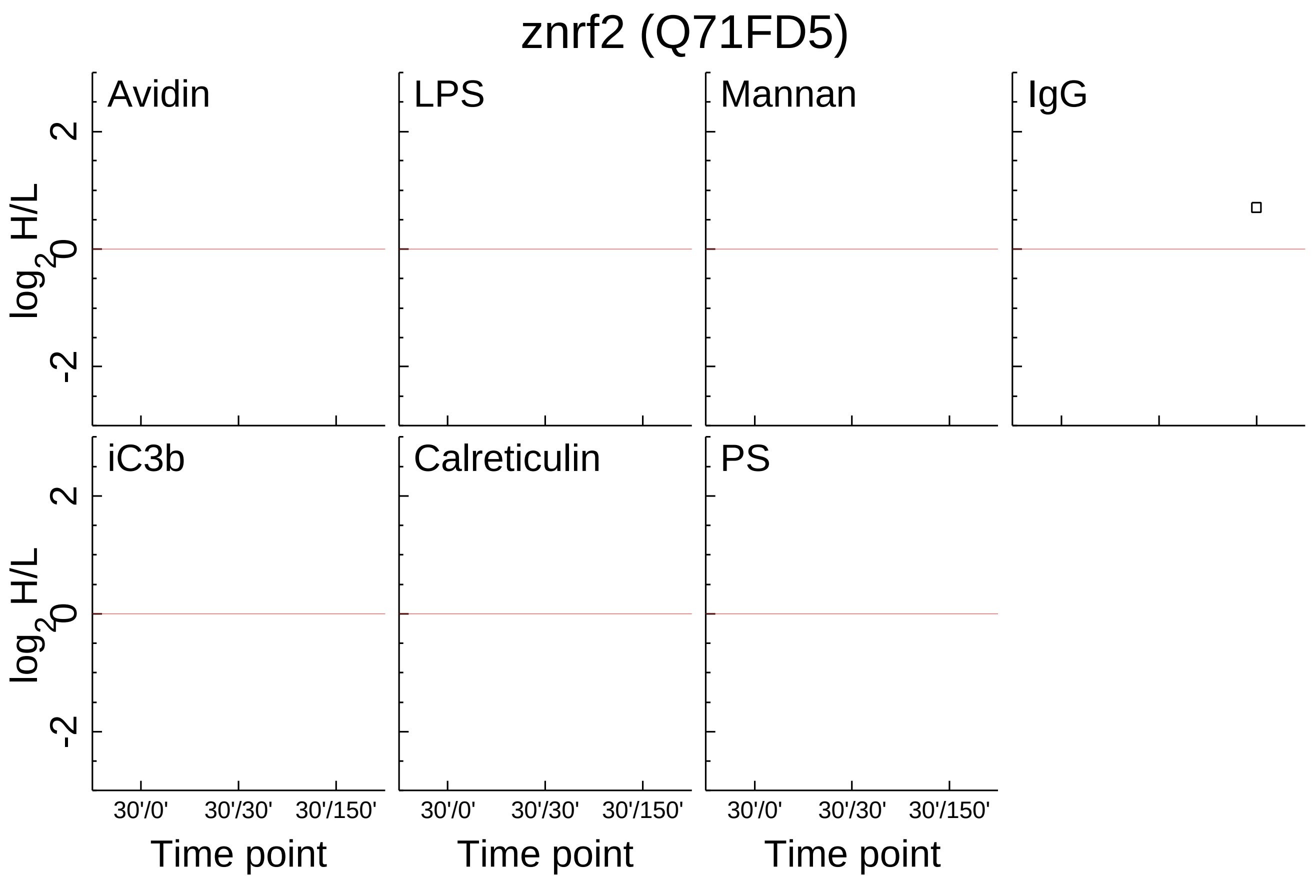
znrf2 (Q71FD5)
Avidin
LPS
Mannan
IgG
iC3b
Calreticulin
PS
log2 H/L
2
0
-2
30'/0'
30'/30'
30'/150'
Time point

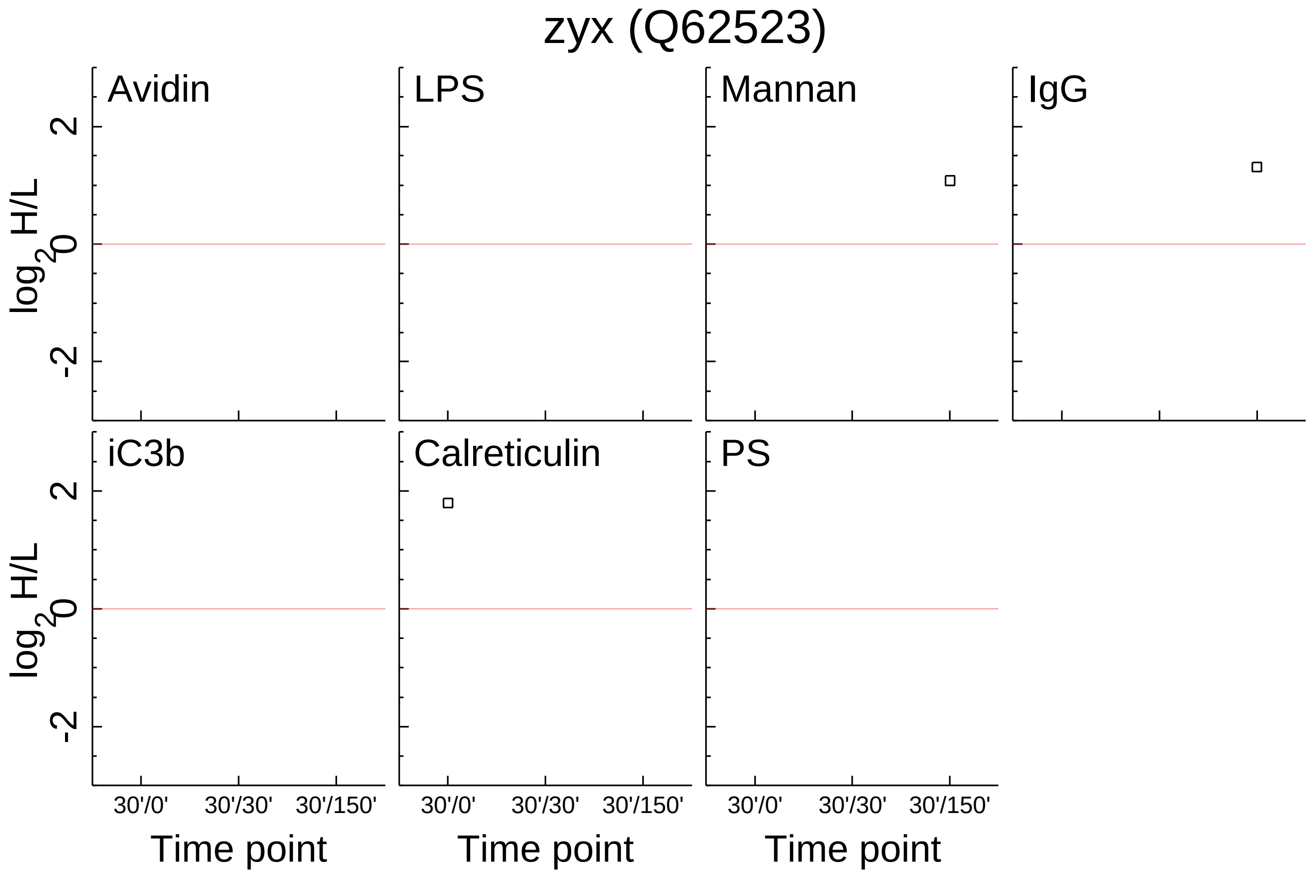
zyx (Q62523)
Avidin
LPS
Mannan
IgG
iC3b
Calreticulin
PS
log2 H/L
2
0
-2
30'/0'
30'/30'
30'/150'
Time point
