# Supplementary material for: Quantitative Proteome Analysis of Temporally Resolved Phagosomes Following Uptake Via Key Phagocytic Receptors
Source: Mol Cell Proteomics. 2015 May;14(5):1334–49. doi: 10.1074/mcp.M114.044594 (PMC4424403; doi:10.1074/mcp.M114.044594)

GO:0000009 alpha-1,6-mannosyltransferase activity (n = 3)

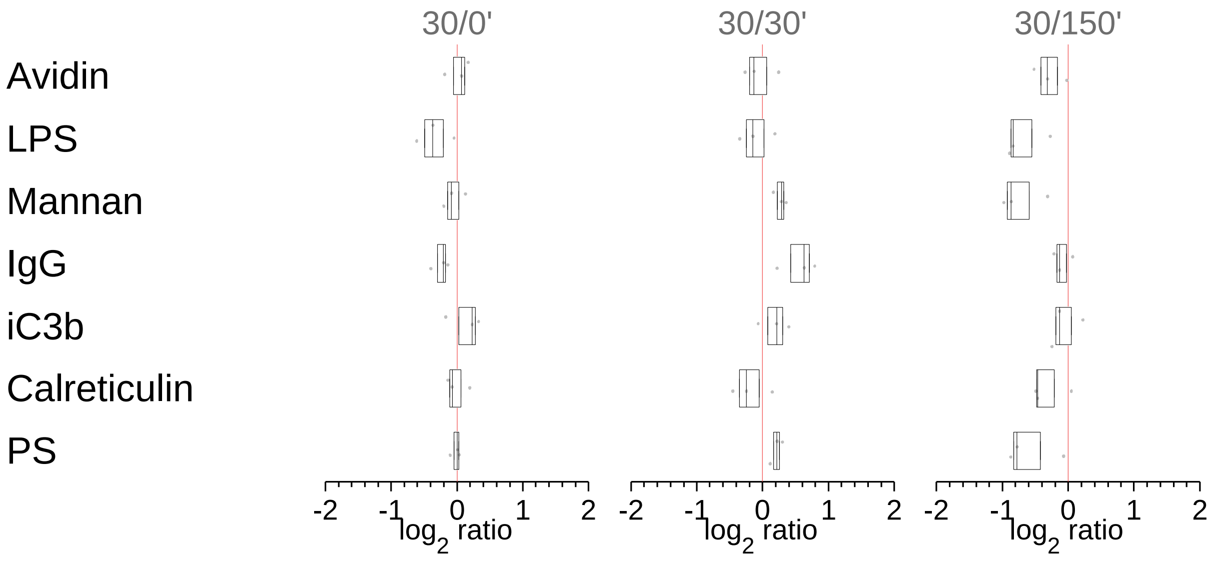

GO:0000026 alpha-1,2-mannosyltransferase activity (n = 3)

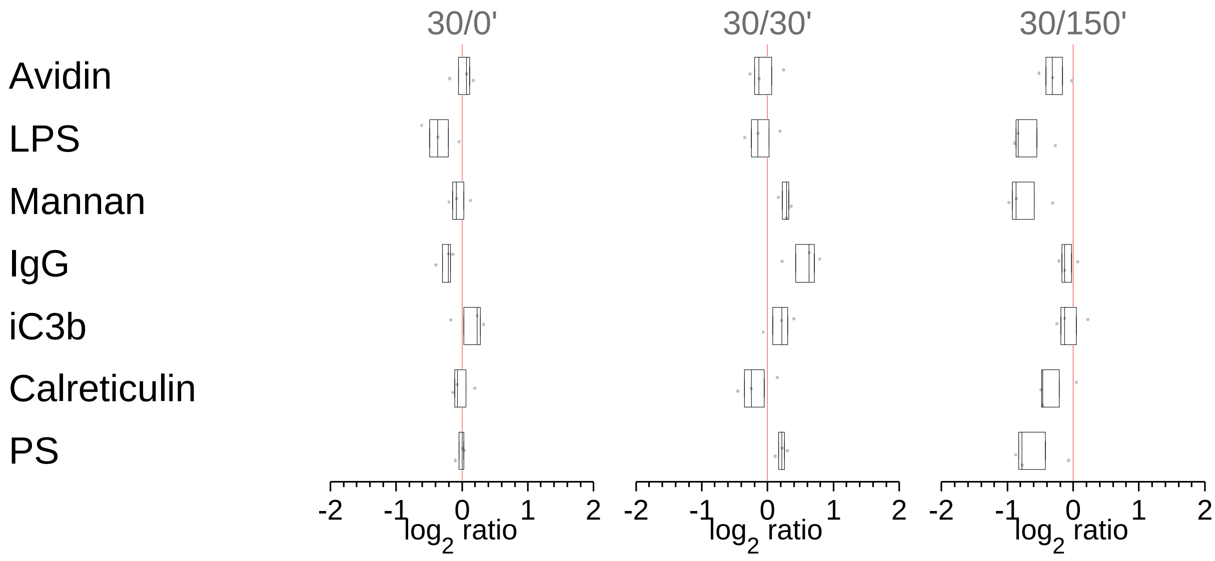

GO:0000028 ribosomal small subunit assembly (n = 6)

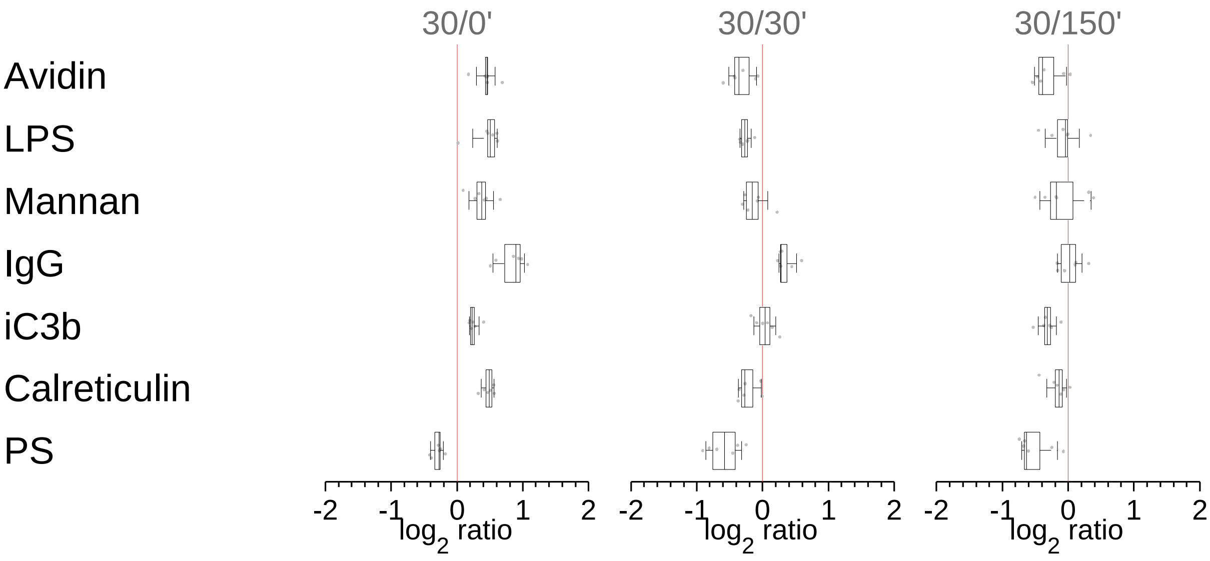

GO:0000030 mannosyltransferase activity (n = 3)

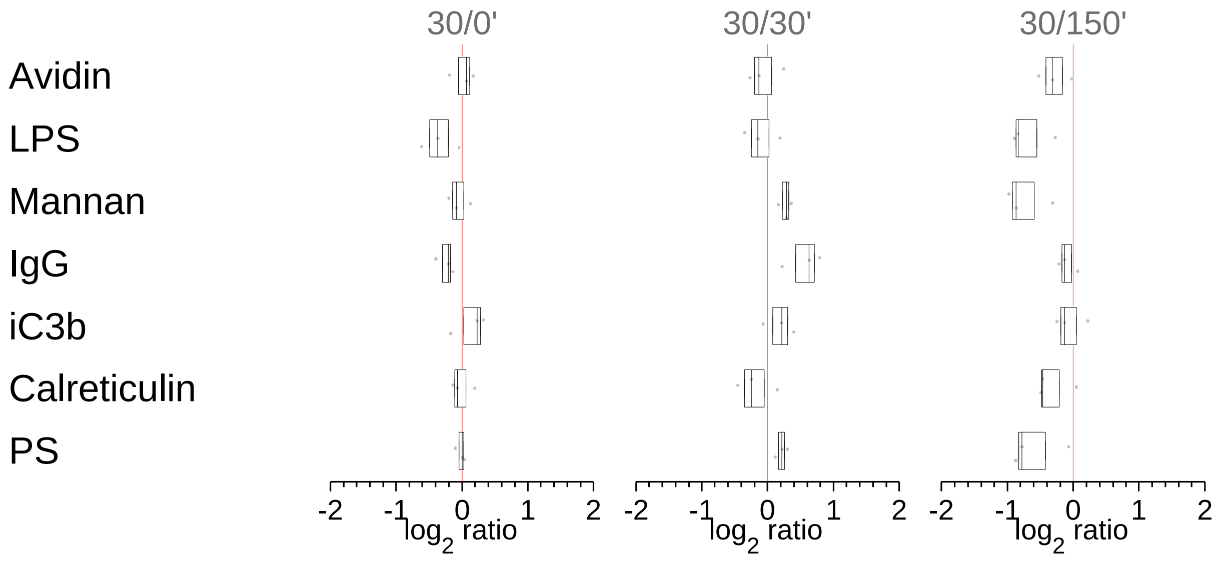

GO:0000033 alpha-1,3-mannosyltransferase activity (n = 4)

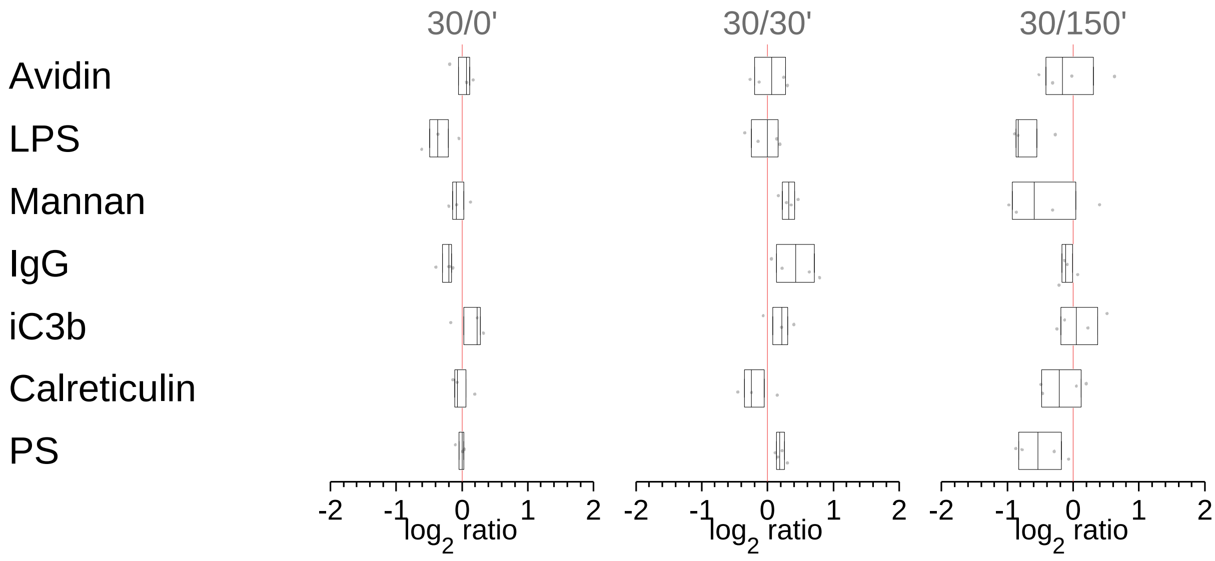

GO:0000045 autophagic vacuole assembly (n = 6)

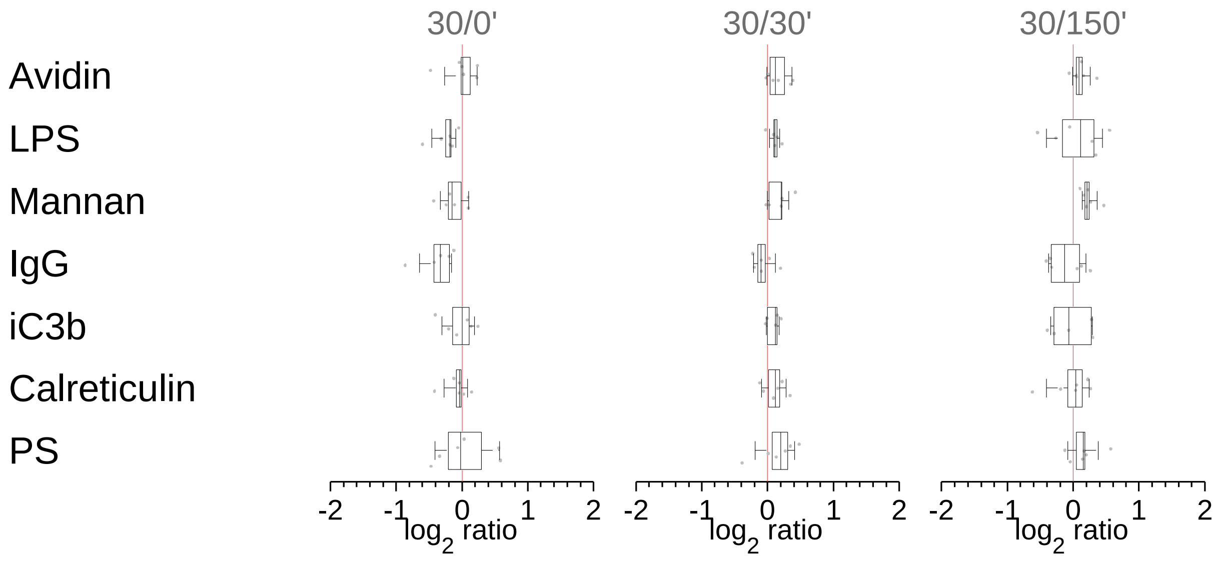

GO:0000046 autophagic vacuole fusion (n = 5)

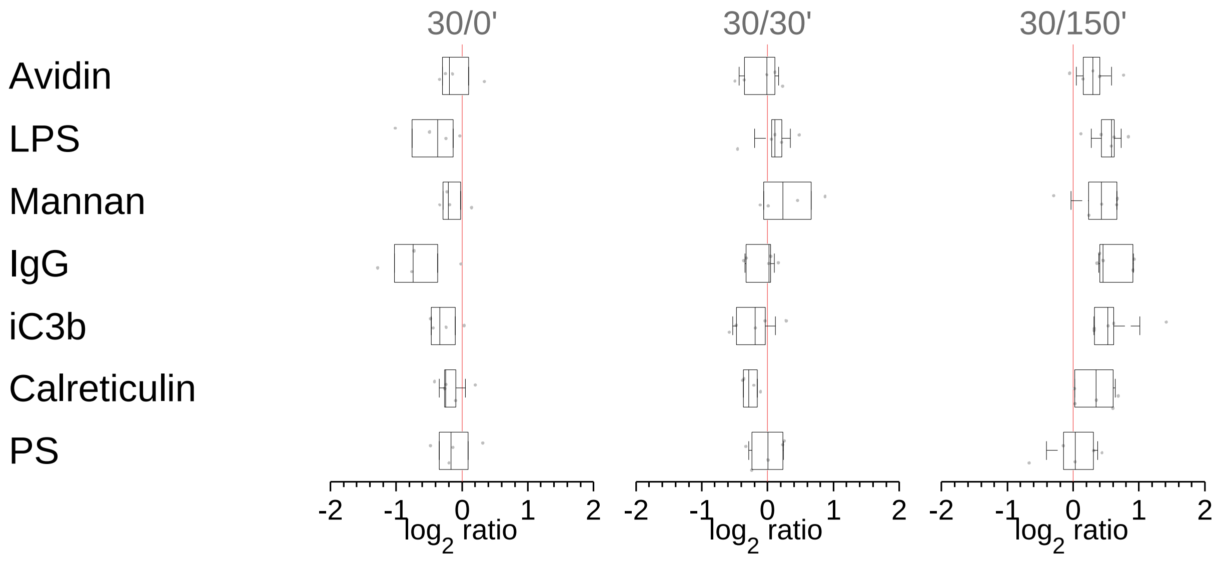

GO:0000056 ribosomal small subunit export from nucleus (n = 3)

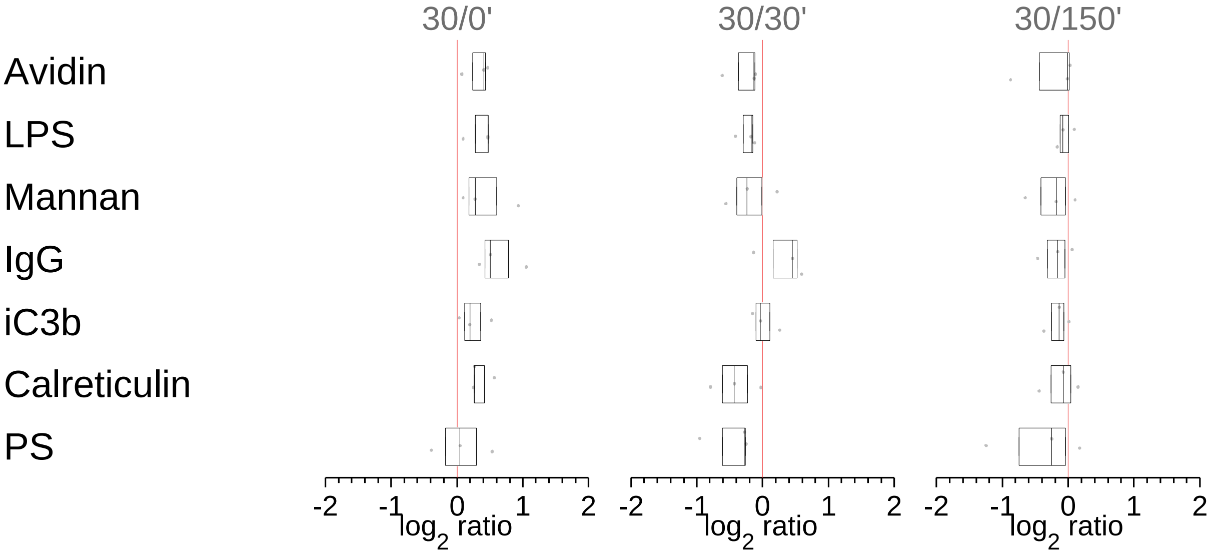

GO:0000060 protein import into nucleus, translocation (n = 3)

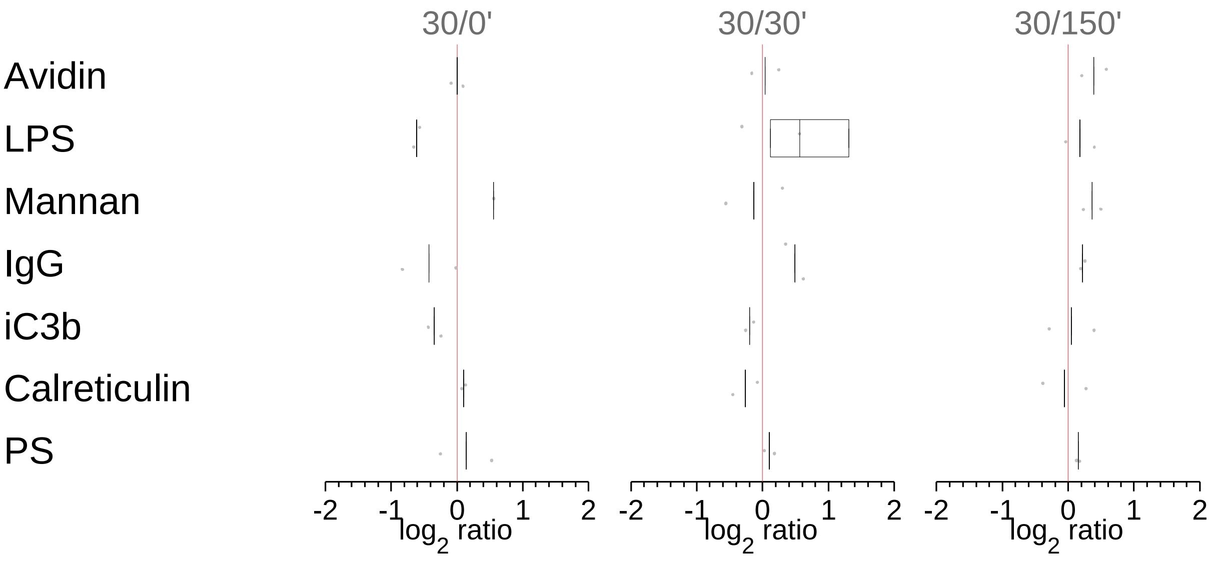

GO:0000062 fatty-acyl-CoA binding (n = 11)

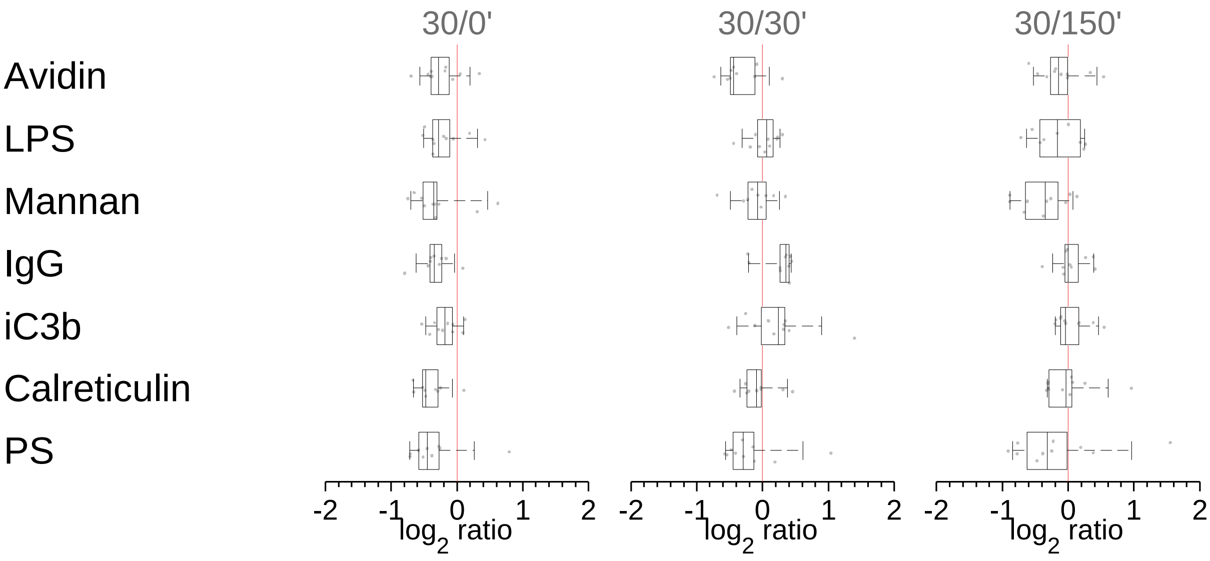

GO:0000082 G1/S transition of mitotic cell cycle (n = 8)

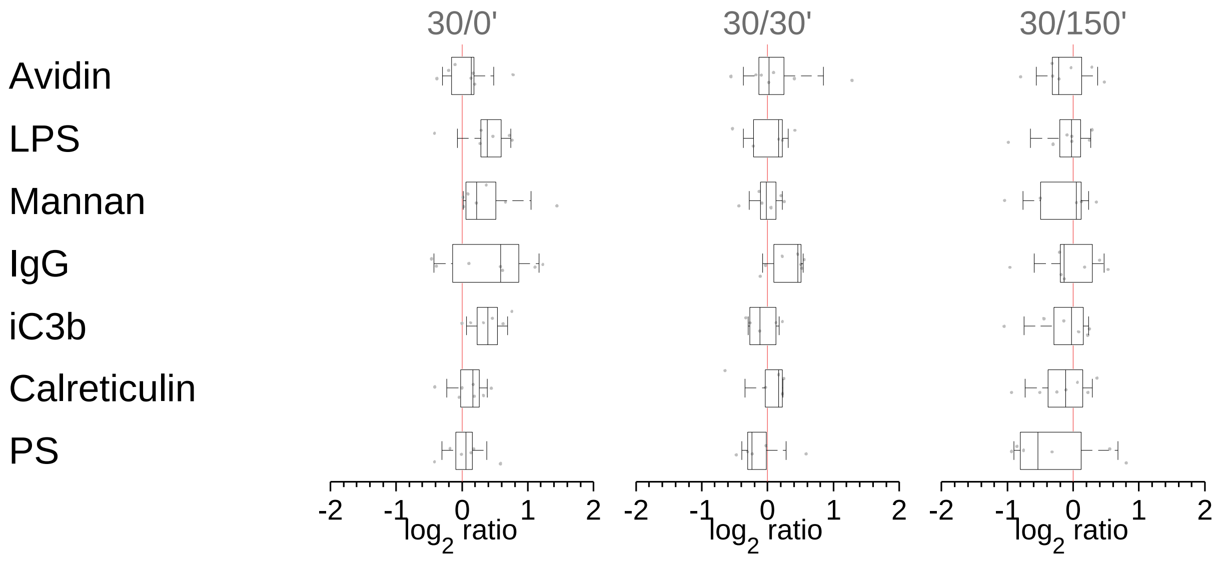

GO:0000118 histone deacetylase complex (n = 3)

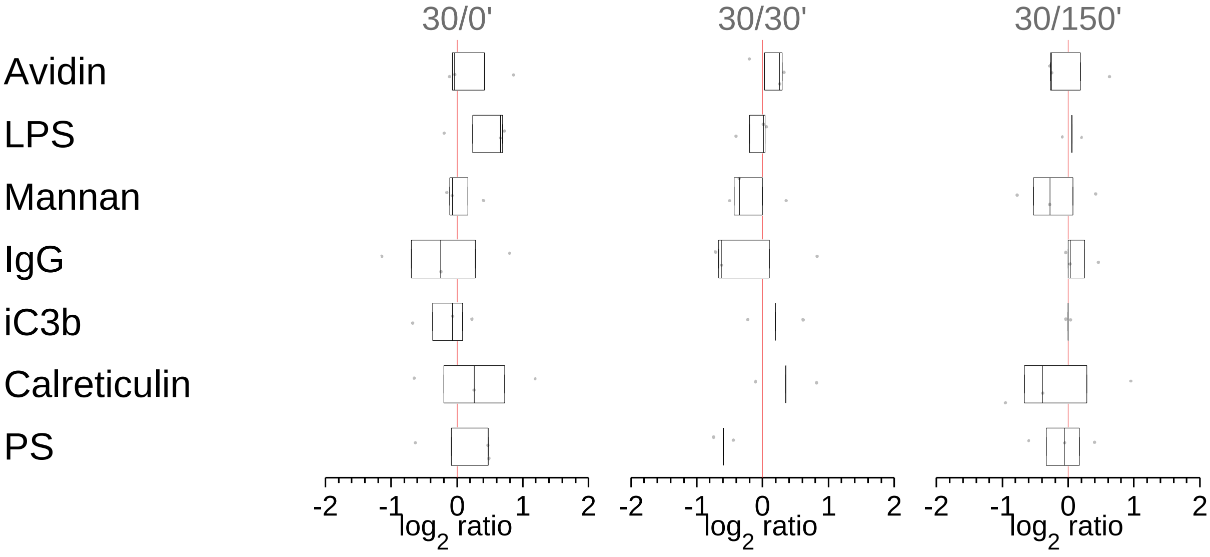

GO:0000122 negative regulation of transcription from RNA polymerase II promoter (n = 34)

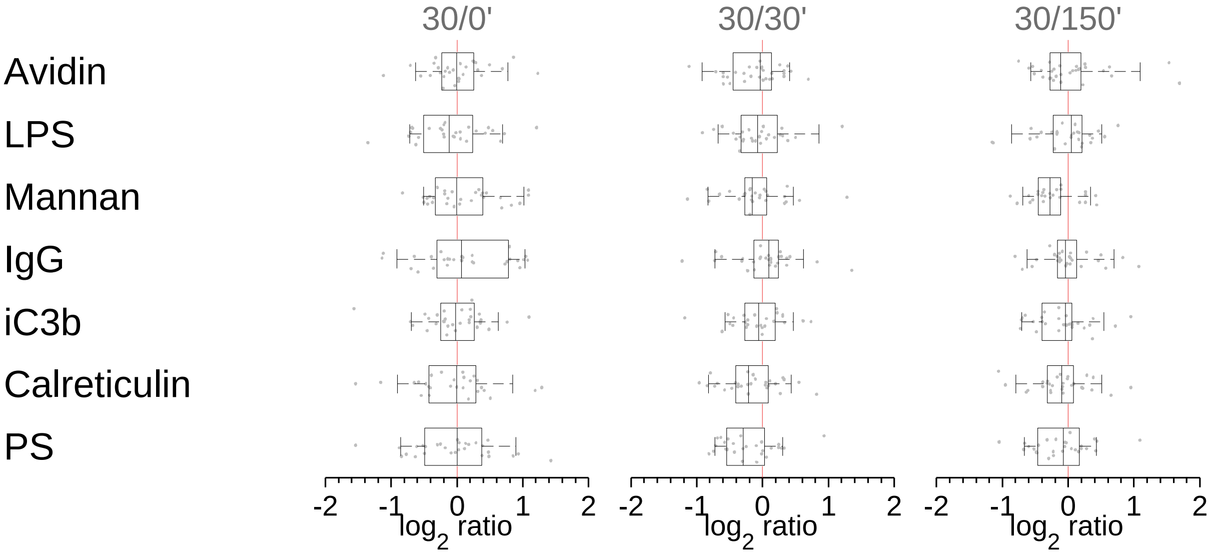

GO:0000137 Golgi cis cisterna (n = 3)

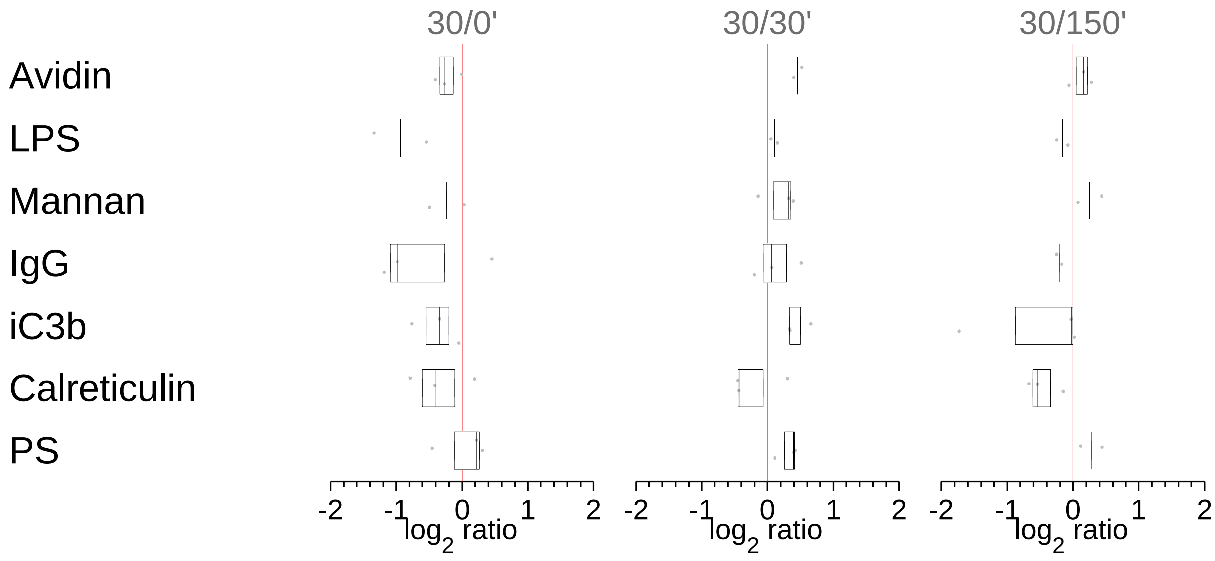

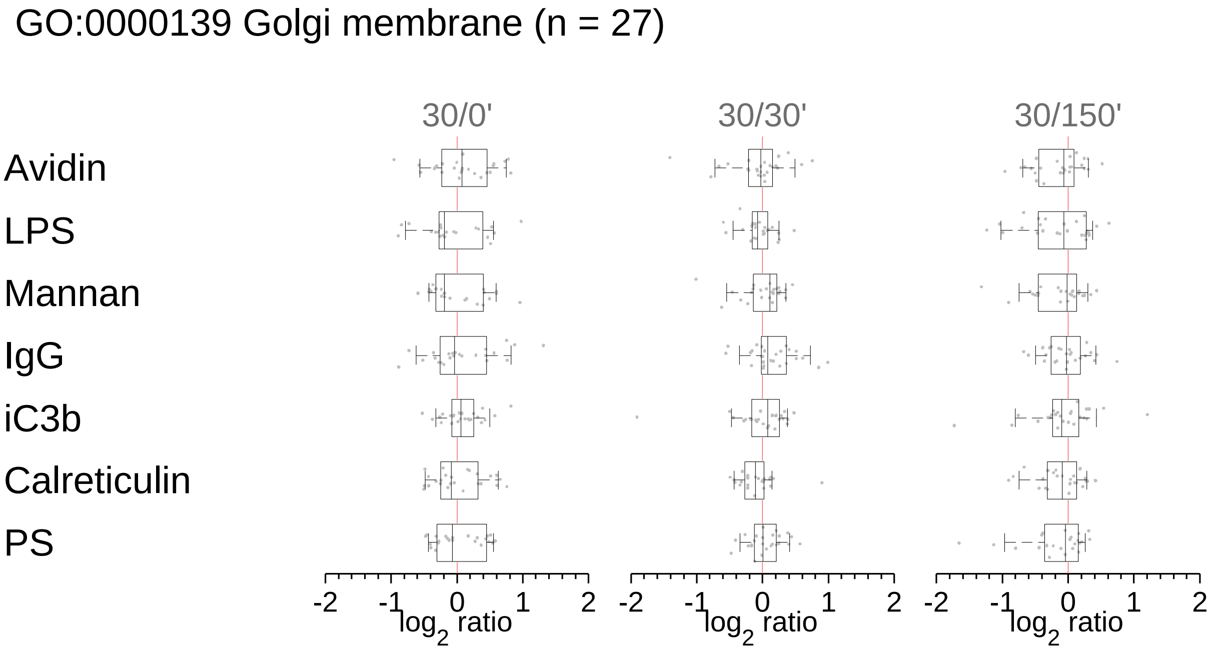
GO:0000139 Golgi membrane (n = 27)
30/0'
30/30'
30/150'
Avidin
LPS
Mannan
IgG
iC3b
Calreticulin
PS
-2
-1
0
1
2
log2 ratio

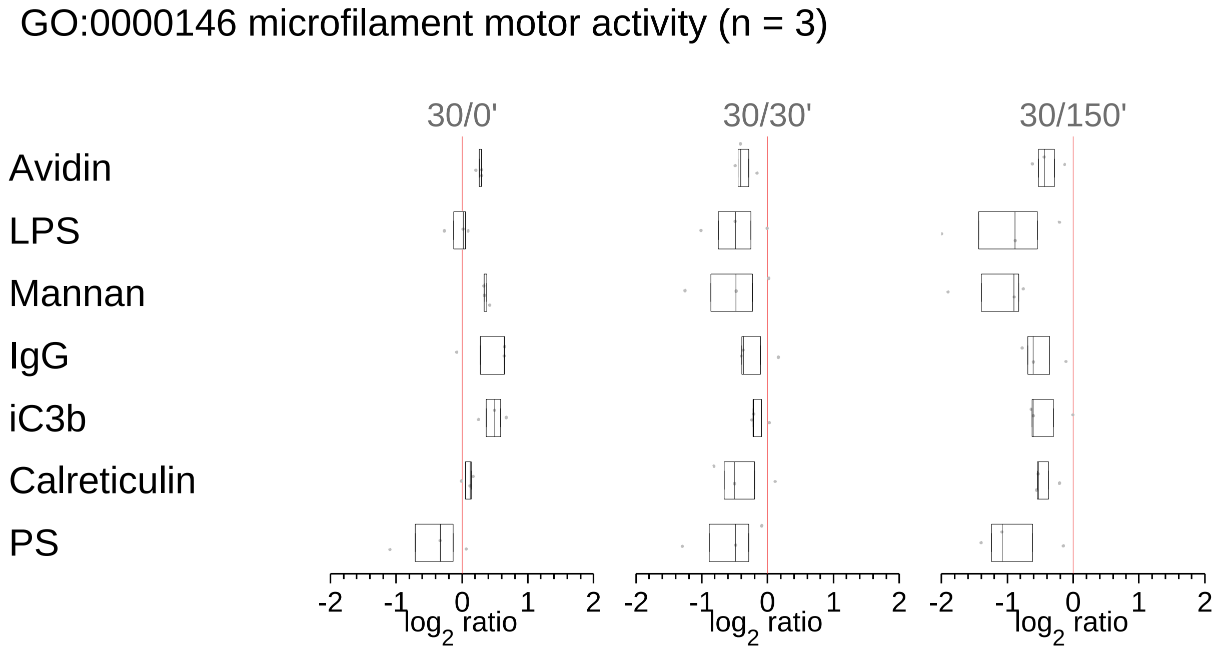
GO:0000146 microfilament motor activity (n = 3)
30/0'
30/30'
30/150'
Avidin
LPS
Mannan
IgG
iC3b
Calreticulin
PS
-2 -1 0 1 2
$\log_2$ ratio
-2 -1 0 1 2
$\log_2$ ratio
-2 -1 0 1 2
$\log_2$ ratio

GO:0000149 SNARE binding (n = 12)

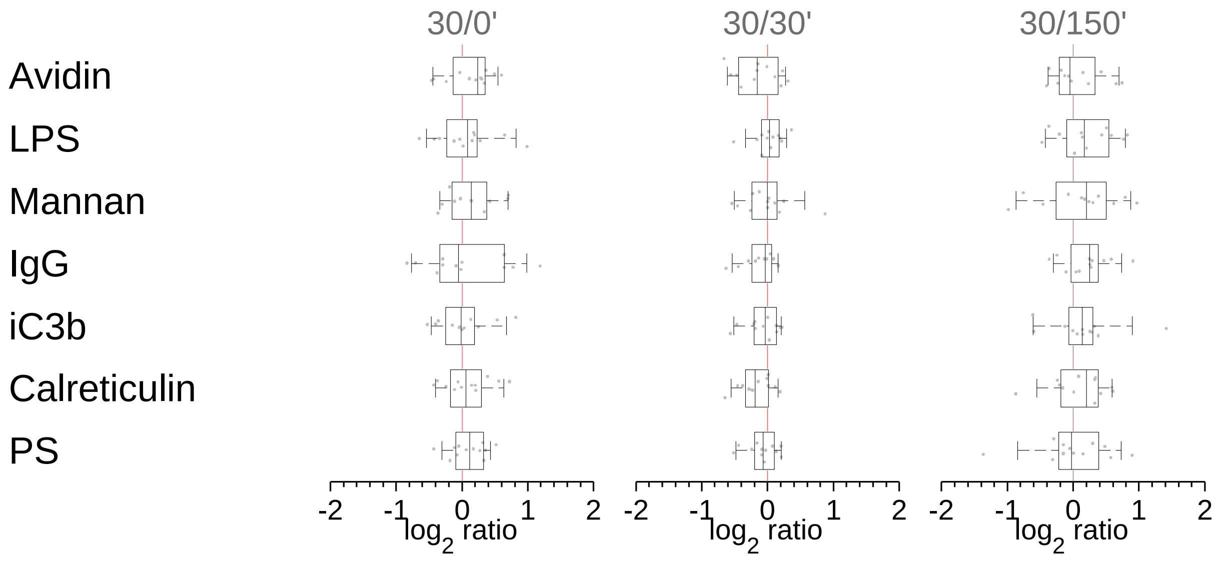

GO:0000151 ubiquitin ligase complex (n = 4)

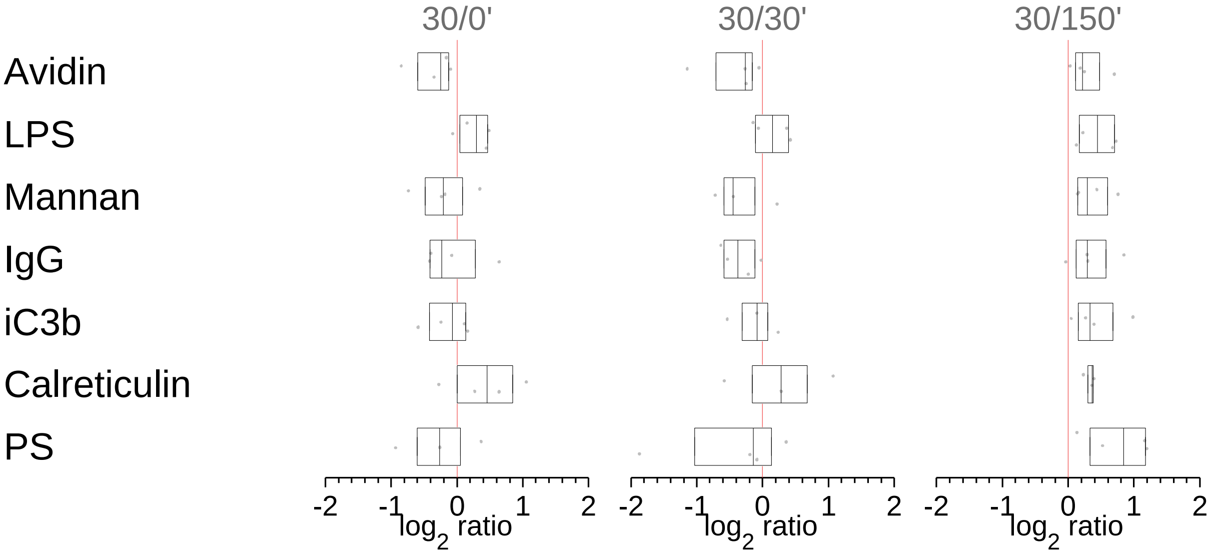

GO:0000159 protein phosphatase type 2A complex (n = 3)

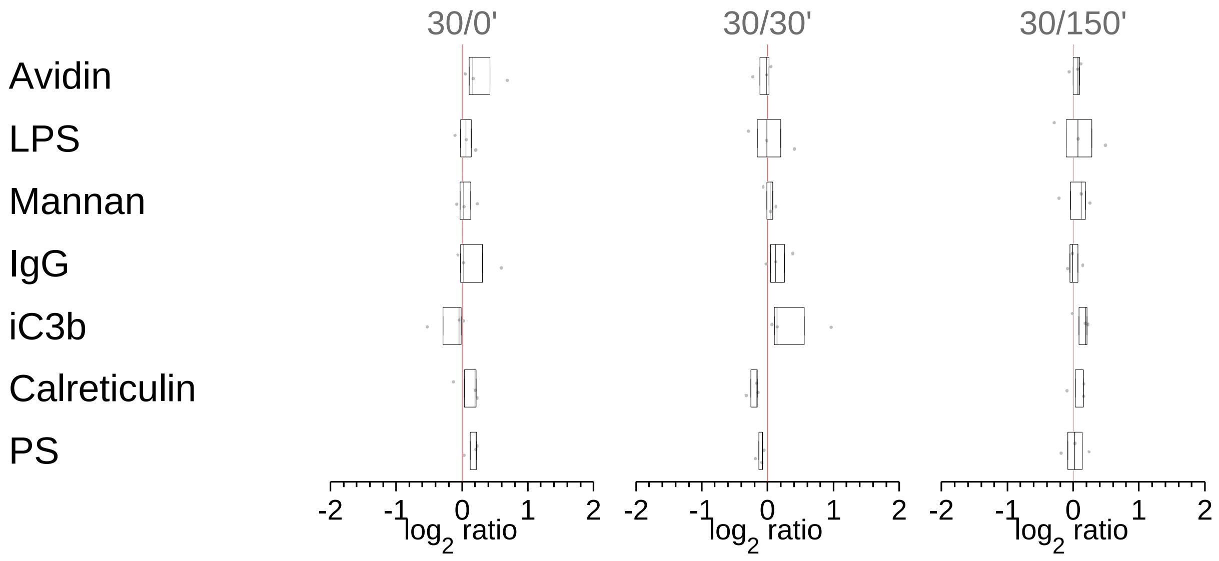

GO:0000165 MAPK cascade (n = 7)

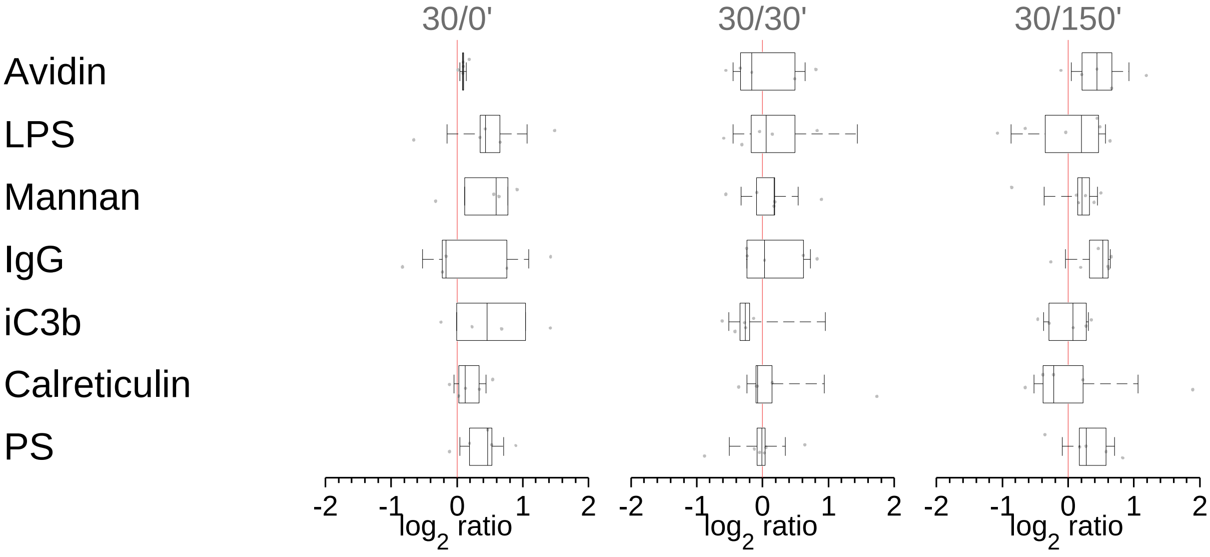

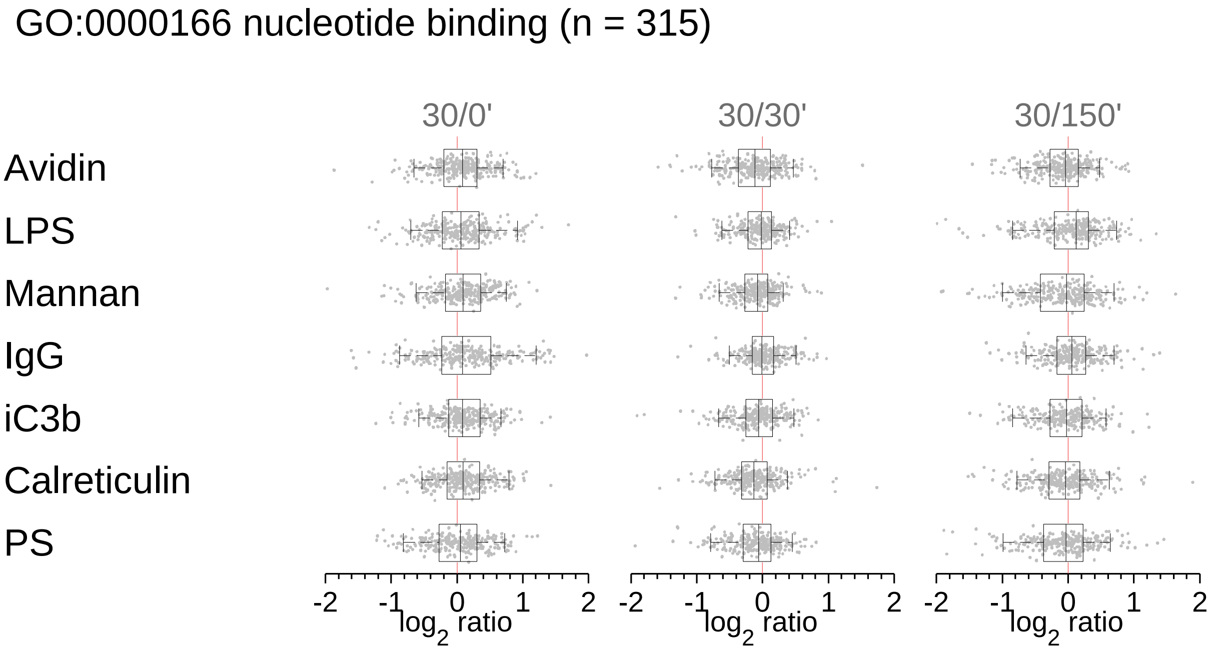
GO:0000166 nucleotide binding (n = 315)
30/0'
30/30'
30/150'
Avidin
LPS
Mannan
IgG
iC3b
Calreticulin
PS
-2
-1
0
1
2
$\log_2$ ratio

GO:0000184 nuclear-transcribed mRNA catabolic process, nonsense-mediated decay (n = 3)

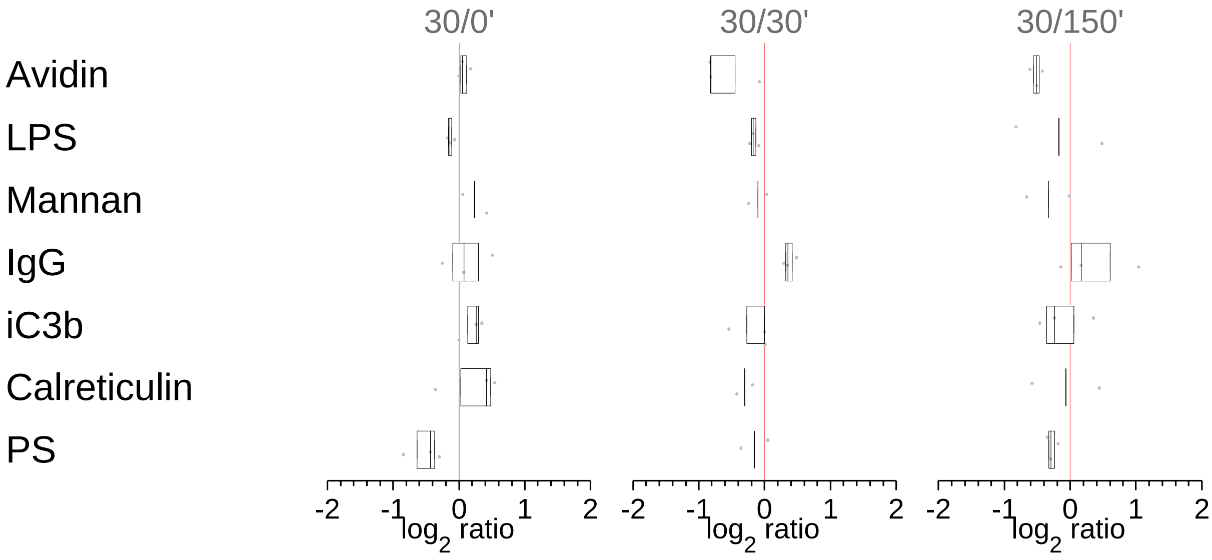

GO:0000186 activation of MAPKK activity (n = 5)

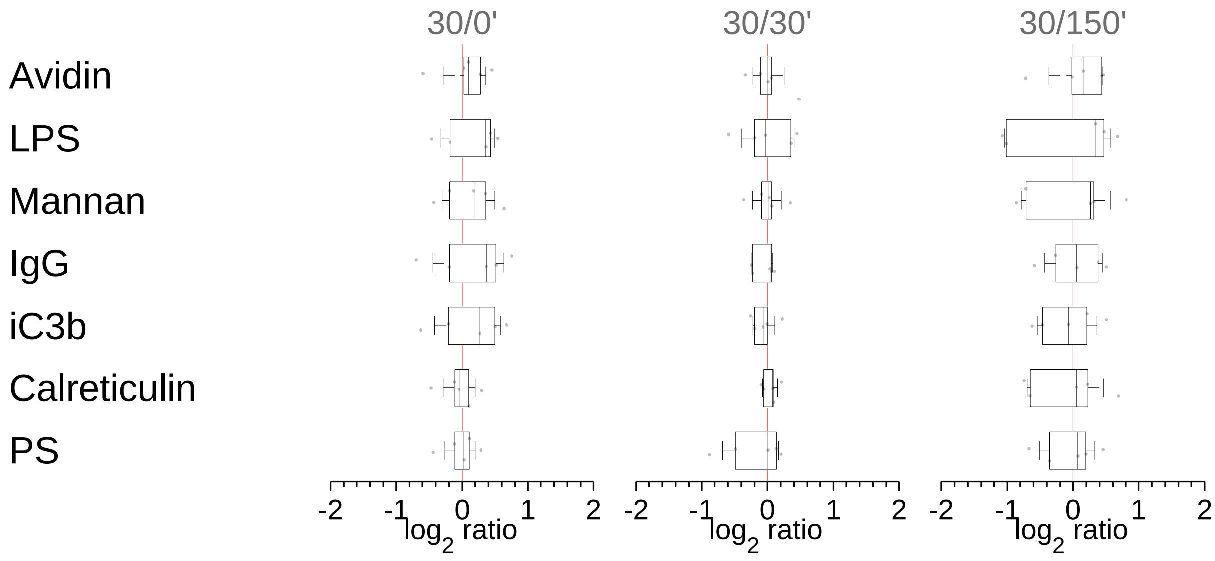

GO:0000187 activation of MAPK activity (n = 10)

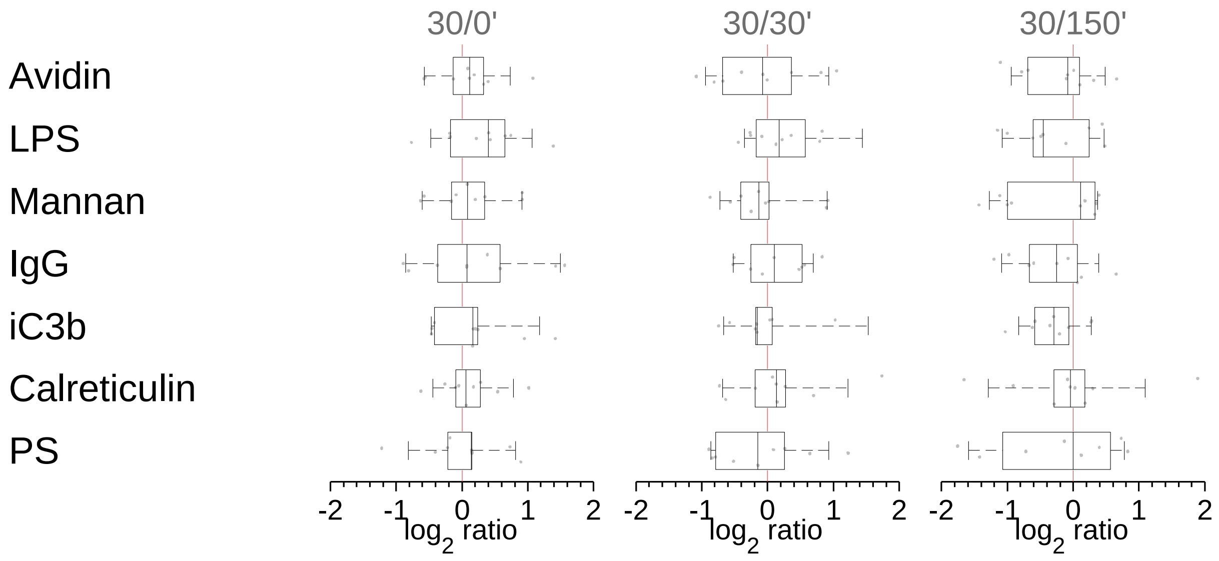

GO:0000209 protein polyubiquitination (n = 4)

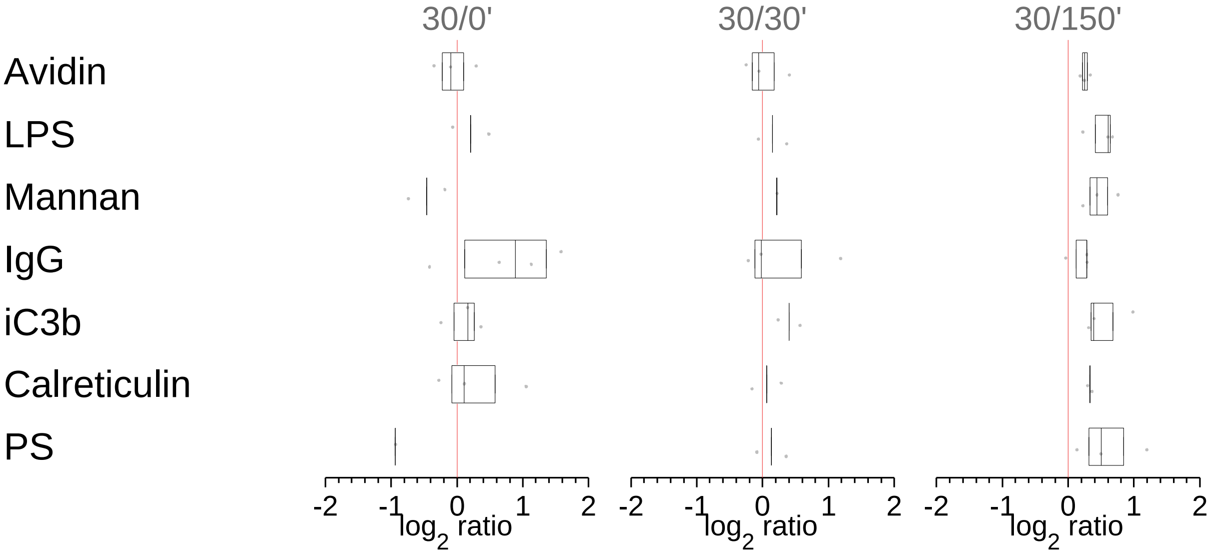

GO:0000226 microtubule cytoskeleton organization (n = 5)

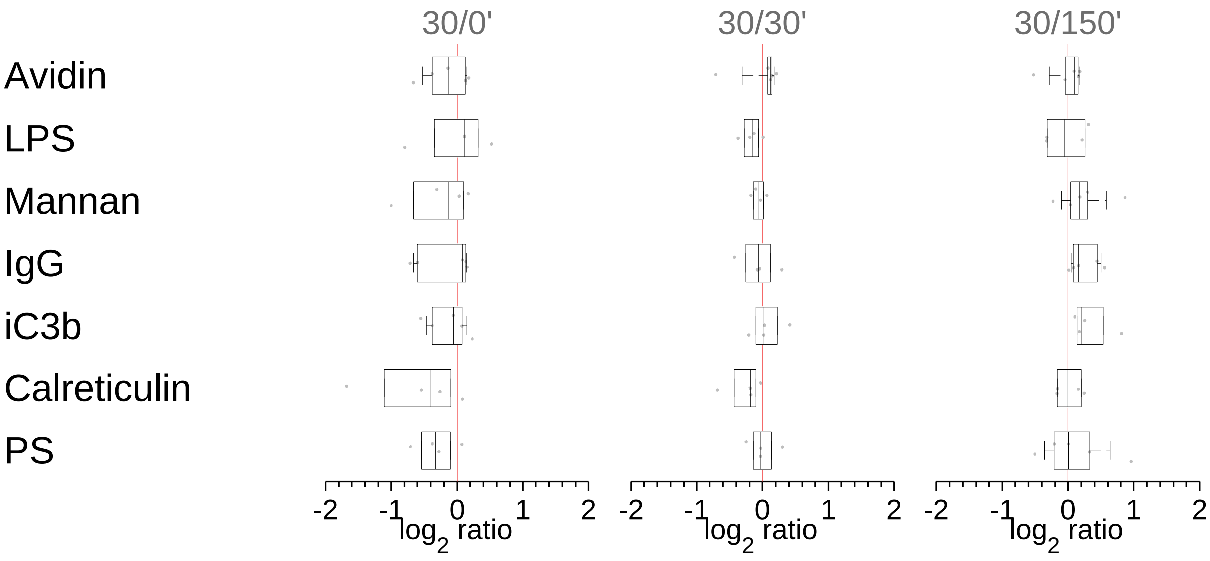

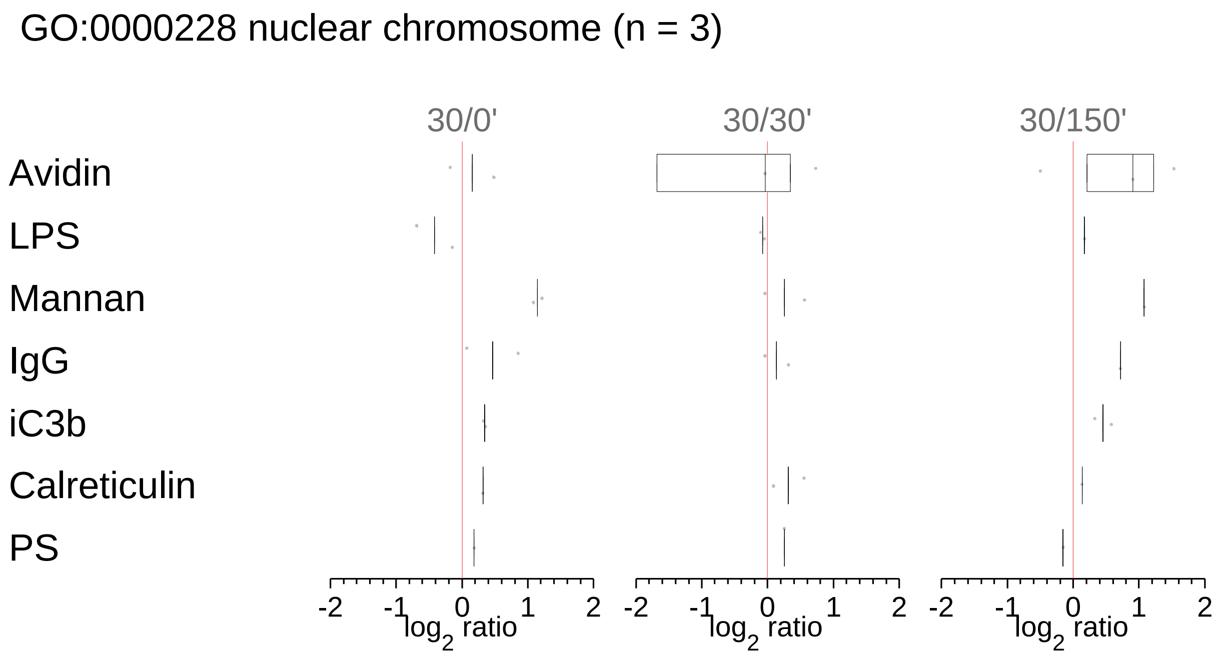
GO:0000228 nuclear chromosome (n = 3)
30/0'
30/30'
30/150'
Avidin
LPS
Mannan
IgG
iC3b
Calreticulin
PS
-2
-1
0
1
2
log2 ratio

GO:0000252 C-3 sterol dehydrogenase (C-4 sterol decarboxylase) activity (n = 3)

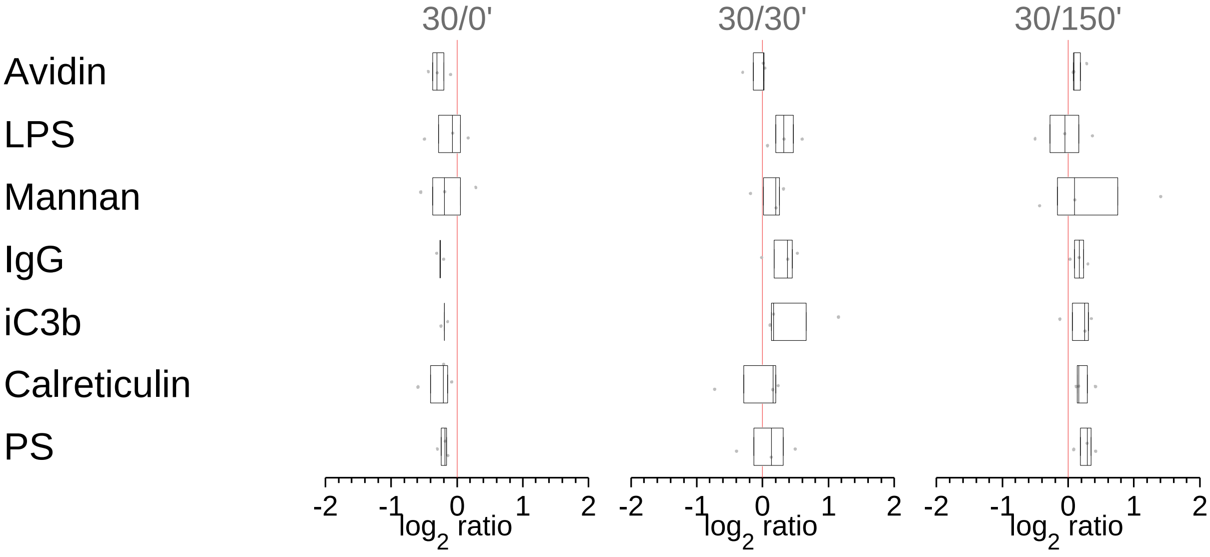

GO:0000275 mitochondrial proton-transporting ATP synthase complex, catalytic core F(1) (n = 4)

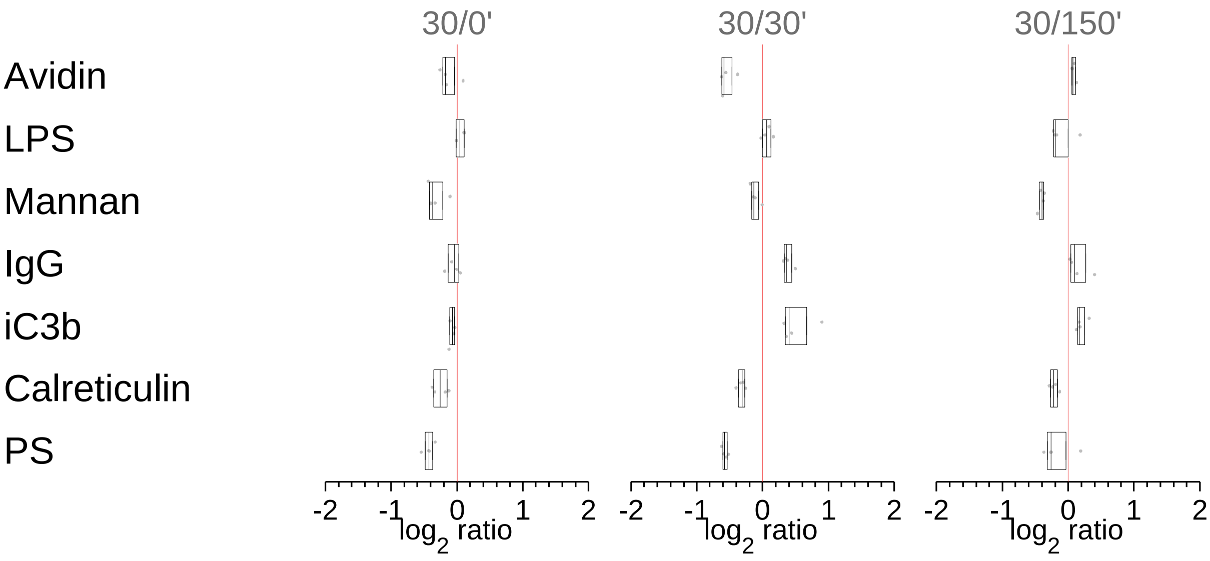

GO:0000276 mitochondrial proton-transporting ATP synthase complex, coupling factor F(o) (n = 7)

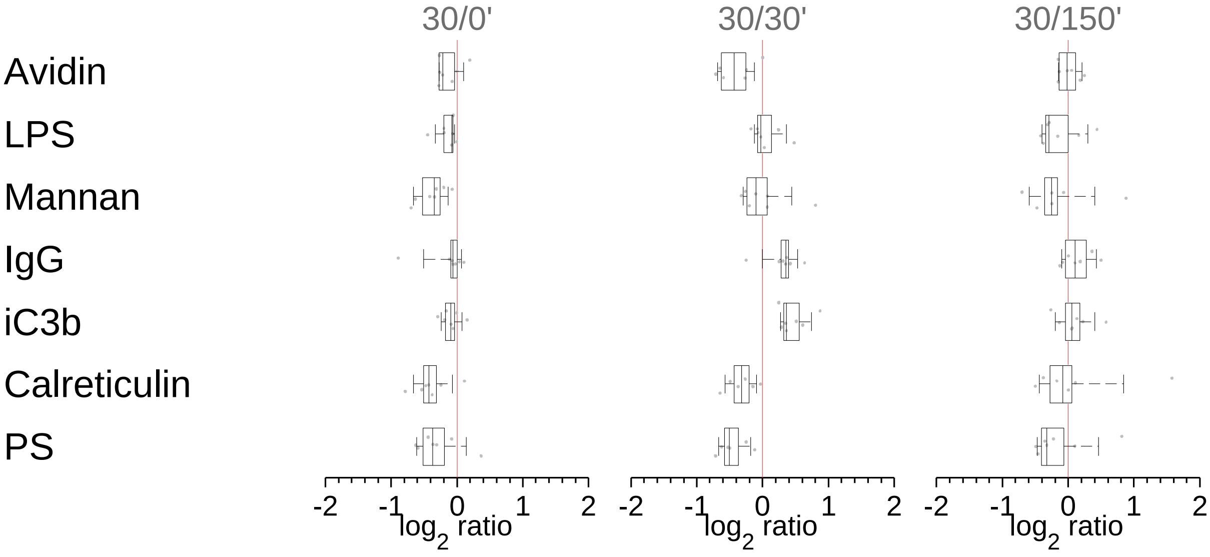

GO:0000281 cytokinesis after mitosis (n = 5)

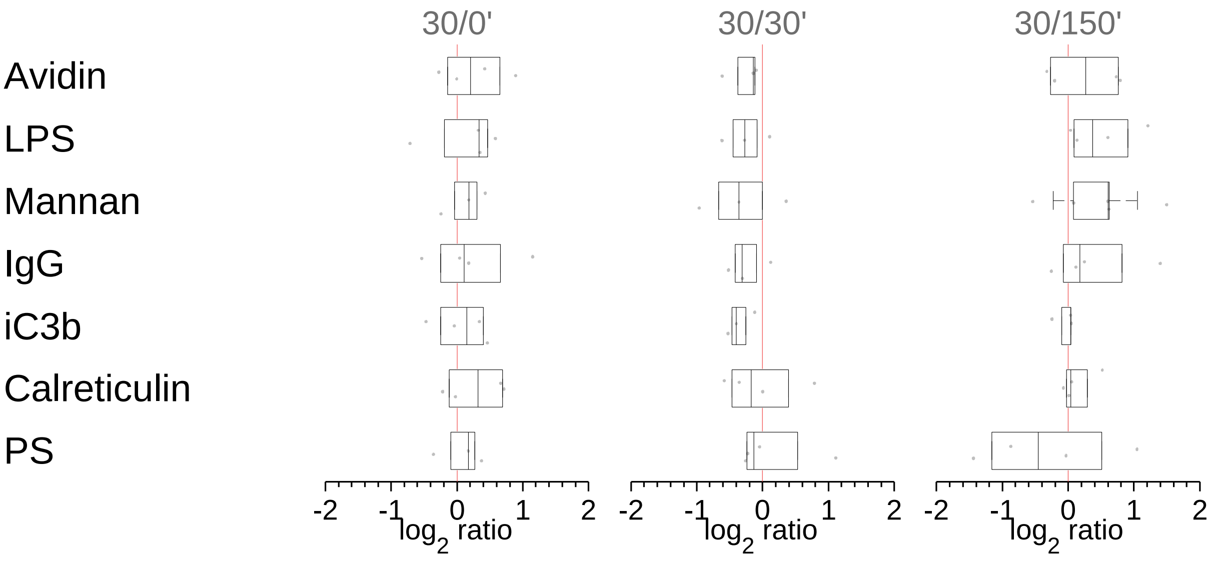

GO:0000287 magnesium ion binding (n = 26)

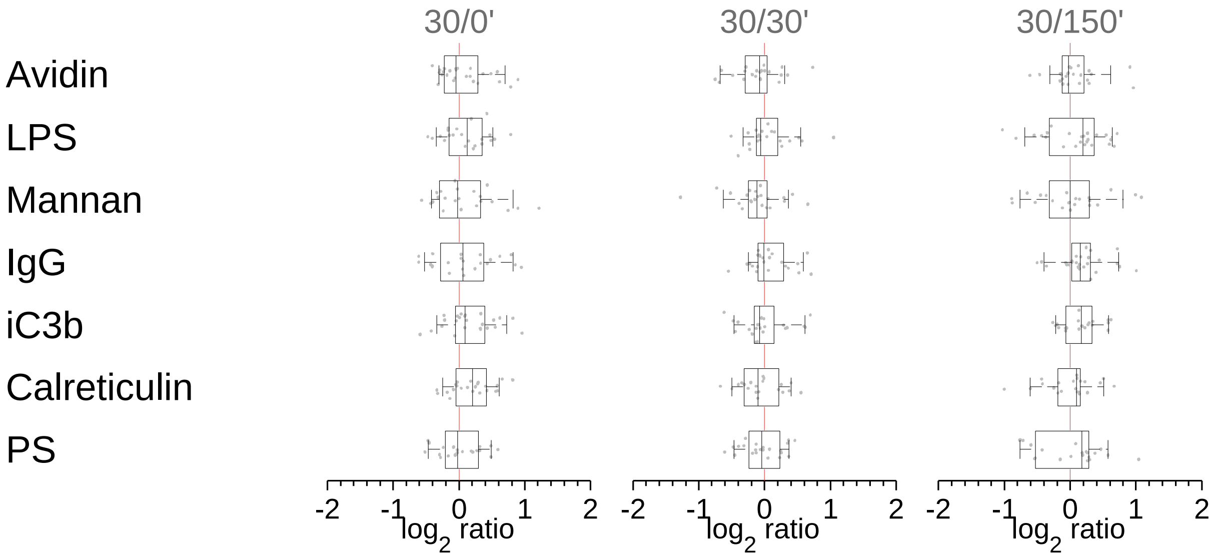

GO:0000302 response to reactive oxygen species (n = 8)

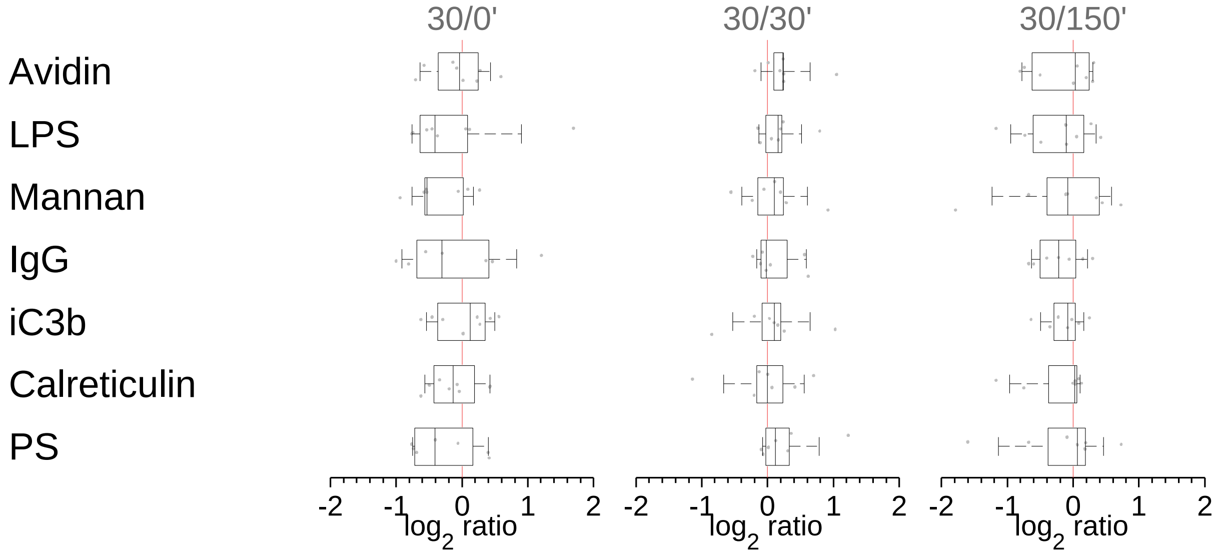

GO:0000380 alternative nuclear mRNA splicing, via spliceosome (n = 4)

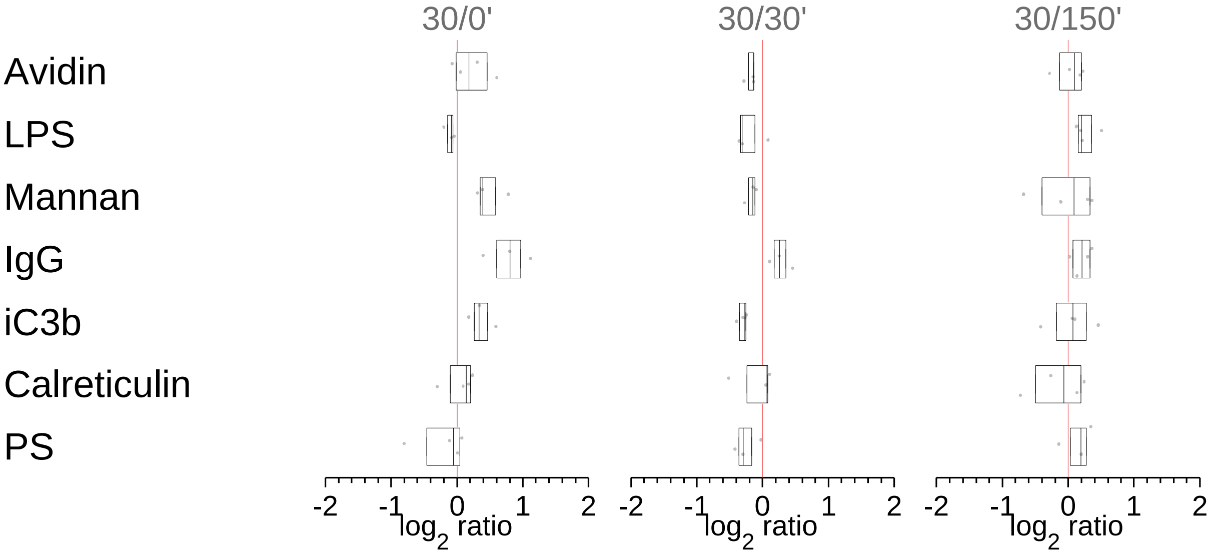

GO:0000381 regulation of alternative nuclear mRNA splicing, via spliceosome (n = 4)

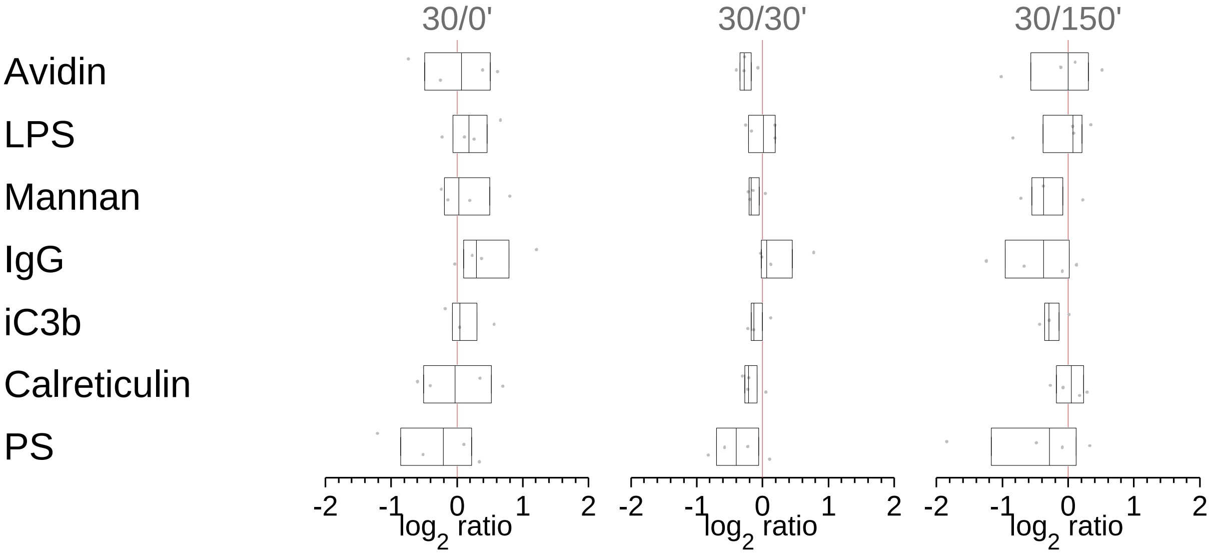

GO:0000387 spliceosomal snRNP assembly (n = 3)

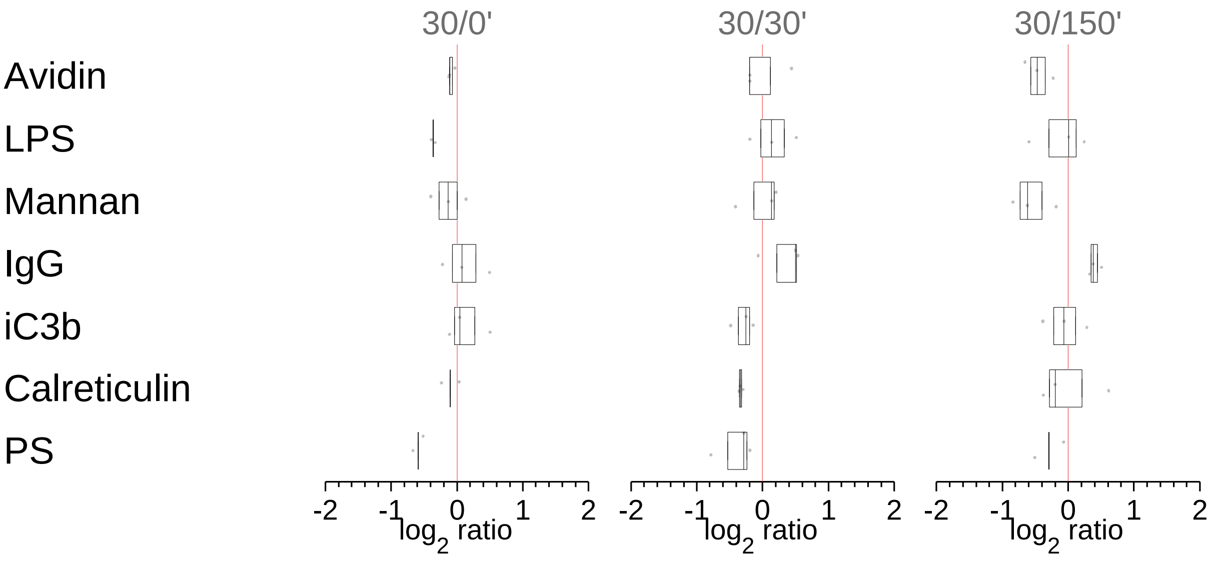

GO:0000398 nuclear mRNA splicing, via spliceosome (n = 8)

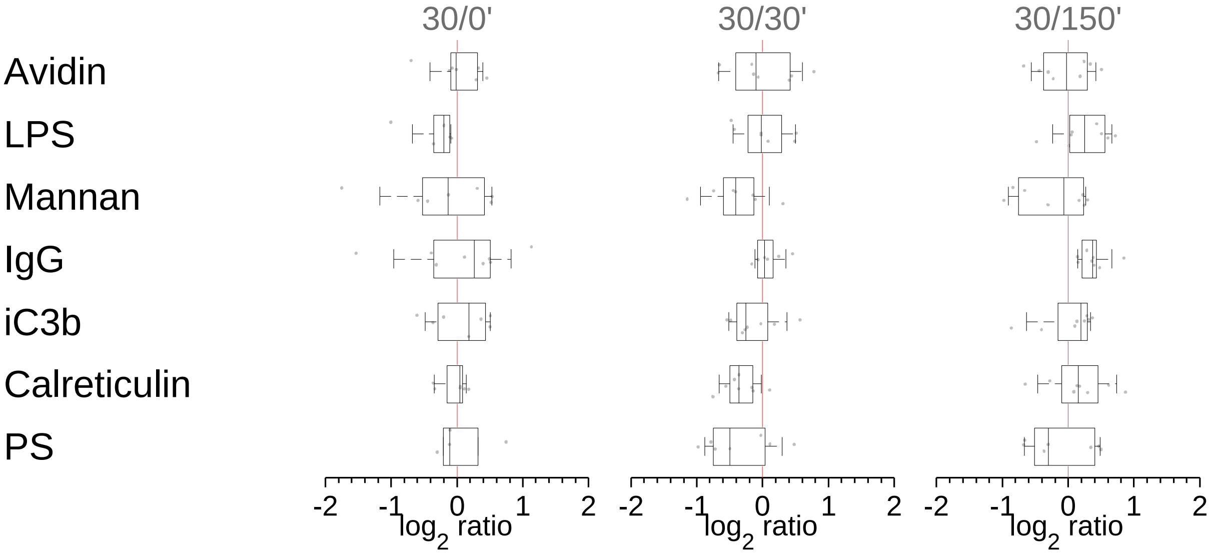

GO:0000413 protein peptidyl-prolyl isomerization (n = 5)

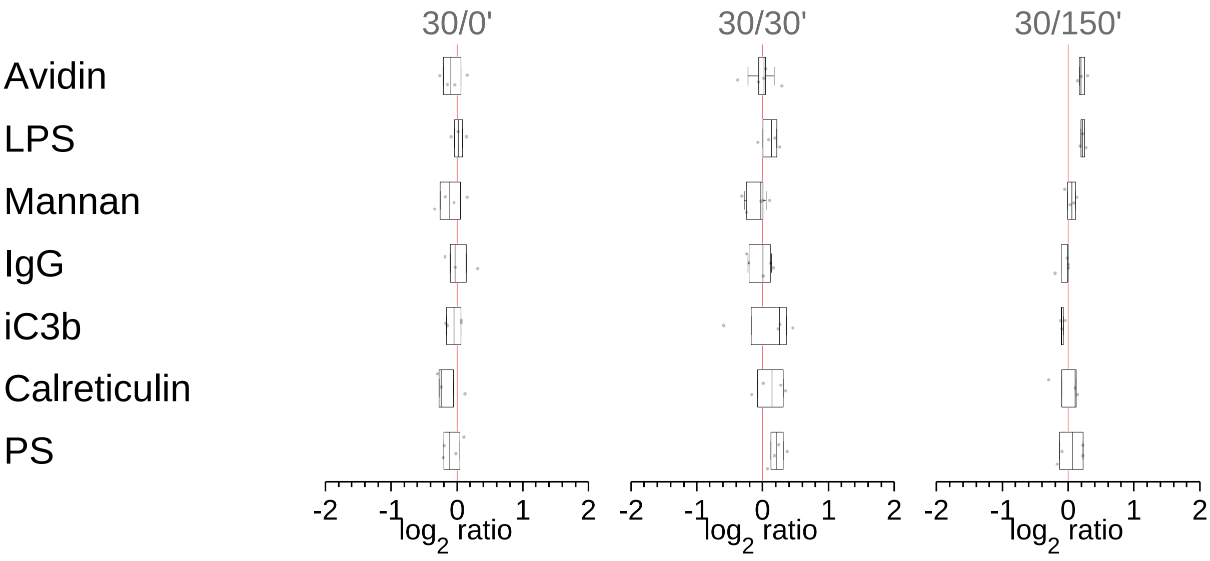

GO:0000421 autophagic vacuole membrane (n = 7)

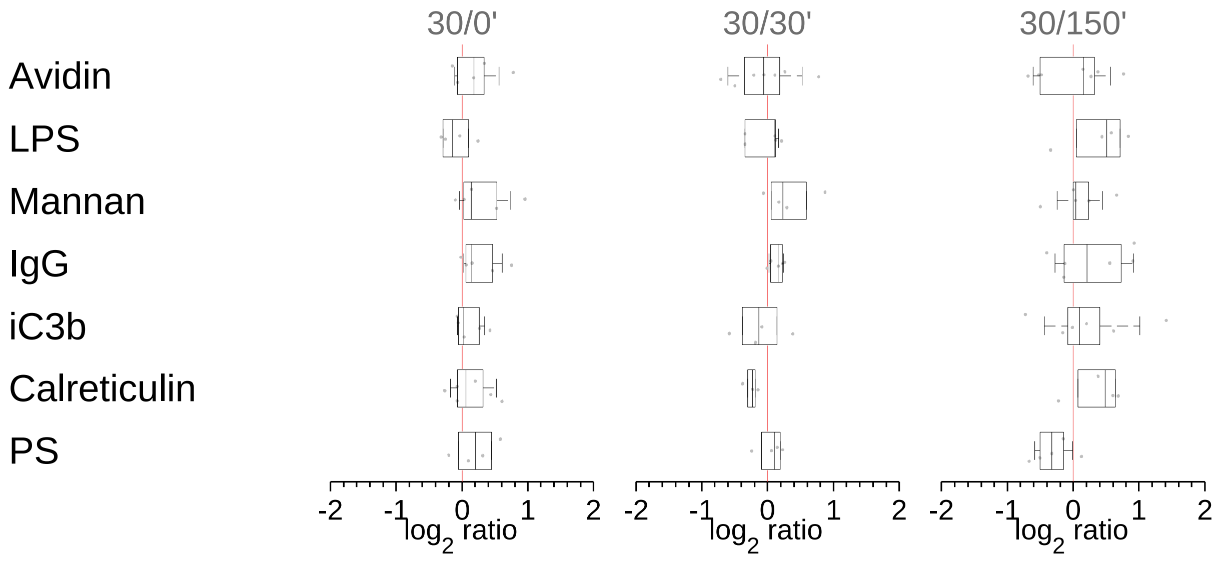

GO:0000502 proteasome complex (n = 12)

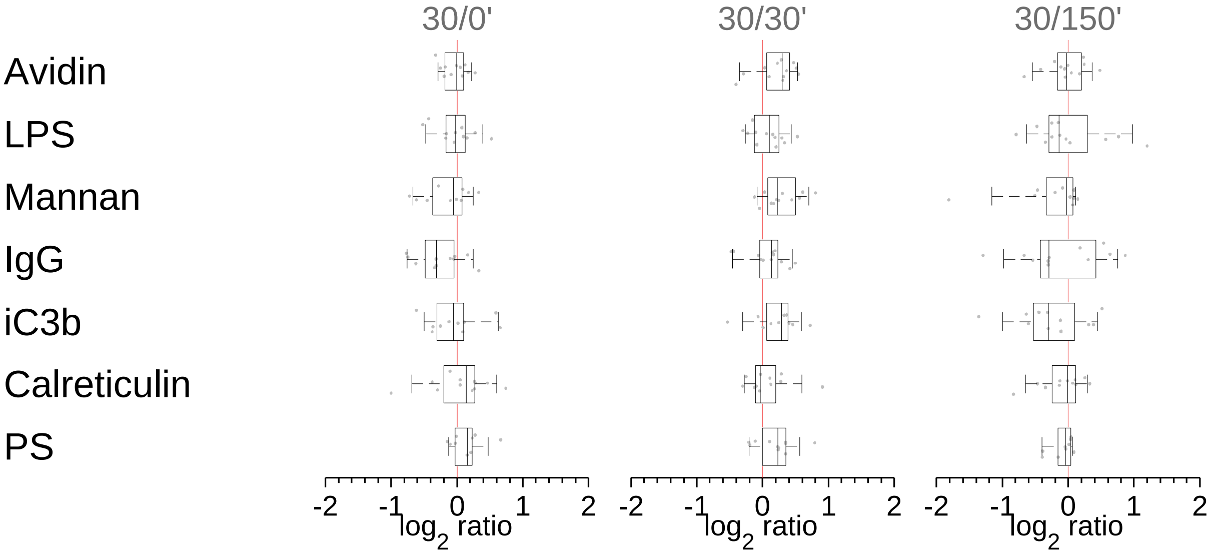

GO:0000737 DNA catabolic process, endonucleolytic (n = 3)

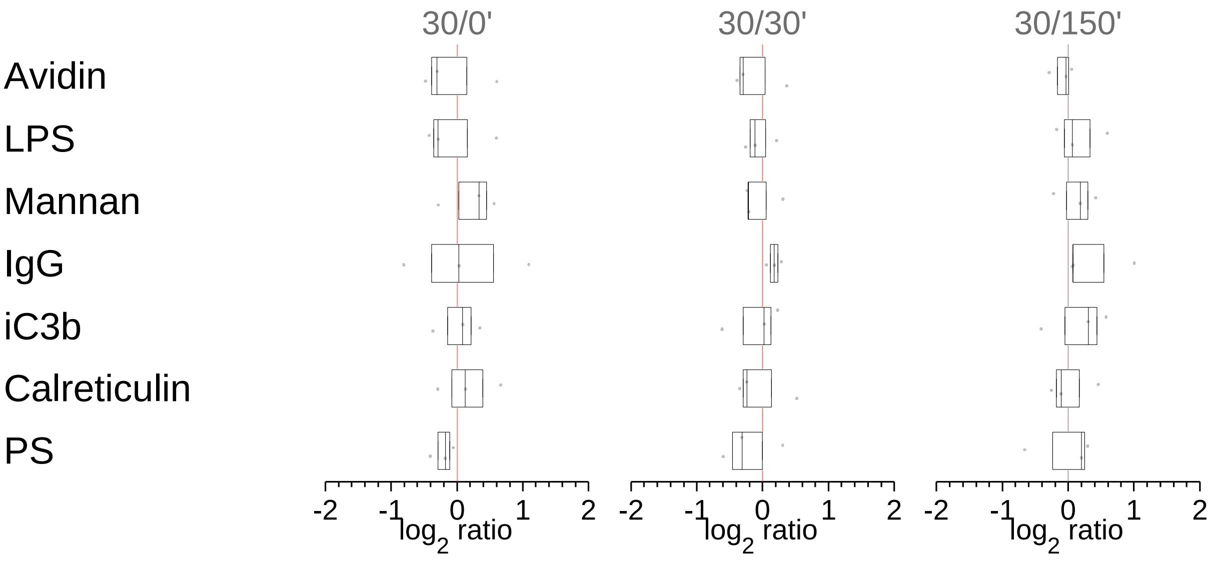

GO:0000775 chromosome, centromeric region (n = 11)

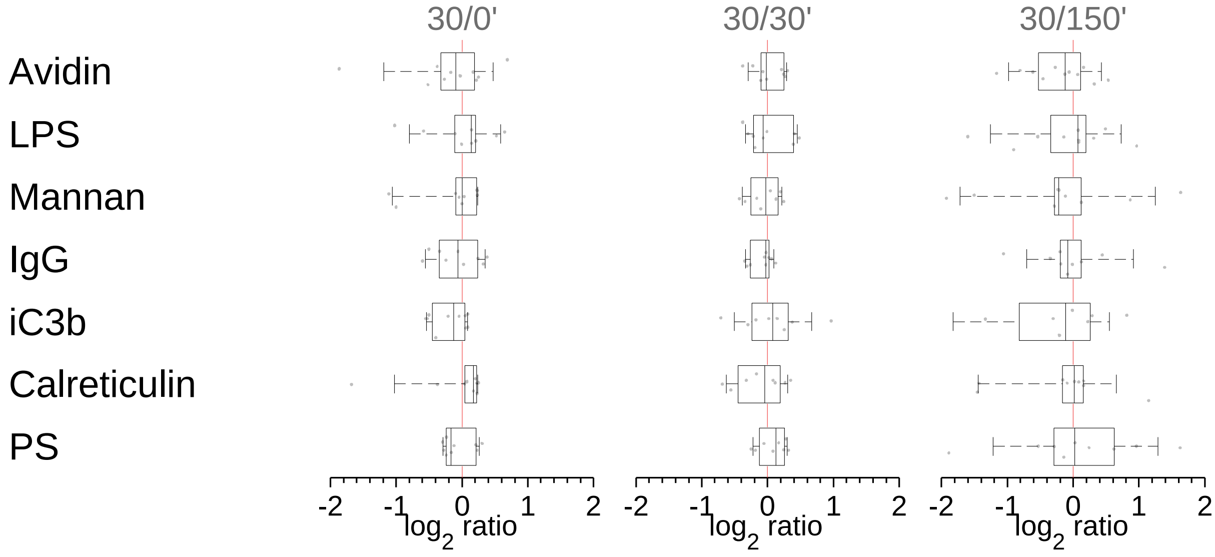

GO:0000776 kinetochore (n = 13)

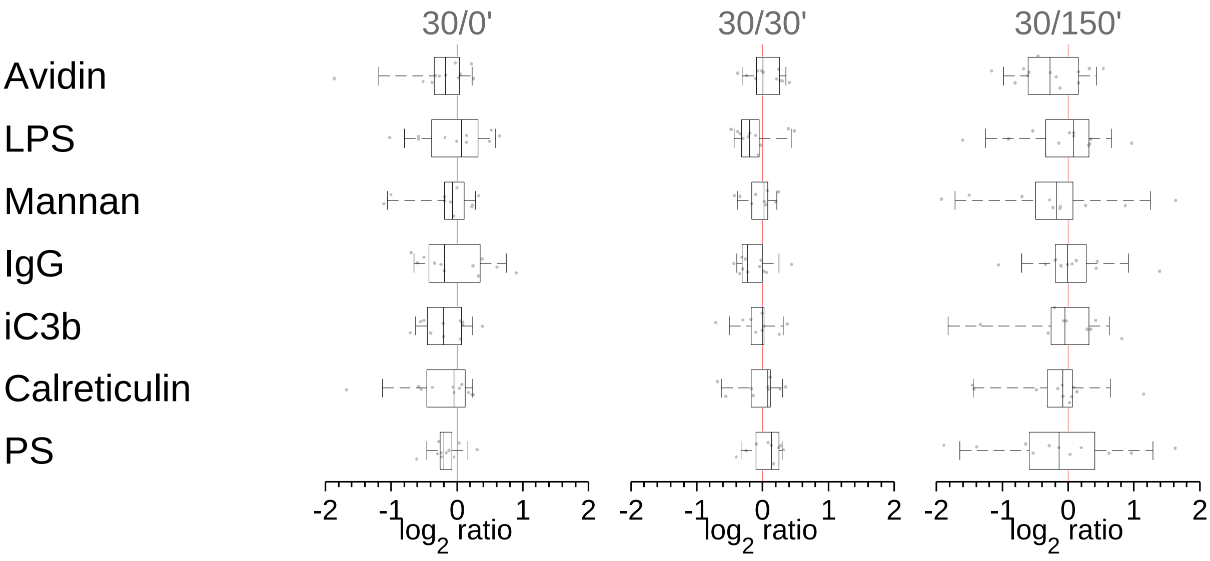

GO:0000785 chromatin (n = 8)

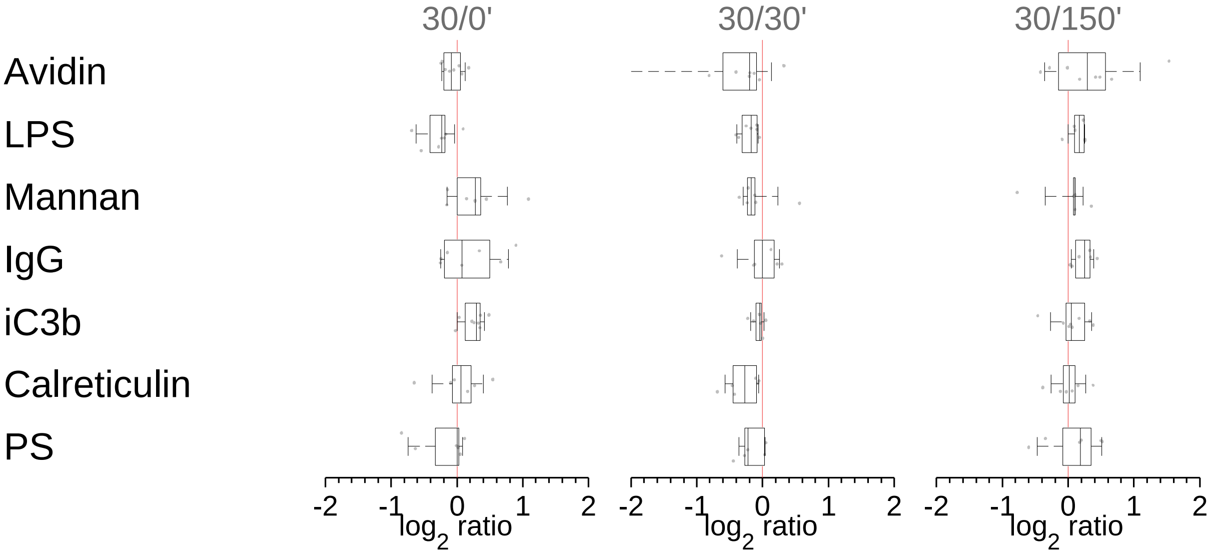

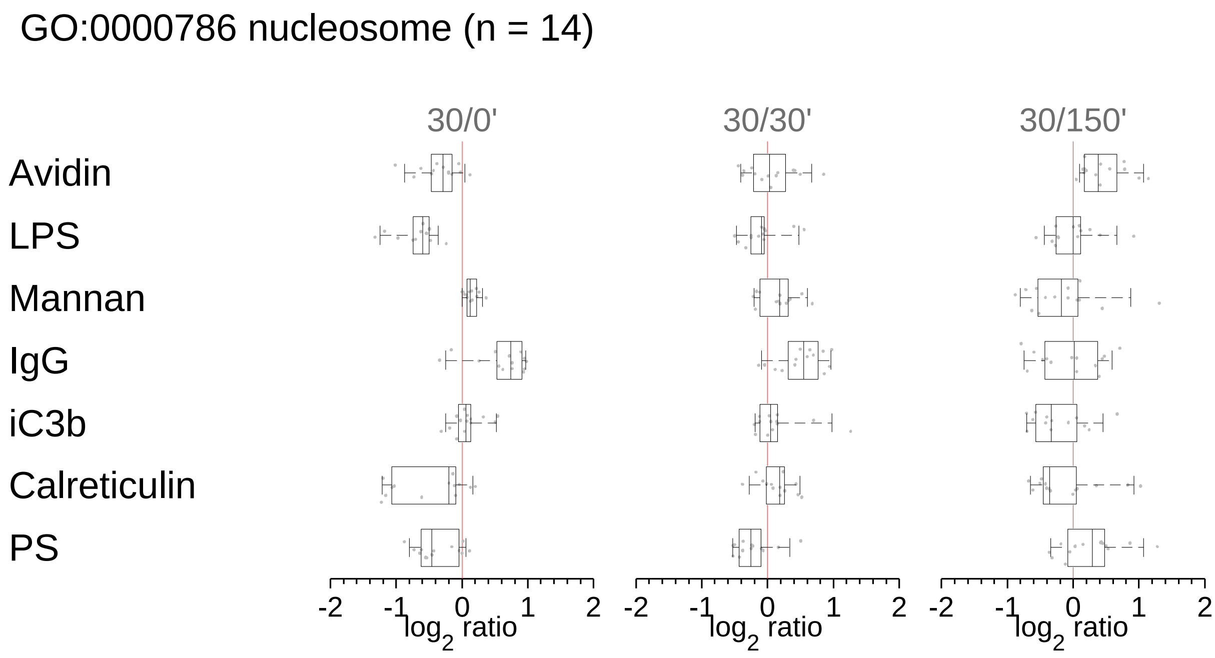
GO:0000786 nucleosome (n = 14)
30/0'
30/30'
30/150'
Avidin
LPS
Mannan
IgG
iC3b
Calreticulin
PS
-2 -1 0 1 2
log2 ratio

GO:0000790 nuclear chromatin (n = 14)

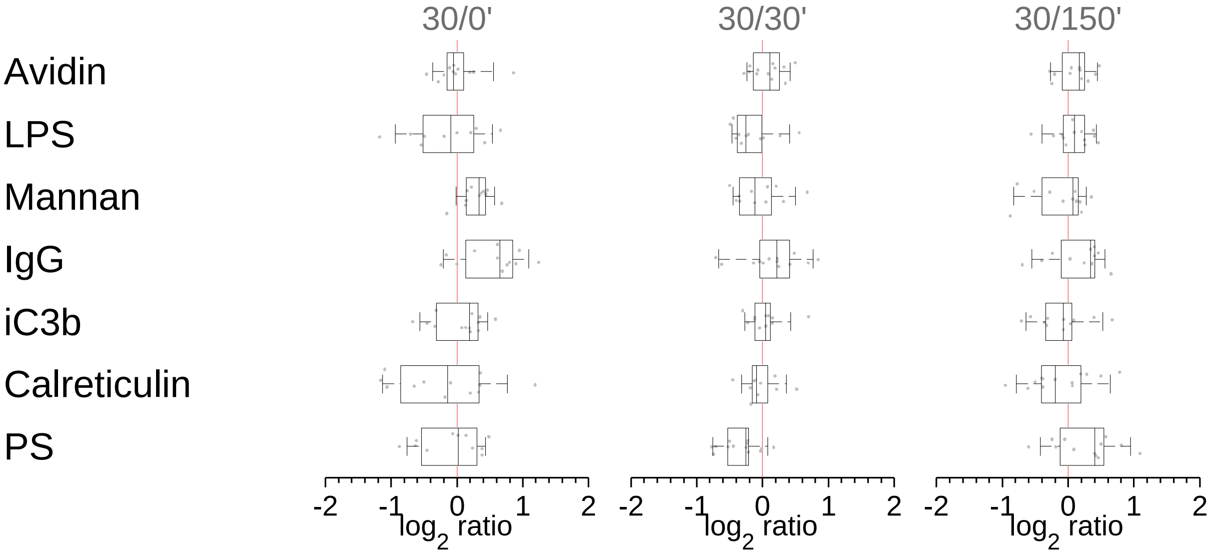

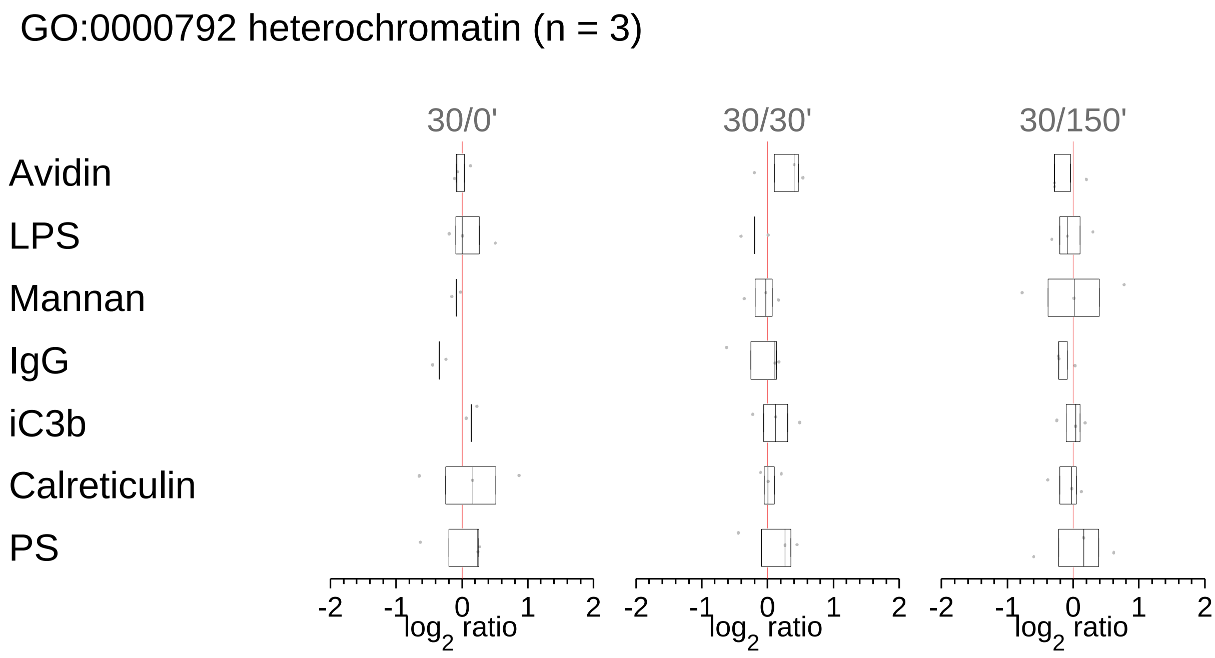
GO:0000792 heterochromatin (n = 3)
30/0'
30/30'
30/150'
Avidin
LPS
Mannan
IgG
iC3b
Calreticulin
PS
-2 -1 0 1 2
log2 ratio
-2 -1 0 1 2
log2 ratio
-2 -1 0 1 2
log2 ratio

GO:0000793 condensed chromosome (n = 6)

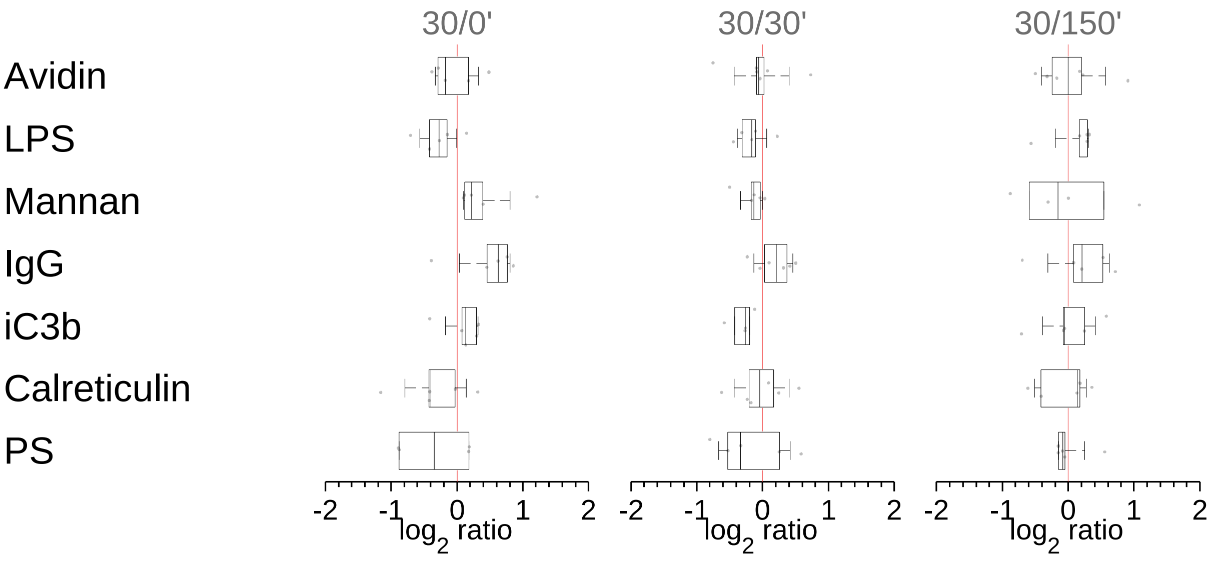

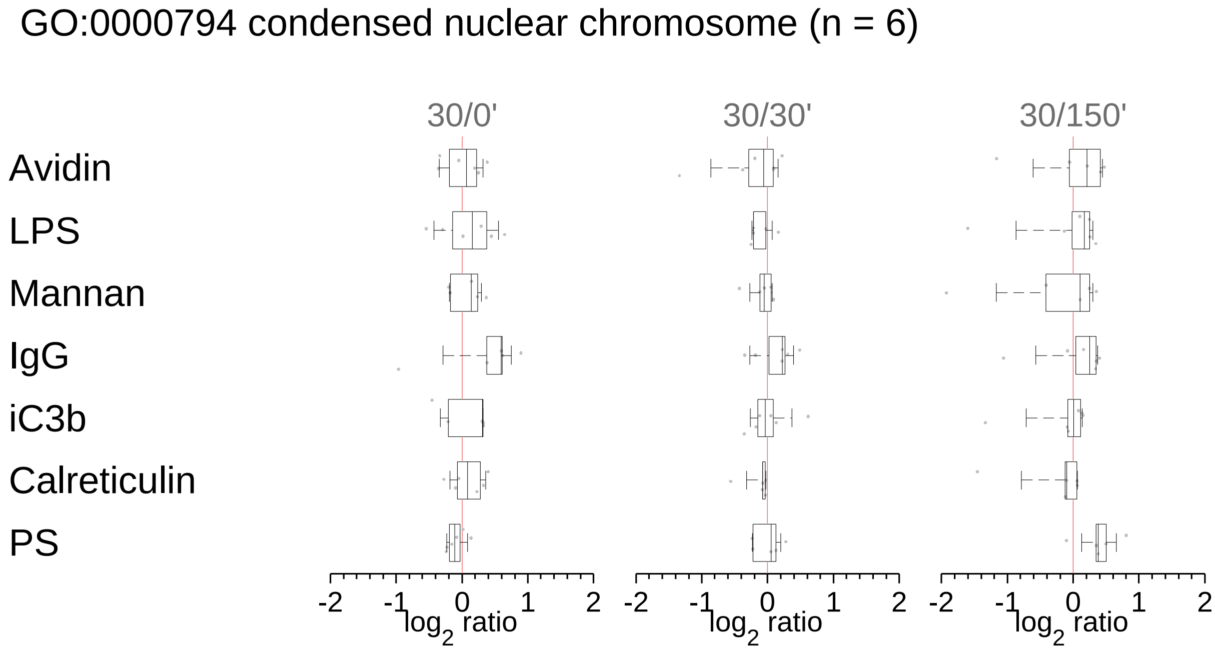
GO:0000794 condensed nuclear chromosome (n = 6)
30/0'
30/30'
30/150'
Avidin
LPS
Mannan
IgG
iC3b
Calreticulin
PS
-2
-1
0
1
2
log2 ratio

GO:0000813 ESCRT I complex (n = 5)

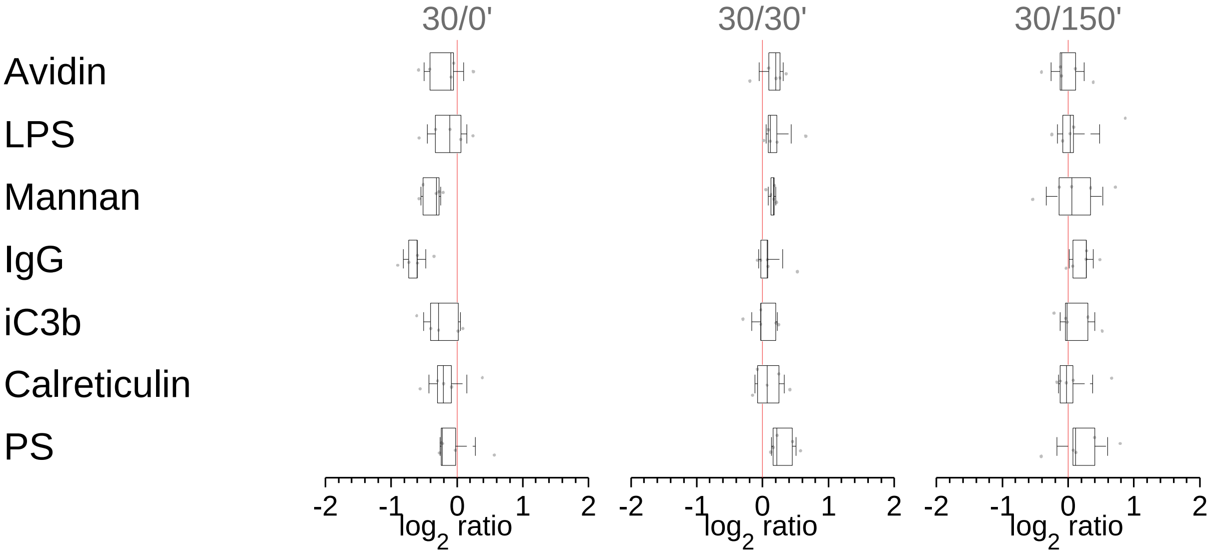

GO:0000902 cell morphogenesis (n = 13)

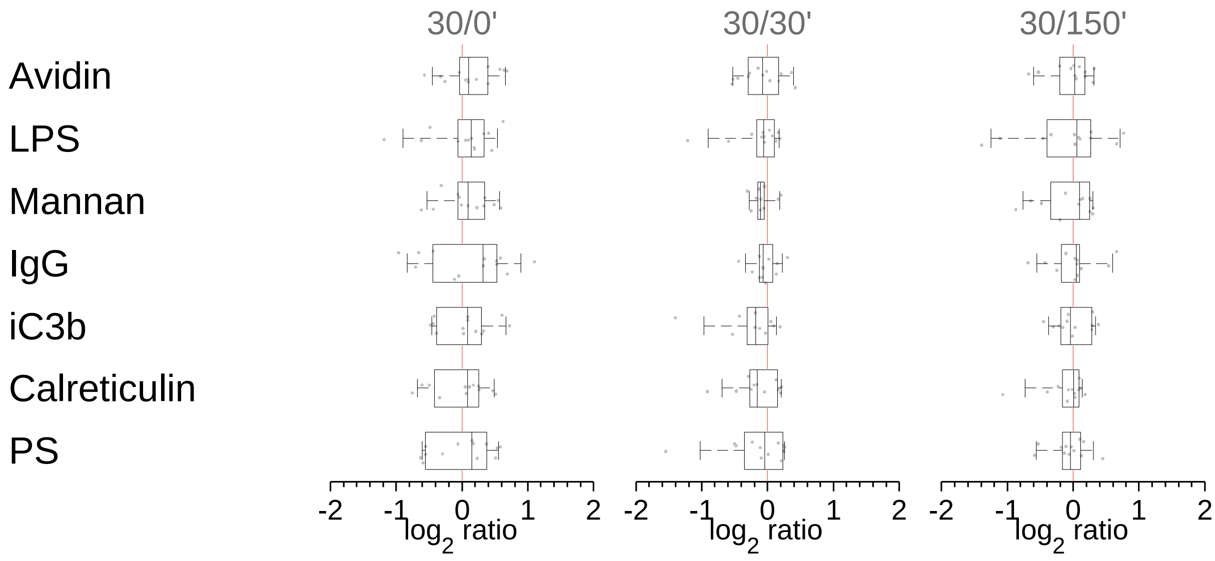

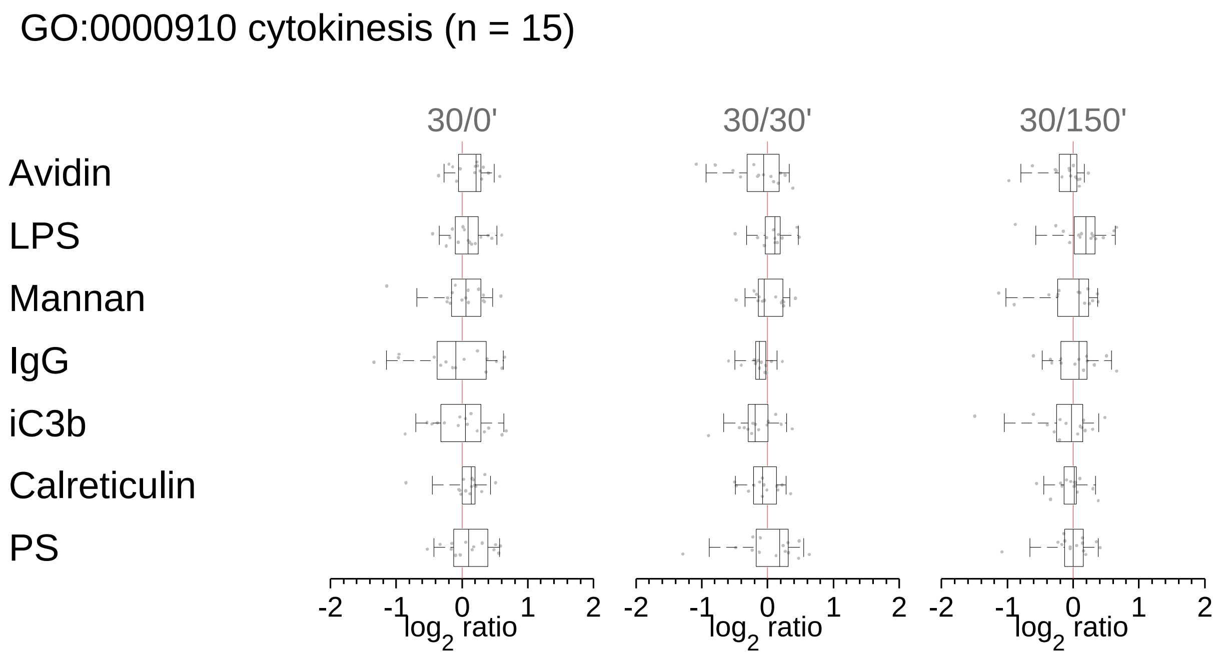
GO:0000910 cytokinesis (n = 15)
30/0'
30/30'
30/150'
Avidin
LPS
Mannan
IgG
iC3b
Calreticulin
PS
-2
-1
0
1
2
log_2 ratio

GO:0000922 spindle pole (n = 5)

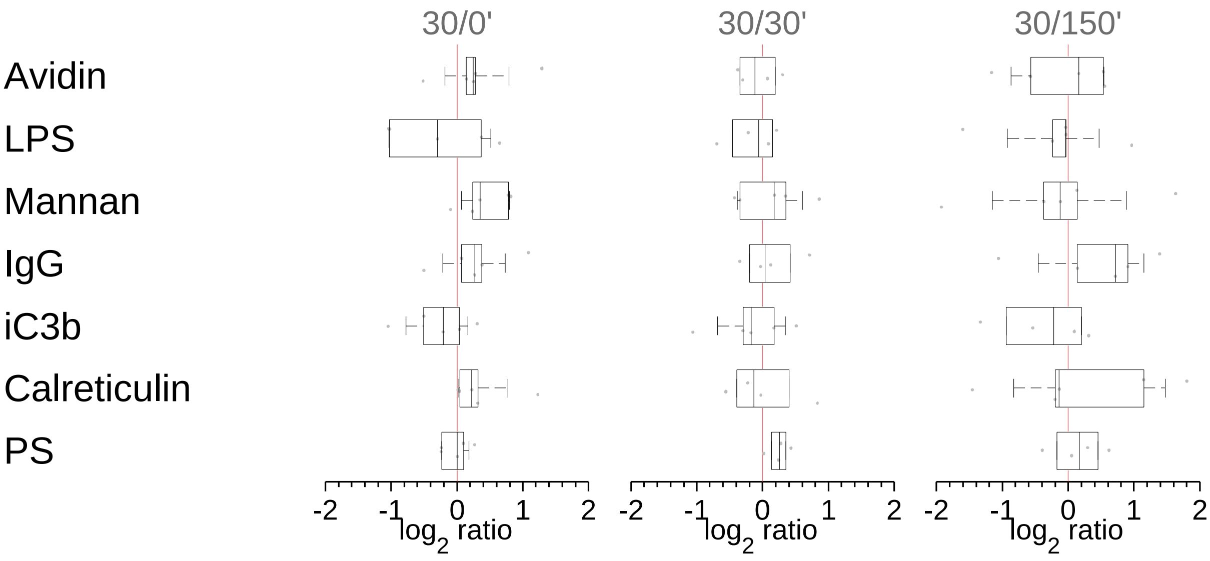

GO:0000932 cytoplasmic mRNA processing body (n = 5)

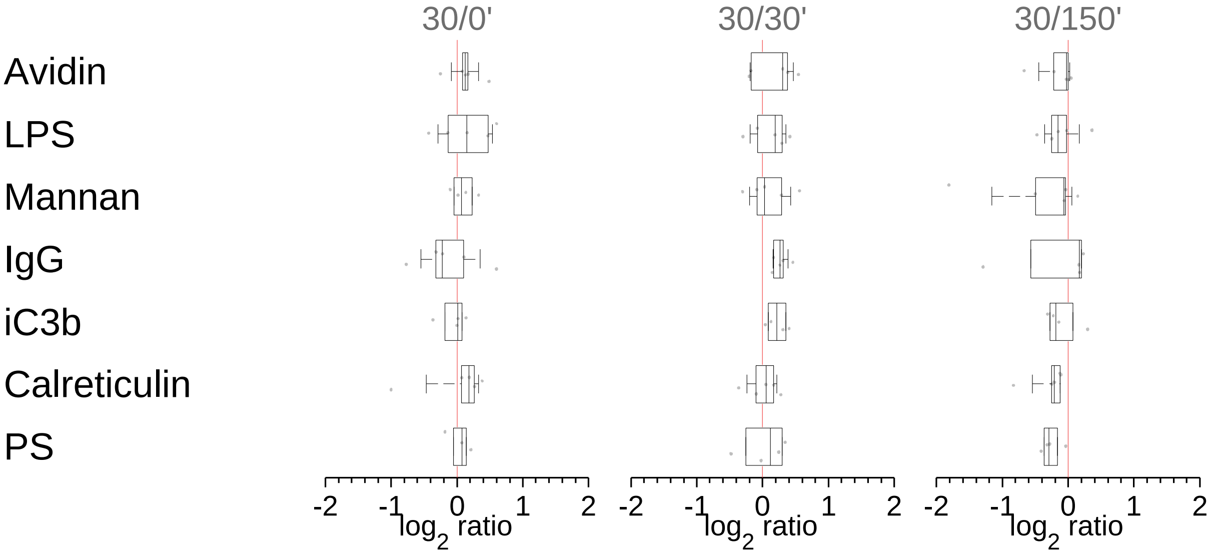

GO:0000978 RNA polymerase II core promoter proximal region sequence-specific DNA binding (n = 9)

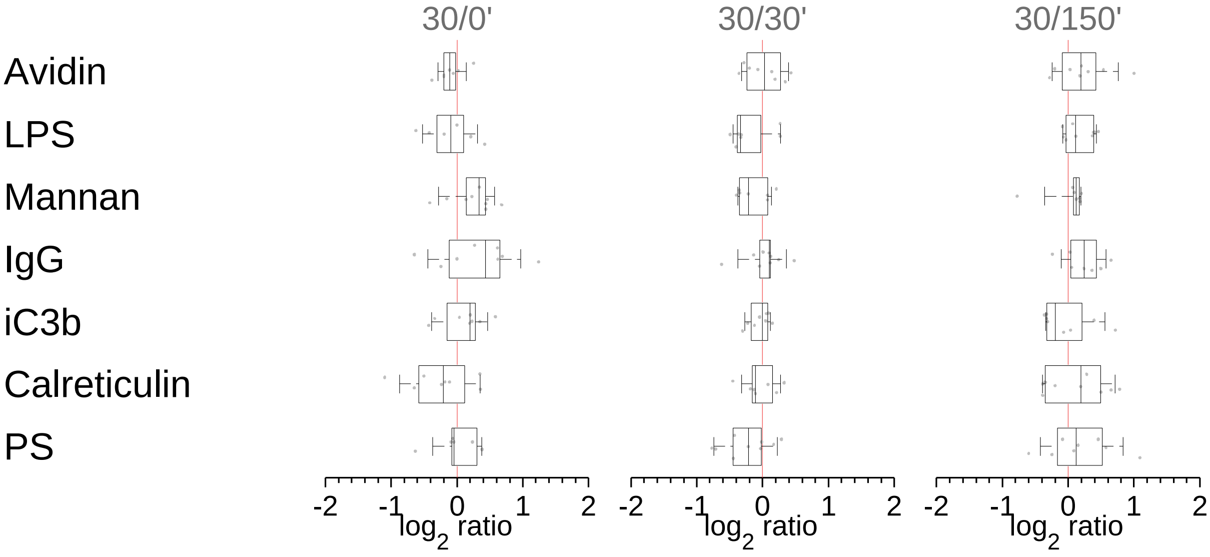

GO:0000980 RNA polymerase II distal enhancer sequence-specific DNA binding (n = 6)

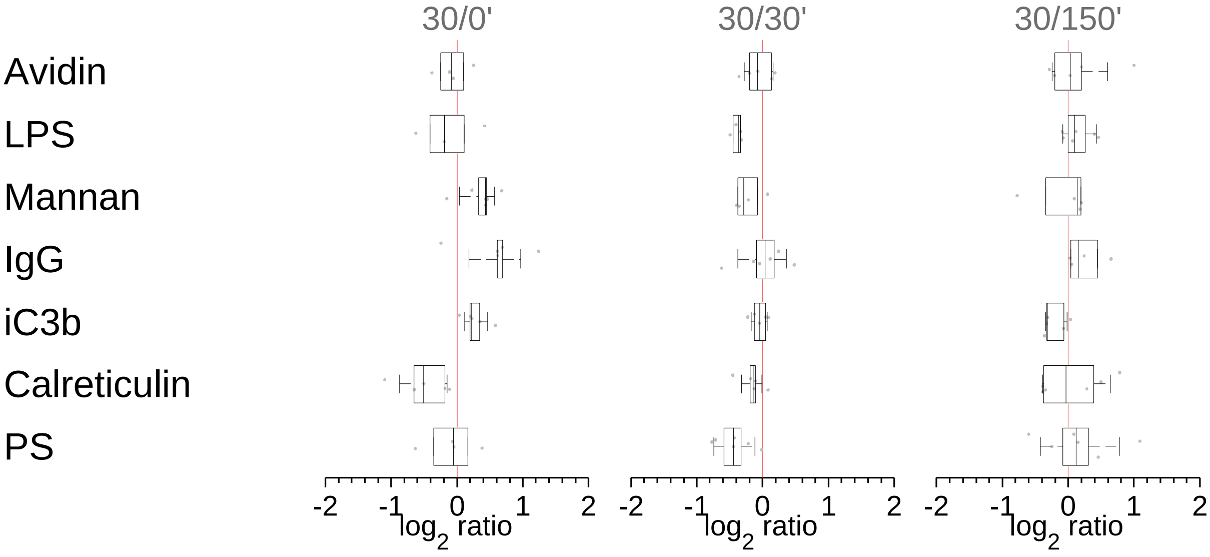

GO:0001047 core promoter binding (n = 4)

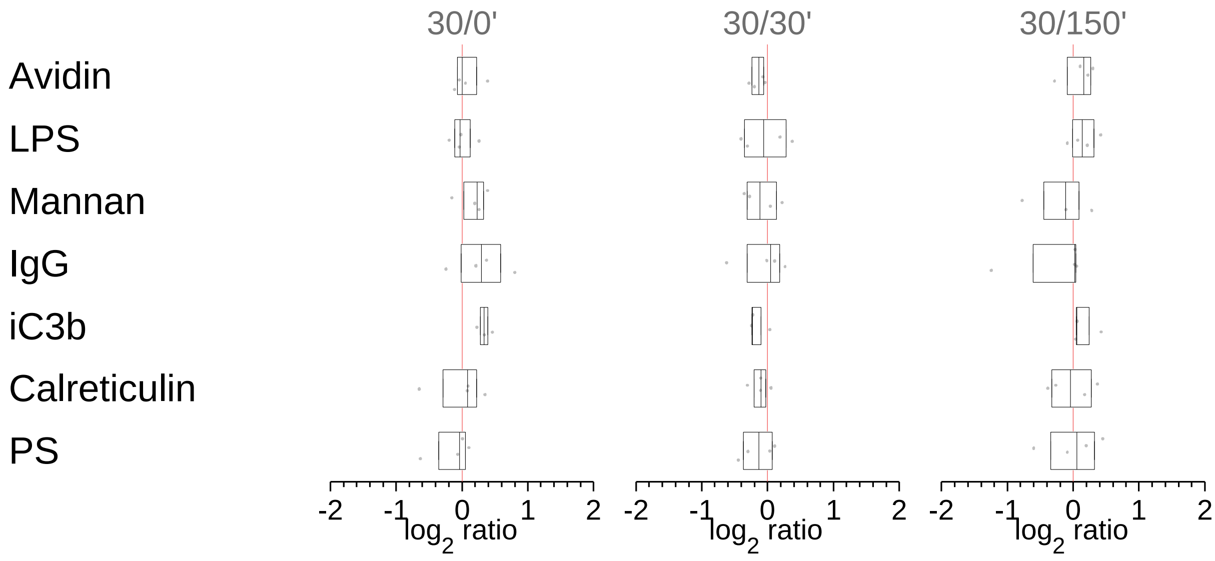

GO:0001085 RNA polymerase II transcription factor binding (n = 6)

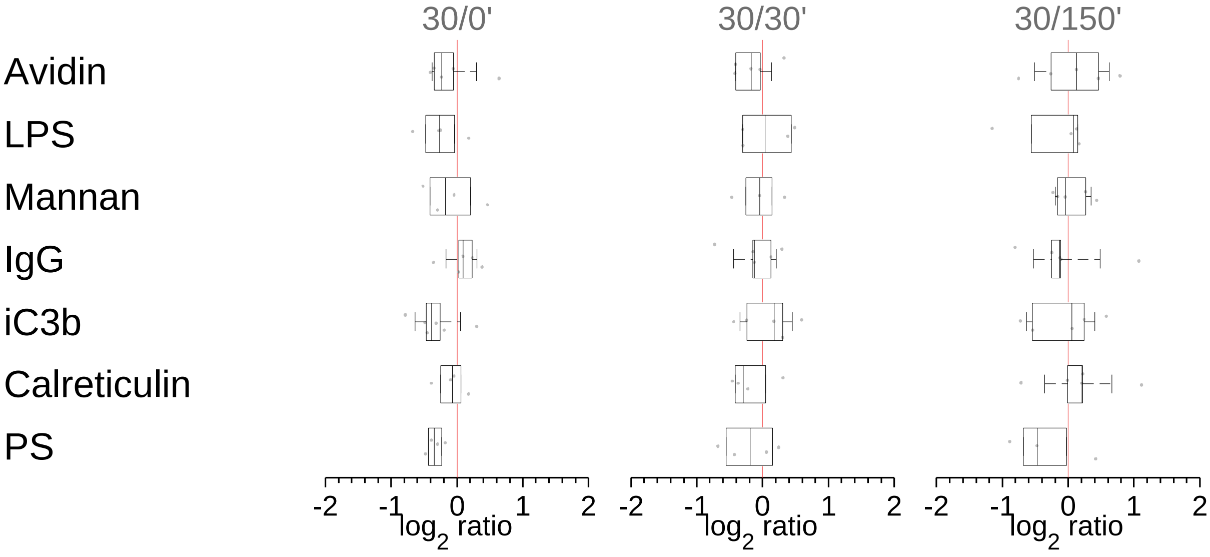

GO:0001205 RNA polymerase II distal enhancer sequence-specific DNA binding transcription factor activity involved in positive regulation of transcription (n = 3)

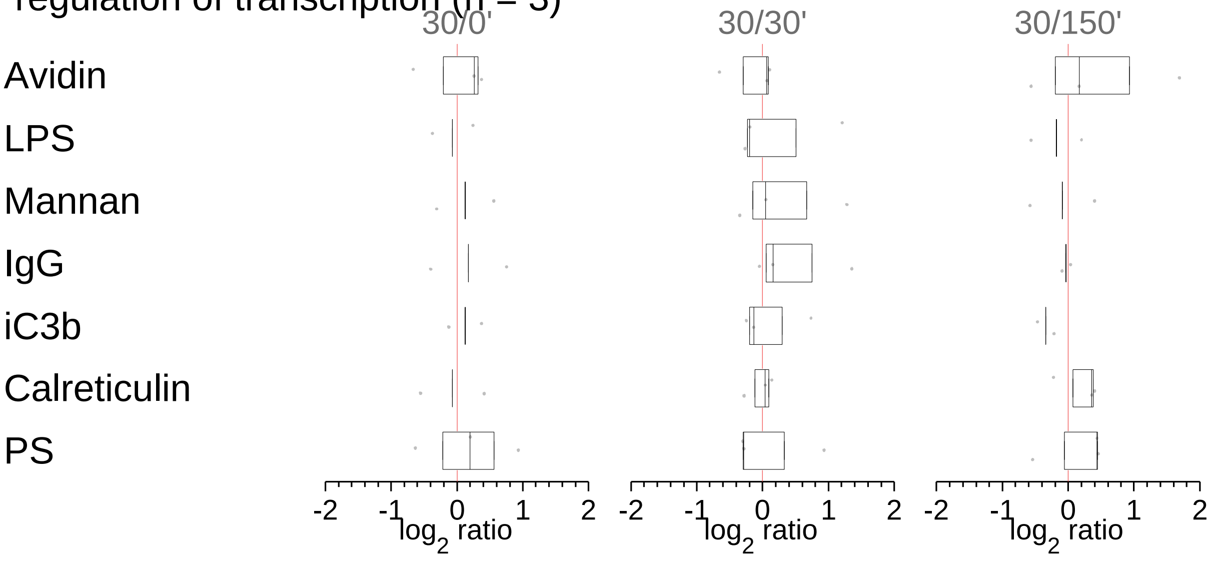

GO:0001501 skeletal system development (n = 9)

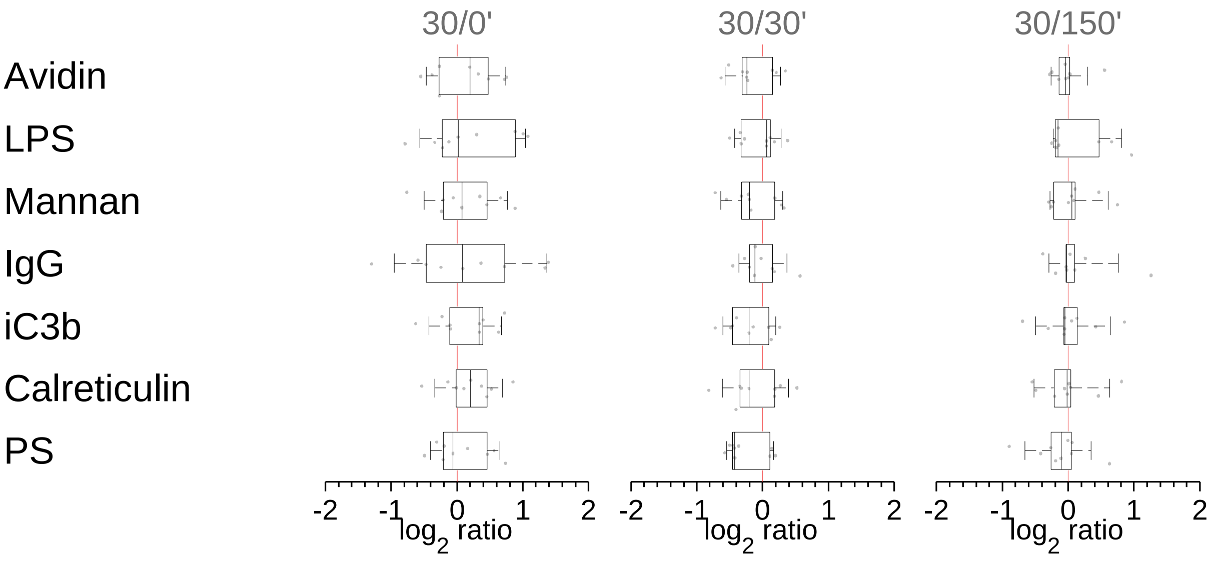

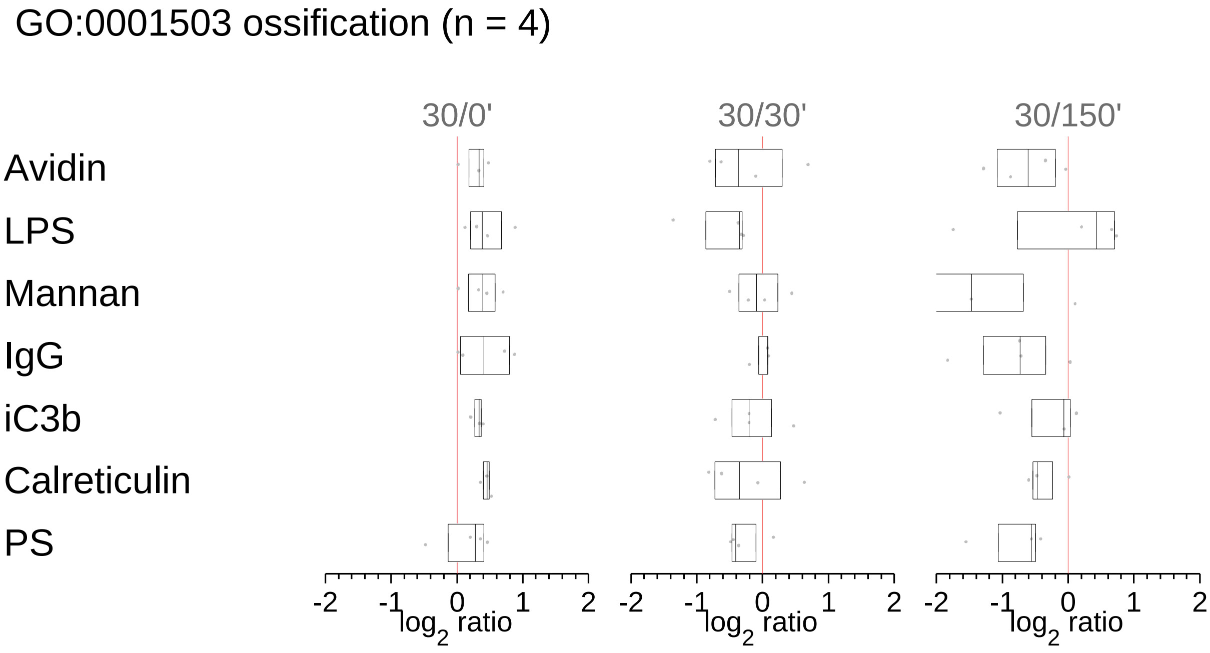
GO:0001503 ossification (n = 4)
30/0'
30/30'
30/150'
Avidin
LPS
Mannan
IgG
iC3b
Calreticulin
PS
-2
-1
0
1
2
$\log_2$ ratio

GO:0001516 prostaglandin biosynthetic process (n = 3)

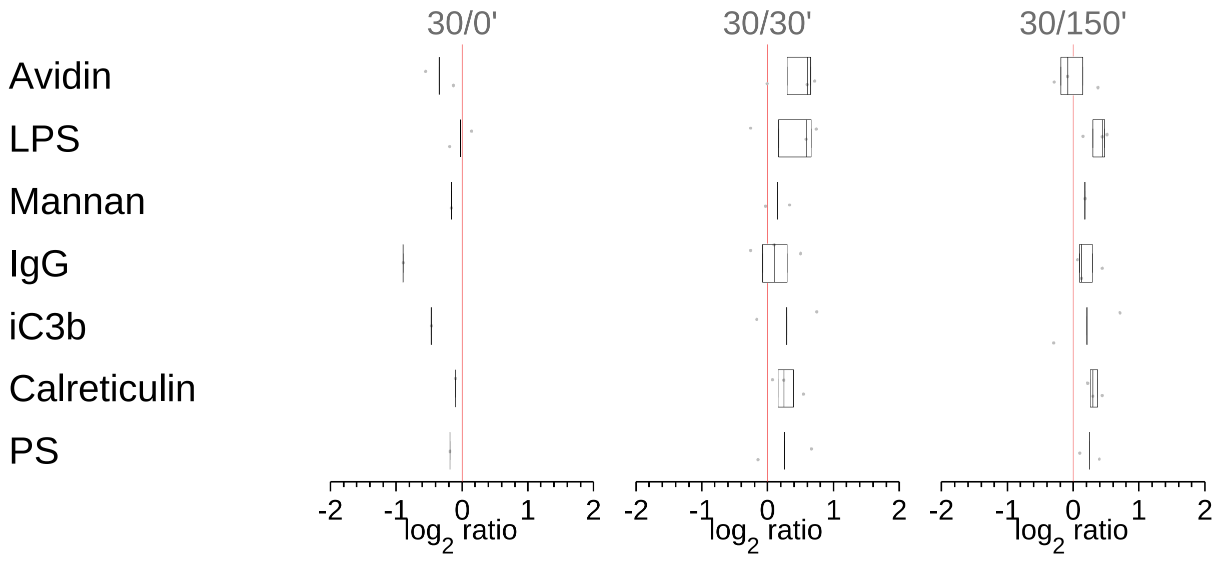

GO:0001525 angiogenesis (n = 26)

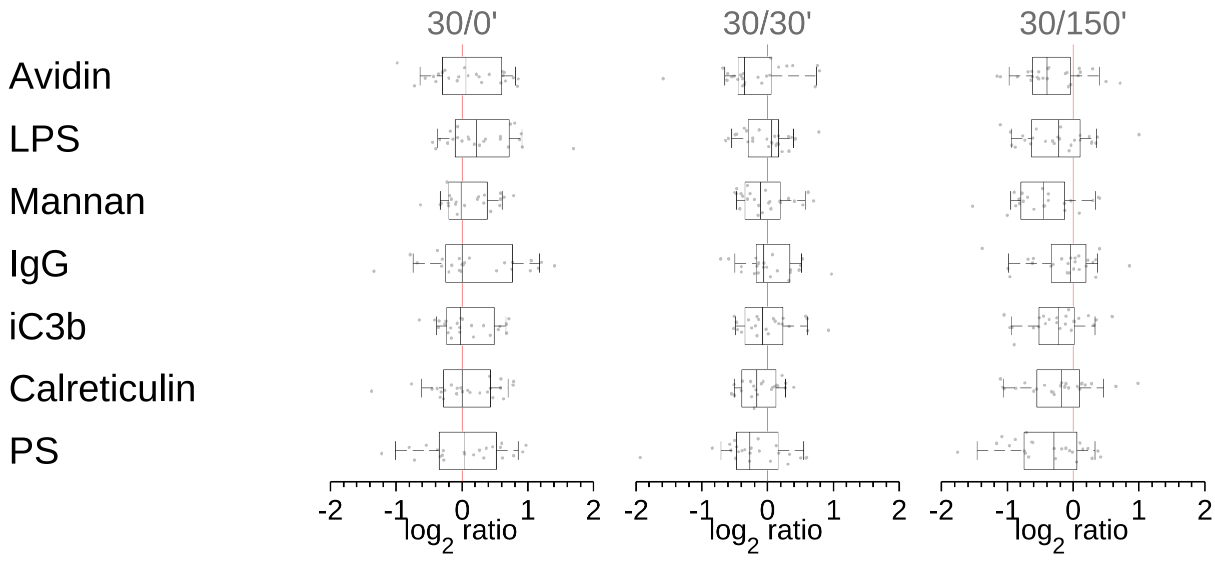

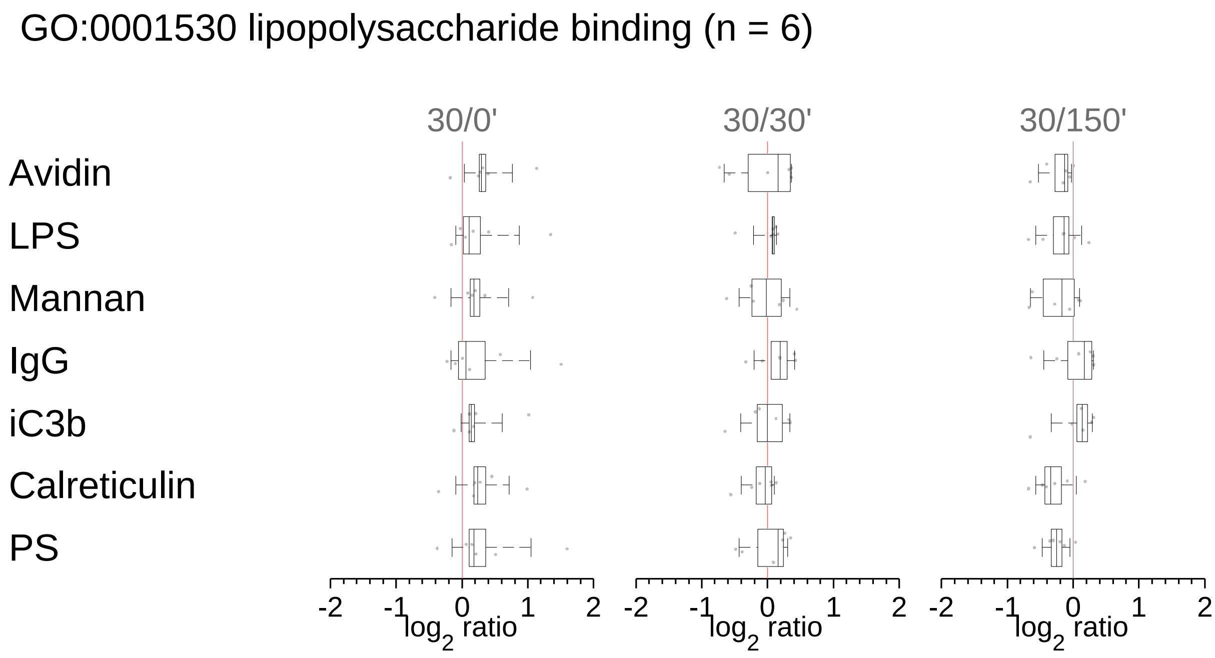
GO:0001530 lipopolysaccharide binding (n = 6)
30/0'
30/30'
30/150'
Avidin
LPS
Mannan
IgG
iC3b
Calreticulin
PS
-2
-1
0
1
2
log2 ratio

GO:0001540 beta-amyloid binding (n = 6)

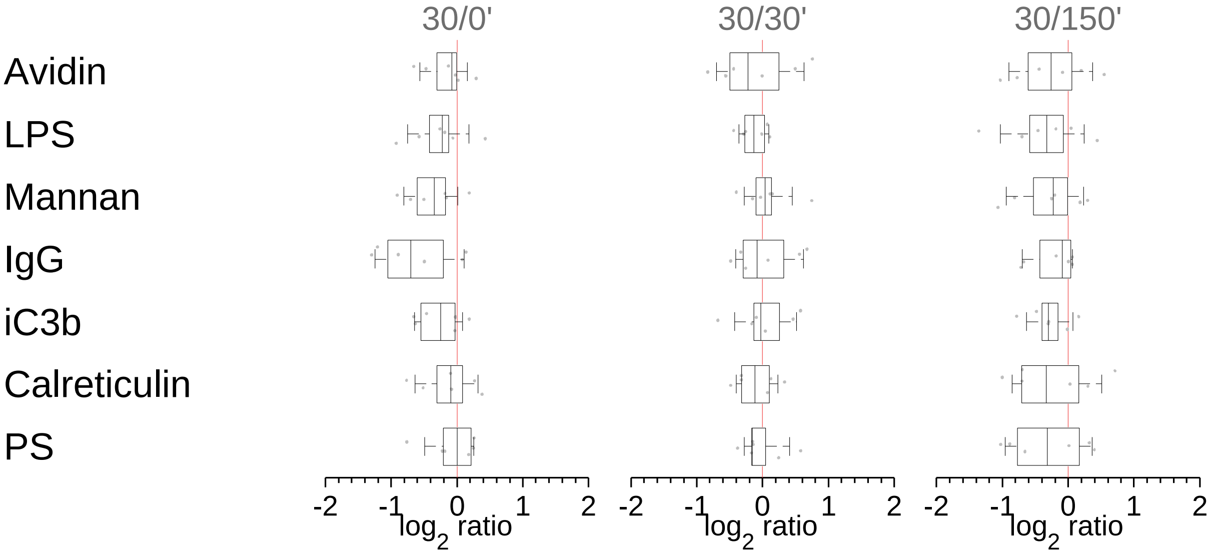

GO:0001558 regulation of cell growth (n = 3)

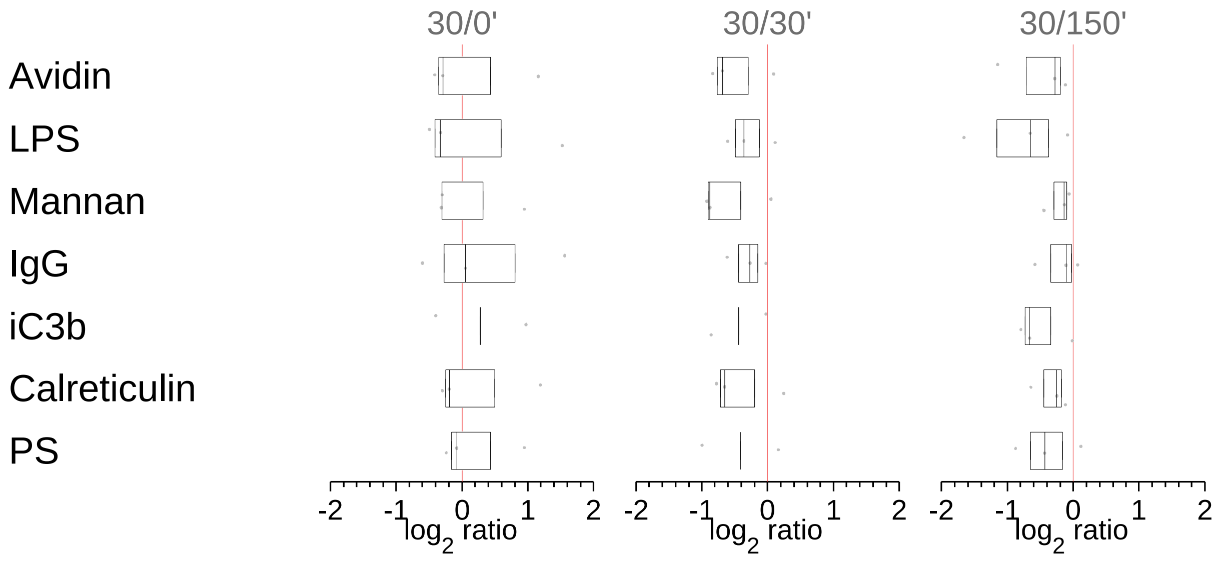

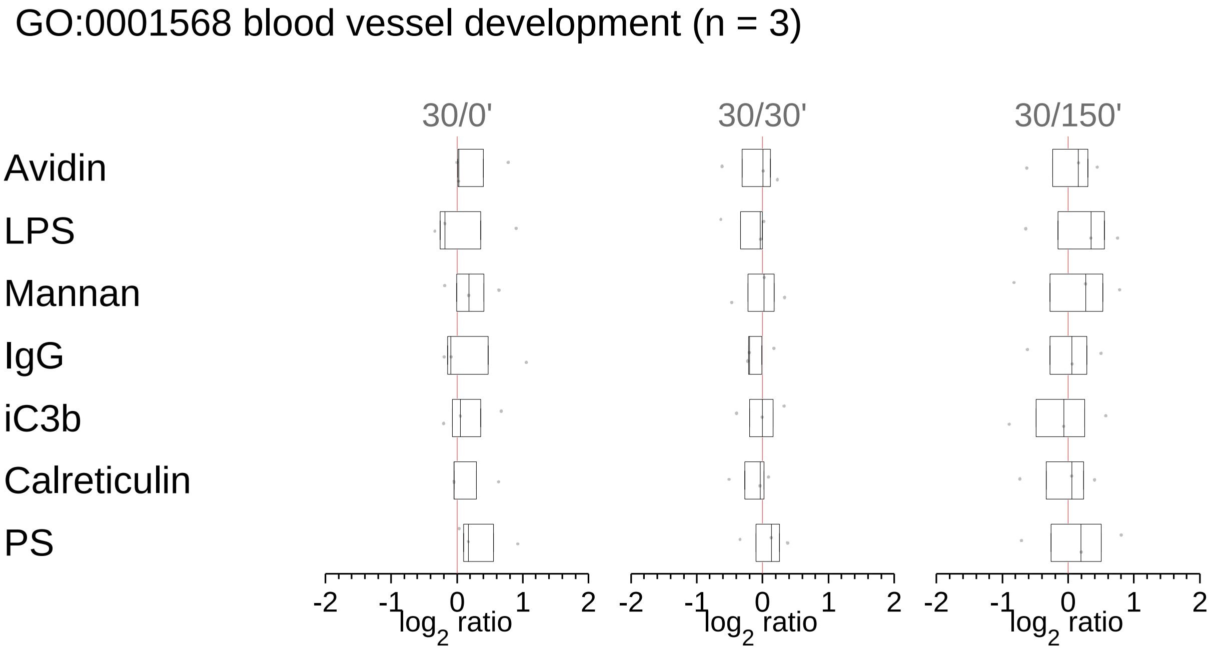
GO:0001568 blood vessel development (n = 3)
30/0'
30/30'
30/150'
Avidin
LPS
Mannan
IgG
iC3b
Calreticulin
PS
-2
-1
0
1
2
log2 ratio

GO:0001570 vasculogenesis (n = 5)

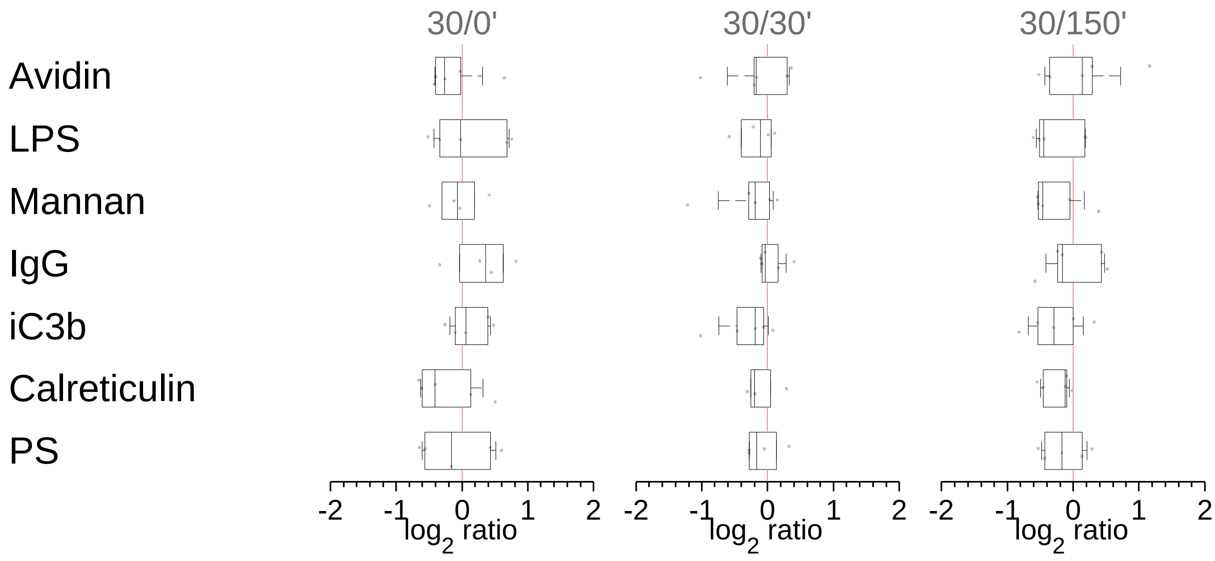

GO:0001649 osteoblast differentiation (n = 17)

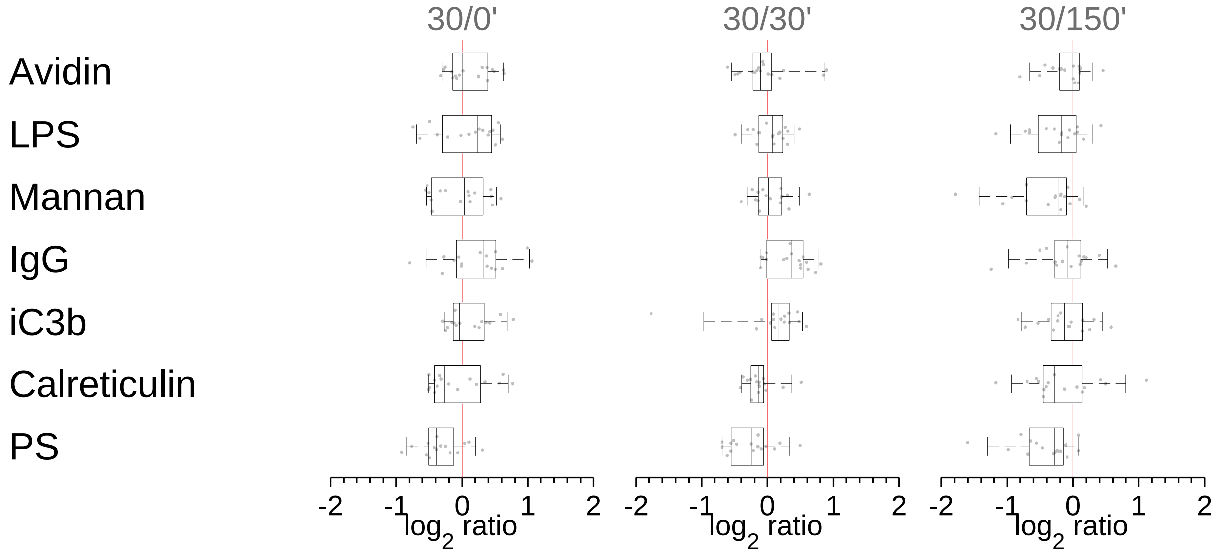

GO:0001659 temperature homeostasis (n = 3)

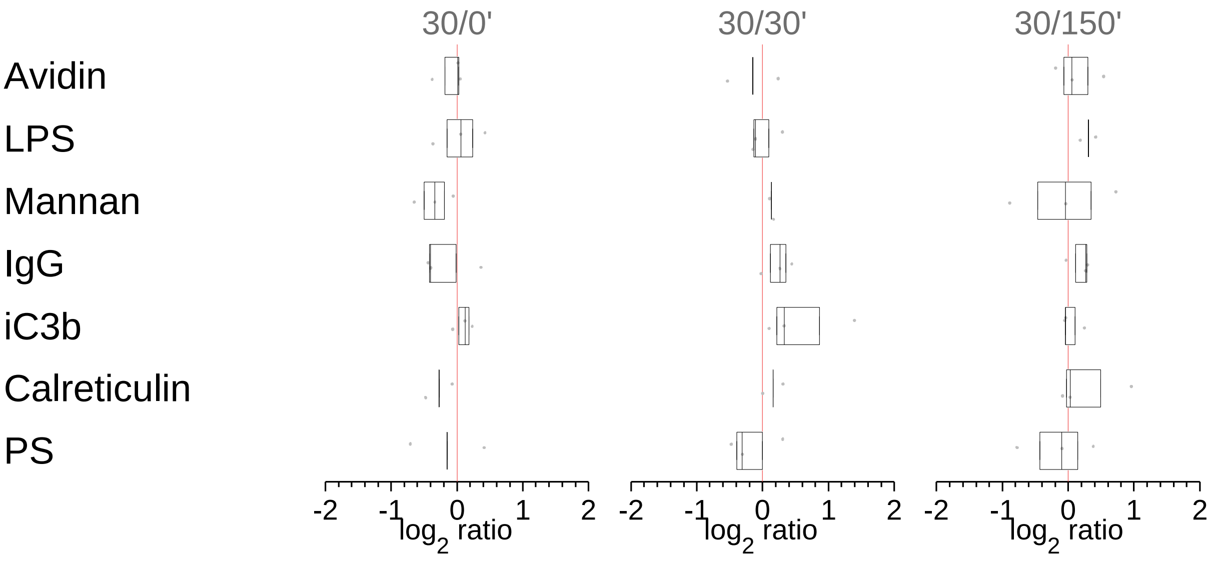

GO:0001662 behavioral fear response (n = 3)

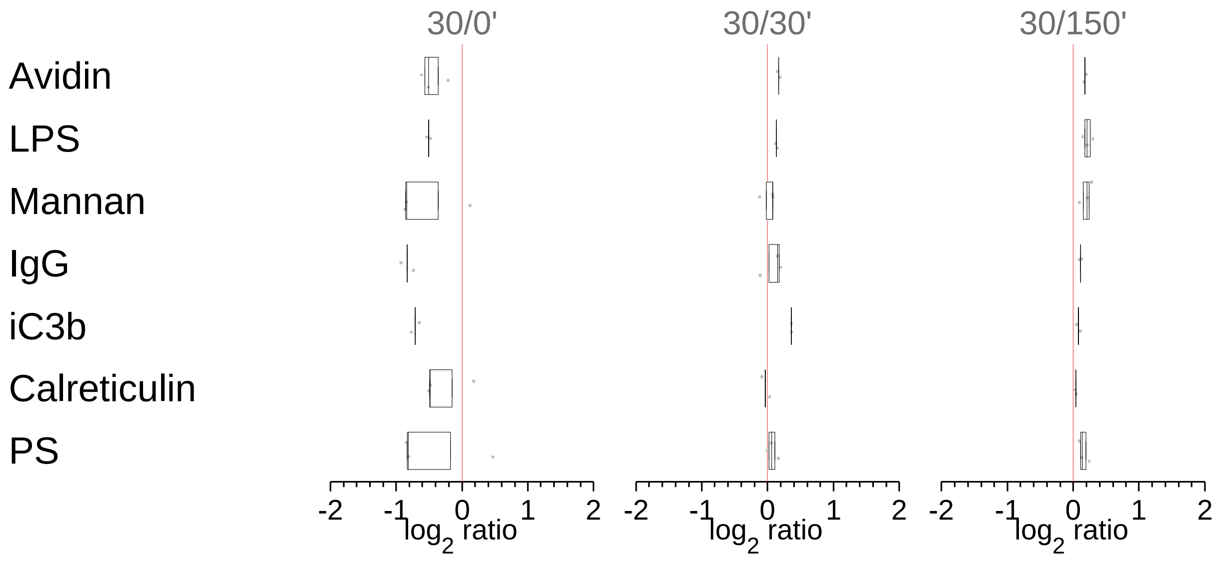

GO:0001664 G-protein coupled receptor binding (n = 6)

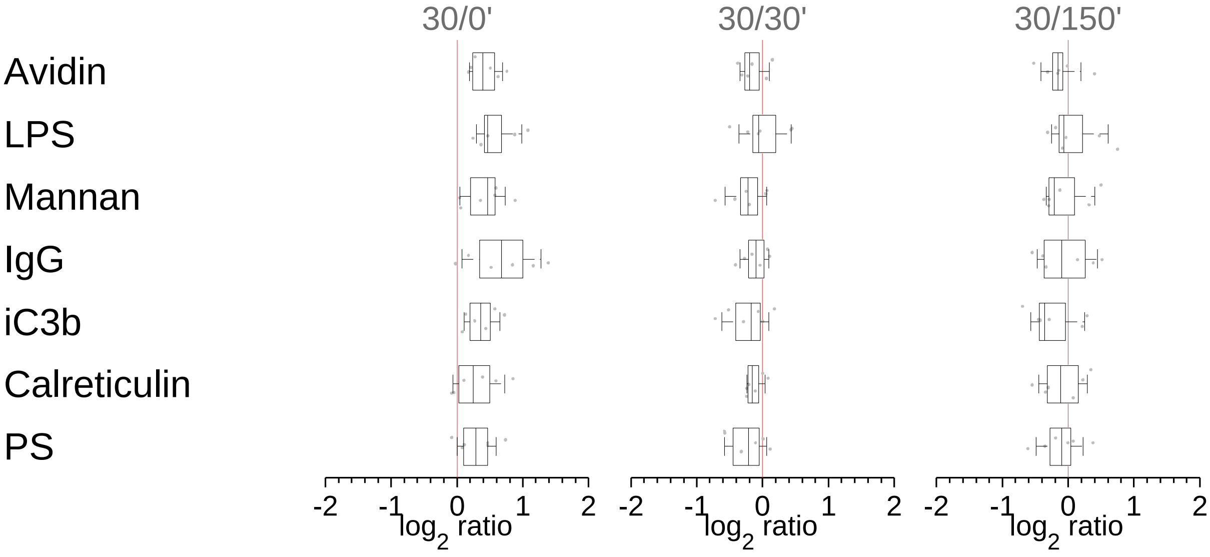

GO:0001666 response to hypoxia (n = 9)

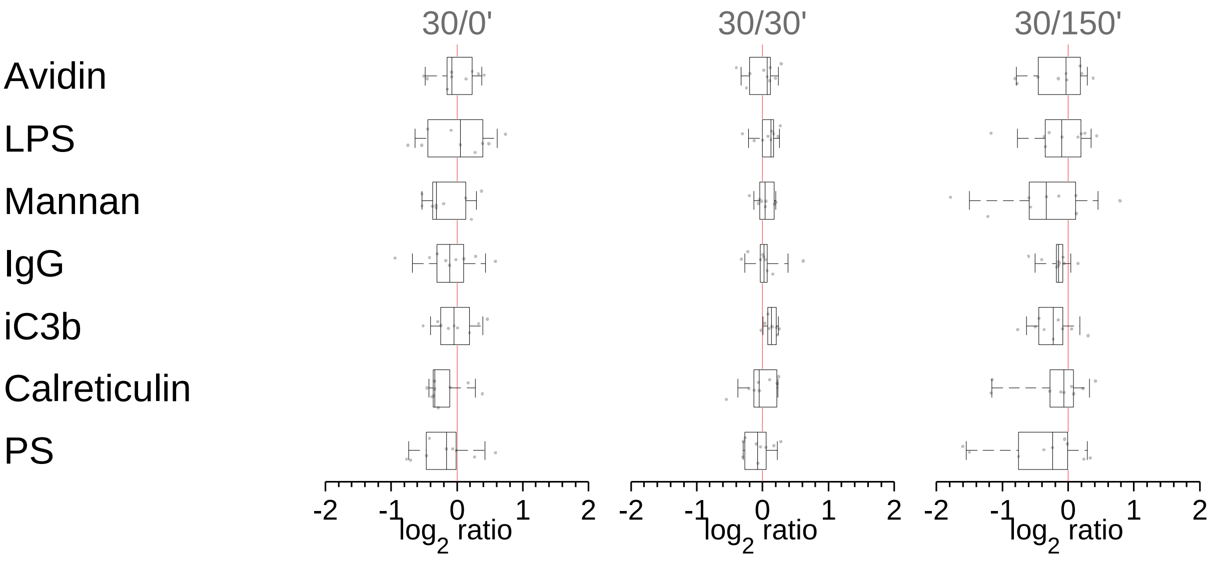

GO:0001669 acrosomal vesicle (n = 10)

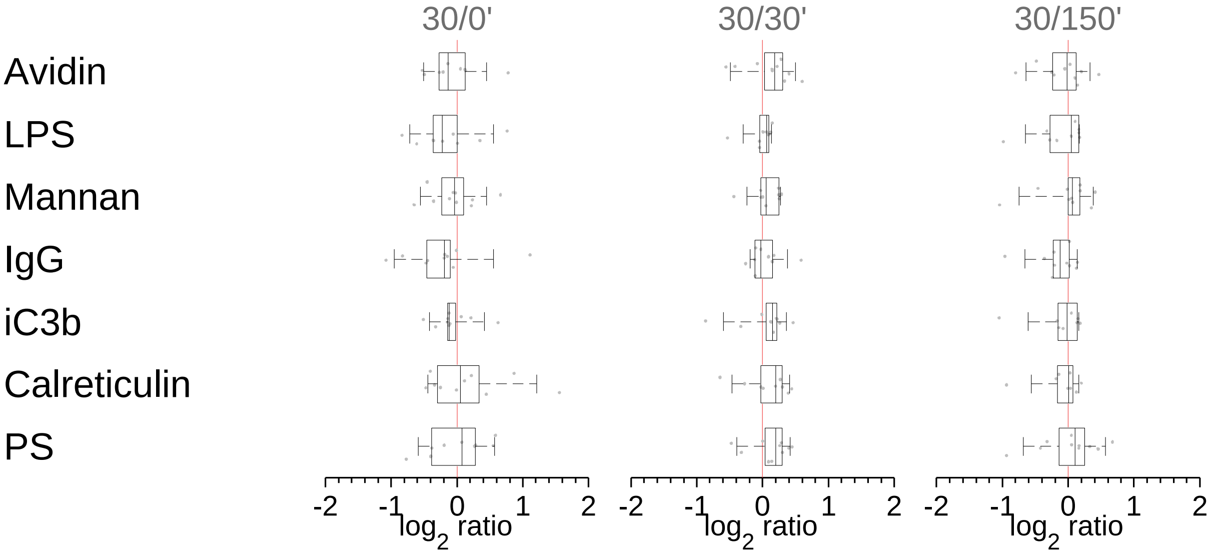

GO:0001671 ATPase activator activity (n = 5)

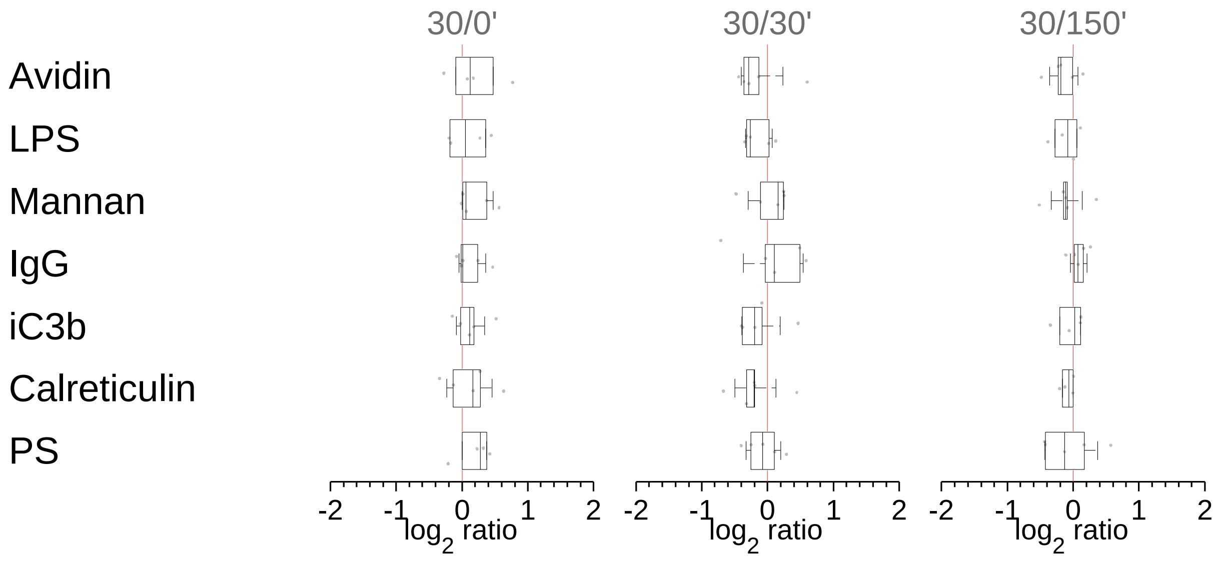

GO:0001676 long-chain fatty acid metabolic process (n = 5)

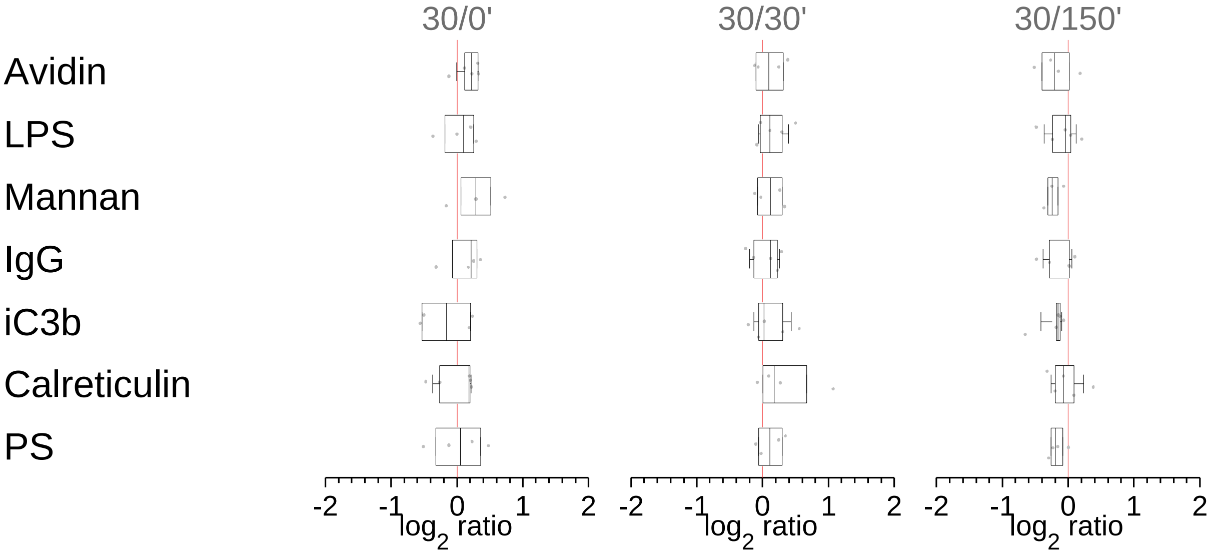

GO:0001701 in utero embryonic development (n = 26)

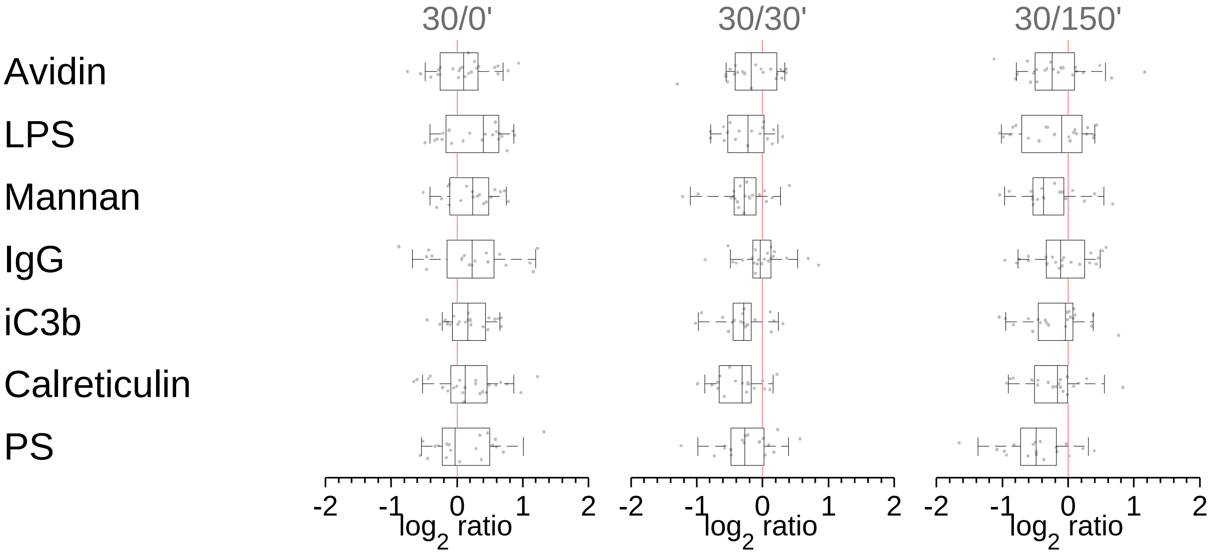

GO:0001707 mesoderm formation (n = 3)

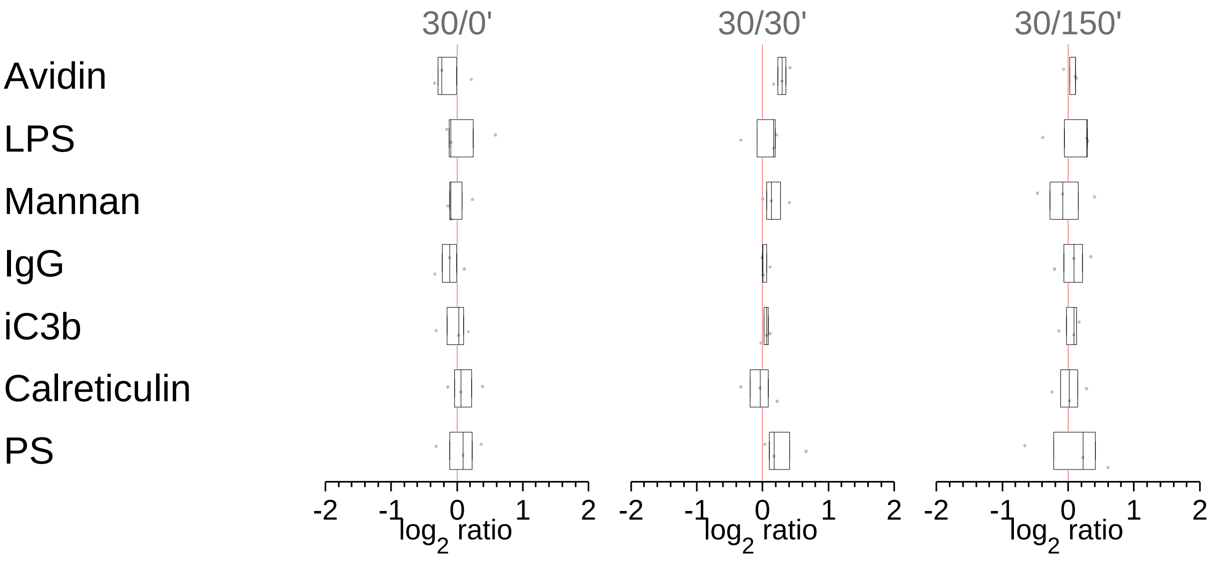

GO:0001708 cell fate specification (n = 4)

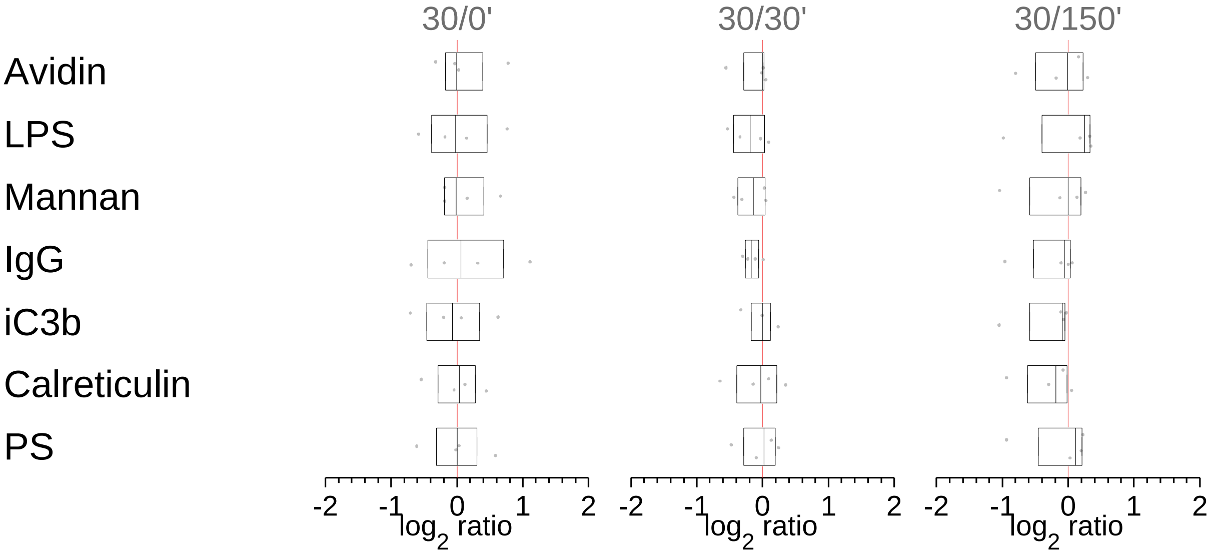

GO:0001725 stress fiber (n = 20)

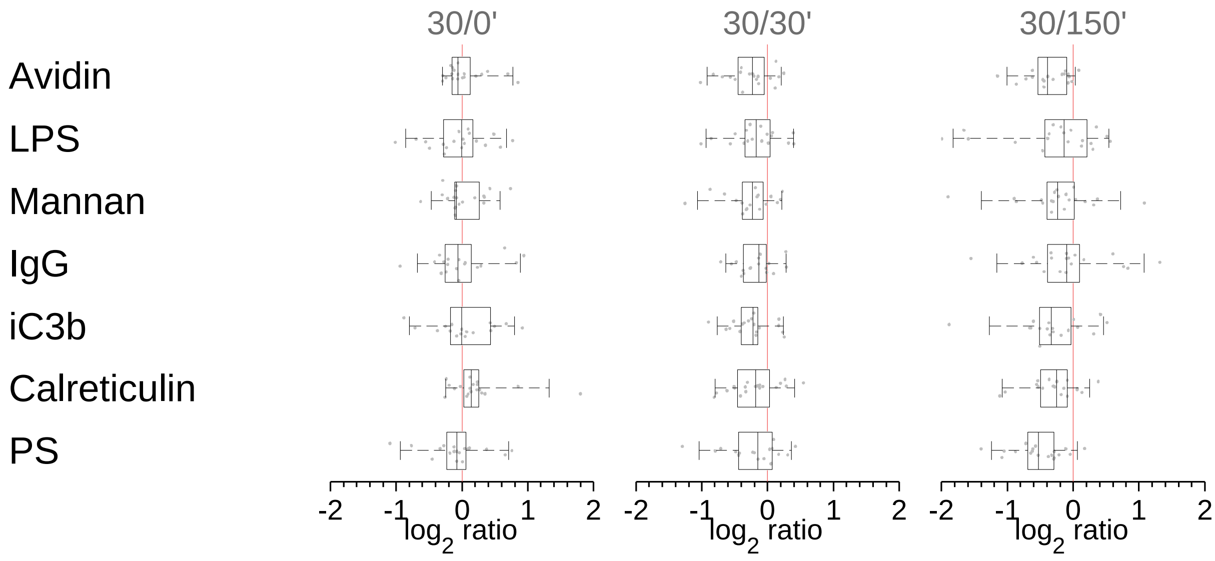

GO:0001726 ruffle (n = 22)

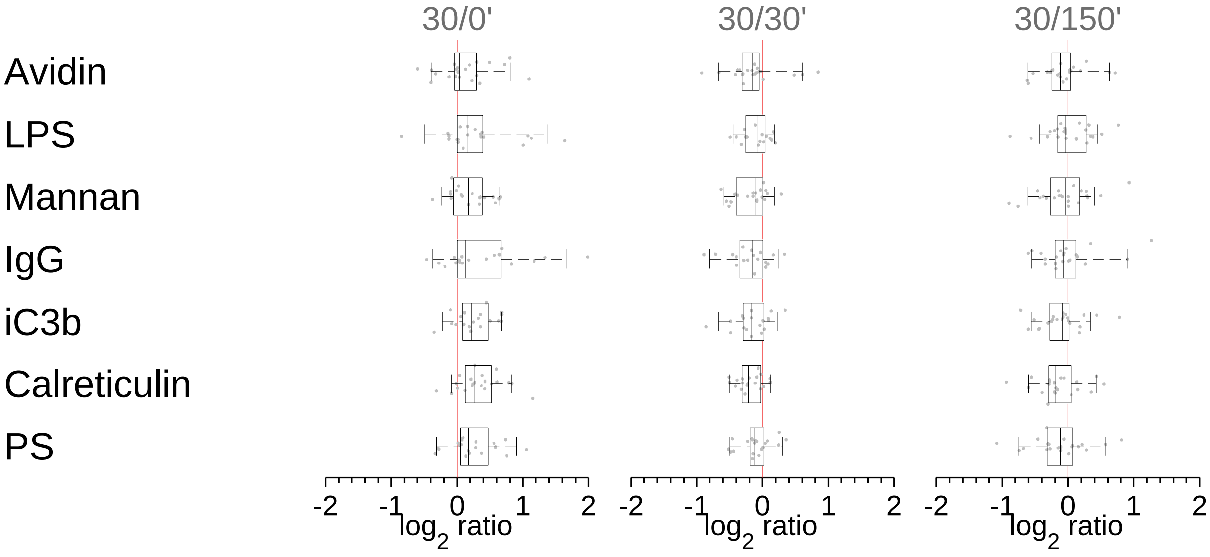

GO:0001750 photoreceptor outer segment (n = 5)

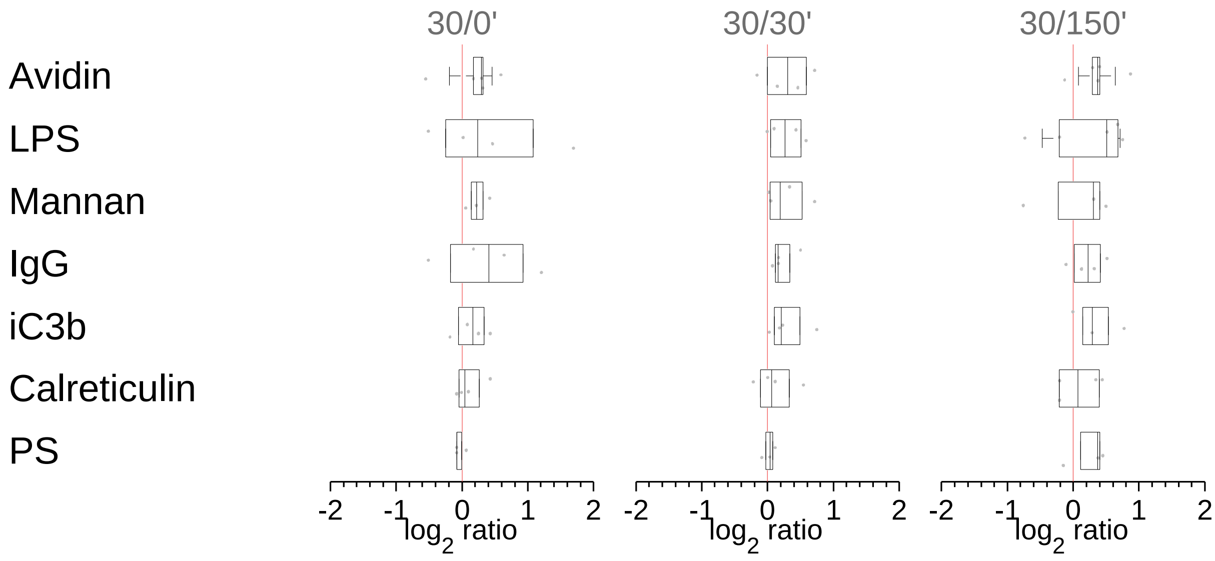

GO:0001756 somitogenesis (n = 4)

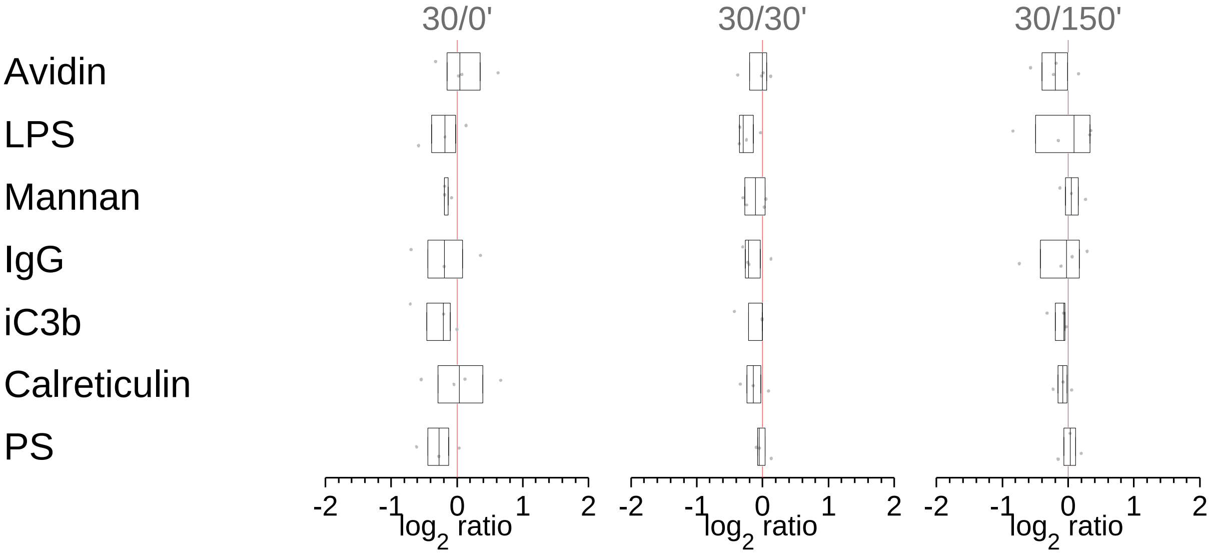

GO:0001764 neuron migration (n = 10)

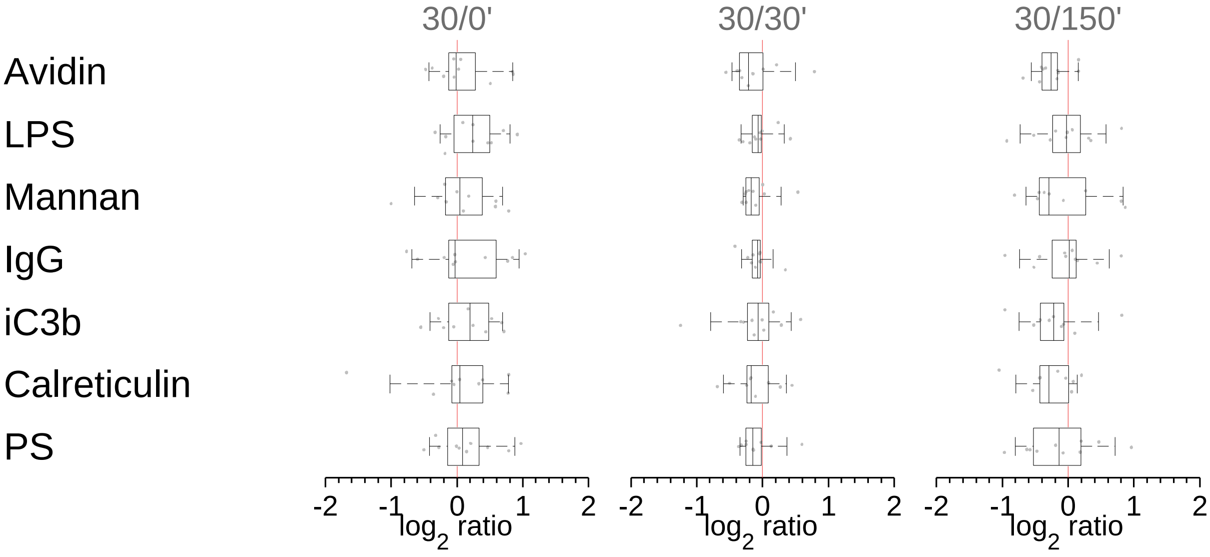

GO:0001765 membrane raft assembly (n = 3)

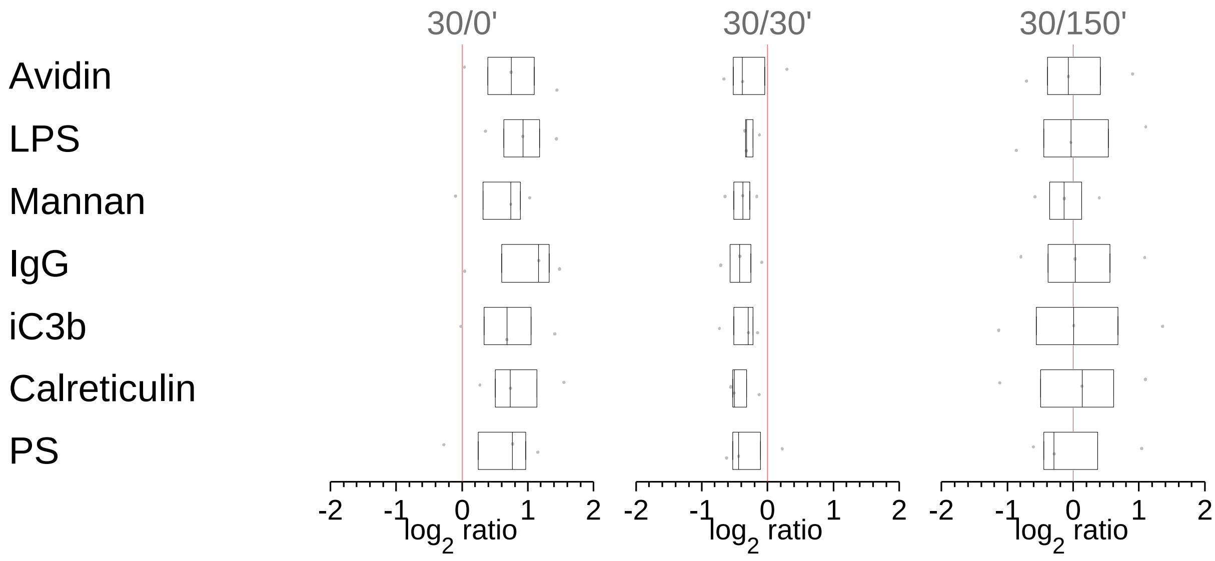

GO:0001772 immunological synapse (n = 10)

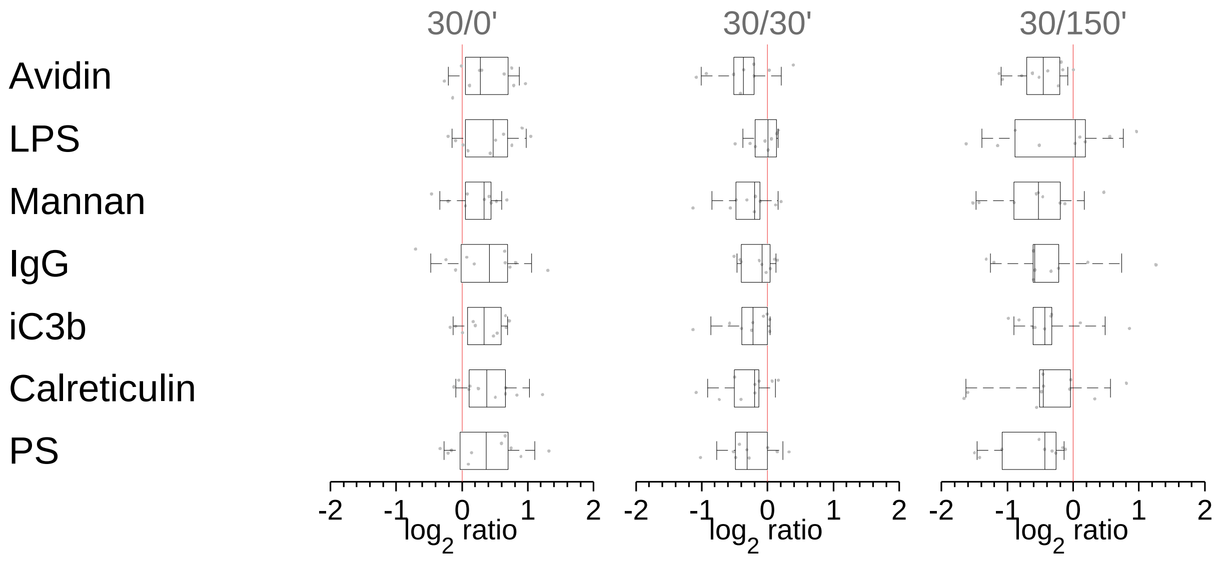

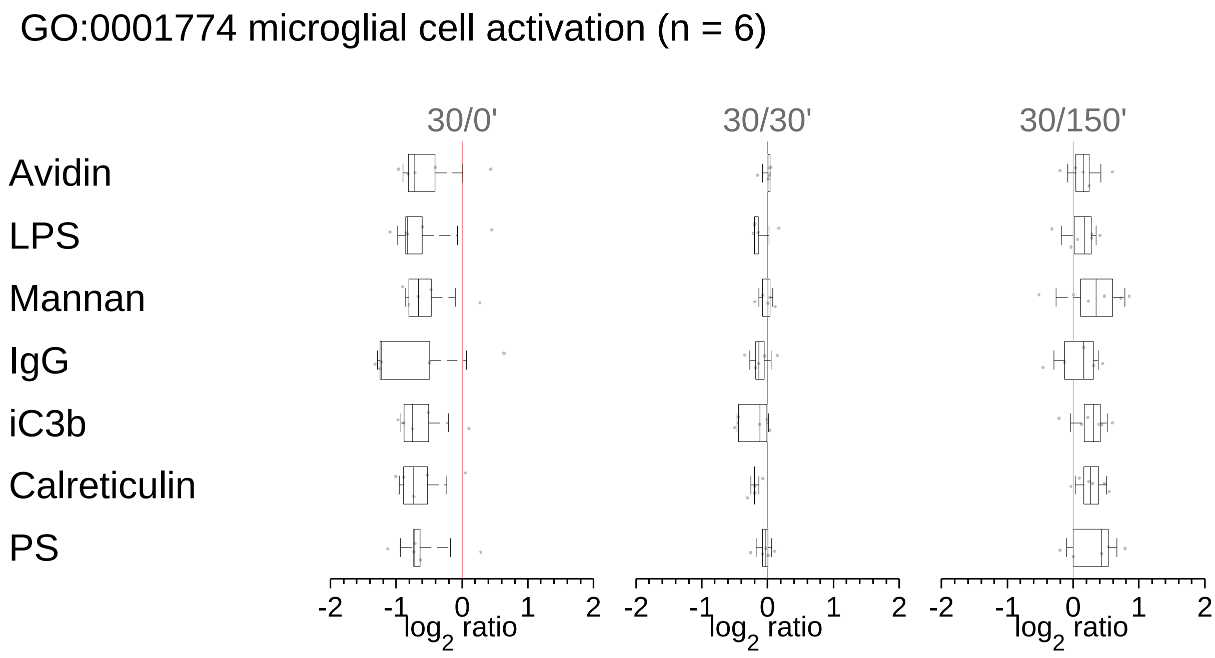
GO:0001774 microglial cell activation (n = 6)
30/0'
30/30'
30/150'
Avidin
LPS
Mannan
IgG
iC3b
Calreticulin
PS
-2 -1 0 1 2
log2 ratio

GO:0001782 B cell homeostasis (n = 4)

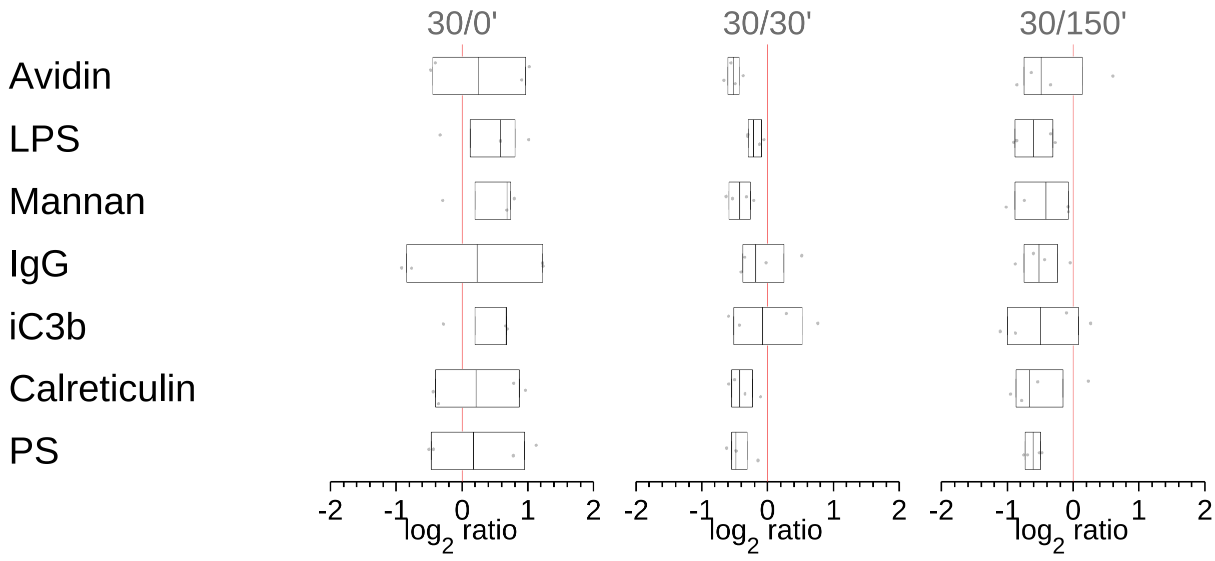

GO:0001784 phosphotyrosine binding (n = 4)

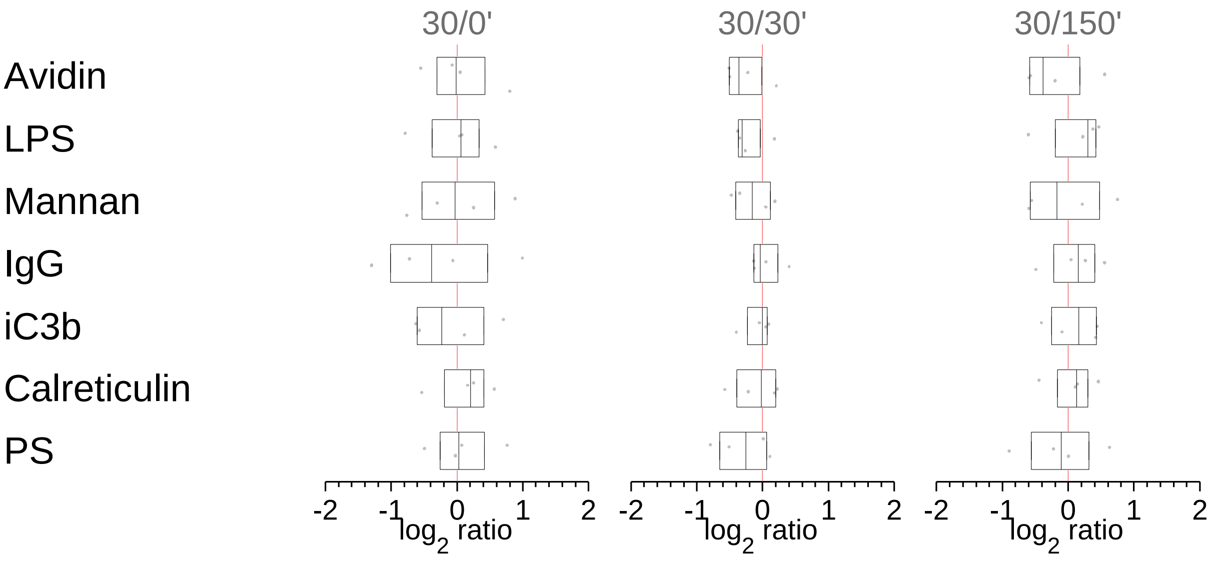

GO:0001786 phosphatidylserine binding (n = 4)

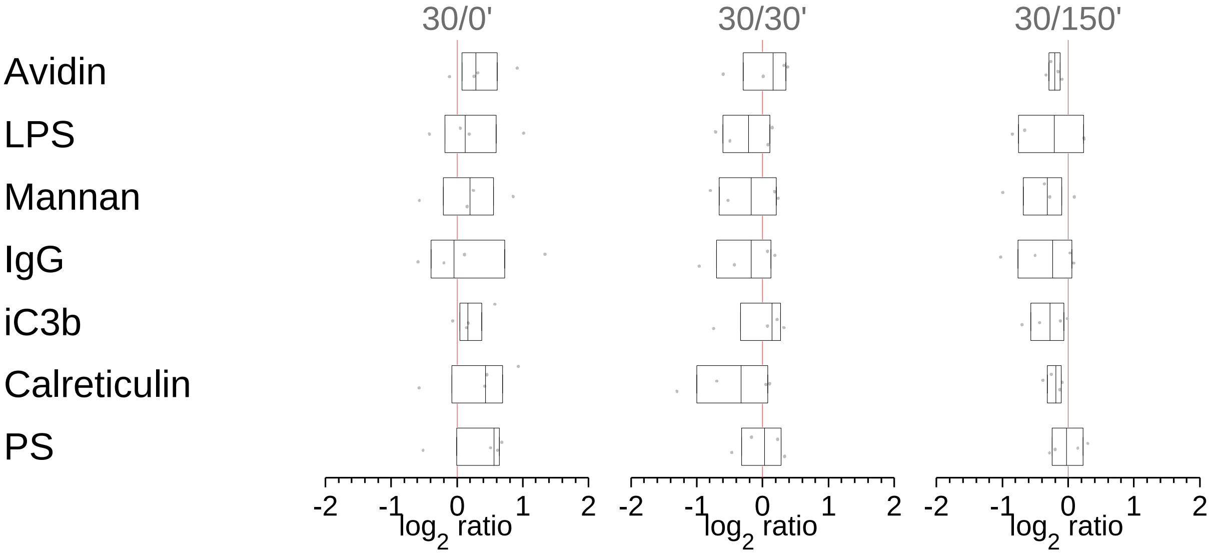

GO:0001798 positive regulation of type IIa hypersensitivity (n = 4)

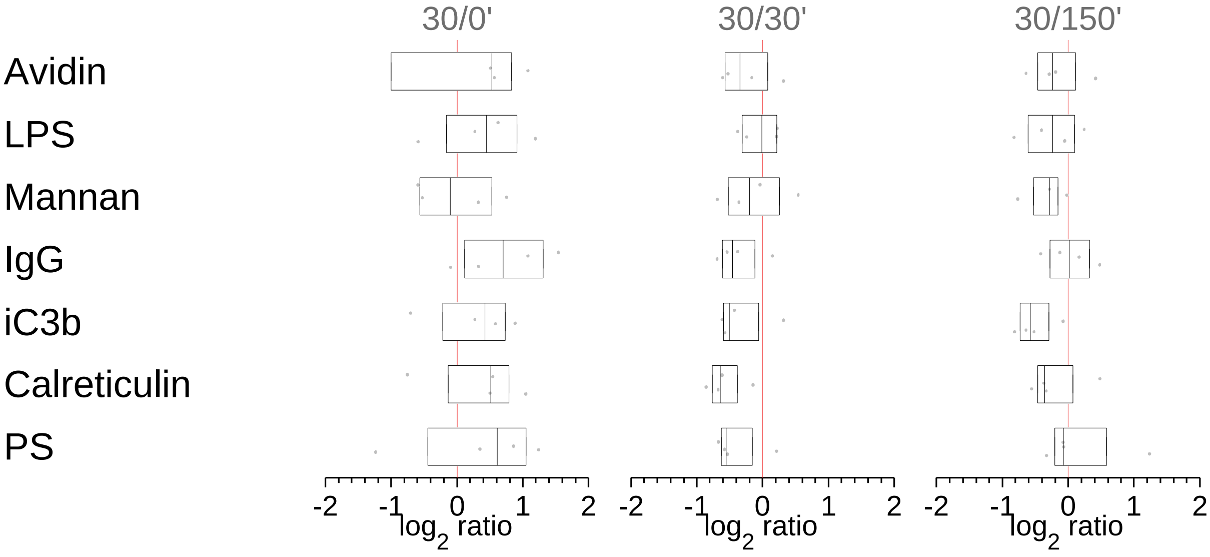

GO:0001805 positive regulation of type III hypersensitivity (n = 3)

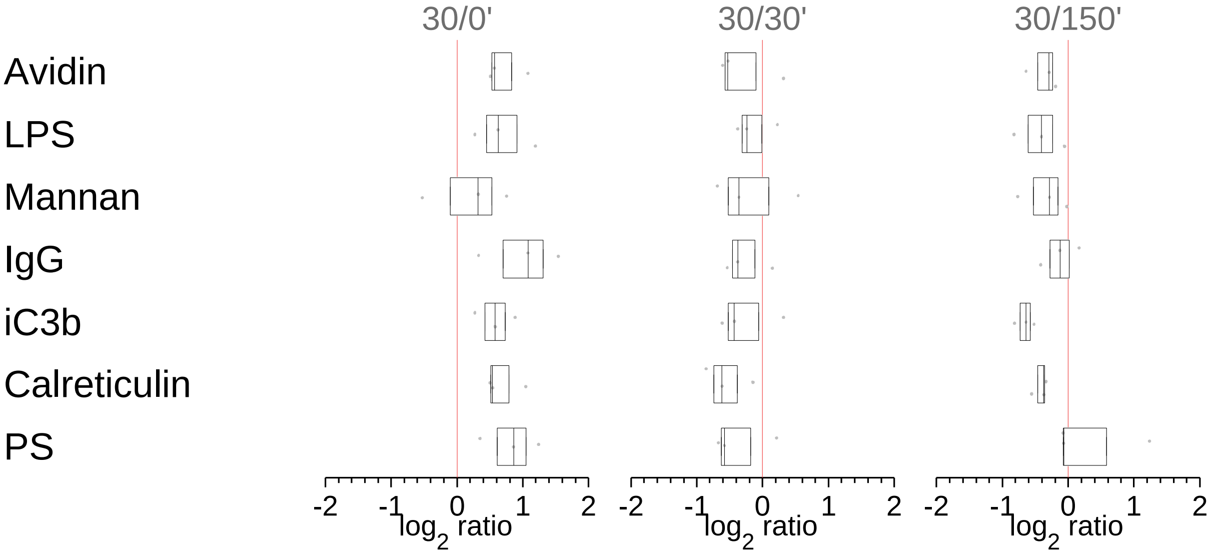

GO:0001816 cytokine production (n = 3)

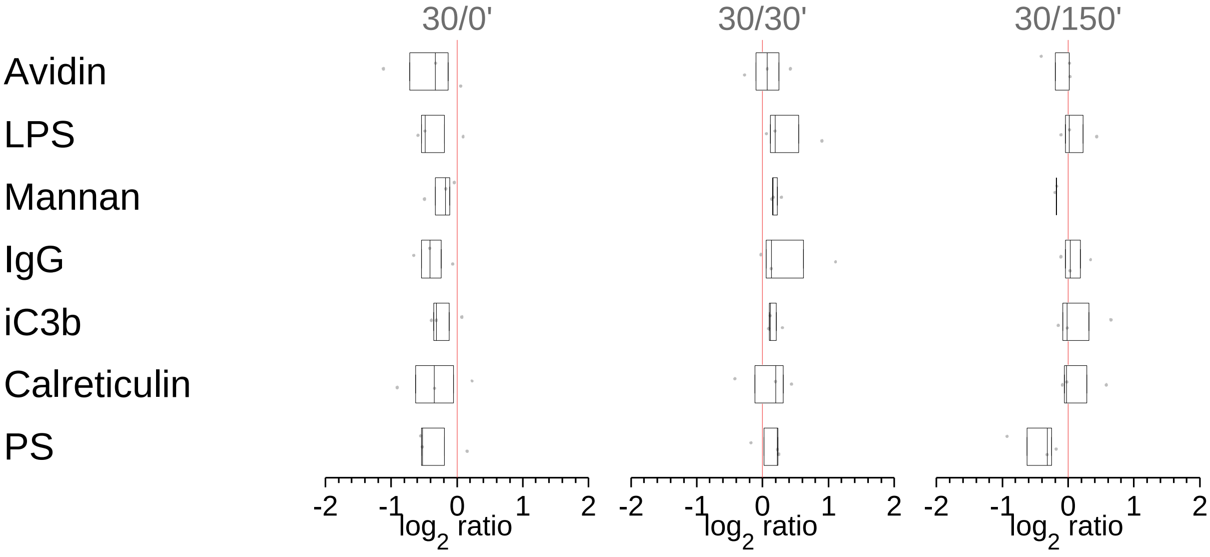

GO:0001819 positive regulation of cytokine production (n = 4)

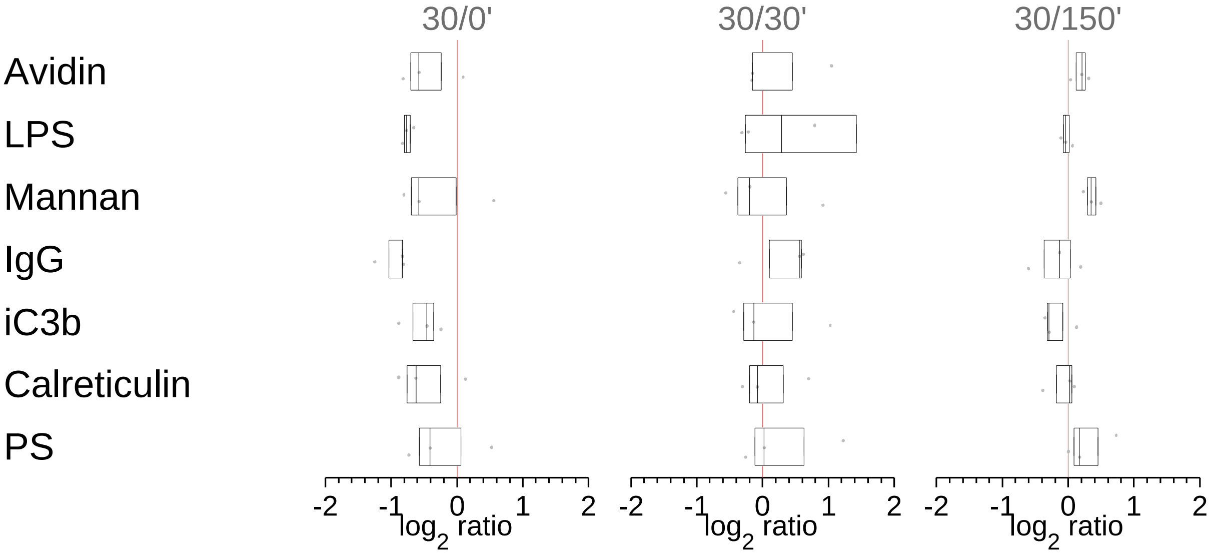

GO:0001822 kidney development (n = 6)

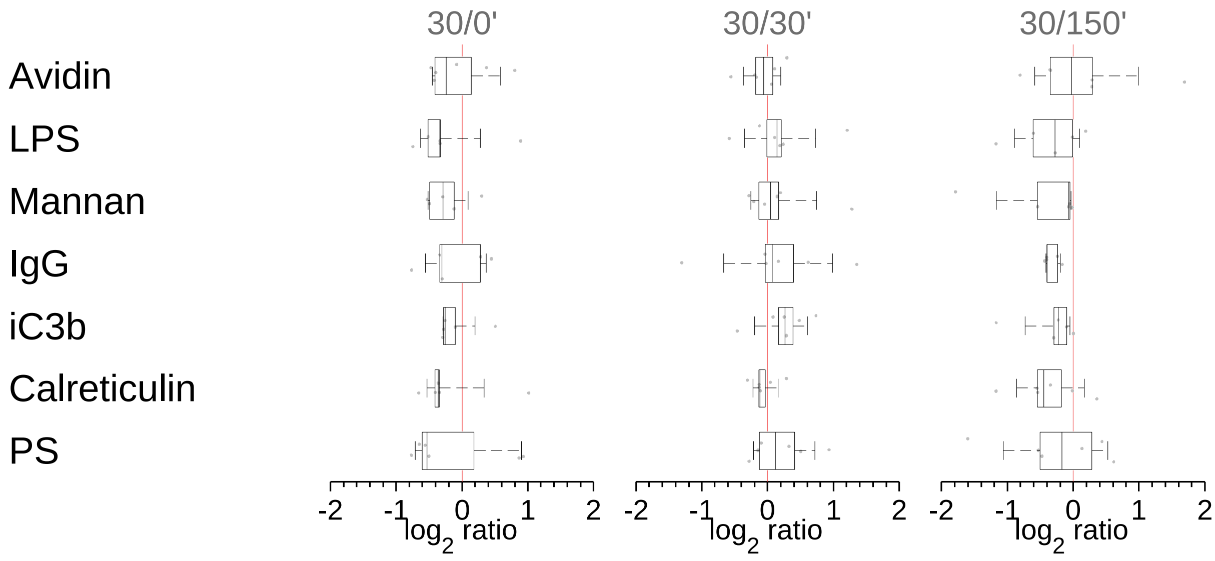

GO:0001824 blastocyst development (n = 3)

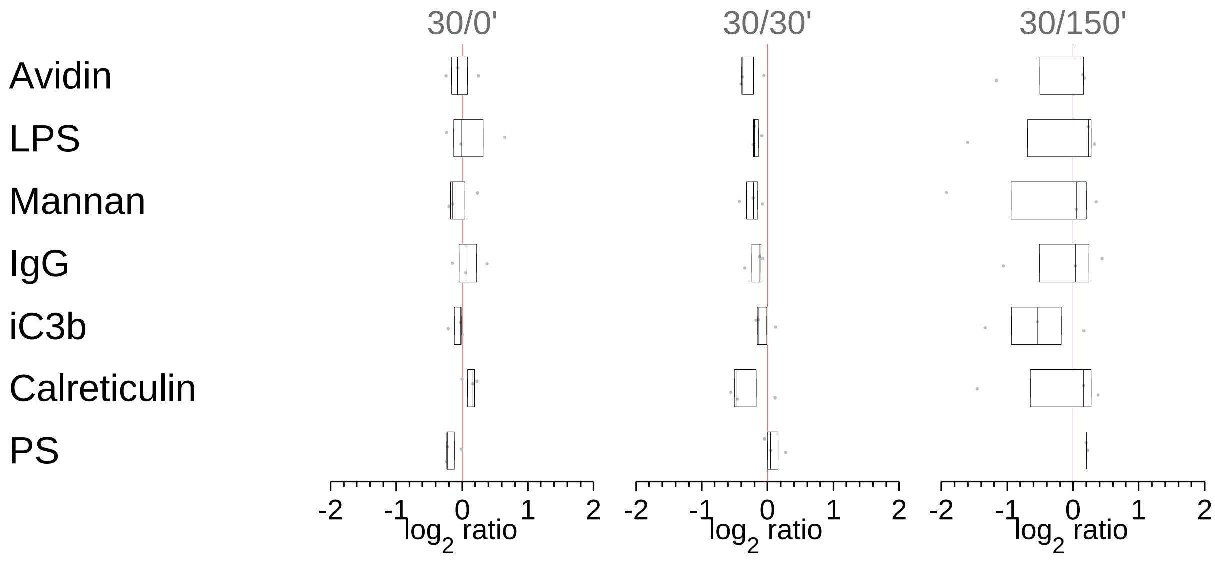

GO:0001836 release of cytochrome c from mitochondria (n = 6)

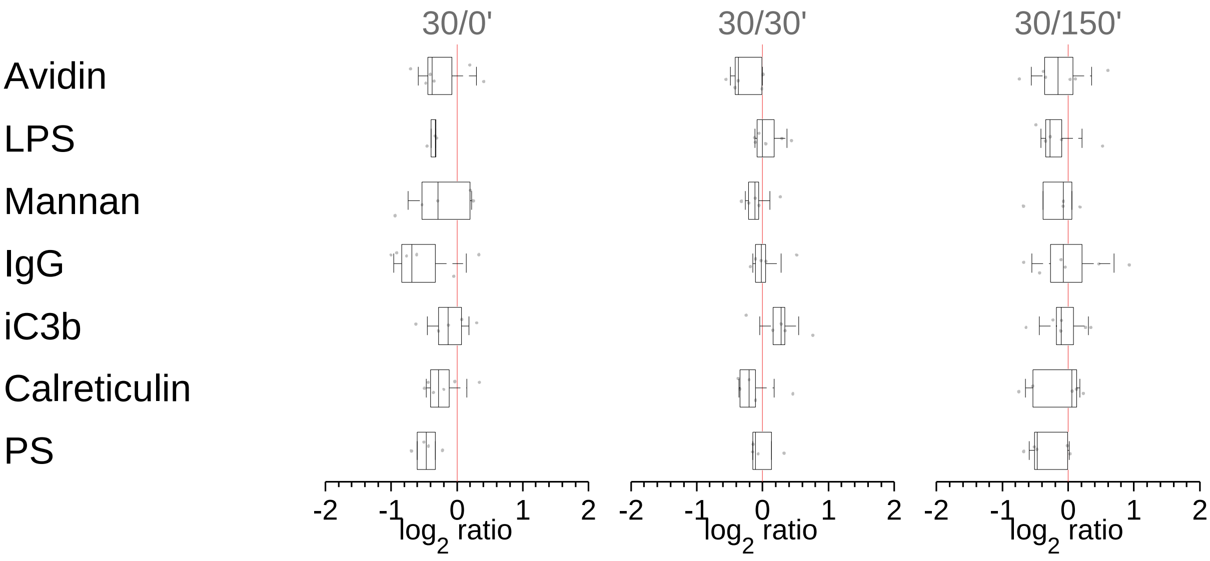

GO:0001837 epithelial to mesenchymal transition (n = 3)

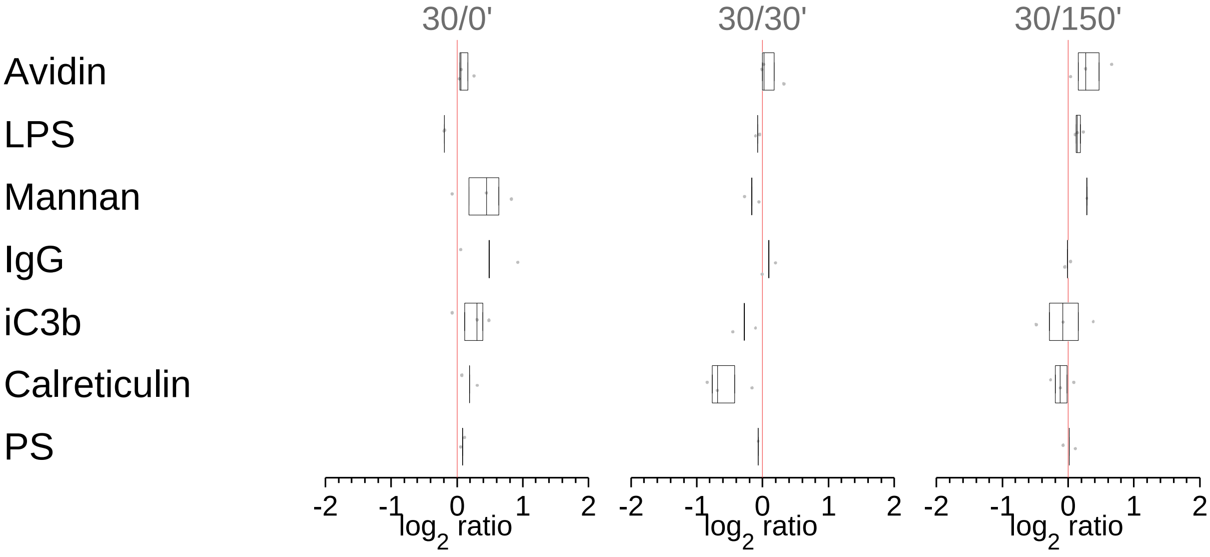

GO:0001843 neural tube closure (n = 8)

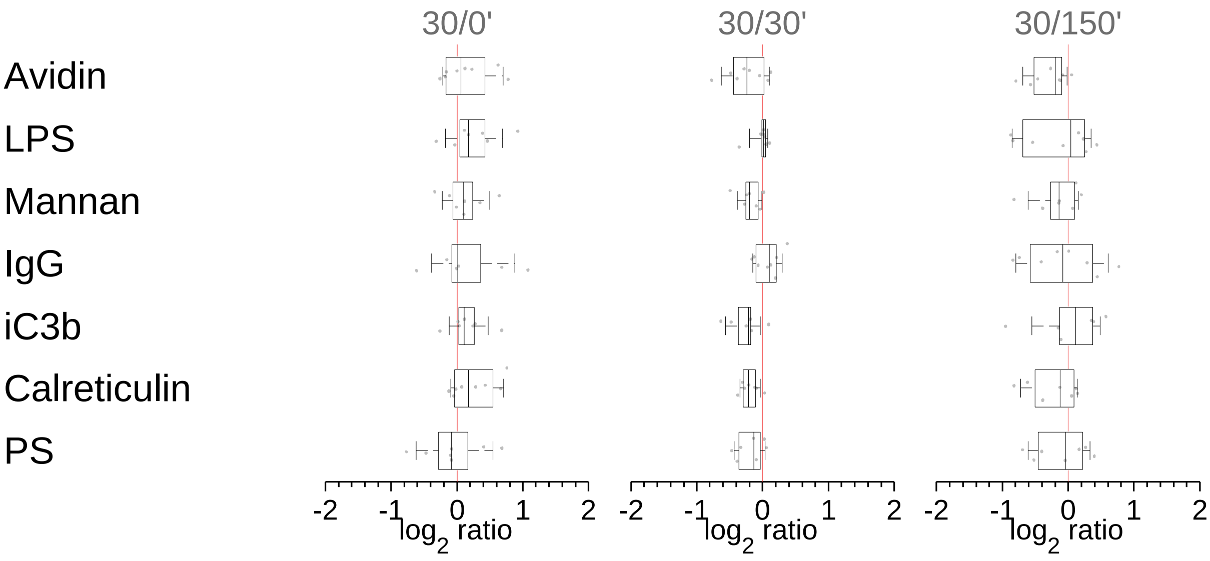

GO:0001889 liver development (n = 8)

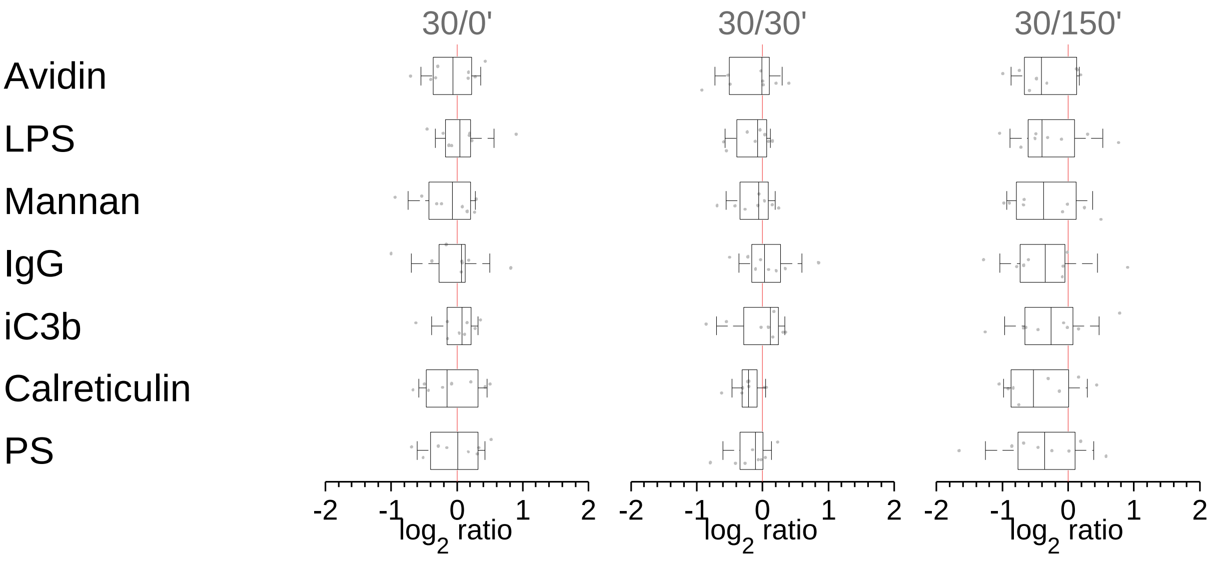

GO:0001890 placenta development (n = 4)

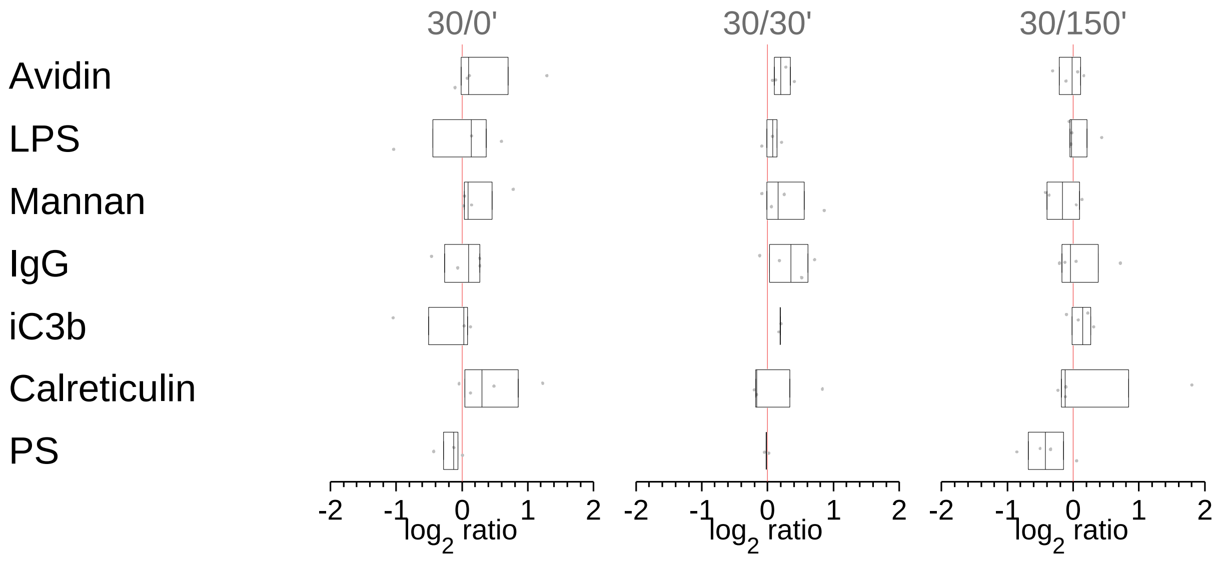

GO:0001891 phagocytic cup (n = 6)

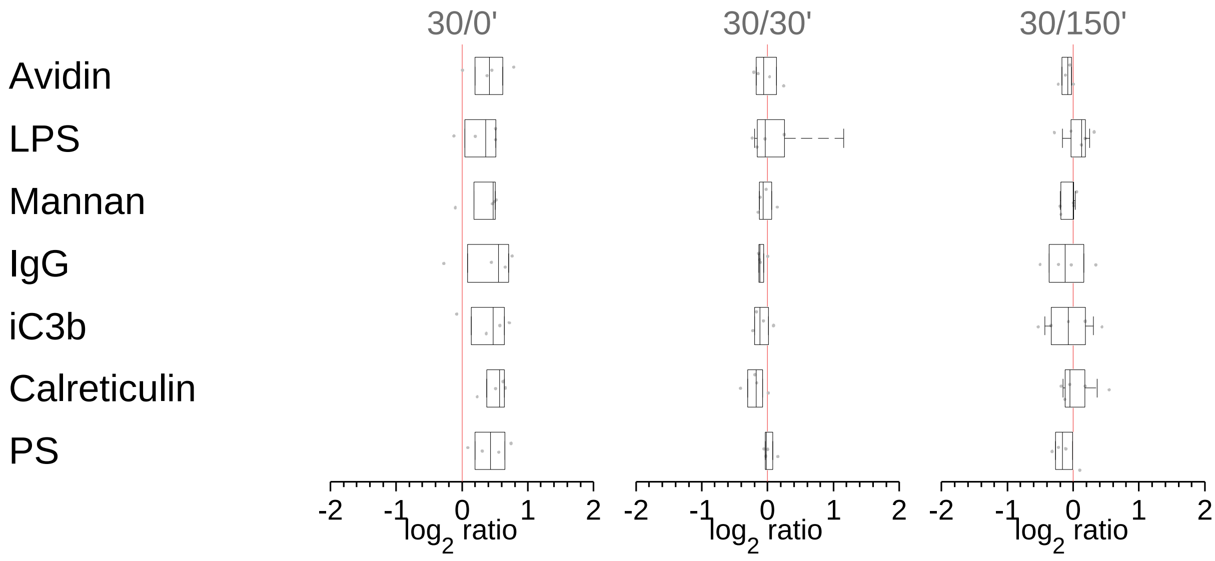

GO:0001894 tissue homeostasis (n = 3)

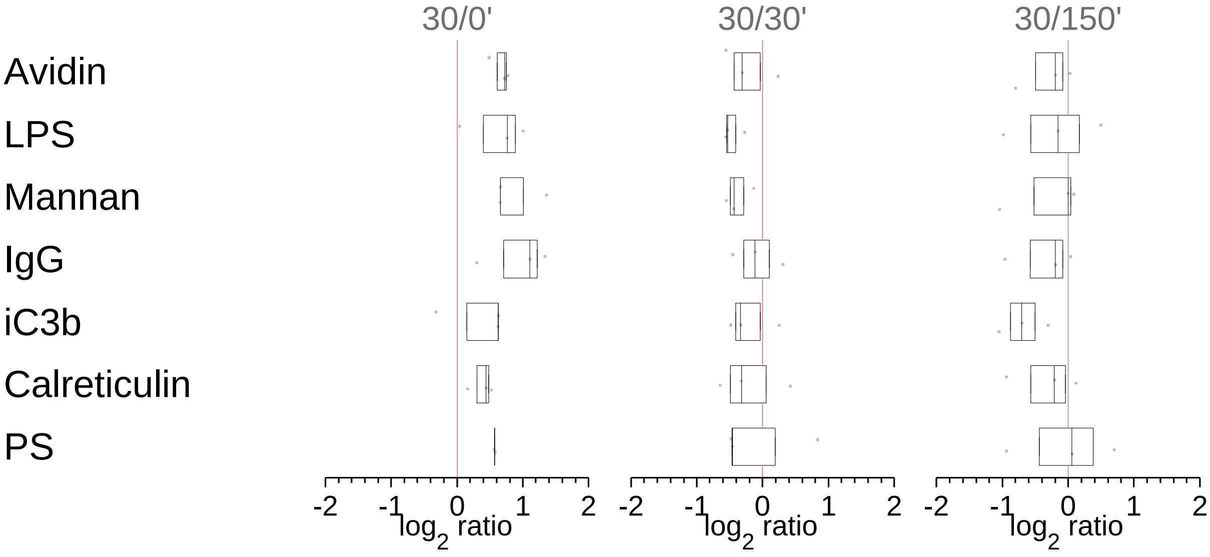

GO:0001913 T cell mediated cytotoxicity (n = 3)

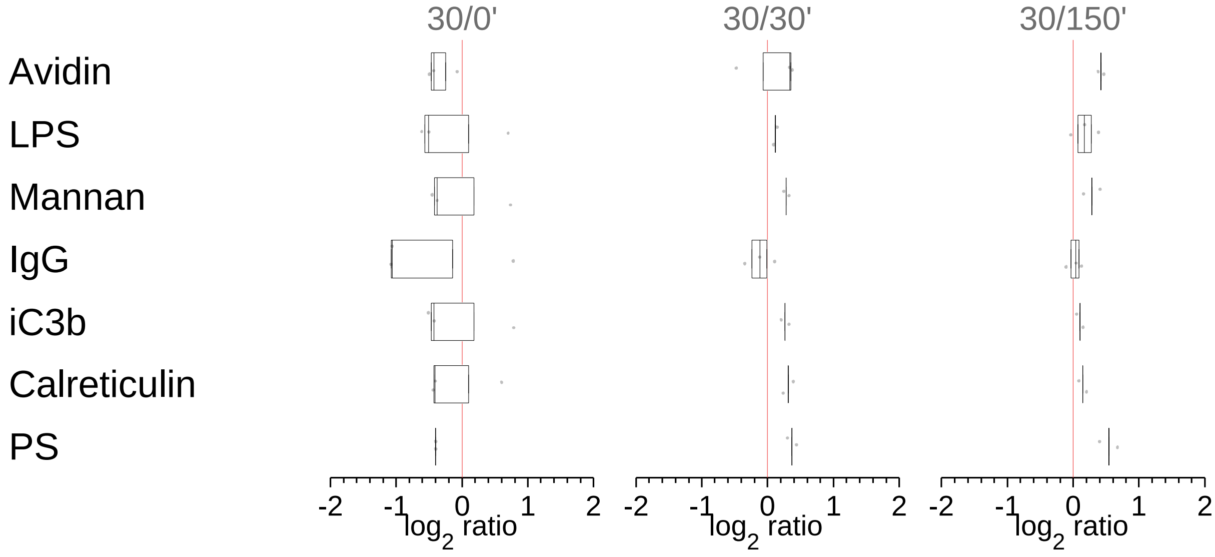

GO:0001916 positive regulation of T cell mediated cytotoxicity
(n = 9)

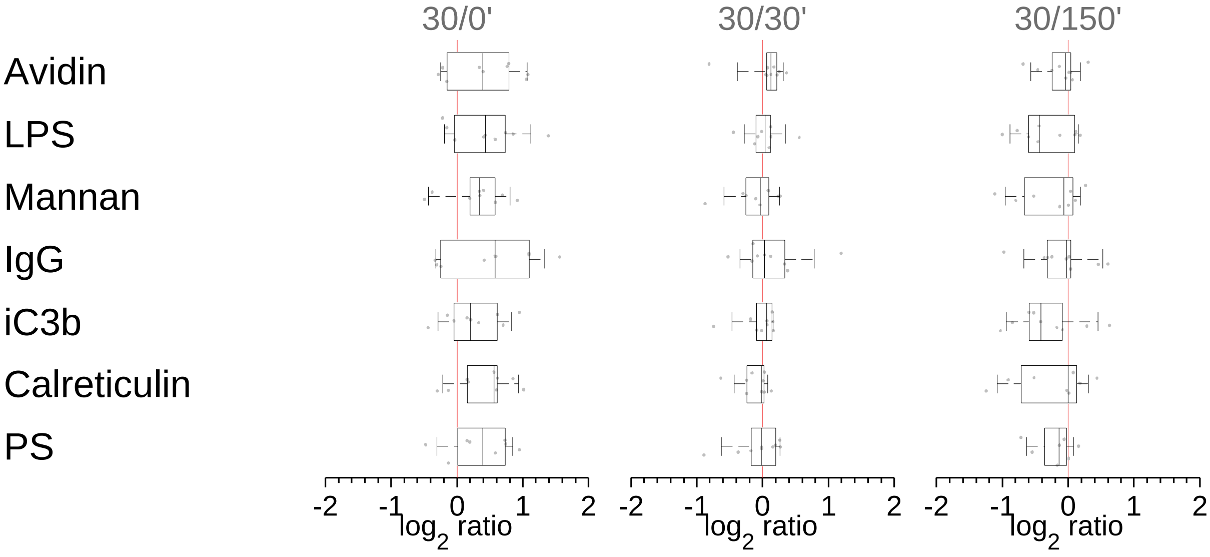

GO:0001919 regulation of receptor recycling (n = 3)

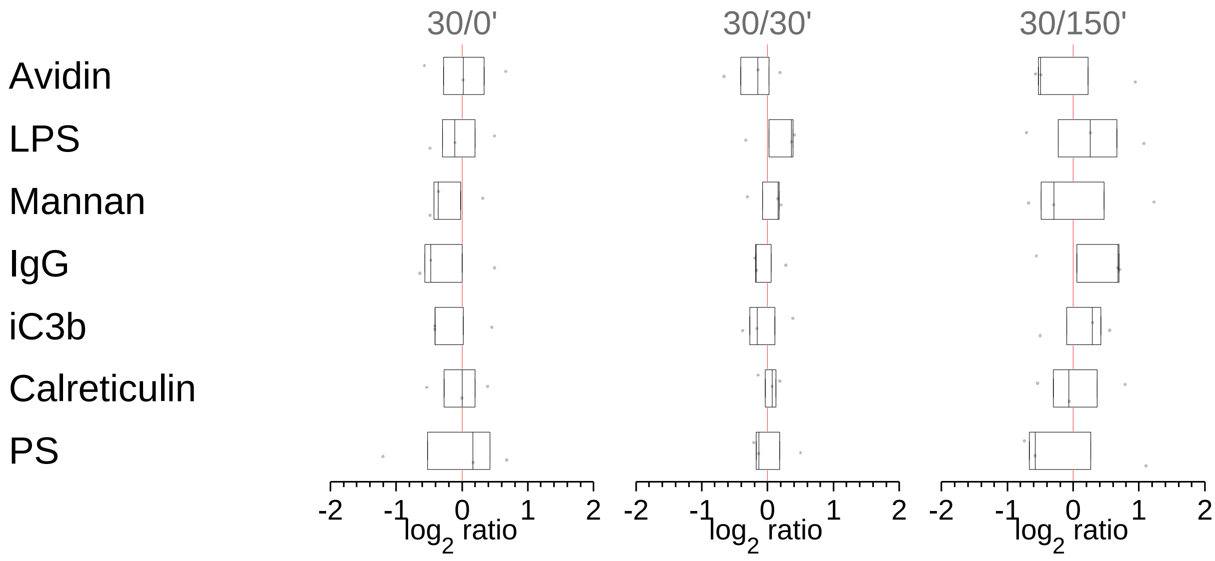

GO:0001921 positive regulation of receptor recycling (n = 7)

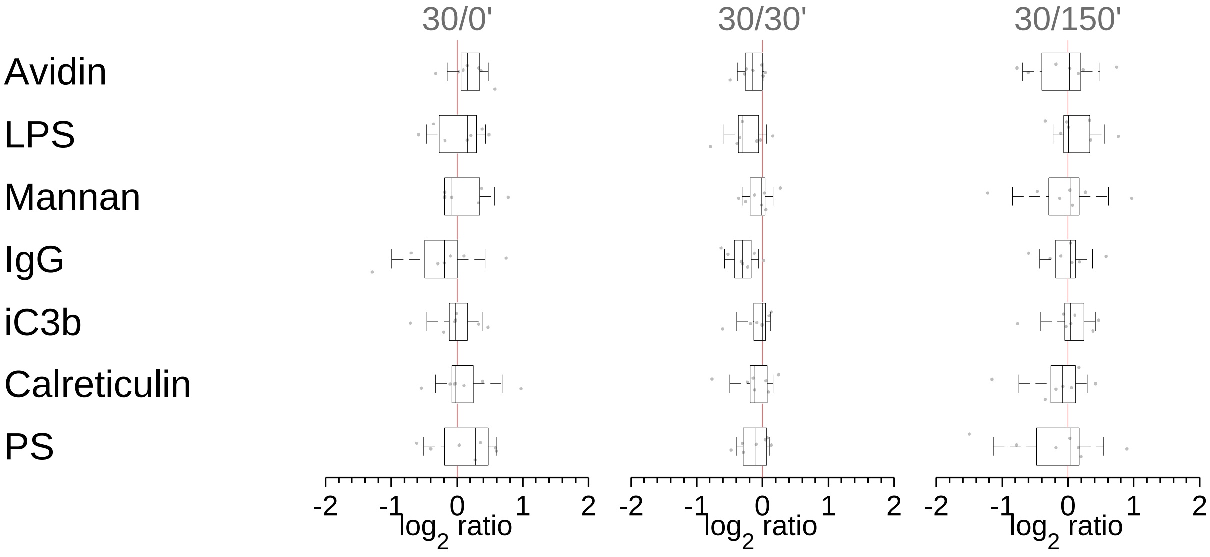

GO:0001931 uropod (n = 4)

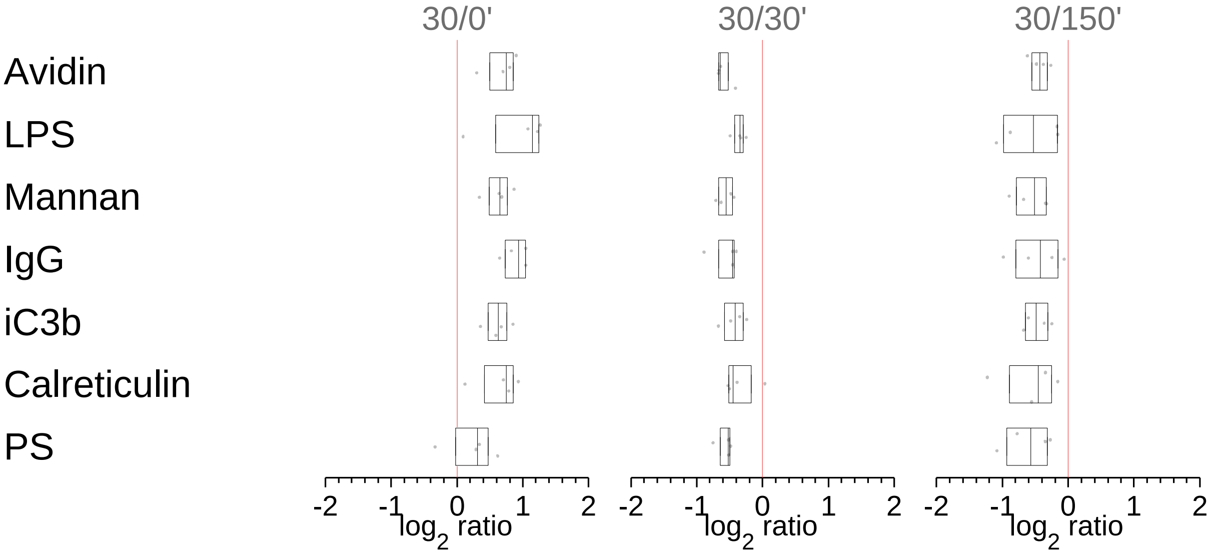

GO:0001932 regulation of protein phosphorylation (n = 6)

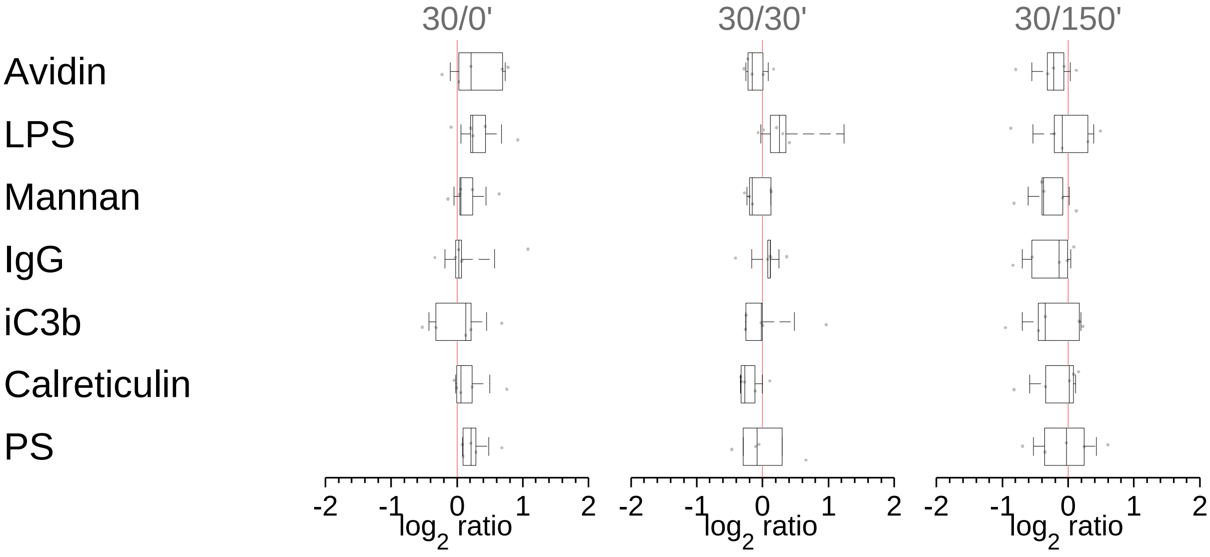

GO:0001933 negative regulation of protein phosphorylation (n = 10)

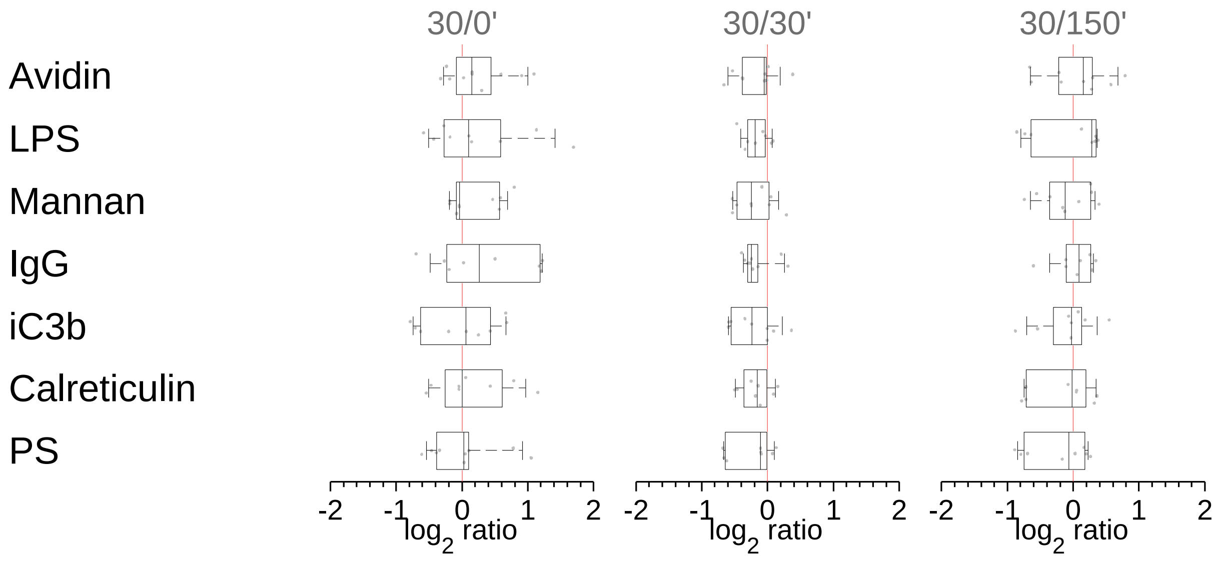

GO:0001934 positive regulation of protein phosphorylation (n = 26)

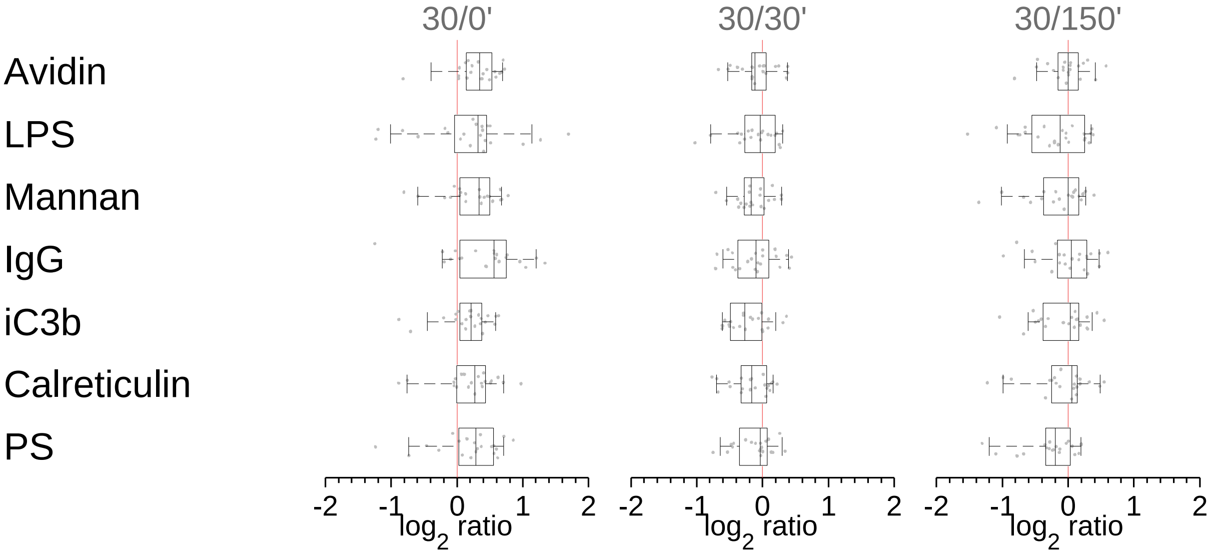

GO:0001937 negative regulation of endothelial cell proliferation
(n = 4)

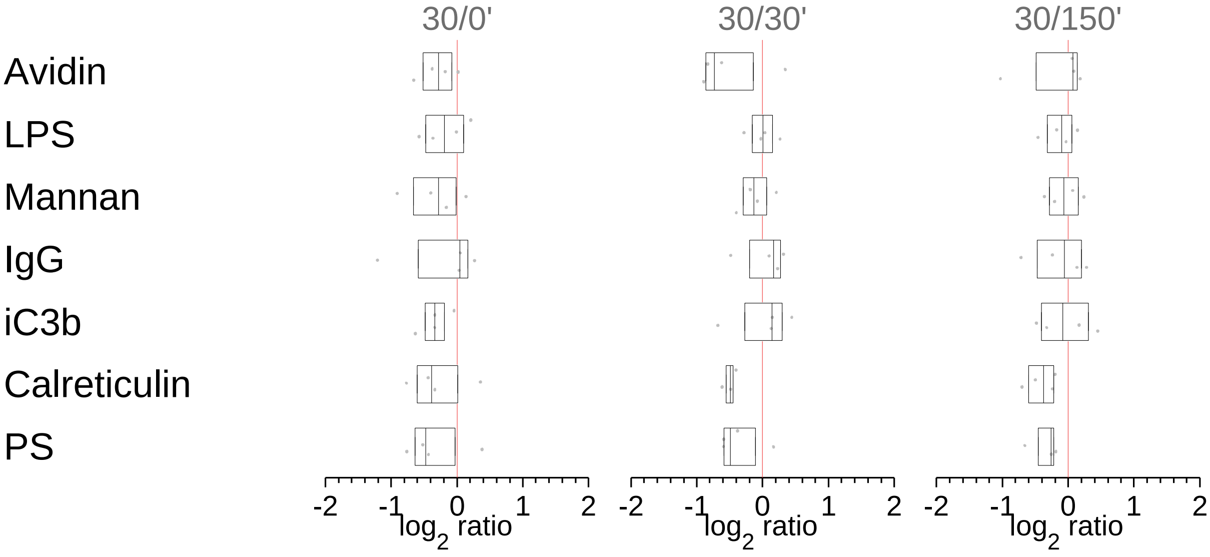

GO:0001938 positive regulation of endothelial cell proliferation
(n = 5)

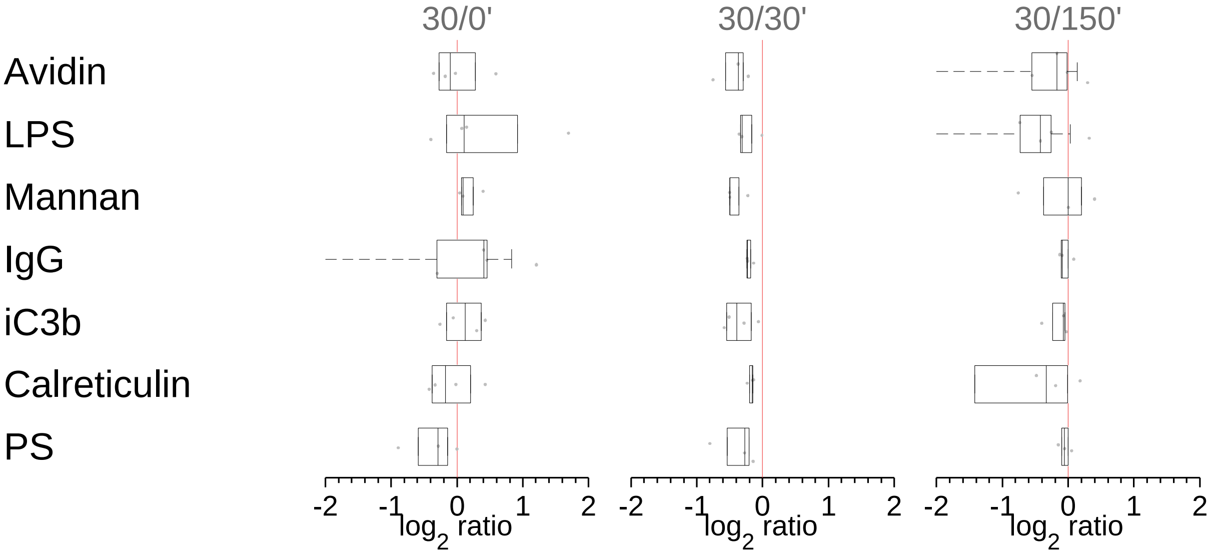

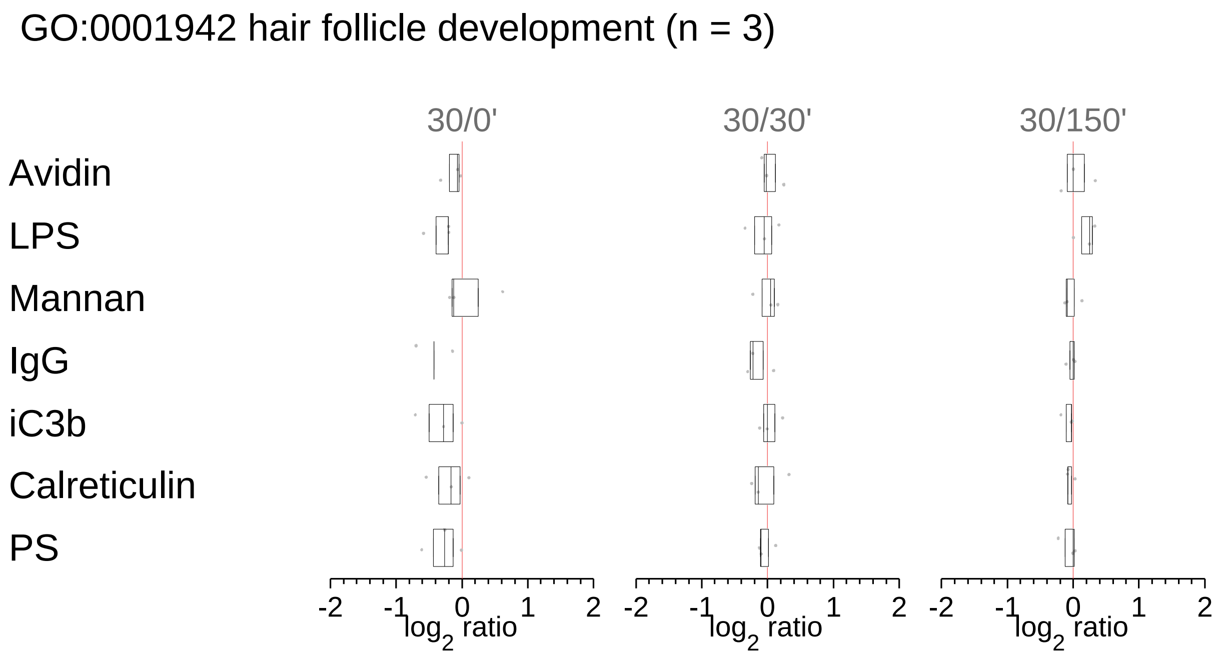
GO:0001942 hair follicle development (n = 3)
30/0'
30/30'
30/150'
Avidin
LPS
Mannan
IgG
iC3b
Calreticulin
PS
-2
-1
0
1
2
log2 ratio

GO:0001948 glycoprotein binding (n = 19)

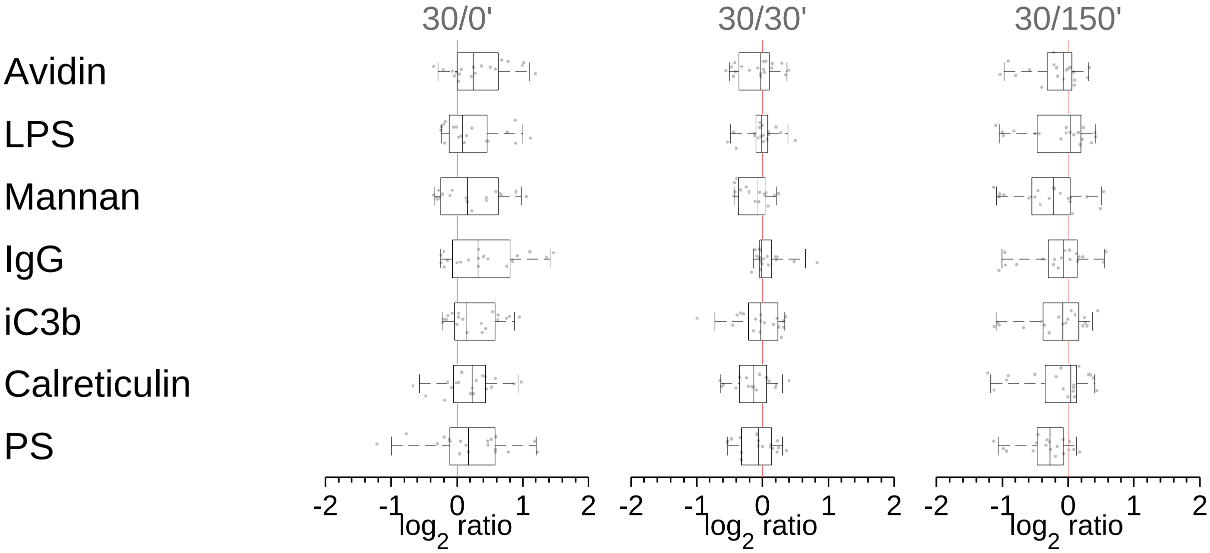

GO:0001954 positive regulation of cell-matrix adhesion (n = 5)

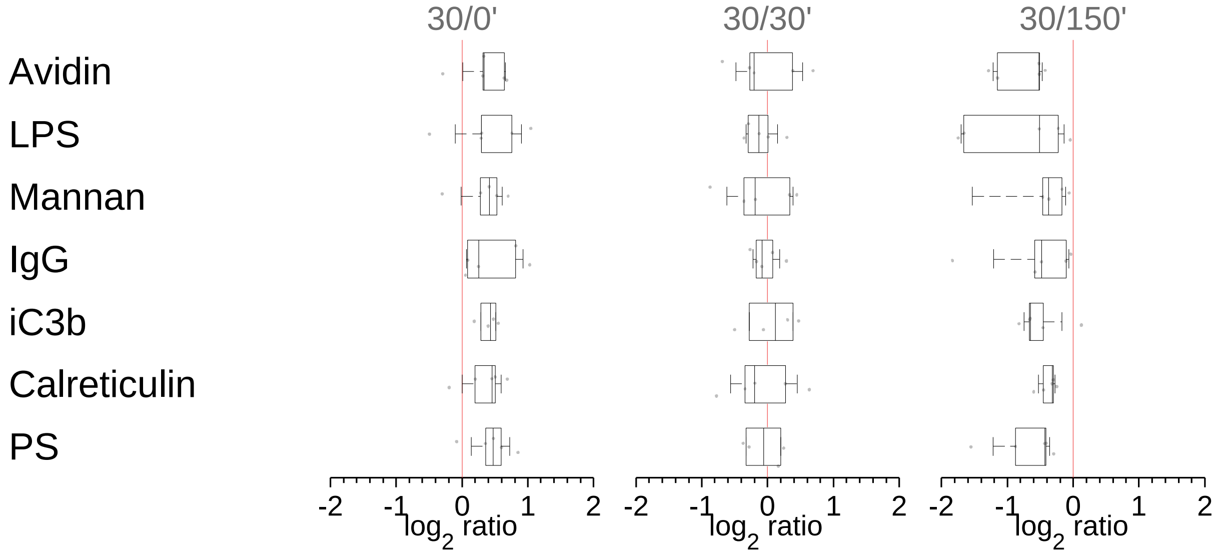

GO:0001962 alpha-1,3-galactosyltransferase activity (n = 3)

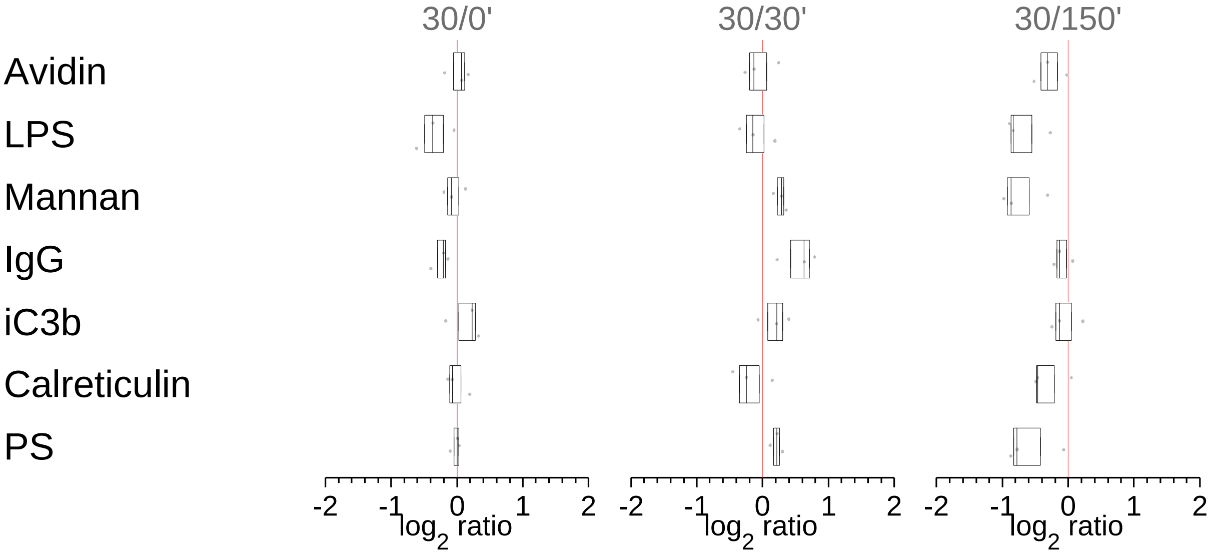

GO:0001965 G-protein alpha-subunit binding (n = 3)

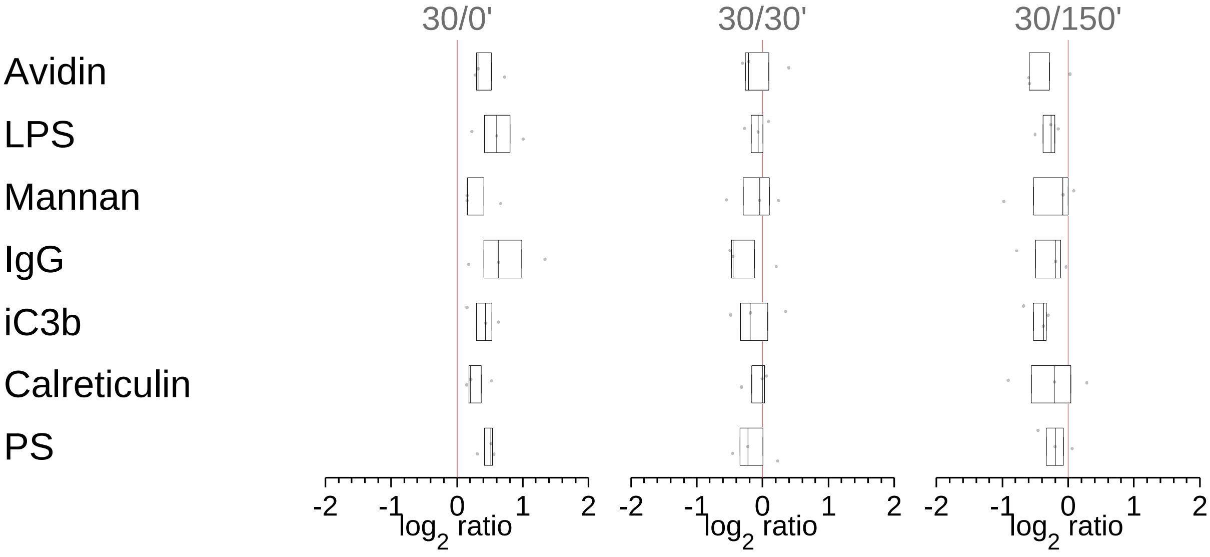

GO:0001968 fibronectin binding (n = 5)

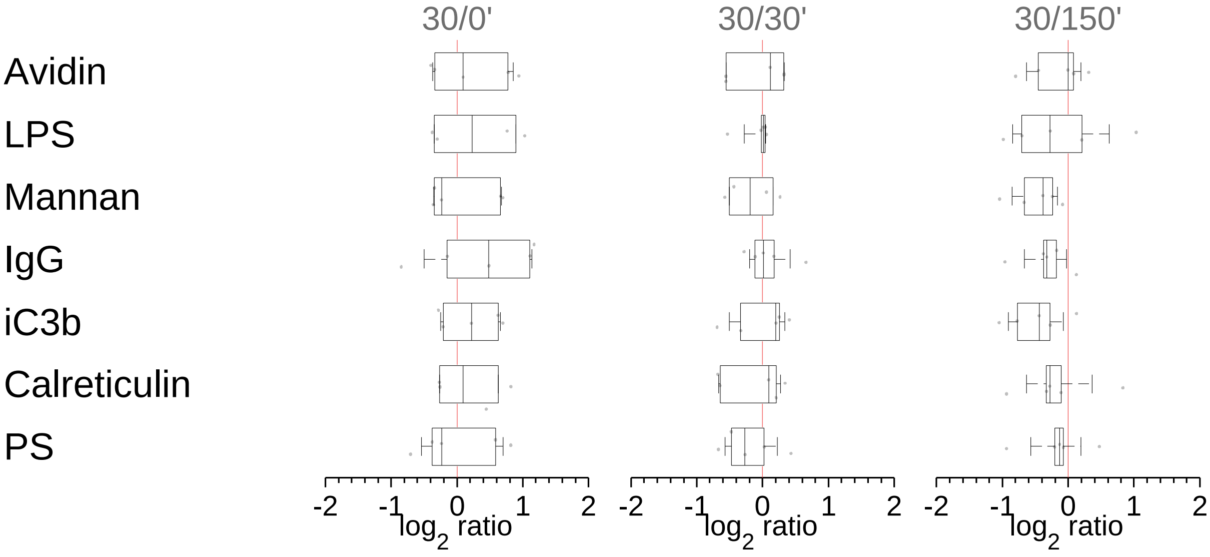

GO:0001974 blood vessel remodeling (n = 4)

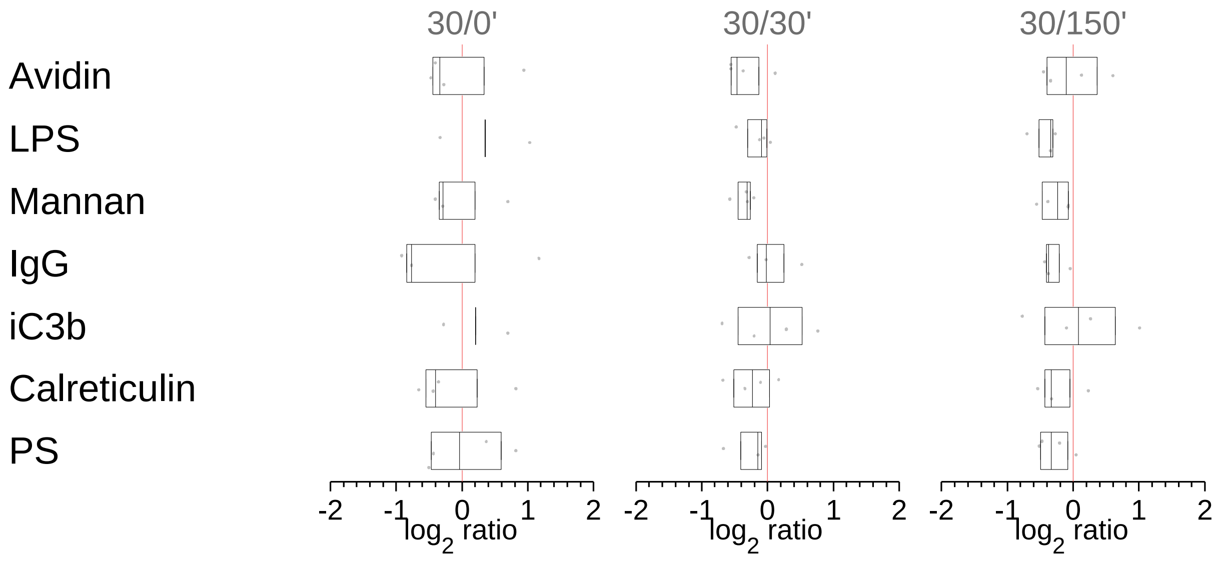

GO:0002020 protease binding (n = 16)

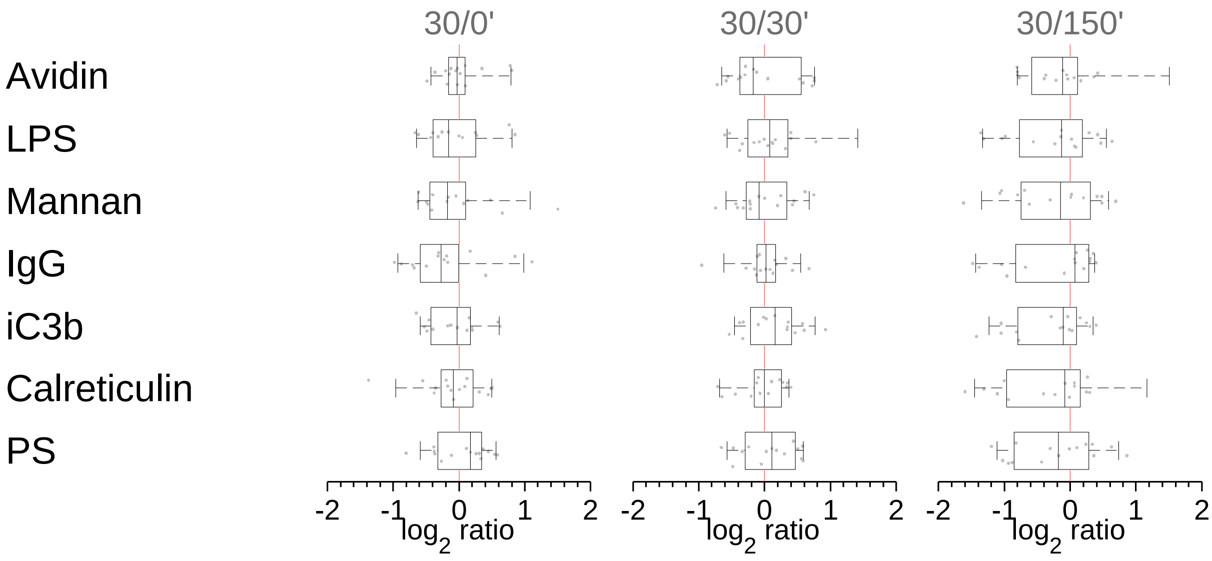

GO:0002026 regulation of the force of heart contraction (n = 5)

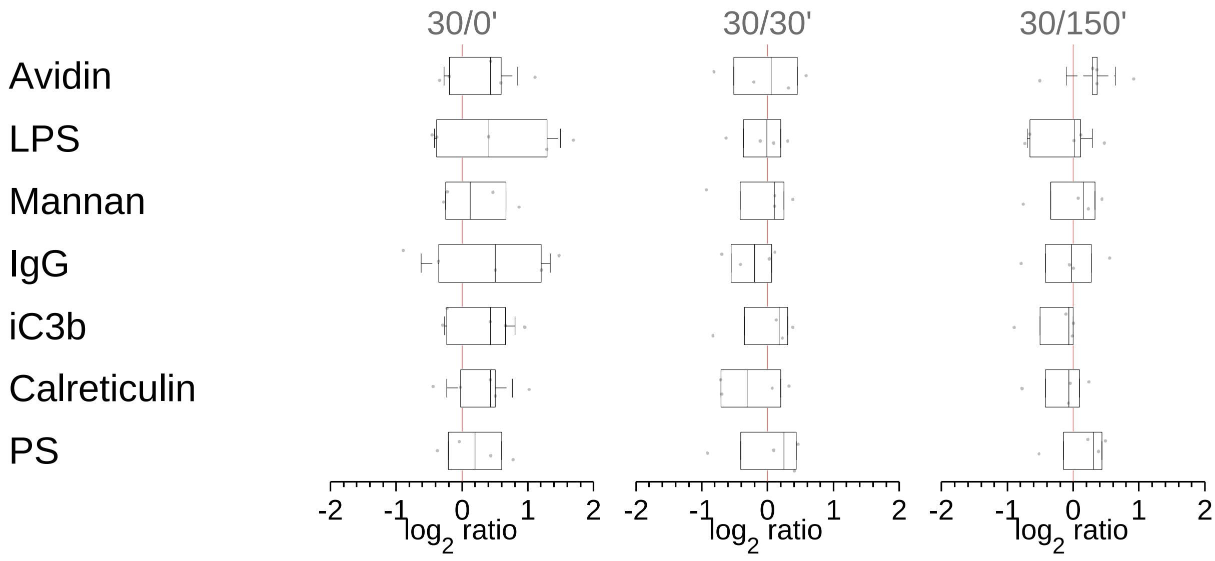

GO:0002028 regulation of sodium ion transport (n = 4)

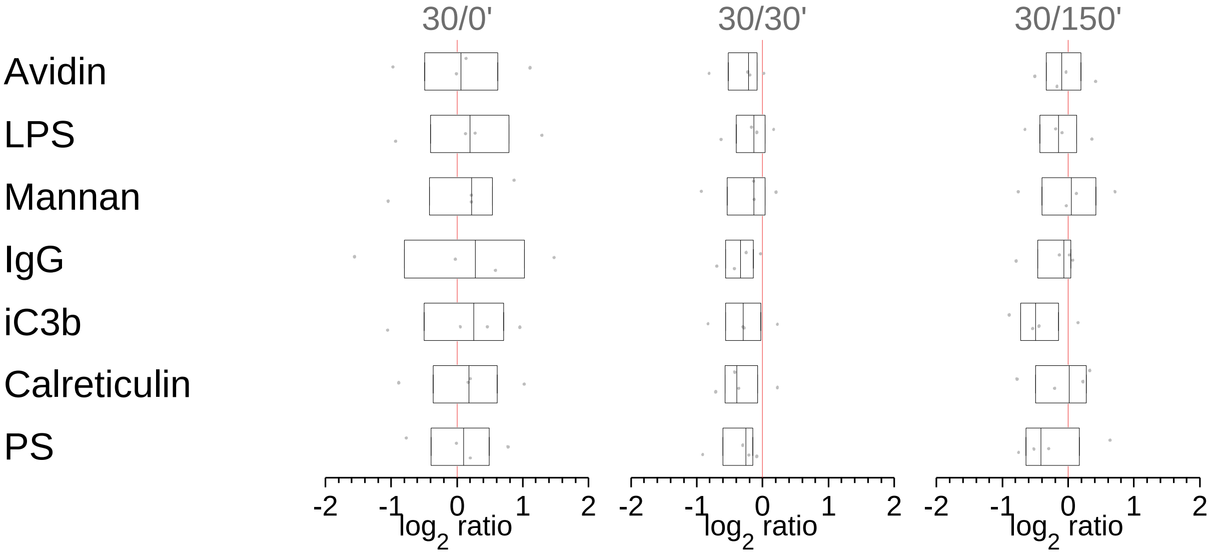

GO:0002039 p53 binding (n = 3)

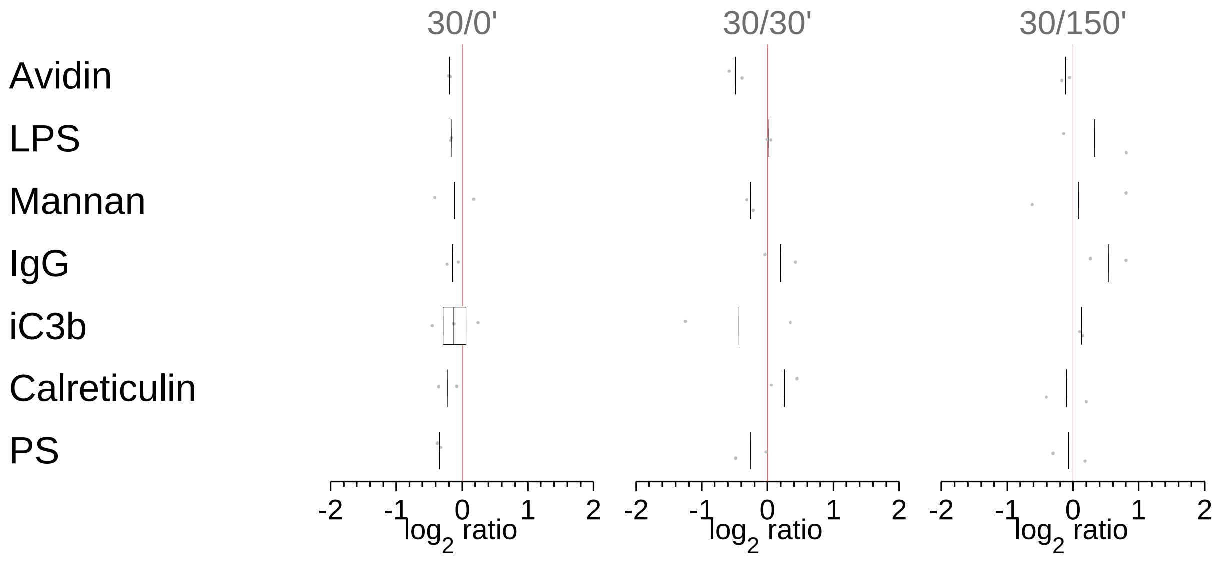

GO:0002042 cell migration involved in sprouting angiogenesis (n = 3)

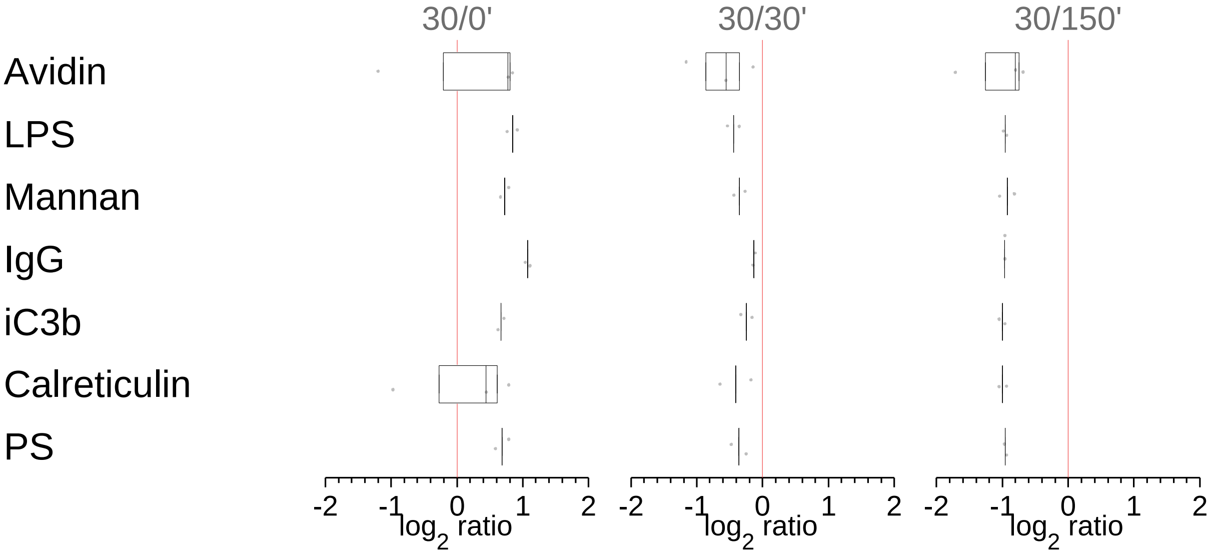

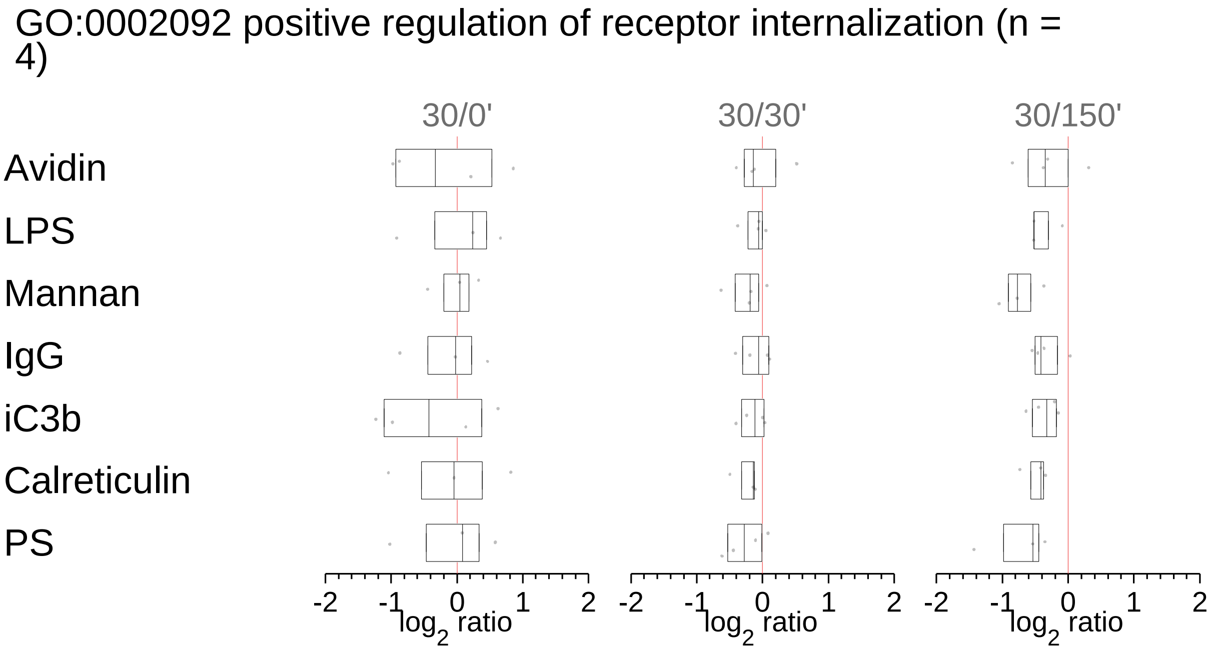

GO:0002092 positive regulation of receptor internalization (n = 4)
30/0'
30/30'
30/150'
Avidin
LPS
Mannan
IgG
iC3b
Calreticulin
PS
-2
-1
0
1
2
log2 ratio

GO:0002102 podosome (n = 6)

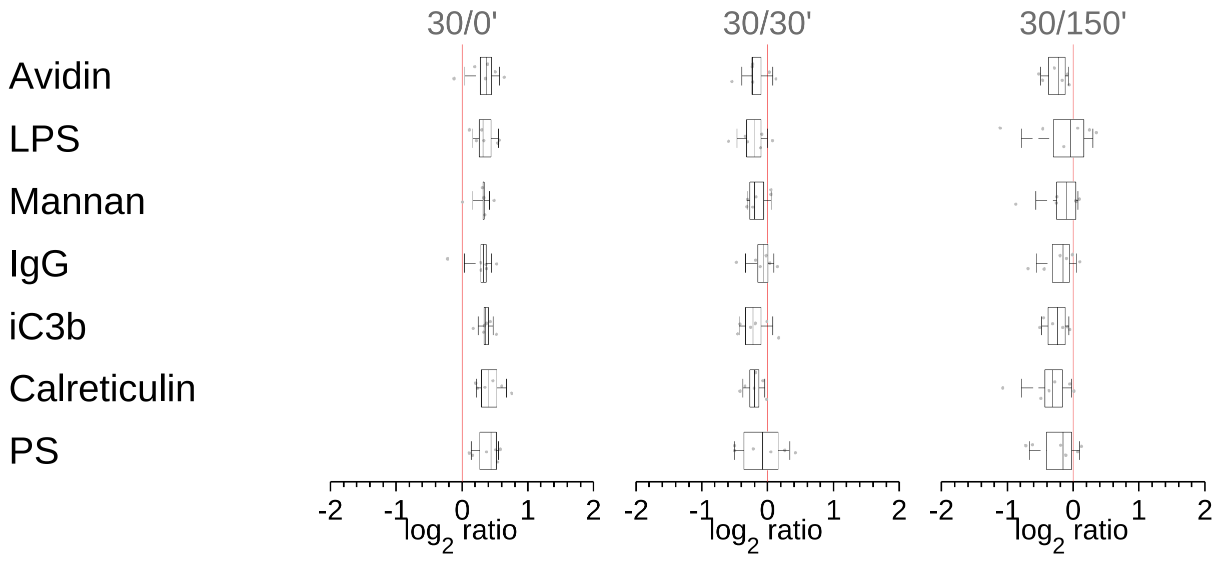

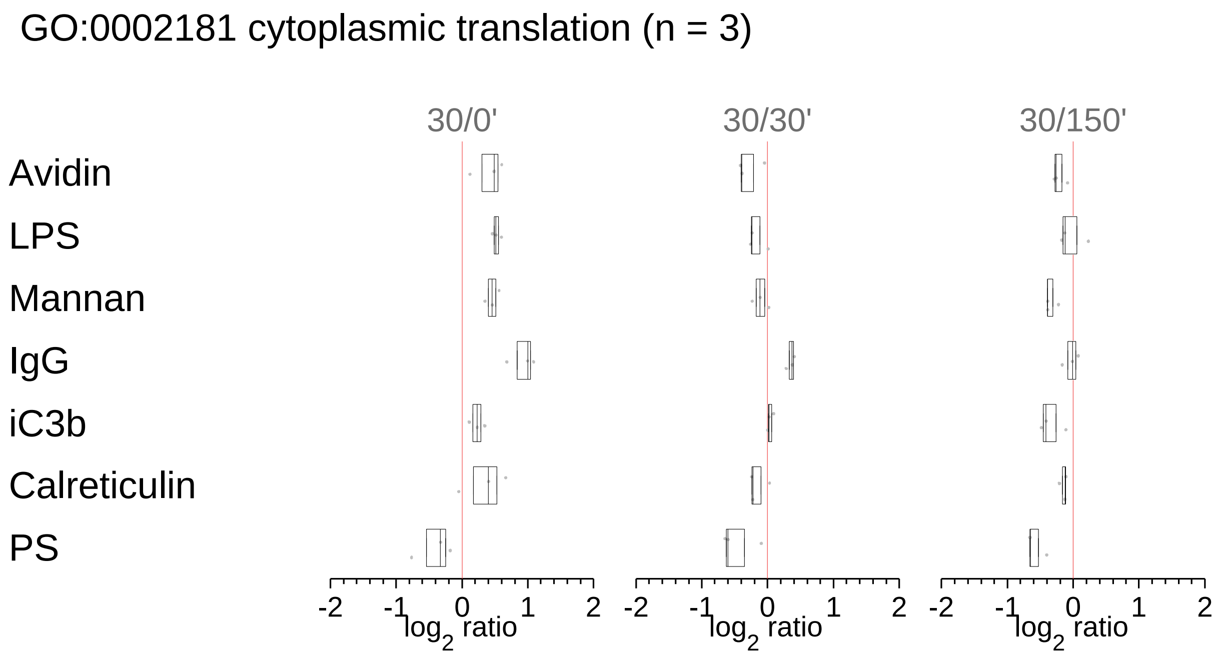

GO:0002181 cytoplasmic translation (n = 3)
30/0'
30/30'
30/150'
Avidin
LPS
Mannan
IgG
iC3b
Calreticulin
PS
-2
-1
0
1
2
log2 ratio

GO:0002199 sperm protein complex (n = 8)

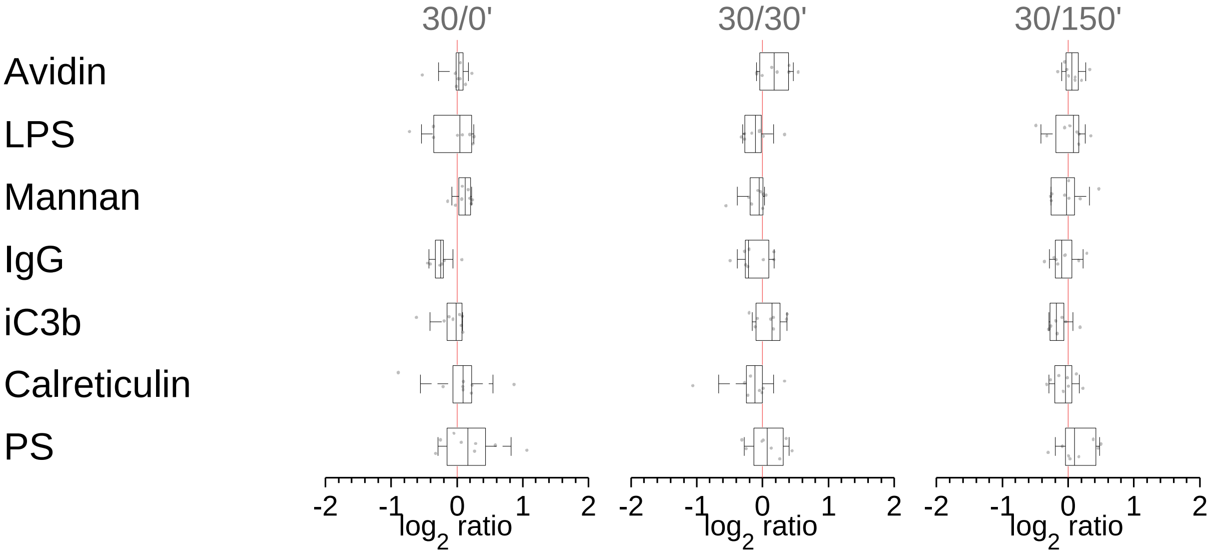

GO:0002221 pattern recognition receptor signaling pathway (n = 3)

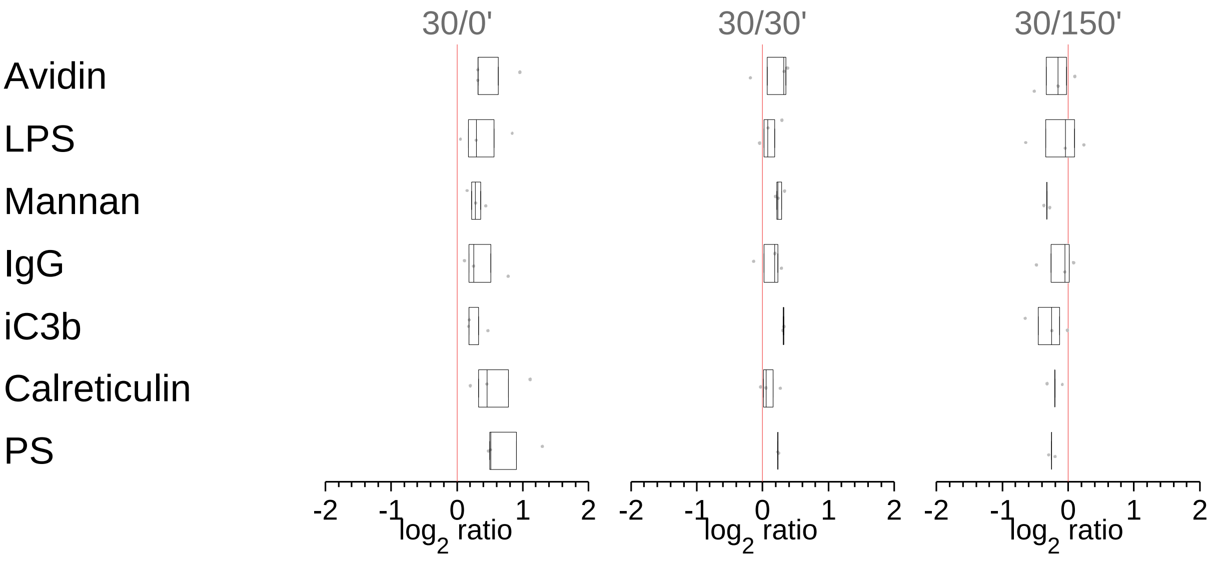

GO:0002224 toll-like receptor signaling pathway (n = 6)

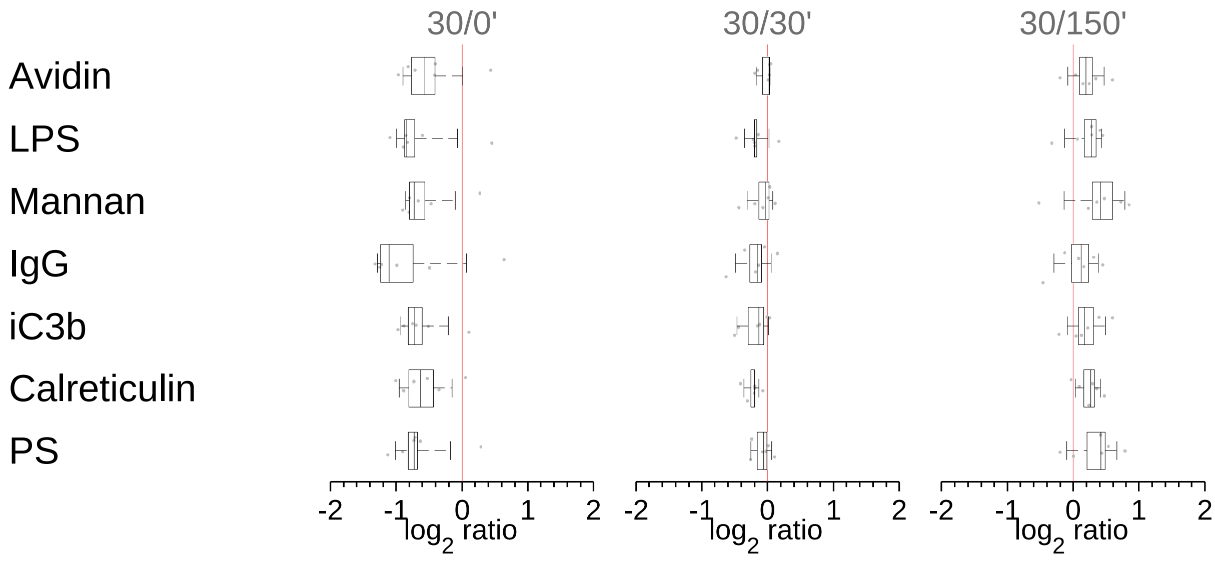

GO:0002237 response to molecule of bacterial origin (n = 6)

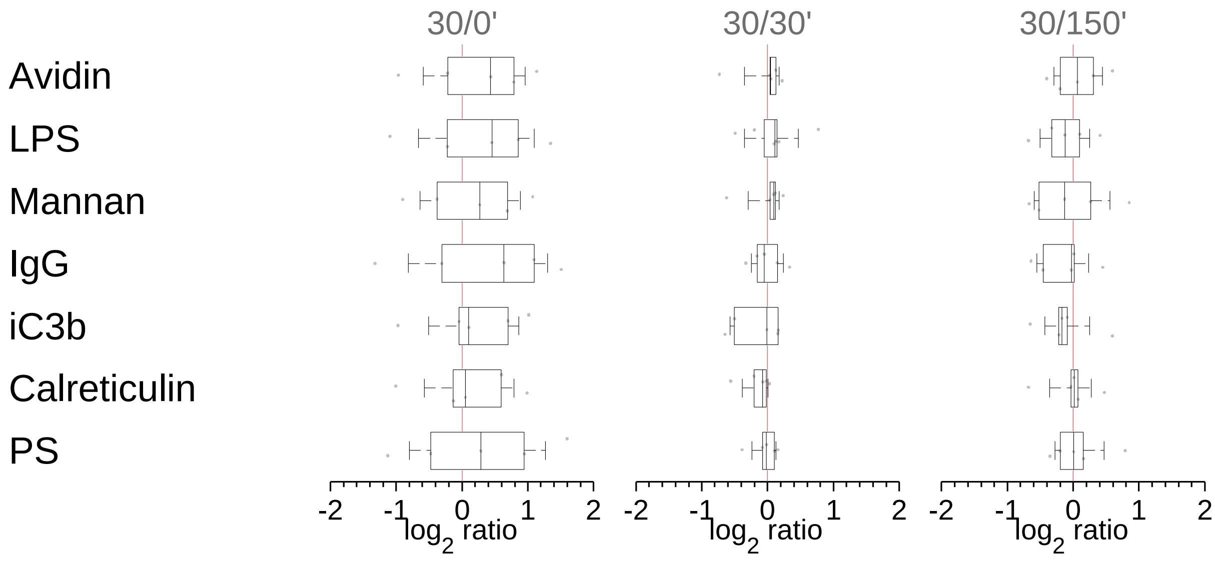

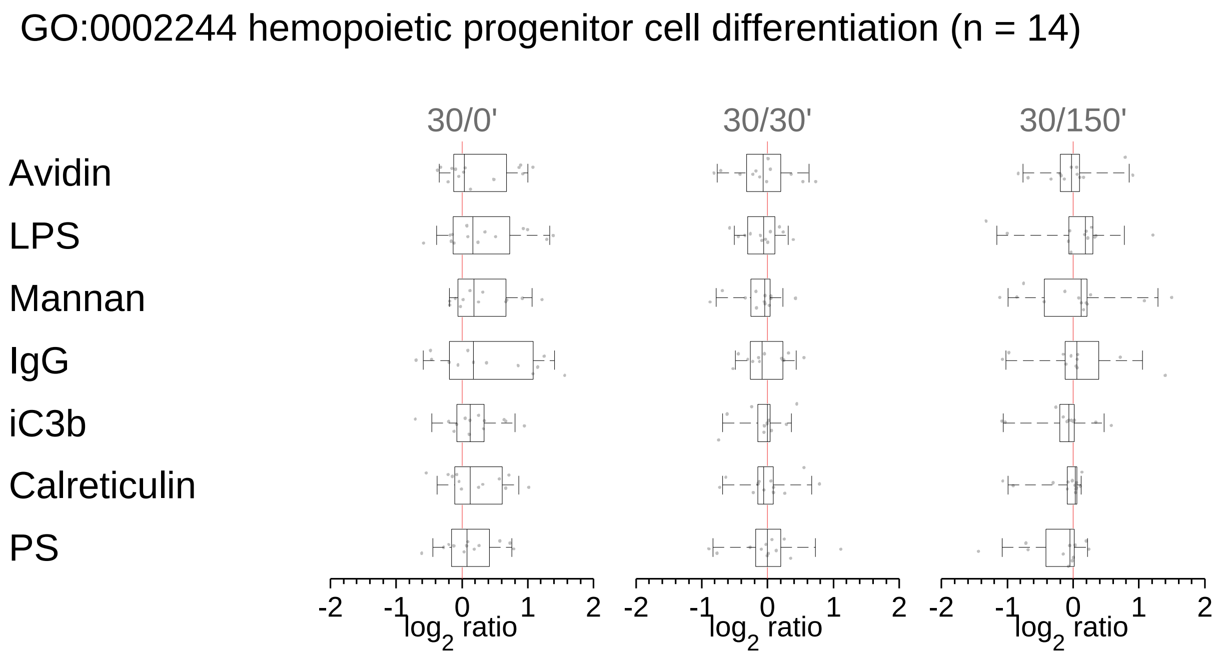
GO:0002244 hemopoietic progenitor cell differentiation (n = 14)
30/0'
30/30'
30/150'
Avidin
LPS
Mannan
IgG
iC3b
Calreticulin
PS
-2
-1
0
1
2
log2 ratio

GO:0002262 myeloid cell homeostasis (n = 4)

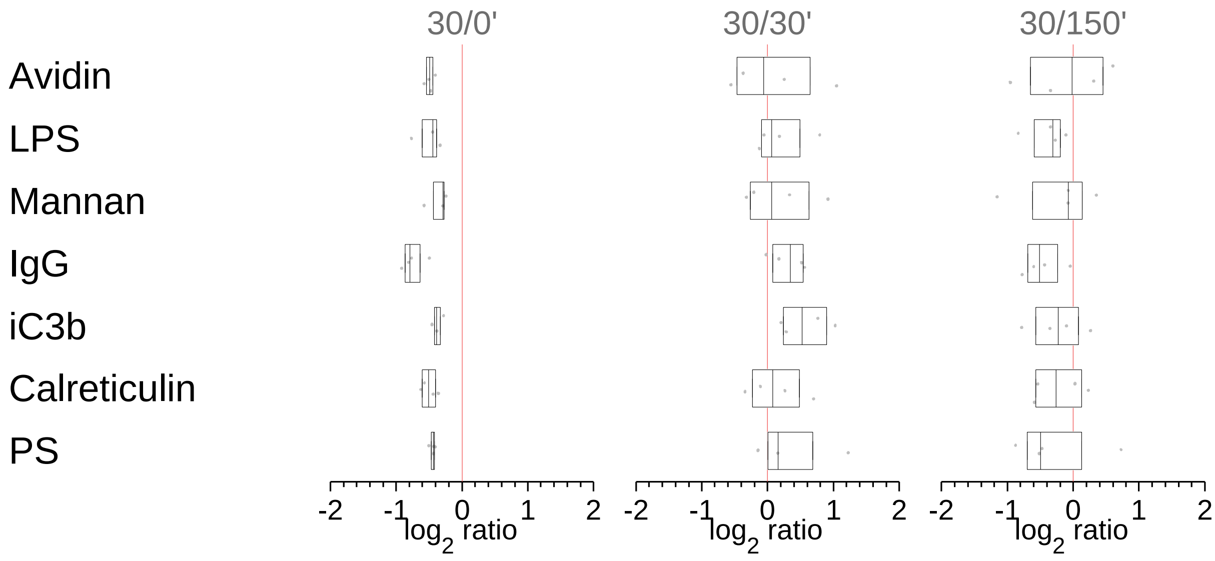

GO:0002283 neutrophil activation involved in immune response (n = 3)

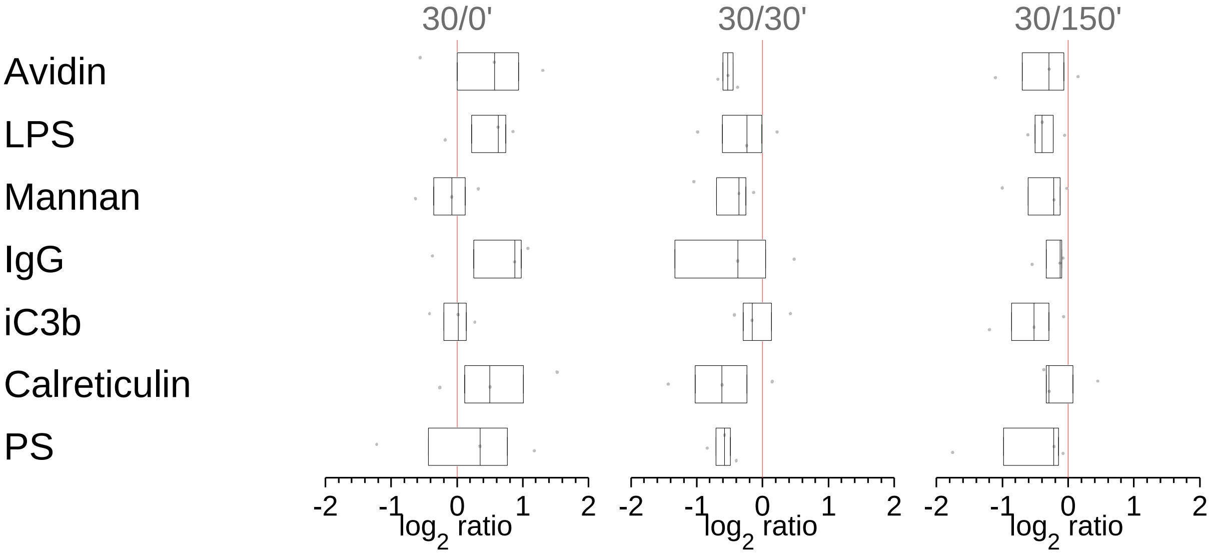

GO:0002286 T cell activation involved in immune response (n = 3)

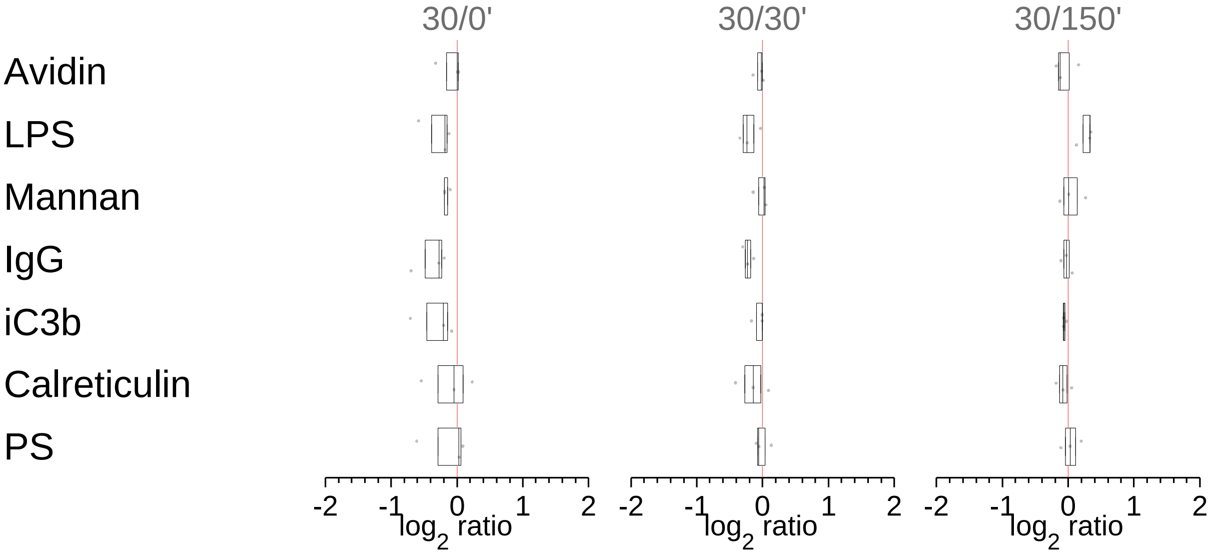

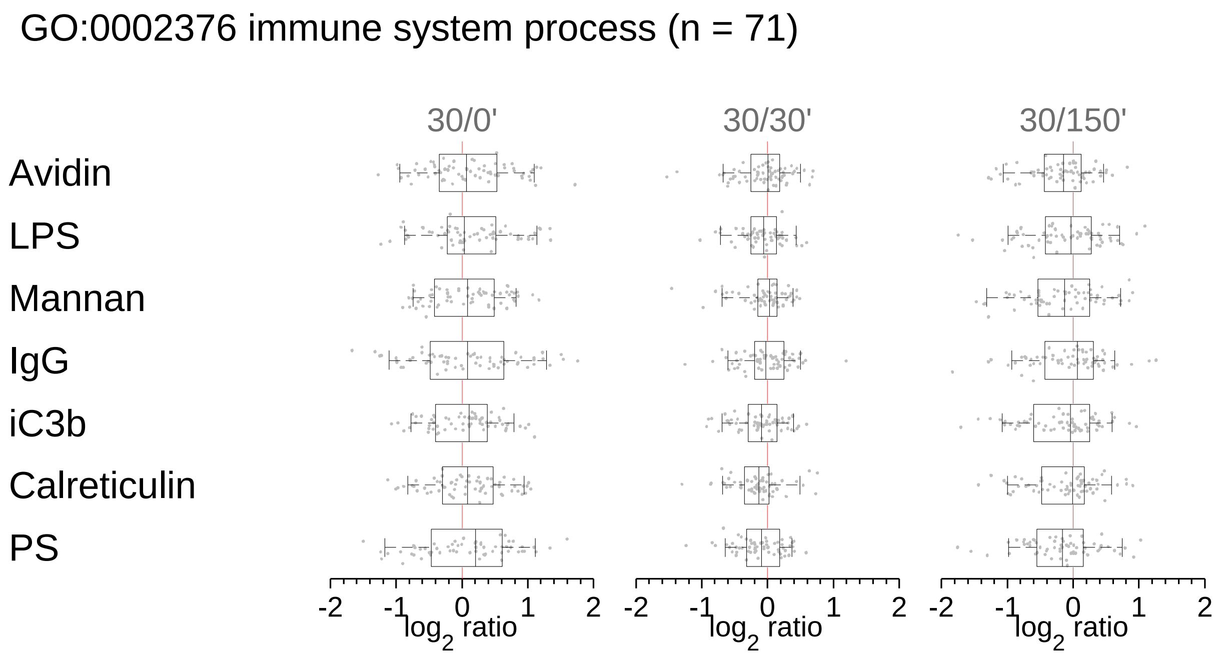

GO:0002376 immune system process (n = 71)
30/0'
30/30'
30/150'
Avidin
LPS
Mannan
IgG
iC3b
Calreticulin
PS
-2
-1
0
1
2
log2 ratio

GO:0002474 antigen processing and presentation of peptide antigen via MHC class I (n = 4)

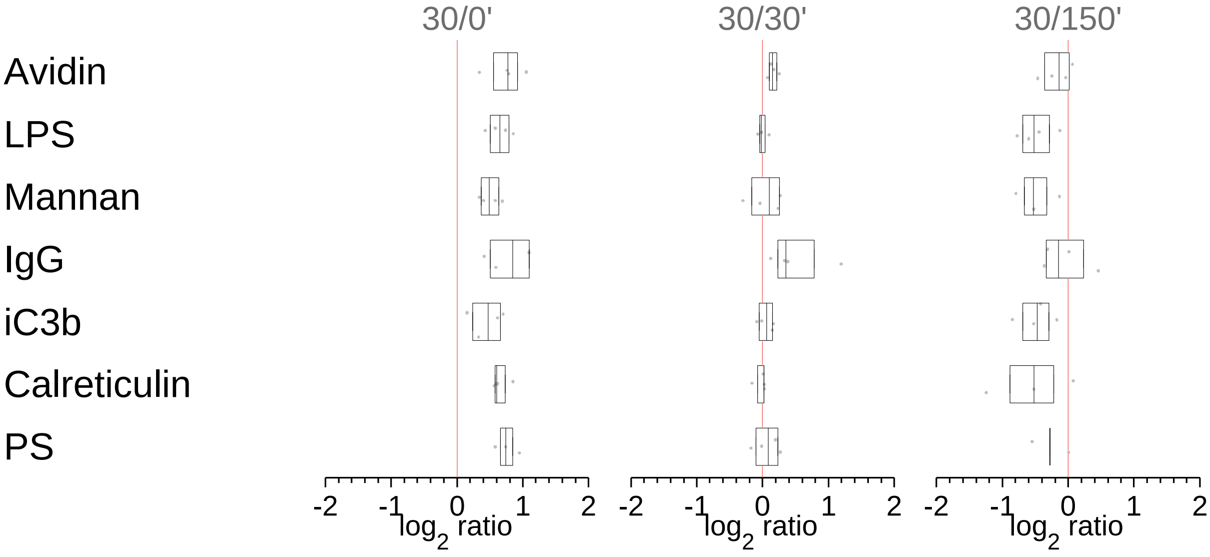

GO:0002523 leukocyte migration involved in inflammatory response
(n = 3)

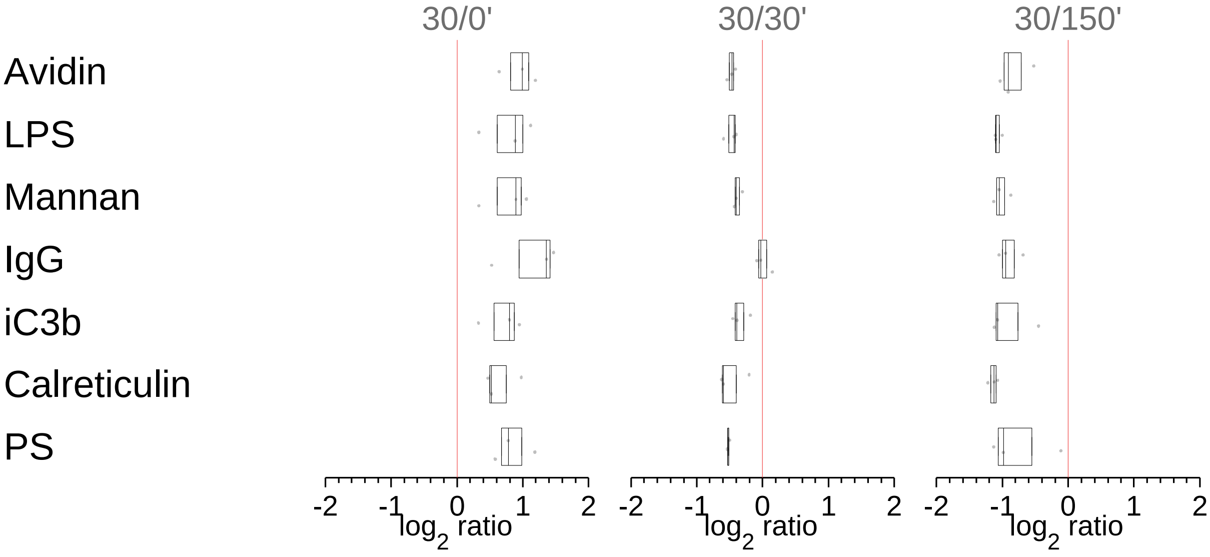

GO:0002526 acute inflammatory response (n = 3)

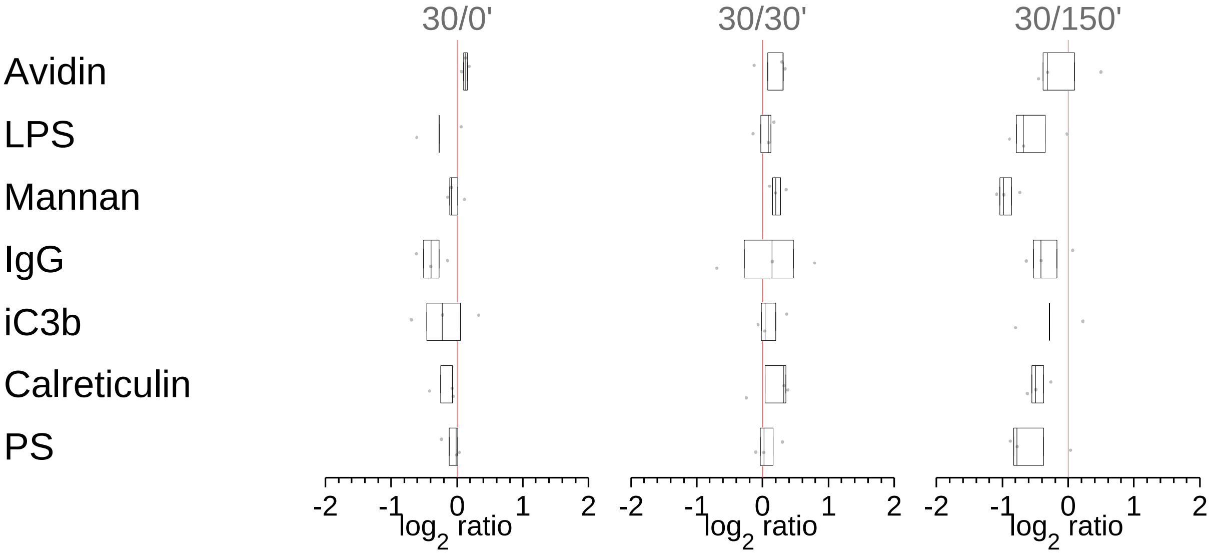

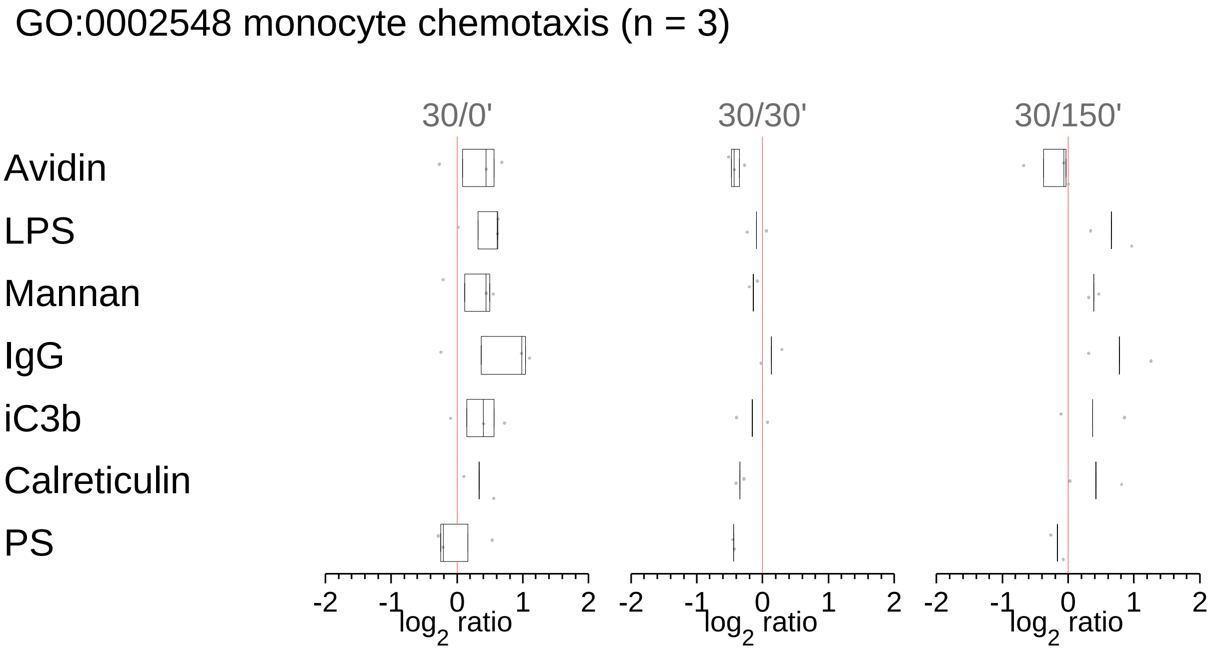
GO:0002548 monocyte chemotaxis (n = 3)
30/0'
30/30'
30/150'
Avidin
LPS
Mannan
IgG
iC3b
Calreticulin
PS
-2 -1 0 1 2
log2 ratio

GO:0002675 positive regulation of acute inflammatory response (n = 3)

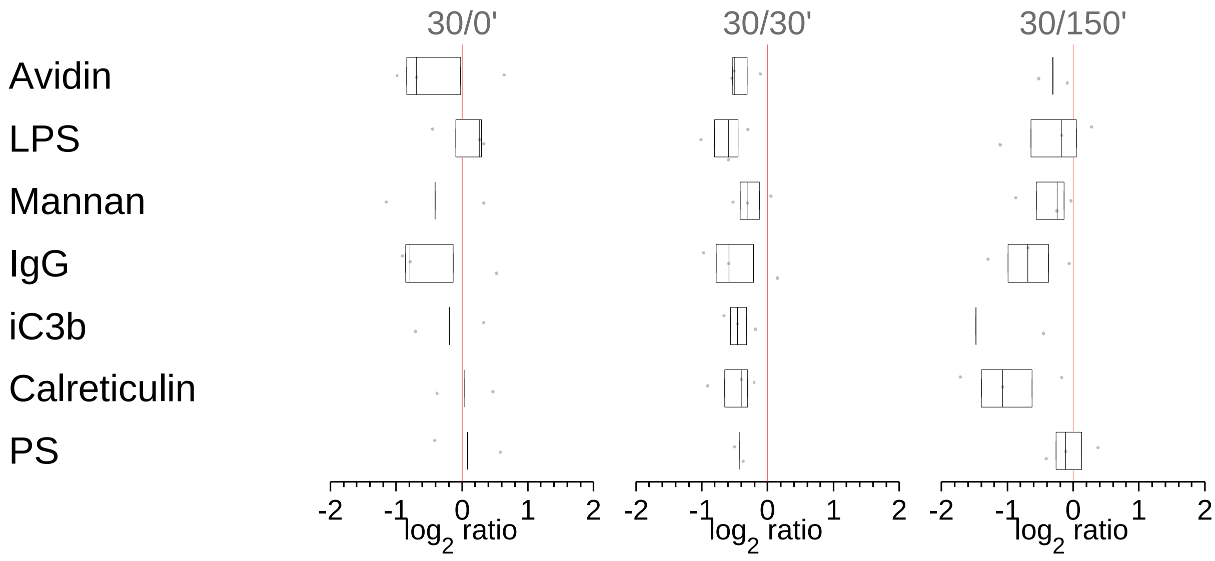

GO:0002755 MyD88-dependent toll-like receptor signaling pathway
(n = 6)

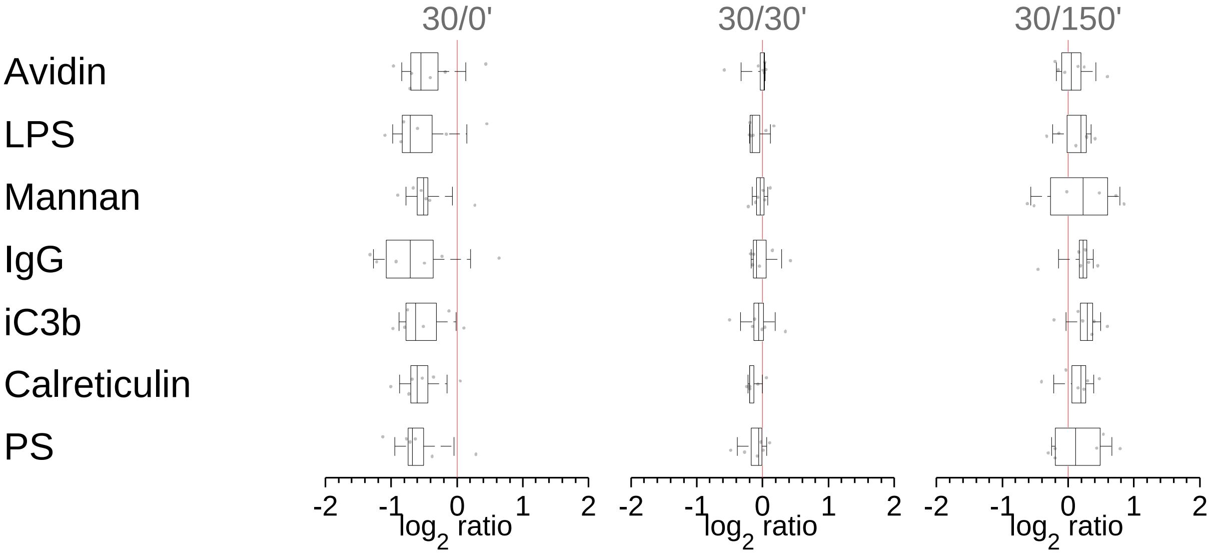

GO:0002821 positive regulation of adaptive immune response (n = 3)

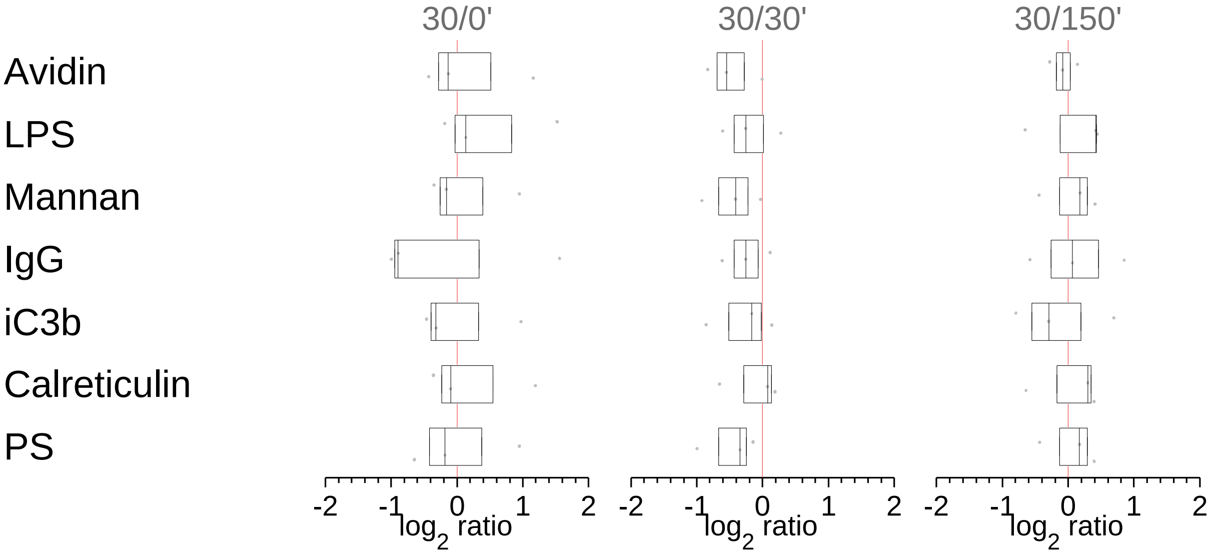

GO:0002862 negative regulation of inflammatory response to antigenic stimulus (n = 3)

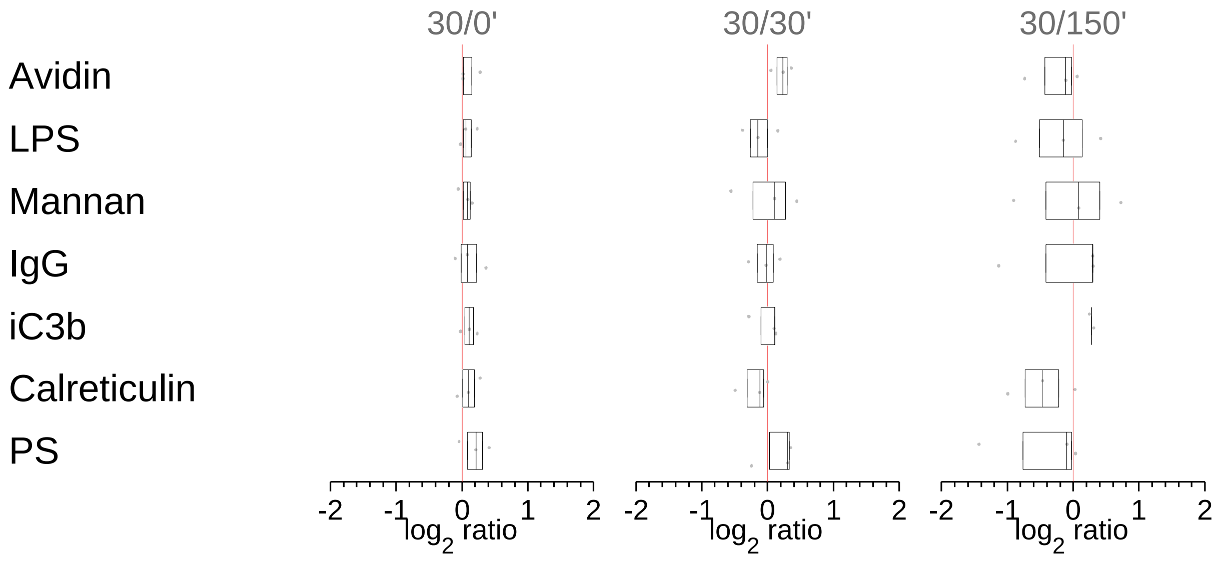

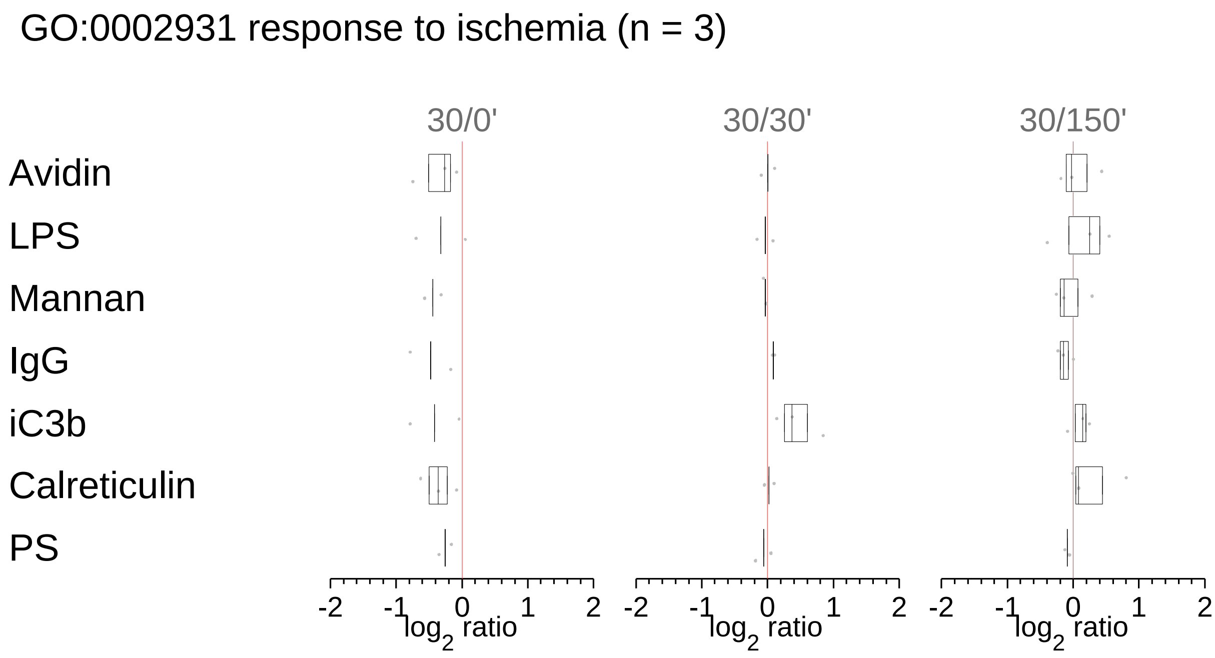

GO:0002931 response to ischemia (n = 3)
30/0'
30/30'
30/150'
Avidin
LPS
Mannan
IgG
iC3b
Calreticulin
PS
-2
-1
0
1
2
log2 ratio

GO:0003007 heart morphogenesis (n = 3)

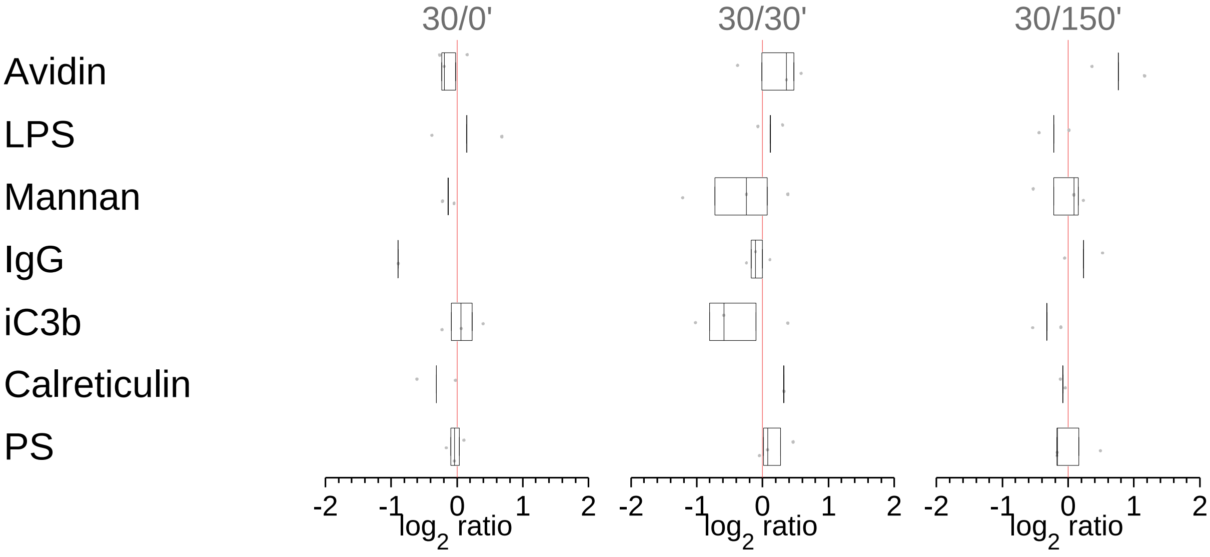

GO:0003333 amino acid transmembrane transport (n = 9)

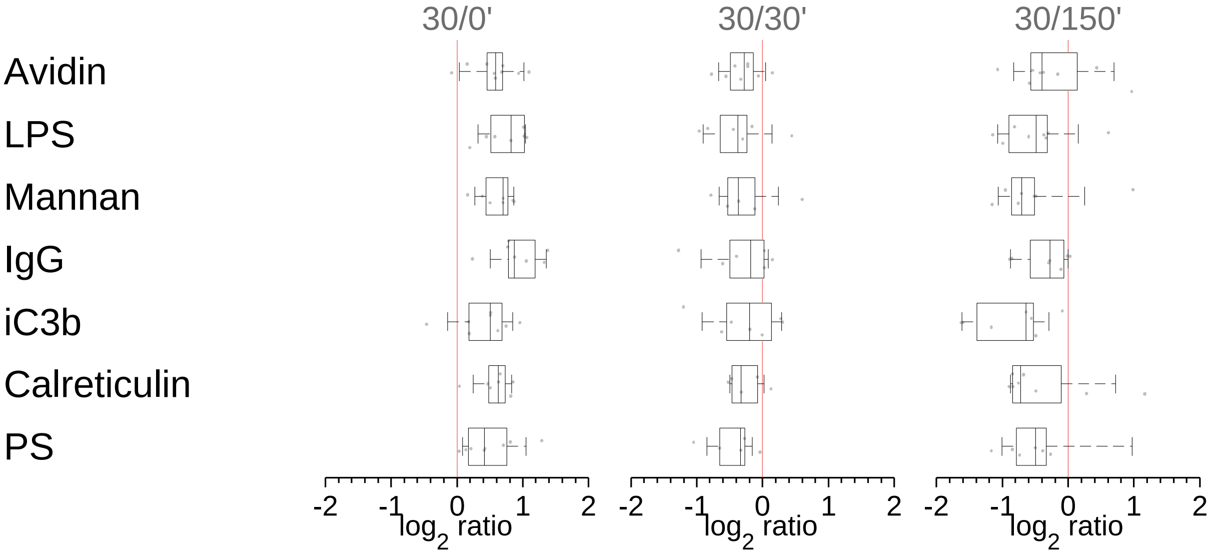

GO:0003674 molecular_function (n = 218)

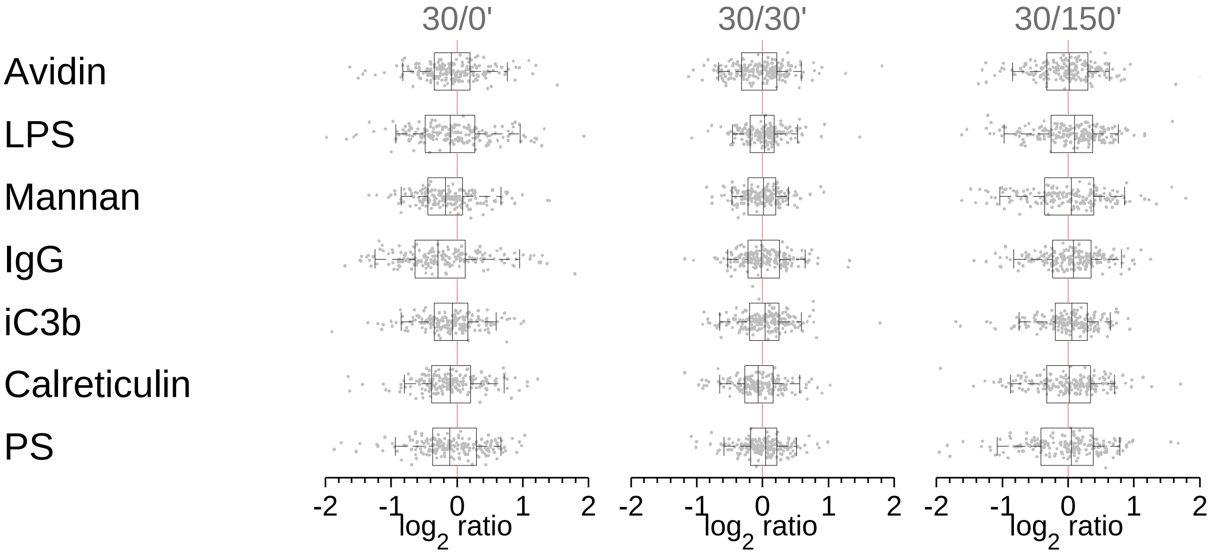

GO:0003676 nucleic acid binding (n = 69)

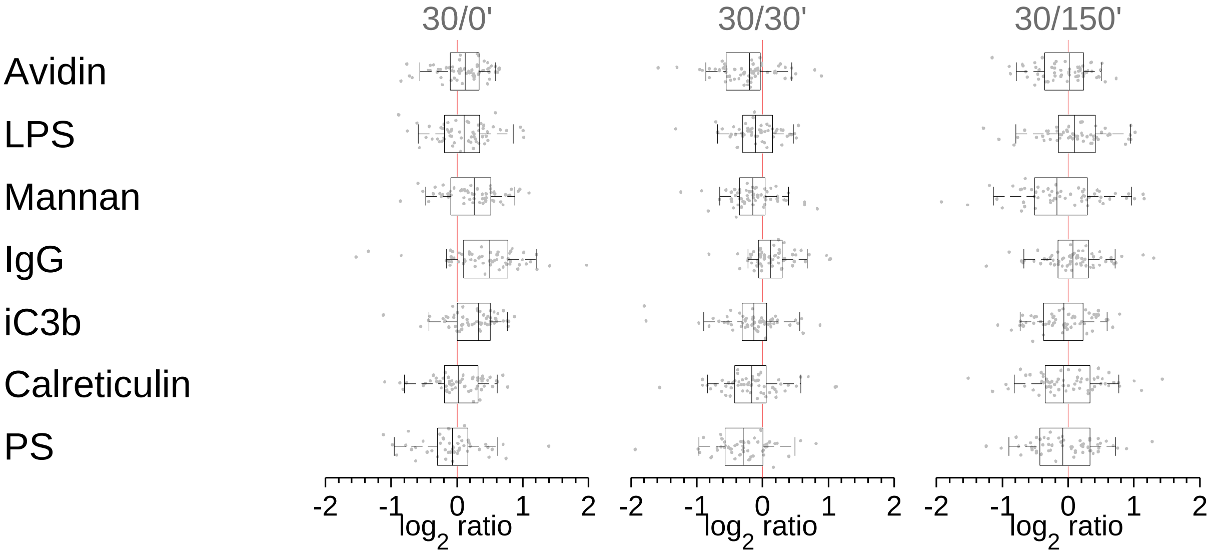

GO:0003677 DNA binding (n = 84)

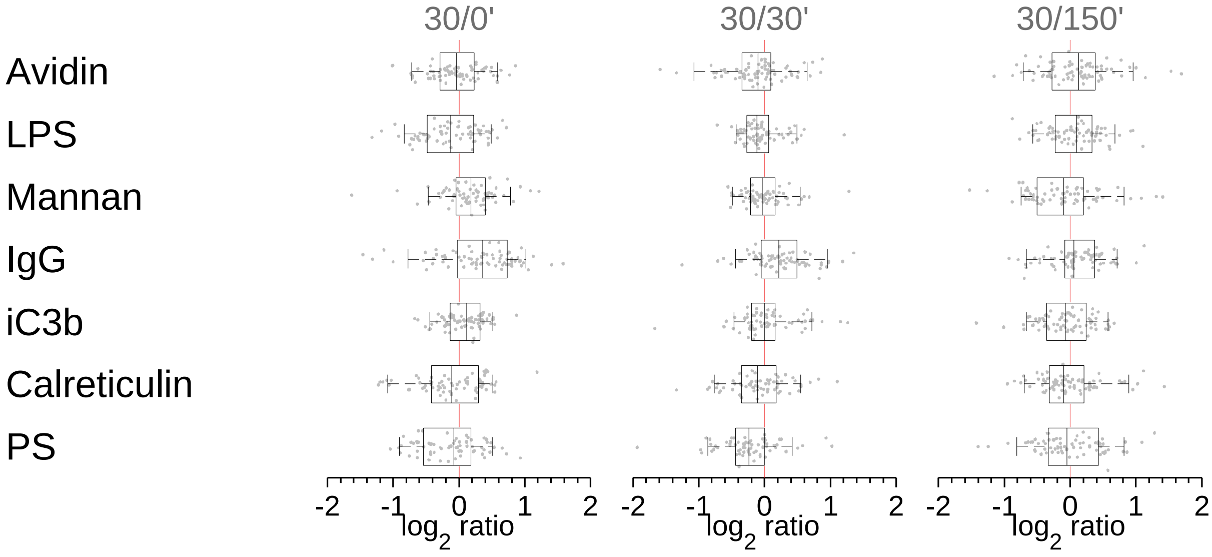

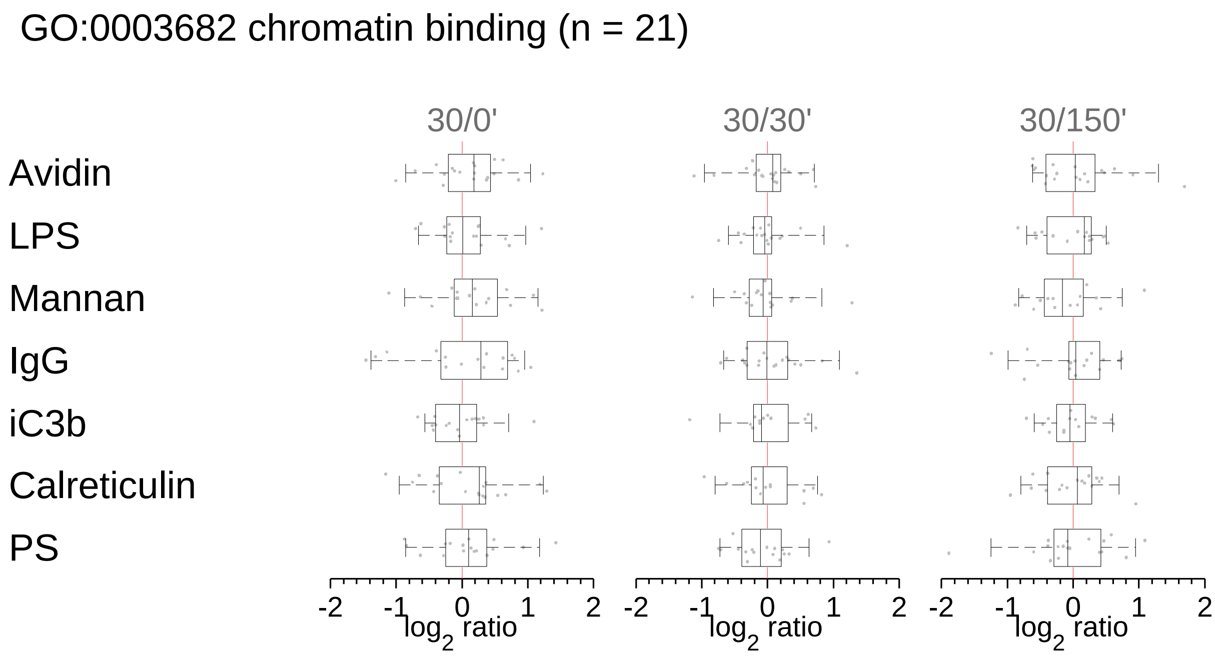
GO:0003682 chromatin binding (n = 21)
30/0'
30/30'
30/150'
Avidin
LPS
Mannan
IgG
iC3b
Calreticulin
PS
-2
-1
0
1
2
log2 ratio

GO:0003684 damaged DNA binding (n = 4)

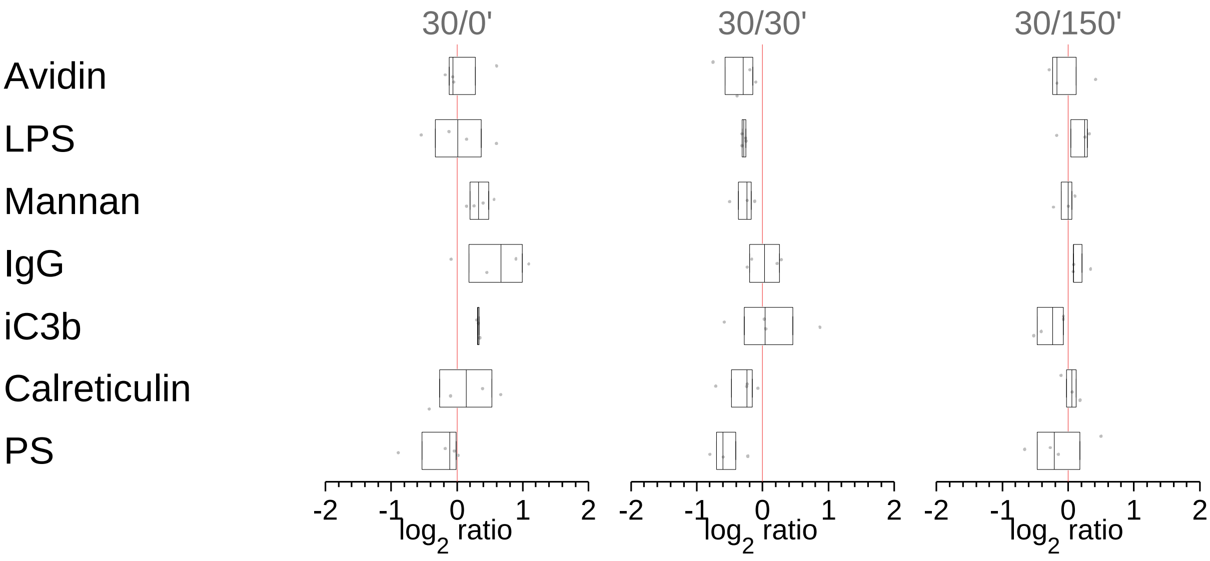

GO:0003690 double-stranded DNA binding (n = 3)

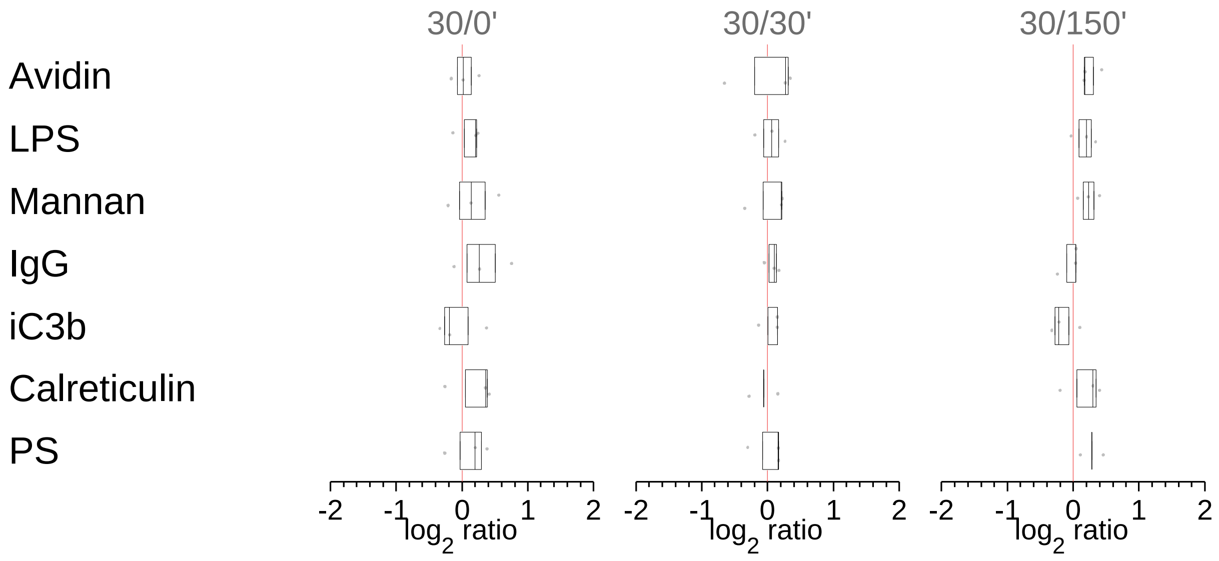

GO:0003697 single-stranded DNA binding (n = 7)

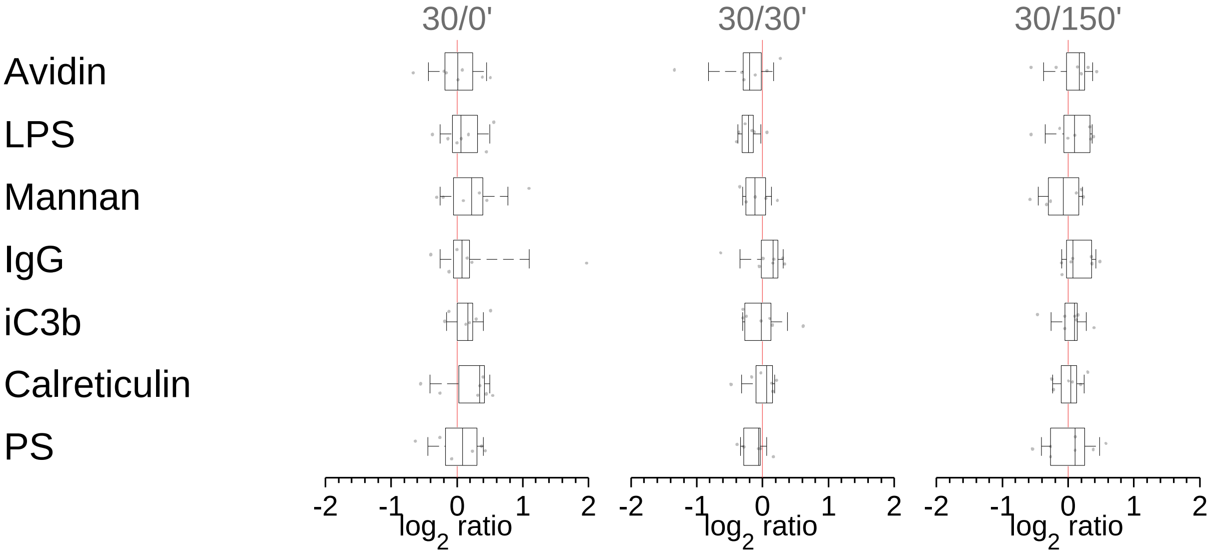

GO:0003700 sequence-specific DNA binding transcription factor activity (n = 10)

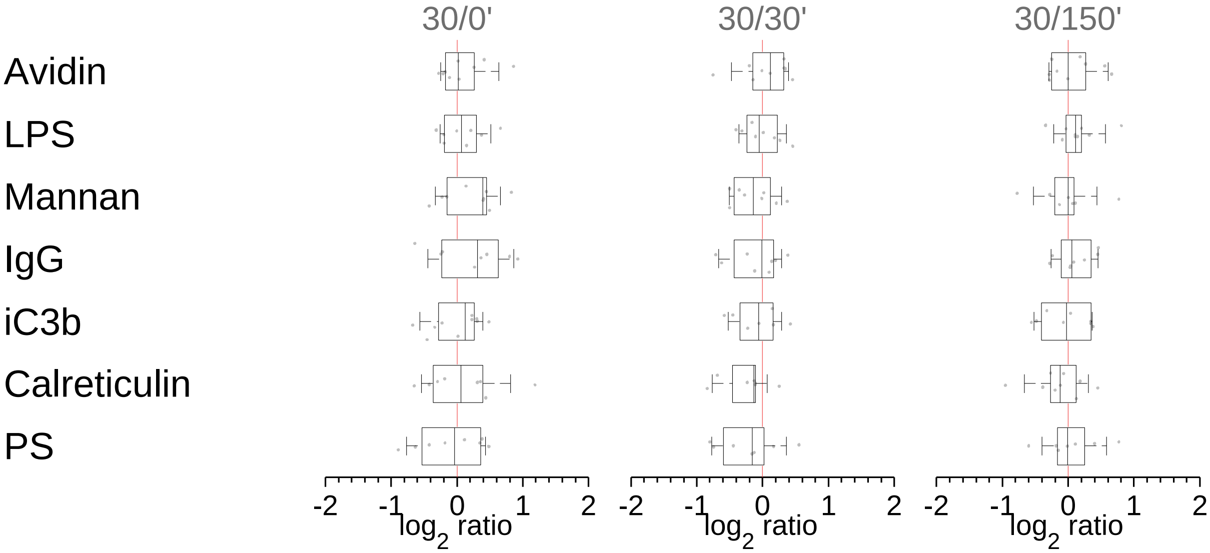

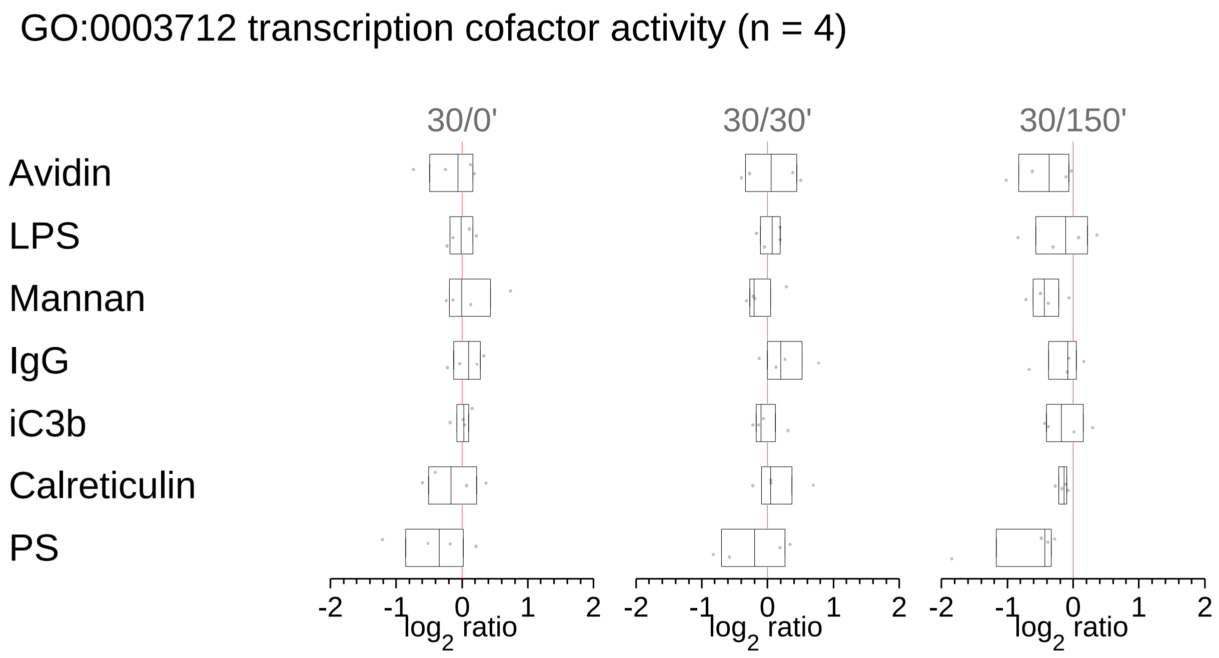
GO:0003712 transcription cofactor activity (n = 4)
30/0'
30/30'
30/150'
Avidin
LPS
Mannan
IgG
iC3b
Calreticulin
PS
-2
-1
0
1
2
log2 ratio

GO:0003713 transcription coactivator activity (n = 12)

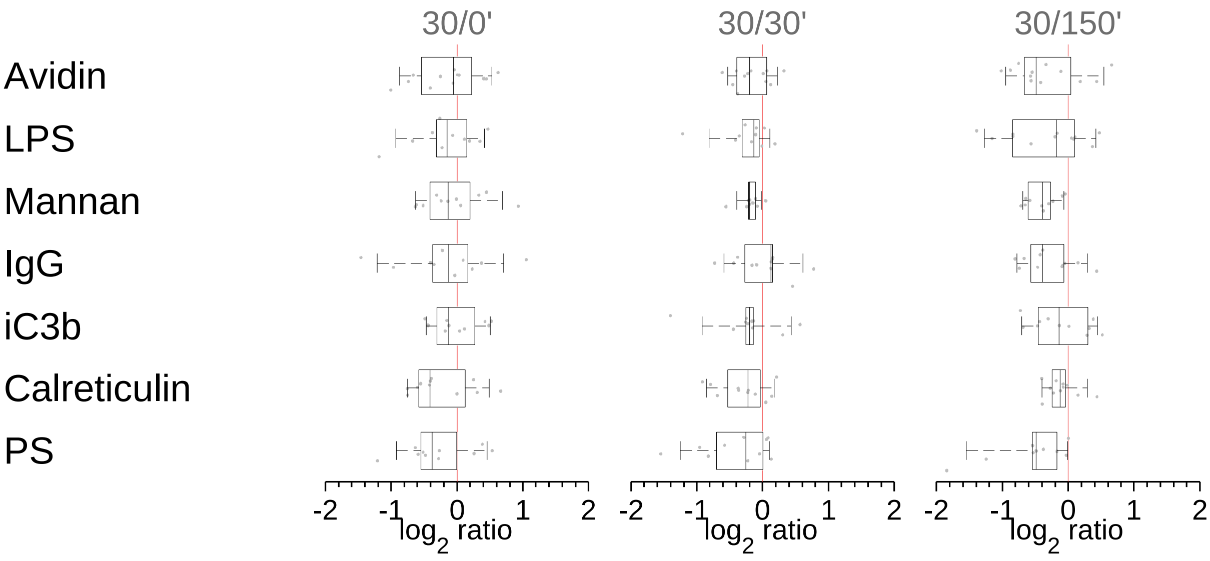

GO:0003714 transcription corepressor activity (n = 9)

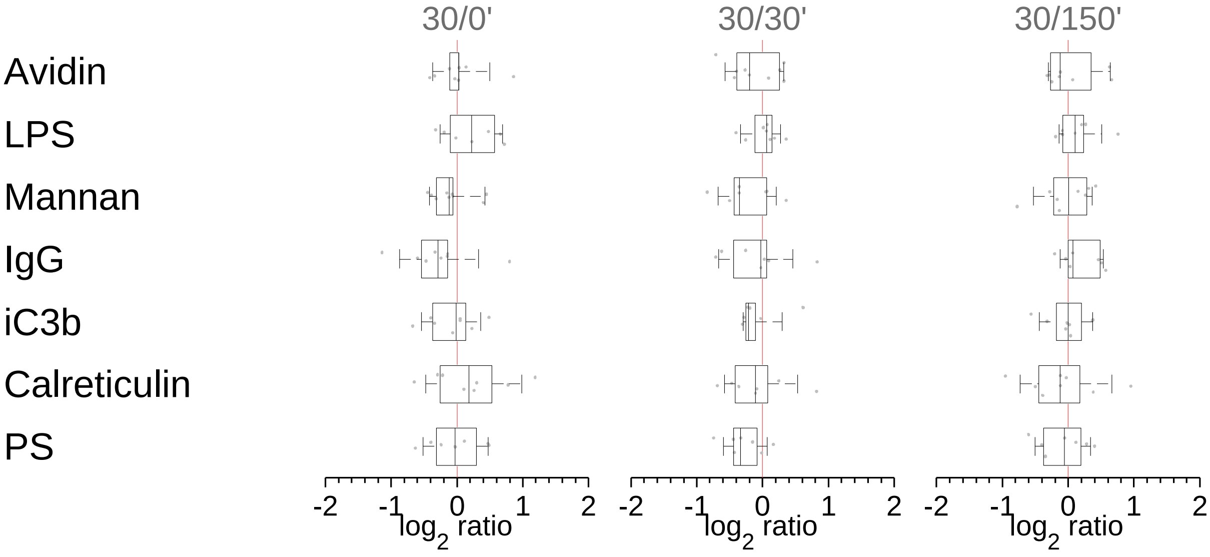

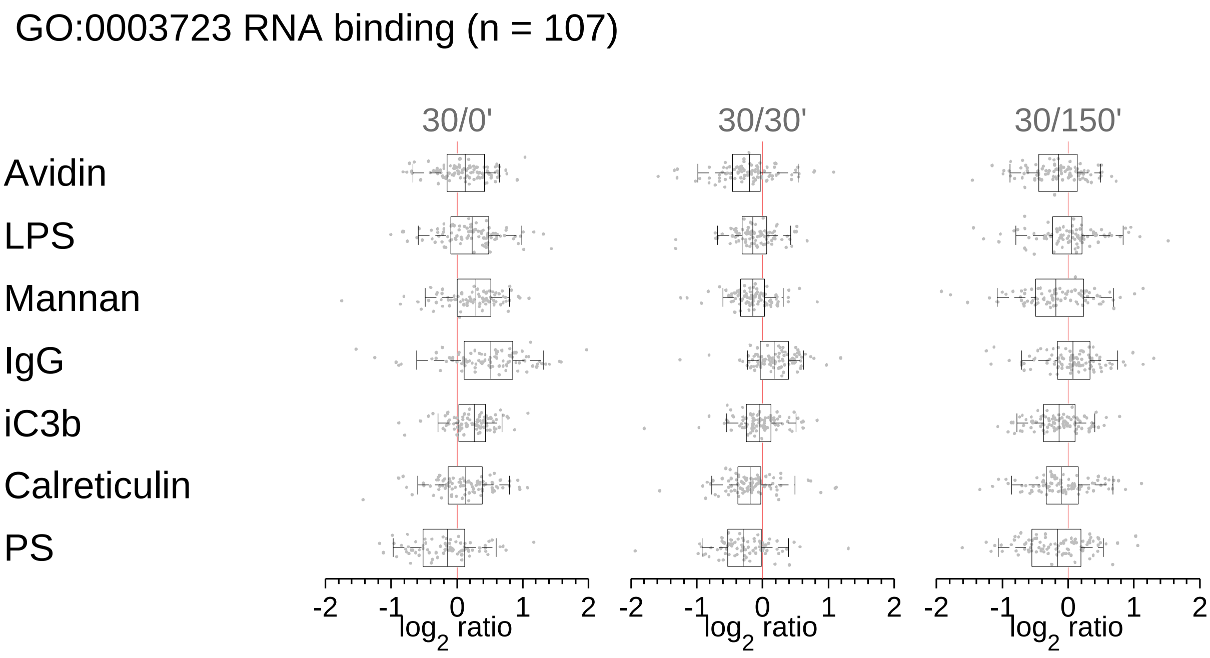
GO:0003723 RNA binding (n = 107)
30/0'
30/30'
30/150'
Avidin
LPS
Mannan
IgG
iC3b
Calreticulin
PS
-2 -1 0 1 2
log2 ratio

GO:0003725 double-stranded RNA binding (n = 20)

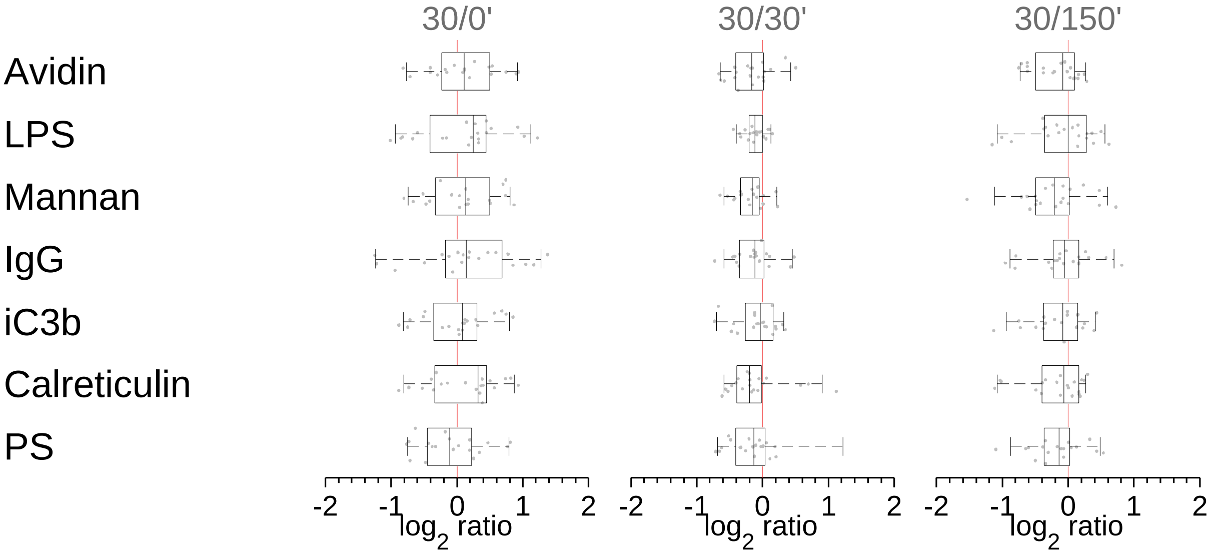

GO:0003727 single-stranded RNA binding (n = 5)

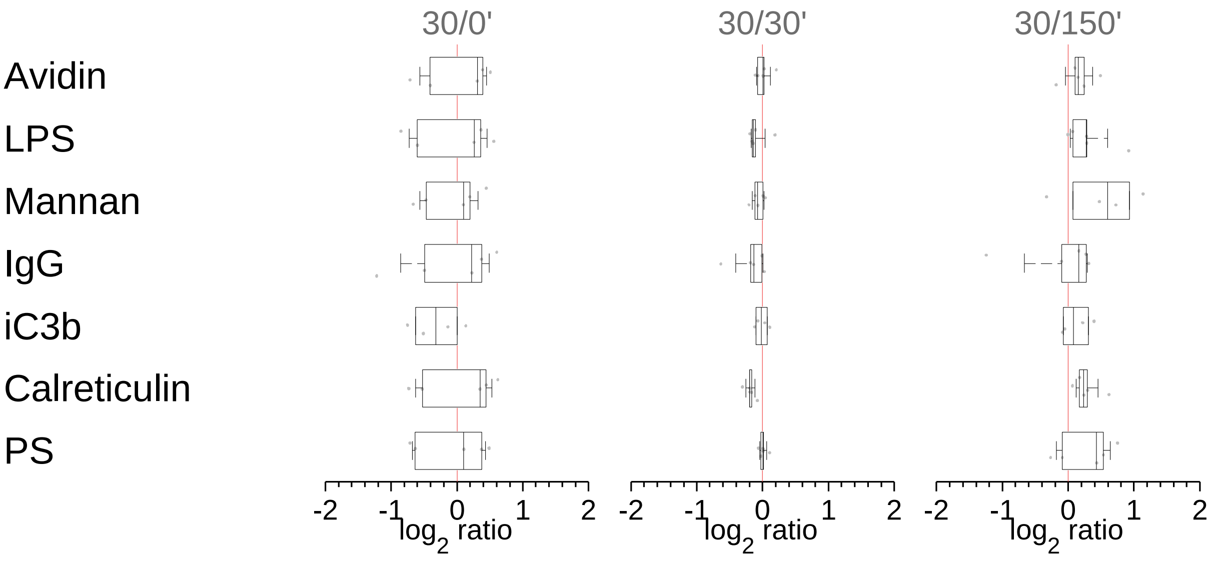

GO:0003729 mRNA binding (n = 14)

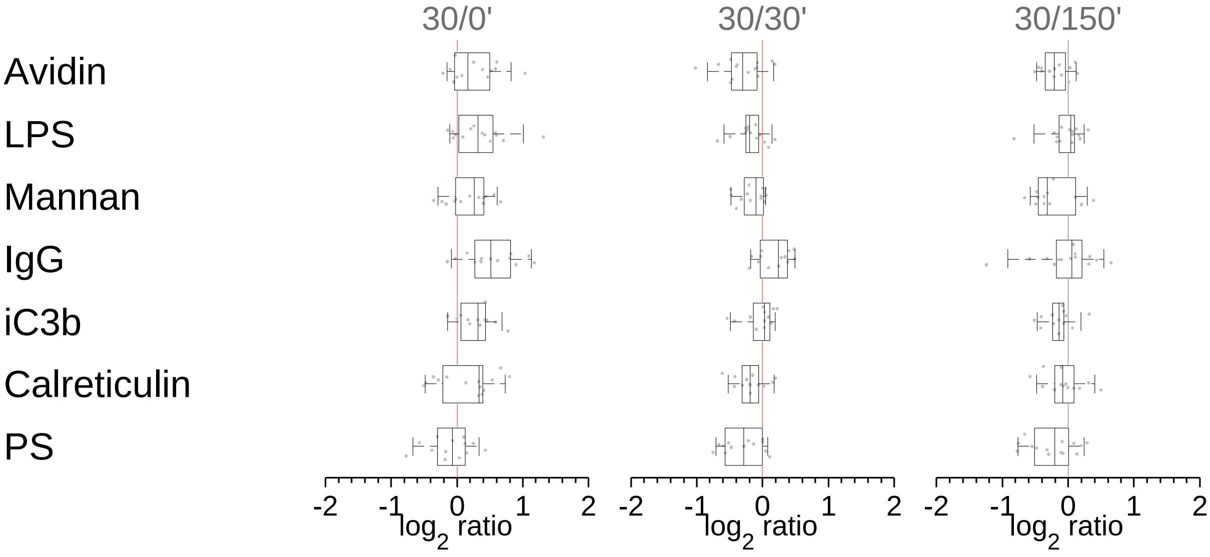

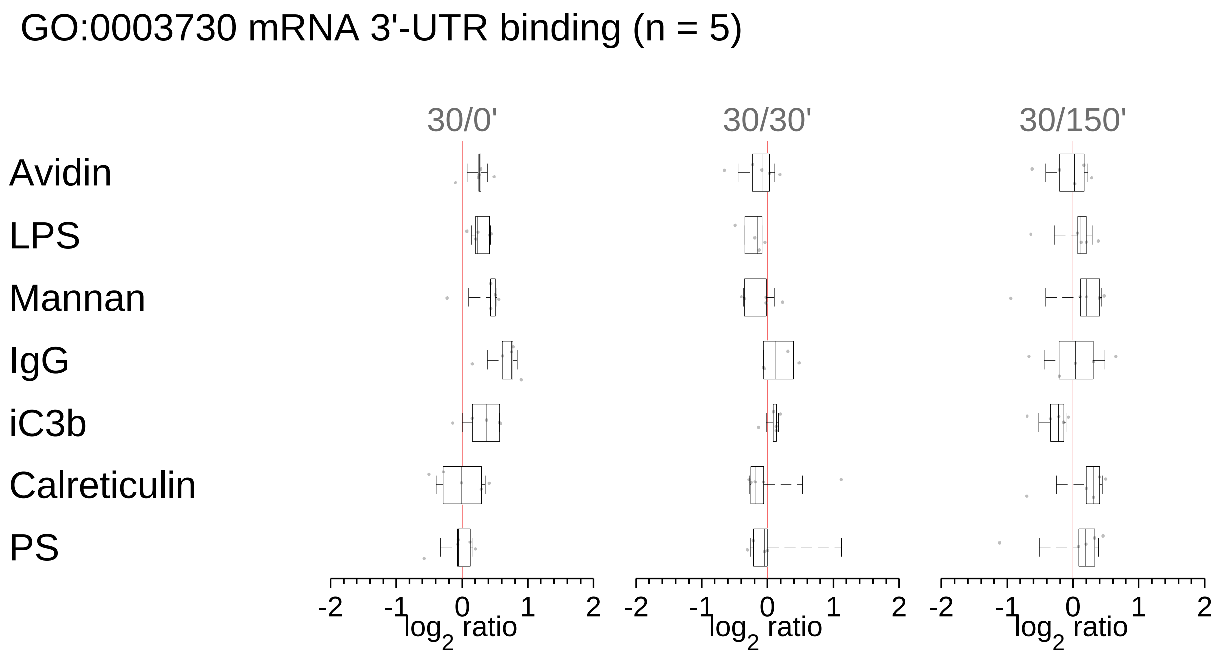
GO:0003730 mRNA 3'-UTR binding (n = 5)
30/0'
30/30'
30/150'
Avidin
LPS
Mannan
IgG
iC3b
Calreticulin
PS
-2
-1
0
1
2
log2 ratio

GO:0003735 structural constituent of ribosome (n = 52)

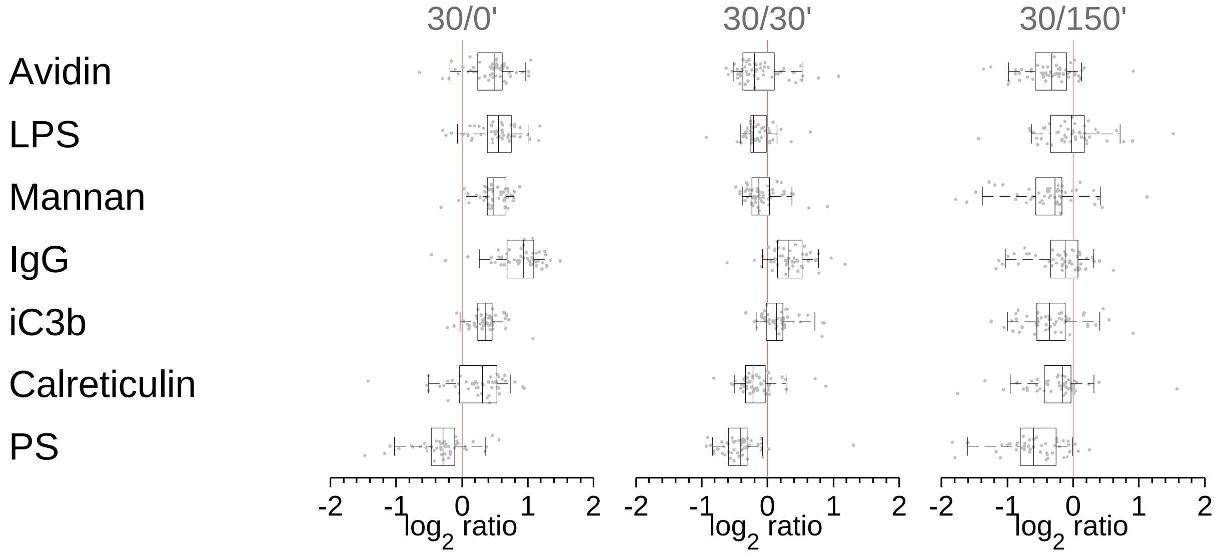

GO:0003743 translation initiation factor activity (n = 12)

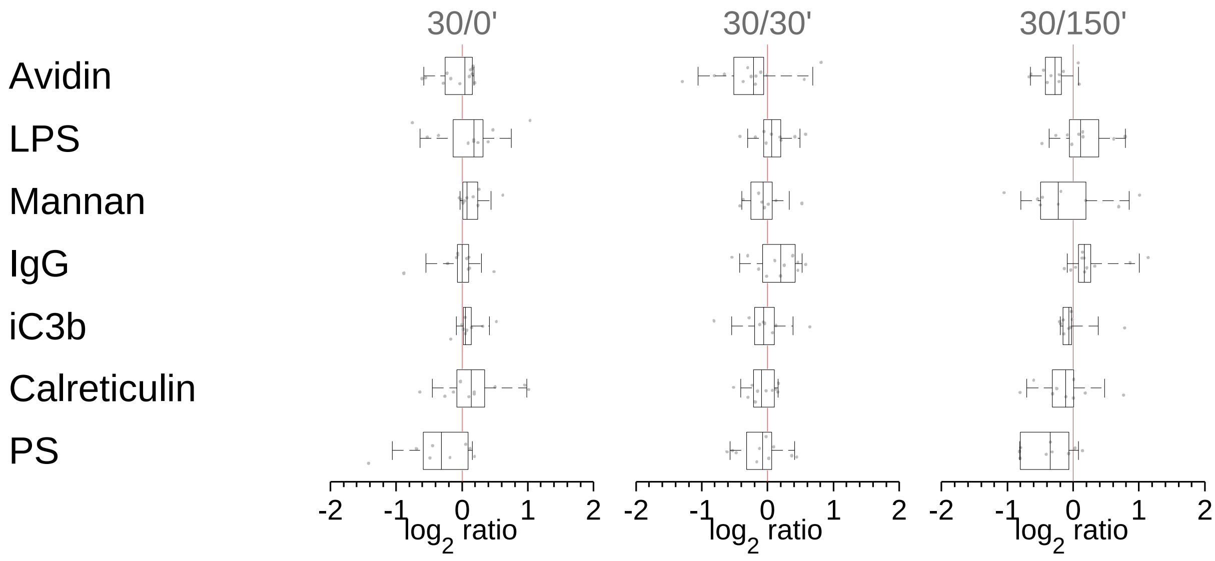

GO:0003746 translation elongation factor activity (n = 7)

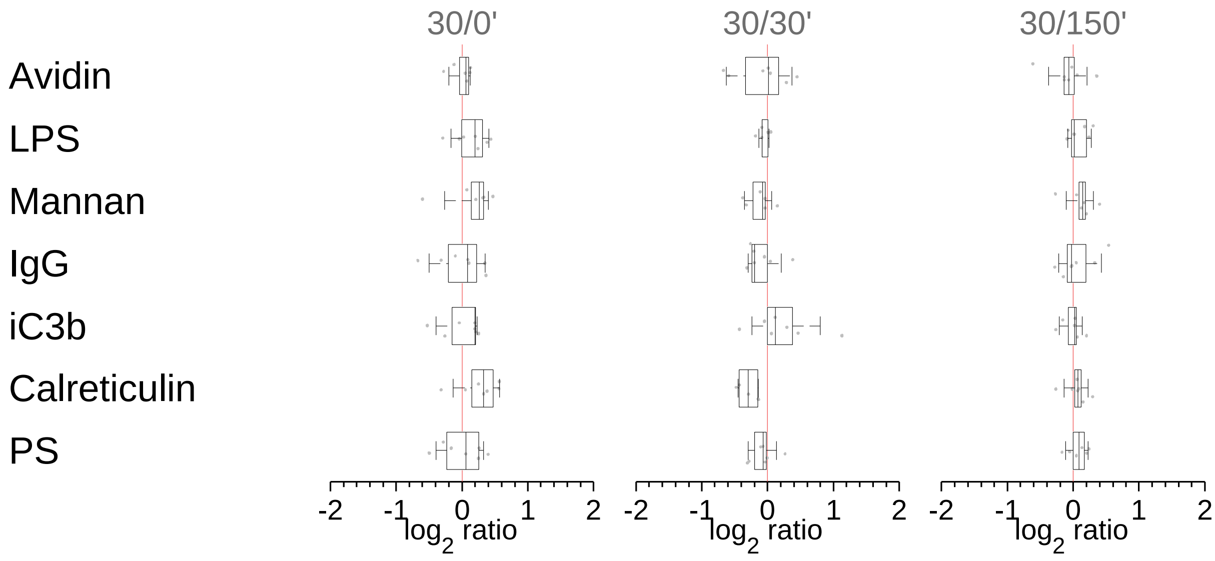

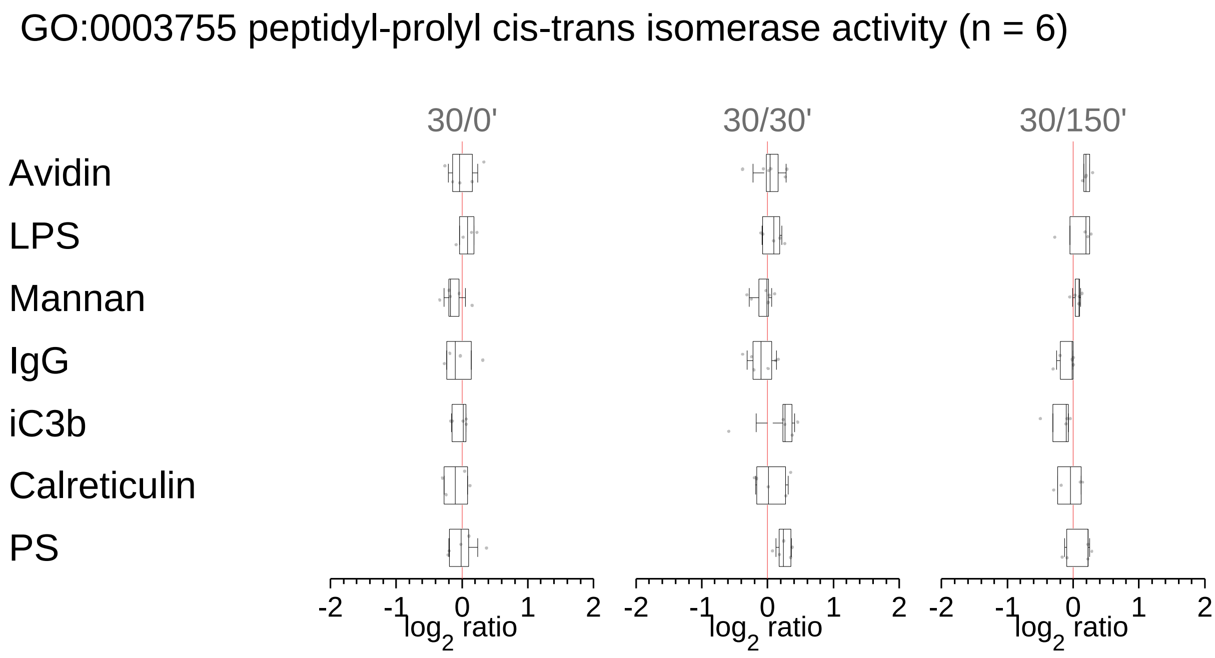

GO:0003755 peptidyl-prolyl cis-trans isomerase activity (n = 6)
30/0'
30/30'
30/150'
Avidin
LPS
Mannan
IgG
iC3b
Calreticulin
PS
-2 -1 0 1 2
log2 ratio
-2 -1 0 1 2
log2 ratio
-2 -1 0 1 2
log2 ratio

GO:0003756 protein disulfide isomerase activity (n = 9)

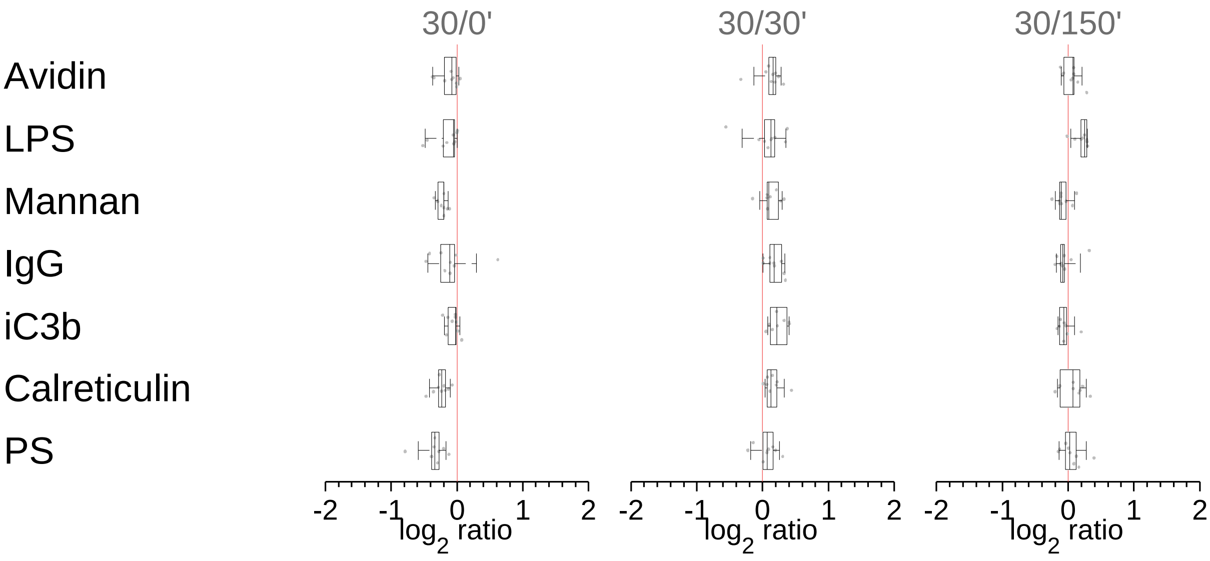

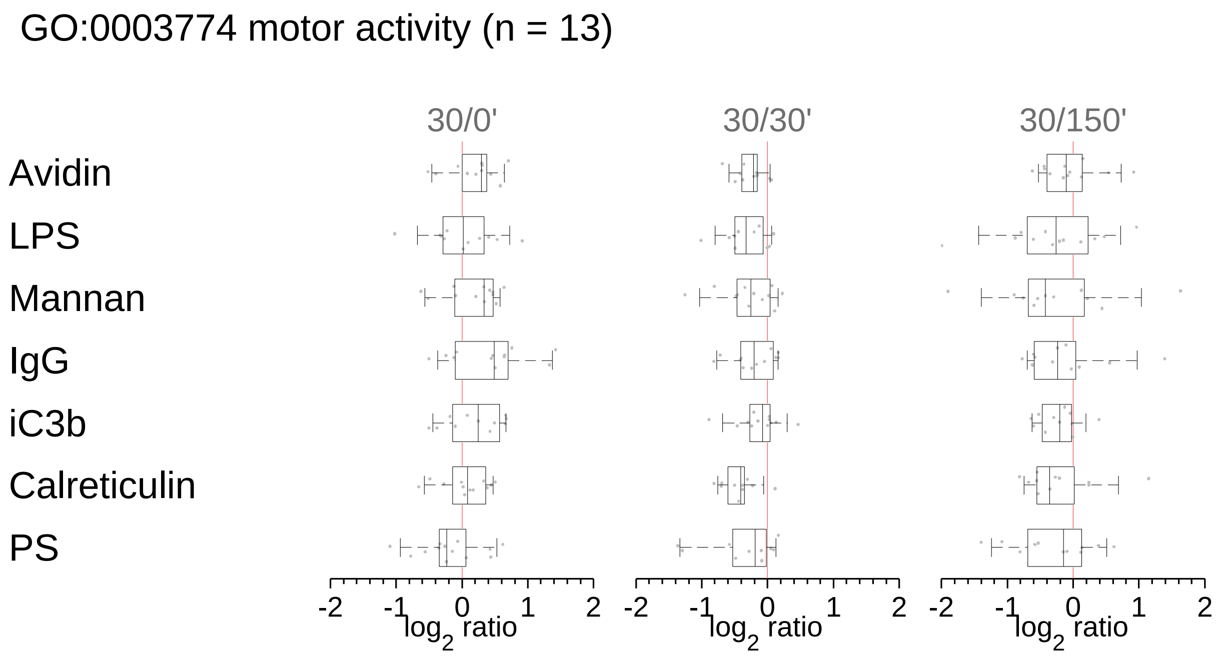
GO:0003774 motor activity (n = 13)
30/0'
30/30'
30/150'
Avidin
LPS
Mannan
IgG
iC3b
Calreticulin
PS
-2
-1
0
1
2
log2 ratio

GO:0003777 microtubule motor activity (n = 6)

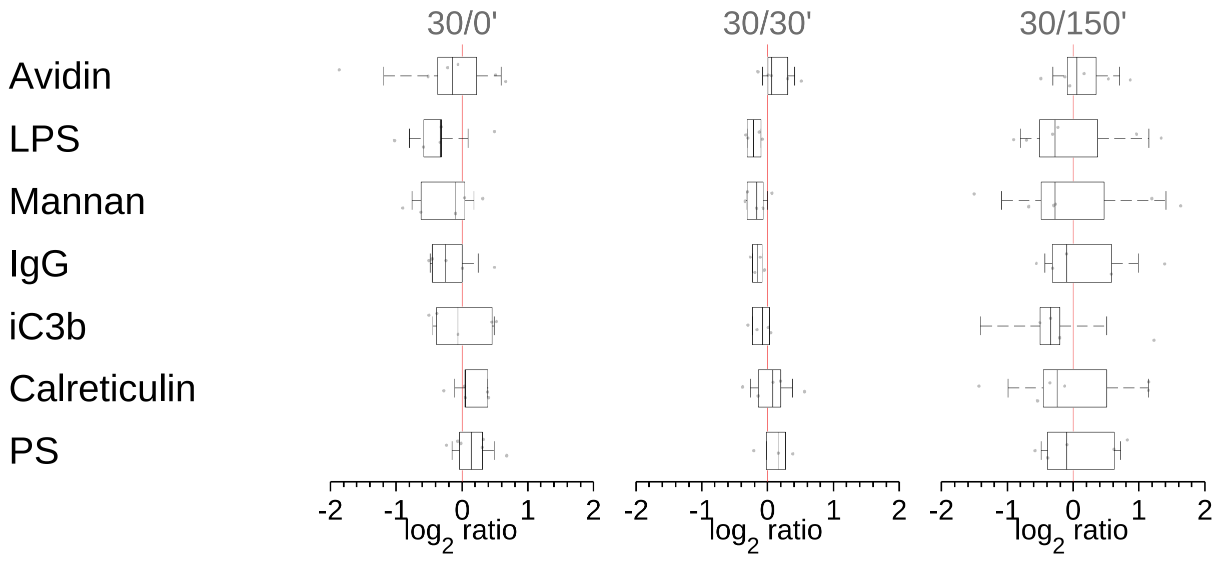

GO:0003779 actin binding (n = 82)

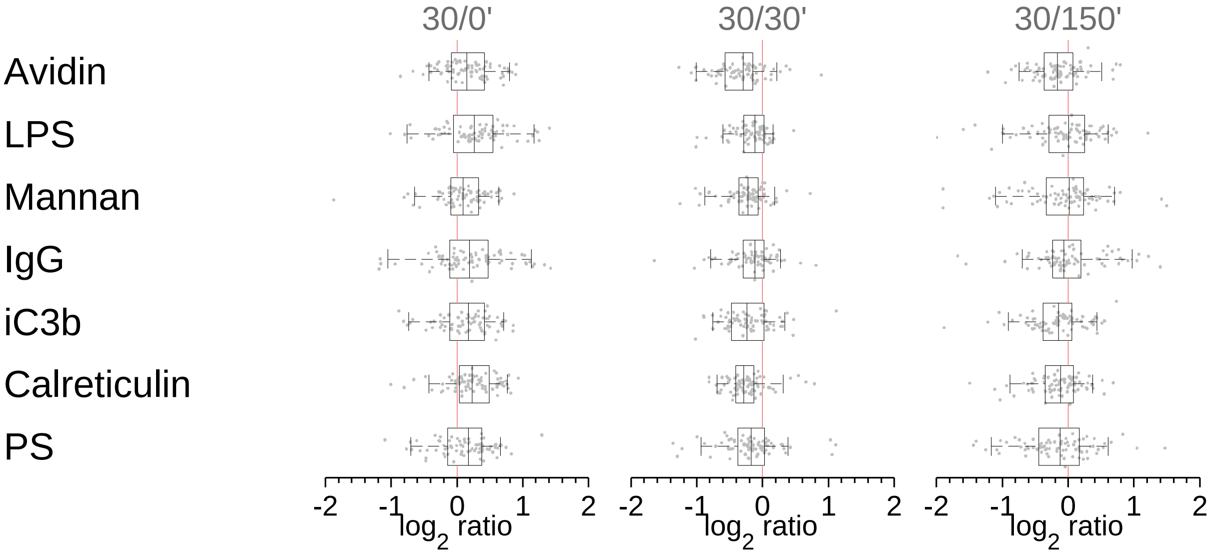

GO:0003785 actin monomer binding (n = 3)

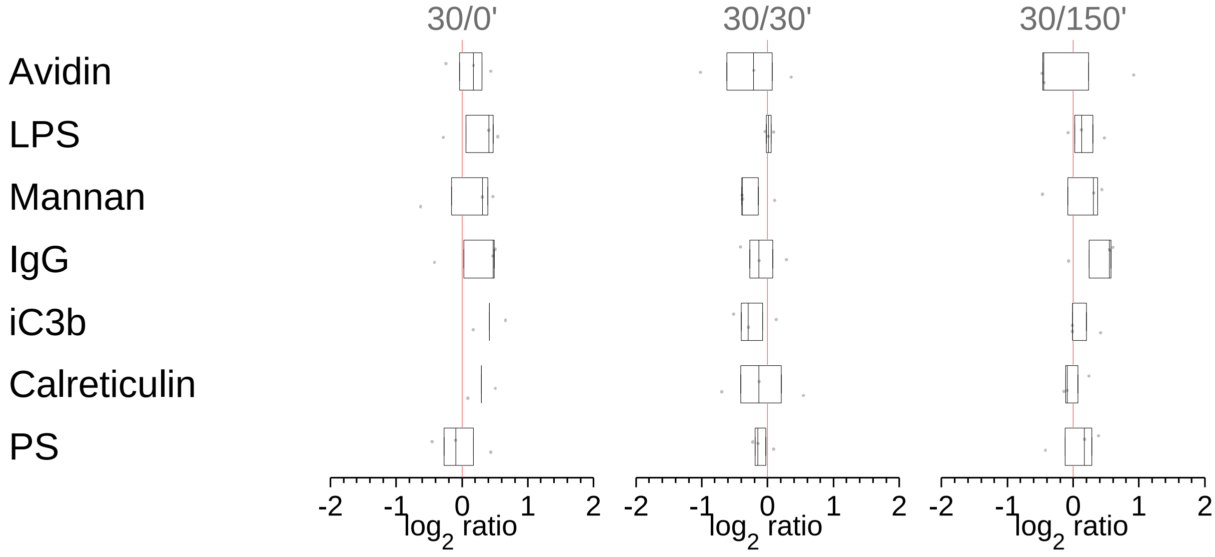

GO:0003823 antigen binding (n = 3)

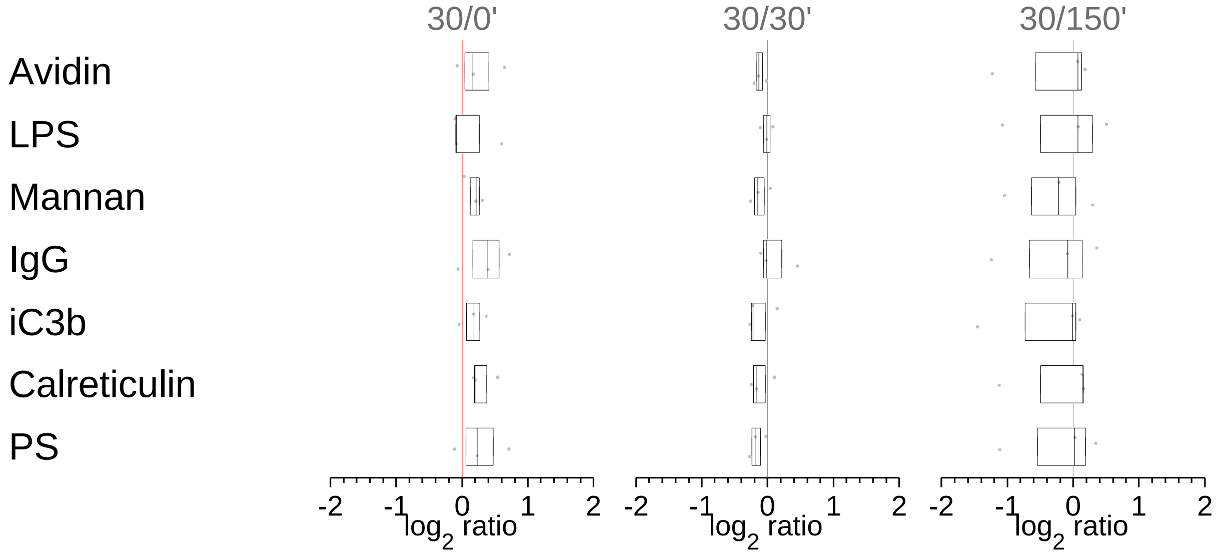

GO:0003824 catalytic activity (n = 77)

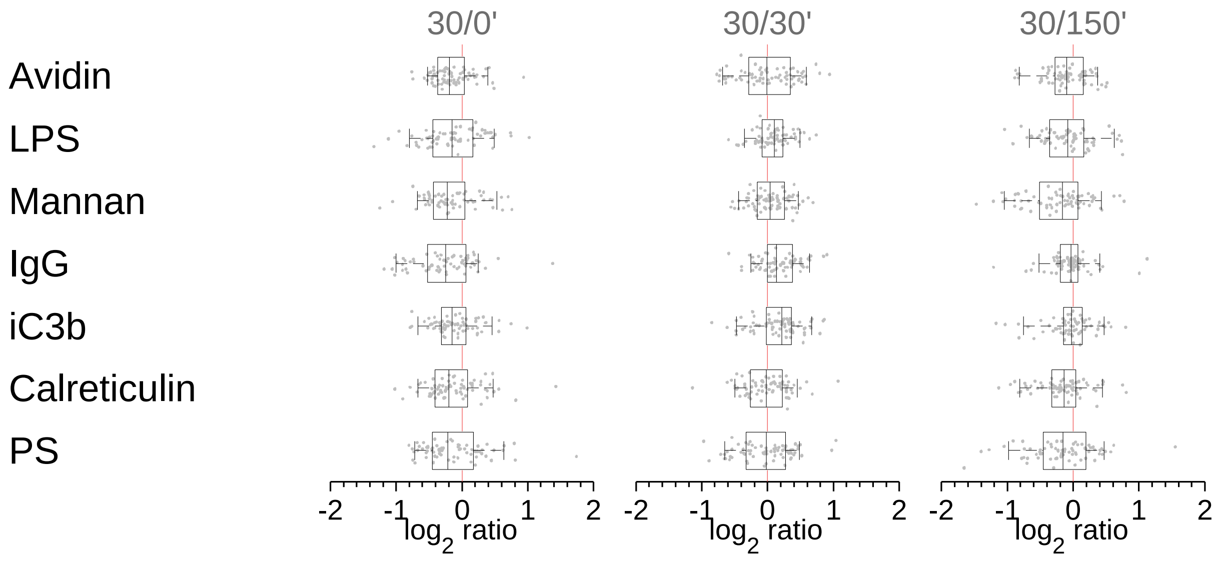

GO:0003857 3-hydroxyacyl-CoA dehydrogenase activity (n = 4)

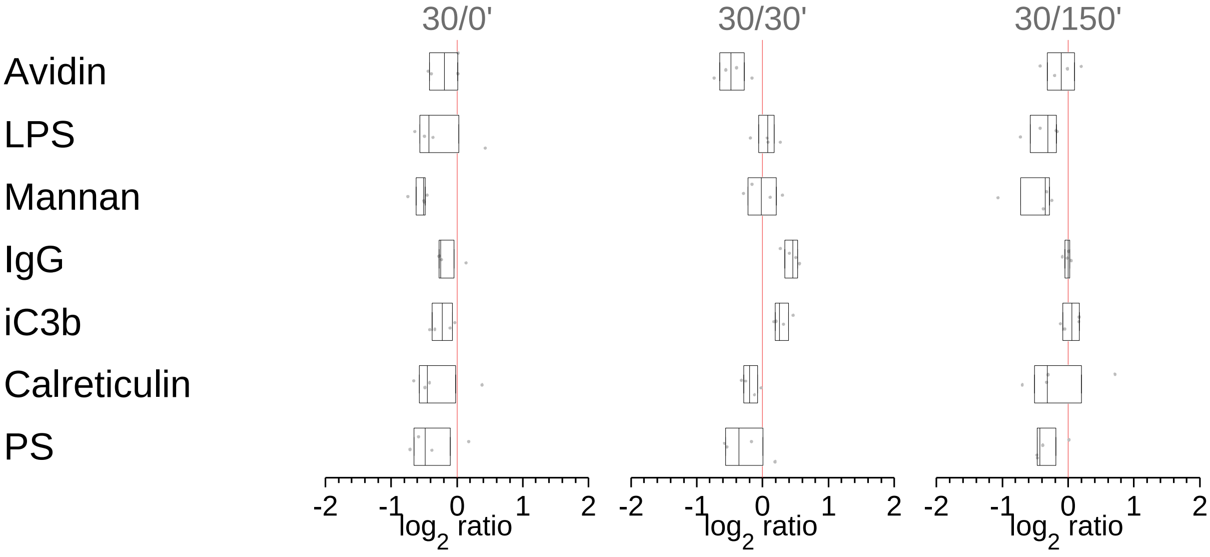

GO:0003923 GPI-anchor transamidase activity (n = 4)

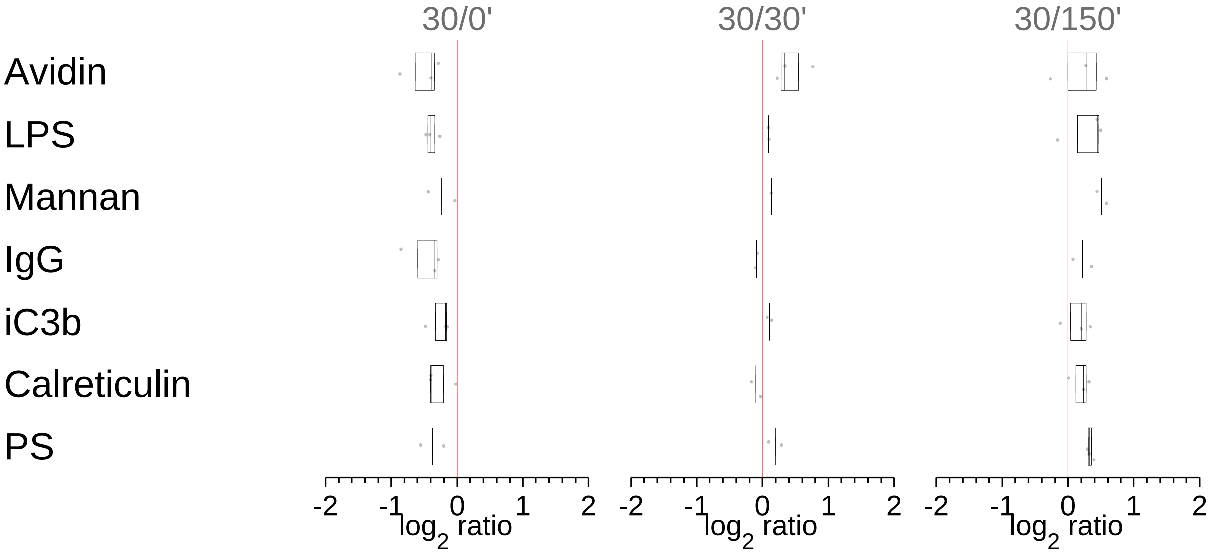

GO:0003924 GTPase activity (n = 64)

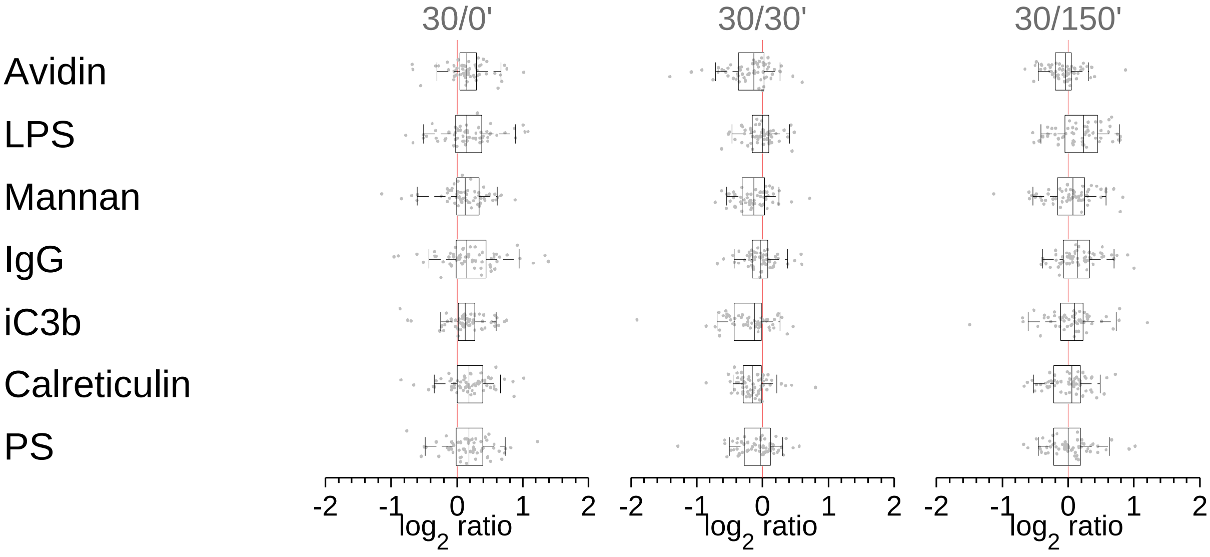

GO:0003950 NAD+ ADP-ribosyltransferase activity (n = 3)

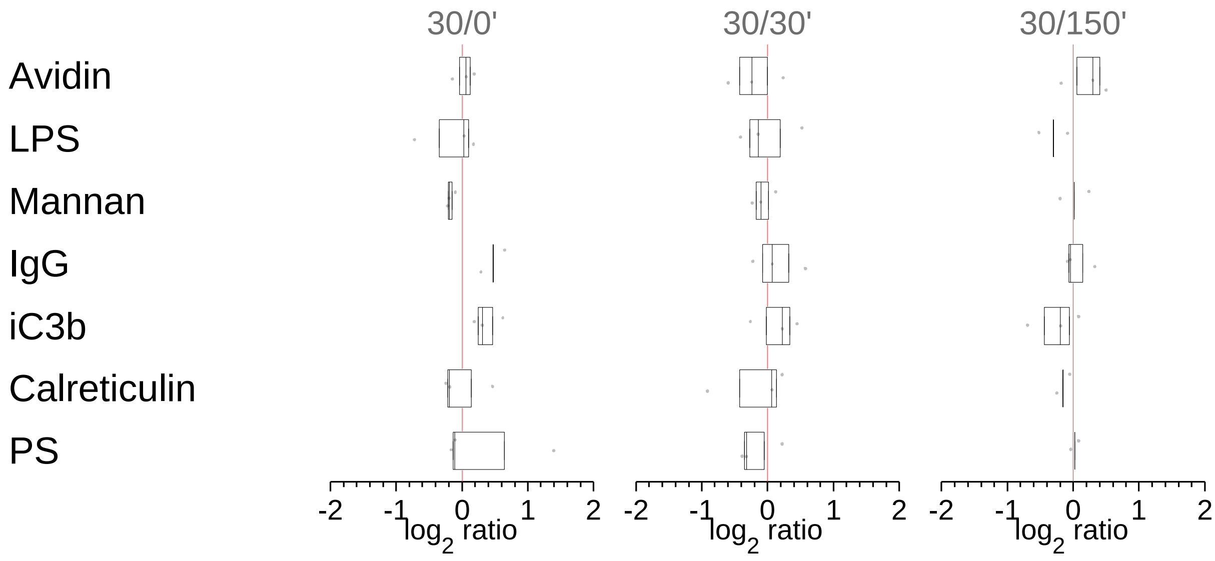

GO:0003954 NADH dehydrogenase activity (n = 5)

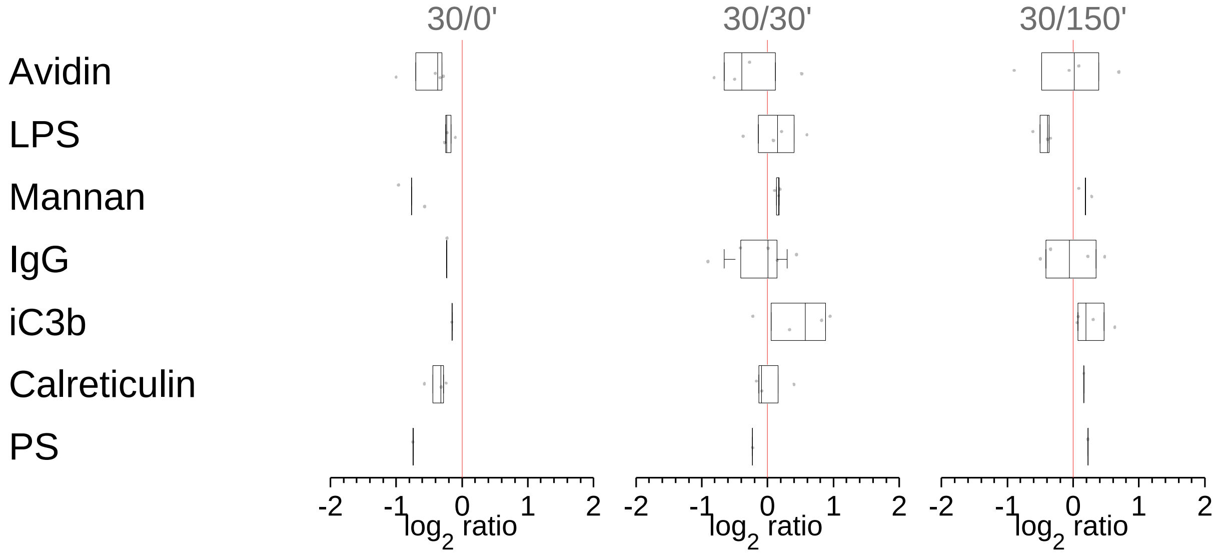

GO:0003980 UDP-glucose:glycoprotein glucosyltransferase activity
(n = 3)

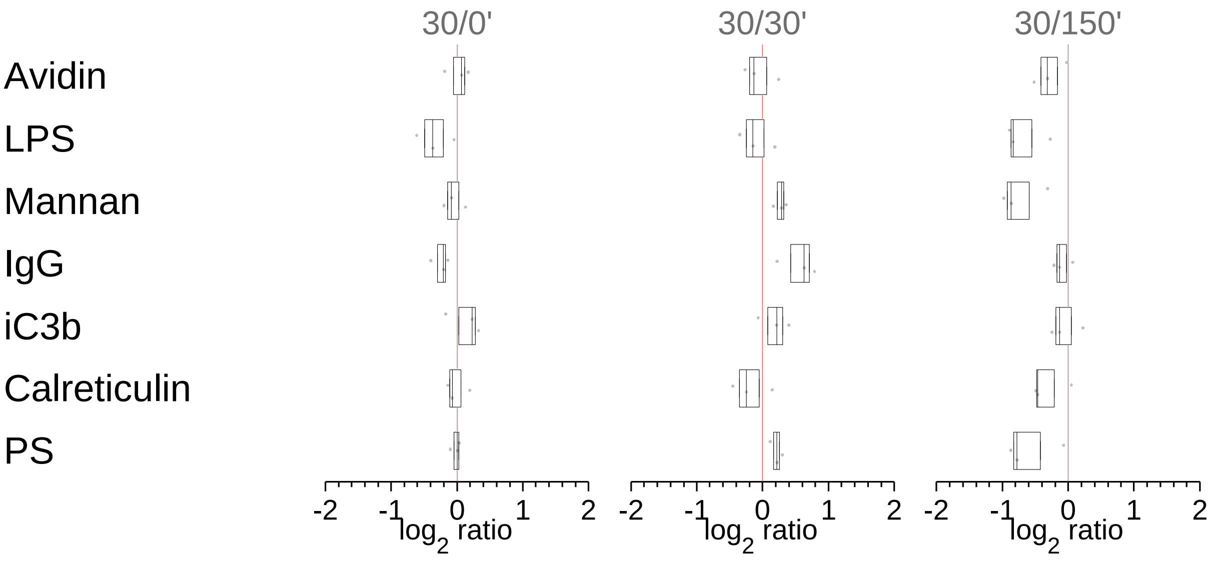

GO:0003988 acetyl-CoA C-acyltransferase activity (n = 4)

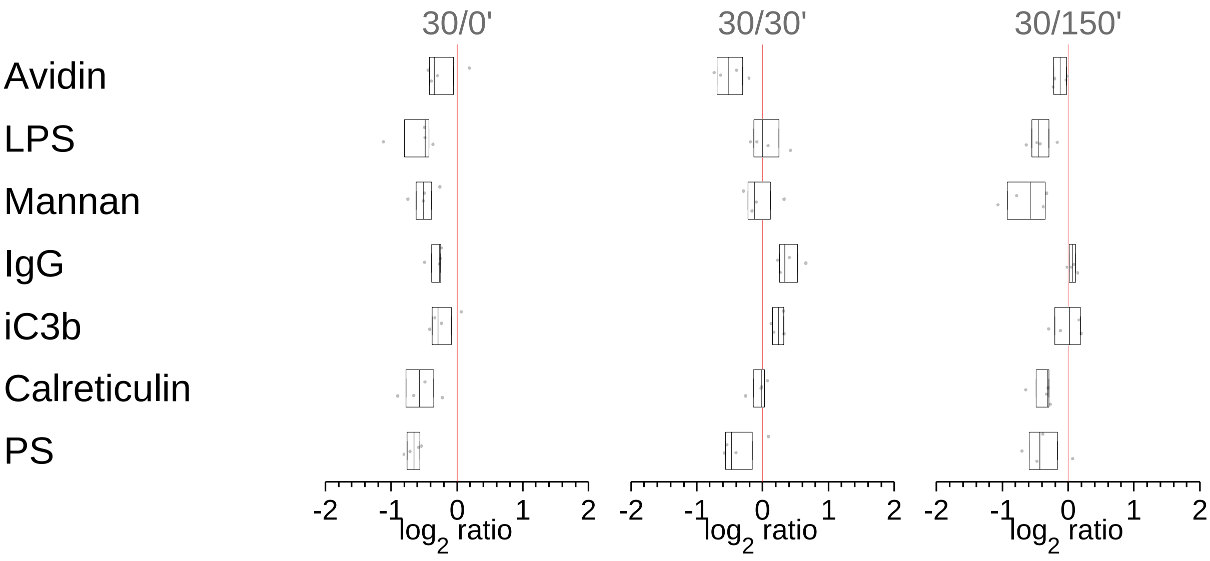

GO:0003995 acyl-CoA dehydrogenase activity (n = 5)

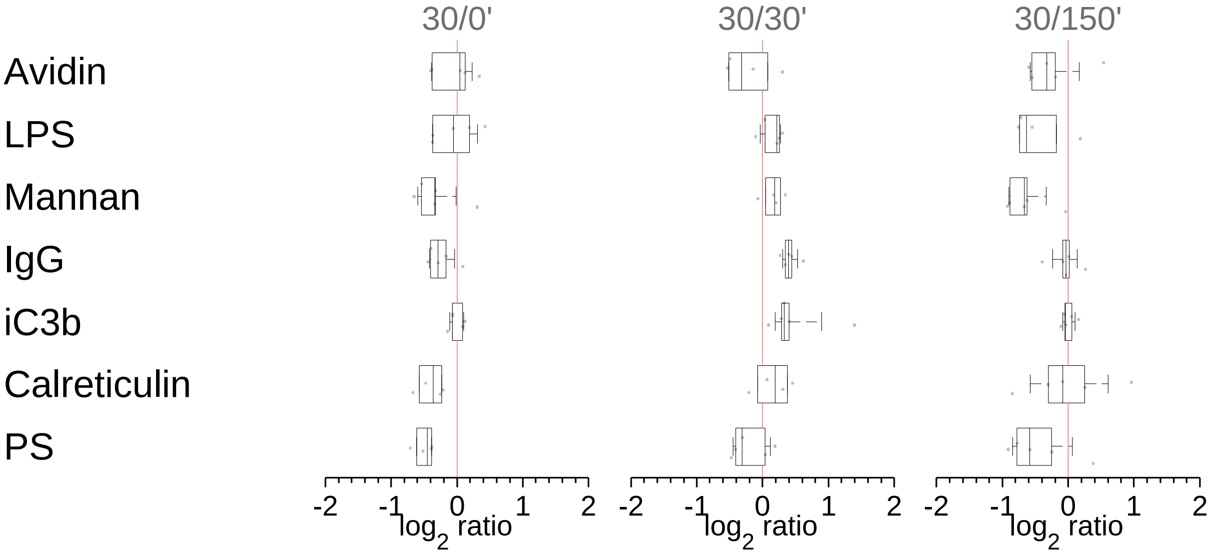

GO:0004004 ATP-dependent RNA helicase activity (n = 4)

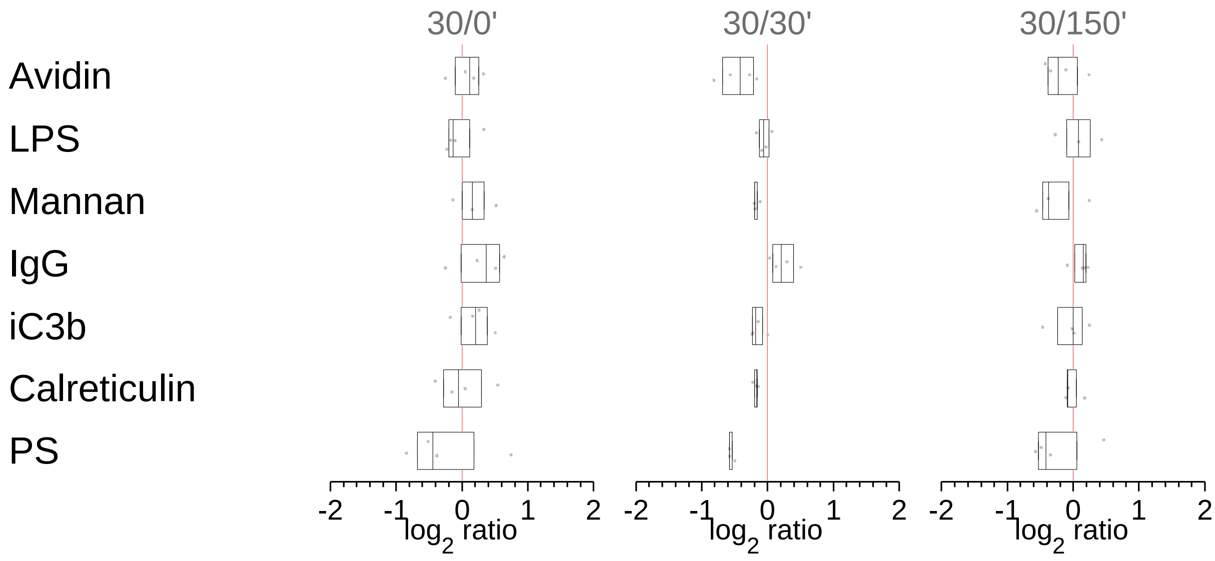

GO:0004012 phospholipid-translocating ATPase activity (n = 4)

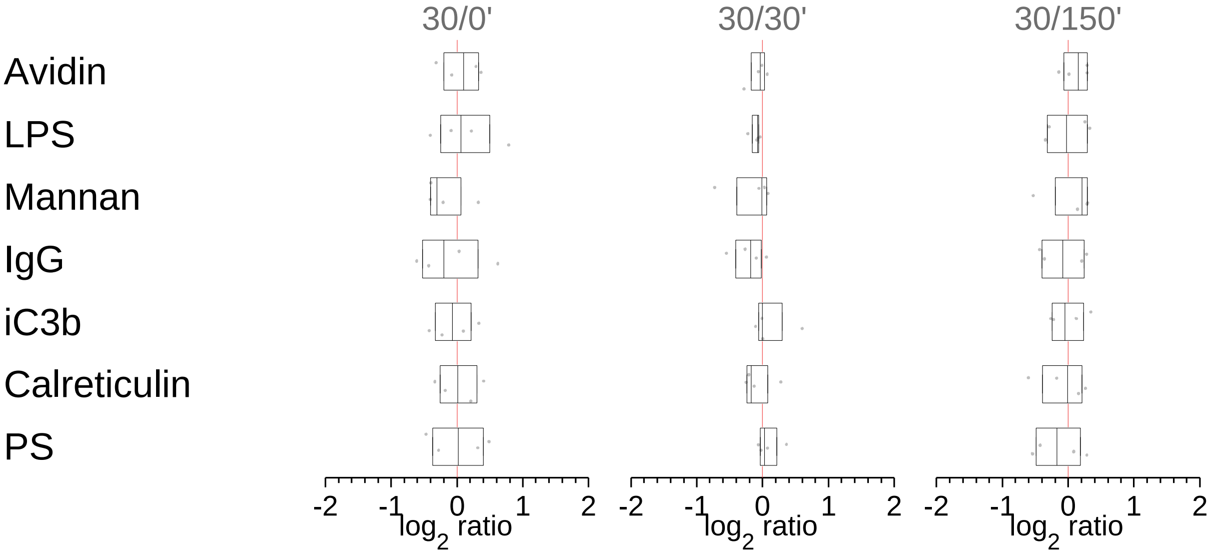

GO:0004029 aldehyde dehydrogenase (NAD) activity (n = 3)

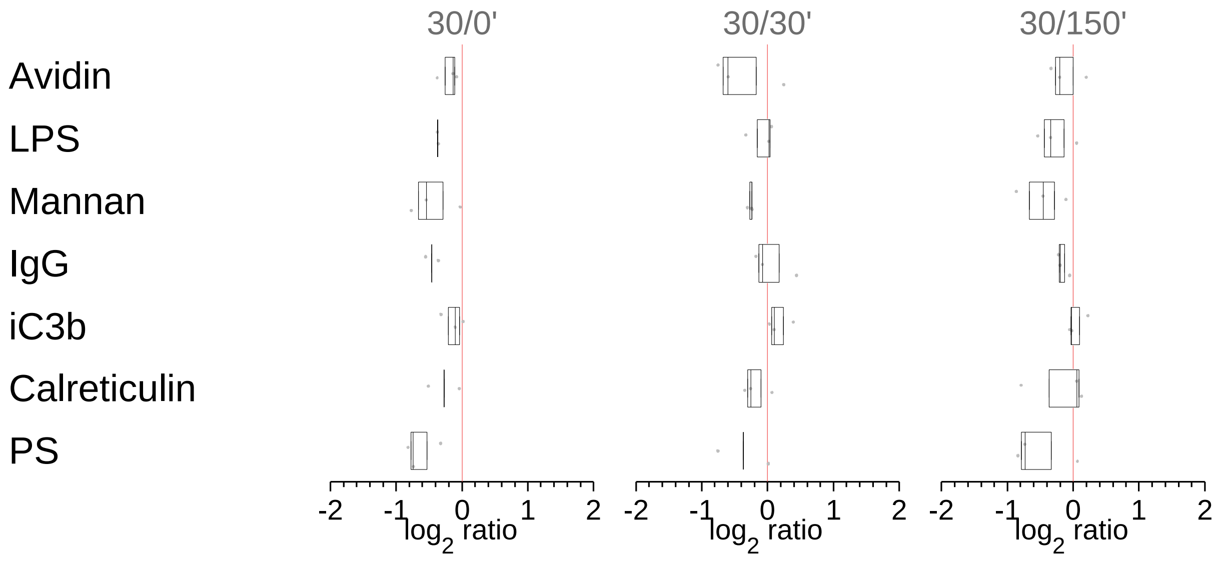

GO:0004033 aldo-keto reductase (NADP) activity (n = 3)

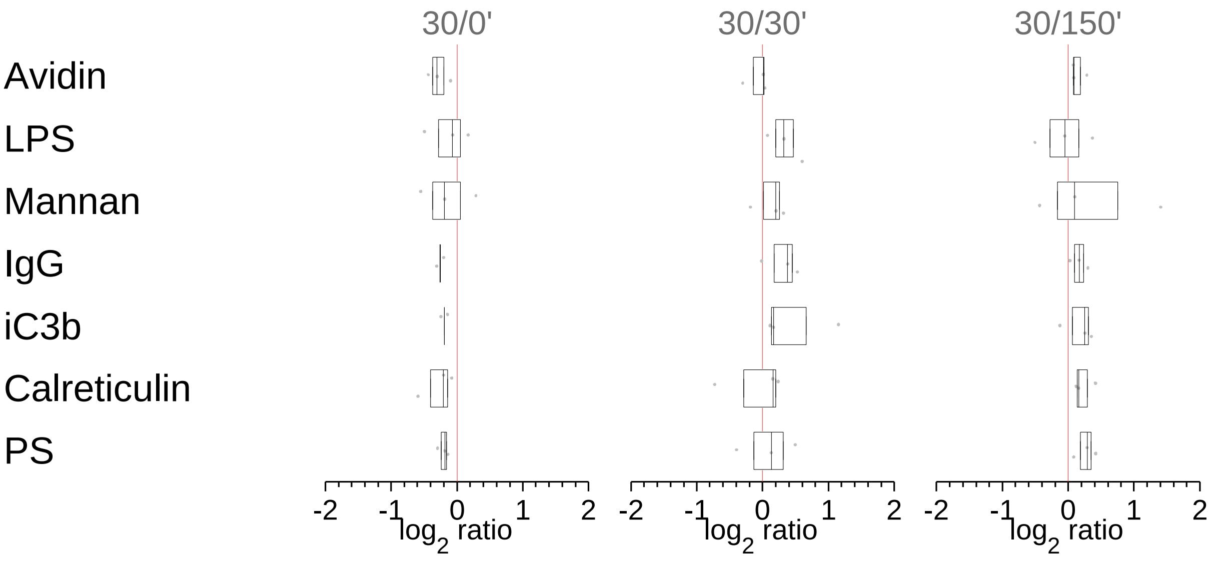

GO:0004064 arylesterase activity (n = 3)

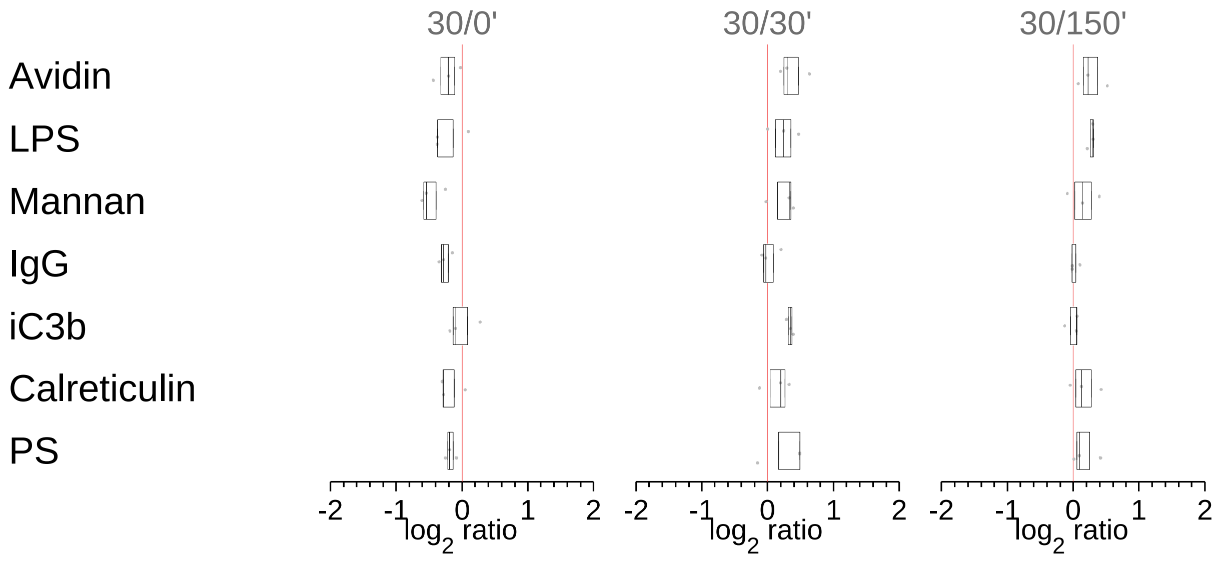

GO:0004129 cytochrome-c oxidase activity (n = 9)

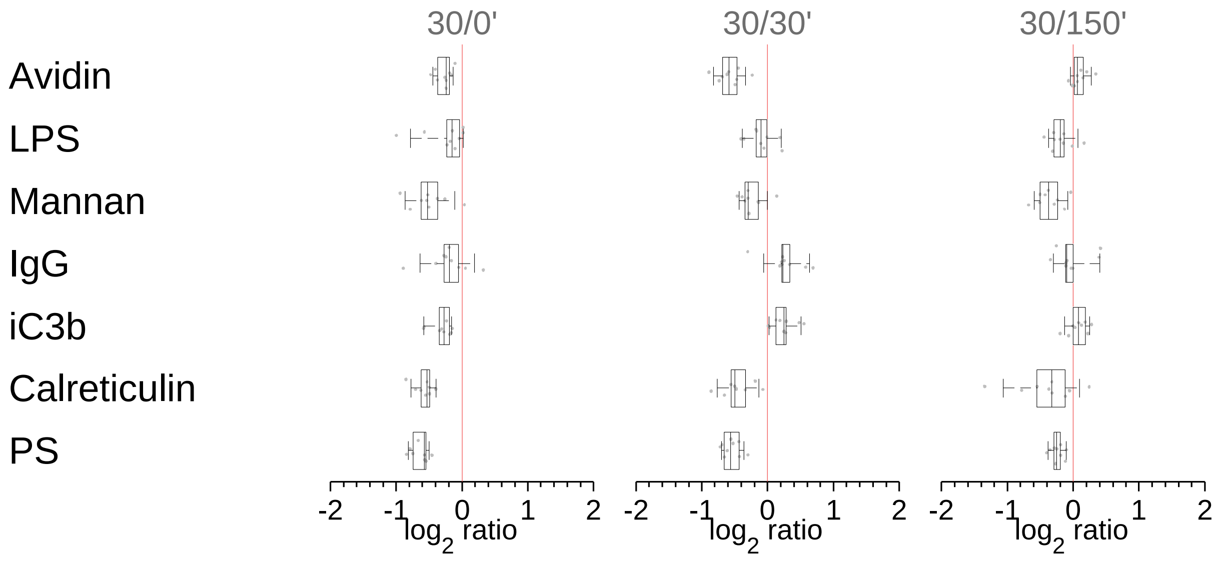

GO:0004147 dihydrolipoamide branched chain acyltransferase activity (n = 3)

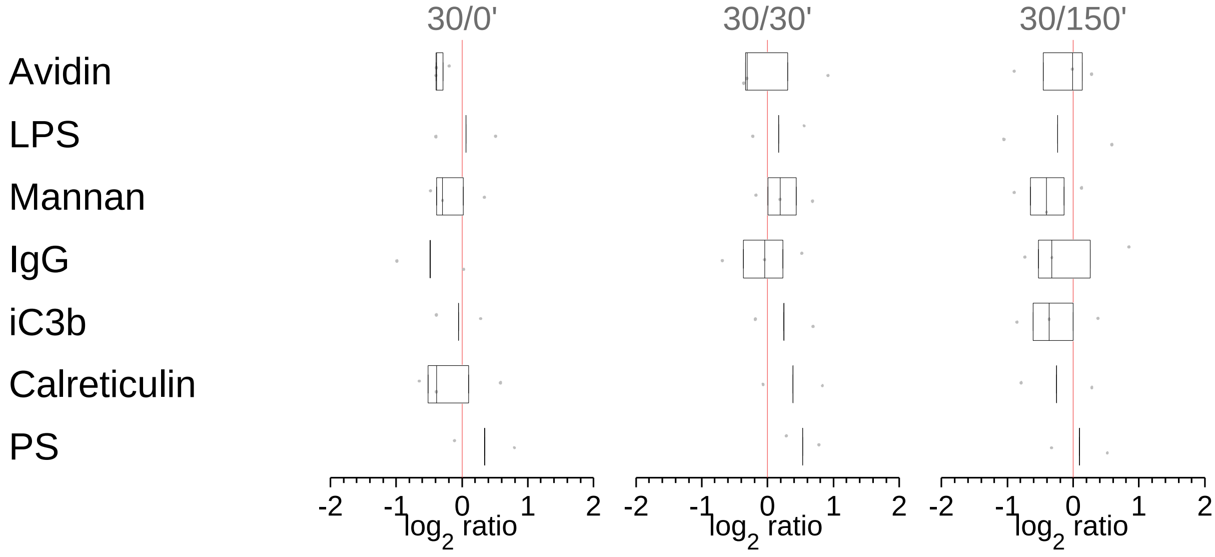

GO:0004174 electron-transferring-flavoprotein dehydrogenase activity (n = 3)

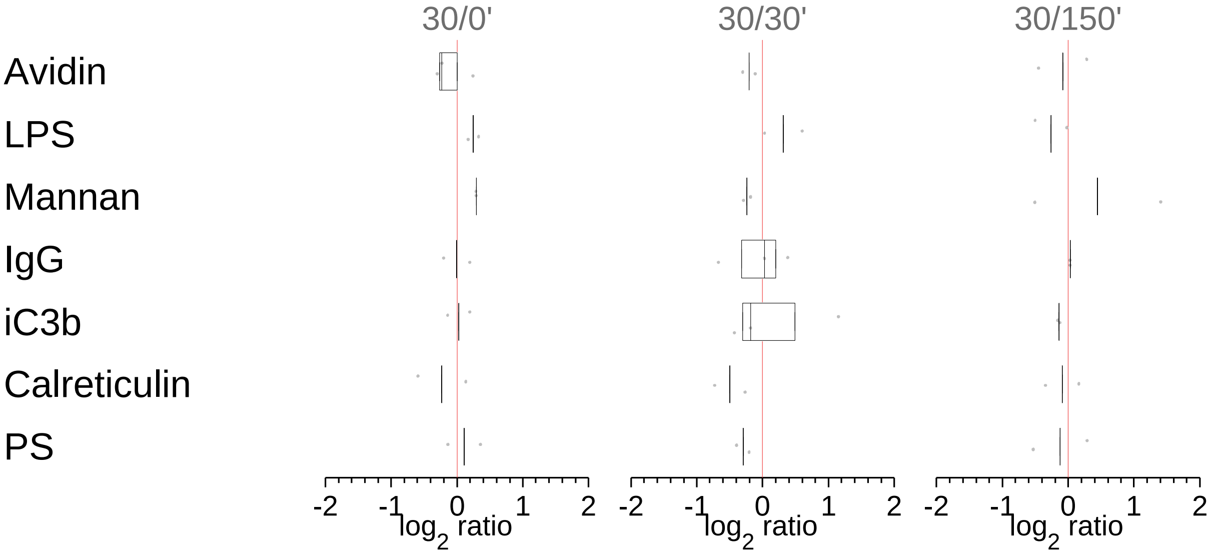

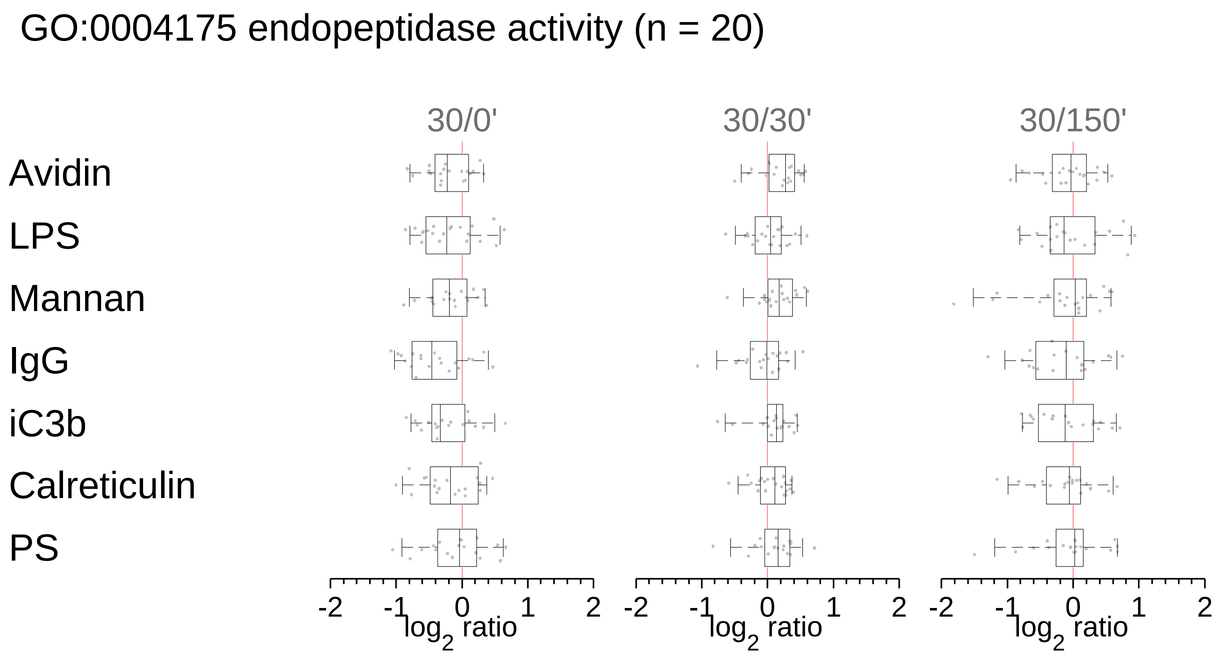
GO:0004175 endopeptidase activity (n = 20)
30/0'
30/30'
30/150'
Avidin
LPS
Mannan
IgG
iC3b
Calreticulin
PS
-2 -1 0 1 2
log2 ratio

GO:0004177 aminopeptidase activity (n = 8)

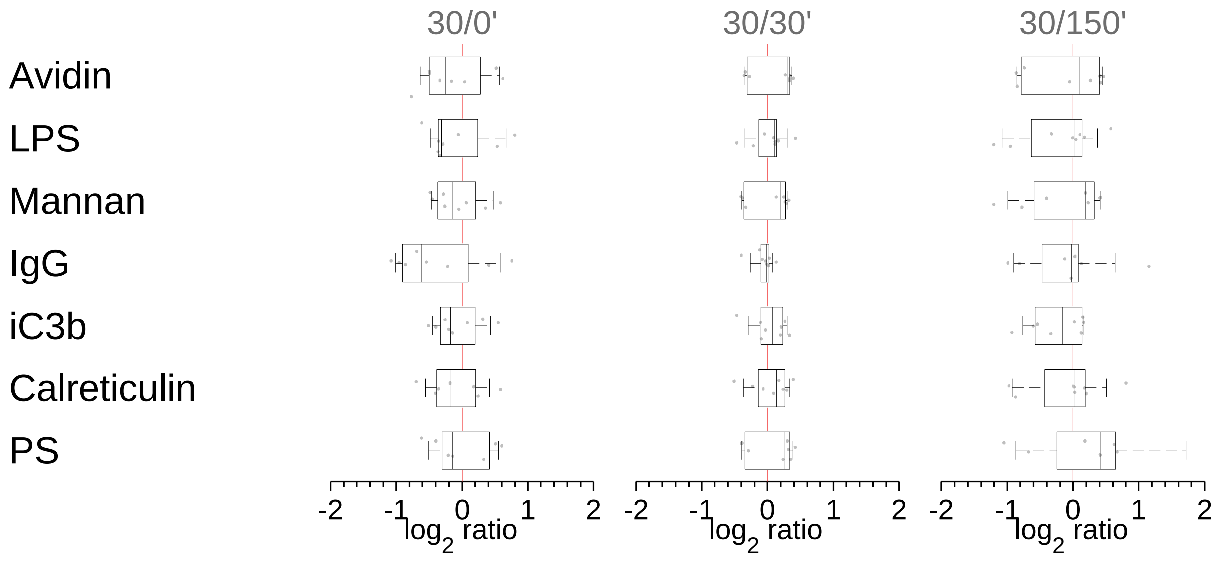

GO:0004180 carboxypeptidase activity (n = 6)

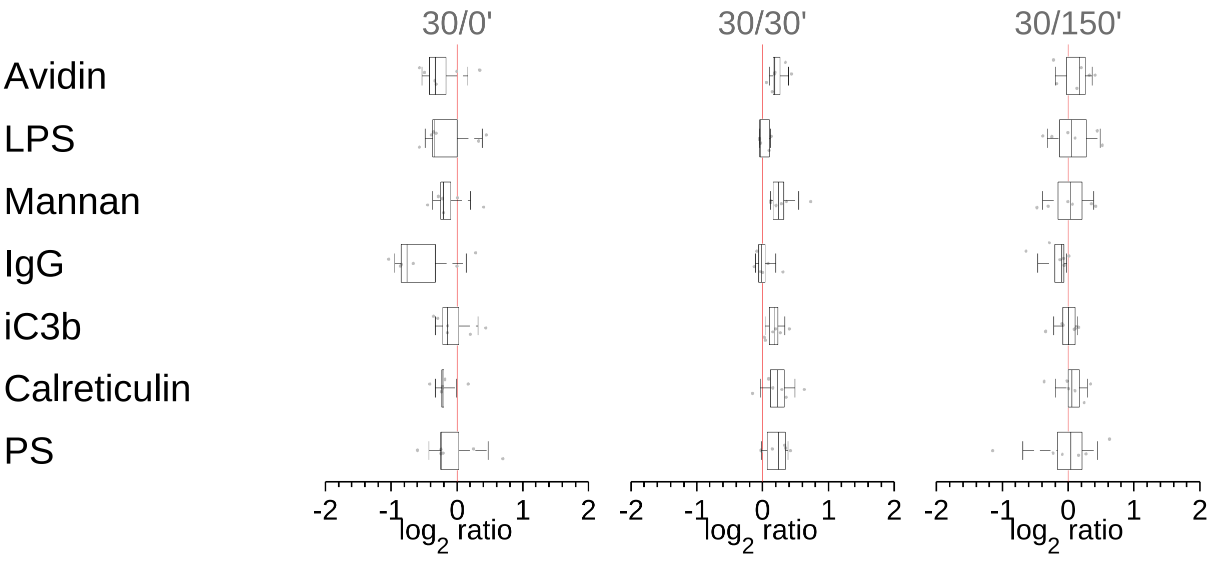

GO:0004185 serine-type carboxypeptidase activity (n = 3)

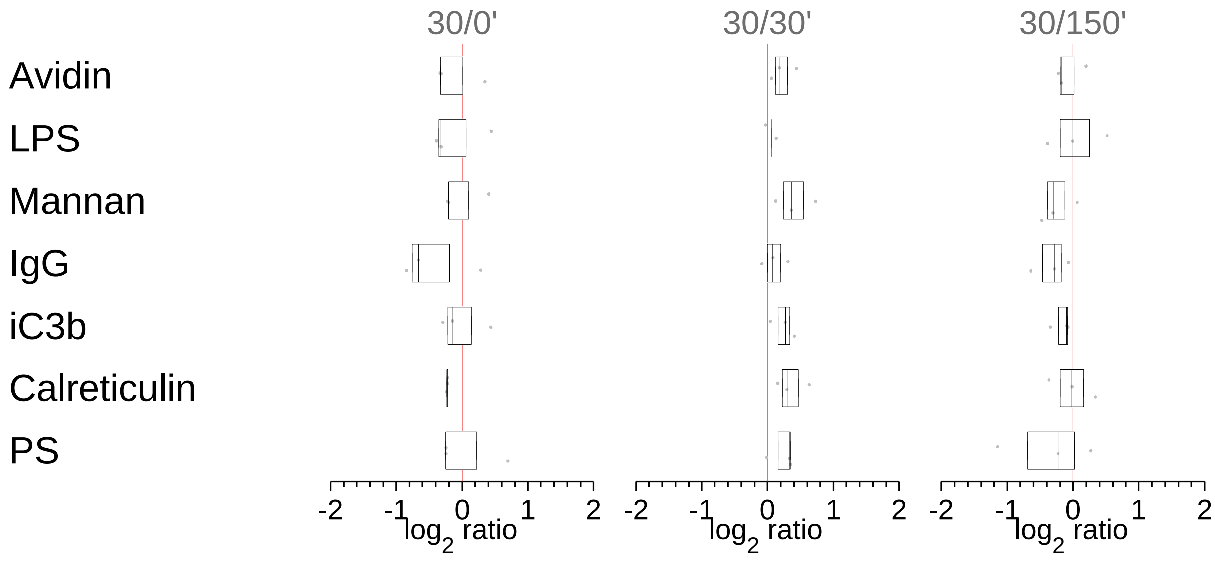

GO:0004190 aspartic-type endopeptidase activity (n = 7)

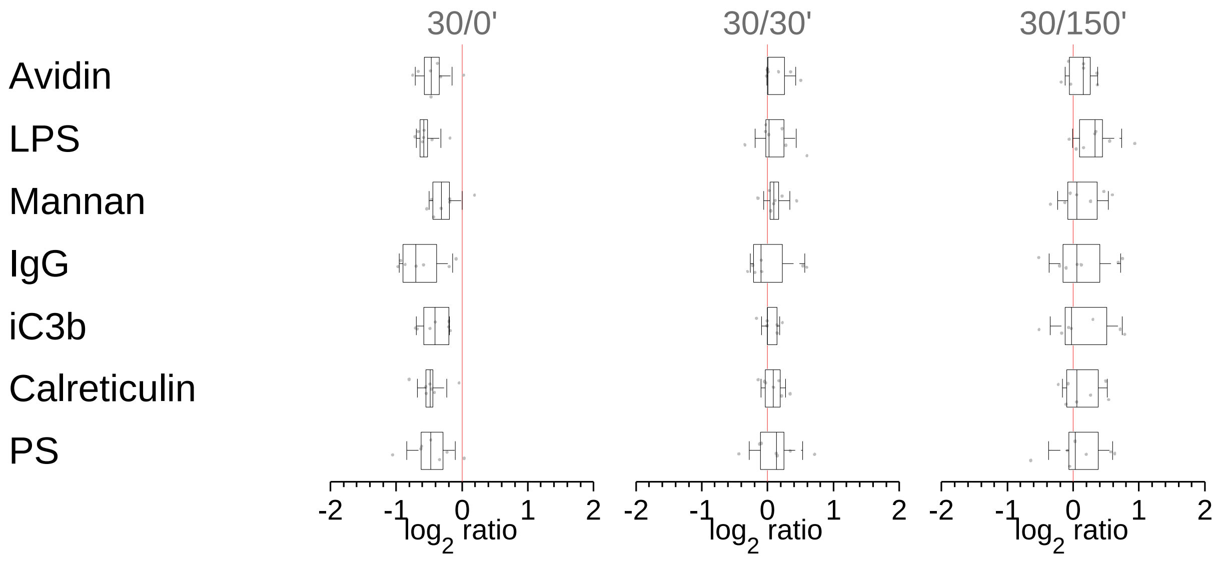

GO:0004197 cysteine-type endopeptidase activity (n = 7)

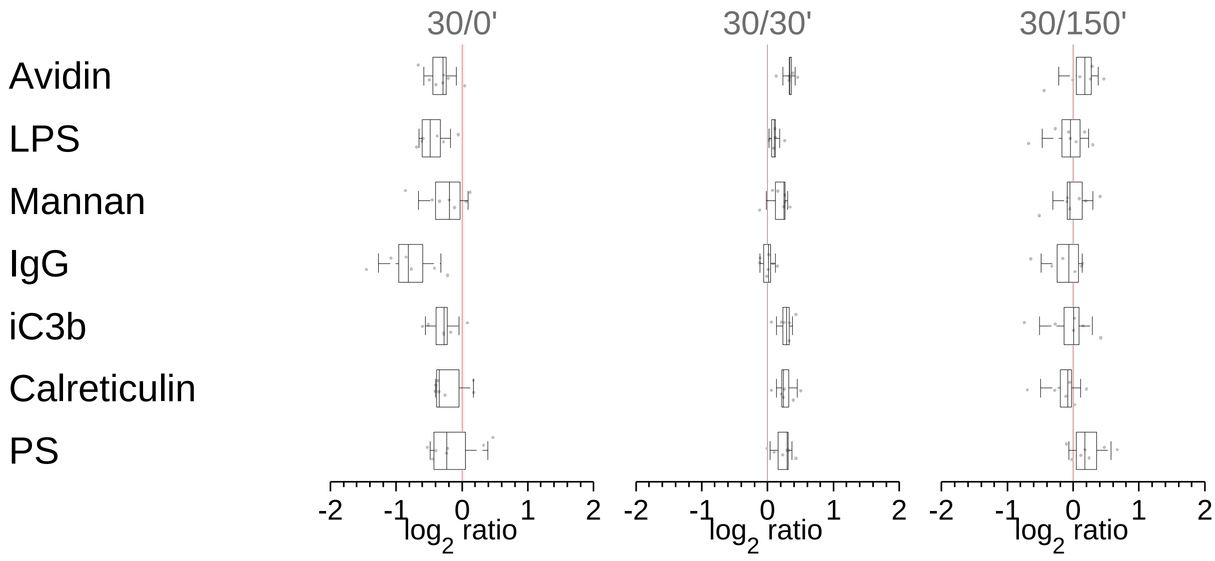

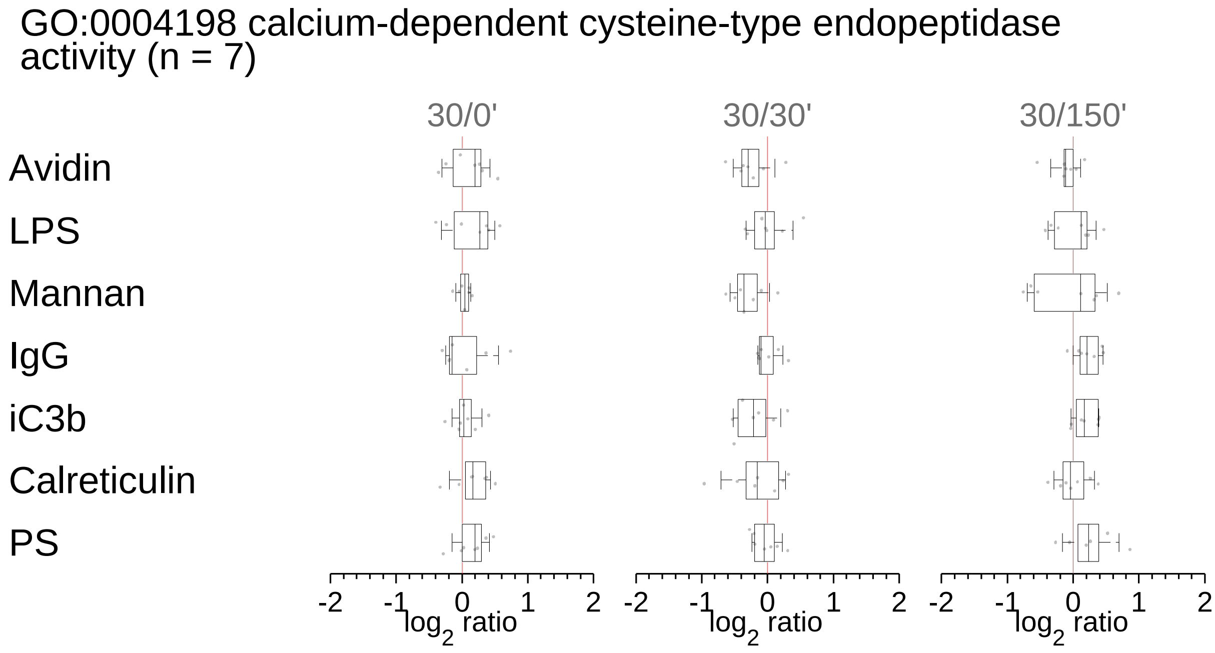

GO:0004198 calcium-dependent cysteine-type endopeptidase activity (n = 7)
30/0'
30/30'
30/150'
Avidin
LPS
Mannan
IgG
iC3b
Calreticulin
PS
-2
-1
0
1
2
log2 ratio

GO:0004222 metalloendopeptidase activity (n = 7)

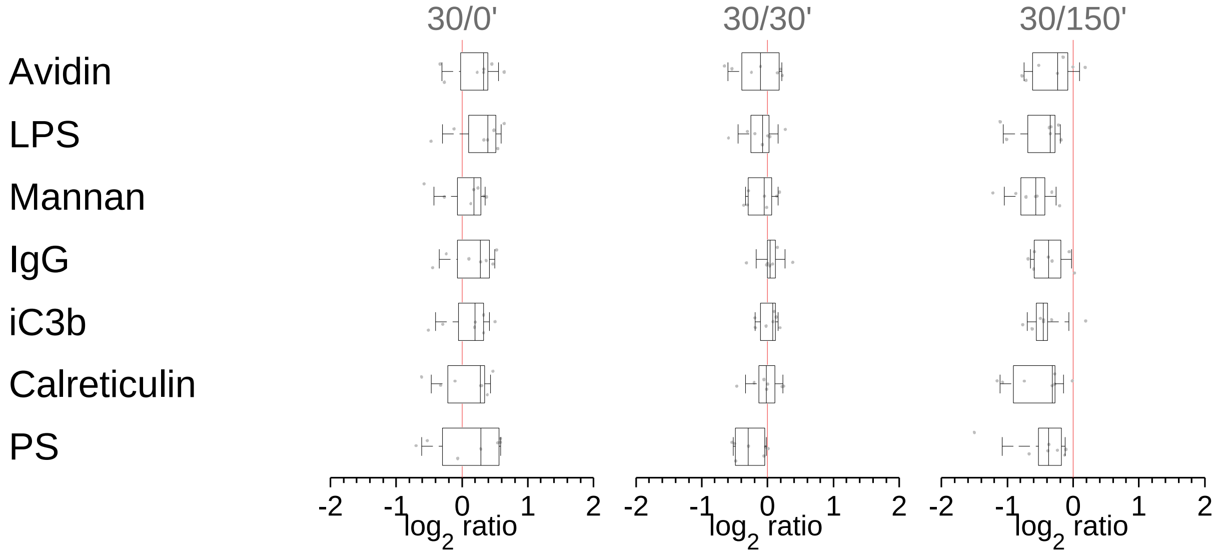

GO:0004252 serine-type endopeptidase activity (n = 5)

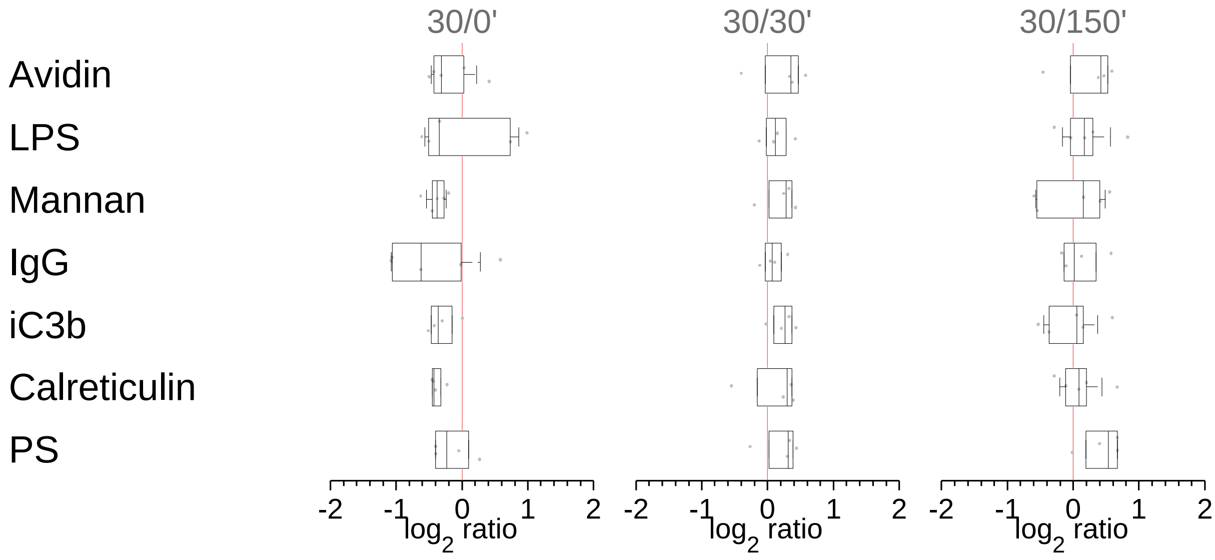

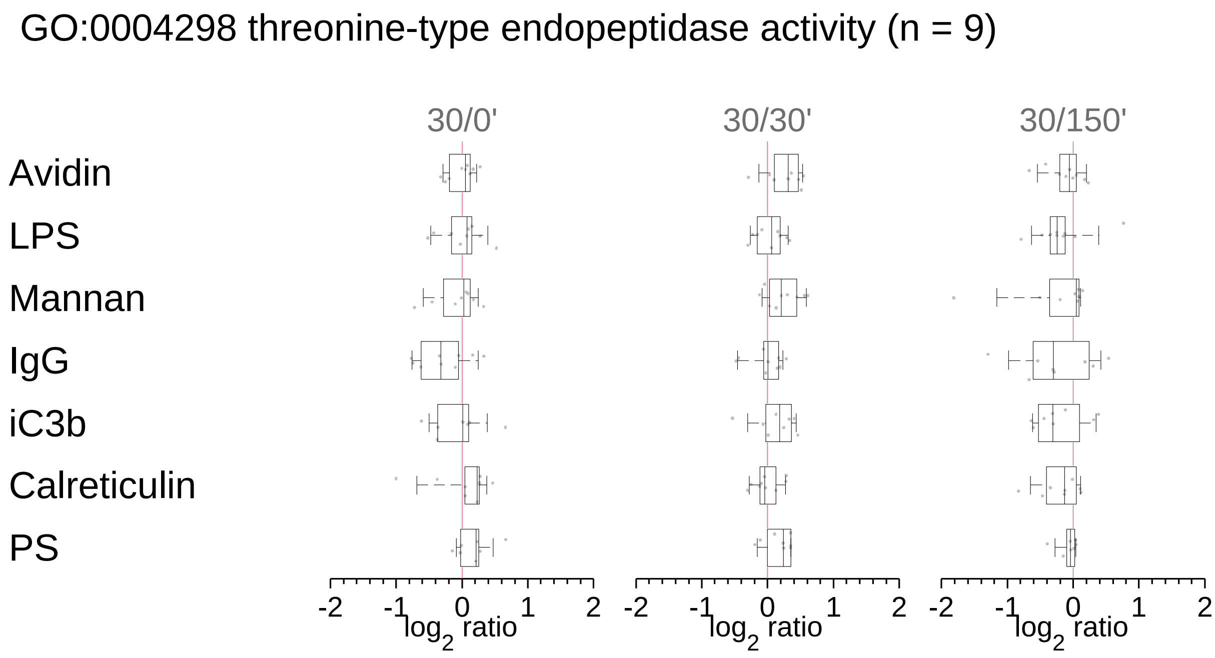
GO:0004298 threonine-type endopeptidase activity (n = 9)
30/0'
30/30'
30/150'
Avidin
LPS
Mannan
IgG
iC3b
Calreticulin
PS
-2
-1
0
1
2
log2 ratio

GO:0004300 enoyl-CoA hydratase activity (n = 3)

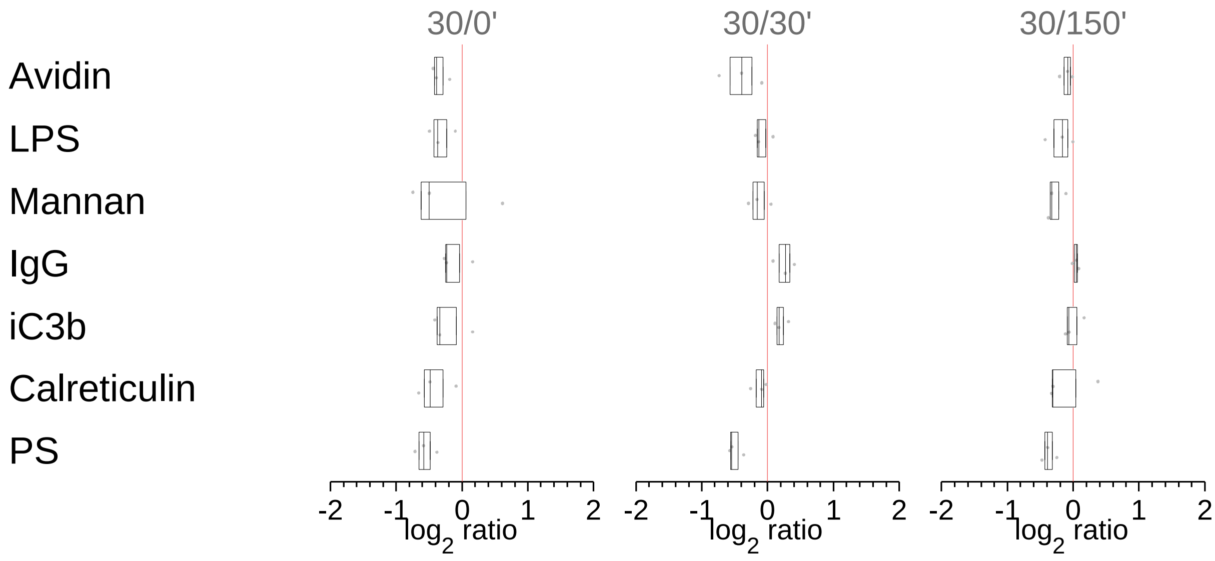

GO:0004303 estradiol 17-beta-dehydrogenase activity (n = 3)

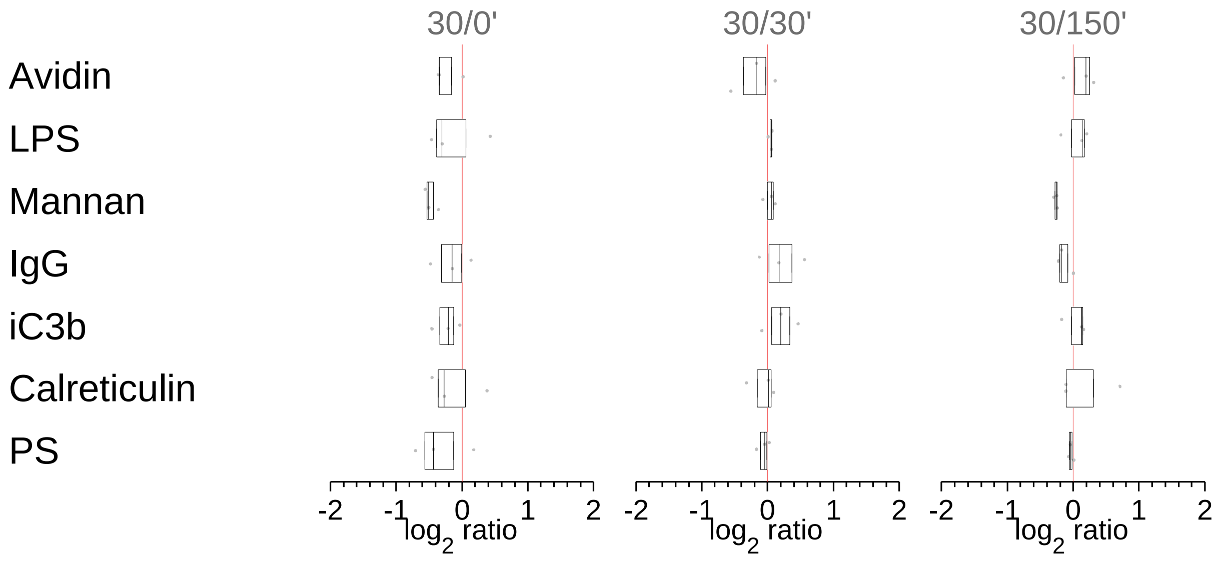

GO:0004364 glutathione transferase activity (n = 3)

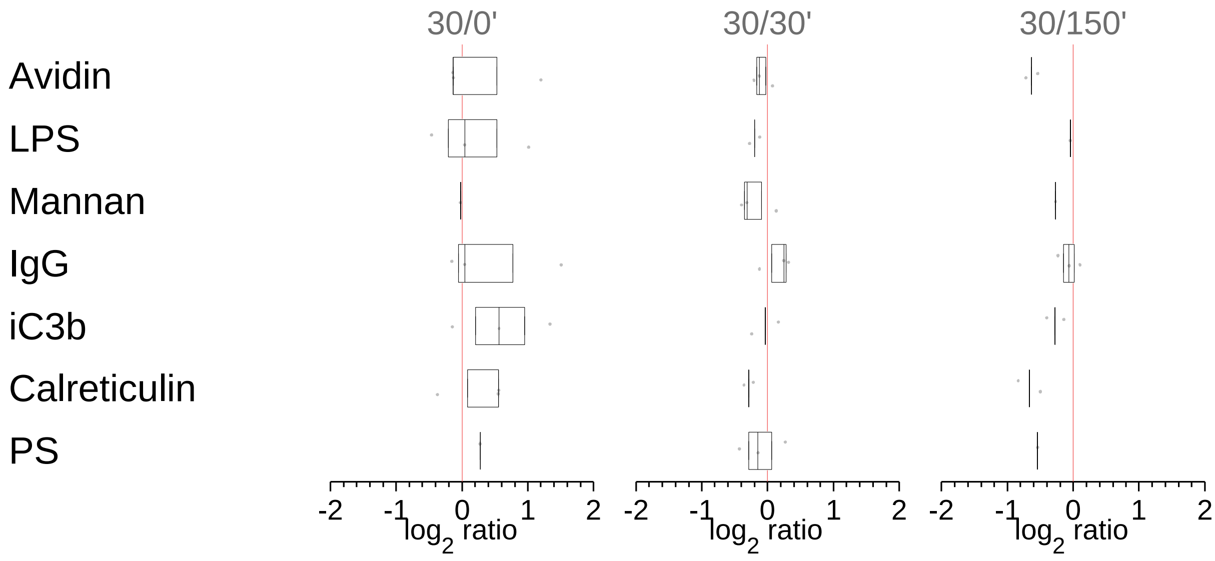

GO:0004376 glycolipid mannosyltransferase activity (n = 3)

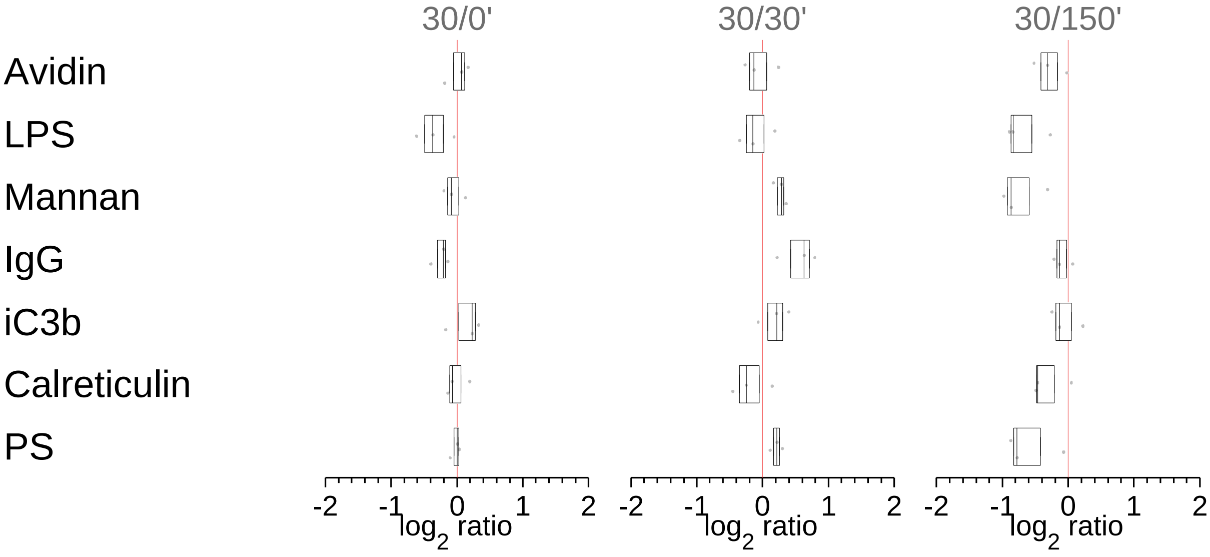

GO:0004386 helicase activity (n = 18)

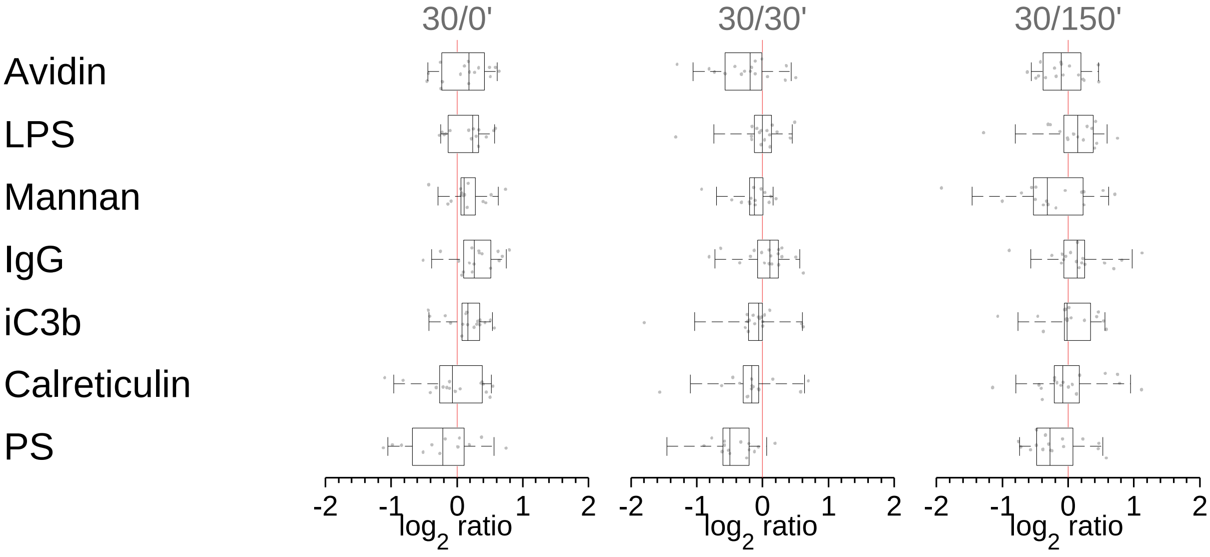

GO:0004407 histone deacetylase activity (n = 3)

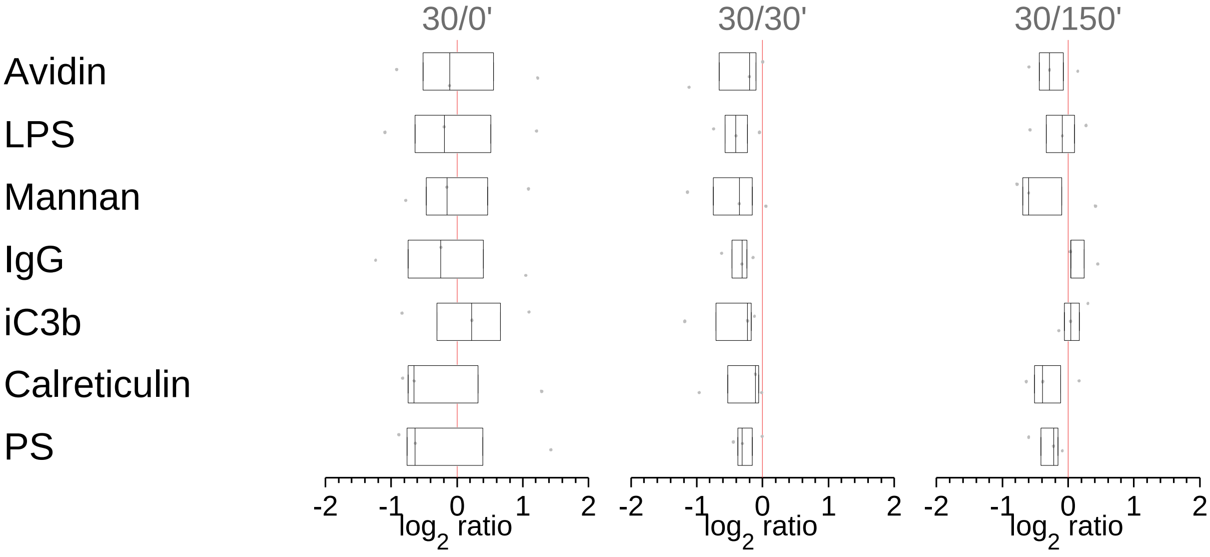

GO:0004448 isocitrate dehydrogenase activity (n = 3)

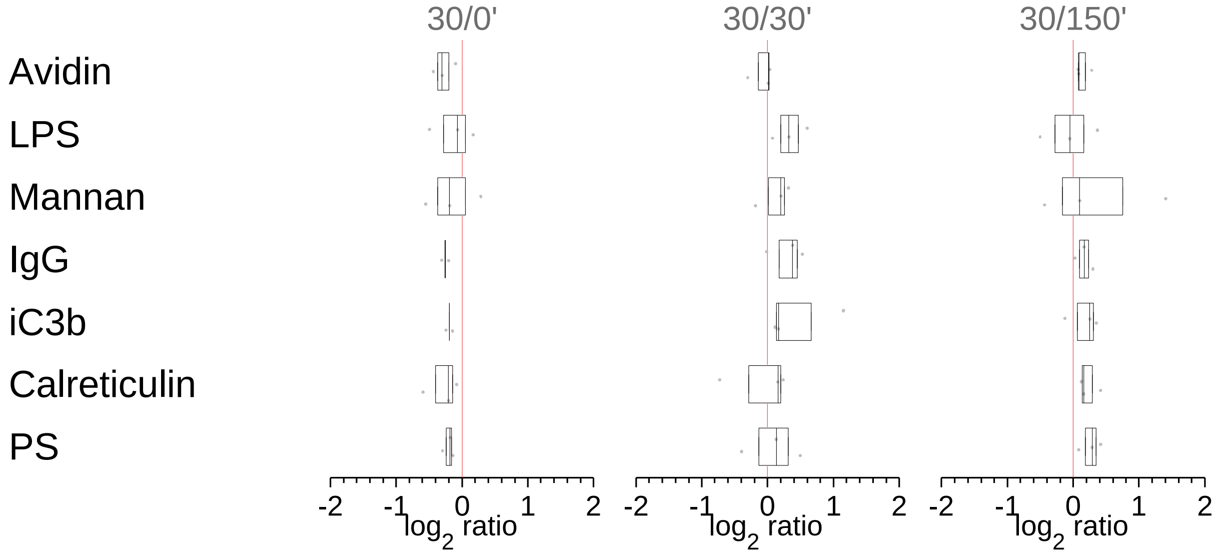

GO:0004467 long-chain fatty acid-CoA ligase activity (n = 3)

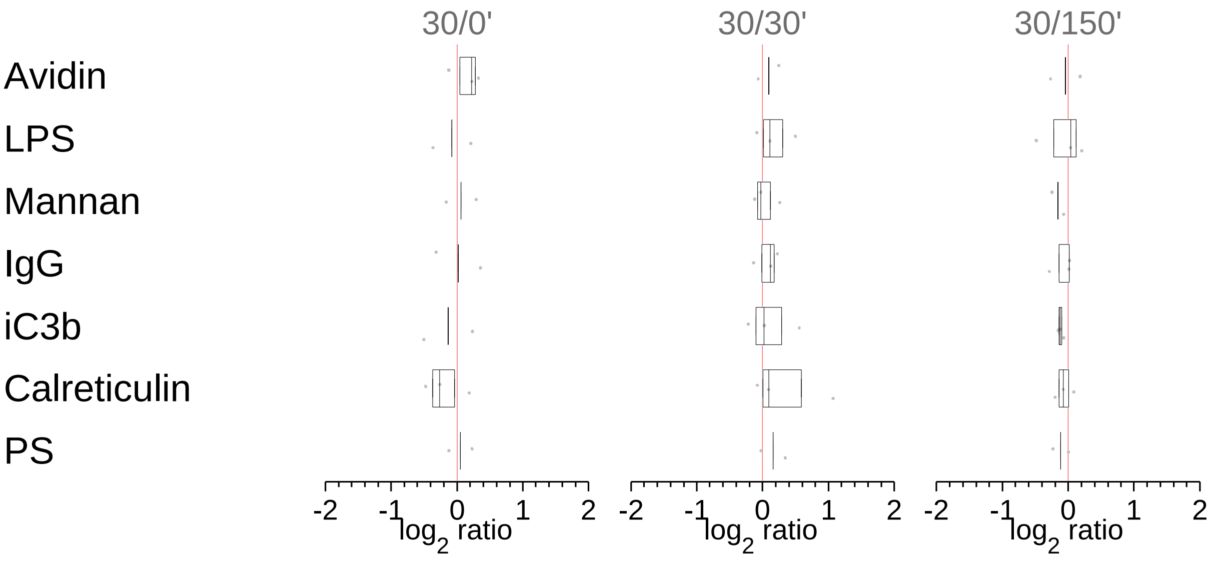

GO:0004495 mevaldate reductase activity (n = 3)

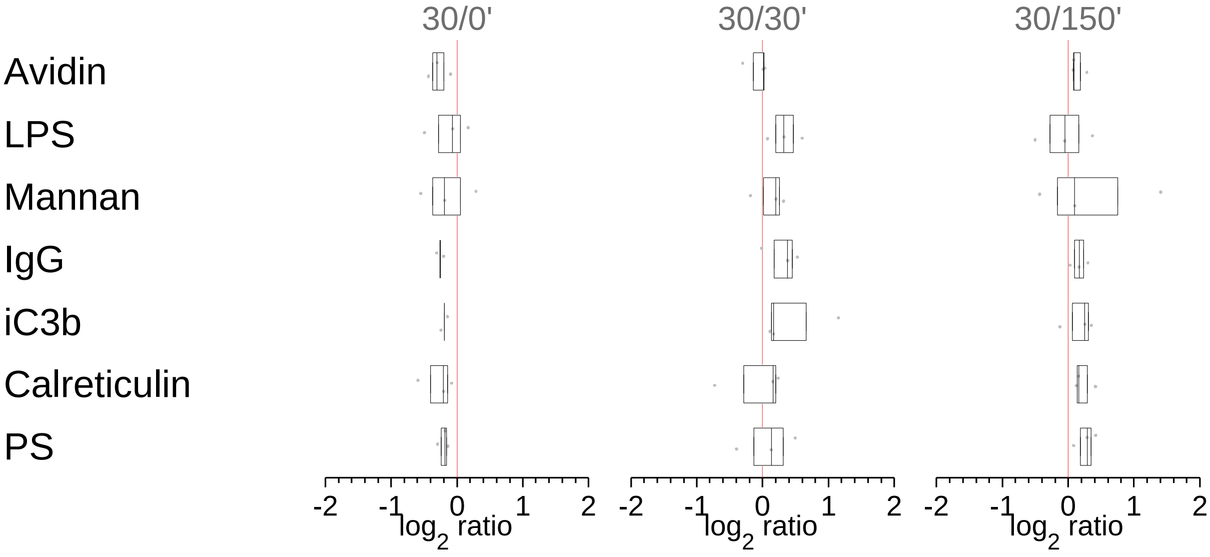

GO:0004497 monooxygenase activity (n = 6)

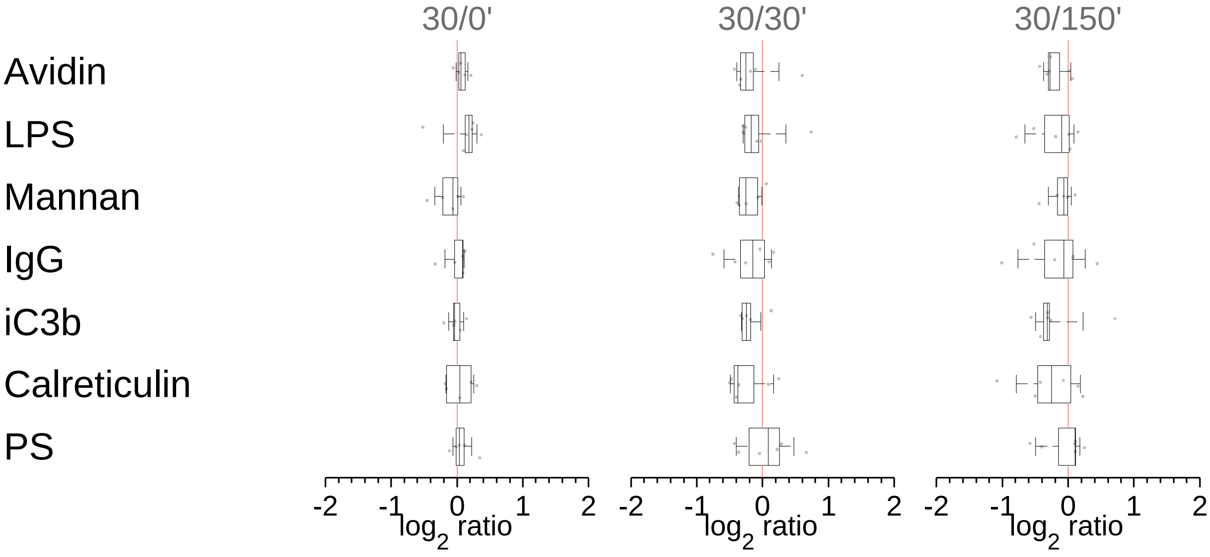

GO:0004518 nuclease activity (n = 13)

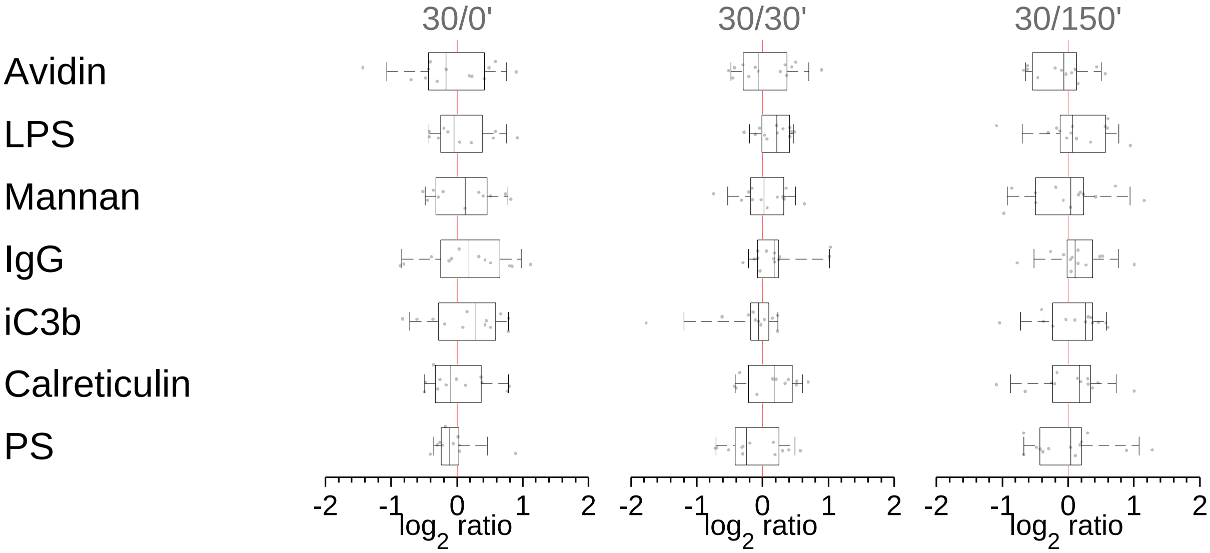

GO:0004519 endonuclease activity (n = 7)

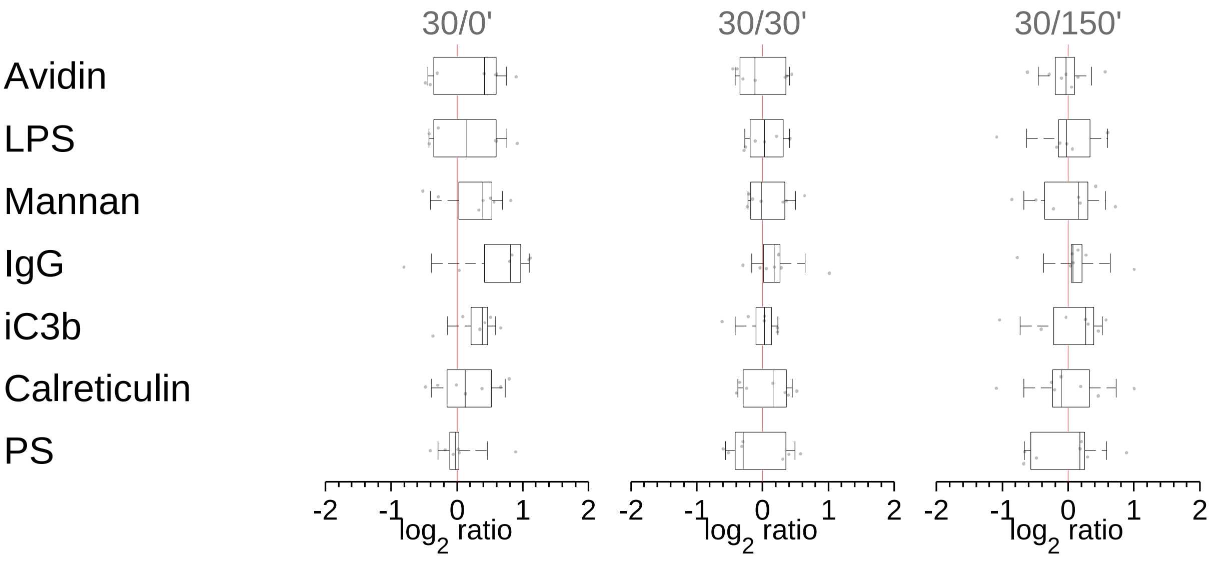

GO:0004527 exonuclease activity (n = 4)

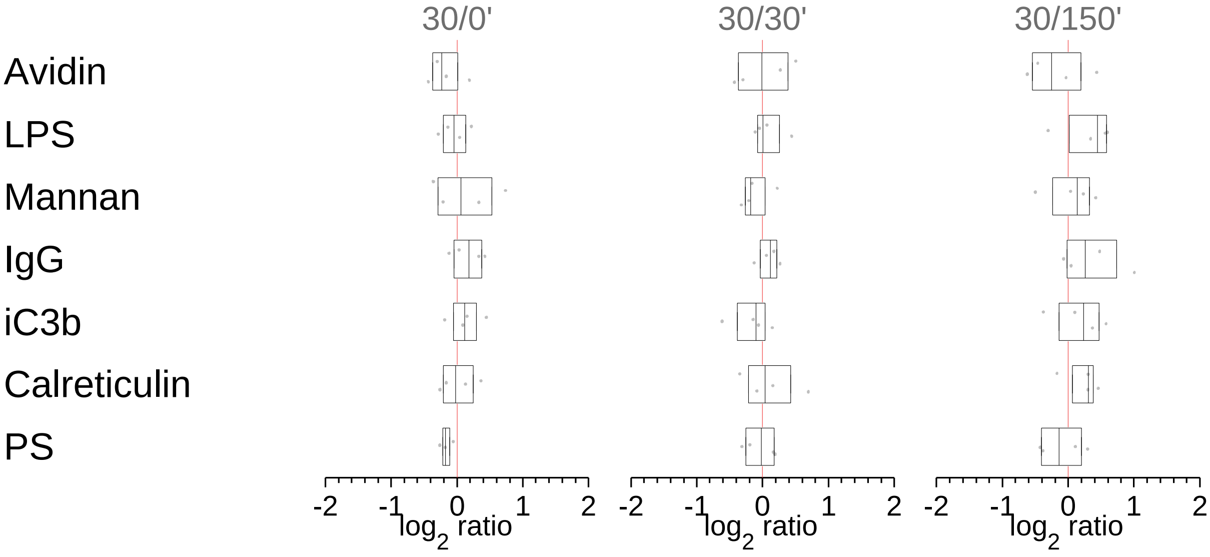

GO:0004553 hydrolase activity, hydrolyzing O-glycosyl compounds
(n = 17)

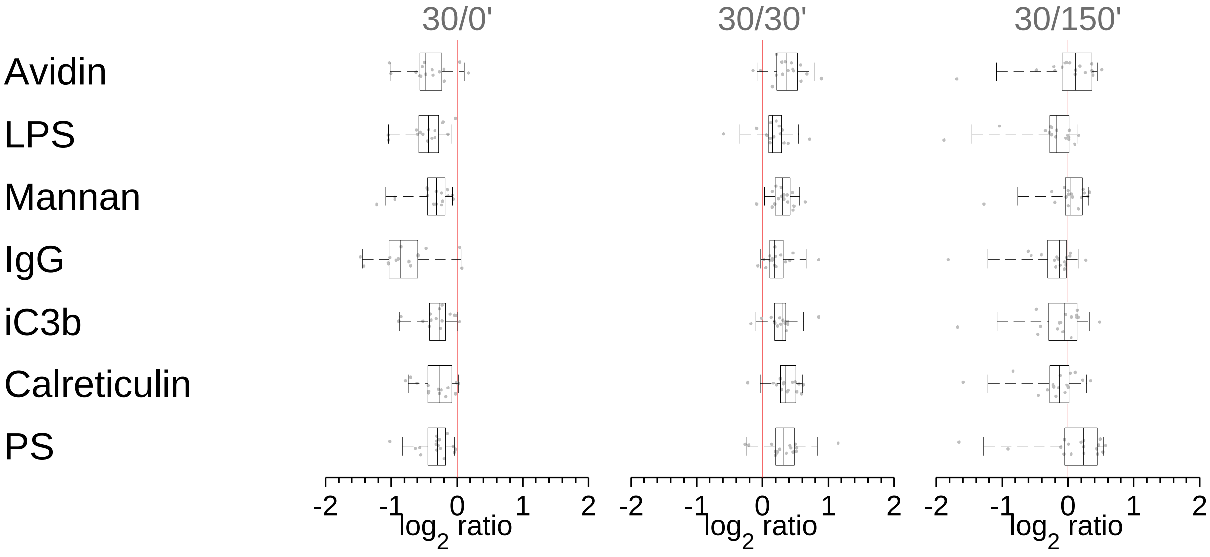

GO:0004559 alpha-mannosidase activity (n = 3)

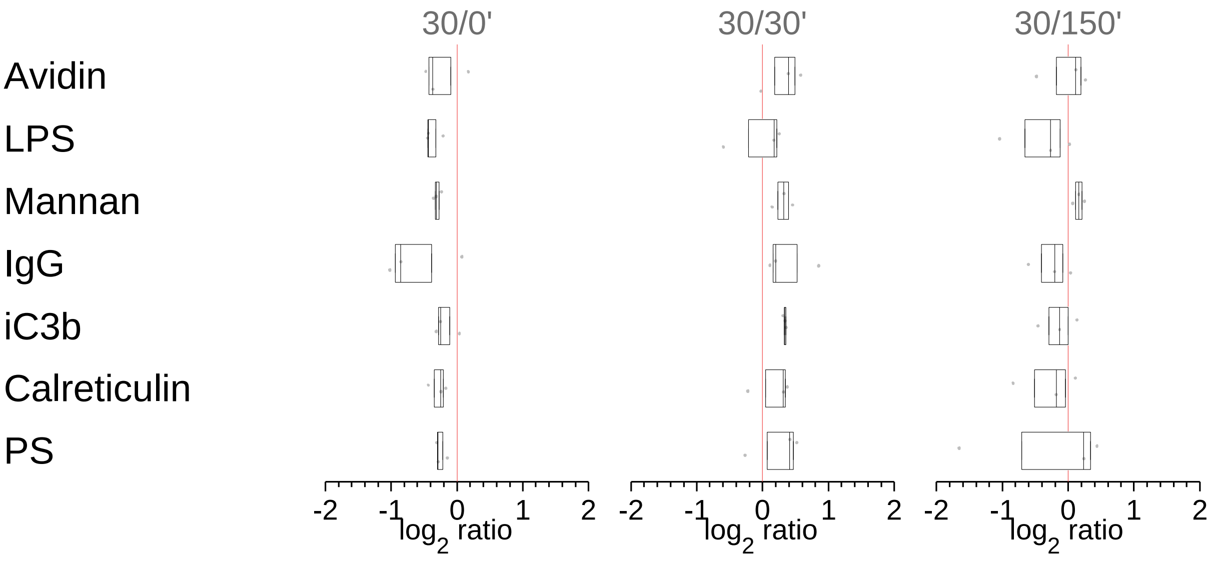

GO:0004563 beta-N-acetylhexosaminidase activity (n = 4)

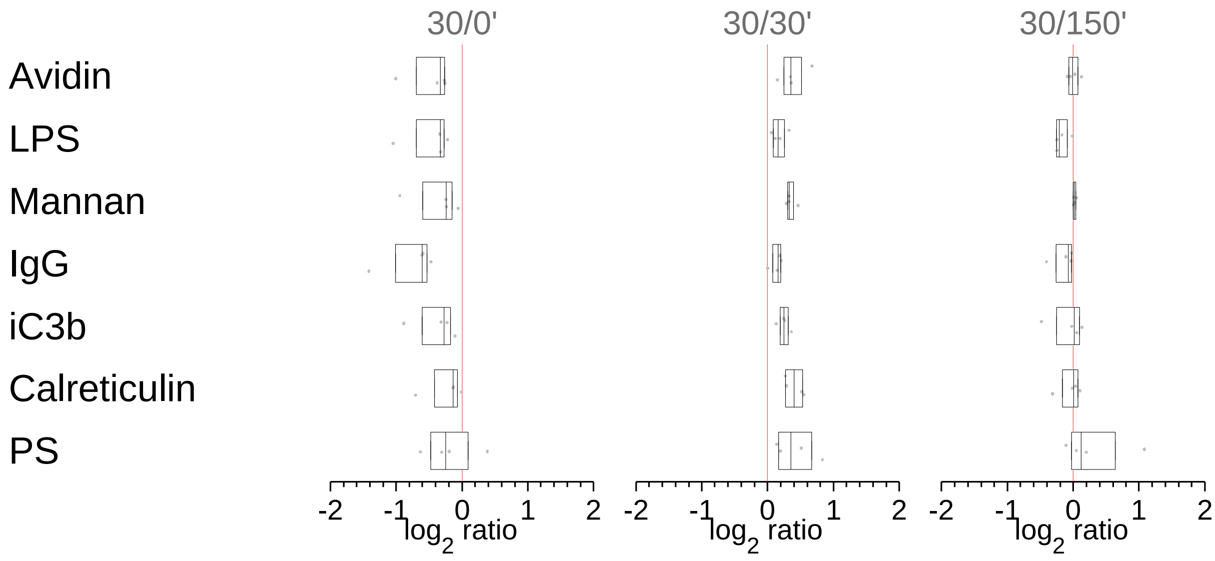

GO:0004568 chitinase activity (n = 3)

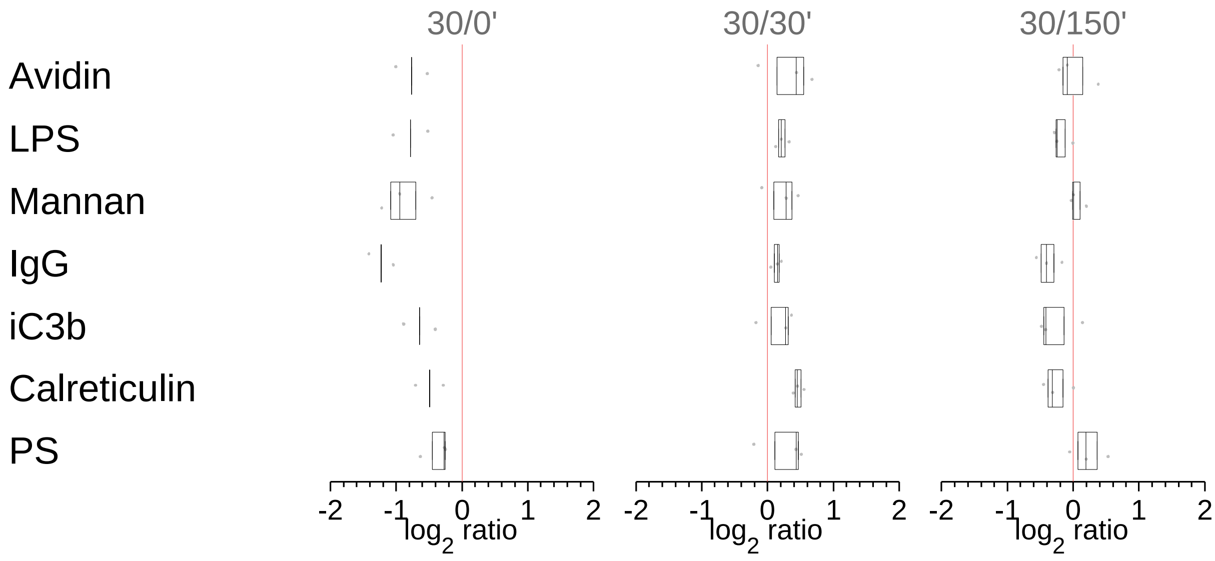

GO:0004576 oligosaccharyl transferase activity (n = 4)

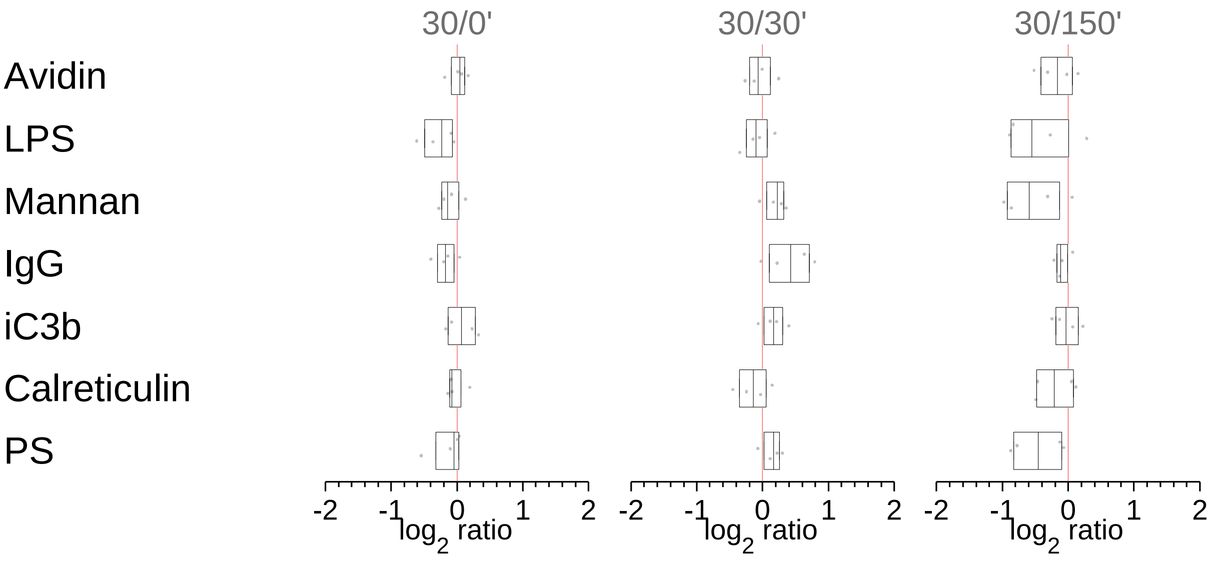

GO:0004579 dolichyl-diphosphooligosaccharide-protein glycotransferase activity (n = 4)

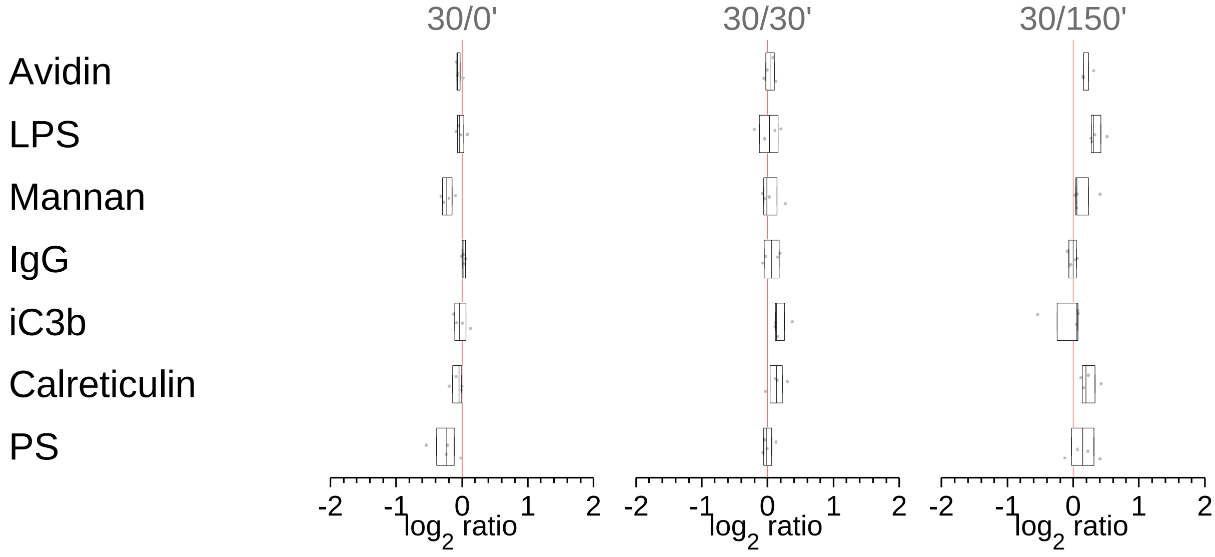

GO:0004583 dolichyl-phosphate-glucose-glycolipid alpha-glucosyltransferase activity (n = 3)

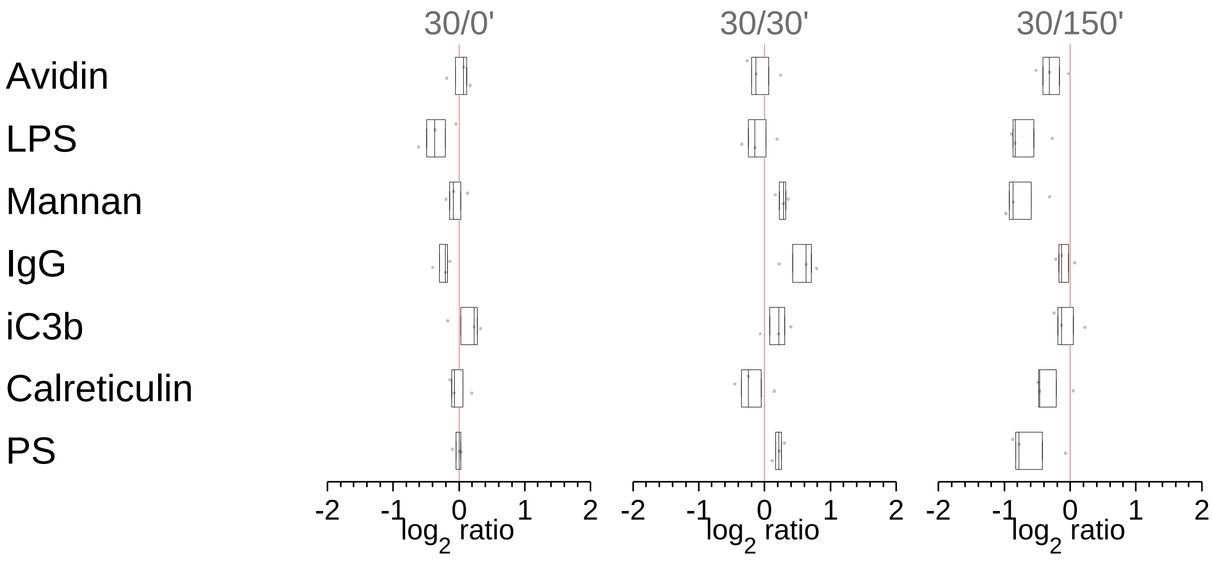

GO:0004601 peroxidase activity (n = 10)

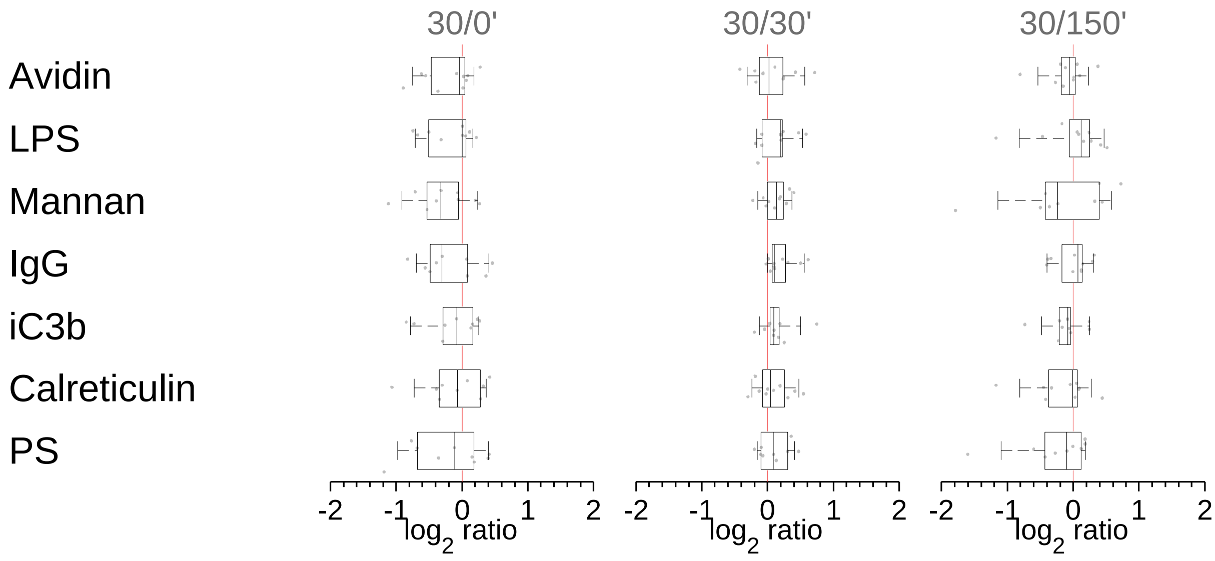

GO:0004602 glutathione peroxidase activity (n = 3)

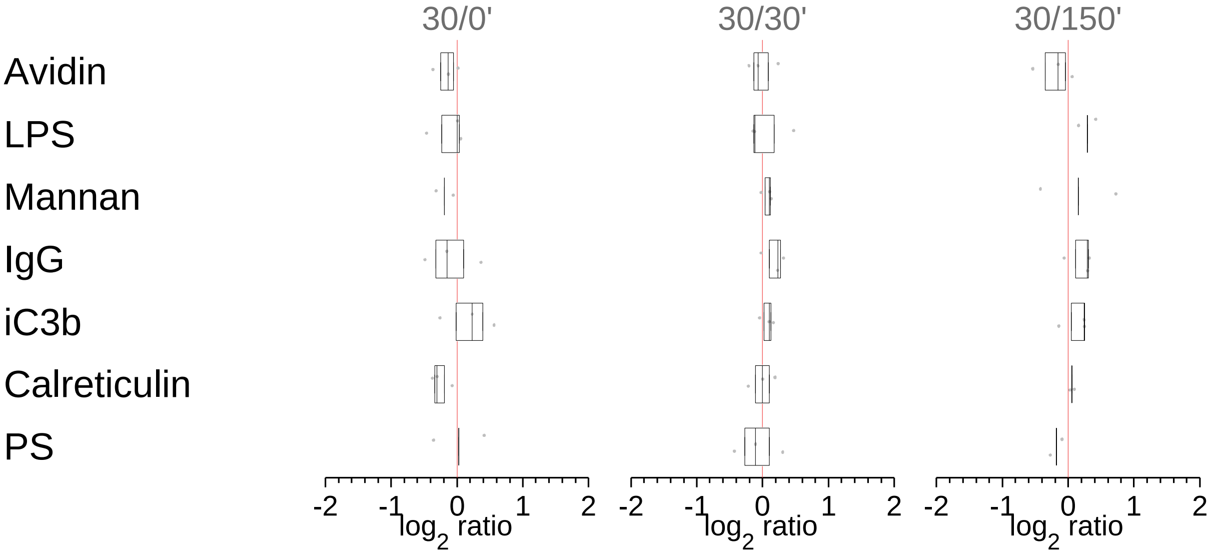

GO:0004620 phospholipase activity (n = 4)

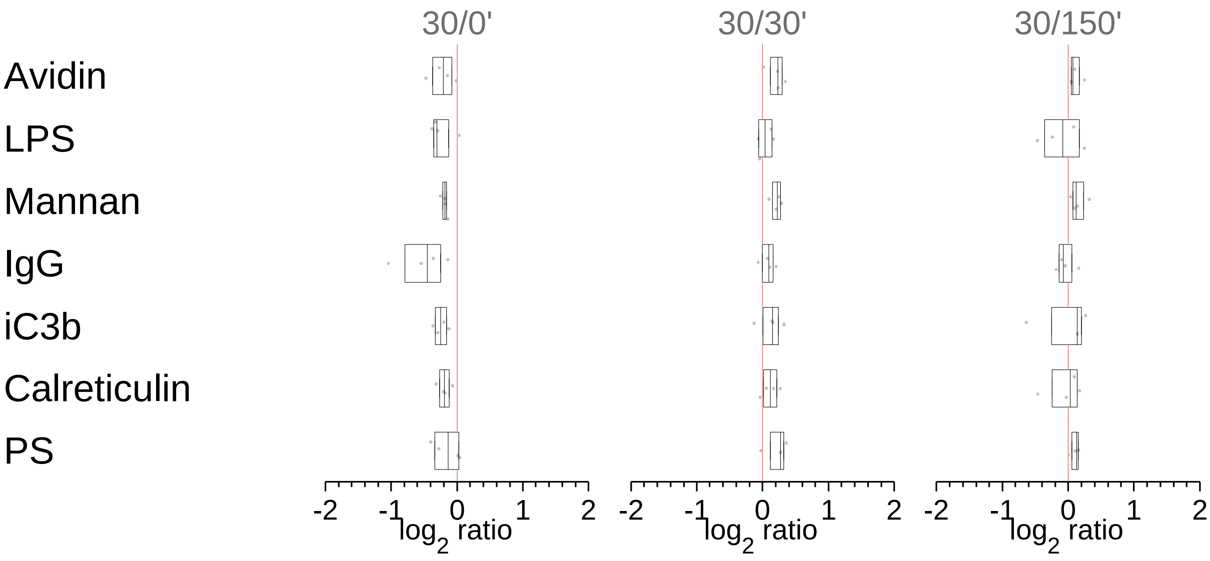

GO:0004630 phospholipase D activity (n = 5)

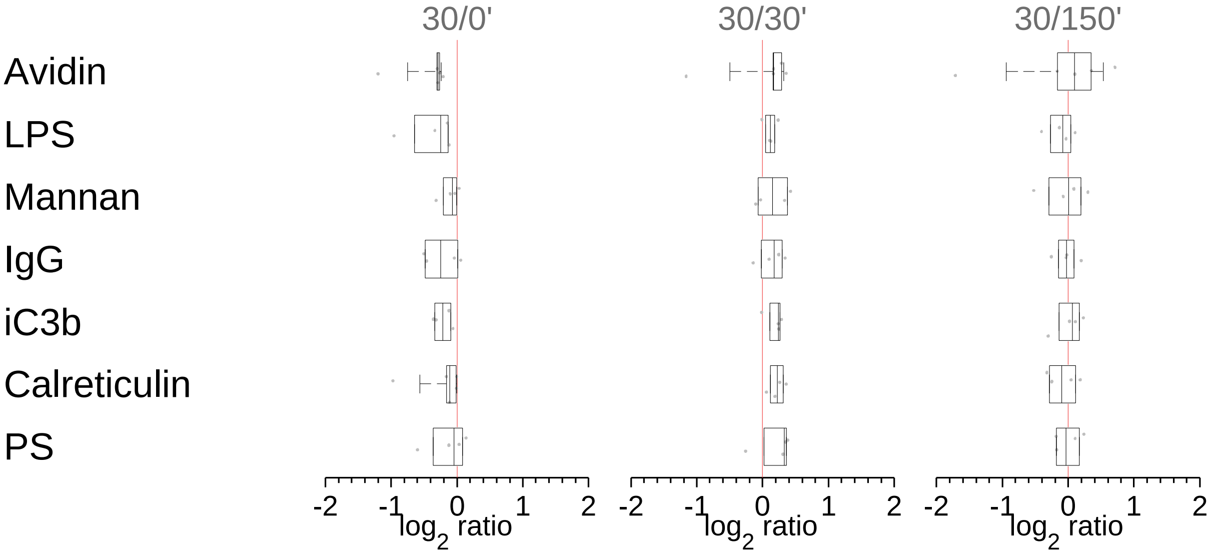

GO:0004672 protein kinase activity (n = 51)

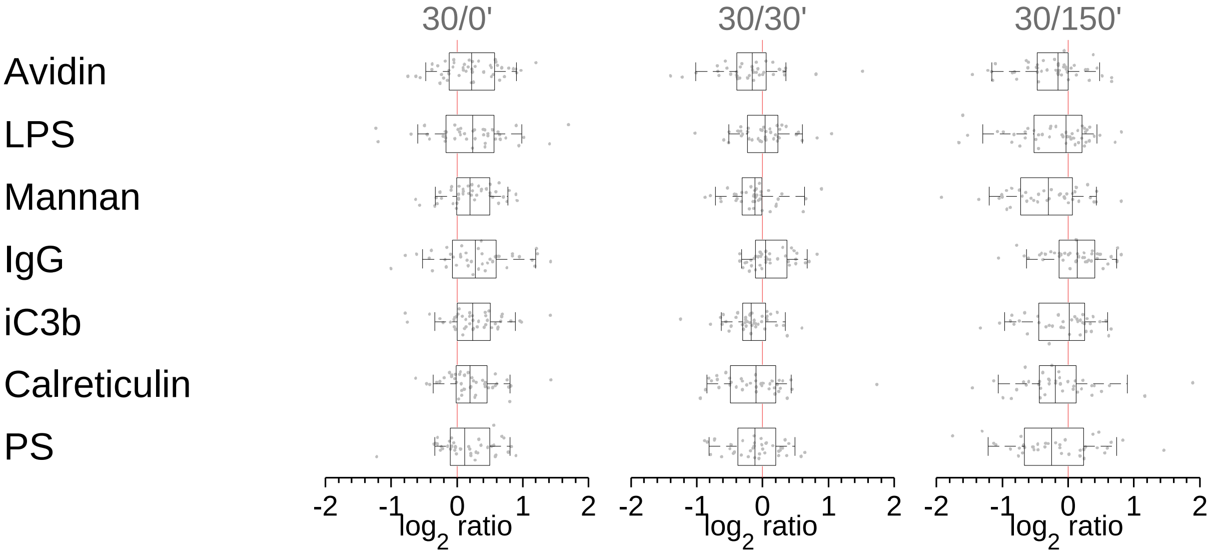

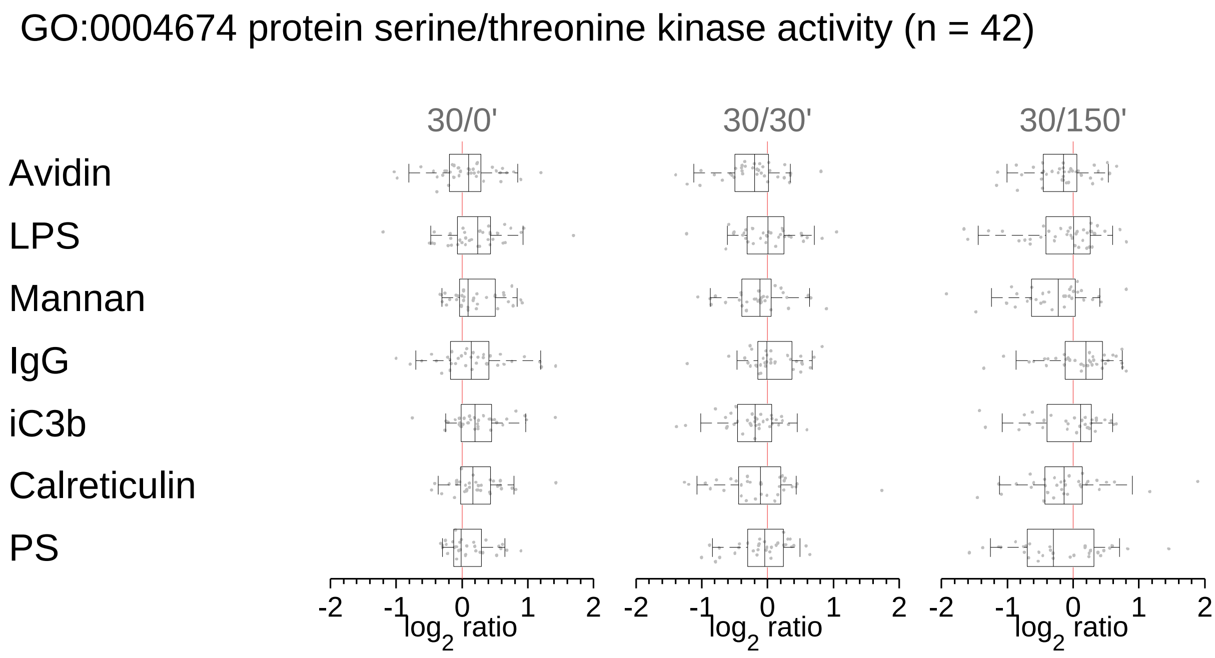

GO:0004674 protein serine/threonine kinase activity (n = 42)
30/0'
30/30'
30/150'
Avidin
LPS
Mannan
IgG
iC3b
Calreticulin
PS
-2
-1
0
1
2
log2 ratio

GO:0004693 cyclin-dependent protein kinase activity (n = 5)

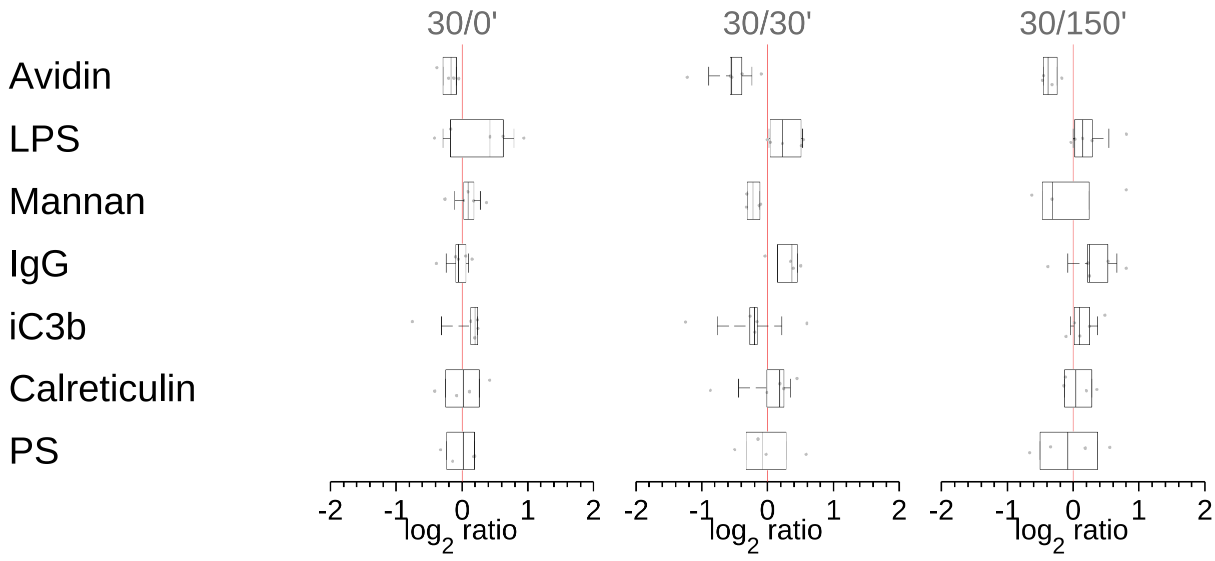

GO:0004697 protein kinase C activity (n = 3)

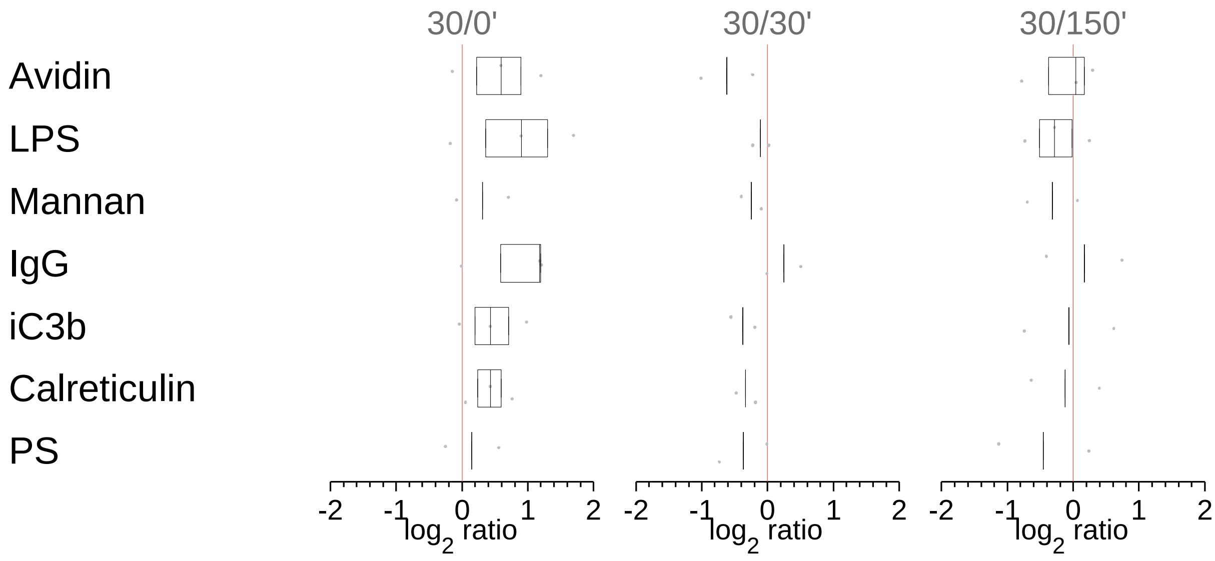

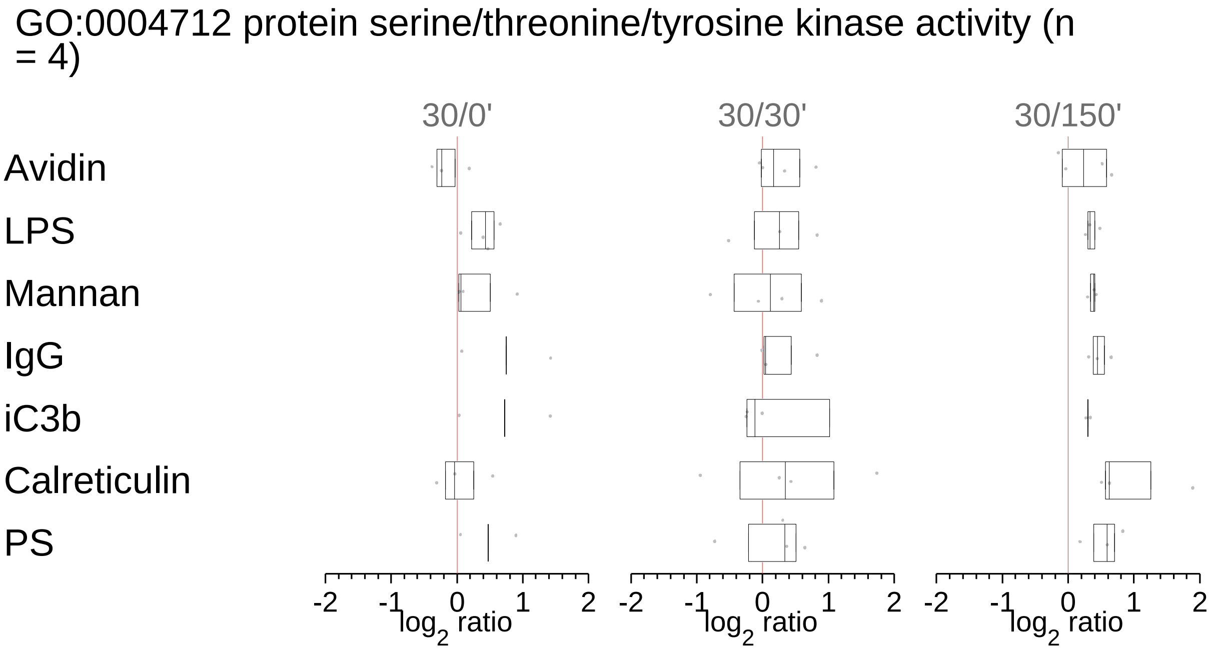
GO:0004712 protein serine/threonine/tyrosine kinase activity (n = 4)
30/0'
30/30'
30/150'
Avidin
LPS
Mannan
IgG
iC3b
Calreticulin
PS
-2
-1
0
1
2
log2 ratio

GO:0004713 protein tyrosine kinase activity (n = 14)

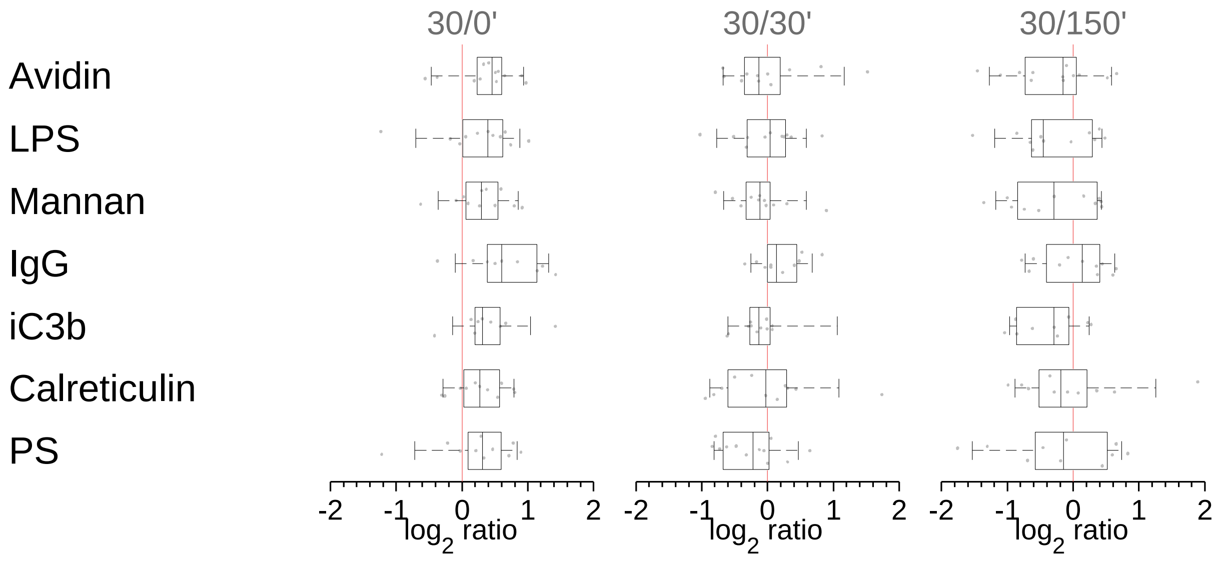

GO:0004715 non-membrane spanning protein tyrosine kinase activity (n = 8)

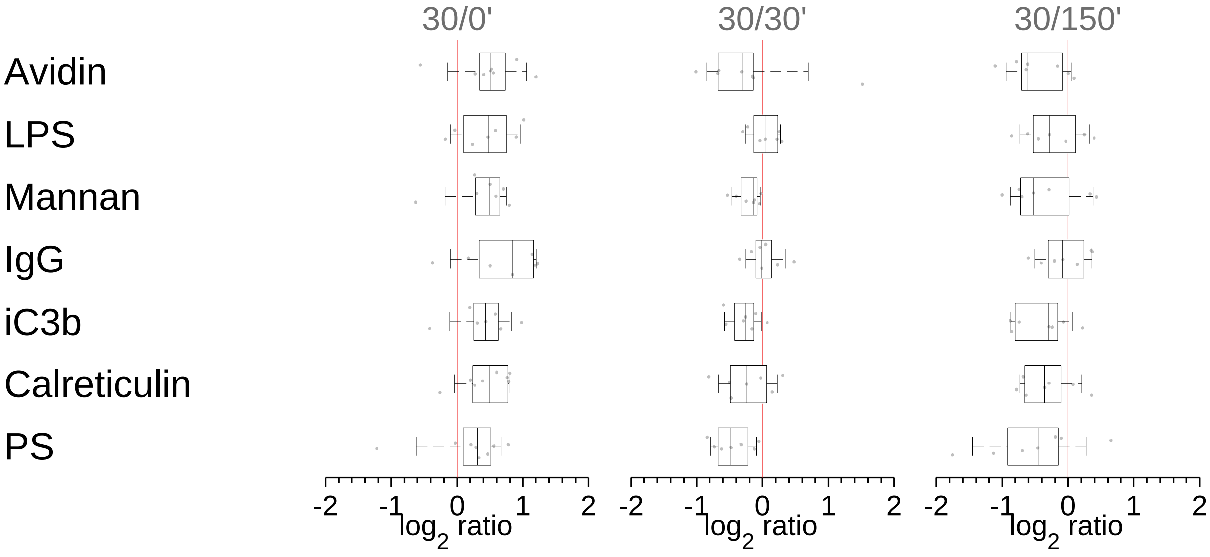

GO:0004721 phosphoprotein phosphatase activity (n = 20)

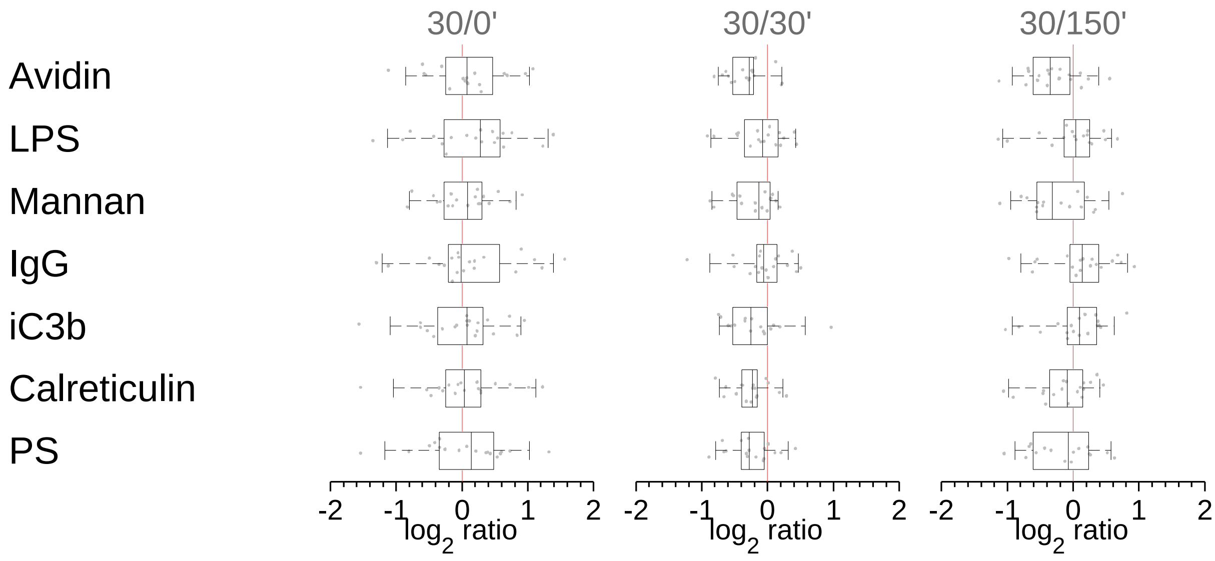

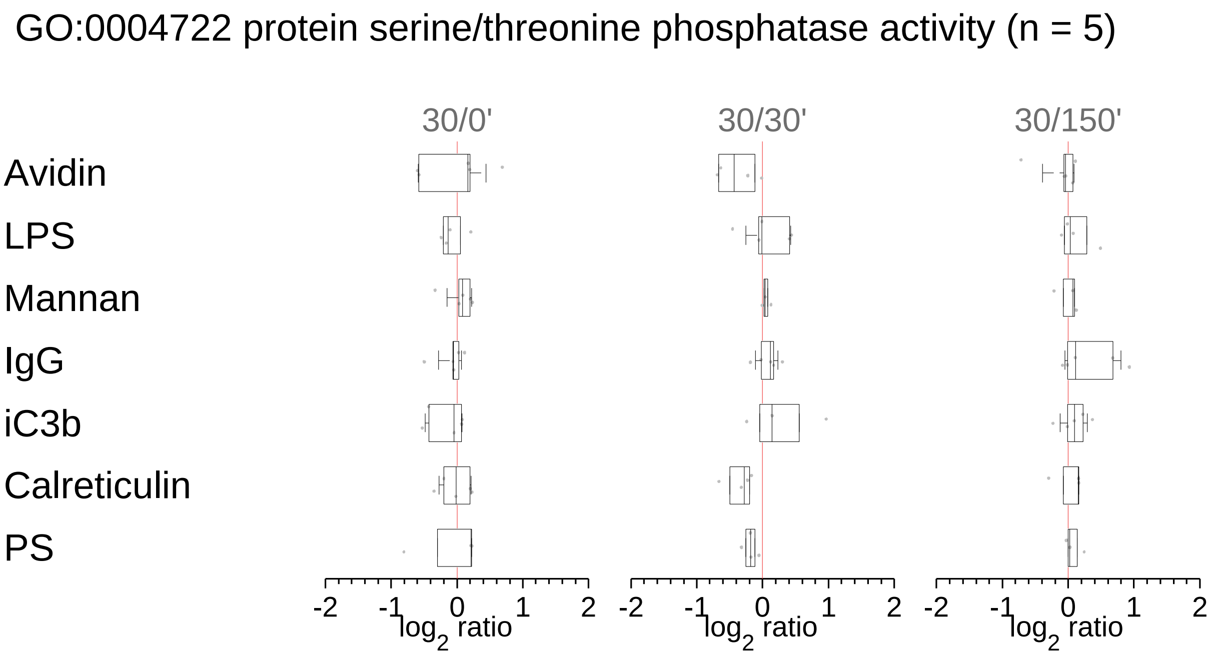

GO:0004722 protein serine/threonine phosphatase activity (n = 5)
30/0'
30/30'
30/150'
Avidin
LPS
Mannan
IgG
iC3b
Calreticulin
PS
-2 -1 0 1 2
log2 ratio

GO:0004725 protein tyrosine phosphatase activity (n = 13)

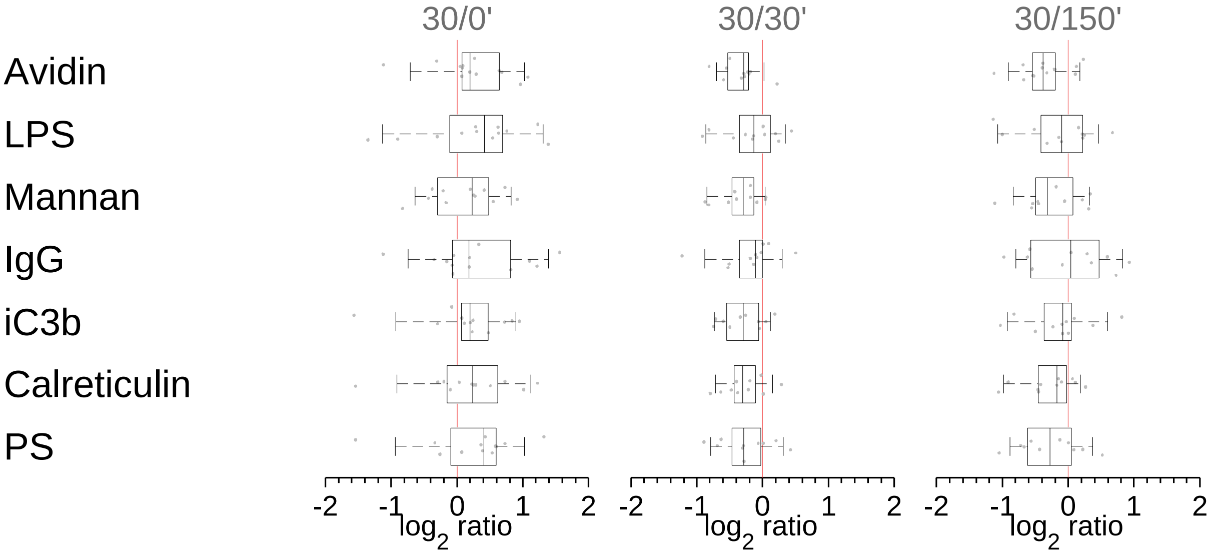

GO:0004767 sphingomyelin phosphodiesterase activity (n = 3)

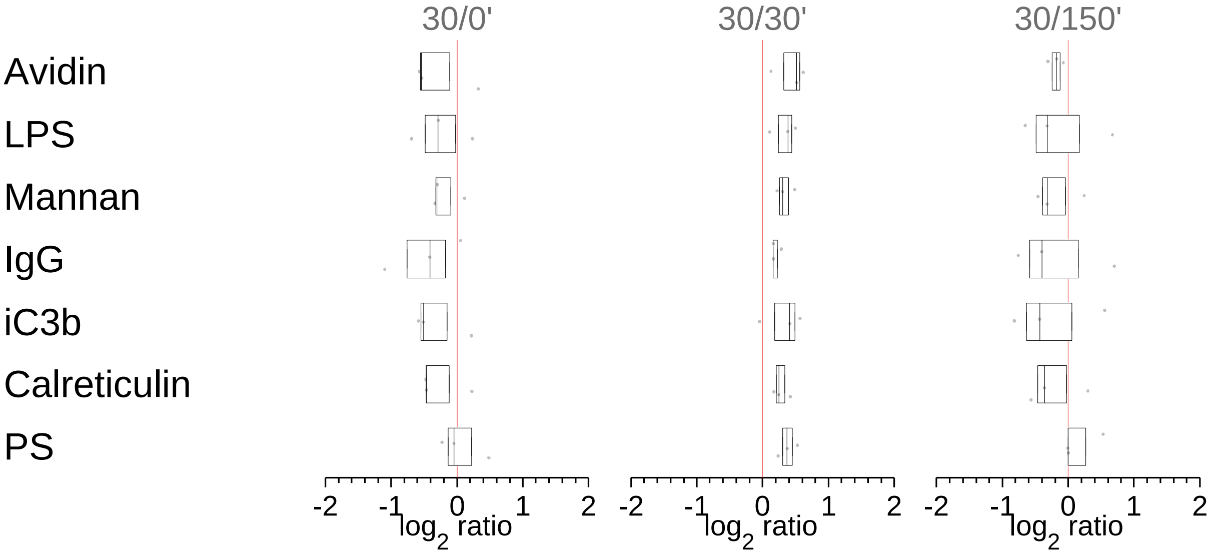

GO:0004772 sterol O-acyltransferase activity (n = 3)

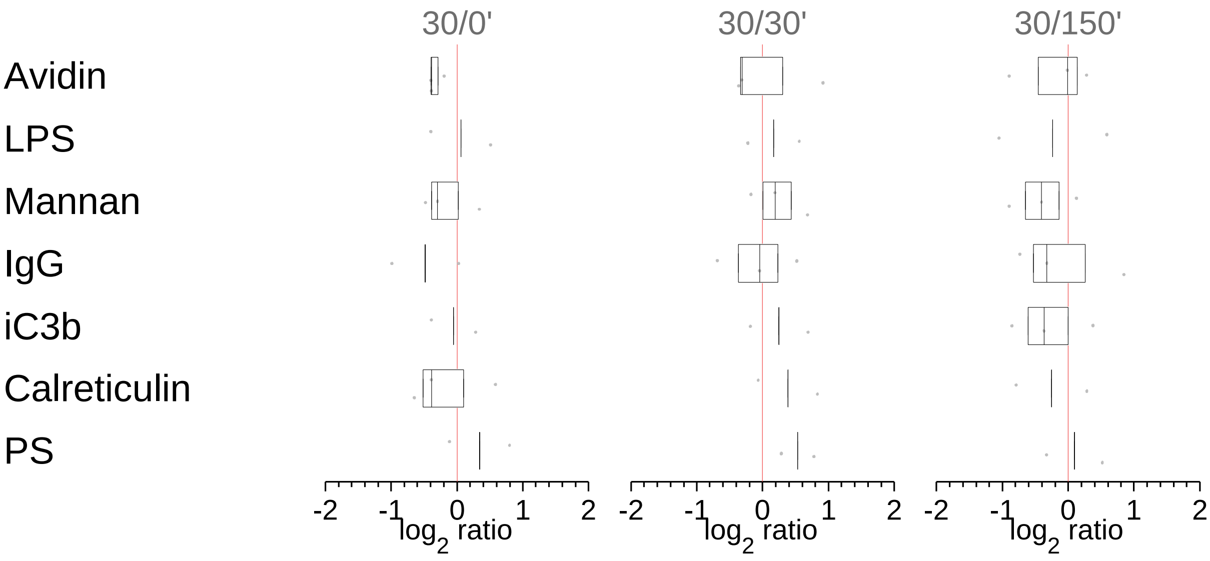

GO:0004812 aminoacyl-tRNA ligase activity (n = 3)

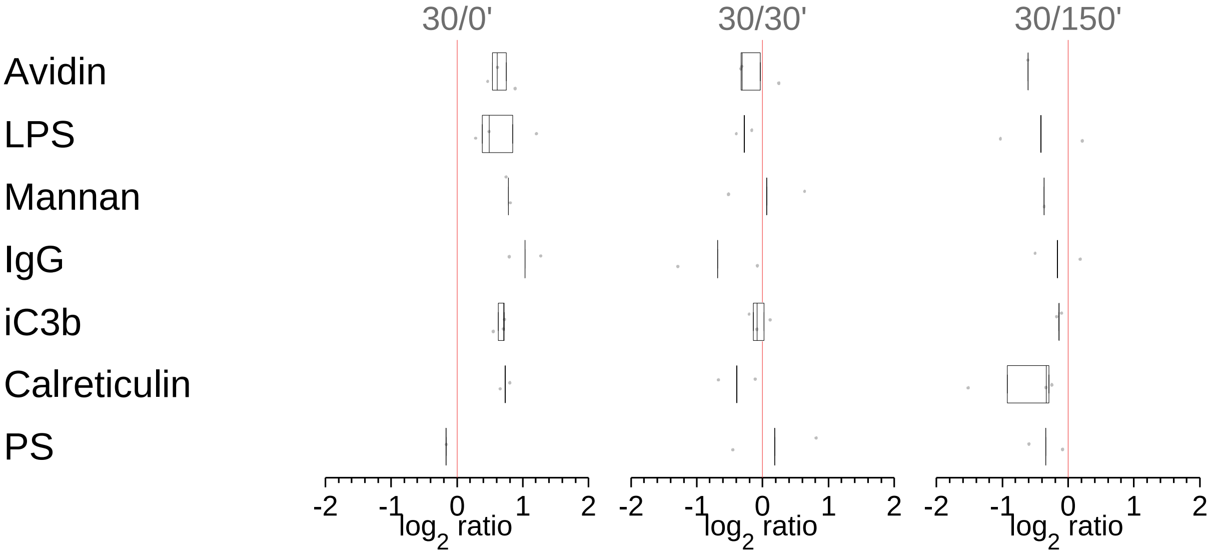

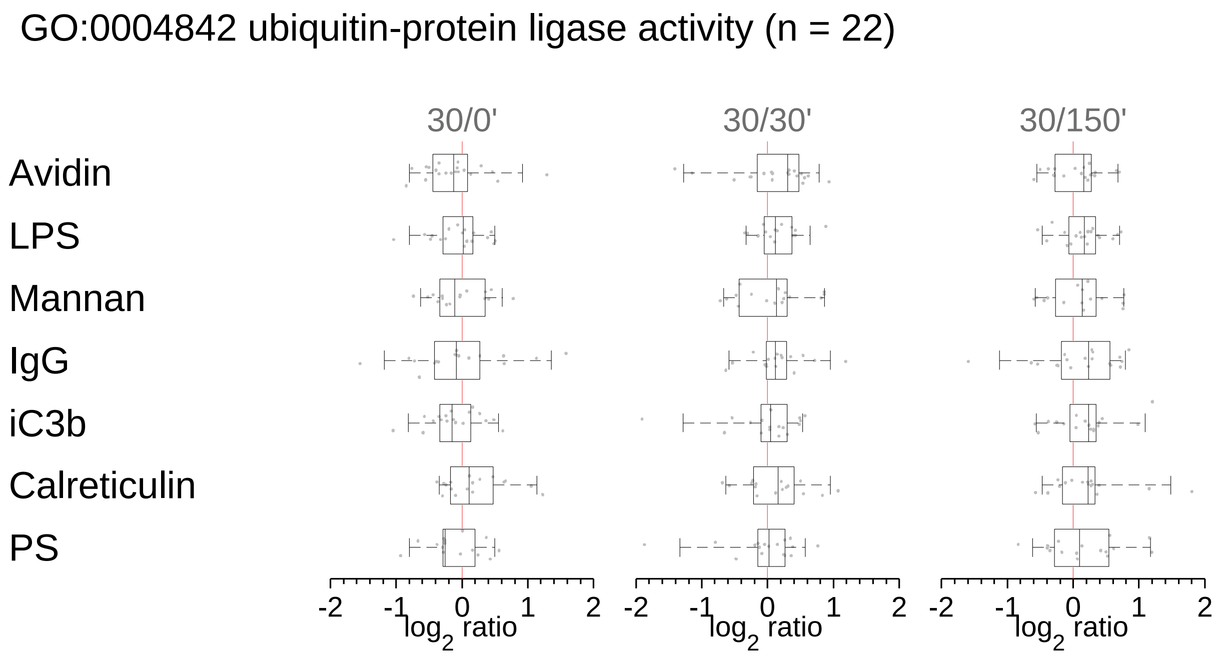

GO:0004842 ubiquitin-protein ligase activity (n = 22)
30/0'
30/30'
30/150'
Avidin
LPS
Mannan
IgG
iC3b
Calreticulin
PS
-2
-1
0
1
2
log2 ratio

GO:0004857 enzyme inhibitor activity (n = 3)

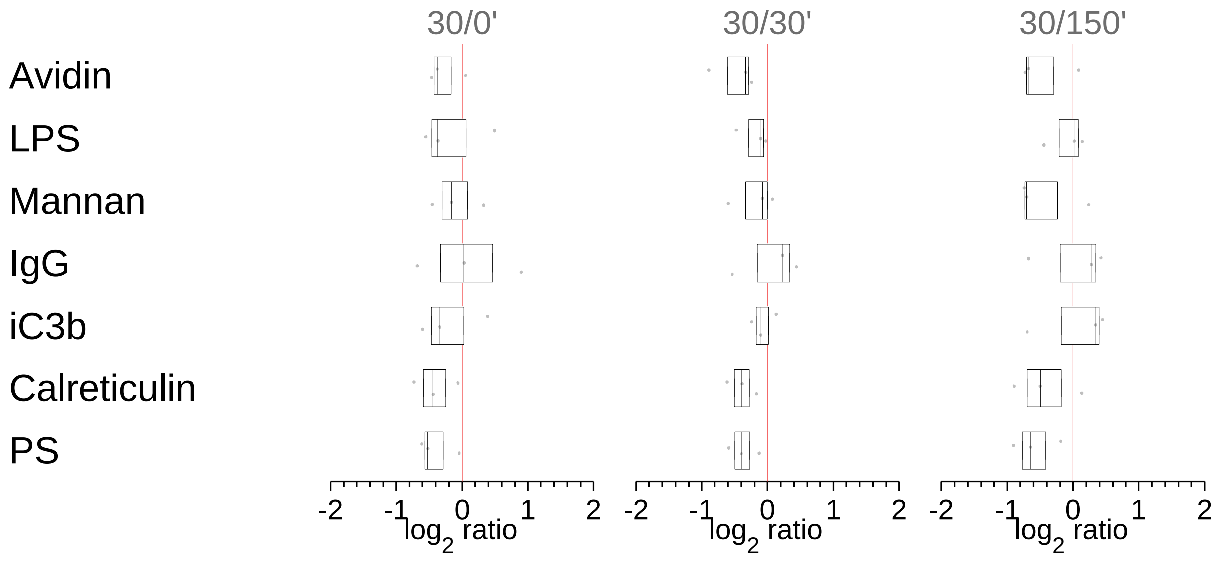

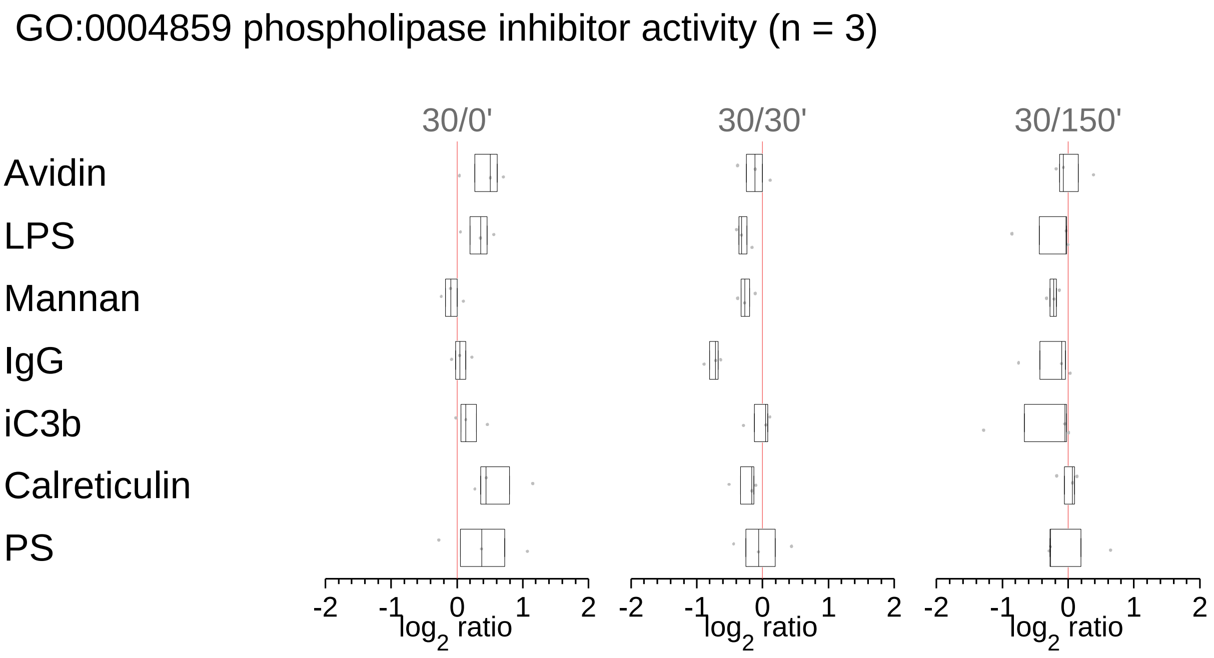

GO:0004859 phospholipase inhibitor activity (n = 3)
30/0'
30/30'
30/150'
Avidin
LPS
Mannan
IgG
iC3b
Calreticulin
PS
-2 -1 0 1 2
log2 ratio
-2 -1 0 1 2
log2 ratio
-2 -1 0 1 2
log2 ratio

GO:0004860 protein kinase inhibitor activity (n = 4)

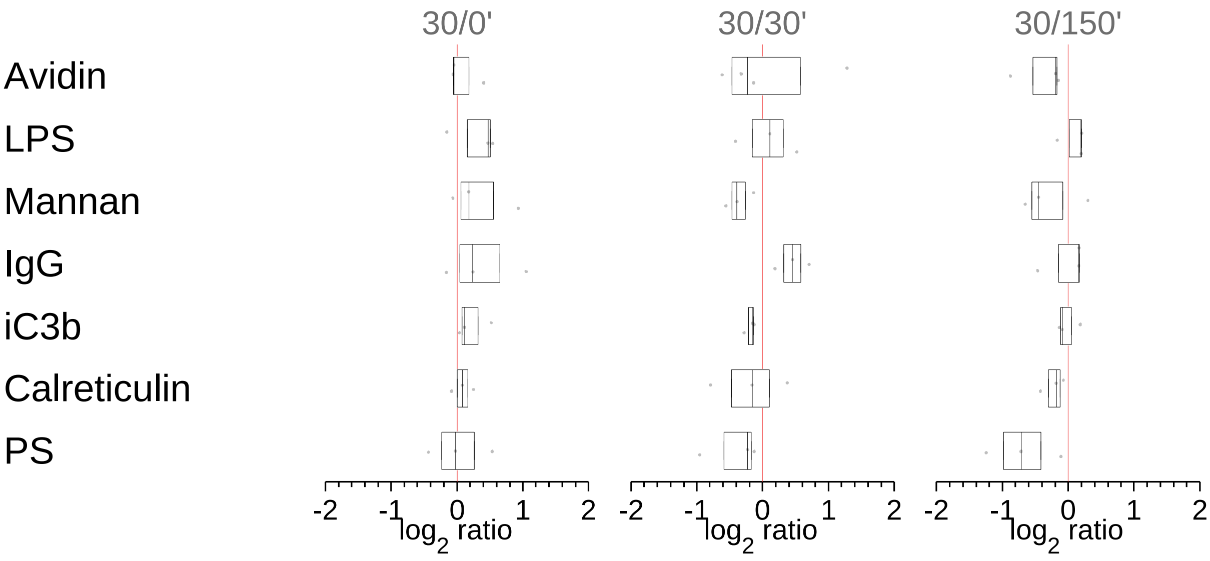

GO:0004866 endopeptidase inhibitor activity (n = 4)

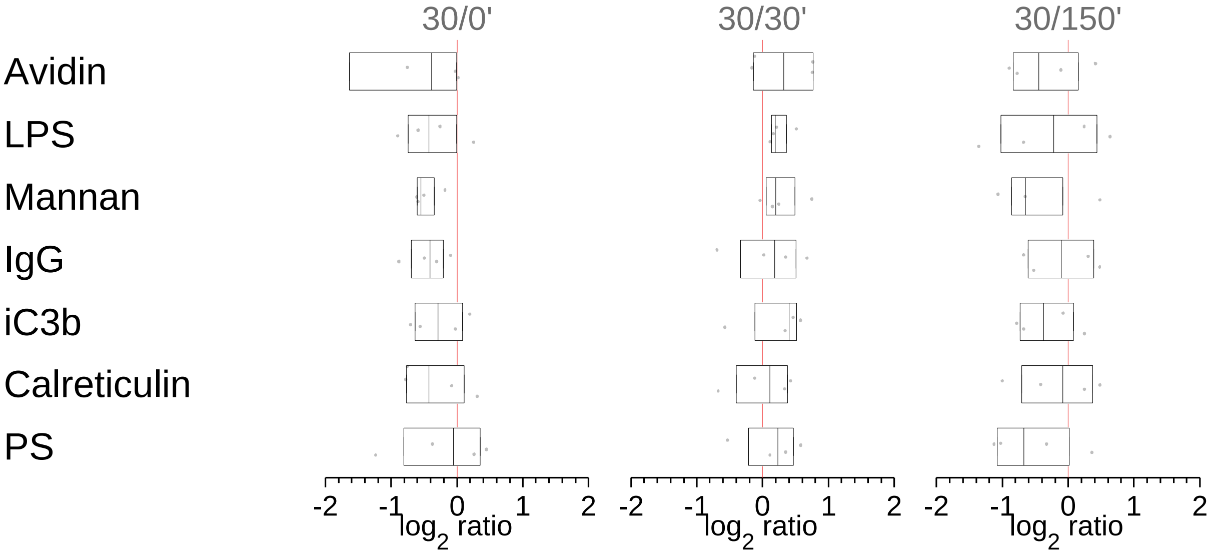

GO:0004867 serine-type endopeptidase inhibitor activity (n = 4)

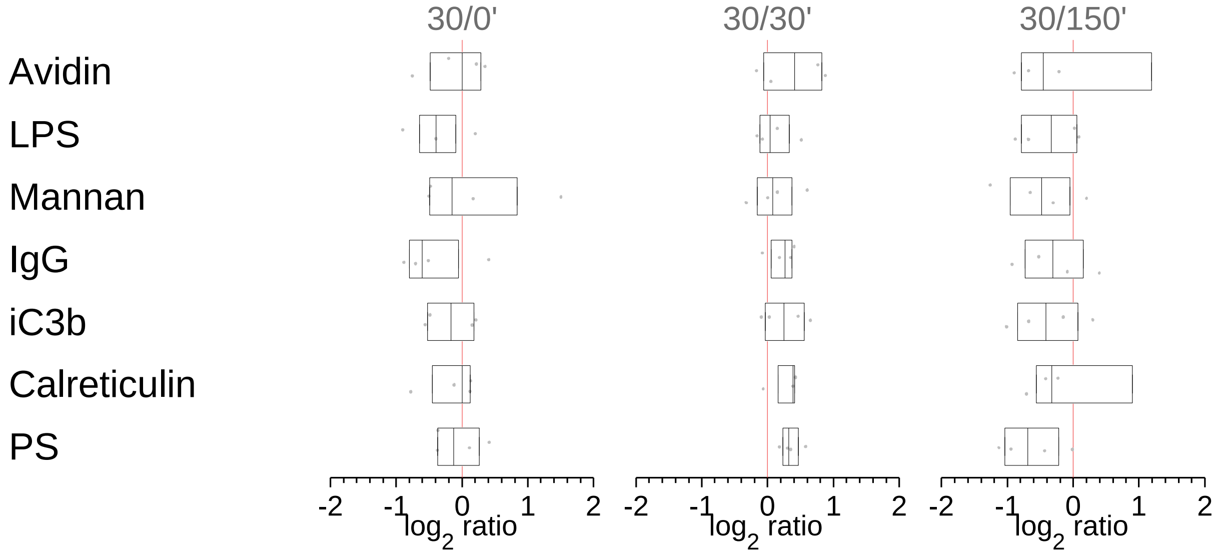

GO:0004869 cysteine-type endopeptidase inhibitor activity (n = 5)

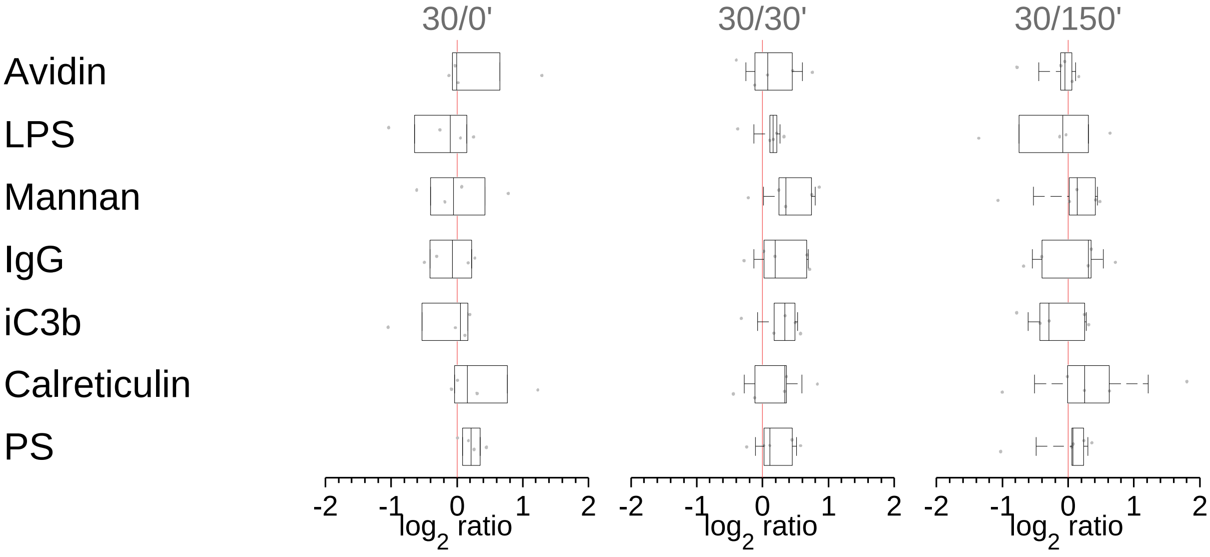

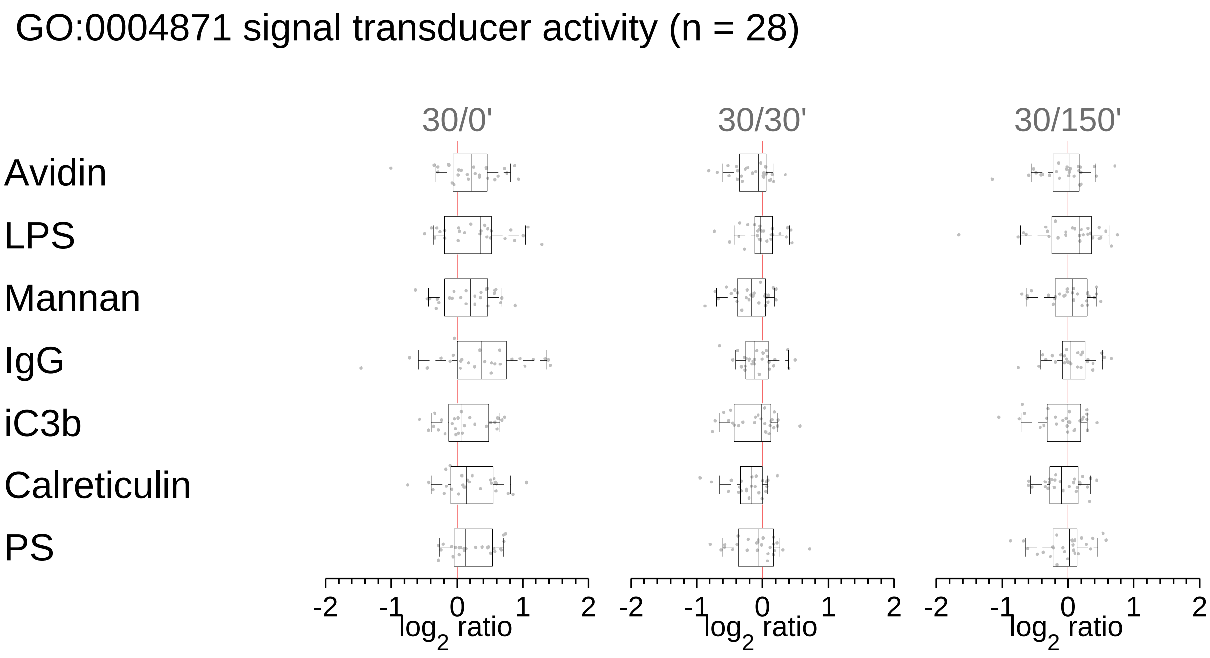
GO:0004871 signal transducer activity (n = 28)
30/0'
30/30'
30/150'
Avidin
LPS
Mannan
IgG
iC3b
Calreticulin
PS
-2
-1
0
1
2
log2 ratio

GO:0004872 receptor activity (n = 24)

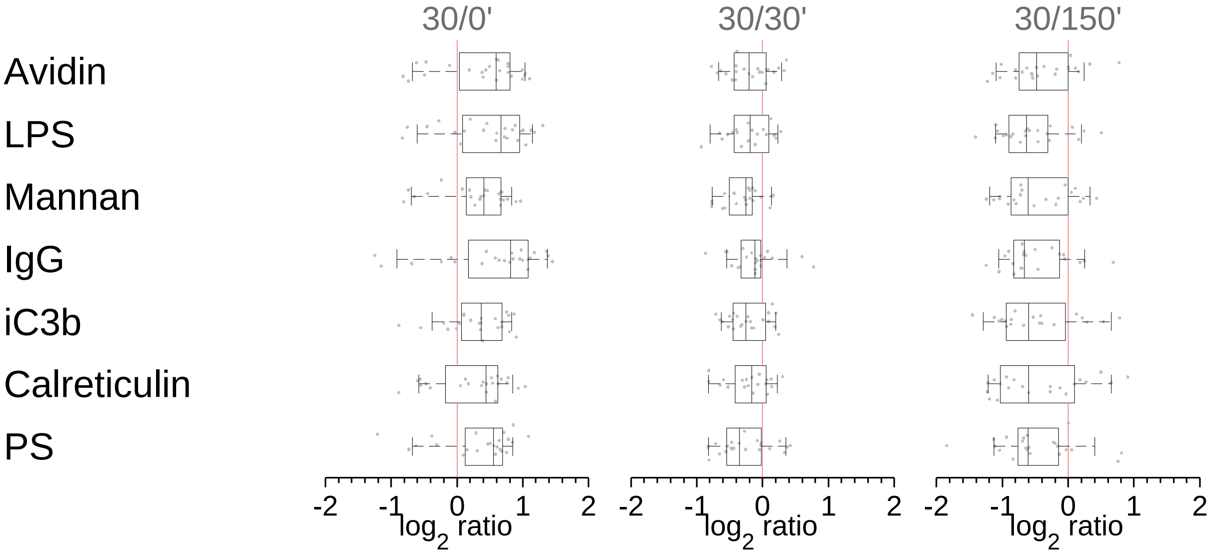

GO:0004888 transmembrane signaling receptor activity (n = 11)

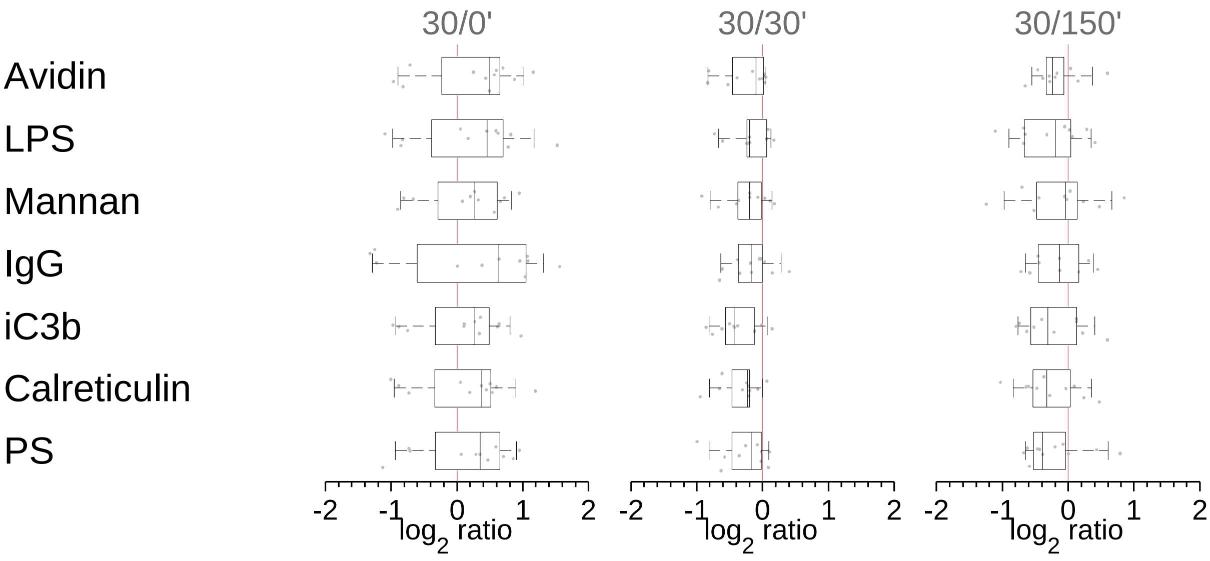

GO:0004896 cytokine receptor activity (n = 3)

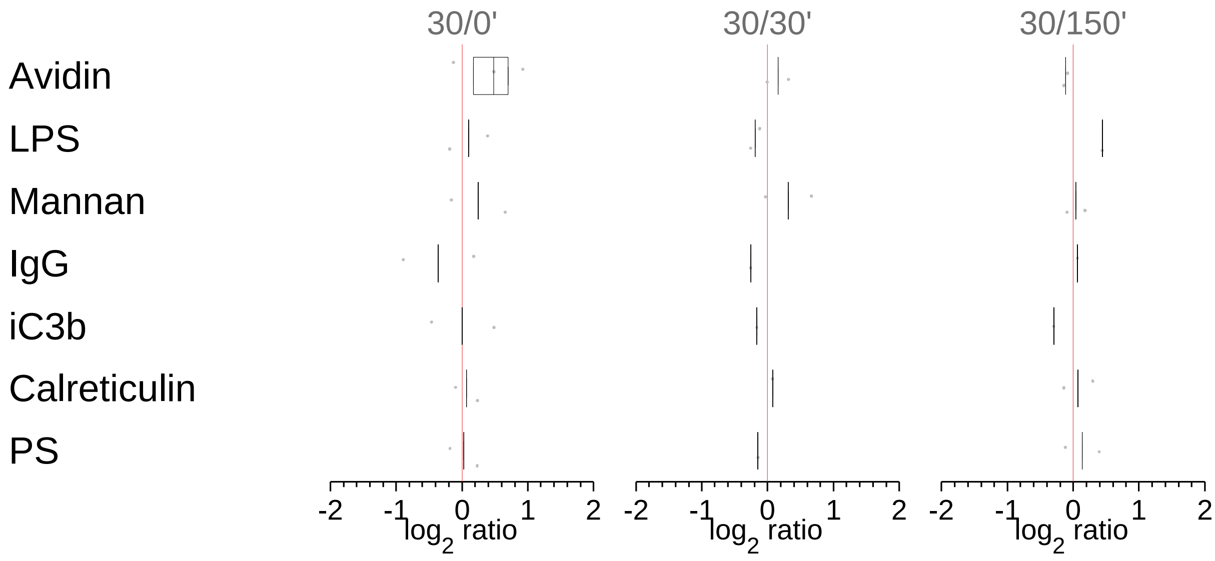

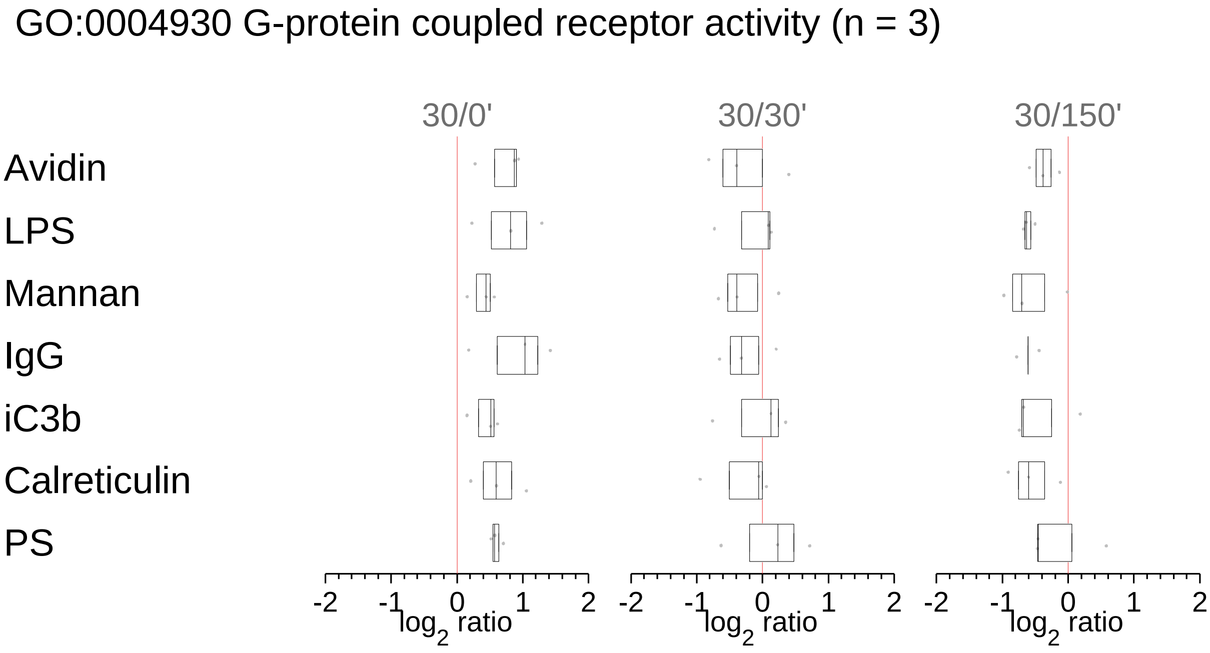
GO:0004930 G-protein coupled receptor activity (n = 3)
30/0'
30/30'
30/150'
Avidin
LPS
Mannan
IgG
iC3b
Calreticulin
PS
-2 -1 0 1 2
log2 ratio
-2 -1 0 1 2
log2 ratio
-2 -1 0 1 2
log2 ratio

GO:0005041 low-density lipoprotein receptor activity (n = 4)

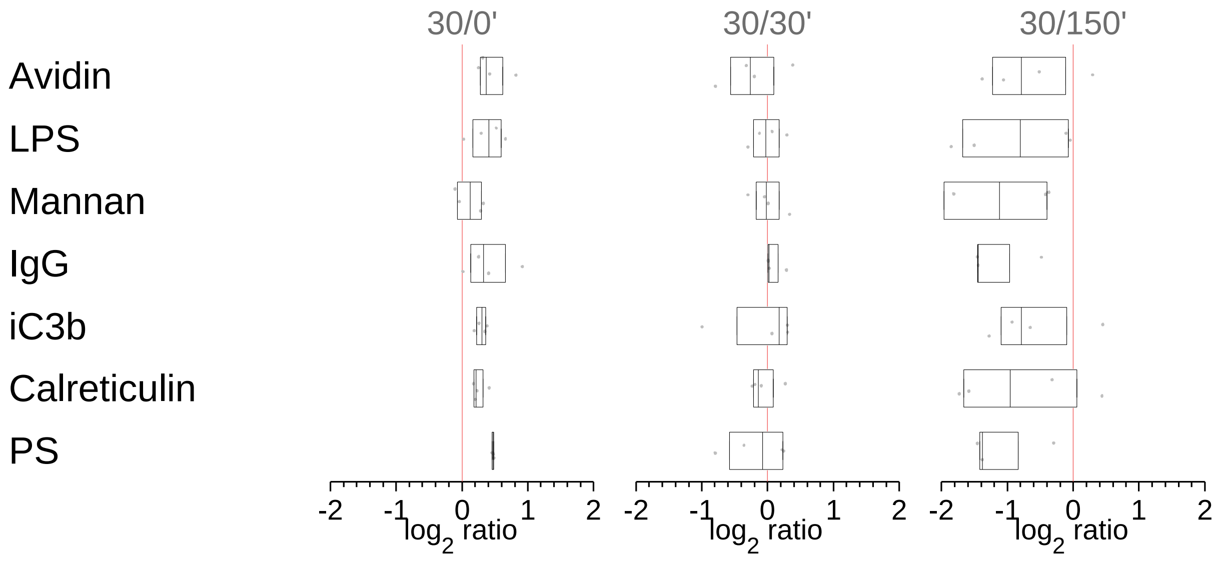

GO:0005044 scavenger receptor activity (n = 4)

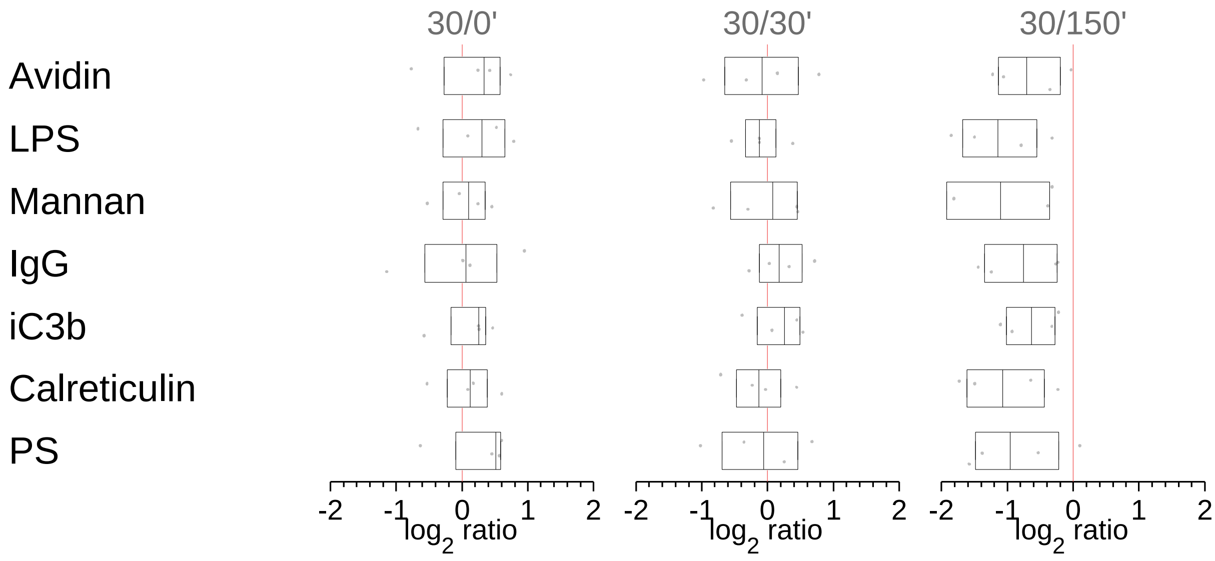

GO:0005057 receptor signaling protein activity (n = 3)

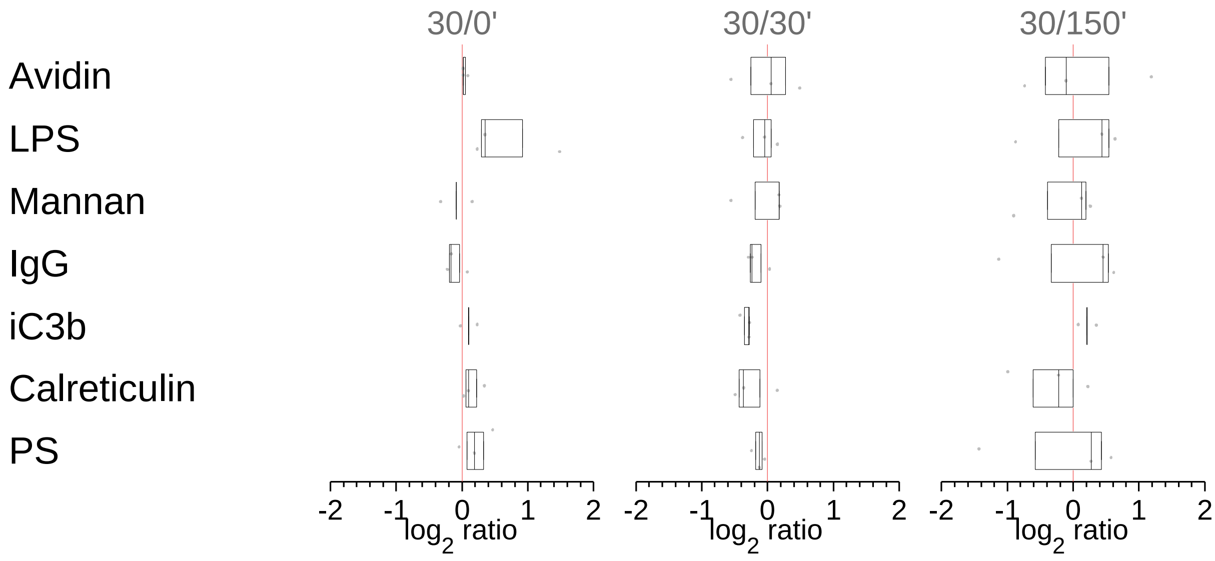

GO:0005070 SH3/SH2 adaptor activity (n = 3)

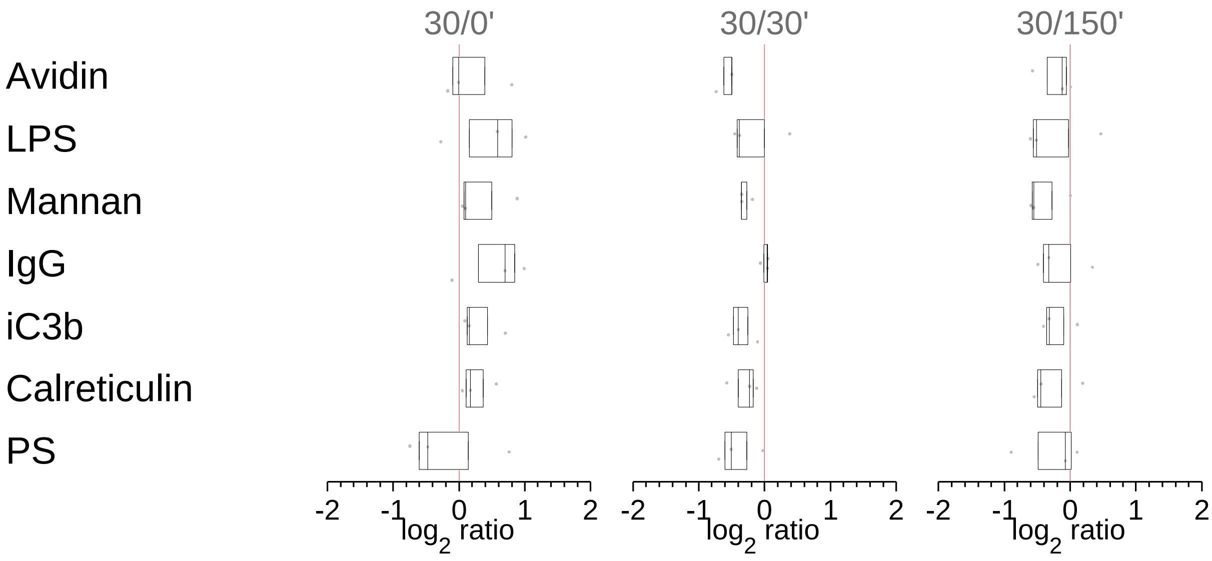

GO:0005080 protein kinase C binding (n = 15)

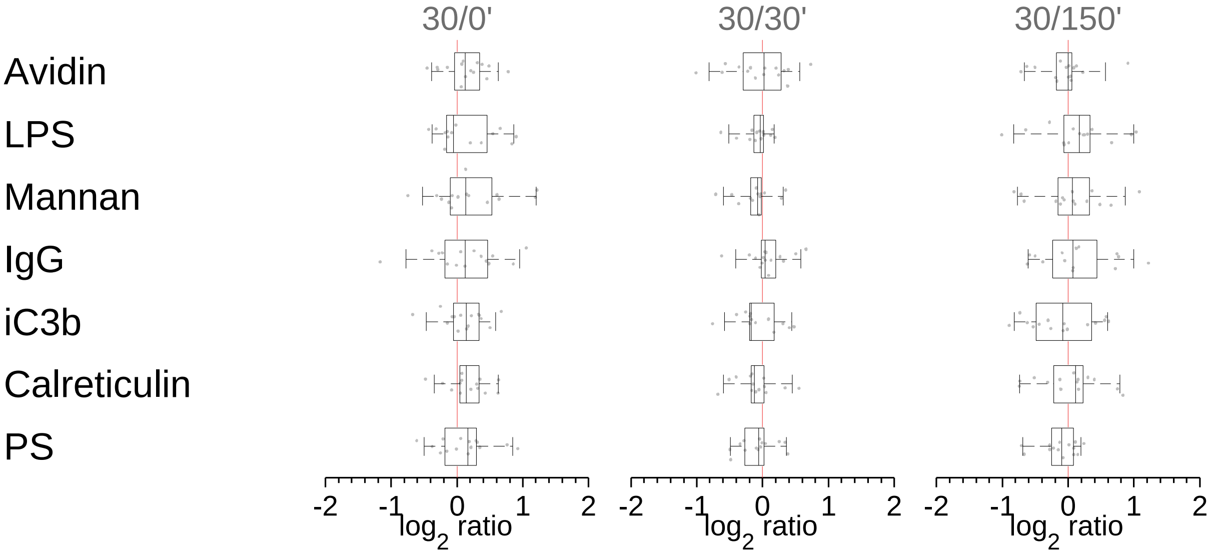

GO:0005085 guanyl-nucleotide exchange factor activity (n = 13)

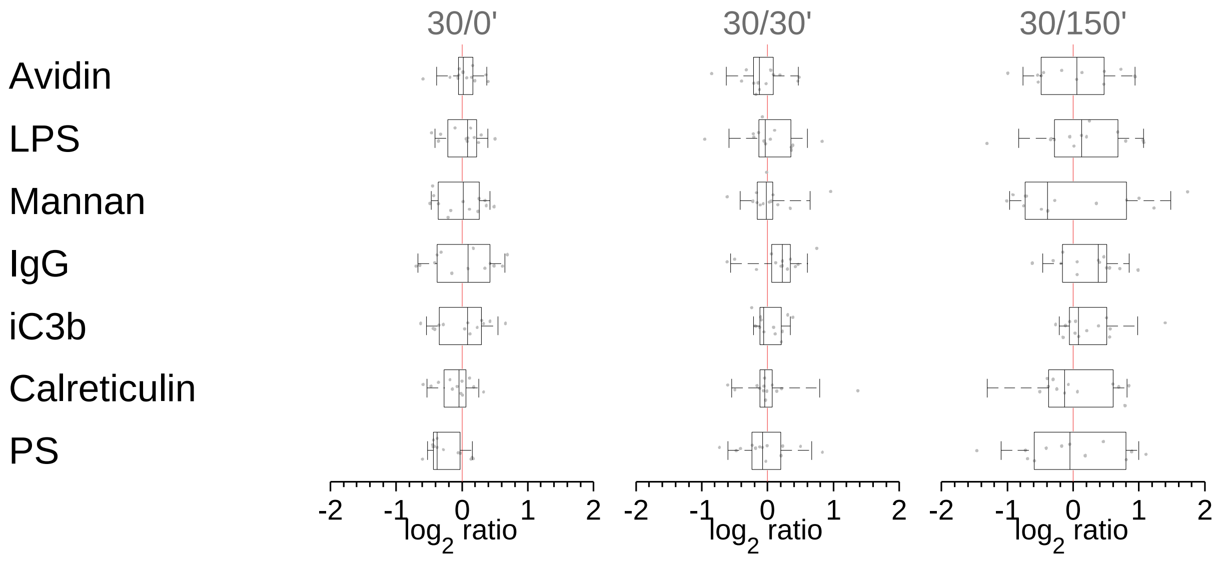

GO:0005089 Rho guanyl-nucleotide exchange factor activity (n = 6)

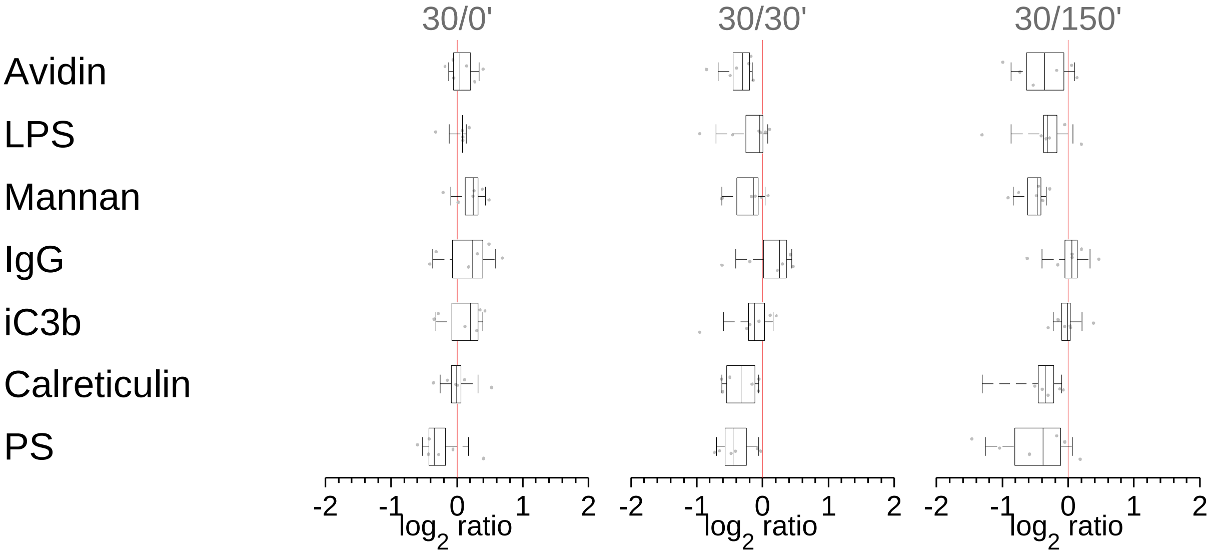

GO:0005096 GTPase activator activity (n = 37)

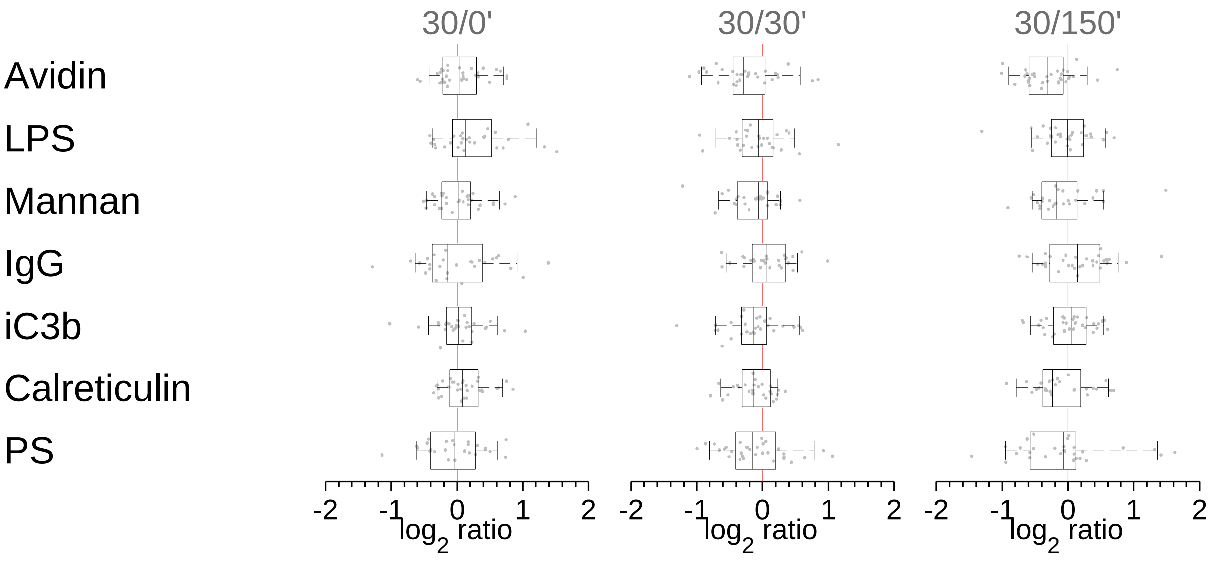

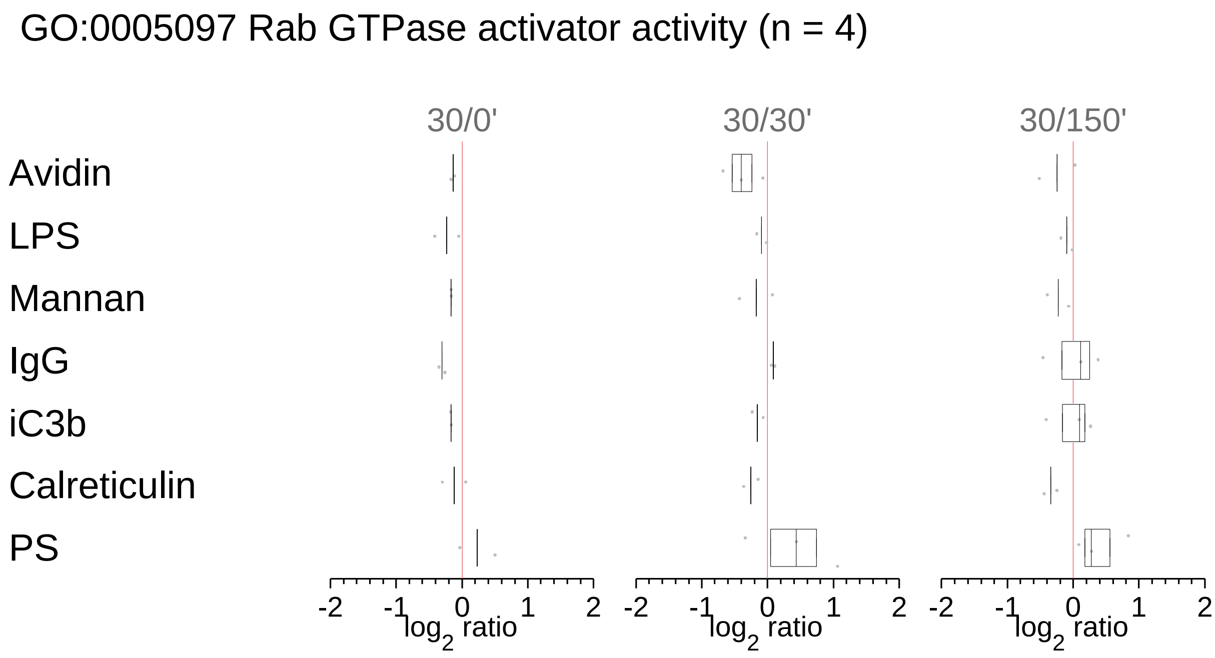
GO:0005097 Rab GTPase activator activity (n = 4)
30/0'
30/30'
30/150'
Avidin
LPS
Mannan
IgG
iC3b
Calreticulin
PS
-2
-1
0
1
2
log2 ratio

GO:0005099 Ras GTPase activator activity (n = 4)

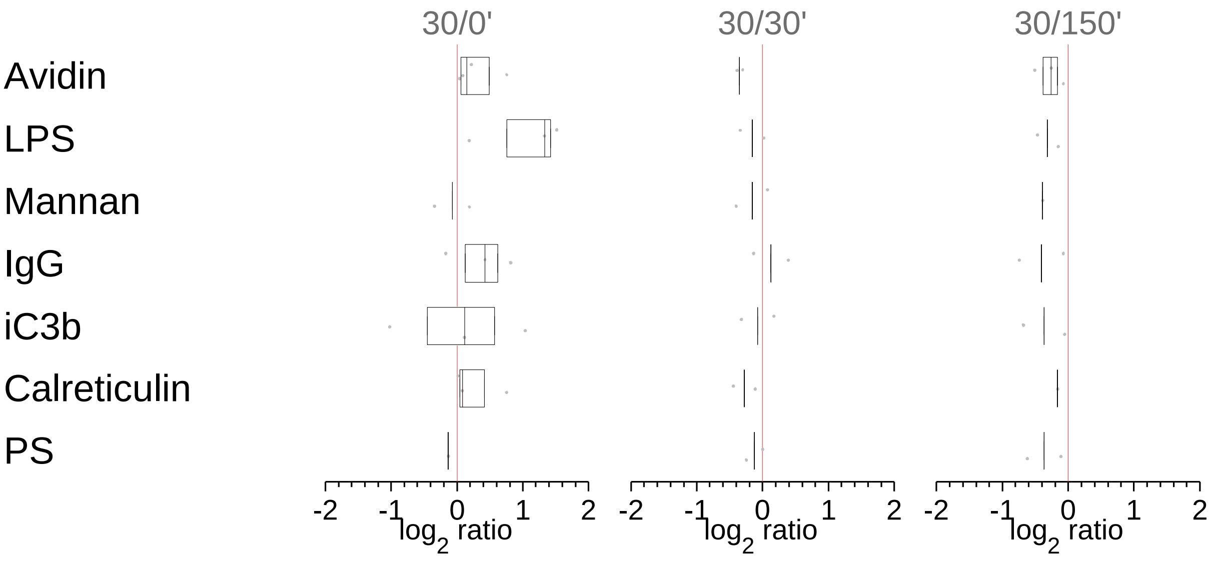

GO:0005100 Rho GTPase activator activity (n = 3)

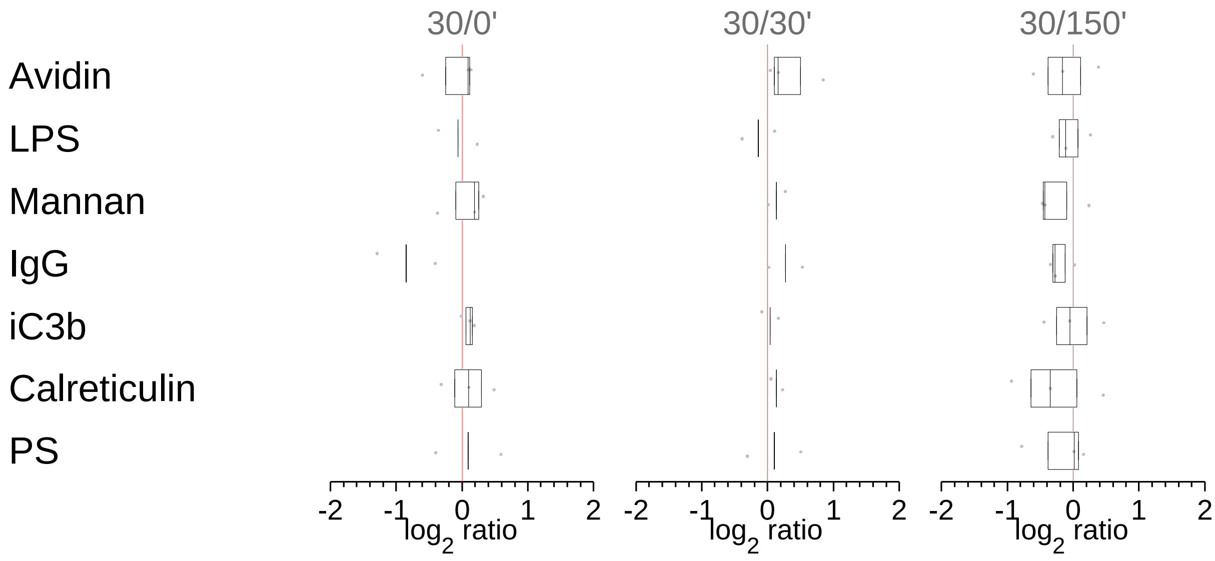

GO:0005102 receptor binding (n = 46)

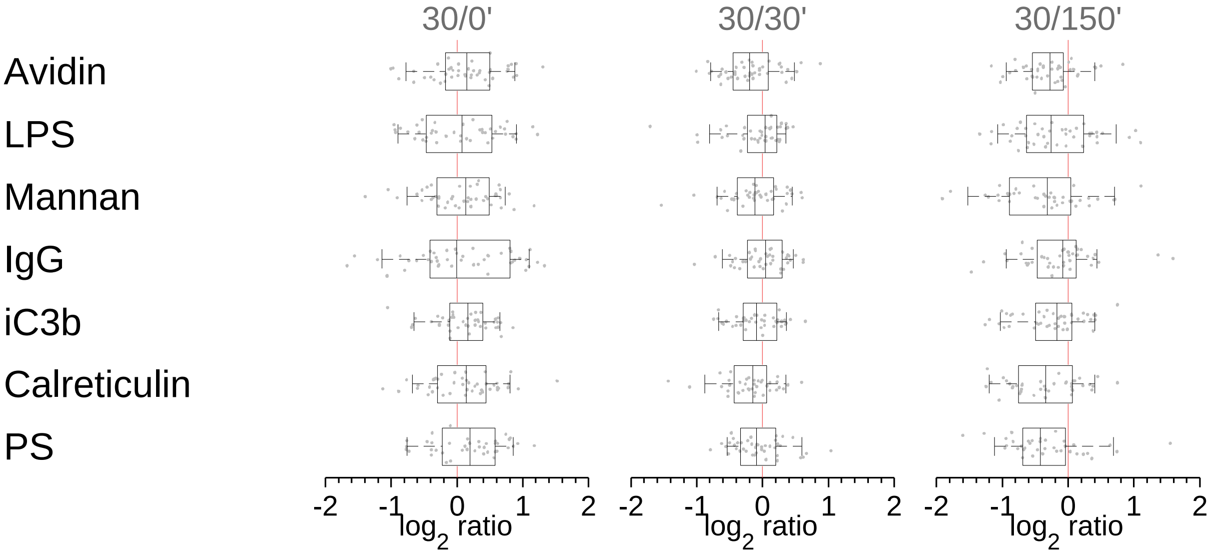

GO:0005125 cytokine activity (n = 8)

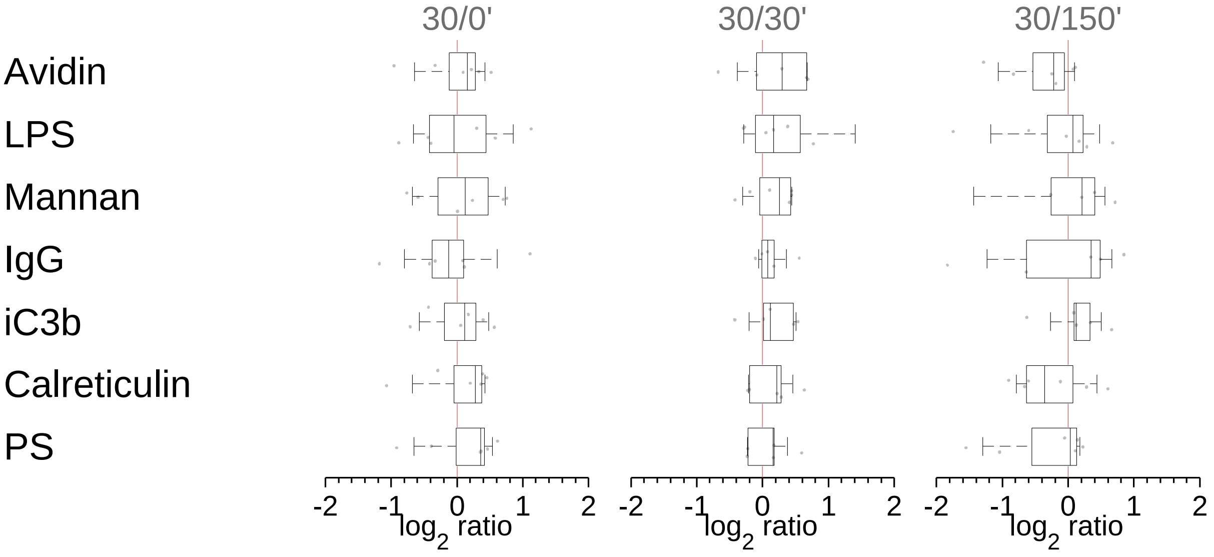

GO:0005154 epidermal growth factor receptor binding (n = 3)

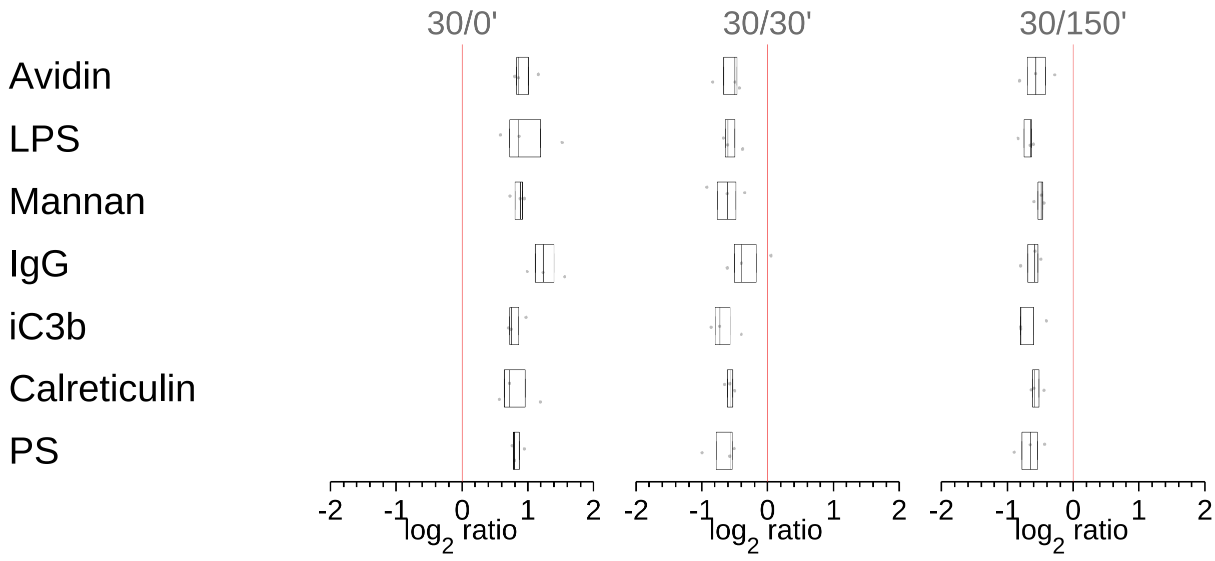

GO:0005158 insulin receptor binding (n = 6)

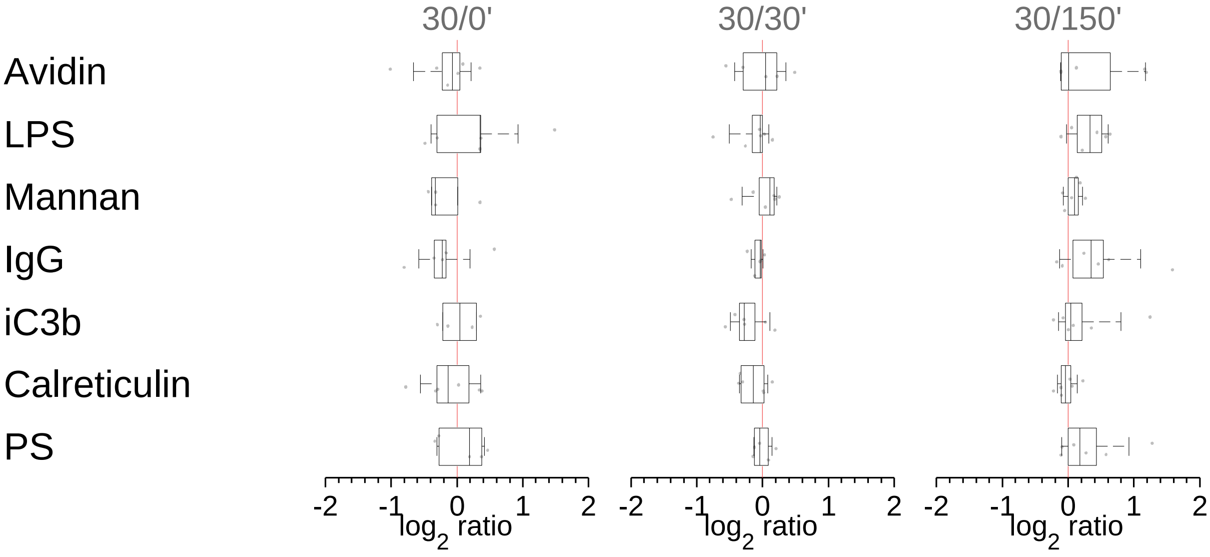

GO:0005164 tumor necrosis factor receptor binding (n = 5)

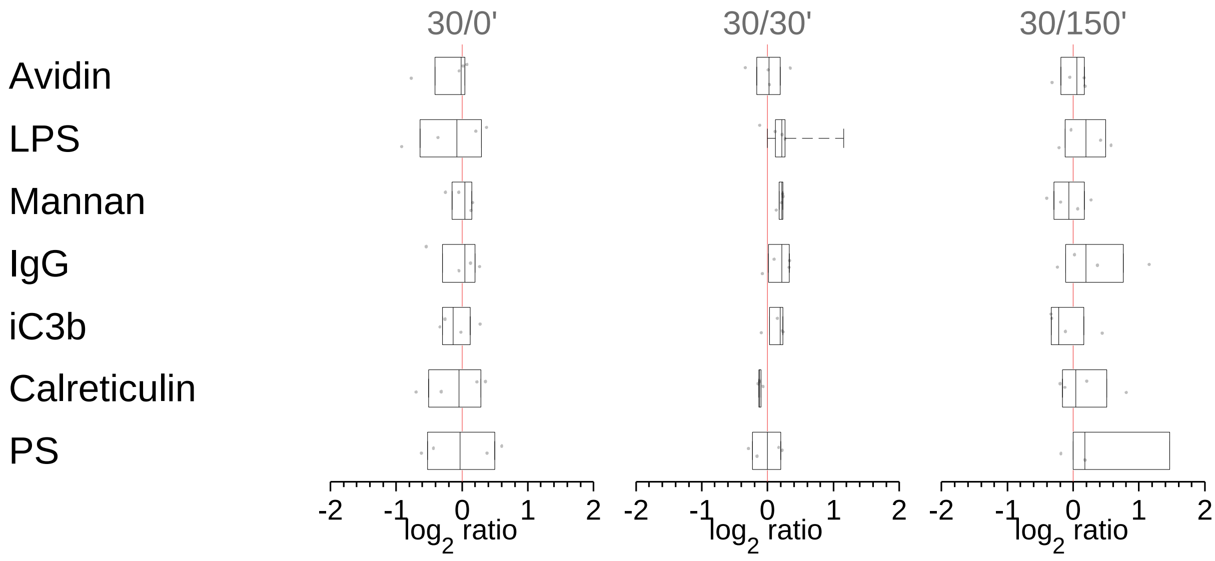

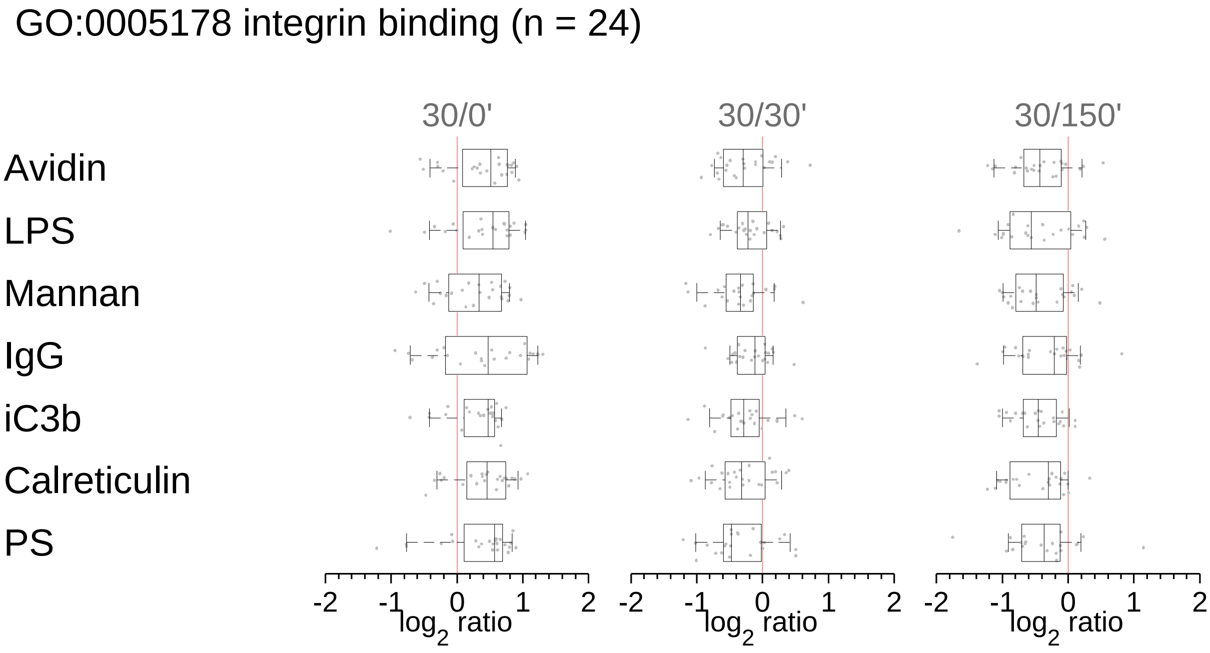
GO:0005178 integrin binding (n = 24)
30/0'
30/30'
30/150'
Avidin
LPS
Mannan
IgG
iC3b
Calreticulin
PS
-2
-1
0
1
2
log2 ratio

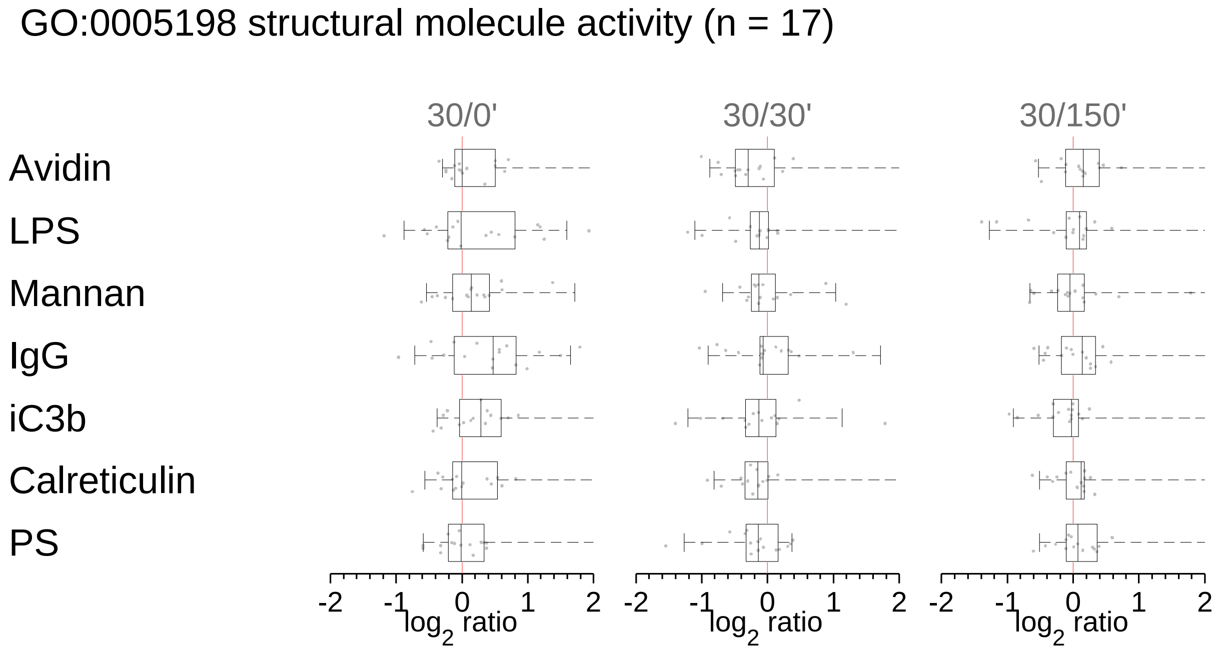

GO:0005198 structural molecule activity (n = 17)
30/0'
30/30'
30/150'
Avidin
LPS
Mannan
IgG
iC3b
Calreticulin
PS
-2 -1 0 1 2
log2 ratio

GO:0005200 structural constituent of cytoskeleton (n = 9)

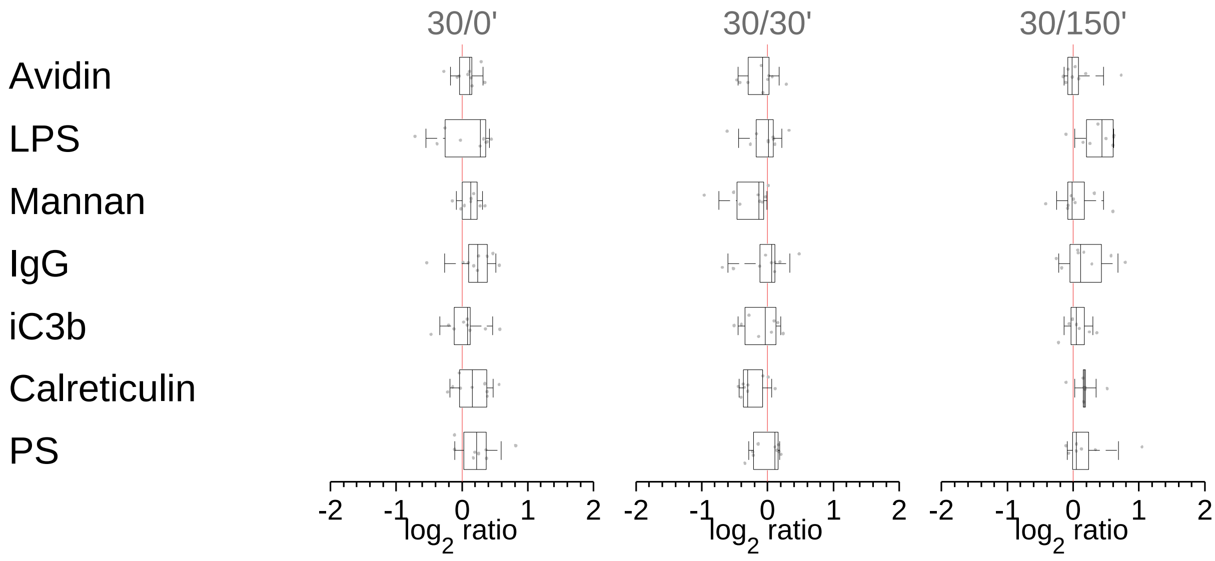

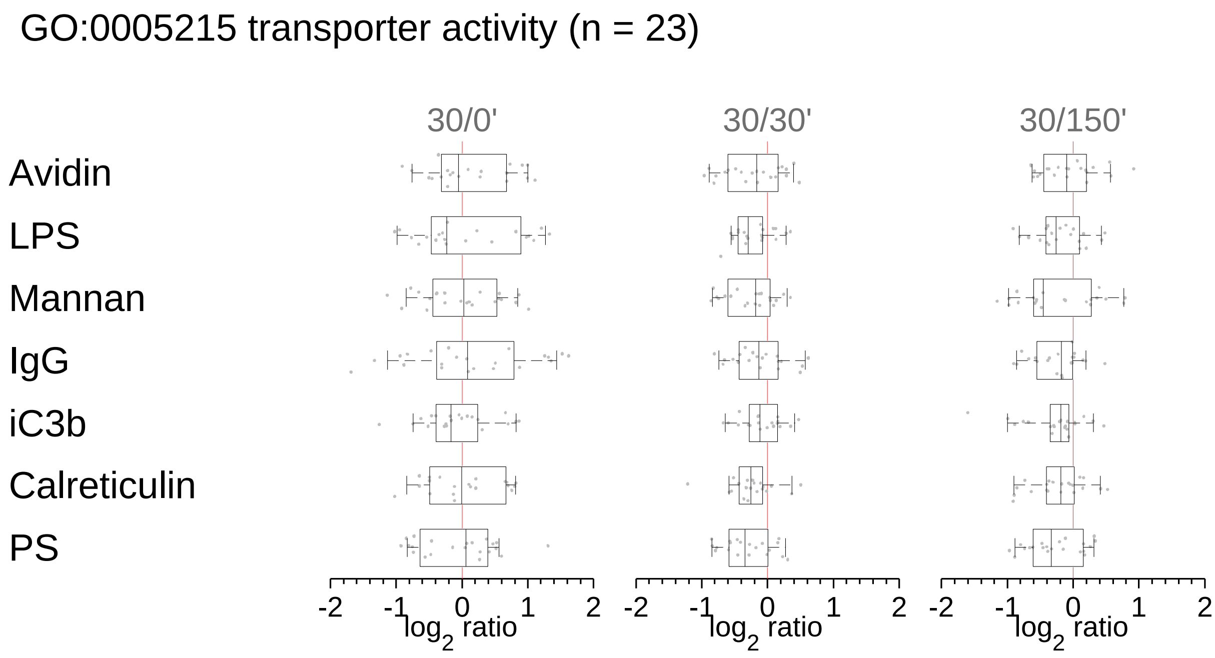
GO:0005215 transporter activity (n = 23)
30/0'
30/30'
30/150'
Avidin
LPS
Mannan
IgG
iC3b
Calreticulin
PS
-2
-1
0
1
2
log2 ratio

GO:0005216 ion channel activity (n = 10)

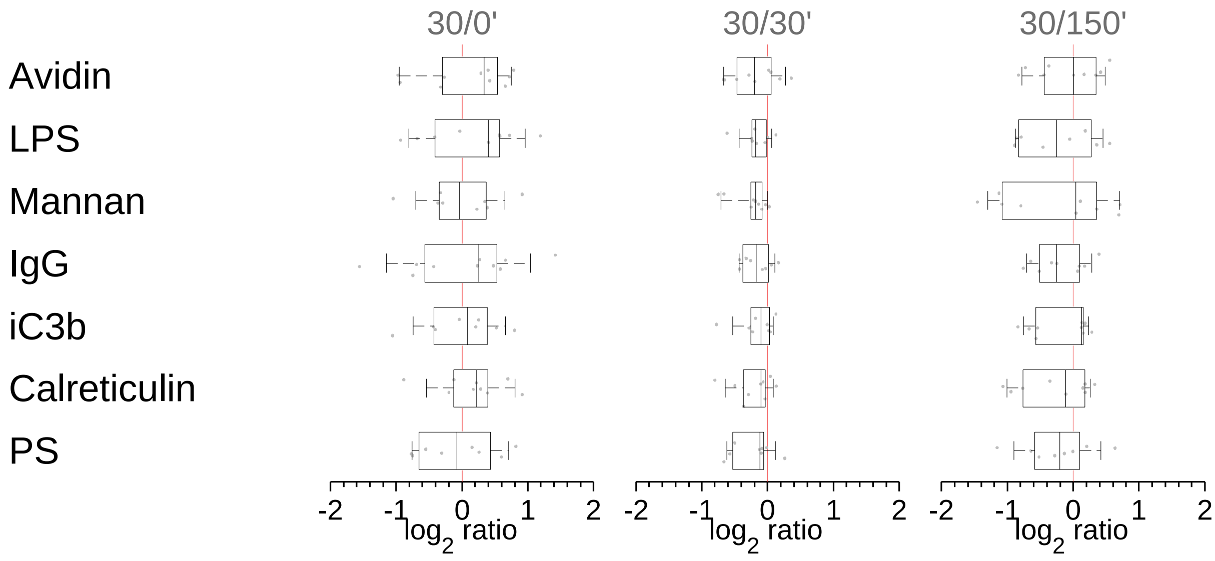

GO:0005244 voltage-gated ion channel activity (n = 7)

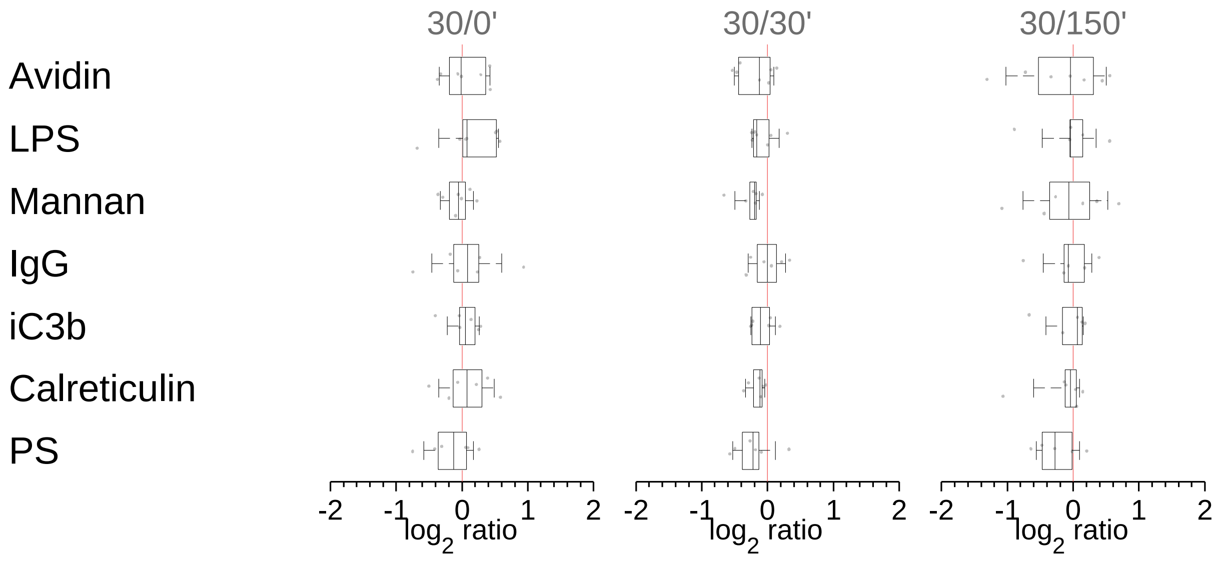

GO:0005245 voltage-gated calcium channel activity (n = 3)

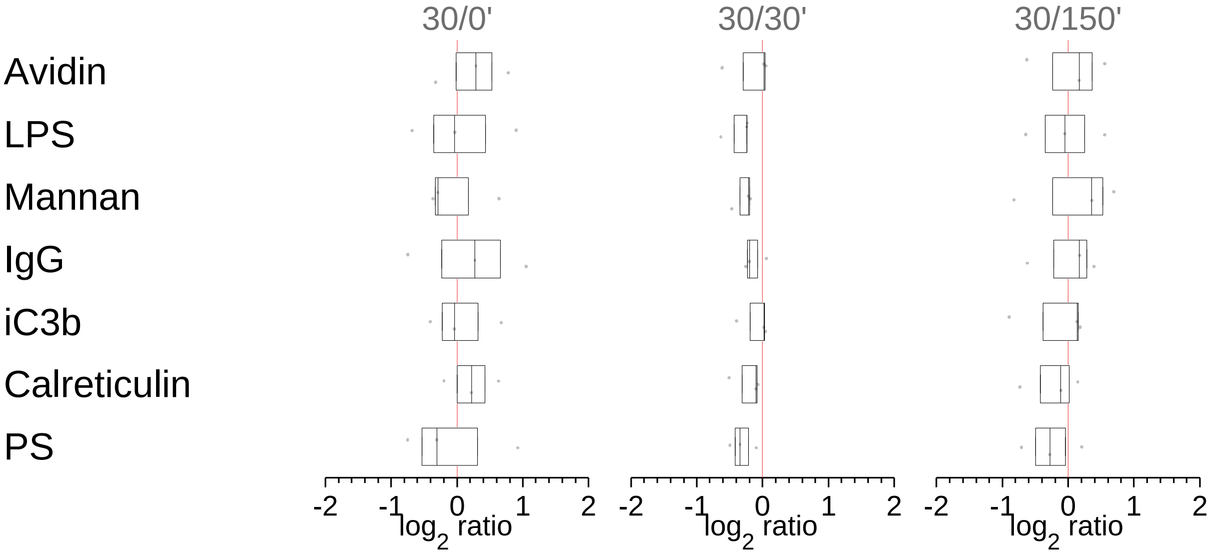

GO:0005247 voltage-gated chloride channel activity (n = 5)

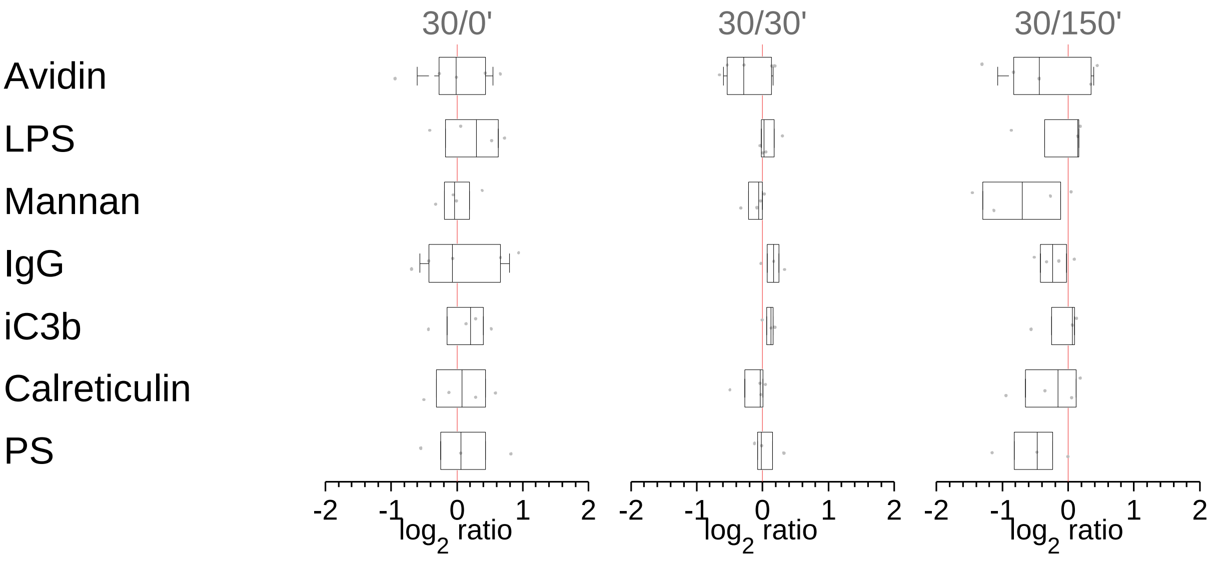

GO:0005254 chloride channel activity (n = 4)

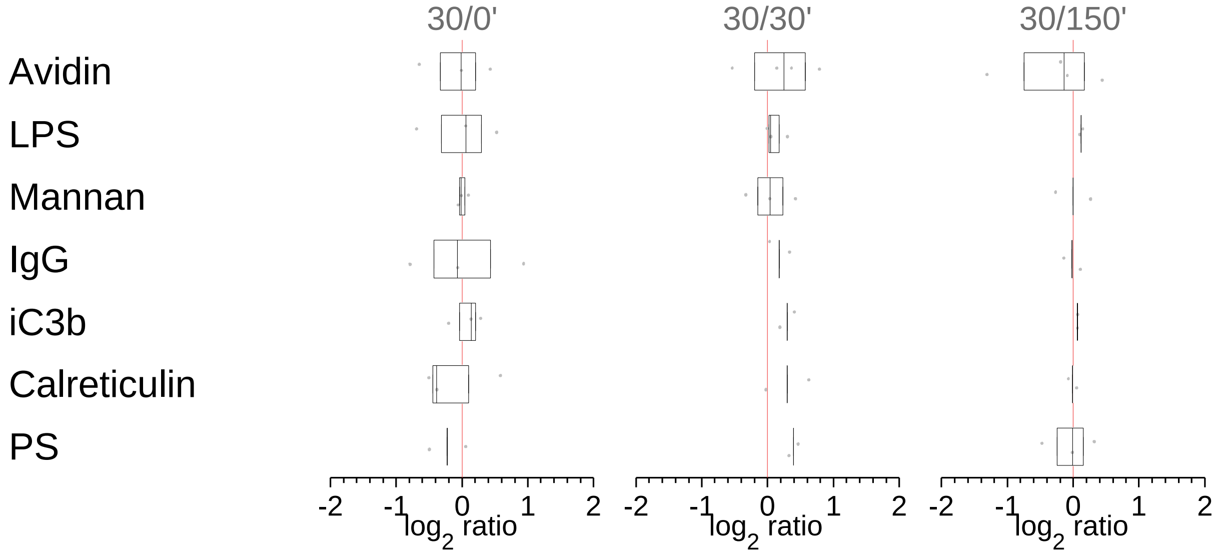

GO:0005262 calcium channel activity (n = 4)

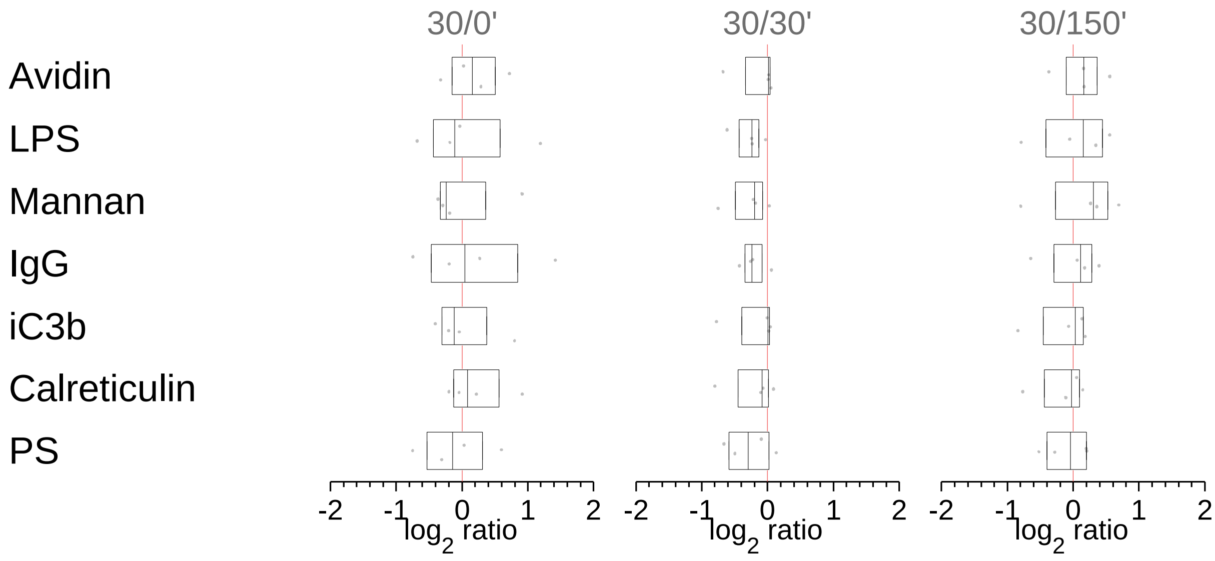

GO:0005313 L-glutamate transmembrane transporter activity (n = 3)

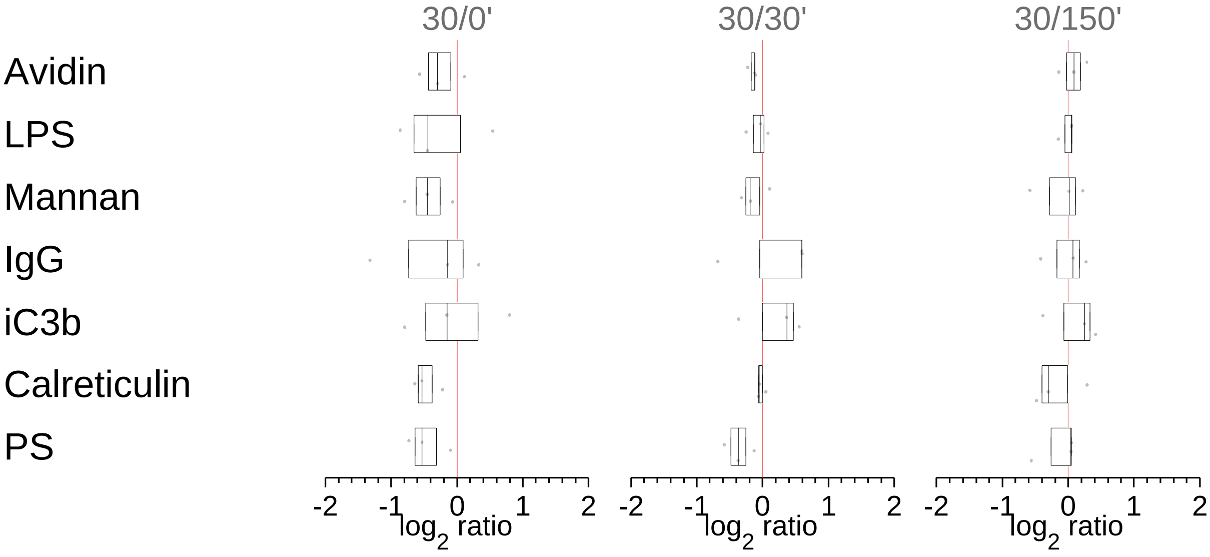

GO:0005391 sodium:potassium-exchanging ATPase activity (n = 3)

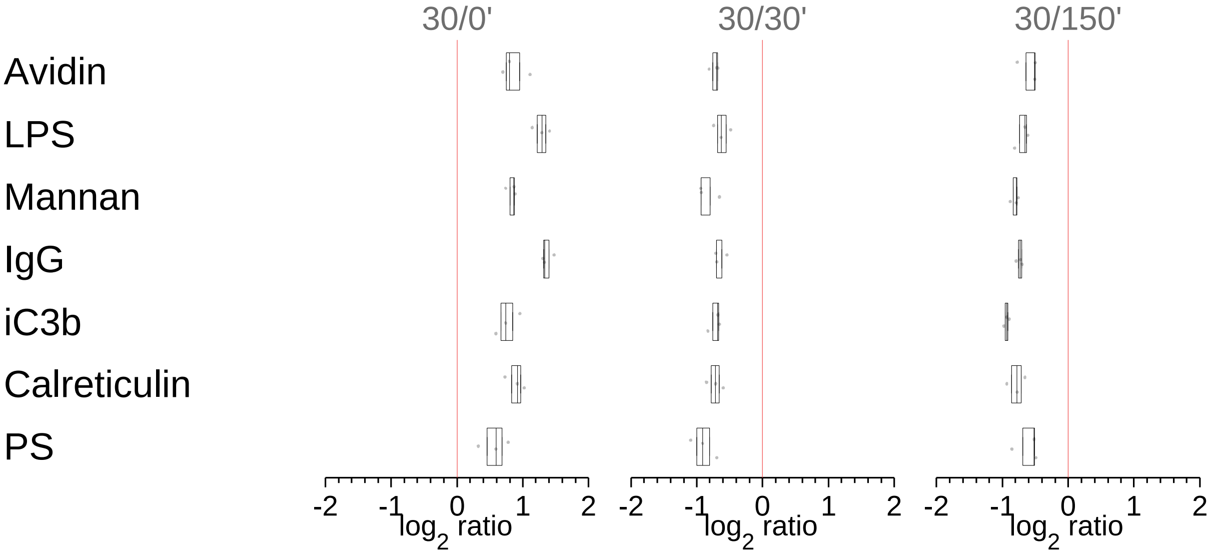

GO:0005484 SNAP receptor activity (n = 17)

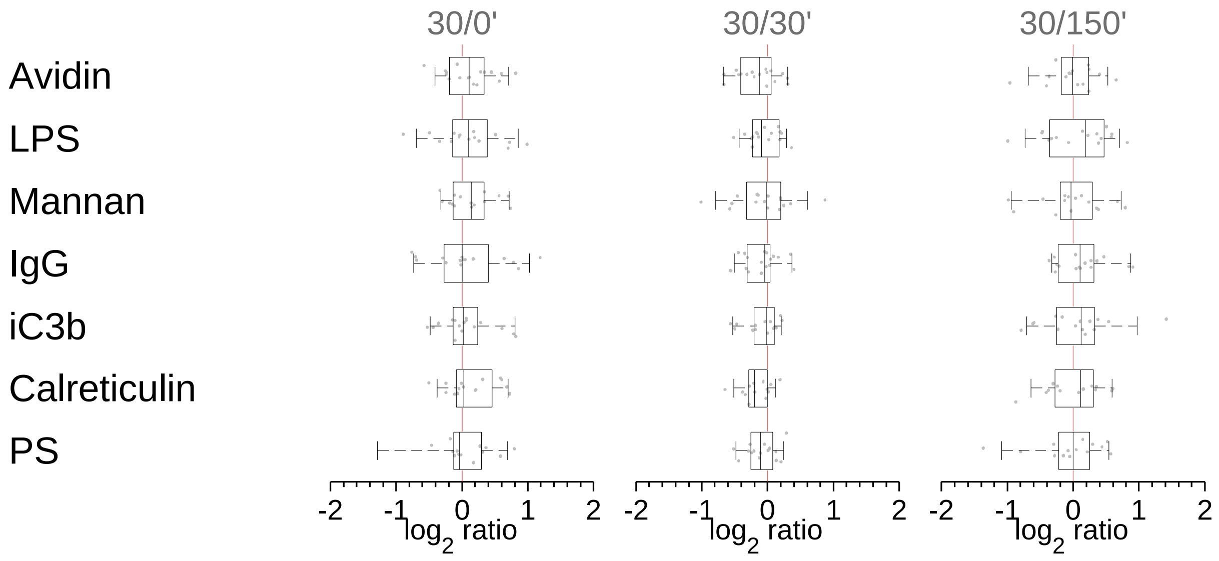

GO:0005496 steroid binding (n = 5)

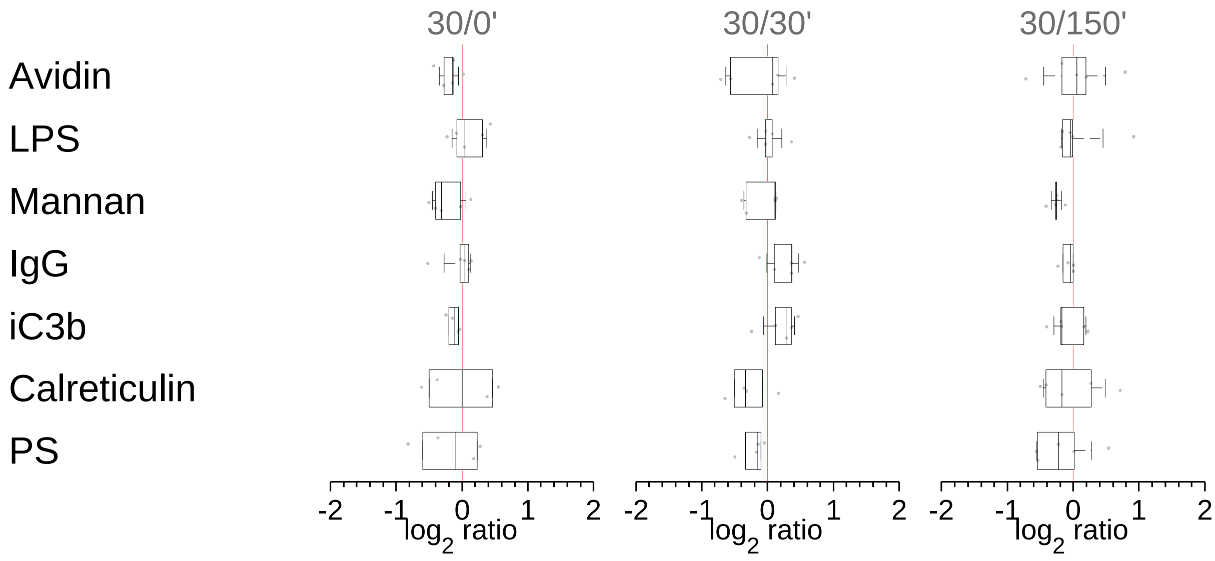

GO:0005504 fatty acid binding (n = 3)

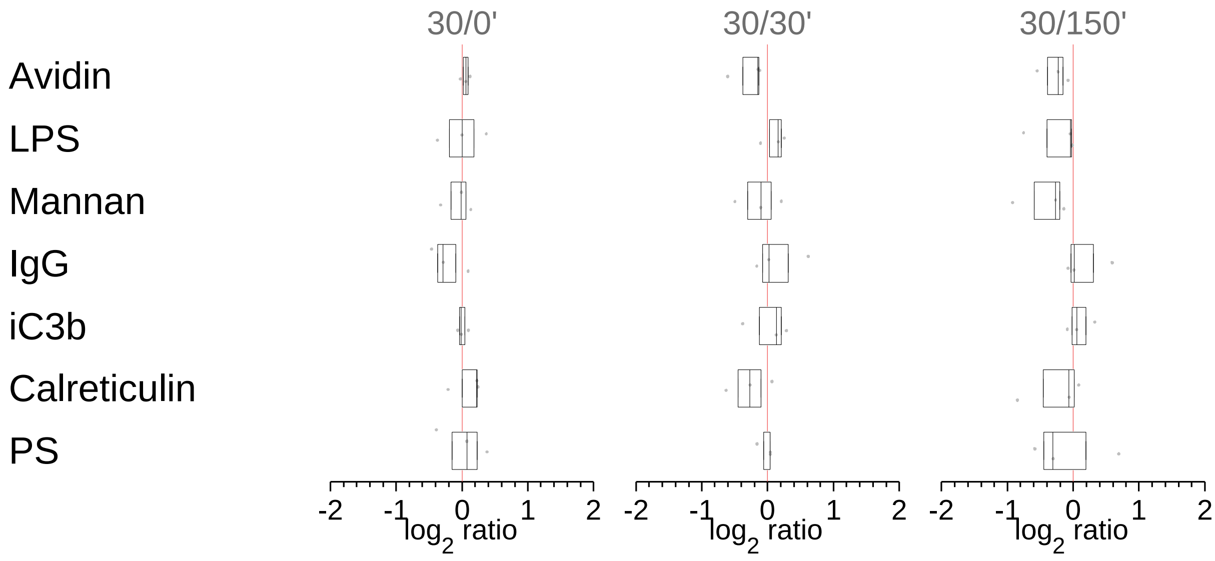

GO:0005506 iron ion binding (n = 10)

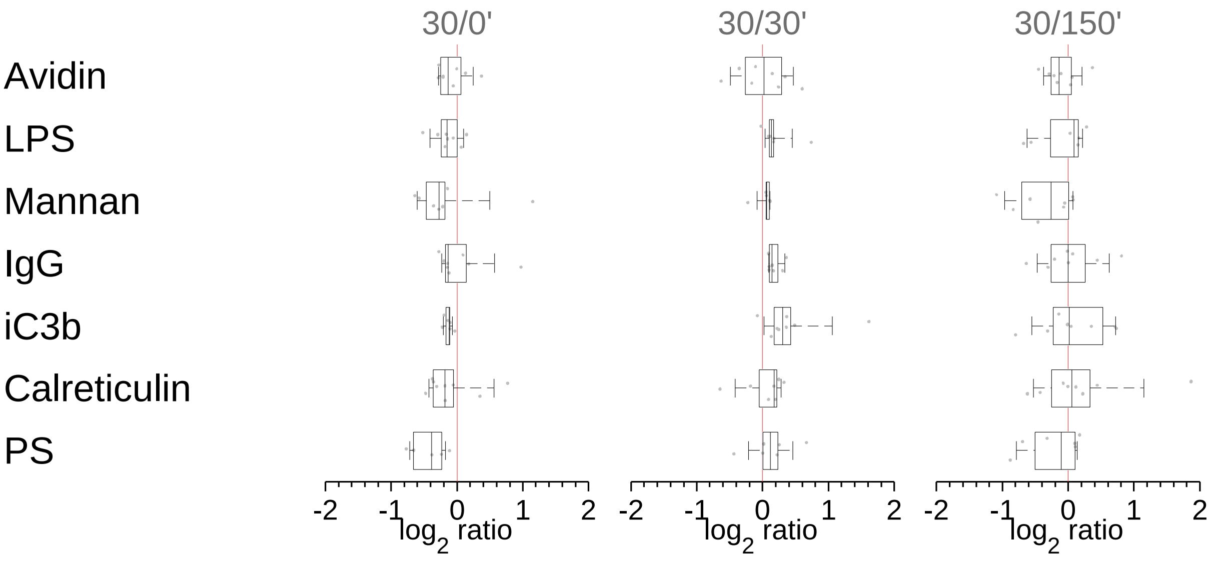

GO:0005507 copper ion binding (n = 5)

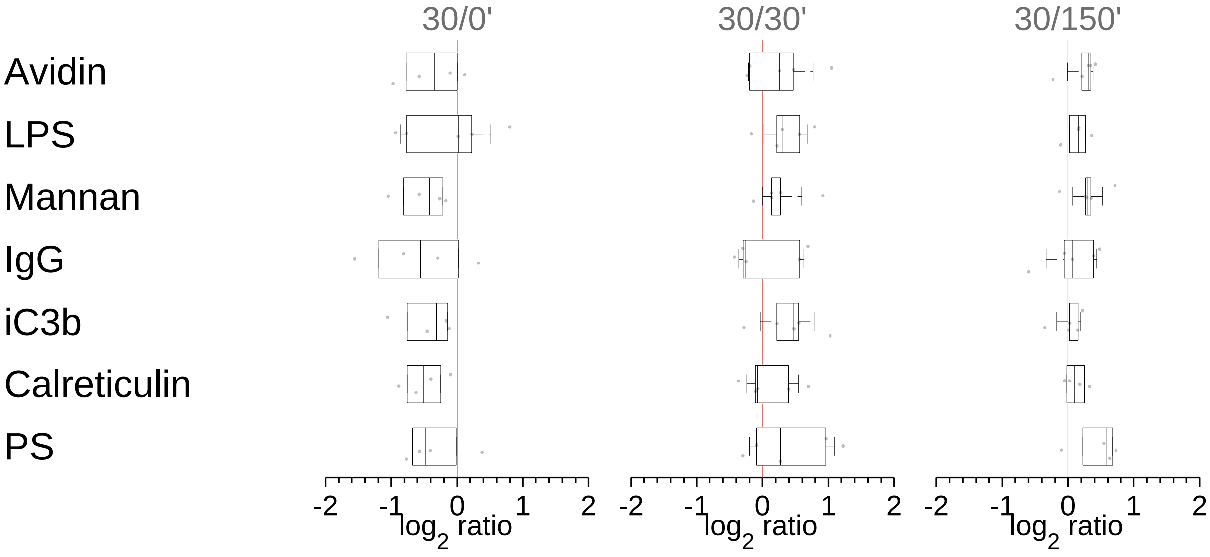

GO:0005509 calcium ion binding (n = 72)

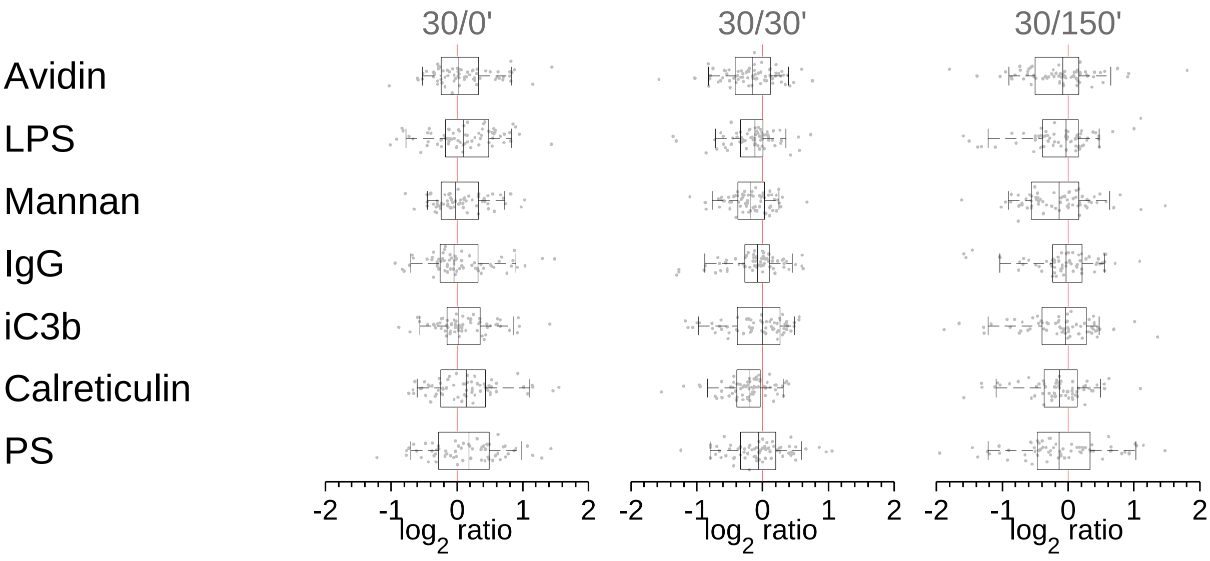

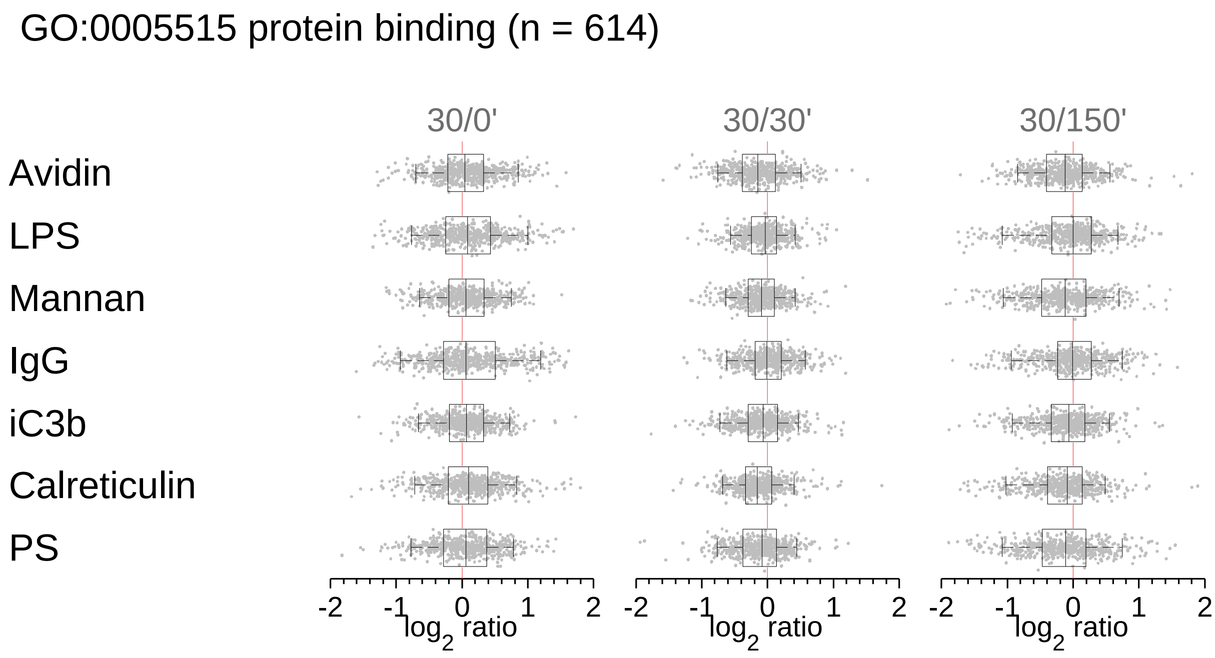
GO:0005515 protein binding (n = 614)
30/0'
30/30'
30/150'
Avidin
LPS
Mannan
IgG
iC3b
Calreticulin
PS
-2
-1
0
1
2
log2 ratio

GO:0005516 calmodulin binding (n = 20)

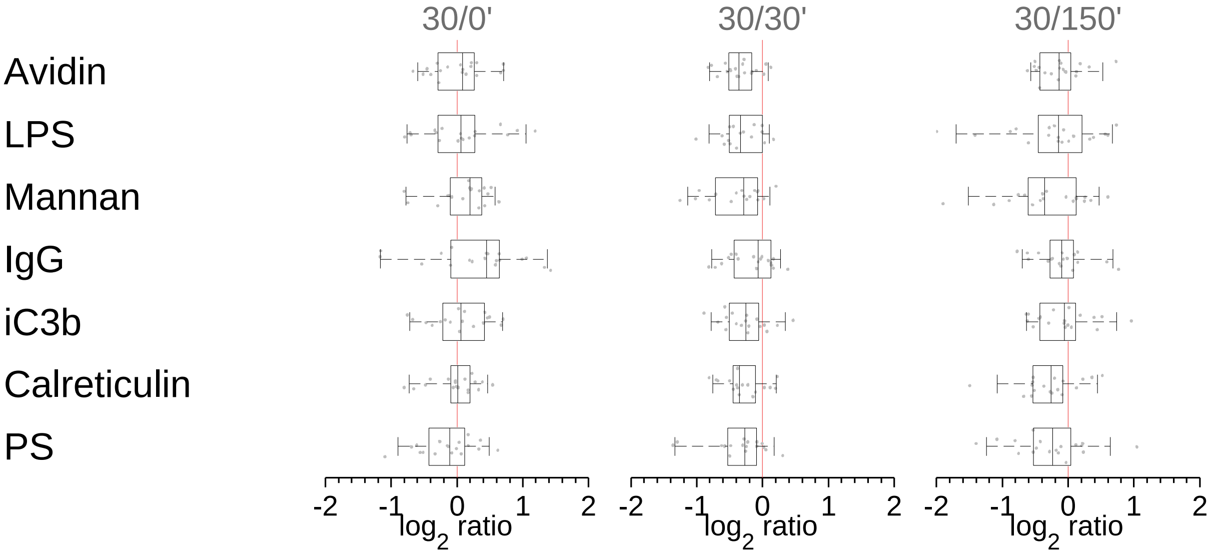

GO:0005518 collagen binding (n = 7)

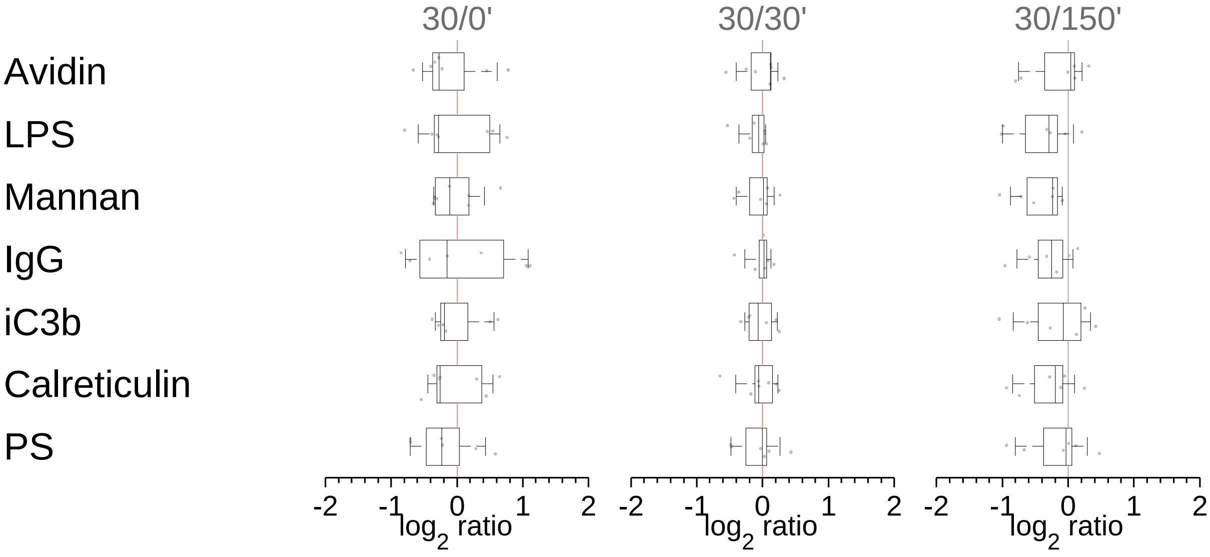

GO:0005522 profilin binding (n = 5)

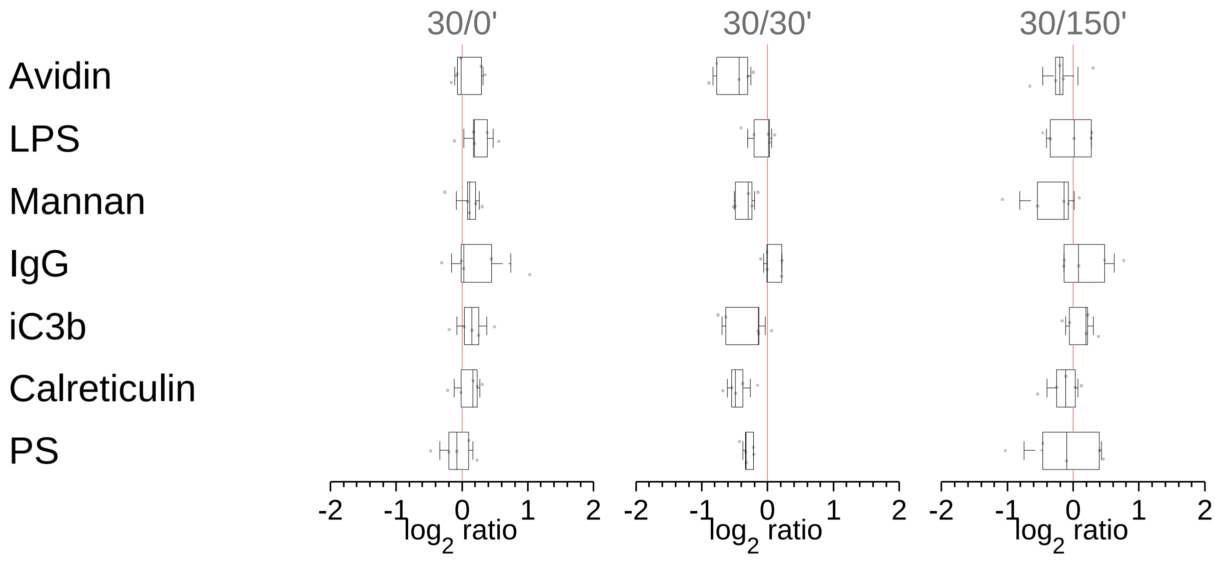

GO:0005524 ATP binding (n = 185)

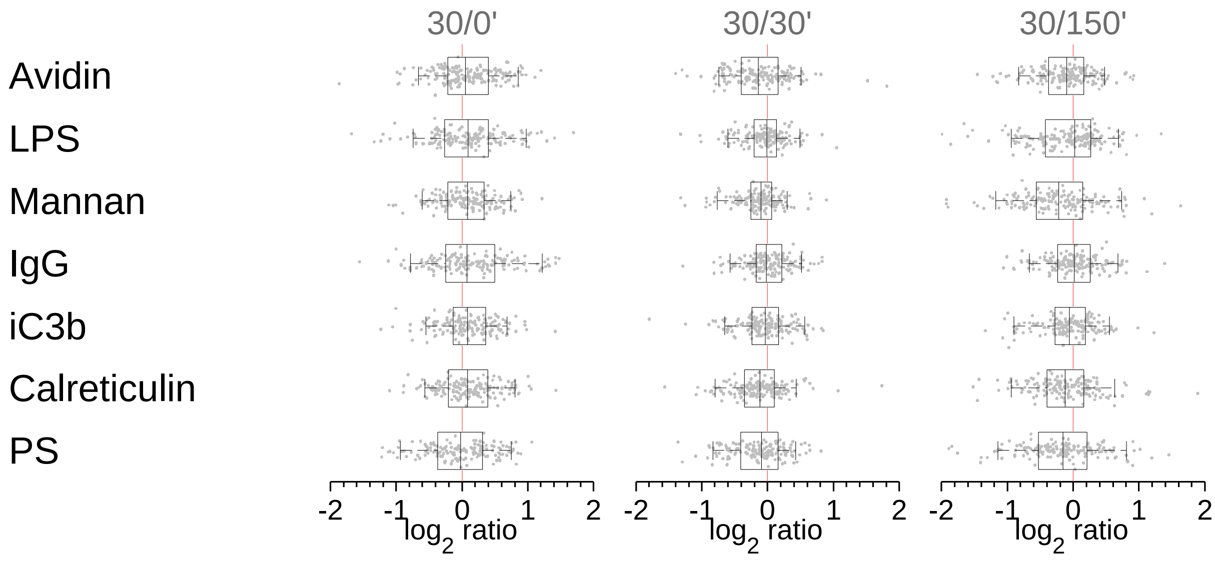

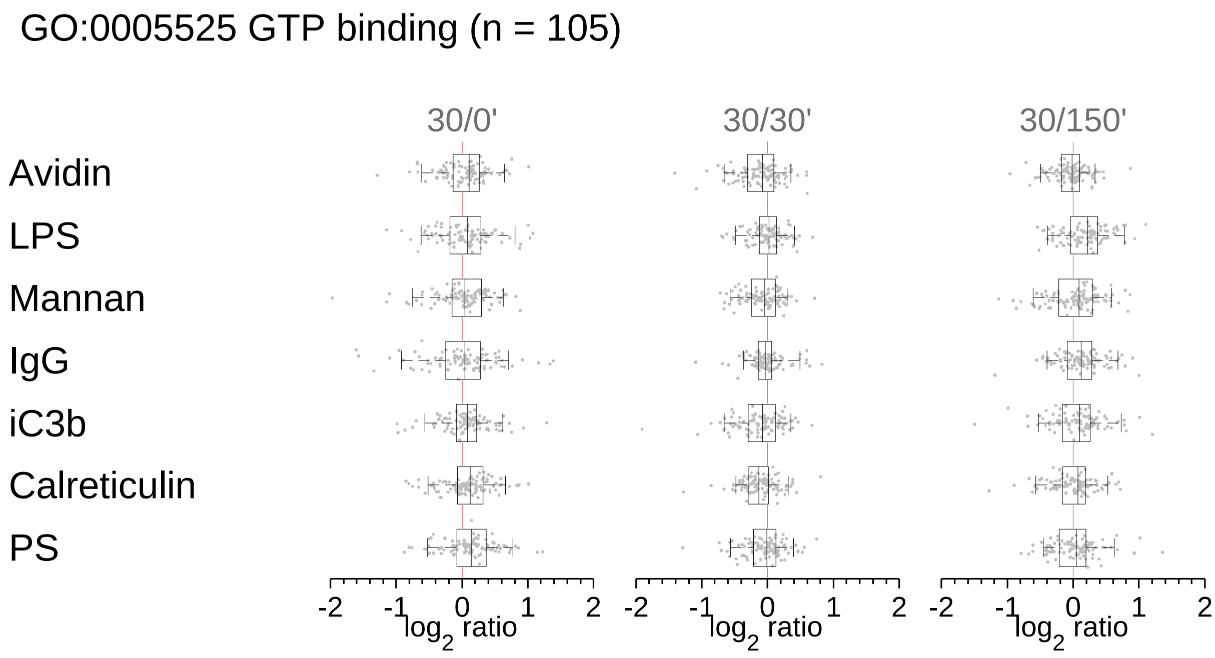
GO:0005525 GTP binding (n = 105)
30/0'
30/30'
30/150'
Avidin
LPS
Mannan
IgG
iC3b
Calreticulin
PS
-2
-1
0
1
2
log2 ratio

GO:0005528 FK506 binding (n = 3)

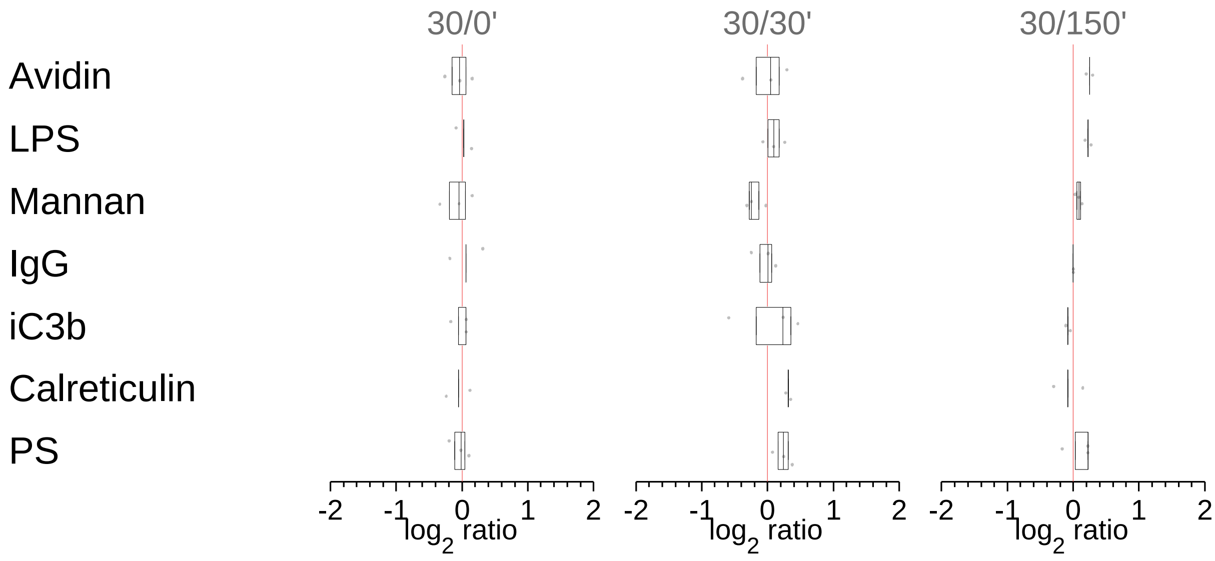

GO:0005536 glucose binding (n = 4)

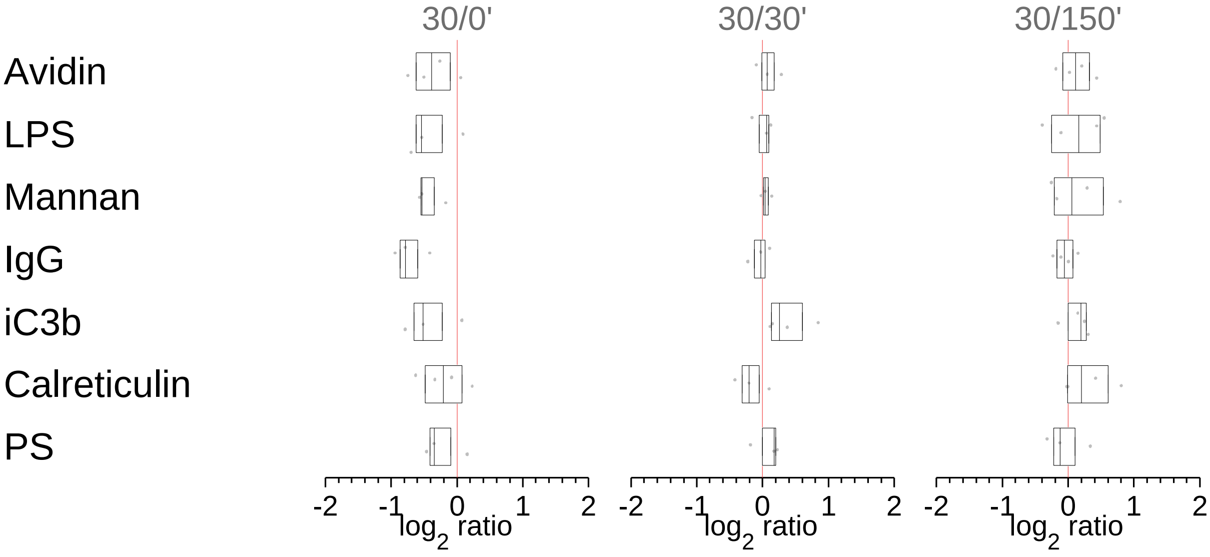

GO:0005537 mannose binding (n = 7)

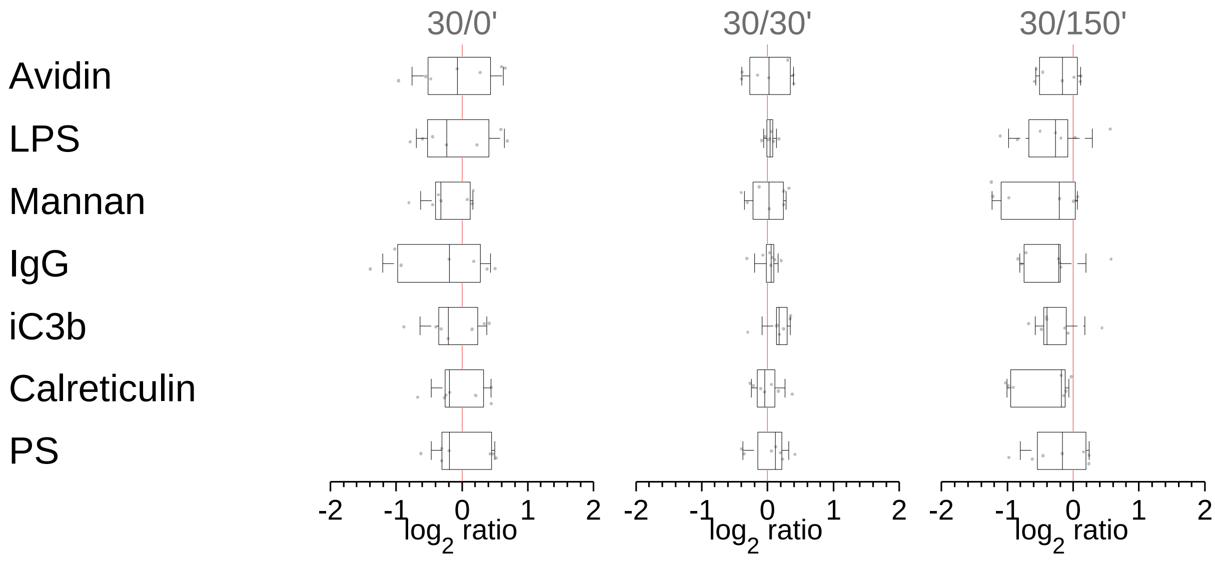

GO:0005543 phospholipid binding (n = 10)

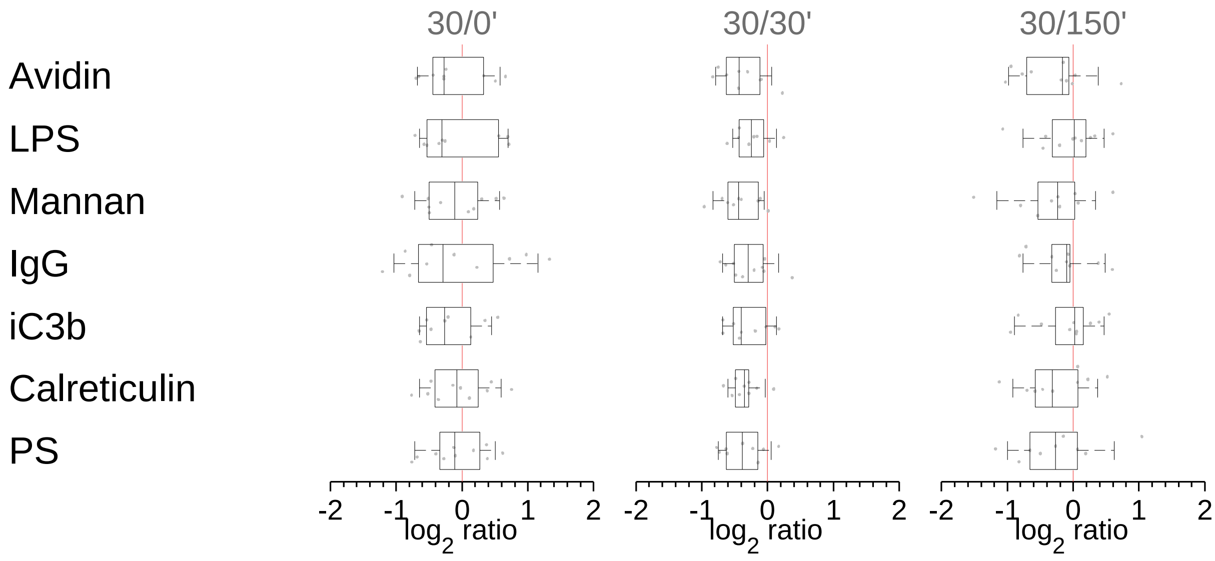

GO:0005544 calcium-dependent phospholipid binding (n = 9)

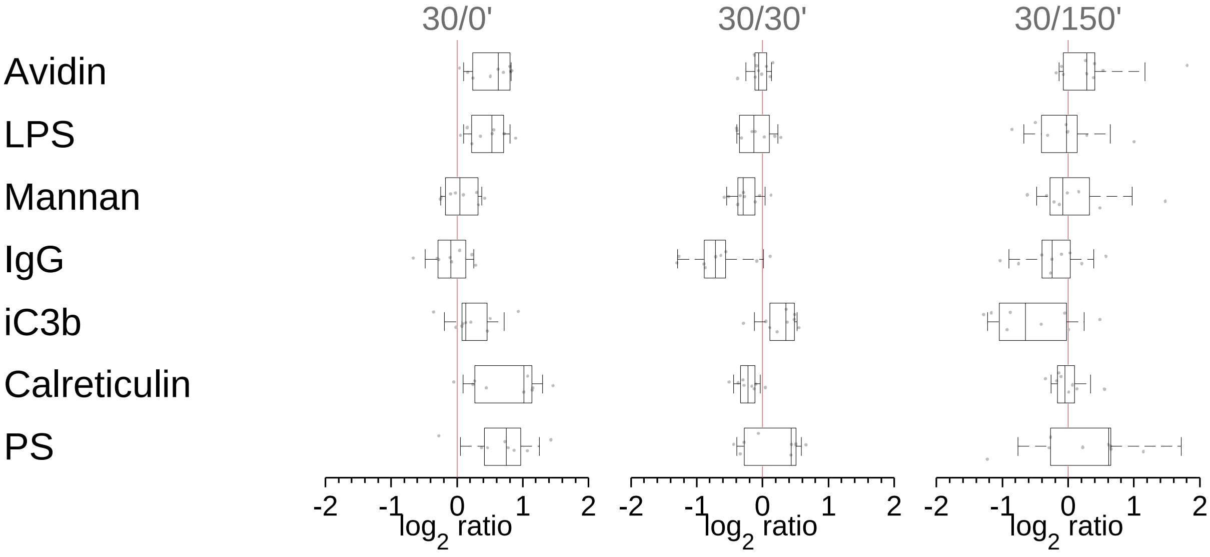

GO:0005545 1-phosphatidylinositol binding (n = 3)

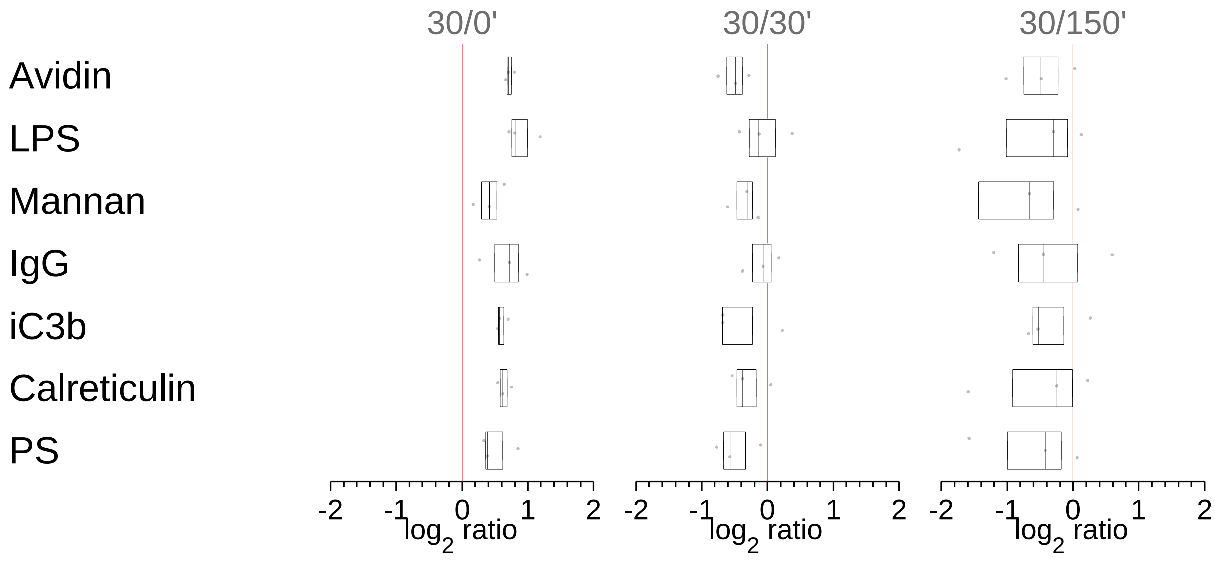

GO:0005546 phosphatidylinositol-4,5-bisphosphate binding (n = 4)

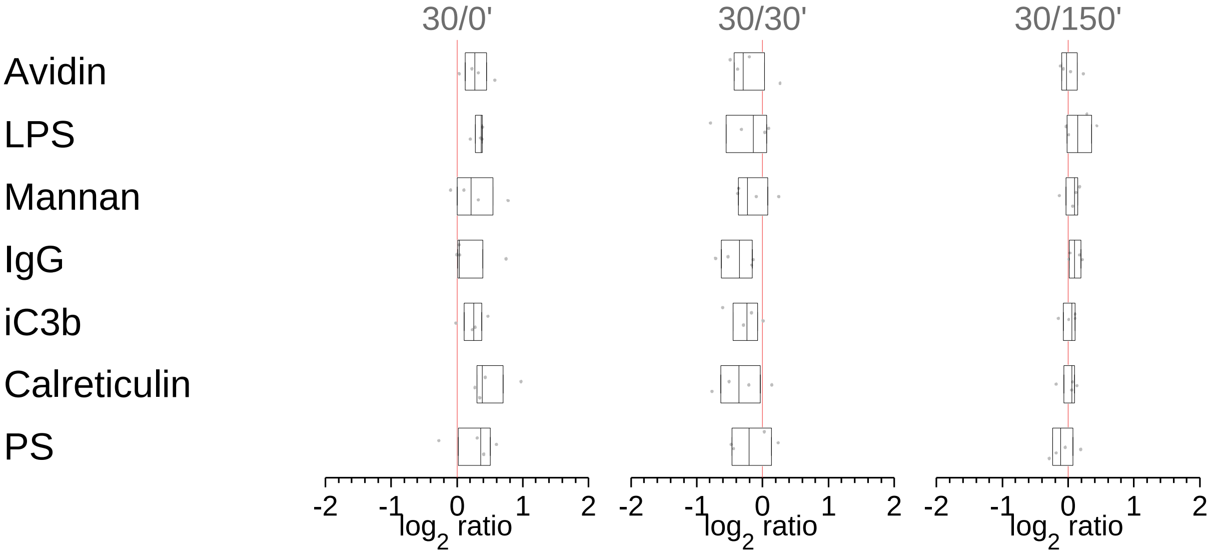

GO:0005547 phosphatidylinositol-3,4,5-trisphosphate binding (n = 5)

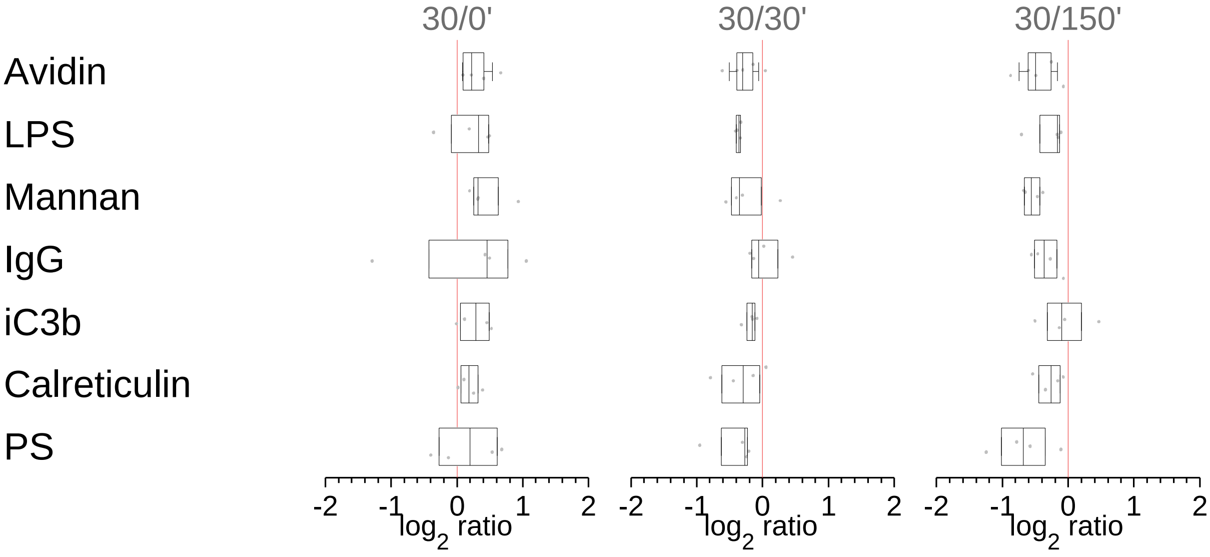

GO:0005575 cellular_component (n = 110)

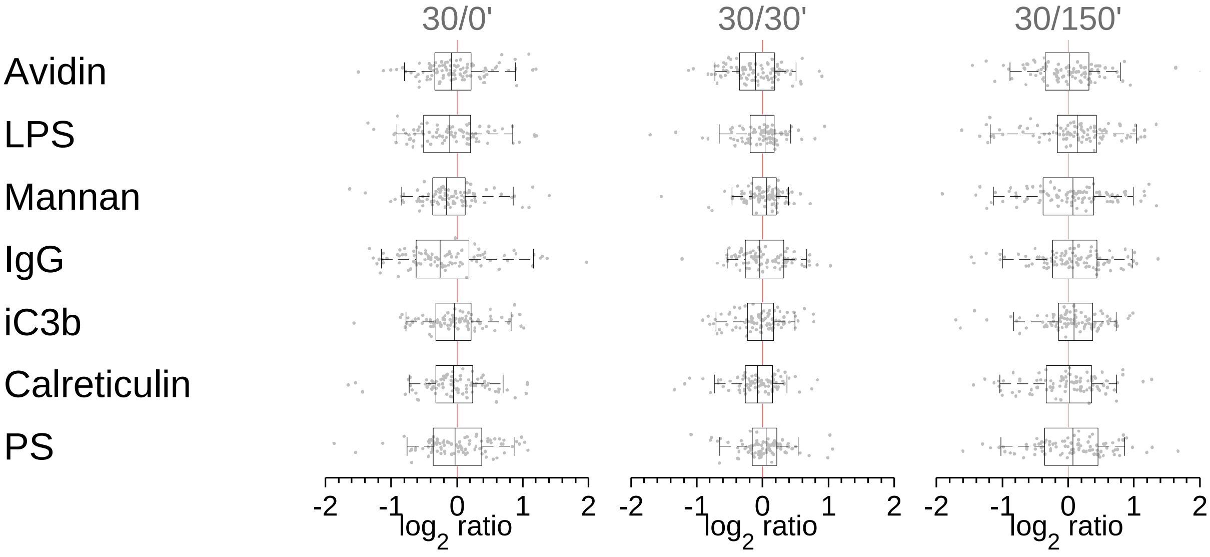

GO:0005576 extracellular region (n = 115)

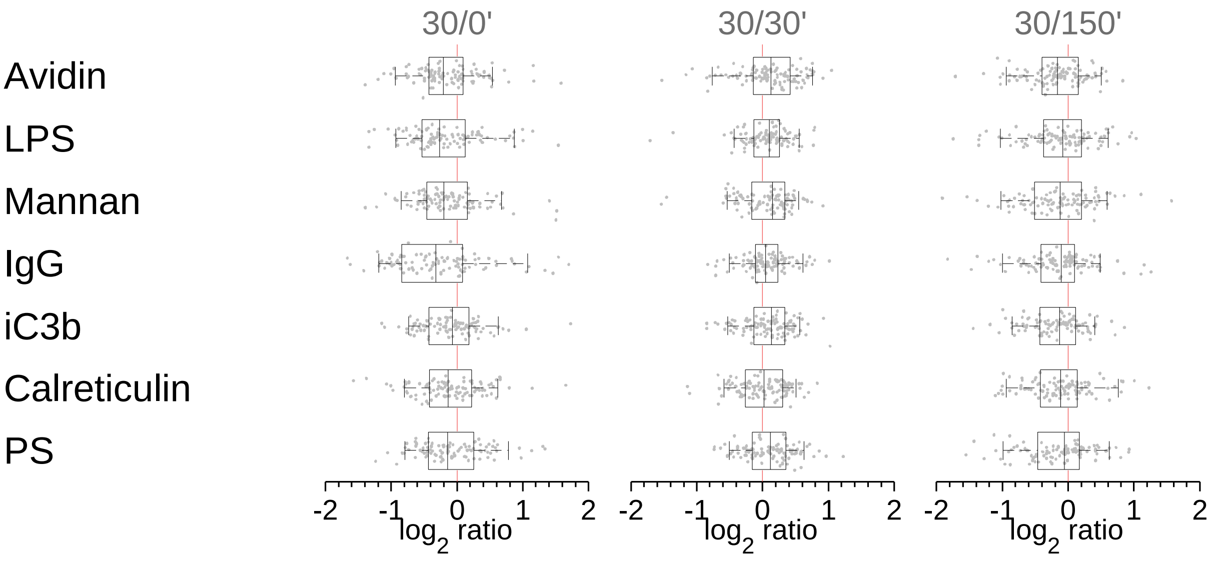

GO:0005578 proteinaceous extracellular matrix (n = 12)

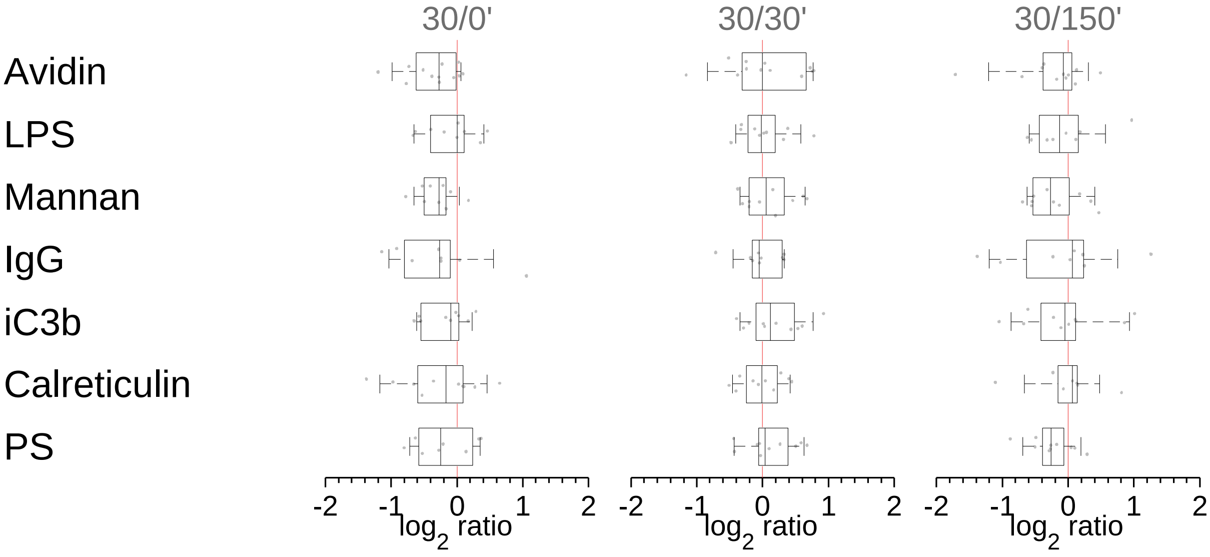

GO:0005581 collagen (n = 6)

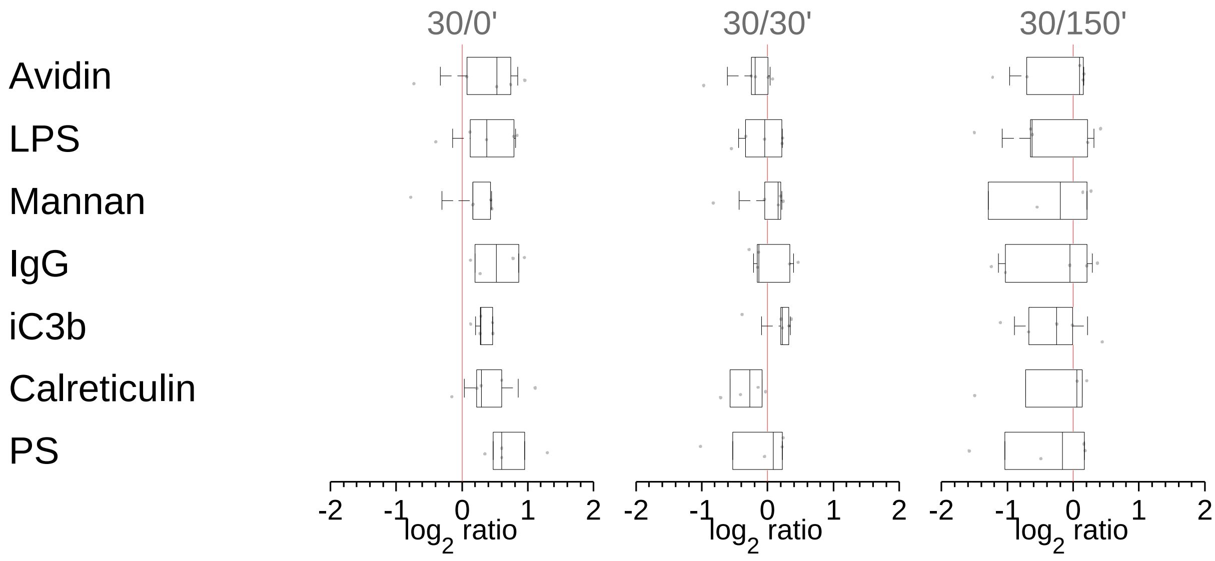

GO:0005604 basement membrane (n = 10)

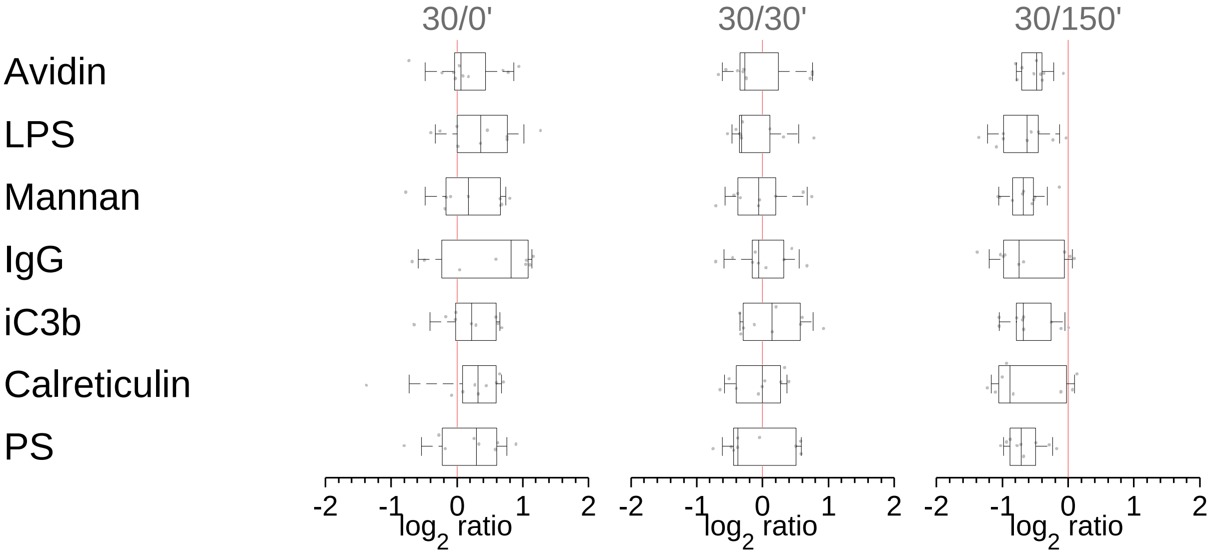

GO:0005605 basal lamina (n = 4)

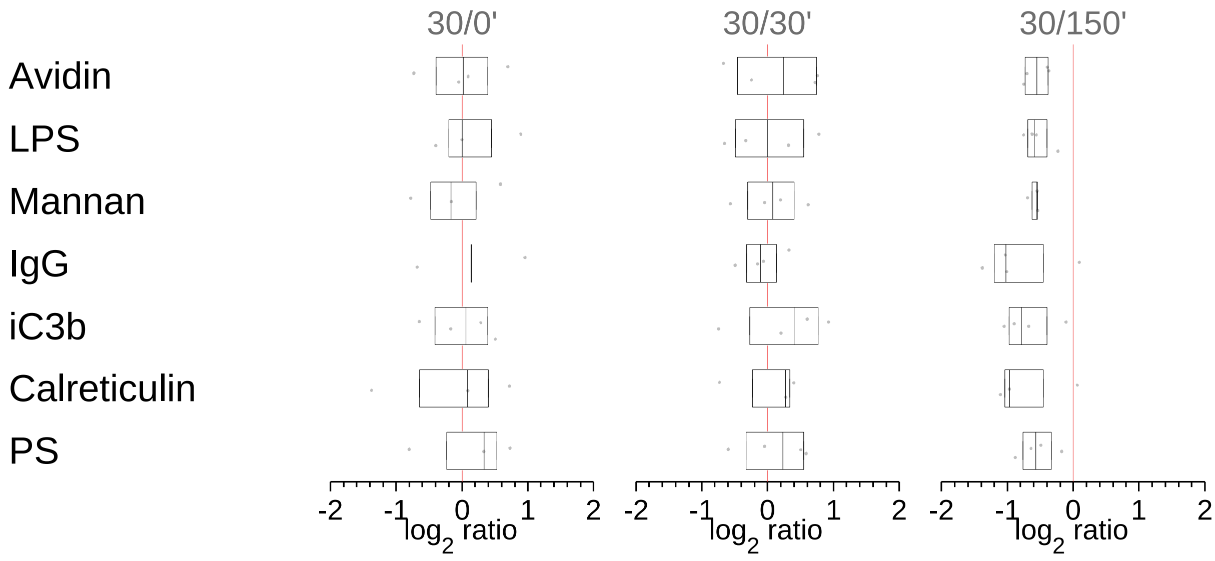

GO:0005615 extracellular space (n = 123)

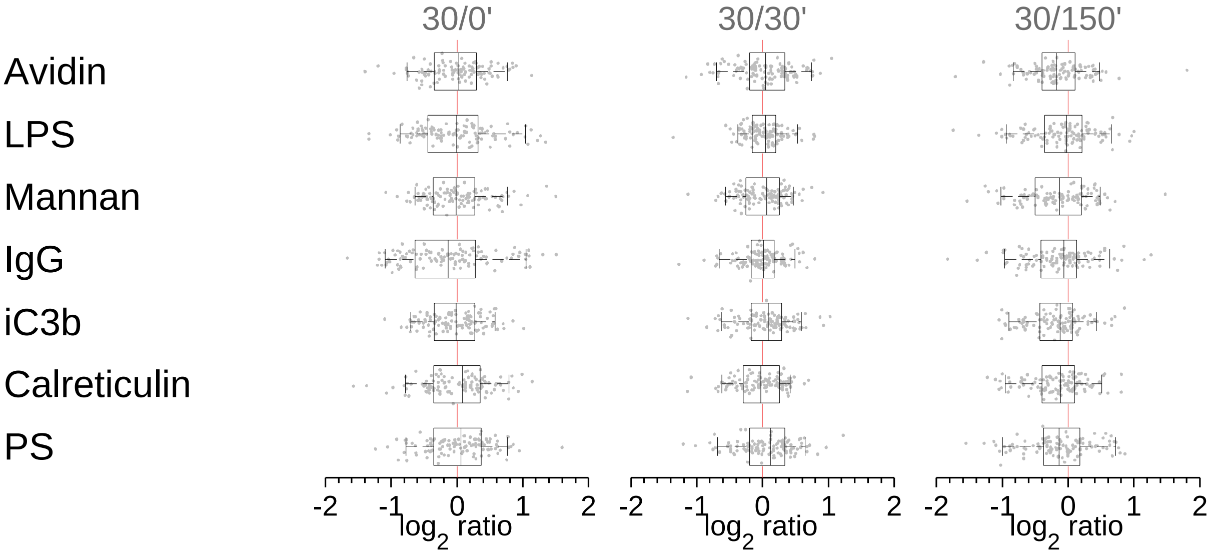

GO:0005622 intracellular (n = 246)

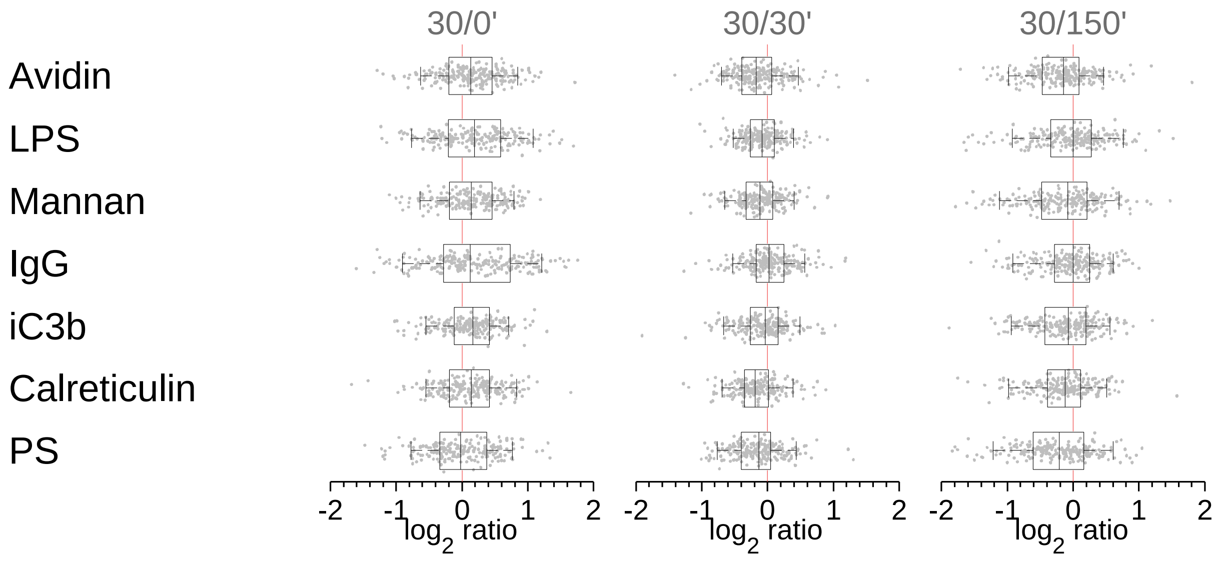

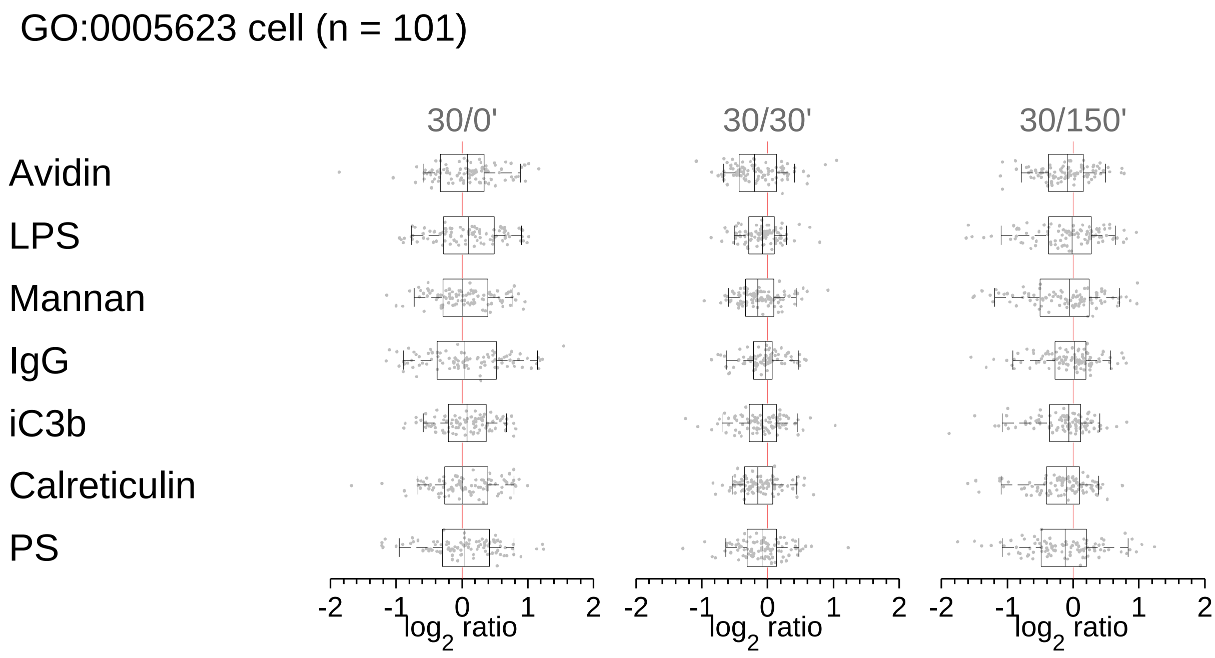
GO:0005623 cell (n = 101)
30/0'
30/30'
30/150'
Avidin
LPS
Mannan
IgG
iC3b
Calreticulin
PS
-2
-1
0
1
2
log2 ratio

GO:0005634 nucleus (n = 543)

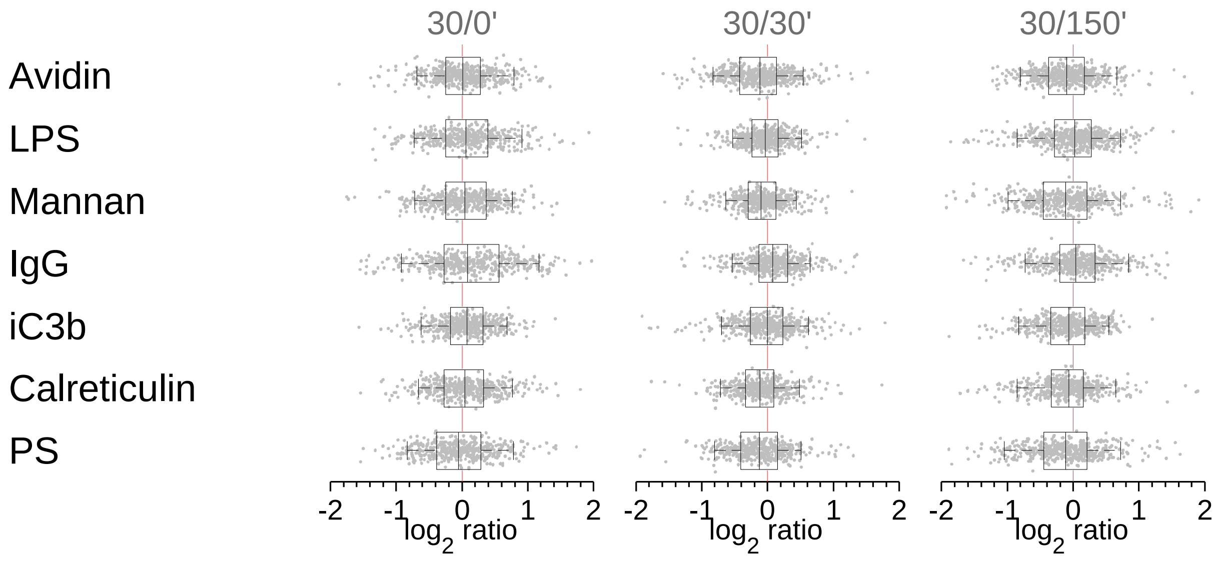

GO:0005635 nuclear envelope (n = 29)

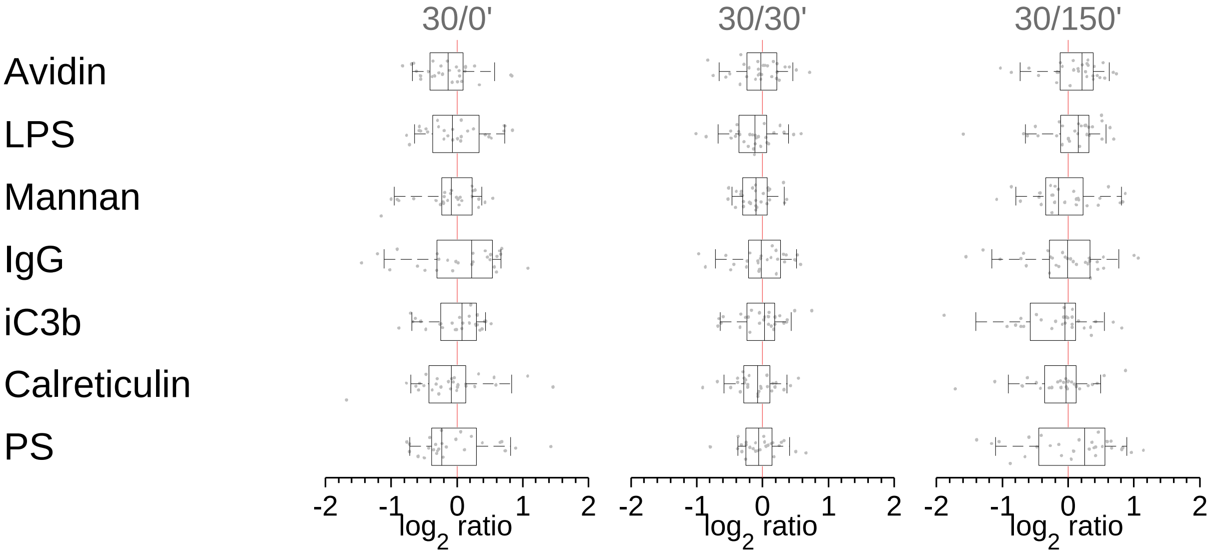

GO:0005637 nuclear inner membrane (n = 8)

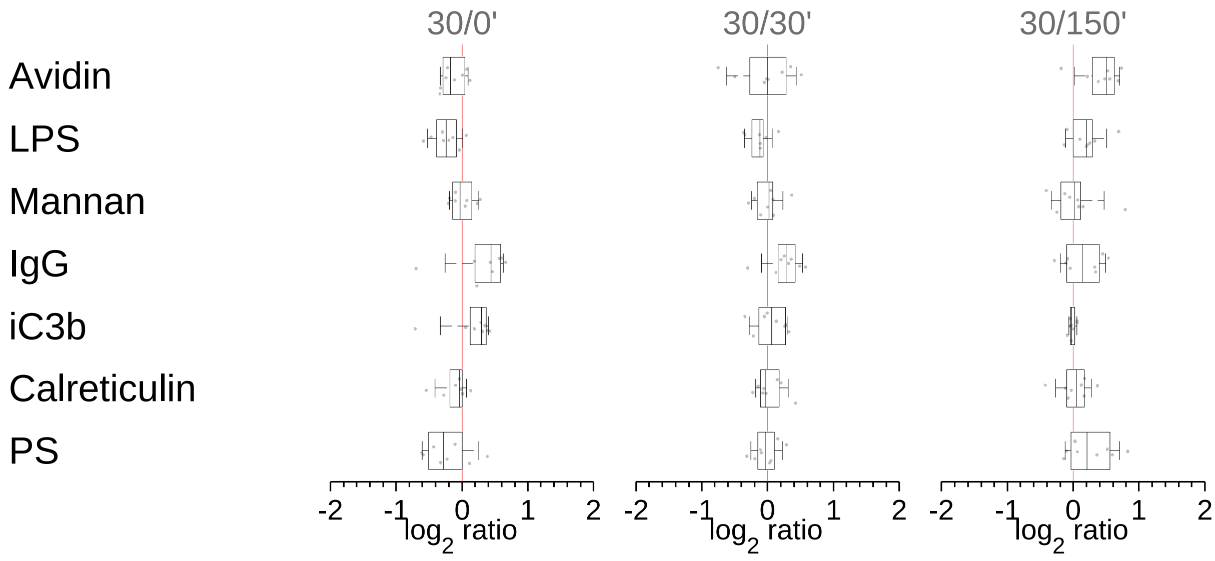

GO:0005638 lamin filament (n = 4)

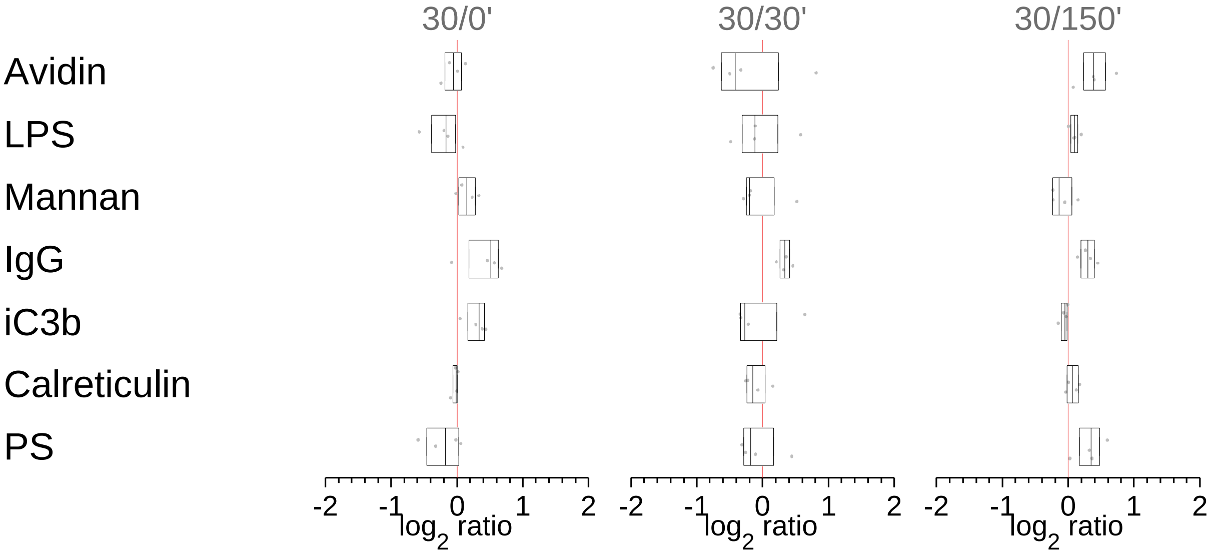

GO:0005639 integral to nuclear inner membrane (n = 3)

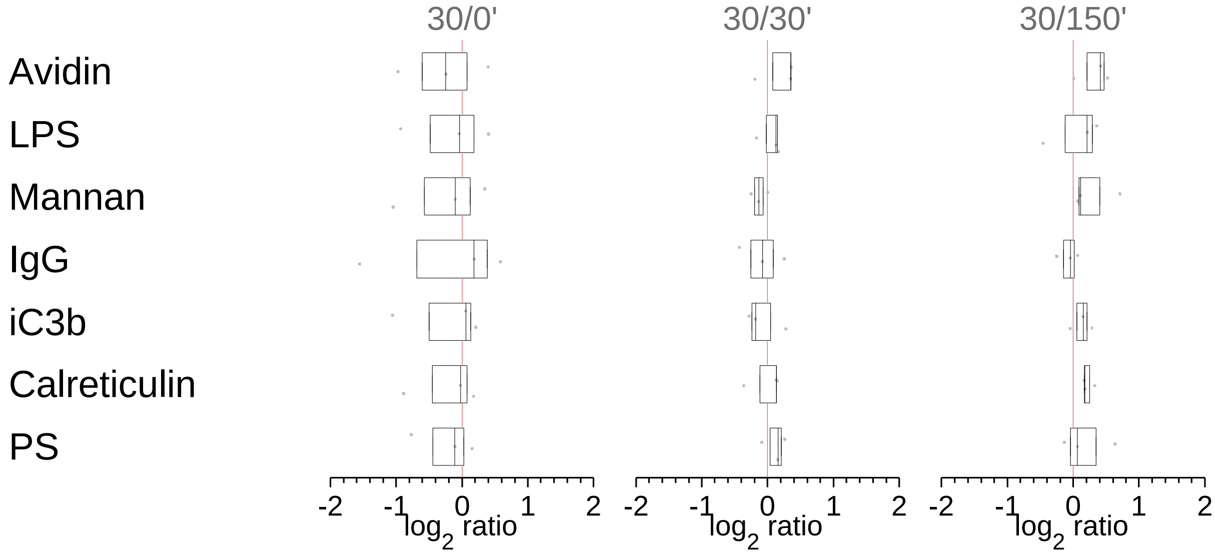

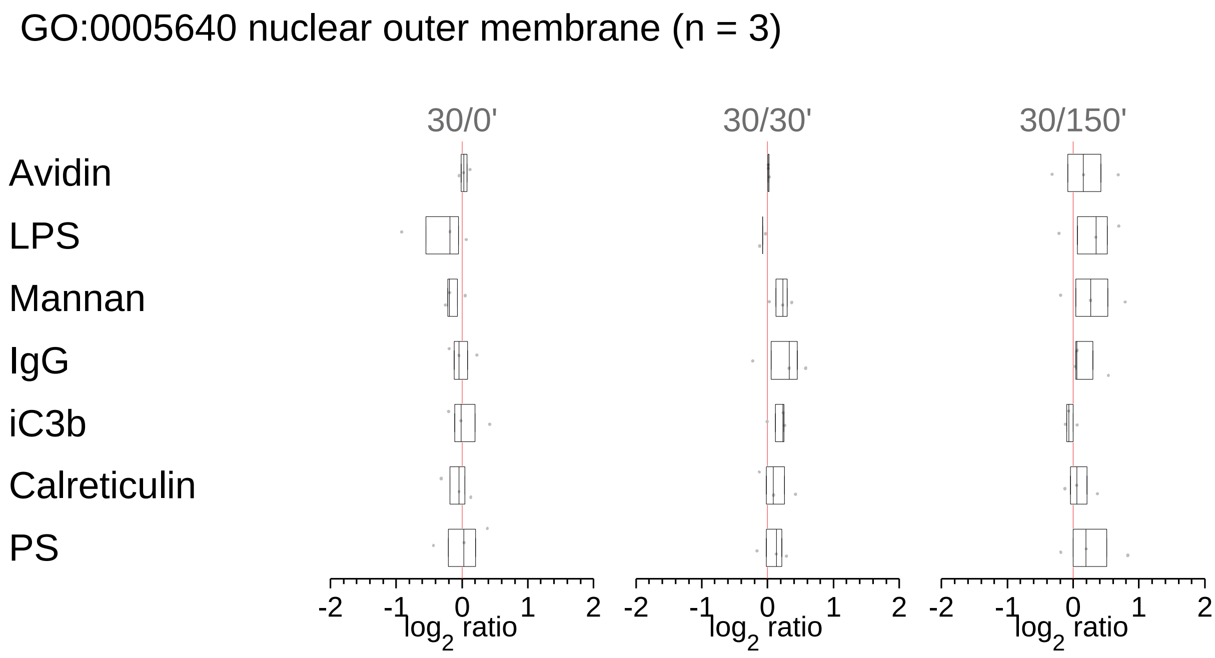
GO:0005640 nuclear outer membrane (n = 3)
30/0'
30/30'
30/150'
Avidin
LPS
Mannan
IgG
iC3b
Calreticulin
PS
-2
-1
0
1
2
log2 ratio

GO:0005641 nuclear envelope lumen (n = 5)

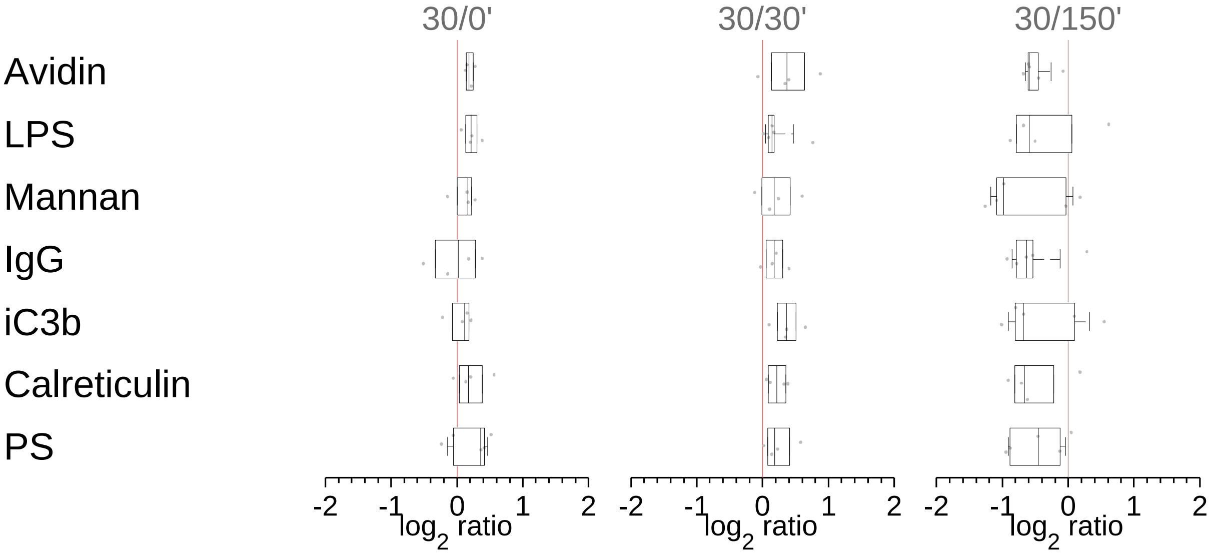

GO:0005643 nuclear pore (n = 8)

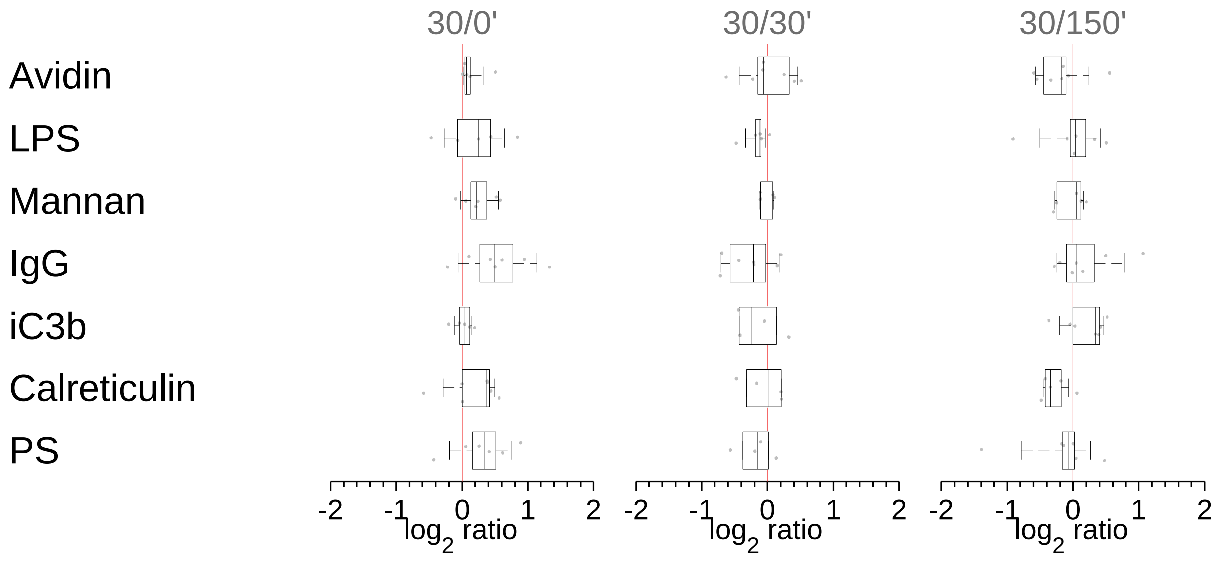

GO:0005652 nuclear lamina (n = 3)

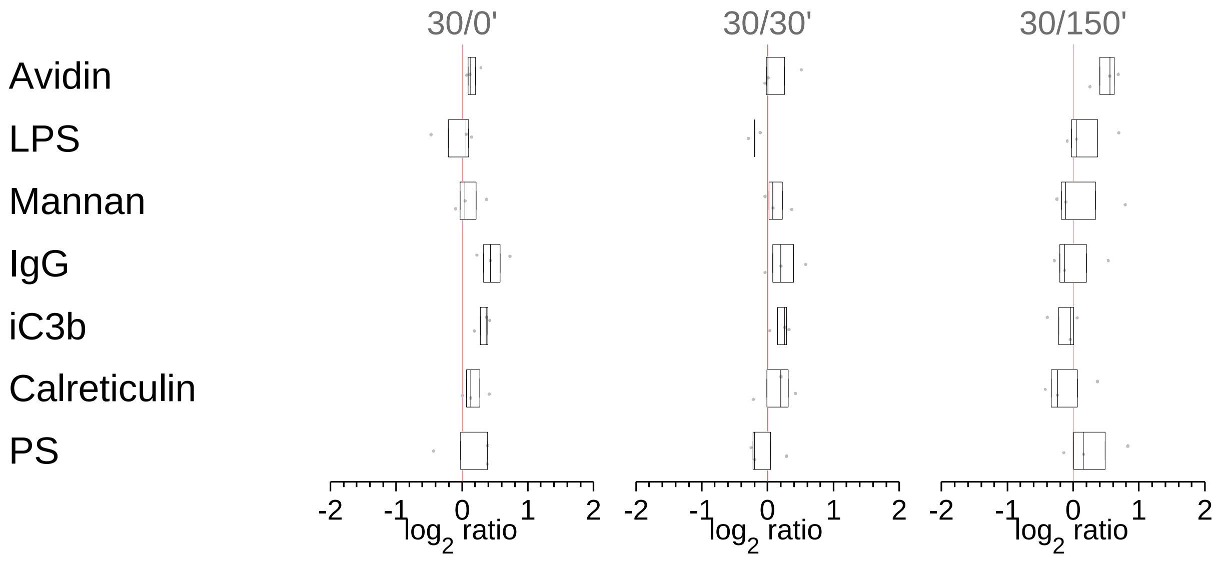

GO:0005654 nucleoplasm (n = 18)

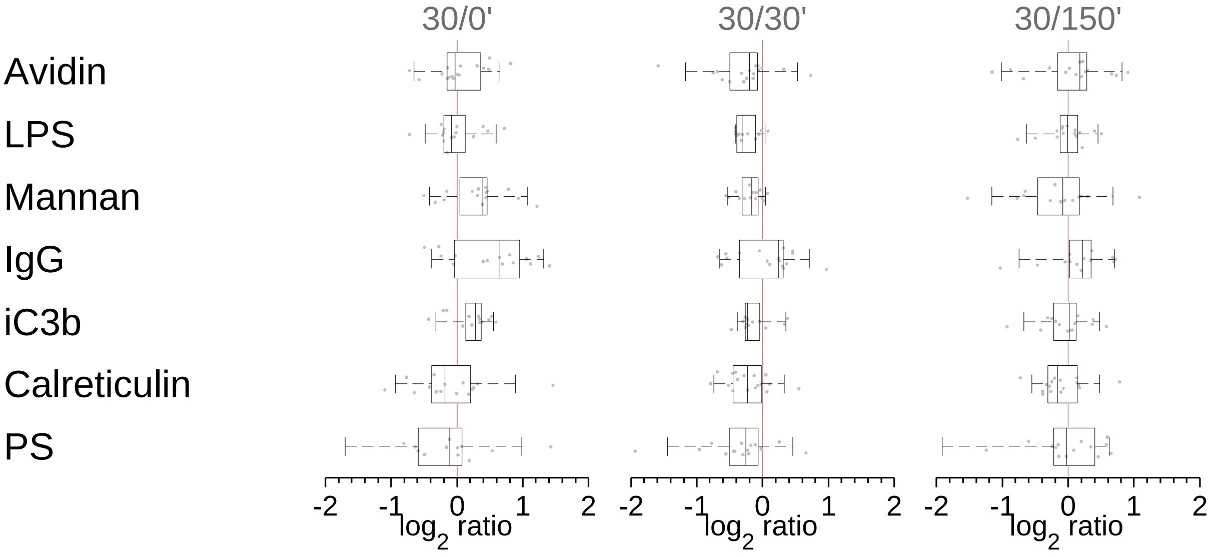

GO:0005667 transcription factor complex (n = 12)

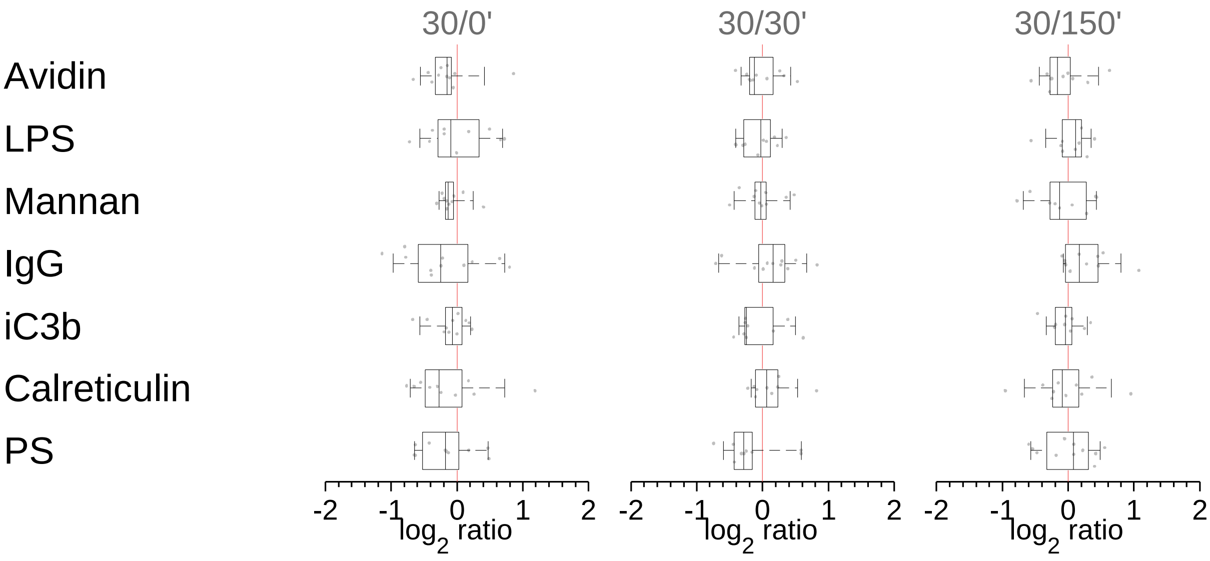

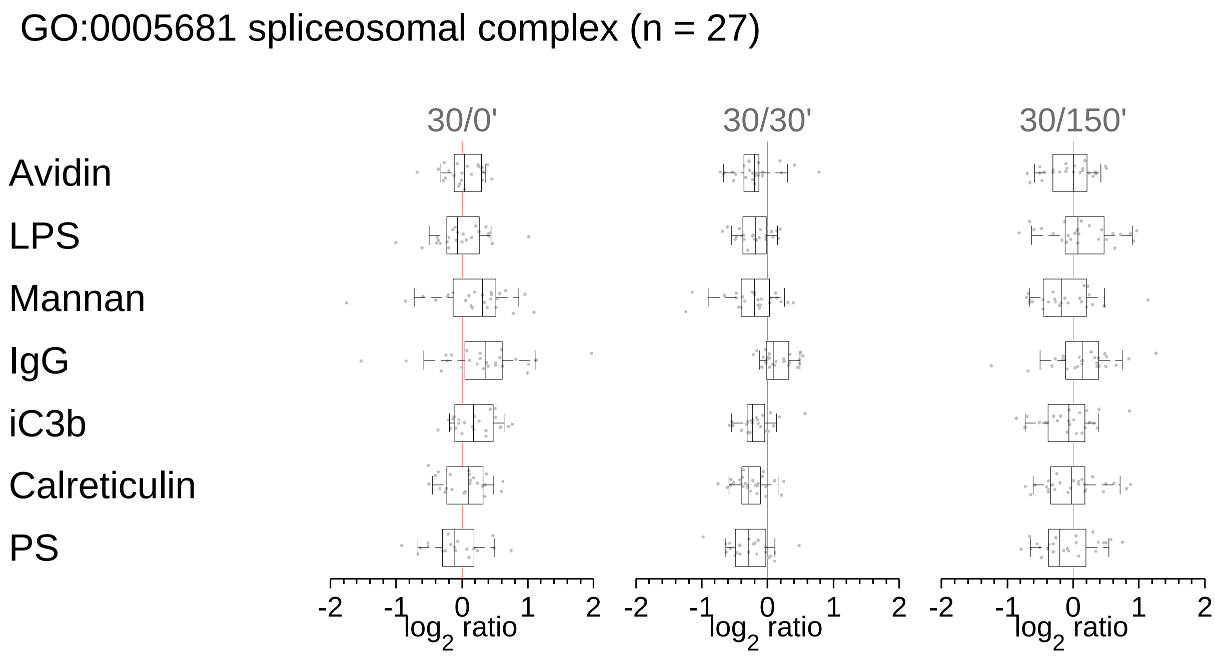
GO:0005681 spliceosomal complex (n = 27)
30/0'
30/30'
30/150'
Avidin
LPS
Mannan
IgG
iC3b
Calreticulin
PS
-2 -1 0 1 2
log2 ratio

GO:0005685 U1 snRNP (n = 3)

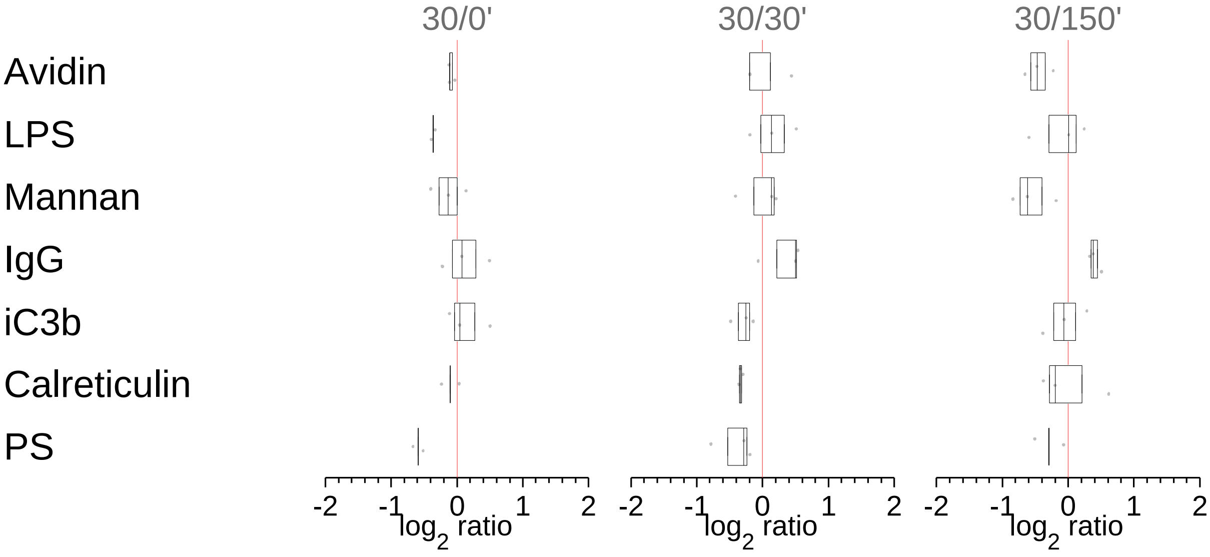

GO:0005687 U4 snRNP (n = 4)

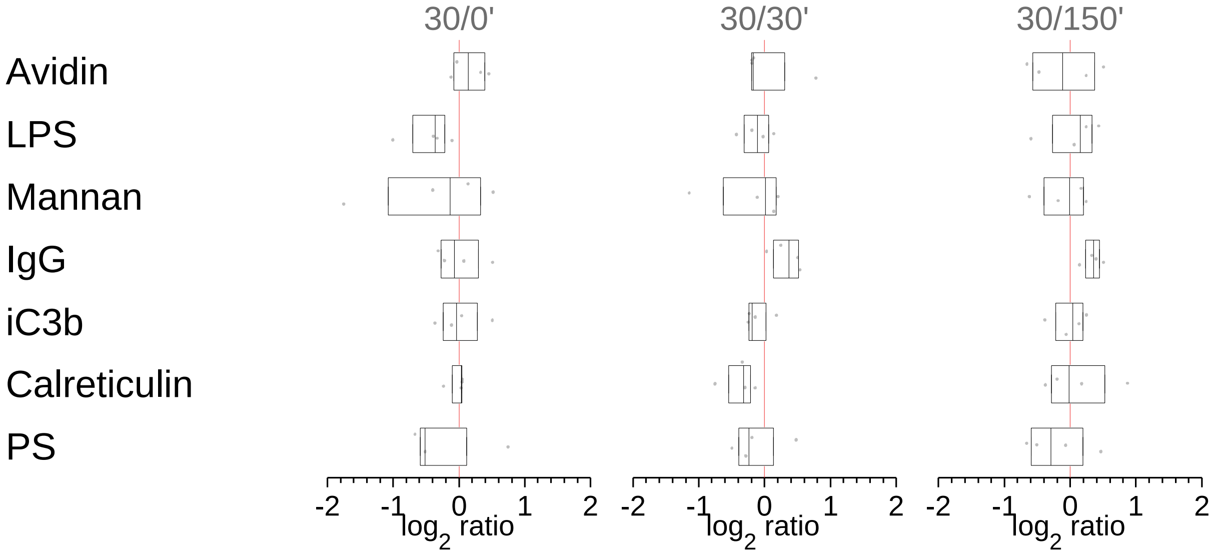

GO:0005689 U12-type spliceosomal complex (n = 4)

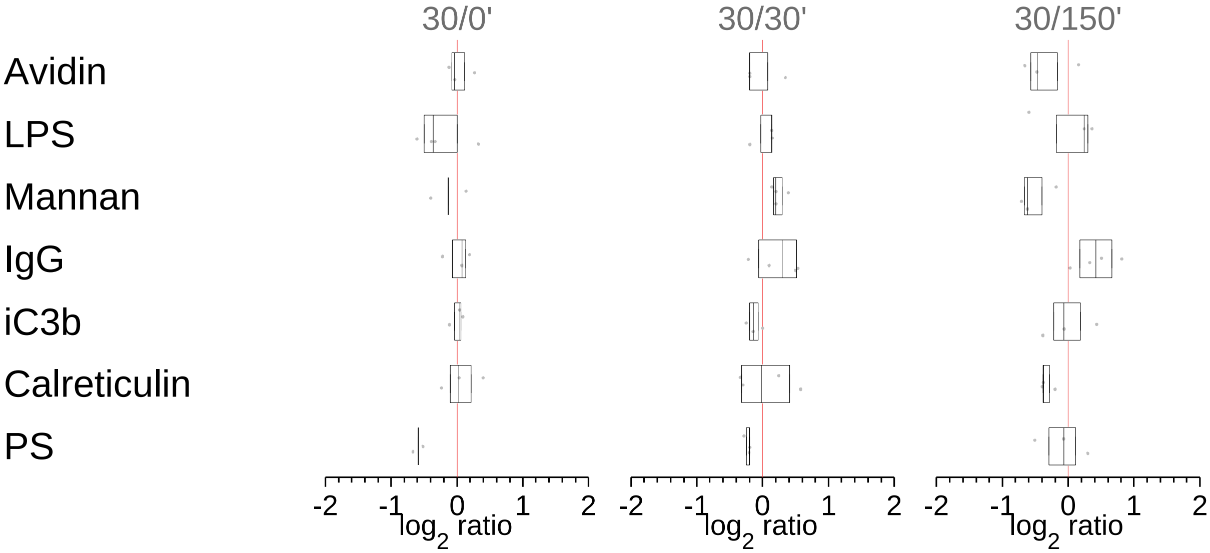

GO:0005694 chromosome (n = 32)

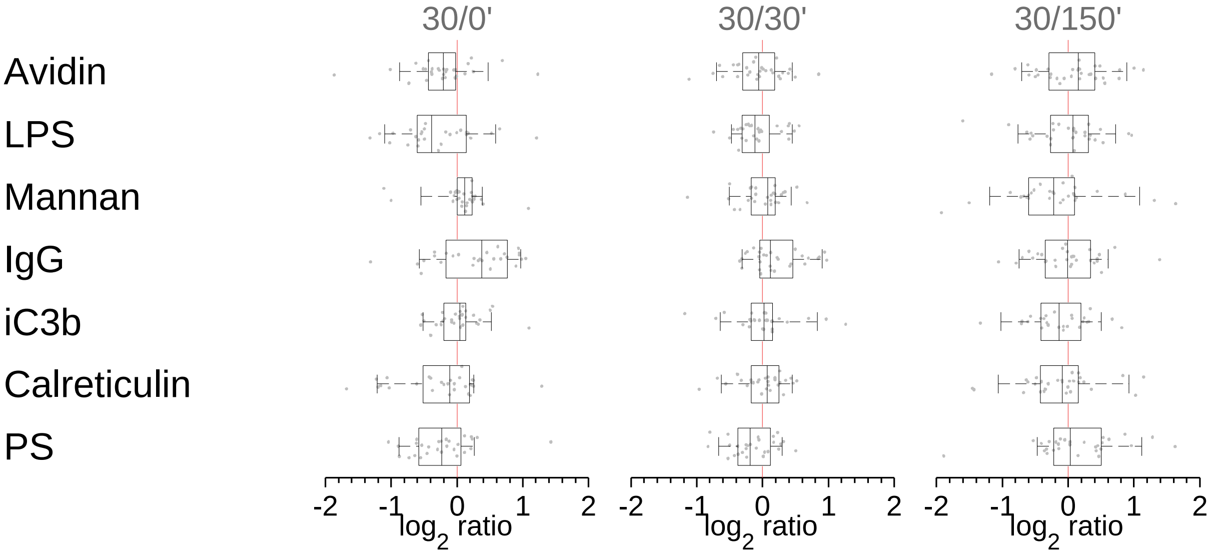

GO:0005719 nuclear euchromatin (n = 7)

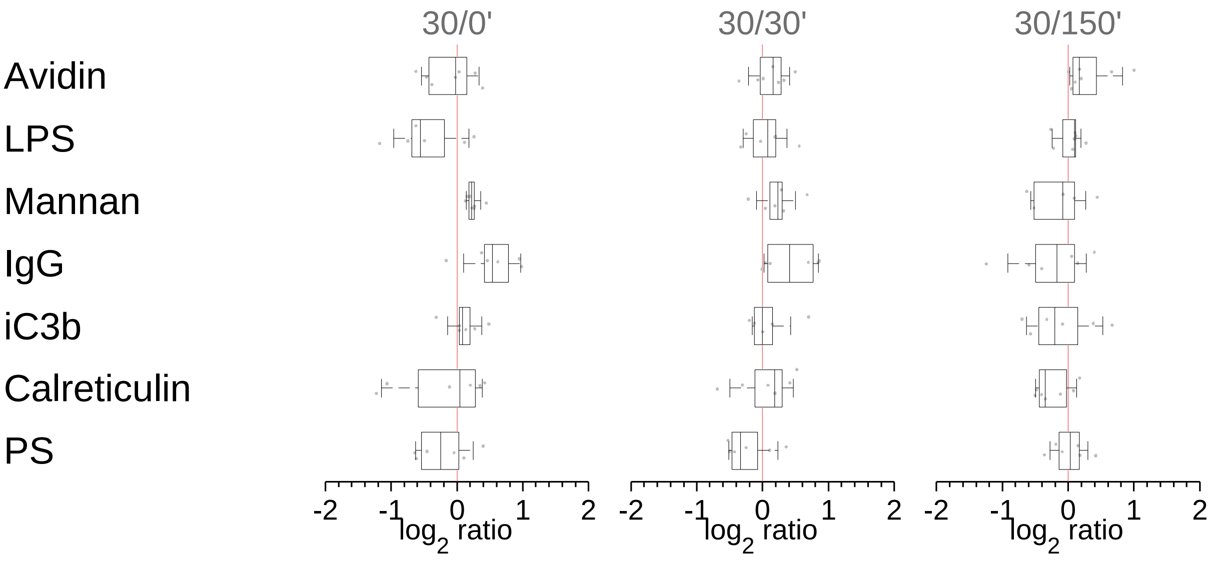

GO:0005720 nuclear heterochromatin (n = 4)

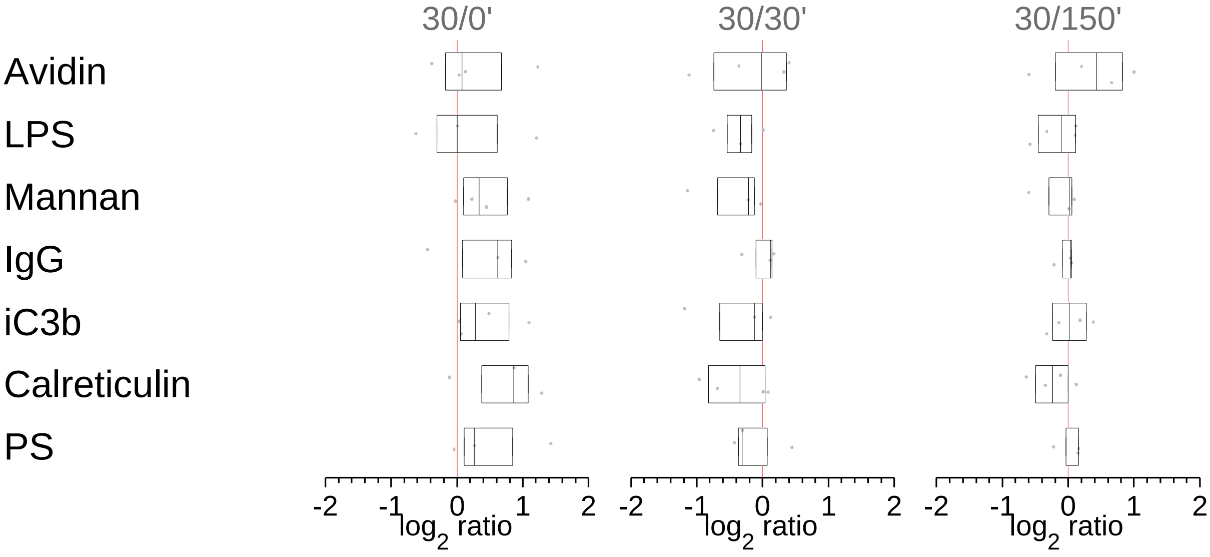

GO:0005730 nucleolus (n = 75)

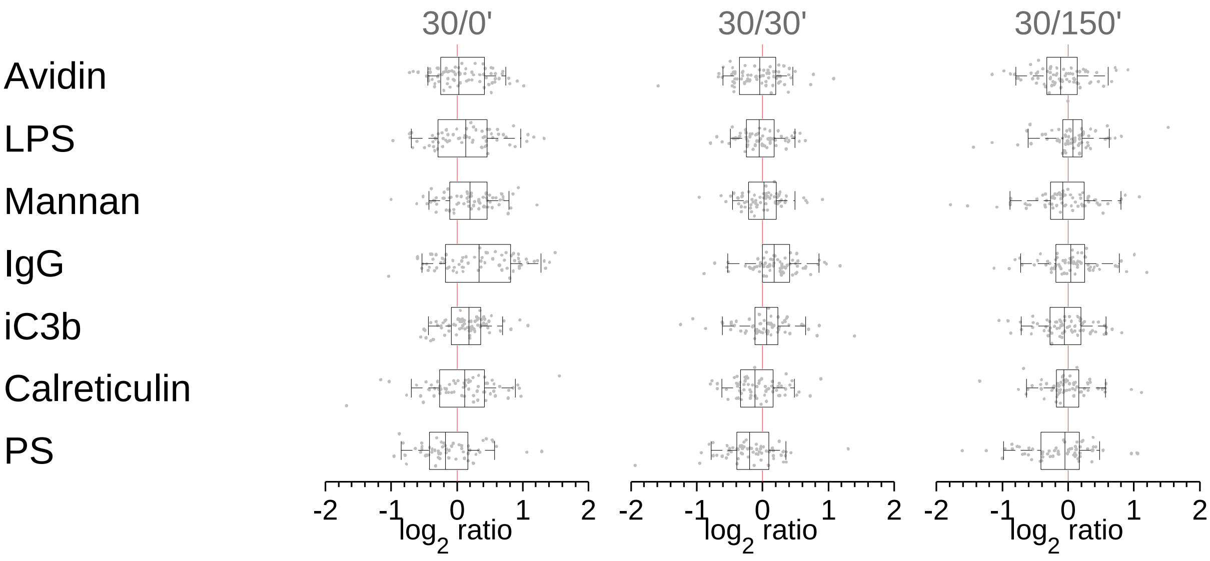

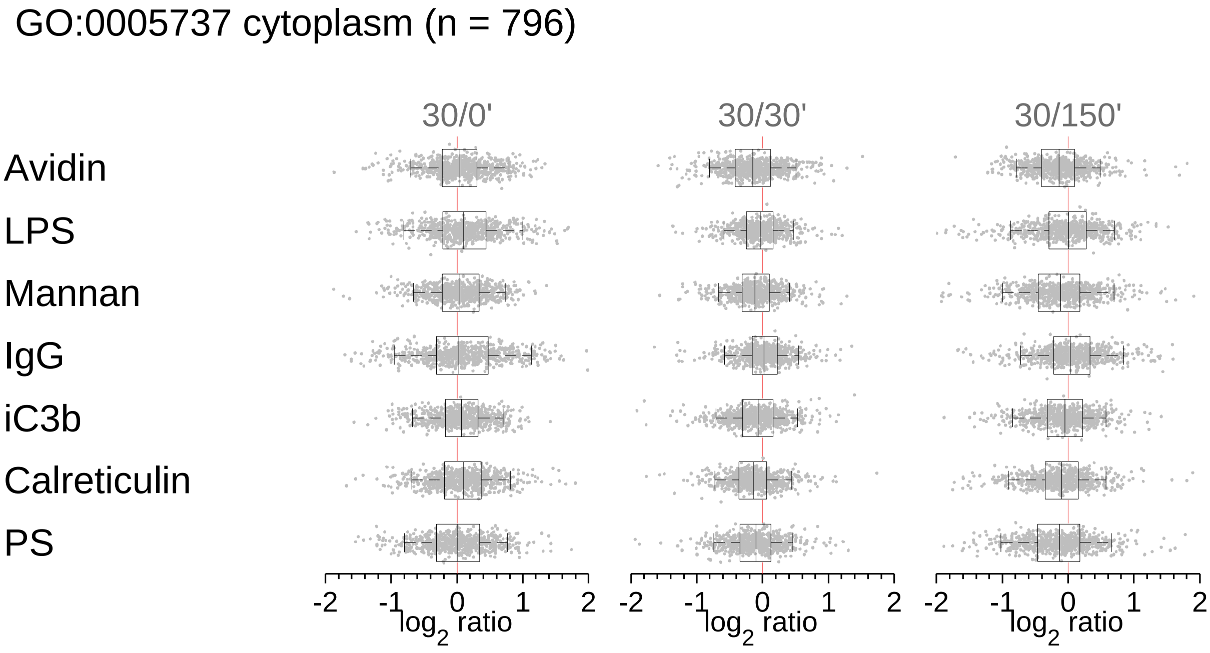
GO:0005737 cytoplasm (n = 796)
30/0'
30/30'
30/150'
Avidin
LPS
Mannan
IgG
iC3b
Calreticulin
PS
-2
-1
0
1
2
log2 ratio

GO:0005739 mitochondrion (n = 280)

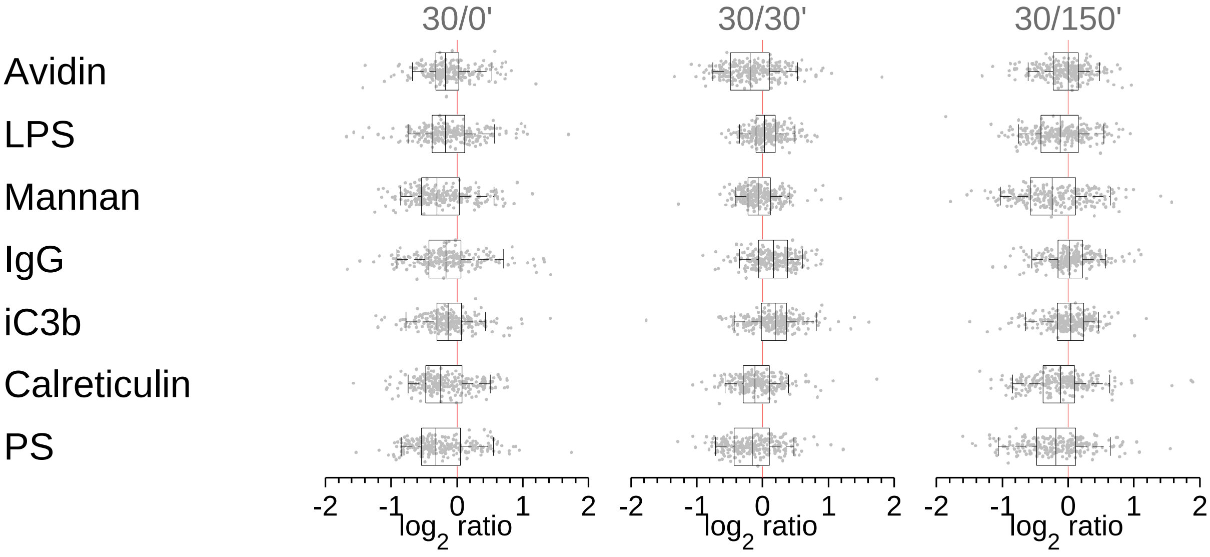

GO:0005740 mitochondrial envelope (n = 5)

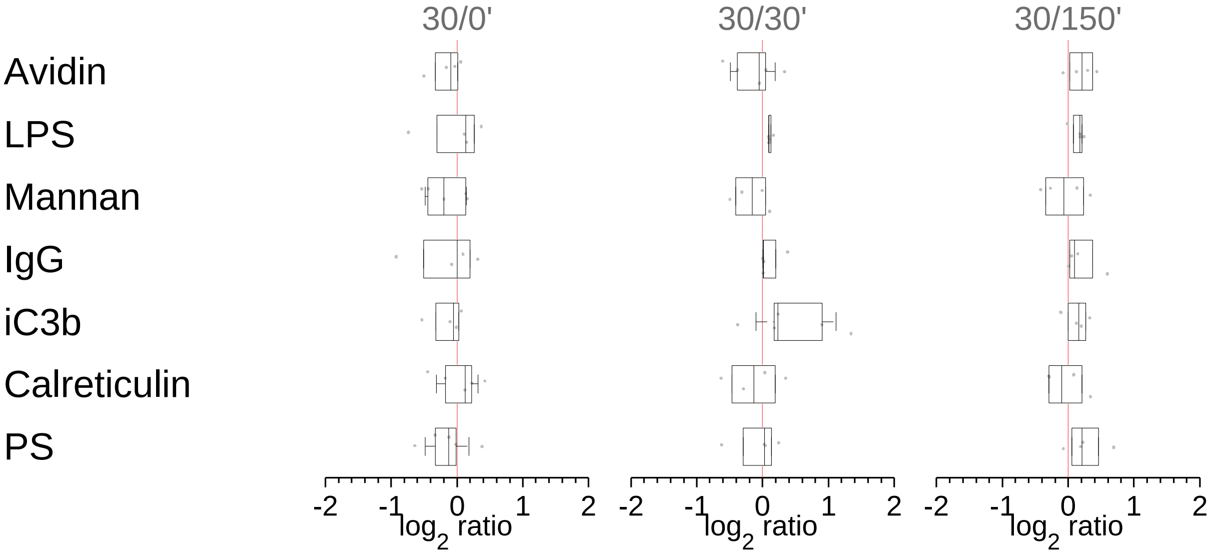

GO:0005741 mitochondrial outer membrane (n = 37)

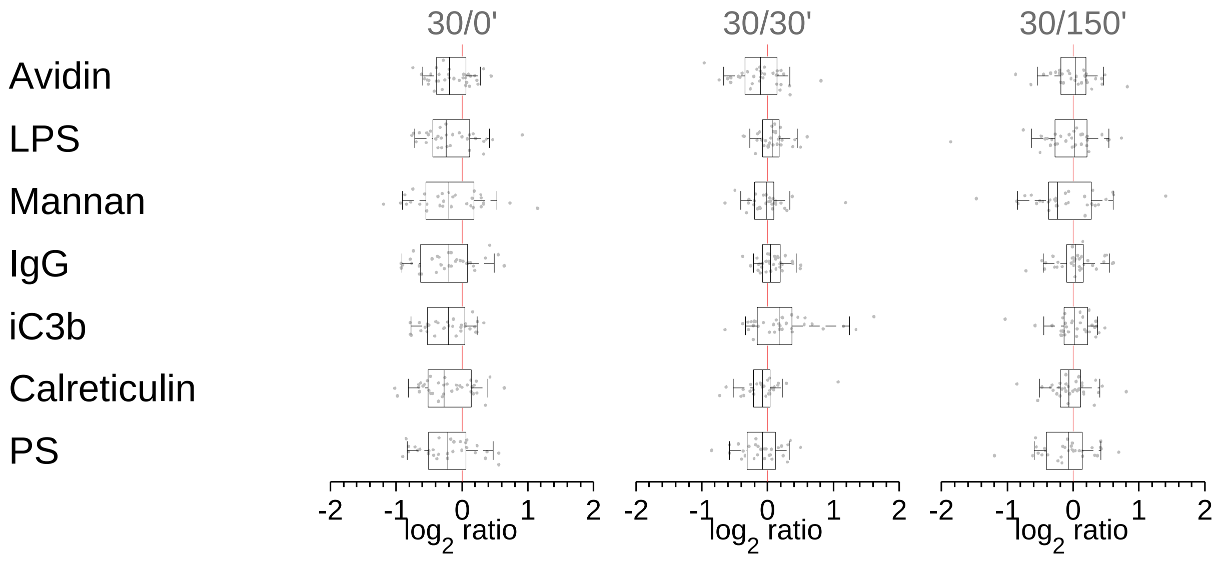

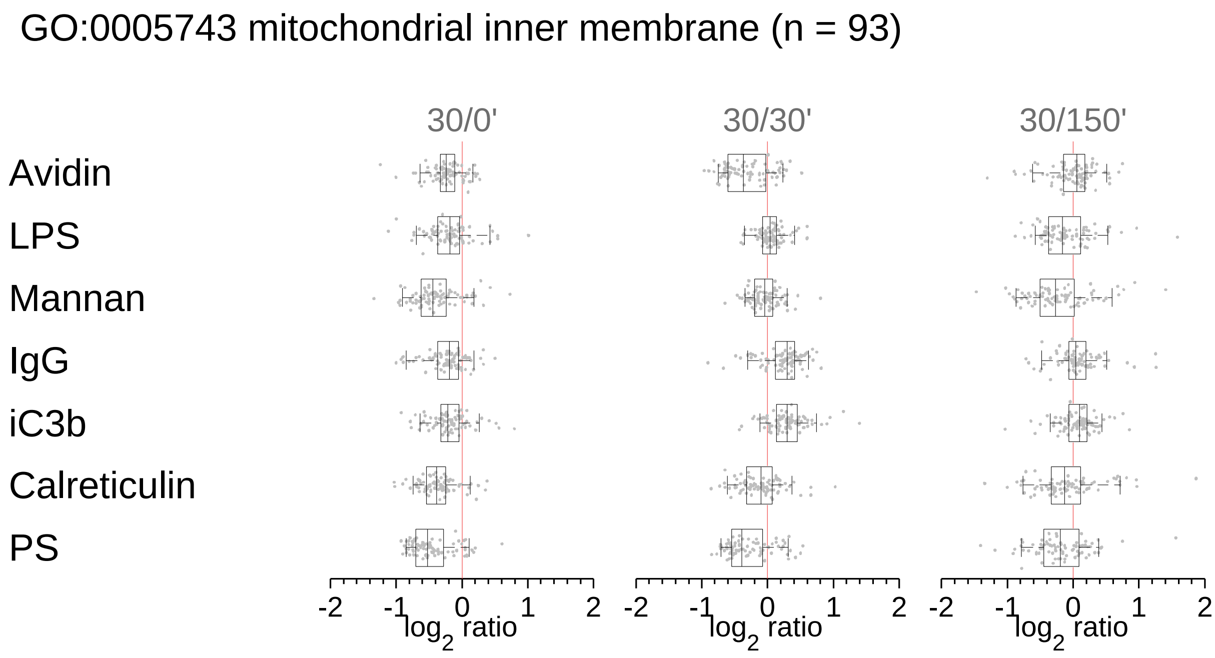

GO:0005743 mitochondrial inner membrane (n = 93)
30/0'
30/30'
30/150'
Avidin
LPS
Mannan
IgG
iC3b
Calreticulin
PS
-2
-1
0
1
2
log2 ratio

GO:0005747 mitochondrial respiratory chain complex I (n = 9)

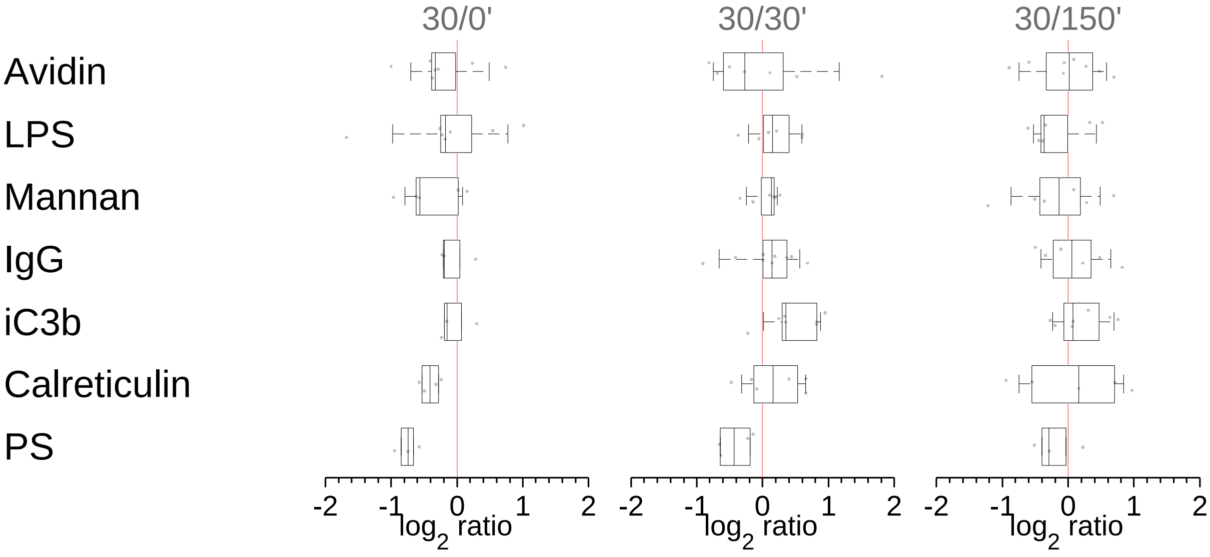

GO:0005750 mitochondrial respiratory chain complex III (n = 5)

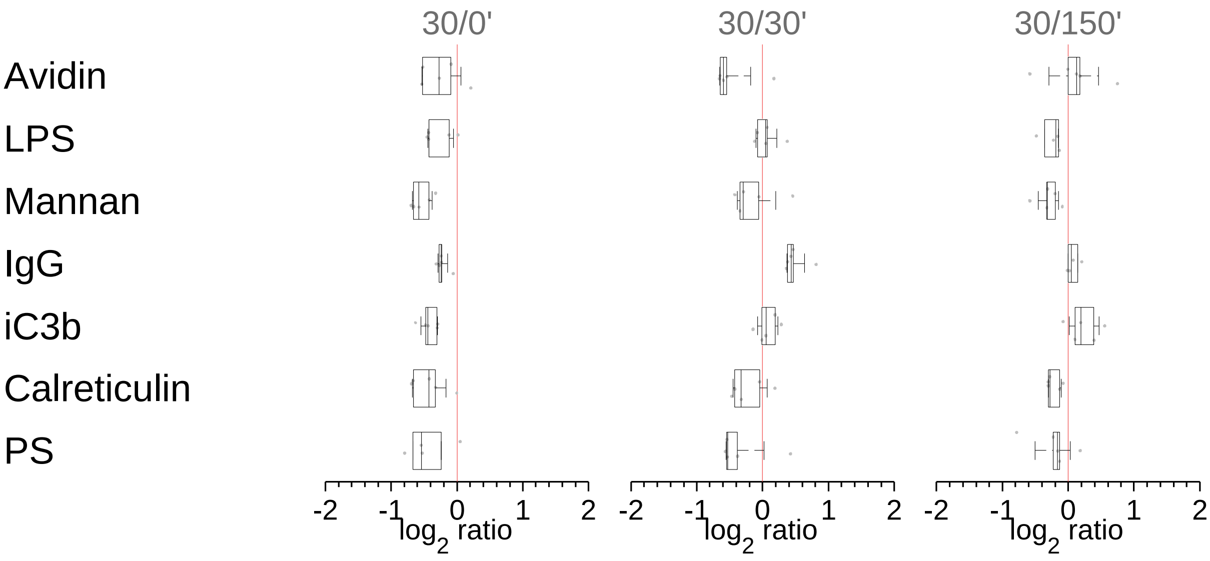

GO:0005753 mitochondrial proton-transporting ATP synthase complex (n = 13)

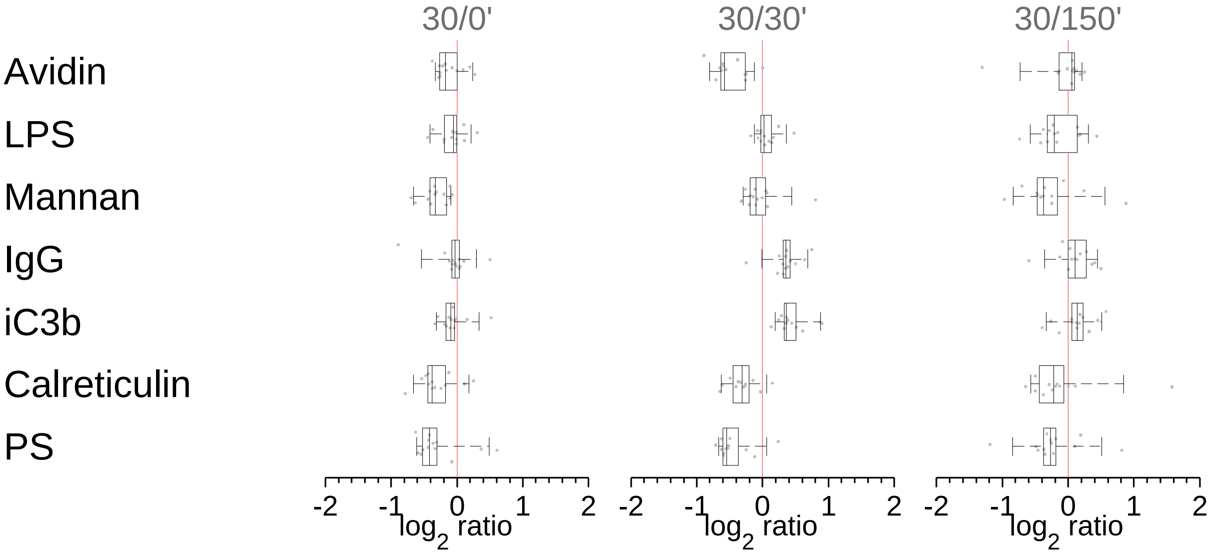

GO:0005758 mitochondrial intermembrane space (n = 6)

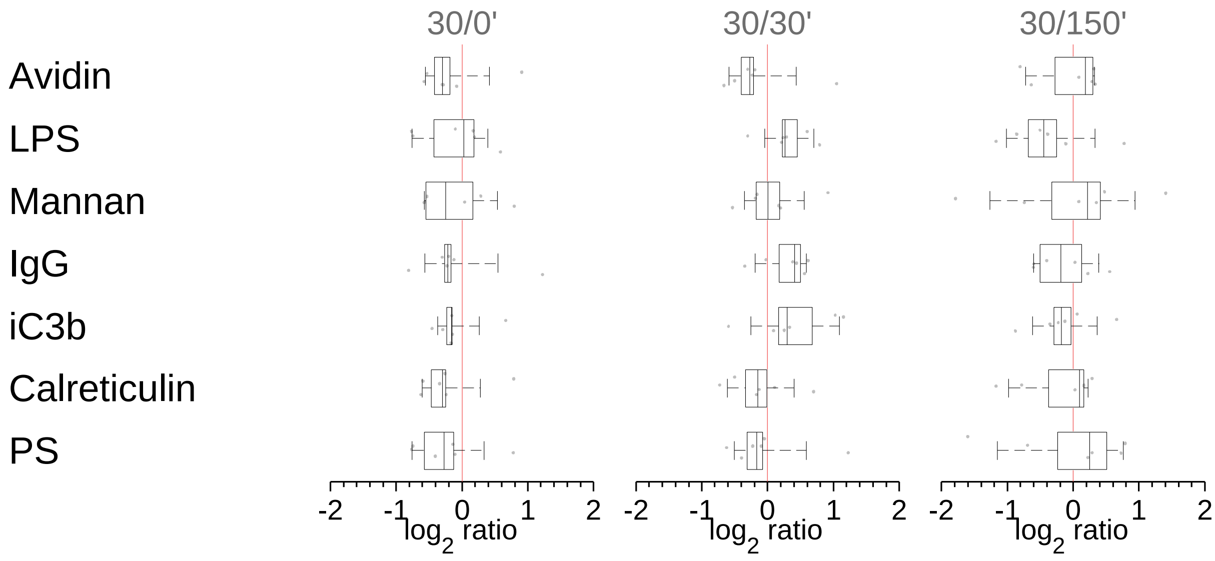

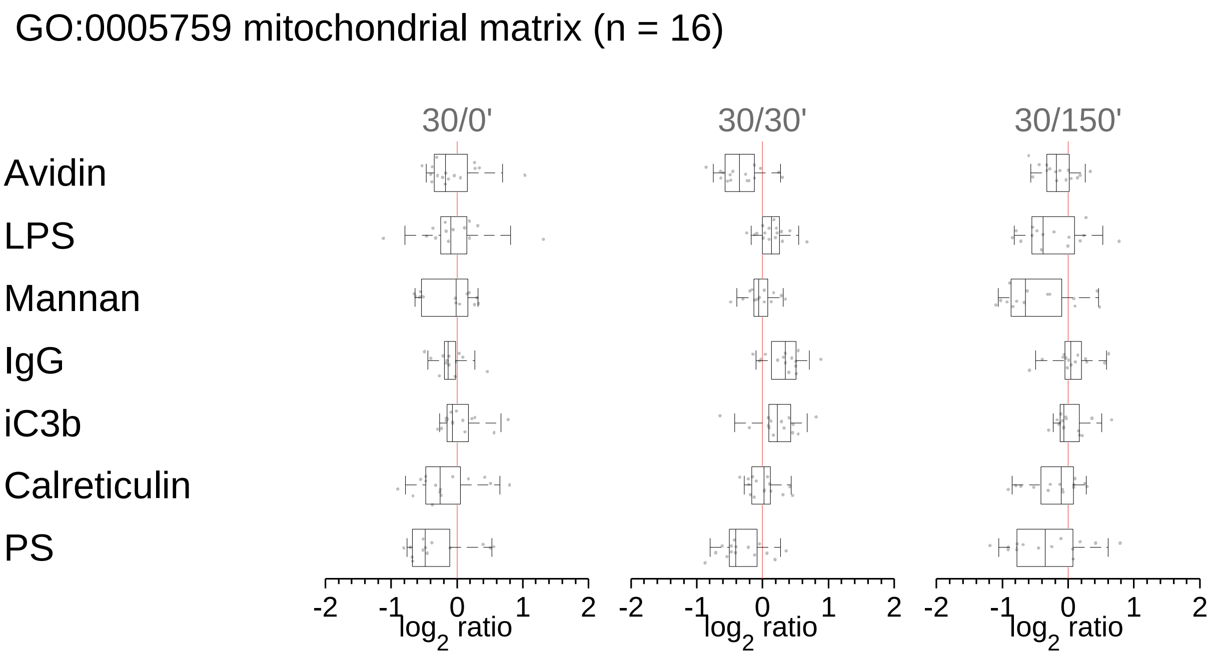

GO:0005759 mitochondrial matrix (n = 16)
30/0'
30/30'
30/150'
Avidin
LPS
Mannan
IgG
iC3b
Calreticulin
PS
-2
-1
0
1
2
log2 ratio

GO:0005764 lysosome (n = 163)

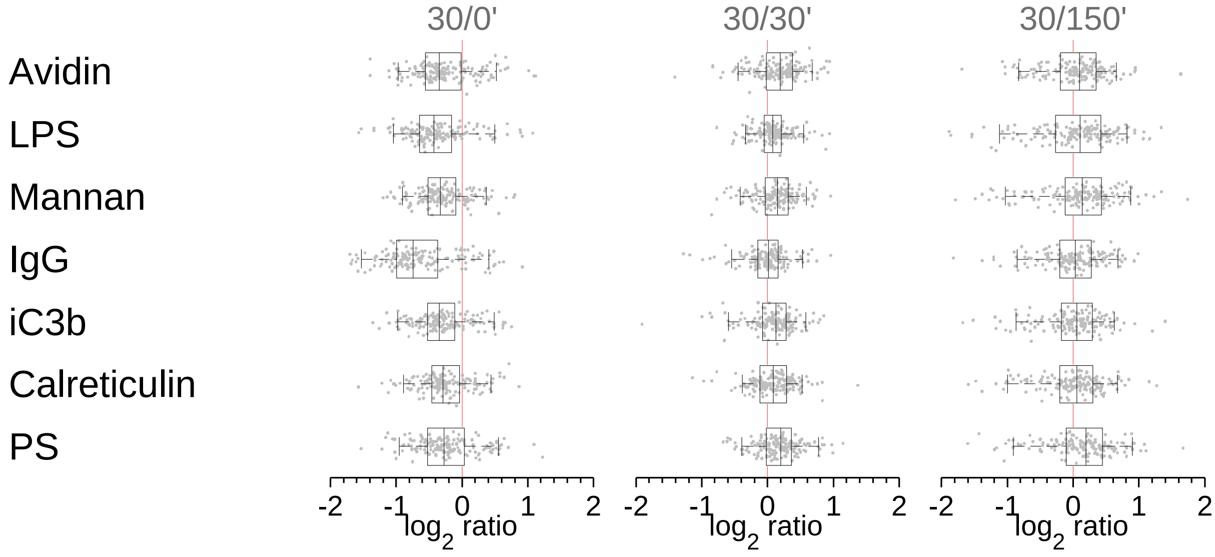

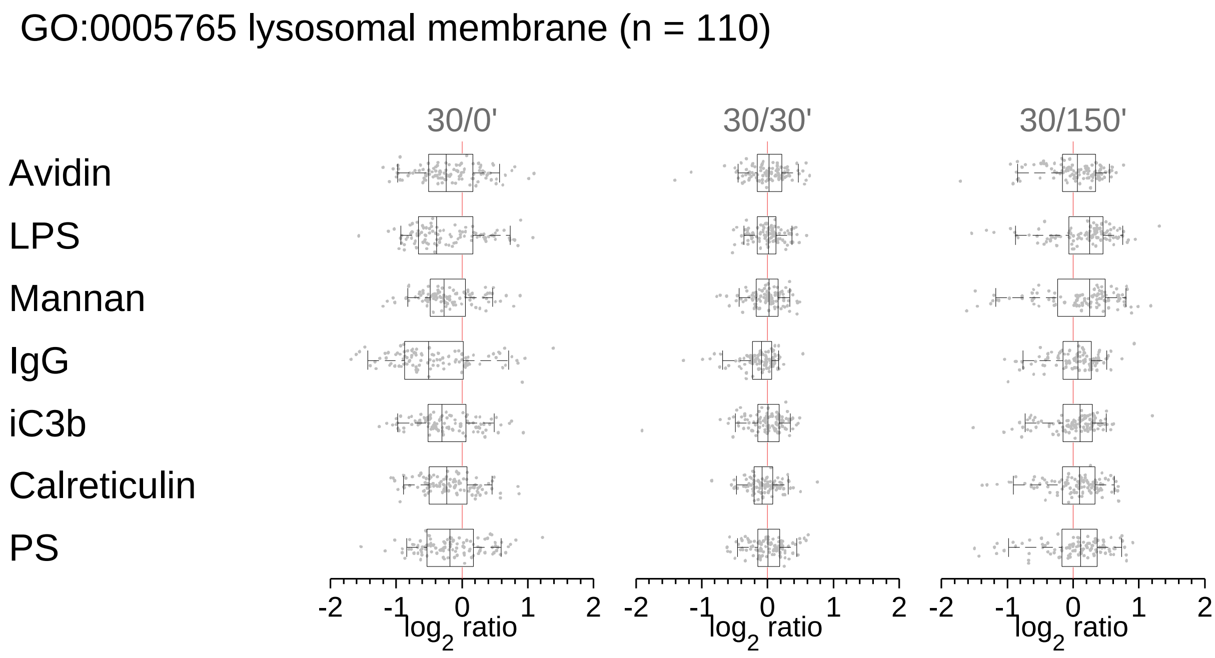
GO:0005765 lysosomal membrane (n = 110)
30/0'
30/30'
30/150'
Avidin
LPS
Mannan
IgG
iC3b
Calreticulin
PS
-2
-1
0
1
2
log2 ratio

GO:0005768 endosome (n = 187)

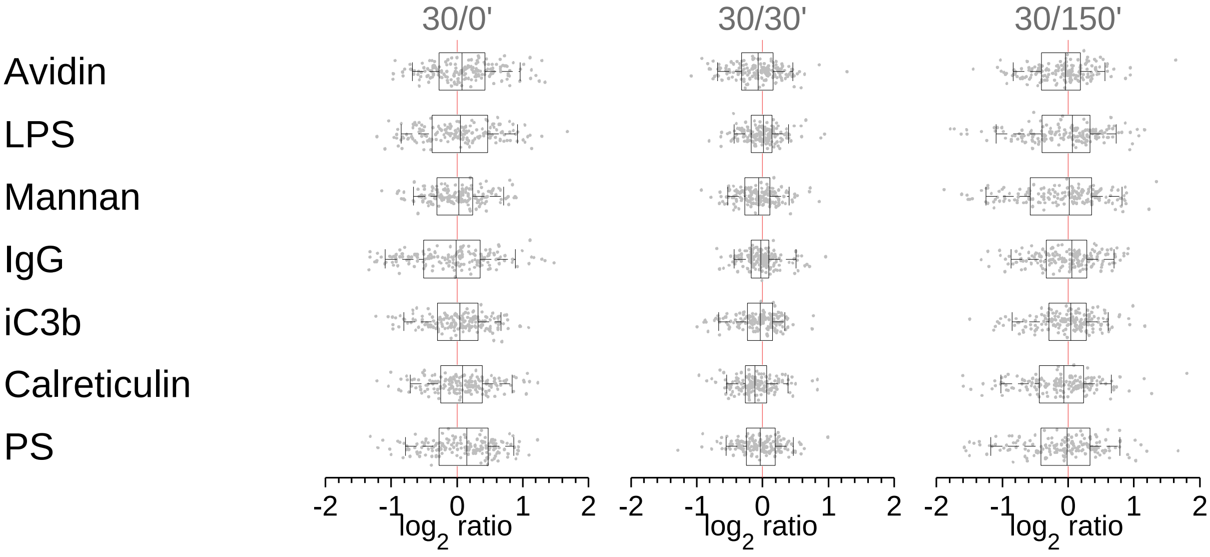

GO:0005769 early endosome (n = 52)

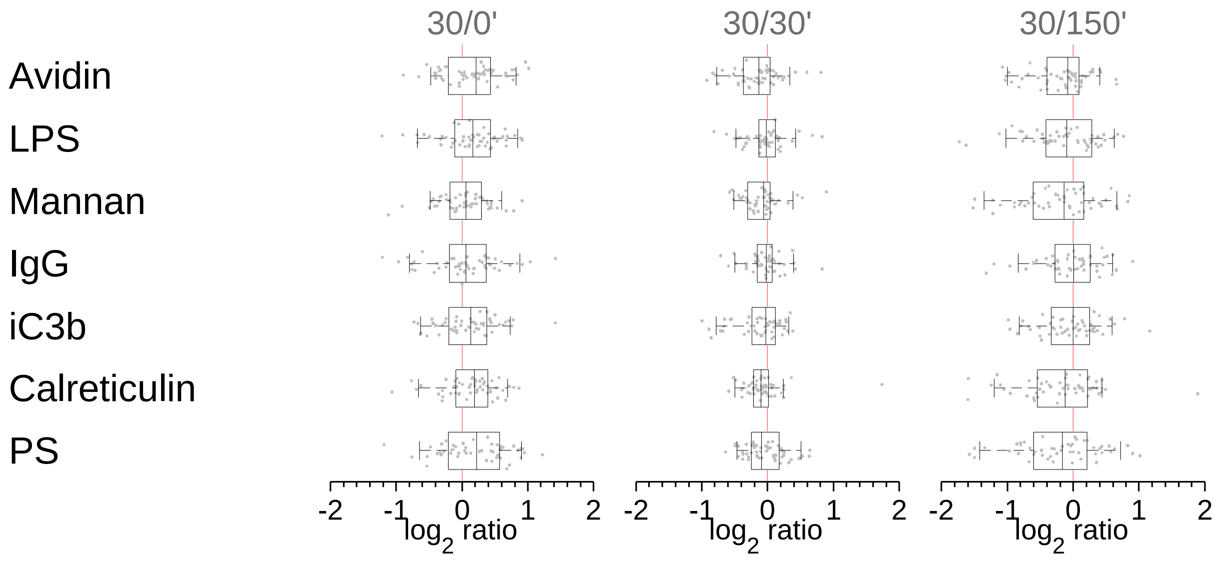

GO:0005770 late endosome (n = 43)

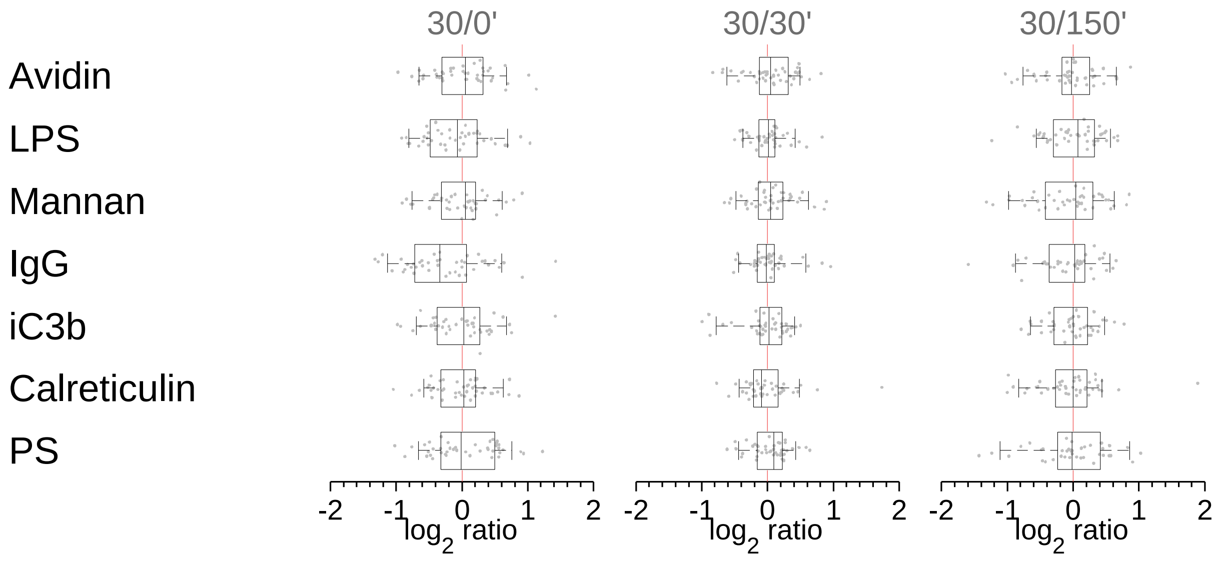

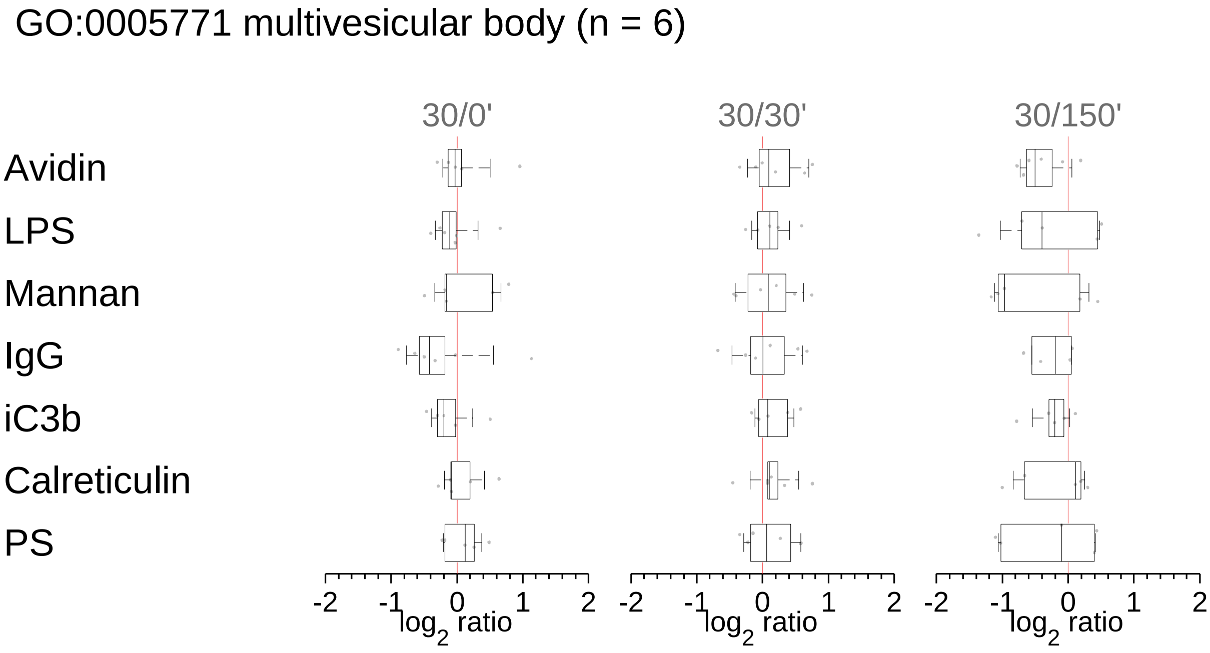

GO:0005771 multivesicular body (n = 6)
30/0'
30/30'
30/150'
Avidin
LPS
Mannan
IgG
iC3b
Calreticulin
PS
-2 -1 0 1 2
log2 ratio

GO:0005773 vacuole (n = 8)

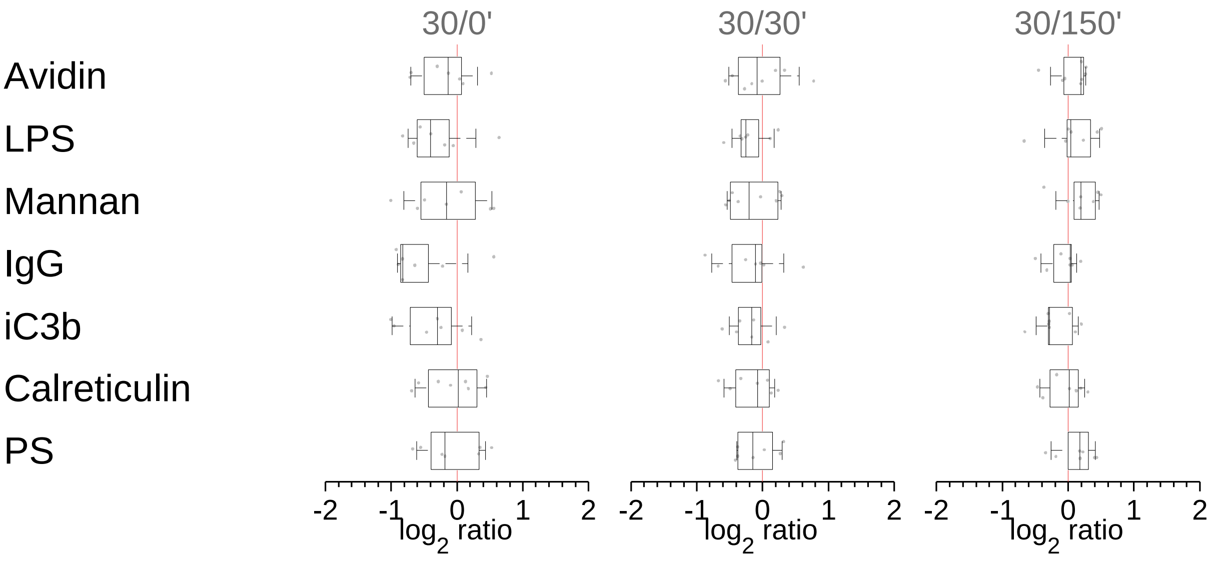

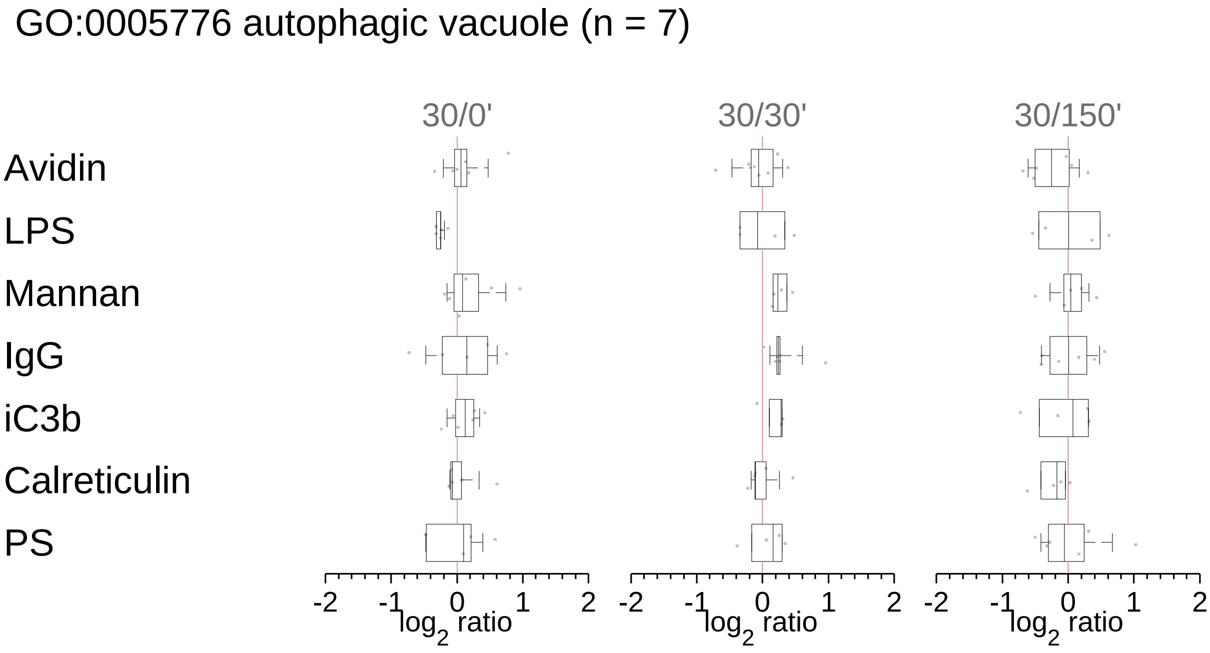

GO:0005776 autophagic vacuole (n = 7)
30/0'
30/30'
30/150'
Avidin
LPS
Mannan
IgG
iC3b
Calreticulin
PS
-2 -1 0 1 2
log2 ratio

GO:0005777 peroxisome (n = 29)

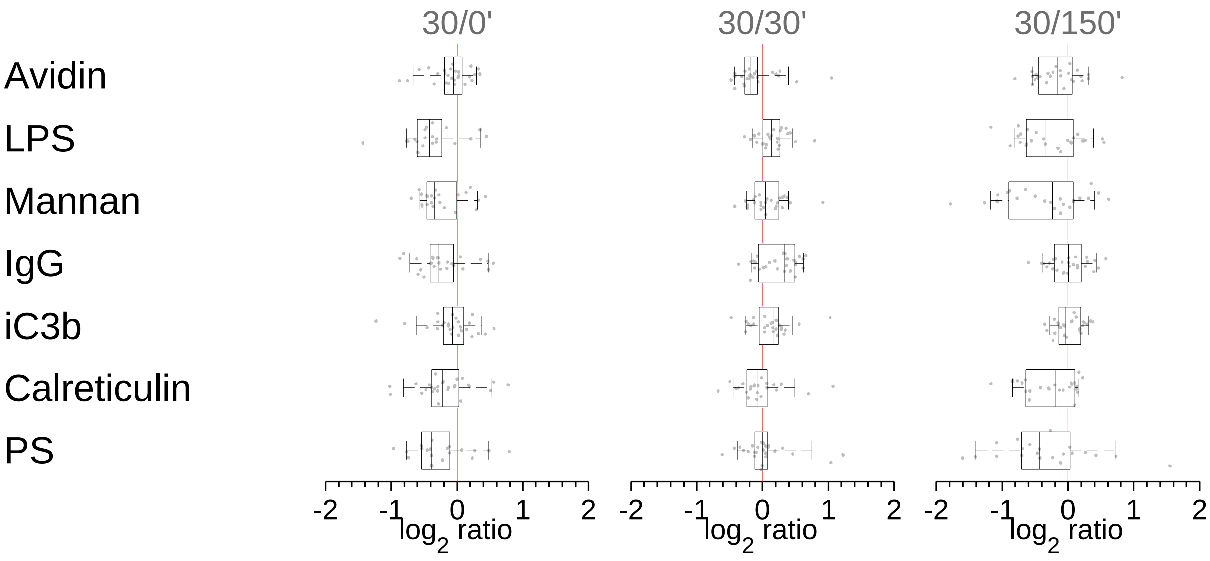

GO:0005778 peroxisomal membrane (n = 13)

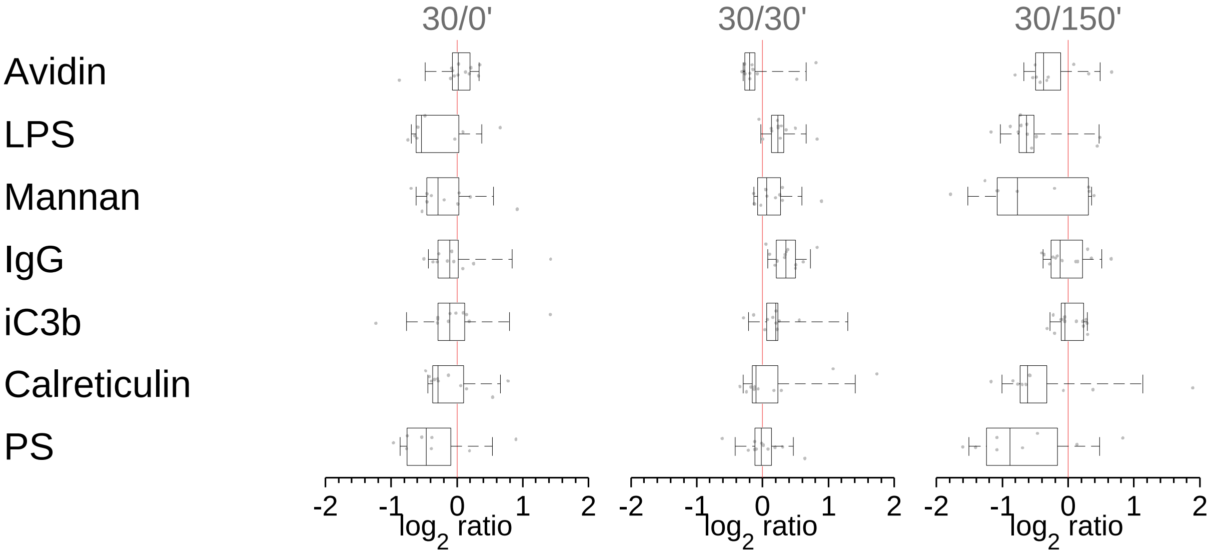

GO:0005782 peroxisomal matrix (n = 4)

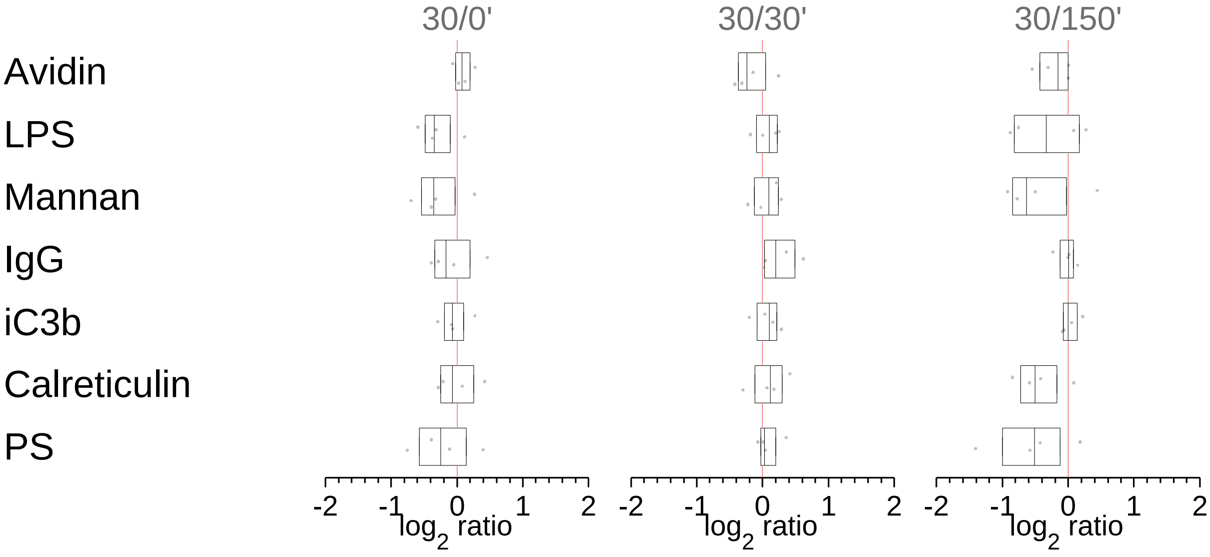

GO:0005783 endoplasmic reticulum (n = 238)

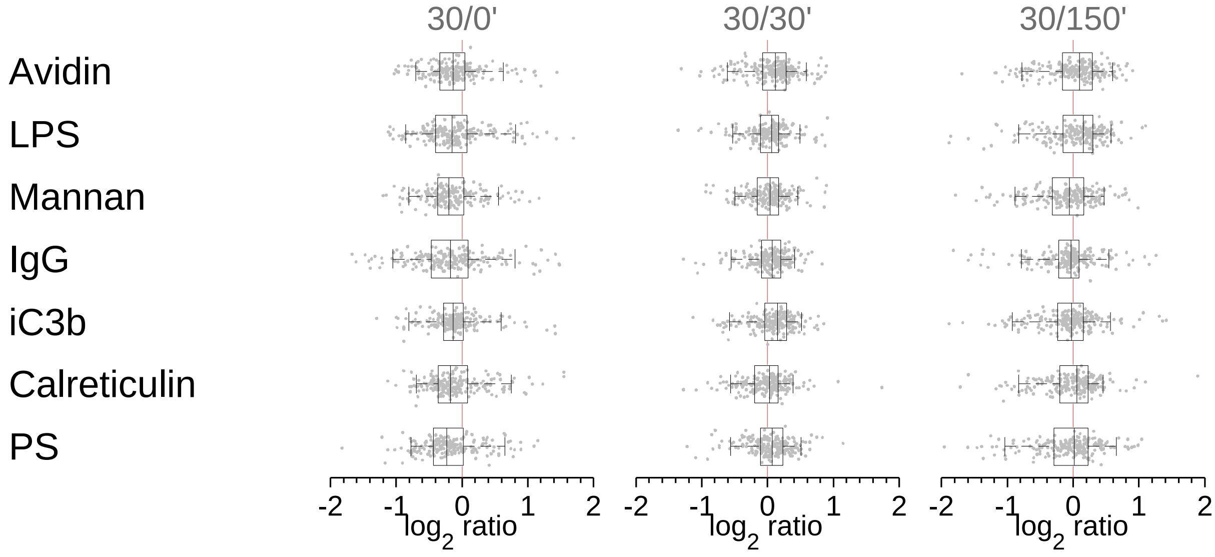

GO:0005786 signal recognition particle, endoplasmic reticulum targeting (n = 3)

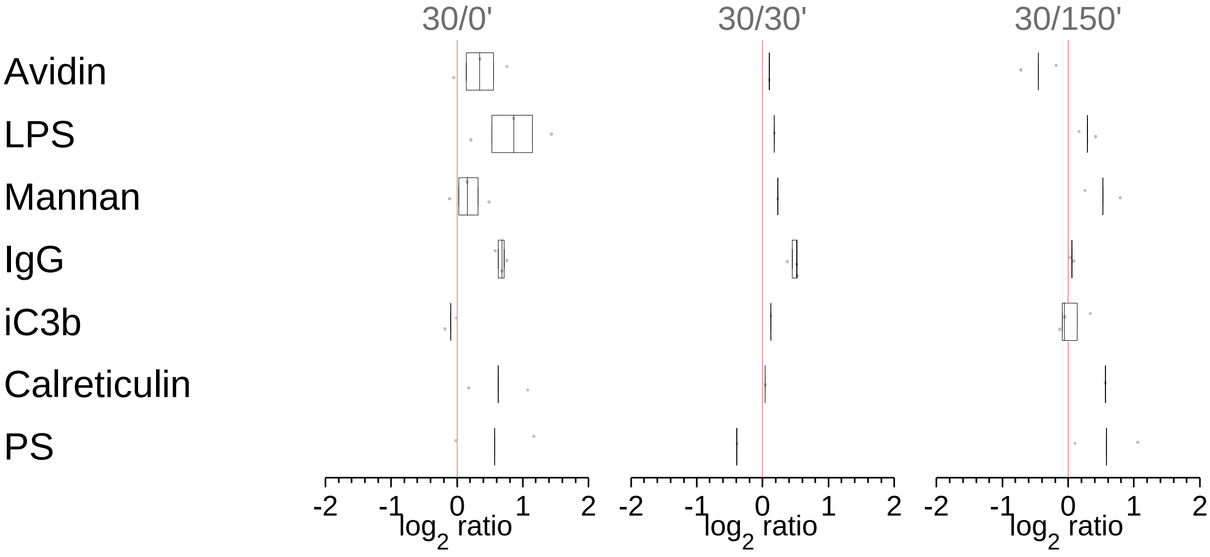

GO:0005788 endoplasmic reticulum lumen (n = 18)

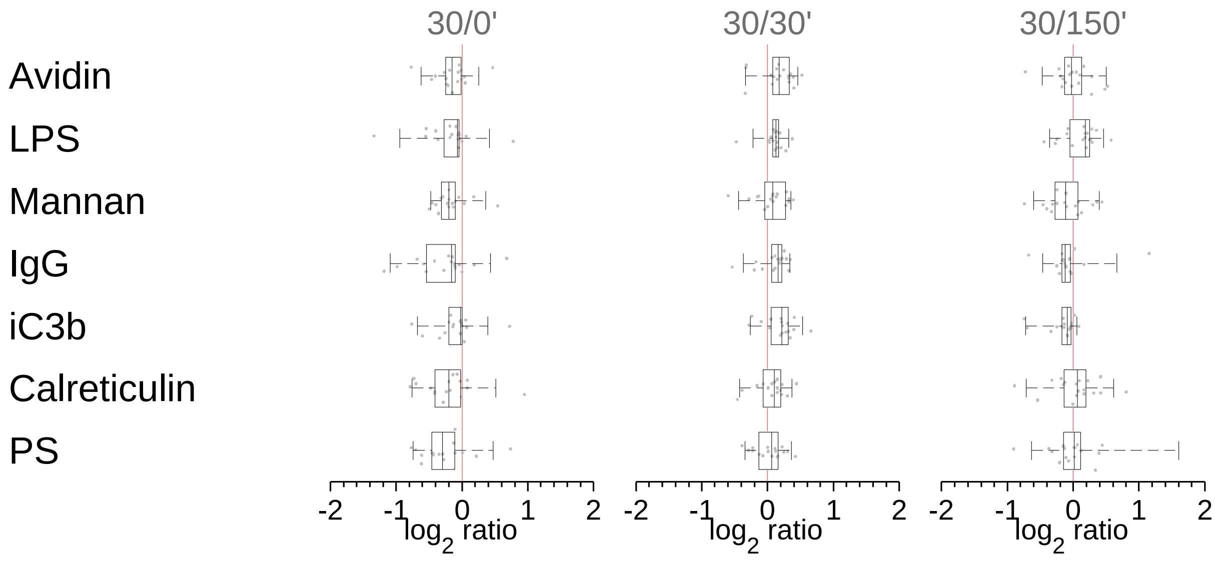

GO:0005789 endoplasmic reticulum membrane (n = 42)

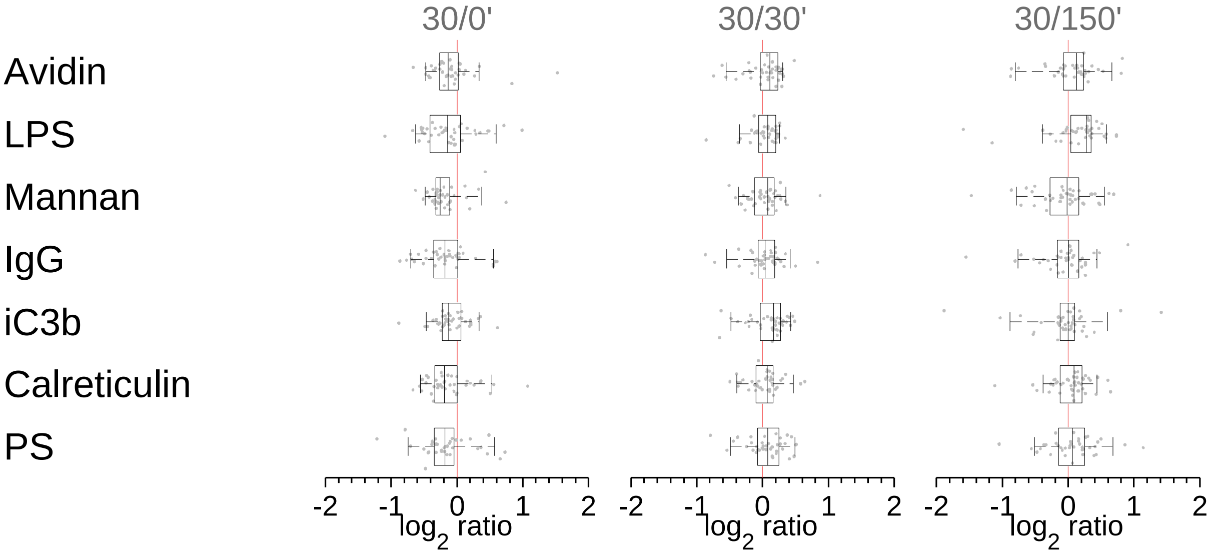

GO:0005790 smooth endoplasmic reticulum (n = 16)

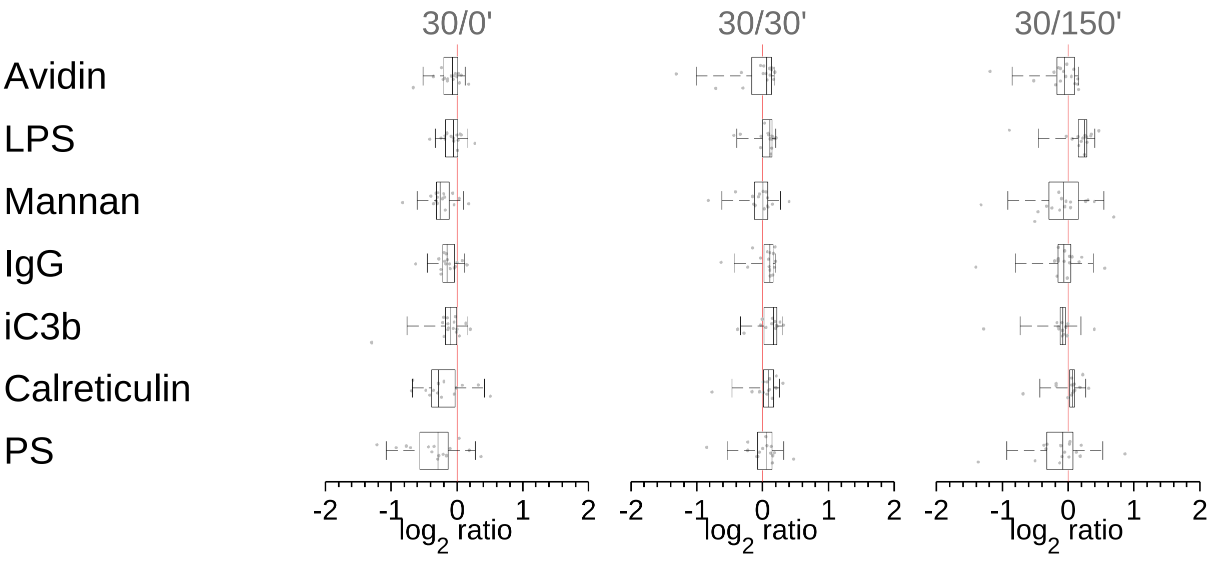

GO:0005791 rough endoplasmic reticulum (n = 12)

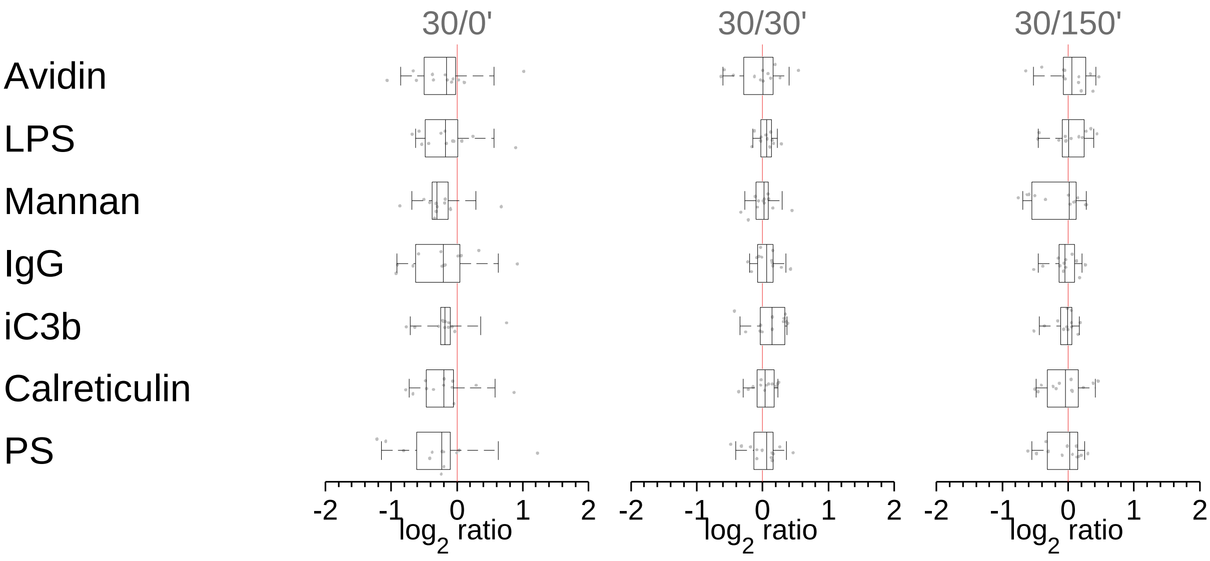

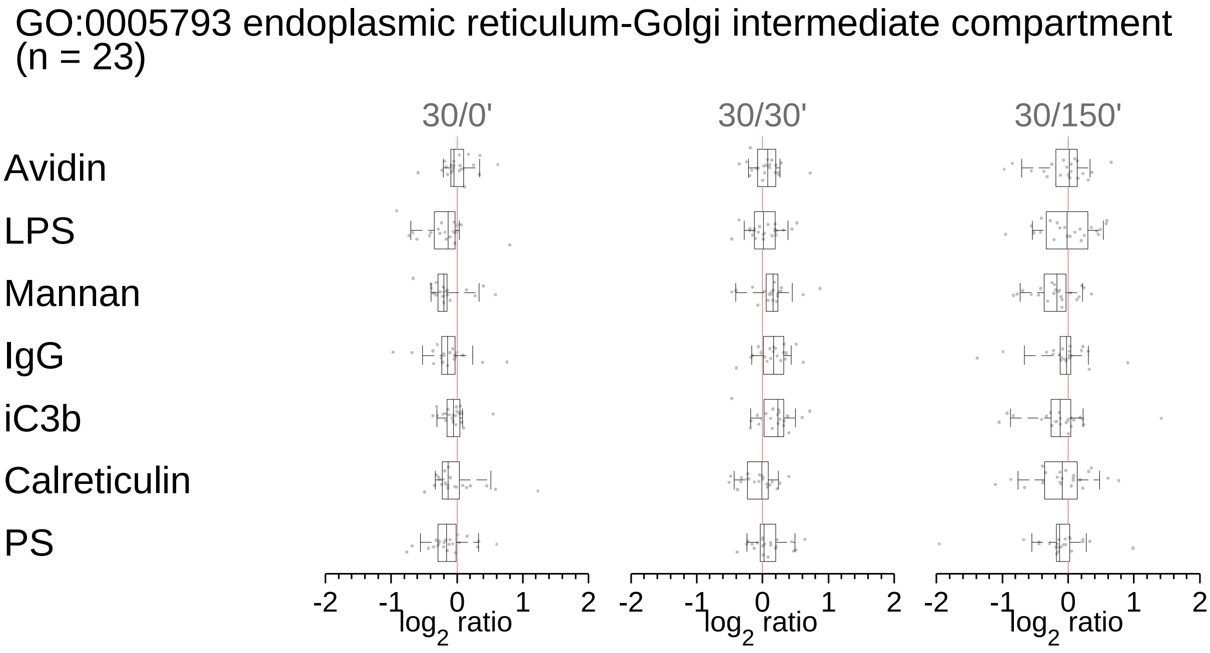
GO:0005793 endoplasmic reticulum-Golgi intermediate compartment
(n = 23)
30/0'
30/30'
30/150'
Avidin
LPS
Mannan
IgG
iC3b
Calreticulin
PS
-2
-1
0
1
2
log2 ratio

GO:0005794 Golgi apparatus (n = 203)

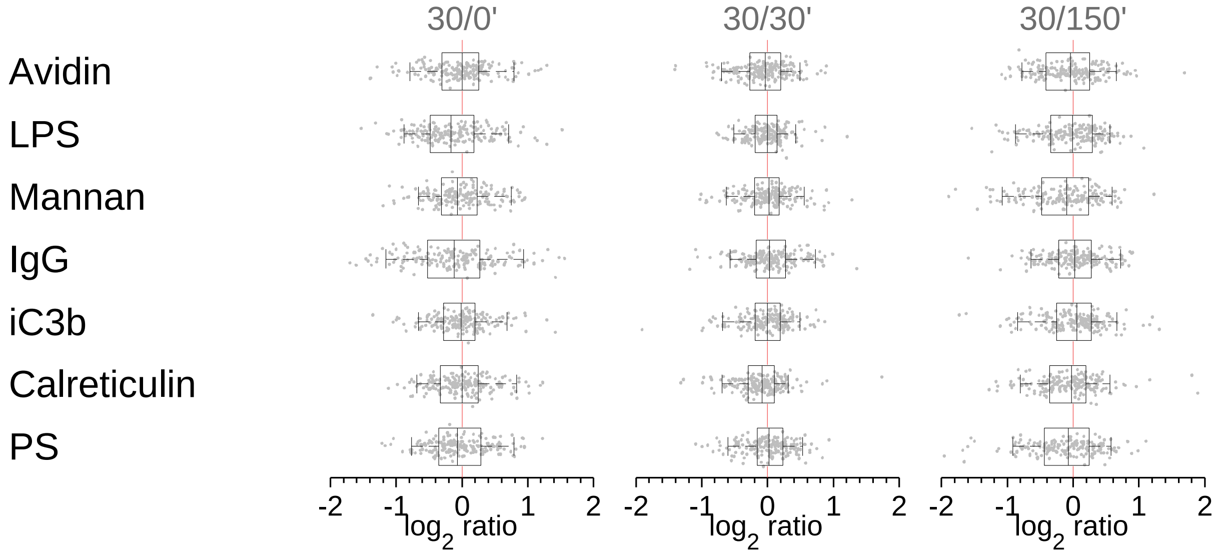

GO:0005795 Golgi stack (n = 9)

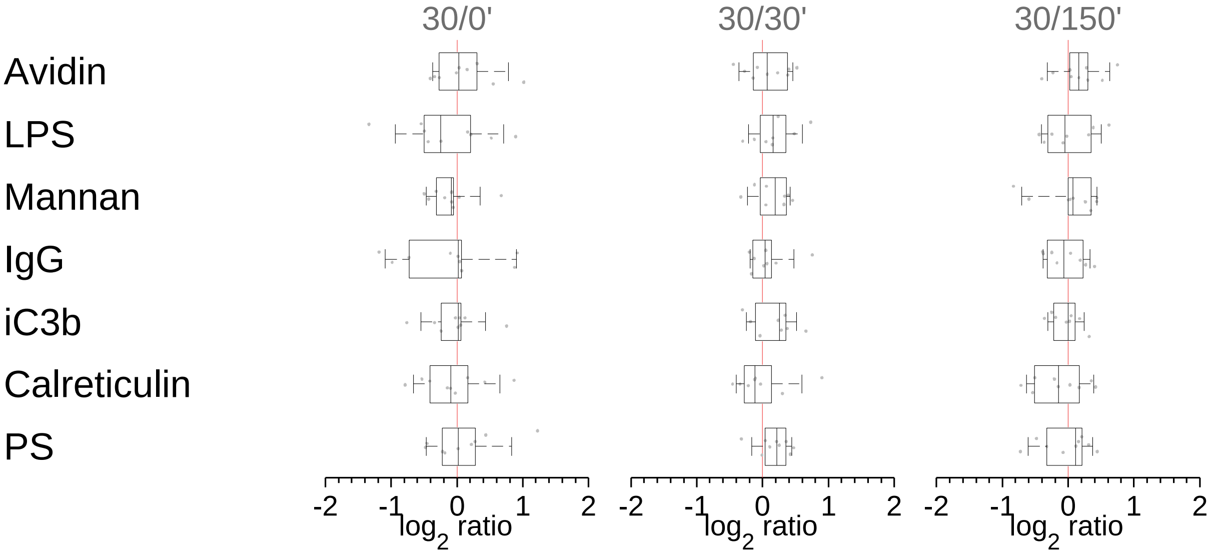

GO:0005797 Golgi medial cisterna (n = 4)

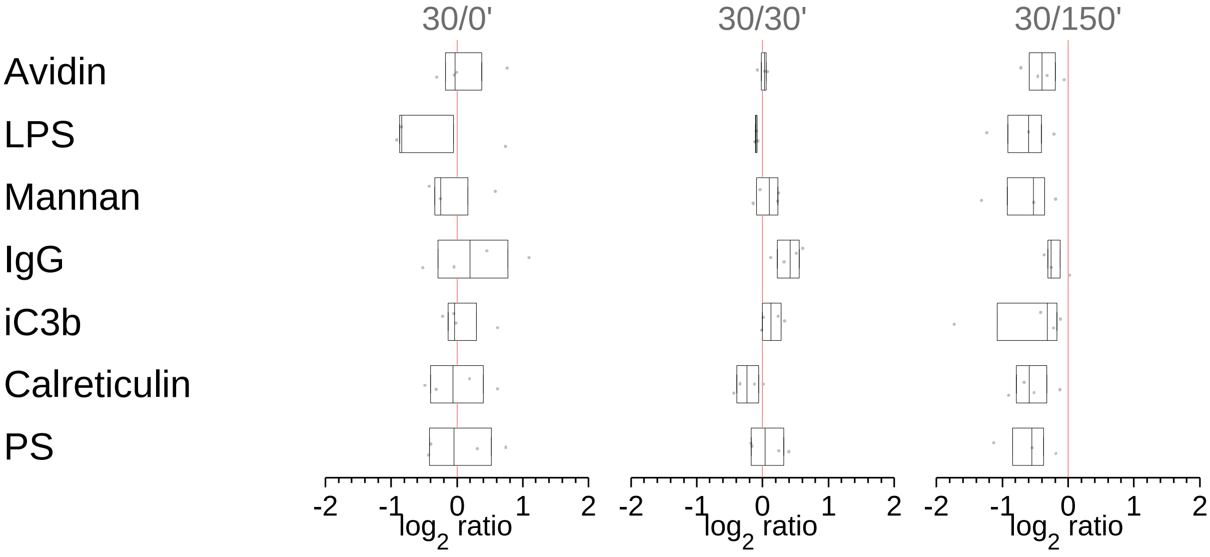

GO:0005798 Golgi-associated vesicle (n = 4)

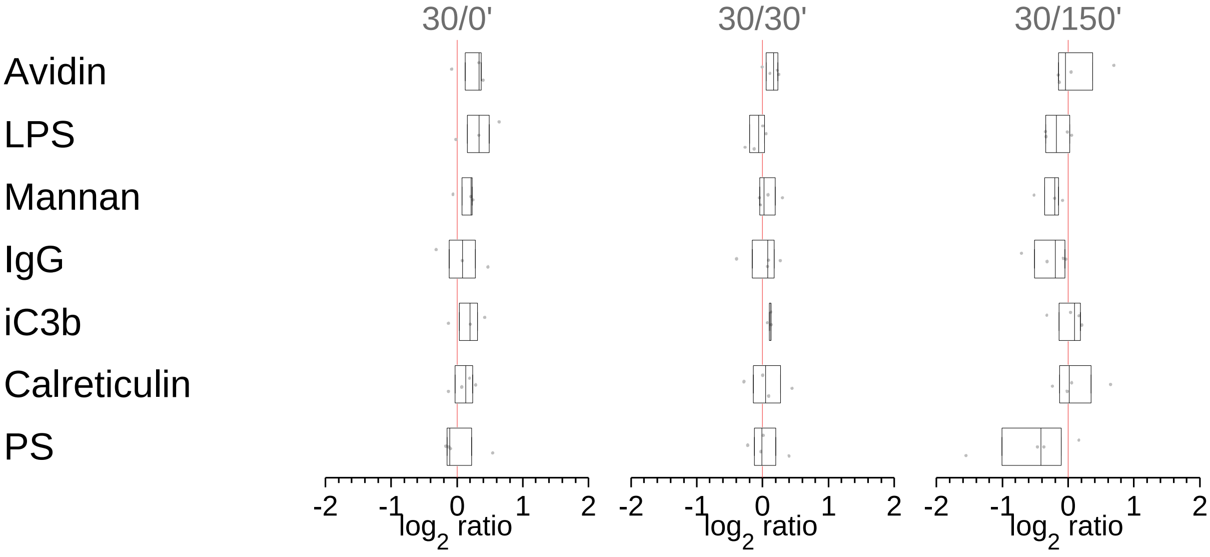

GO:0005801 cis-Golgi network (n = 7)

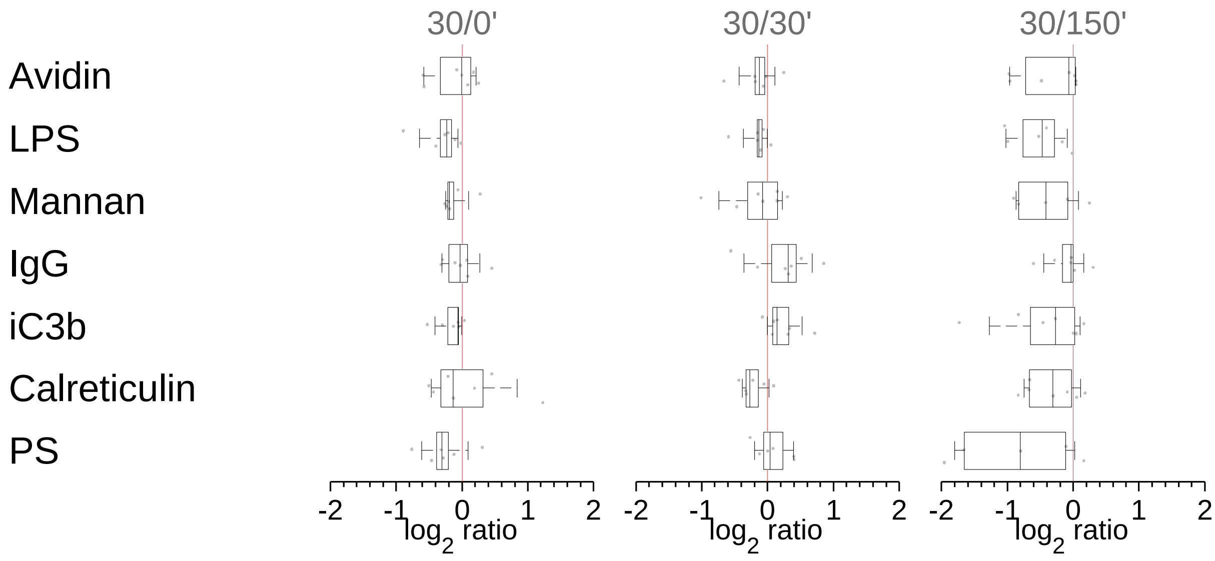

GO:0005802 trans-Golgi network (n = 35)

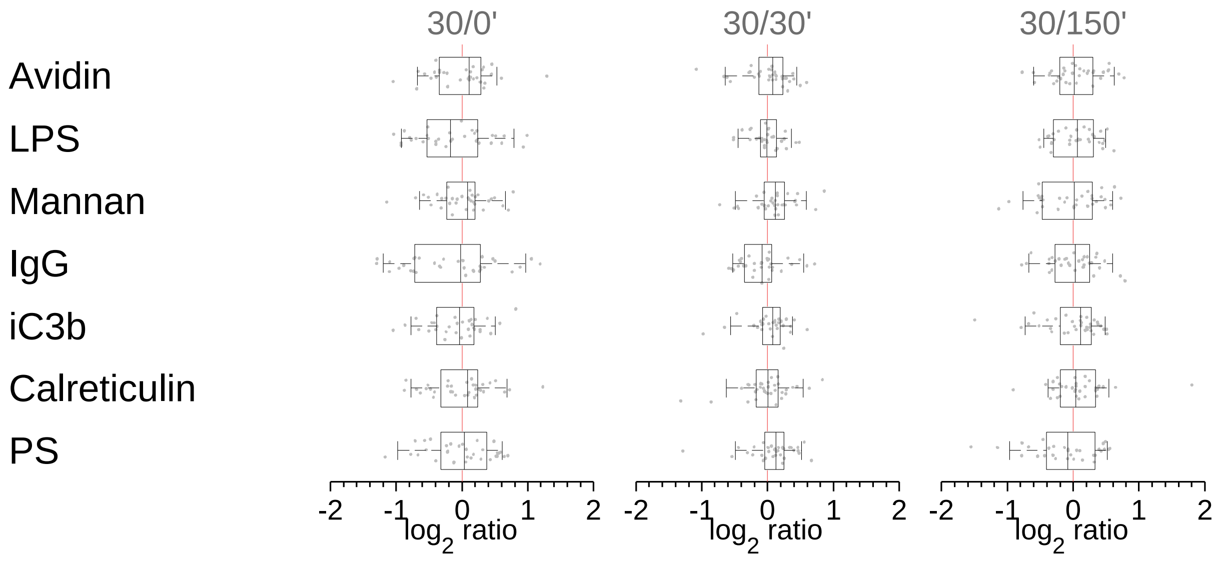

GO:0005811 lipid particle (n = 19)

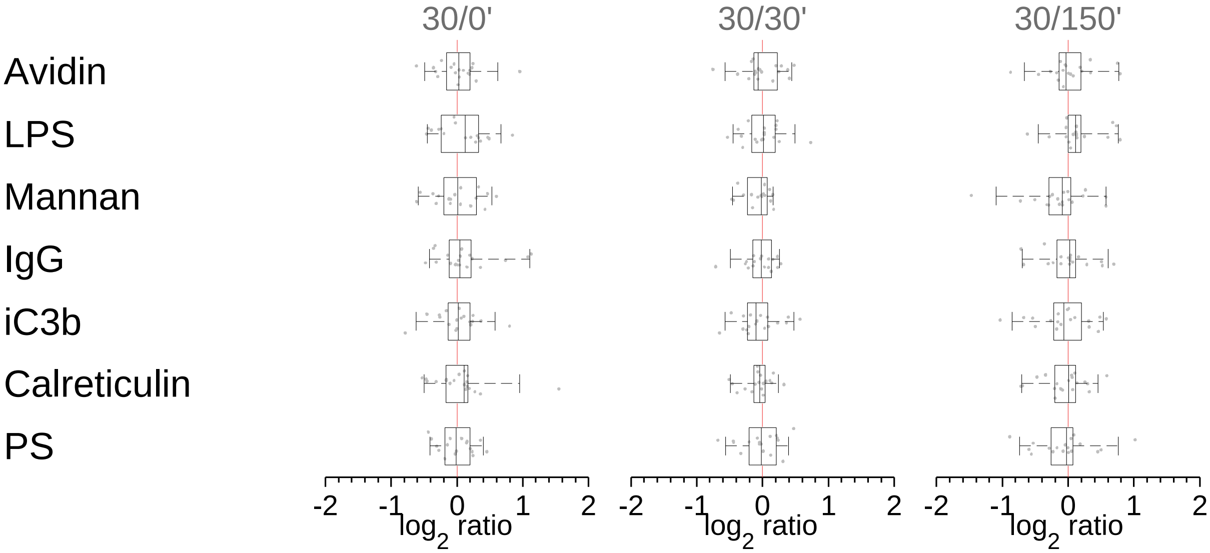

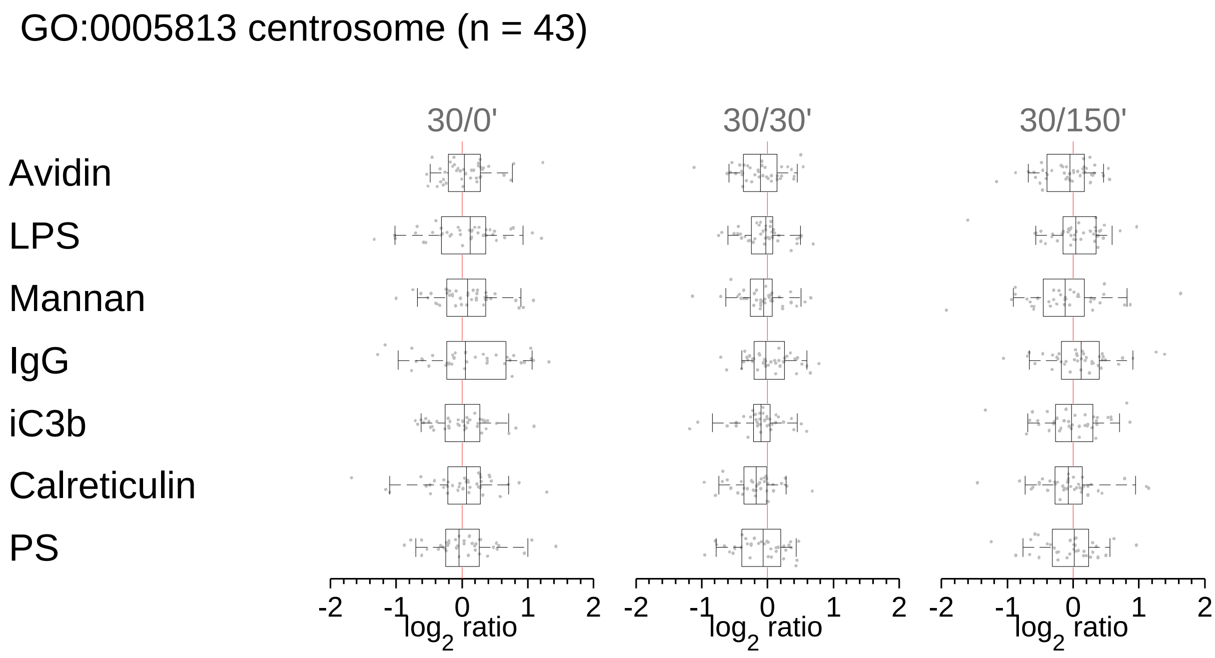
GO:0005813 centrosome (n = 43)
30/0'
30/30'
30/150'
Avidin
LPS
Mannan
IgG
iC3b
Calreticulin
PS
-2
-1
0
1
2
log2 ratio

GO:0005814 centriole (n = 5)

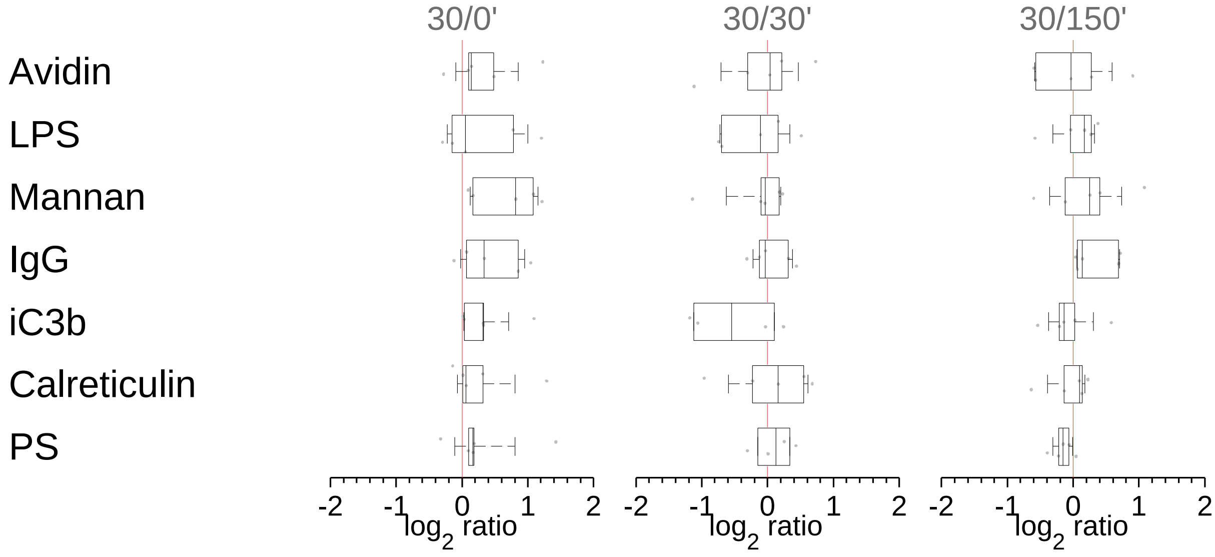

GO:0005815 microtubule organizing center (n = 9)

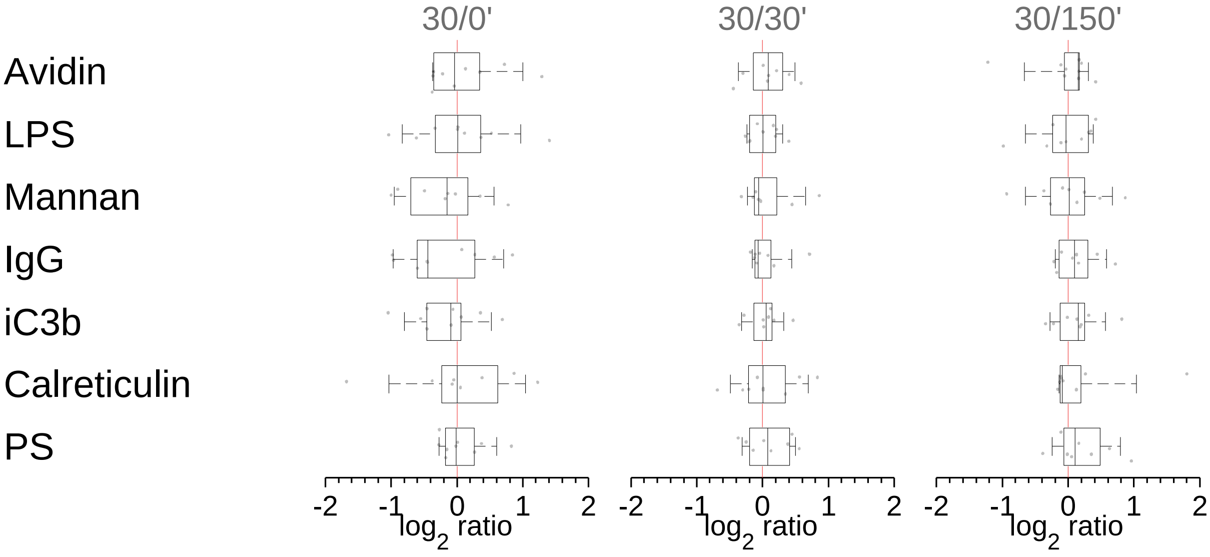

GO:0005819 spindle (n = 8)

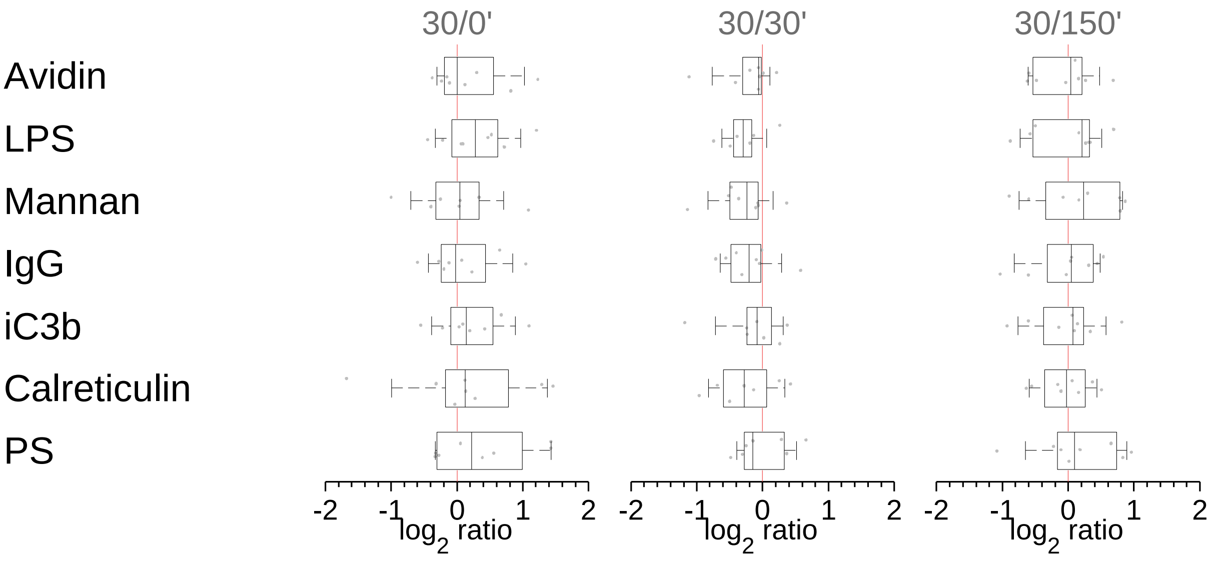

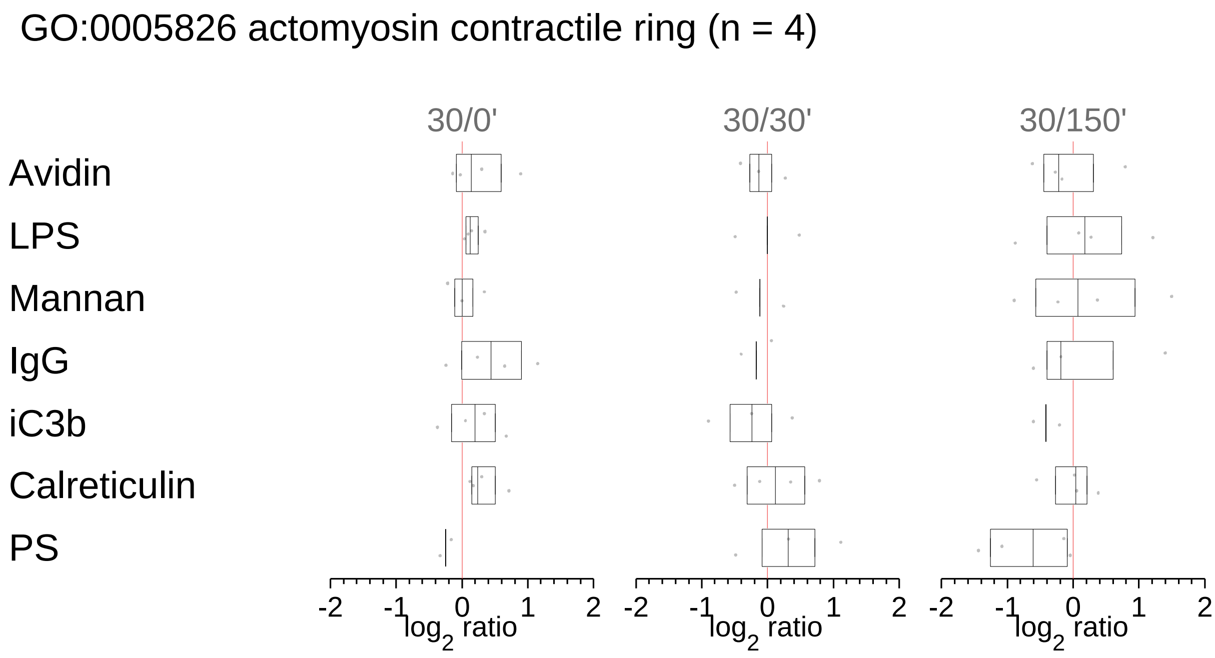
GO:0005826 actomyosin contractile ring (n = 4)
30/0'
30/30'
30/150'
Avidin
LPS
Mannan
IgG
iC3b
Calreticulin
PS
-2 -1 0 1 2
log2 ratio

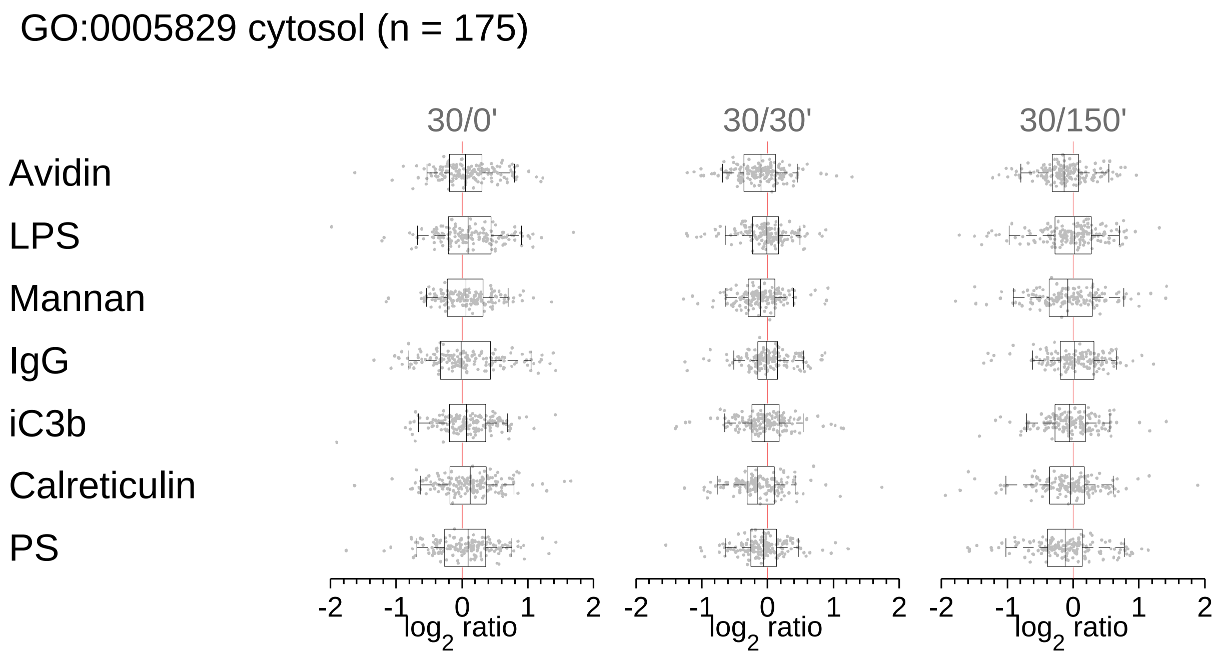
GO:0005829 cytosol (n = 175)
30/0'
30/30'
30/150'
Avidin
LPS
Mannan
IgG
iC3b
Calreticulin
PS
-2
-1
0
1
2
log2 ratio

GO:0005832 chaperonin-containing T-complex (n = 8)

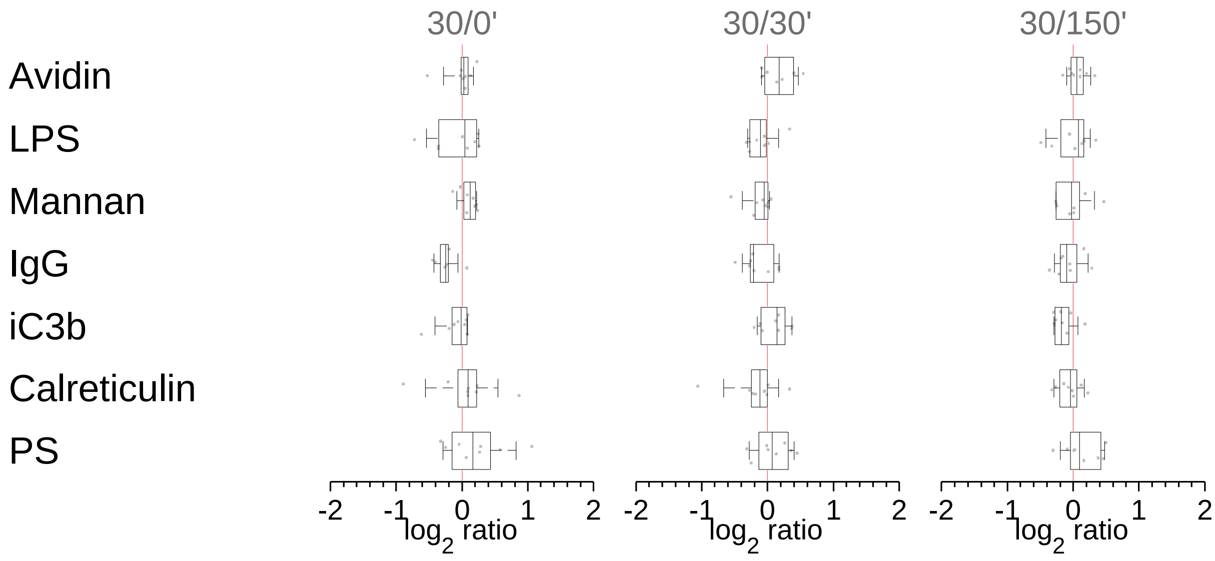

GO:0005834 heterotrimeric G-protein complex (n = 10)

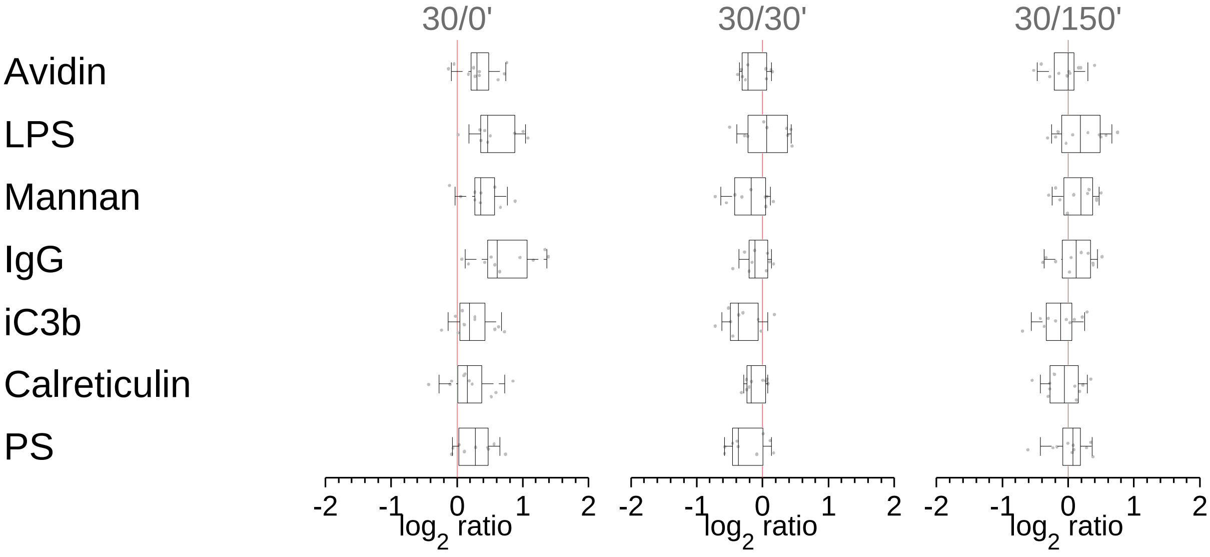

GO:0005839 proteasome core complex (n = 9)

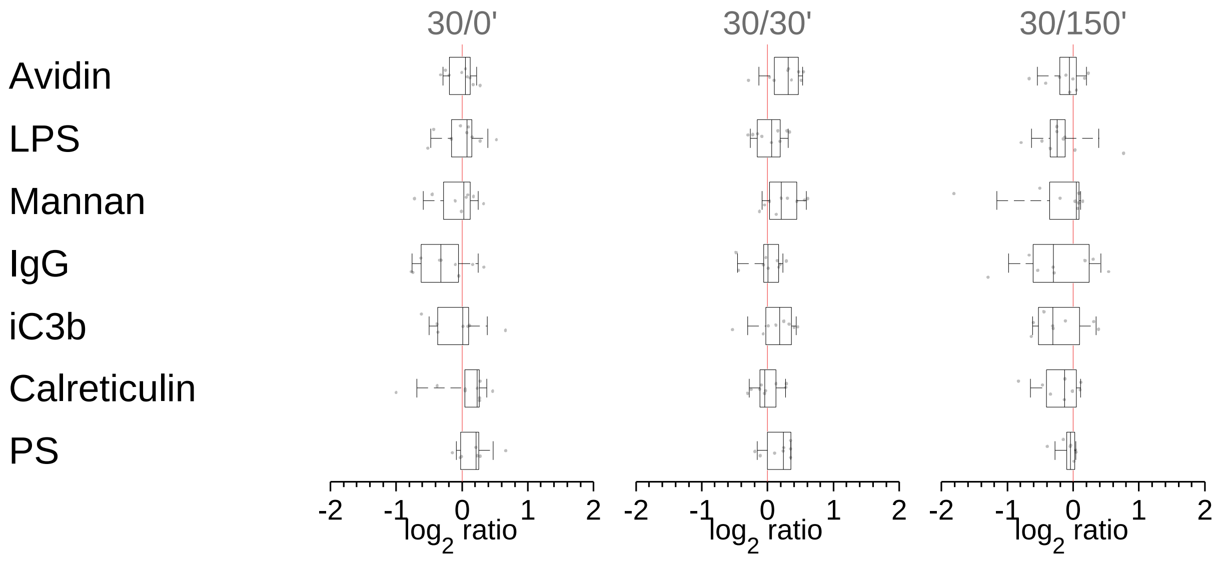

GO:0005840 ribosome (n = 54)

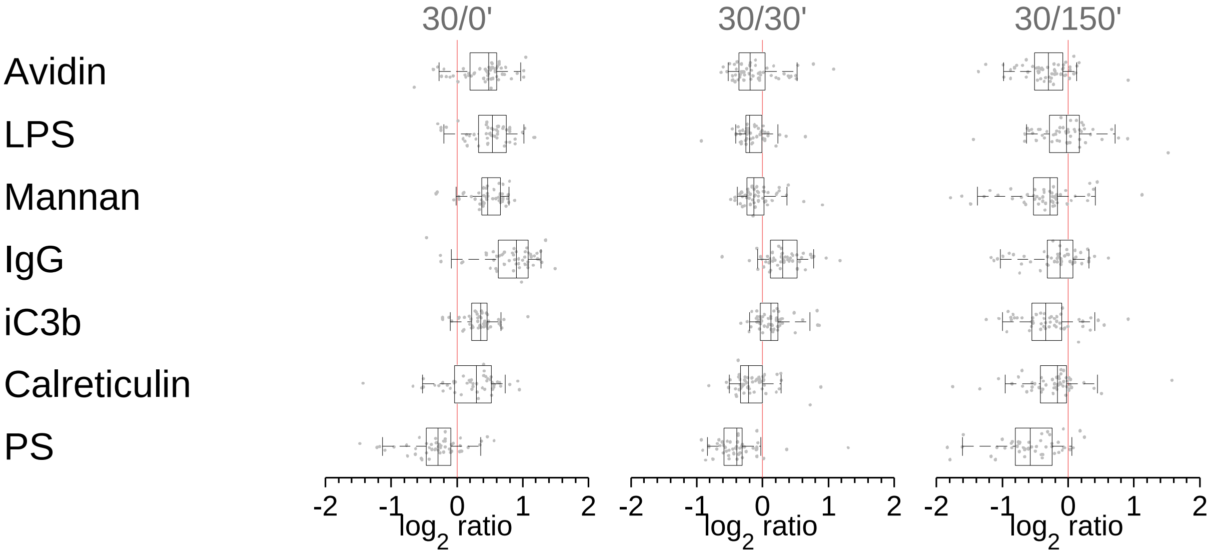

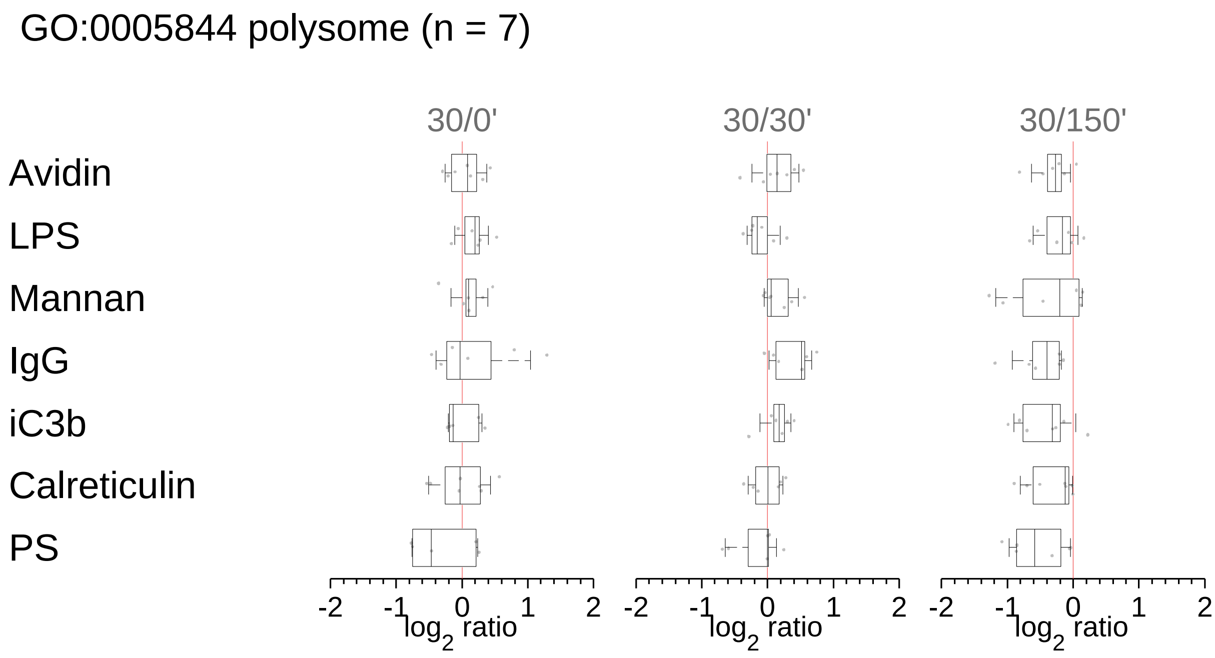
GO:0005844 polysome (n = 7)
30/0'
30/30'
30/150'
Avidin
LPS
Mannan
IgG
iC3b
Calreticulin
PS
-2
-1
0
1
2
log2 ratio

GO:0005845 mRNA cap binding complex (n = 3)

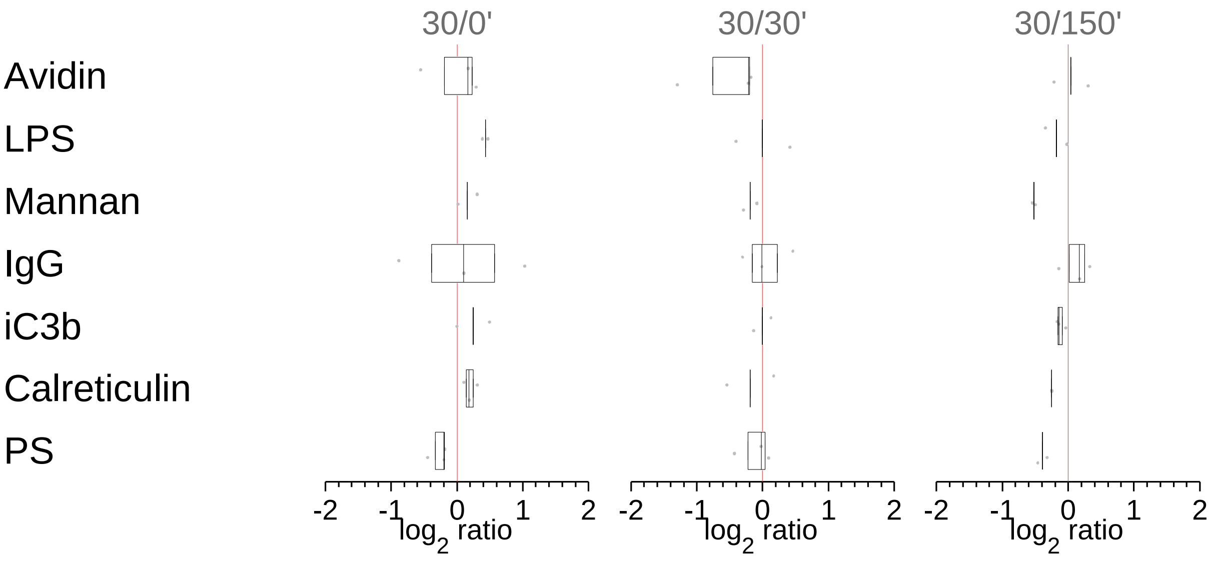

GO:0005852 eukaryotic translation initiation factor 3 complex (n = 3)

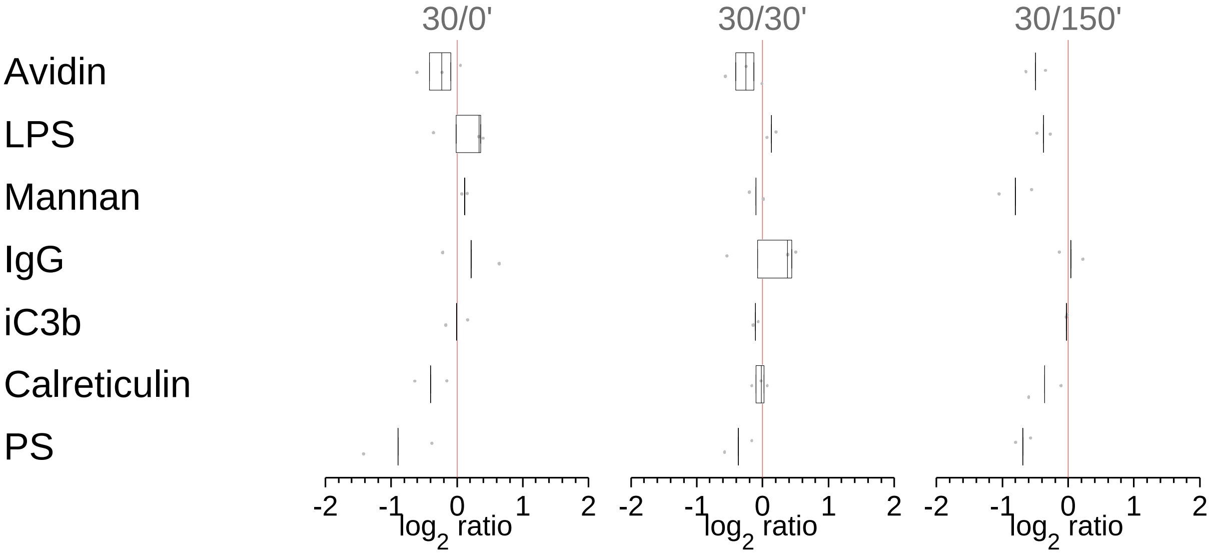

GO:0005856 cytoskeleton (n = 152)

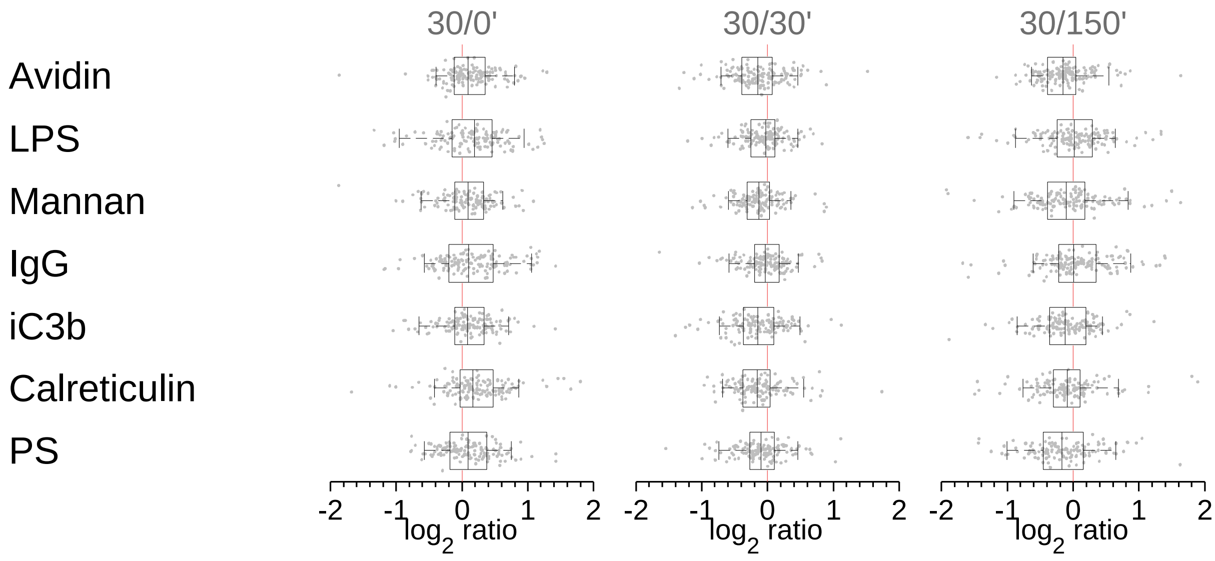

GO:0005868 cytoplasmic dynein complex (n = 3)

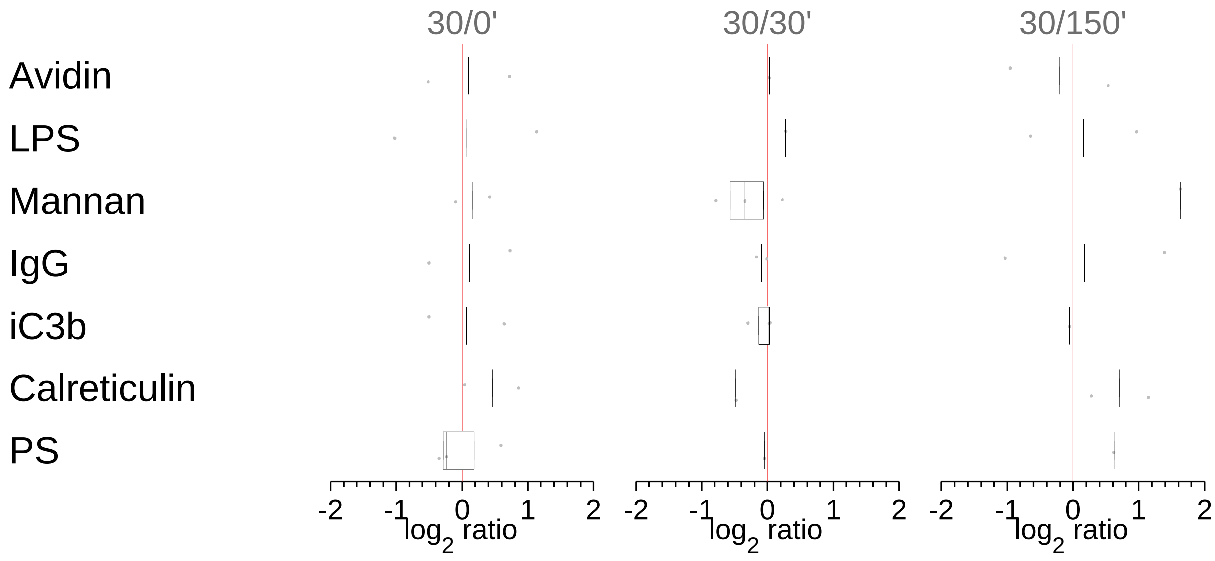

GO:0005871 kinesin complex (n = 6)

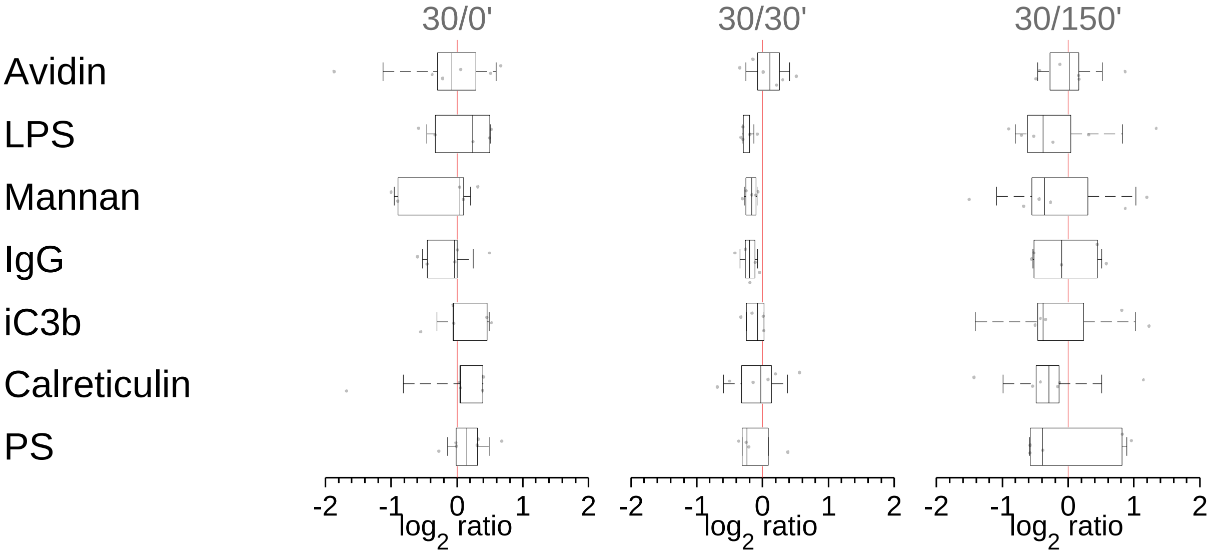

GO:0005874 microtubule (n = 50)

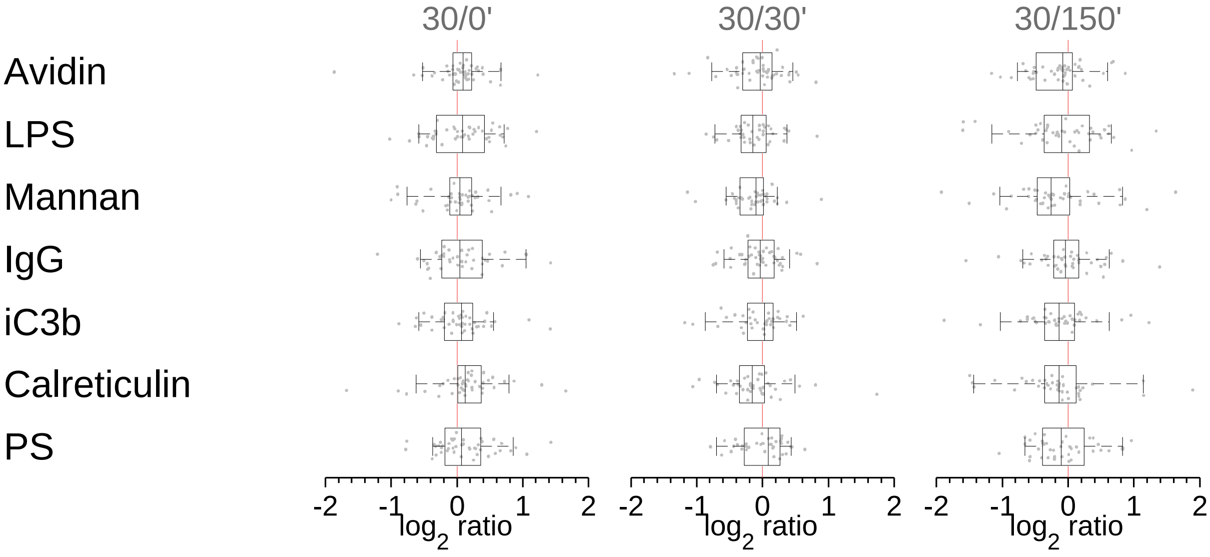

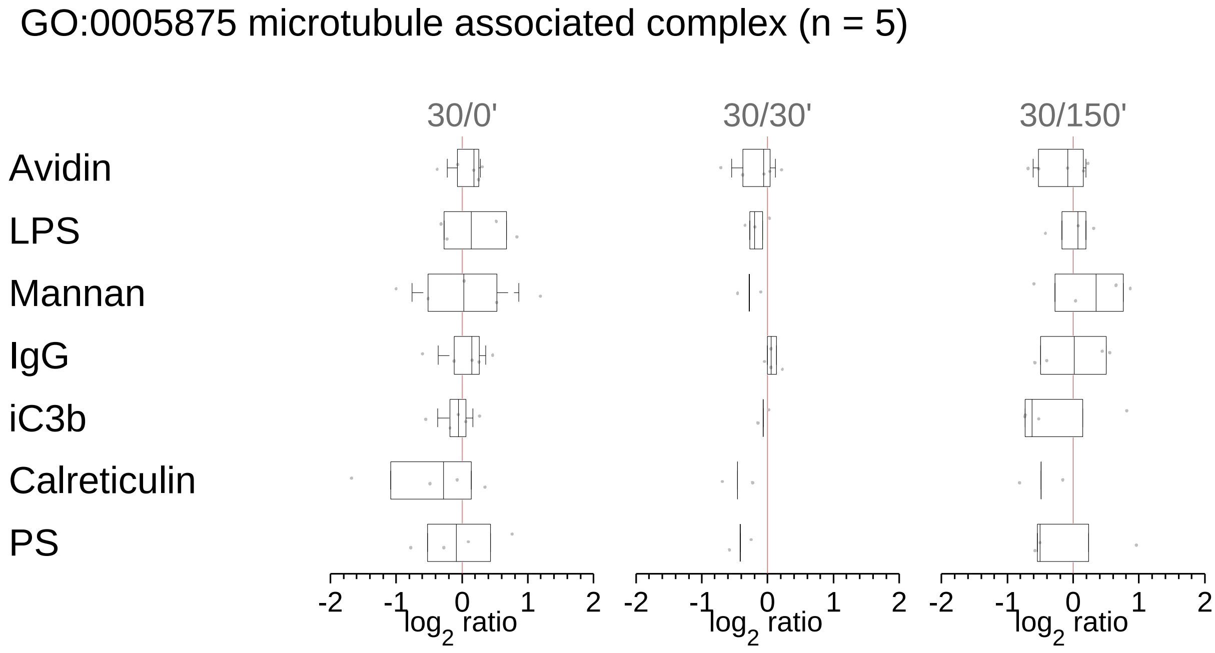

GO:0005875 microtubule associated complex (n = 5)
30/0'
30/30'
30/150'
Avidin
LPS
Mannan
IgG
iC3b
Calreticulin
PS
-2
-1
0
1
2
log2 ratio

GO:0005876 spindle microtubule (n = 4)

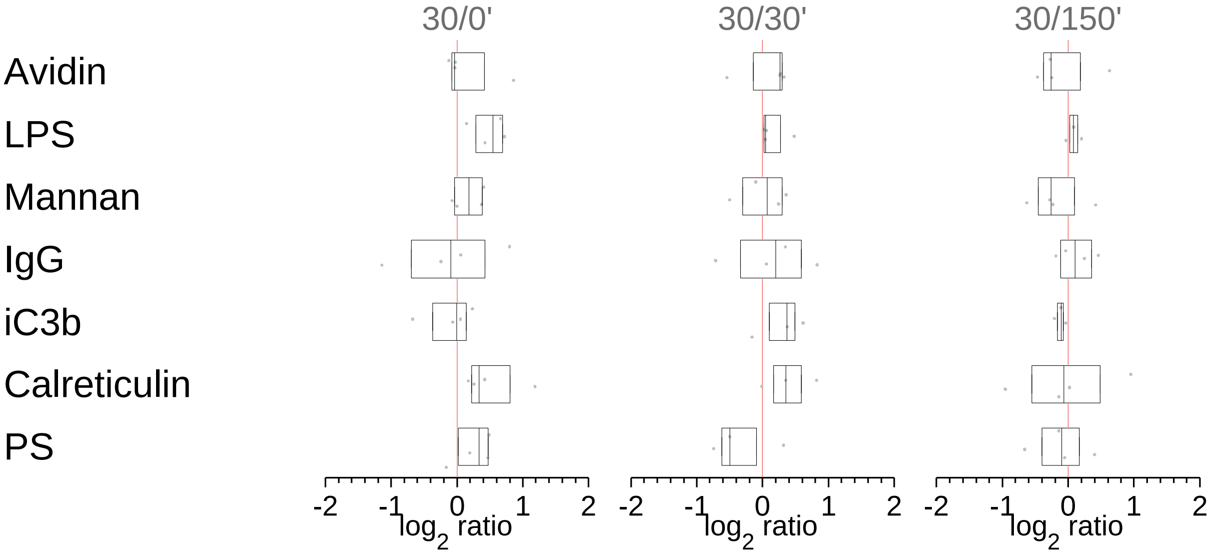

GO:0005881 cytoplasmic microtubule (n = 4)

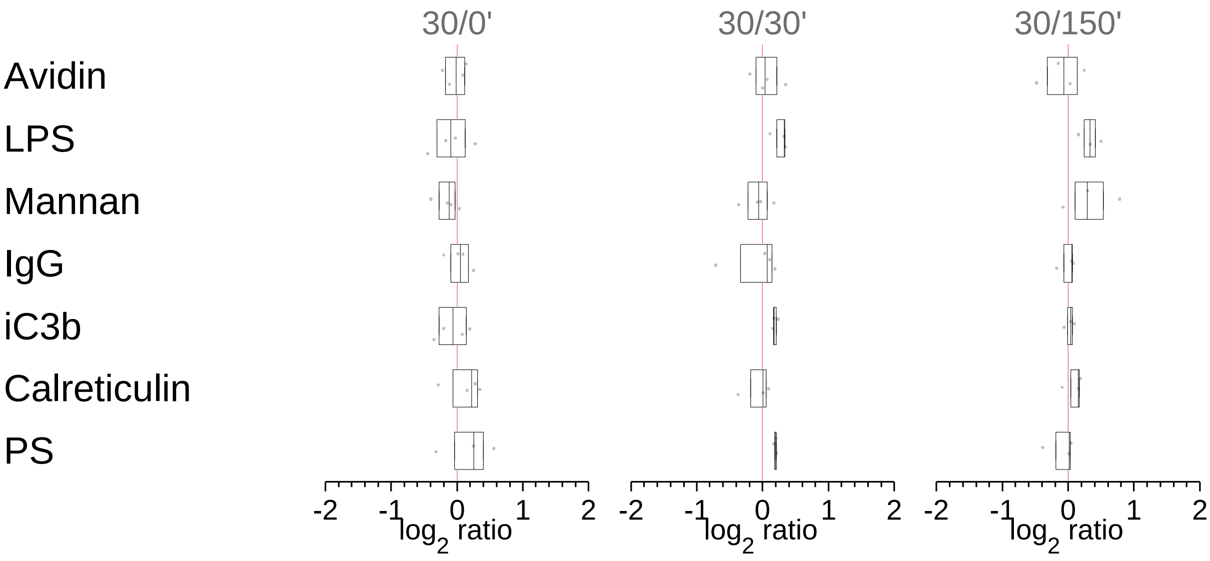

GO:0005882 intermediate filament (n = 11)

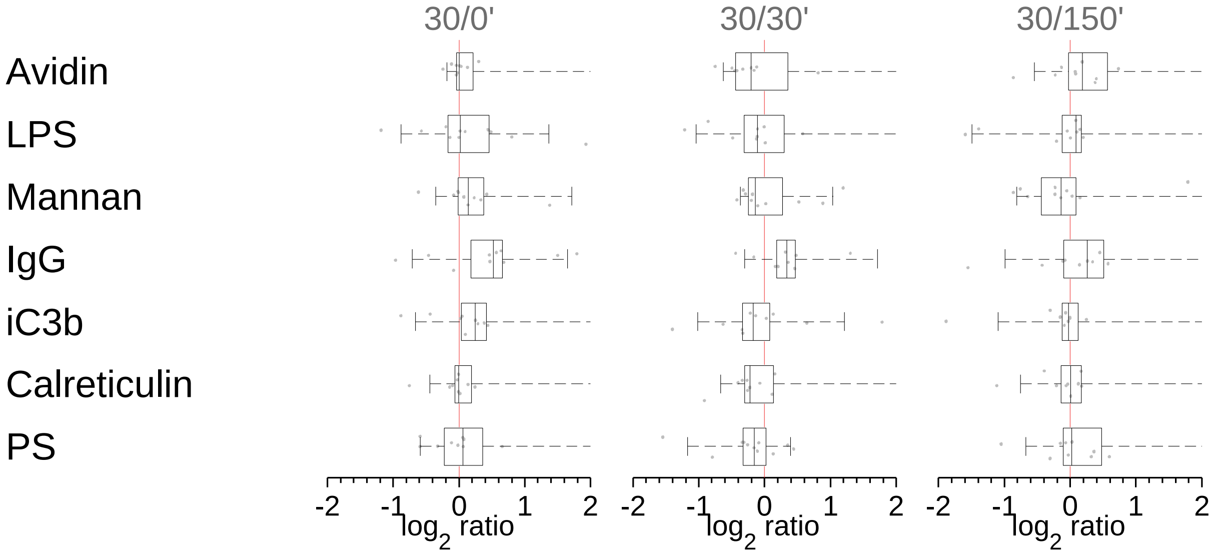

GO:0005884 actin filament (n = 18)

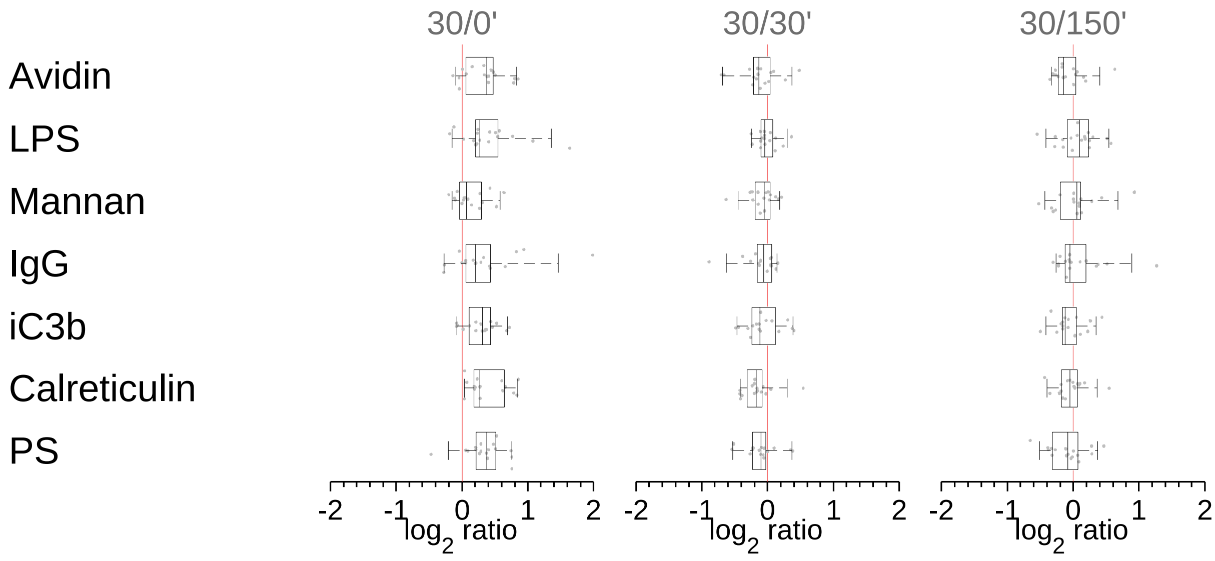

GO:0005885 Arp2/3 protein complex (n = 5)

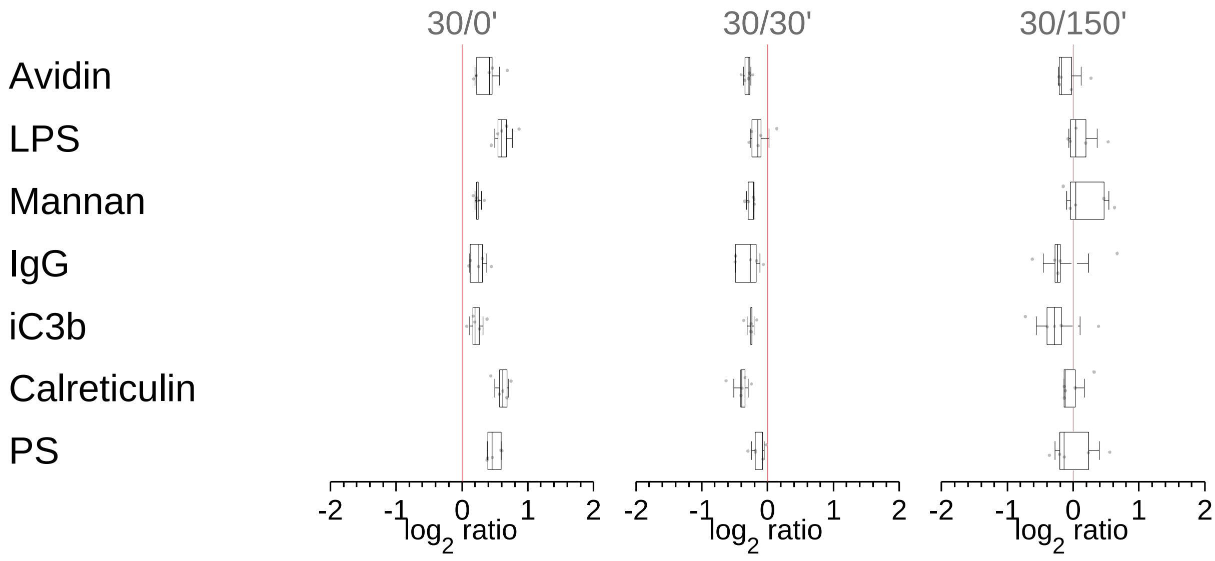

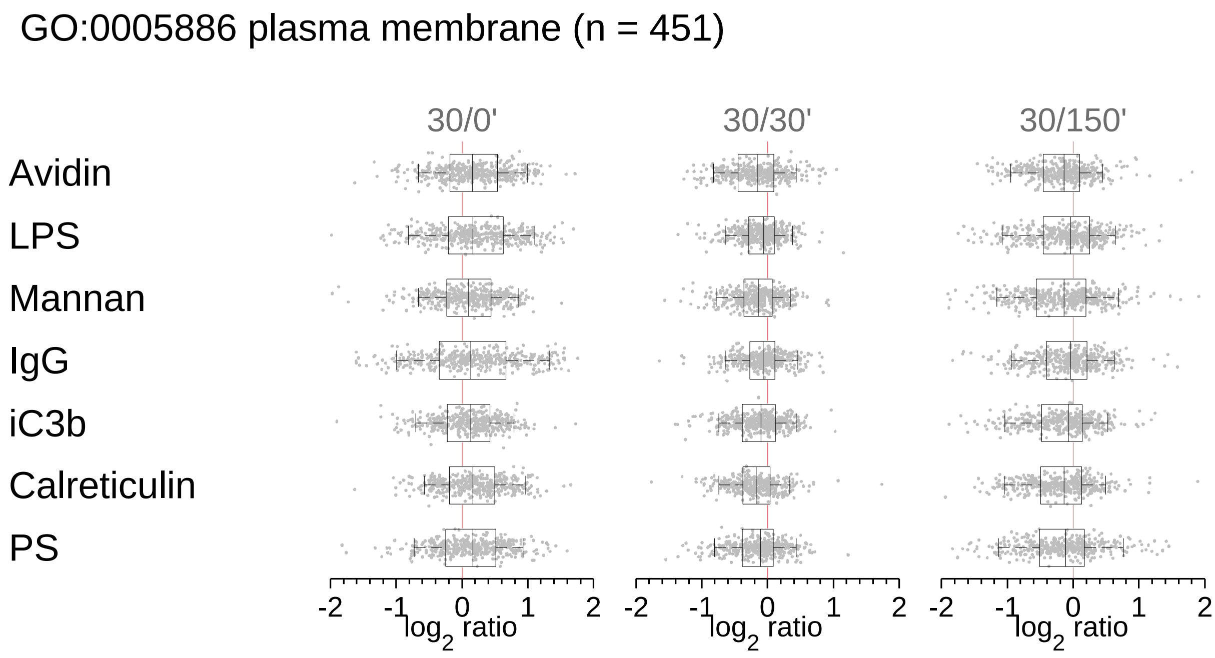
GO:0005886 plasma membrane (n = 451)
30/0'
30/30'
30/150'
Avidin
LPS
Mannan
IgG
iC3b
Calreticulin
PS
-2 -1 0 1 2
log2 ratio

GO:0005887 integral to plasma membrane (n = 50)

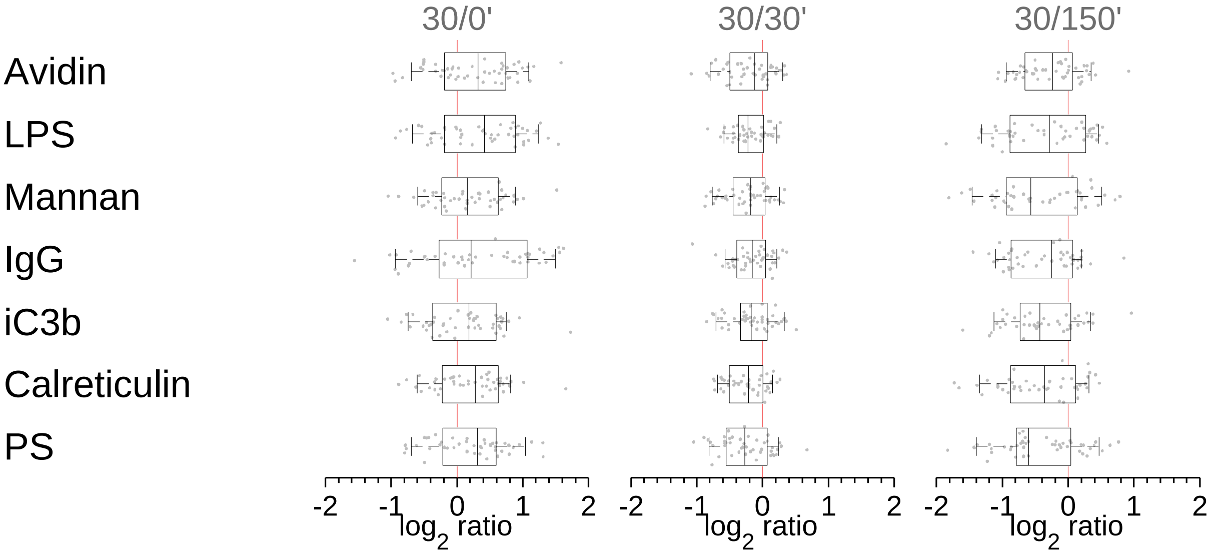

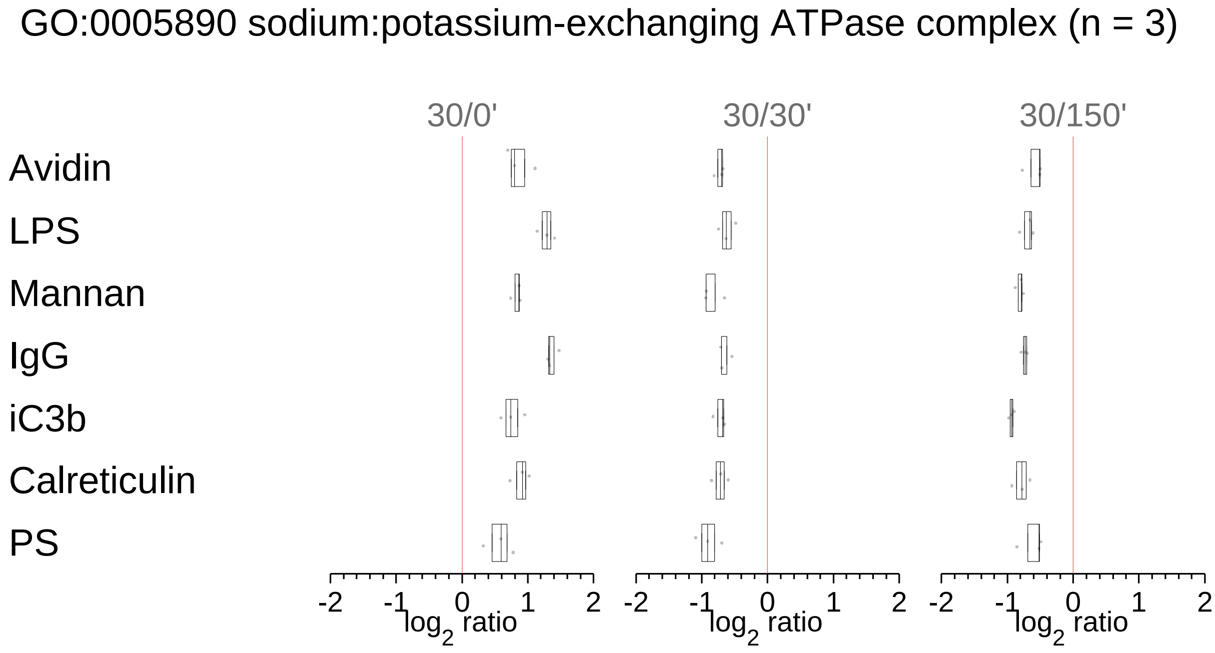
GO:0005890 sodium:potassium-exchanging ATPase complex (n = 3)
30/0'
30/30'
30/150'
Avidin
LPS
Mannan
IgG
iC3b
Calreticulin
PS
-2 -1 0 1 2
log2 ratio
-2 -1 0 1 2
log2 ratio
-2 -1 0 1 2
log2 ratio

GO:0005901 caveola (n = 15)

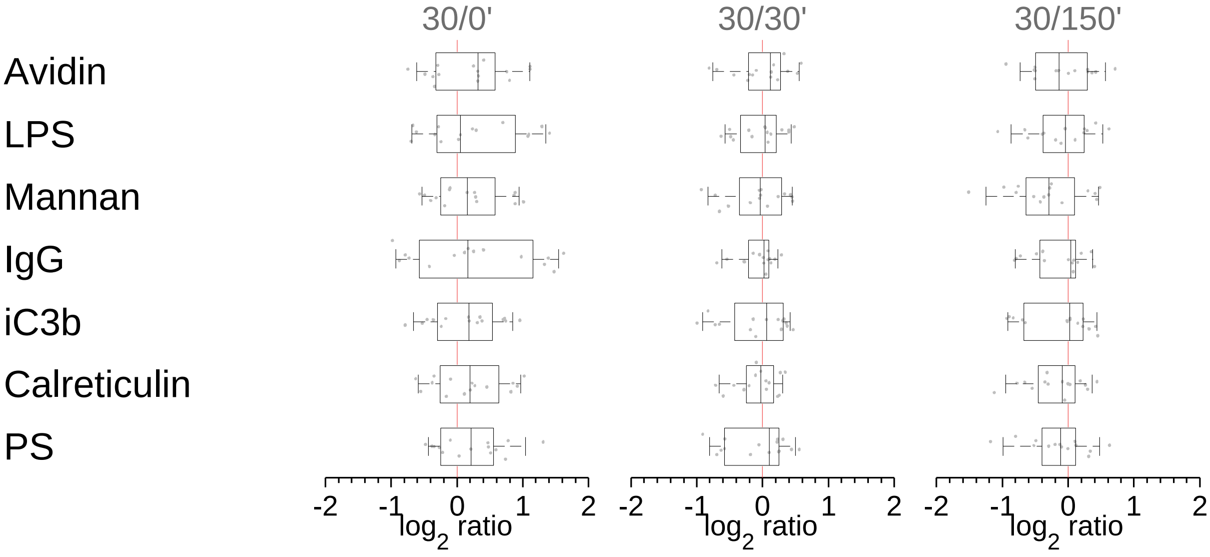

GO:0005902 microvillus (n = 13)

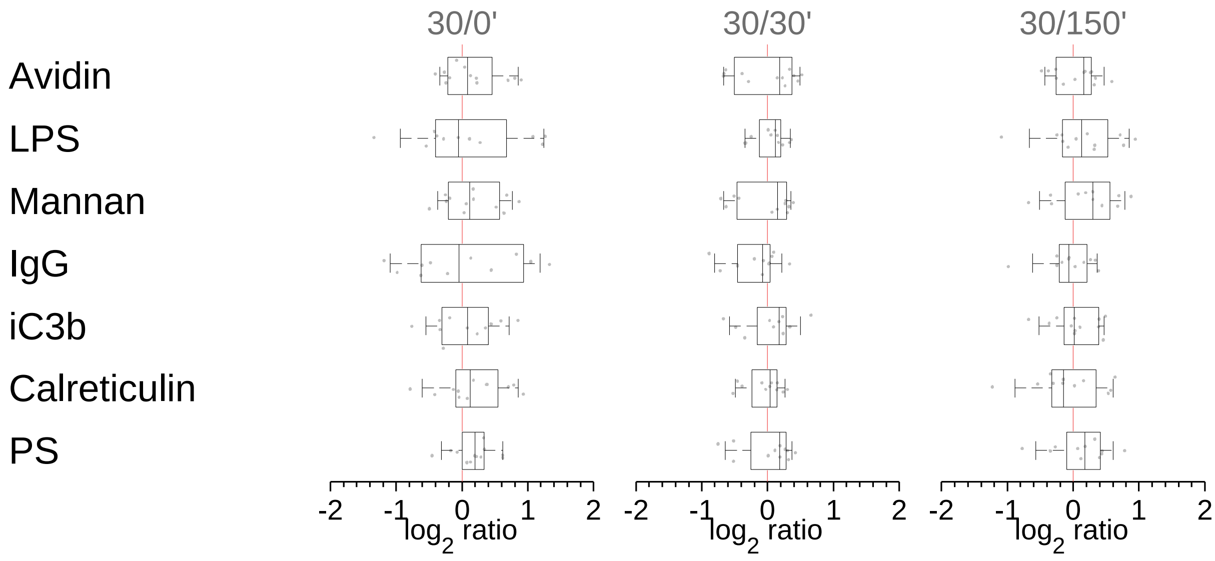

GO:0005905 coated pit (n = 21)

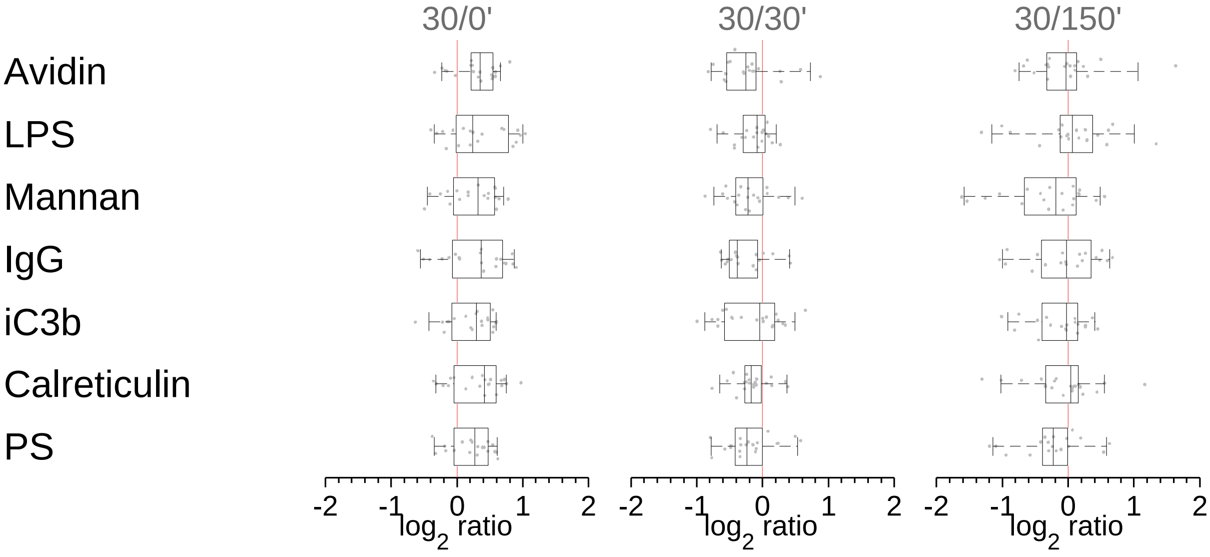

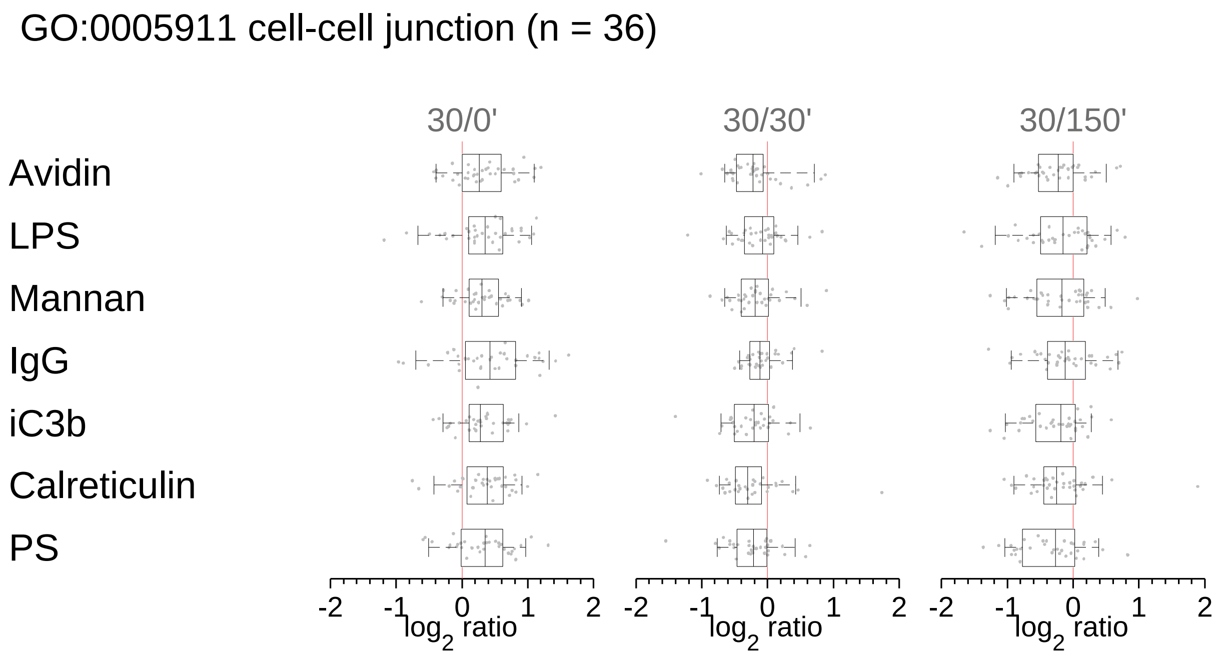
GO:0005911 cell-cell junction (n = 36)
30/0'
30/30'
30/150'
Avidin
LPS
Mannan
IgG
iC3b
Calreticulin
PS
-2
-1
0
1
2
log2 ratio

GO:0005912 adherens junction (n = 5)

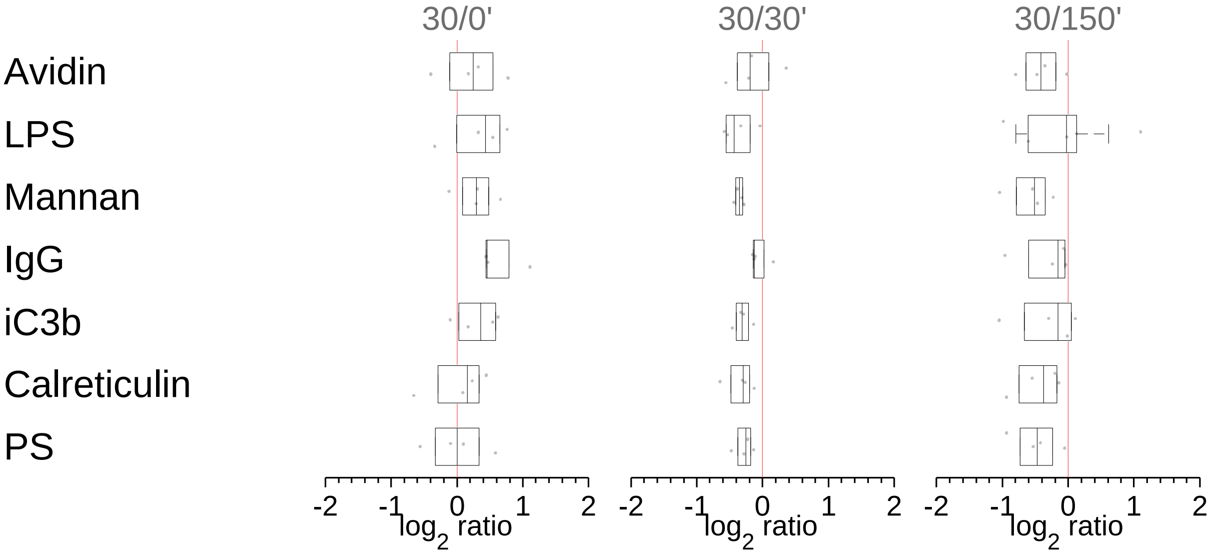

GO:0005913 cell-cell adherens junction (n = 7)

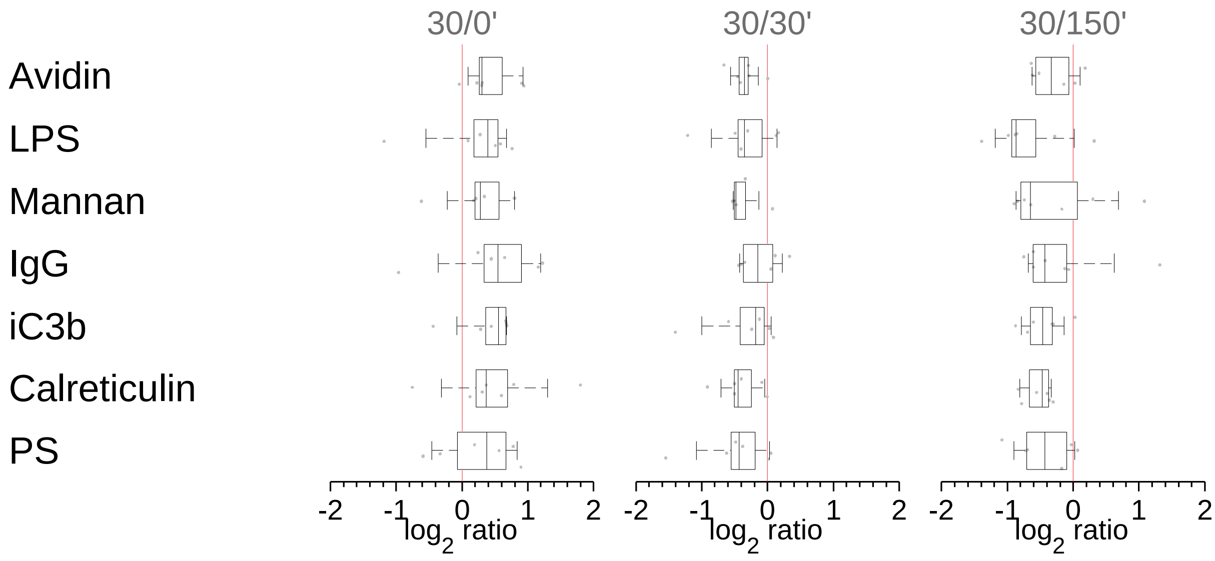

GO:0005923 tight junction (n = 10)

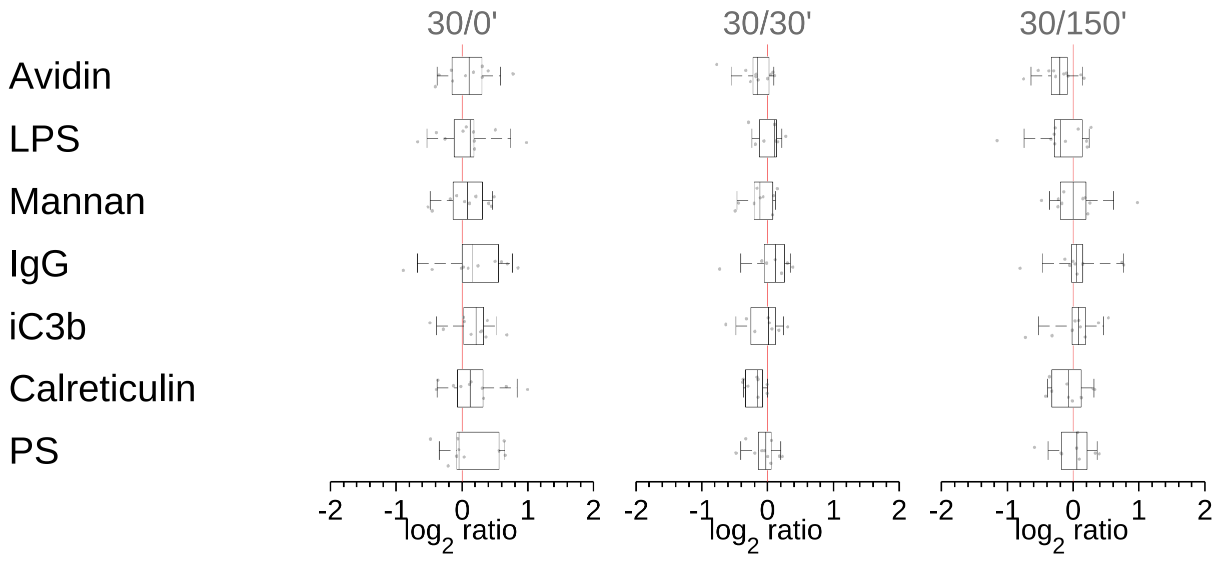

GO:0005925 focal adhesion (n = 27)

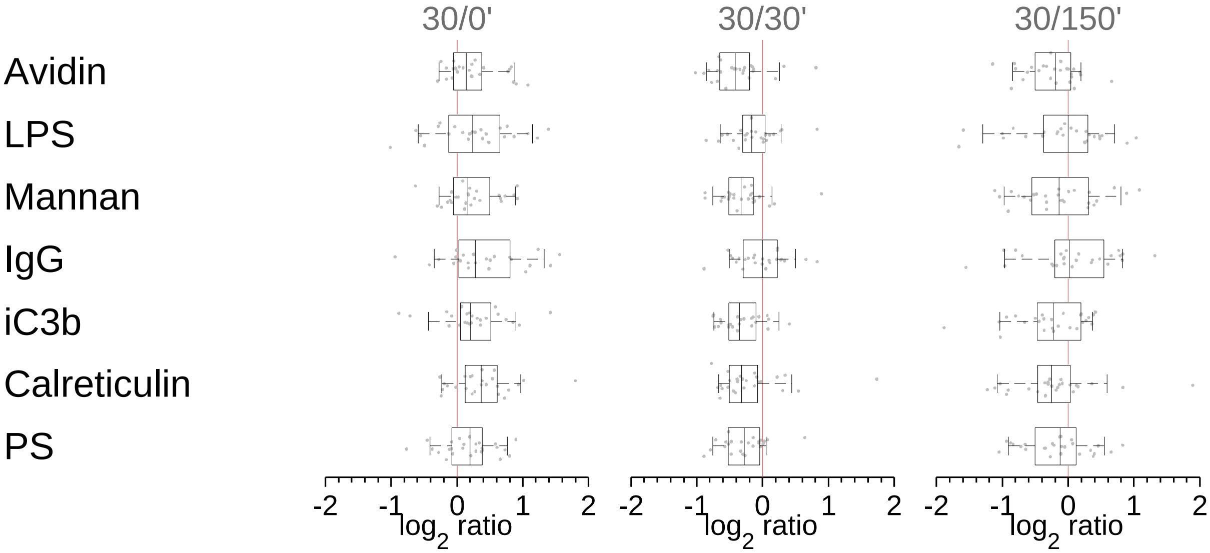

GO:0005929 cilium (n = 12)

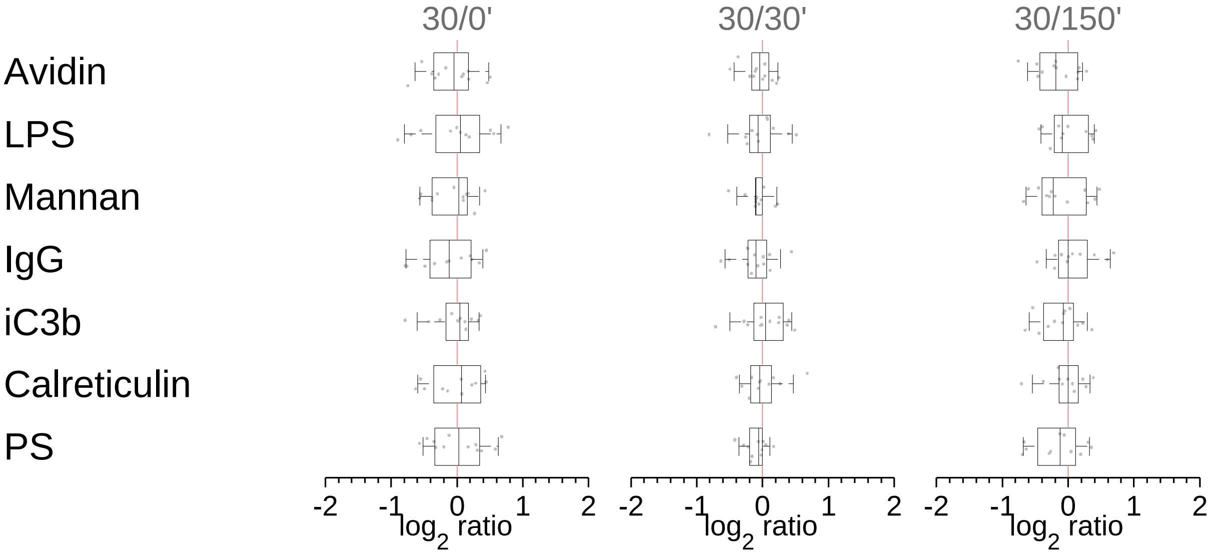

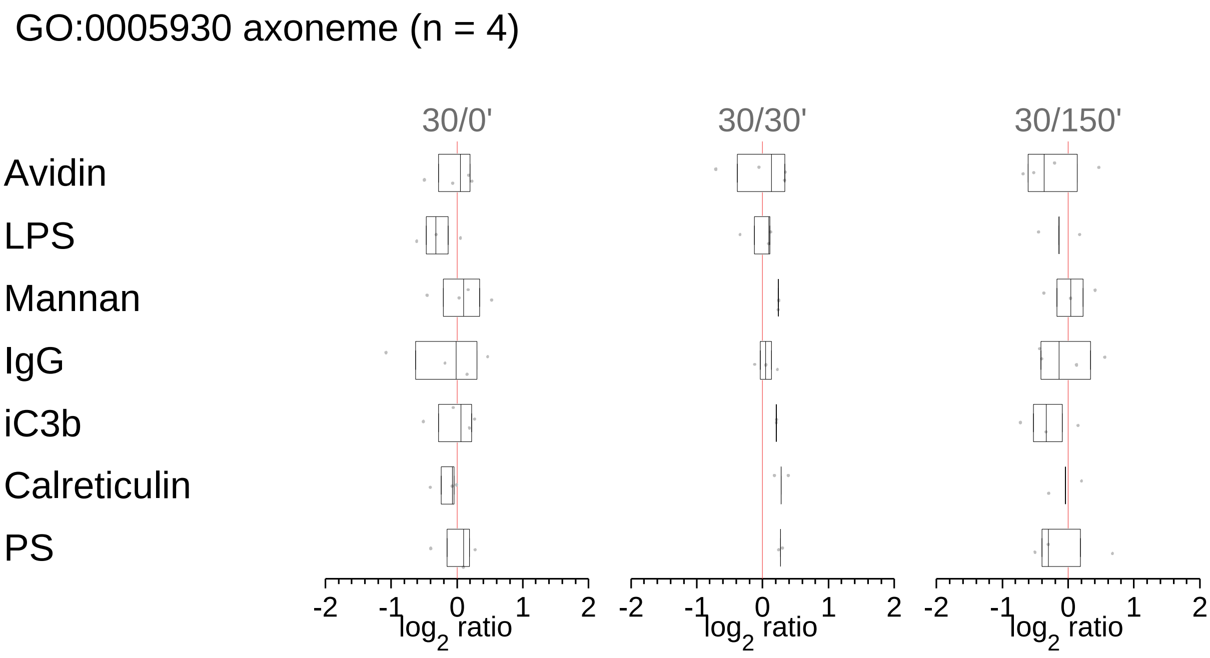
GO:0005930 axoneme (n = 4)
30/0'
30/30'
30/150'
Avidin
LPS
Mannan
IgG
iC3b
Calreticulin
PS
-2 -1 0 1 2
log2 ratio
-2 -1 0 1 2
log2 ratio
-2 -1 0 1 2
log2 ratio

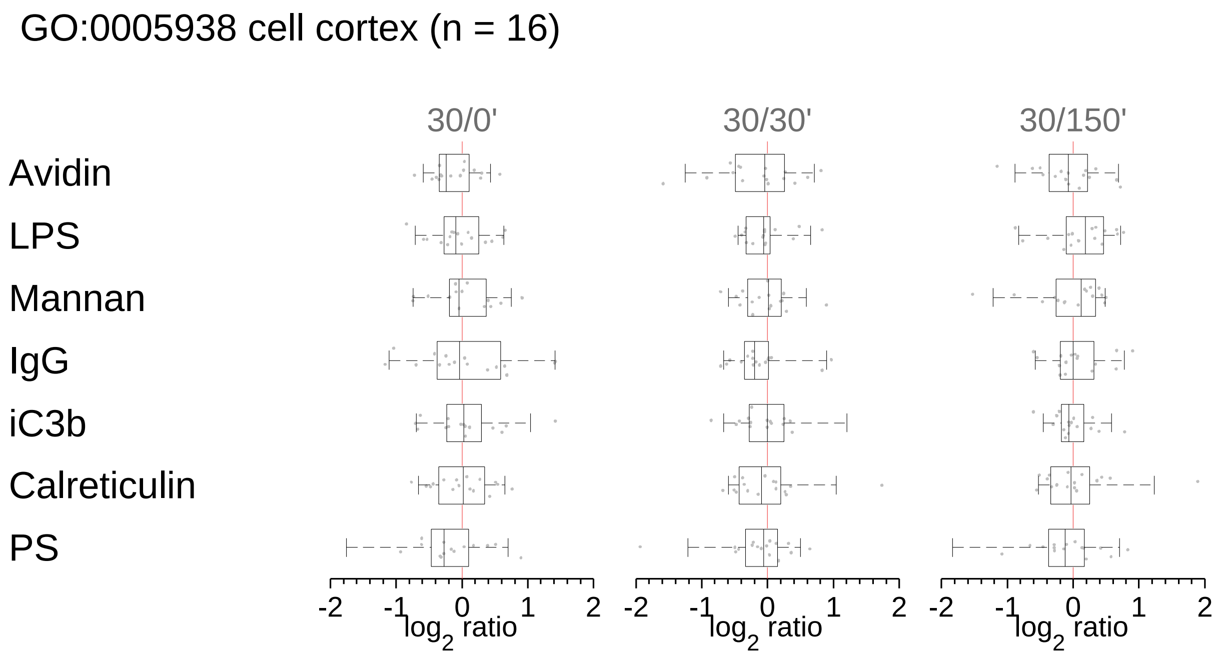
GO:0005938 cell cortex (n = 16)
30/0'
30/30'
30/150'
Avidin
LPS
Mannan
IgG
iC3b
Calreticulin
PS
-2 -1 0 1 2
log2 ratio

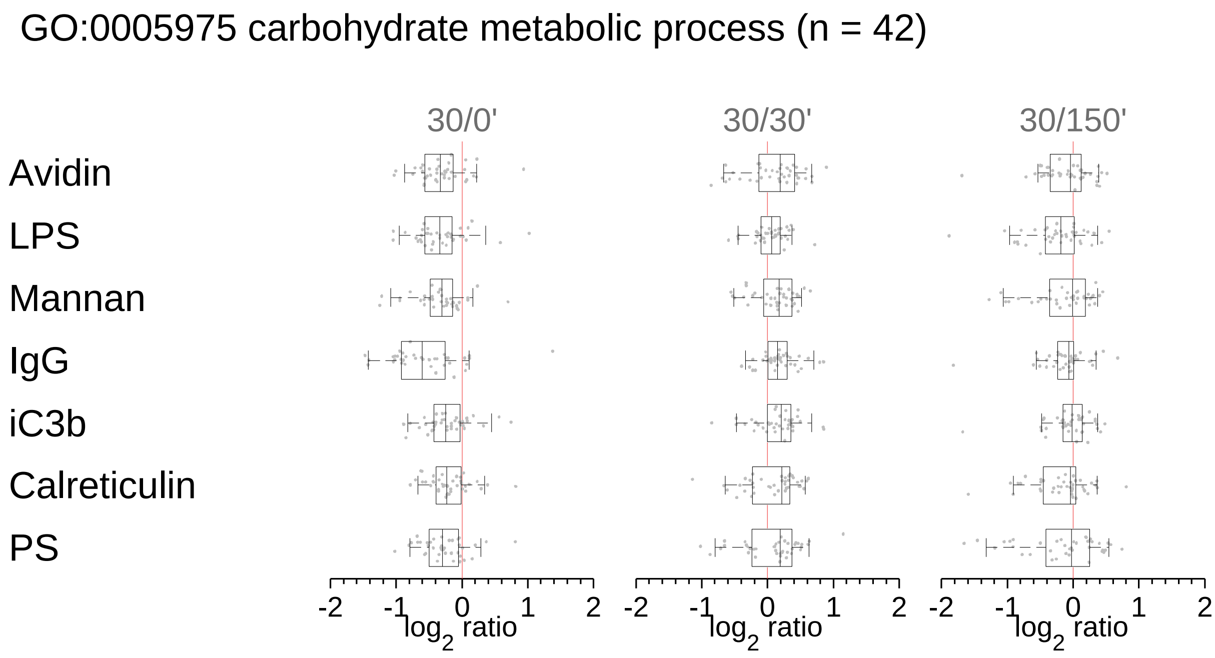
GO:0005975 carbohydrate metabolic process (n = 42)
30/0'
30/30'
30/150'
Avidin
LPS
Mannan
IgG
iC3b
Calreticulin
PS
-2
-1
0
1
2
log2 ratio

GO:0005977 glycogen metabolic process (n = 3)

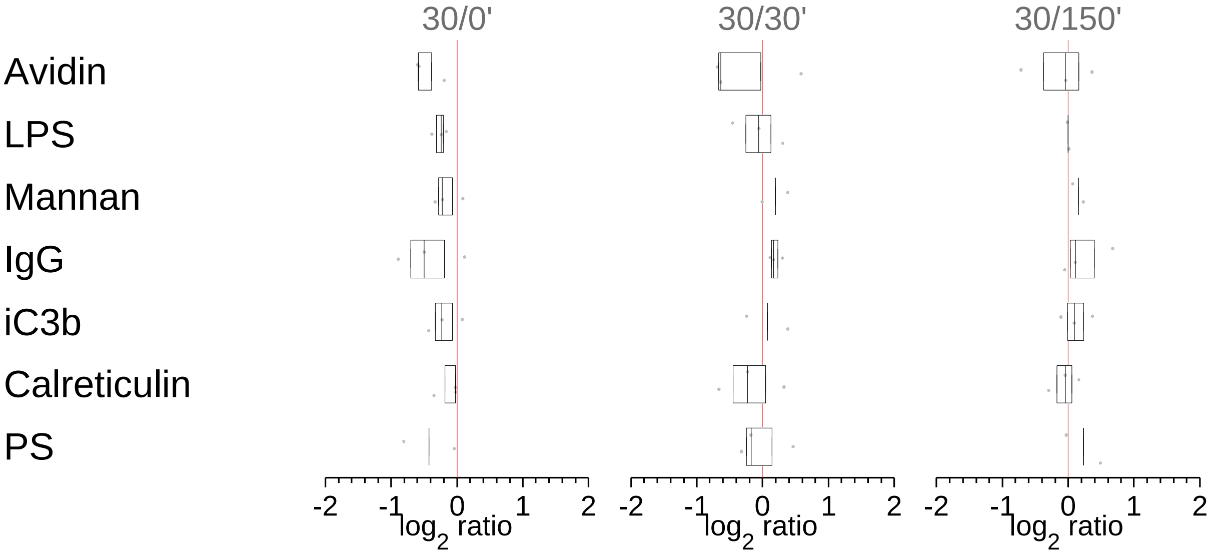

GO:0006006 glucose metabolic process (n = 10)

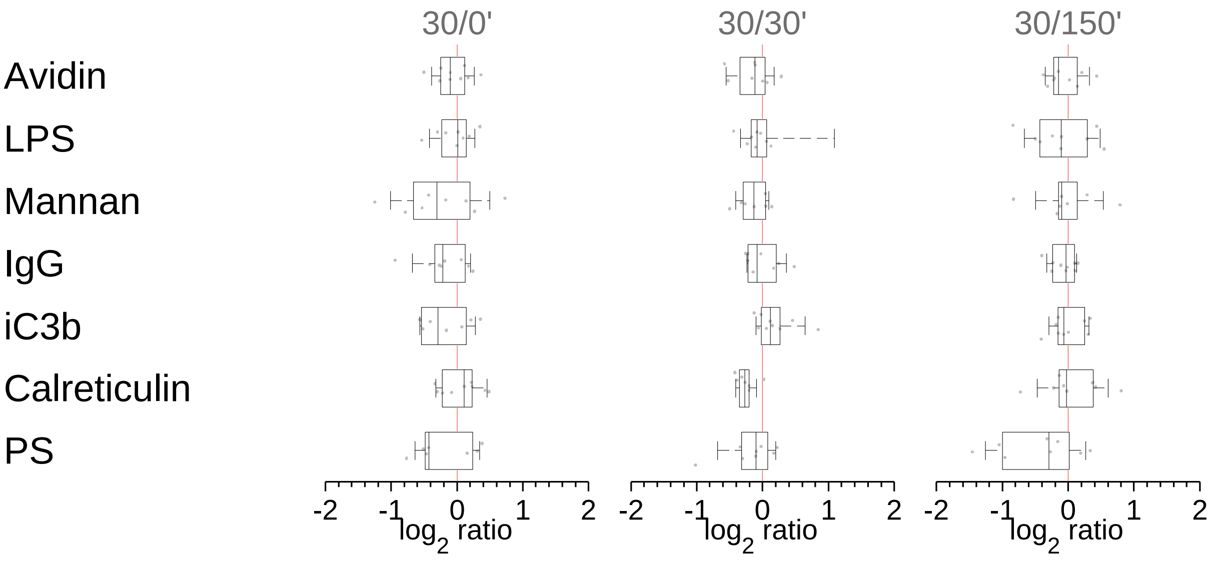

GO:0006013 mannose metabolic process (n = 3)

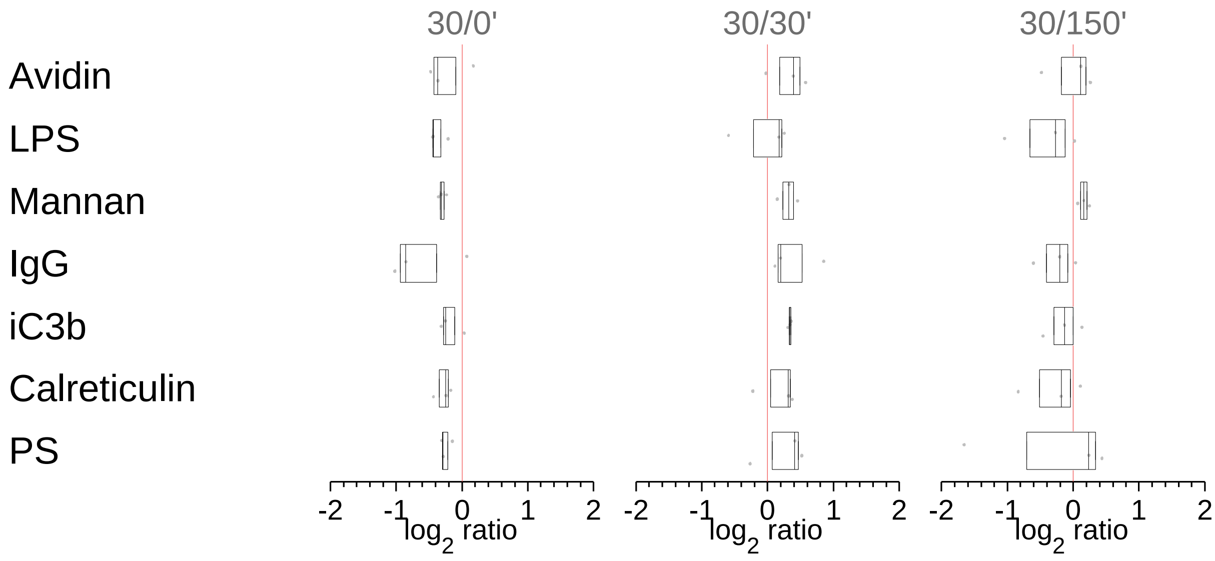

GO:0006032 chitin catabolic process (n = 3)

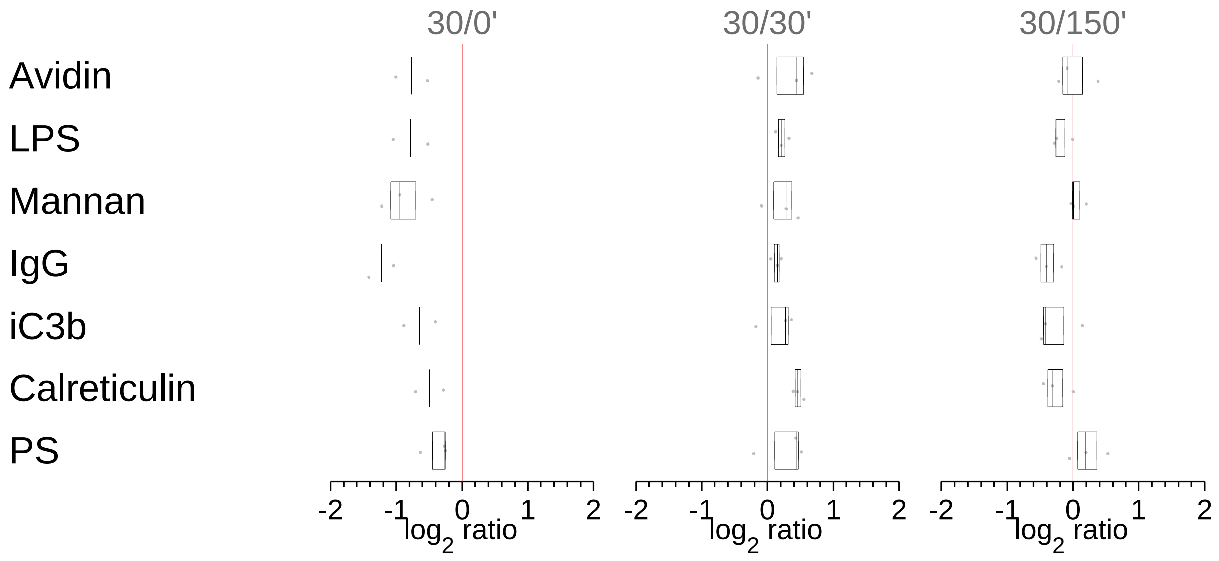

GO:0006081 cellular aldehyde metabolic process (n = 3)

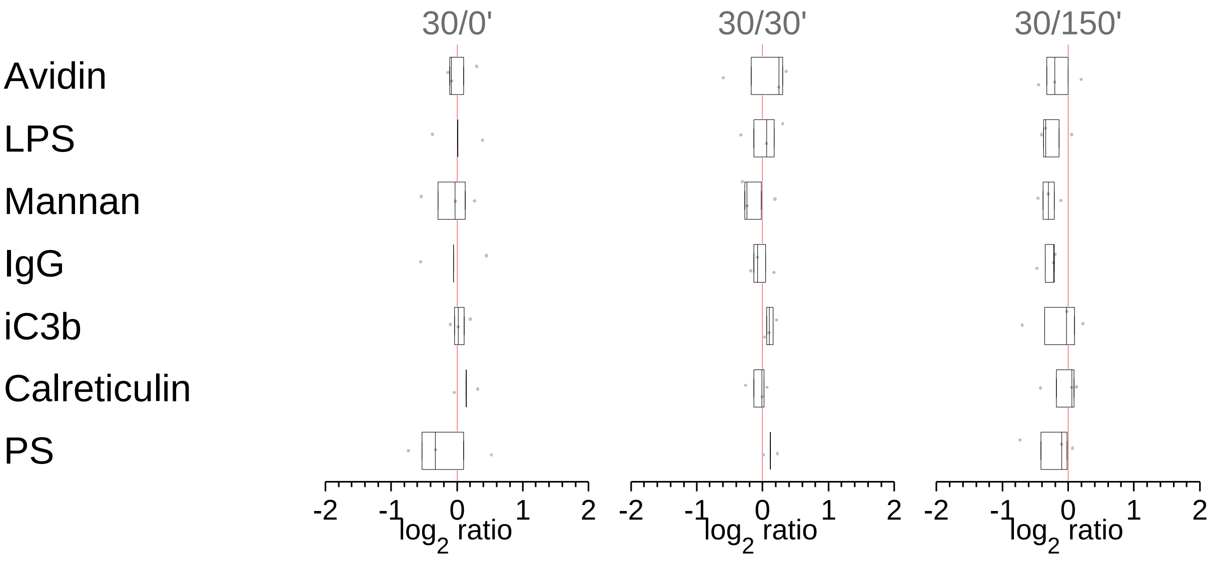

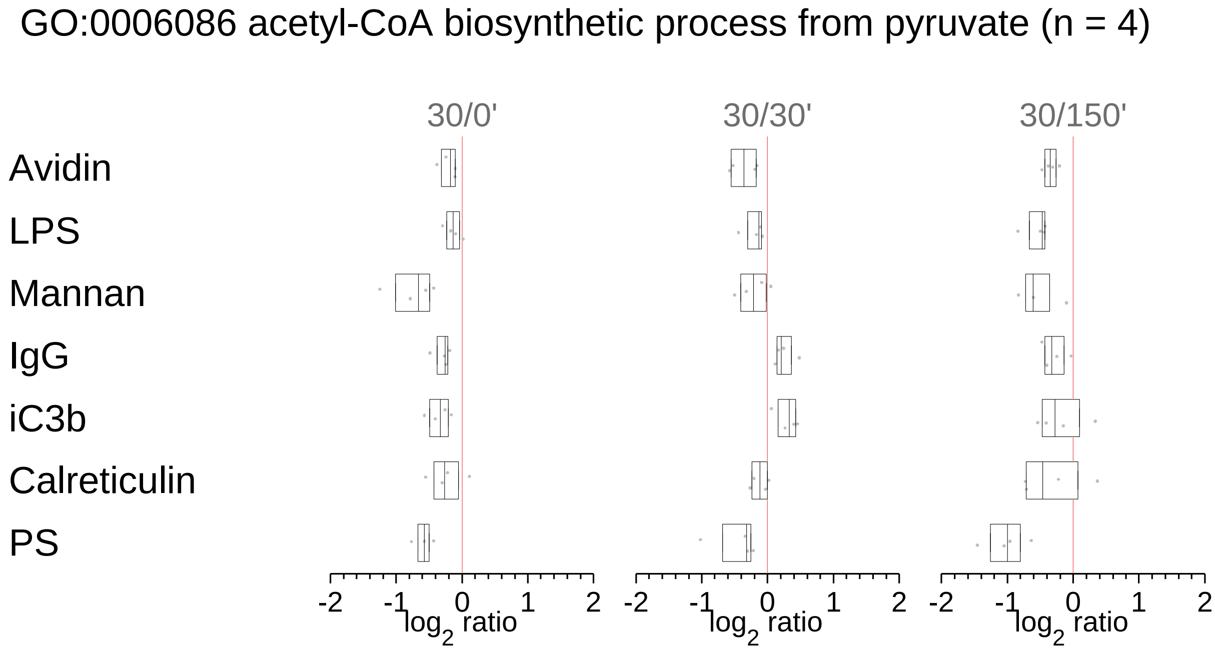
GO:0006086 acetyl-CoA biosynthetic process from pyruvate (n = 4)
30/0'
30/30'
30/150'
Avidin
LPS
Mannan
IgG
iC3b
Calreticulin
PS
-2
-1
0
1
2
log2 ratio

GO:0006094 gluconeogenesis (n = 5)

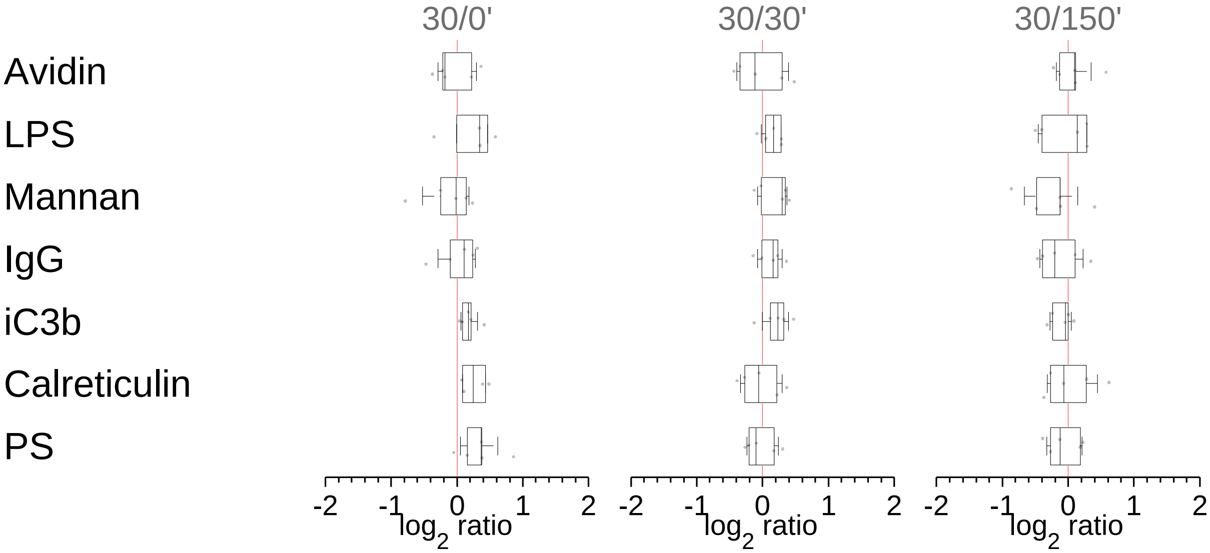

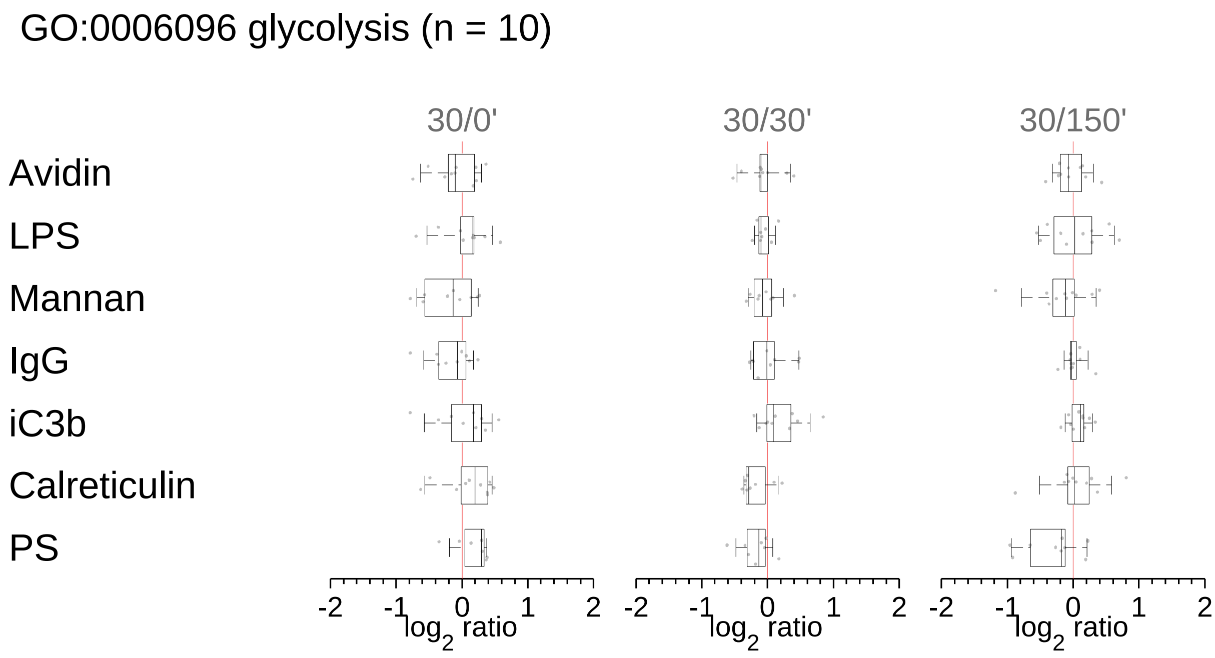

GO:0006096 glycolysis (n = 10)
30/0'
30/30'
30/150'
Avidin
LPS
Mannan
IgG
iC3b
Calreticulin
PS
-2 -1 0 1 2
log2 ratio
-2 -1 0 1 2
log2 ratio
-2 -1 0 1 2
log2 ratio

GO:0006098 pentose-phosphate shunt (n = 4)

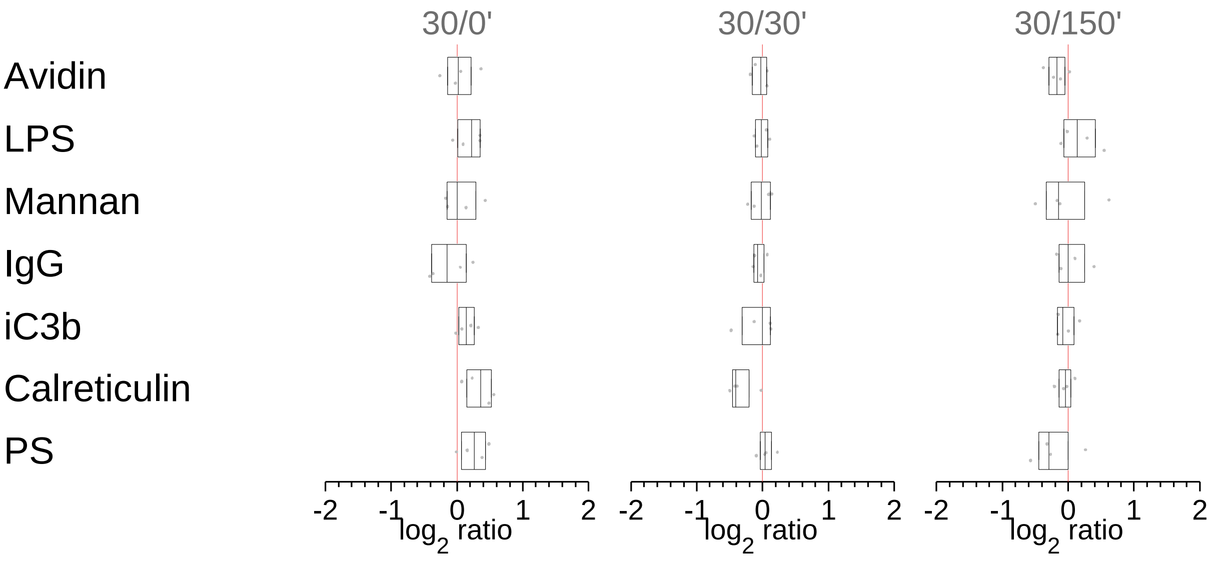

GO:0006099 tricarboxylic acid cycle (n = 18)

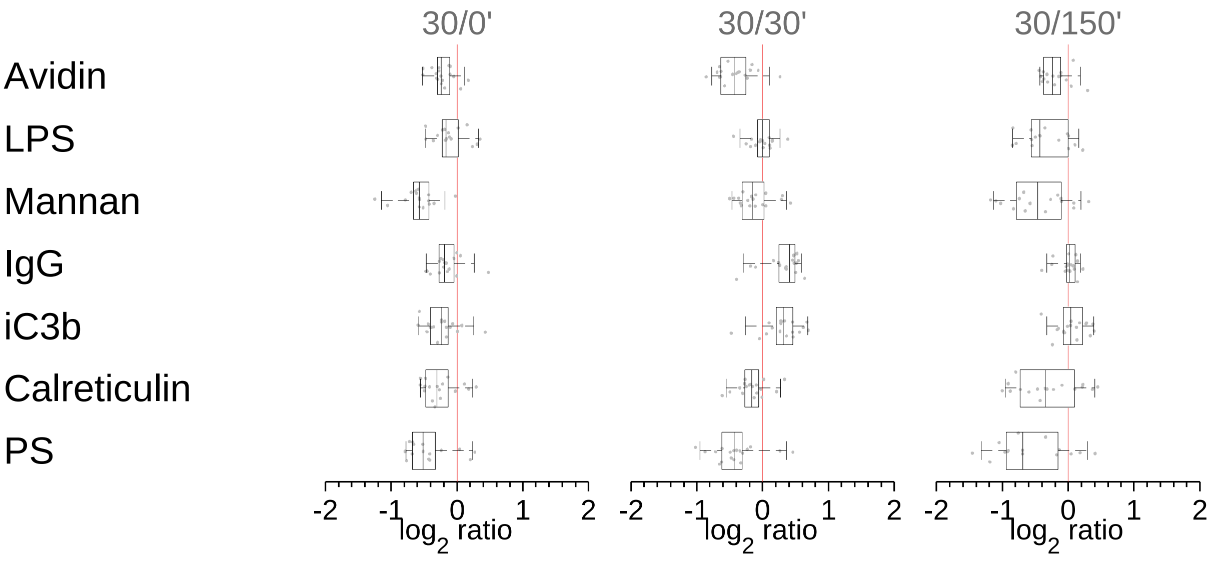

GO:0006102 isocitrate metabolic process (n = 5)

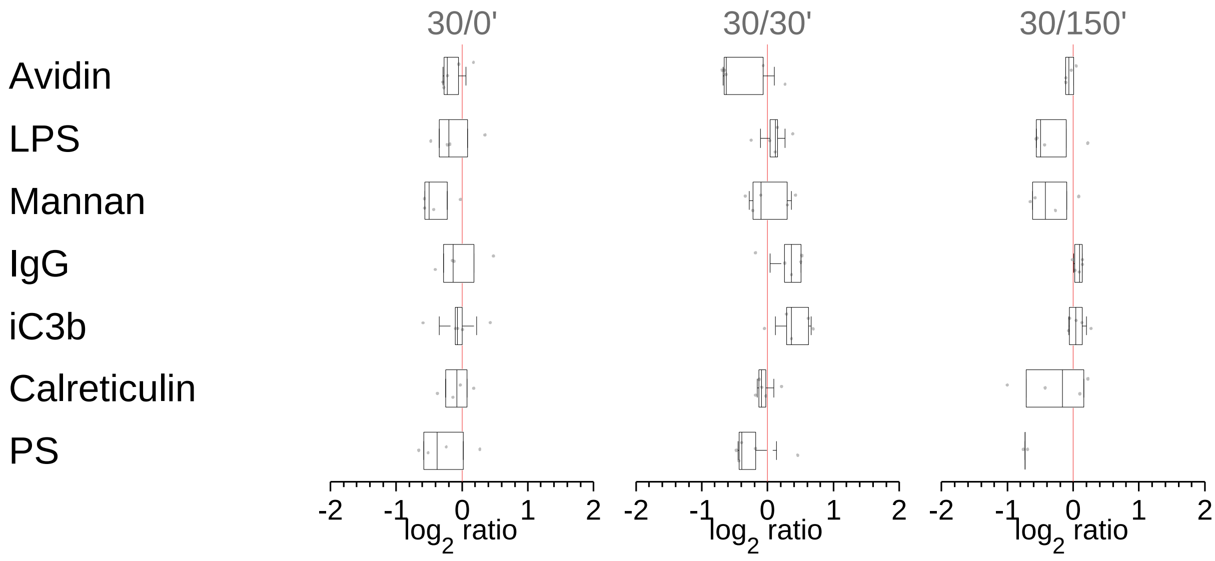

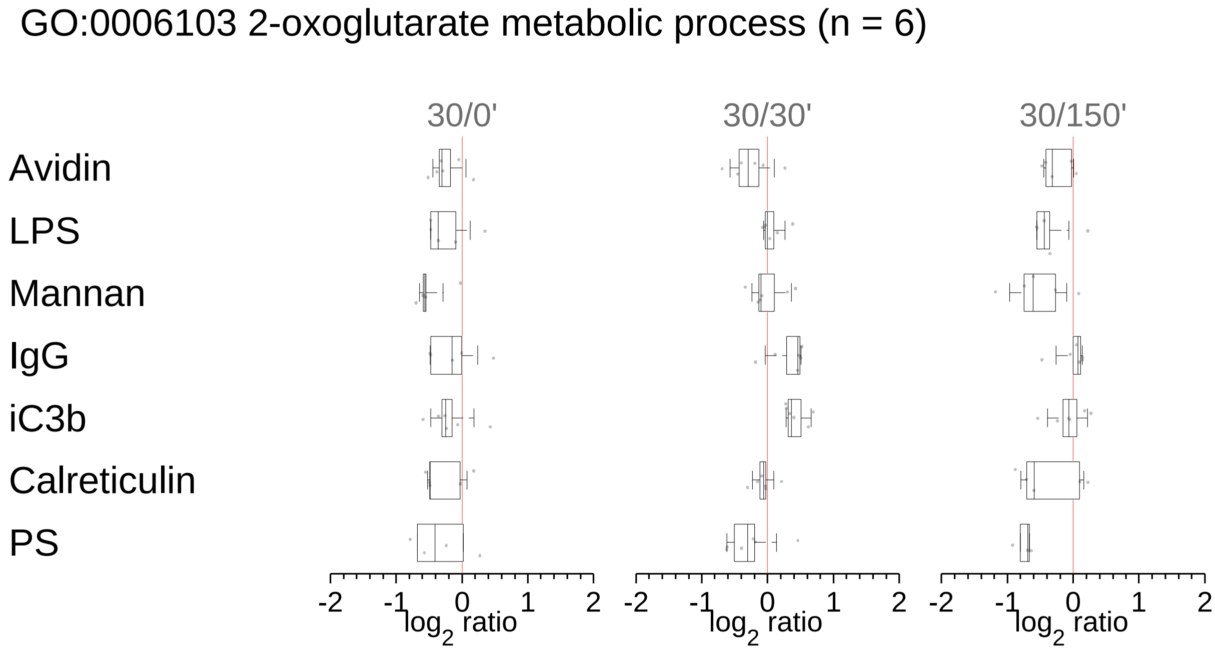
GO:0006103 2-oxoglutarate metabolic process (n = 6)
30/0'
30/30'
30/150'
Avidin
LPS
Mannan
IgG
iC3b
Calreticulin
PS
-2 -1 0 1 2
log2 ratio

GO:0006104 succinyl-CoA metabolic process (n = 3)

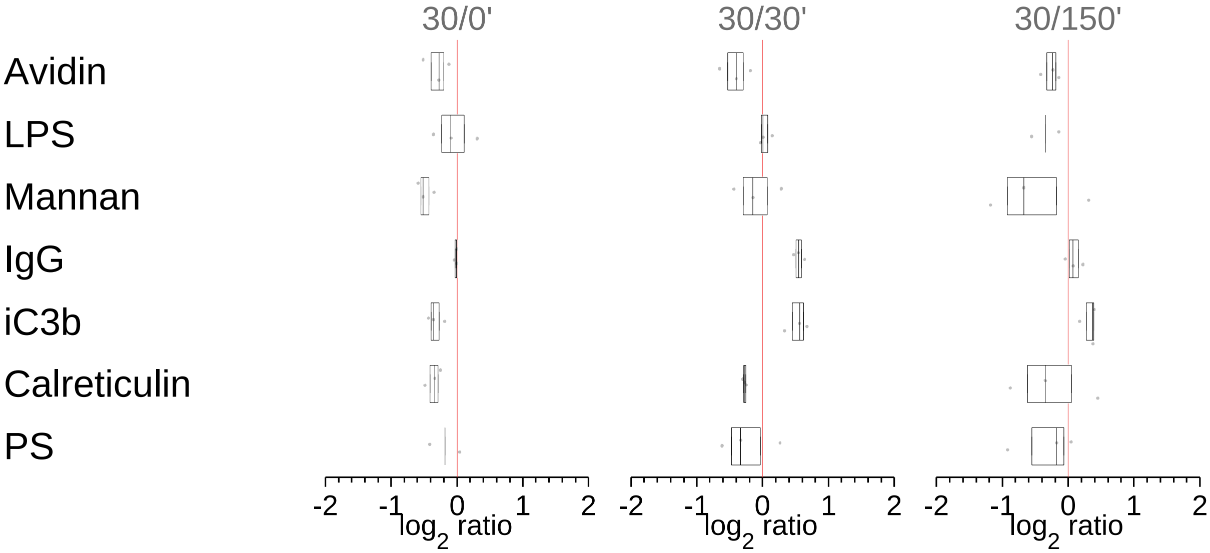

GO:0006105 succinate metabolic process (n = 4)

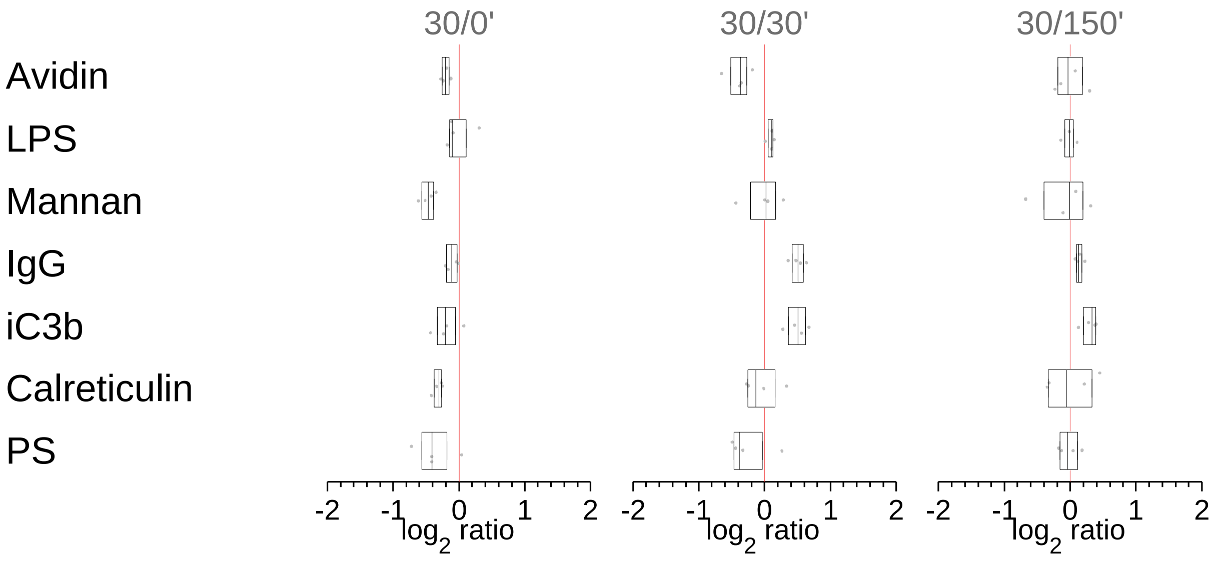

GO:0006107 oxaloacetate metabolic process (n = 5)

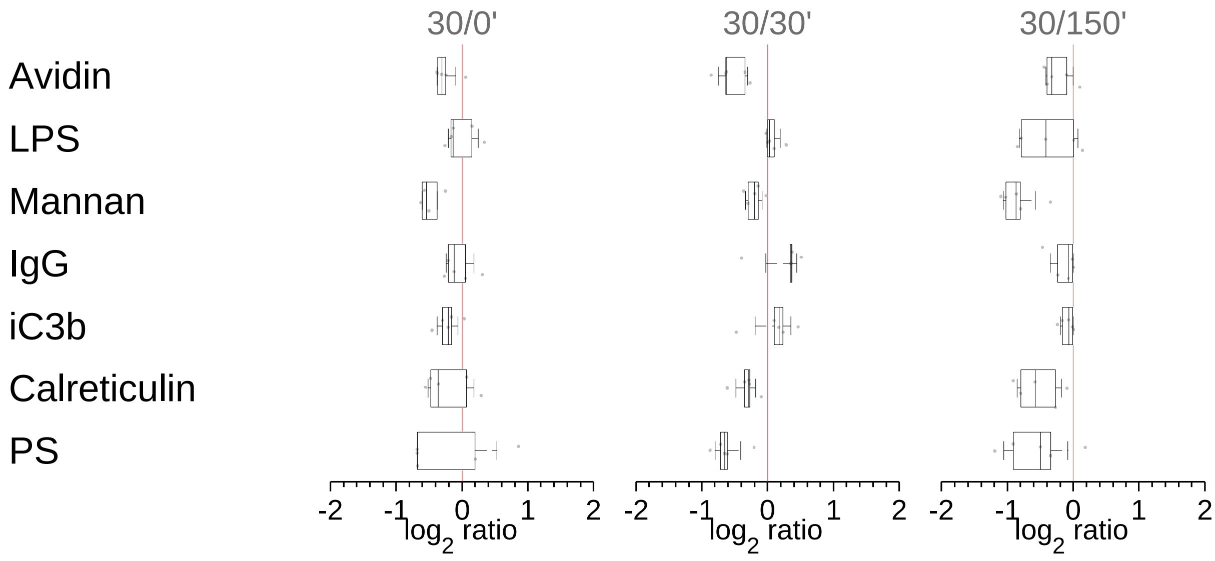

GO:0006108 malate metabolic process (n = 4)

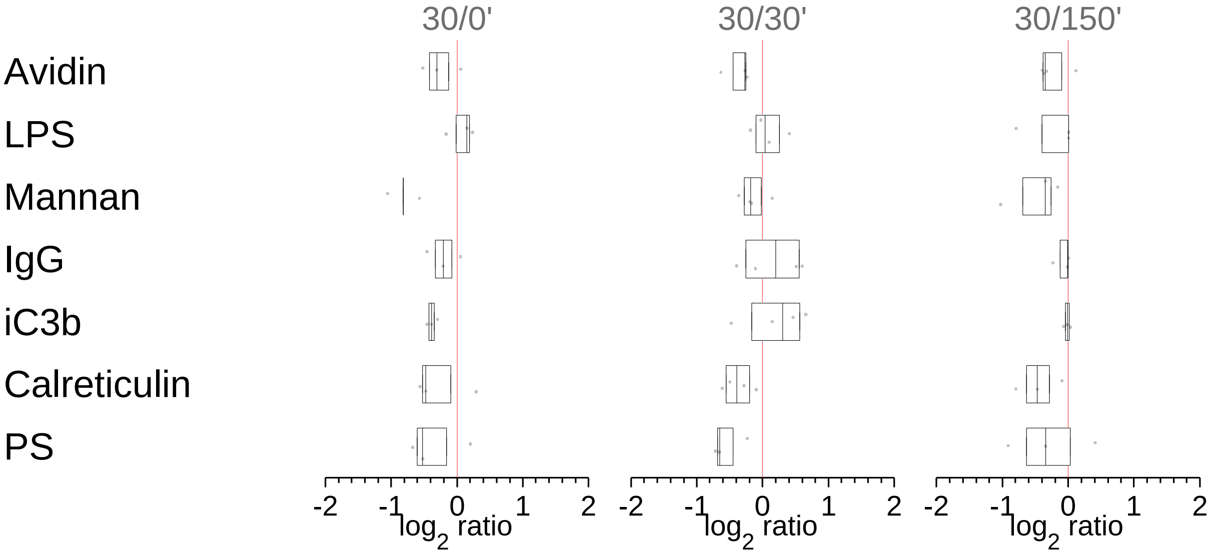

GO:0006120 mitochondrial electron transport, NADH to ubiquinone
(n = 3)

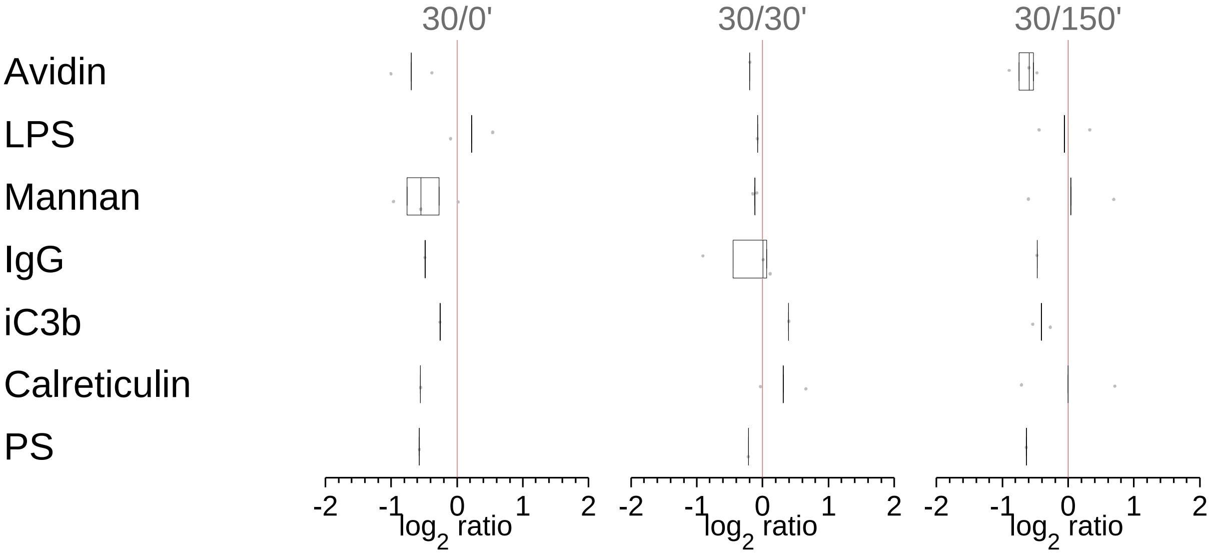

GO:0006122 mitochondrial electron transport, ubiquinol to cytochrome c (n = 3)

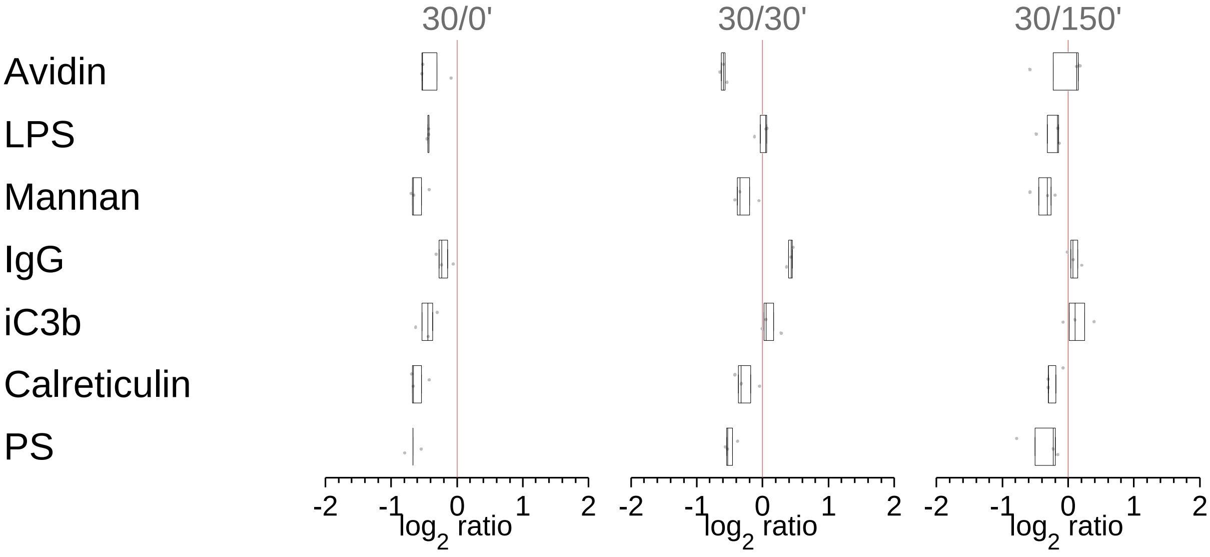

GO:0006139 nucleobase-containing compound metabolic process (n = 3)

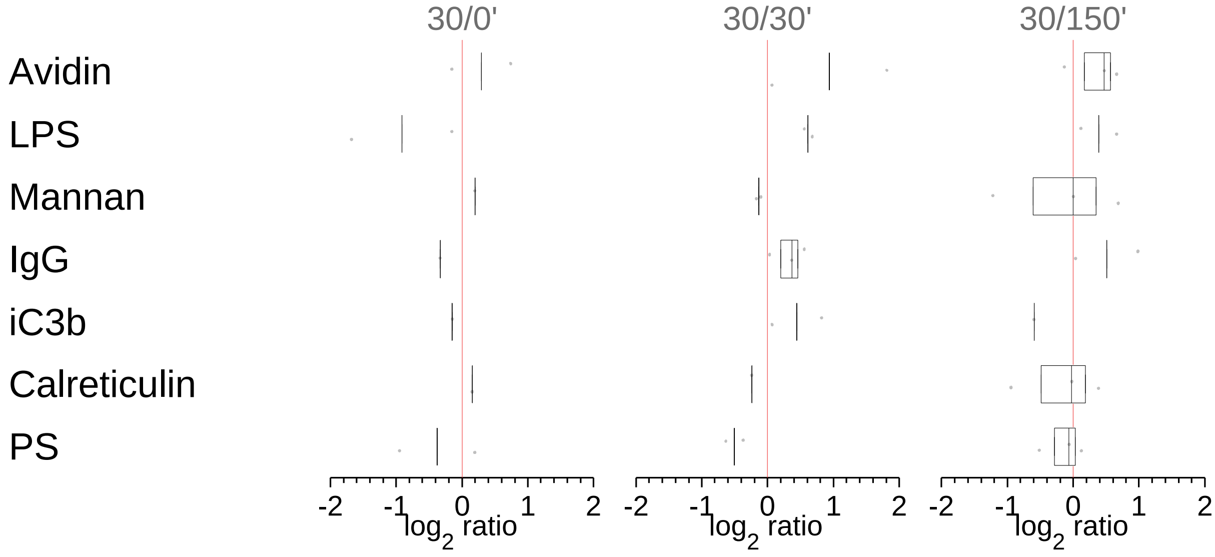

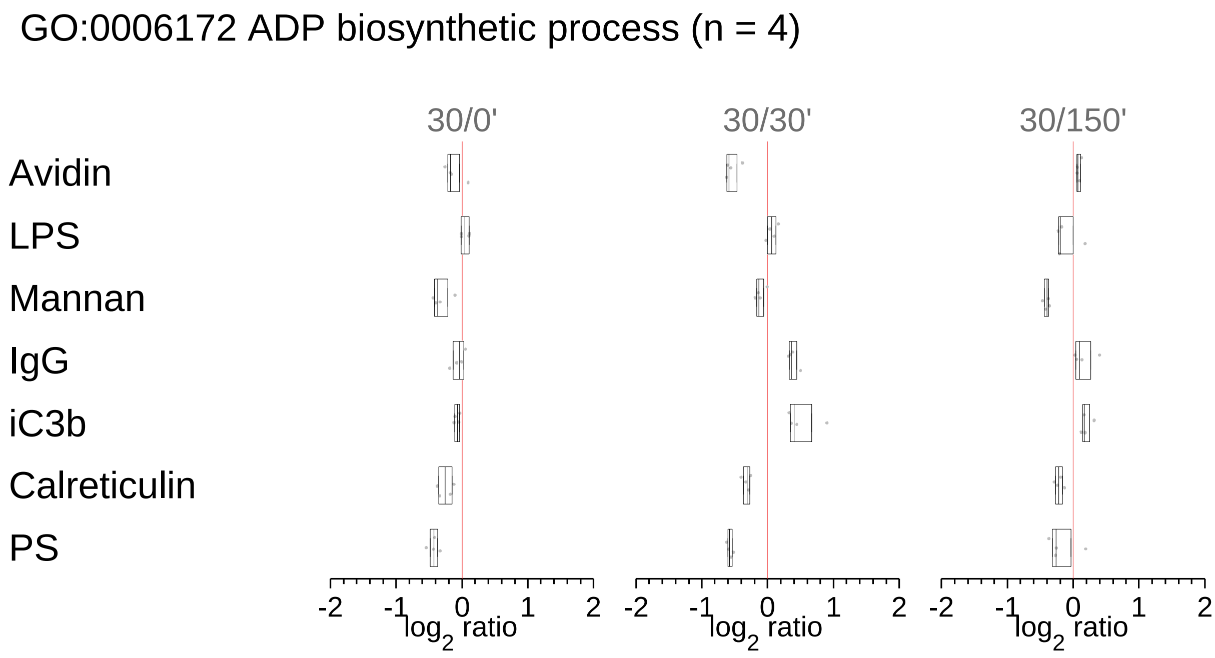

GO:0006172 ADP biosynthetic process (n = 4)
30/0'
30/30'
30/150'
Avidin
LPS
Mannan
IgG
iC3b
Calreticulin
PS
-2
-1
0
1
2
log2 ratio

GO:0006183 GTP biosynthetic process (n = 3)

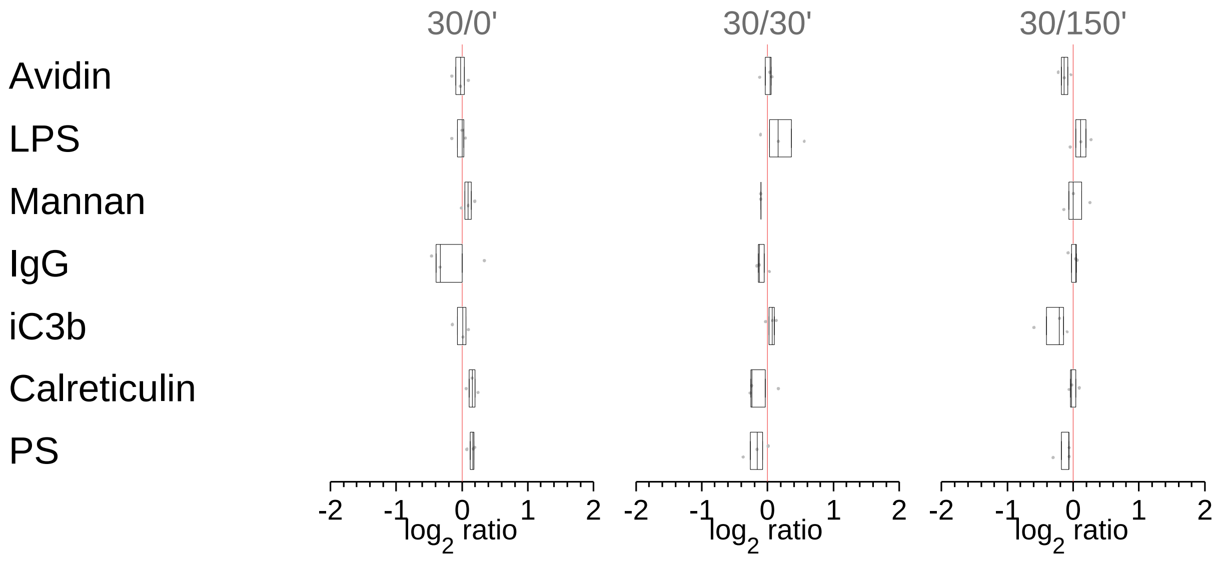

GO:0006184 GTP catabolic process (n = 59)

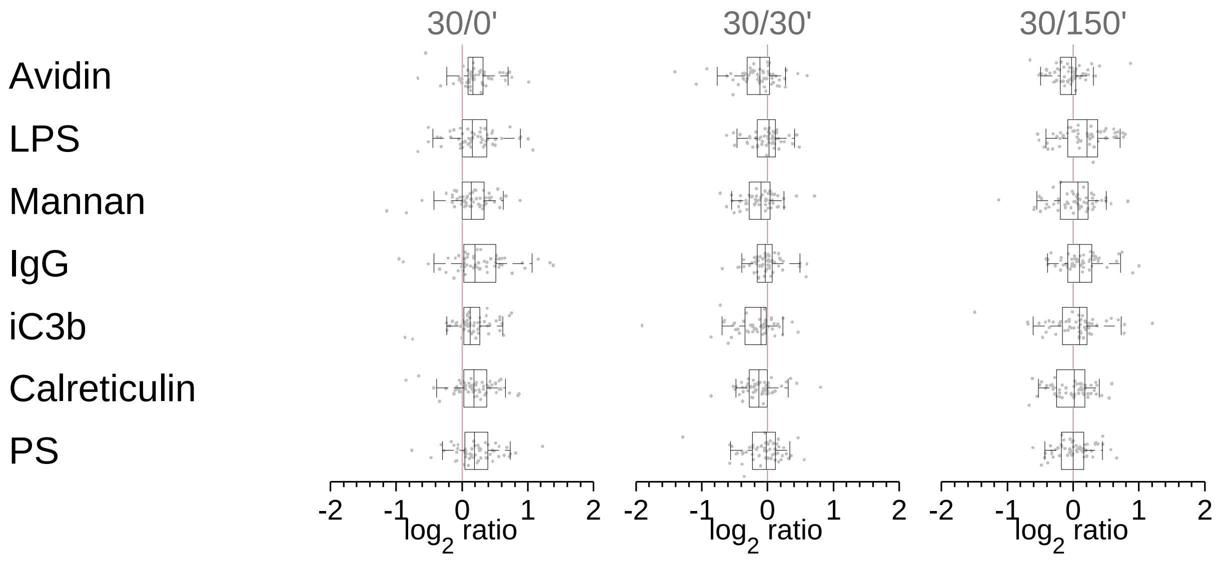

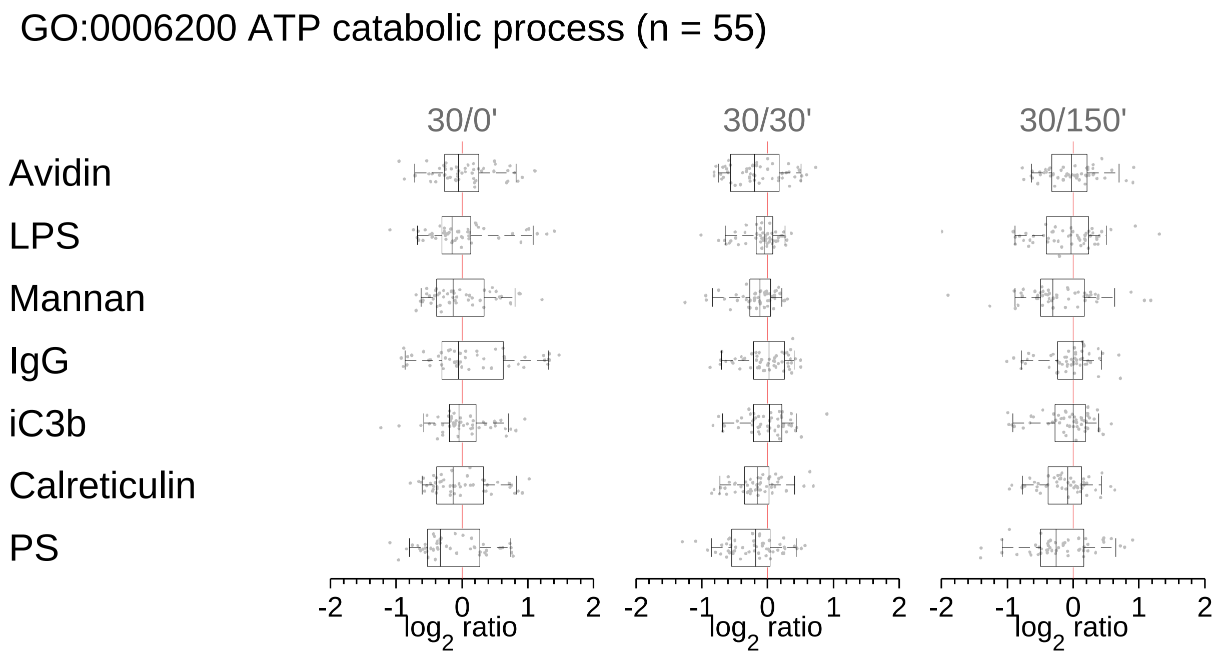
GO:0006200 ATP catabolic process (n = 55)
30/0'
30/30'
30/150'
Avidin
LPS
Mannan
IgG
iC3b
Calreticulin
PS
-2 -1 0 1 2
log2 ratio

GO:0006259 DNA metabolic process (n = 4)

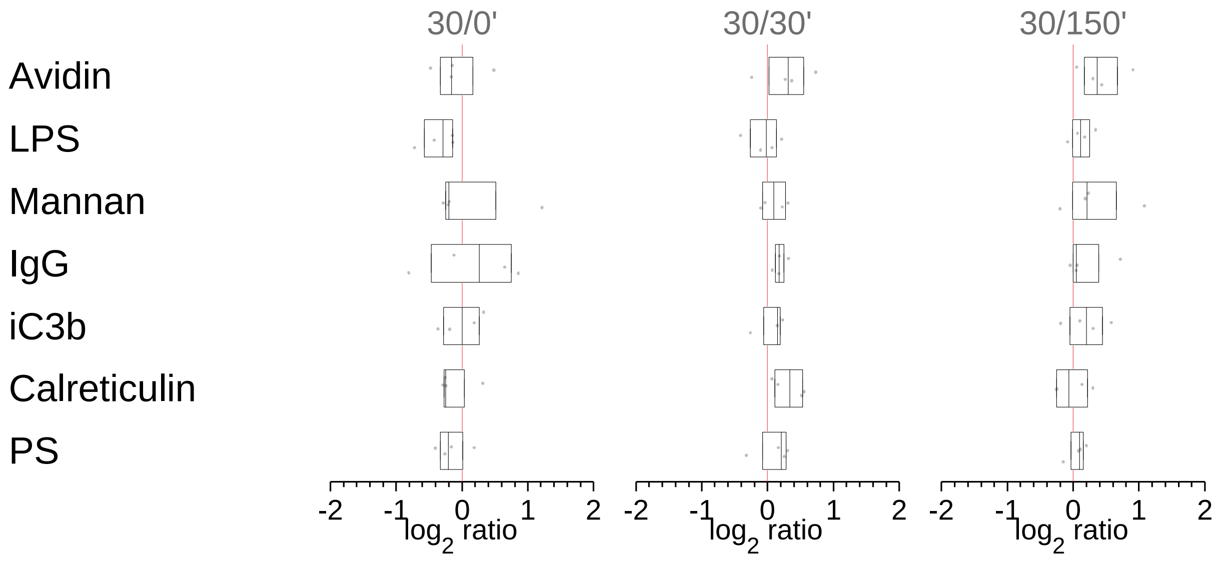

GO:0006260 DNA replication (n = 7)

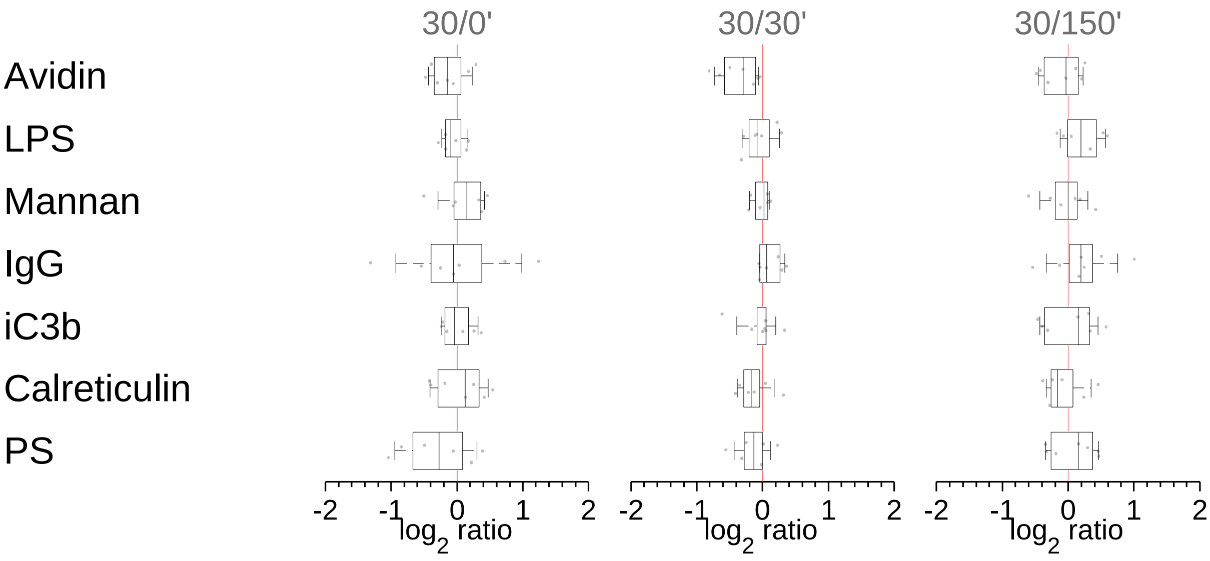

GO:0006281 DNA repair (n = 23)

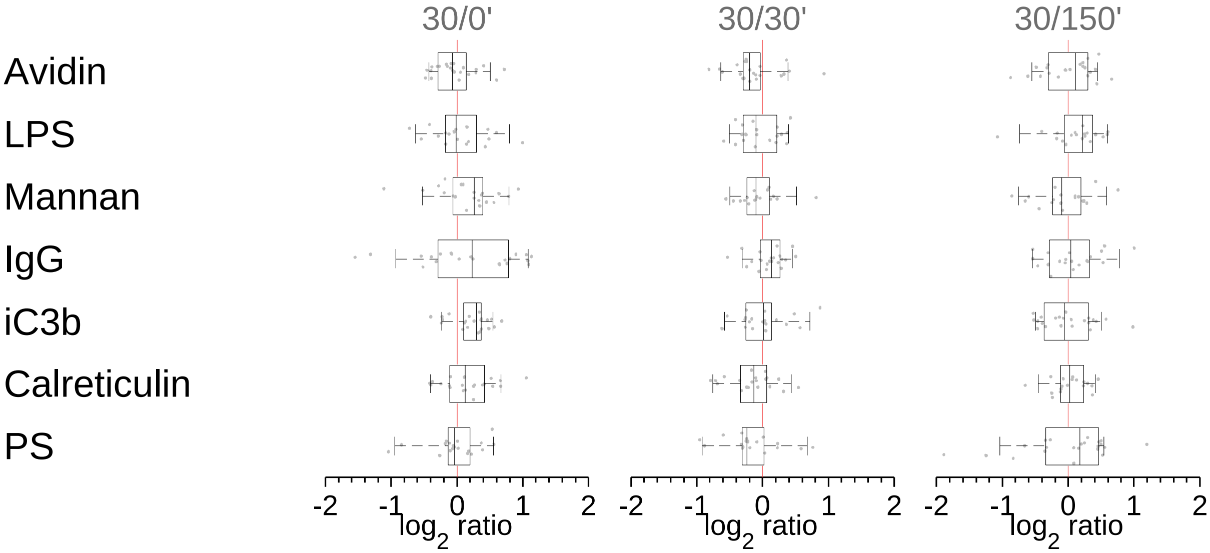

GO:0006302 double-strand break repair (n = 5)

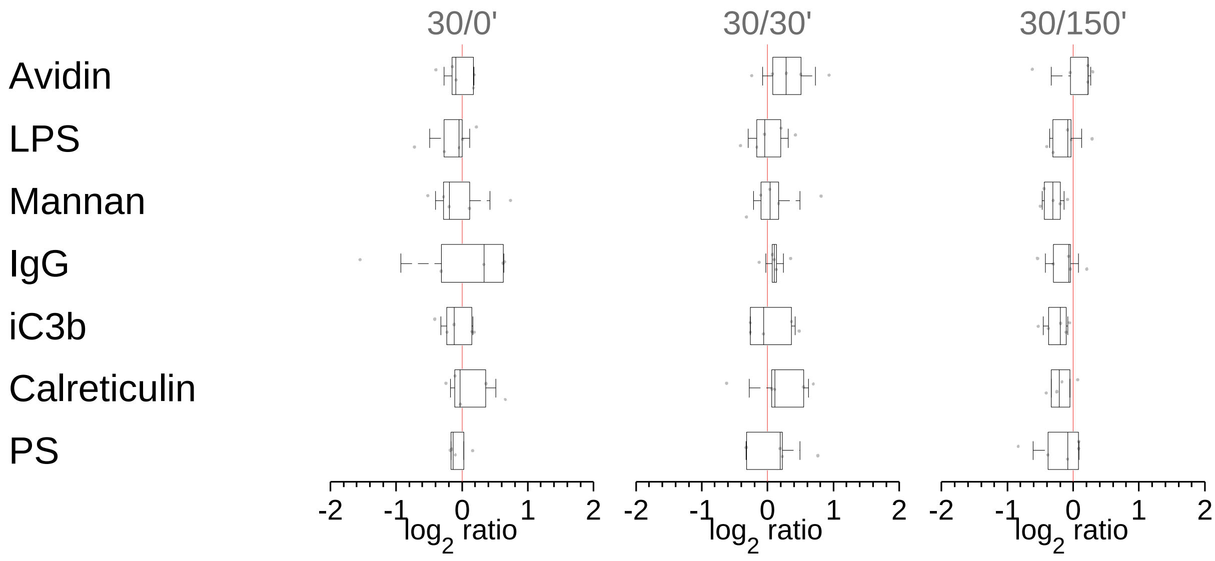

GO:0006310 DNA recombination (n = 4)

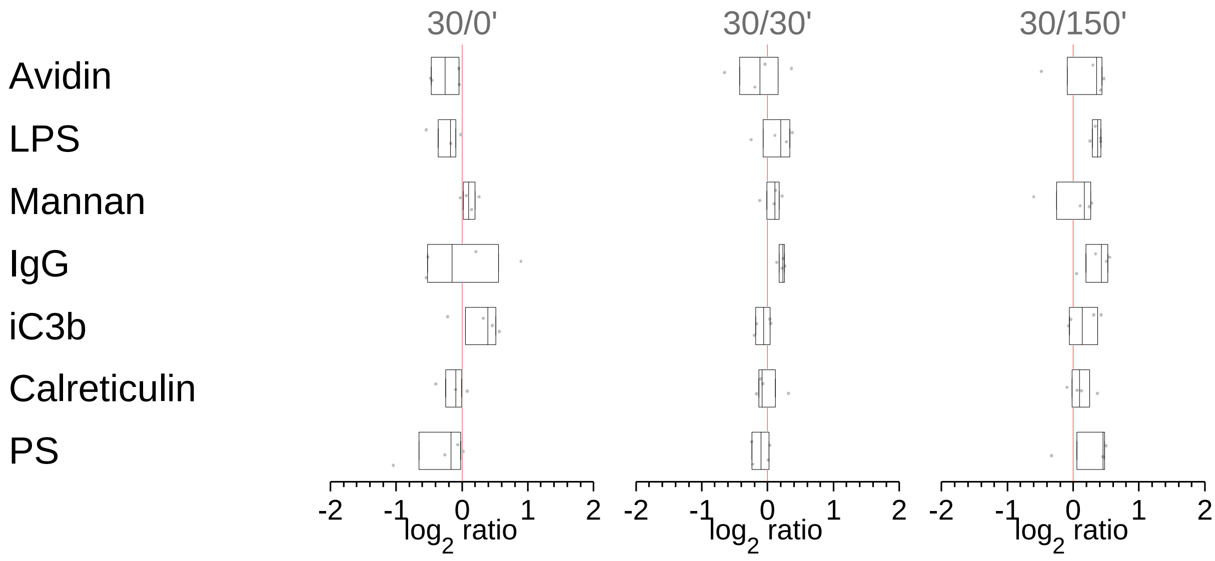

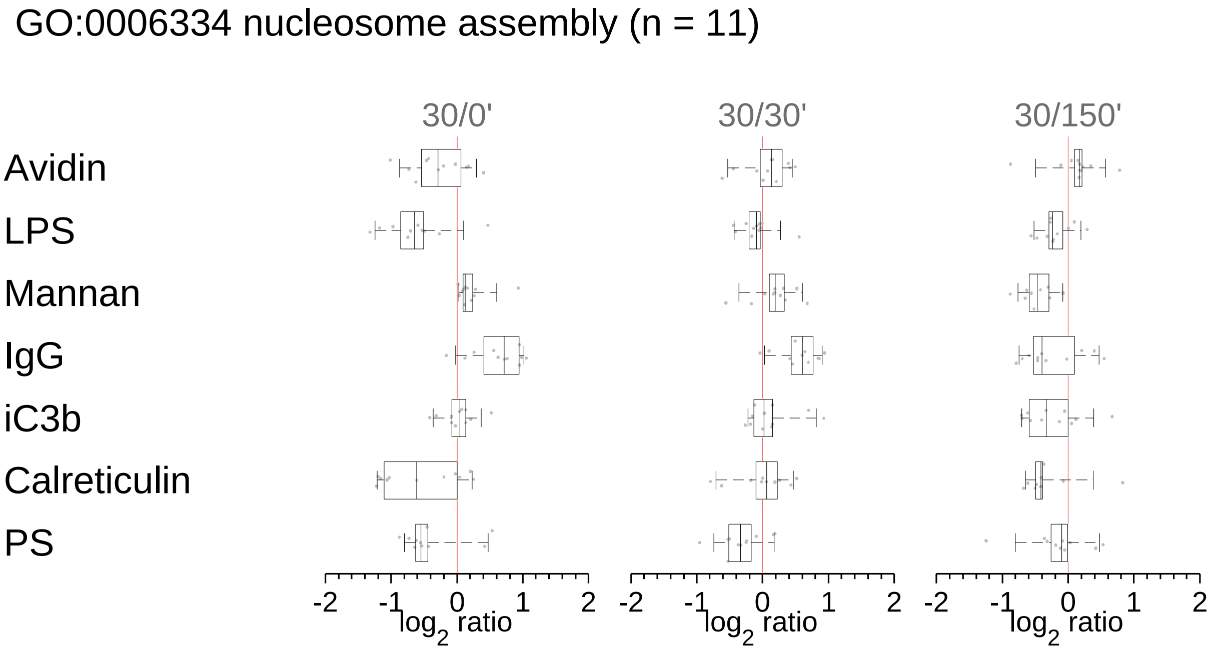
GO:0006334 nucleosome assembly (n = 11)
30/0'
30/30'
30/150'
Avidin
LPS
Mannan
IgG
iC3b
Calreticulin
PS
-2
-1
0
1
2
log2 ratio

GO:0006338 chromatin remodeling (n = 3)

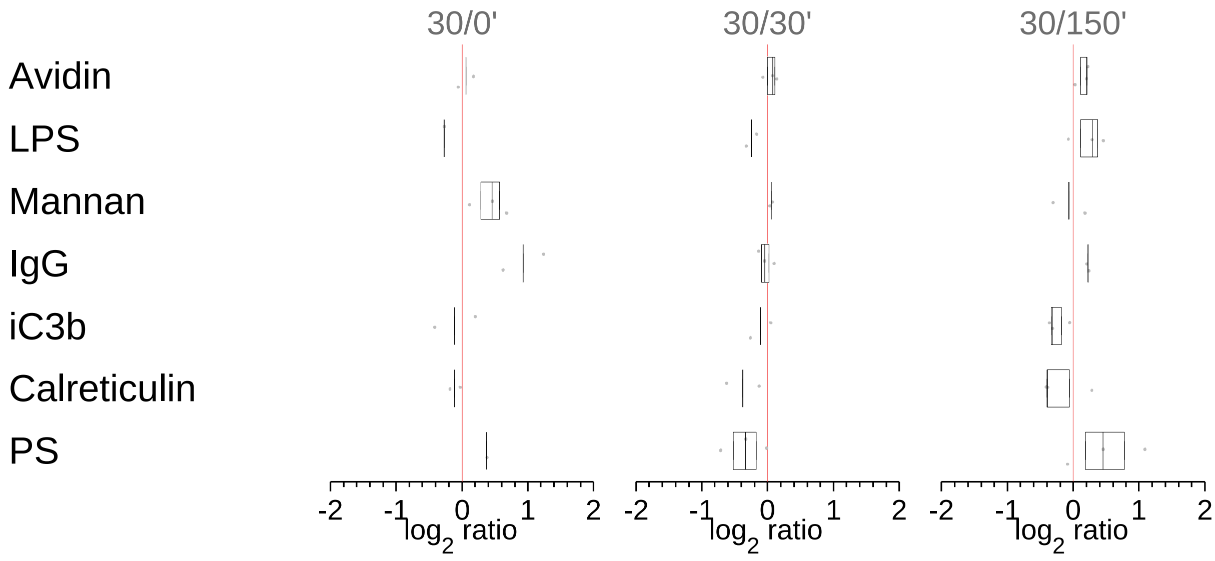

GO:0006351 transcription, DNA-dependent (n = 72)

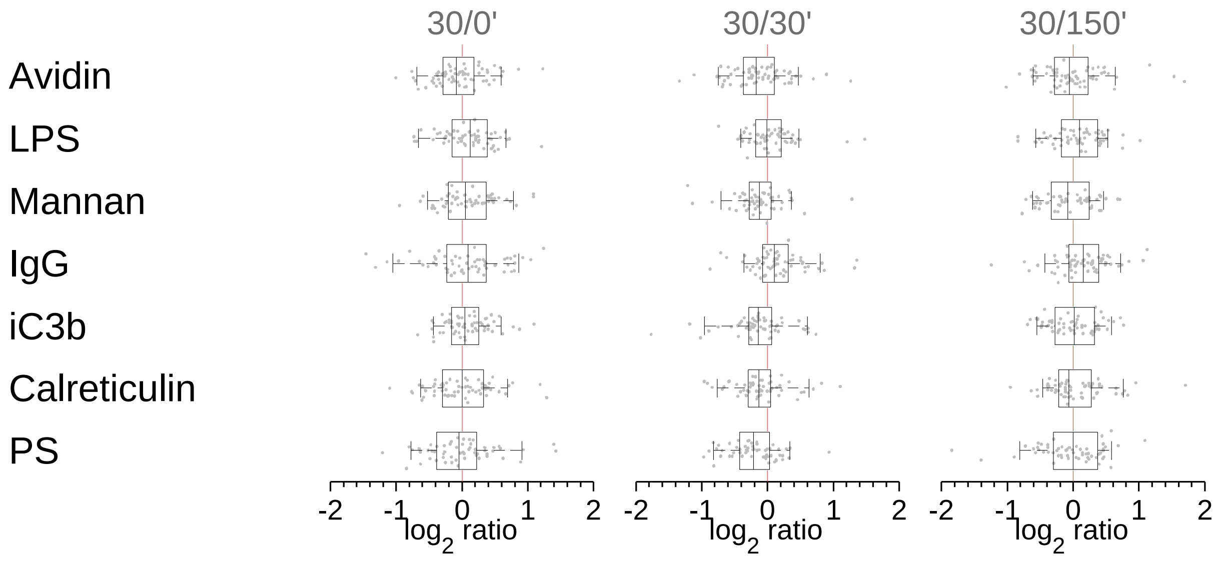

GO:0006355 regulation of transcription, DNA-dependent (n = 73)

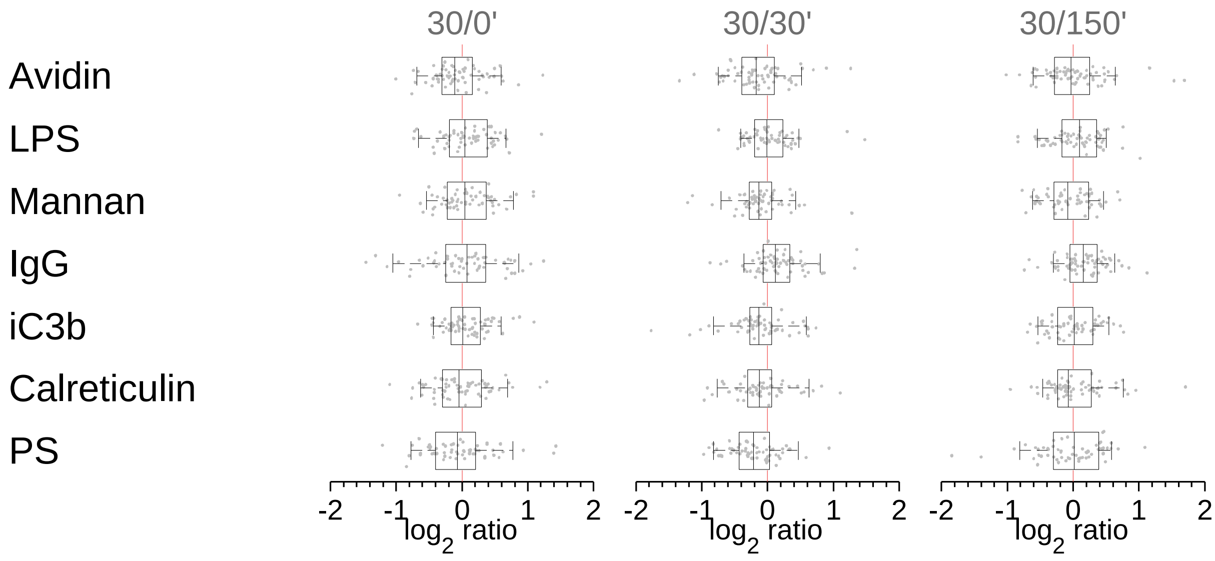

GO:0006357 regulation of transcription from RNA polymerase II promoter (n = 13)

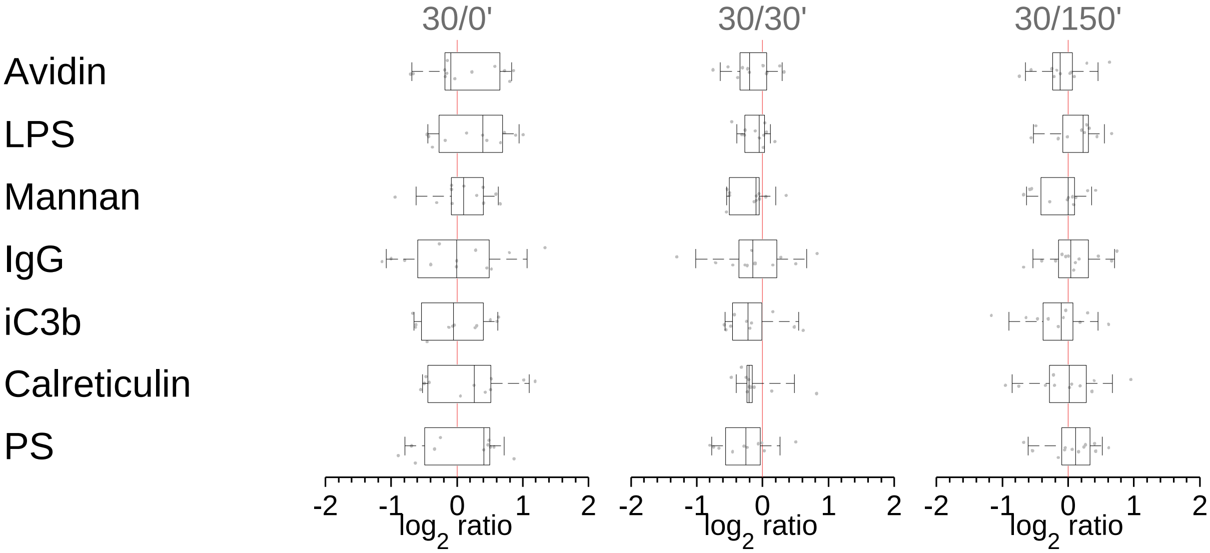

GO:0006364 rRNA processing (n = 14)

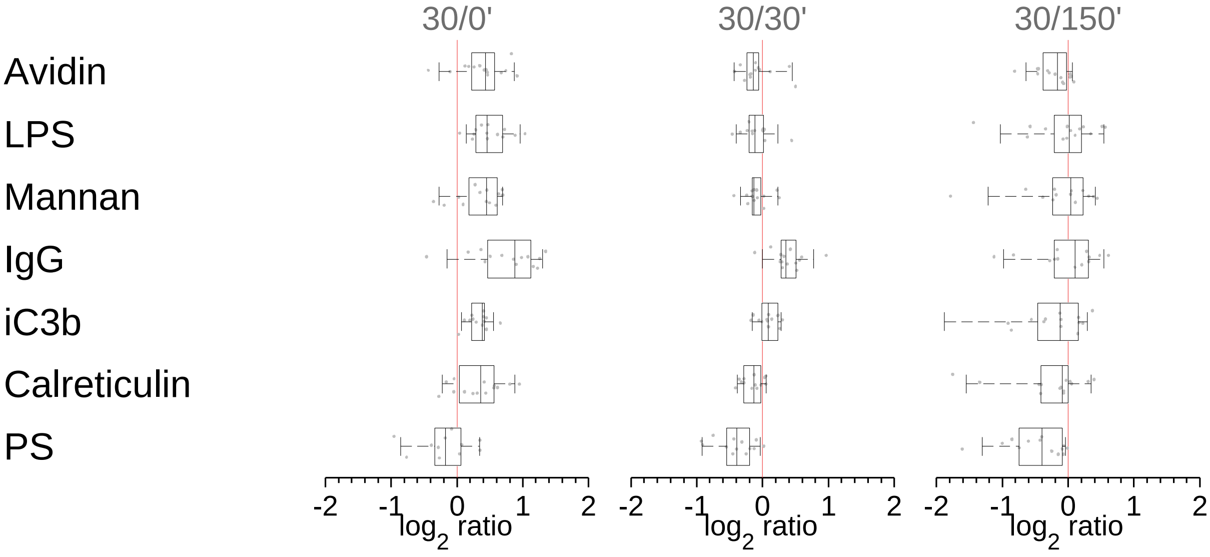

GO:0006366 transcription from RNA polymerase II promoter (n = 17)

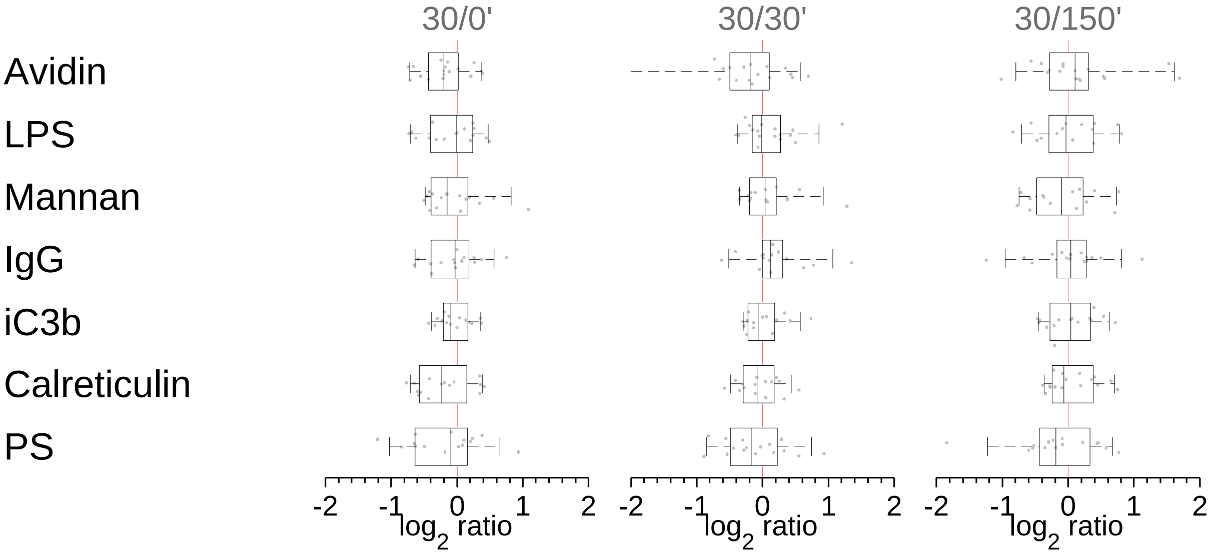

GO:0006367 transcription initiation from RNA polymerase II promoter (n = 3)

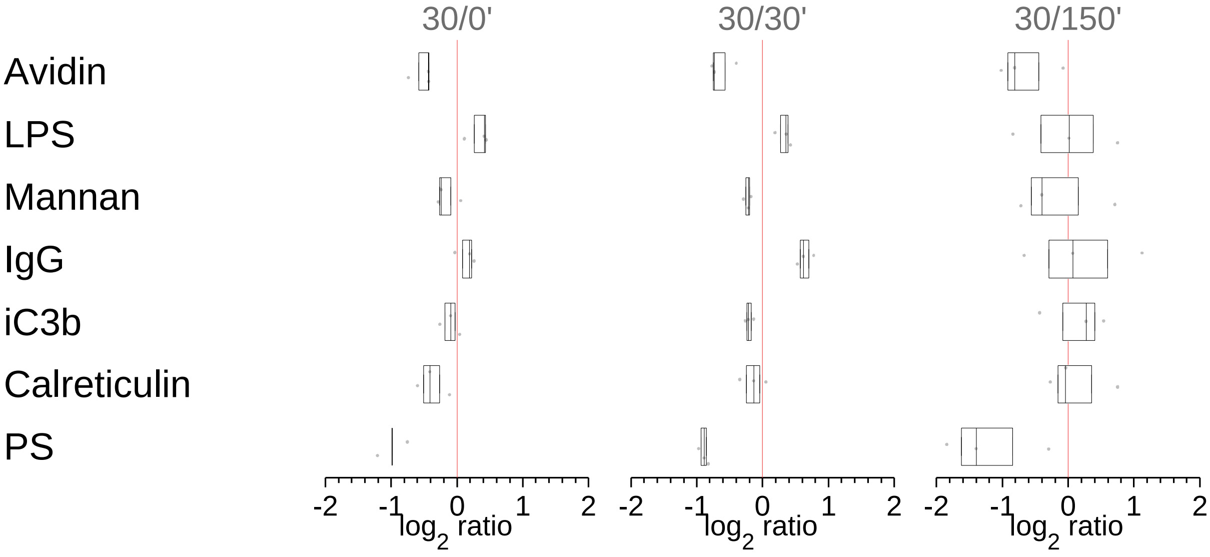

GO:0006396 RNA processing (n = 3)

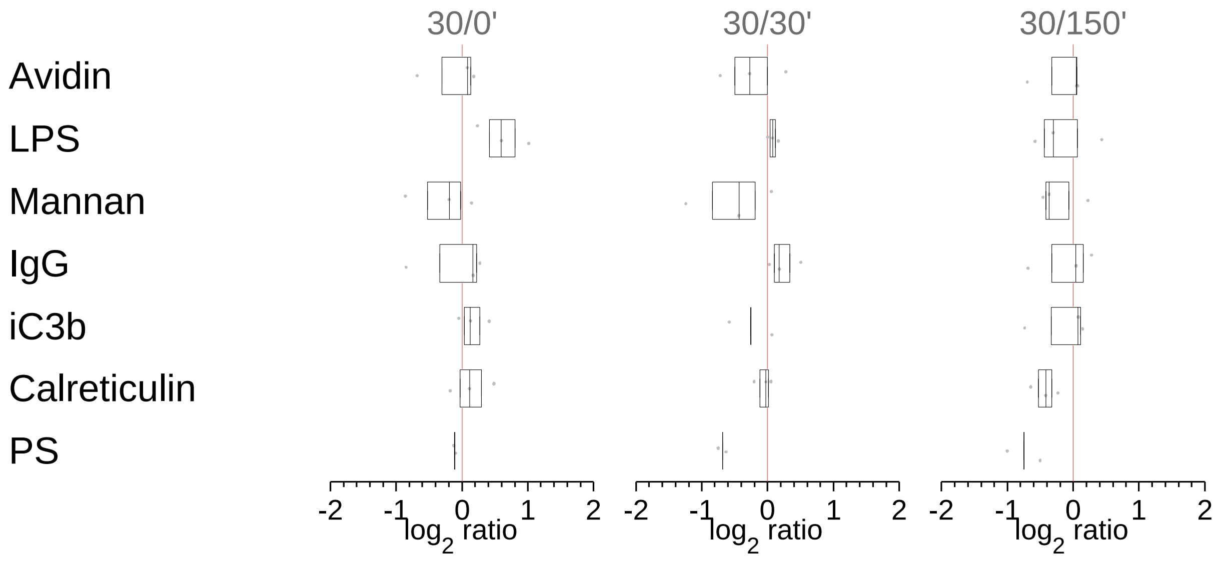

GO:0006397 mRNA processing (n = 47)

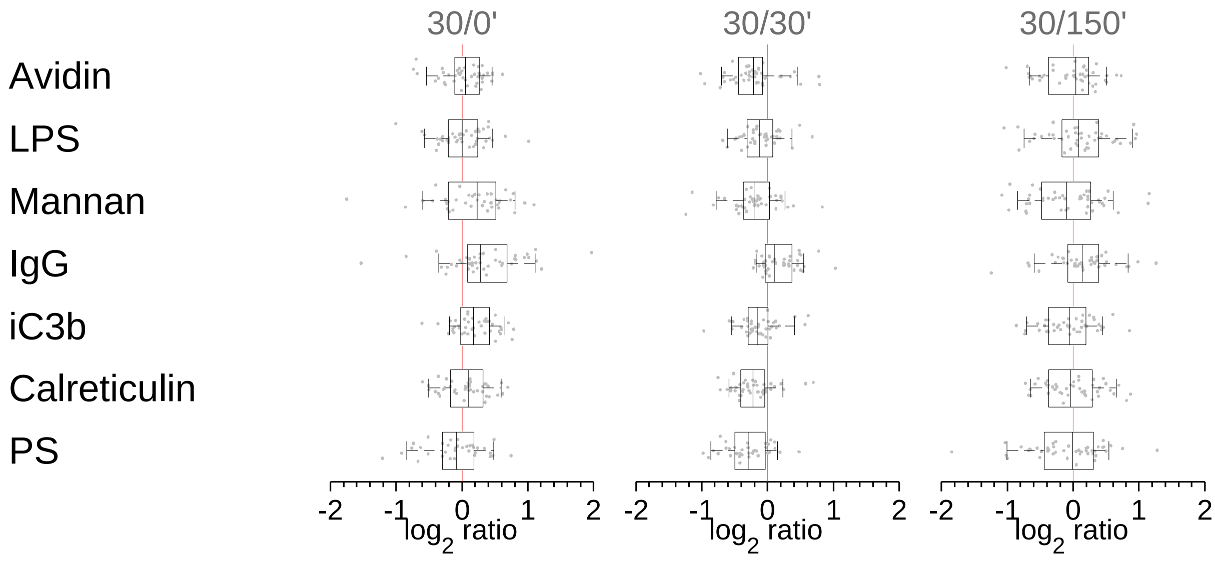

GO:0006406 mRNA export from nucleus (n = 3)

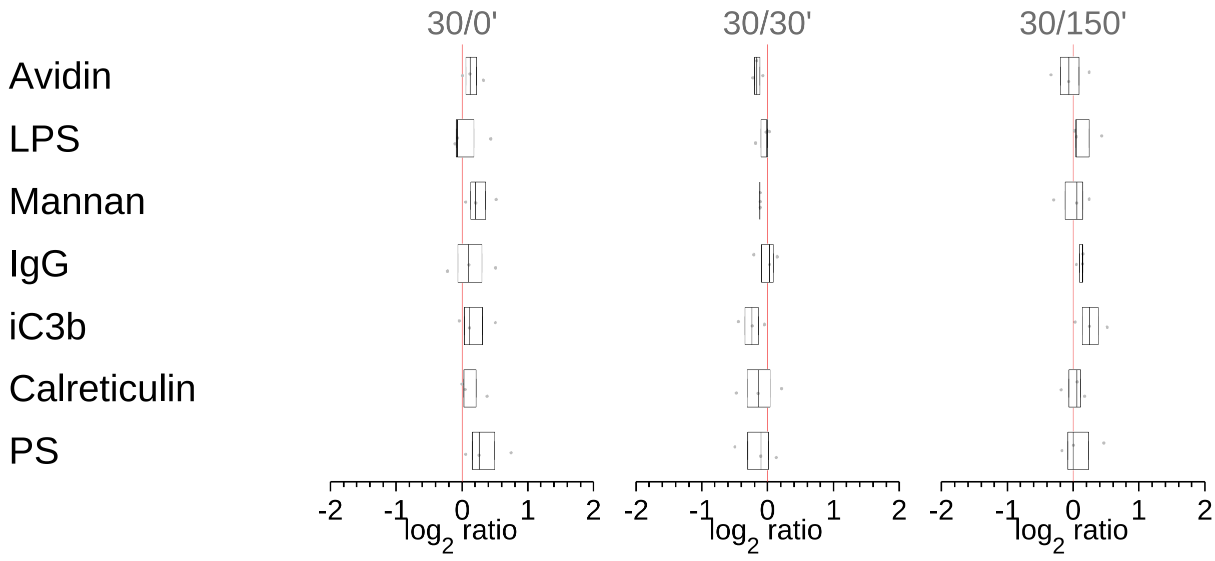

GO:0006407 rRNA export from nucleus (n = 3)

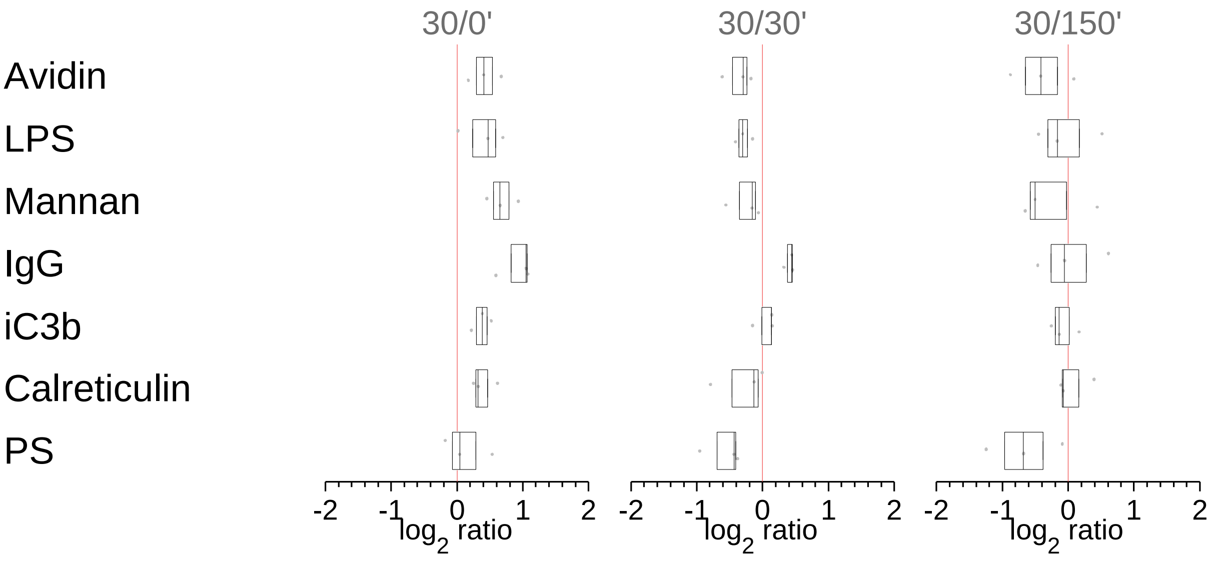

GO:0006412 translation (n = 71)

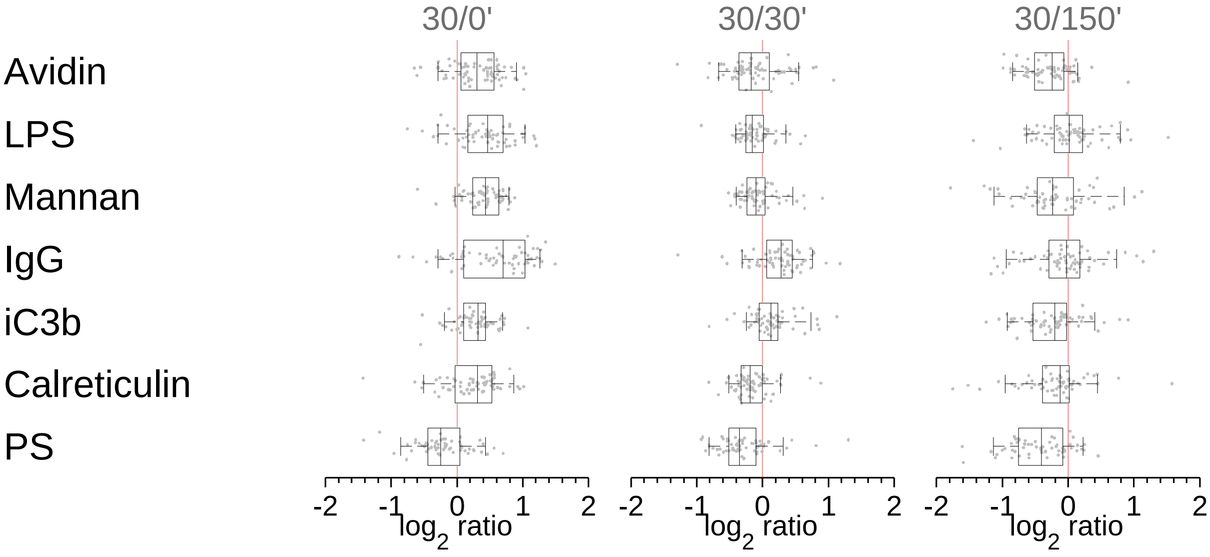

GO:0006413 translational initiation (n = 12)

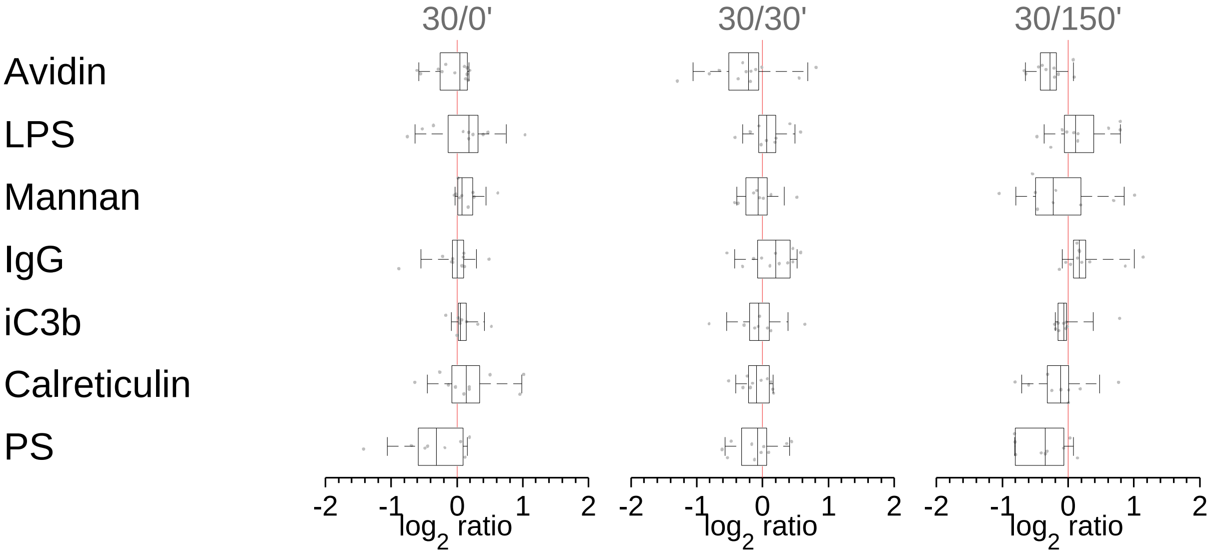

GO:0006414 translational elongation (n = 13)

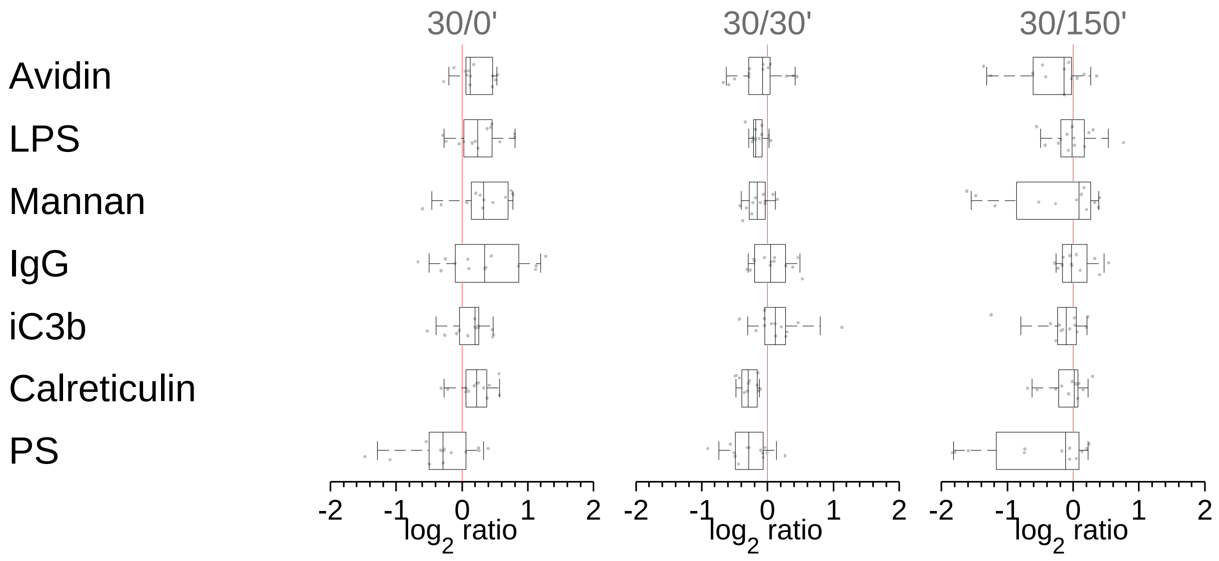

GO:0006417 regulation of translation (n = 18)

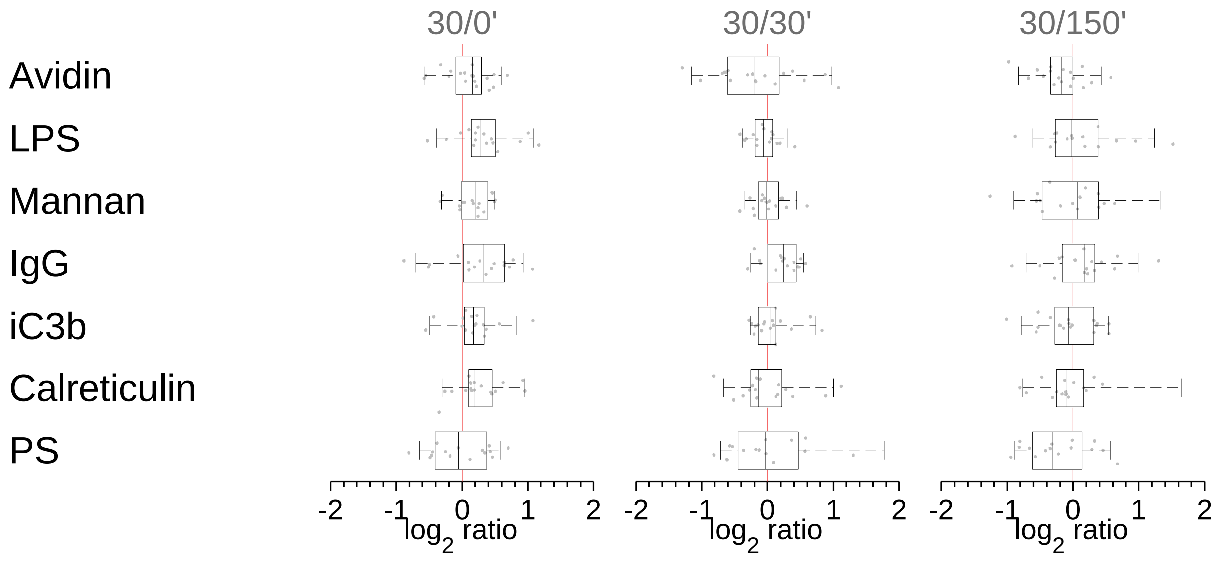

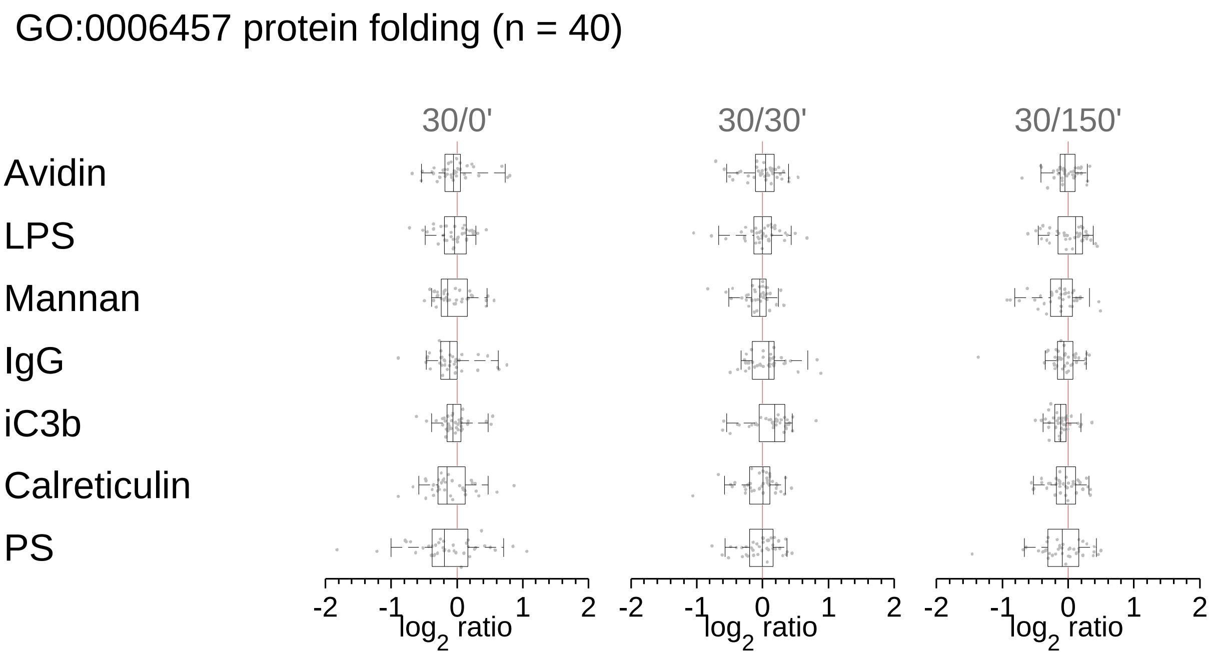
GO:0006457 protein folding (n = 40)
30/0'
30/30'
30/150'
Avidin
LPS
Mannan
IgG
iC3b
Calreticulin
PS
-2 -1 0 1 2
log2 ratio

GO:0006461 protein complex assembly (n = 12)

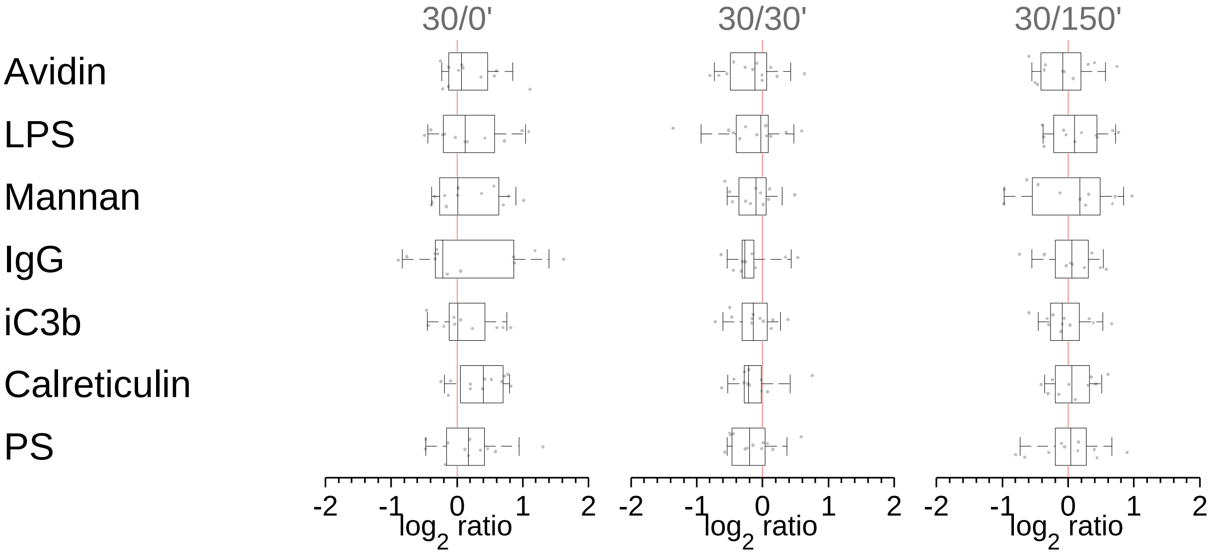

GO:0006464 cellular protein modification process (n = 7)

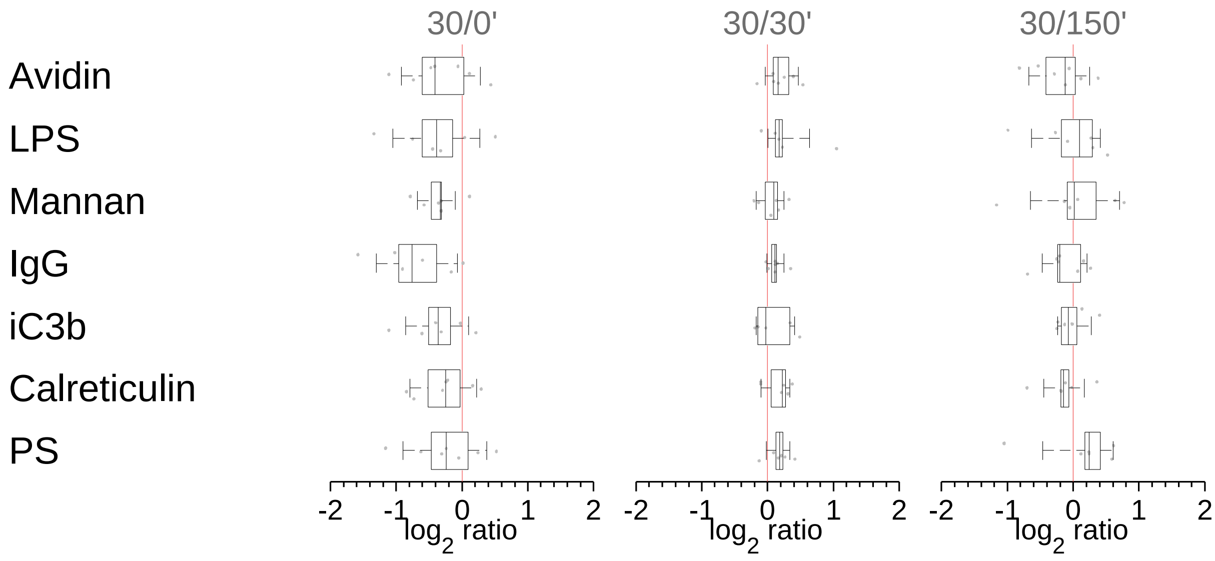

GO:0006465 signal peptide processing (n = 3)

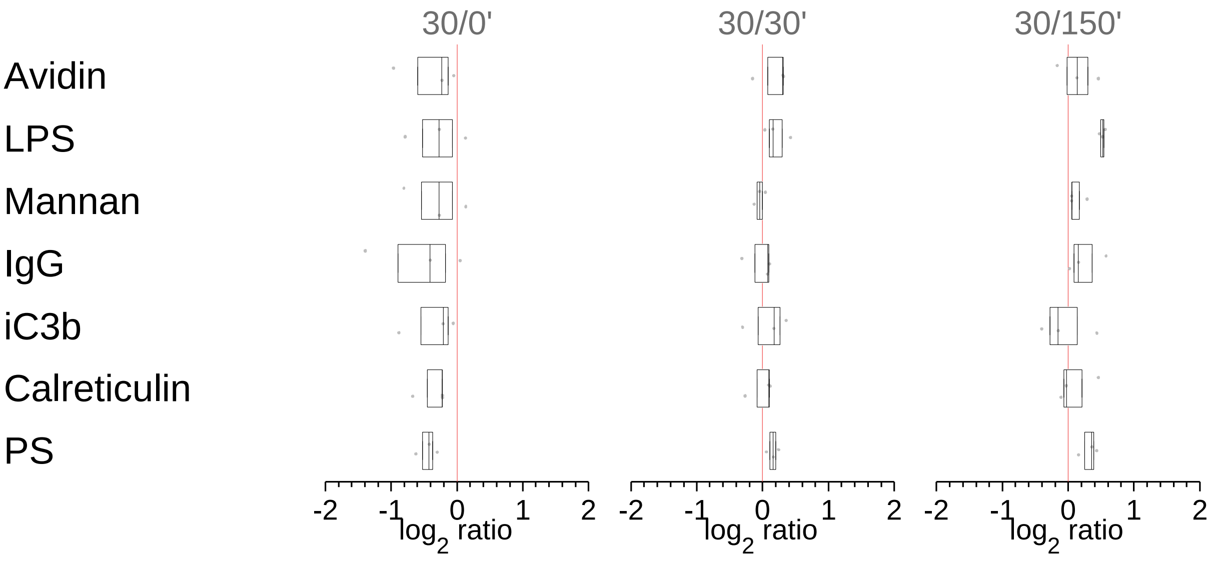

GO:0006468 protein phosphorylation (n = 60)

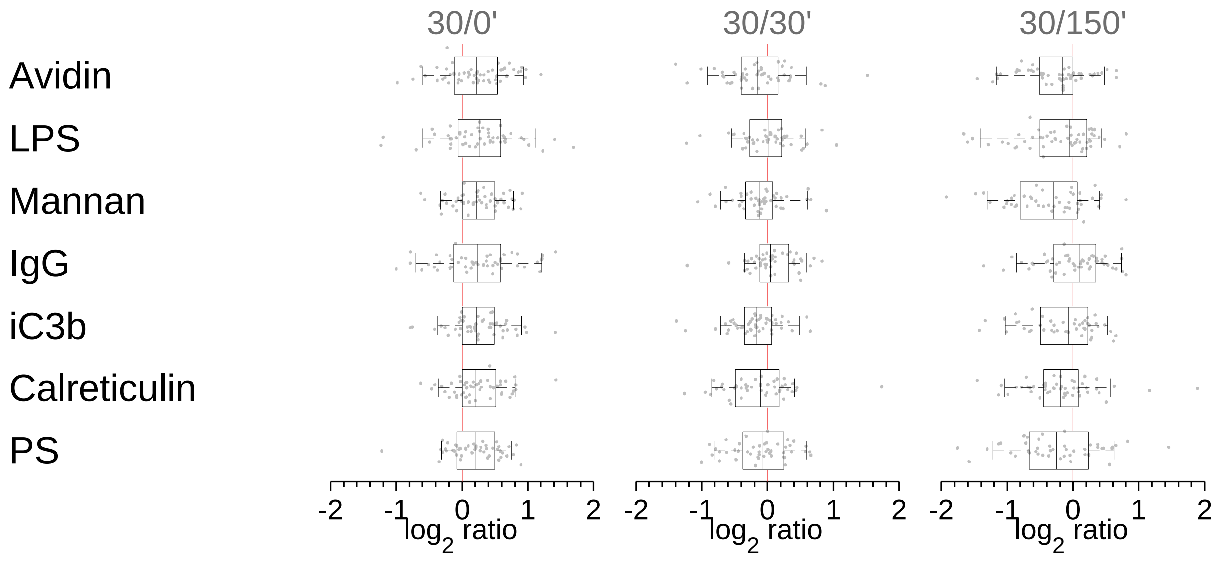

GO:0006469 negative regulation of protein kinase activity (n = 7)

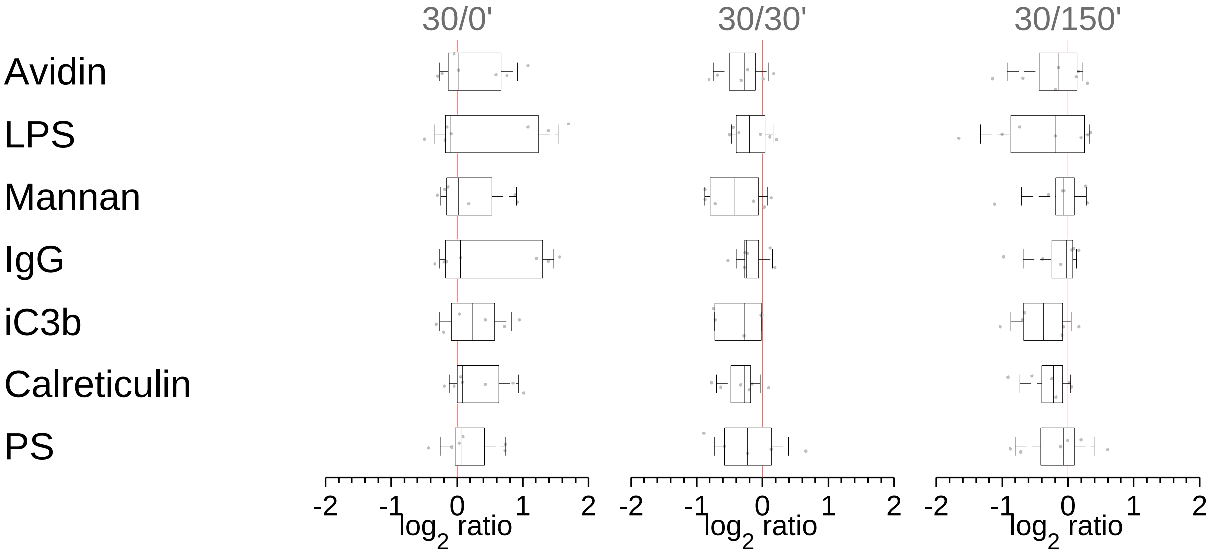

GO:0006470 protein dephosphorylation (n = 20)

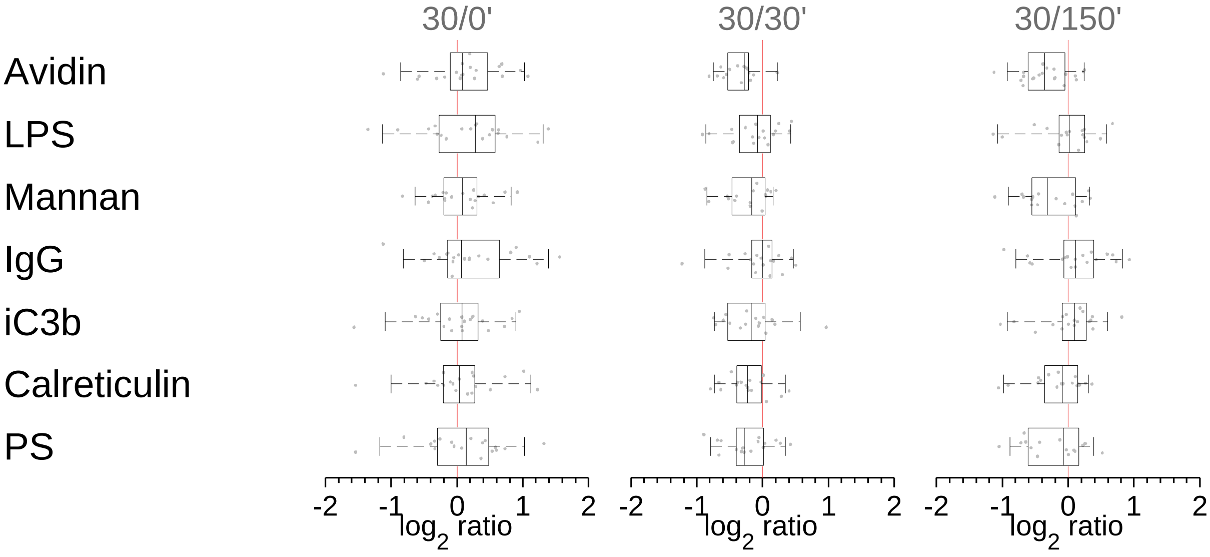

GO:0006486 protein glycosylation (n = 8)

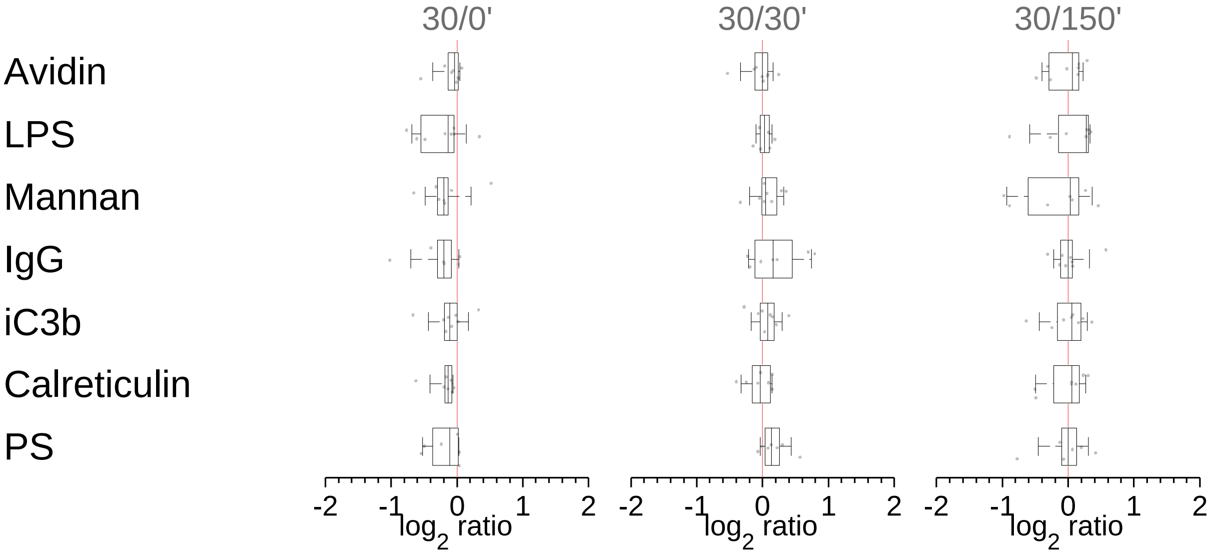

GO:0006487 protein N-linked glycosylation (n = 4)

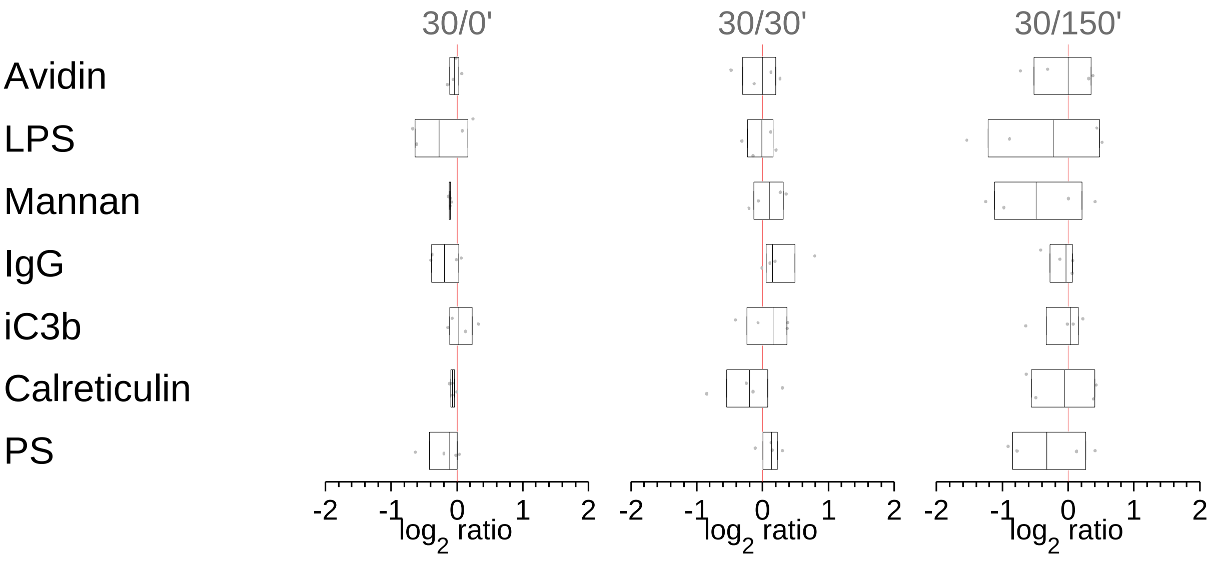

GO:0006491 N-glycan processing (n = 3)

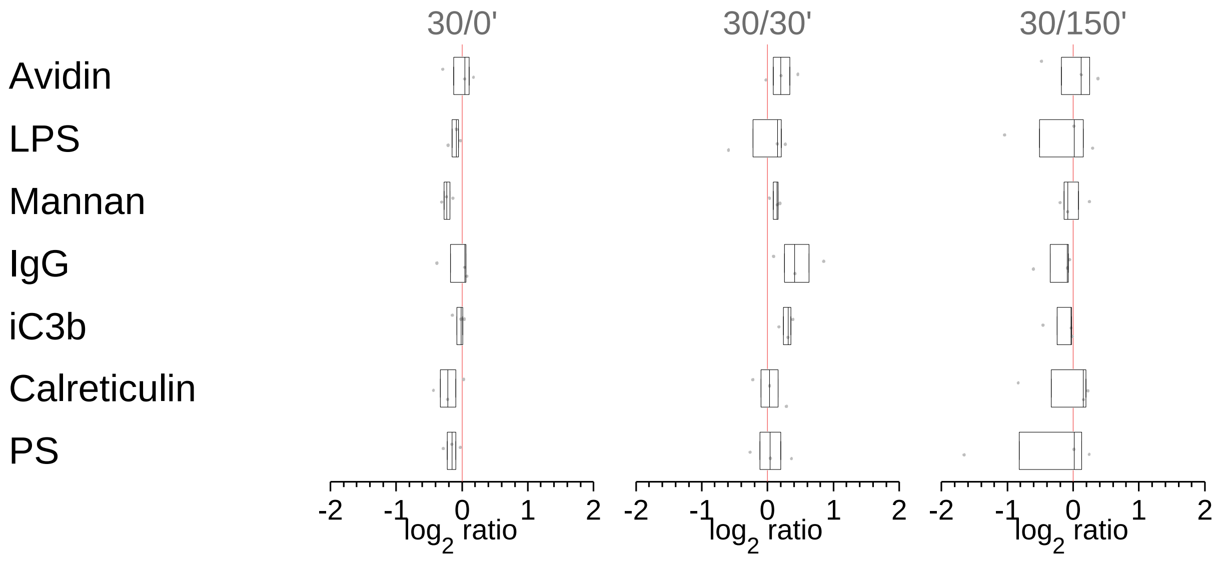

GO:0006506 GPI anchor biosynthetic process (n = 3)

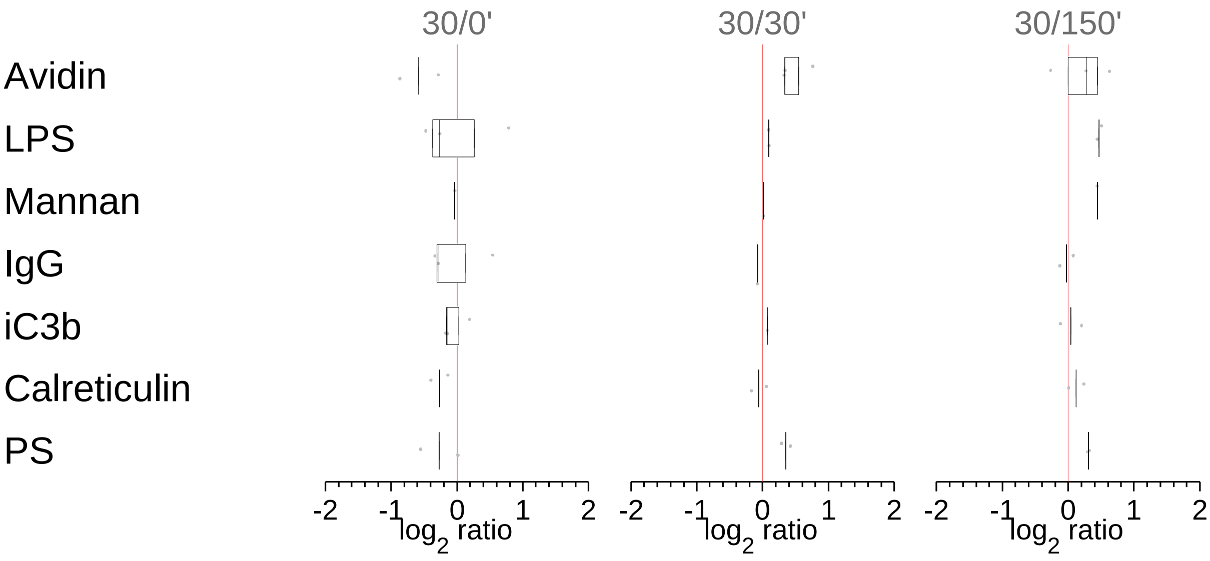

GO:0006508 proteolysis (n = 67)

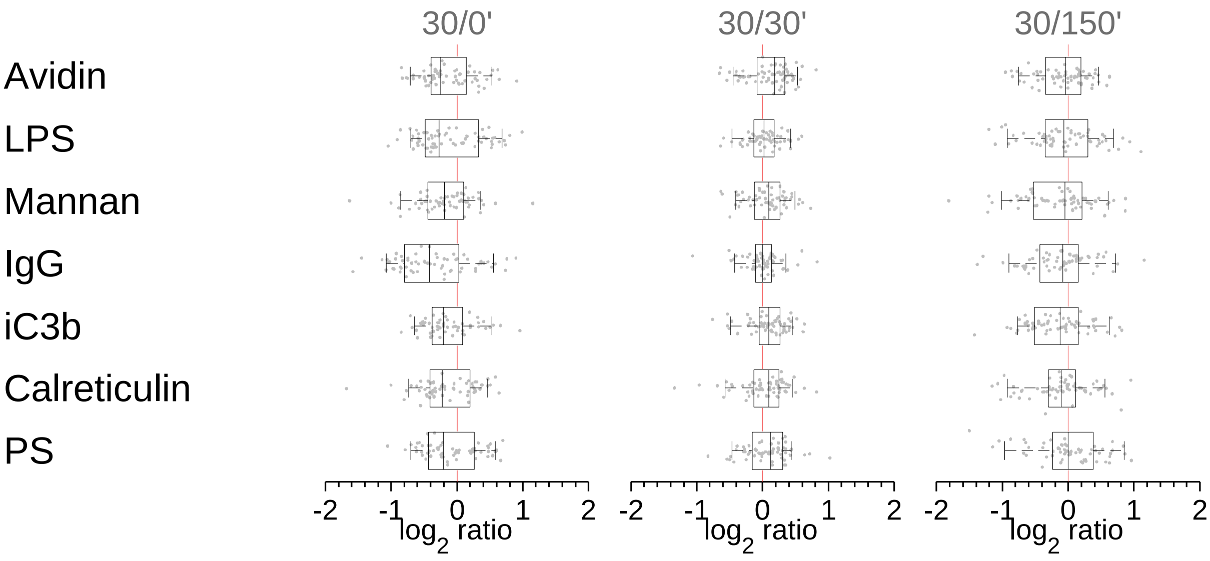

GO:0006509 membrane protein ectodomain proteolysis (n = 12)

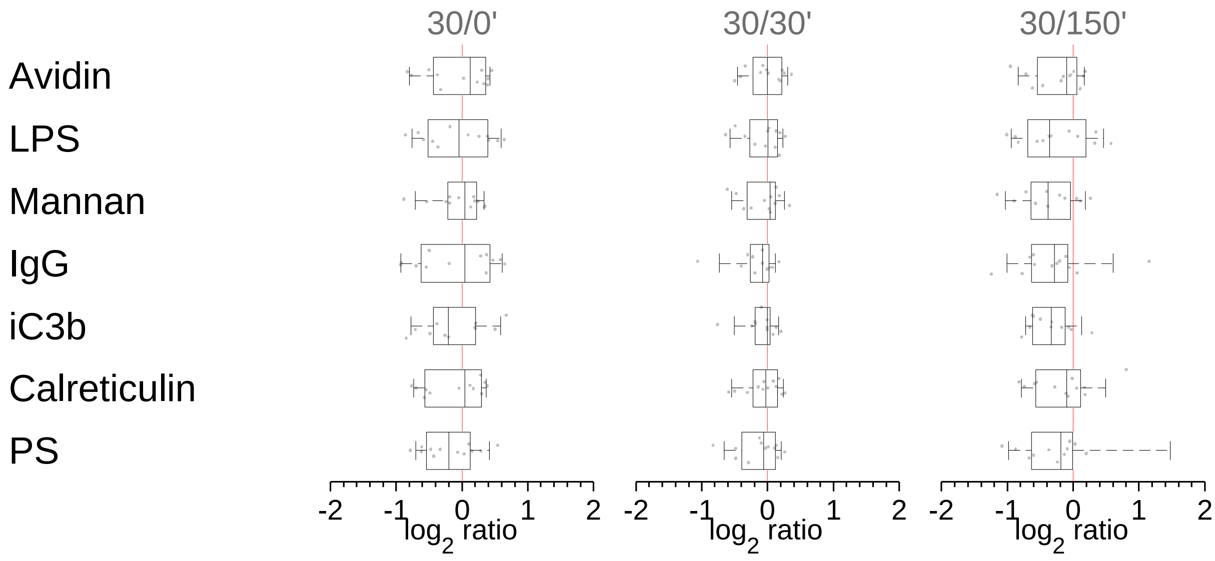

GO:0006511 ubiquitin-dependent protein catabolic process (n = 13)

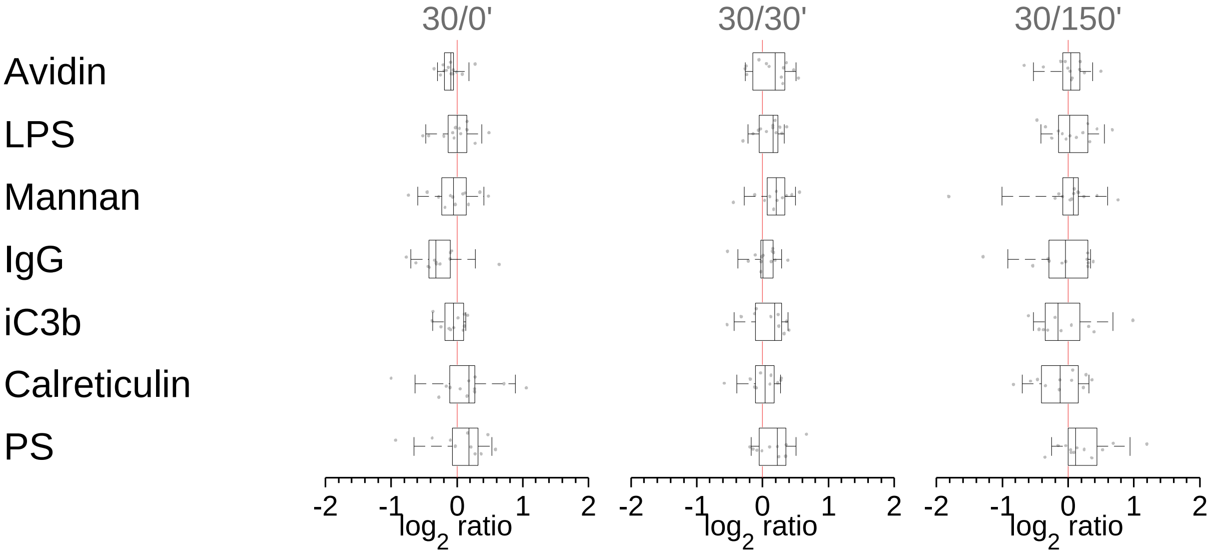

GO:0006513 protein monoubiquitination (n = 3)

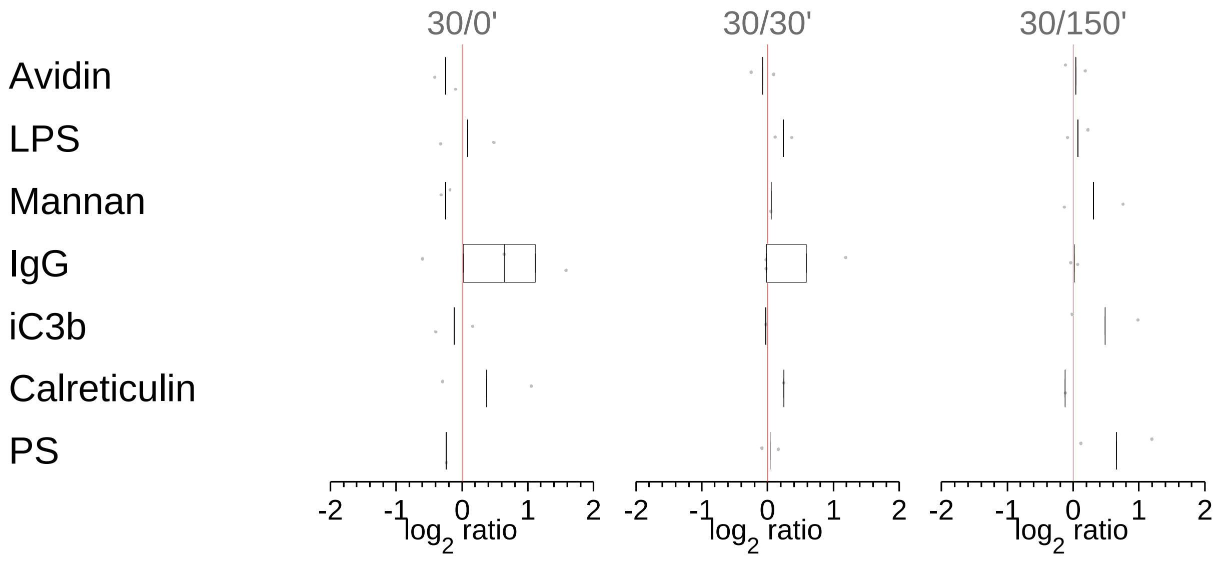

GO:0006520 cellular amino acid metabolic process (n = 3)

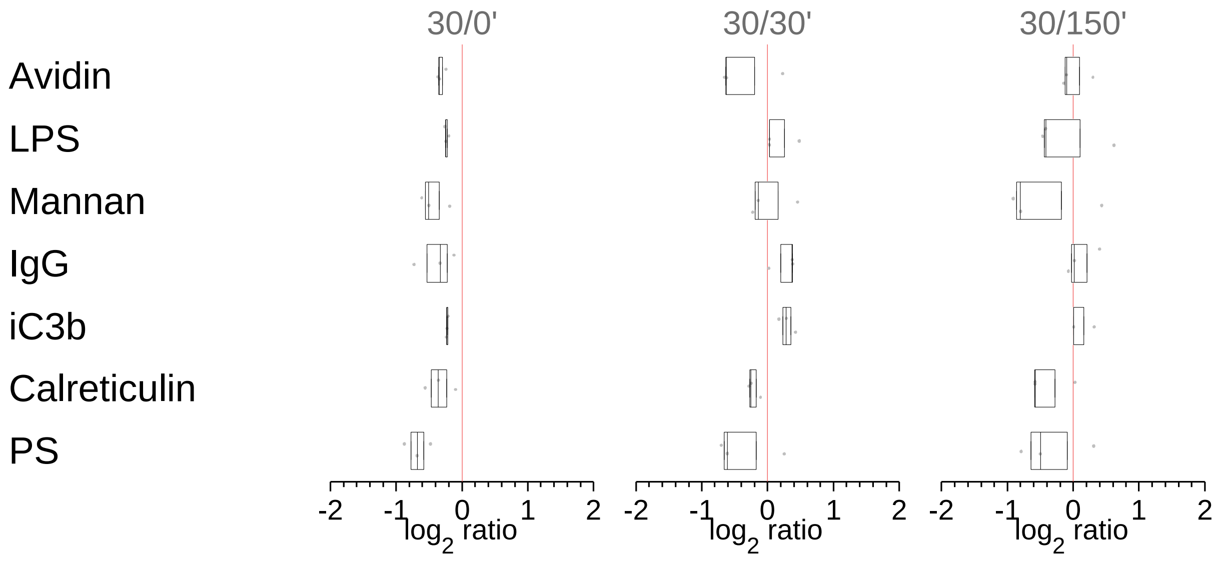

GO:0006541 glutamine metabolic process (n = 3)

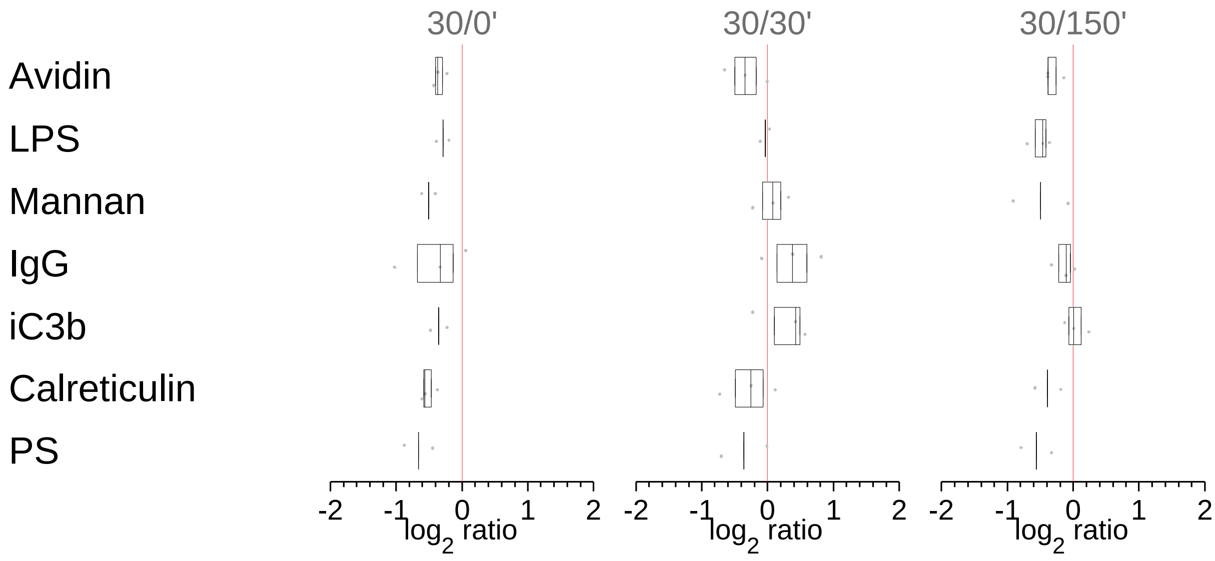

GO:0006605 protein targeting (n = 14)

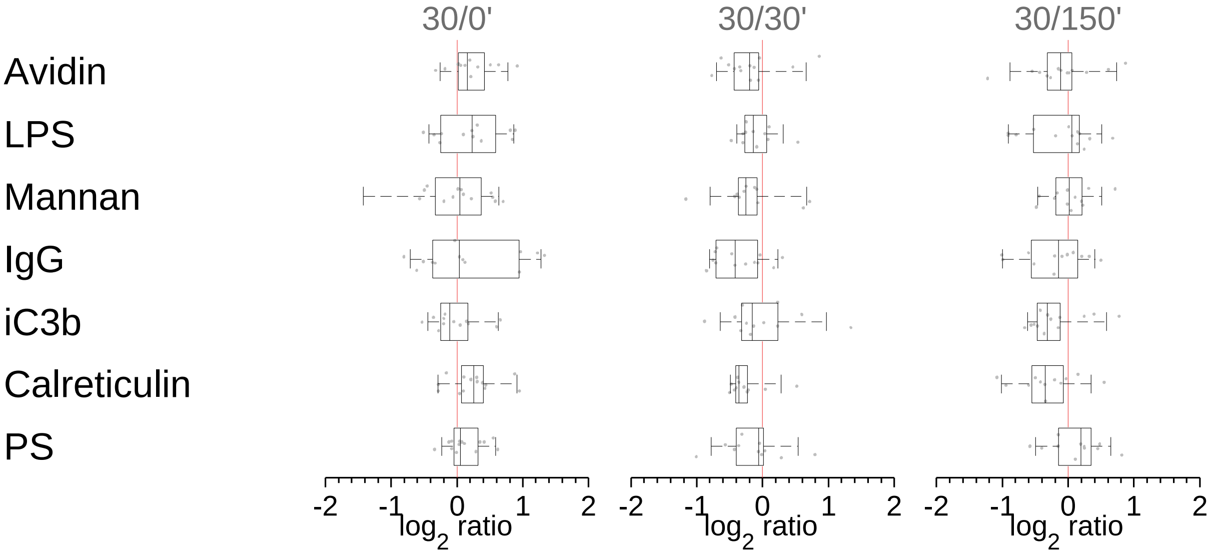

GO:0006606 protein import into nucleus (n = 4)

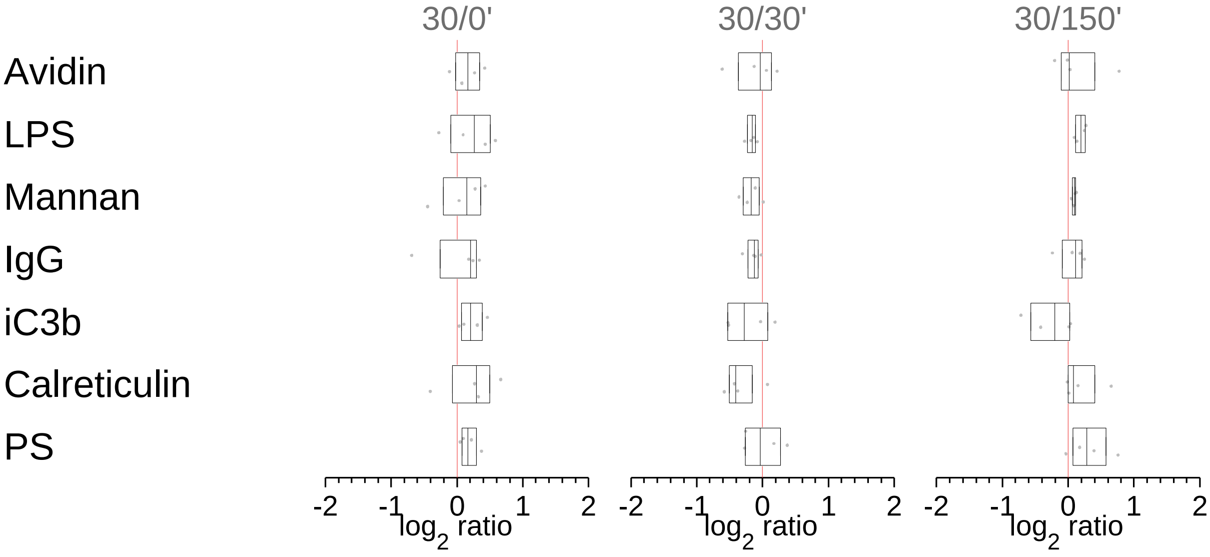

GO:0006611 protein export from nucleus (n = 4)

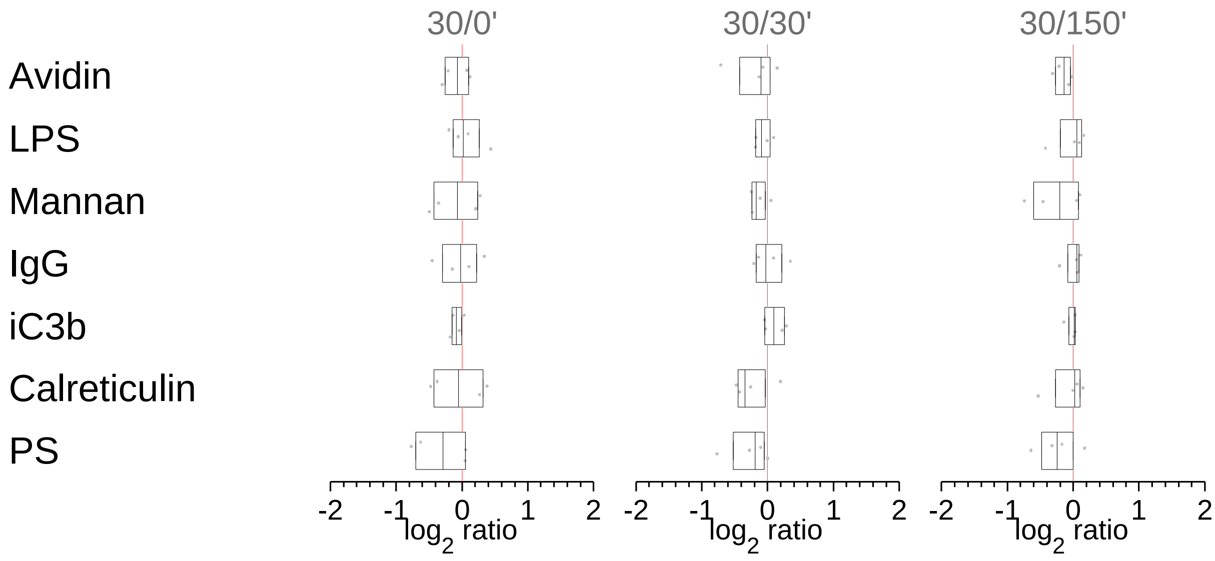

GO:0006614 SRP-dependent cotranslational protein targeting to membrane (n = 4)

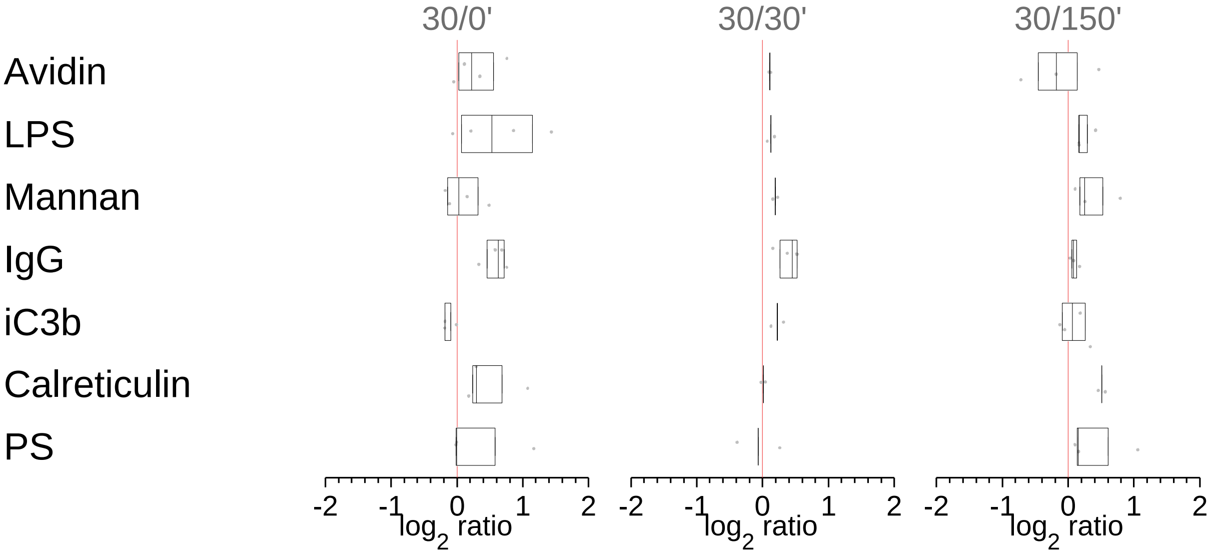

GO:0006620 posttranslational protein targeting to membrane (n = 5)

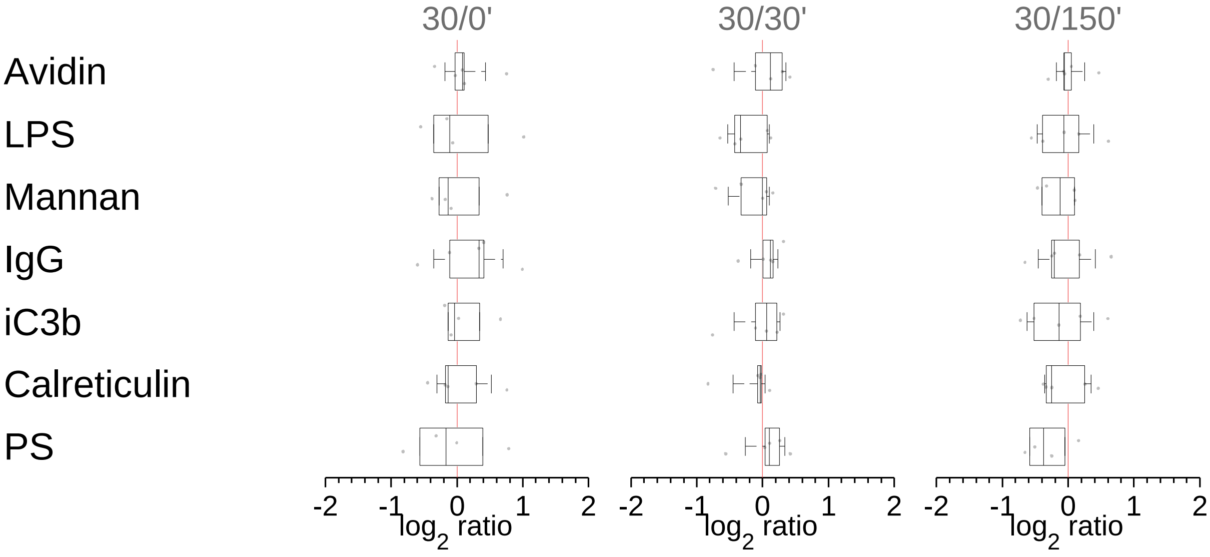

GO:0006622 protein targeting to lysosome (n = 4)

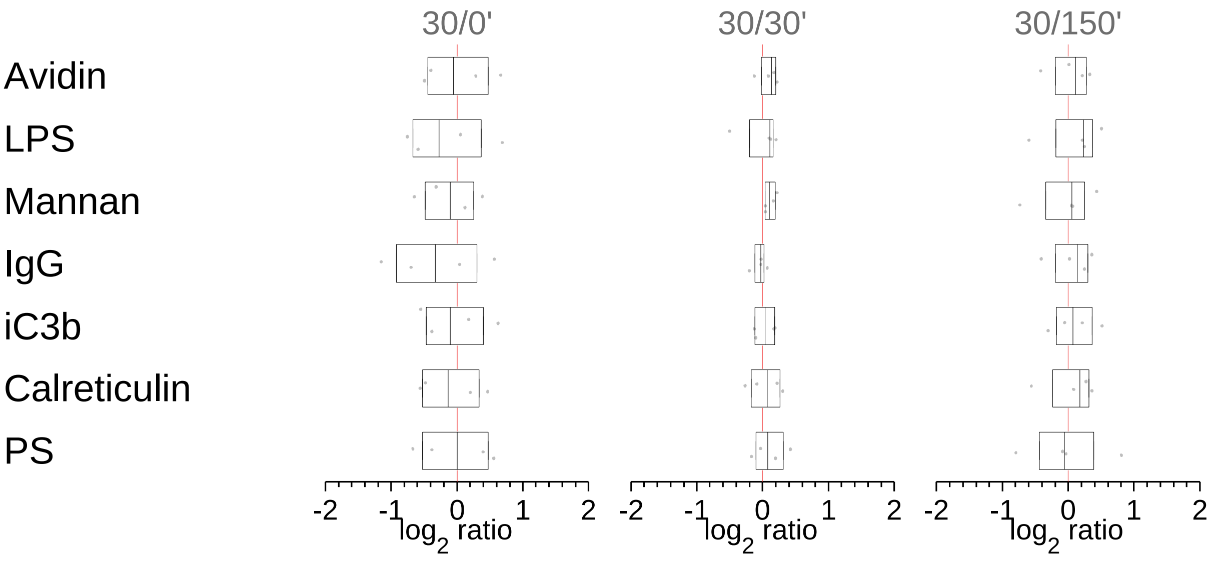

GO:0006626 protein targeting to mitochondrion (n = 4)

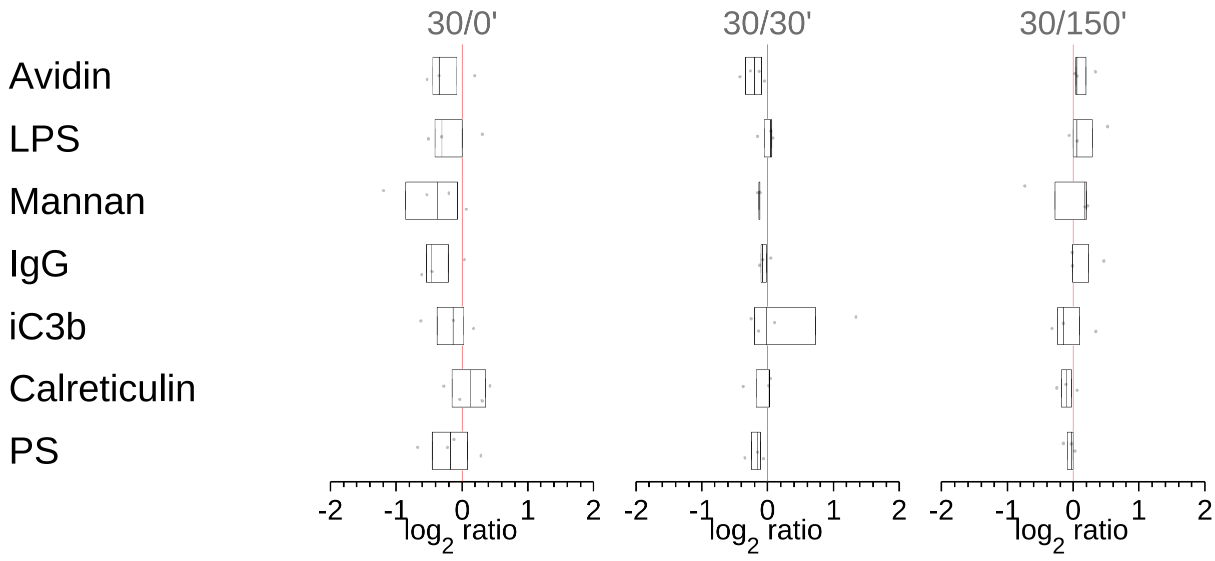

GO:0006629 lipid metabolic process (n = 74)

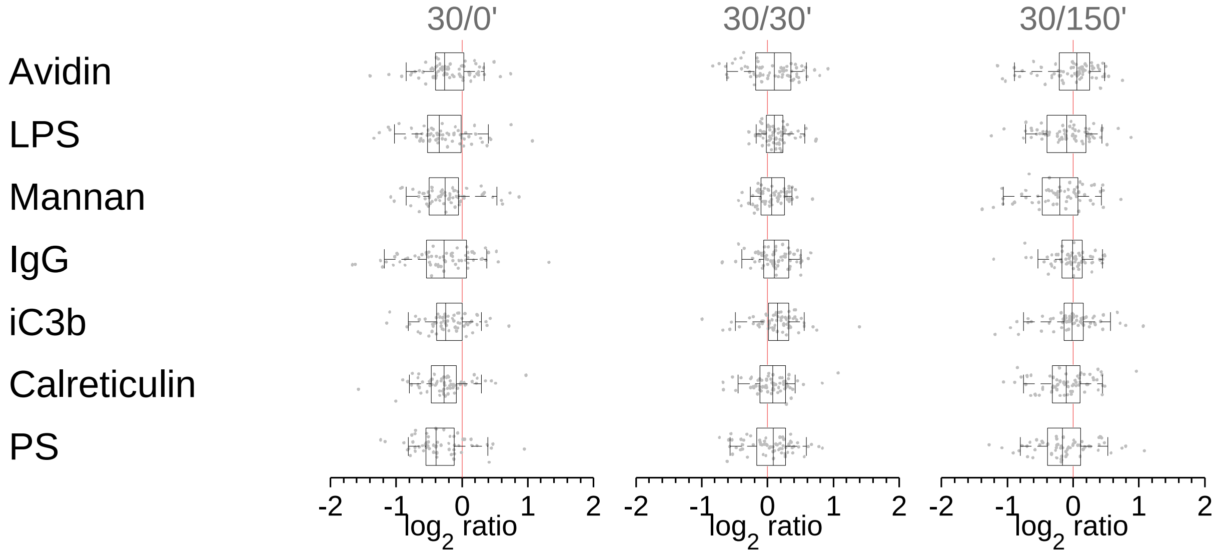

GO:0006631 fatty acid metabolic process (n = 28)

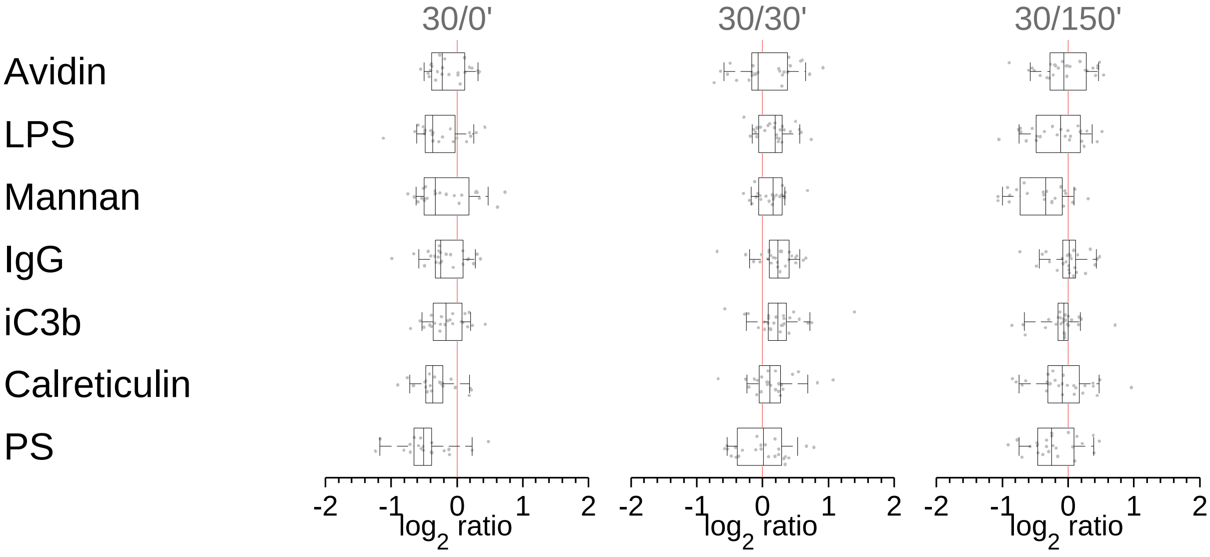

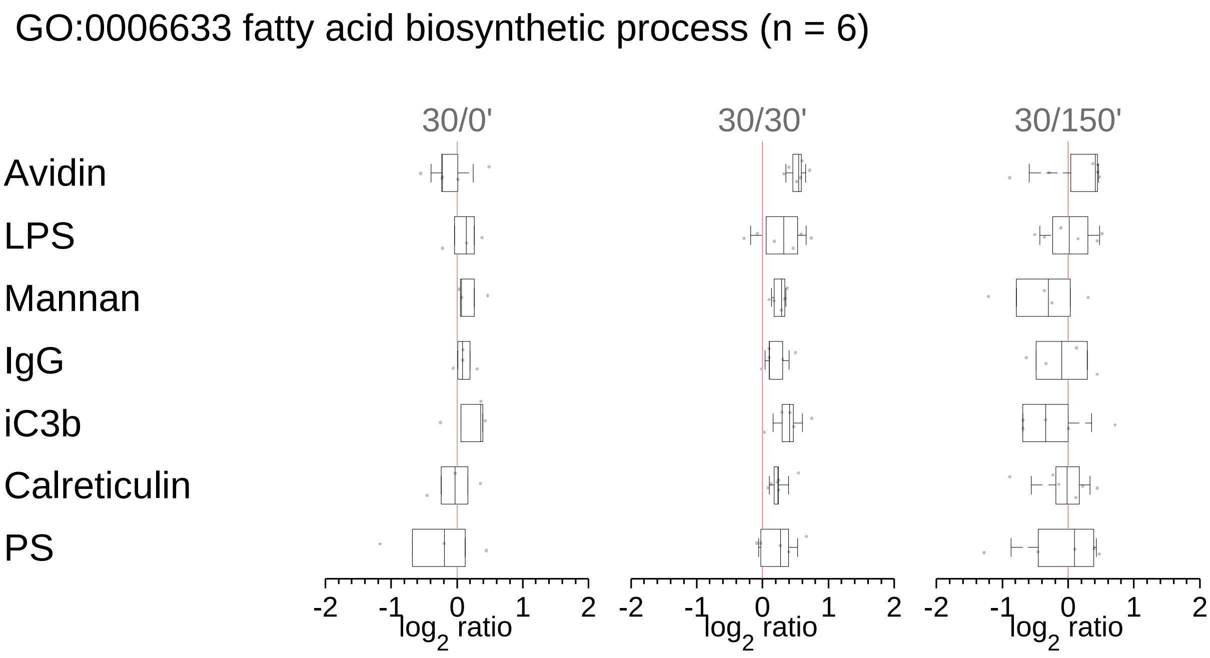
GO:0006633 fatty acid biosynthetic process (n = 6)
30/0'
30/30'
30/150'
Avidin
LPS
Mannan
IgG
iC3b
Calreticulin
PS
-2
-1
0
1
2
log2 ratio
log2 ratio
log2 ratio

GO:0006635 fatty acid beta-oxidation (n = 11)

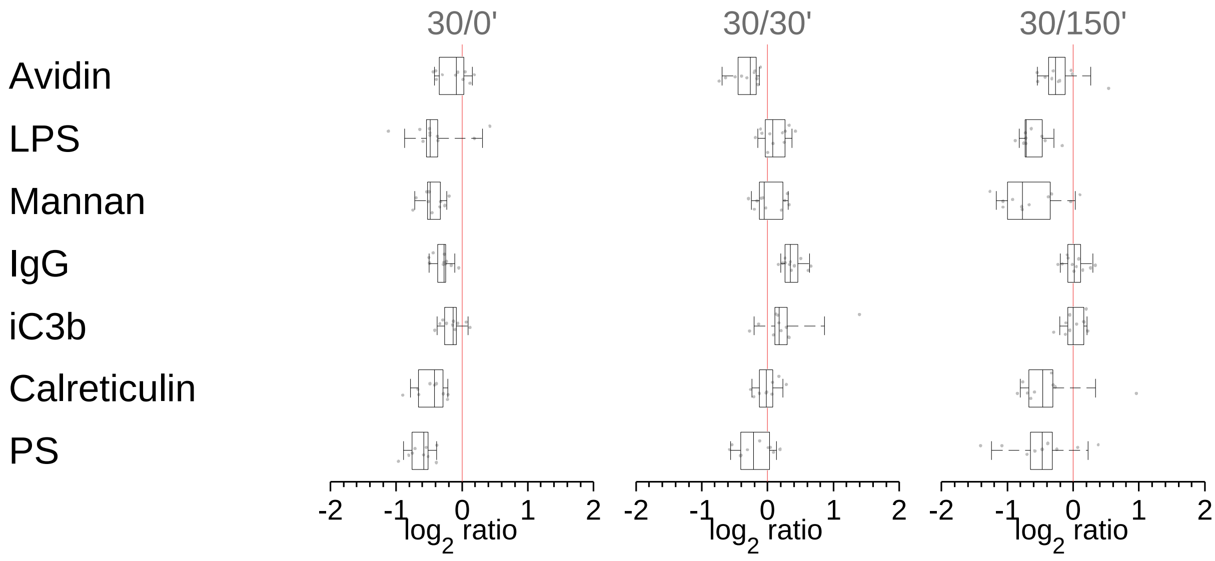

GO:0006641 triglyceride metabolic process (n = 9)

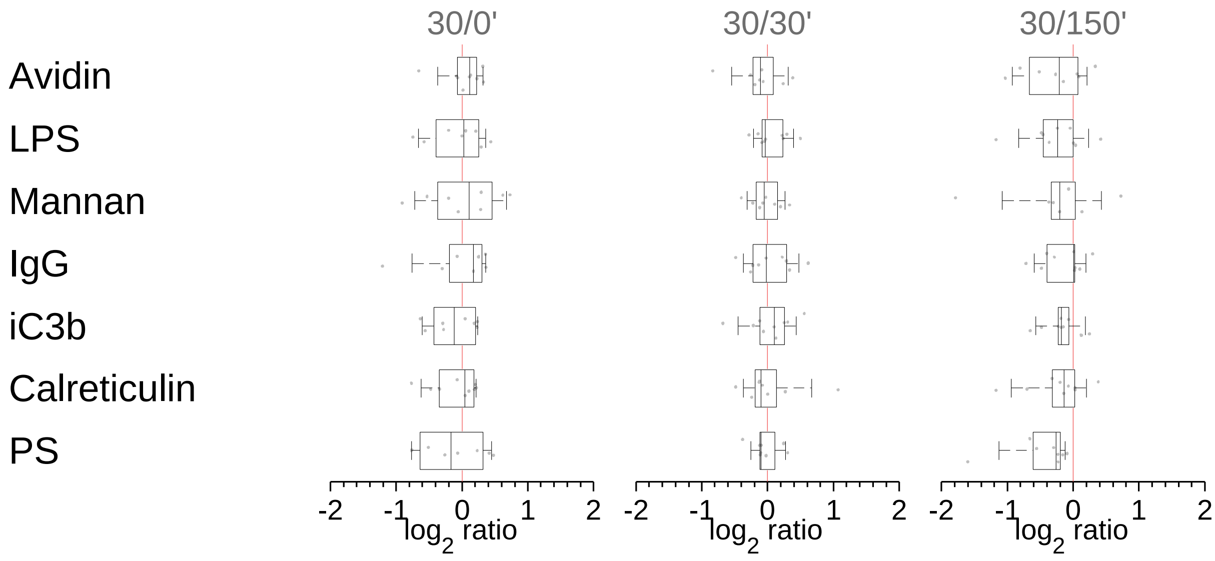

GO:0006661 phosphatidylinositol biosynthetic process (n = 3)

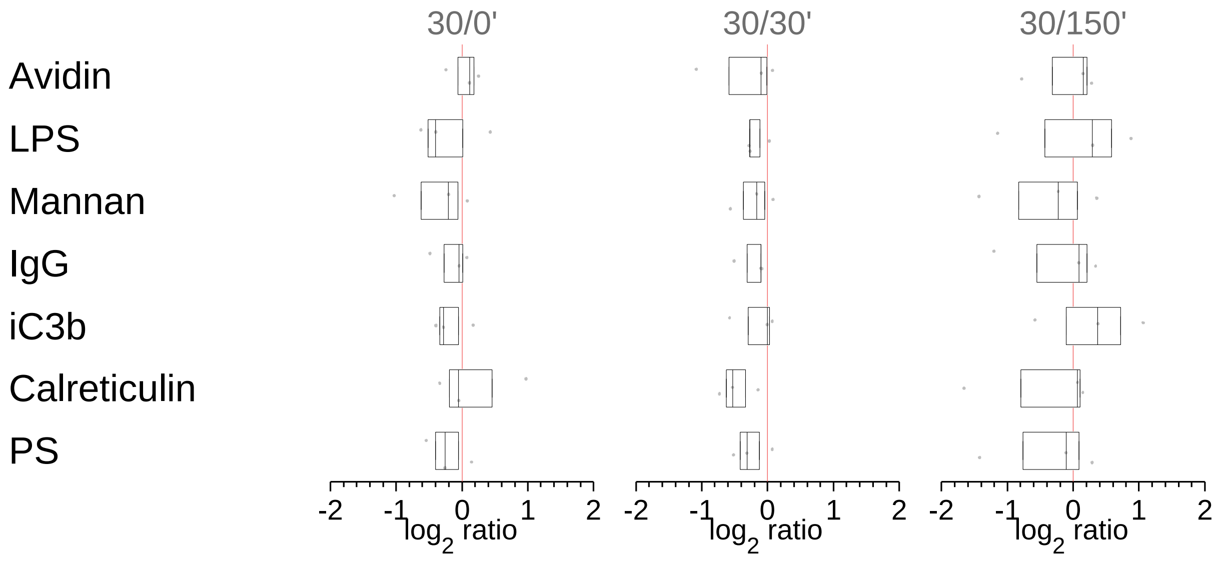

GO:0006665 sphingolipid metabolic process (n = 8)

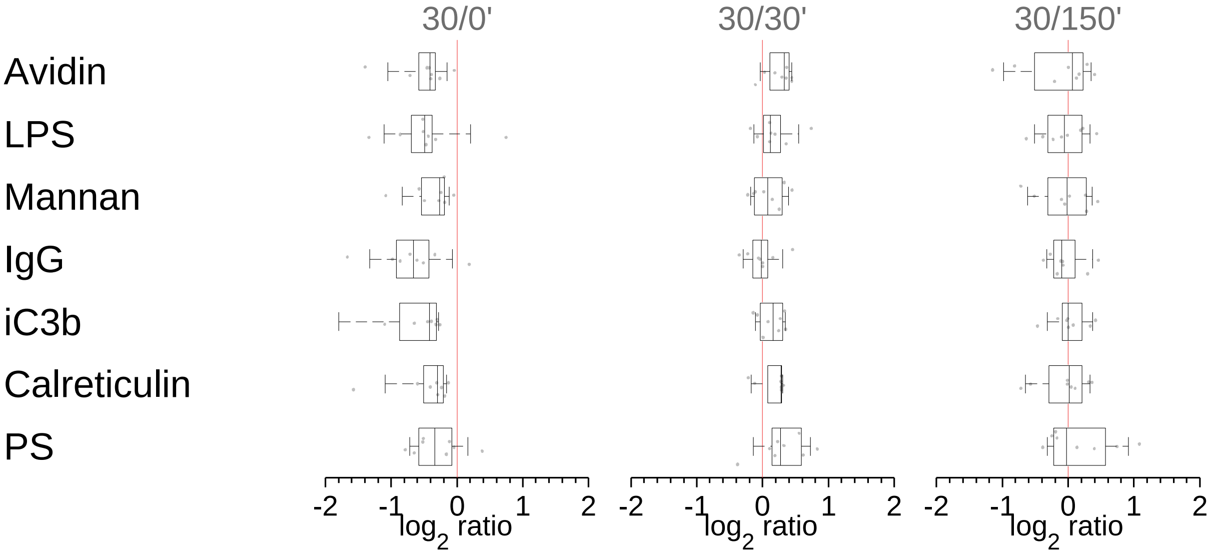

GO:0006672 ceramide metabolic process (n = 3)

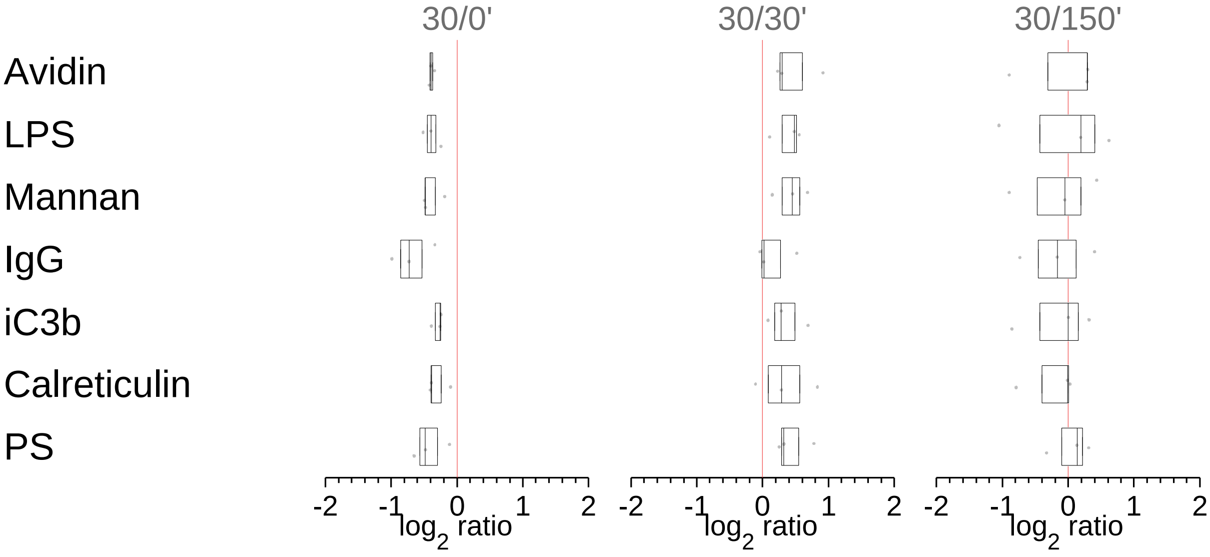

GO:0006685 sphingomyelin catabolic process (n = 3)

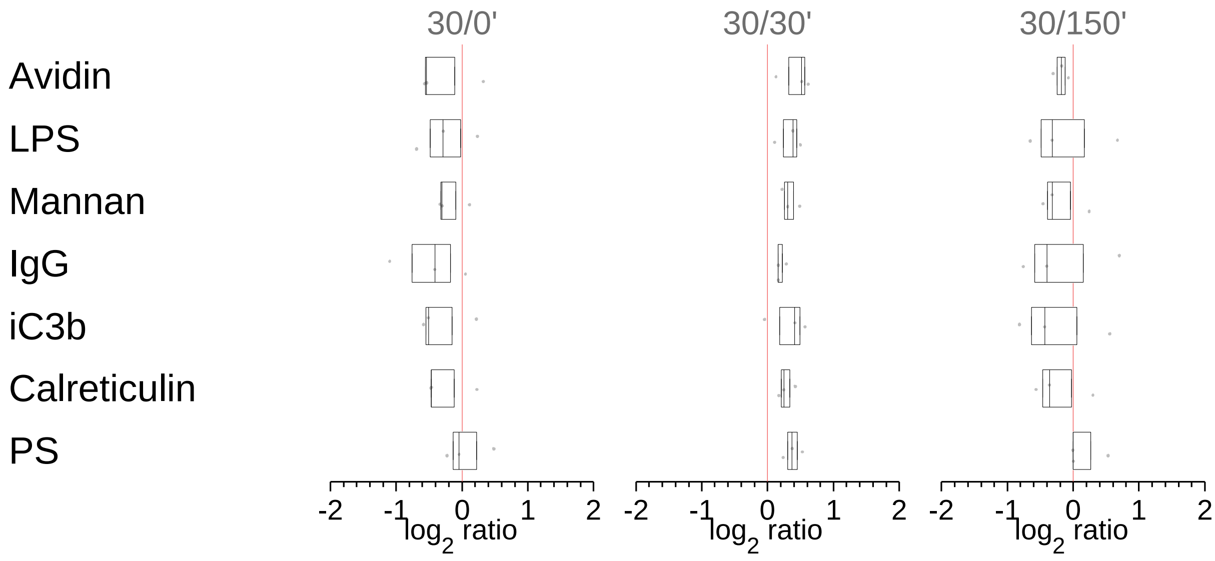

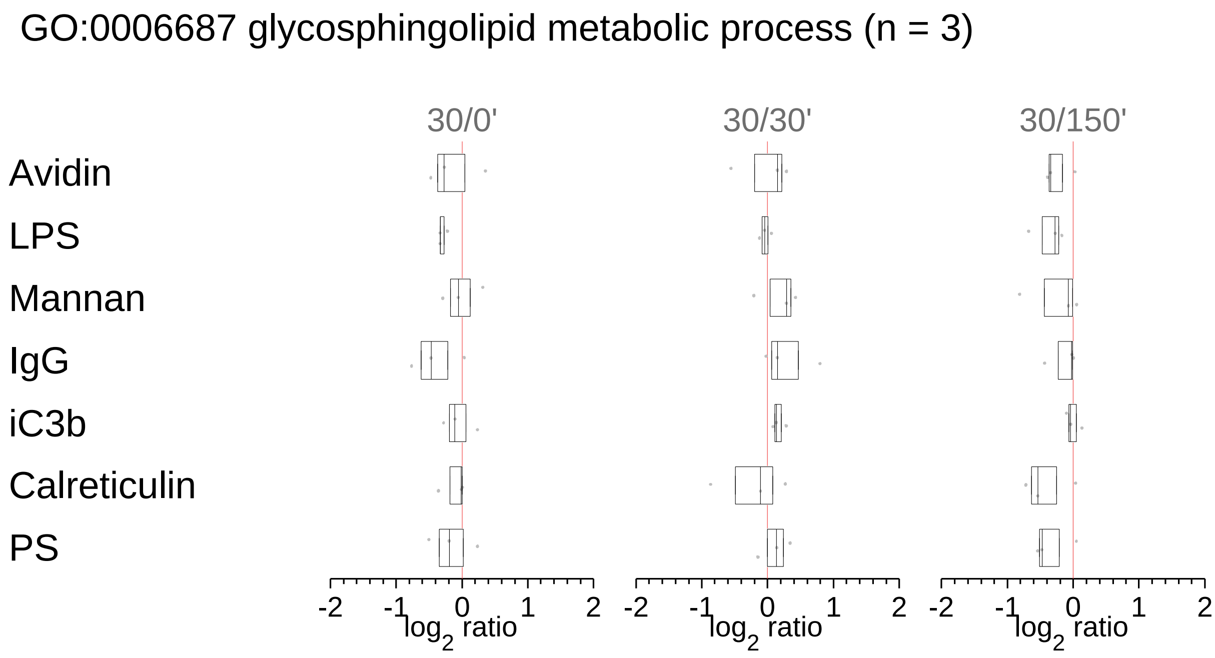

GO:0006687 glycosphingolipid metabolic process (n = 3)
30/0'
30/30'
30/150'
Avidin
LPS
Mannan
IgG
iC3b
Calreticulin
PS
-2
-1
0
1
2
log2 ratio

GO:0006689 ganglioside catabolic process (n = 3)

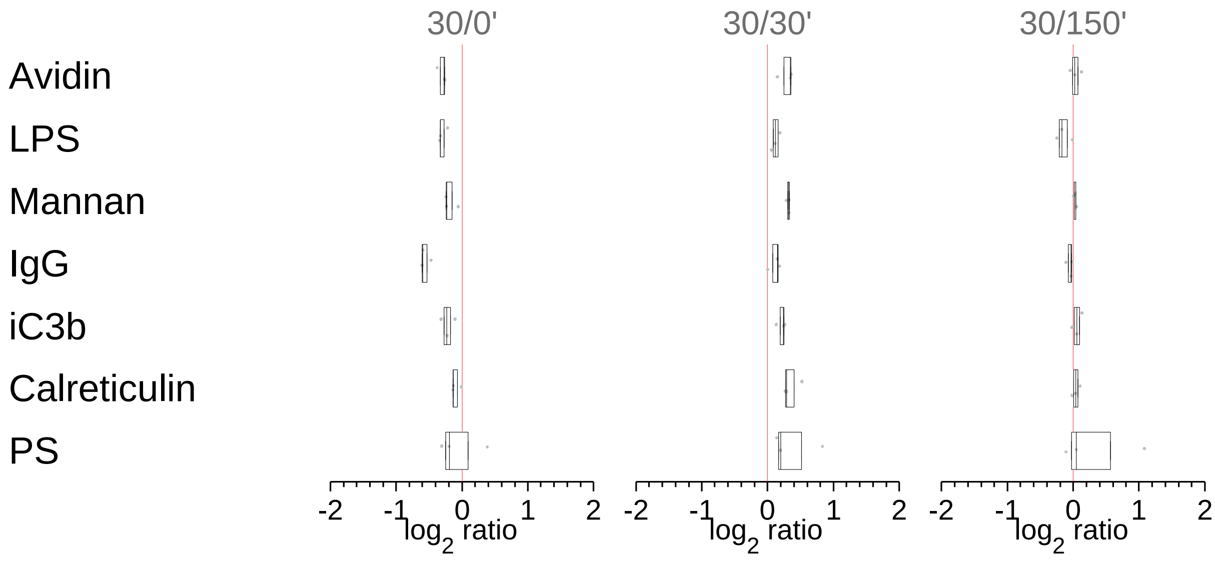

GO:0006691 leukotriene metabolic process (n = 4)

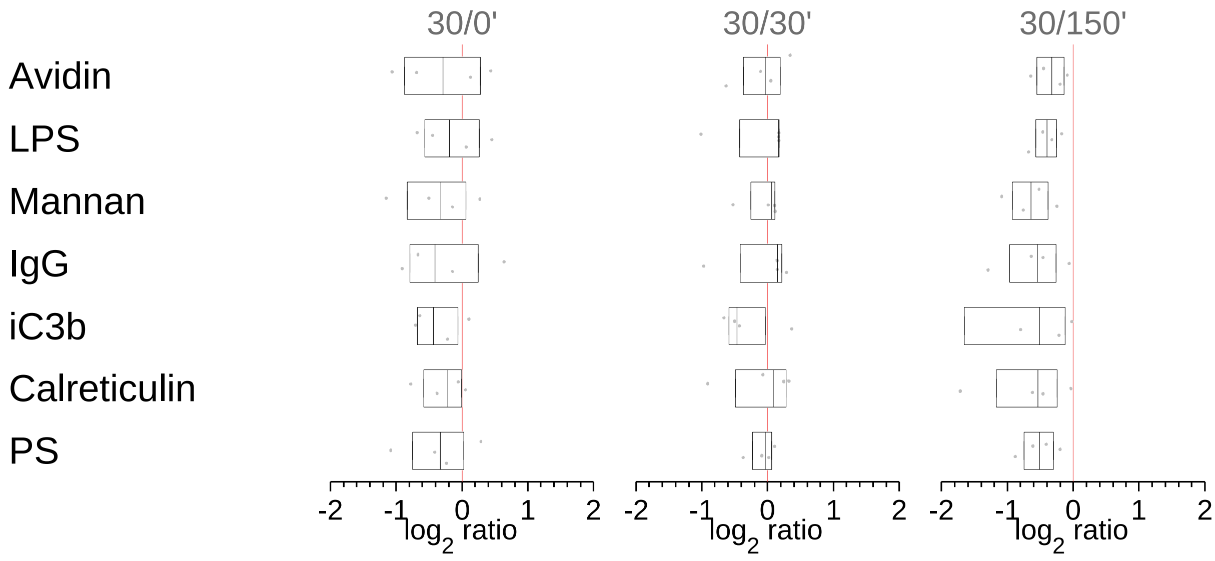

GO:0006694 steroid biosynthetic process (n = 11)

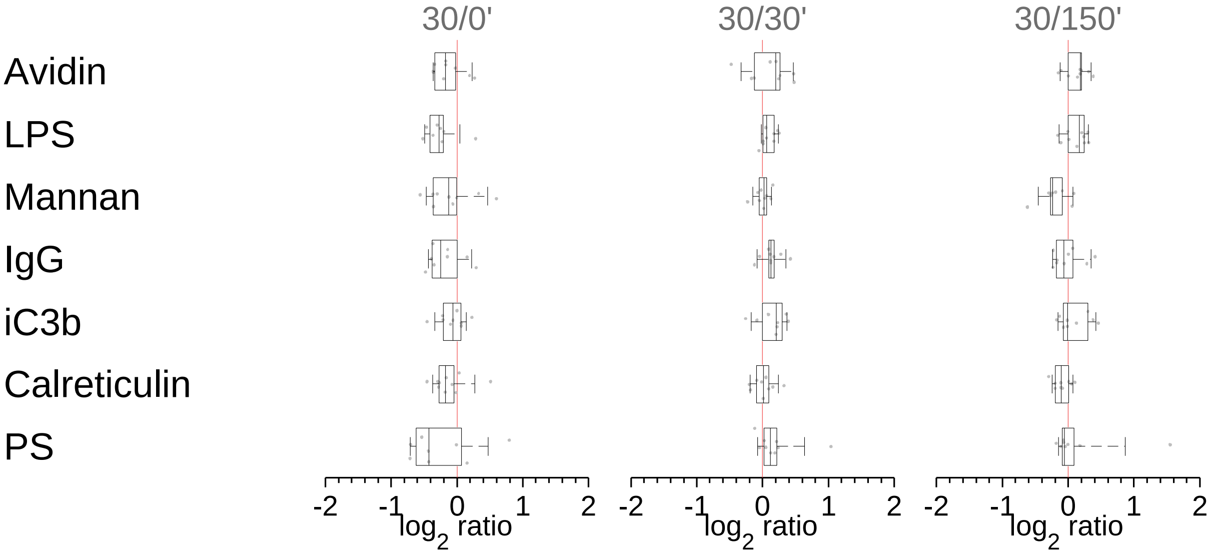

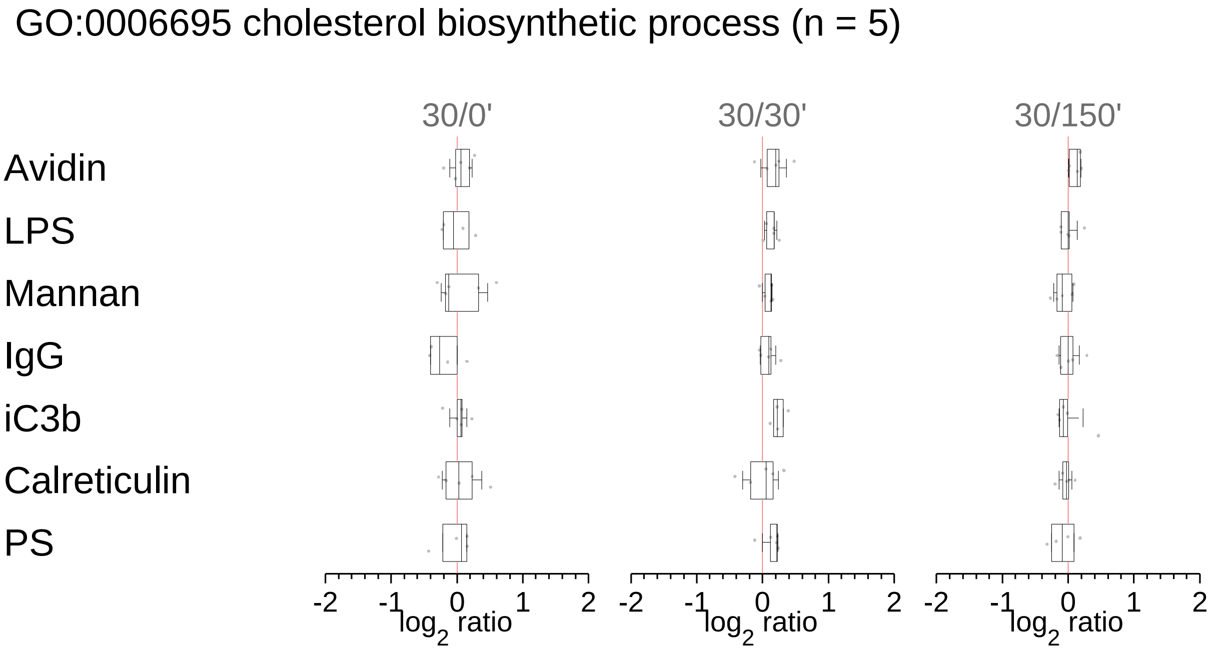

GO:0006695 cholesterol biosynthetic process (n = 5)
30/0'
30/30'
30/150'
Avidin
LPS
Mannan
IgG
iC3b
Calreticulin
PS
-2 -1 0 1 2
log2 ratio

GO:0006734 NADH metabolic process (n = 7)

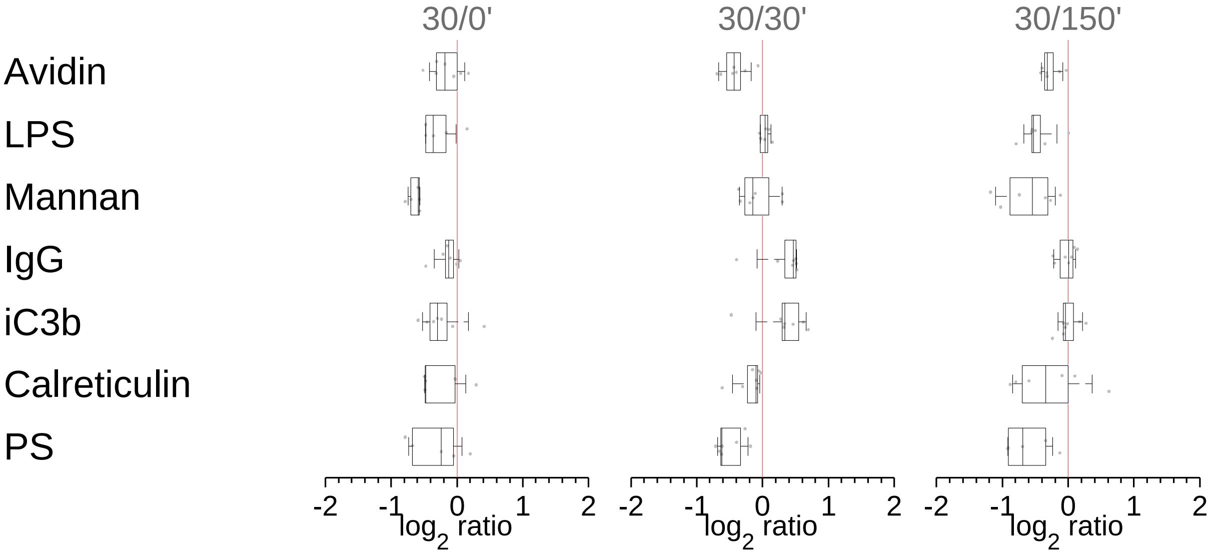

GO:0006749 glutathione metabolic process (n = 9)

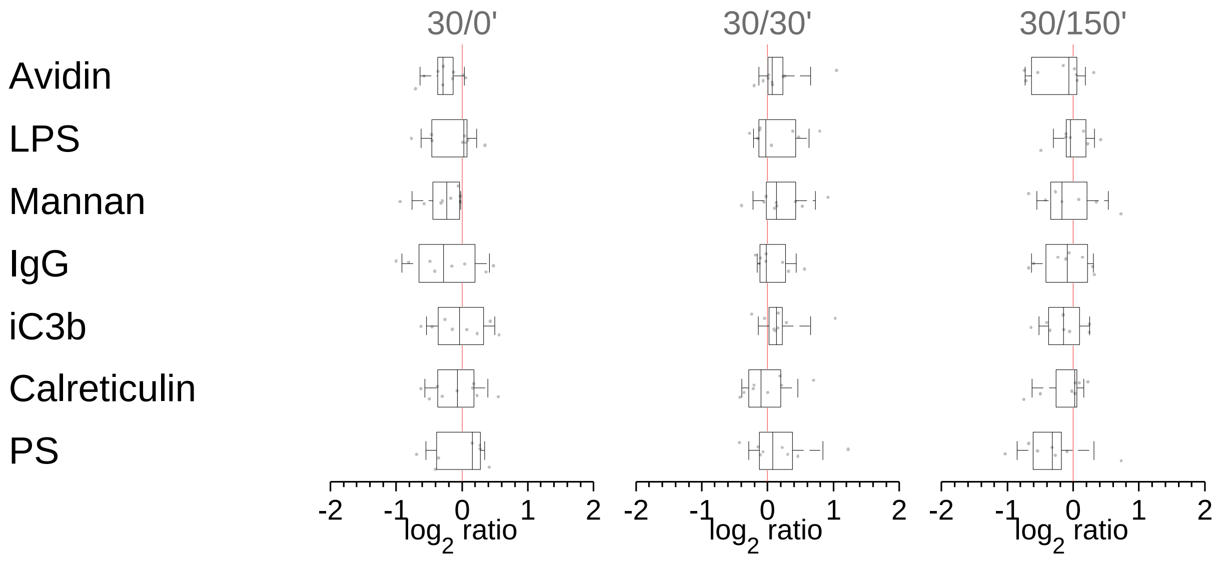

GO:0006754 ATP biosynthetic process (n = 12)

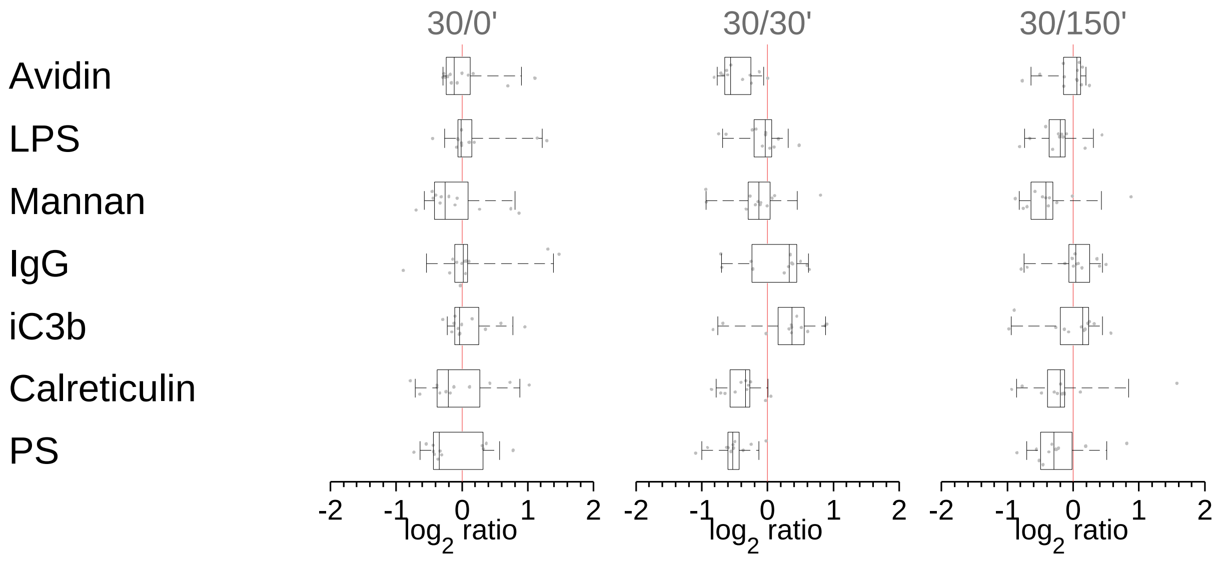

GO:0006783 heme biosynthetic process (n = 3)

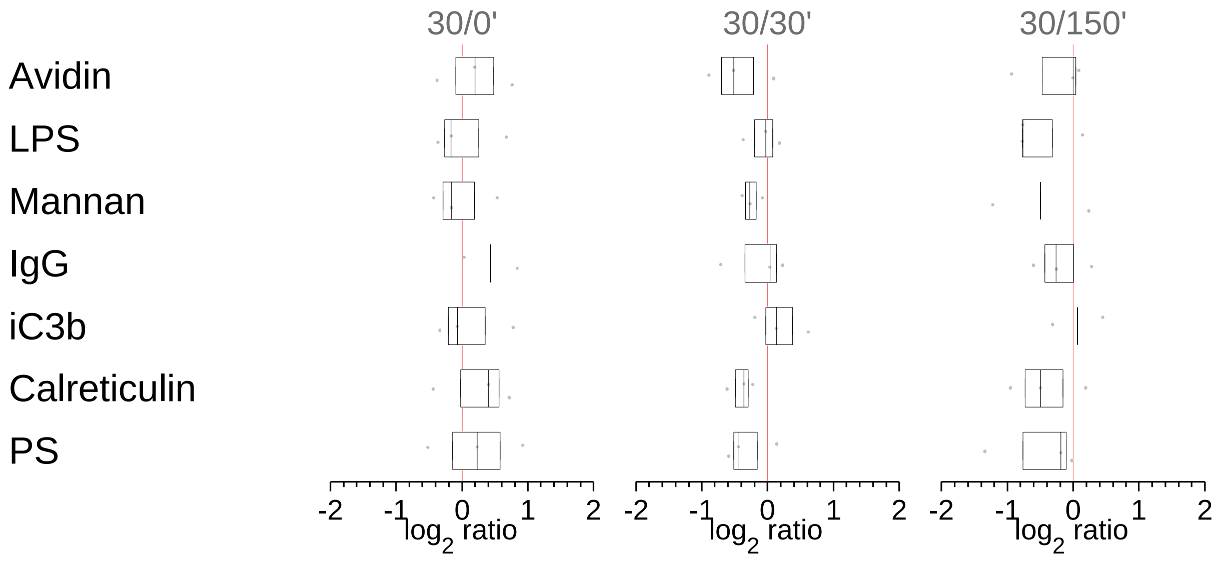

GO:0006801 superoxide metabolic process (n = 6)

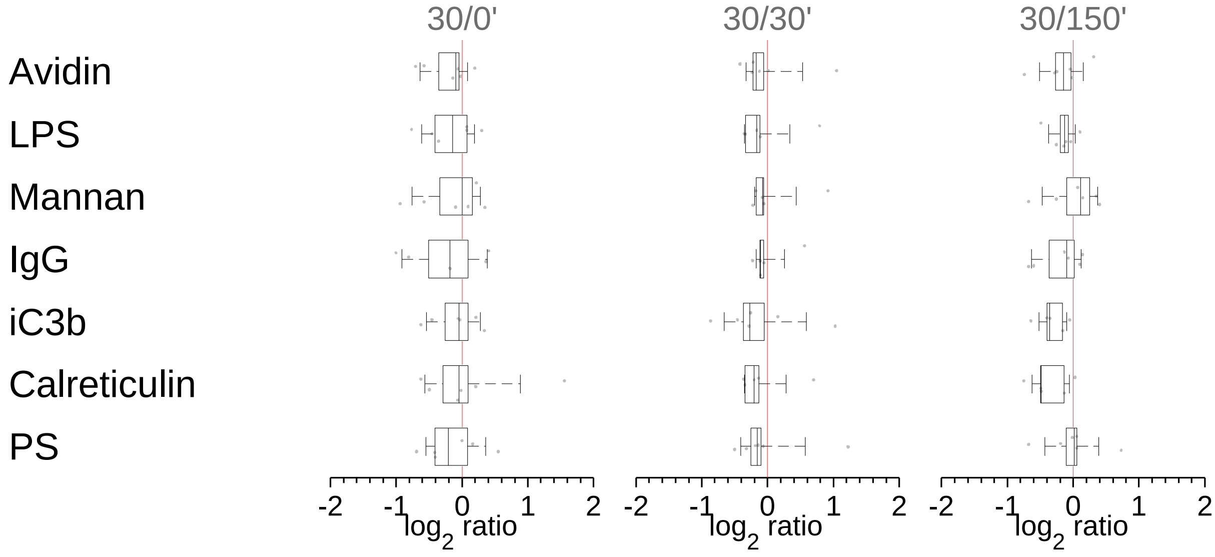

GO:0006807 nitrogen compound metabolic process (n = 6)

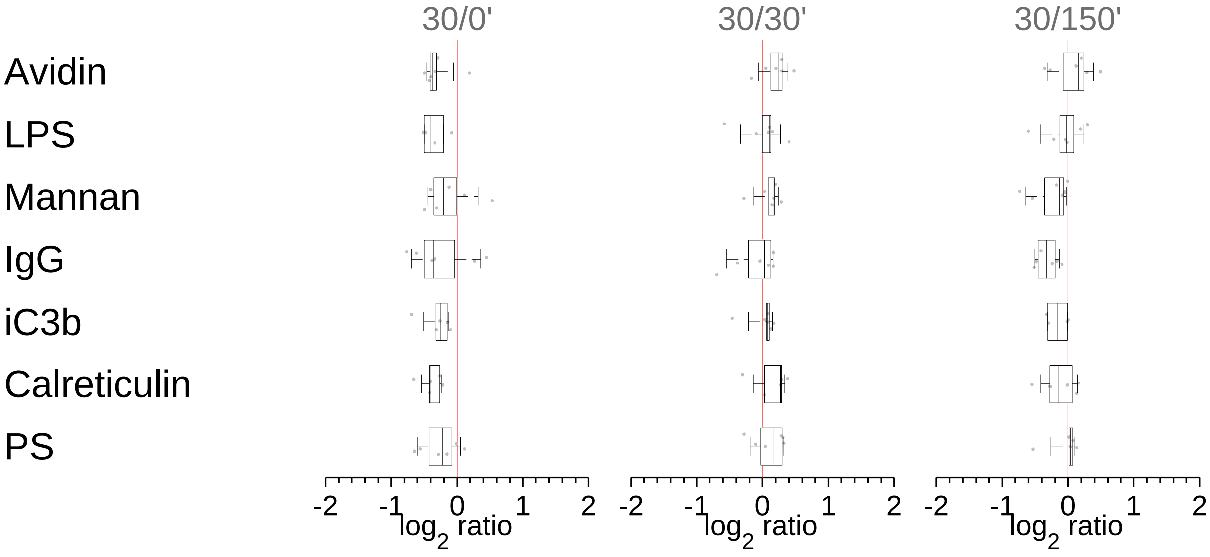

GO:0006810 transport (n = 401)

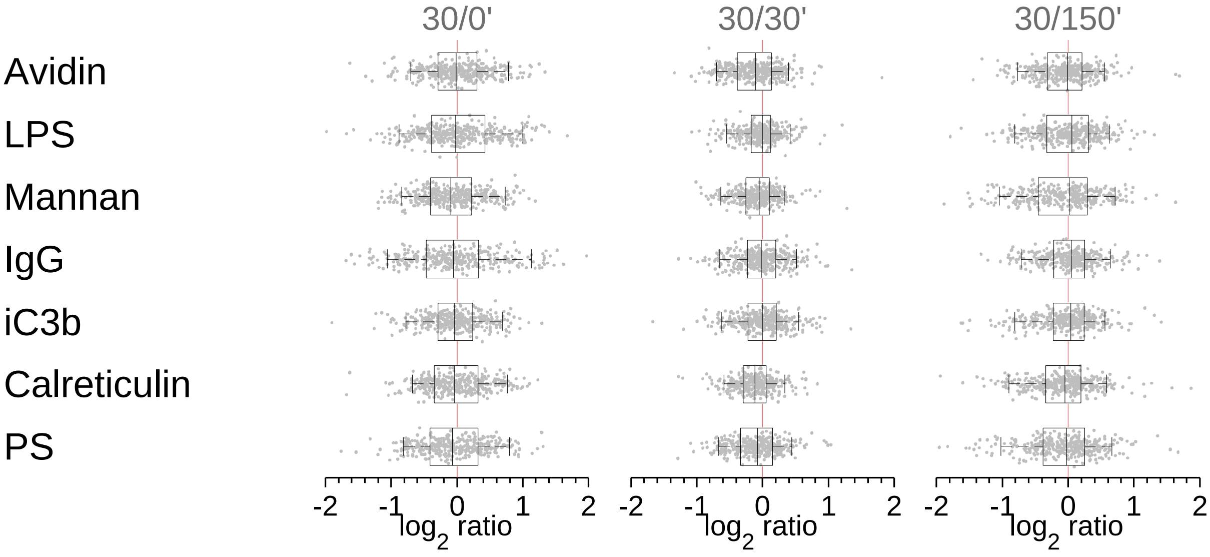

GO:0006811 ion transport (n = 76)

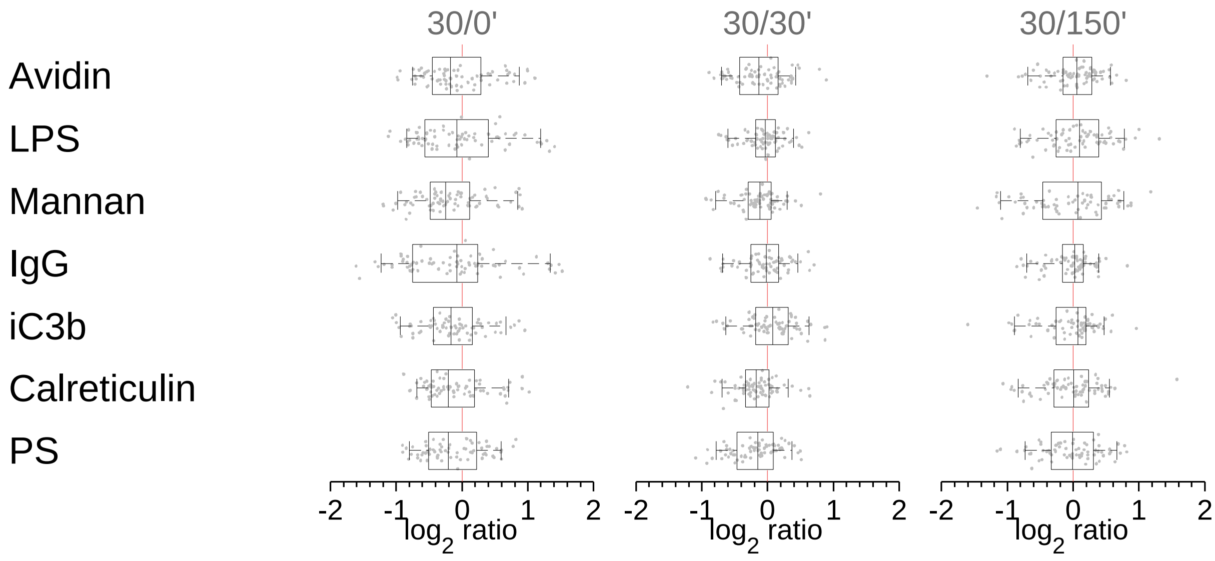

GO:0006812 cation transport (n = 18)

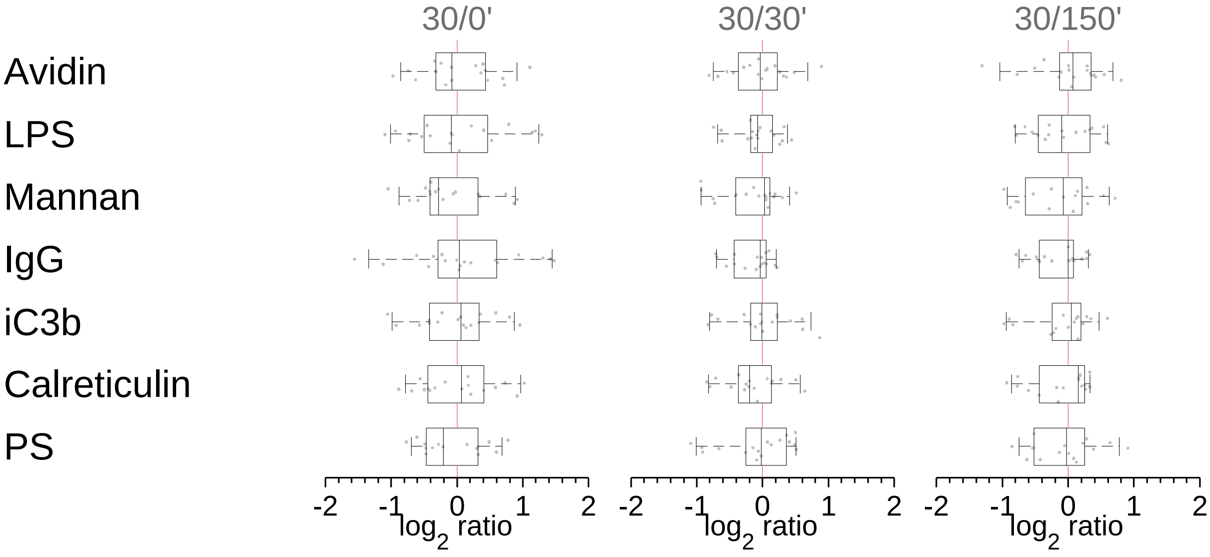

GO:0006813 potassium ion transport (n = 11)

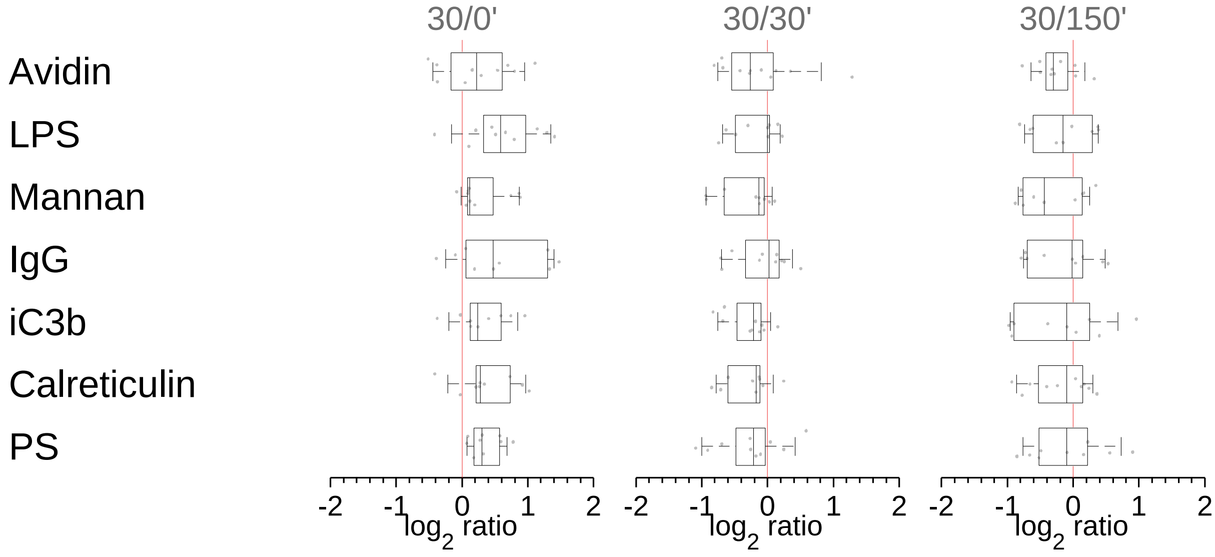

GO:0006814 sodium ion transport (n = 9)

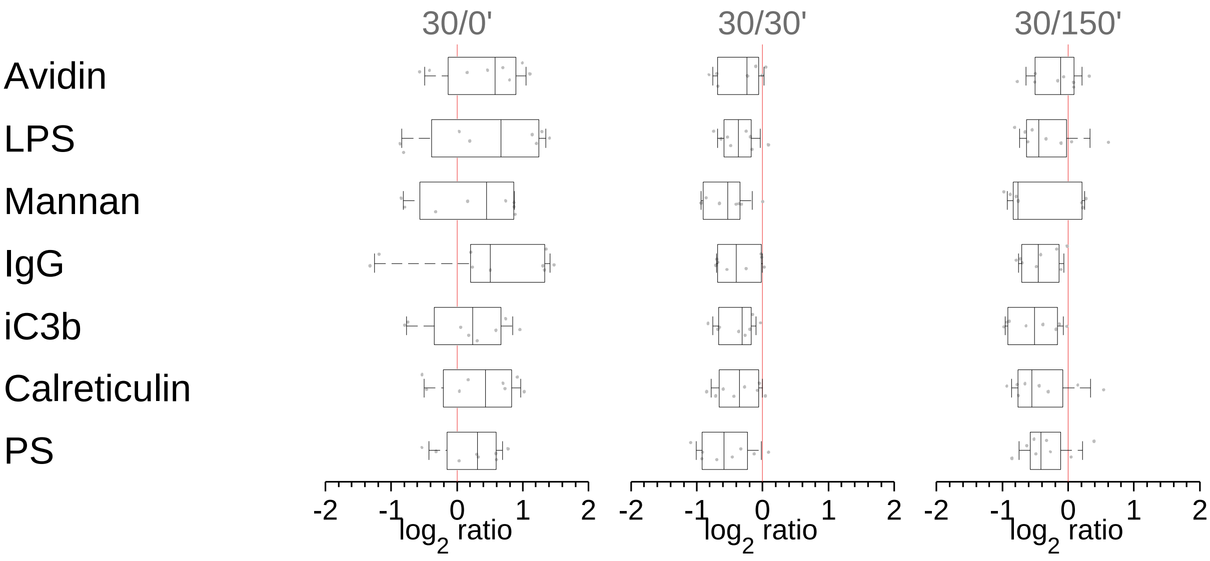

GO:0006816 calcium ion transport (n = 15)

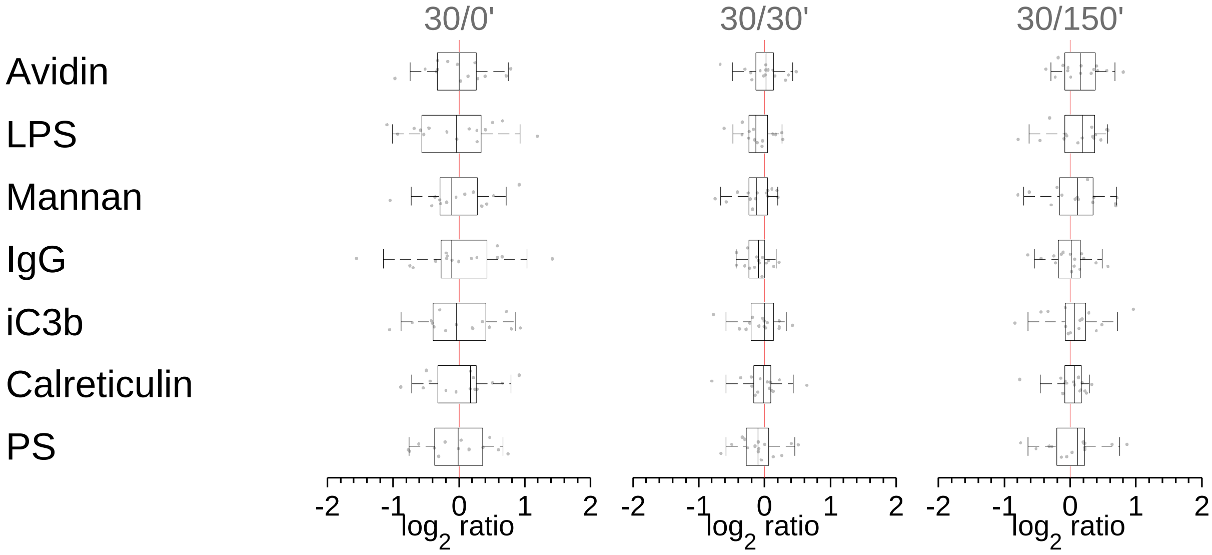

GO:0006820 anion transport (n = 6)

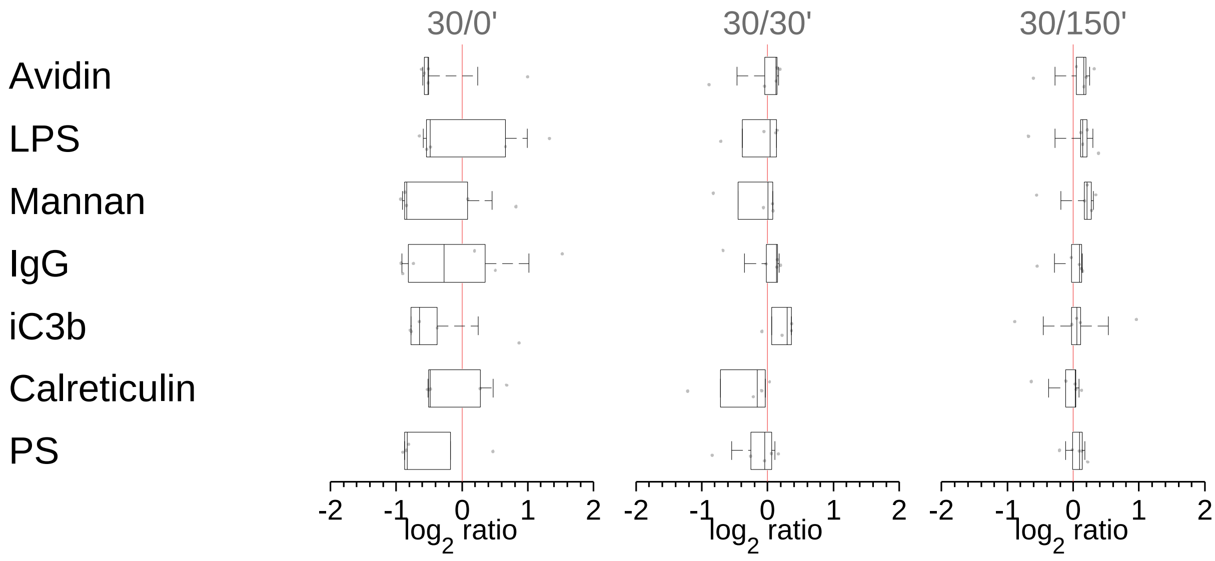

GO:0006821 chloride transport (n = 9)

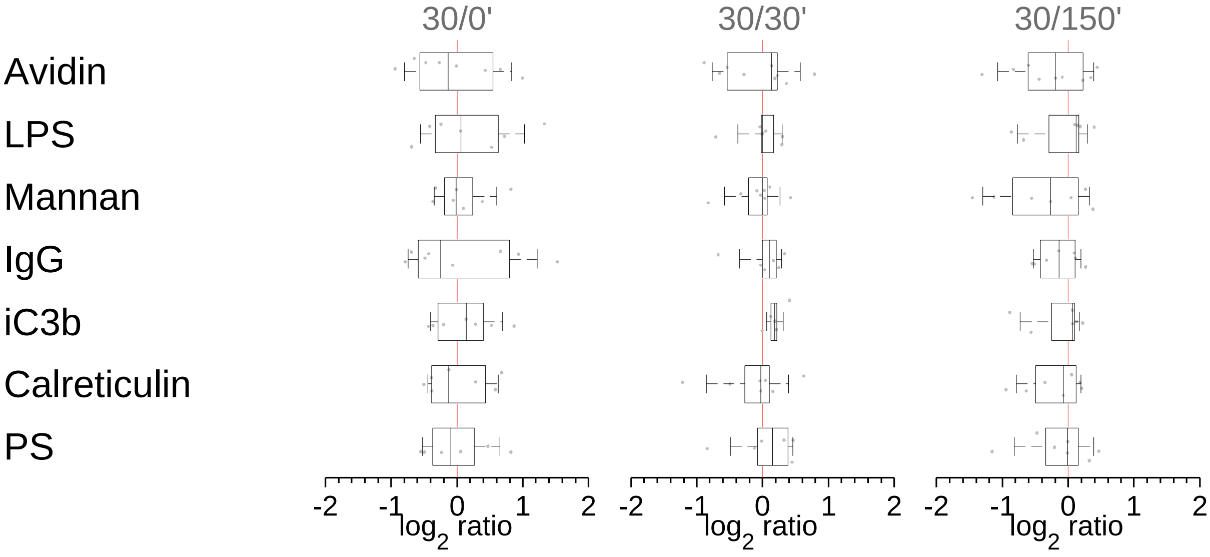

GO:0006826 iron ion transport (n = 4)

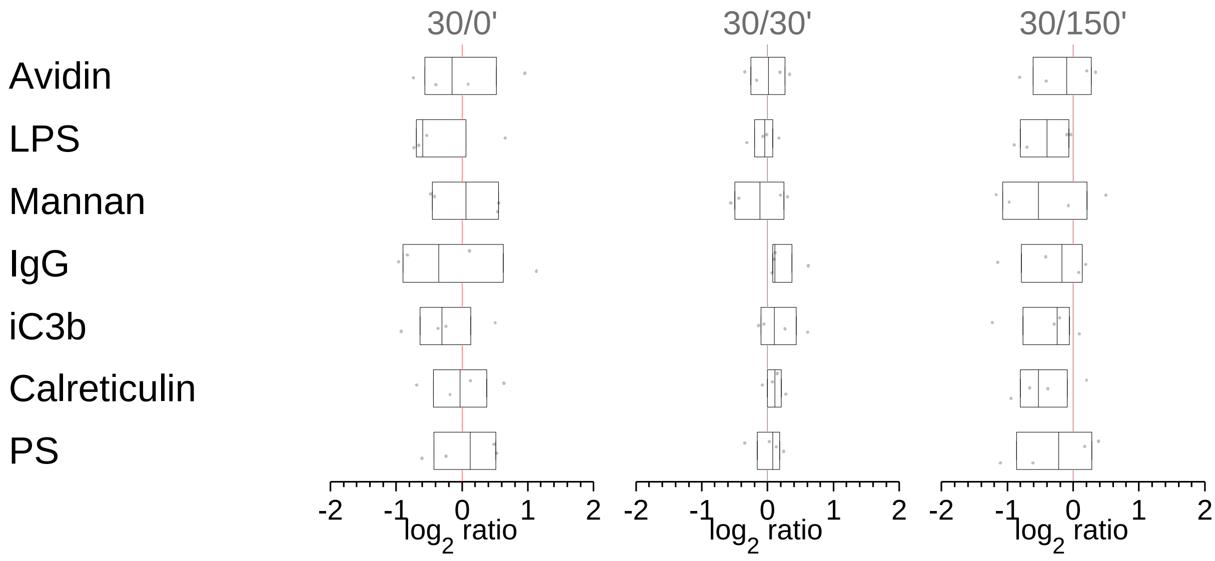

GO:0006829 zinc ion transport (n = 4)

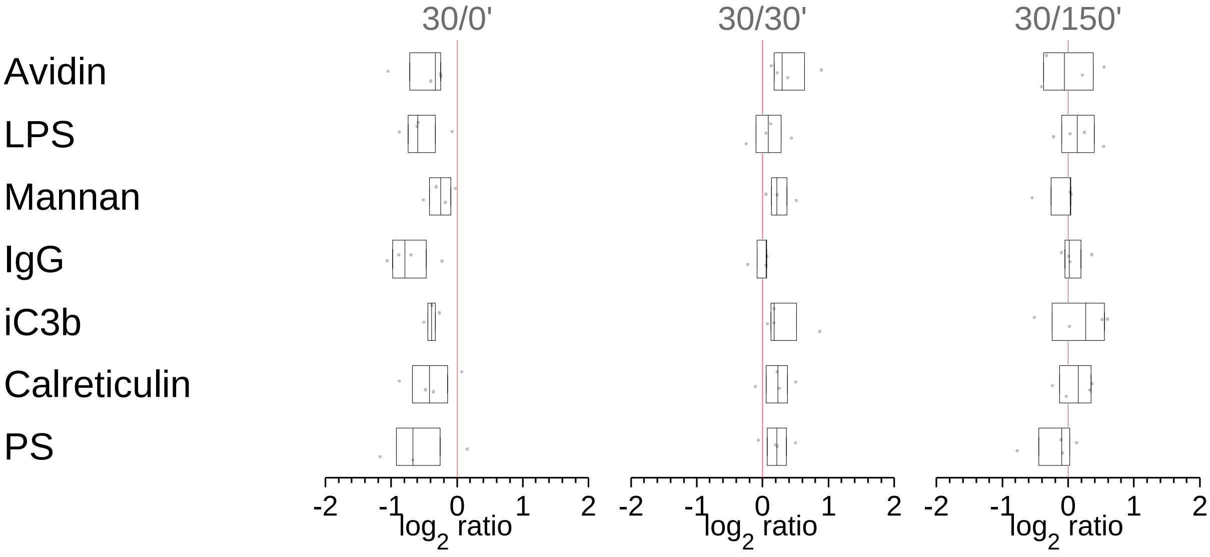

GO:0006836 neurotransmitter transport (n = 3)

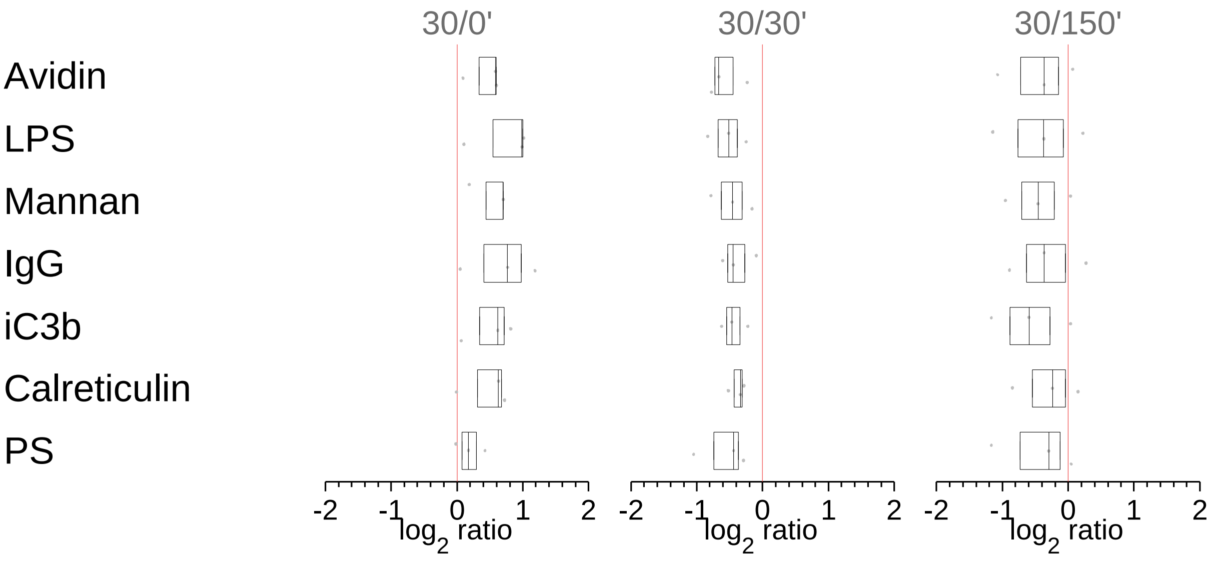

GO:0006839 mitochondrial transport (n = 4)

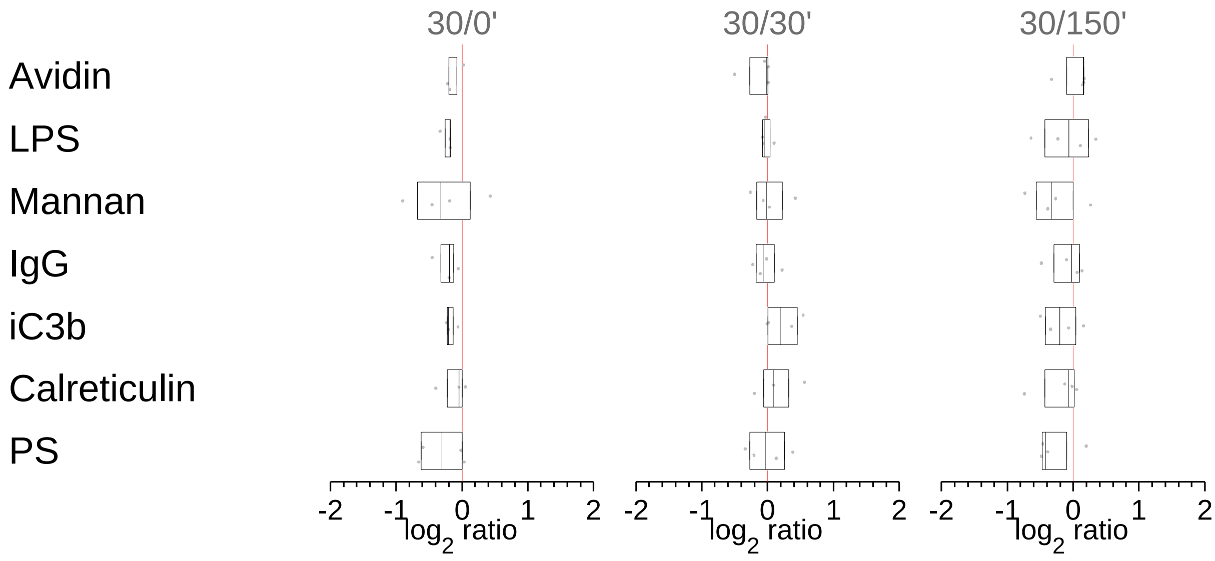

GO:0006855 drug transmembrane transport (n = 8)

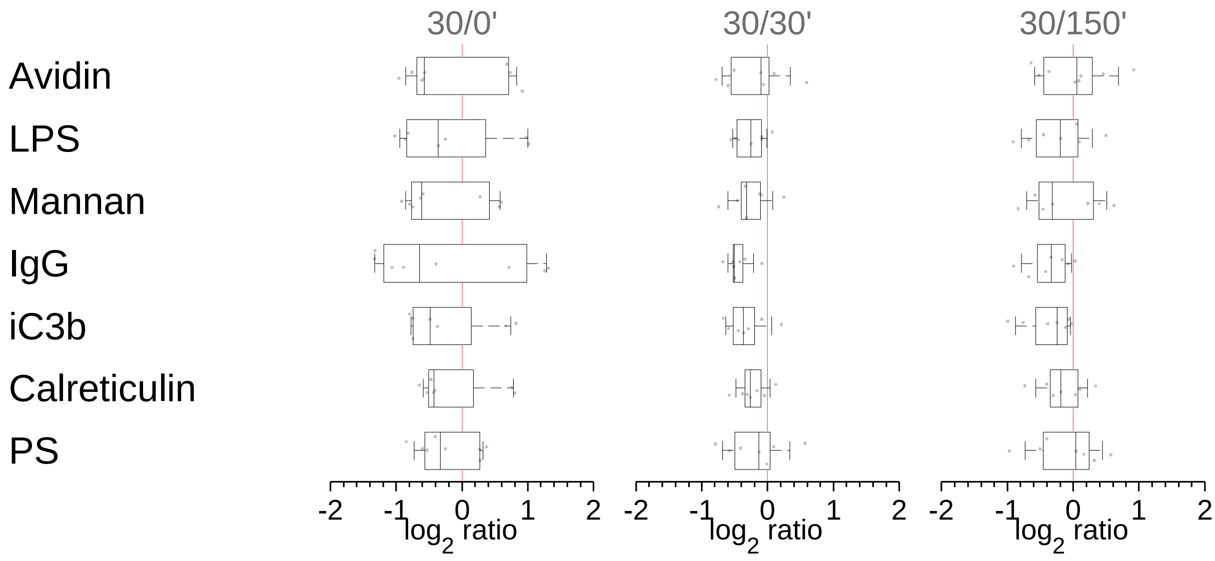

GO:0006865 amino acid transport (n = 11)

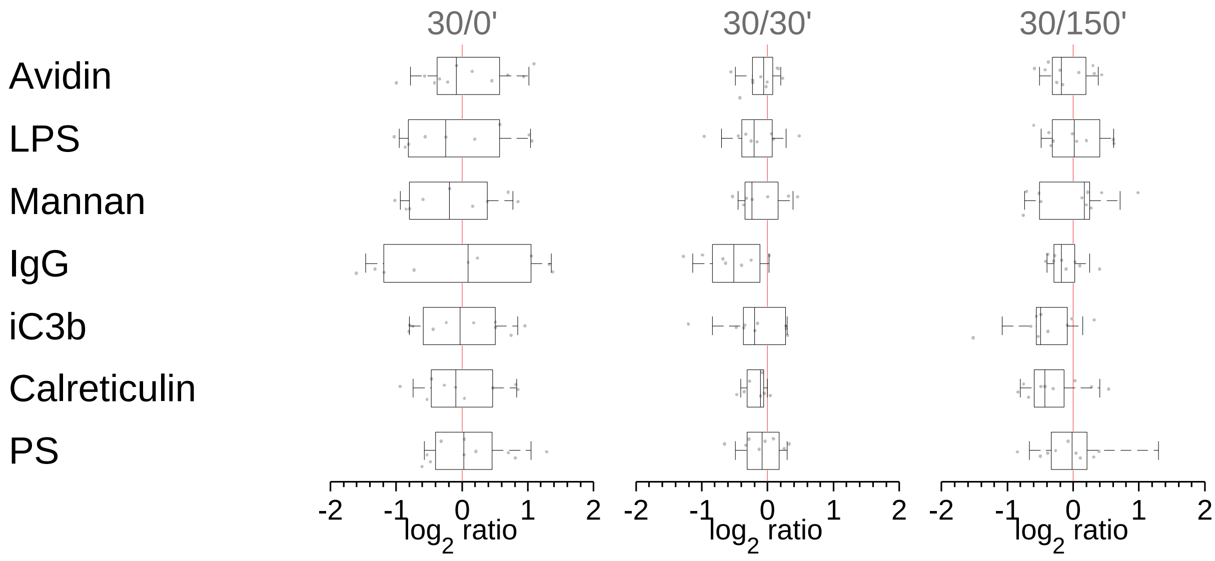

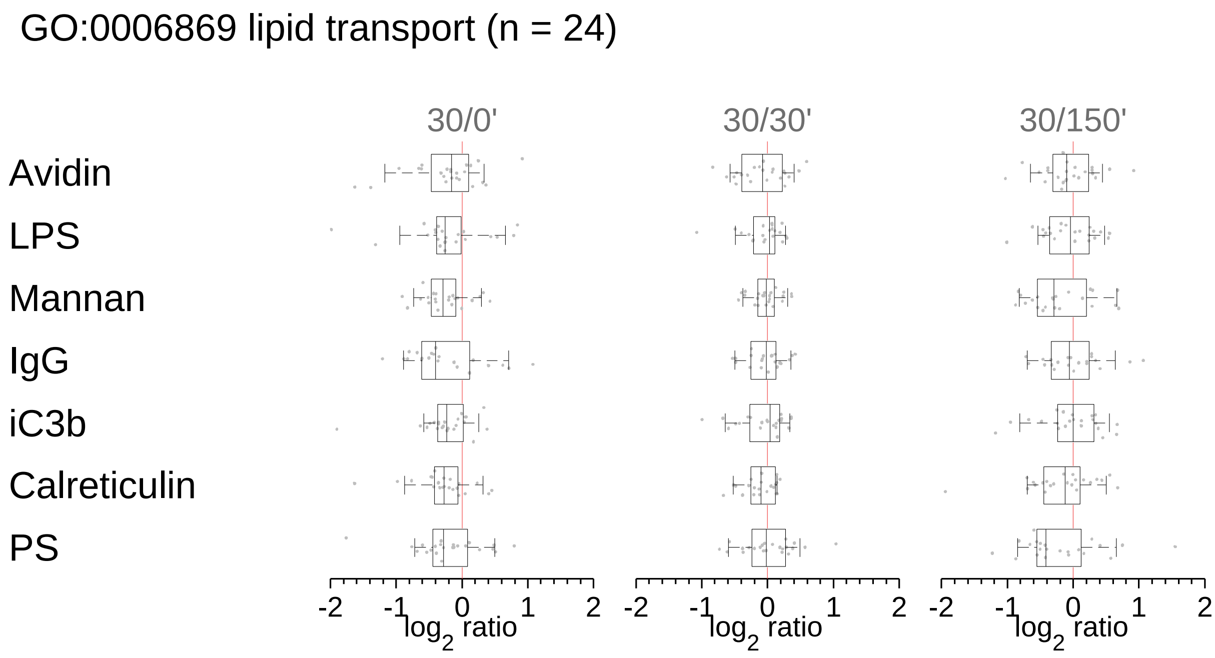
GO:0006869 lipid transport (n = 24)
30/0'
30/30'
30/150'
Avidin
LPS
Mannan
IgG
iC3b
Calreticulin
PS
-2 -1 0 1 2
log2 ratio

GO:0006874 cellular calcium ion homeostasis (n = 12)

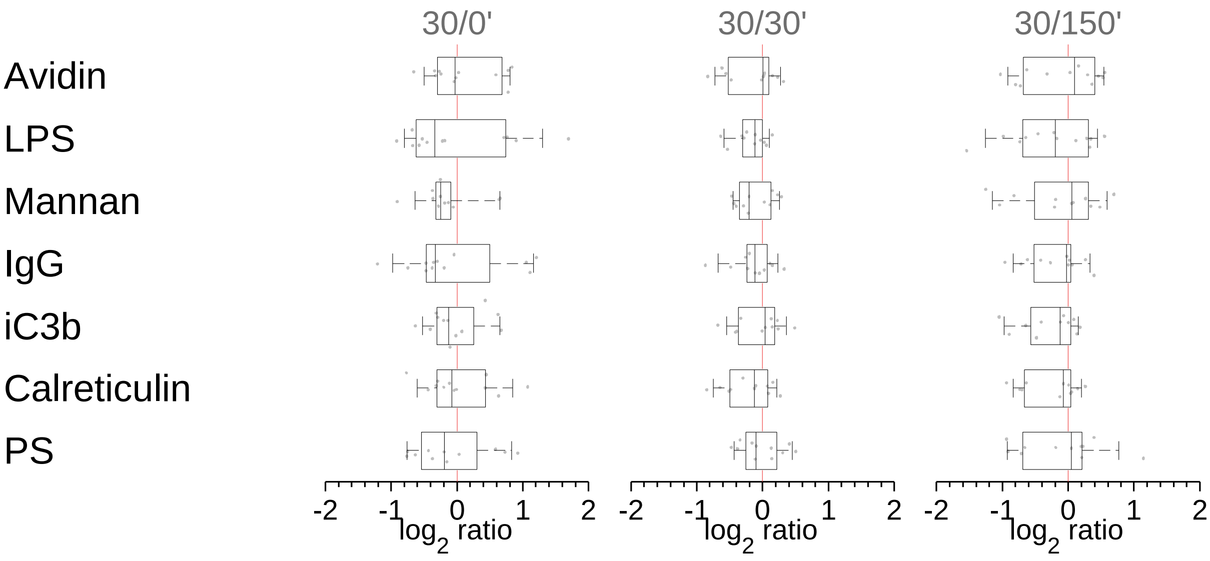

GO:0006878 cellular copper ion homeostasis (n = 5)

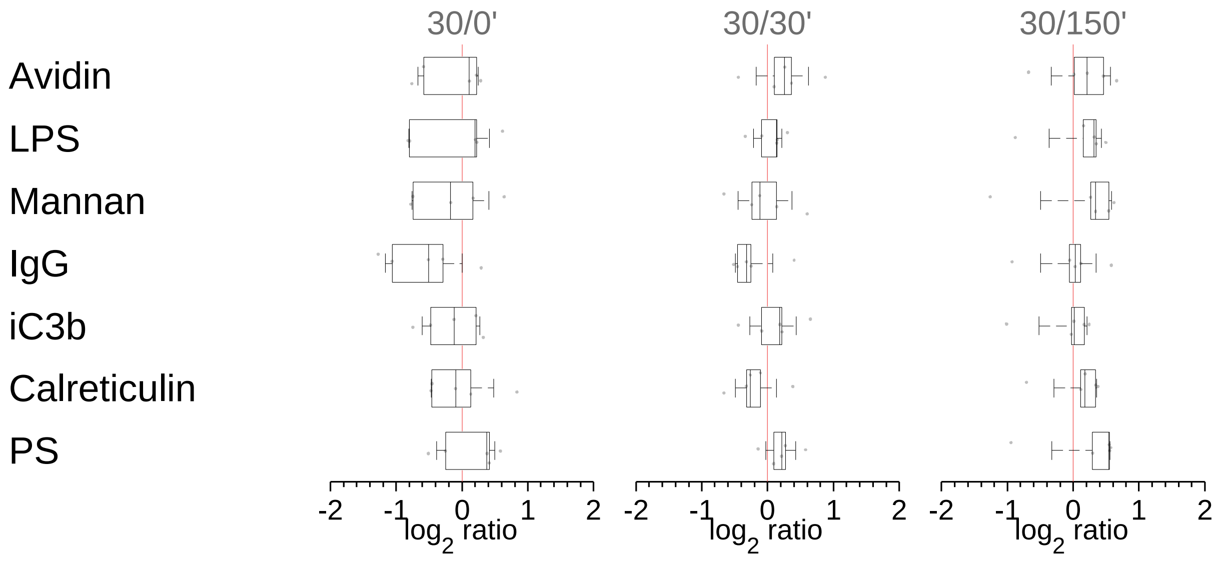

GO:0006879 cellular iron ion homeostasis (n = 5)

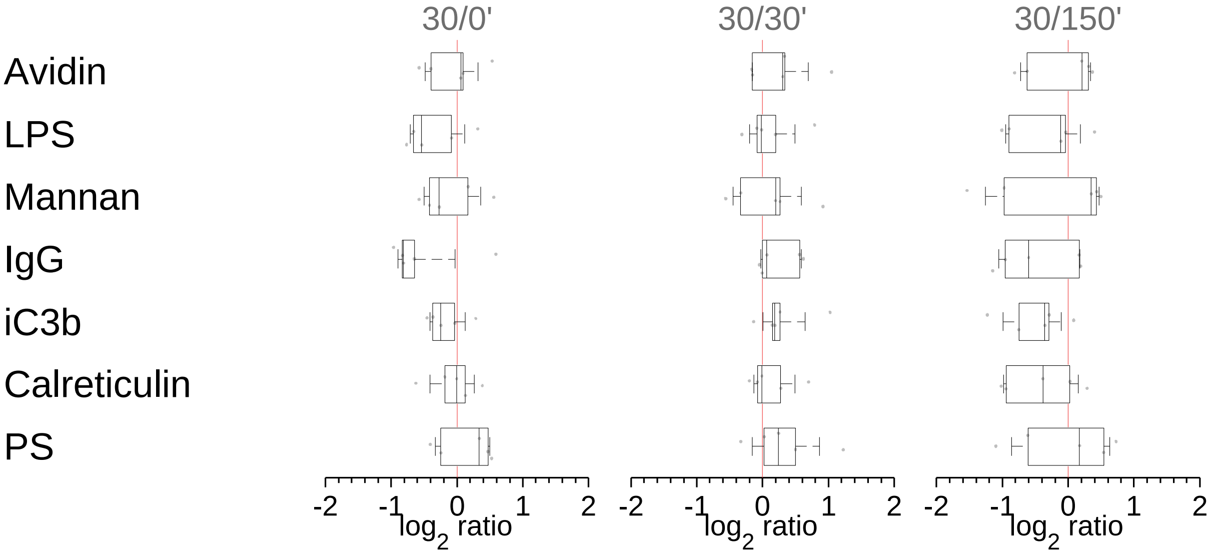

GO:0006884 cell volume homeostasis (n = 4)

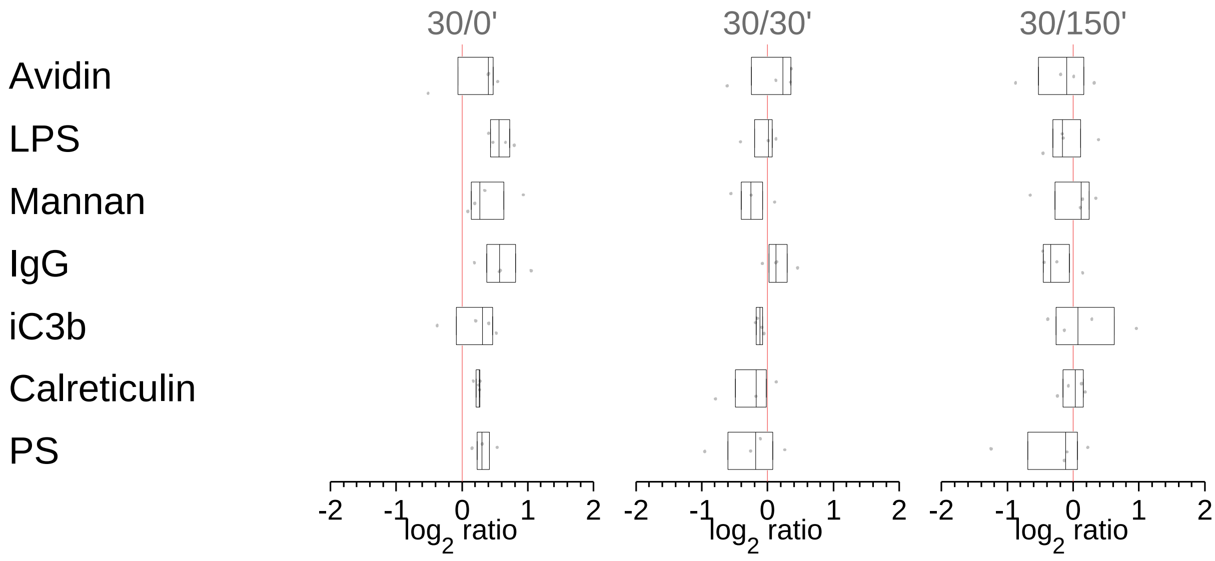

GO:0006886 intracellular protein transport (n = 76)

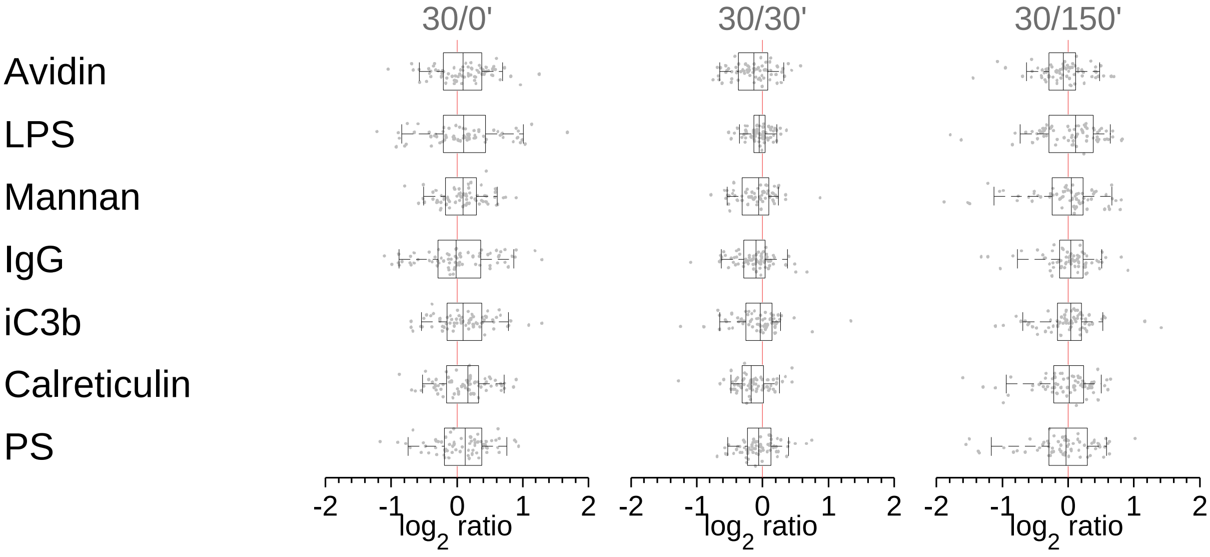

GO:0006887 exocytosis (n = 21)

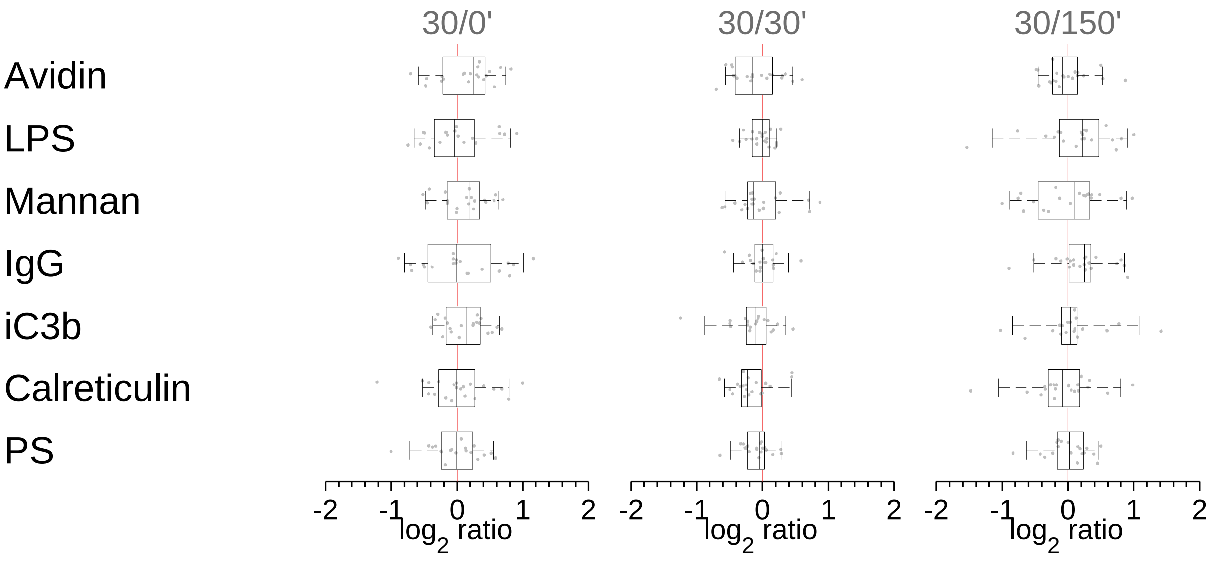

GO:0006888 ER to Golgi vesicle-mediated transport (n = 14)

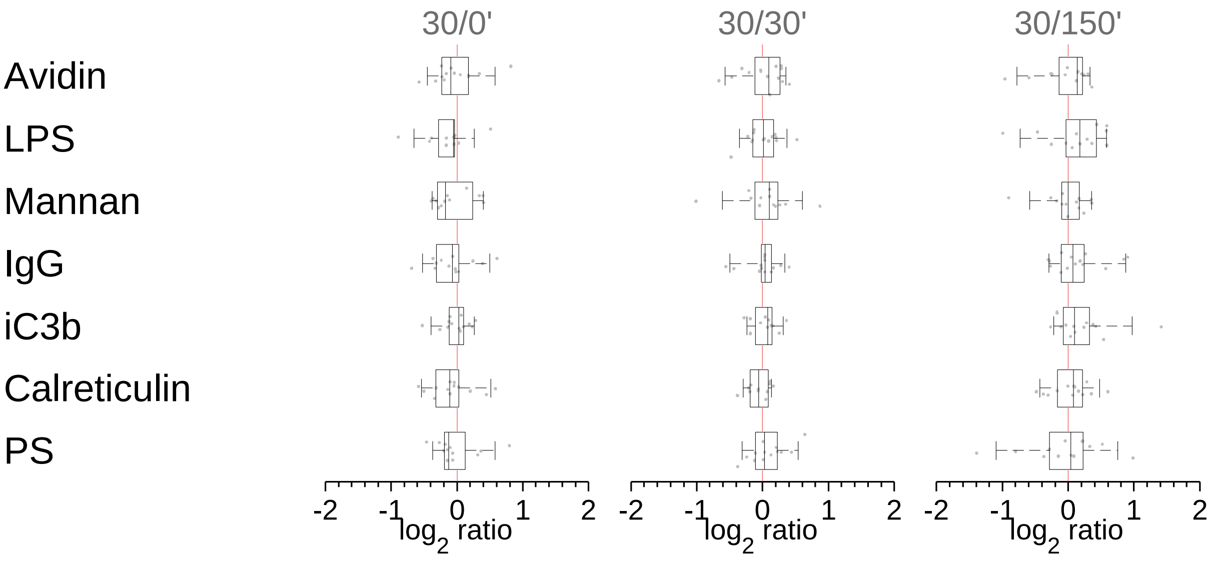

GO:0006890 retrograde vesicle-mediated transport, Golgi to ER (n = 3)

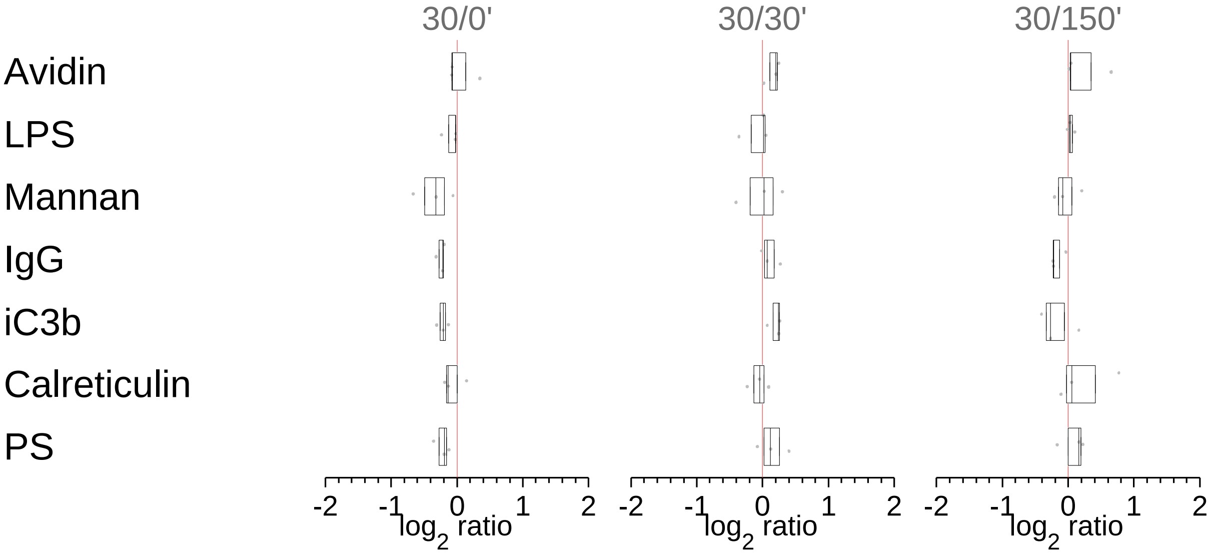

GO:0006891 intra-Golgi vesicle-mediated transport (n = 4)

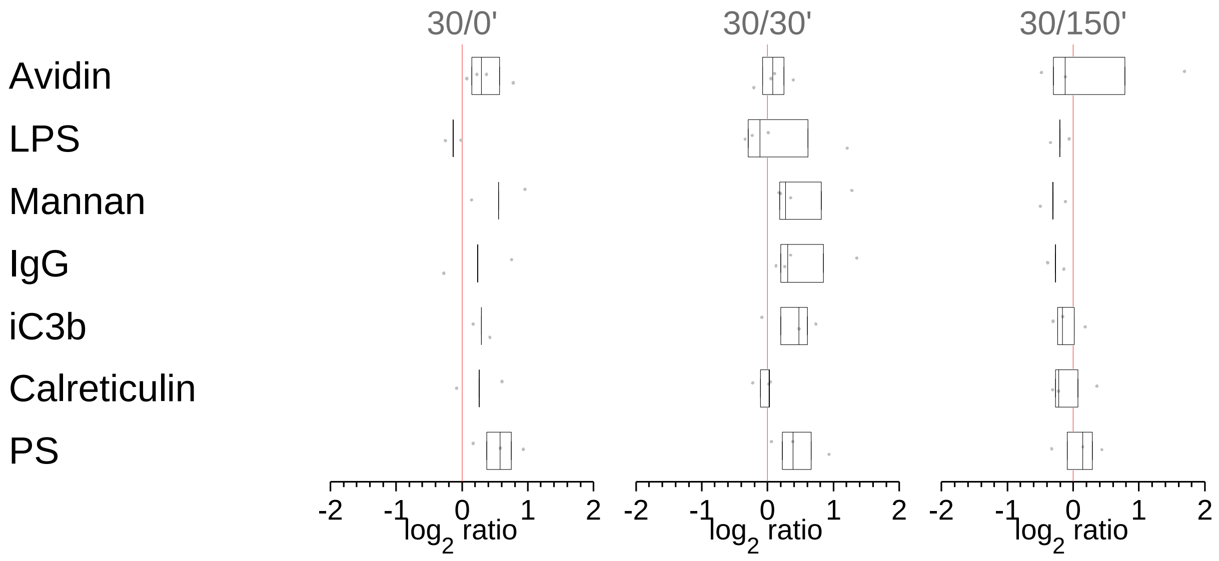

GO:0006897 endocytosis (n = 55)

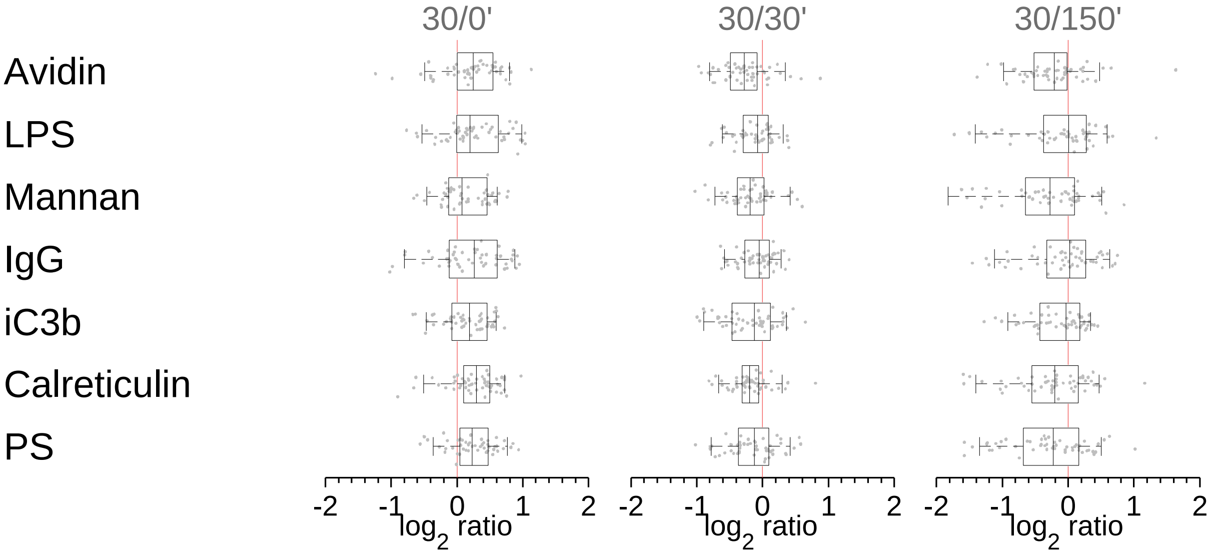

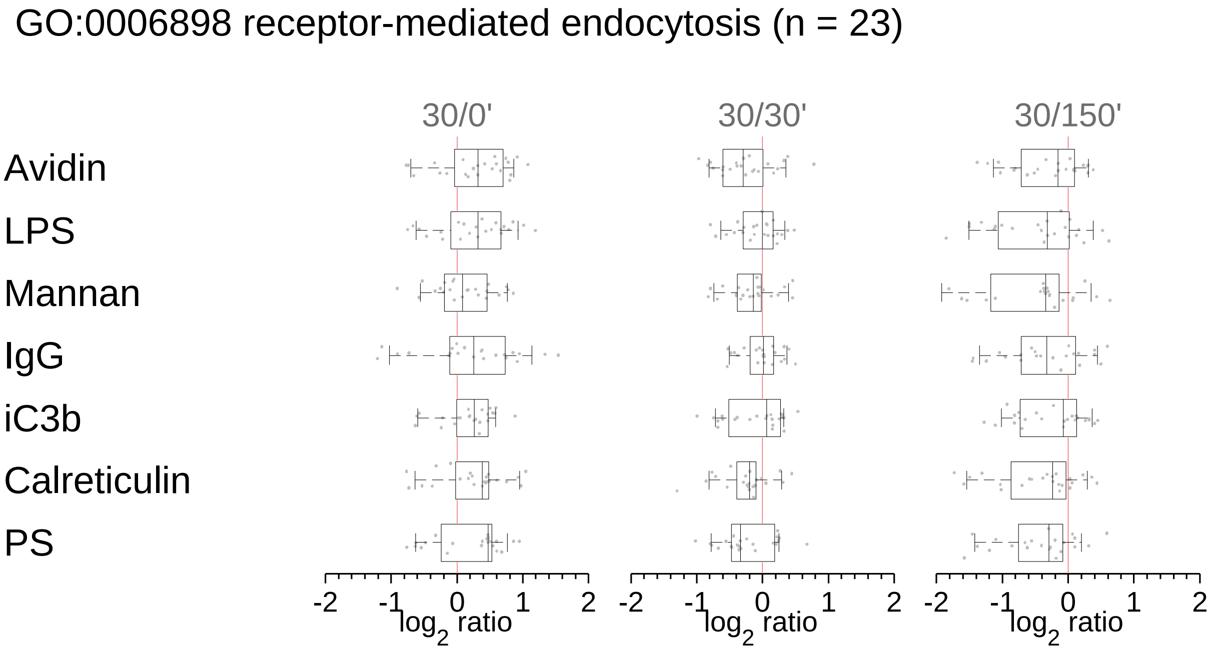
GO:0006898 receptor-mediated endocytosis (n = 23)
30/0'
30/30'
30/150'
Avidin
LPS
Mannan
IgG
iC3b
Calreticulin
PS
-2
-1
0
1
2
log2 ratio

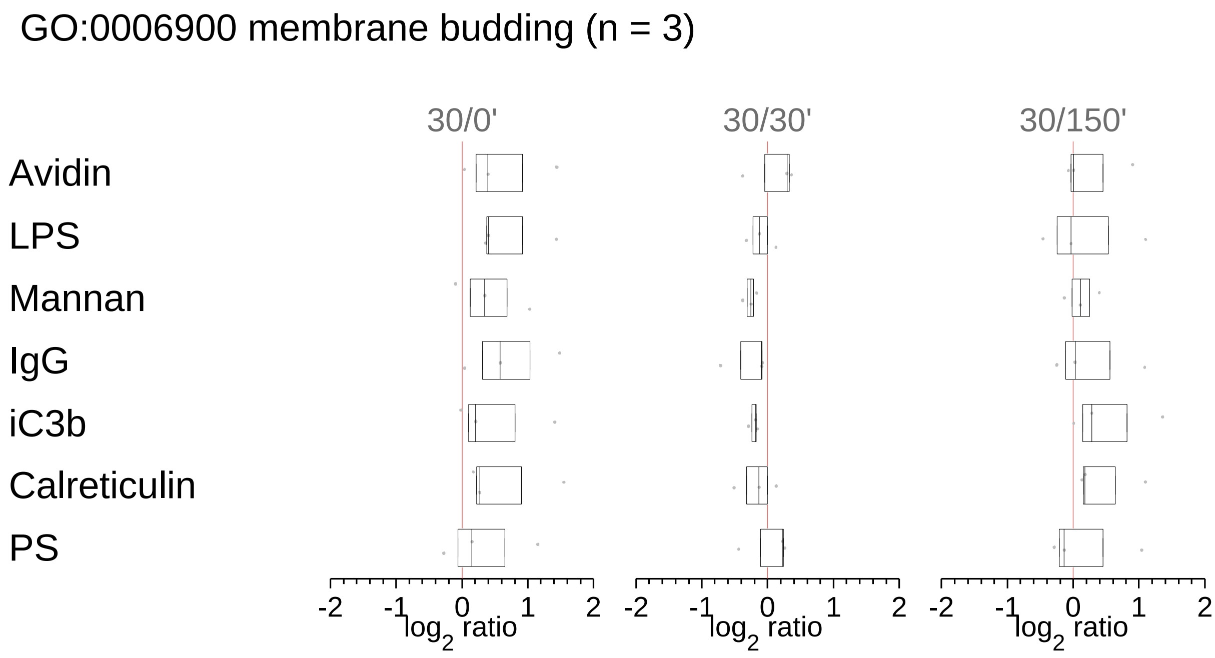

GO:0006900 membrane budding (n = 3)
30/0'
30/30'
30/150'
Avidin
LPS
Mannan
IgG
iC3b
Calreticulin
PS
-2
-1
0
1
2
log2 ratio

GO:0006904 vesicle docking involved in exocytosis (n = 10)

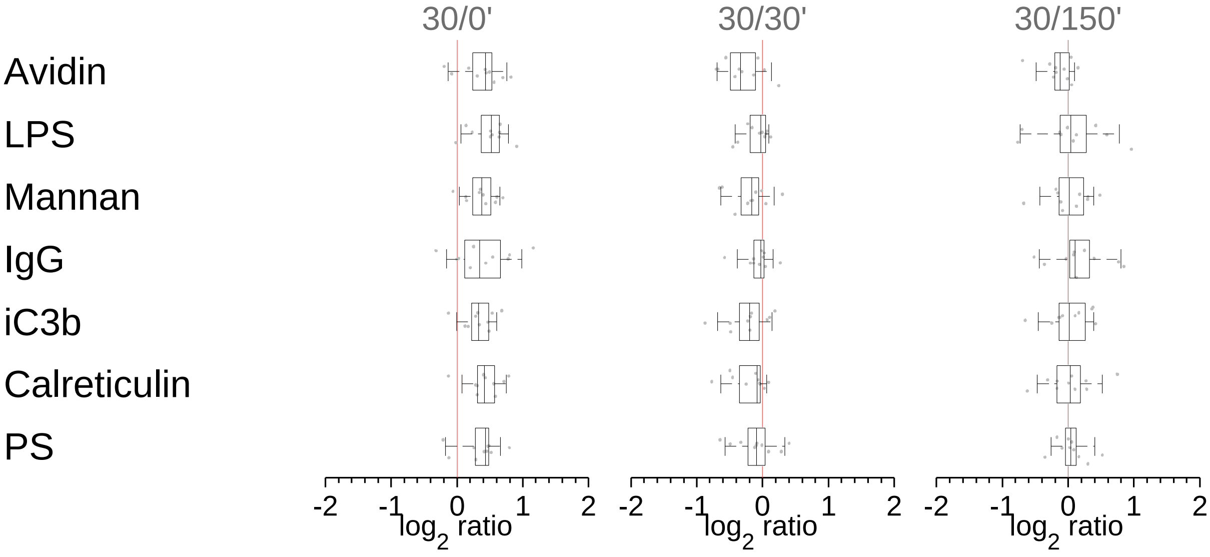

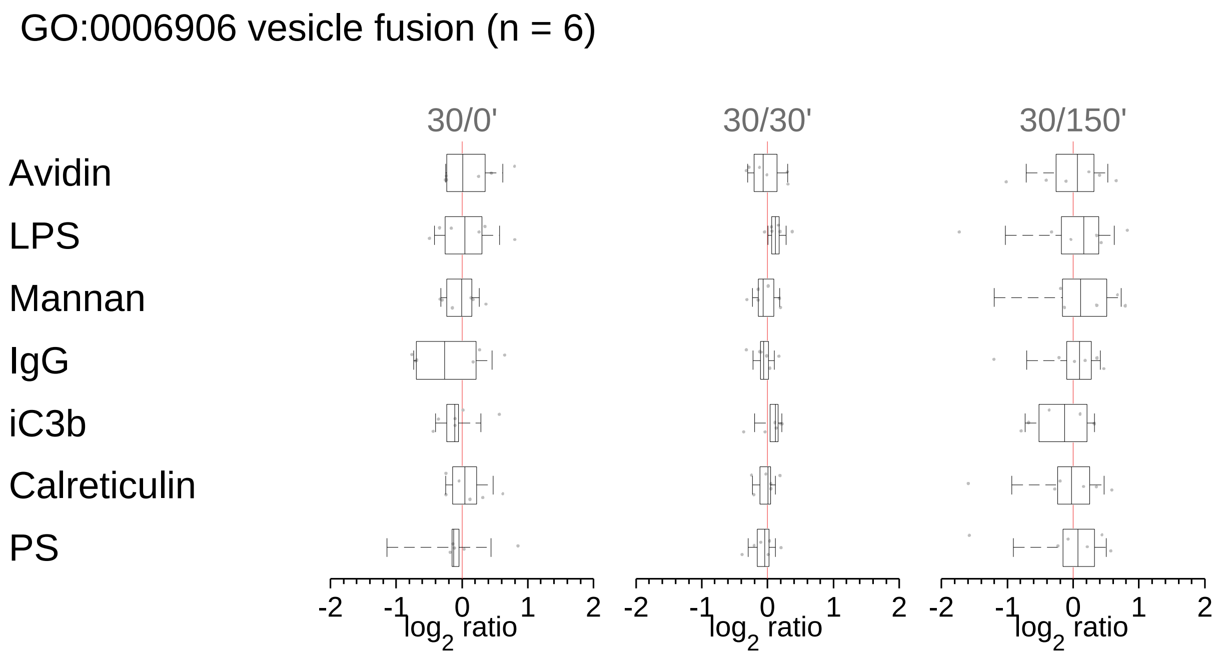
GO:0006906 vesicle fusion (n = 6)
30/0'
30/30'
30/150'
Avidin
LPS
Mannan
IgG
iC3b
Calreticulin
PS
-2
-1
0
1
2
log2 ratio

GO:0006907 pinocytosis (n = 4)

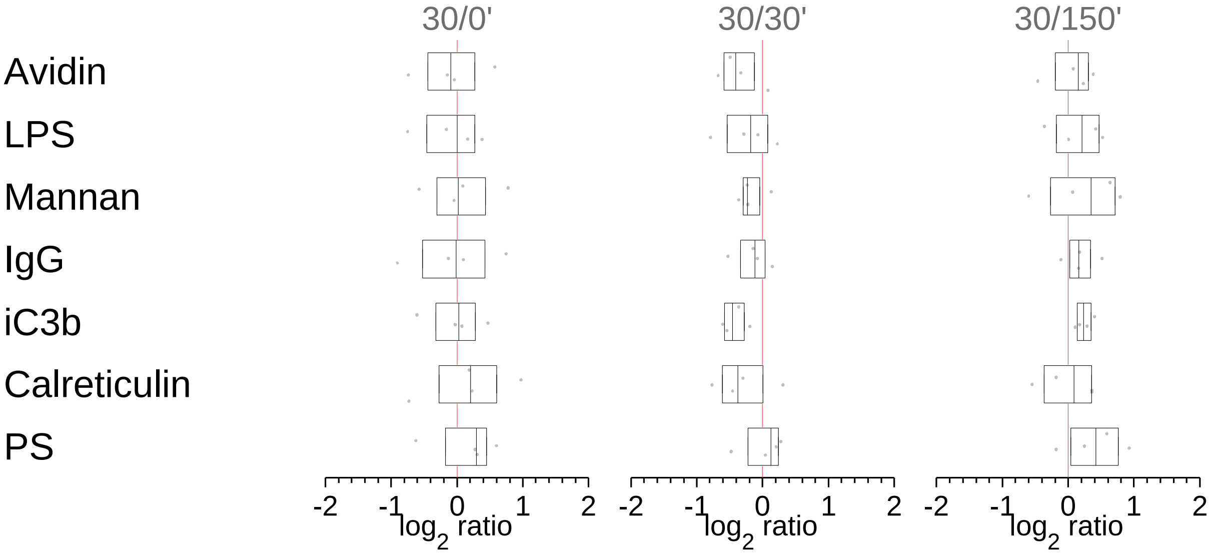

GO:0006909 phagocytosis (n = 9)

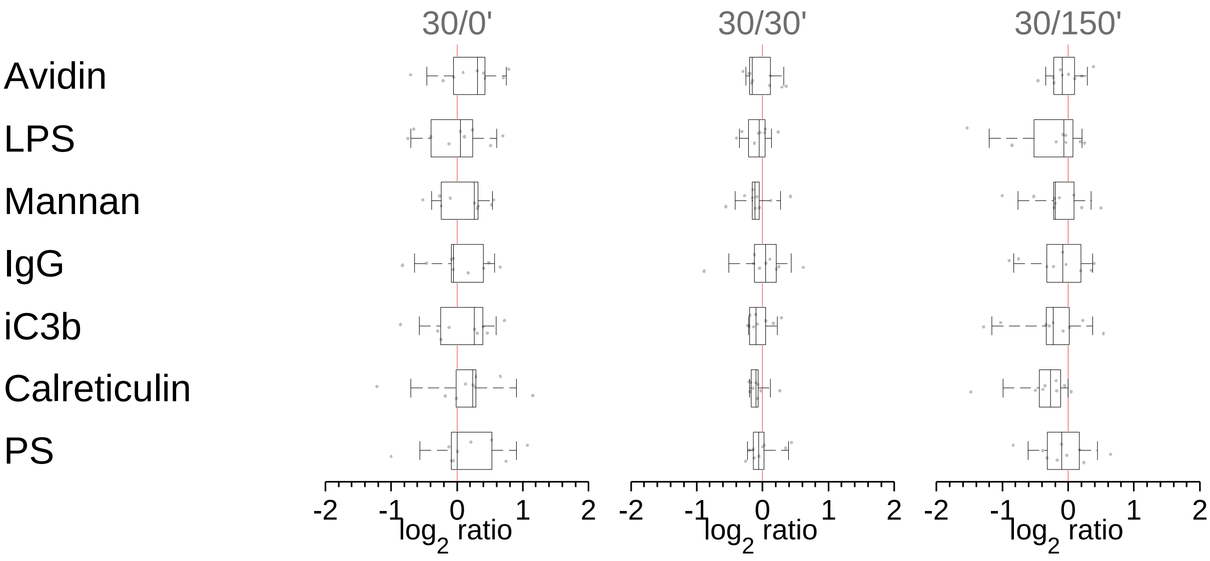

GO:0006910 phagocytosis, recognition (n = 8)

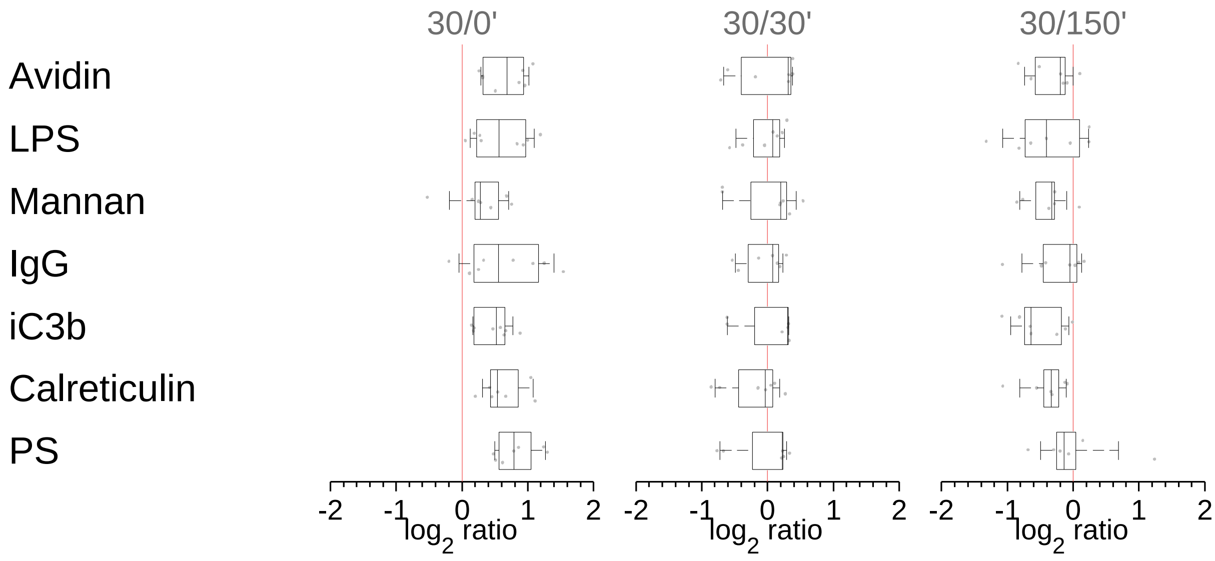

GO:0006911 phagocytosis, engulfment (n = 11)

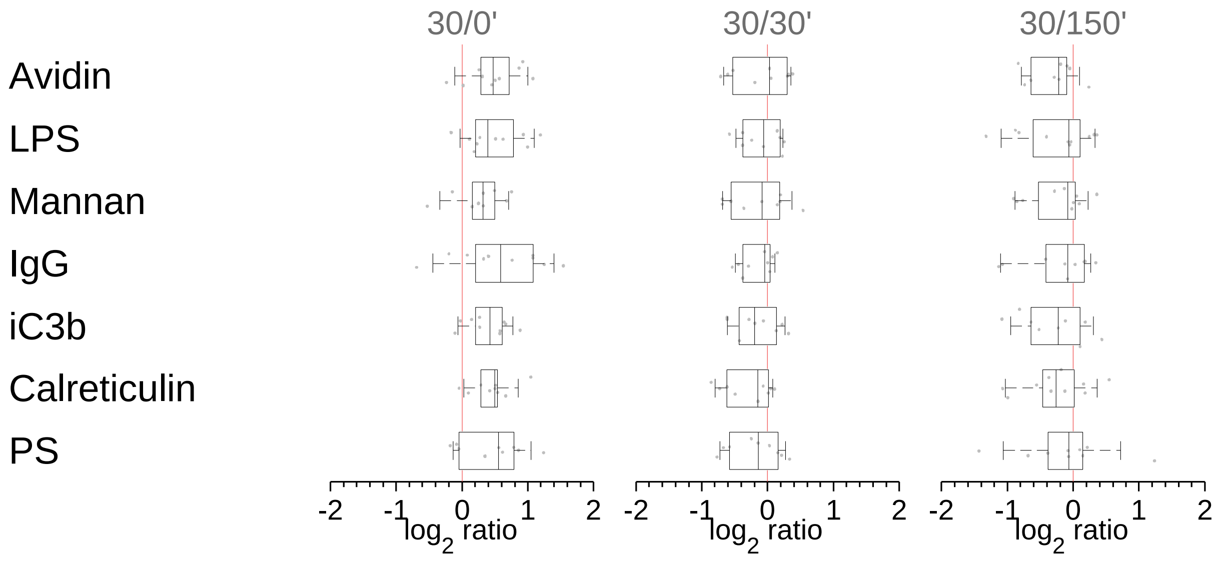

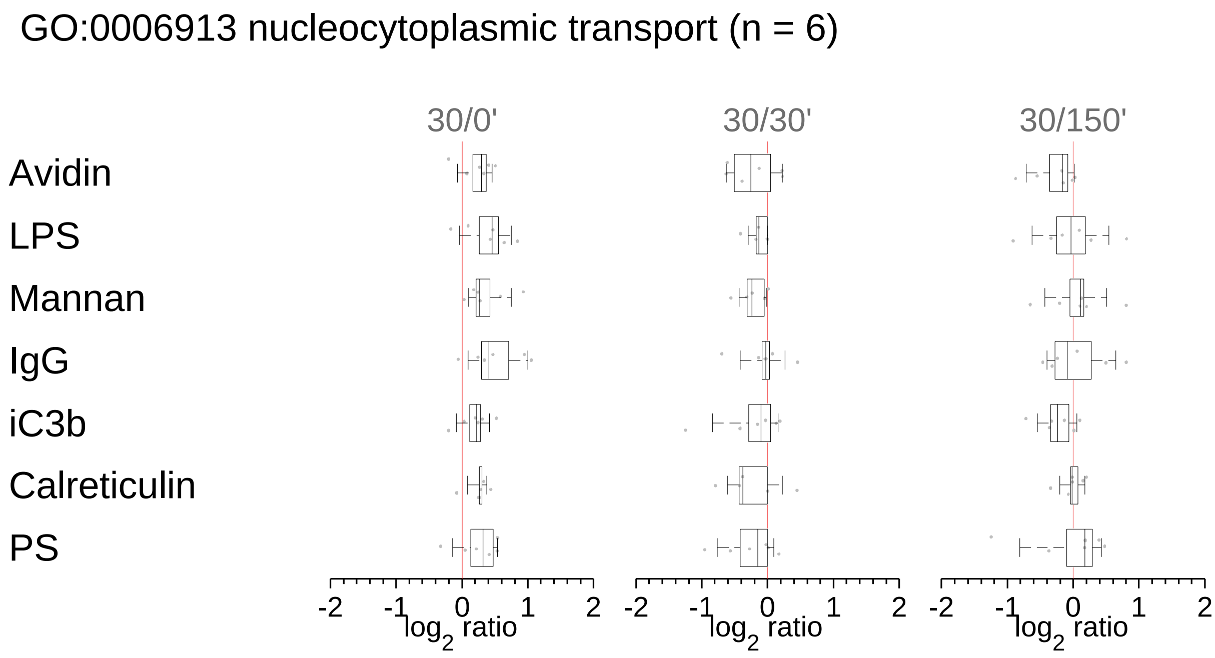
GO:0006913 nucleocytoplasmic transport (n = 6)
30/0'
30/30'
30/150'
Avidin
LPS
Mannan
IgG
iC3b
Calreticulin
PS
-2
-1
0
1
2
log2 ratio

GO:0006914 autophagy (n = 24)

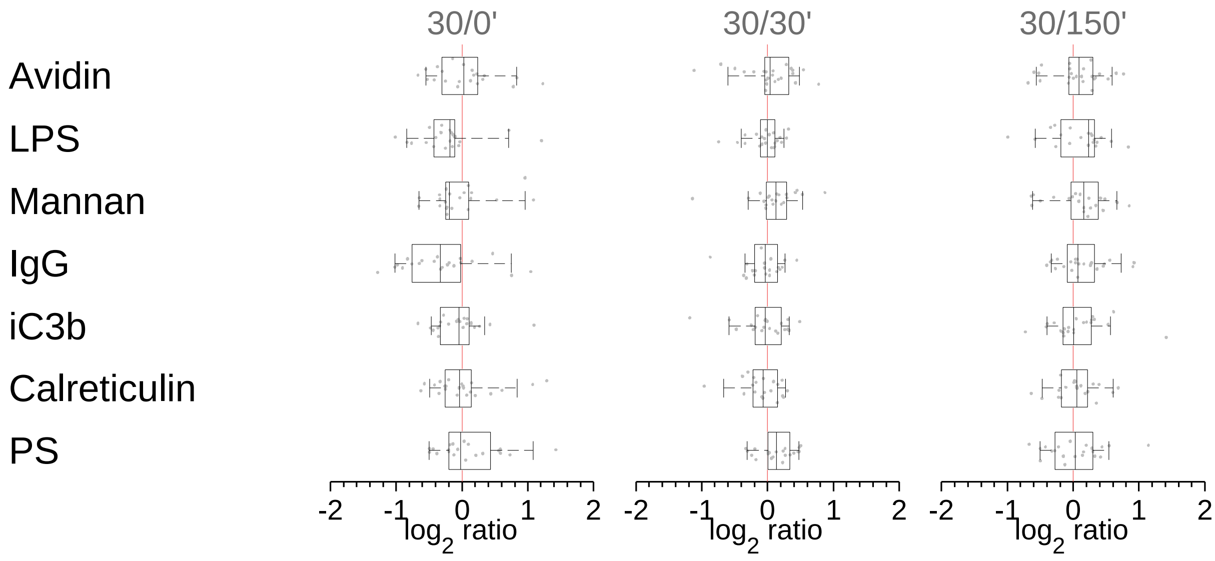

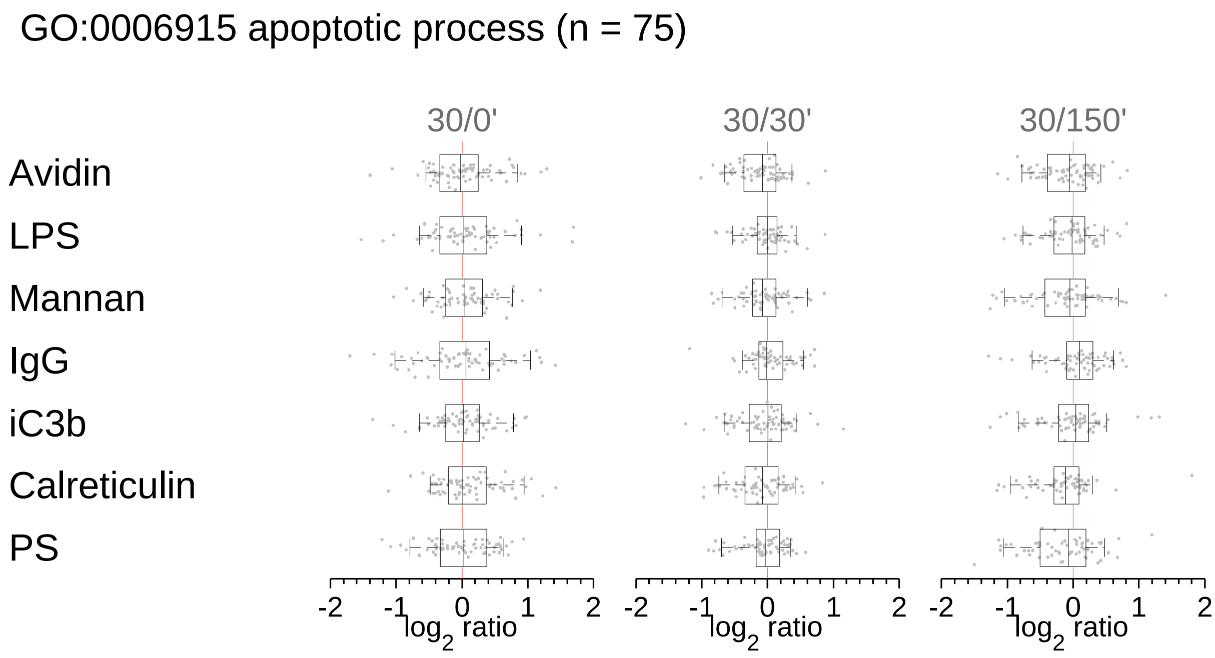
GO:0006915 apoptotic process (n = 75)
30/0'
30/30'
30/150'
Avidin
LPS
Mannan
IgG
iC3b
Calreticulin
PS
-2
-1
0
1
2
log2 ratio

GO:0006919 activation of cysteine-type endopeptidase activity involved in apoptotic process (n = 12)

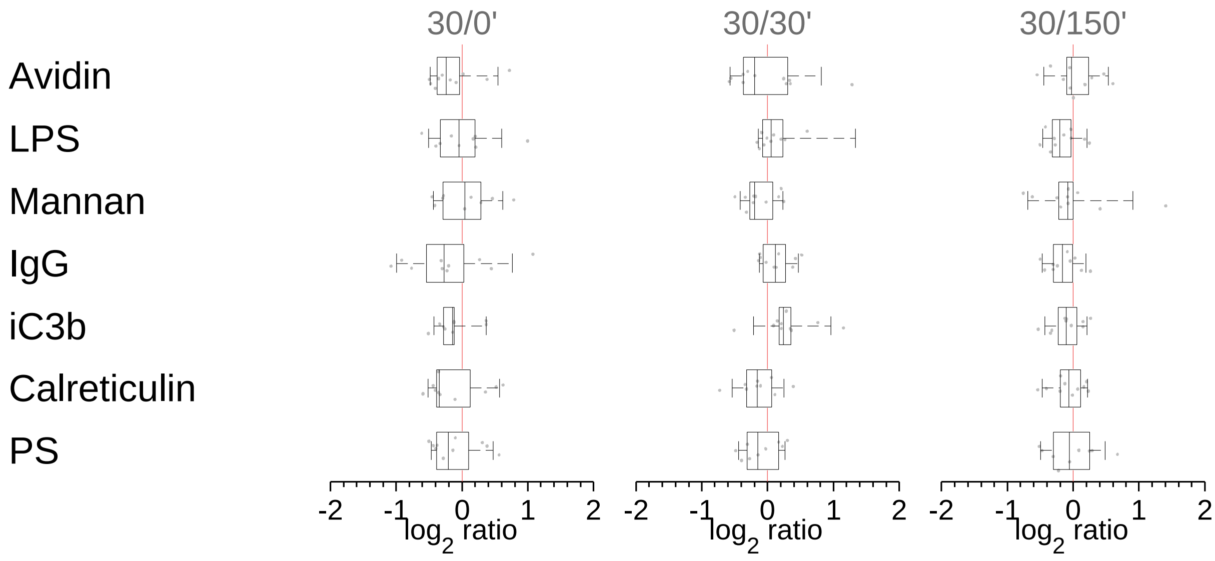

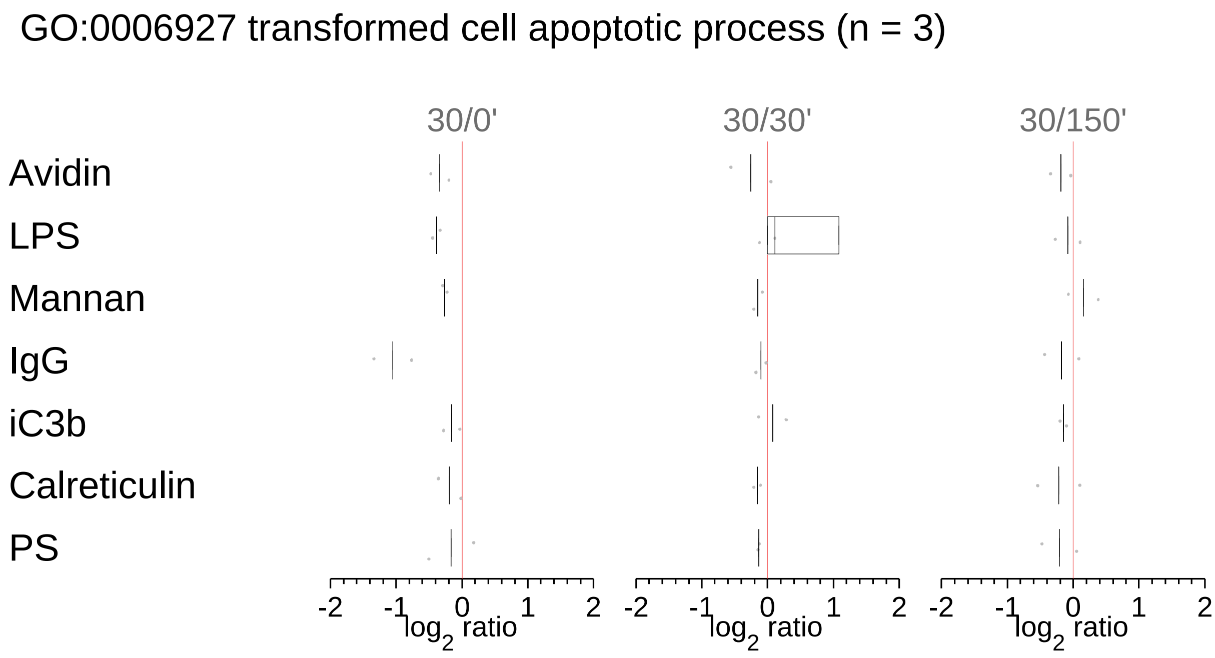

GO:0006927 transformed cell apoptotic process (n = 3)
30/0'
30/30'
30/150'
Avidin
LPS
Mannan
IgG
iC3b
Calreticulin
PS
-2 -1 0 1 2
log2 ratio

GO:0006928 cellular component movement (n = 10)

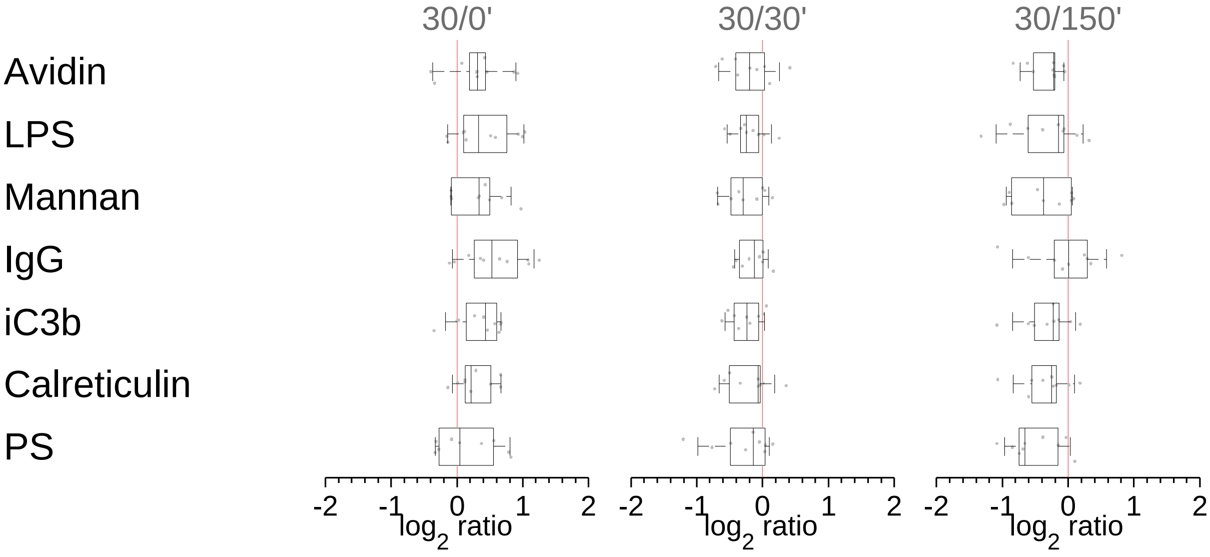

GO:0006935 chemotaxis (n = 11)

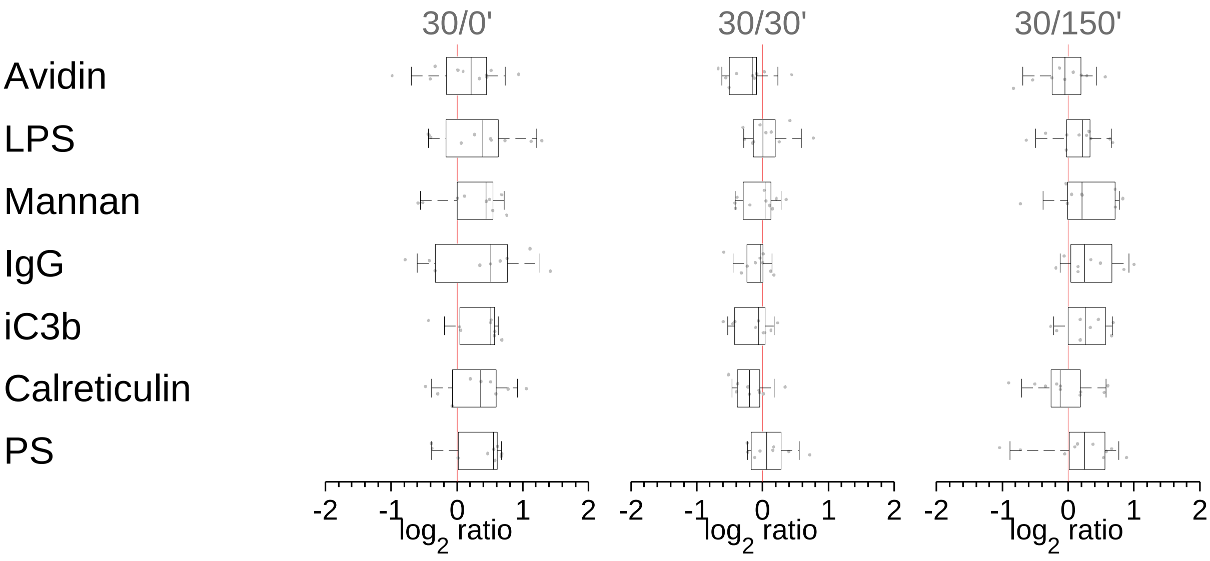

GO:0006936 muscle contraction (n = 4)

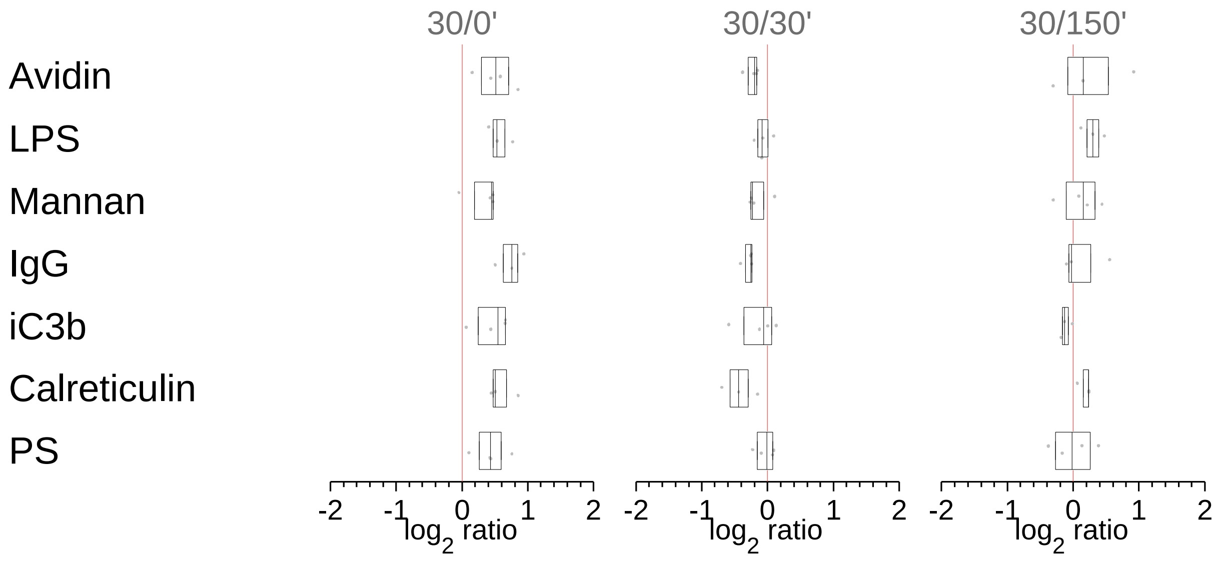

GO:0006937 regulation of muscle contraction (n = 3)

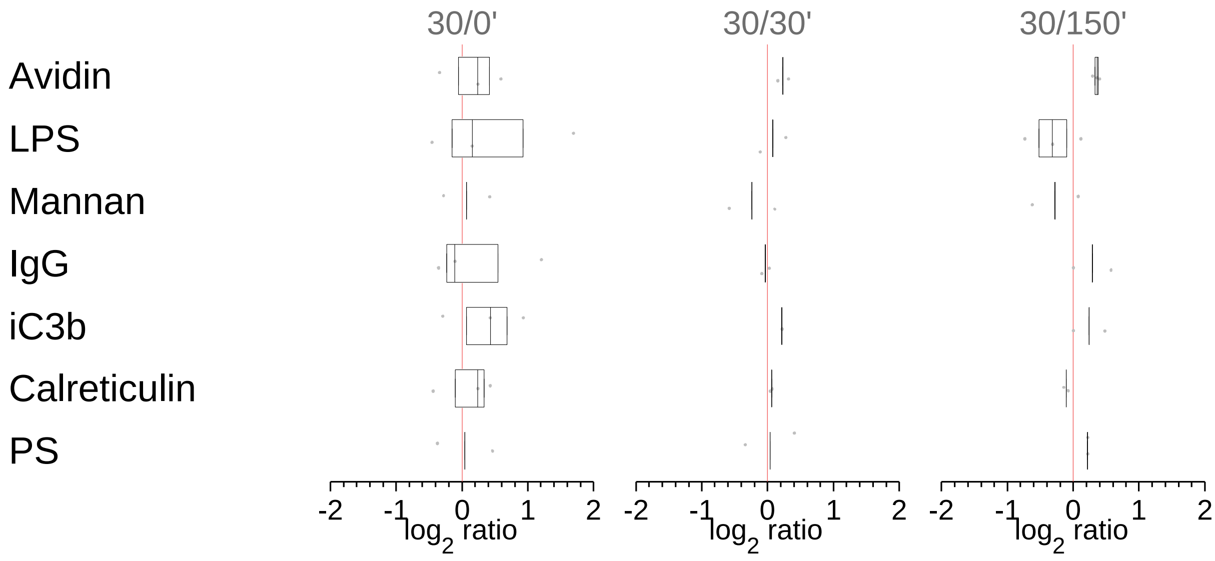

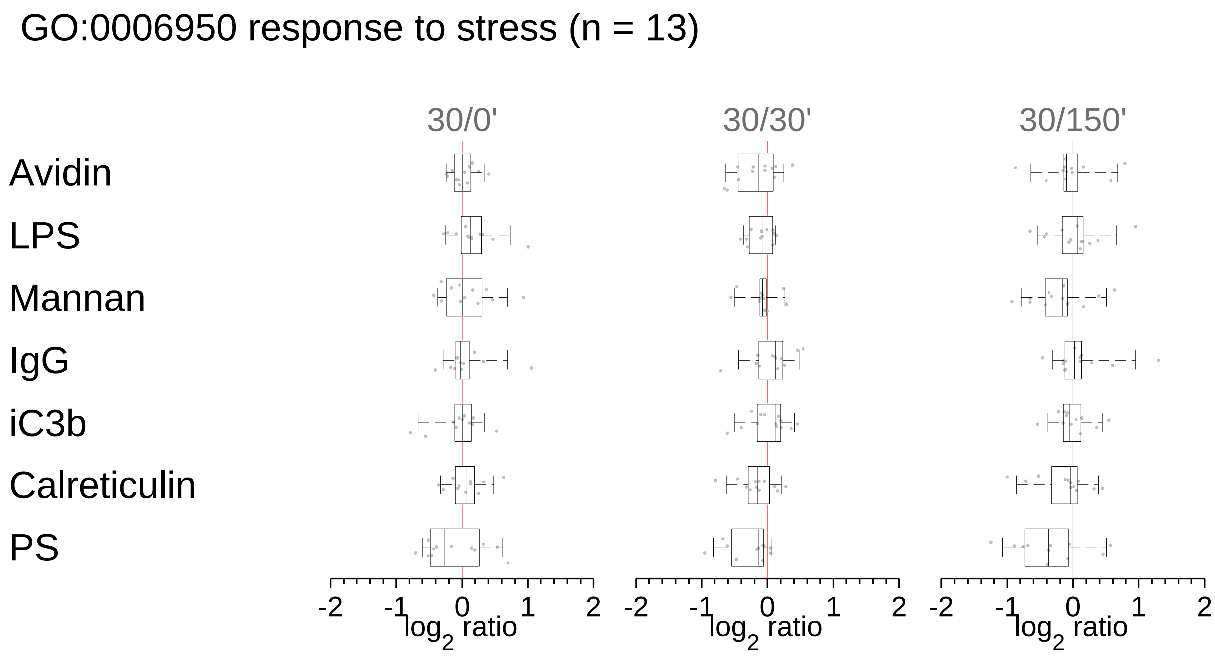
GO:0006950 response to stress (n = 13)
30/0'
30/30'
30/150'
Avidin
LPS
Mannan
IgG
iC3b
Calreticulin
PS
-2 -1 0 1 2
log2 ratio

GO:0006952 defense response (n = 10)

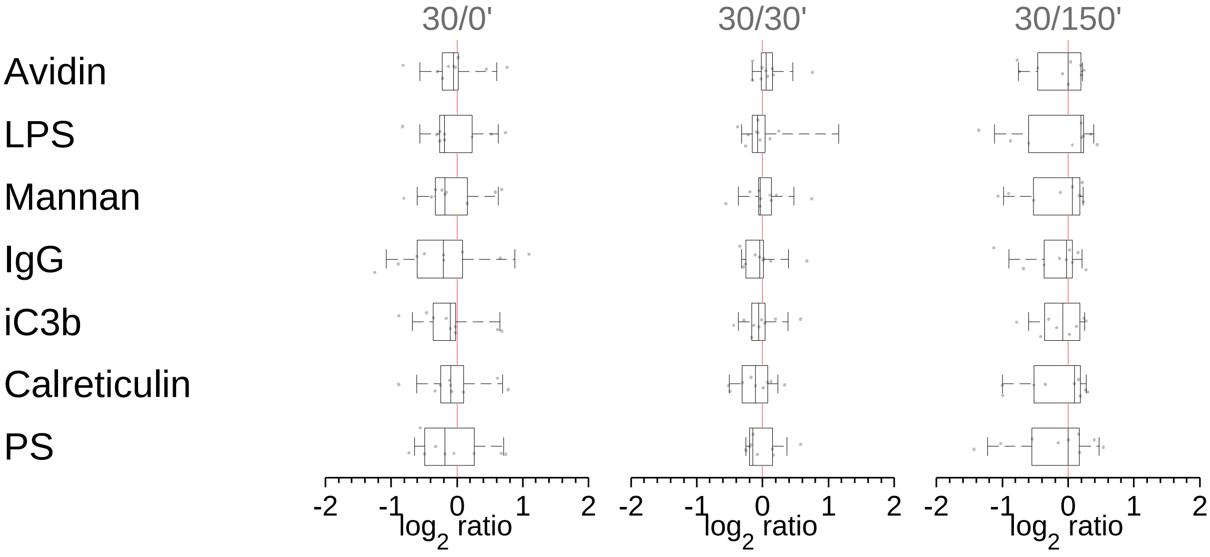

GO:0006954 inflammatory response (n = 38)

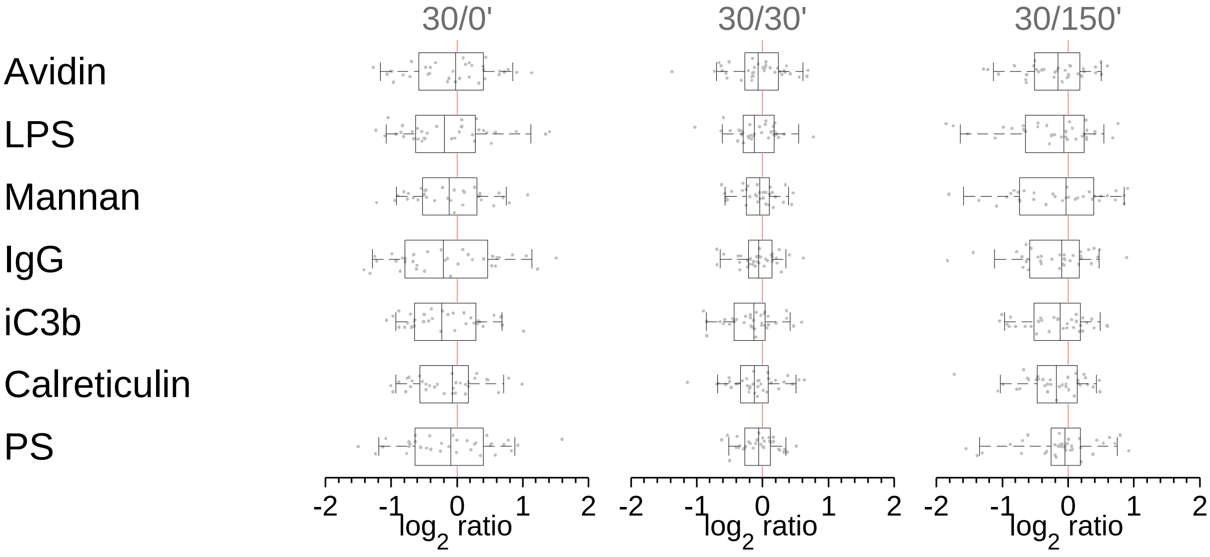

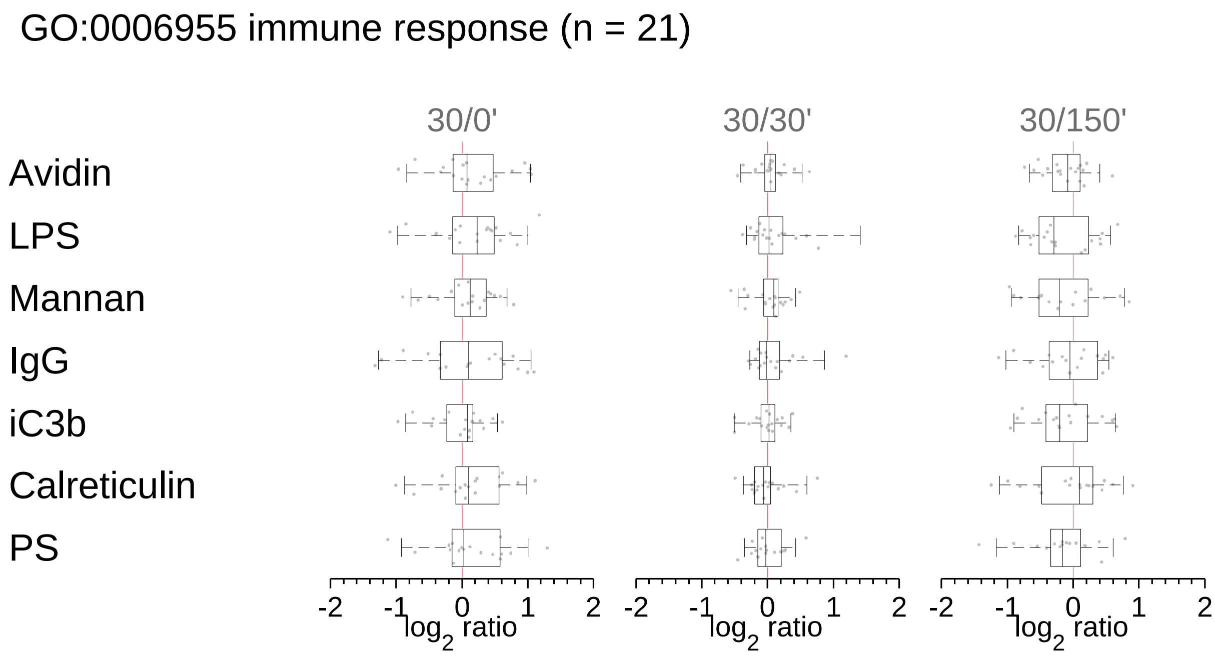
GO:0006955 immune response (n = 21)
30/0'
30/30'
30/150'
Avidin
LPS
Mannan
IgG
iC3b
Calreticulin
PS
-2 -1 0 1 2
log2 ratio

GO:0006958 complement activation, classical pathway (n = 5)

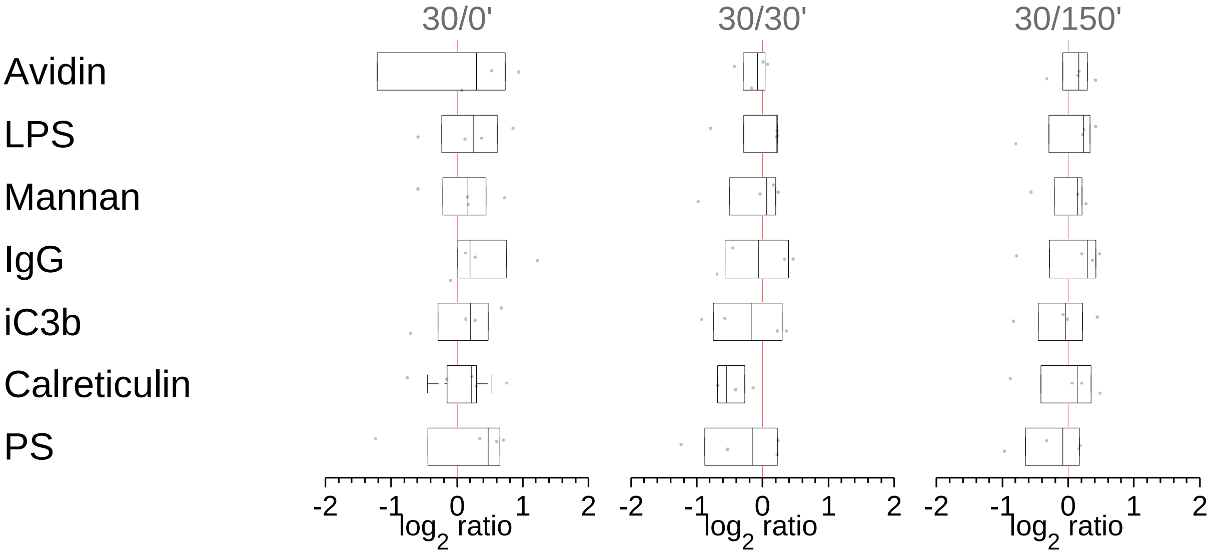

GO:0006968 cellular defense response (n = 3)

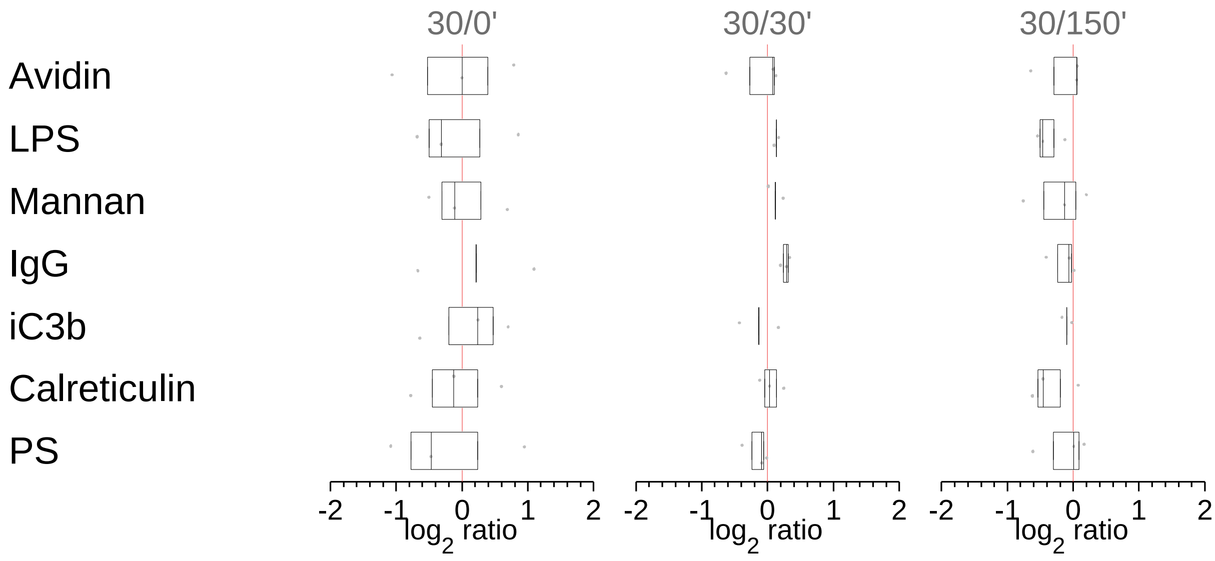

GO:0006974 response to DNA damage stimulus (n = 24)

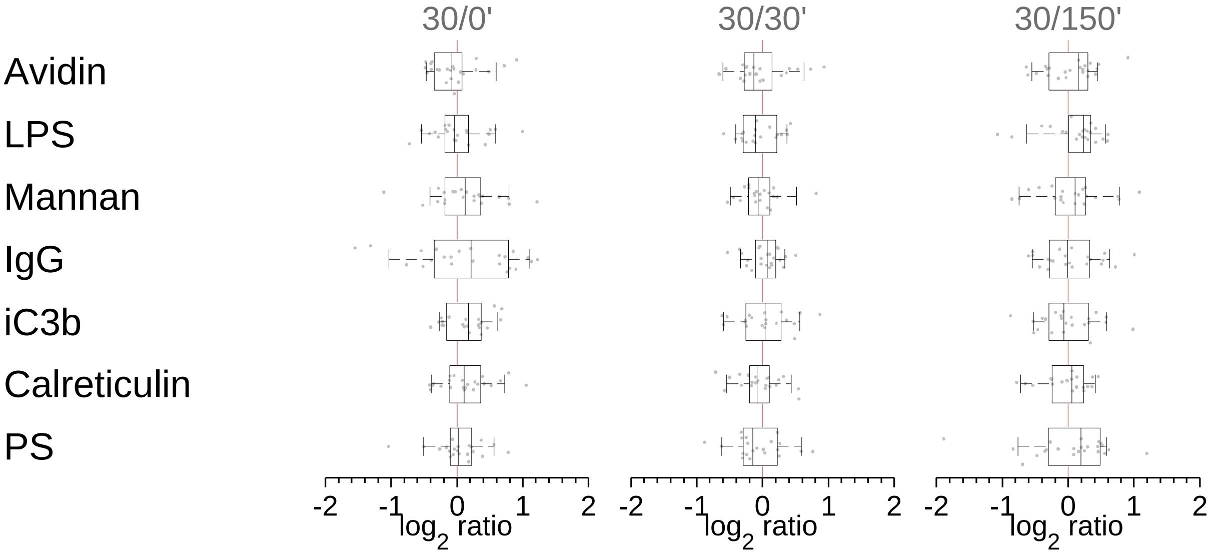

GO:0006979 response to oxidative stress (n = 29)

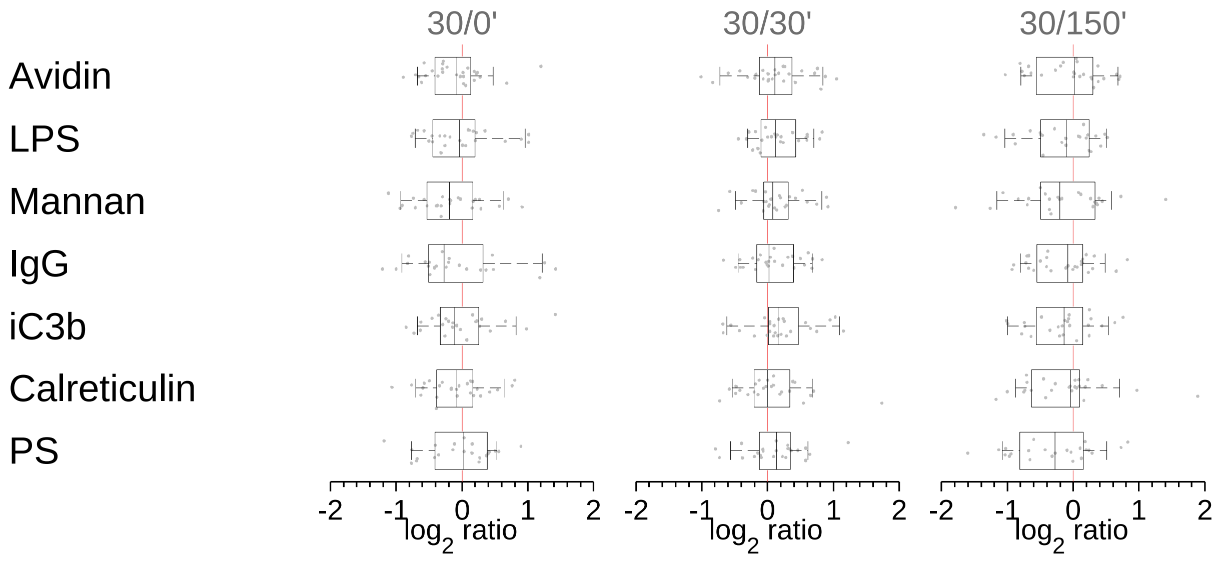

GO:0006986 response to unfolded protein (n = 8)

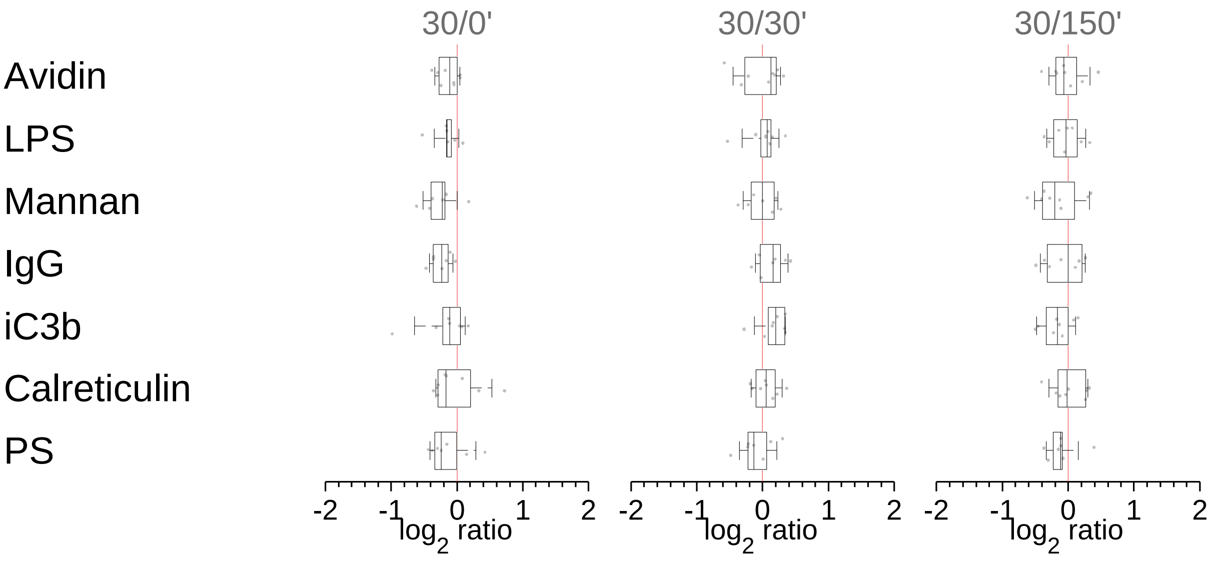

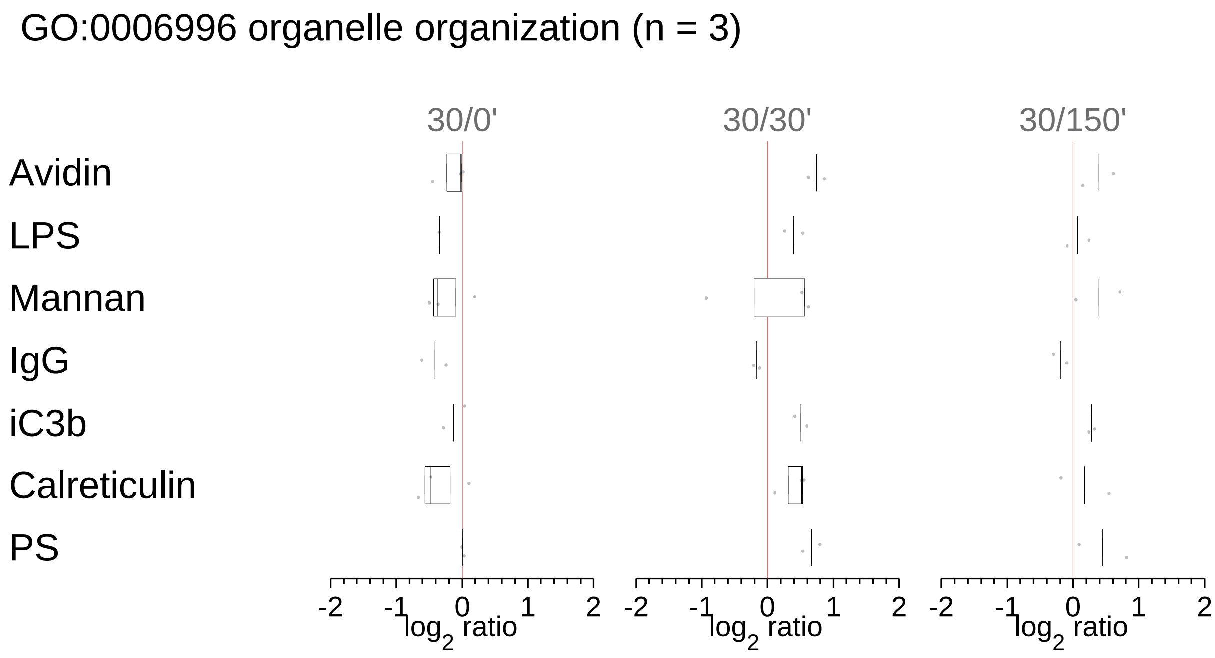

GO:0006996 organelle organization (n = 3)
30/0'
30/30'
30/150'
Avidin
LPS
Mannan
IgG
iC3b
Calreticulin
PS
-2
-1
0
1
2
log_2 ratio

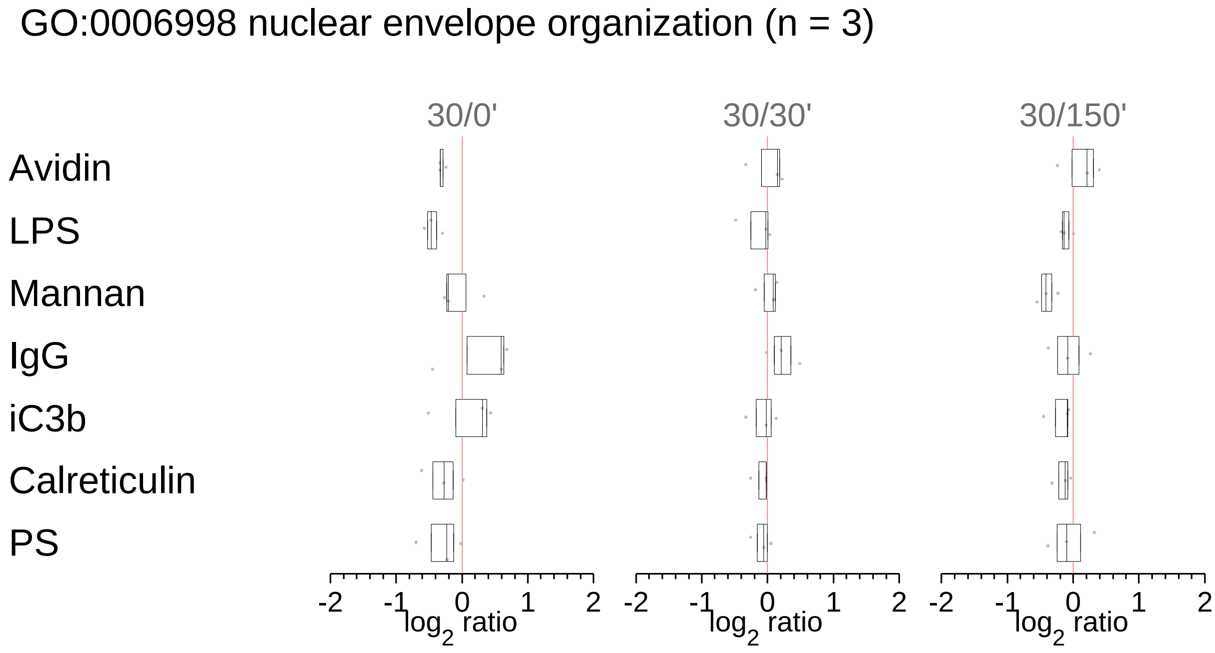
GO:0006998 nuclear envelope organization (n = 3)
30/0'
30/30'
30/150'
Avidin
LPS
Mannan
IgG
iC3b
Calreticulin
PS
-2
-1
0
1
2
log2 ratio

GO:0007005 mitochondrion organization (n = 6)

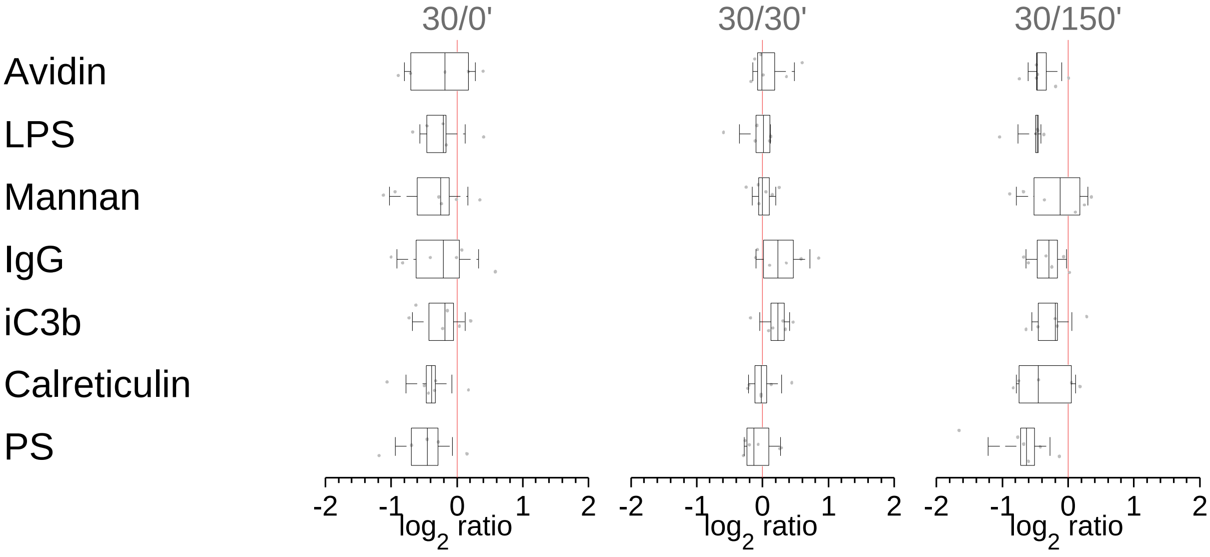

GO:0007010 cytoskeleton organization (n = 19)

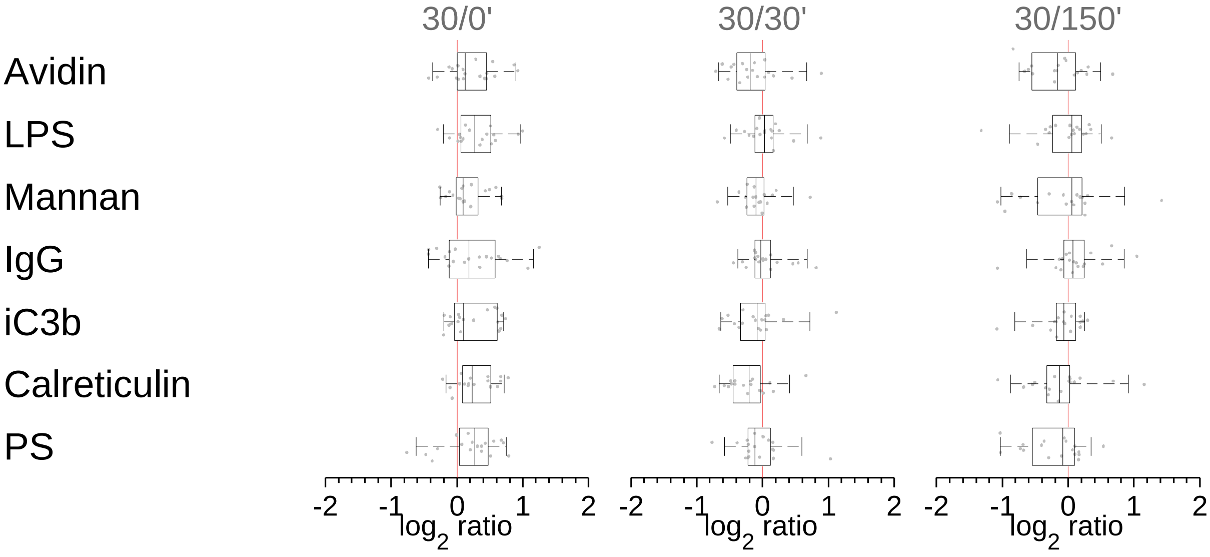

GO:0007015 actin filament organization (n = 13)

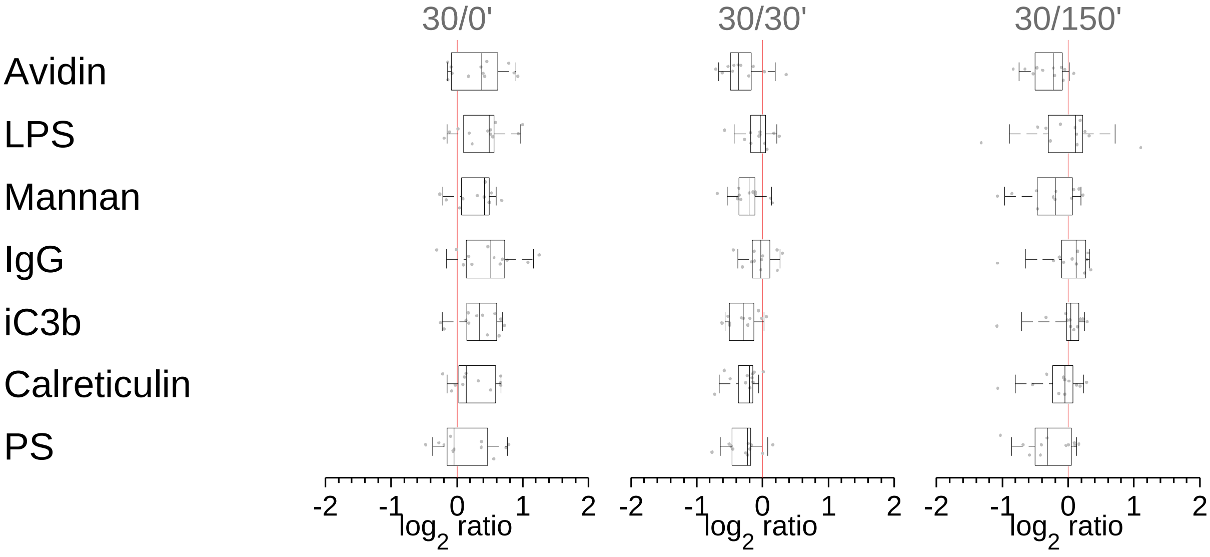

GO:0007017 microtubule-based process (n = 8)

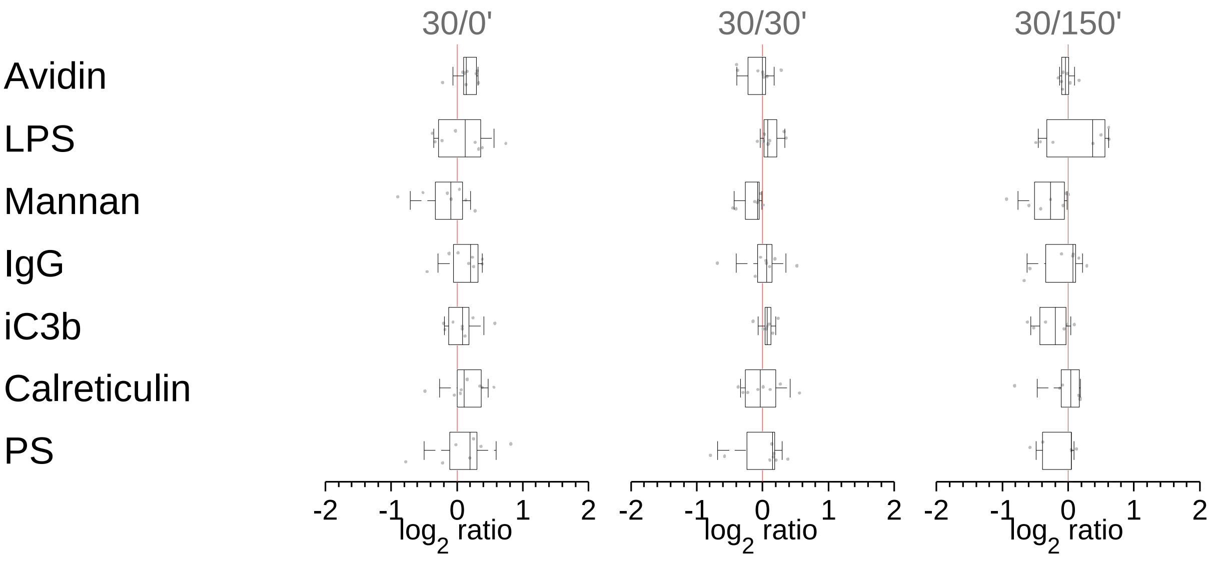

GO:0007018 microtubule-based movement (n = 7)

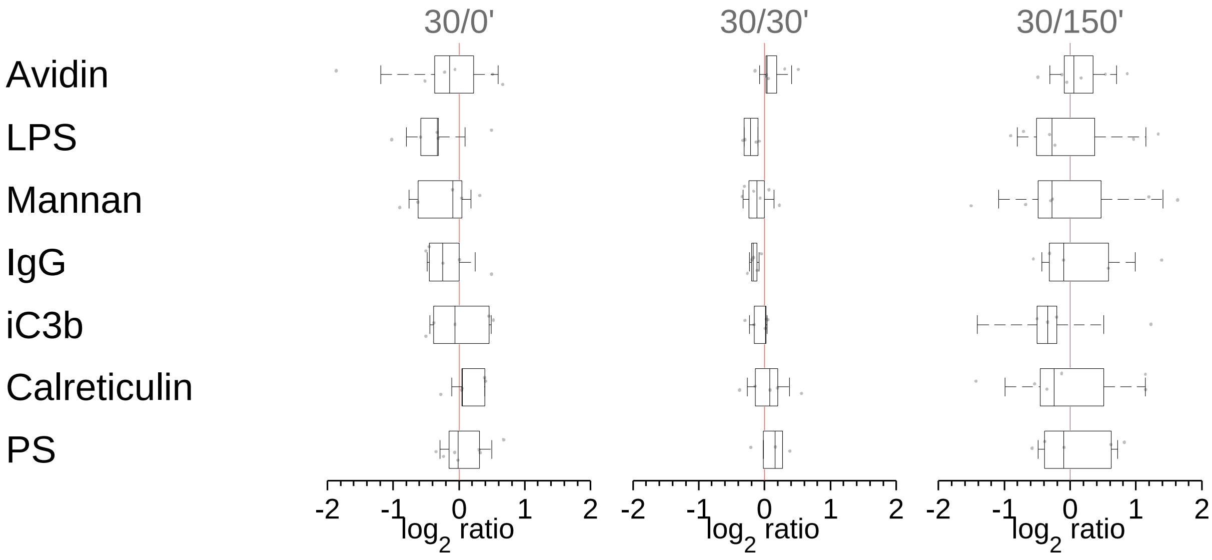

GO:0007029 endoplasmic reticulum organization (n = 4)

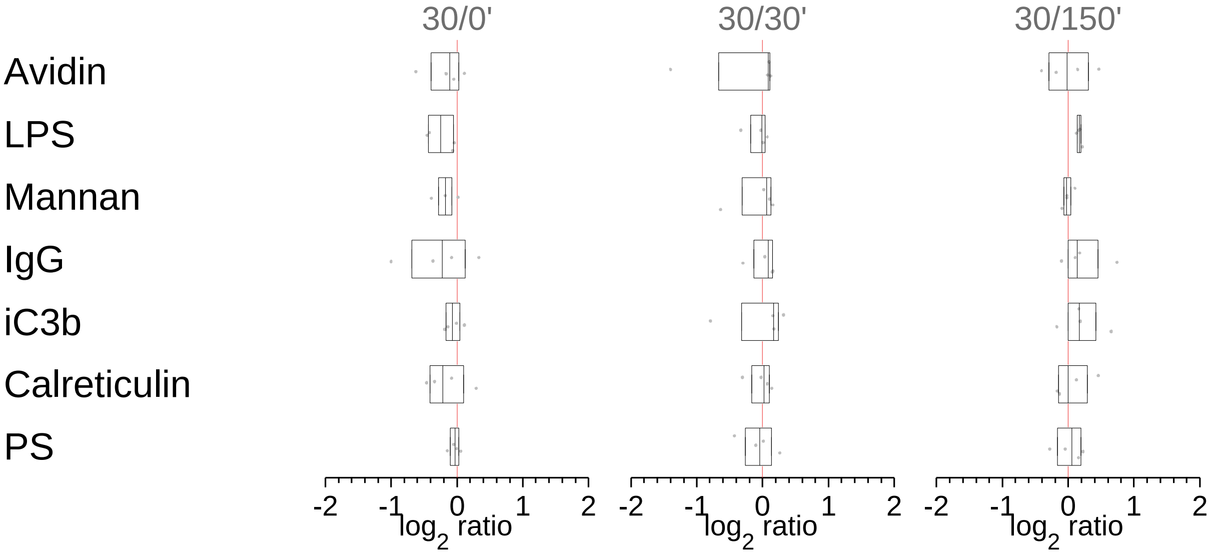

GO:0007030 Golgi organization (n = 12)

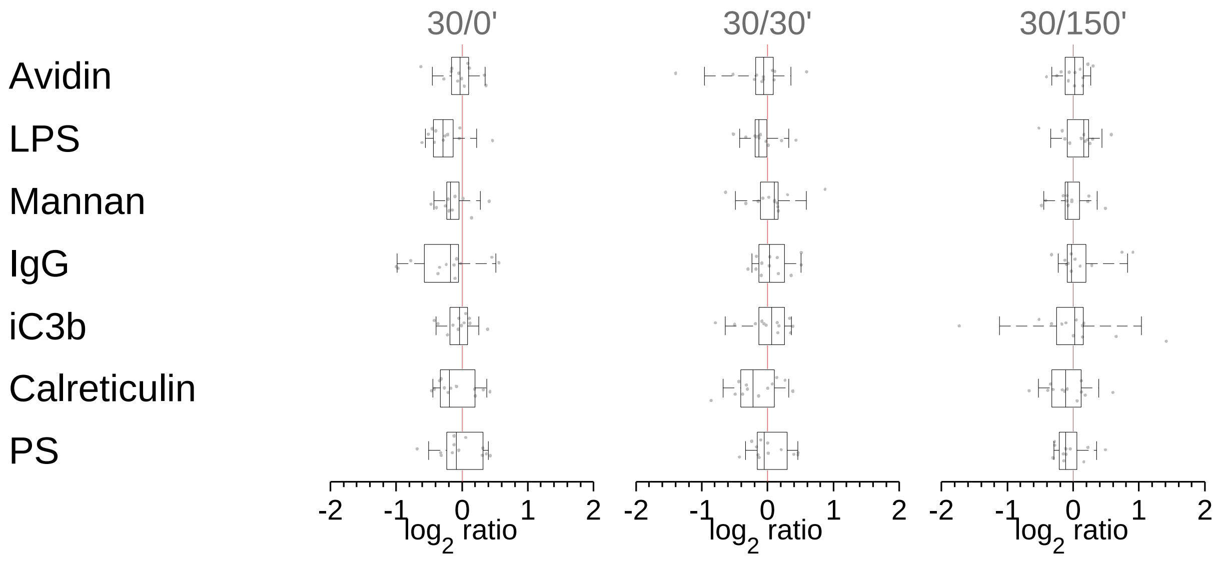

GO:0007031 peroxisome organization (n = 4)

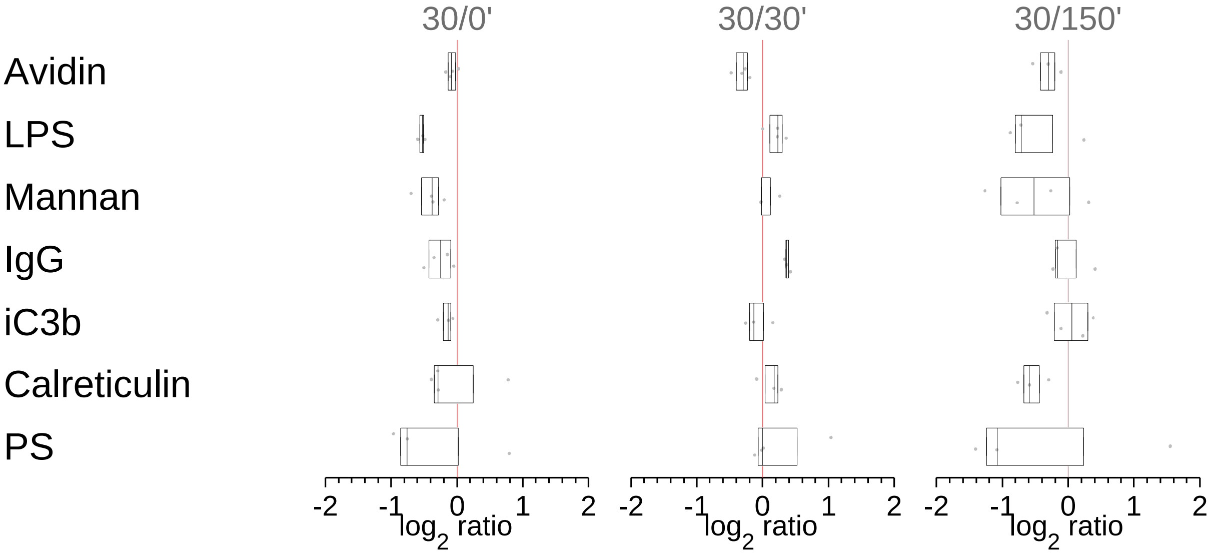

GO:0007032 endosome organization (n = 7)

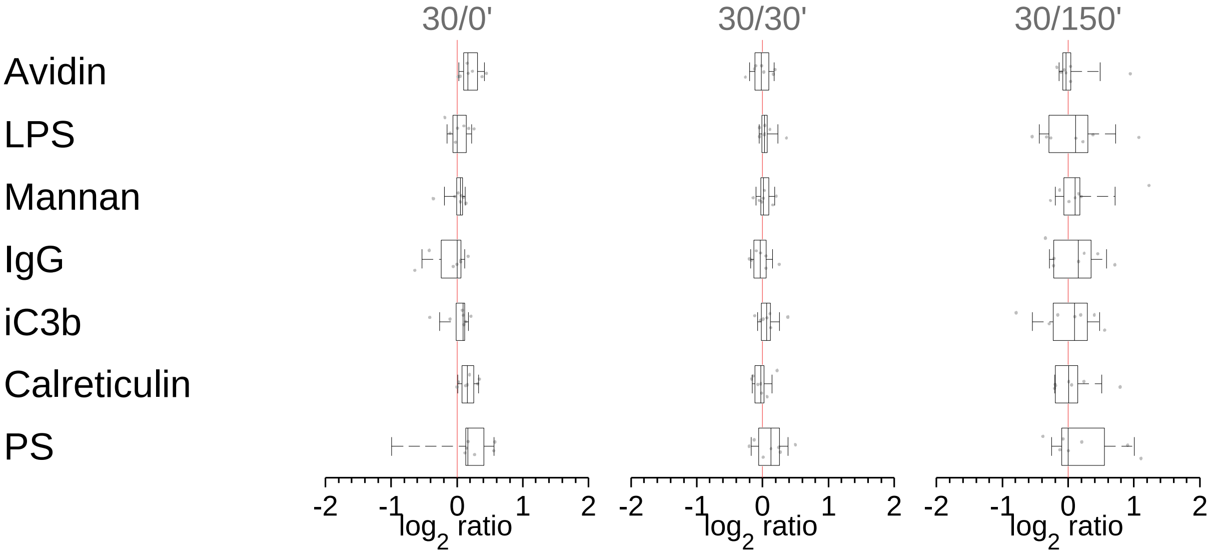

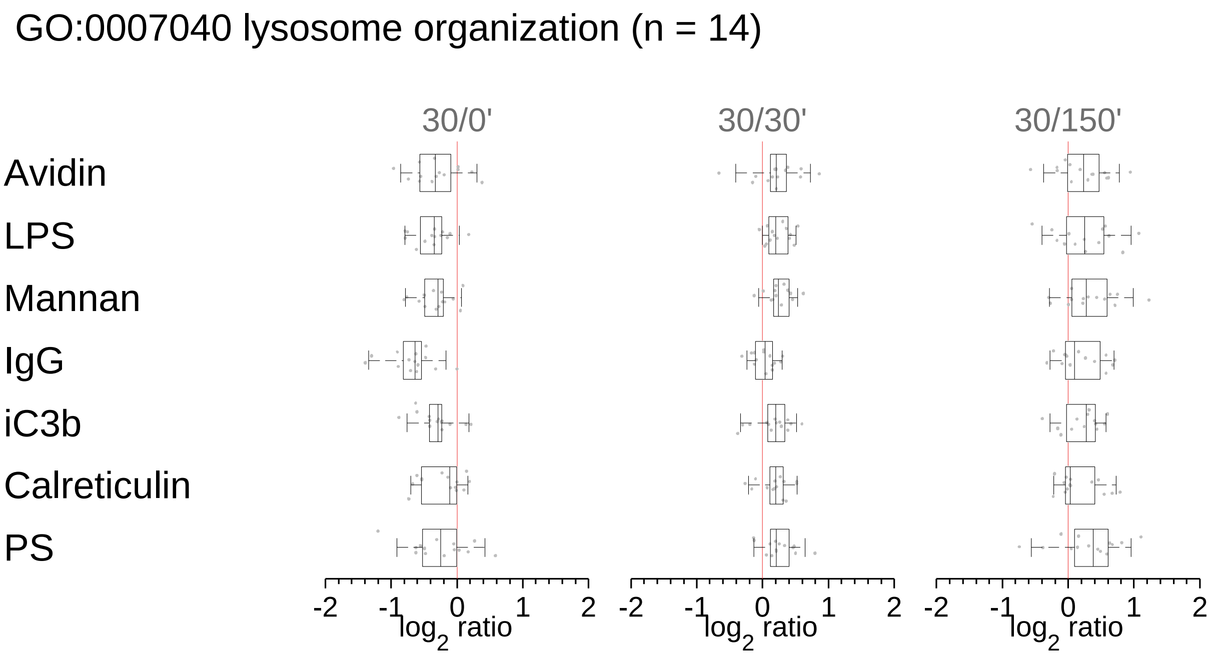

GO:0007040 lysosome organization (n = 14)
30/0'
30/30'
30/150'
Avidin
LPS
Mannan
IgG
iC3b
Calreticulin
PS
-2
-1
0
1
2
log_2 ratio

GO:0007042 lysosomal lumen acidification (n = 4)

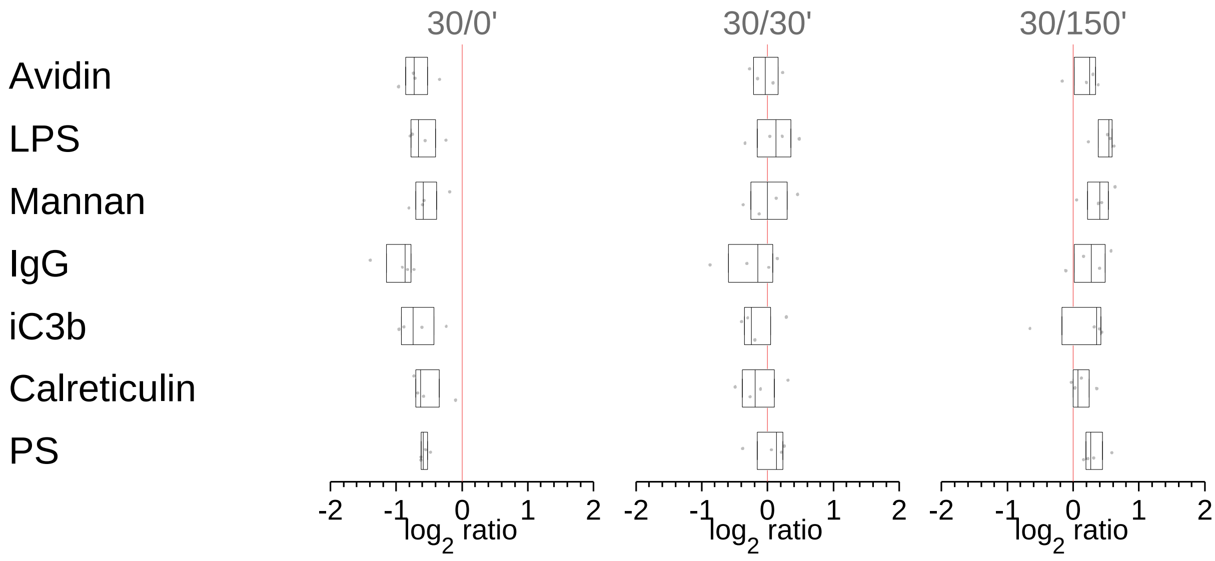

GO:0007044 cell-substrate junction assembly (n = 3)

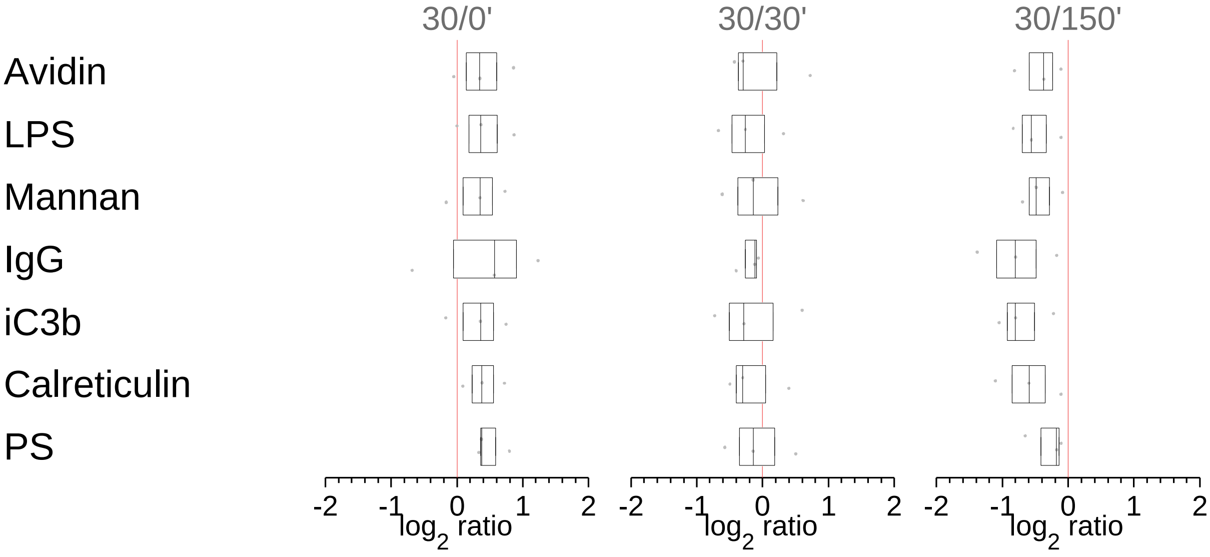

GO:0007049 cell cycle (n = 69)

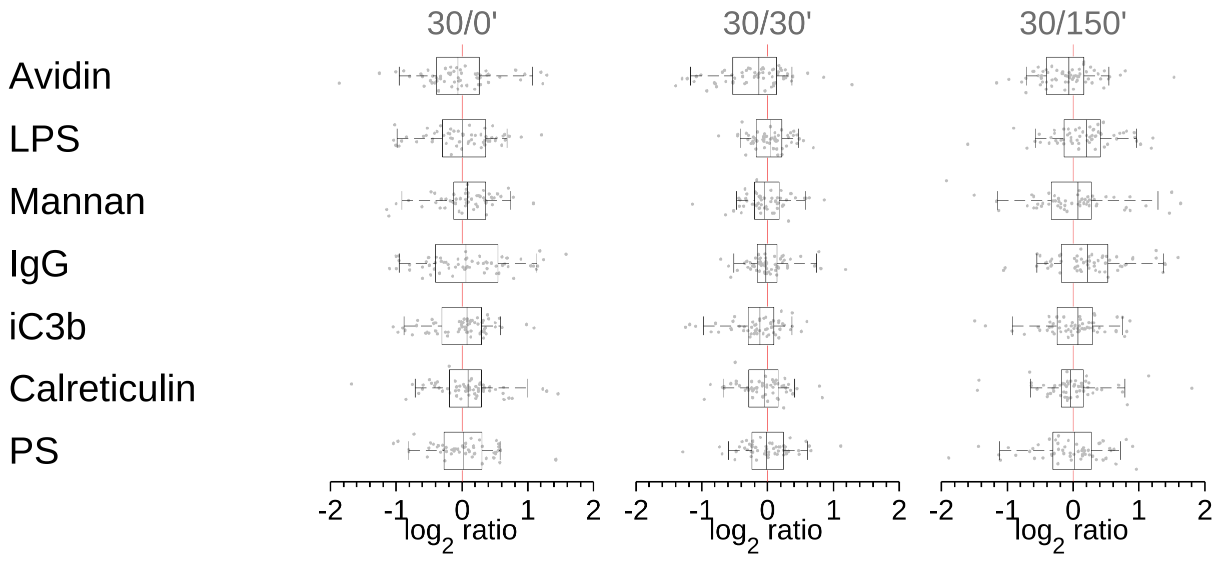

GO:0007050 cell cycle arrest (n = 8)

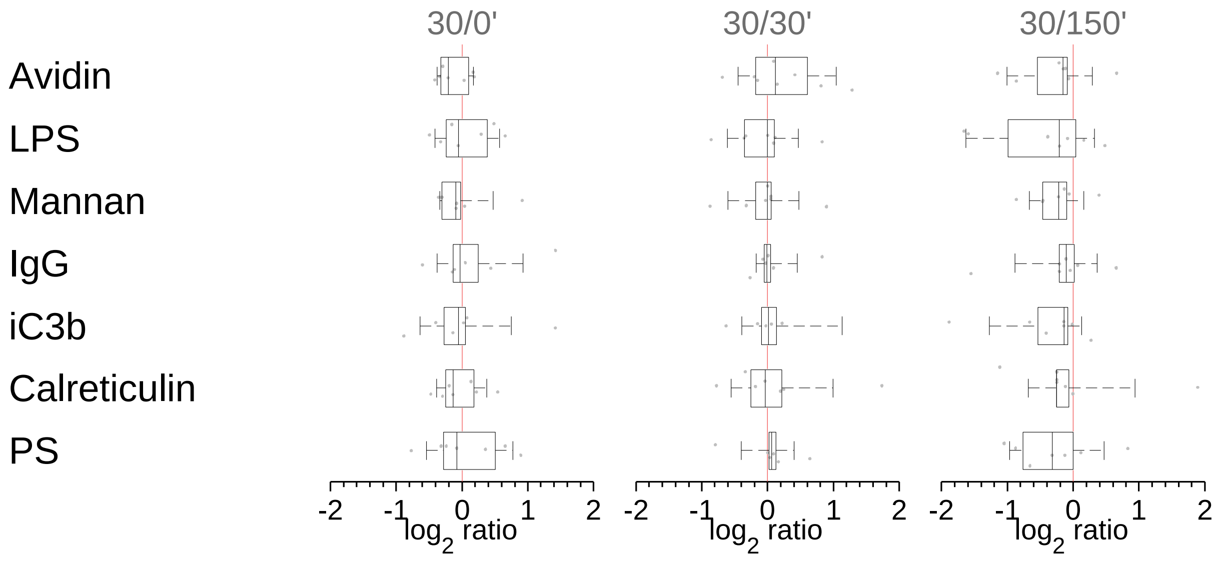

GO:0007052 mitotic spindle organization (n = 3)

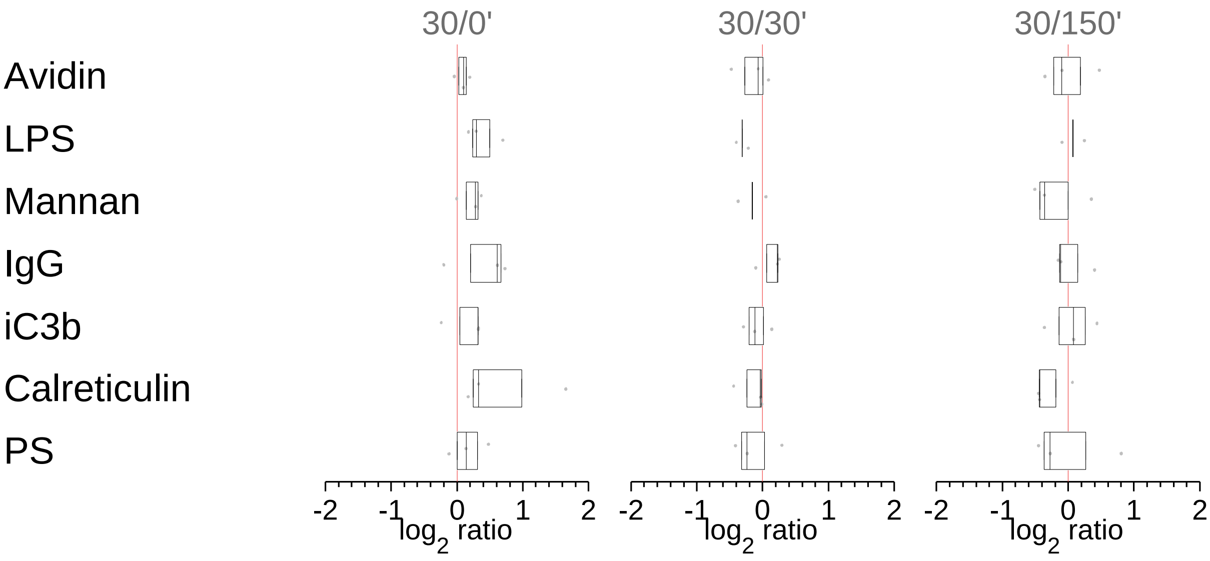

GO:0007059 chromosome segregation (n = 9)

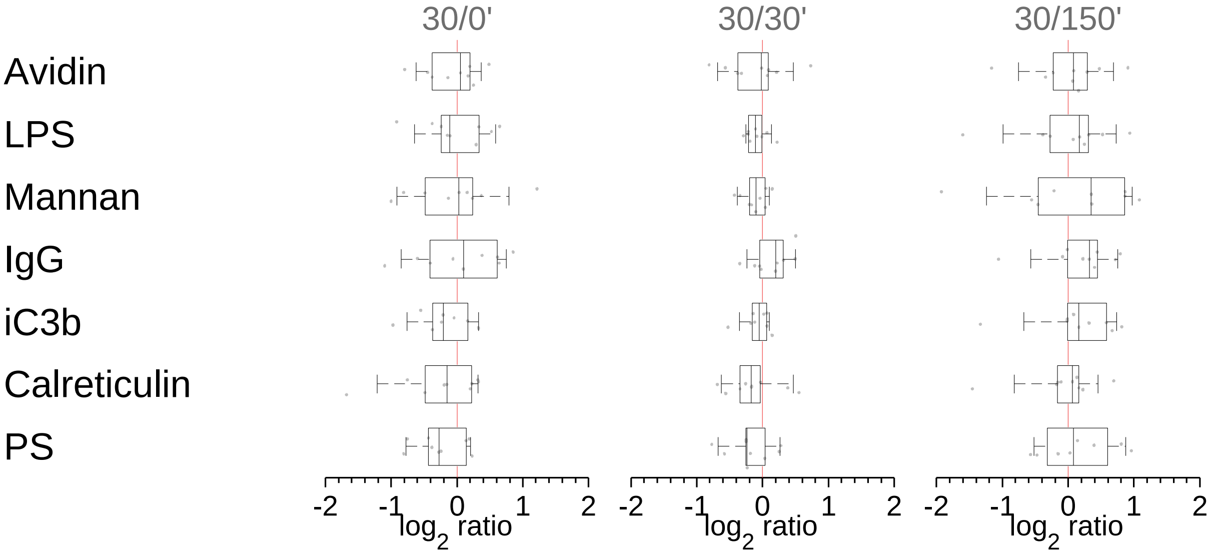

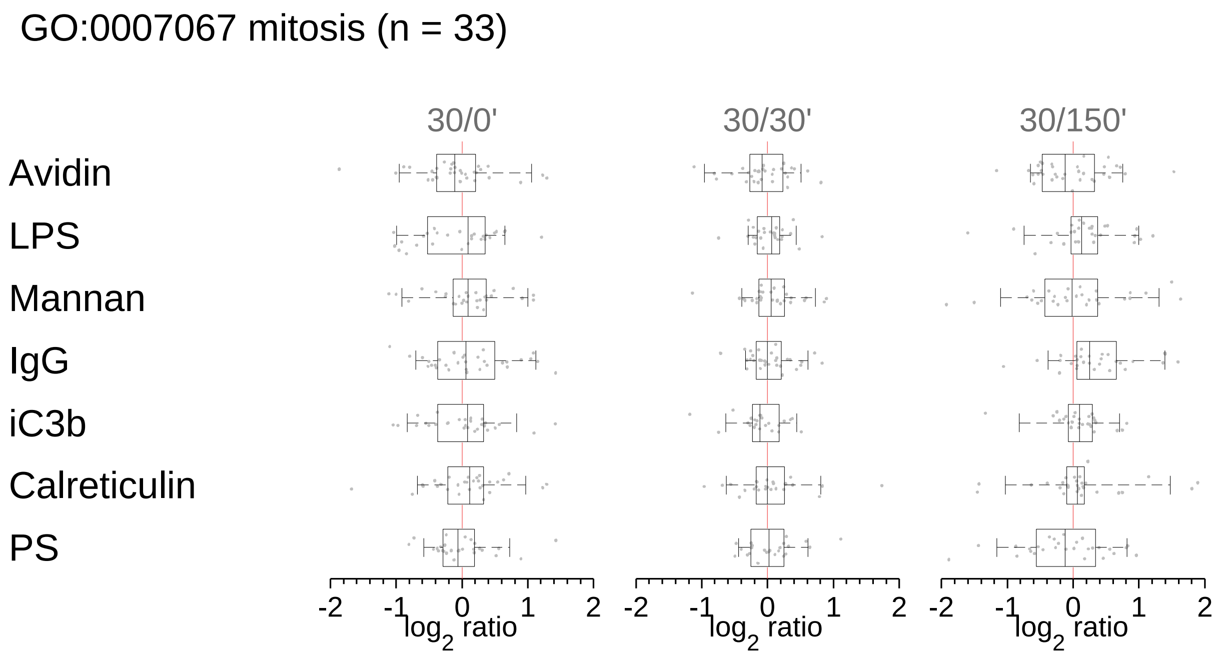

GO:0007067 mitosis (n = 33)
30/0'
30/30'
30/150'
Avidin
LPS
Mannan
IgG
iC3b
Calreticulin
PS
-2
-1
0
1
2
$\log_2$ ratio

GO:0007088 regulation of mitosis (n = 4)

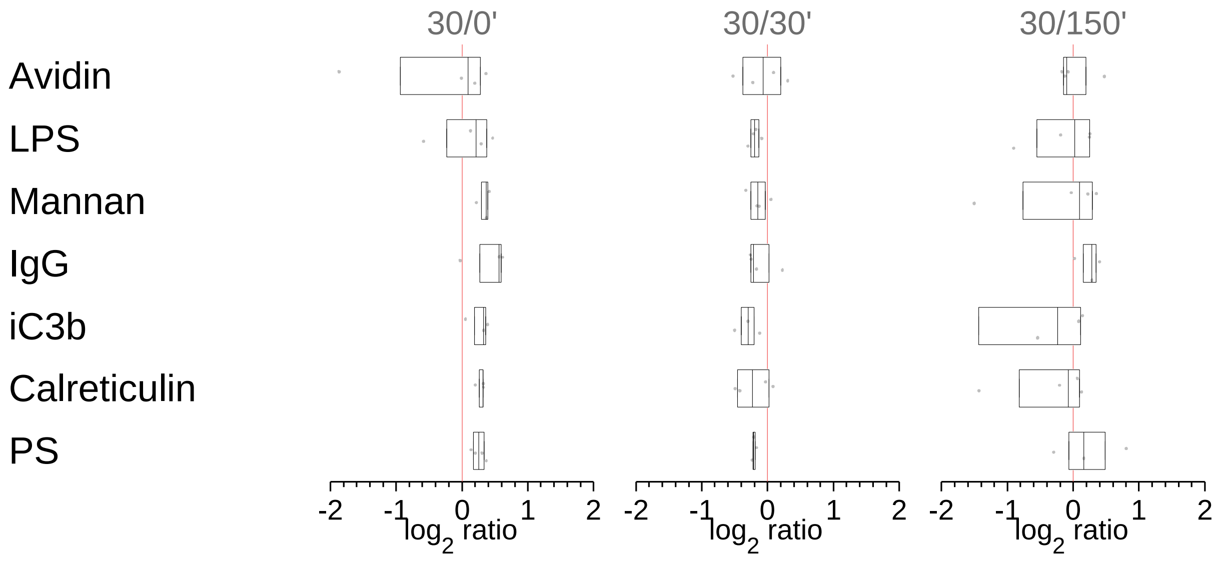

GO:0007093 mitotic cell cycle checkpoint (n = 5)

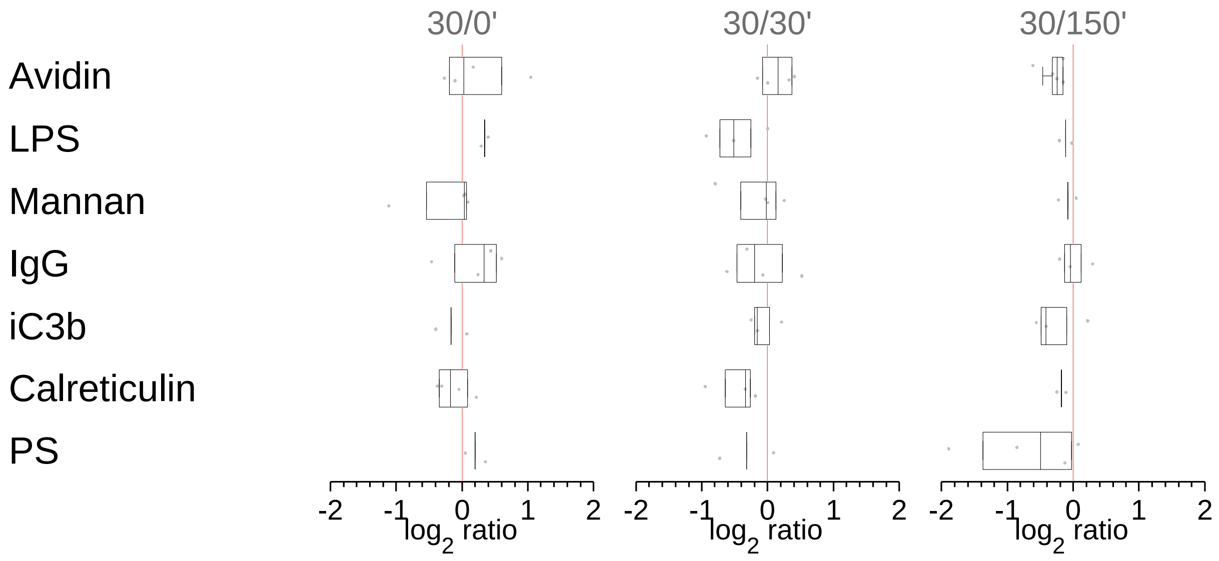

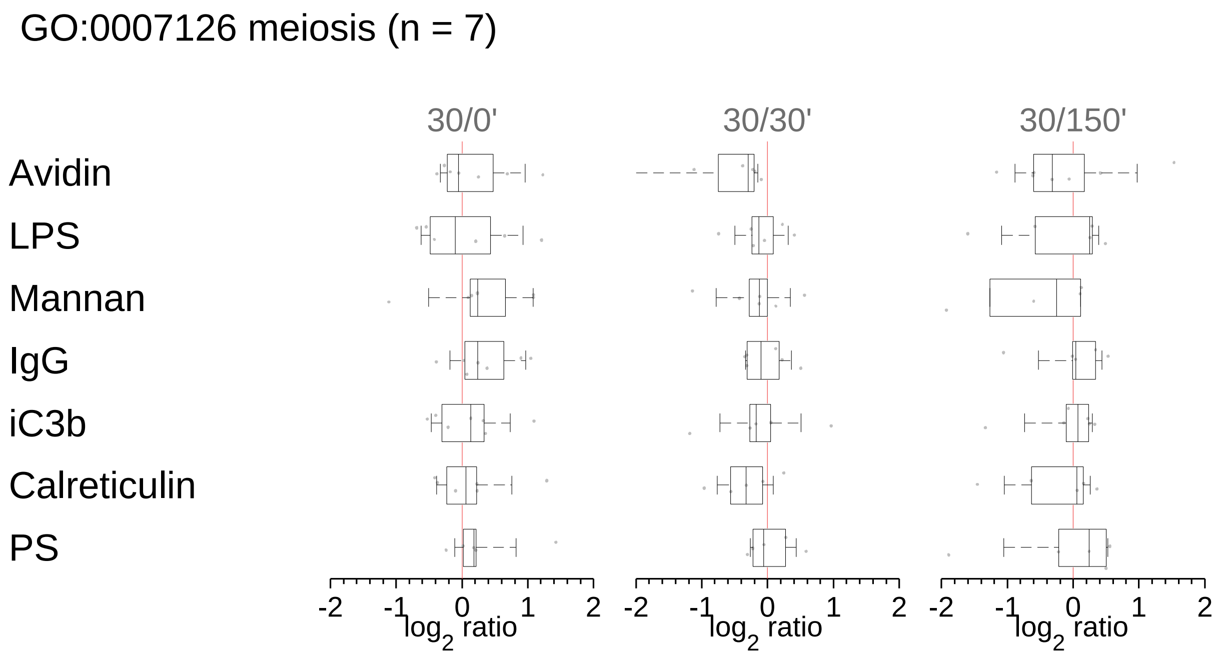
GO:0007126 meiosis (n = 7)
30/0'
30/30'
30/150'
Avidin
LPS
Mannan
IgG
iC3b
Calreticulin
PS
-2 -1 0 1 2
$\log_2$ ratio
-2 -1 0 1 2
$\log_2$ ratio
-2 -1 0 1 2
$\log_2$ ratio

GO:0007155 cell adhesion (n = 54)

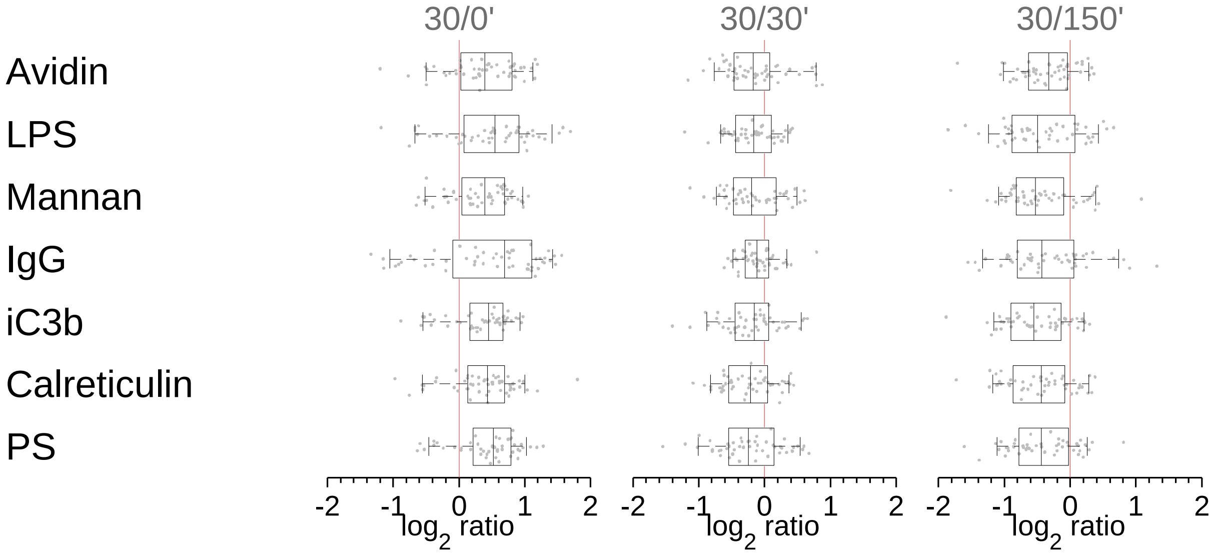

GO:0007157 heterophilic cell-cell adhesion (n = 5)

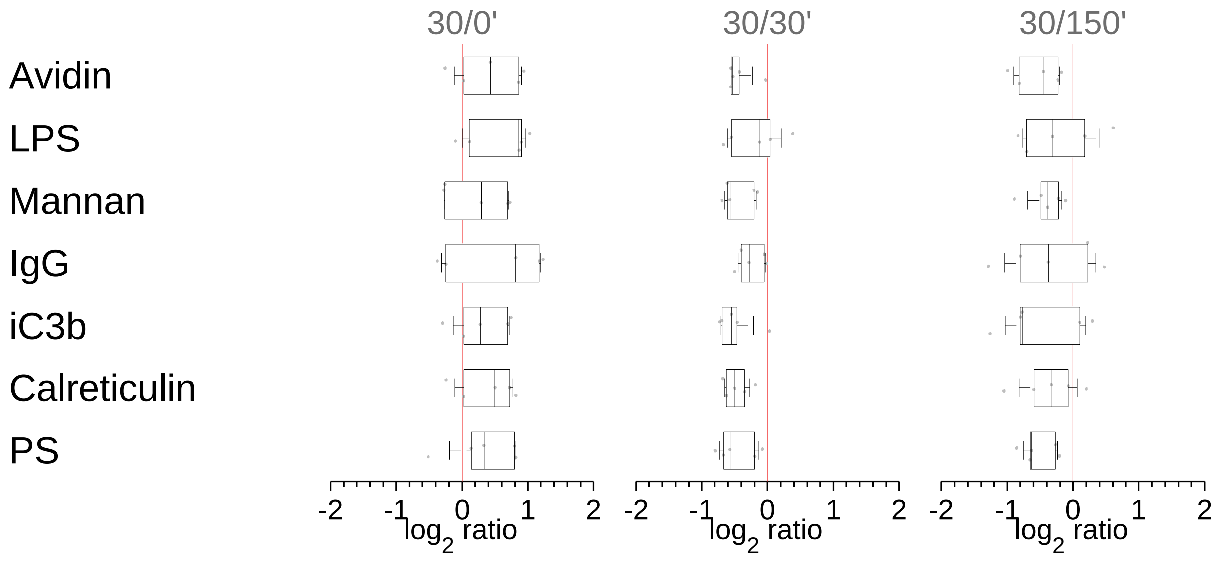

GO:0007159 leukocyte cell-cell adhesion (n = 10)

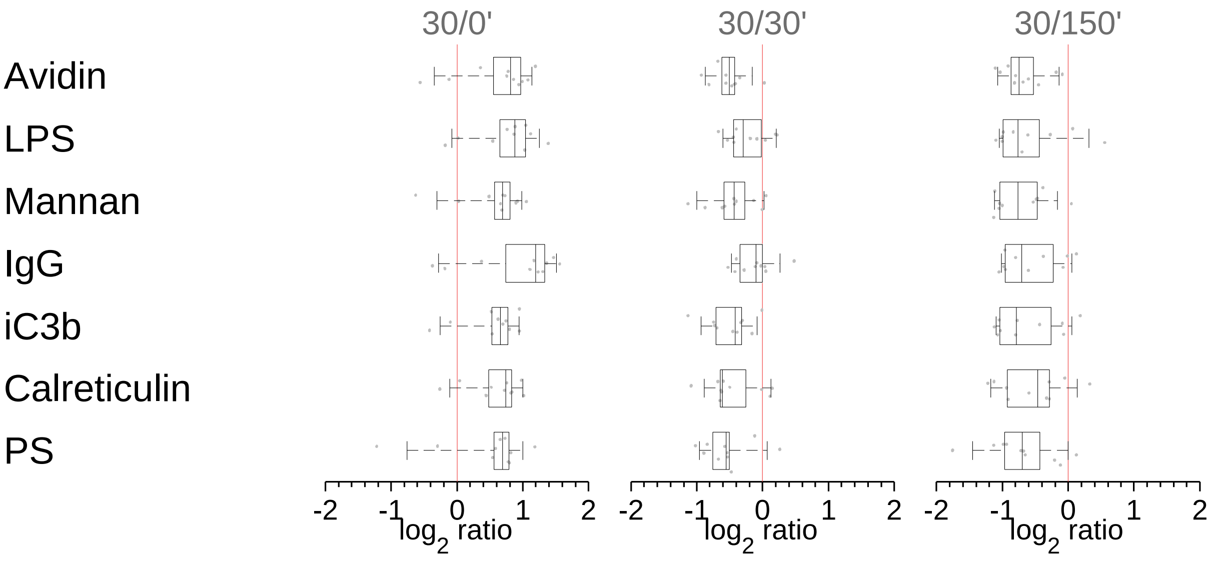

GO:0007160 cell-matrix adhesion (n = 16)

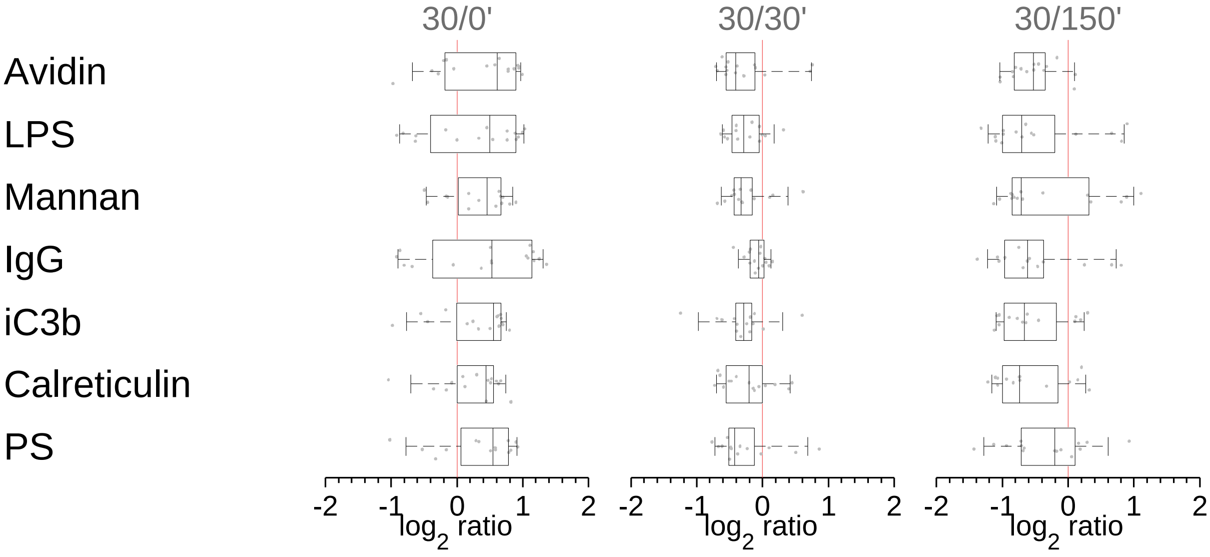

GO:0007162 negative regulation of cell adhesion (n = 6)

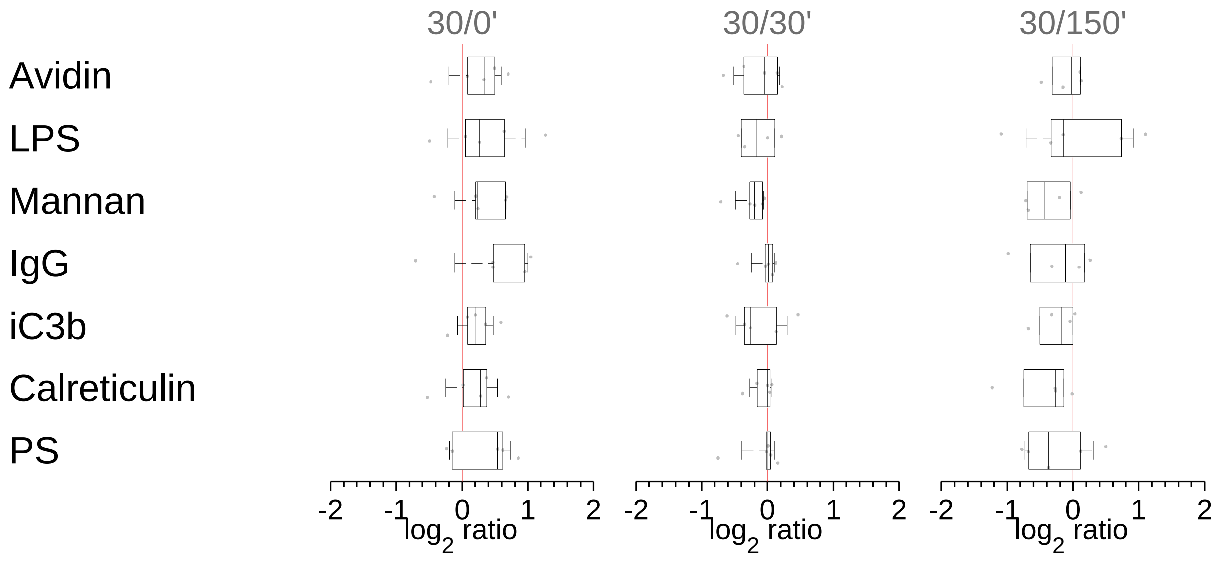

GO:0007163 establishment or maintenance of cell polarity (n = 6)

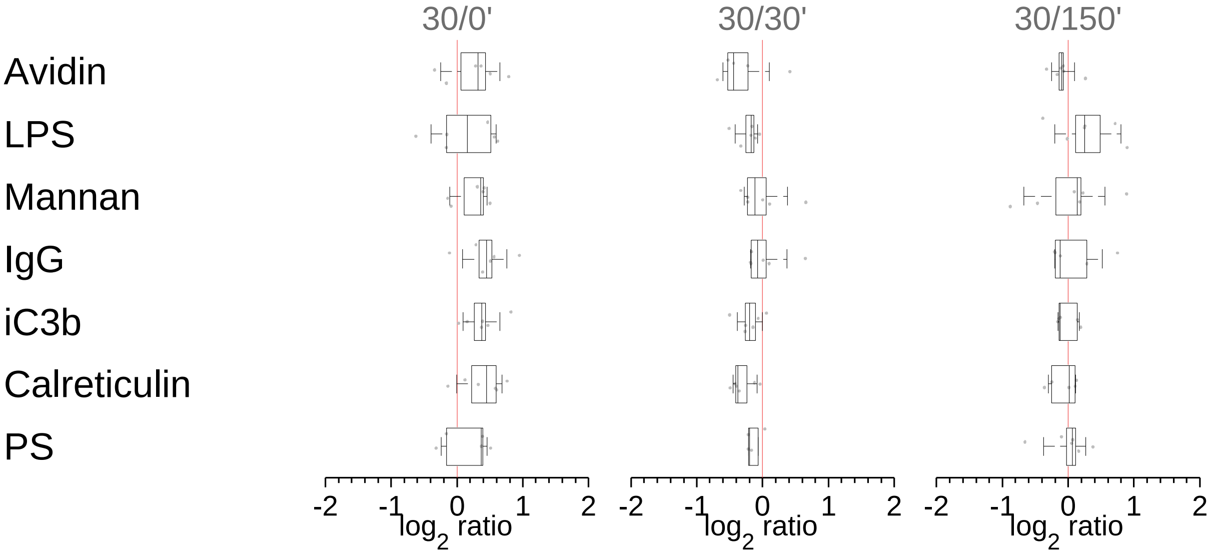

GO:0007165 signal transduction (n = 87)

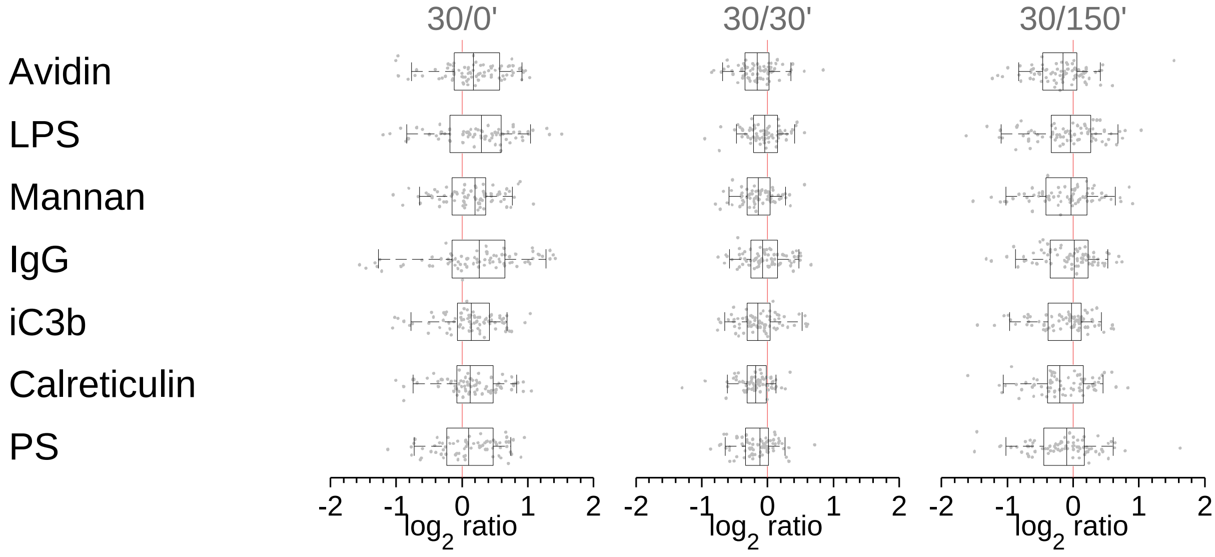

GO:0007166 cell surface receptor signaling pathway (n = 13)

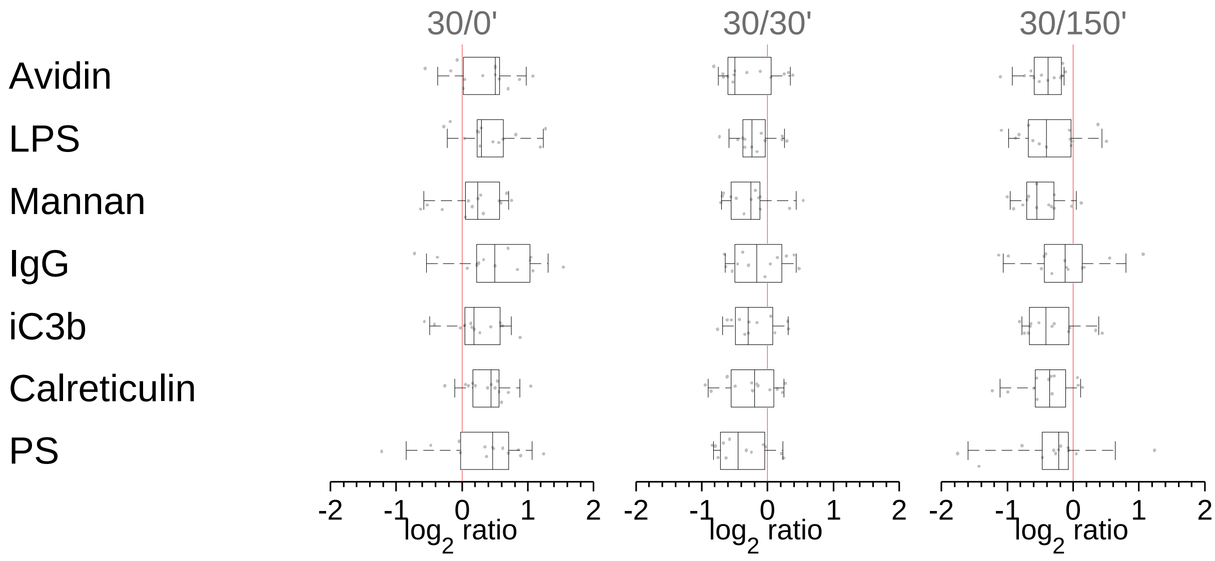

GO:0007169 transmembrane receptor protein tyrosine kinase signaling pathway (n = 4)

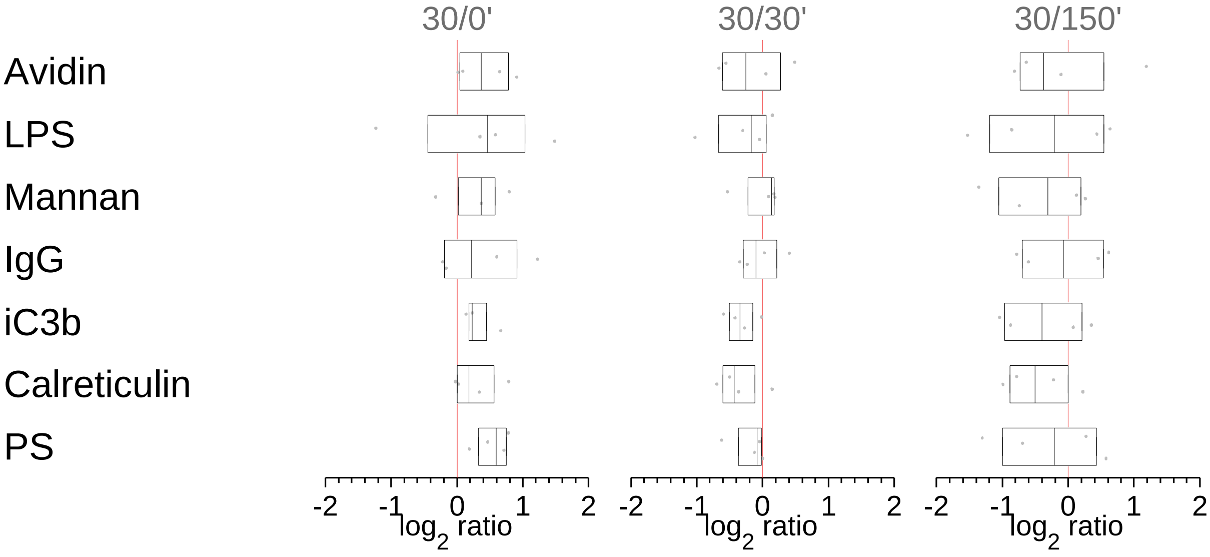

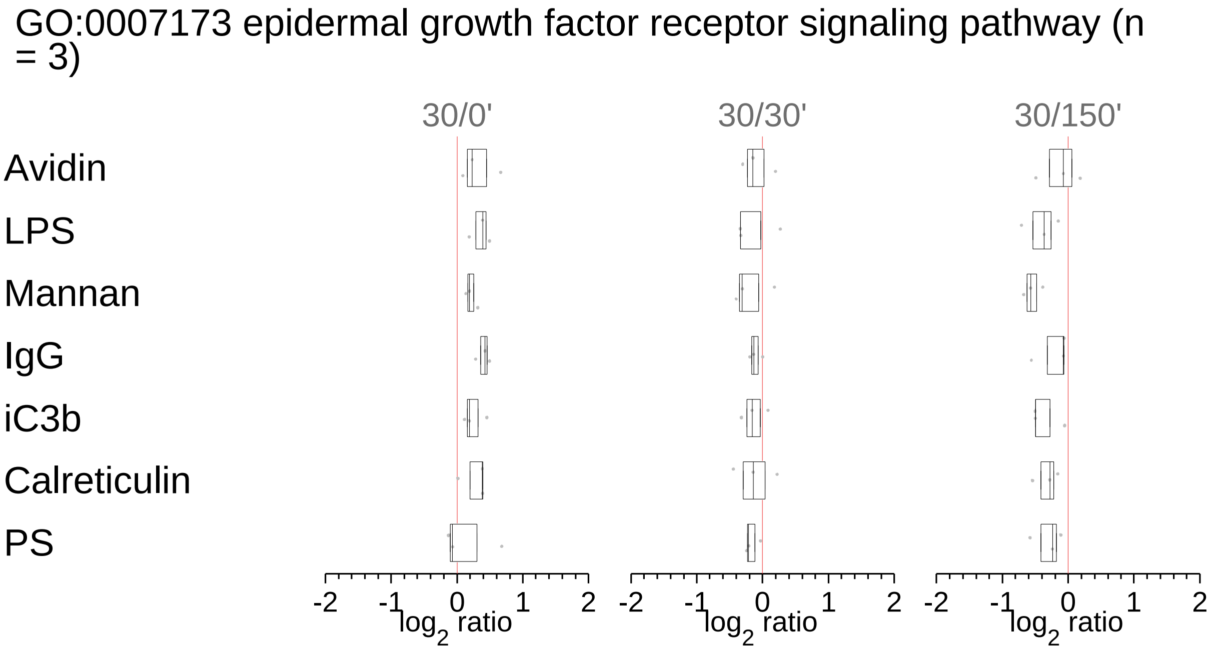
GO:0007173 epidermal growth factor receptor signaling pathway (n = 3)
30/0'
30/30'
30/150'
Avidin
LPS
Mannan
IgG
iC3b
Calreticulin
PS
-2 -1 0 1 2
log2 ratio

GO:0007176 regulation of epidermal growth factor-activated receptor activity (n = 3)

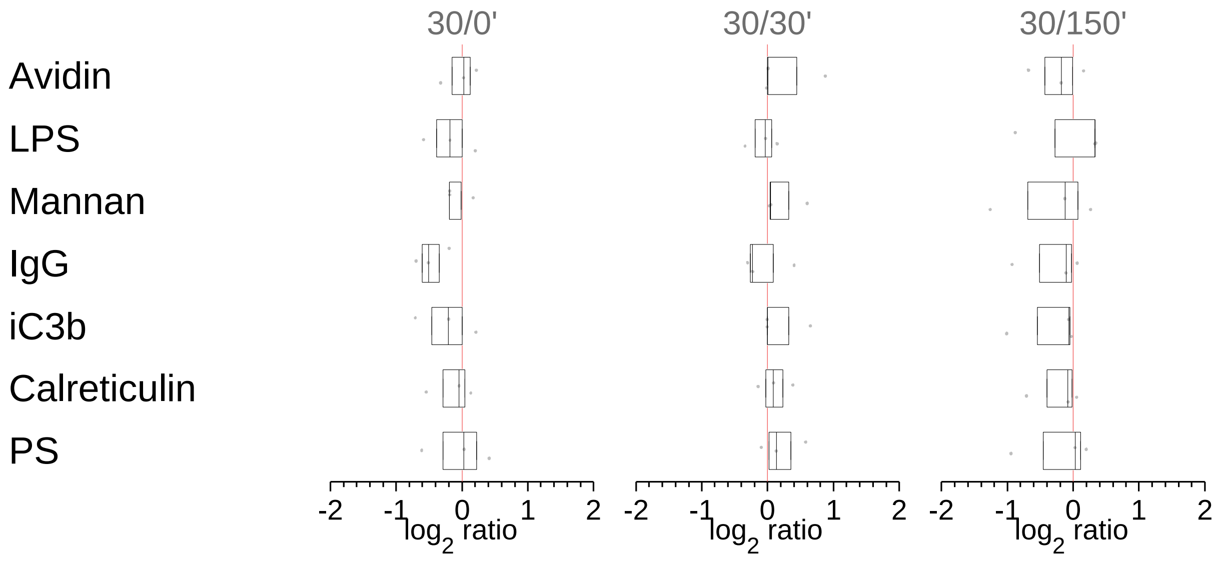

GO:0007179 transforming growth factor beta receptor signaling pathway (n = 4)

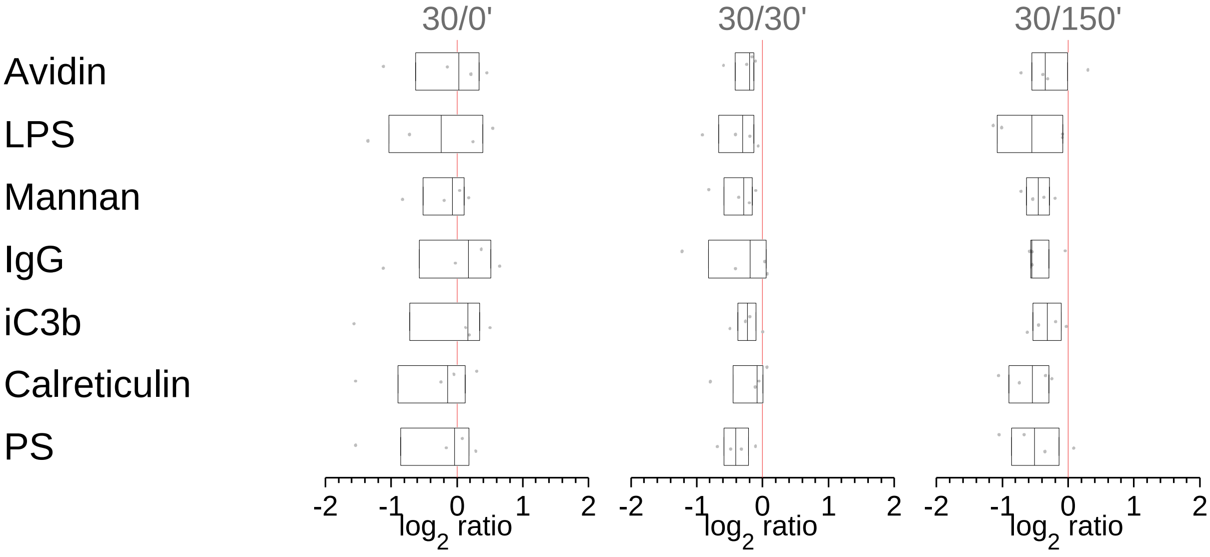

GO:0007186 G-protein coupled receptor signaling pathway (n = 24)

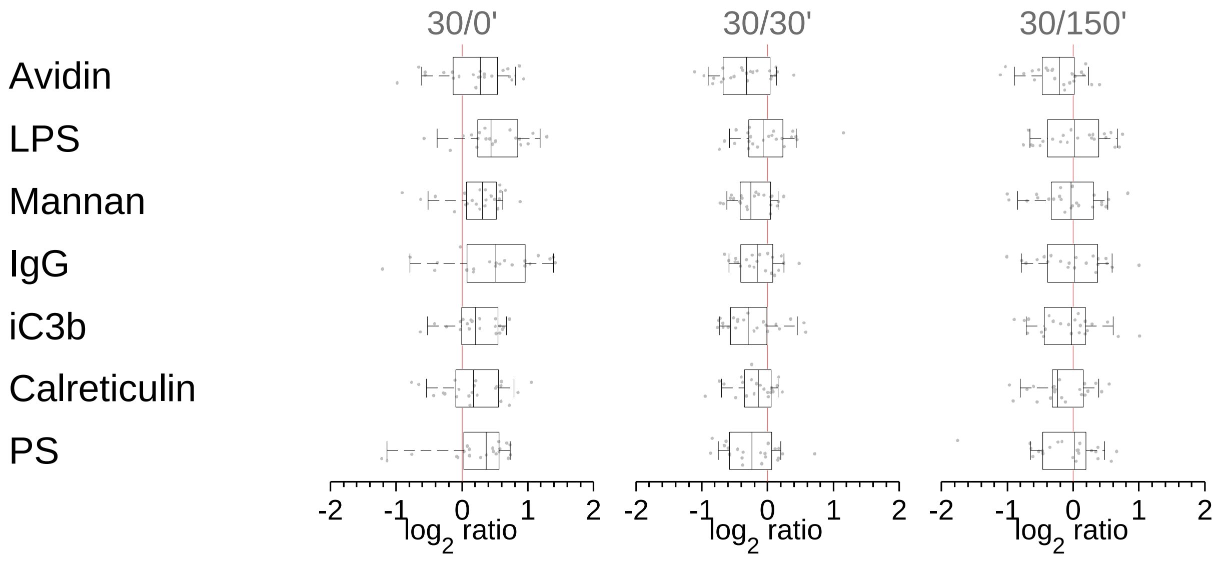

GO:0007188 adenylate cyclase-modulating G-protein coupled receptor signaling pathway (n = 3)

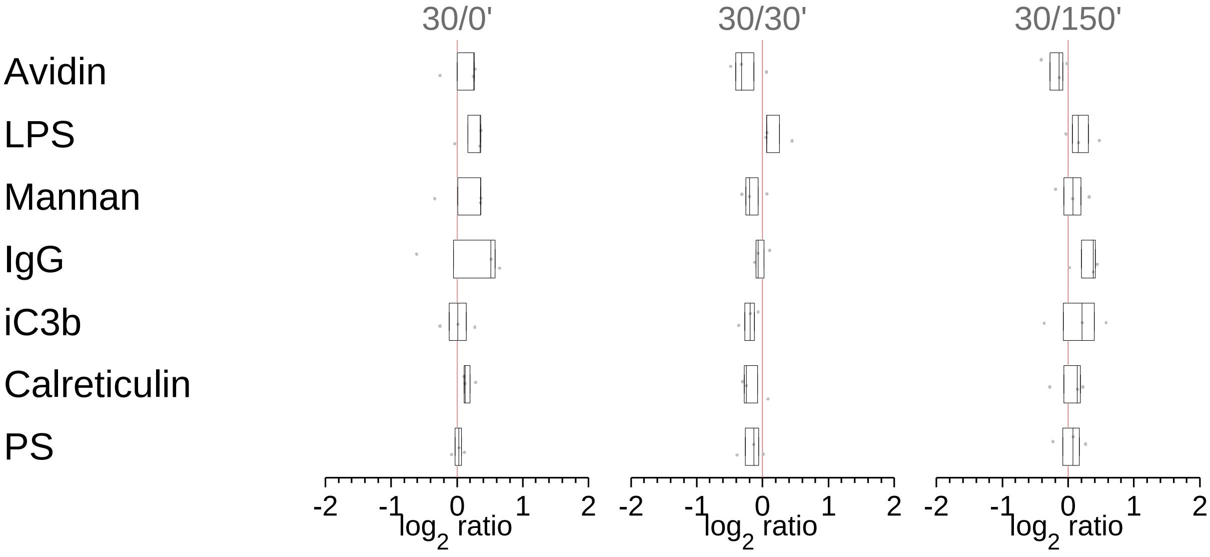

GO:0007189 adenylate cyclase-activating G-protein coupled receptor signaling pathway (n = 3)

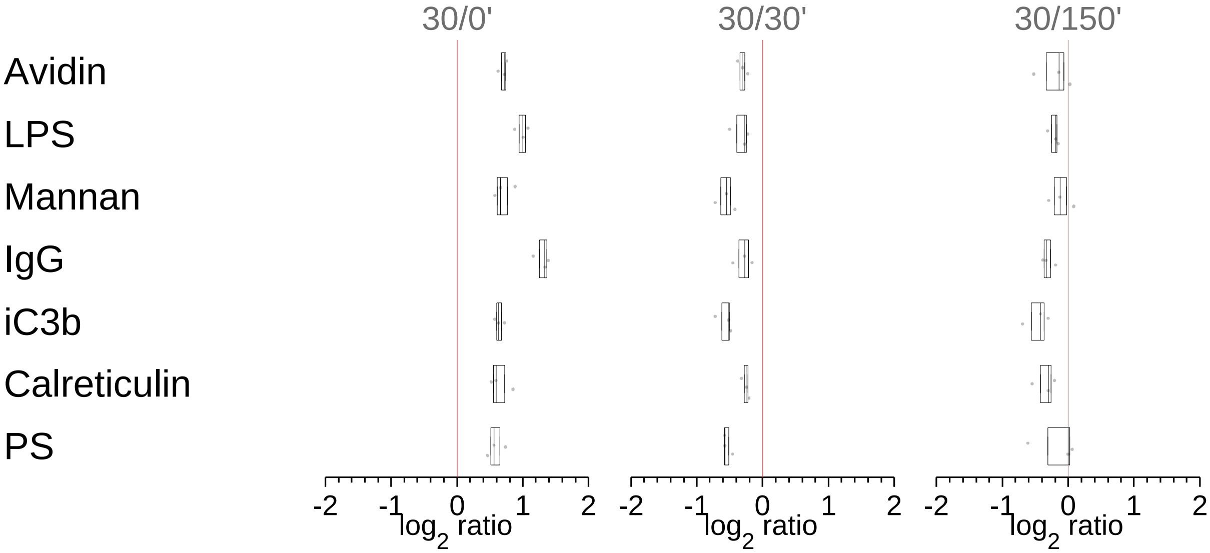

GO:0007200 phospholipase C-activating G-protein coupled receptor signaling pathway (n = 3)

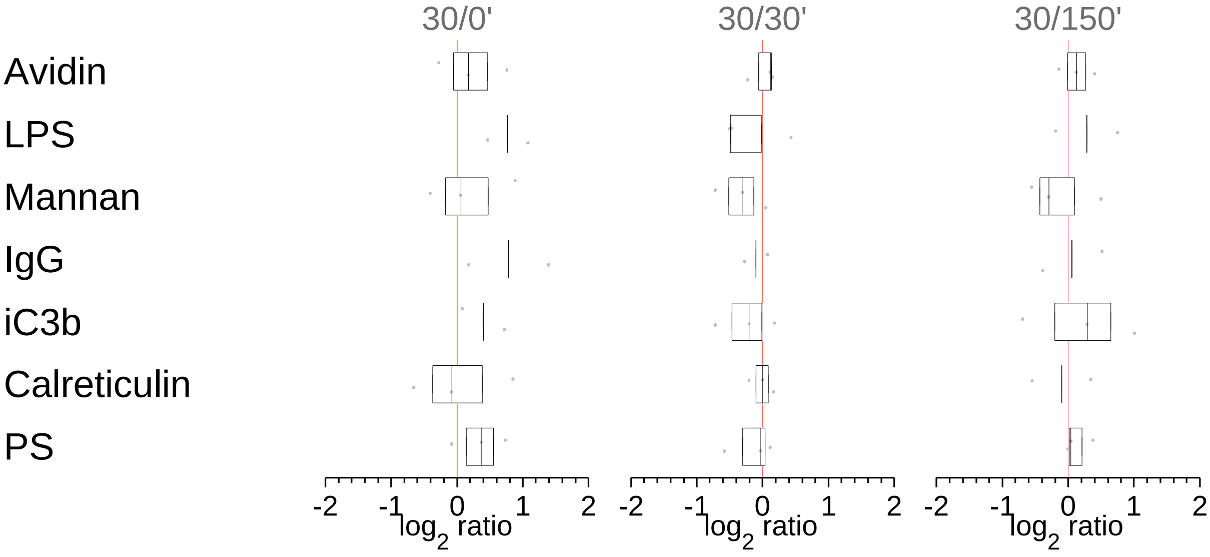

GO:0007204 elevation of cytosolic calcium ion concentration (n = 8)

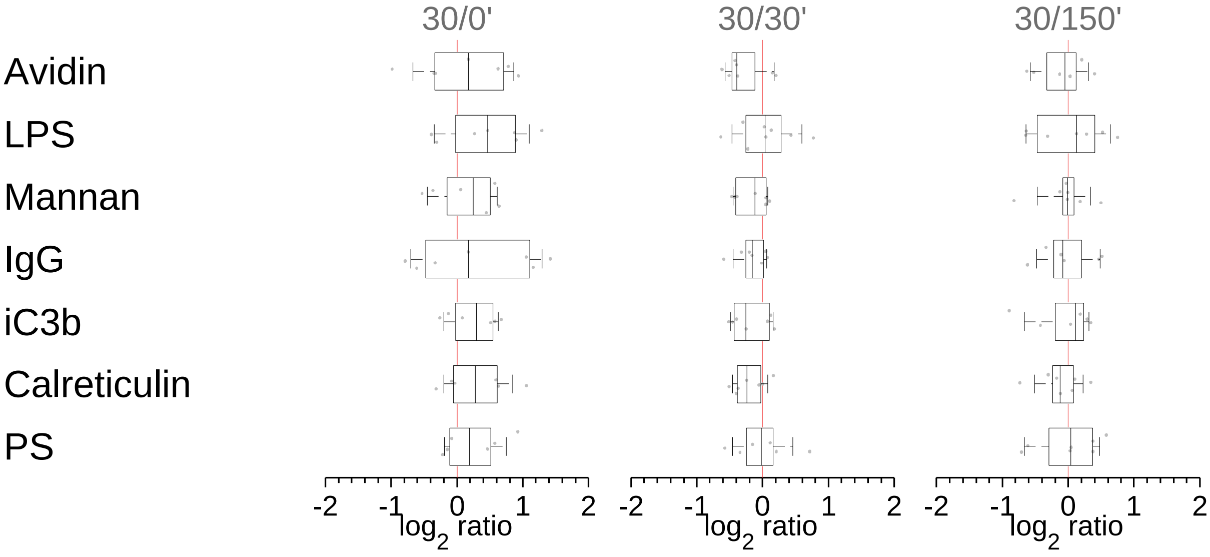

GO:0007219 Notch signaling pathway (n = 8)

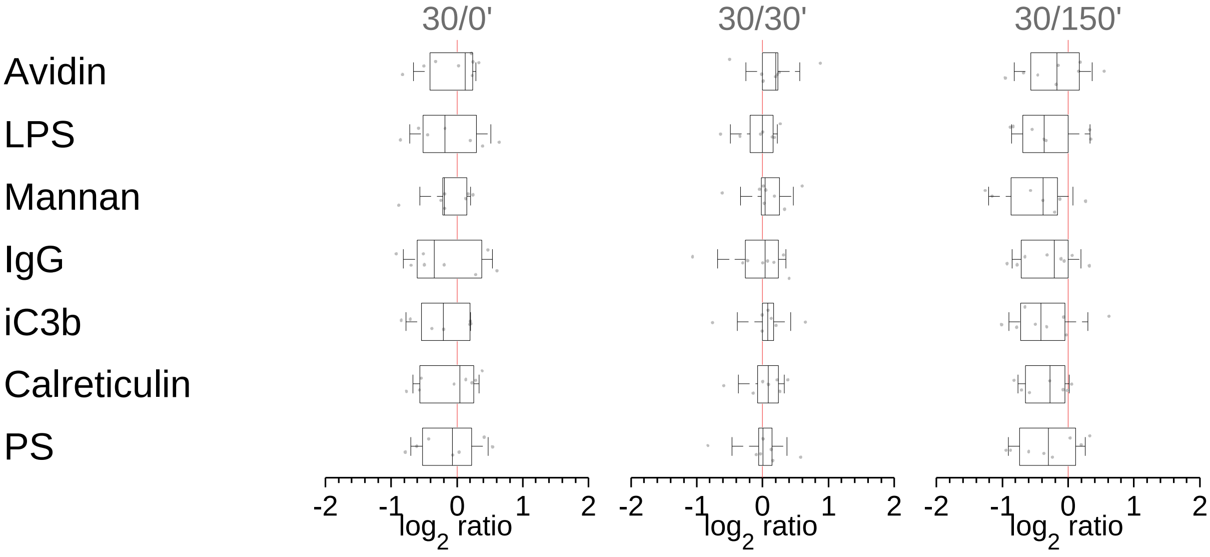

GO:0007220 Notch receptor processing (n = 5)

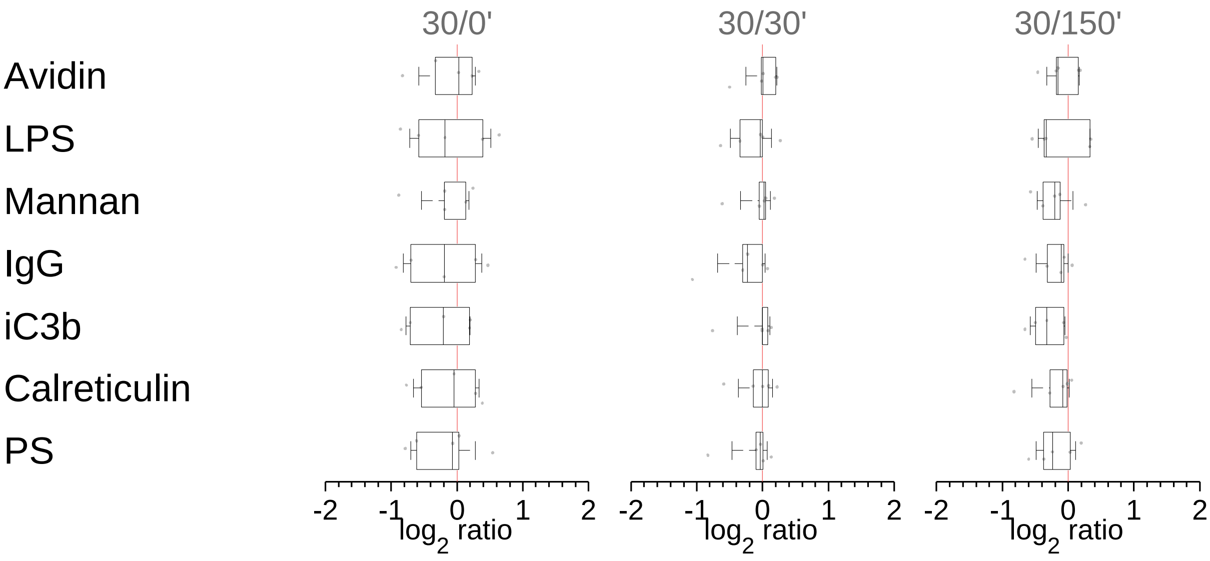

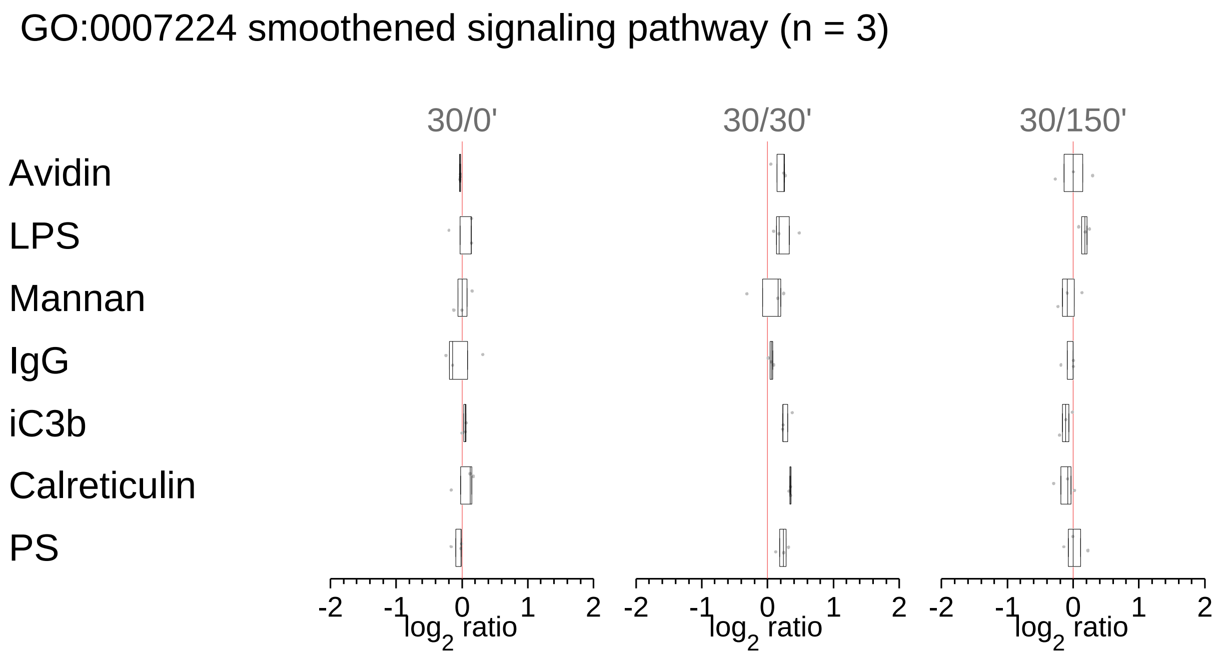
GO:0007224 smoothened signaling pathway (n = 3)
30/0'
30/30'
30/150'
Avidin
LPS
Mannan
IgG
iC3b
Calreticulin
PS
-2
-1
0
1
2
log2 ratio

GO:0007229 integrin-mediated signaling pathway (n = 23)

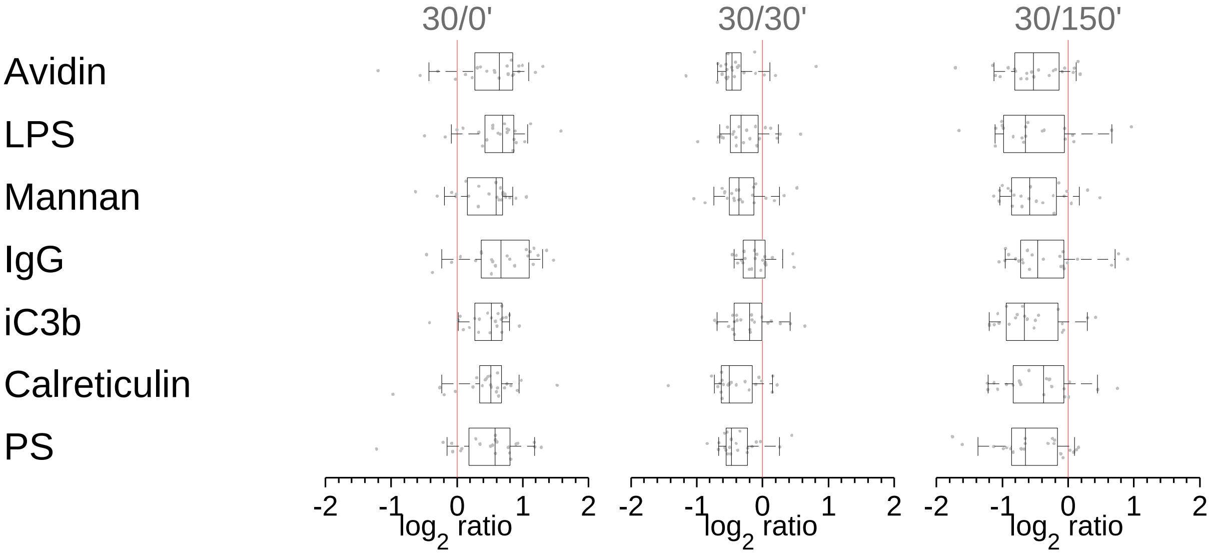

GO:0007249 I-kappaB kinase/NF-kappaB cascade (n = 3)

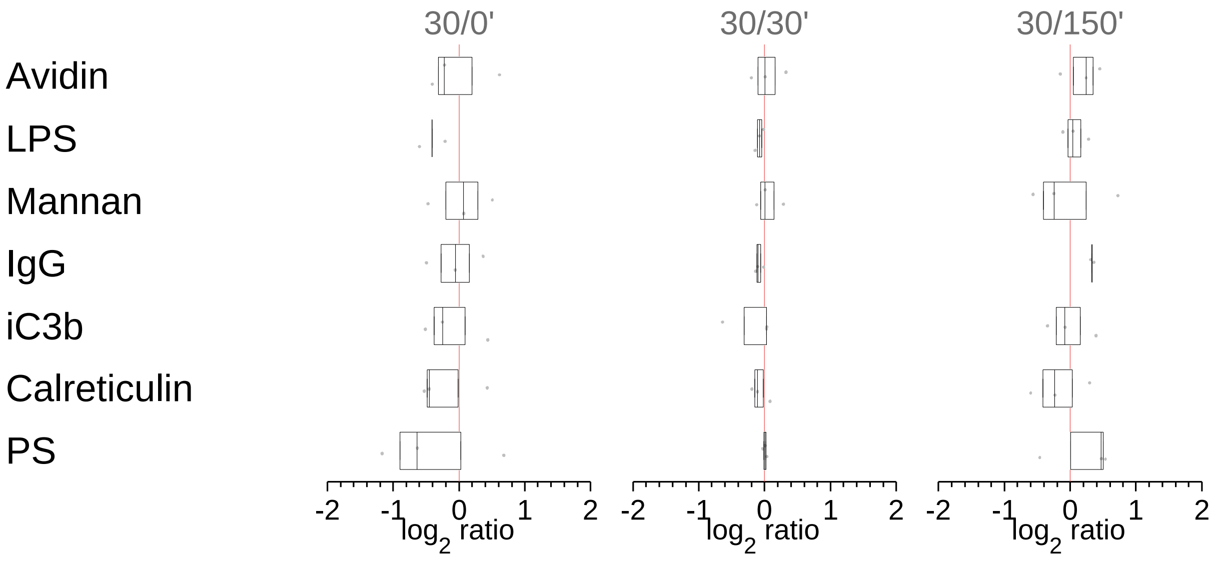

GO:0007252 I-kappaB phosphorylation (n = 4)

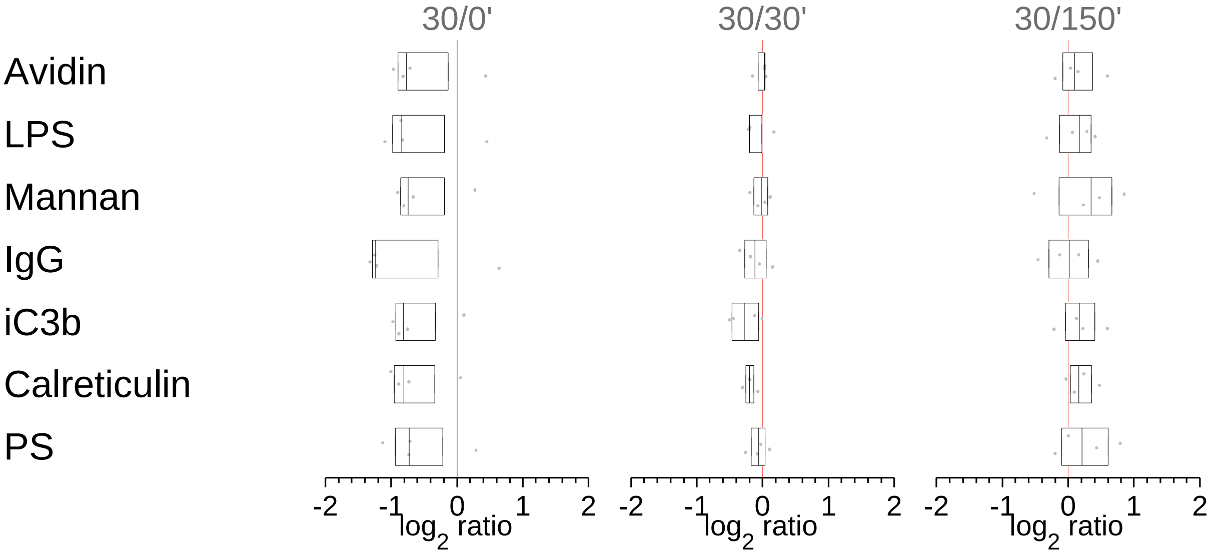

GO:0007254 JNK cascade (n = 4)

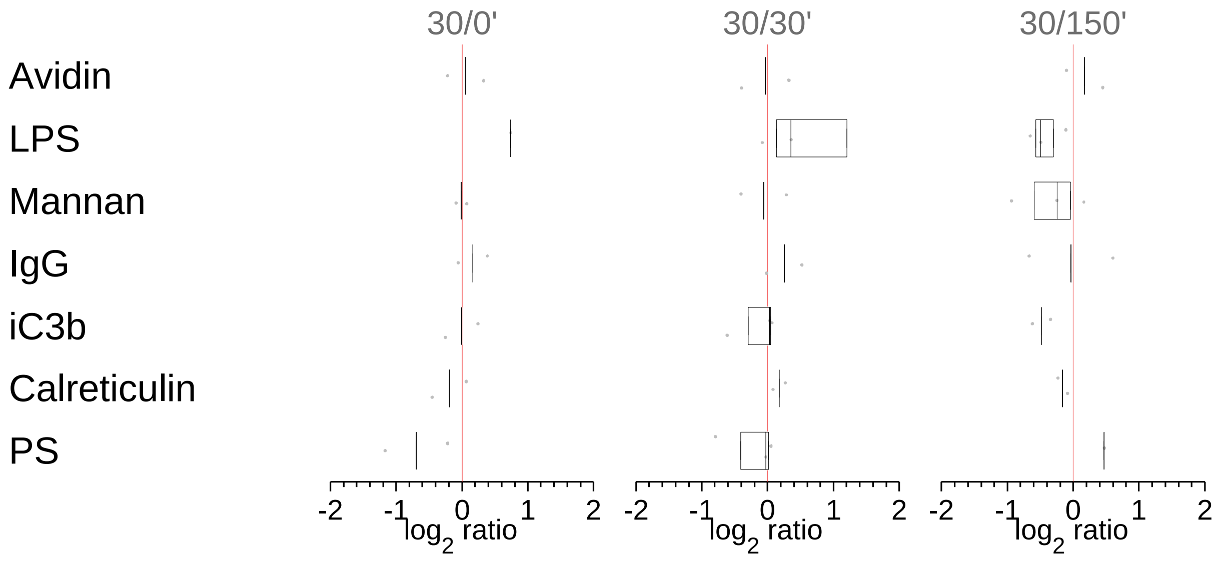

GO:0007257 activation of JUN kinase activity (n = 4)

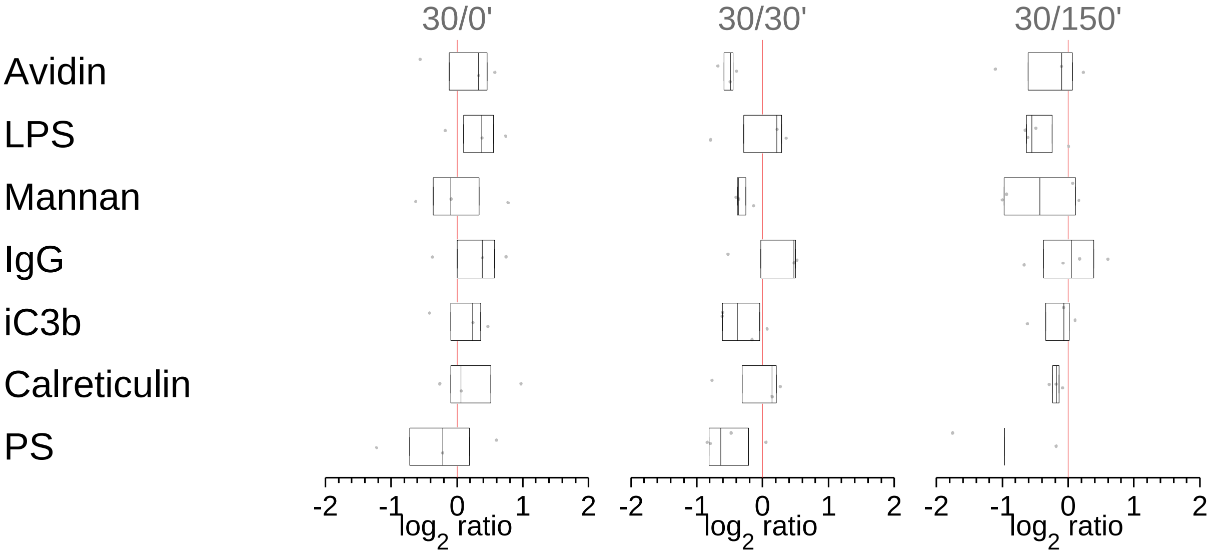

GO:0007264 small GTPase mediated signal transduction (n = 77)

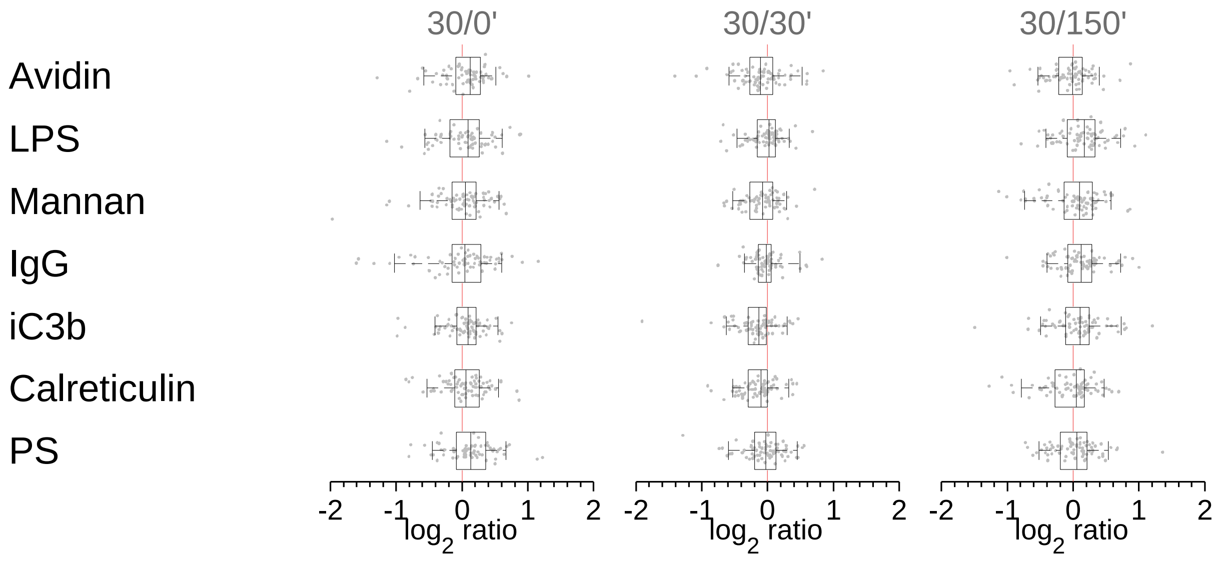

GO:0007265 Ras protein signal transduction (n = 11)

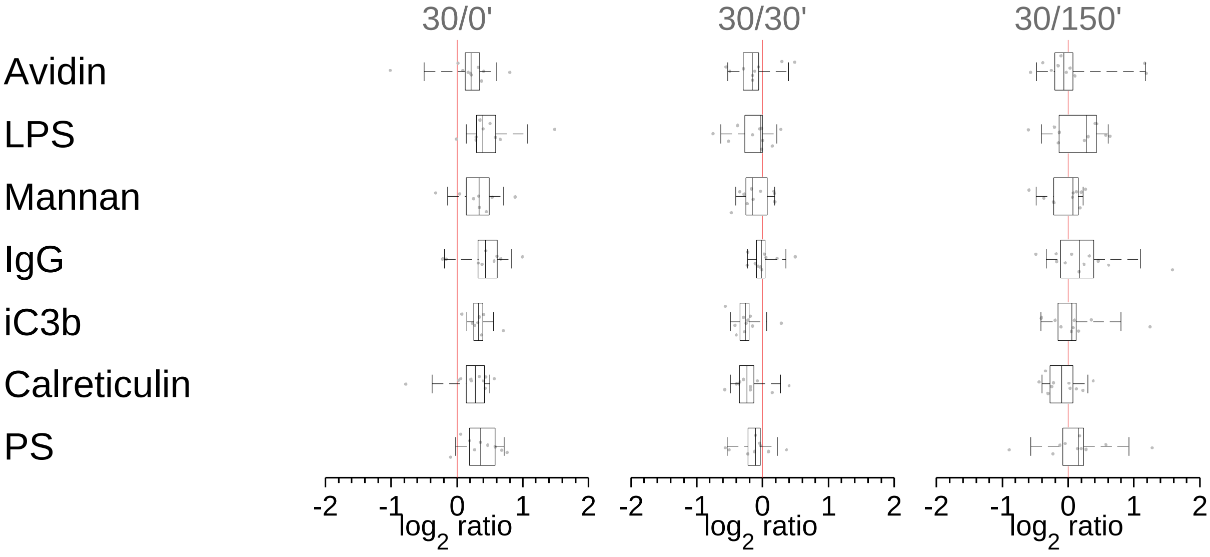

GO:0007266 Rho protein signal transduction (n = 9)

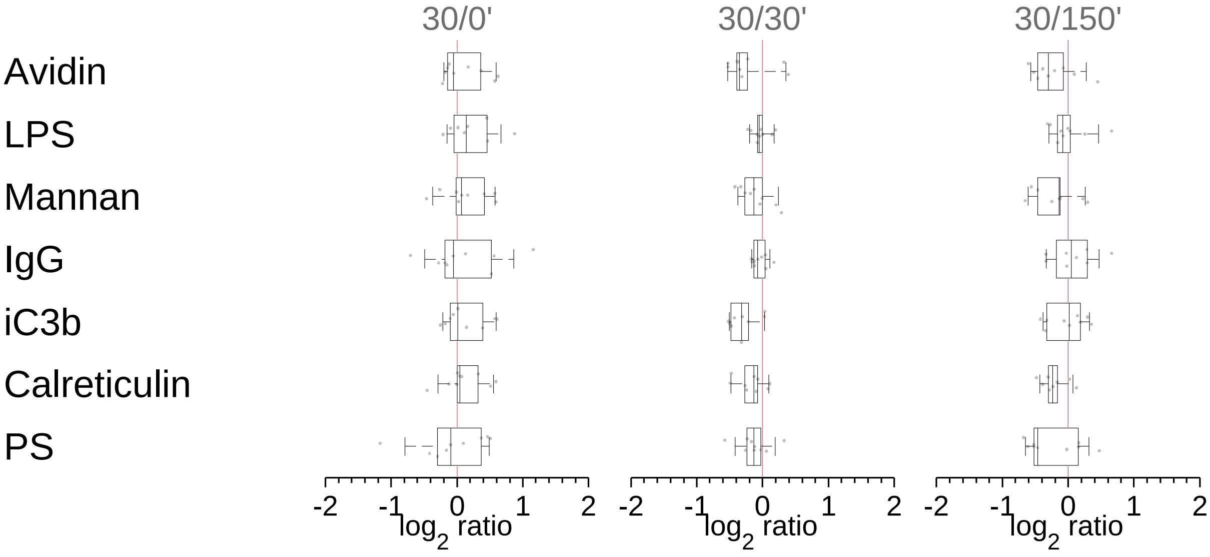

GO:0007268 synaptic transmission (n = 5)

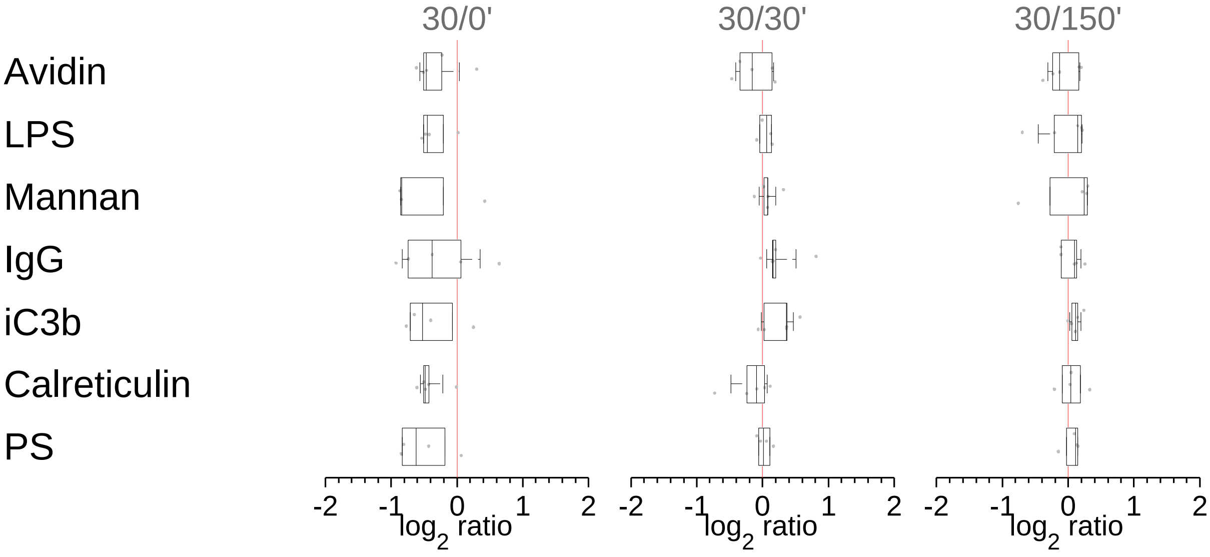

GO:0007269 neurotransmitter secretion (n = 3)

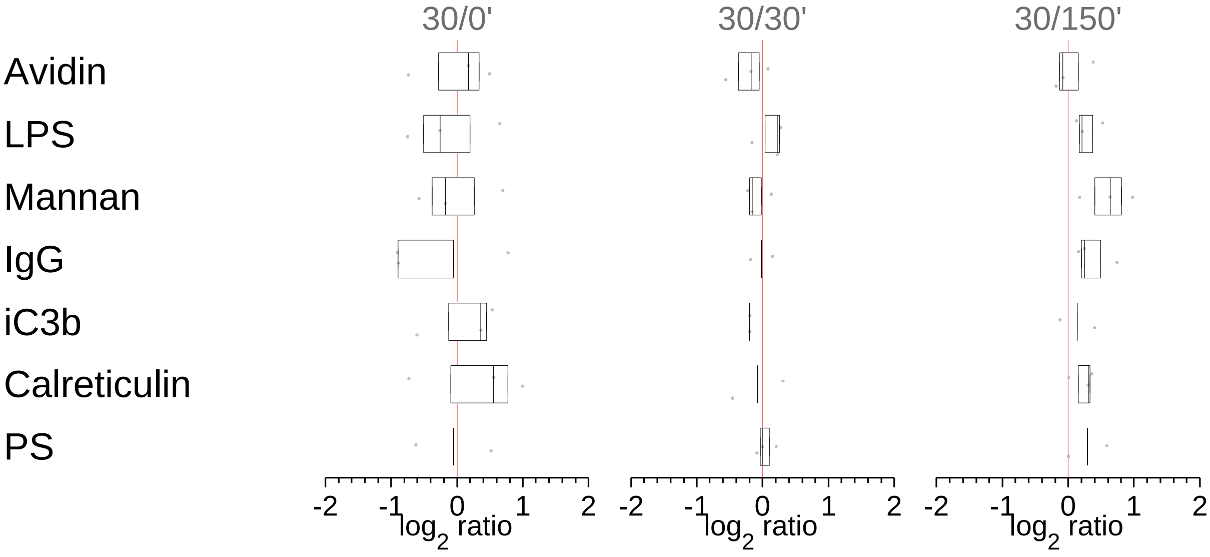

GO:0007275 multicellular organismal development (n = 56)

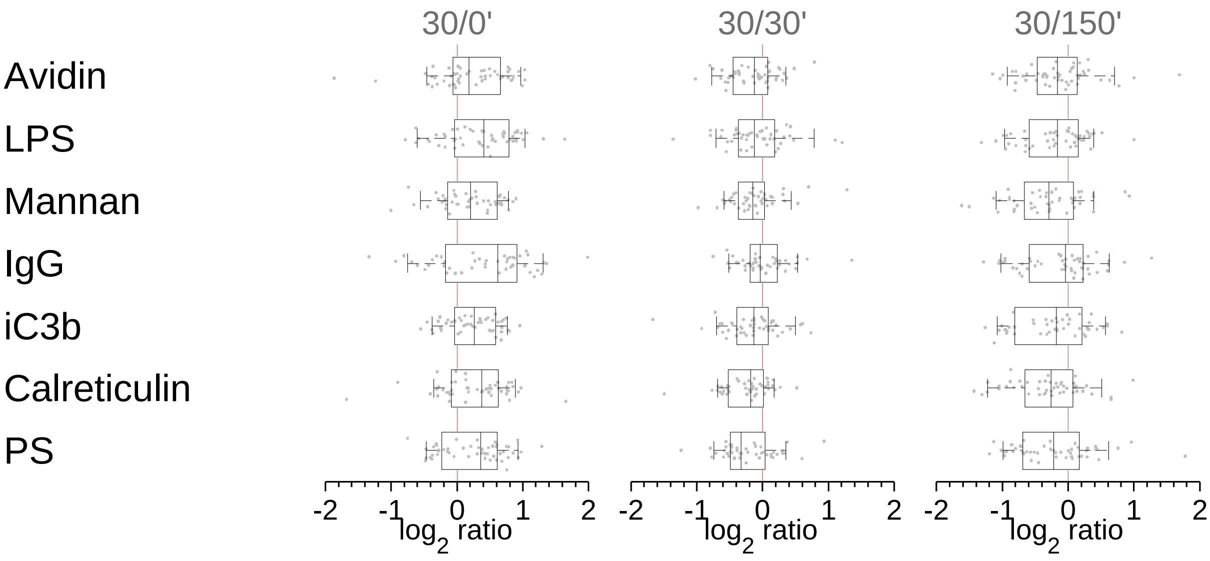

GO:0007283 spermatogenesis (n = 22)

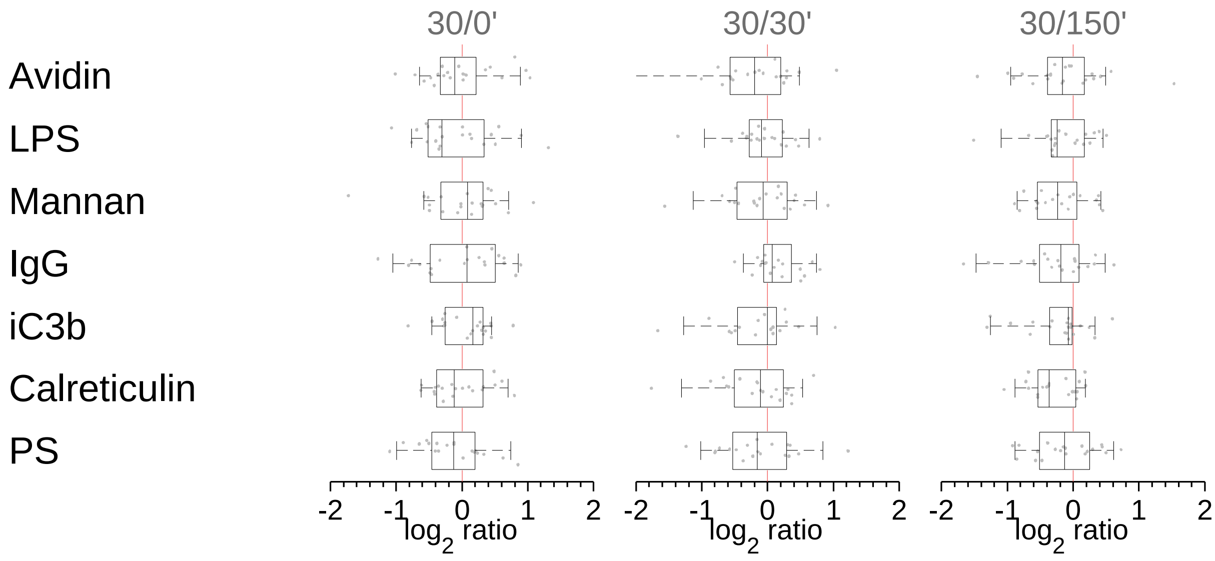

GO:0007338 single fertilization (n = 3)

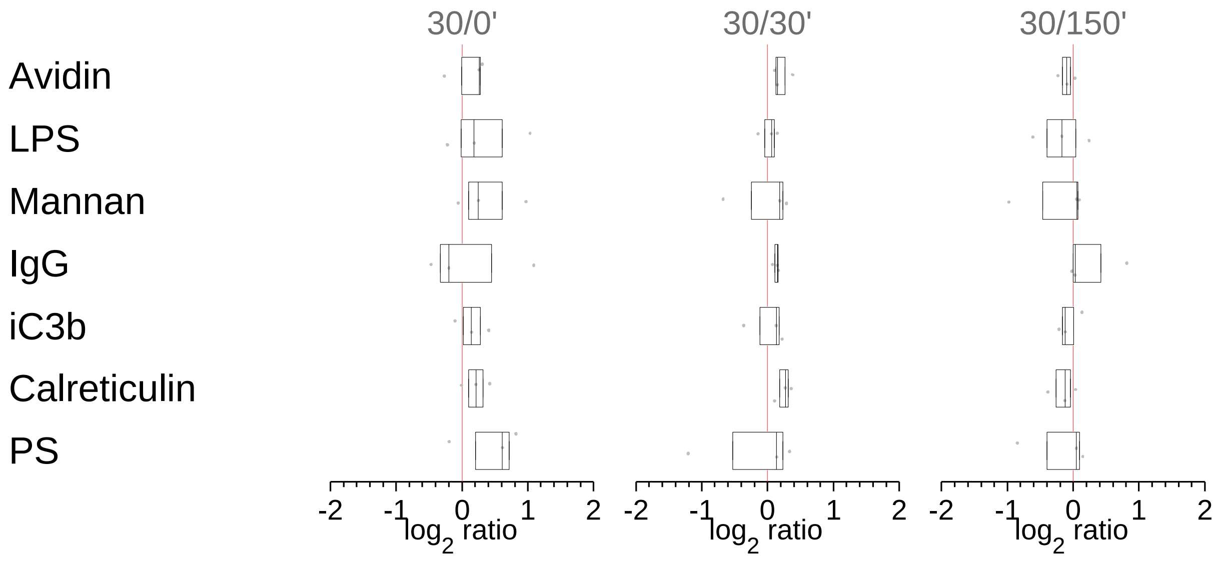

GO:0007339 binding of sperm to zona pellucida (n = 10)

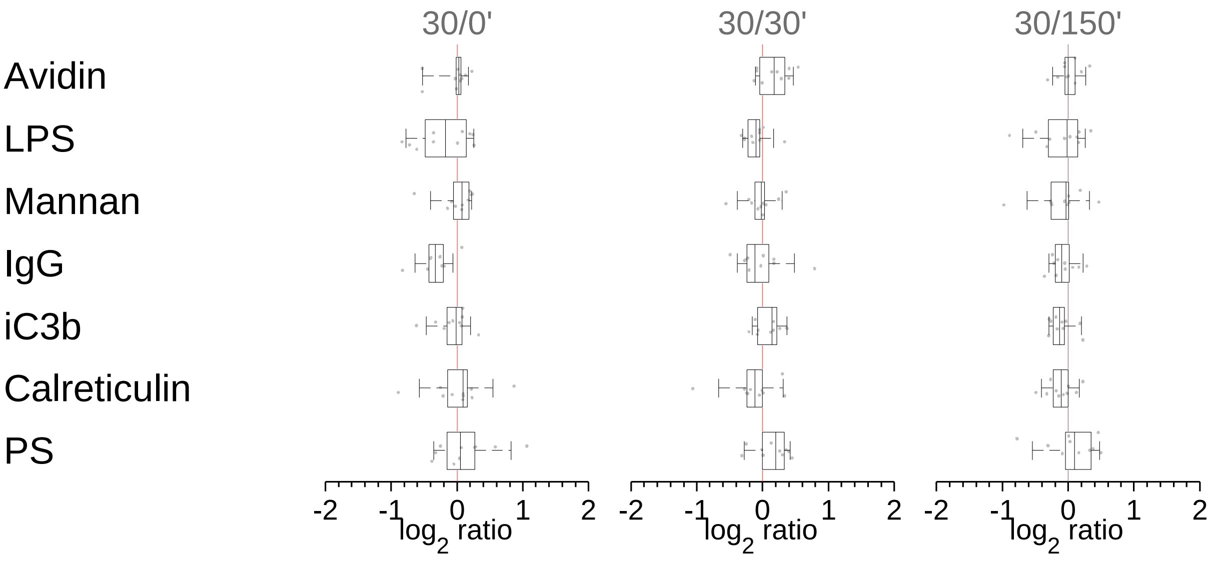

GO:0007369 gastrulation (n = 5)

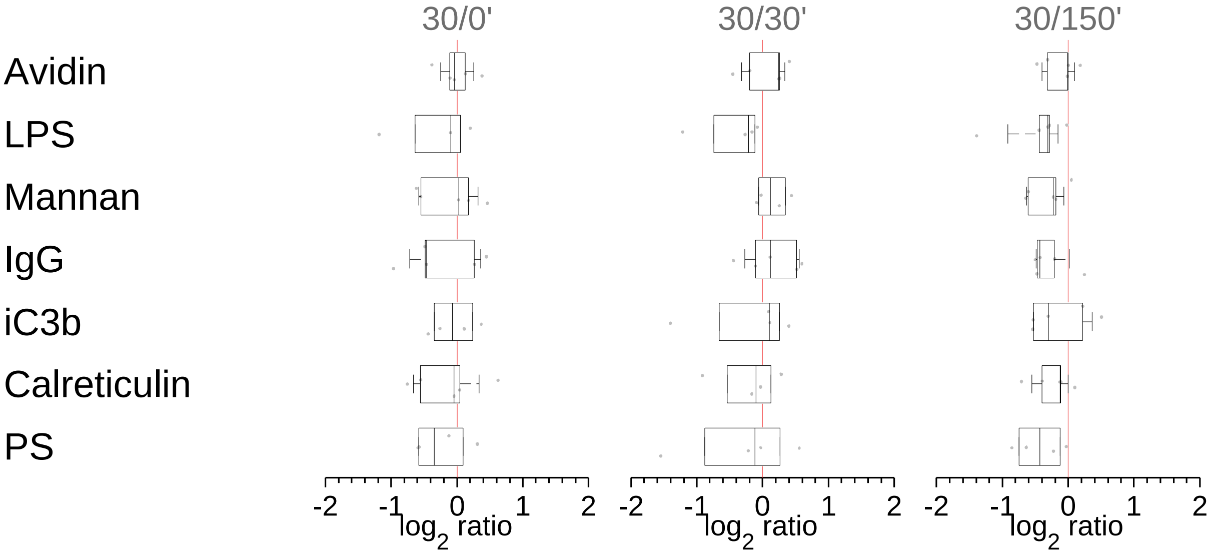

GO:0007399 nervous system development (n = 34)

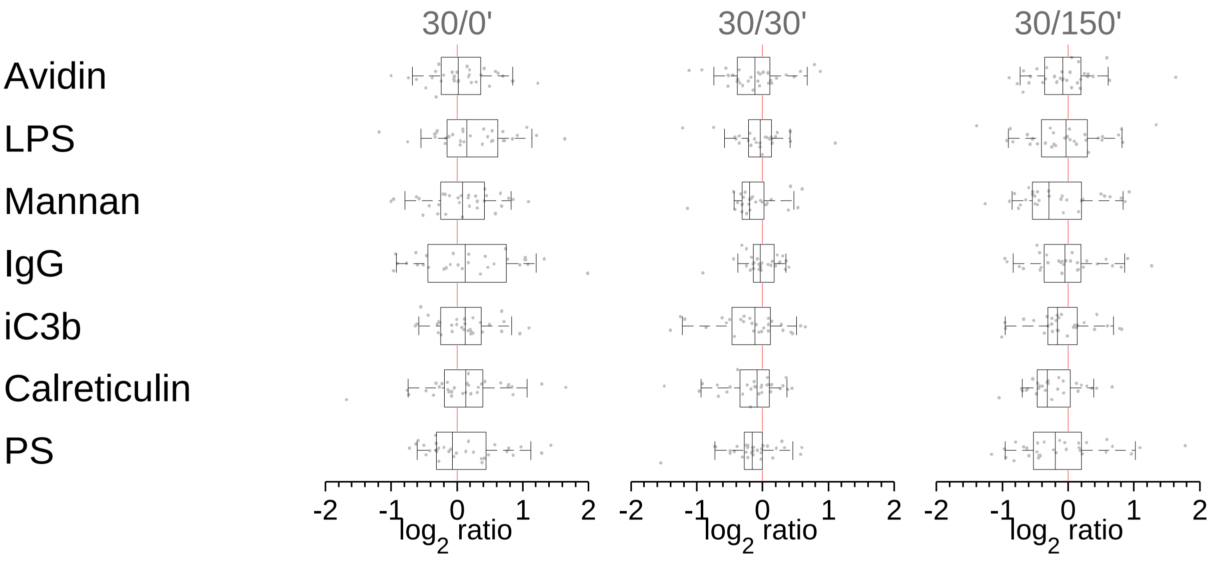

GO:0007405 neuroblast proliferation (n = 5)

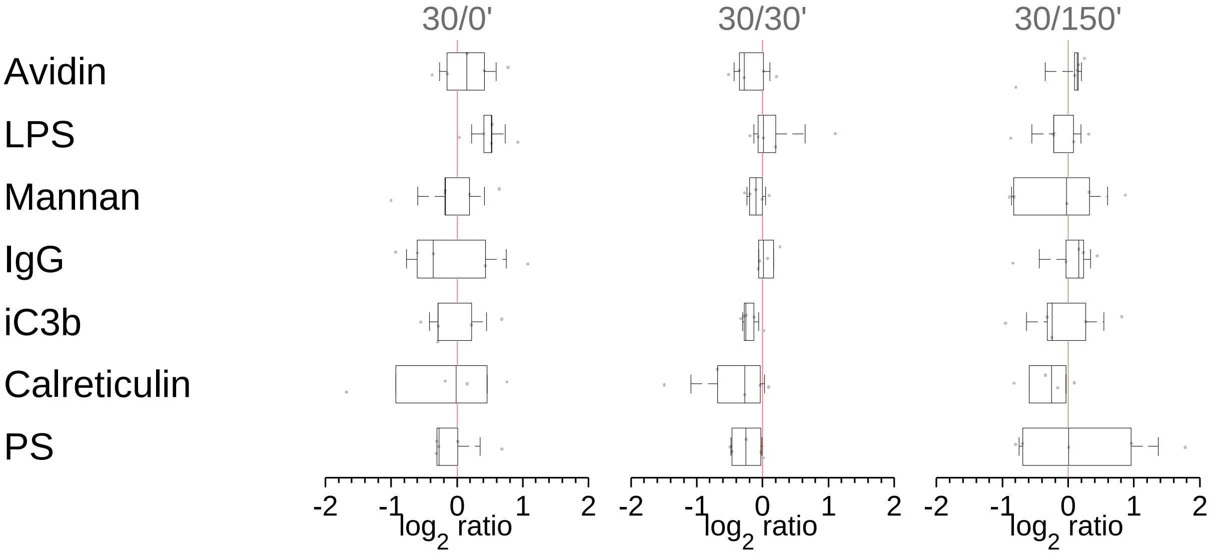

GO:0007409 axonogenesis (n = 14)

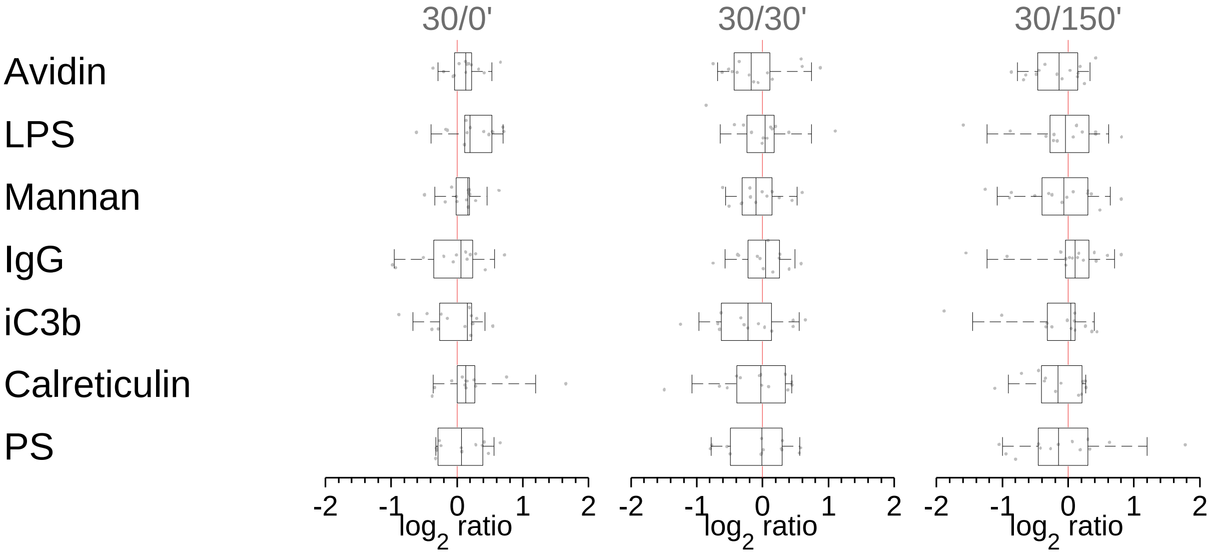

GO:0007411 axon guidance (n = 8)

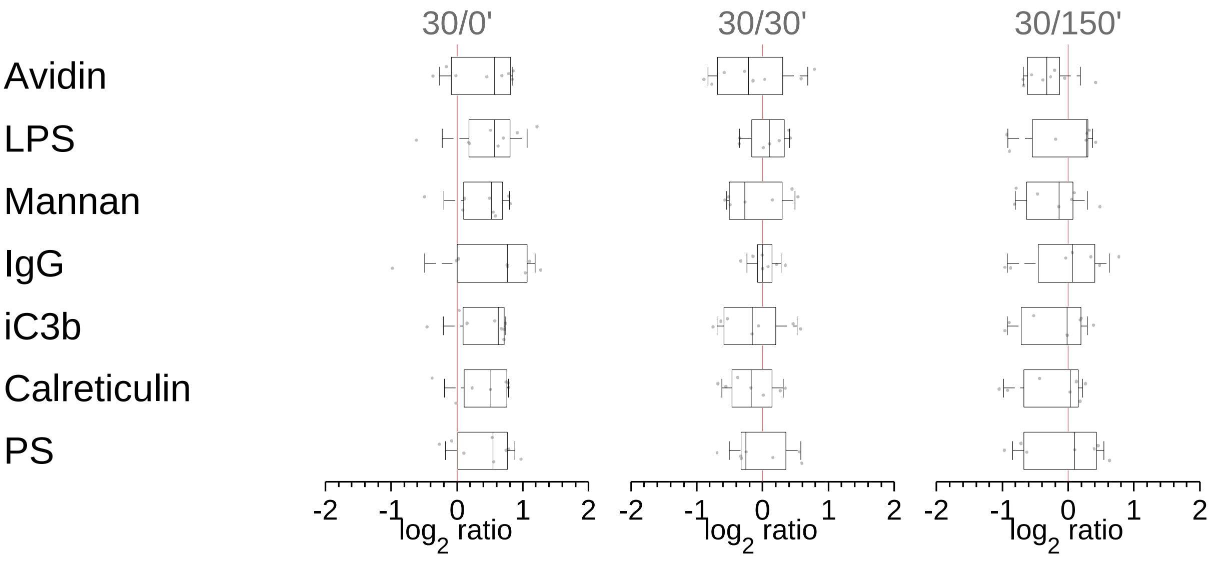

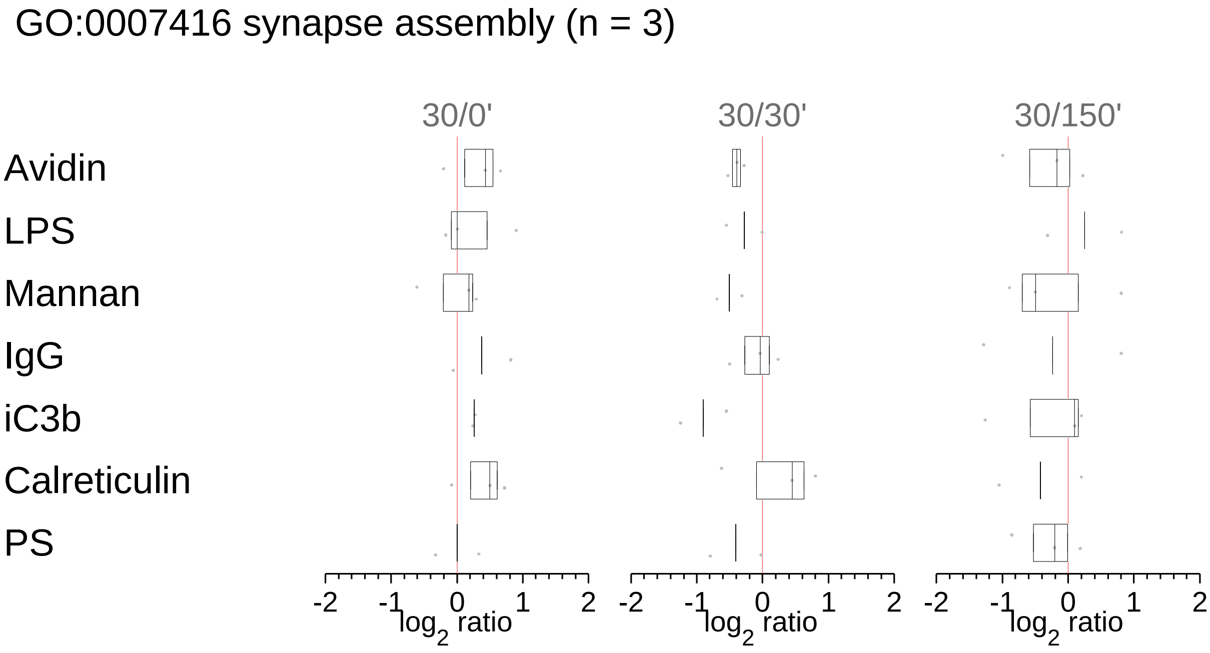
GO:0007416 synapse assembly (n = 3)
30/0'
30/30'
30/150'
Avidin
LPS
Mannan
IgG
iC3b
Calreticulin
PS
-2 -1 0 1 2
log2 ratio

GO:0007417 central nervous system development (n = 4)

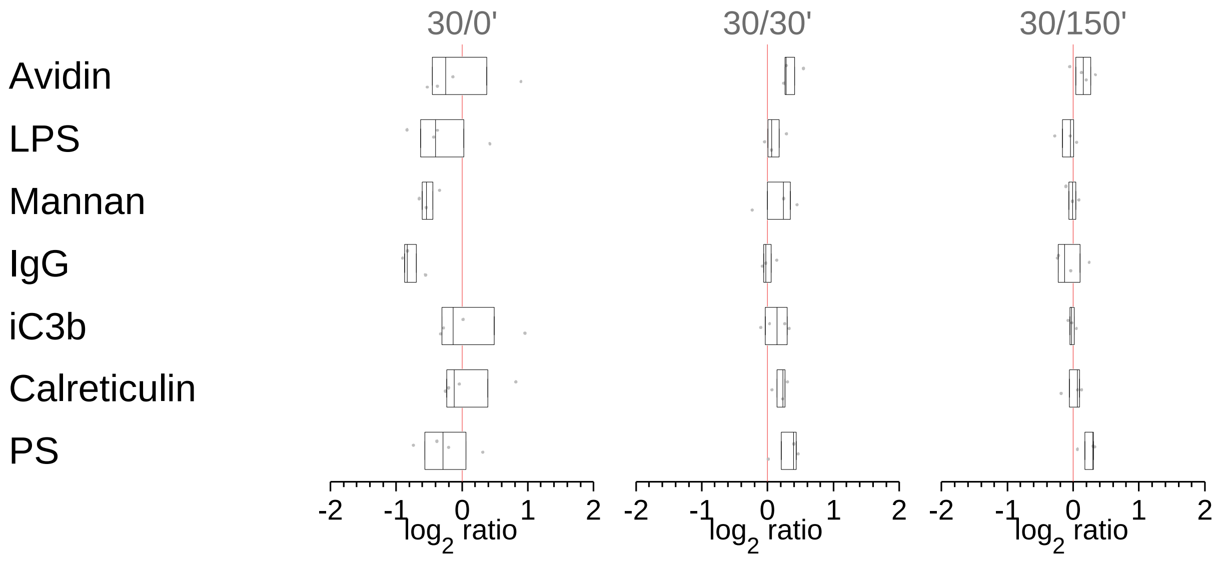

GO:0007420 brain development (n = 9)

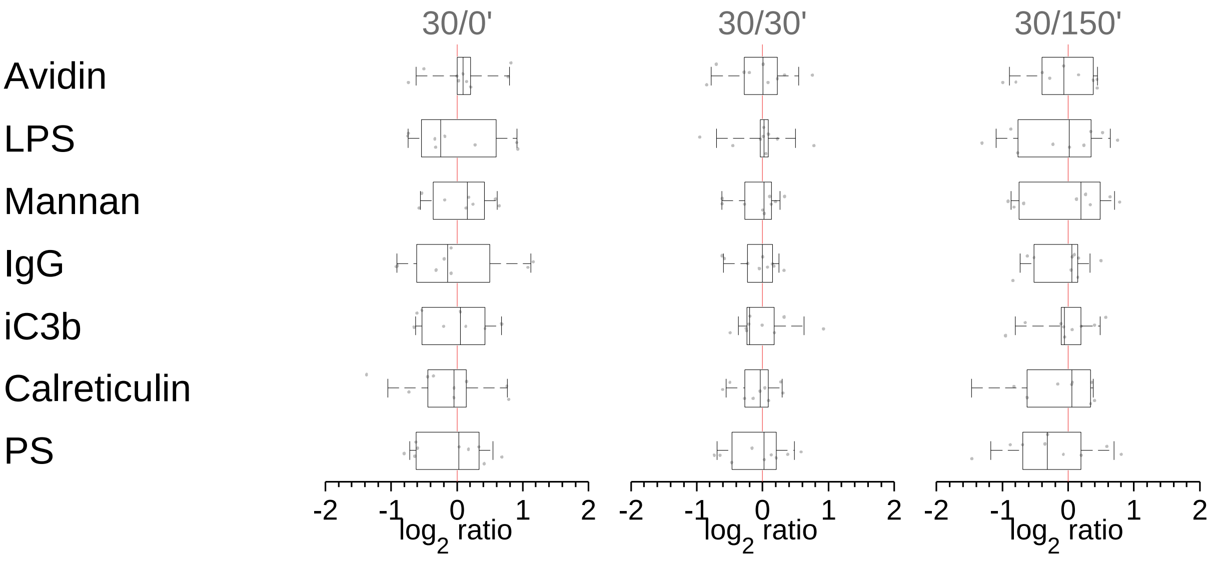

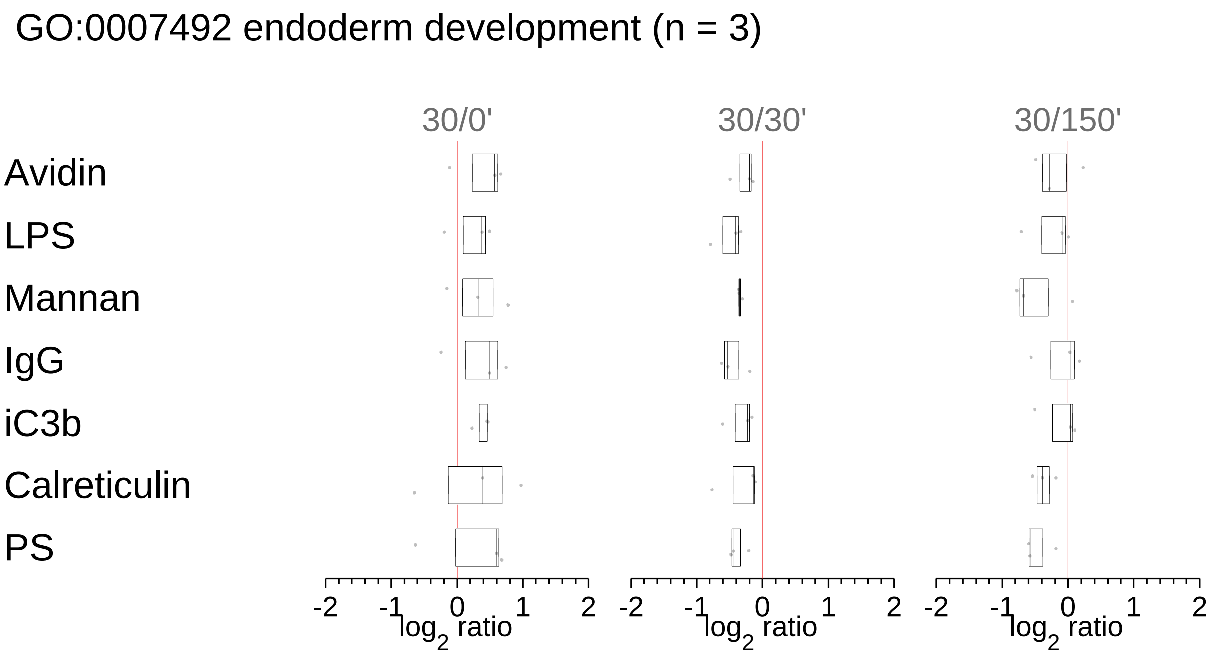

GO:0007492 endoderm development (n = 3)
30/0'
30/30'
30/150'
Avidin
LPS
Mannan
IgG
iC3b
Calreticulin
PS
-2 -1 0 1 2
log2 ratio
-2 -1 0 1 2
log2 ratio
-2 -1 0 1 2
log2 ratio

GO:0007507 heart development (n = 12)

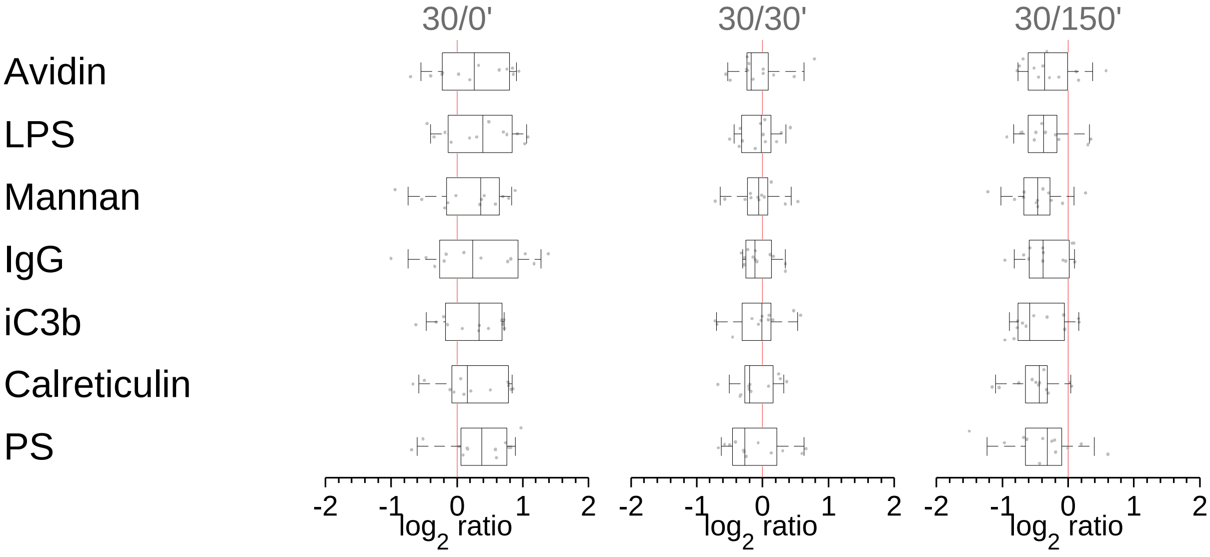

GO:0007517 muscle organ development (n = 3)

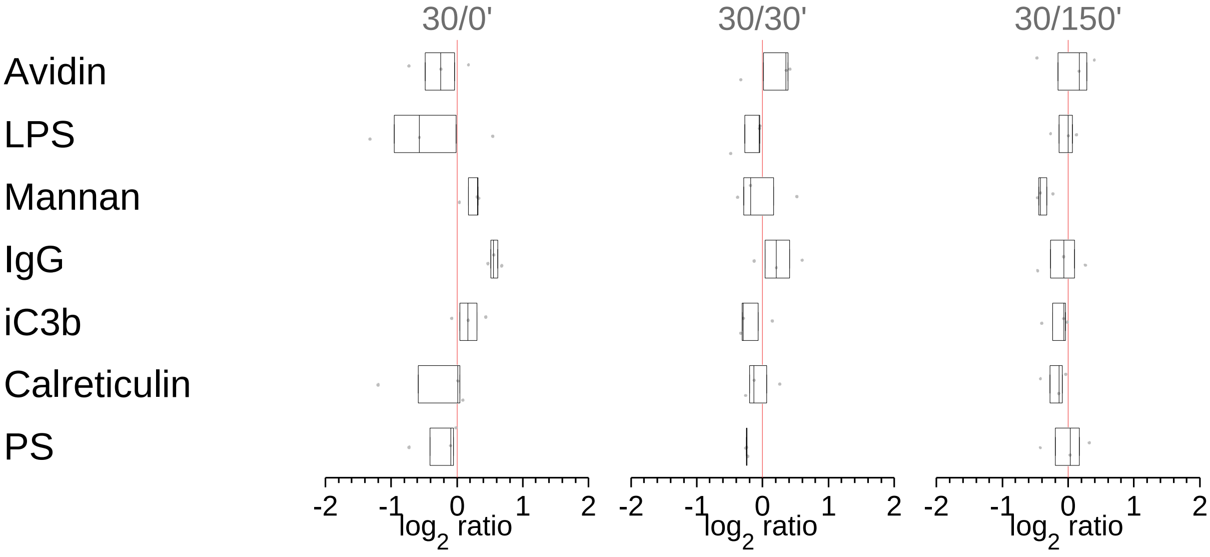

GO:0007519 skeletal muscle tissue development (n = 5)

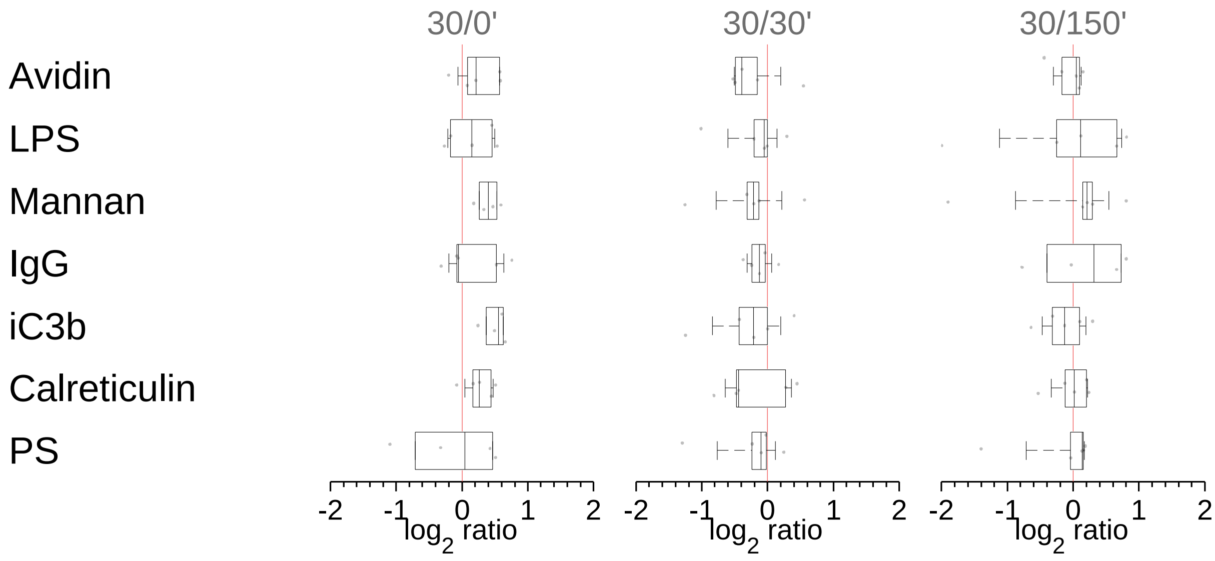

GO:0007565 female pregnancy (n = 3)

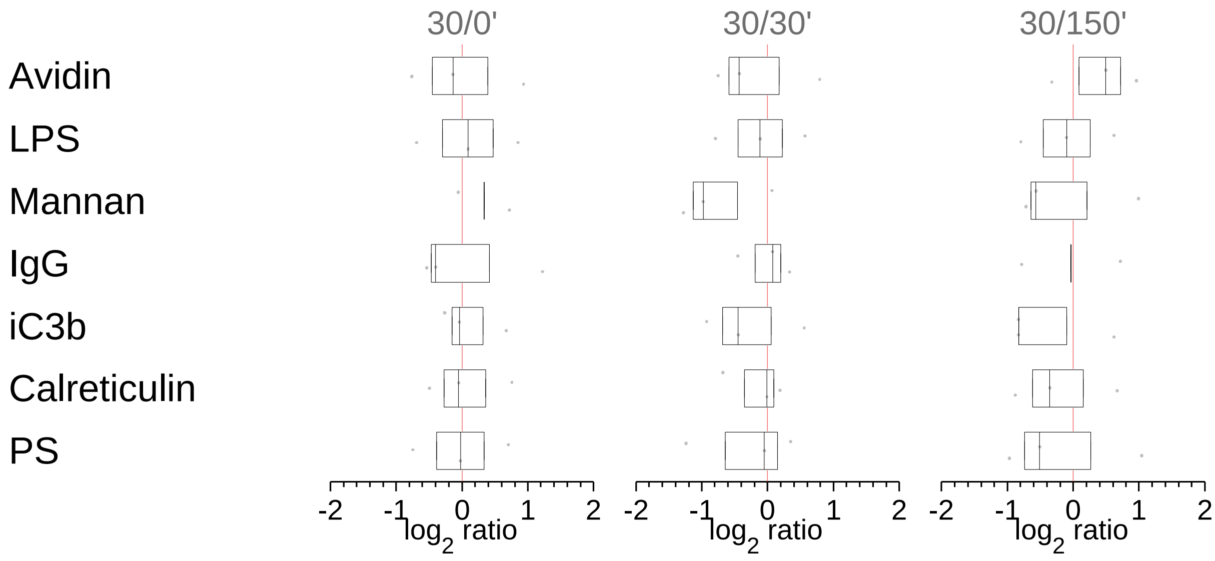

GO:0007566 embryo implantation (n = 6)

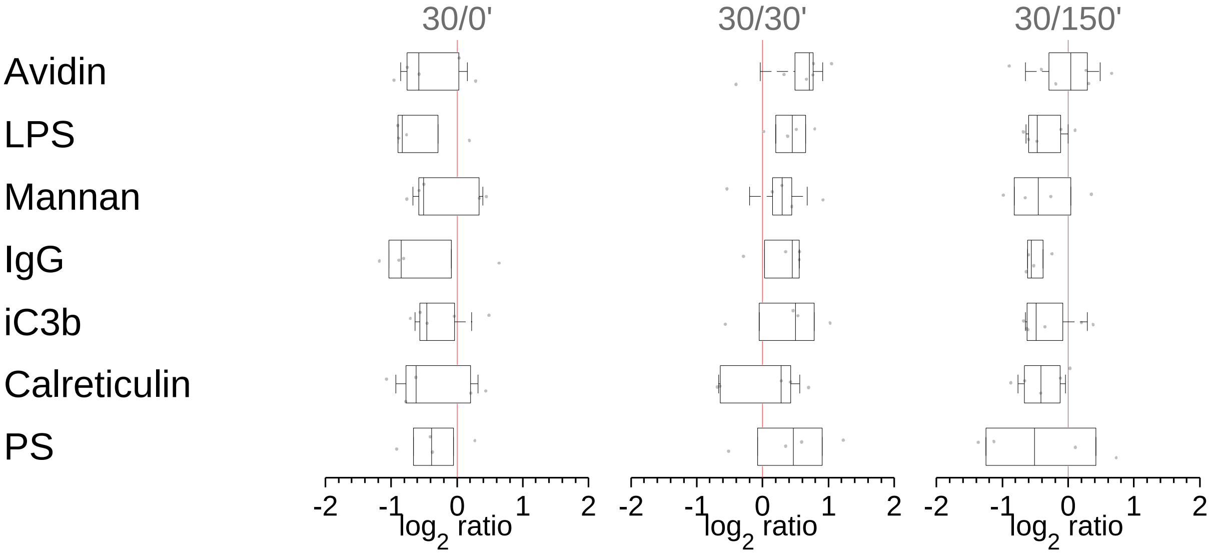

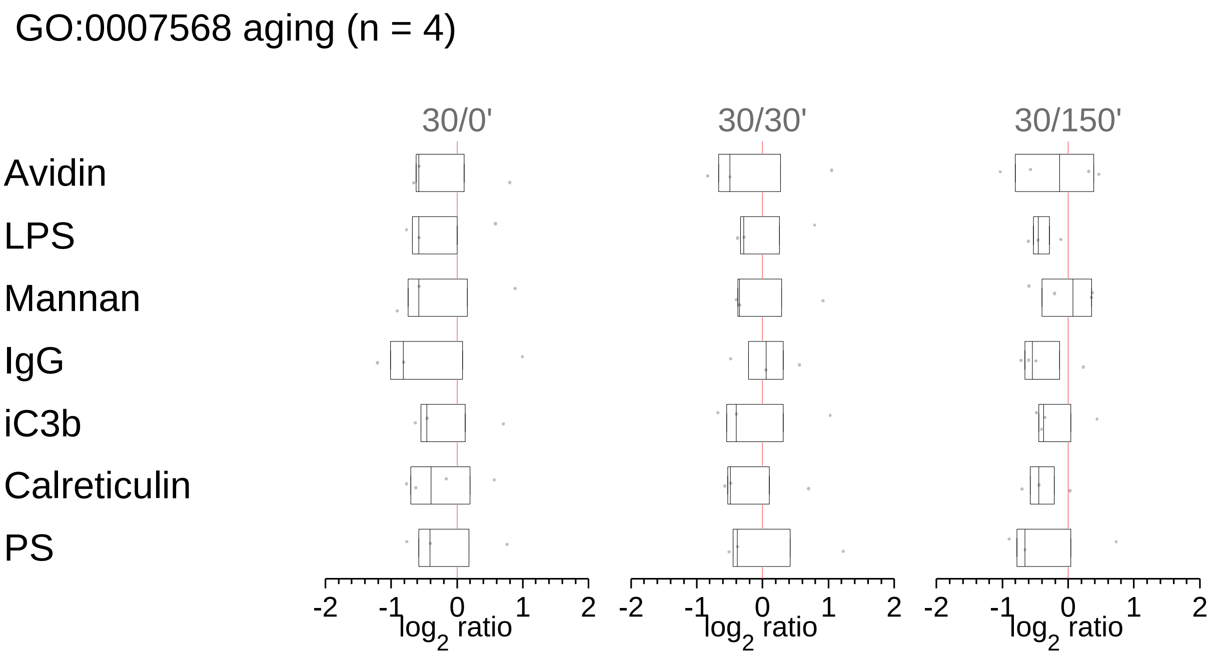
GO:0007568 aging (n = 4)
30/0'
30/30'
30/150'
Avidin
LPS
Mannan
IgG
iC3b
Calreticulin
PS
-2 -1 0 1 2
log2 ratio
-2 -1 0 1 2
log2 ratio
-2 -1 0 1 2
log2 ratio

GO:0007569 cell aging (n = 7)

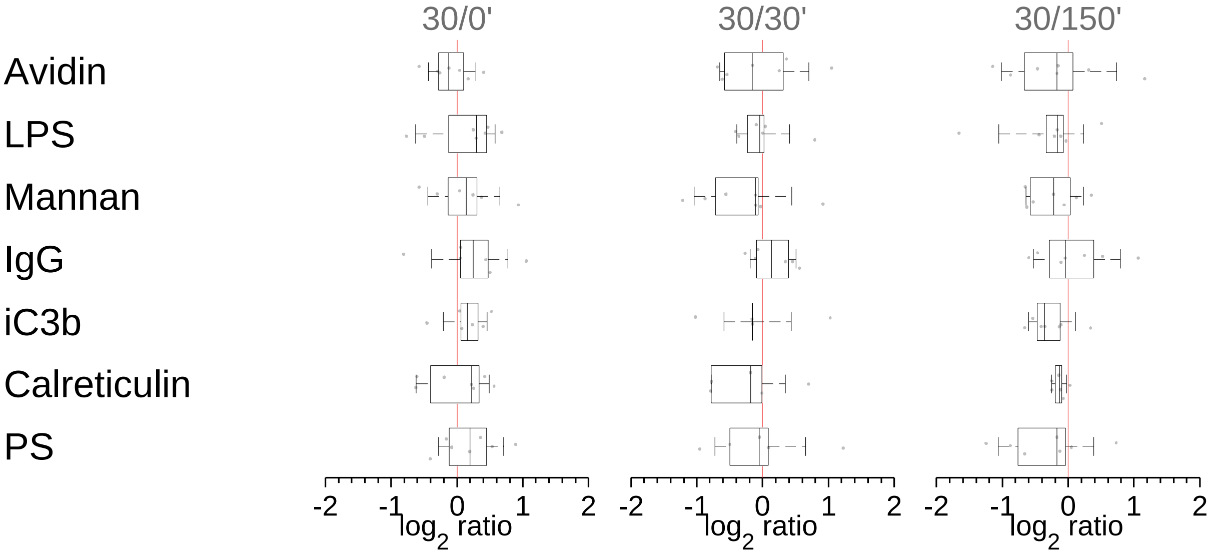

GO:0007584 response to nutrient (n = 5)

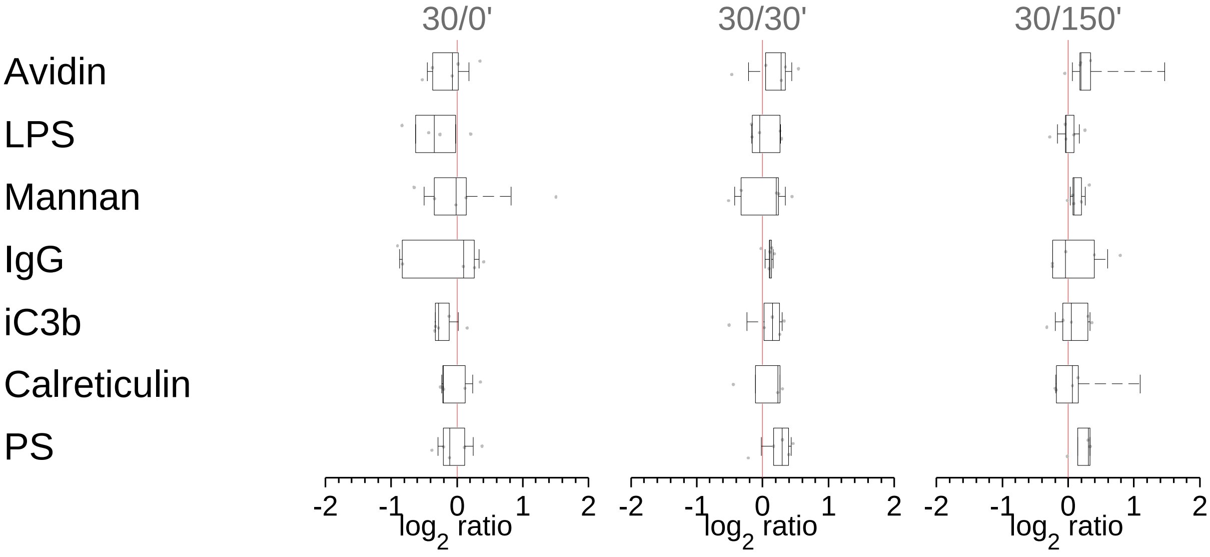

GO:0007596 blood coagulation (n = 10)

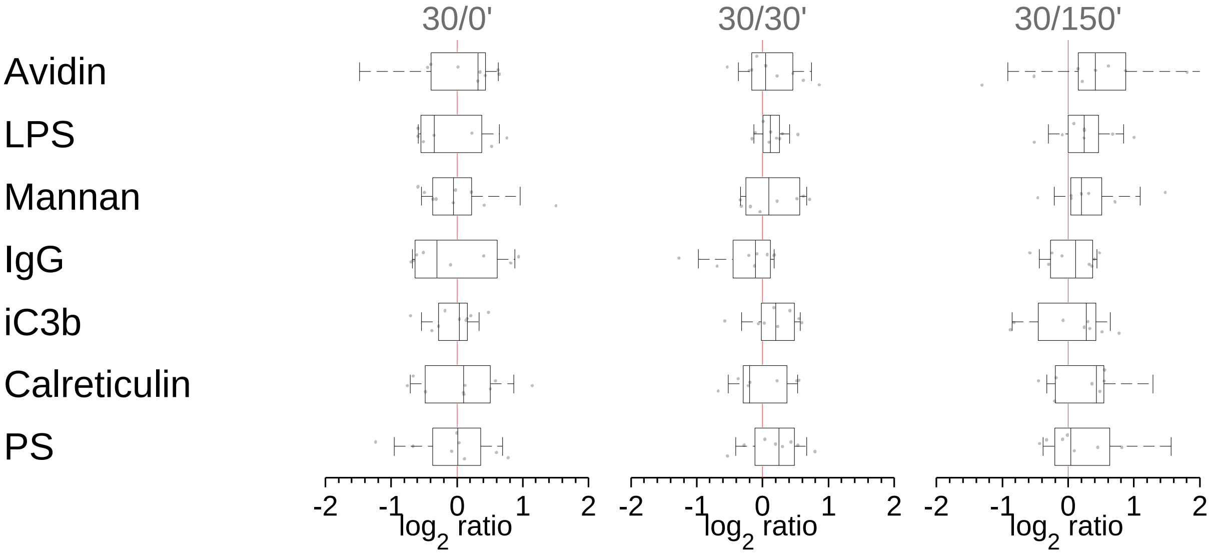

GO:0007599 hemostasis (n = 4)

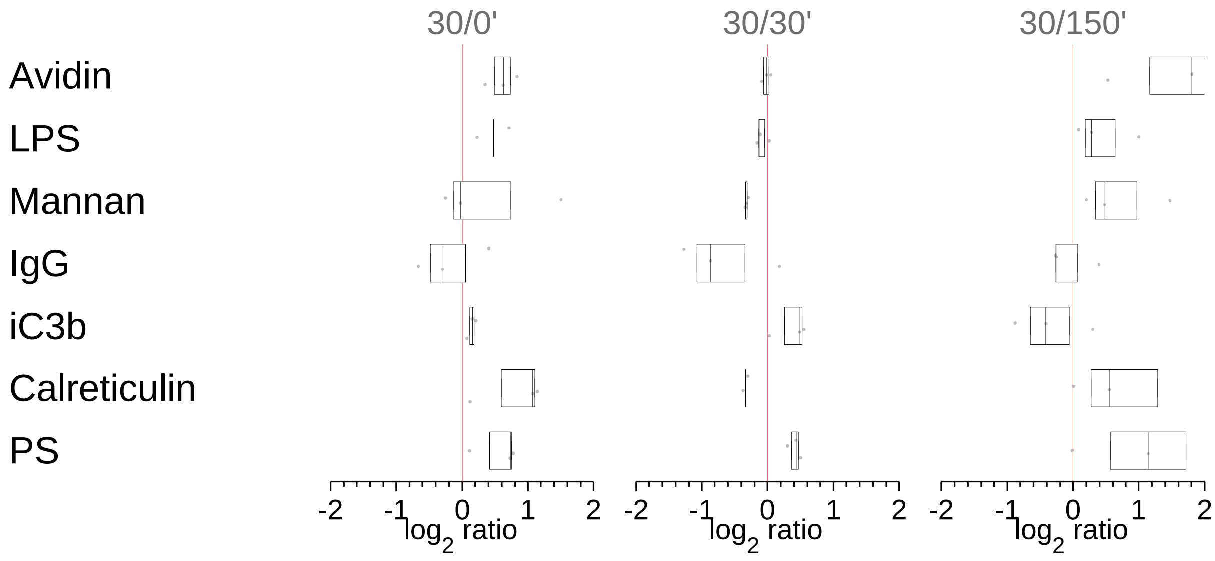

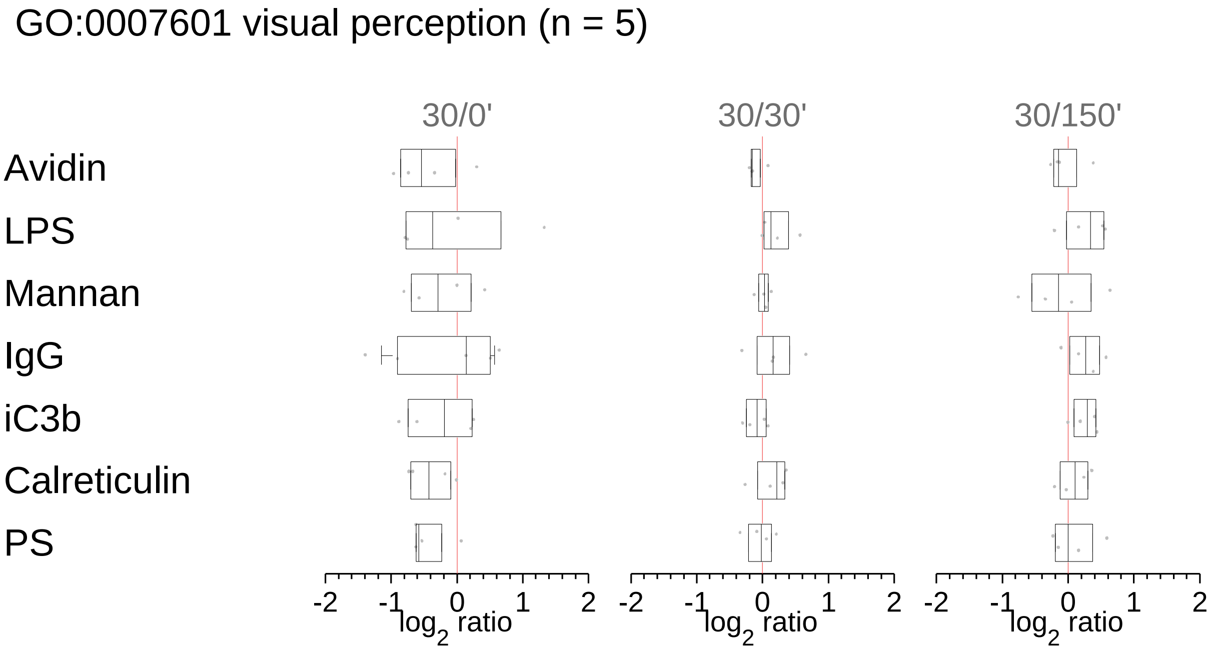

GO:0007601 visual perception (n = 5)
30/0'
30/30'
30/150'
Avidin
LPS
Mannan
IgG
iC3b
Calreticulin
PS
-2
-1
0
1
2
log2 ratio

GO:0007605 sensory perception of sound (n = 12)

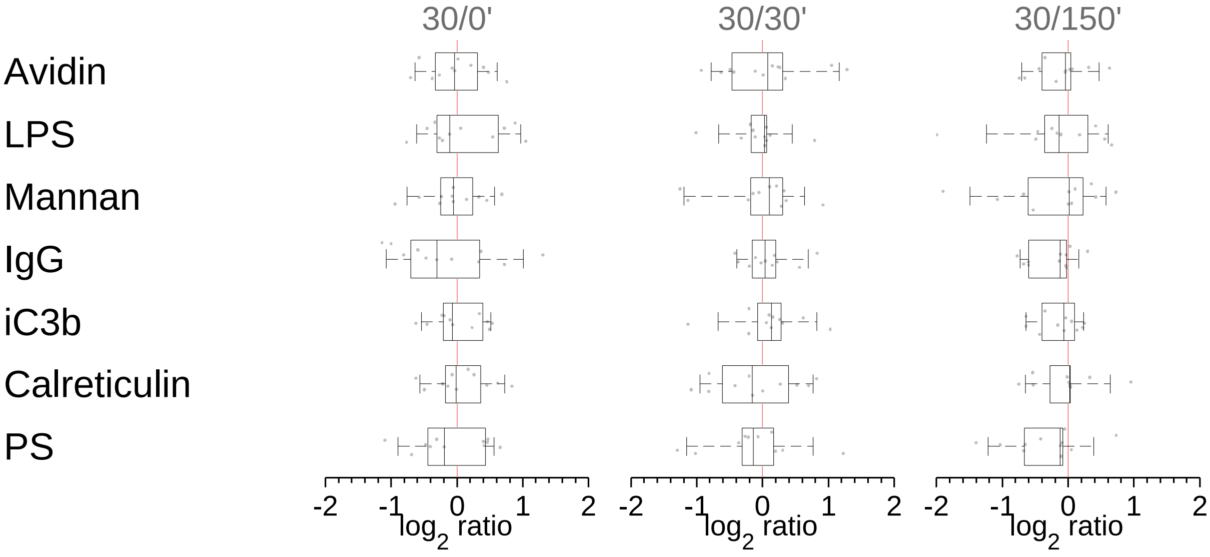

GO:0007611 learning or memory (n = 5)

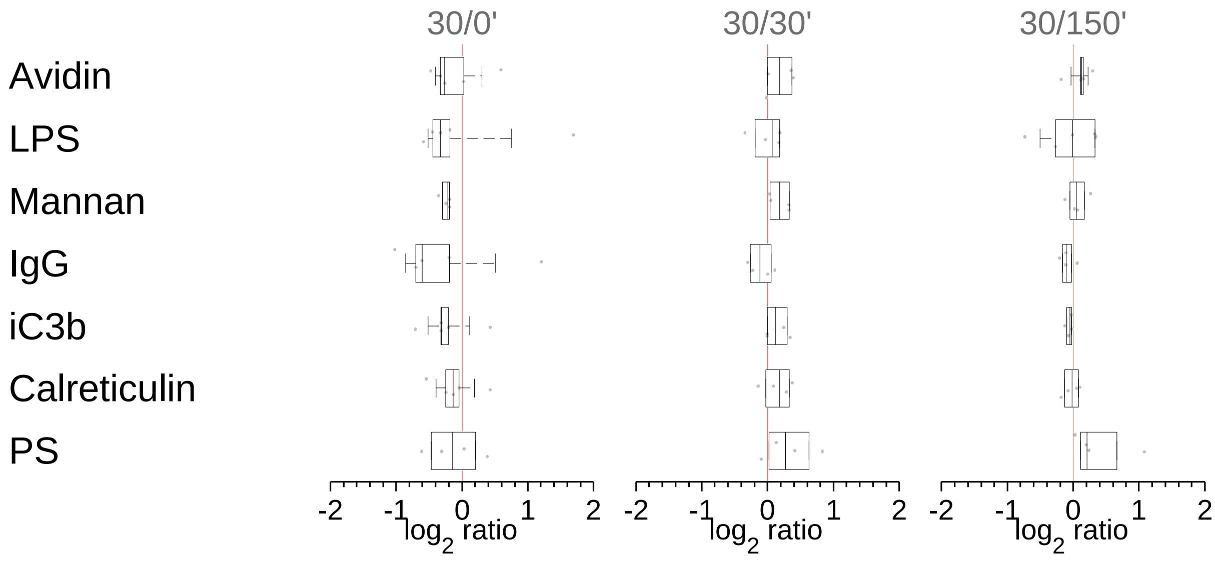

GO:0007612 learning (n = 6)

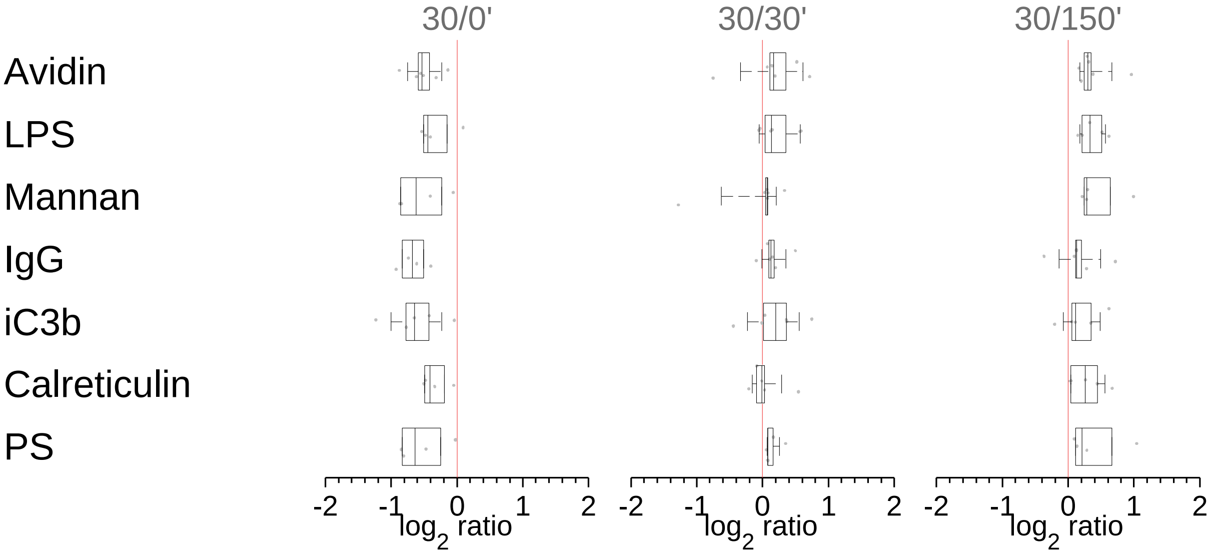

GO:0007613 memory (n = 7)

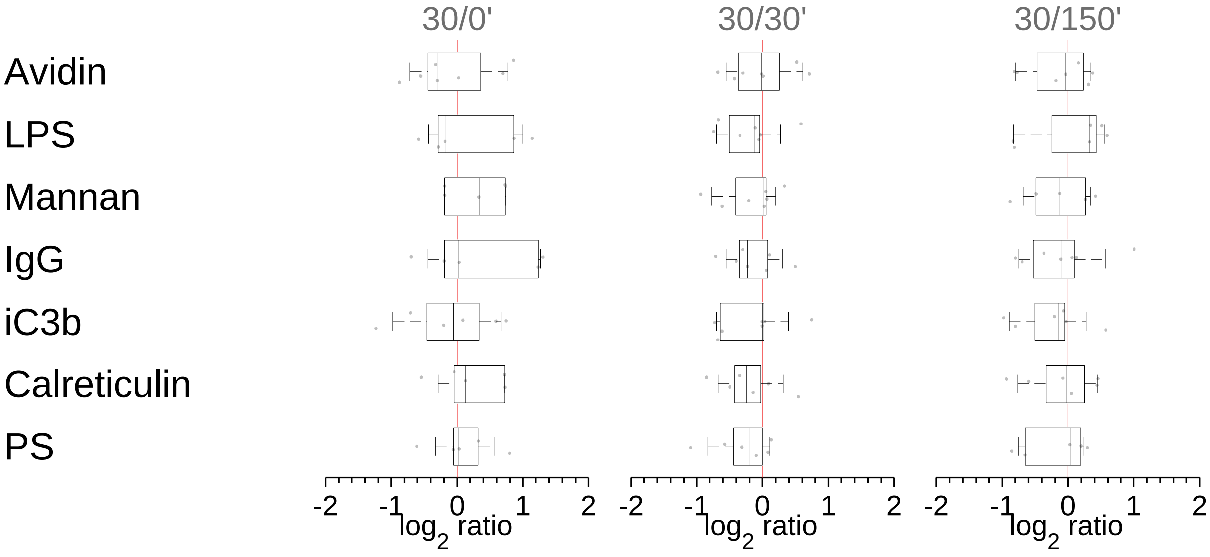

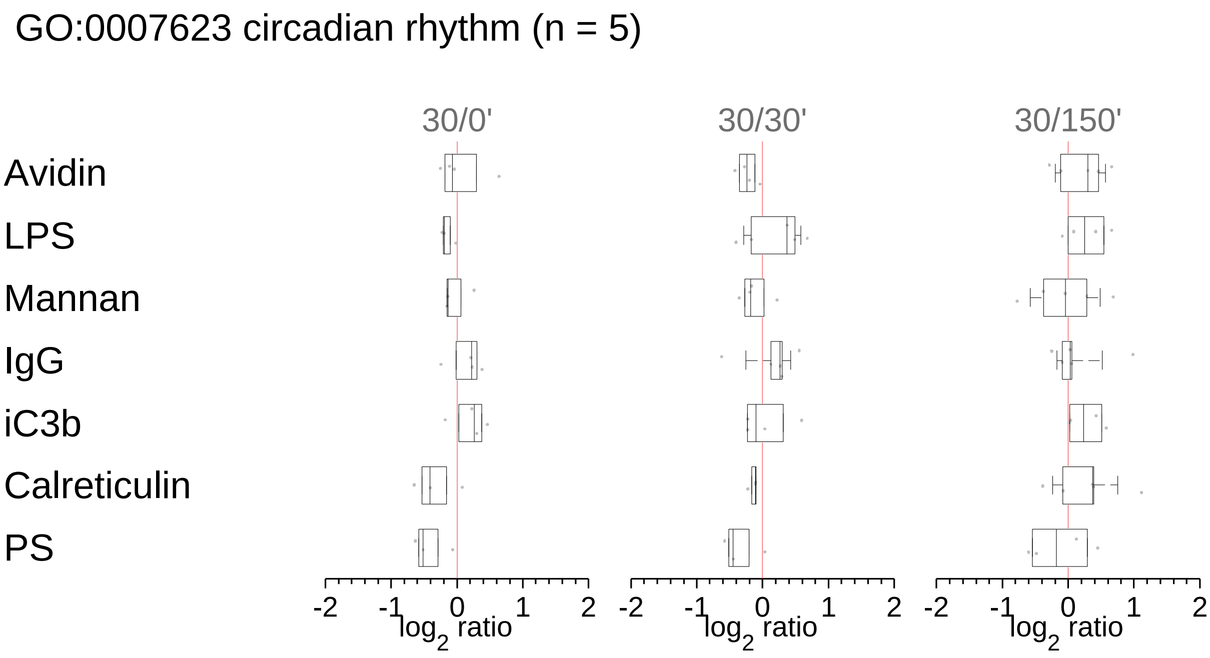
GO:0007623 circadian rhythm (n = 5)
30/0'
30/30'
30/150'
Avidin
LPS
Mannan
IgG
iC3b
Calreticulin
PS
-2
-1
0
1
2
log2 ratio

GO:0007626 locomotory behavior (n = 7)

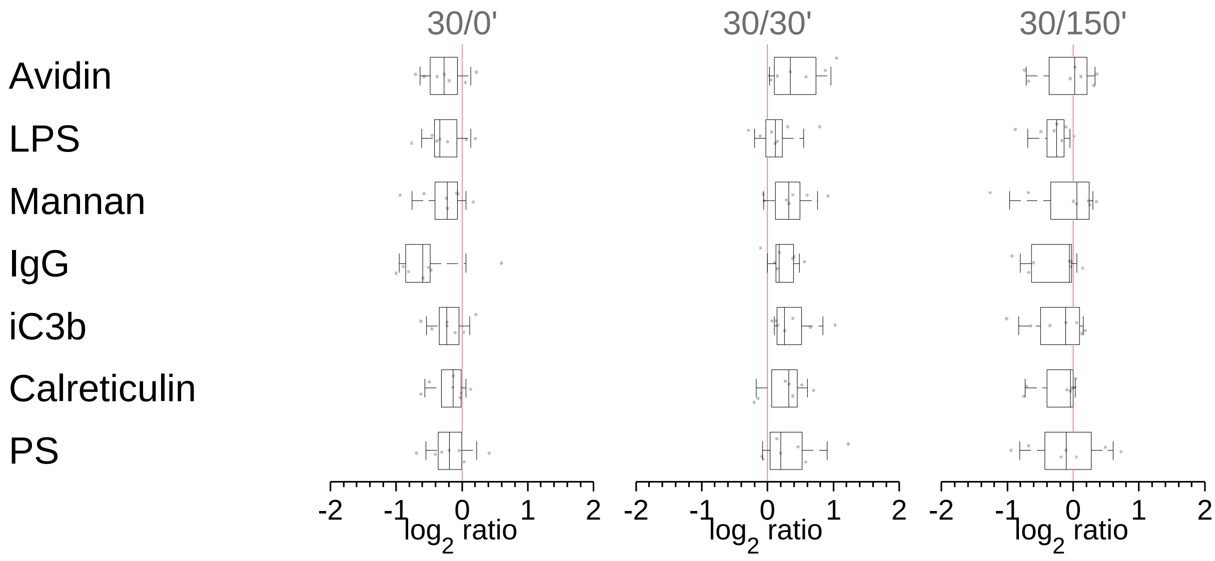

GO:0007628 adult walking behavior (n = 4)

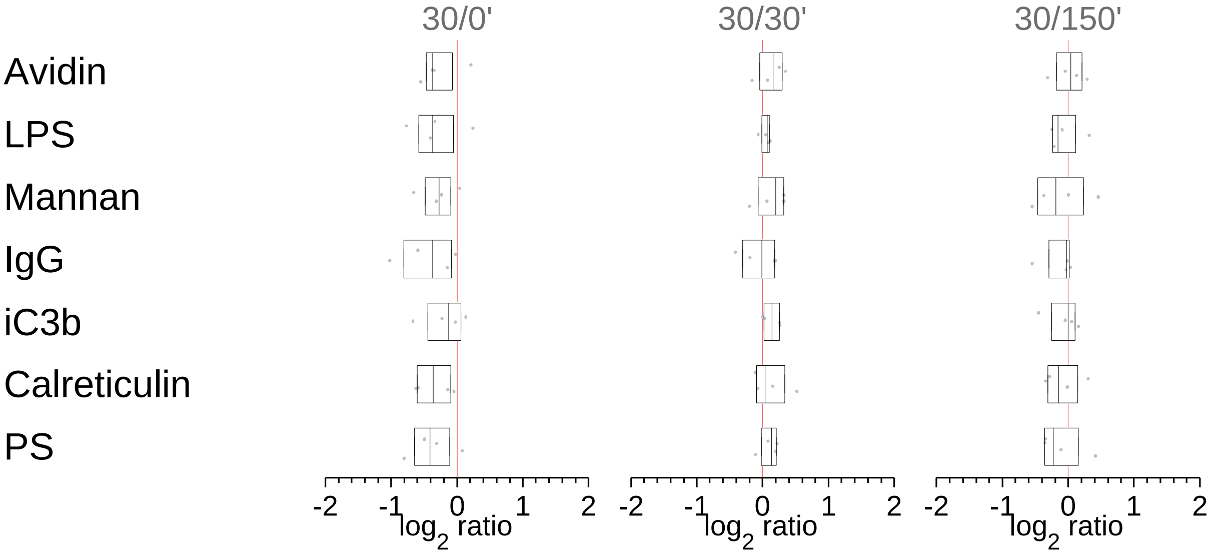

GO:0008013 beta-catenin binding (n = 6)

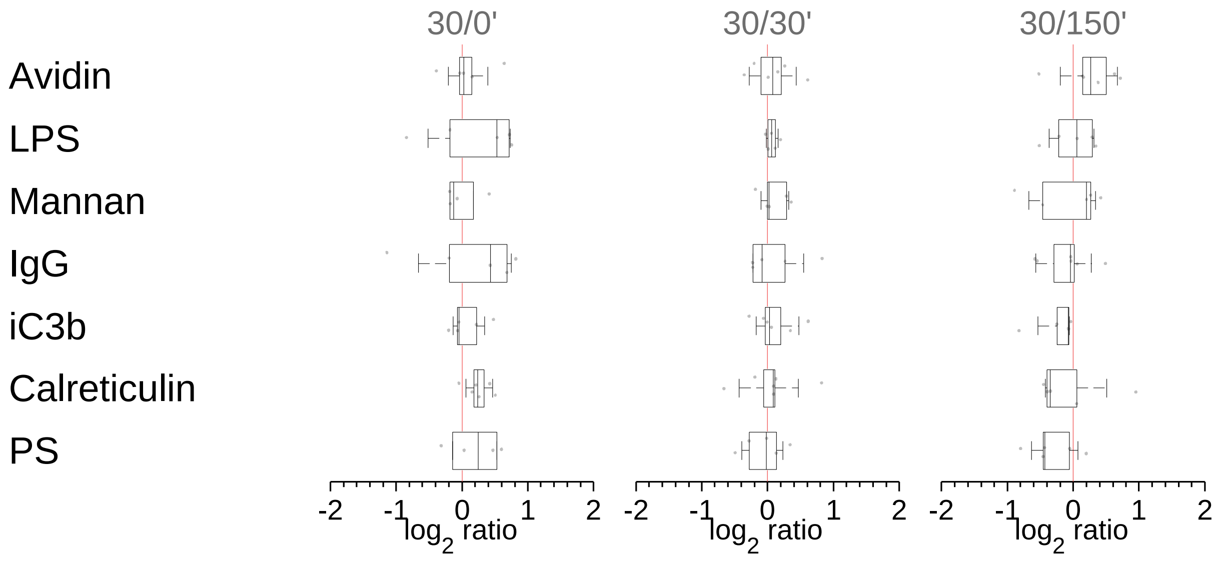

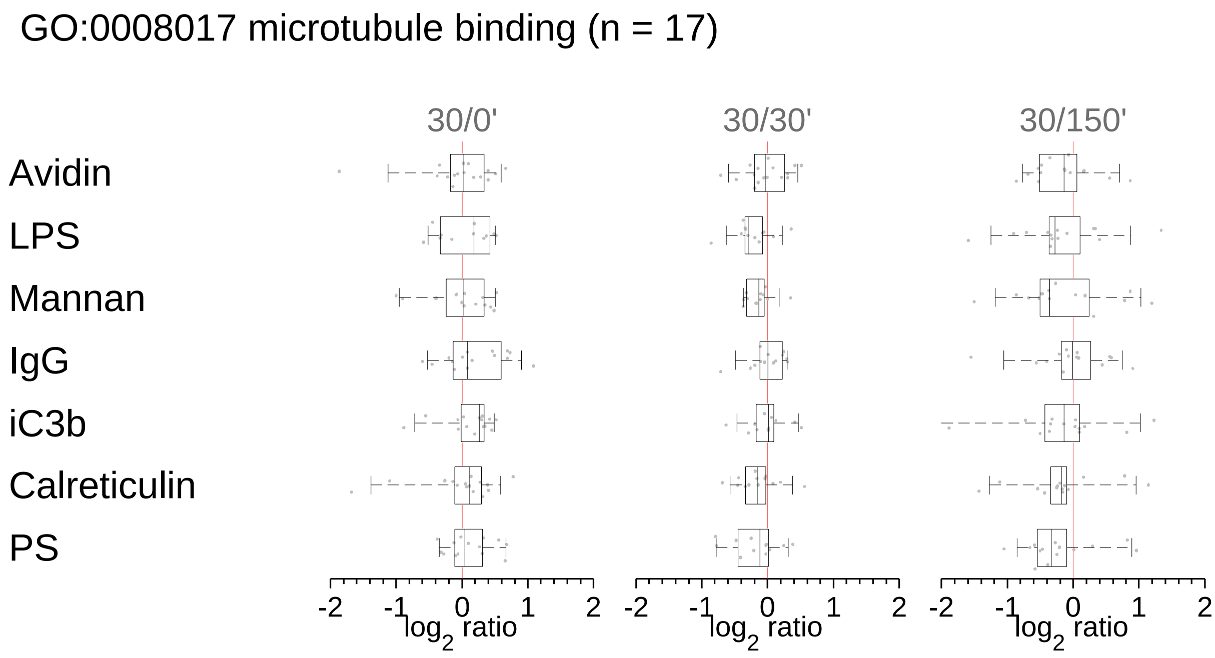
GO:0008017 microtubule binding (n = 17)
30/0'
30/30'
30/150'
Avidin
LPS
Mannan
IgG
iC3b
Calreticulin
PS
-2 -1 0 1 2
log2 ratio
-2 -1 0 1 2
log2 ratio
-2 -1 0 1 2
log2 ratio

GO:0008021 synaptic vesicle (n = 21)

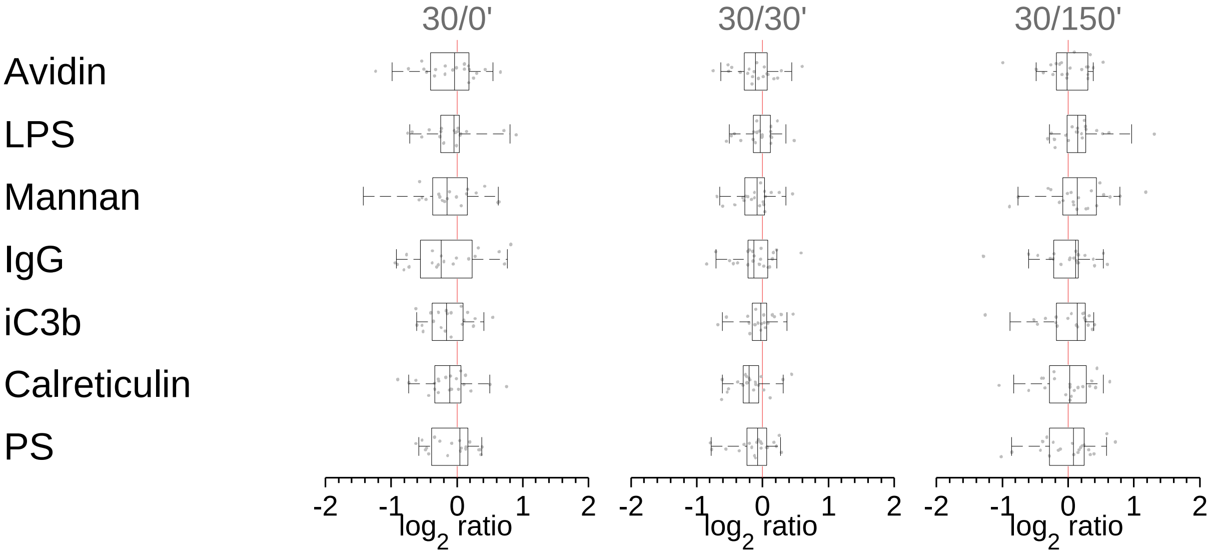

GO:0008022 protein C-terminus binding (n = 29)

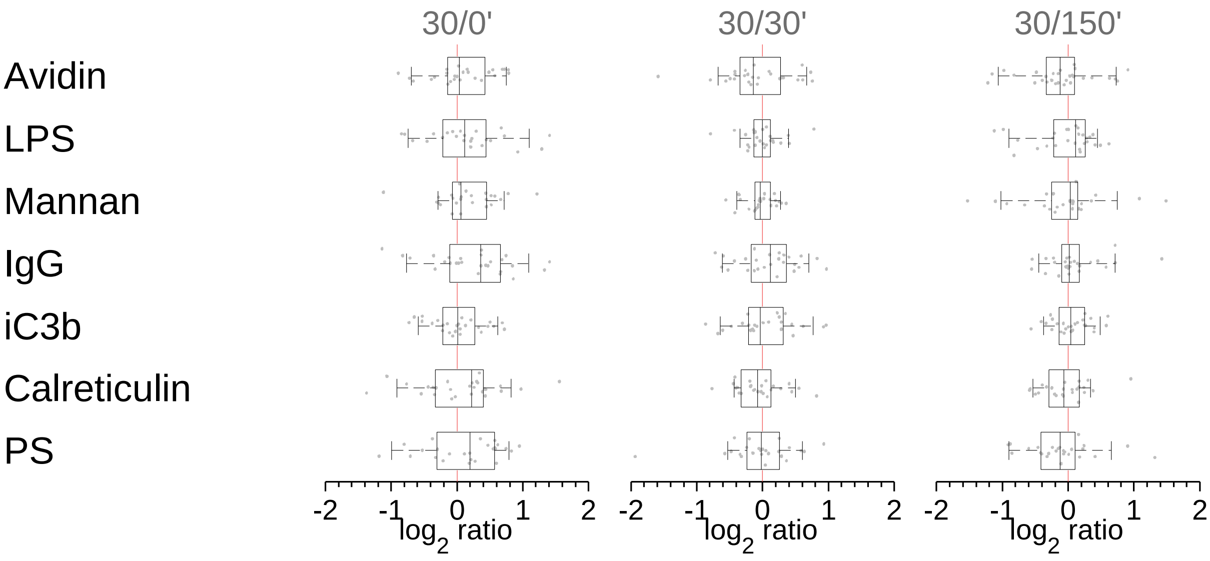

GO:0008026 ATP-dependent helicase activity (n = 12)

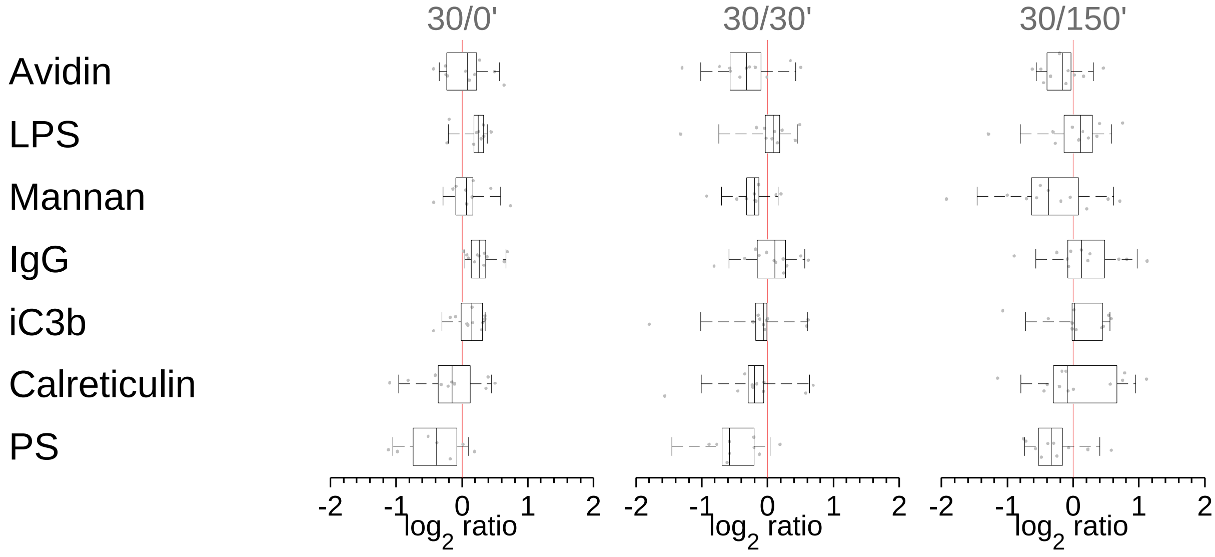

GO:0008033 tRNA processing (n = 3)

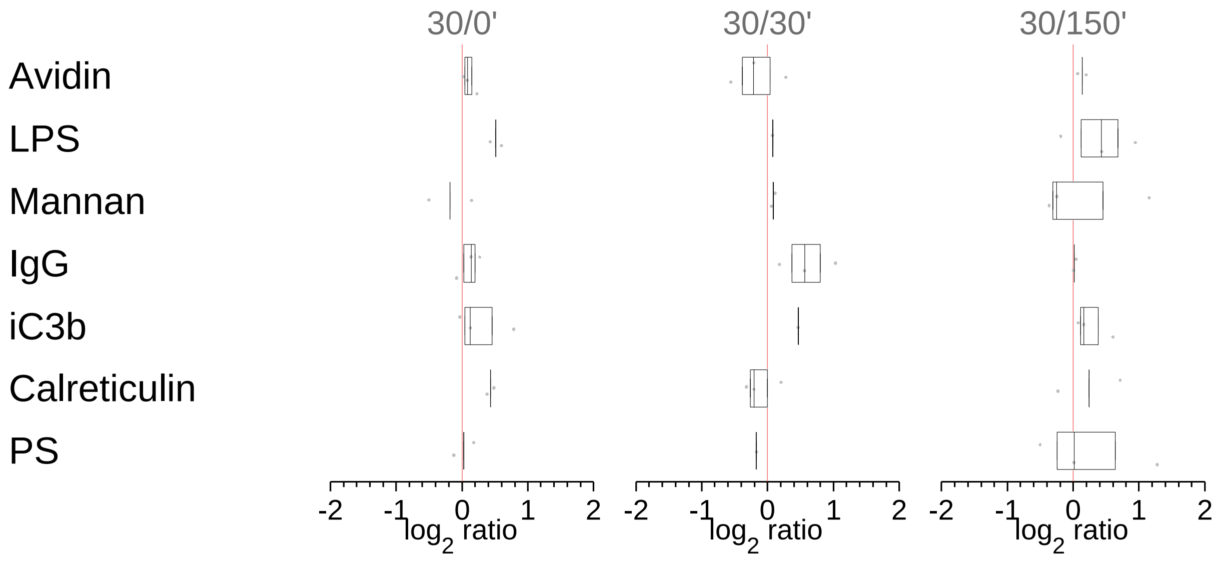

GO:0008047 enzyme activator activity (n = 5)

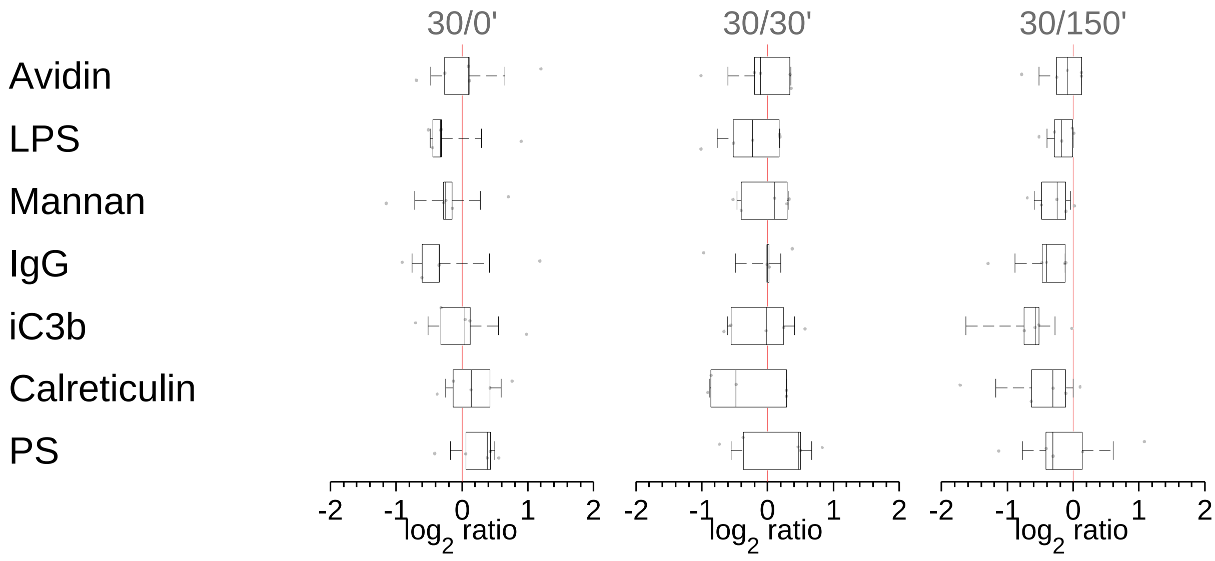

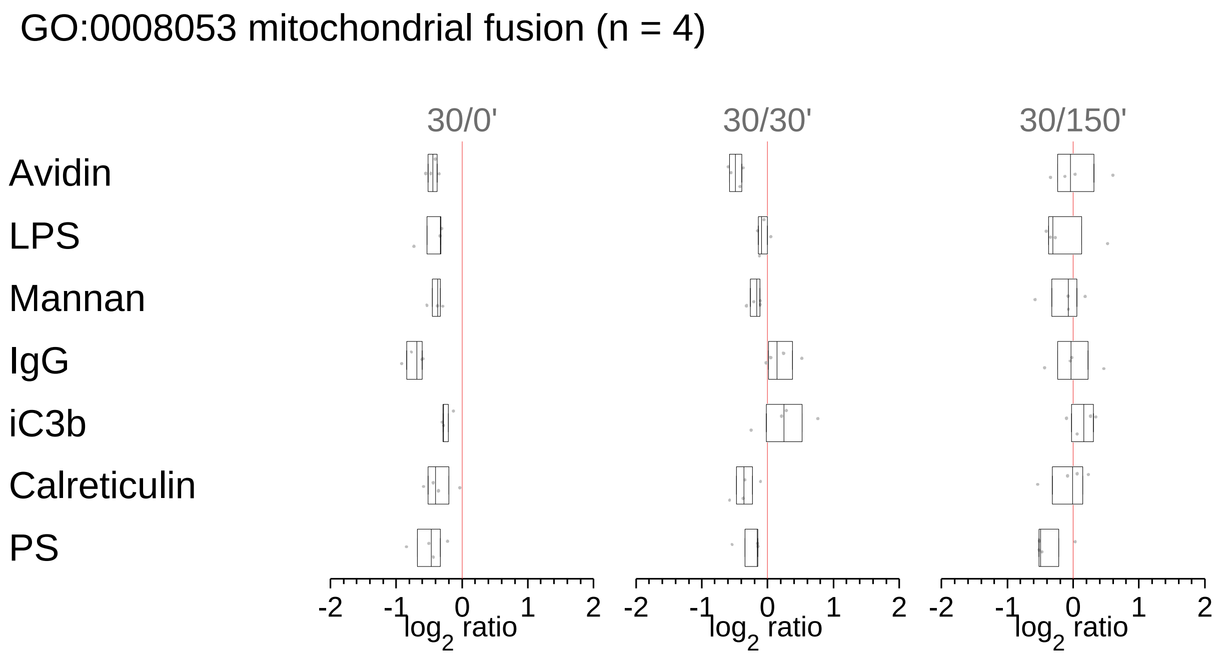
GO:0008053 mitochondrial fusion (n = 4)
30/0'
30/30'
30/150'
Avidin
LPS
Mannan
IgG
iC3b
Calreticulin
PS
-2 -1 0 1 2
log2 ratio

GO:0008060 ARF GTPase activator activity (n = 9)

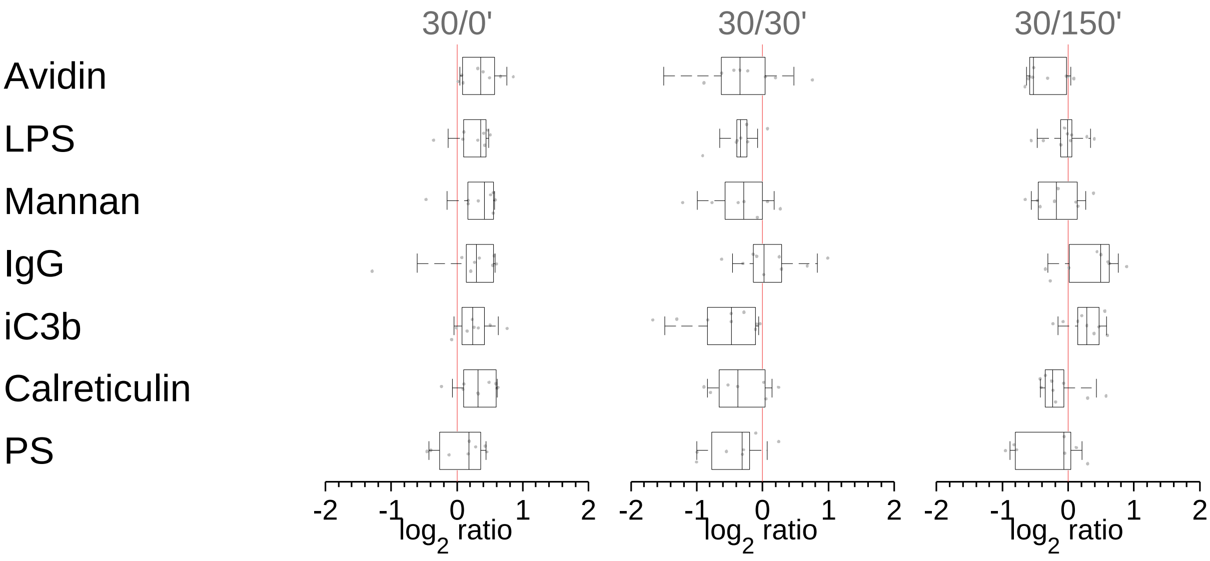

GO:0008080 N-acetyltransferase activity (n = 4)

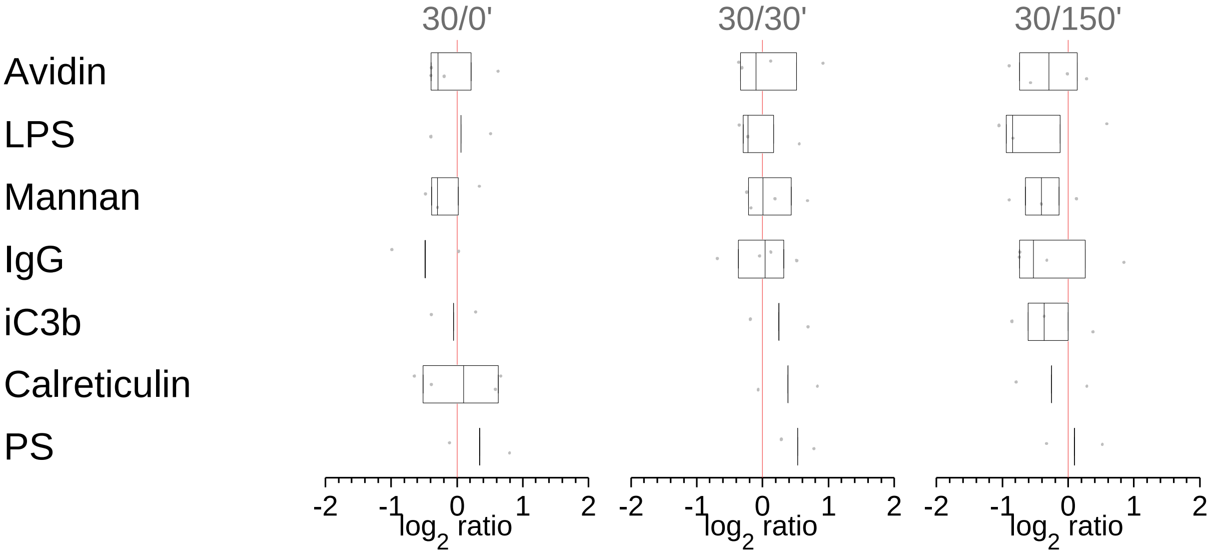

GO:0008083 growth factor activity (n = 3)

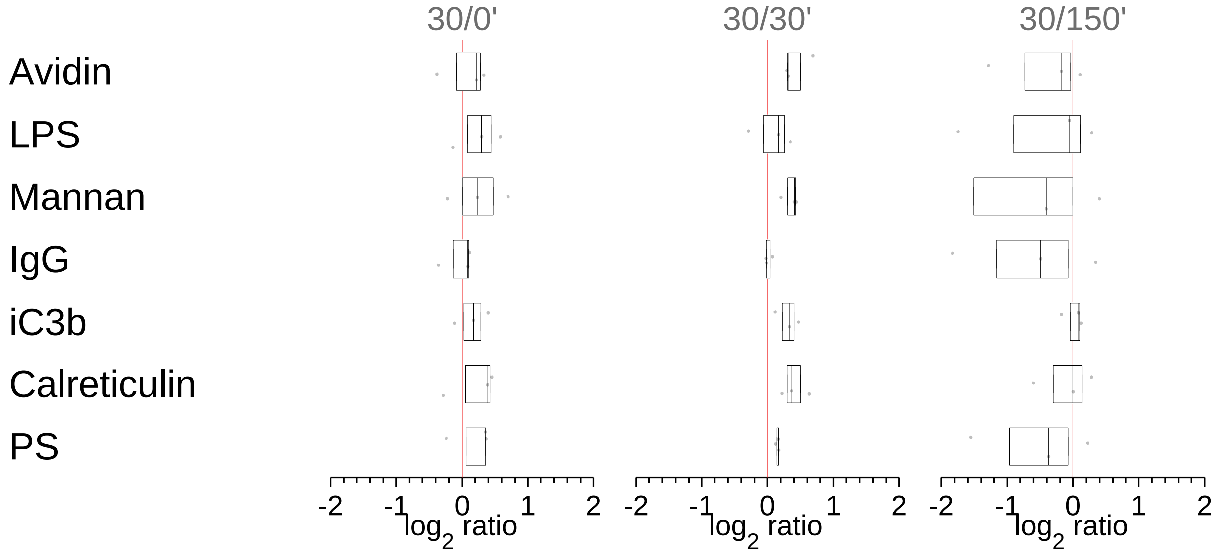

GO:0008089 anterograde axon cargo transport (n = 8)

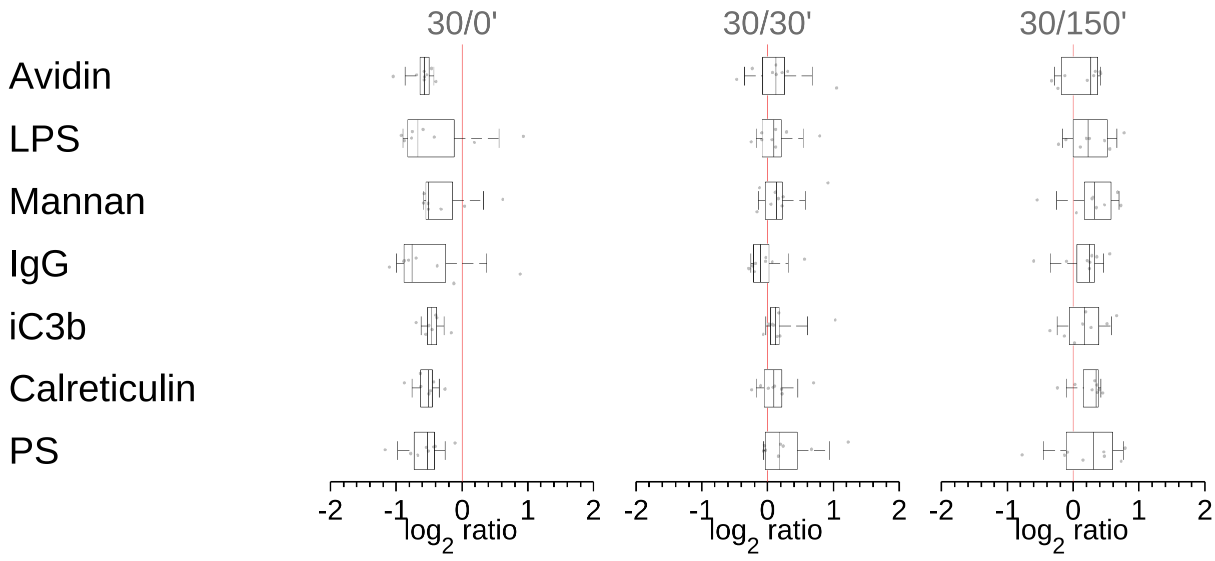

GO:0008090 retrograde axon cargo transport (n = 3)

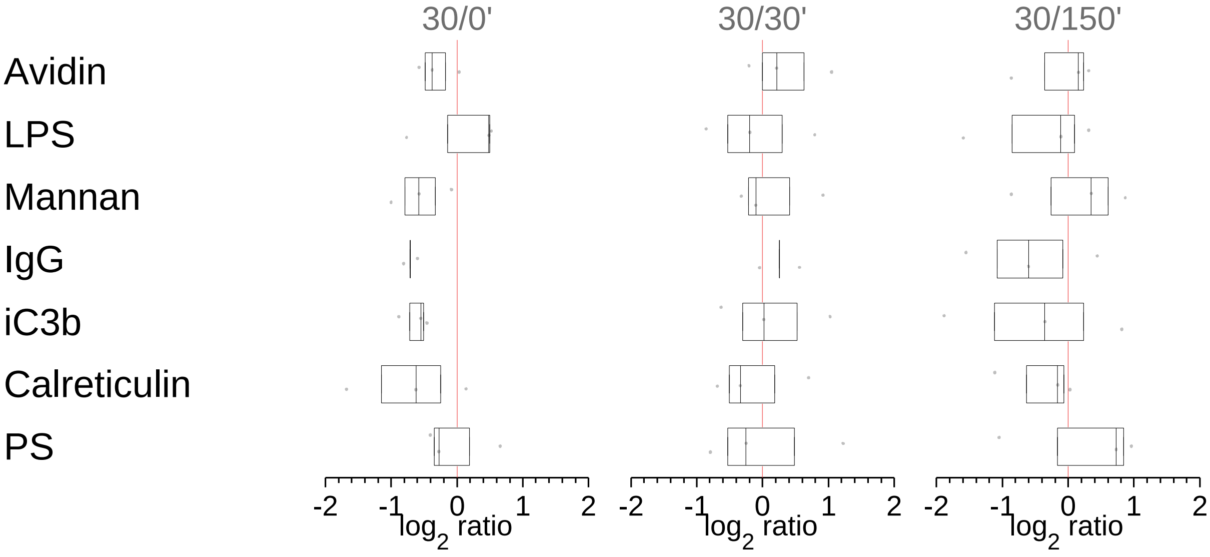

GO:0008092 cytoskeletal protein binding (n = 19)

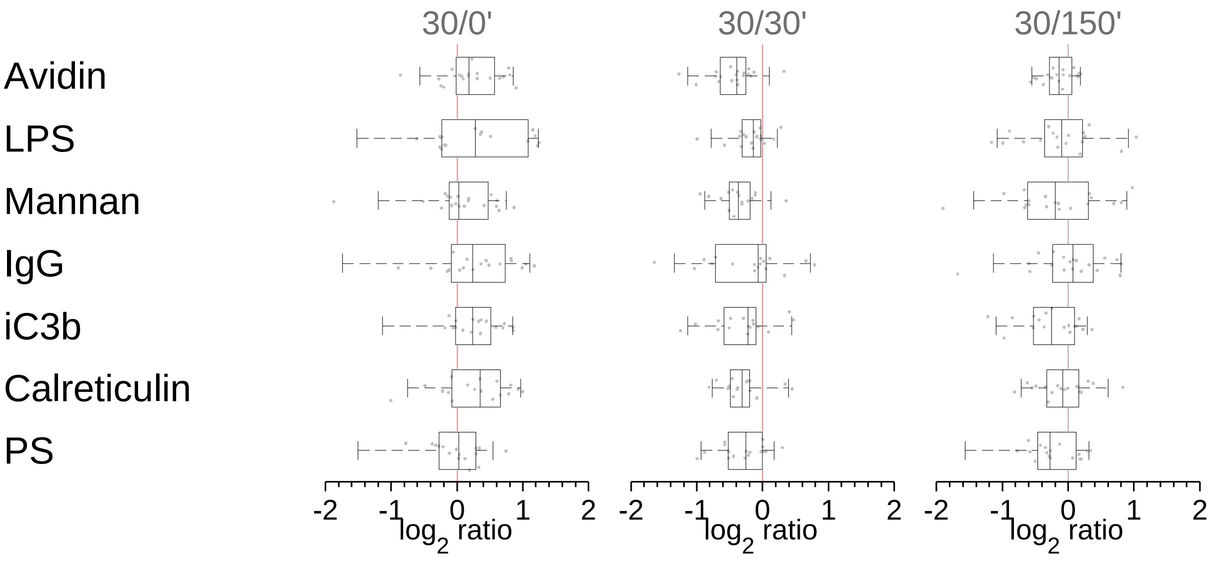

GO:0008094 DNA-dependent ATPase activity (n = 3)

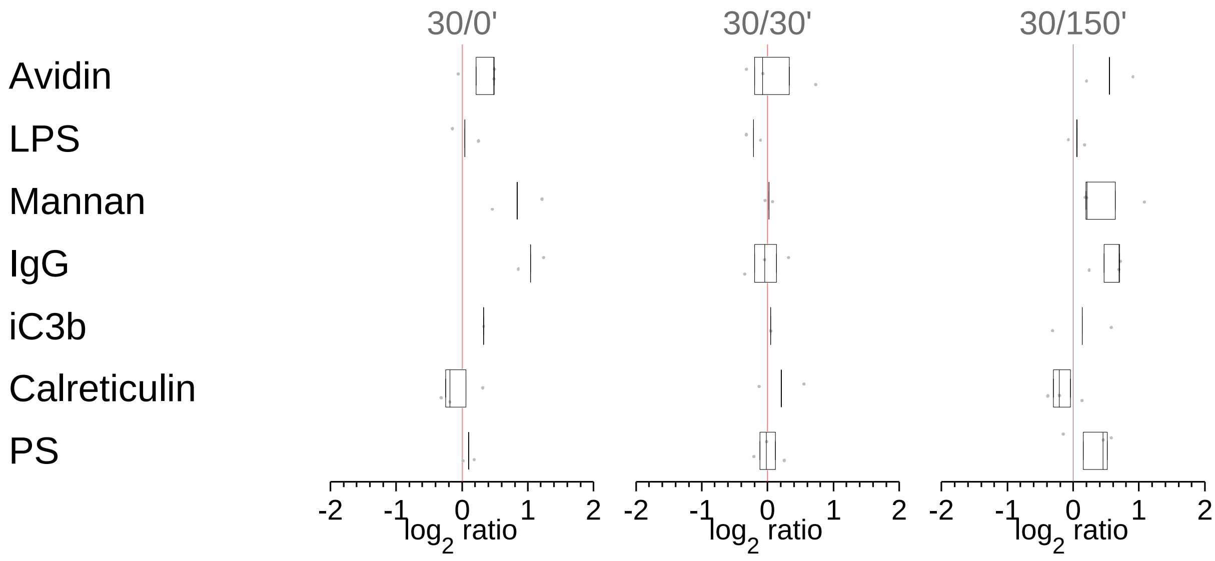

GO:0008104 protein localization (n = 12)

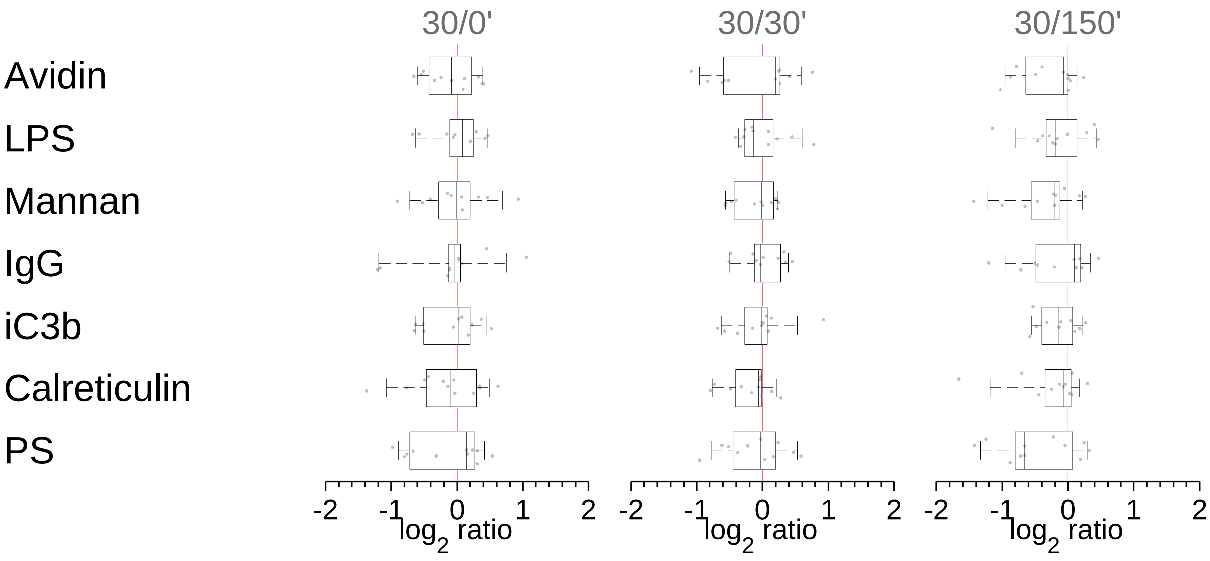

GO:0008134 transcription factor binding (n = 20)

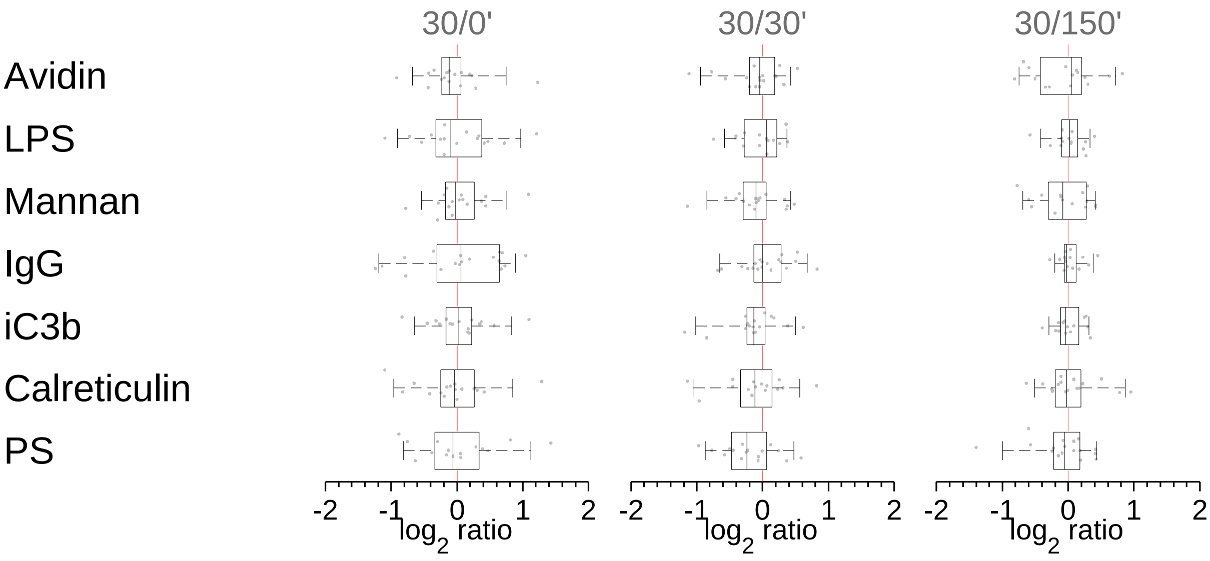

GO:0008137 NADH dehydrogenase (ubiquinone) activity (n = 7)

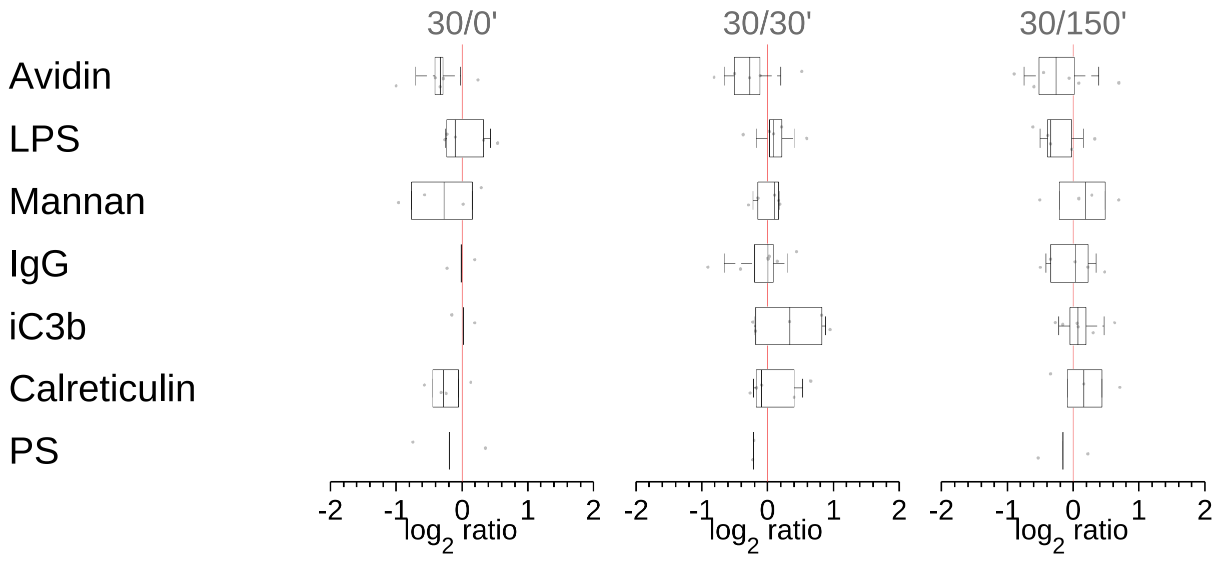

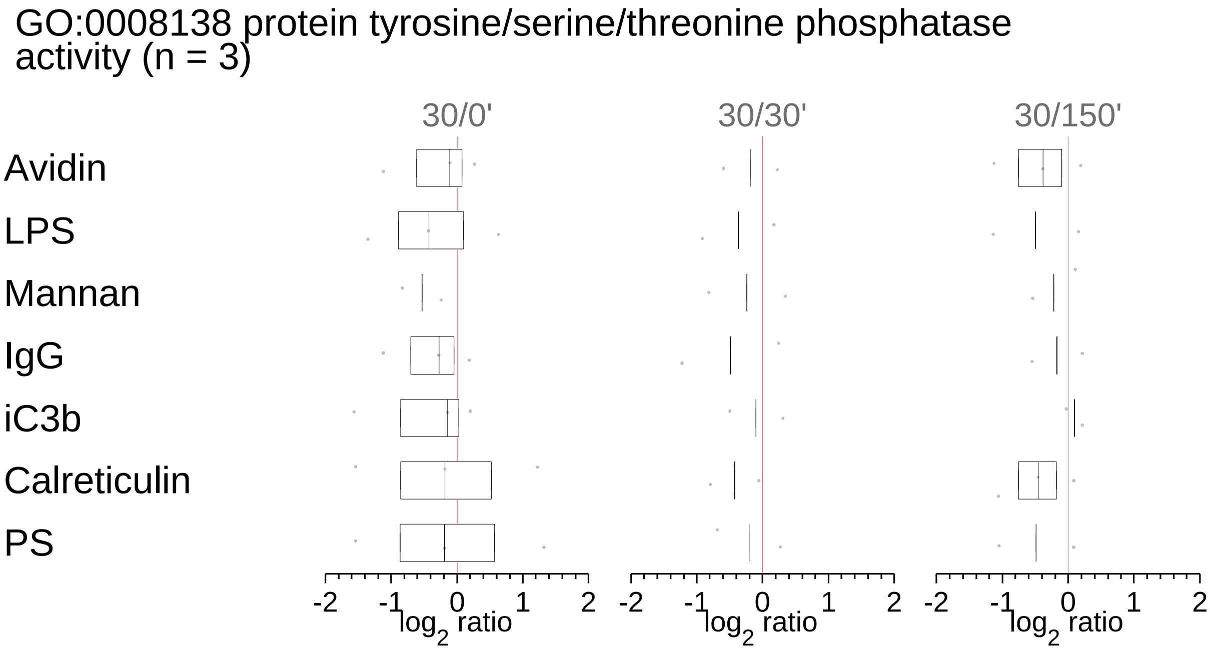
GO:0008138 protein tyrosine/serine/threonine phosphatase
activity (n = 3)
30/0'
30/30'
30/150'
Avidin
LPS
Mannan
IgG
iC3b
Calreticulin
PS
-2
-1
0
1
2
log2 ratio

GO:0008143 poly(A) RNA binding (n = 5)

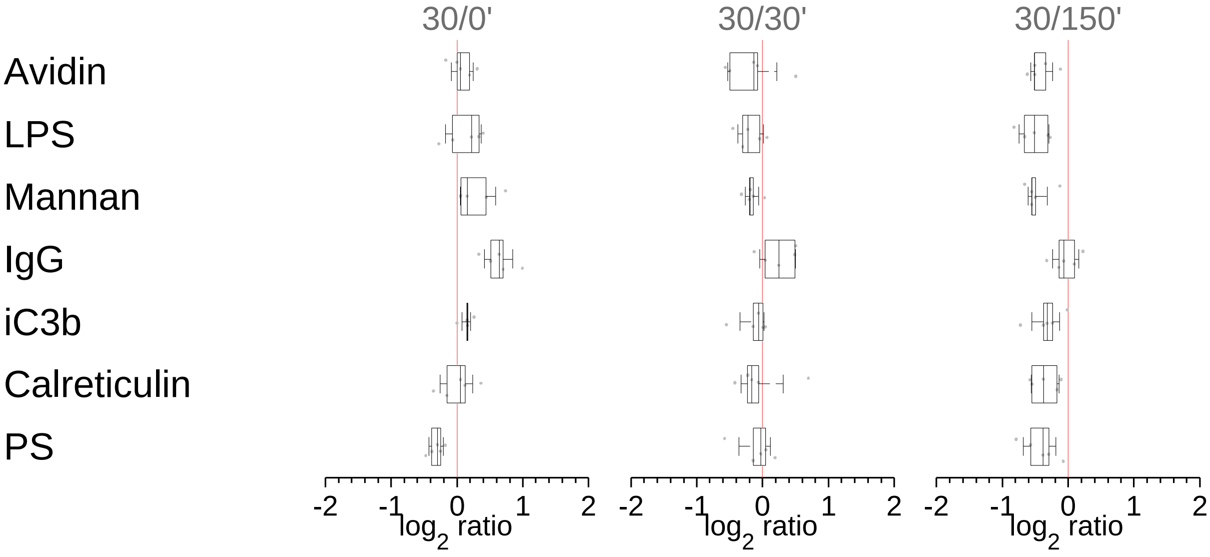

GO:0008144 drug binding (n = 9)

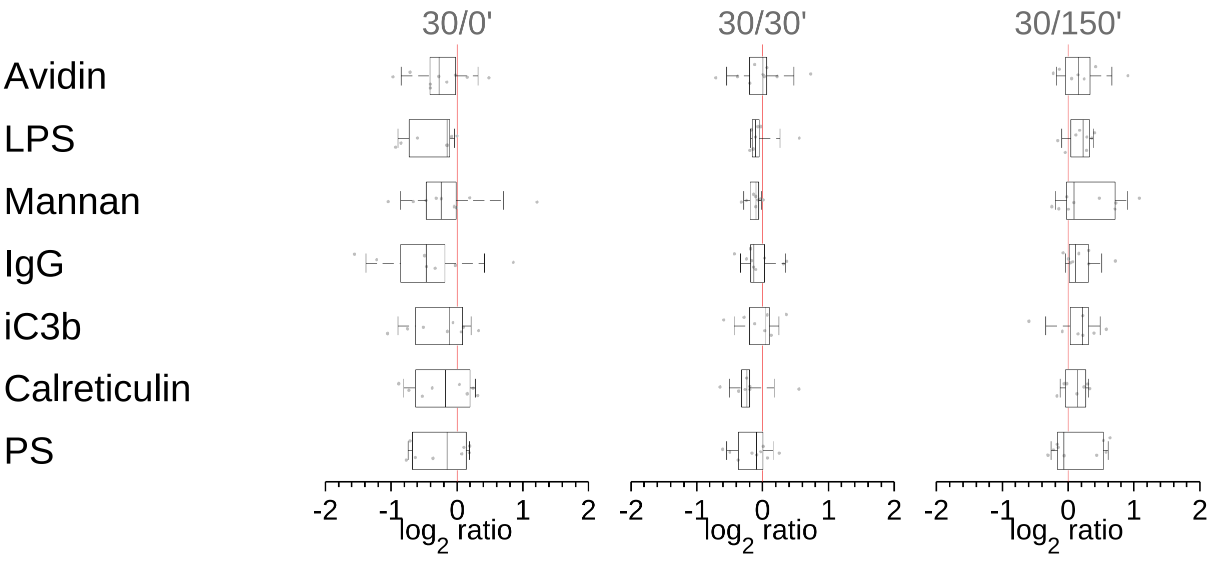

GO:0008150 biological_process (n = 194)

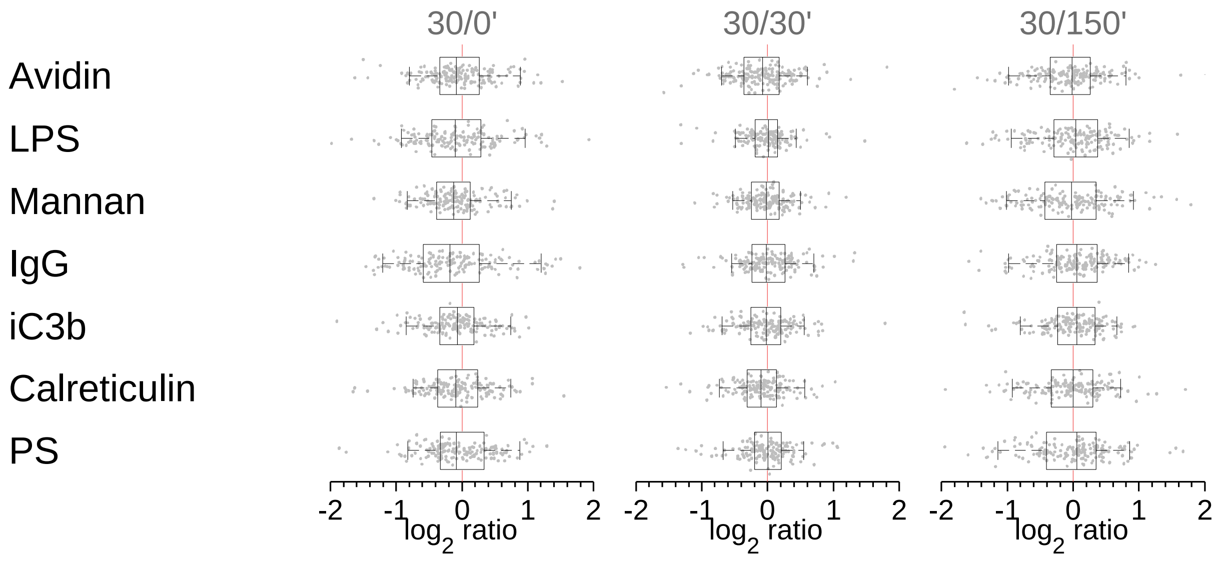

GO:0008152 metabolic process (n = 103)

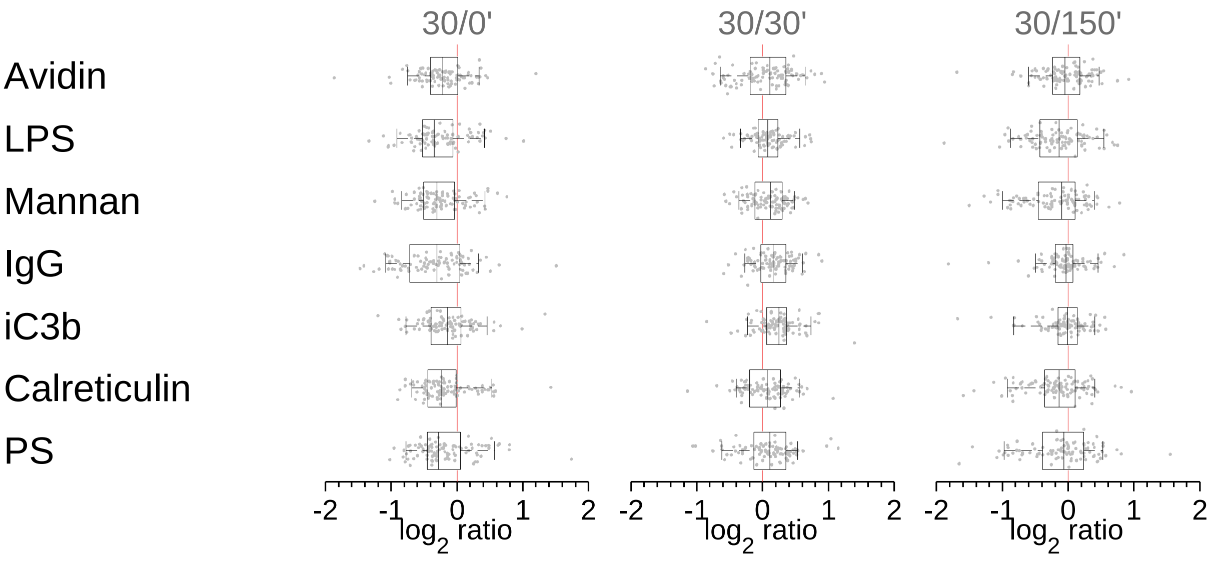

GO:0008154 actin polymerization or depolymerization (n = 5)

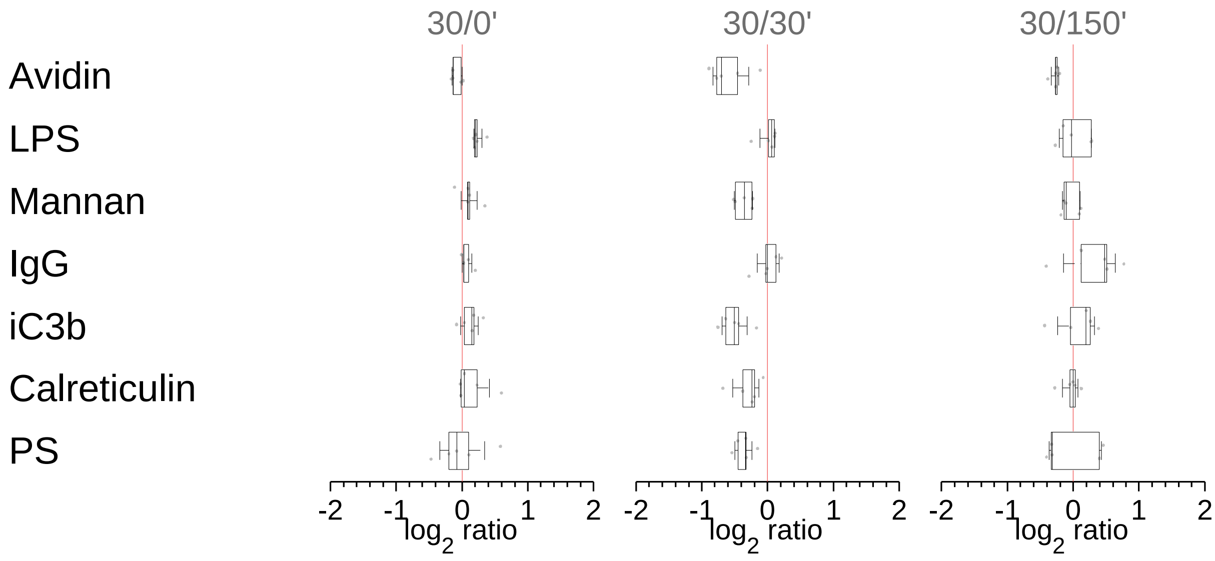

GO:0008168 methyltransferase activity (n = 3)

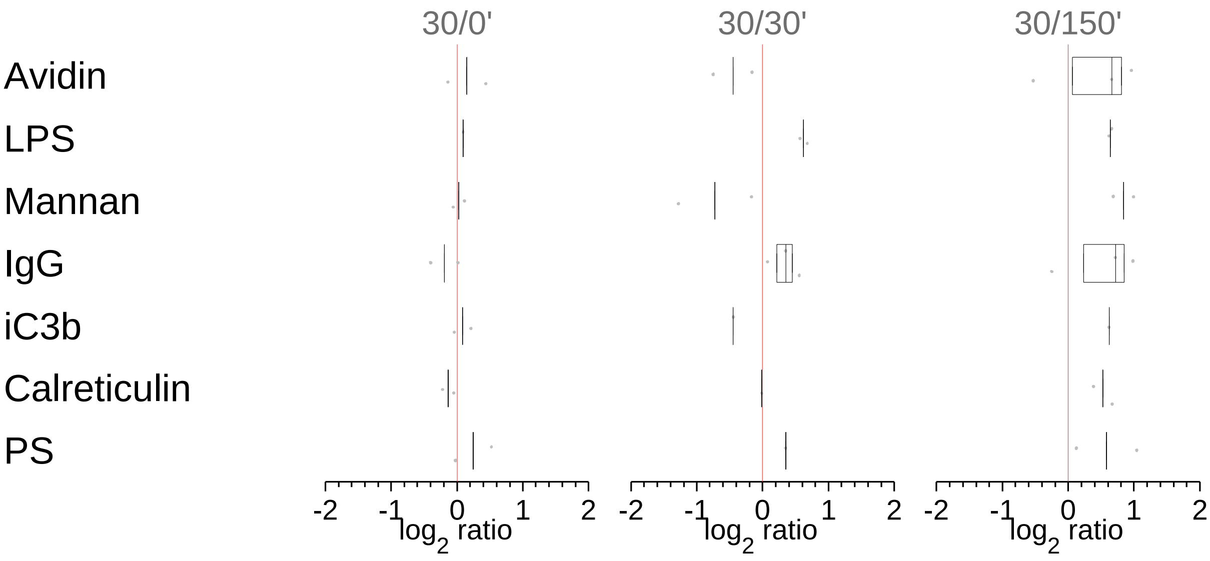

GO:0008180 signalosome (n = 8)

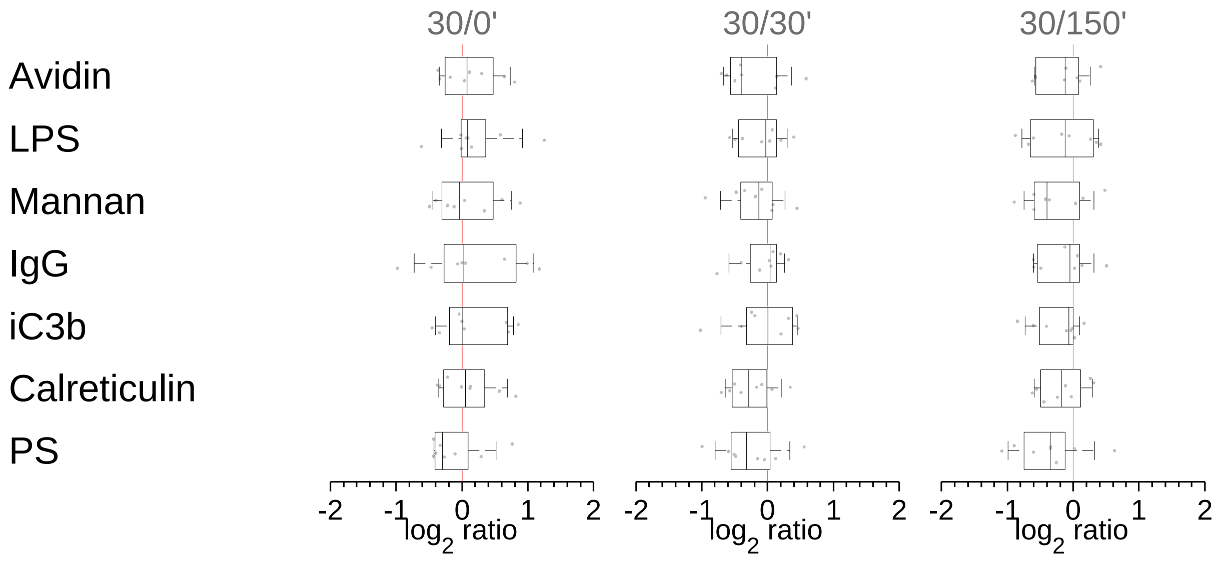

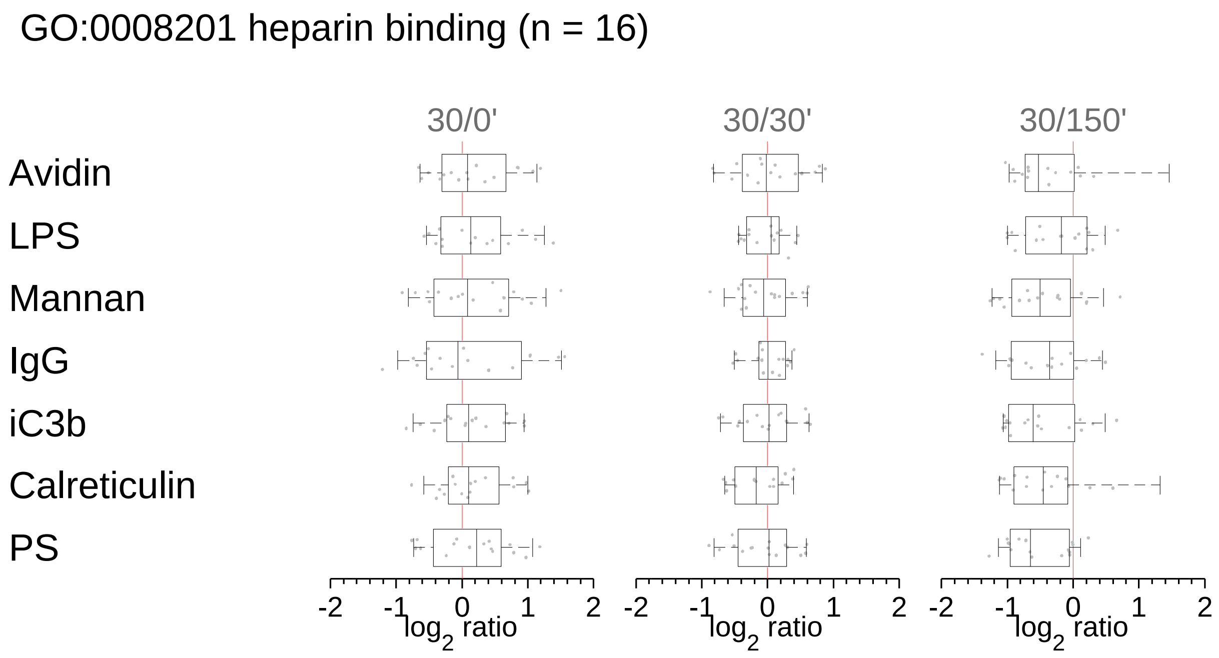
GO:0008201 heparin binding (n = 16)
30/0'
30/30'
30/150'
Avidin
LPS
Mannan
IgG
iC3b
Calreticulin
PS
-2
-1
0
1
2
log2 ratio

GO:0008202 steroid metabolic process (n = 10)

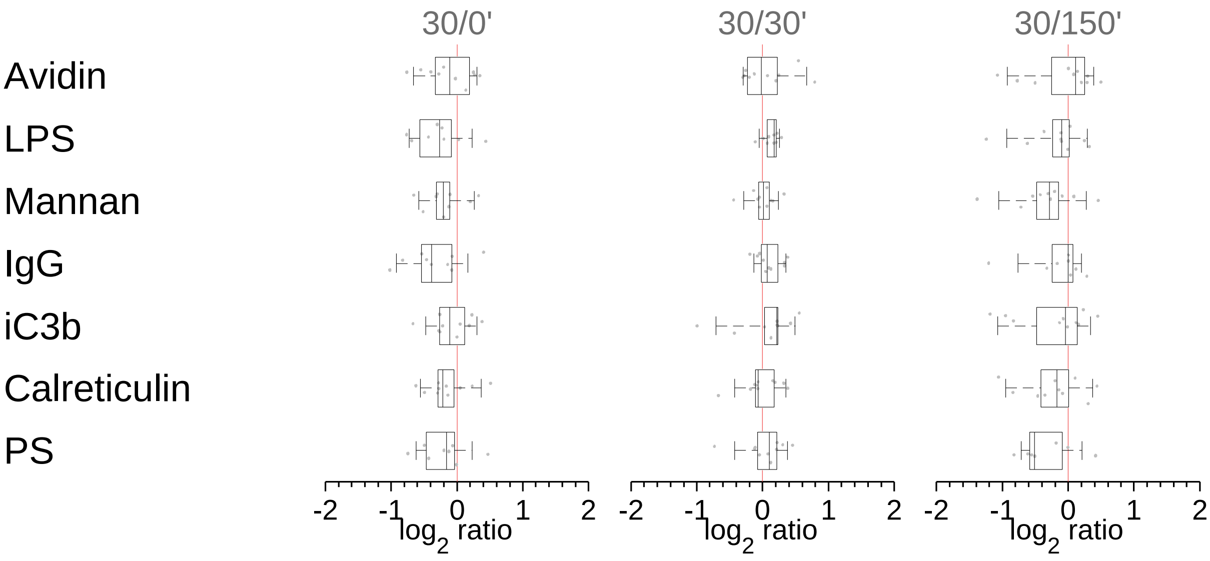

GO:0008203 cholesterol metabolic process (n = 12)

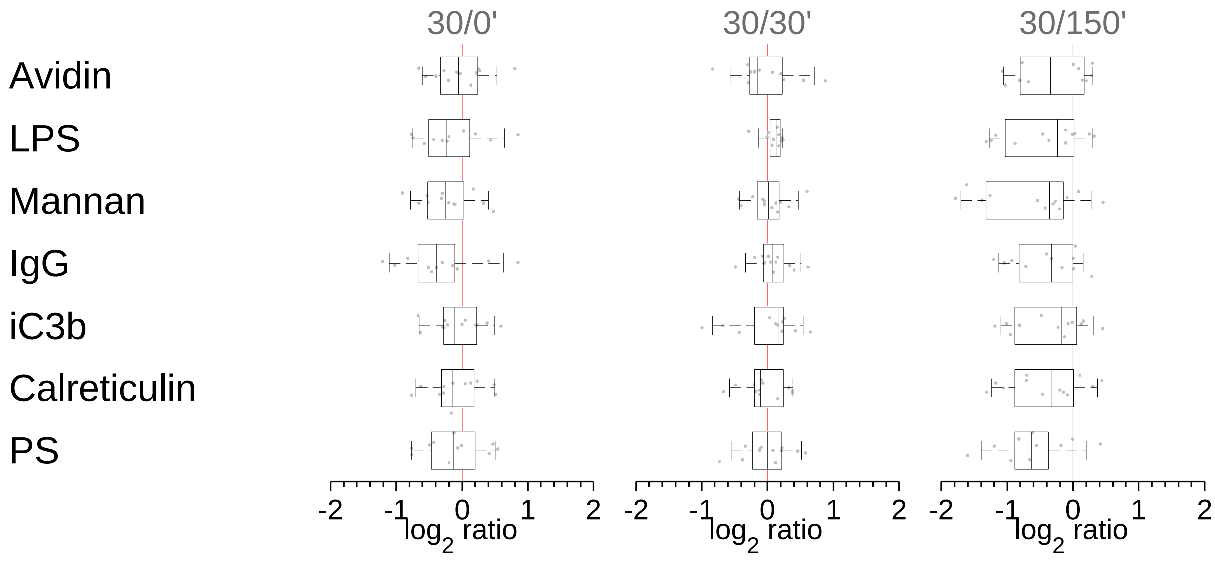

GO:0008209 androgen metabolic process (n = 3)

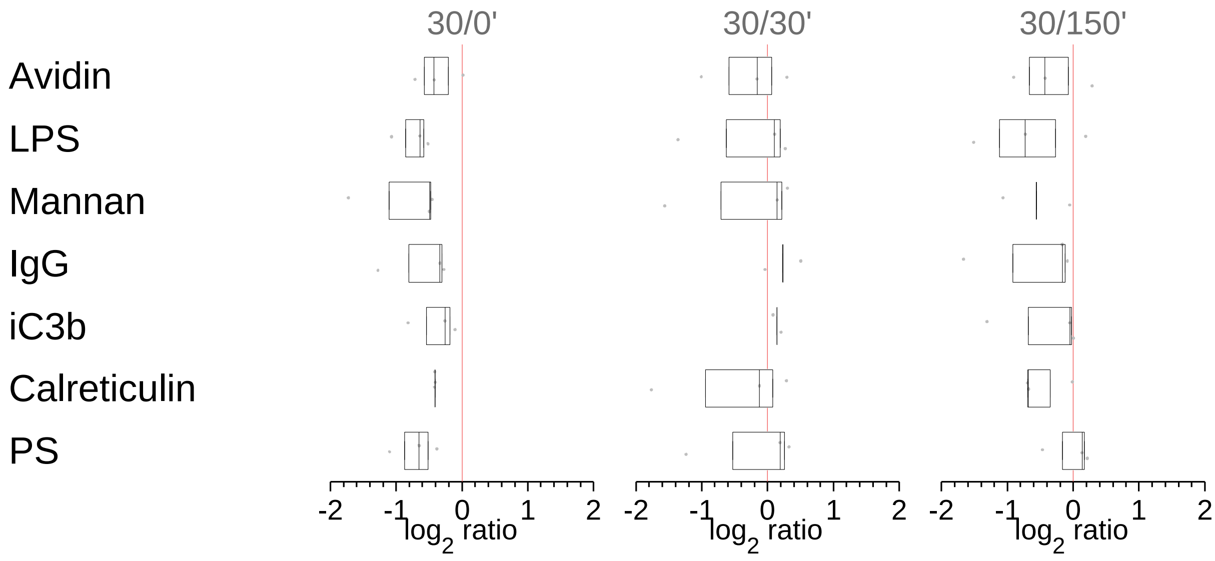

GO:0008210 estrogen metabolic process (n = 4)

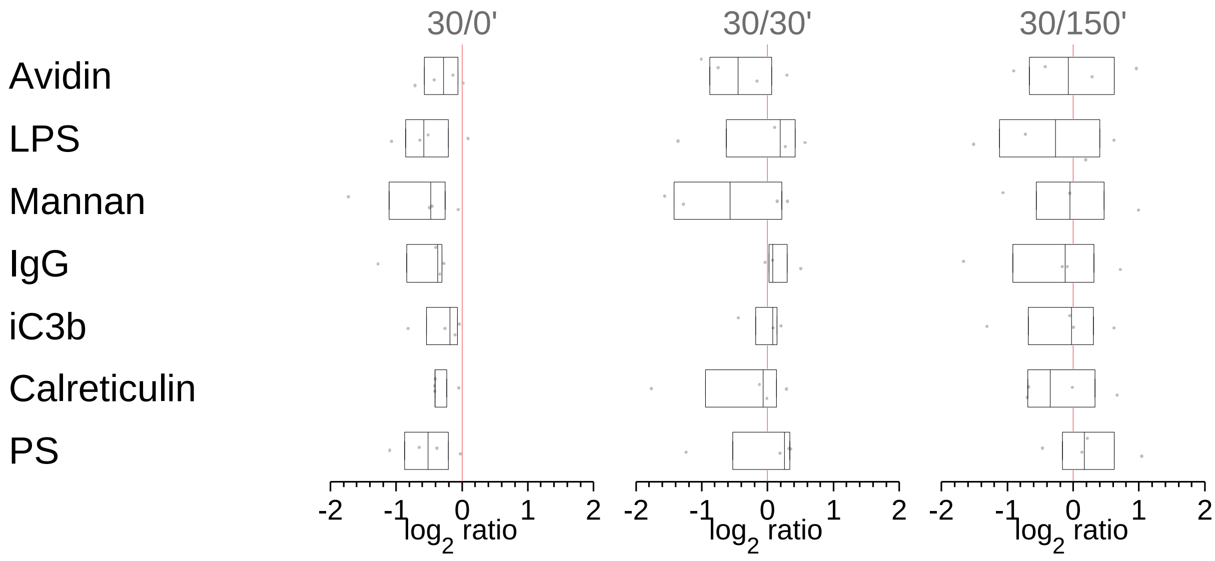

GO:0008217 regulation of blood pressure (n = 11)

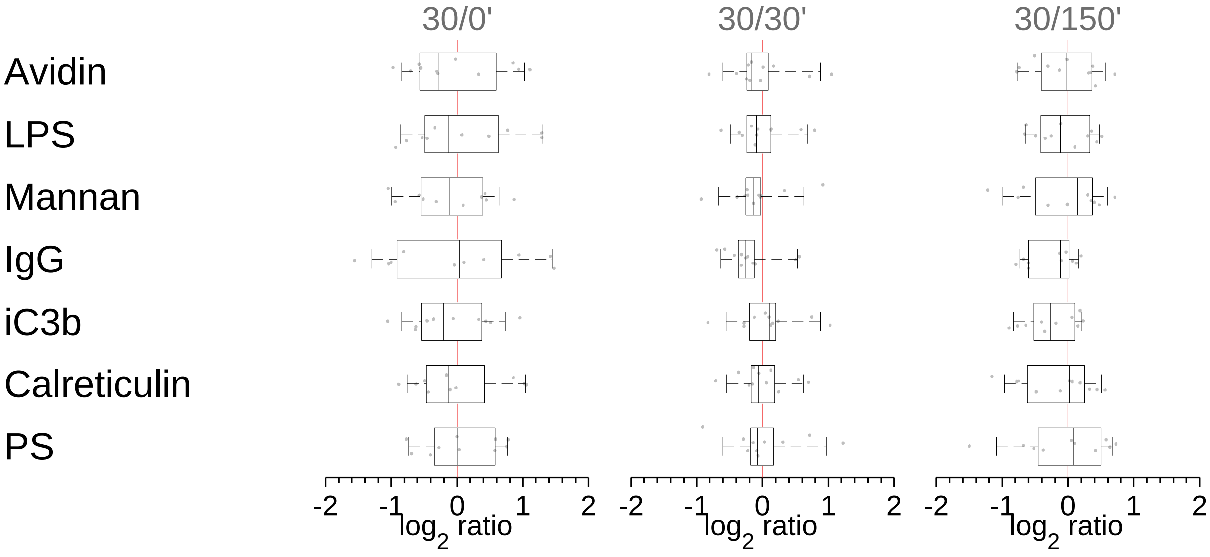

GO:0008219 cell death (n = 11)

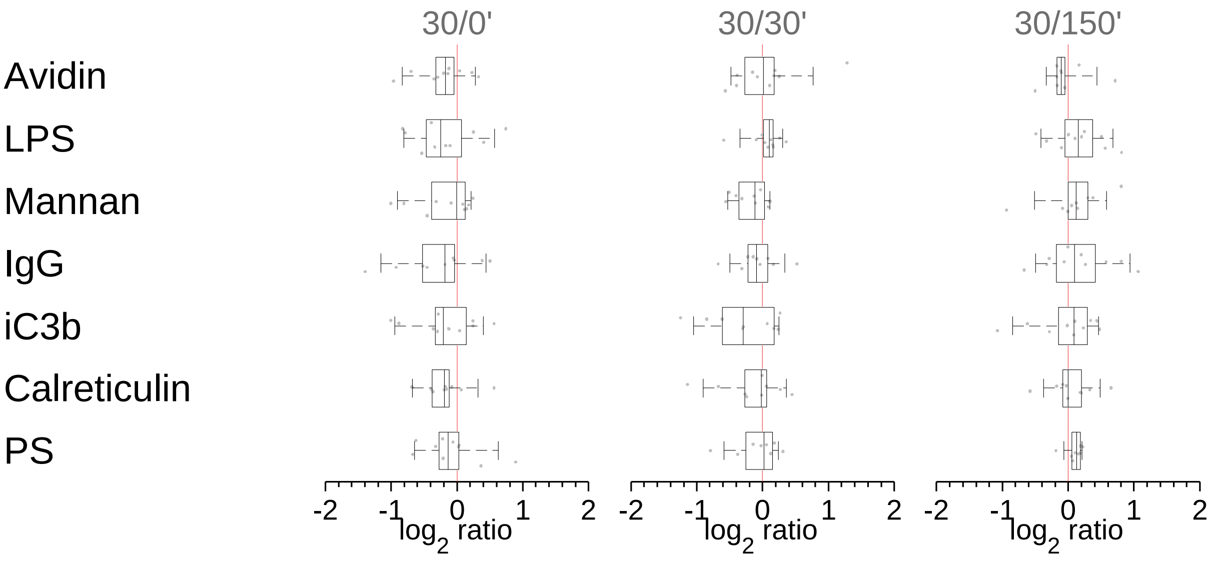

GO:0008233 peptidase activity (n = 57)

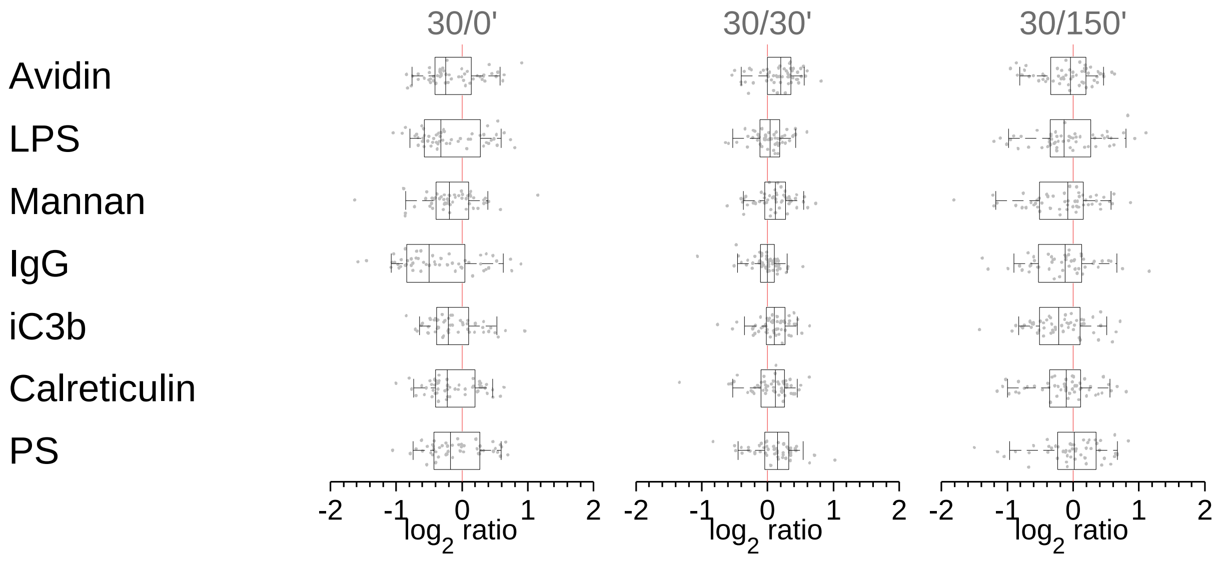

GO:0008234 cysteine-type peptidase activity (n = 16)

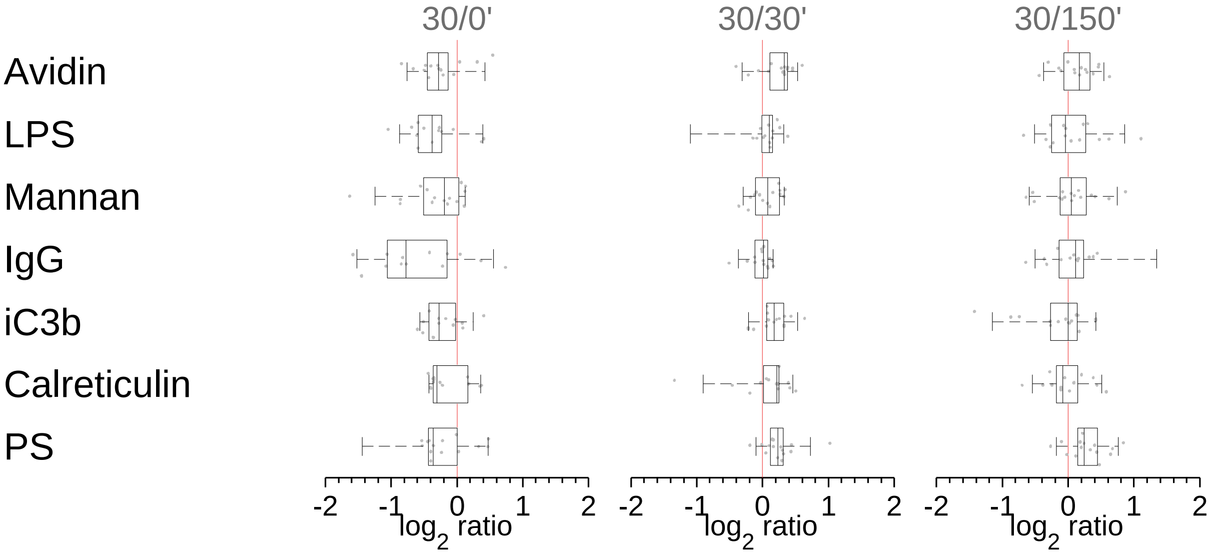

GO:0008236 serine-type peptidase activity (n = 5)

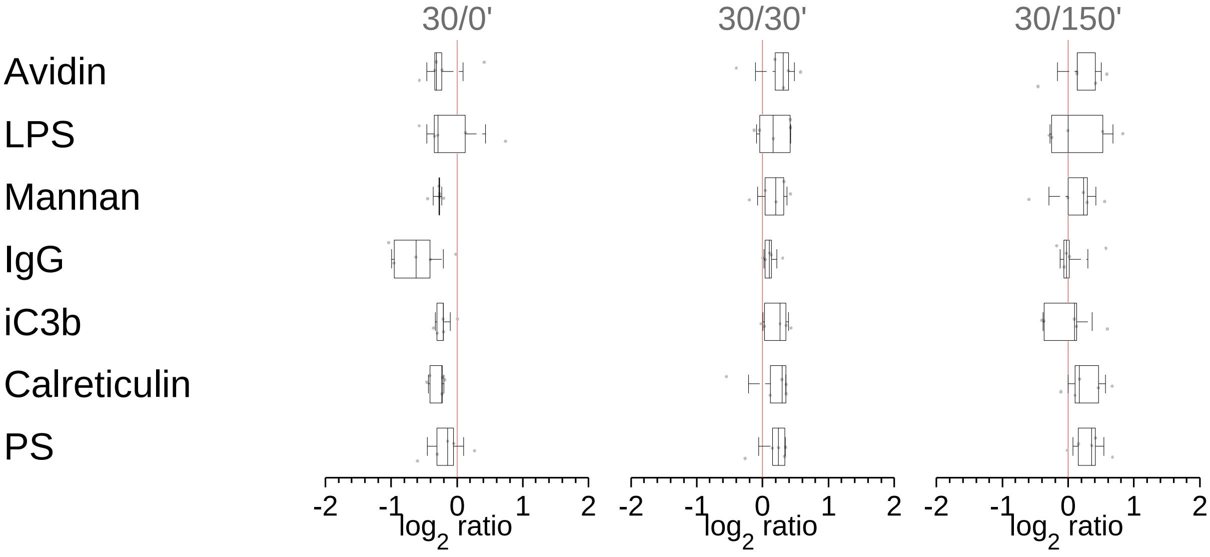

GO:0008237 metallopeptidase activity (n = 15)

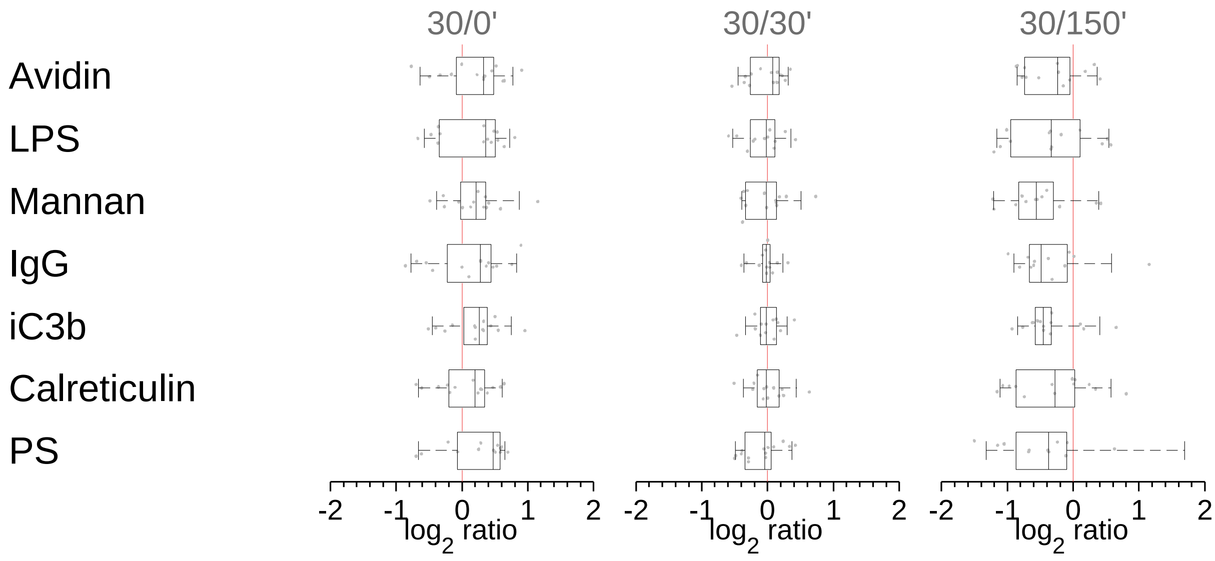

GO:0008250 oligosaccharyltransferase complex (n = 3)

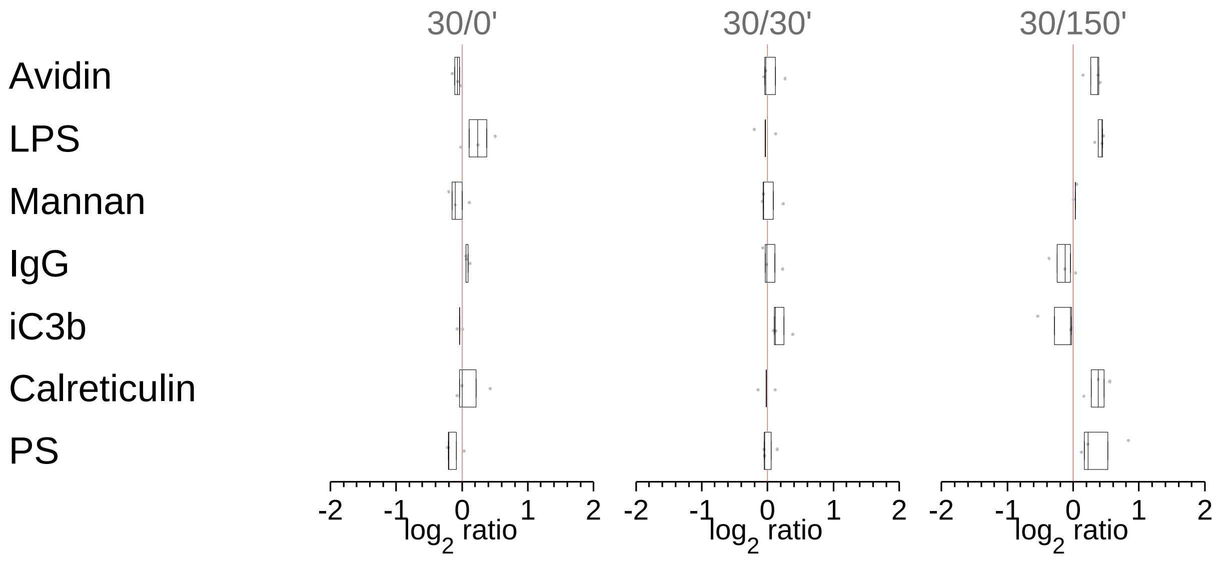

GO:0008266 poly(U) RNA binding (n = 3)

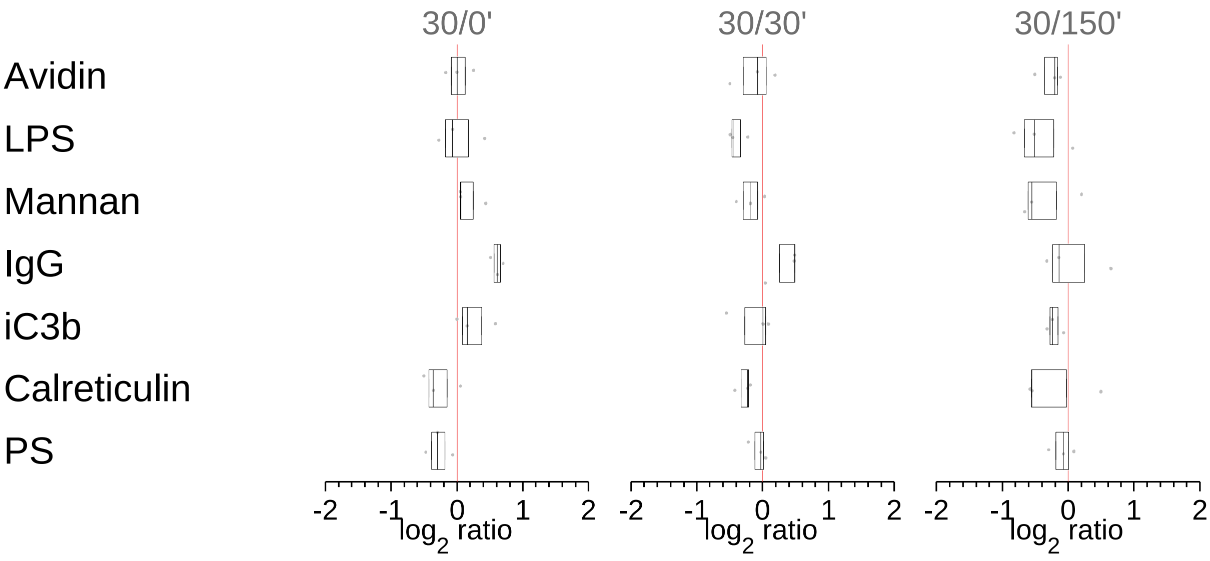

GO:0008270 zinc ion binding (n = 75)

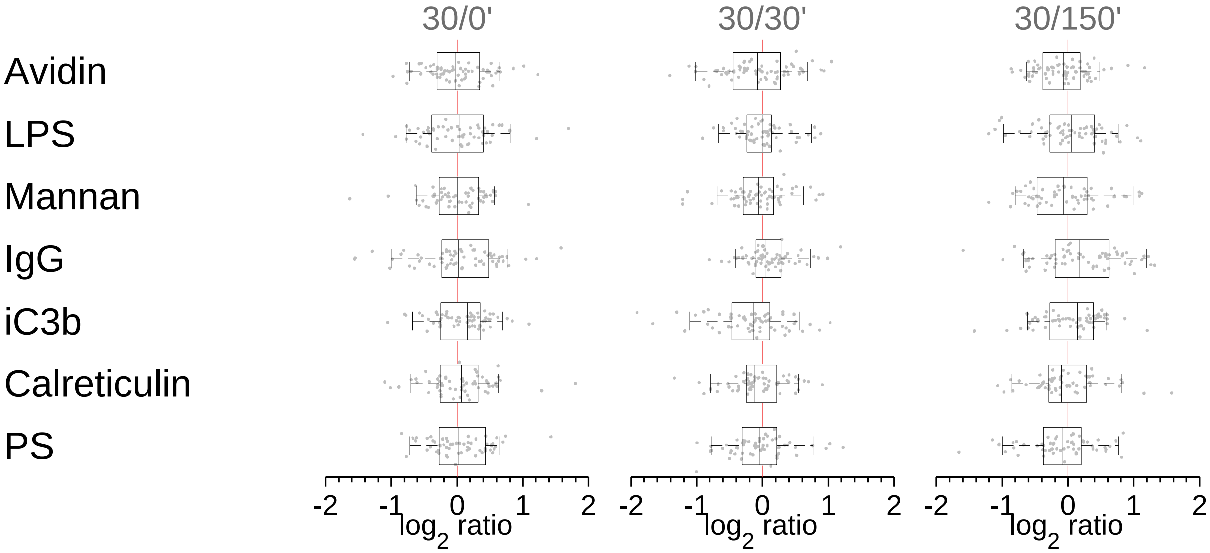

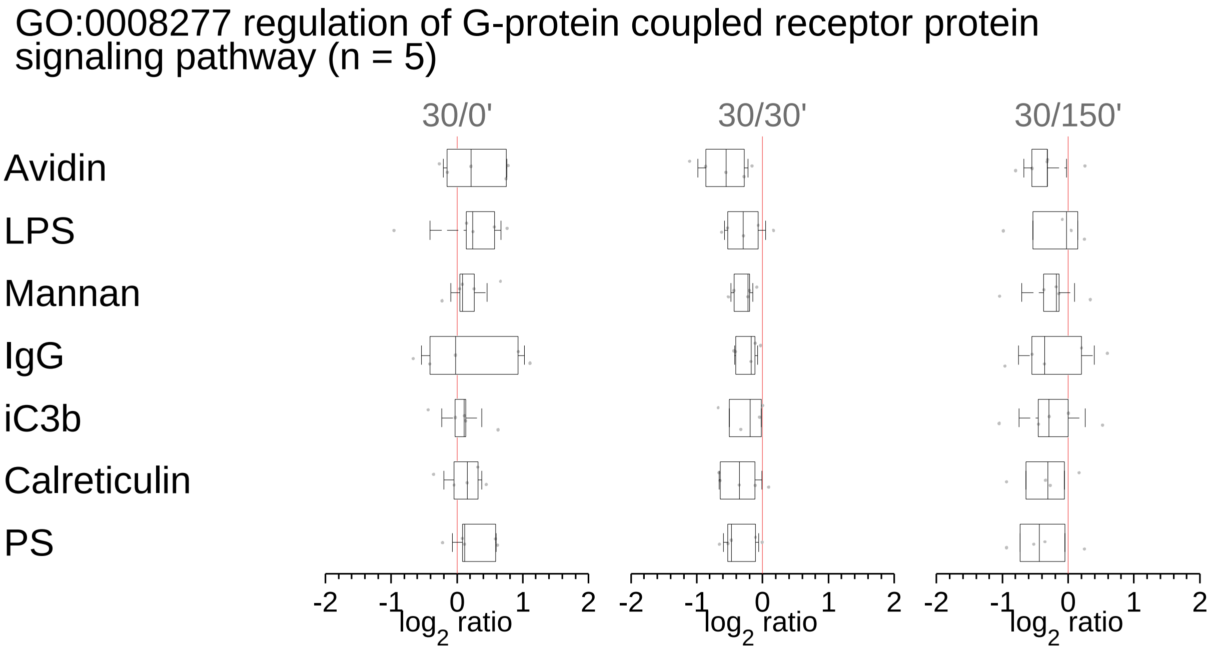
GO:0008277 regulation of G-protein coupled receptor protein signaling pathway (n = 5)
30/0'
30/30'
30/150'
Avidin
LPS
Mannan
IgG
iC3b
Calreticulin
PS
-2 -1 0 1 2
log_2 ratio

GO:0008283 cell proliferation (n = 31)

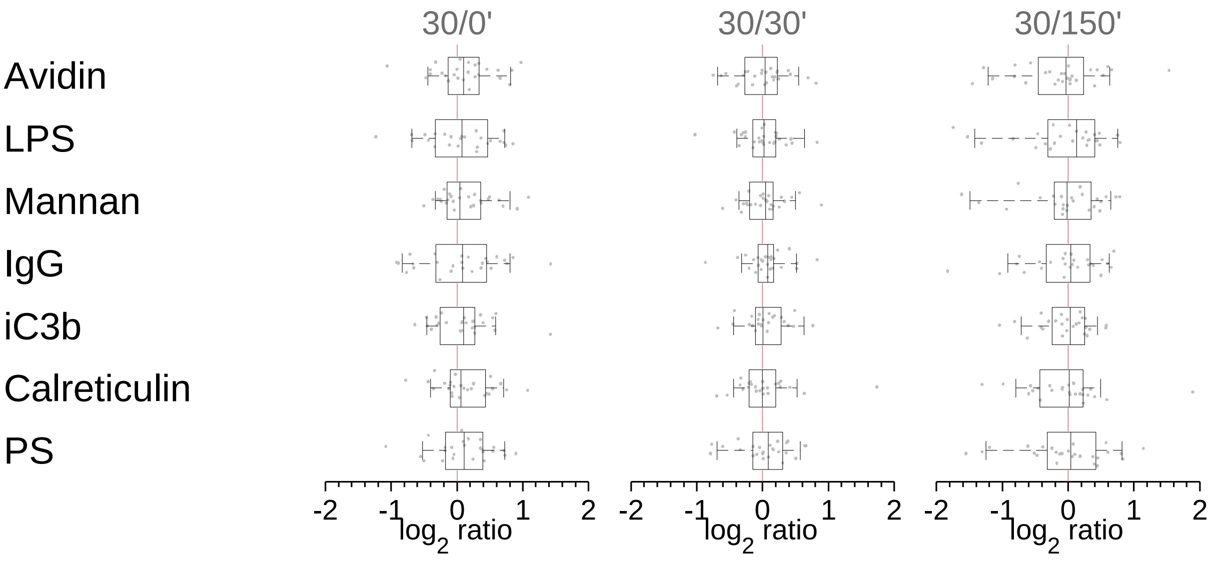

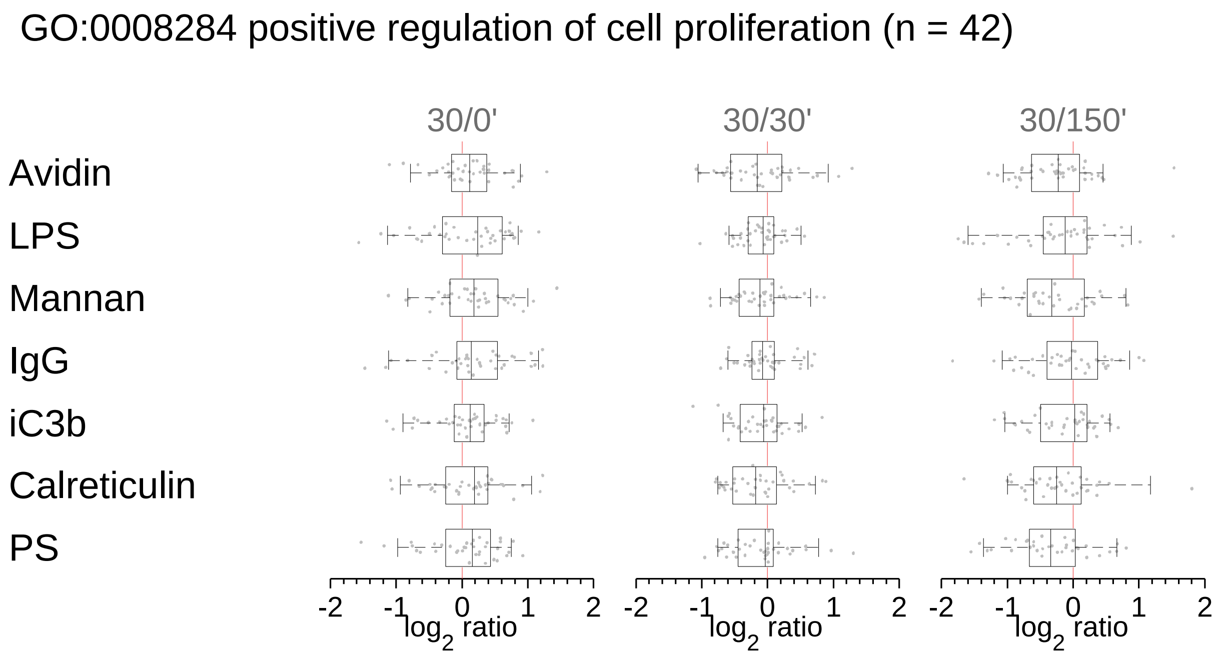
GO:0008284 positive regulation of cell proliferation (n = 42)
30/0'
30/30'
30/150'
Avidin
LPS
Mannan
IgG
iC3b
Calreticulin
PS
-2
-1
0
1
2
log2 ratio

GO:0008285 negative regulation of cell proliferation (n = 32)

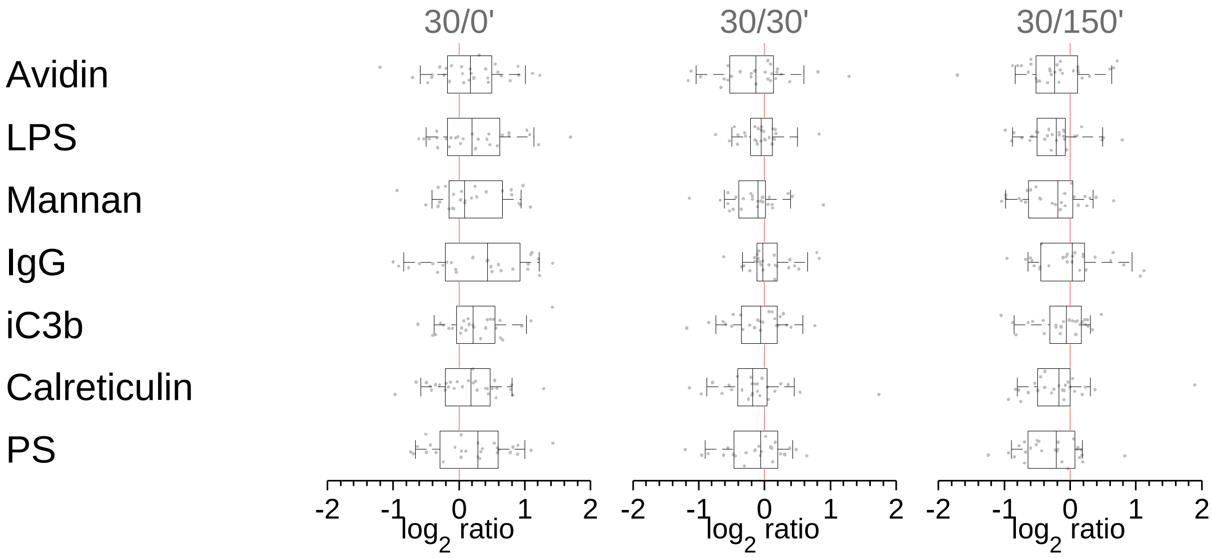

GO:0008286 insulin receptor signaling pathway (n = 6)

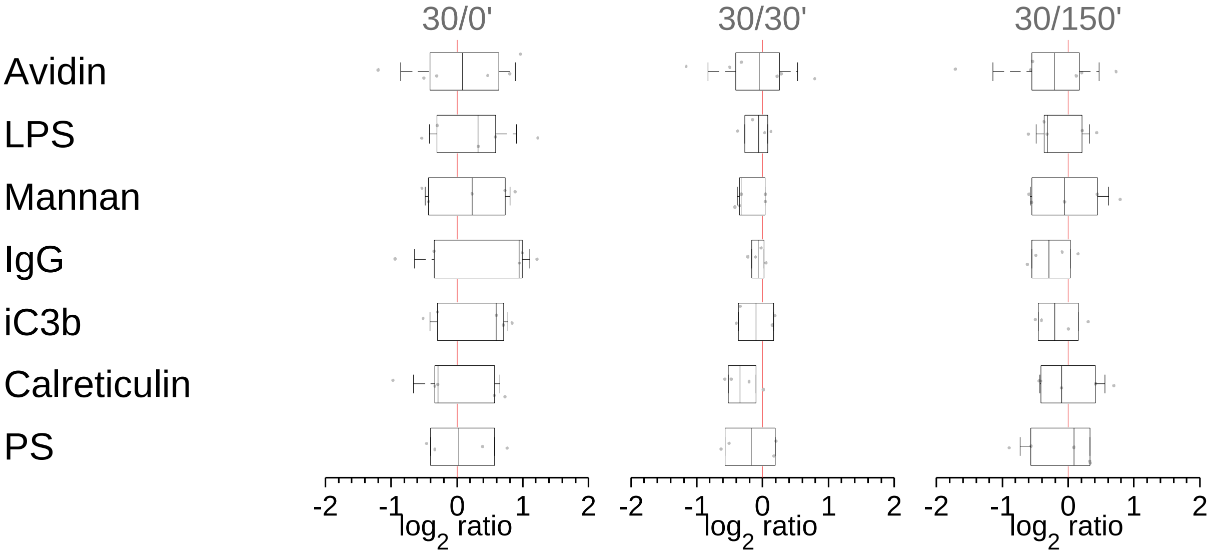

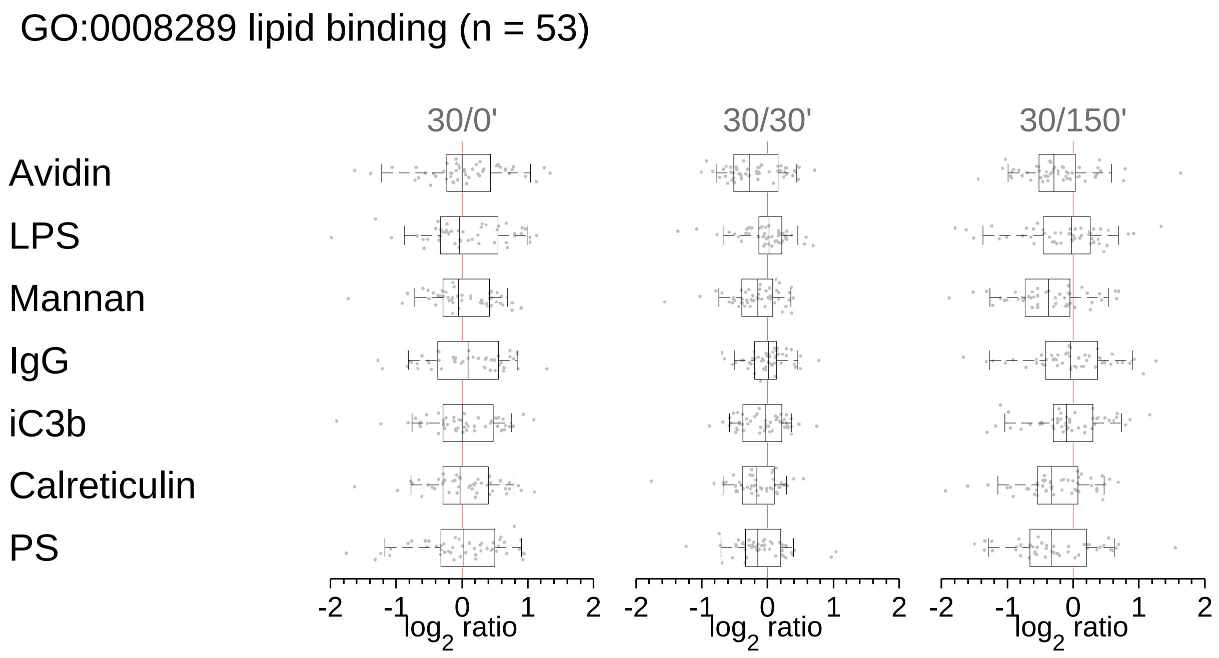
GO:0008289 lipid binding (n = 53)
30/0'
30/30'
30/150'
Avidin
LPS
Mannan
IgG
iC3b
Calreticulin
PS
-2
-1
0
1
2
$\log_2$ ratio

GO:0008290 F-actin capping protein complex (n = 3)

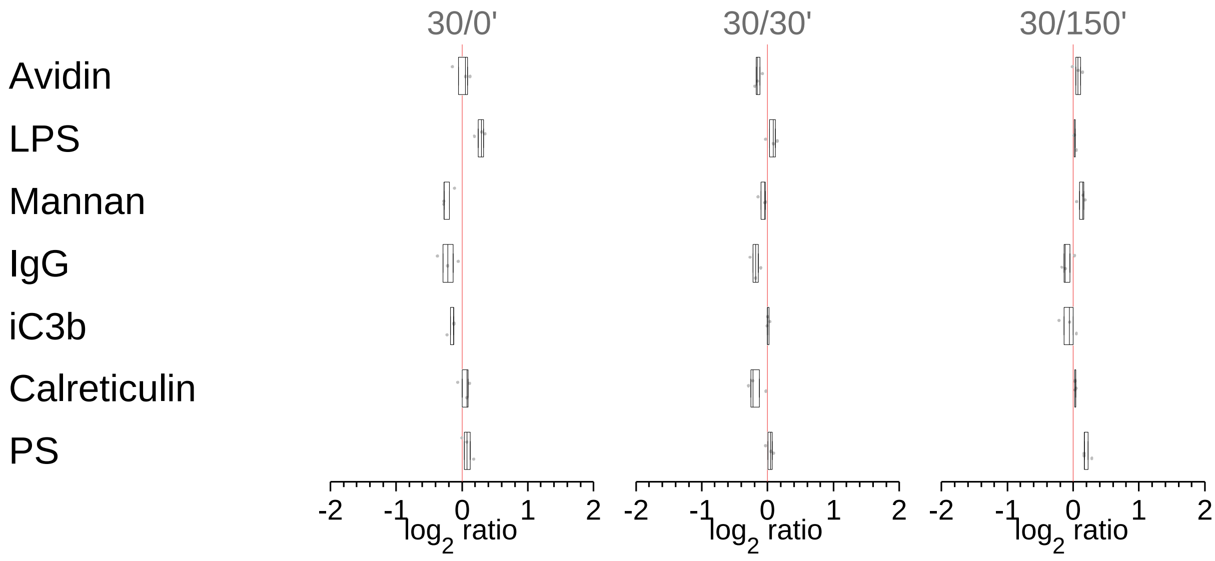

GO:0008301 DNA binding, bending (n = 3)

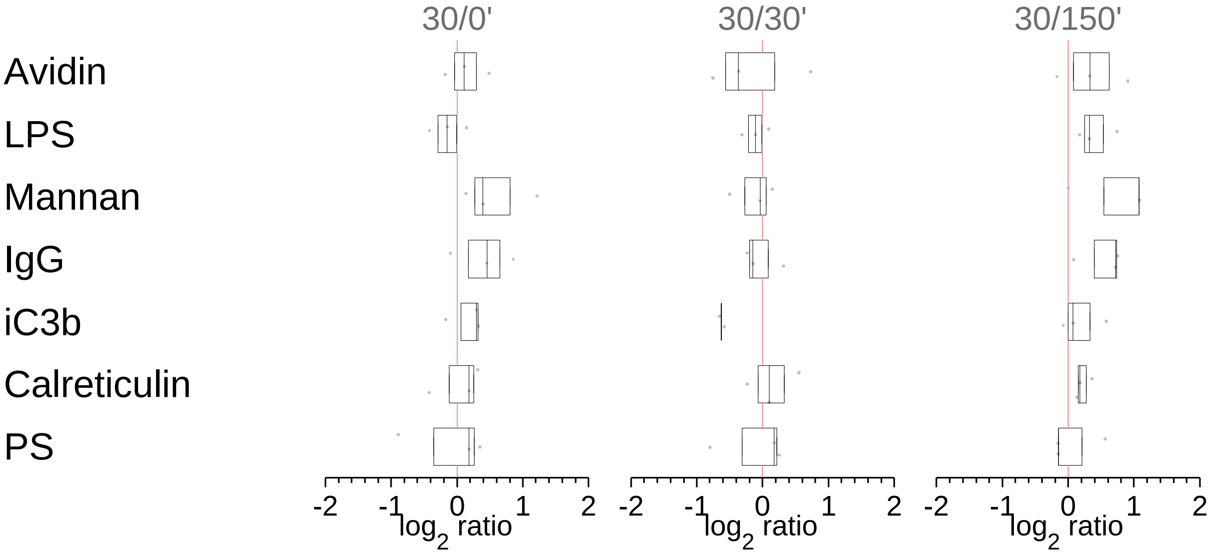

GO:0008305 integrin complex (n = 10)

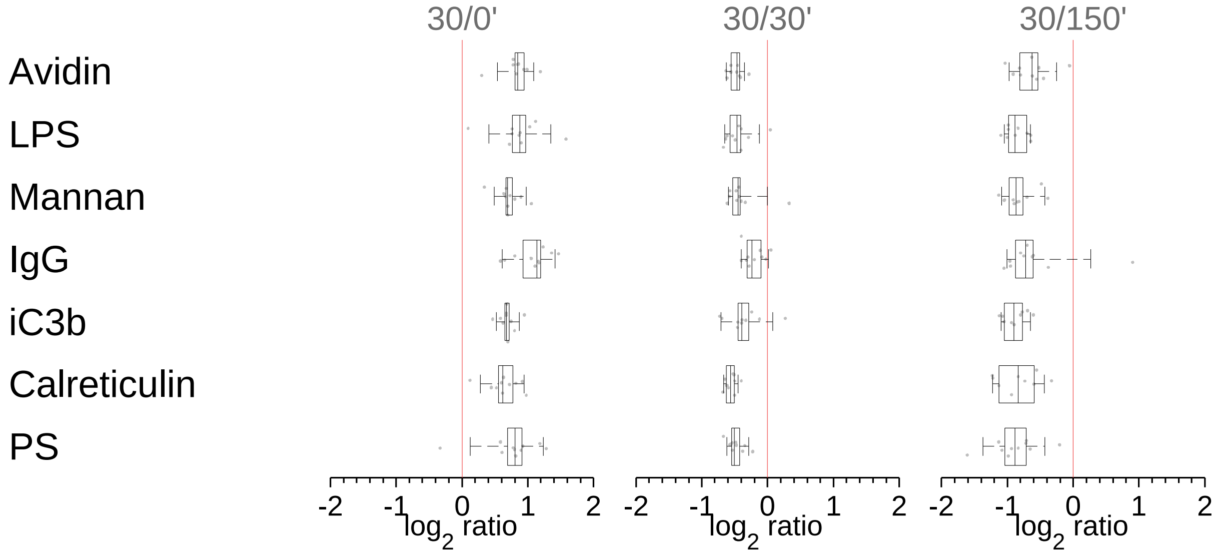

GO:0008306 associative learning (n = 3)

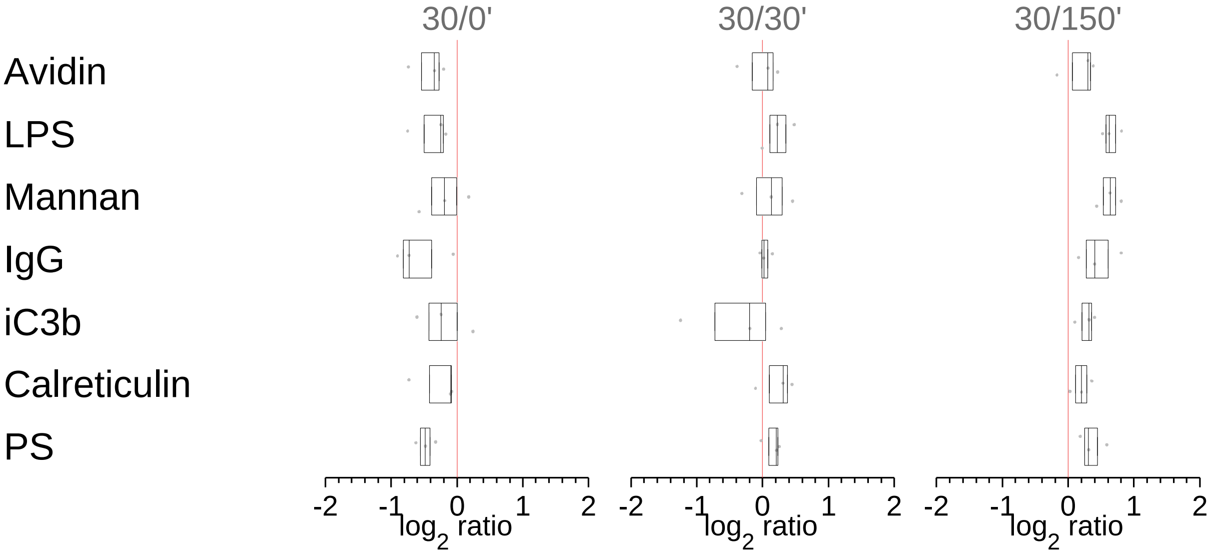

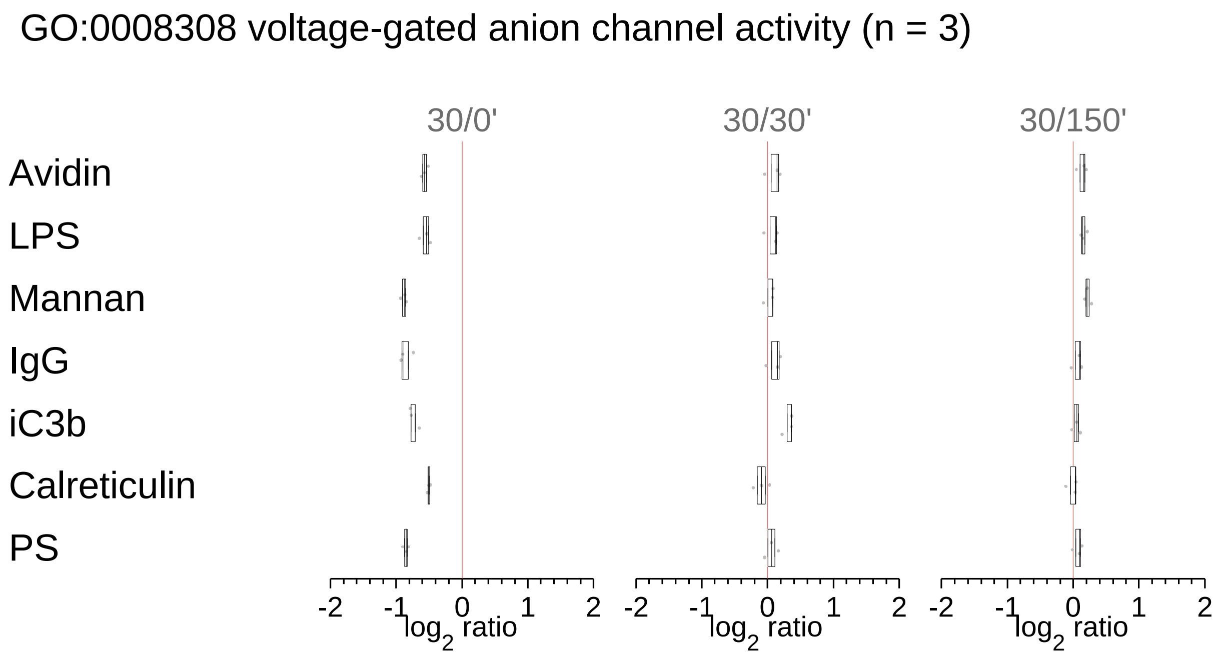

GO:0008308 voltage-gated anion channel activity (n = 3)
30/0'
30/30'
30/150'
Avidin
LPS
Mannan
IgG
iC3b
Calreticulin
PS
-2 -1 0 1 2
log2 ratio

GO:0008312 7S RNA binding (n = 3)

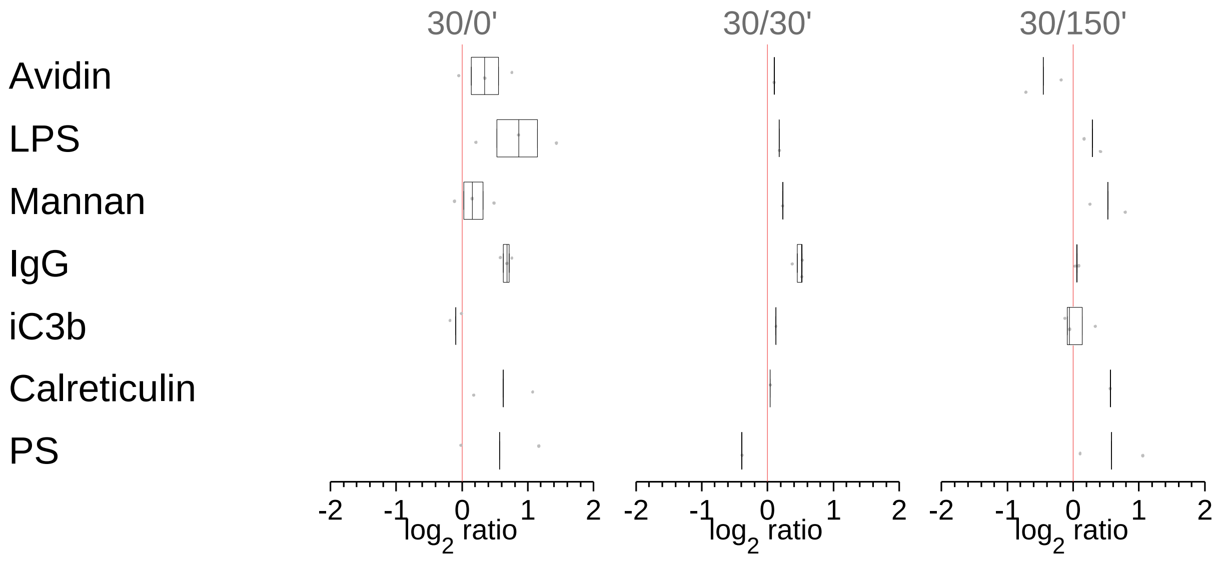

GO:0008324 cation transmembrane transporter activity (n = 3)

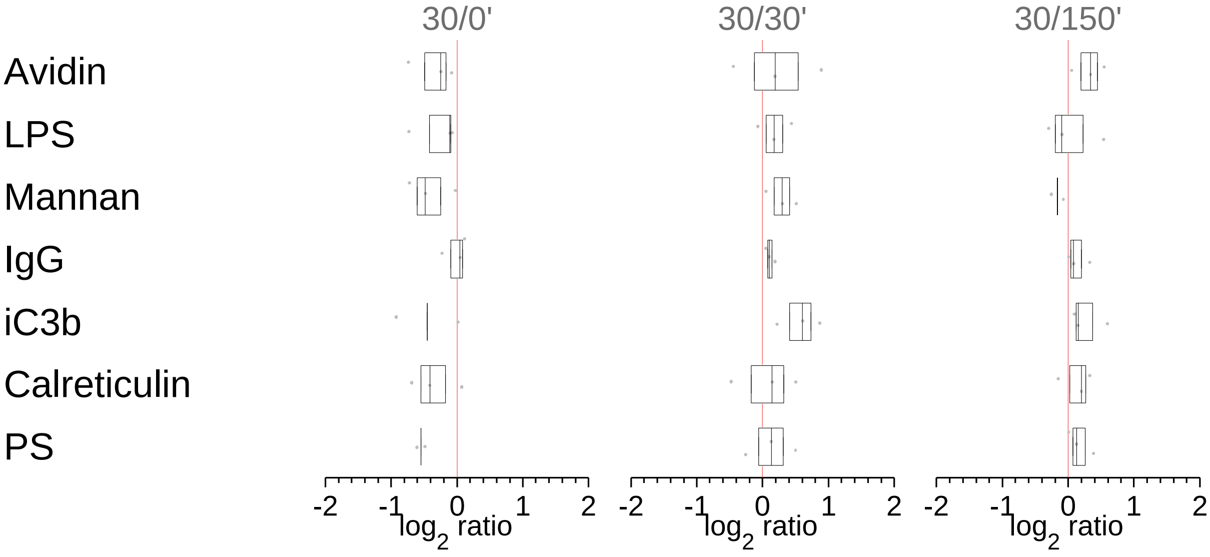

GO:0008333 endosome to lysosome transport (n = 13)

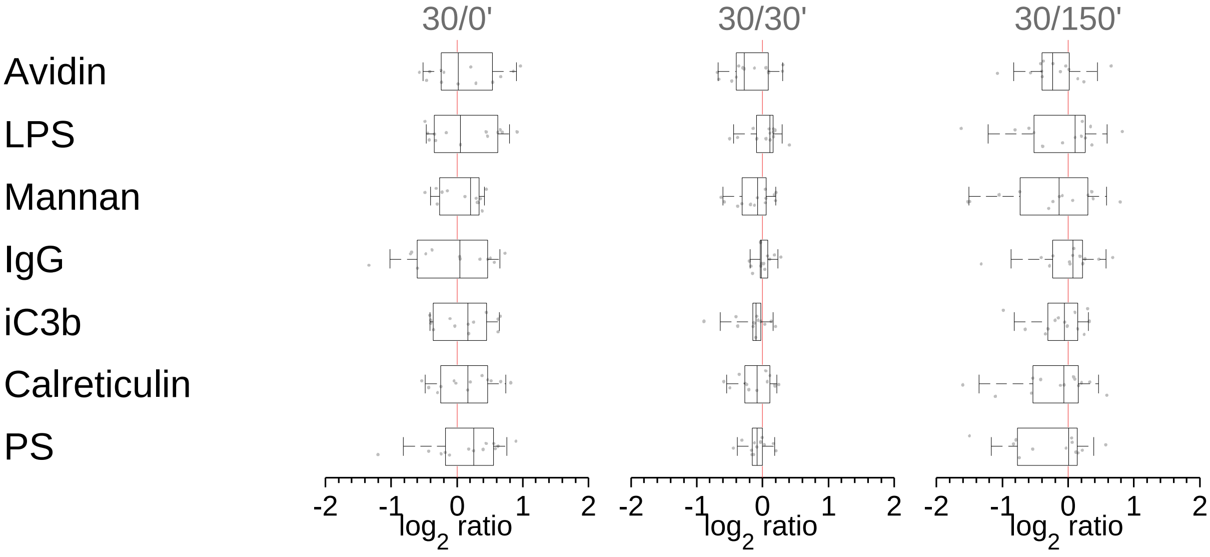

GO:0008344 adult locomotory behavior (n = 5)

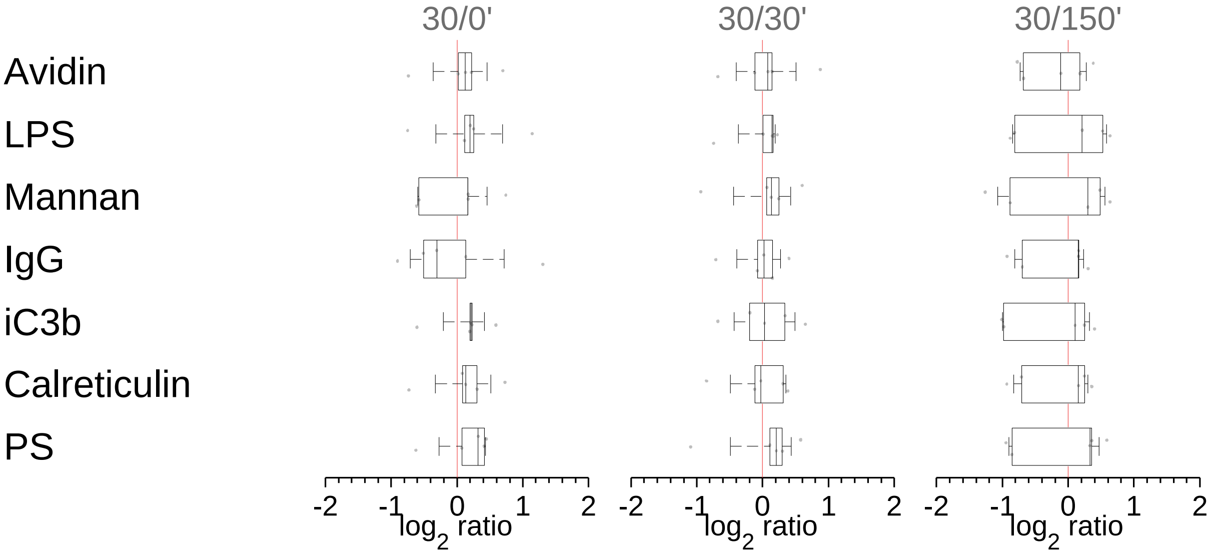

GO:0008360 regulation of cell shape (n = 27)

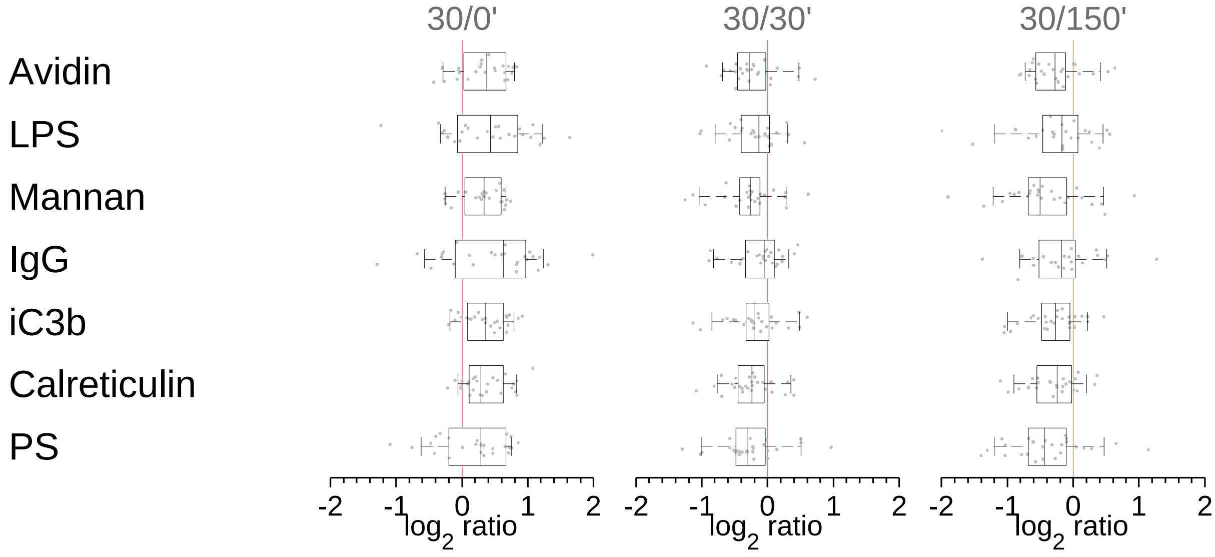

GO:0008361 regulation of cell size (n = 3)

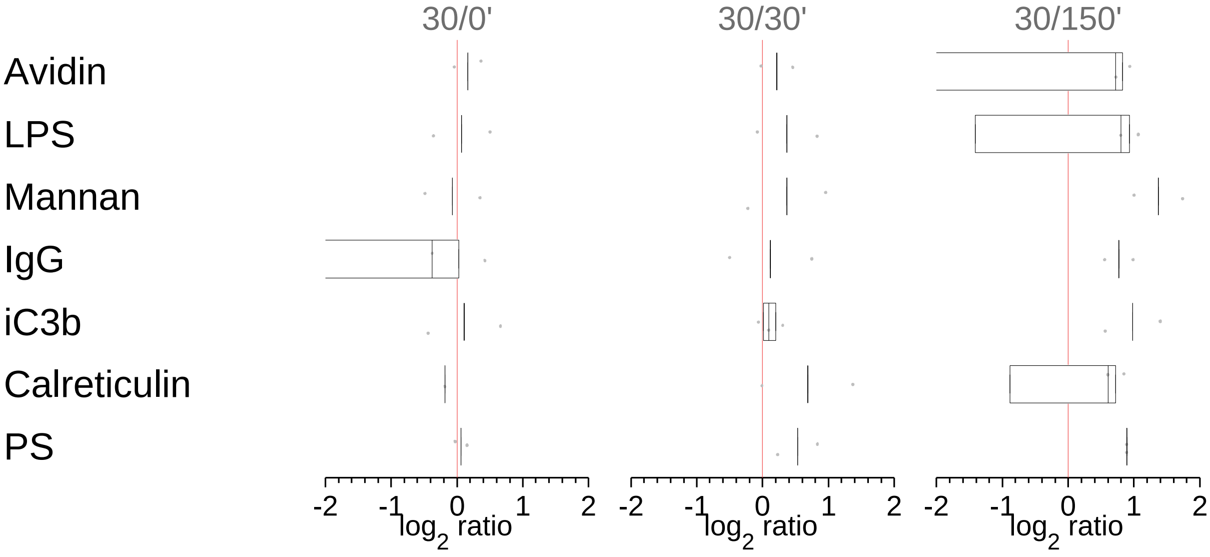

GO:0008374 O-acyltransferase activity (n = 3)

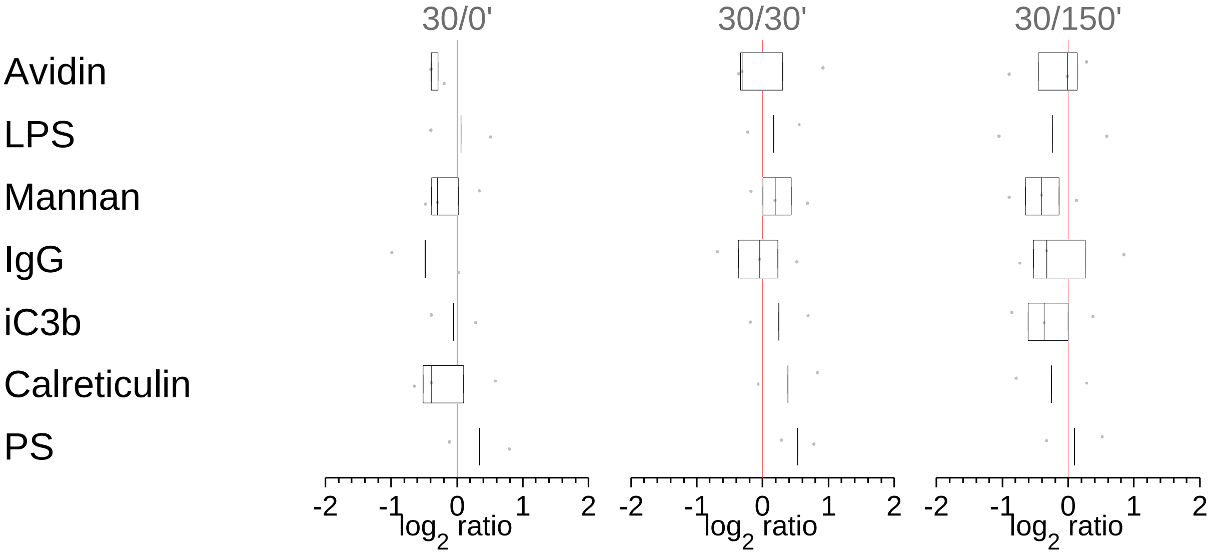

GO:0008375 acetylglucosaminyltransferase activity (n = 4)

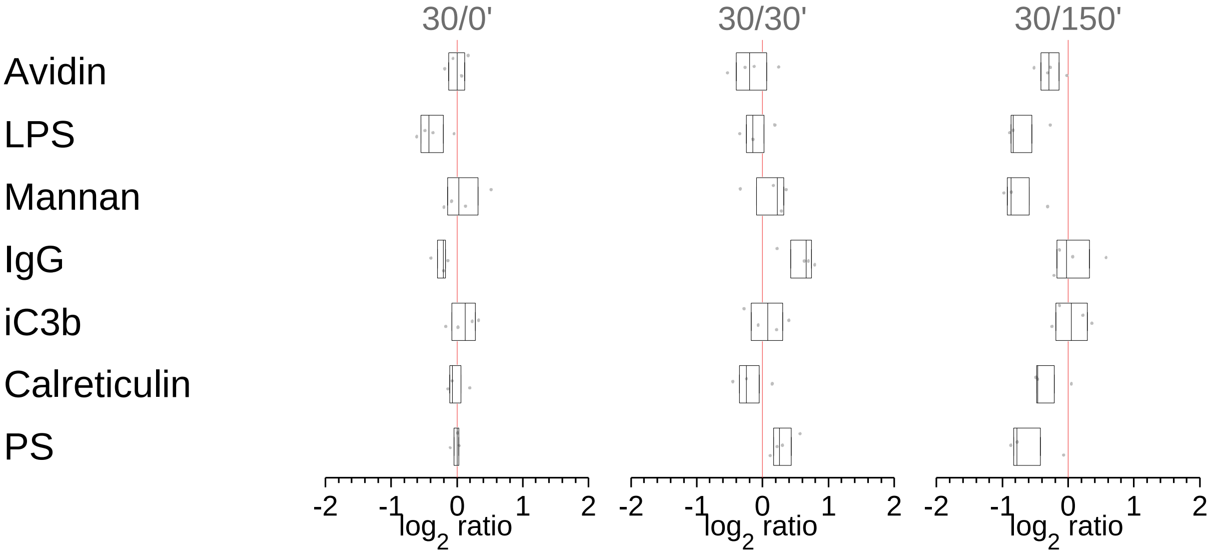

GO:0008376 acetylgalactosaminyltransferase activity (n = 4)

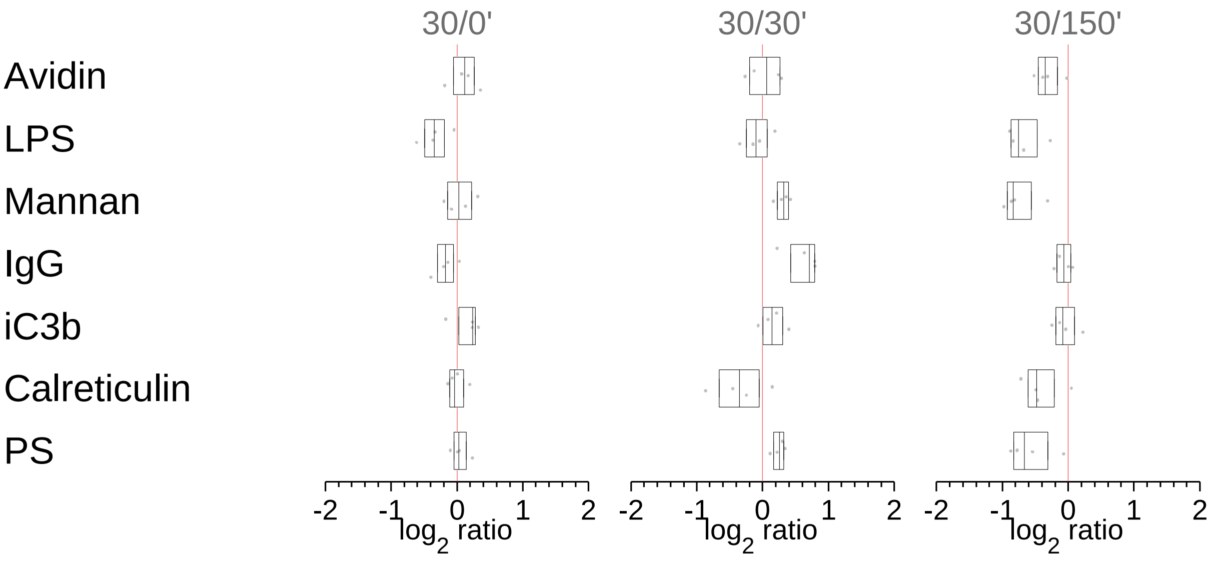

GO:0008378 galactosyltransferase activity (n = 3)

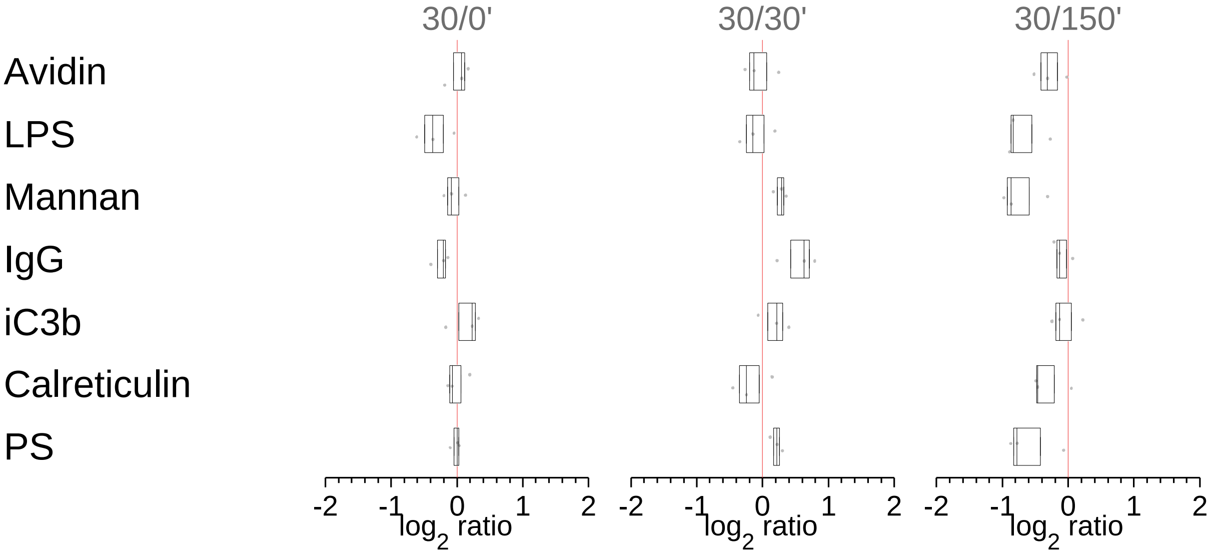

GO:0008379 thioredoxin peroxidase activity (n = 4)

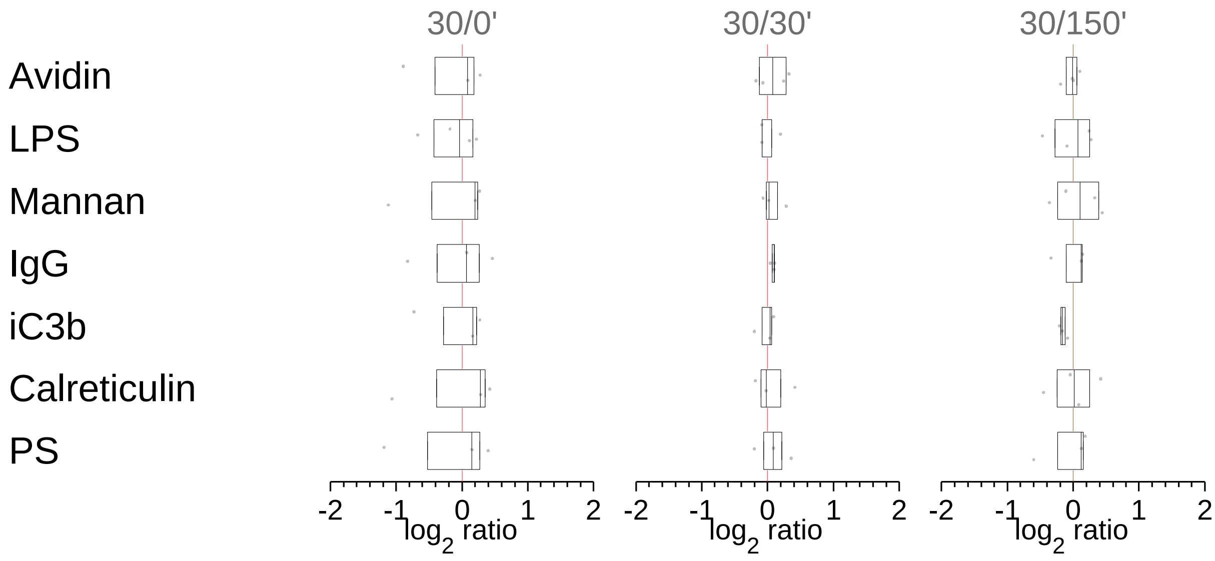

GO:0008380 RNA splicing (n = 41)

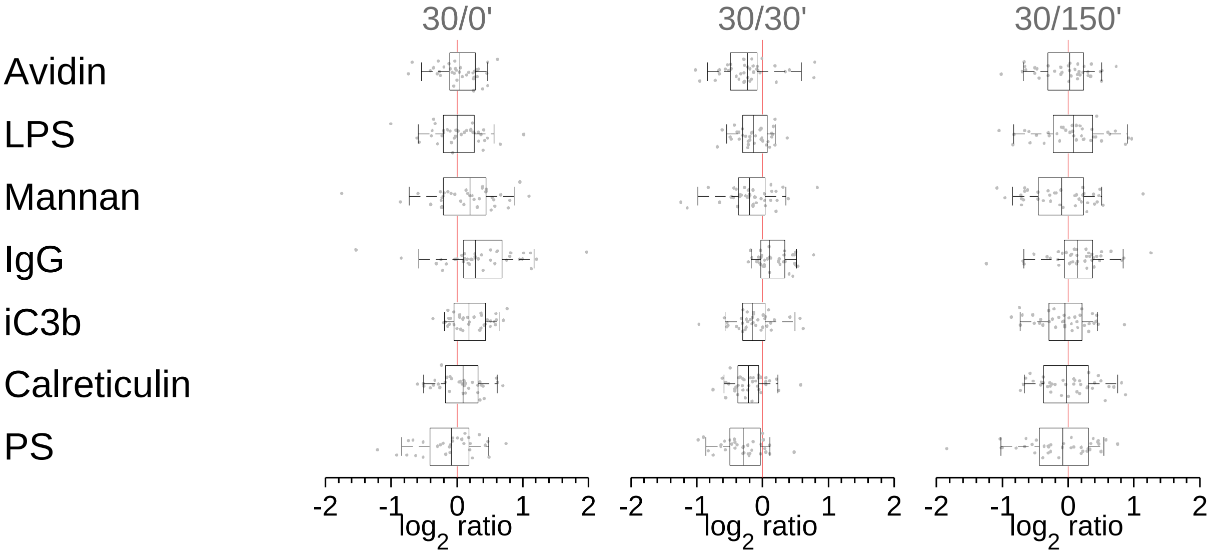

GO:0008417 fucosyltransferase activity (n = 3)

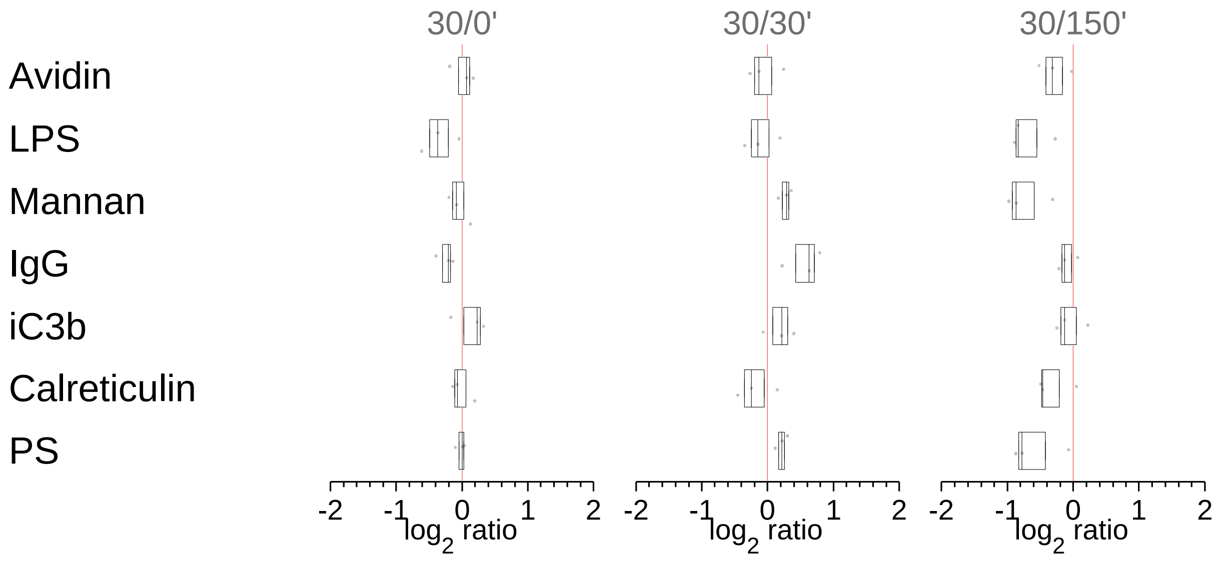

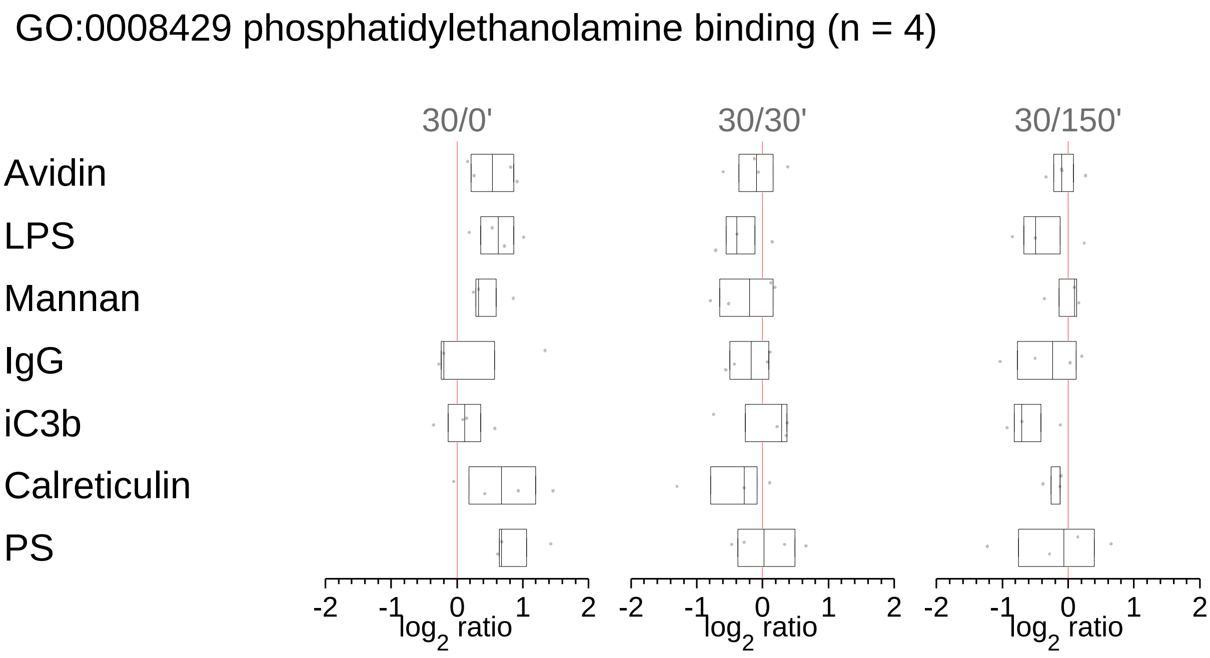
GO:0008429 phosphatidylethanolamine binding (n = 4)
30/0'
30/30'
30/150'
Avidin
LPS
Mannan
IgG
iC3b
Calreticulin
PS
-2 -1 0 1 2
log2 ratio
-2 -1 0 1 2
log2 ratio
-2 -1 0 1 2
log2 ratio

GO:0008430 selenium binding (n = 4)

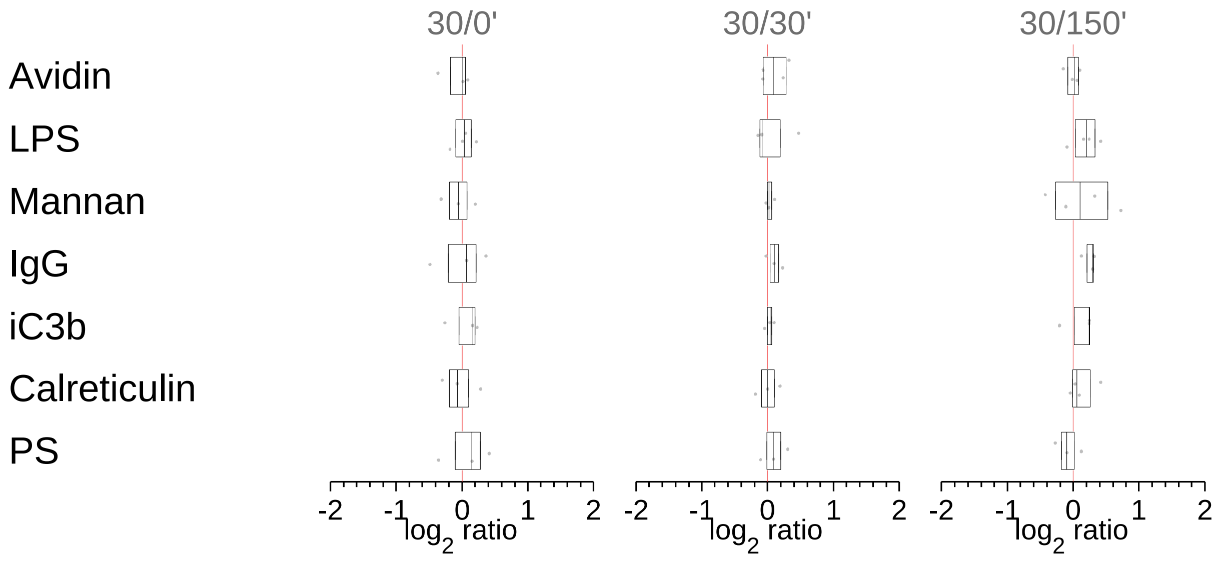

GO:0008483 transaminase activity (n = 4)

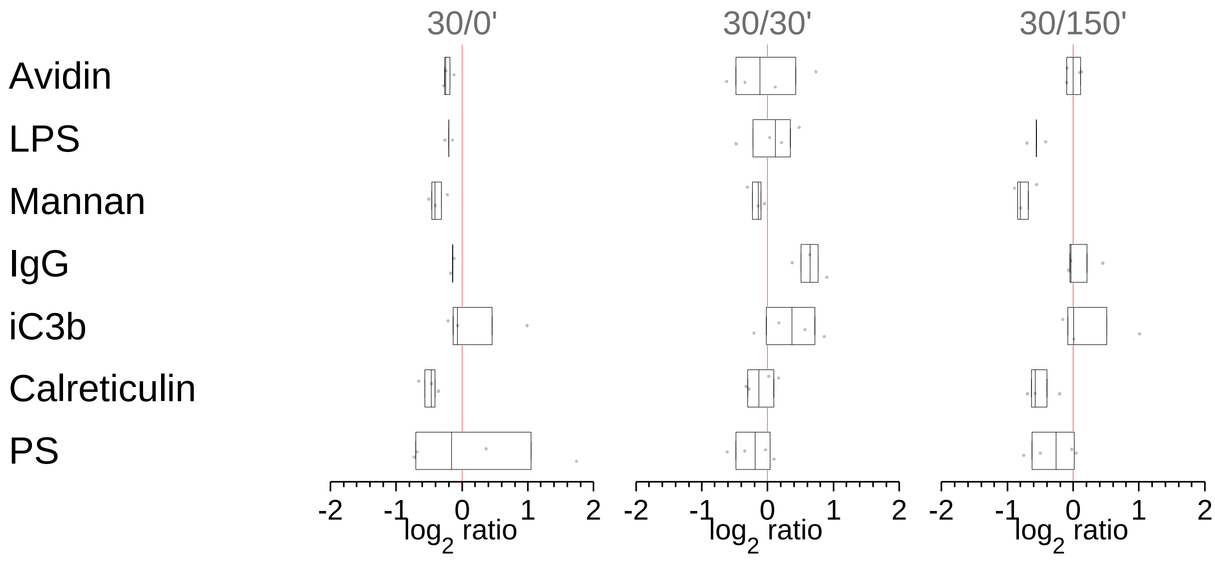

GO:0008484 sulfuric ester hydrolase activity (n = 7)

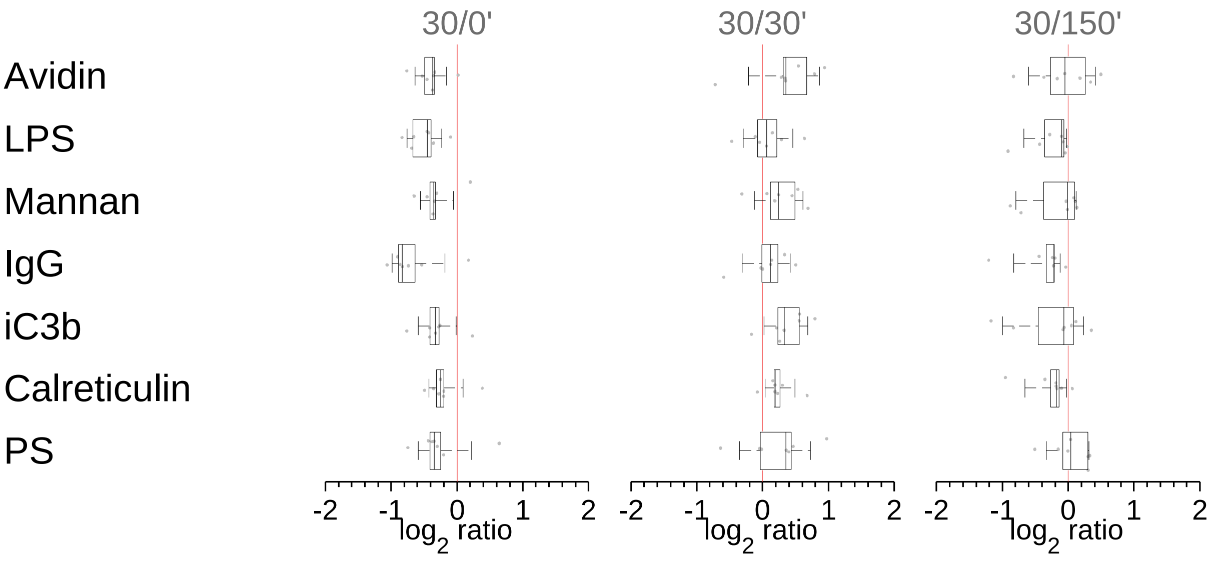

GO:0008494 translation activator activity (n = 4)

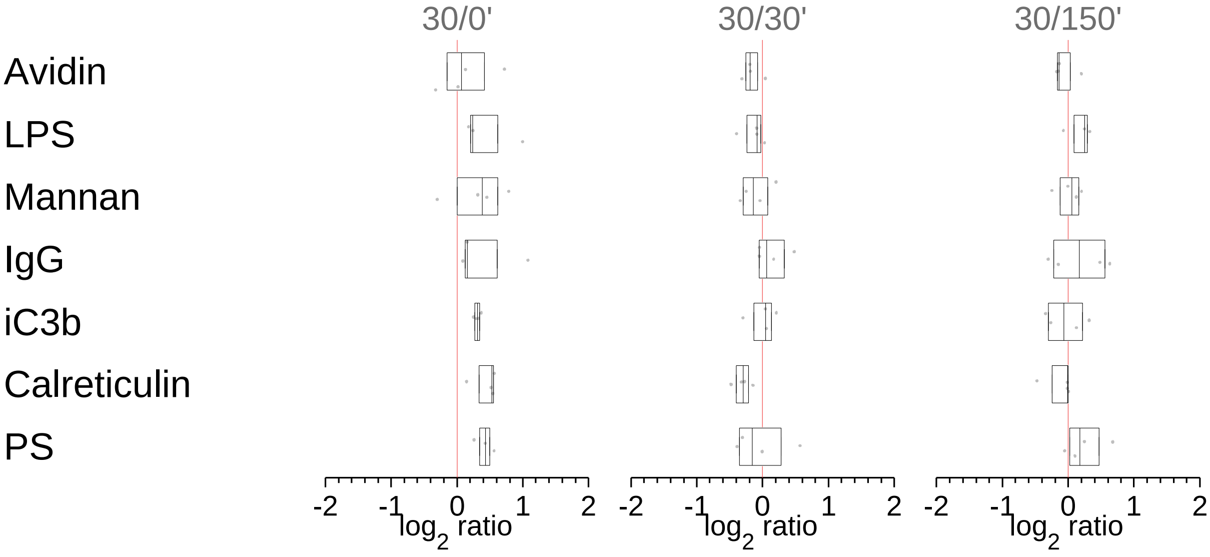

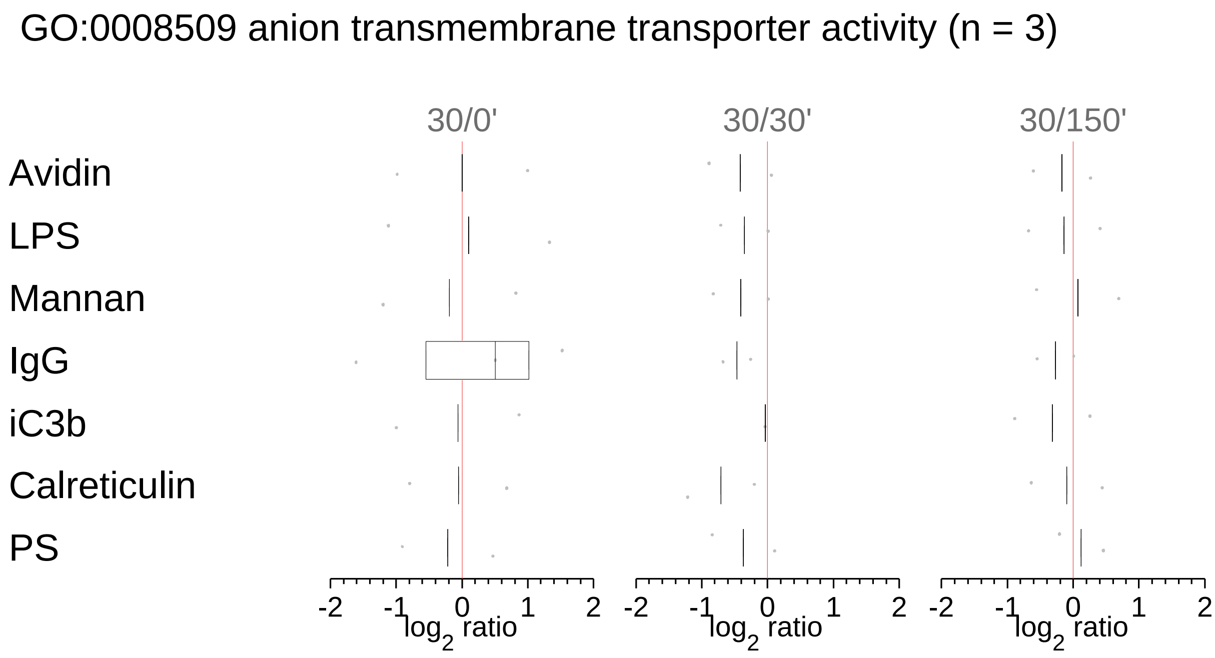
GO:0008509 anion transmembrane transporter activity (n = 3)
30/0'
30/30'
30/150'
Avidin
LPS
Mannan
IgG
iC3b
Calreticulin
PS
-2
-1
0
1
2
log2 ratio

GO:0008542 visual learning (n = 4)

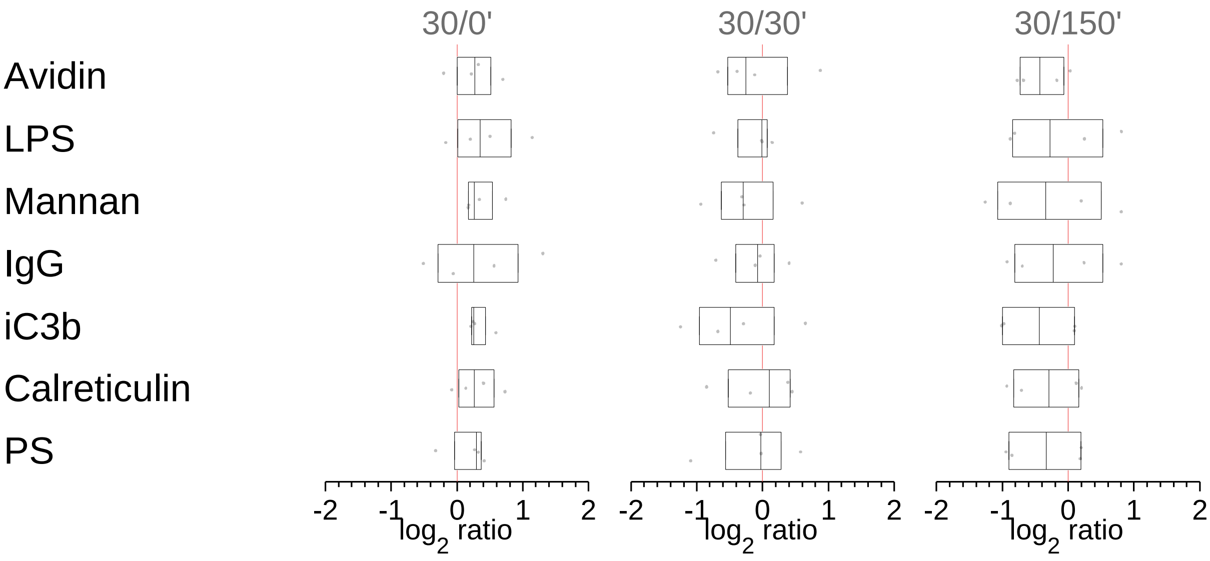

GO:0008543 fibroblast growth factor receptor signaling pathway
(n = 4)

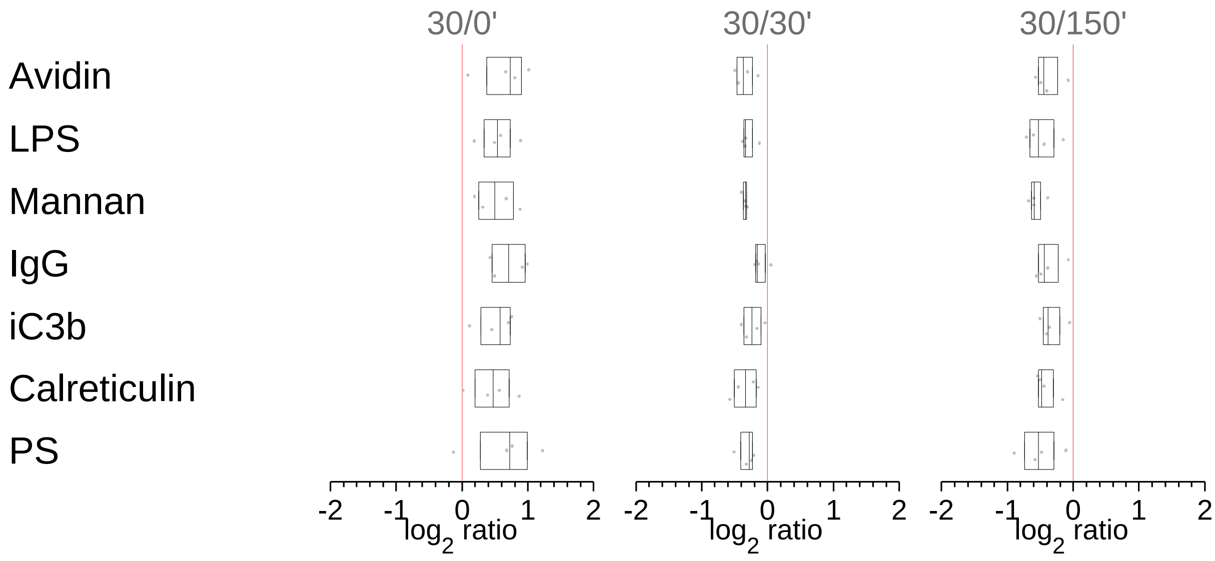

GO:0008553 hydrogen-exporting ATPase activity, phosphorylative mechanism (n = 5)

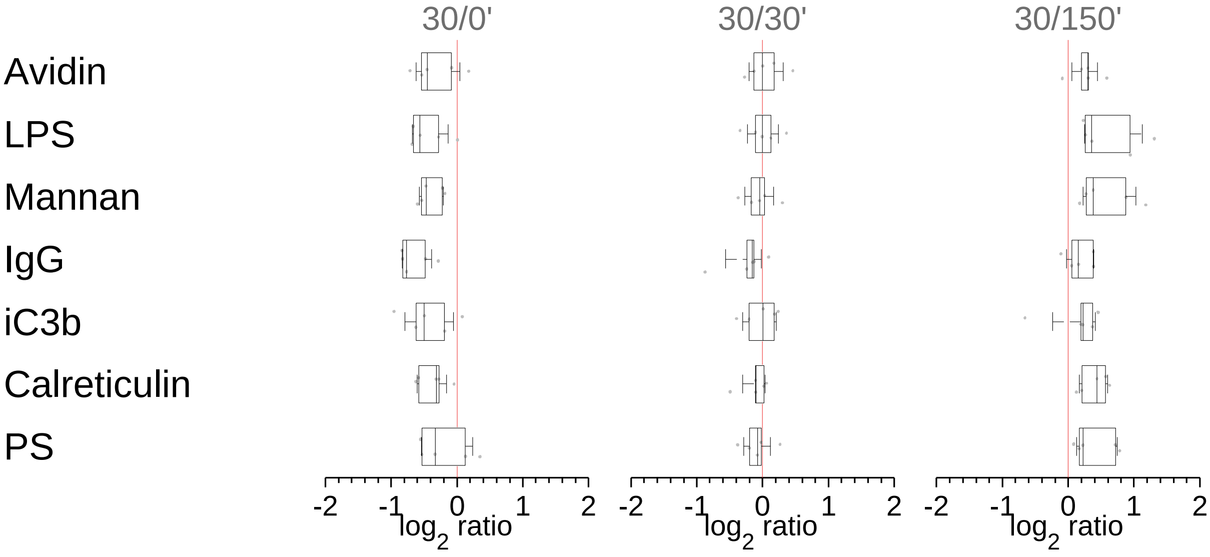

GO:0008559 xenobiotic-transporting ATPase activity (n = 3)

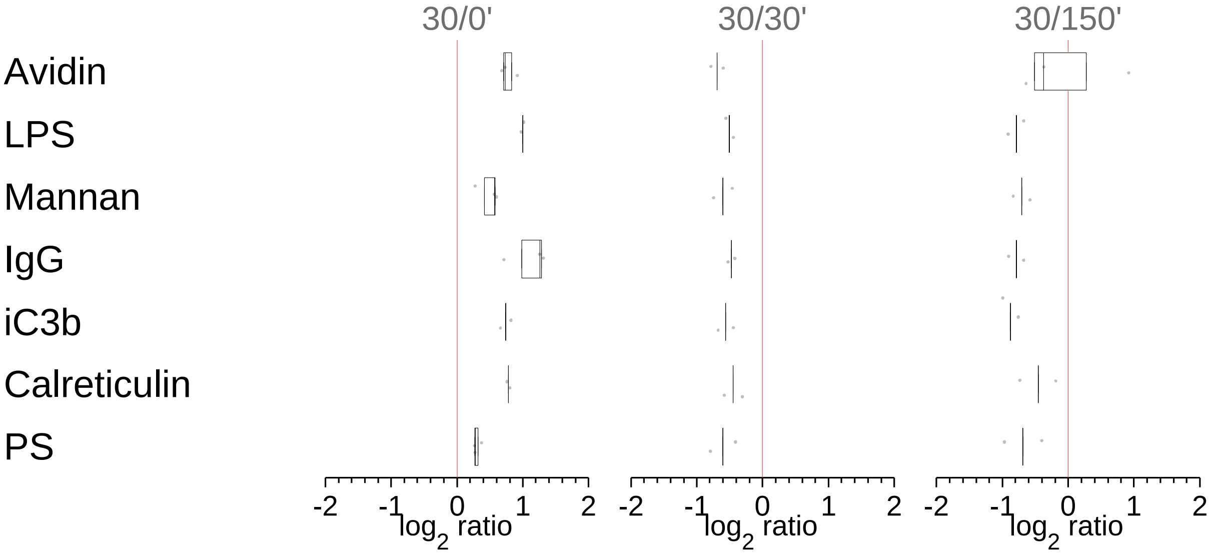

GO:0008565 protein transporter activity (n = 16)

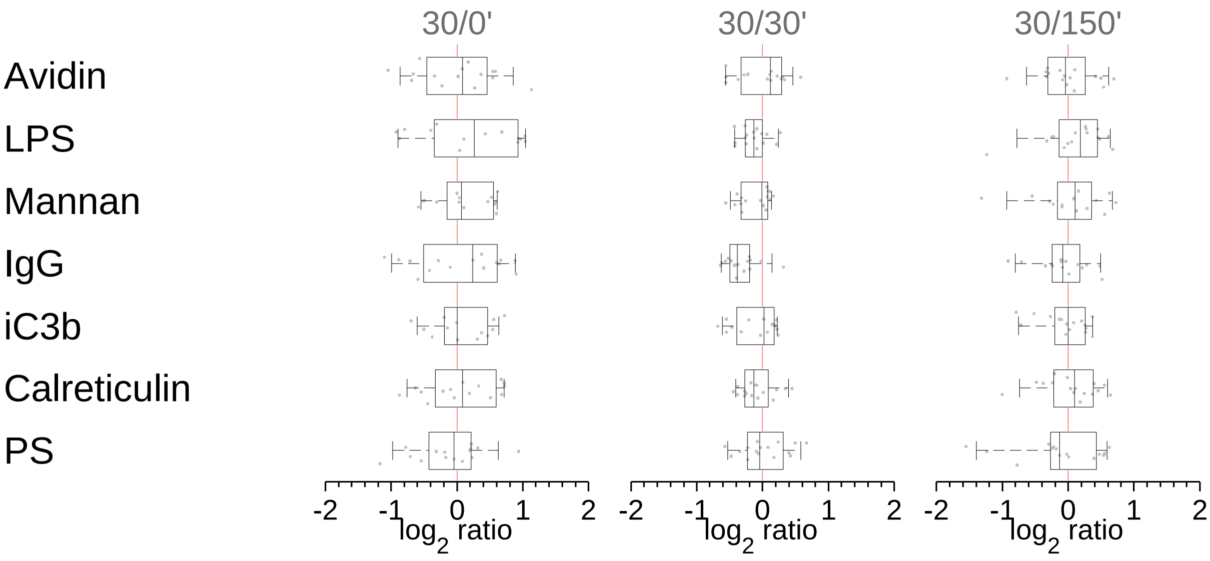

GO:0008584 male gonad development (n = 6)

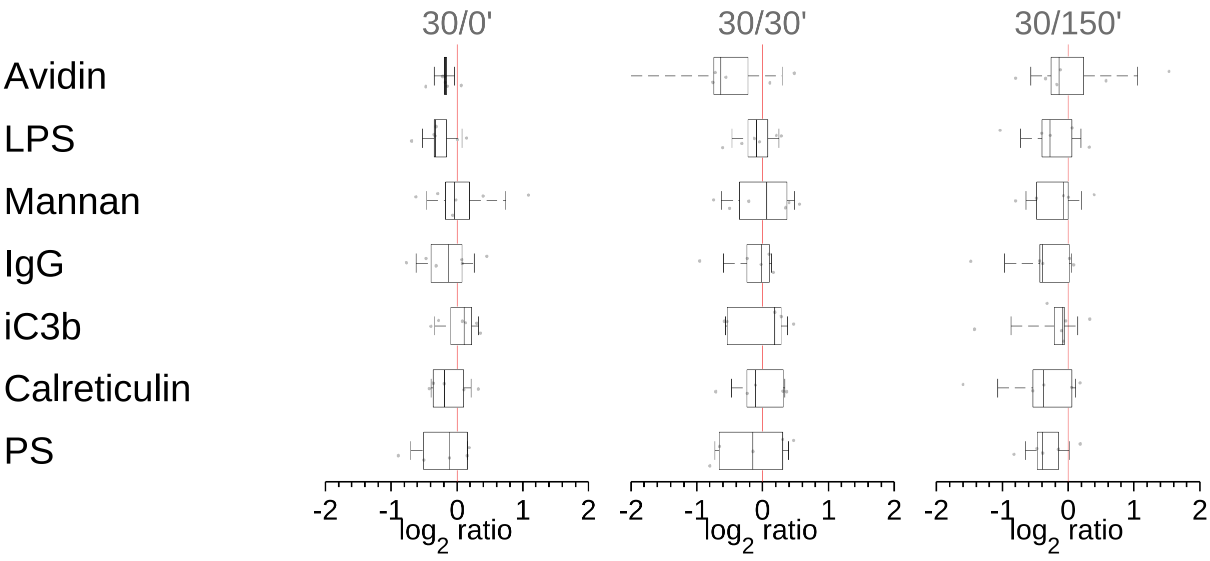

GO:0008585 female gonad development (n = 3)

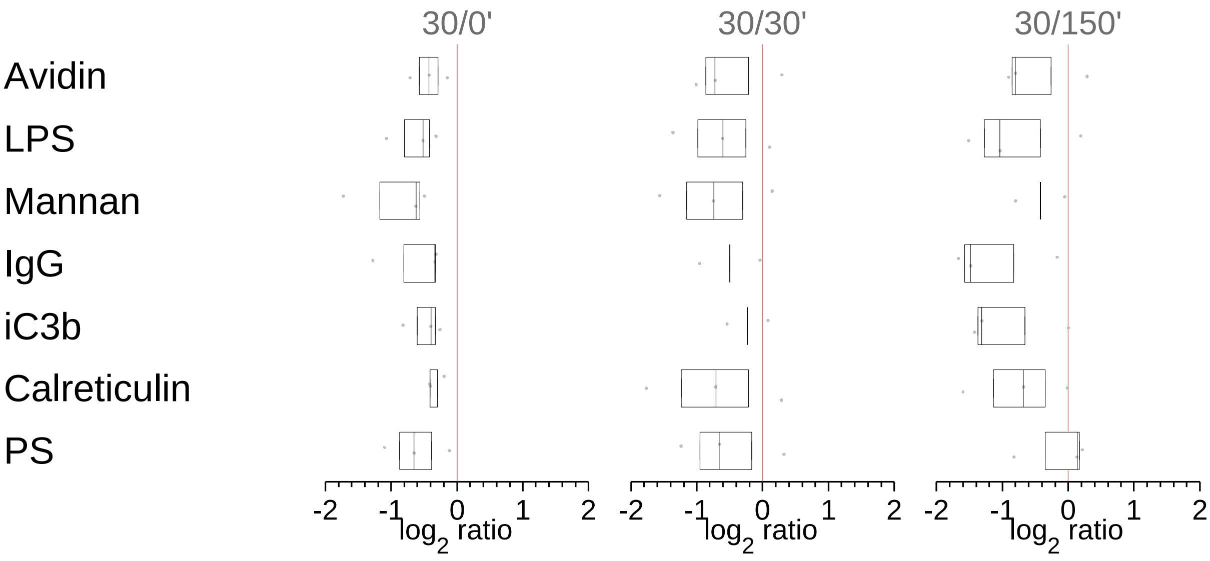

GO:0008610 lipid biosynthetic process (n = 4)

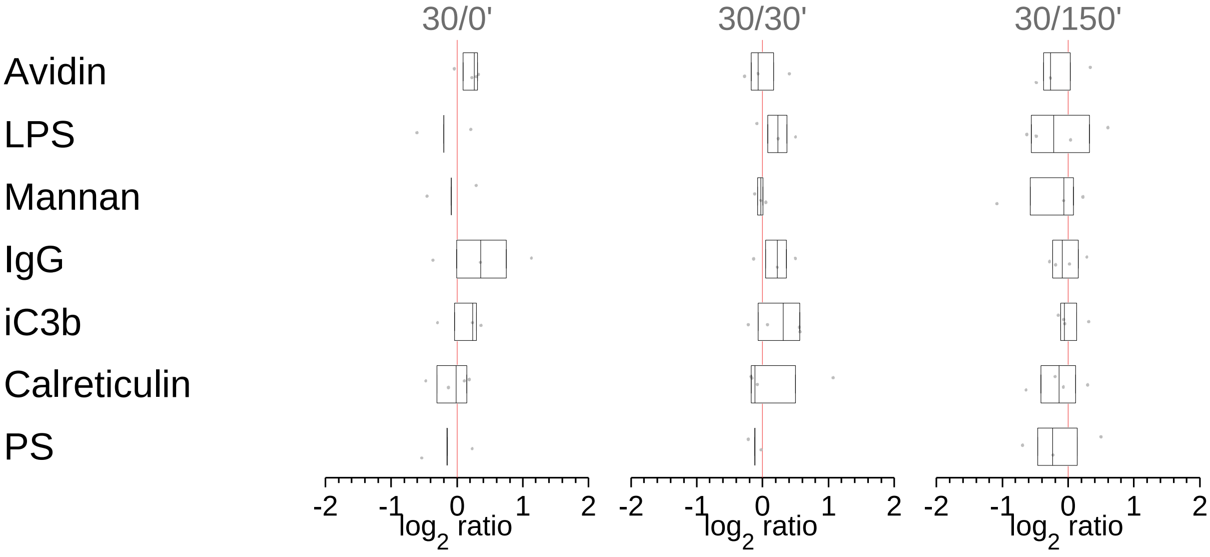

GO:0008625 induction of apoptosis via death domain receptors (n = 6)

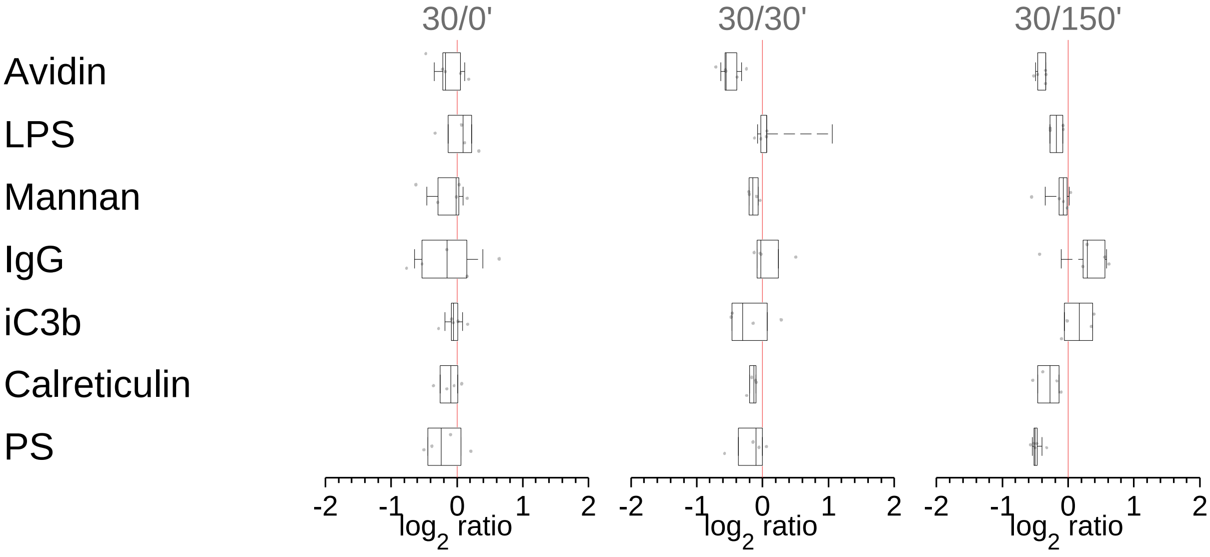

GO:0008630 DNA damage response, signal transduction resulting in induction of apoptosis (n = 9)

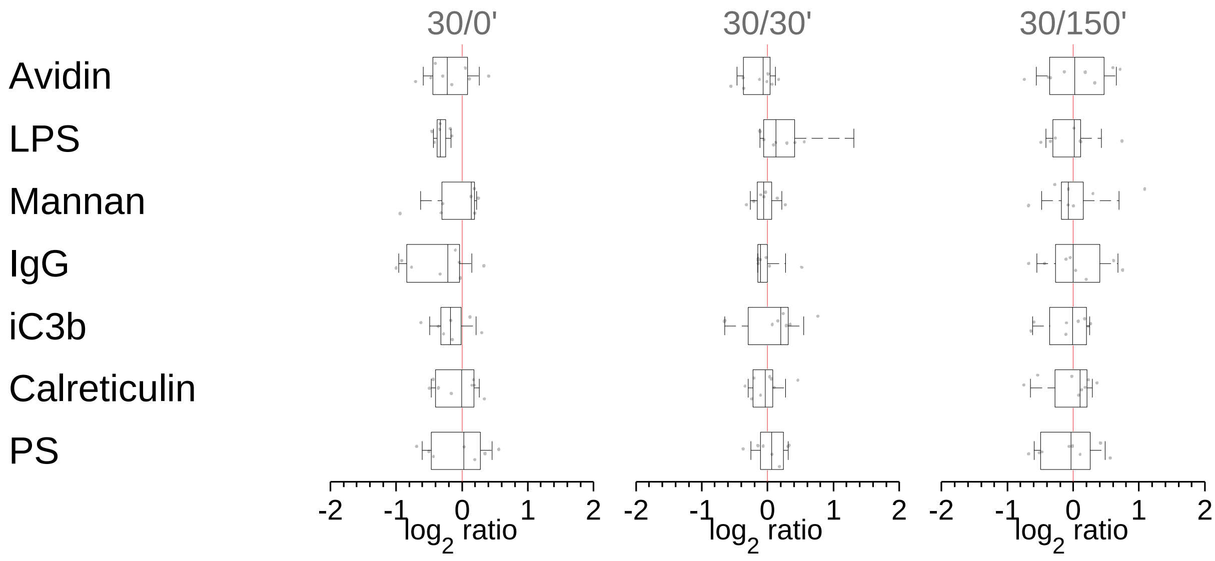

GO:0008631 induction of apoptosis by oxidative stress (n = 4)

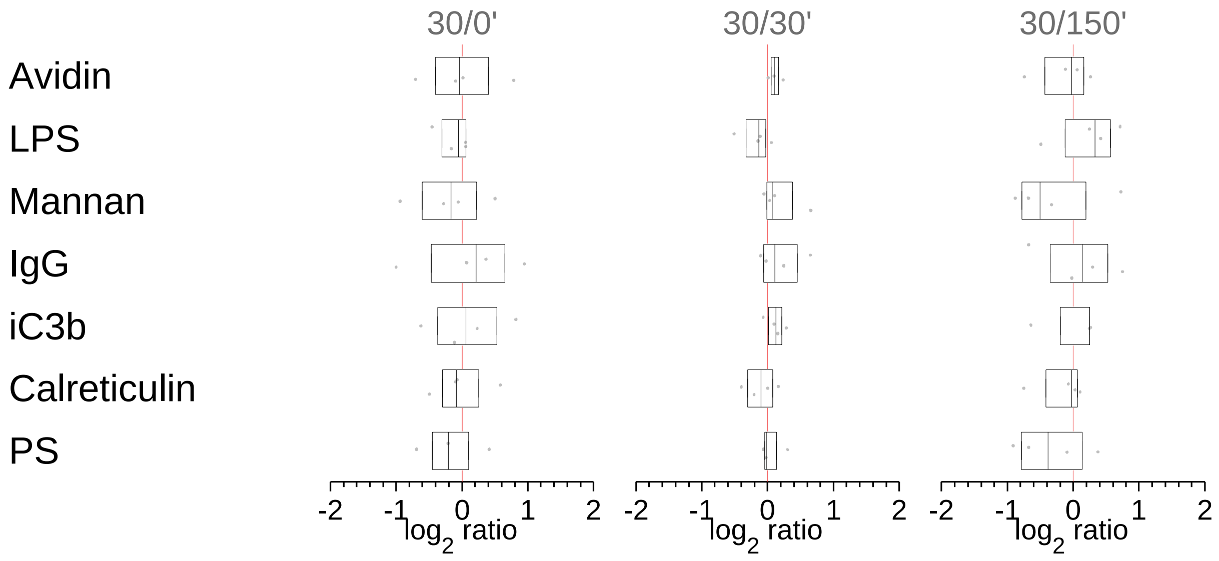

GO:0008637 apoptotic mitochondrial changes (n = 11)

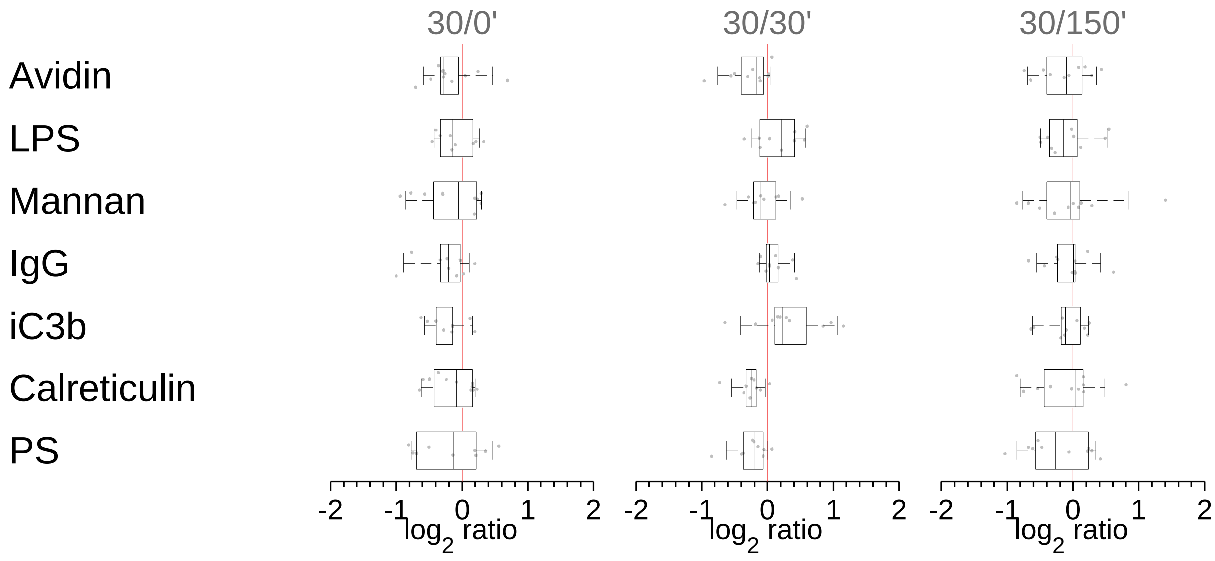

GO:0008643 carbohydrate transport (n = 4)

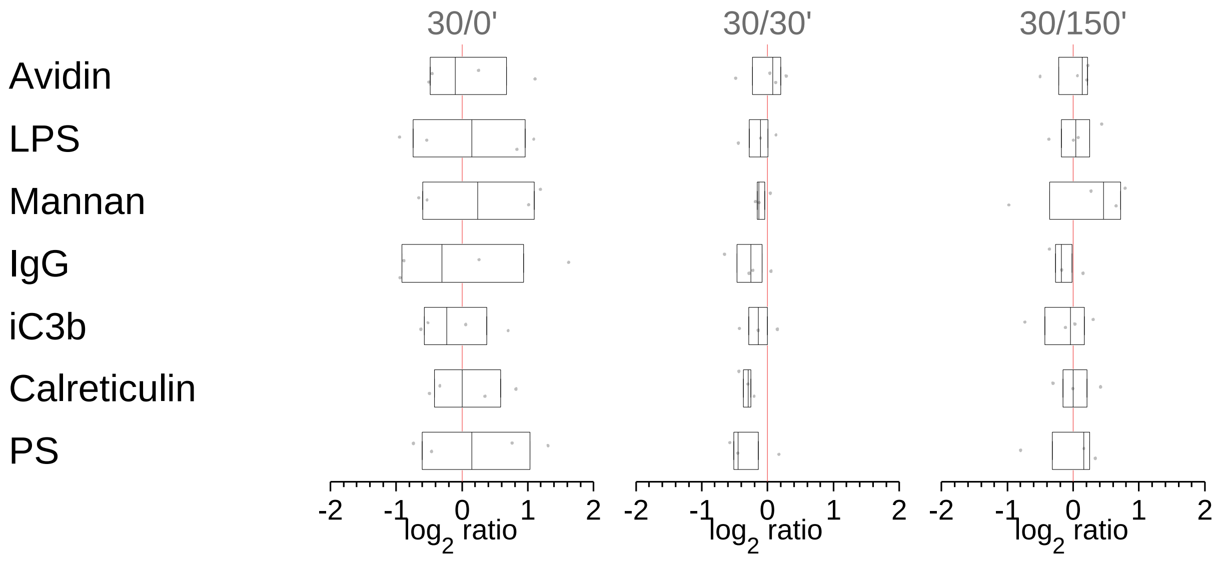

GO:0008654 phospholipid biosynthetic process (n = 10)

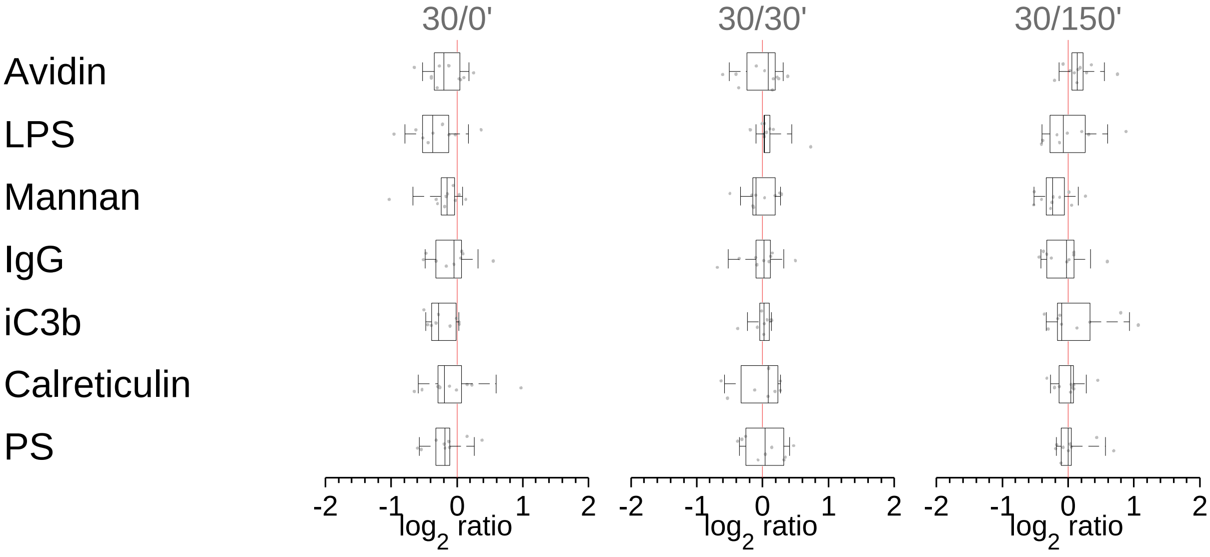

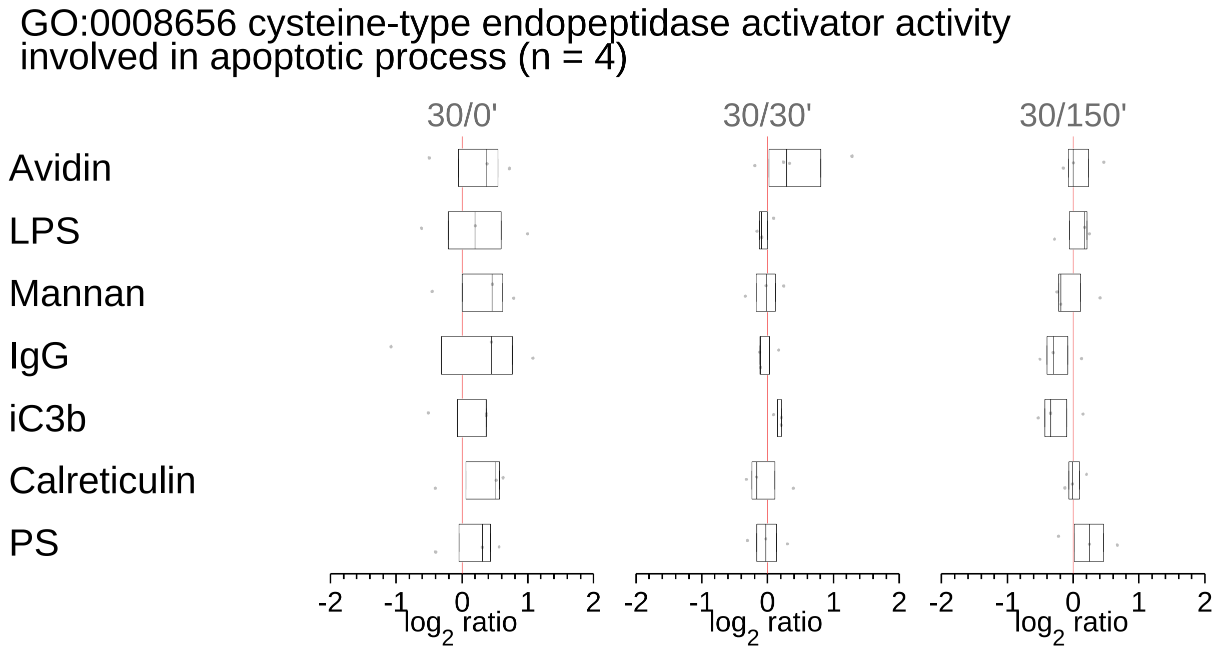
GO:0008656 cysteine-type endopeptidase activator activity
involved in apoptotic process (n = 4)
30/0'
30/30'
30/150'
Avidin
LPS
Mannan
IgG
iC3b
Calreticulin
PS
-2
-1
0
1
2
log2 ratio

GO:0008748 N-ethylmaleimide reductase activity (n = 9)

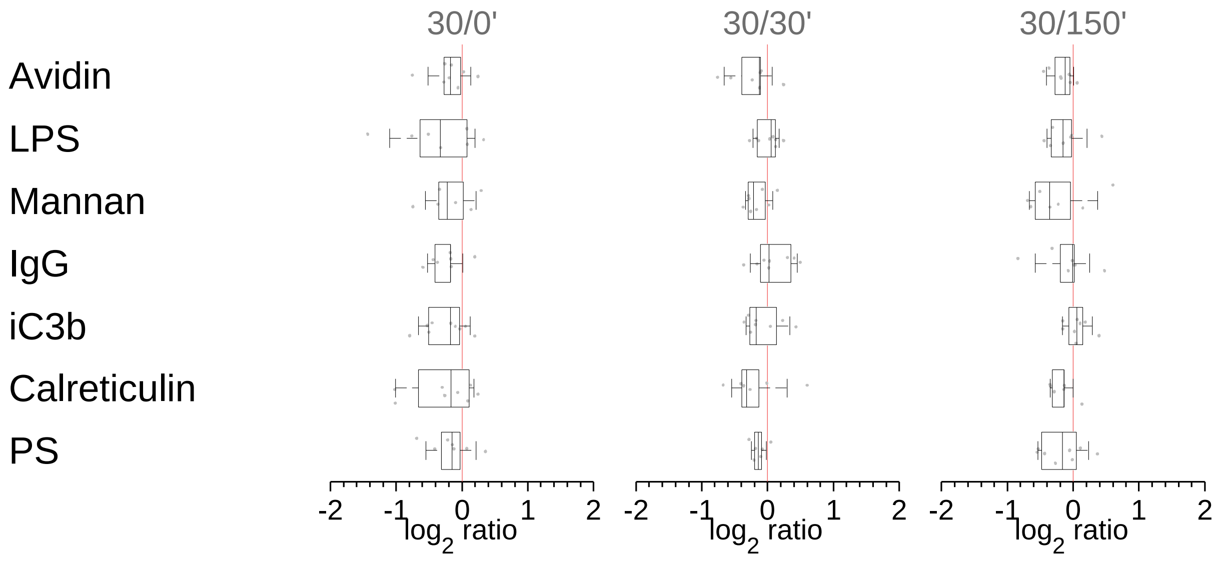

GO:0008755 O antigen polymerase activity (n = 3)

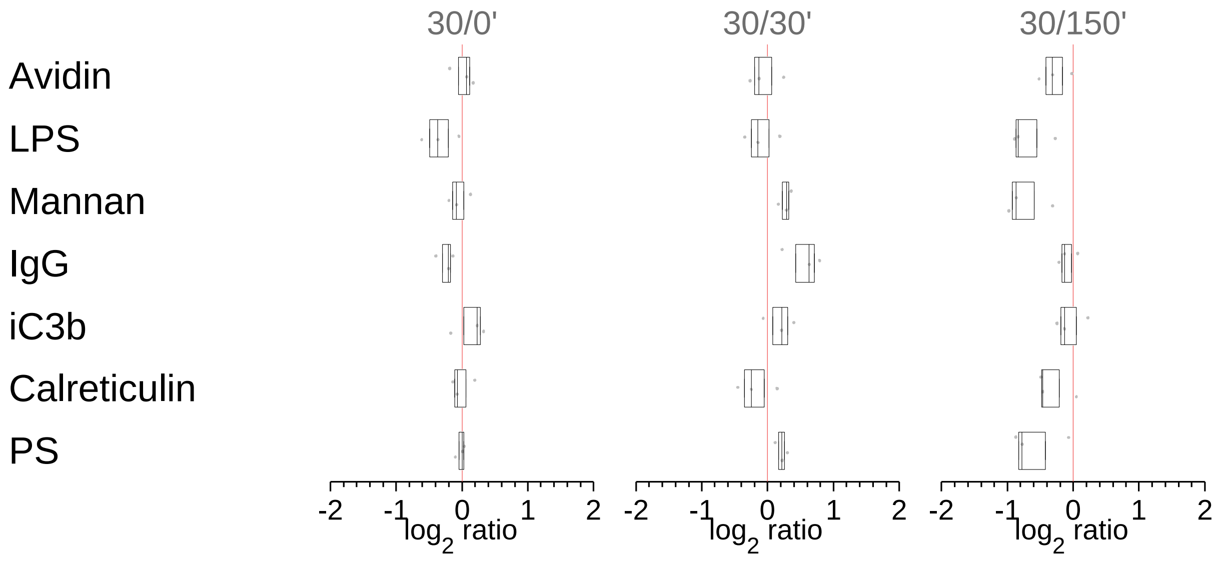

GO:0008766
UDP-N-acetylmuramoylalanyl-D-glutamyl-2,6-diaminopimelate-D-alanyl-D-alanine ligase activity (n = 17)

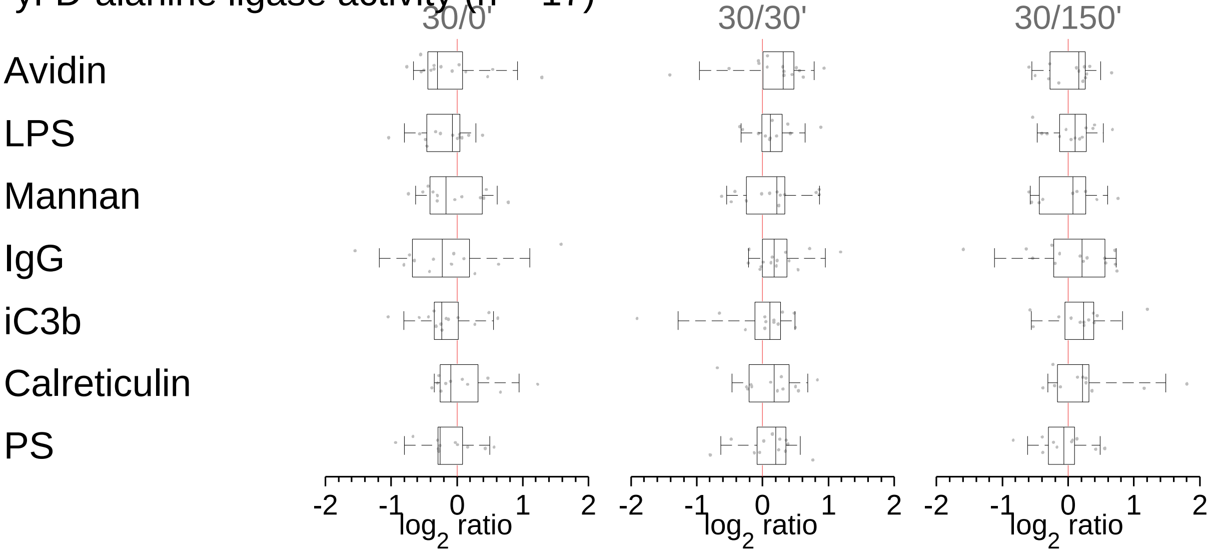

GO:0008875 gluconate dehydrogenase activity (n = 3)

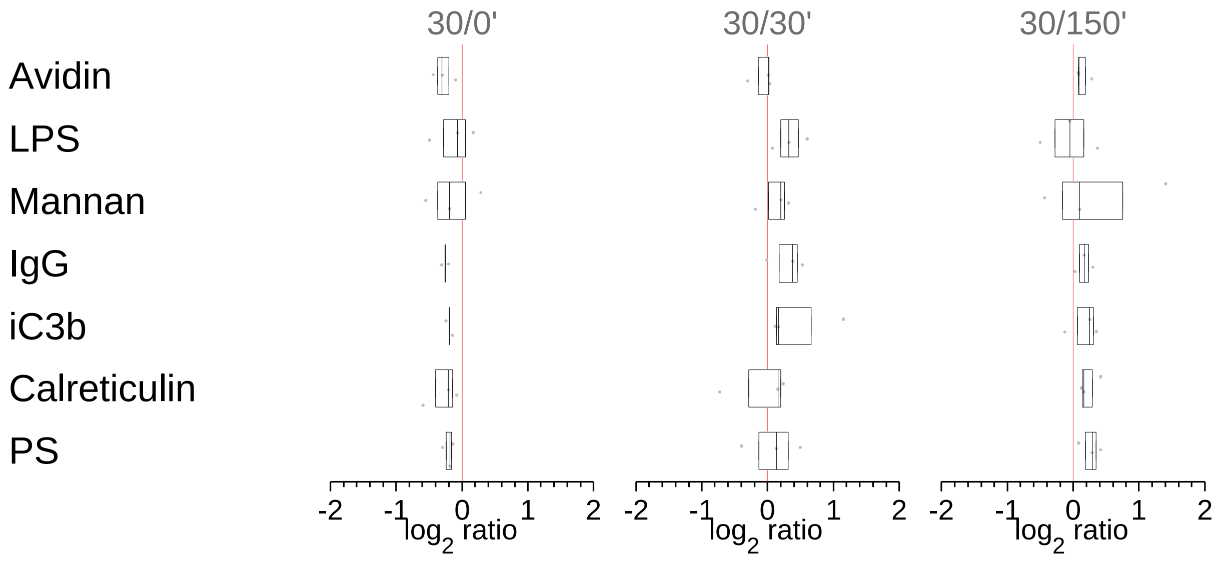

GO:0008921 lipopolysaccharide-1,6-galactosyltransferase activity
(n = 3)

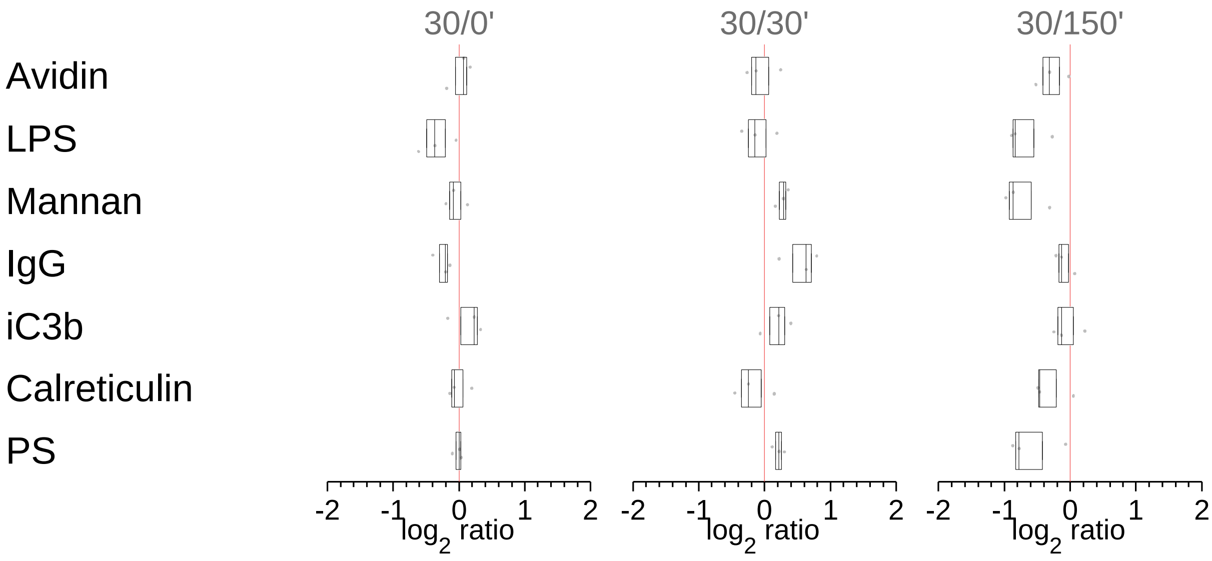

GO:0008951 palmitoleoyl [acyl-carrier-protein]-dependent acyltransferase activity (n = 3)

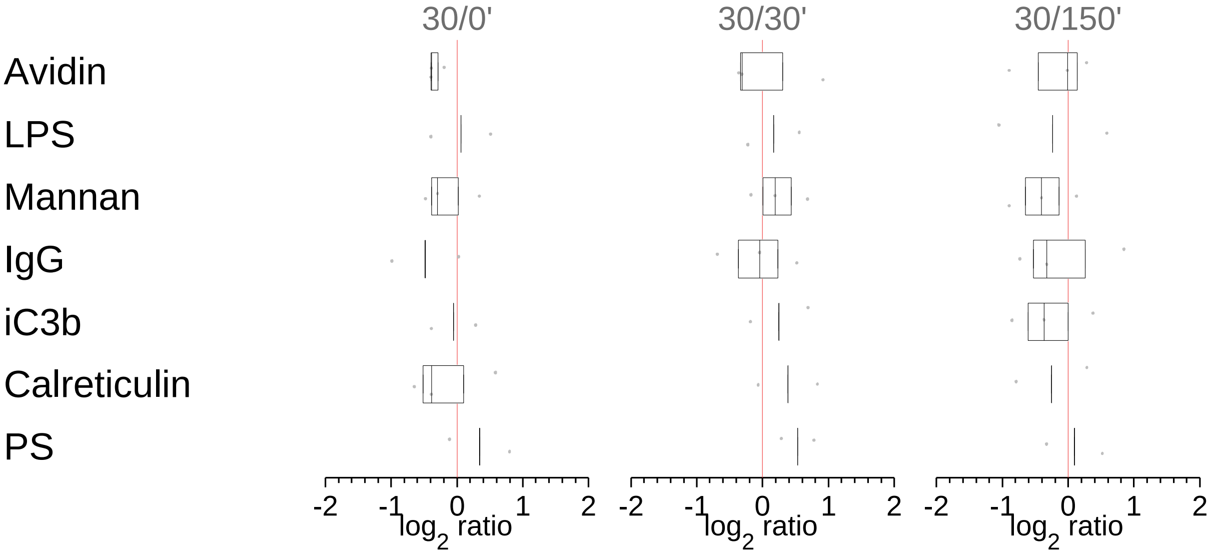

GO:0009055 electron carrier activity (n = 12)

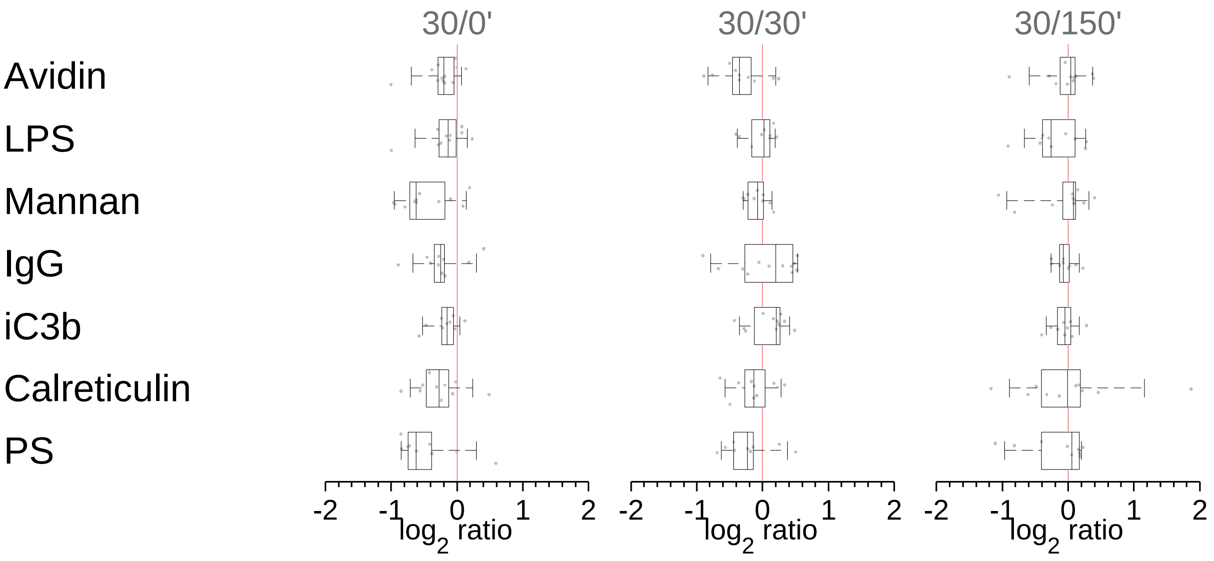

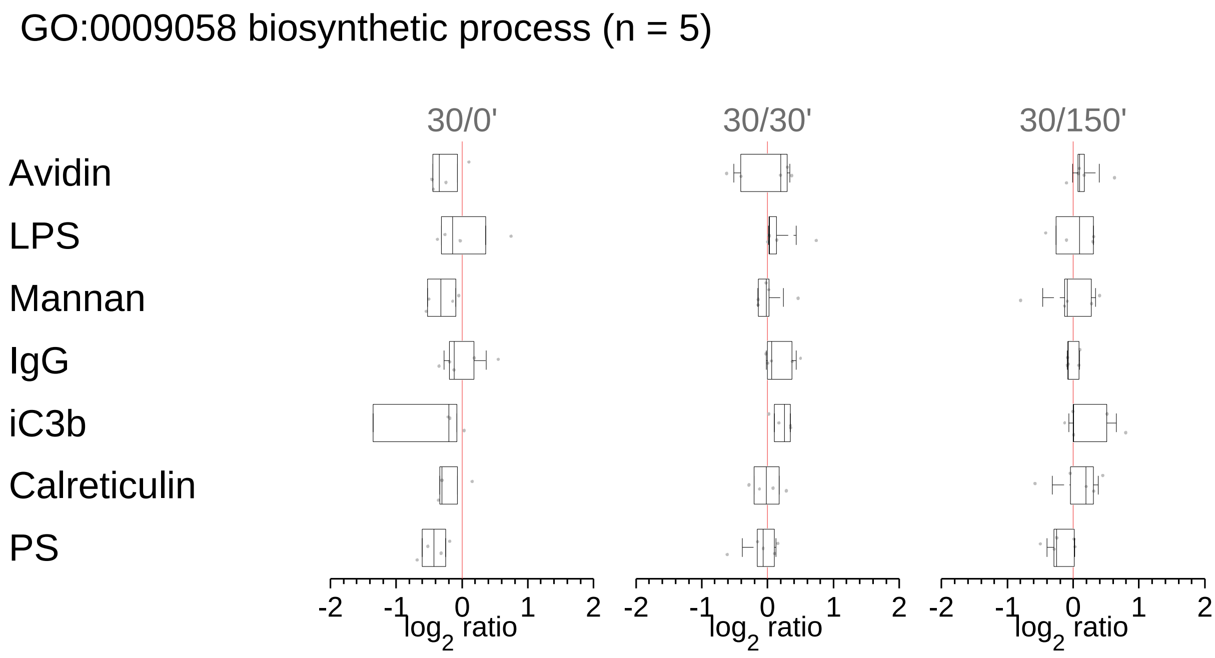

GO:0009058 biosynthetic process (n = 5)
30/0'
30/30'
30/150'
Avidin
LPS
Mannan
IgG
iC3b
Calreticulin
PS
-2 -1 0 1 2
log2 ratio

GO:0009268 response to pH (n = 5)

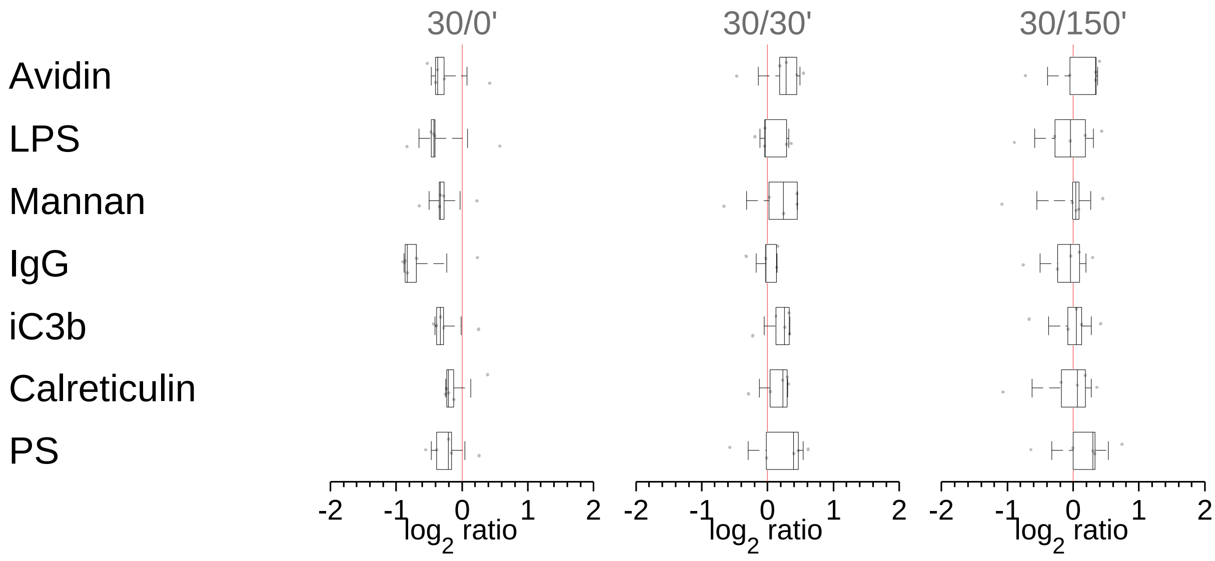

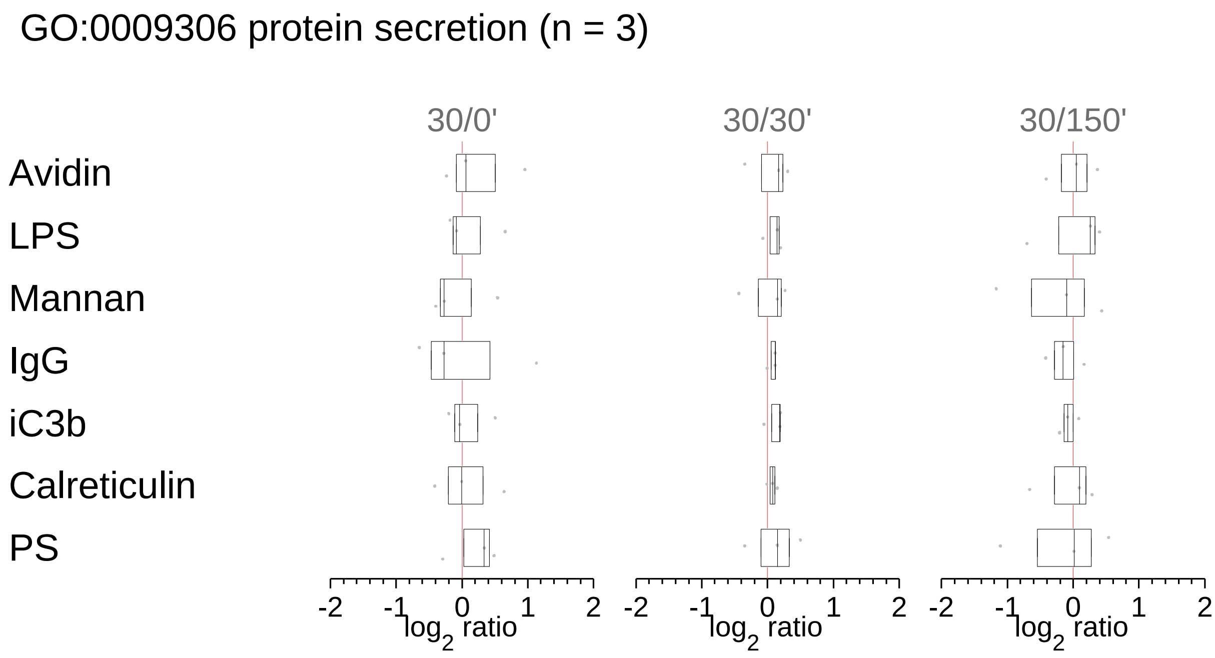
GO:0009306 protein secretion (n = 3)
30/0'
30/30'
30/150'
Avidin
LPS
Mannan
IgG
iC3b
Calreticulin
PS
-2
-1
0
1
2
log2 ratio

GO:0009313 oligosaccharide catabolic process (n = 4)

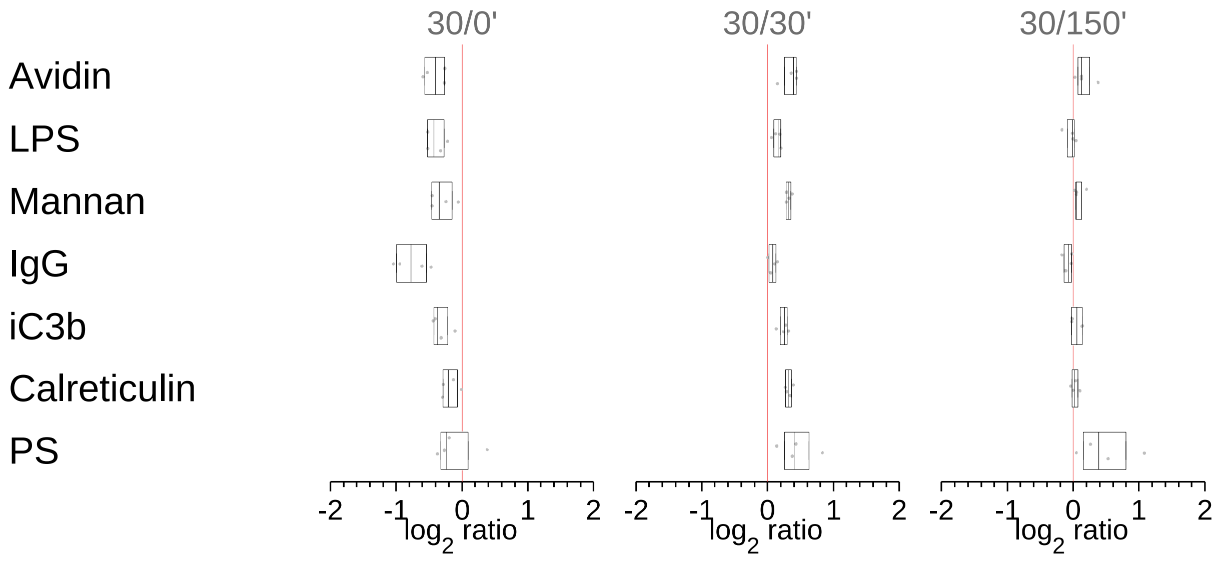

GO:0009408 response to heat (n = 5)

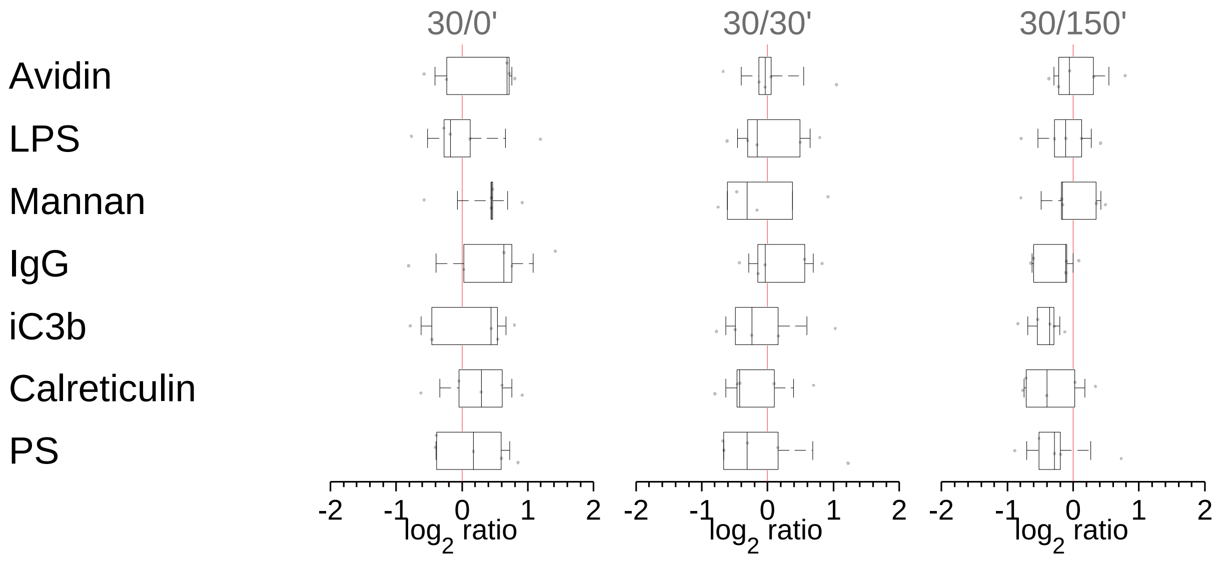

GO:0009437 carnitine metabolic process (n = 3)

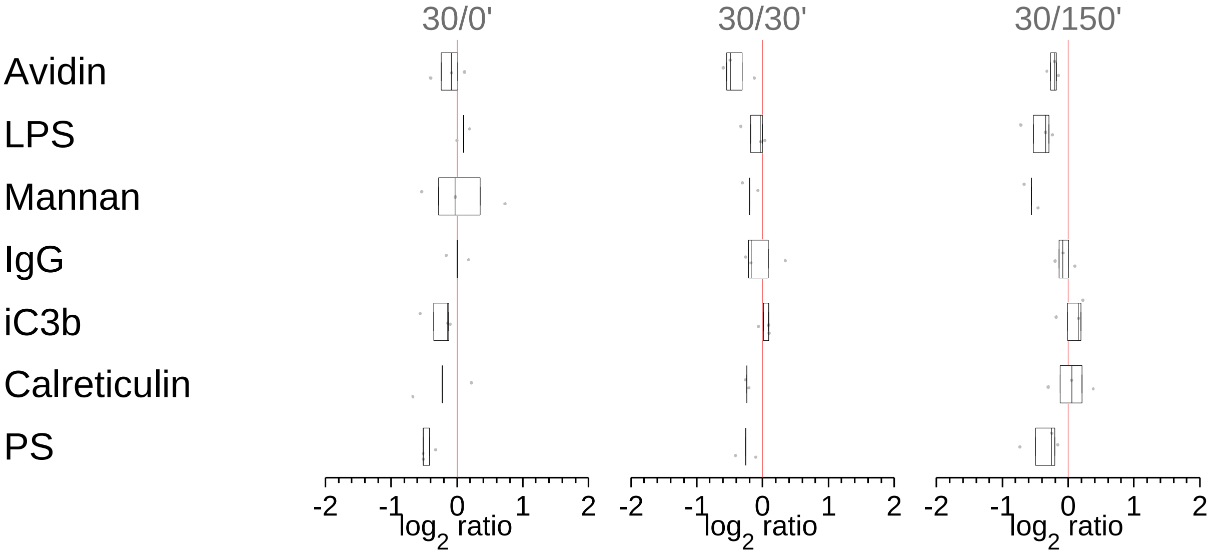

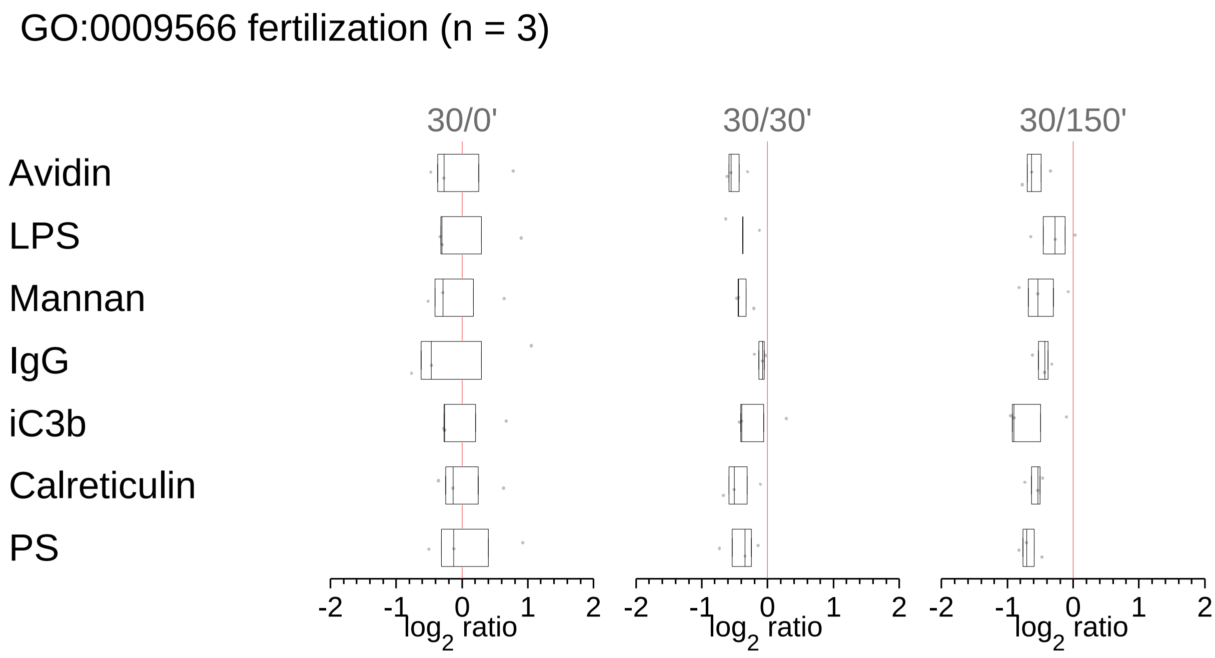

GO:0009566 fertilization (n = 3)
30/0'
30/30'
30/150'
Avidin
LPS
Mannan
IgG
iC3b
Calreticulin
PS
-2
-1
0
1
2
log2 ratio

GO:0009611 response to wounding (n = 4)

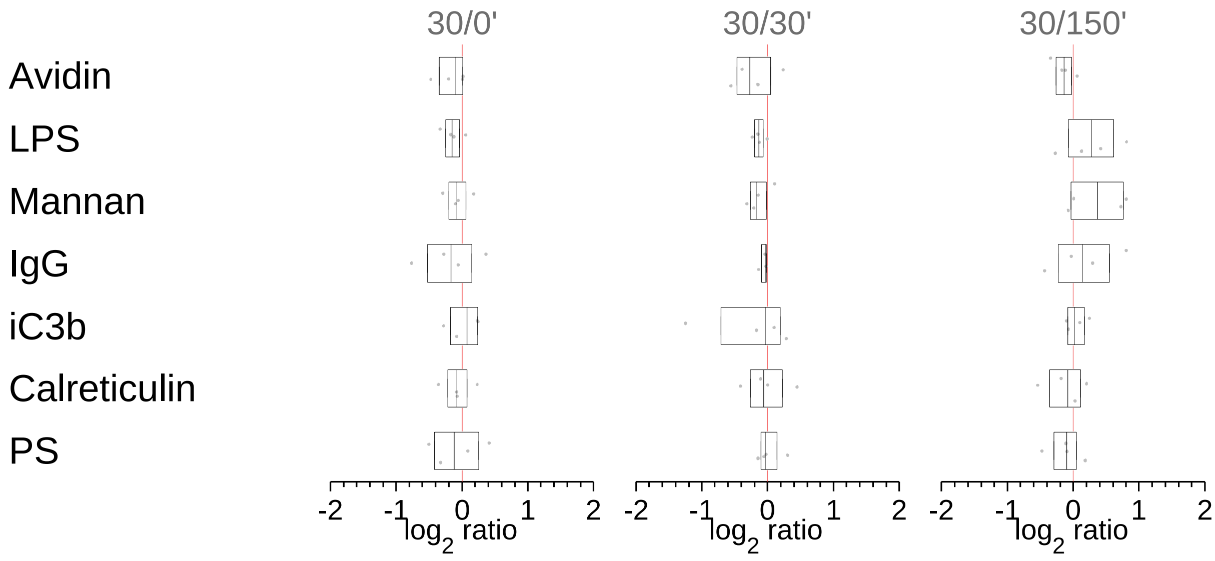

GO:0009612 response to mechanical stimulus (n = 4)

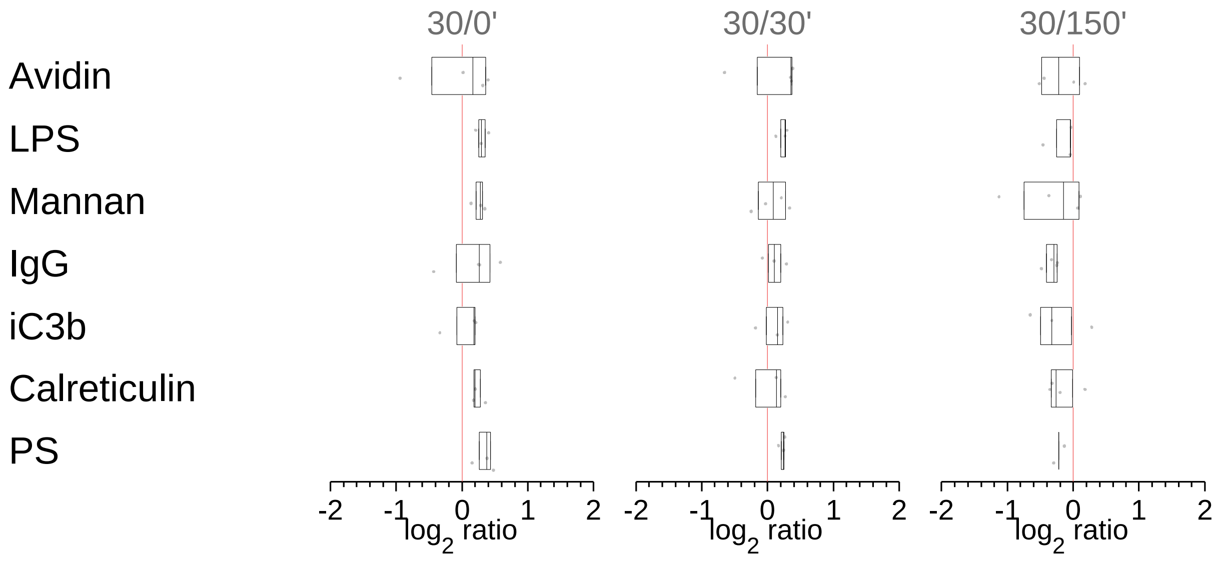

GO:0009615 response to virus (n = 16)

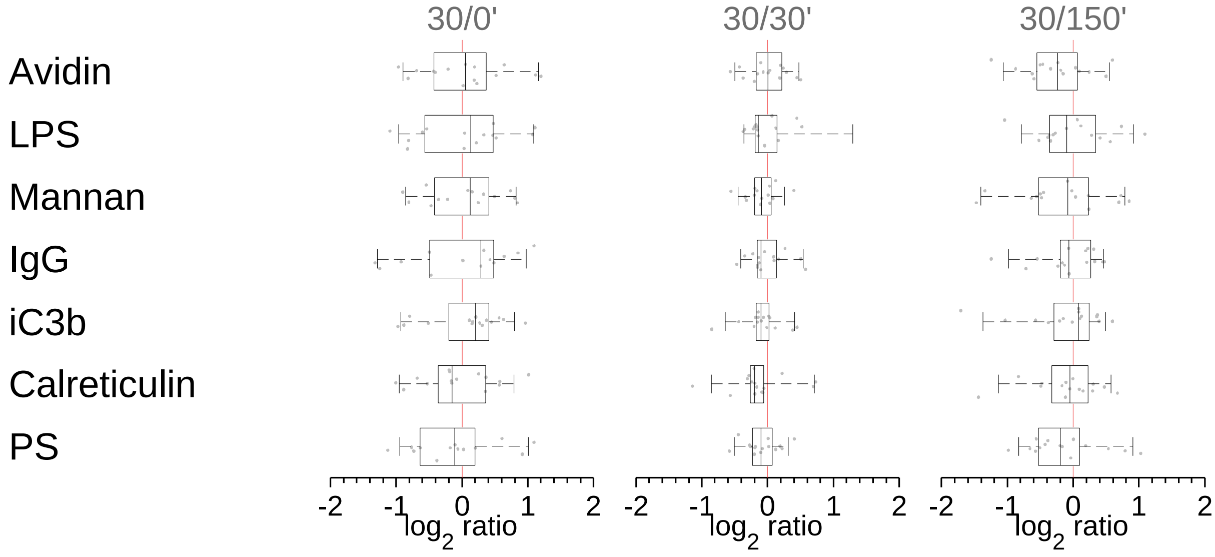

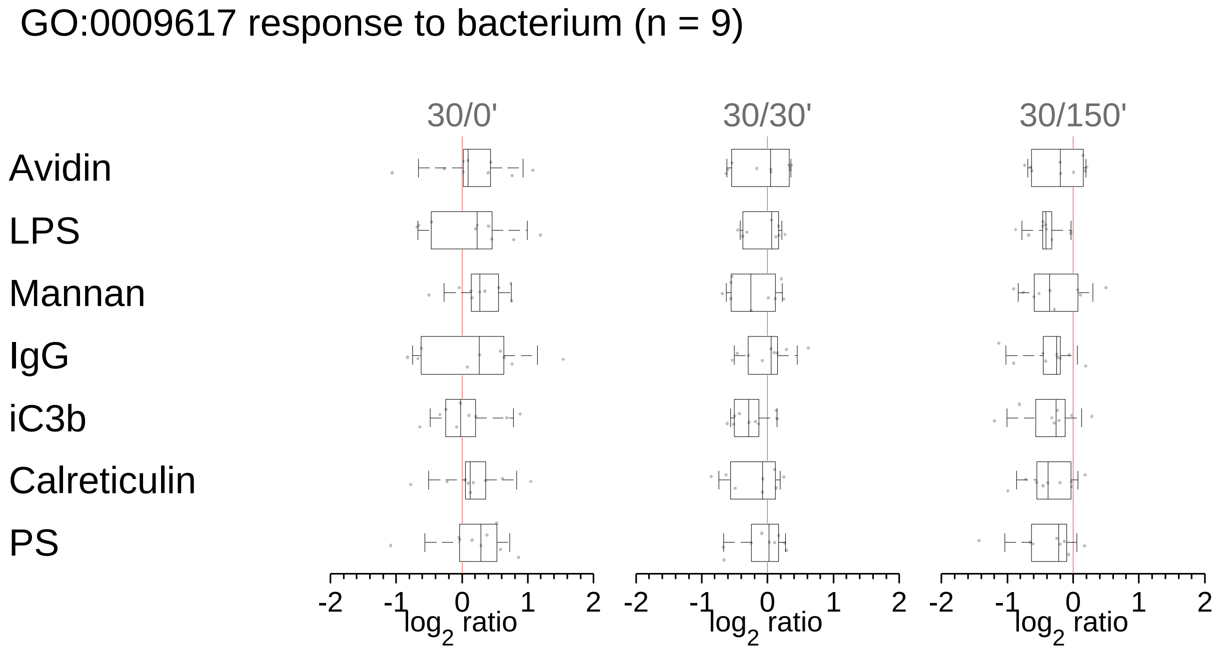

GO:0009617 response to bacterium (n = 9)
30/0'
30/30'
30/150'
Avidin
LPS
Mannan
IgG
iC3b
Calreticulin
PS
-2
-1
0
1
2
log2 ratio

GO:0009636 response to toxin (n = 6)

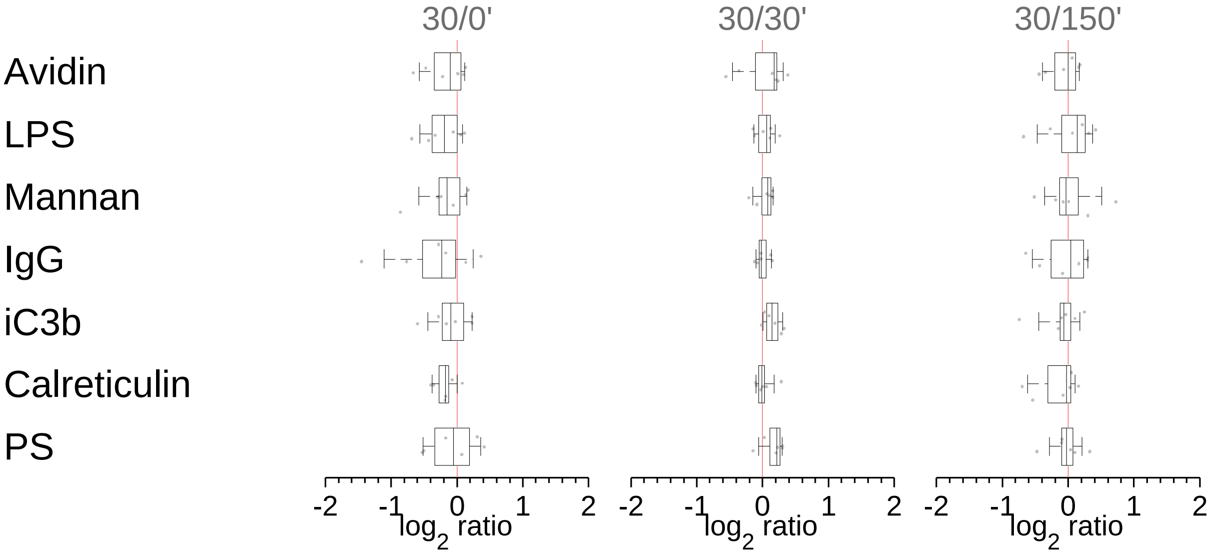

GO:0009749 response to glucose stimulus (n = 3)

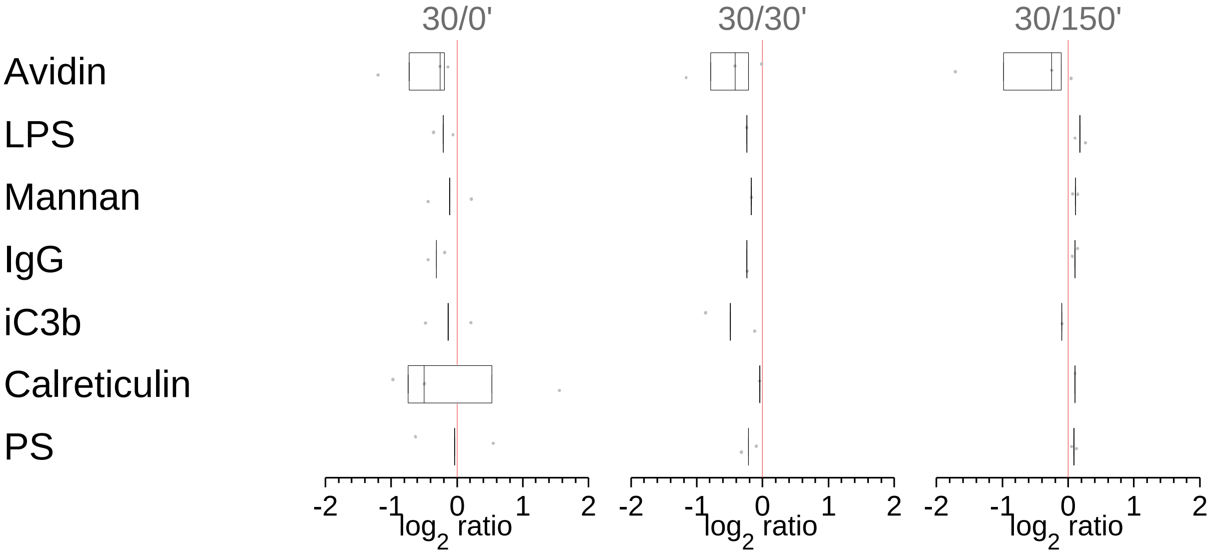

GO:0009790 embryo development (n = 8)

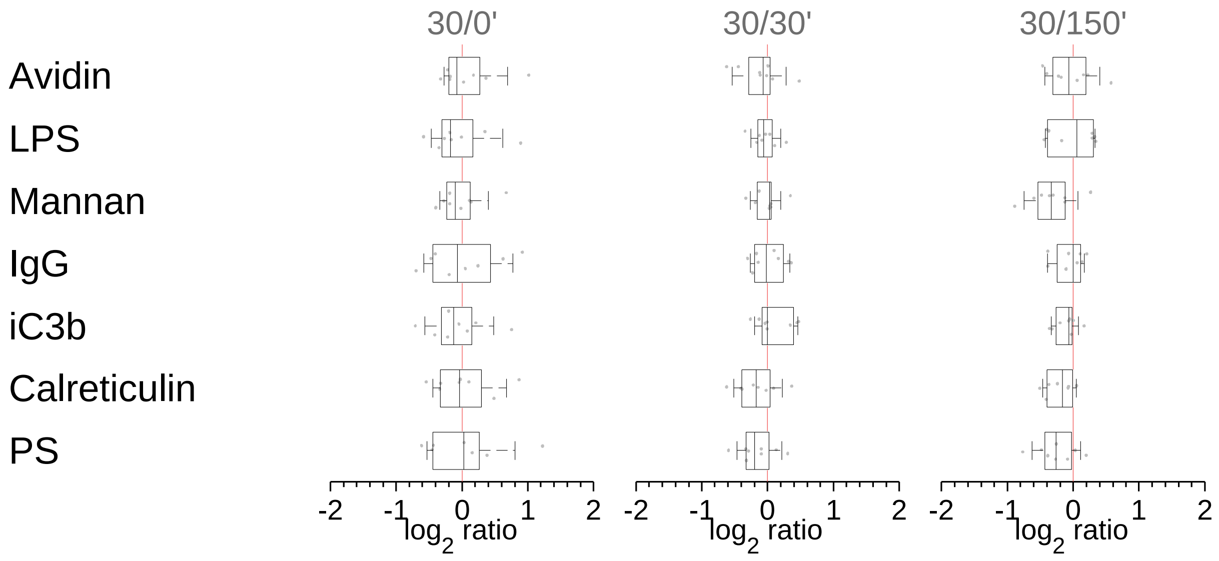

GO:0009791 post-embryonic development (n = 10)

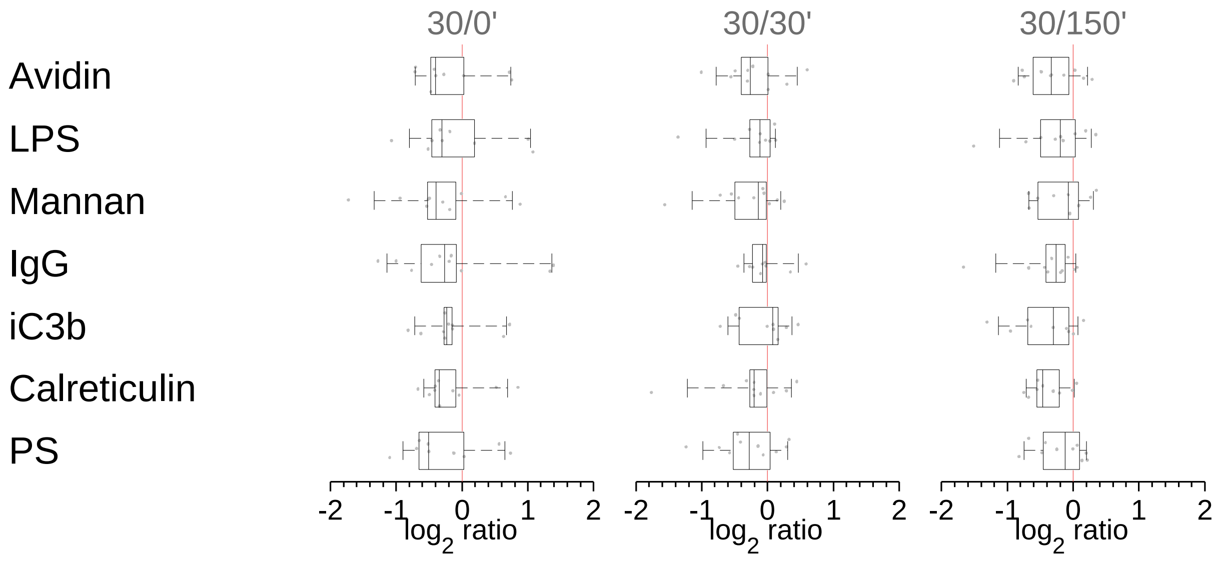

GO:0009838 abscission (n = 3)

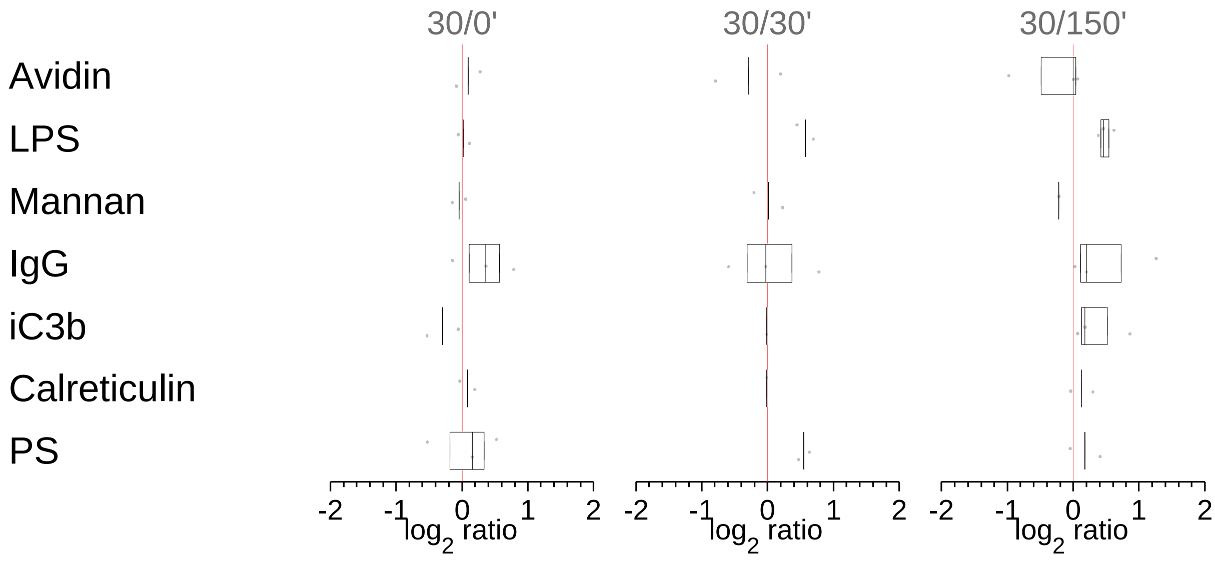

GO:0009887 organ morphogenesis (n = 3)

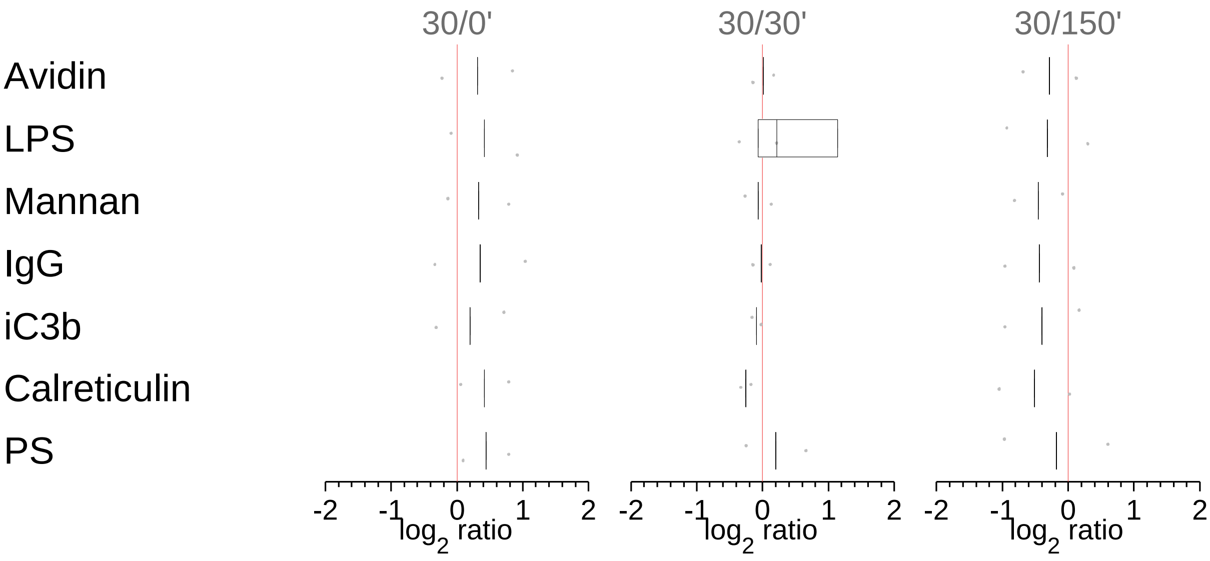

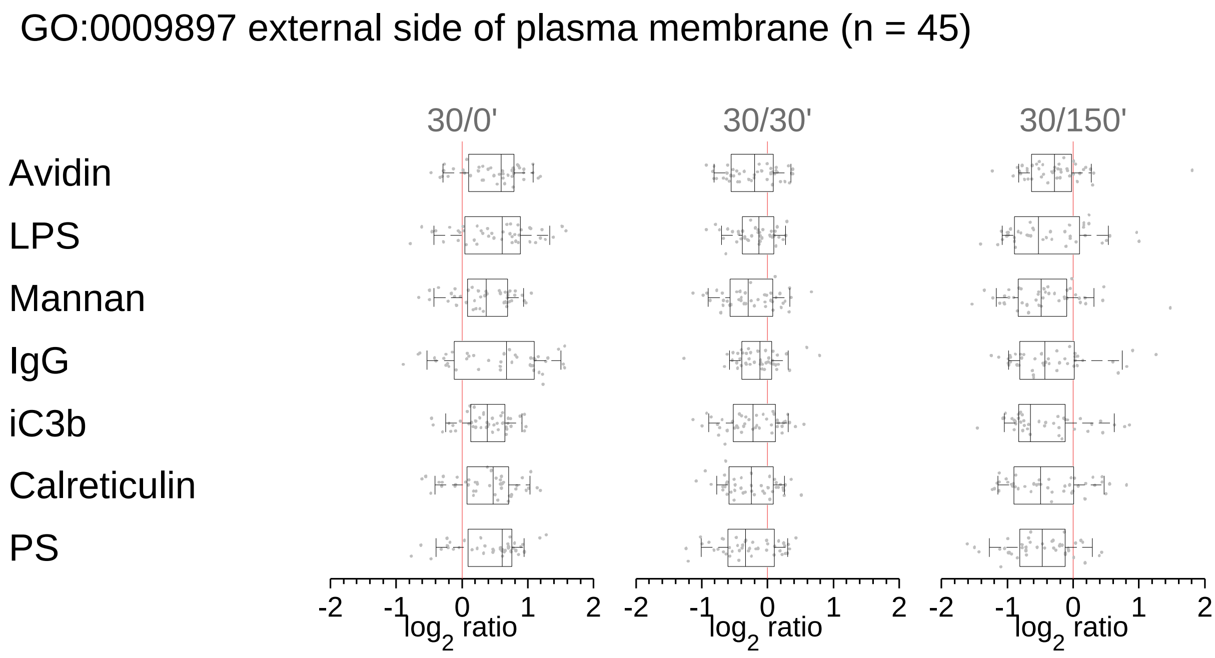
GO:0009897 external side of plasma membrane (n = 45)
30/0'
30/30'
30/150'
Avidin
LPS
Mannan
IgG
iC3b
Calreticulin
PS
-2
-1
0
1
2
log2 ratio

GO:0009898 internal side of plasma membrane (n = 6)

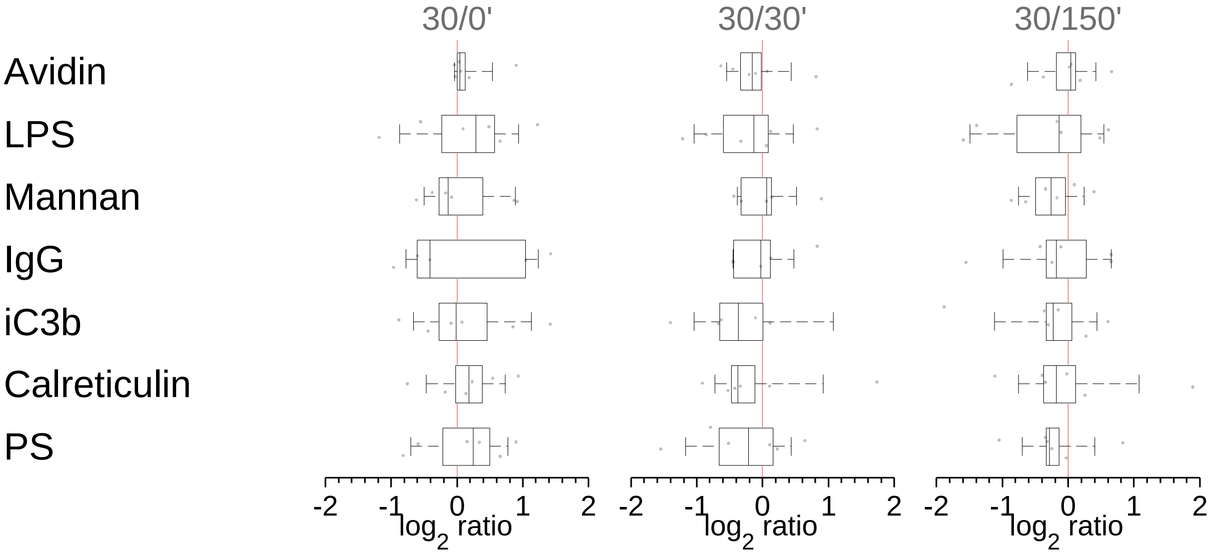

GO:0009925 basal plasma membrane (n = 4)

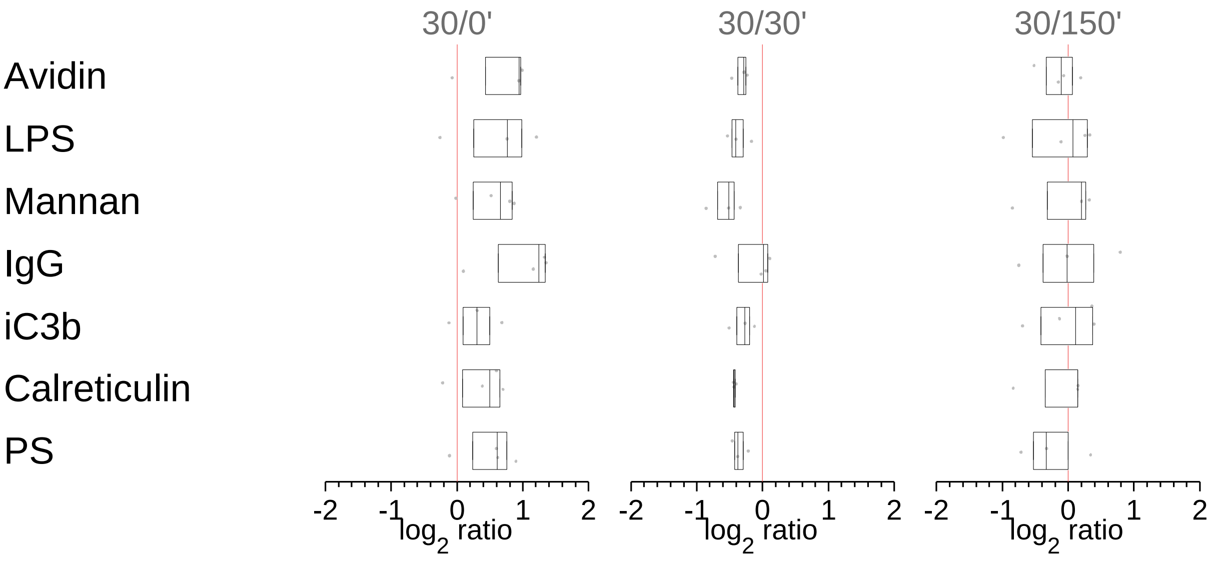

GO:0009966 regulation of signal transduction (n = 3)

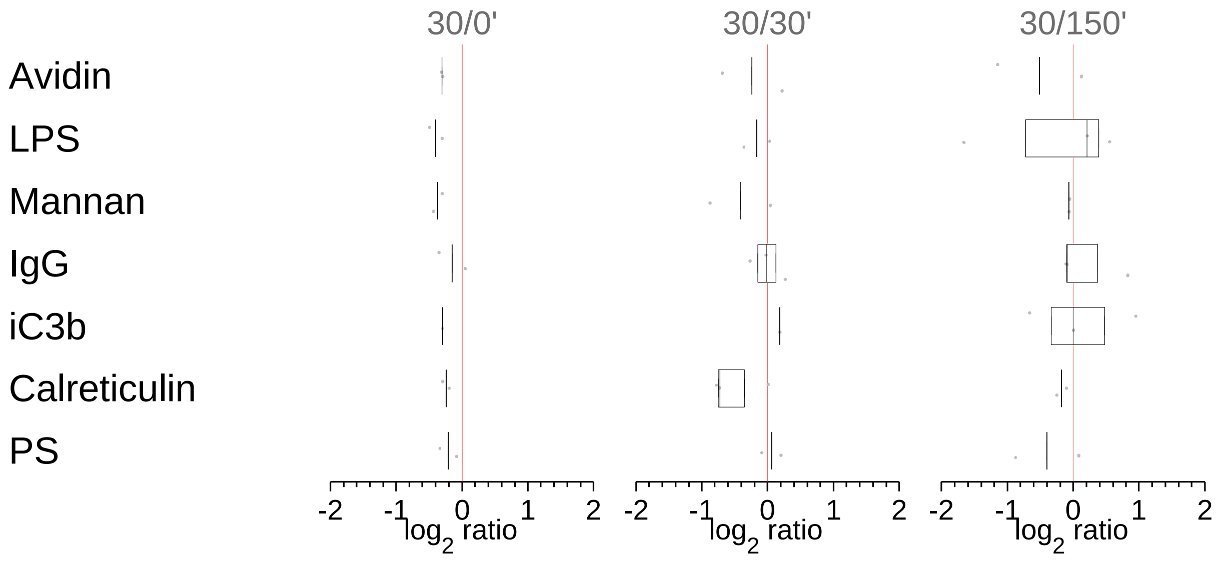

GO:0009967 positive regulation of signal transduction (n = 4)

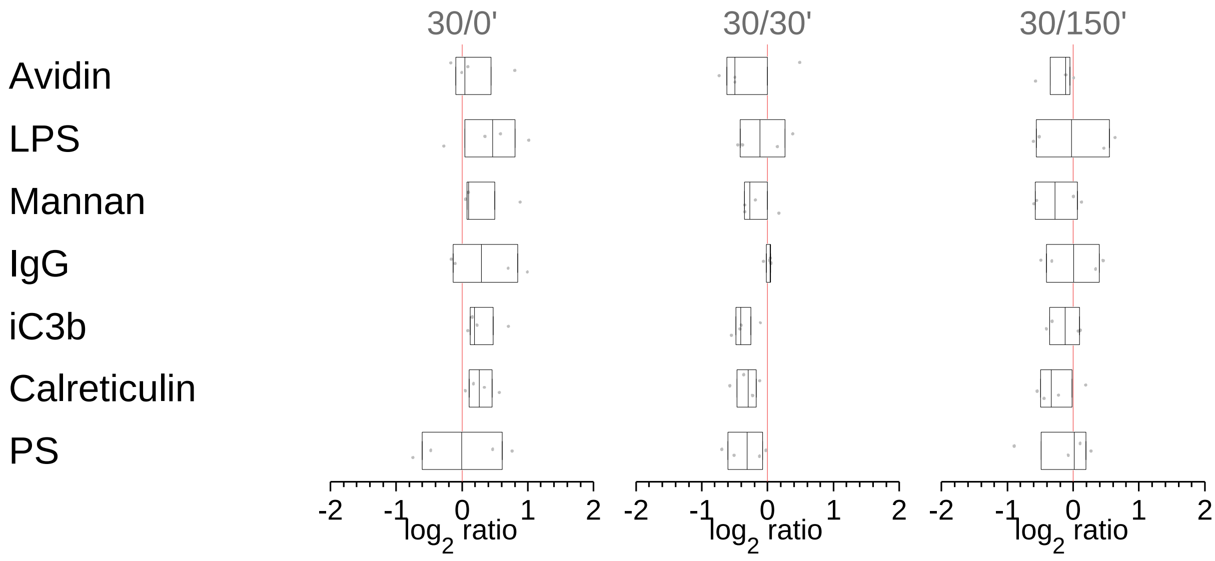

GO:0009968 negative regulation of signal transduction (n = 7)

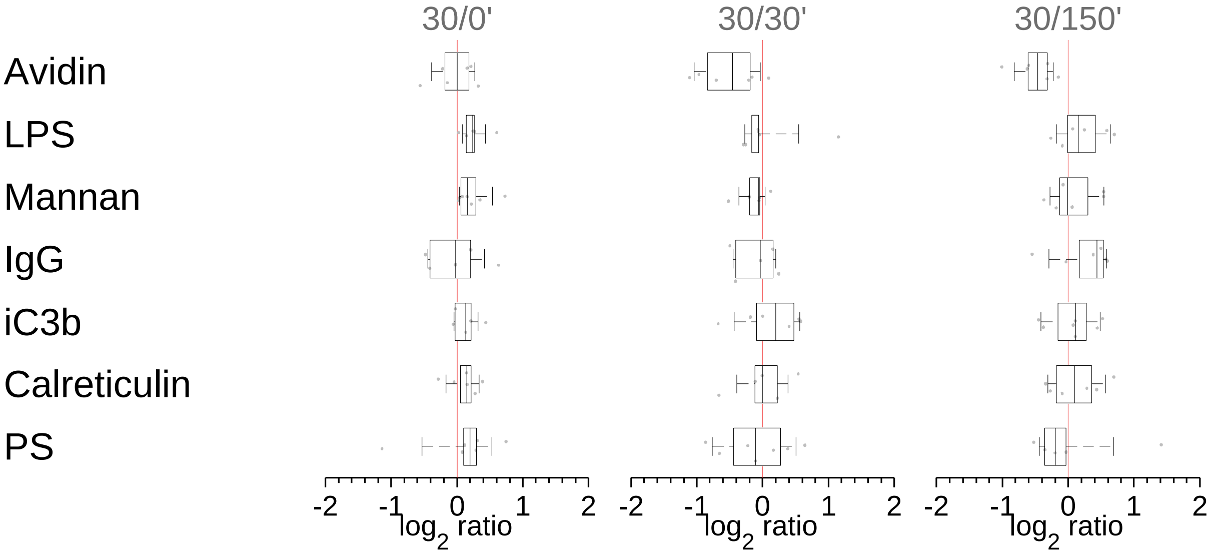

GO:0009986 cell surface (n = 93)

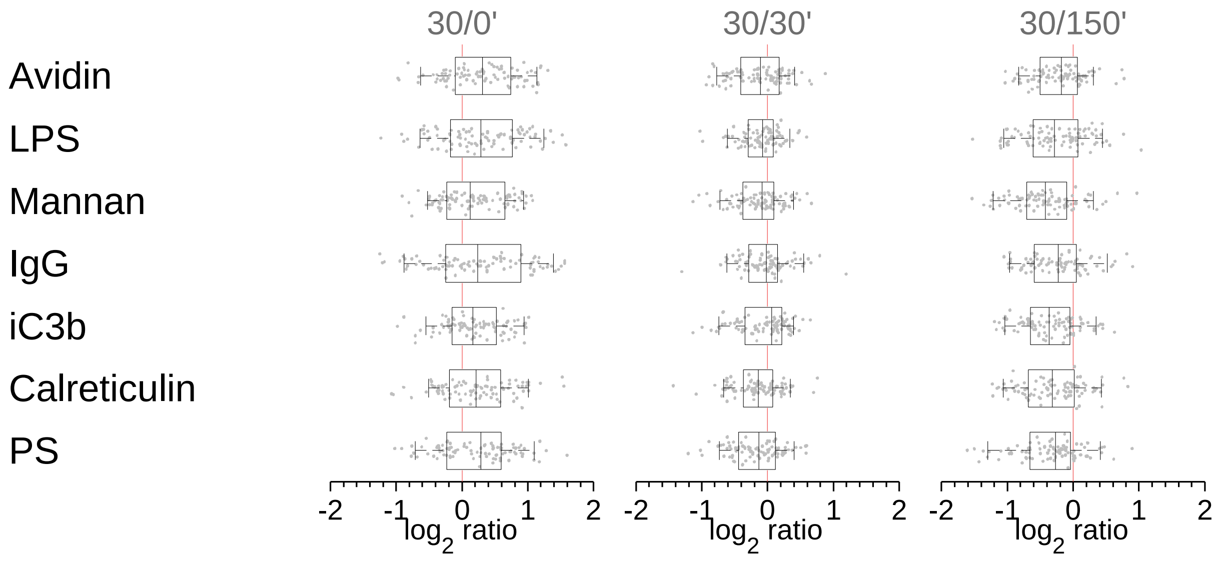

GO:0009987 cellular process (n = 4)

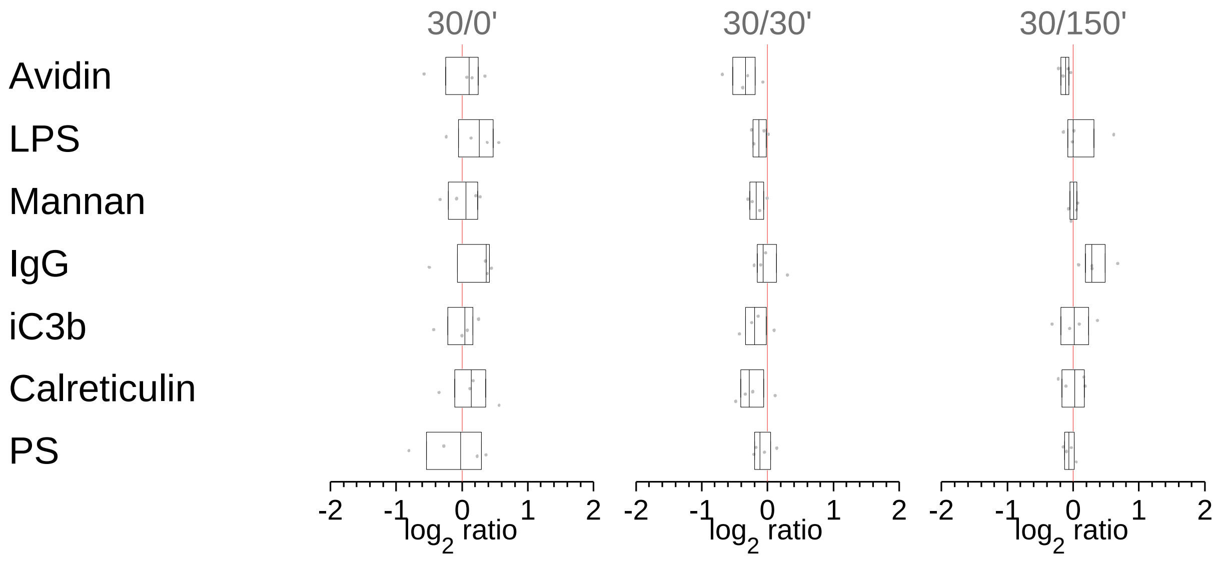

GO:0010008 endosome membrane (n = 22)

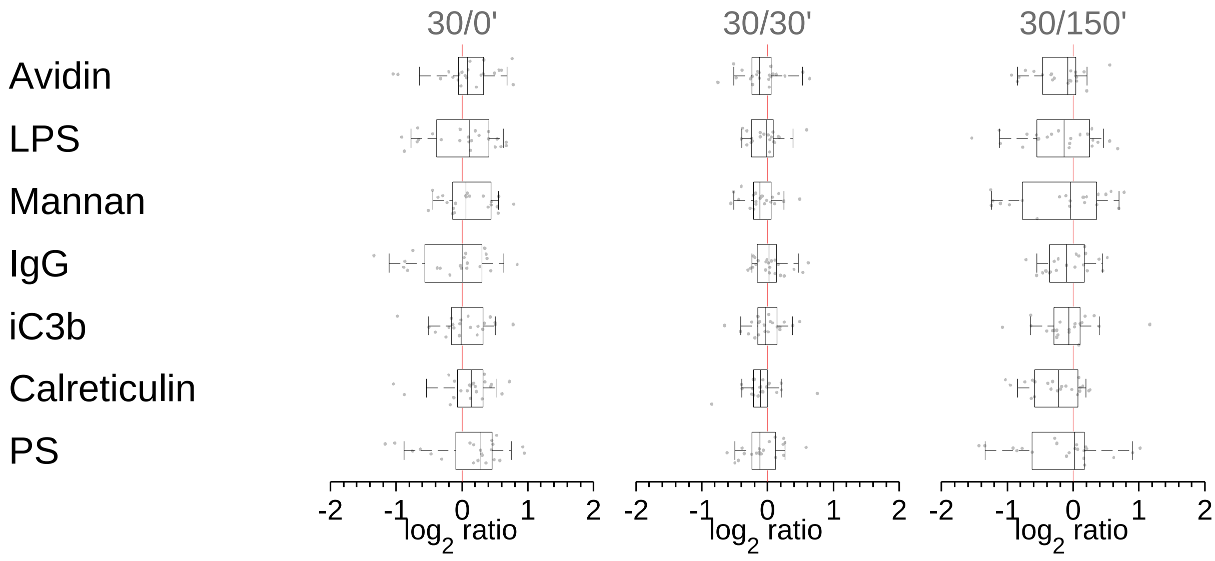

GO:0010033 response to organic substance (n = 9)

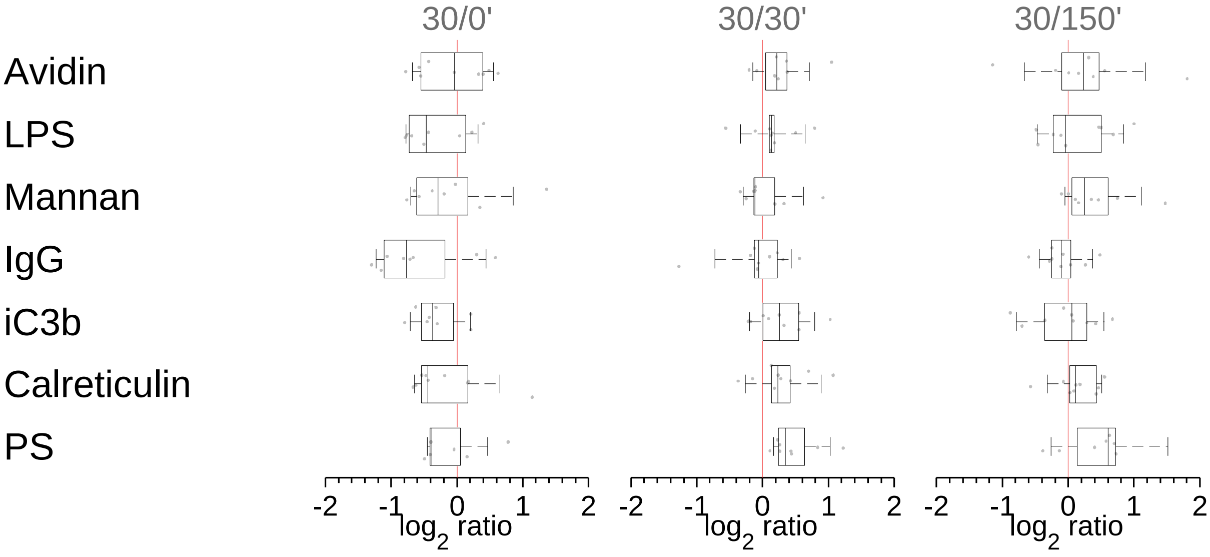

GO:0010043 response to zinc ion (n = 5)

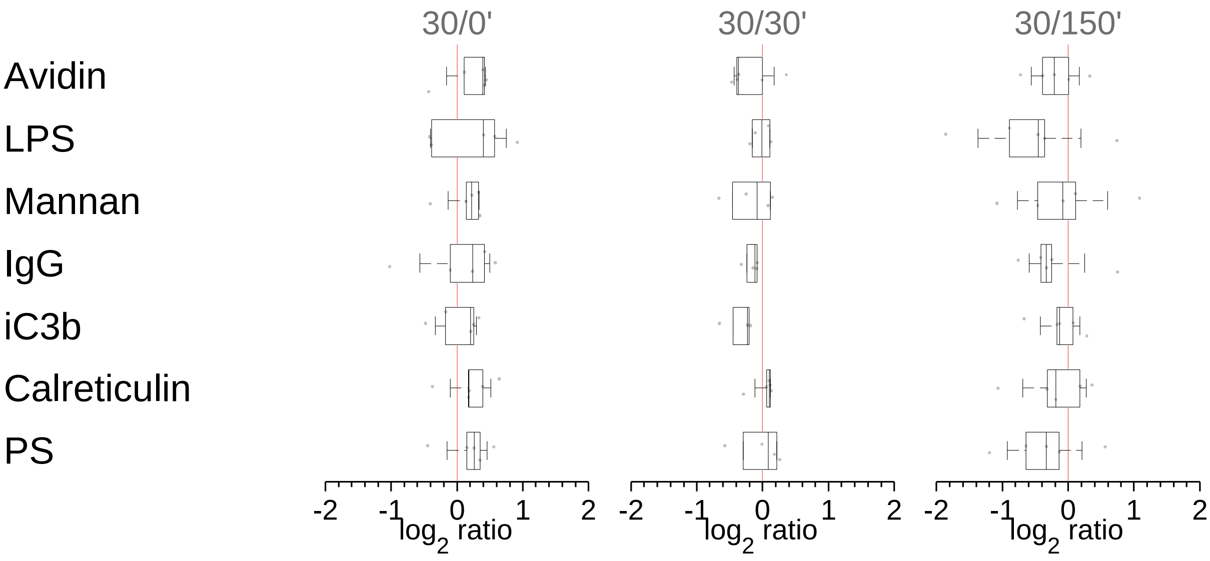

GO:0010181 FMN binding (n = 3)

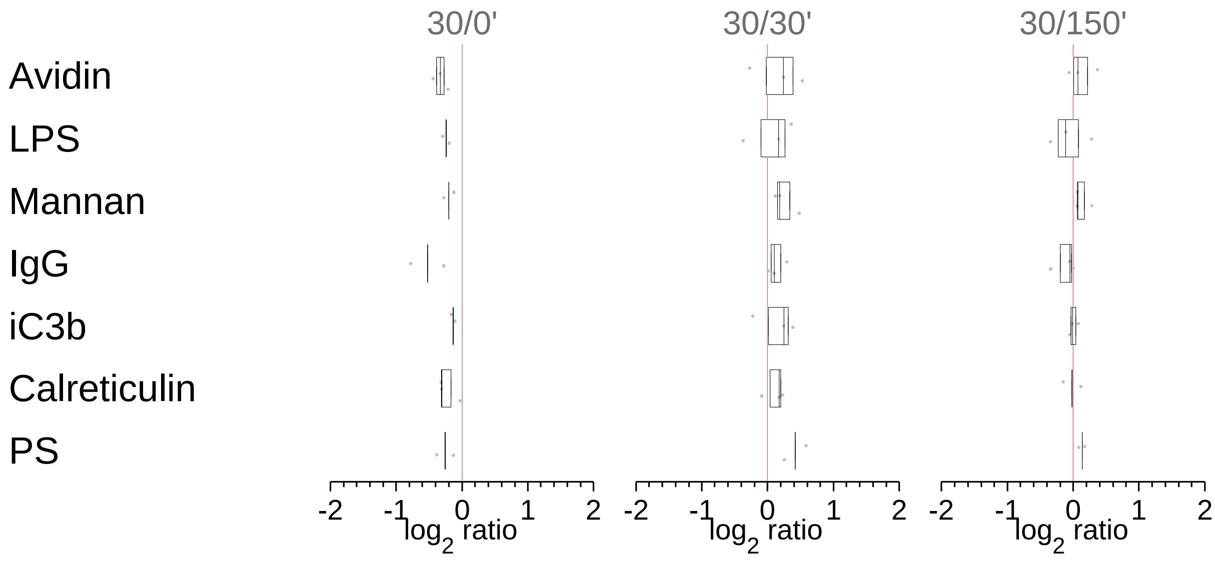

GO:0010296 prenylcysteine methylesterase activity (n = 4)

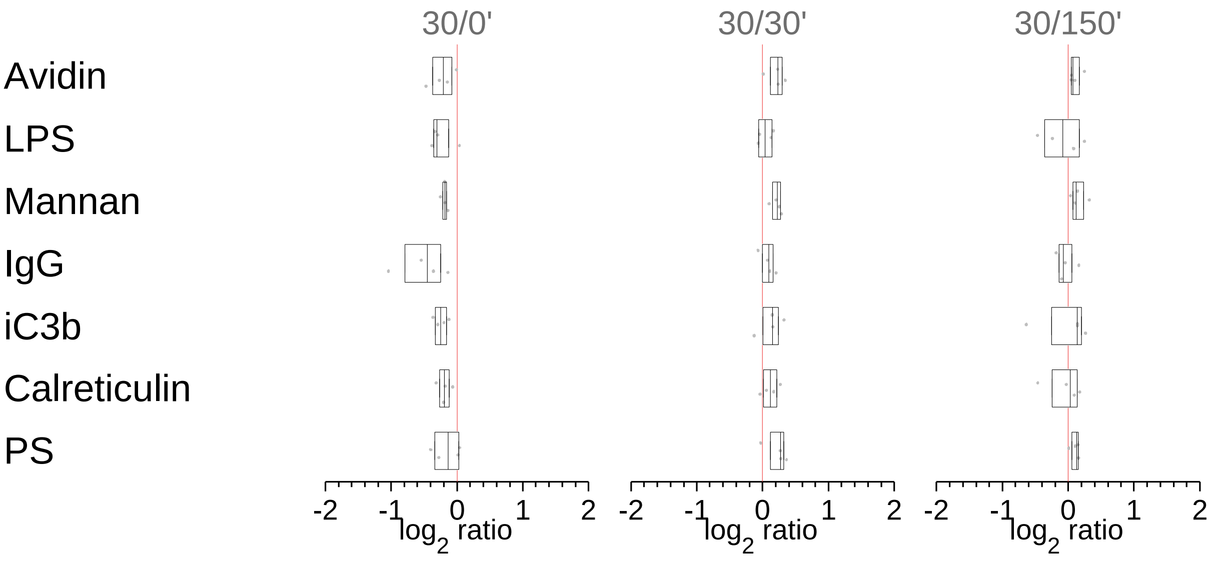

GO:0010332 response to gamma radiation (n = 6)

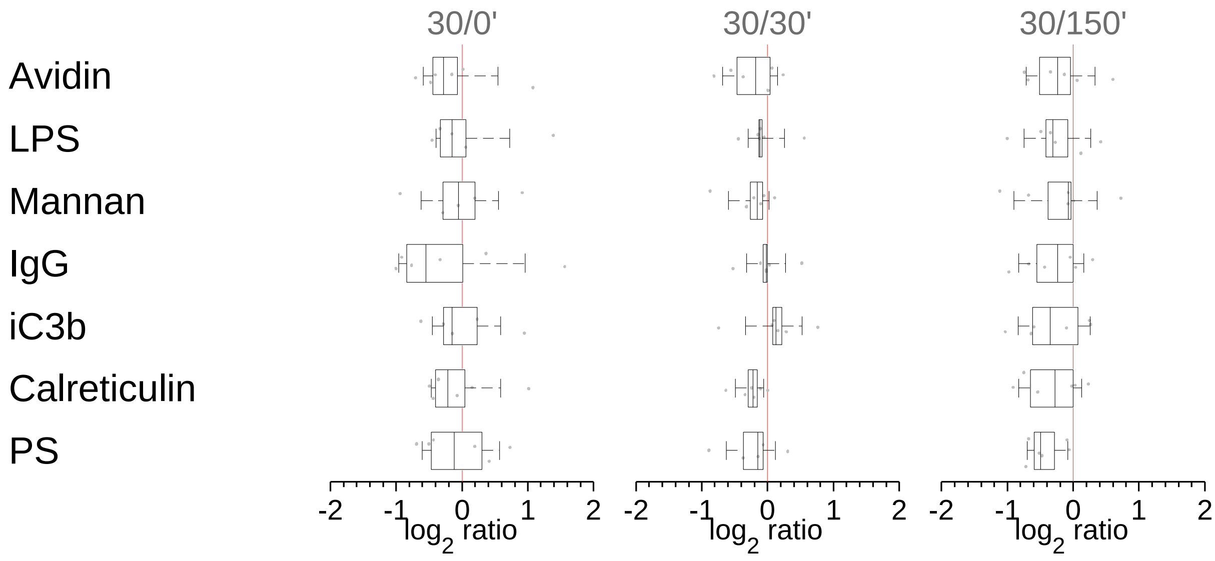

GO:0010466 negative regulation of peptidase activity (n = 9)

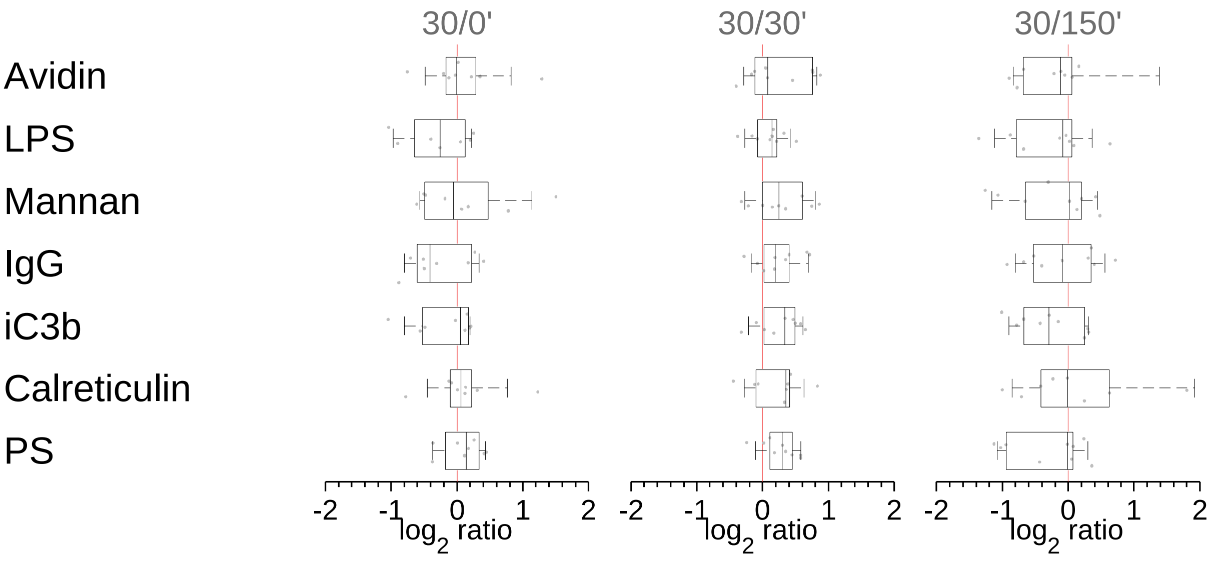

GO:0010468 regulation of gene expression (n = 9)

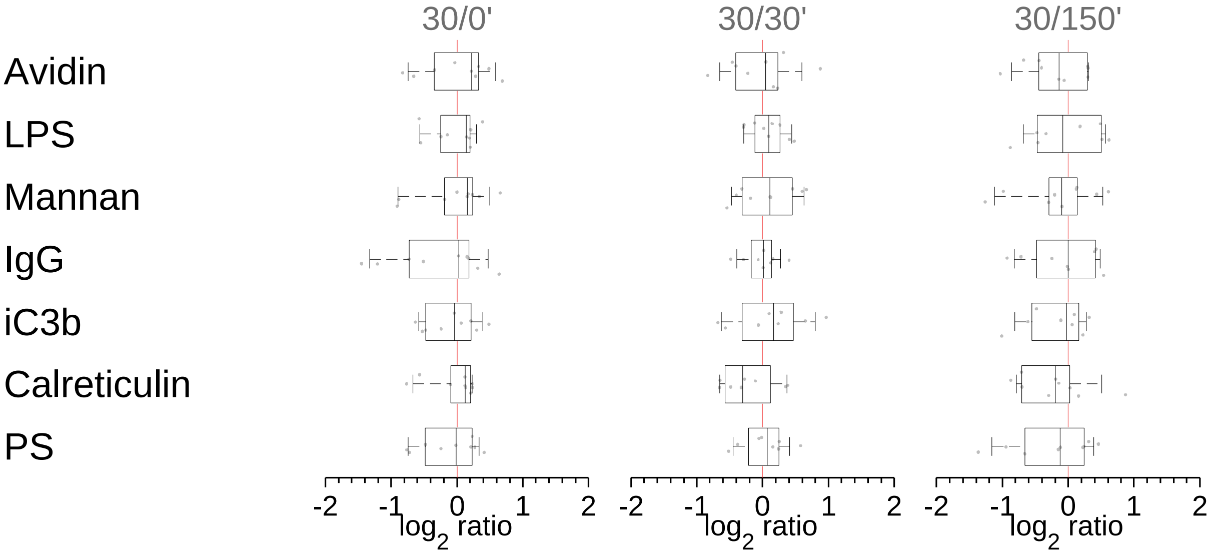

GO:0010494 cytoplasmic stress granule (n = 10)

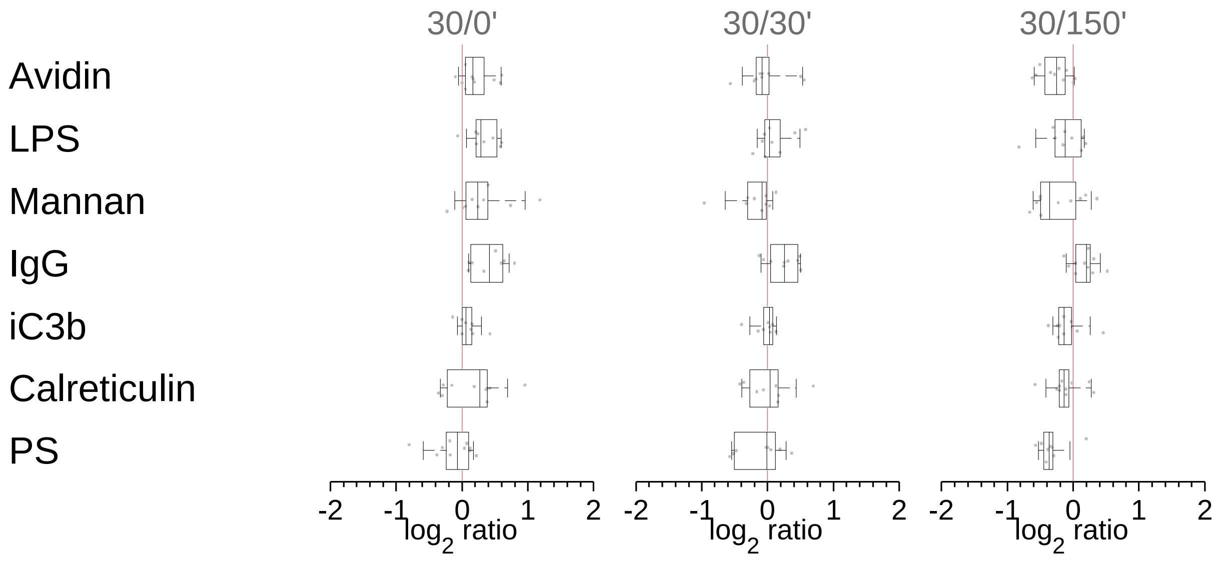

GO:0010524 positive regulation of calcium ion transport into cytosol (n = 5)

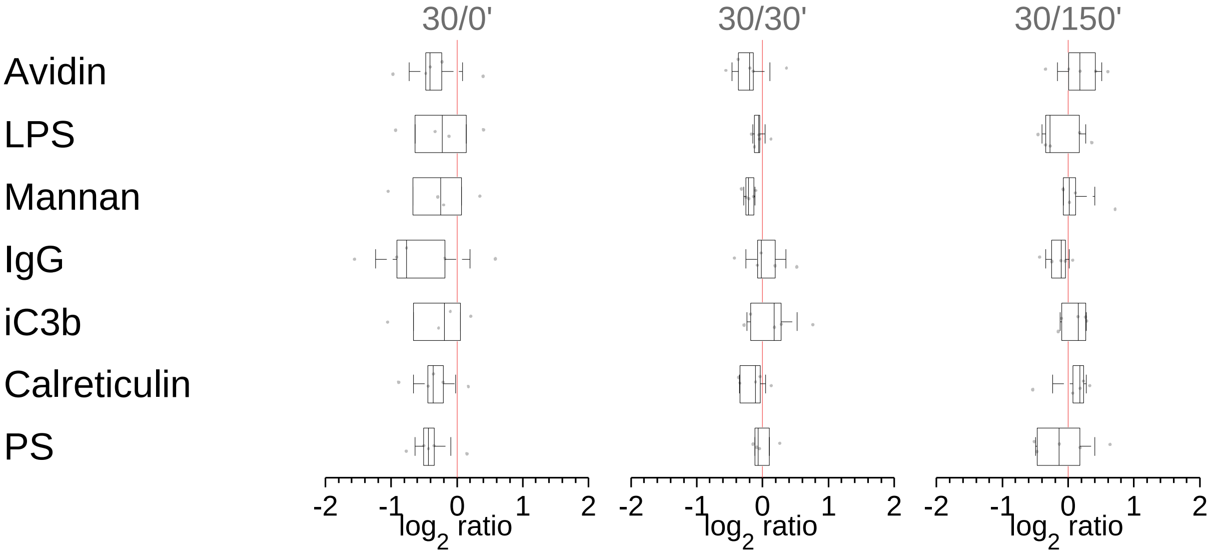

GO:0010575 positive regulation vascular endothelial growth factor production (n = 3)

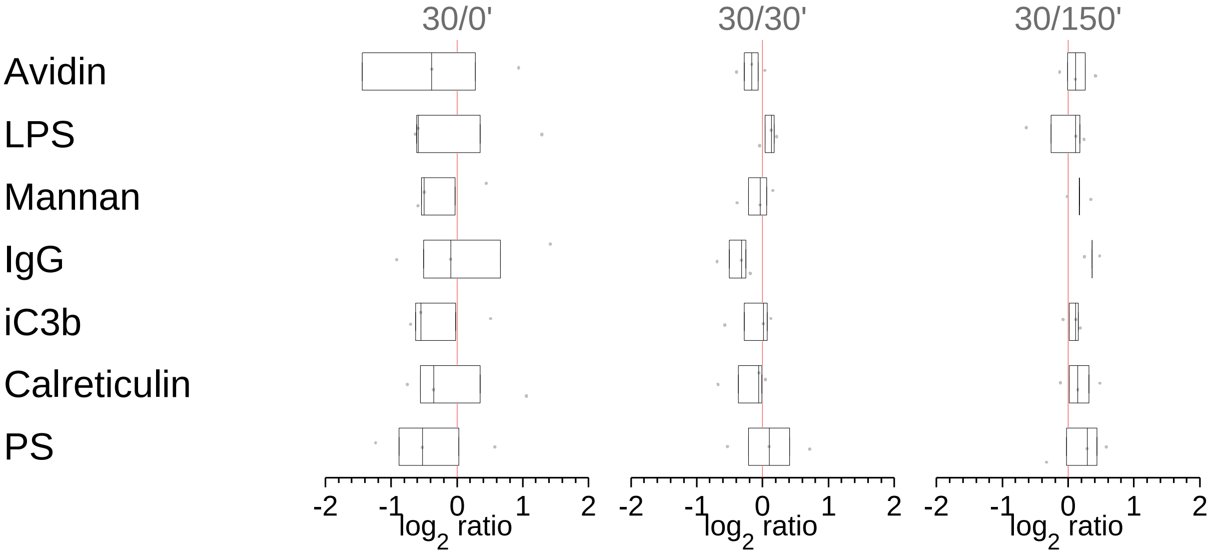

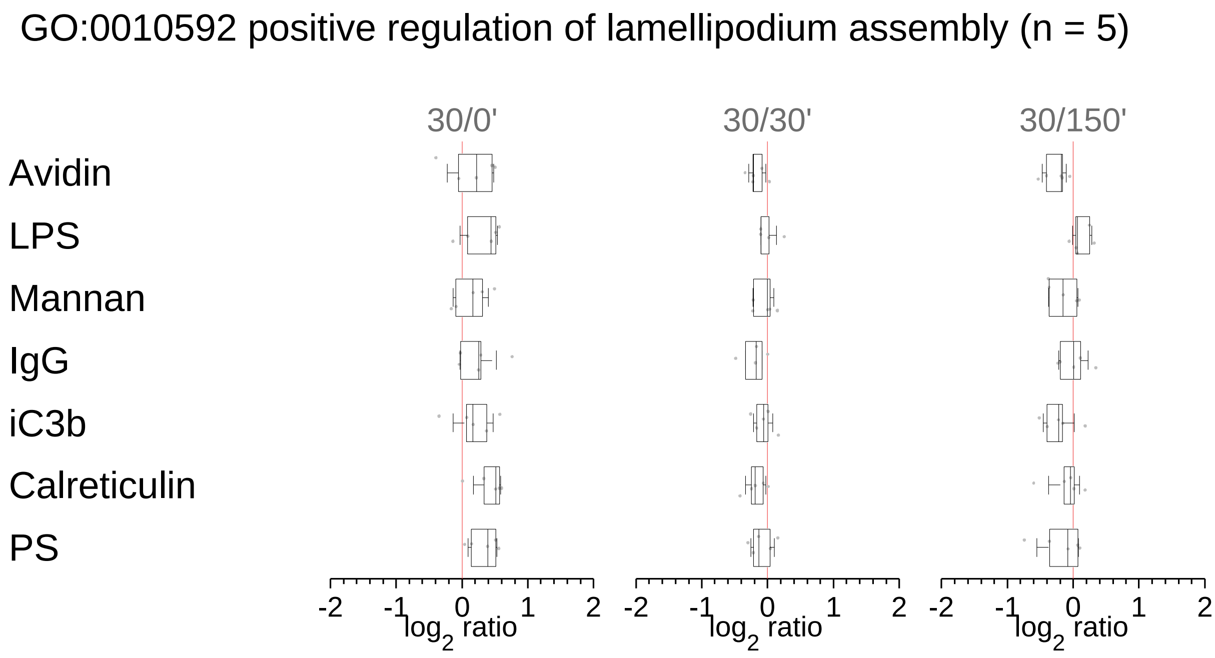
GO:0010592 positive regulation of lamellipodium assembly (n = 5)
30/0'
30/30'
30/150'
Avidin
LPS
Mannan
IgG
iC3b
Calreticulin
PS
-2 -1 0 1 2
log2 ratio

GO:0010595 positive regulation of endothelial cell migration (n = 4)

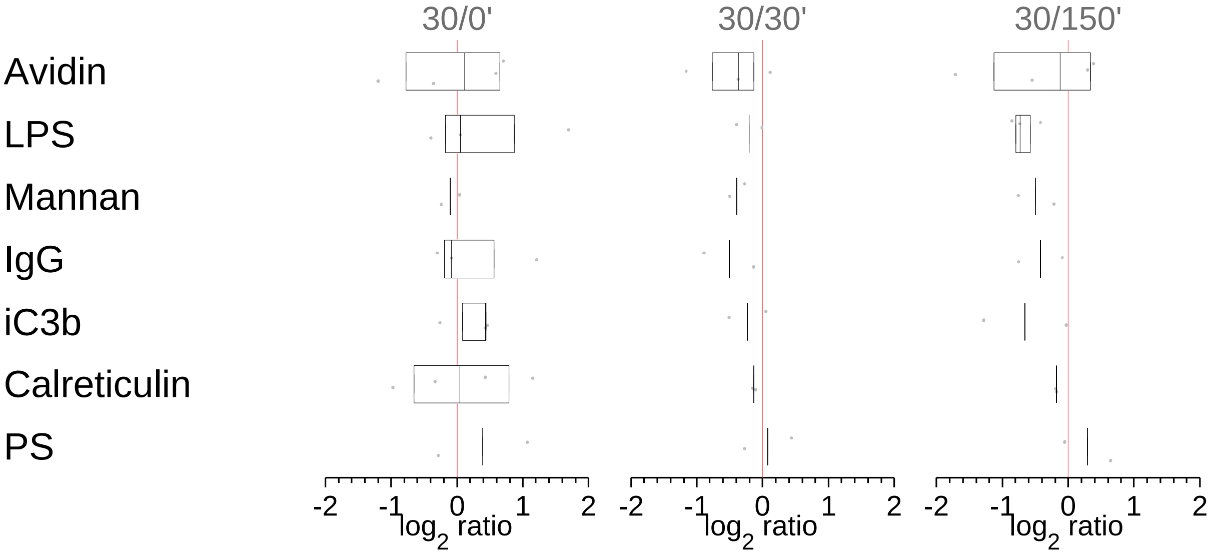

GO:0010613 positive regulation of cardiac muscle hypertrophy (n = 3)

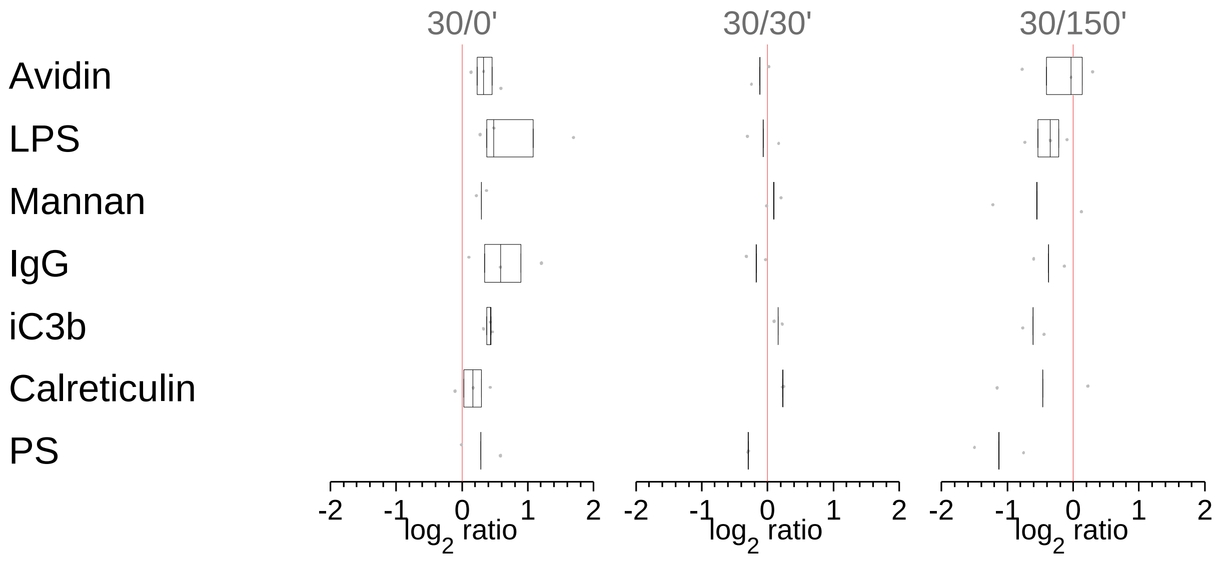

GO:0010628 positive regulation of gene expression (n = 26)

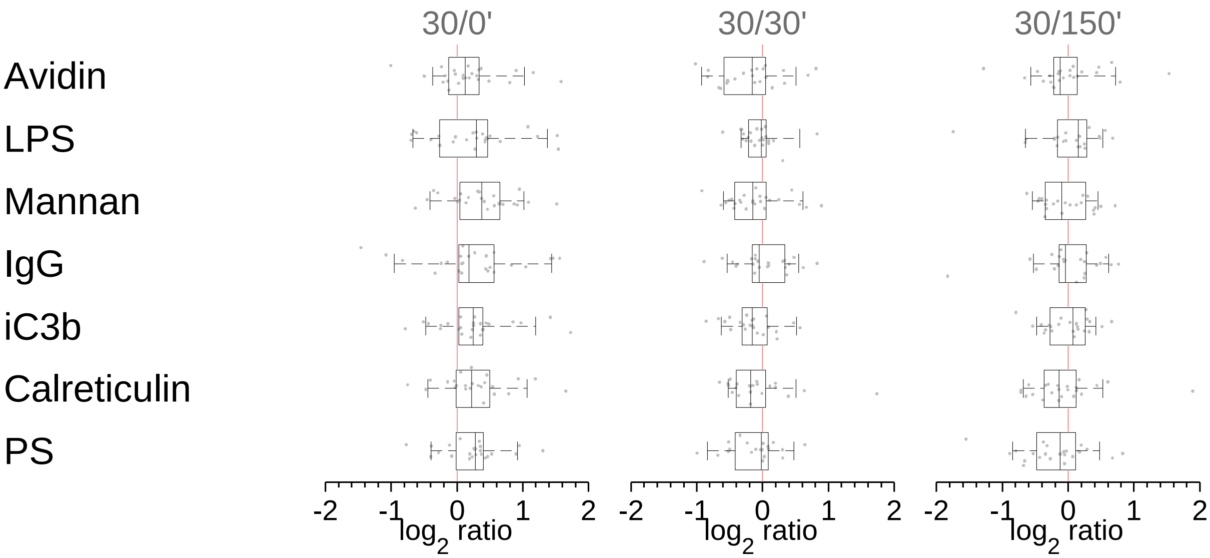

GO:0010629 negative regulation of gene expression (n = 19)

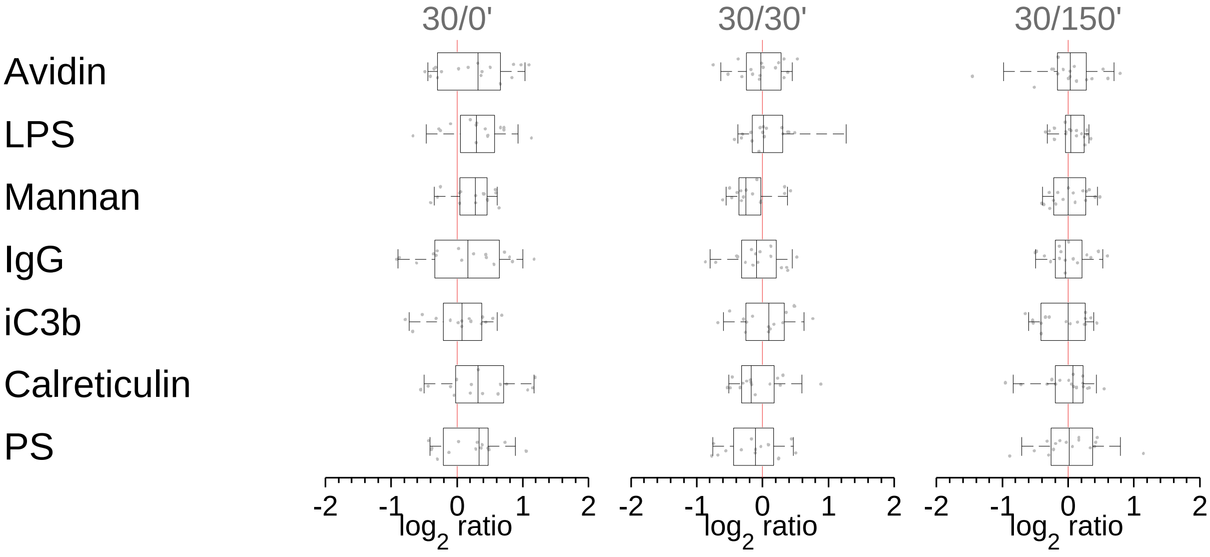

GO:0010634 positive regulation of epithelial cell migration (n = 4)

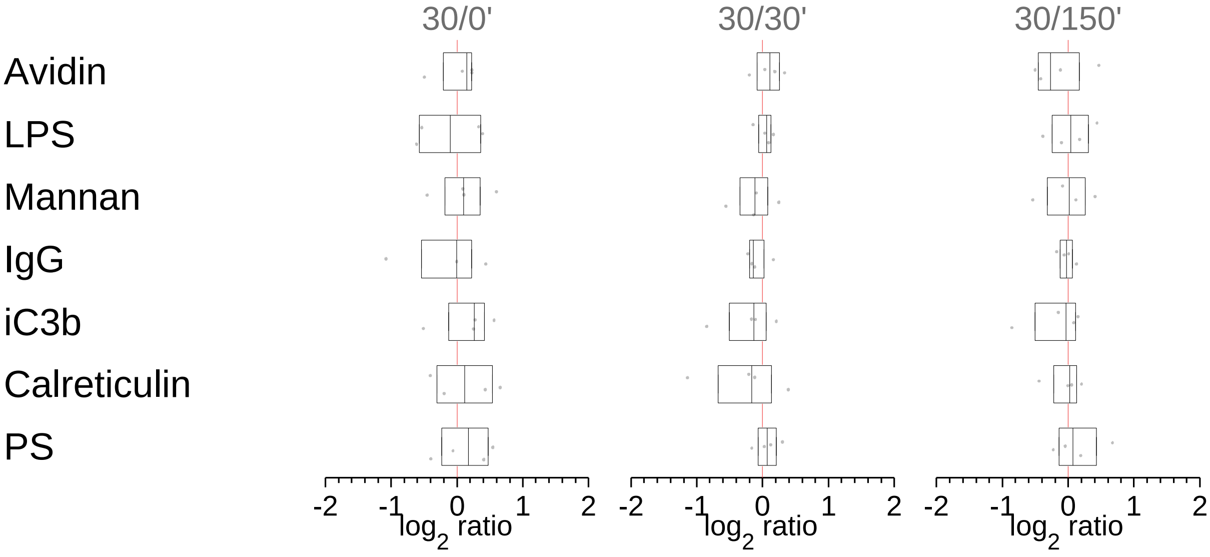

GO:0010638 positive regulation of organelle organization (n = 3)

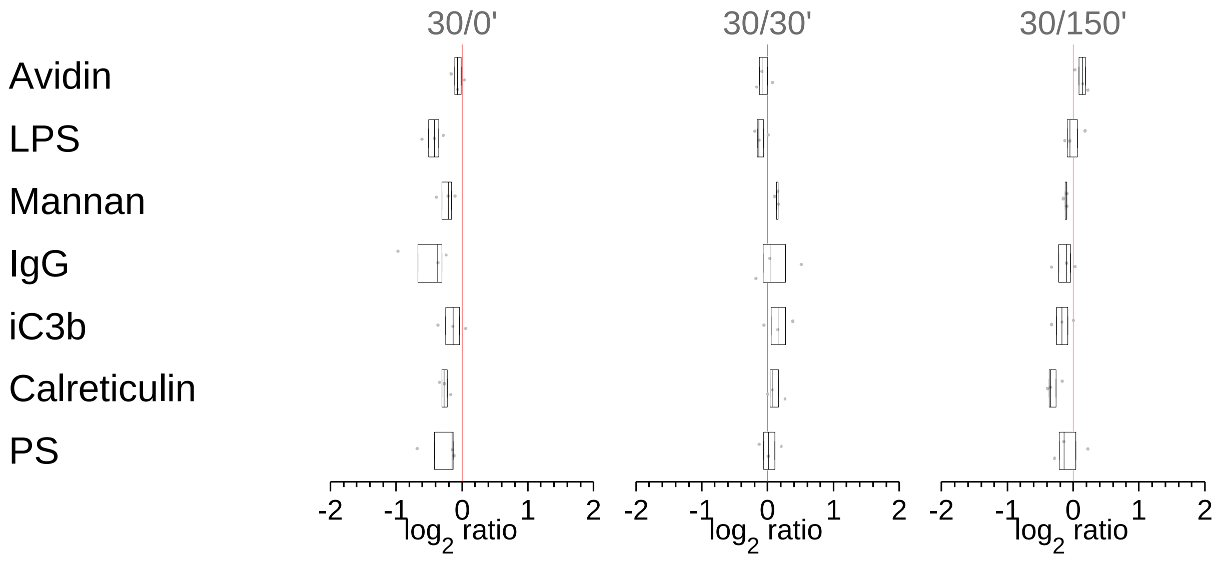

GO:0010718 positive regulation of epithelial to mesenchymal transition (n = 3)

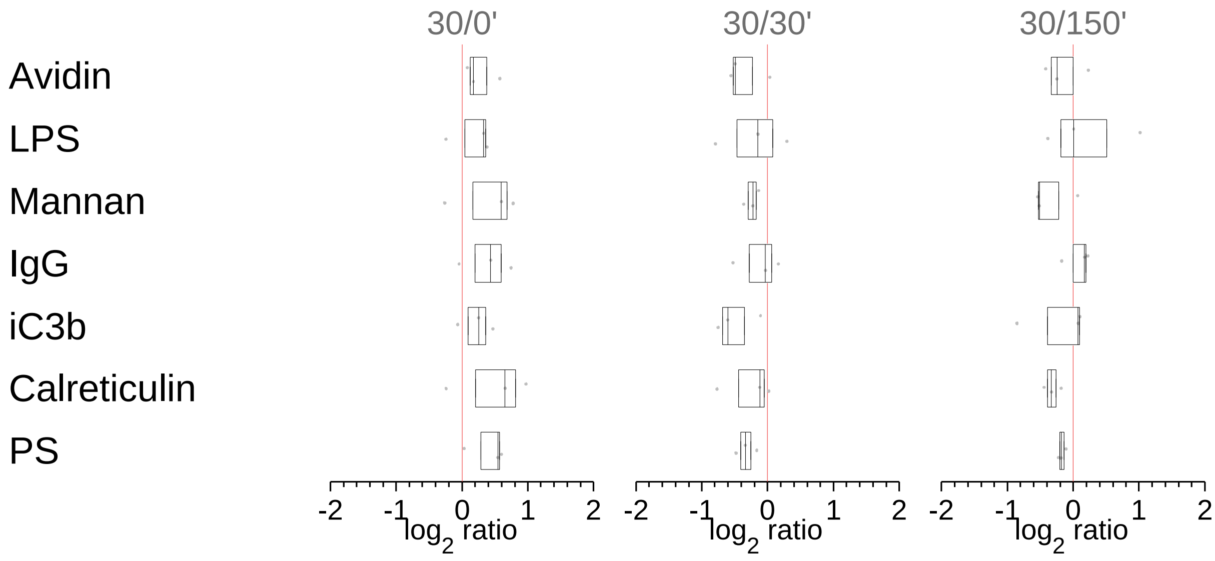

GO:0010744 positive regulation of macrophage derived foam cell differentiation (n = 6)

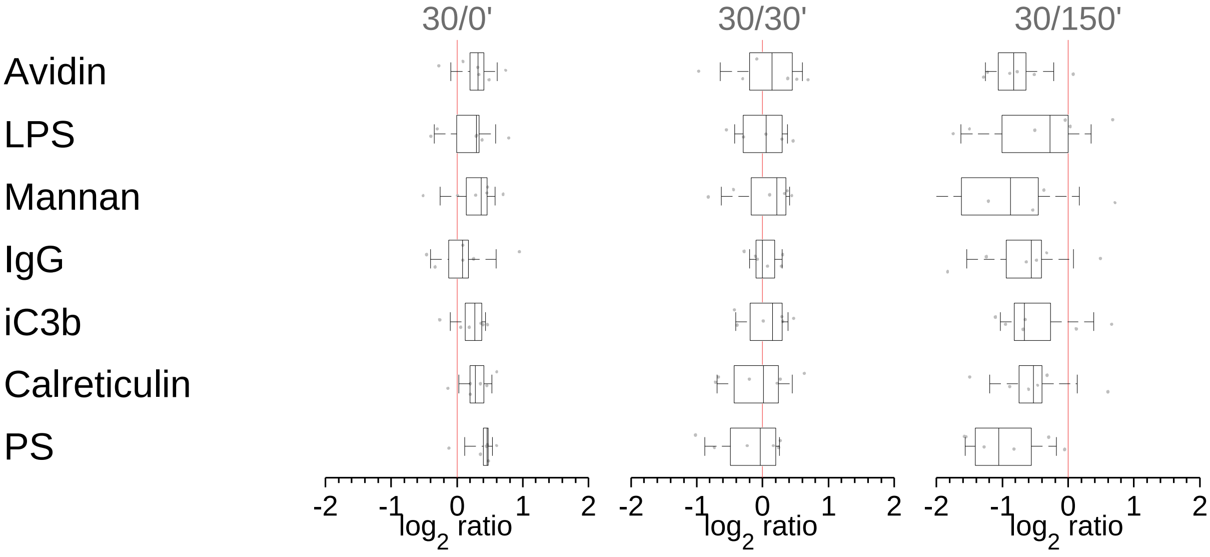

GO:0010811 positive regulation of cell-substrate adhesion (n = 6)

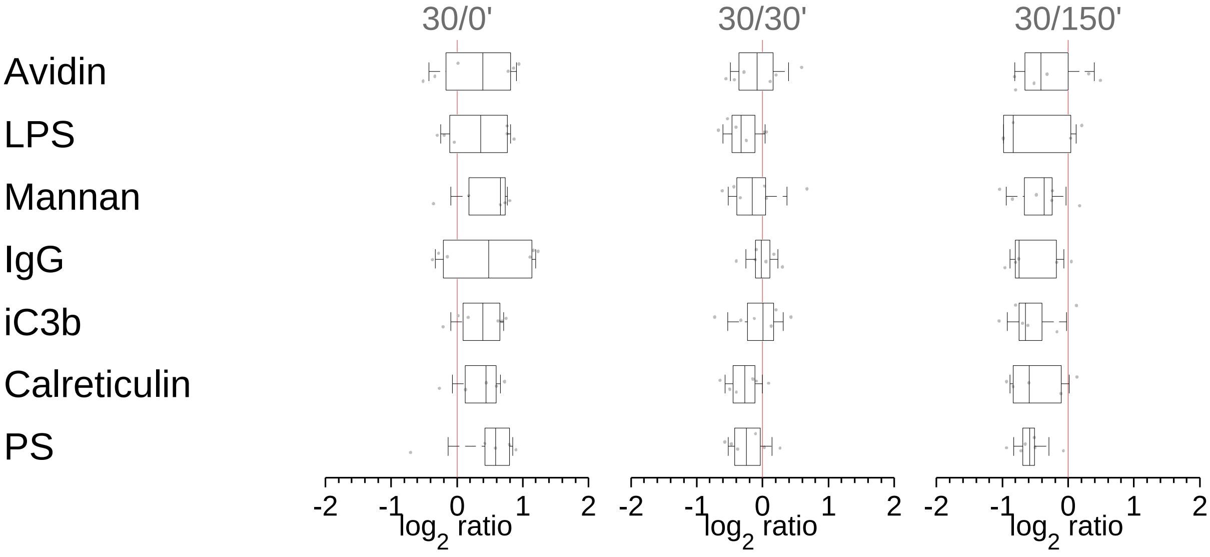

GO:0010867 positive regulation of triglyceride biosynthetic process (n = 4)

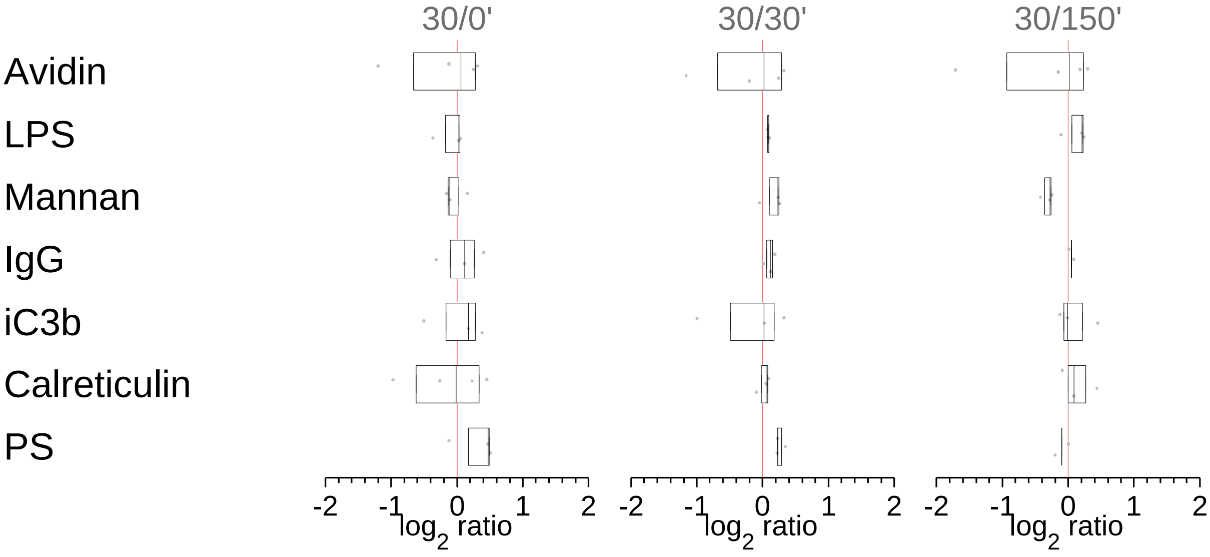

GO:0010886 positive regulation of cholesterol storage (n = 6)

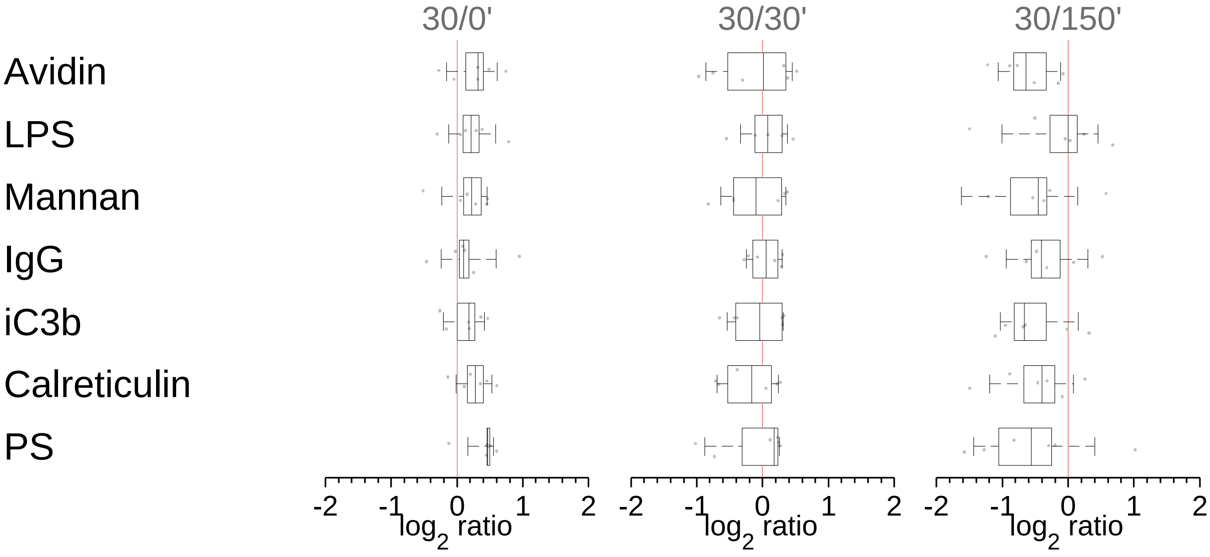

GO:0010923 negative regulation of phosphatase activity (n = 3)

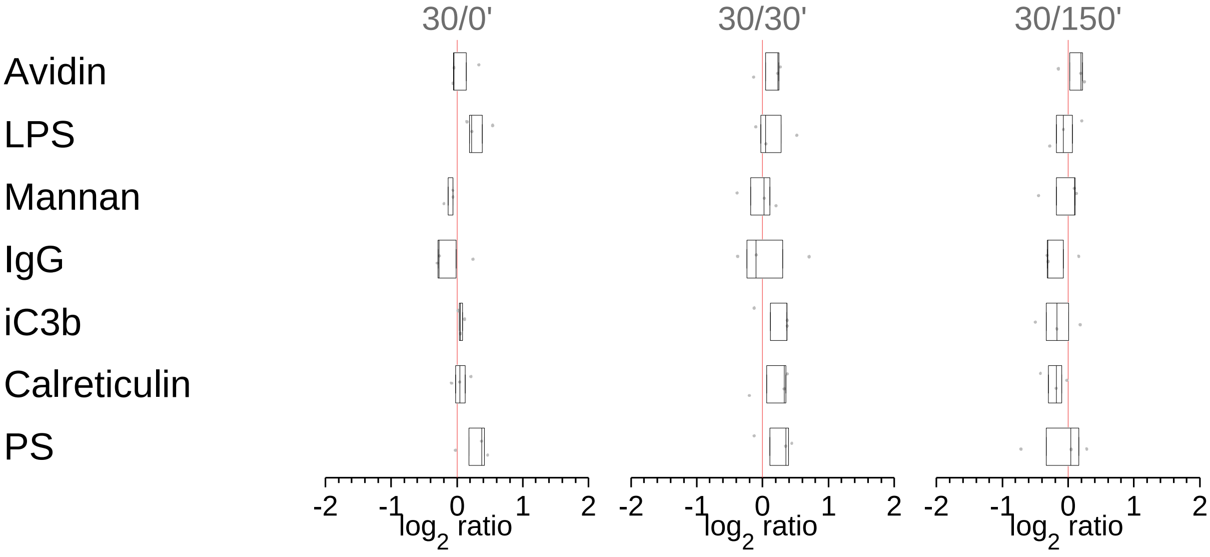

GO:0010942 positive regulation of cell death (n = 3)

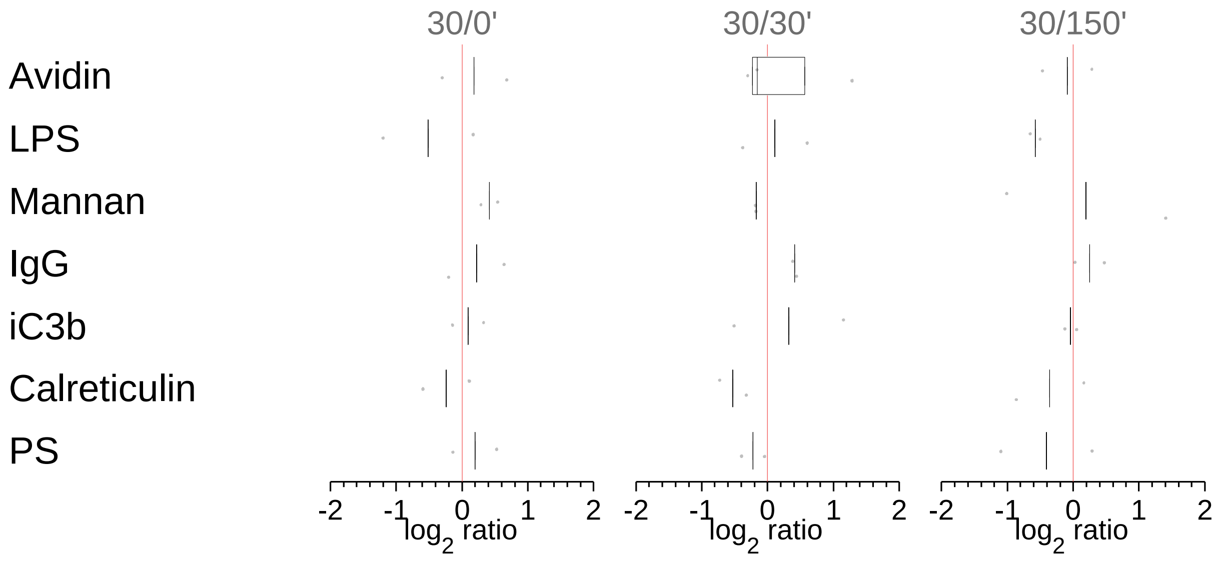

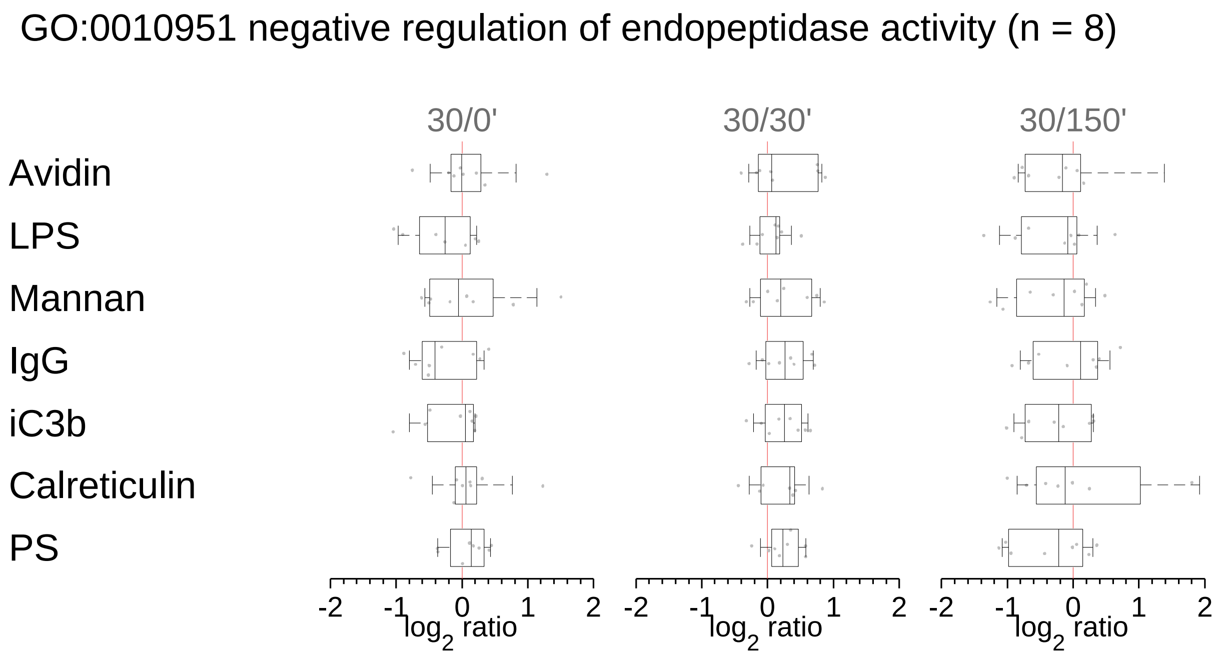

GO:0010951 negative regulation of endopeptidase activity (n = 8)
30/0'
30/30'
30/150'
Avidin
LPS
Mannan
IgG
iC3b
Calreticulin
PS
-2
-1
0
1
2
log2 ratio

GO:0010952 positive regulation of peptidase activity (n = 4)

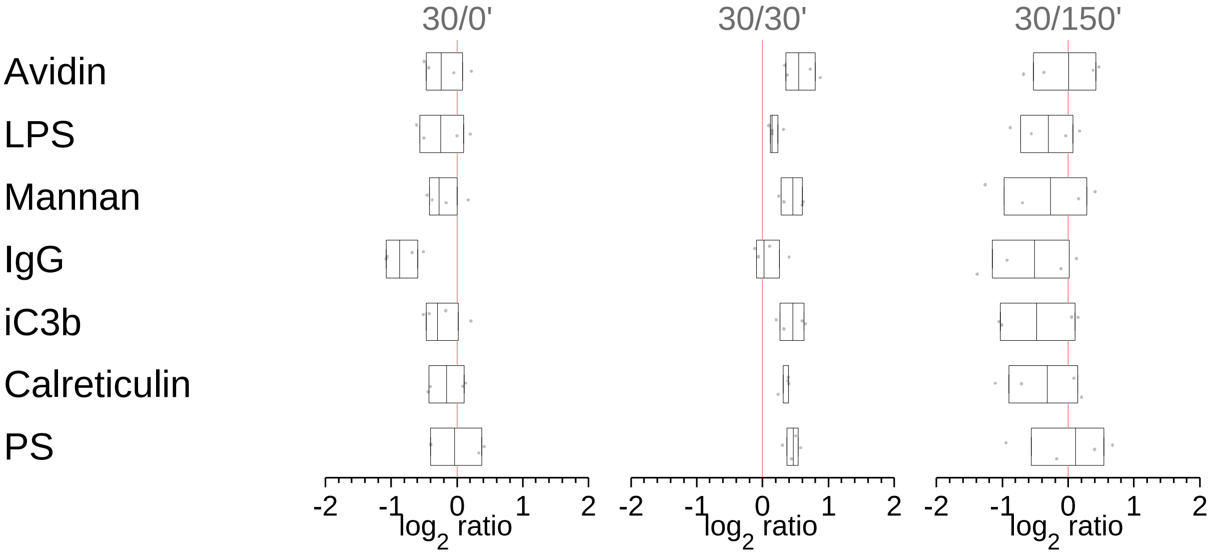

GO:0010976 positive regulation of neuron projection development
(n = 12)

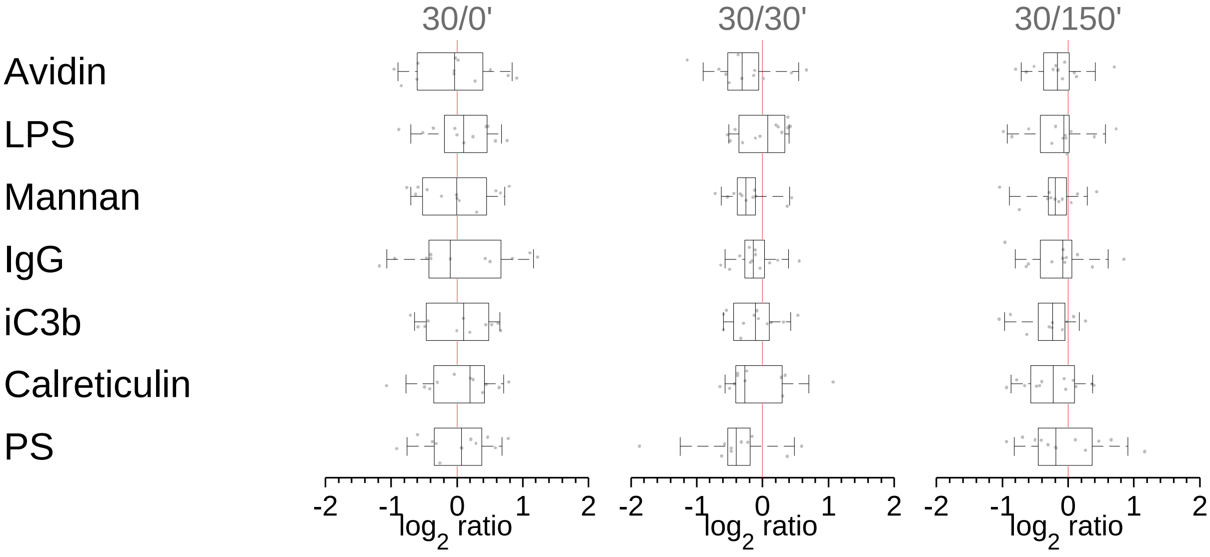

GO:0010977 negative regulation of neuron projection development
(n = 6)

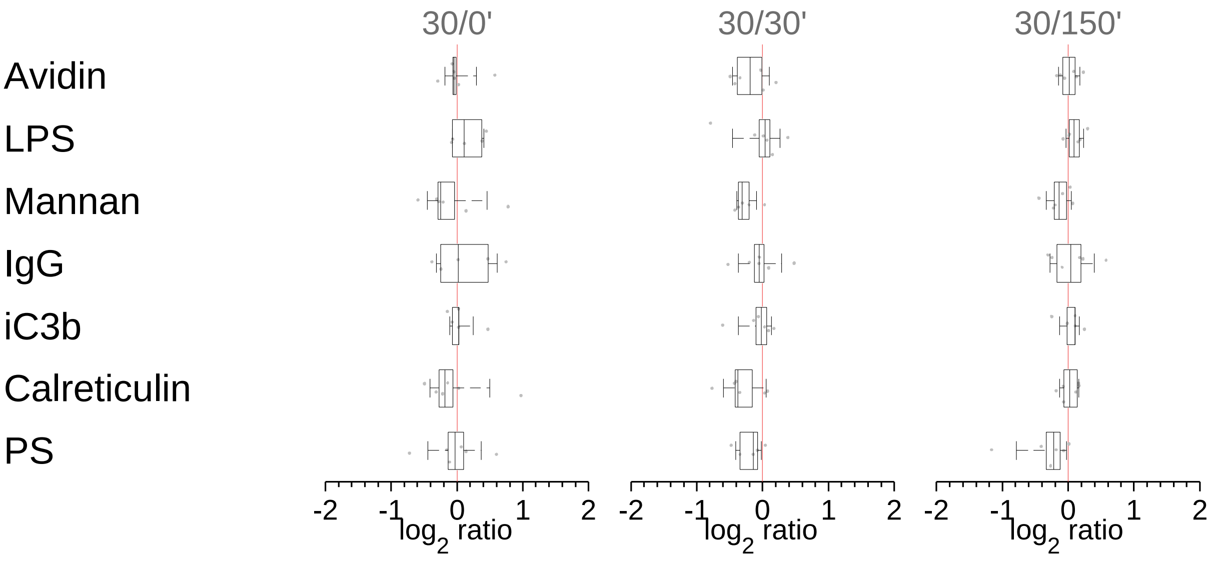

GO:0012501 programmed cell death (n = 5)

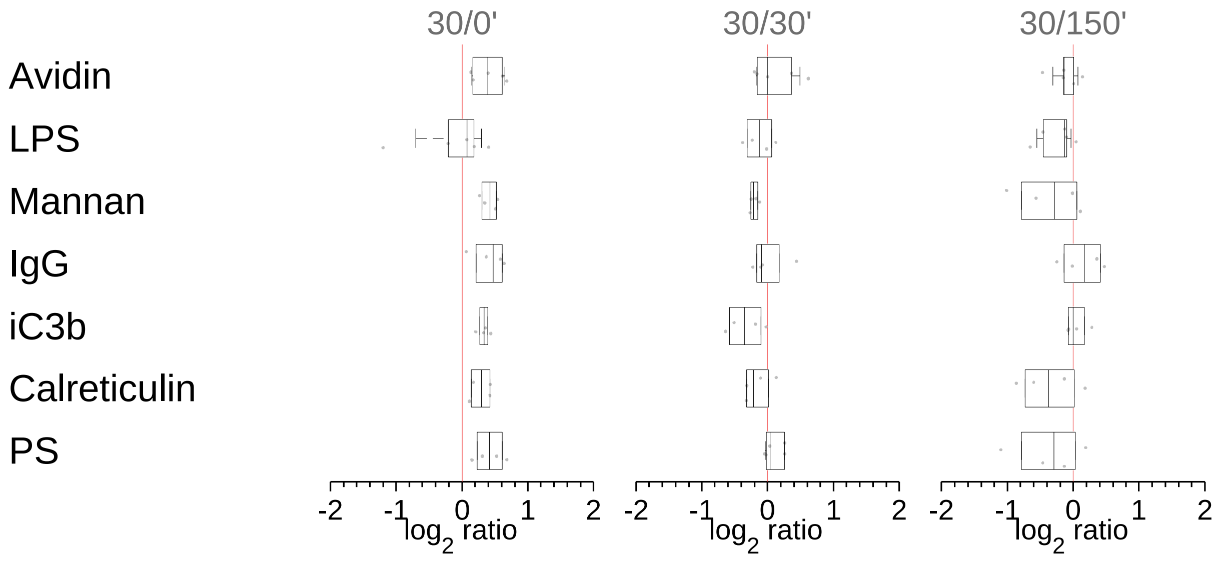

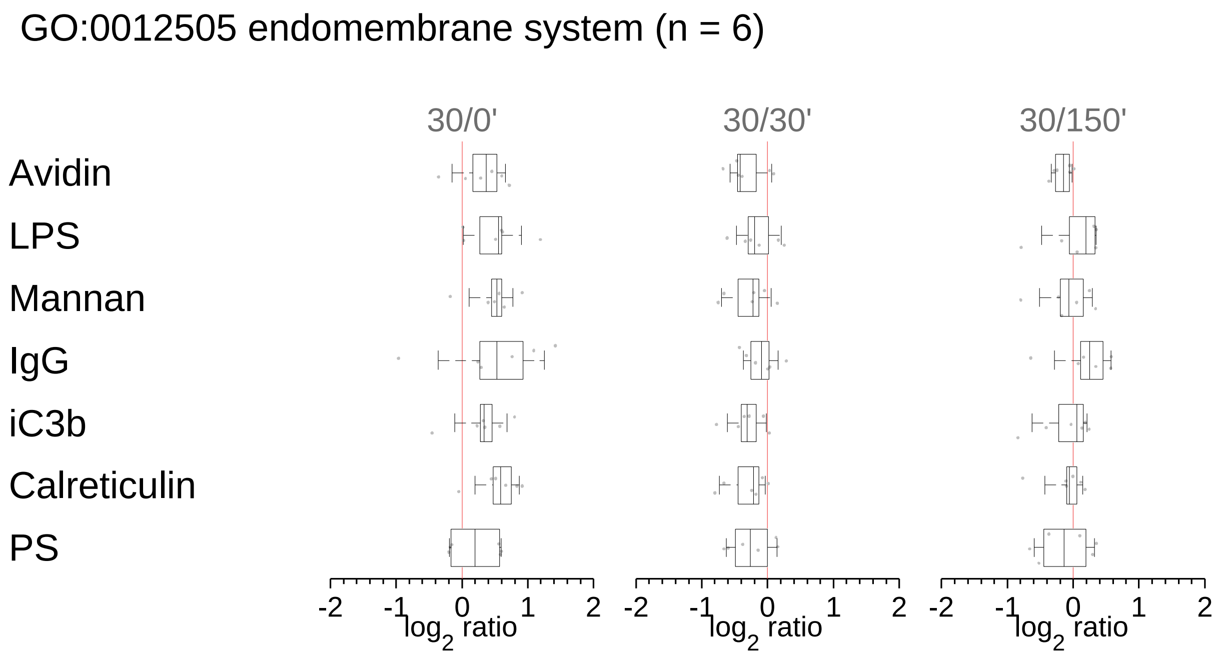
GO:0012505 endomembrane system (n = 6)
30/0'
30/30'
30/150'
Avidin
LPS
Mannan
IgG
iC3b
Calreticulin
PS
-2
-1
0
1
2
log2 ratio

GO:0012506 vesicle membrane (n = 6)

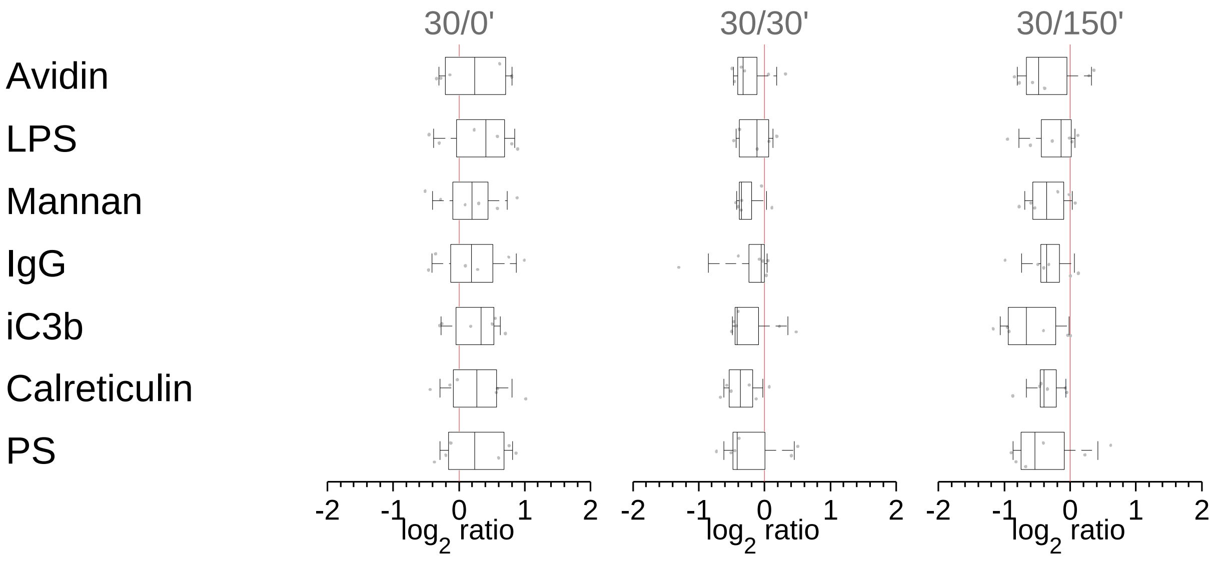

GO:0014065 phosphatidylinositol 3-kinase cascade (n = 3)

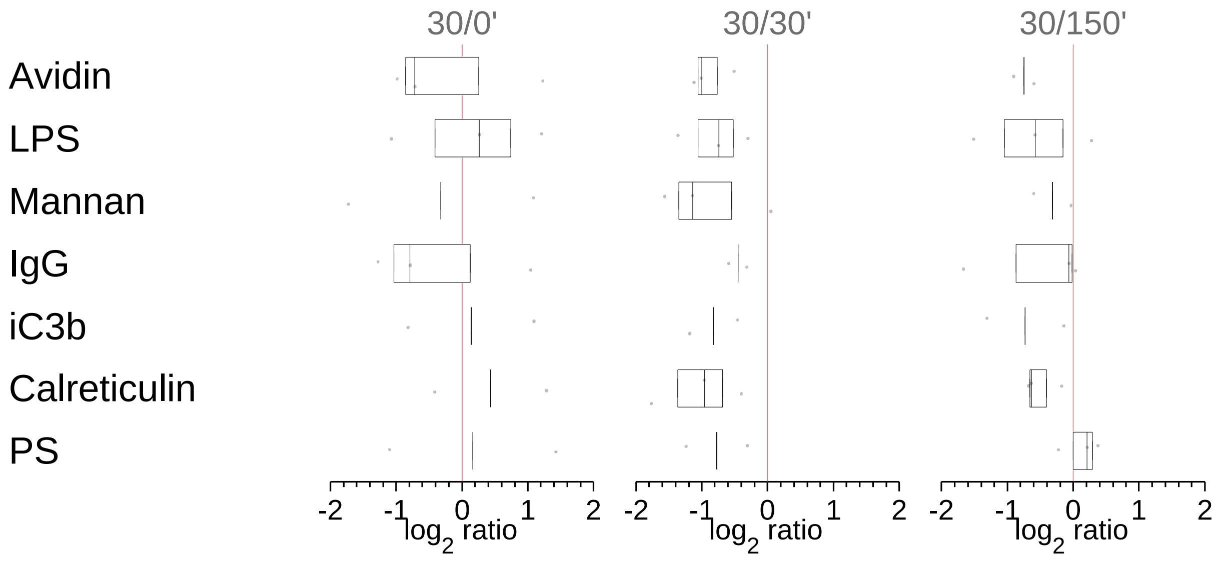

GO:0014068 positive regulation of phosphatidylinositol 3-kinase cascade (n = 7)

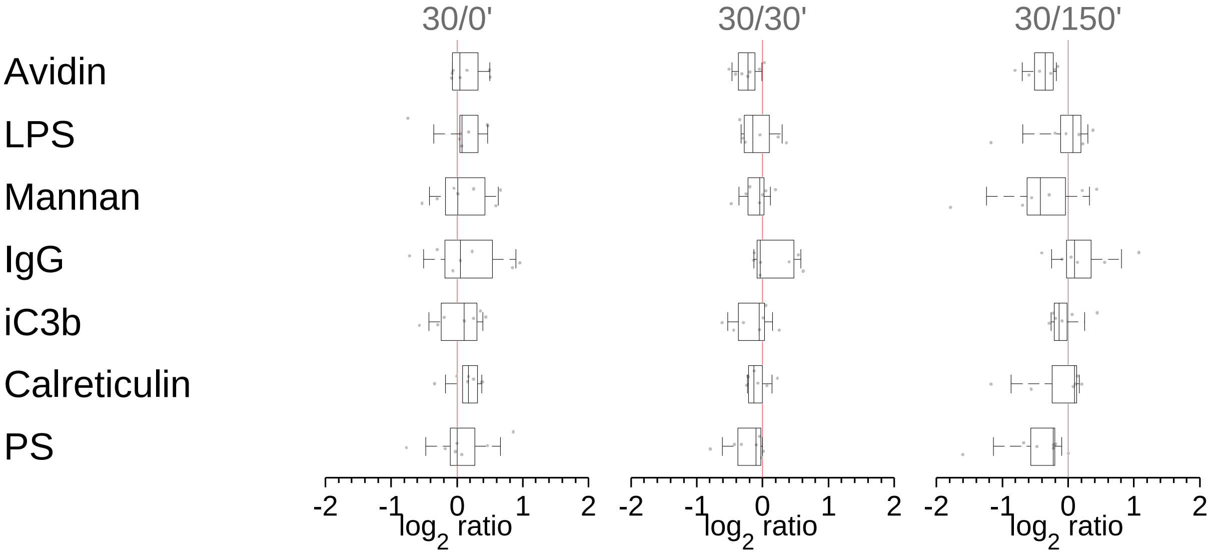

GO:0014069 postsynaptic density (n = 13)

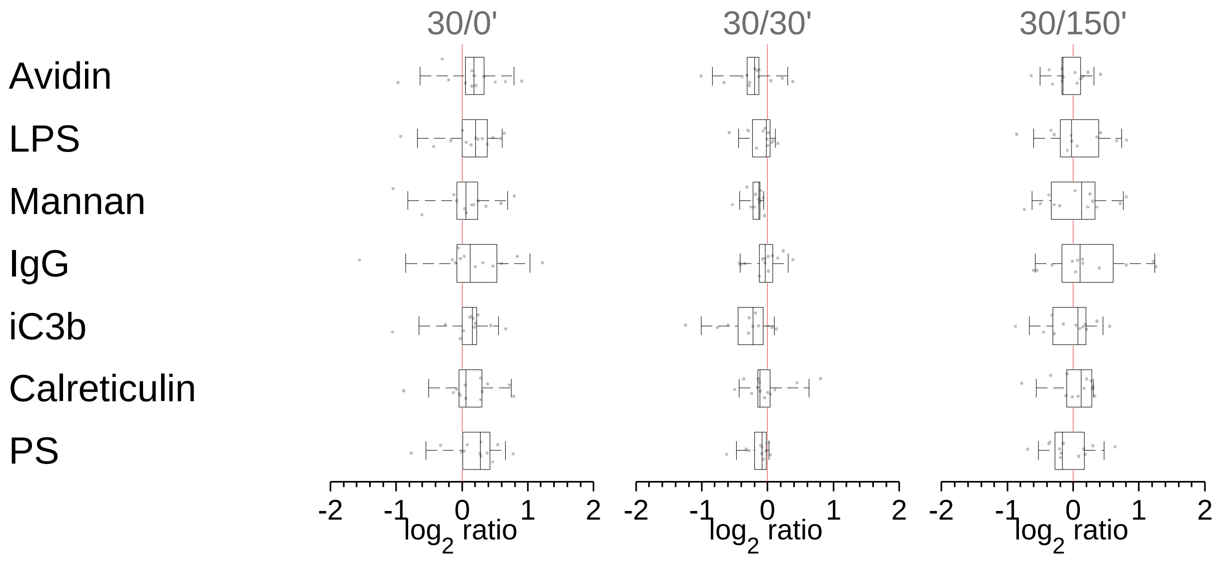

GO:0014070 response to organic cyclic compound (n = 5)

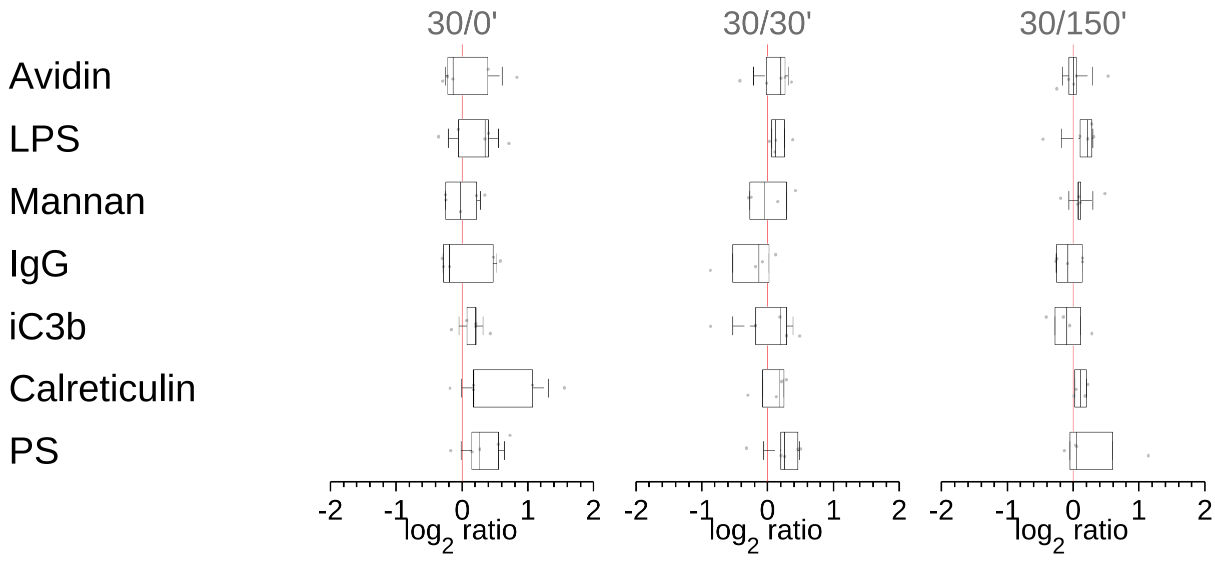

GO:0014704 intercalated disc (n = 7)

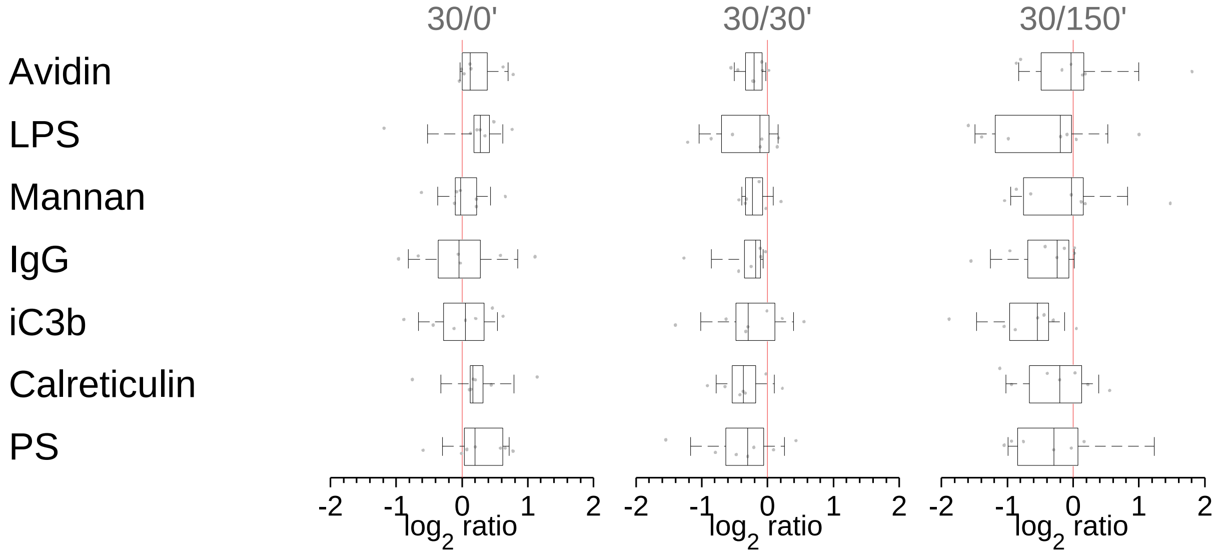

GO:0014911 positive regulation of smooth muscle cell migration
(n = 4)

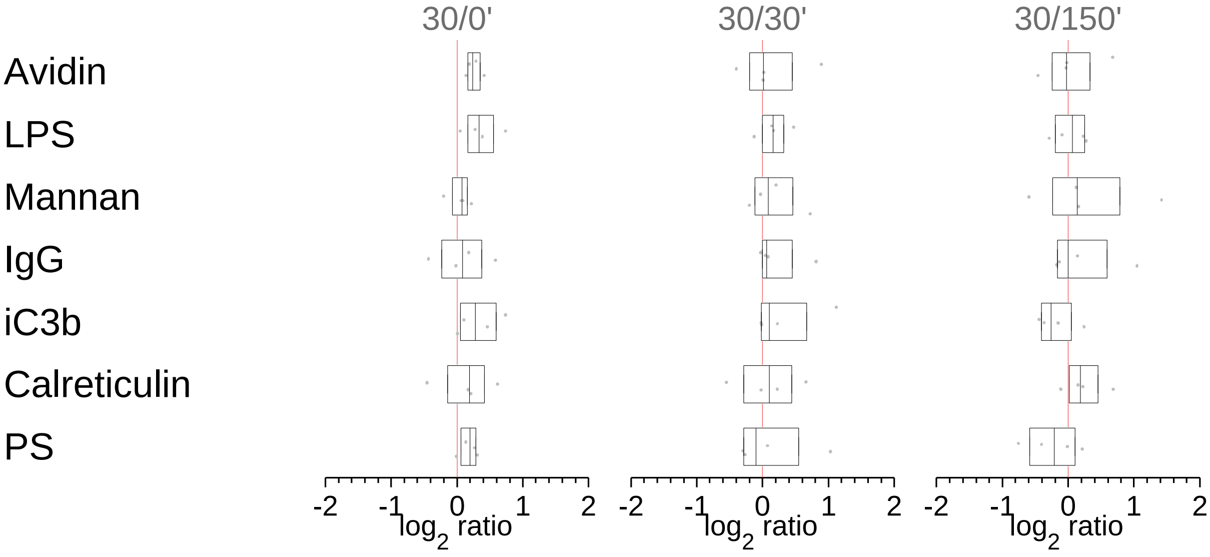

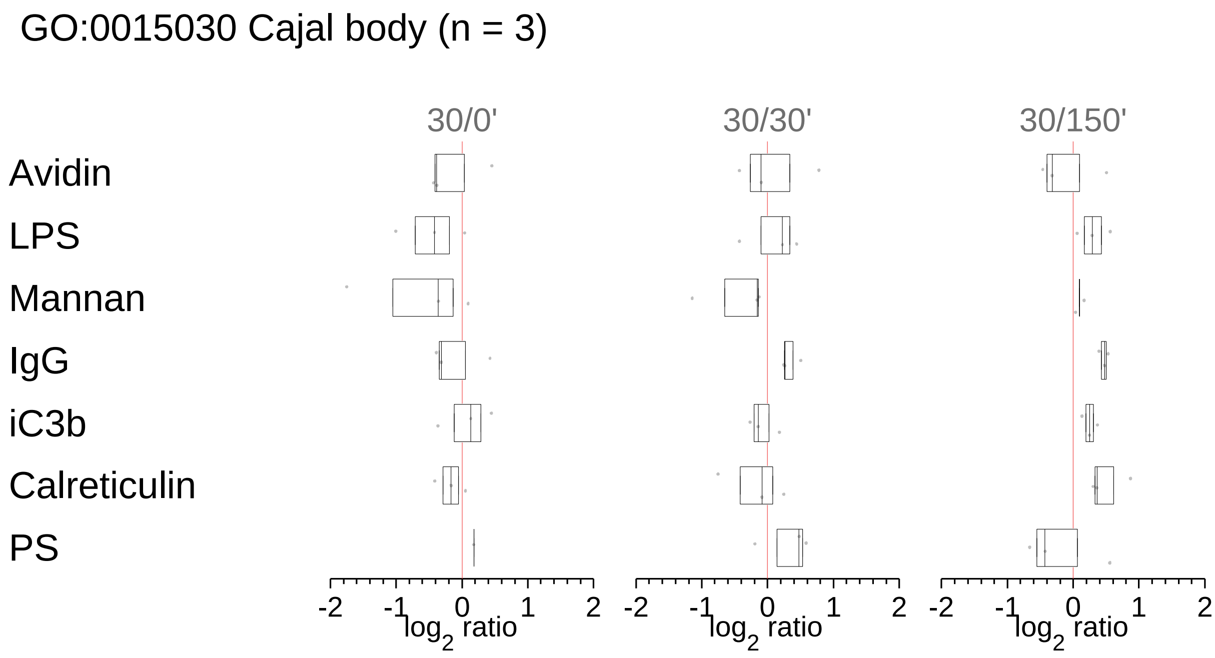
GO:0015030 Cajal body (n = 3)
30/0'
30/30'
30/150'
Avidin
LPS
Mannan
IgG
iC3b
Calreticulin
PS
-2
-1
0
1
2
log2 ratio

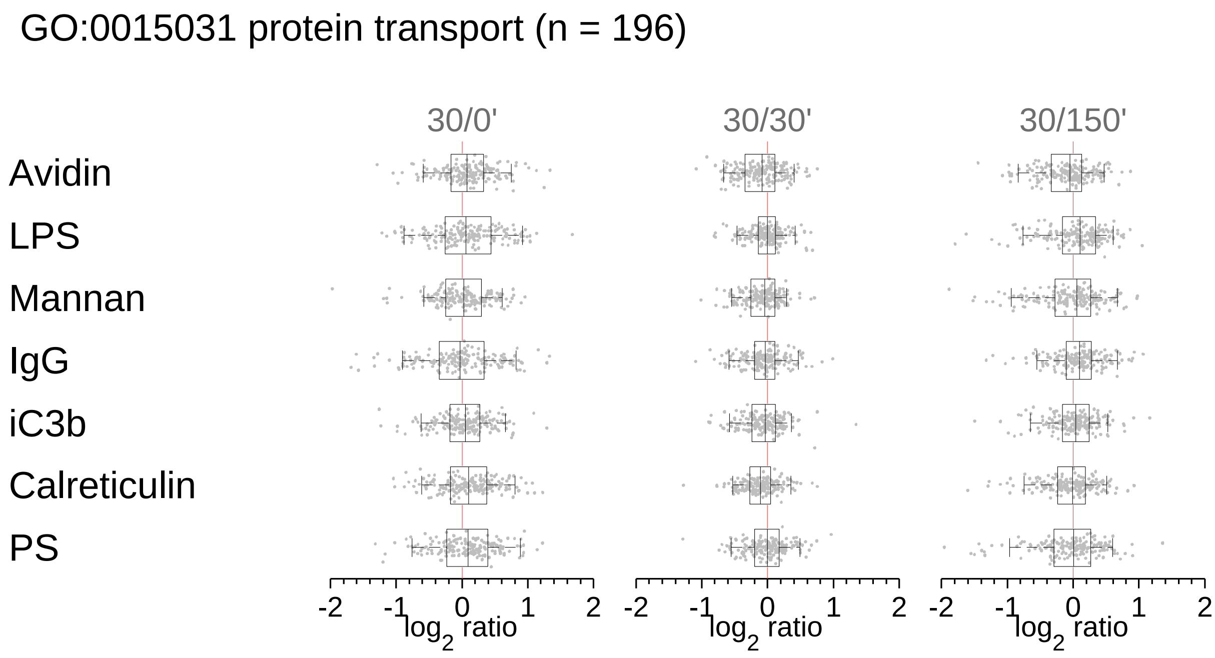
GO:0015031 protein transport (n = 196)
30/0'
30/30'
30/150'
Avidin
LPS
Mannan
IgG
iC3b
Calreticulin
PS
-2
-1
0
1
2
log2 ratio

GO:0015035 protein disulfide oxidoreductase activity (n = 3)

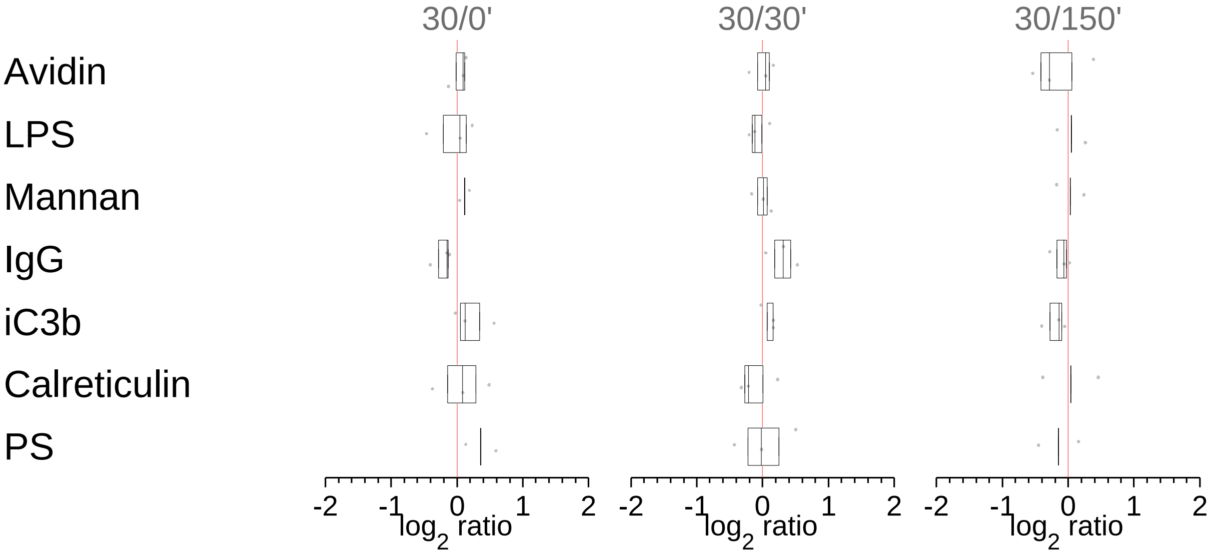

GO:0015078 hydrogen ion transmembrane transporter activity (n = 12)

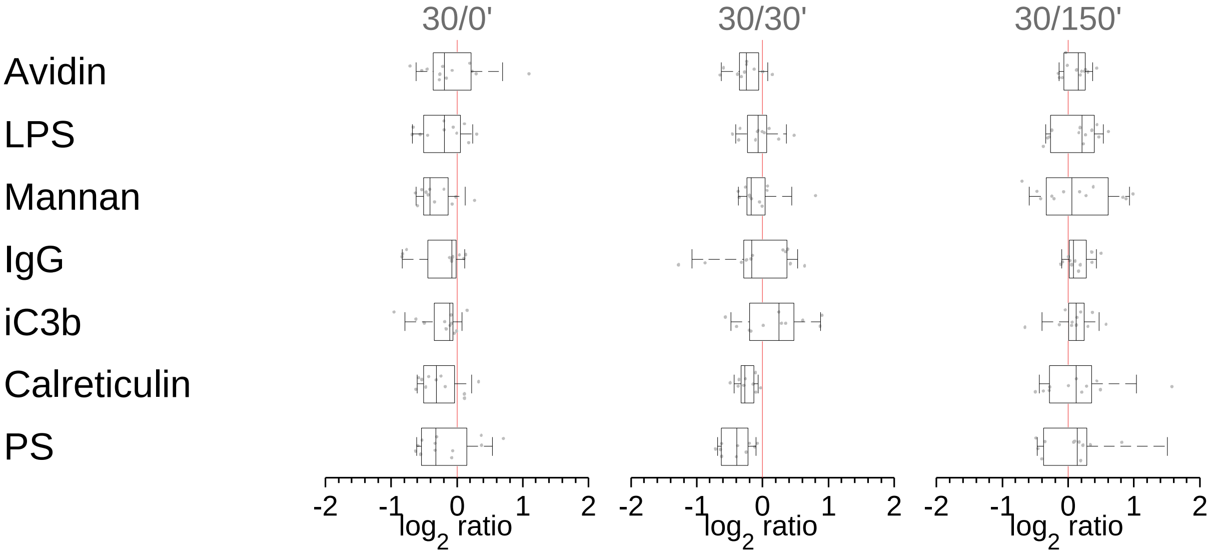

GO:0015171 amino acid transmembrane transporter activity (n = 5)

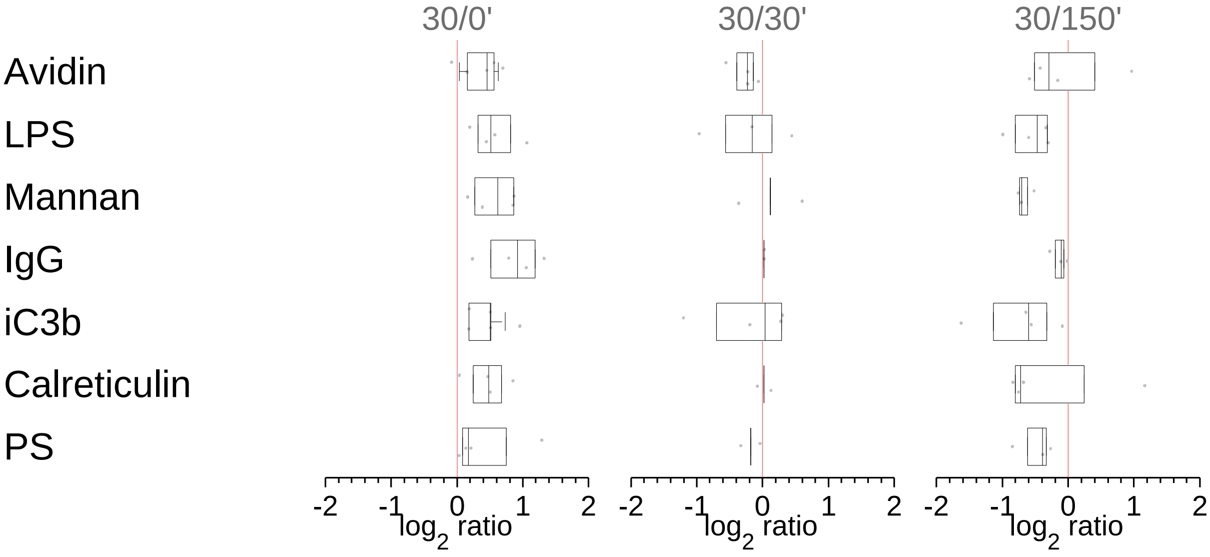

GO:0015175 neutral amino acid transmembrane transporter activity
(n = 3)

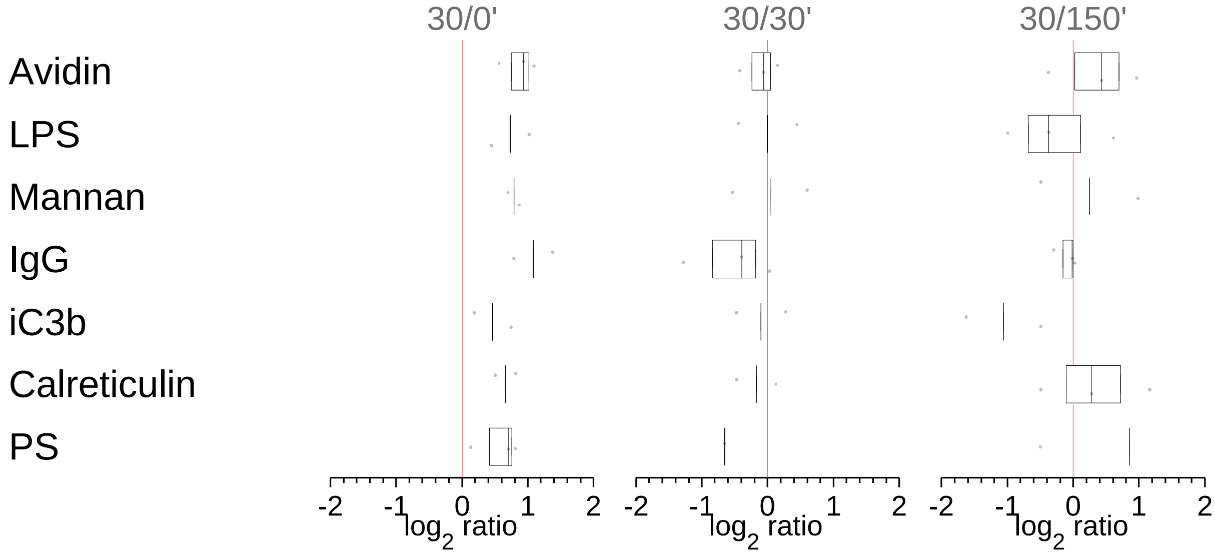

GO:0015179 L-amino acid transmembrane transporter activity (n = 4)

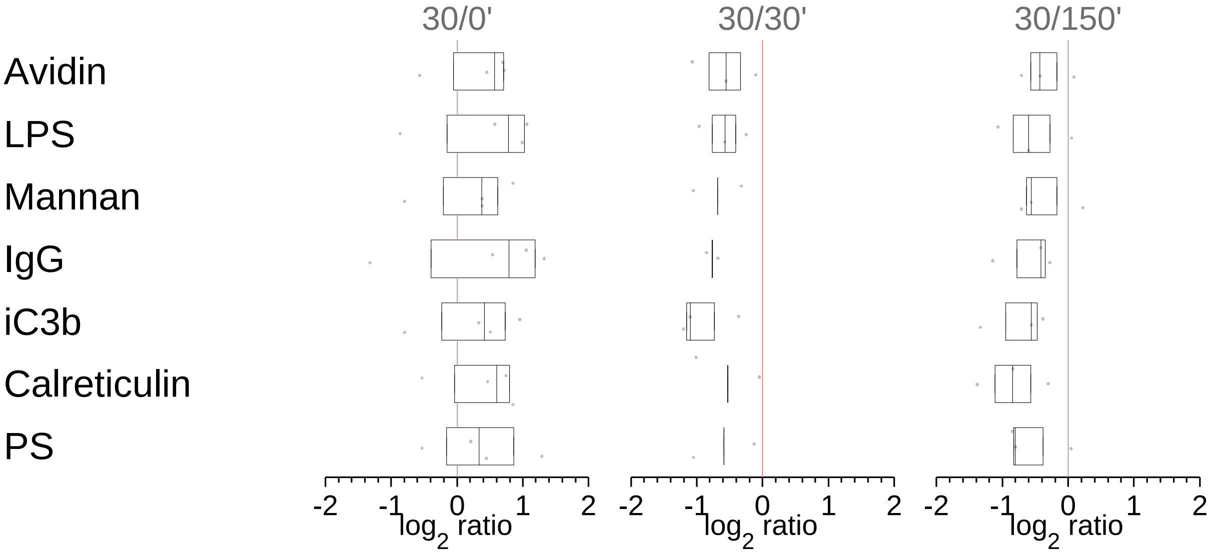

GO:0015183 L-aspartate transmembrane transporter activity (n = 3)

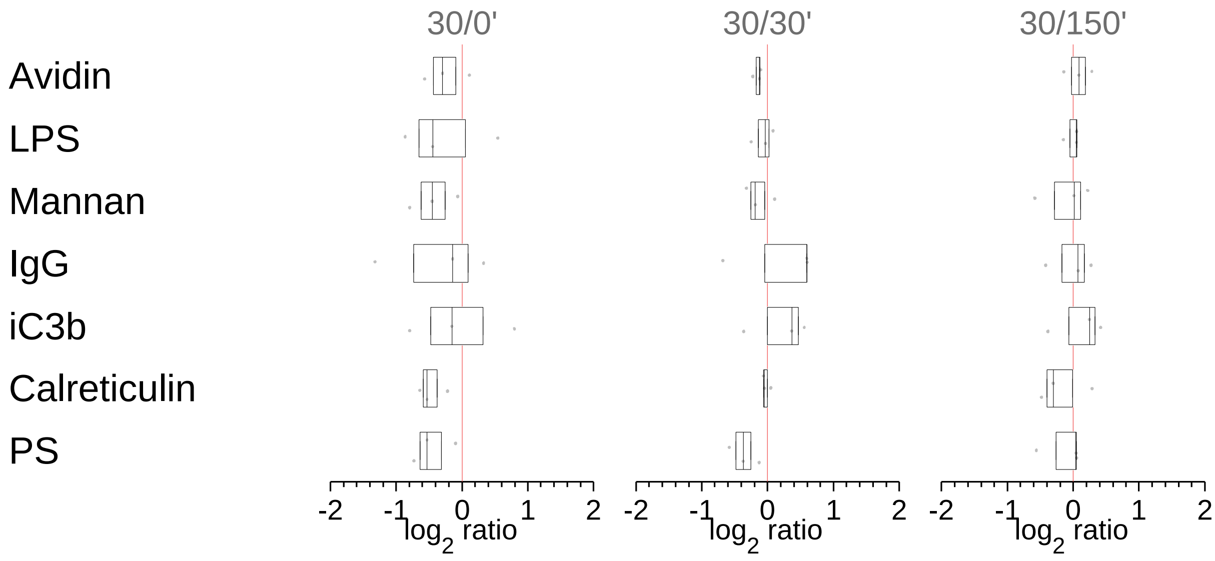

GO:0015194 L-serine transmembrane transporter activity (n = 3)

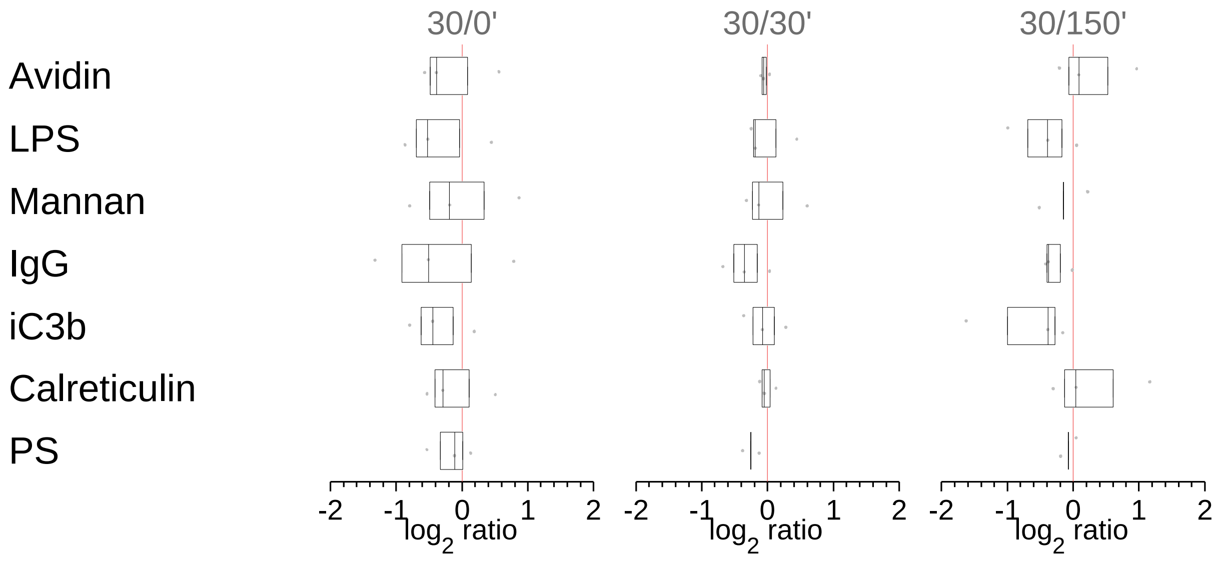

GO:0015238 drug transmembrane transporter activity (n = 4)

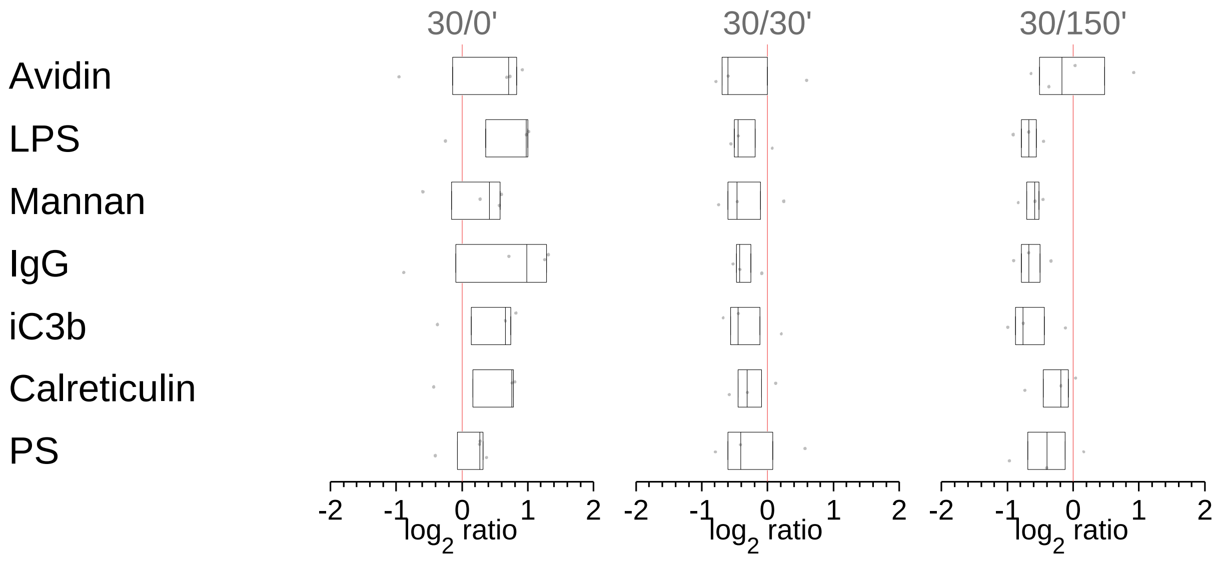

GO:0015288 porin activity (n = 4)

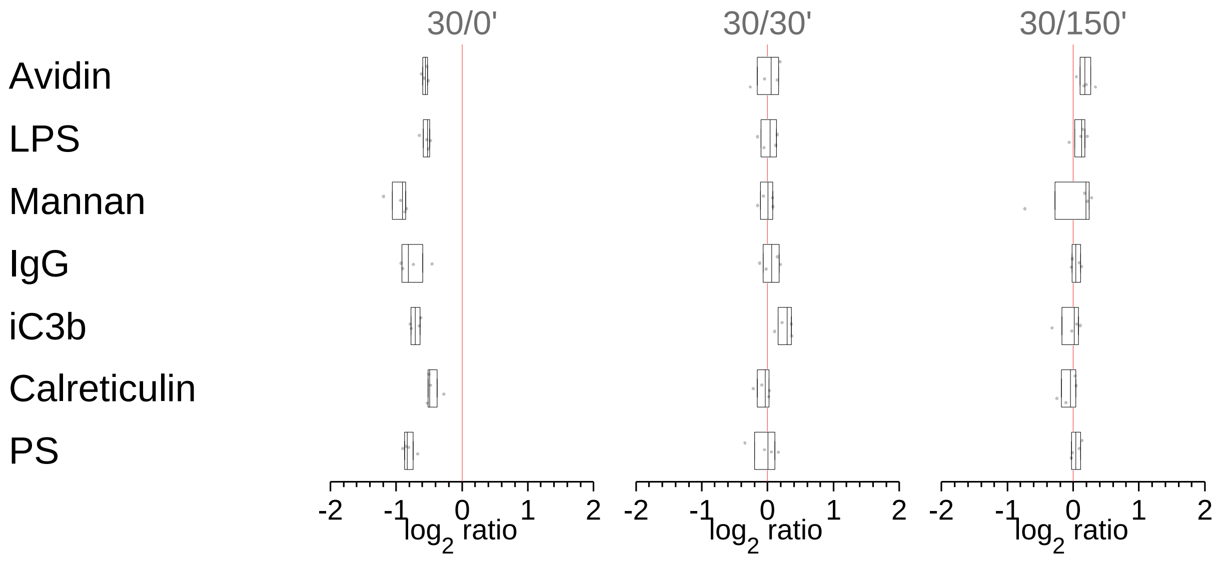

GO:0015293 symporter activity (n = 14)

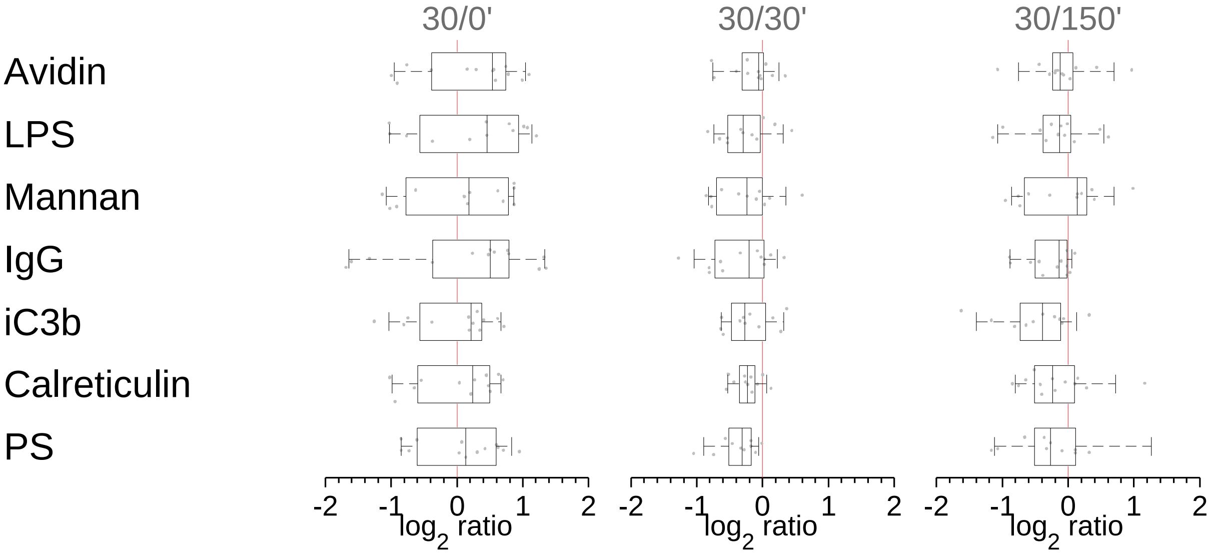

GO:0015297 antiporter activity (n = 6)

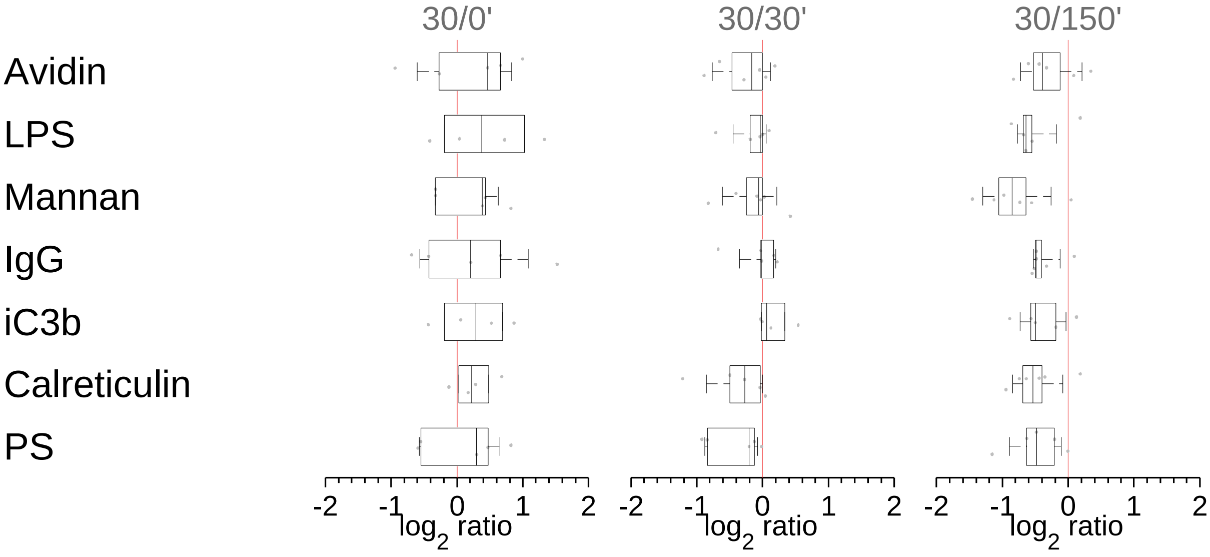

GO:0015485 cholesterol binding (n = 5)

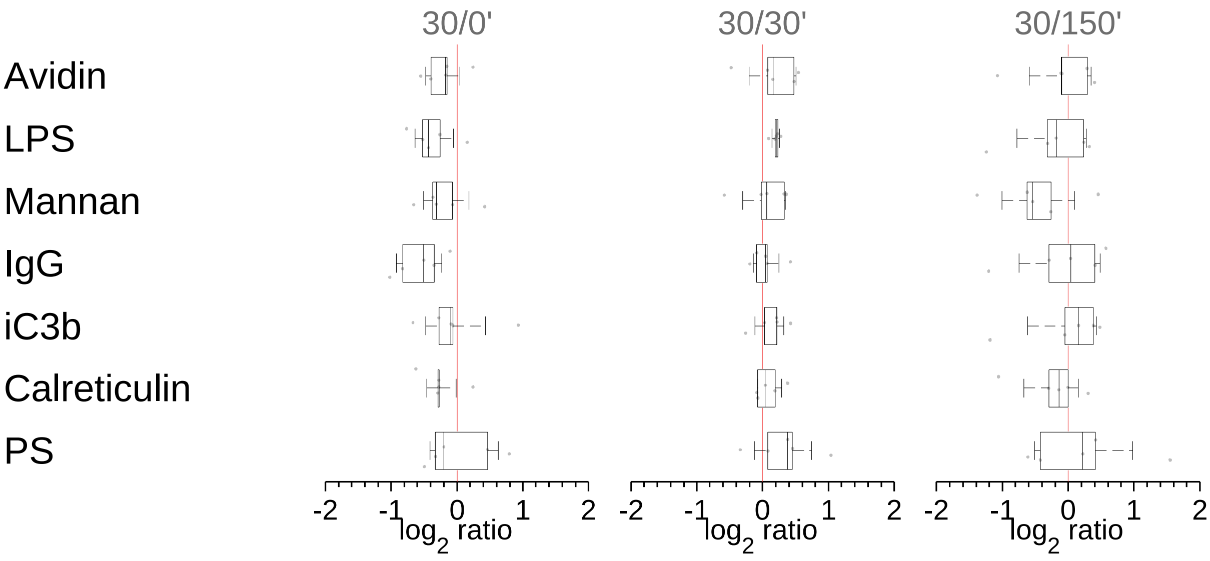

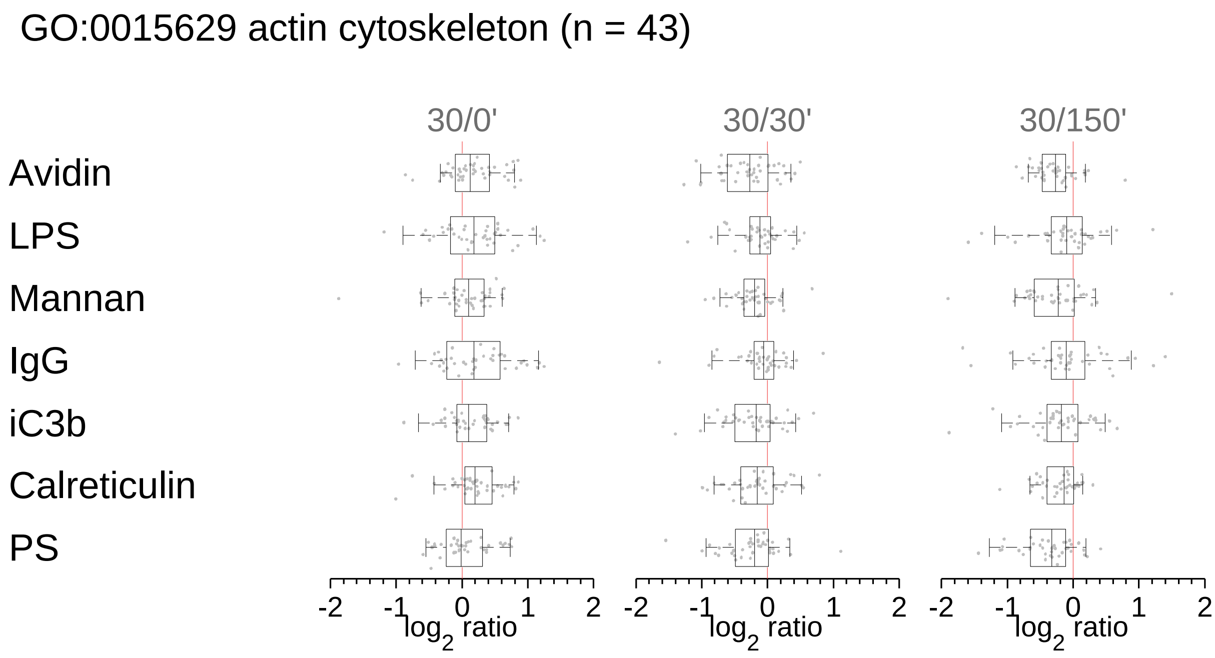
GO:0015629 actin cytoskeleton (n = 43)
30/0'
30/30'
30/150'
Avidin
LPS
Mannan
IgG
iC3b
Calreticulin
PS
-2
-1
0
1
2
log2 ratio

GO:0015630 microtubule cytoskeleton (n = 15)

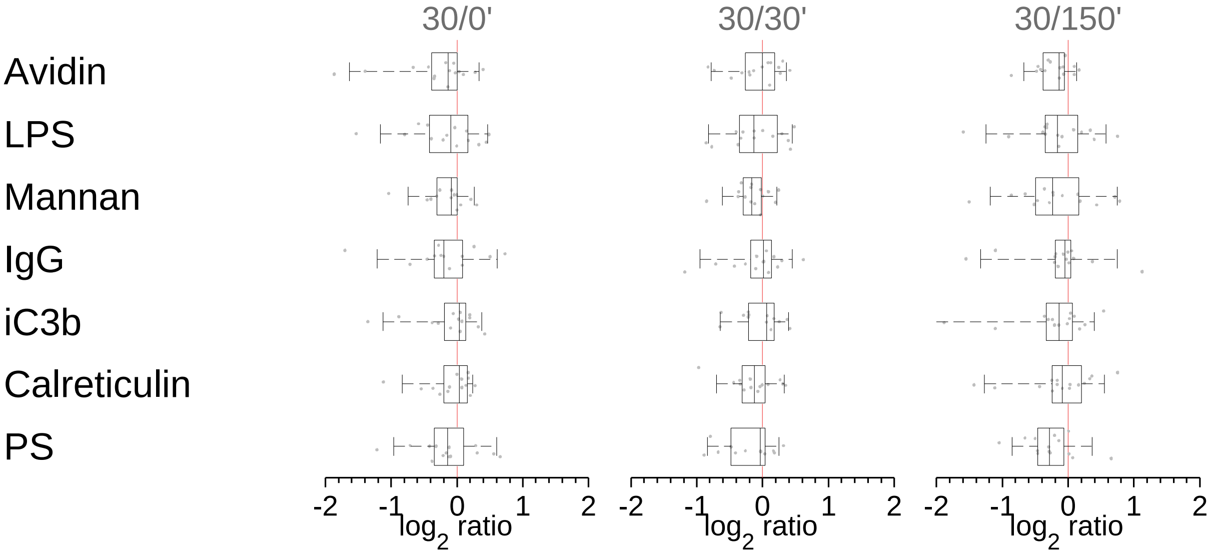

GO:0015631 tubulin binding (n = 5)

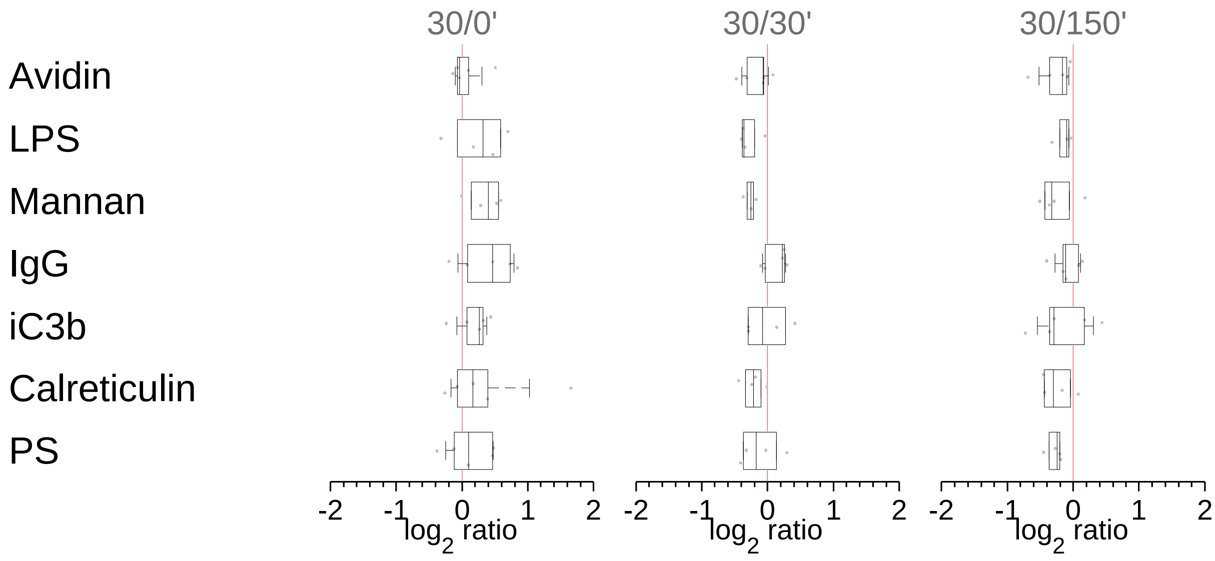

GO:0015711 organic anion transport (n = 3)

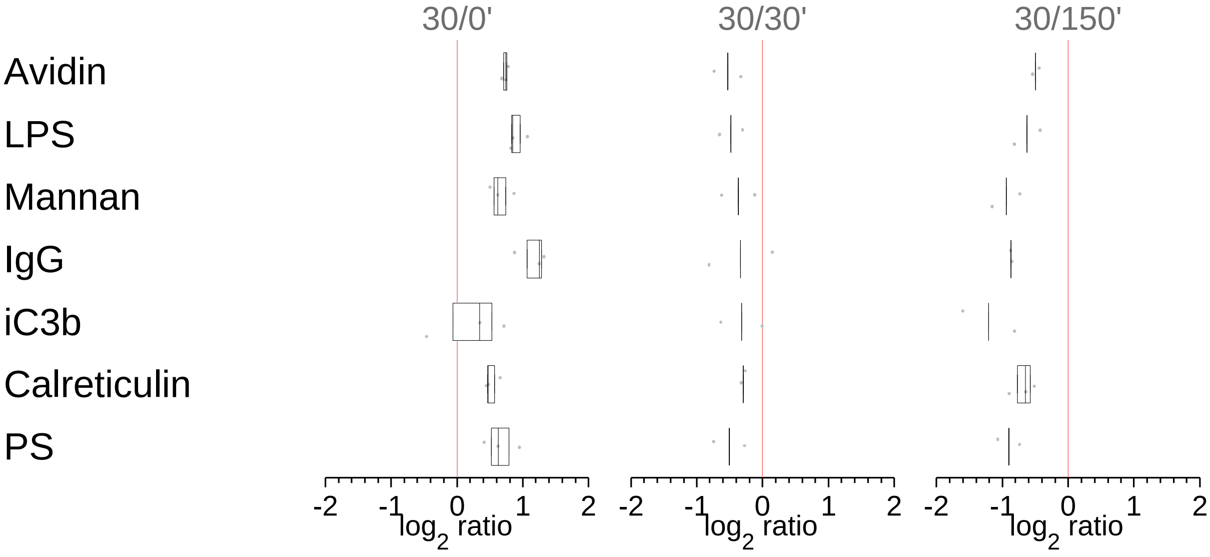

GO:0015758 glucose transport (n = 3)

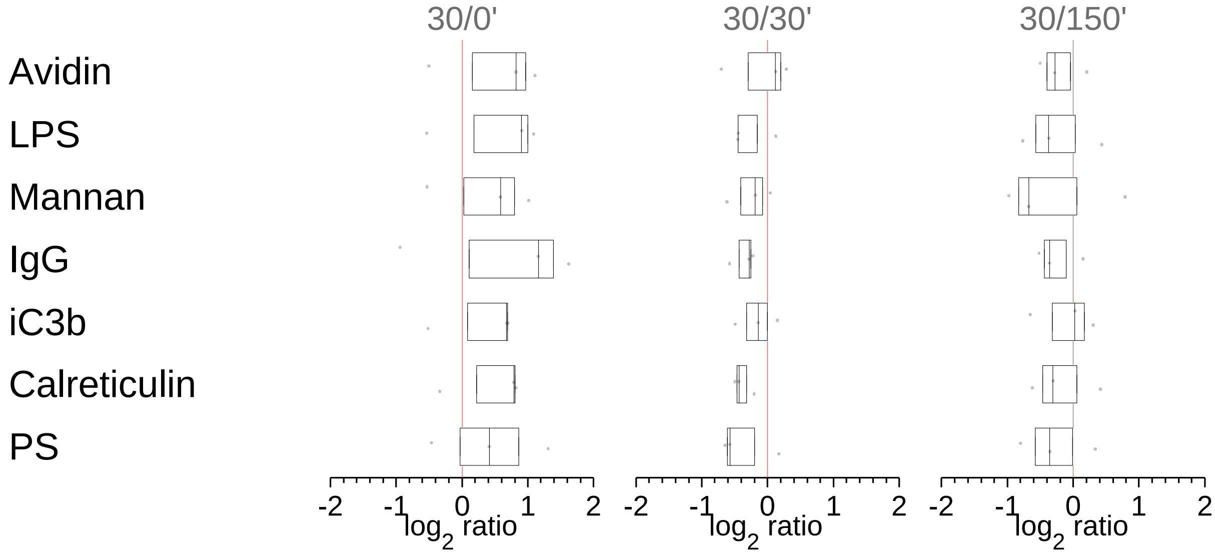

GO:0015804 neutral amino acid transport (n = 3)

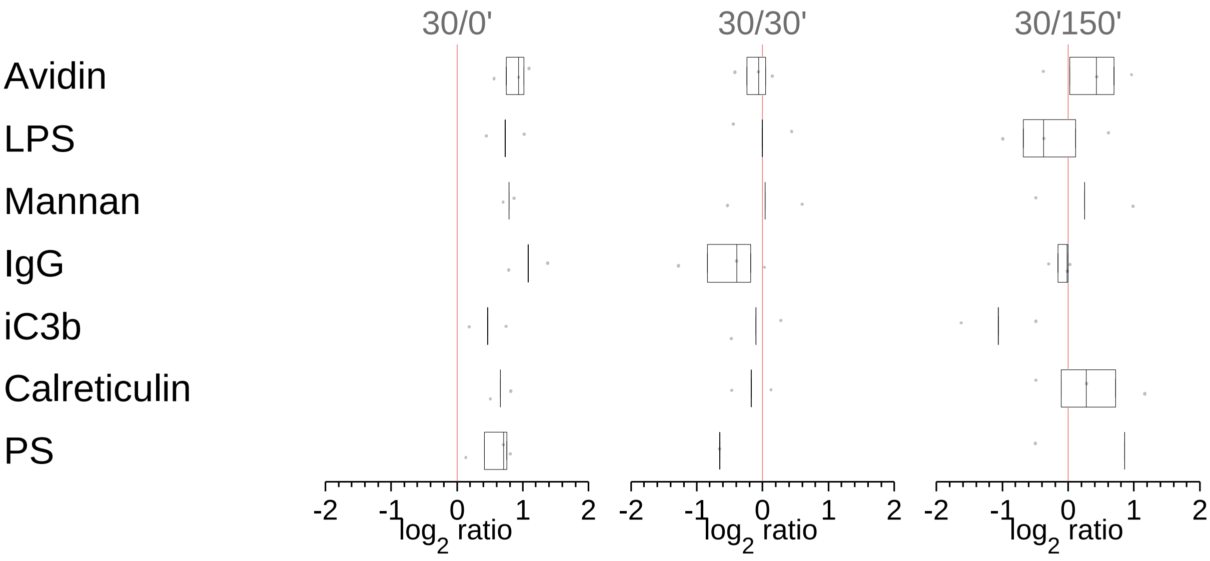

GO:0015807 L-amino acid transport (n = 3)

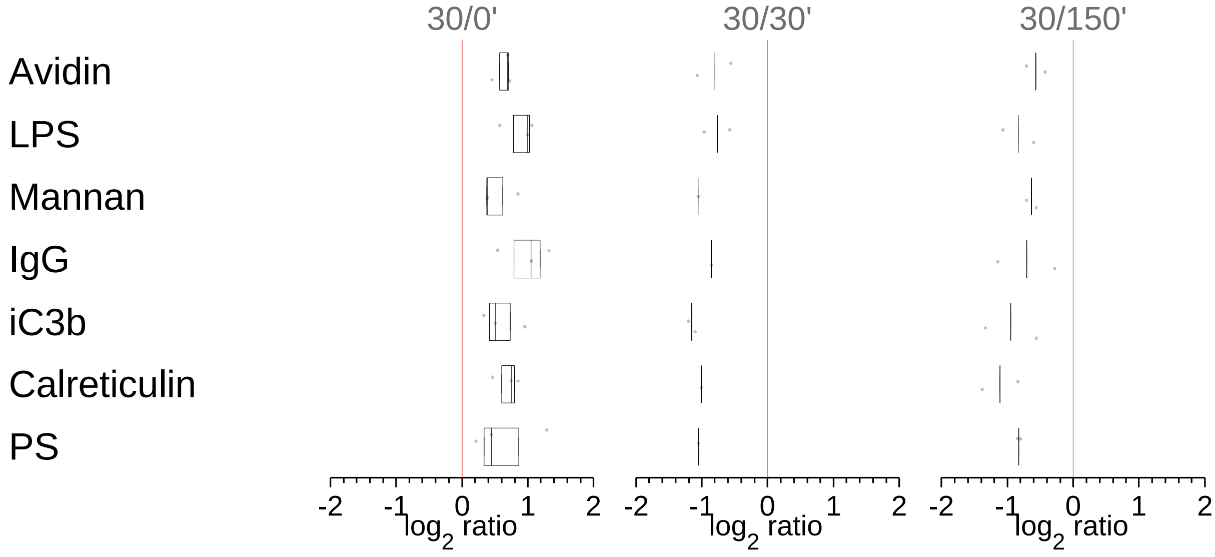

GO:0015809 arginine transport (n = 3)

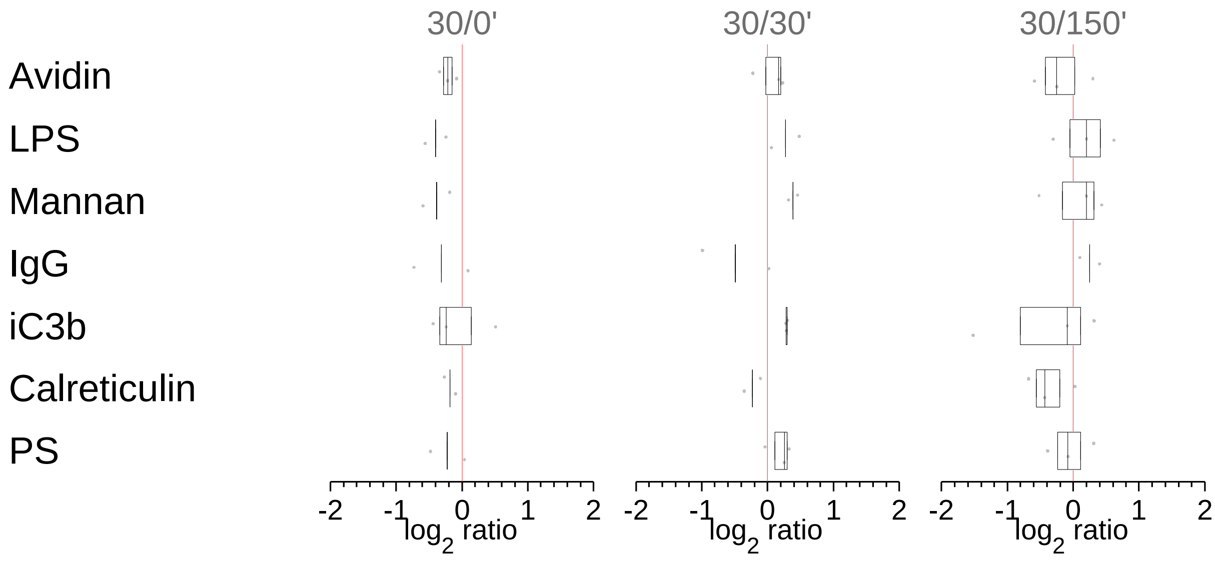

GO:0015810 aspartate transport (n = 4)

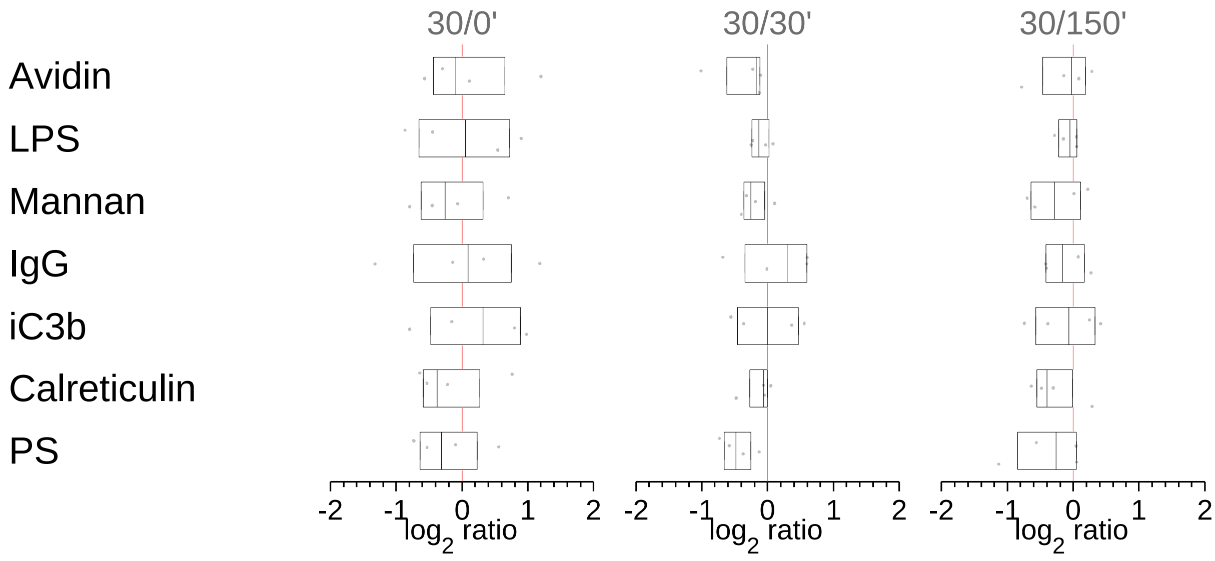

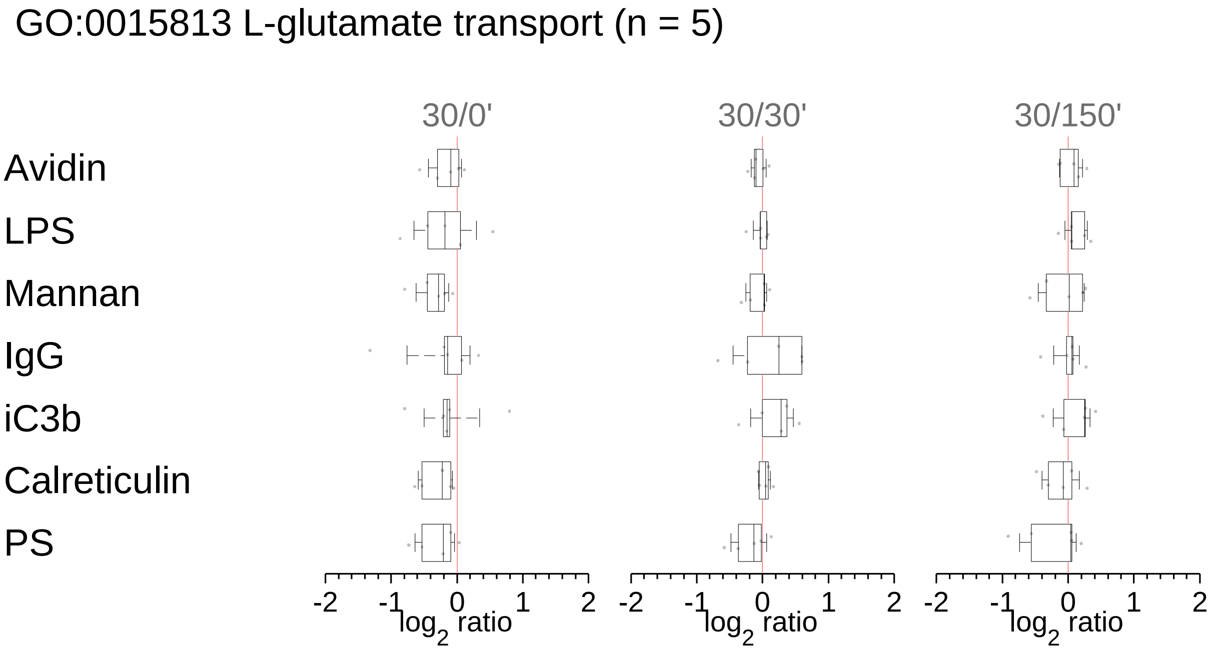

GO:0015813 L-glutamate transport (n = 5)
30/0'
30/30'
30/150'
Avidin
LPS
Mannan
IgG
iC3b
Calreticulin
PS
-2 -1 0 1 2
log2 ratio

GO:0015825 L-serine transport (n = 3)

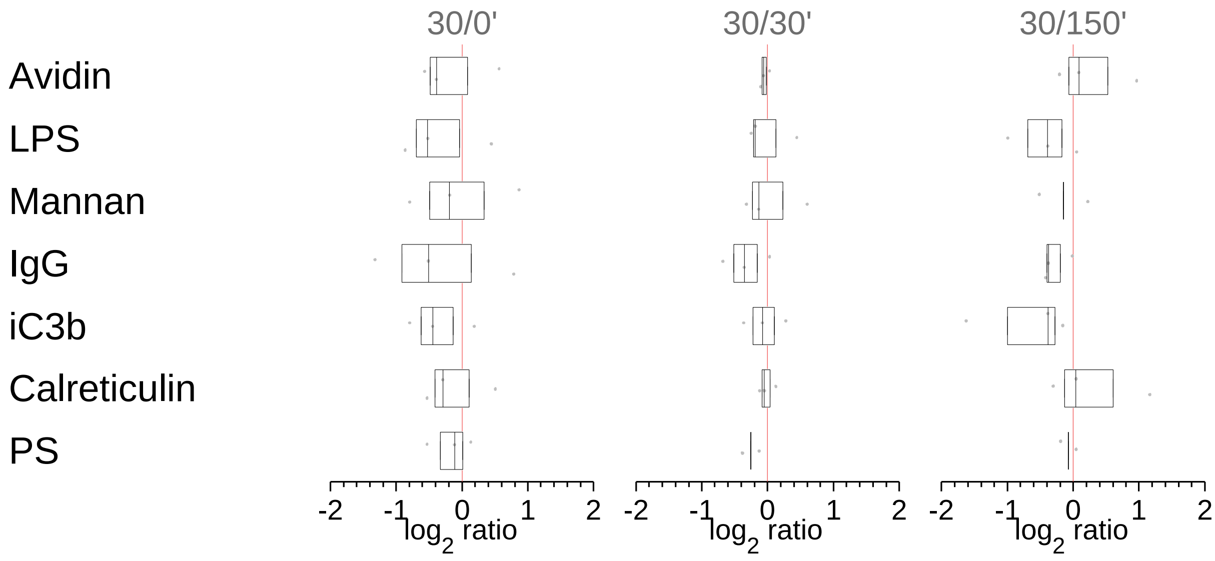

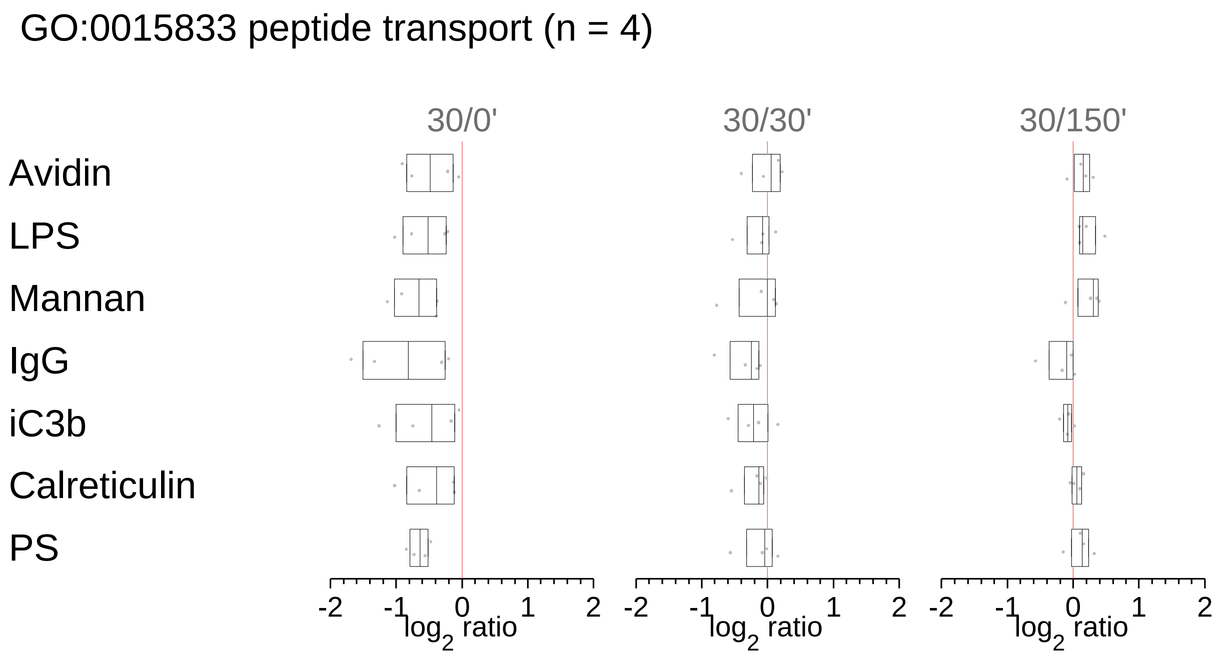
GO:0015833 peptide transport (n = 4)
30/0'
30/30'
30/150'
Avidin
LPS
Mannan
IgG
iC3b
Calreticulin
PS
-2
-1
0
1
2
log2 ratio

GO:0015908 fatty acid transport (n = 4)

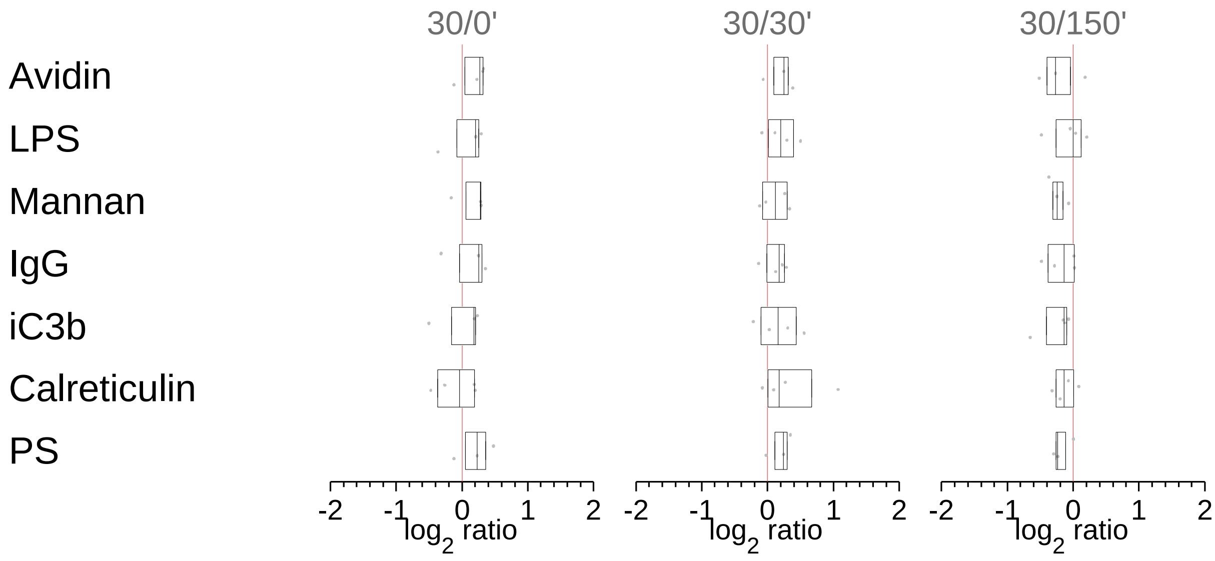

GO:0015914 phospholipid transport (n = 9)

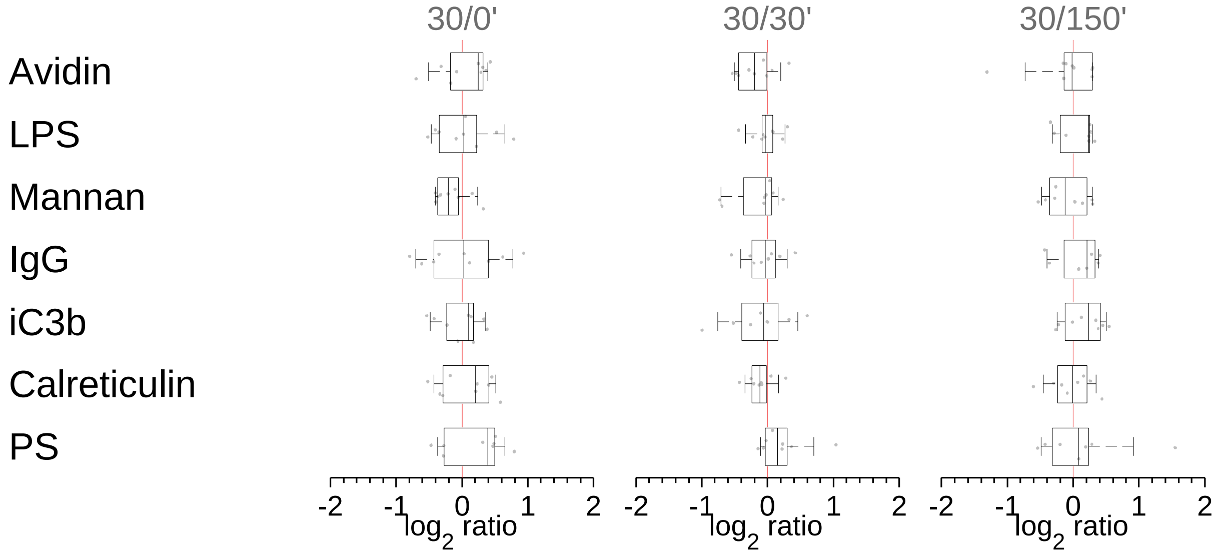

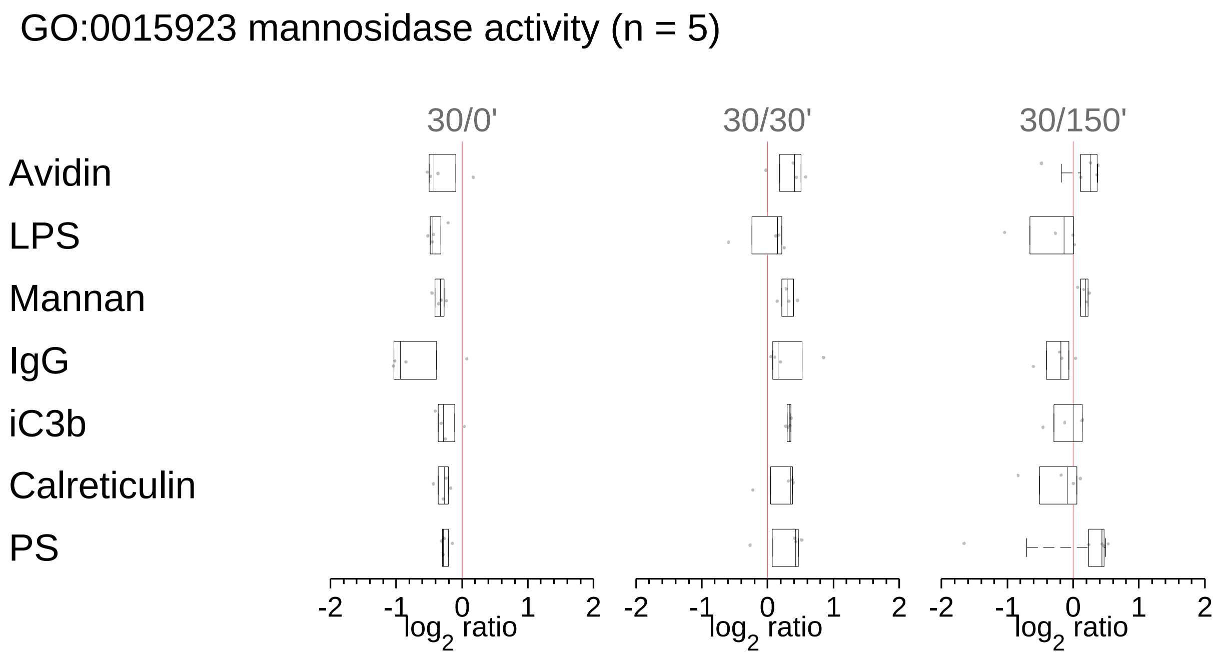
GO:0015923 mannosidase activity (n = 5)
30/0'
30/30'
30/150'
Avidin
LPS
Mannan
IgG
iC3b
Calreticulin
PS
-2 -1 0 1 2
log2 ratio

GO:0015926 glucosidase activity (n = 3)

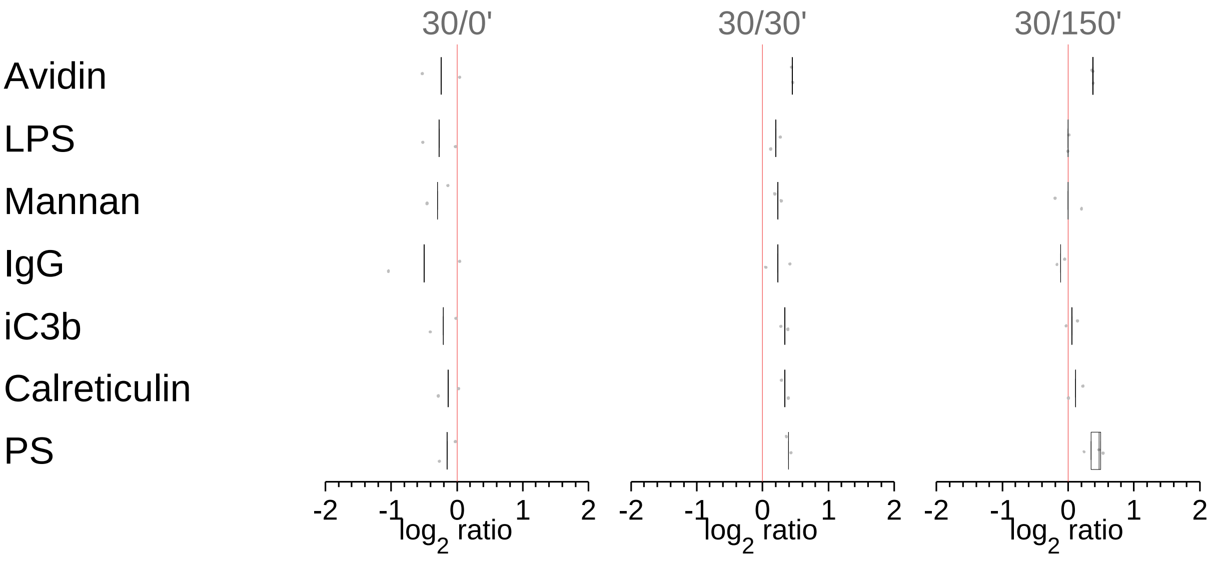

GO:0015928 fucosidase activity (n = 3)

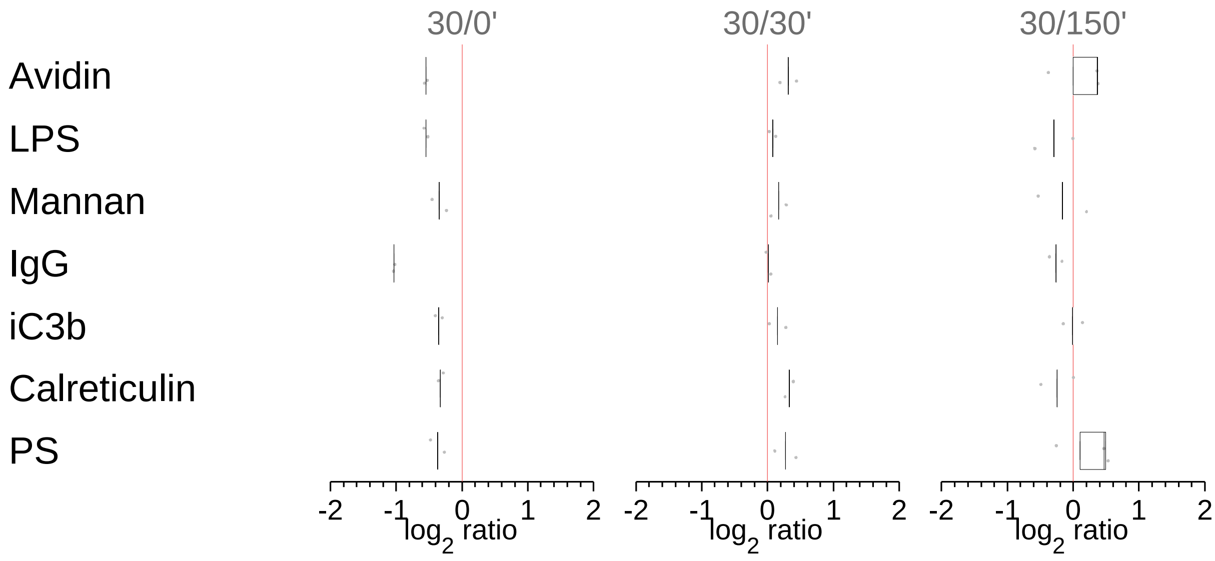

GO:0015929 hexosaminidase activity (n = 3)

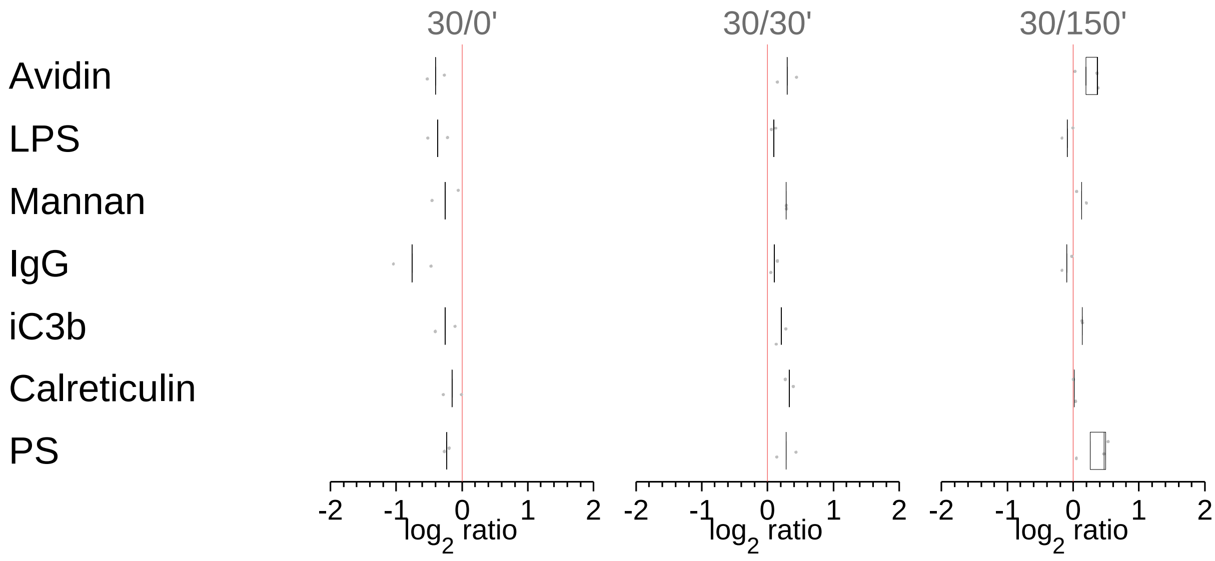

GO:0015934 large ribosomal subunit (n = 6)

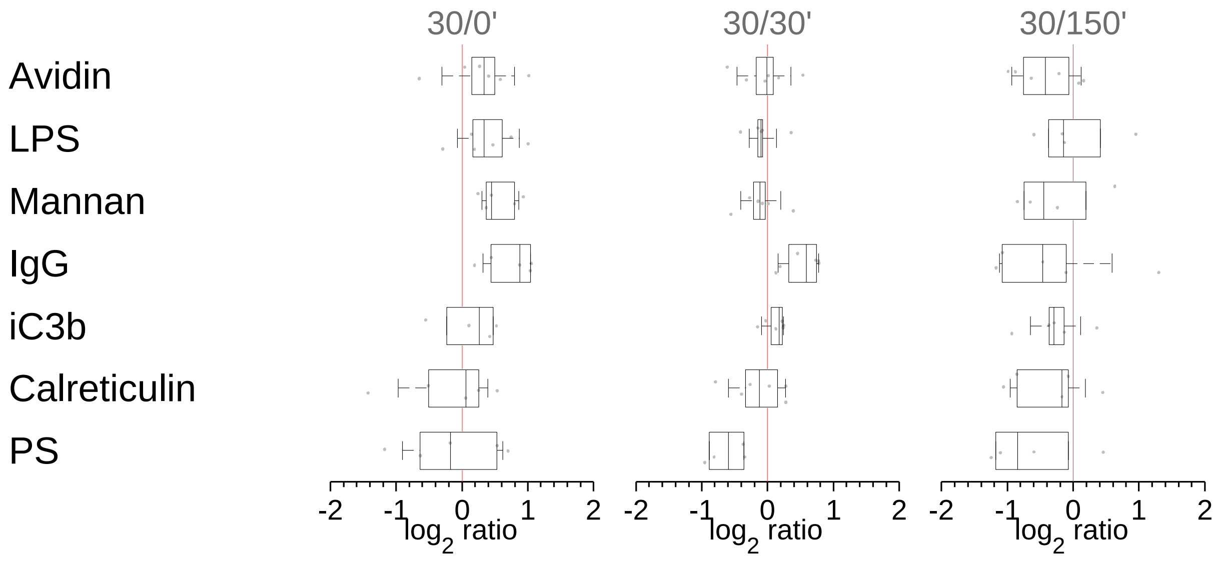

GO:0015935 small ribosomal subunit (n = 18)

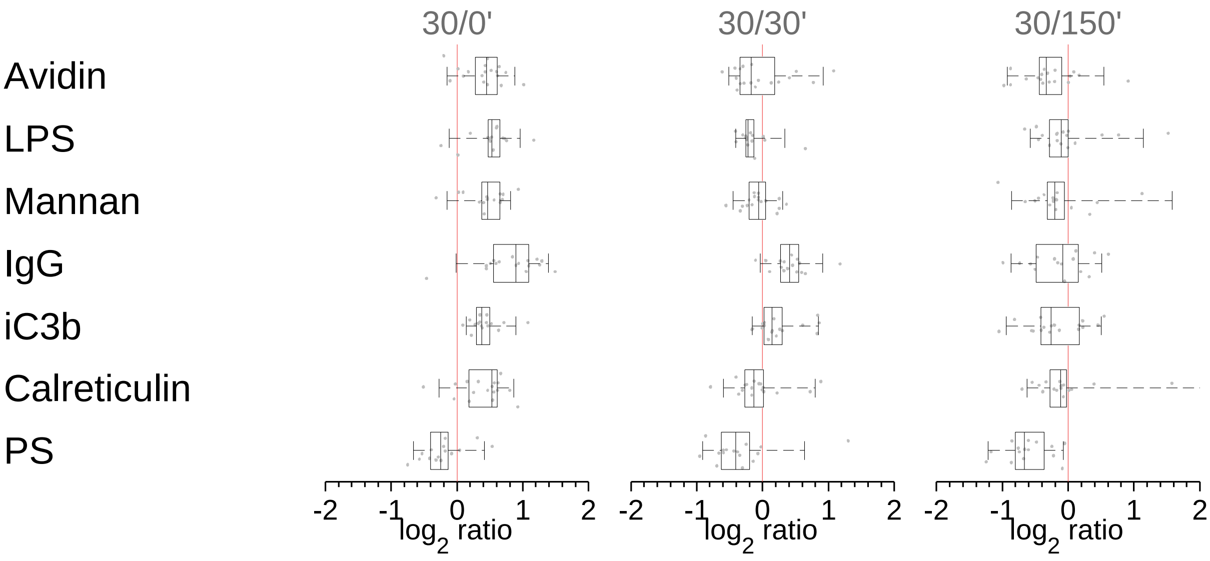

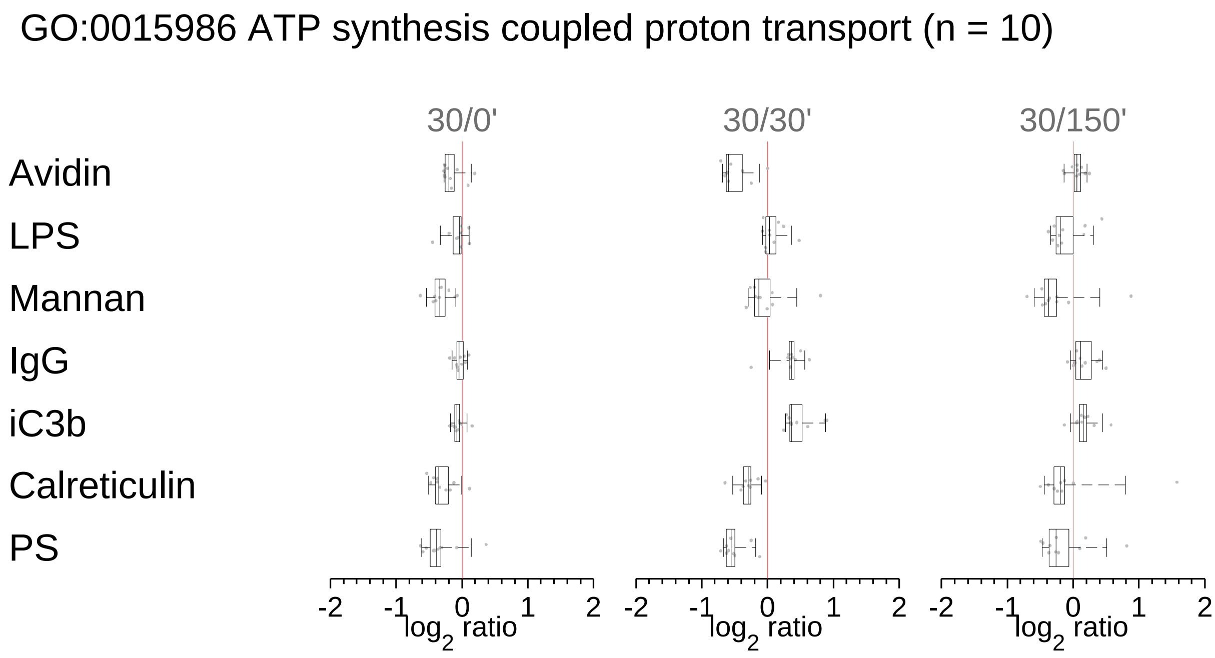
GO:0015986 ATP synthesis coupled proton transport (n = 10)
30/0'
30/30'
30/150'
Avidin
LPS
Mannan
IgG
iC3b
Calreticulin
PS
-2 -1 0 1 2
log2 ratio

GO:0015991 ATP hydrolysis coupled proton transport (n = 13)

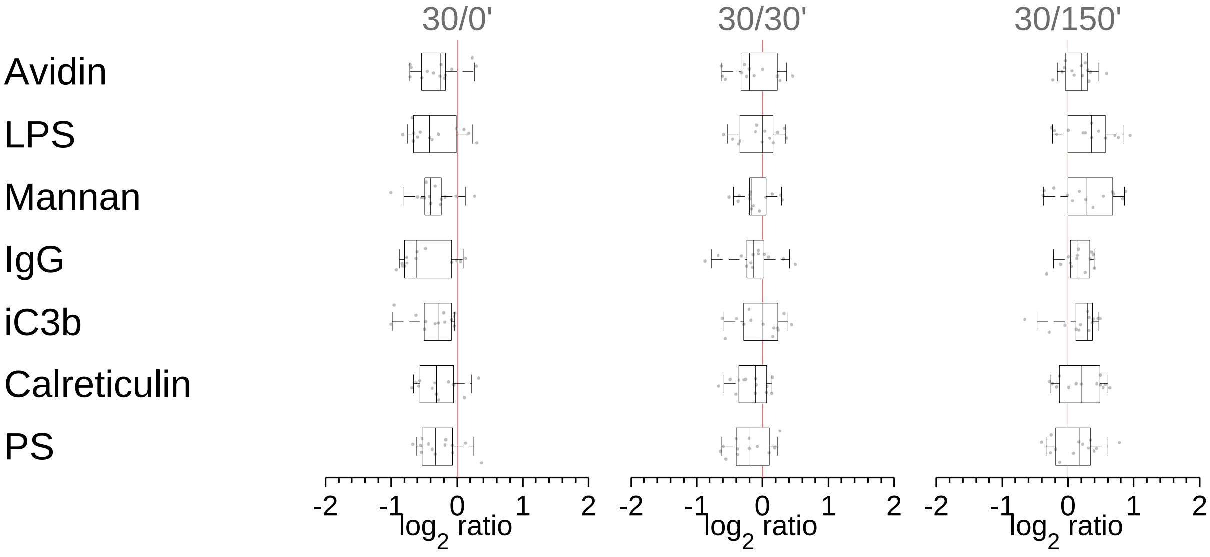

GO:0015992 proton transport (n = 26)

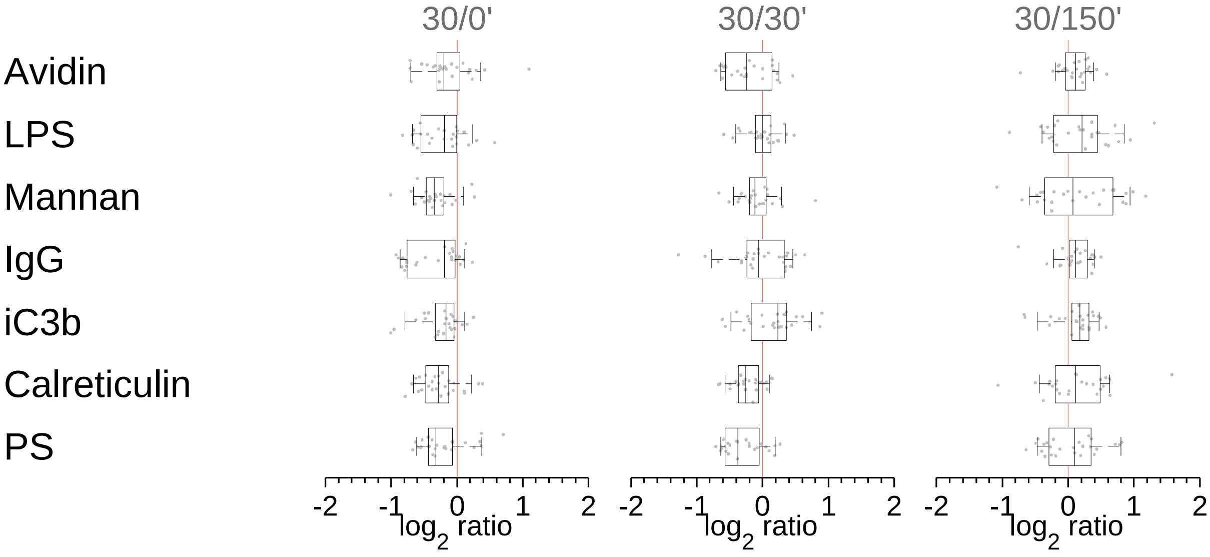

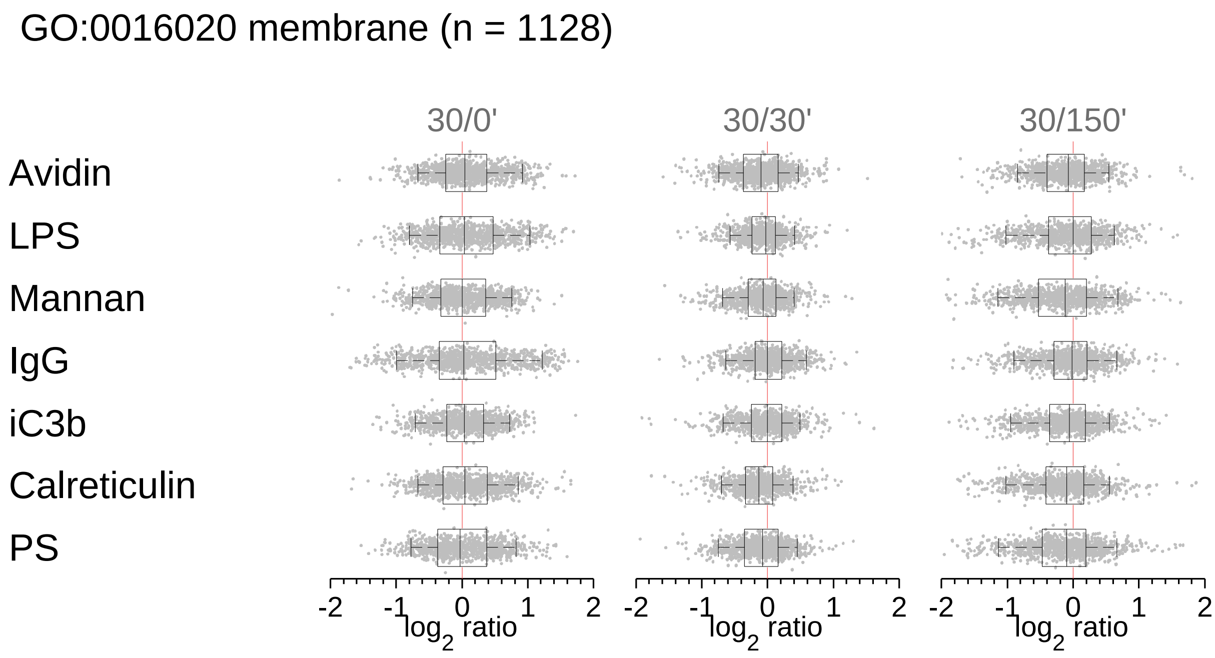

GO:0016020 membrane (n = 1128)
30/0'
30/30'
30/150'
Avidin
LPS
Mannan
IgG
iC3b
Calreticulin
PS
-2
-1
0
1
2
log2 ratio

GO:0016021 integral to membrane (n = 501)

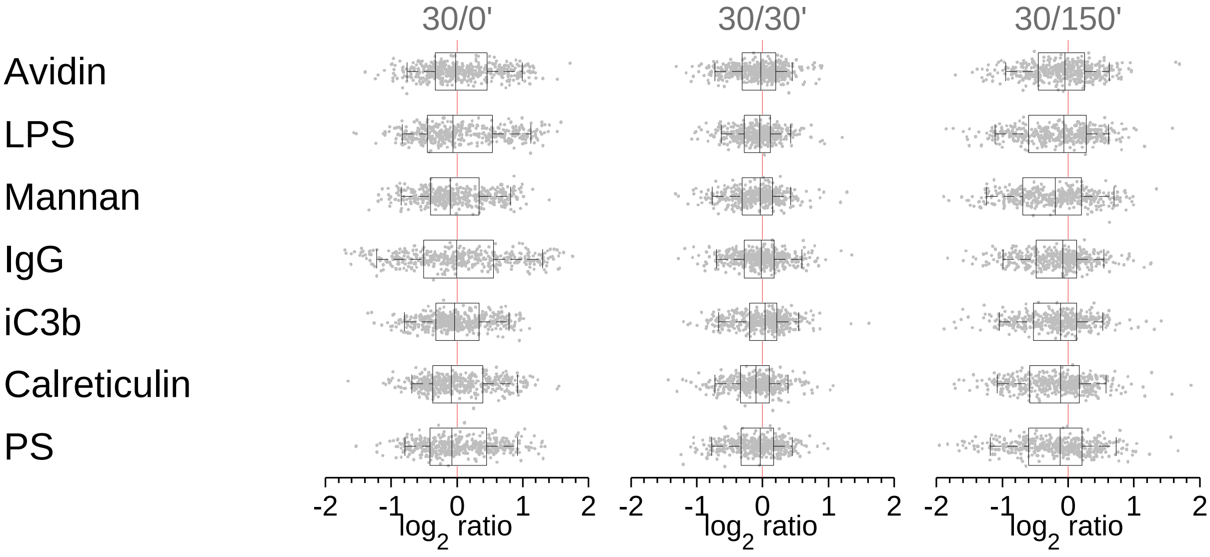

GO:0016023 cytoplasmic membrane-bounded vesicle (n = 22)

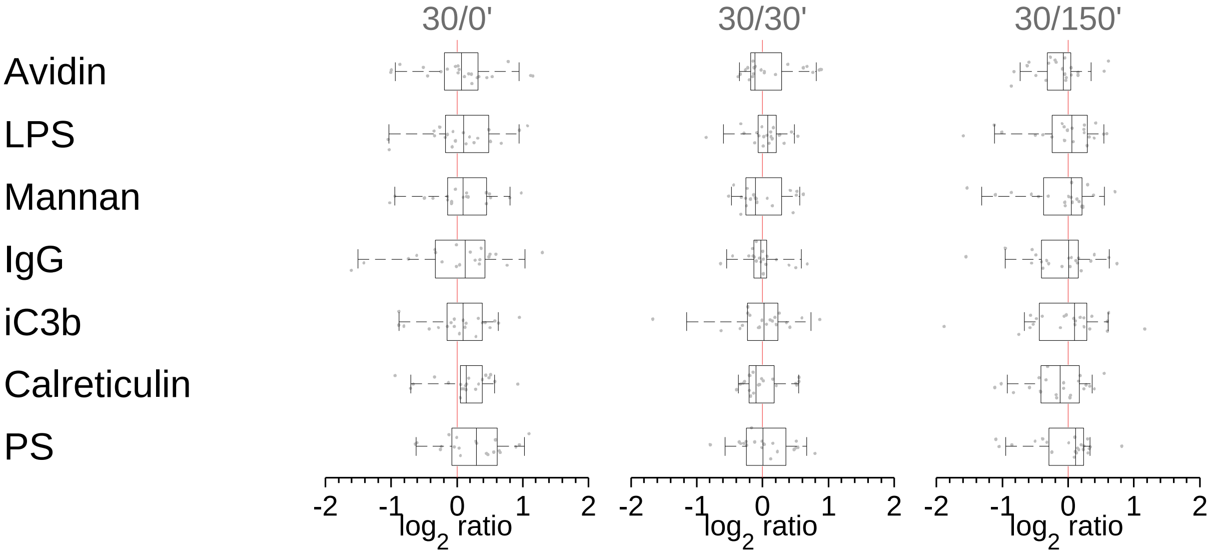

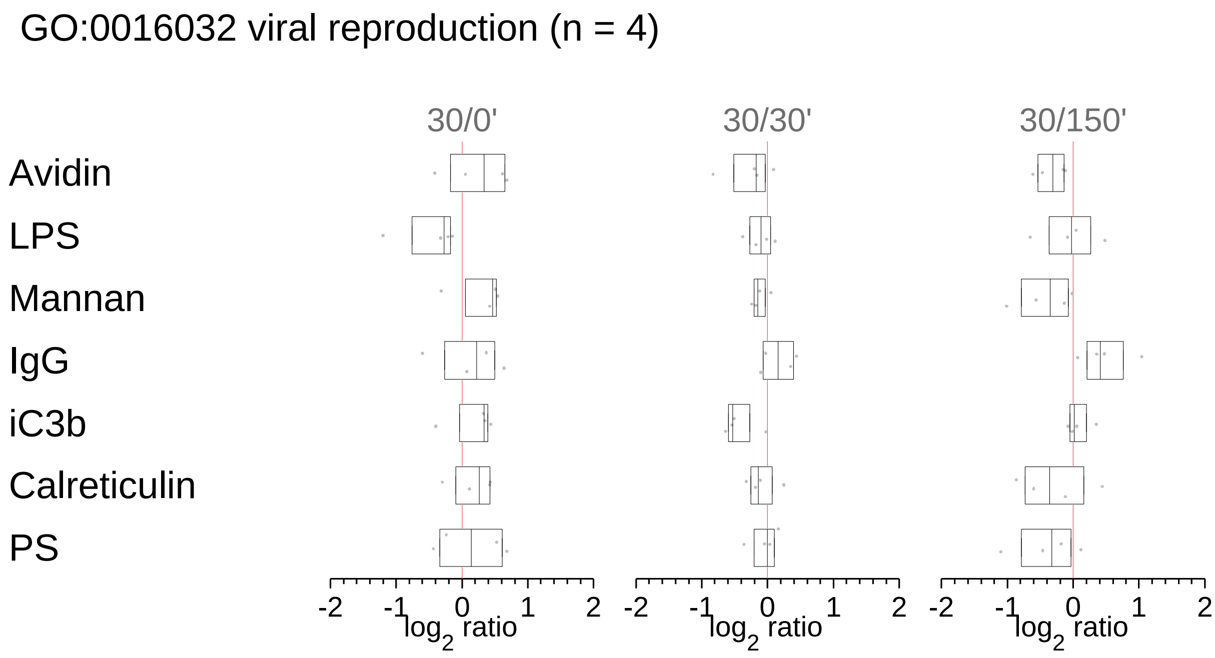
GO:0016032 viral reproduction (n = 4)
30/0'
30/30'
30/150'
Avidin
LPS
Mannan
IgG
iC3b
Calreticulin
PS
-2 -1 0 1 2
log2 ratio

GO:0016042 lipid catabolic process (n = 14)

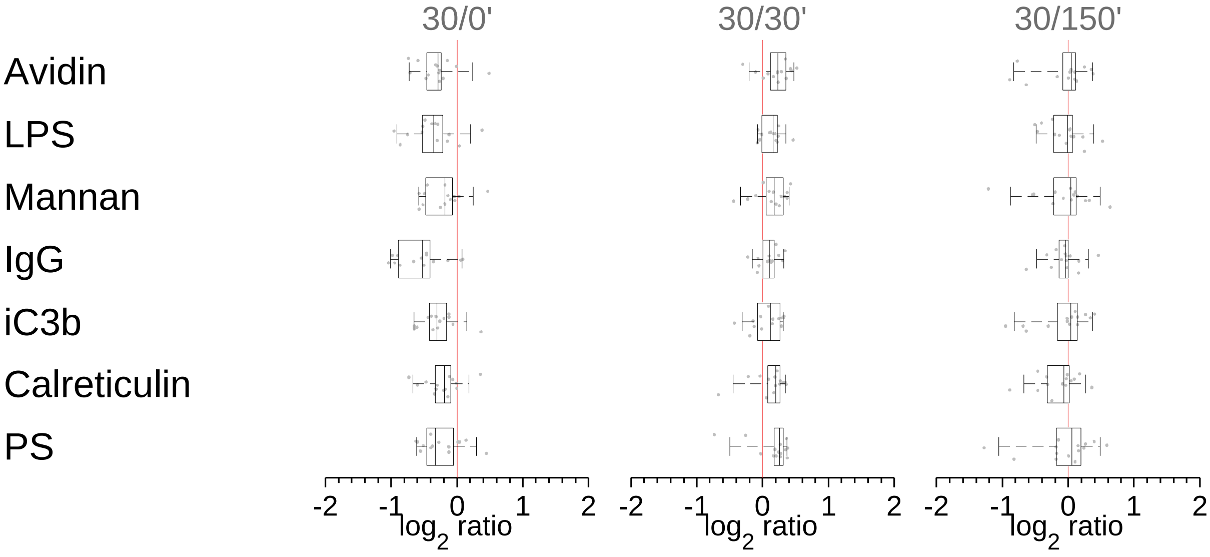

GO:0016043 cellular component organization (n = 4)

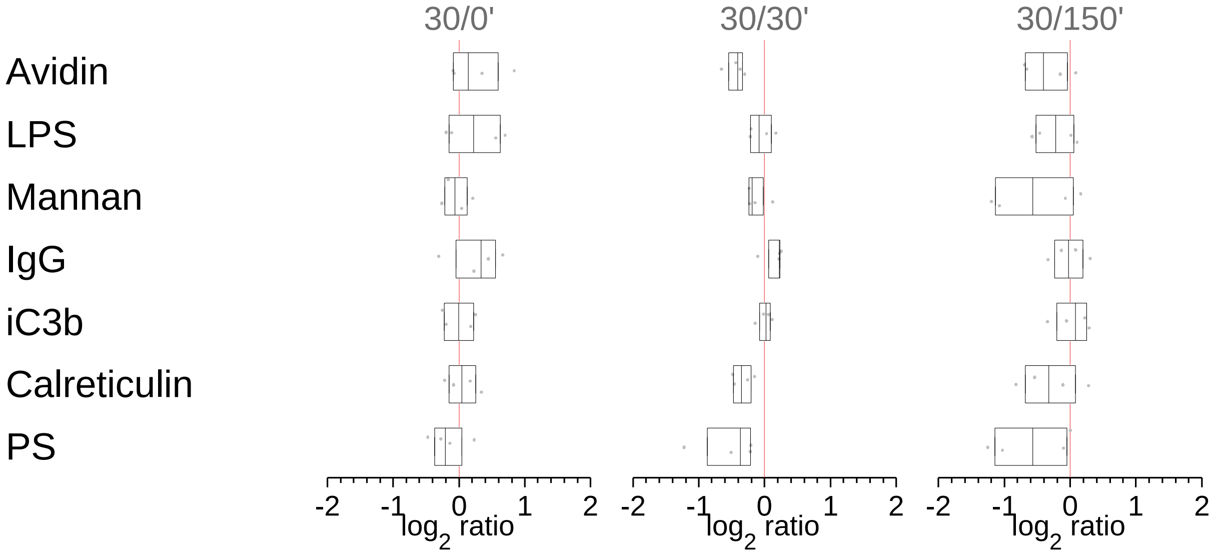

GO:0016049 cell growth (n = 6)

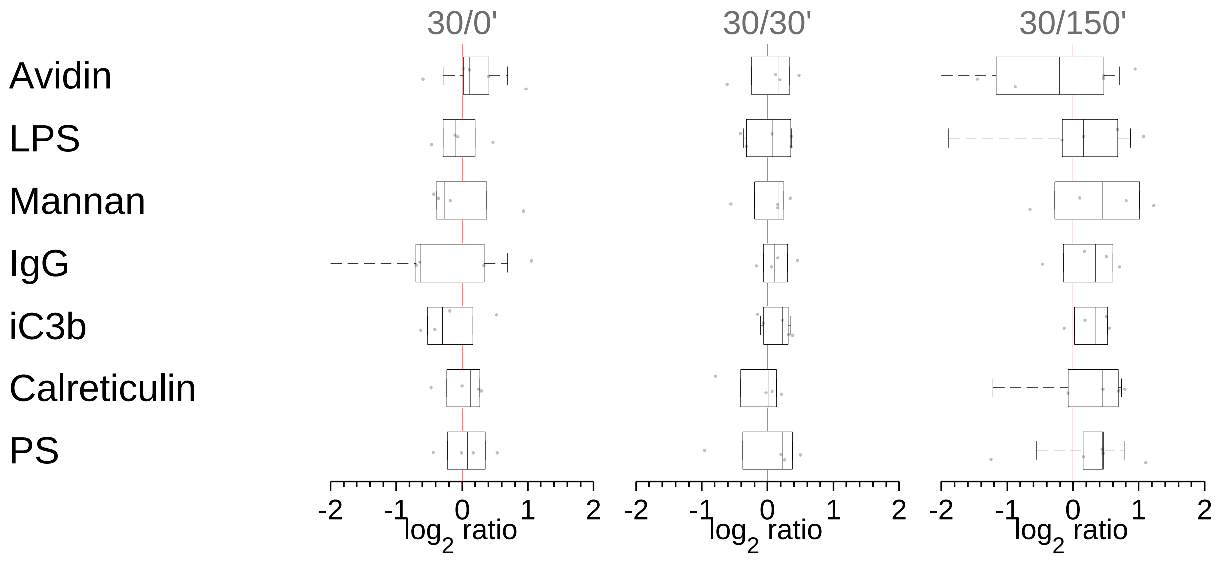

GO:0016055 Wnt receptor signaling pathway (n = 13)

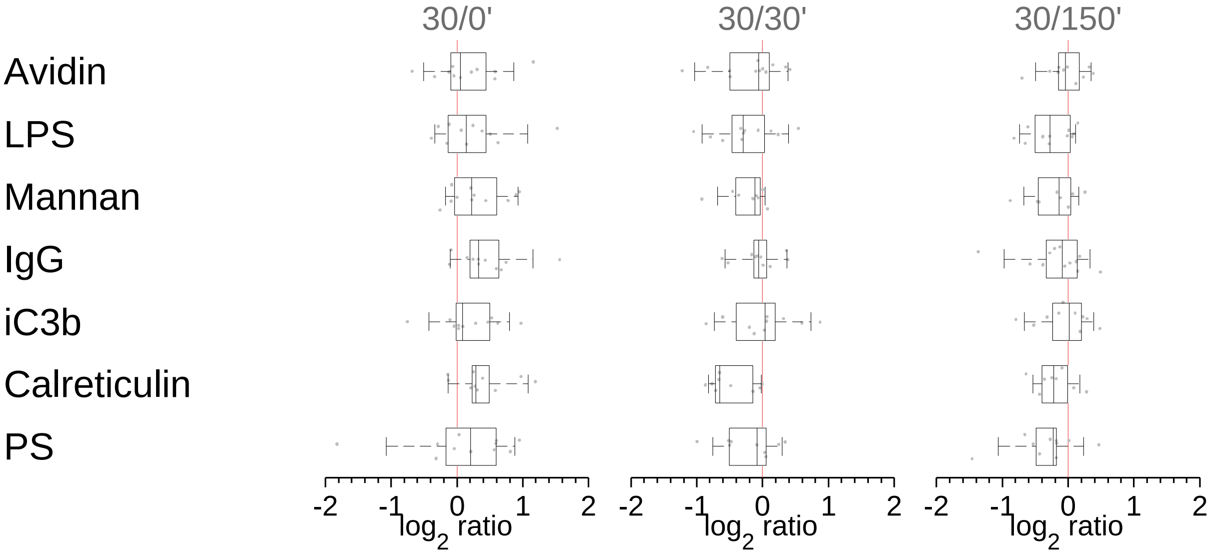

GO:0016064 immunoglobulin mediated immune response (n = 6)

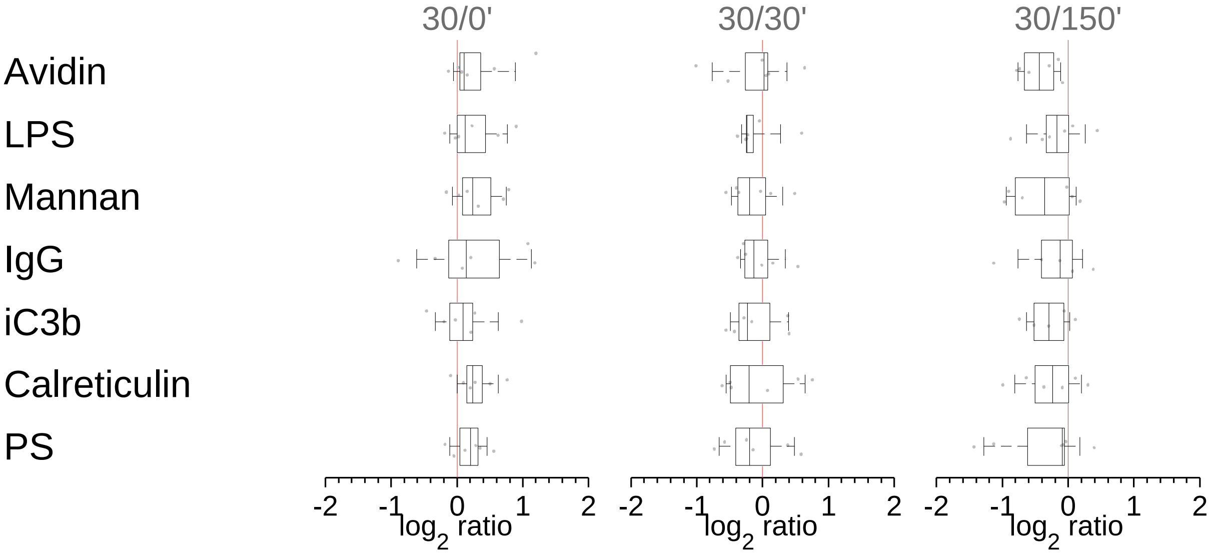

GO:0016079 synaptic vesicle exocytosis (n = 6)

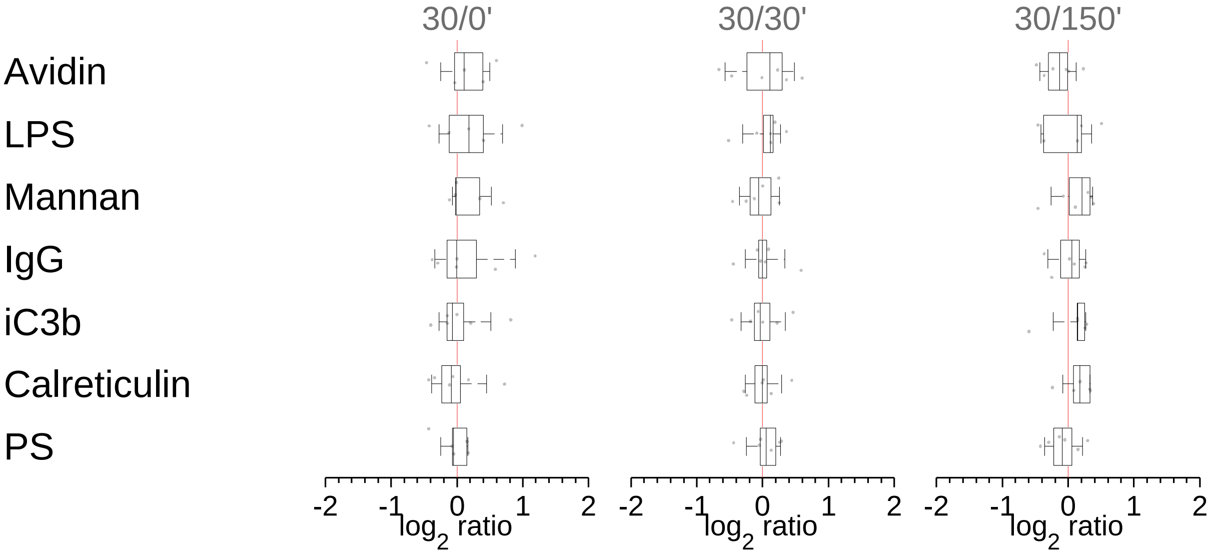

GO:0016126 sterol biosynthetic process (n = 3)

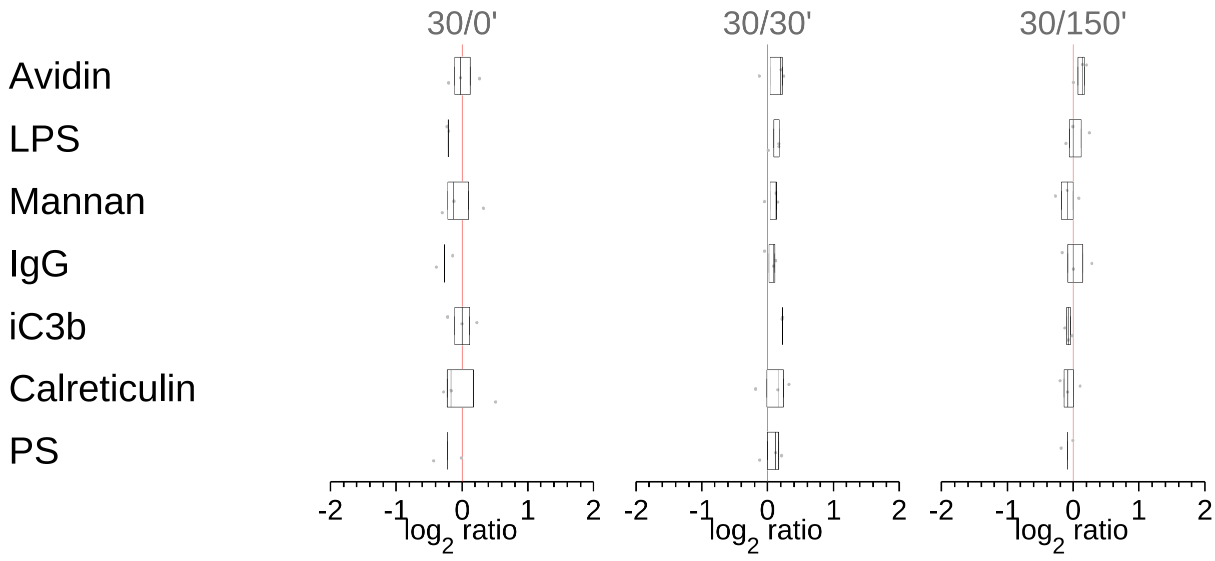

GO:0016139 glycoside catabolic process (n = 4)

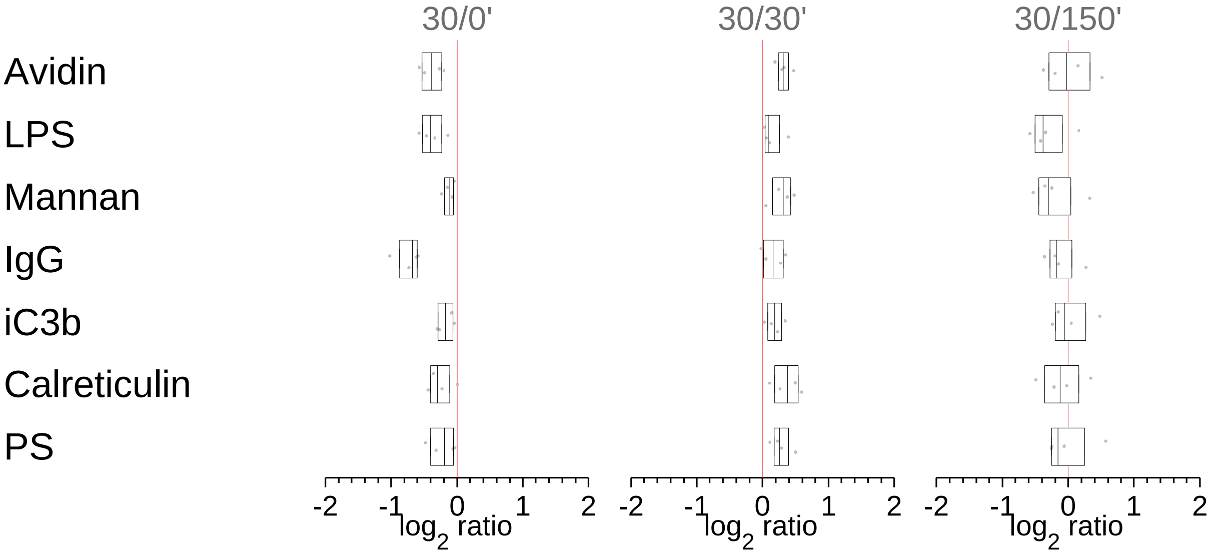

GO:0016175 superoxide-generating NADPH oxidase activity (n = 4)

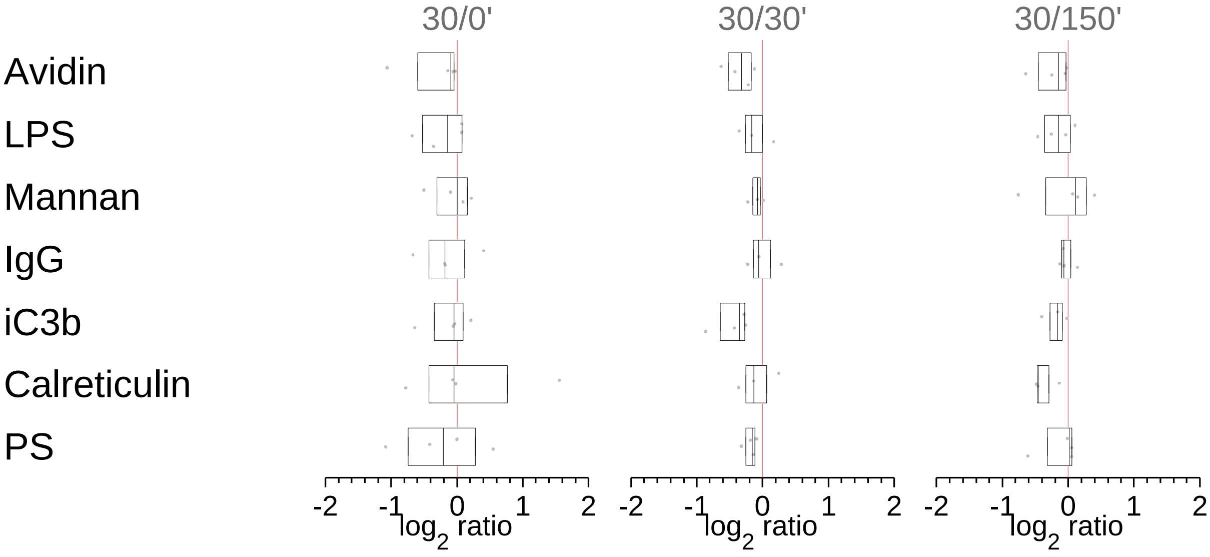

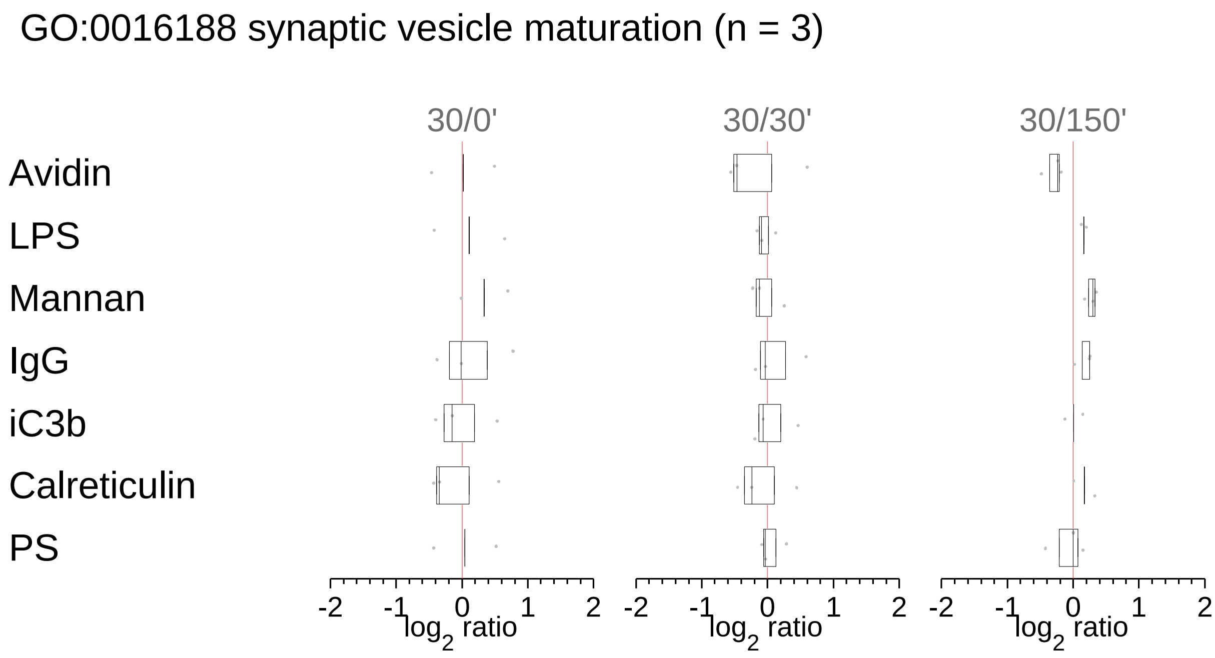
GO:0016188 synaptic vesicle maturation (n = 3)
30/0'
30/30'
30/150'
Avidin
LPS
Mannan
IgG
iC3b
Calreticulin
PS
-2
-1
0
1
2
log2 ratio

GO:0016192 vesicle-mediated transport (n = 85)

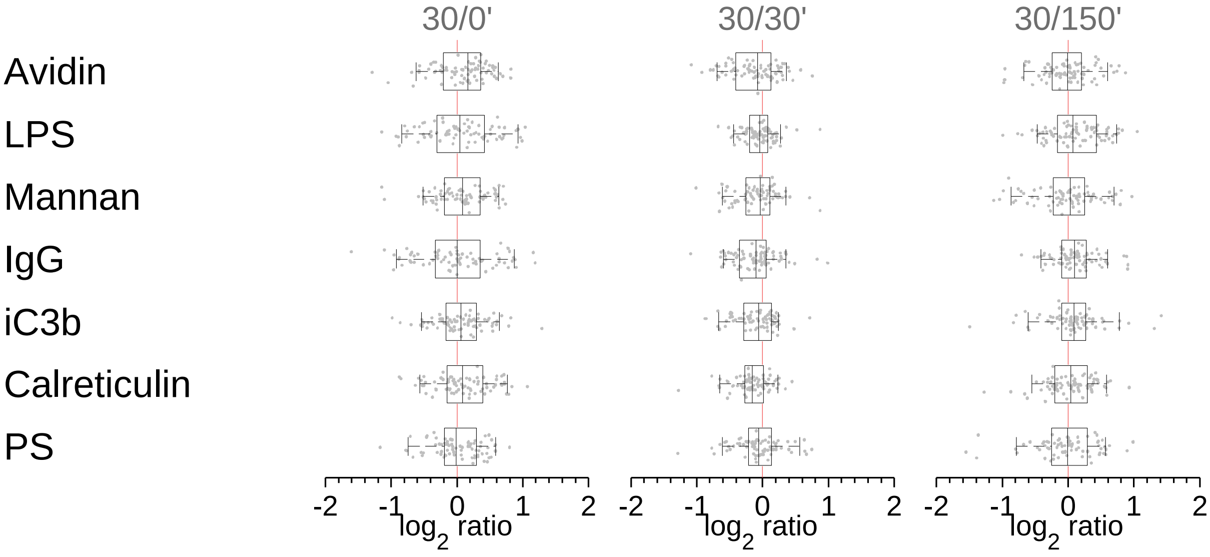

GO:0016197 endosomal transport (n = 14)

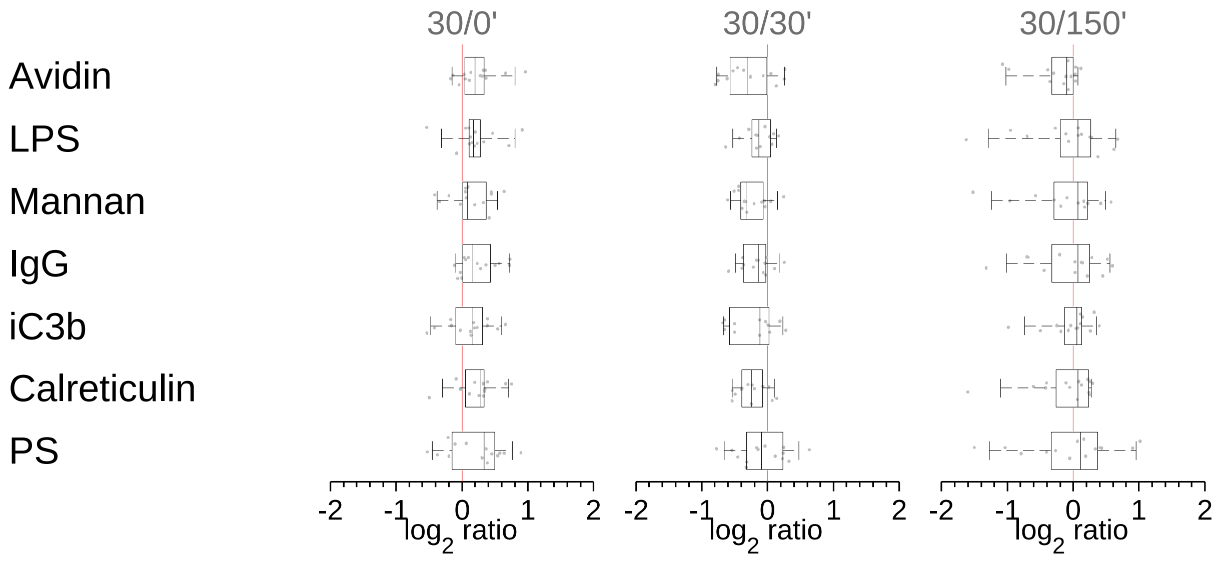

GO:0016209 antioxidant activity (n = 8)

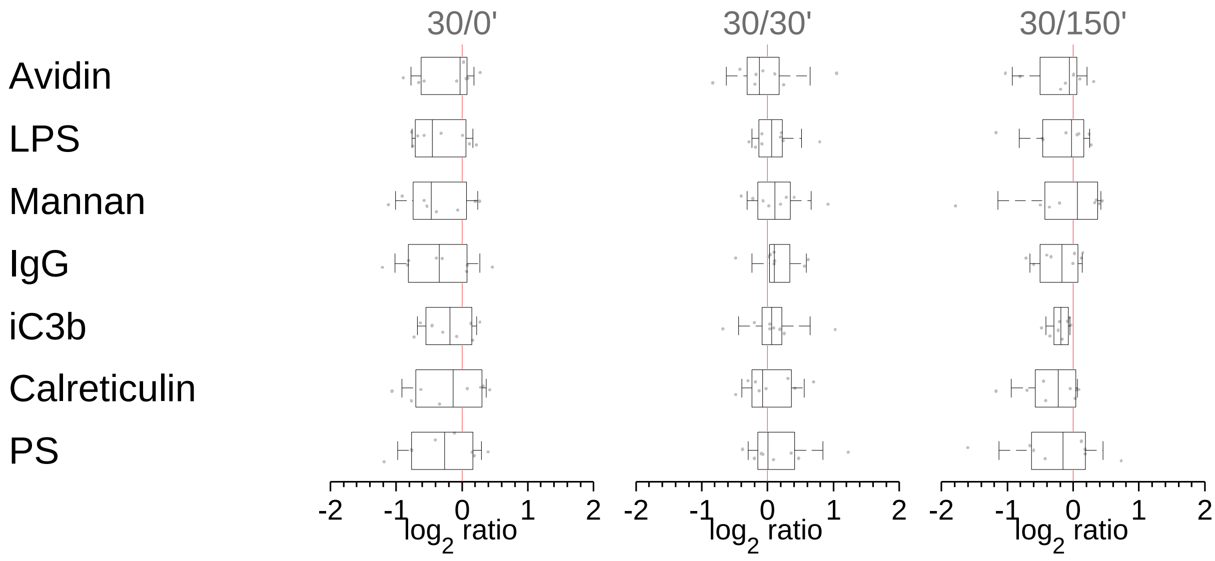

GO:0016229 steroid dehydrogenase activity (n = 4)

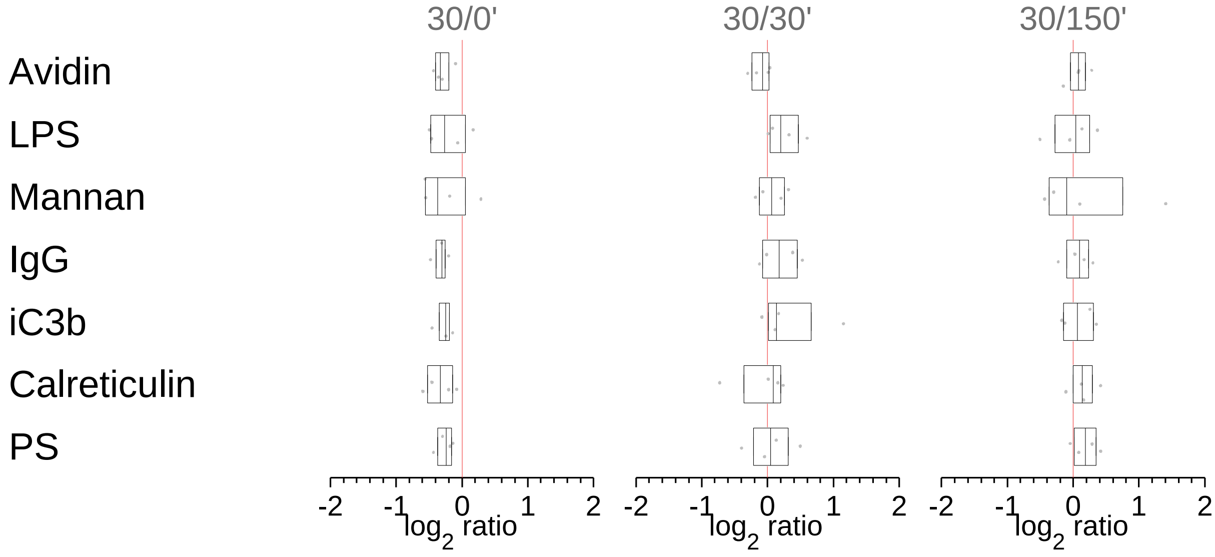

GO:0016235 aggresome (n = 3)

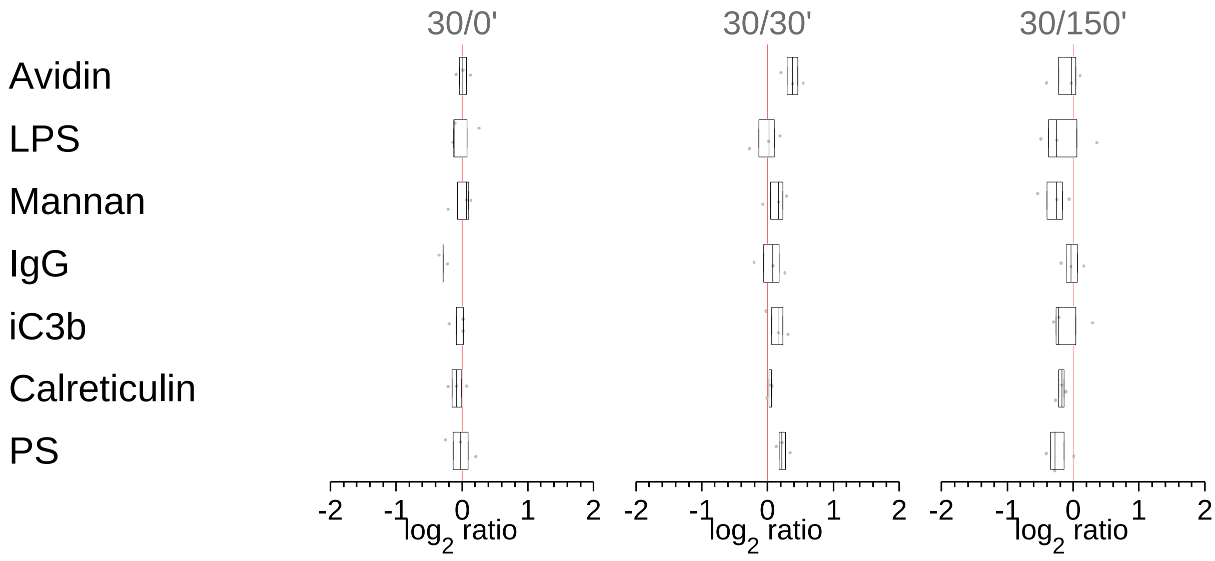

GO:0016281 eukaryotic translation initiation factor 4F complex
(n = 3)

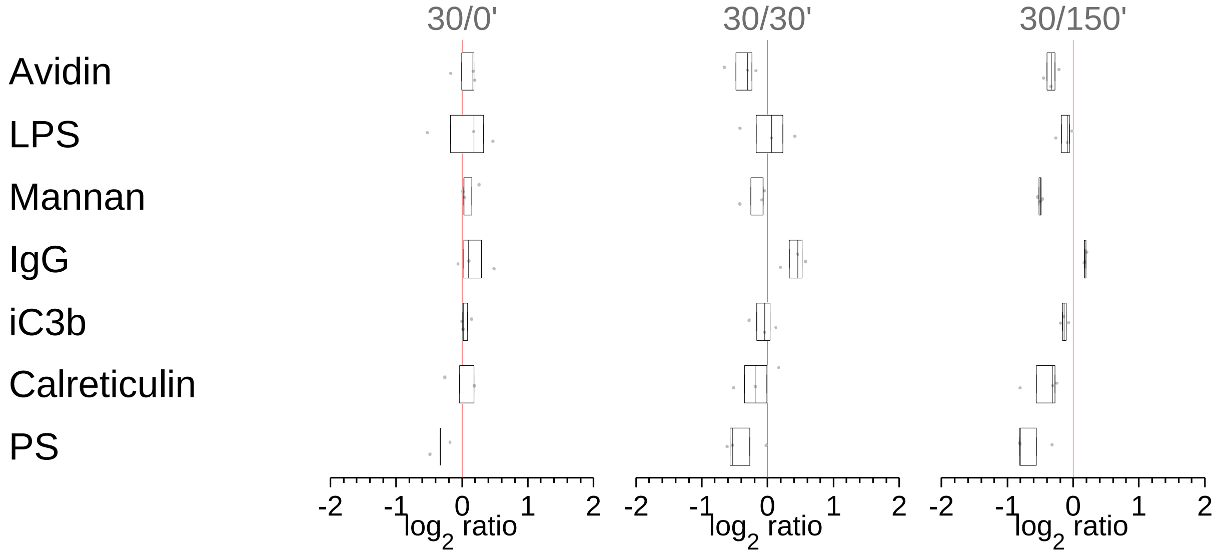

GO:0016289 CoA hydrolase activity (n = 3)

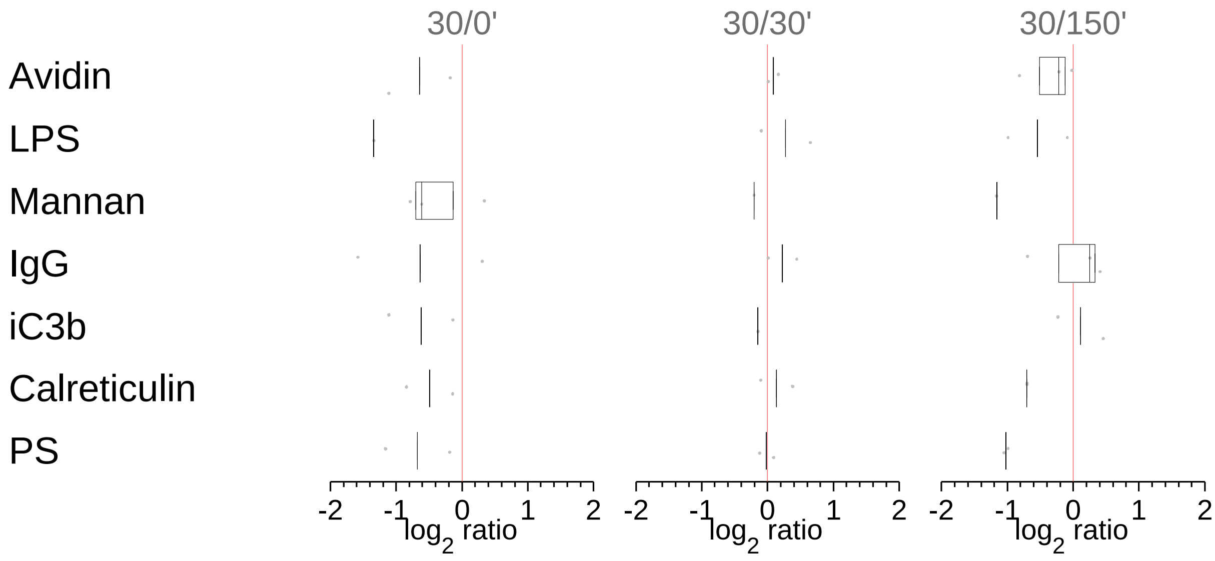

GO:0016298 lipase activity (n = 5)

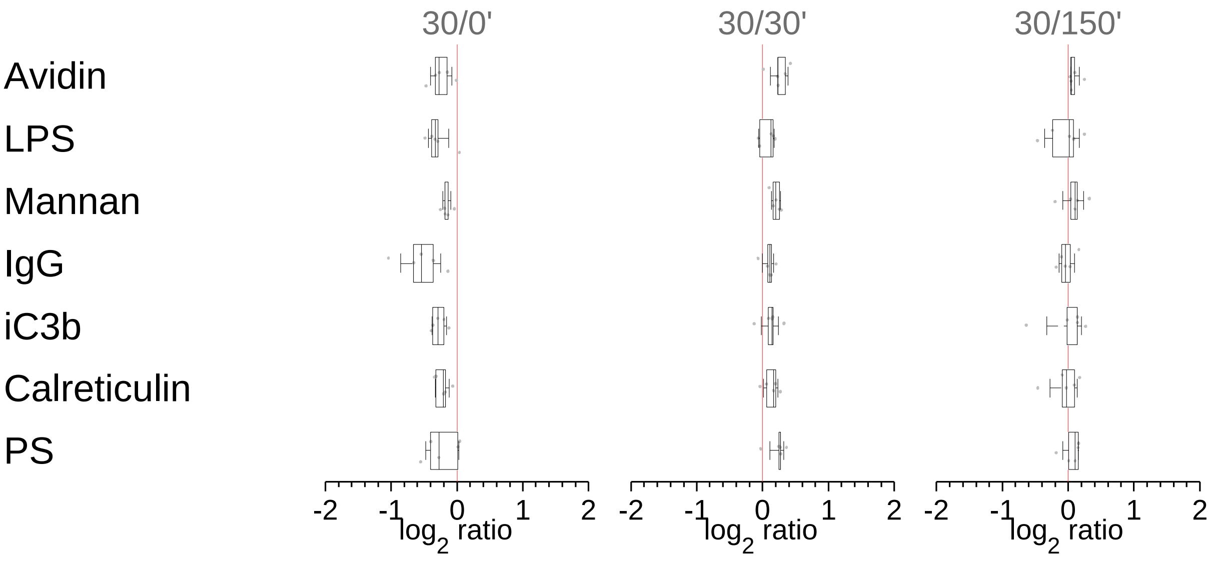

GO:0016301 kinase activity (n = 66)

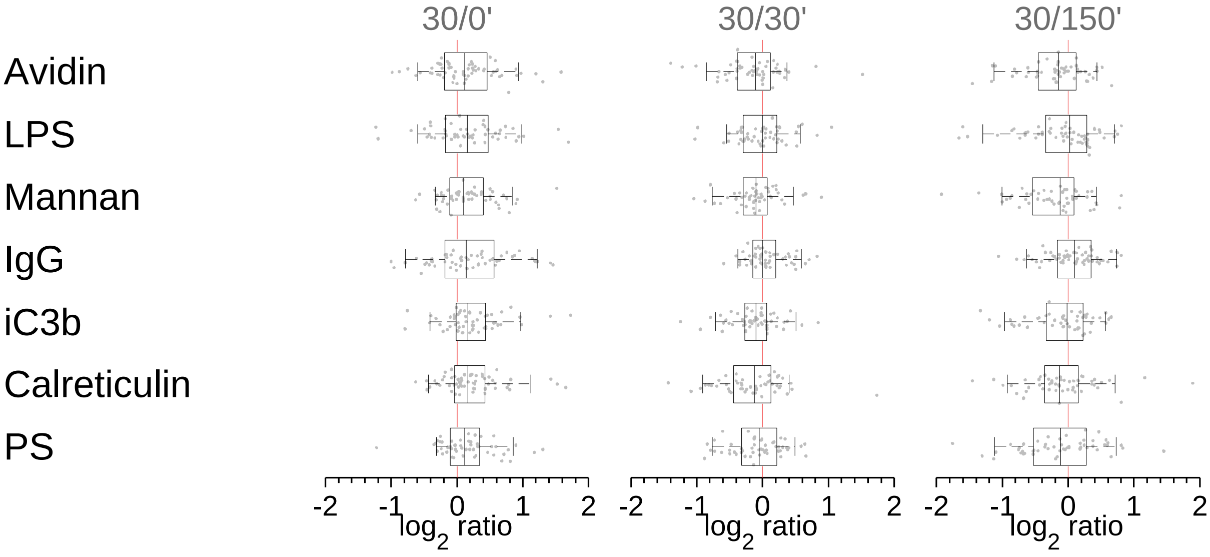

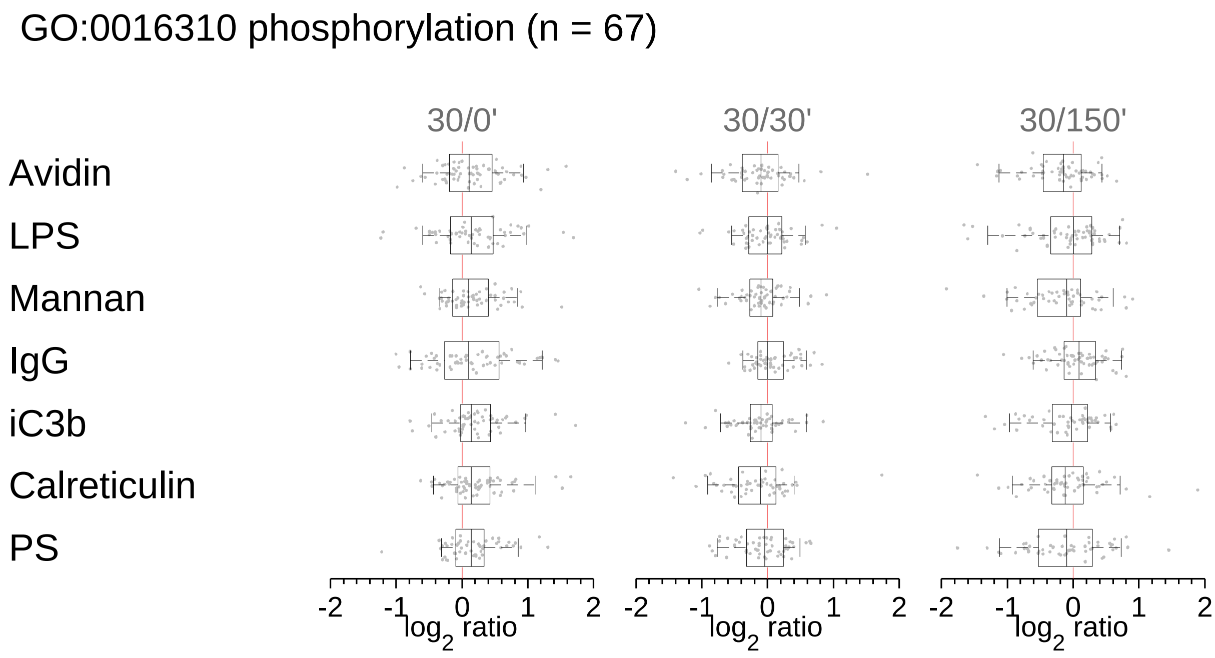

GO:0016310 phosphorylation (n = 67)
30/0'
30/30'
30/150'
Avidin
LPS
Mannan
IgG
iC3b
Calreticulin
PS
-2
-1
0
1
2
log2 ratio

GO:0016311 dephosphorylation (n = 18)

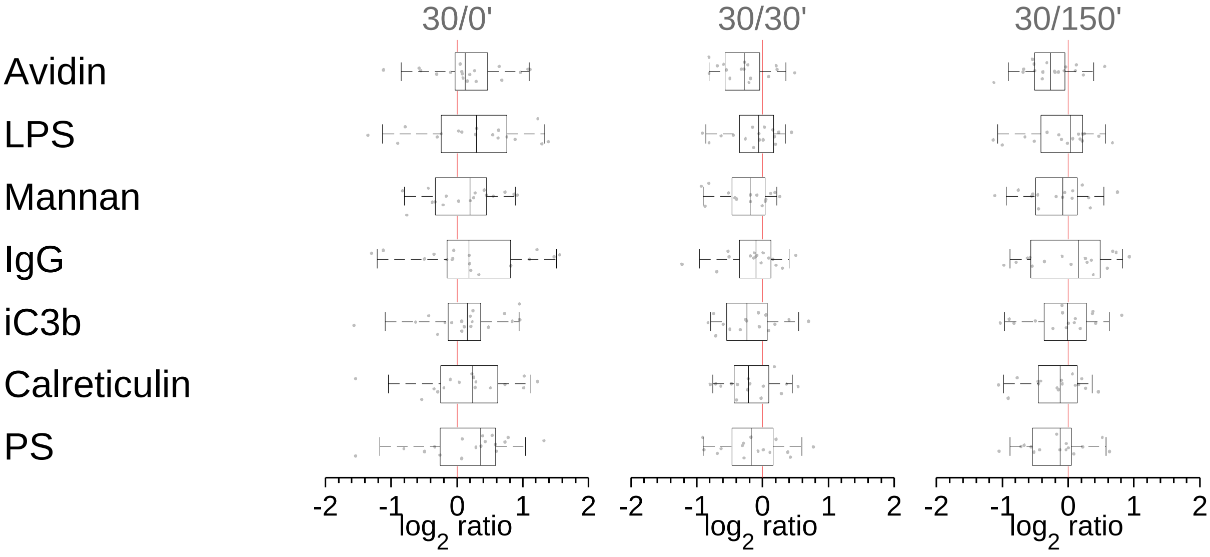

GO:0016322 neuron remodeling (n = 3)

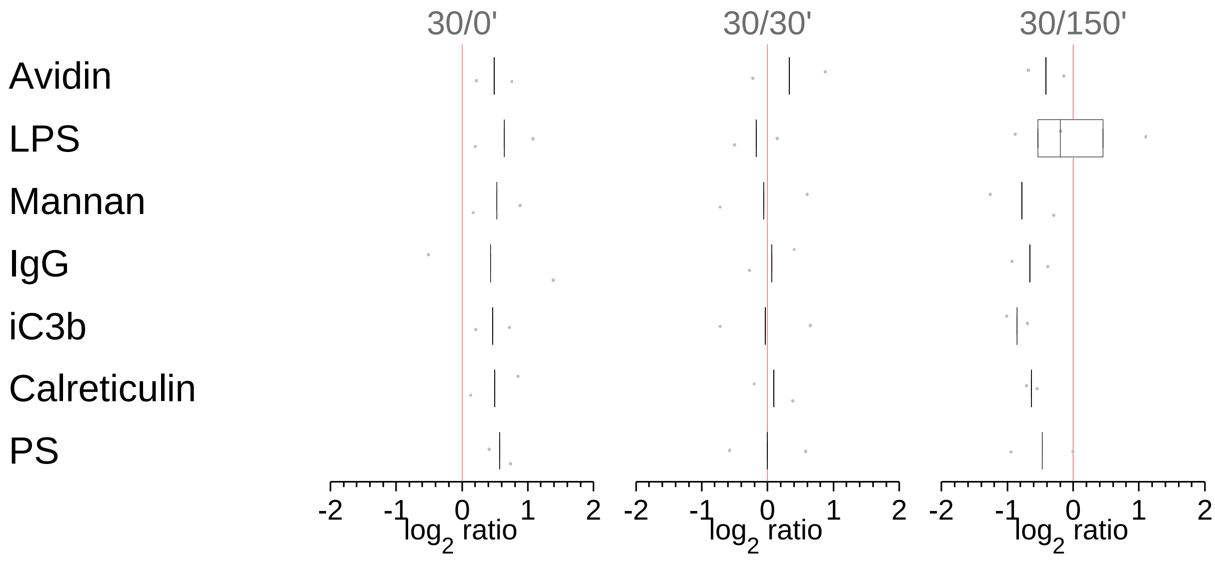

GO:0016323 basolateral plasma membrane (n = 27)

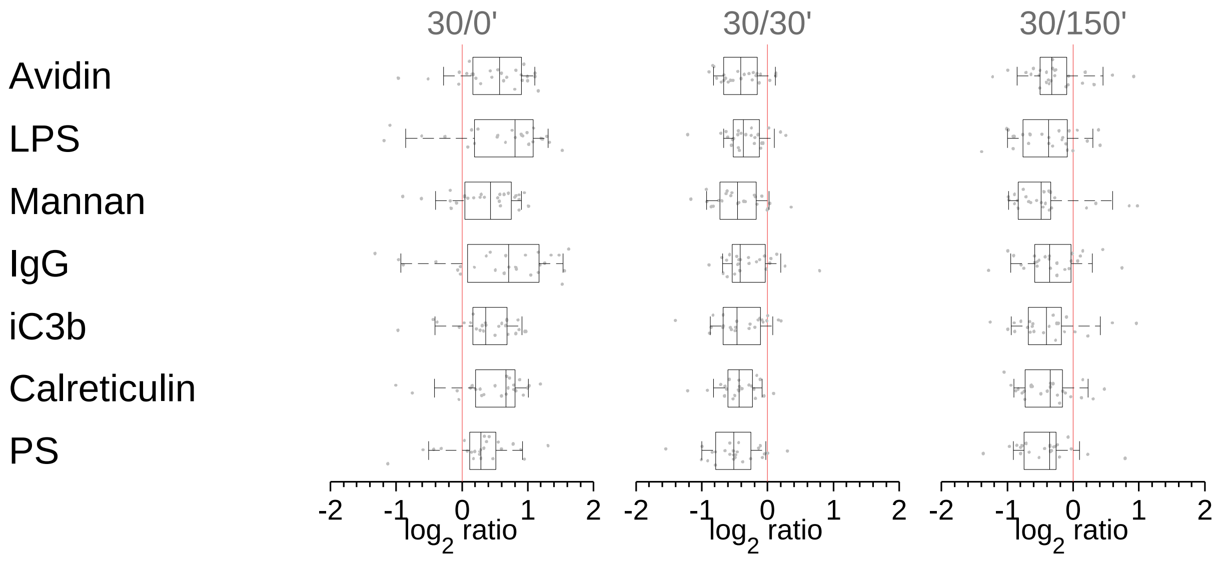

GO:0016324 apical plasma membrane (n = 42)

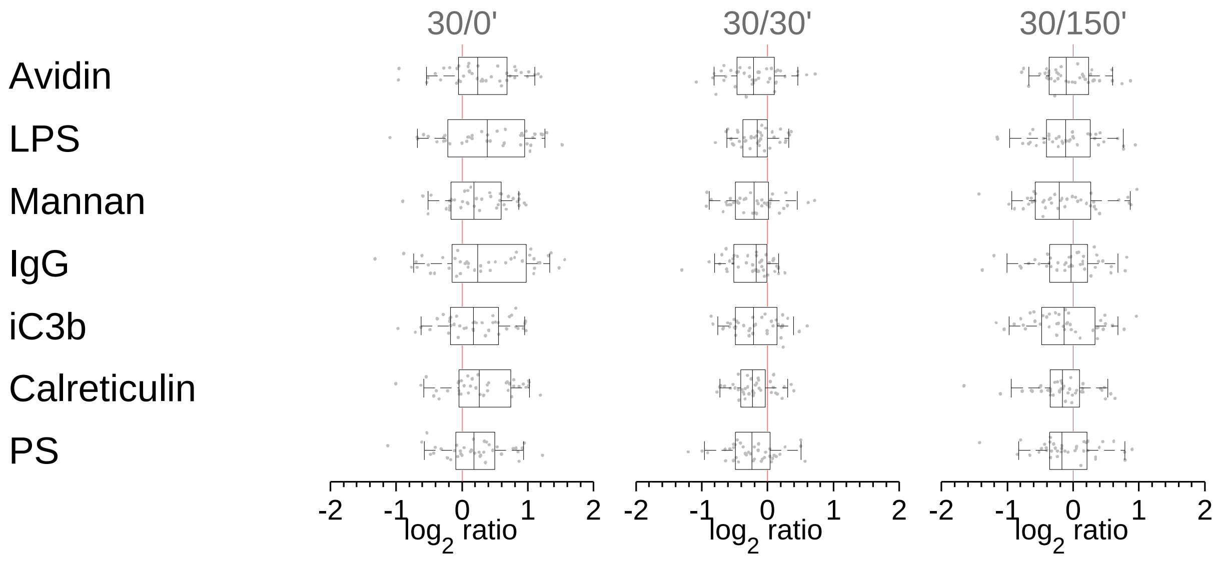

GO:0016328 lateral plasma membrane (n = 6)

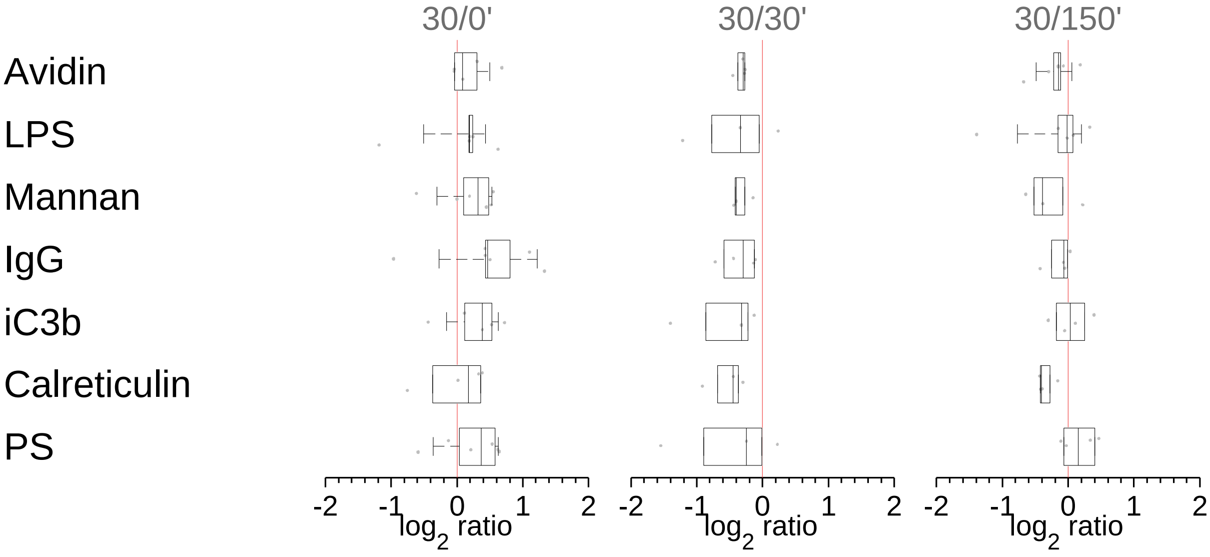

GO:0016337 cell-cell adhesion (n = 17)

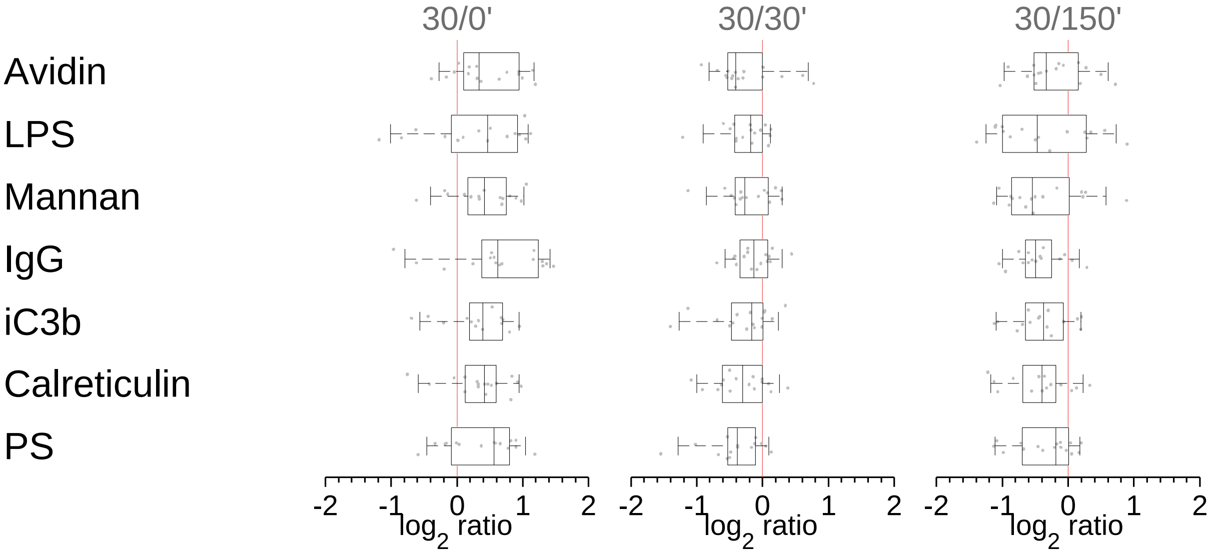

GO:0016358 dendrite development (n = 4)

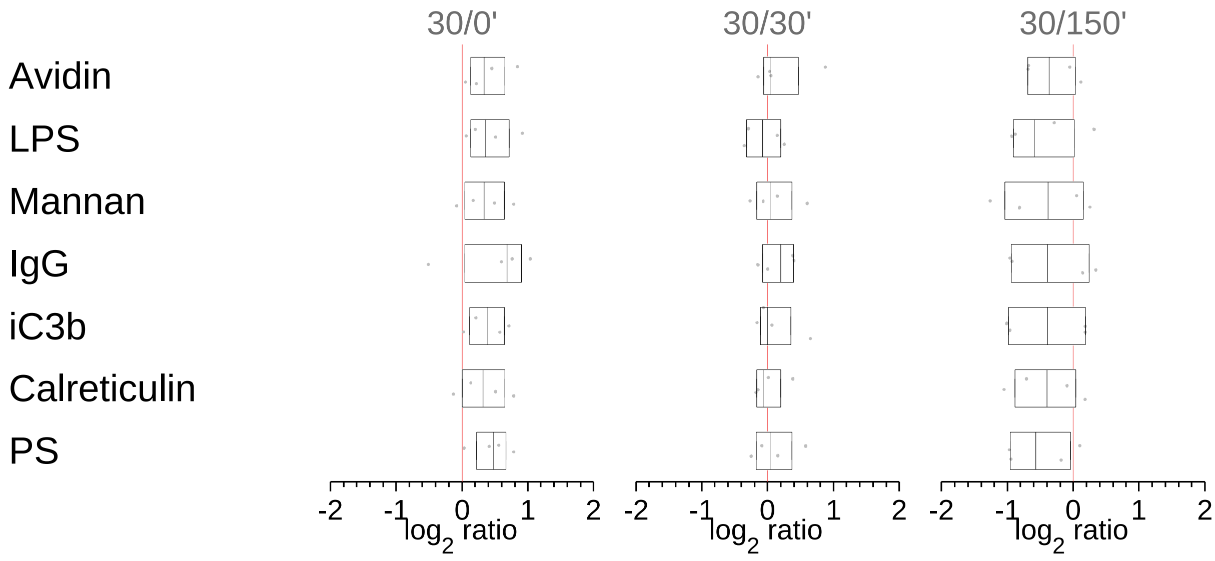

GO:0016363 nuclear matrix (n = 12)

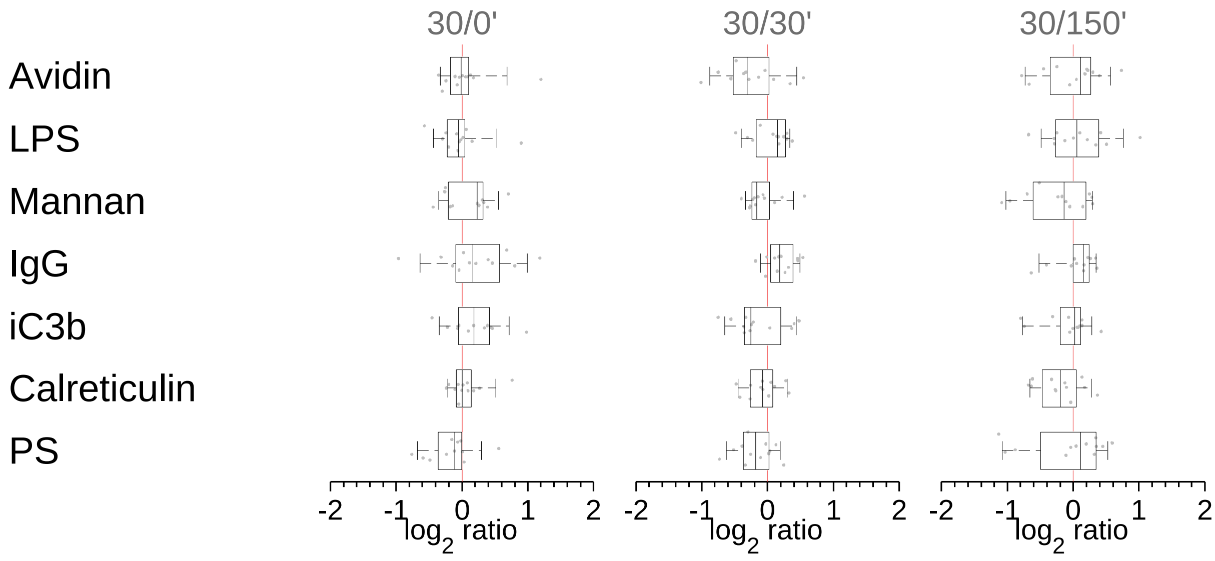

GO:0016406 carnitine O-acyltransferase activity (n = 3)

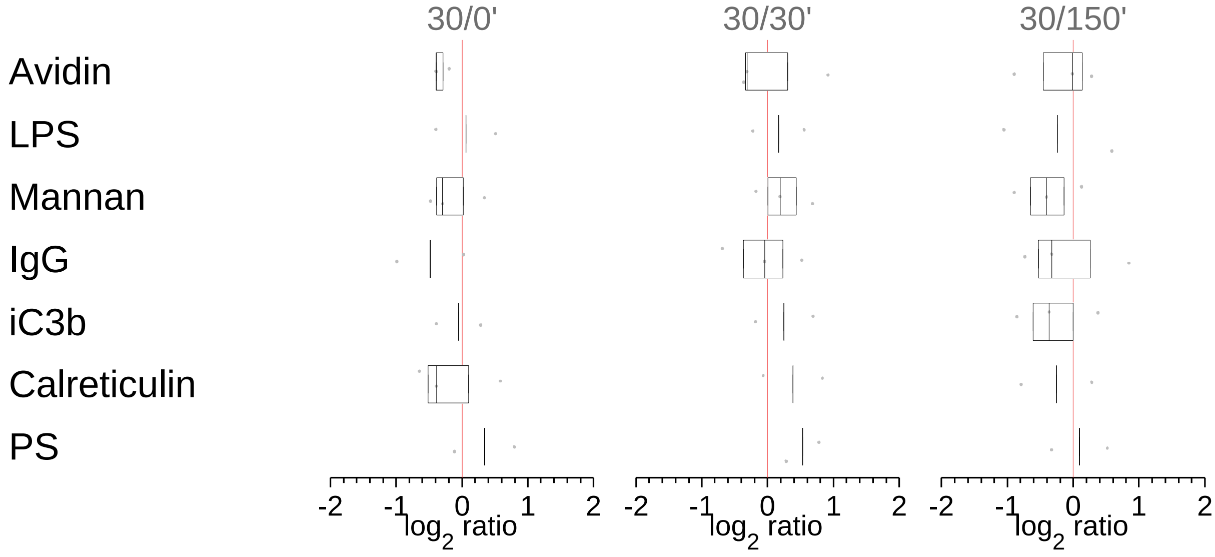

GO:0016407 acetyltransferase activity (n = 3)

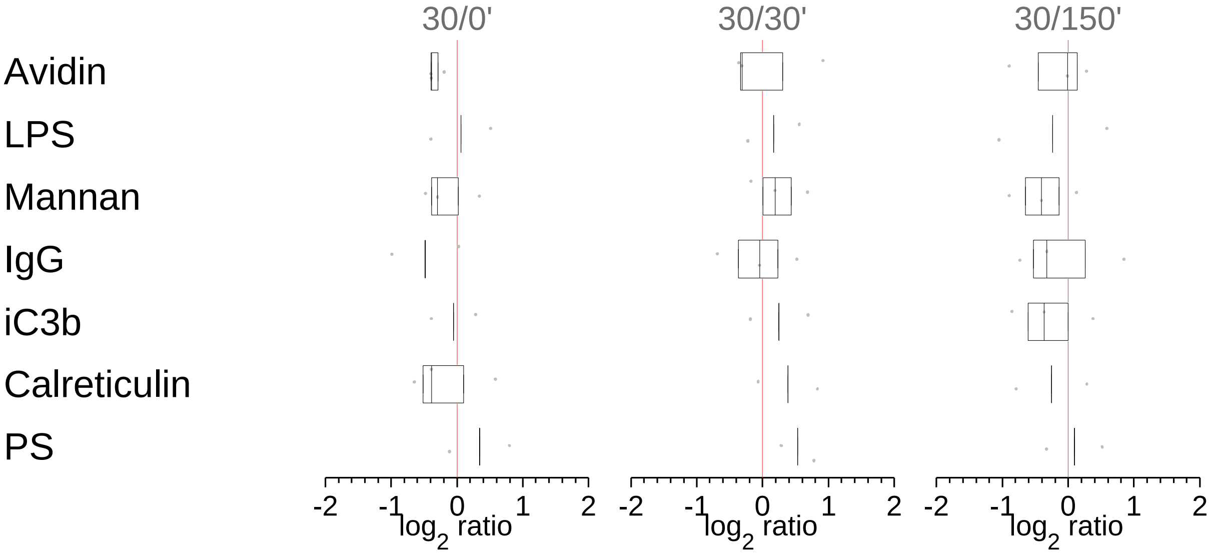

GO:0016408 C-acyltransferase activity (n = 3)

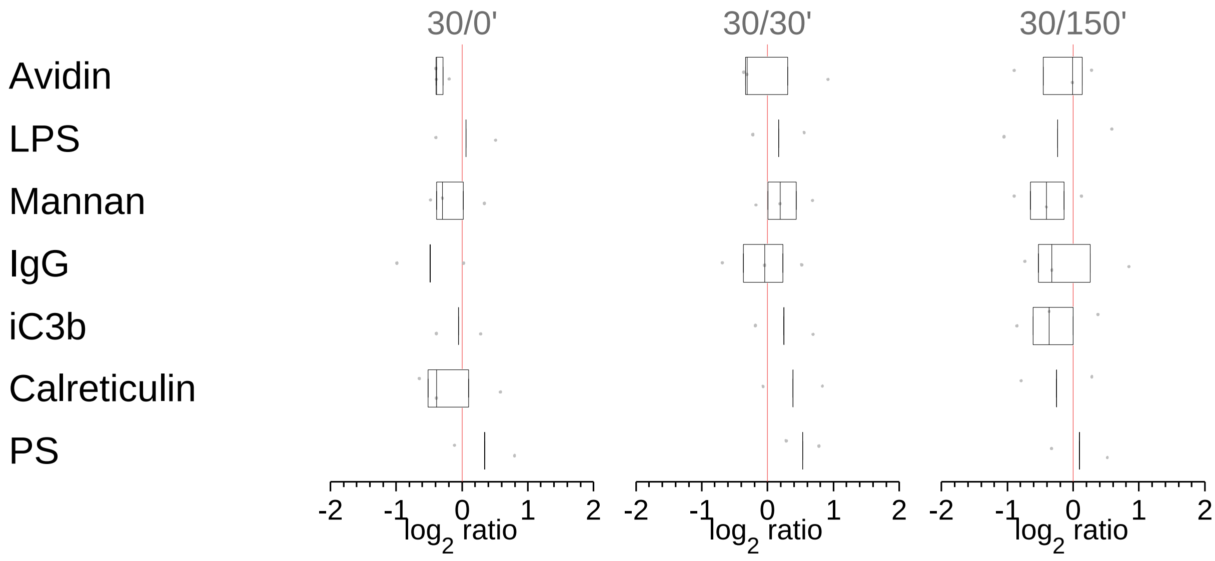

GO:0016409 palmitoyltransferase activity (n = 4)

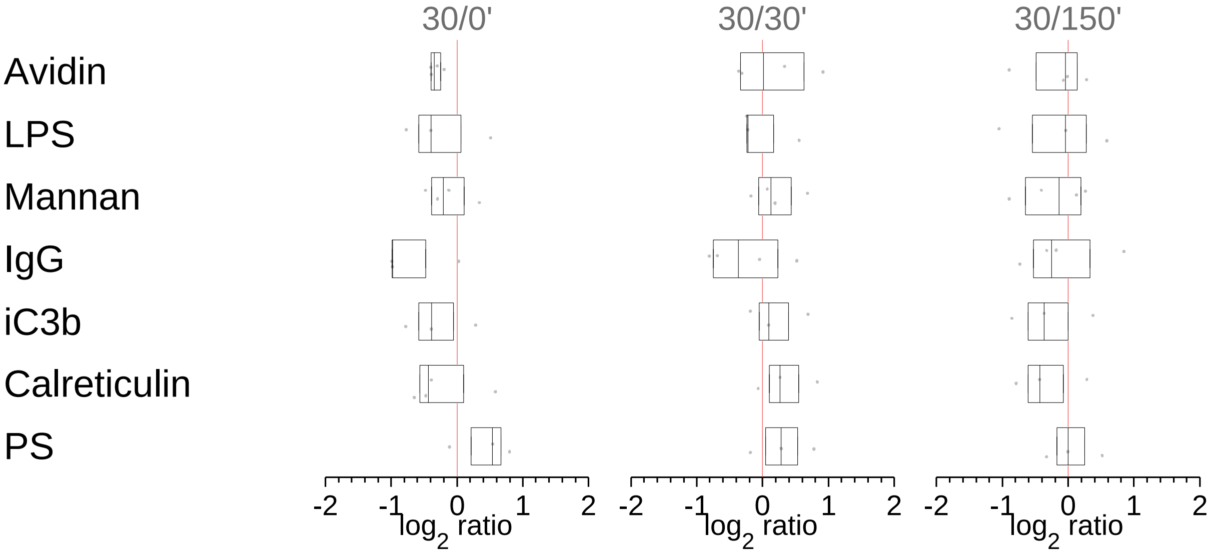

GO:0016410 N-acyltransferase activity (n = 3)

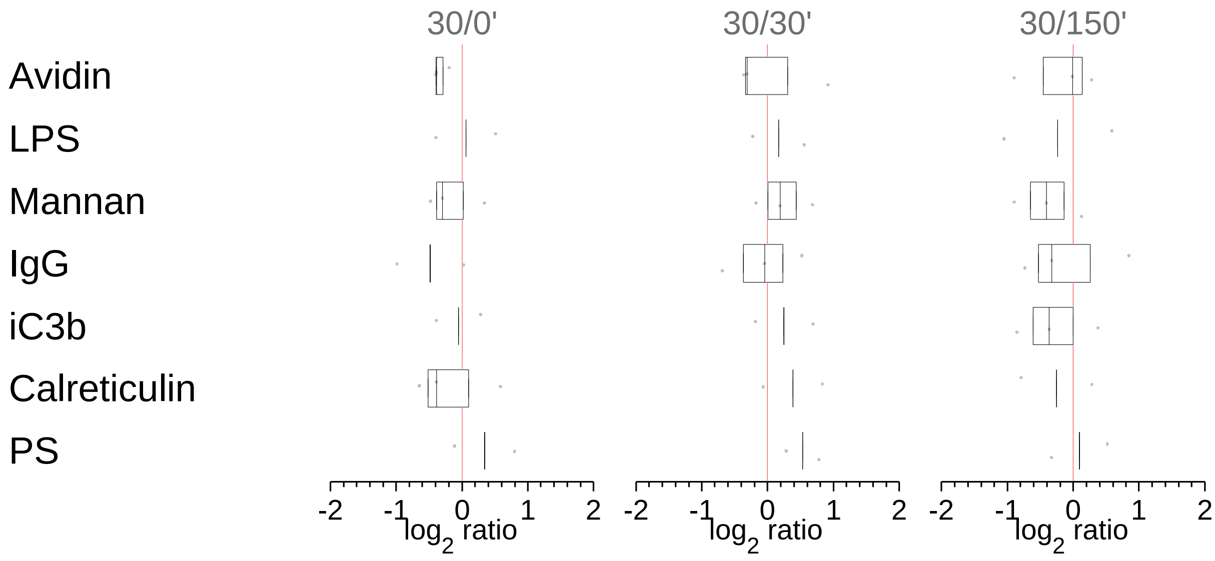

GO:0016411 acylglycerol O-acyltransferase activity (n = 3)

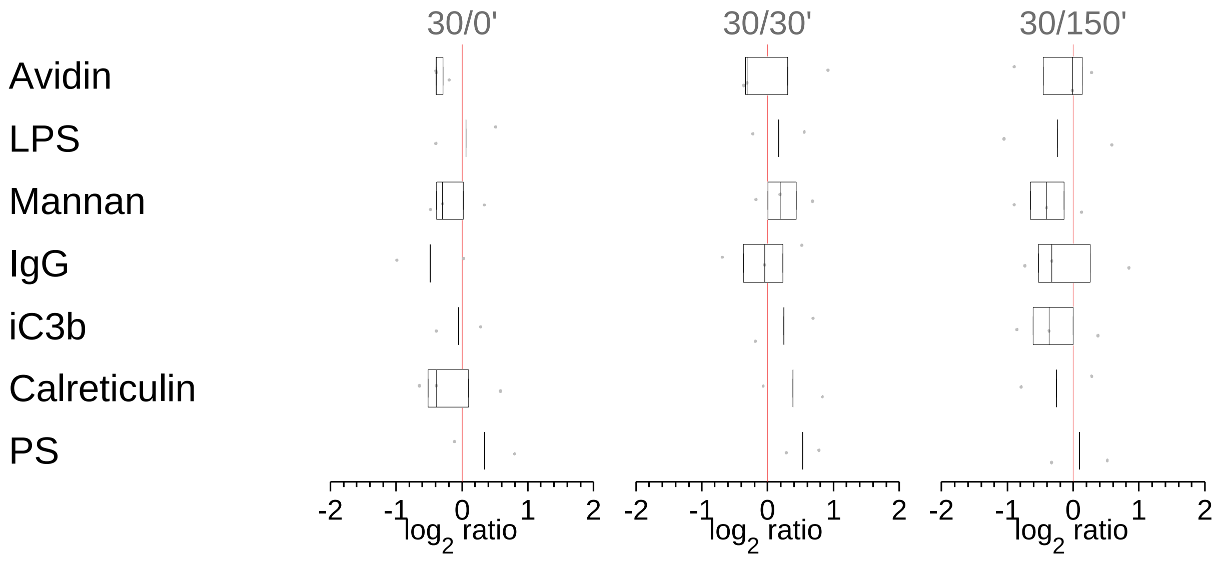

GO:0016412 serine O-acyltransferase activity (n = 3)

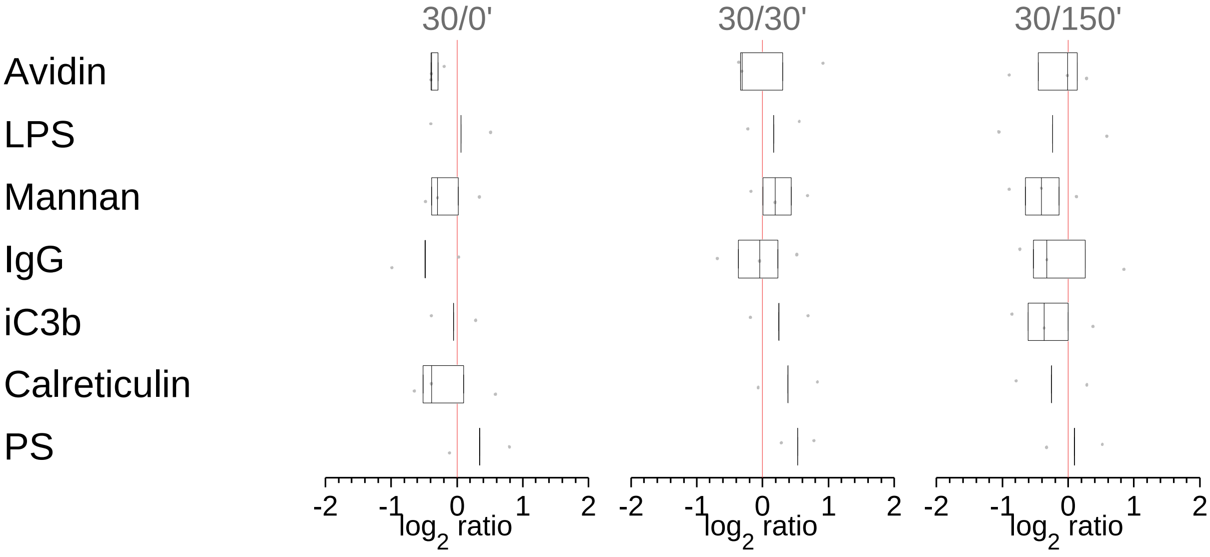

GO:0016413 O-acetyltransferase activity (n = 3)

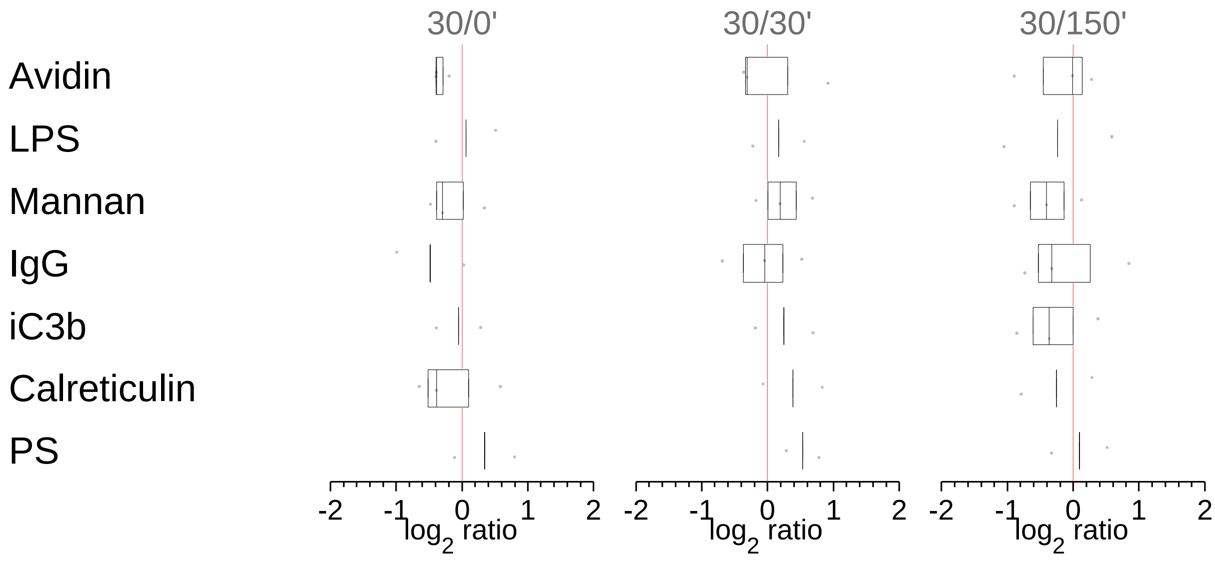

GO:0016414 O-octanoyltransferase activity (n = 3)

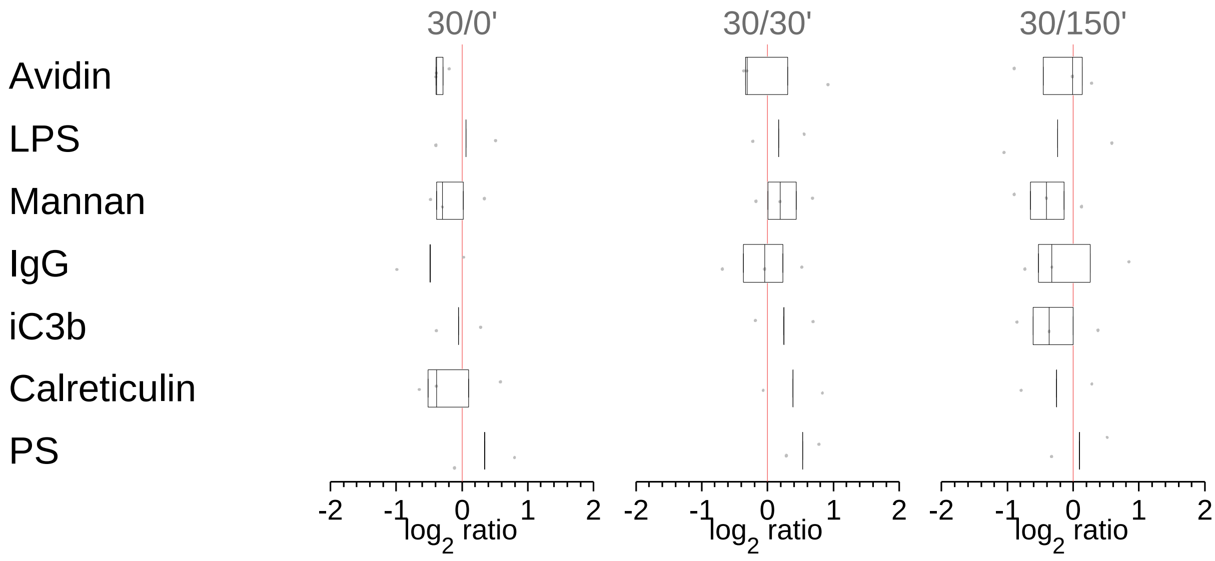

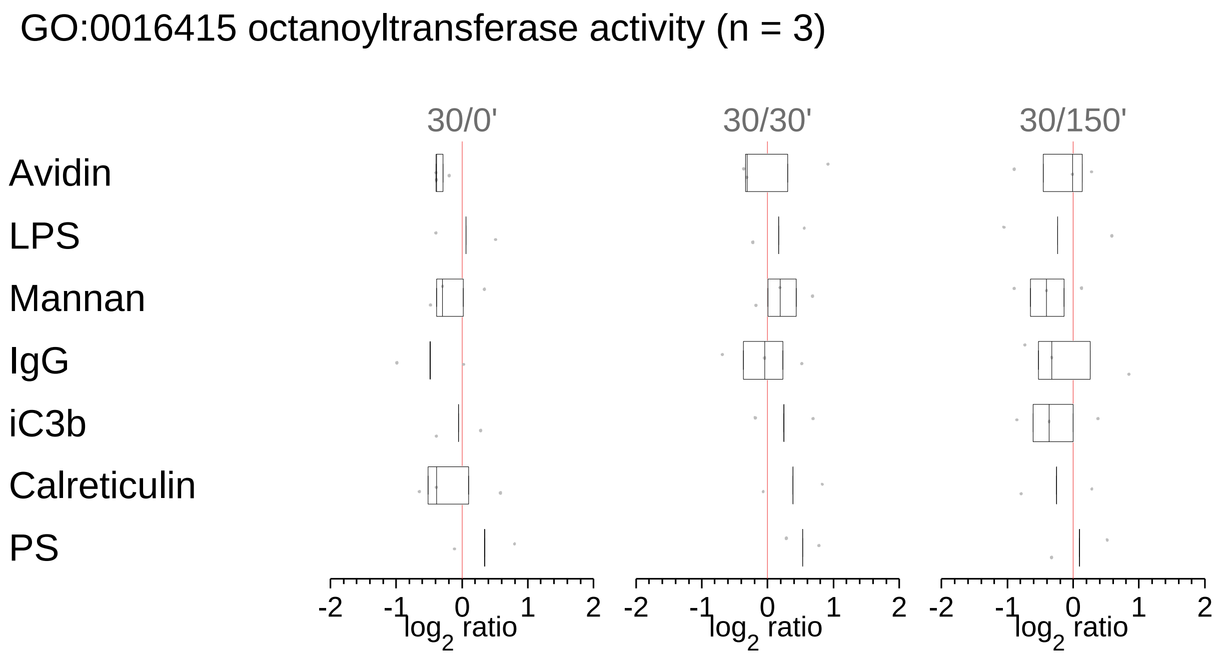
GO:0016415 octanoyltransferase activity (n = 3)
30/0'
30/30'
30/150'
Avidin
LPS
Mannan
IgG
iC3b
Calreticulin
PS
-2 -1 0 1 2
log2 ratio

GO:0016416 O-palmitoyltransferase activity (n = 3)

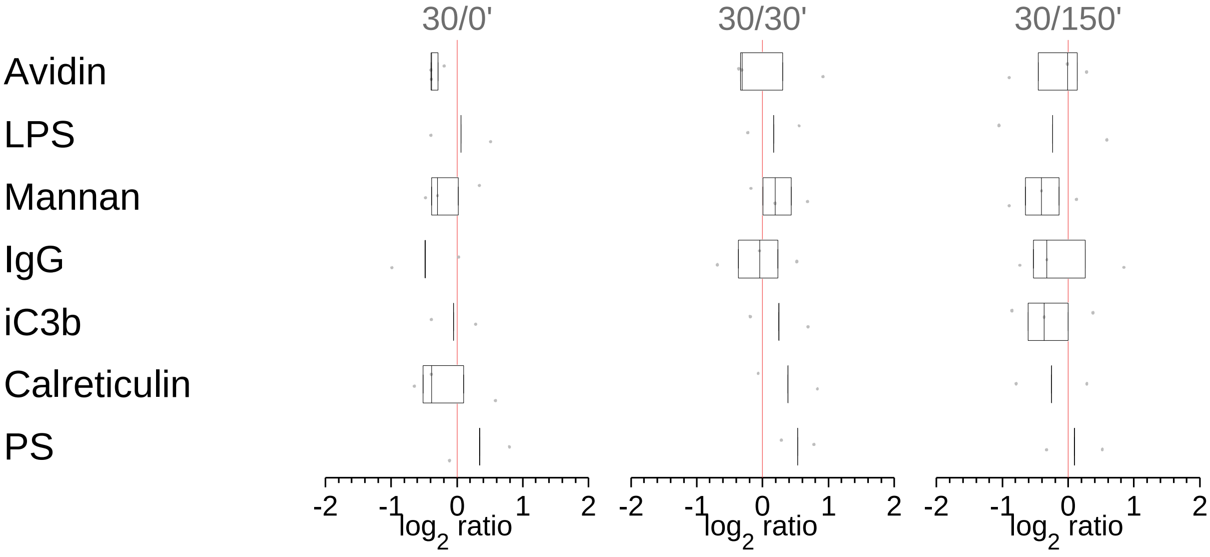

GO:0016417 S-acyltransferase activity (n = 3)

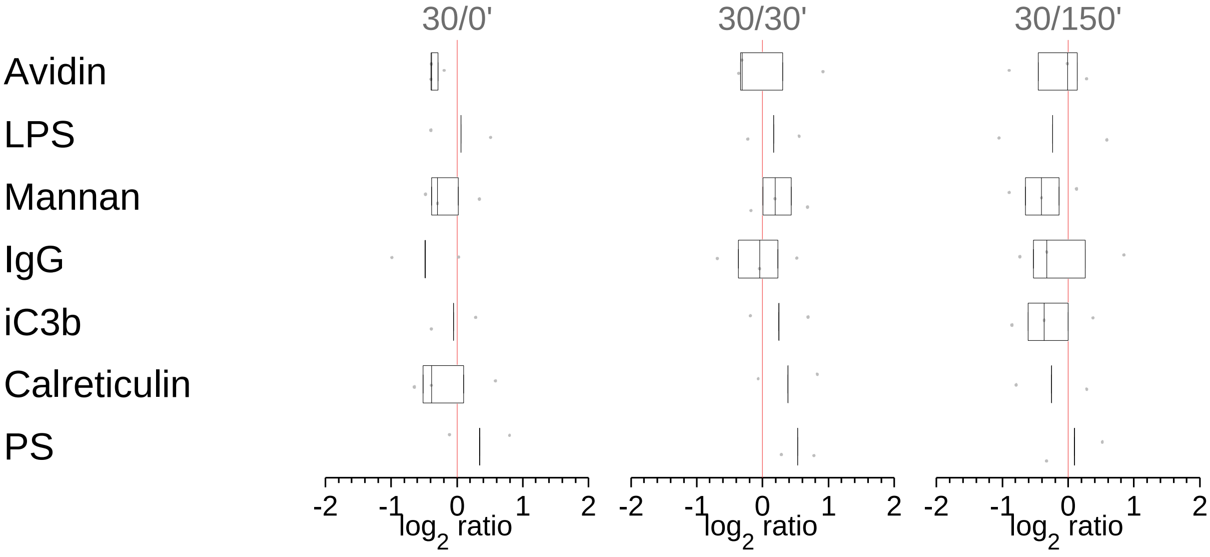

GO:0016418 S-acetyltransferase activity (n = 3)

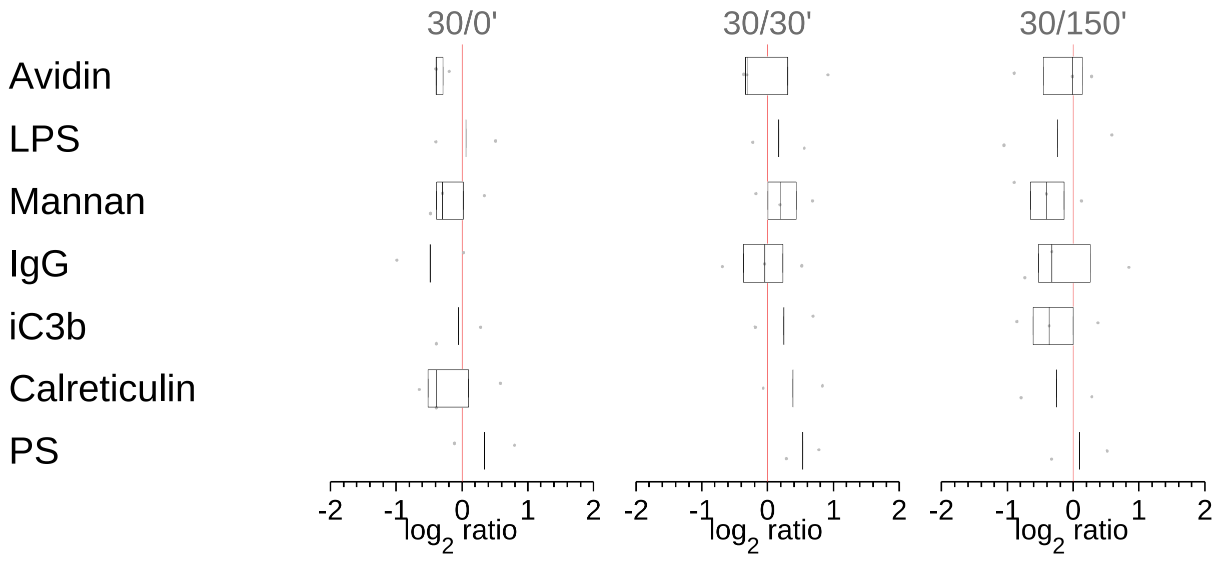

GO:0016419 S-malonyltransferase activity (n = 3)

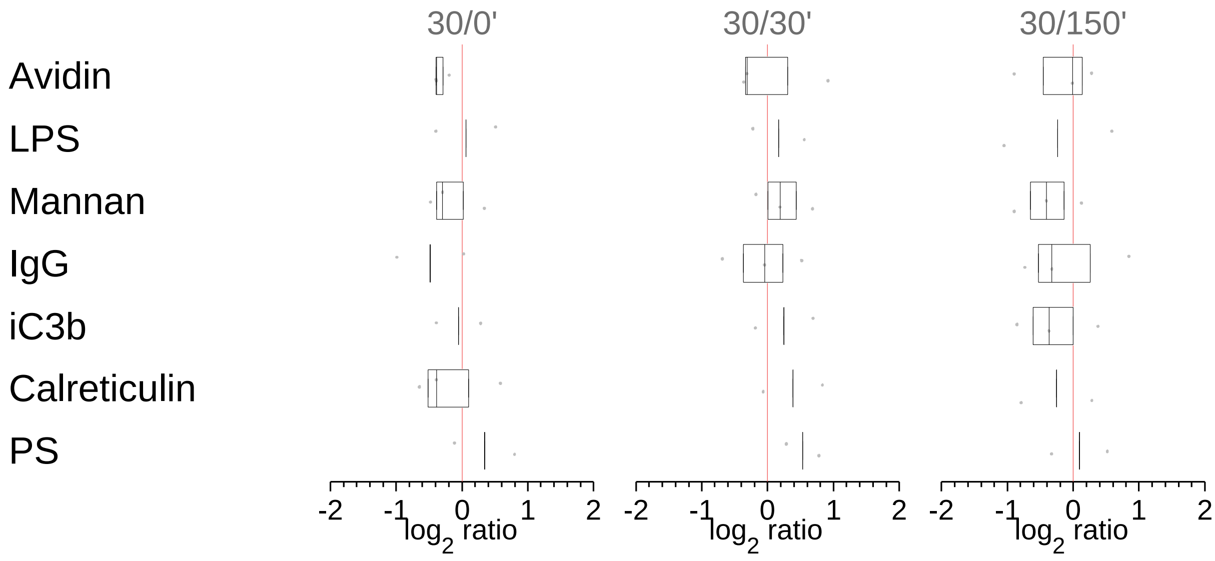

GO:0016420 malonyltransferase activity (n = 3)

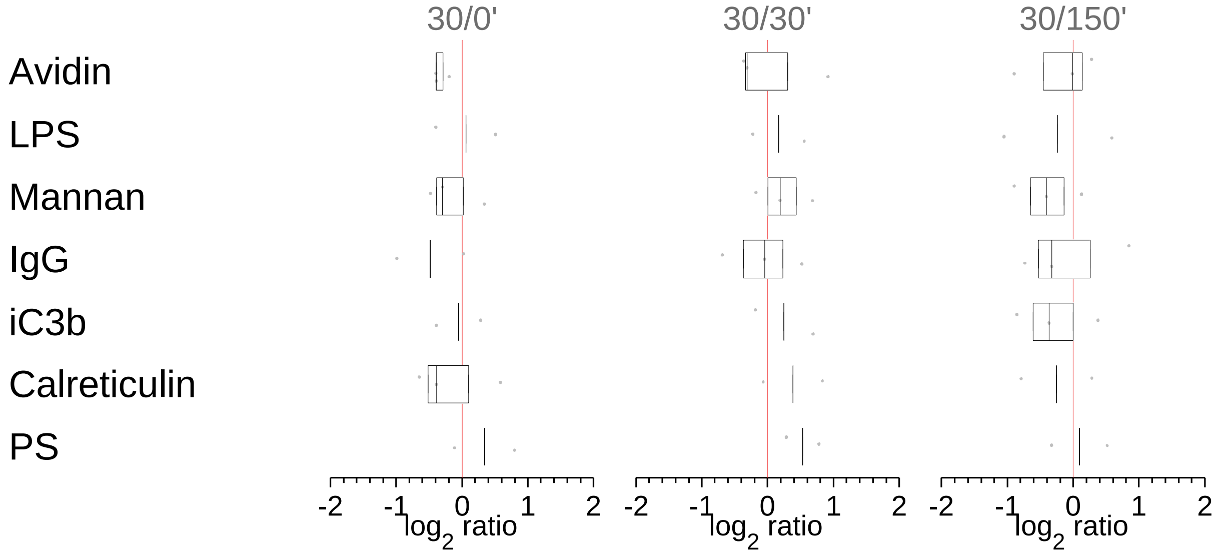

GO:0016454 C-palmitoyltransferase activity (n = 3)

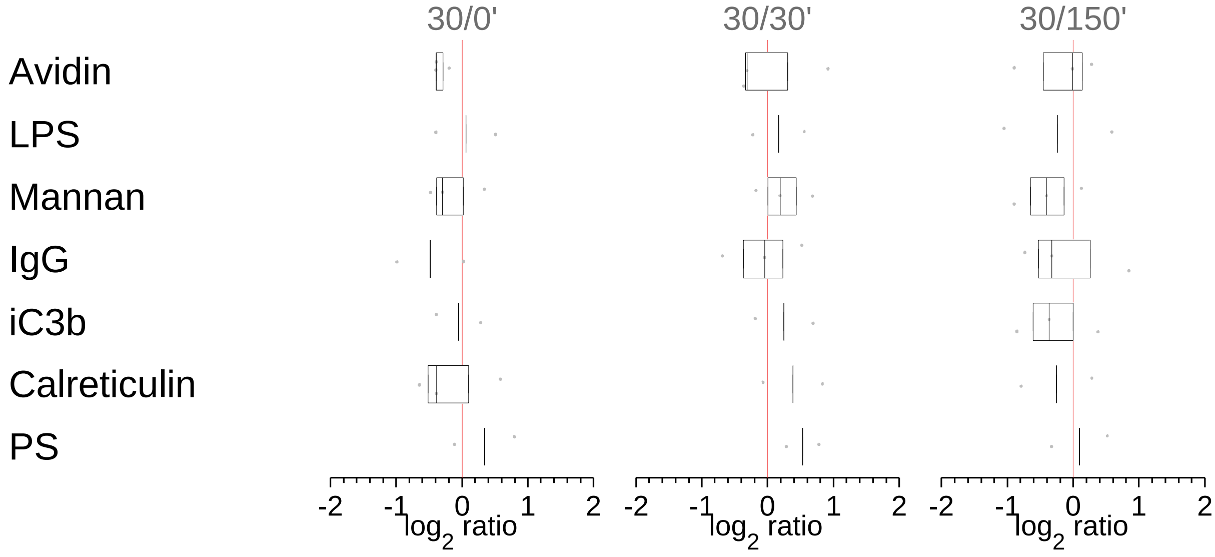

GO:0016459 myosin complex (n = 11)

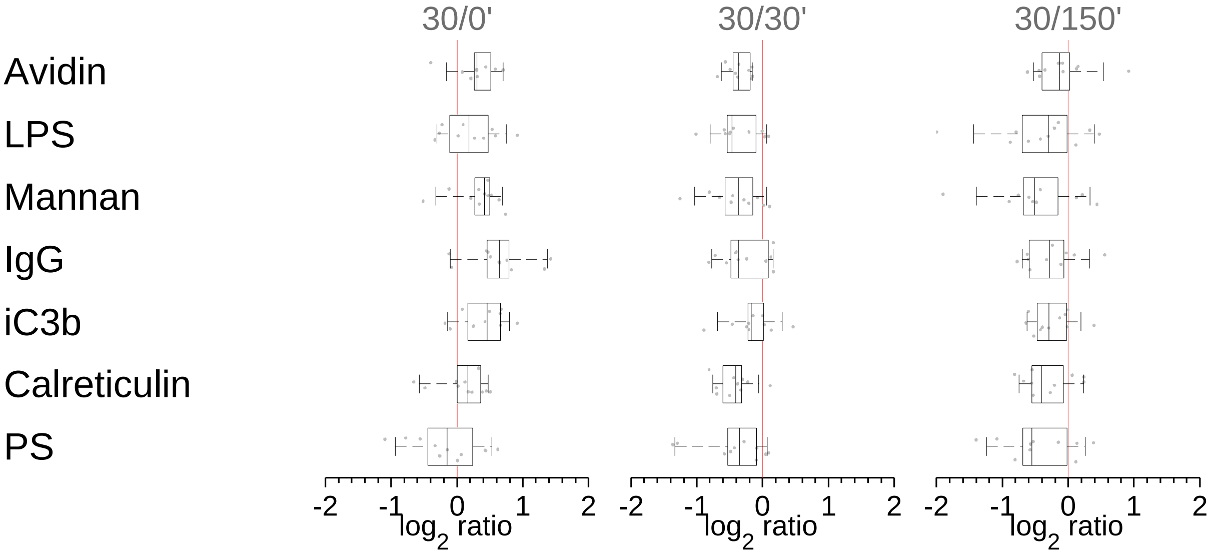

GO:0016460 myosin II complex (n = 3)

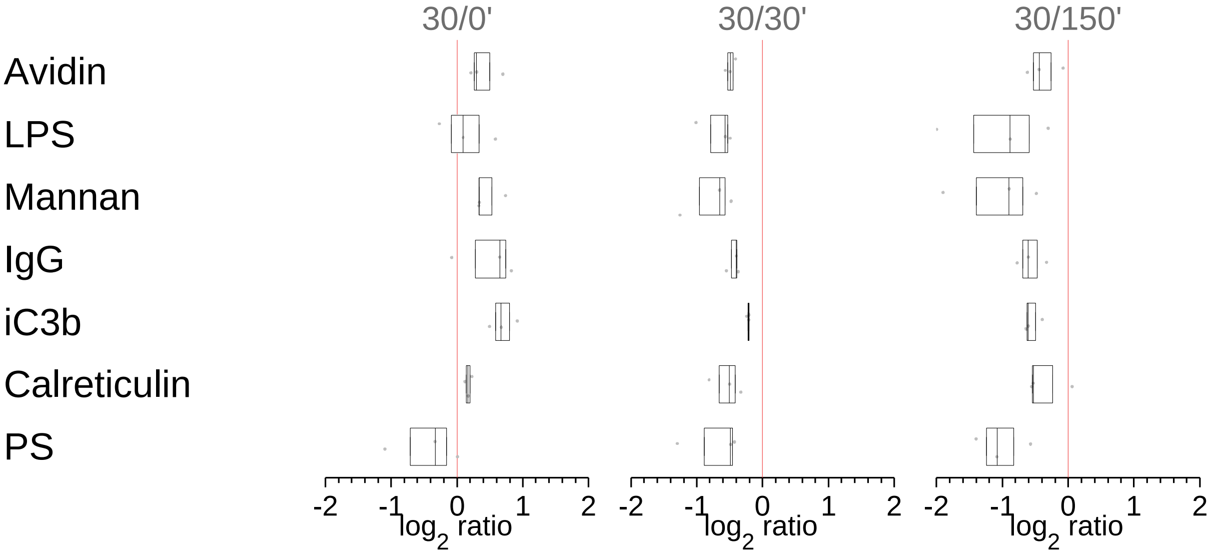

GO:0016471 vacuolar proton-transporting V-type ATPase complex (n = 6)

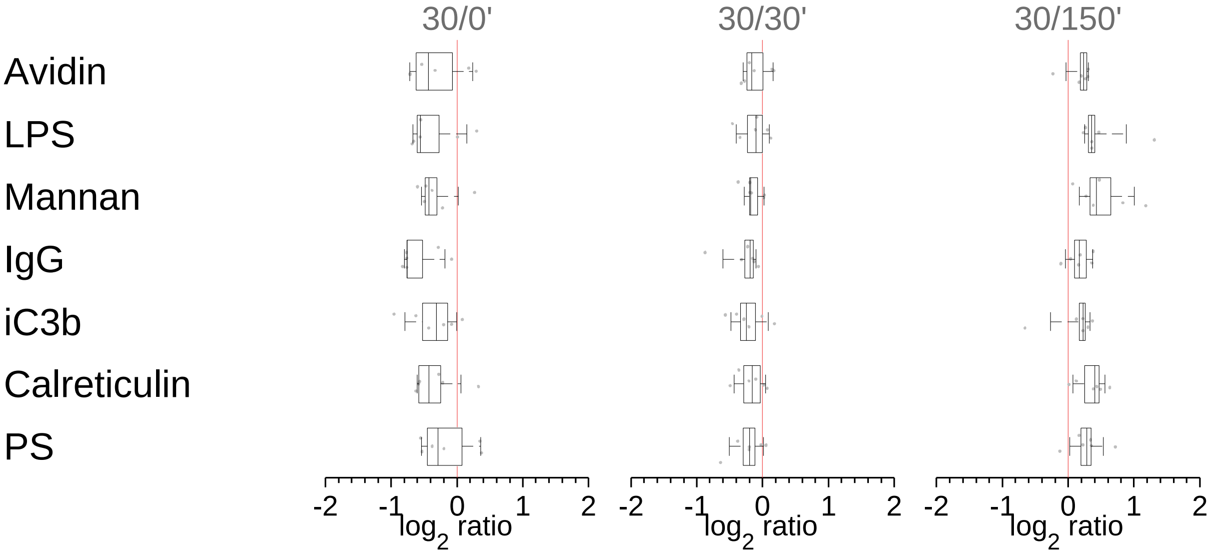

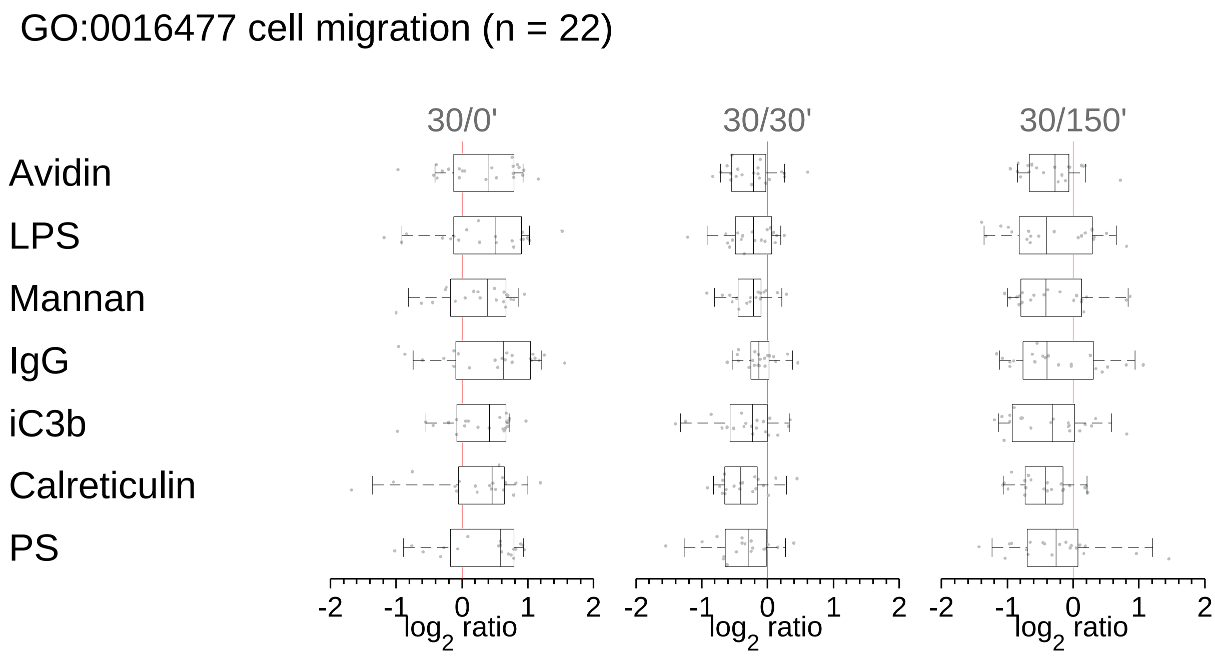
GO:0016477 cell migration (n = 22)
30/0'
30/30'
30/150'
Avidin
LPS
Mannan
IgG
iC3b
Calreticulin
PS
-2
-1
0
1
2
log2 ratio

GO:0016485 protein processing (n = 9)

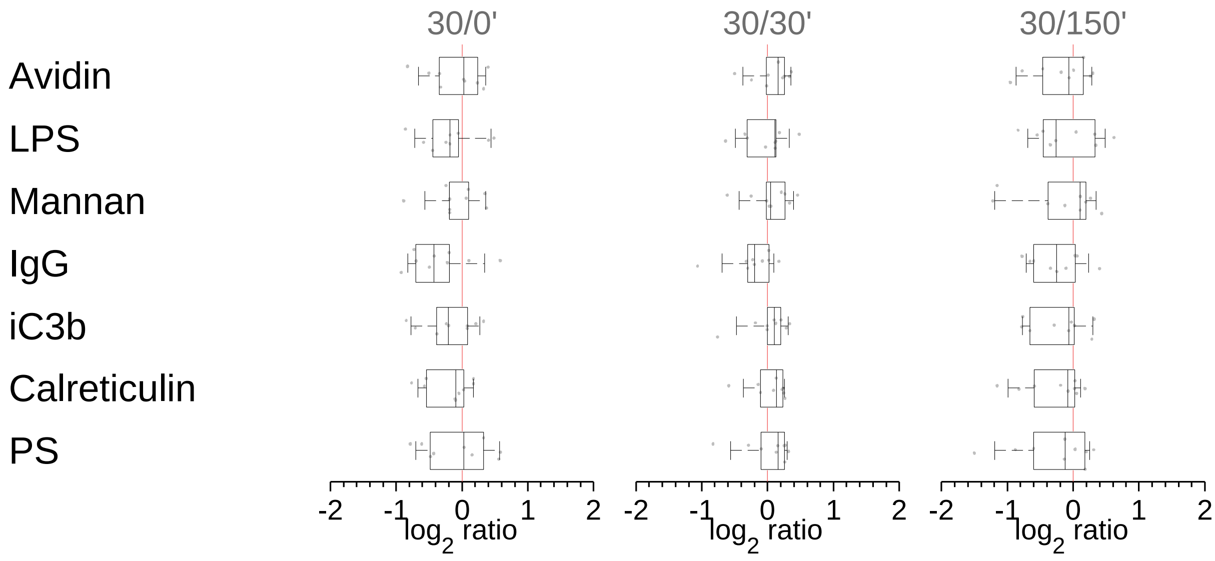

GO:0016491 oxidoreductase activity (n = 106)

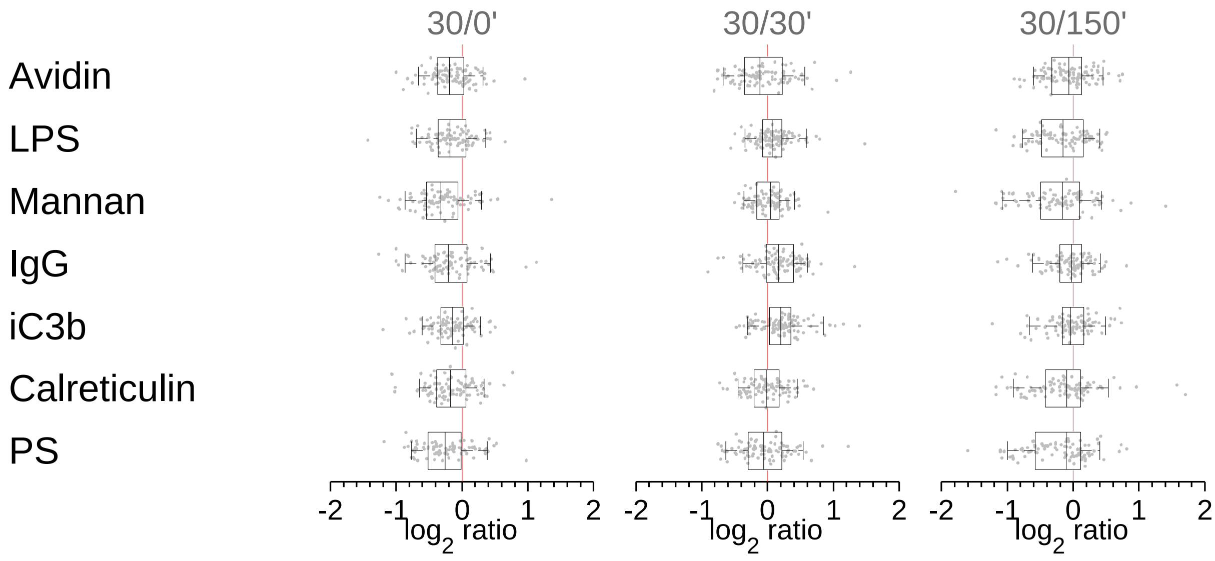

GO:0016508 long-chain-enoyl-CoA hydratase activity (n = 3)

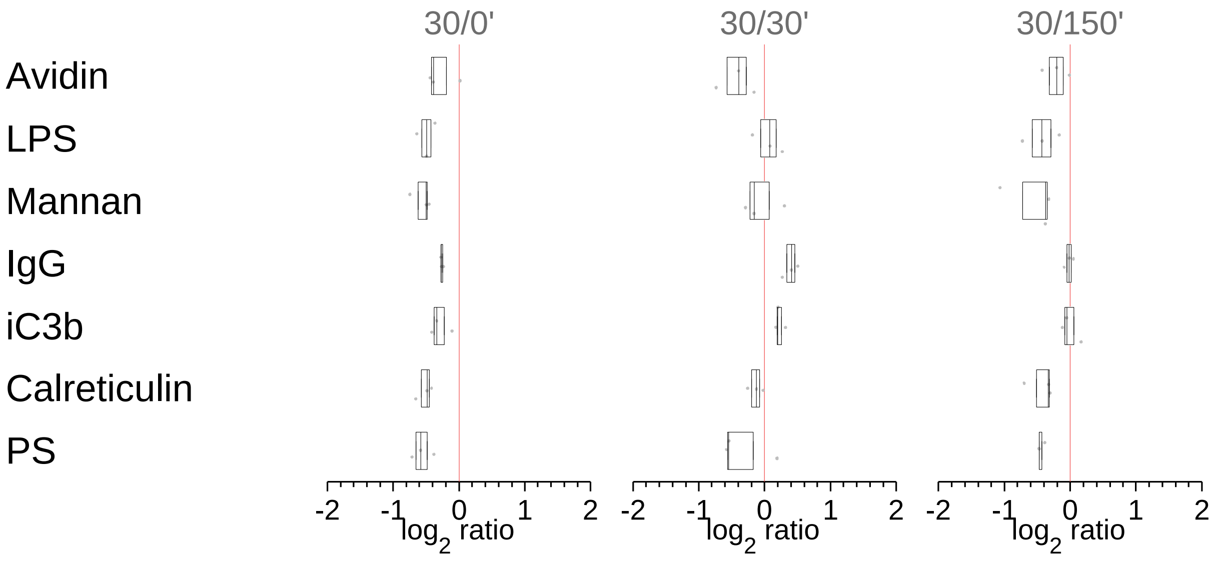

GO:0016525 negative regulation of angiogenesis (n = 7)

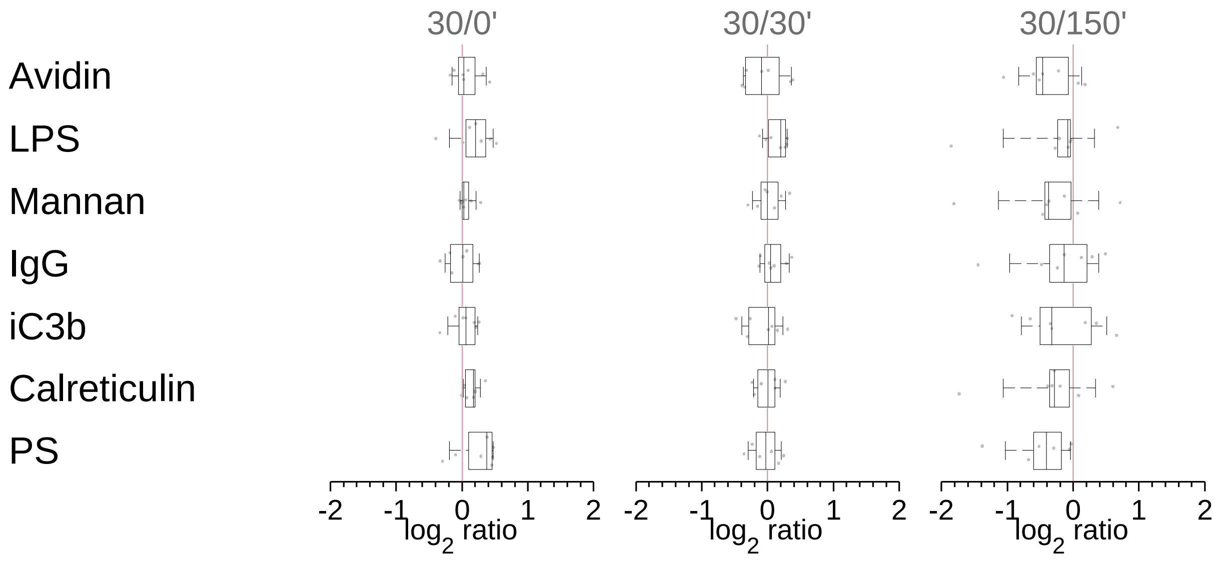

GO:0016529 sarcoplasmic reticulum (n = 8)

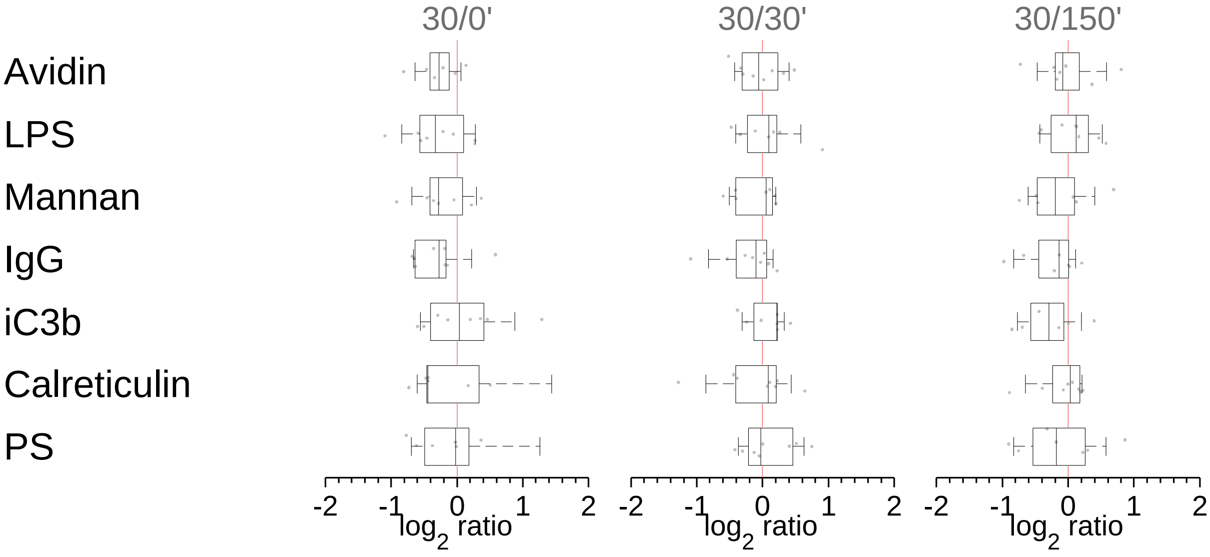

GO:0016540 protein autoprocessing (n = 4)

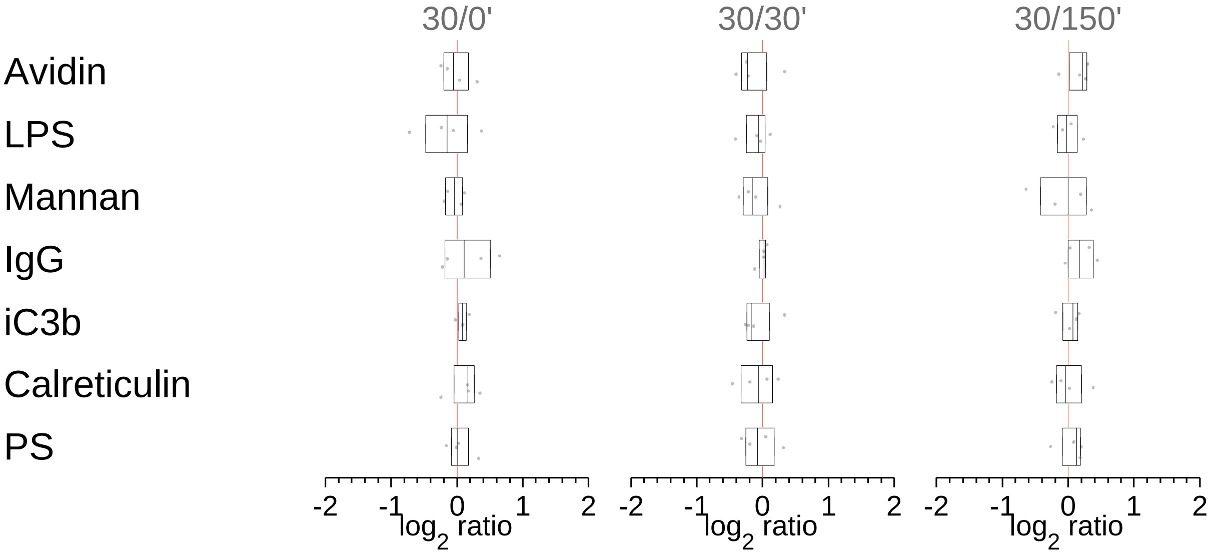

GO:0016567 protein ubiquitination (n = 21)

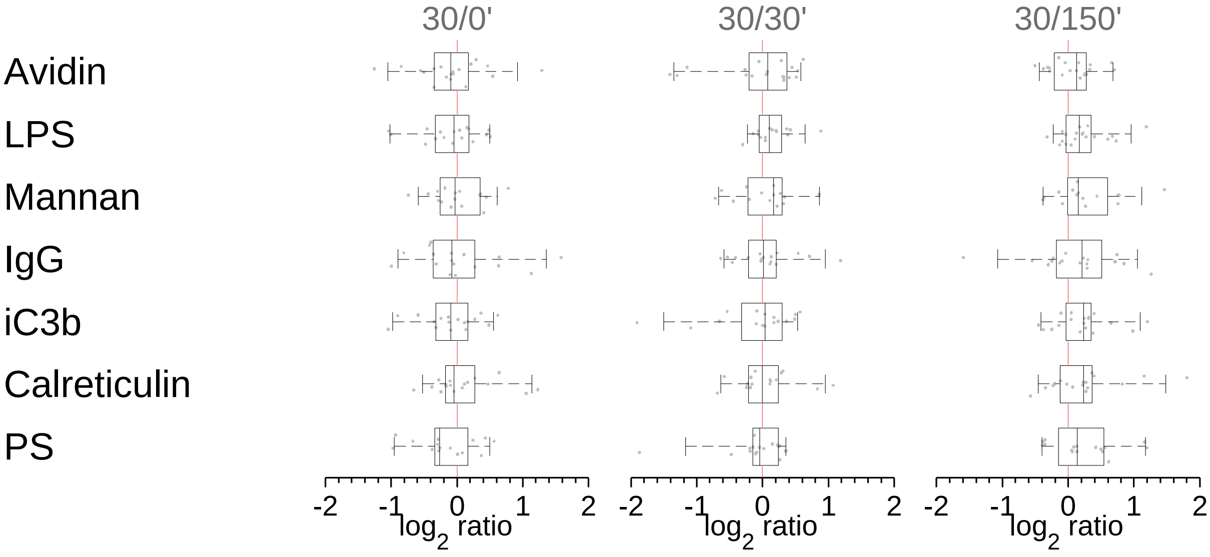

GO:0016568 chromatin modification (n = 17)

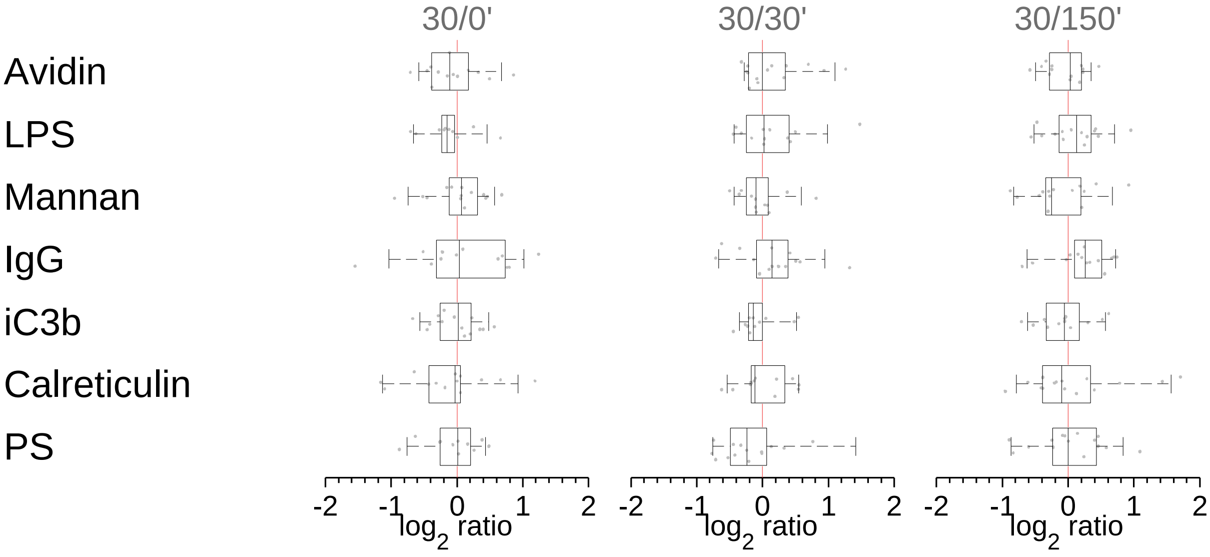

GO:0016575 histone deacetylation (n = 3)

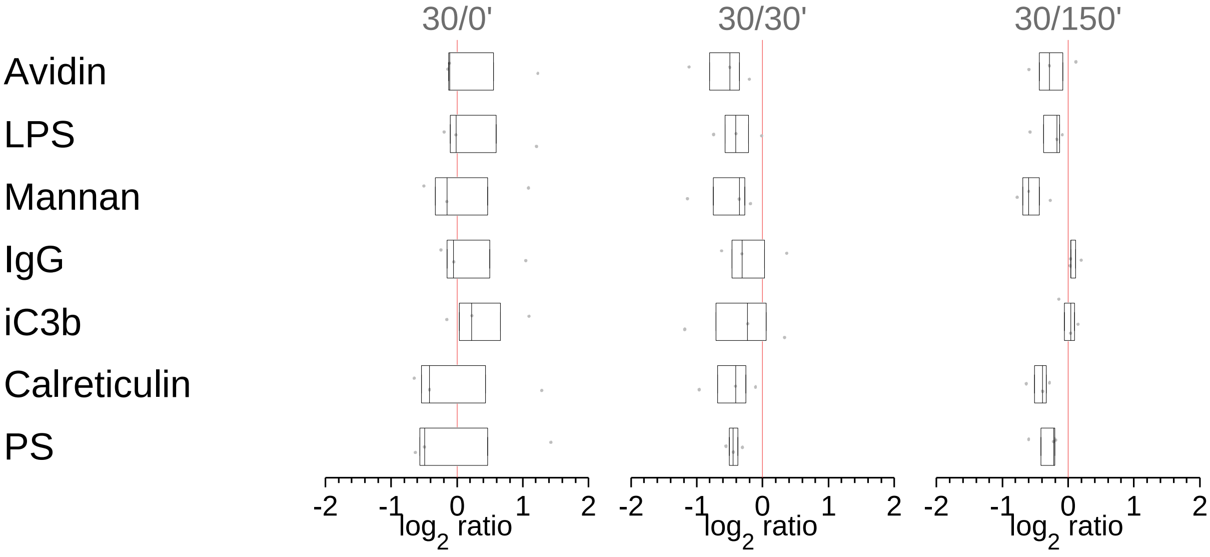

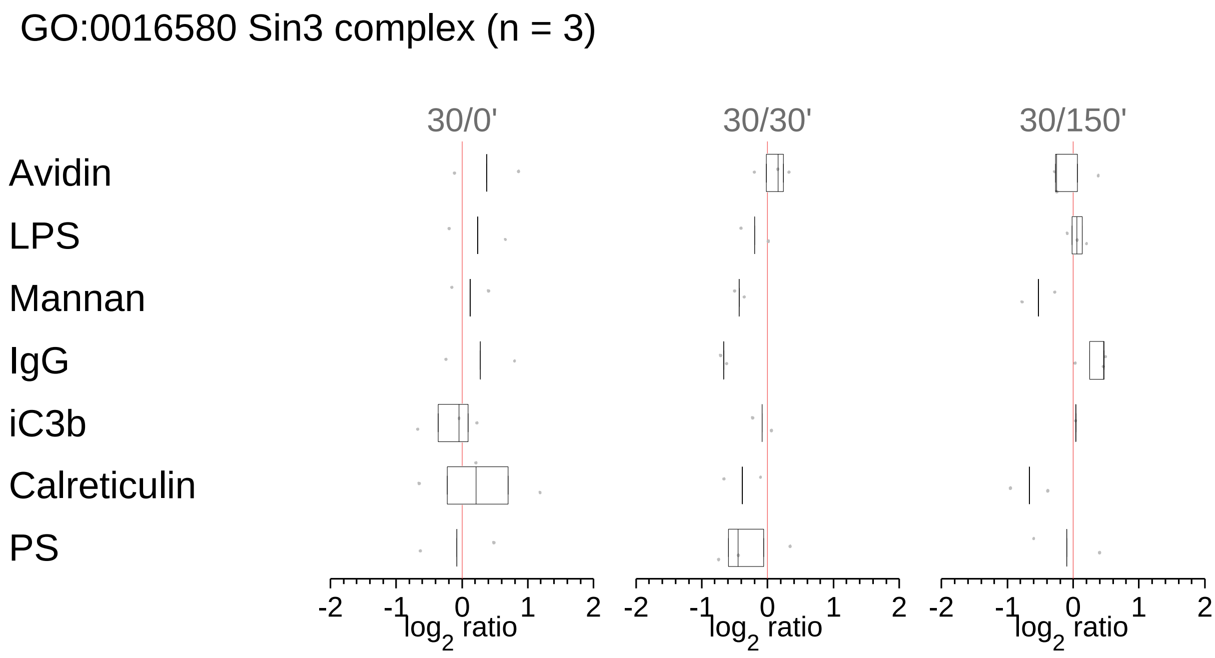
GO:0016580 Sin3 complex (n = 3)
30/0'
30/30'
30/150'
Avidin
LPS
Mannan
IgG
iC3b
Calreticulin
PS
-2 -1 0 1 2
log2 ratio
-2 -1 0 1 2
log2 ratio
-2 -1 0 1 2
log2 ratio

GO:0016581 NuRD complex (n = 4)

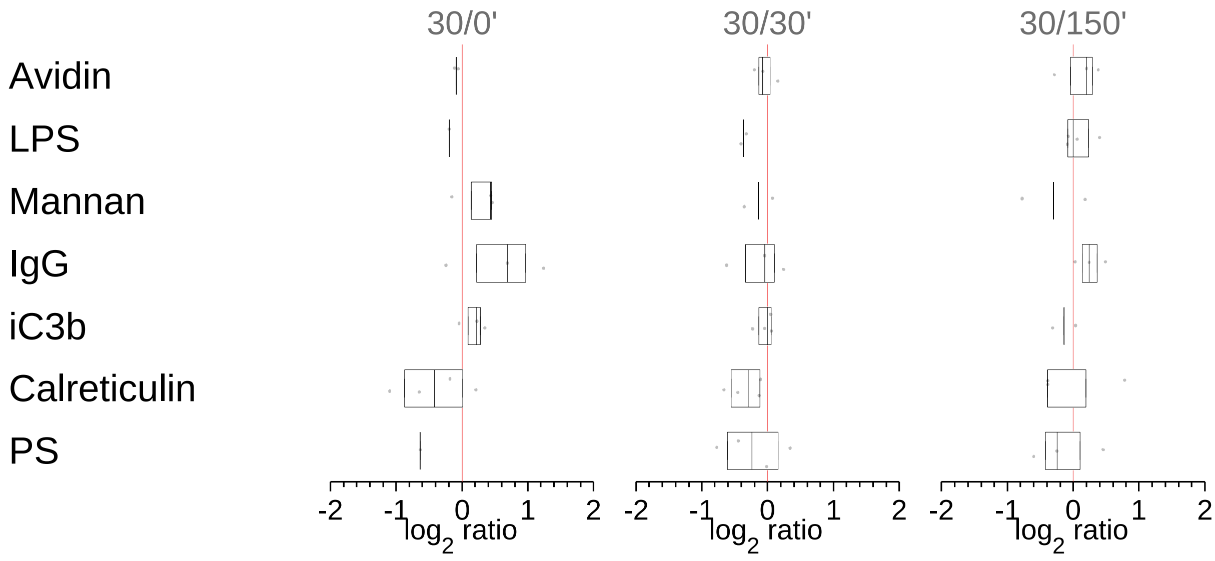

GO:0016584 nucleosome positioning (n = 4)

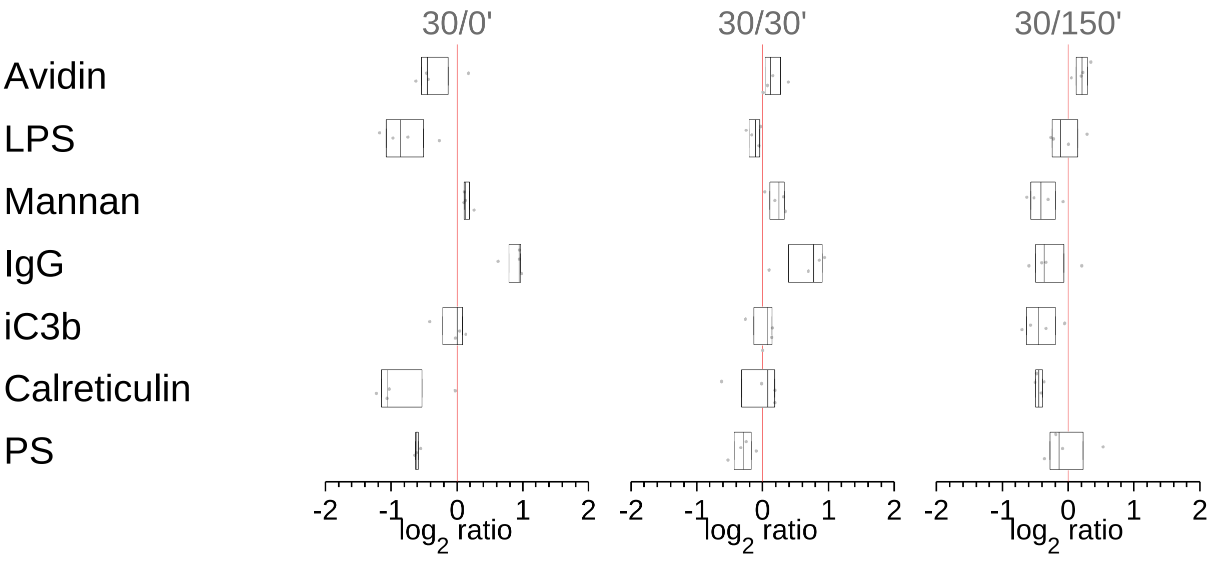

GO:0016600 flotillin complex (n = 3)

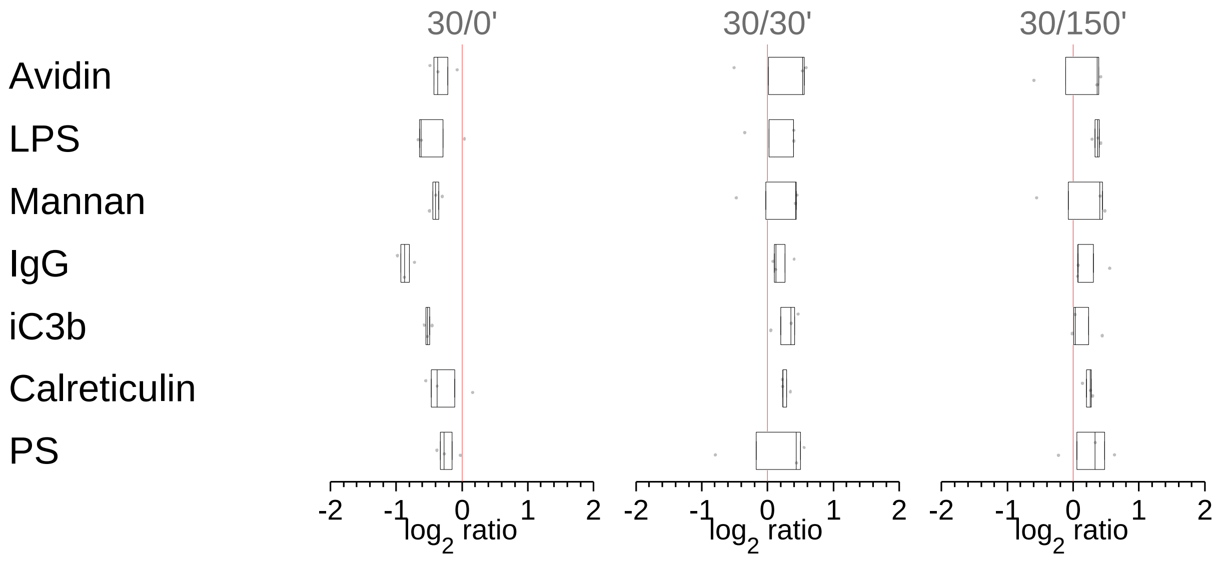

GO:0016601 Rac protein signal transduction (n = 6)

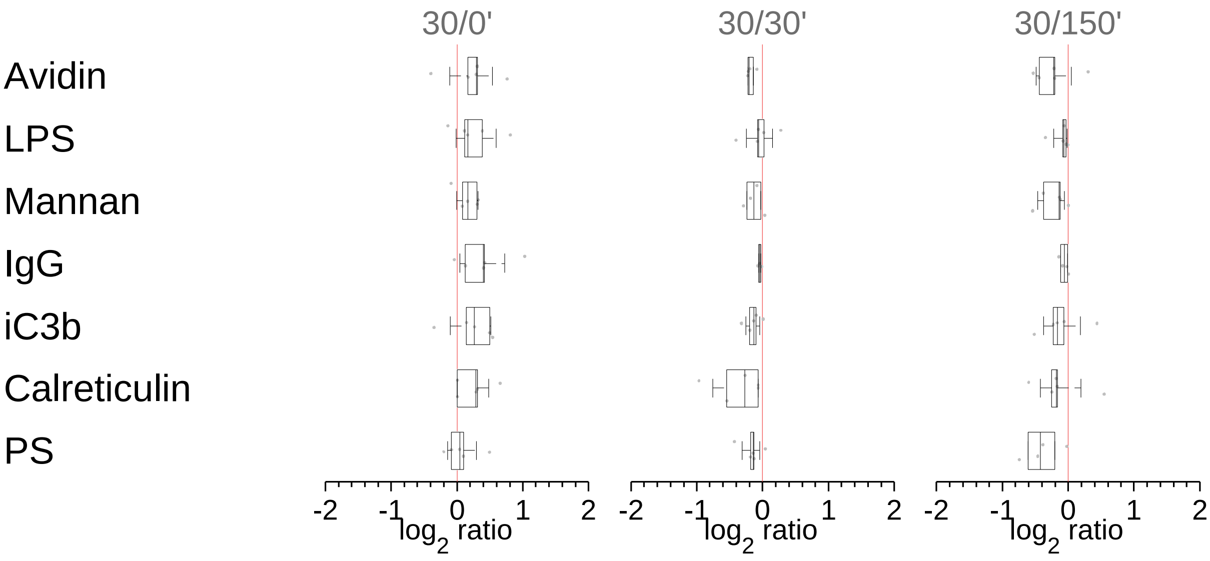

GO:0016604 nuclear body (n = 3)

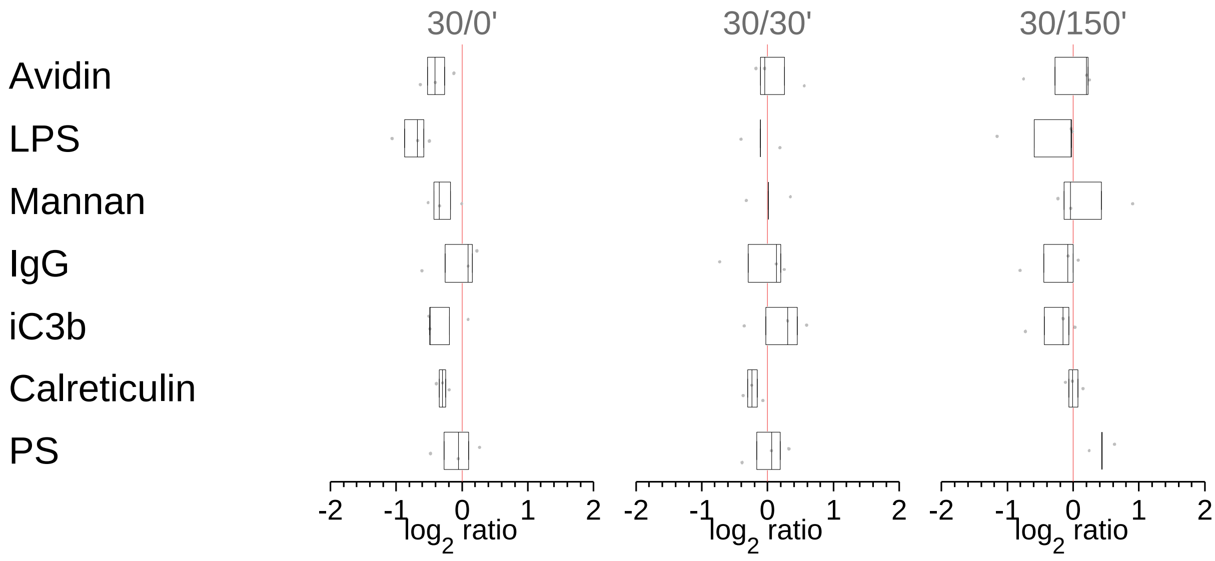

GO:0016605 PML body (n = 3)

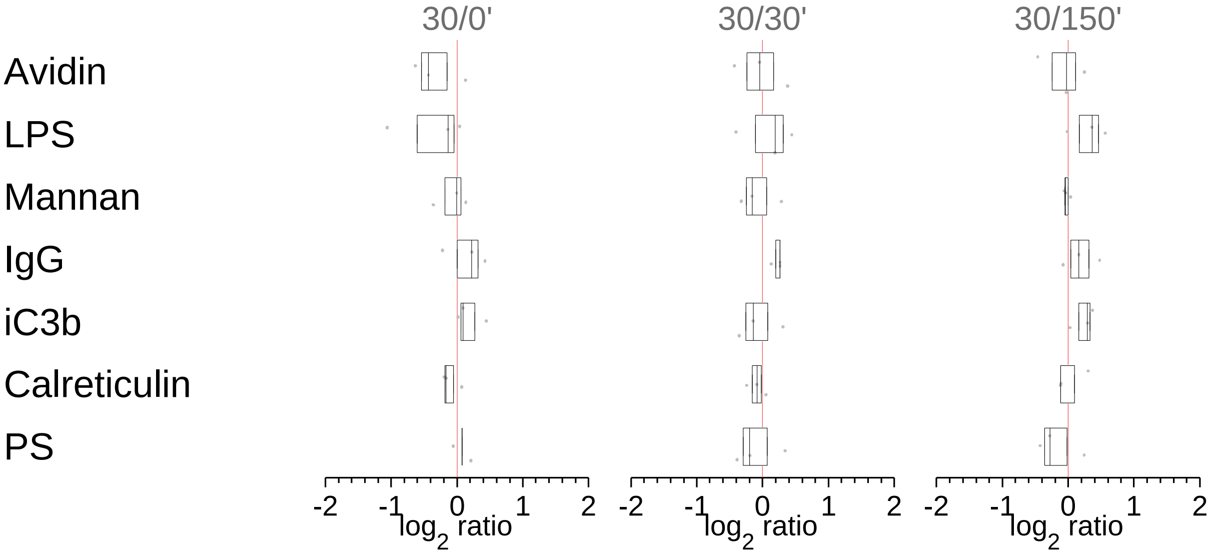

GO:0016607 nuclear speck (n = 11)

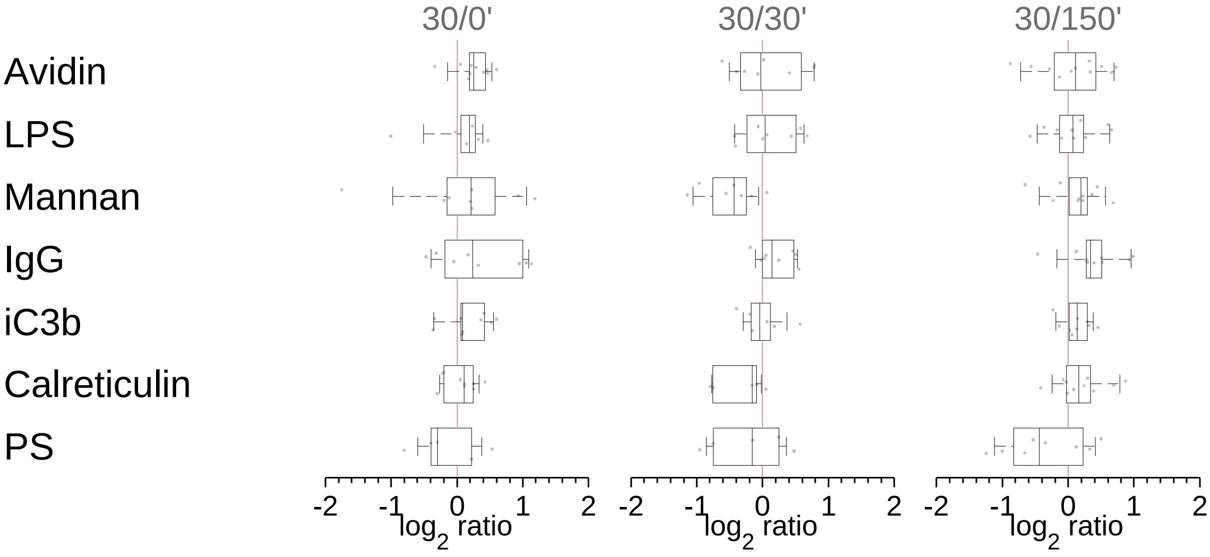

GO:0016616 oxidoreductase activity, acting on the CH-OH group of donors, NAD or NADP as acceptor (n = 10)

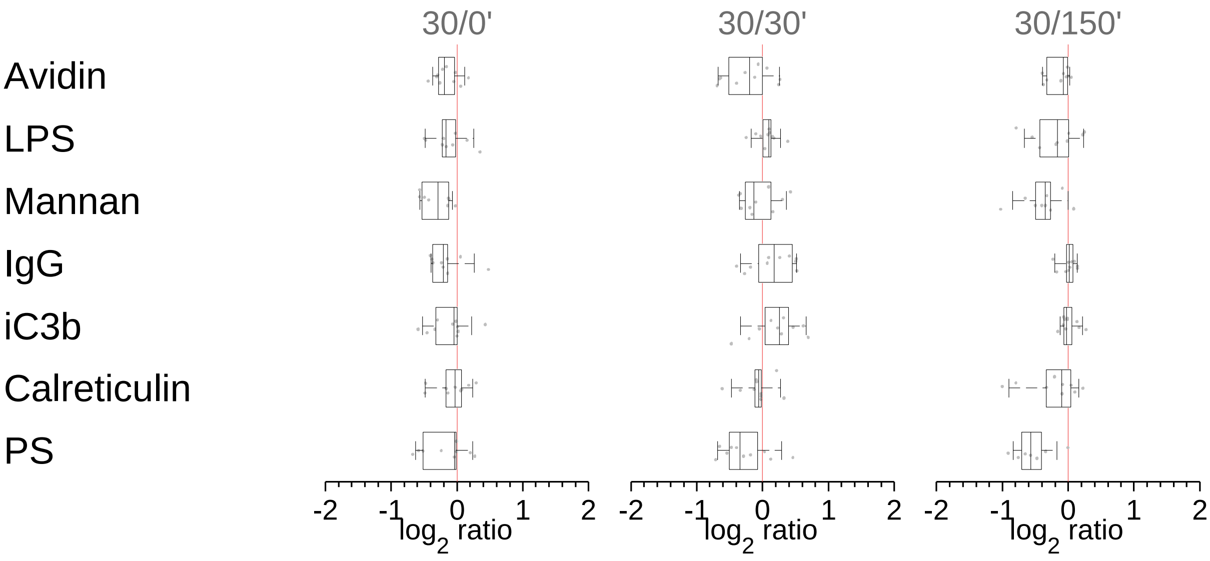

GO:0016620 oxidoreductase activity, acting on the aldehyde or oxo group of donors, NAD or NADP as acceptor (n = 4)

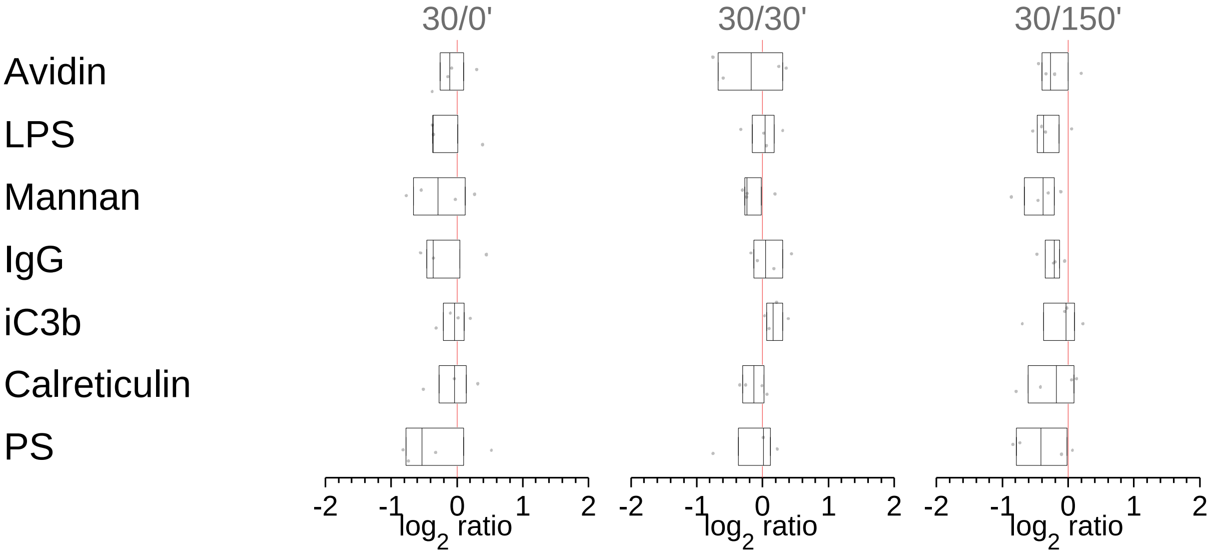

GO:0016627 oxidoreductase activity, acting on the CH-CH group of donors (n = 7)

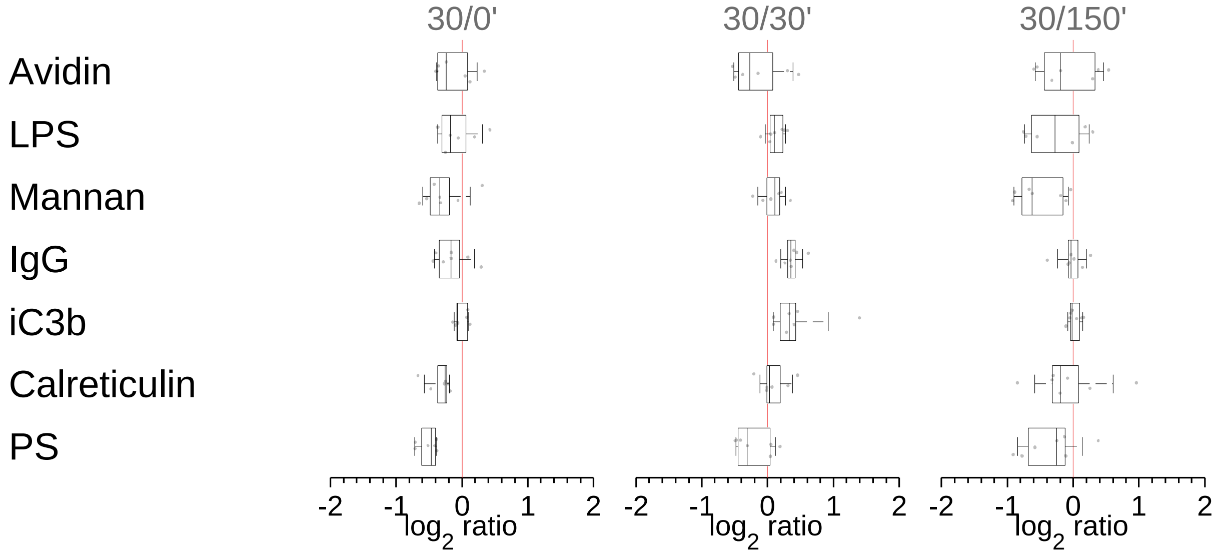

GO:0016651 oxidoreductase activity, acting on NADH or NADPH (n = 5)

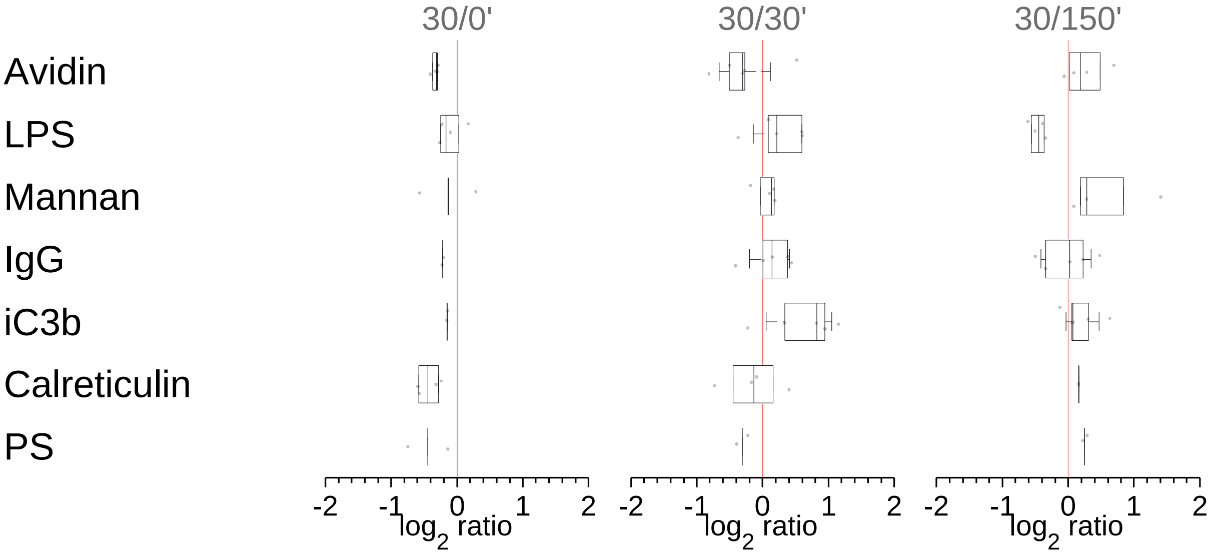

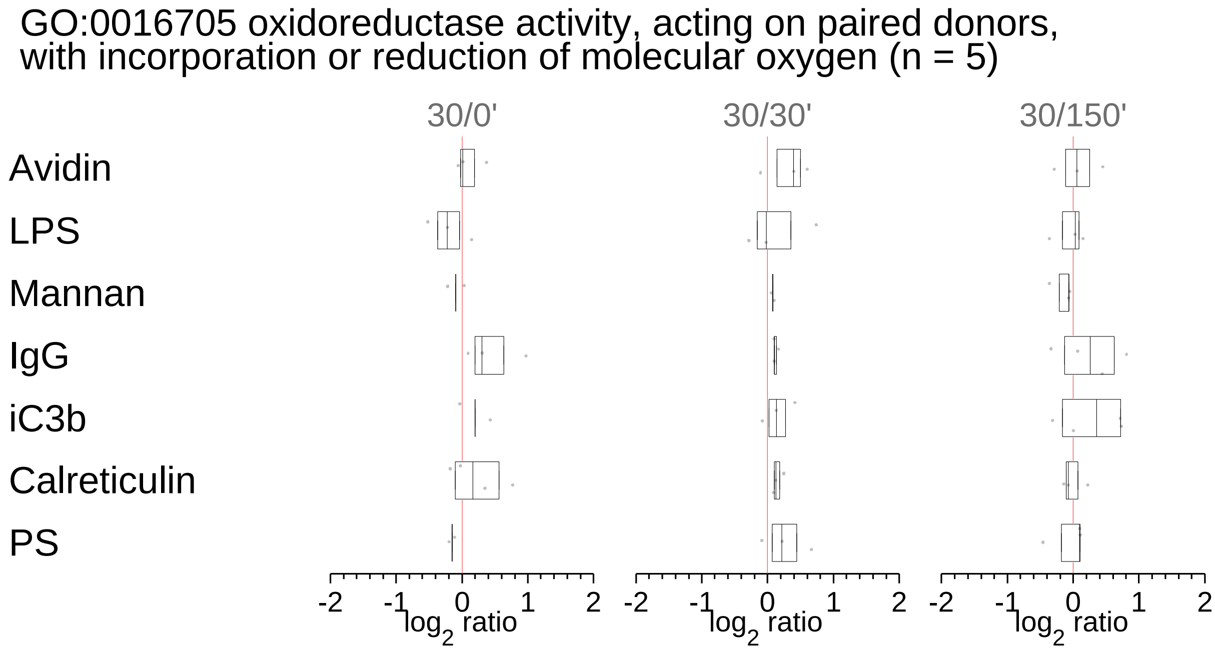

GO:0016705 oxidoreductase activity, acting on paired donors,
with incorporation or reduction of molecular oxygen (n = 5)
30/0'
30/30'
30/150'
Avidin
LPS
Mannan
IgG
iC3b
Calreticulin
PS
-2
-1
0
1
2
log2 ratio

GO:0016740 transferase activity (n = 128)

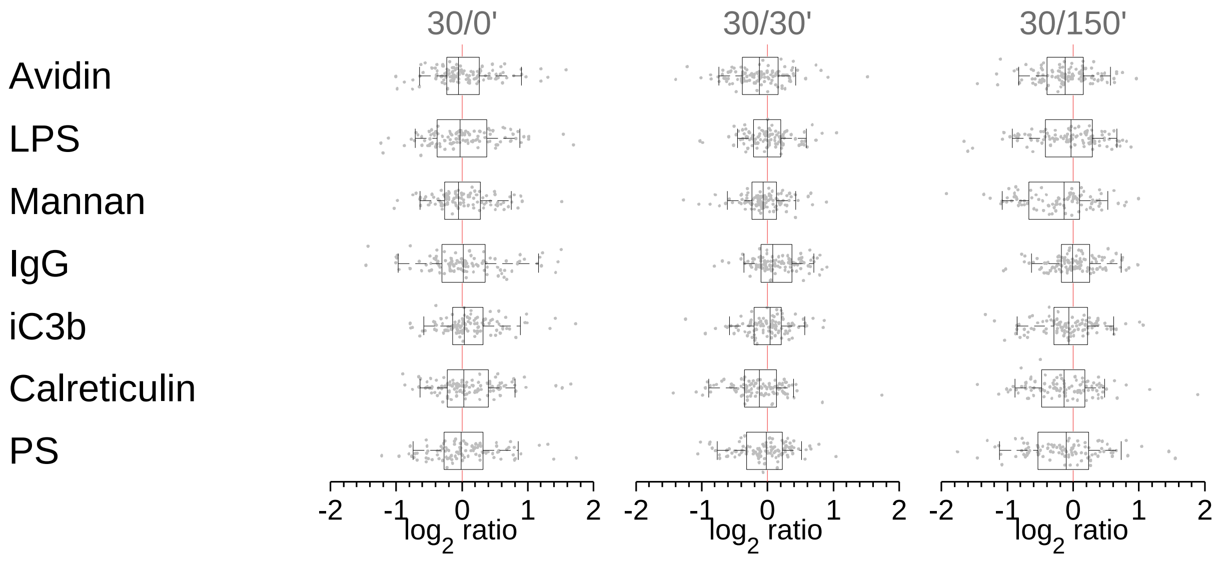

GO:0016746 transferase activity, transferring acyl groups (n = 20)

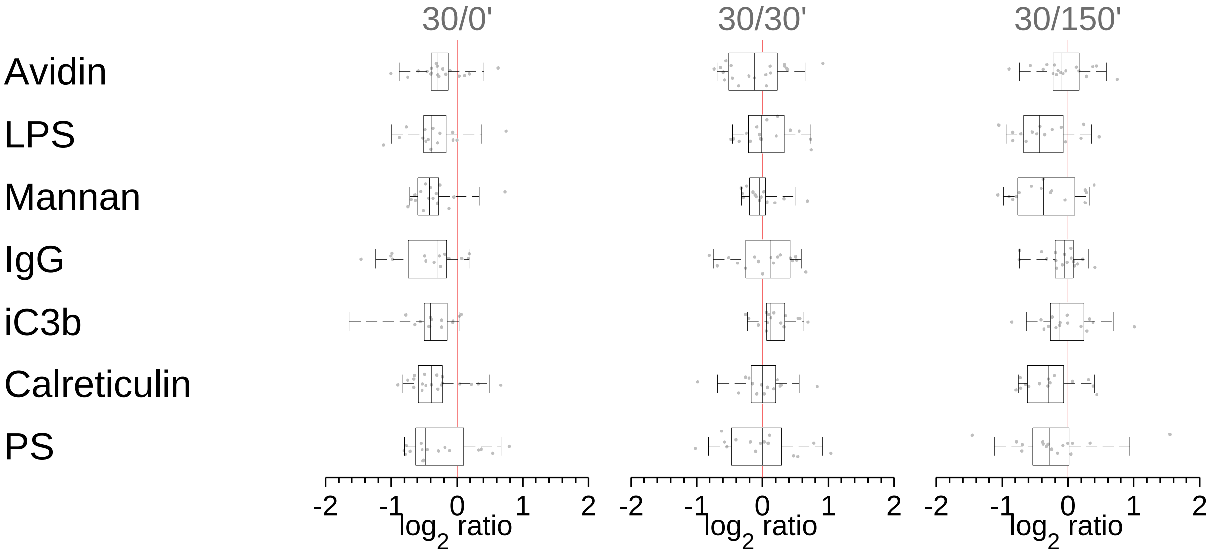

GO:0016747 transferase activity, transferring acyl groups other than amino-acyl groups (n = 5)

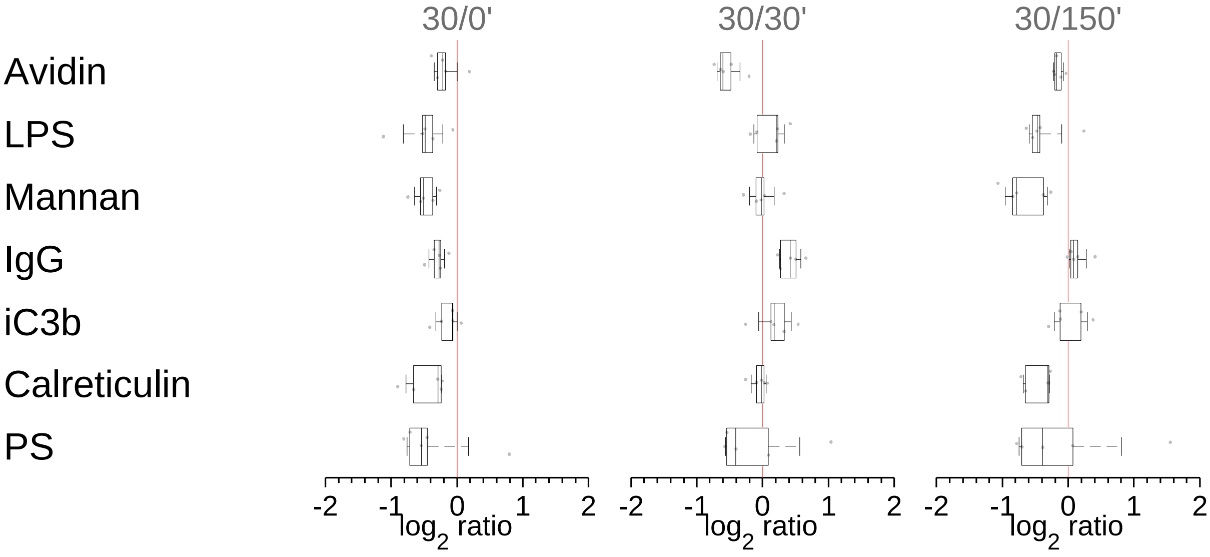

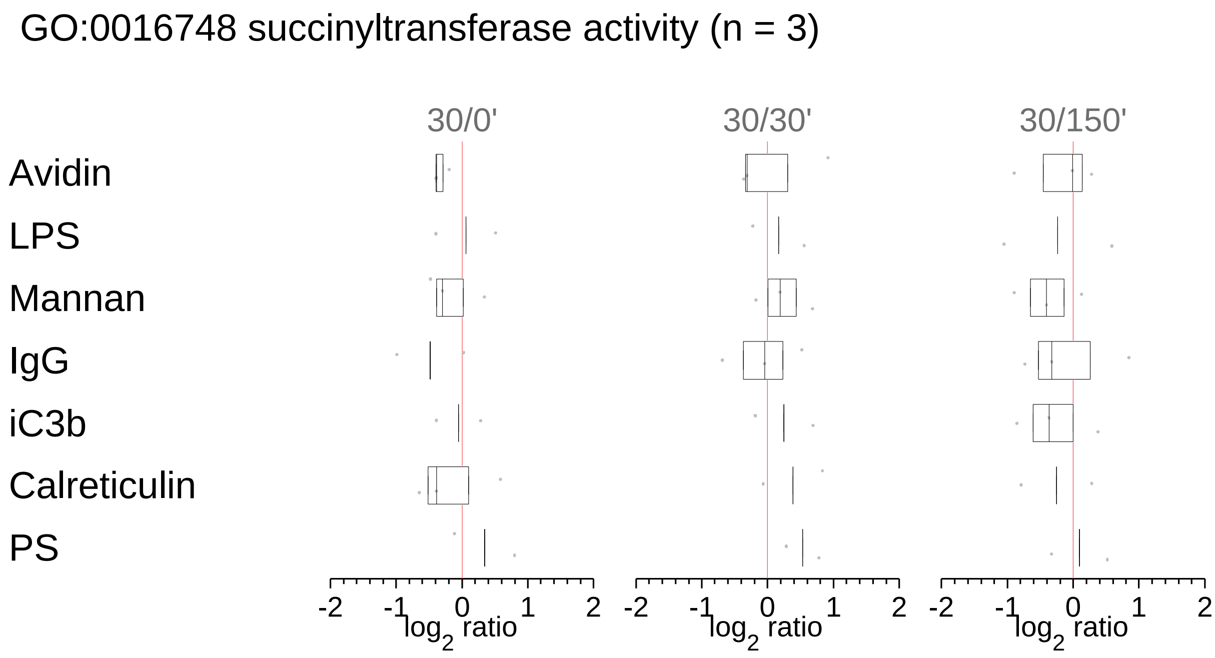

GO:0016748 succinyltransferase activity (n = 3)
30/0'
30/30'
30/150'
Avidin
LPS
Mannan
IgG
iC3b
Calreticulin
PS
-2 -1 0 1 2
log2 ratio
-2 -1 0 1 2
log2 ratio
-2 -1 0 1 2
log2 ratio

GO:0016749 N-succinyltransferase activity (n = 3)

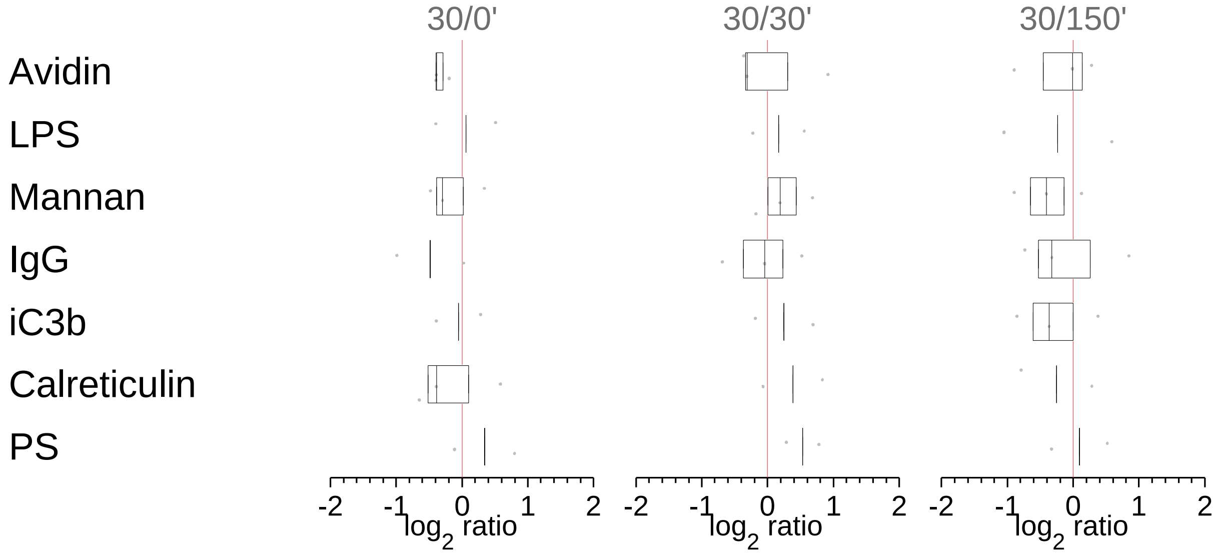

GO:0016750 O-succinyltransferase activity (n = 3)

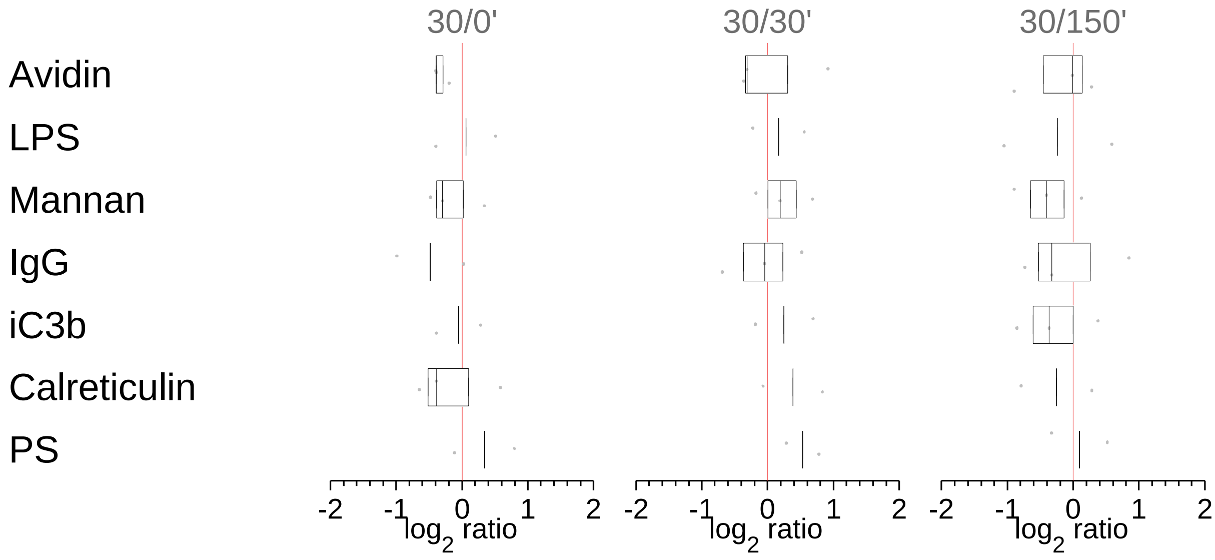

GO:0016751 S-succinyltransferase activity (n = 3)

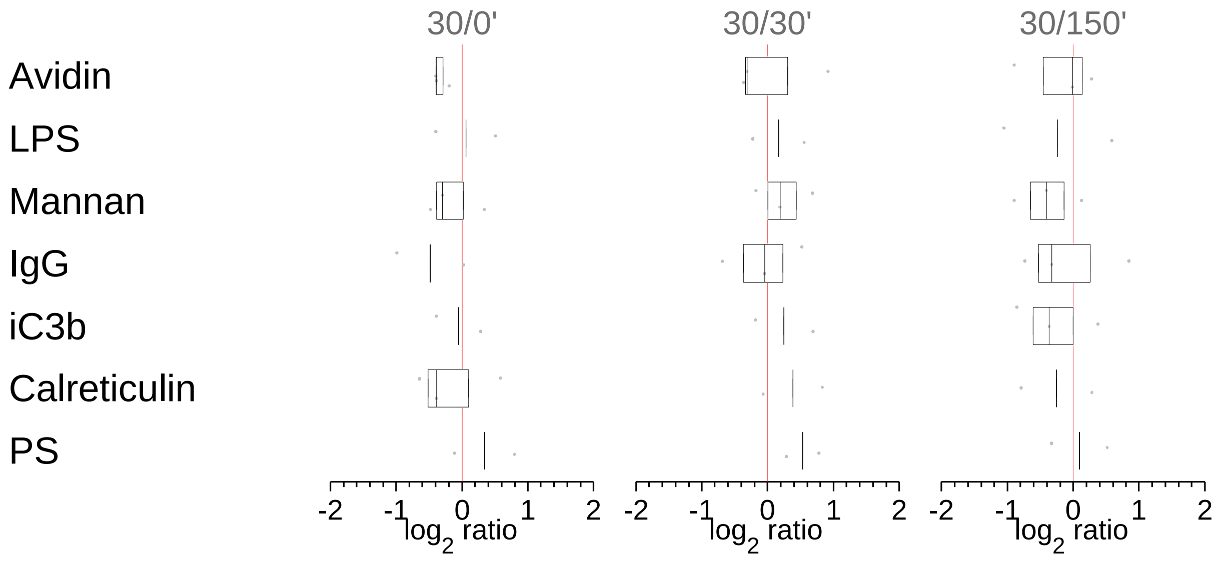

GO:0016752 sinapoyltransferase activity (n = 3)

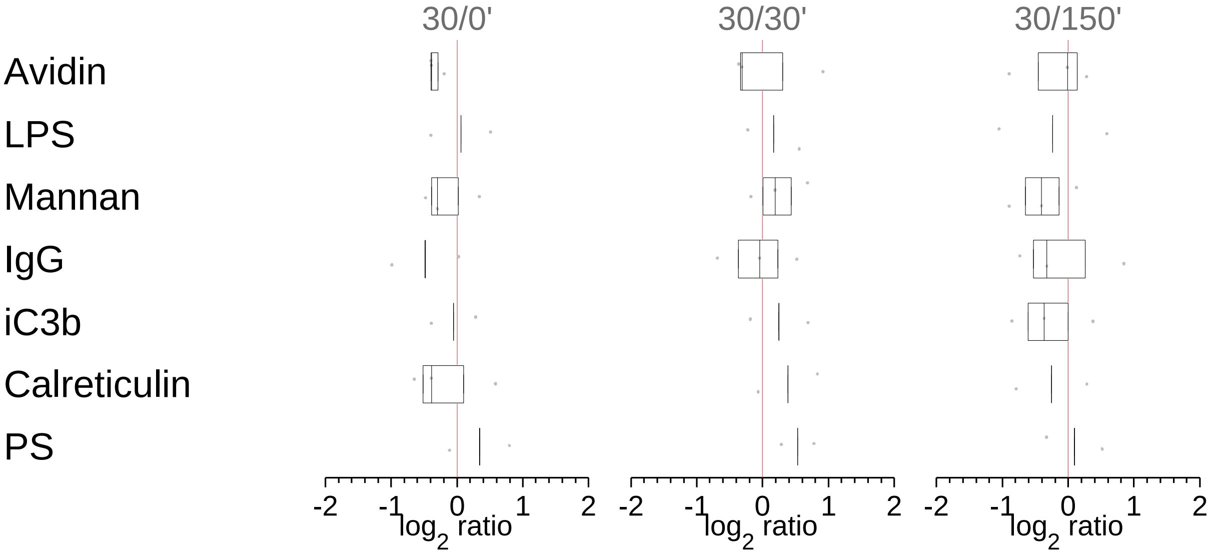

GO:0016753 O-sinapoyltransferase activity (n = 3)

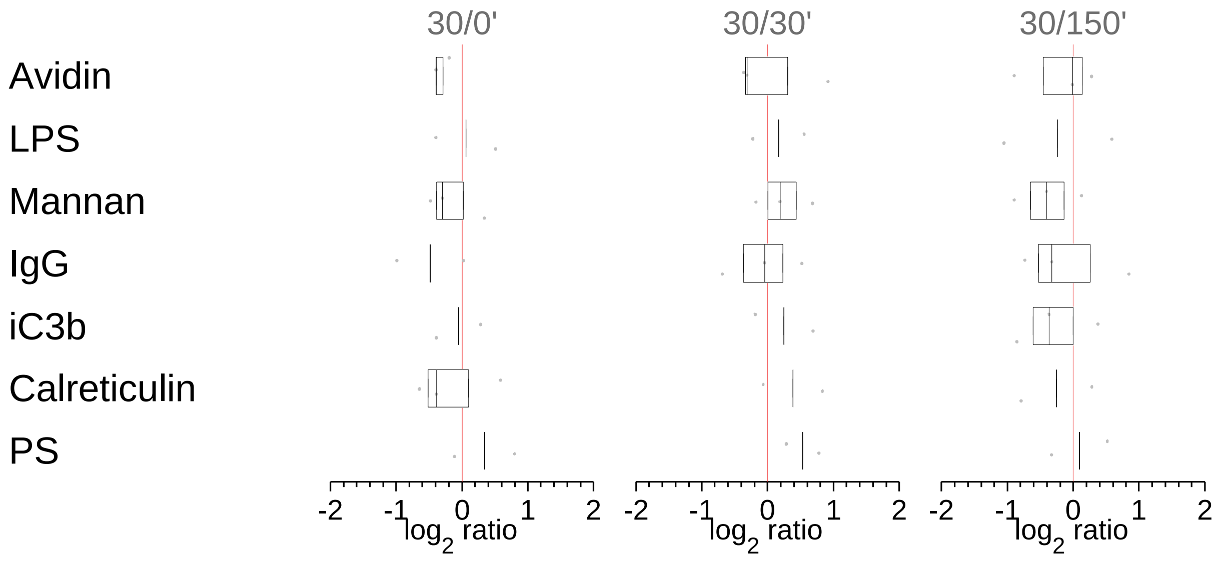

GO:0016757 transferase activity, transferring glycosyl groups (n = 20)

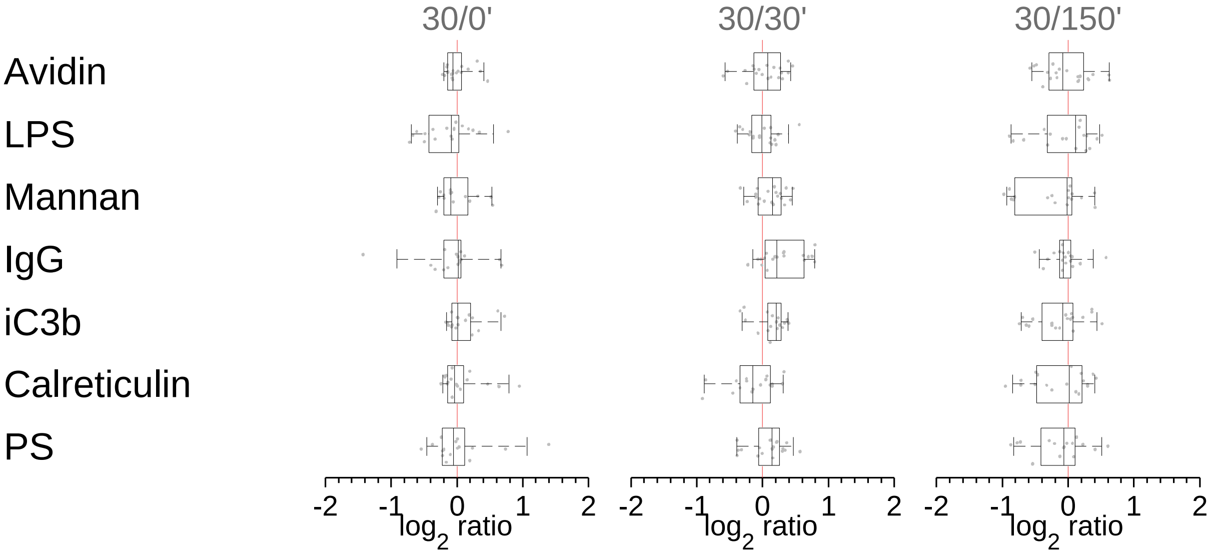

GO:0016758 transferase activity, transferring hexosyl groups (n = 3)

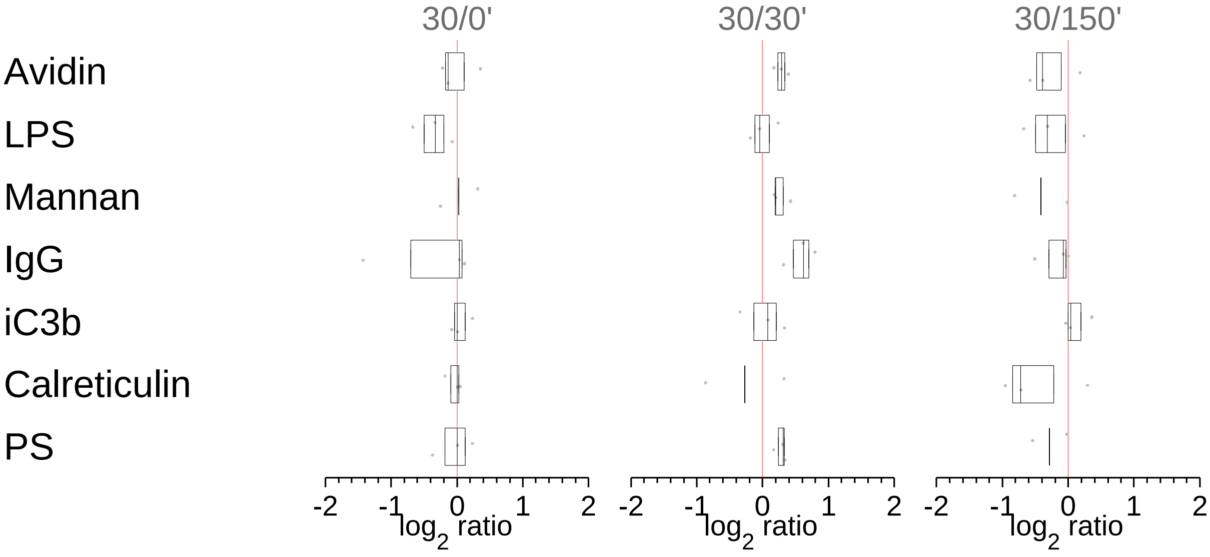

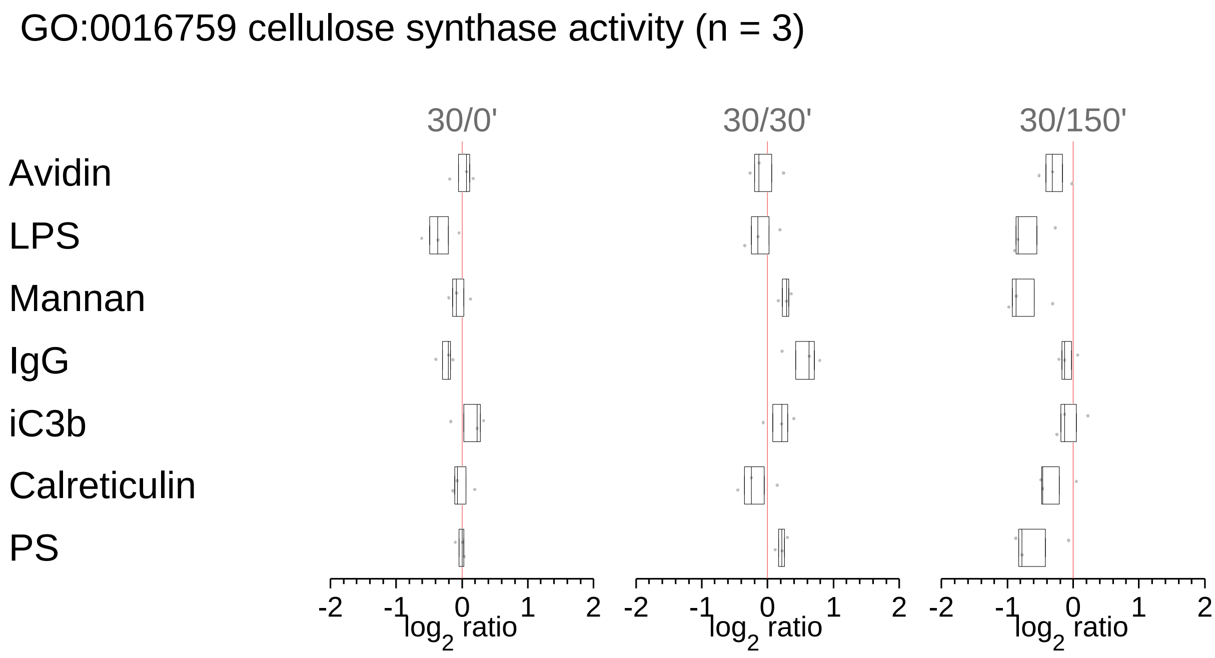

GO:0016759 cellulose synthase activity (n = 3)
30/0'
30/30'
30/150'
Avidin
LPS
Mannan
IgG
iC3b
Calreticulin
PS
-2
-1
0
1
2
log2 ratio

GO:0016772 transferase activity, transferring phosphorus-containing groups (n = 53)

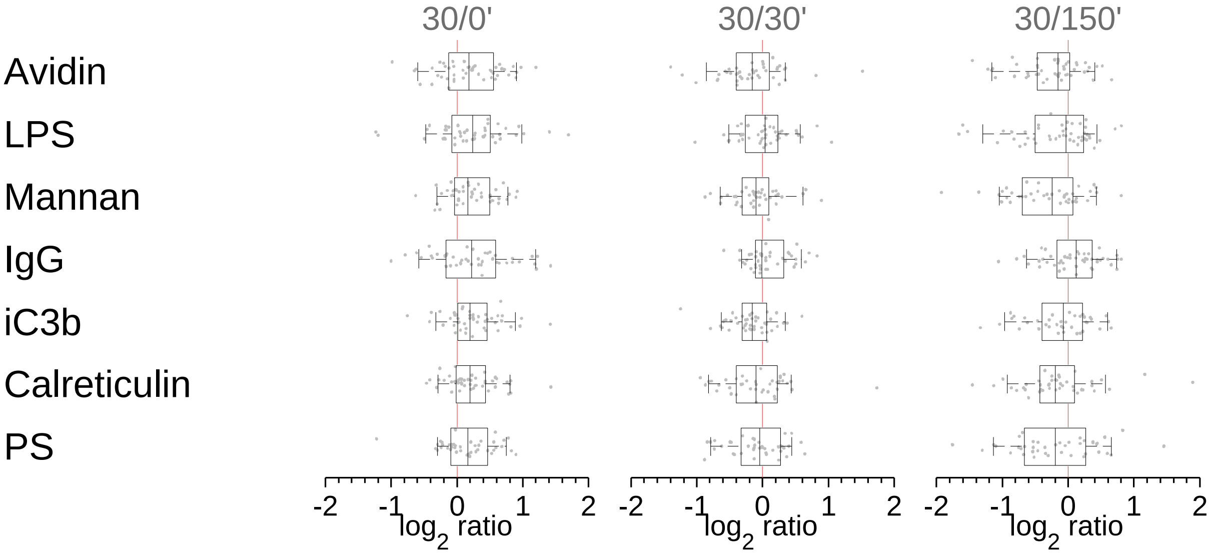

GO:0016773 phosphotransferase activity, alcohol group as acceptor (n = 5)

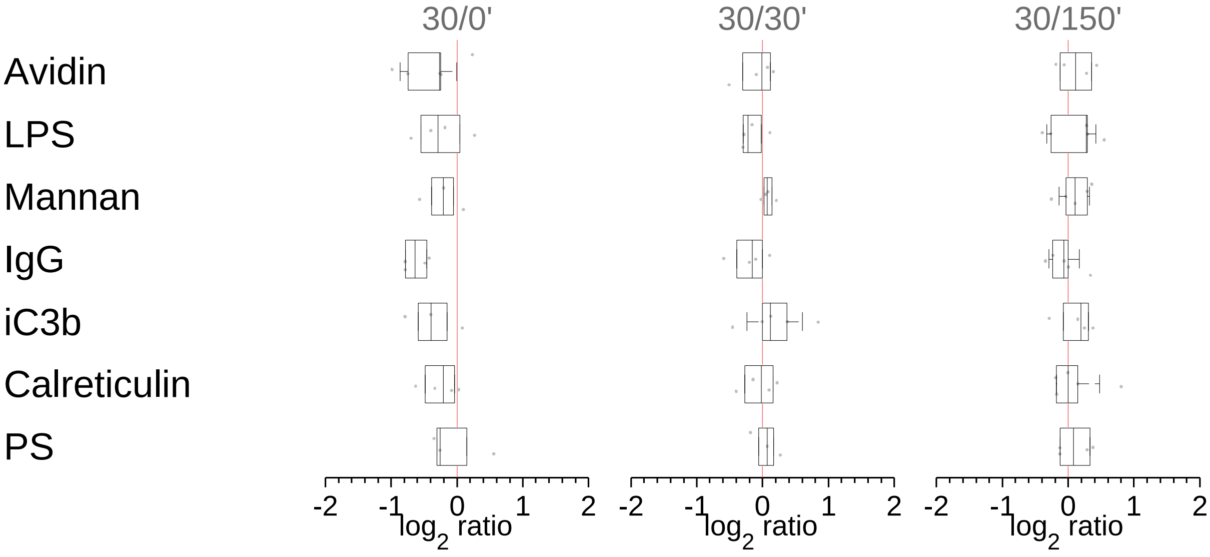

GO:0016779 nucleotidyltransferase activity (n = 3)

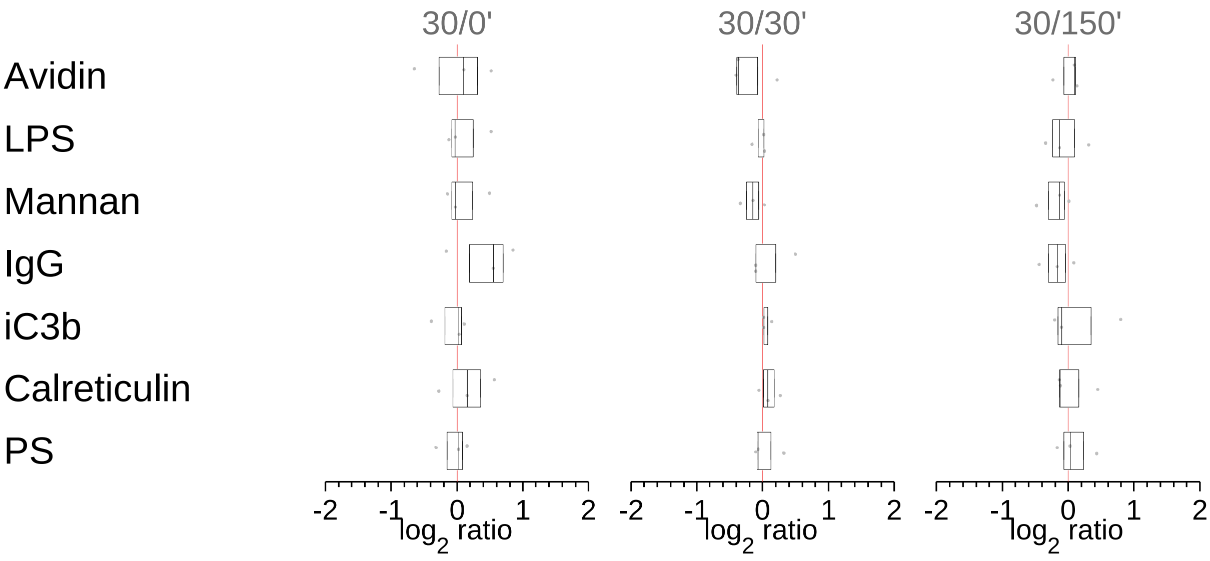

GO:0016787 hydrolase activity (n = 210)

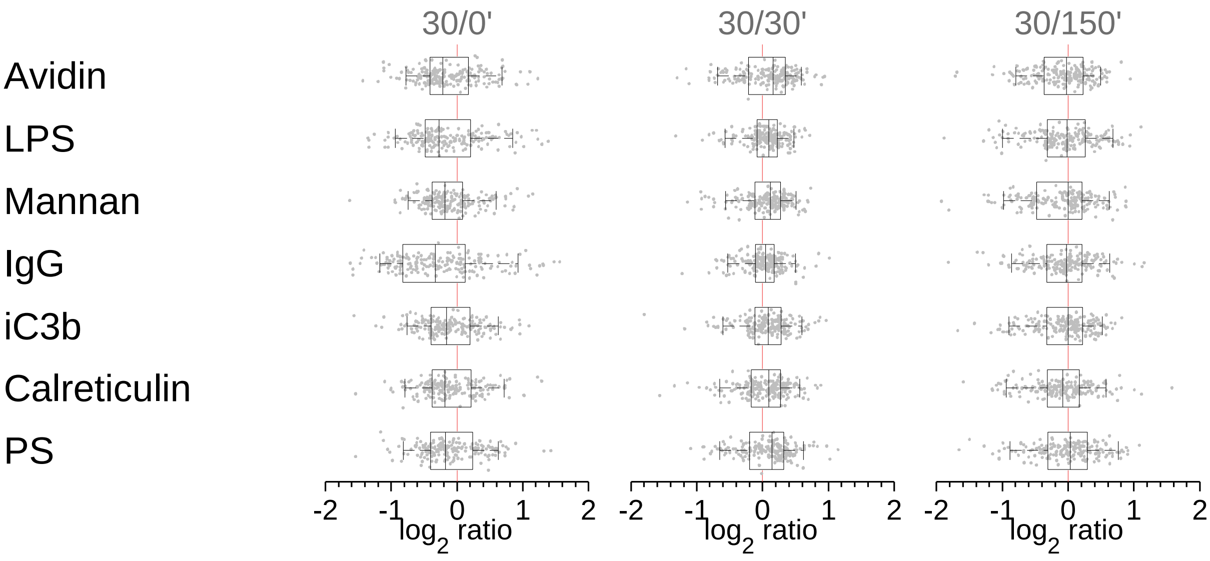

GO:0016788 hydrolase activity, acting on ester bonds (n = 6)

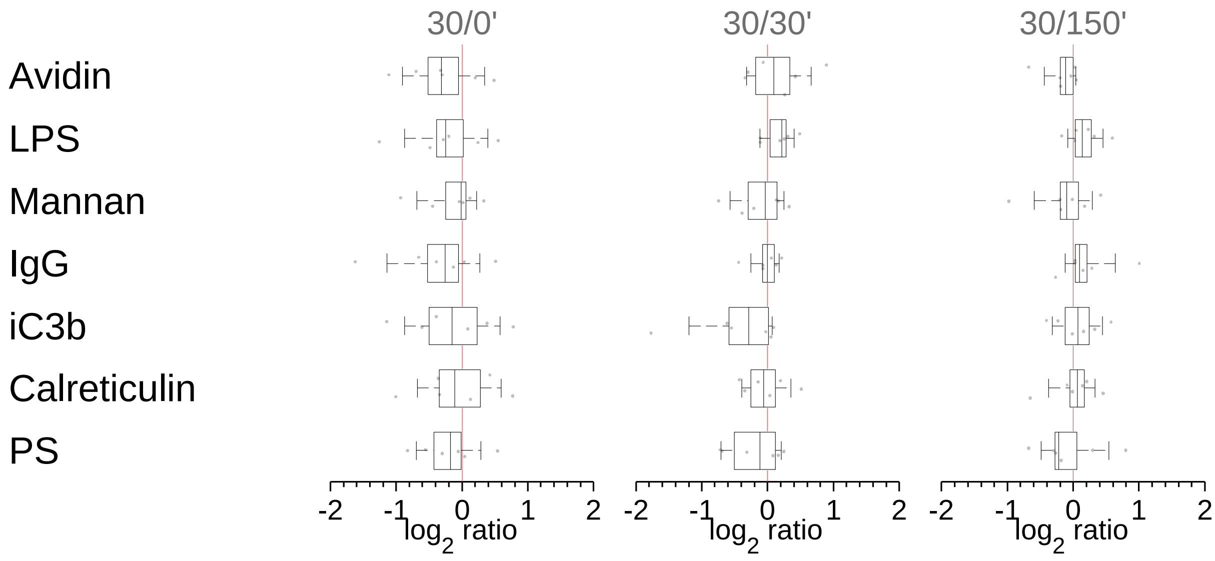

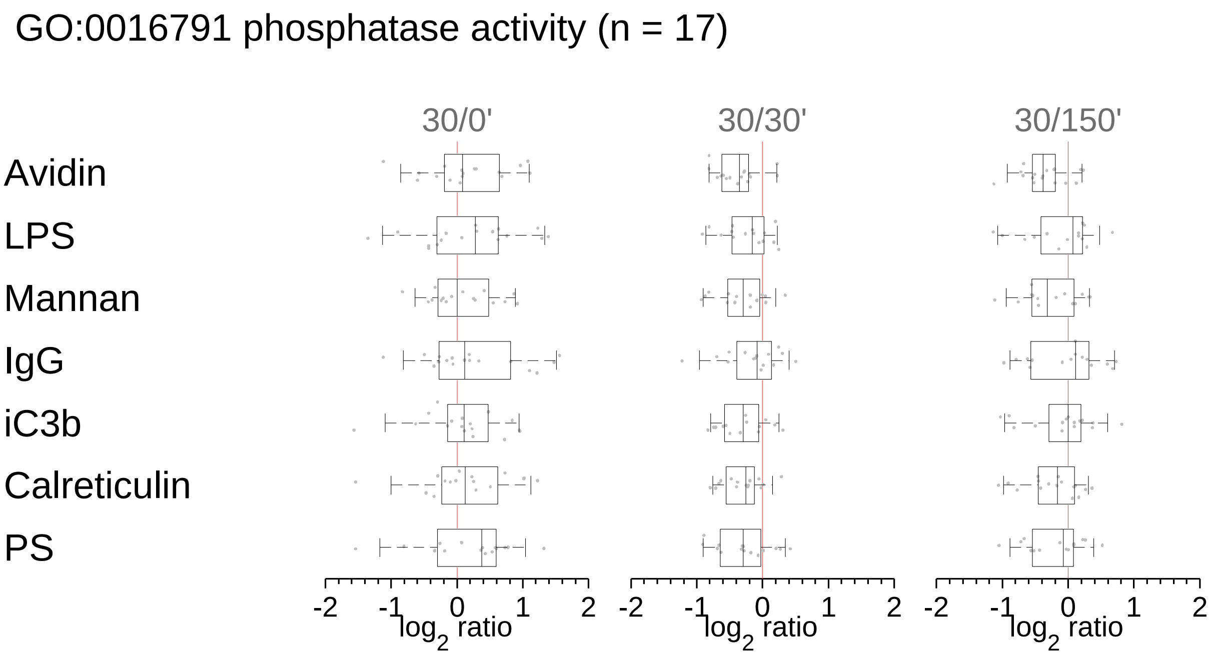
GO:0016791 phosphatase activity (n = 17)
30/0'
30/30'
30/150'
Avidin
LPS
Mannan
IgG
iC3b
Calreticulin
PS
-2
-1
0
1
2
log2 ratio

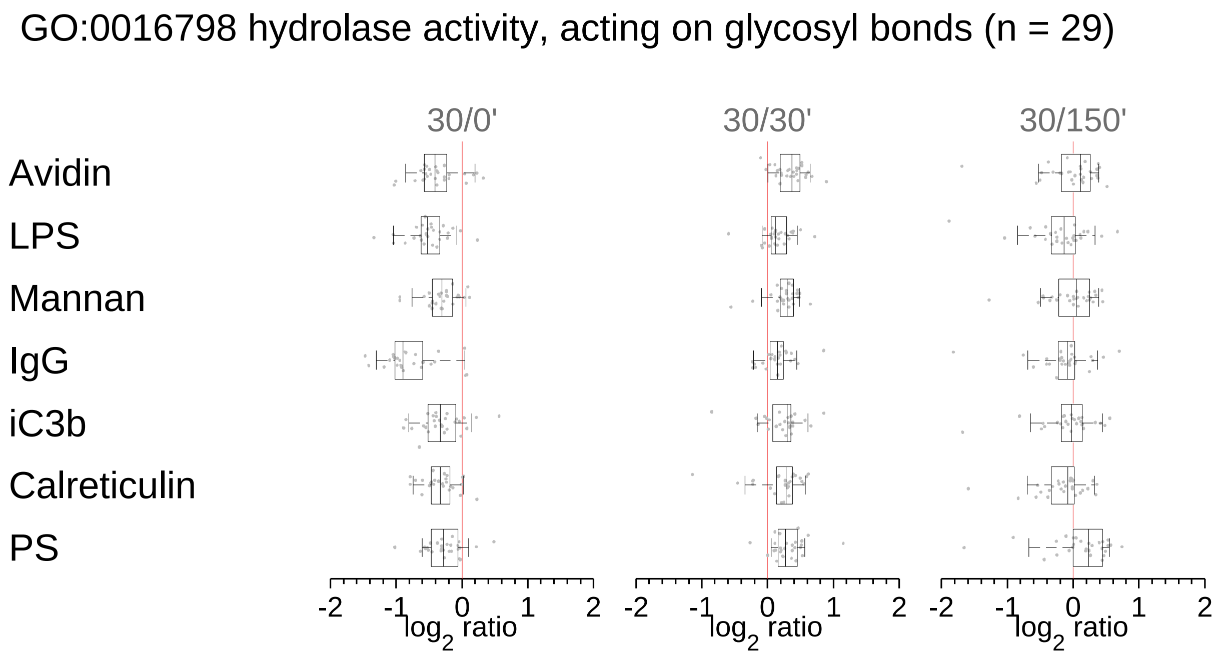
GO:0016798 hydrolase activity, acting on glycosyl bonds (n = 29)
30/0'
30/30'
30/150'
Avidin
LPS
Mannan
IgG
iC3b
Calreticulin
PS
-2
-1
0
1
2
log2 ratio

GO:0016805 dipeptidase activity (n = 3)

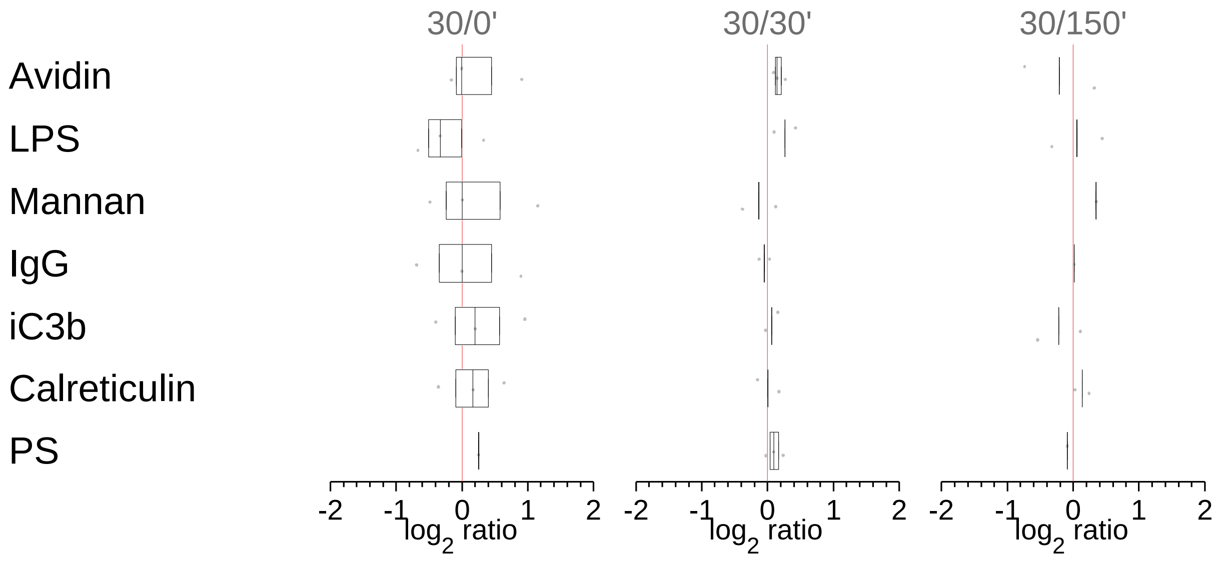

GO:0016810 hydrolase activity, acting on carbon-nitrogen (but not peptide) bonds (n = 5)

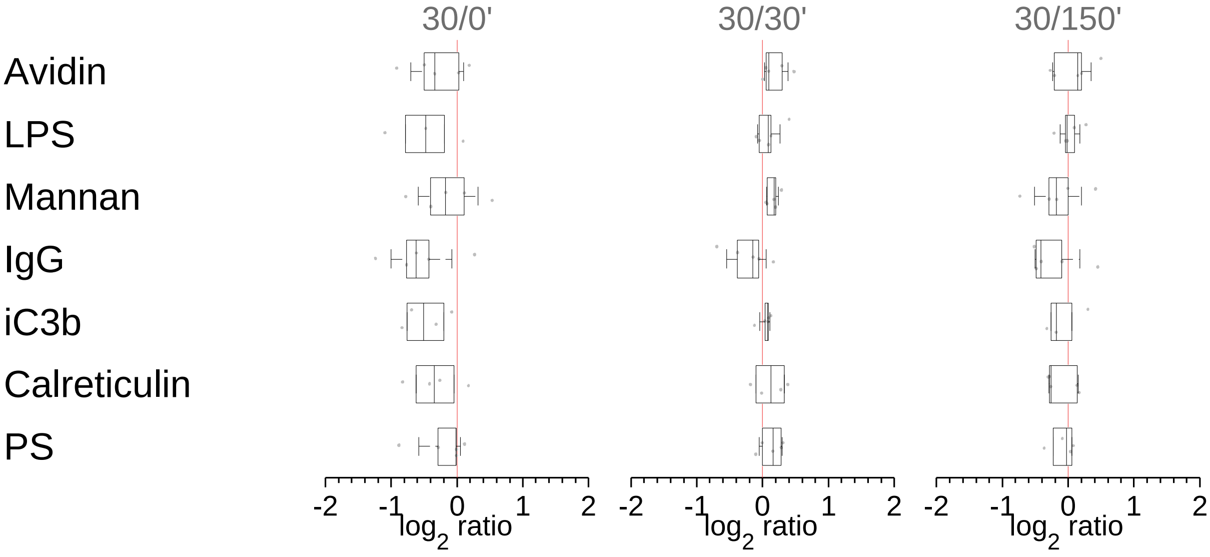

GO:0016811 hydrolase activity, acting on carbon-nitrogen (but not peptide) bonds, in linear amides (n = 4)

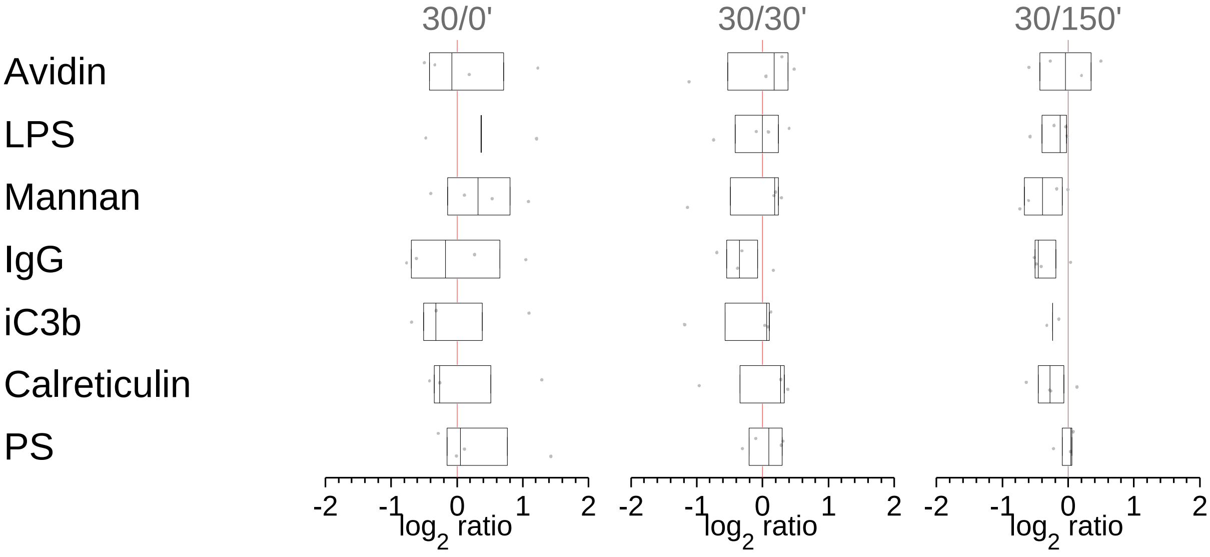

GO:0016817 hydrolase activity, acting on acid anhydrides (n = 3)

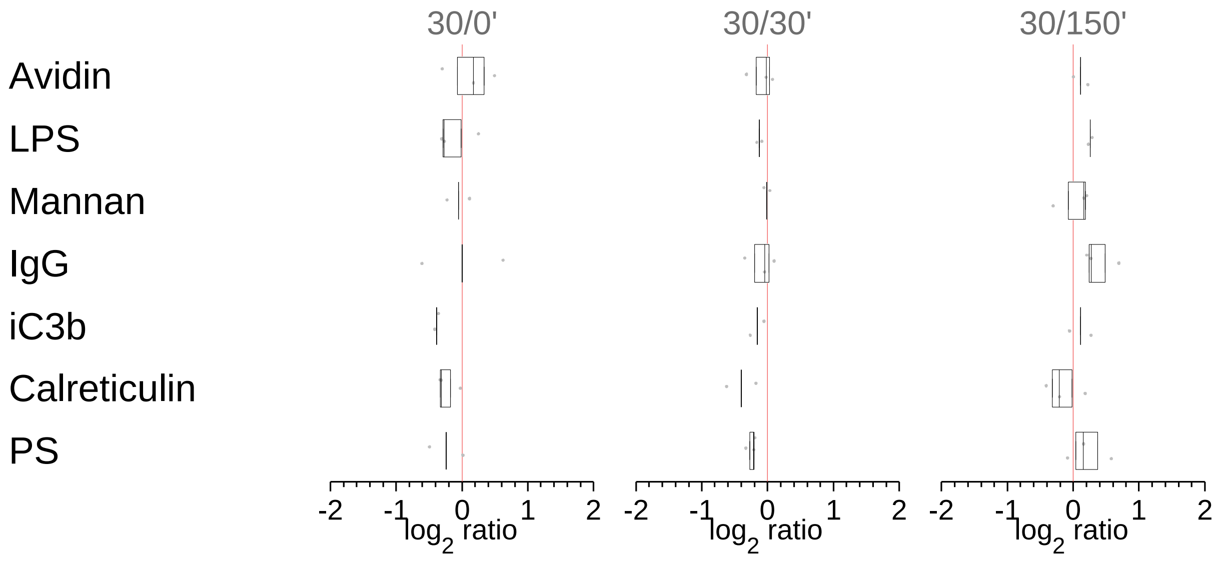

GO:0016818 hydrolase activity, acting on acid anhydrides, in phosphorus-containing anhydrides (n = 3)

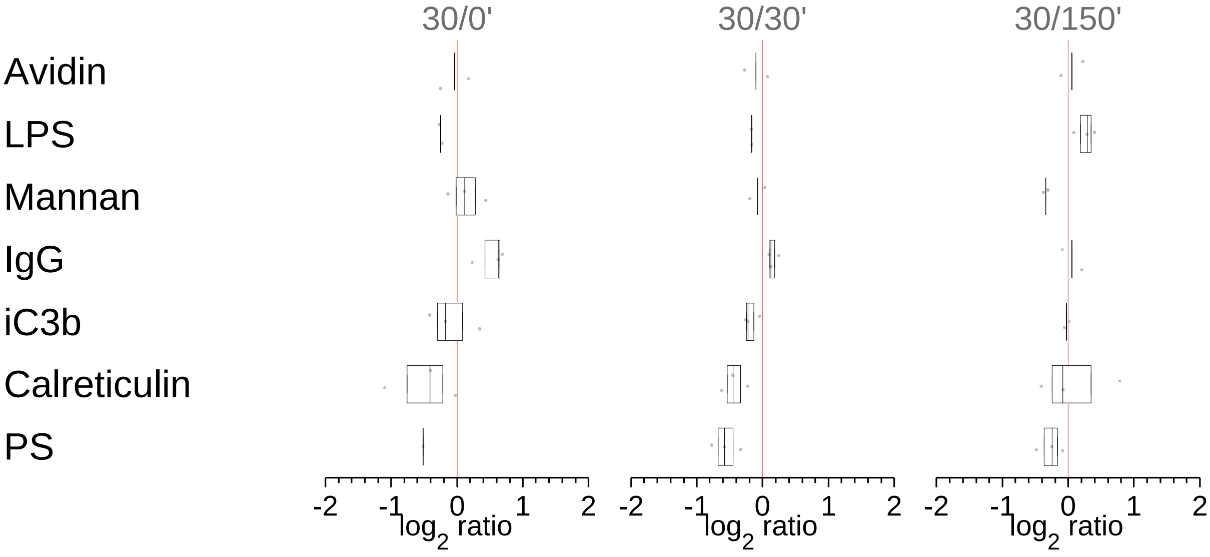

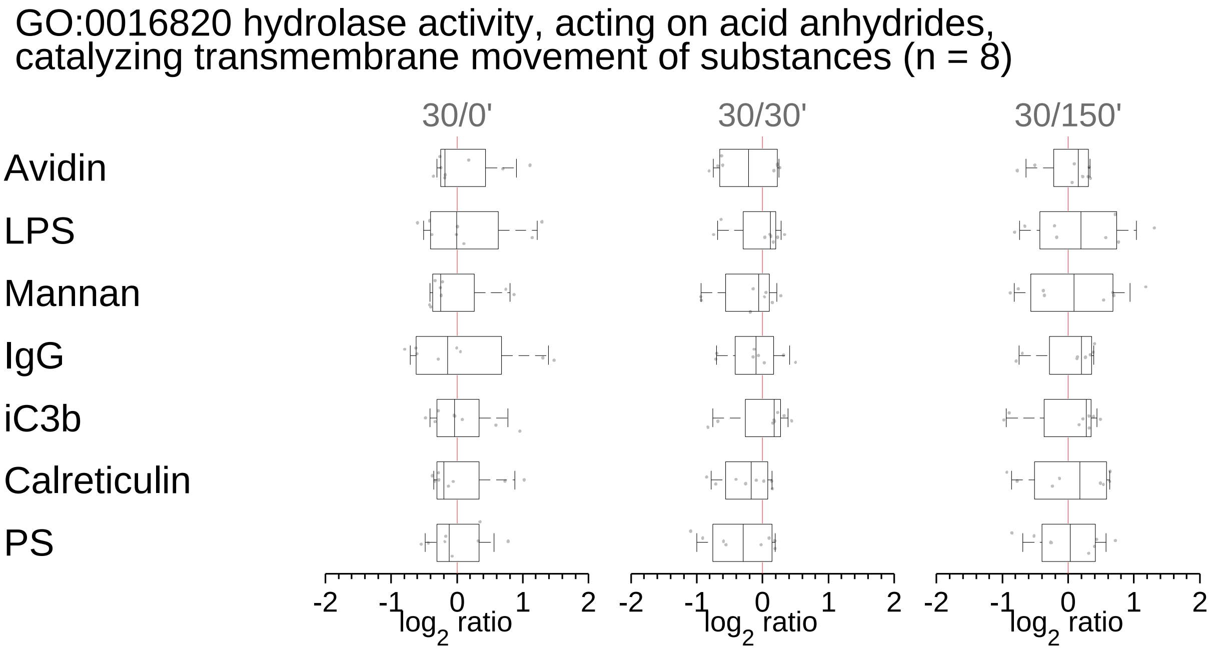

GO:0016820 hydrolase activity, acting on acid anhydrides,
catalyzing transmembrane movement of substances (n = 8)
30/0'
30/30'
30/150'
Avidin
LPS
Mannan
IgG
iC3b
Calreticulin
PS
-2 -1 0 1 2
log2 ratio

GO:0016829 lyase activity (n = 12)

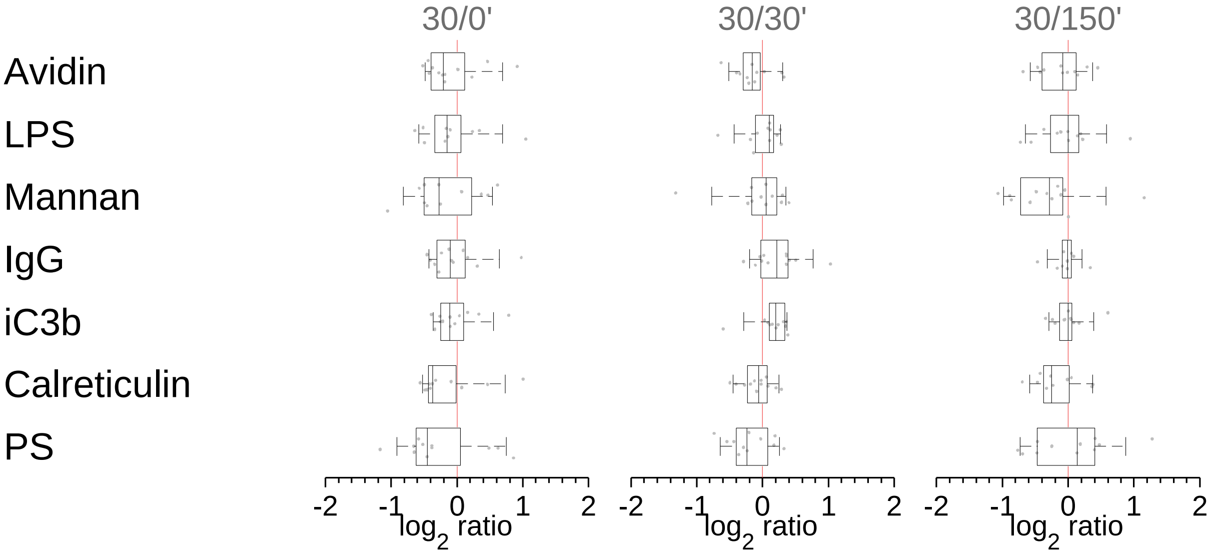

GO:0016831 carboxy-lyase activity (n = 3)

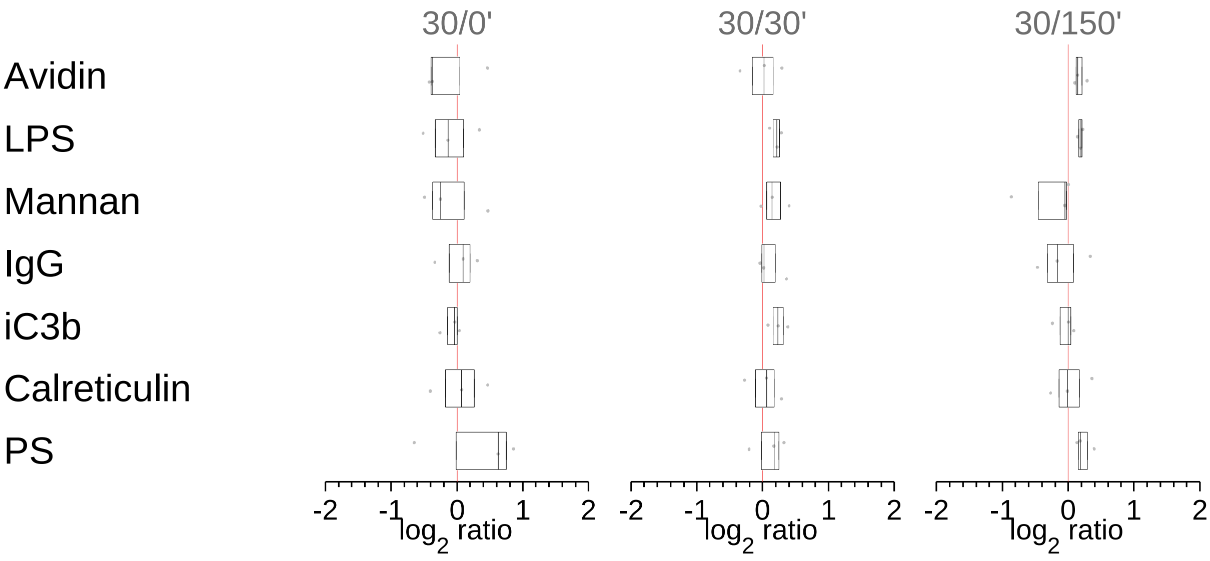

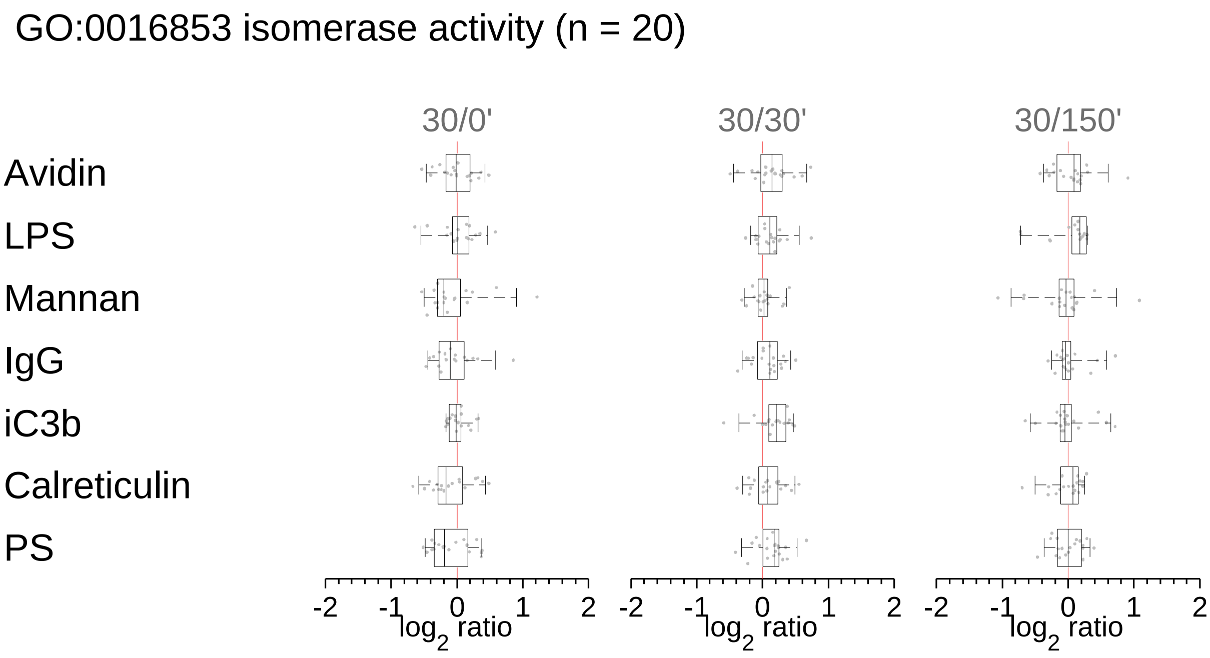
GO:0016853 isomerase activity (n = 20)
30/0'
30/30'
30/150'
Avidin
LPS
Mannan
IgG
iC3b
Calreticulin
PS
-2
-1
0
1
2
log2 ratio

GO:0016874 ligase activity (n = 33)

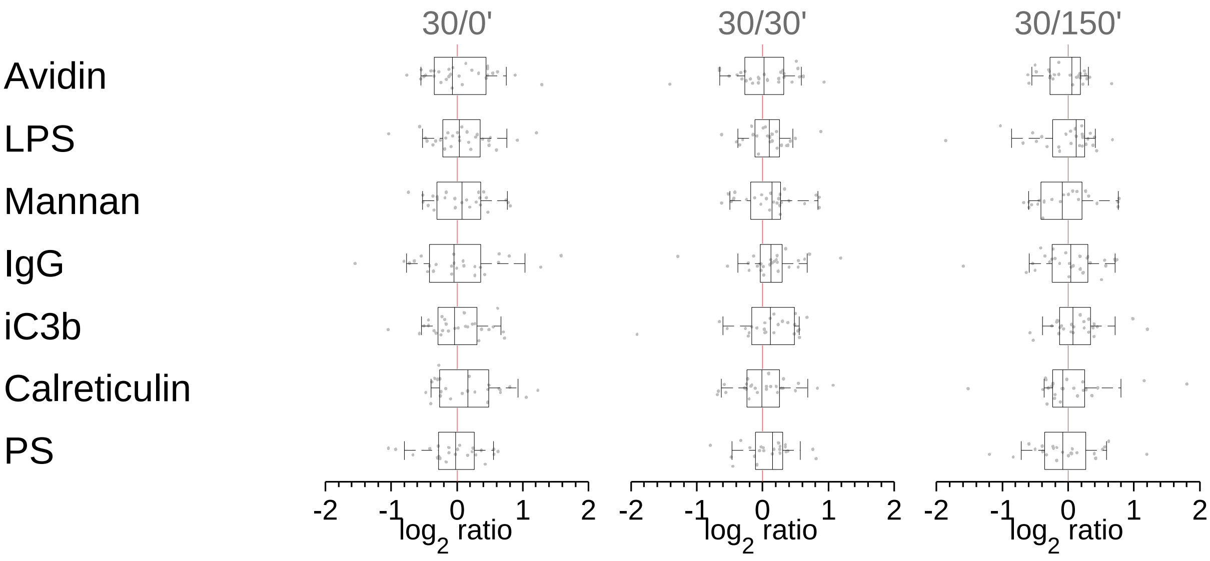

GO:0016881 acid-amino acid ligase activity (n = 4)

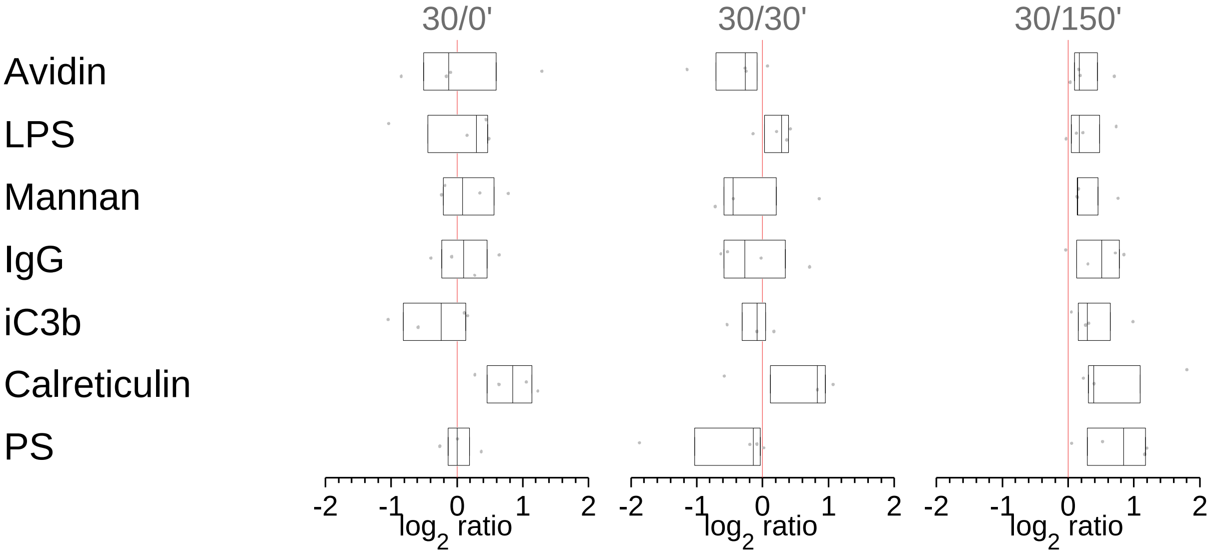

GO:0016887 ATPase activity (n = 37)

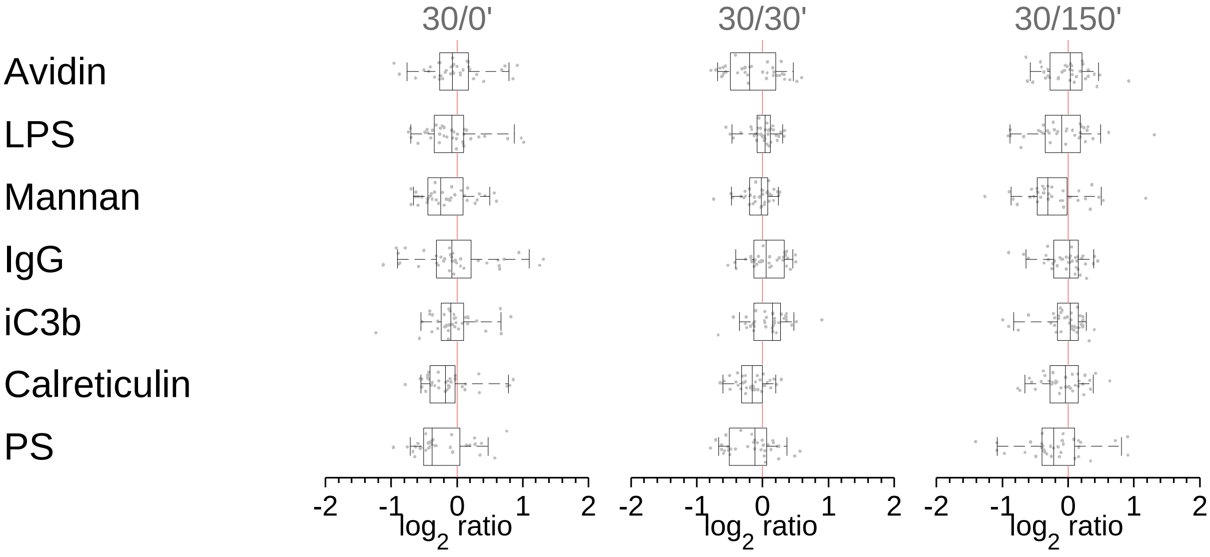

GO:0016936 galactoside binding (n = 4)

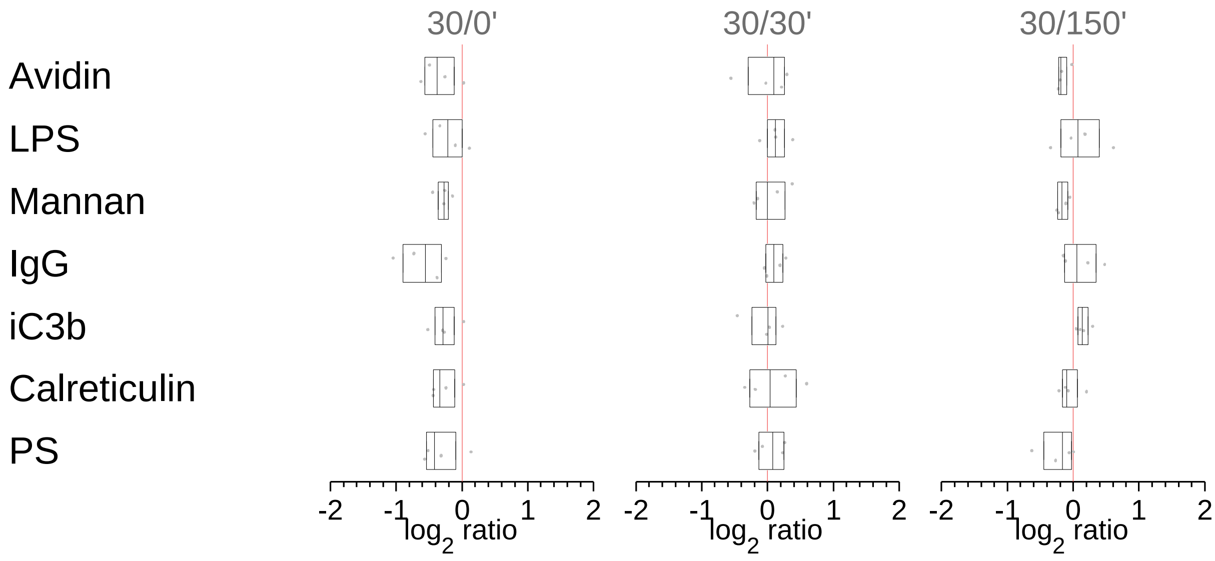

GO:0017048 Rho GTPase binding (n = 12)

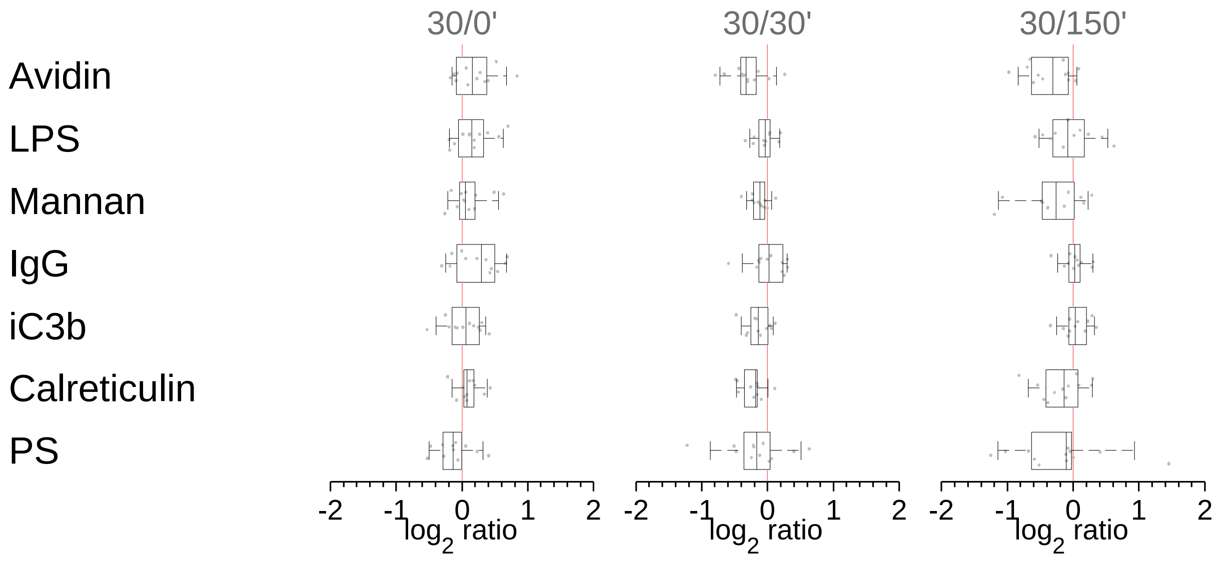

GO:0017053 transcriptional repressor complex (n = 7)

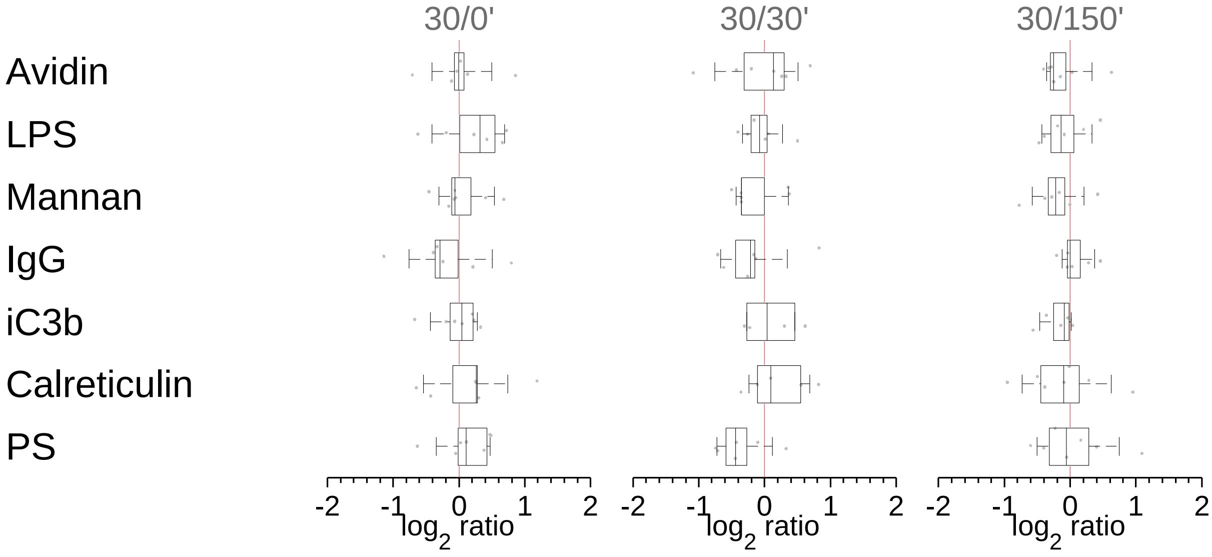

GO:0017075 syntaxin-1 binding (n = 6)

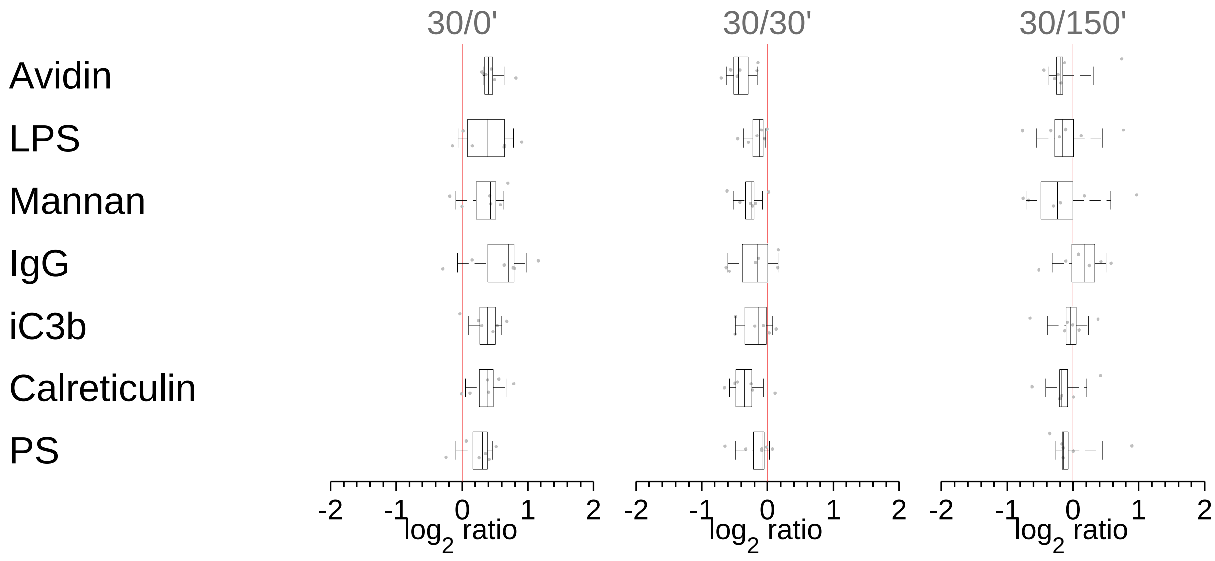

GO:0017080 sodium channel regulator activity (n = 3)

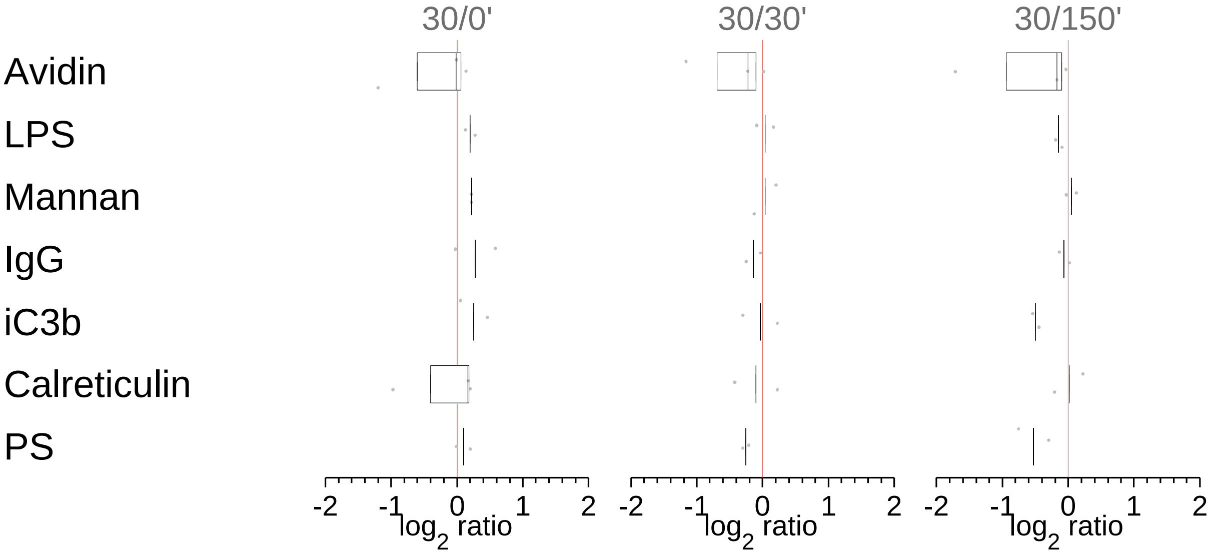

GO:0017124 SH3 domain binding (n = 35)

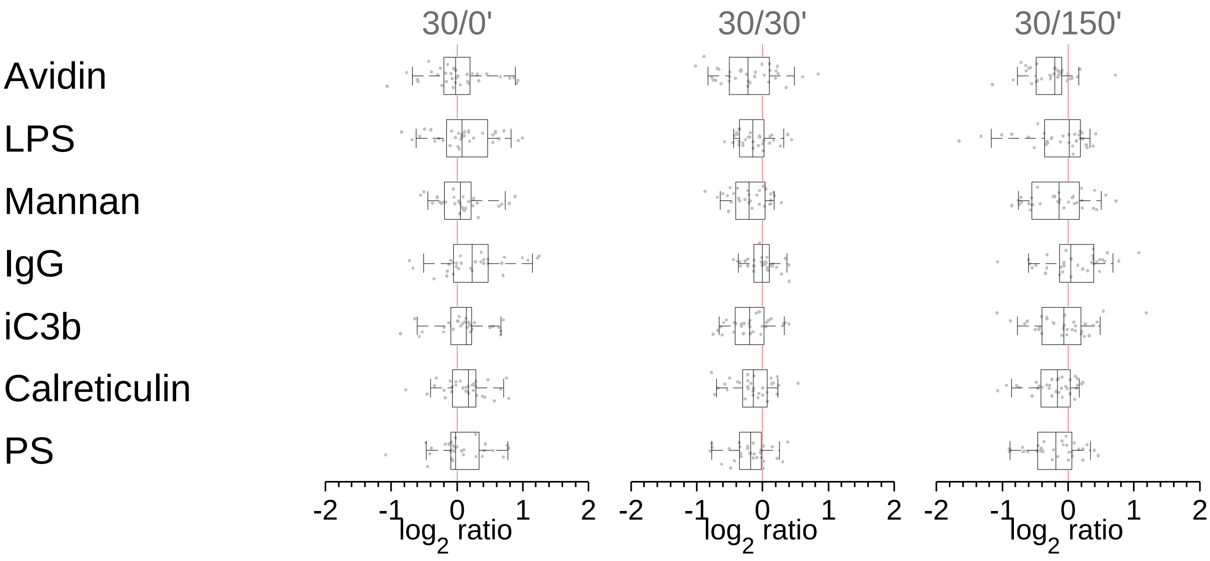

GO:0017133 mitochondrial electron transfer flavoprotein complex
(n = 3)

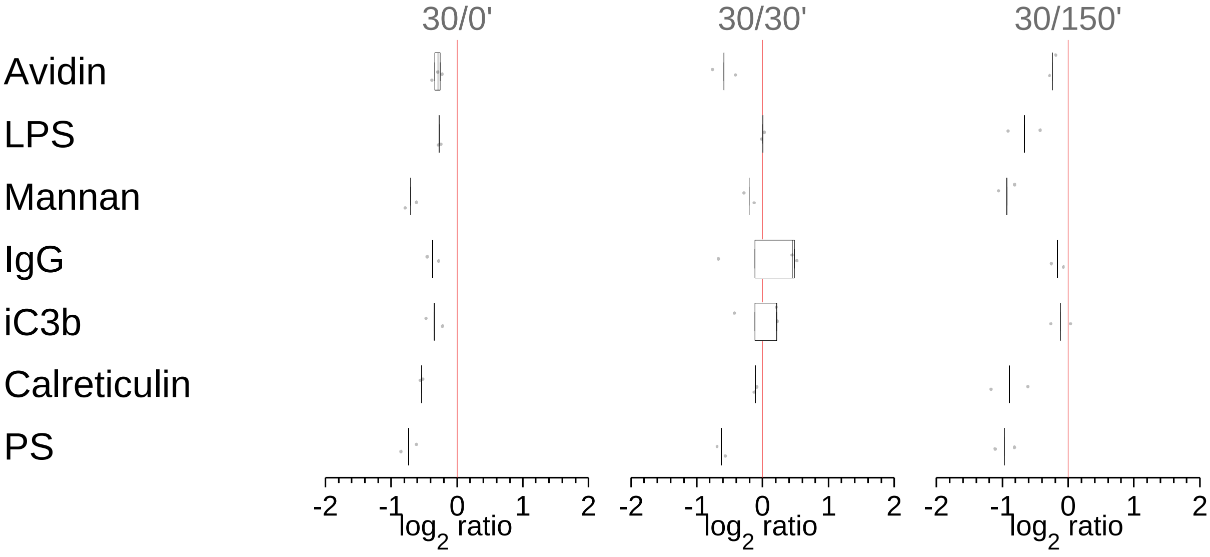

GO:0017134 fibroblast growth factor binding (n = 4)

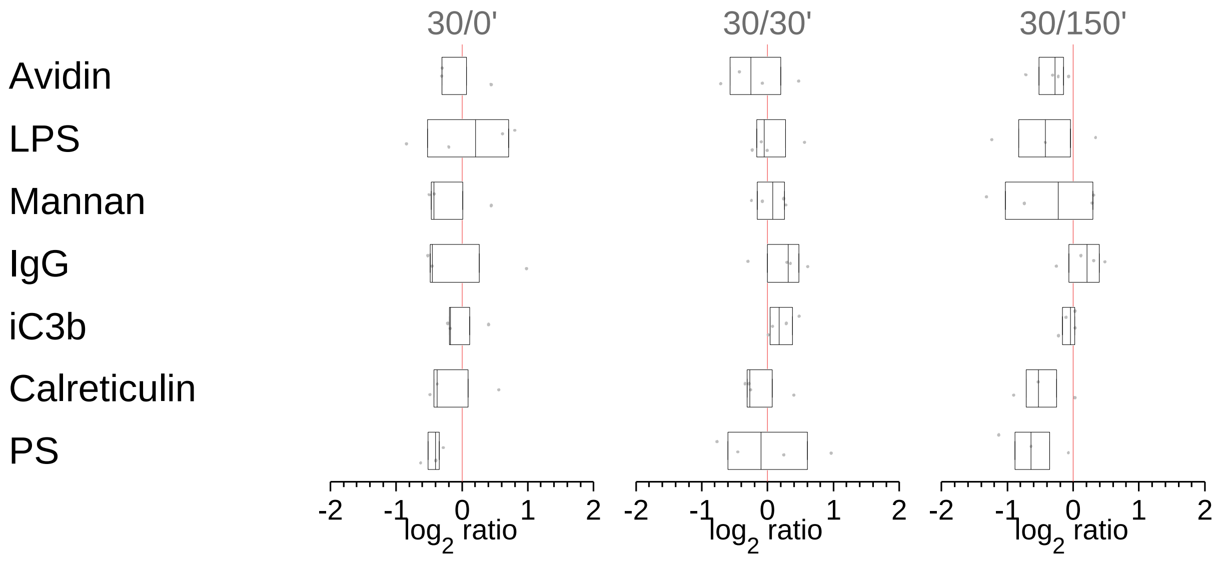

GO:0017137 Rab GTPase binding (n = 11)

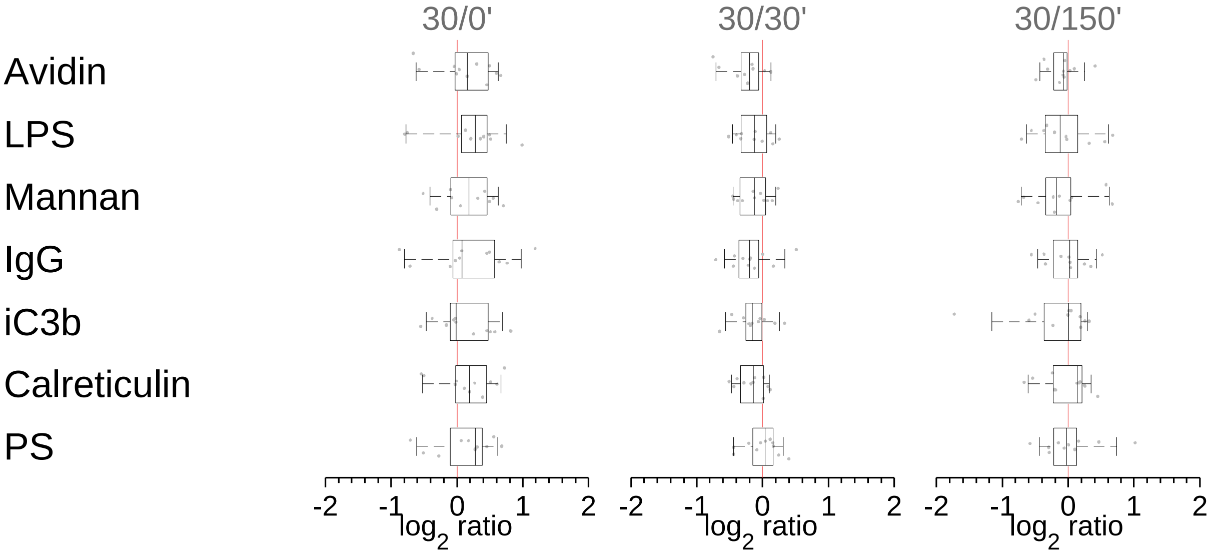

GO:0017148 negative regulation of translation (n = 7)

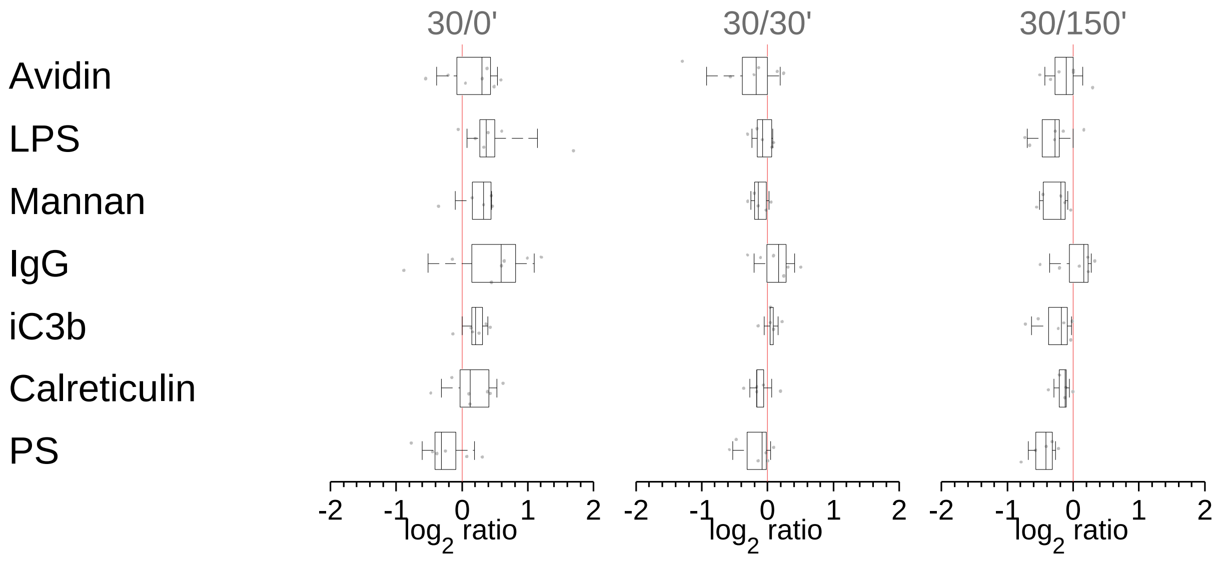

GO:0017154 semaphorin receptor activity (n = 4)

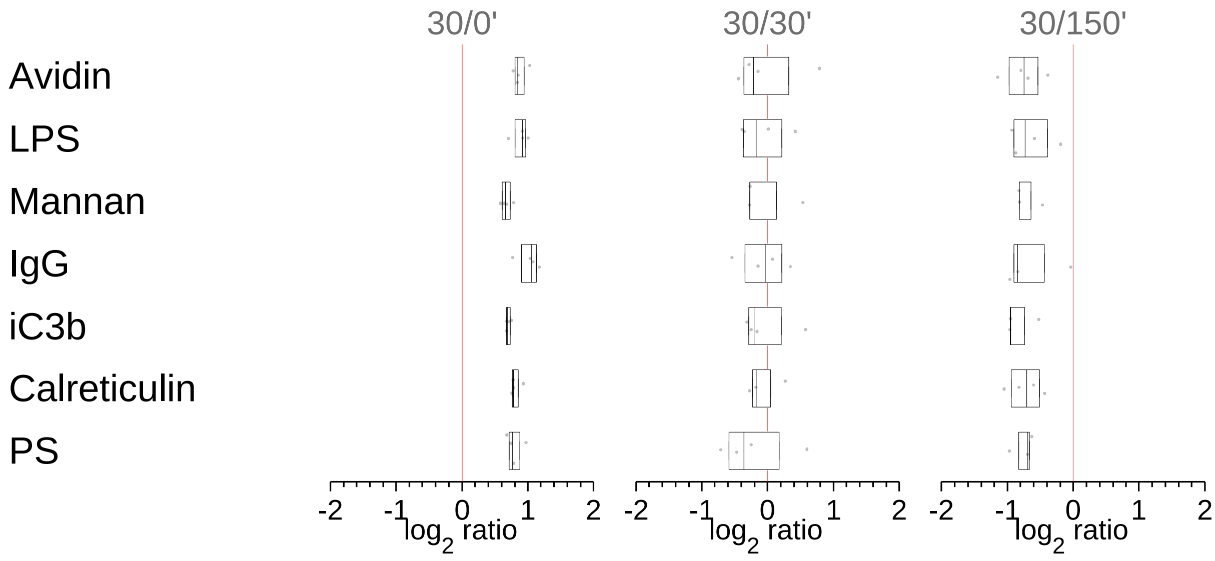

GO:0017156 calcium ion-dependent exocytosis (n = 3)

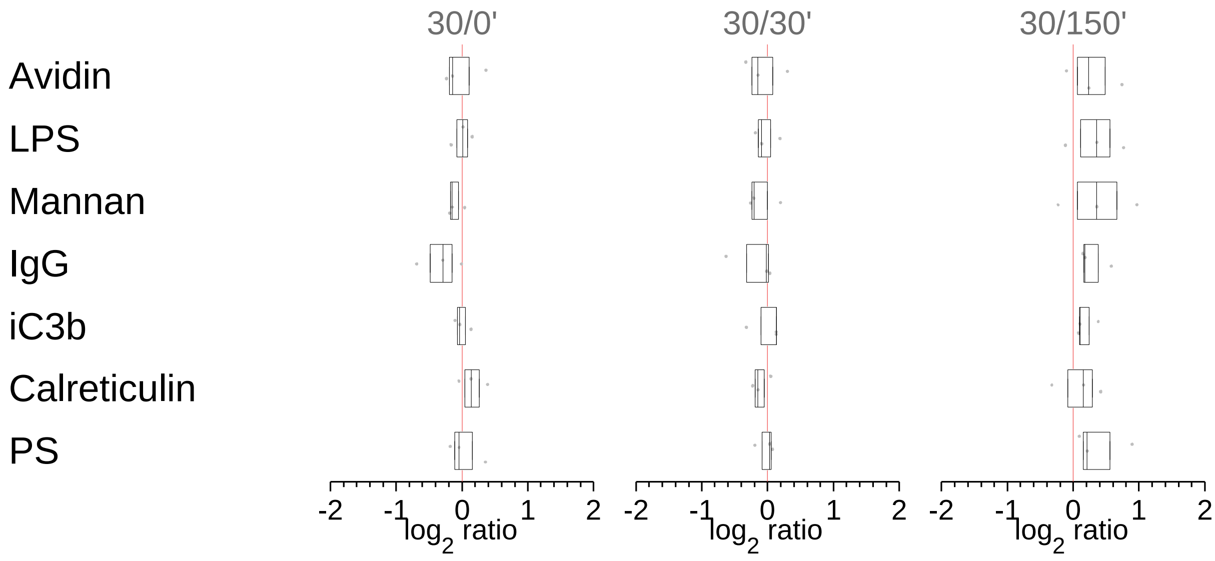

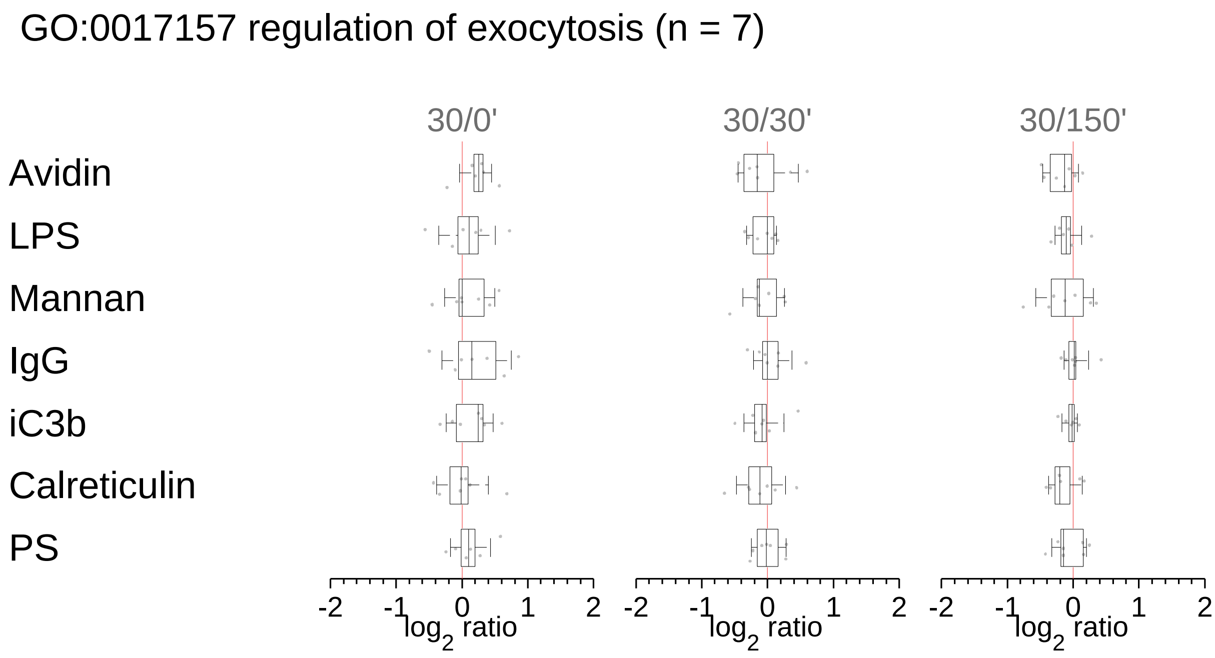

GO:0017157 regulation of exocytosis (n = 7)
30/0'
30/30'
30/150'
Avidin
LPS
Mannan
IgG
iC3b
Calreticulin
PS
-2
-1
0
1
2
log2 ratio

GO:0017166 vinculin binding (n = 3)

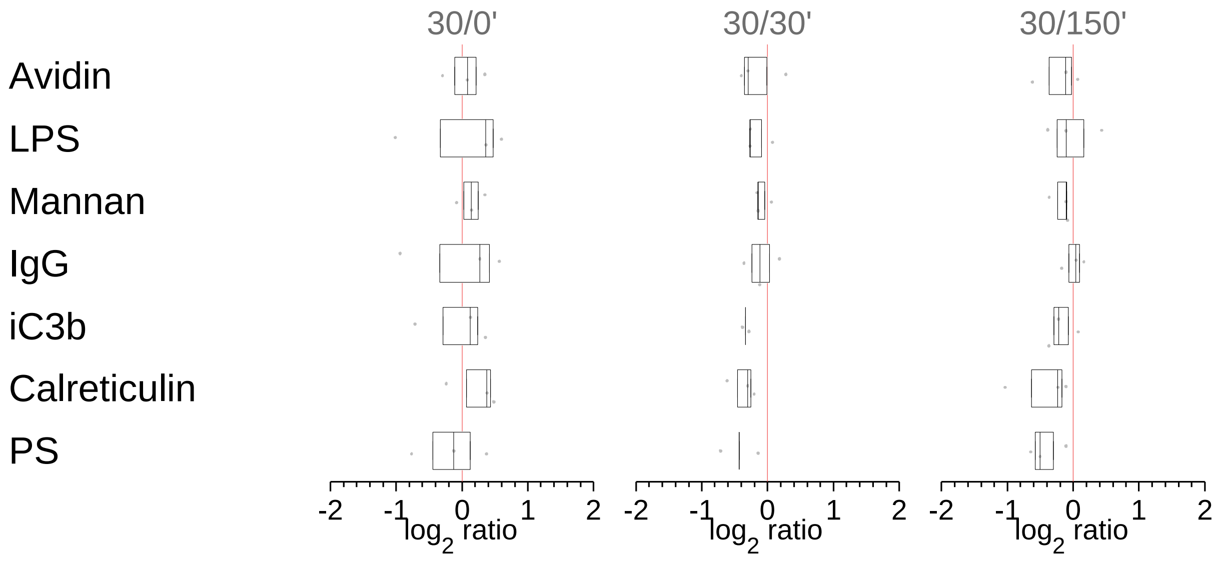

GO:0018030 peptidyl-lysine N6-myristoyltransferase activity (n = 3)

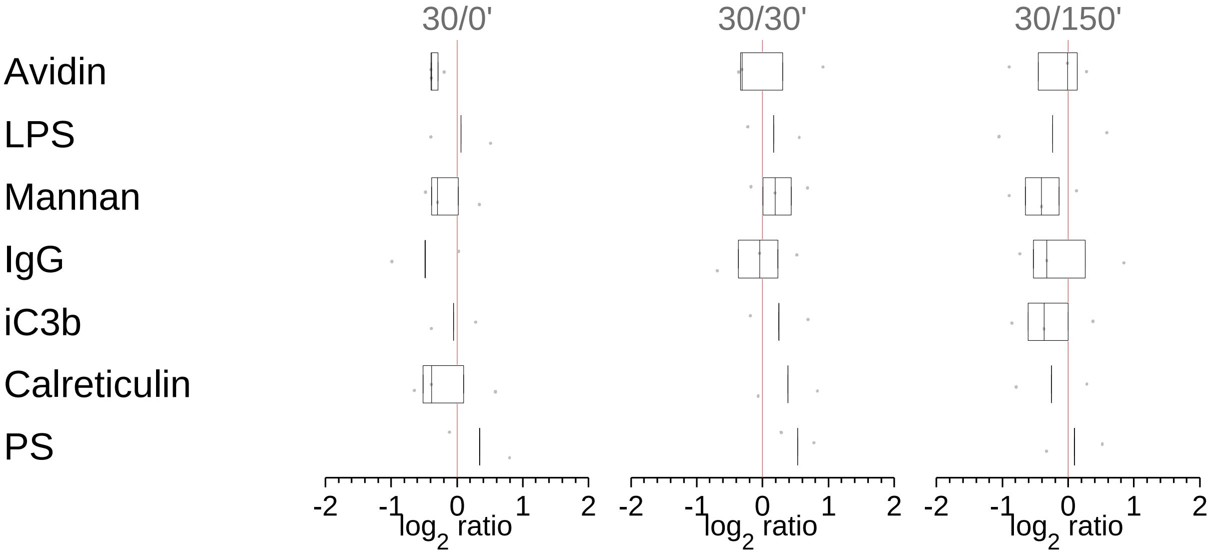

GO:0018031 peptidyl-lysine N6-palmitoyltransferase activity (n = 3)

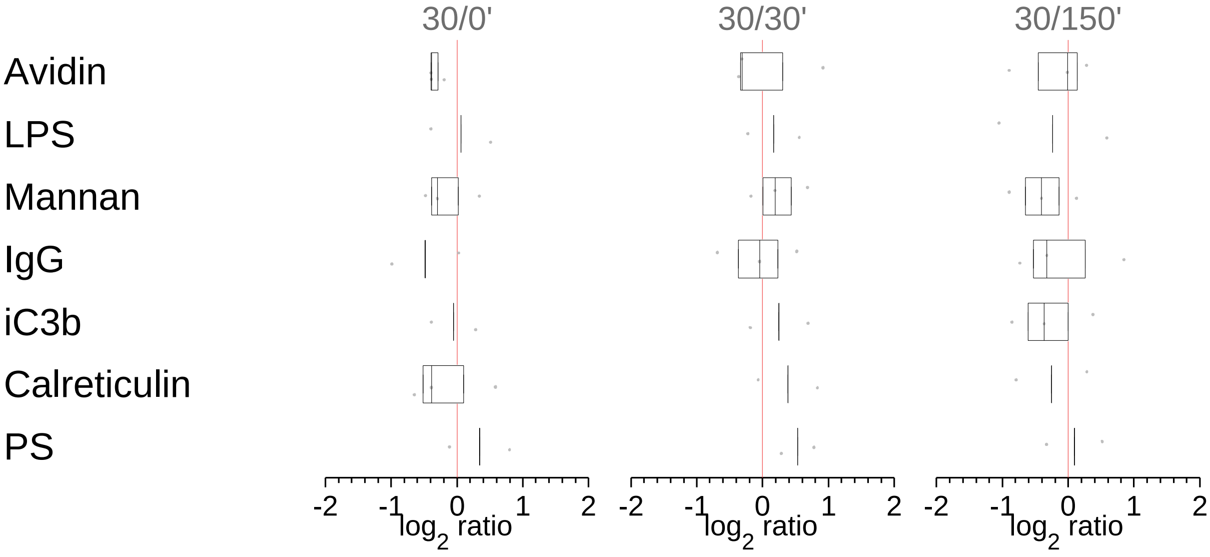

GO:0018105 peptidyl-serine phosphorylation (n = 8)

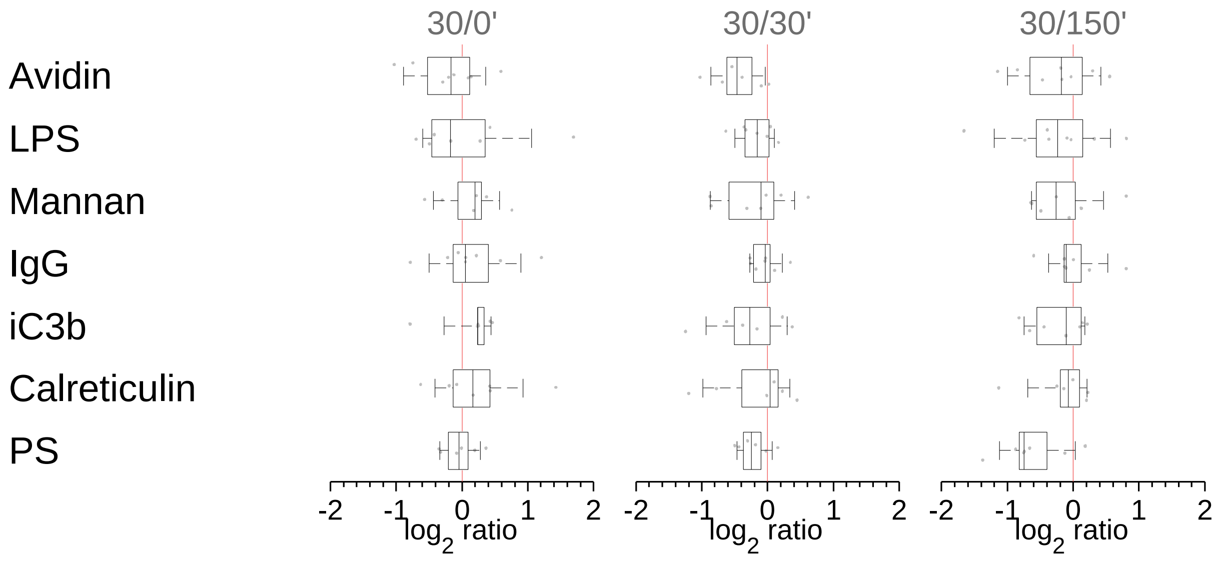

GO:0018107 peptidyl-threonine phosphorylation (n = 6)

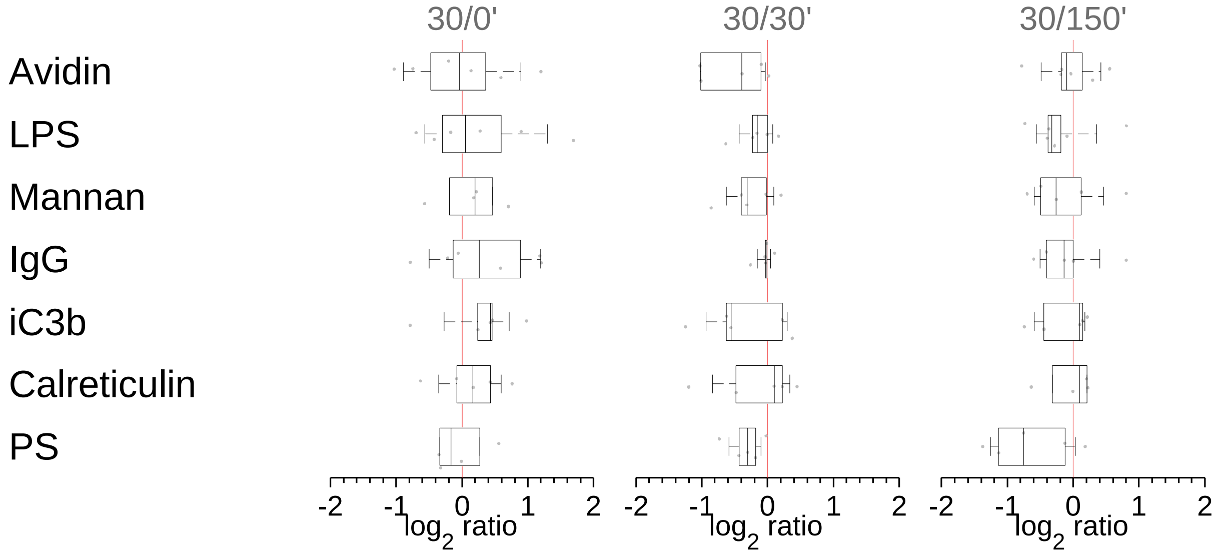

GO:0018108 peptidyl-tyrosine phosphorylation (n = 13)

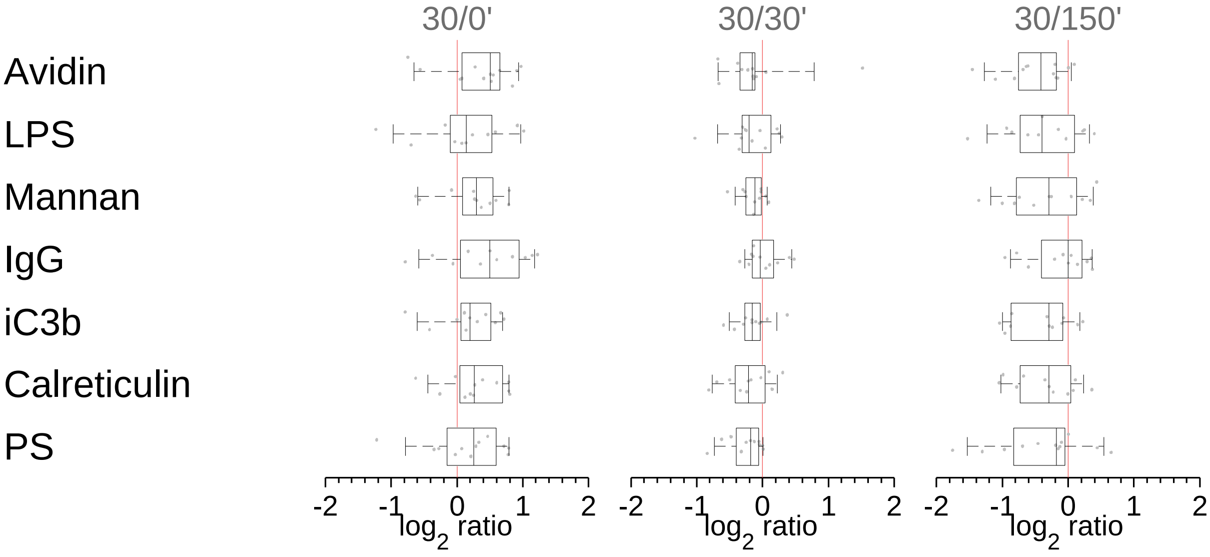

GO:0018149 peptide cross-linking (n = 4)

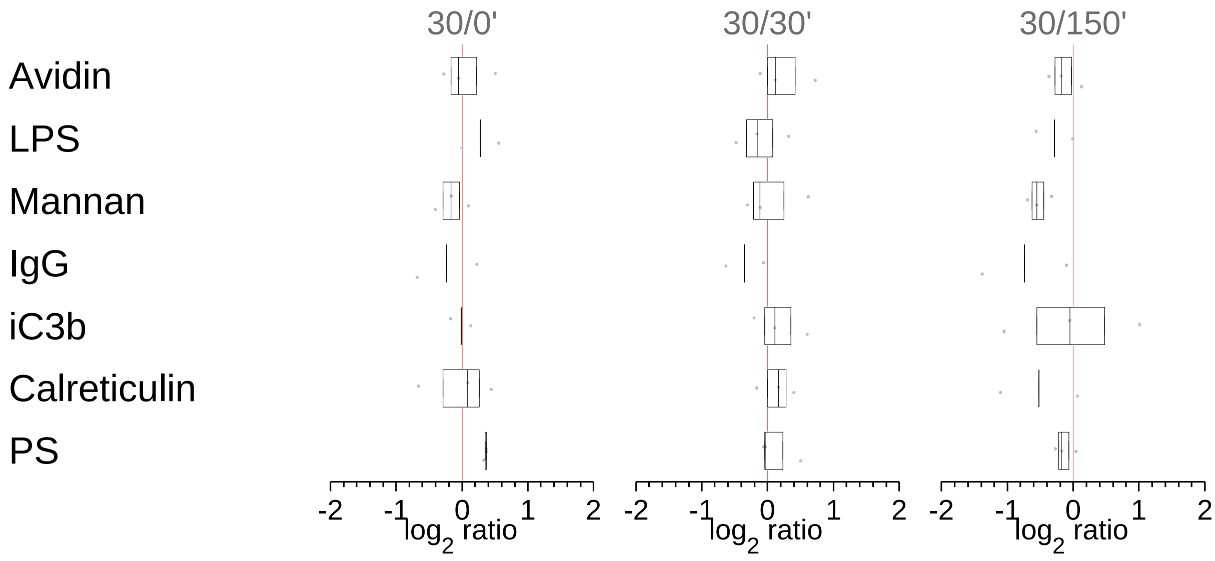

GO:0018169 ribosomal S6-glutamic acid ligase activity (n = 17)

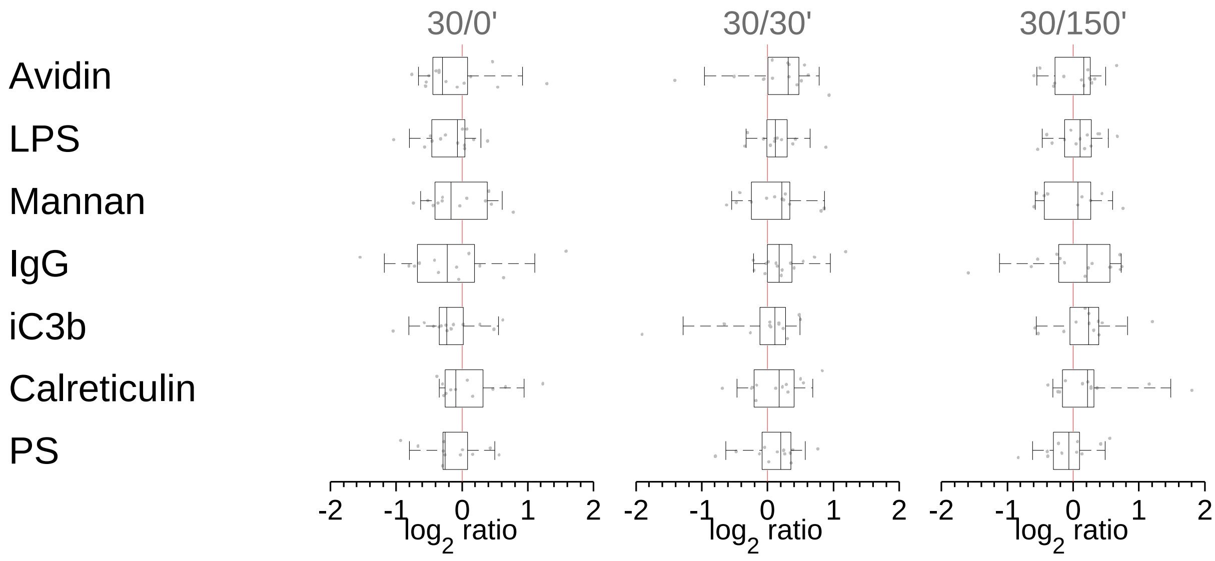

GO:0018279 protein N-linked glycosylation via asparagine (n = 4)

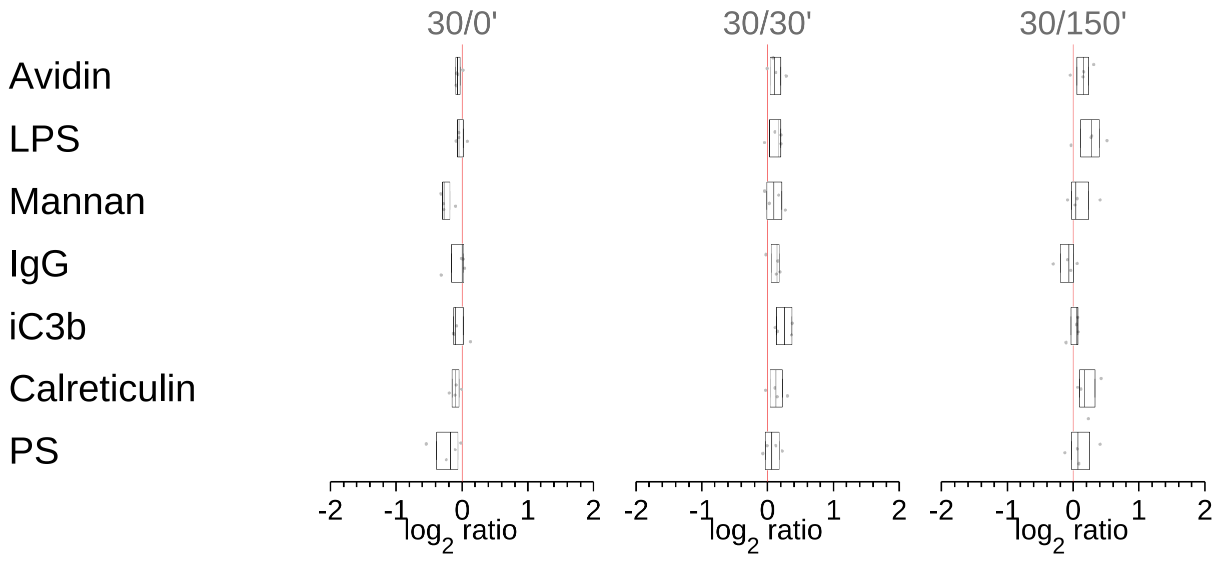

GO:0018451 epoxide dehydrogenase activity (n = 3)

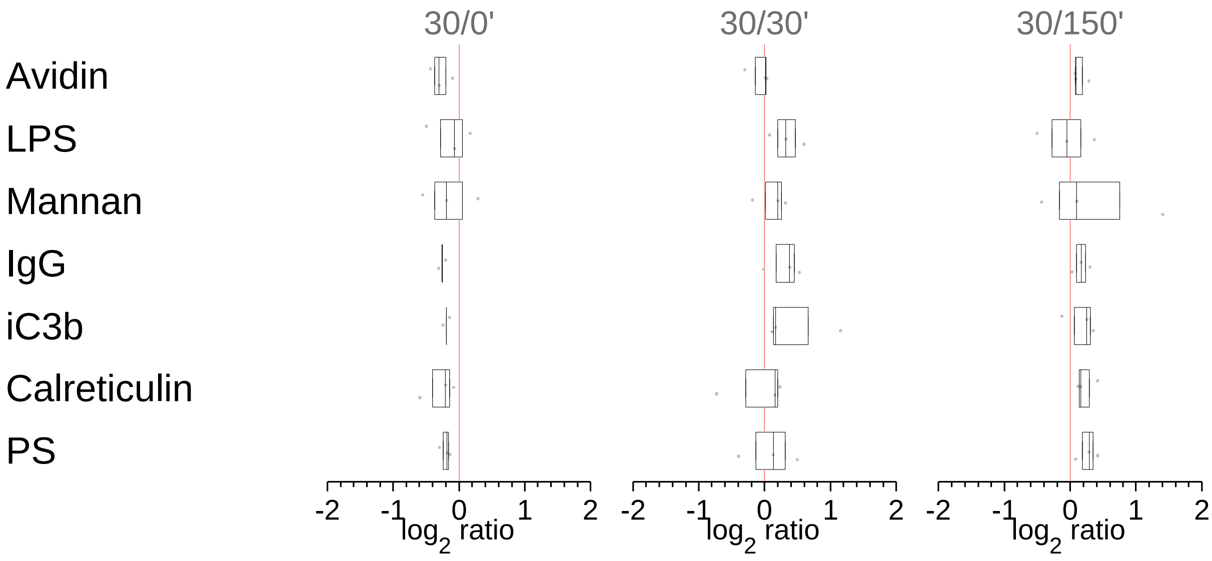

GO:0018452 5-exo-hydroxycamphor dehydrogenase activity (n = 3)

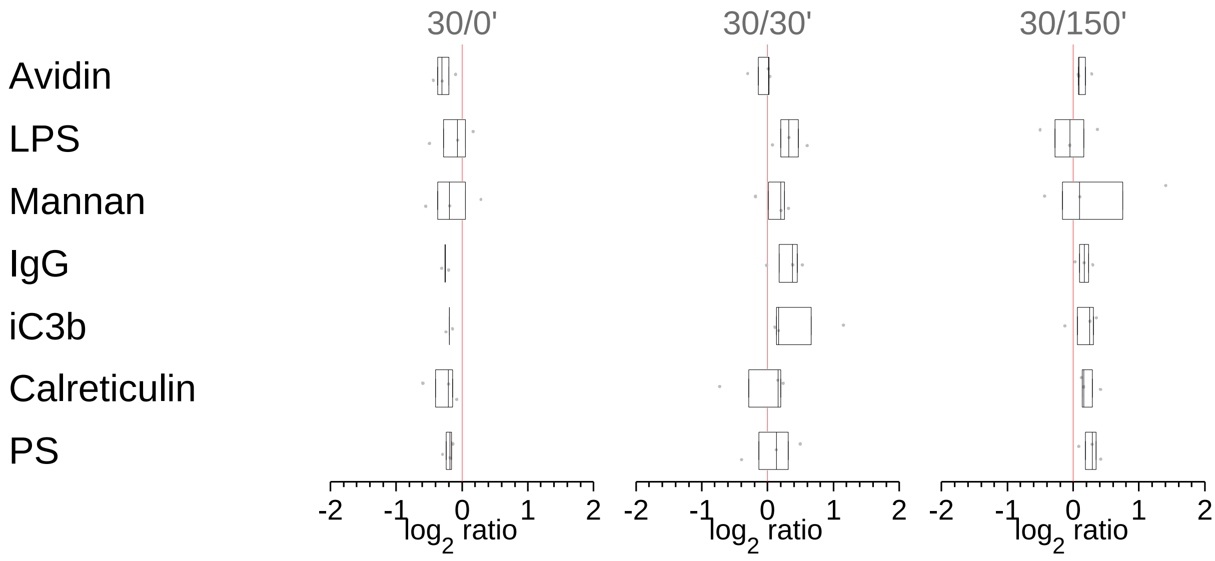

GO:0018453 2-hydroxytetrahydrofuran dehydrogenase activity (n = 3)

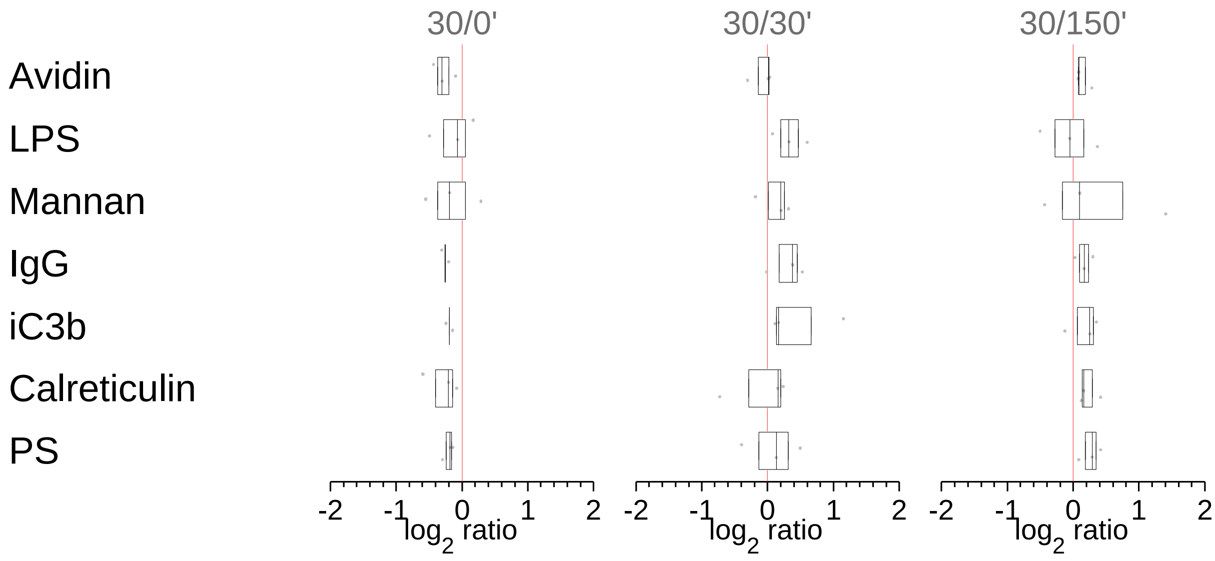

GO:0018711 benzoyl acetate-CoA thiolase activity (n = 3)

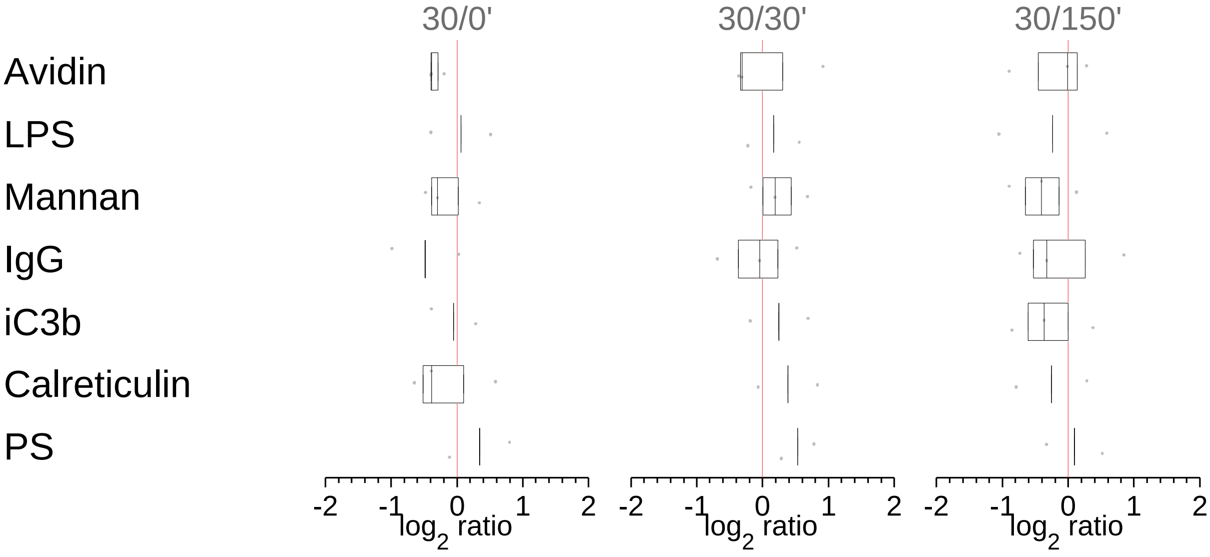

GO:0018712 3-hydroxybutyryl-CoA thiolase activity (n = 3)

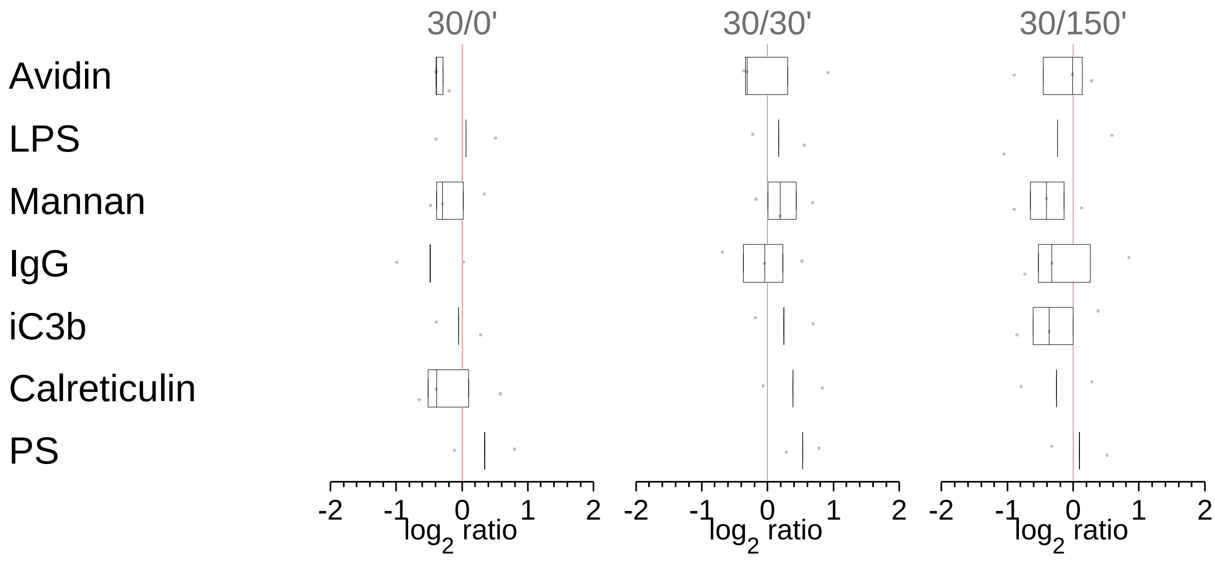

GO:0018713 3-ketopimelyl-CoA thiolase activity (n = 3)

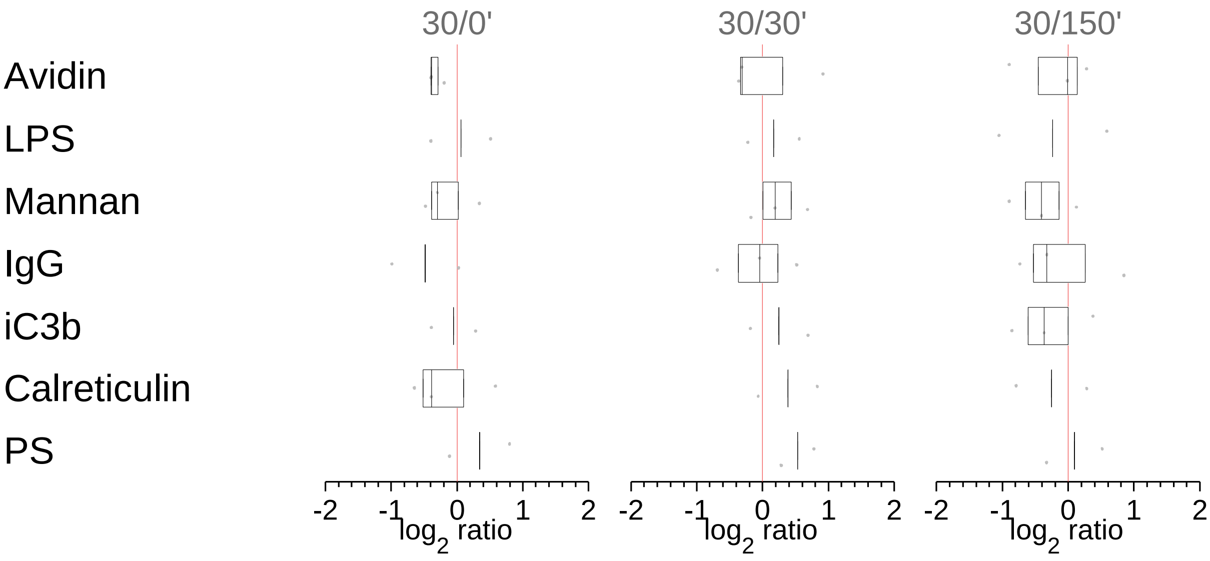

GO:0018715 9-phenanthrol UDP-glucuronosyltransferase activity (n = 3)

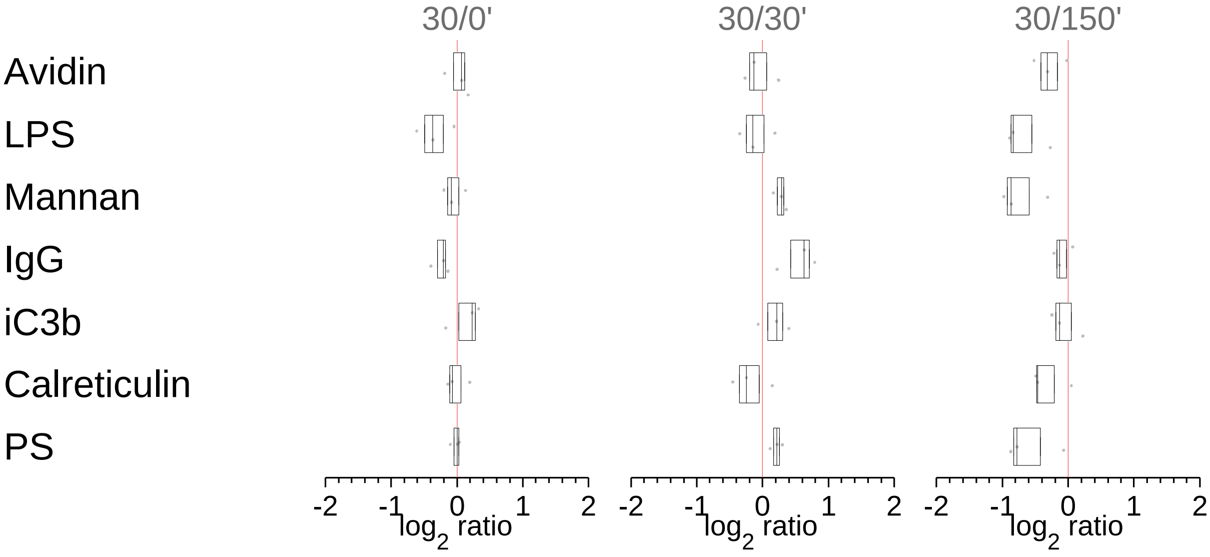

GO:0018716 1-phenanthrol glycosyltransferase activity (n = 3)

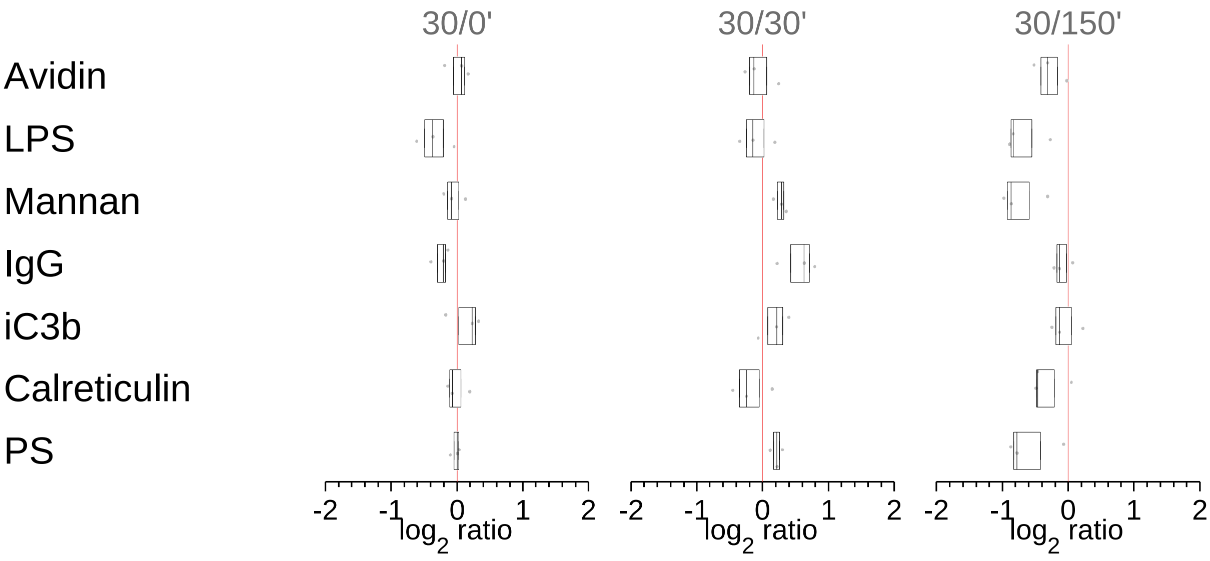

GO:0018717 9-phenanthrol glycosyltransferase activity (n = 3)

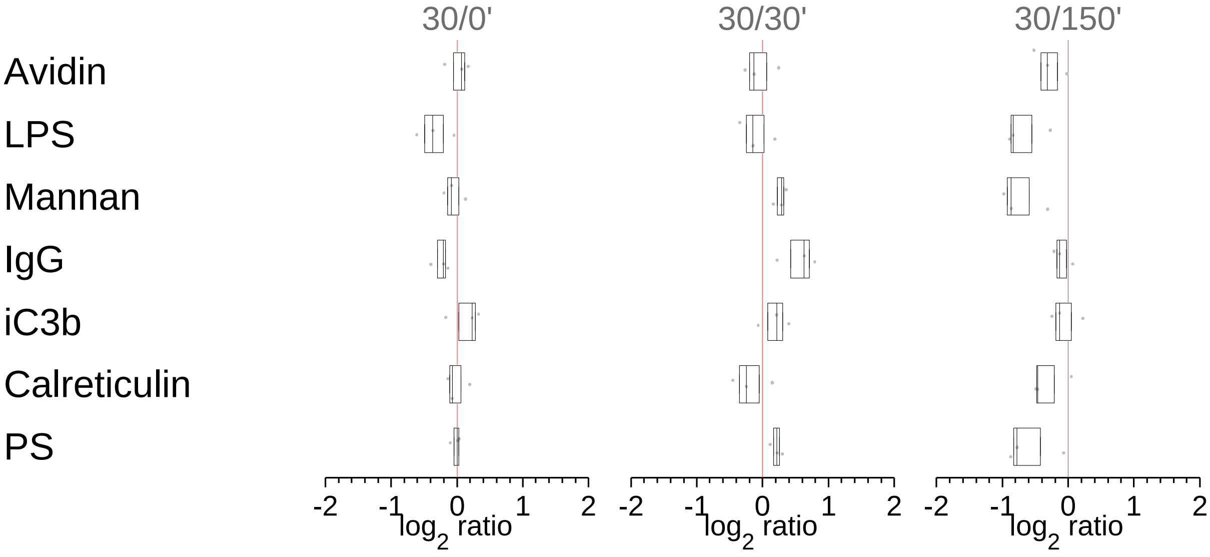

GO:0018718 1,2-dihydroxy-phenanthrene glycosyltransferase activity (n = 3)

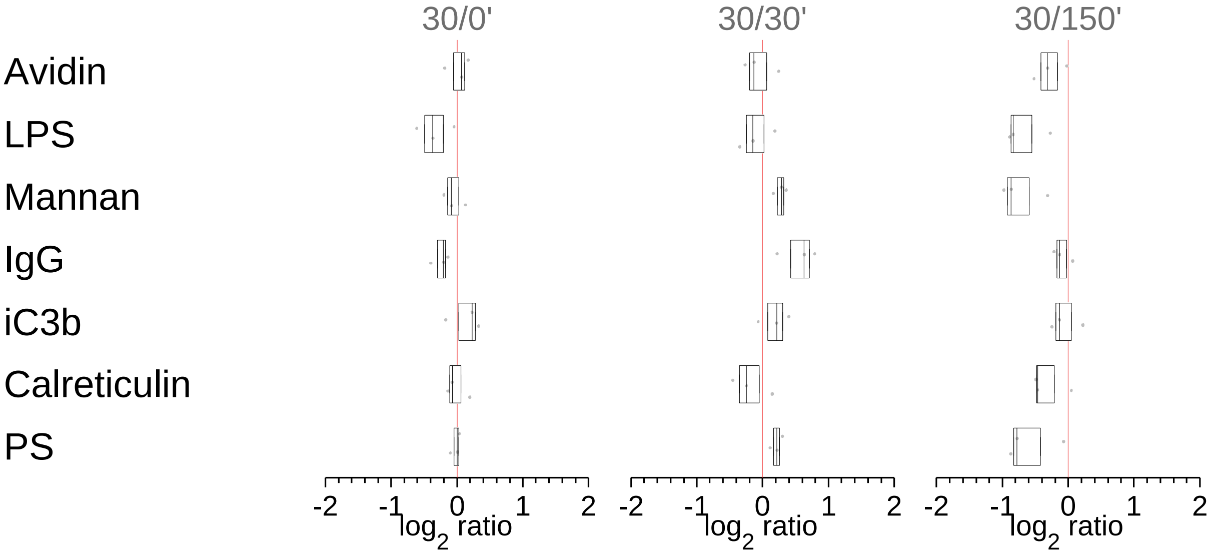

GO:0018731 1-oxa-2-oxocycloheptane lactonase activity (n = 4)

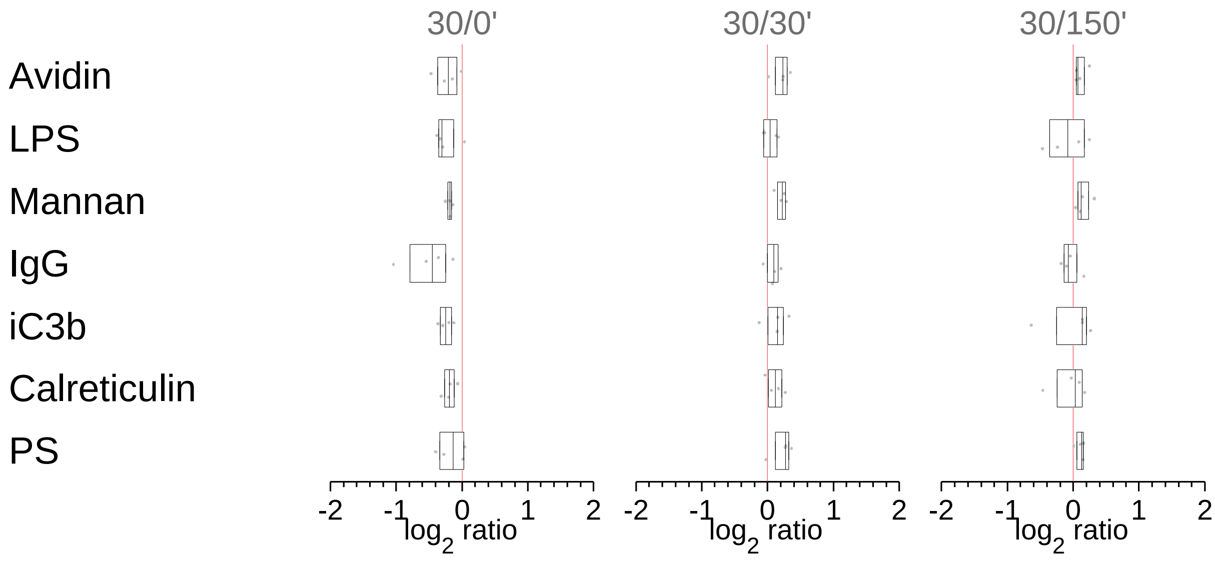

GO:0018732 sulfolactone hydrolase activity (n = 4)

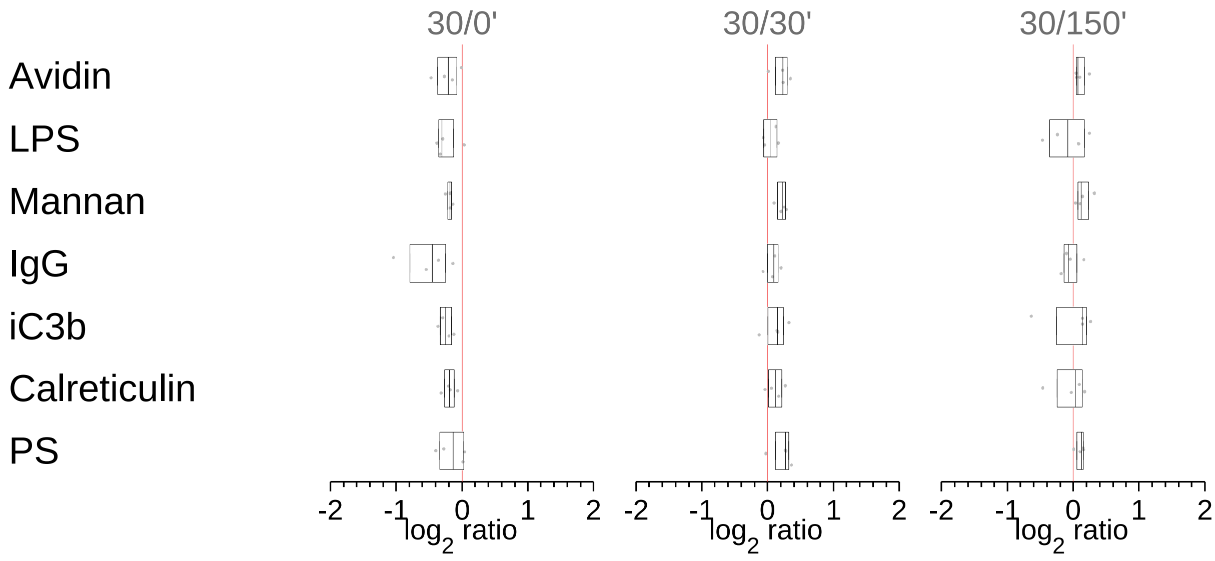

GO:0018733 3,4-dihydrocoumarin hydrolase activity (n = 5)

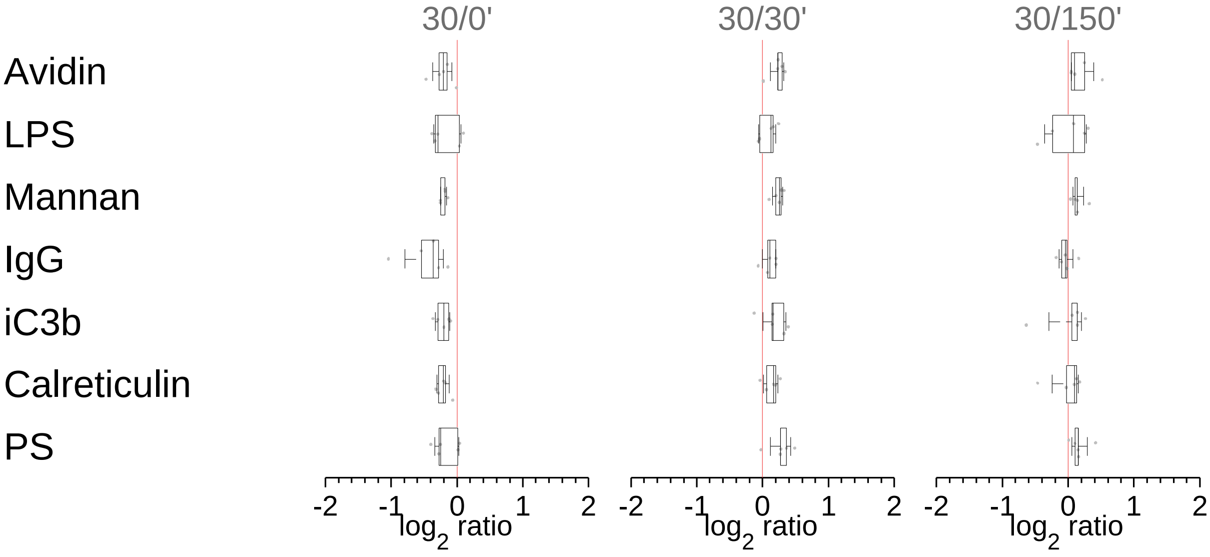

GO:0018734 butyrolactone hydrolase activity (n = 4)

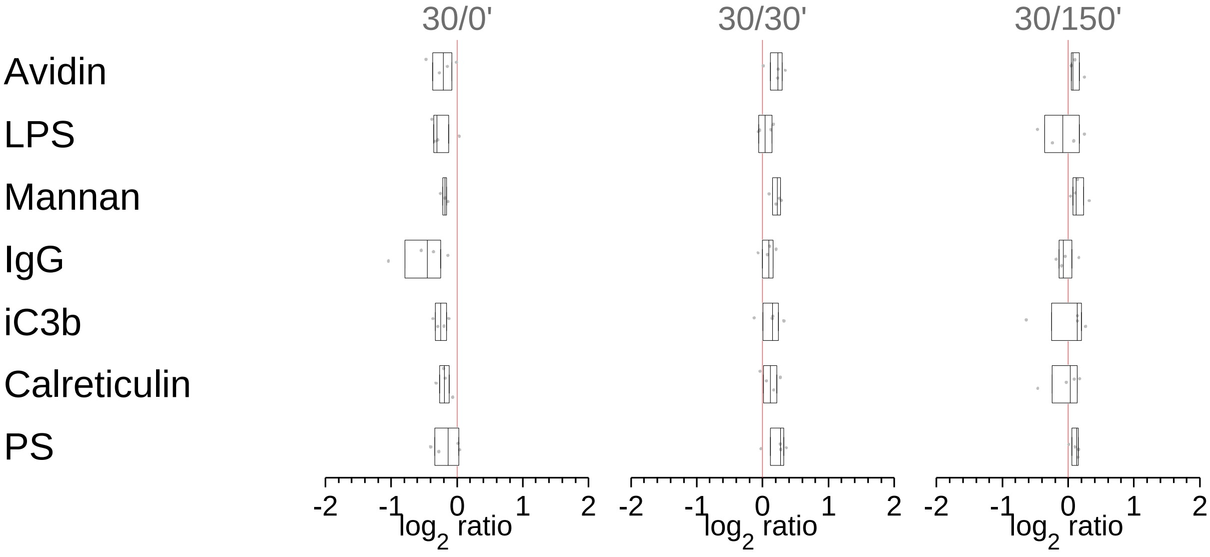

GO:0019001 guanyl nucleotide binding (n = 7)

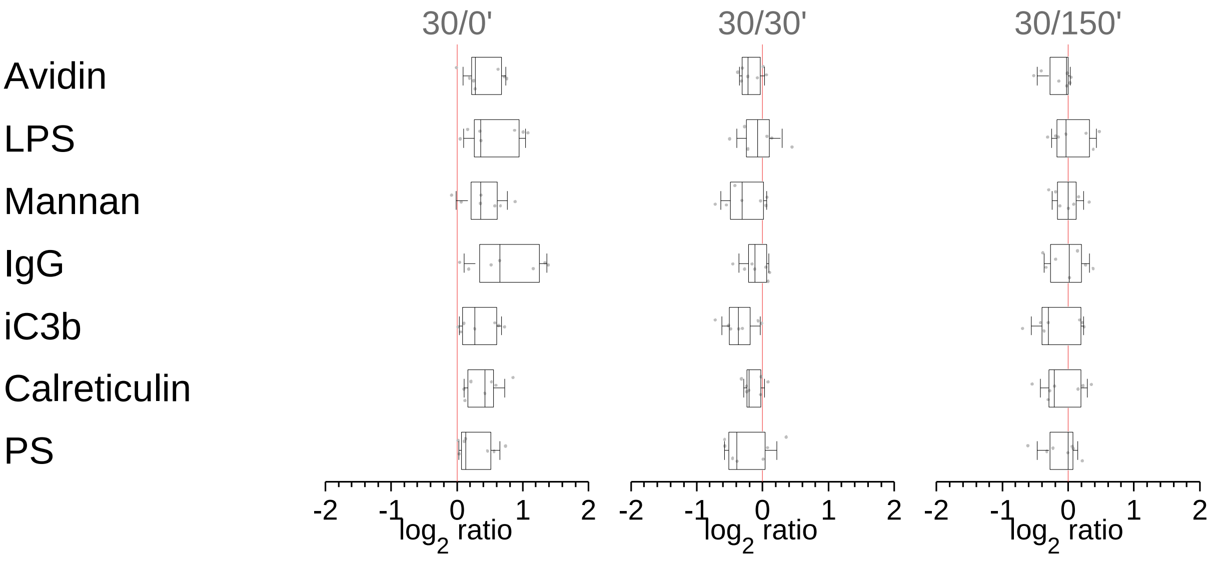

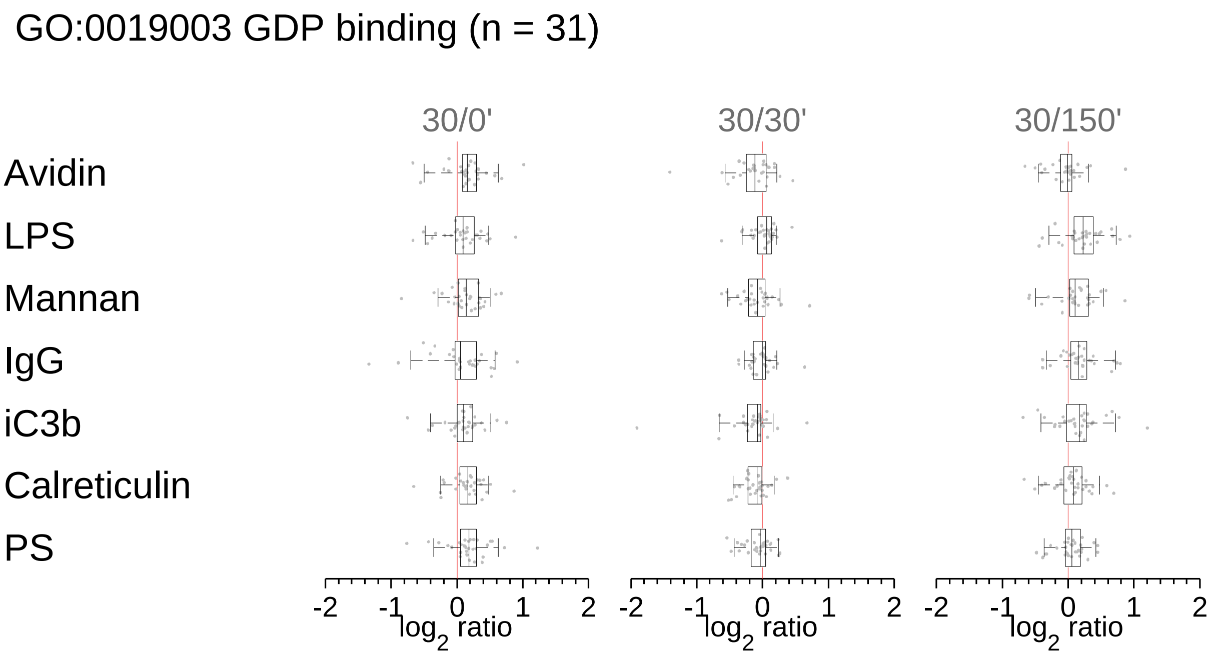
GO:0019003 GDP binding (n = 31)
30/0'
30/30'
30/150'
Avidin
LPS
Mannan
IgG
iC3b
Calreticulin
PS
-2
-1
0
1
2
log2 ratio

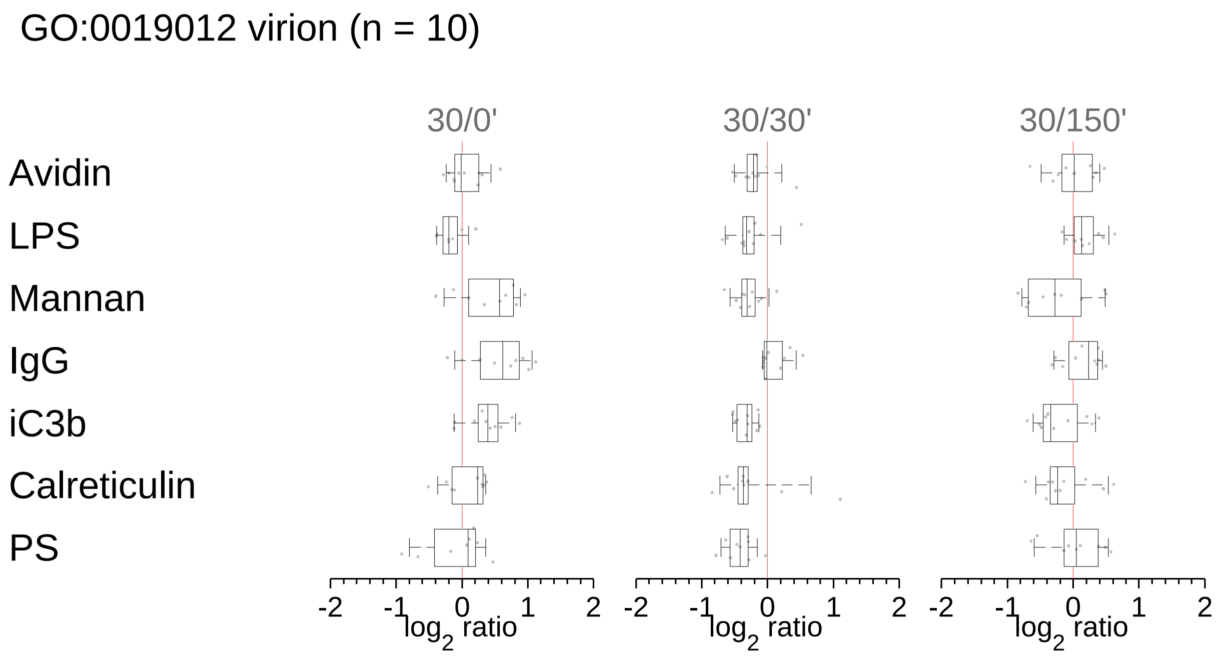
GO:0019012 virion (n = 10)
30/0'
30/30'
30/150'
Avidin
LPS
Mannan
IgG
iC3b
Calreticulin
PS
-2 -1 0 1 2
$\log_2$ ratio

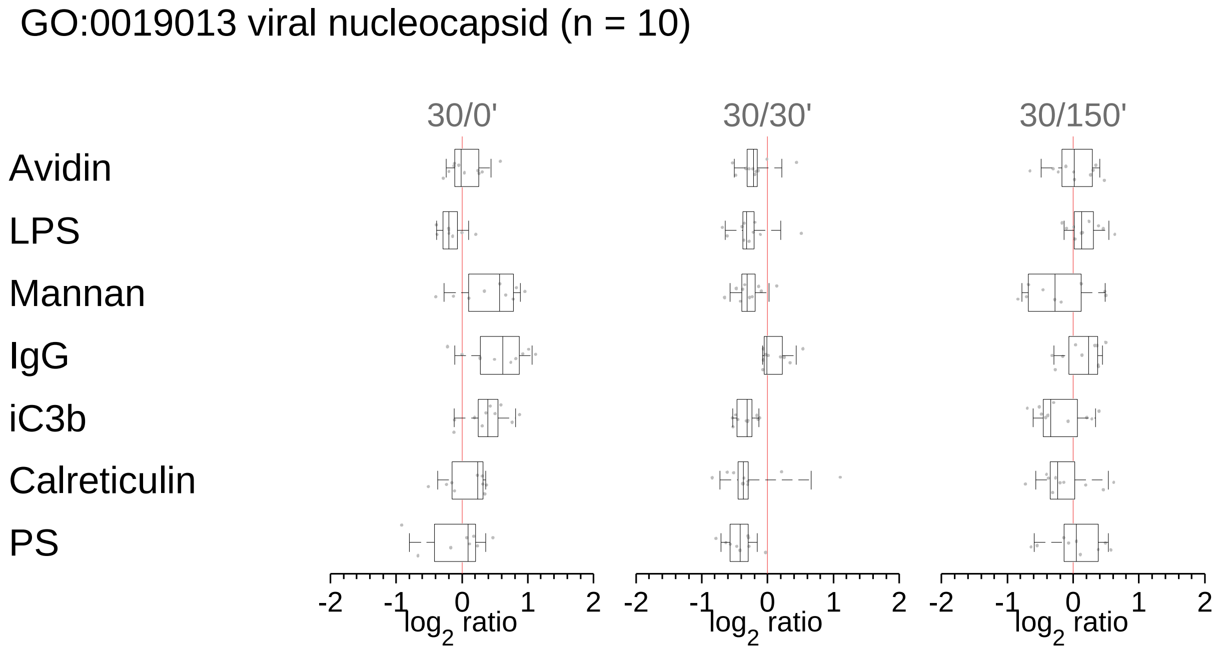

GO:0019013 viral nucleocapsid (n = 10)
30/0'
30/30'
30/150'
Avidin
LPS
Mannan
IgG
iC3b
Calreticulin
PS
-2 -1 0 1 2
log2 ratio

GO:0019105 N-palmitoyltransferase activity (n = 3)

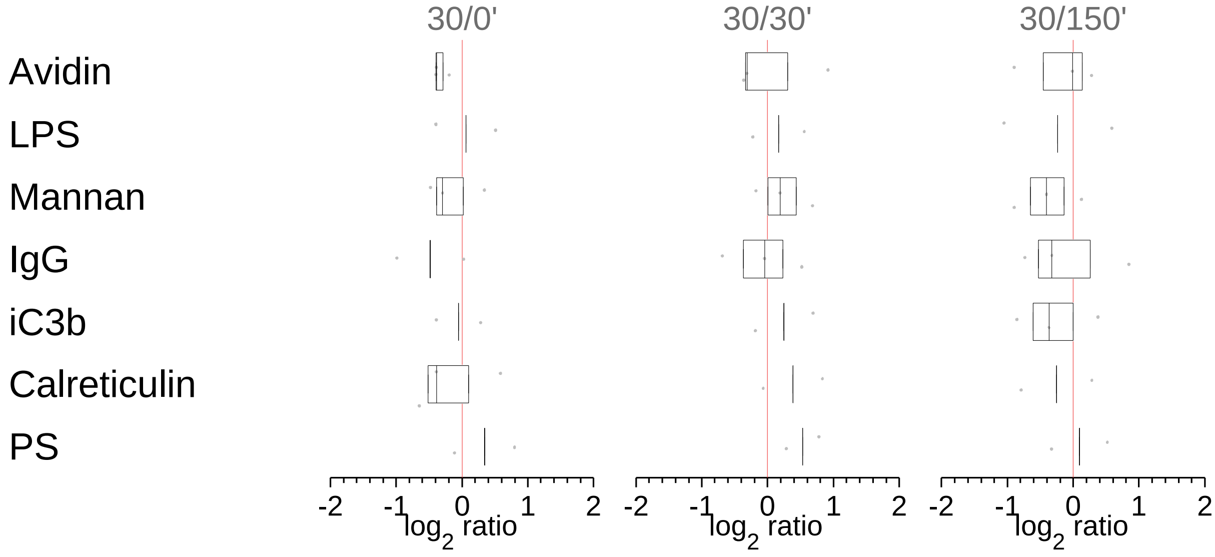

GO:0019107 myristoyltransferase activity (n = 4)

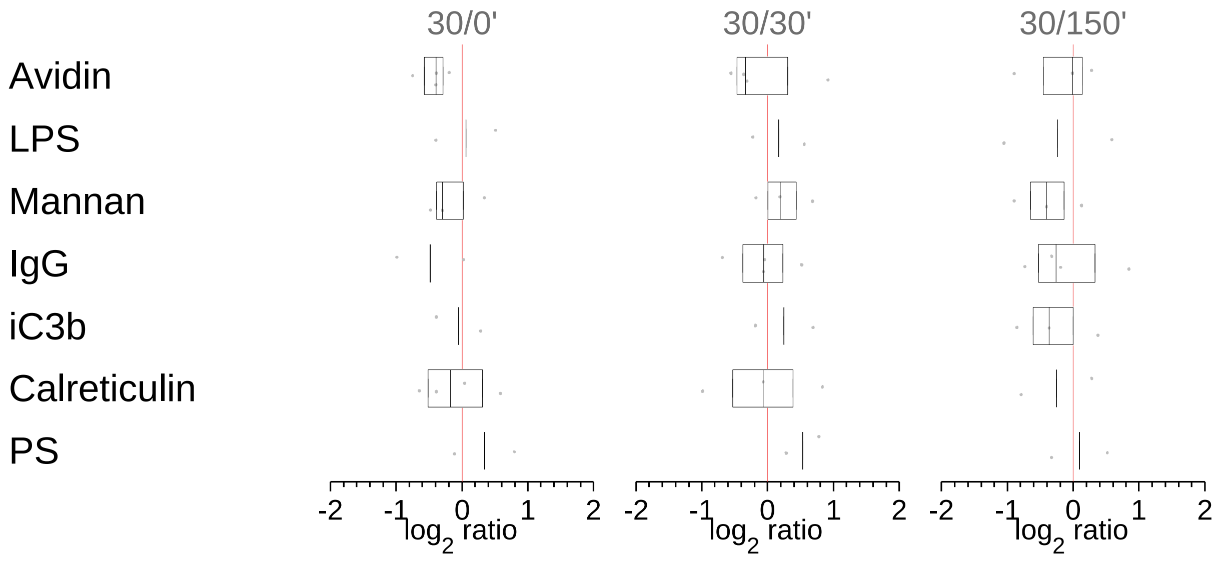

GO:0019112 phenanthrol glycosyltransferase activity (n = 3)

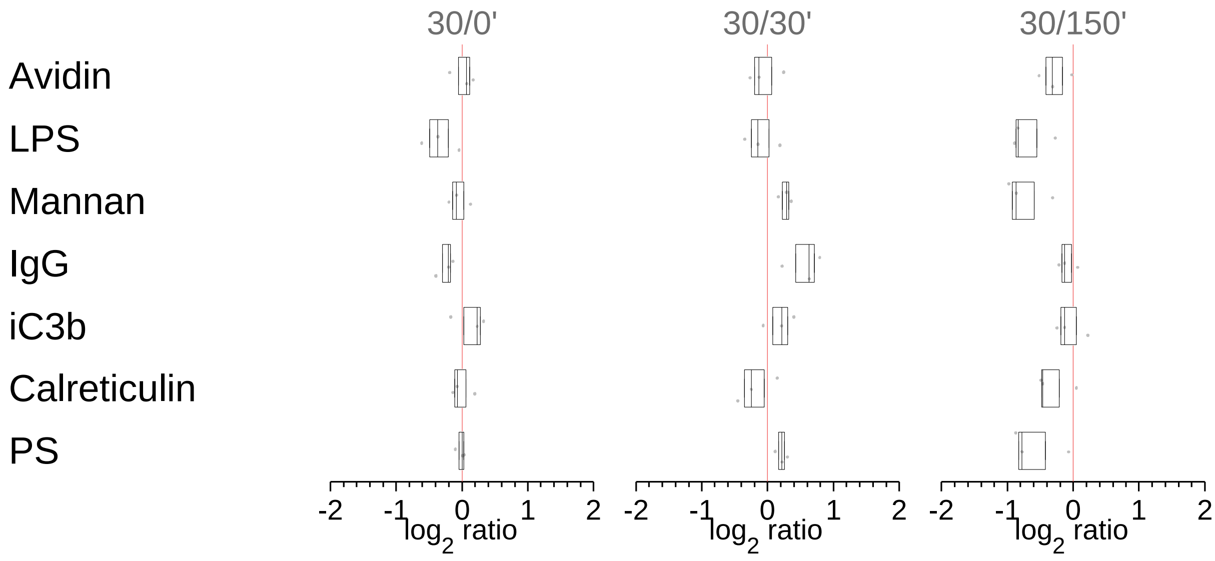

GO:0019152 acetoin dehydrogenase activity (n = 3)

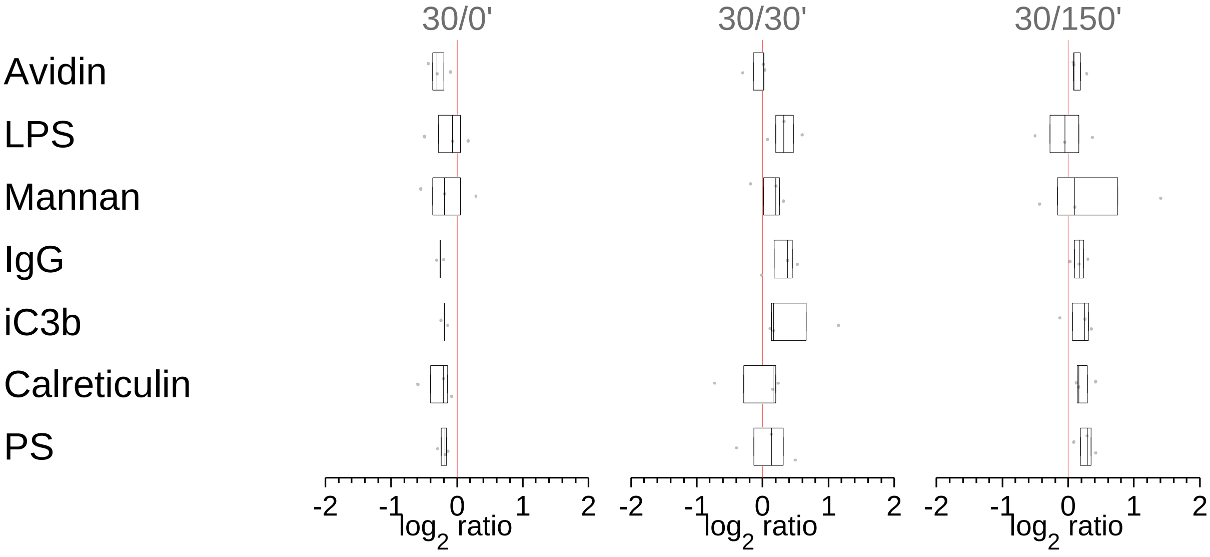

GO:0019186 acyl-CoA N-acyltransferase activity (n = 3)

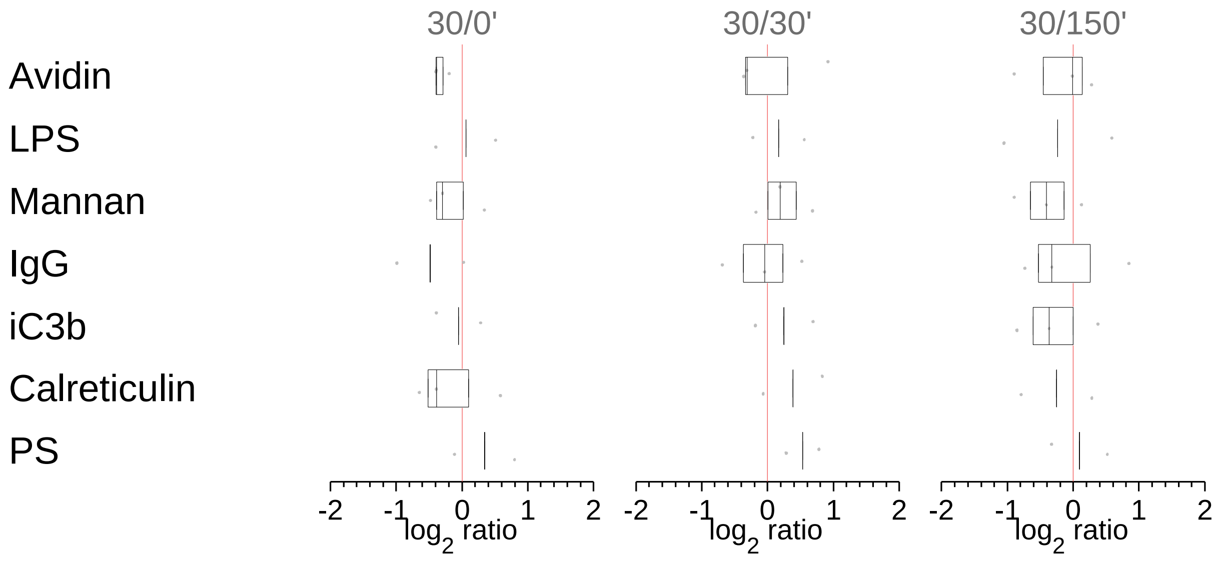

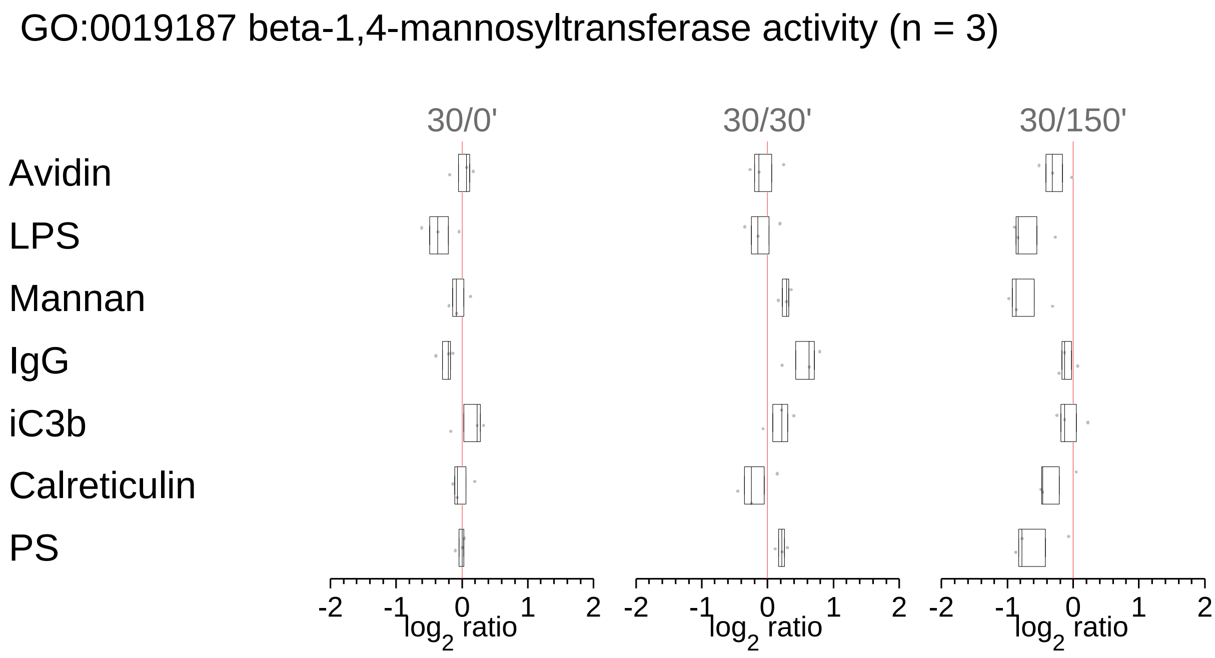
GO:0019187 beta-1,4-mannosyltransferase activity (n = 3)
30/0'
30/30'
30/150'
Avidin
LPS
Mannan
IgG
iC3b
Calreticulin
PS
-2 -1 0 1 2
log2 ratio
-2 -1 0 1 2
log2 ratio
-2 -1 0 1 2
log2 ratio

GO:0019221 cytokine-mediated signaling pathway (n = 10)

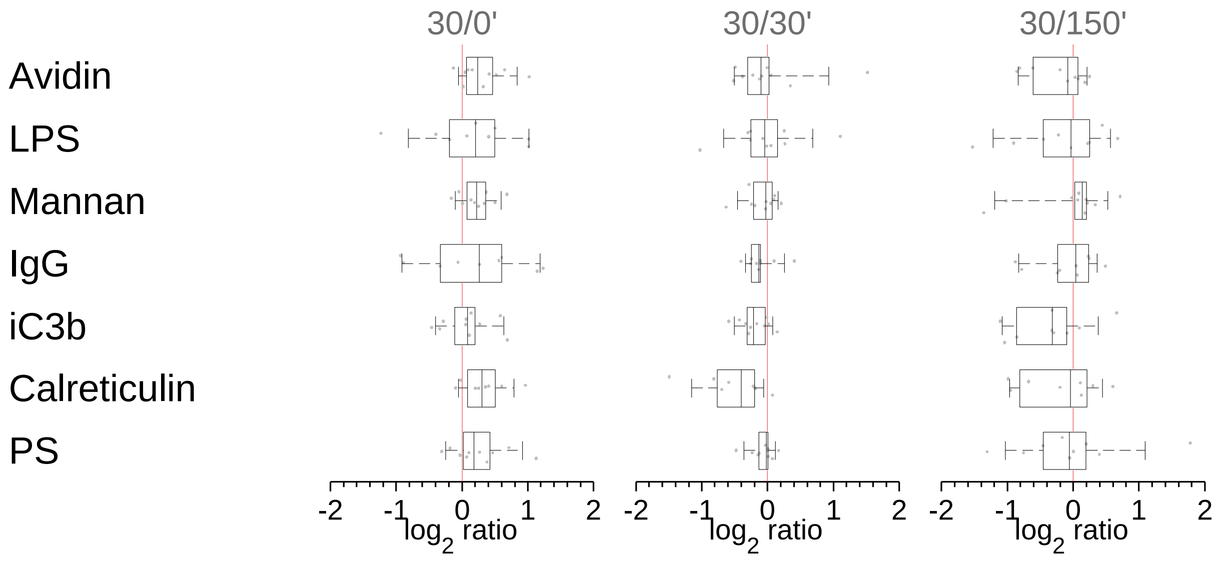

GO:0019233 sensory perception of pain (n = 5)

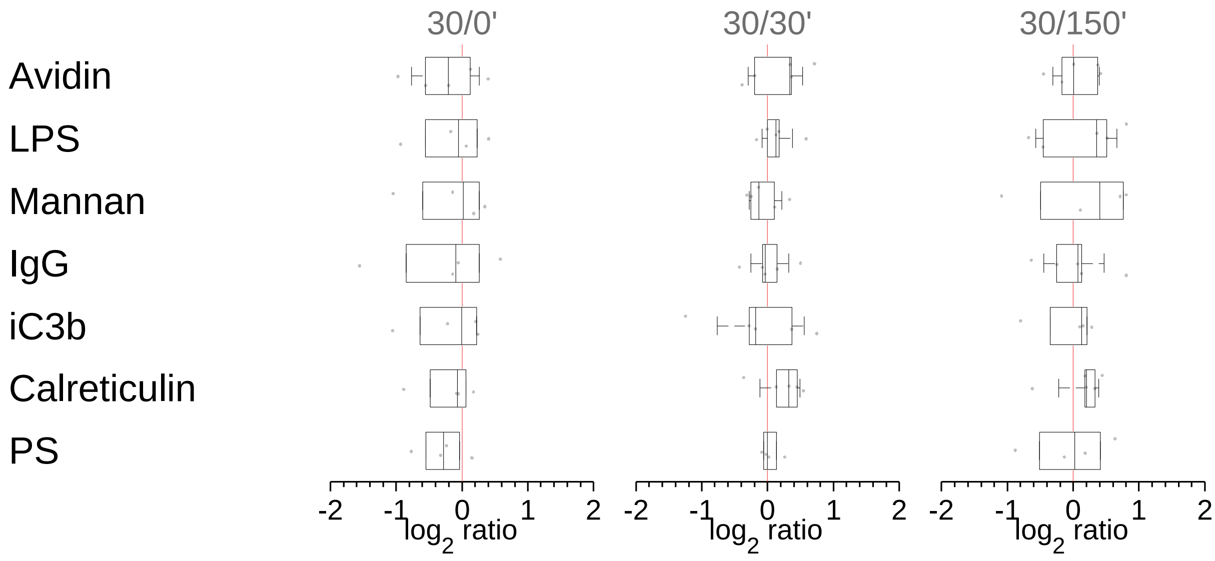

GO:0019370 leukotriene biosynthetic process (n = 3)

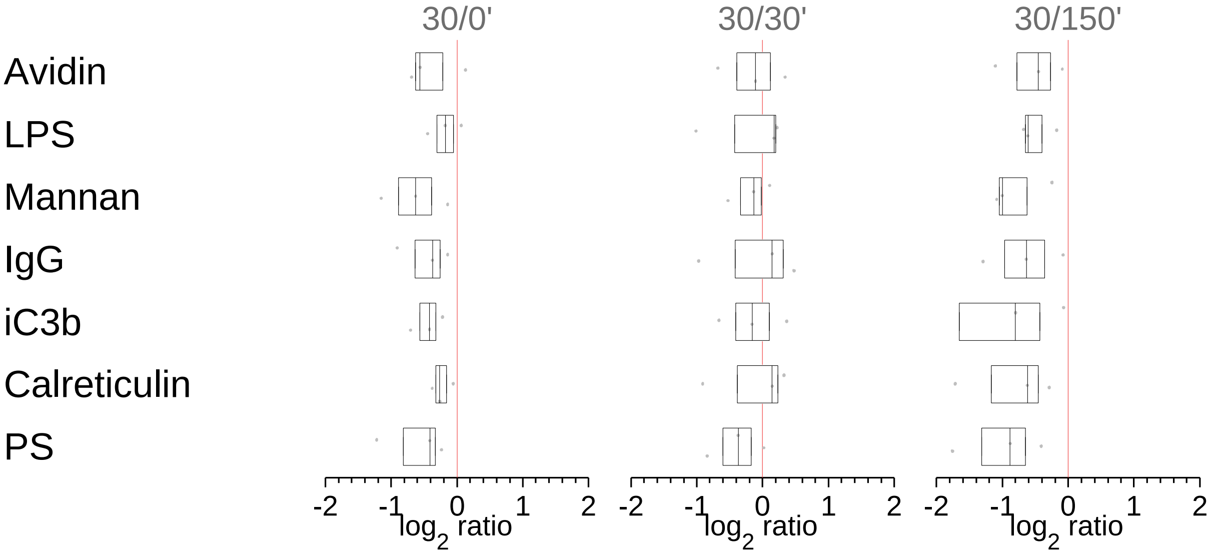

GO:0019430 removal of superoxide radicals (n = 5)

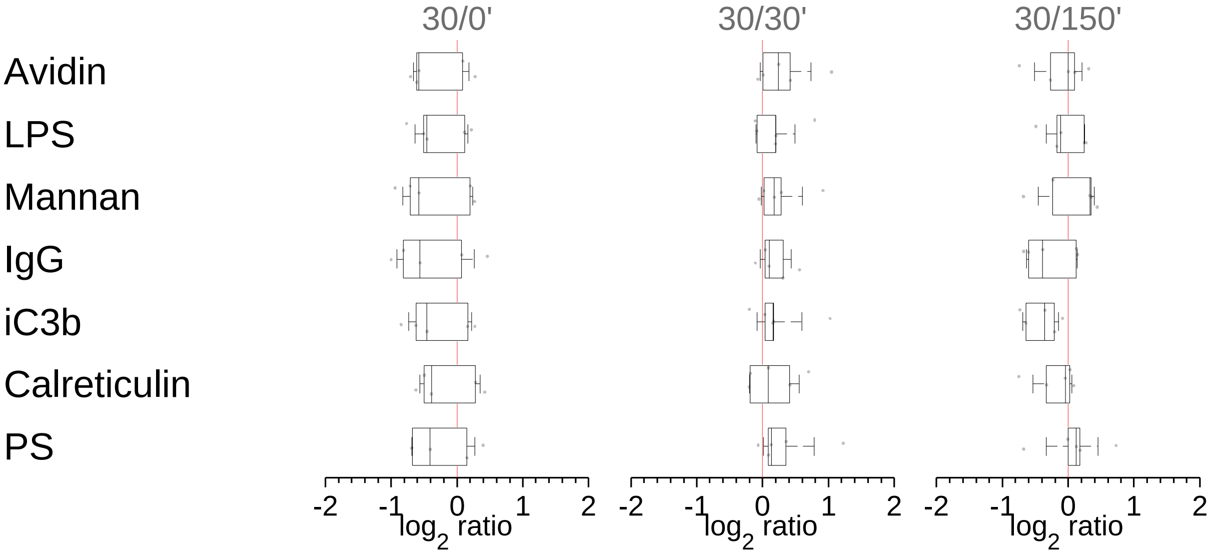

GO:0019439 aromatic compound catabolic process (n = 4)

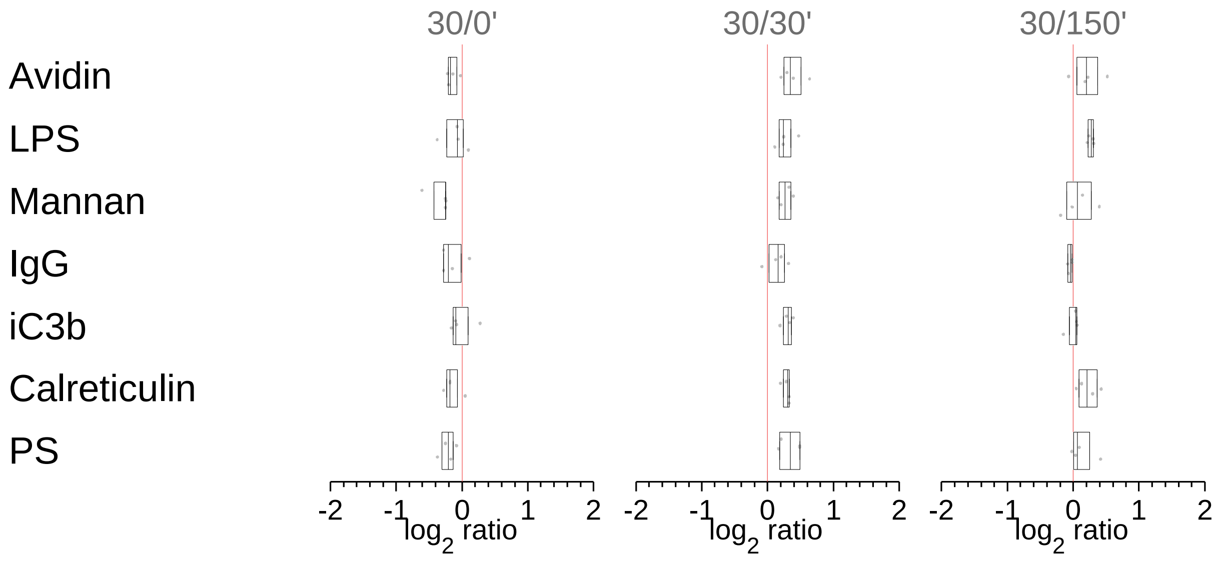

GO:0019705 protein-cysteine S-myristoyltransferase activity (n = 3)

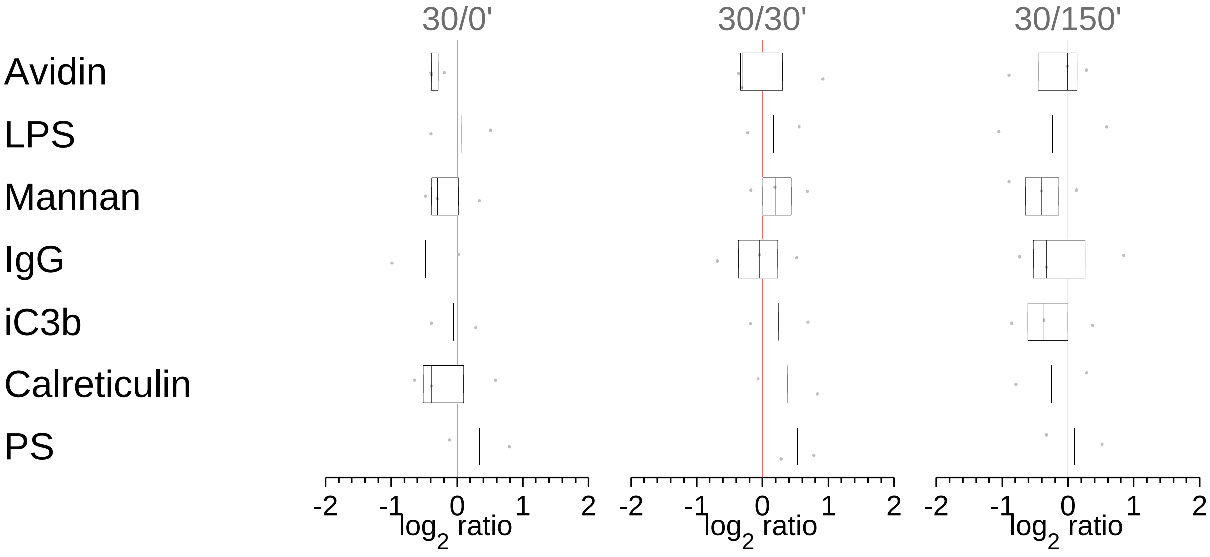

GO:0019707 protein-cysteine S-acyltransferase activity (n = 3)

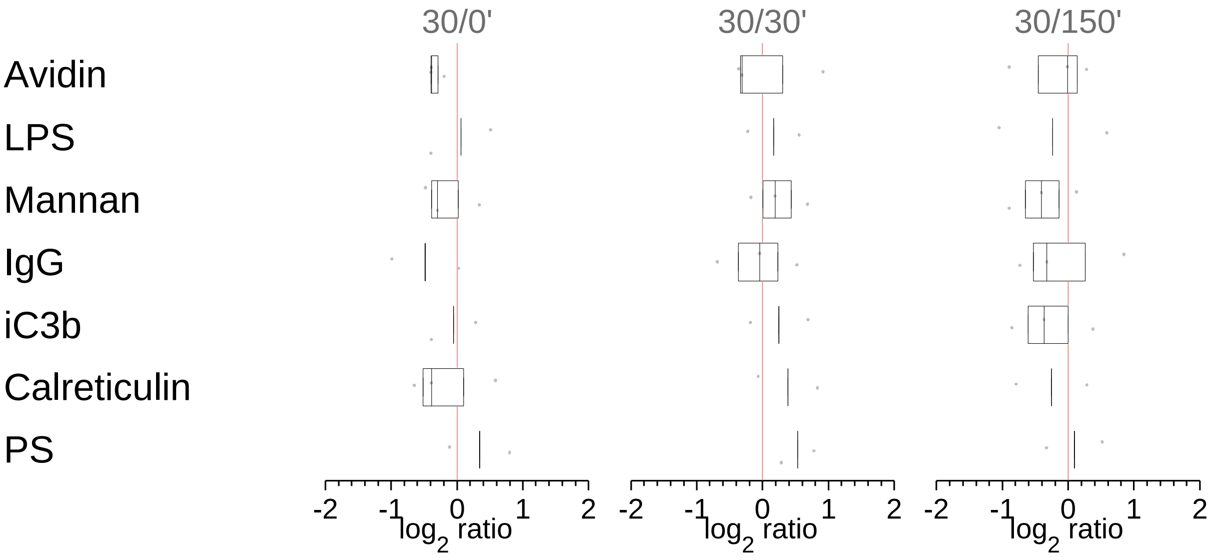

GO:0019773 proteasome core complex, alpha-subunit complex (n = 5)

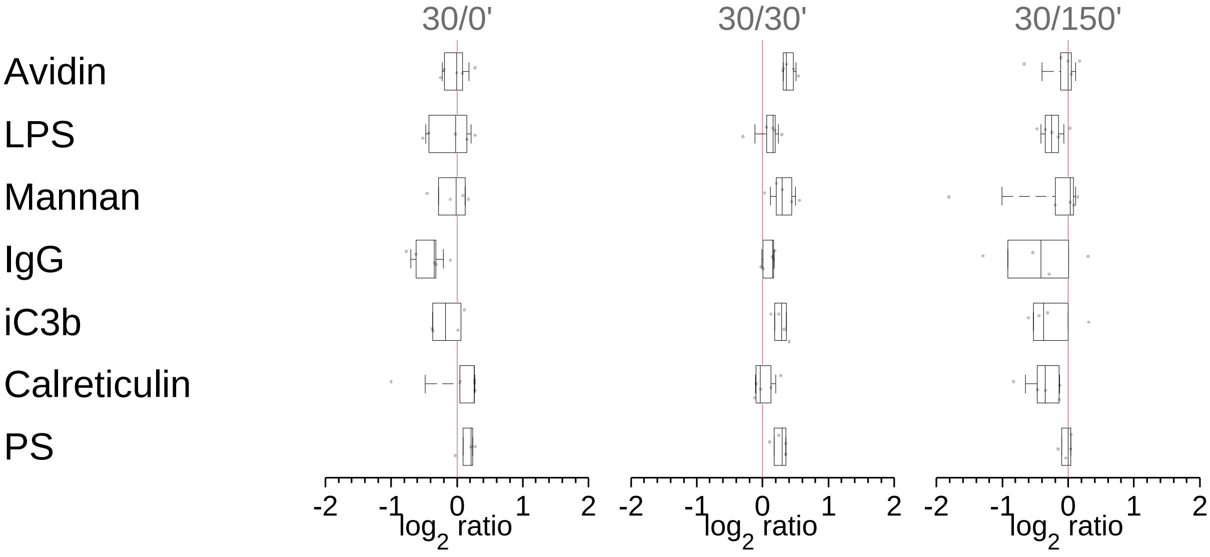

GO:0019827 stem cell maintenance (n = 3)

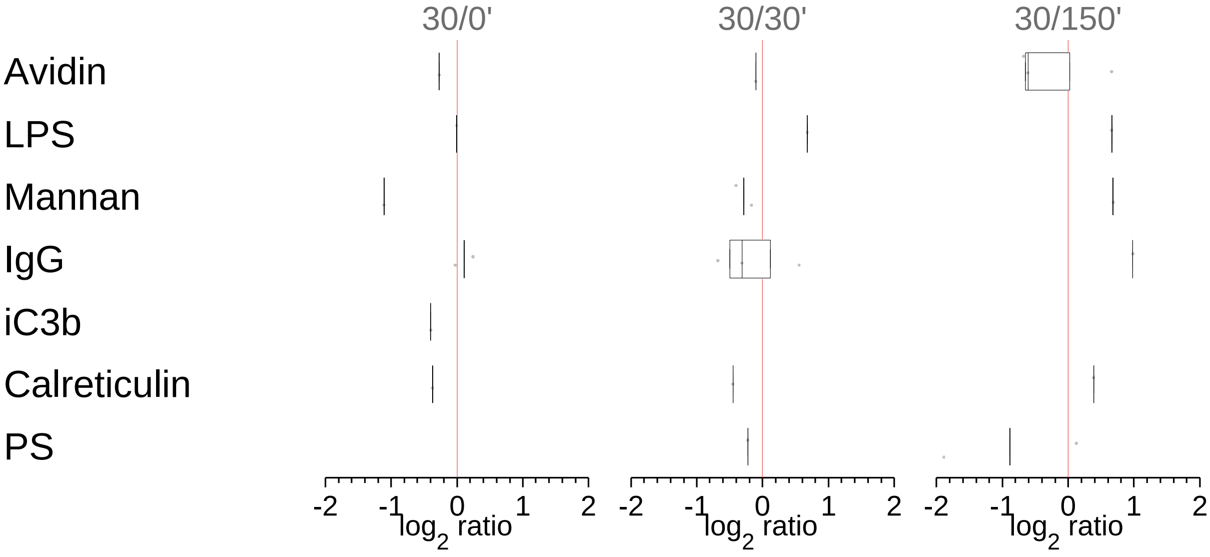

GO:0019829 cation-transporting ATPase activity (n = 9)

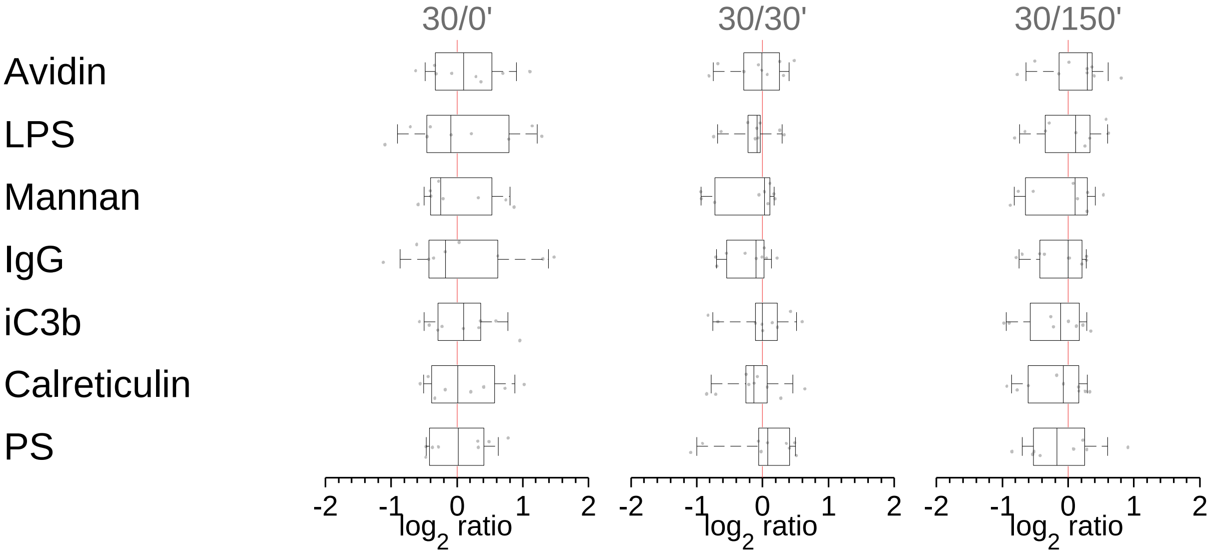

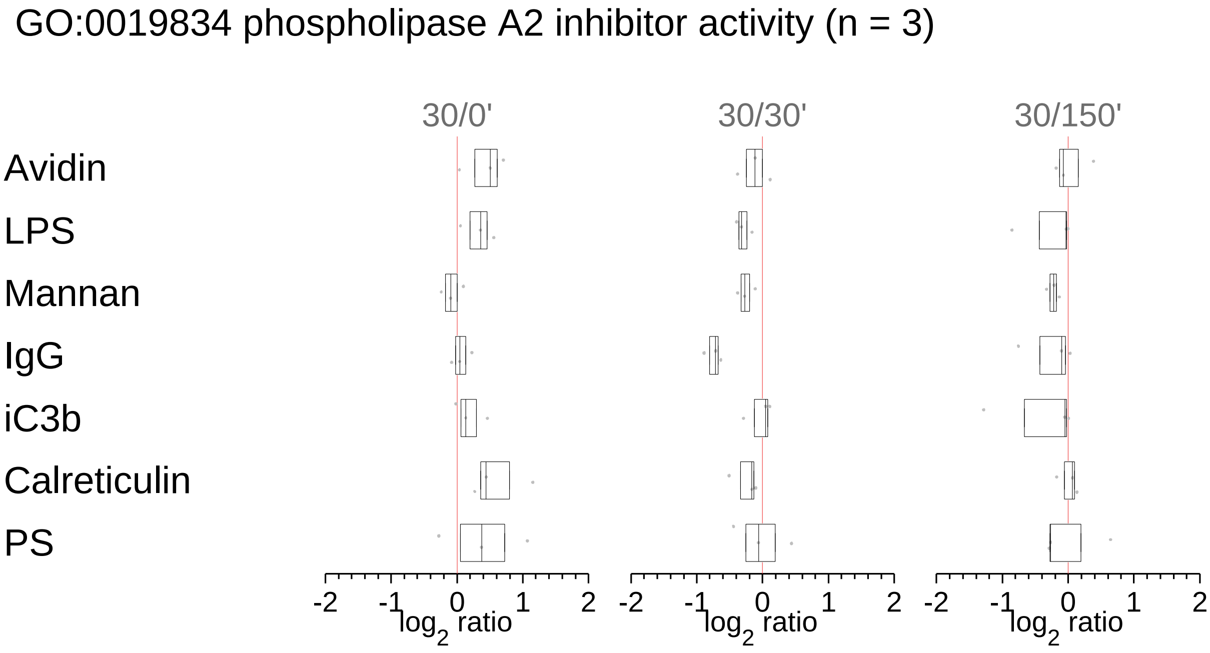

GO:0019834 phospholipase A2 inhibitor activity (n = 3)
30/0'
30/30'
30/150'
Avidin
LPS
Mannan
IgG
iC3b
Calreticulin
PS
-2 -1 0 1 2
log2 ratio

GO:0019835 cytolysis (n = 3)

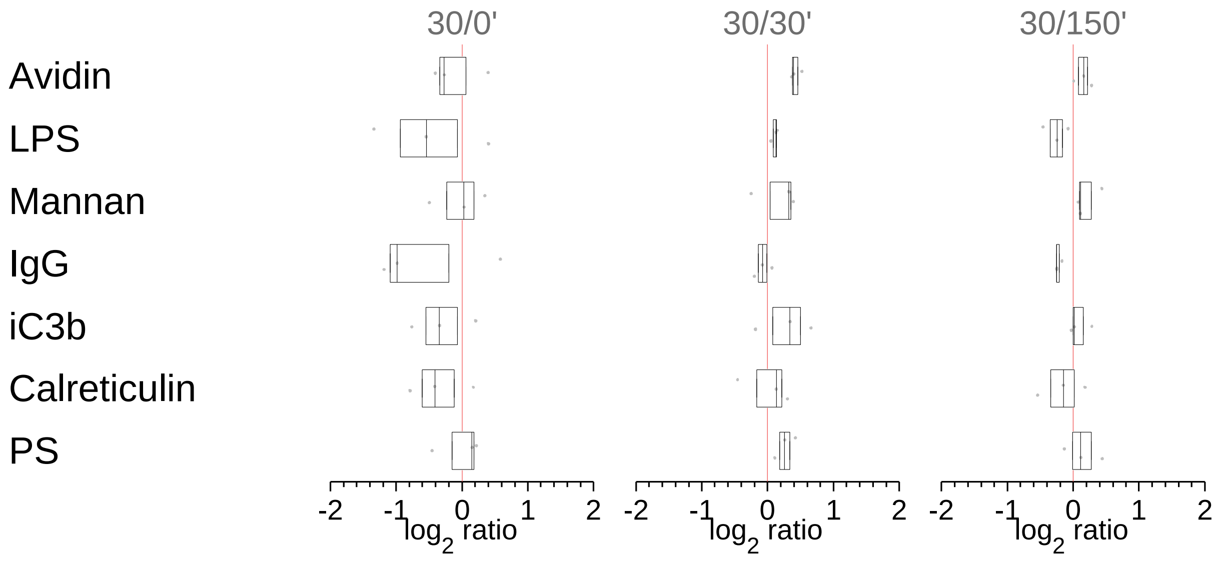

GO:0019843 rRNA binding (n = 10)

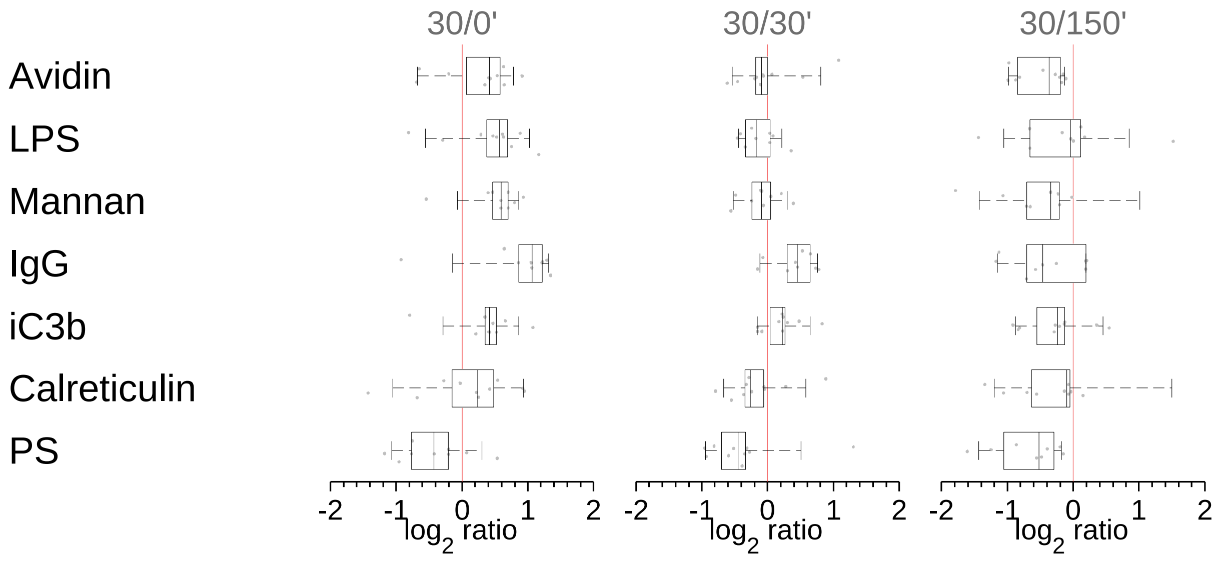

GO:0019864 IgG binding (n = 5)

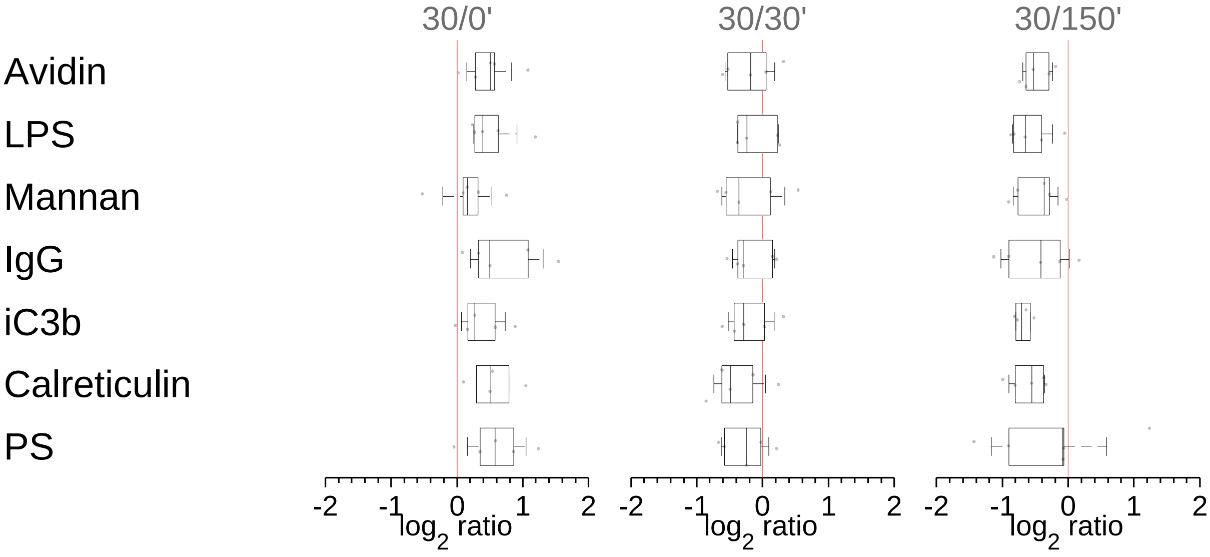

GO:0019882 antigen processing and presentation (n = 19)

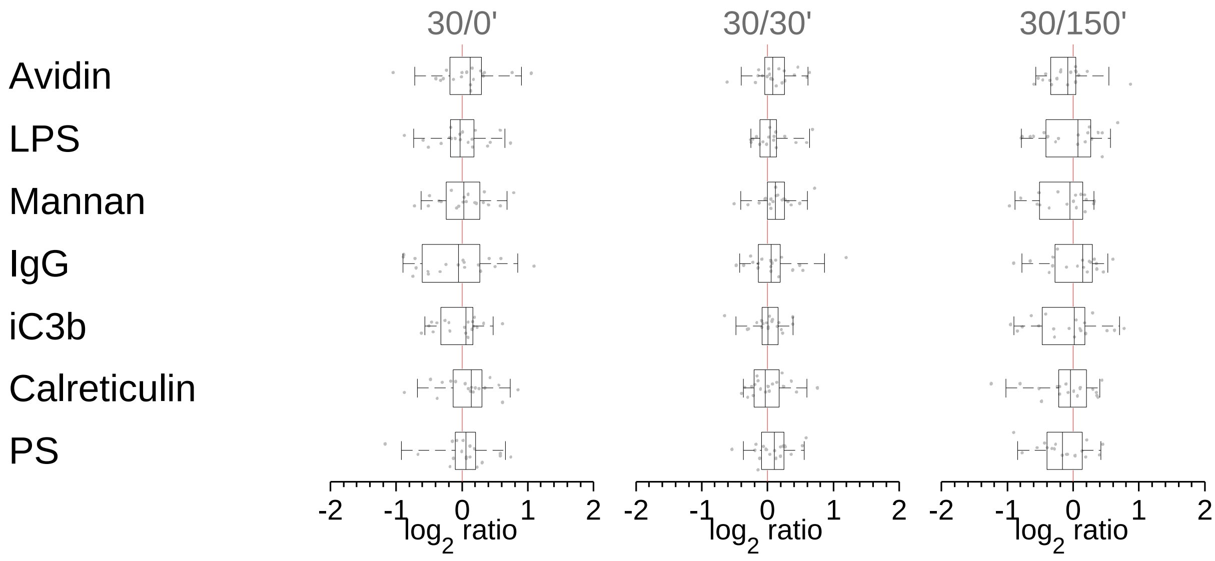

GO:0019885 antigen processing and presentation of endogenous peptide antigen via MHC class I (n = 3)

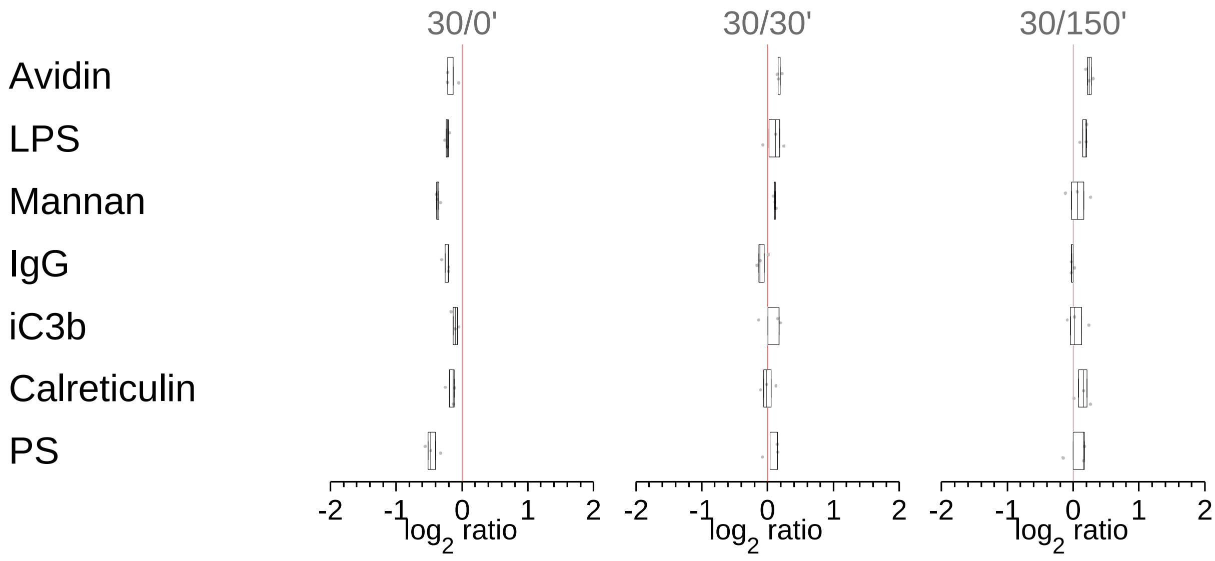

GO:0019886 antigen processing and presentation of exogenous peptide antigen via MHC class II (n = 7)

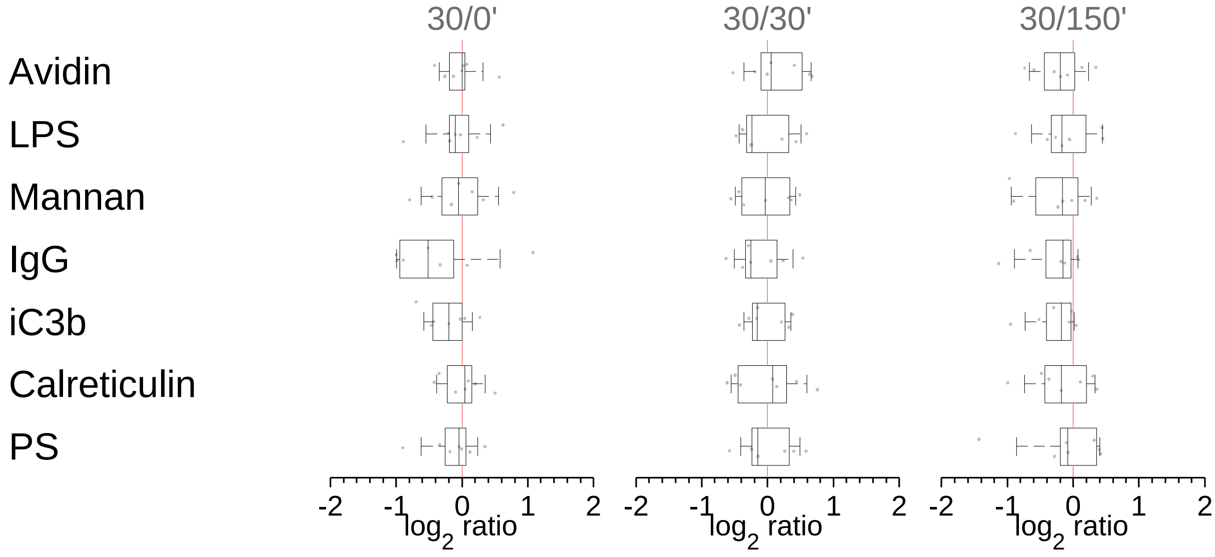

GO:0019897 extrinsic to plasma membrane (n = 7)

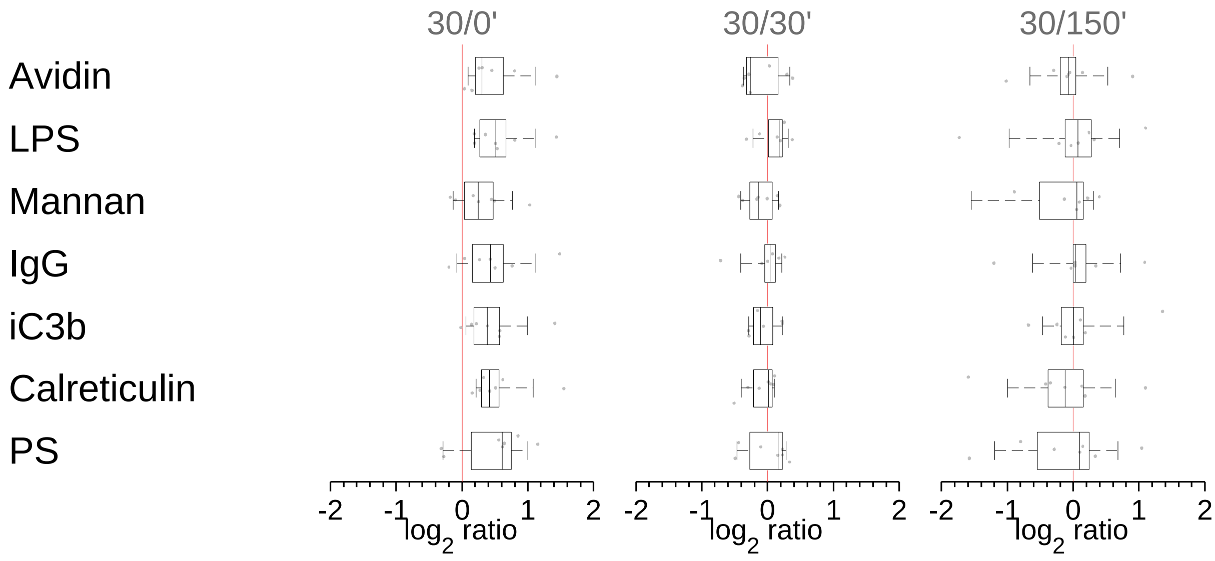

GO:0019898 extrinsic to membrane (n = 8)

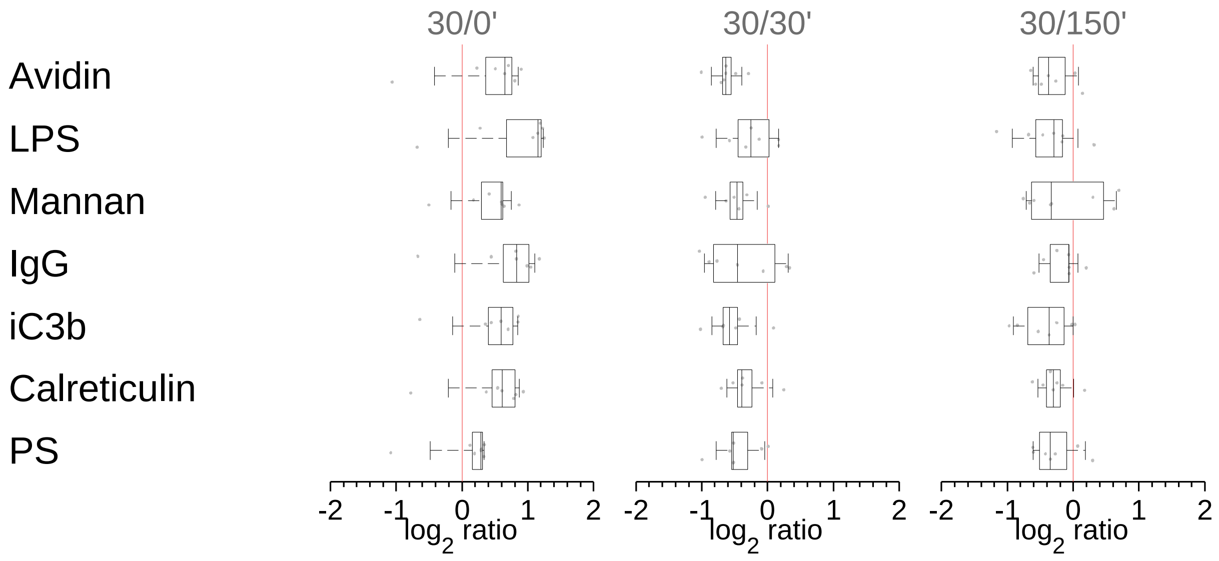

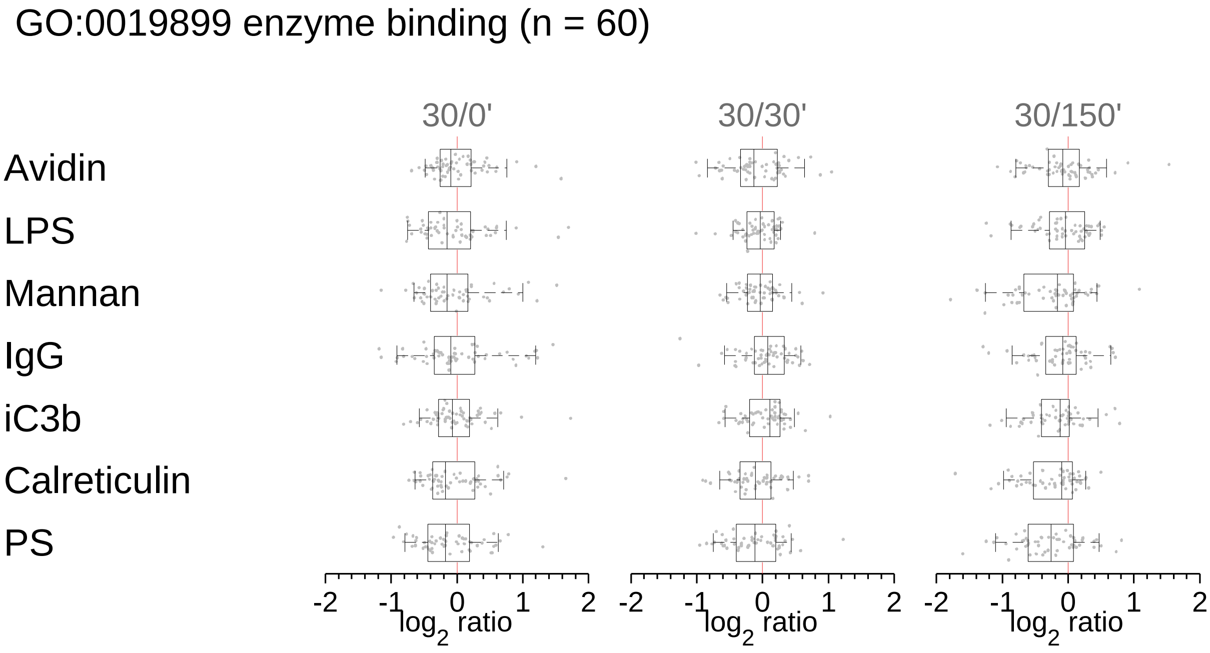
GO:0019899 enzyme binding (n = 60)
30/0'
30/30'
30/150'
Avidin
LPS
Mannan
IgG
iC3b
Calreticulin
PS
-2
-1
0
1
2
log2 ratio

GO:0019900 kinase binding (n = 10)

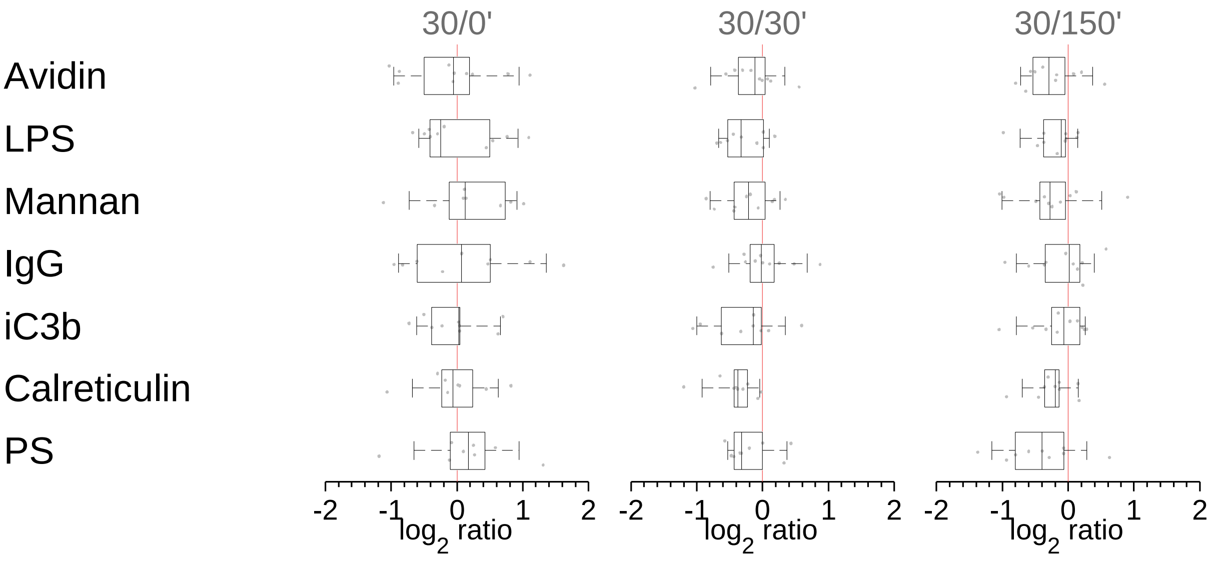

GO:0019901 protein kinase binding (n = 57)

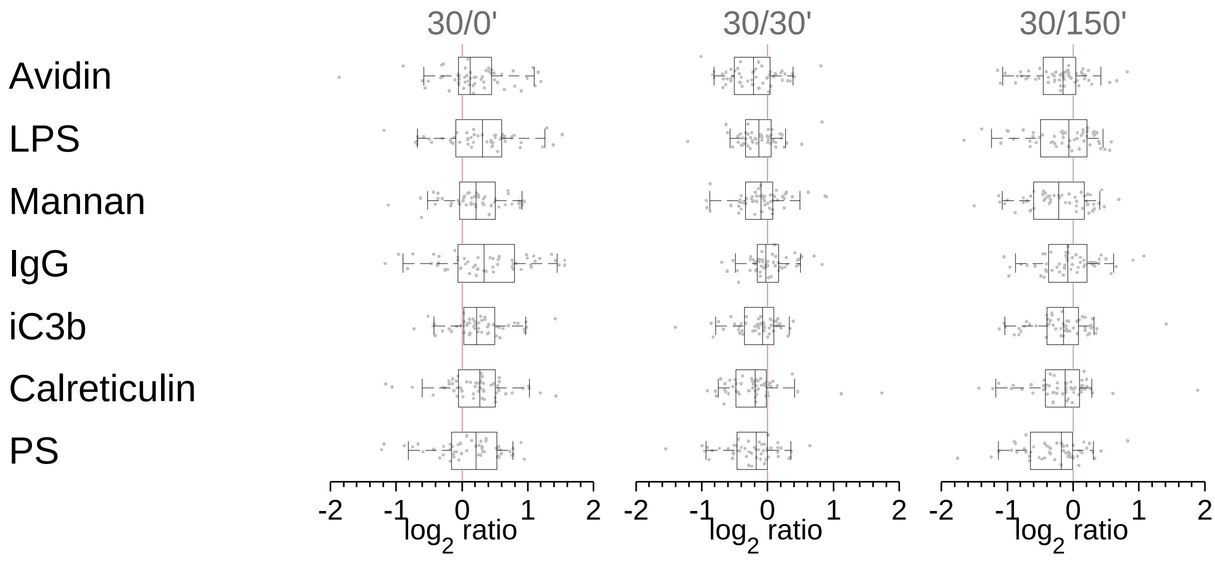

GO:0019902 phosphatase binding (n = 6)

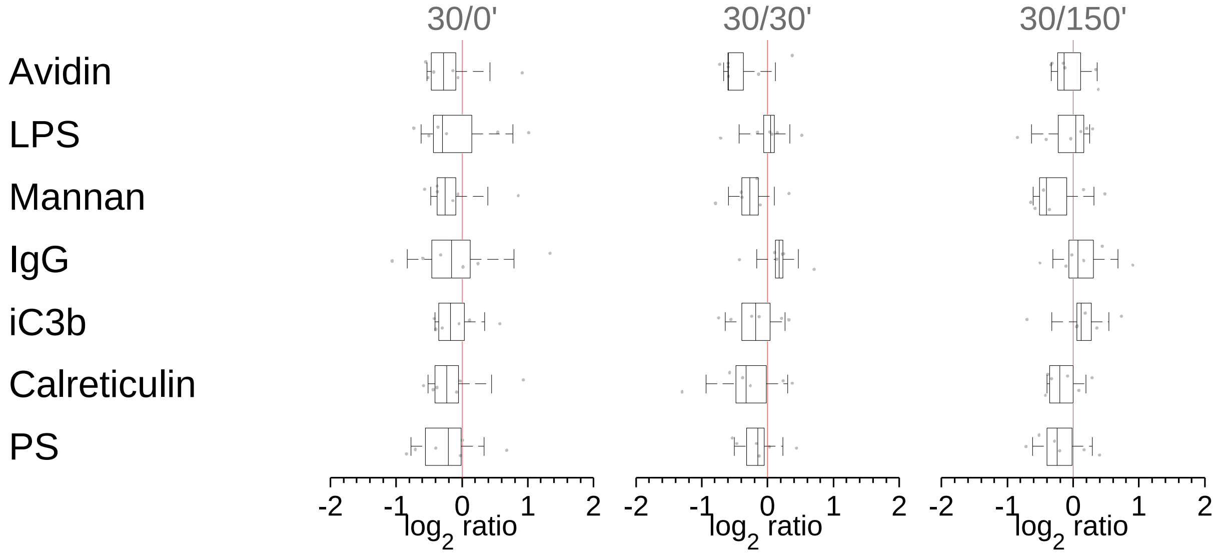

GO:0019903 protein phosphatase binding (n = 21)

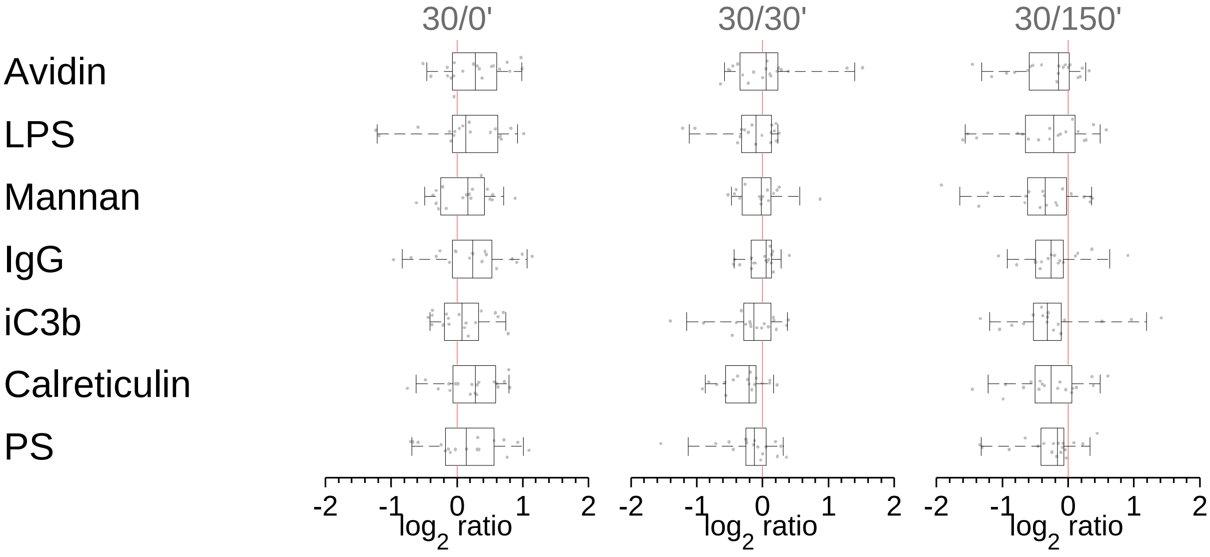

GO:0019904 protein domain specific binding (n = 53)

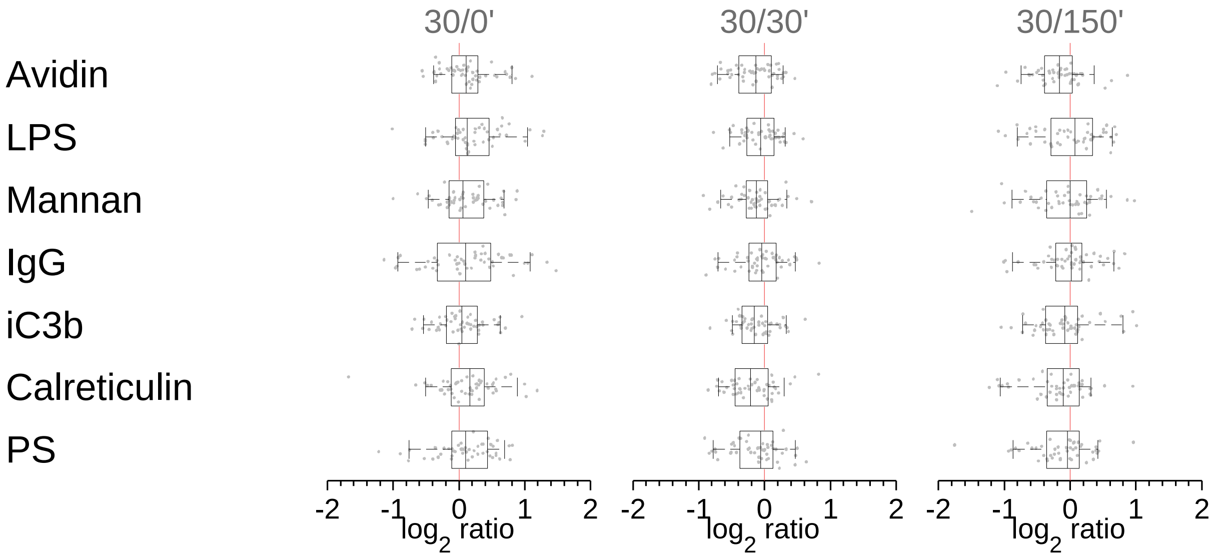

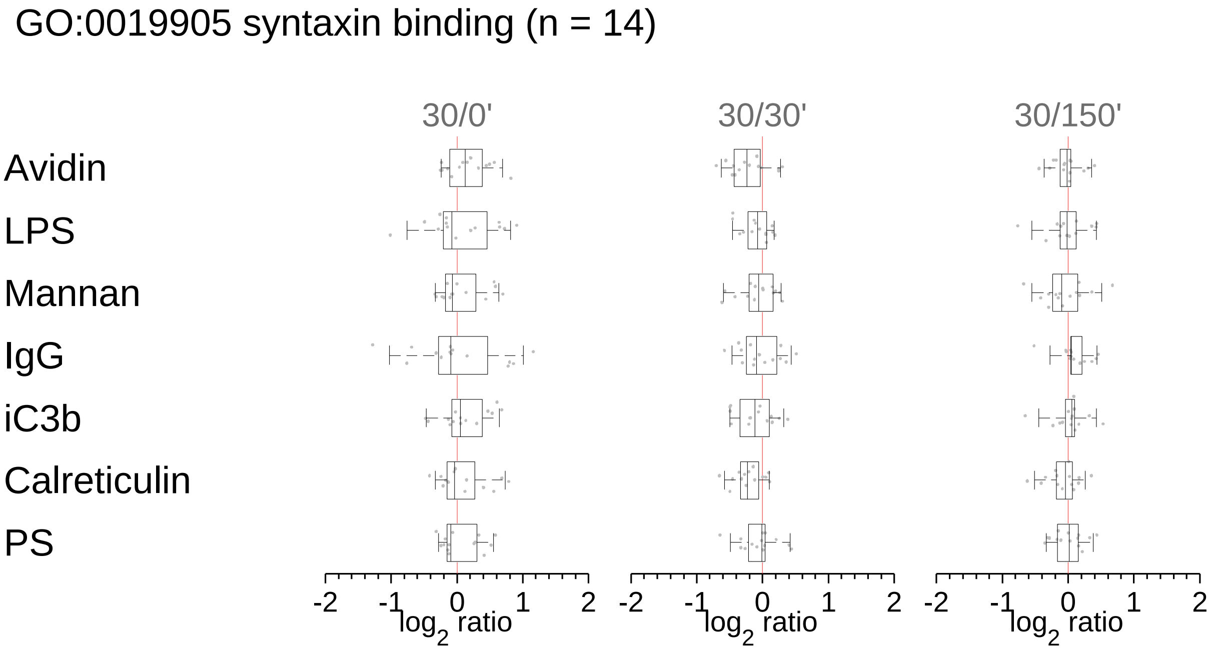
GO:0019905 syntaxin binding (n = 14)
30/0'
30/30'
30/150'
Avidin
LPS
Mannan
IgG
iC3b
Calreticulin
PS
-2 -1 0 1 2
log2 ratio

GO:0019915 lipid storage (n = 5)

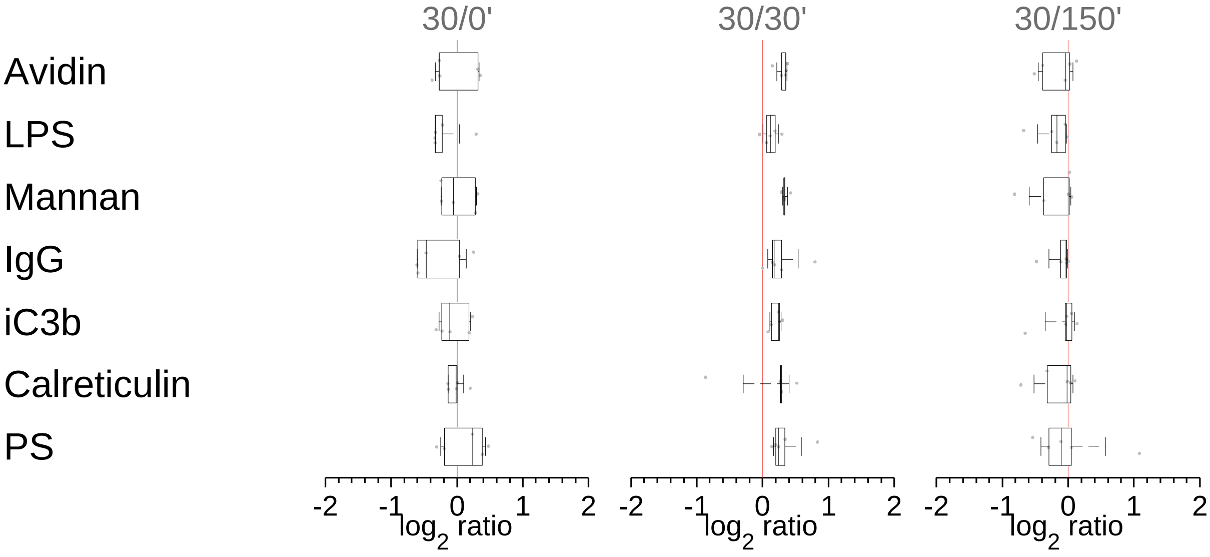

GO:0019953 sexual reproduction (n = 3)

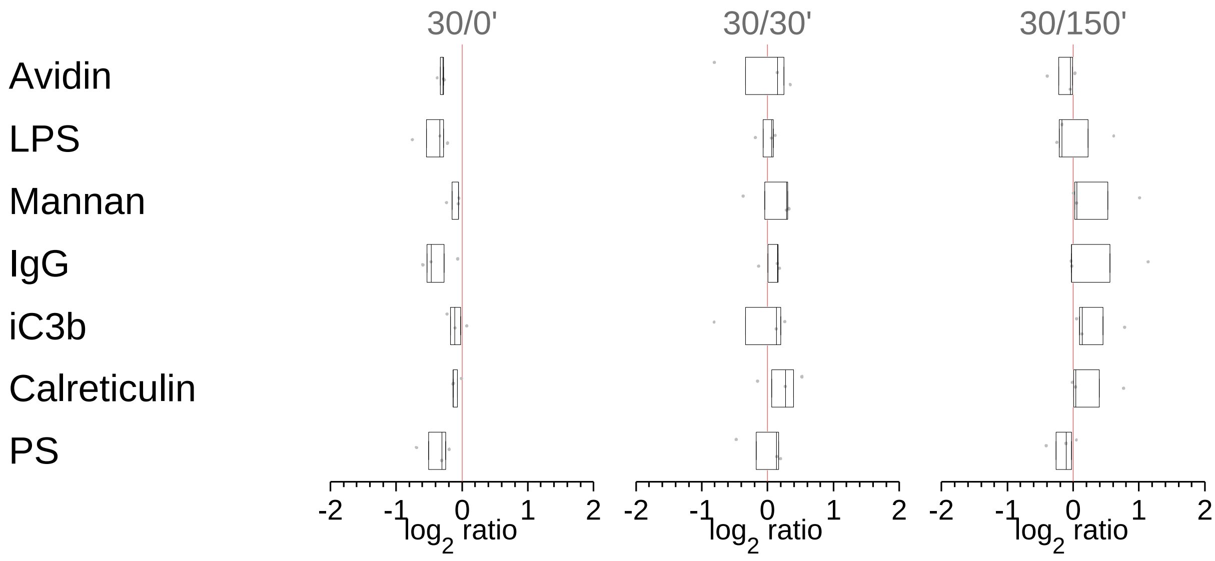

GO:0019955 cytokine binding (n = 4)

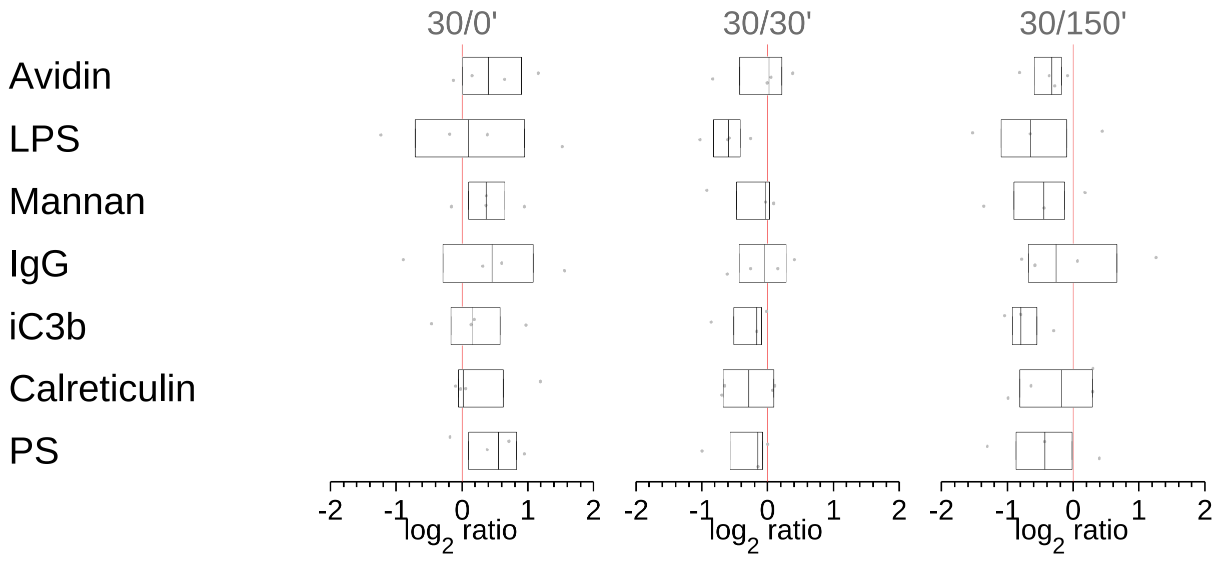

GO:0020037 heme binding (n = 15)

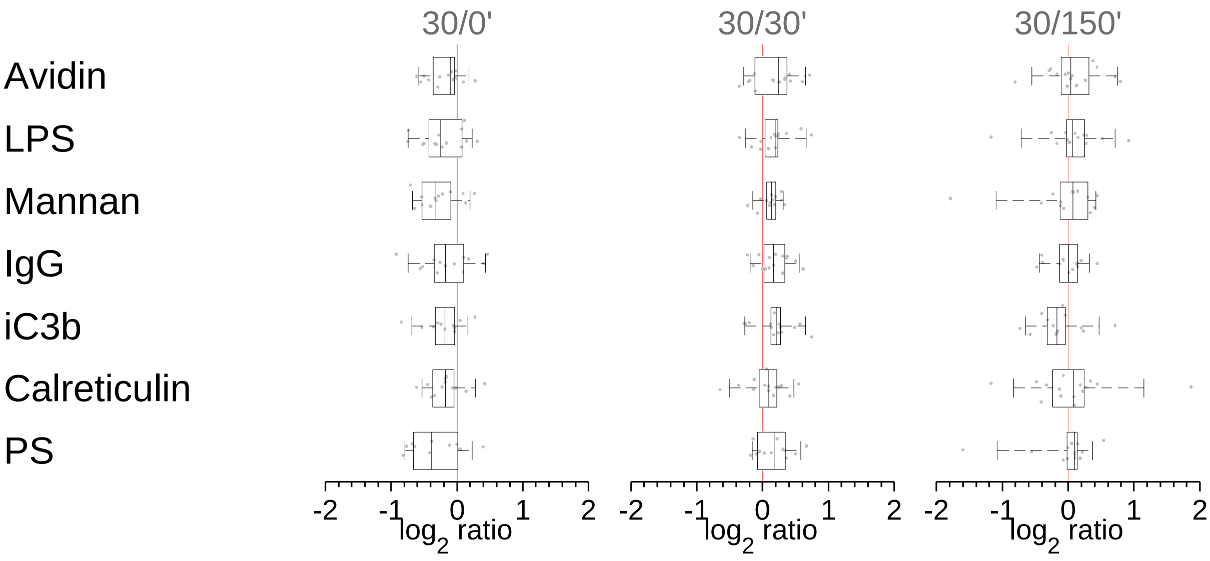

GO:0021675 nerve development (n = 3)

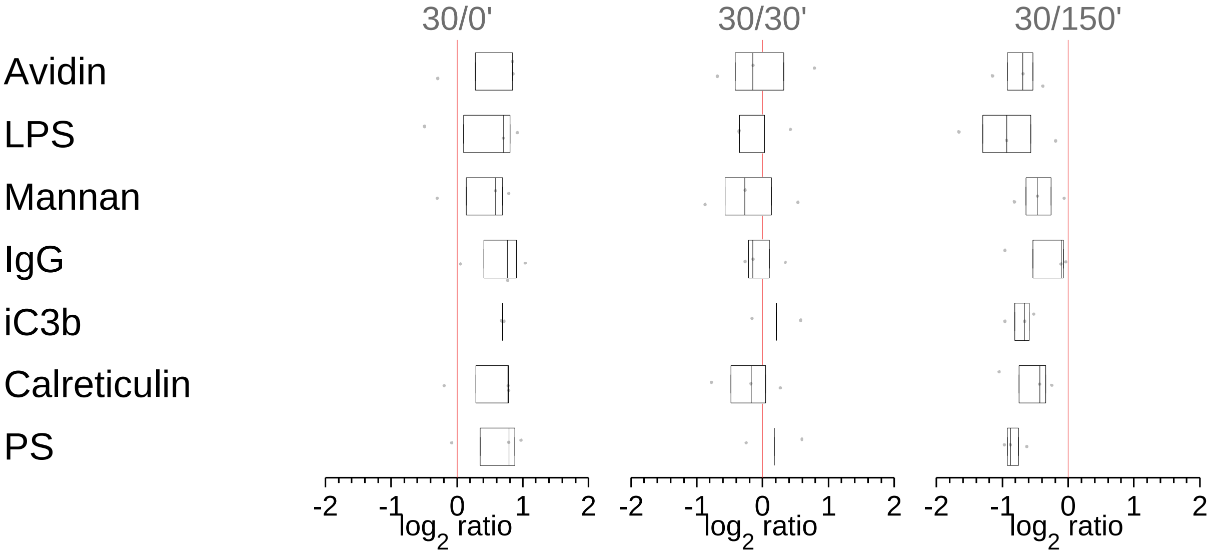

GO:0021766 hippocampus development (n = 3)

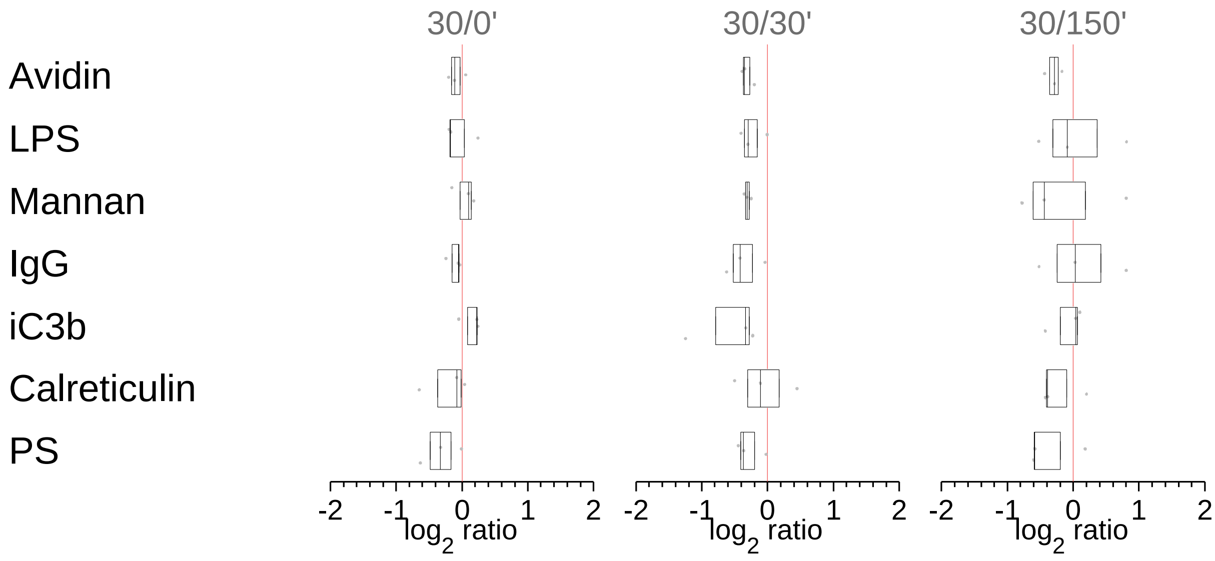

GO:0021904 dorsal/ventral neural tube patterning (n = 4)

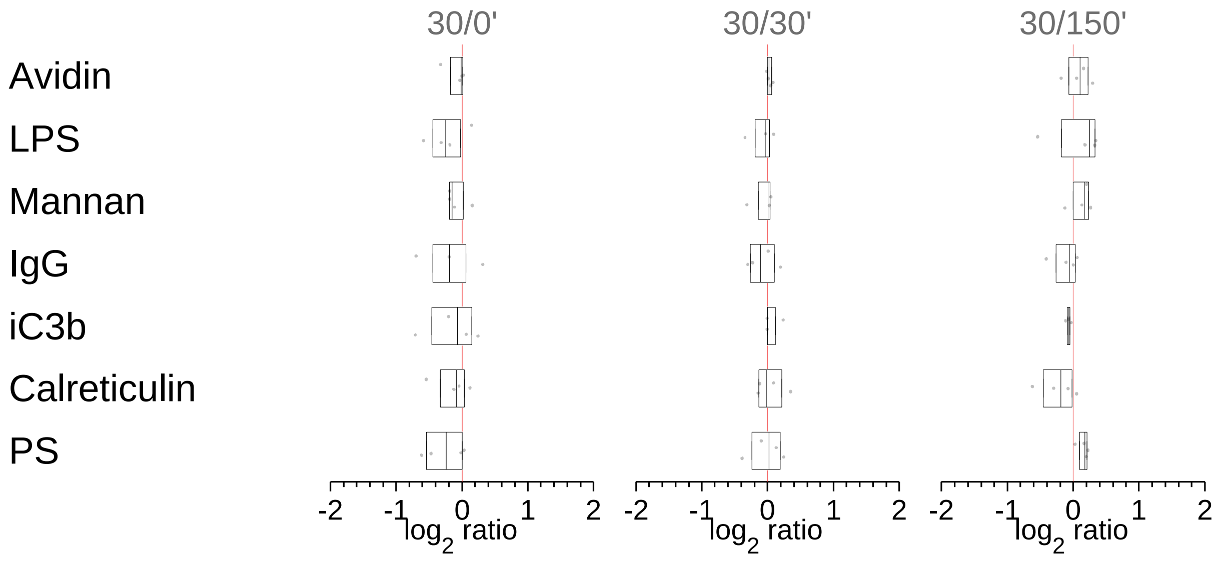

GO:0021987 cerebral cortex development (n = 5)

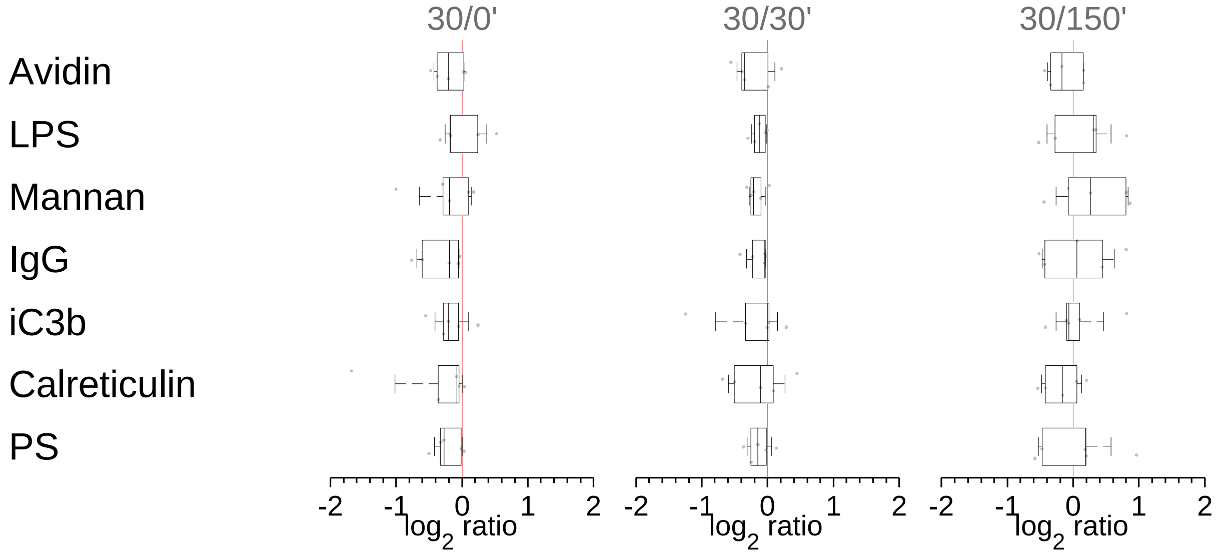

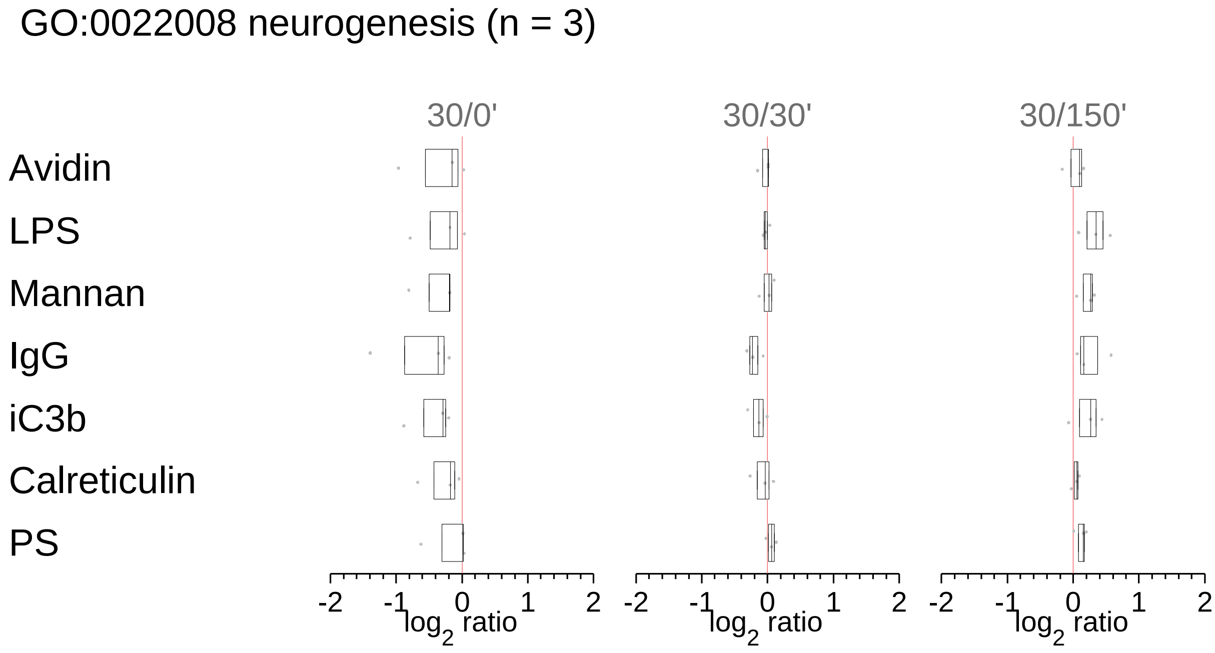
GO:0022008 neurogenesis (n = 3)
30/0'
30/30'
30/150'
Avidin
LPS
Mannan
IgG
iC3b
Calreticulin
PS
-2
-1
0
1
2
$\log_2$ ratio

GO:0022625 cytosolic large ribosomal subunit (n = 24)

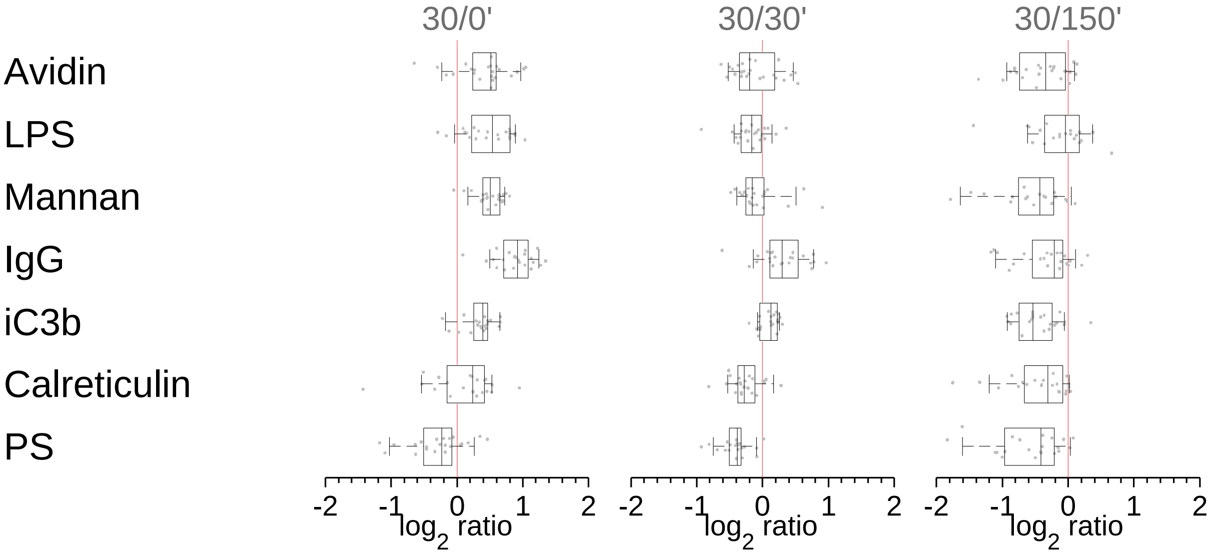

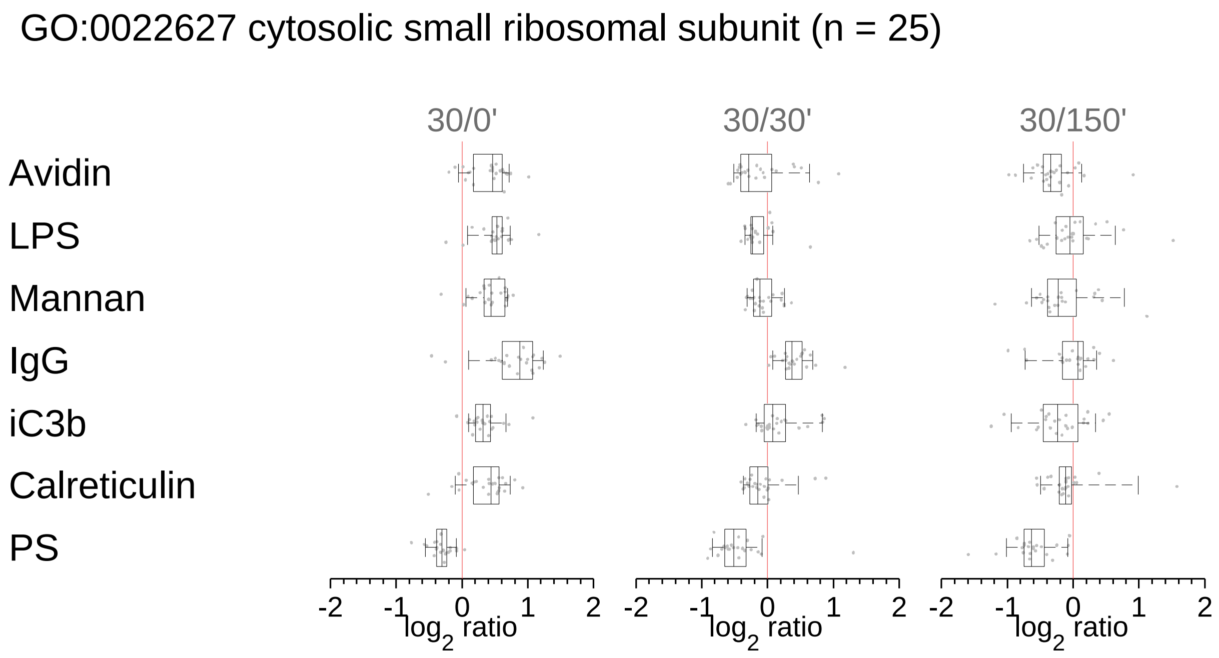

GO:0022627 cytosolic small ribosomal subunit (n = 25)
30/0'
30/30'
30/150'
Avidin
LPS
Mannan
IgG
iC3b
Calreticulin
PS
-2
-1
0
1
2
log2 ratio

GO:0022900 electron transport chain (n = 3)

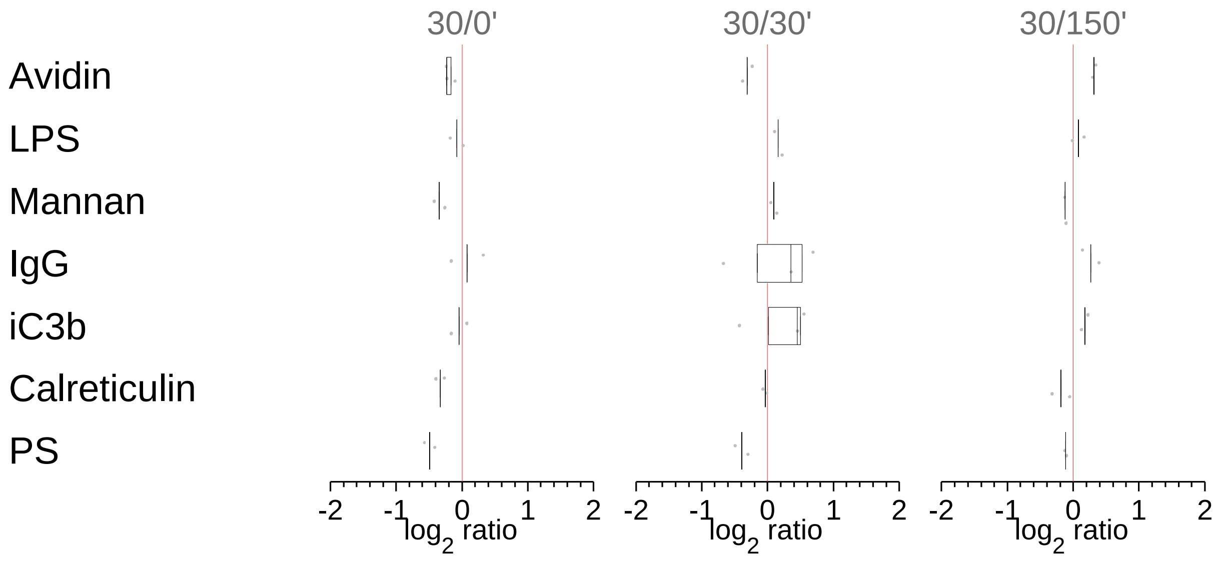

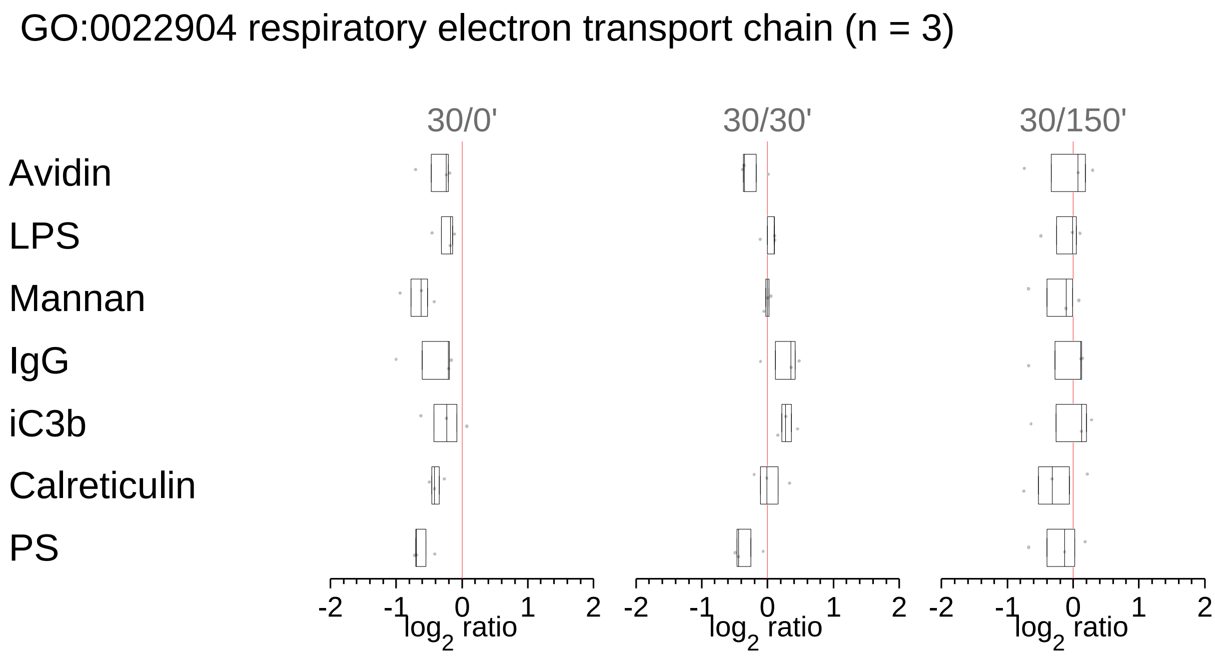
GO:0022904 respiratory electron transport chain (n = 3)
30/0'
30/30'
30/150'
Avidin
LPS
Mannan
IgG
iC3b
Calreticulin
PS
-2
-1
0
1
2
log2 ratio

GO:0023014 signal transduction by phosphorylation (n = 6)

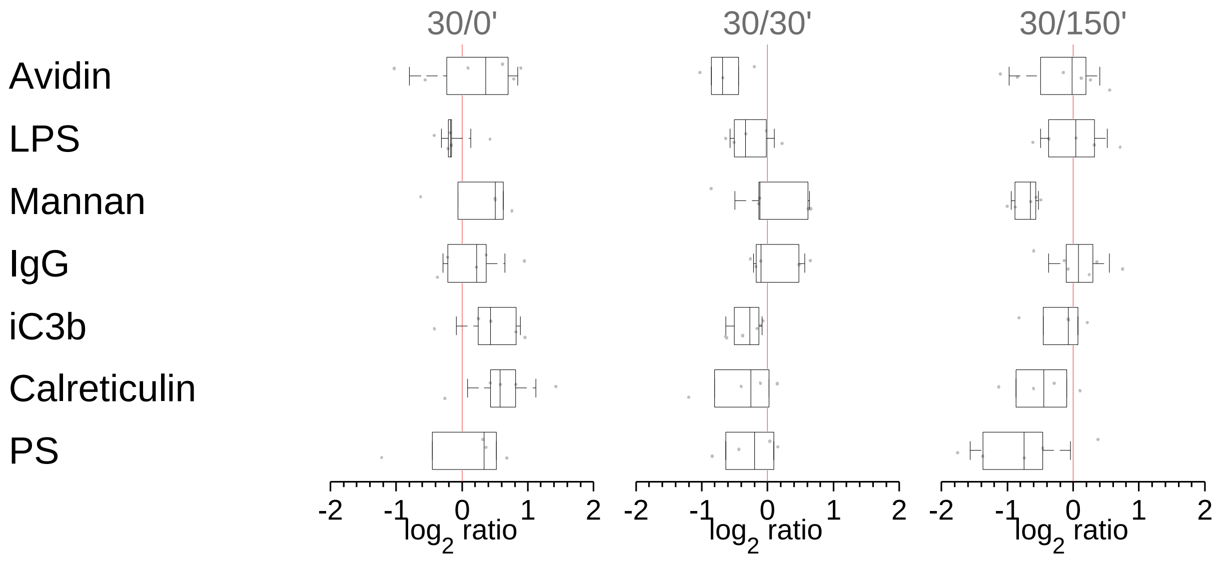

GO:0023021 termination of signal transduction (n = 3)

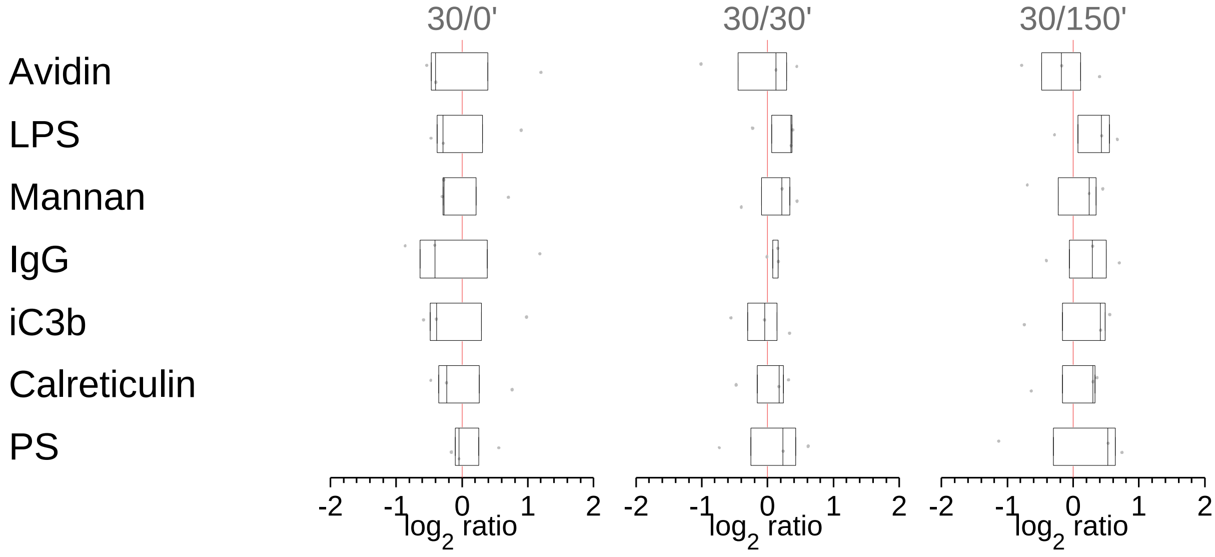

GO:0023026 MHC class II protein complex binding (n = 10)

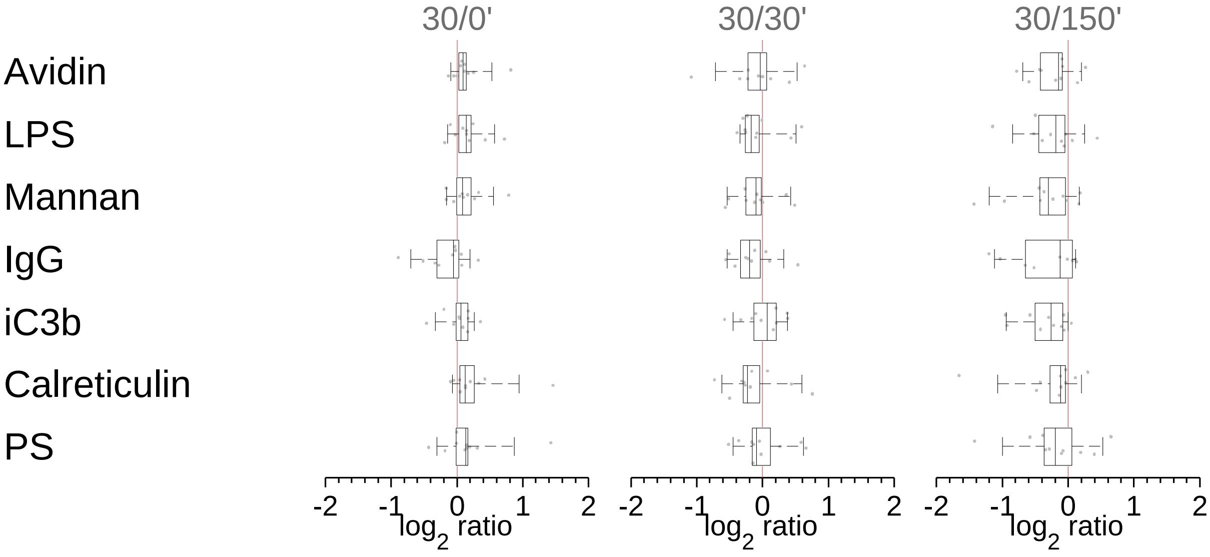

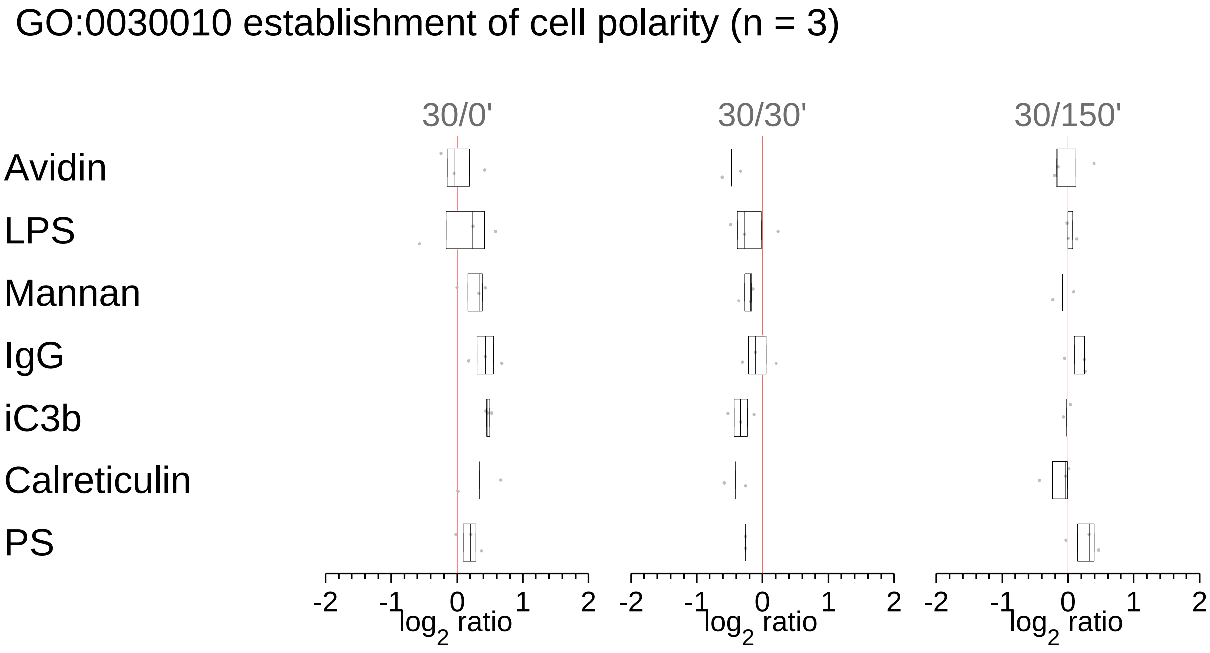
GO:0030010 establishment of cell polarity (n = 3)
30/0'
30/30'
30/150'
Avidin
LPS
Mannan
IgG
iC3b
Calreticulin
PS
-2
-1
0
1
2
log2 ratio

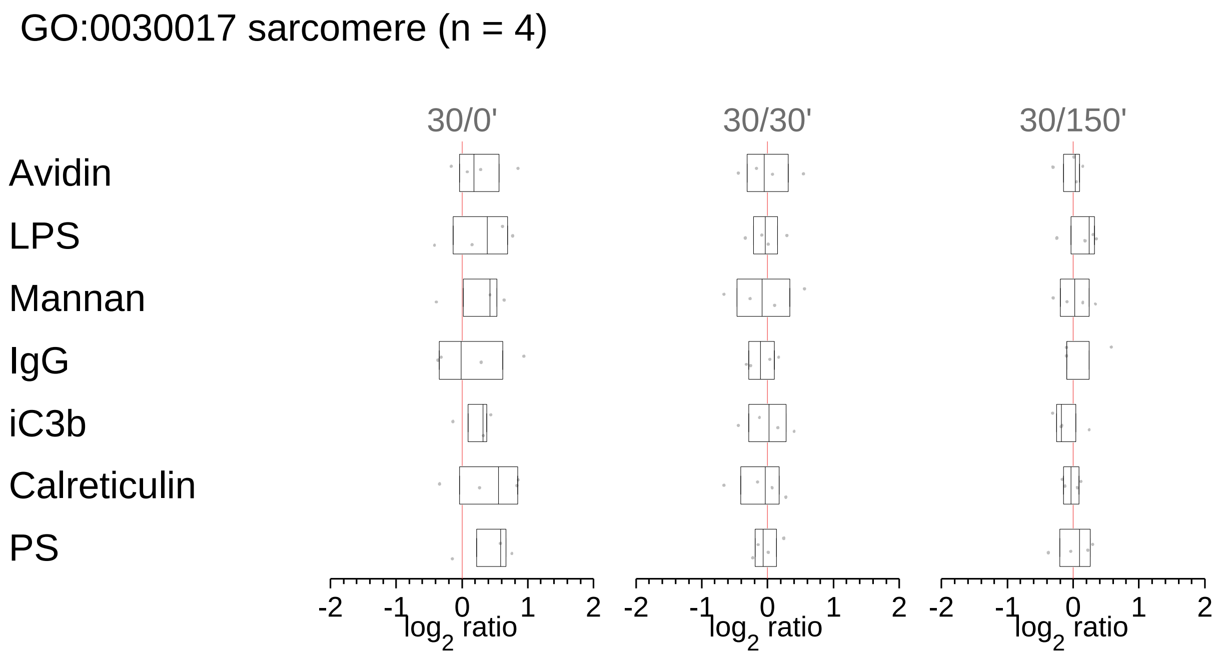
GO:0030017 sarcomere (n = 4)
30/0'
30/30'
30/150'
Avidin
LPS
Mannan
IgG
iC3b
Calreticulin
PS
-2 -1 0 1 2
log2 ratio

GO:0030018 Z disc (n = 15)

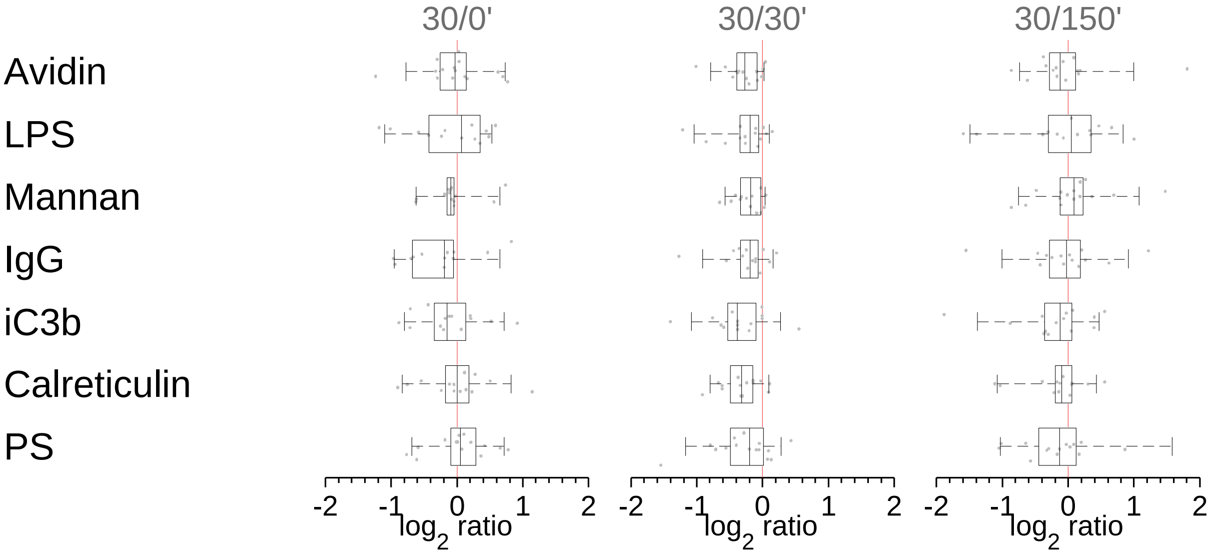

GO:0030027 lamellipodium (n = 27)

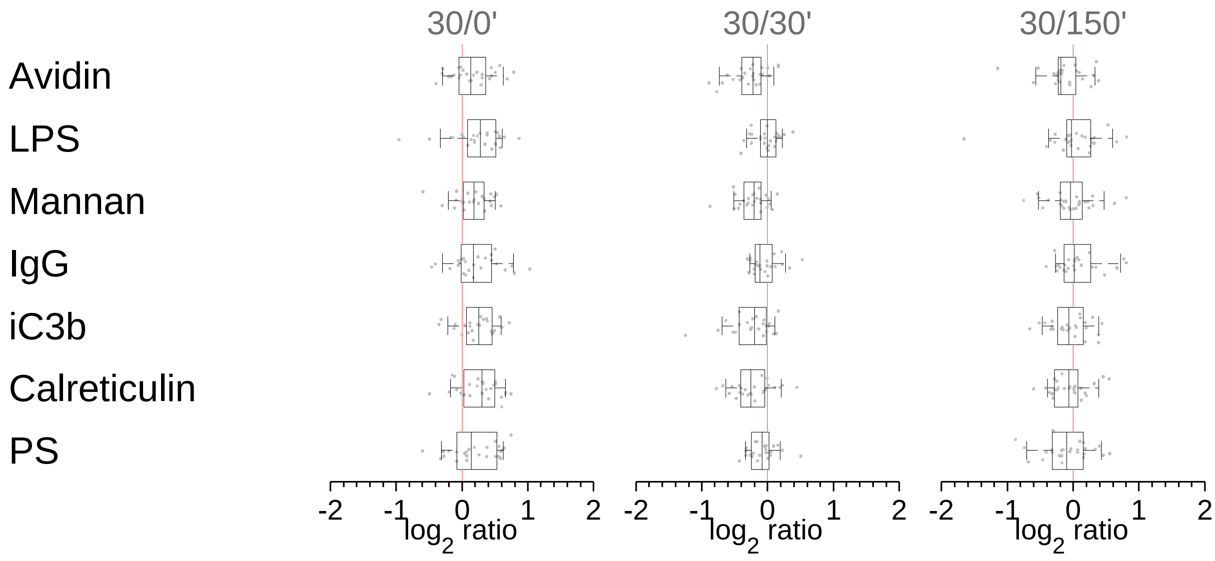

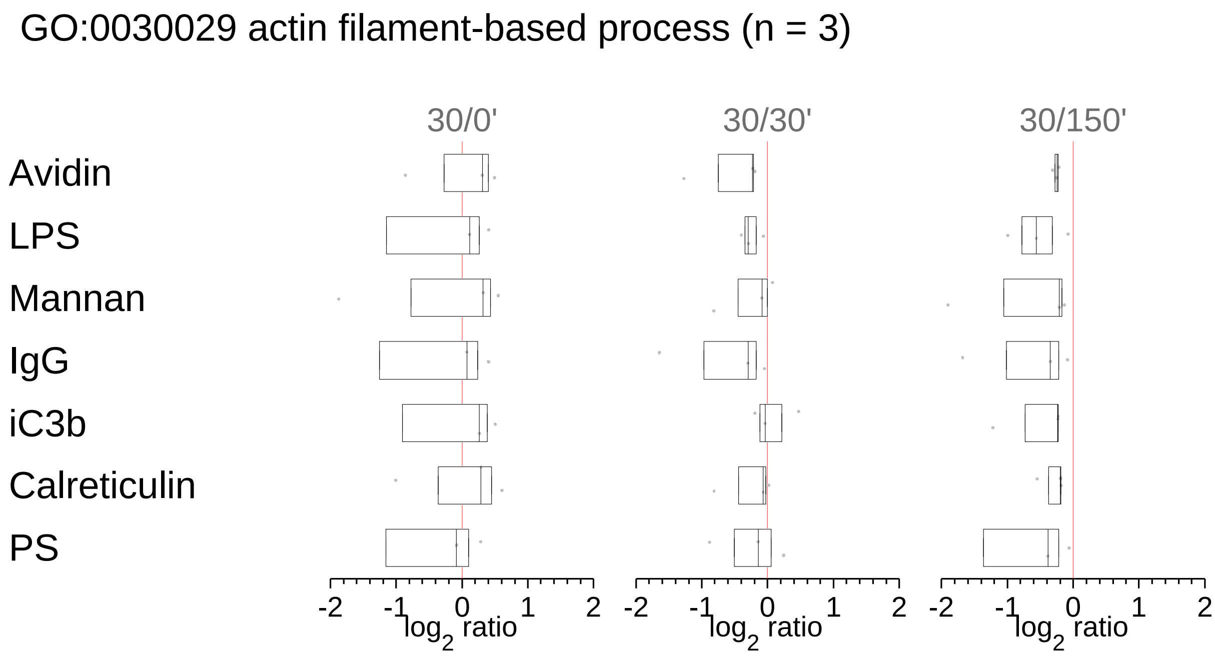
GO:0030029 actin filament-based process (n = 3)
30/0'
30/30'
30/150'
Avidin
LPS
Mannan
IgG
iC3b
Calreticulin
PS
-2
-1
0
1
2
log2 ratio

GO:0030030 cell projection organization (n = 12)

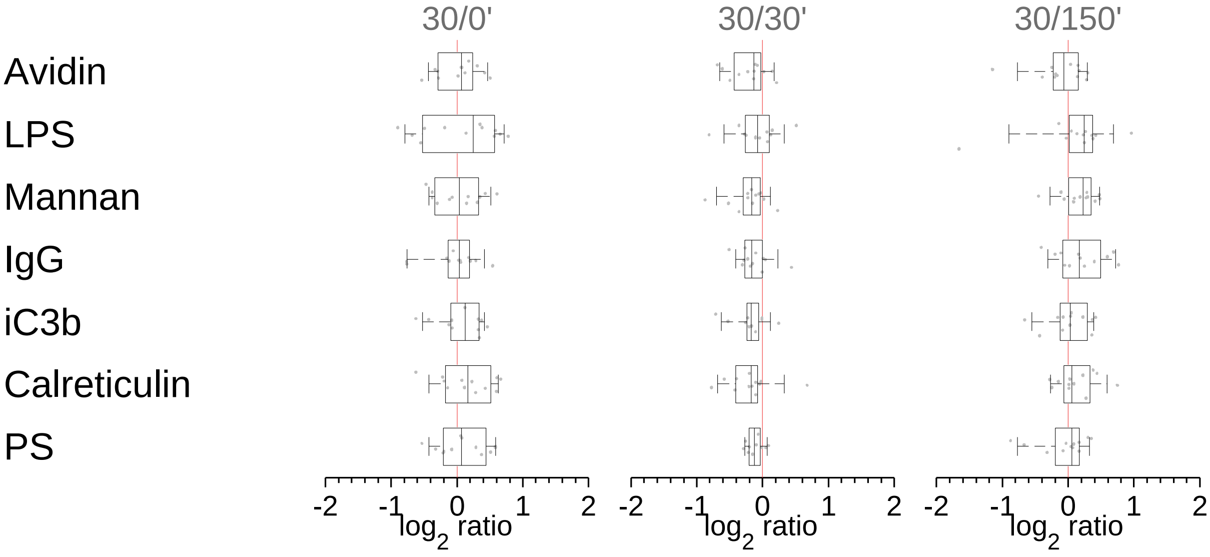

GO:0030031 cell projection assembly (n = 3)

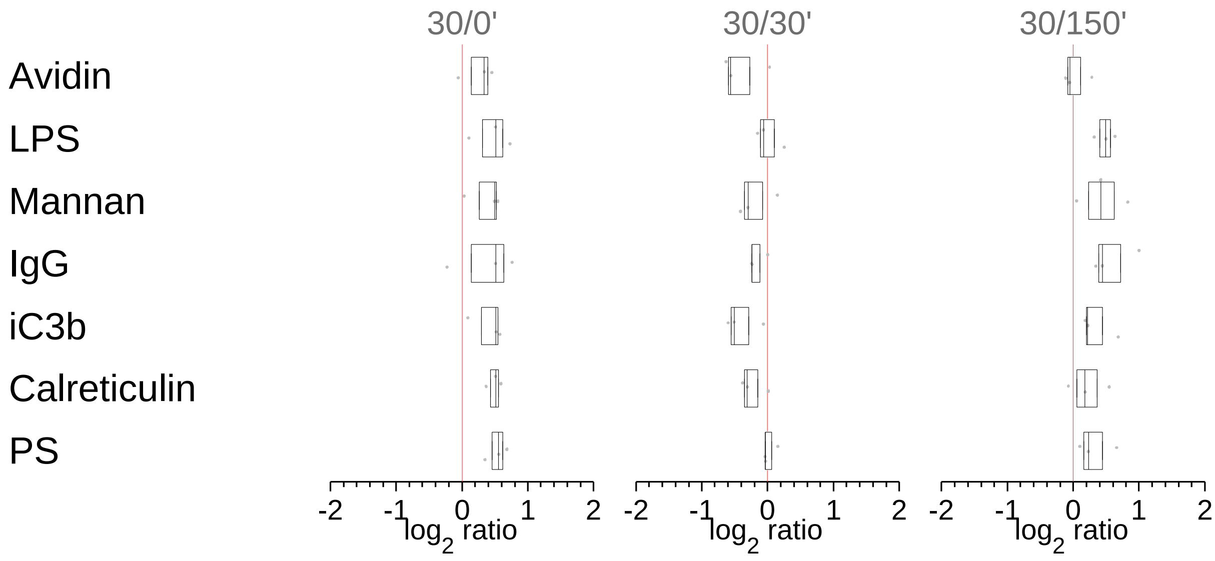

GO:0030032 lamellipodium assembly (n = 6)

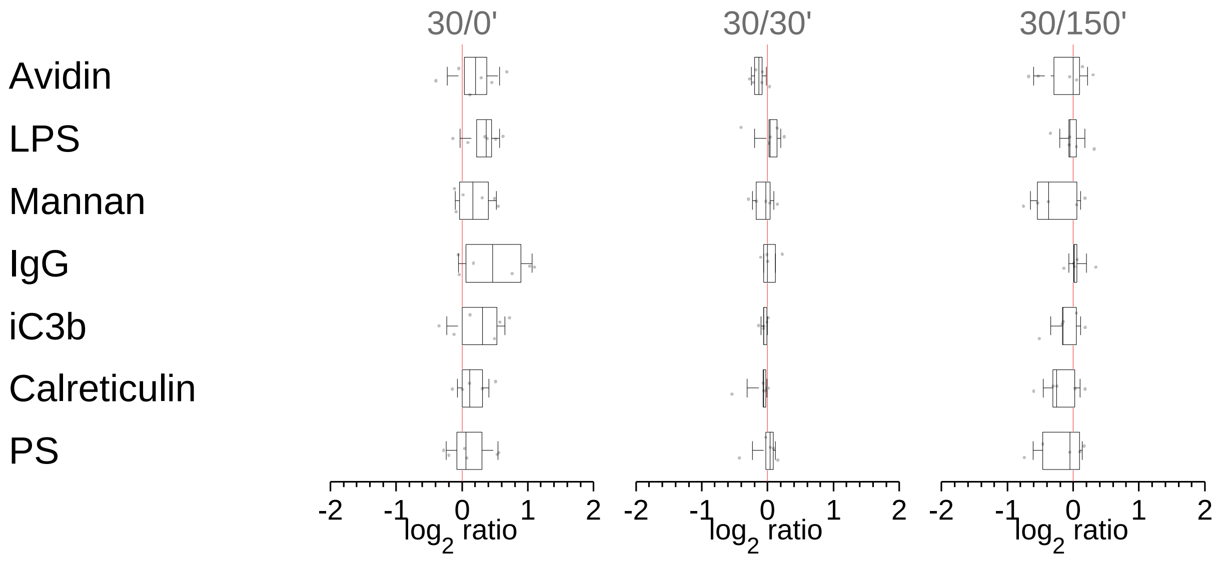

GO:0030036 actin cytoskeleton organization (n = 35)

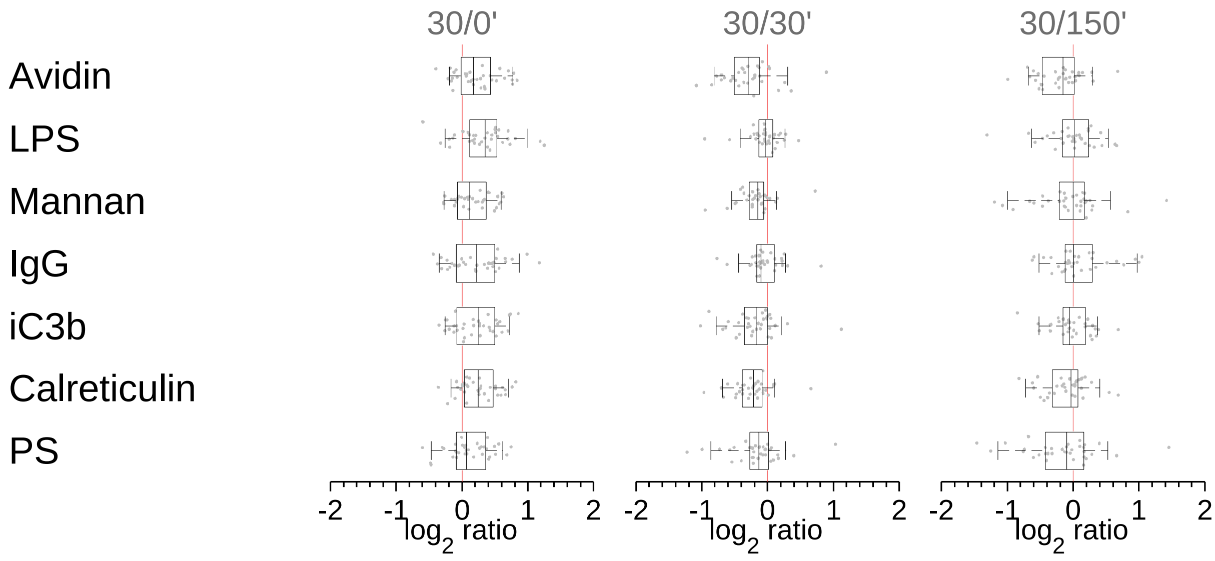

GO:0030041 actin filament polymerization (n = 10)

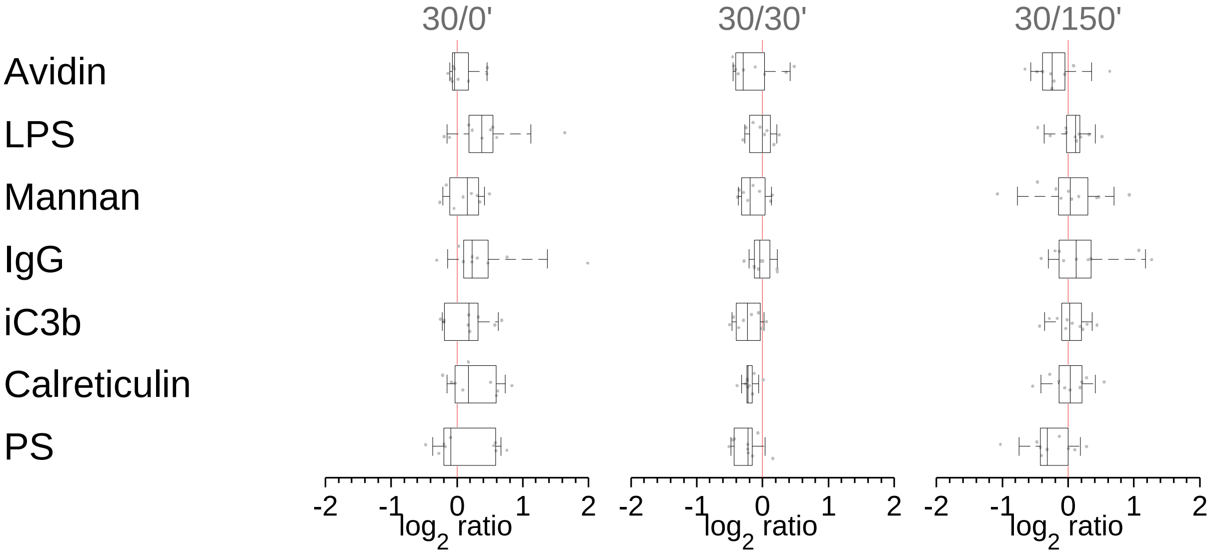

GO:0030048 actin filament-based movement (n = 8)

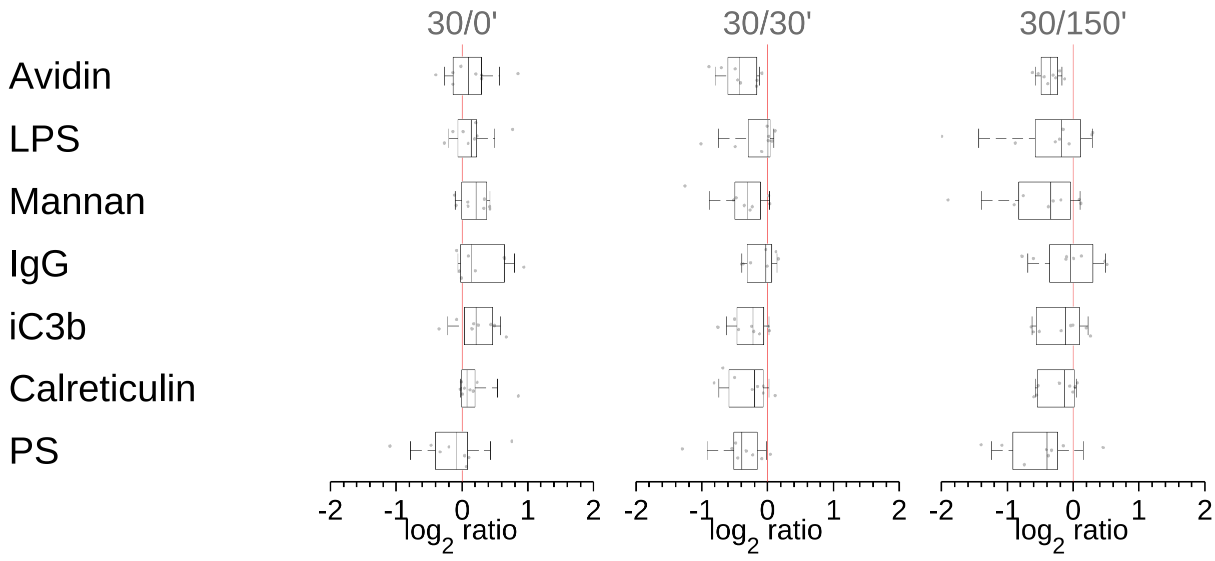

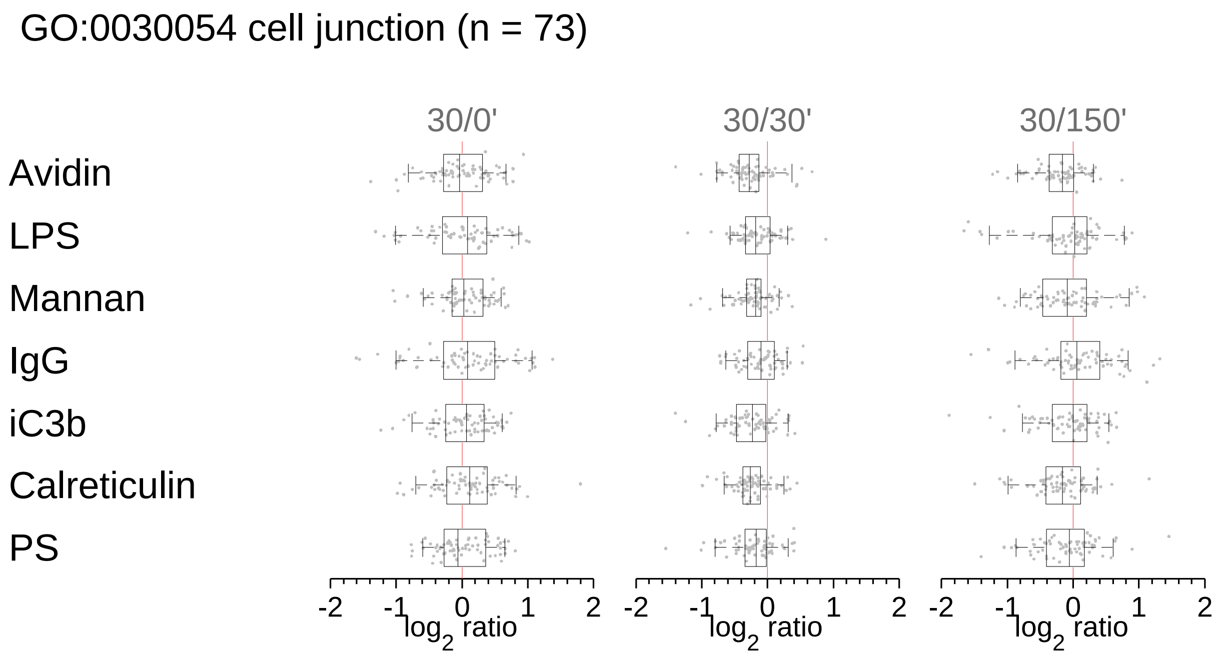

GO:0030054 cell junction (n = 73)
30/0'
30/30'
30/150'
Avidin
LPS
Mannan
IgG
iC3b
Calreticulin
PS
-2
-1
0
1
2
log2 ratio

GO:0030056 hemidesmosome (n = 6)

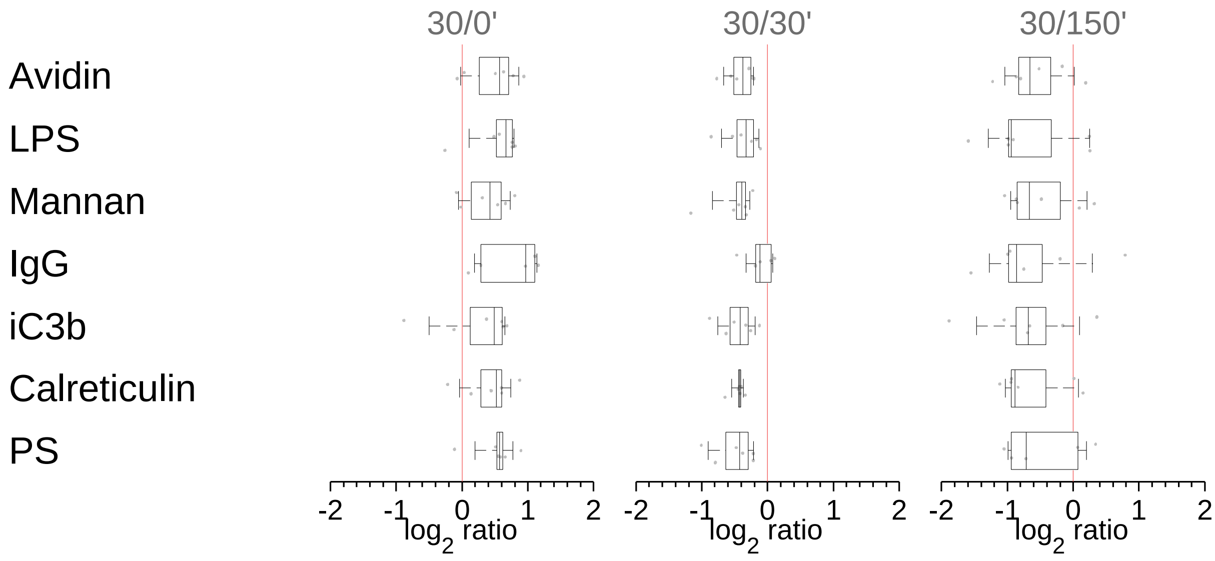

GO:0030057 desmosome (n = 3)

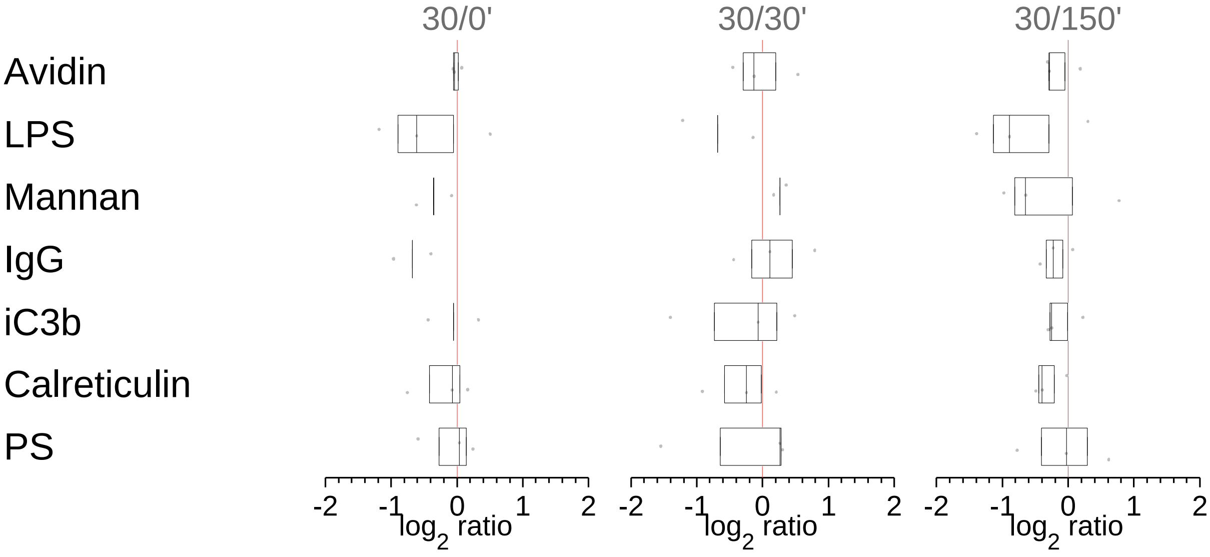

GO:0030061 mitochondrial crista (n = 3)

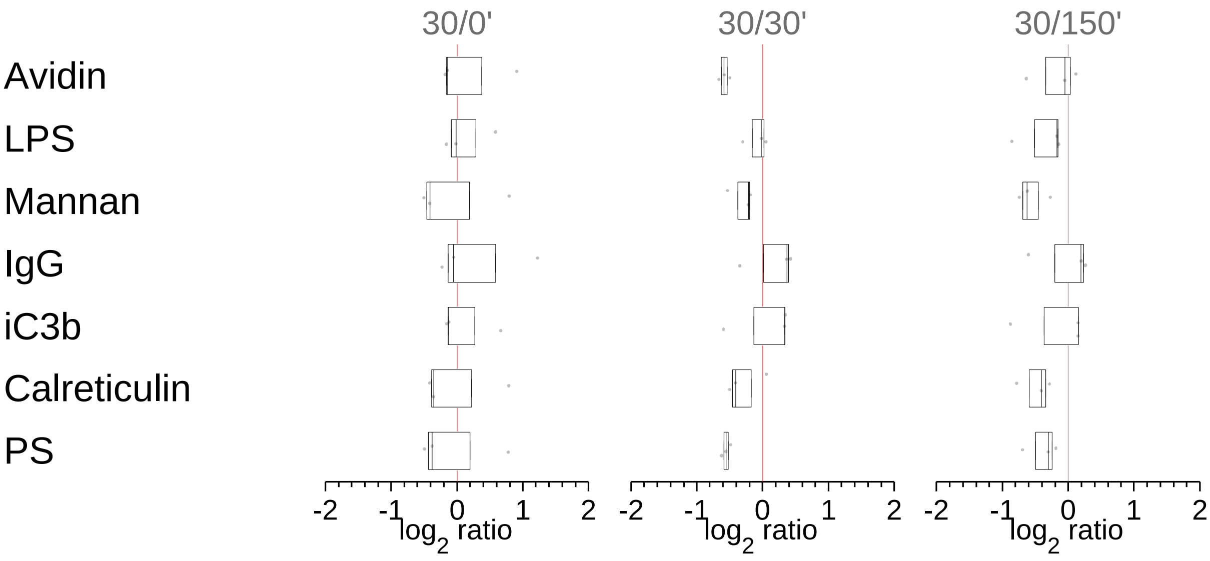

GO:0030073 insulin secretion (n = 3)

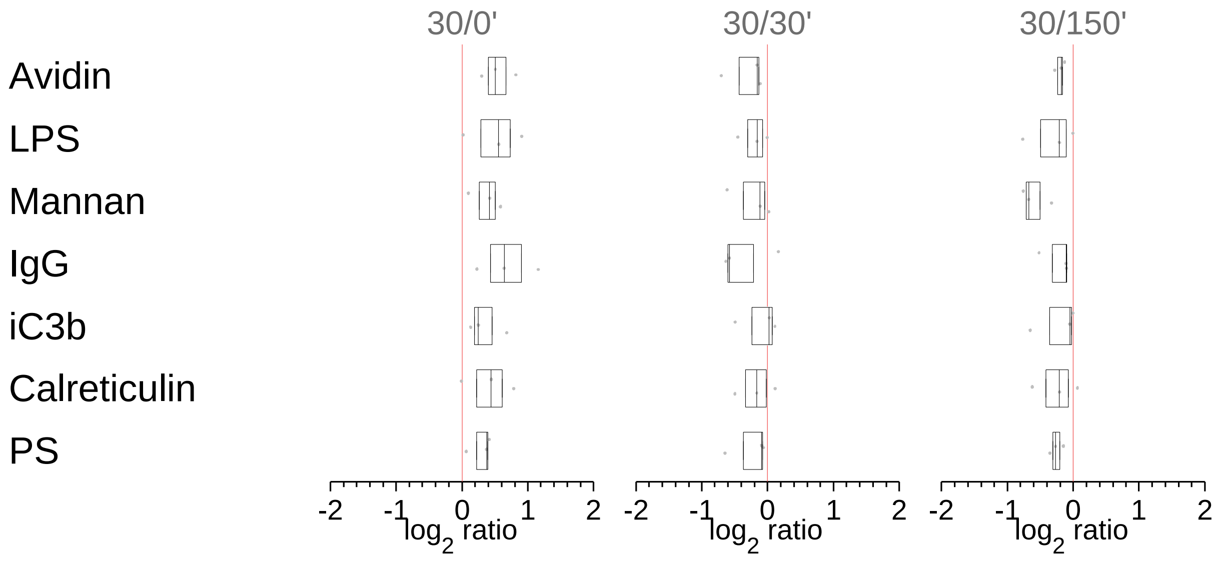

GO:0030097 hemopoiesis (n = 8)

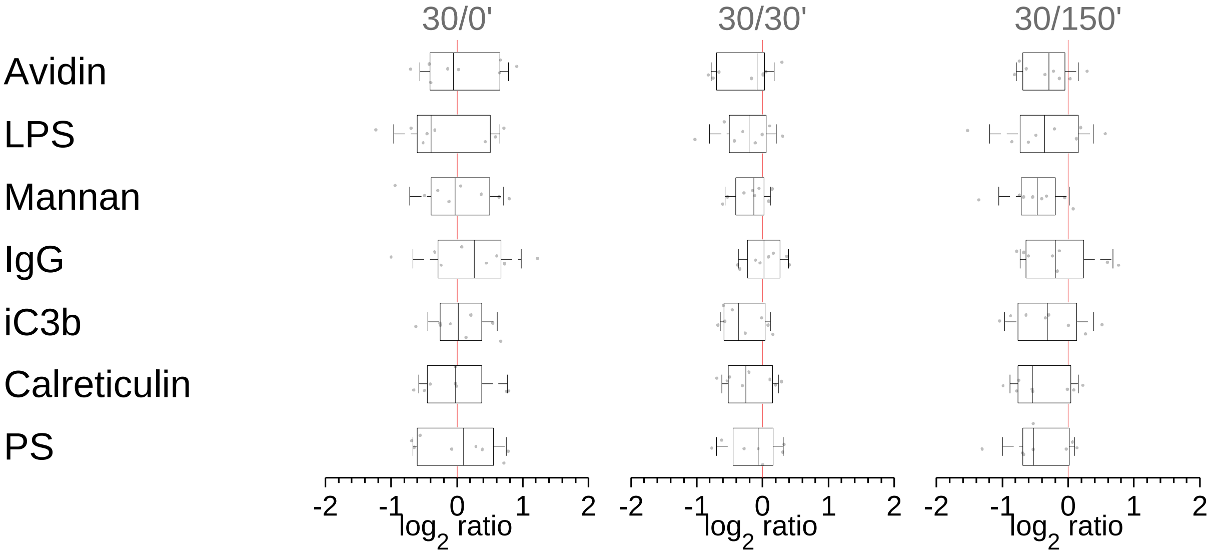

GO:0030100 regulation of endocytosis (n = 8)

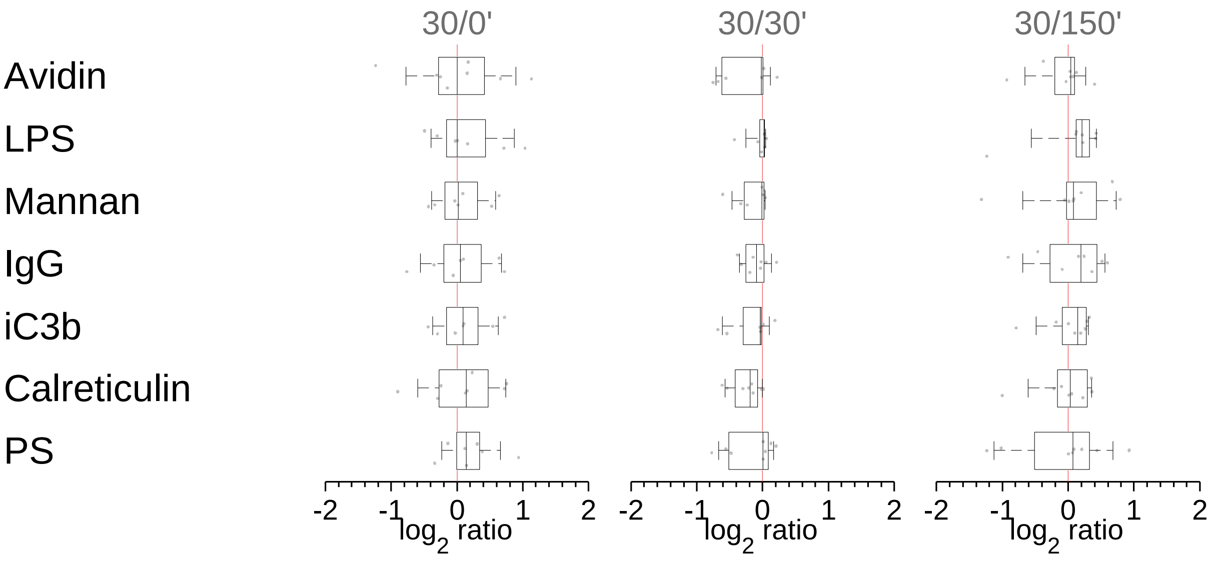

GO:0030117 membrane coat (n = 15)

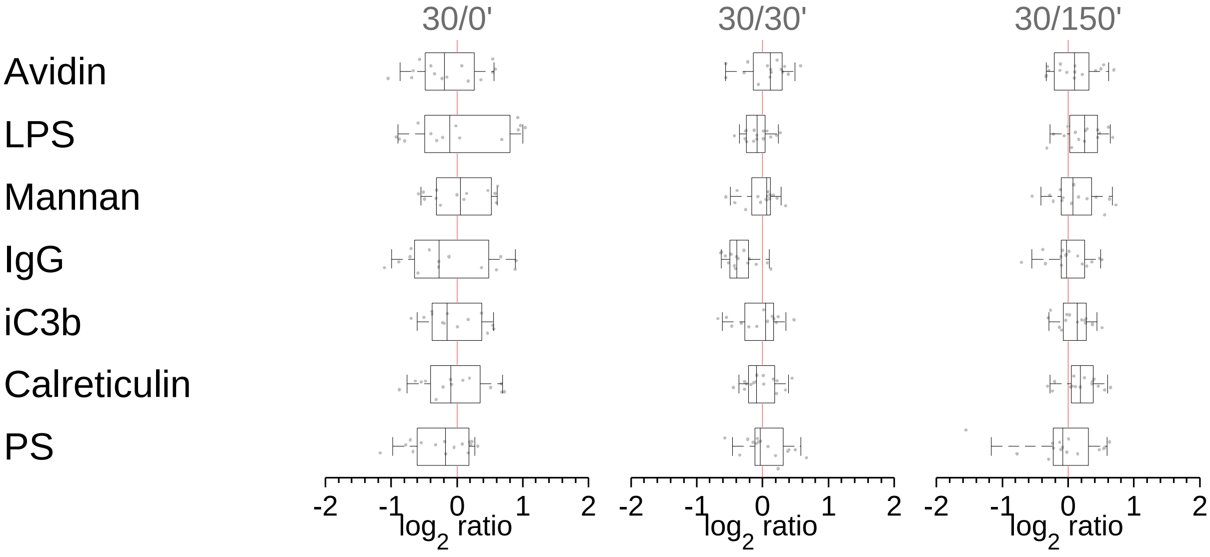

GO:0030118 clathrin coat (n = 4)

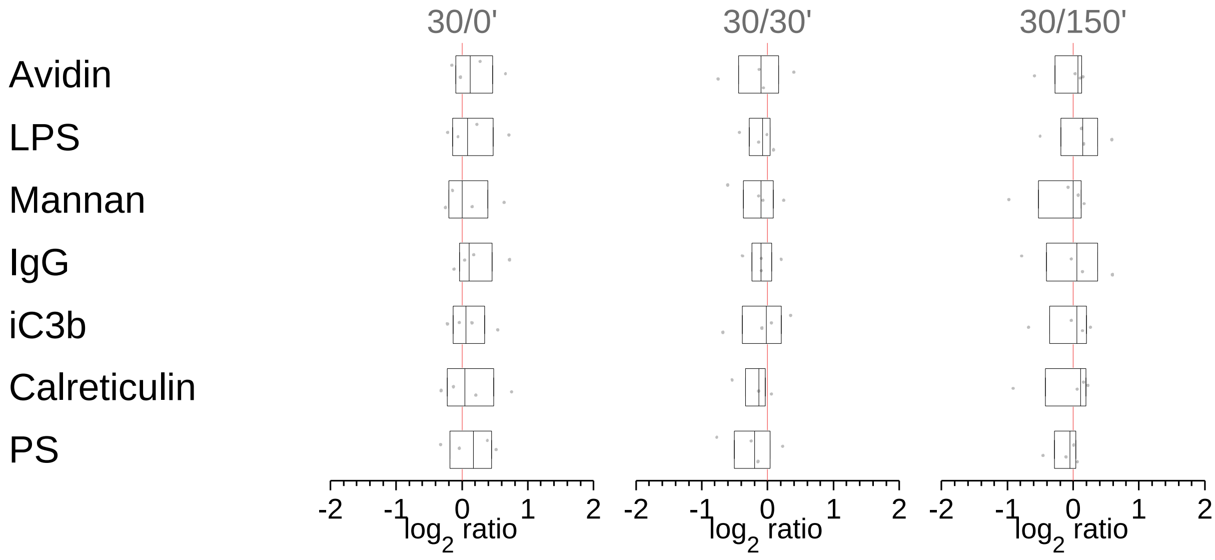

GO:0030123 AP-3 adaptor complex (n = 3)

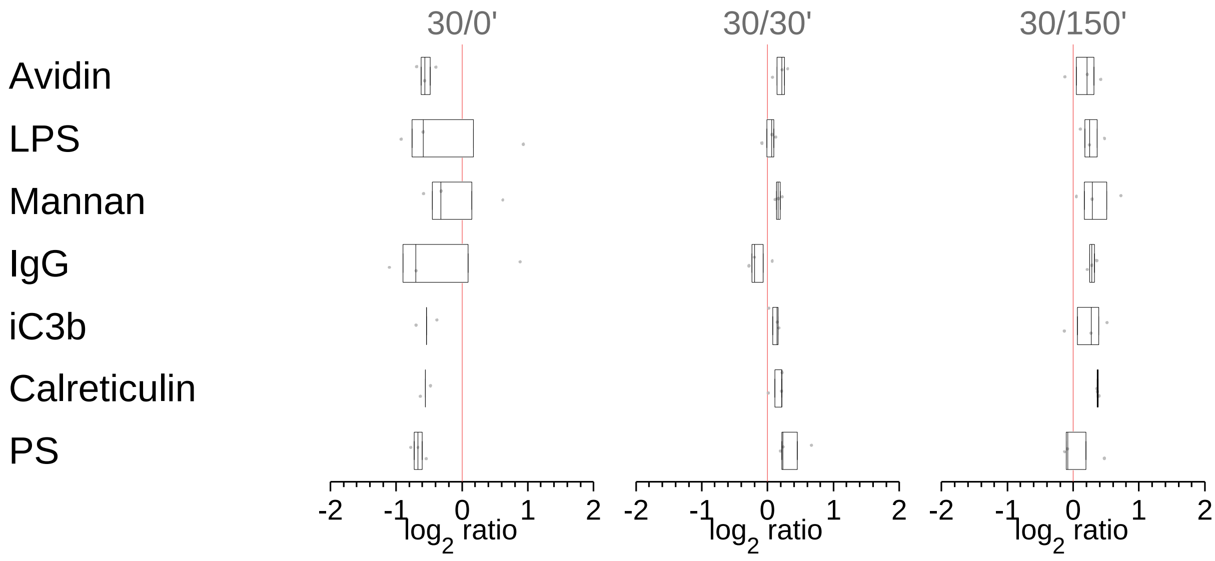

GO:0030130 clathrin coat of trans-Golgi network vesicle (n = 3)

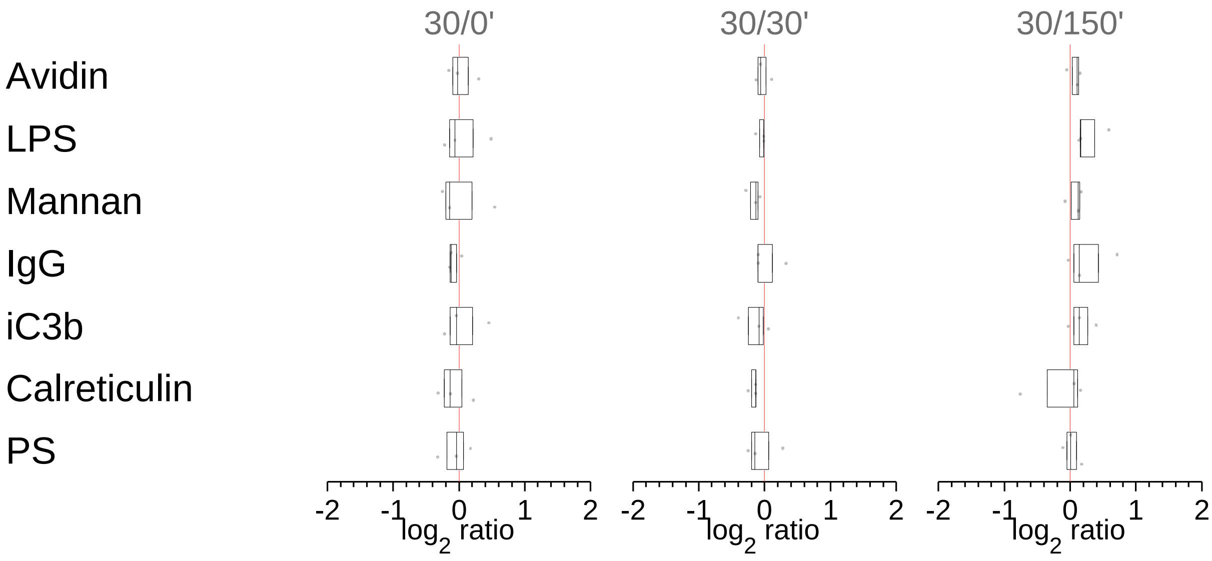

GO:0030131 clathrin adaptor complex (n = 9)

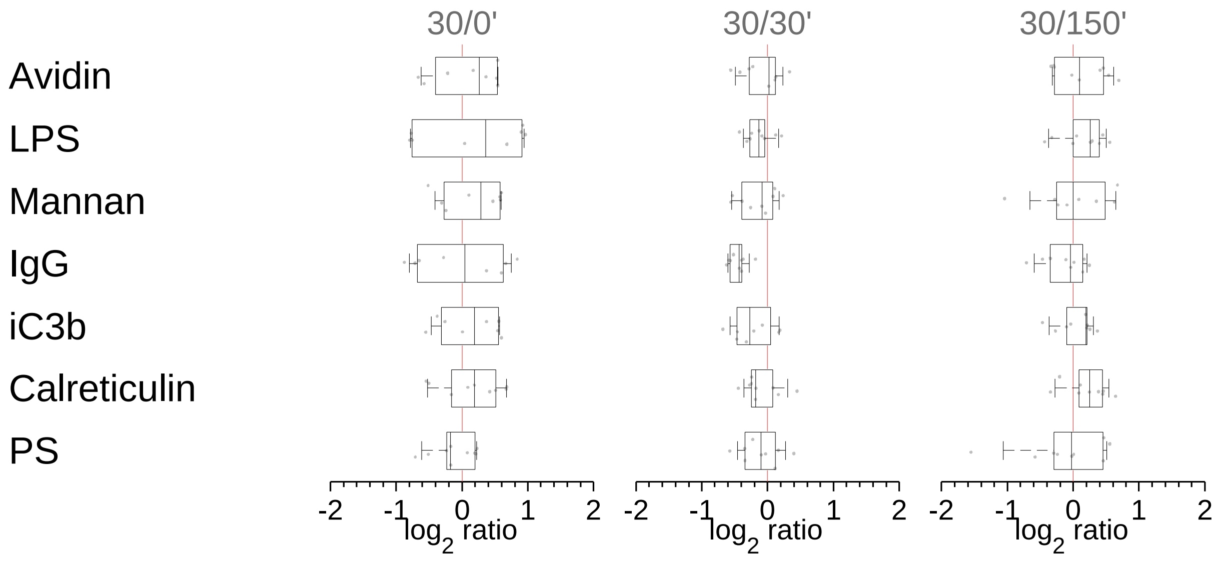

GO:0030132 clathrin coat of coated pit (n = 5)

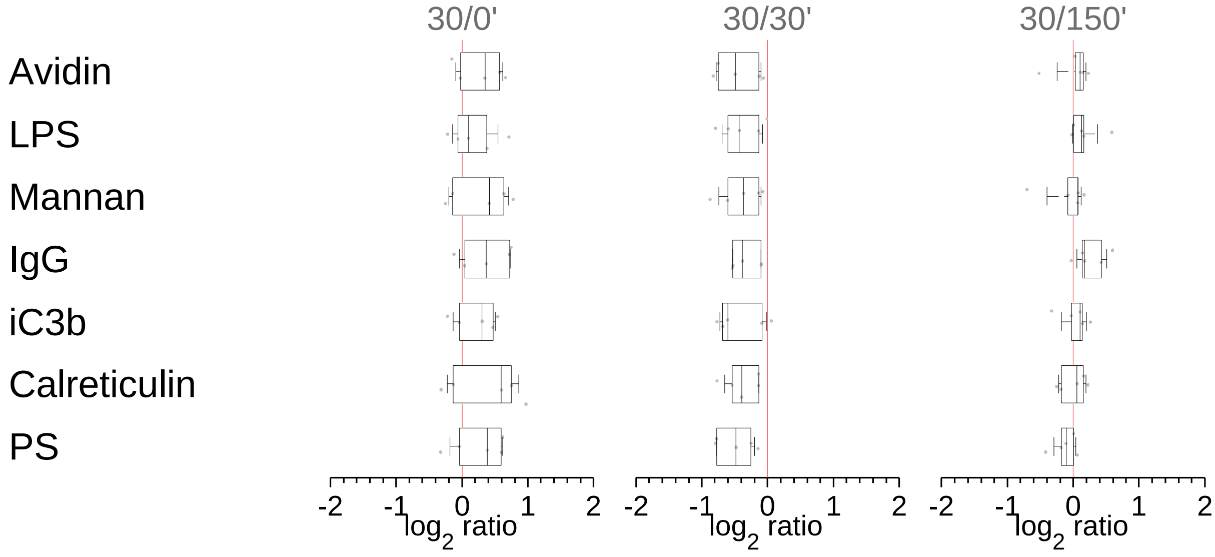

GO:0030133 transport vesicle (n = 10)

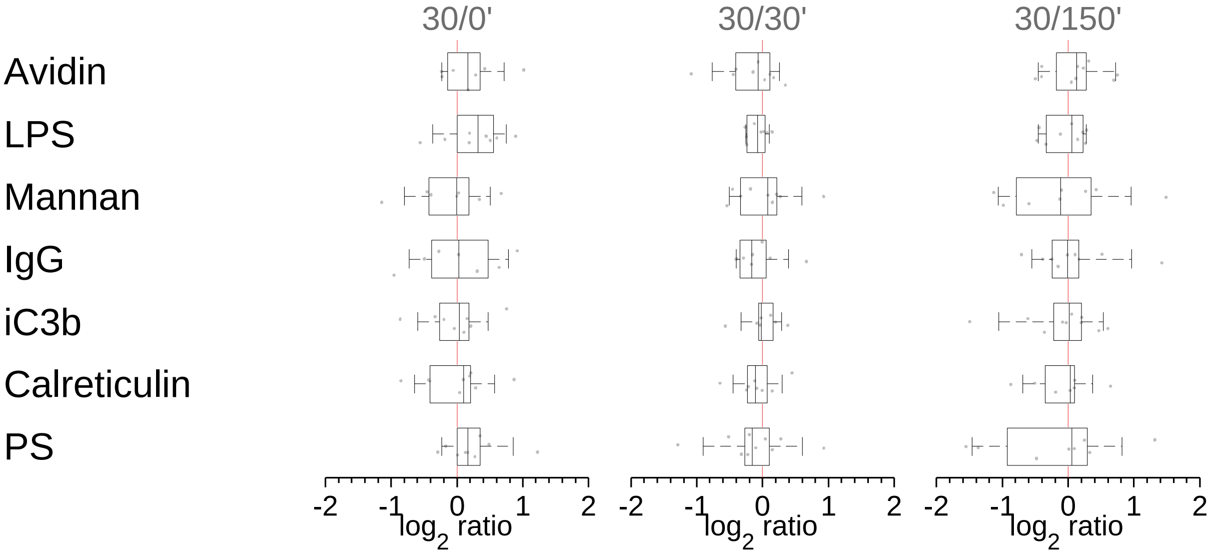

GO:0030134 ER to Golgi transport vesicle (n = 4)

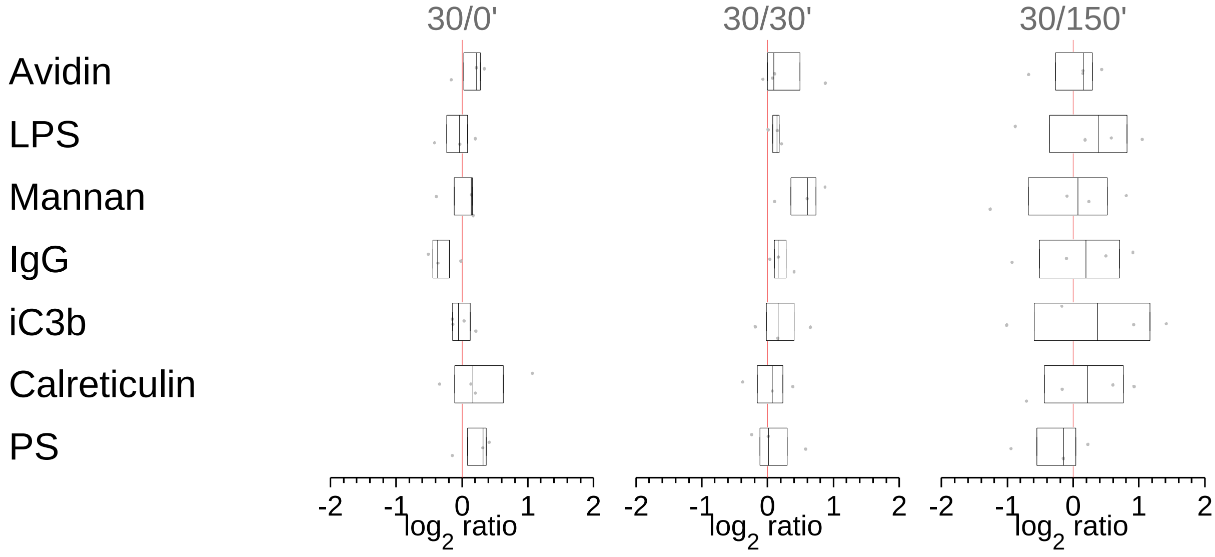

GO:0030136 clathrin-coated vesicle (n = 12)

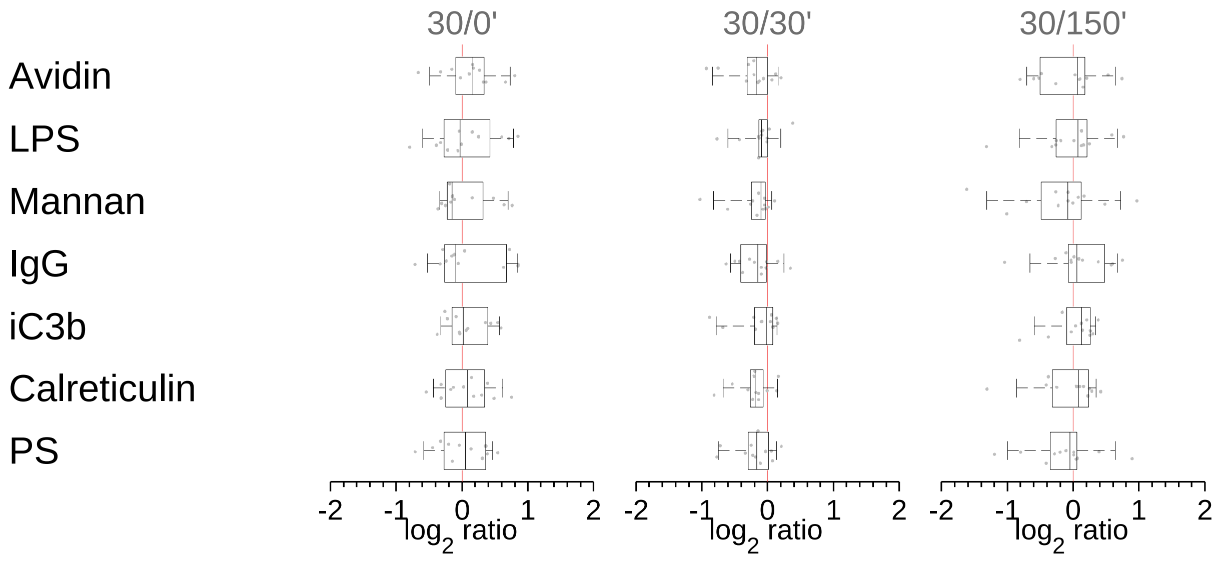

GO:0030139 endocytic vesicle (n = 13)

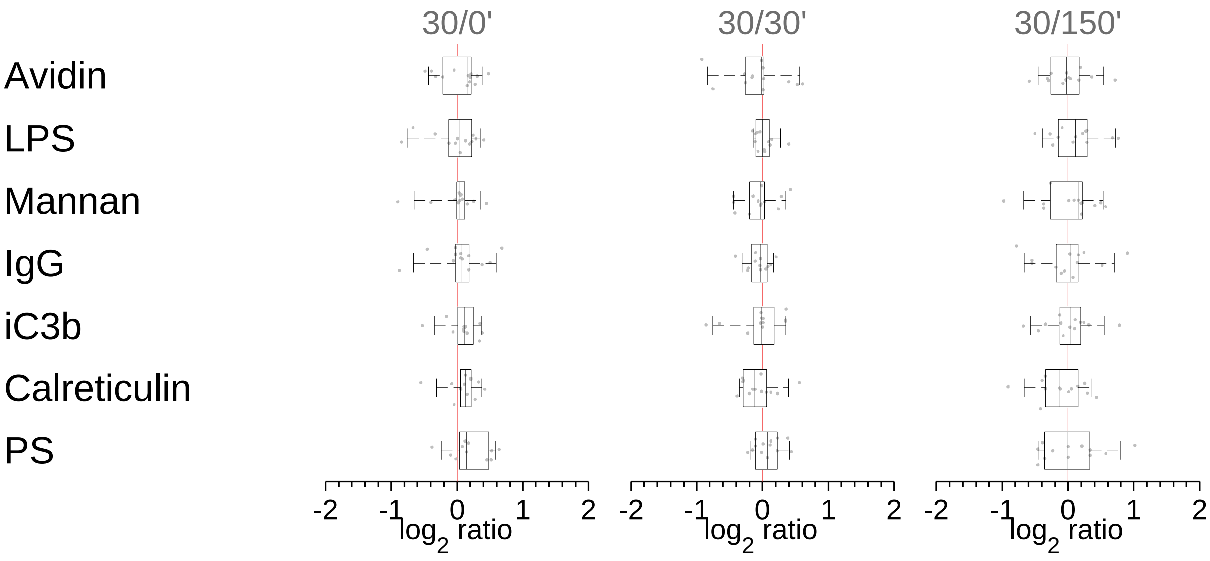

GO:0030140 trans-Golgi network transport vesicle (n = 6)

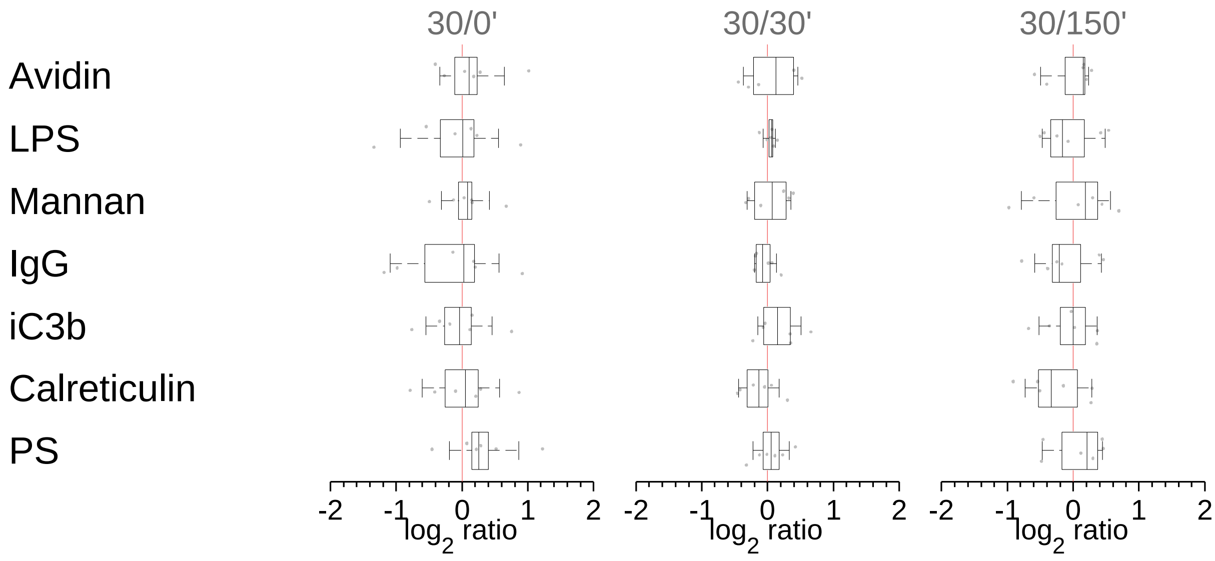

GO:0030141 secretory granule (n = 25)

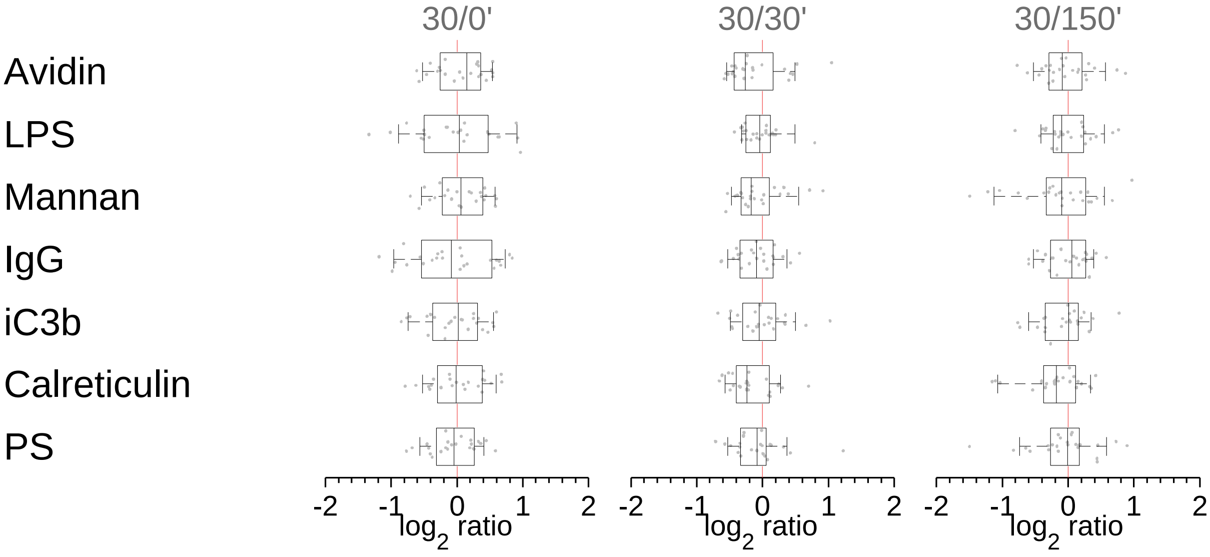

GO:0030145 manganese ion binding (n = 7)

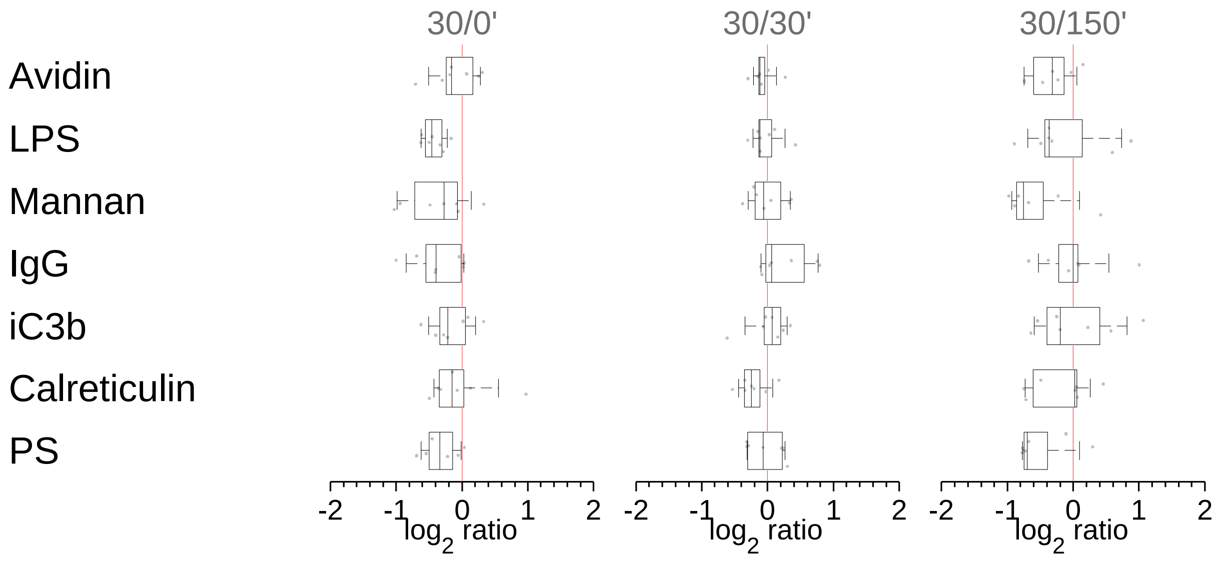

GO:0030148 sphingolipid biosynthetic process (n = 3)

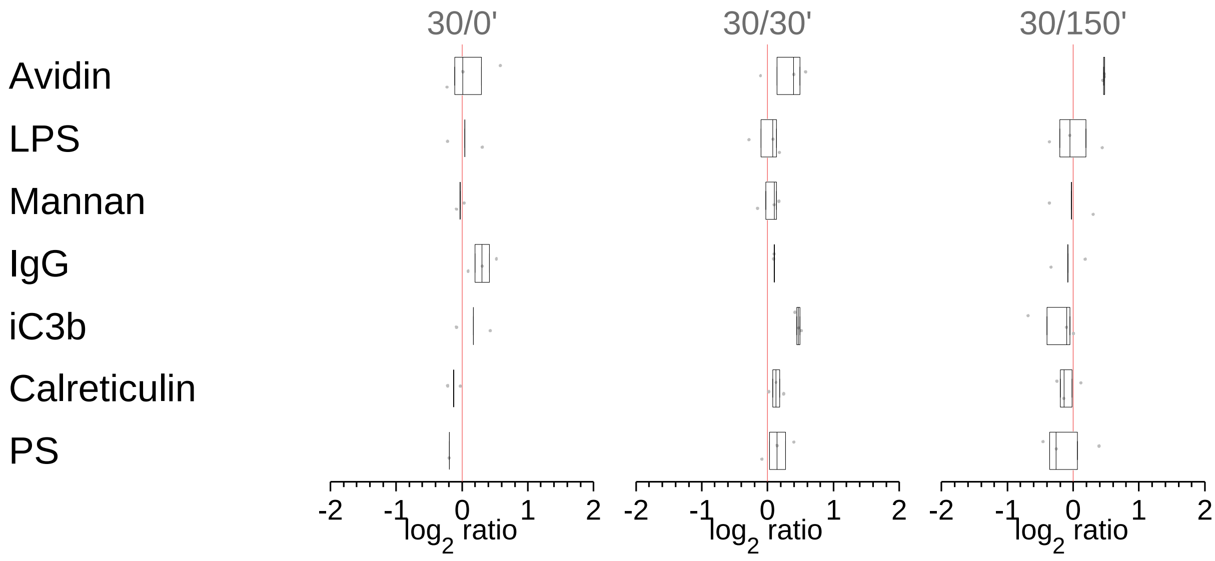

GO:0030154 cell differentiation (n = 51)

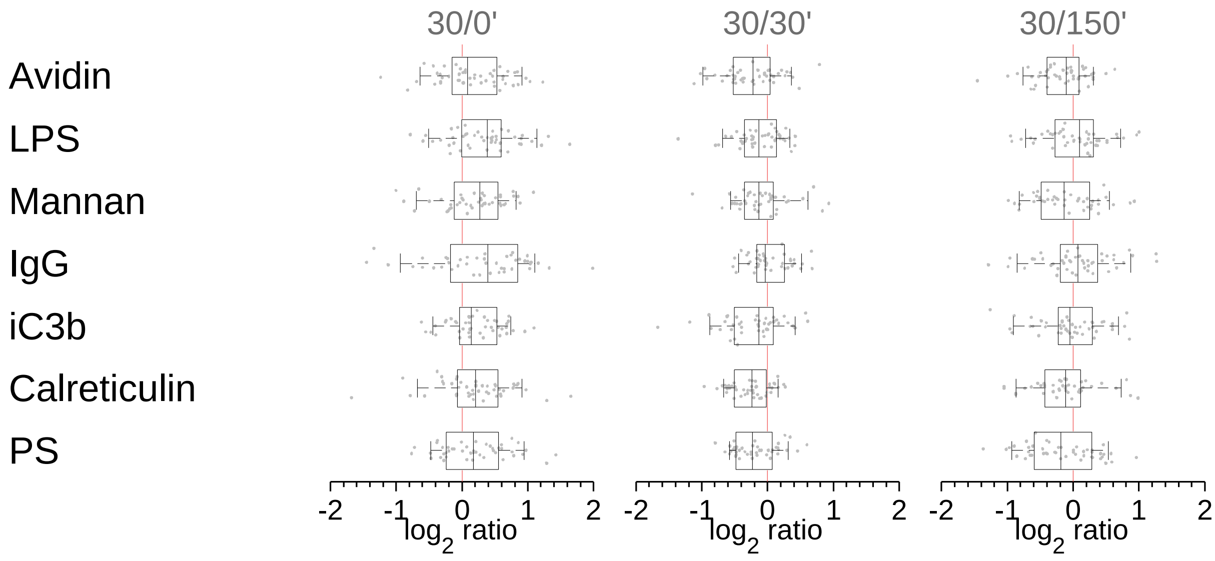

GO:0030155 regulation of cell adhesion (n = 6)

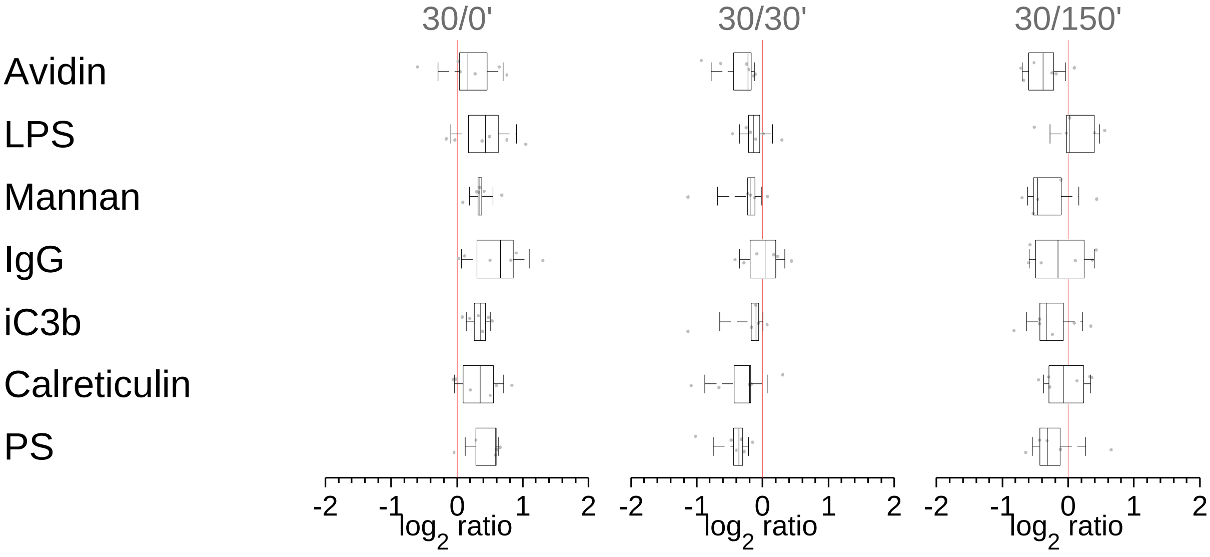

GO:0030162 regulation of proteolysis (n = 3)

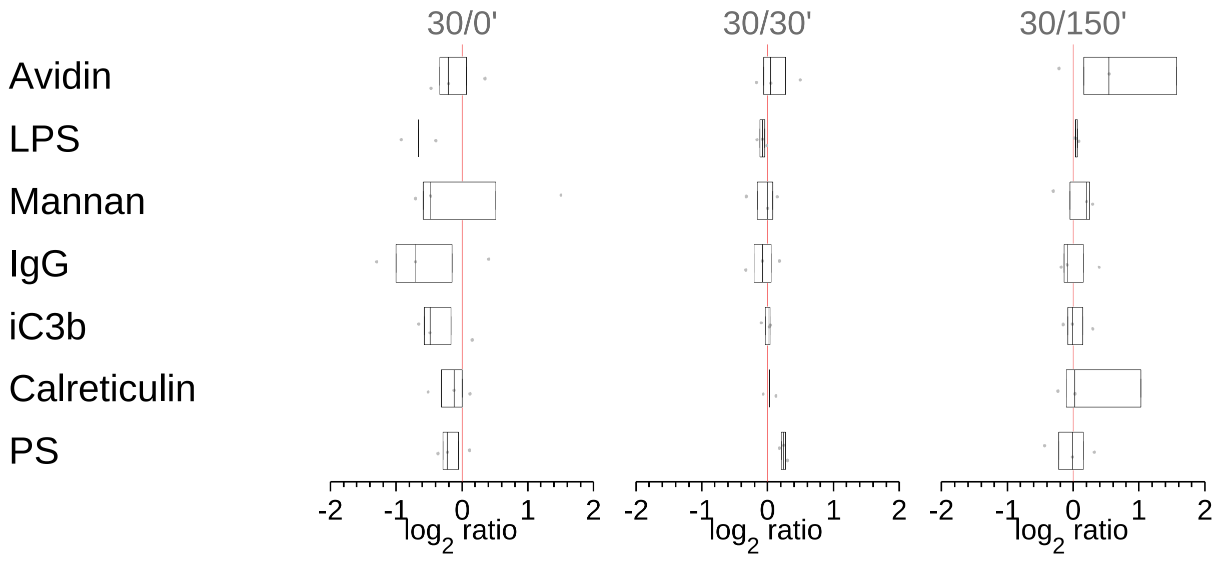

GO:0030163 protein catabolic process (n = 3)

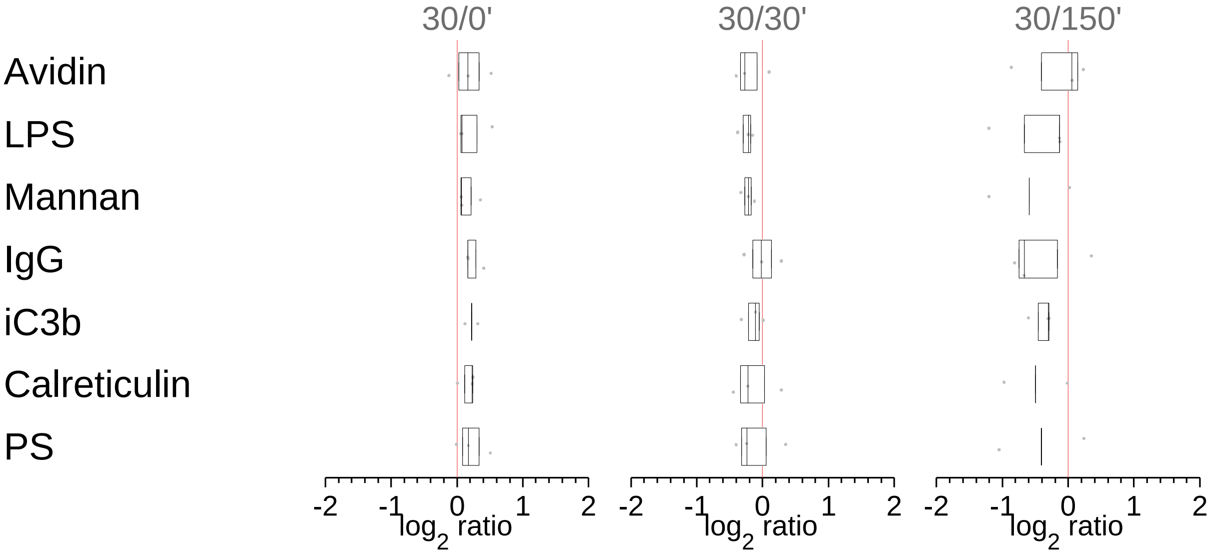

GO:0030165 PDZ domain binding (n = 8)

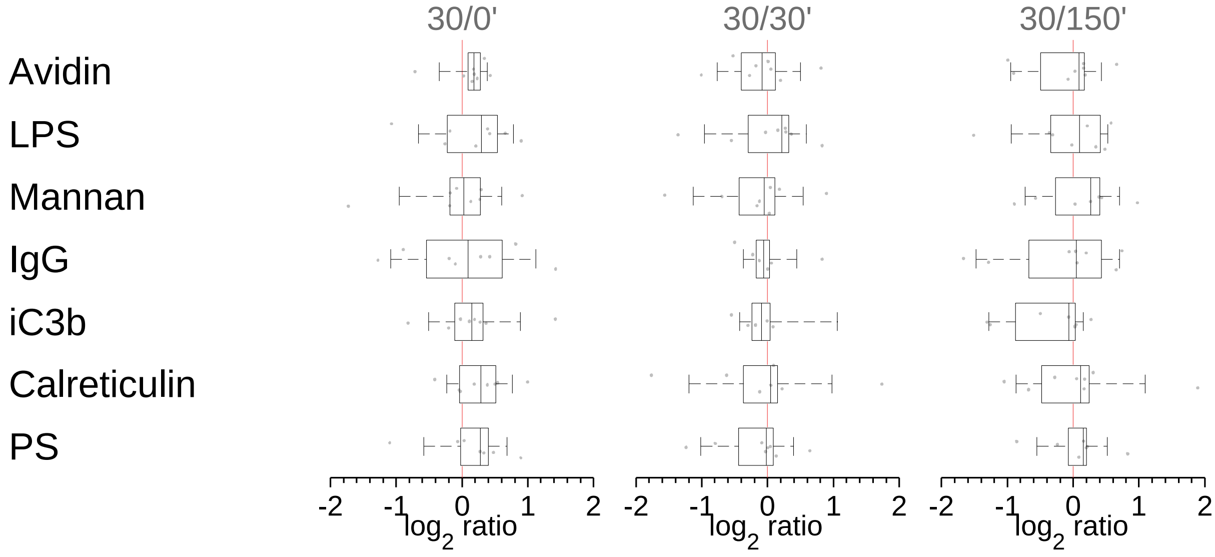

GO:0030168 platelet activation (n = 4)

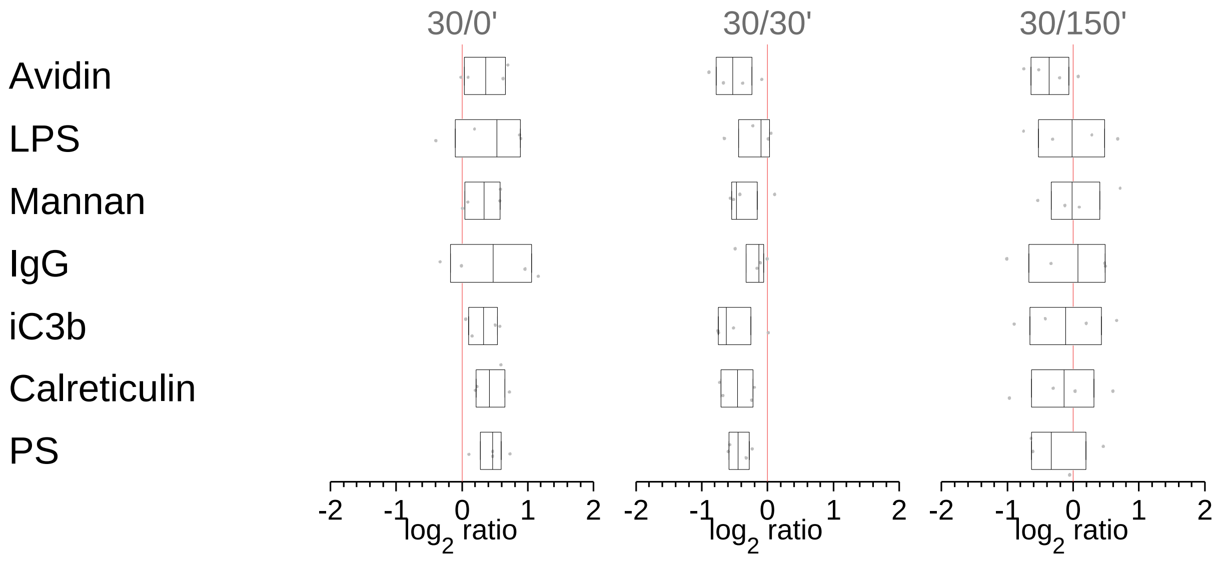

GO:0030169 low-density lipoprotein particle binding (n = 6)

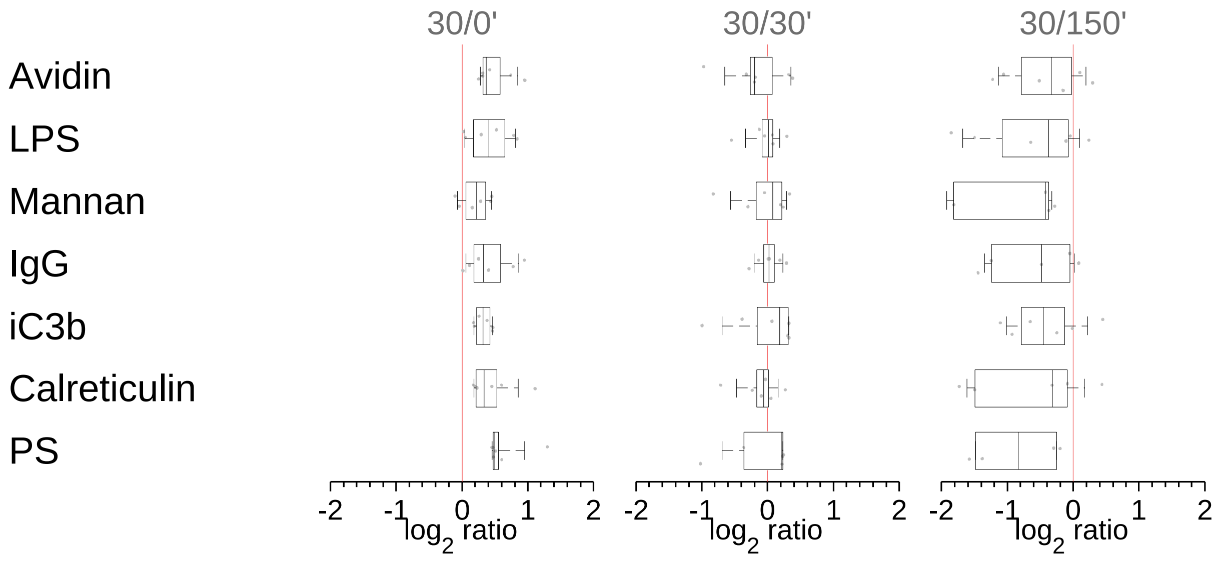

GO:0030170 pyridoxal phosphate binding (n = 6)

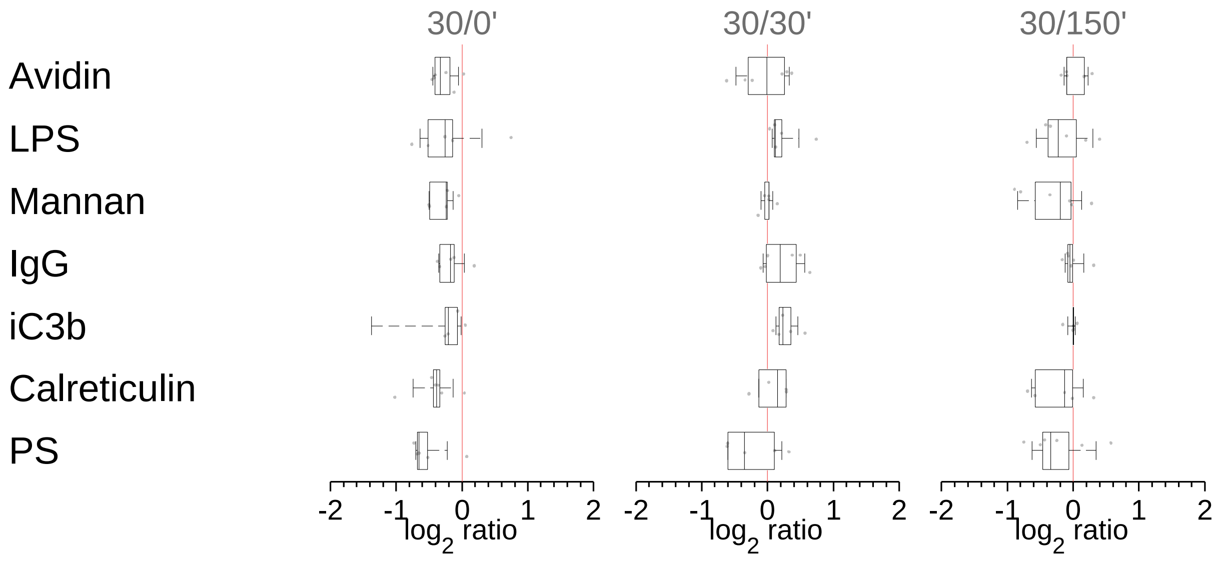

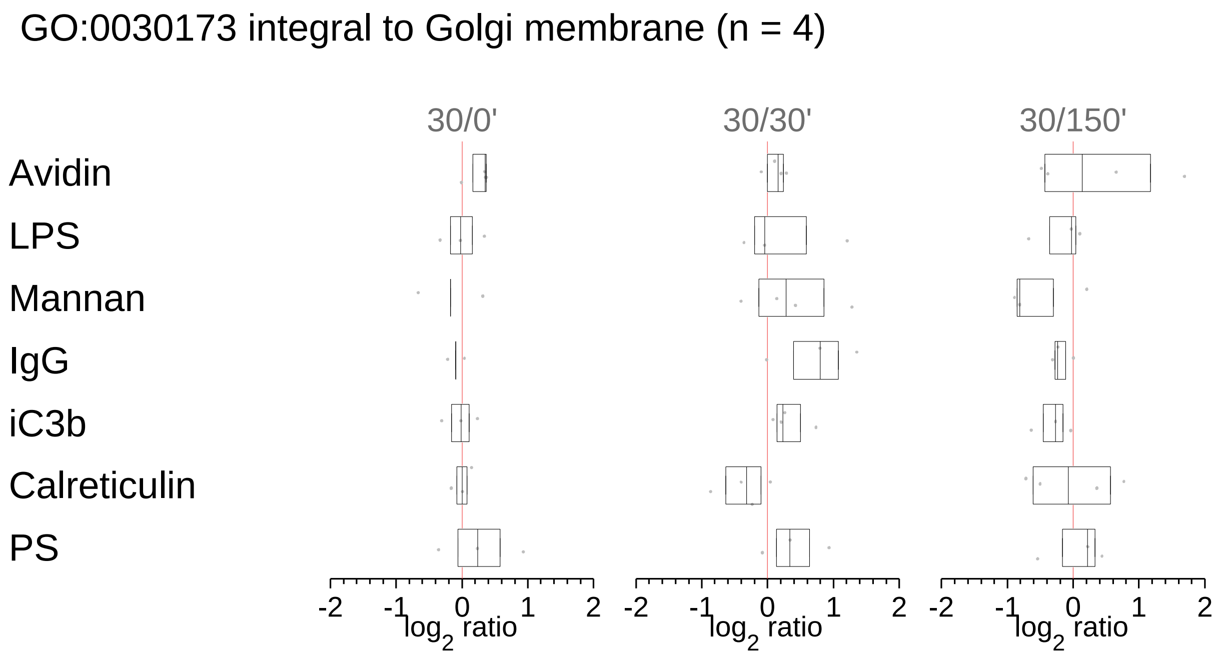
GO:0030173 integral to Golgi membrane (n = 4)
30/0'
30/30'
30/150'
Avidin
LPS
Mannan
IgG
iC3b
Calreticulin
PS
-2 -1 0 1 2
log2 ratio

GO:0030175 filopodium (n = 10)

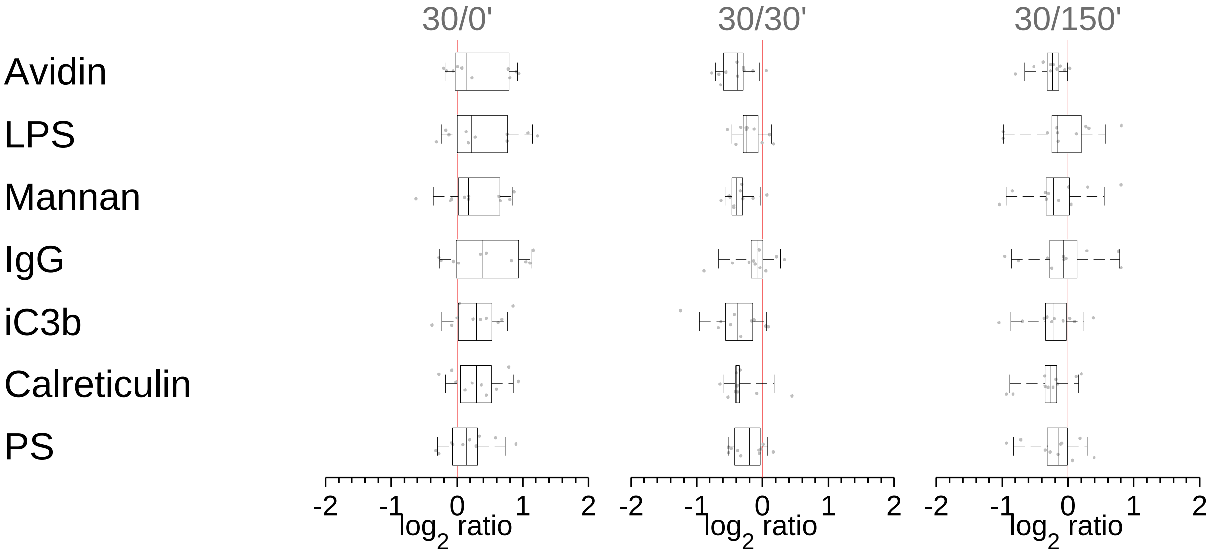

GO:0030176 integral to endoplasmic reticulum membrane (n = 13)

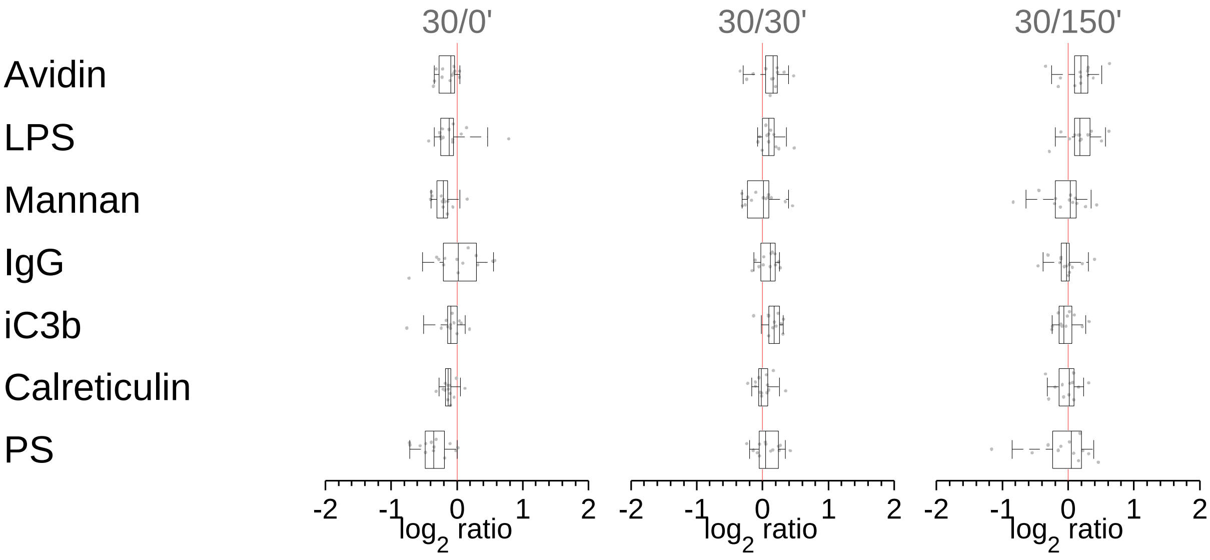

GO:0030177 positive regulation of Wnt receptor signaling pathway
(n = 5)

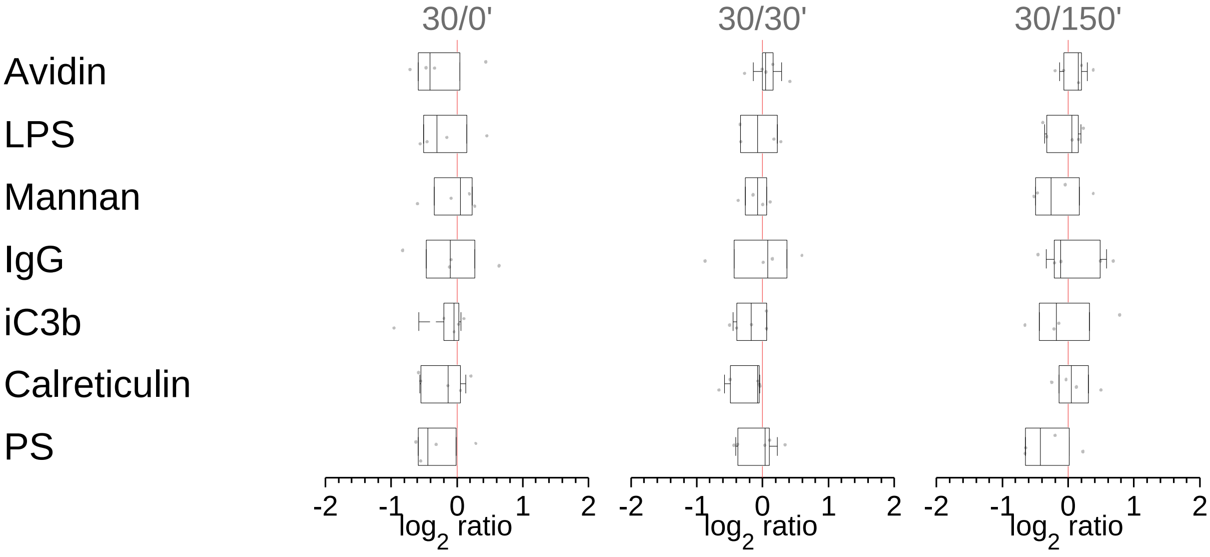

GO:0030182 neuron differentiation (n = 9)

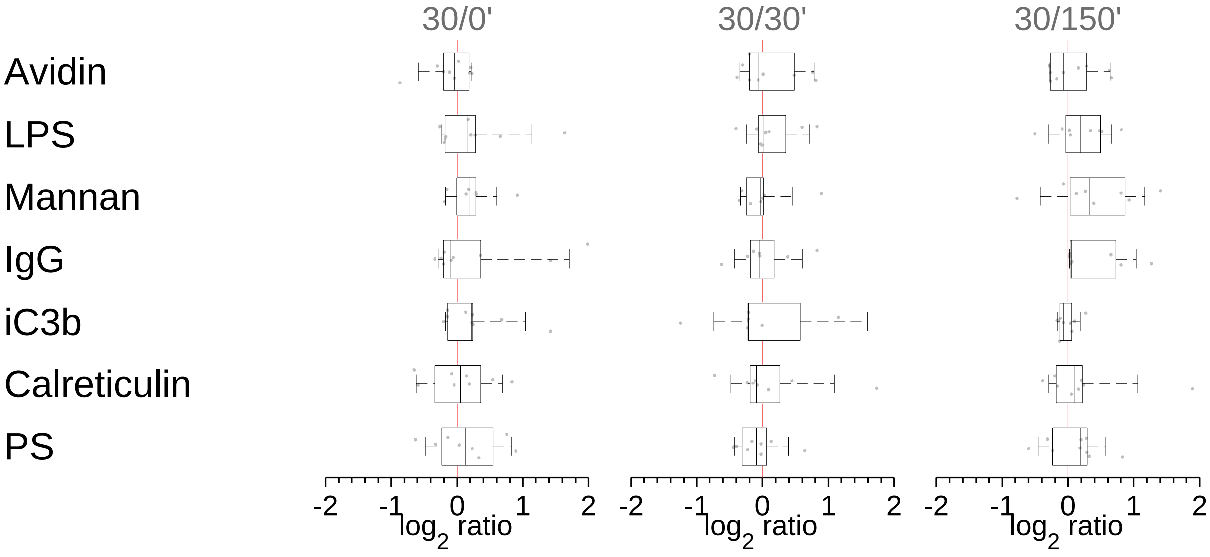

GO:0030183 B cell differentiation (n = 3)

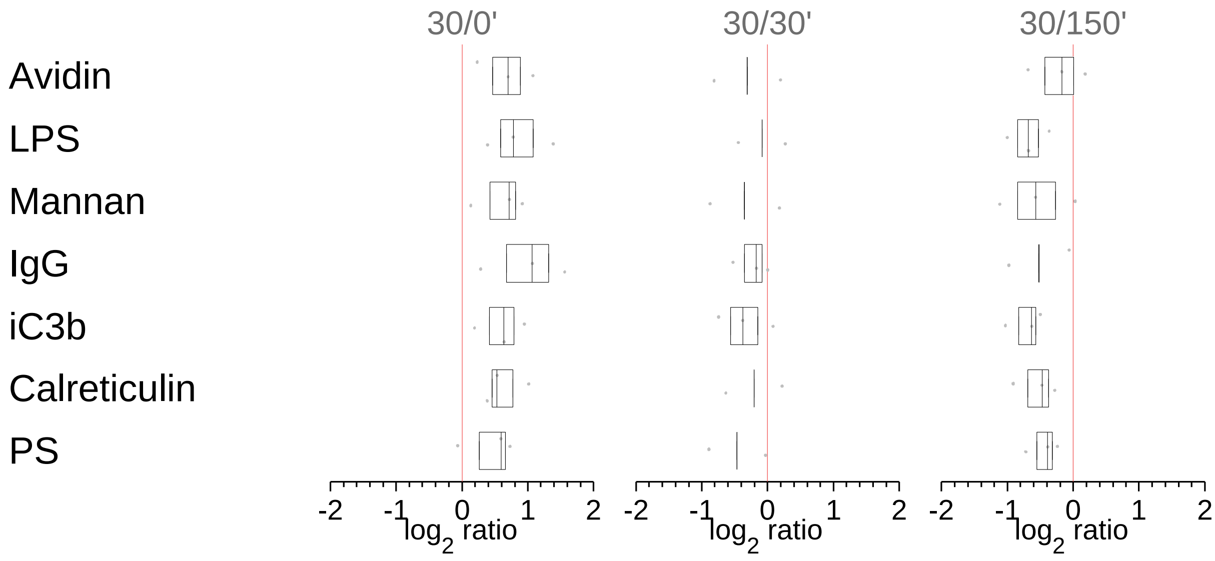

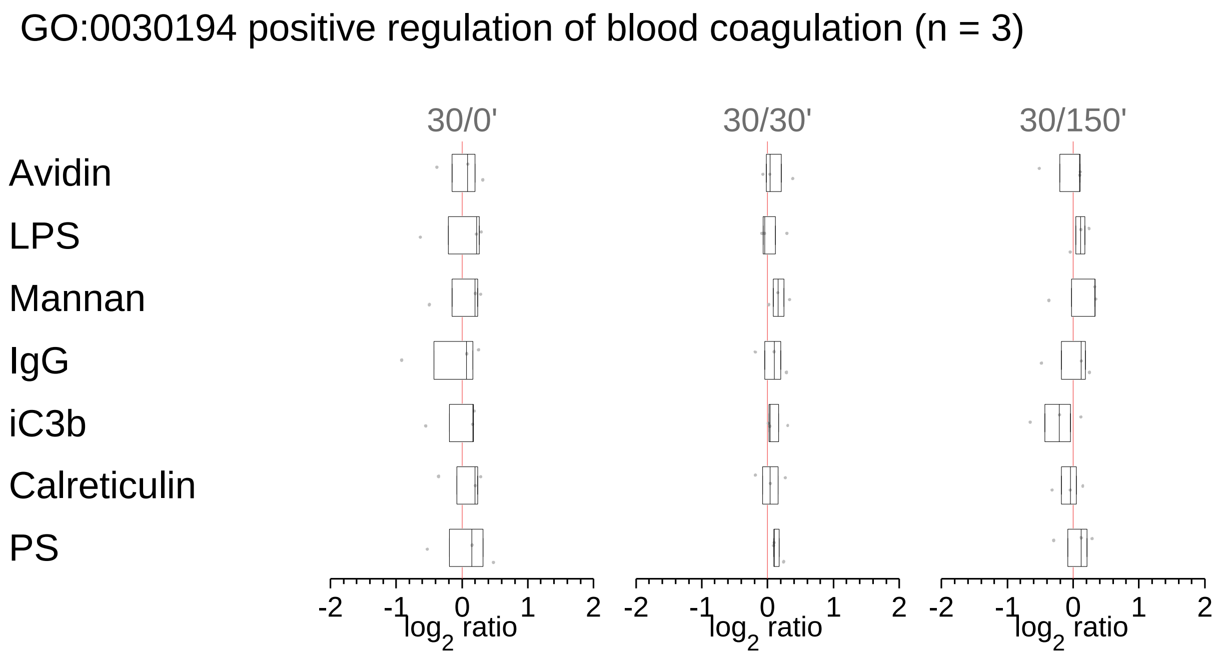
GO:0030194 positive regulation of blood coagulation (n = 3)
30/0'
30/30'
30/150'
Avidin
LPS
Mannan
IgG
iC3b
Calreticulin
PS
-2 -1 0 1 2
log2 ratio

GO:0030198 extracellular matrix organization (n = 10)

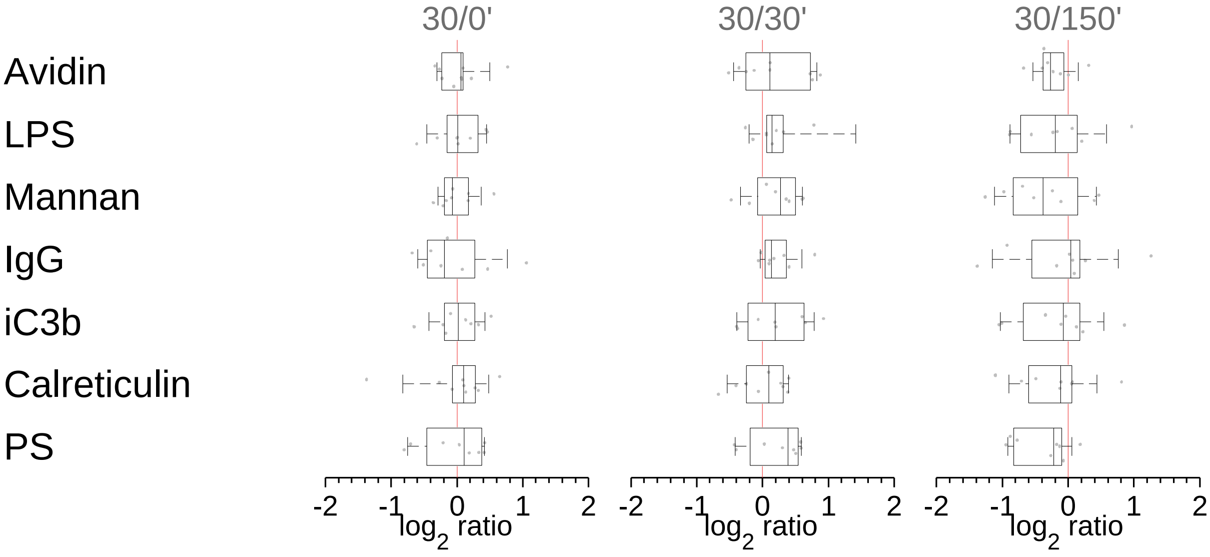

GO:0030199 collagen fibril organization (n = 3)

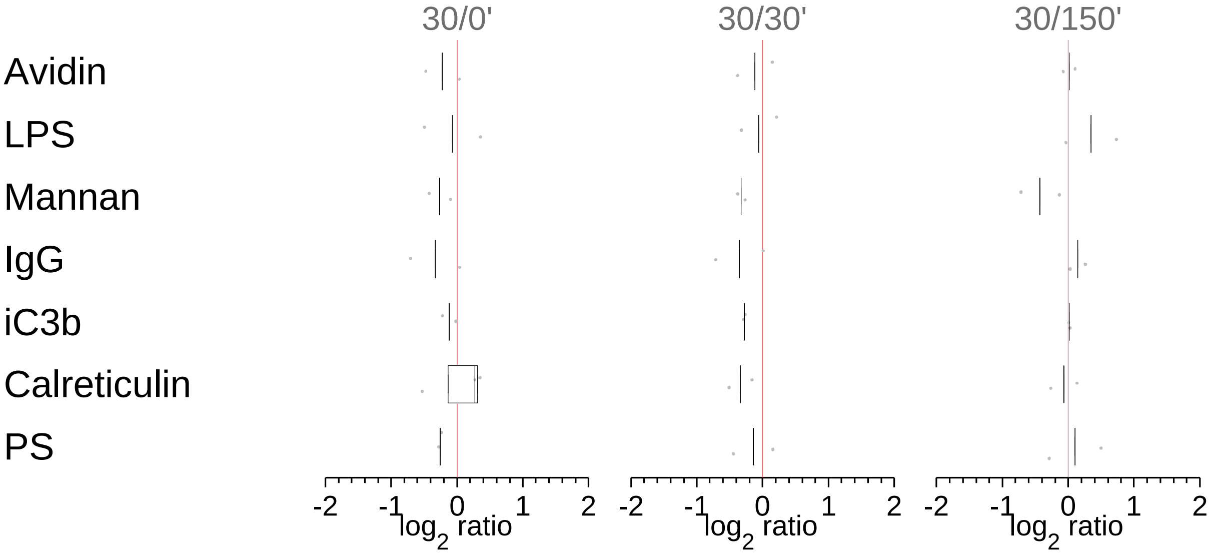

GO:0030203 glycosaminoglycan metabolic process (n = 3)

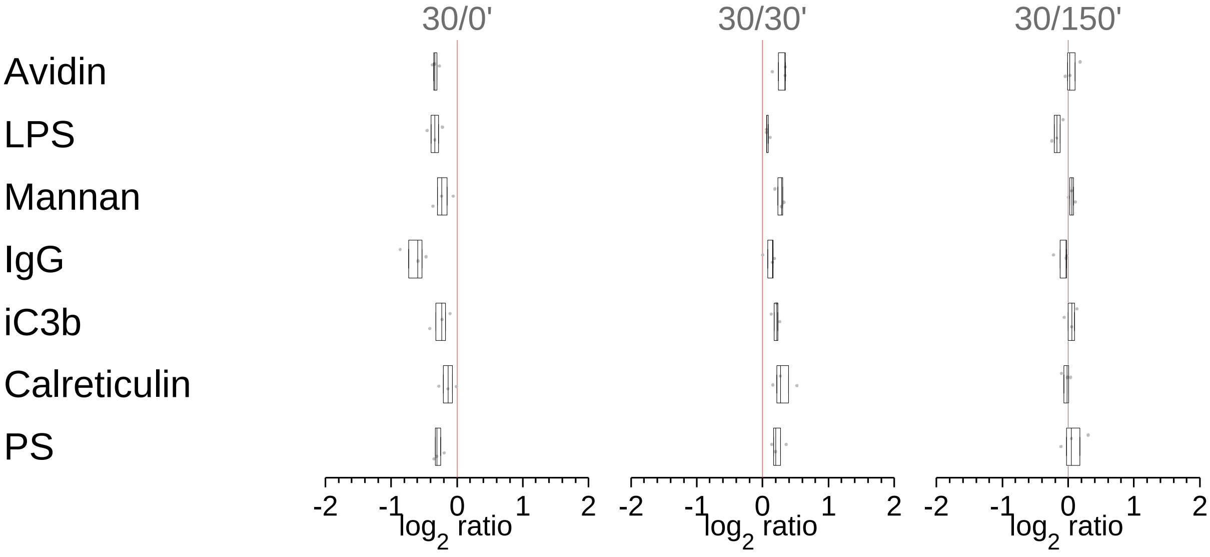

GO:0030216 keratinocyte differentiation (n = 6)

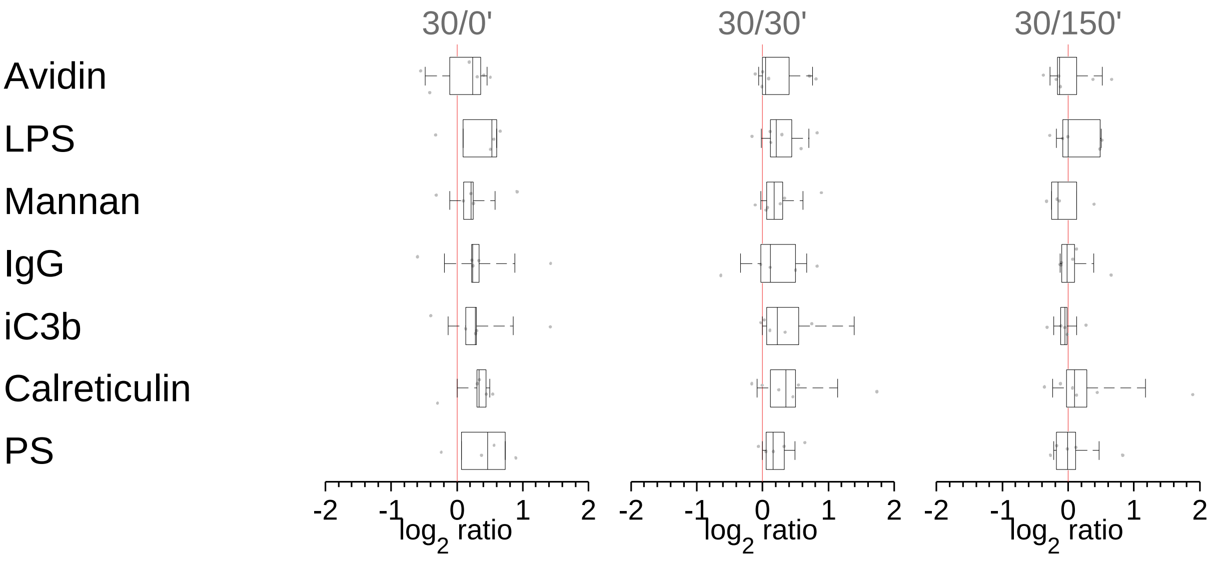

GO:0030218 erythrocyte differentiation (n = 8)

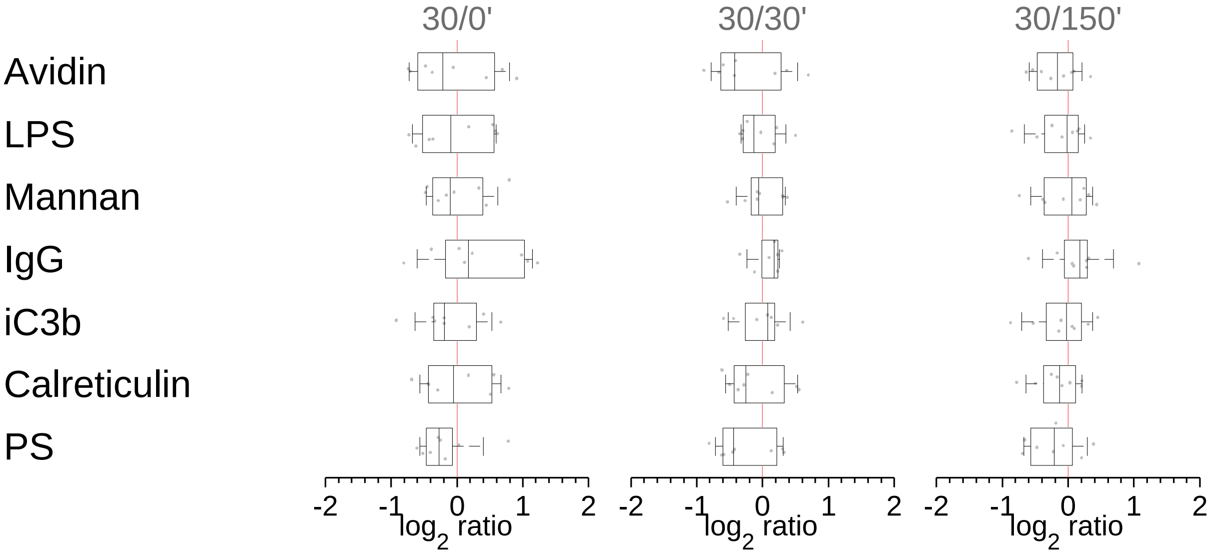

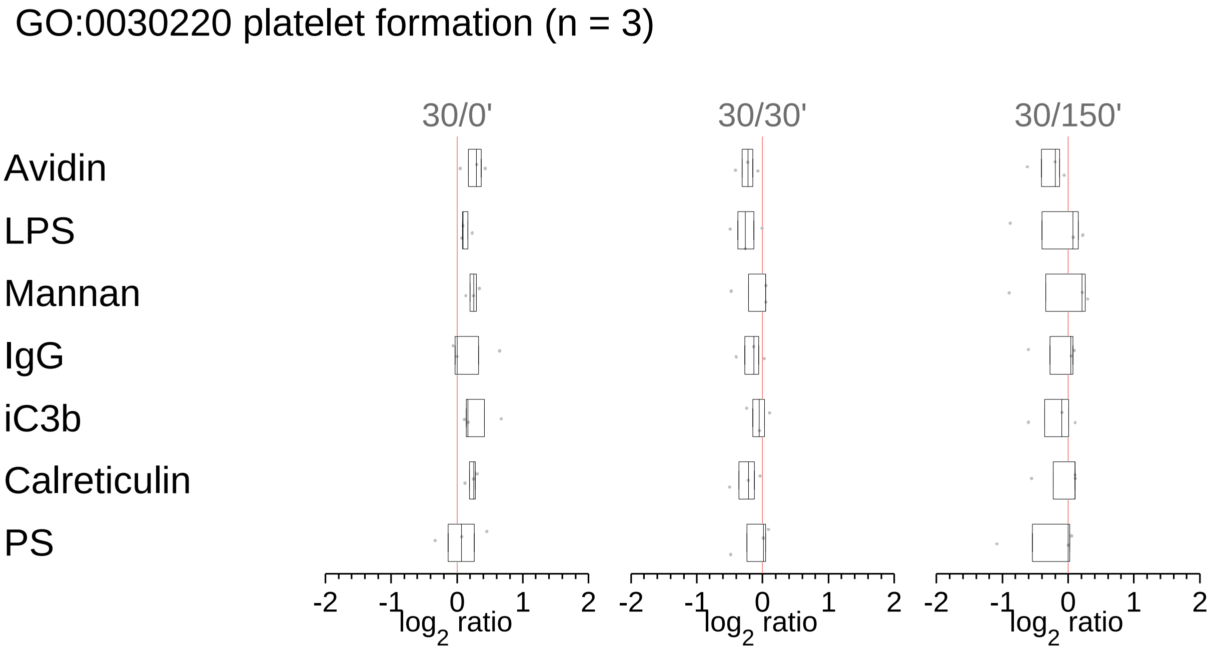
GO:0030220 platelet formation (n = 3)
30/0'
30/30'
30/150'
Avidin
LPS
Mannan
IgG
iC3b
Calreticulin
PS
-2
-1
0
1
2
$\log_2$ ratio

GO:0030246 carbohydrate binding (n = 28)

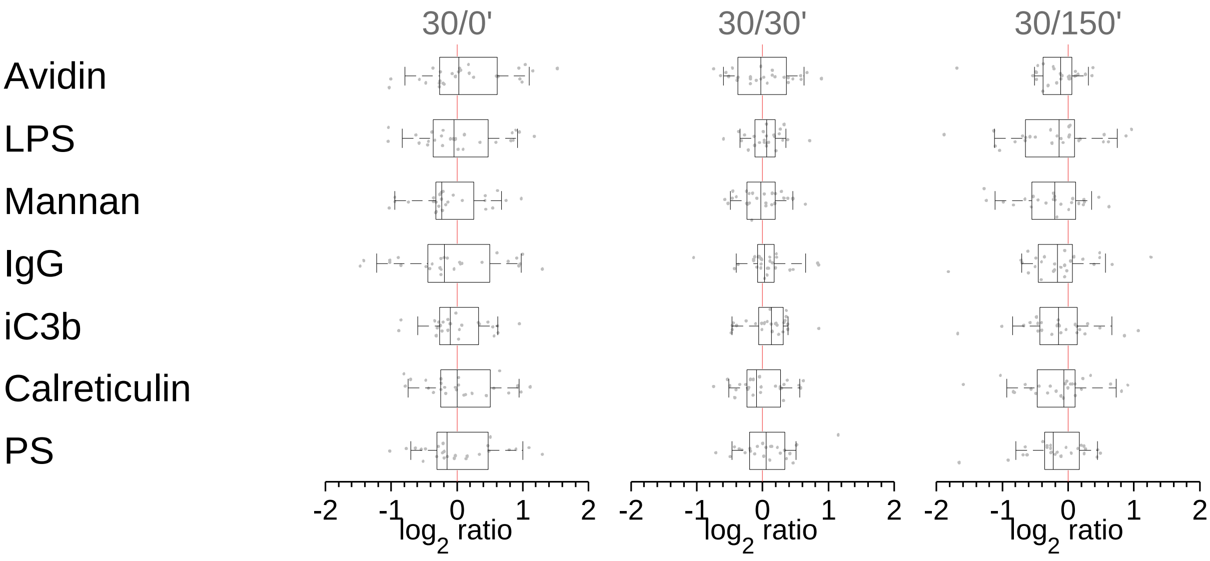

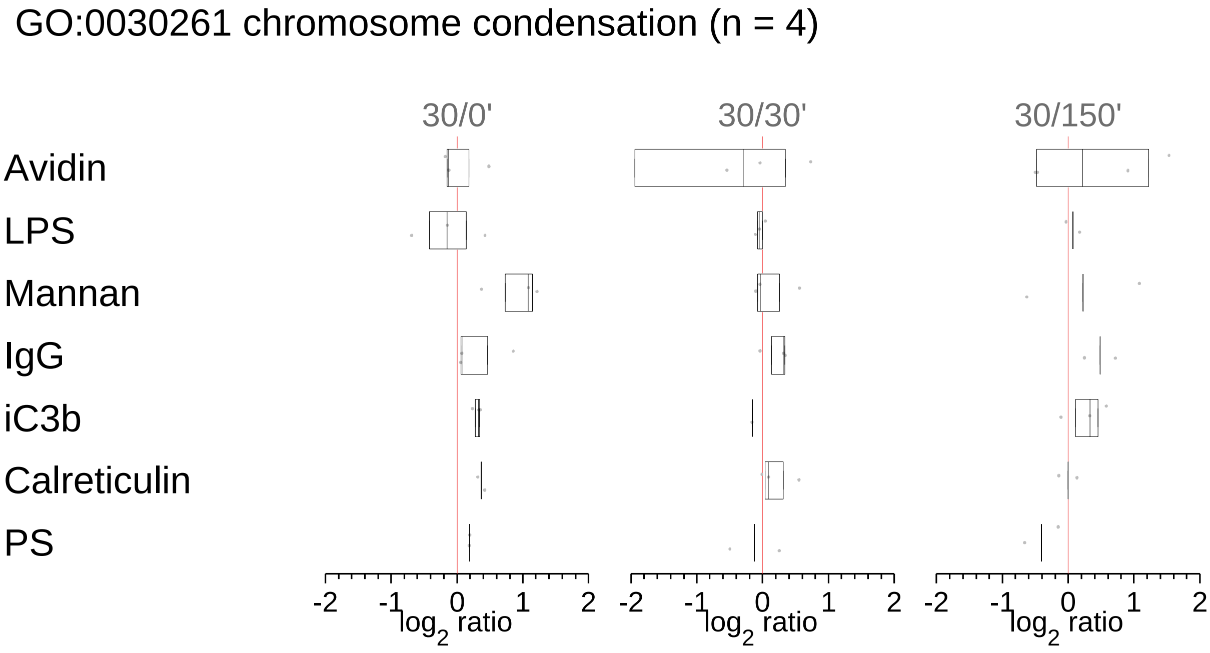
GO:0030261 chromosome condensation (n = 4)
30/0'
30/30'
30/150'
Avidin
LPS
Mannan
IgG
iC3b
Calreticulin
PS
-2
-1
0
1
2
log2 ratio

GO:0030276 clathrin binding (n = 6)

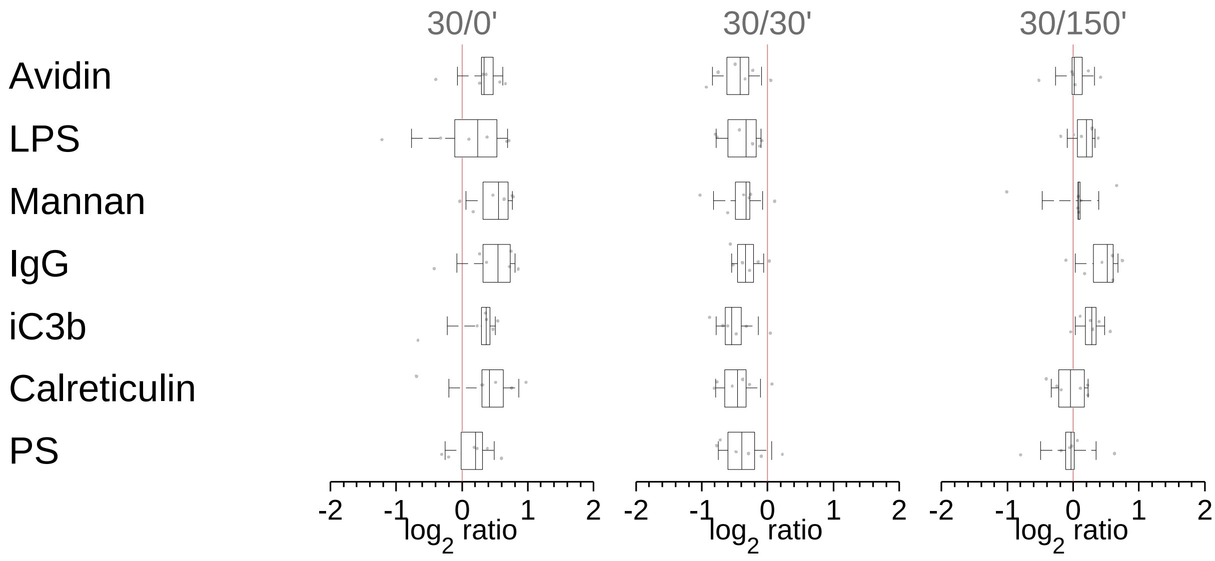

GO:0030286 dynein complex (n = 4)

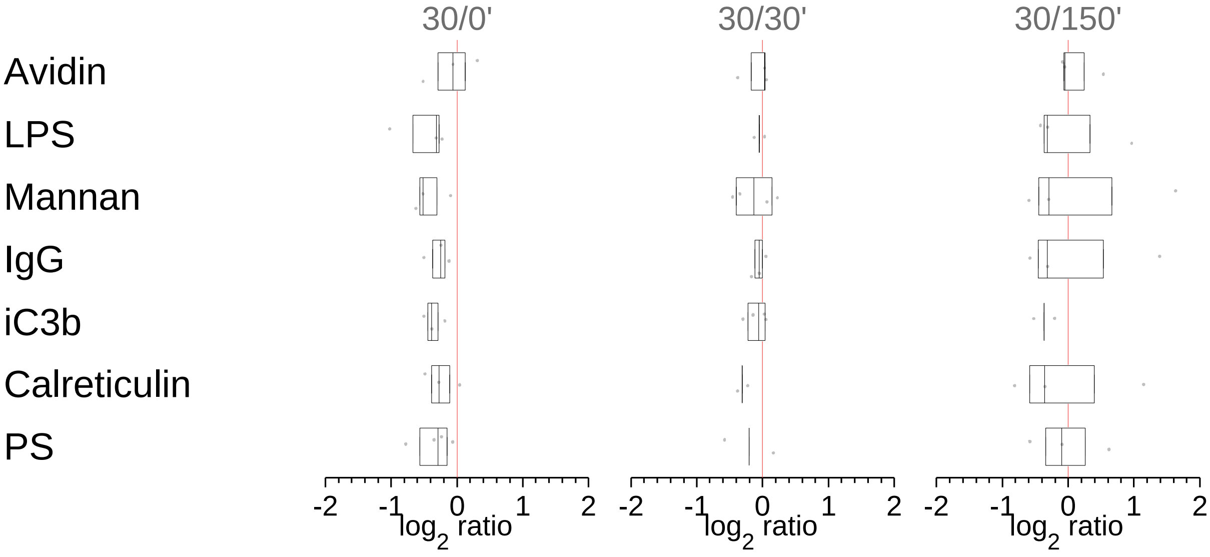

GO:0030301 cholesterol transport (n = 8)

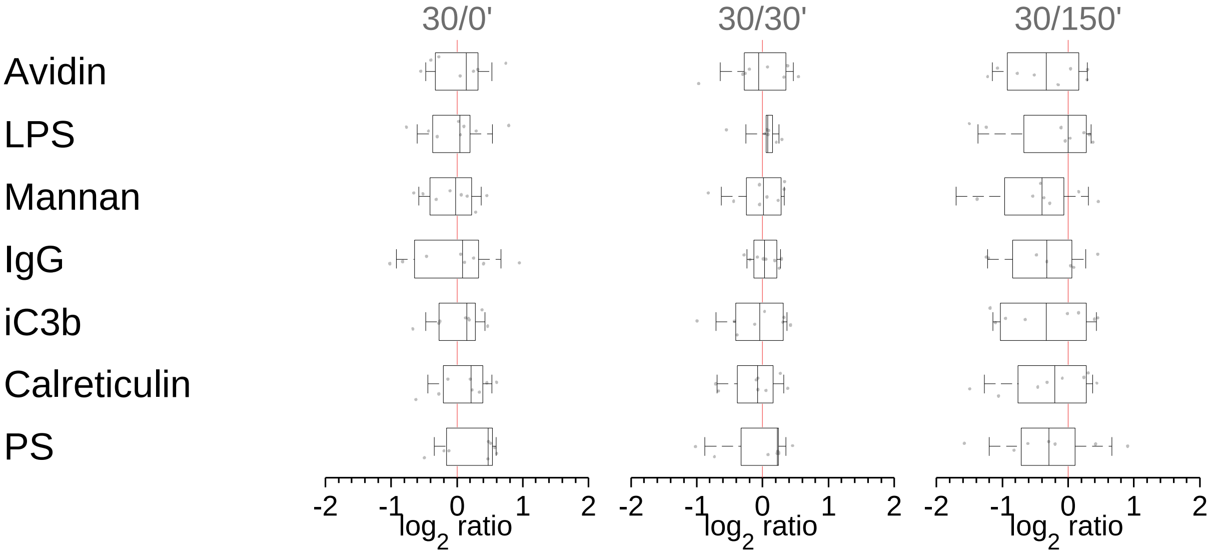

GO:0030307 positive regulation of cell growth (n = 11)

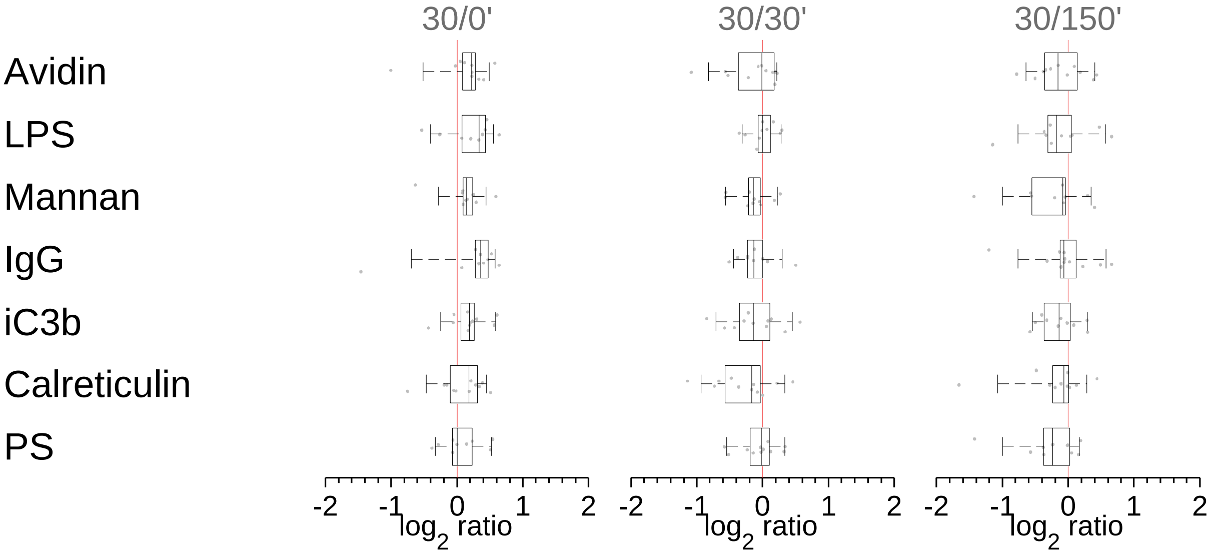

GO:0030308 negative regulation of cell growth (n = 11)

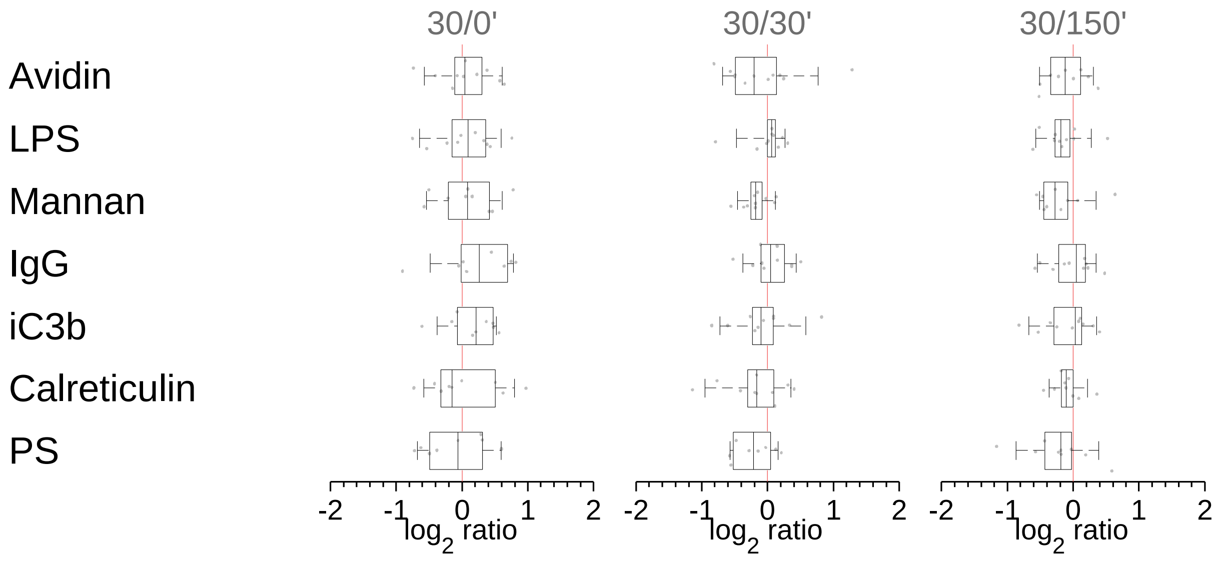

GO:0030315 T-tubule (n = 8)

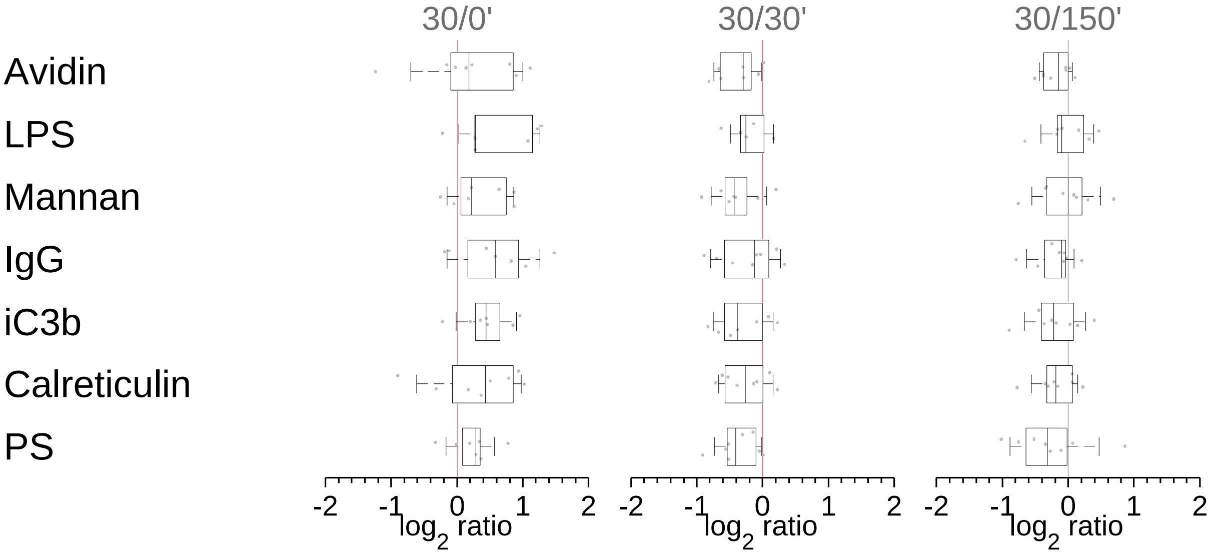

GO:0030316 osteoclast differentiation (n = 5)

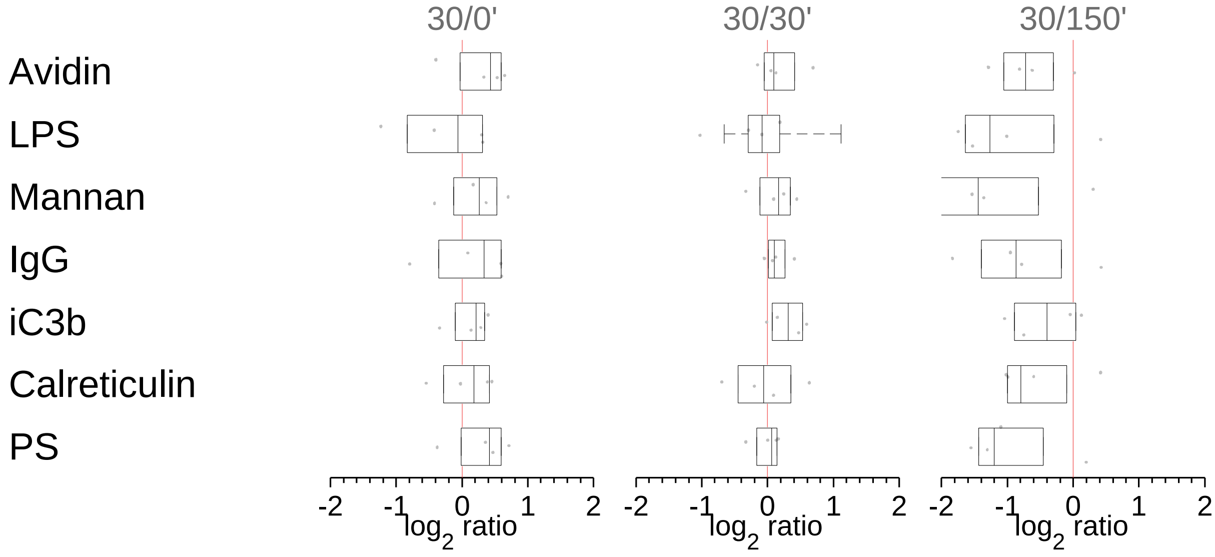

GO:0030317 sperm motility (n = 3)

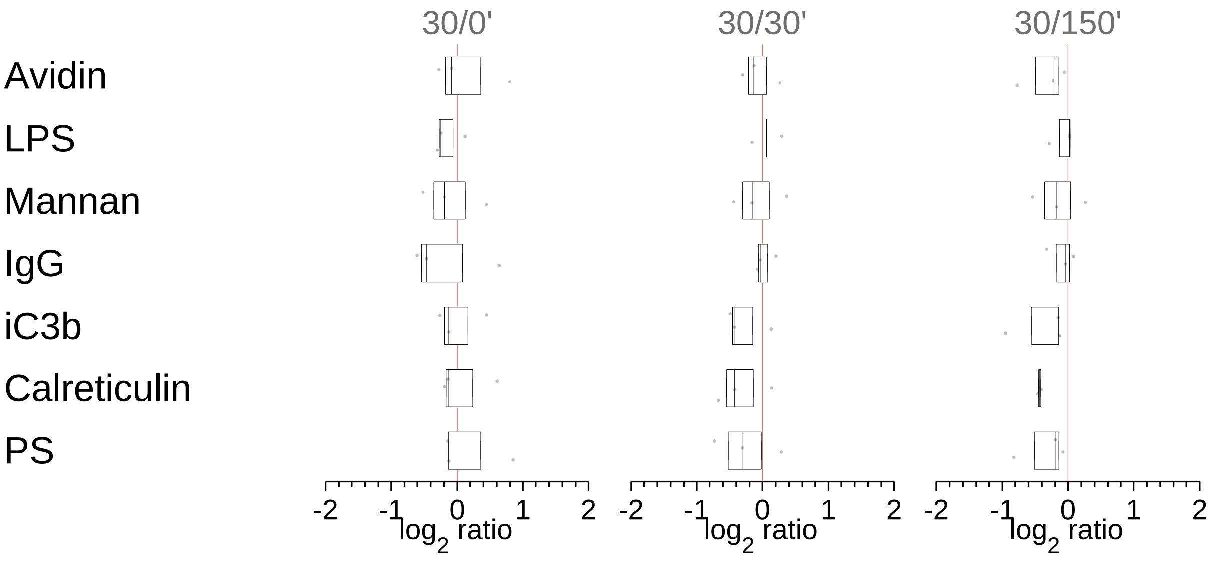

GO:0030318 melanocyte differentiation (n = 5)

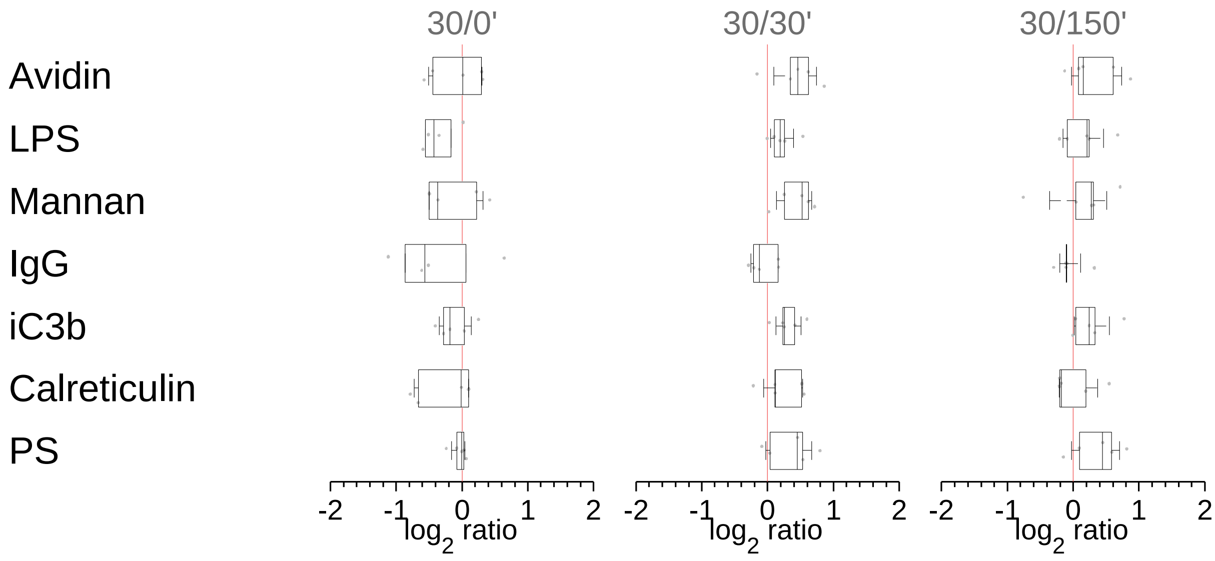

GO:0030324 lung development (n = 8)

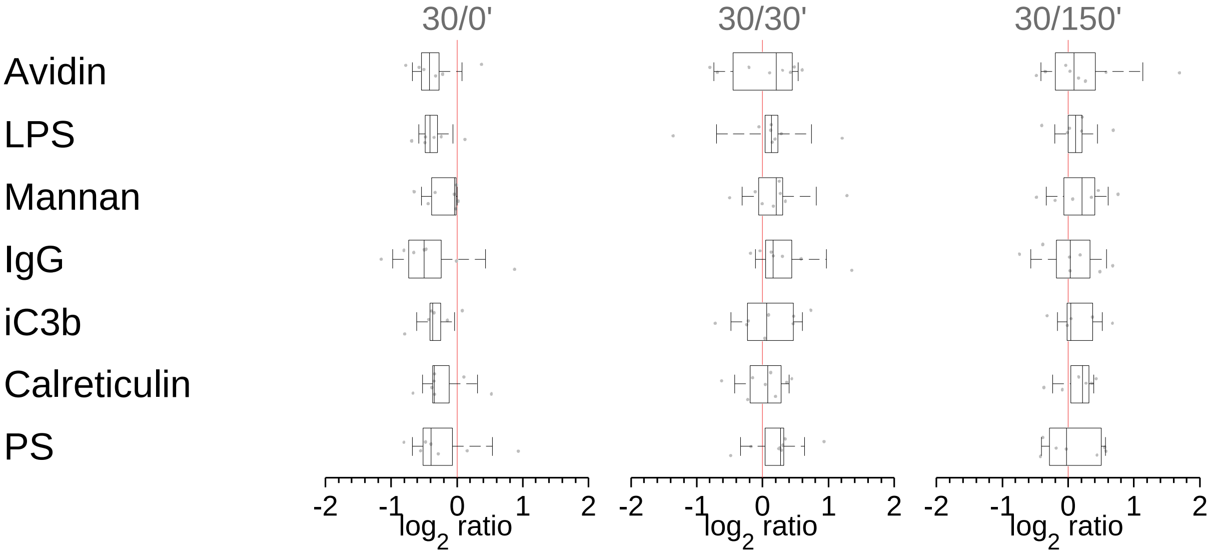

GO:0030331 estrogen receptor binding (n = 5)

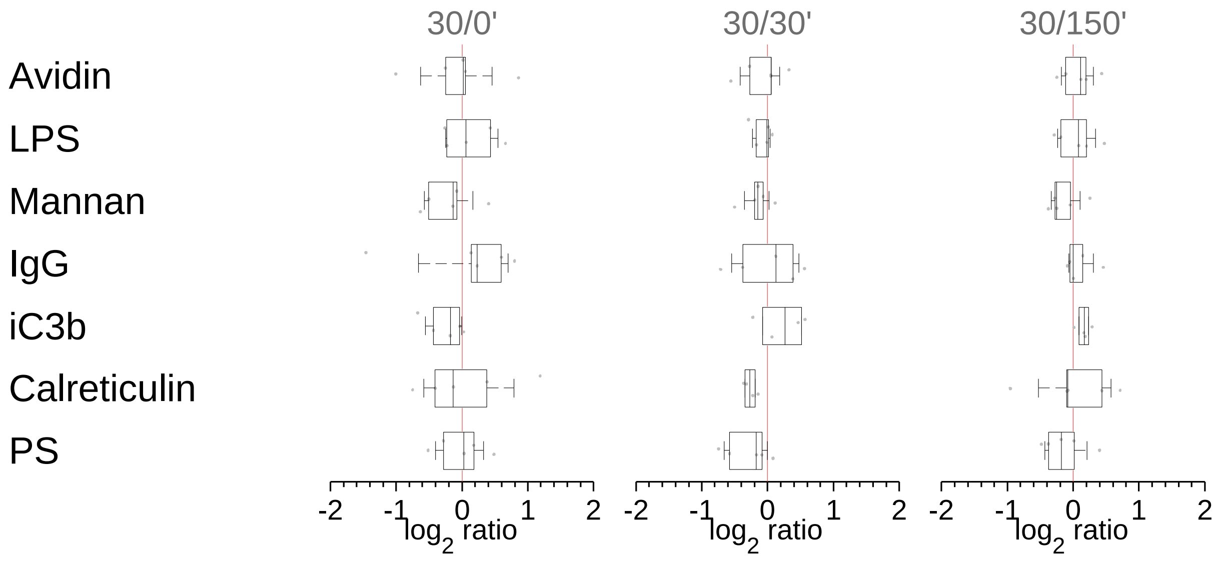

GO:0030332 cyclin binding (n = 4)

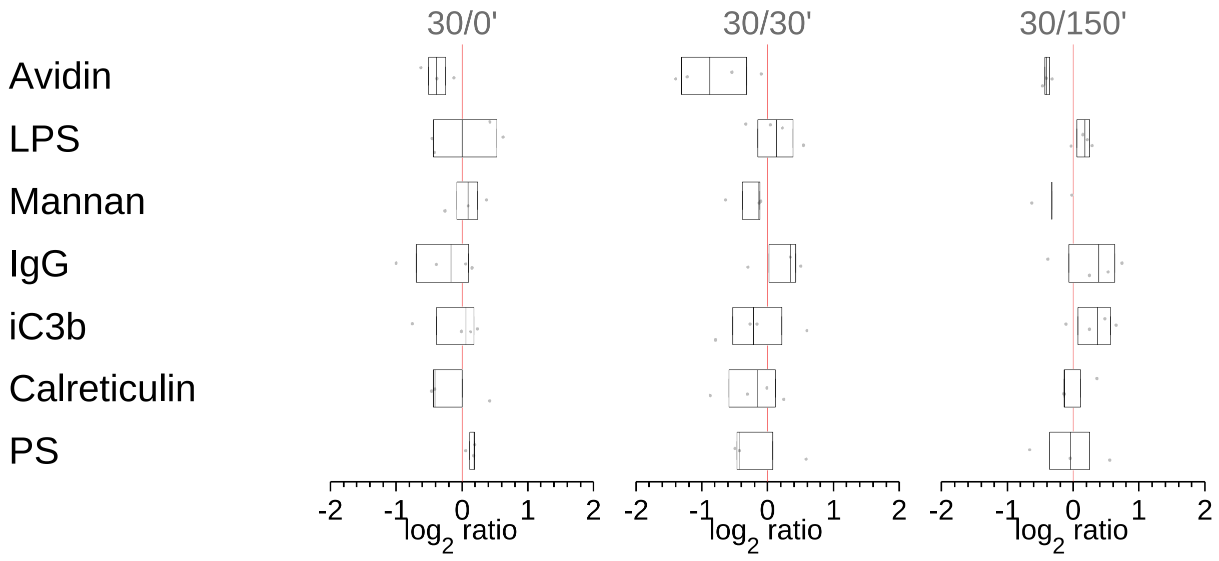

GO:0030334 regulation of cell migration (n = 8)

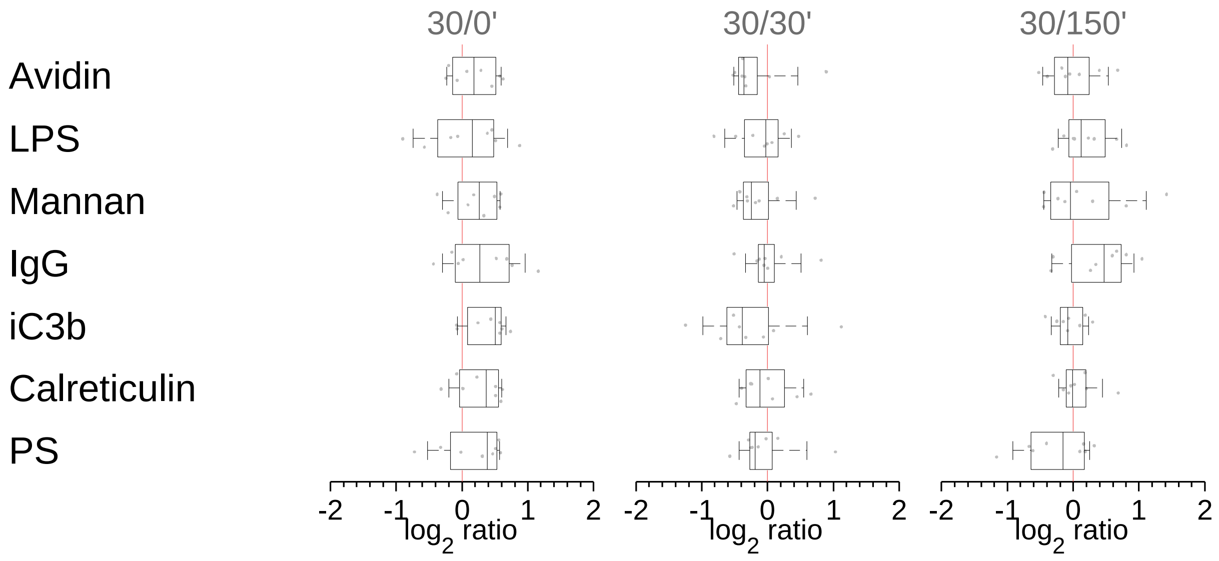

GO:0030335 positive regulation of cell migration (n = 30)

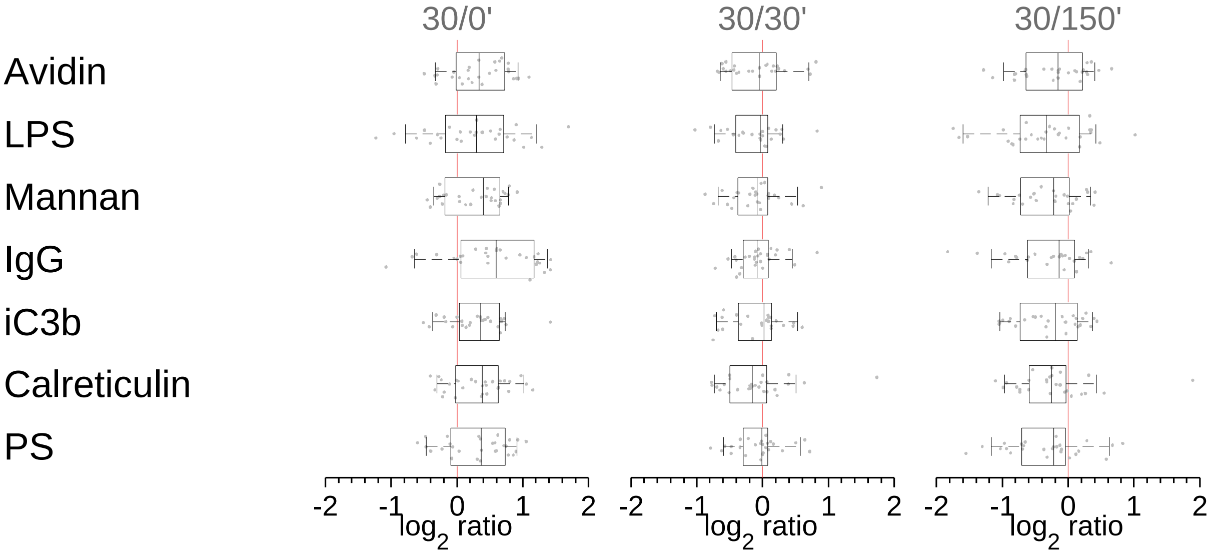

GO:0030336 negative regulation of cell migration (n = 12)

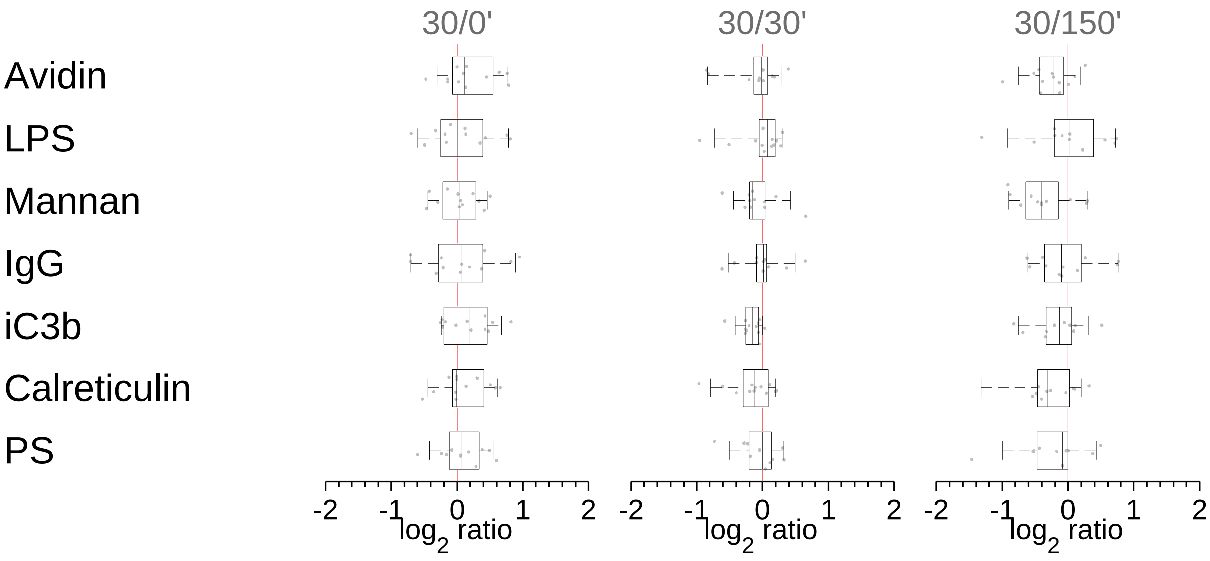

GO:0030374 ligand-dependent nuclear receptor transcription coactivator activity (n = 6)

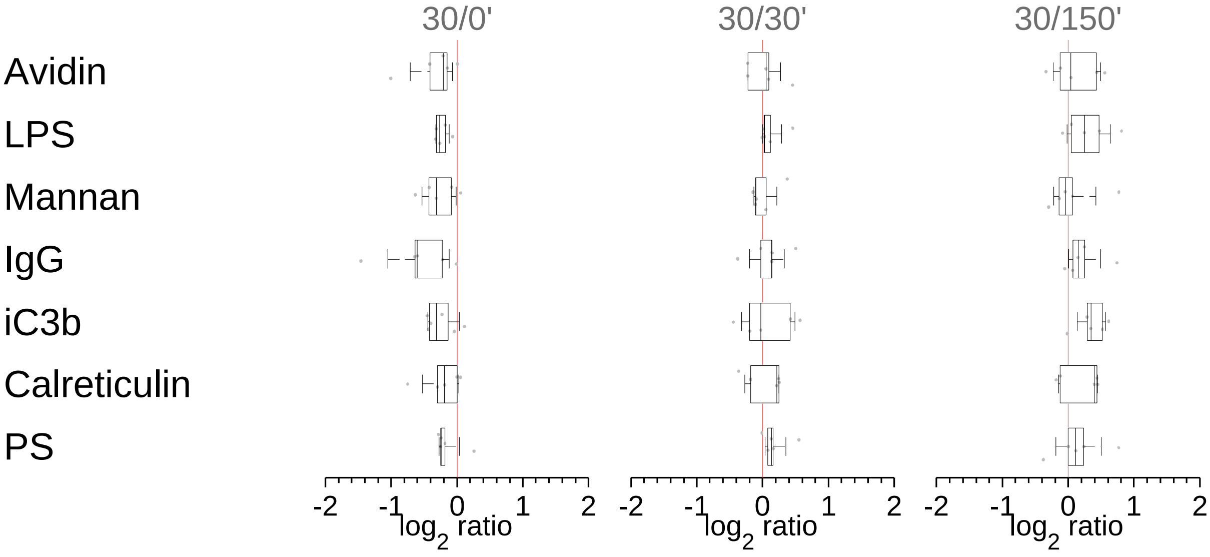

GO:0030414 peptidase inhibitor activity (n = 9)

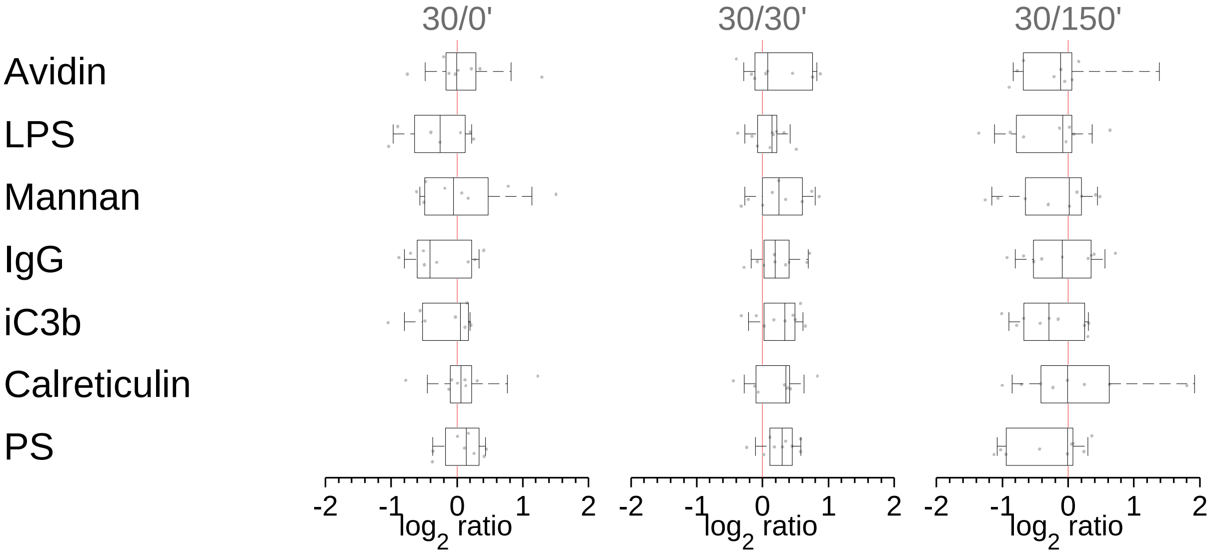

GO:0030424 axon (n = 39)

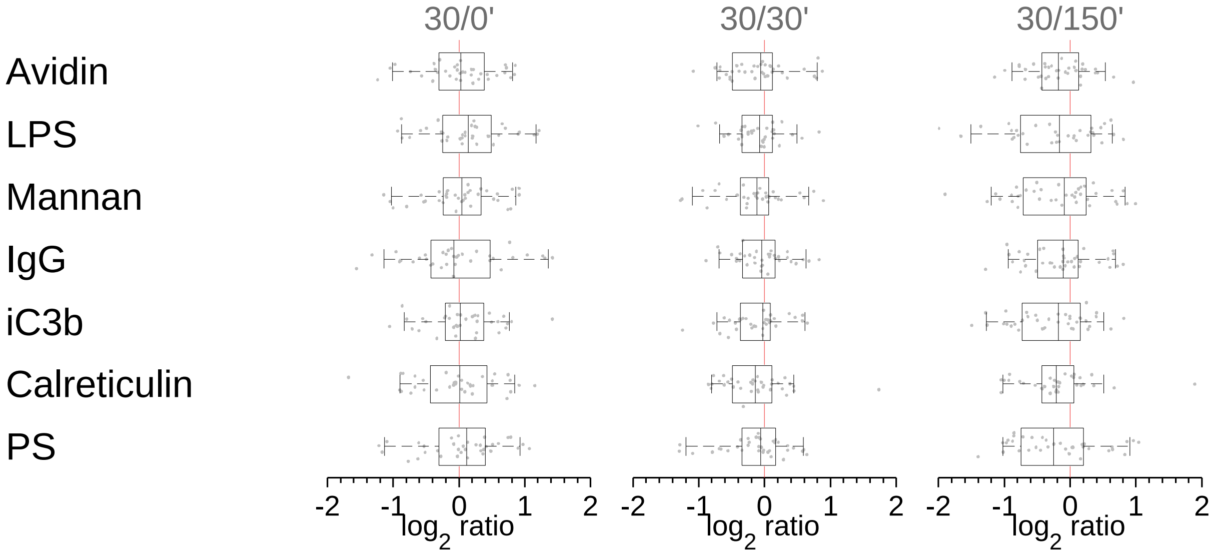

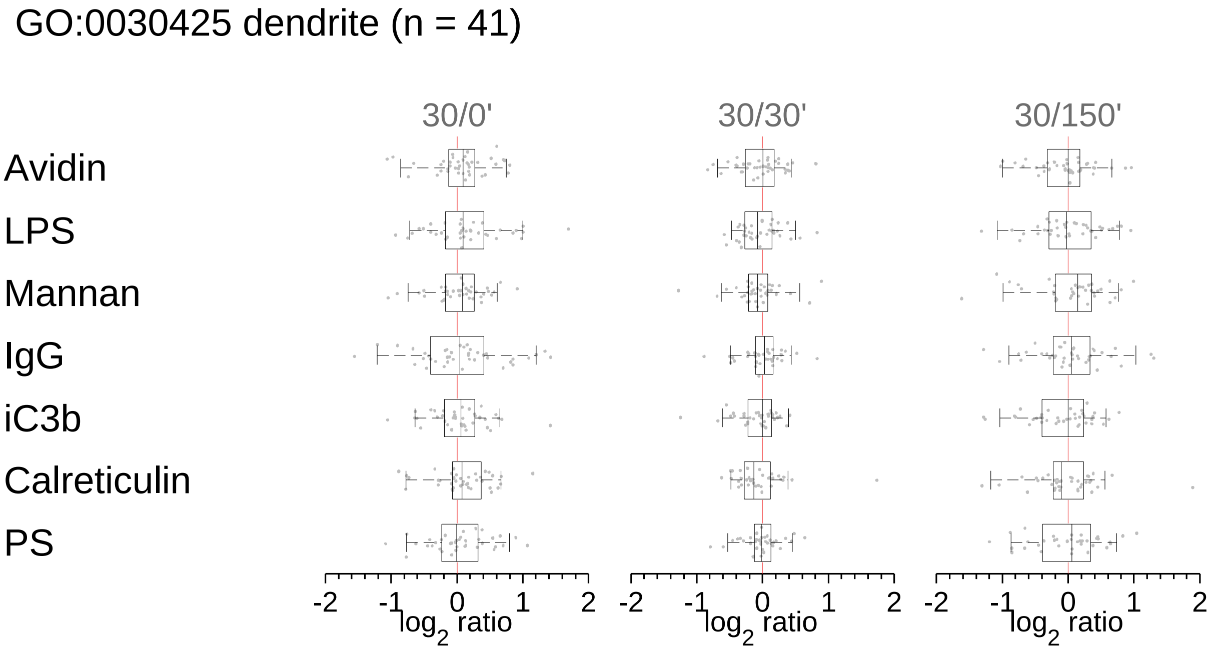
GO:0030425 dendrite (n = 41)
30/0'
30/30'
30/150'
Avidin
LPS
Mannan
IgG
iC3b
Calreticulin
PS
-2
-1
0
1
2
log2 ratio

GO:0030426 growth cone (n = 14)

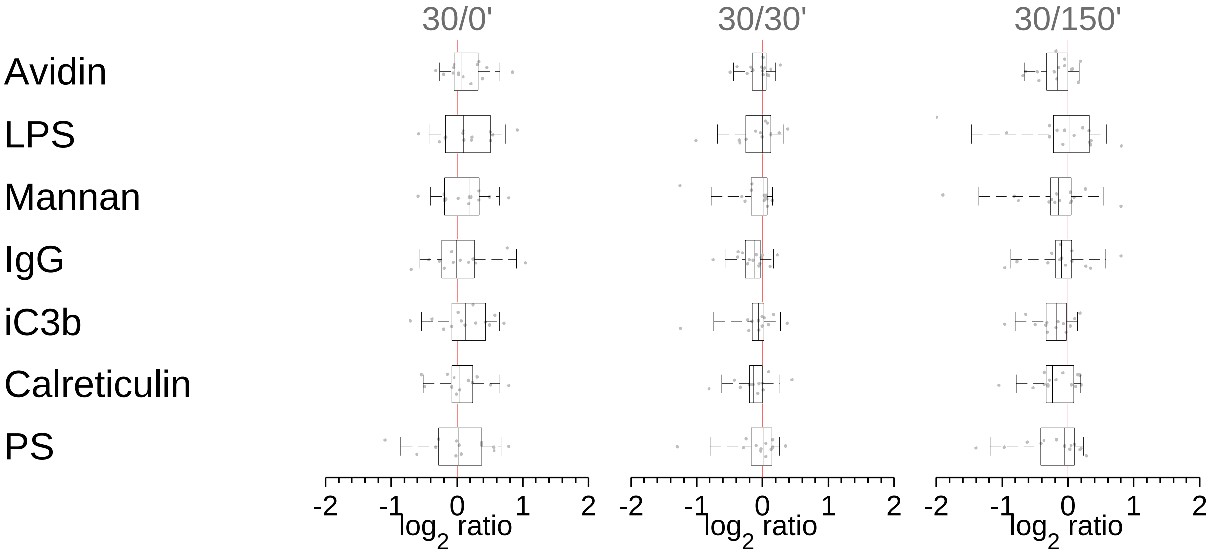

GO:0030433 ER-associated protein catabolic process (n = 5)

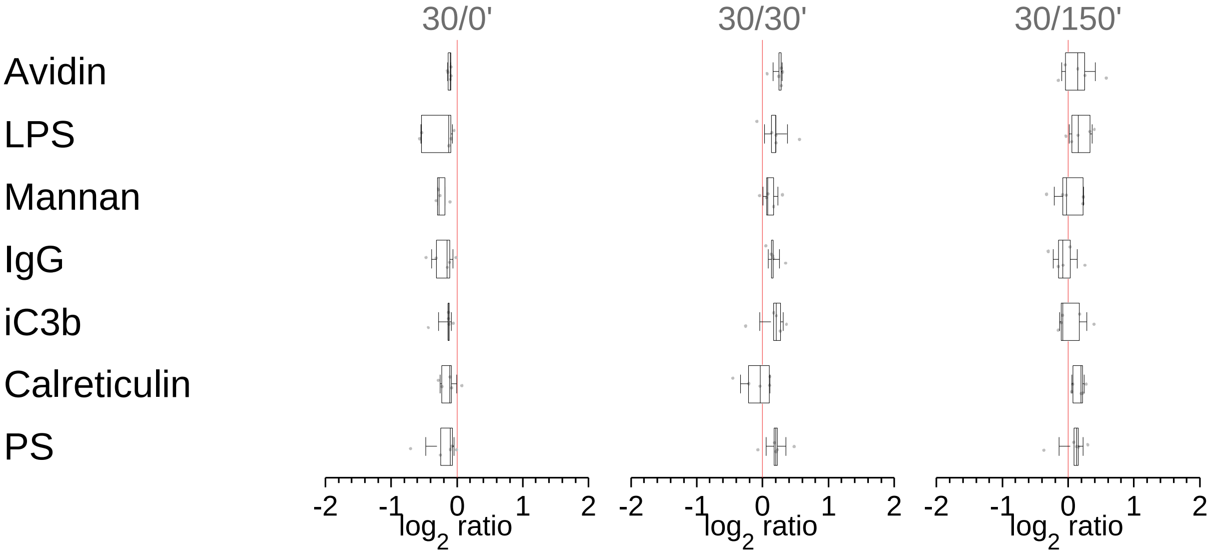

GO:0030496 midbody (n = 31)

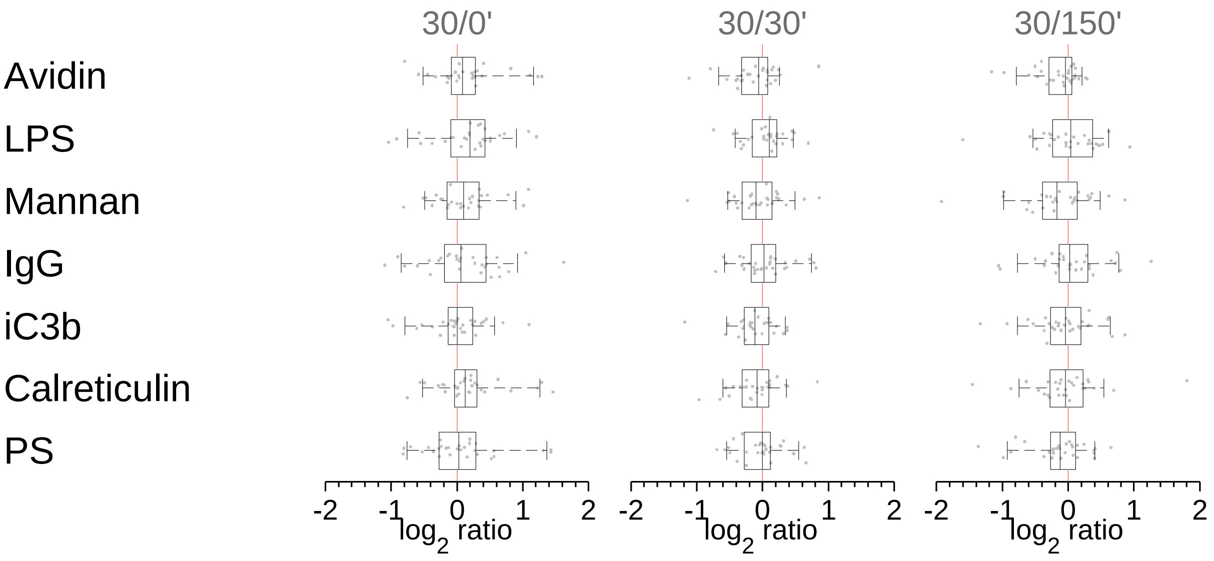

GO:0030500 regulation of bone mineralization (n = 3)

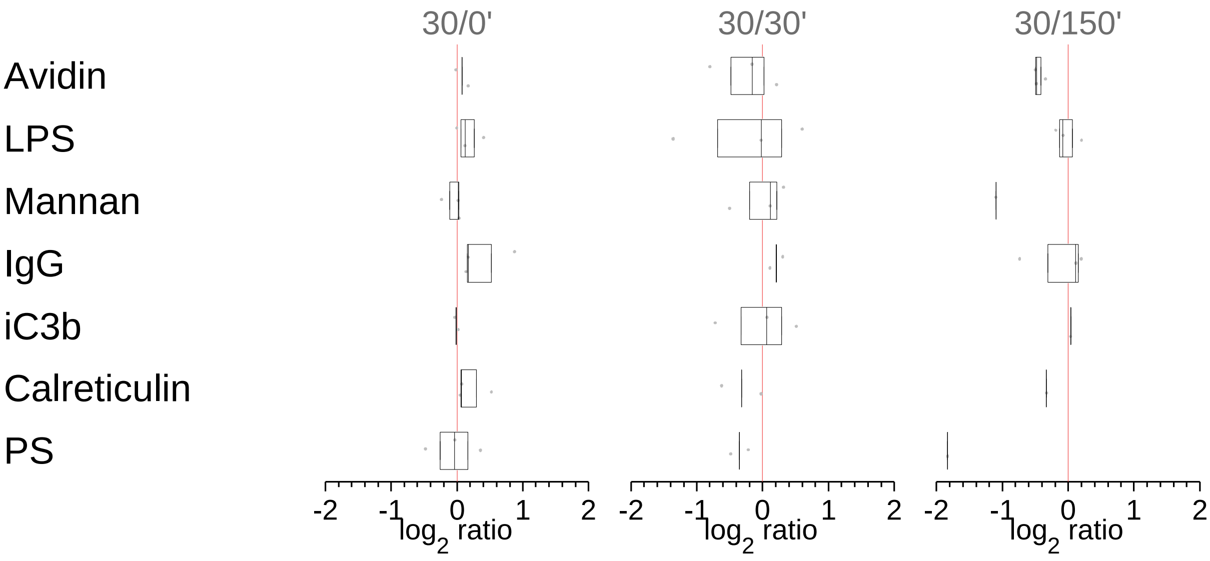

GO:0030501 positive regulation of bone mineralization (n = 3)

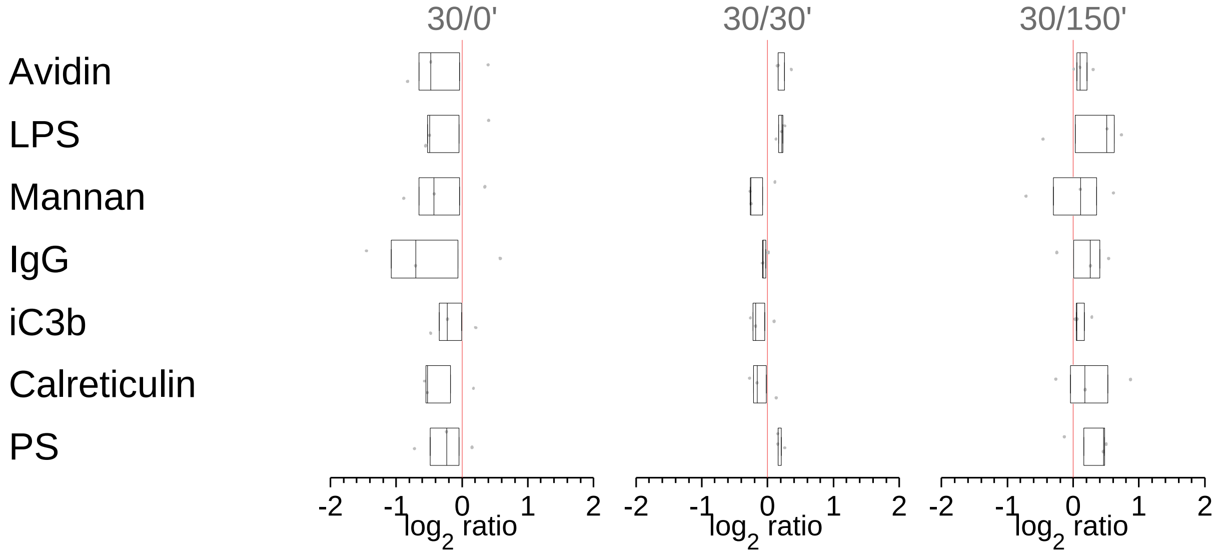

GO:0030506 ankyrin binding (n = 5)

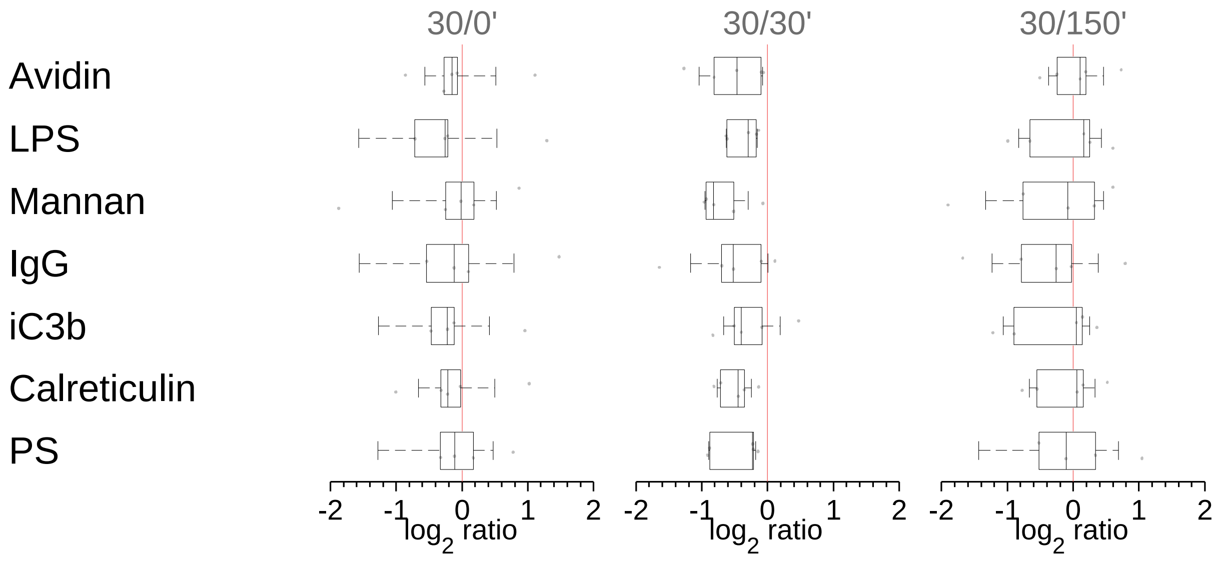

GO:0030507 spectrin binding (n = 4)

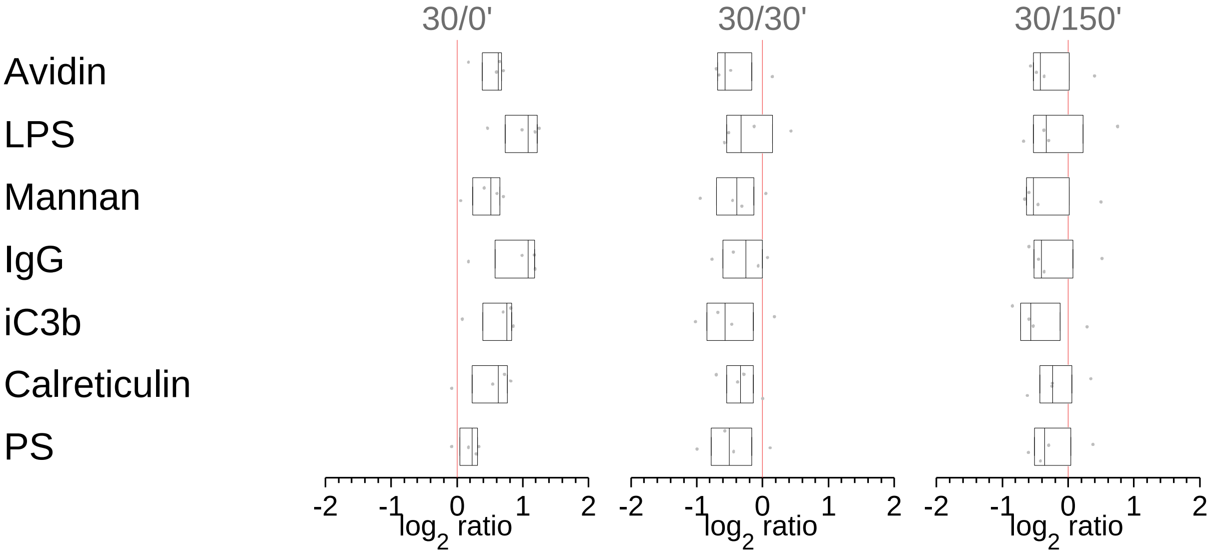

GO:0030511 positive regulation of transforming growth factor beta receptor signaling pathway (n = 3)

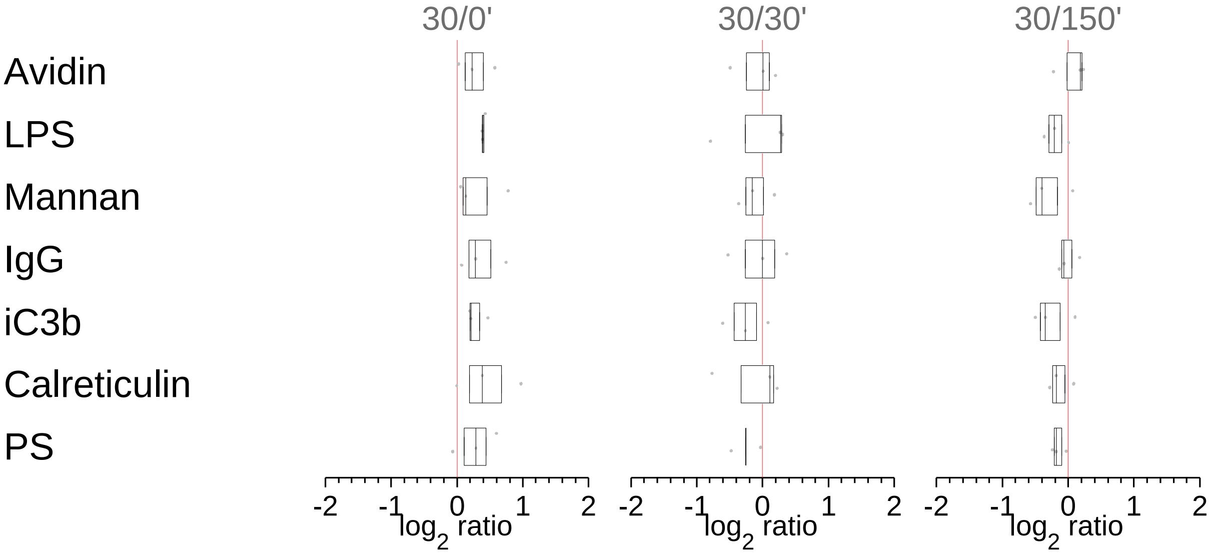

GO:0030512 negative regulation of transforming growth factor beta receptor signaling pathway (n = 4)

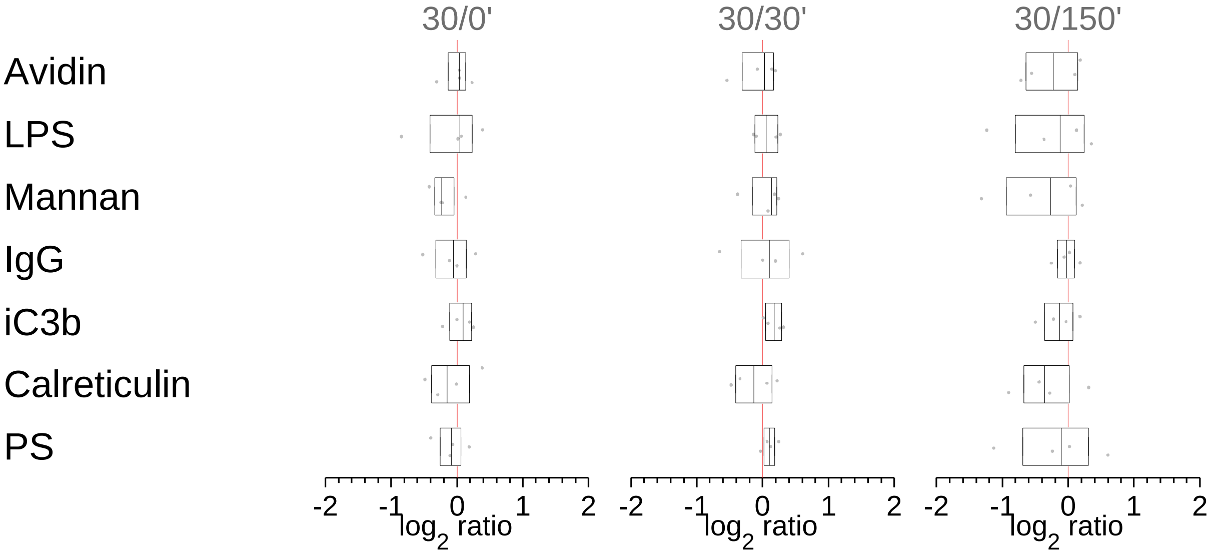

GO:0030513 positive regulation of BMP signaling pathway (n = 3)

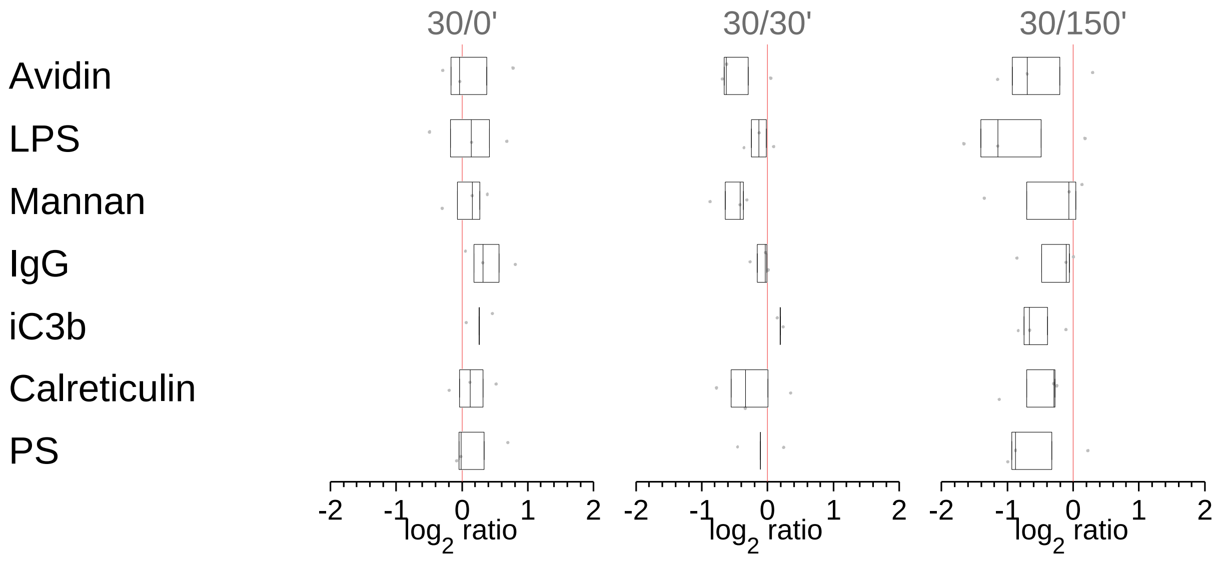

GO:0030517 negative regulation of axon extension (n = 3)

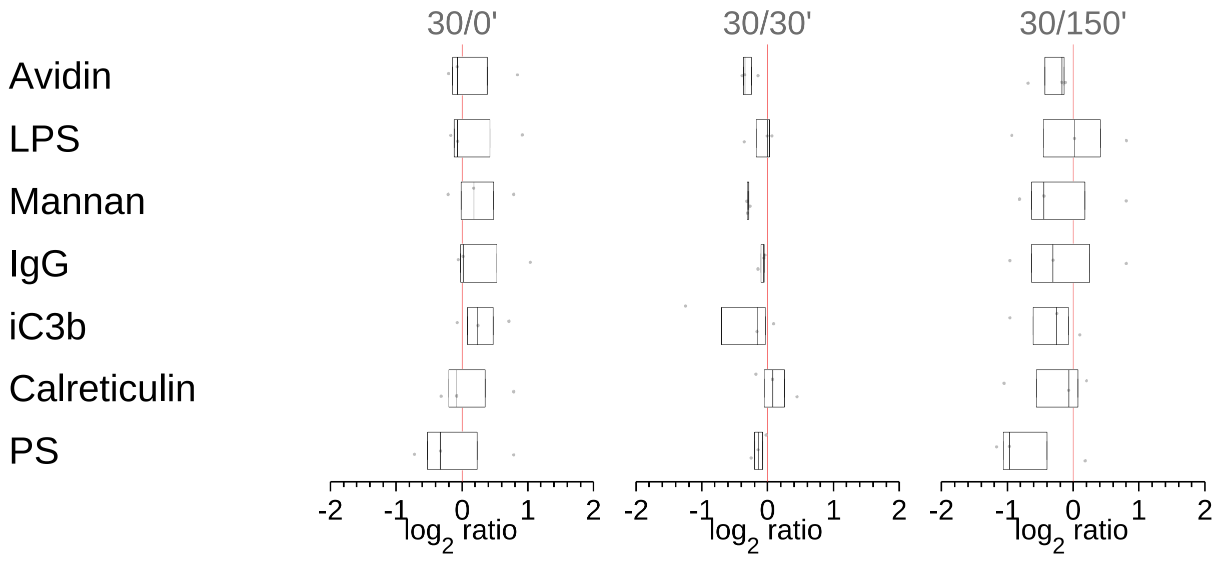

GO:0030521 androgen receptor signaling pathway (n = 3)

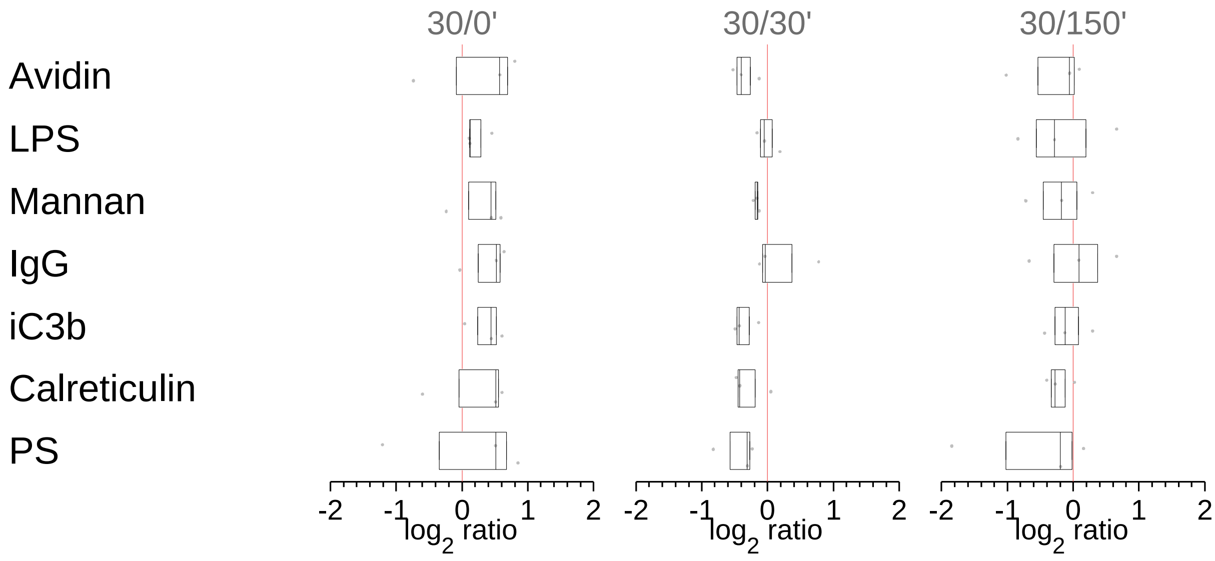

GO:0030523 dihydrolipoamide S-acyltransferase activity (n = 3)

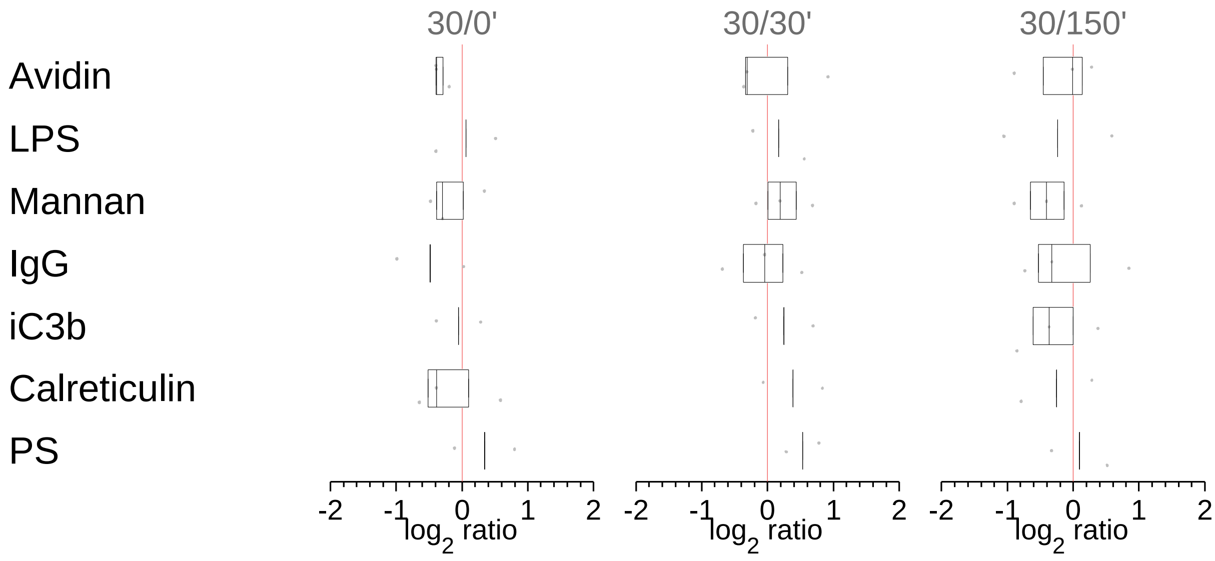

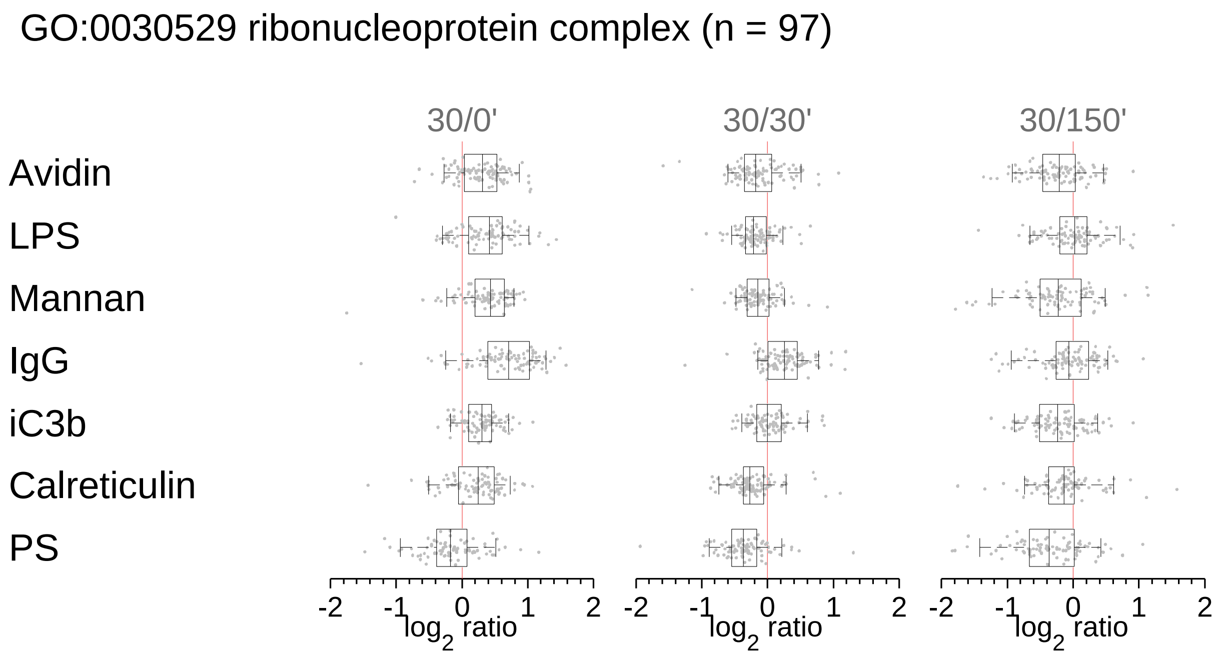
GO:0030529 ribonucleoprotein complex (n = 97)
30/0'
30/30'
30/150'
Avidin
LPS
Mannan
IgG
iC3b
Calreticulin
PS
-2
-1
0
1
2
log2 ratio

GO:0030544 Hsp70 protein binding (n = 6)

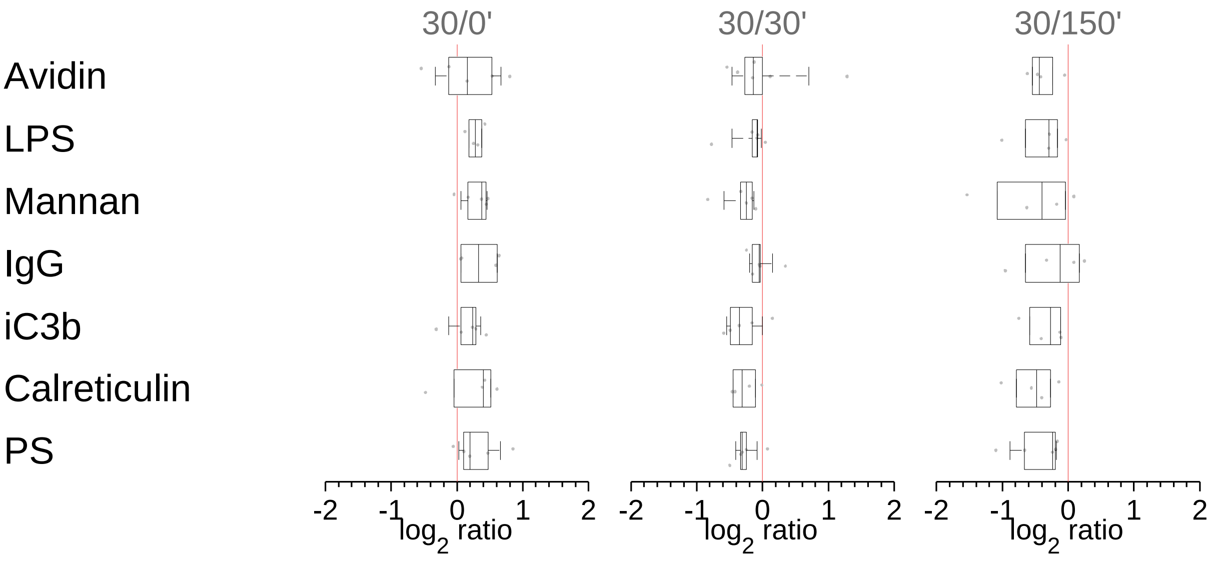

GO:0030554 adenyl nucleotide binding (n = 3)

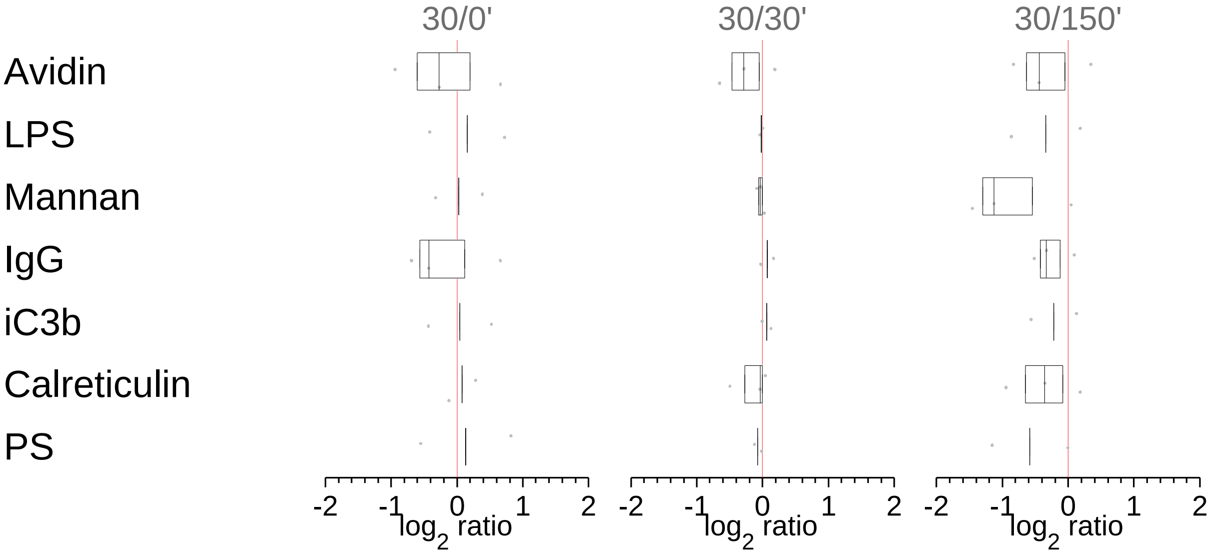

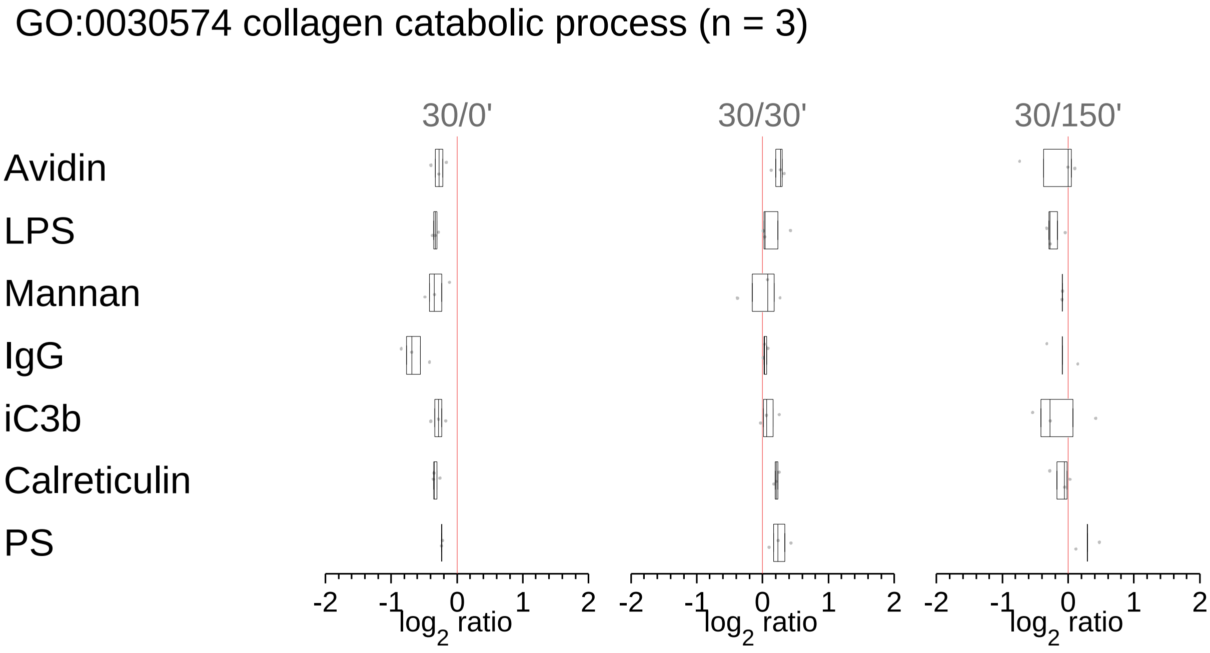
GO:0030574 collagen catabolic process (n = 3)
30/0'
30/30'
30/150'
Avidin
LPS
Mannan
IgG
iC3b
Calreticulin
PS
-2 -1 0 1 2
log2 ratio

GO:0030593 neutrophil chemotaxis (n = 8)

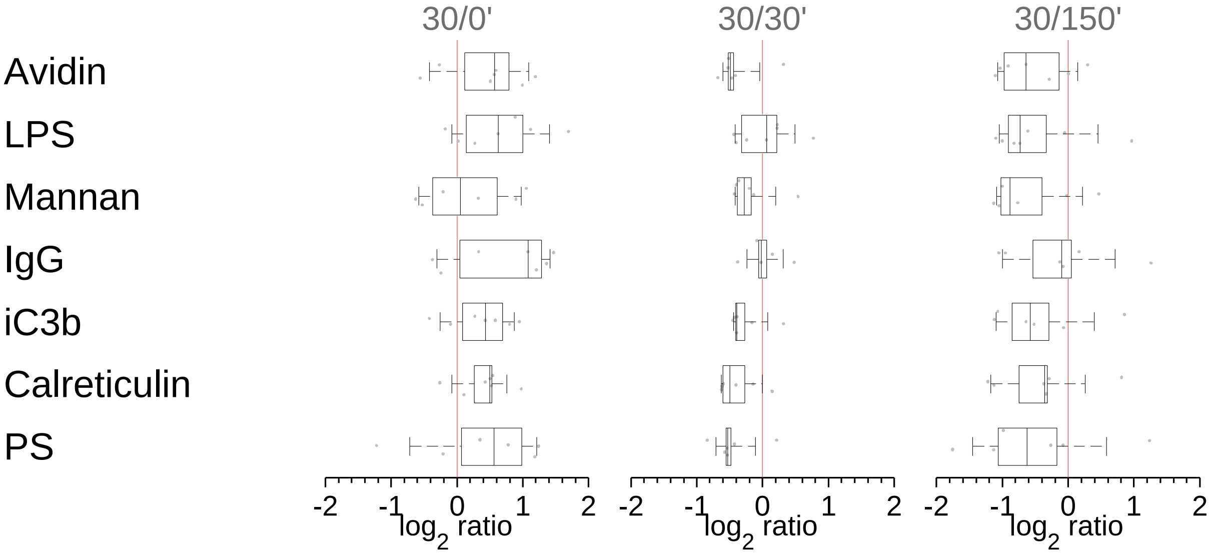

GO:0030595 leukocyte chemotaxis (n = 4)

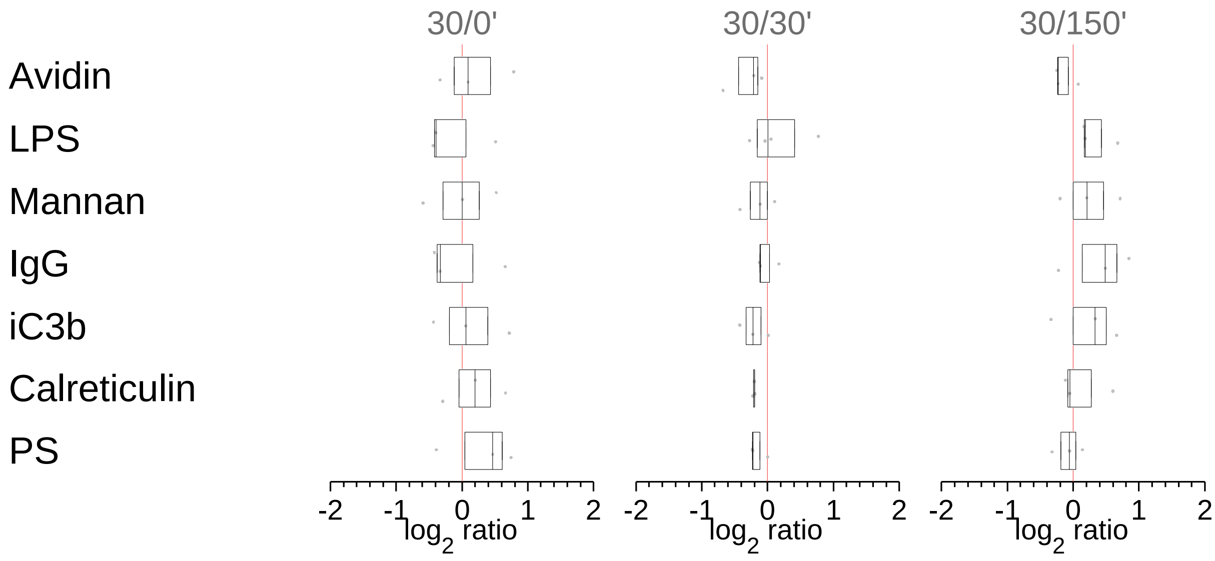

GO:0030665 clathrin coated vesicle membrane (n = 3)

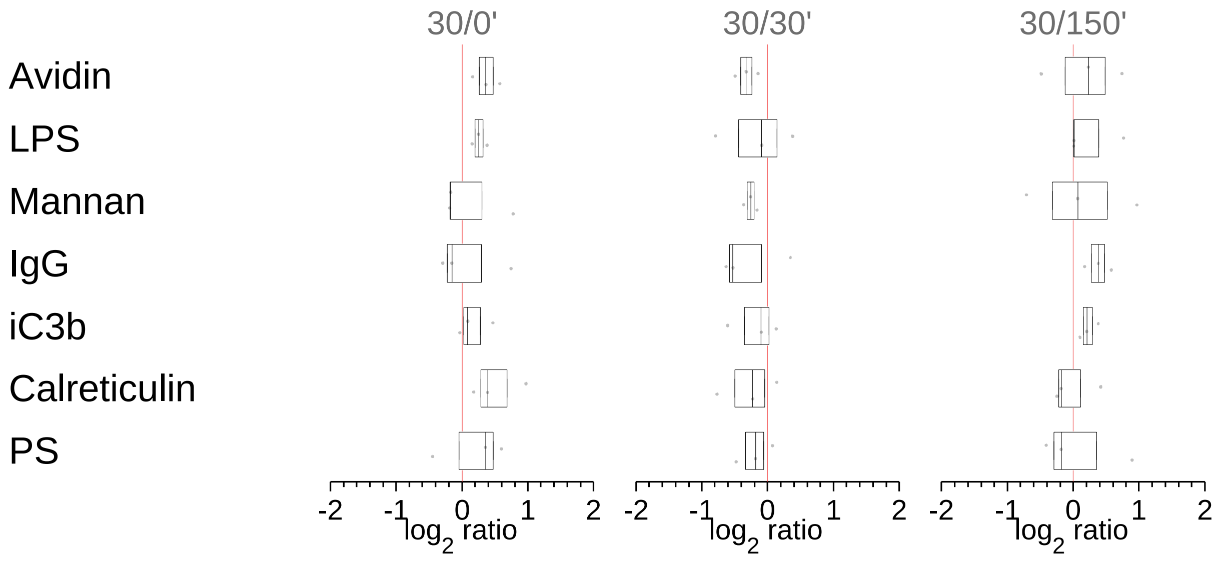

GO:0030667 secretory granule membrane (n = 3)

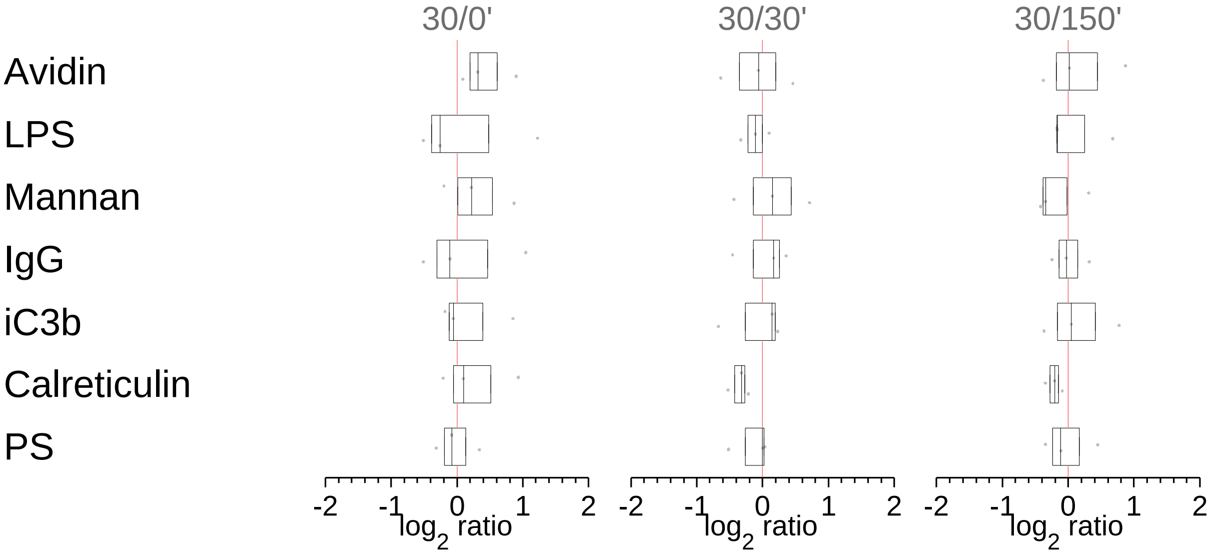

GO:0030670 phagocytic vesicle membrane (n = 3)

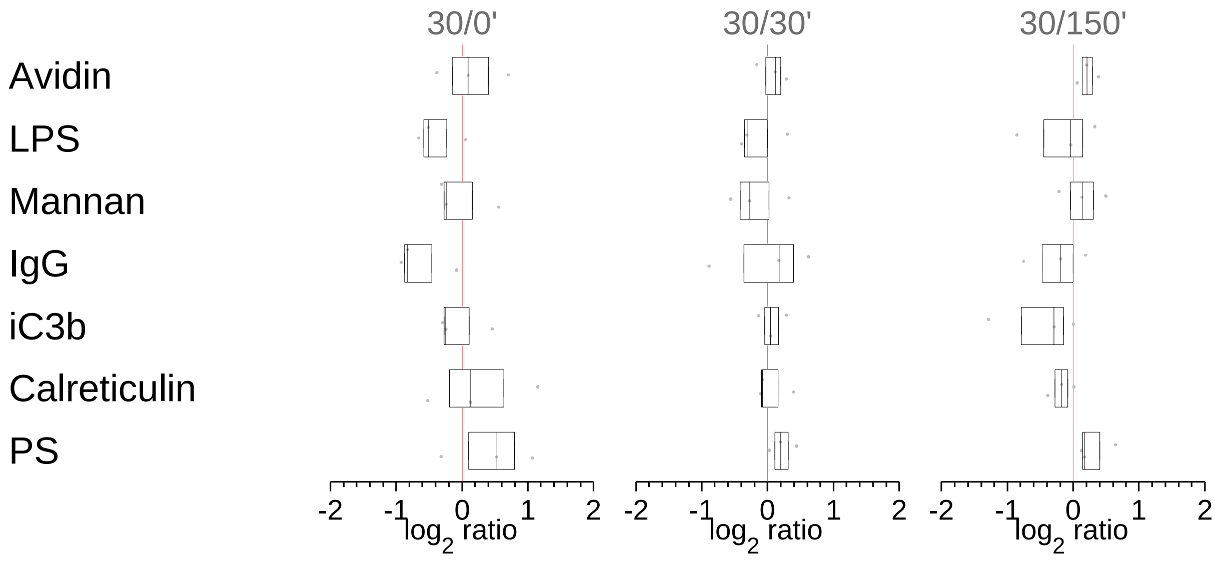

GO:0030672 synaptic vesicle membrane (n = 3)

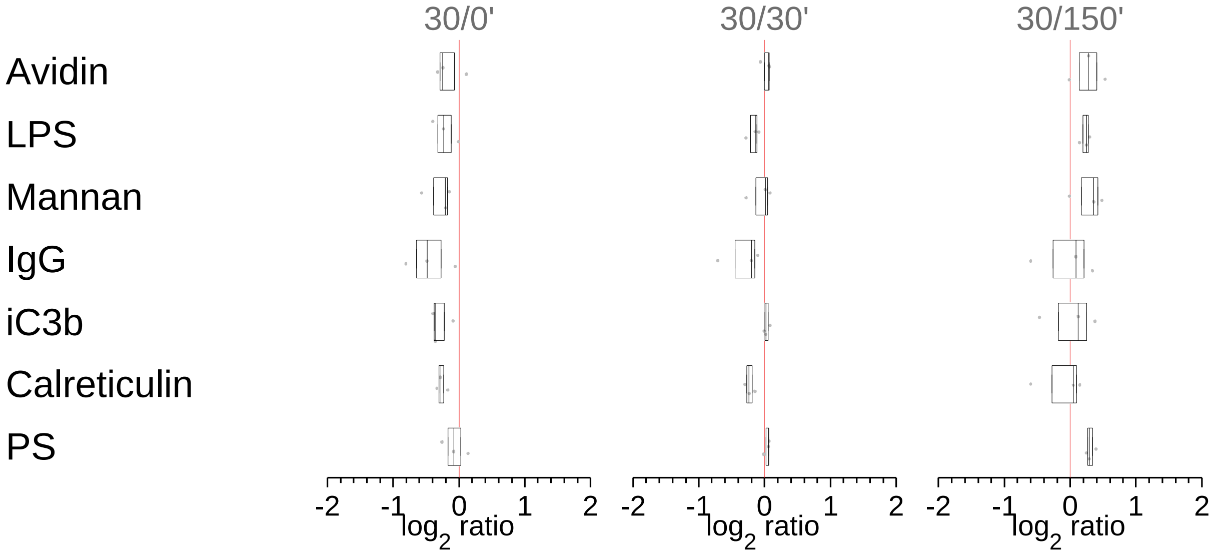

GO:0030674 protein binding, bridging (n = 3)

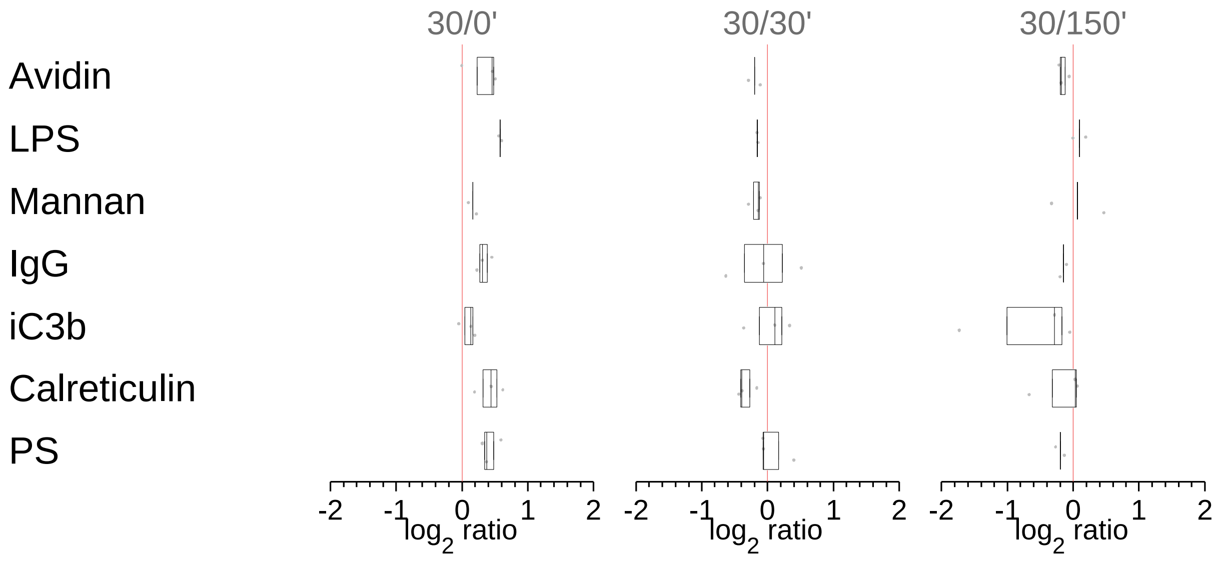

GO:0030675 Rac GTPase activator activity (n = 3)

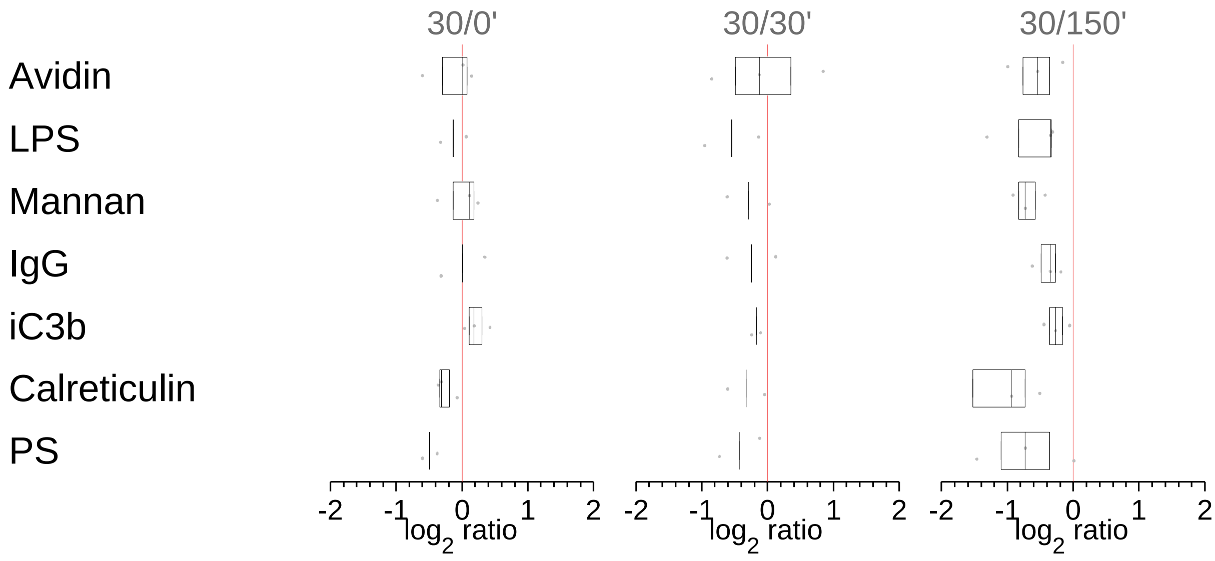

GO:0030742 GTP-dependent protein binding (n = 7)

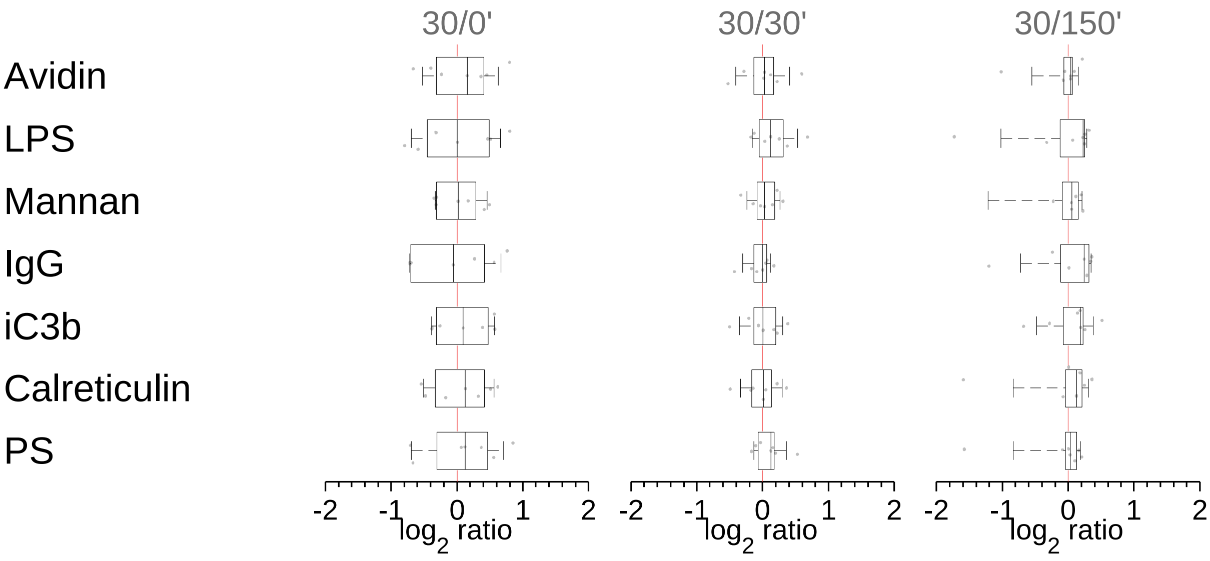

GO:0030819 positive regulation of cAMP biosynthetic process (n = 4)

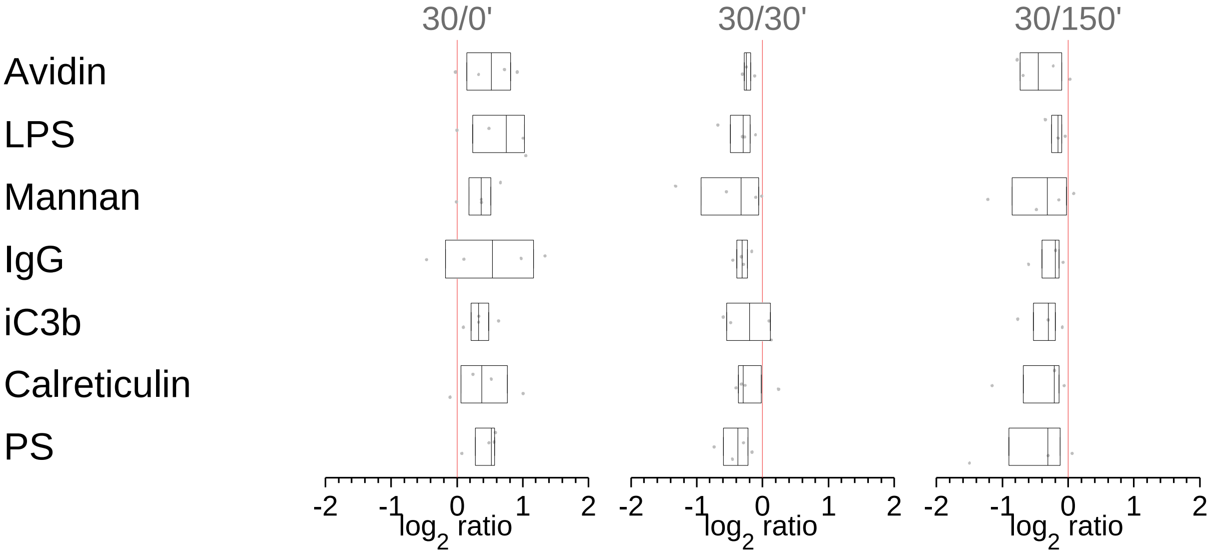

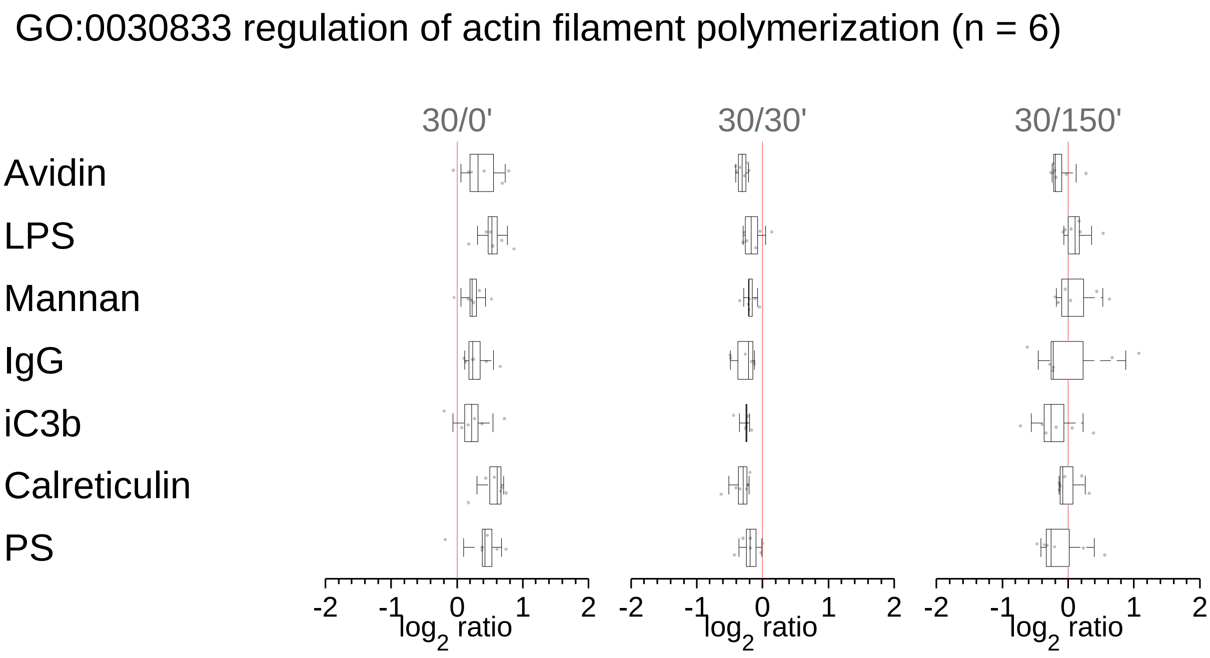
GO:0030833 regulation of actin filament polymerization (n = 6)
30/0'
30/30'
30/150'
Avidin
LPS
Mannan
IgG
iC3b
Calreticulin
PS
-2 -1 0 1 2
log2 ratio

GO:0030837 negative regulation of actin filament polymerization
(n = 3)

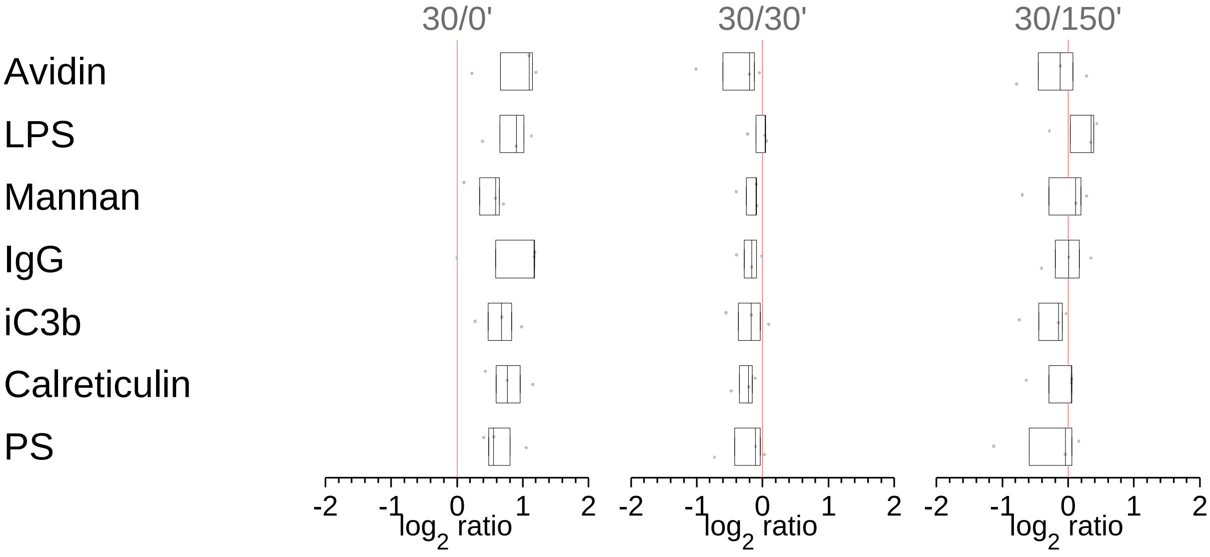

GO:0030838 positive regulation of actin filament polymerization
(n = 11)

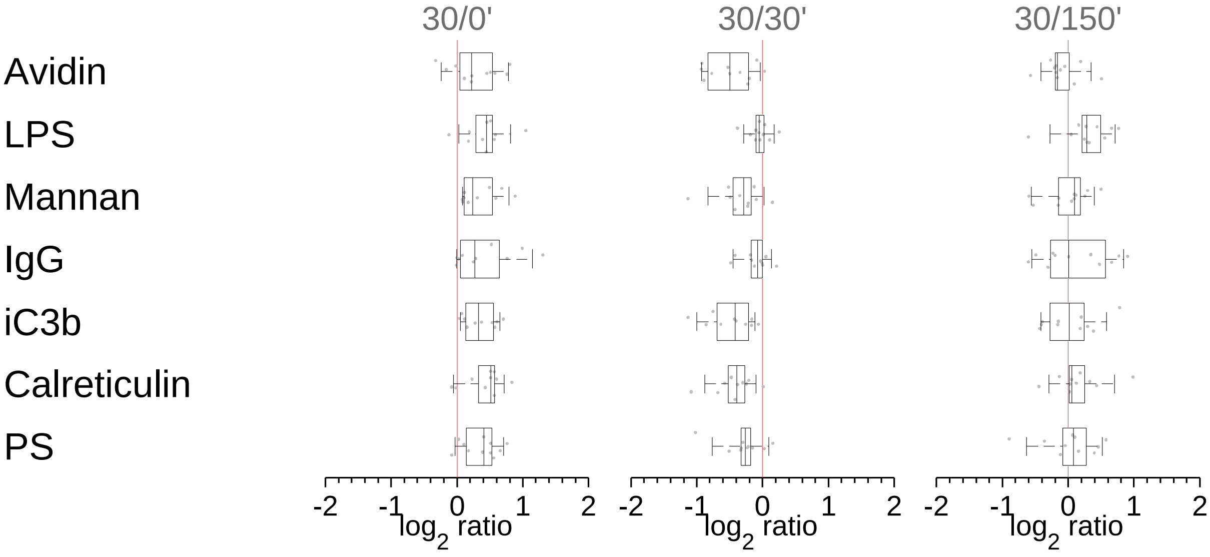

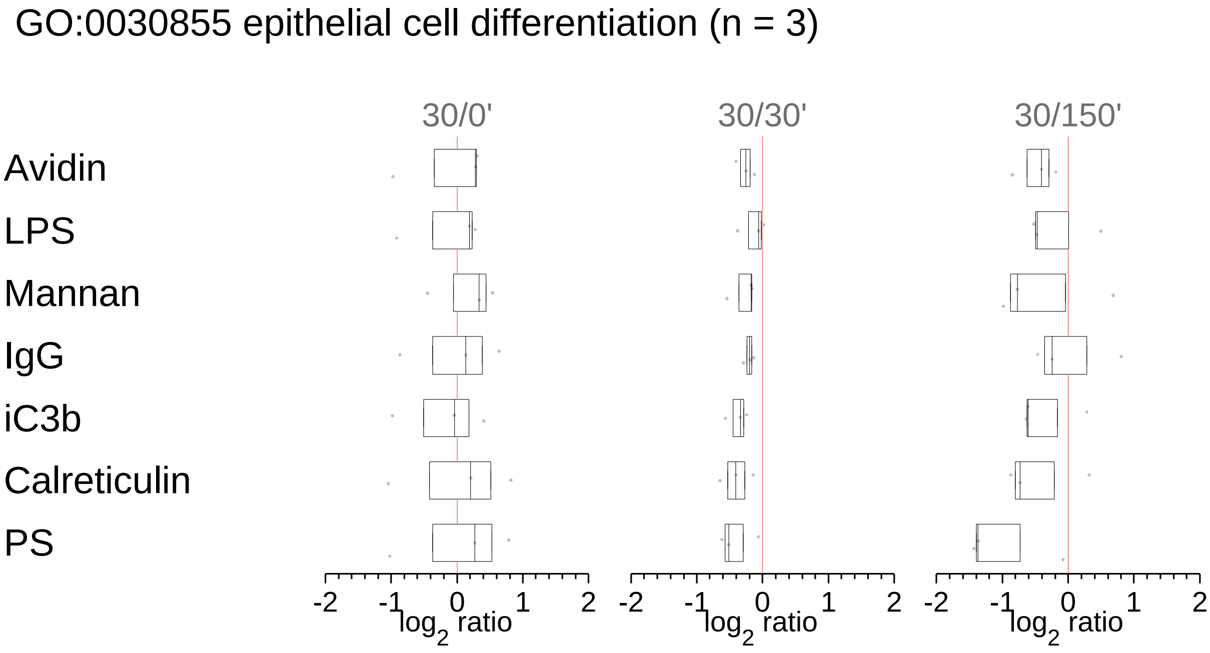
GO:0030855 epithelial cell differentiation (n = 3)
30/0'
30/30'
30/150'
Avidin
LPS
Mannan
IgG
iC3b
Calreticulin
PS
-2
-1
0
1
2
log2 ratio

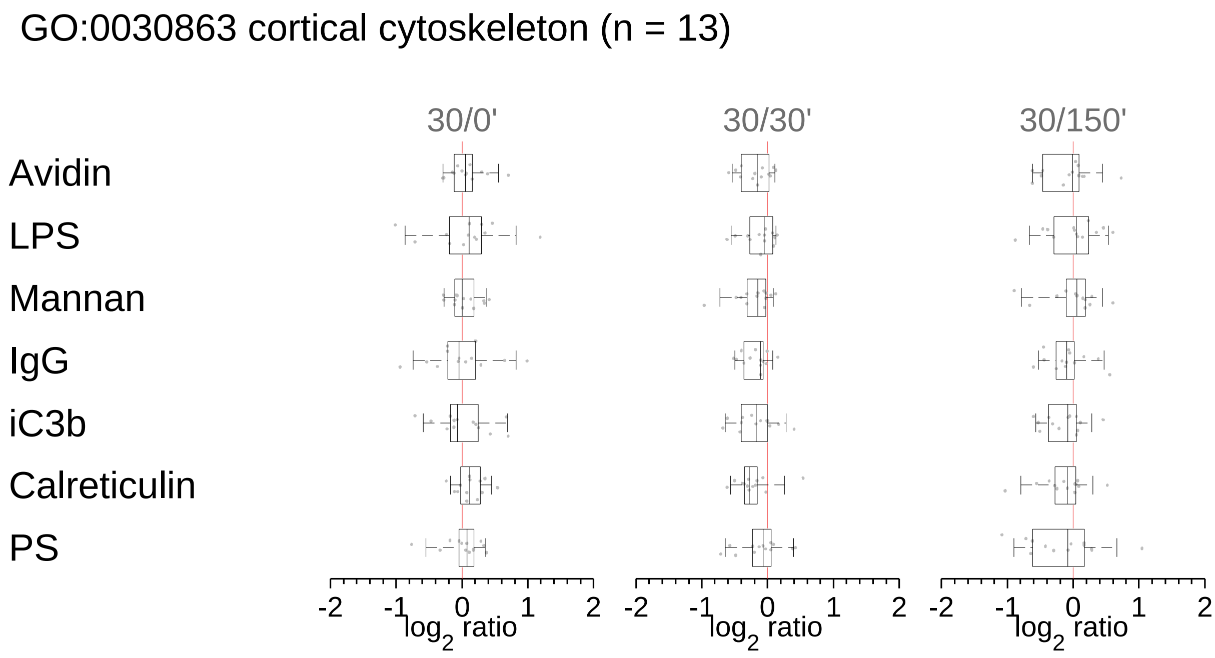
GO:0030863 cortical cytoskeleton (n = 13)
30/0'
30/30'
30/150'
Avidin
LPS
Mannan
IgG
iC3b
Calreticulin
PS
-2
-1
0
1
2
log2 ratio

GO:0030864 cortical actin cytoskeleton (n = 9)

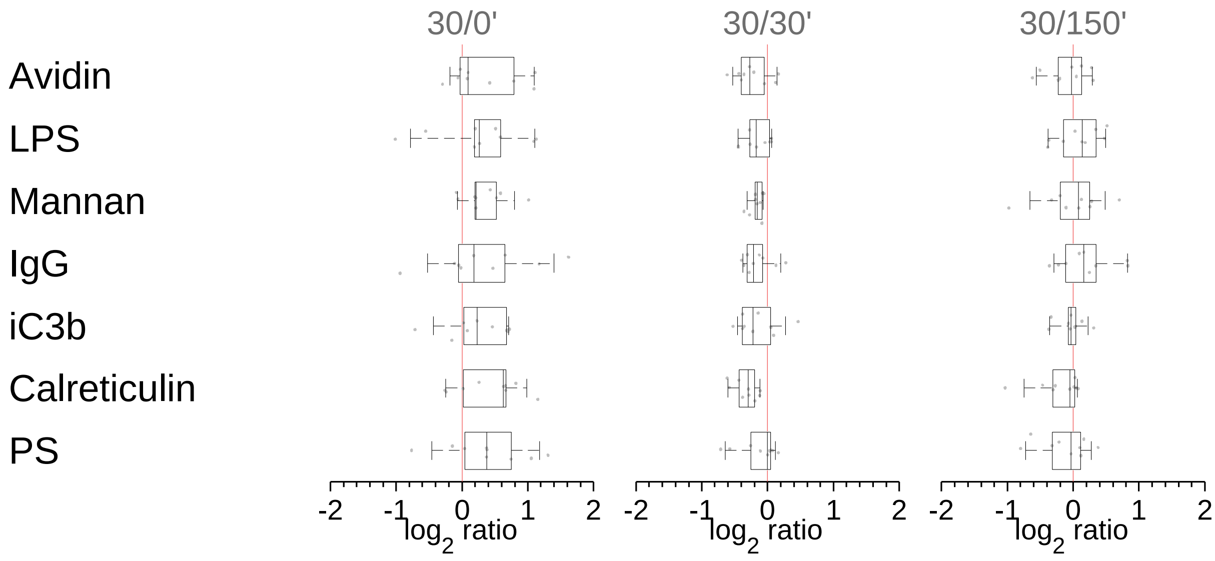

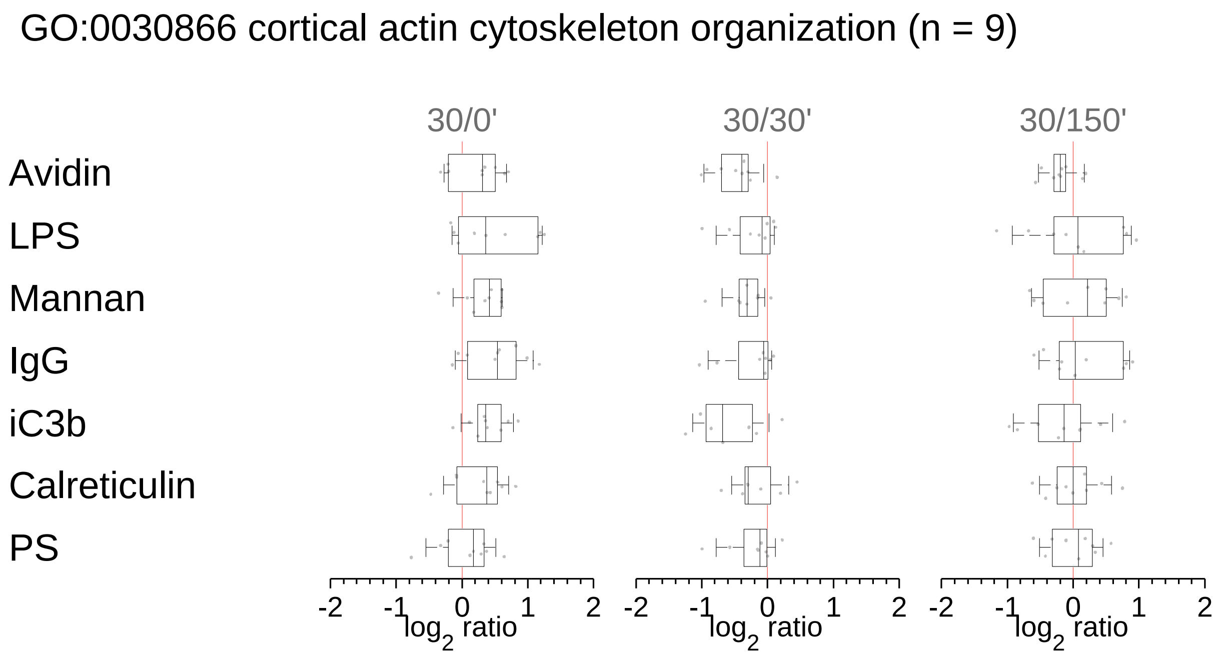
GO:0030866 cortical actin cytoskeleton organization (n = 9)
30/0'
30/30'
30/150'
Avidin
LPS
Mannan
IgG
iC3b
Calreticulin
PS
-2
-1
0
1
2
log2 ratio

GO:0030889 negative regulation of B cell proliferation (n = 4)

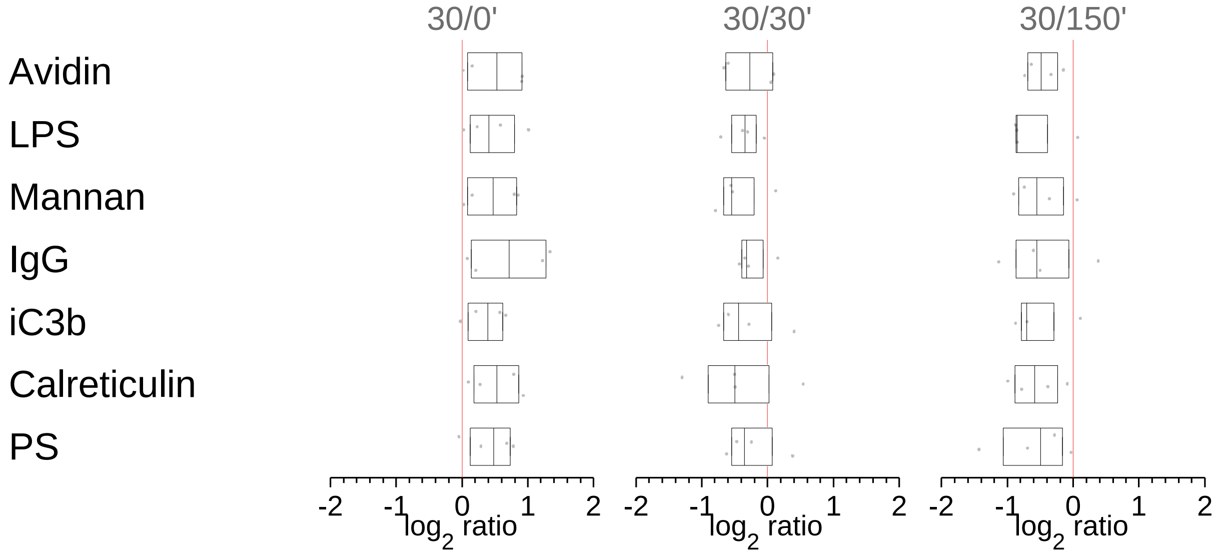

GO:0030890 positive regulation of B cell proliferation (n = 4)

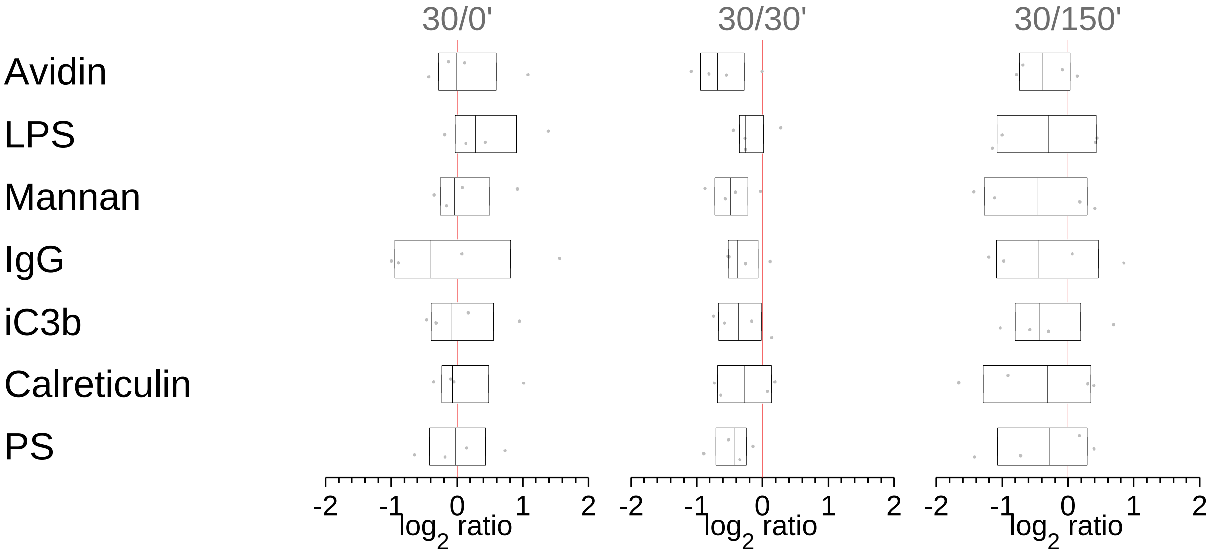

GO:0030897 HOPS complex (n = 8)

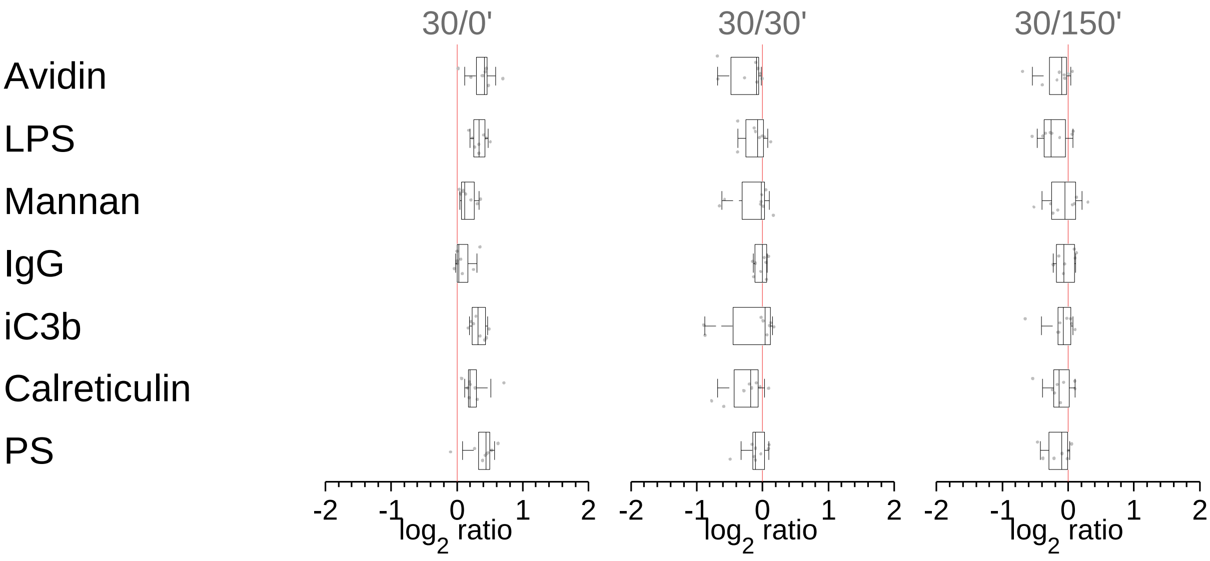

GO:0030898 actin-dependent ATPase activity (n = 3)

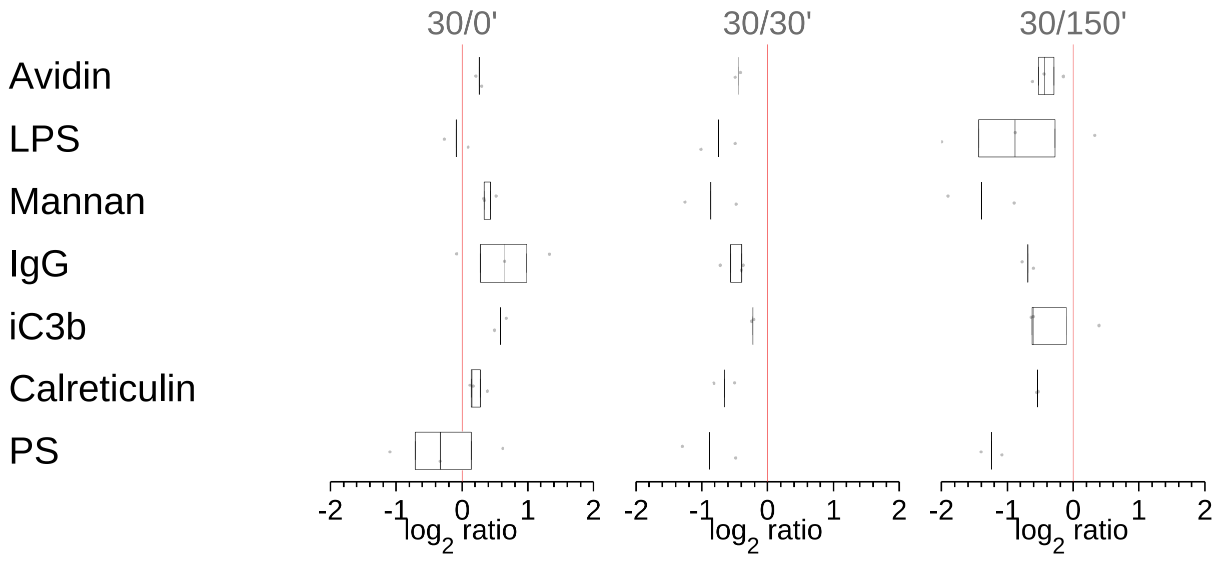

GO:0030900 forebrain development (n = 8)

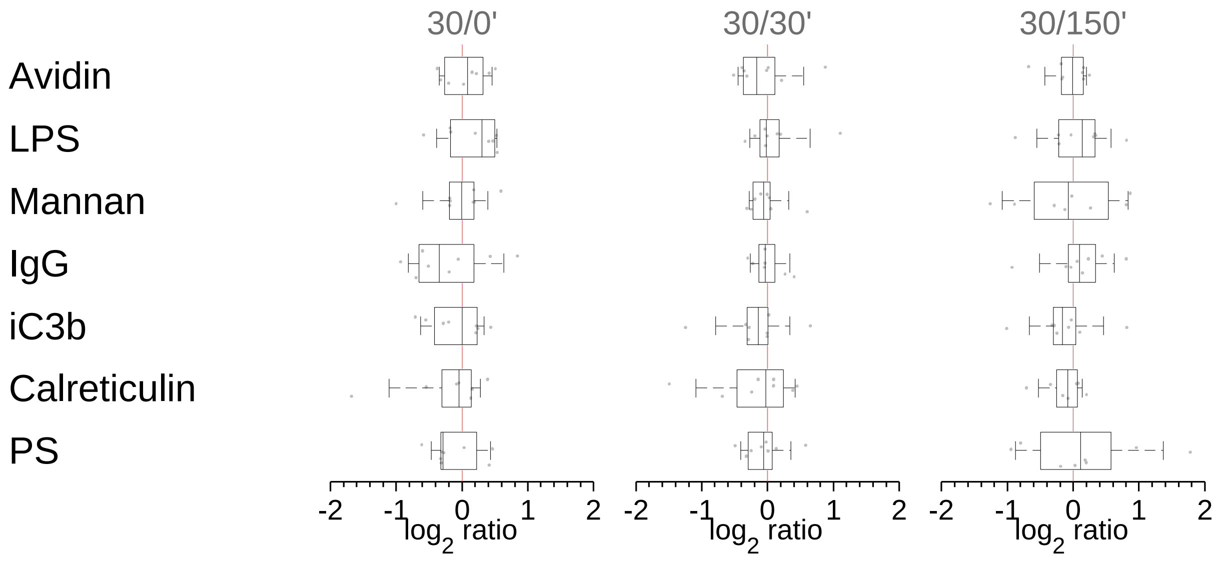

GO:0030904 retromer complex (n = 6)

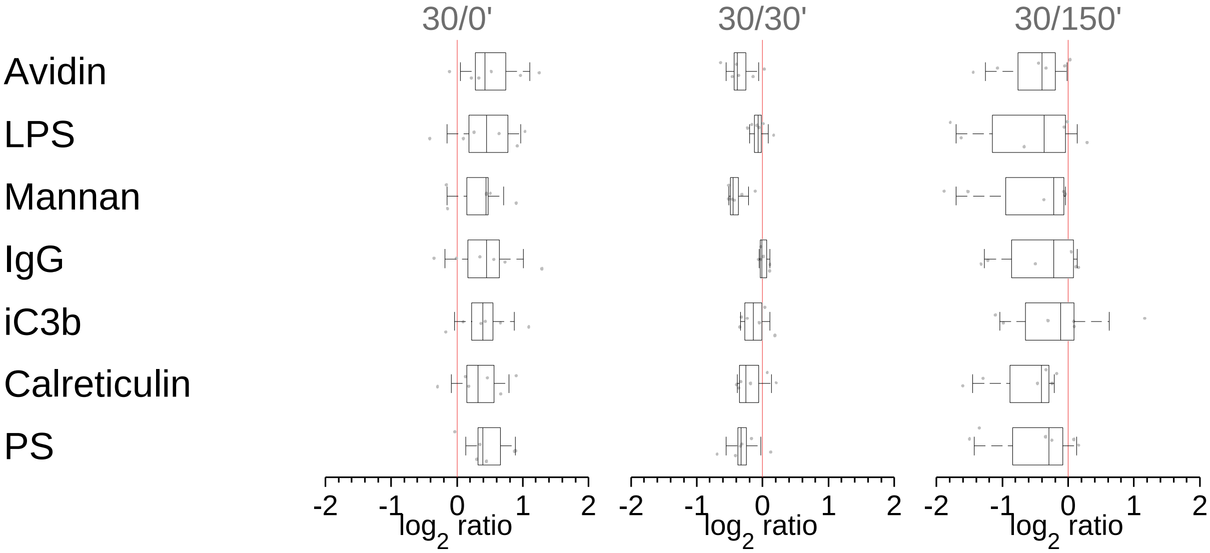

GO:0030955 potassium ion binding (n = 3)

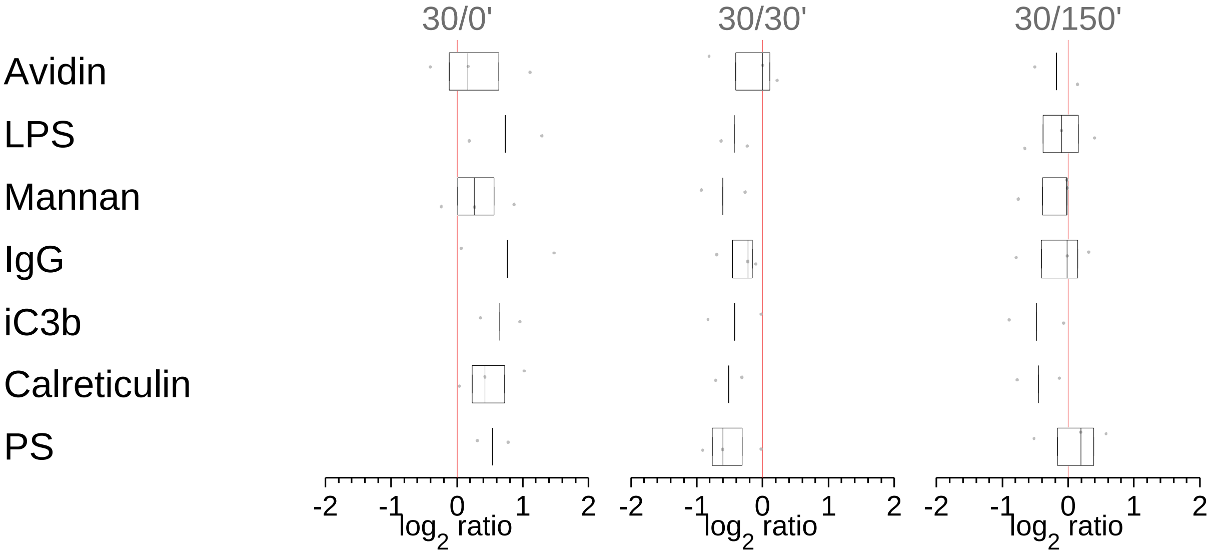

GO:0030968 endoplasmic reticulum unfolded protein response (n = 3)

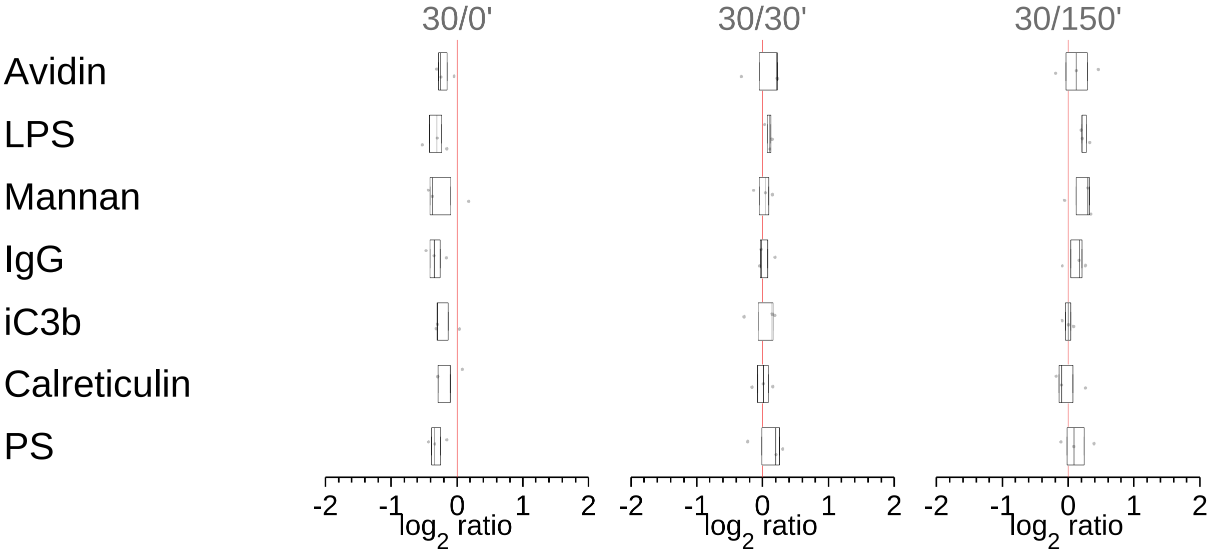

GO:0030971 receptor tyrosine kinase binding (n = 6)

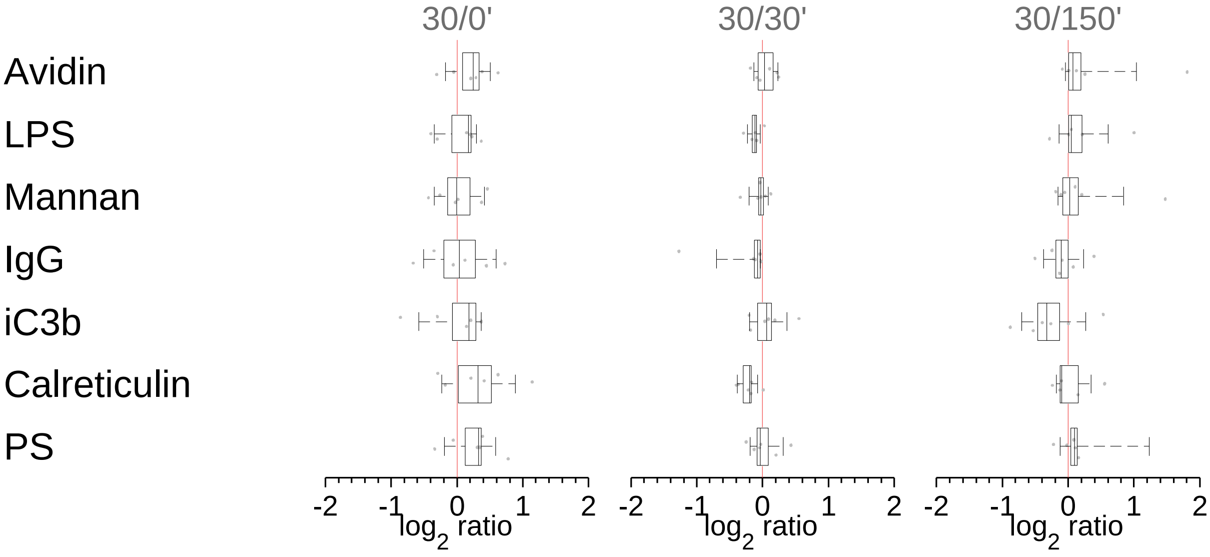

GO:0030976 thiamine pyrophosphate binding (n = 3)

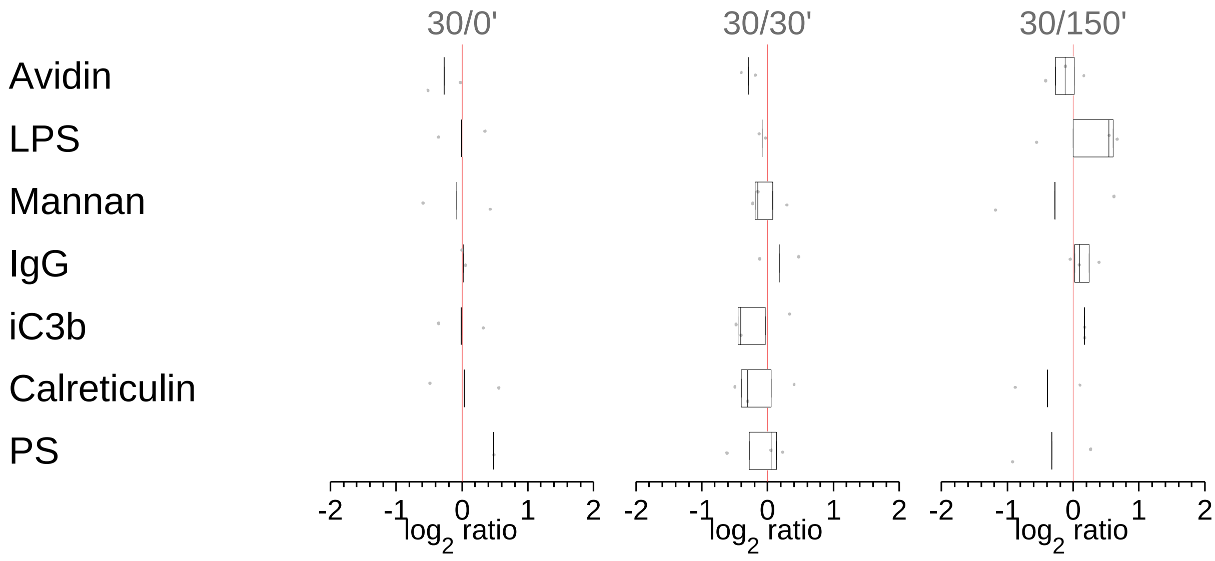

GO:0030984 kininogen binding (n = 3)

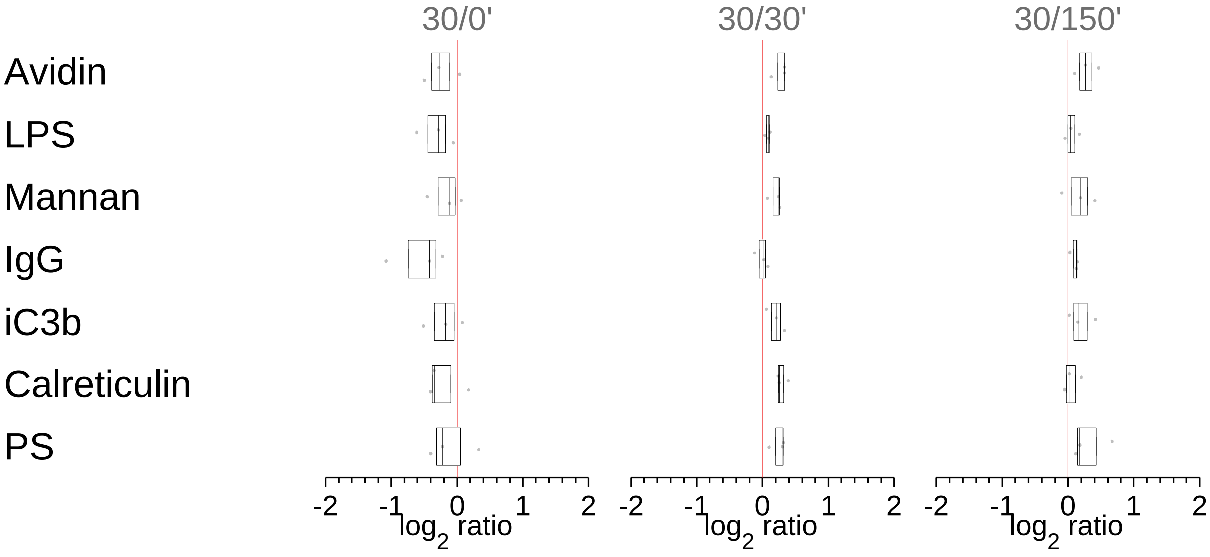

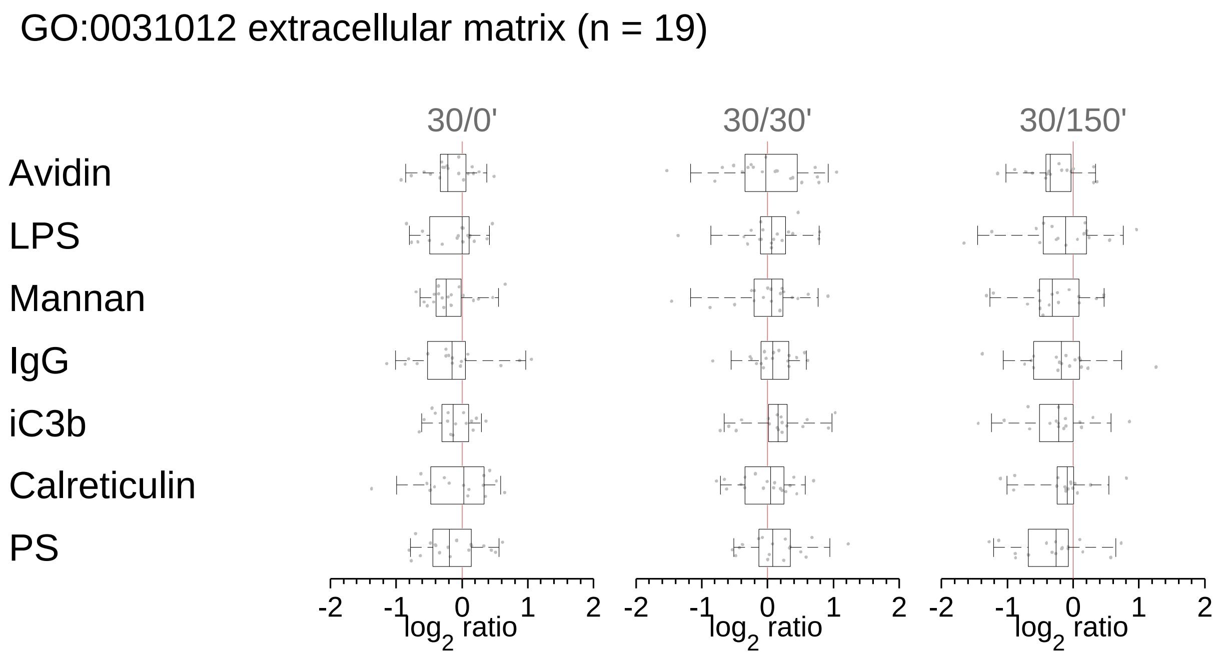
GO:0031012 extracellular matrix (n = 19)
30/0'
30/30'
30/150'
Avidin
LPS
Mannan
IgG
iC3b
Calreticulin
PS
-2
-1
0
1
2
log2 ratio

GO:0031032 actomyosin structure organization (n = 5)

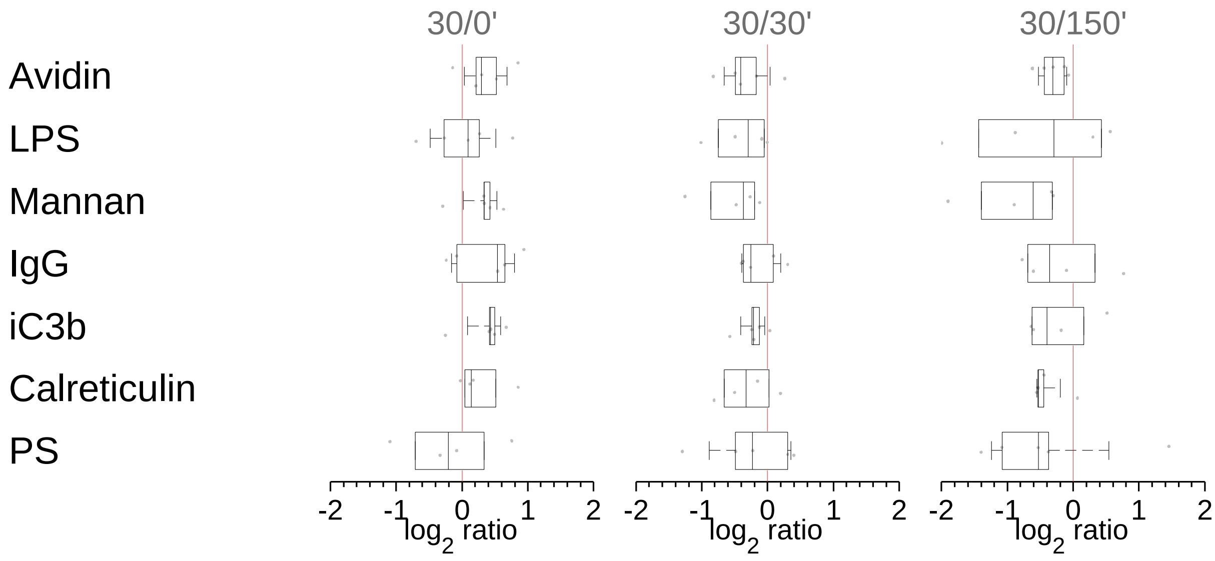

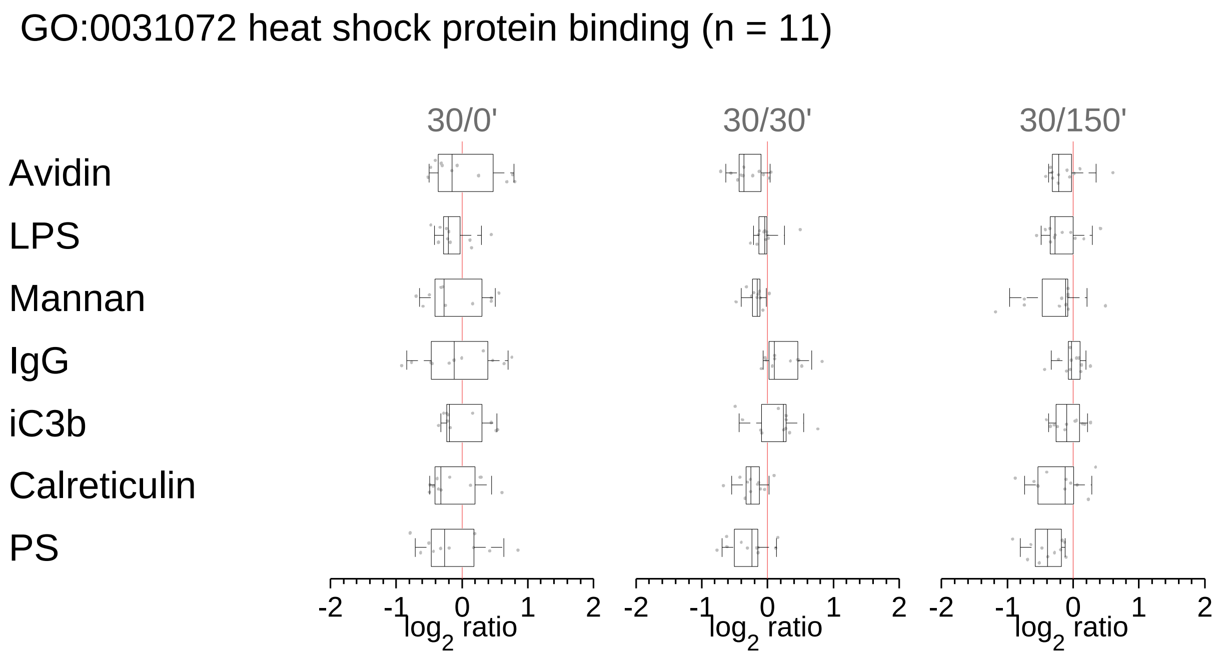
GO:0031072 heat shock protein binding (n = 11)
30/0'
30/30'
30/150'
Avidin
LPS
Mannan
IgG
iC3b
Calreticulin
PS
-2
-1
0
1
2
log2 ratio

GO:0031083 BLOC-1 complex (n = 4)

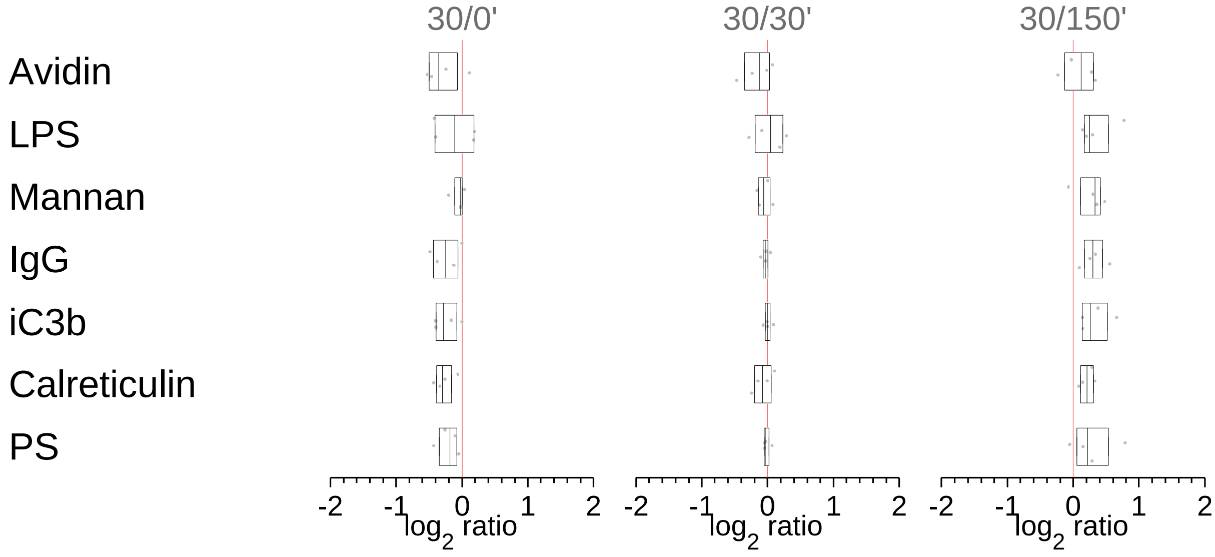

GO:0031091 platelet alpha granule (n = 4)

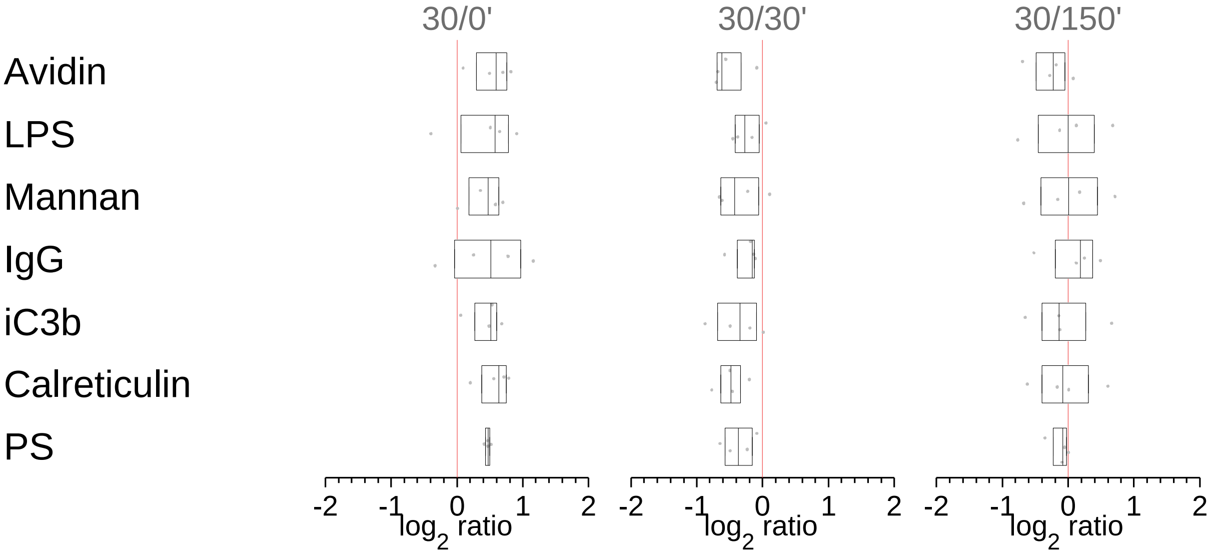

GO:0031105 septin complex (n = 4)

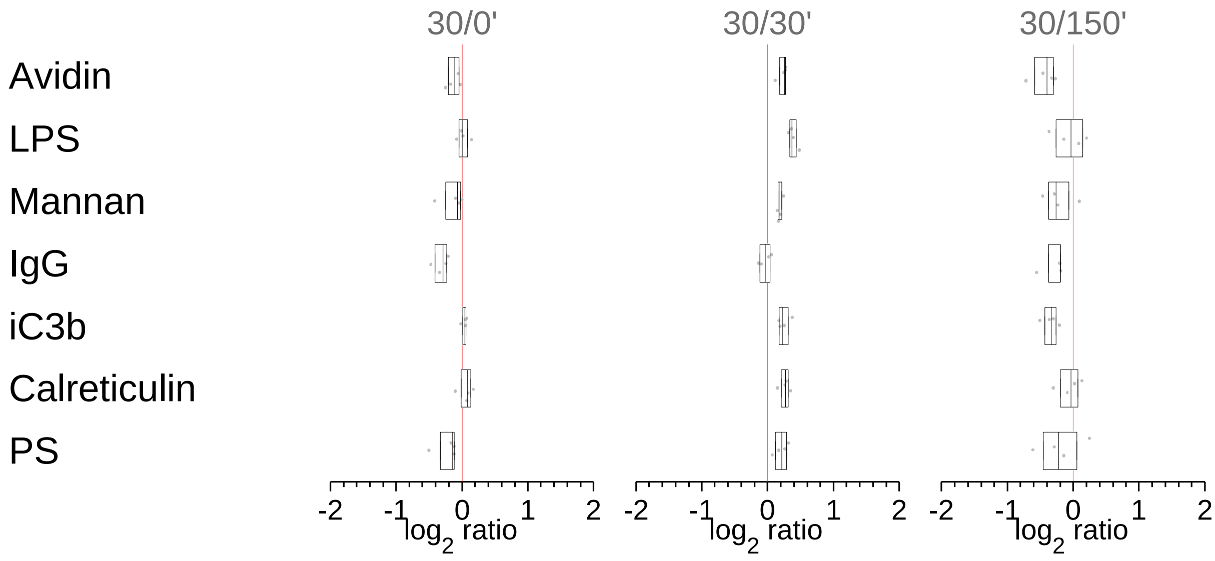

GO:0031110 regulation of microtubule polymerization or depolymerization (n = 3)

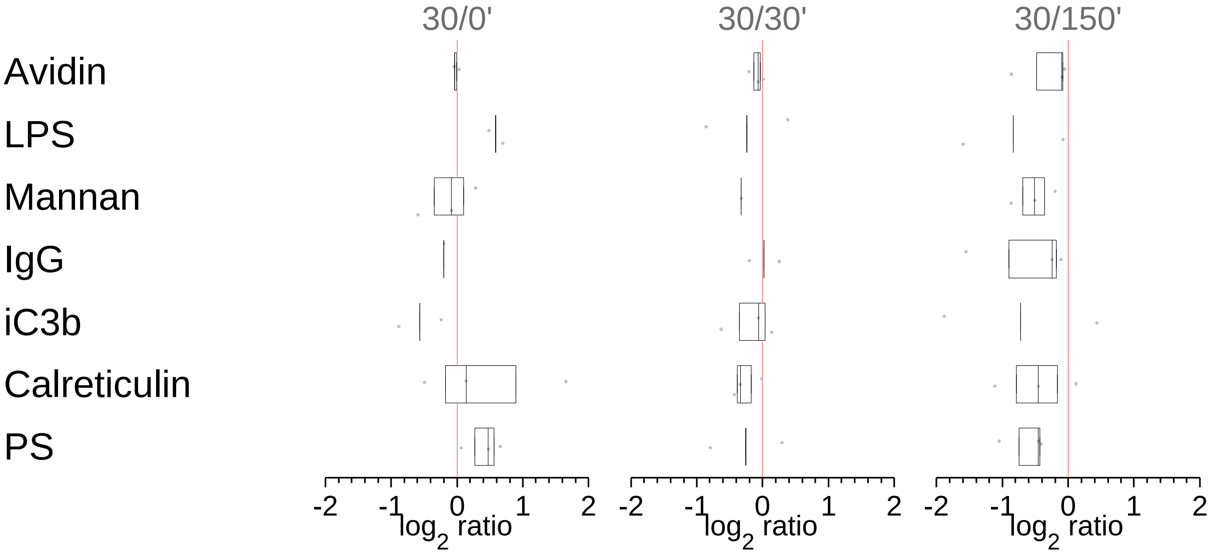

GO:0031115 negative regulation of microtubule polymerization (n = 4)

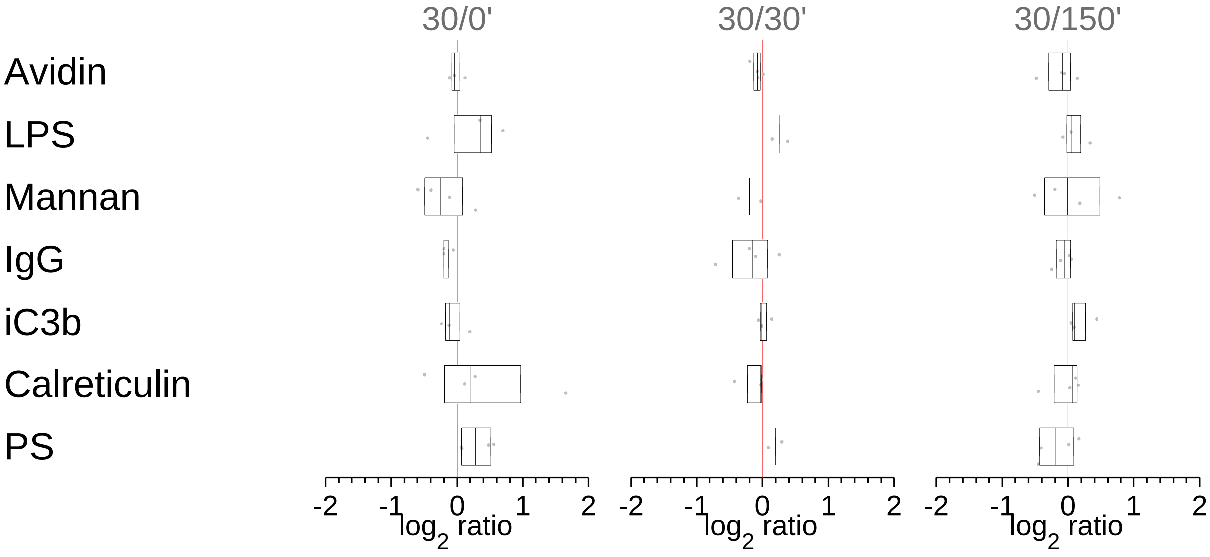

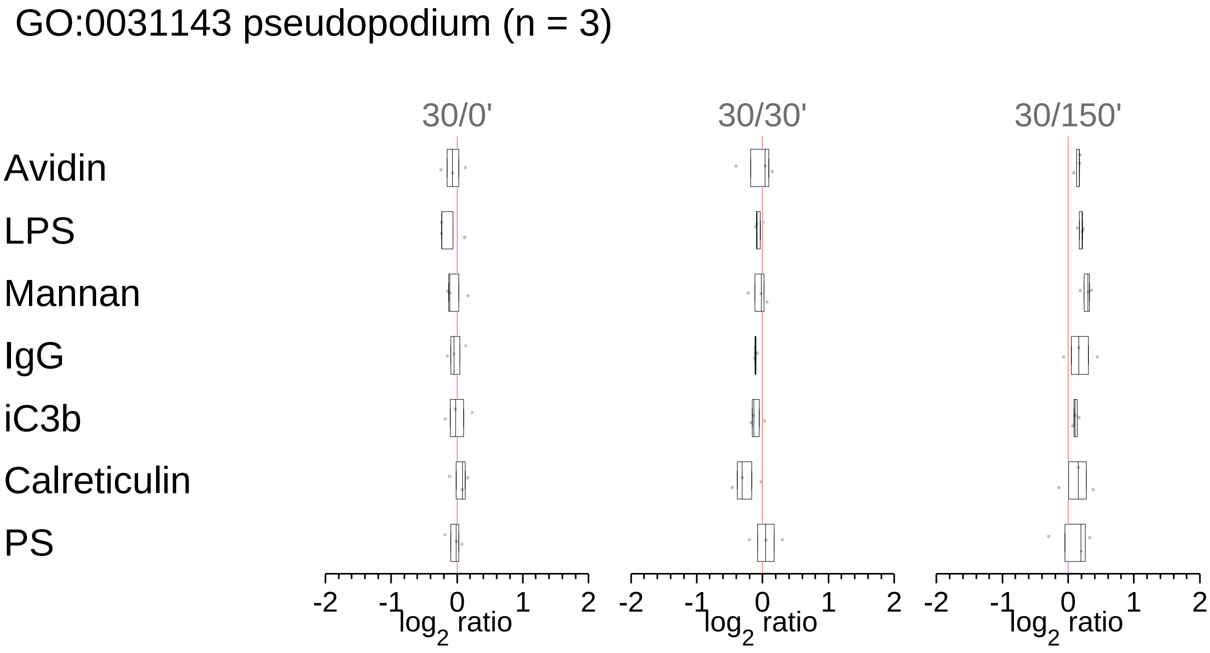
GO:0031143 pseudopodium (n = 3)
30/0'
30/30'
30/150'
Avidin
LPS
Mannan
IgG
iC3b
Calreticulin
PS
-2
-1
0
1
2
log2 ratio

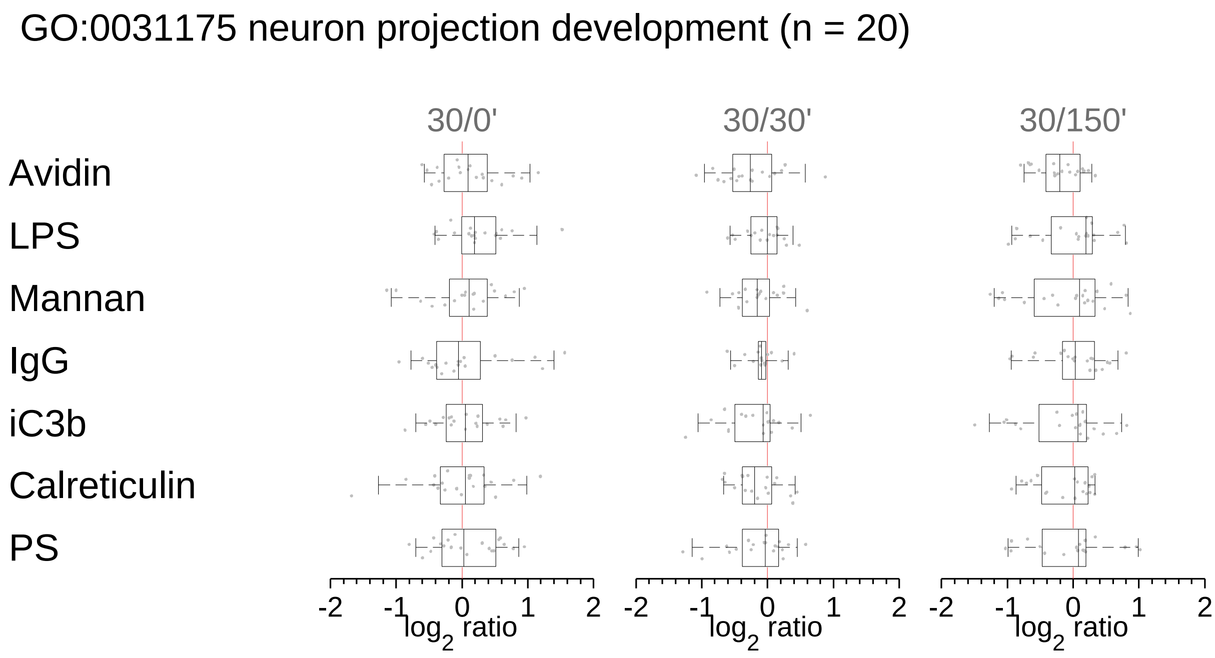
GO:0031175 neuron projection development (n = 20)
30/0'
30/30'
30/150'
Avidin
LPS
Mannan
IgG
iC3b
Calreticulin
PS
-2
-1
0
1
2
$\log_2$ ratio

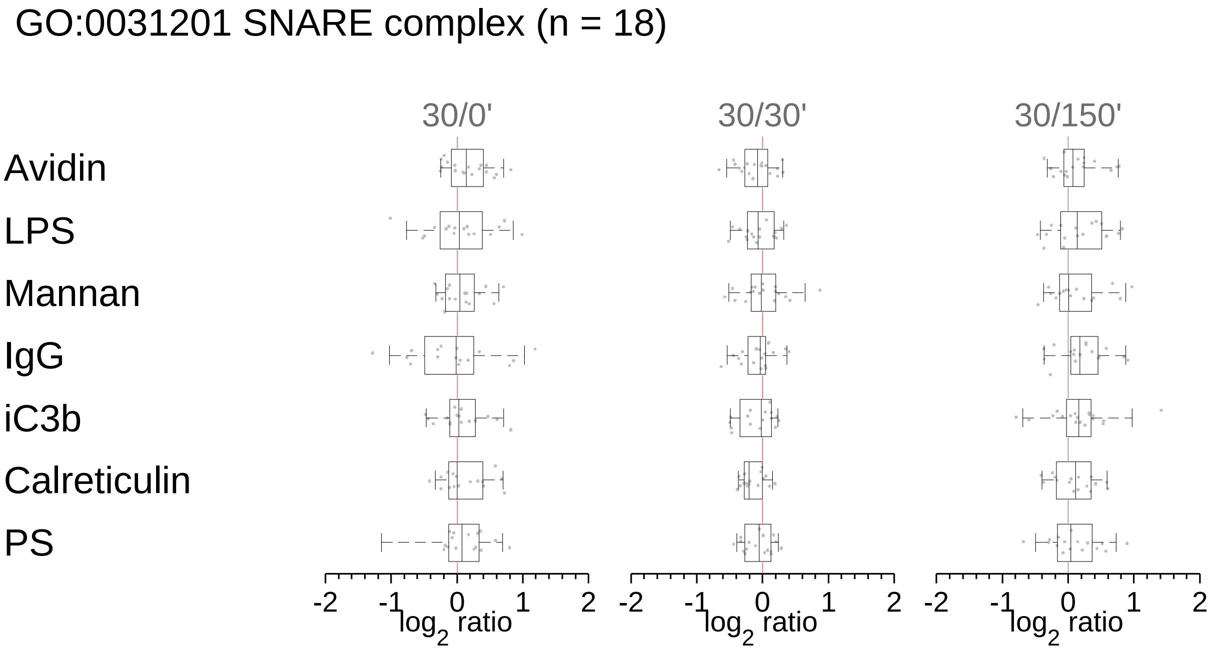

GO:0031201 SNARE complex (n = 18)
30/0'
30/30'
30/150'
Avidin
LPS
Mannan
IgG
iC3b
Calreticulin
PS
-2 -1 0 1 2
log2 ratio

GO:0031209 SCAR complex (n = 3)

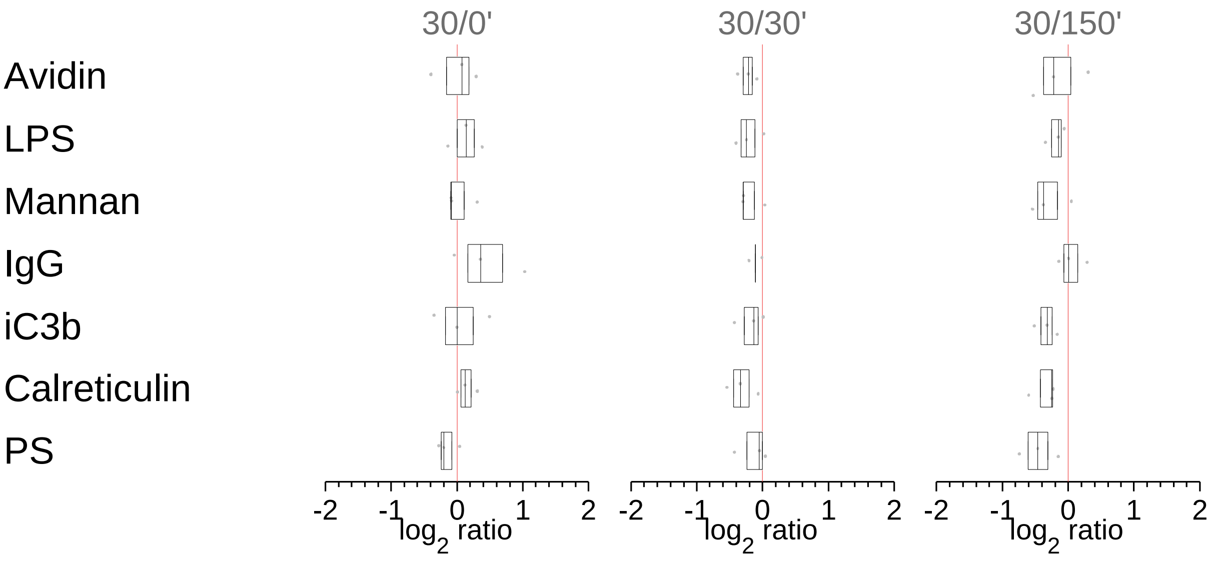

GO:0031225 anchored to membrane (n = 13)

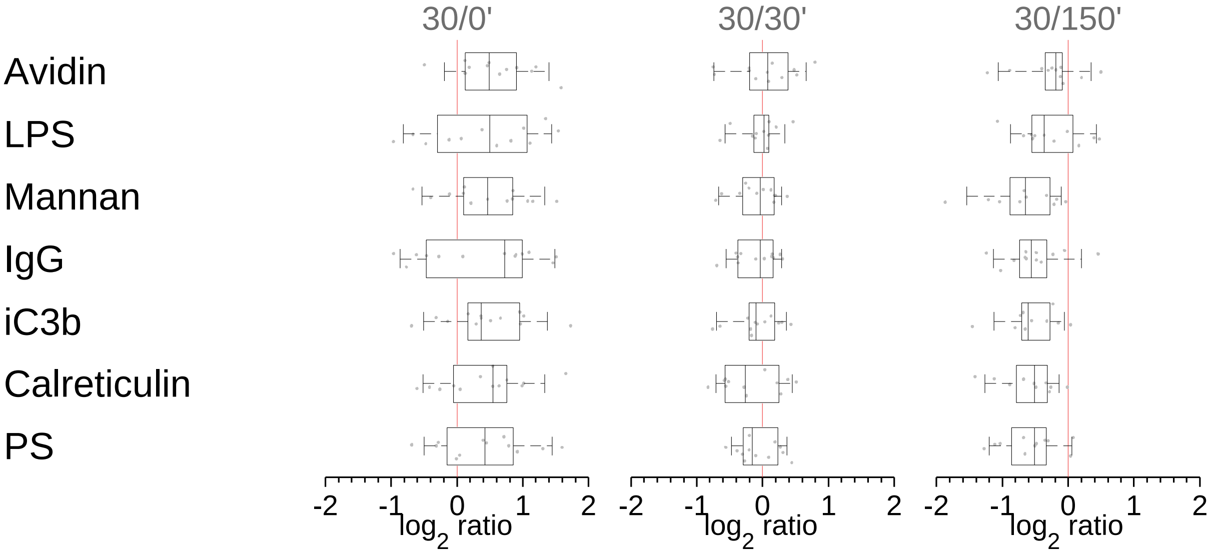

GO:0031234 extrinsic to internal side of plasma membrane (n = 7)

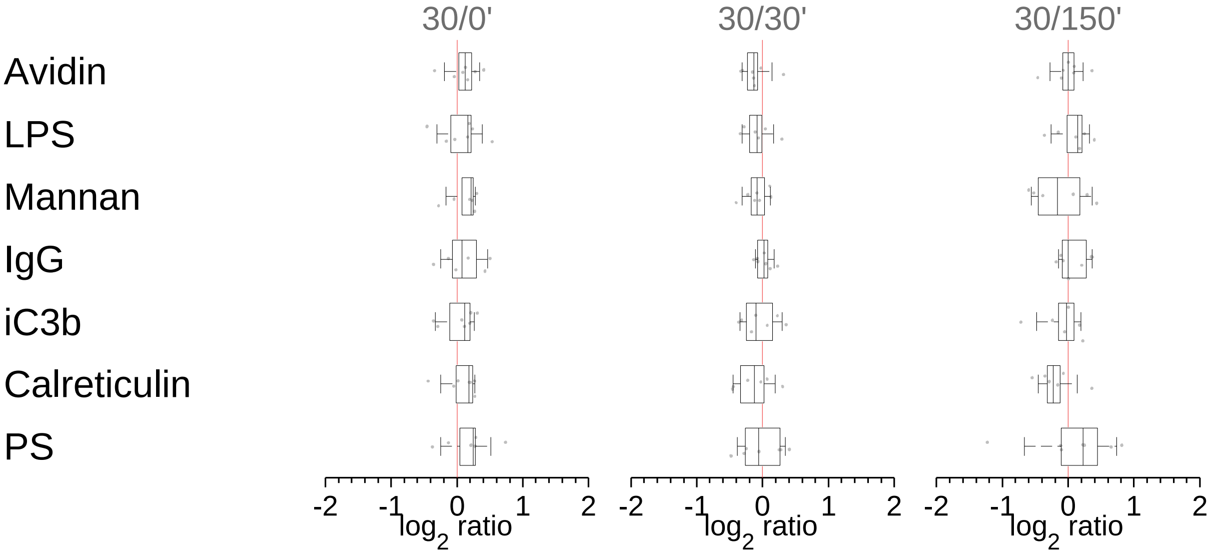

GO:0031252 cell leading edge (n = 15)

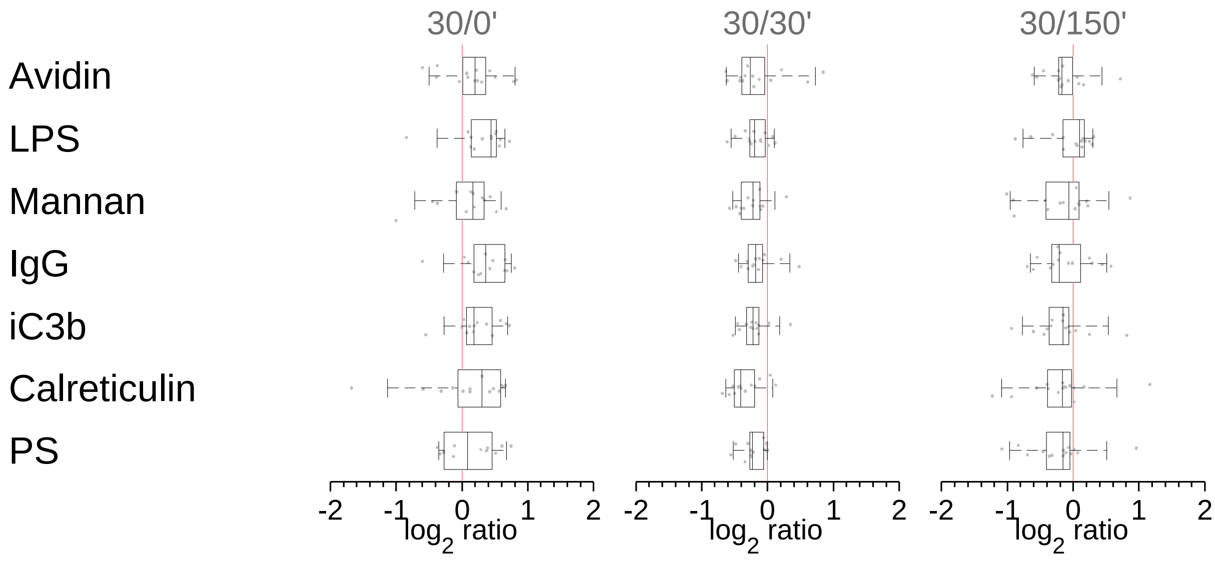

GO:0031278 alpha-1,2-galactosyltransferase activity (n = 3)

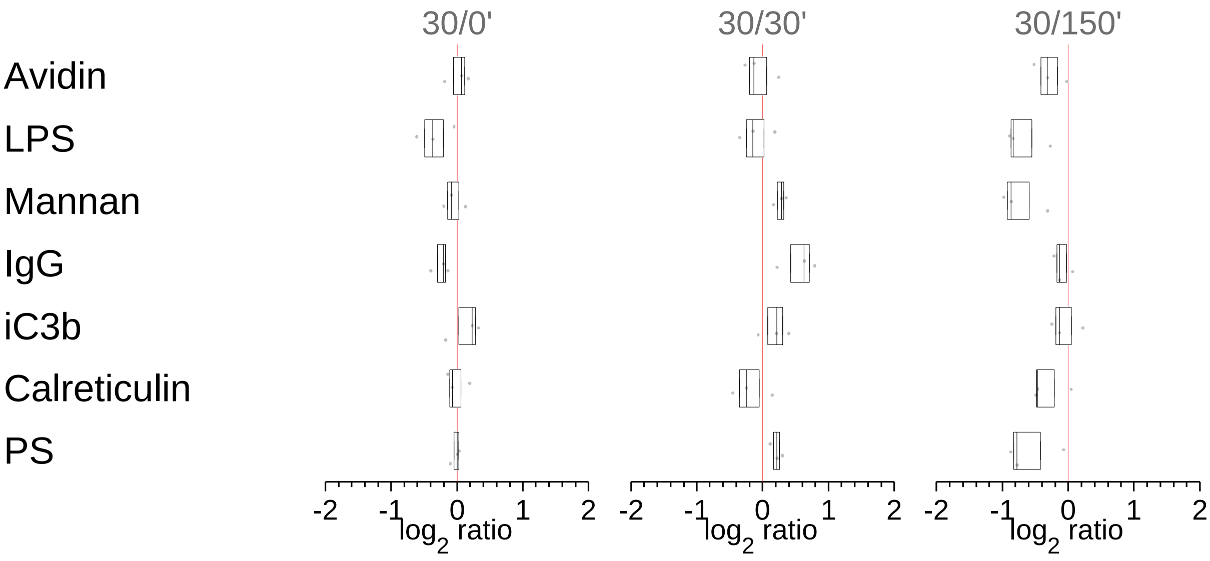

GO:0031295 T cell costimulation (n = 3)

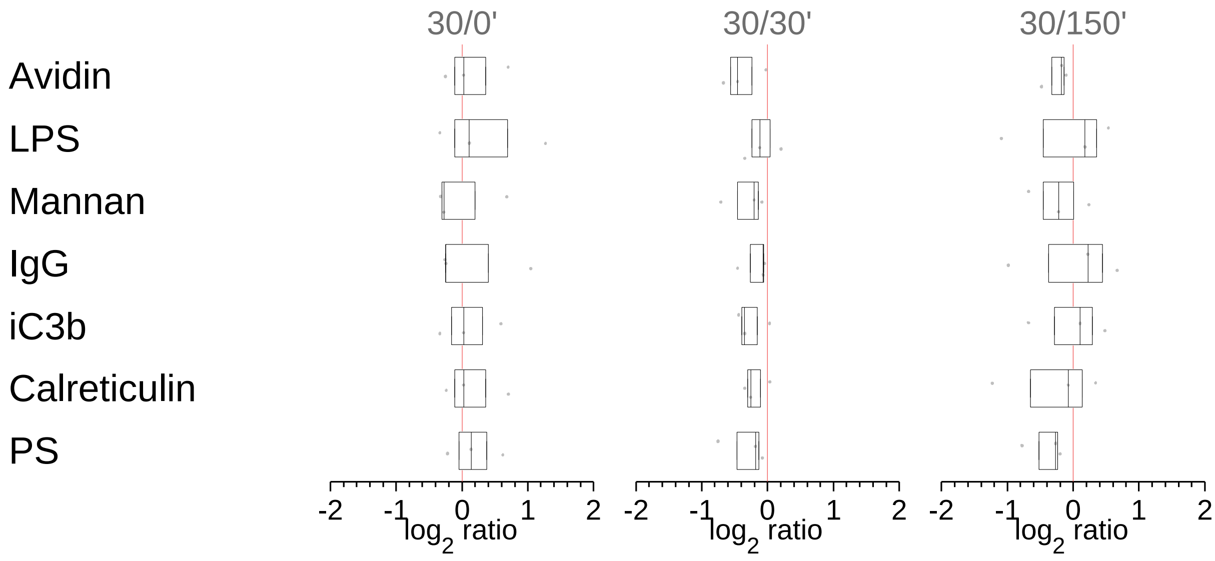

GO:0031301 integral to organelle membrane (n = 3)

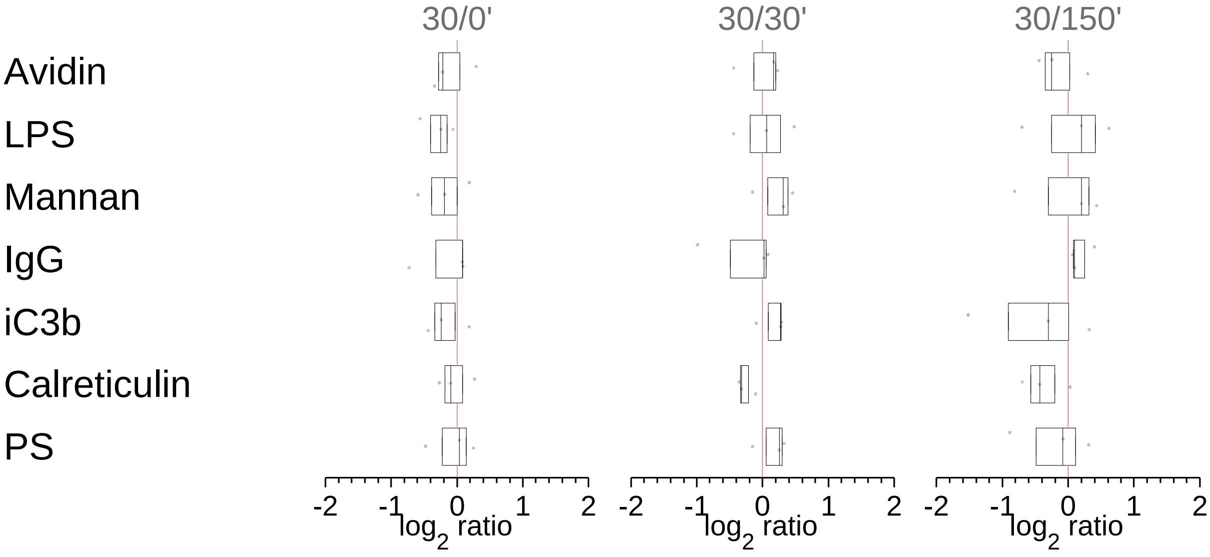

GO:0031307 integral to mitochondrial outer membrane (n = 5)

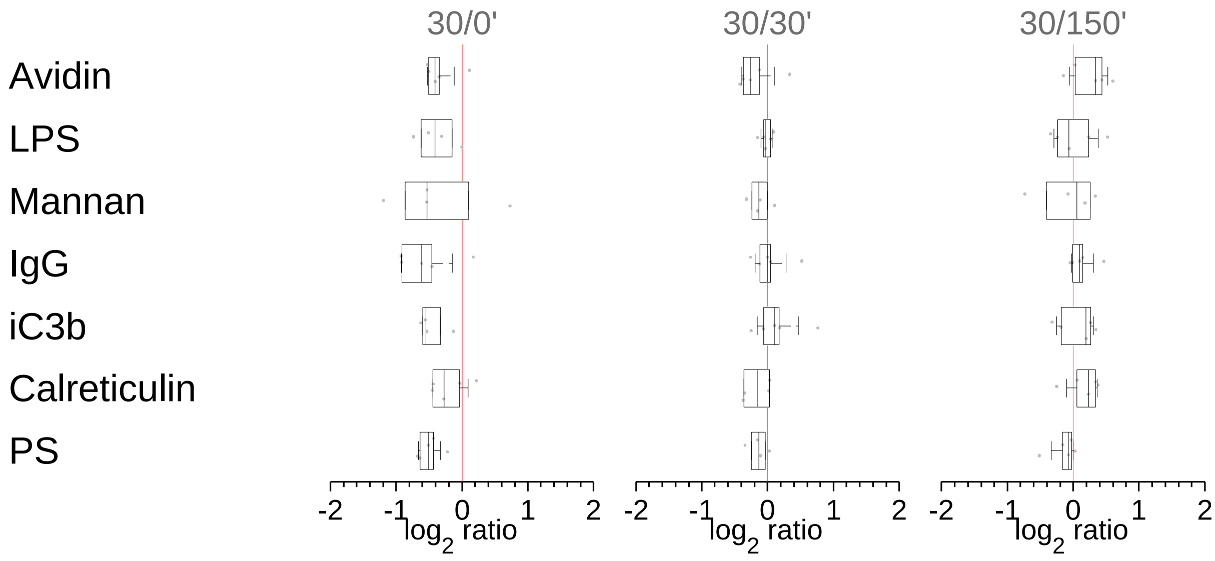

GO:0031333 negative regulation of protein complex assembly (n = 3)

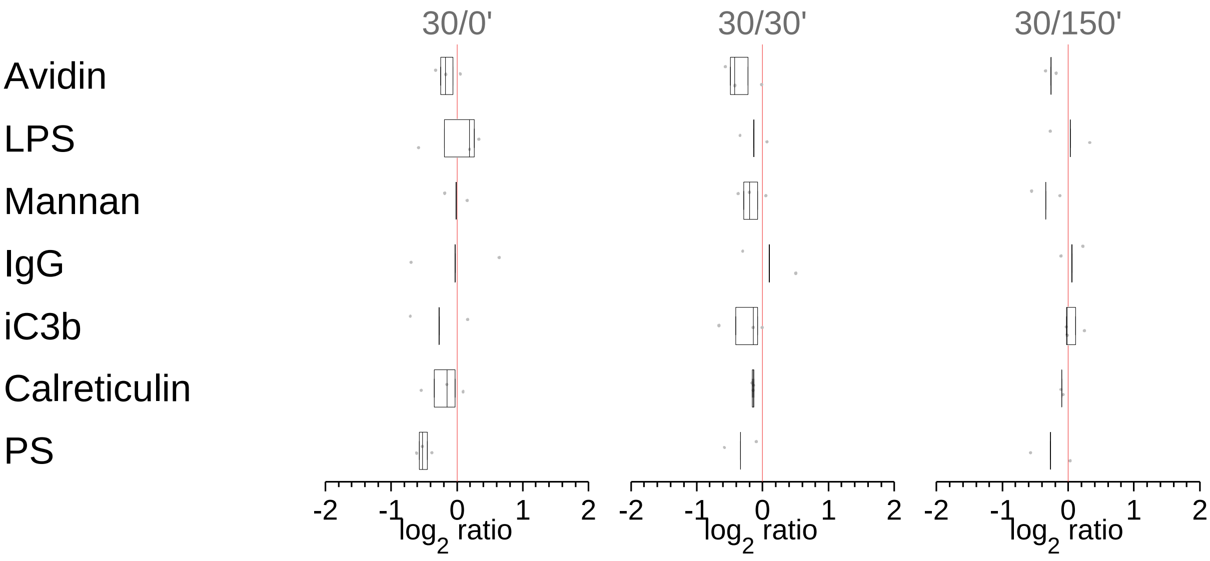

GO:0031369 translation initiation factor binding (n = 3)

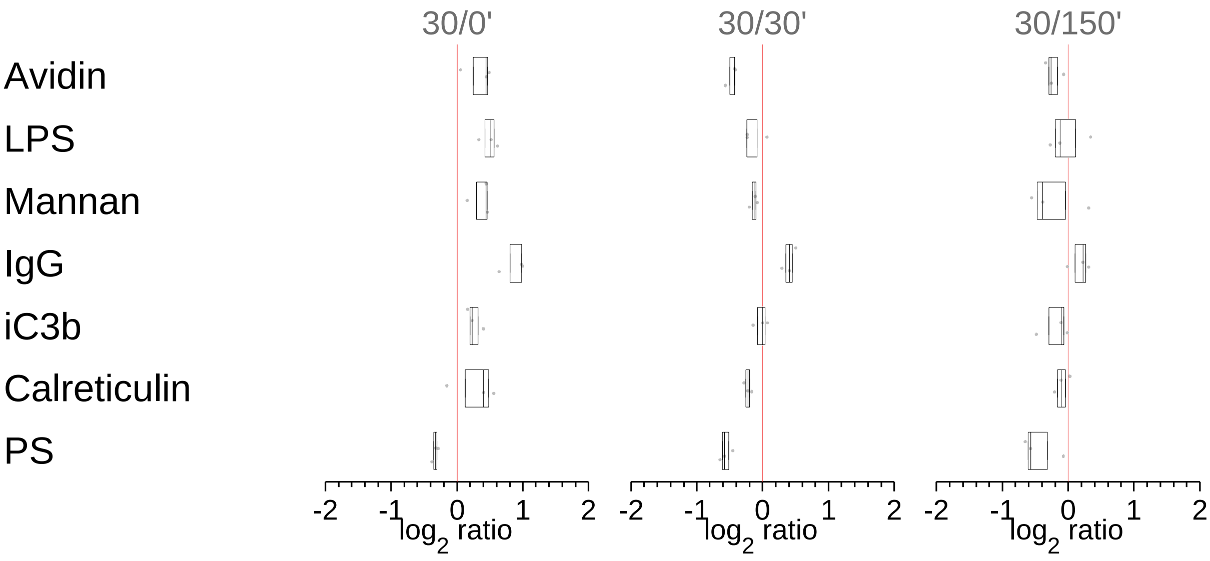

GO:0031397 negative regulation of protein ubiquitination (n = 4)

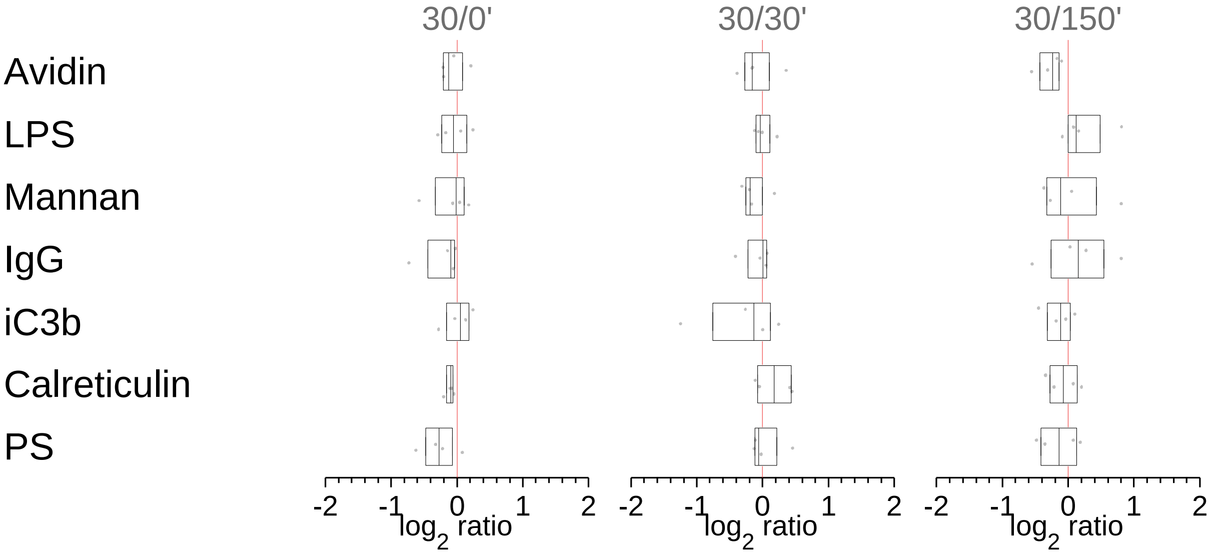

GO:0031398 positive regulation of protein ubiquitination (n = 5)

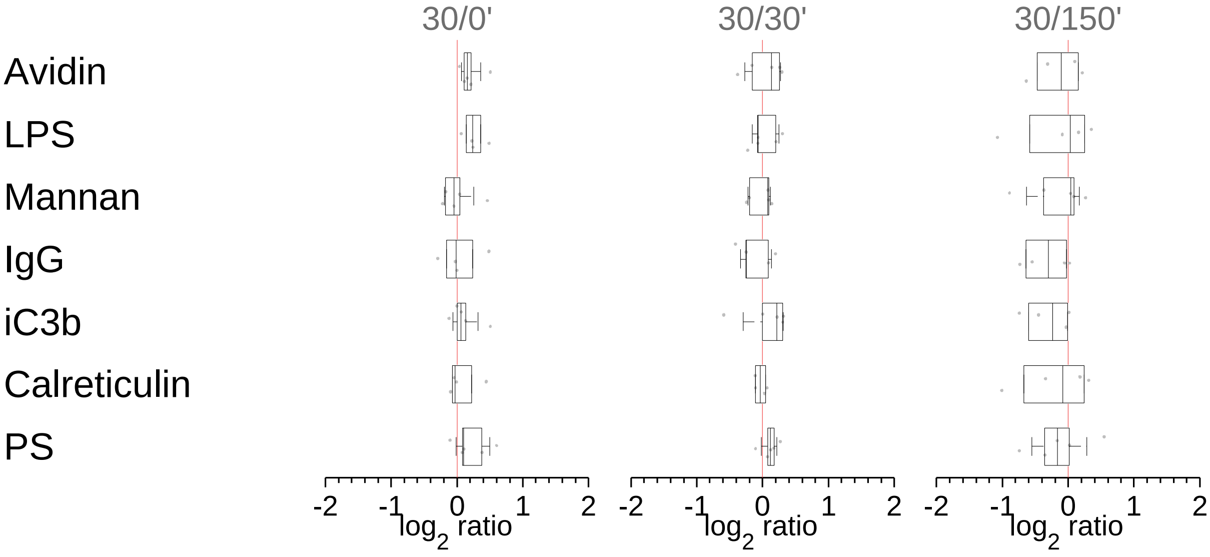

GO:0031410 cytoplasmic vesicle (n = 129)

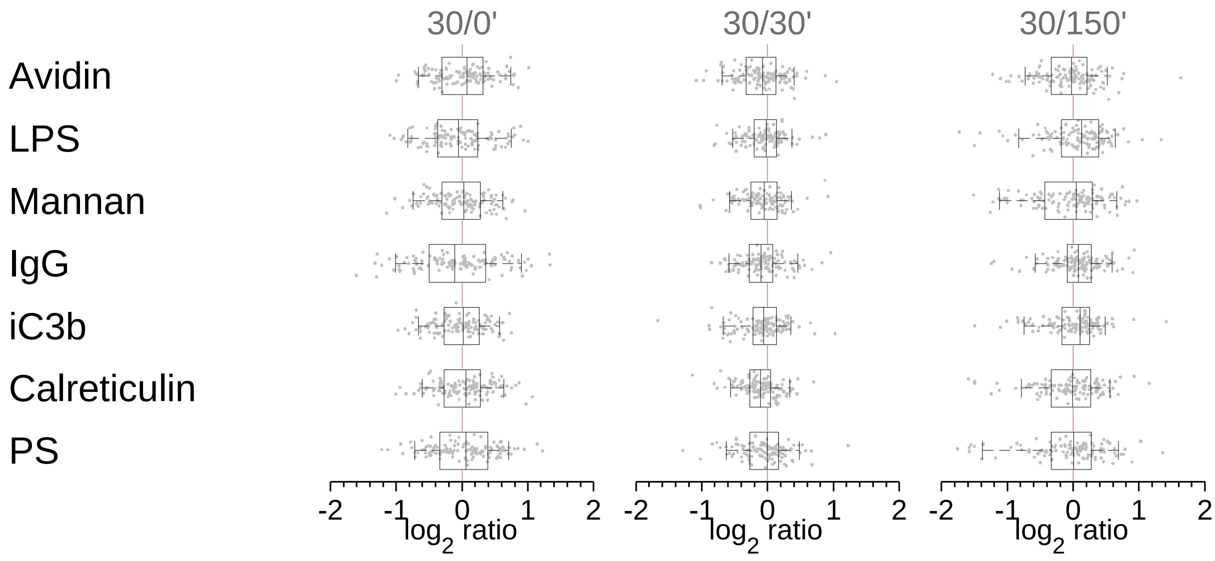

GO:0031435 mitogen-activated protein kinase kinase kinase binding (n = 5)

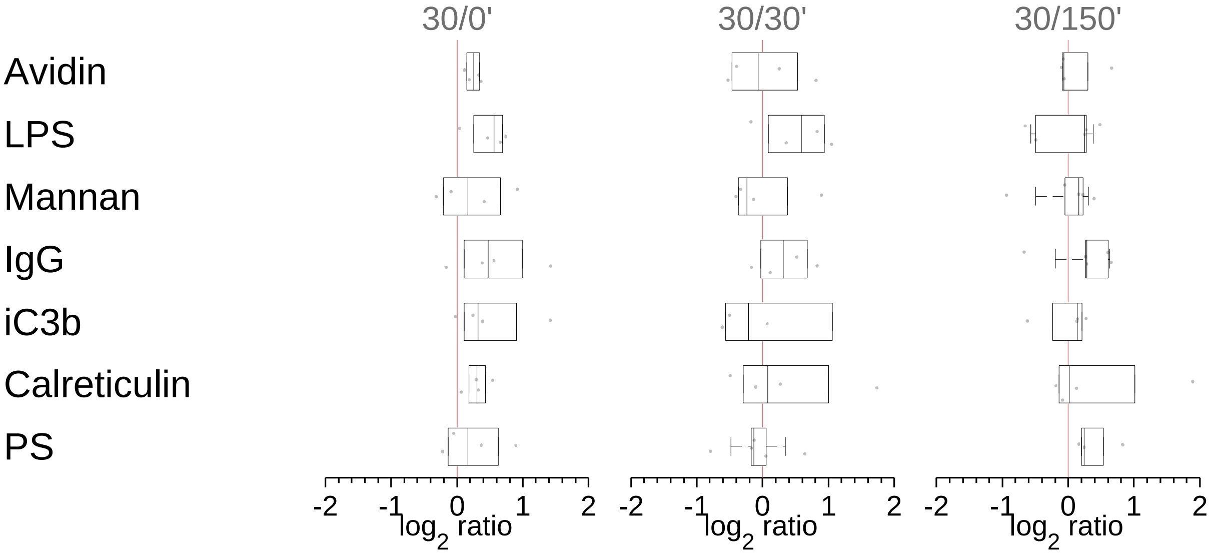

GO:0031490 chromatin DNA binding (n = 5)

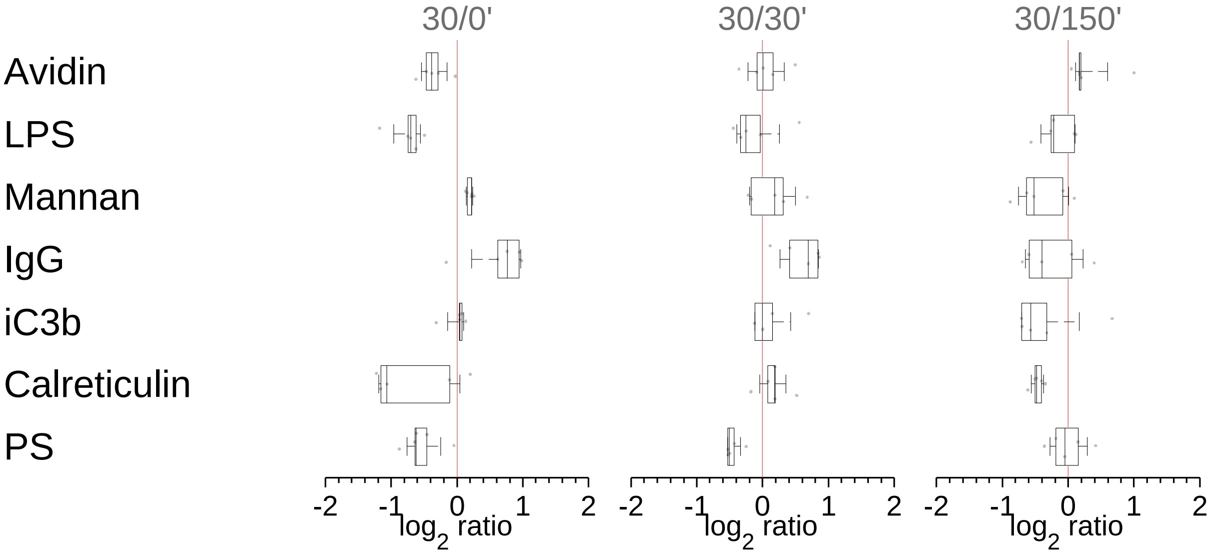

GO:0031492 nucleosomal DNA binding (n = 7)

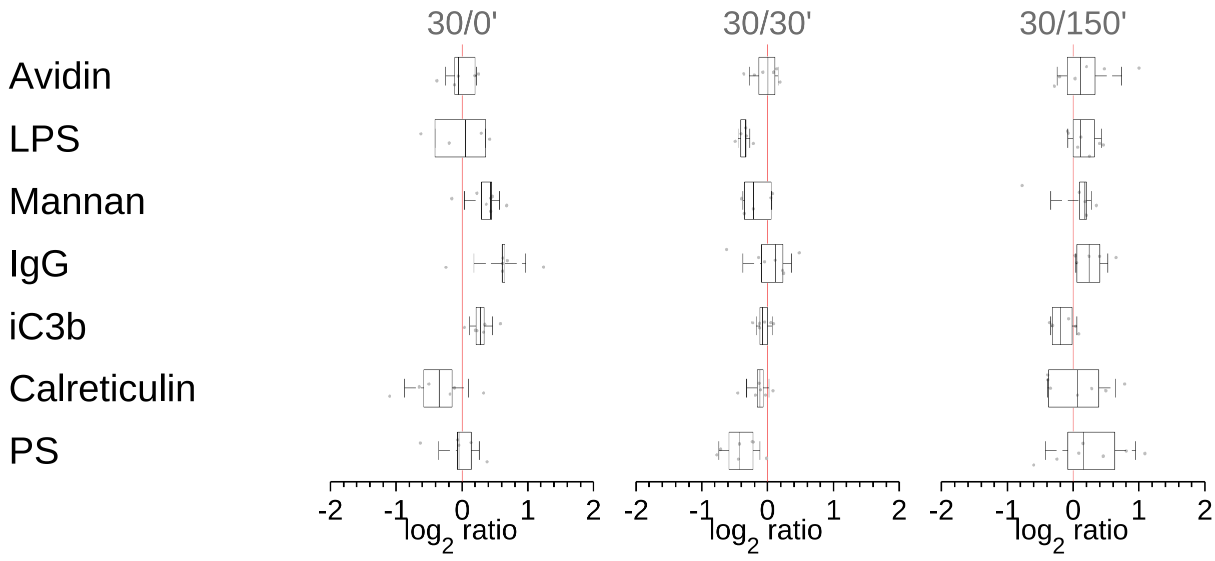

GO:0031519 PcG protein complex (n = 3)

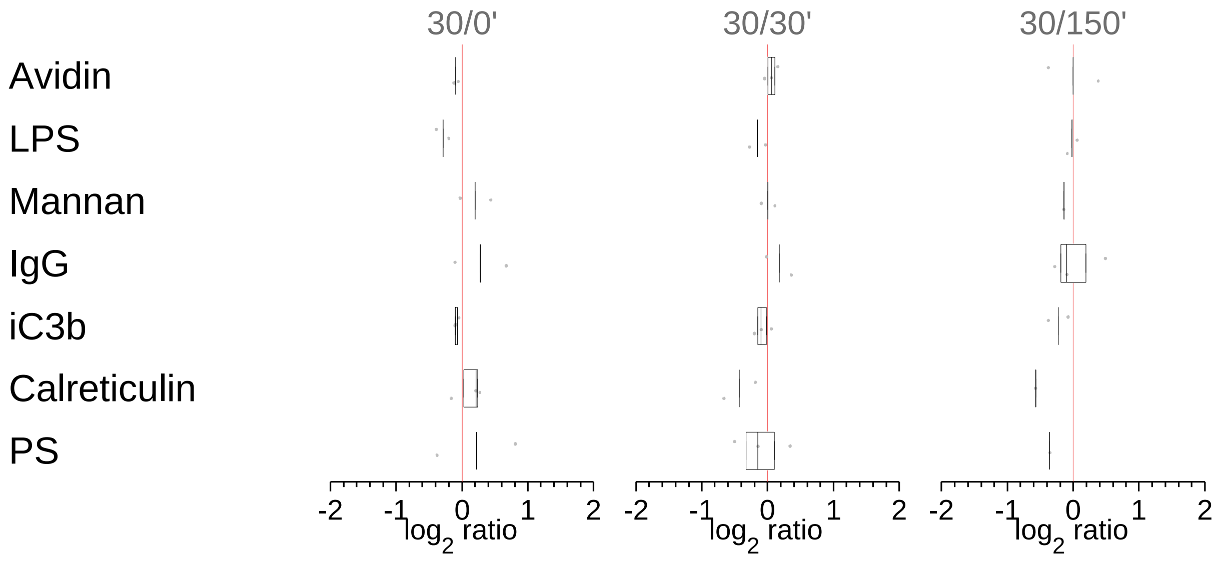

GO:0031526 brush border membrane (n = 4)

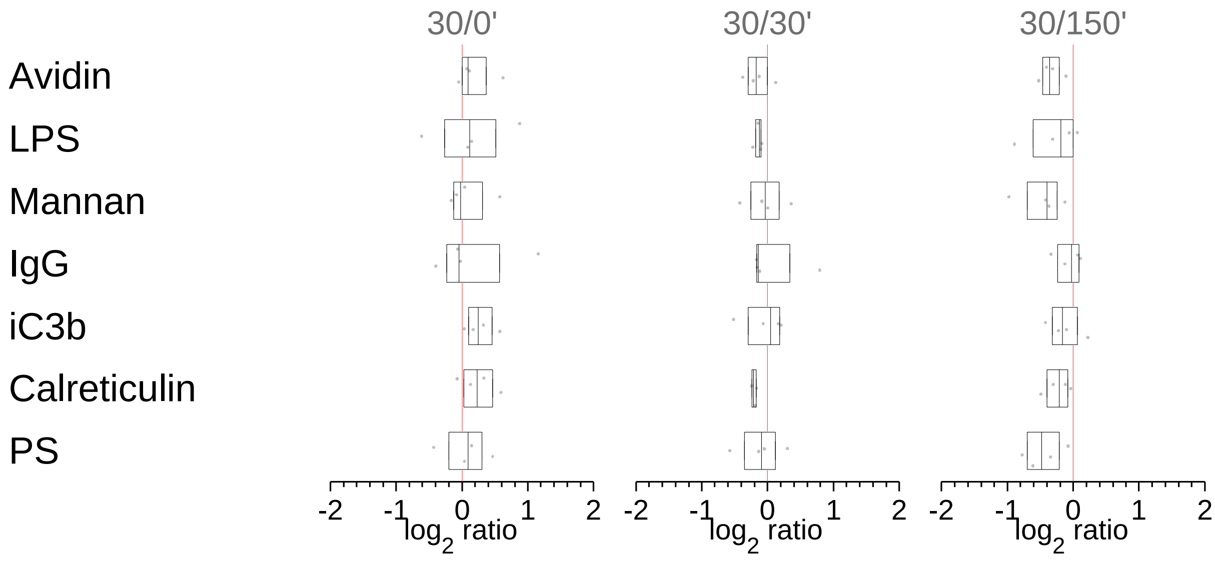

GO:0031529 ruffle organization (n = 6)

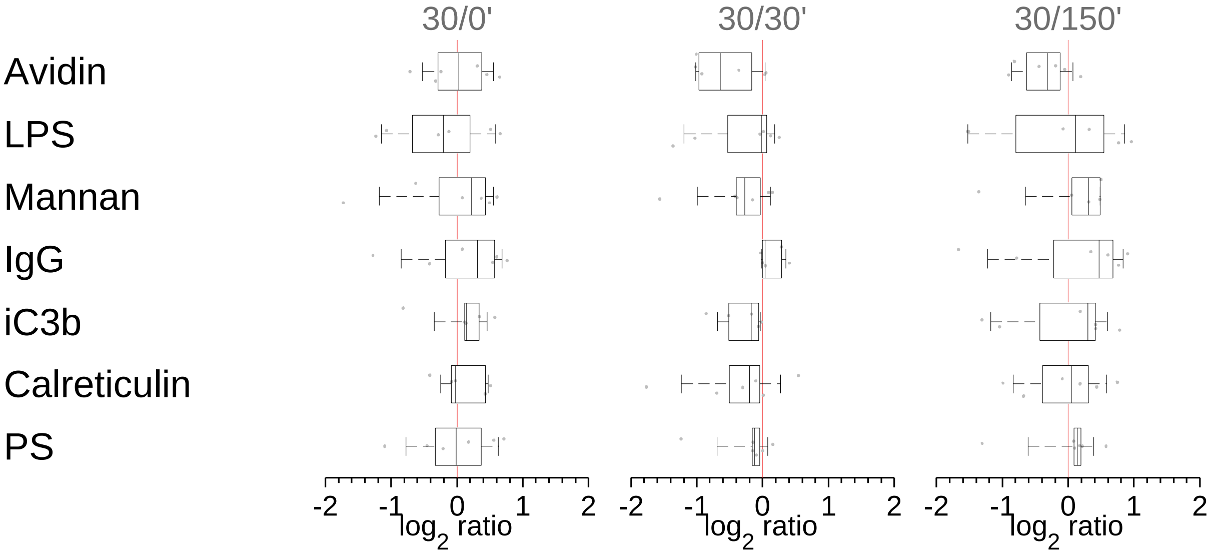

GO:0031532 actin cytoskeleton reorganization (n = 8)

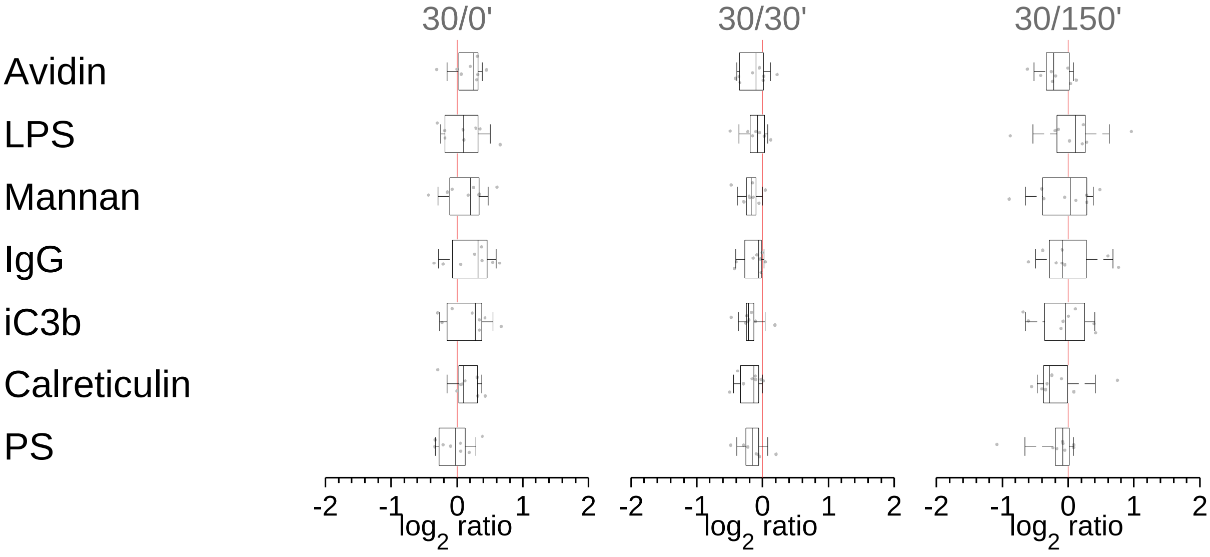

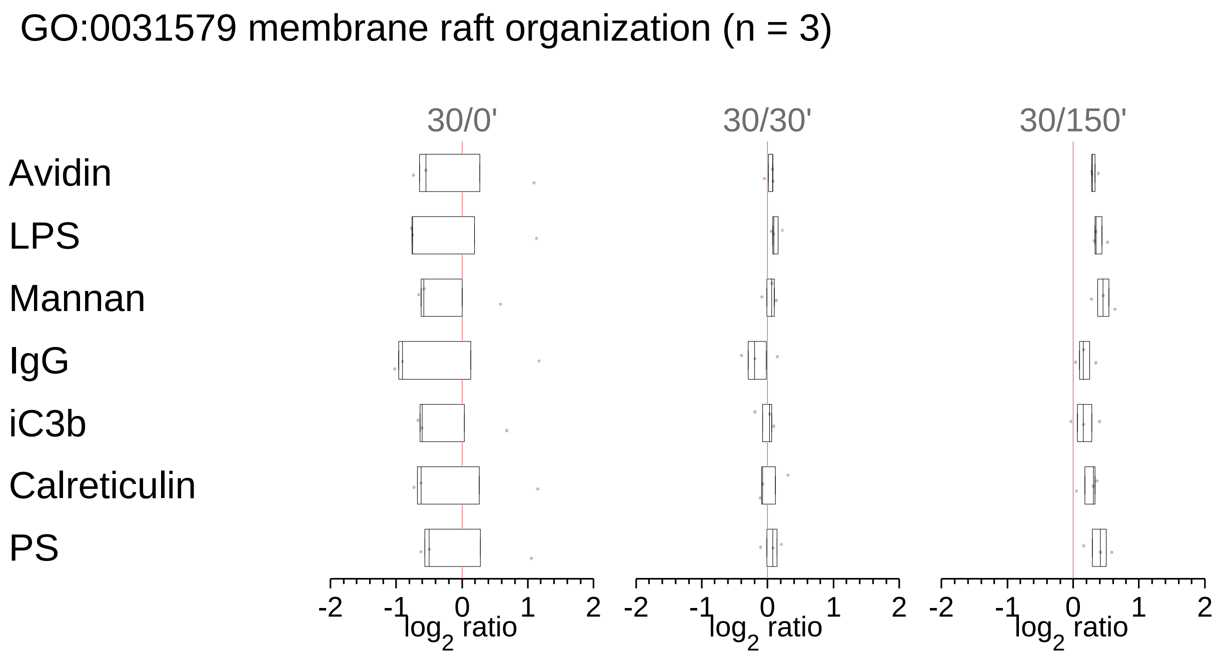

GO:0031579 membrane raft organization (n = 3)
30/0'
30/30'
30/150'
Avidin
LPS
Mannan
IgG
iC3b
Calreticulin
PS
-2 -1 0 1 2
log2 ratio

GO:0031589 cell-substrate adhesion (n = 5)

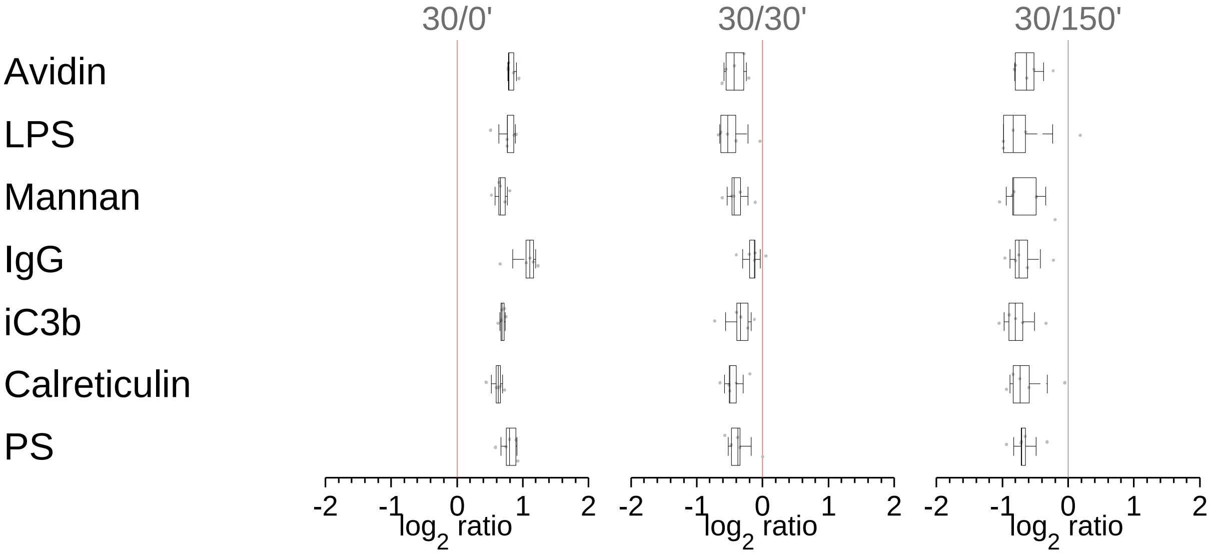

GO:0031594 neuromuscular junction (n = 9)

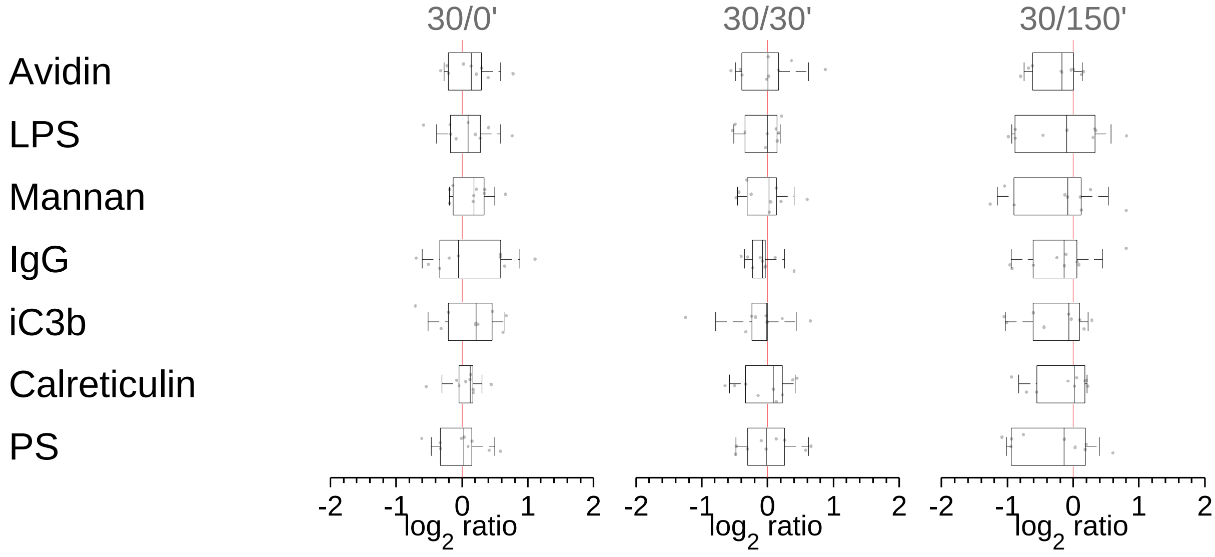

GO:0031616 spindle pole centrosome (n = 3)

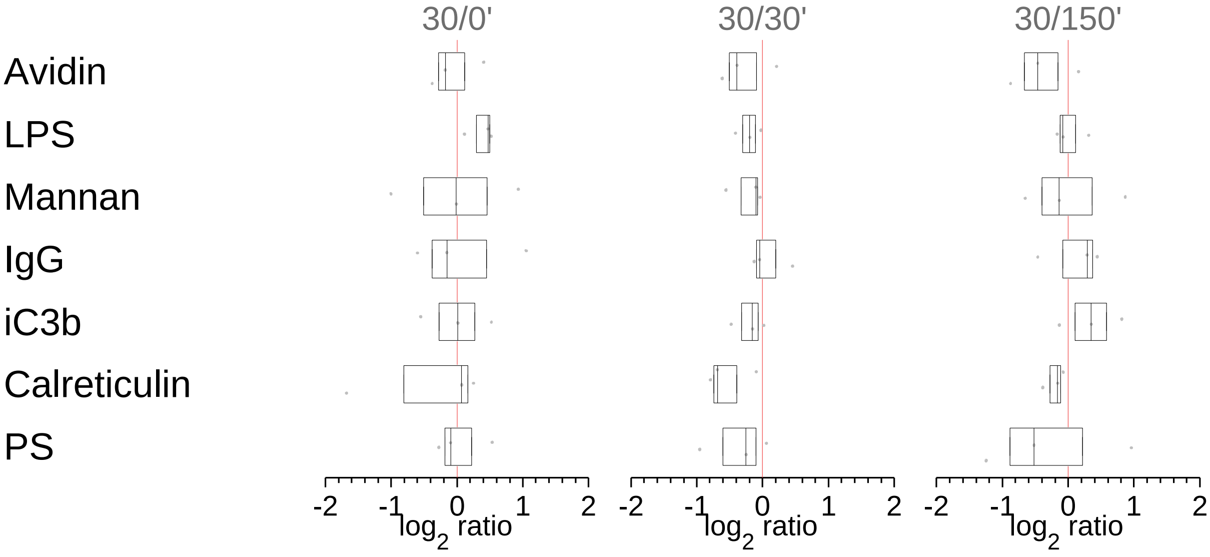

GO:0031623 receptor internalization (n = 7)

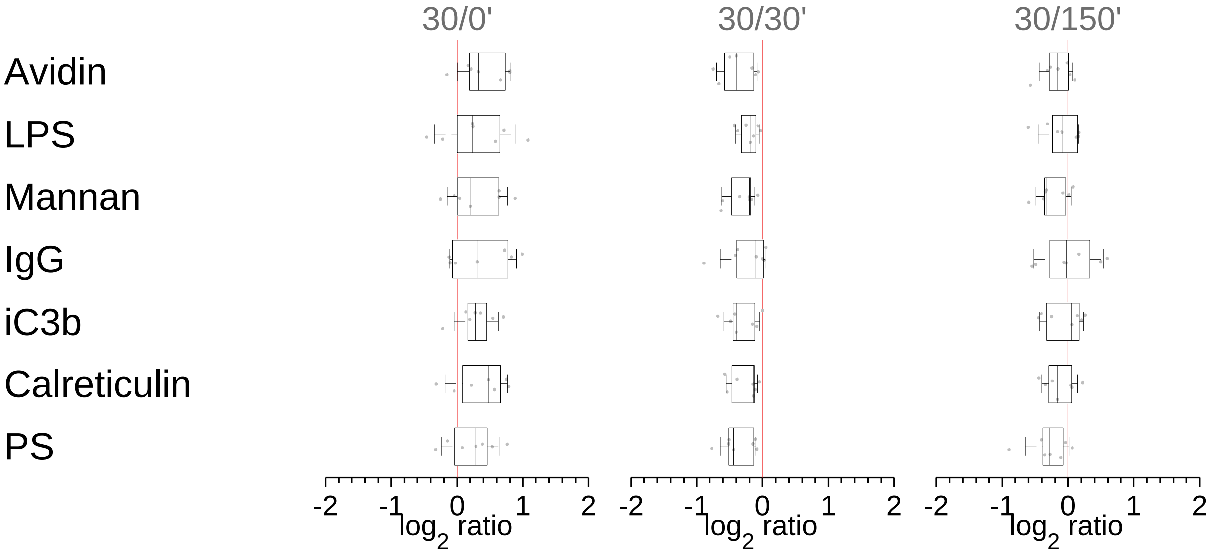

GO:0031625 ubiquitin protein ligase binding (n = 20)

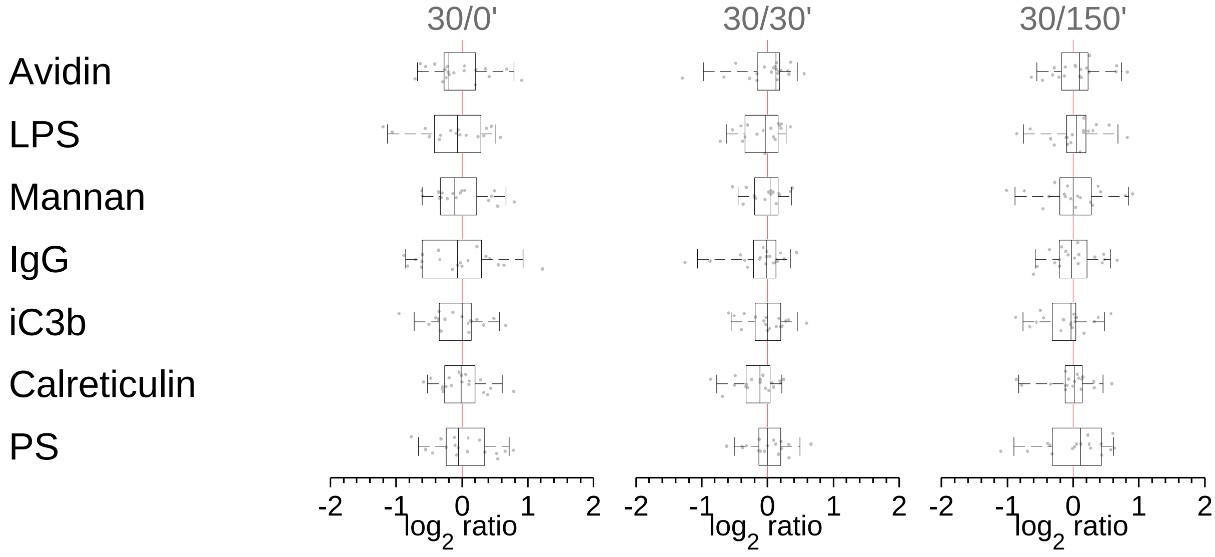

GO:0031647 regulation of protein stability (n = 4)

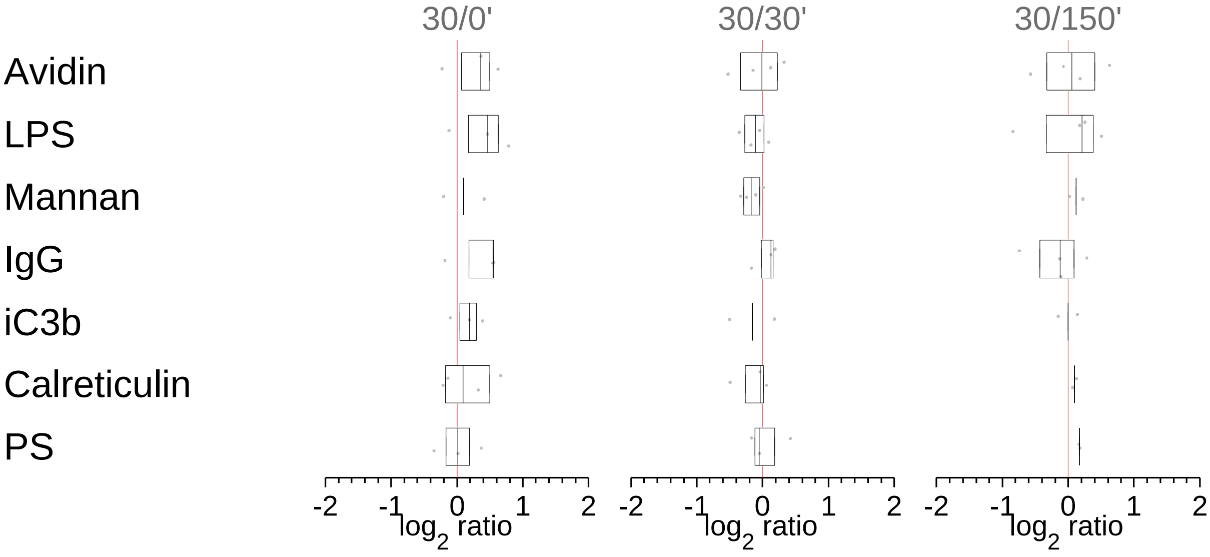

GO:0031648 protein destabilization (n = 3)

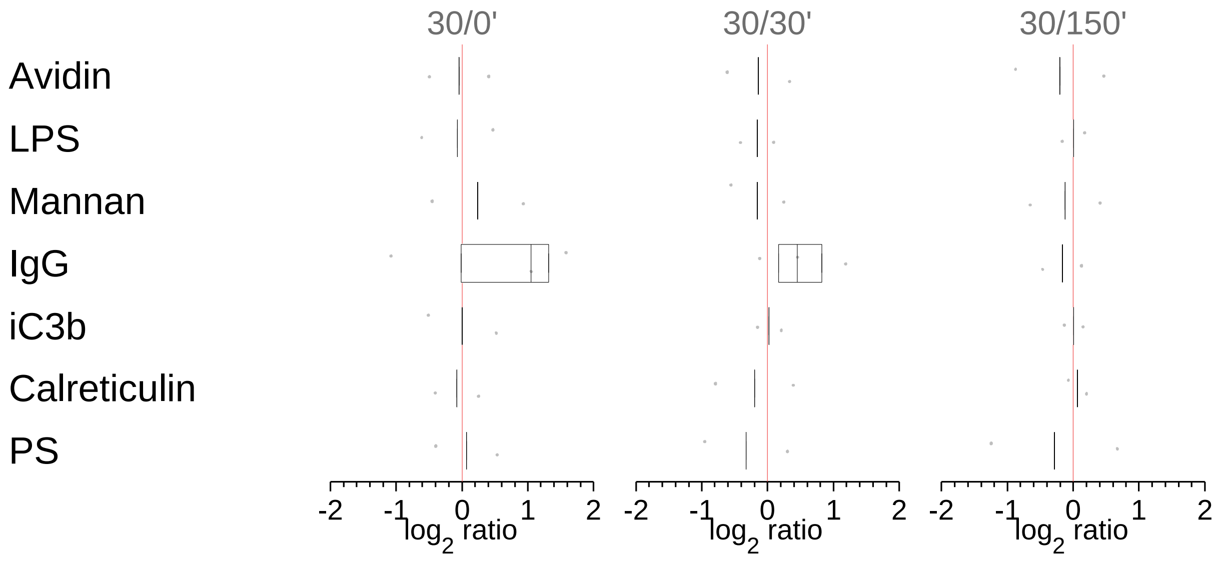

GO:0031663 lipopolysaccharide-mediated signaling pathway (n = 5)

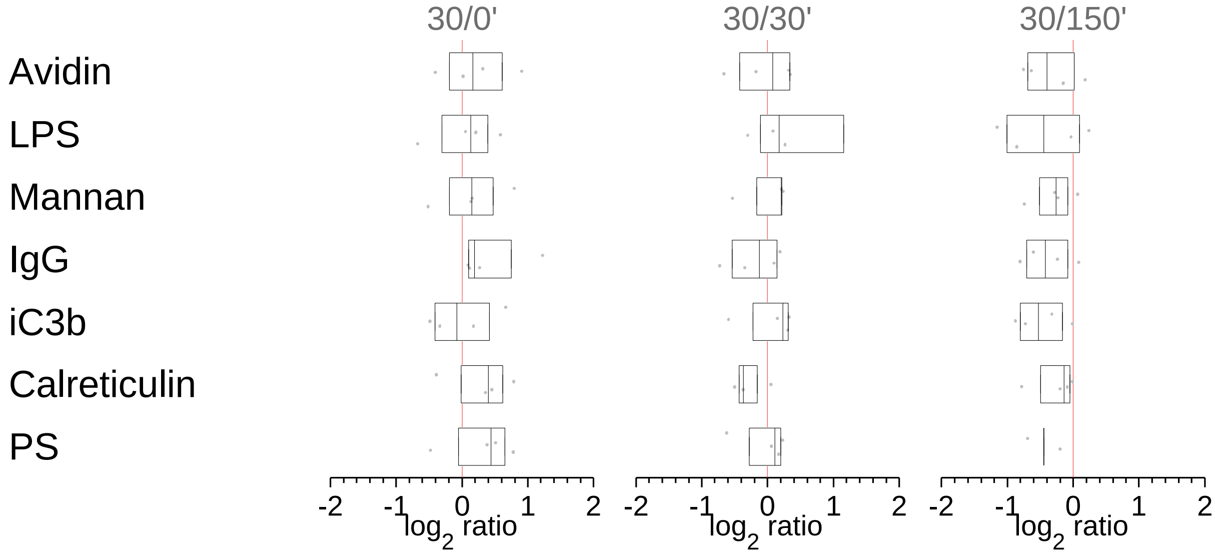

GO:0031666 positive regulation of lipopolysaccharide-mediated signaling pathway (n = 3)

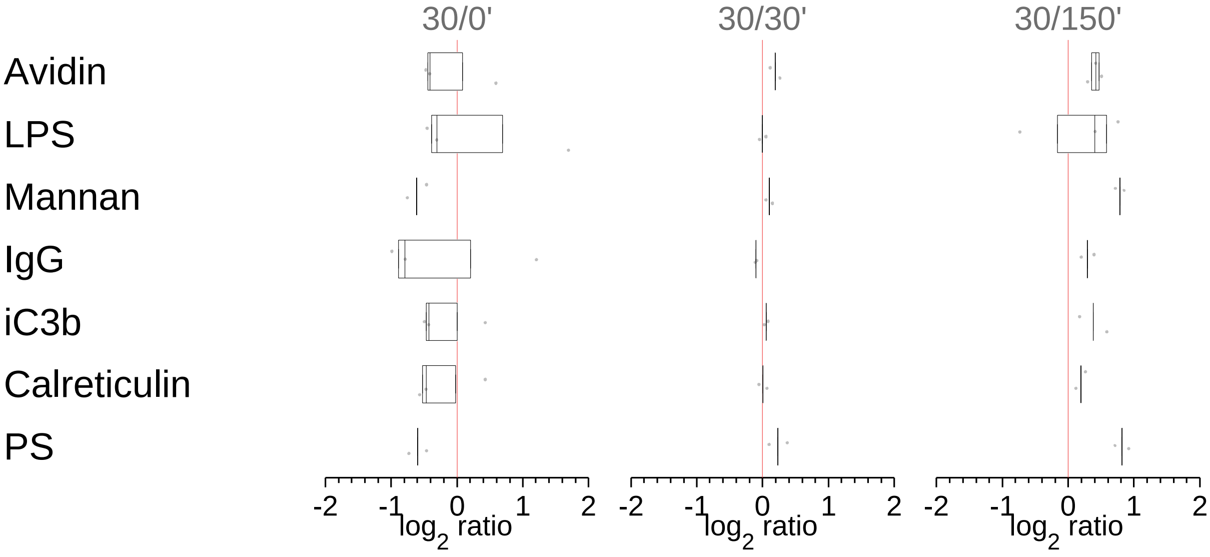

GO:0031668 cellular response to extracellular stimulus (n = 4)

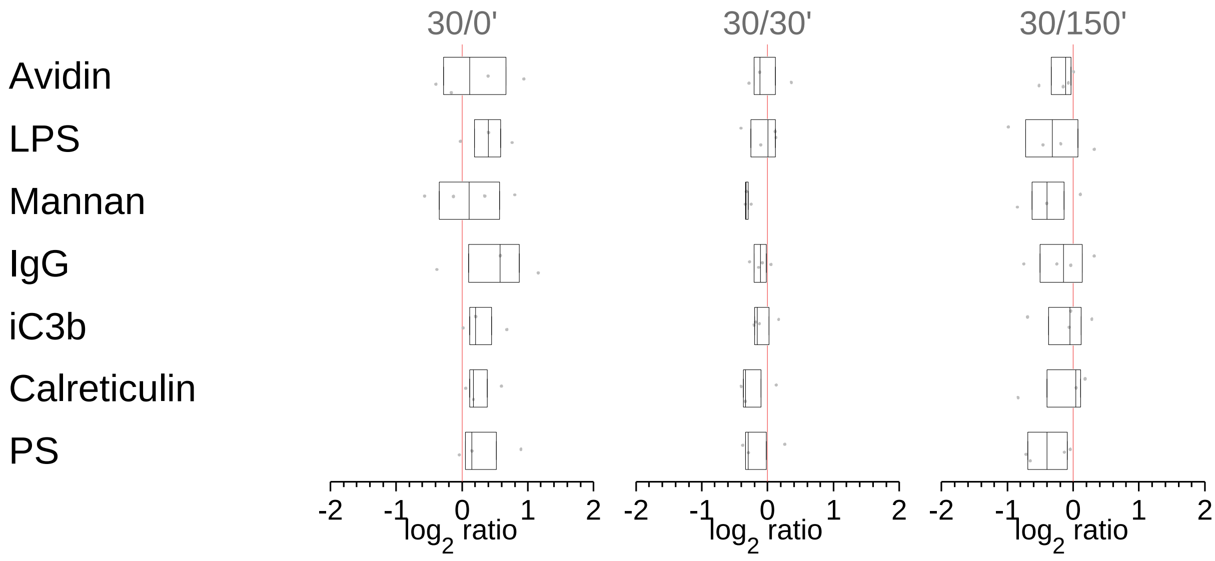

GO:0031674 I band (n = 3)

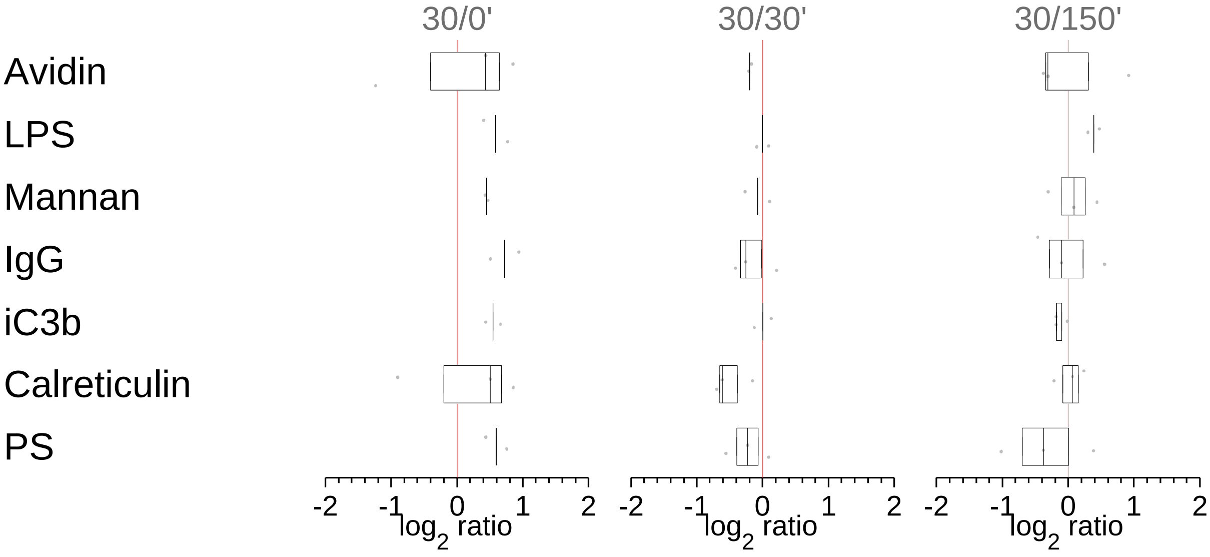

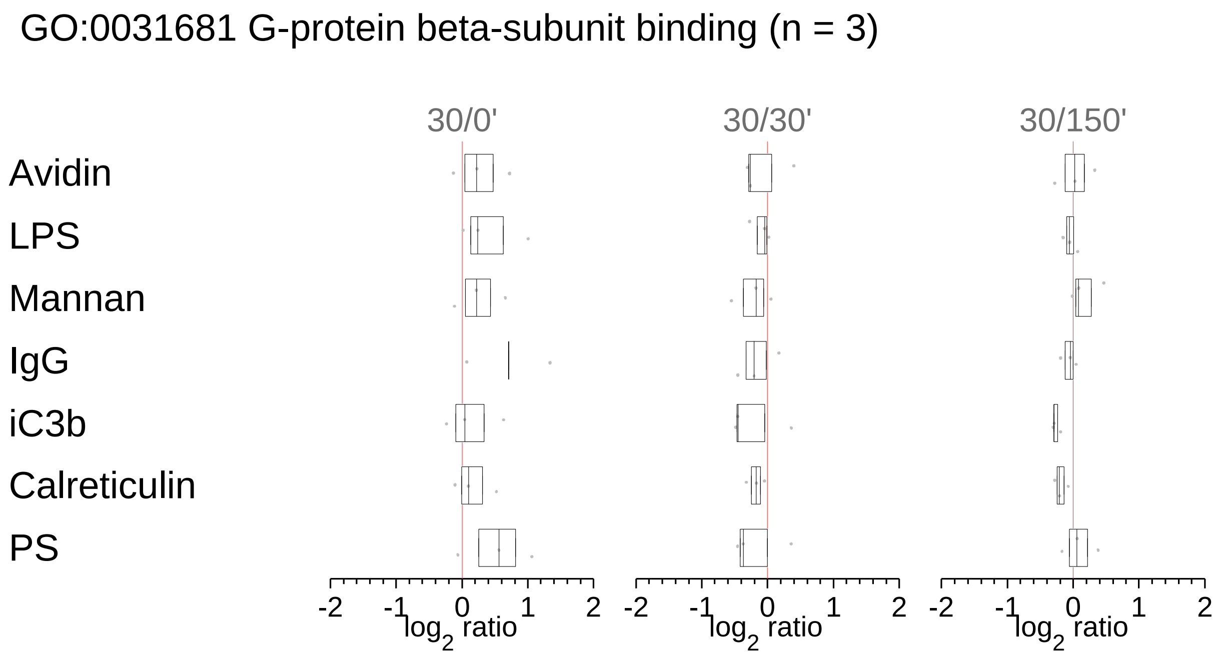
GO:0031681 G-protein beta-subunit binding (n = 3)
30/0'
30/30'
30/150'
Avidin
LPS
Mannan
IgG
iC3b
Calreticulin
PS
-2
-1
0
1
2
log2 ratio

GO:0031683 G-protein beta/gamma-subunit complex binding (n = 5)

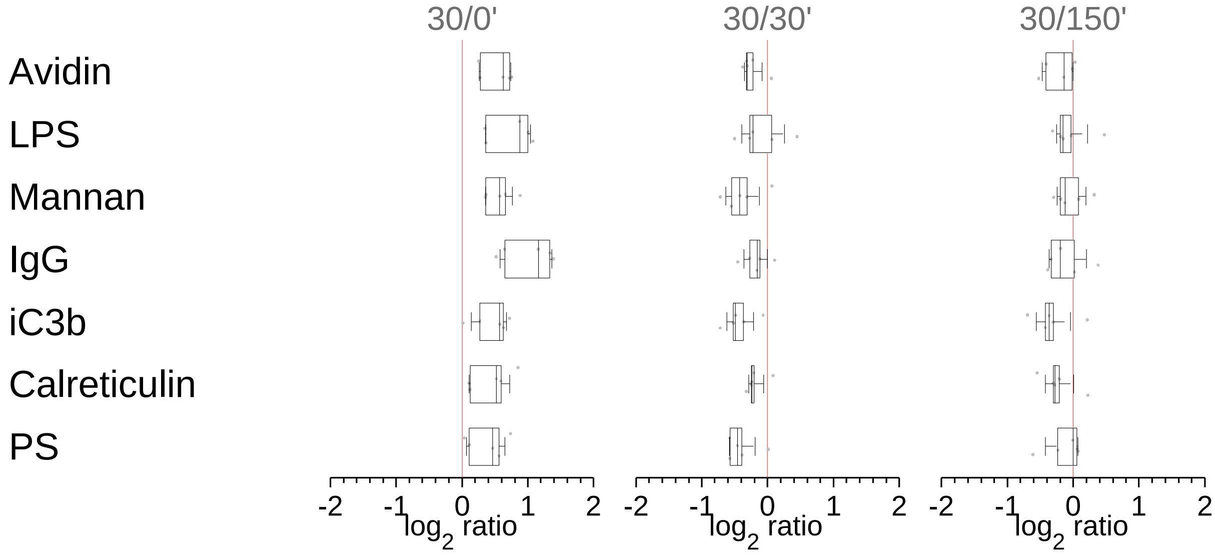

GO:0031702 type 1 angiotensin receptor binding (n = 4)

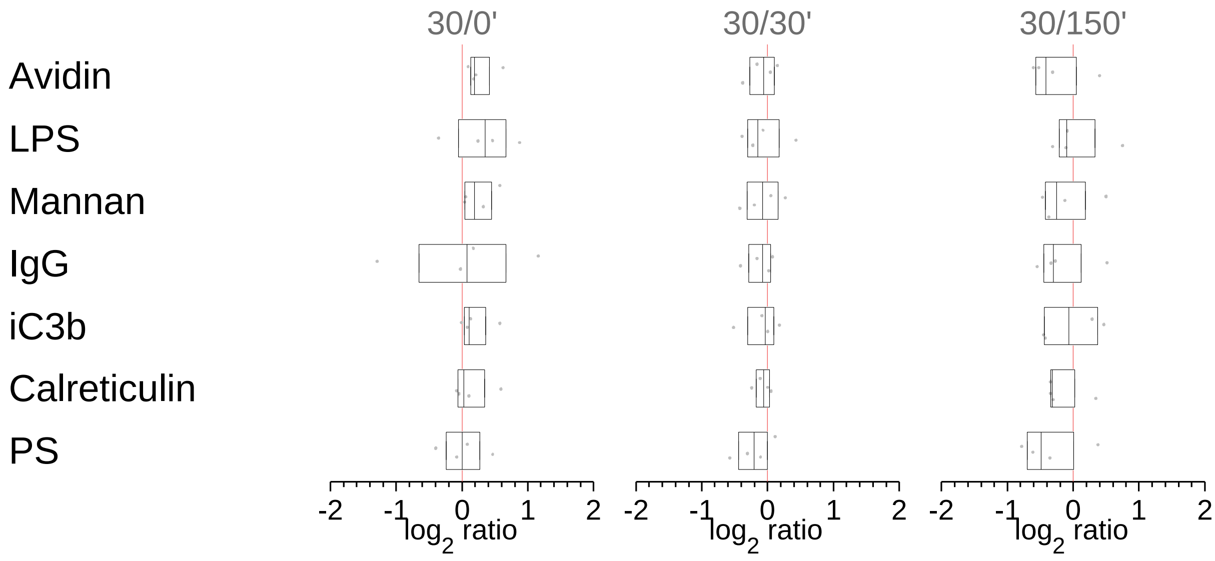

GO:0031748 D1 dopamine receptor binding (n = 4)

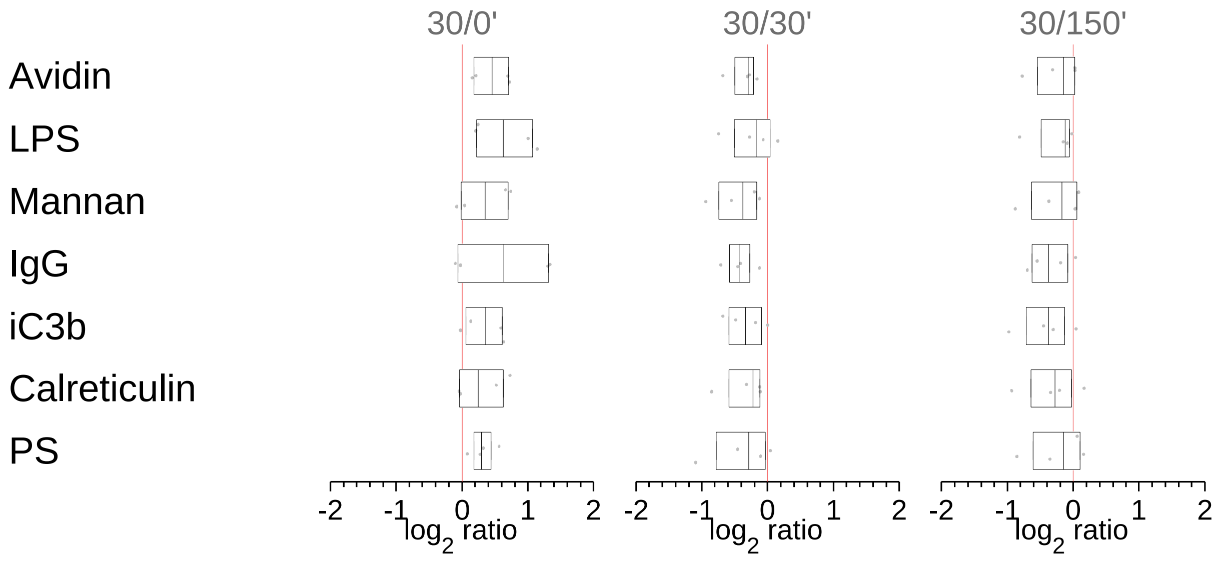

GO:0031901 early endosome membrane (n = 7)

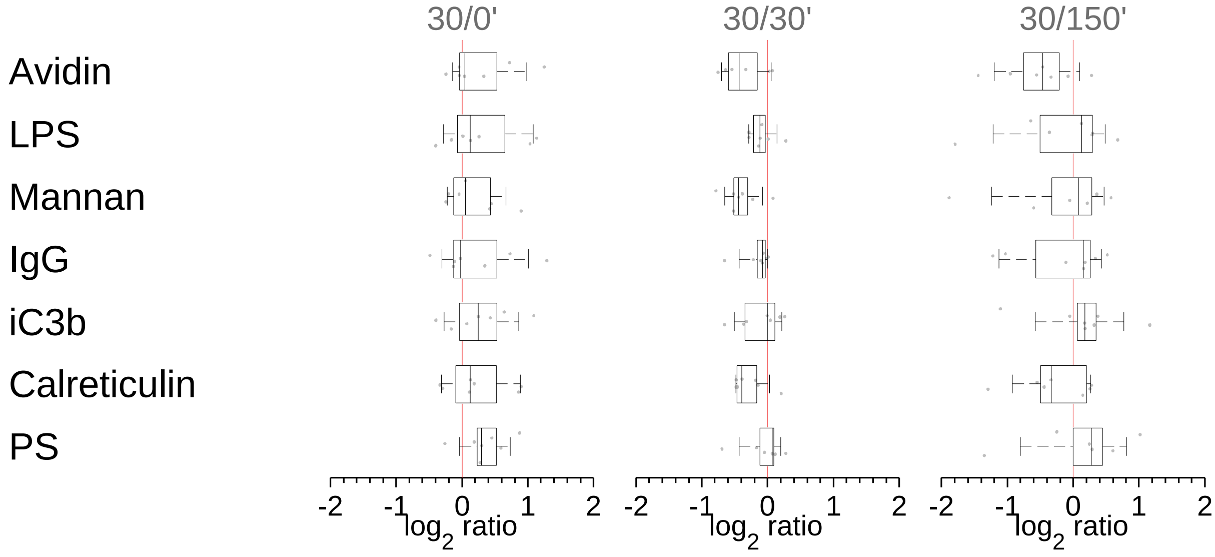

GO:0031902 late endosome membrane (n = 12)

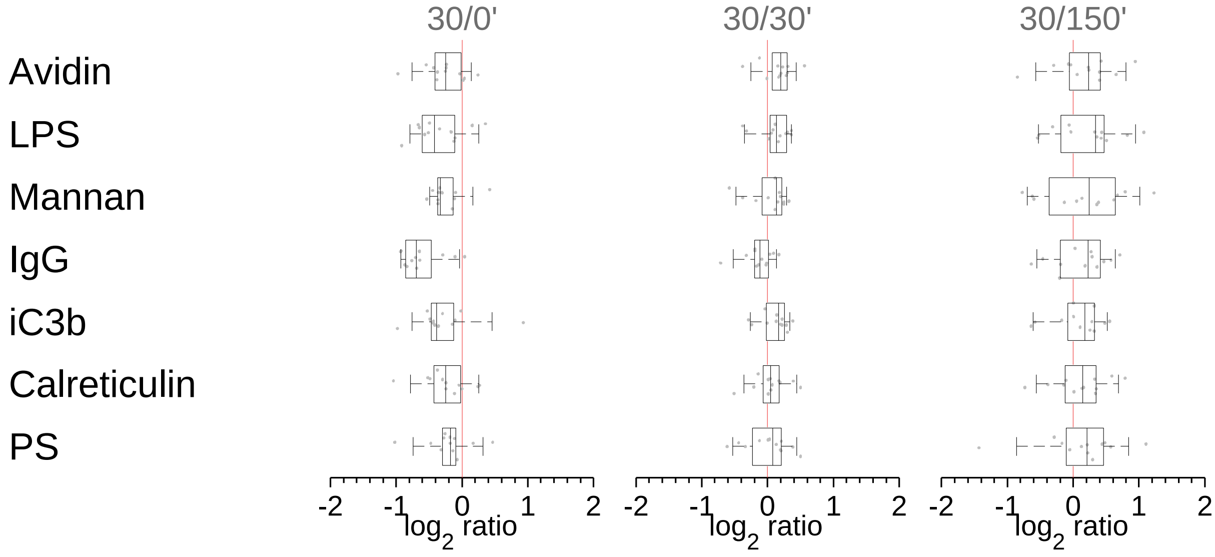

GO:0031929 TOR signaling cascade (n = 3)

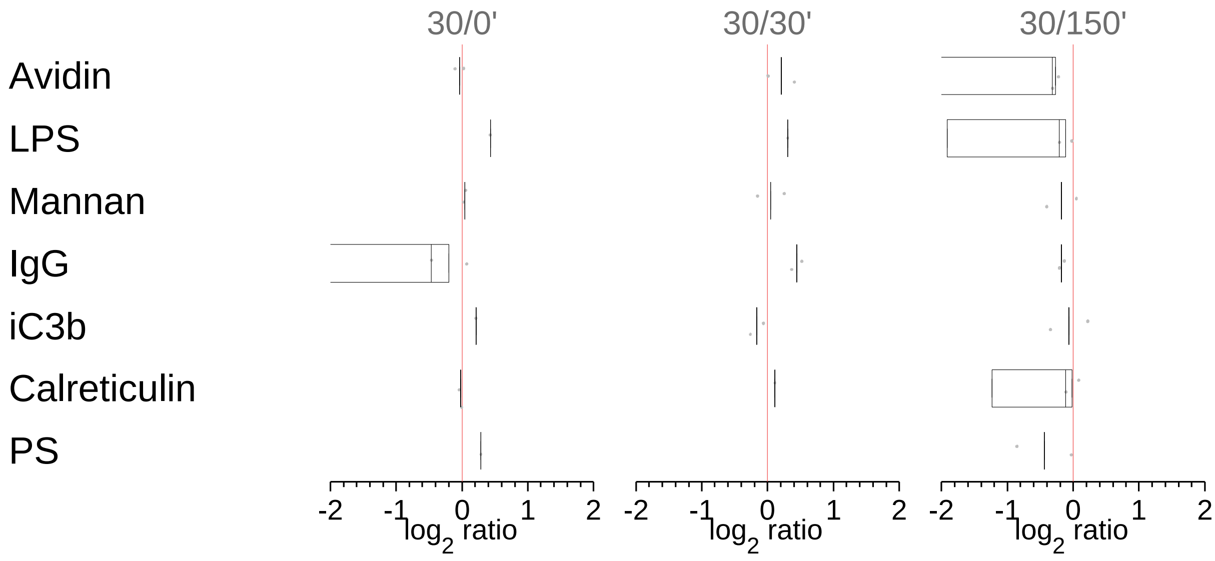

GO:0031941 filamentous actin (n = 6)

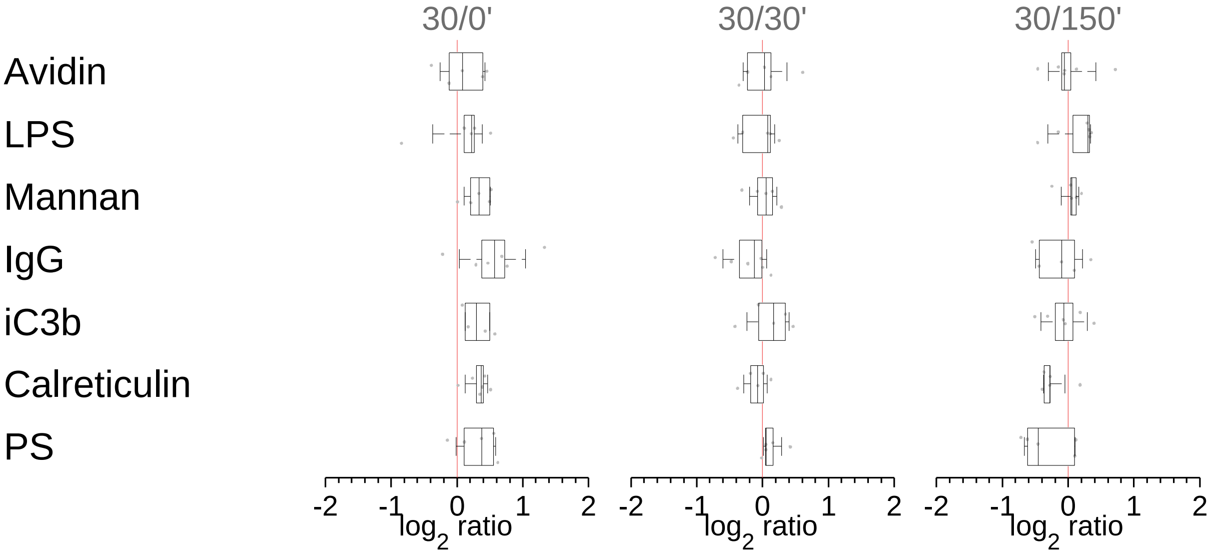

GO:0031954 positive regulation of protein autophosphorylation (n = 3)

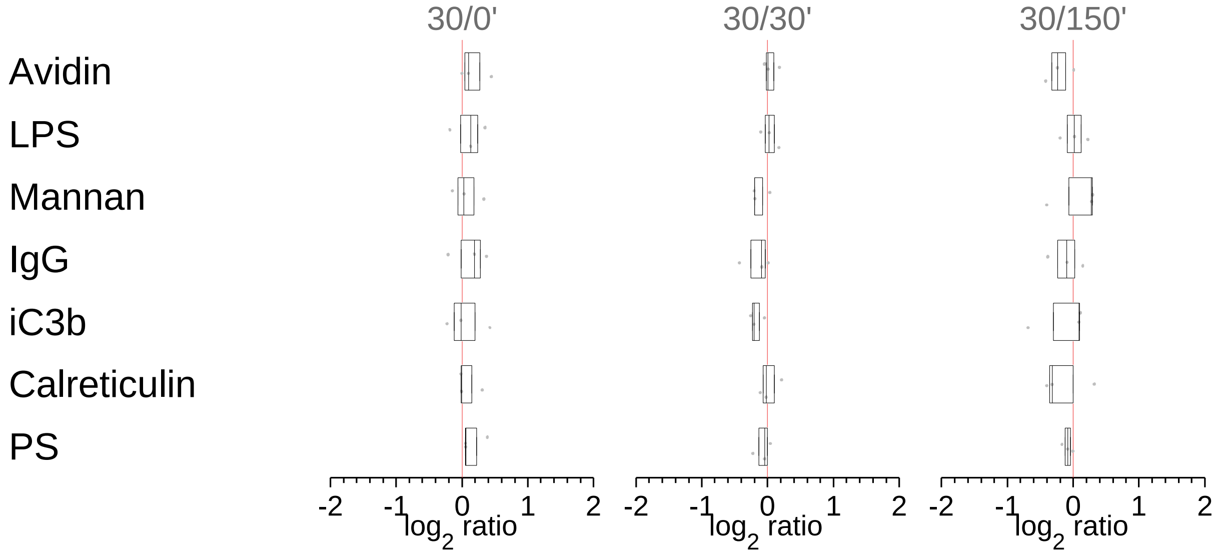

GO:0031965 nuclear membrane (n = 20)

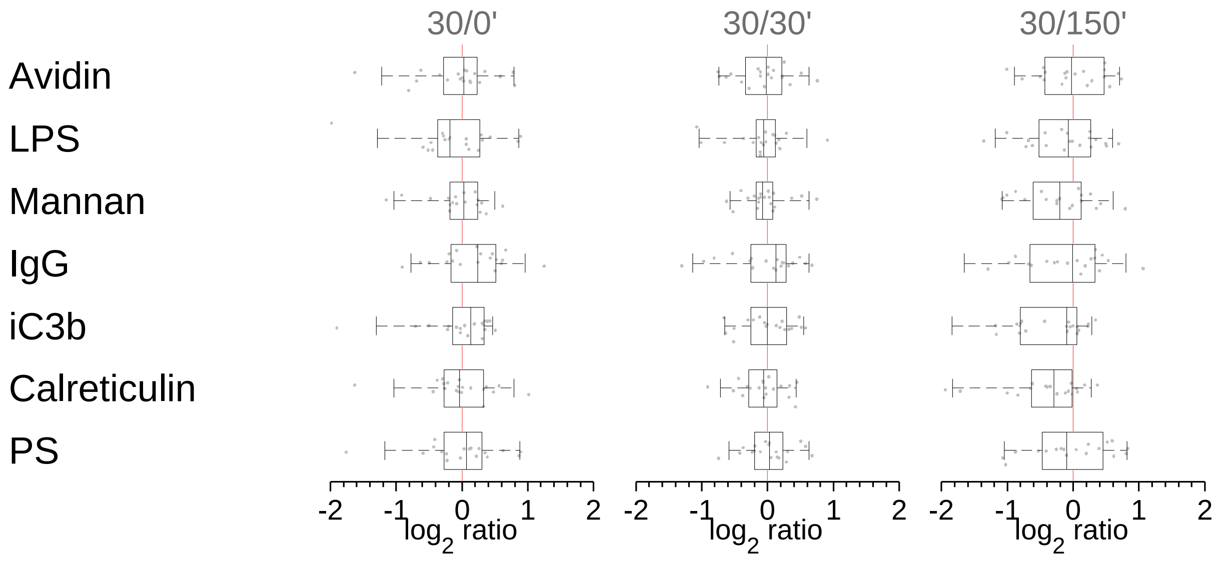

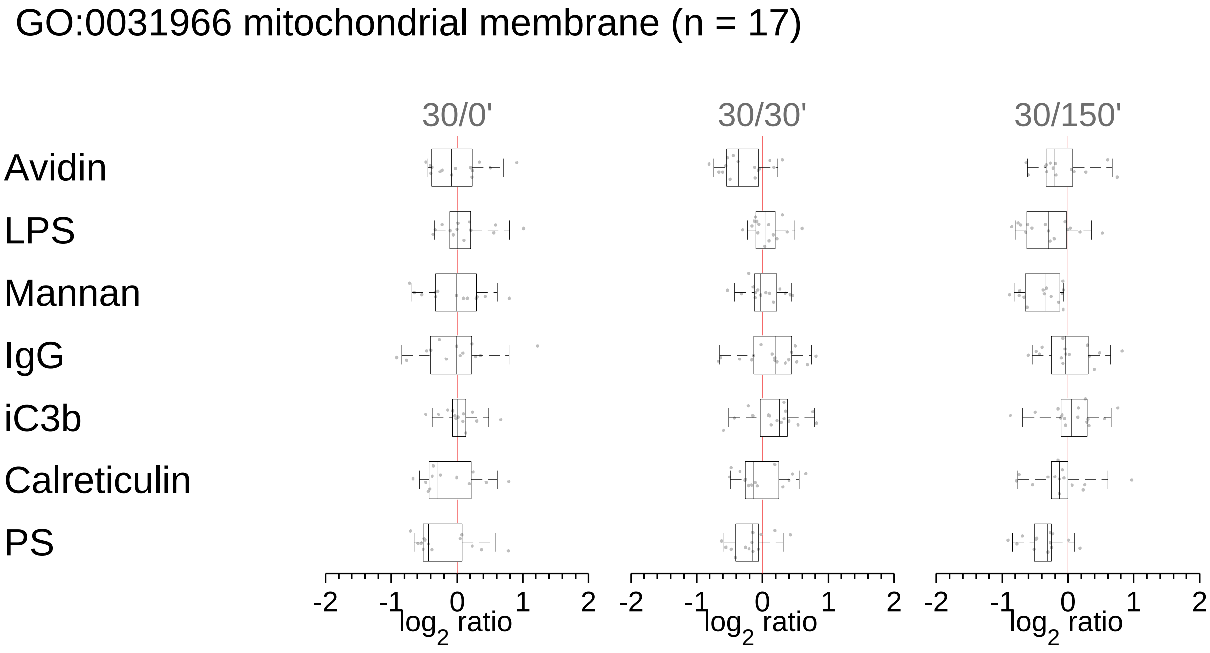
GO:0031966 mitochondrial membrane (n = 17)
30/0'
30/30'
30/150'
Avidin
LPS
Mannan
IgG
iC3b
Calreticulin
PS
-2 -1 0 1 2
log2 ratio

GO:0031982 vesicle (n = 23)

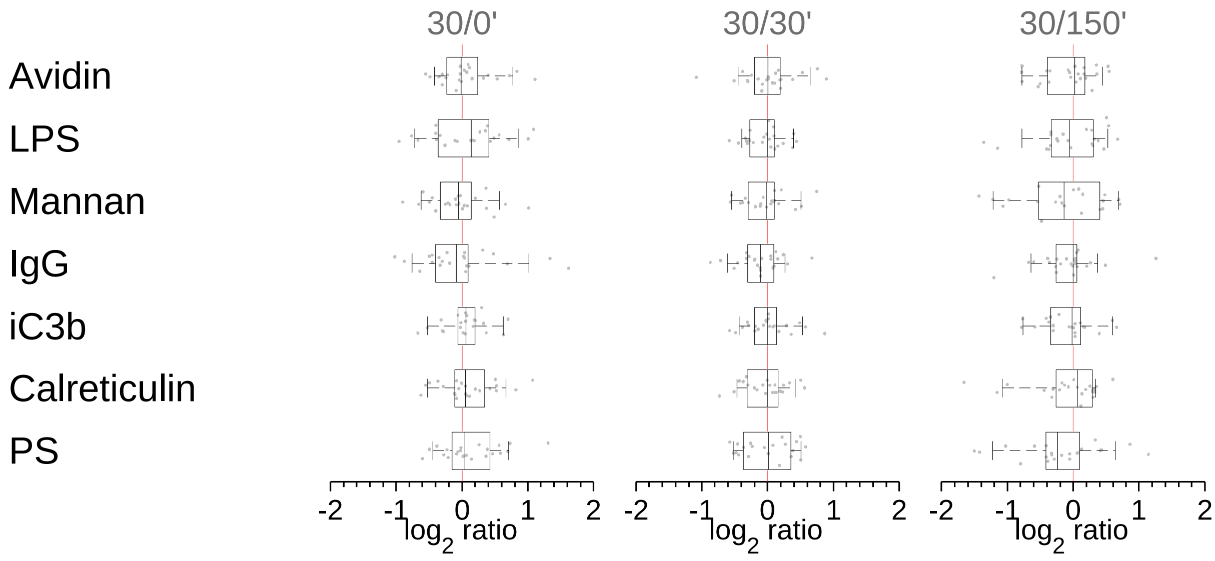

GO:0031985 Golgi cisterna (n = 4)

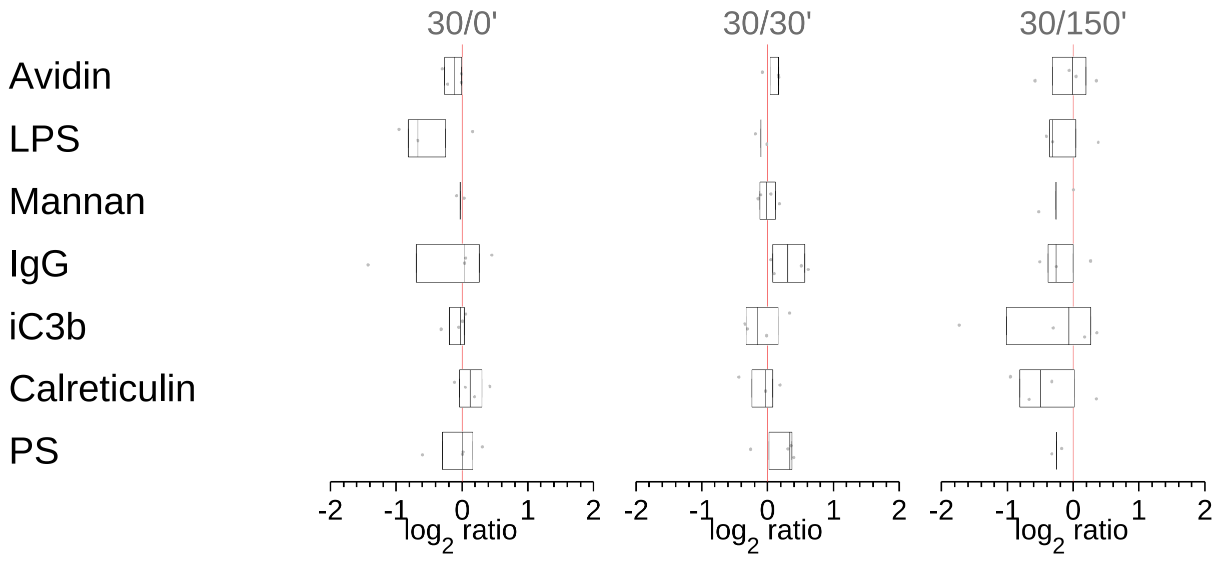

GO:0032006 regulation of TOR signaling cascade (n = 3)

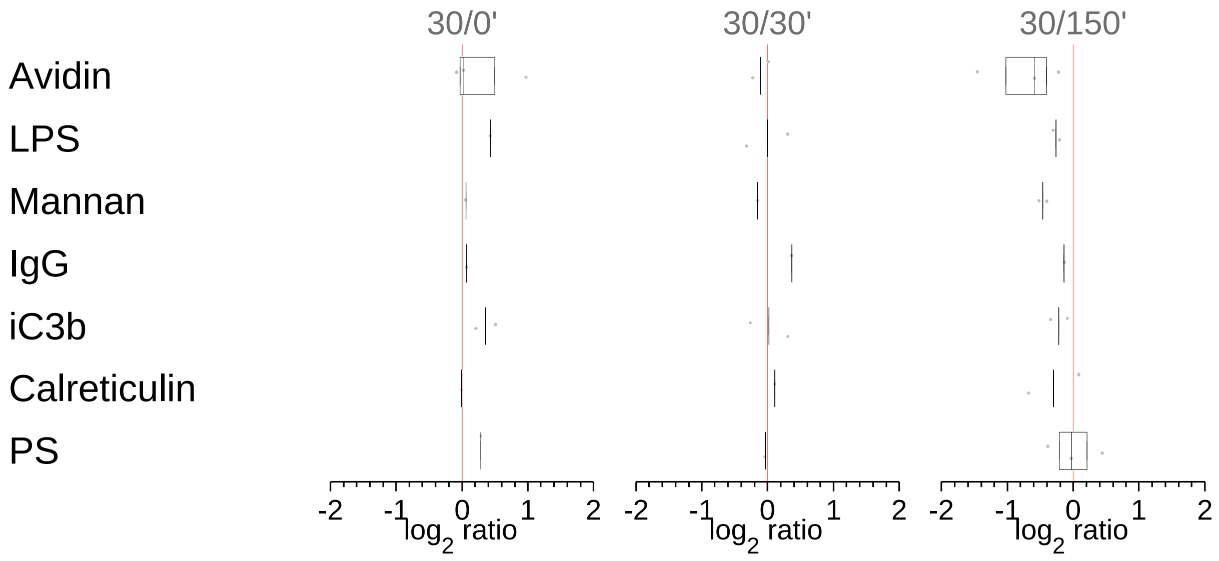

GO:0032008 positive regulation of TOR signaling cascade (n = 8)

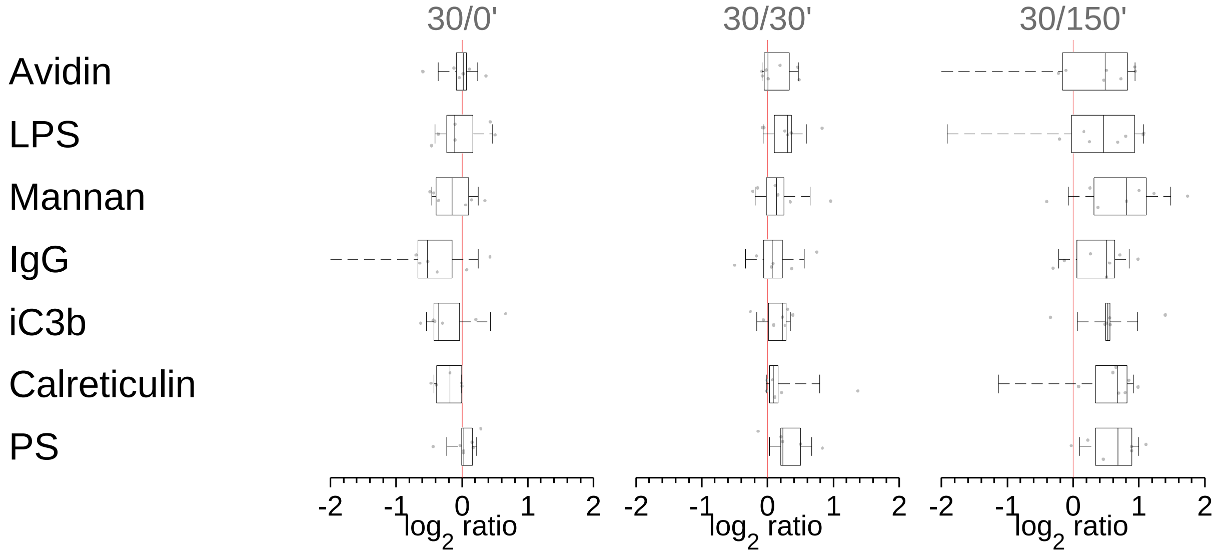

GO:0032009 early phagosome (n = 4)

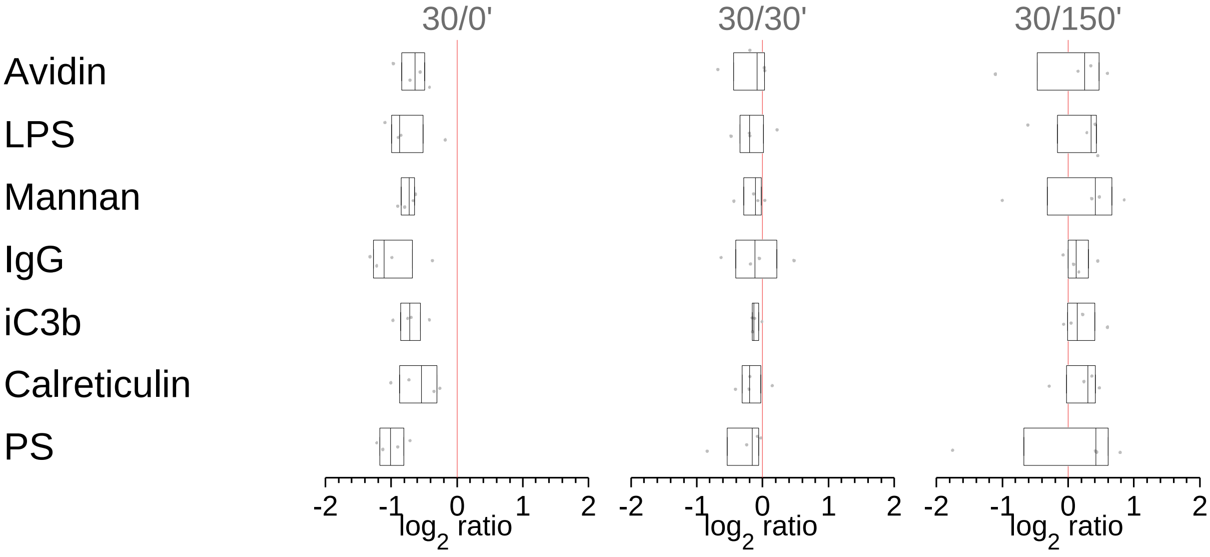

GO:0032024 positive regulation of insulin secretion (n = 3)

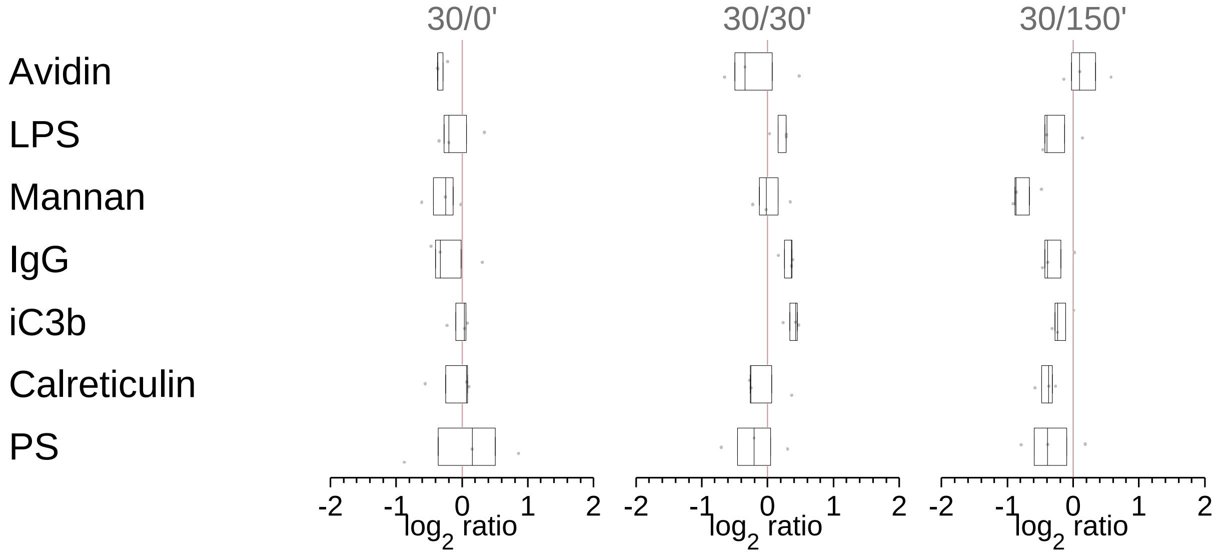

GO:0032088 negative regulation of NF-kappaB transcription factor activity (n = 11)

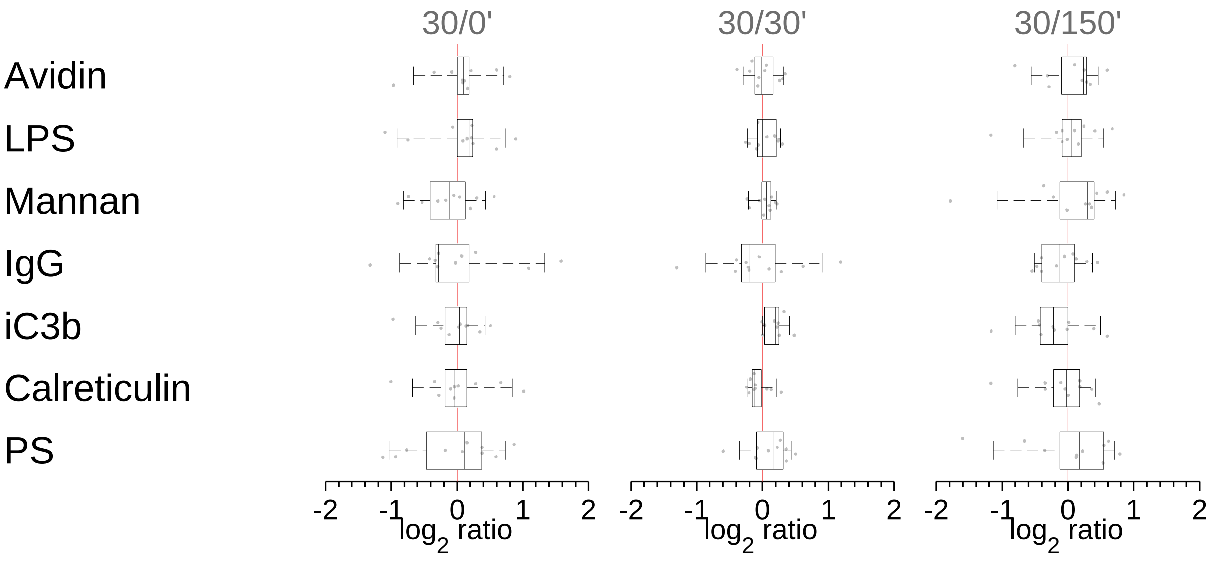

GO:0032091 negative regulation of protein binding (n = 7)

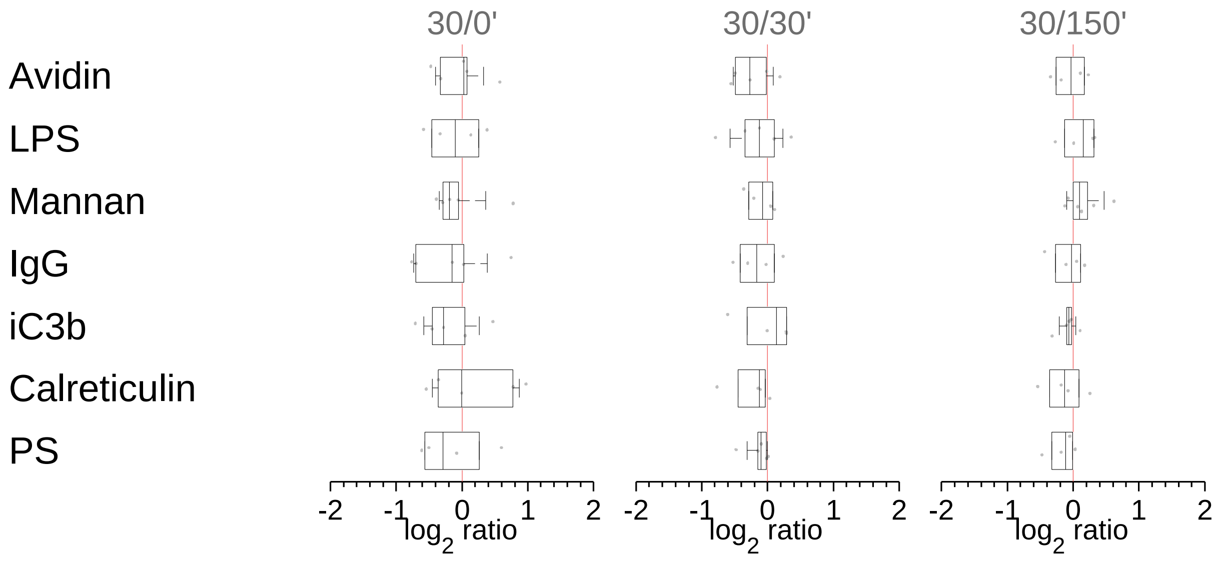

GO:0032092 positive regulation of protein binding (n = 8)

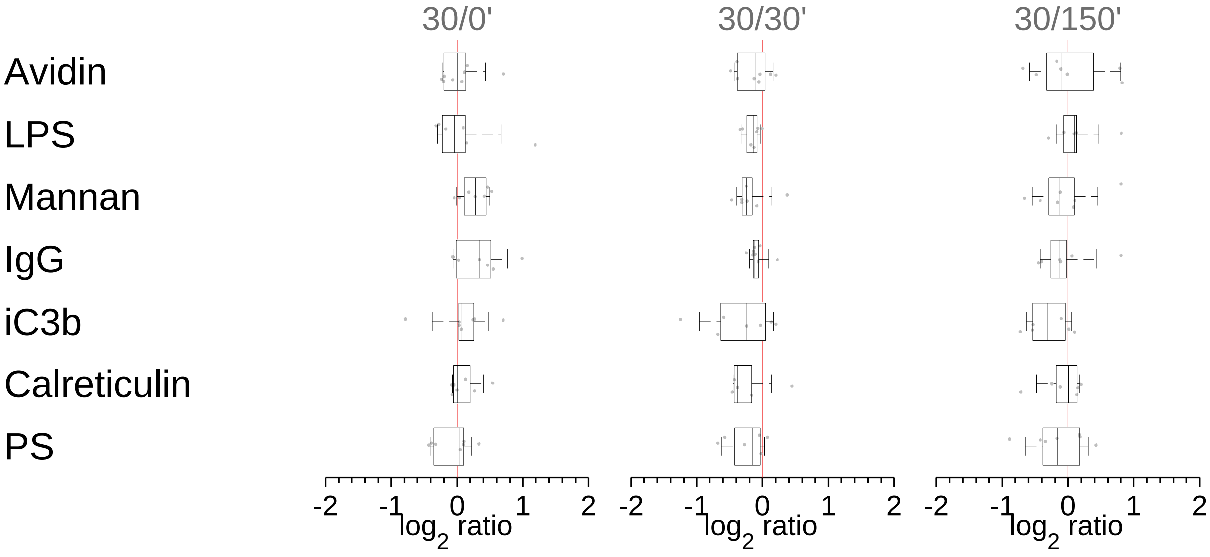

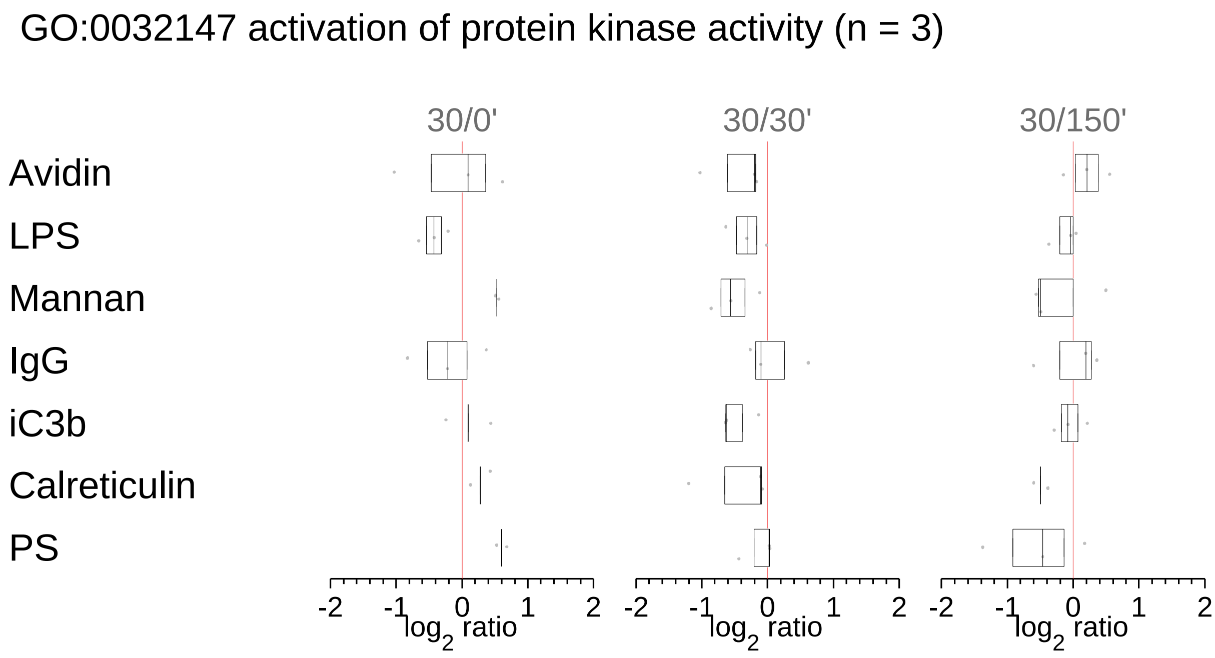
GO:0032147 activation of protein kinase activity (n = 3)
30/0'
30/30'
30/150'
Avidin
LPS
Mannan
IgG
iC3b
Calreticulin
PS
-2 -1 0 1 2
log2 ratio

GO:0032154 cleavage furrow (n = 11)

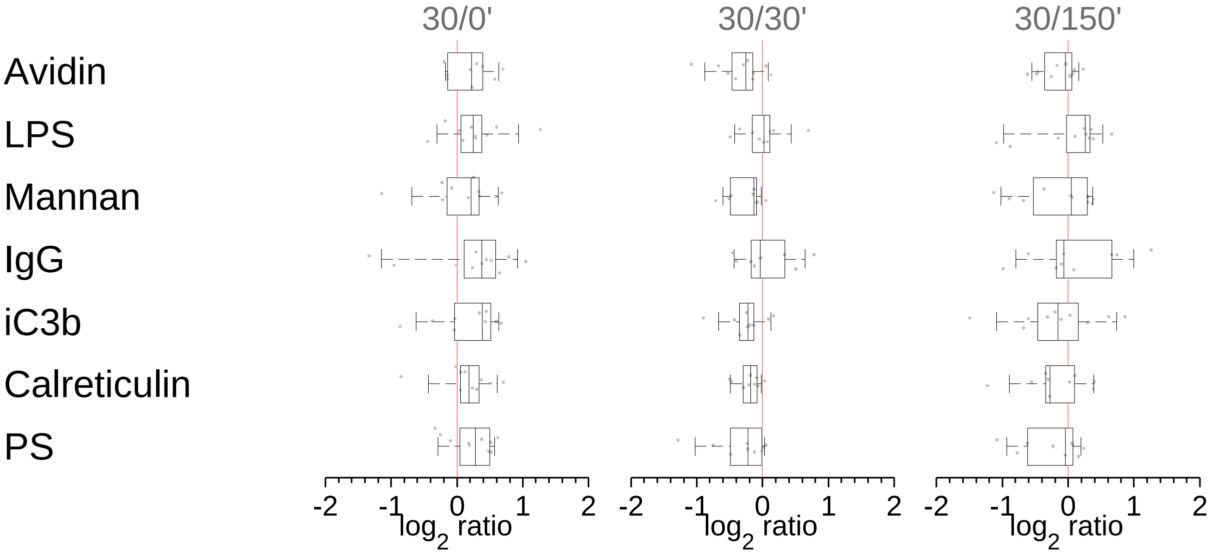

GO:0032216 glucosaminyl-phosphotidylinositol O-acyltransferase activity (n = 3)

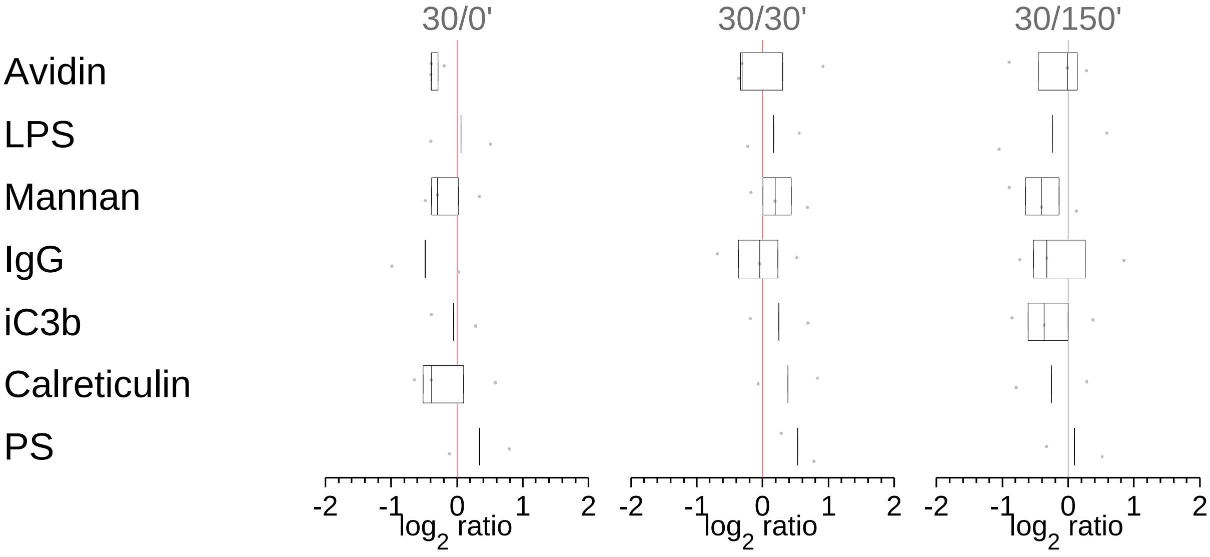

GO:0032228 regulation of synaptic transmission, GABAergic (n = 3)

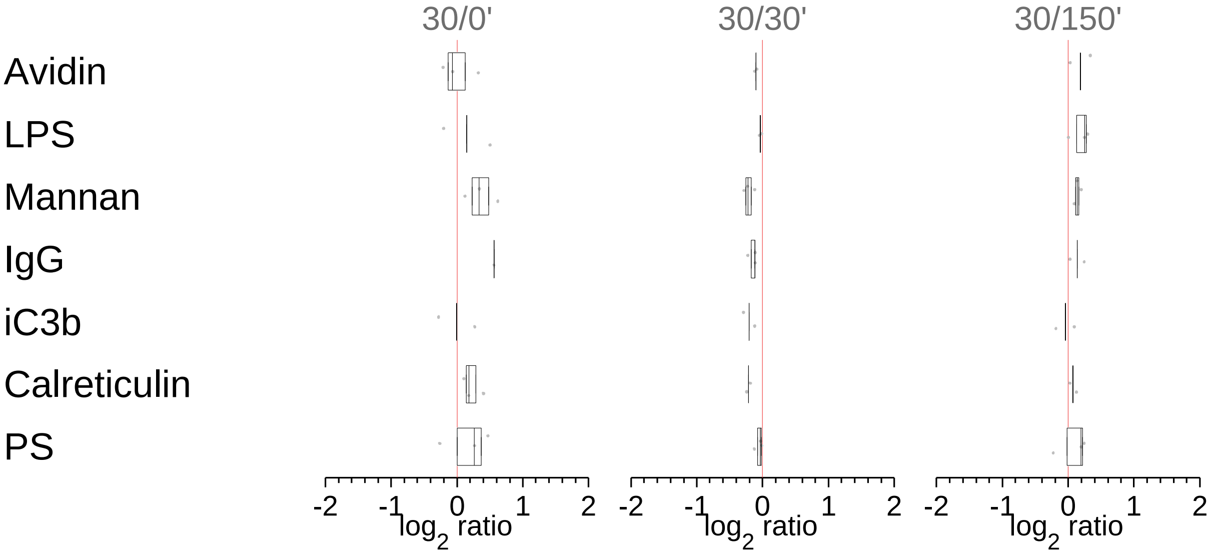

GO:0032259 methylation (n = 3)

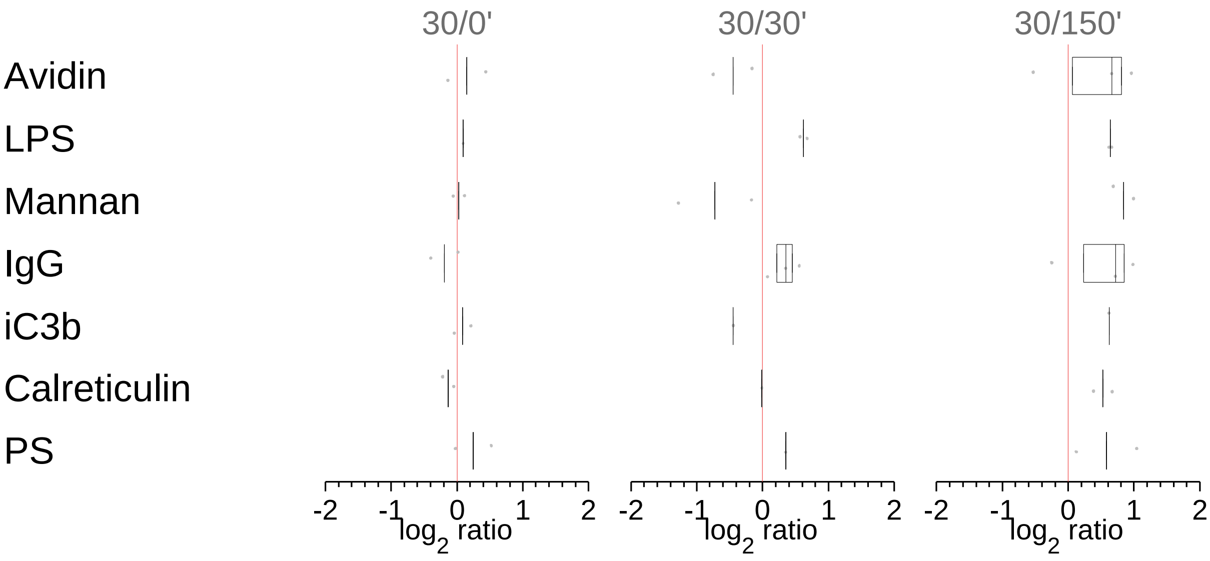

GO:0032266 phosphatidylinositol-3-phosphate binding (n = 6)

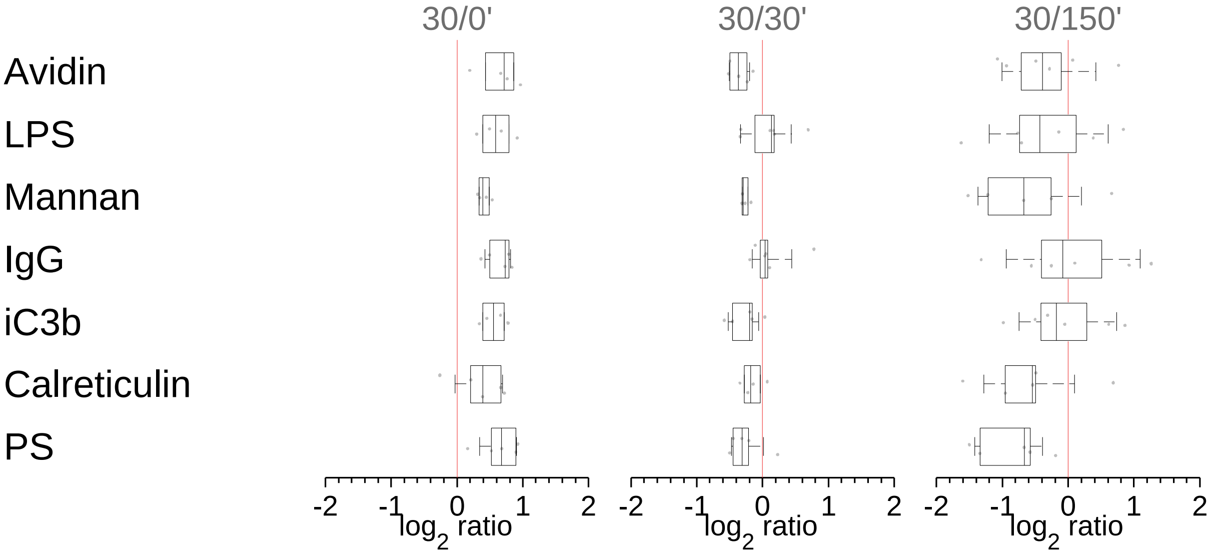

GO:0032312 regulation of ARF GTPase activity (n = 9)

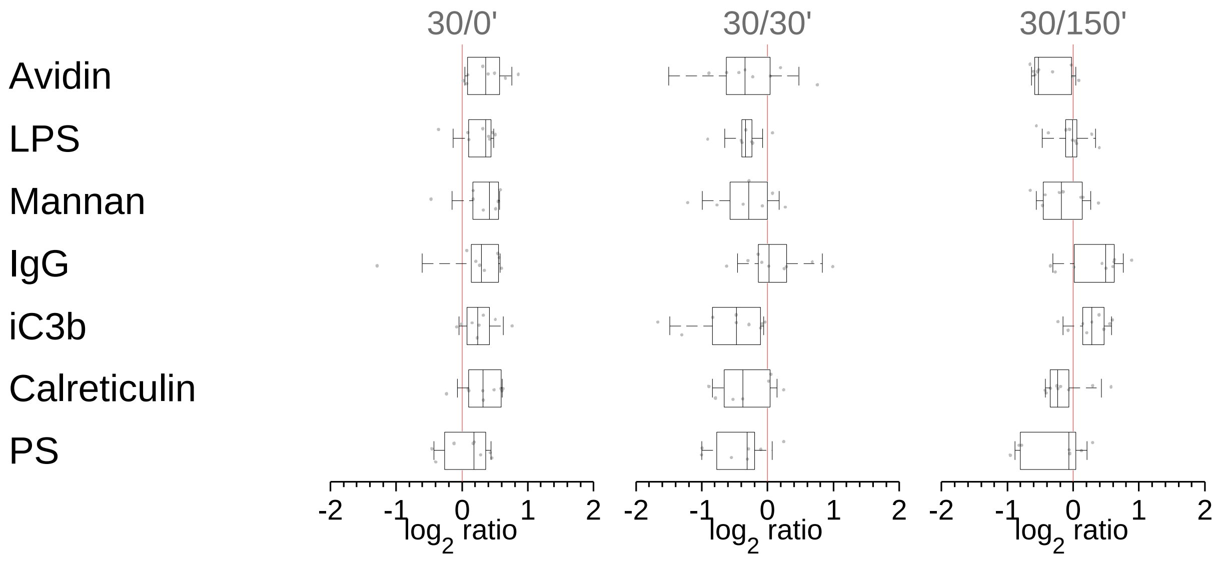

GO:0032313 regulation of Rab GTPase activity (n = 3)

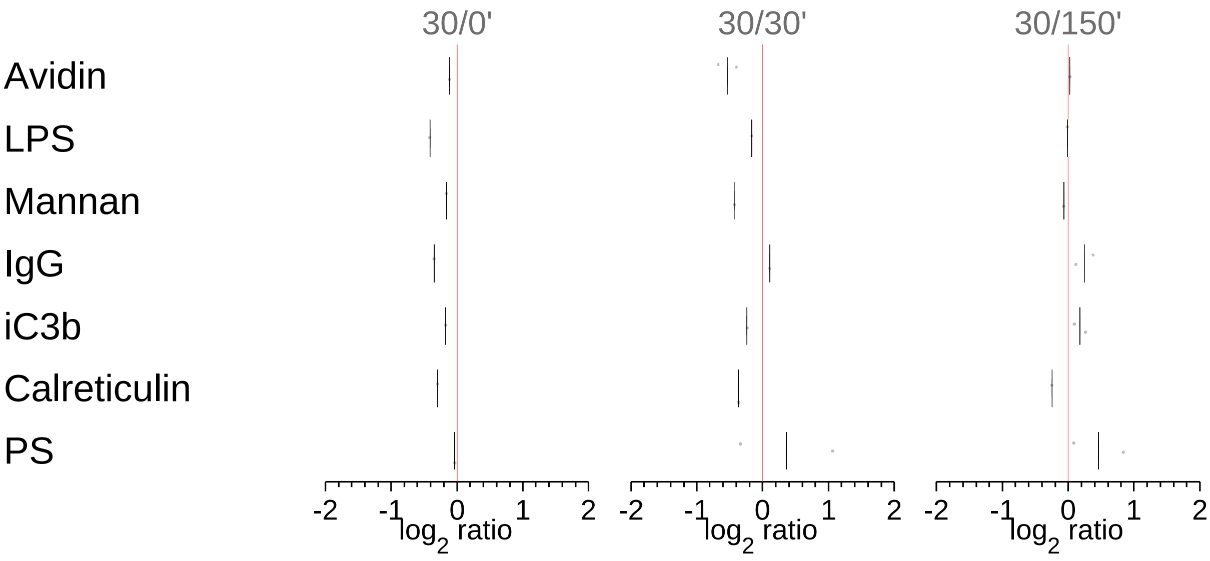

GO:0032320 positive regulation of Ras GTPase activity (n = 4)

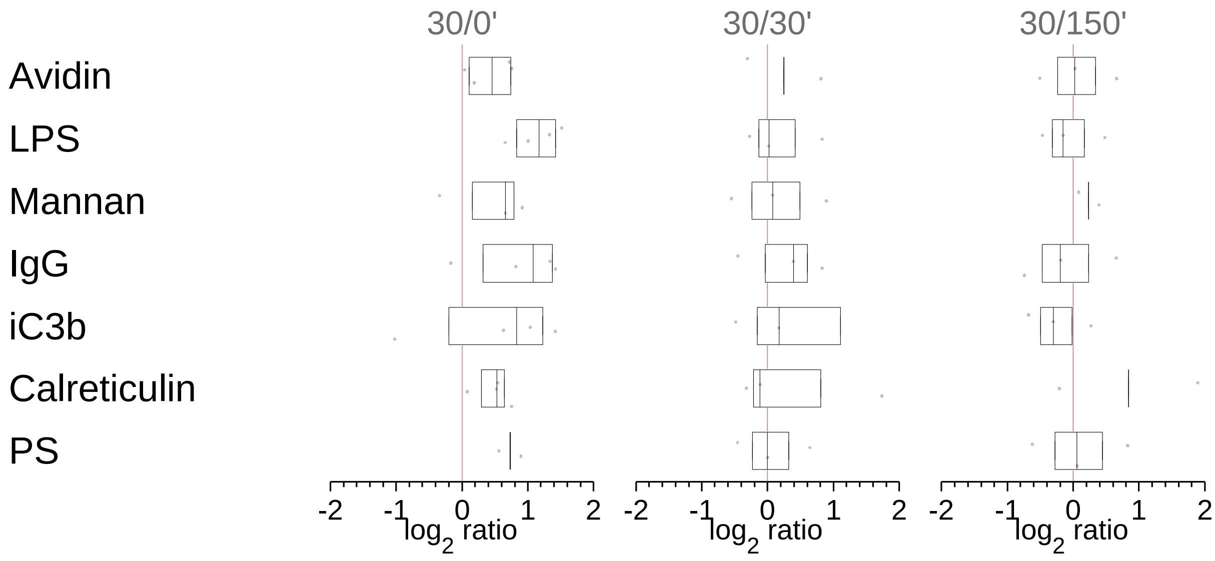

GO:0032321 positive regulation of Rho GTPase activity (n = 5)

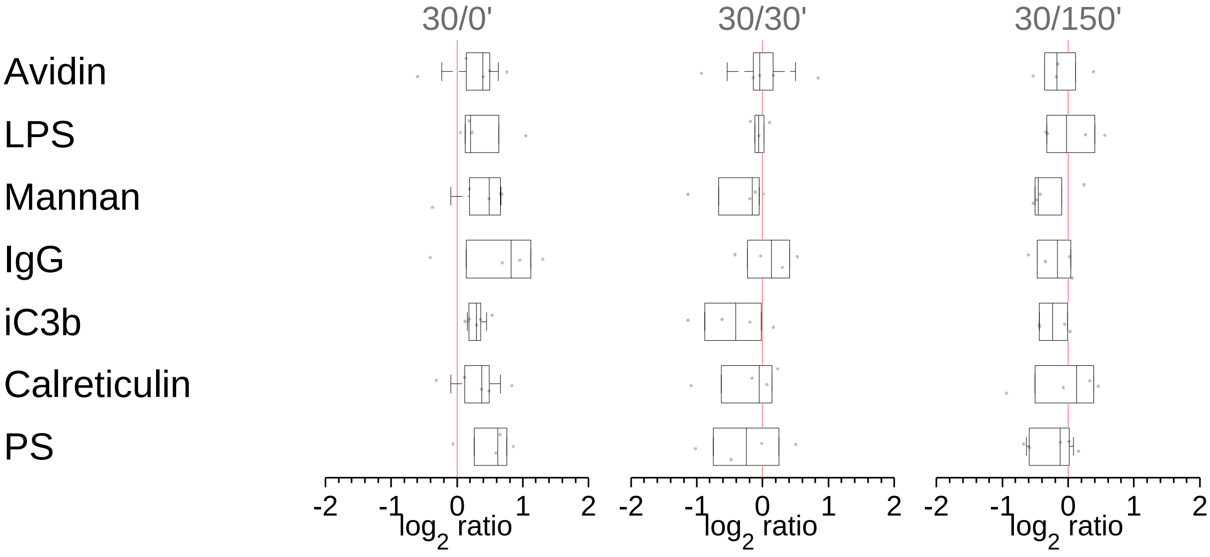

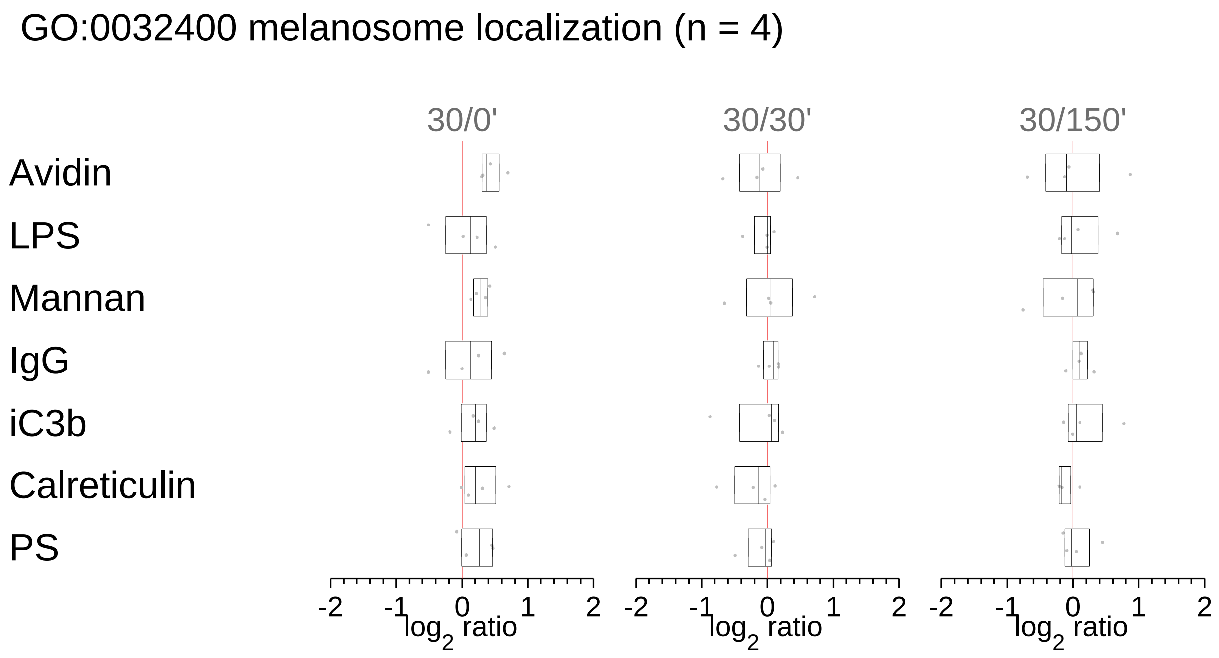
GO:0032400 melanosome localization (n = 4)
30/0'
30/30'
30/150'
Avidin
LPS
Mannan
IgG
iC3b
Calreticulin
PS
-2
-1
0
1
2
log_2 ratio

GO:0032402 melanosome transport (n = 6)

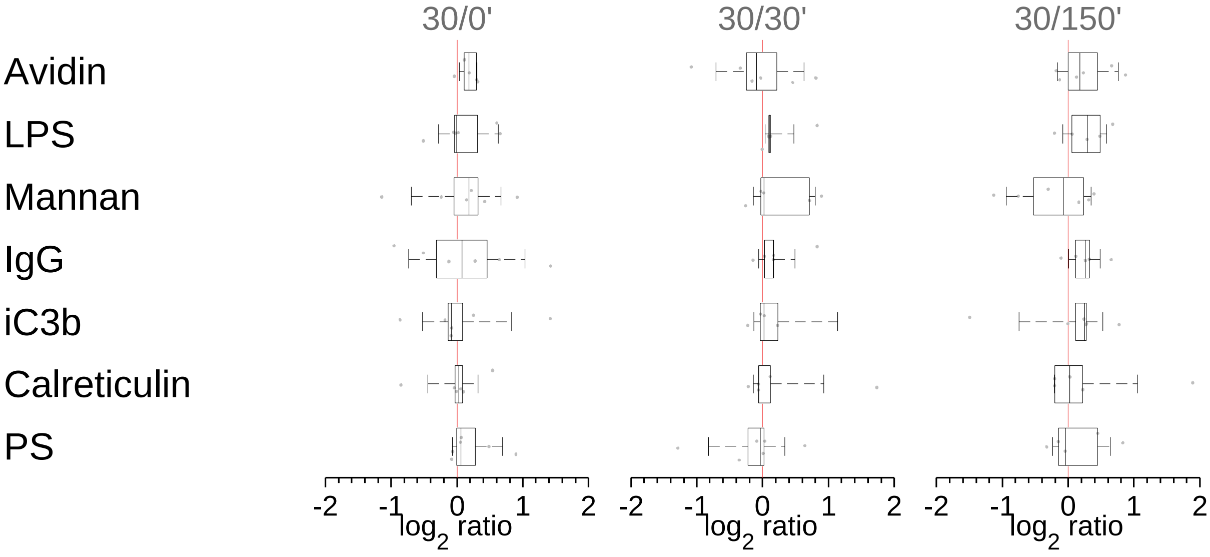

GO:0032403 protein complex binding (n = 84)

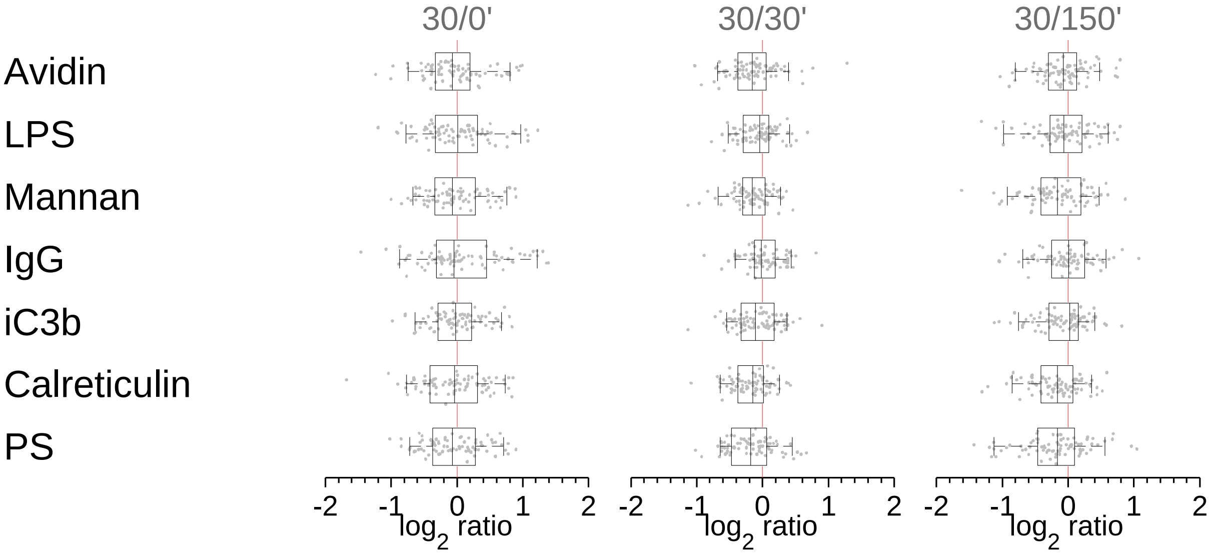

GO:0032418 lysosome localization (n = 5)

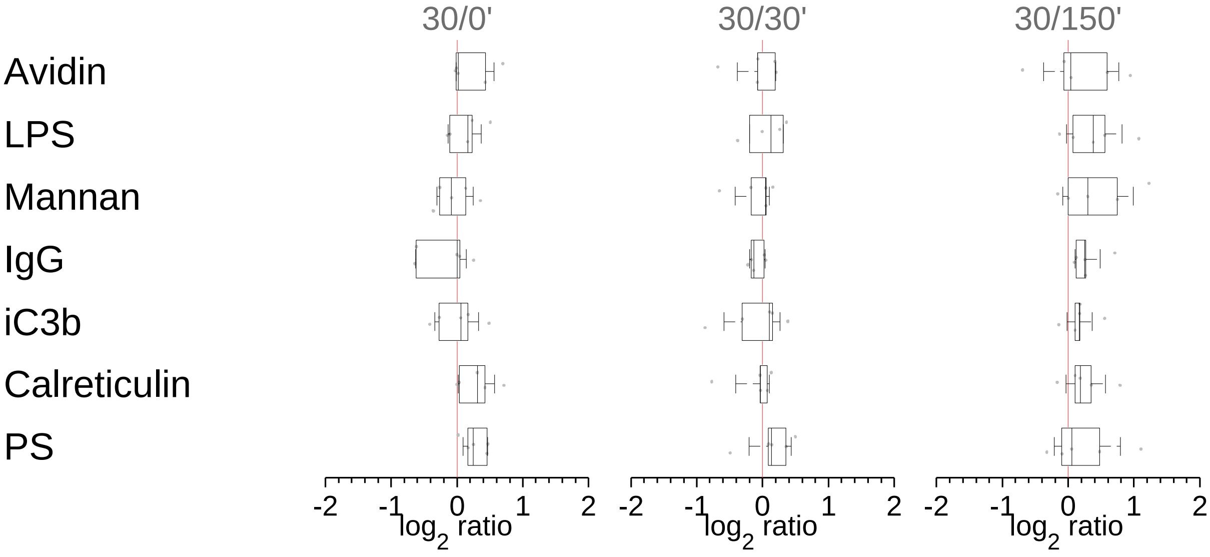

GO:0032436 positive regulation of proteasomal ubiquitin-dependent protein catabolic process (n = 8)

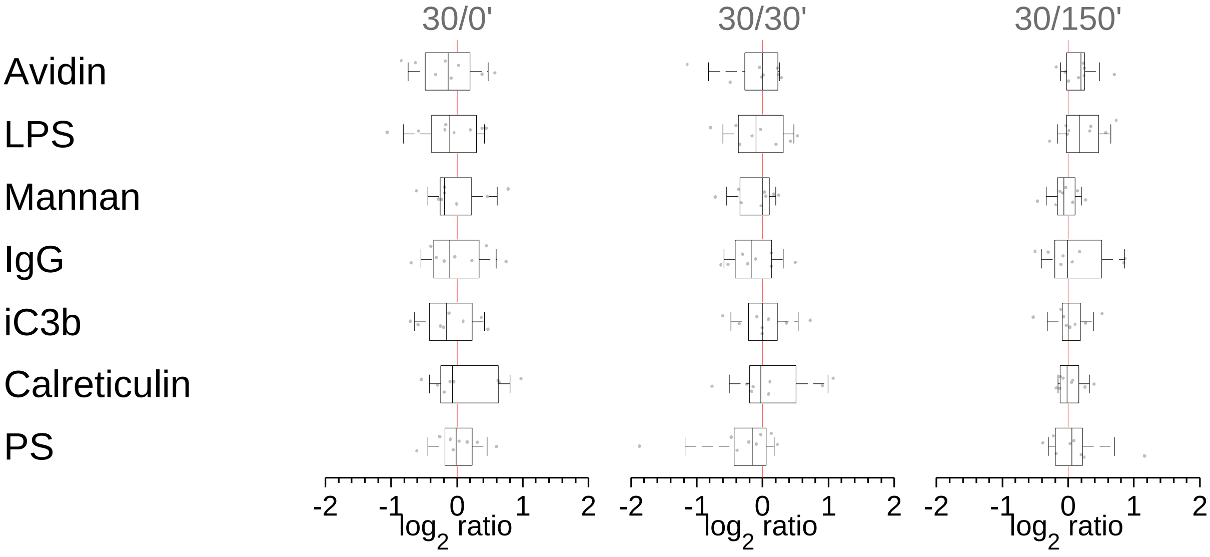

GO:0032438 melanosome organization (n = 3)

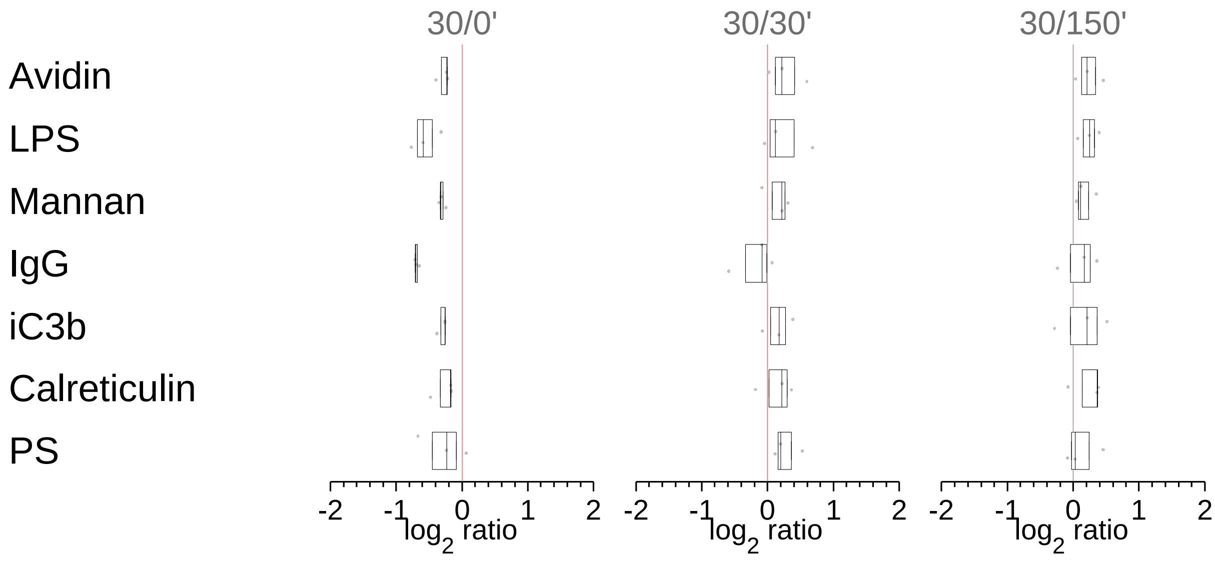

GO:0032442 phenylcoumaran benzylic ether reductase activity (n = 3)

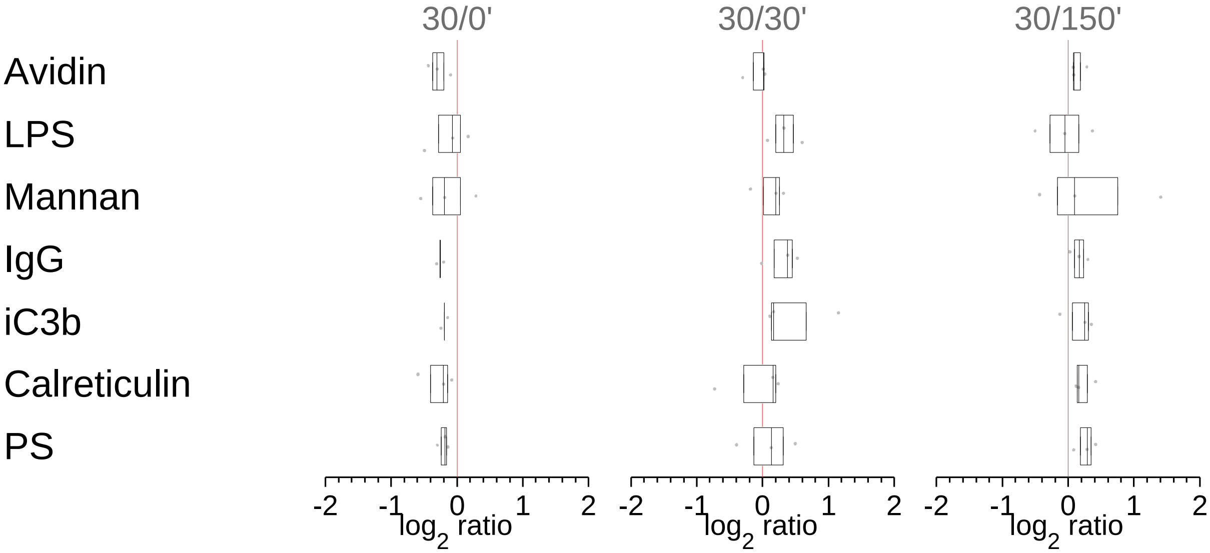

GO:0032456 endocytic recycling (n = 6)

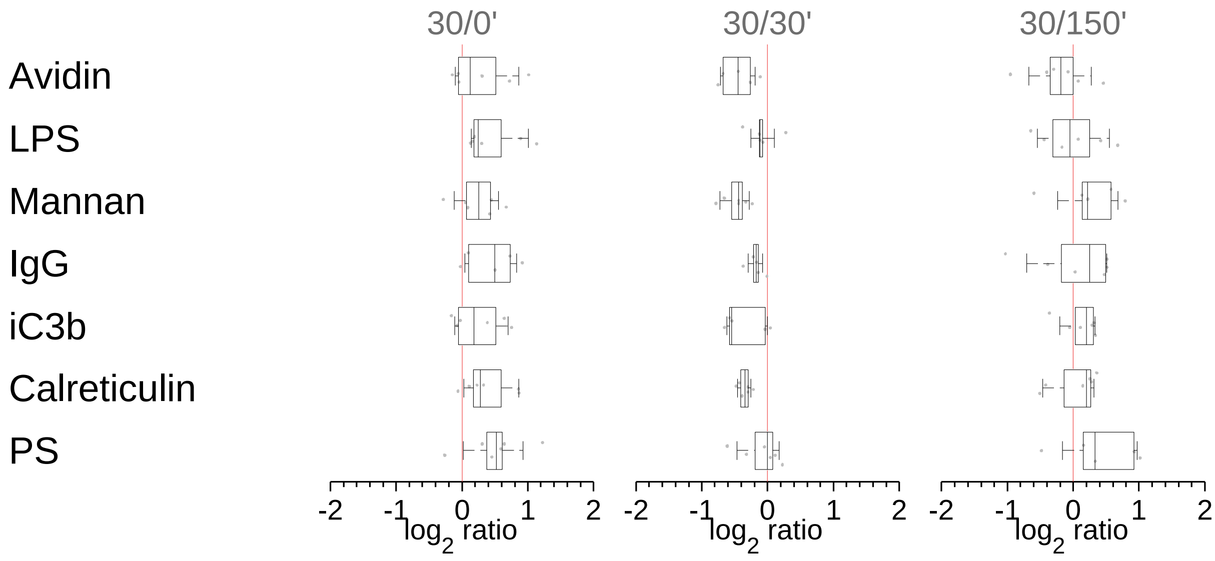

GO:0032465 regulation of cytokinesis (n = 3)

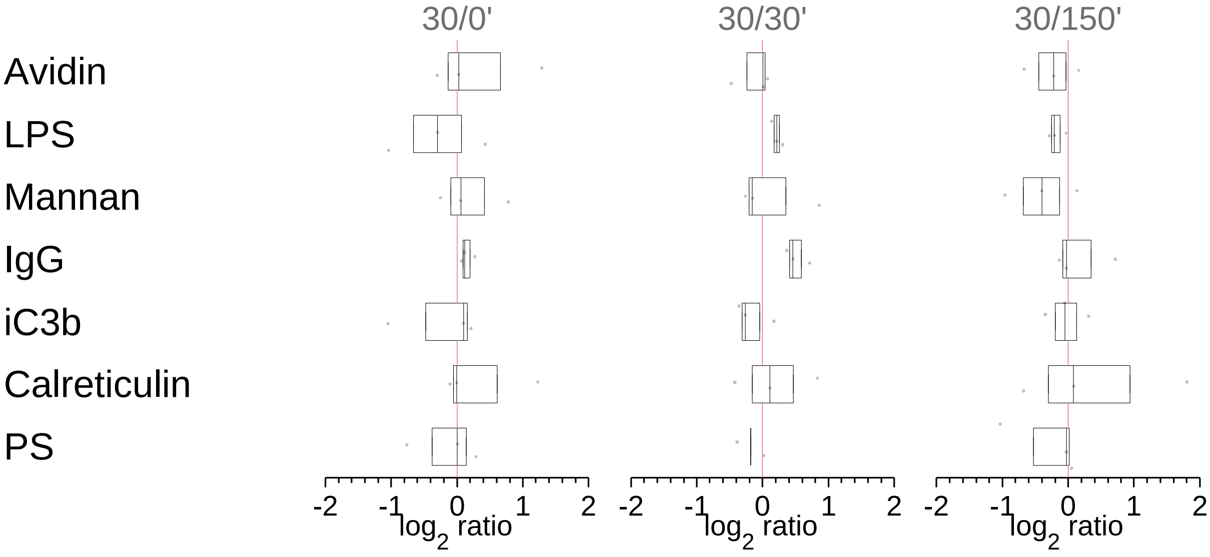

GO:0032471 reduction of endoplasmic reticulum calcium ion concentration (n = 5)

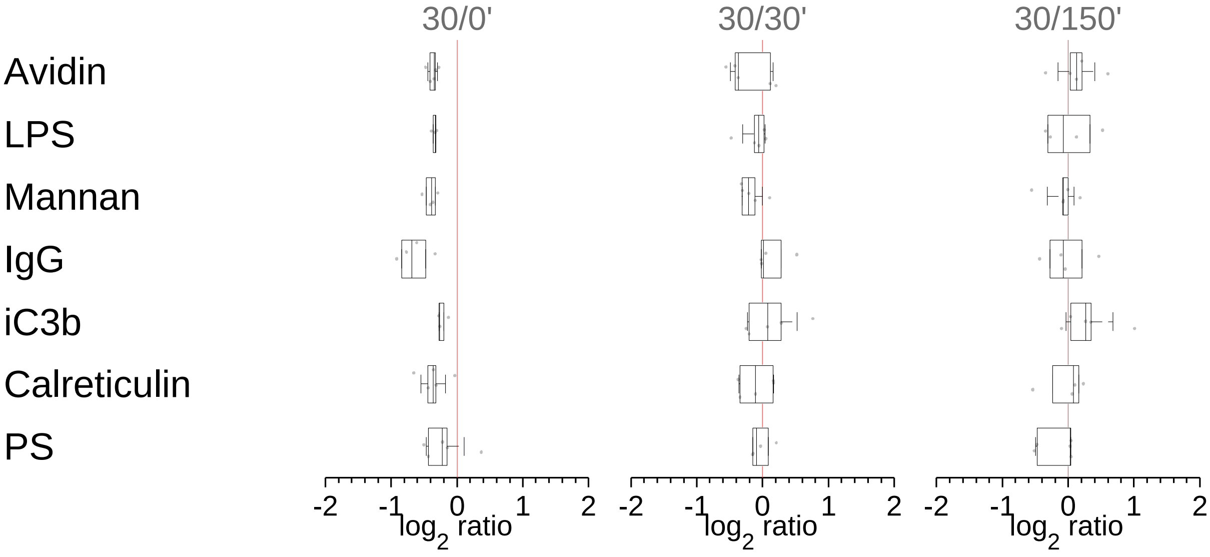

GO:0032481 positive regulation of type I interferon production
(n = 4)

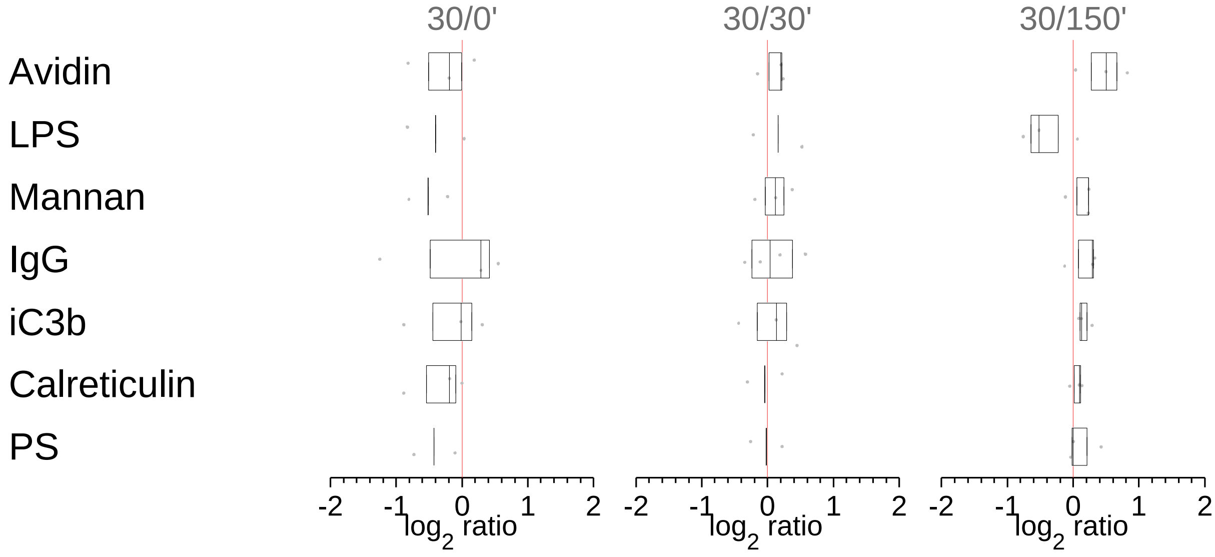

GO:0032486 Rap protein signal transduction (n = 5)

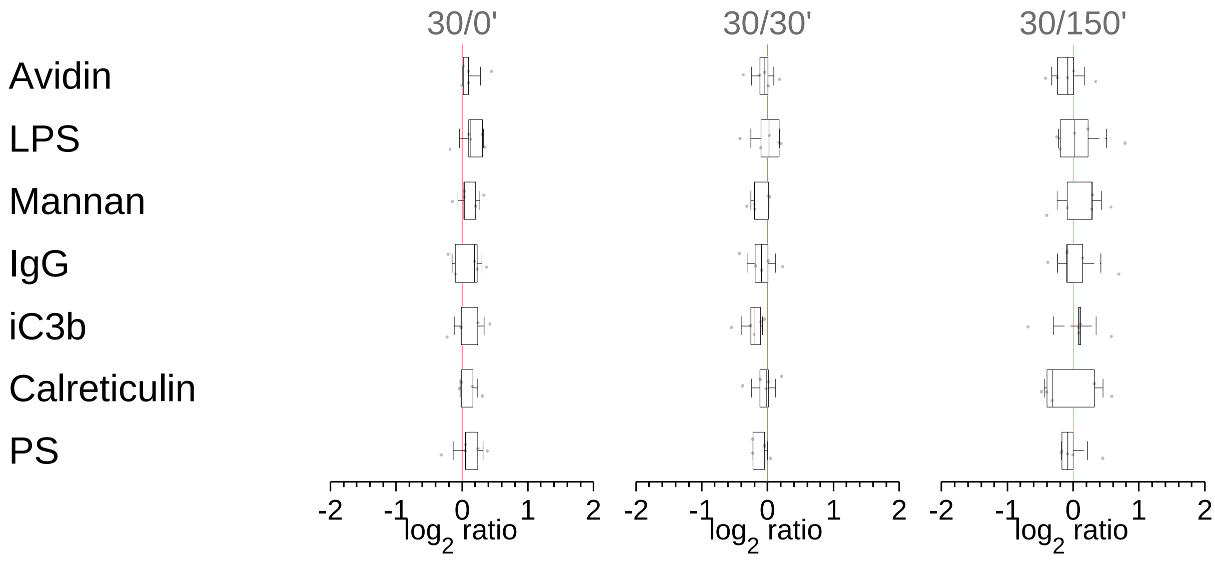

GO:0032496 response to lipopolysaccharide (n = 10)

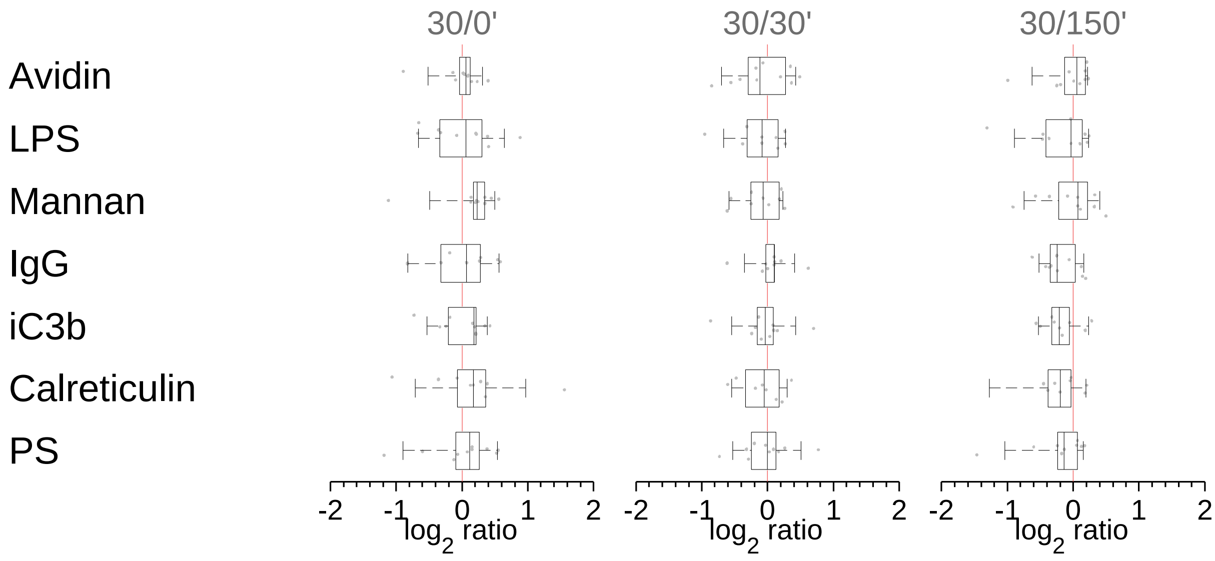

GO:0032508 DNA duplex unwinding (n = 4)

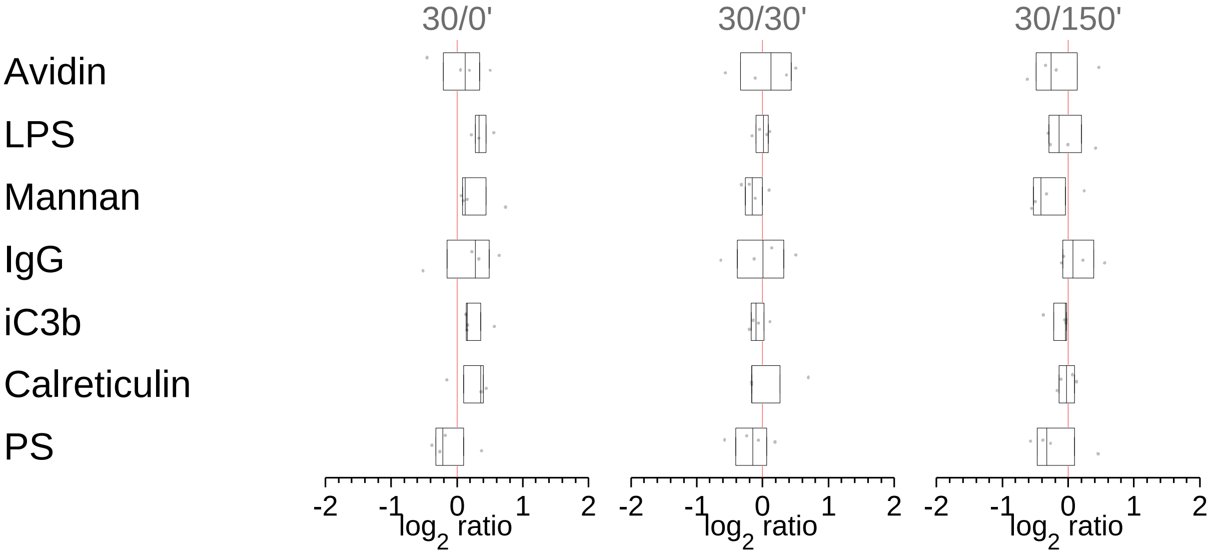

GO:0032526 response to retinoic acid (n = 4)

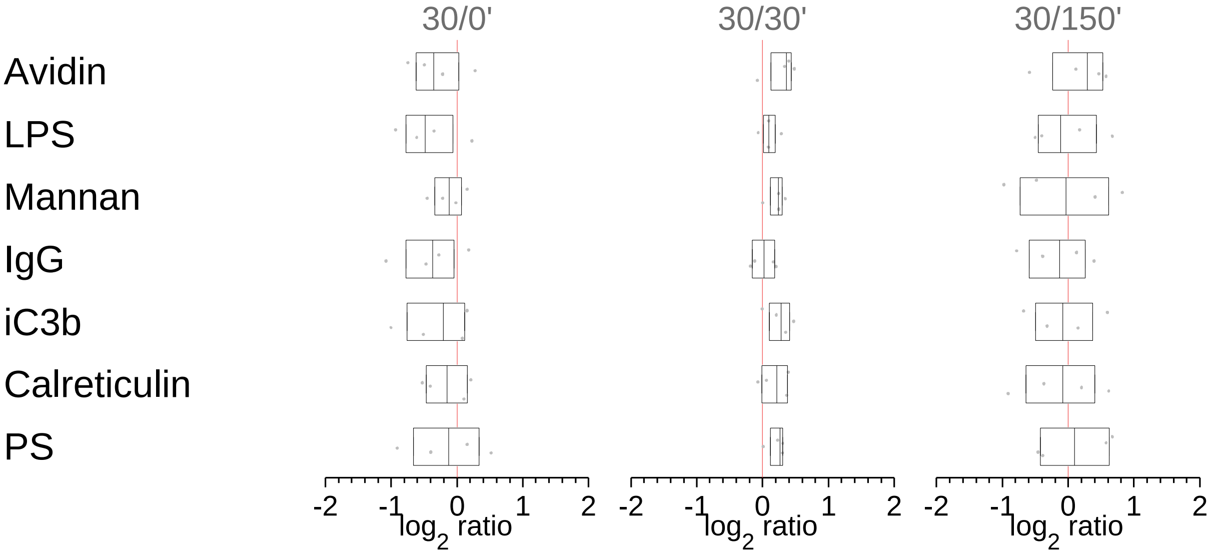

GO:0032564 dATP binding (n = 3)

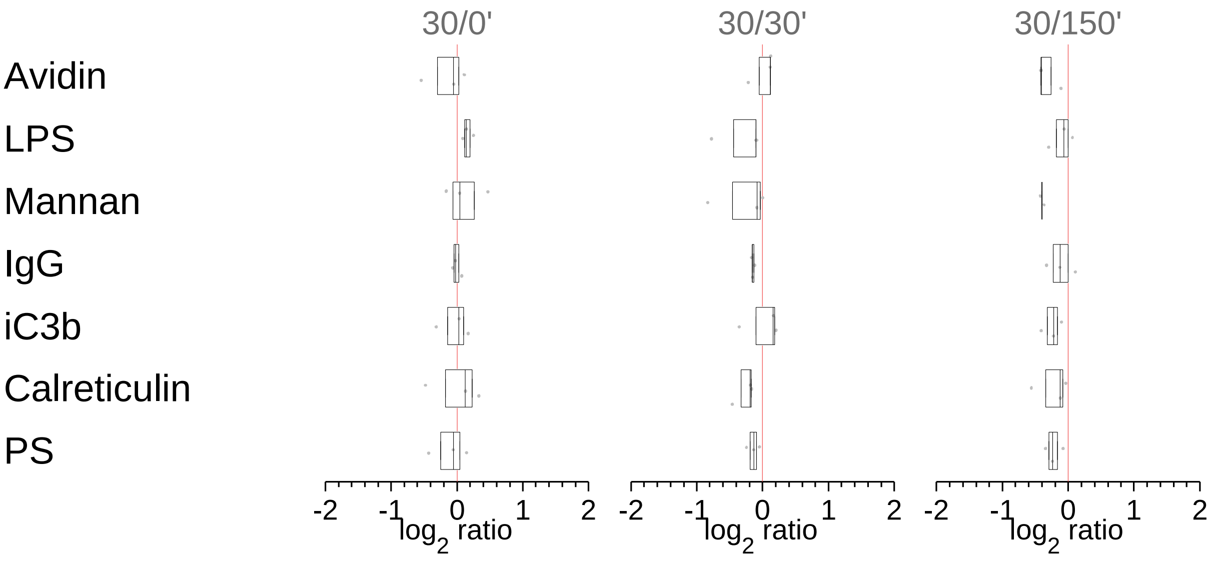

GO:0032587 ruffle membrane (n = 12)

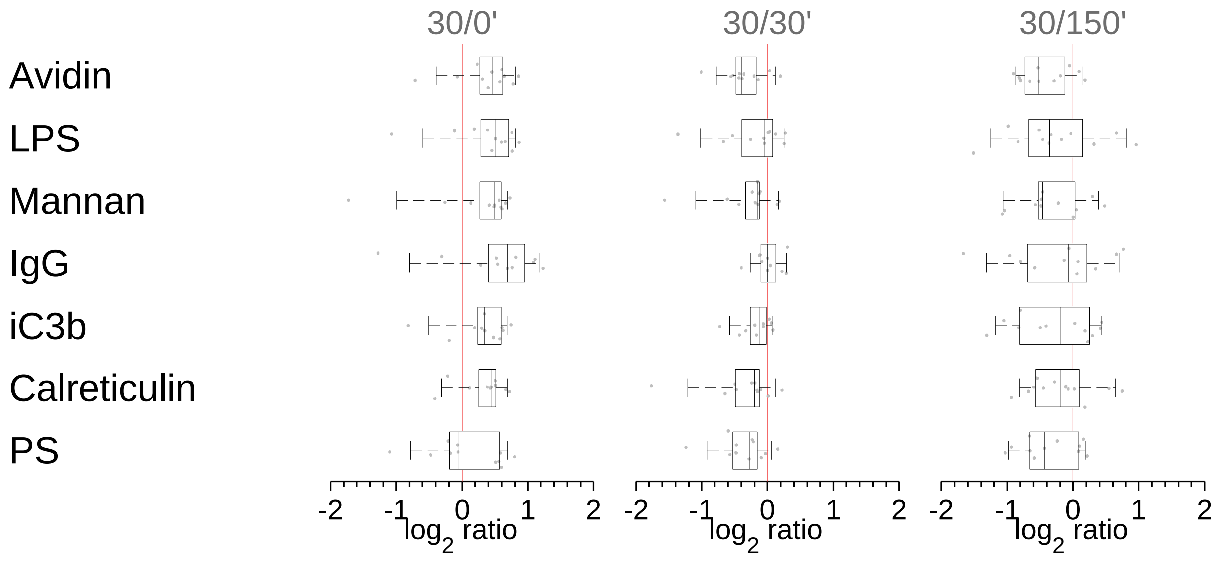

GO:0032588 trans-Golgi network membrane (n = 6)

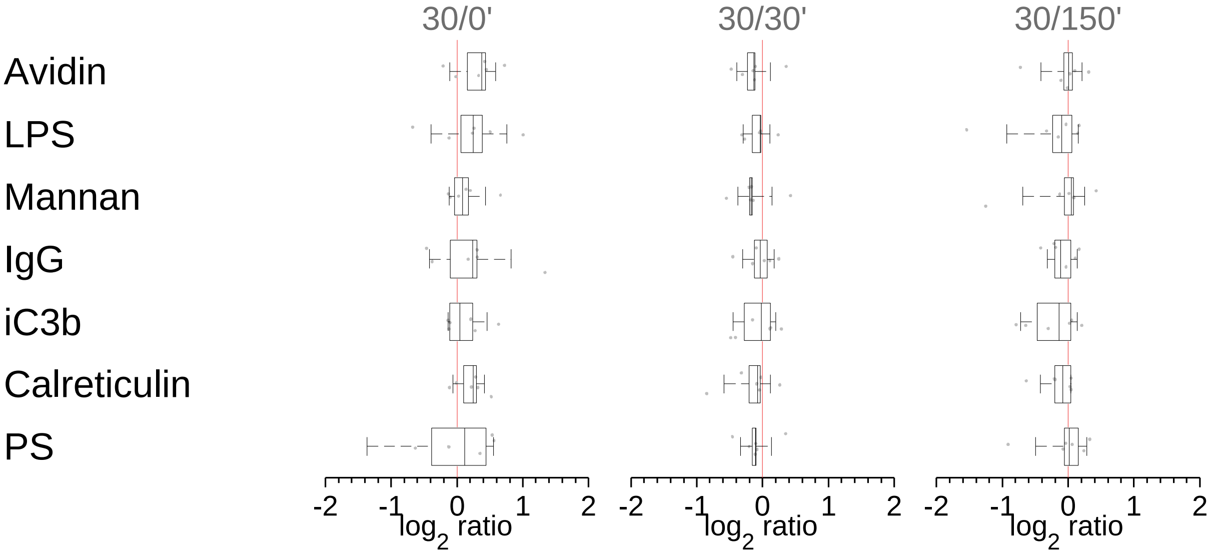

GO:0032593 insulin-responsive compartment (n = 4)

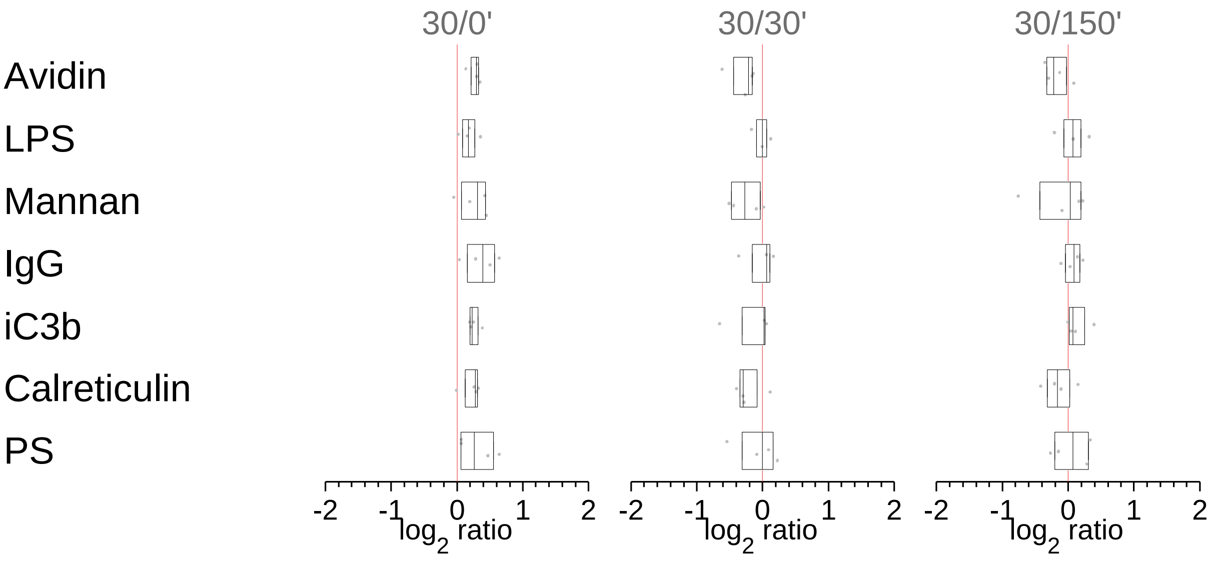

GO:0032695 negative regulation of interleukin-12 production (n = 3)

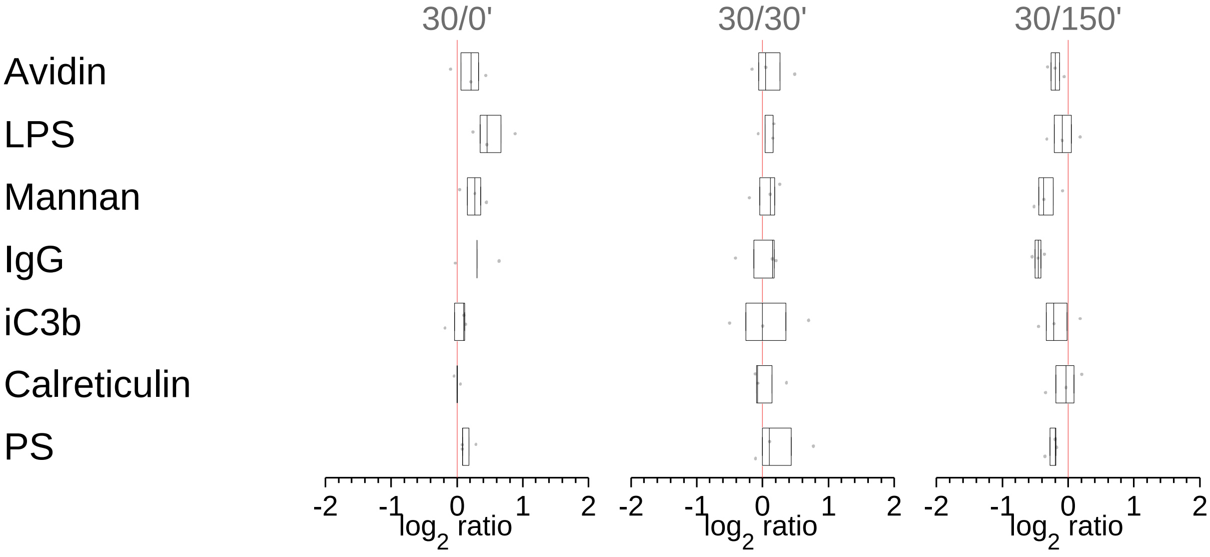

GO:0032715 negative regulation of interleukin-6 production (n = 5)

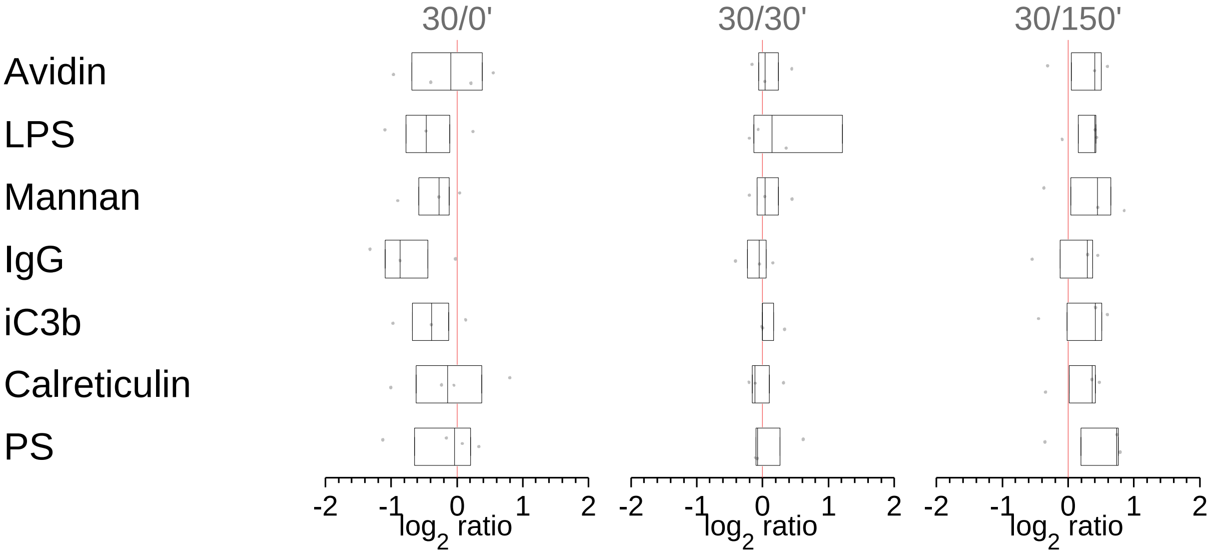

GO:0032722 positive regulation of chemokine production (n = 6)

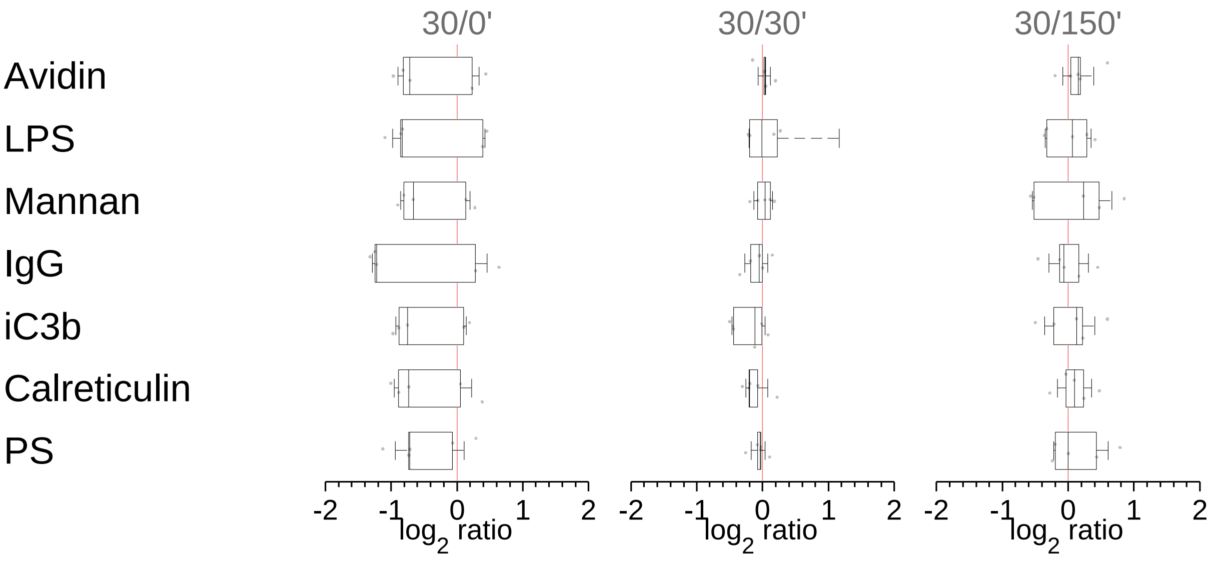

GO:0032727 positive regulation of interferon-alpha production (n = 3)

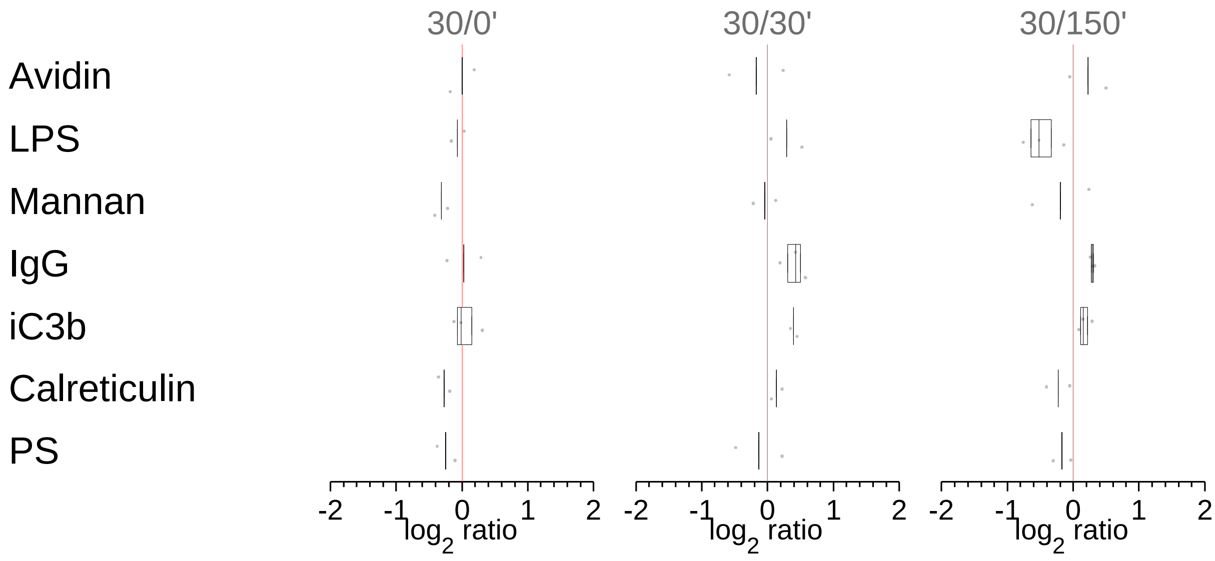

GO:0032728 positive regulation of interferon-beta production (n = 5)

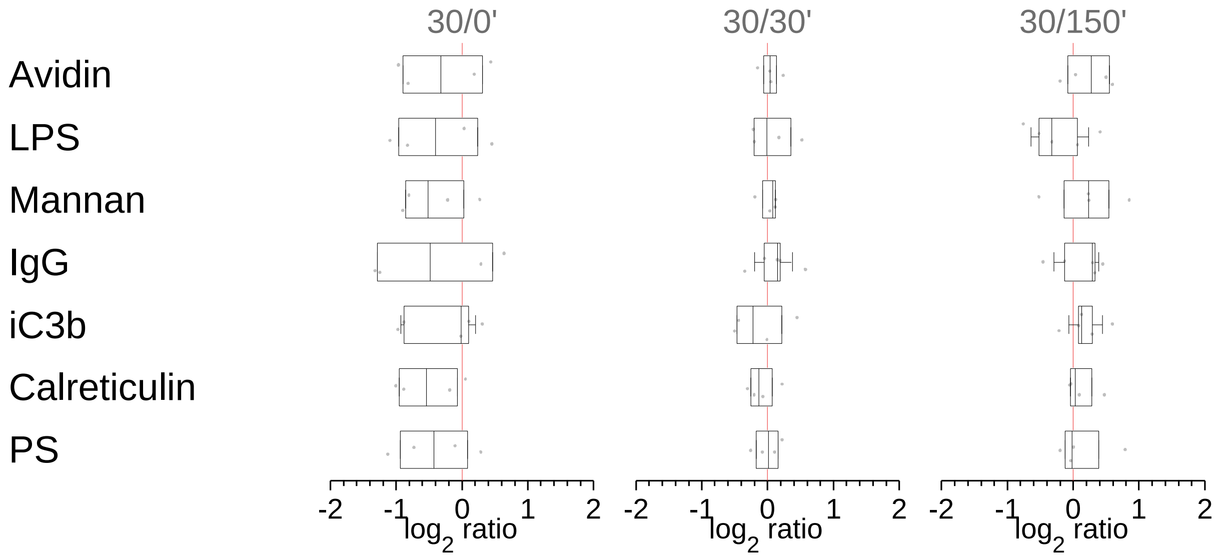

GO:0032729 positive regulation of interferon-gamma production (n = 4)

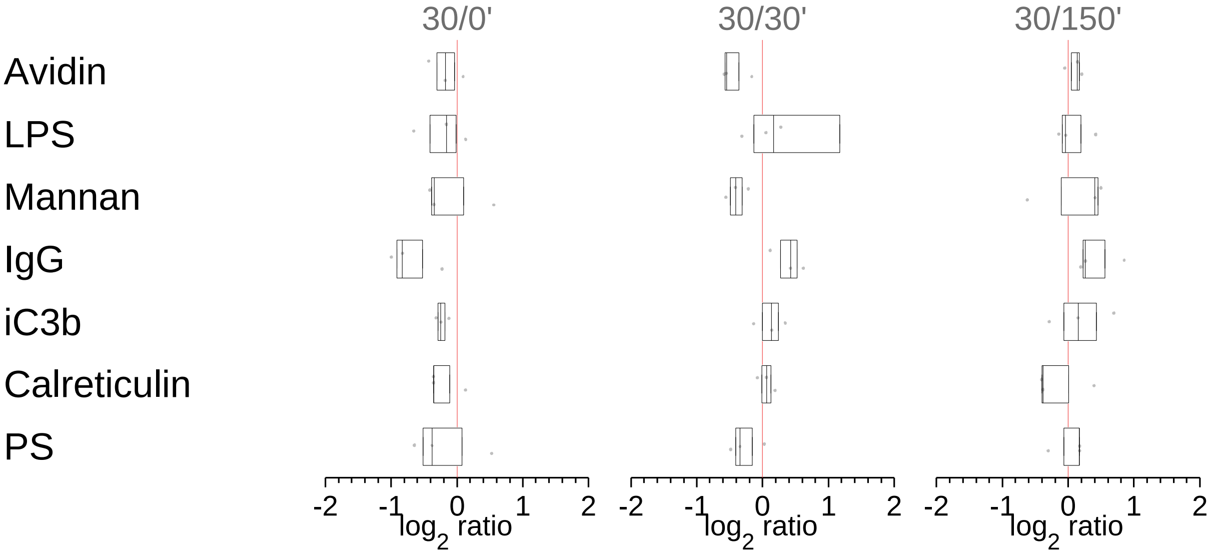

GO:0032733 positive regulation of interleukin-10 production (n = 5)

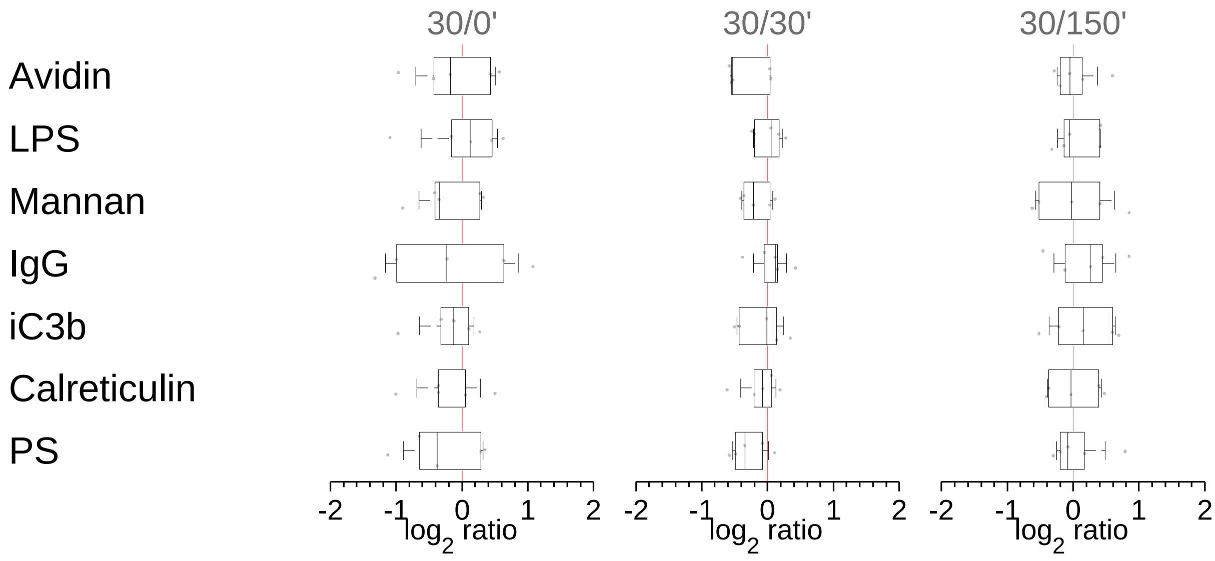

GO:0032735 positive regulation of interleukin-12 production (n = 5)

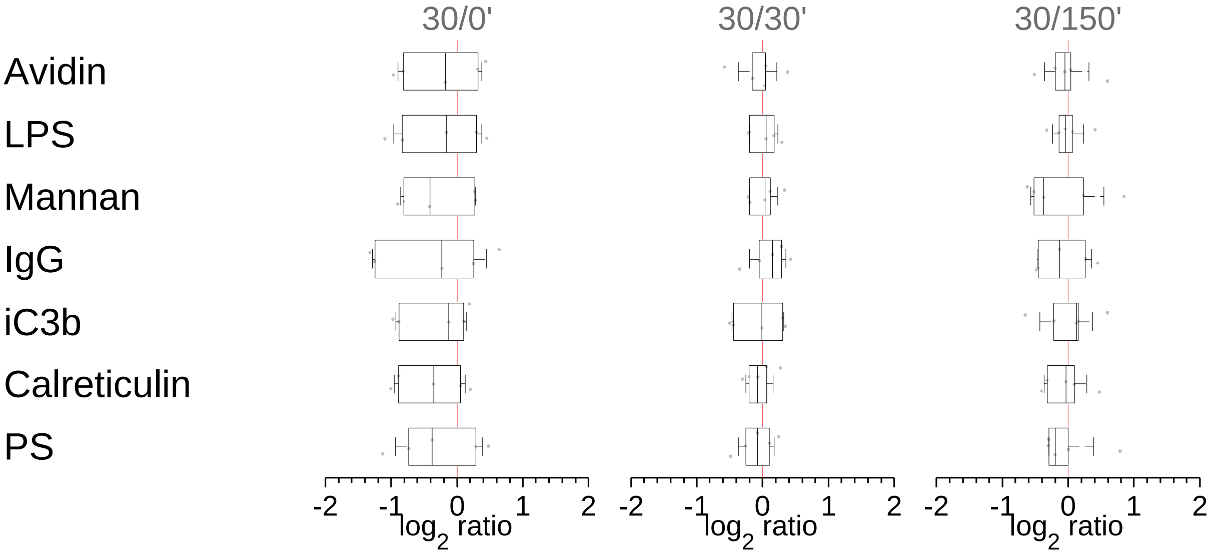

GO:0032741 positive regulation of interleukin-18 production (n = 3)

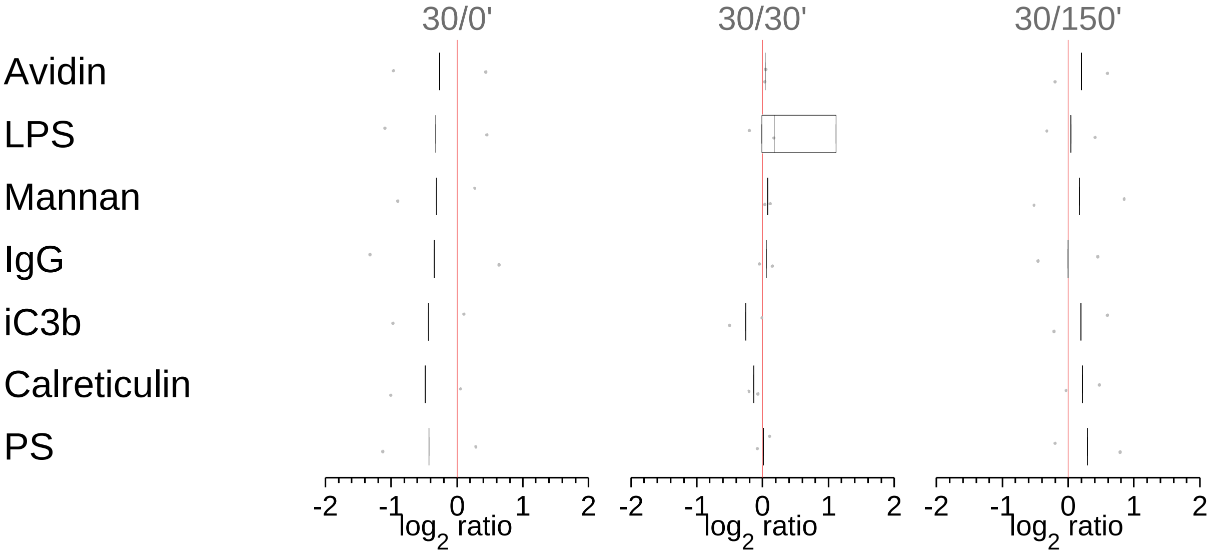

GO:0032755 positive regulation of interleukin-6 production (n = 11)

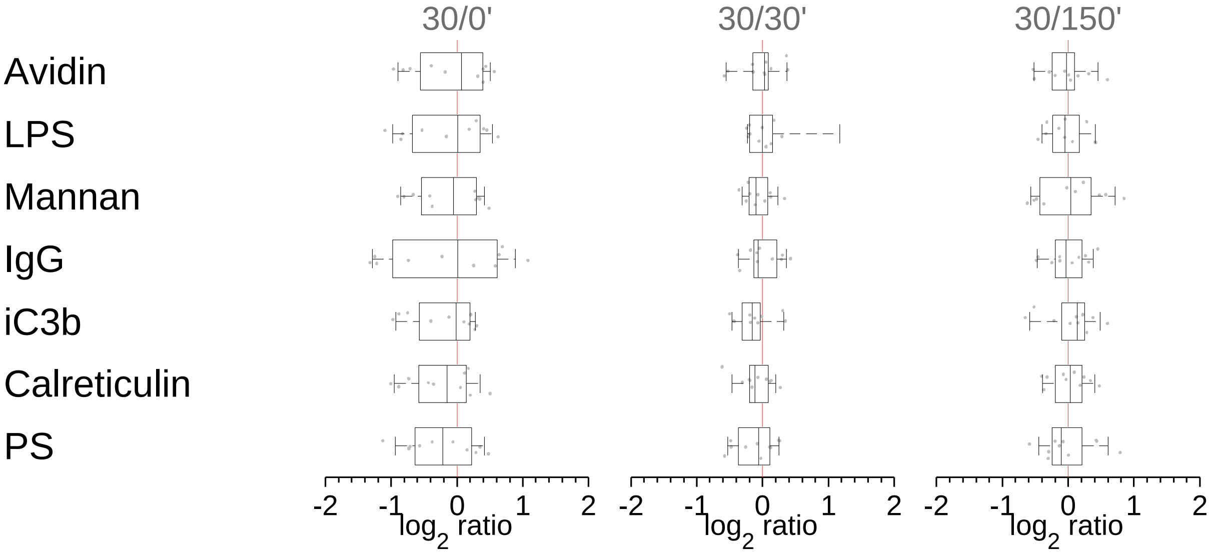

GO:0032757 positive regulation of interleukin-8 production (n = 6)

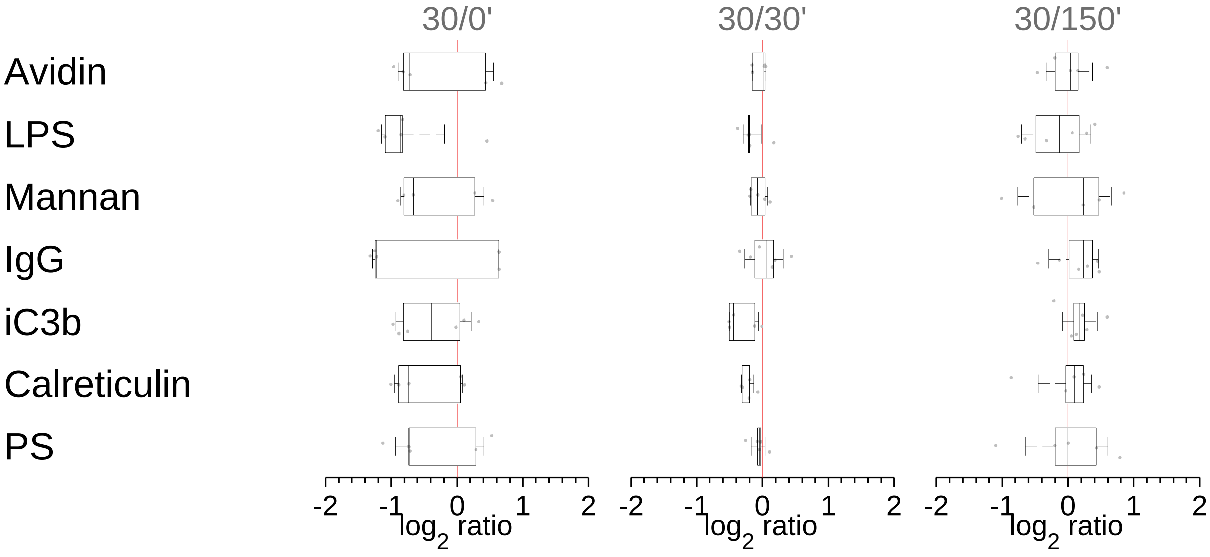

GO:0032760 positive regulation of tumor necrosis factor production (n = 11)

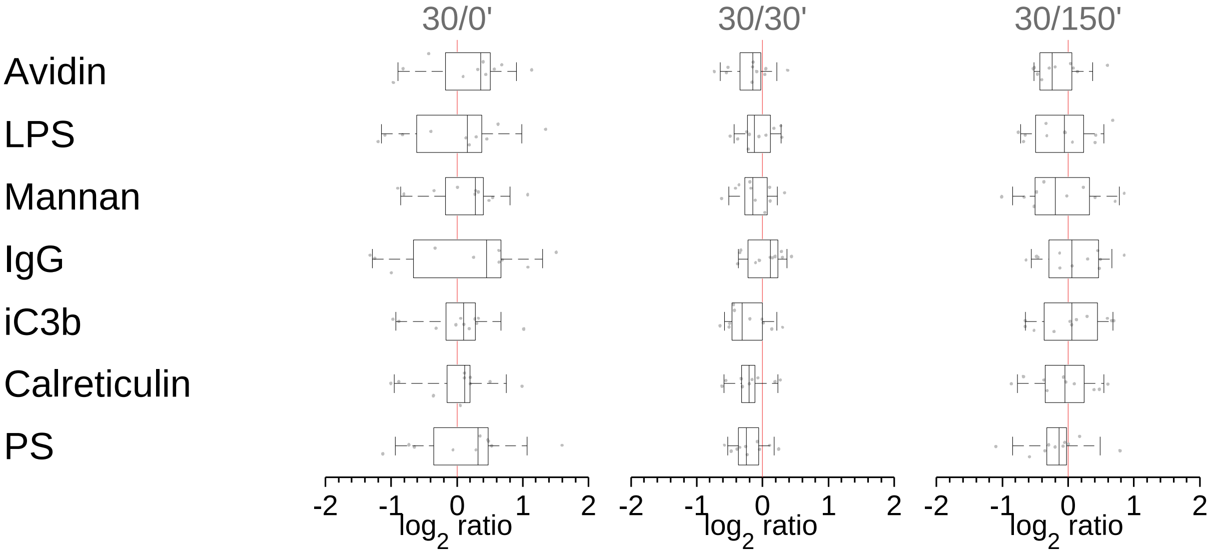

GO:0032781 positive regulation of ATPase activity (n = 7)

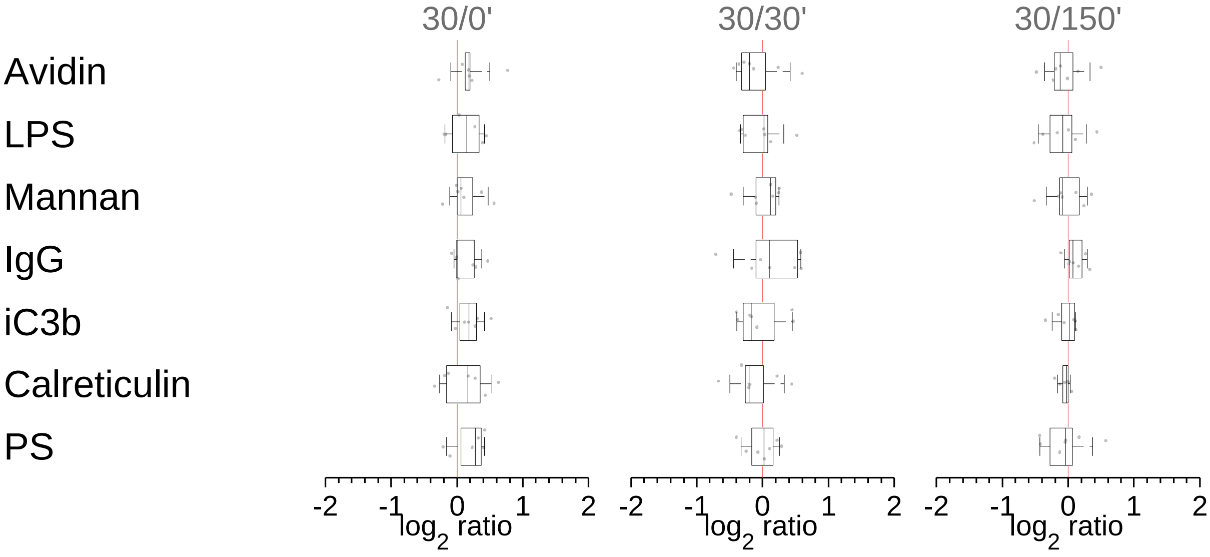

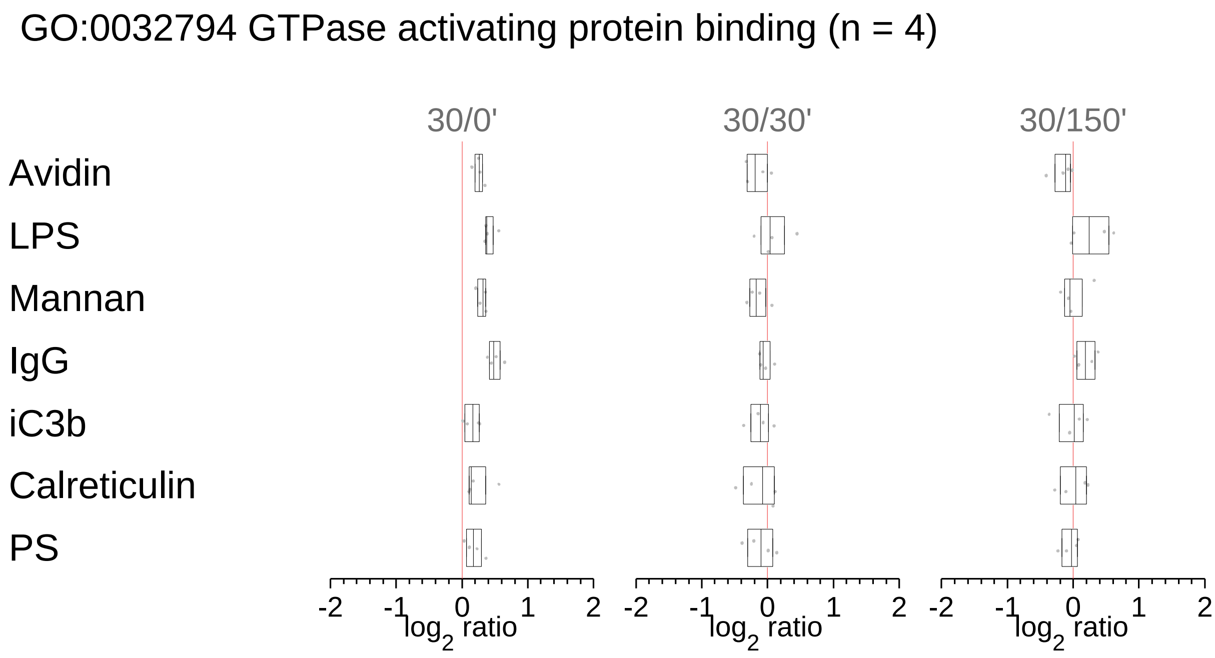
GO:0032794 GTPase activating protein binding (n = 4)
30/0'
30/30'
30/150'
Avidin
LPS
Mannan
IgG
iC3b
Calreticulin
PS
-2
-1
0
1
2
log2 ratio

GO:0032801 receptor catabolic process (n = 4)

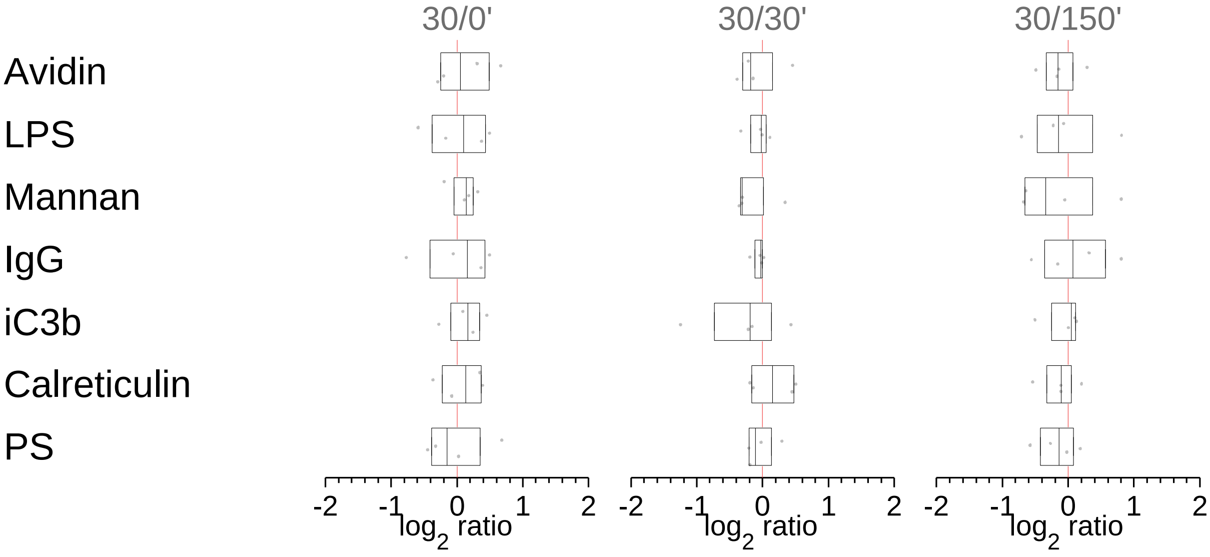

GO:0032839 dendrite cytoplasm (n = 5)

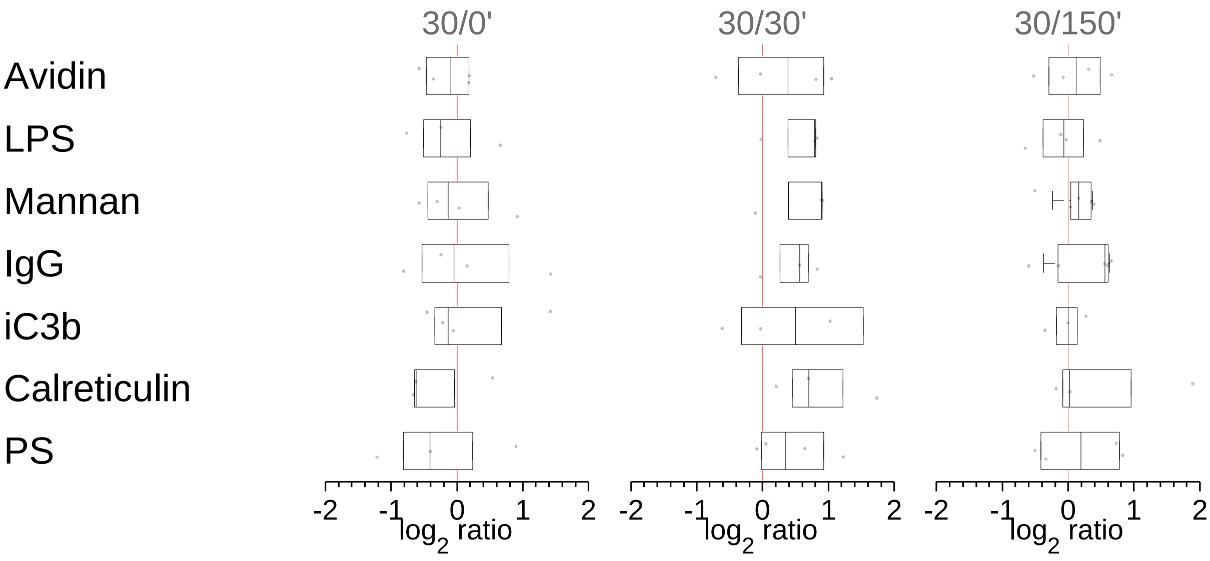

GO:0032855 positive regulation of Rac GTPase activity (n = 6)

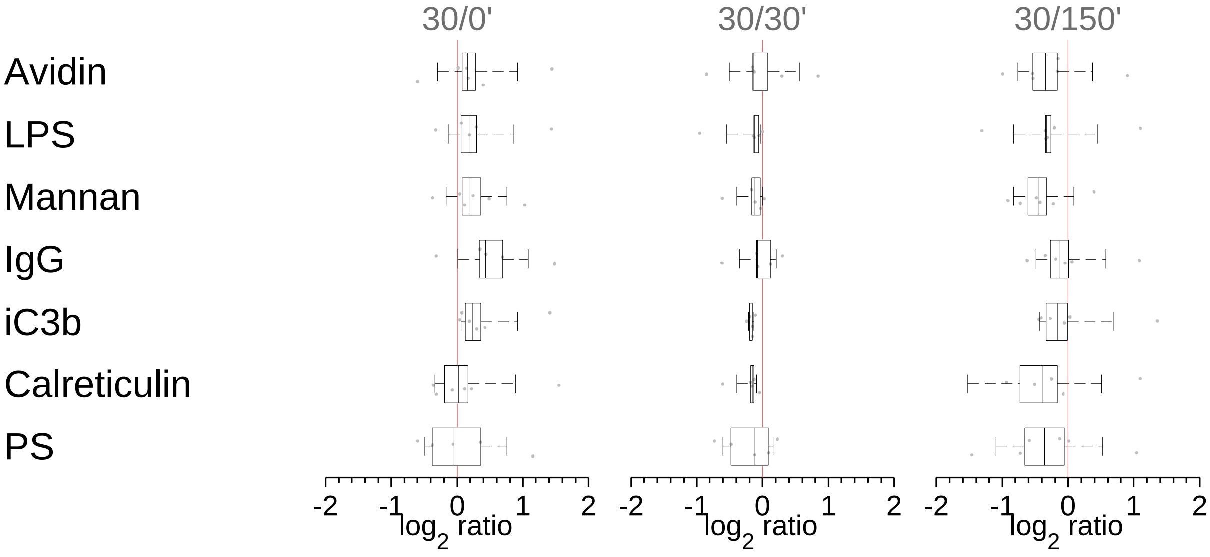

GO:0032866 D-xylose:NADP reductase activity (n = 3)

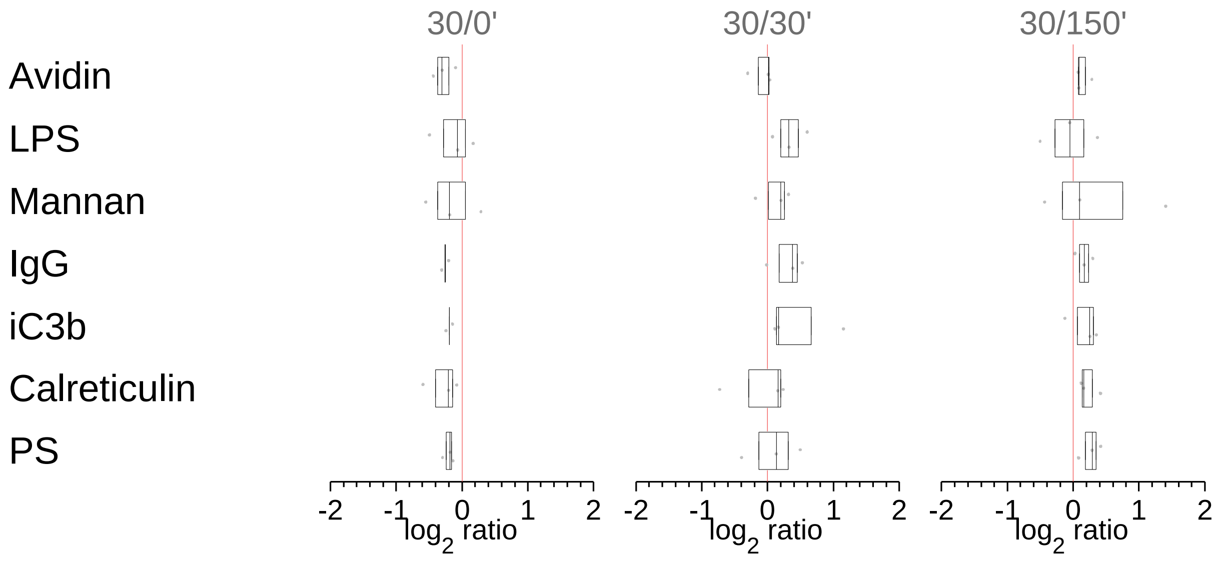

GO:0032867 L-arabinose:NADP reductase activity (n = 3)

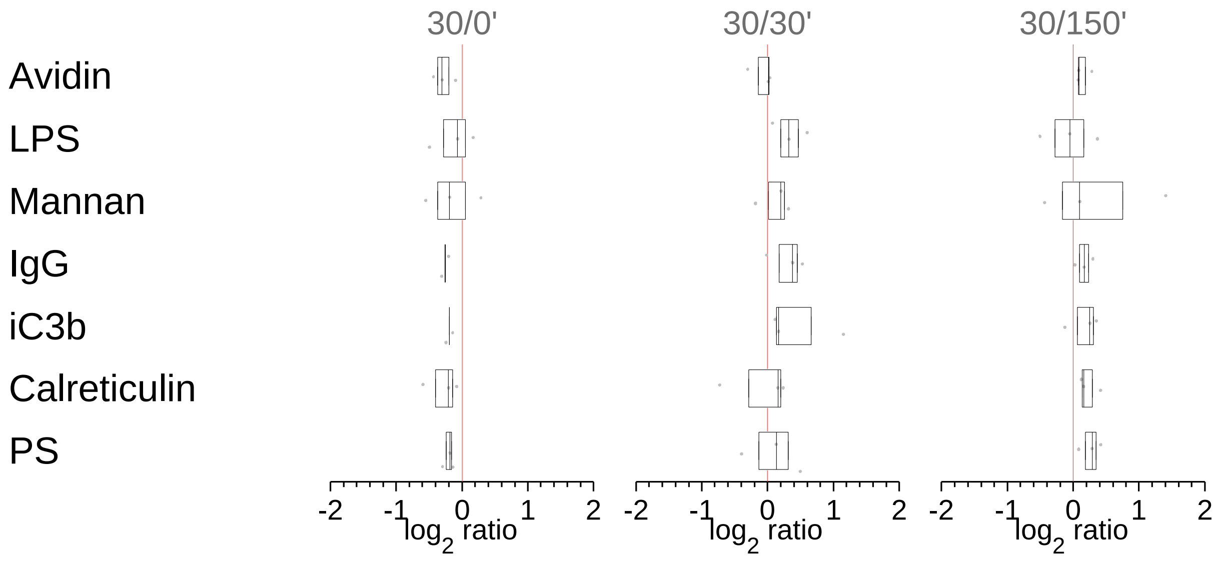

GO:0032868 response to insulin stimulus (n = 4)

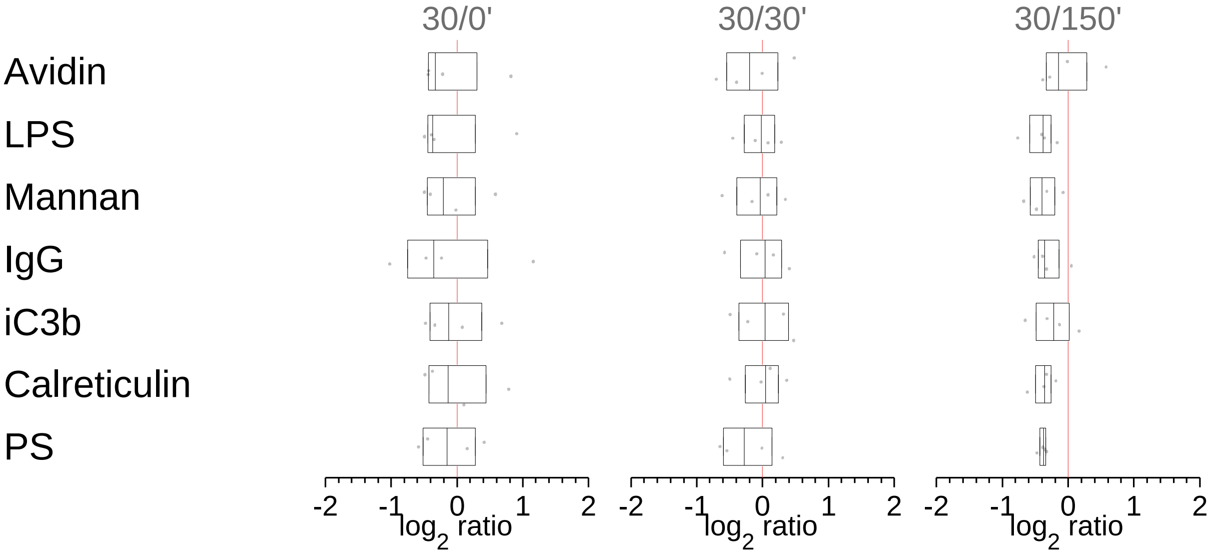

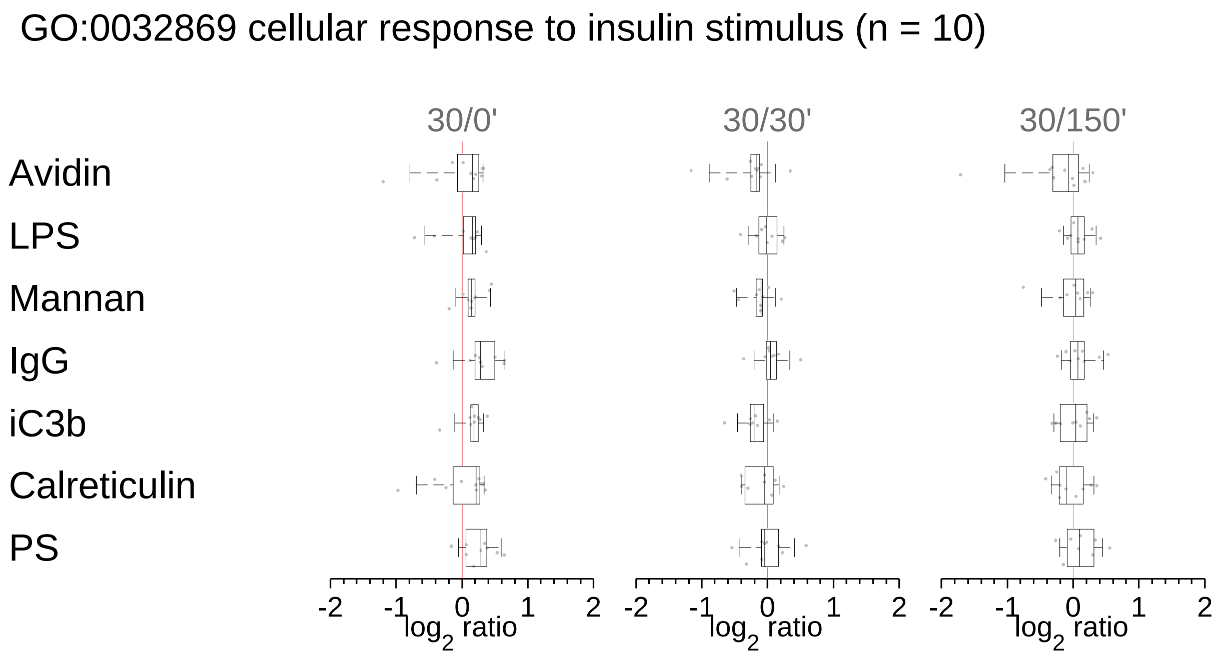

GO:0032869 cellular response to insulin stimulus (n = 10)
30/0'
30/30'
30/150'
Avidin
LPS
Mannan
IgG
iC3b
Calreticulin
PS
-2 -1 0 1 2
log2 ratio

GO:0032880 regulation of protein localization (n = 8)

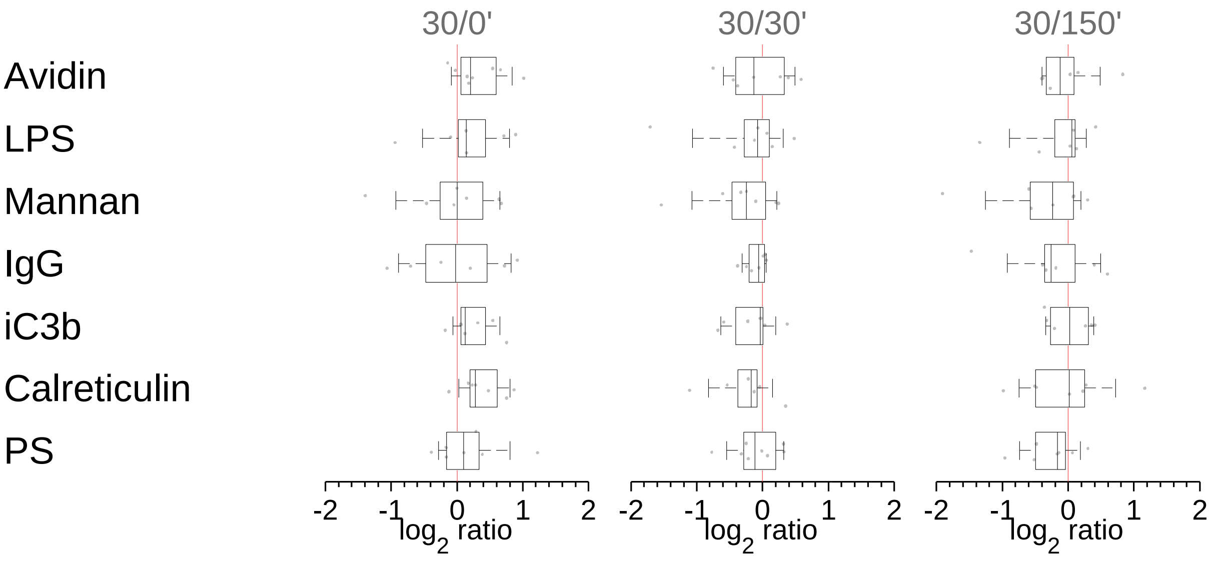

GO:0032897 negative regulation of viral transcription (n = 3)

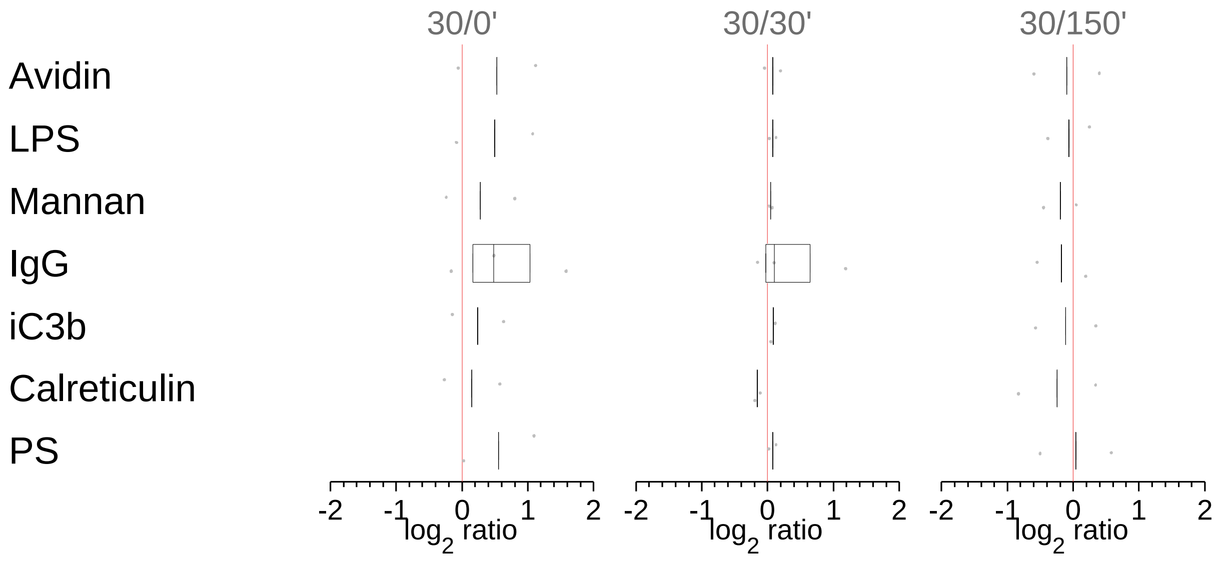

GO:0032922 circadian regulation of gene expression (n = 4)

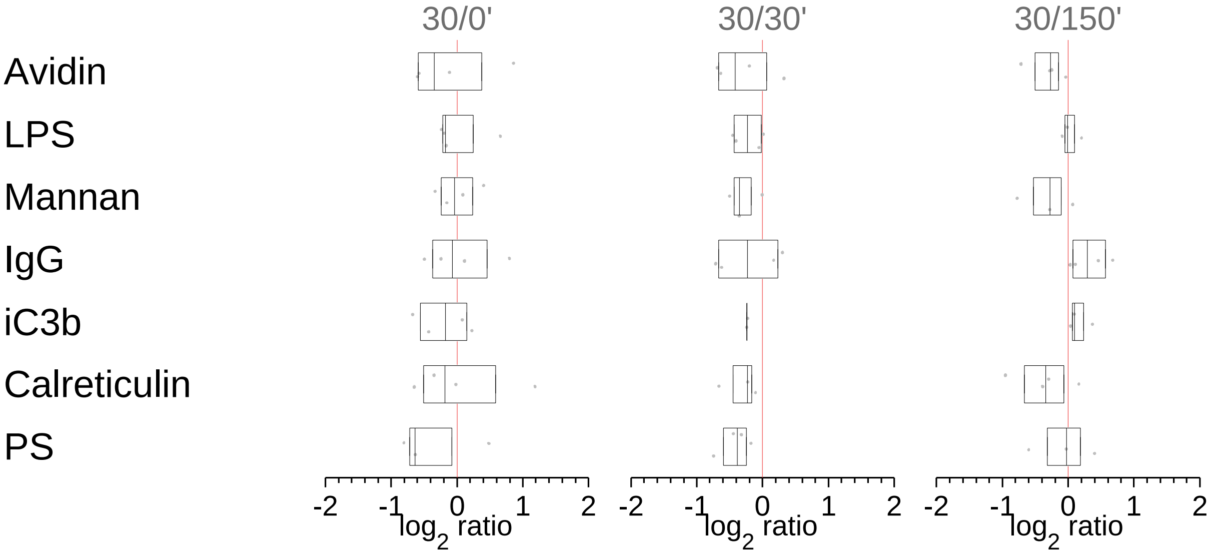

GO:0032947 protein complex scaffold (n = 8)

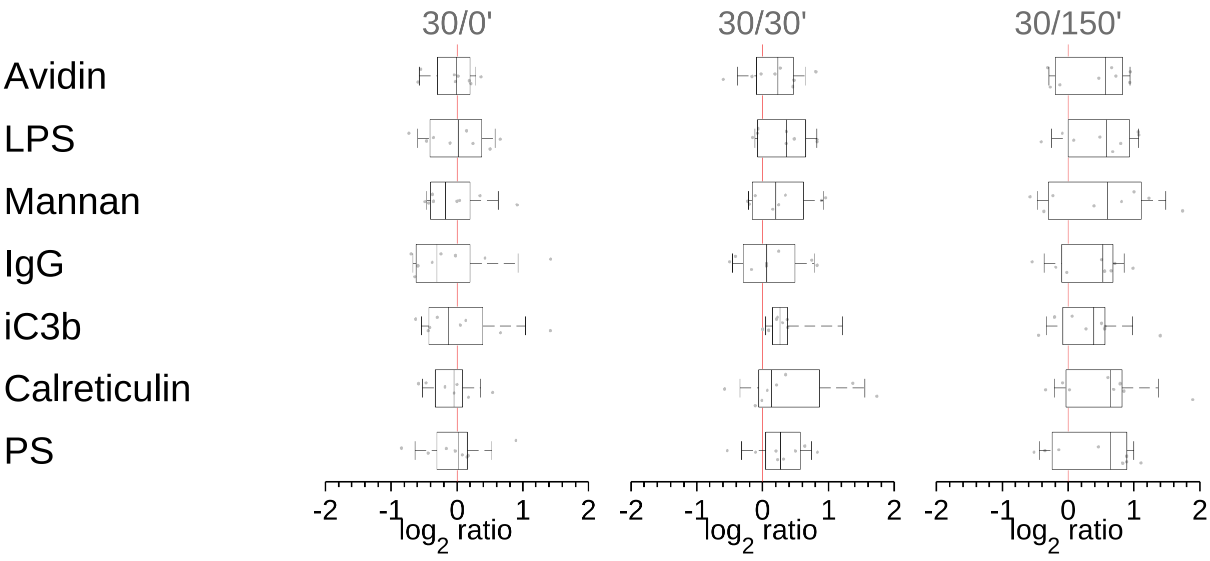

GO:0032956 regulation of actin cytoskeleton organization (n = 6)

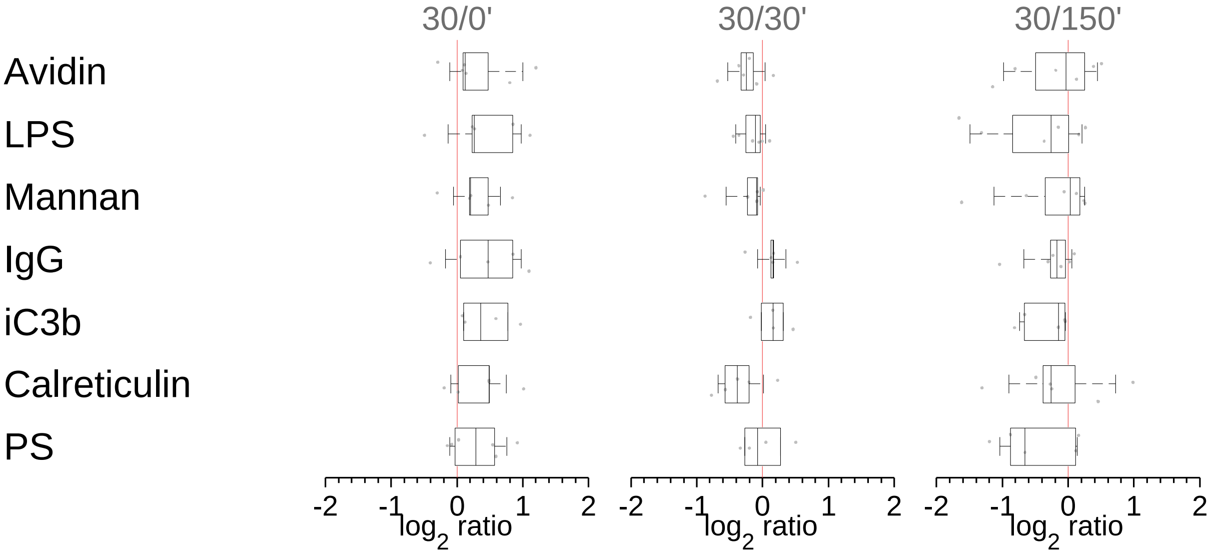

GO:0032968 positive regulation of transcription elongation from RNA polymerase II promoter (n = 3)

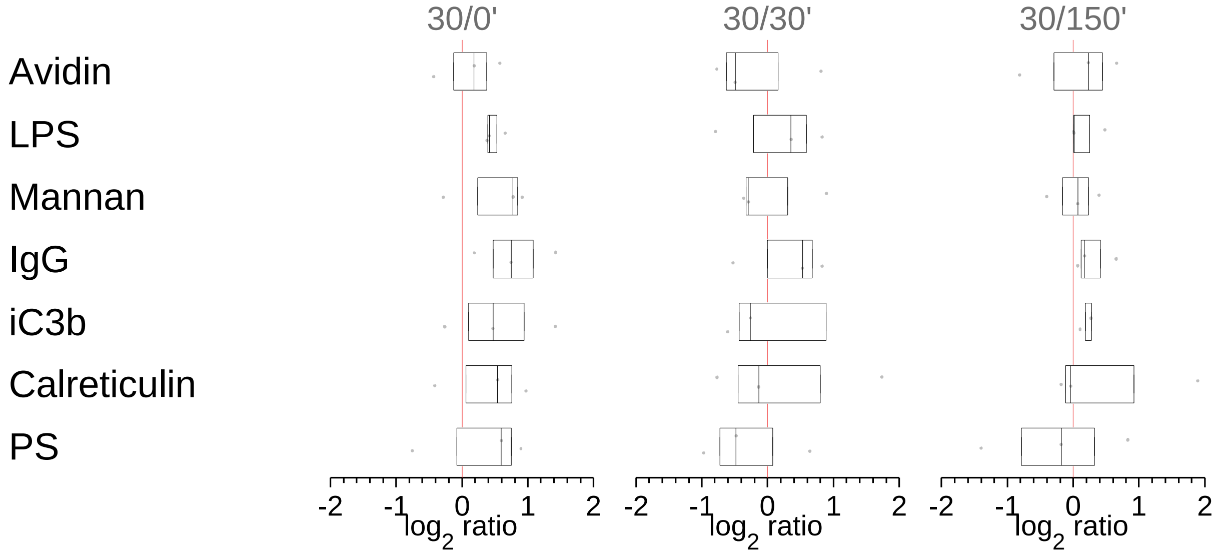

GO:0033077 T cell differentiation in thymus (n = 4)

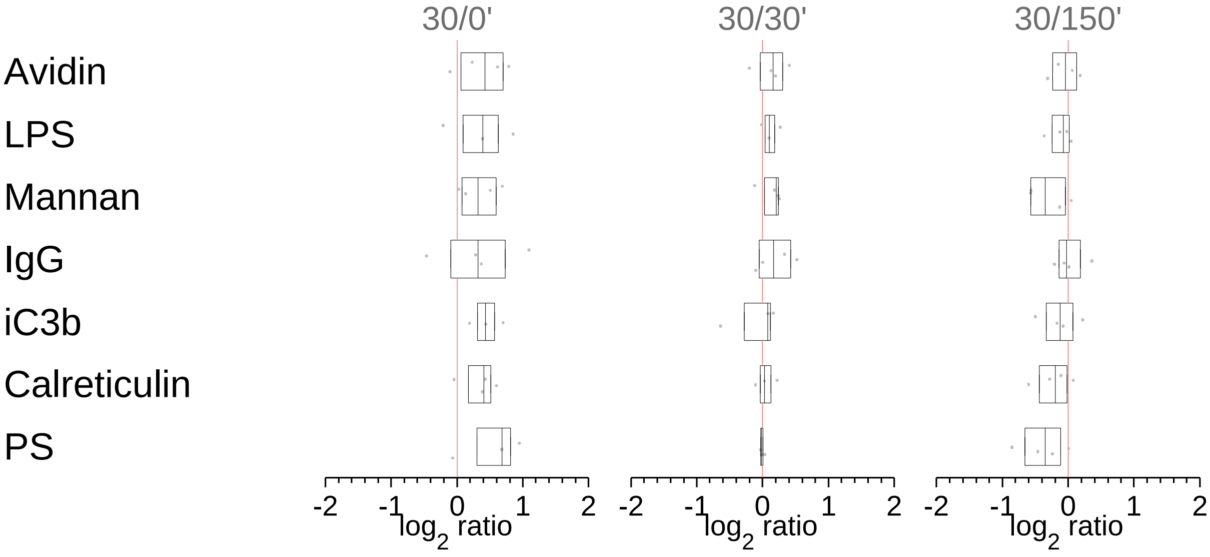

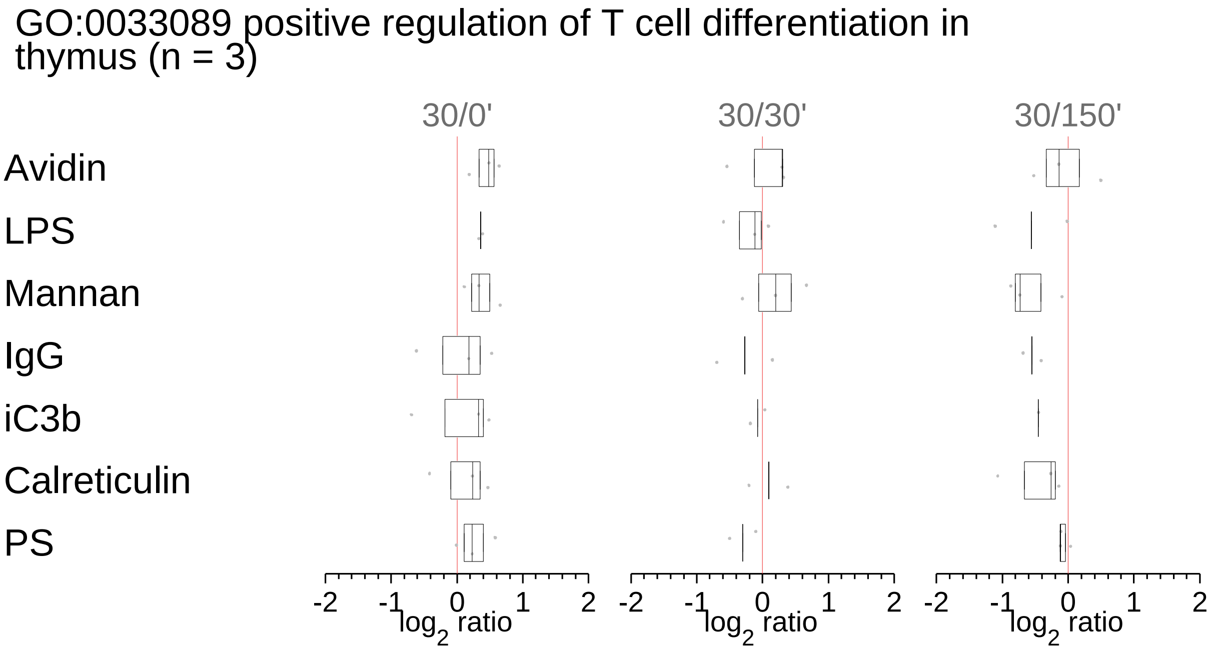
GO:0033089 positive regulation of T cell differentiation in thymus (n = 3)
30/0'
30/30'
30/150'
Avidin
LPS
Mannan
IgG
iC3b
Calreticulin
PS
-2
-1
0
1
2
log2 ratio

GO:0033119 negative regulation of RNA splicing (n = 3)

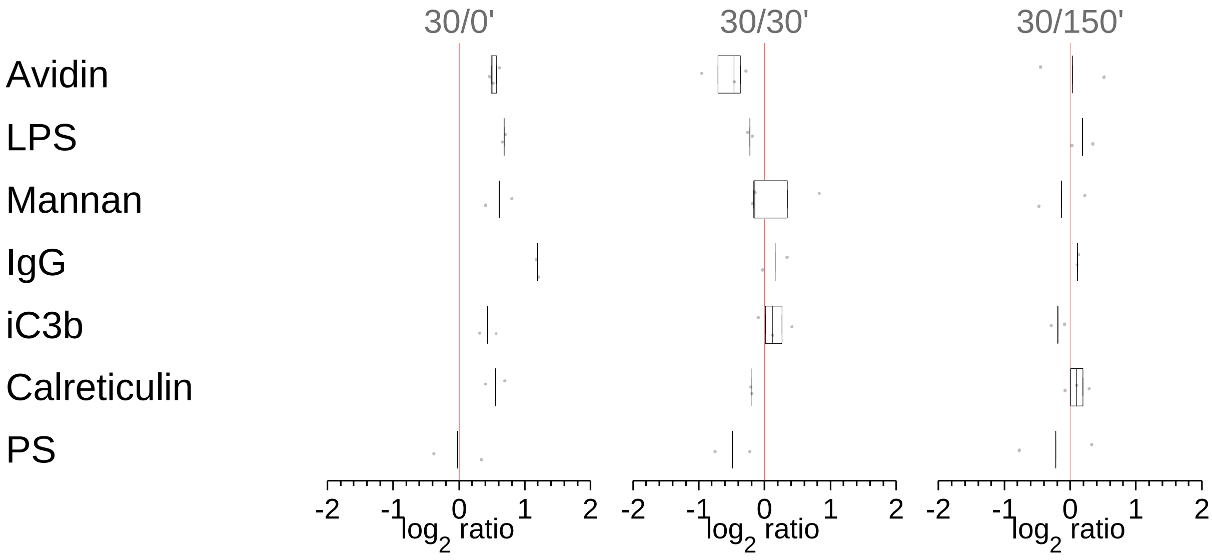

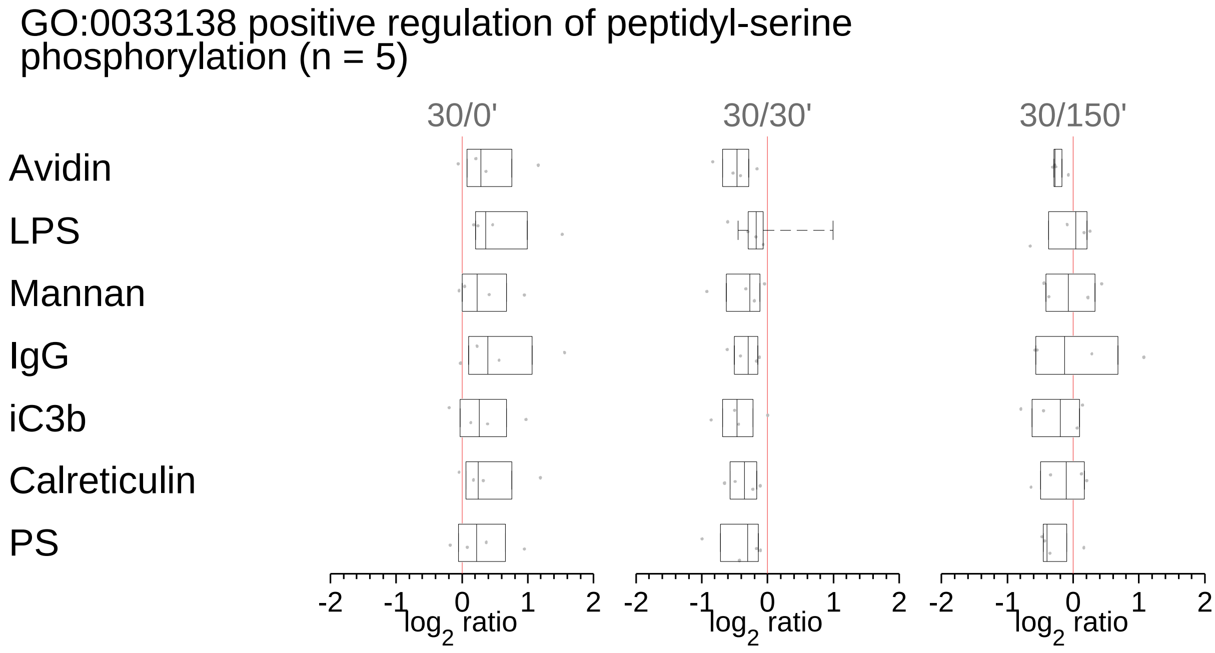
GO:0033138 positive regulation of peptidyl-serine
phosphorylation (n = 5)
30/0'
30/30'
30/150'
Avidin
LPS
Mannan
IgG
iC3b
Calreticulin
PS
-2 -1 0 1 2
log2 ratio

GO:0033144 negative regulation of intracellular steroid hormone receptor signaling pathway (n = 3)

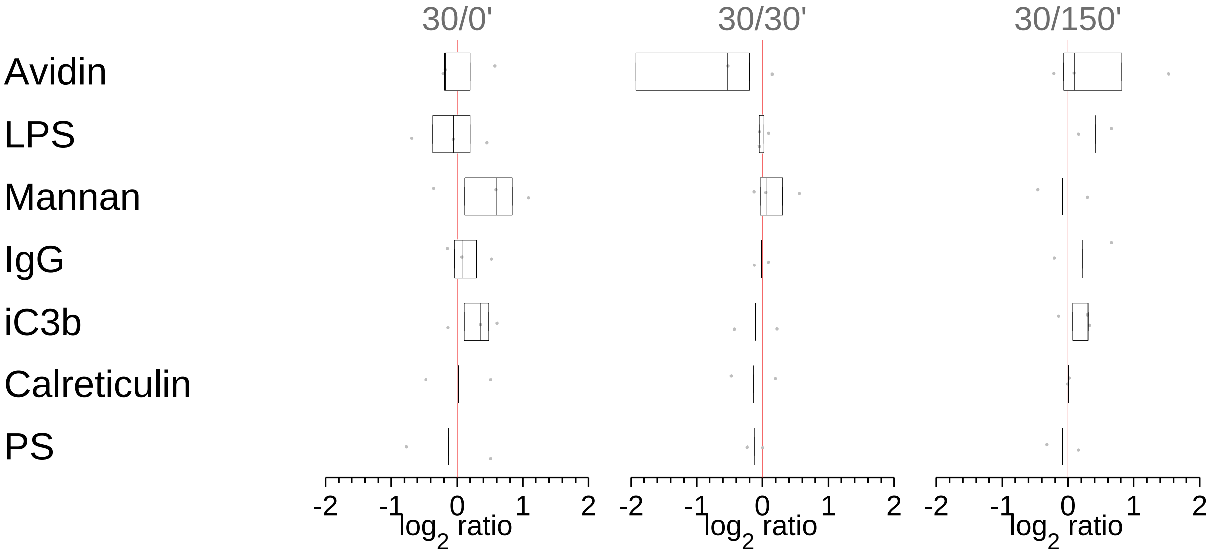

GO:0033160 positive regulation of protein import into nucleus, translocation (n = 5)

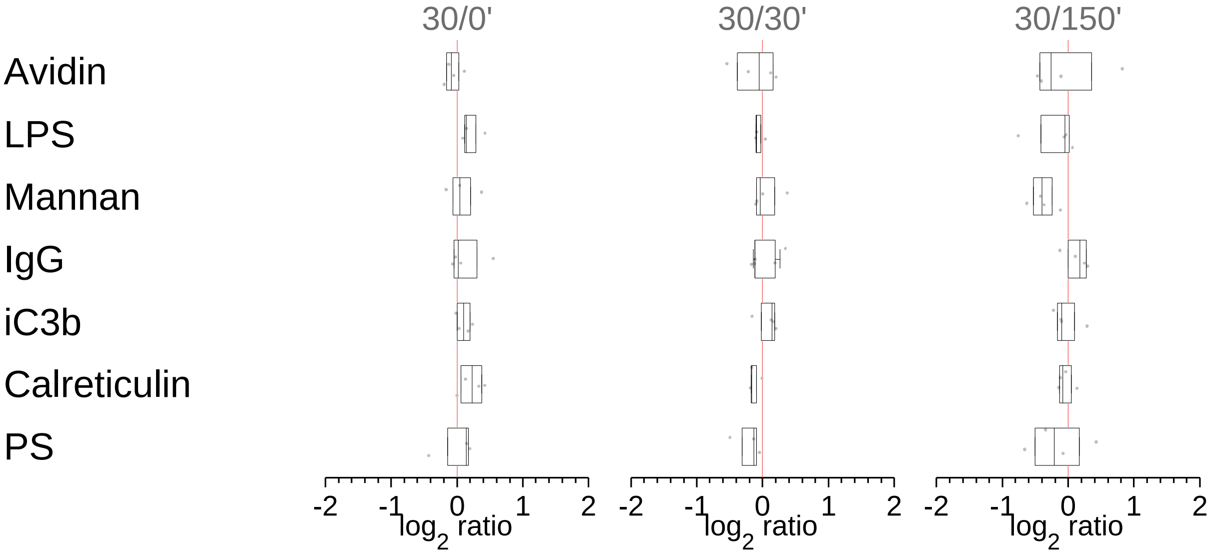

GO:0033178 proton-transporting two-sector ATPase complex, catalytic domain (n = 5)

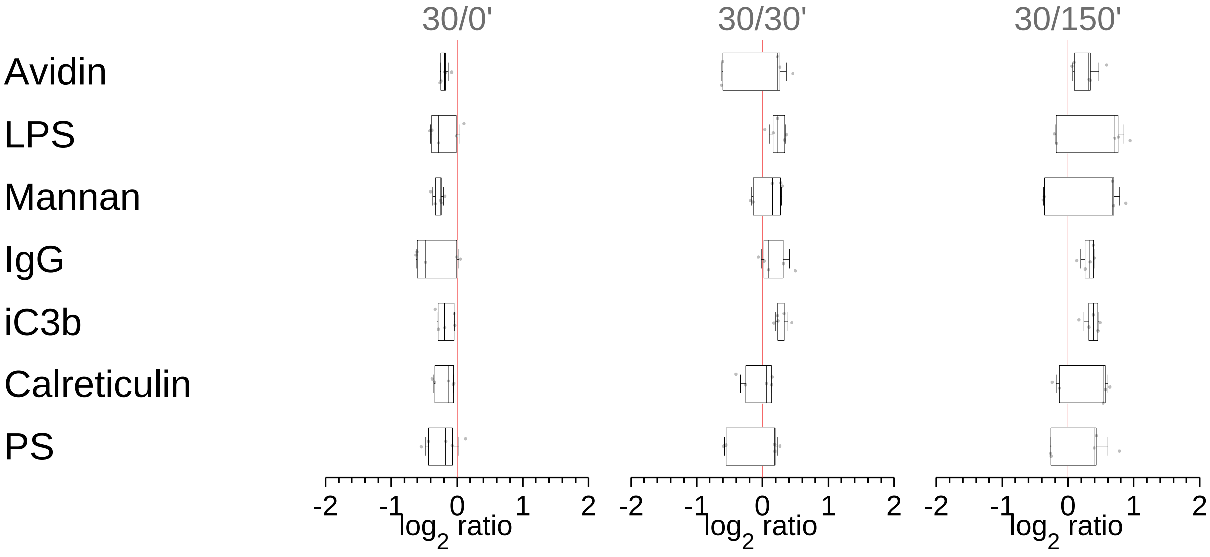

GO:0033179 proton-transporting V-type ATPase, V0 domain (n = 4)

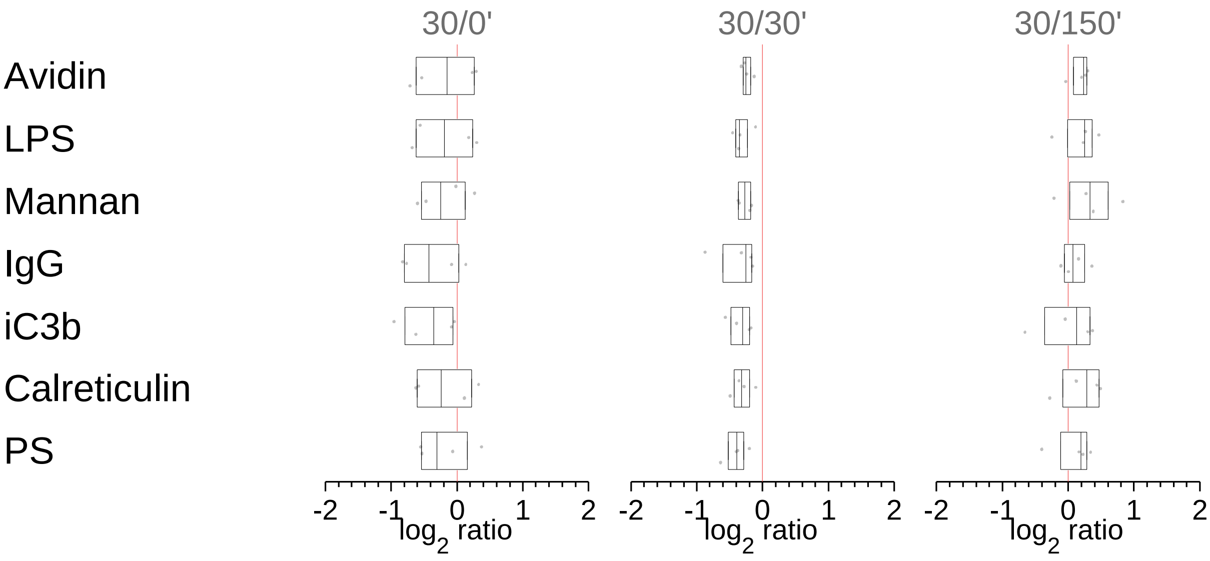

GO:0033180 proton-transporting V-type ATPase, V1 domain (n = 5)

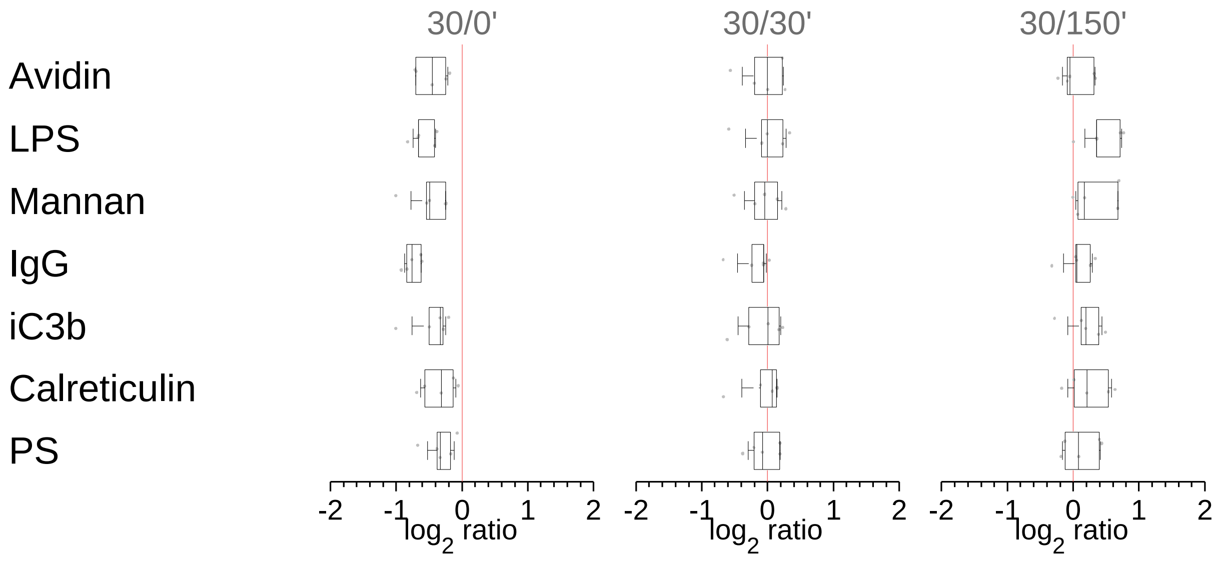

GO:0033198 response to ATP (n = 3)

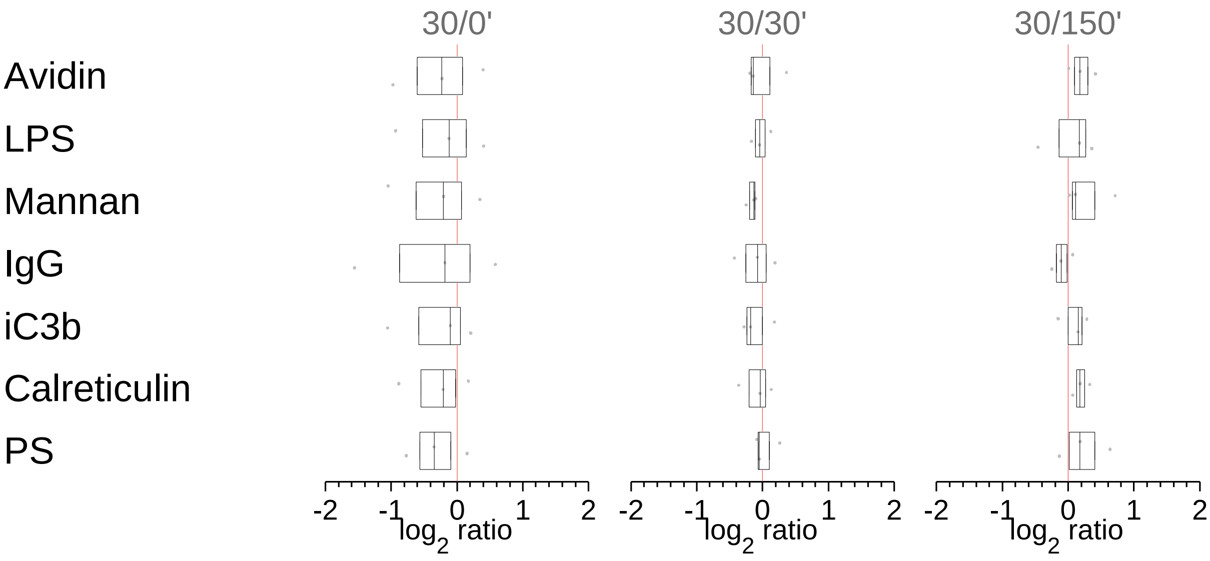

GO:0033209 tumor necrosis factor-mediated signaling pathway (n = 3)

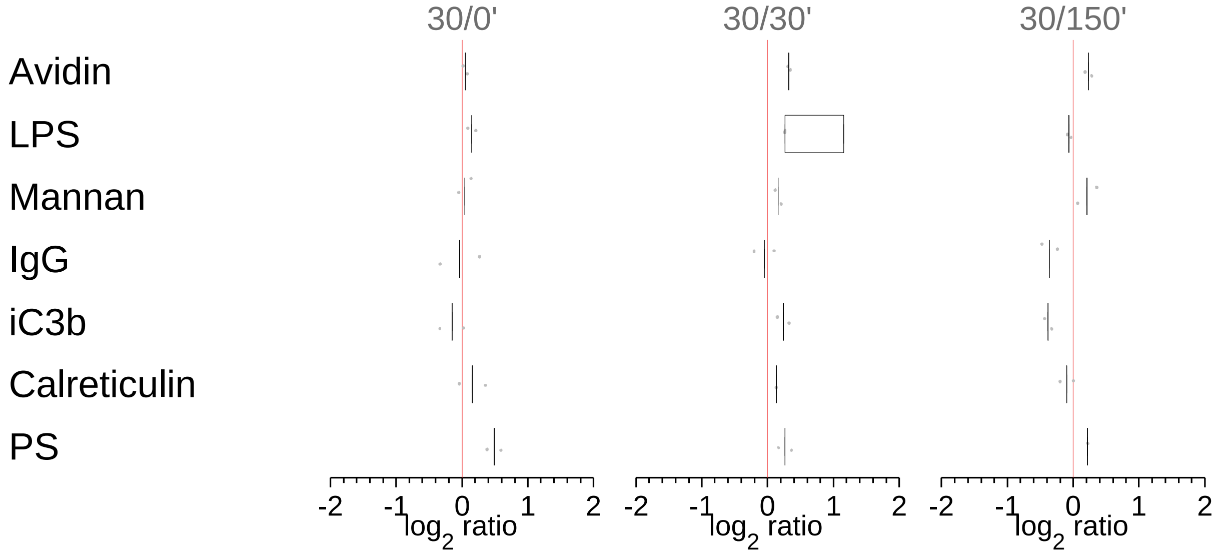

GO:0033327 Leydig cell differentiation (n = 3)

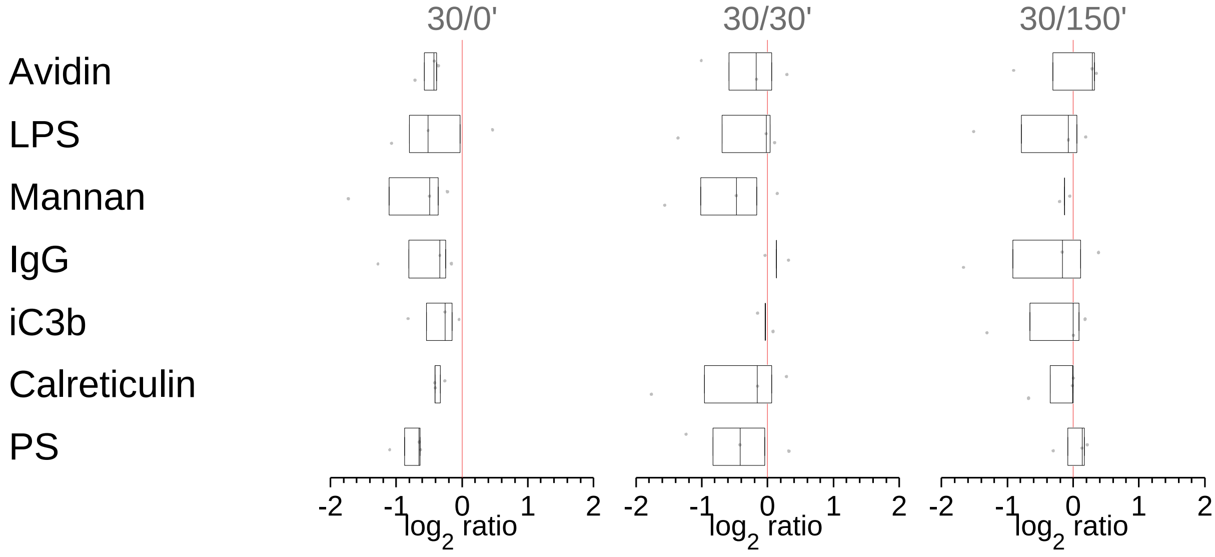

GO:0033344 cholesterol efflux (n = 6)

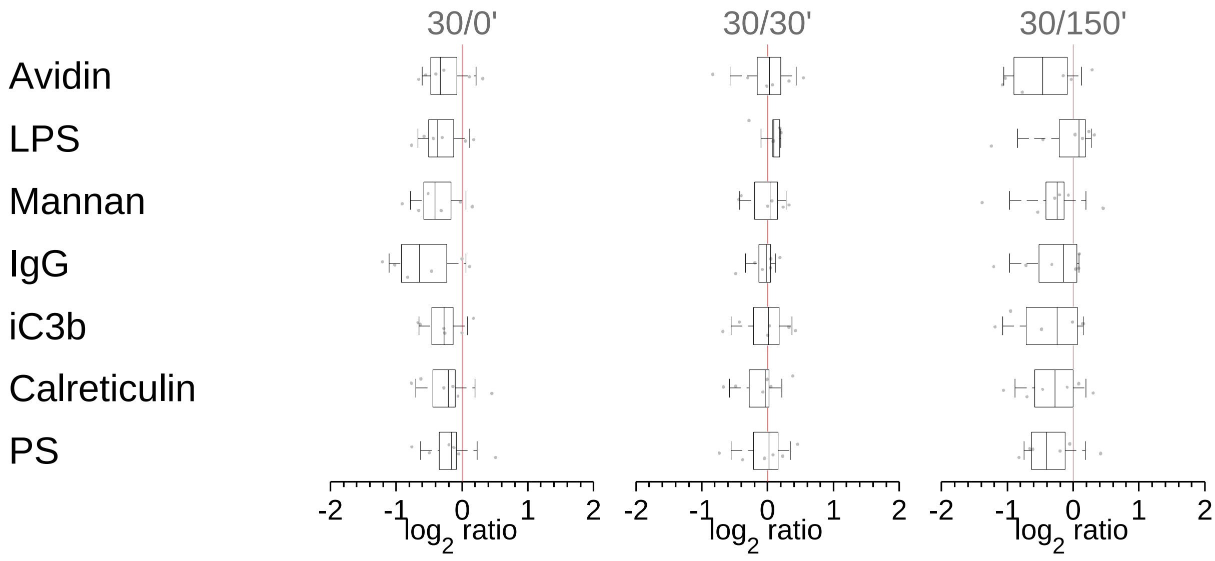

GO:0033539 fatty acid beta-oxidation using acyl-CoA dehydrogenase (n = 5)

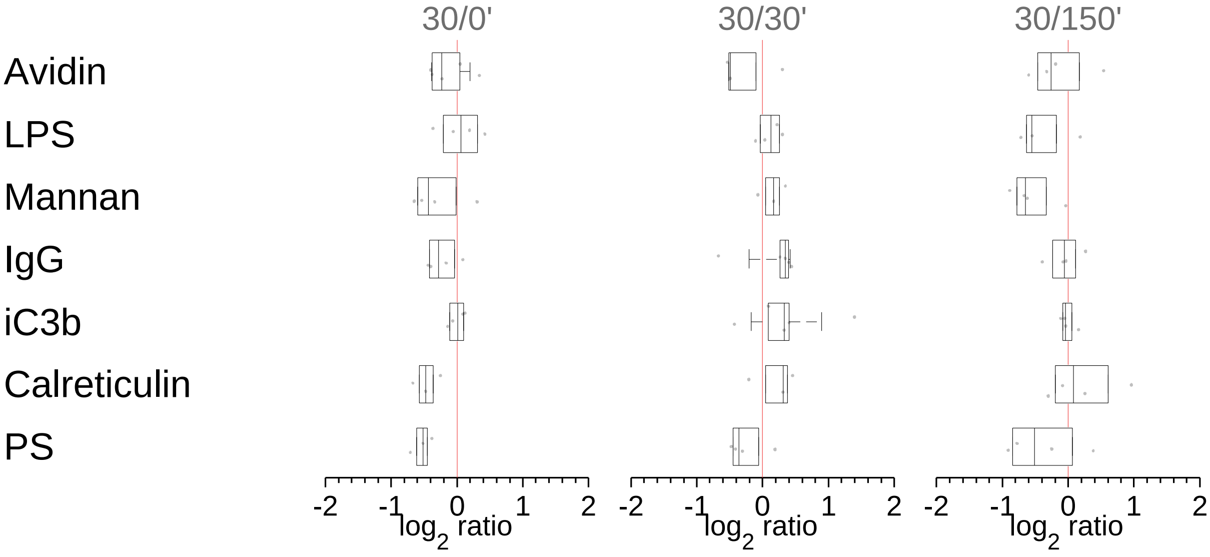

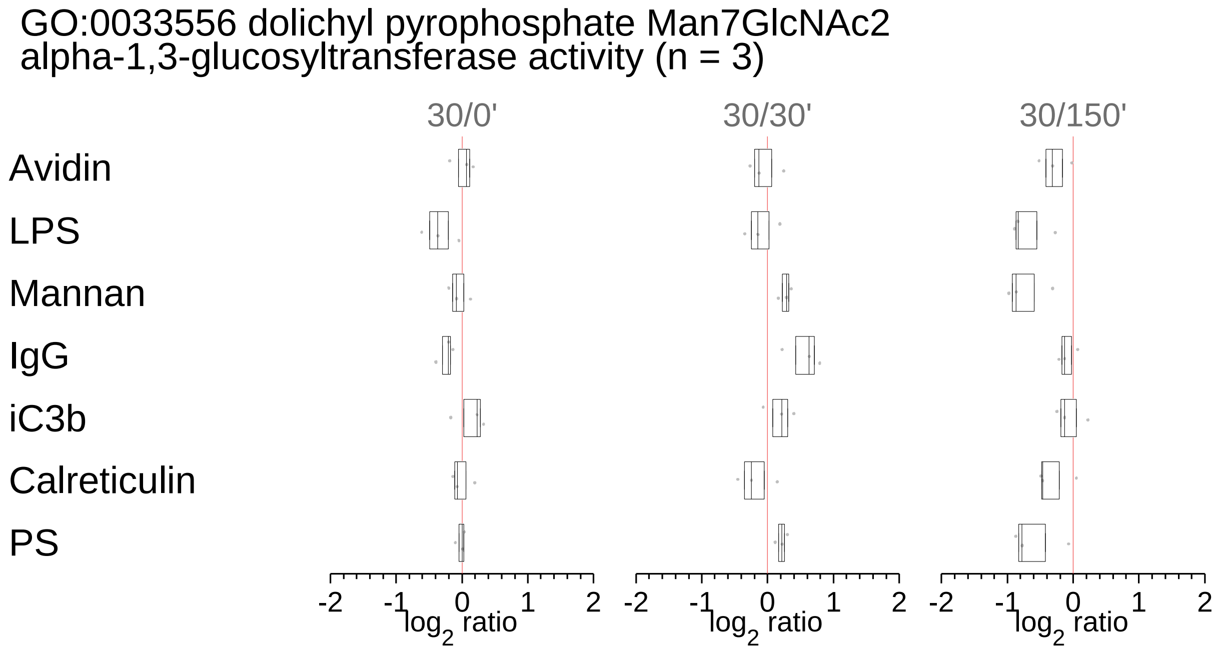
GO:0033556 dolichyl pyrophosphate Man7GlcNAc2
alpha-1,3-glucosyltransferase activity (n = 3)
30/0'
30/30'
30/150'
Avidin
LPS
Mannan
IgG
iC3b
Calreticulin
PS
-2
-1
0
1
2
log2 ratio

GO:0033572 transferrin transport (n = 4)

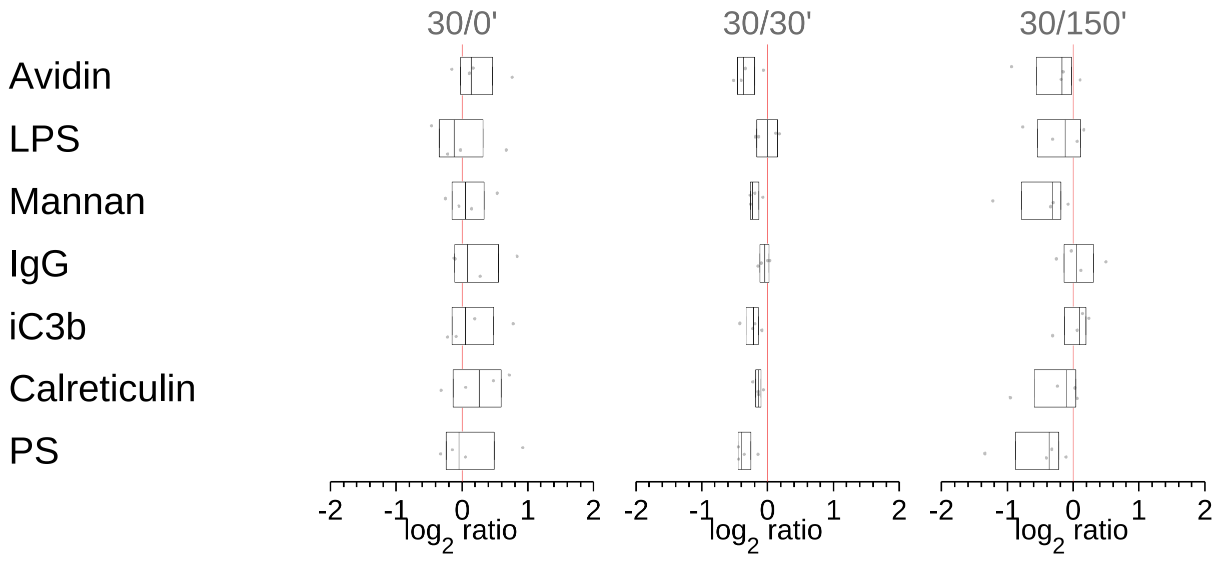

GO:0033619 membrane protein proteolysis (n = 3)

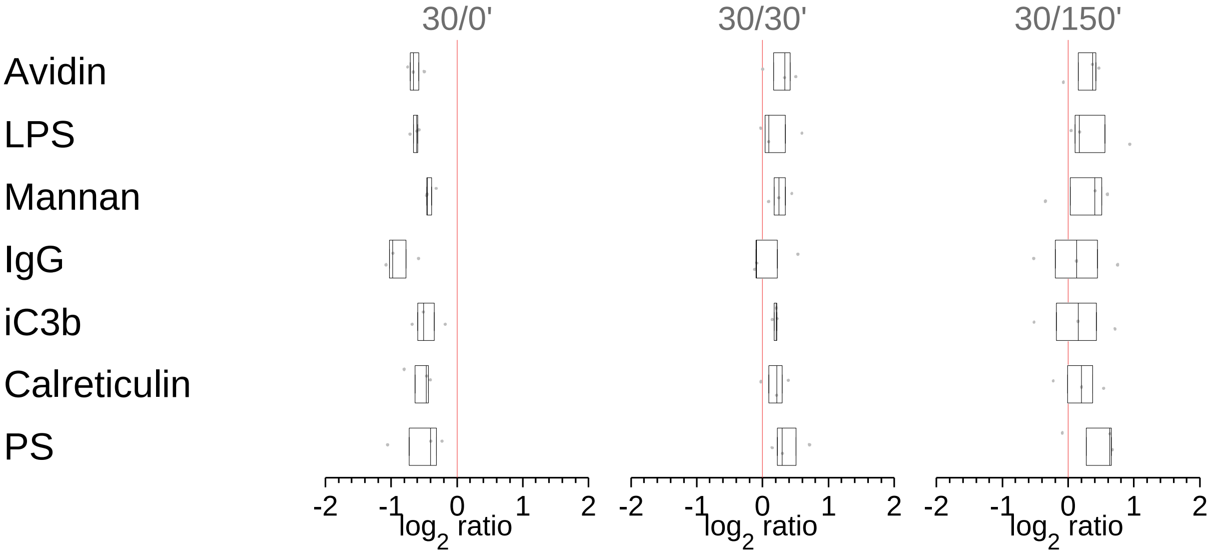

GO:0033627 cell adhesion mediated by integrin (n = 4)

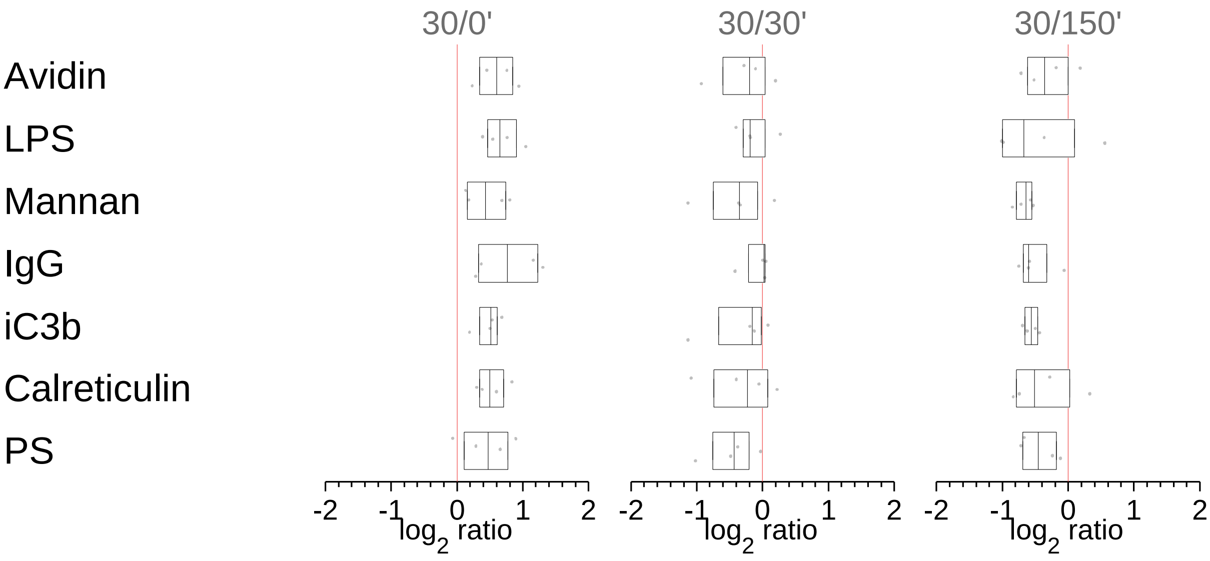

GO:0033630 positive regulation of cell adhesion mediated by integrin (n = 4)

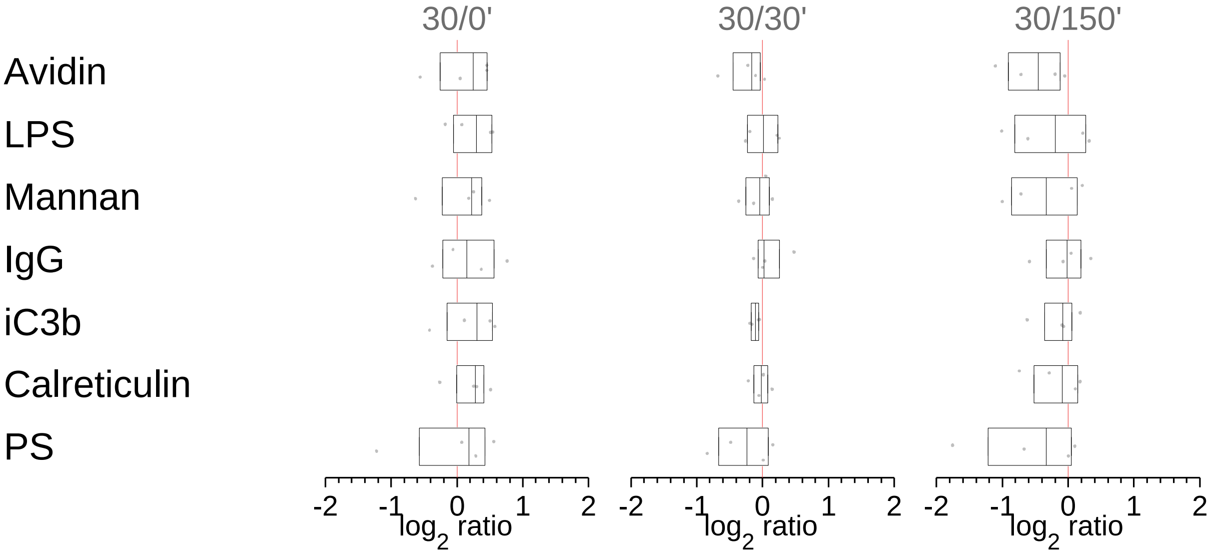

GO:0033673 negative regulation of kinase activity (n = 4)

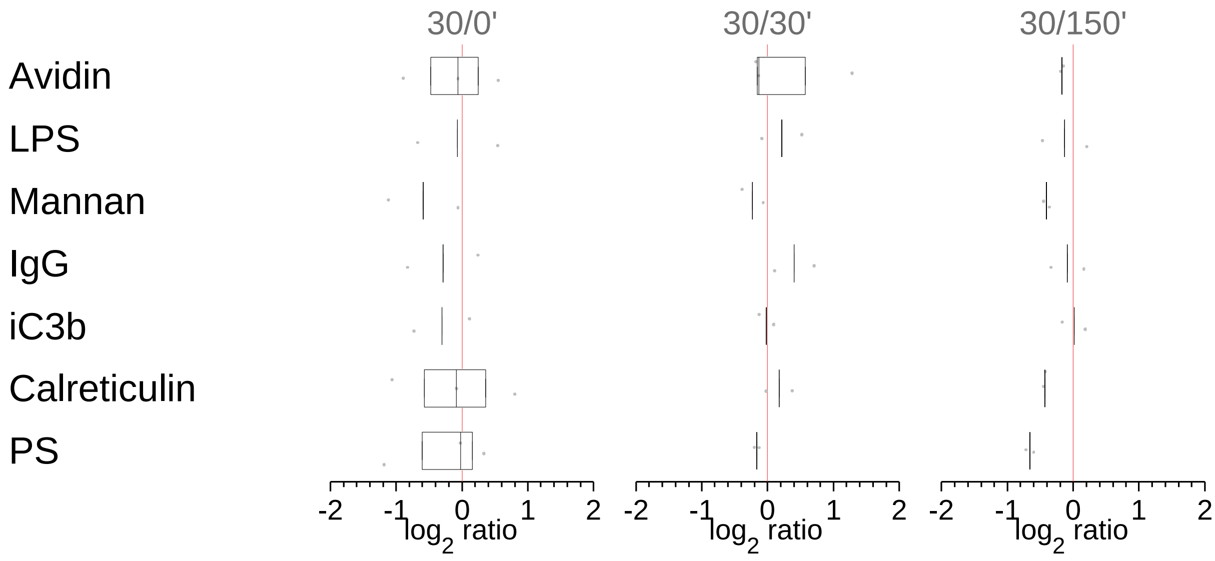

GO:0033709 D-arabinitol dehydrogenase, D-ribulose forming (NADP+) activity (n = 3)

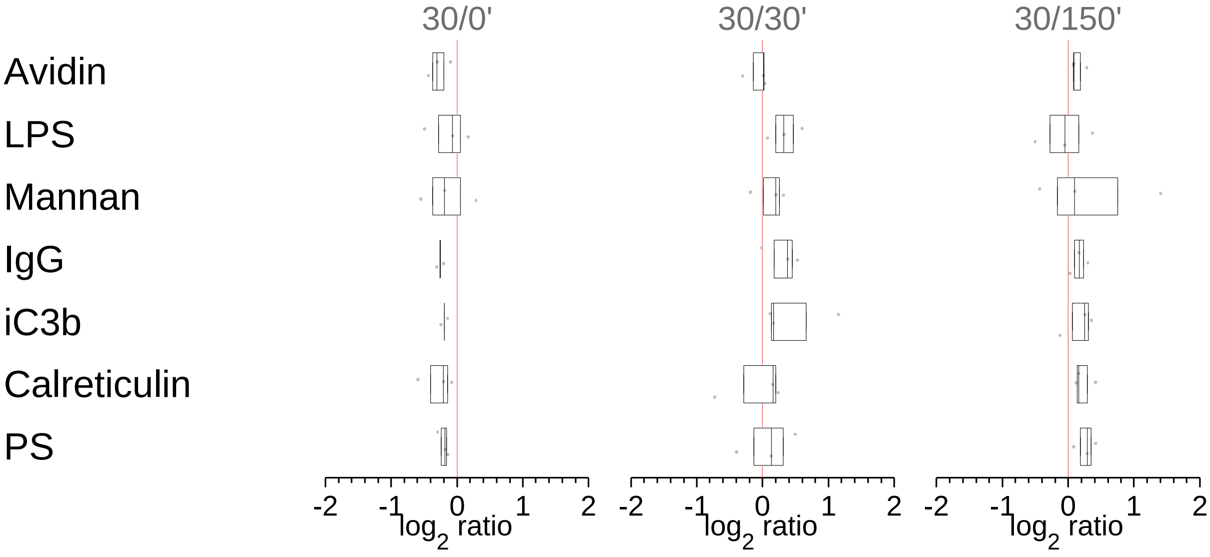

GO:0033764 steroid dehydrogenase activity, acting on the CH-OH group of donors, NAD or NADP as acceptor (n = 3)

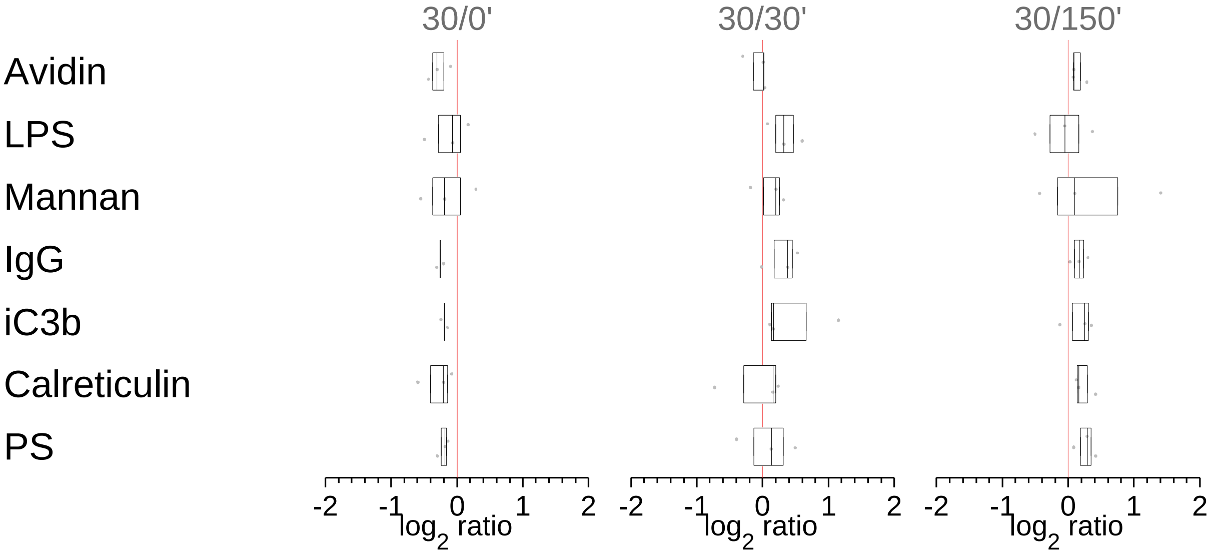

GO:0033765 steroid dehydrogenase activity, acting on the CH-CH group of donors (n = 3)

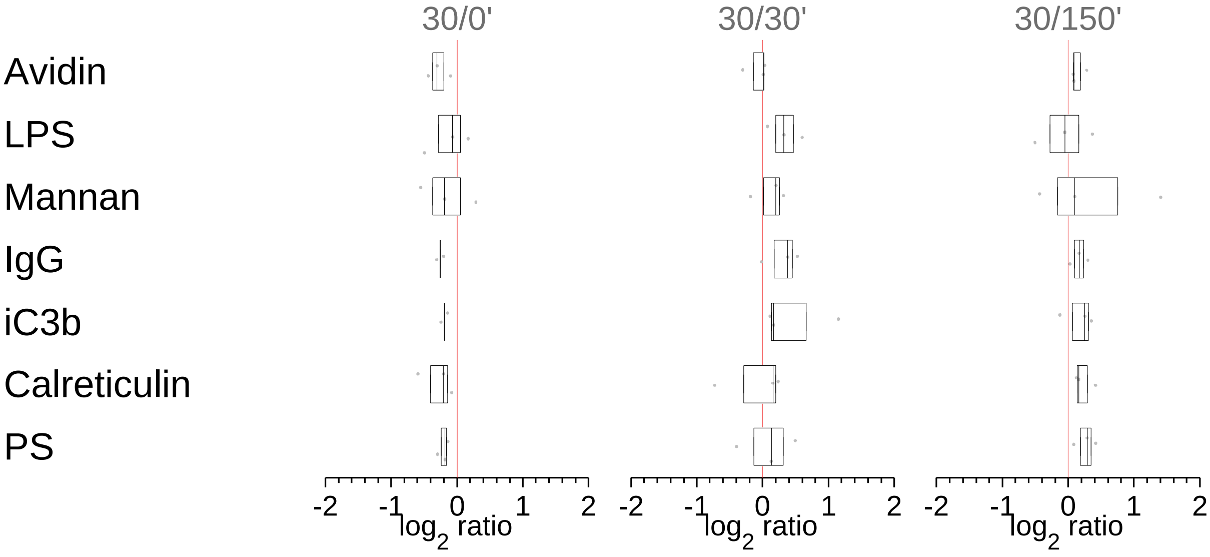

GO:0034063 stress granule assembly (n = 4)

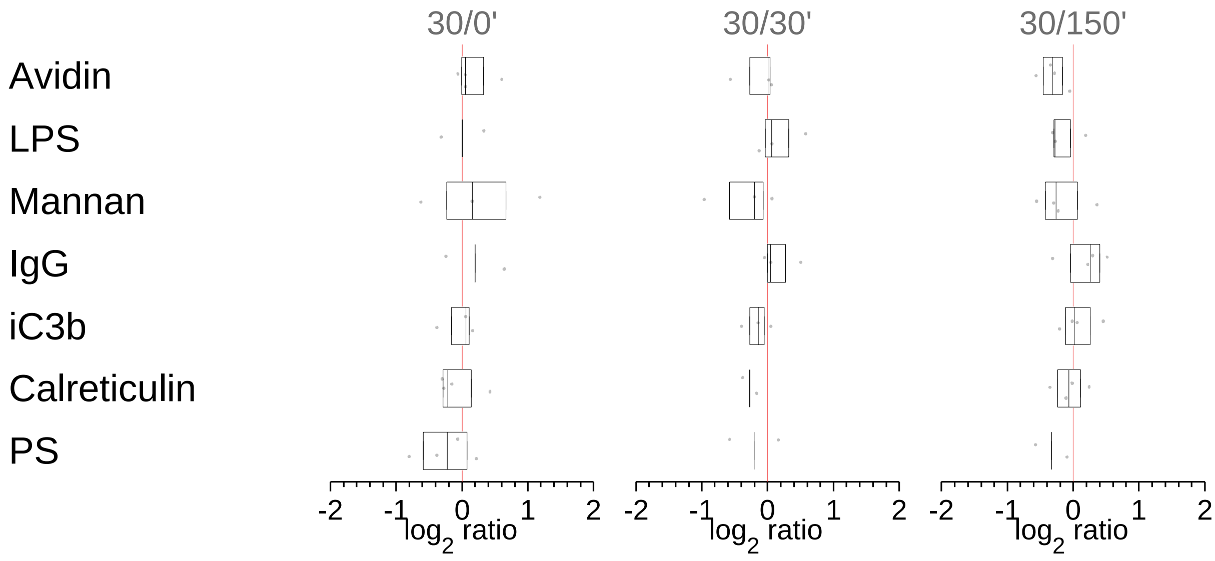

GO:0034097 response to cytokine stimulus (n = 5)

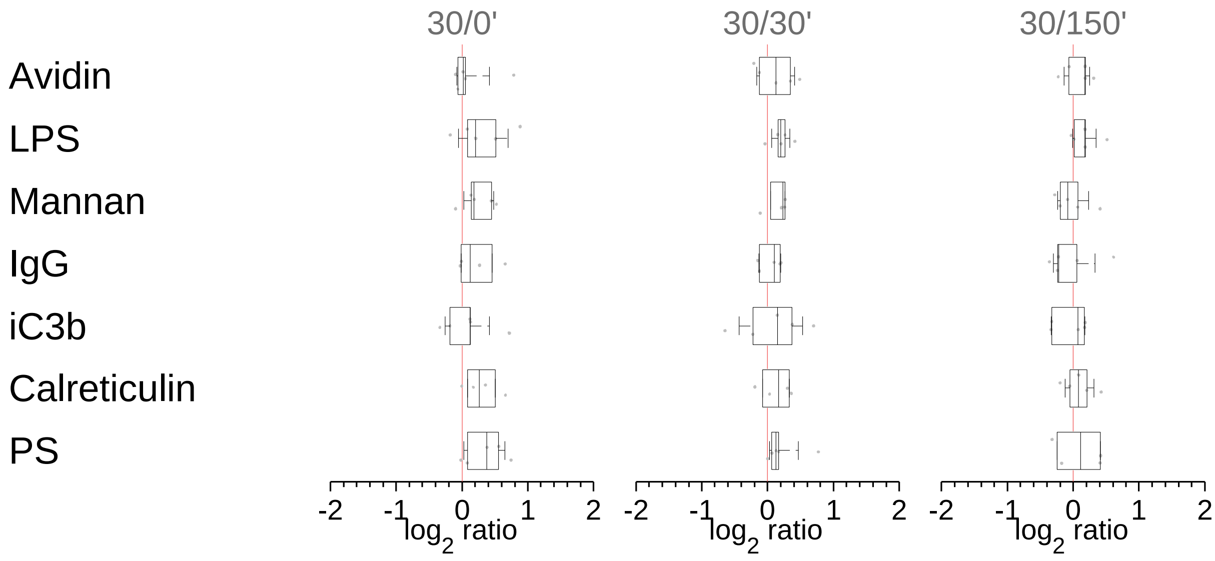

GO:0034101 erythrocyte homeostasis (n = 4)

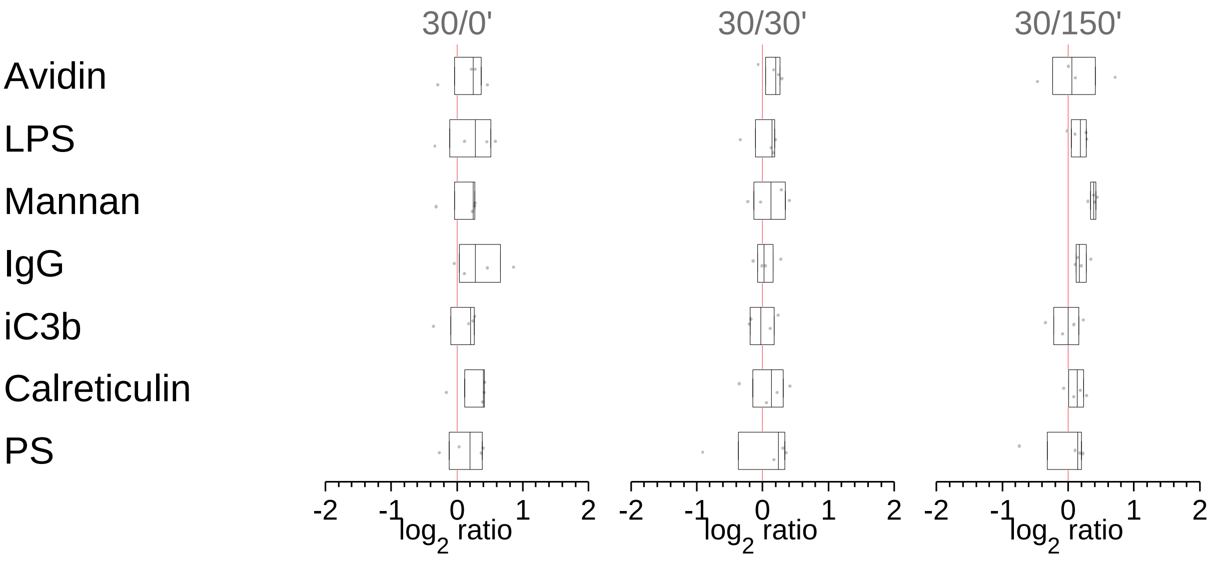

GO:0034122 negative regulation of toll-like receptor signaling pathway (n = 3)

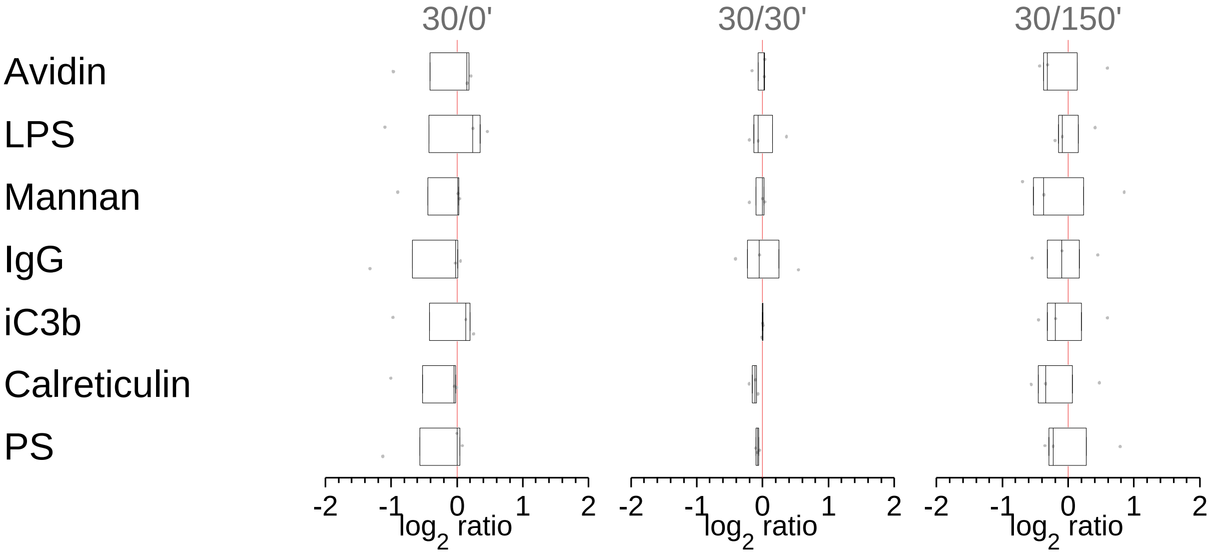

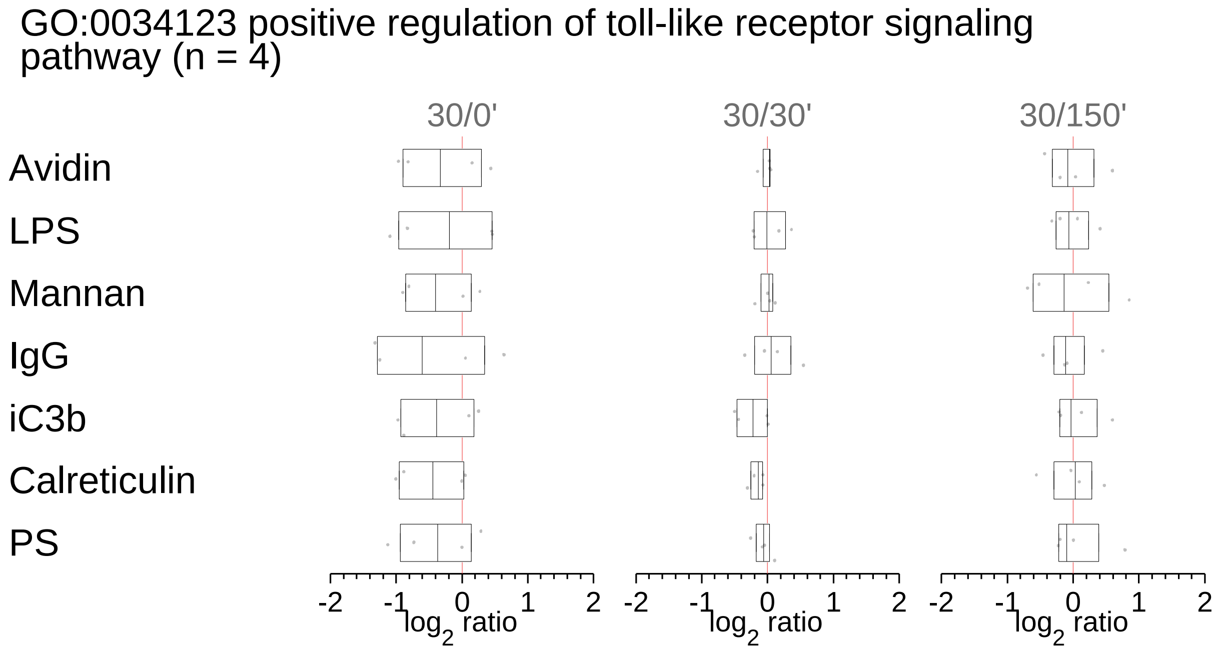
GO:0034123 positive regulation of toll-like receptor signaling pathway (n = 4)
30/0'
30/30'
30/150'
Avidin
LPS
Mannan
IgG
iC3b
Calreticulin
PS
-2
-1
0
1
2
log2 ratio

GO:0034138 toll-like receptor 3 signaling pathway (n = 3)

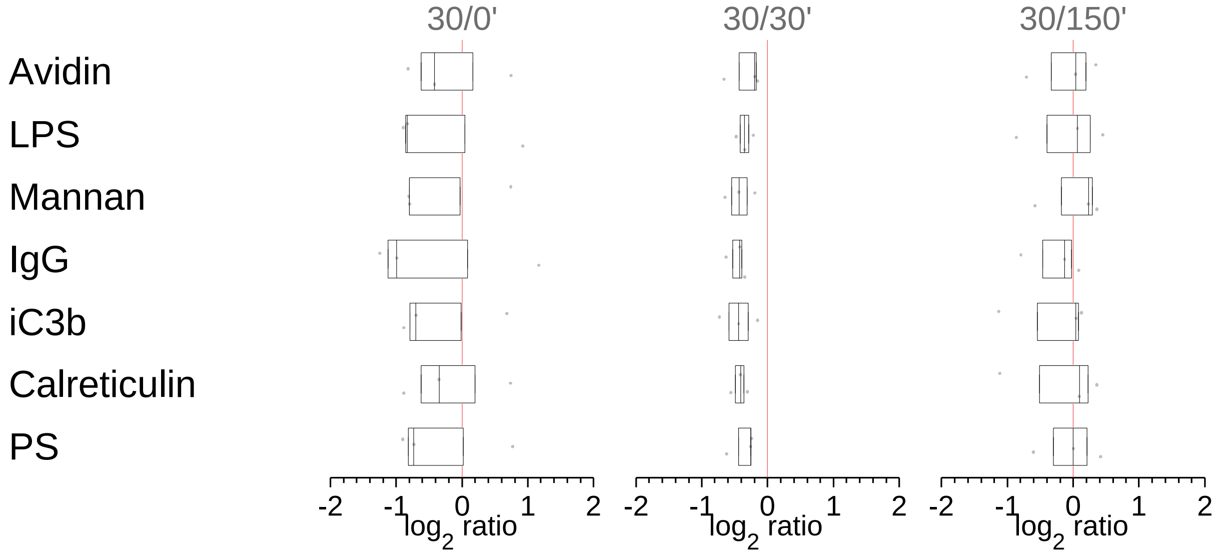

GO:0034154 toll-like receptor 7 signaling pathway (n = 3)

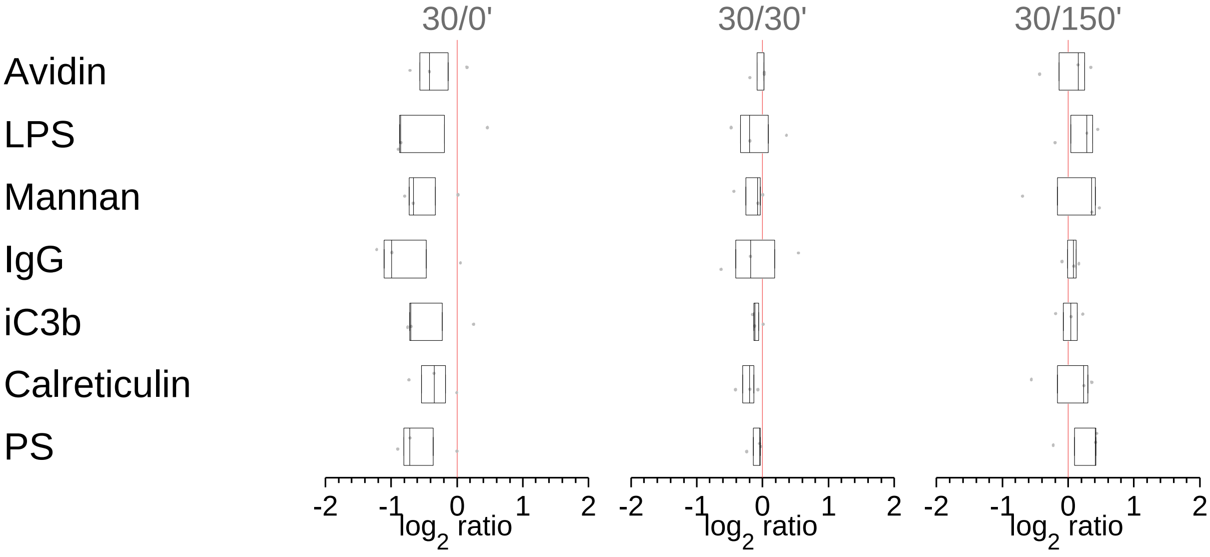

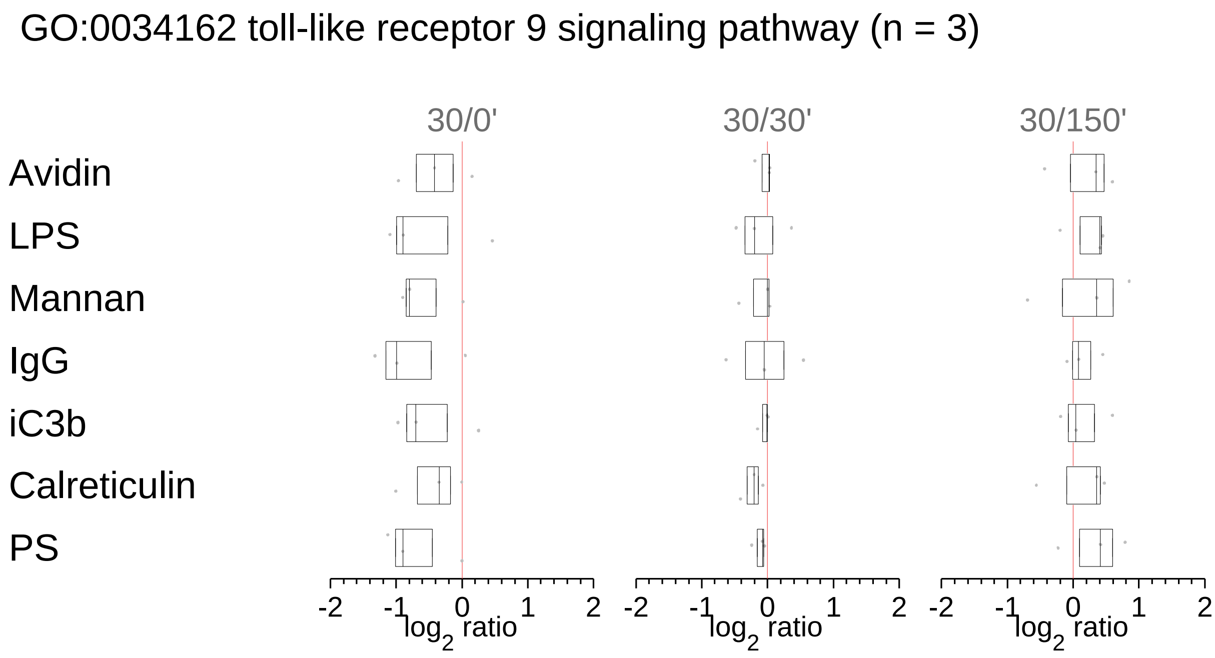
GO:0034162 toll-like receptor 9 signaling pathway (n = 3)
30/0'
30/30'
30/150'
Avidin
LPS
Mannan
IgG
iC3b
Calreticulin
PS
-2
-1
0
1
2
log2 ratio

GO:0034185 apolipoprotein binding (n = 4)

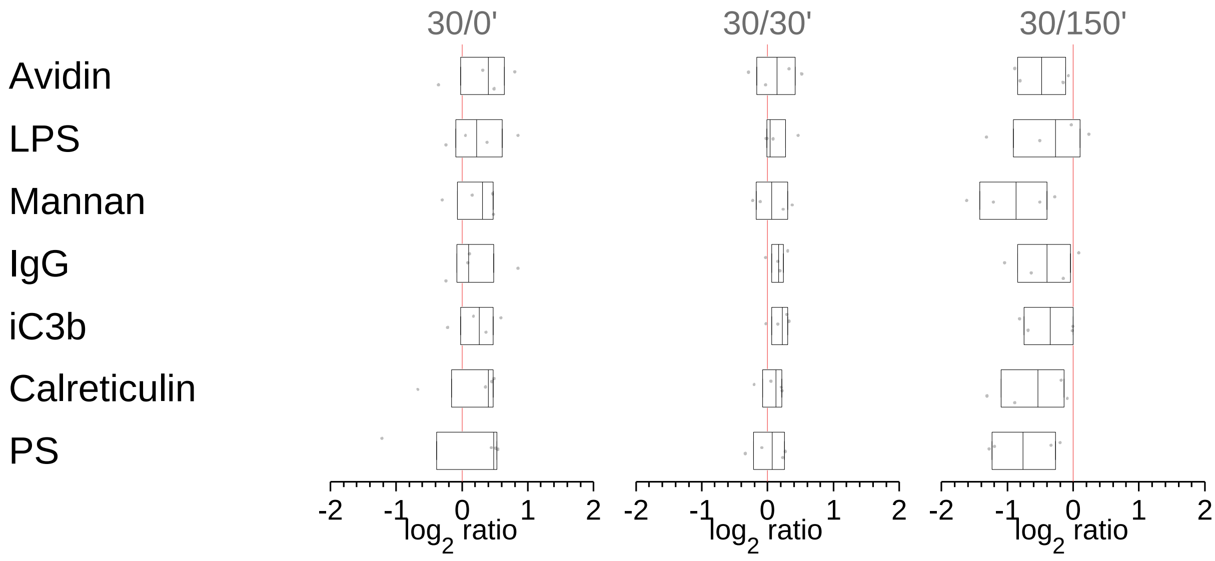

GO:0034220 ion transmembrane transport (n = 20)

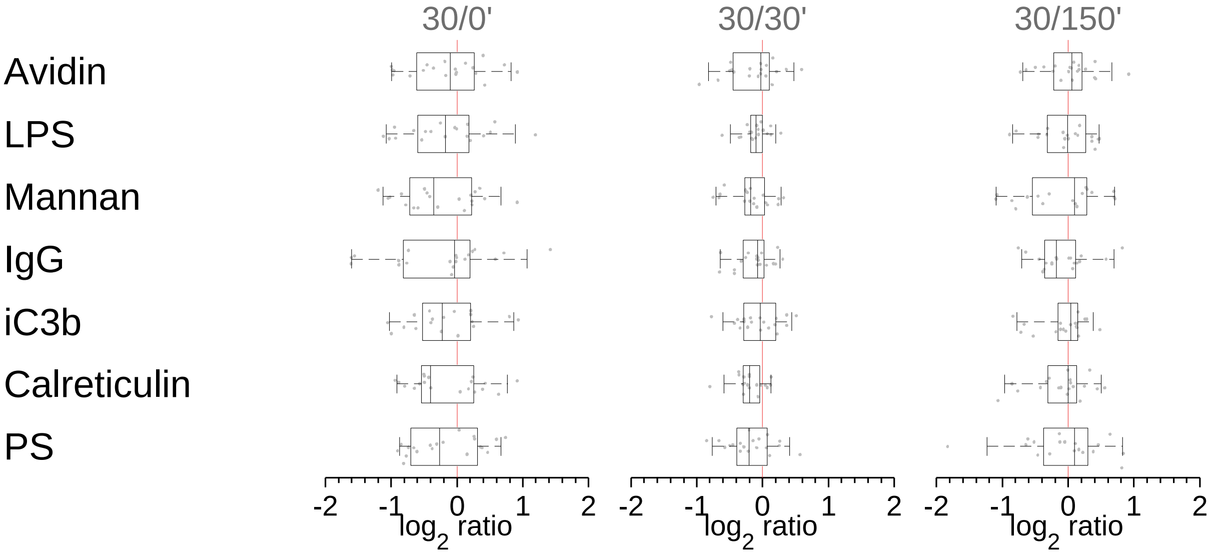

GO:0034314 Arp2/3 complex-mediated actin nucleation (n = 3)

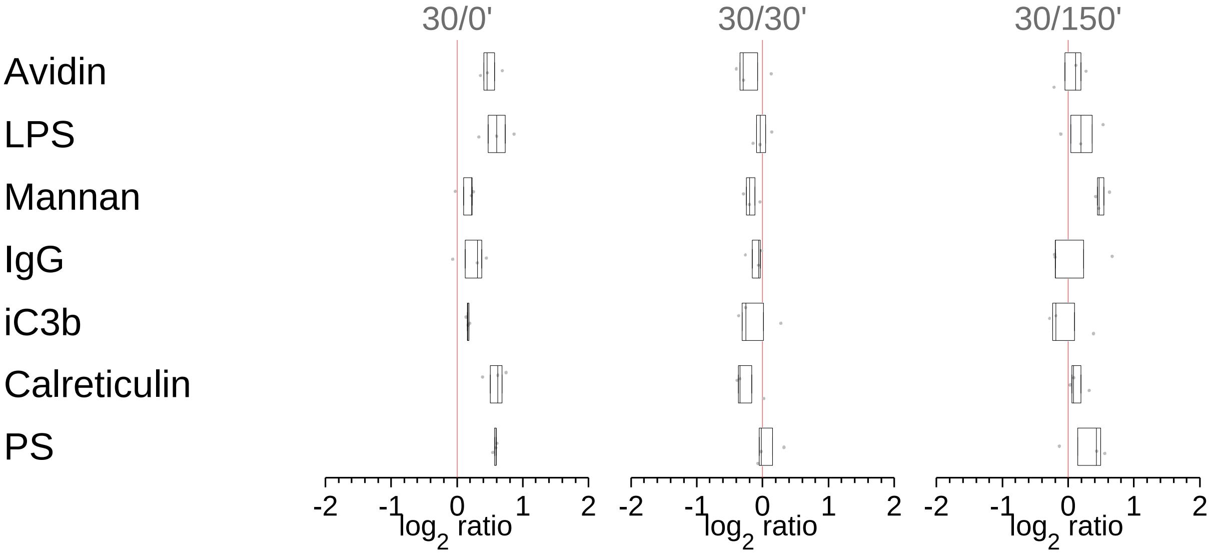

GO:0034332 adherens junction organization (n = 4)

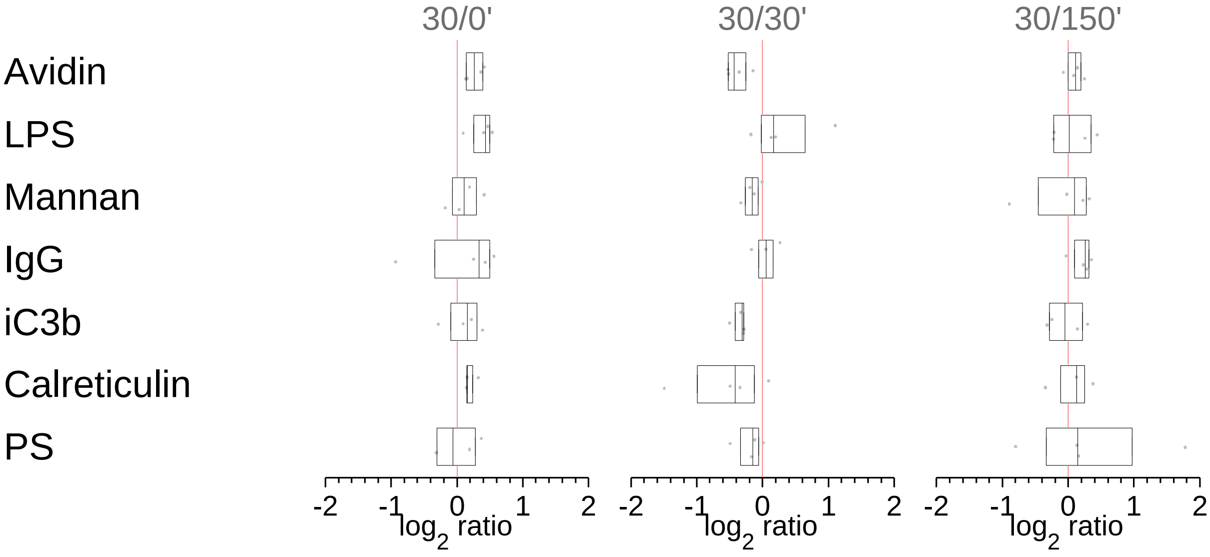

GO:0034341 response to interferon-gamma (n = 4)

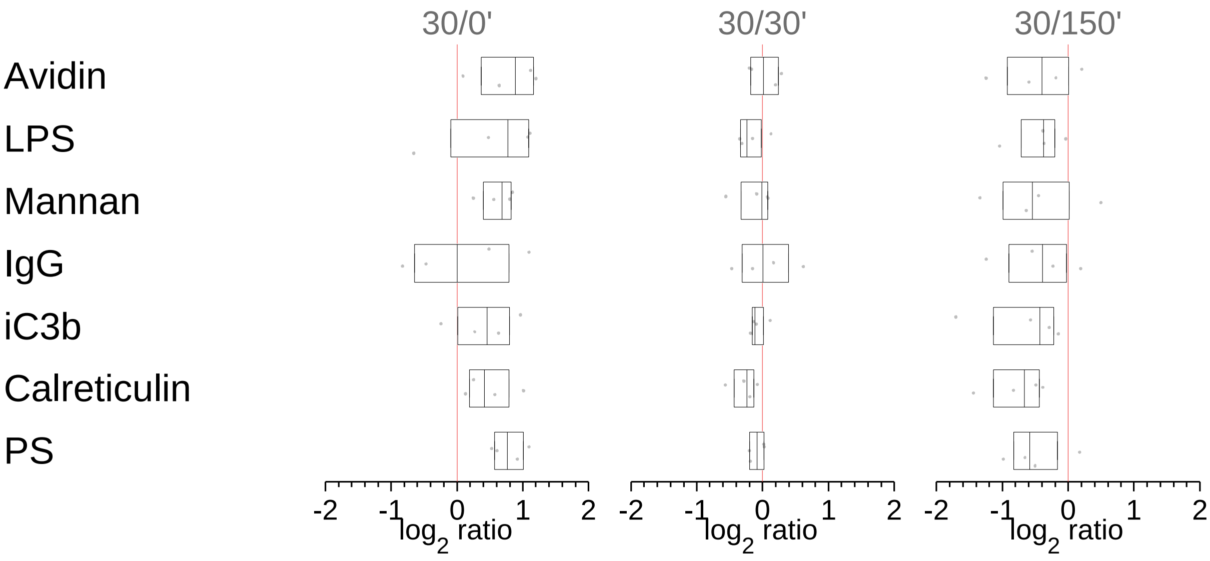

GO:0034361 very-low-density lipoprotein particle (n = 5)

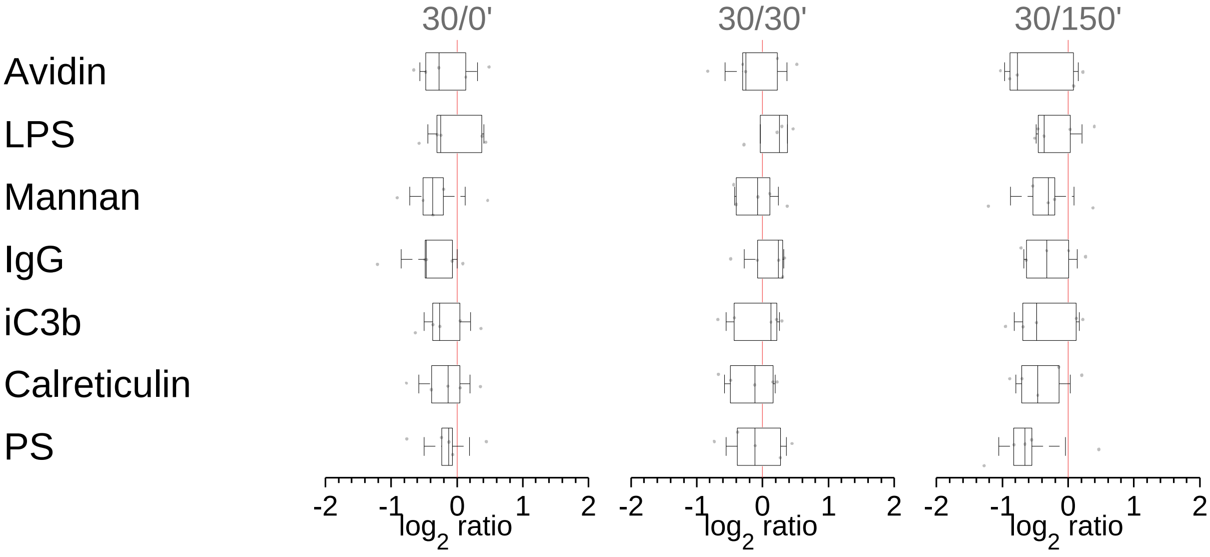

GO:0034362 low-density lipoprotein particle (n = 6)

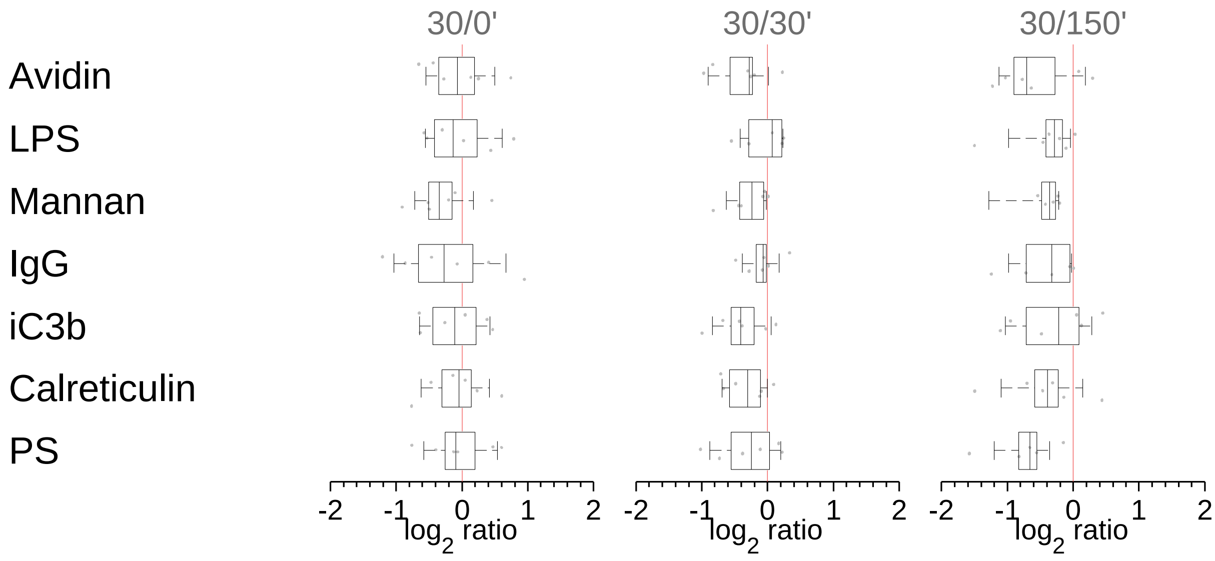

GO:0034364 high-density lipoprotein particle (n = 3)

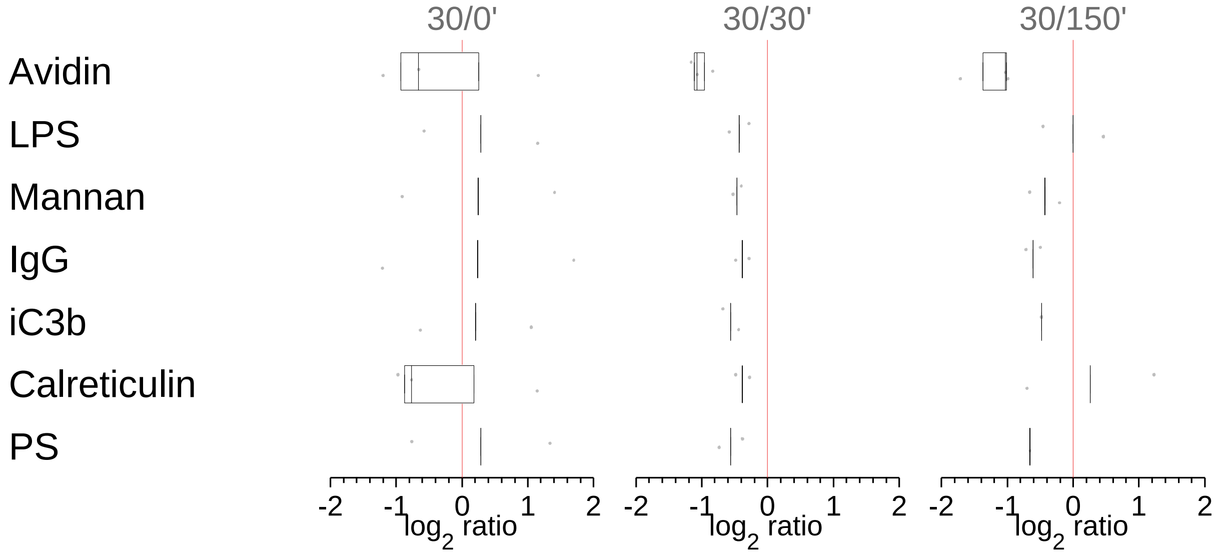

GO:0034374 low-density lipoprotein particle remodeling (n = 4)

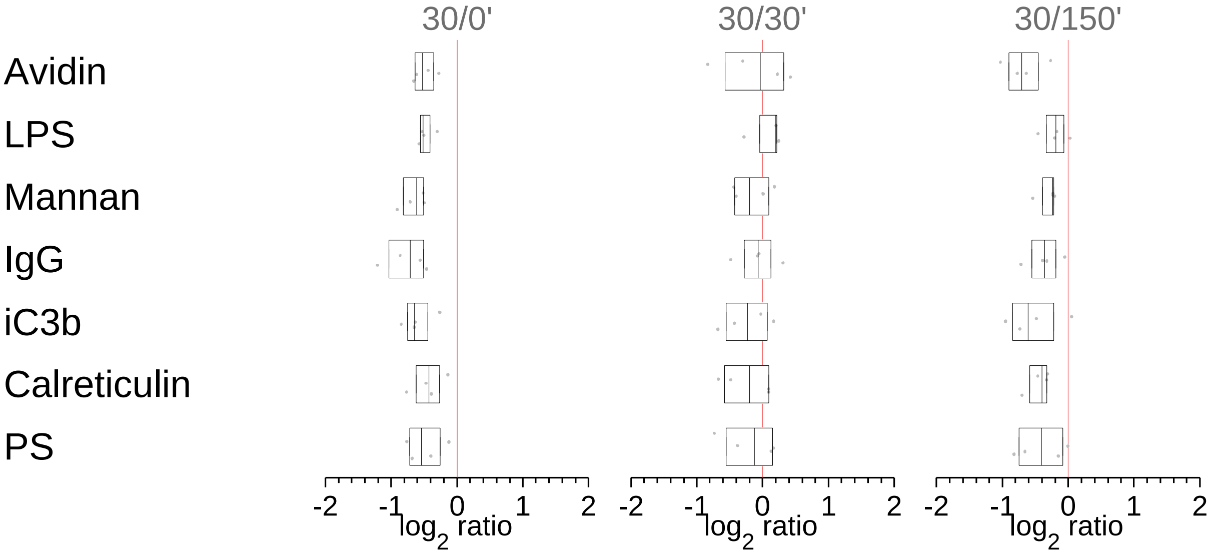

GO:0034383 low-density lipoprotein particle clearance (n = 5)

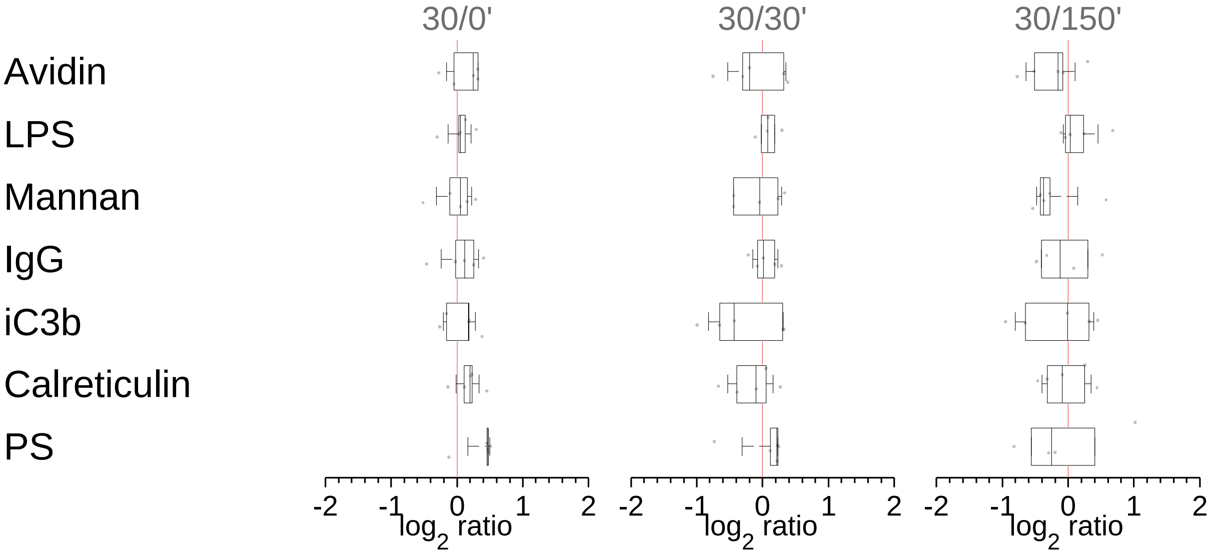

GO:0034389 lipid particle organization (n = 3)

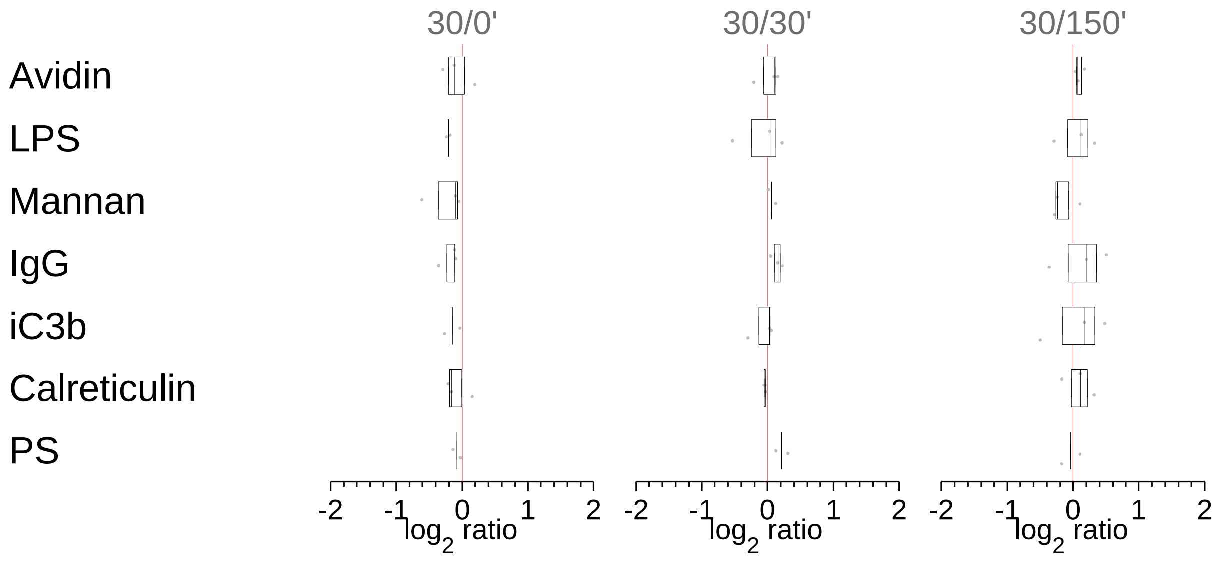

GO:0034394 protein localization at cell surface (n = 3)

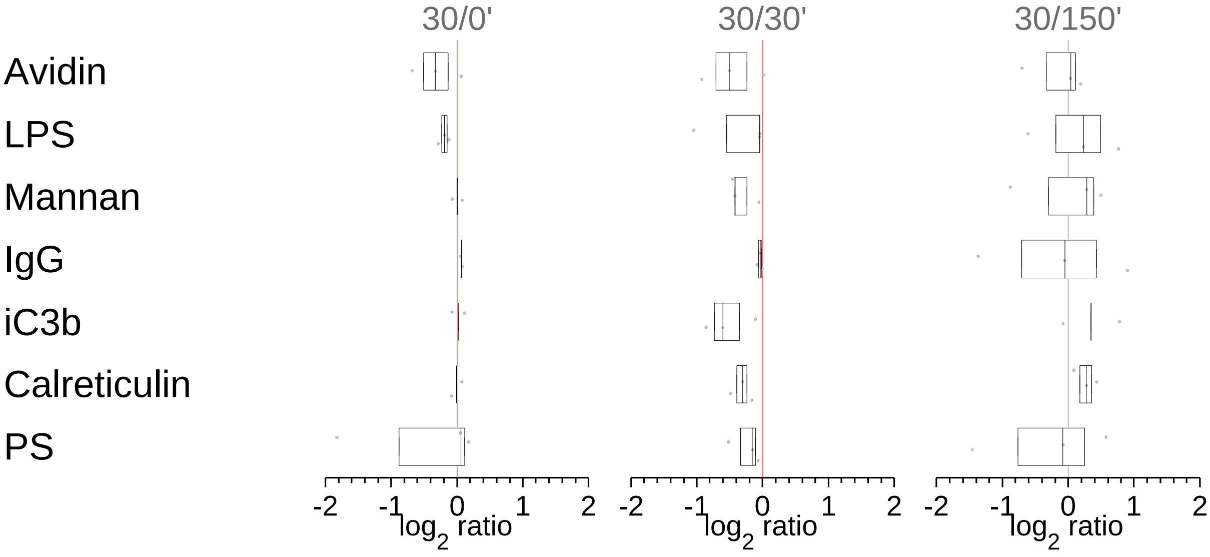

GO:0034446 substrate adhesion-dependent cell spreading (n = 6)

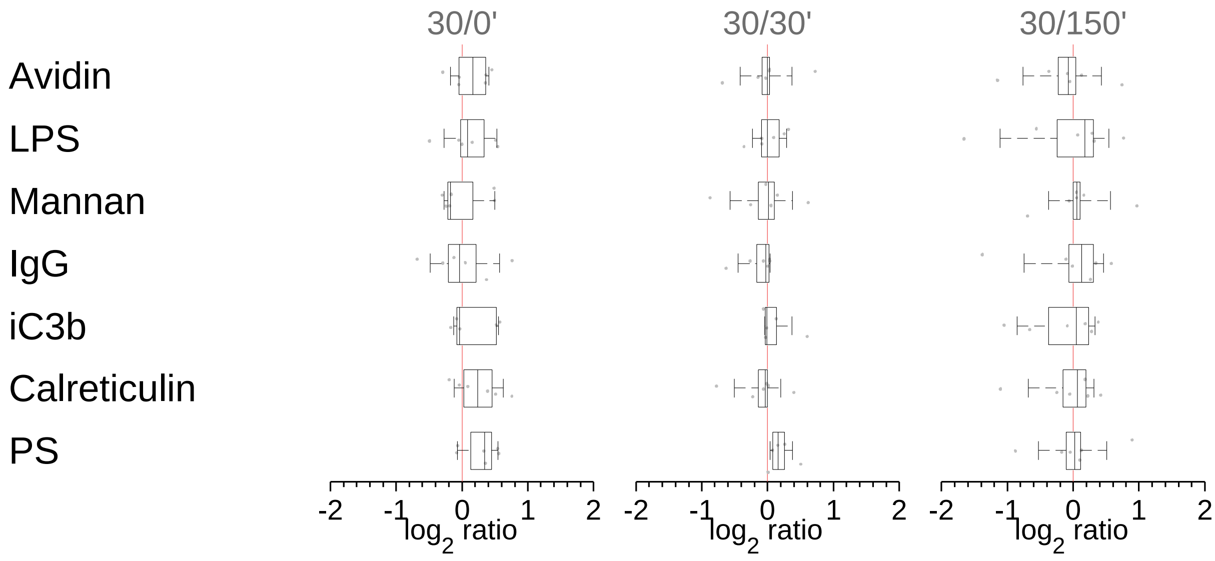

GO:0034504 protein localization to nucleus (n = 6)

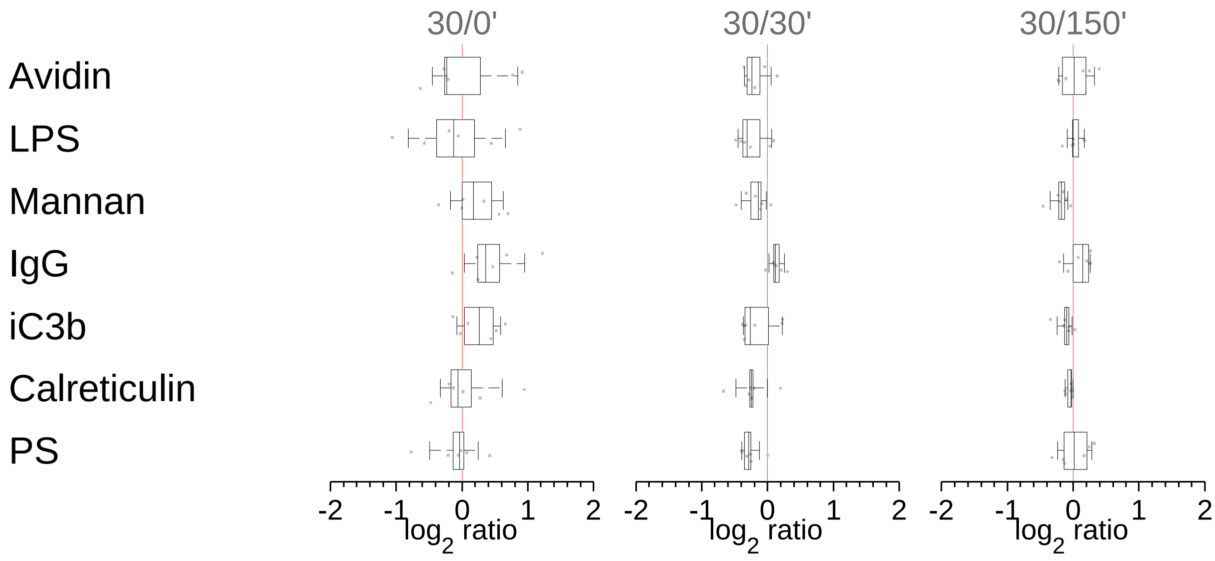

GO:0034599 cellular response to oxidative stress (n = 6)

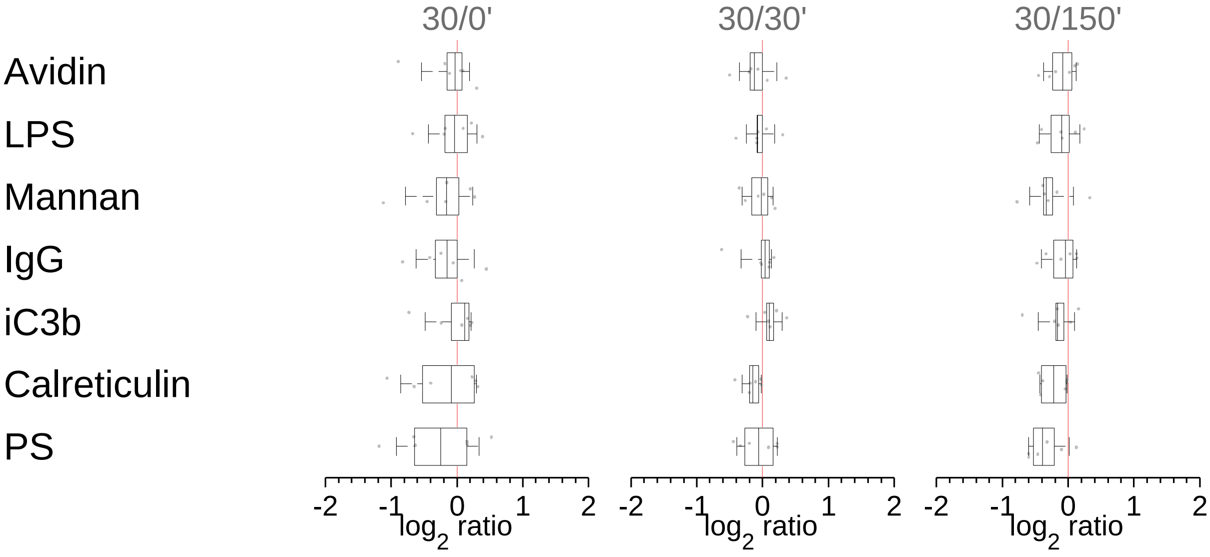

GO:0034605 cellular response to heat (n = 3)

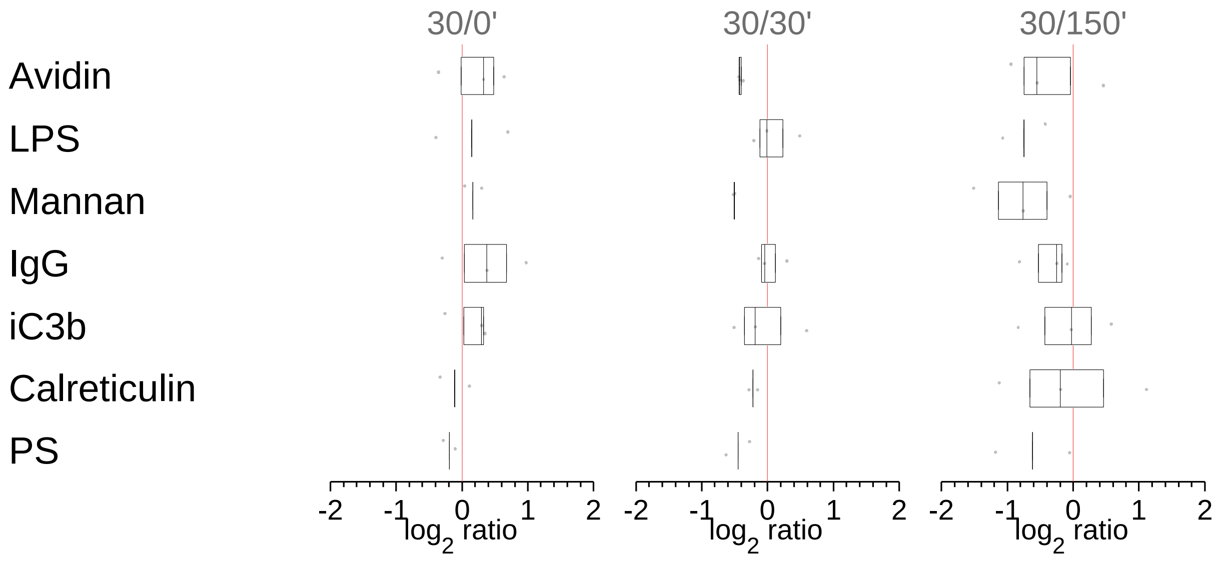

GO:0034612 response to tumor necrosis factor (n = 5)

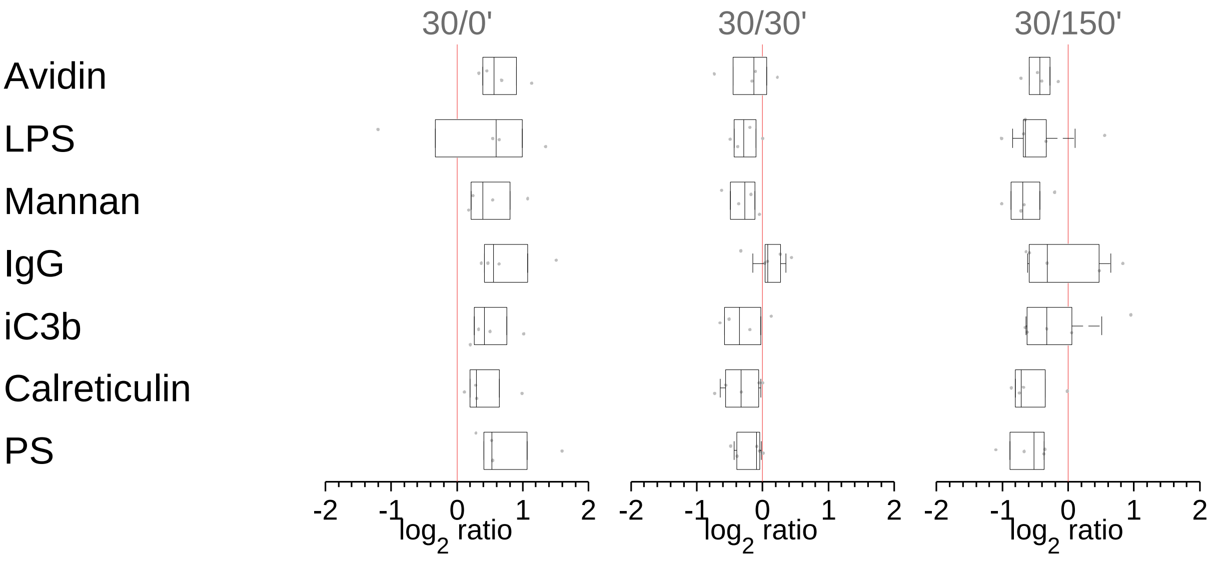

GO:0034613 cellular protein localization (n = 9)

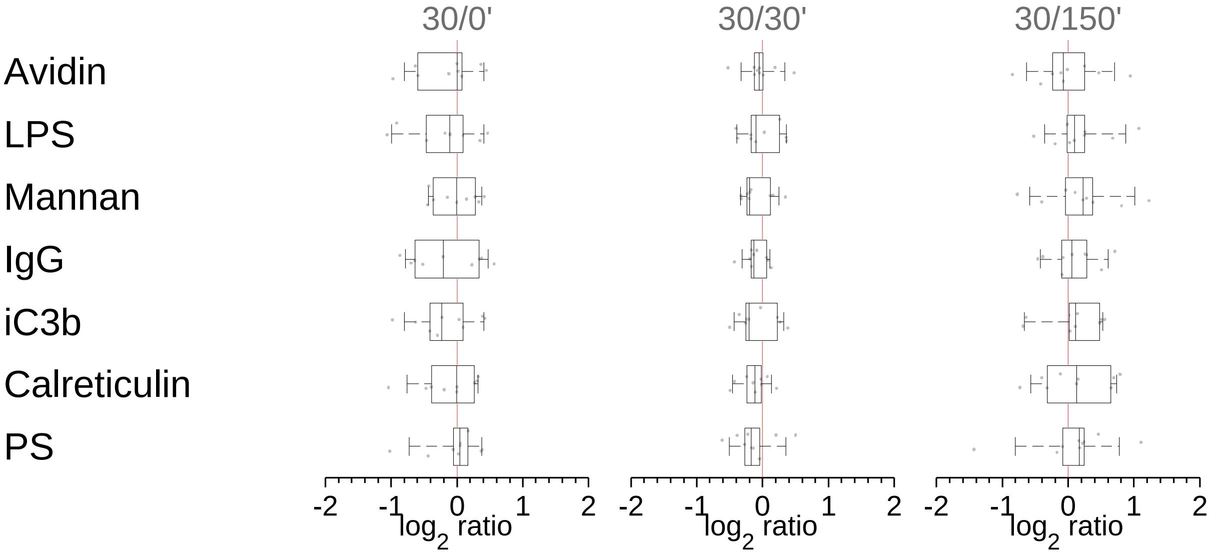

GO:0034707 chloride channel complex (n = 4)

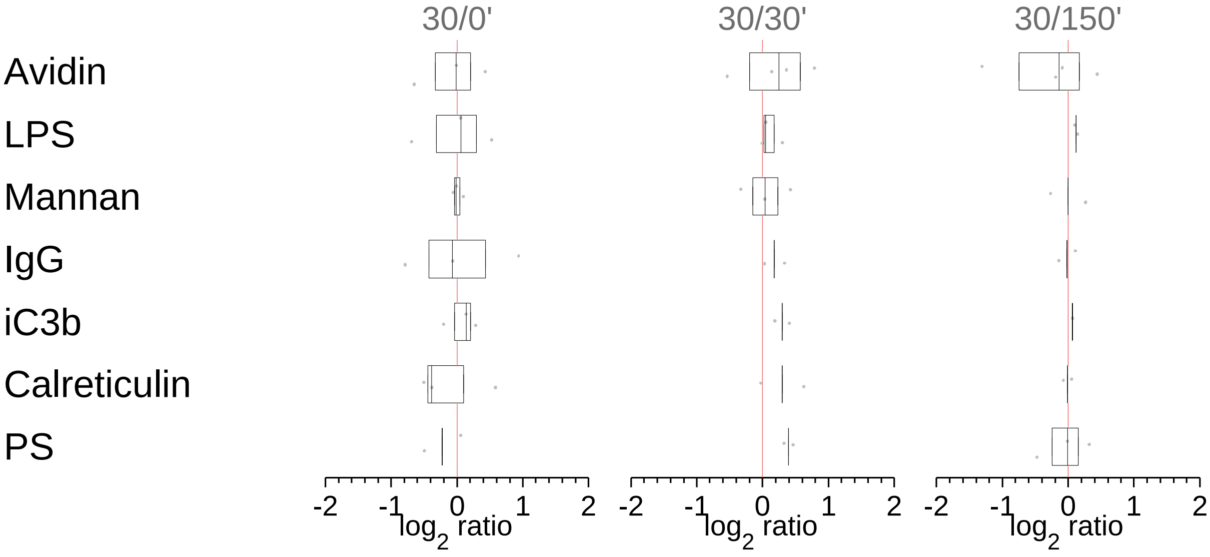

GO:0034737 ergosterol O-acyltransferase activity (n = 3)

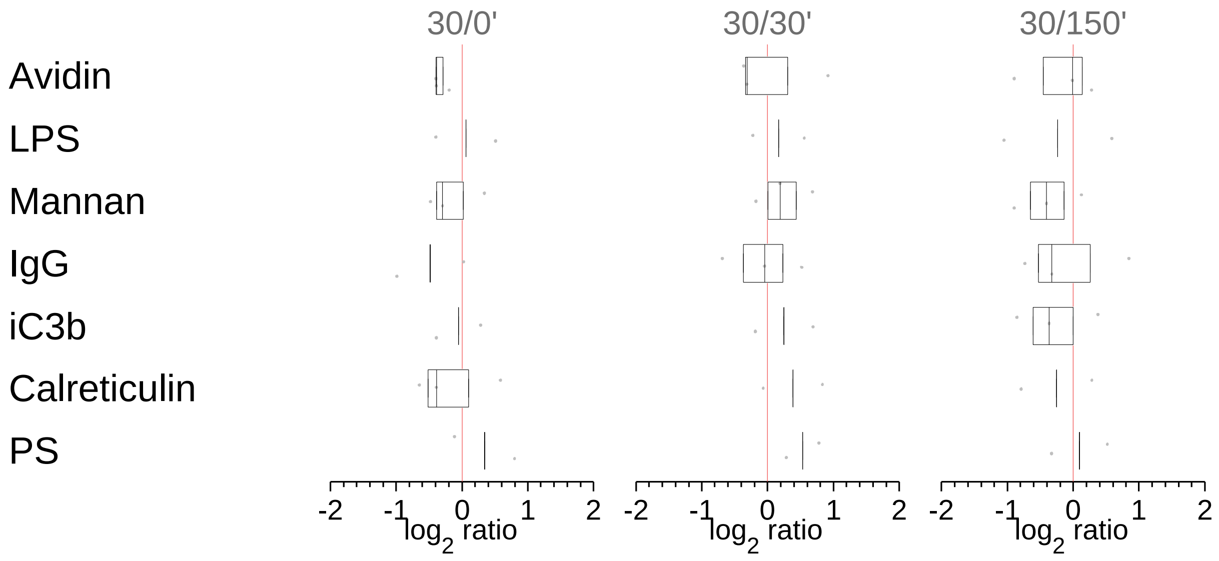

GO:0034738 lanosterol O-acyltransferase activity (n = 3)

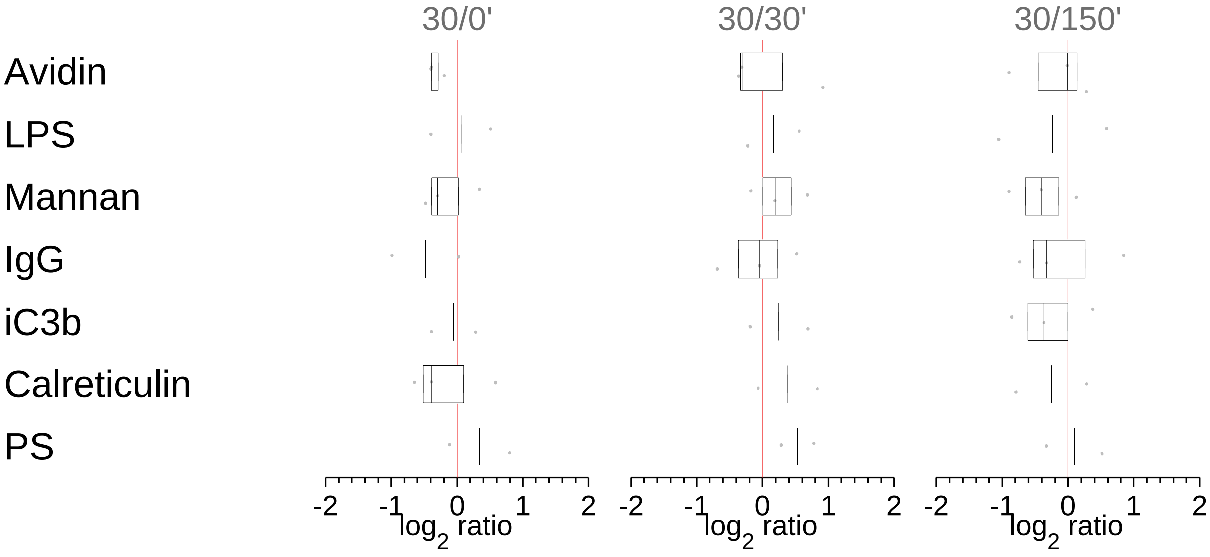

GO:0034765 regulation of ion transmembrane transport (n = 12)

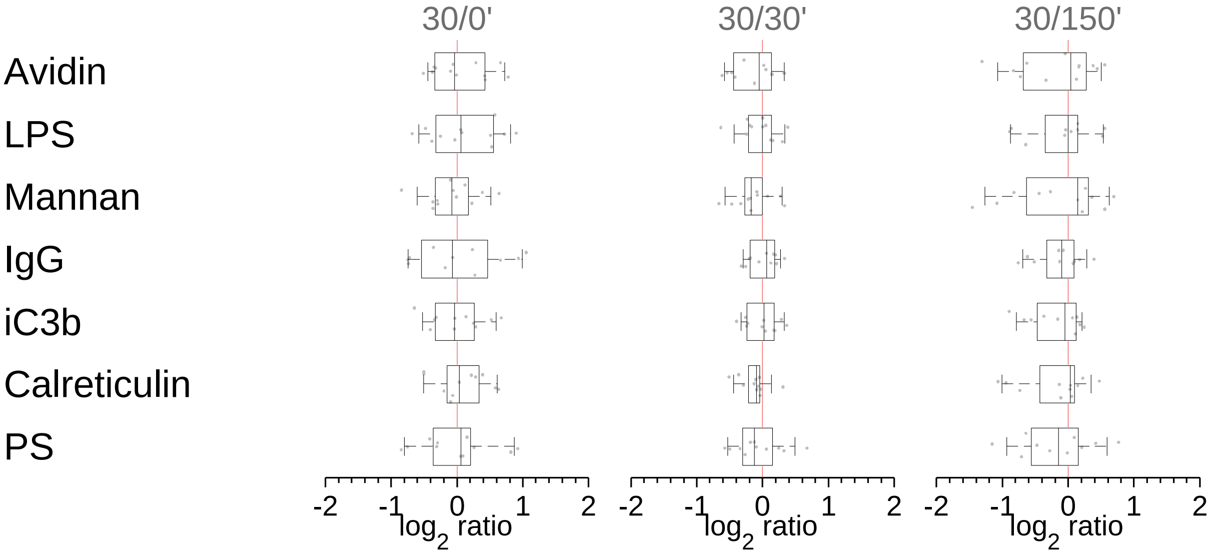

GO:0034831 (R)-(-)-1,2,3,4-tetrahydronaphthol dehydrogenase activity (n = 3)

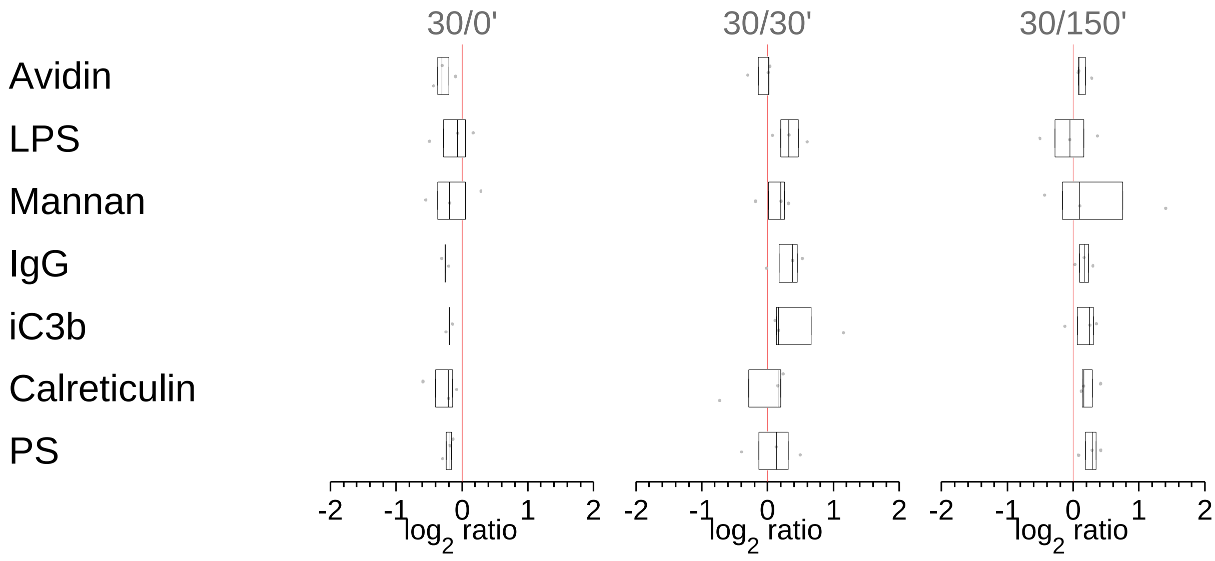

GO:0034840 3-hydroxymenthone dehydrogenase activity (n = 3)

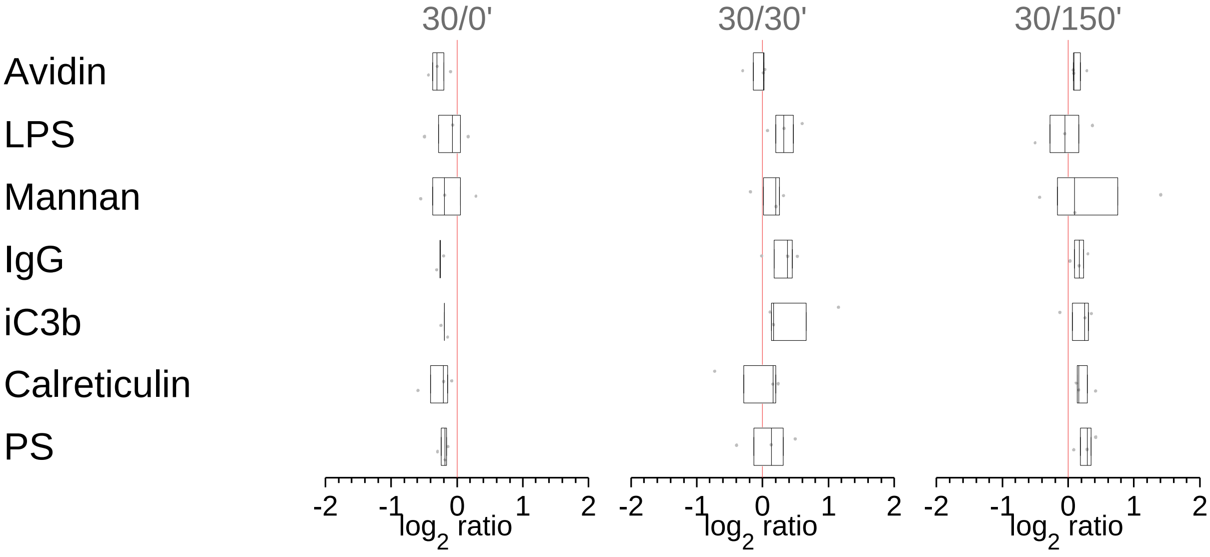

GO:0034843 2-oxoglutaryl-CoA thioesterase activity (n = 3)

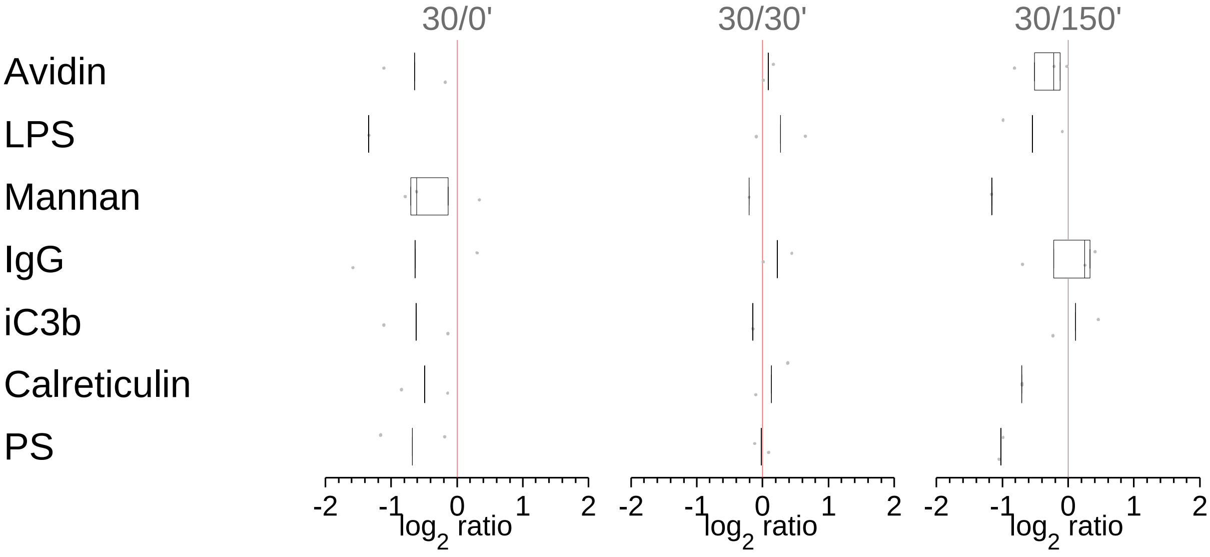

GO:0034848 naphthyl-2-oxomethyl-succinyl-CoA succinyl transferase activity (n = 3)

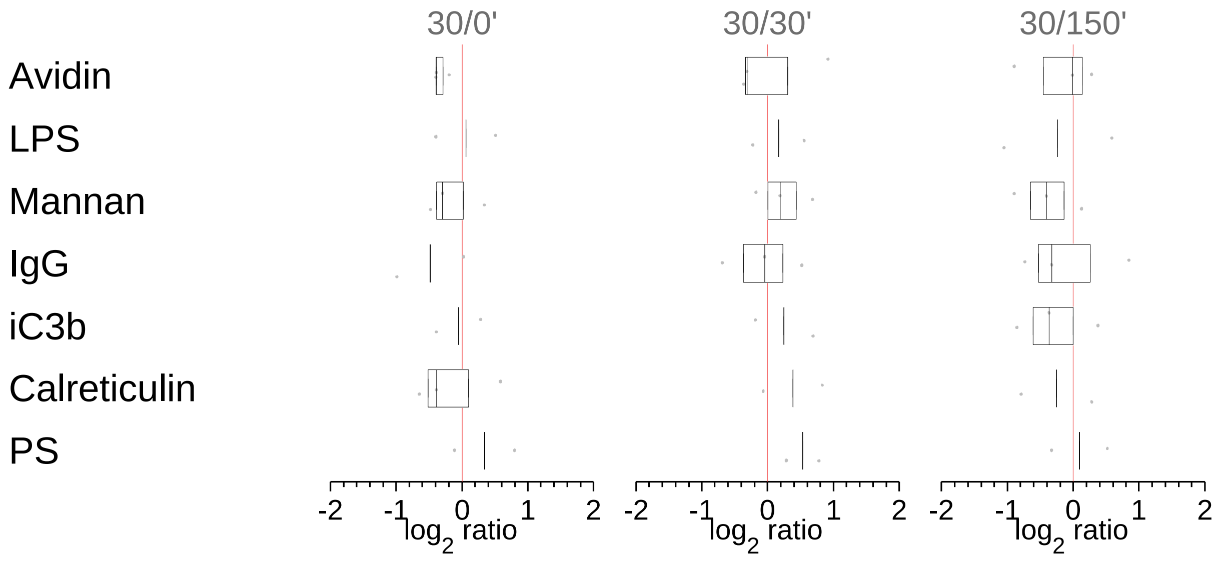

GO:0034851 2,4,4-trimethyl-3-oxopentanoyl-CoA 2-C-propanoyl transferase activity (n = 3)

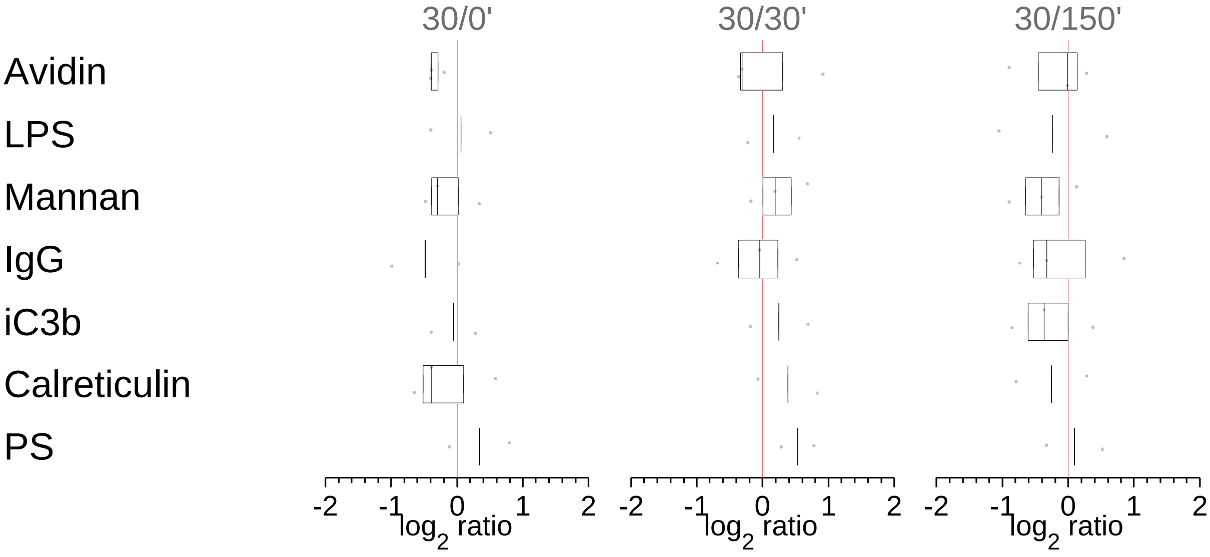

GO:0034869 2,4,4-trimethyl-3-oxopentanoyl-CoA thioesterase activity (n = 3)

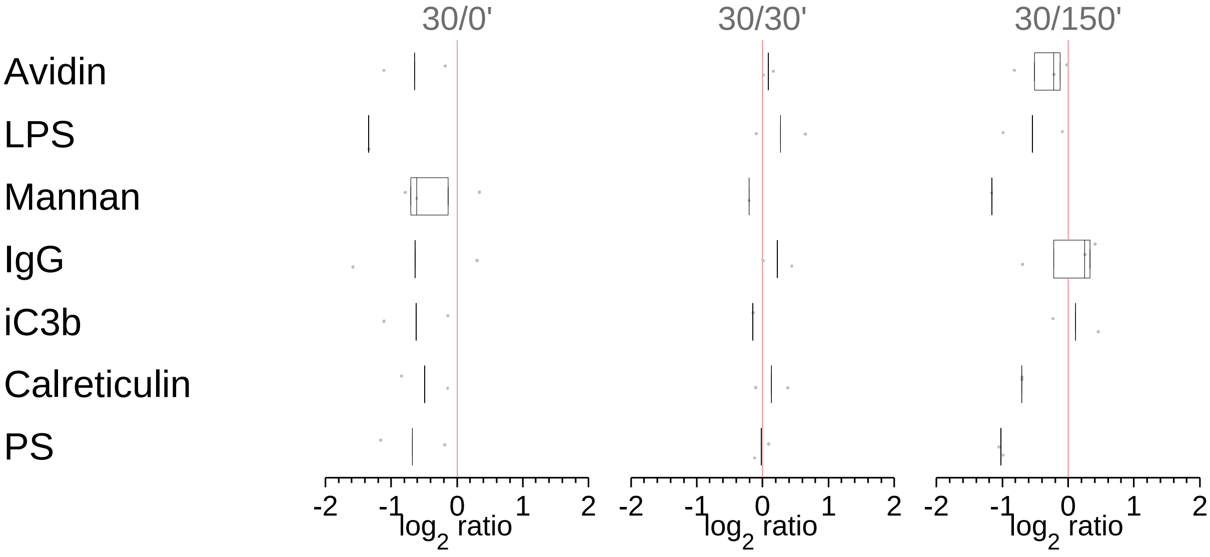

GO:0034892 endosulfan lactone lactonase activity (n = 4)

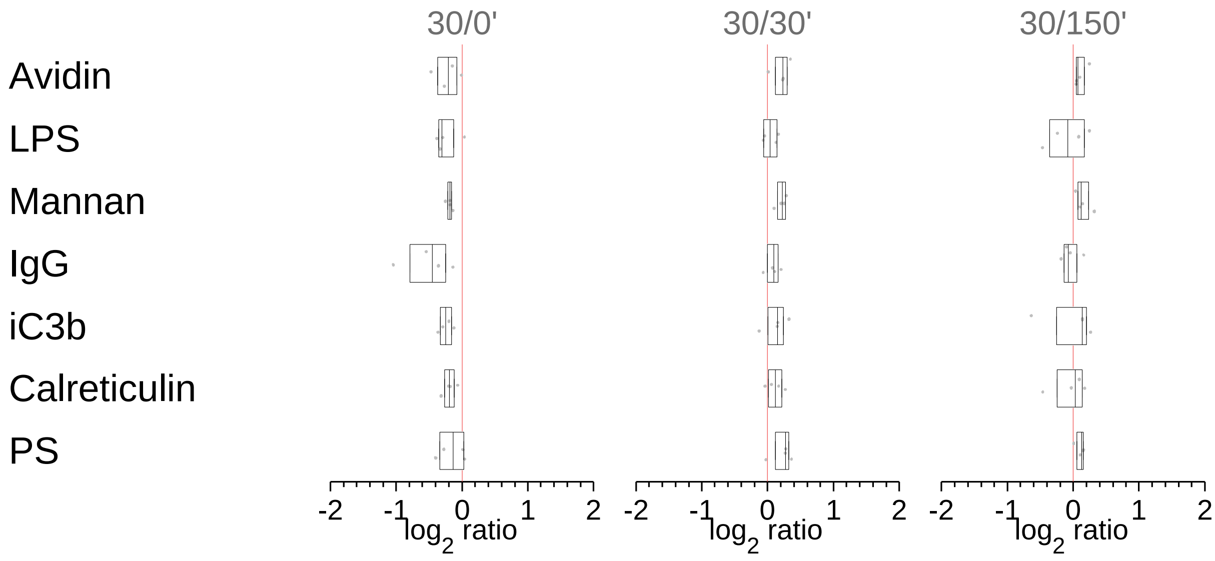

GO:0034915 2-methylhexanoyl-CoA C-acetyltransferase activity (n = 3)

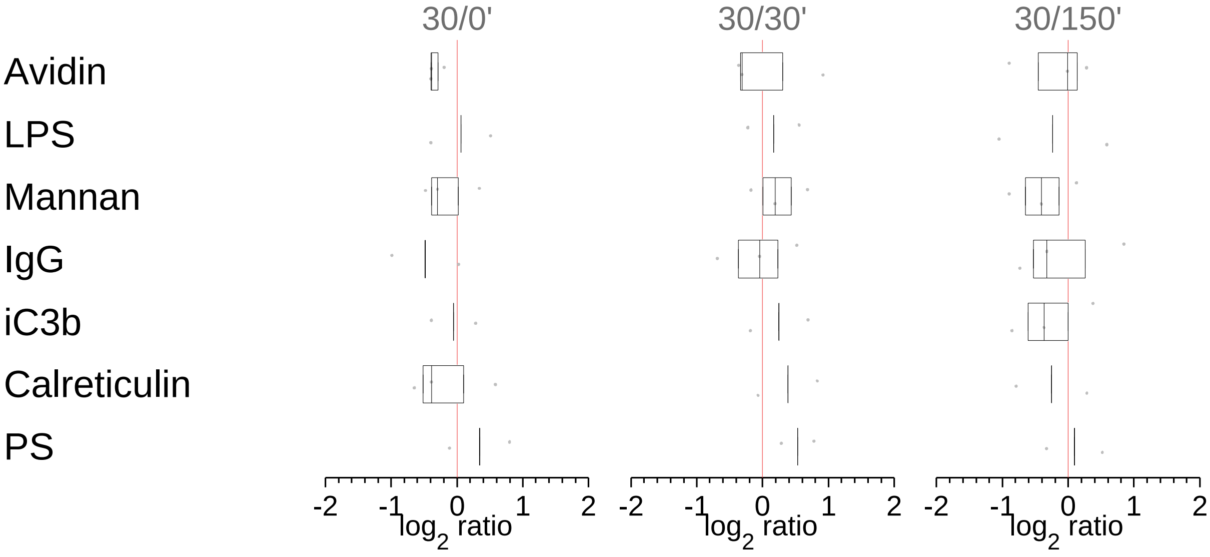

GO:0034919 butyryl-CoA 2-C-propionyltransferase activity (n = 3)

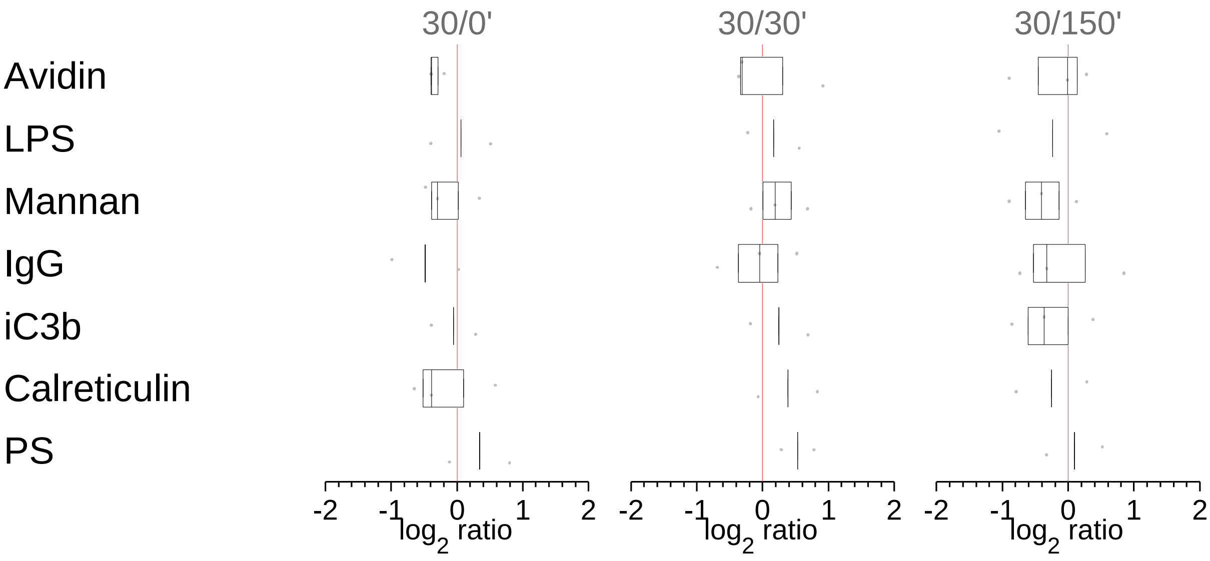

GO:0034945 2,6-dimethyl-5-methylene-3-oxo-heptanoyl-CoA C-acetyltransferase activity (n = 3)

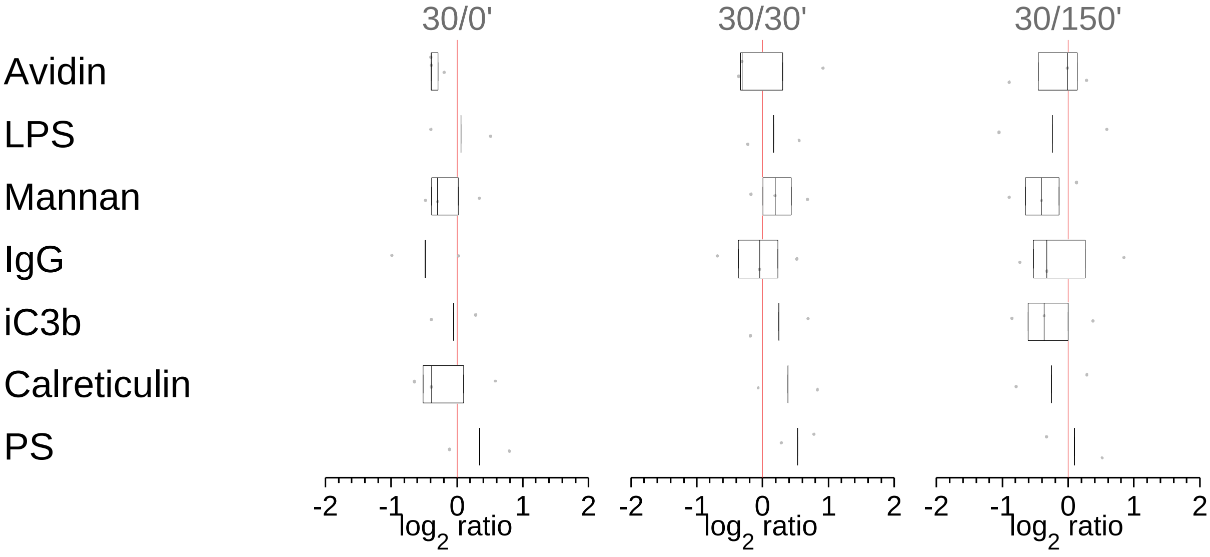

GO:0034946 3-isopropylbut-3-enoyl-CoA thioesterase activity (n = 3)

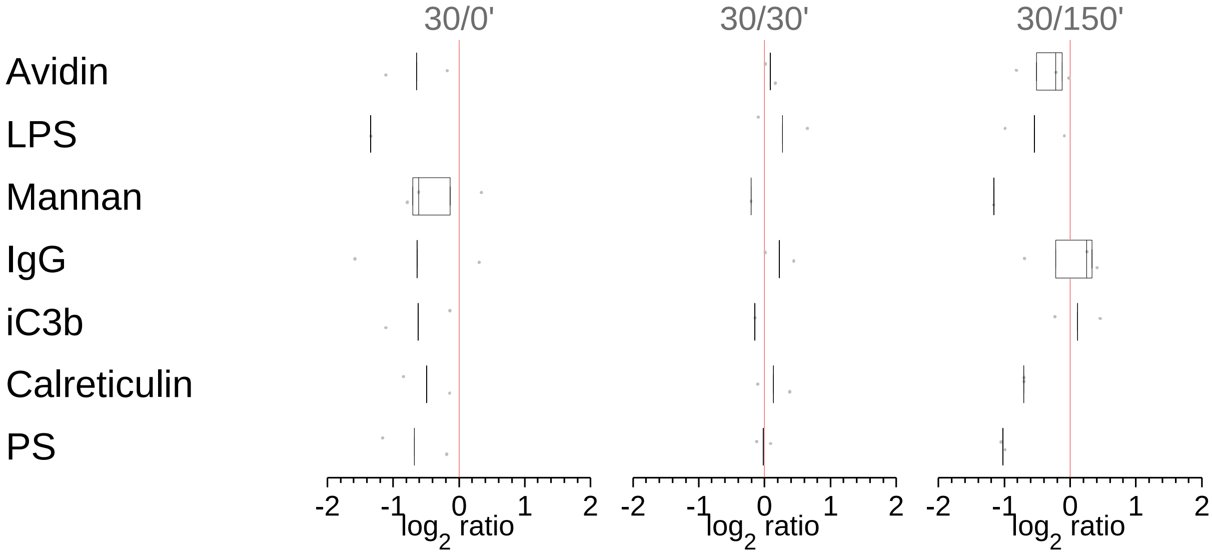

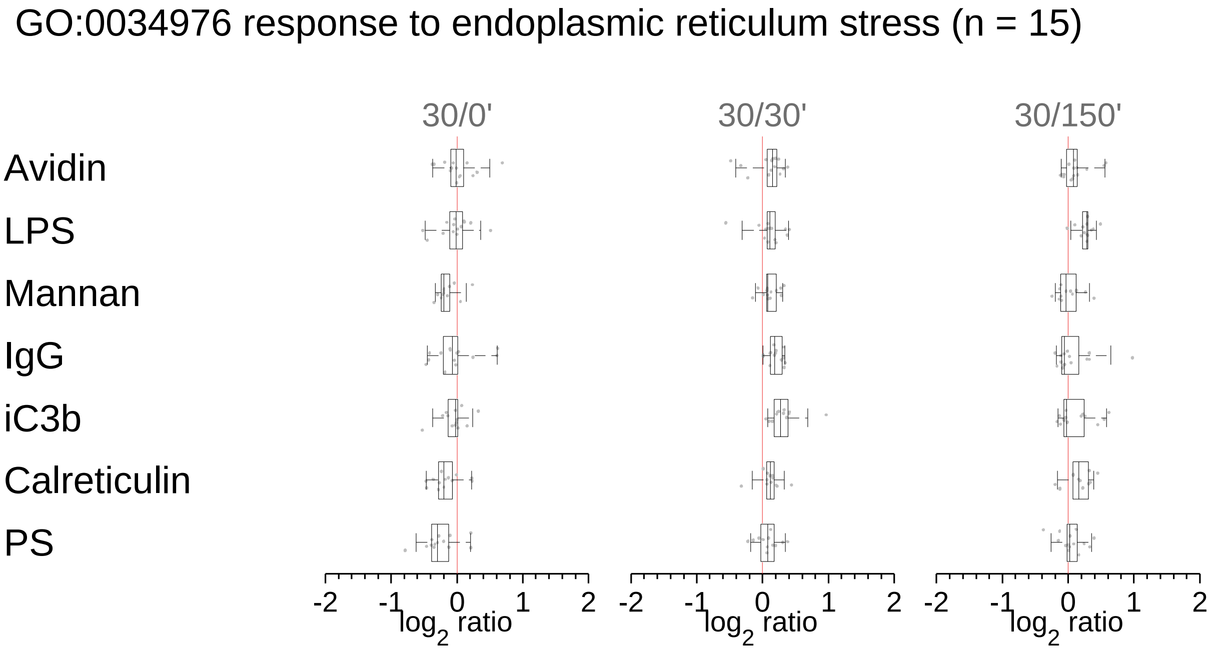

GO:0034976 response to endoplasmic reticulum stress (n = 15)
30/0'
30/30'
30/150'
Avidin
LPS
Mannan
IgG
iC3b
Calreticulin
PS
-2
-1
0
1
2
log2 ratio

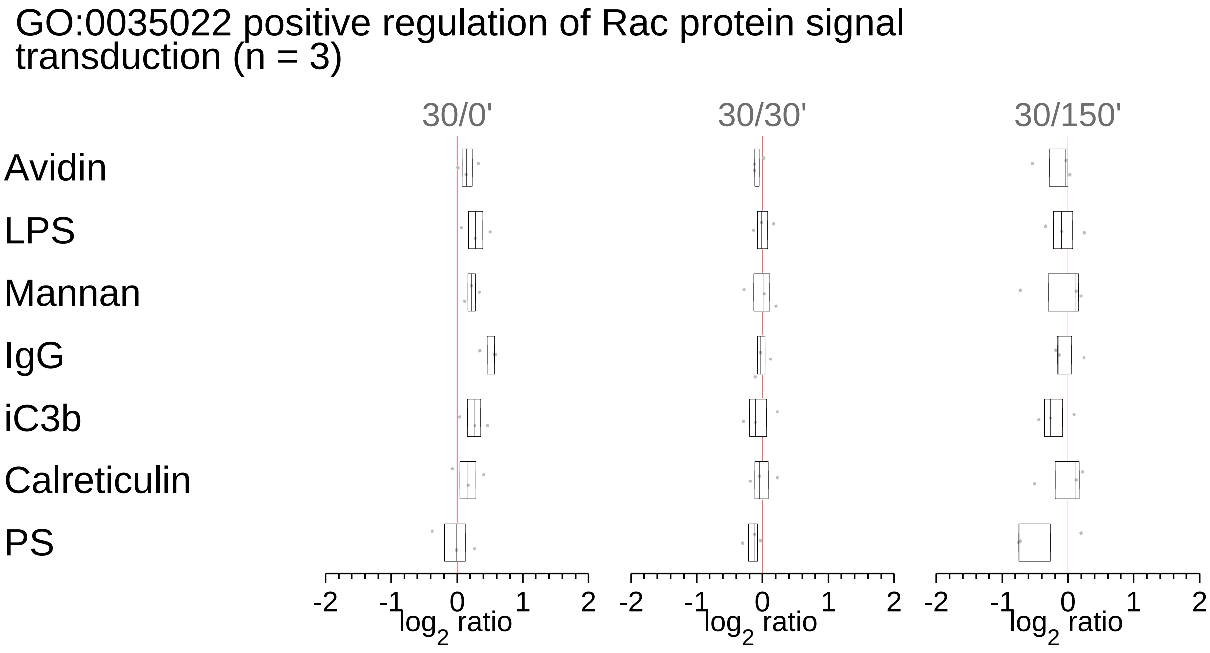
GO:0035022 positive regulation of Rac protein signal transduction (n = 3)
30/0'
30/30'
30/150'
Avidin
LPS
Mannan
IgG
iC3b
Calreticulin
PS
-2
-1
0
1
2
log2 ratio

GO:0035023 regulation of Rho protein signal transduction (n = 7)

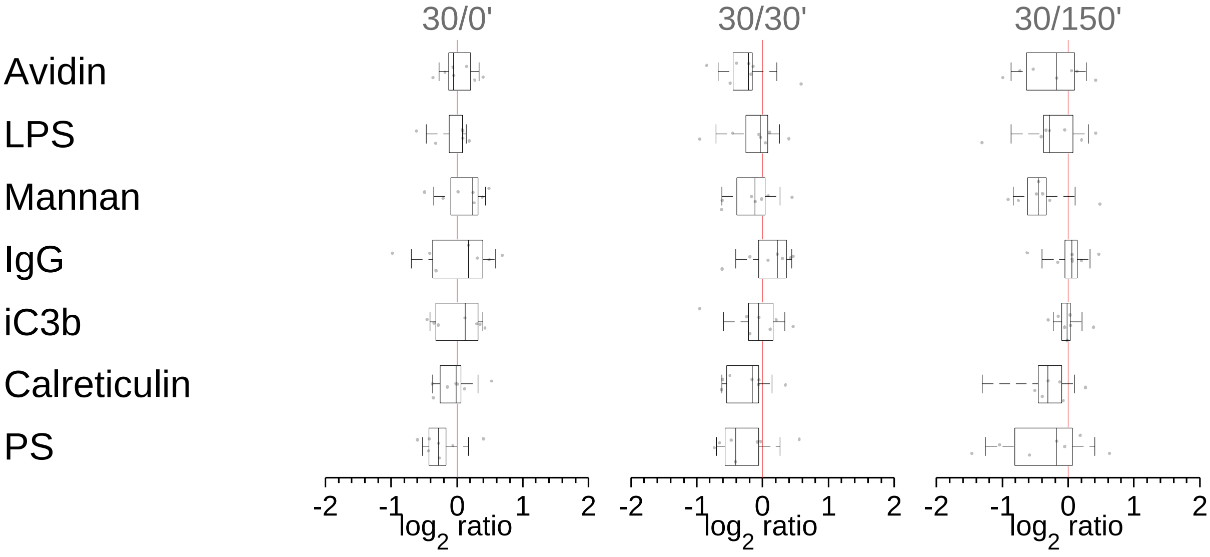

GO:0035091 phosphatidylinositol binding (n = 27)

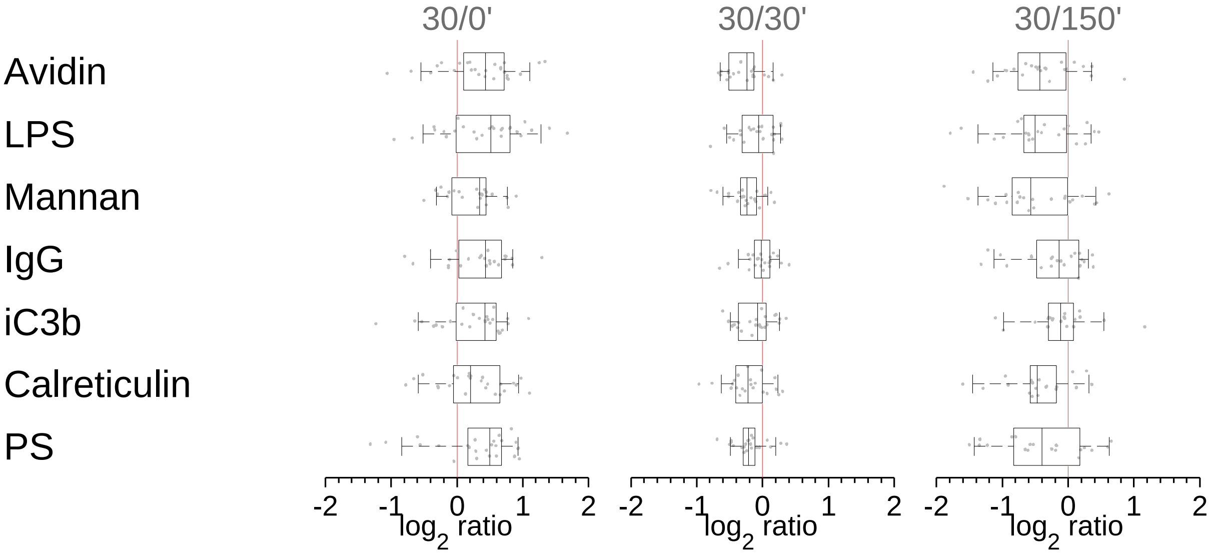

GO:0035108 limb morphogenesis (n = 4)

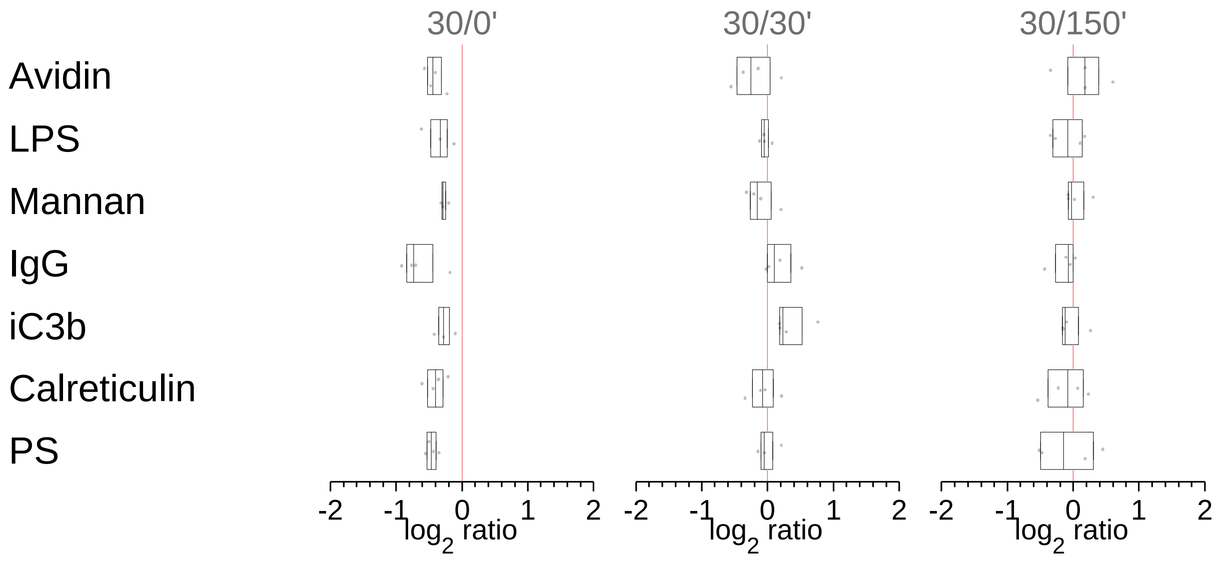

GO:0035235 ionotropic glutamate receptor signaling pathway (n = 3)

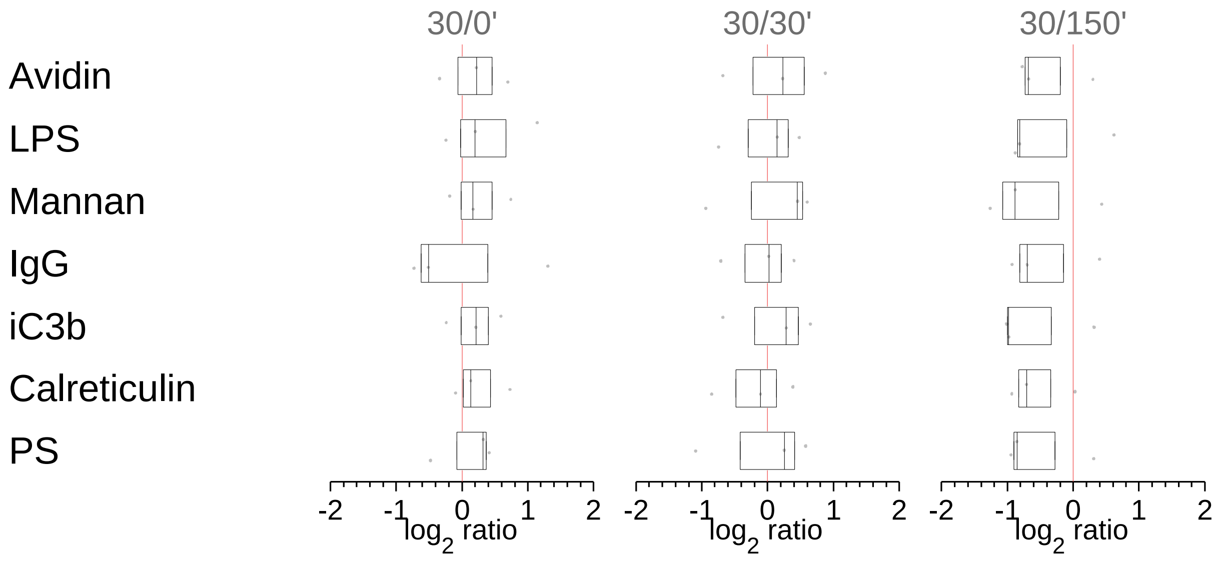

GO:0035253 ciliary rootlet (n = 4)

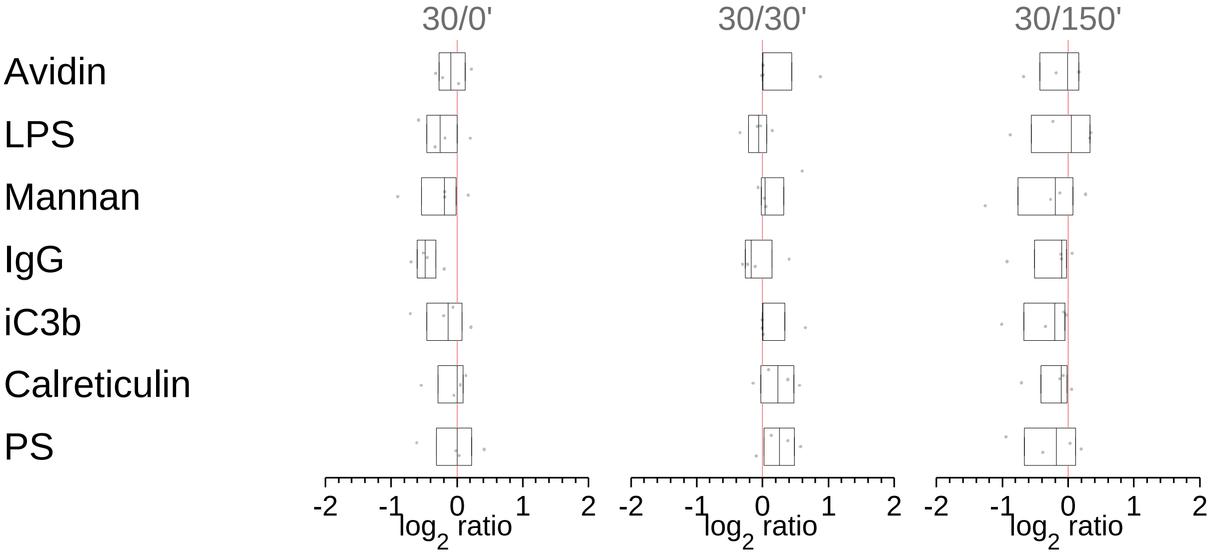

GO:0035255 ionotropic glutamate receptor binding (n = 4)

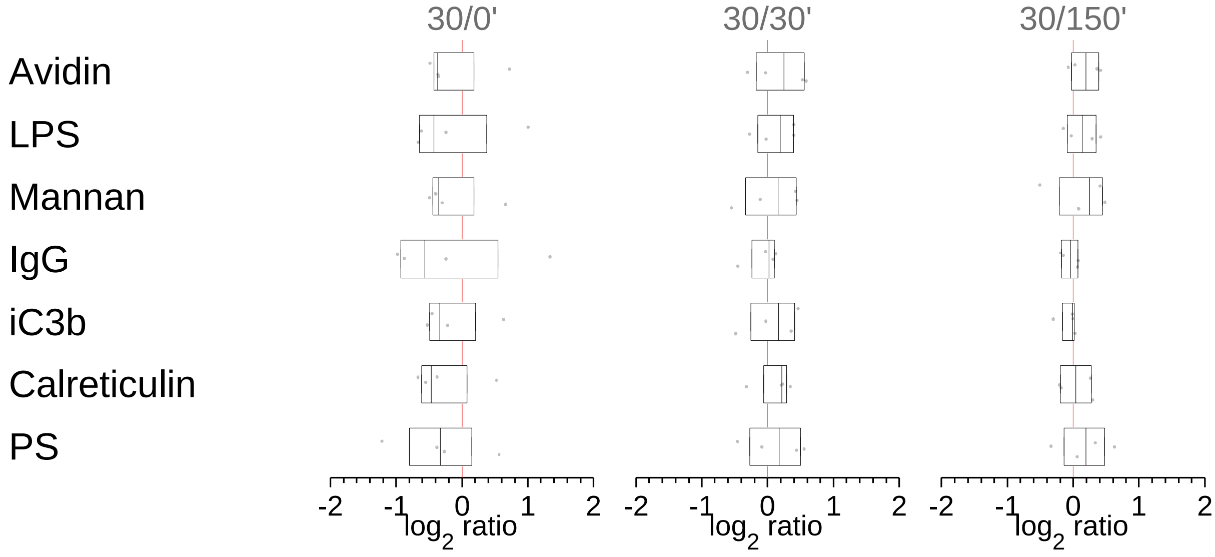

GO:0035264 multicellular organism growth (n = 7)

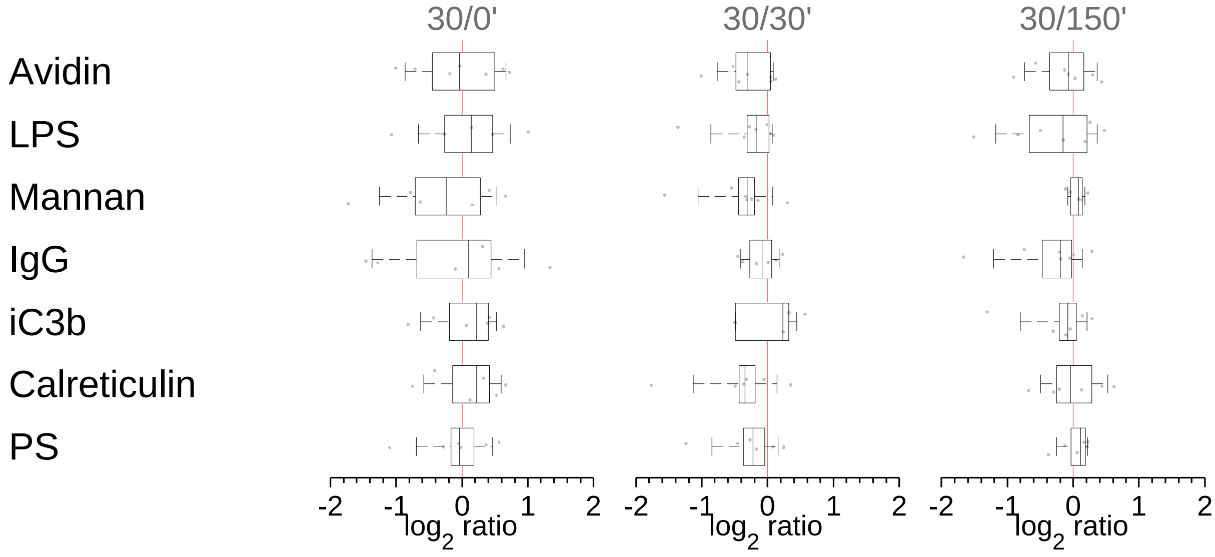

GO:0035307 positive regulation of protein dephosphorylation (n = 3)

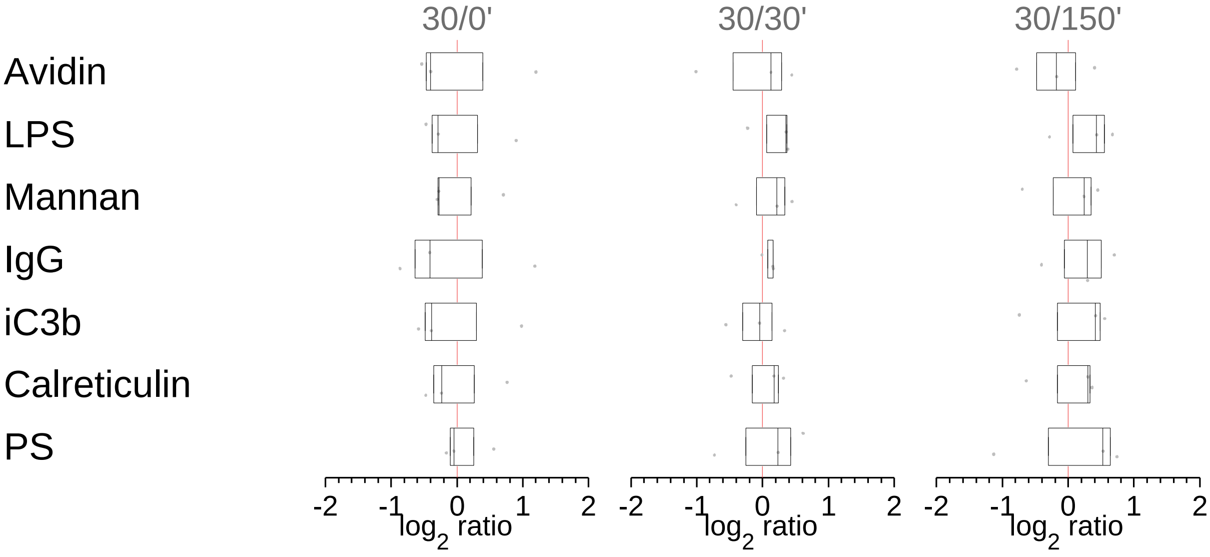

GO:0035308 negative regulation of protein dephosphorylation (n = 3)

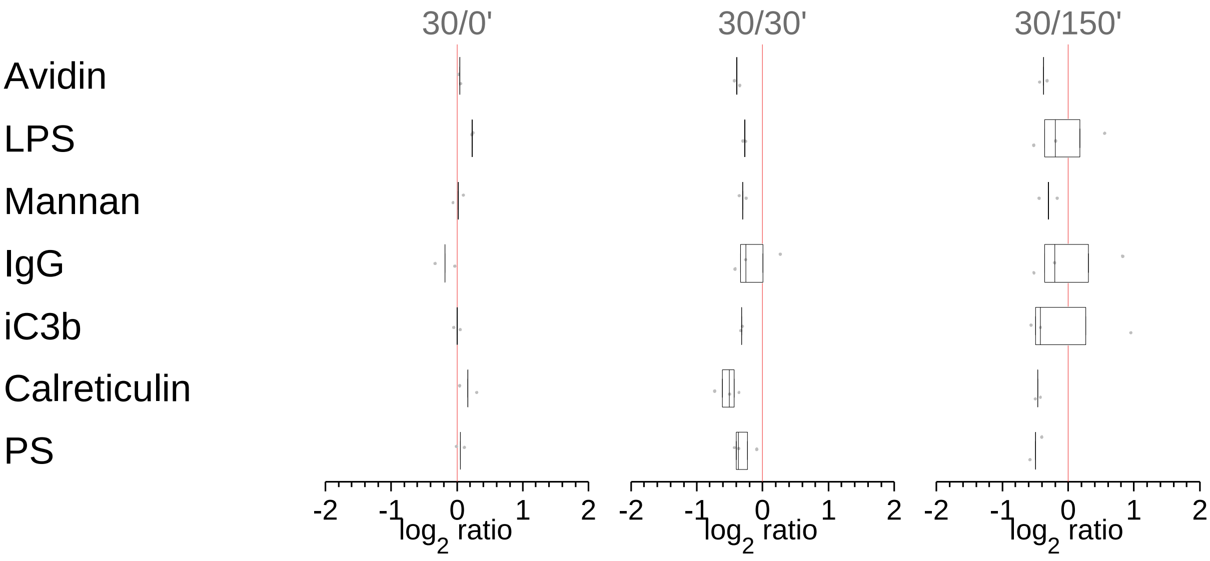

GO:0035335 peptidyl-tyrosine dephosphorylation (n = 11)

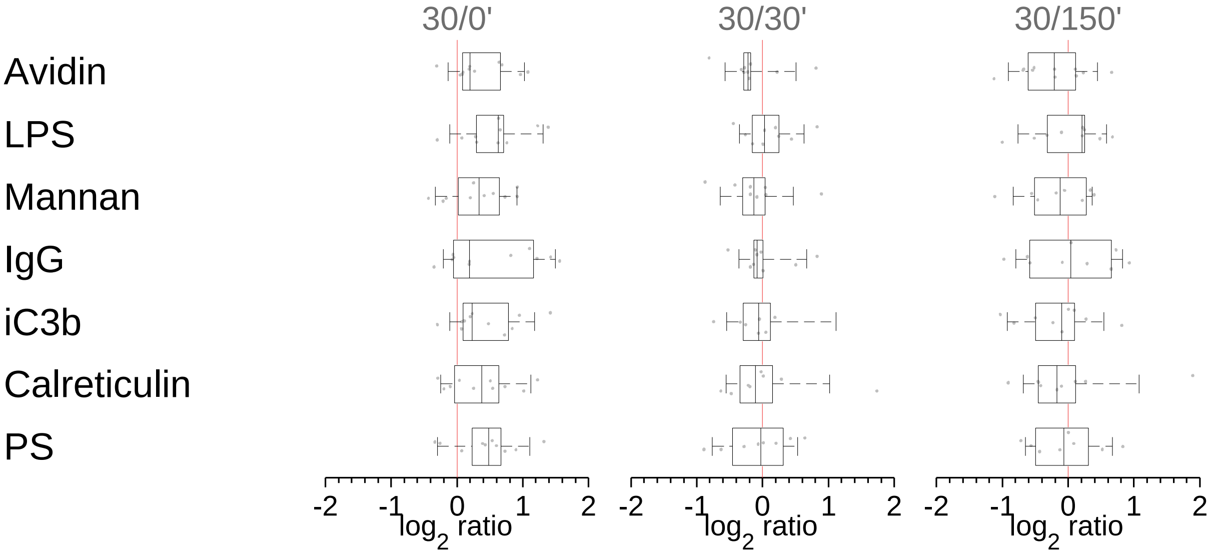

GO:0035371 microtubule plus end (n = 3)

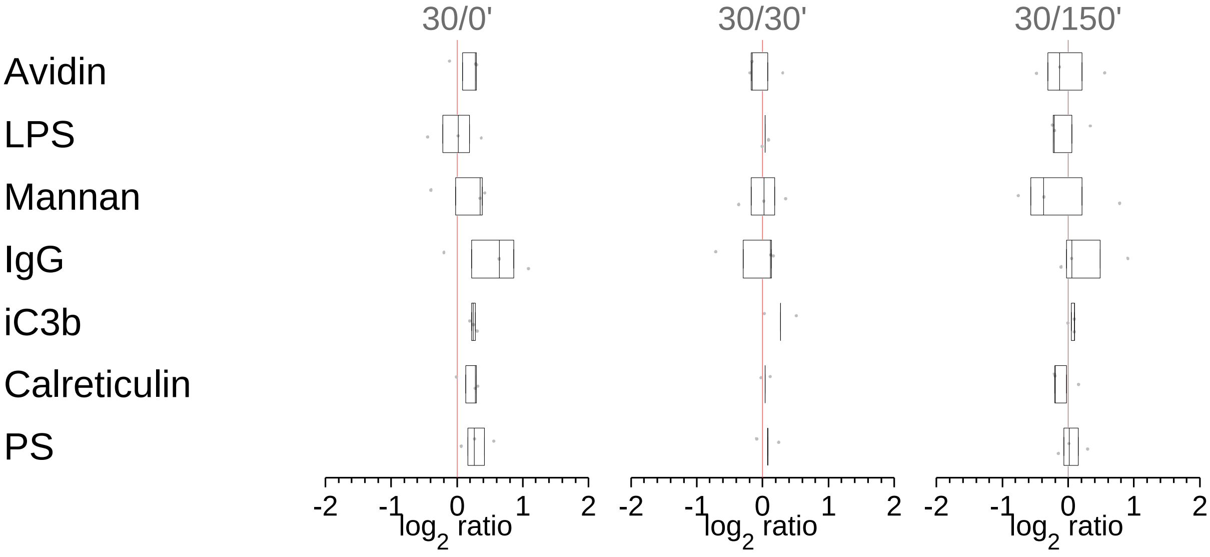

GO:0035380 very long-chain-3-hydroxyacyl-CoA dehydrogenase activity (n = 3)

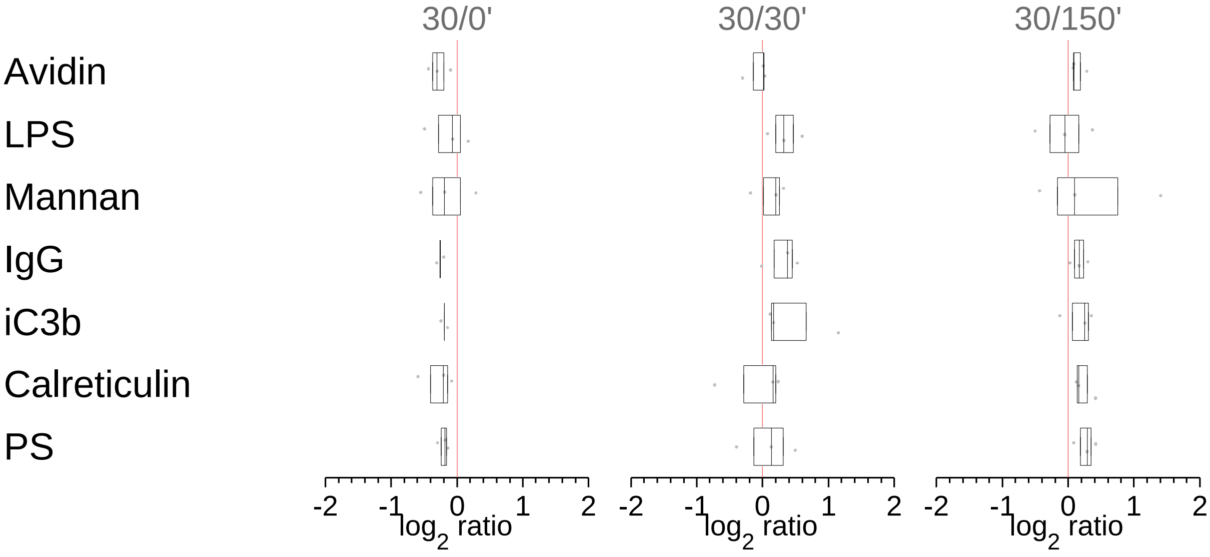

GO:0035410 dihydrotestosterone 17-beta-dehydrogenase activity (n = 3)

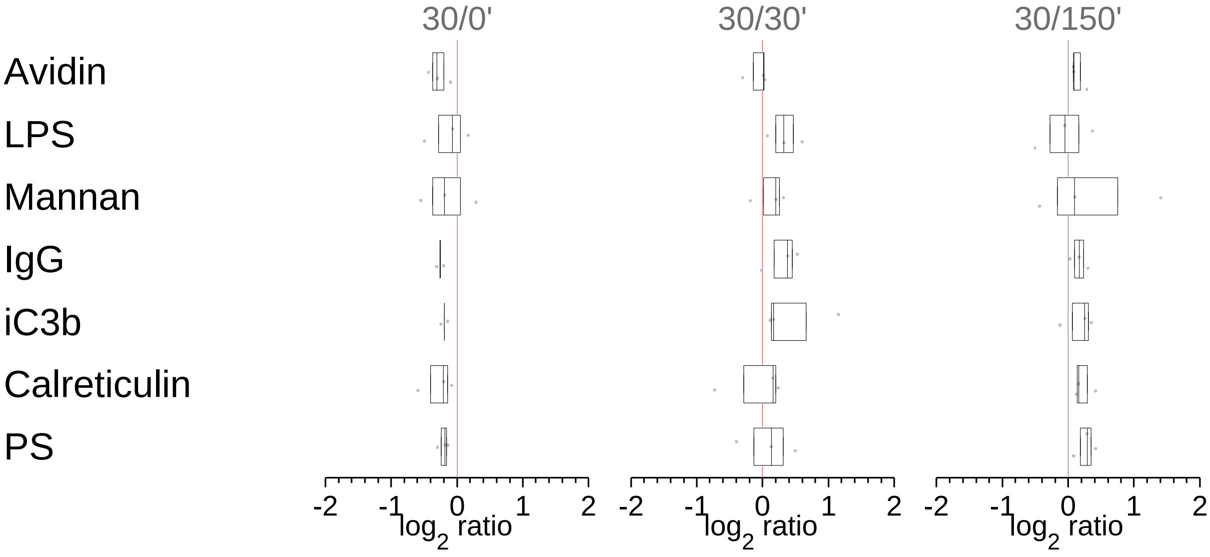

GO:0035455 response to interferon-alpha (n = 3)

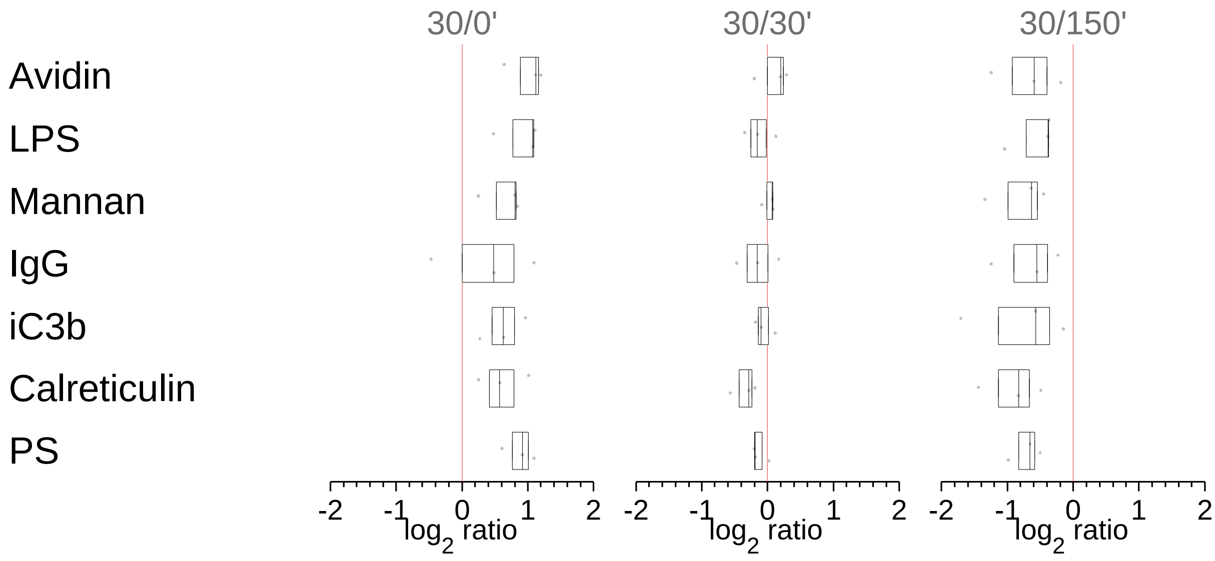

GO:0035456 response to interferon-beta (n = 4)

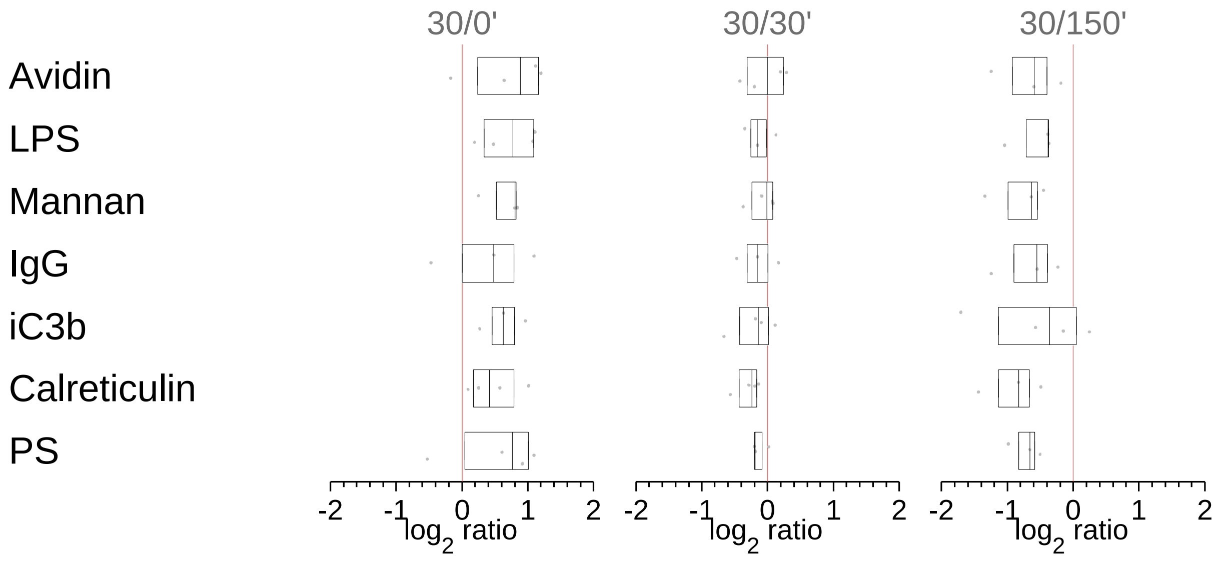

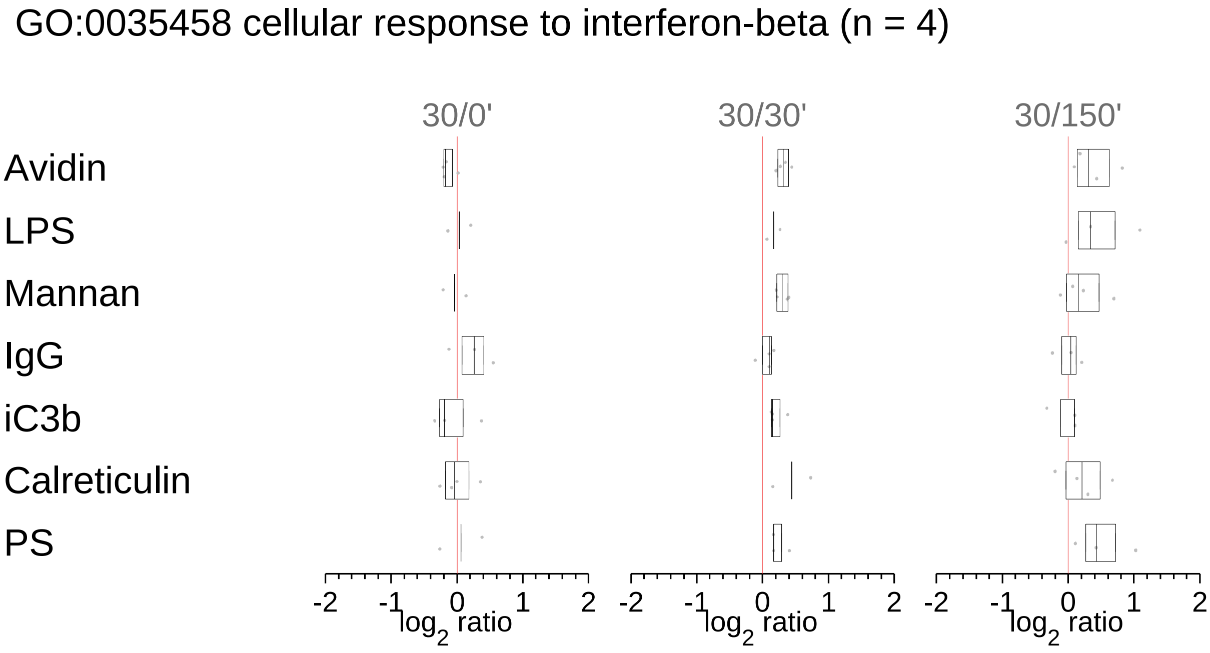

GO:0035458 cellular response to interferon-beta (n = 4)
30/0'
30/30'
30/150'
Avidin
LPS
Mannan
IgG
iC3b
Calreticulin
PS
-2
-1
0
1
2
log2 ratio

GO:0035460 L-ascorbate 6-phosphate lactonase activity (n = 4)

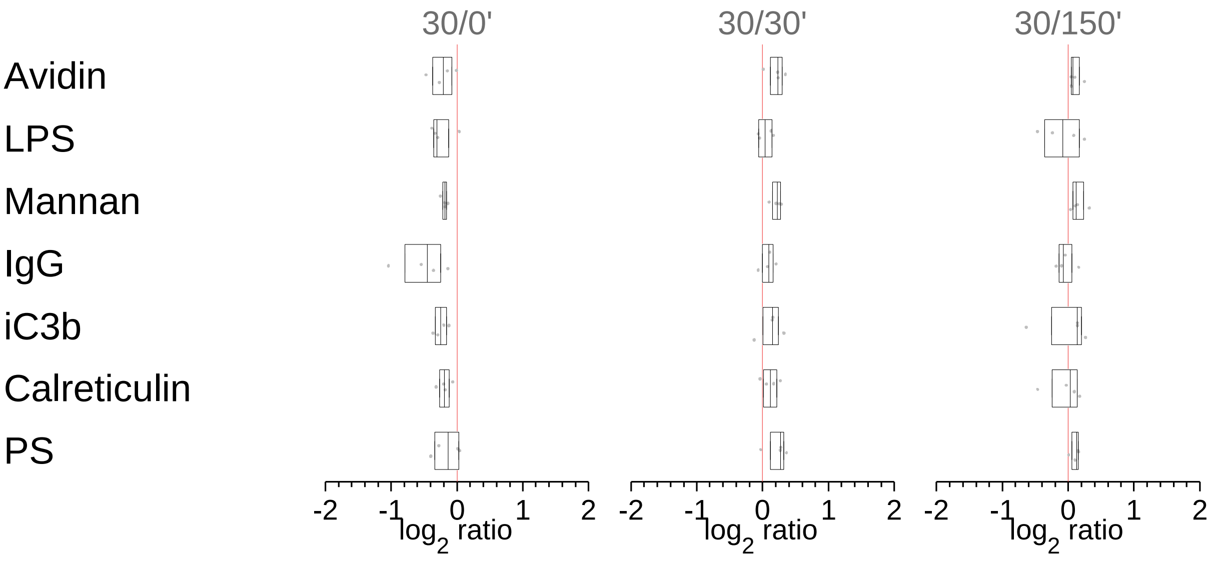

GO:0035473 lipase binding (n = 3)

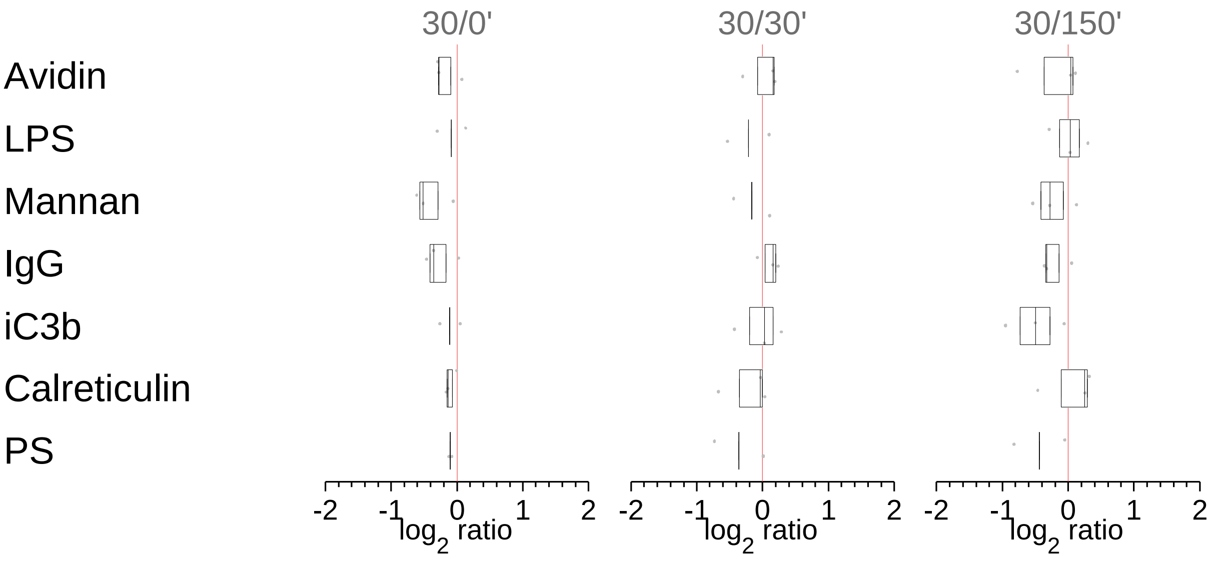

GO:0035493 SNARE complex assembly (n = 5)

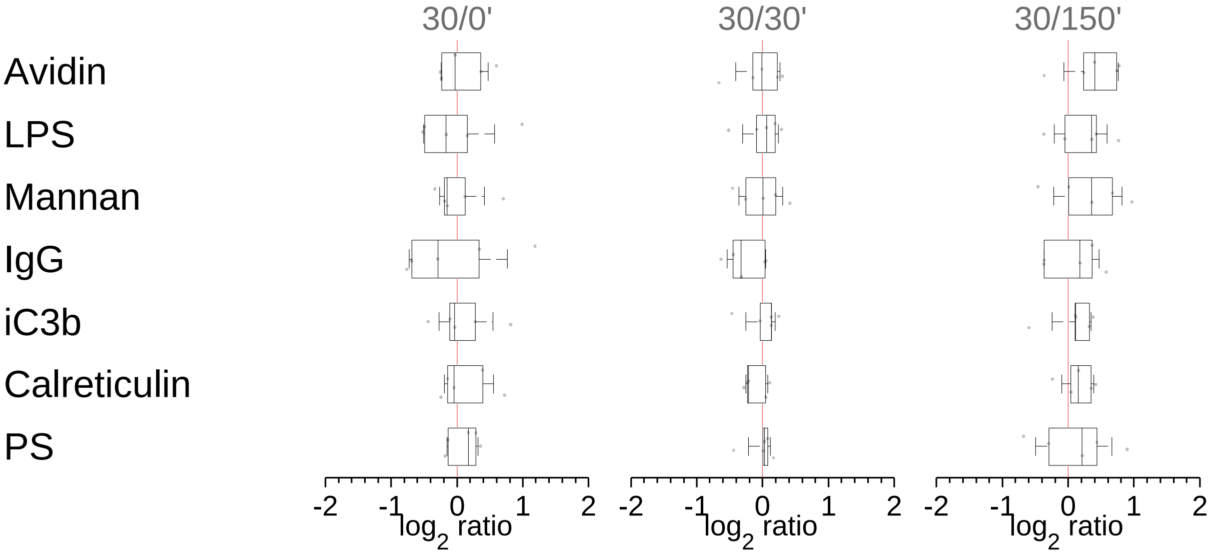

GO:0035496 lipopolysaccharide-1,5-galactosyltransferase activity
(n = 3)

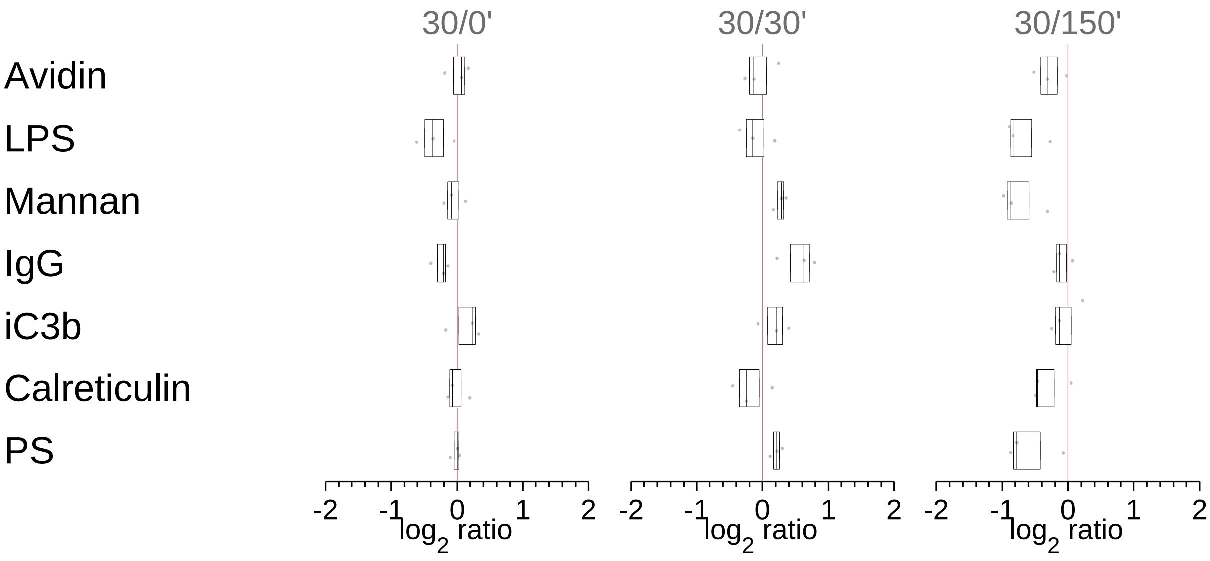

GO:0035556 intracellular signal transduction (n = 40)

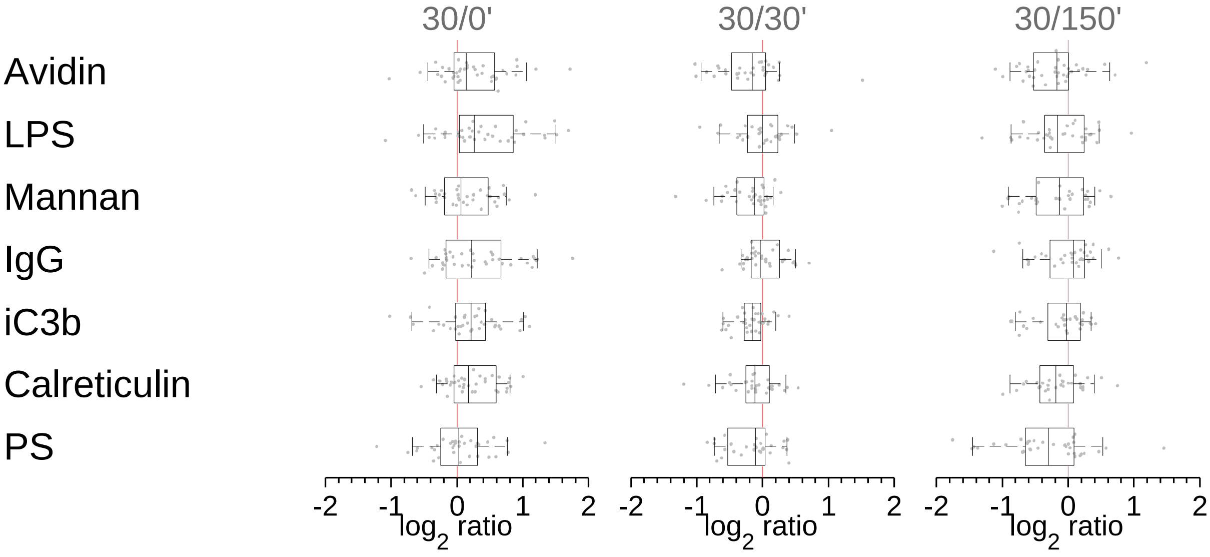

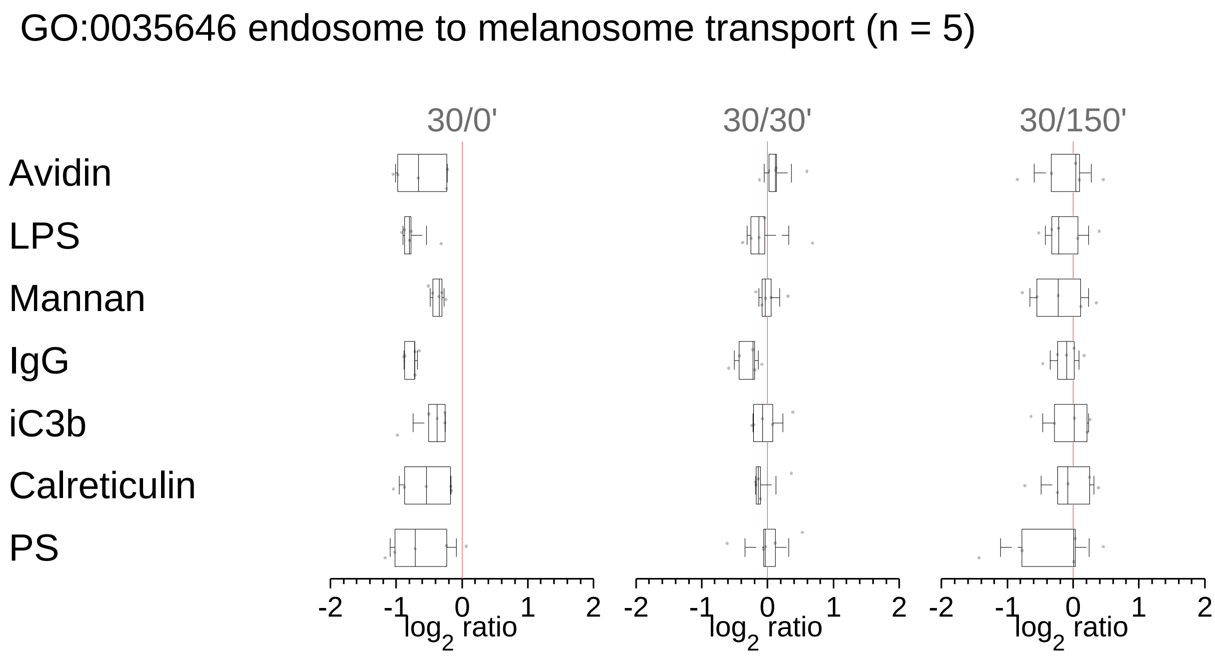
GO:0035646 endosome to melanosome transport (n = 5)
30/0'
30/30'
30/150'
Avidin
LPS
Mannan
IgG
iC3b
Calreticulin
PS
-2
-1
0
1
2
log2 ratio

GO:0035725 sodium ion transmembrane transport (n = 5)

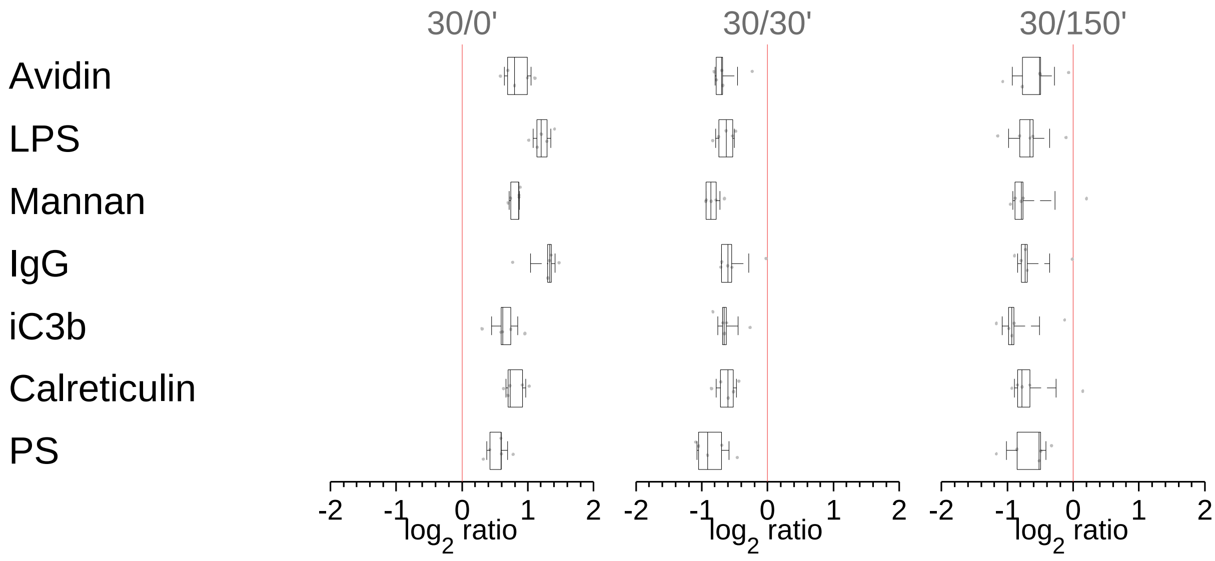

GO:0035749 myelin sheath adaxonal region (n = 3)

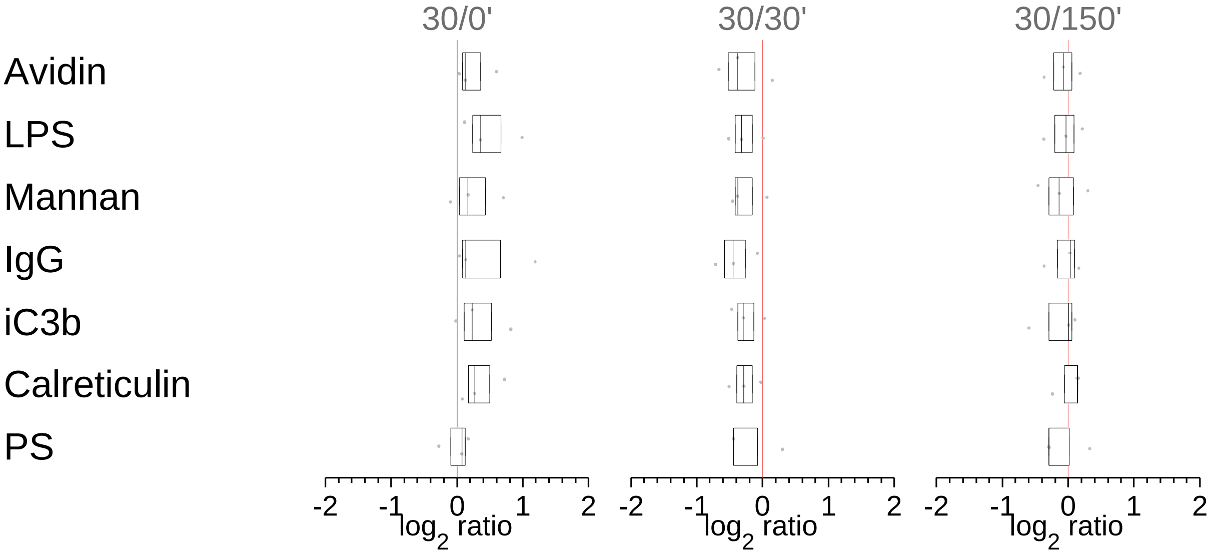

GO:0035774 positive regulation of insulin secretion involved in cellular response to glucose stimulus (n = 3)

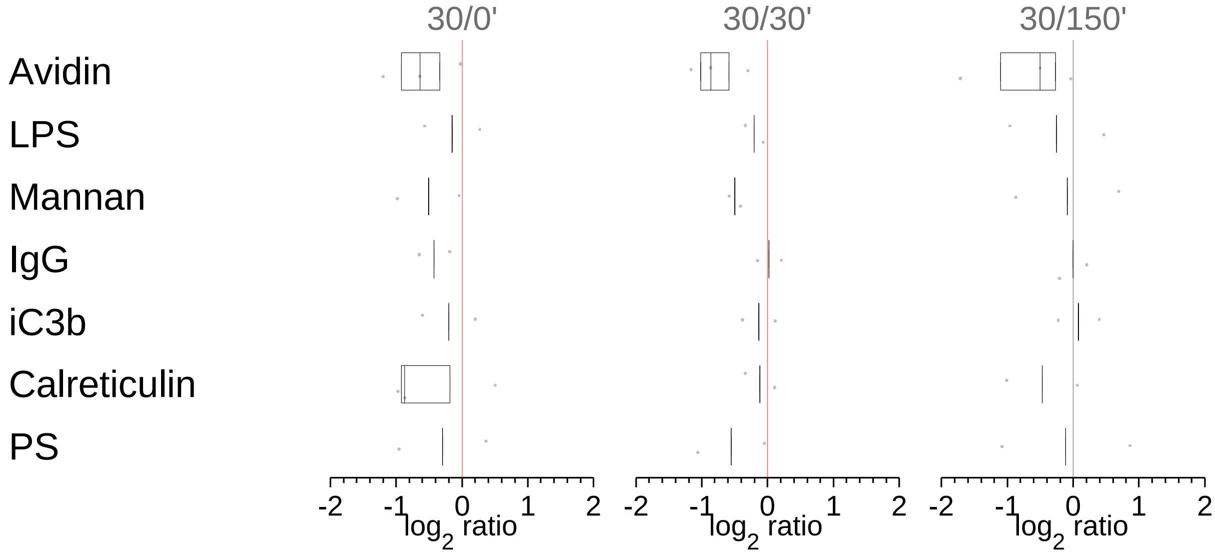

GO:0035814 negative regulation of renal sodium excretion (n = 3)

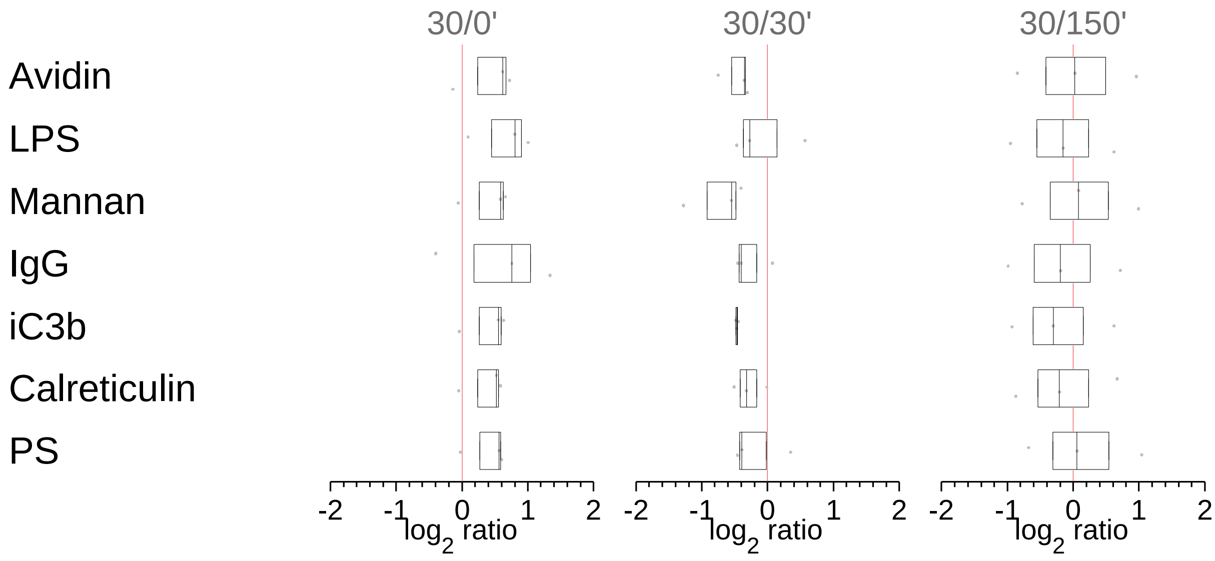

GO:0035855 megakaryocyte development (n = 4)

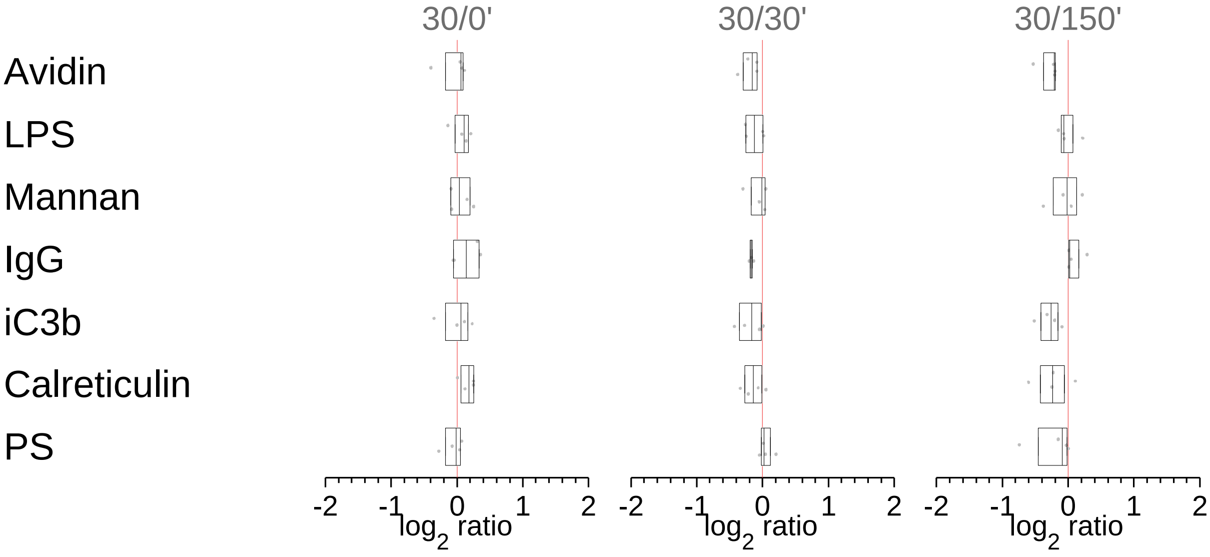

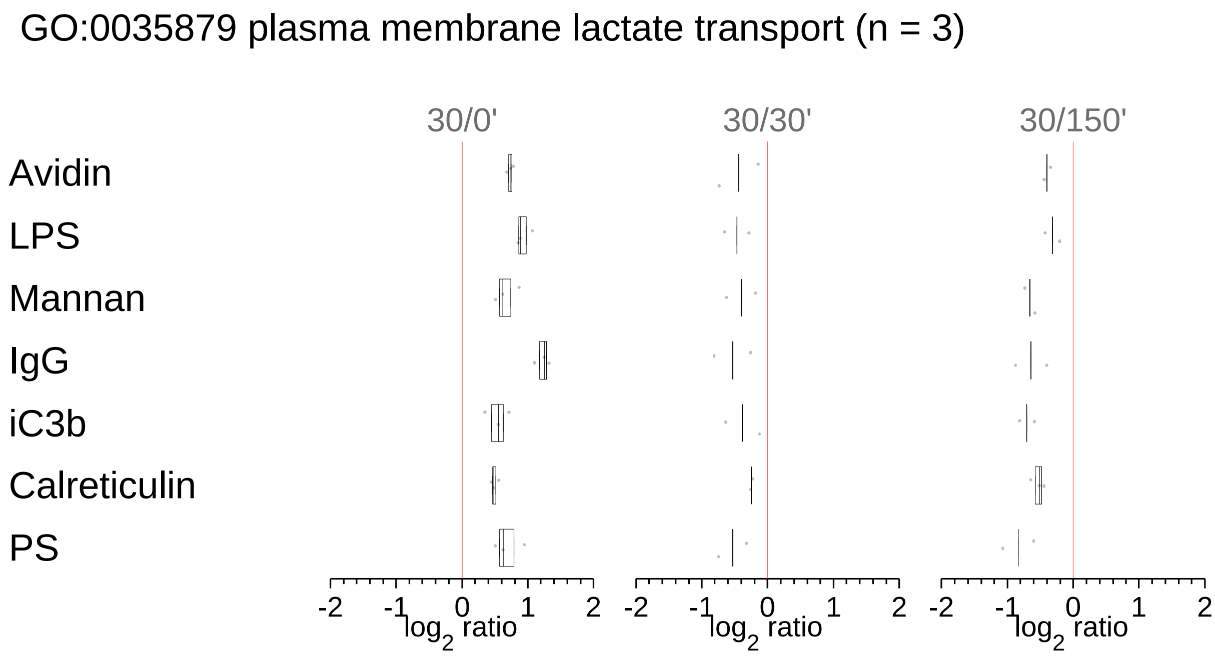

GO:0035879 plasma membrane lactate transport (n = 3)
30/0'
30/30'
30/150'
Avidin
LPS
Mannan
IgG
iC3b
Calreticulin
PS
-2 -1 0 1 2
log2 ratio

GO:0036010 protein localization in endosome (n = 5)

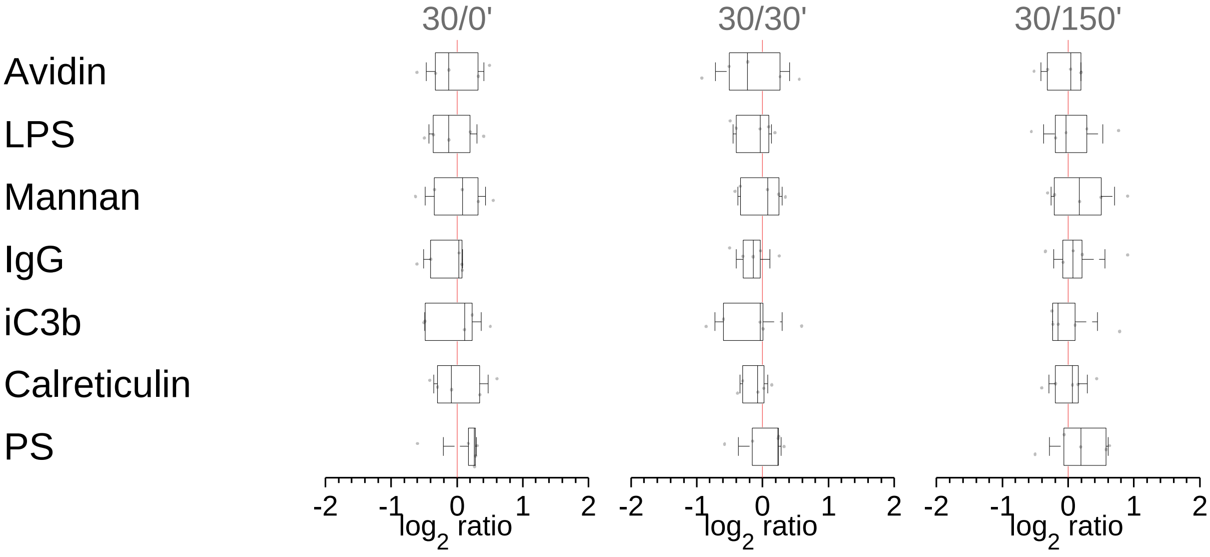

GO:0036064 cilium basal body (n = 6)

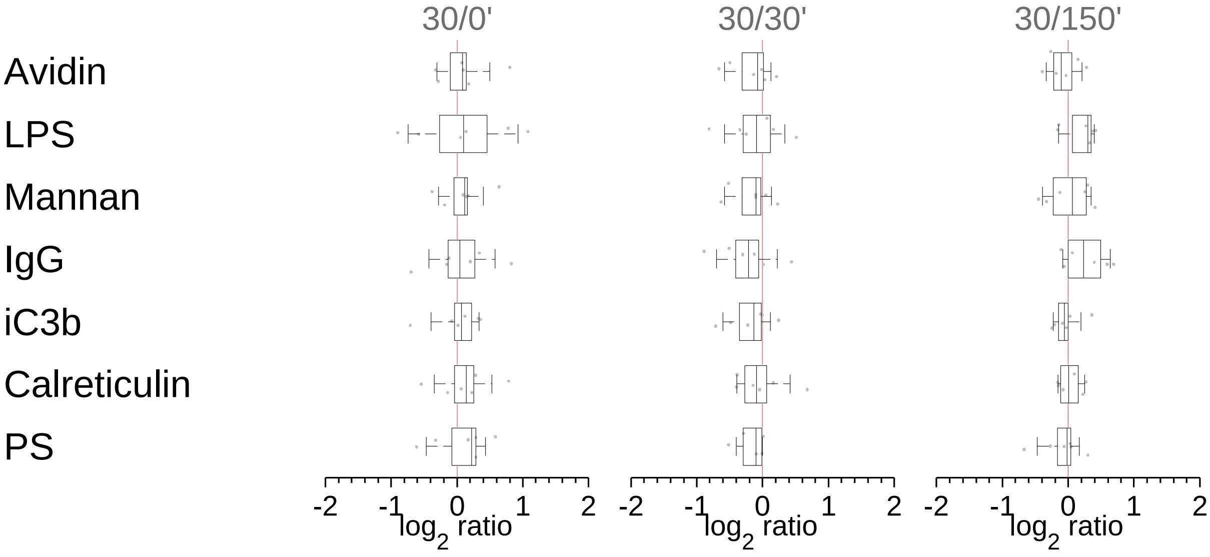

GO:0036289 peptidyl-serine autophosphorylation (n = 3)

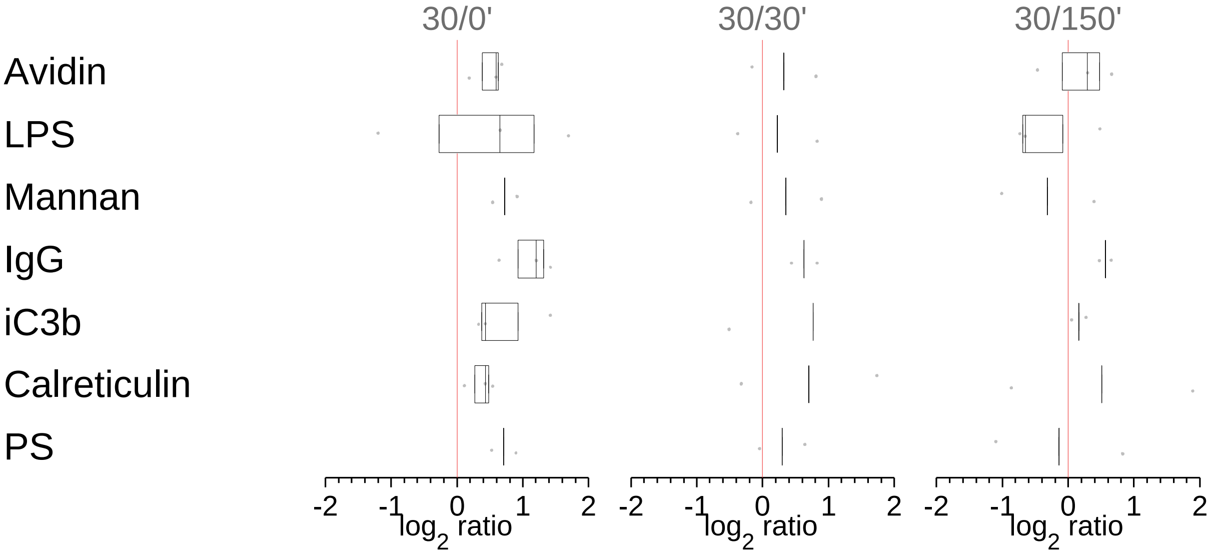

GO:0036464 N/A (n = 13)

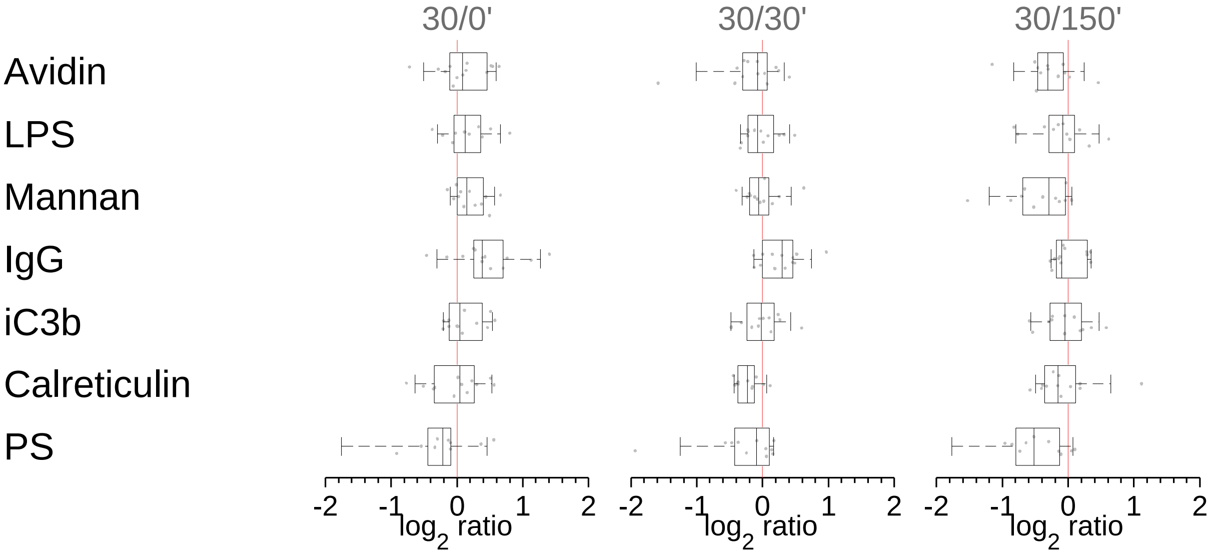

GO:0038032 termination of G-protein coupled receptor signaling pathway (n = 7)

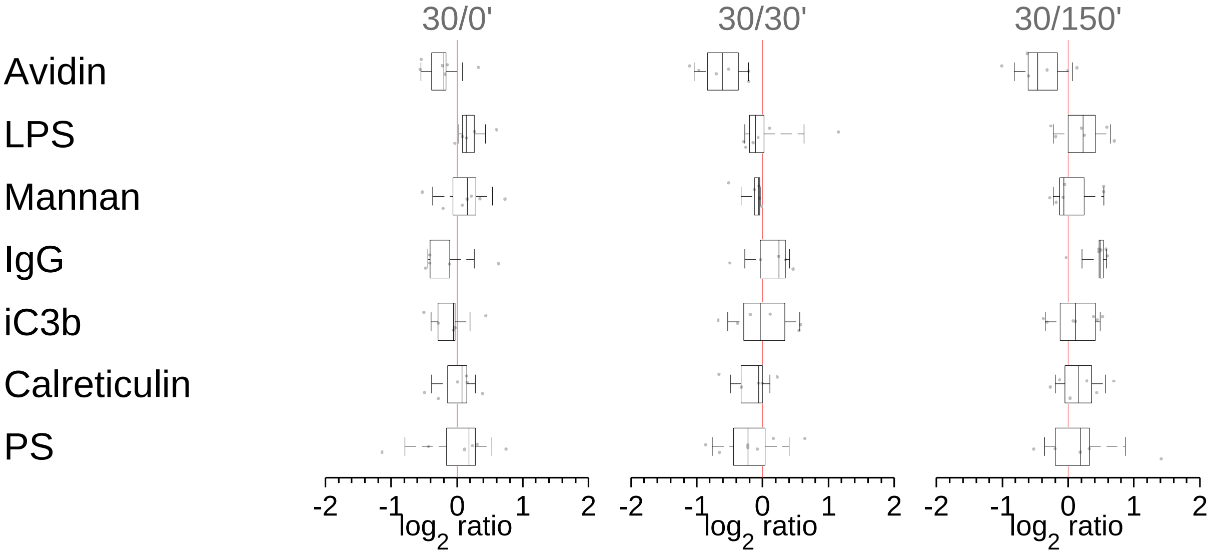

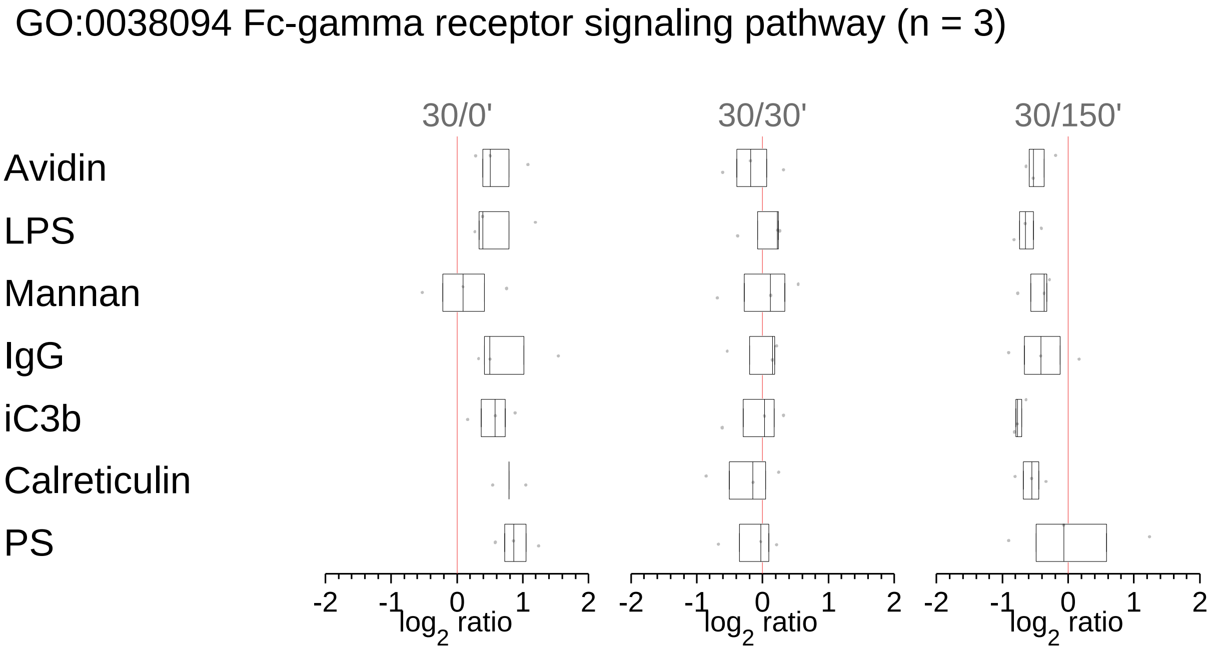

GO:0038094 Fc-gamma receptor signaling pathway (n = 3)
30/0'
30/30'
30/150'
Avidin
LPS
Mannan
IgG
iC3b
Calreticulin
PS
-2
-1
0
1
2
log2 ratio

GO:0040008 regulation of growth (n = 8)

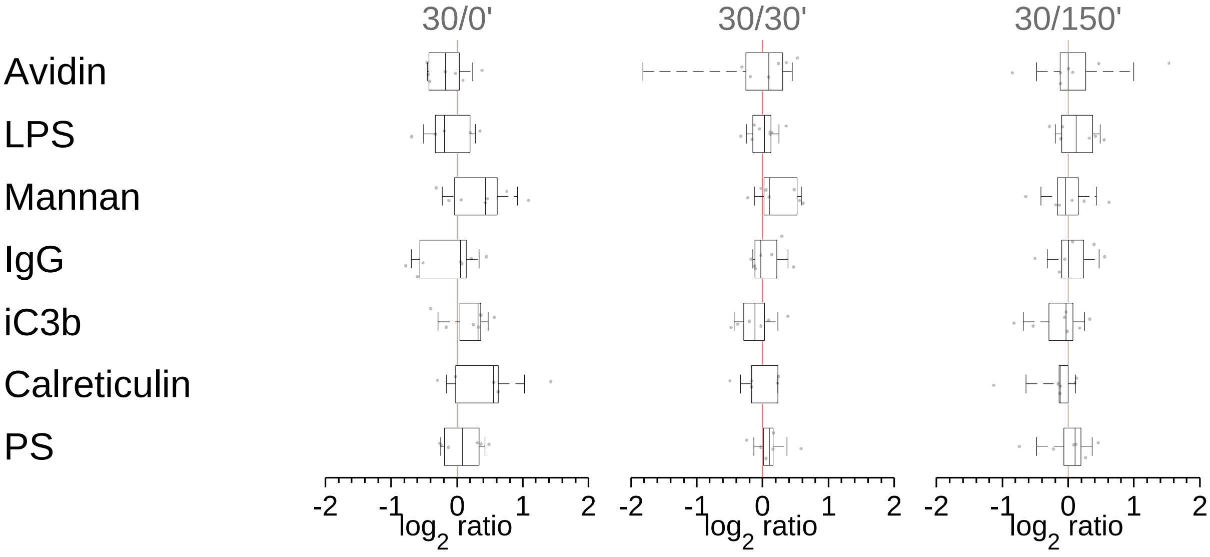

GO:0040014 regulation of multicellular organism growth (n = 6)

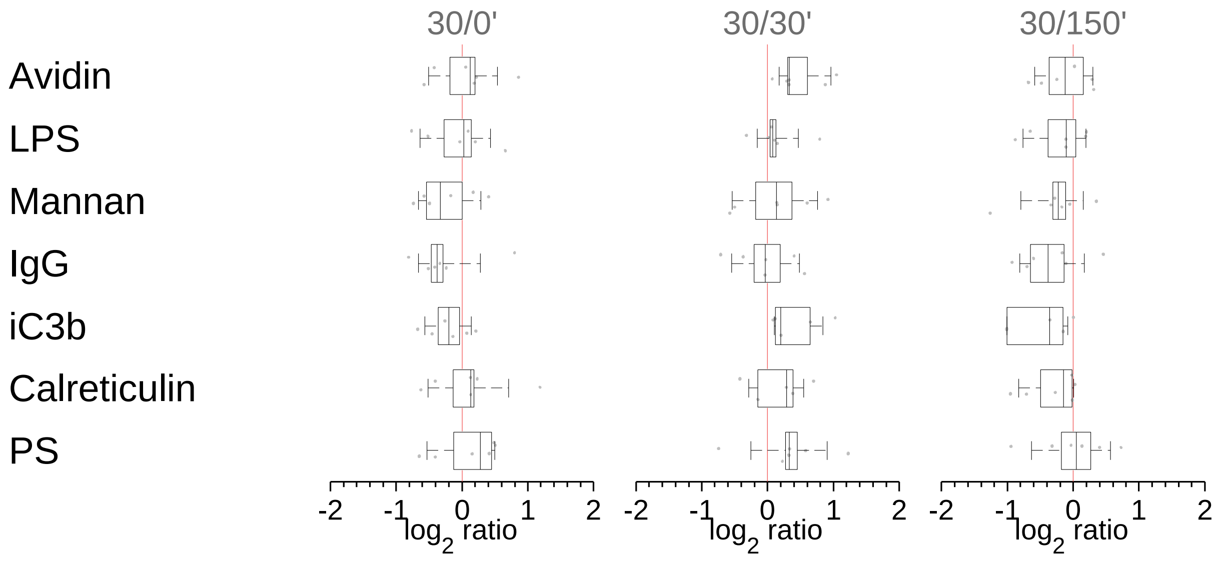

GO:0040018 positive regulation of multicellular organism growth
(n = 3)

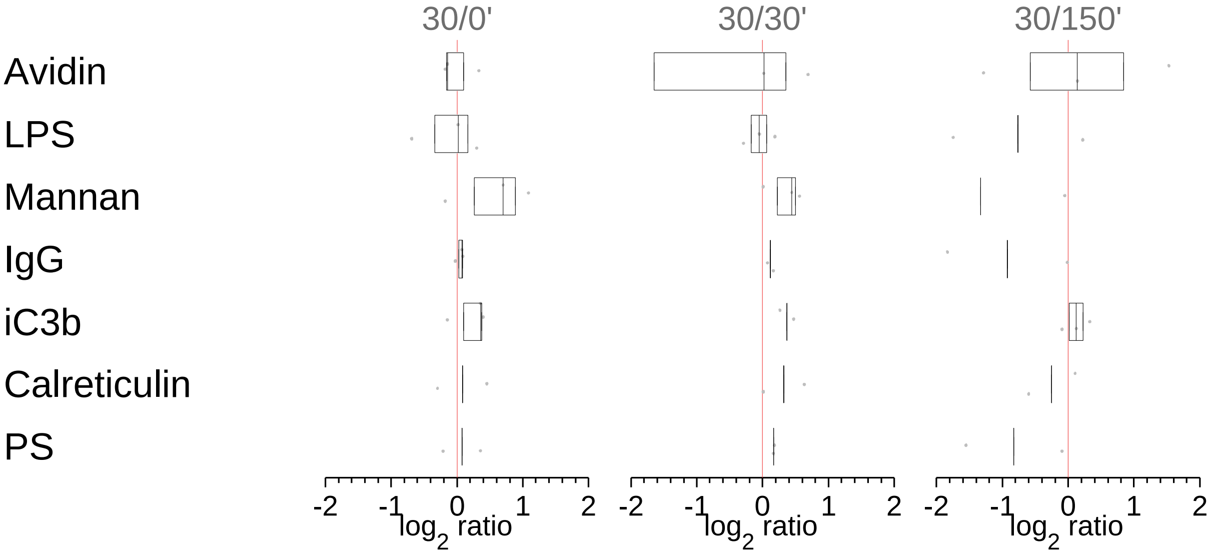

GO:0042026 protein refolding (n = 5)

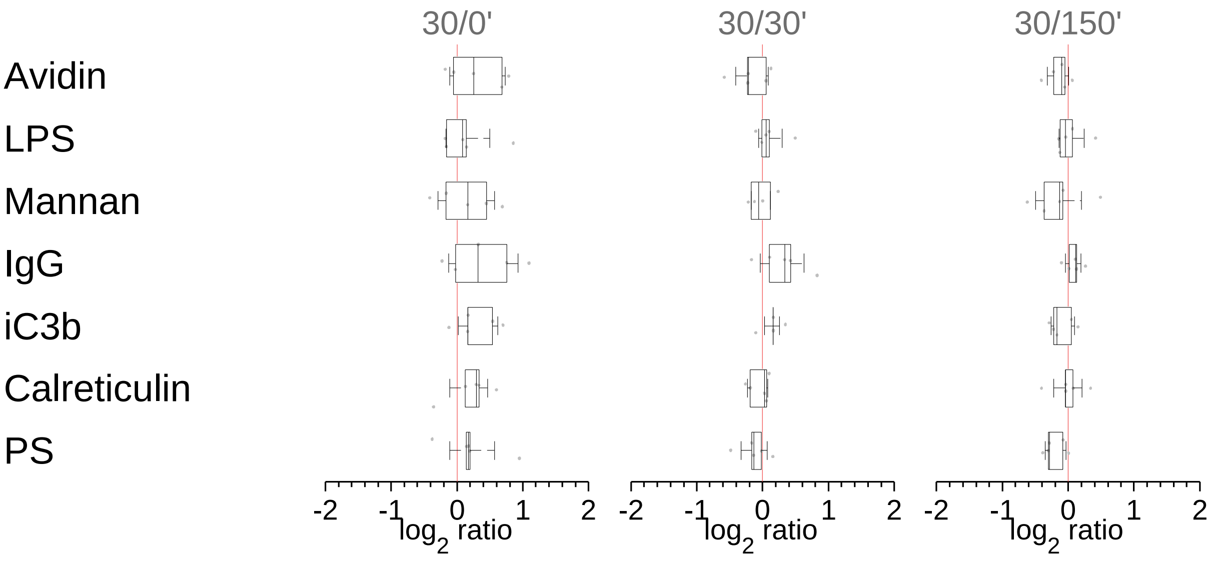

GO:0042060 wound healing (n = 8)

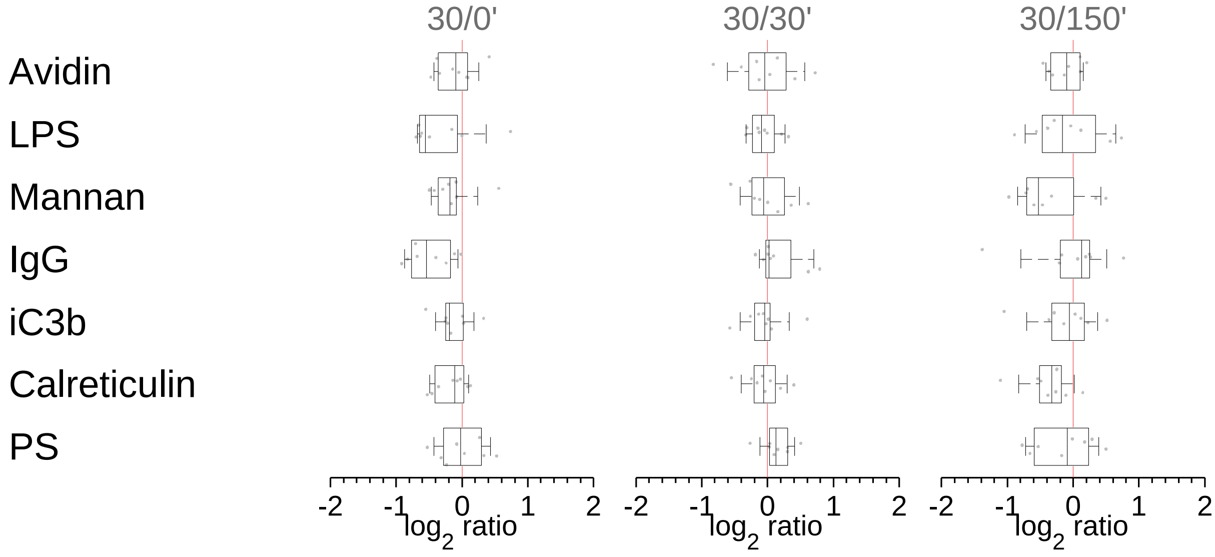

GO:0042098 T cell proliferation (n = 8)

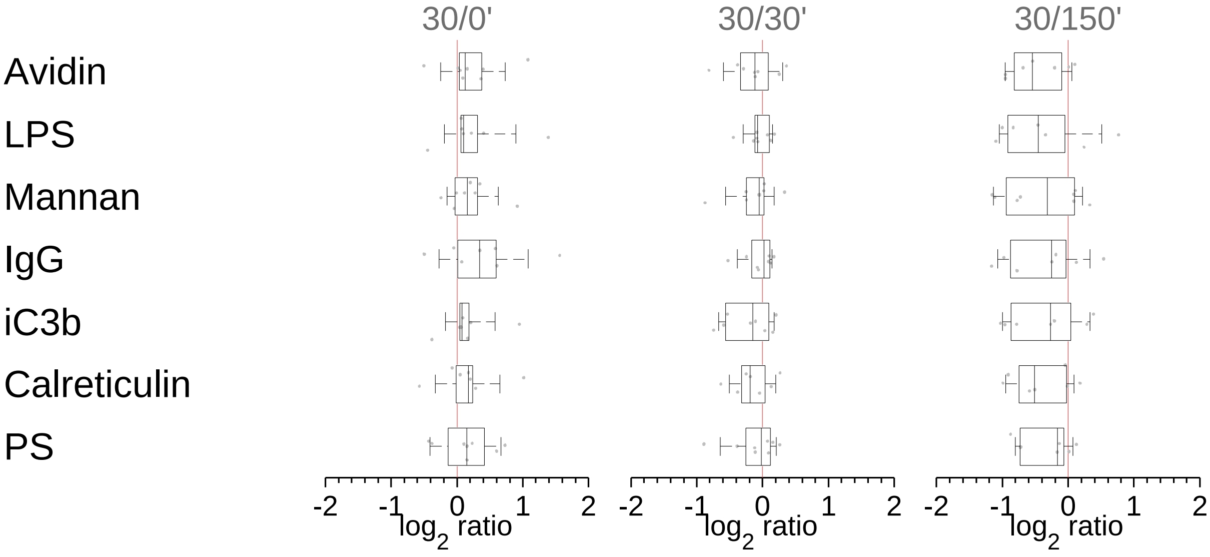

GO:0042100 B cell proliferation (n = 3)

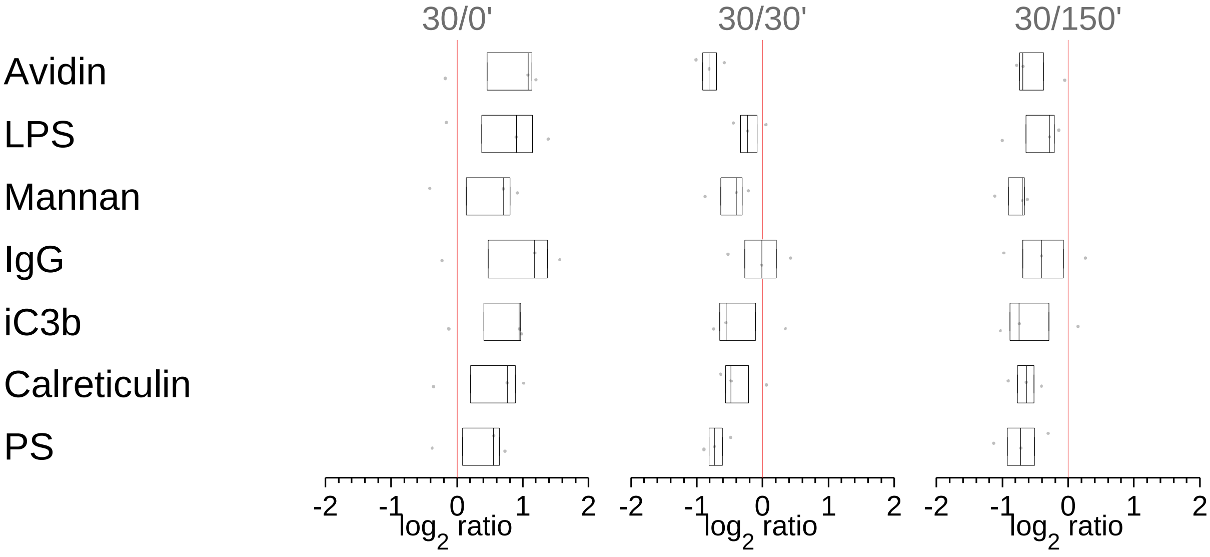

GO:0042102 positive regulation of T cell proliferation (n = 7)

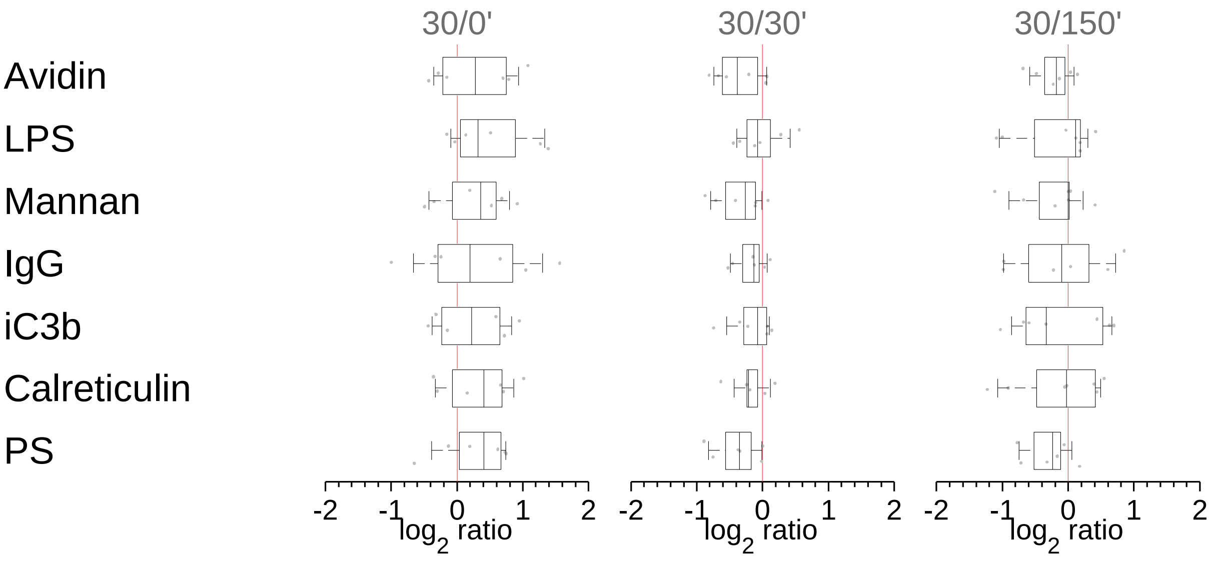

GO:0042110 T cell activation (n = 6)

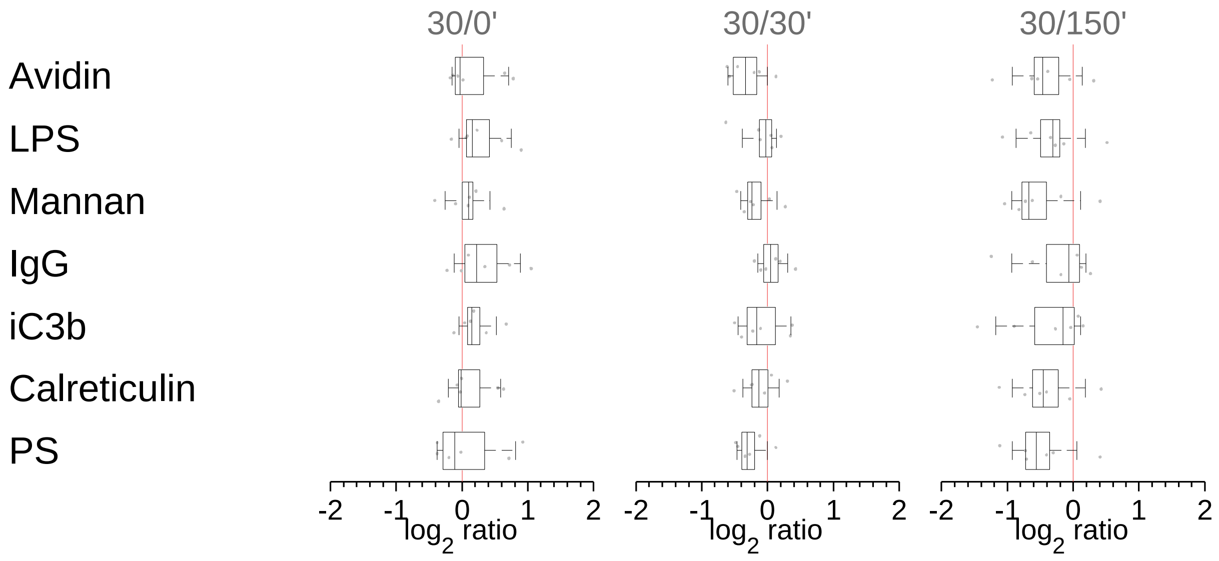

GO:0042113 B cell activation (n = 5)

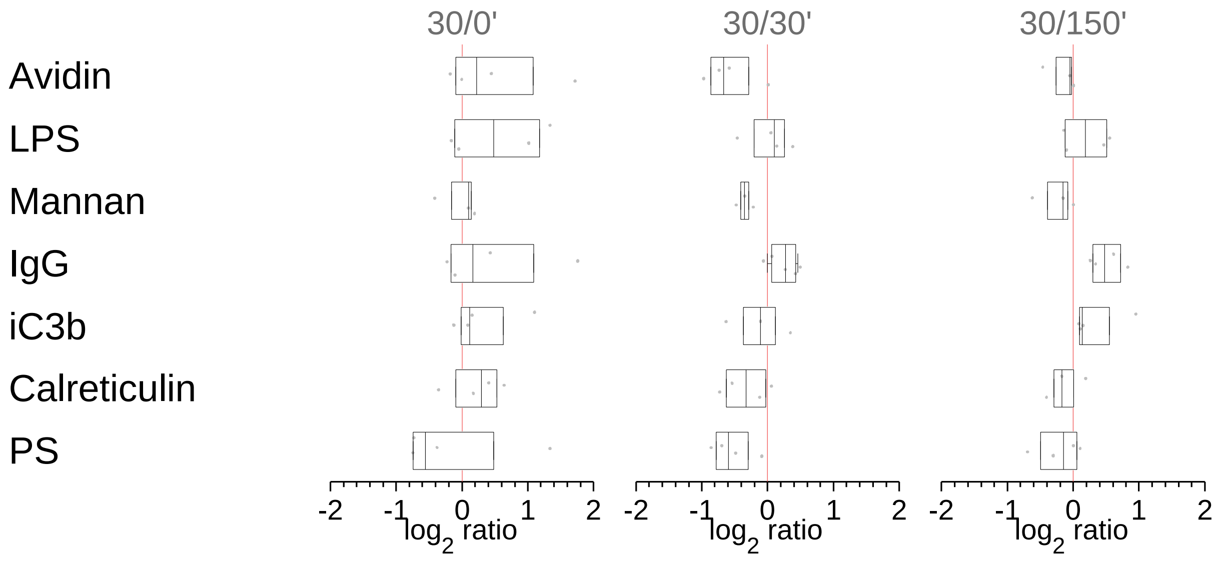

GO:0042127 regulation of cell proliferation (n = 14)

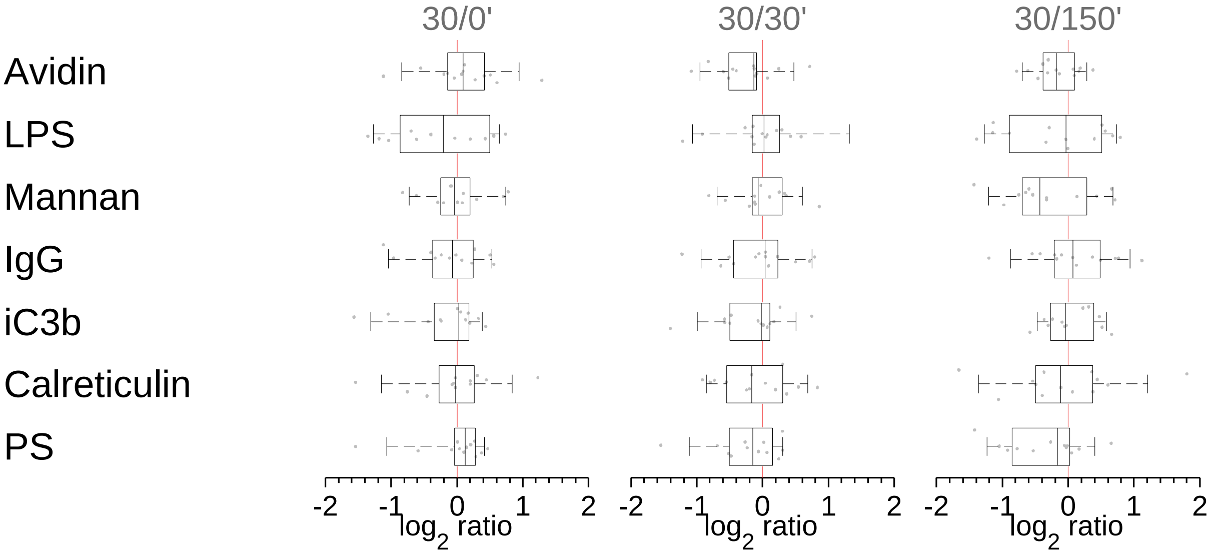

GO:0042147 retrograde transport, endosome to Golgi (n = 14)

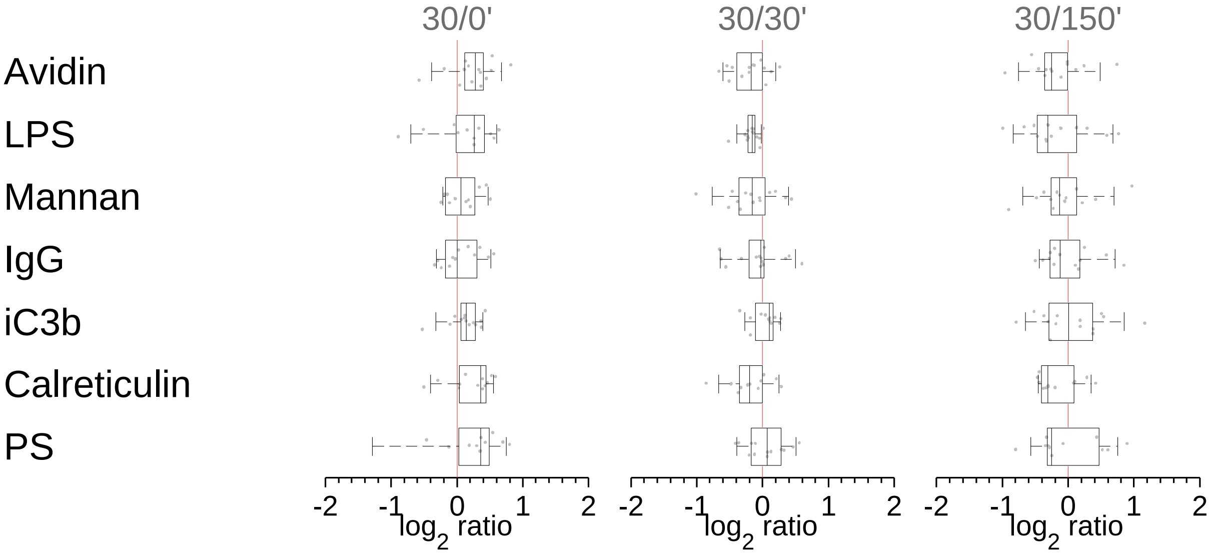

GO:0042157 lipoprotein metabolic process (n = 3)

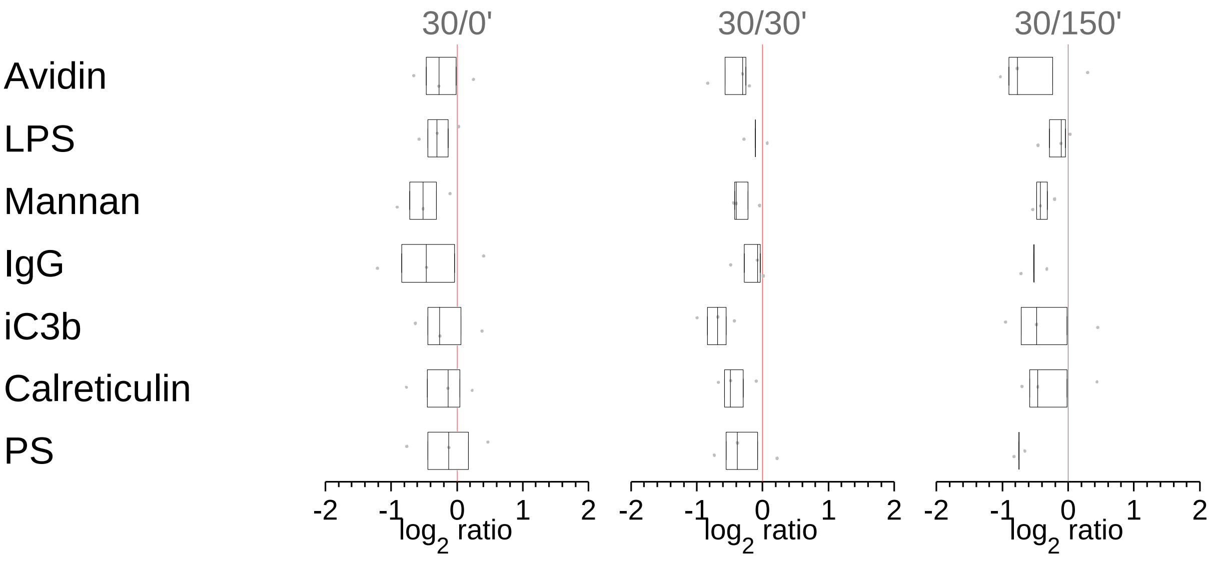

GO:0042159 lipoprotein catabolic process (n = 3)

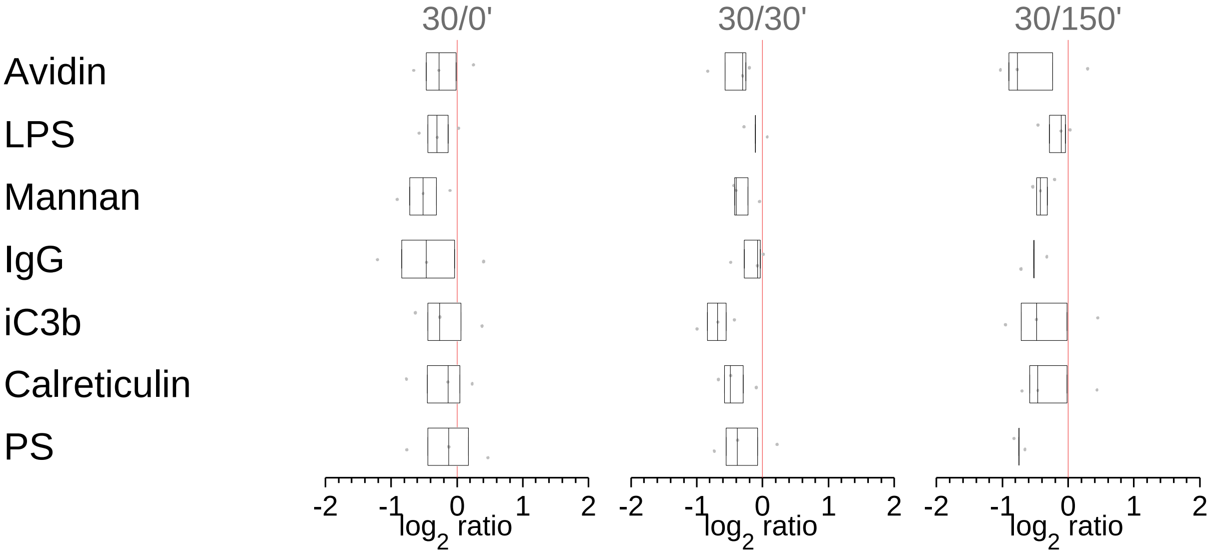

GO:0042169 SH2 domain binding (n = 7)

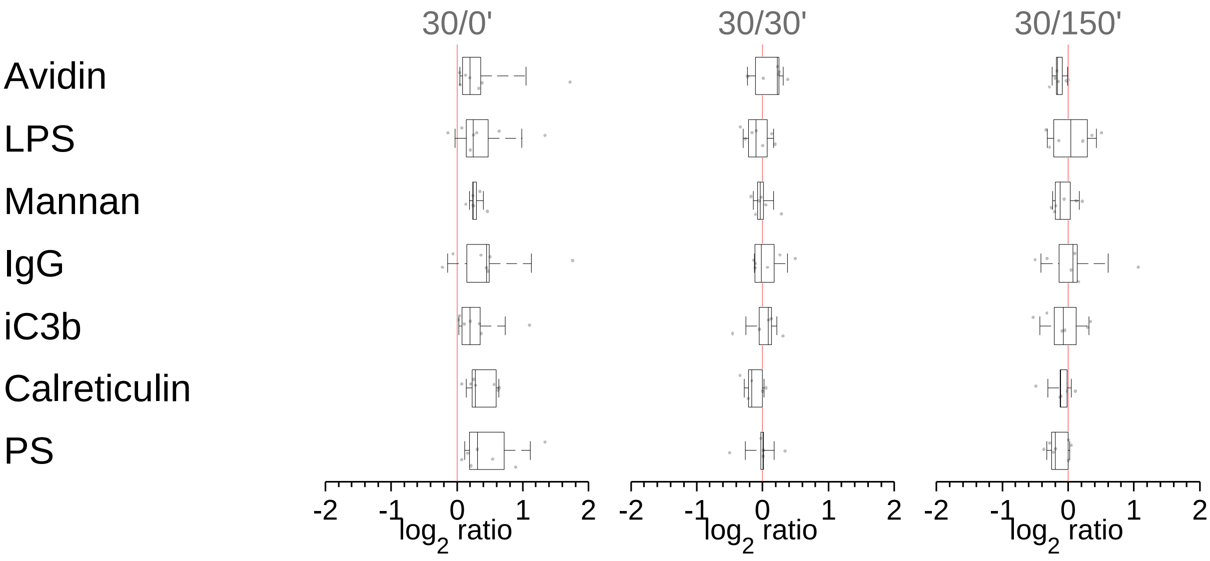

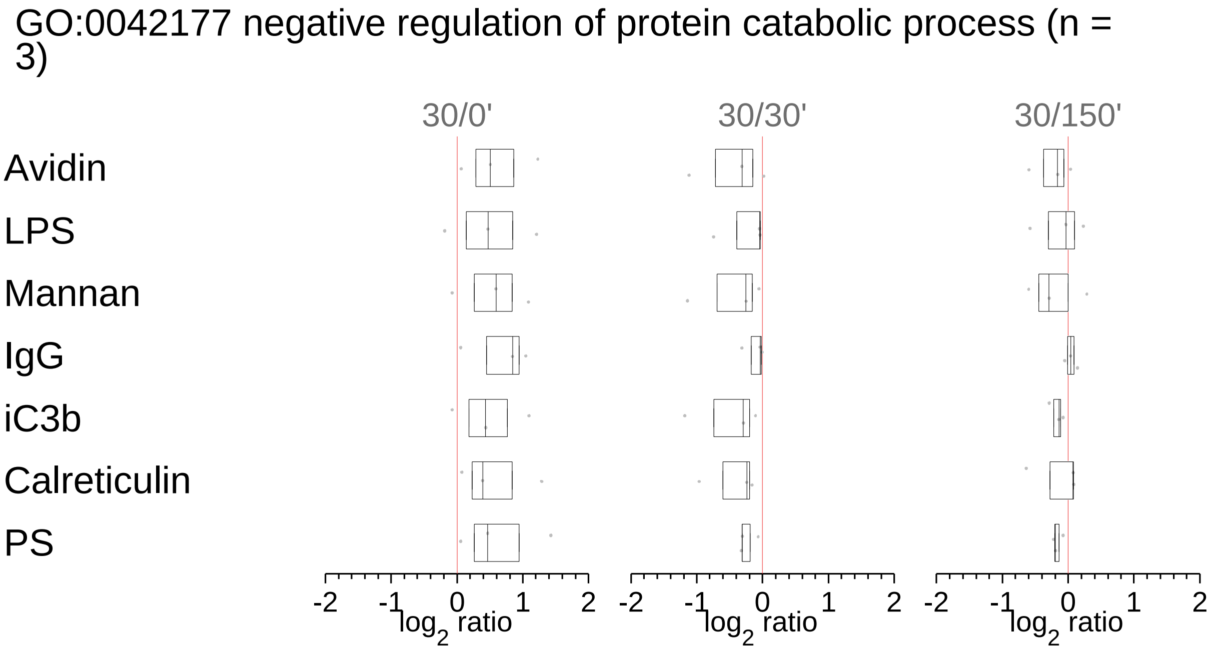
GO:0042177 negative regulation of protein catabolic process (n = 3)
30/0'
30/30'
30/150'
Avidin
LPS
Mannan
IgG
iC3b
Calreticulin
PS
-2 -1 0 1 2
log2 ratio

GO:0042254 ribosome biogenesis (n = 4)

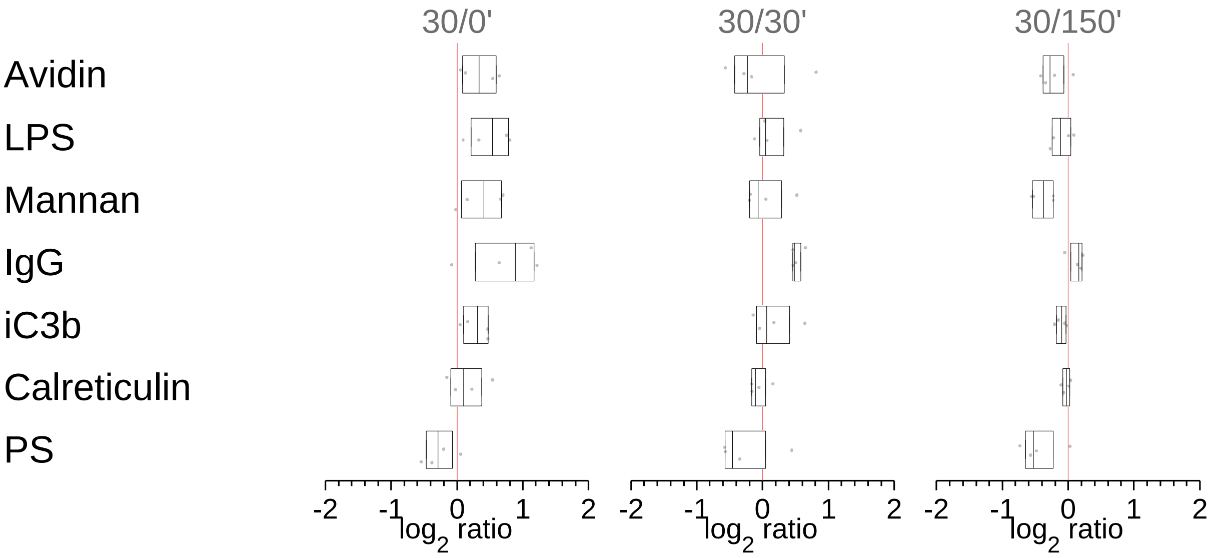

GO:0042267 natural killer cell mediated cytotoxicity (n = 3)

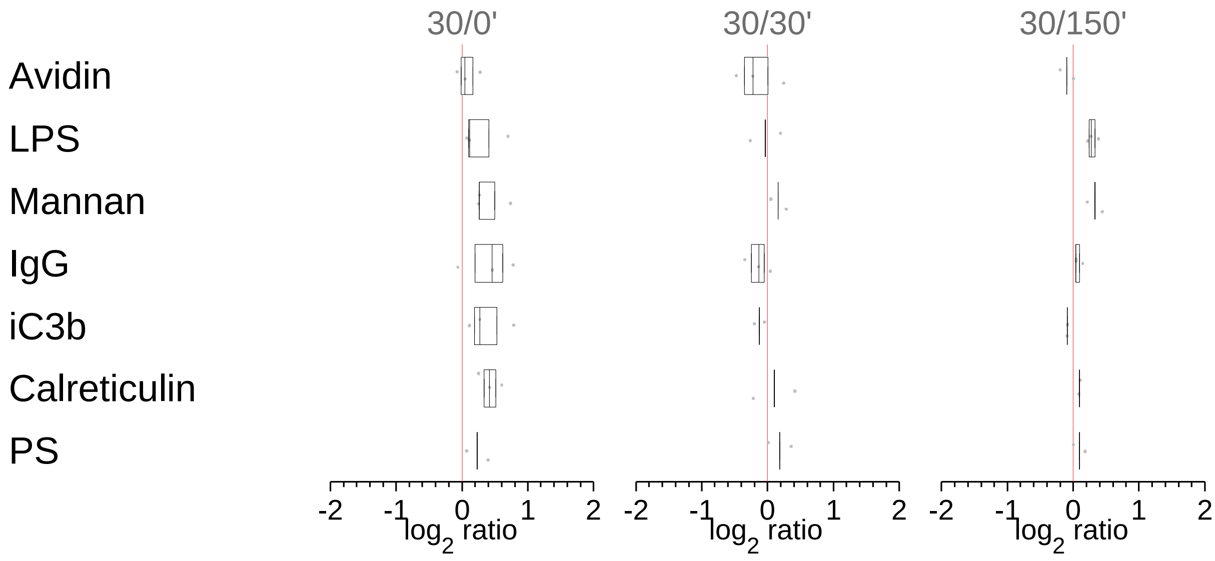

GO:0042273 ribosomal large subunit biogenesis (n = 5)

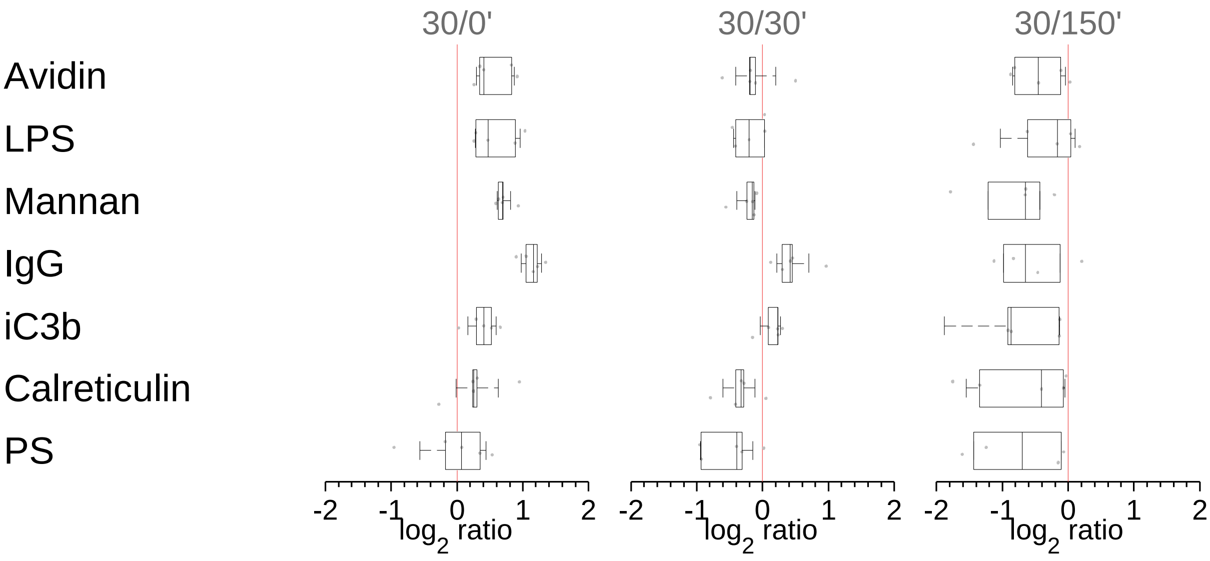

GO:0042274 ribosomal small subunit biogenesis (n = 10)

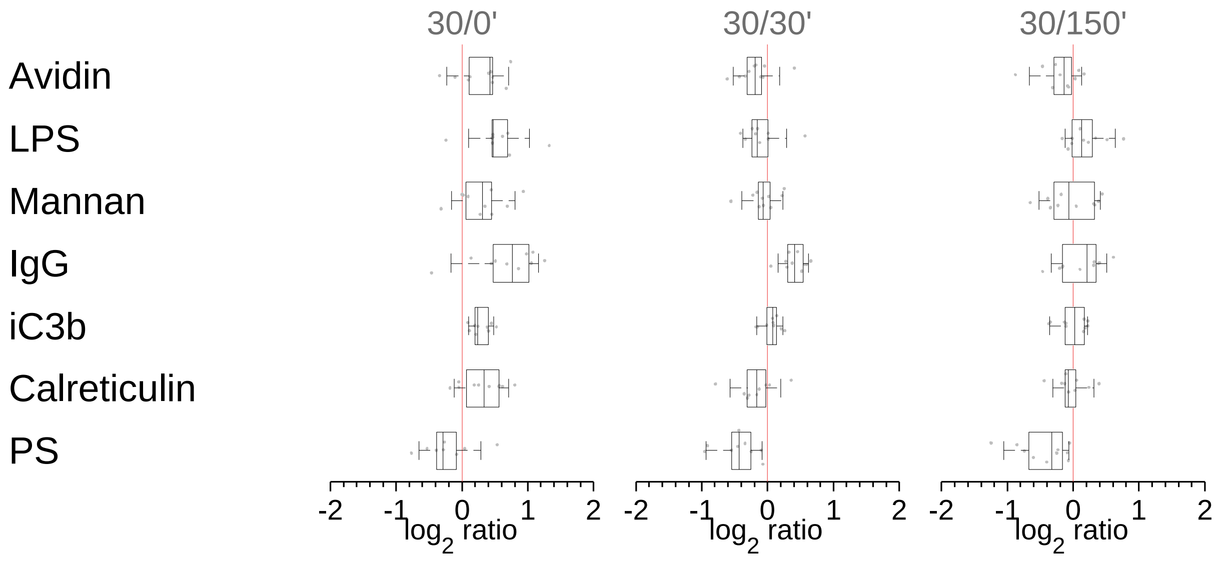

GO:0042277 peptide binding (n = 16)

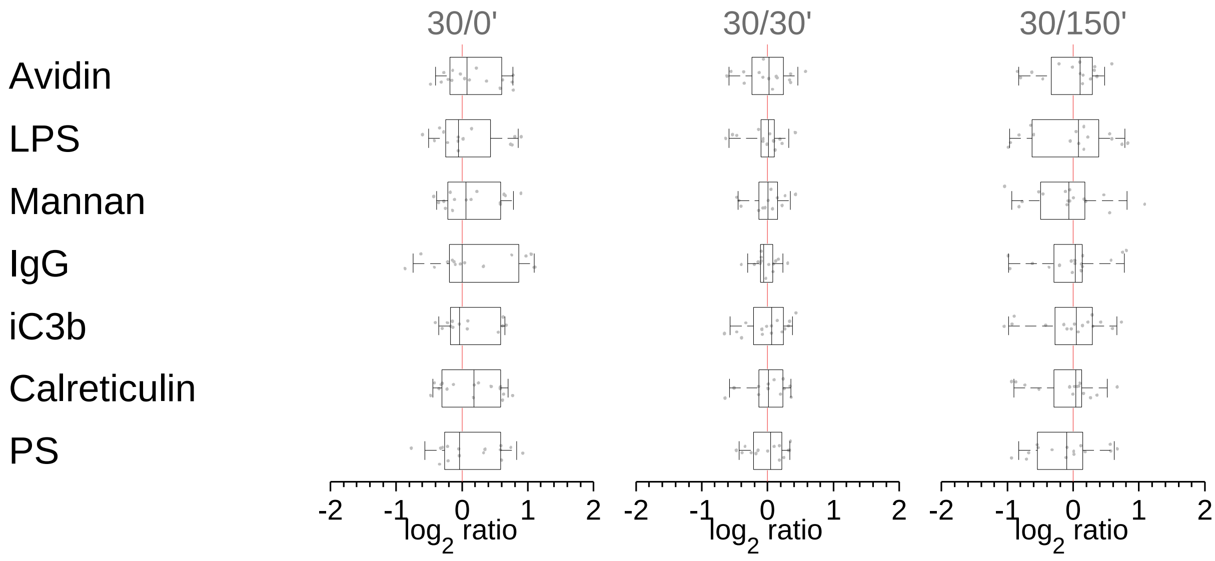

GO:0042281 dolichyl pyrophosphate Man9GlcNAc2 alpha-1,3-glucosyltransferase activity (n = 3)

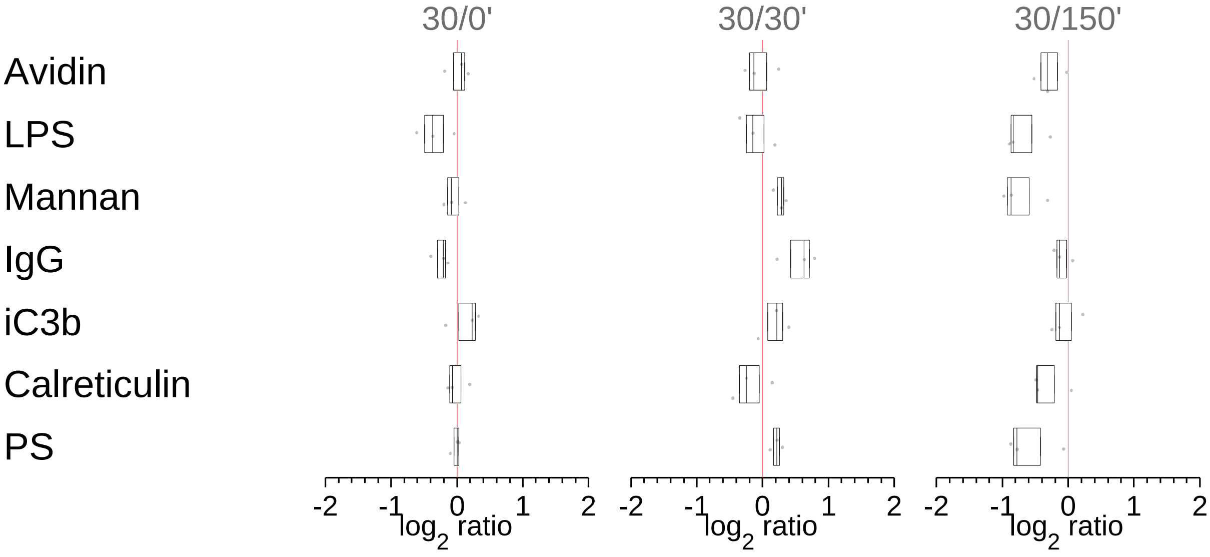

GO:0042283 dolichyl pyrophosphate Glc1Man9GlcNAc2 alpha-1,3-glucosyltransferase activity (n = 3)

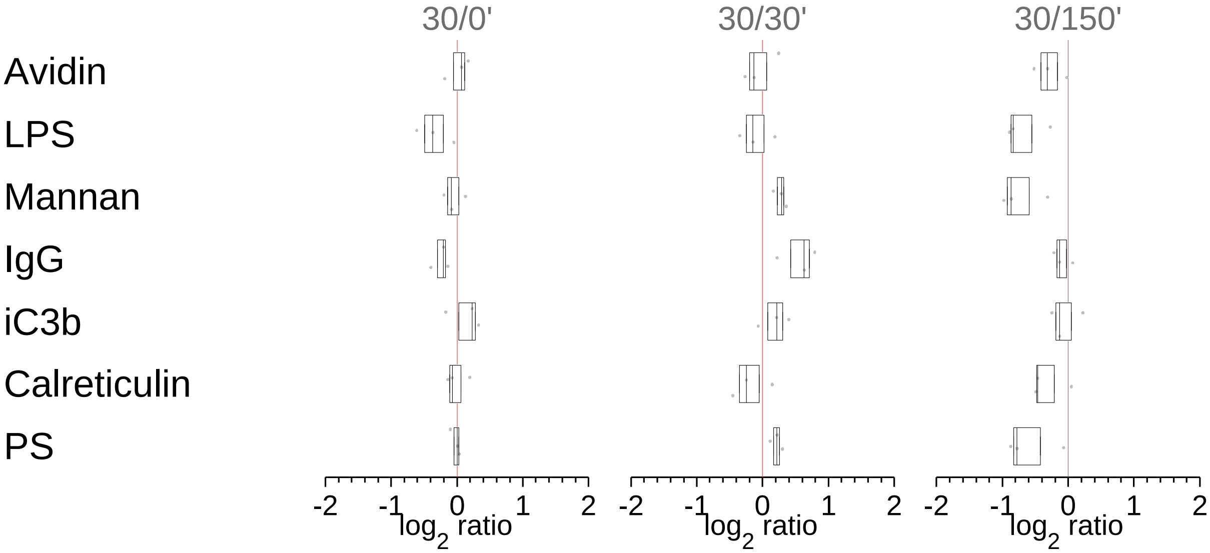

GO:0042288 MHC class I protein binding (n = 7)

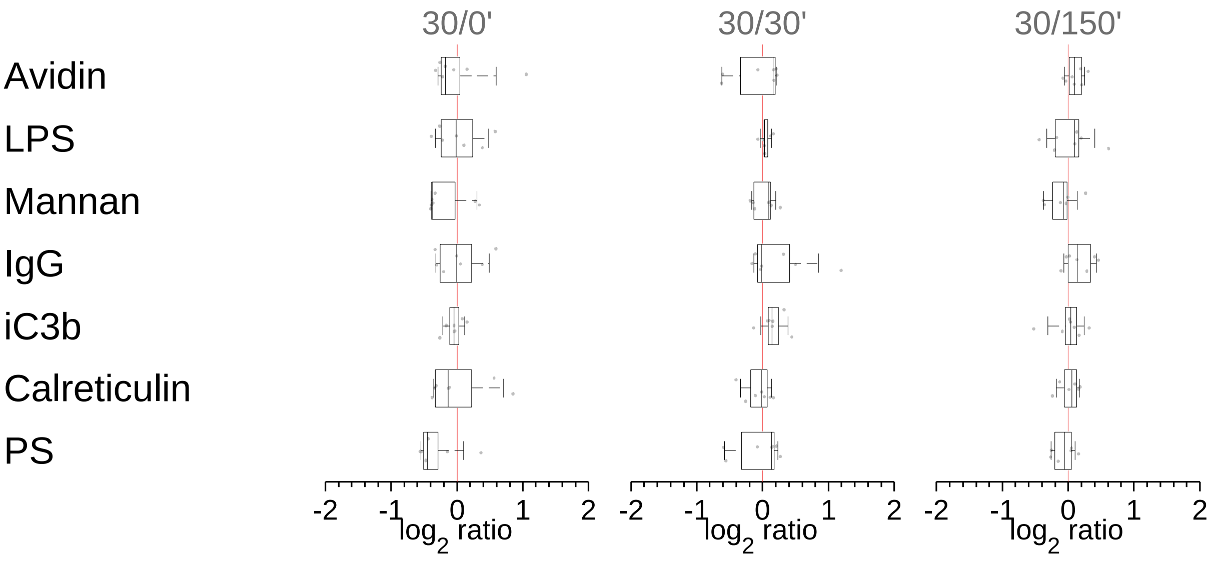

GO:0042301 phosphate ion binding (n = 3)

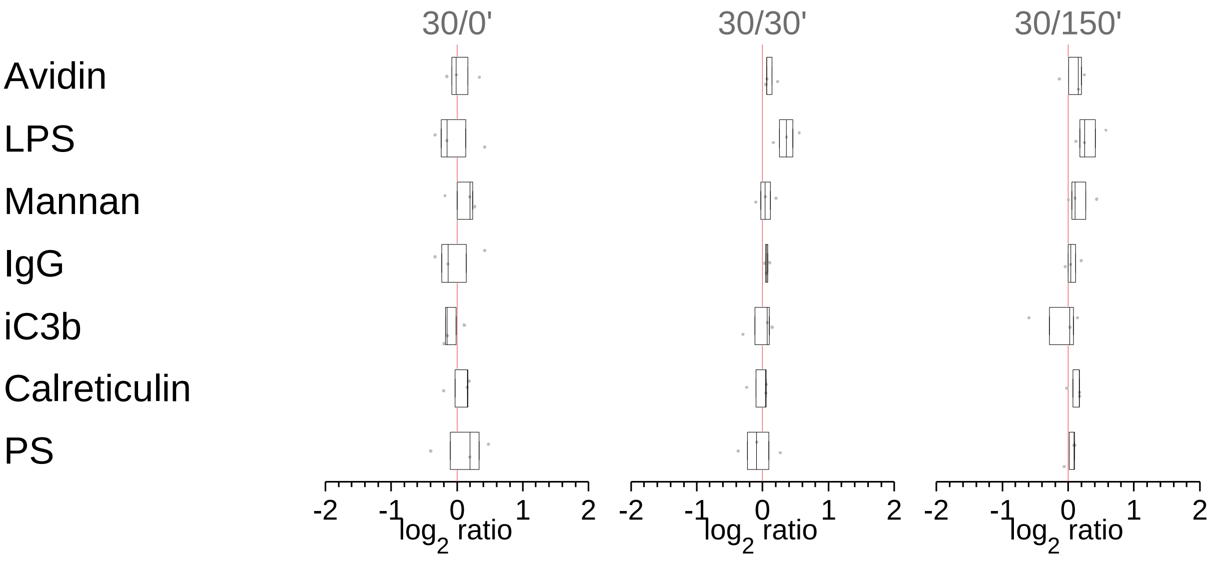

GO:0042311 vasodilation (n = 5)

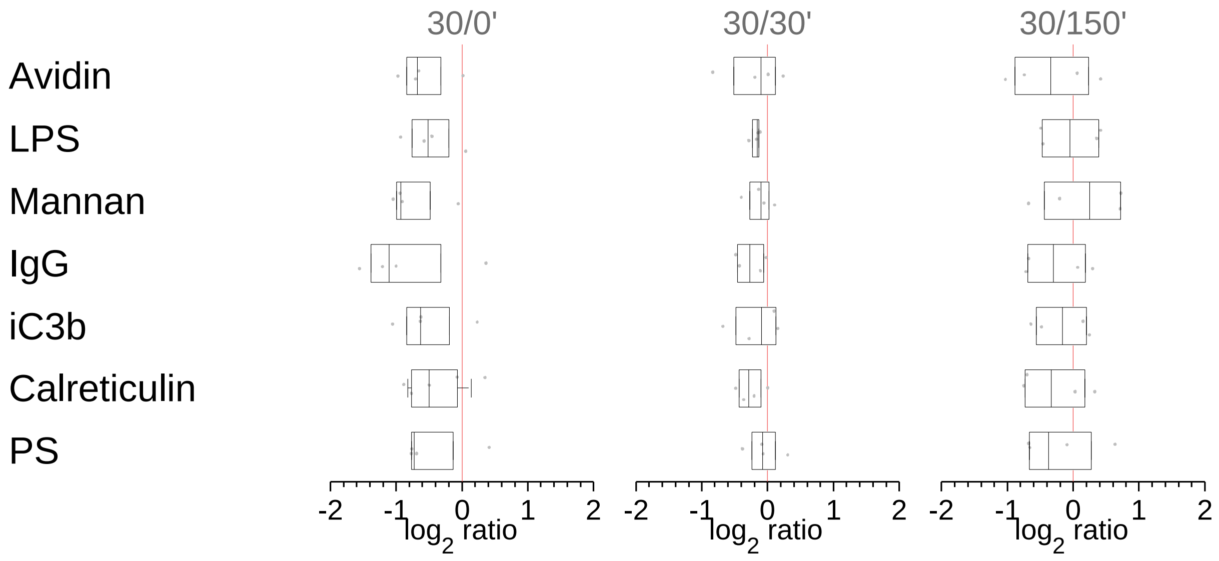

GO:0042327 positive regulation of phosphorylation (n = 7)

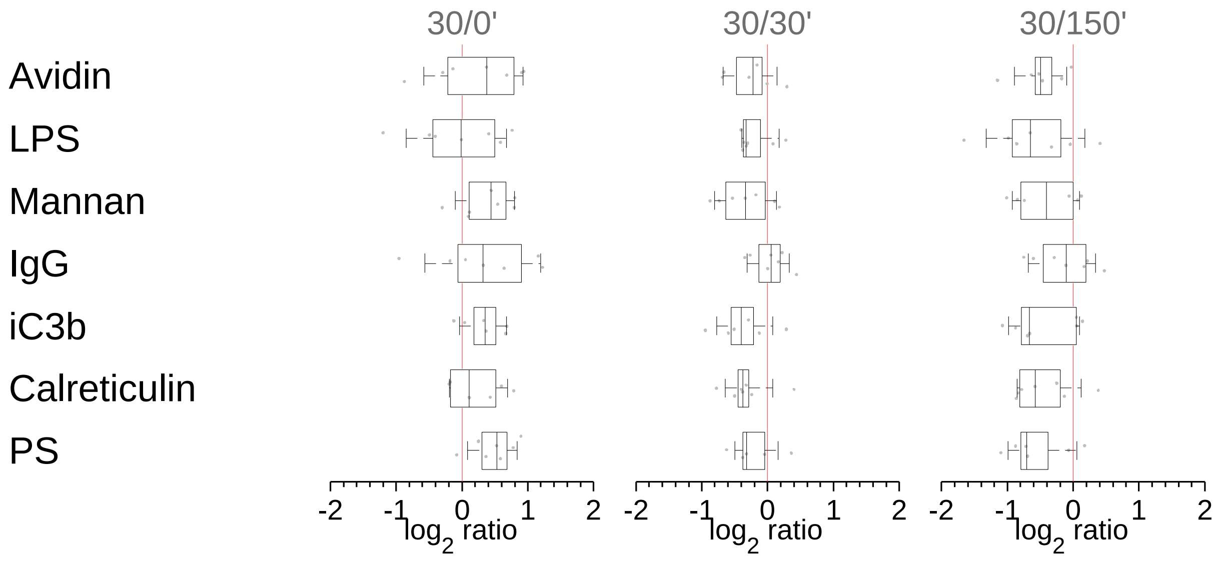

GO:0042346 positive regulation of NF-kappaB import into nucleus
(n = 5)

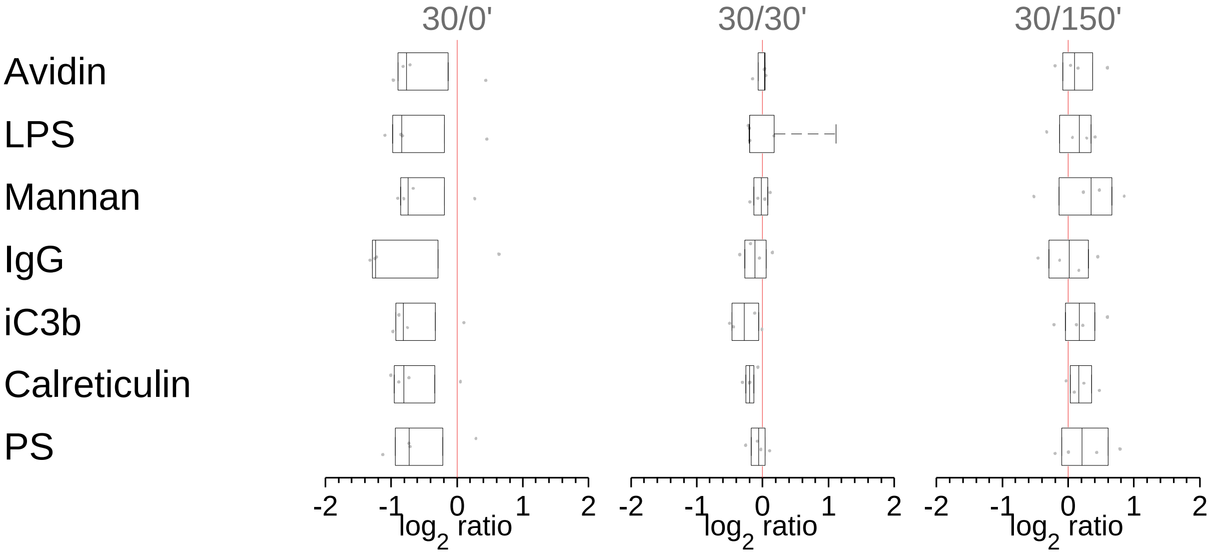

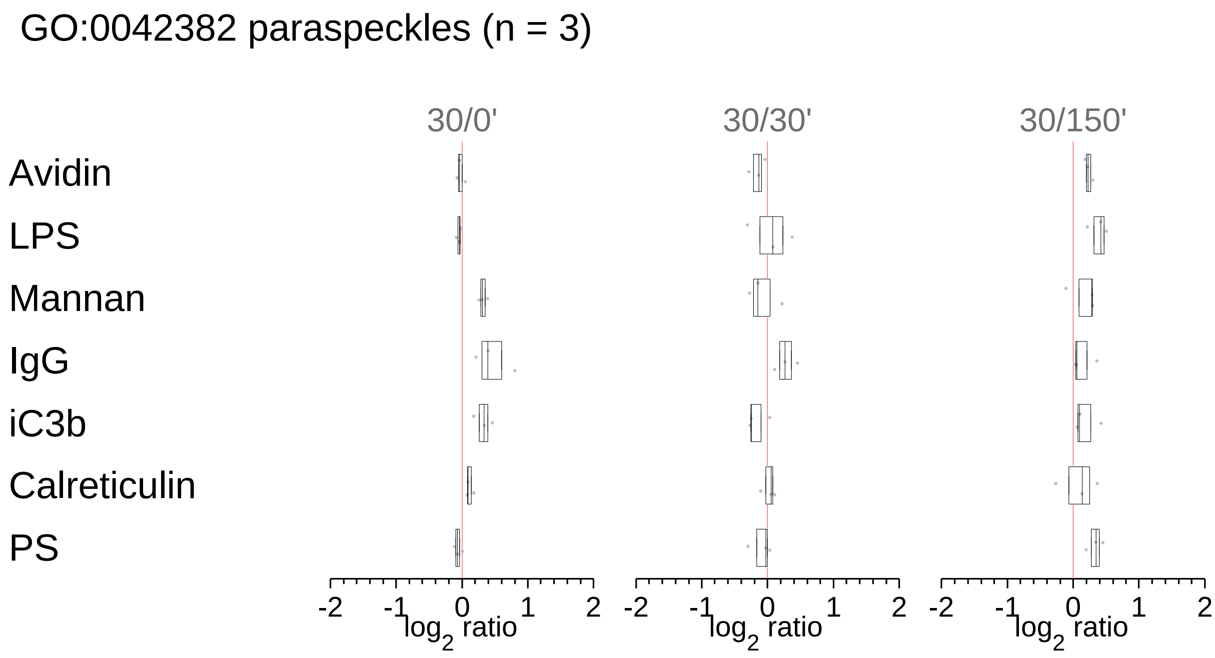
GO:0042382 paraspeckles (n = 3)
30/0'
30/30'
30/150'
Avidin
LPS
Mannan
IgG
iC3b
Calreticulin
PS
-2
-1
0
1
2
log2 ratio

GO:0042383 sarcolemma (n = 16)

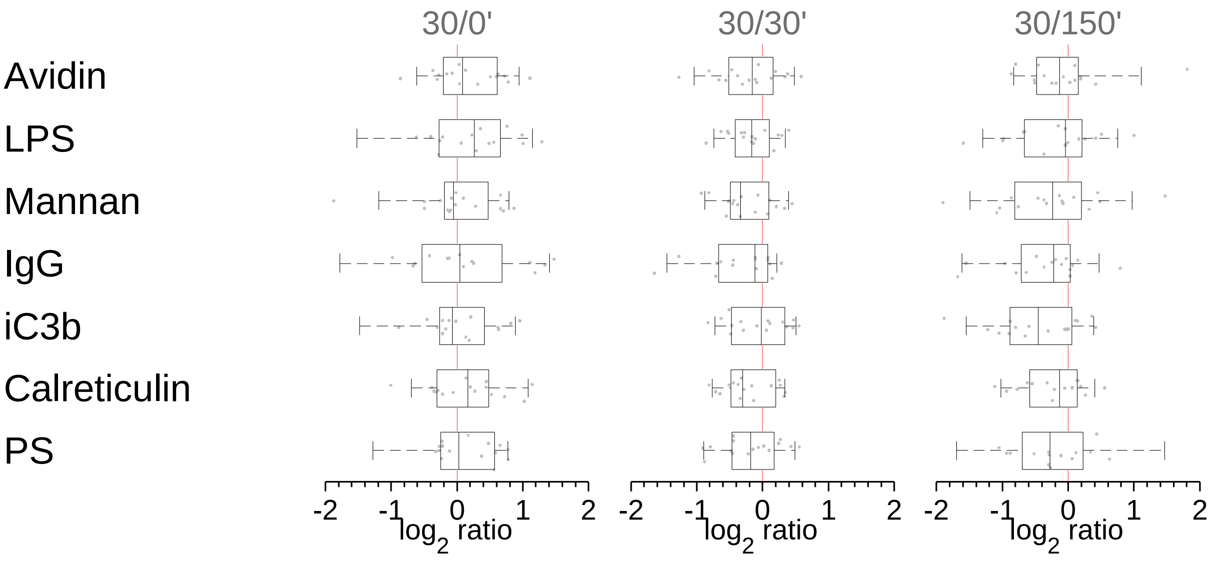

GO:0042384 cilium assembly (n = 5)

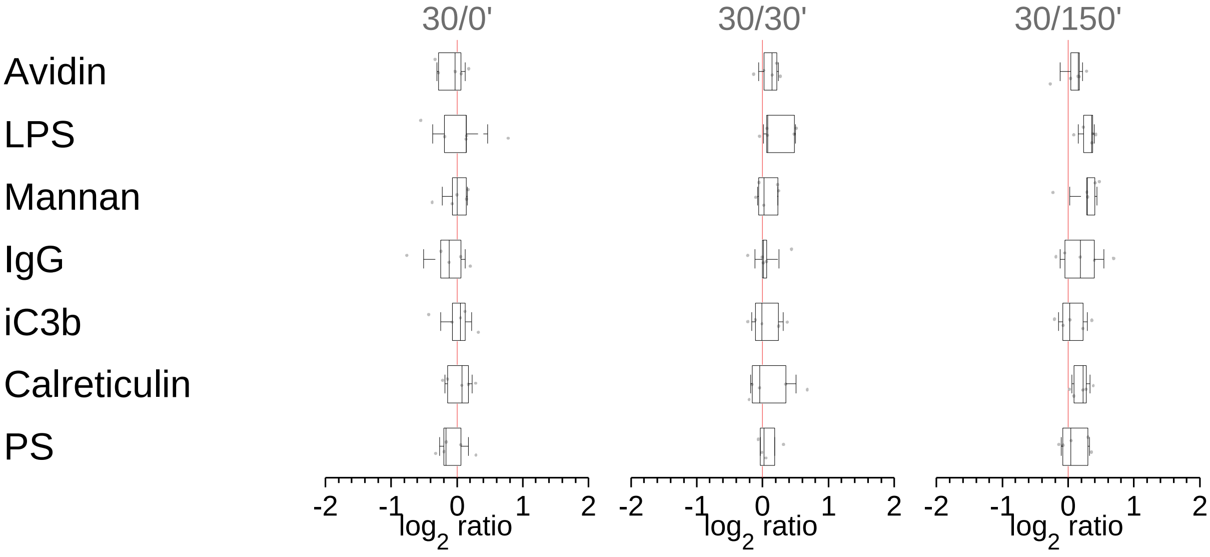

GO:0042391 regulation of membrane potential (n = 3)

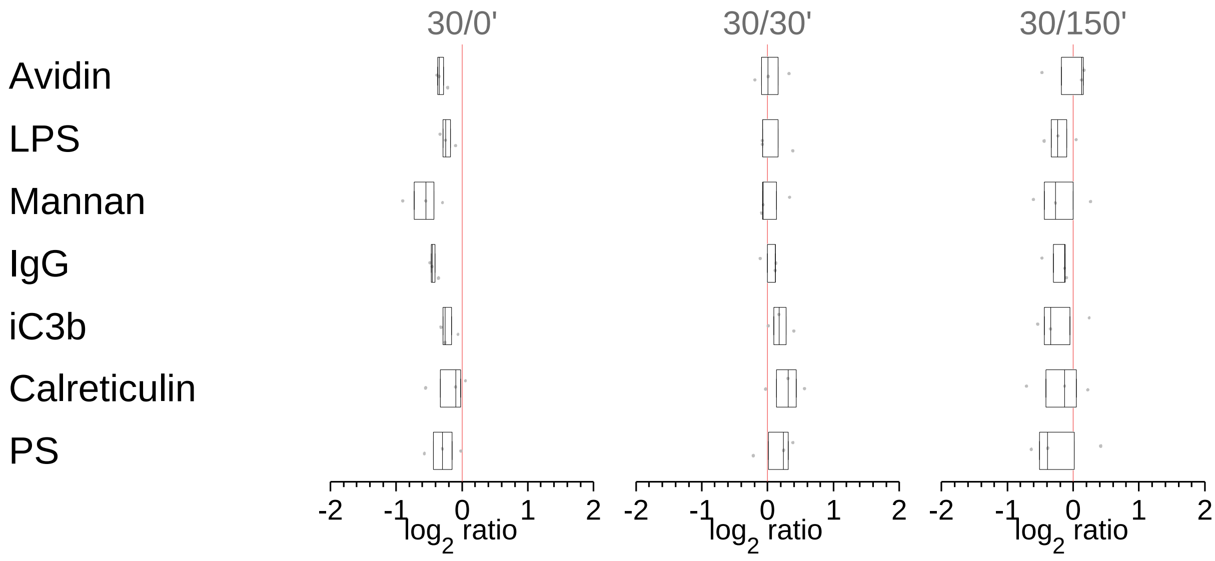

GO:0042393 histone binding (n = 10)

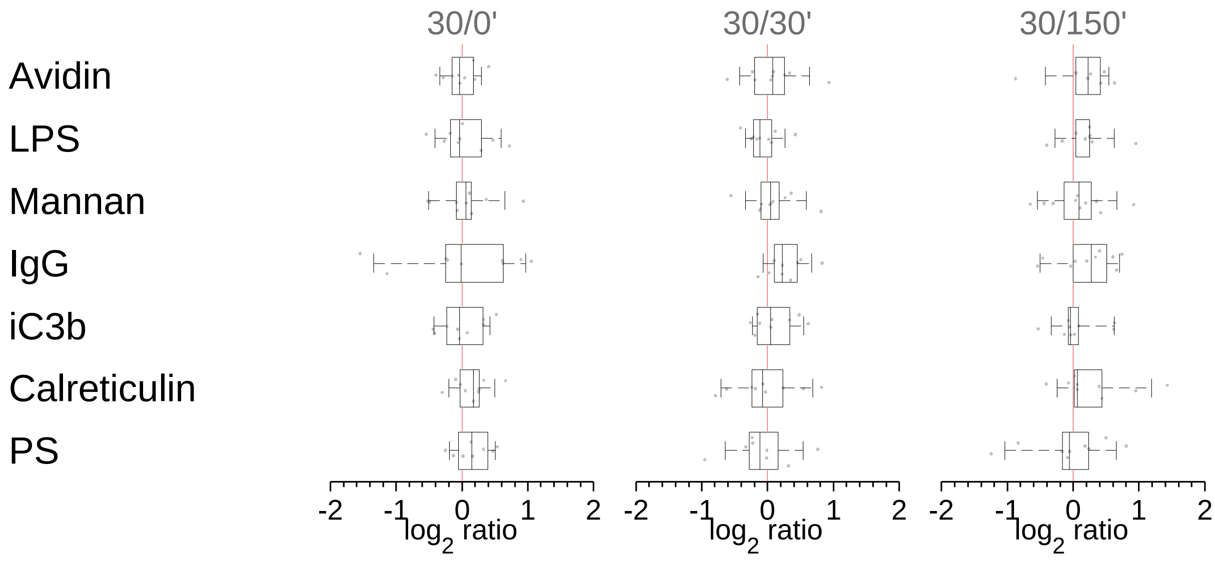

GO:0042470 melanosome (n = 6)

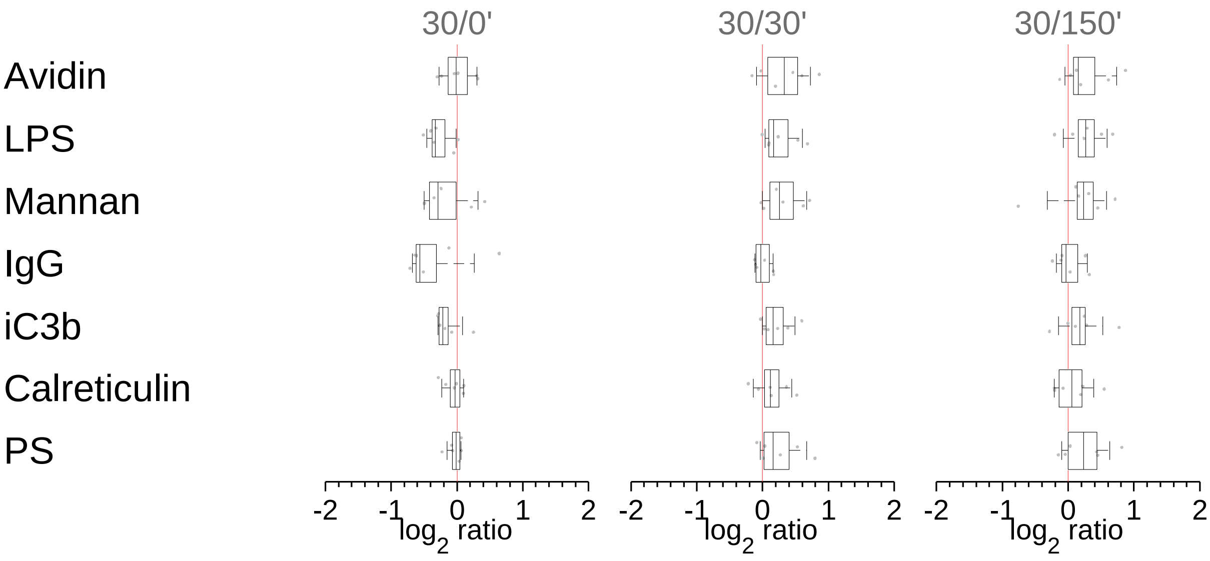

GO:0042475 odontogenesis of dentin-containing tooth (n = 3)

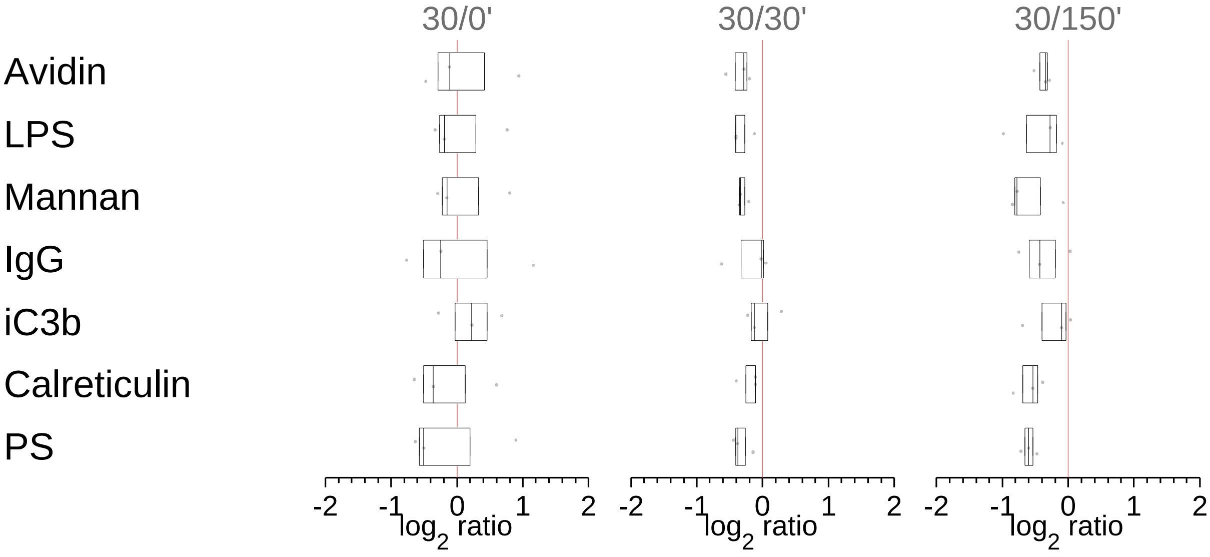

GO:0042493 response to drug (n = 21)

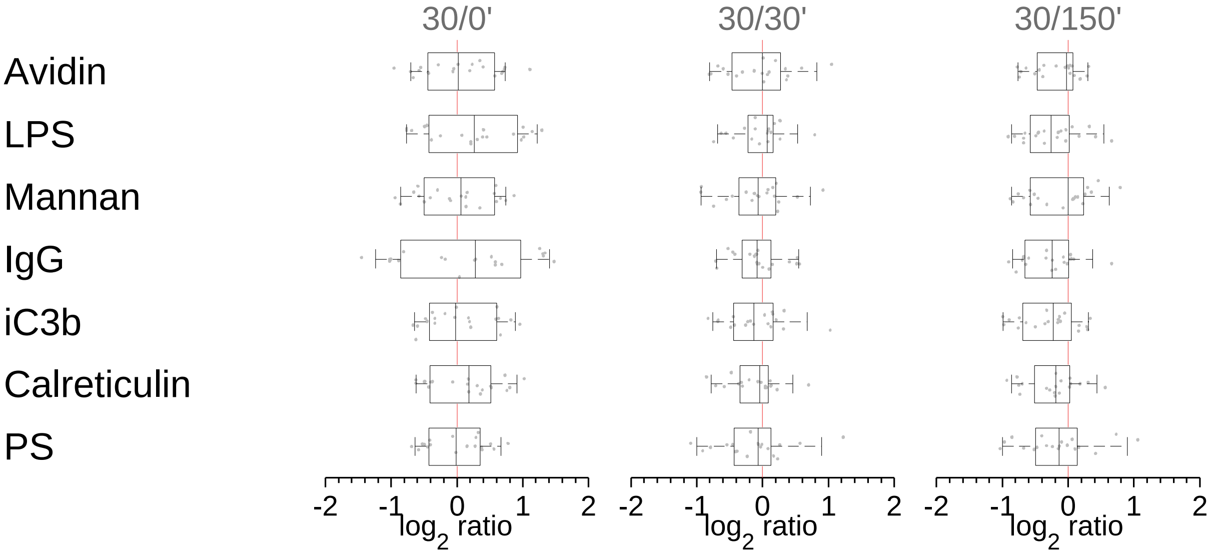

GO:0042500 aspartic endopeptidase activity, intramembrane cleaving (n = 5)

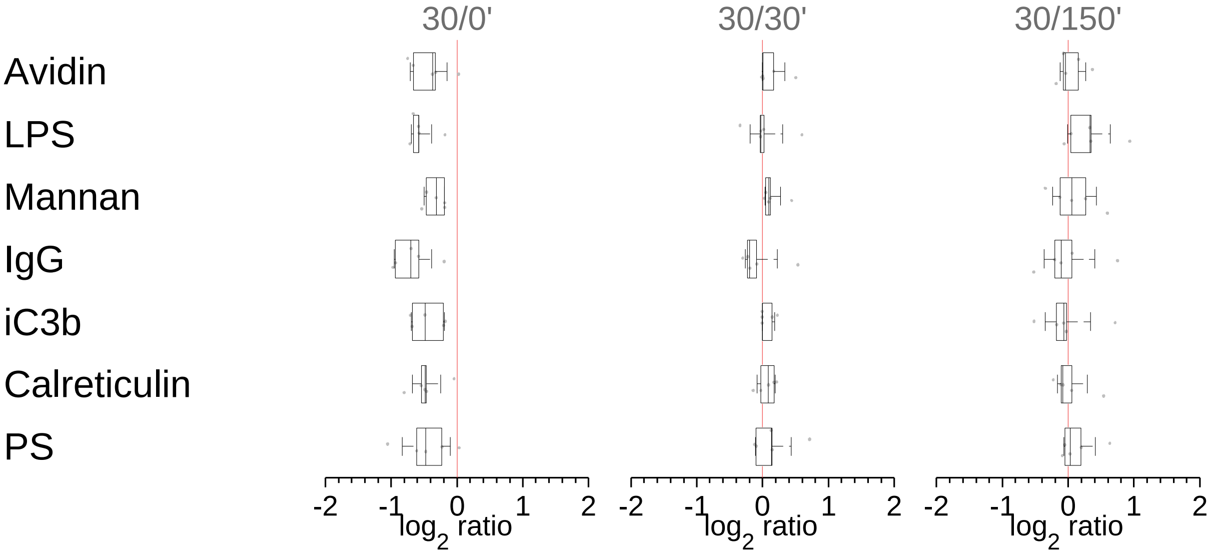

GO:0042535 positive regulation of tumor necrosis factor biosynthetic process (n = 3)

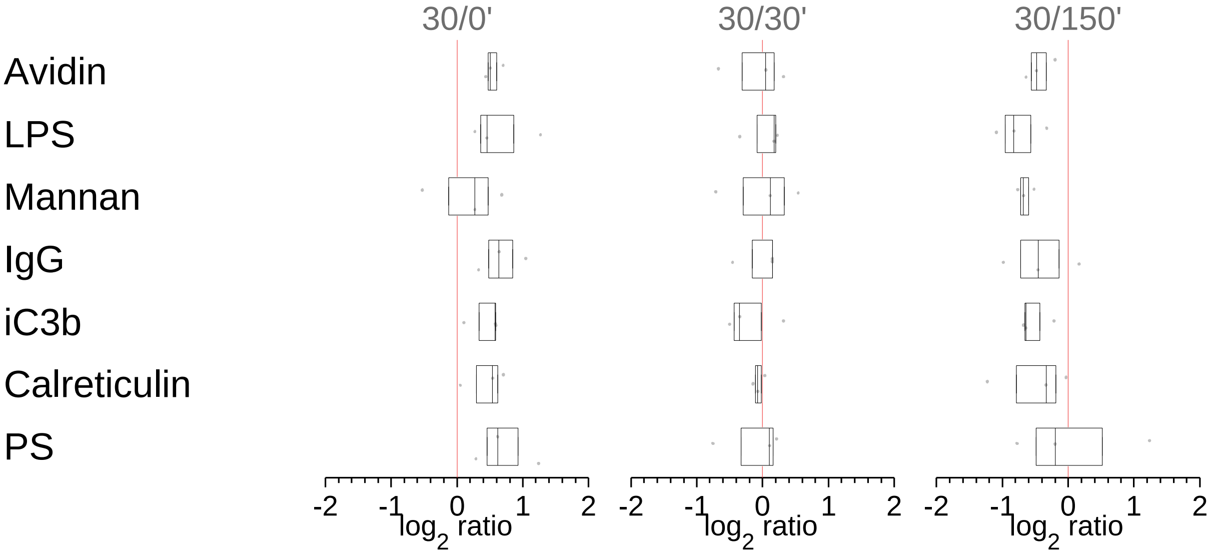

GO:0042542 response to hydrogen peroxide (n = 8)

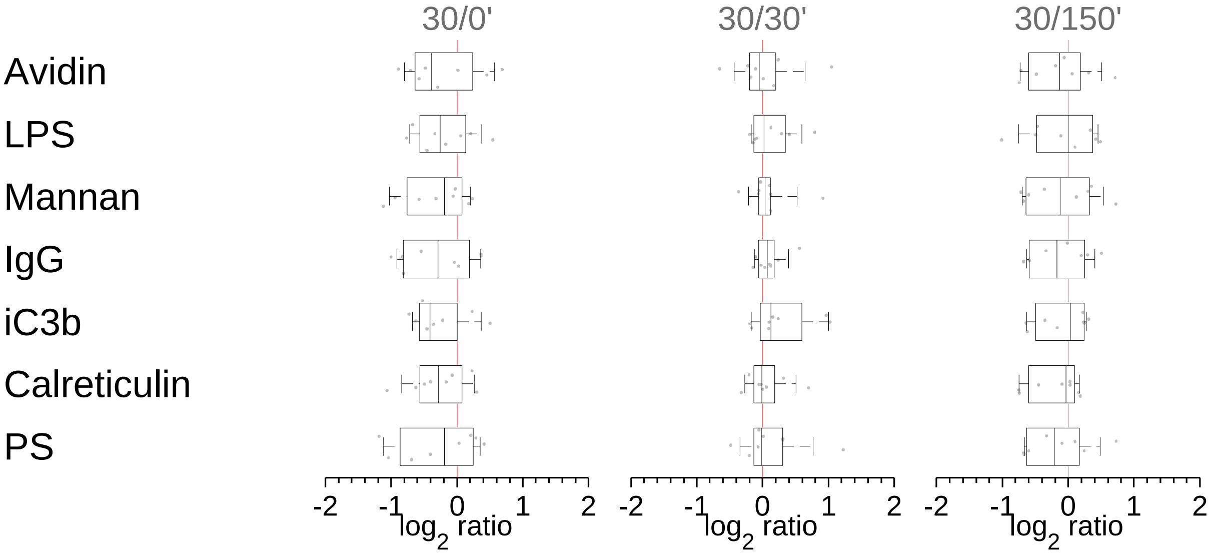

GO:0042552 myelination (n = 7)

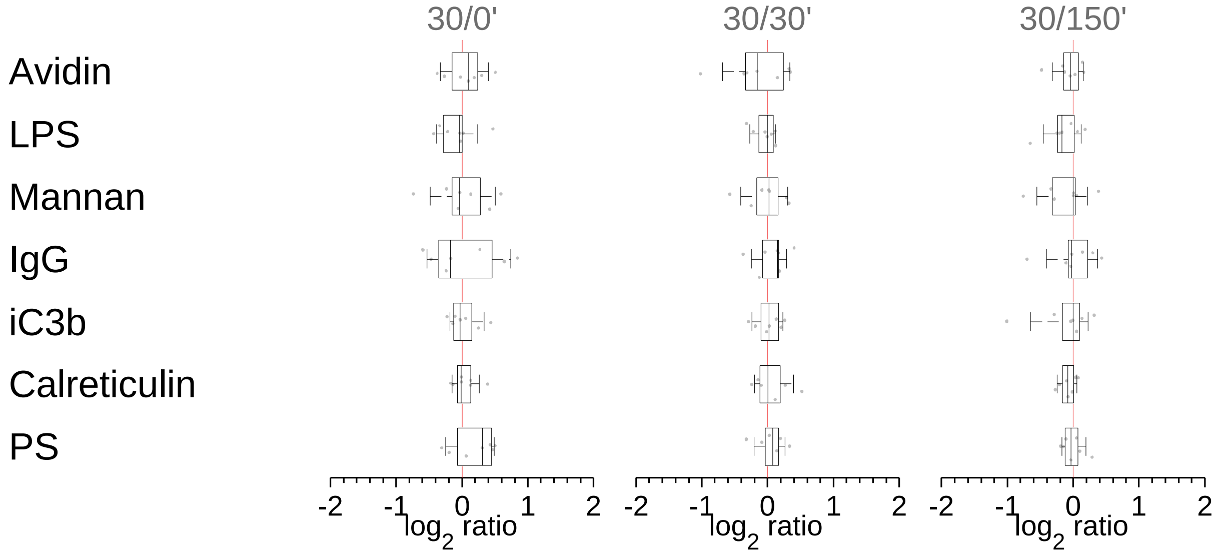

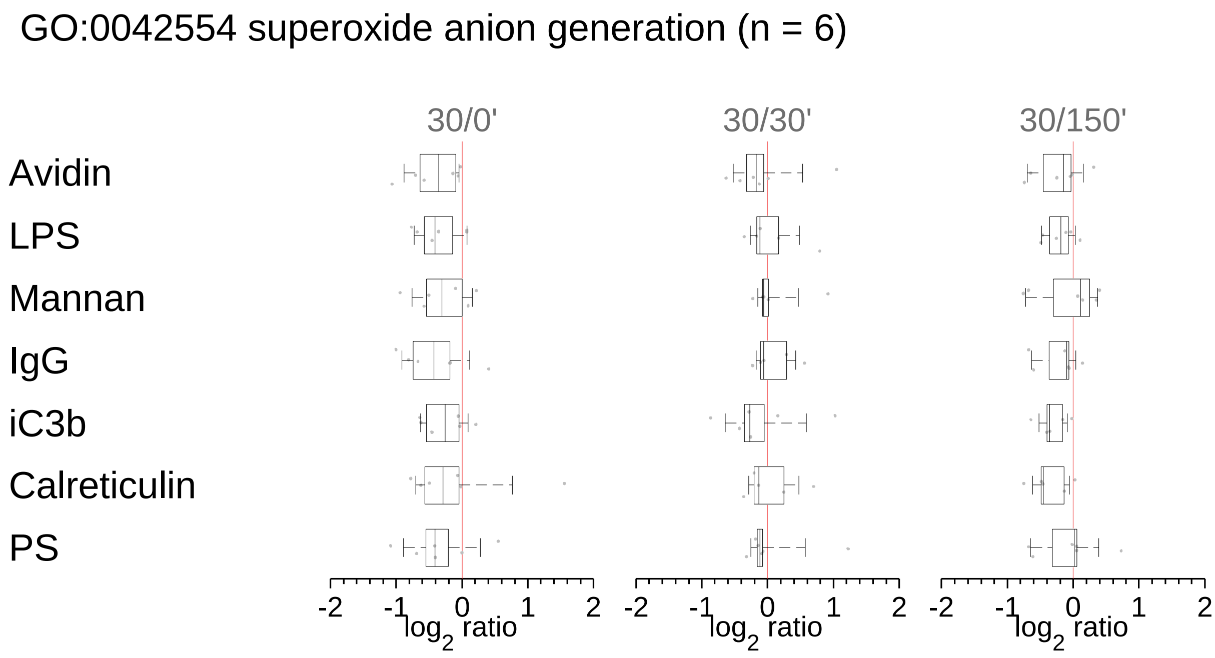
GO:0042554 superoxide anion generation (n = 6)
30/0'
30/30'
30/150'
Avidin
LPS
Mannan
IgG
iC3b
Calreticulin
PS
-2
-1
0
1
2
log2 ratio

GO:0042574 retinal metabolic process (n = 3)

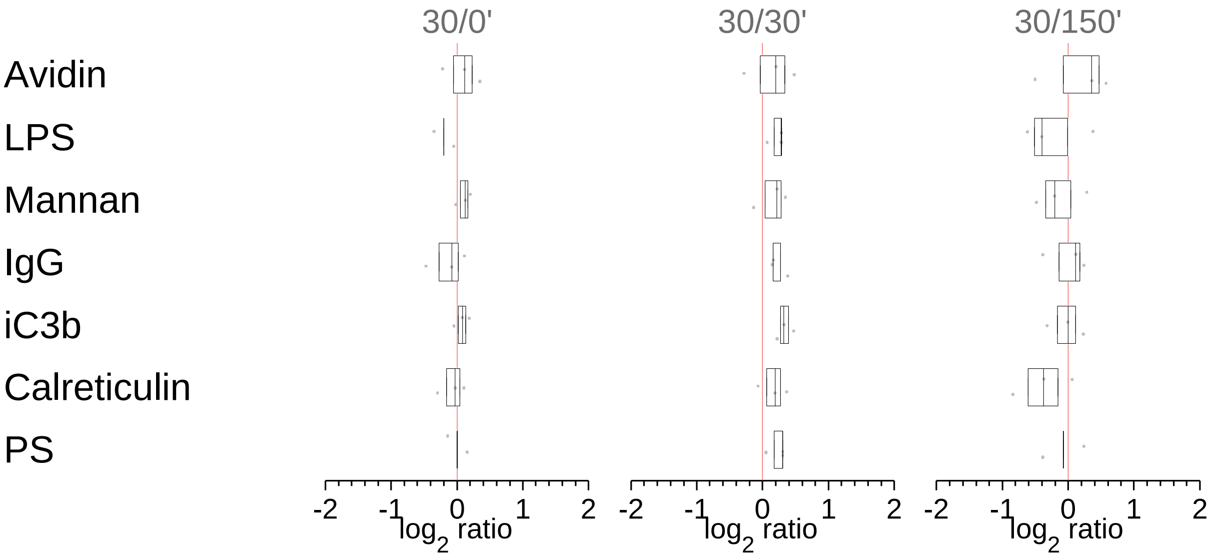

GO:0042578 phosphoric ester hydrolase activity (n = 4)

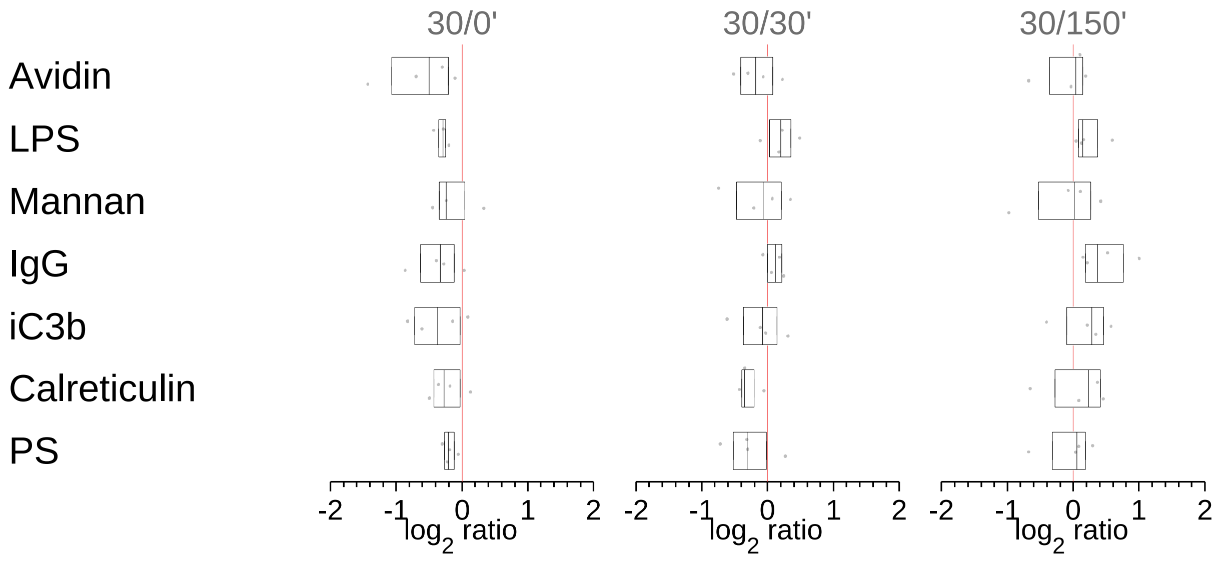

GO:0042581 specific granule (n = 8)

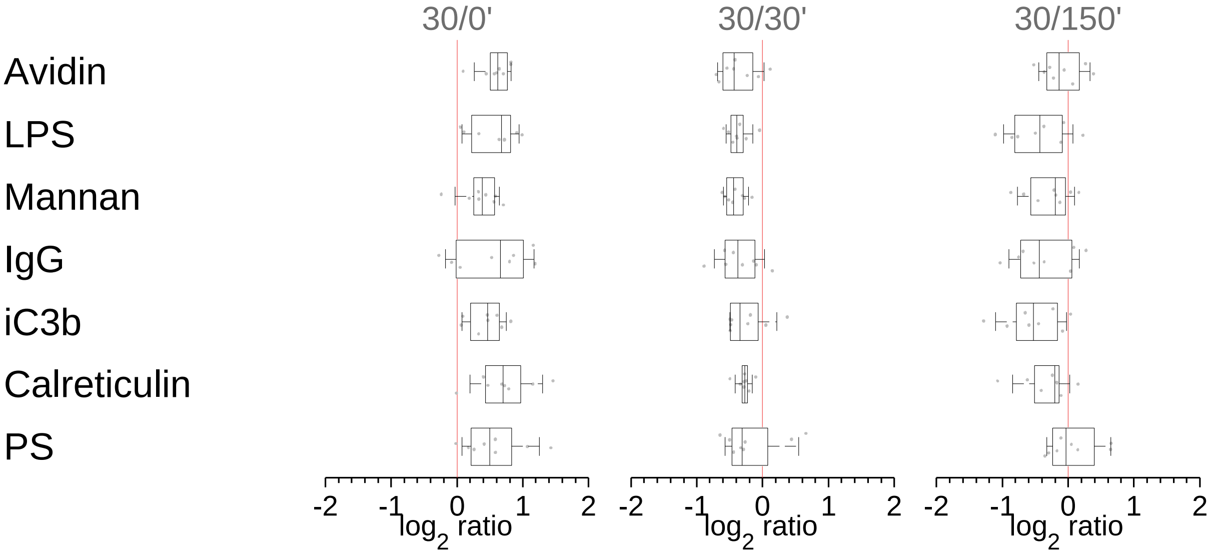

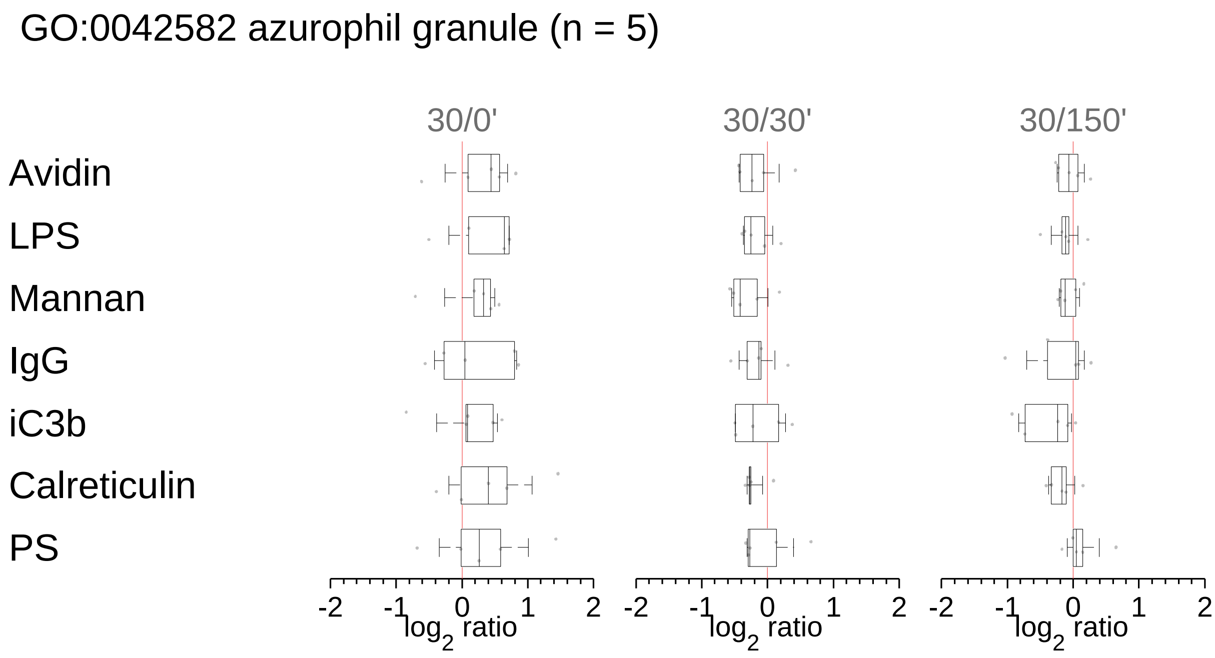
GO:0042582 azurophil granule (n = 5)
30/0'
30/30'
30/150'
Avidin
LPS
Mannan
IgG
iC3b
Calreticulin
PS
-2 -1 0 1 2
log2 ratio

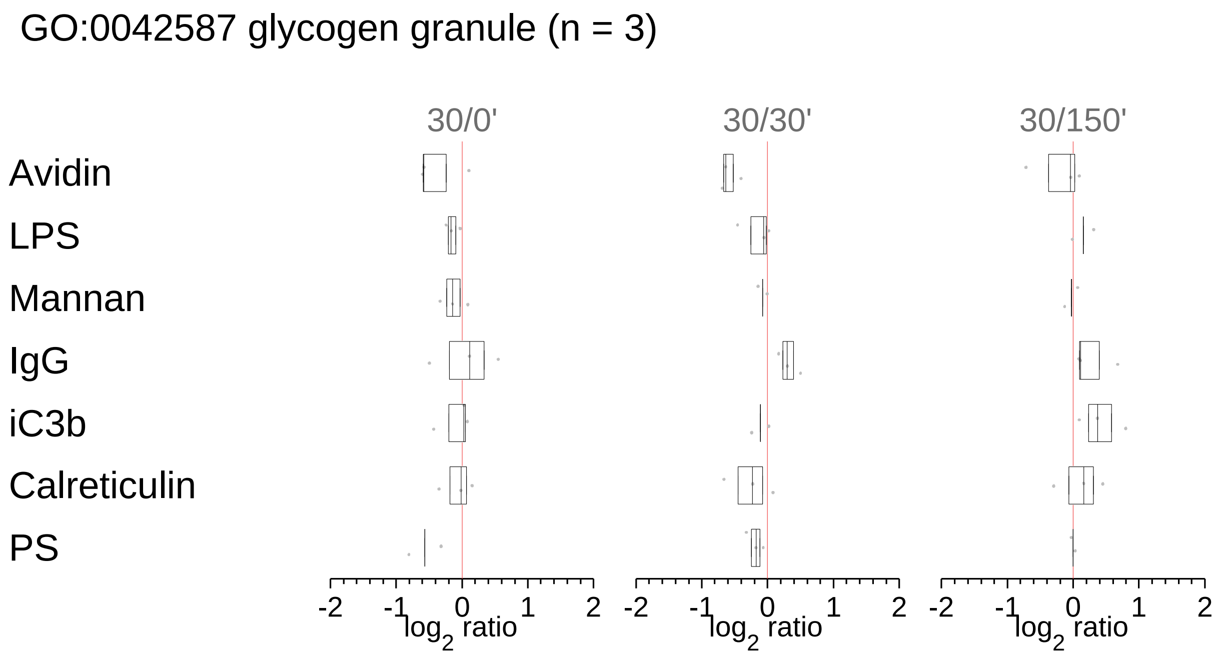
GO:0042587 glycogen granule (n = 3)
30/0'
30/30'
30/150'
Avidin
LPS
Mannan
IgG
iC3b
Calreticulin
PS
-2 -1 0 1 2
log_2 ratio

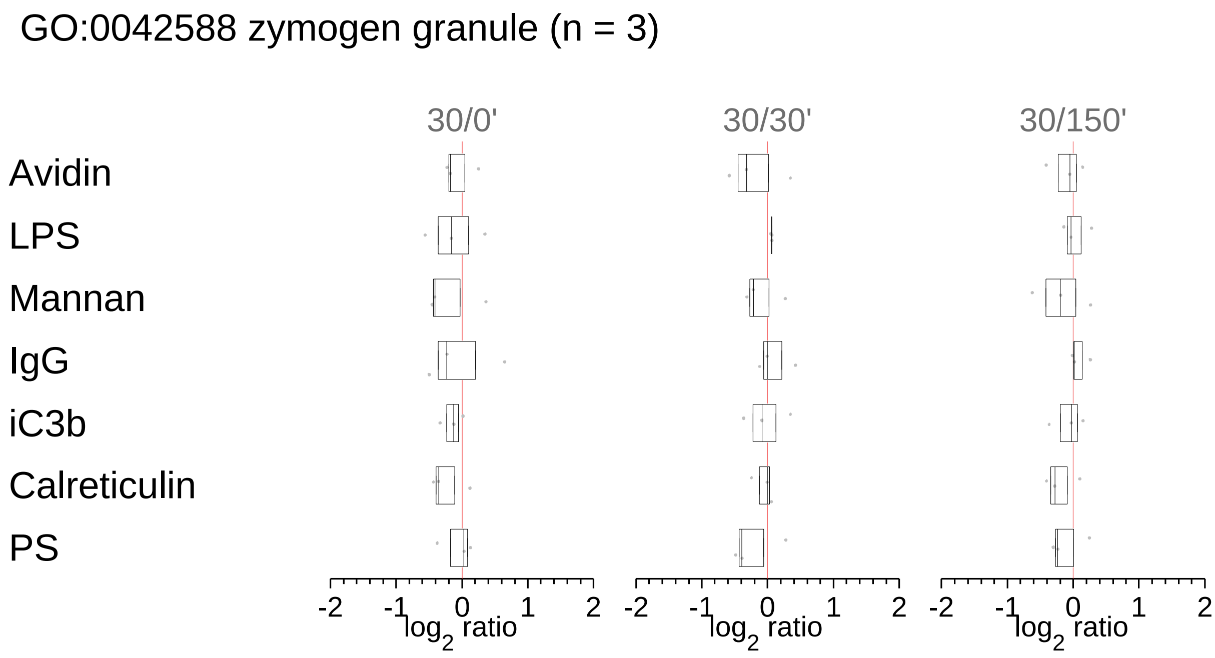
GO:0042588 zymogen granule (n = 3)
30/0'
30/30'
30/150'
Avidin
LPS
Mannan
IgG
iC3b
Calreticulin
PS
-2 -1 0 1 2
log2 ratio

GO:0042589 zymogen granule membrane (n = 3)

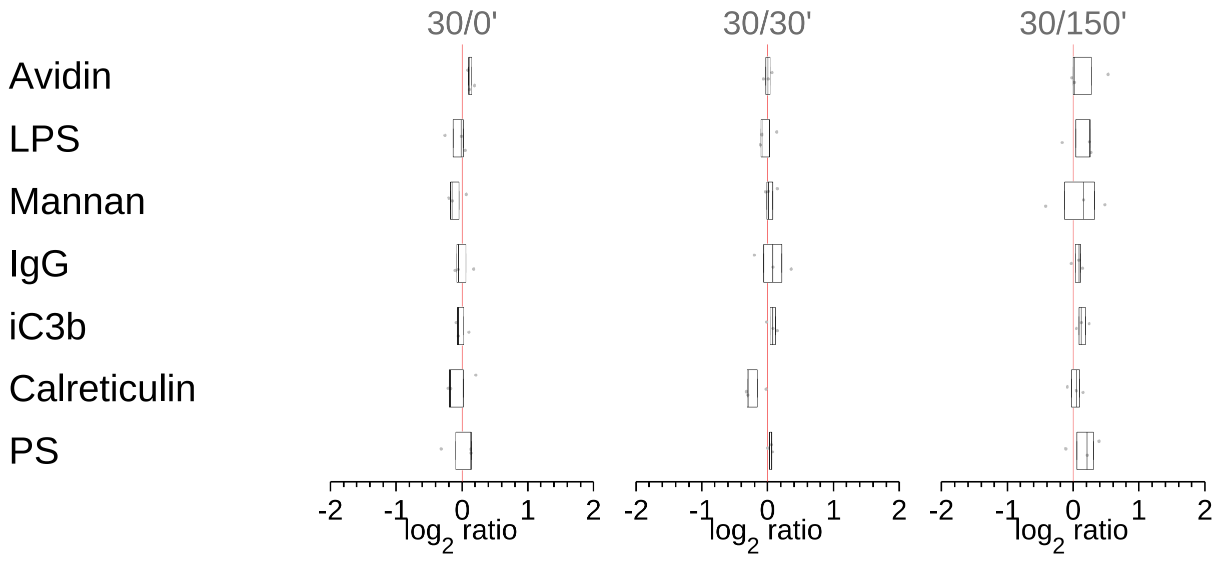

GO:0042590 antigen processing and presentation of exogenous peptide antigen via MHC class I (n = 4)

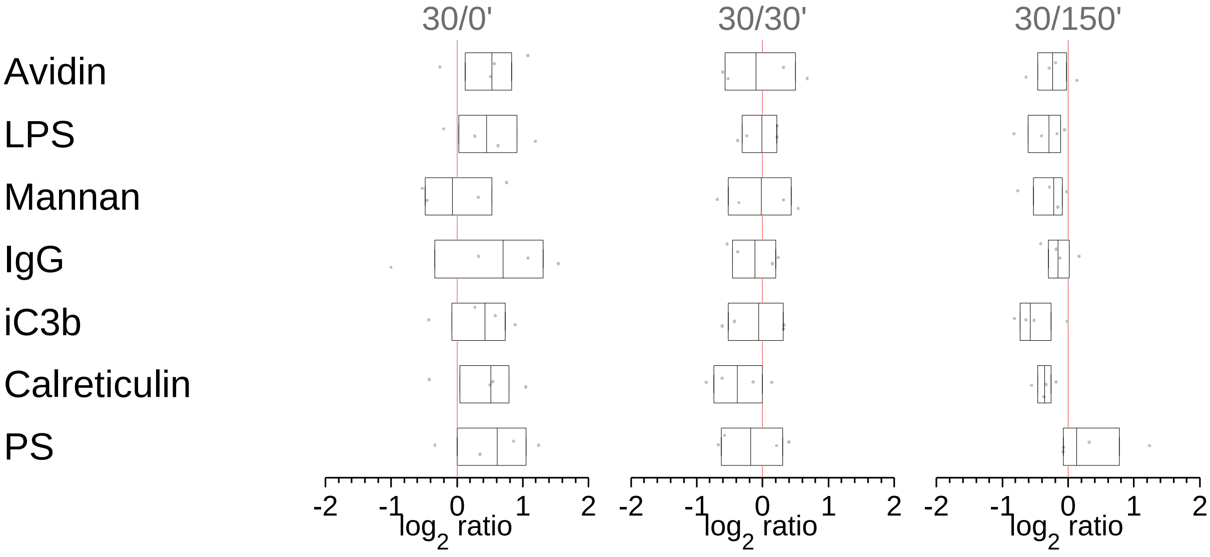

GO:0042593 glucose homeostasis (n = 7)

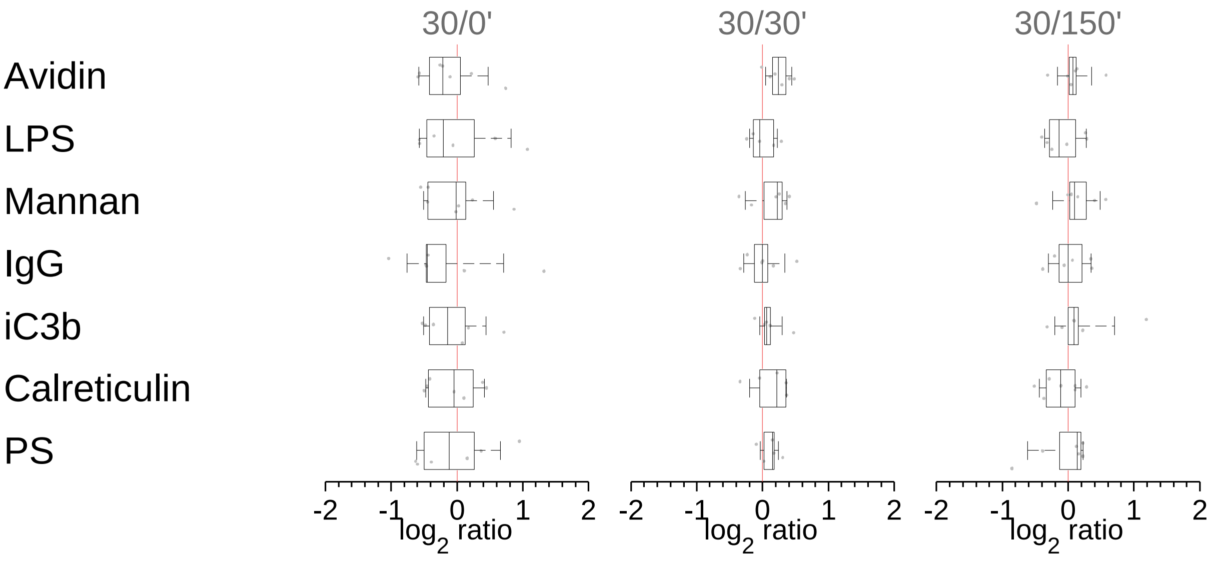

GO:0042605 peptide antigen binding (n = 5)

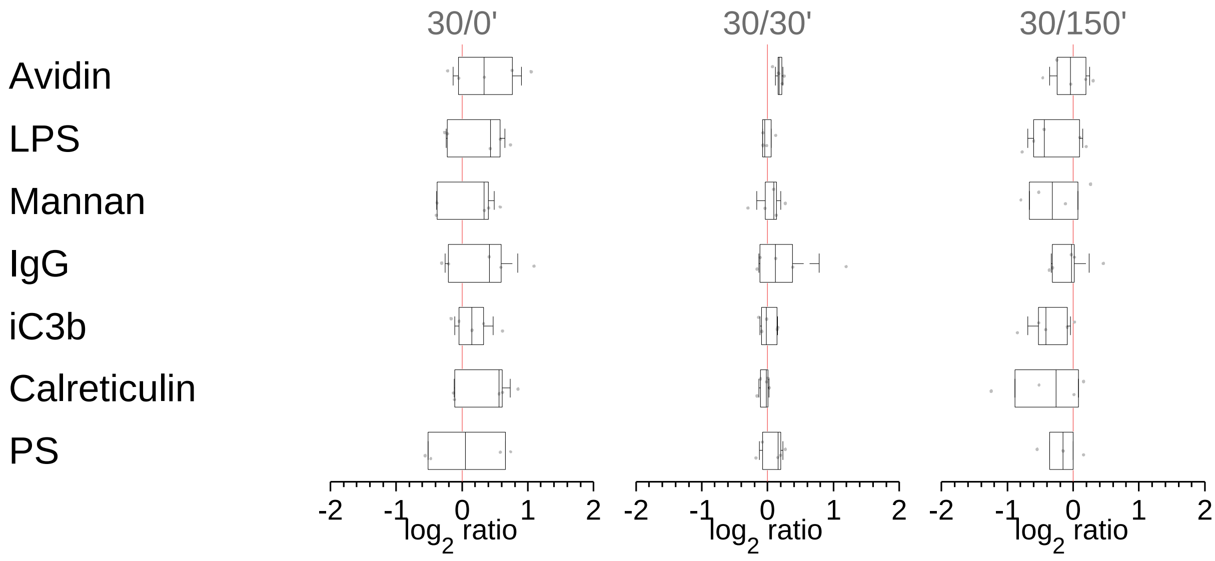

GO:0042608 T cell receptor binding (n = 4)

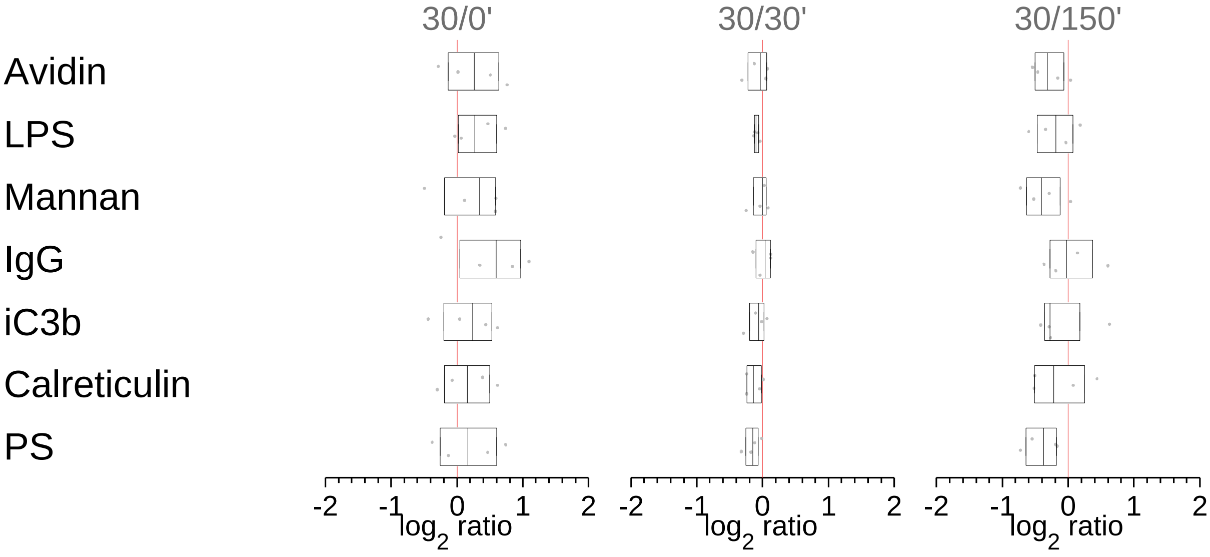

GO:0042612 MHC class I protein complex (n = 4)

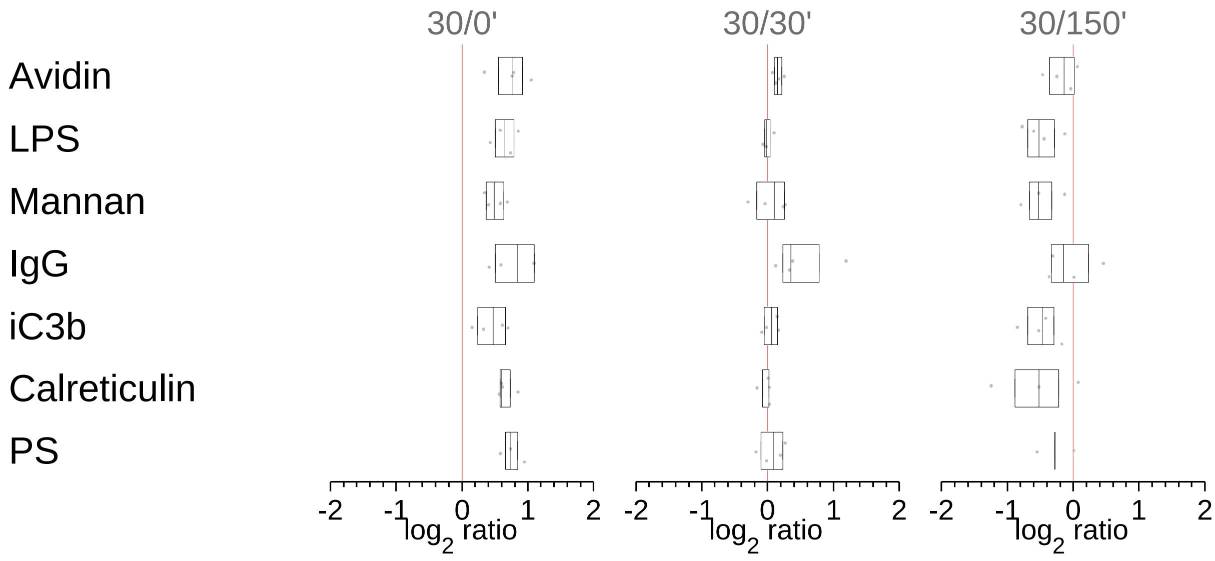

GO:0042613 MHC class II protein complex (n = 3)

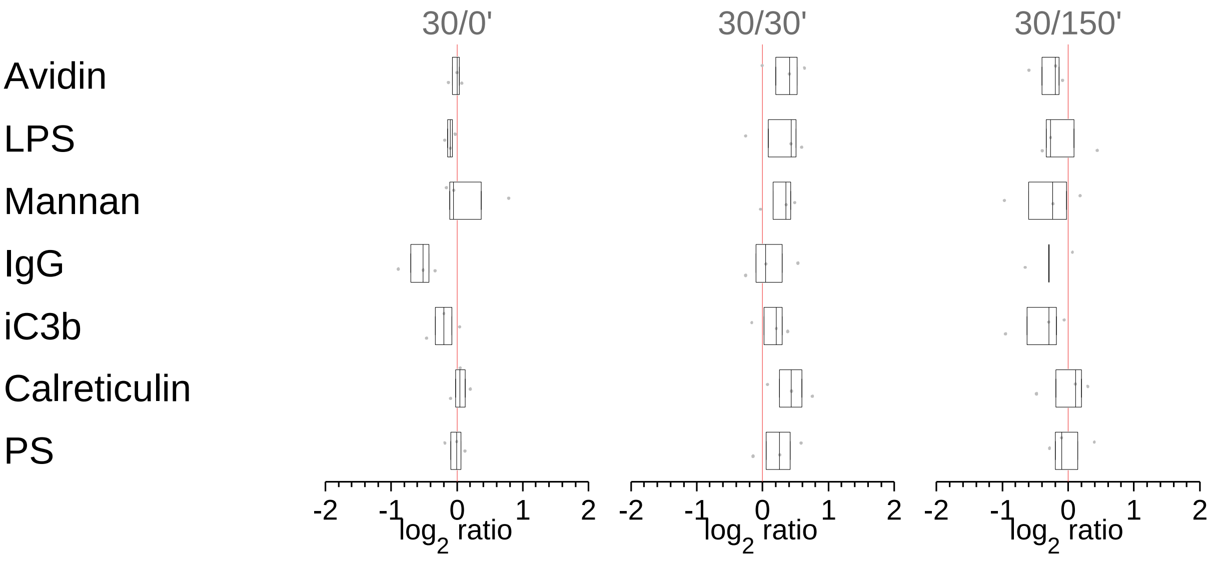

GO:0042623 ATPase activity, coupled (n = 3)

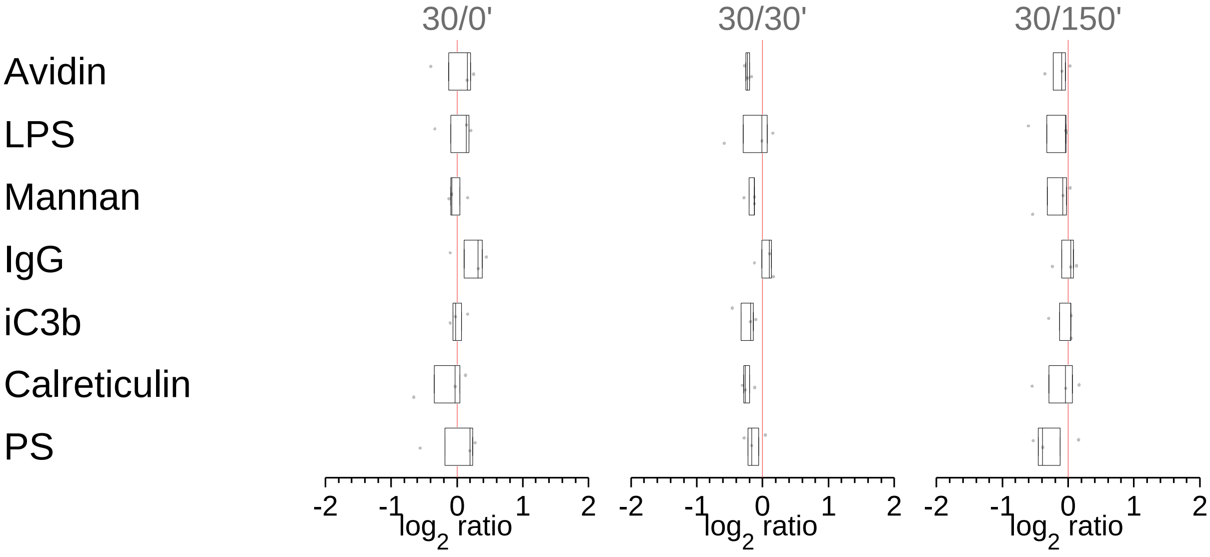

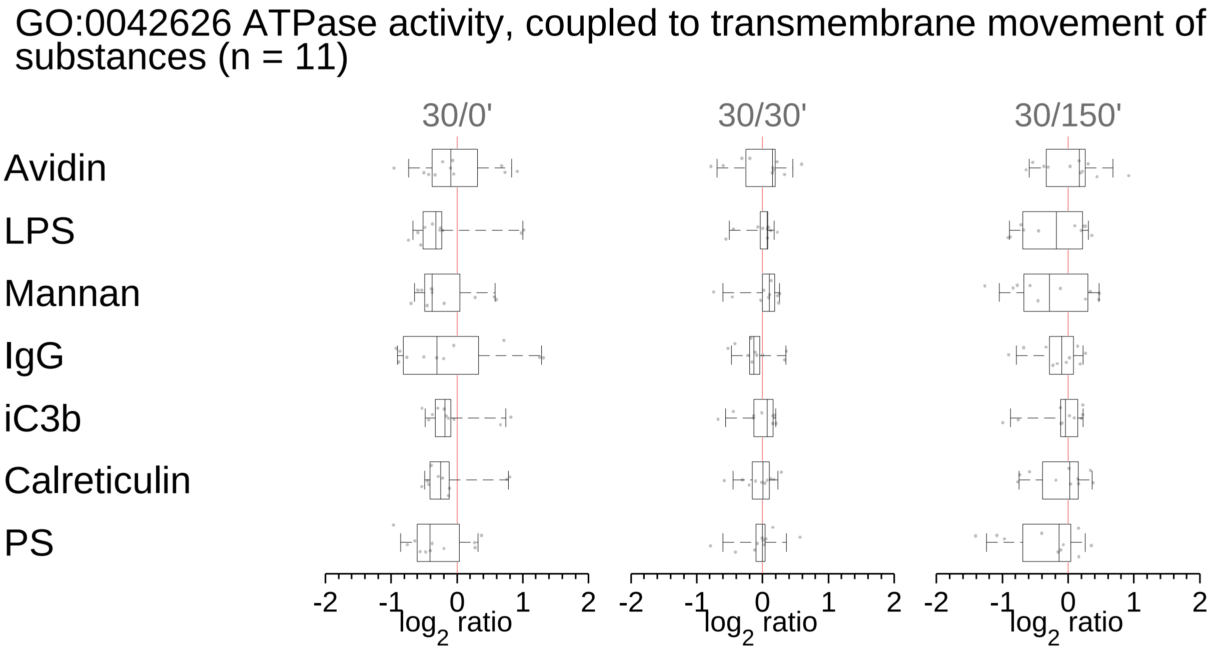
GO:0042626 ATPase activity, coupled to transmembrane movement of substances (n = 11)
30/0'
30/30'
30/150'
Avidin
LPS
Mannan
IgG
iC3b
Calreticulin
PS
-2
-1
0
1
2
log2 ratio

GO:0042627 chylomicron (n = 4)

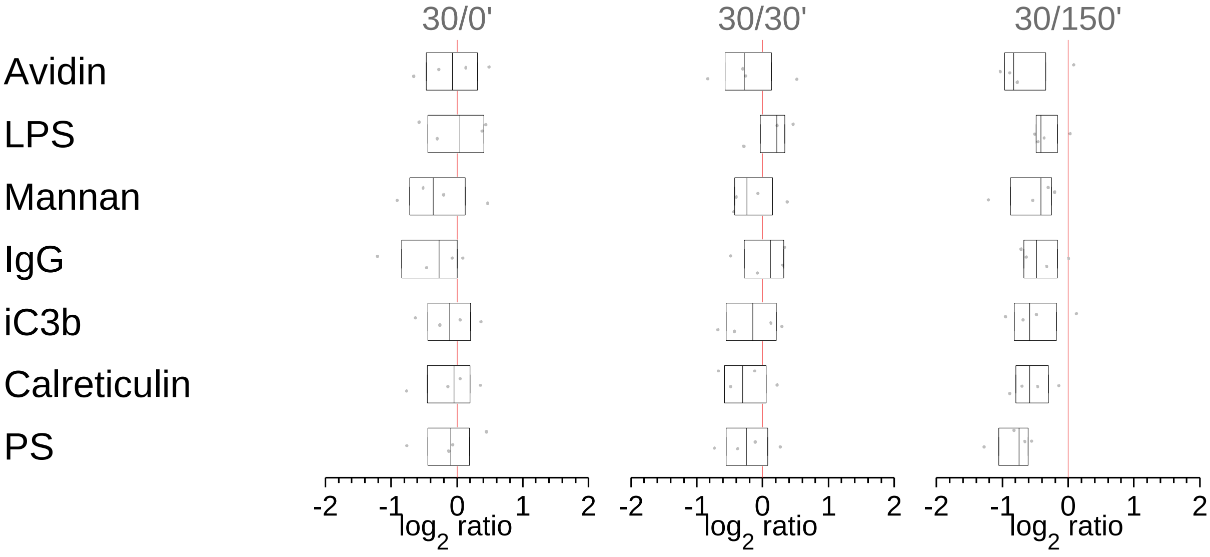

GO:0042629 mast cell granule (n = 5)

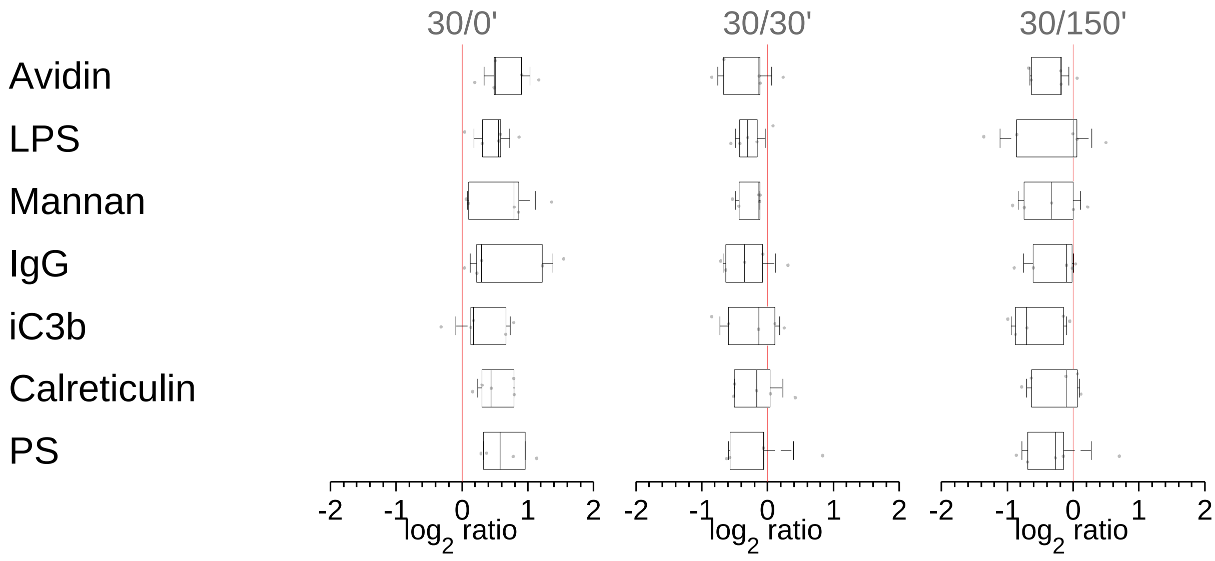

GO:0042632 cholesterol homeostasis (n = 10)

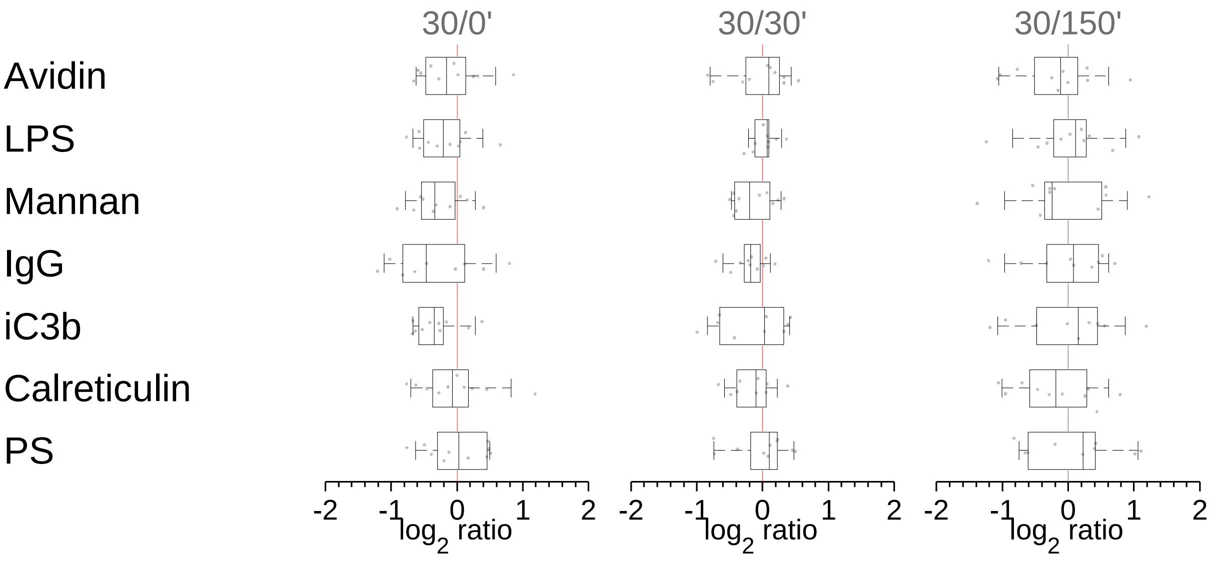

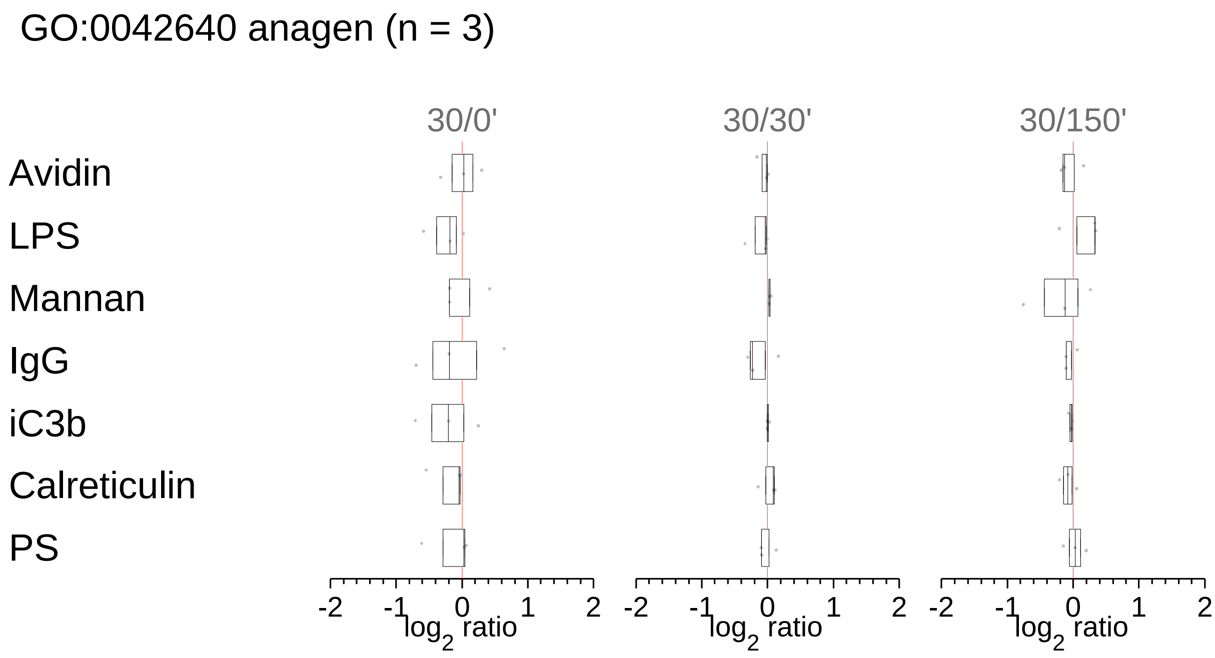
GO:0042640 anagen (n = 3)
30/0'
30/30'
30/150'
Avidin
LPS
Mannan
IgG
iC3b
Calreticulin
PS
-2
-1
0
1
2
log2 ratio

GO:0042641 actomyosin (n = 4)

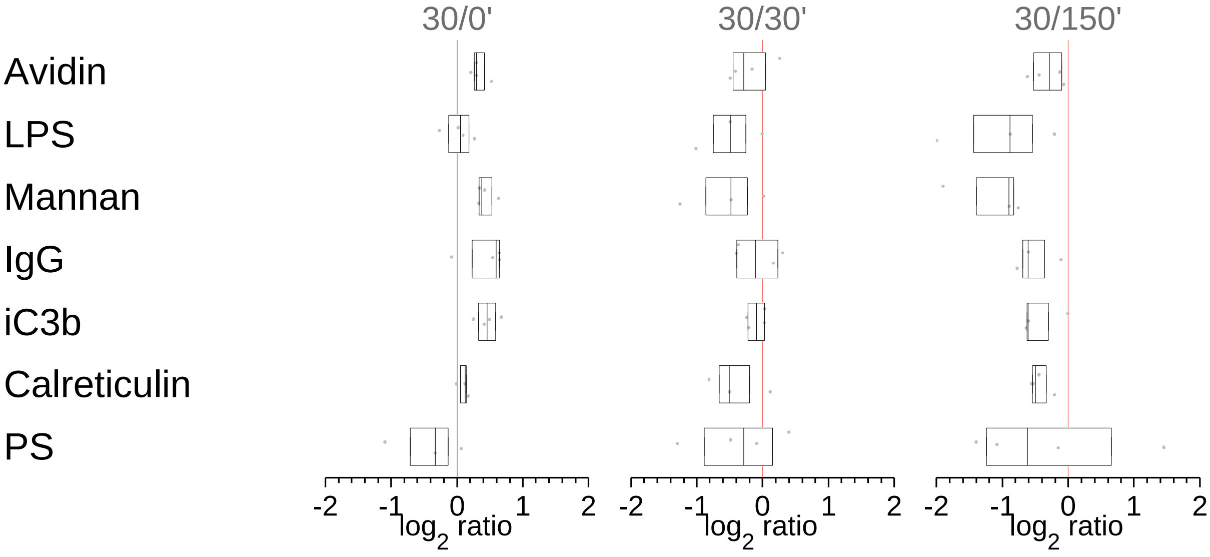

GO:0042645 mitochondrial nucleoid (n = 12)

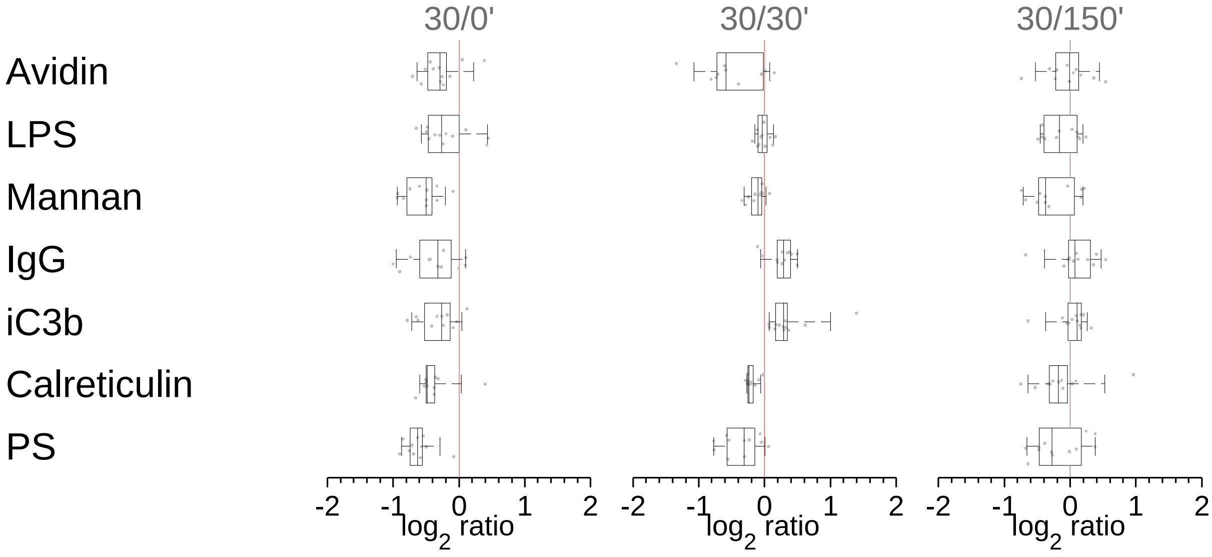

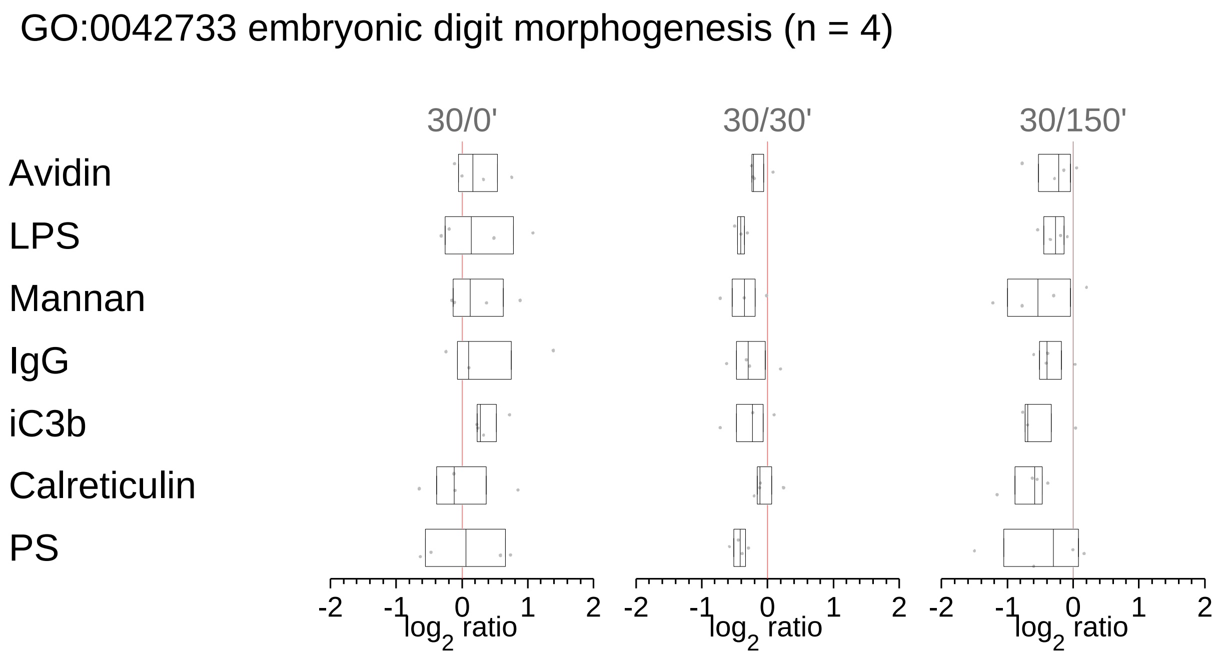
GO:0042733 embryonic digit morphogenesis (n = 4)
30/0'
30/30'
30/150'
Avidin
LPS
Mannan
IgG
iC3b
Calreticulin
PS
-2 -1 0 1 2
log2 ratio

GO:0042734 presynaptic membrane (n = 3)

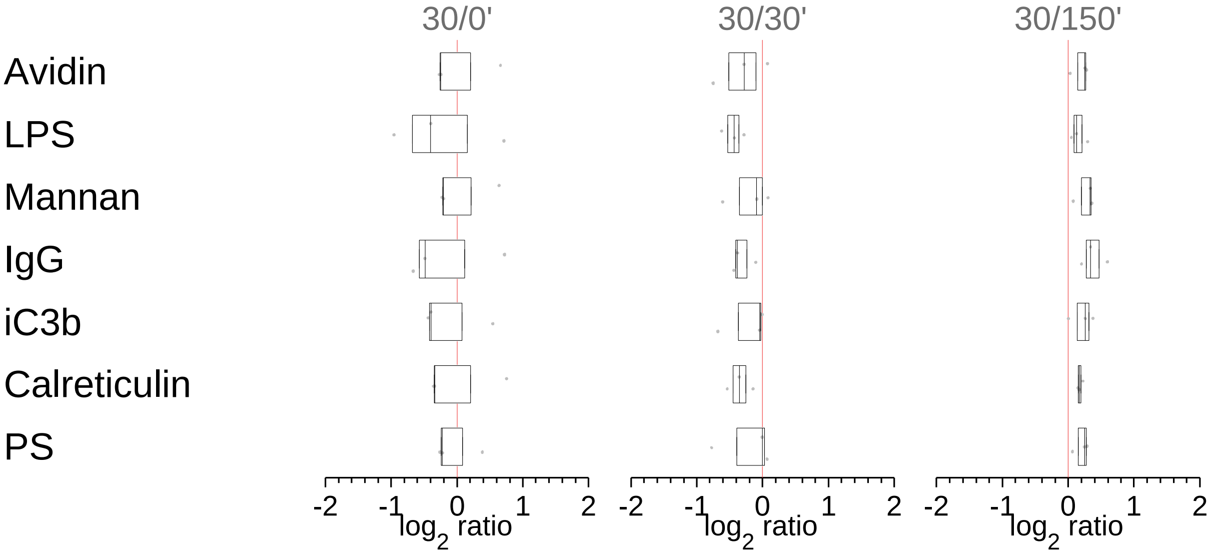

GO:0042742 defense response to bacterium (n = 15)

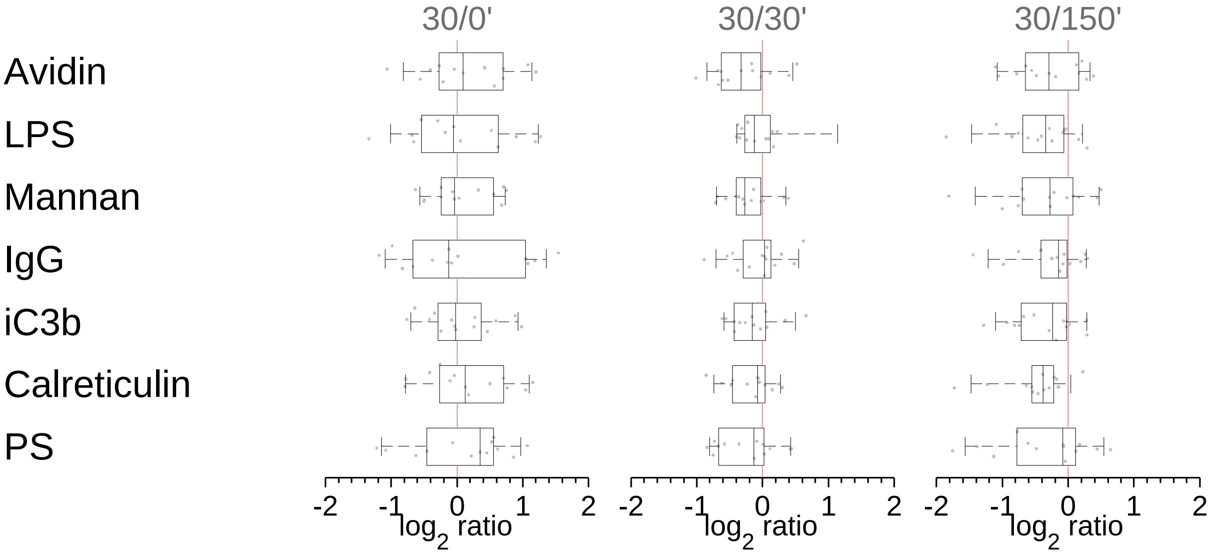

GO:0042744 hydrogen peroxide catabolic process (n = 8)

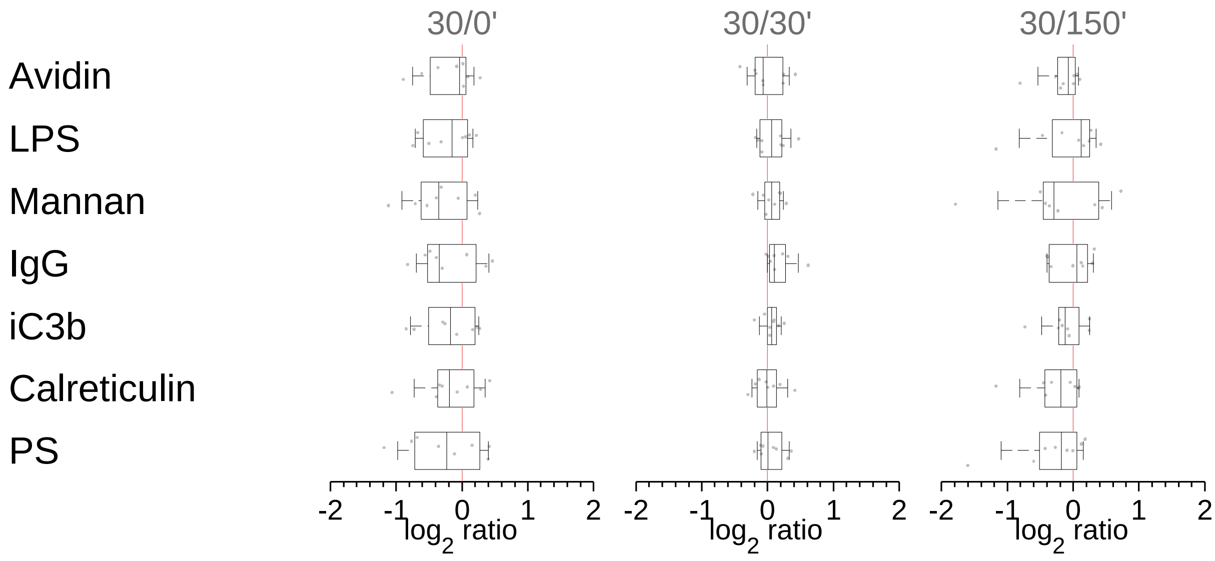

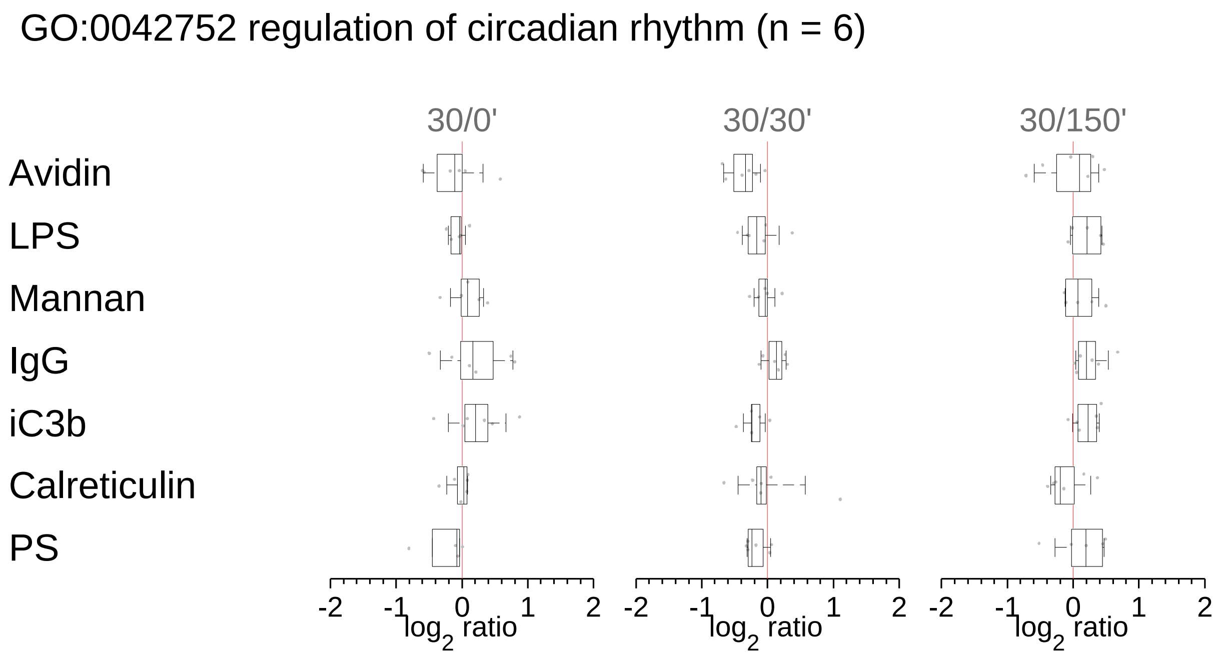

GO:0042752 regulation of circadian rhythm (n = 6)
30/0'
30/30'
30/150'
Avidin
LPS
Mannan
IgG
iC3b
Calreticulin
PS
-2
-1
0
1
2
log2 ratio

GO:0042760 very long-chain fatty acid catabolic process (n = 3)

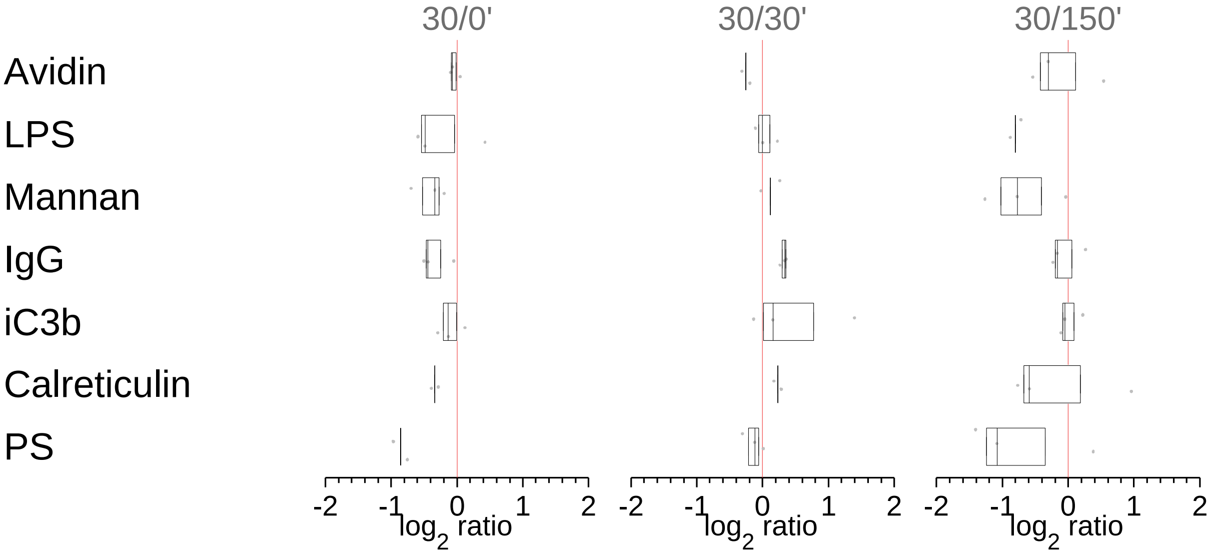

GO:0042787 protein ubiquitination involved in ubiquitin-dependent protein catabolic process (n = 4)

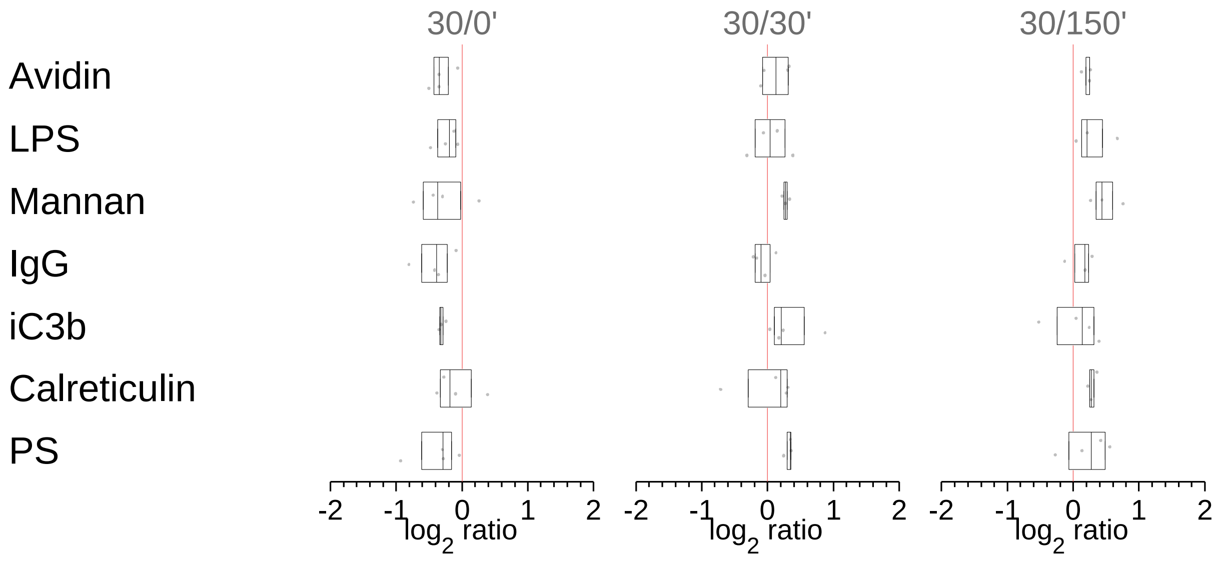

GO:0042802 identical protein binding (n = 84)

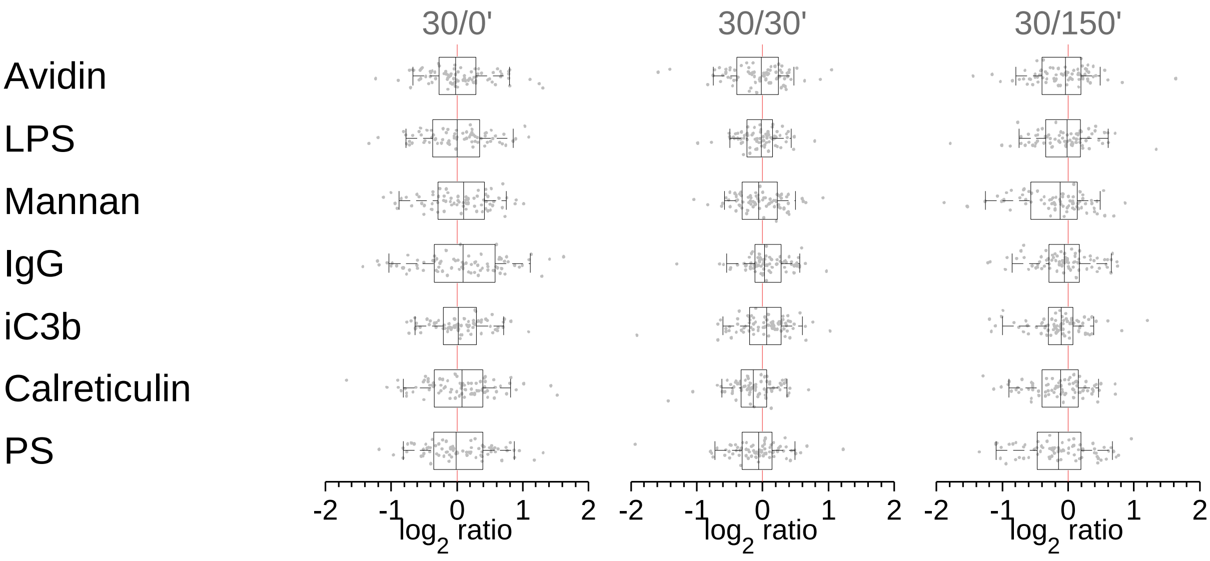

GO:0042803 protein homodimerization activity (n = 99)

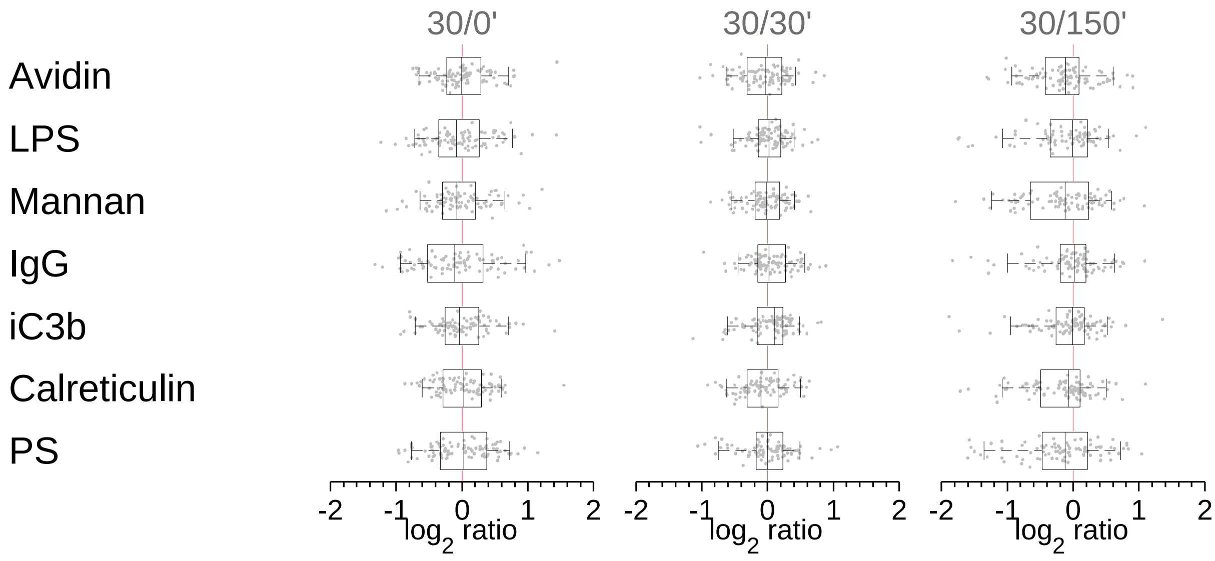

GO:0042824 MHC class I peptide loading complex (n = 3)

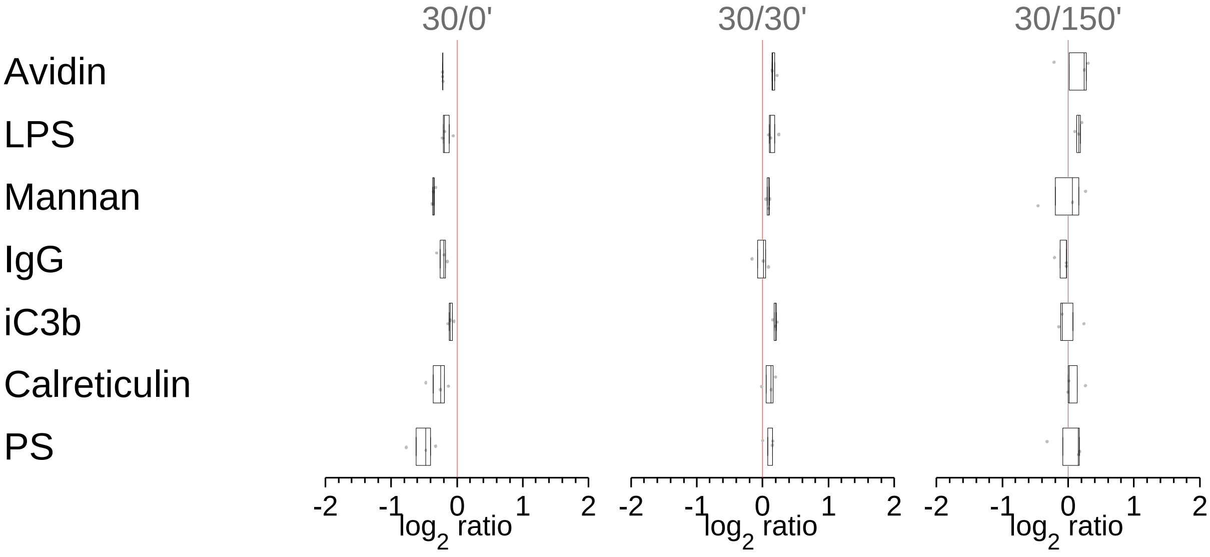

GO:0042825 TAP complex (n = 3)

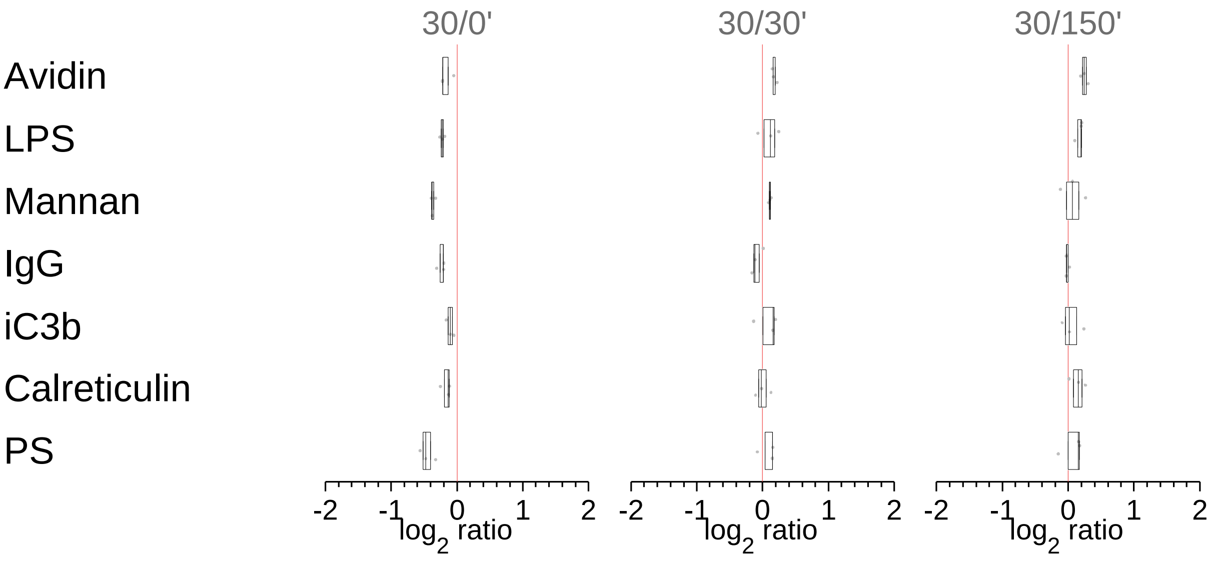

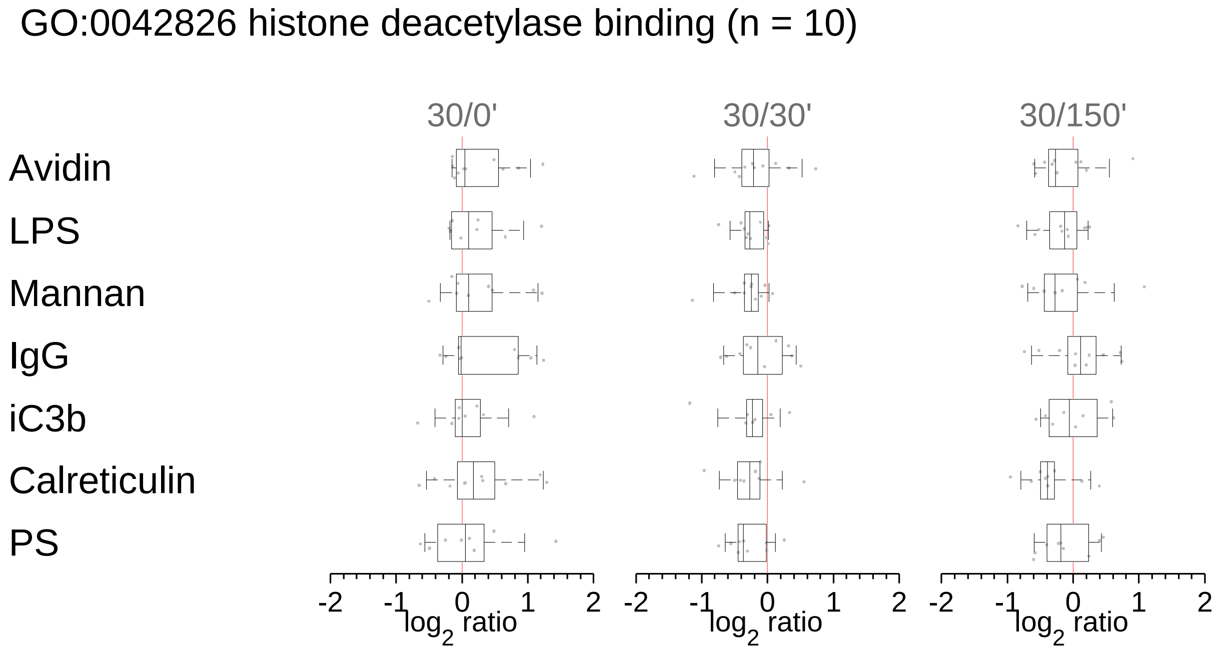

GO:0042826 histone deacetylase binding (n = 10)
30/0'
30/30'
30/150'
Avidin
LPS
Mannan
IgG
iC3b
Calreticulin
PS
-2
-1
0
1
2
log2 ratio

GO:0042908 xenobiotic transport (n = 3)

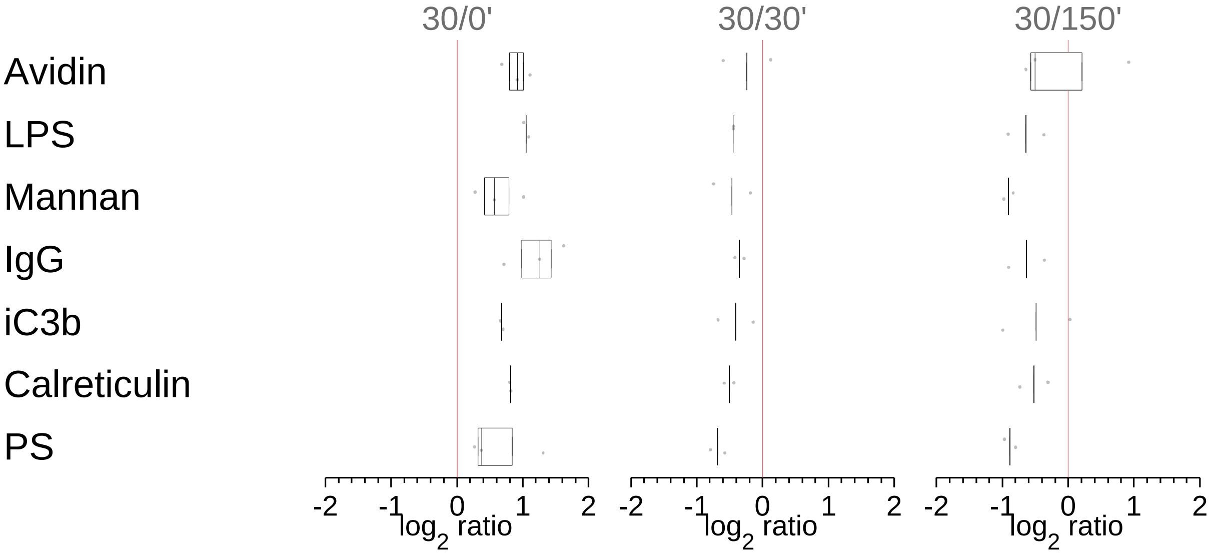

GO:0042953 lipoprotein transport (n = 3)

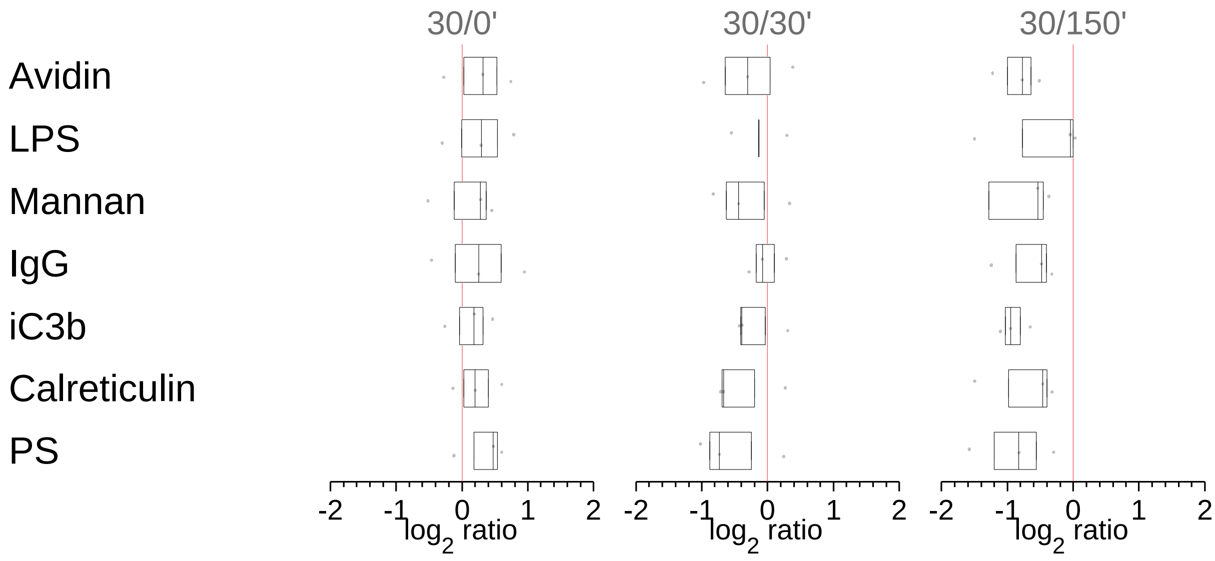

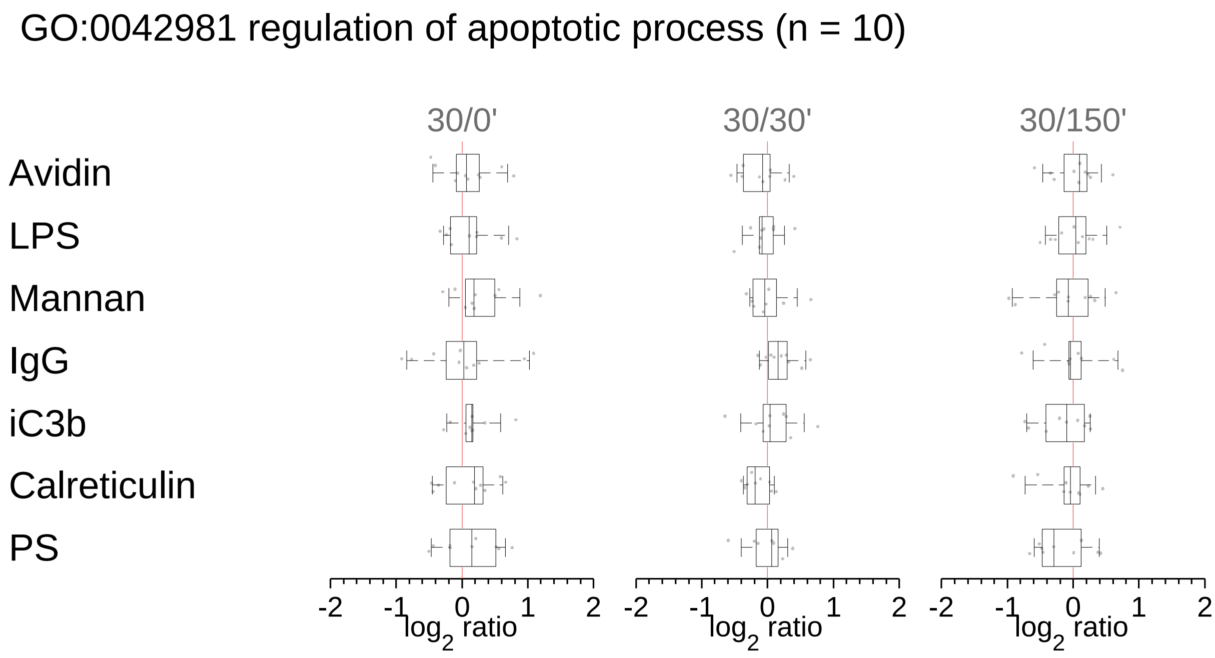
GO:0042981 regulation of apoptotic process (n = 10)
30/0'
30/30'
30/150'
Avidin
LPS
Mannan
IgG
iC3b
Calreticulin
PS
-2
-1
0
1
2
log2 ratio

GO:0042987 amyloid precursor protein catabolic process (n = 4)

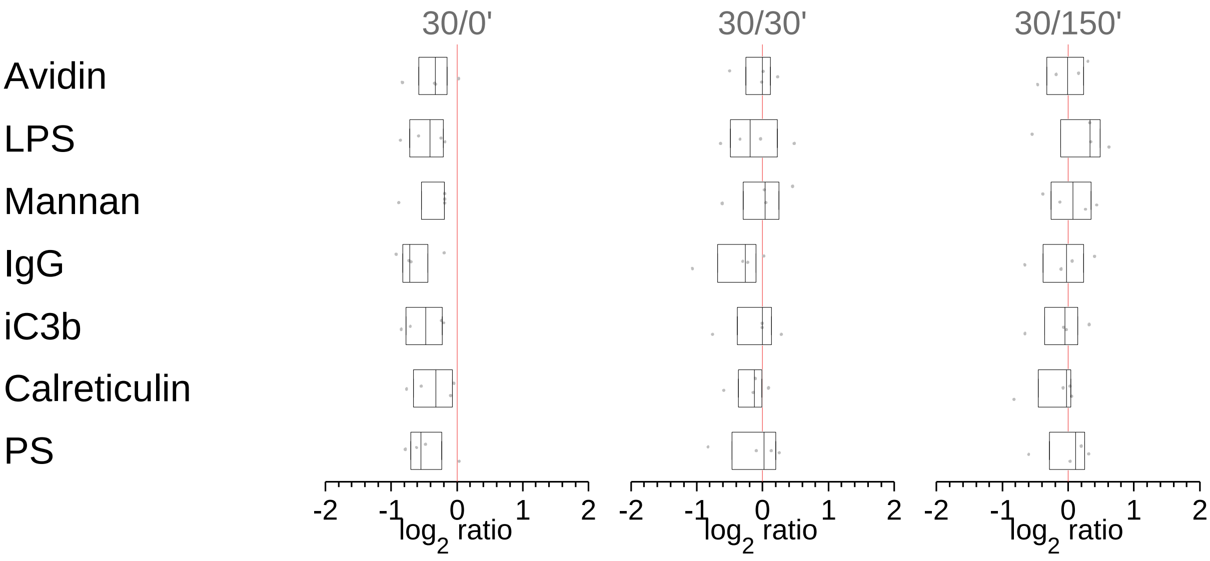

GO:0042993 positive regulation of transcription factor import into nucleus (n = 5)

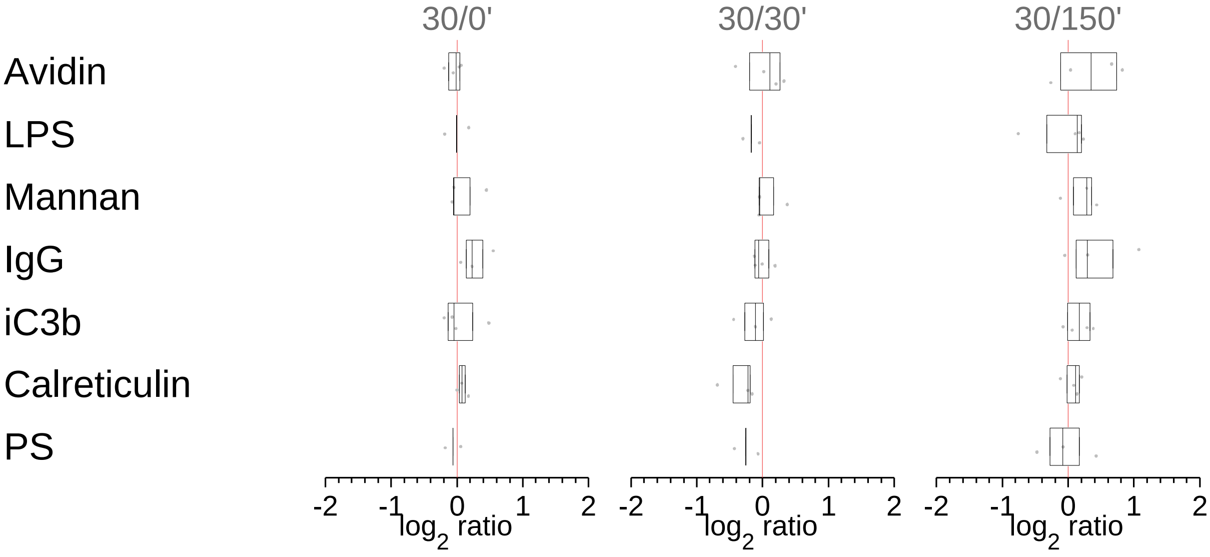

GO:0042995 cell projection (n = 82)

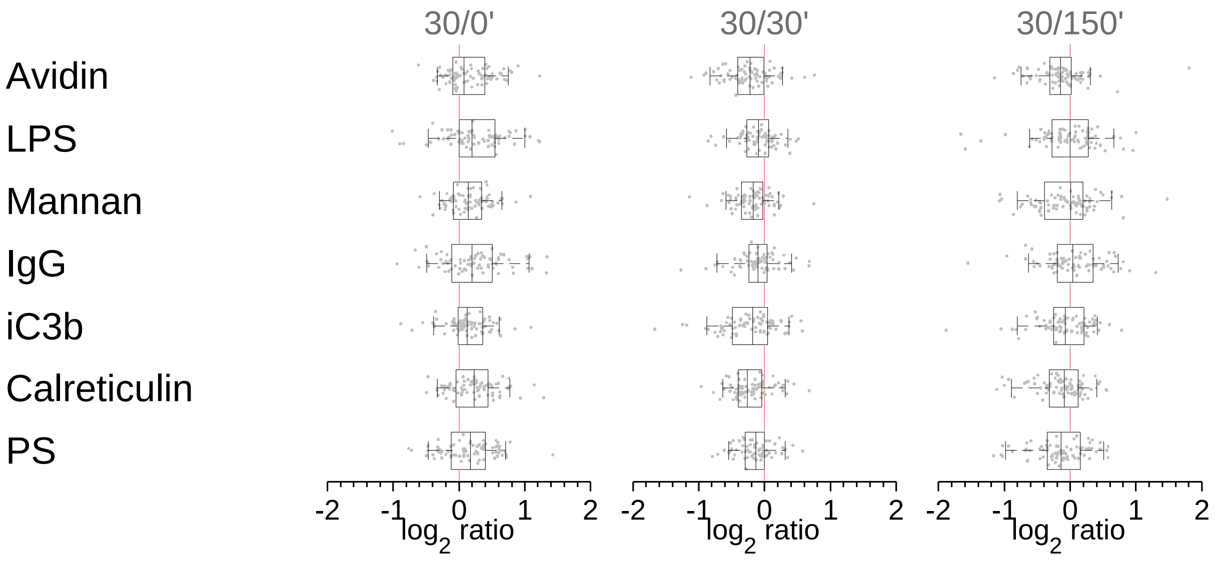

GO:0043001 Golgi to plasma membrane protein transport (n = 9)

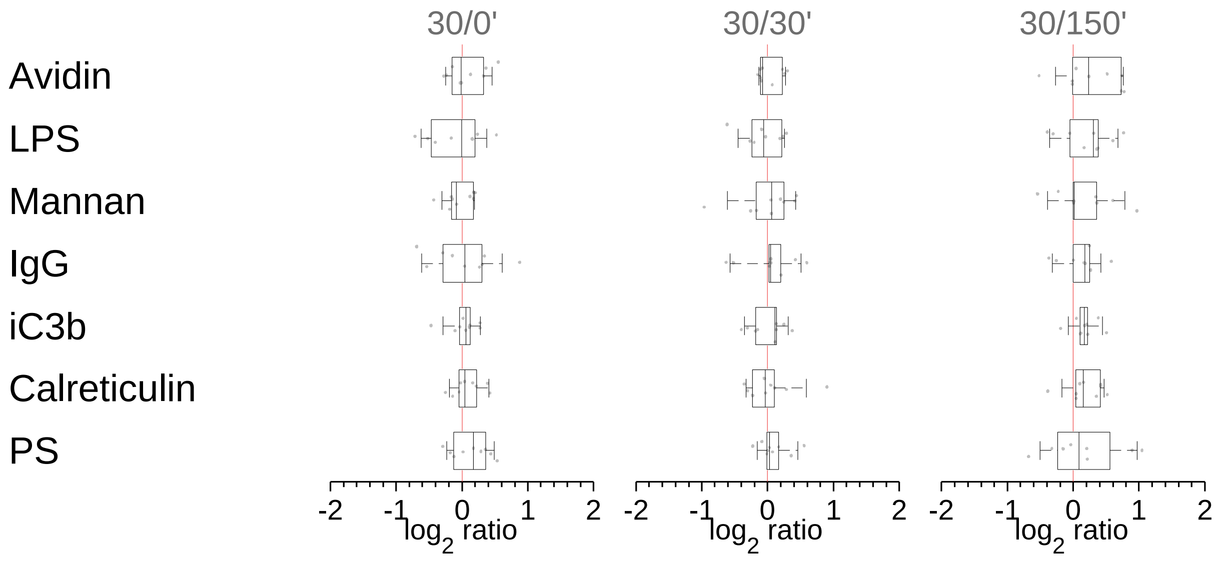

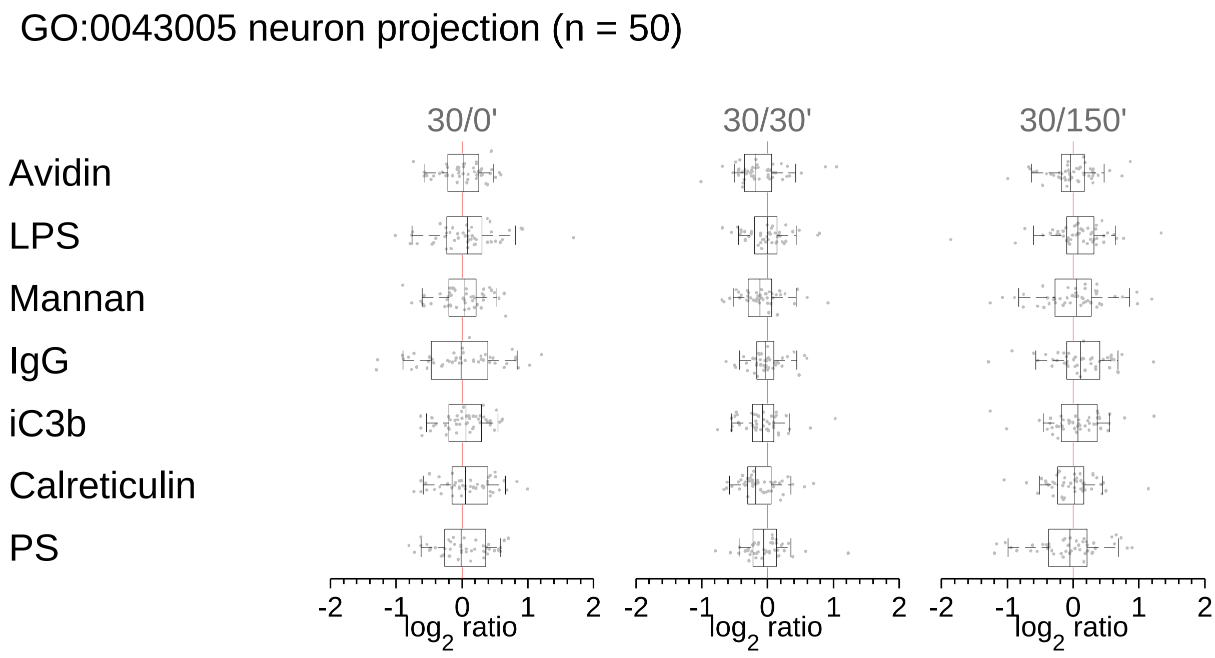
GO:0043005 neuron projection (n = 50)
30/0'
30/30'
30/150'
Avidin
LPS
Mannan
IgG
iC3b
Calreticulin
PS
-2
-1
0
1
2
log2 ratio

GO:0043008 ATP-dependent protein binding (n = 4)

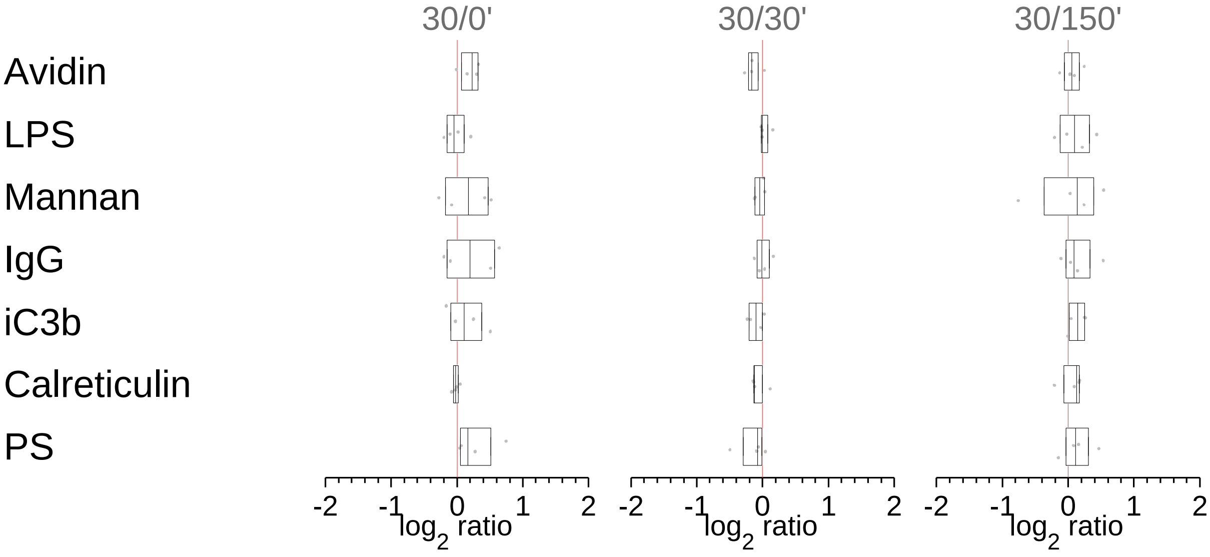

GO:0043014 alpha-tubulin binding (n = 6)

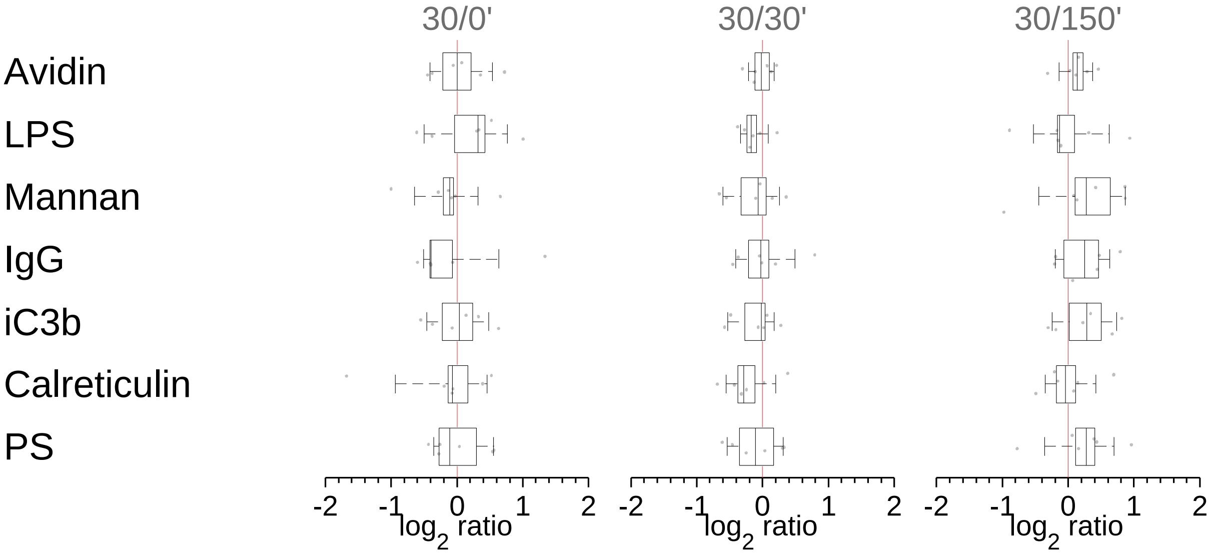

GO:0043020 NADPH oxidase complex (n = 3)

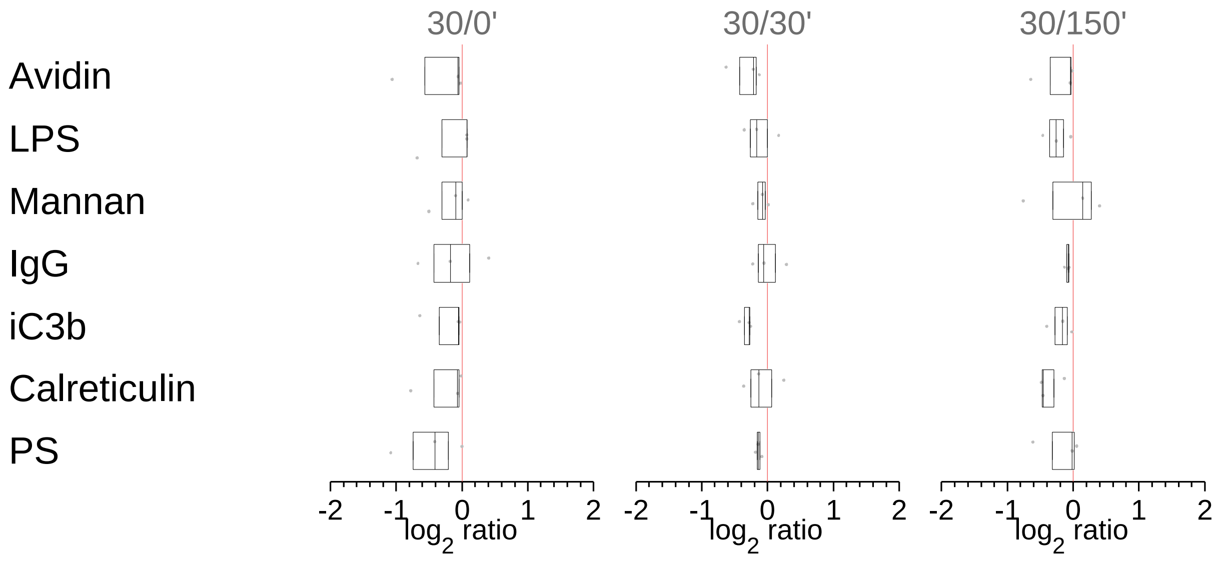

GO:0043021 ribonucleoprotein complex binding (n = 6)

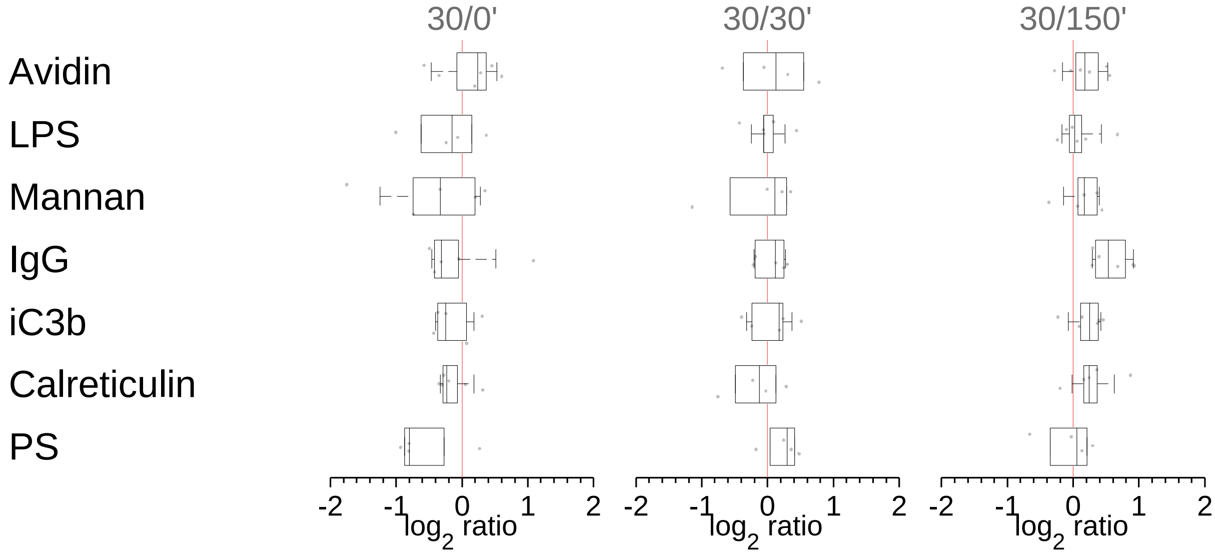

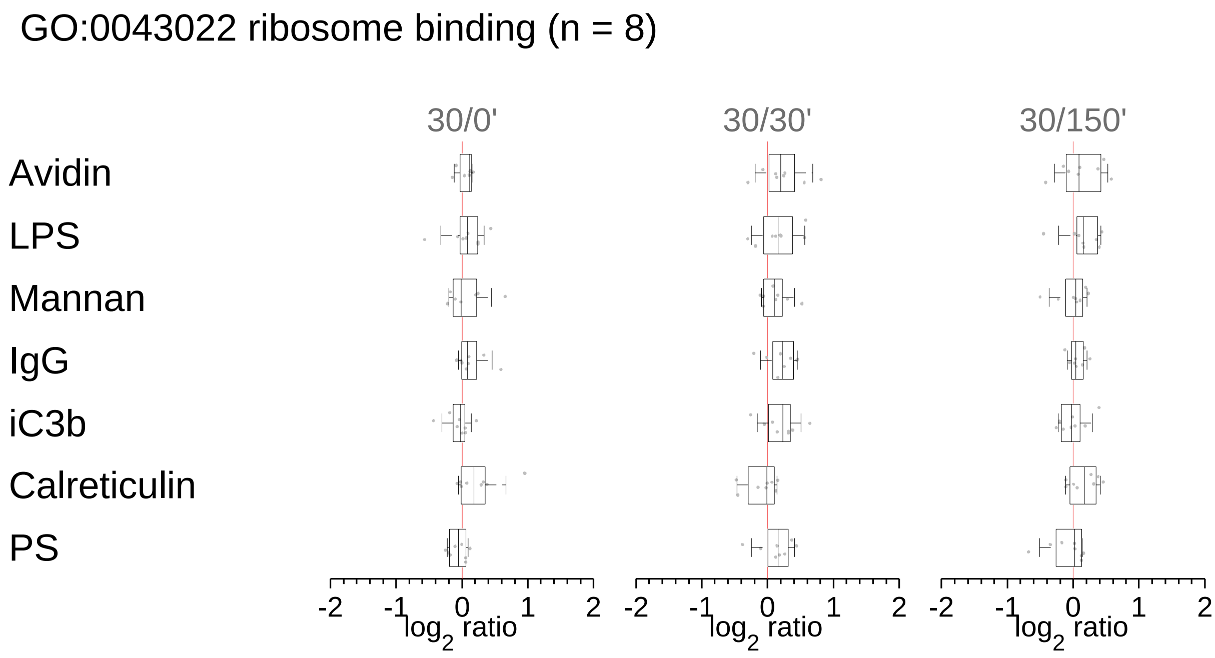

GO:0043022 ribosome binding (n = 8)
30/0'
30/30'
30/150'
Avidin
LPS
Mannan
IgG
iC3b
Calreticulin
PS
-2
-1
0
1
2
log2 ratio

GO:0043025 neuronal cell body (n = 62)

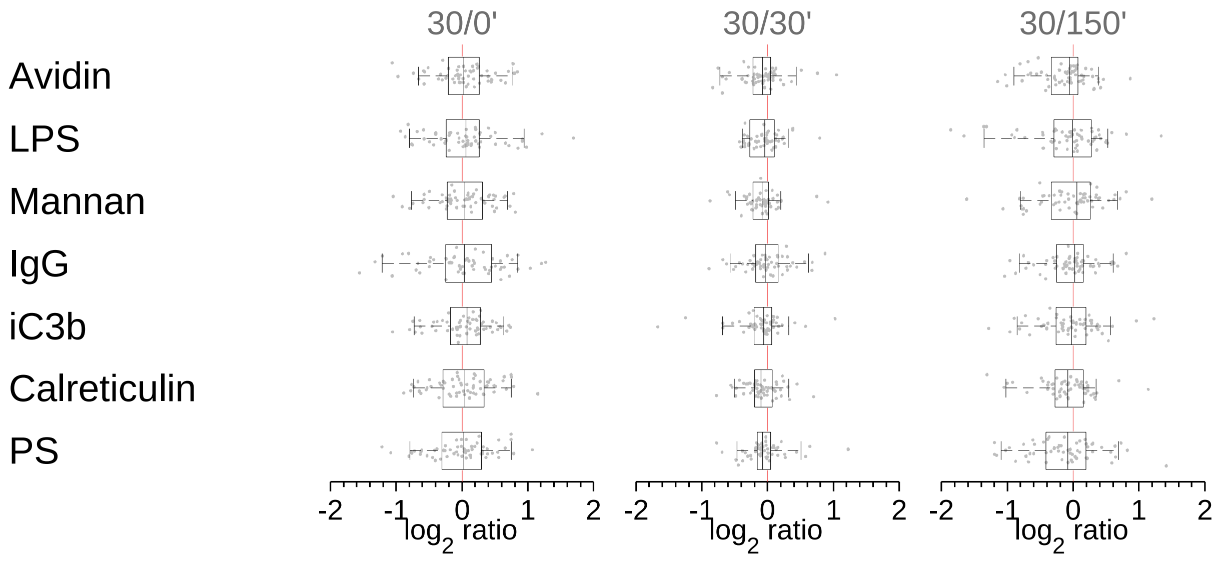

GO:0043027 cysteine-type endopeptidase inhibitor activity involved in apoptotic process (n = 3)

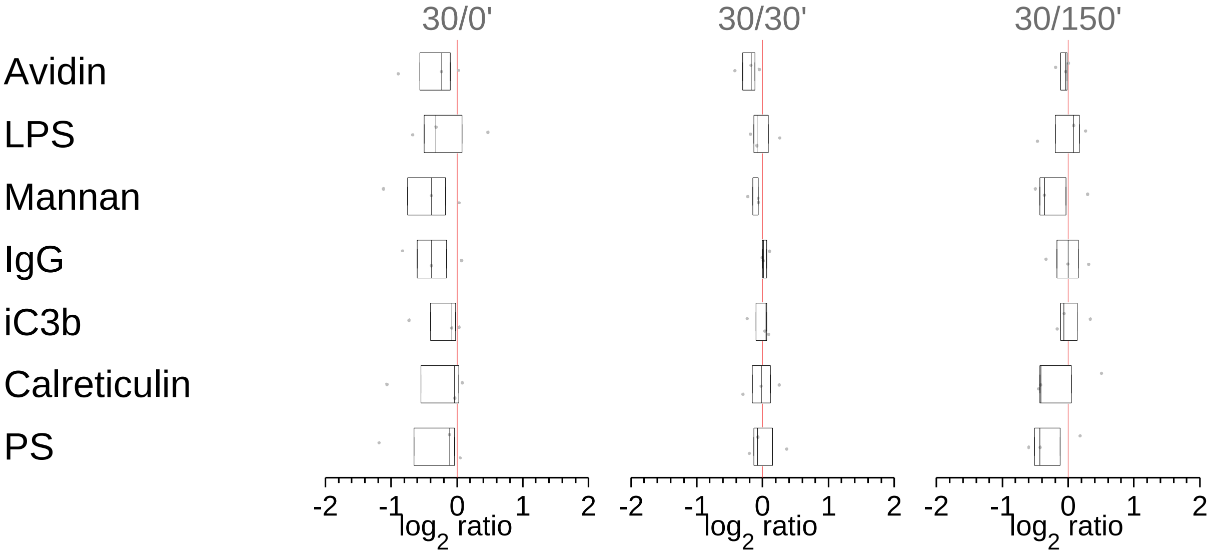

GO:0043029 T cell homeostasis (n = 3)

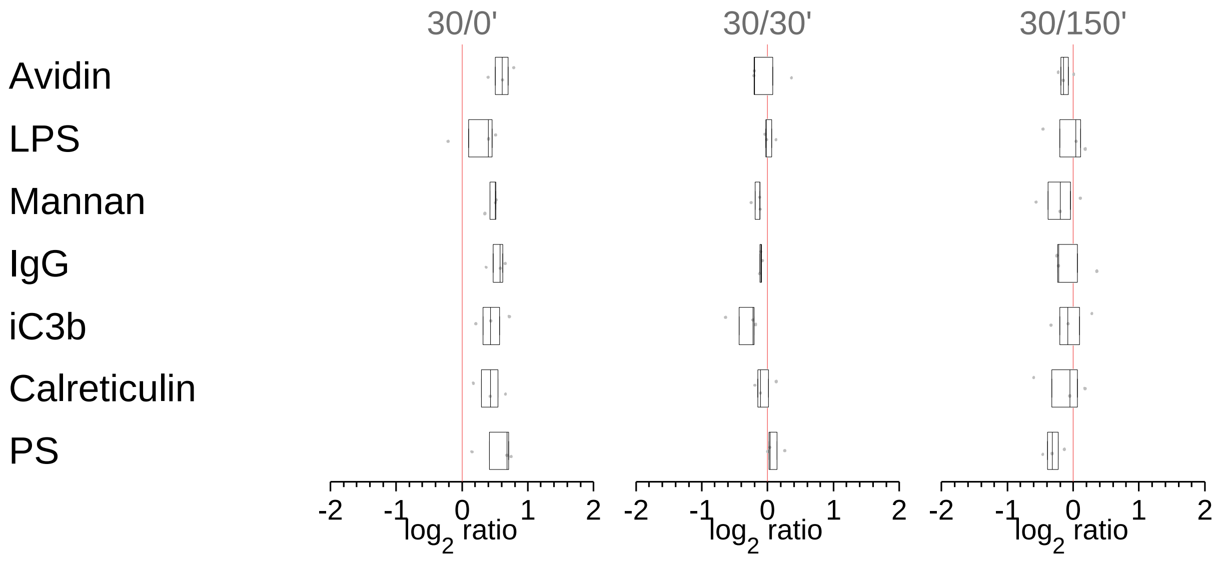

GO:0043044 ATP-dependent chromatin remodeling (n = 6)

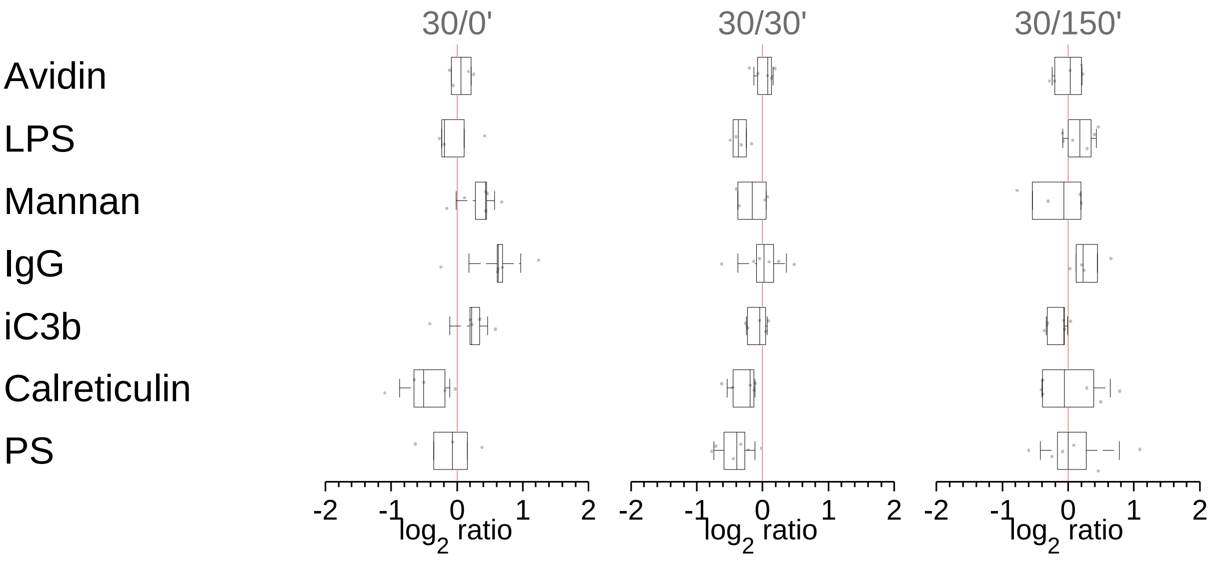

GO:0043065 positive regulation of apoptotic process (n = 36)

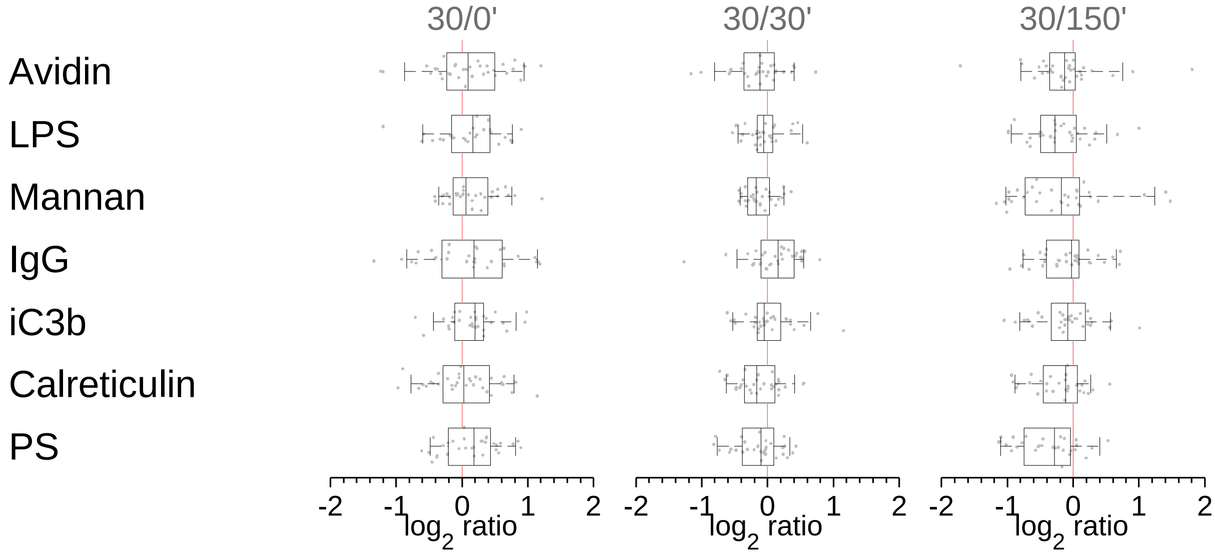

GO:0043066 negative regulation of apoptotic process (n = 57)

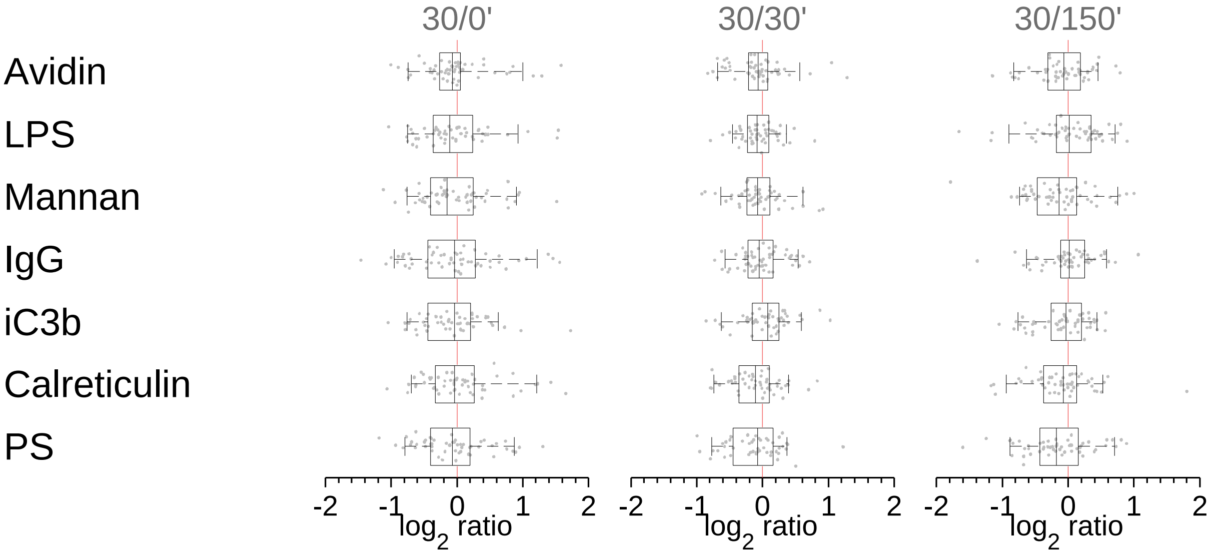

GO:0043068 positive regulation of programmed cell death (n = 3)

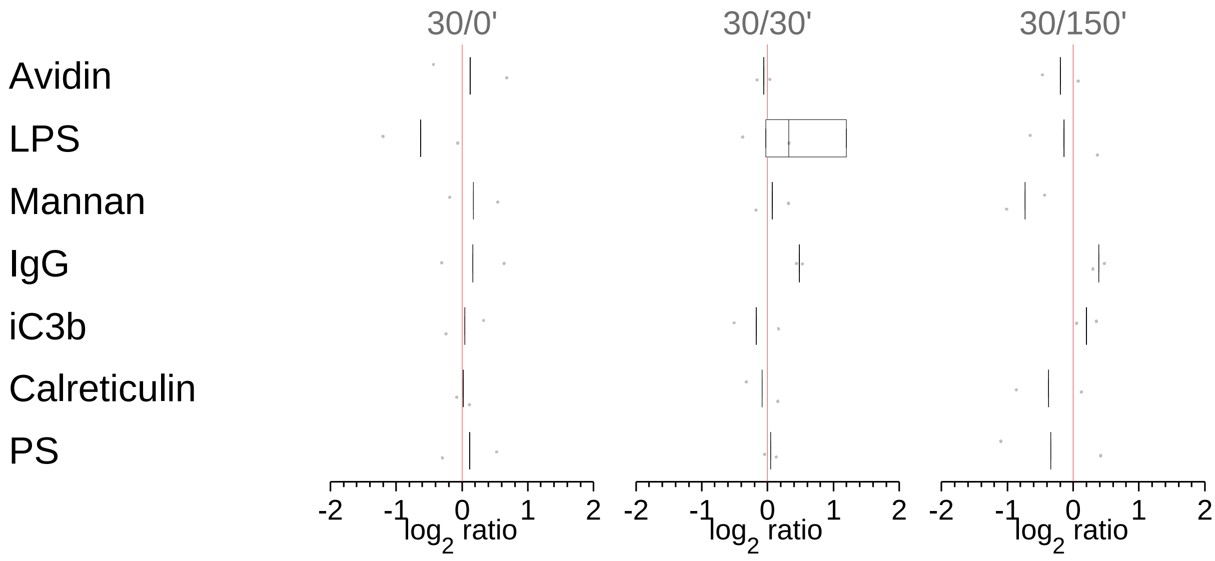

GO:0043085 positive regulation of catalytic activity (n = 14)

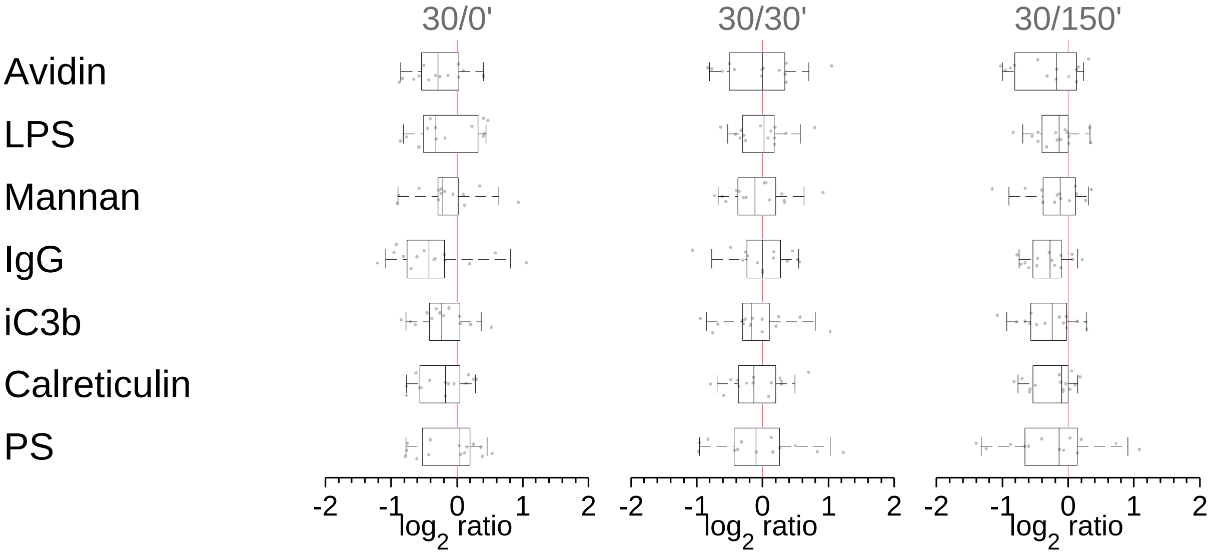

GO:0043086 negative regulation of catalytic activity (n = 9)

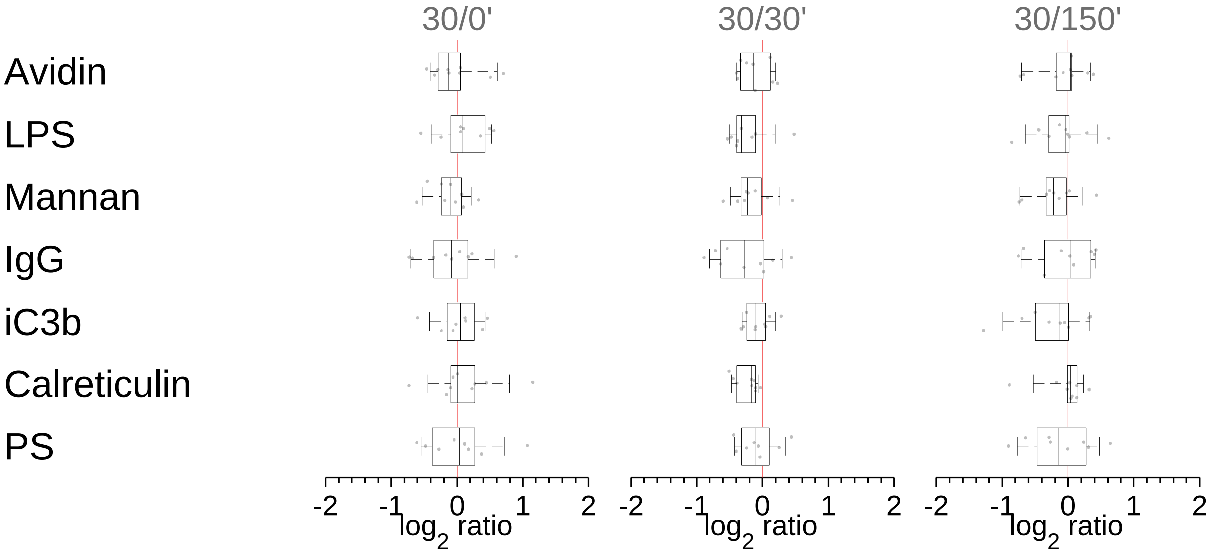

GO:0043122 regulation of I-kappaB kinase/NF-kappaB cascade (n = 3)

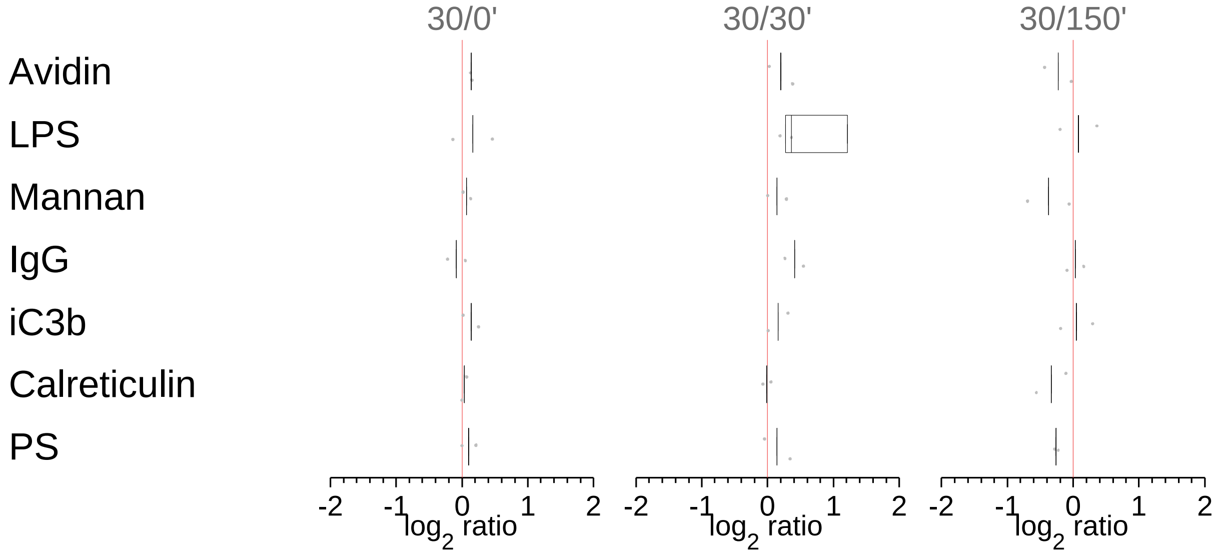

GO:0043123 positive regulation of I-kappaB kinase/NF-kappaB cascade (n = 25)

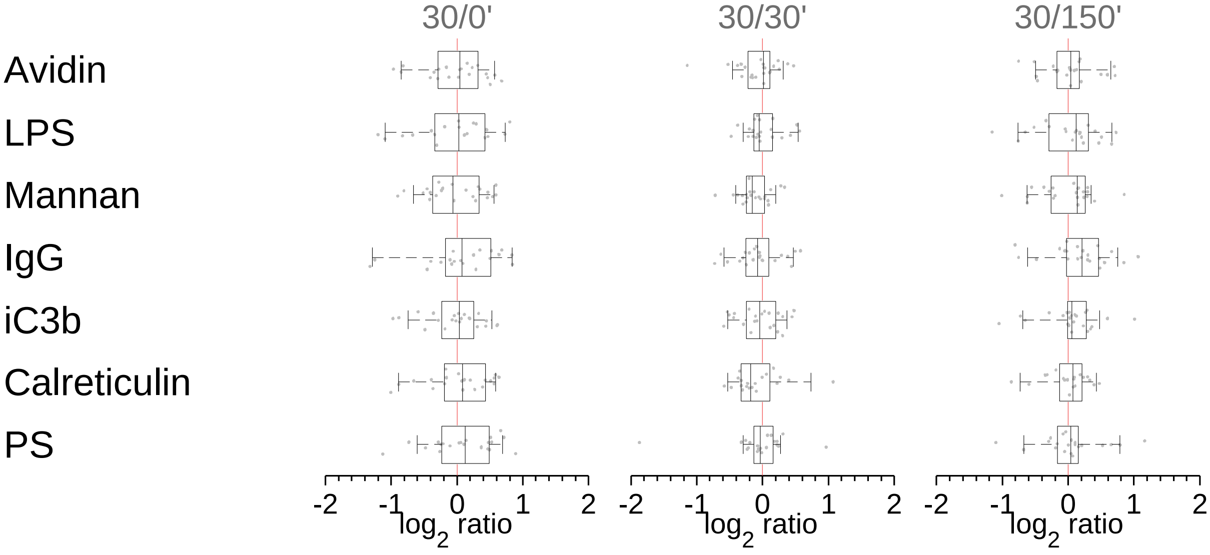

GO:0043124 negative regulation of I-kappaB kinase/NF-kappaB cascade (n = 4)

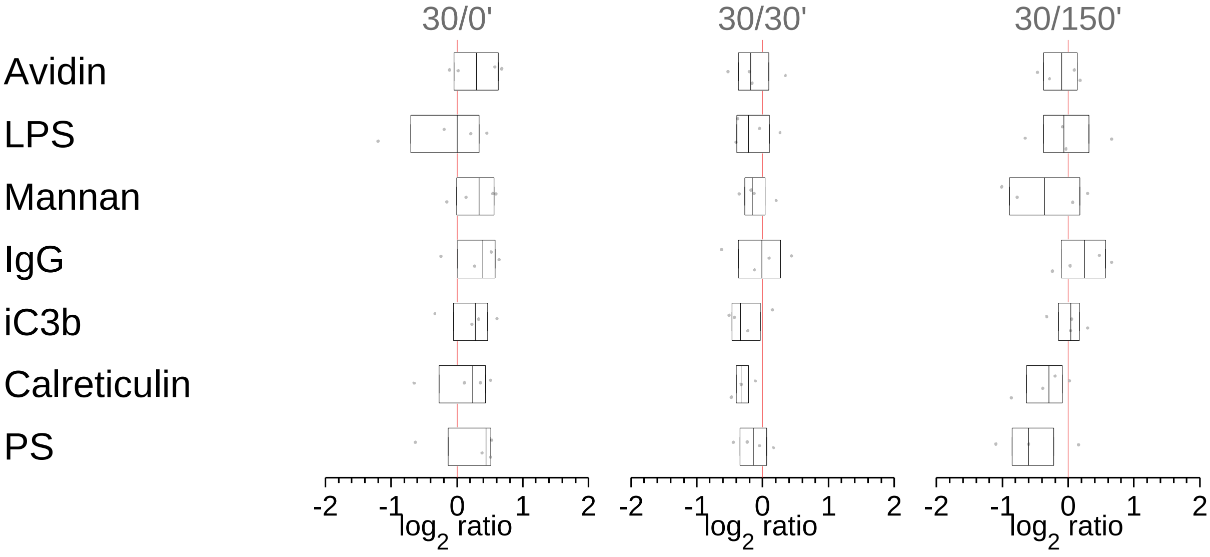

GO:0043130 ubiquitin binding (n = 8)

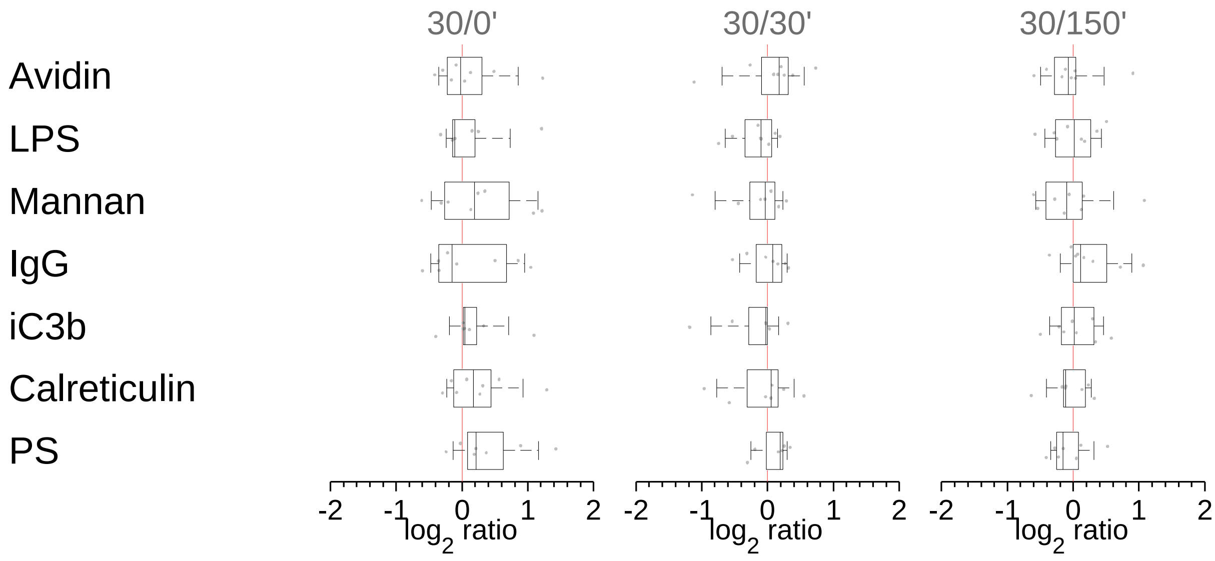

GO:0043154 negative regulation of cysteine-type endopeptidase activity involved in apoptotic process (n = 14)

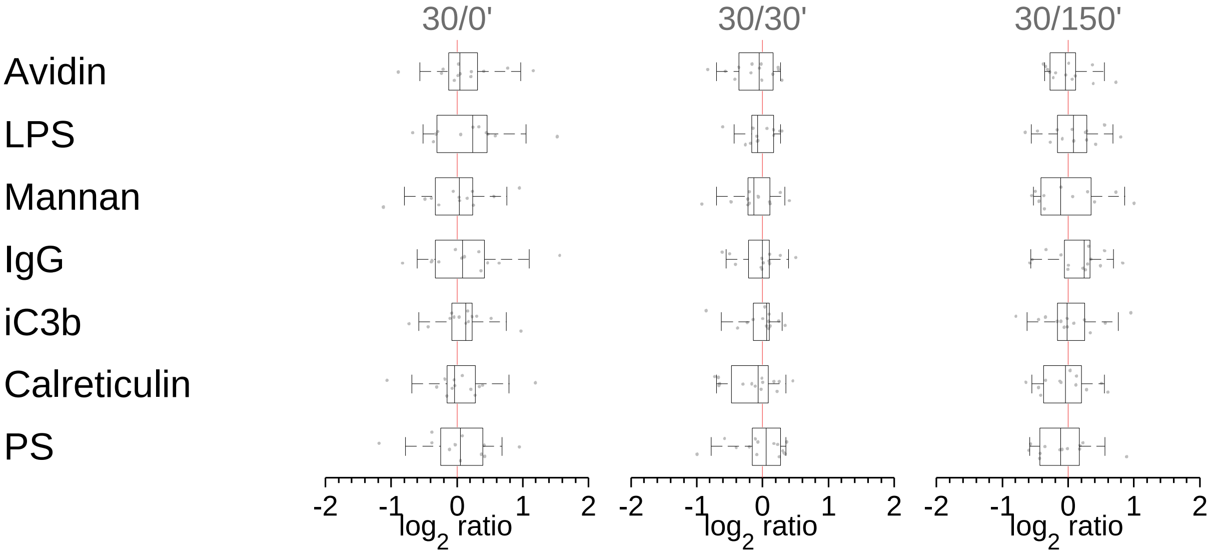

GO:0043161 proteasomal ubiquitin-dependent protein catabolic process (n = 12)

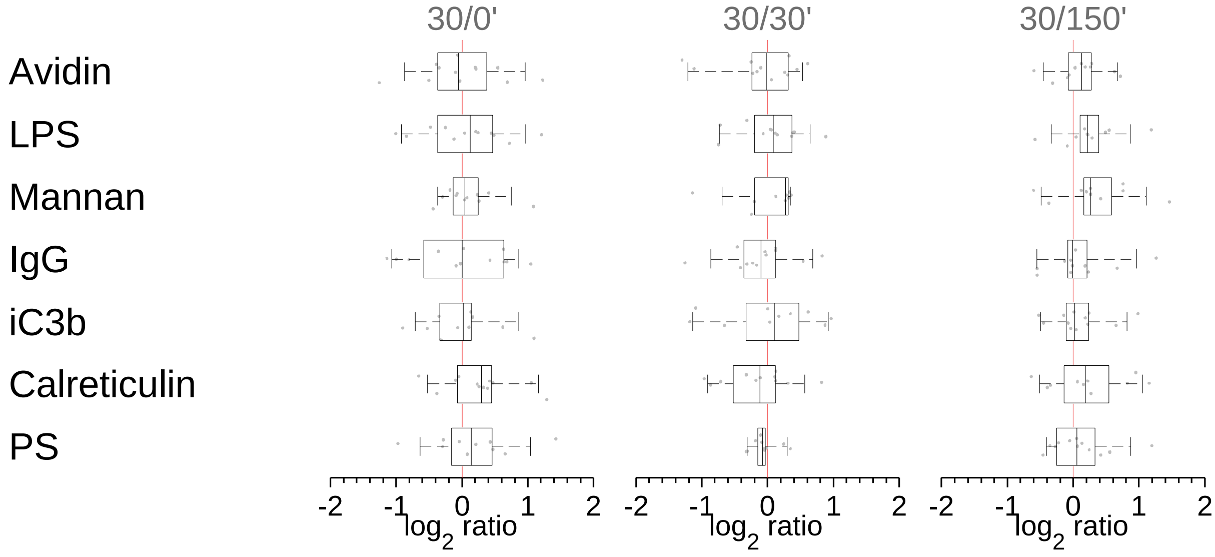

GO:0043162 ubiquitin-dependent protein catabolic process via the multivesicular body sorting pathway (n = 3)

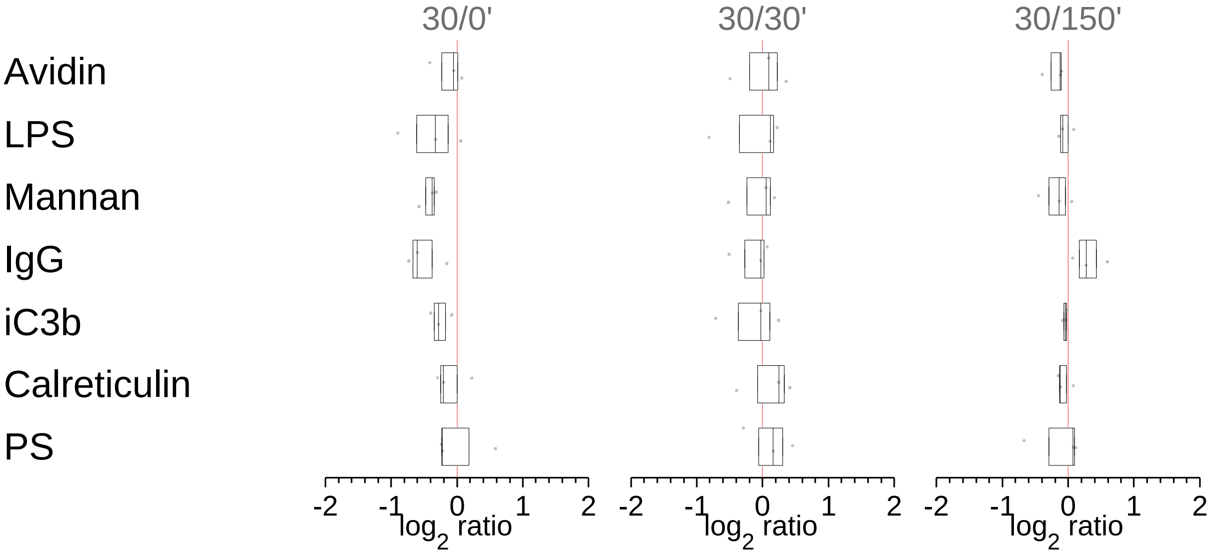

GO:0043195 terminal button (n = 7)

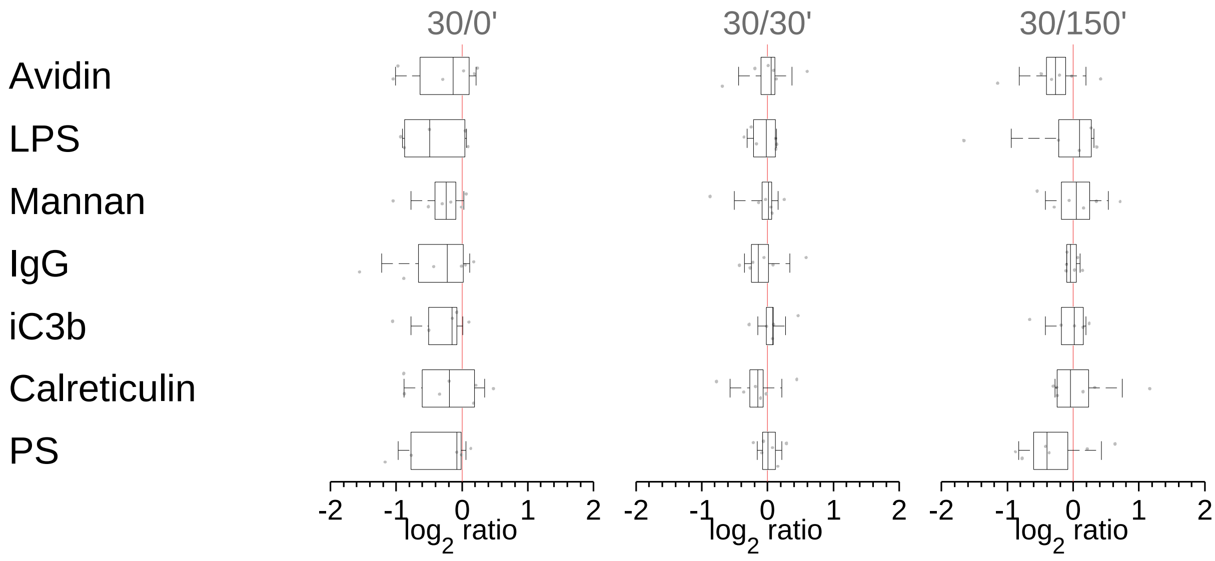

GO:0043197 dendritic spine (n = 12)

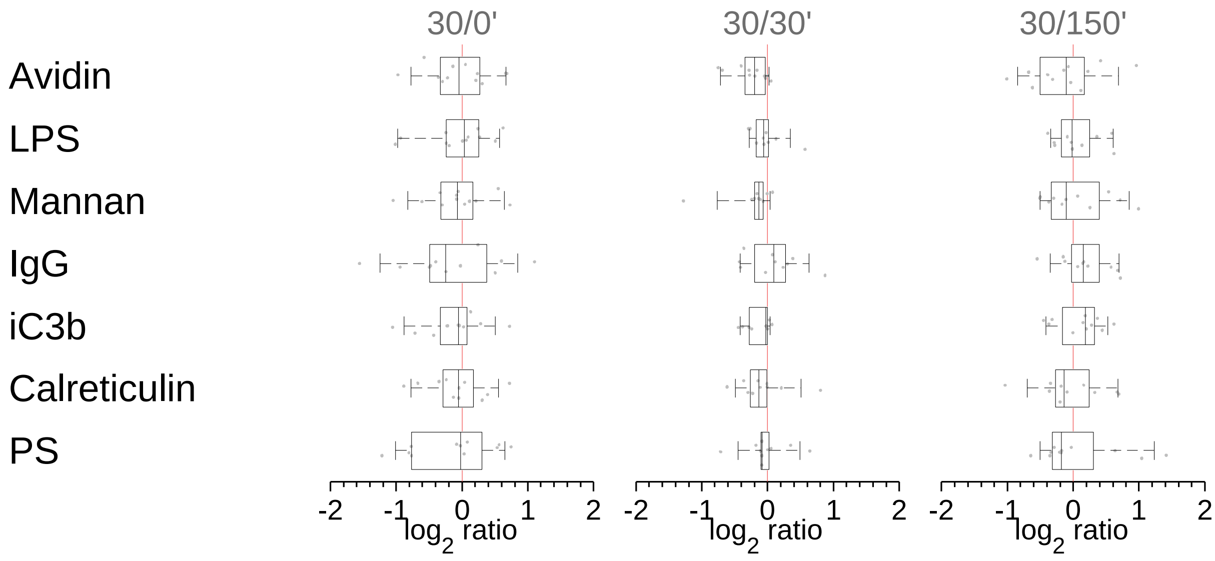

GO:0043198 dendritic shaft (n = 6)

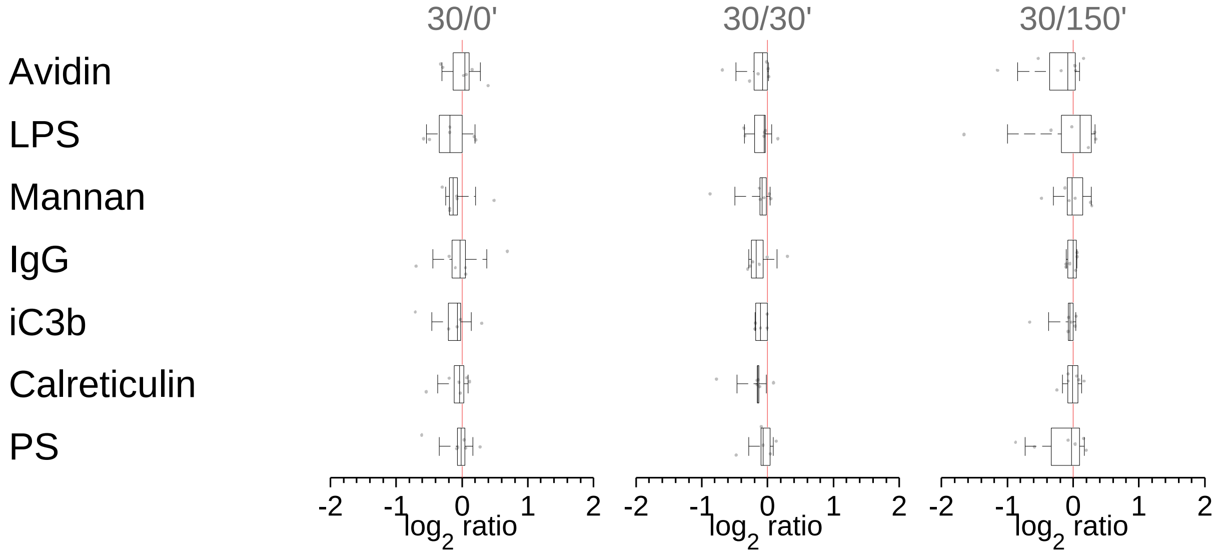

GO:0043202 lysosomal lumen (n = 3)

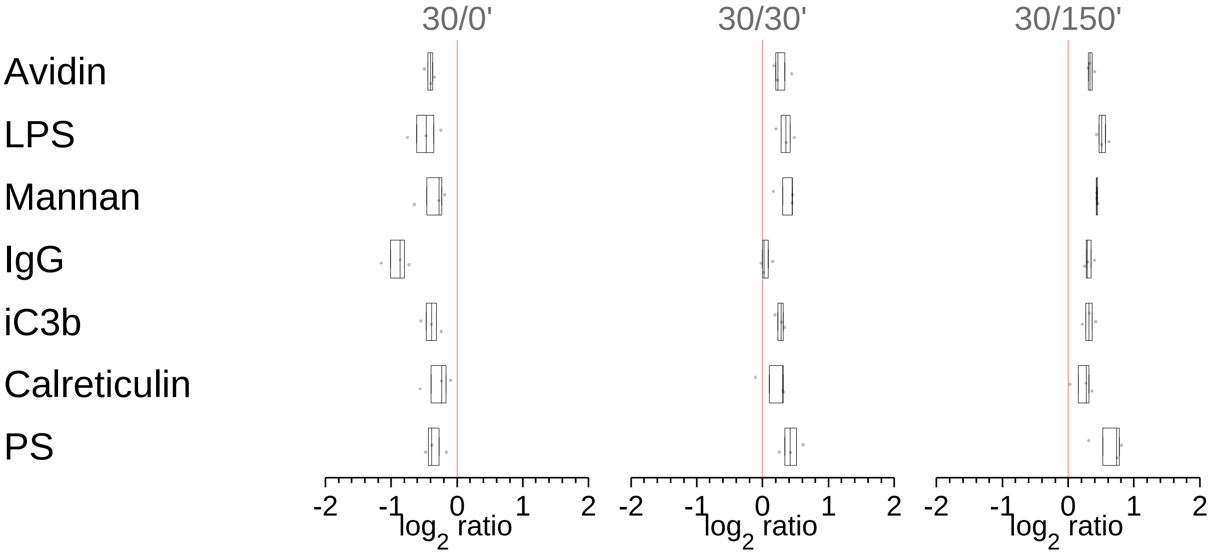

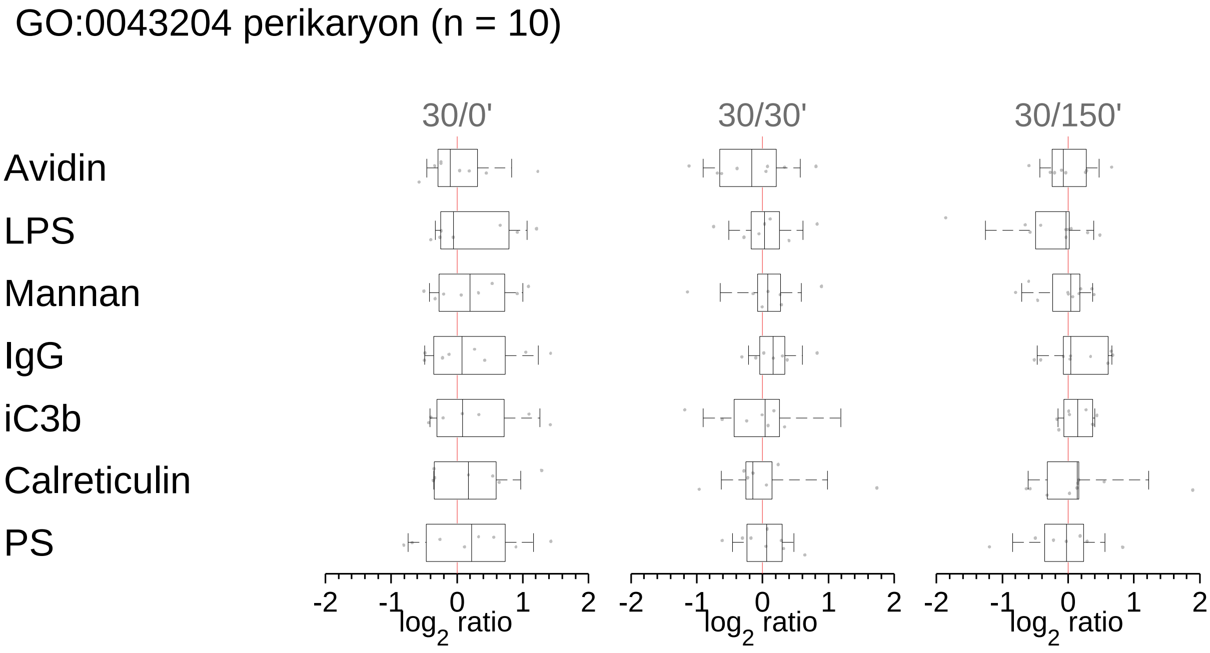
GO:0043204 perikaryon (n = 10)
30/0'
30/30'
30/150'
Avidin
LPS
Mannan
IgG
iC3b
Calreticulin
PS
-2
-1
0
1
2
log2 ratio

GO:0043206 fibril organization (n = 3)

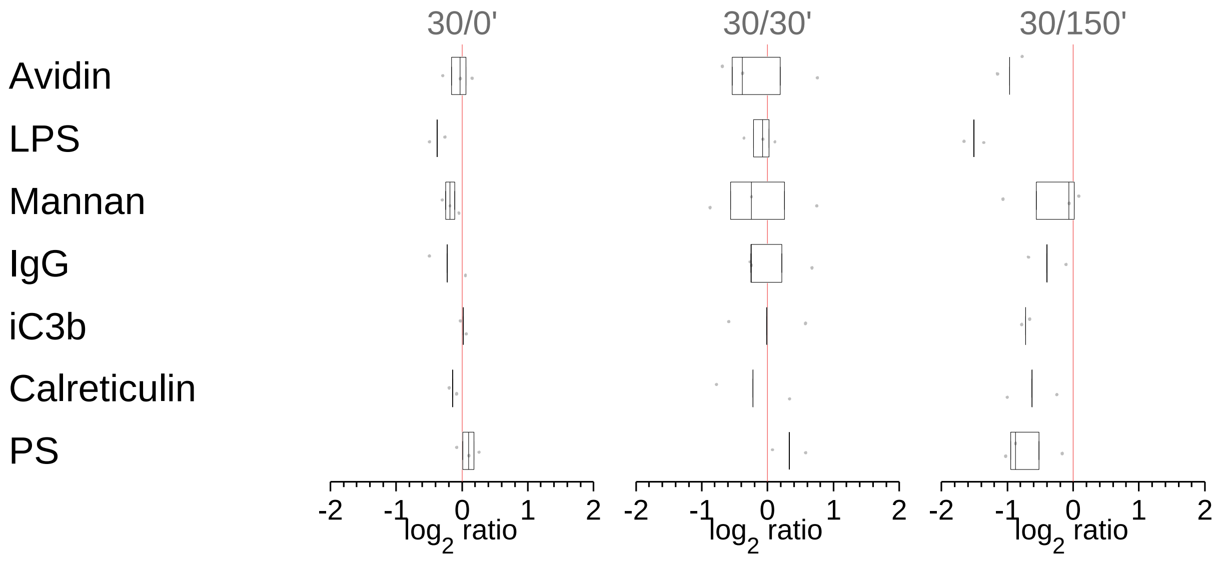

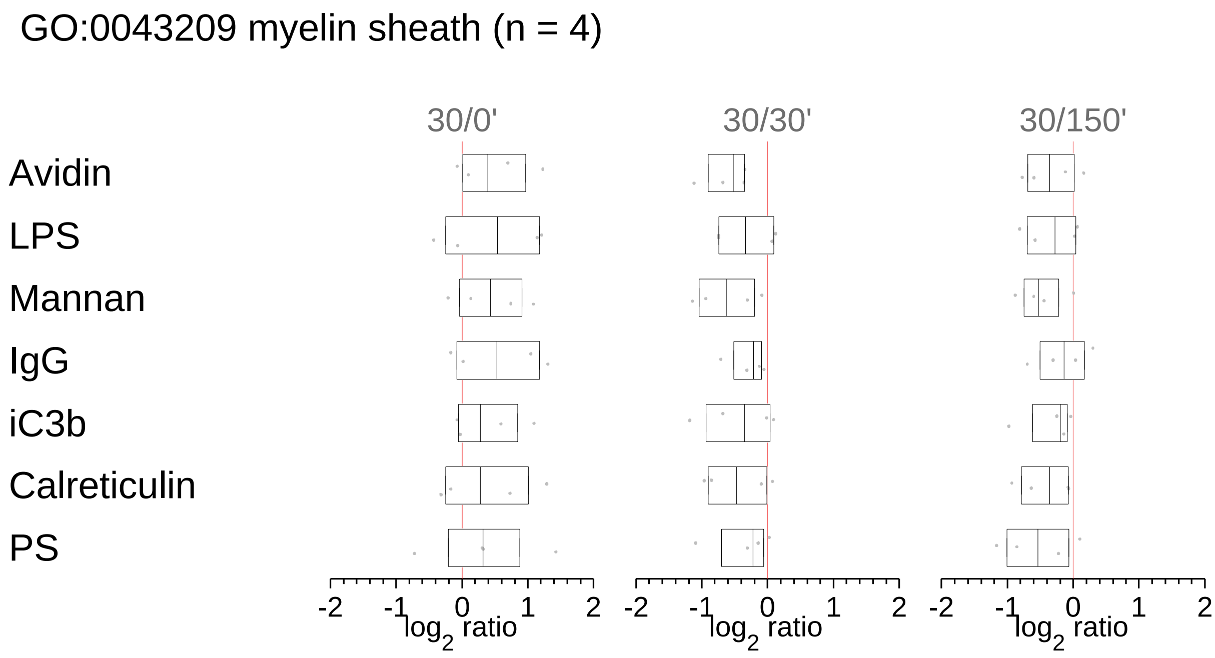
GO:0043209 myelin sheath (n = 4)
30/0'
30/30'
30/150'
Avidin
LPS
Mannan
IgG
iC3b
Calreticulin
PS
-2
-1
0
1
2
log2 ratio

GO:0043220 Schmidt-Lanterman incisure (n = 3)

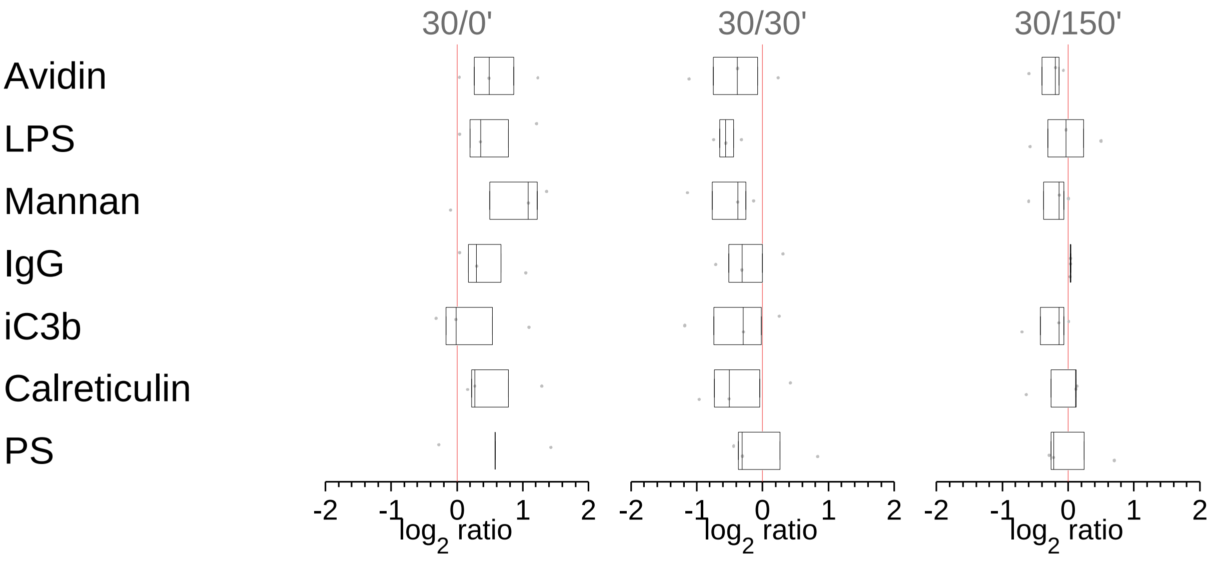

GO:0043231 intracellular membrane-bounded organelle (n = 128)

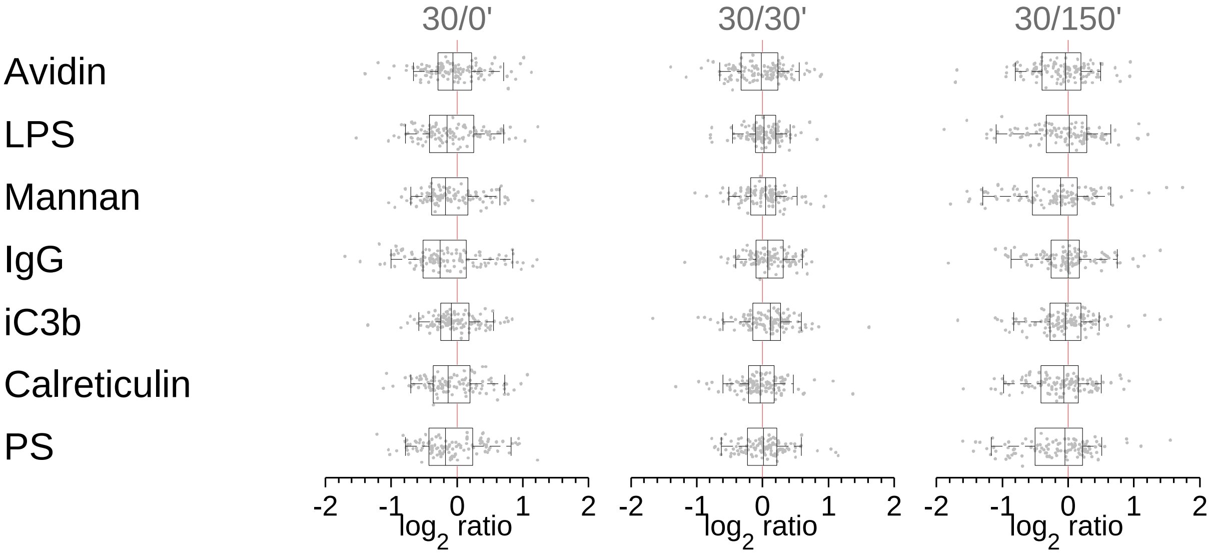

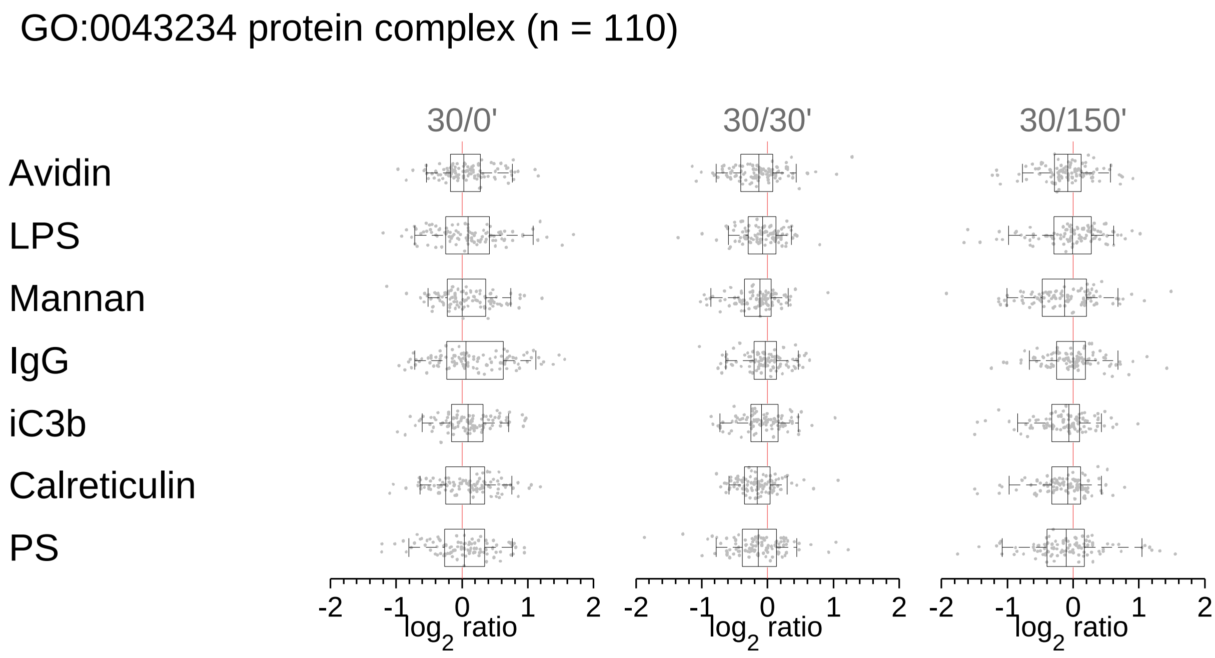
GO:0043234 protein complex (n = 110)
30/0'
30/30'
30/150'
Avidin
LPS
Mannan
IgG
iC3b
Calreticulin
PS
-2
-1
0
1
2
log2 ratio

GO:0043235 receptor complex (n = 17)

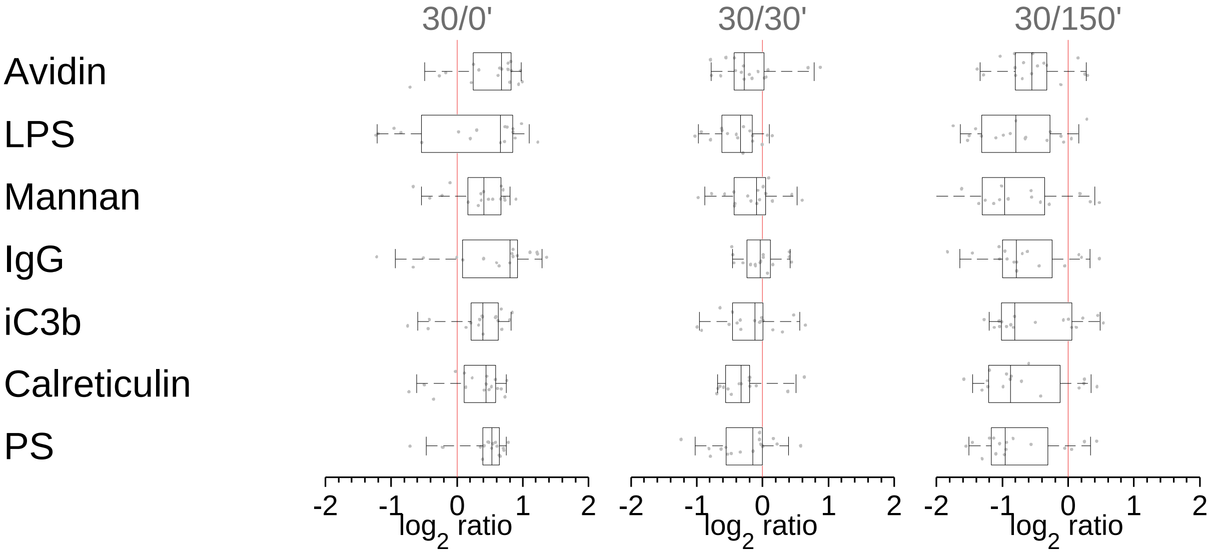

GO:0043236 laminin binding (n = 7)

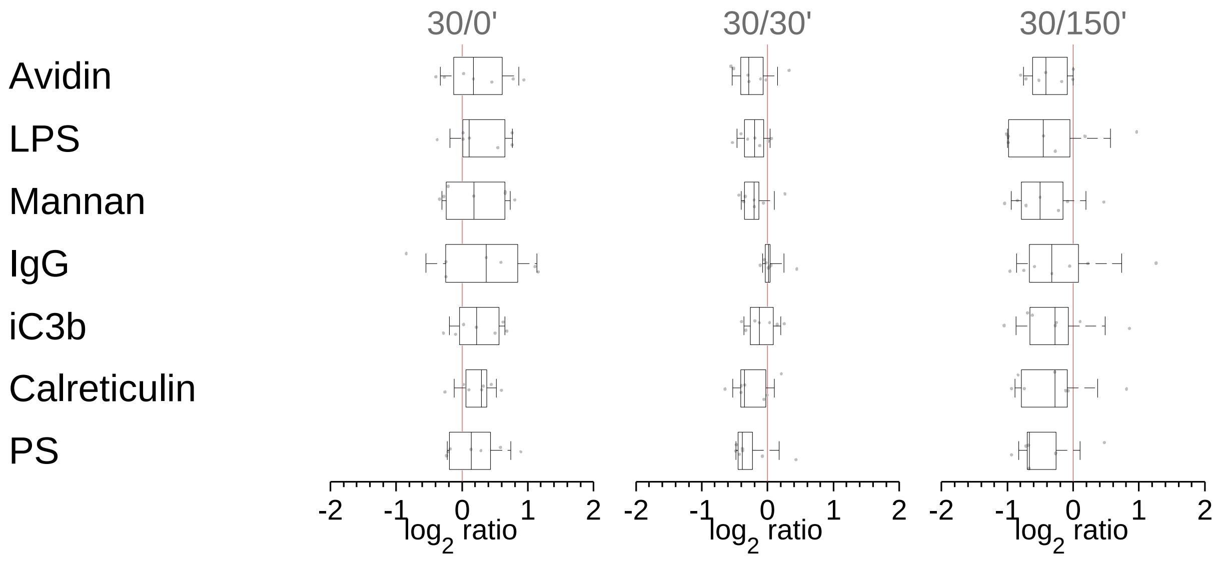

GO:0043277 apoptotic cell clearance (n = 6)

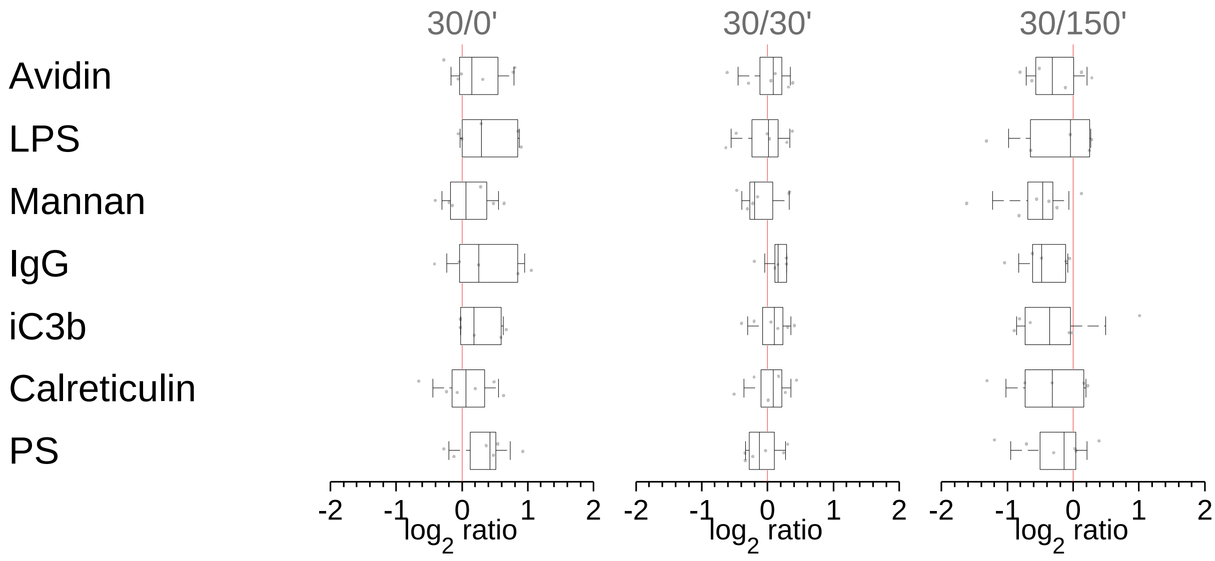

GO:0043280 positive regulation of cysteine-type endopeptidase activity involved in apoptotic process (n = 6)

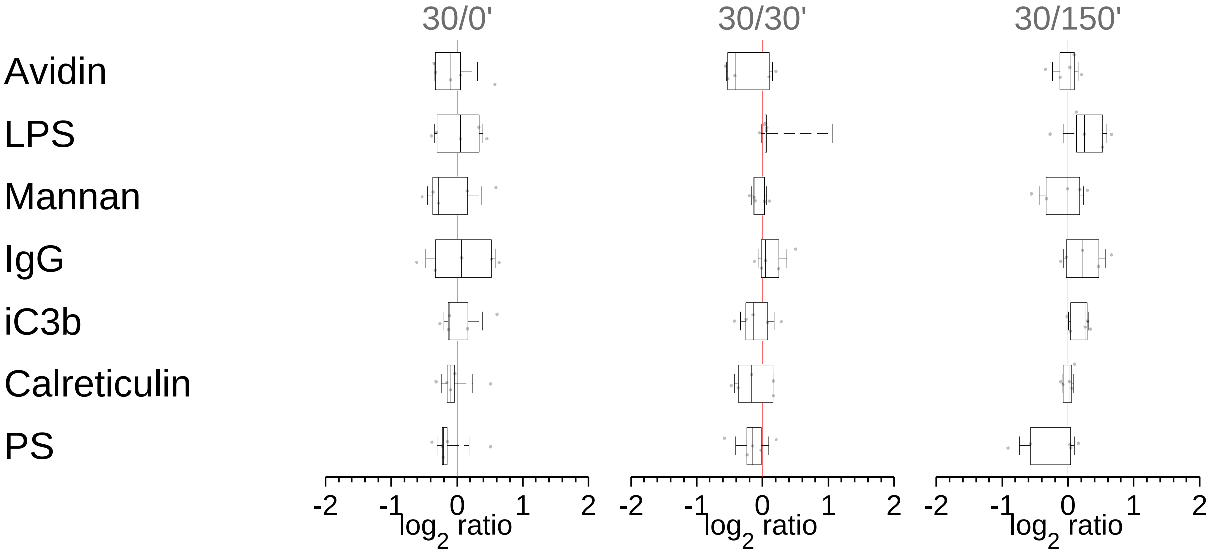

GO:0043292 contractile fiber (n = 5)

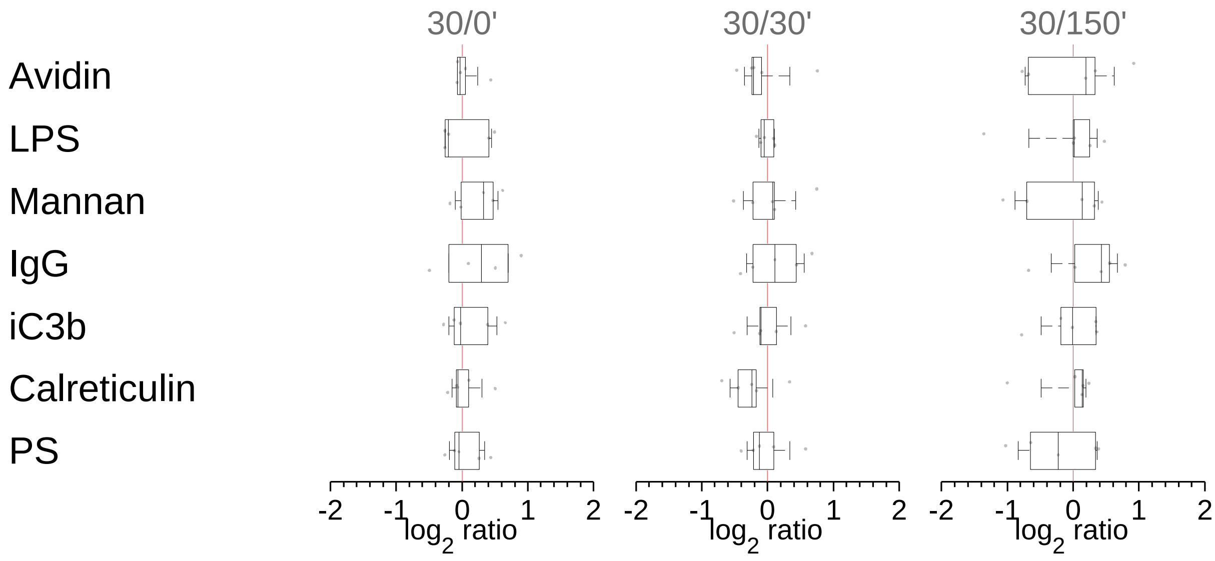

GO:0043295 glutathione binding (n = 3)

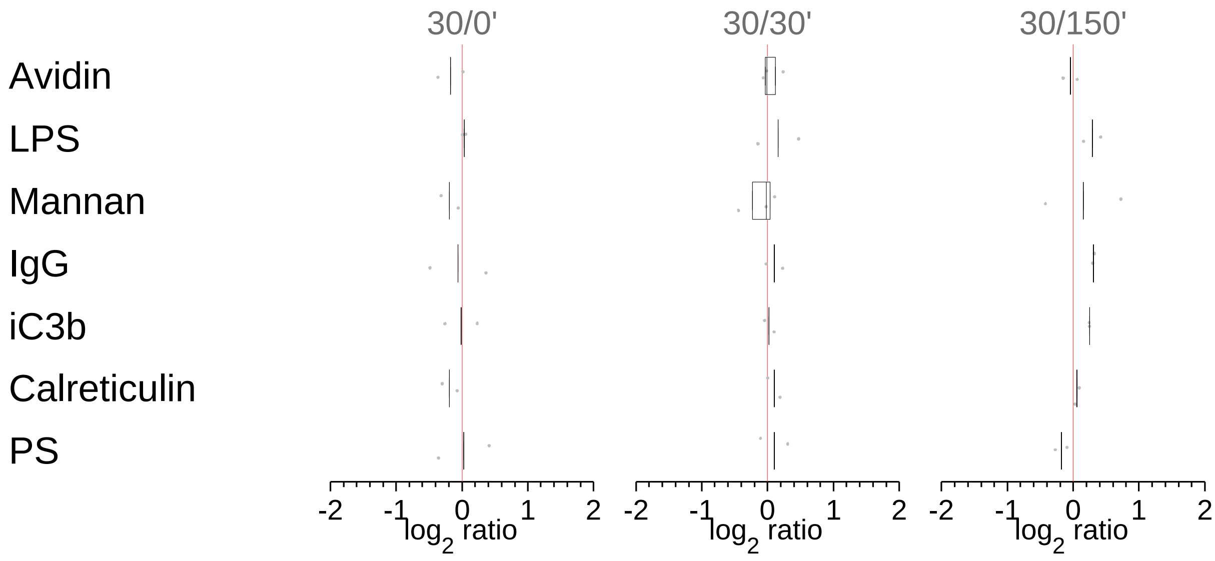

GO:0043304 regulation of mast cell degranulation (n = 4)

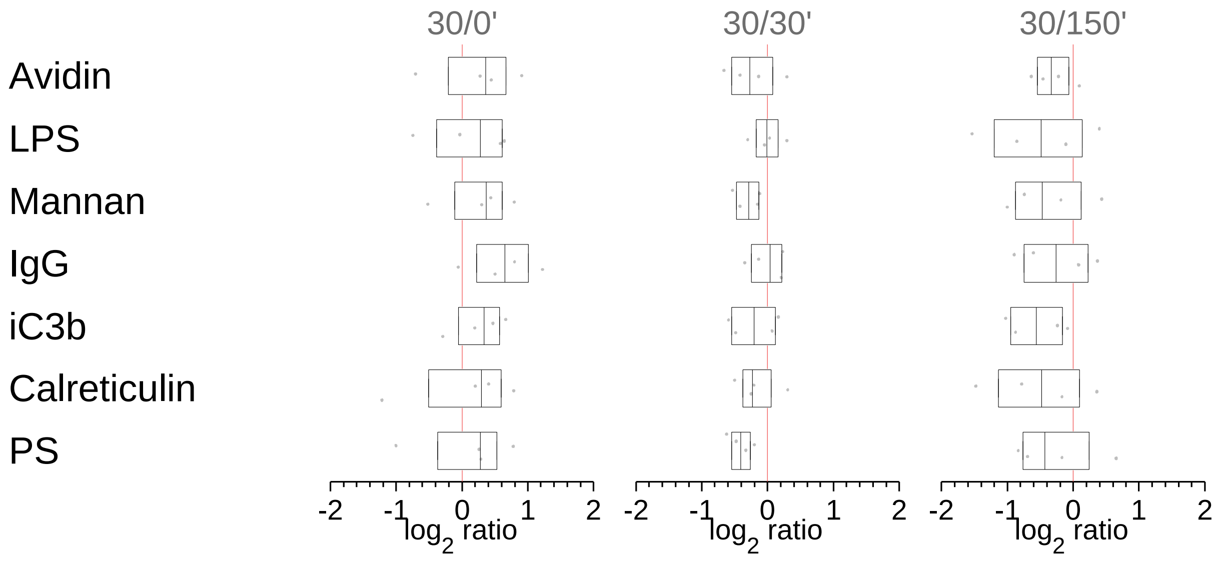

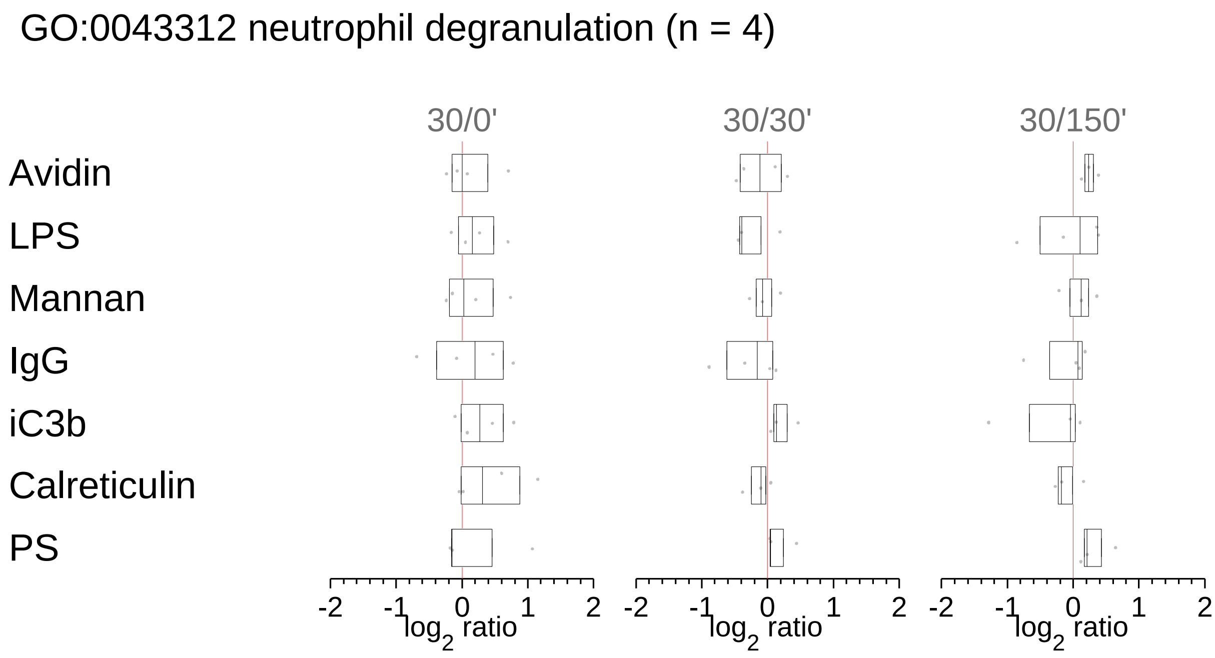
GO:0043312 neutrophil degranulation (n = 4)
30/0'
30/30'
30/150'
Avidin
LPS
Mannan
IgG
iC3b
Calreticulin
PS
-2
-1
0
1
2
log2 ratio

GO:0043320 natural killer cell degranulation (n = 3)

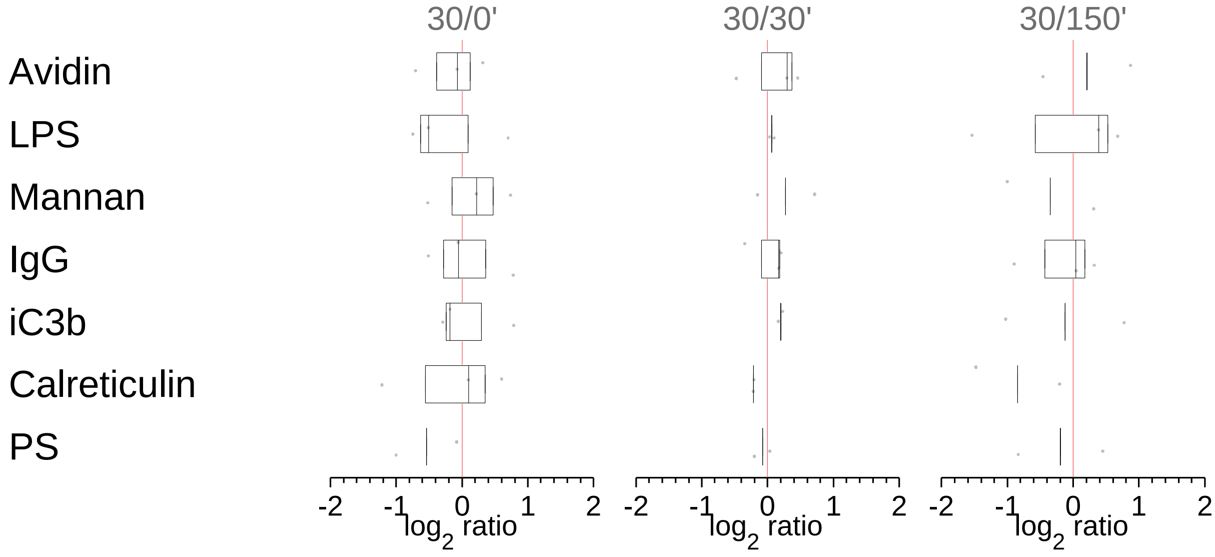

GO:0043325 phosphatidylinositol-3,4-bisphosphate binding (n = 4)

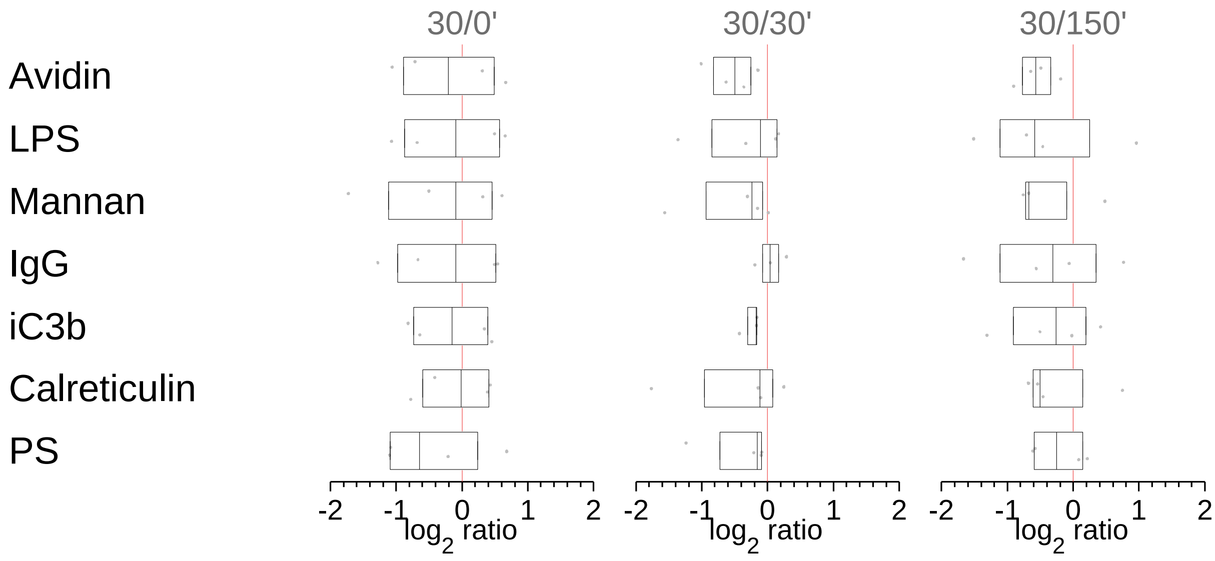

GO:0043330 response to exogenous dsRNA (n = 5)

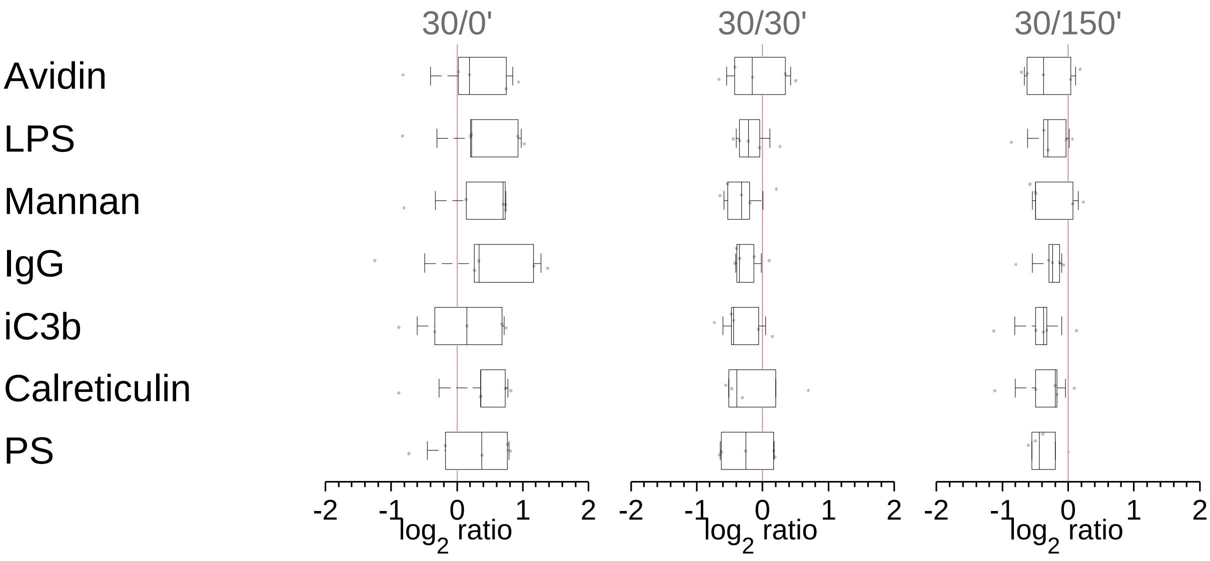

GO:0043392 negative regulation of DNA binding (n = 3)

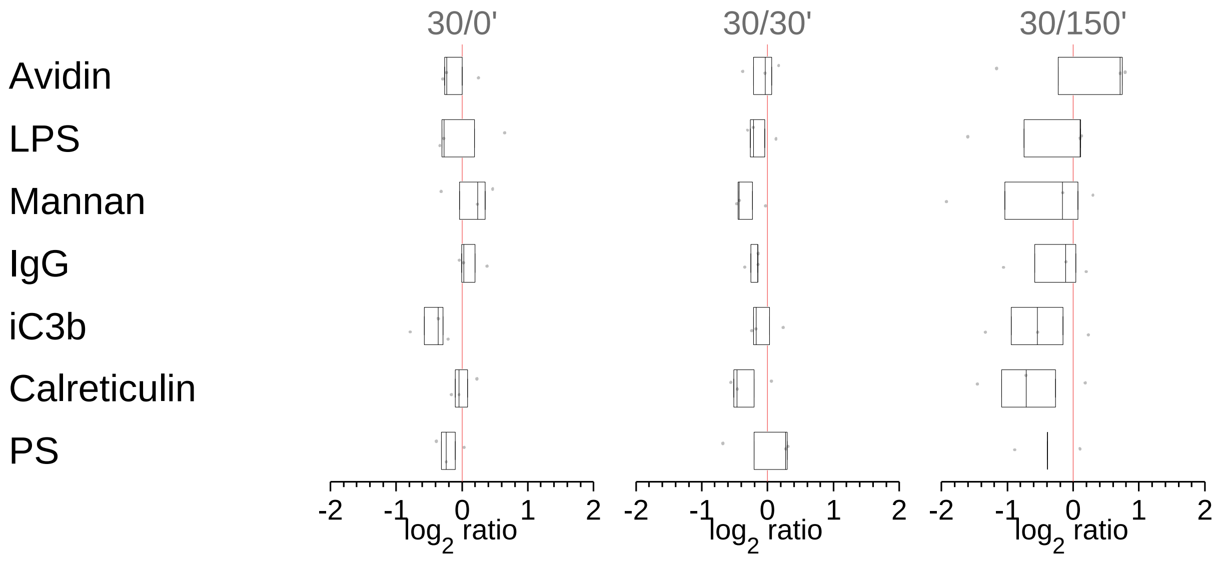

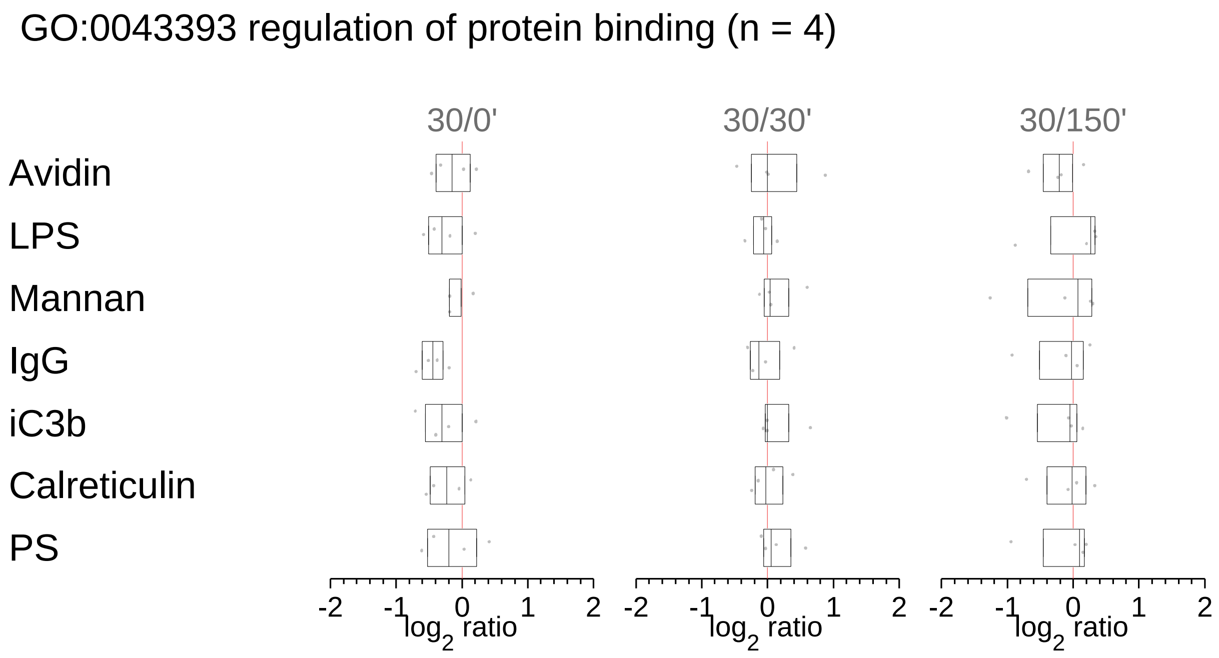
GO:0043393 regulation of protein binding (n = 4)
30/0'
30/30'
30/150'
Avidin
LPS
Mannan
IgG
iC3b
Calreticulin
PS
-2 -1 0 1 2
log2 ratio

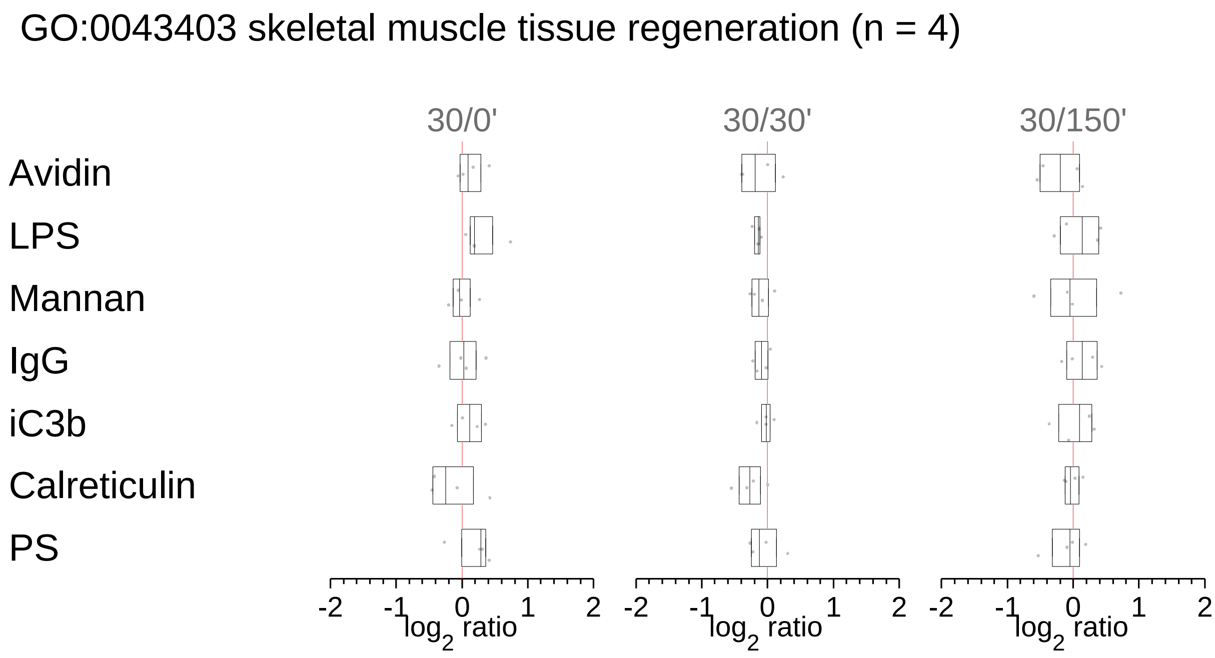

GO:0043403 skeletal muscle tissue regeneration (n = 4)
30/0'
30/30'
30/150'
Avidin
LPS
Mannan
IgG
iC3b
Calreticulin
PS
-2
-1
0
1
2
log2 ratio

GO:0043406 positive regulation of MAP kinase activity (n = 8)

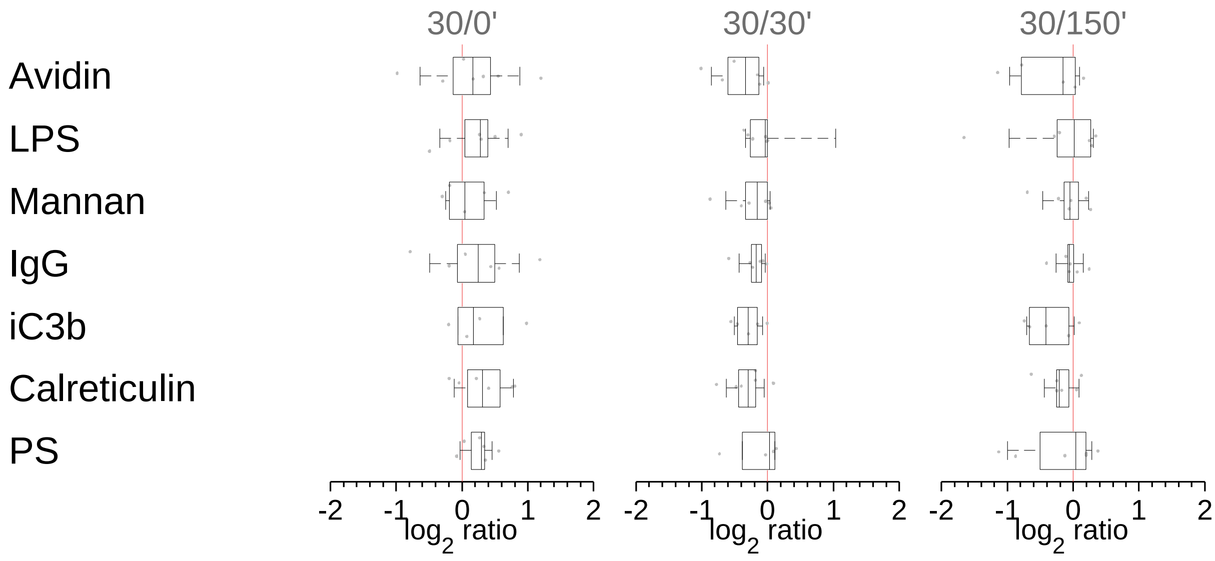

GO:0043407 negative regulation of MAP kinase activity (n = 10)

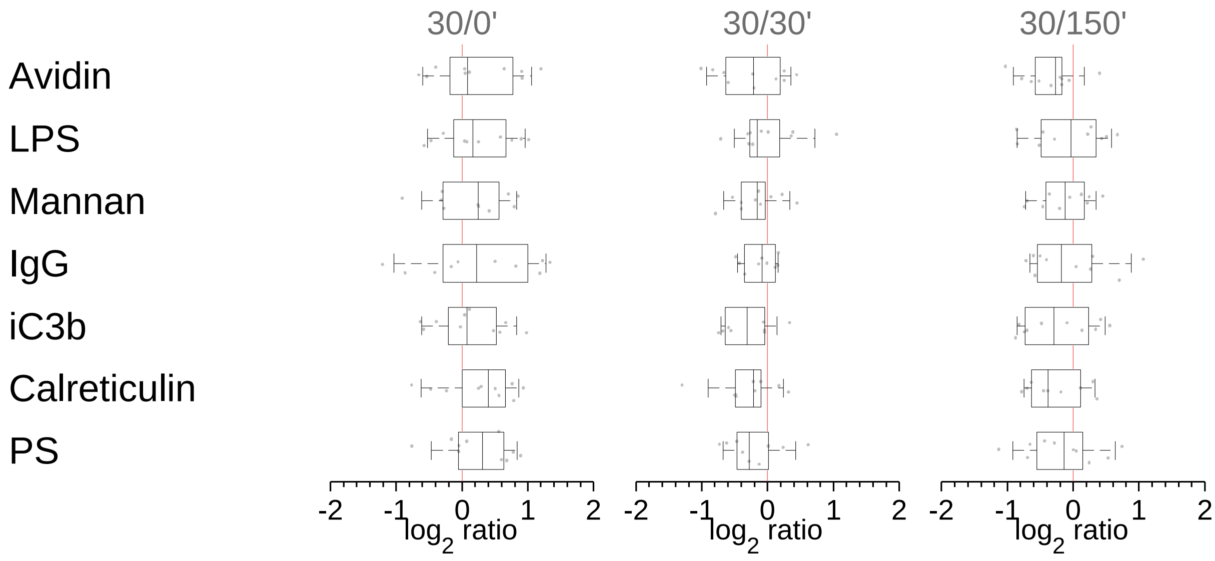

GO:0043408 regulation of MAPK cascade (n = 6)

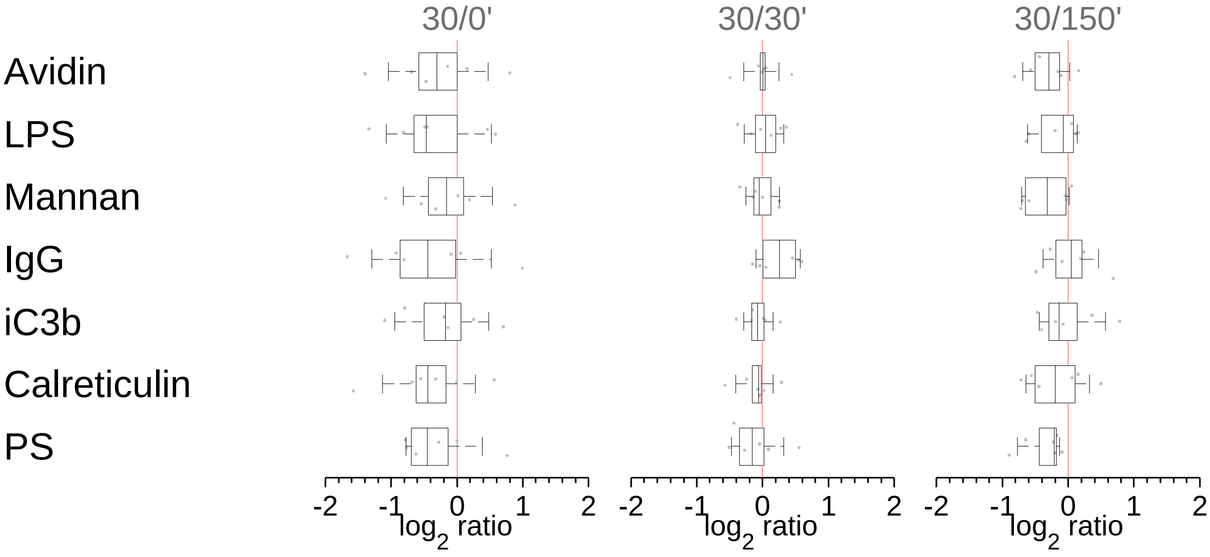

GO:0043409 negative regulation of MAPK cascade (n = 3)

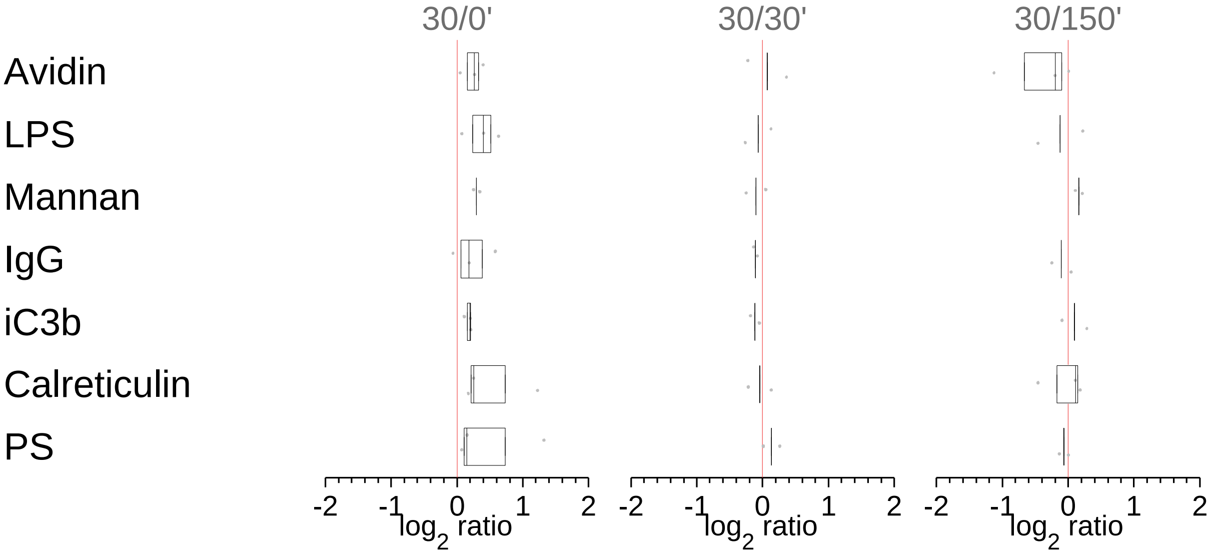

GO:0043410 positive regulation of MAPK cascade (n = 11)

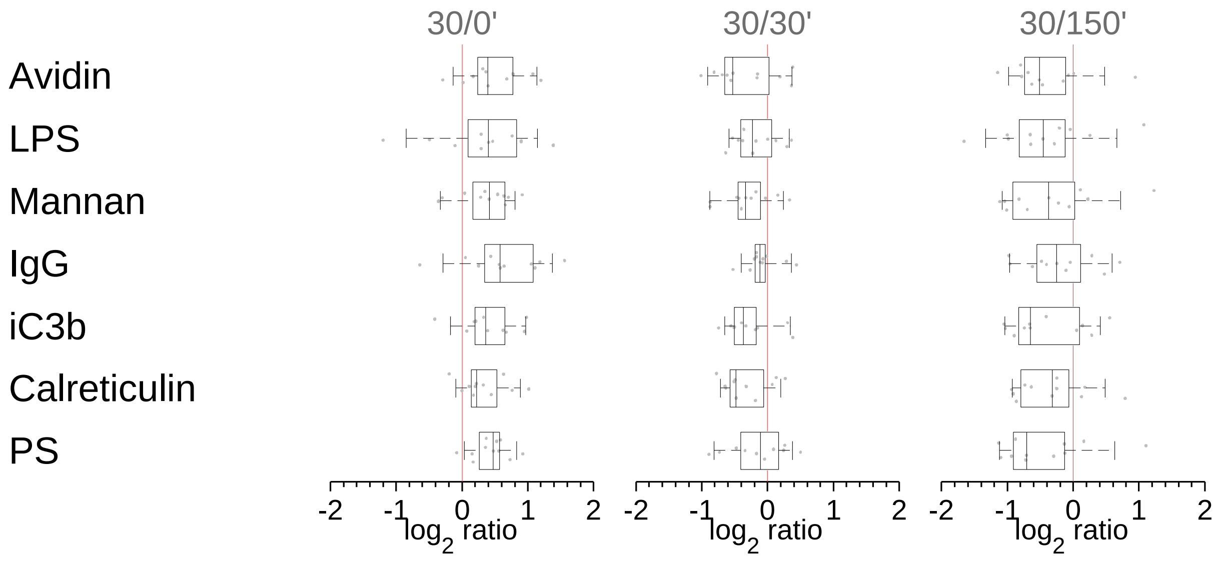

GO:0043433 negative regulation of sequence-specific DNA binding transcription factor activity (n = 5)

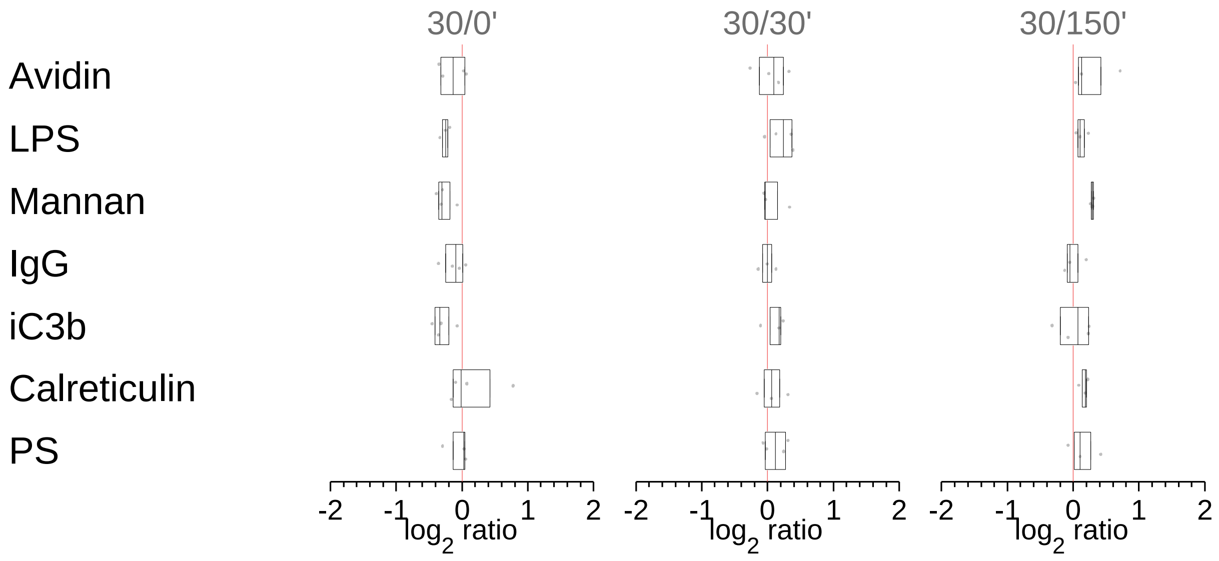

GO:0043434 response to peptide hormone stimulus (n = 3)

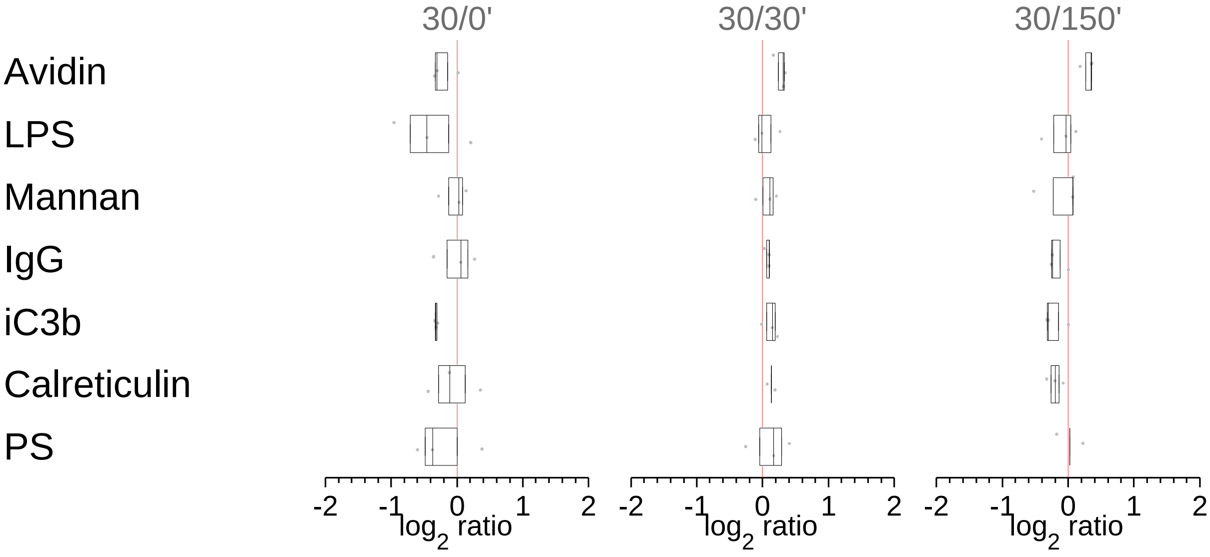

GO:0043473 pigmentation (n = 7)

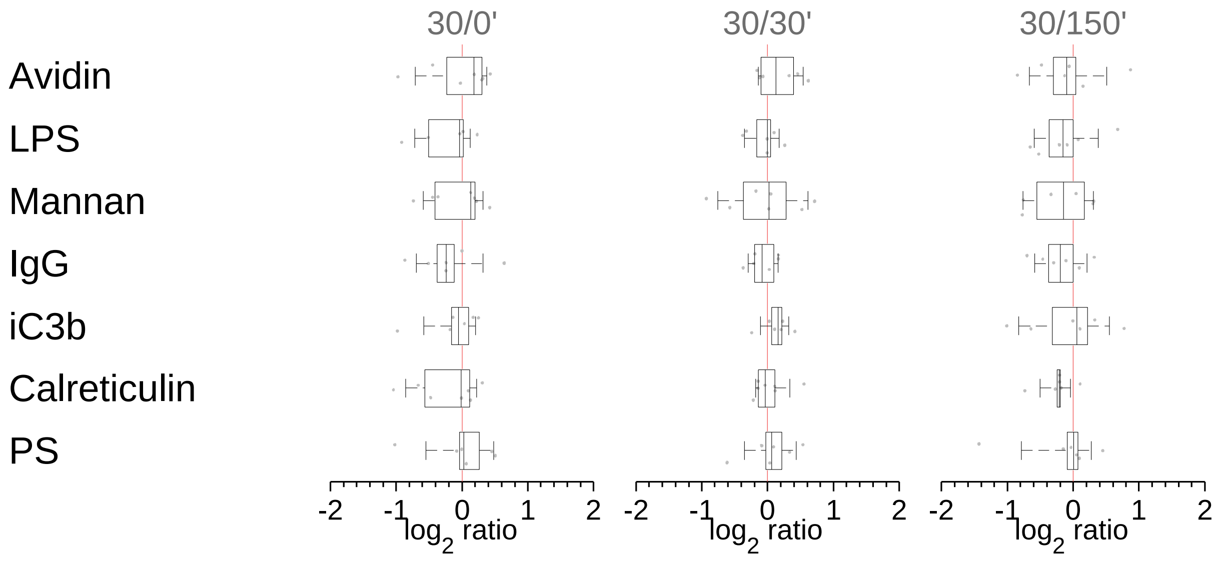

GO:0043484 regulation of RNA splicing (n = 3)

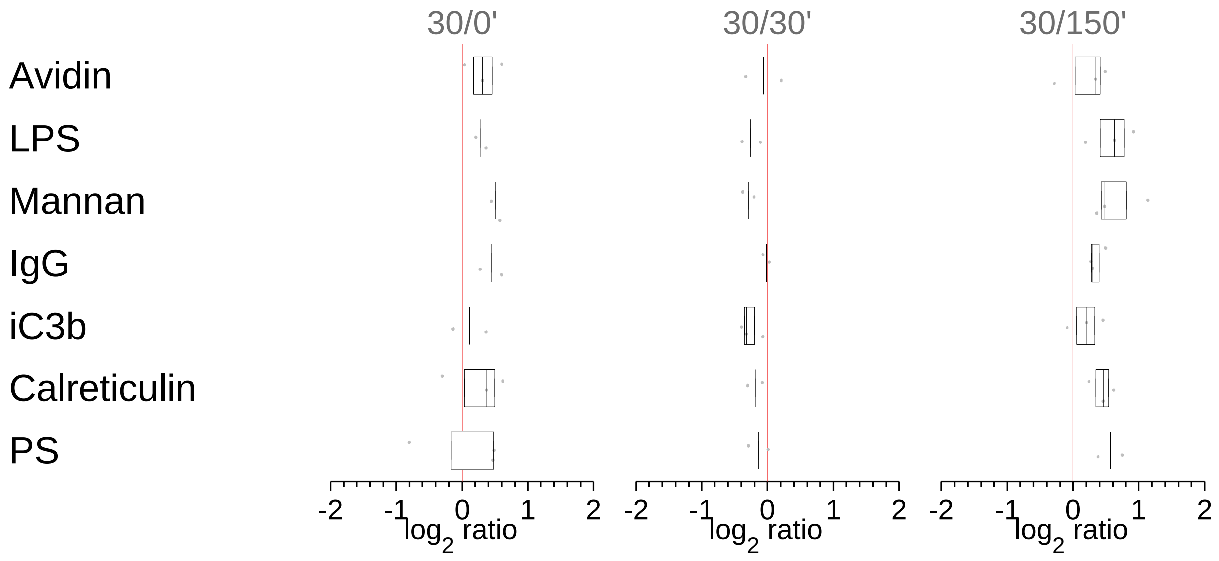

GO:0043491 protein kinase B signaling cascade (n = 3)

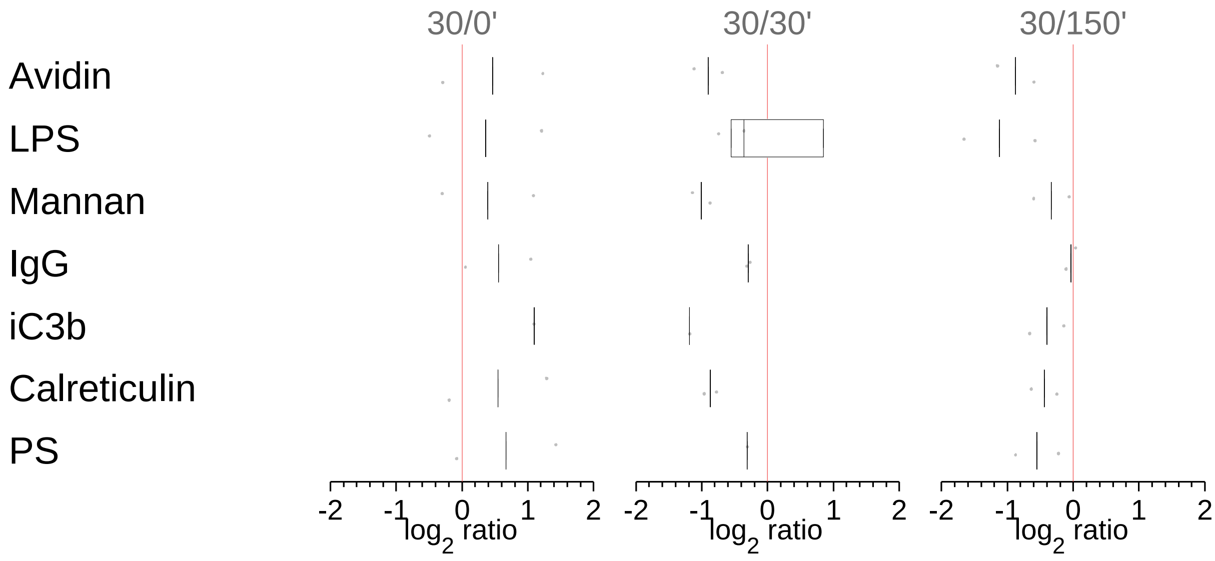

GO:0043497 regulation of protein heterodimerization activity (n = 3)

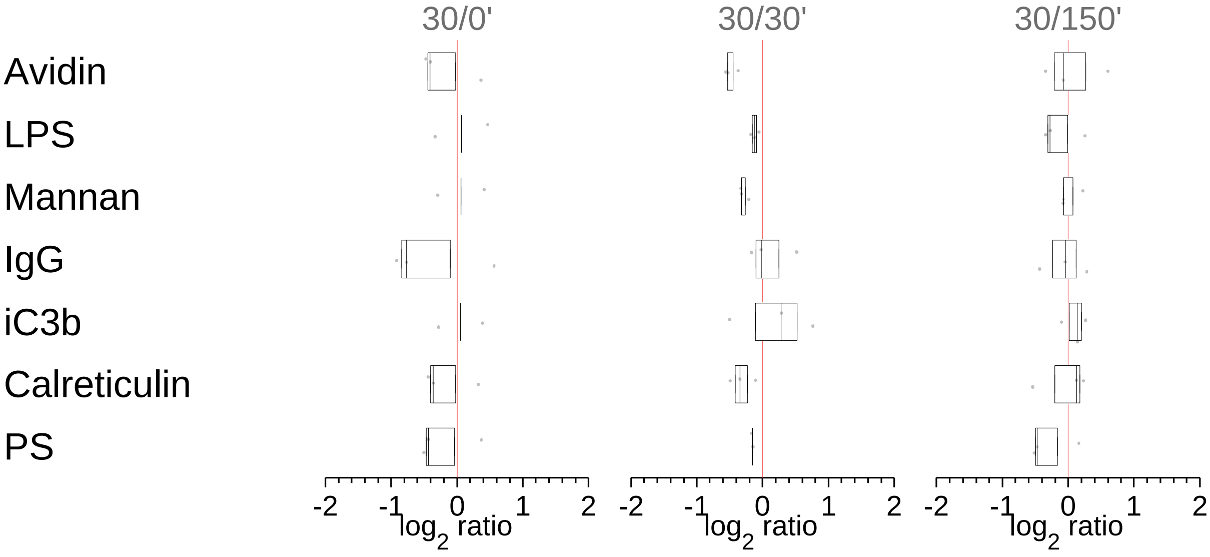

GO:0043507 positive regulation of JUN kinase activity (n = 4)

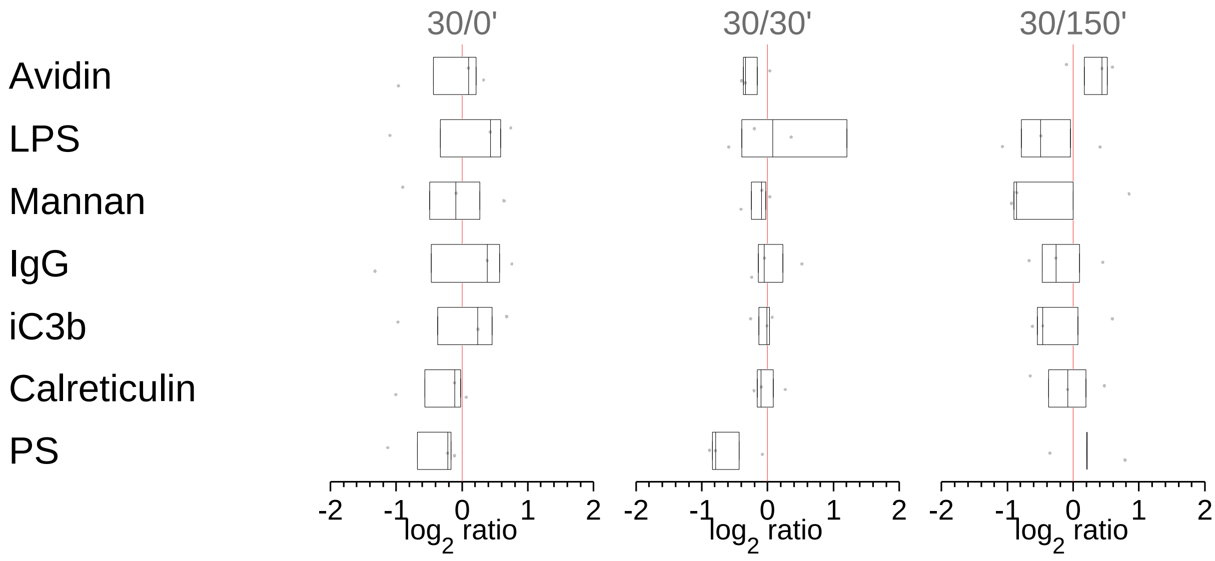

GO:0043518 negative regulation of DNA damage response, signal transduction by p53 class mediator (n = 3)

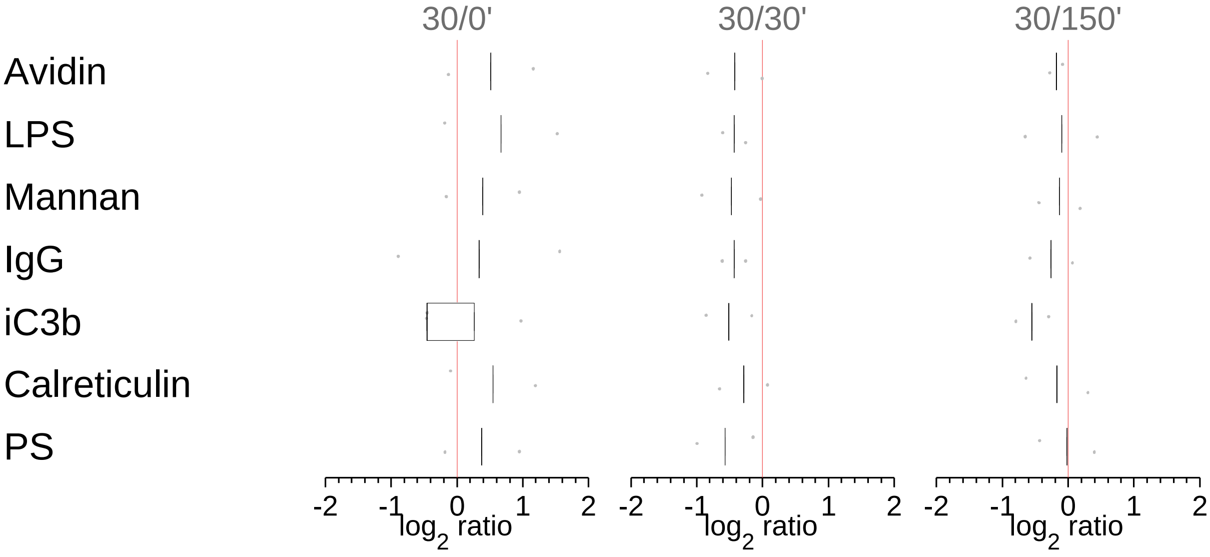

GO:0043523 regulation of neuron apoptotic process (n = 5)

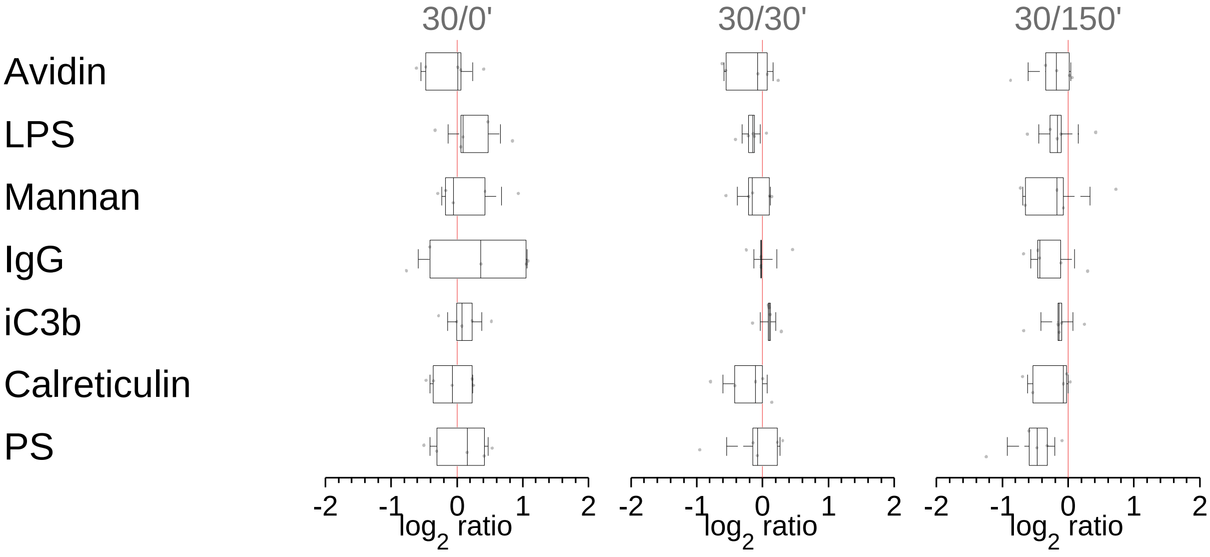

GO:0043524 negative regulation of neuron apoptotic process (n = 29)

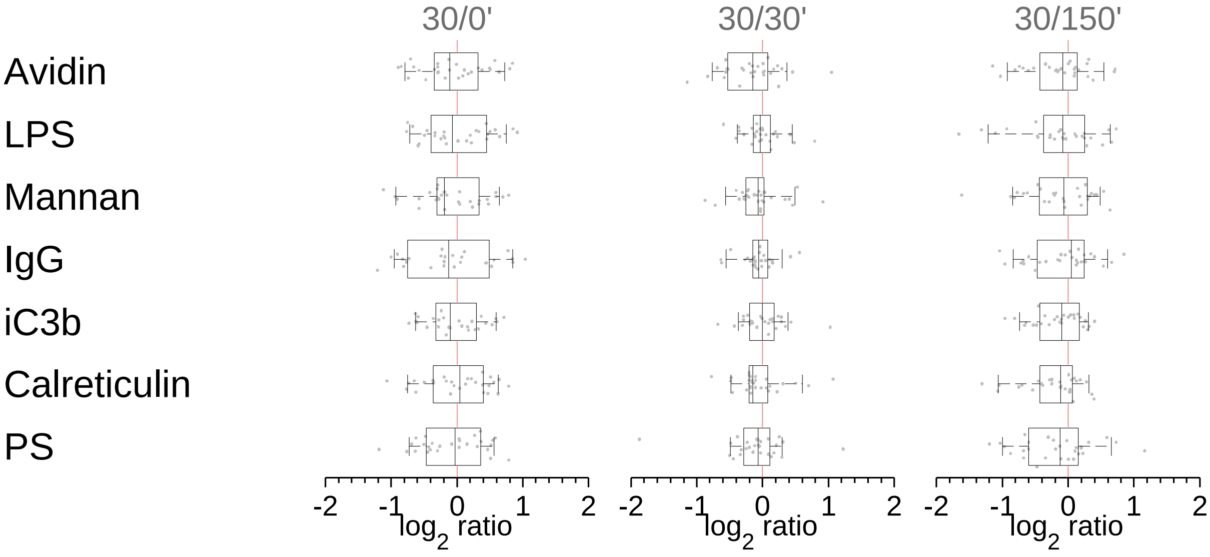

GO:0043525 positive regulation of neuron apoptotic process (n = 8)

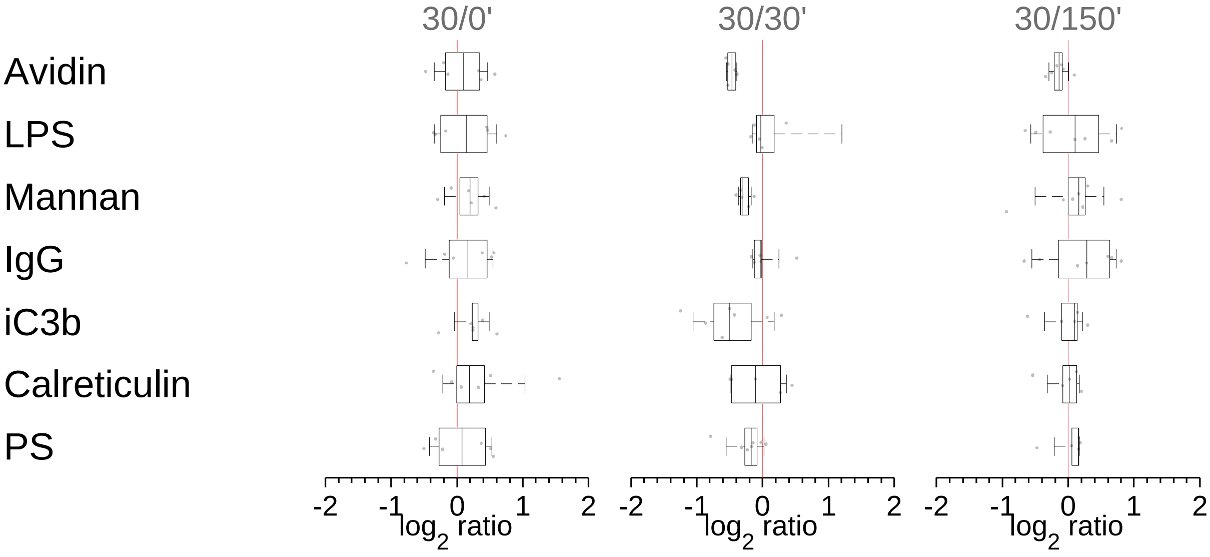

GO:0043531 ADP binding (n = 10)

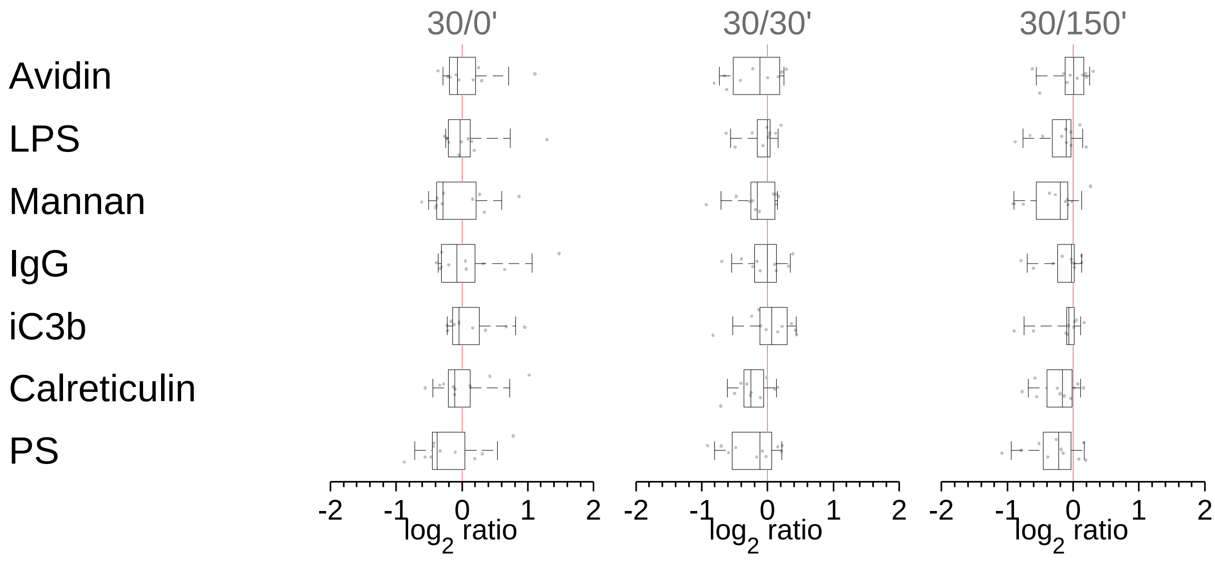

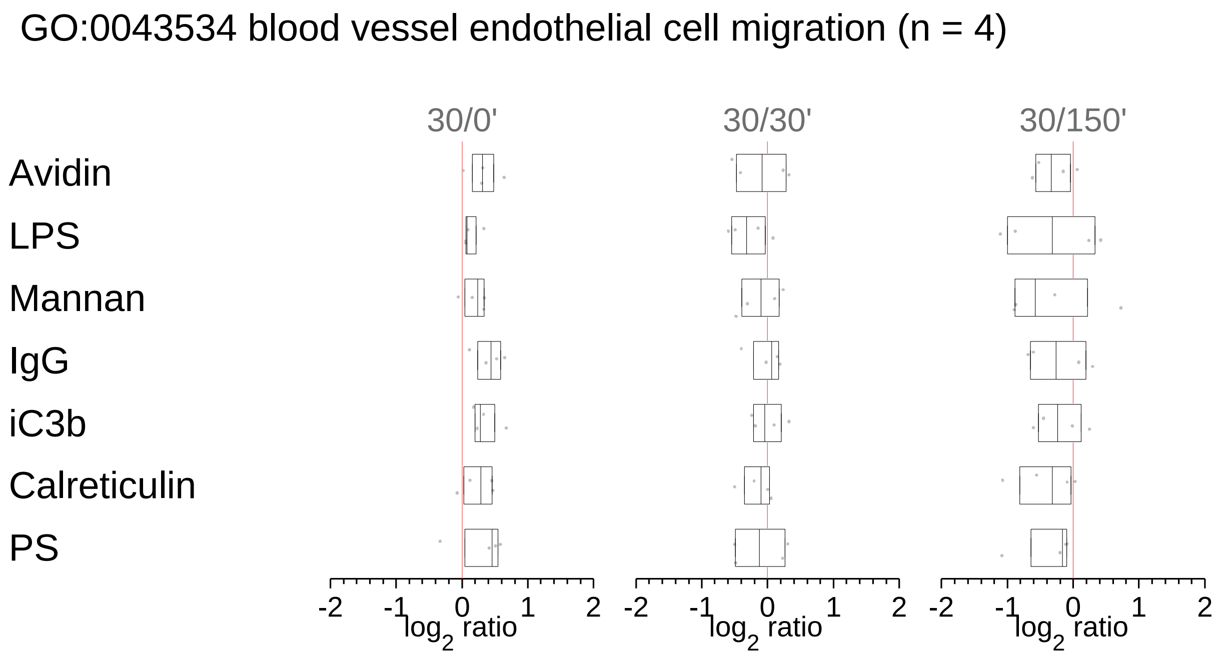
GO:0043534 blood vessel endothelial cell migration (n = 4)
30/0'
30/30'
30/150'
Avidin
LPS
Mannan
IgG
iC3b
Calreticulin
PS
-2
-1
0
1
2
log2 ratio

GO:0043539 protein serine/threonine kinase activator activity (n = 3)

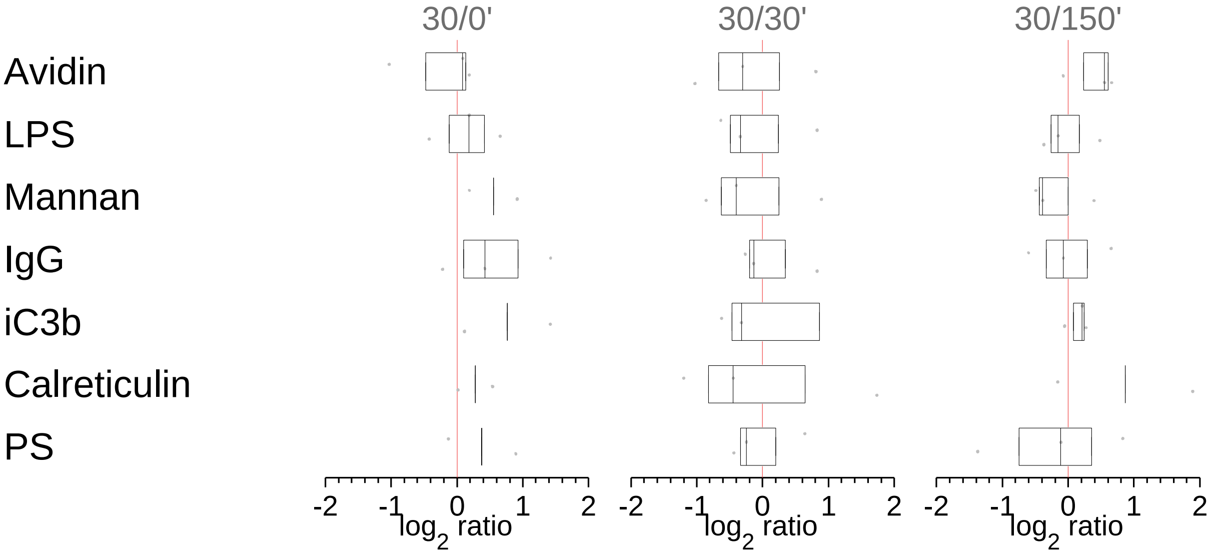

GO:0043547 positive regulation of GTPase activity (n = 21)

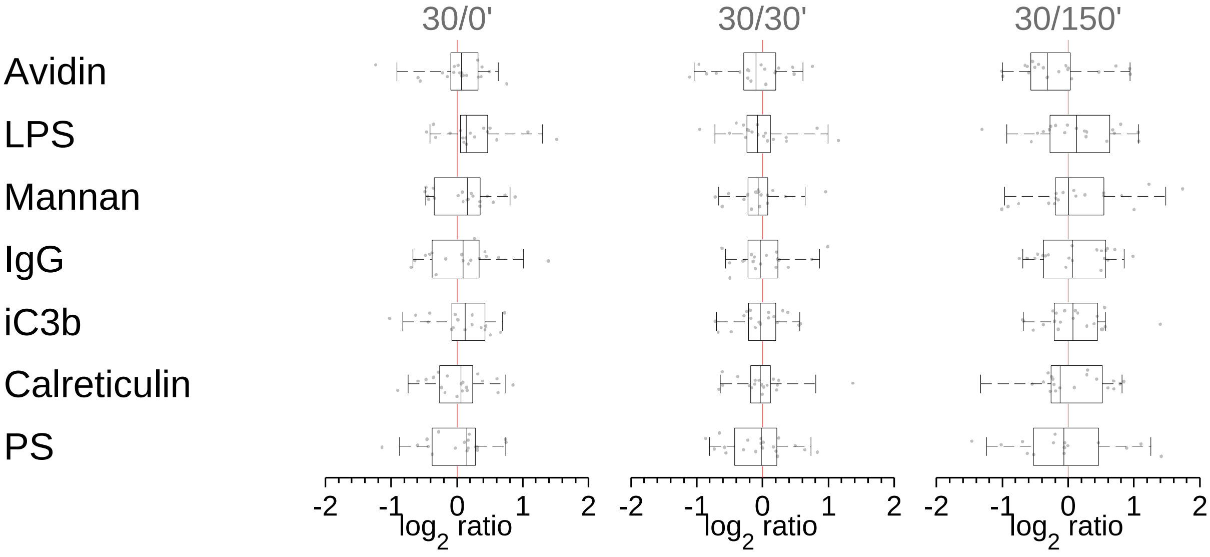

GO:0043548 phosphatidylinositol 3-kinase binding (n = 3)

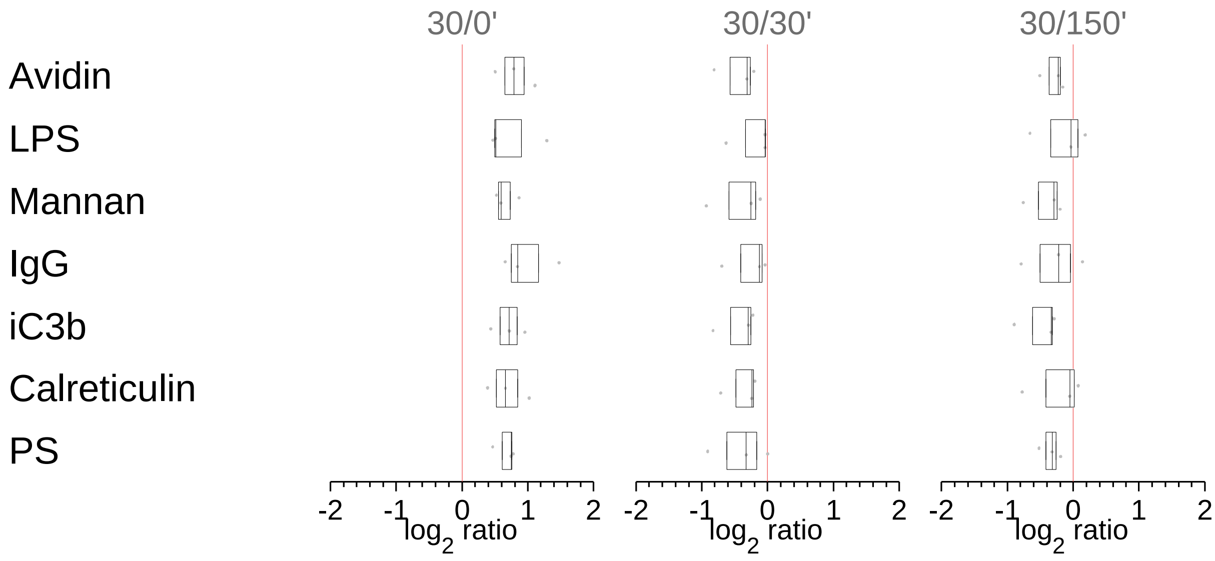

GO:0043552 positive regulation of phosphatidylinositol 3-kinase activity (n = 3)

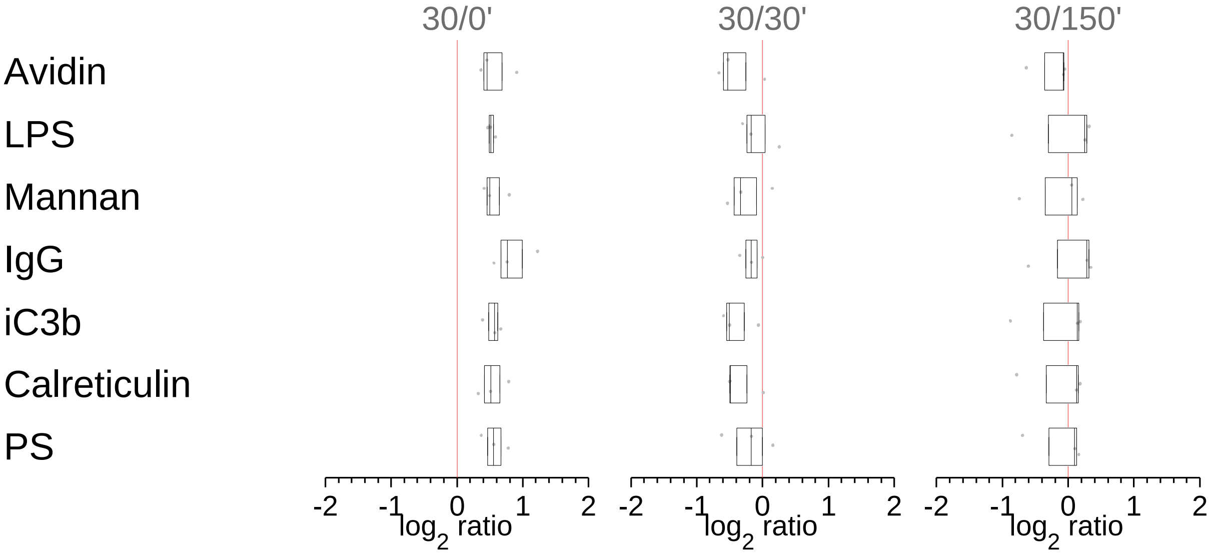

GO:0043565 sequence-specific DNA binding (n = 6)

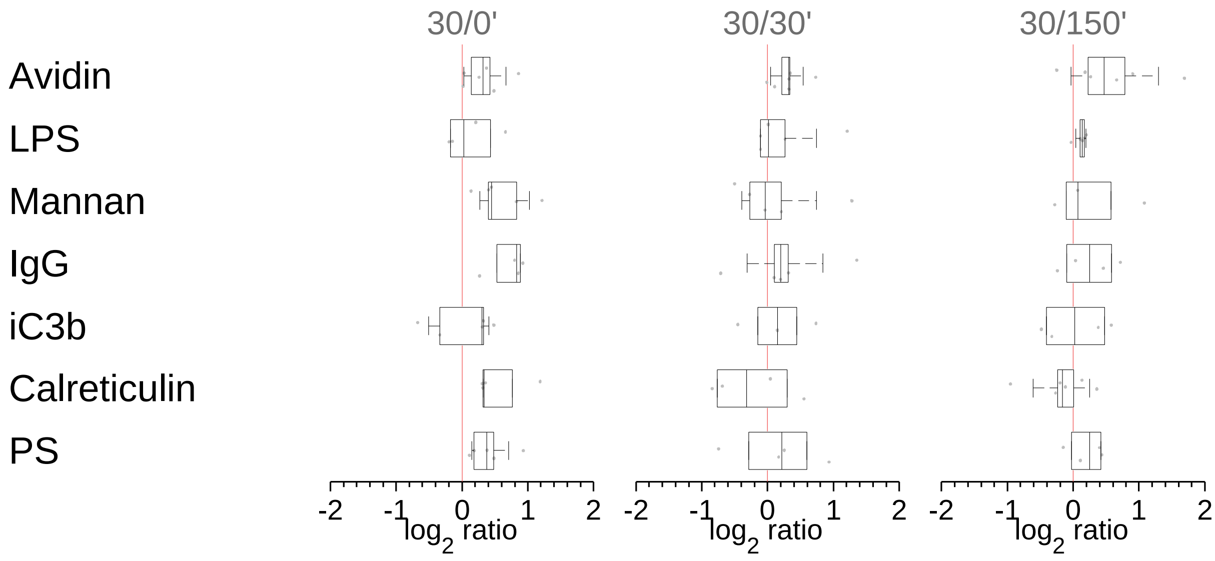

GO:0043588 skin development (n = 5)

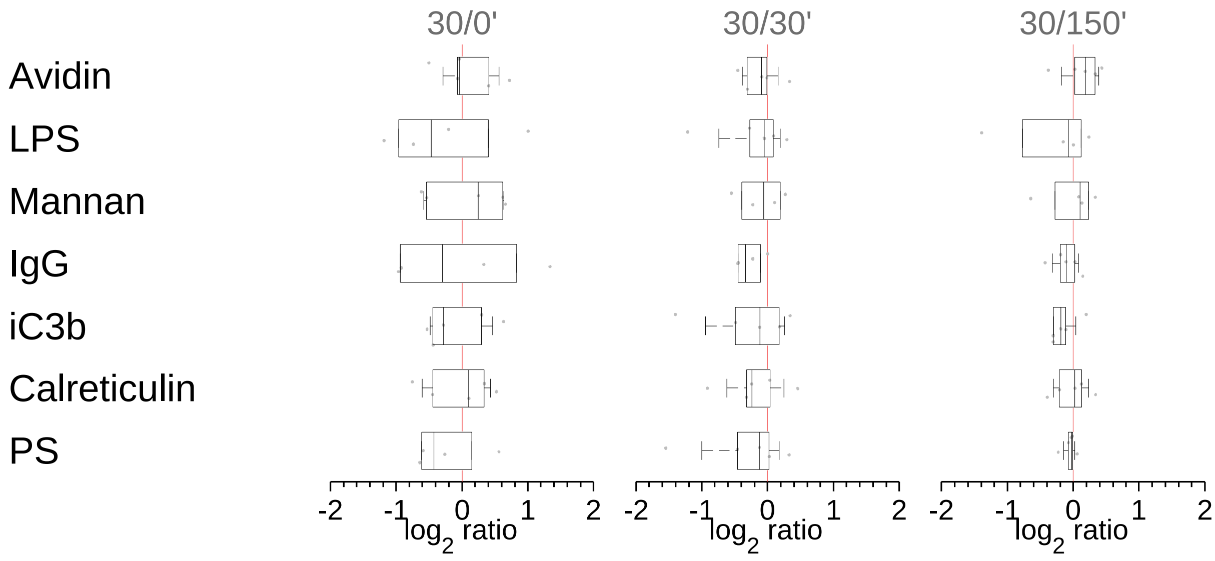

GO:0043589 skin morphogenesis (n = 3)

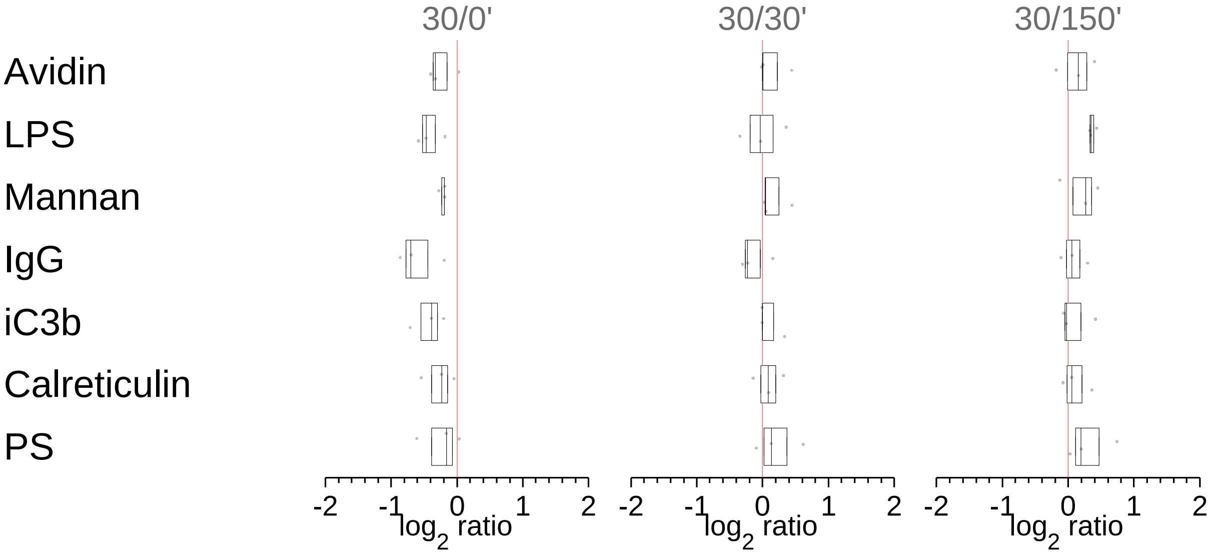

GO:0043621 protein self-association (n = 10)

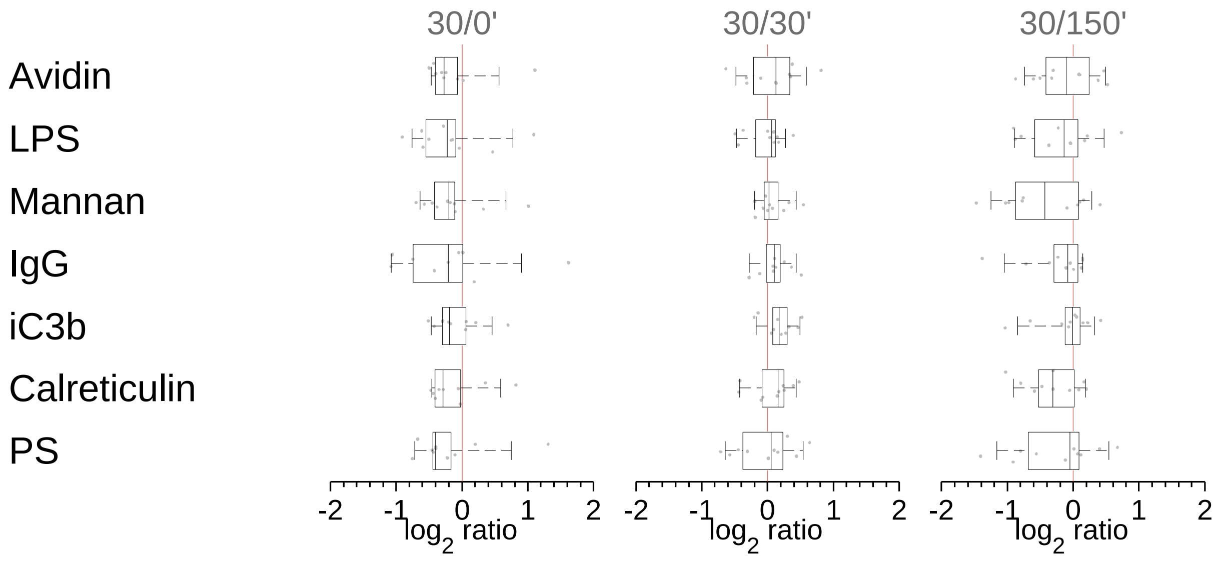

GO:0043627 response to estrogen stimulus (n = 5)

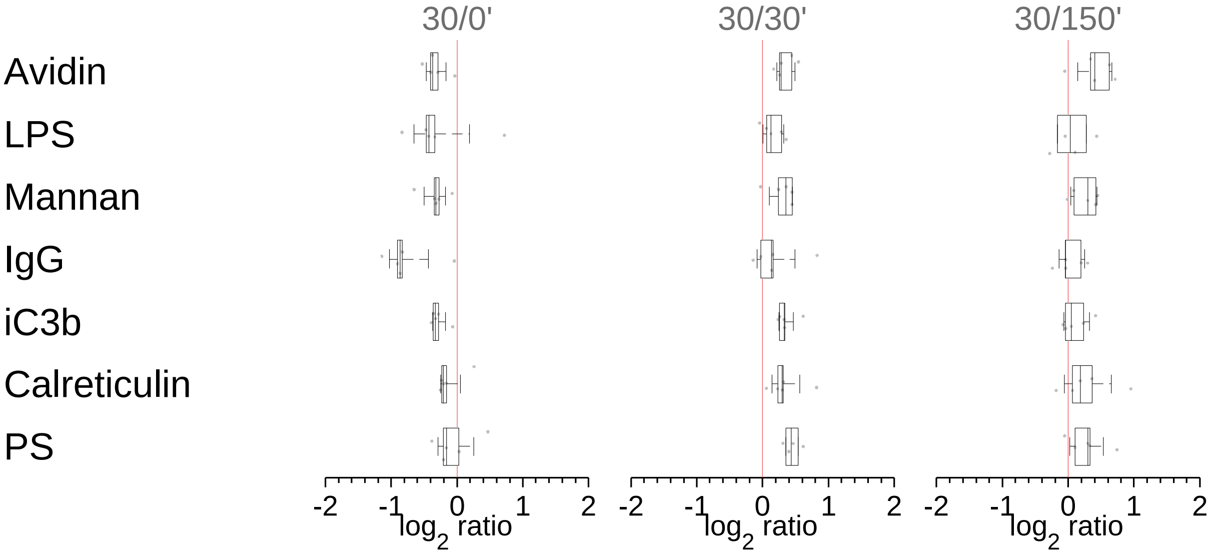

GO:0043679 axon terminus (n = 6)

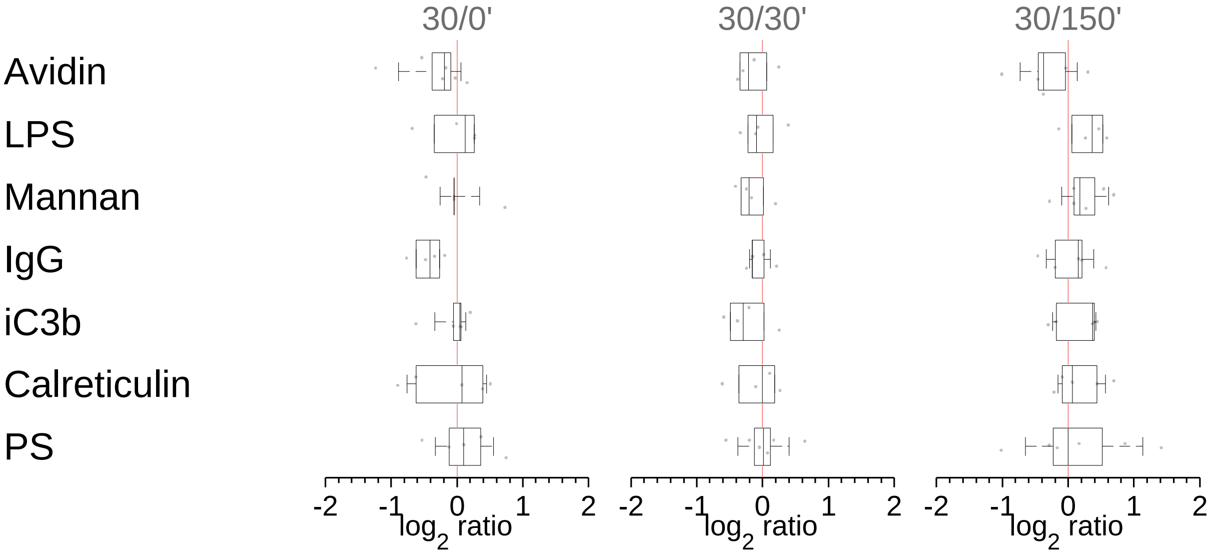

GO:0043713 (R)-2-hydroxyisocaproate dehydrogenase activity (n = 3)

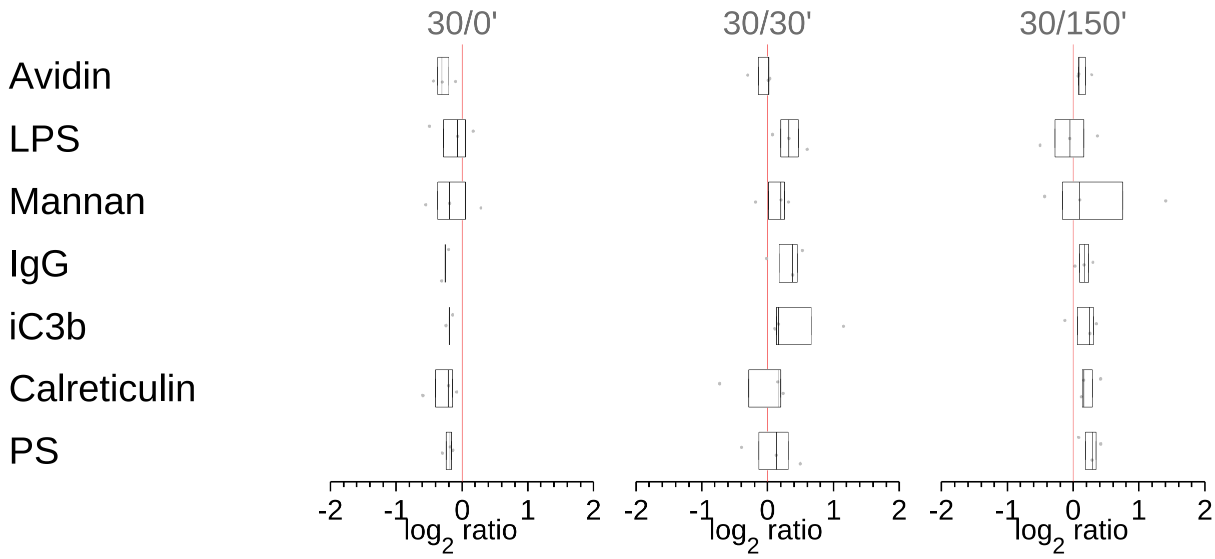

GO:0043738 reduced coenzyme F420 dehydrogenase activity (n = 9)

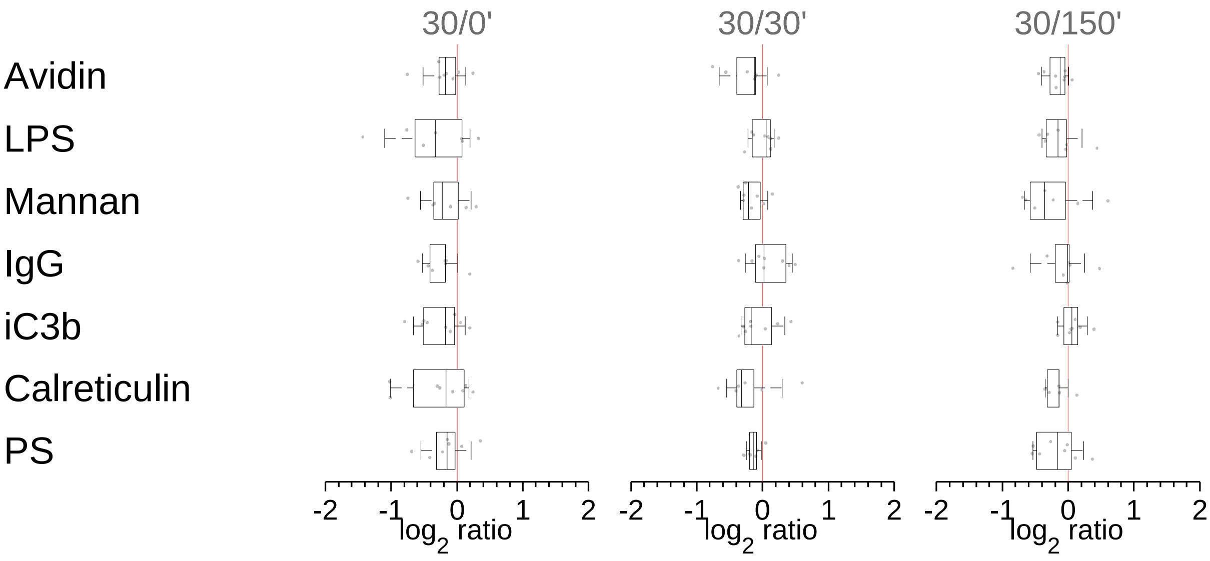

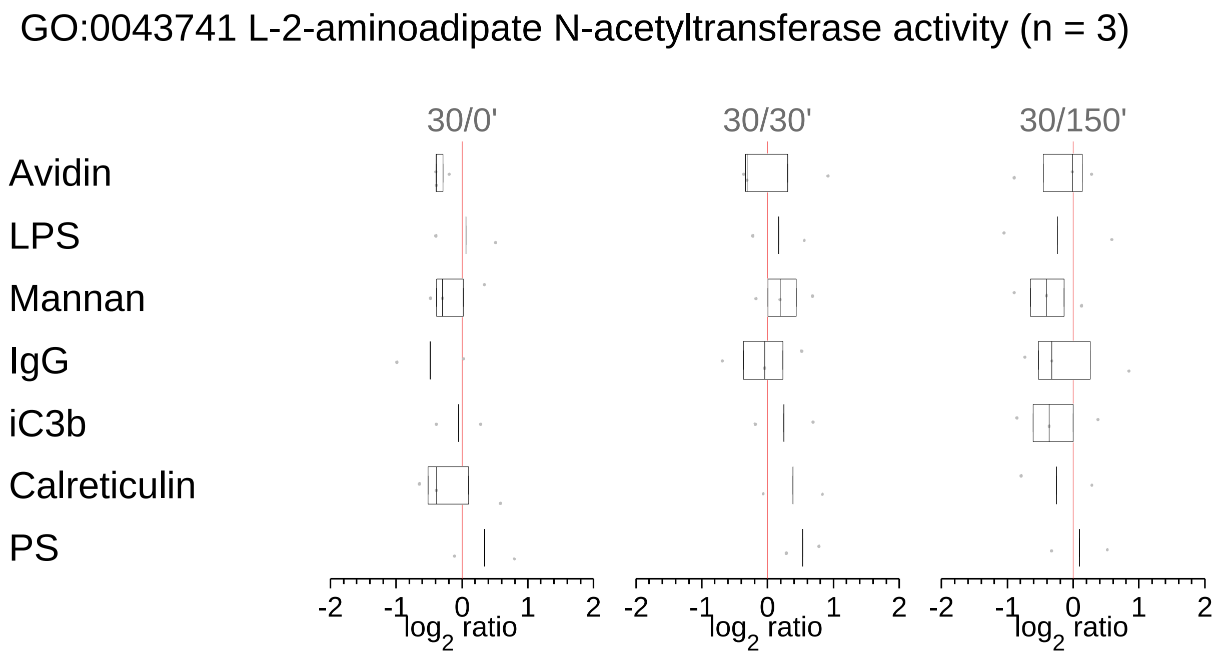
GO:0043741 L-2-aminoadipate N-acetyltransferase activity (n = 3)
30/0'
30/30'
30/150'
Avidin
LPS
Mannan
IgG
iC3b
Calreticulin
PS
-2
-1
0
1
2
log2 ratio

GO:0043764 UDP-3-O-[3-hydroxymyristoyl] glucosamine N-acyltransferase activity (n = 3)

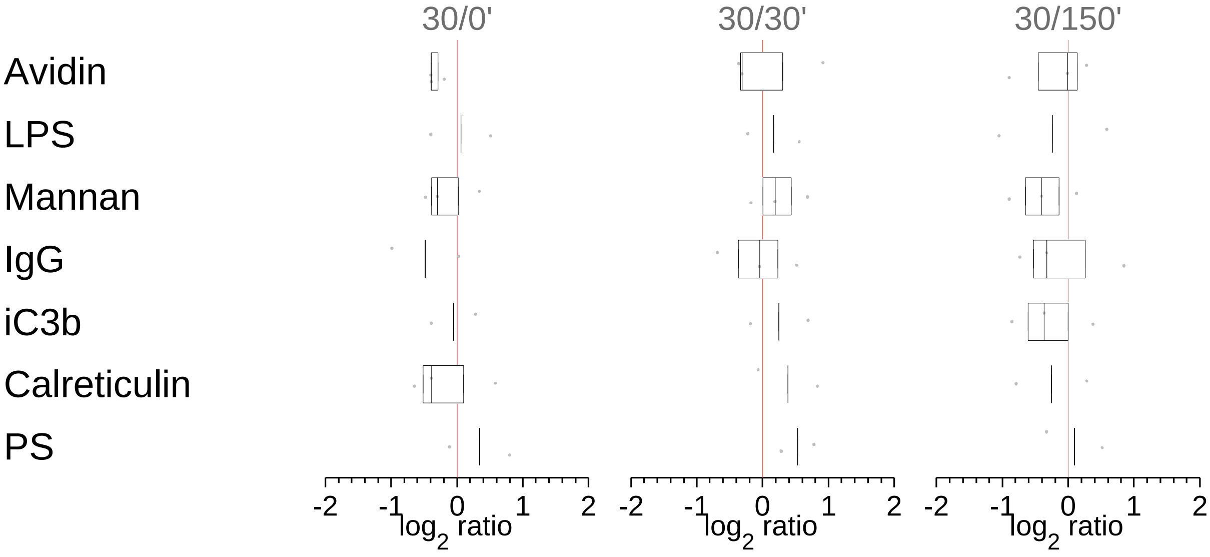

GO:0043765 T/G mismatch-specific endonuclease activity (n = 3)

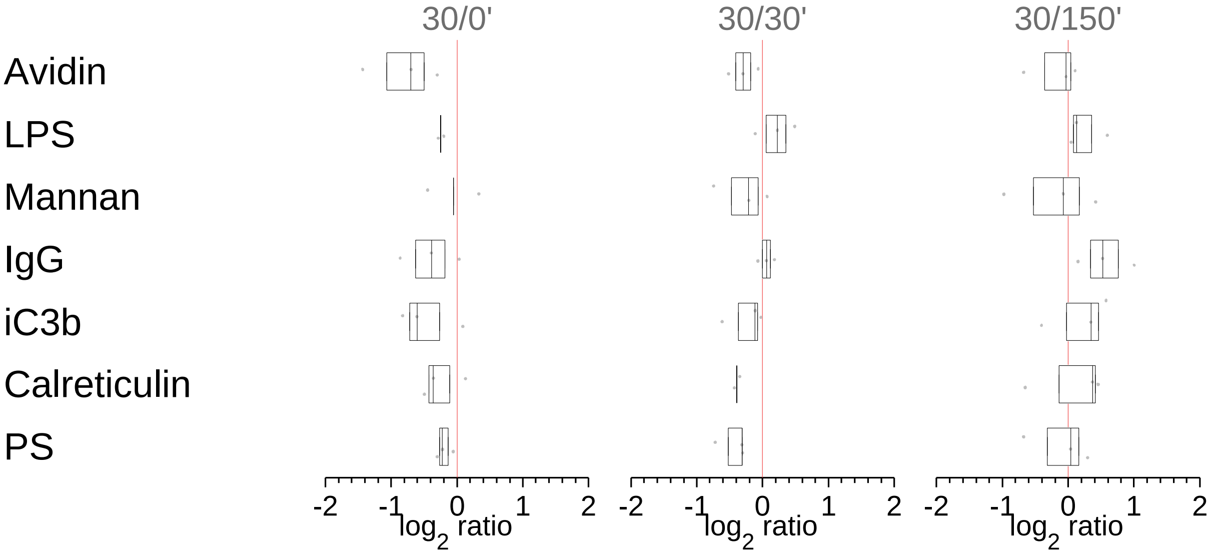

GO:0043773 coenzyme F420-0 gamma-glutamyl ligase activity (n = 17)

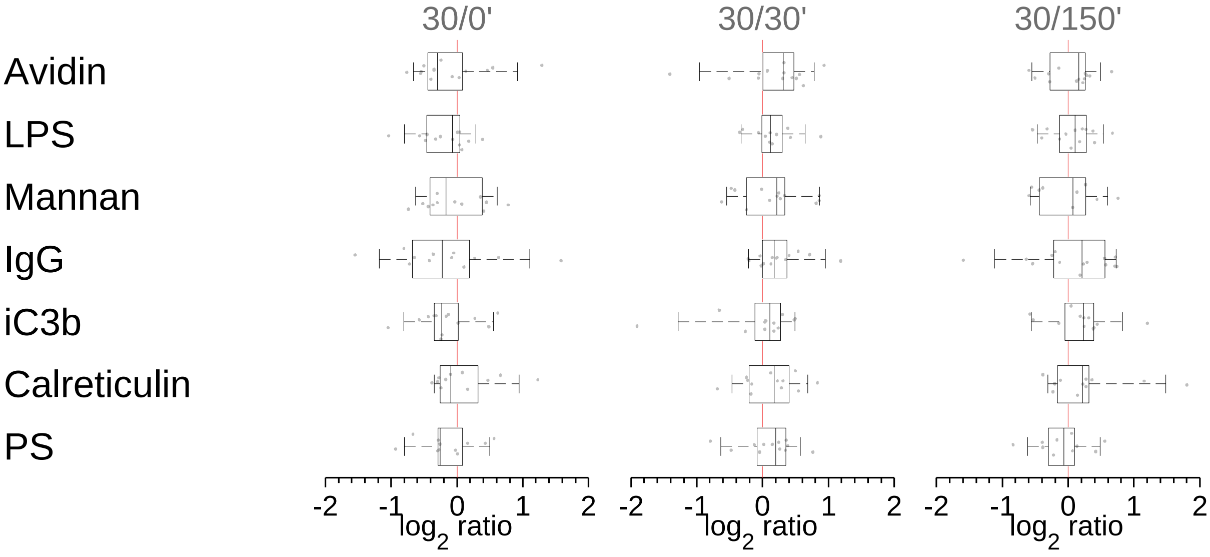

GO:0043774 coenzyme F420-2 alpha-glutamyl ligase activity (n = 17)

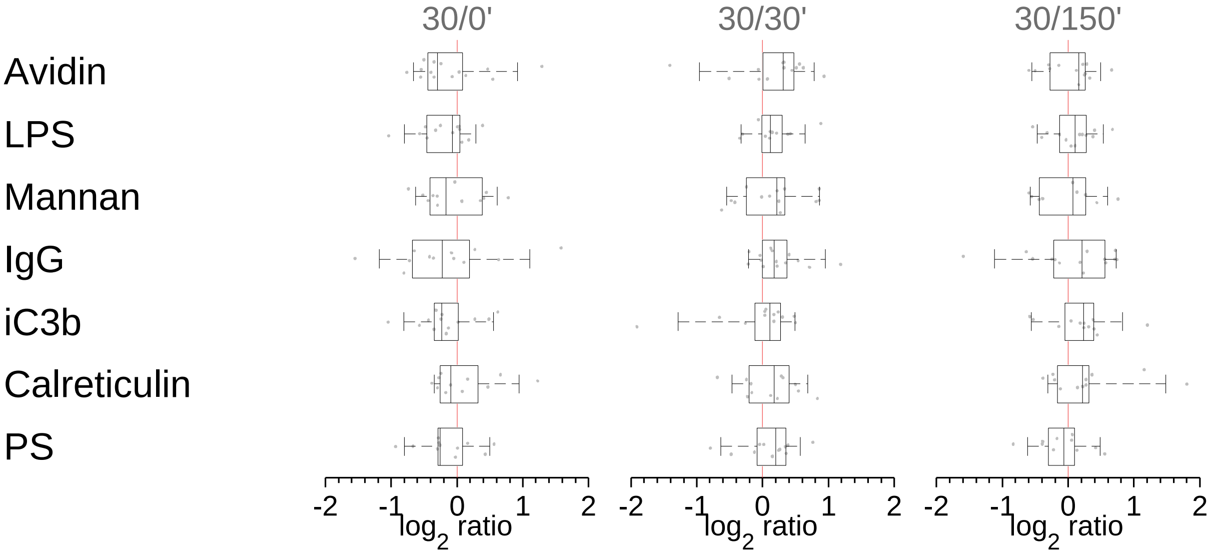

GO:0043806 keto acid formate lyase activity (n = 3)

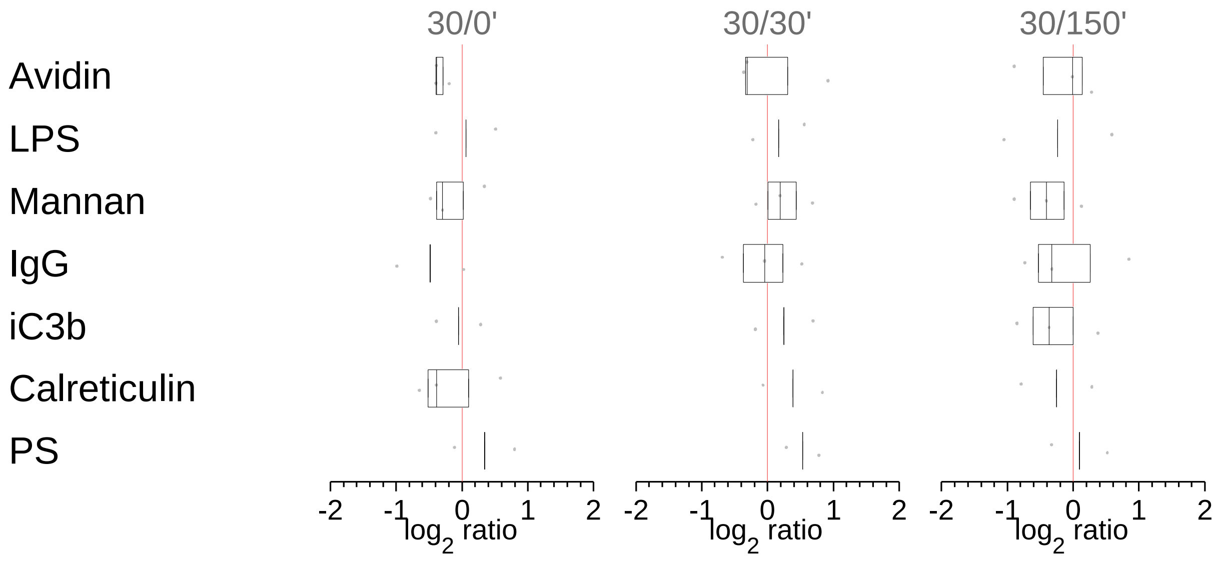

GO:0043826 sulfur oxygenase reductase activity (n = 9)

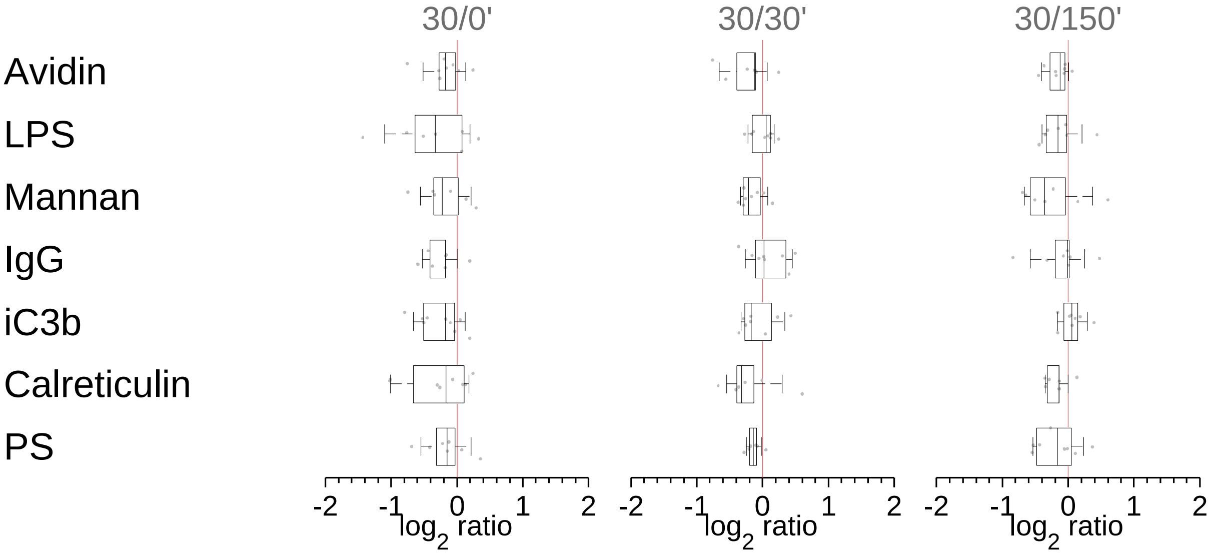

GO:0043849 Ras palmitoyltransferase activity (n = 3)

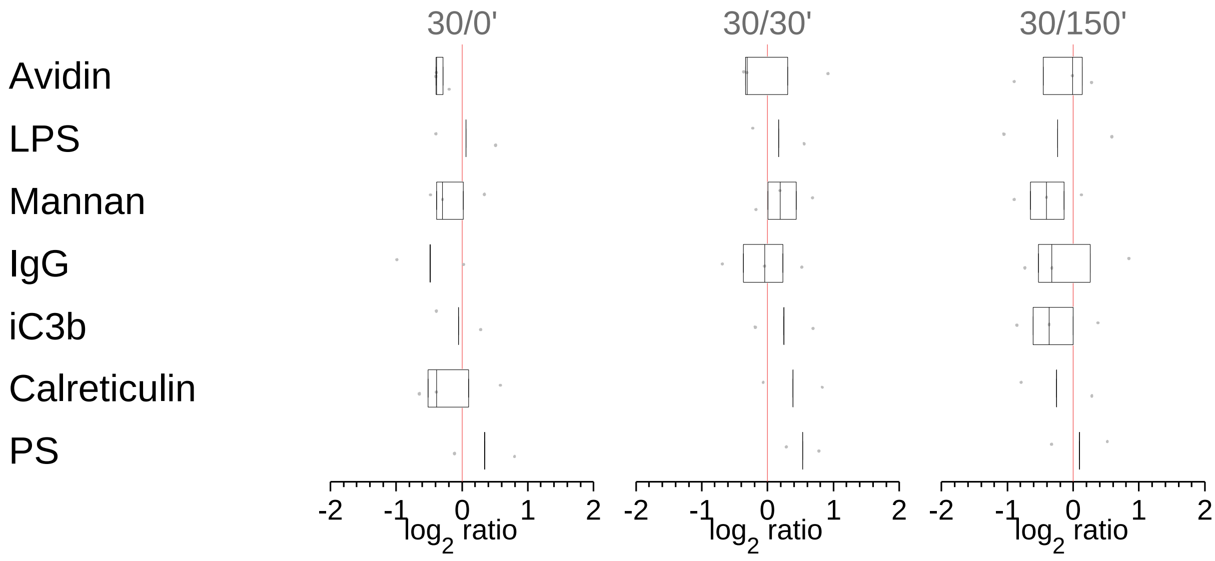

GO:0043883 malolactic enzyme activity (n = 9)

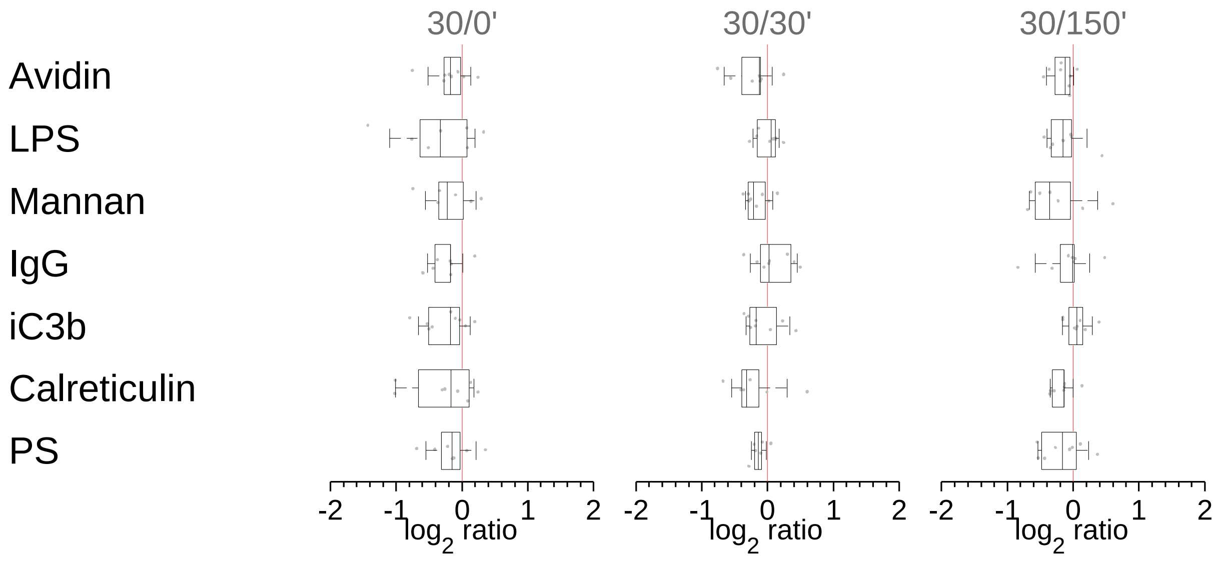

GO:0043905 Ser-tRNA(Thr) hydrolase activity (n = 4)

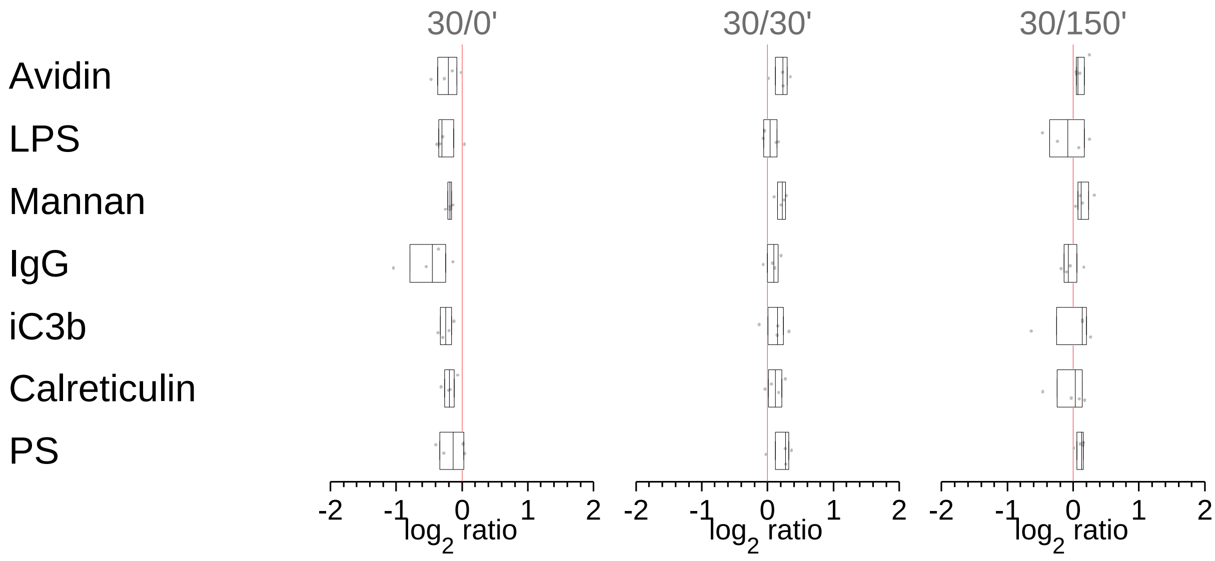

GO:0043906 Ala-tRNA(Pro) hydrolase activity (n = 4)

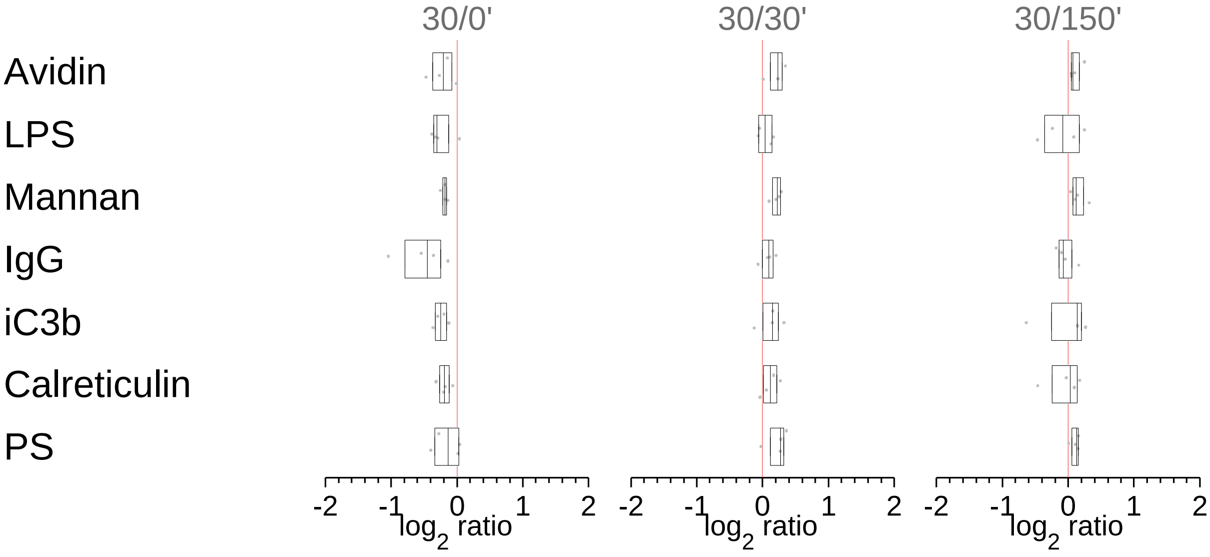

GO:0043907 Cys-tRNA(Pro) hydrolase activity (n = 4)

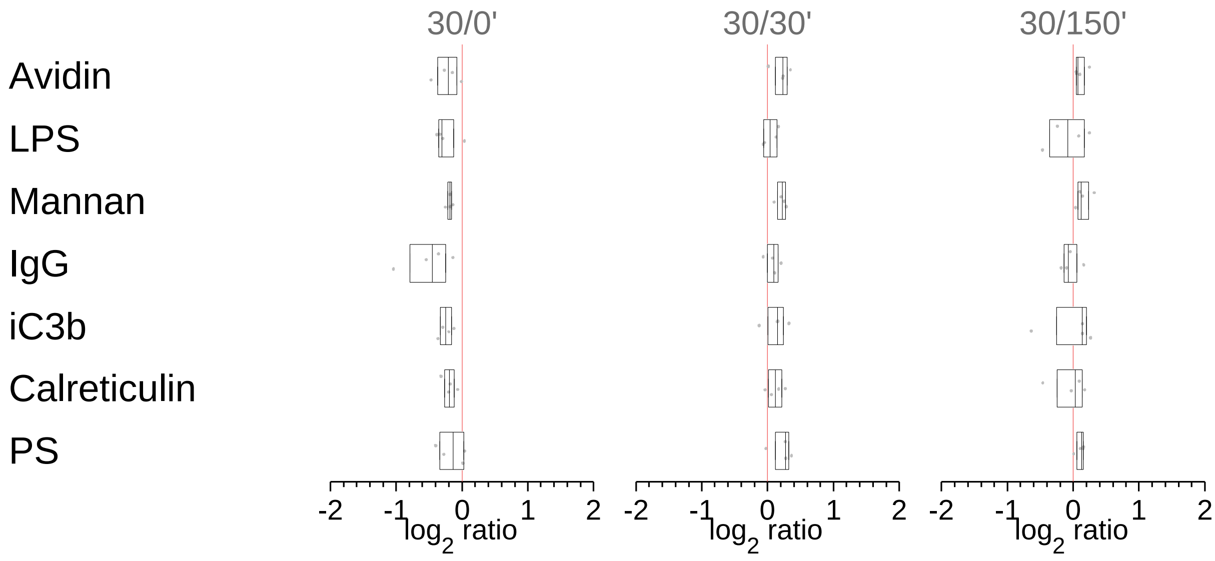

GO:0043908 Ser(Gly)-tRNA(Ala) hydrolase activity (n = 4)

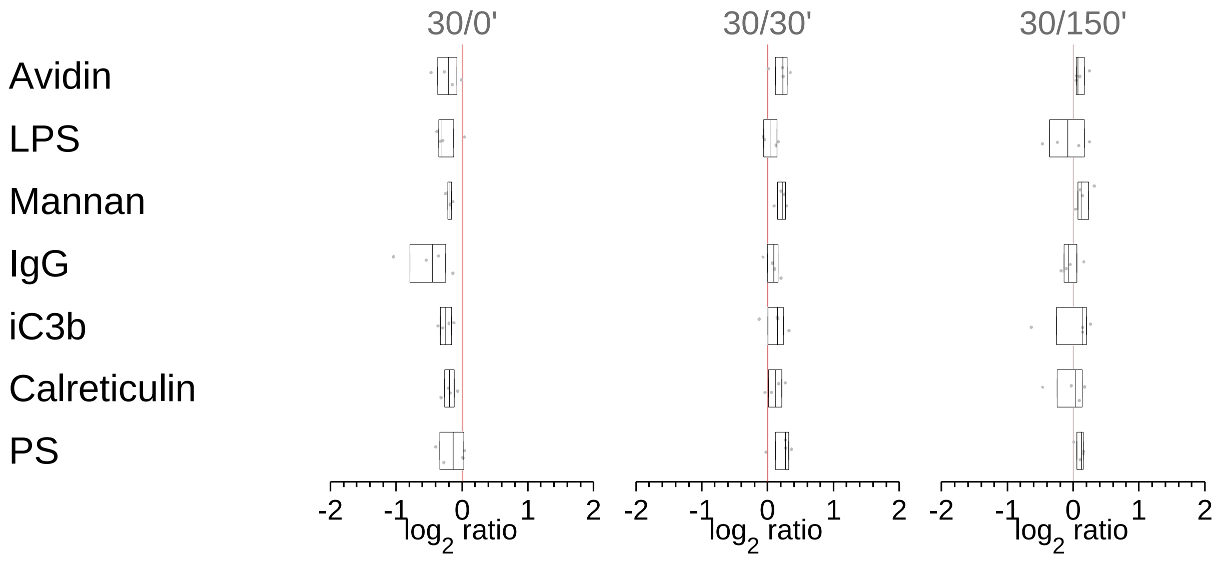

GO:0043914 NADPH:sulfur oxidoreductase activity (n = 9)

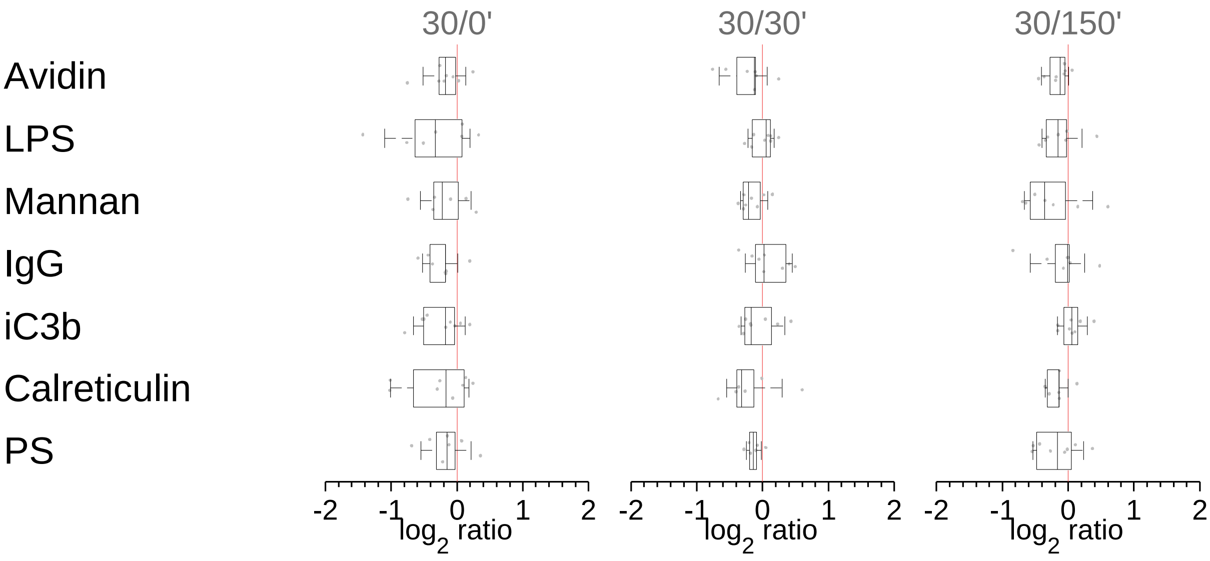

GO:0044070 regulation of anion transport (n = 6)

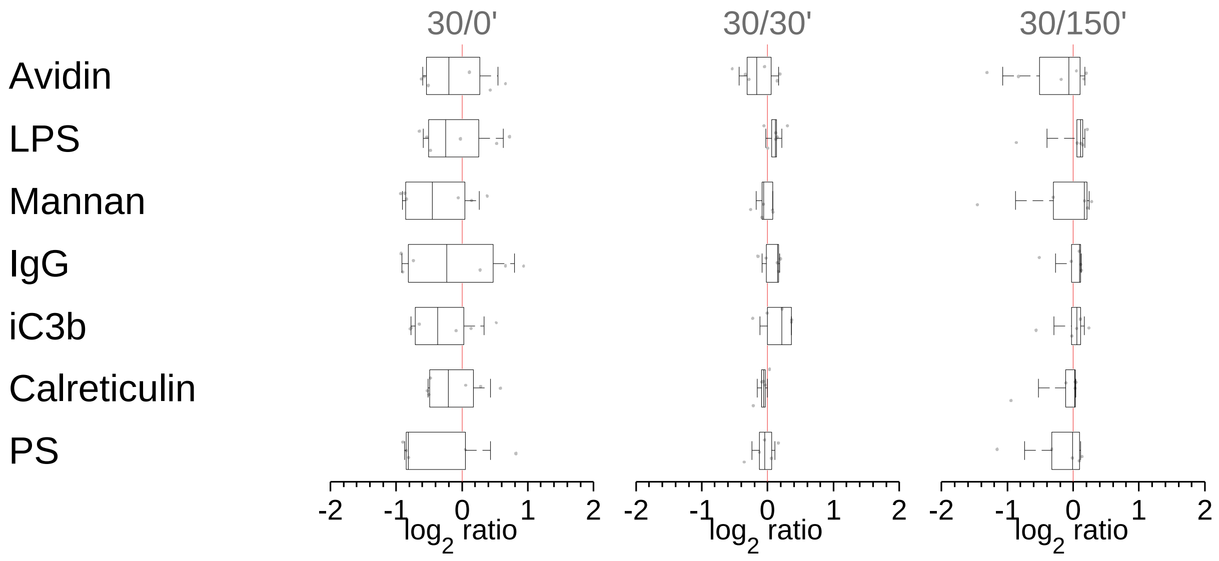

GO:0044103 L-arabinose 1-dehydrogenase (NADP+) activity (n = 3)

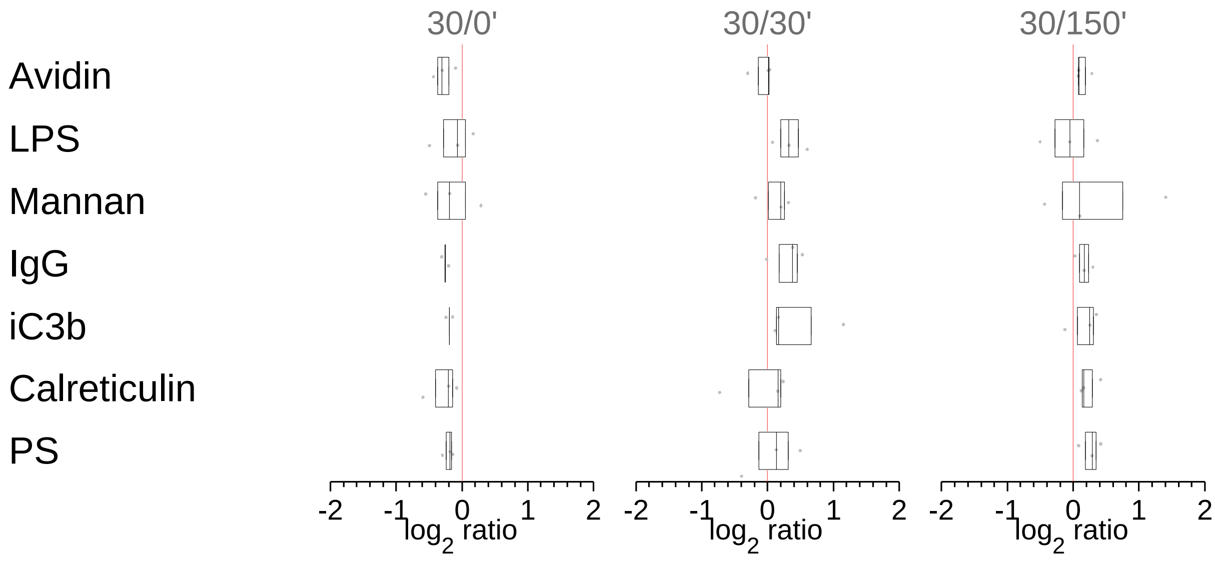

GO:0044105 L-xylulose reductase (NAD+) activity (n = 3)

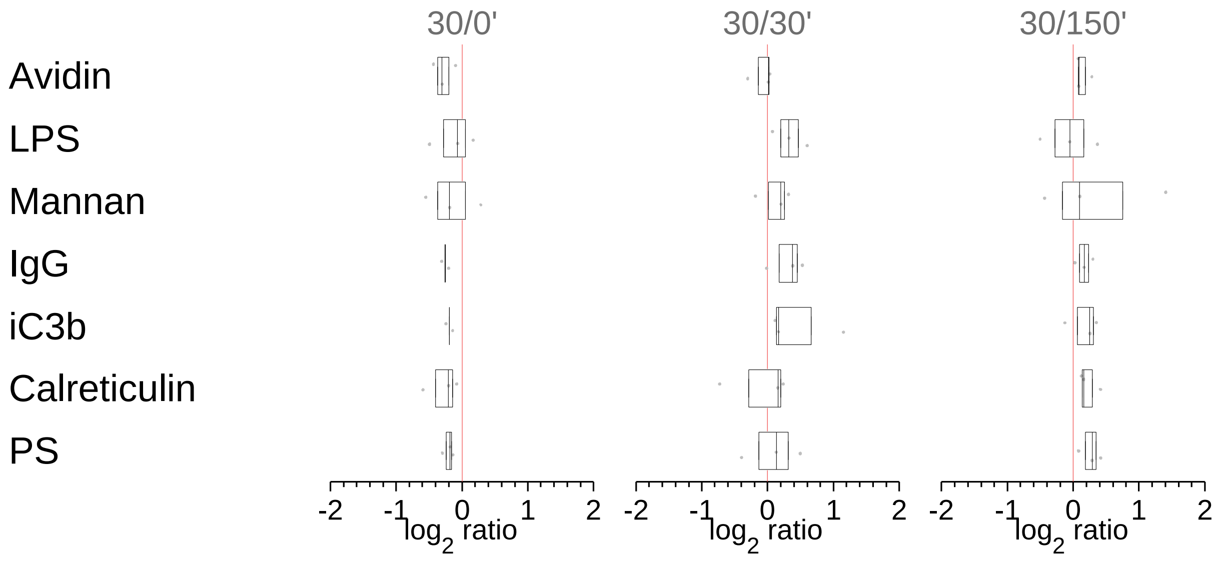

GO:0044130 negative regulation of growth of symbiont in host (n = 4)

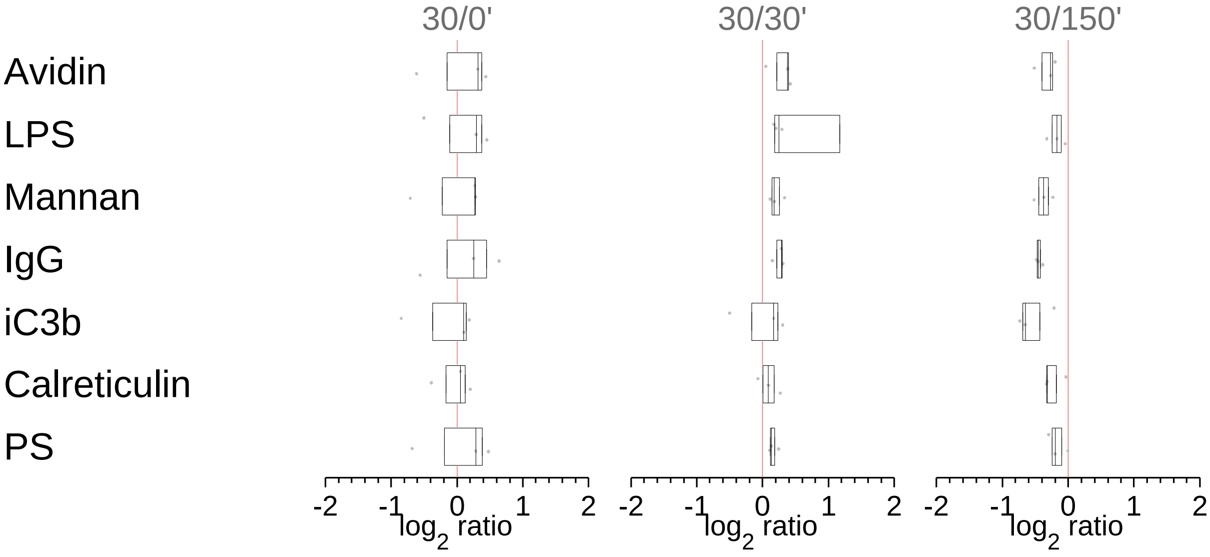

GO:0044212 transcription regulatory region DNA binding (n = 12)

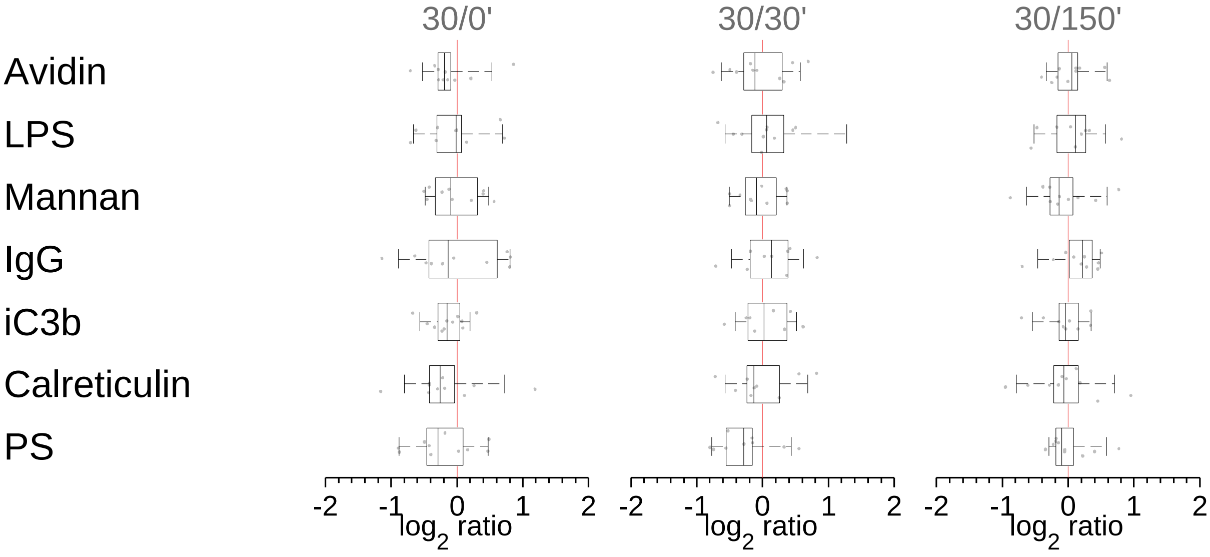

GO:0044233 ER-mitochondrion membrane contact site (n = 4)

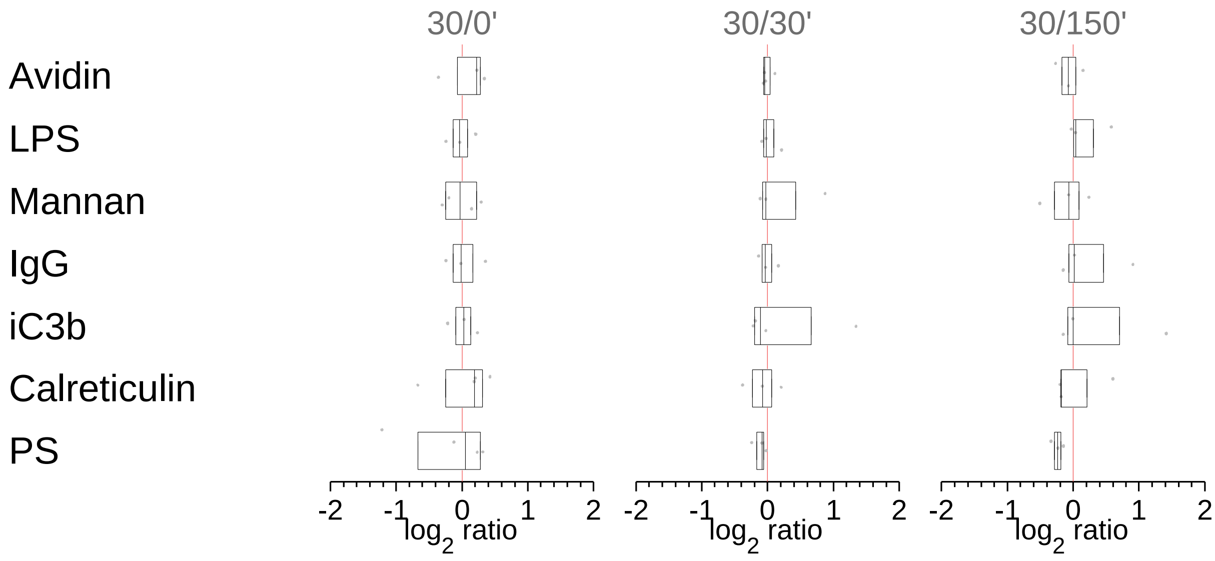

GO:0044257 cellular protein catabolic process (n = 3)

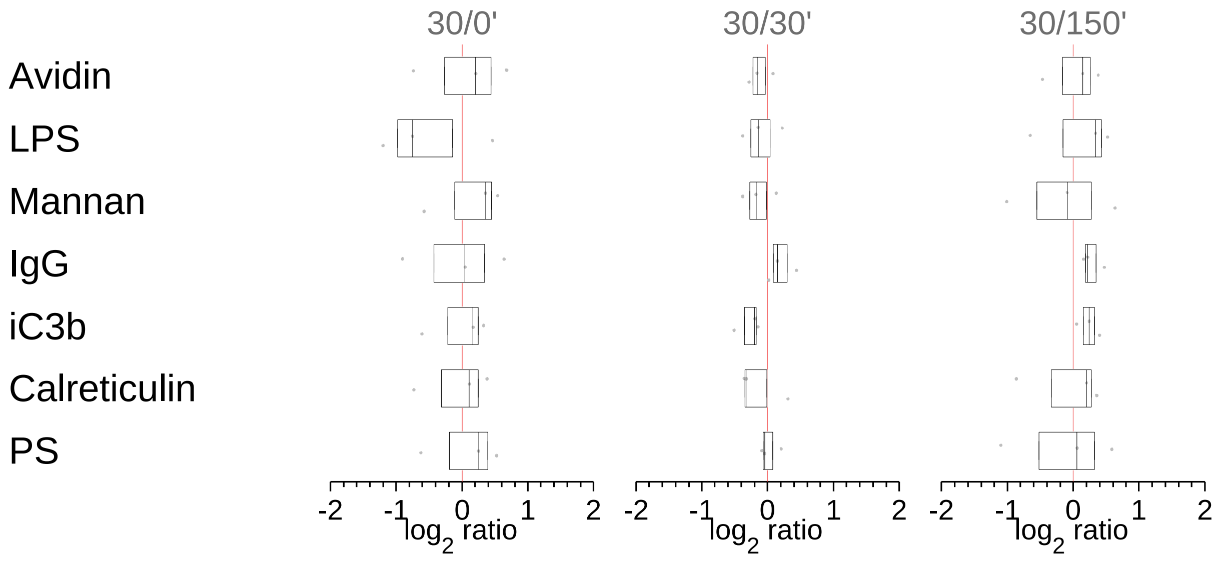

GO:0044262 cellular carbohydrate metabolic process (n = 4)

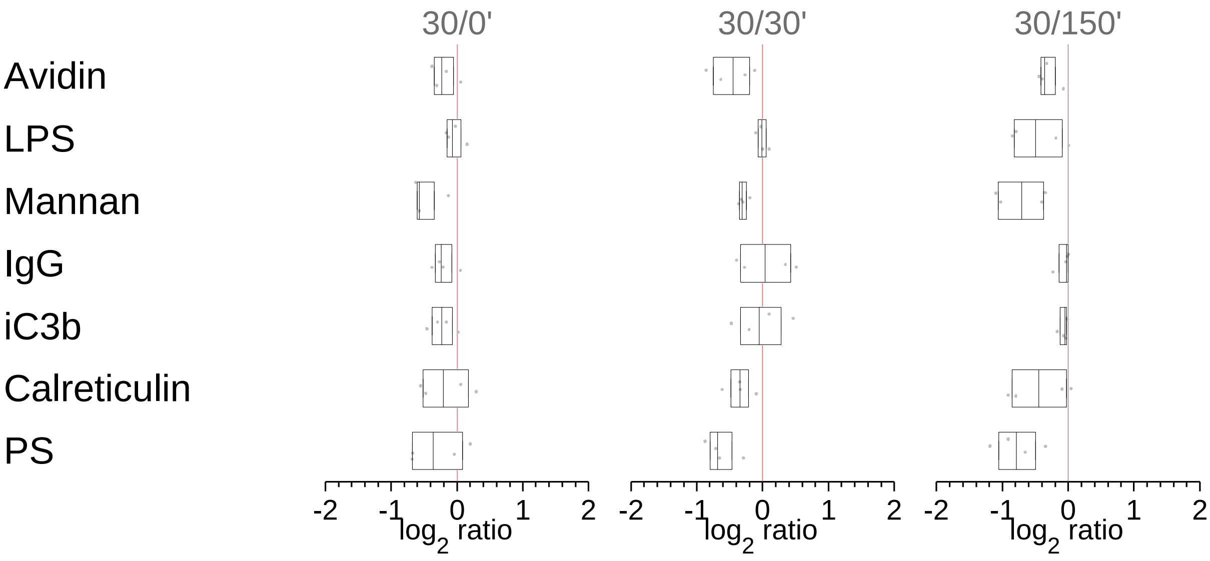

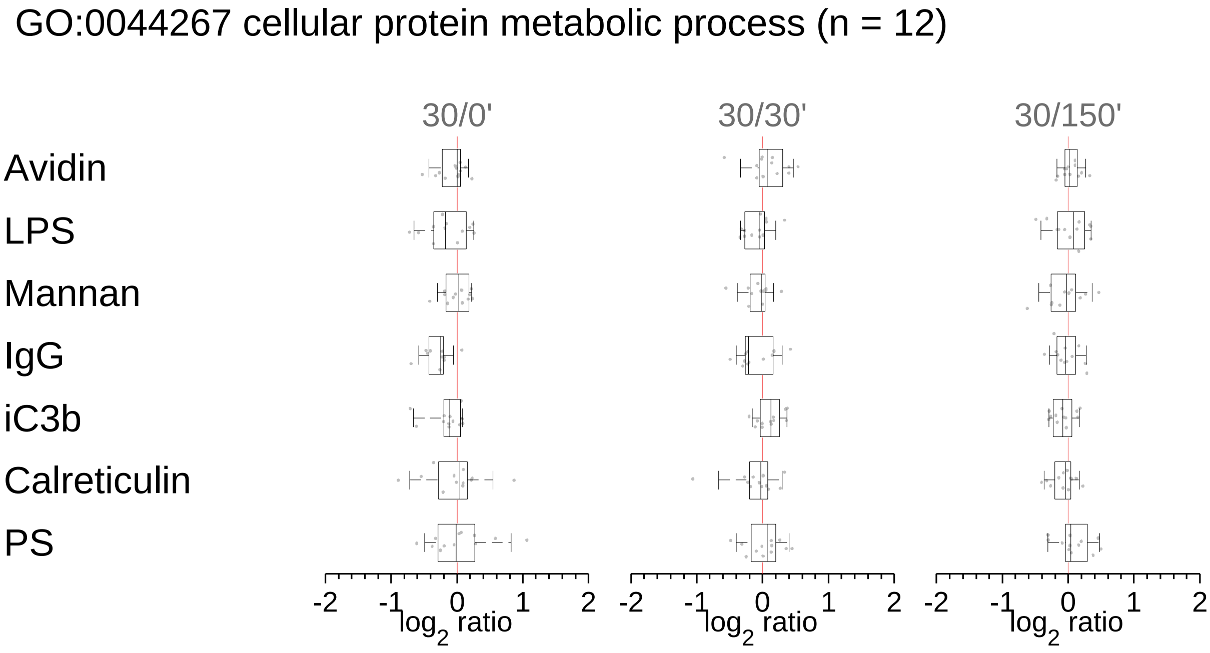

GO:0044267 cellular protein metabolic process (n = 12)
30/0'
30/30'
30/150'
Avidin
LPS
Mannan
IgG
iC3b
Calreticulin
PS
-2
-1
0
1
2
log2 ratio

GO:0044297 cell body (n = 24)

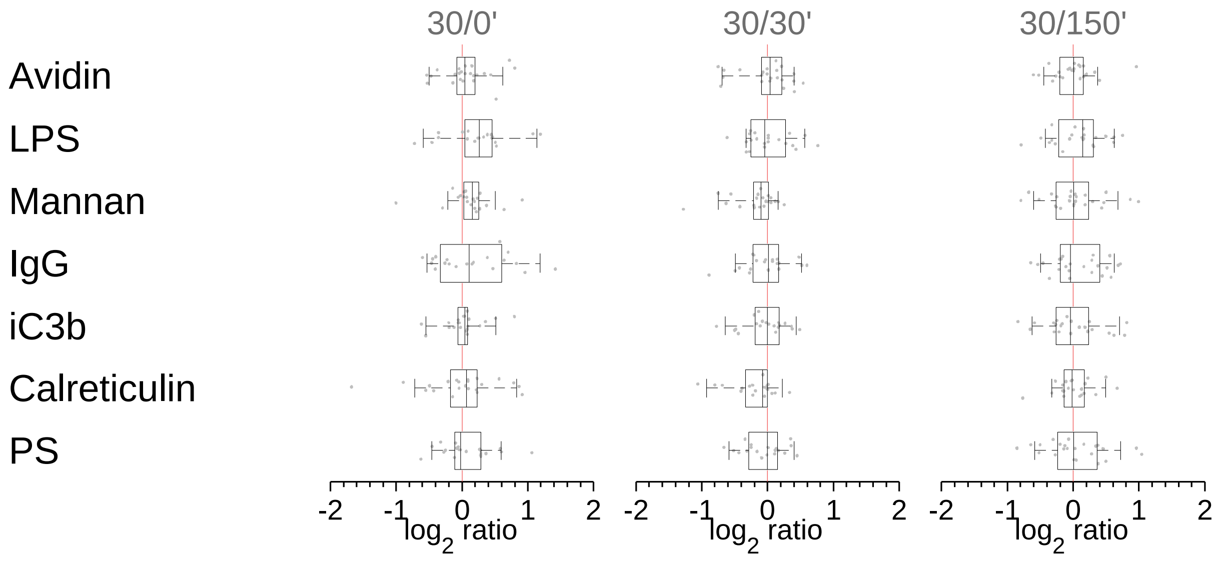

GO:0044325 ion channel binding (n = 12)

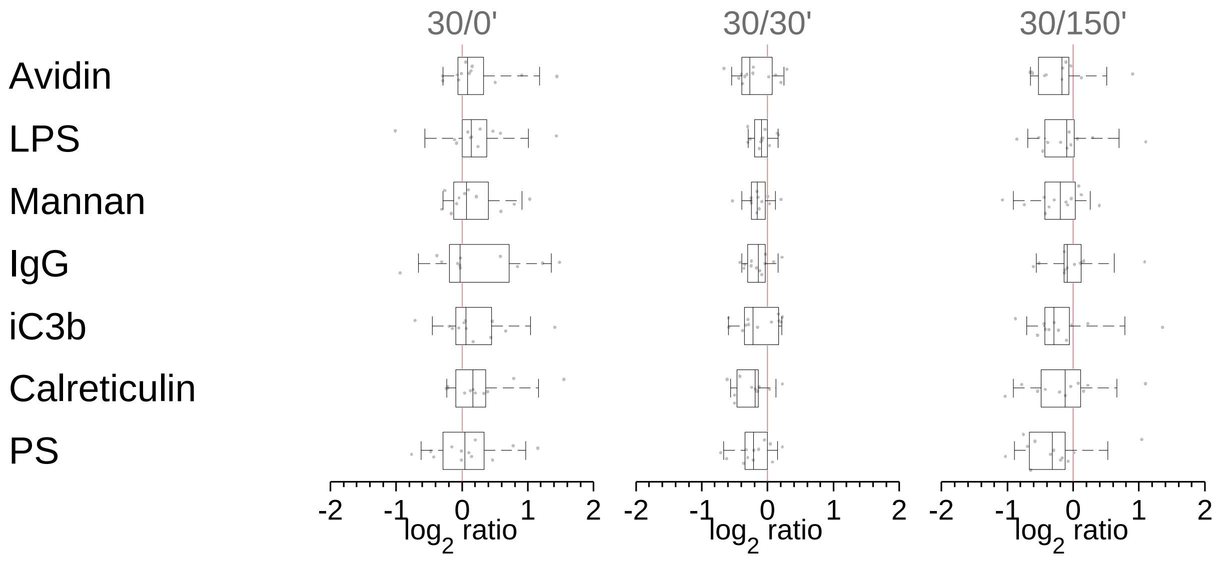

GO:0044344 cellular response to fibroblast growth factor stimulus (n = 3)

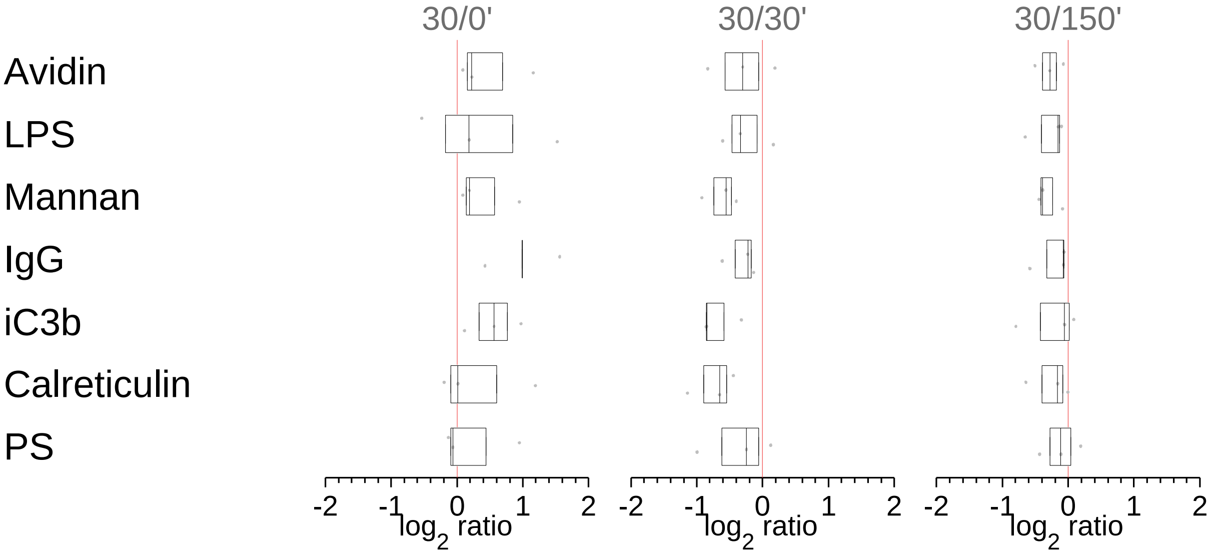

GO:0044466 glutaryl-CoA hydrolase activity (n = 3)

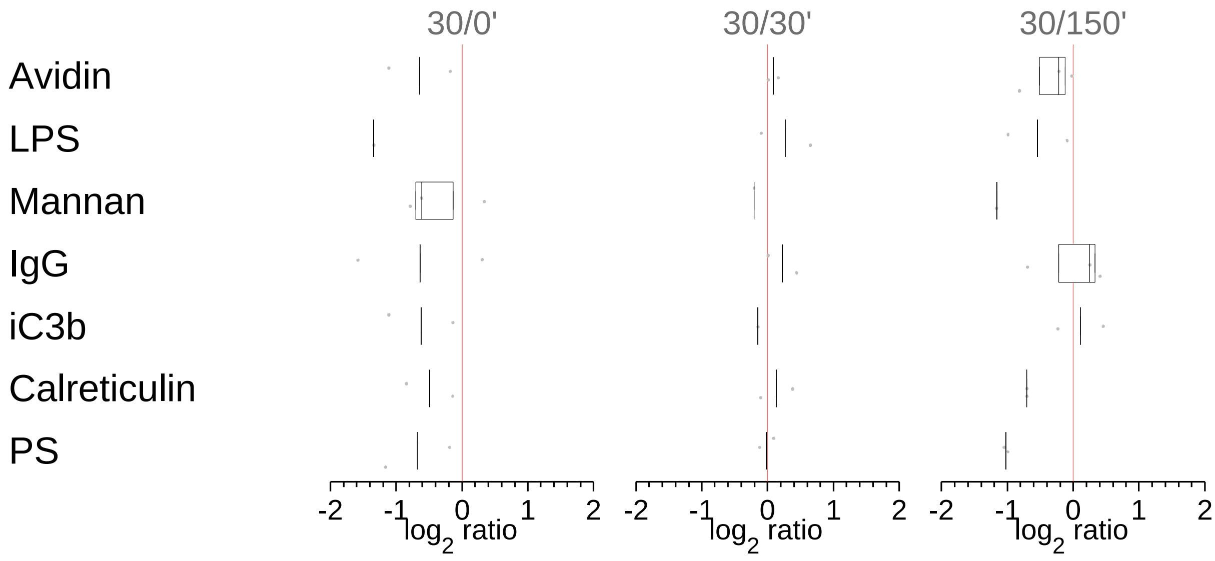

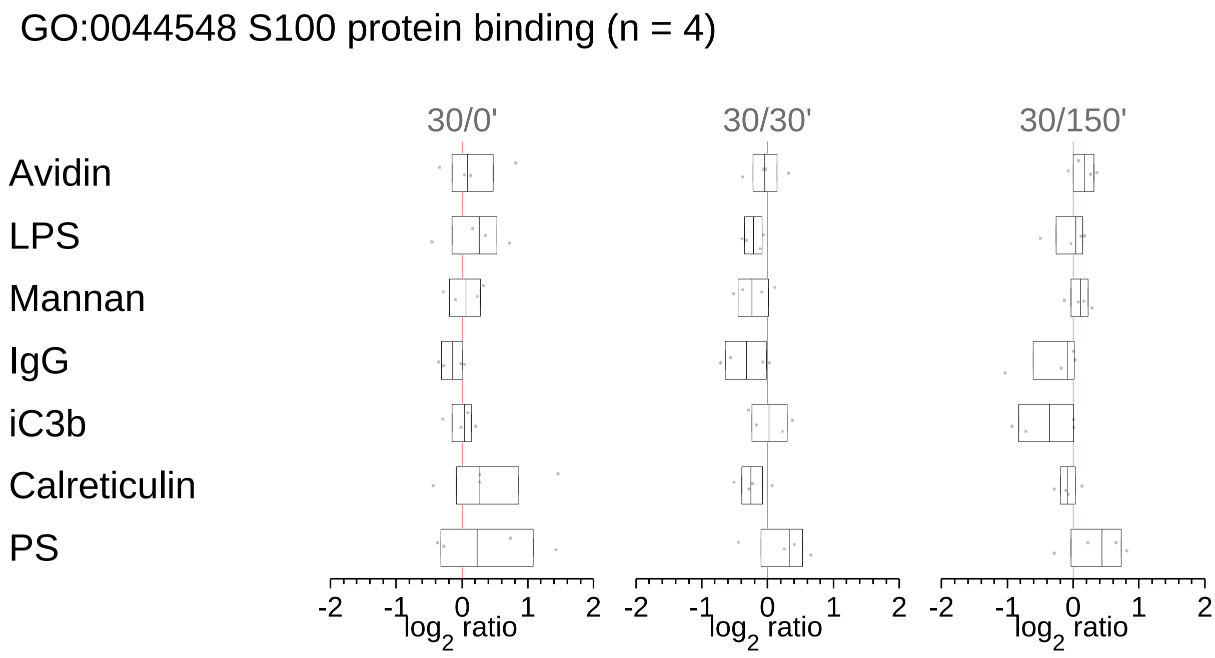
GO:0044548 S100 protein binding (n = 4)
30/0'
30/30'
30/150'
Avidin
LPS
Mannan
IgG
iC3b
Calreticulin
PS
-2
-1
0
1
2
log2 ratio

GO:0044822 N/A (n = 252)

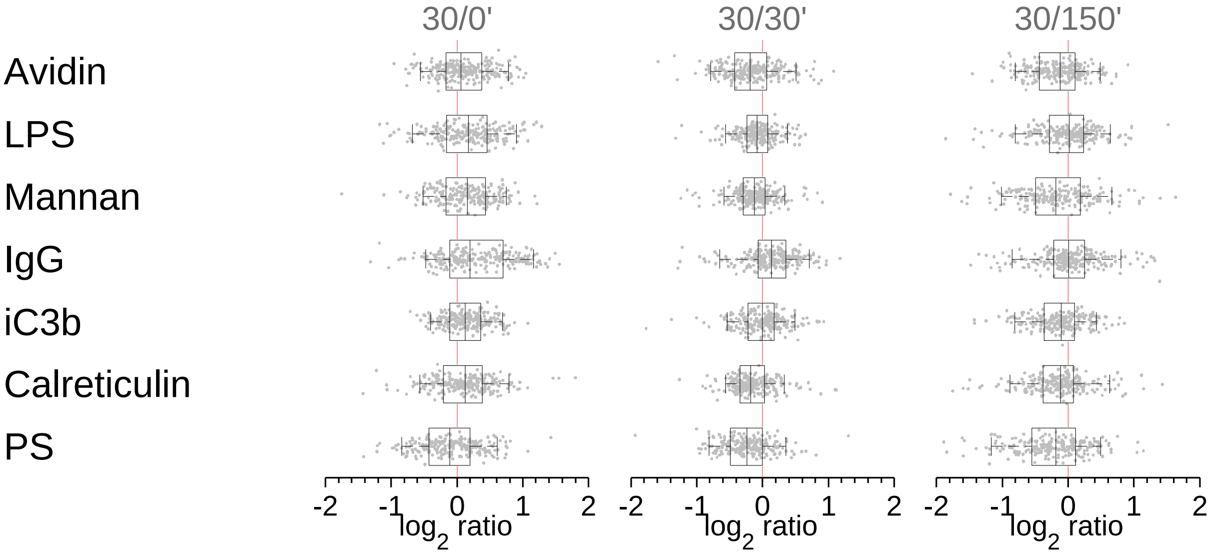

GO:0044824 N/A (n = 3)

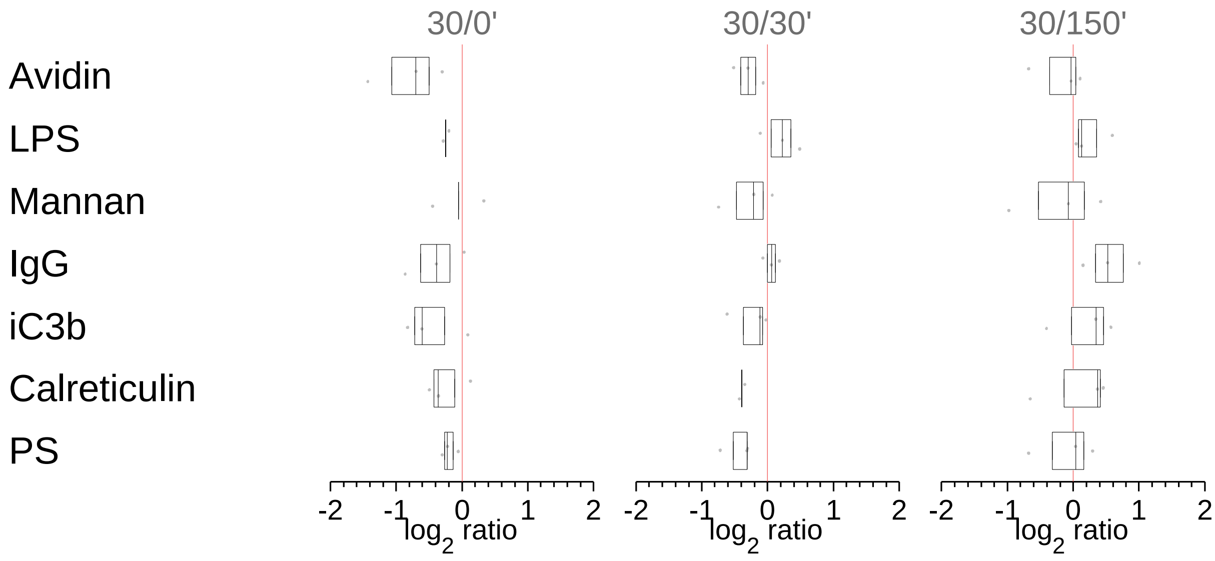

GO:0045022 early endosome to late endosome transport (n = 5)

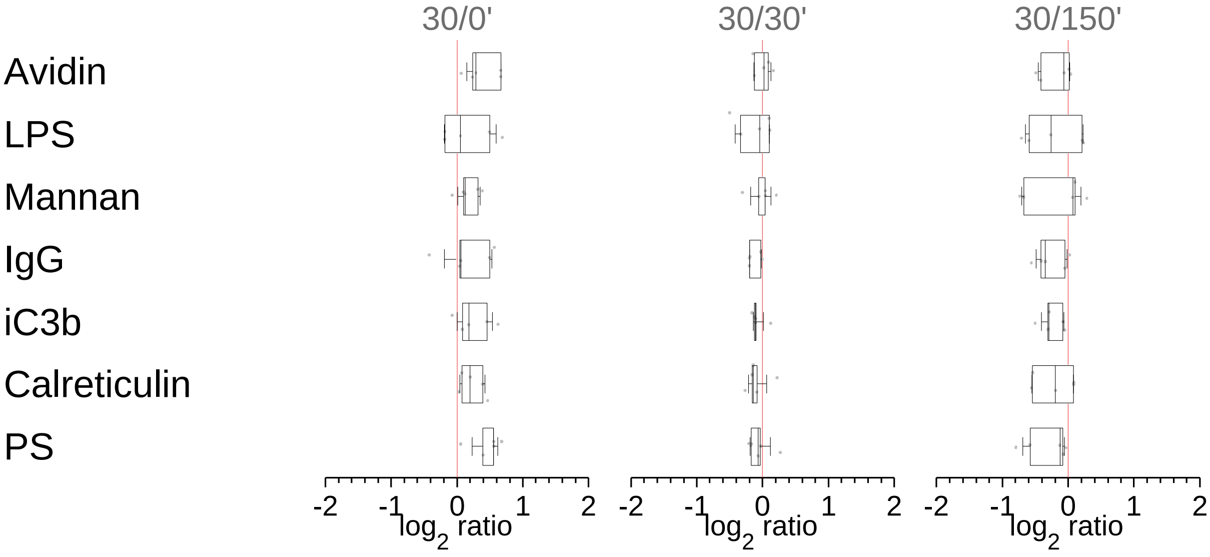

GO:0045040 protein import into mitochondrial outer membrane (n = 3)

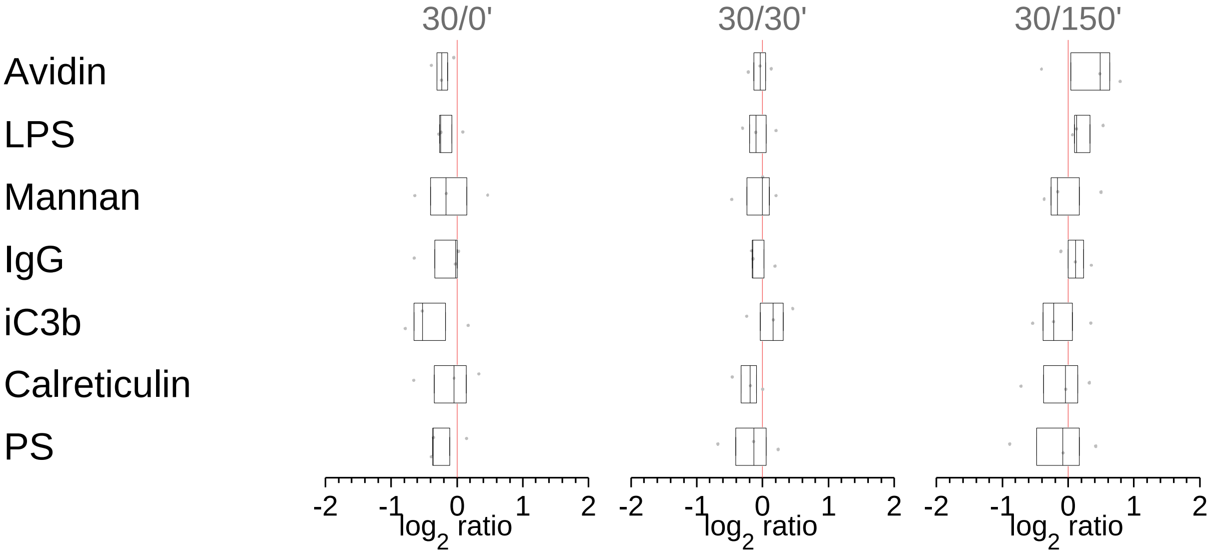

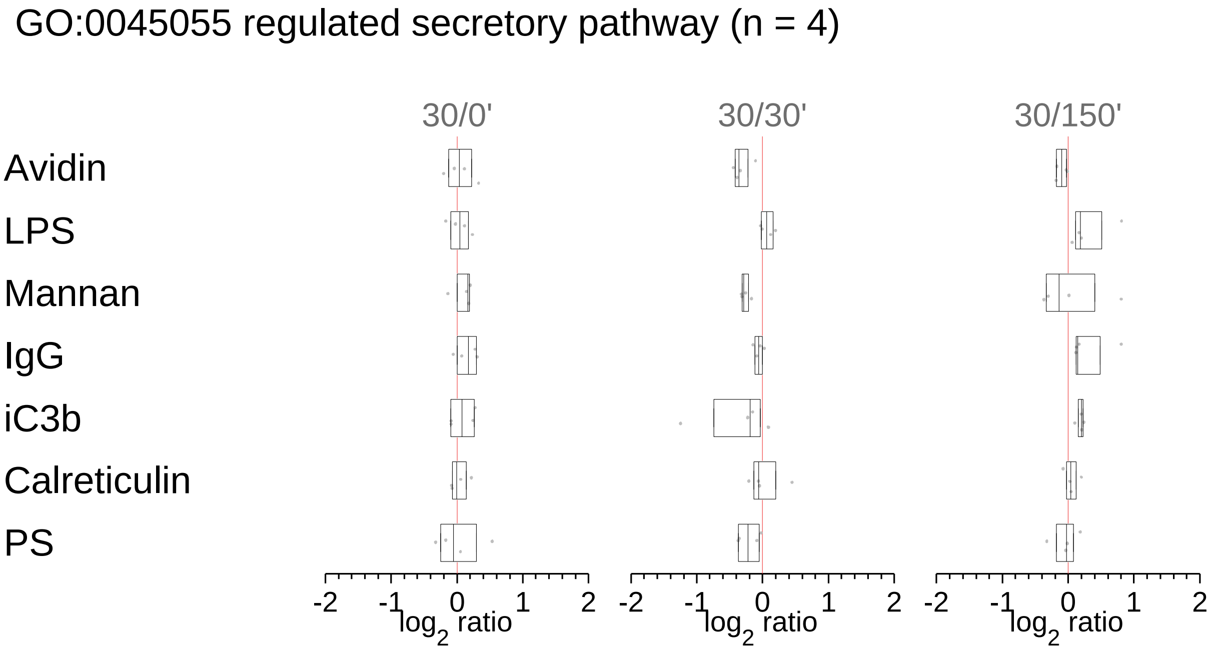
GO:0045055 regulated secretory pathway (n = 4)
30/0'
30/30'
30/150'
Avidin
LPS
Mannan
IgG
iC3b
Calreticulin
PS
-2 -1 0 1 2
log2 ratio

GO:0045059 positive thymic T cell selection (n = 5)

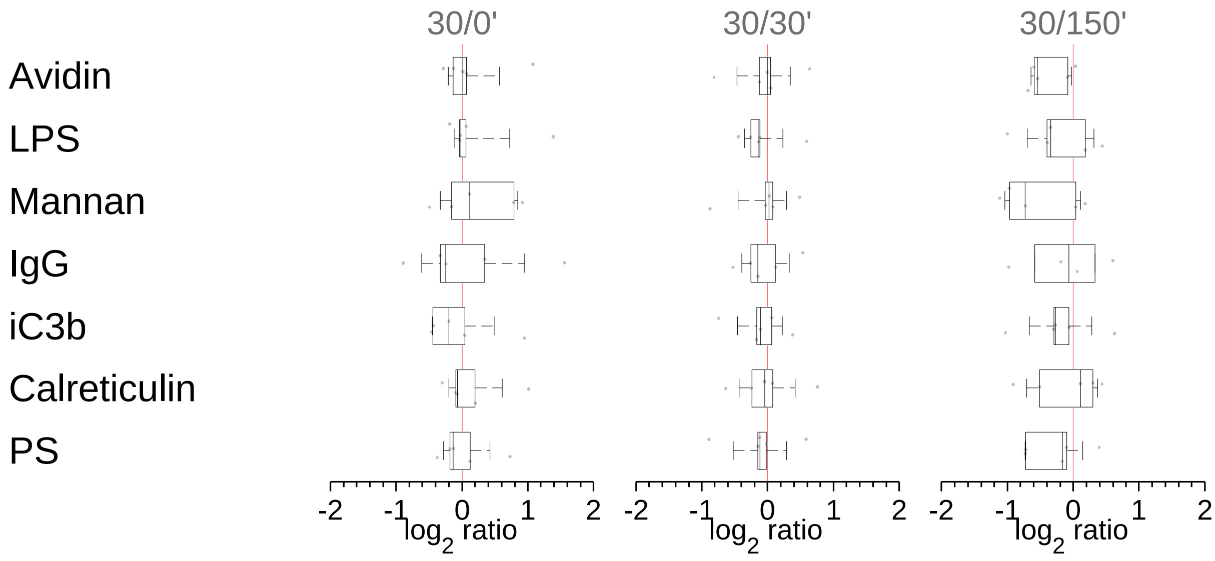

GO:0045060 negative thymic T cell selection (n = 4)

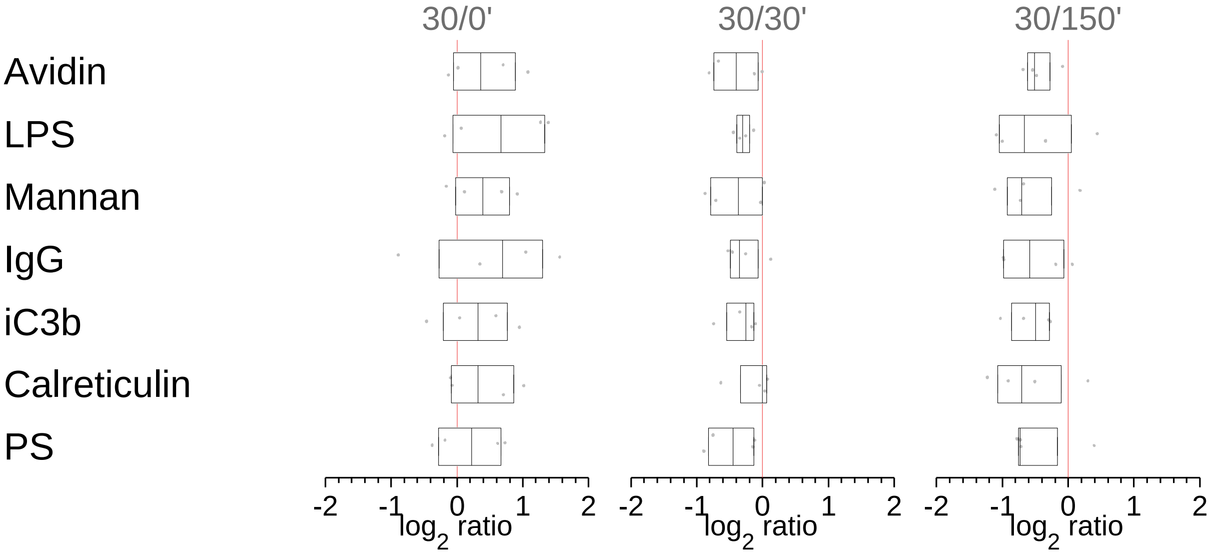

GO:0045071 negative regulation of viral genome replication (n = 10)

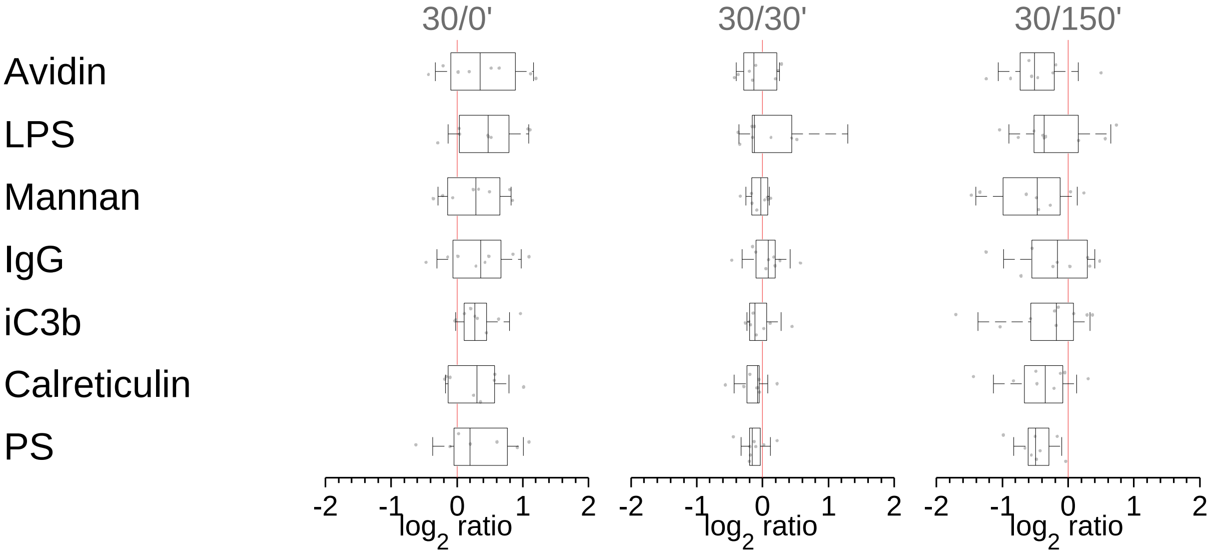

GO:0045078 positive regulation of interferon-gamma biosynthetic process (n = 5)

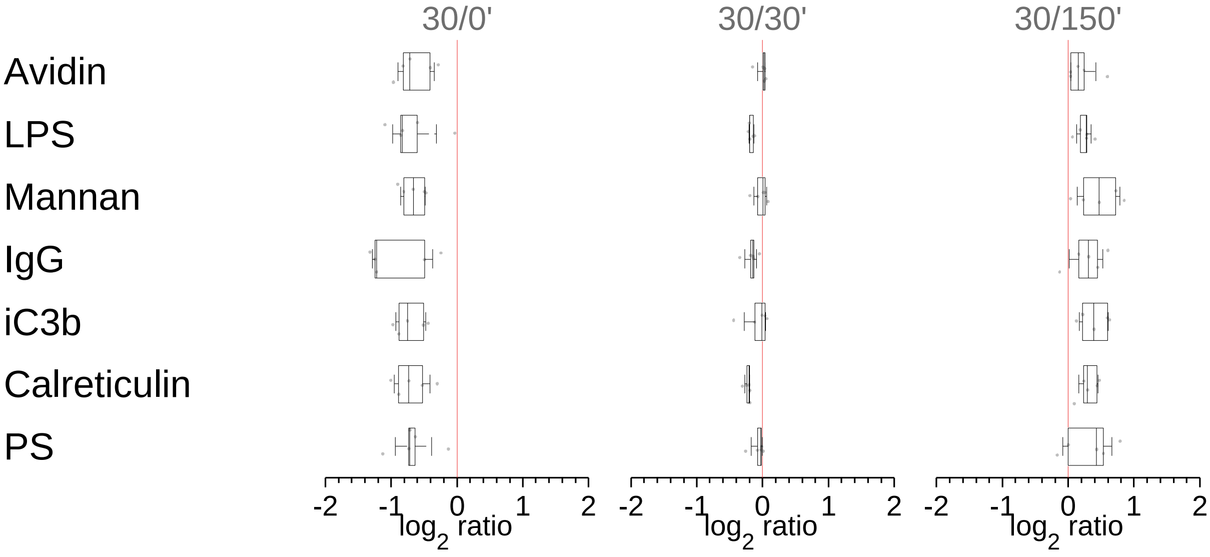

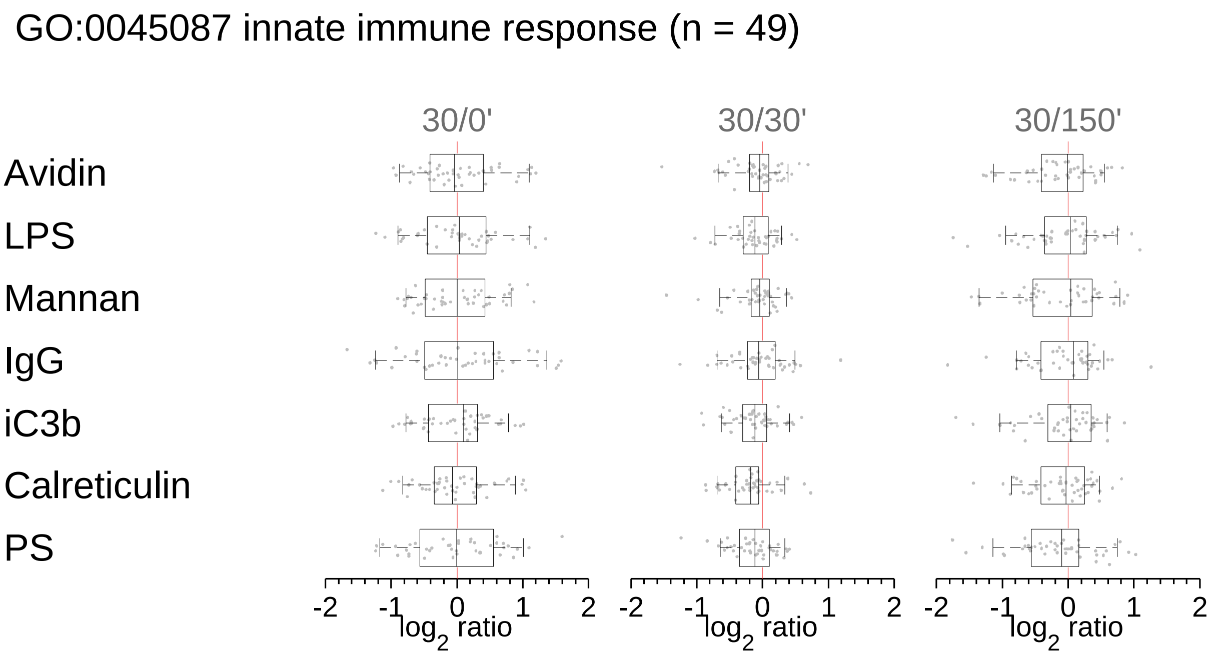
GO:0045087 innate immune response (n = 49)
30/0'
30/30'
30/150'
Avidin
LPS
Mannan
IgG
iC3b
Calreticulin
PS
-2
-1
0
1
2
log2 ratio

GO:0045109 intermediate filament organization (n = 3)

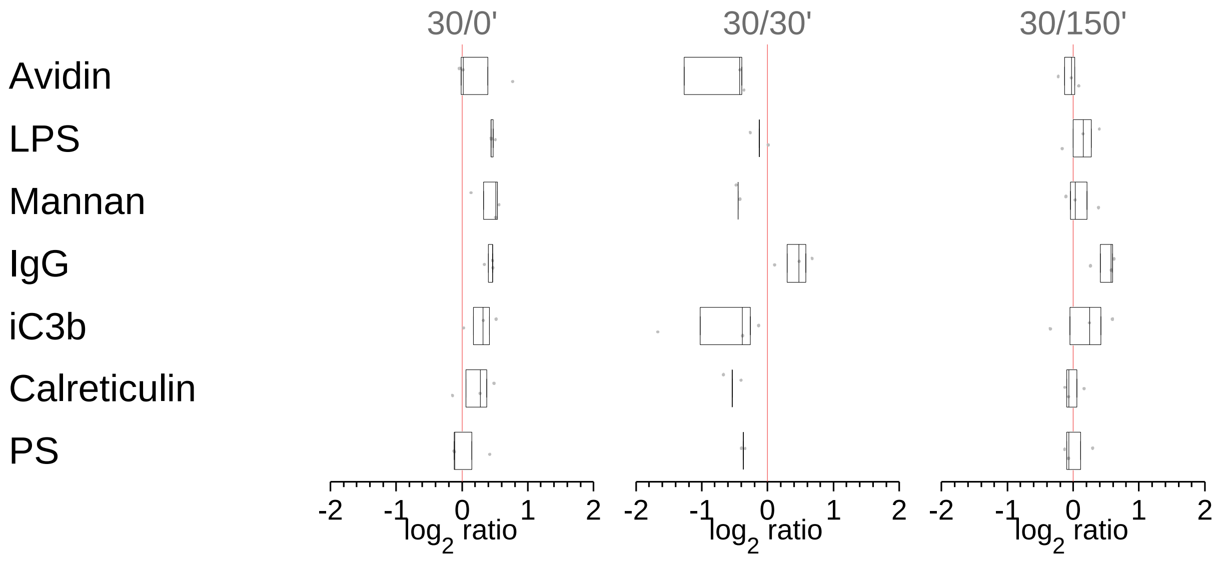

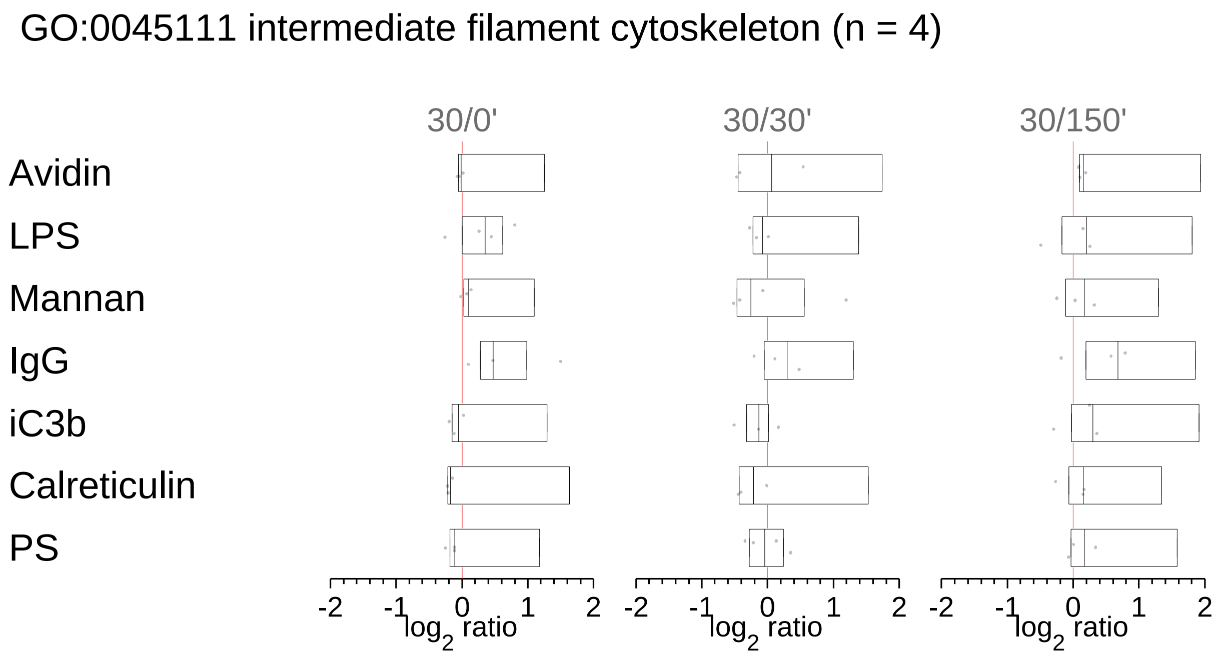

GO:0045111 intermediate filament cytoskeleton (n = 4)
30/0'
30/30'
30/150'
Avidin
LPS
Mannan
IgG
iC3b
Calreticulin
PS
-2 -1 0 1 2
log_2 ratio

GO:0045121 membrane raft (n = 46)

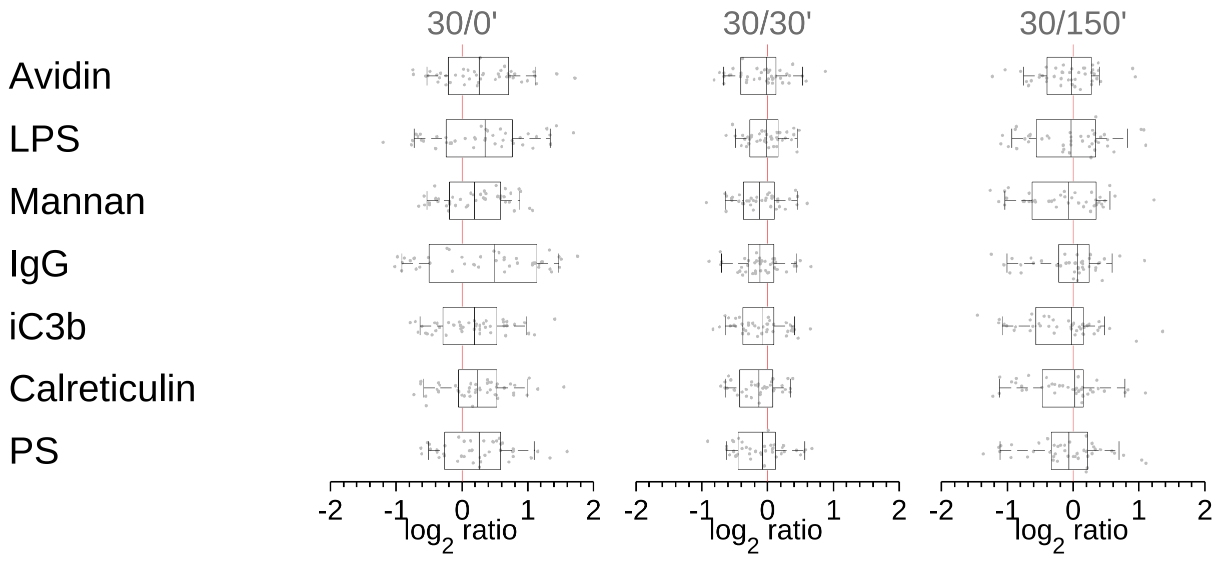

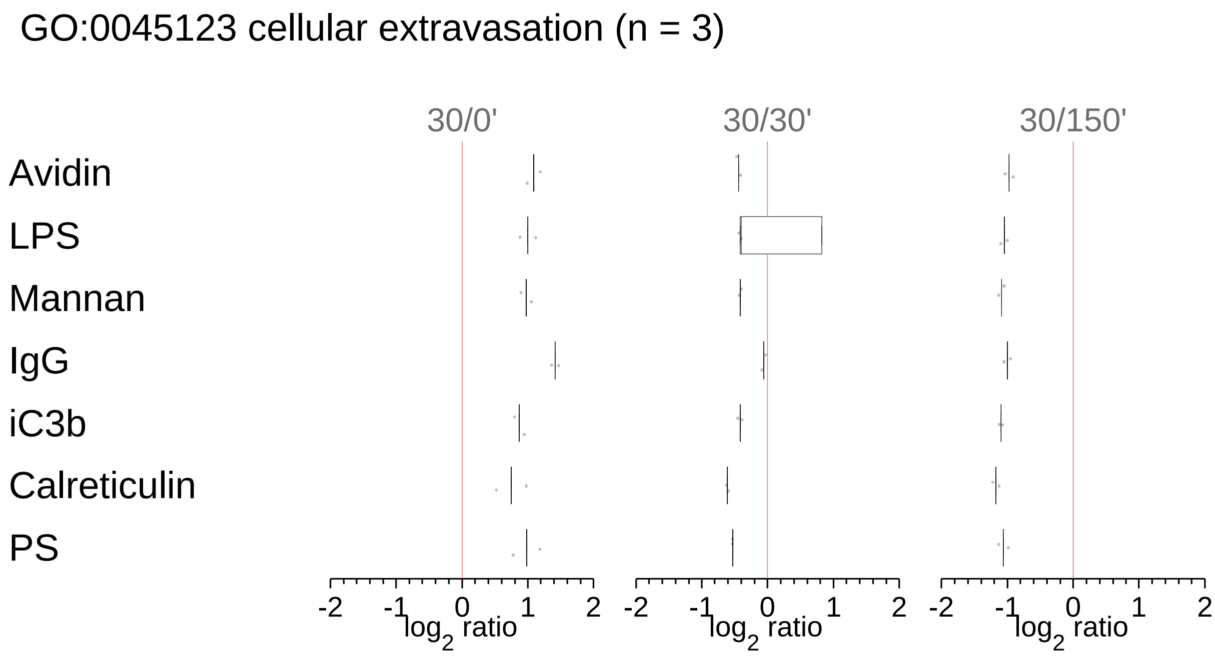
GO:0045123 cellular extravasation (n = 3)
30/0'
30/30'
30/150'
Avidin
LPS
Mannan
IgG
iC3b
Calreticulin
PS
-2
-1
0
1
2
log2 ratio

GO:0045140 inositol phosphoceramide synthase activity (n = 3)

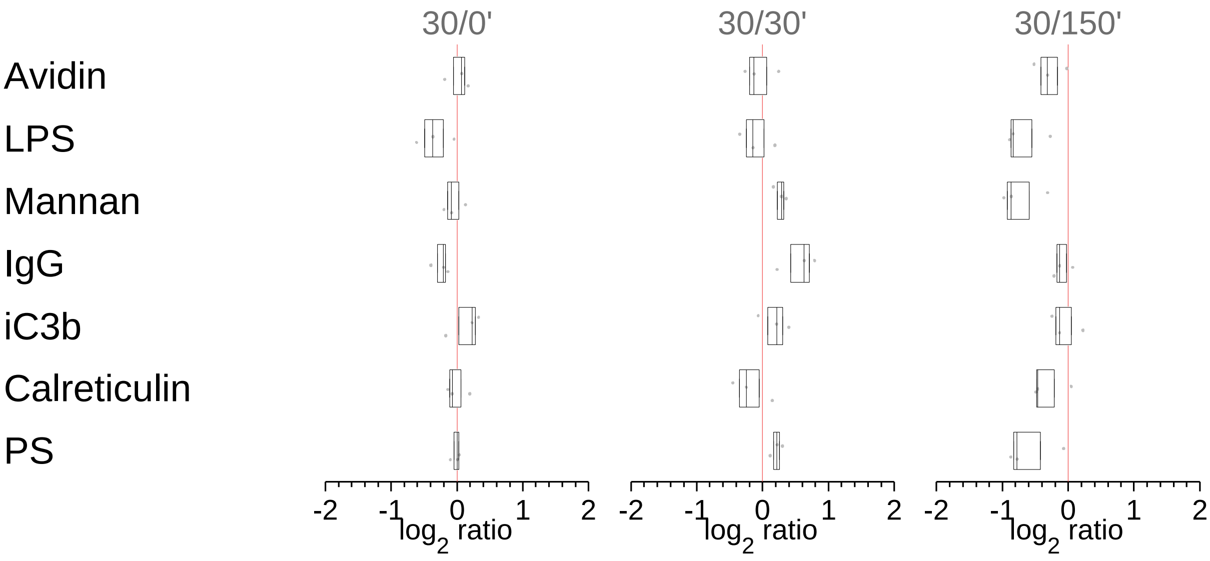

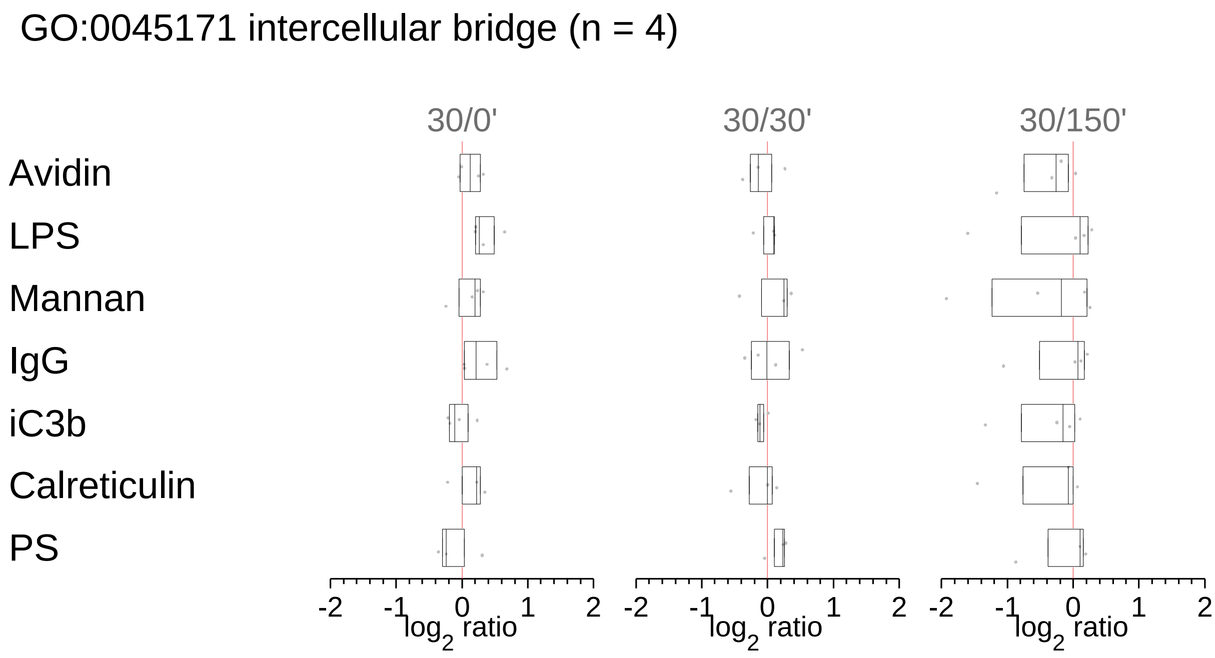

GO:0045171 intercellular bridge (n = 4)
30/0'
30/30'
30/150'
Avidin
LPS
Mannan
IgG
iC3b
Calreticulin
PS
-2
-1
0
1
2
log2 ratio

GO:0045176 apical protein localization (n = 3)

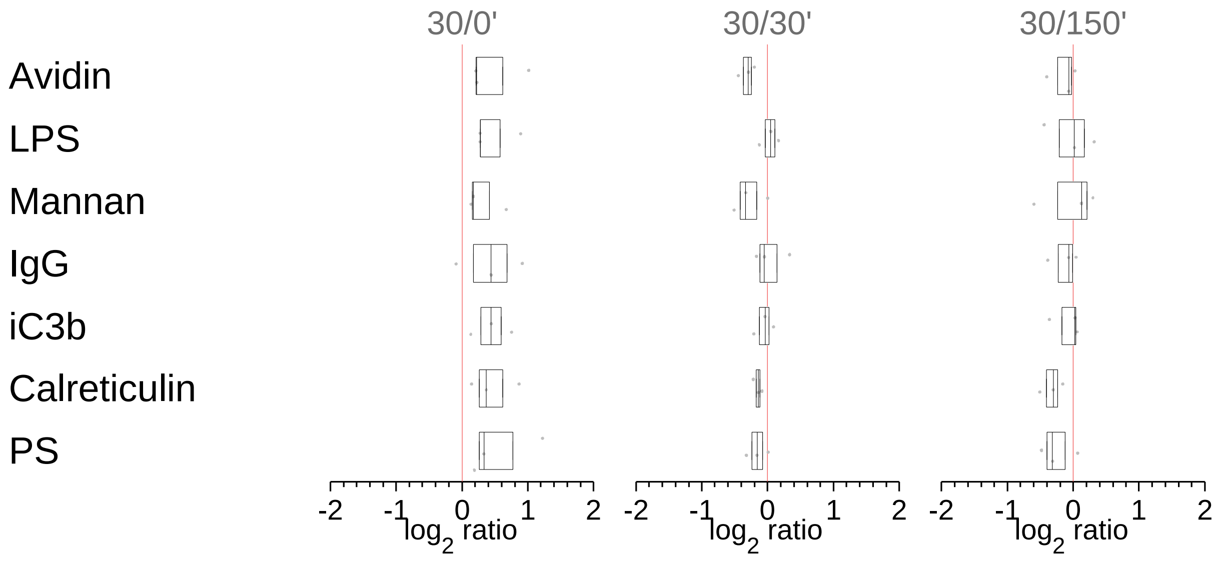

GO:0045177 apical part of cell (n = 19)

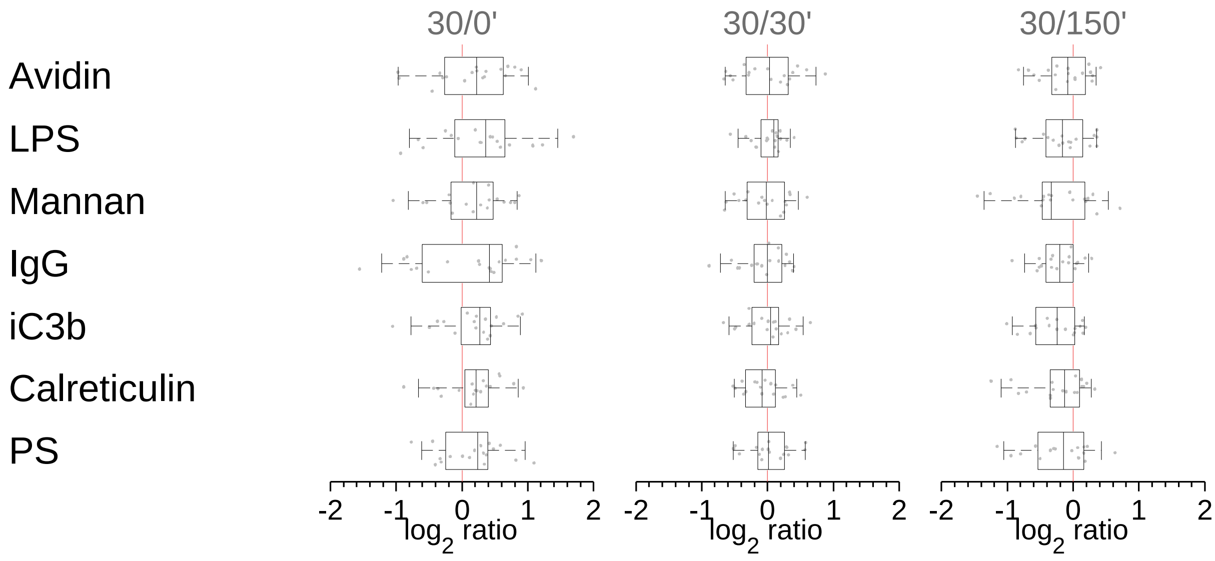

GO:0045178 basal part of cell (n = 3)

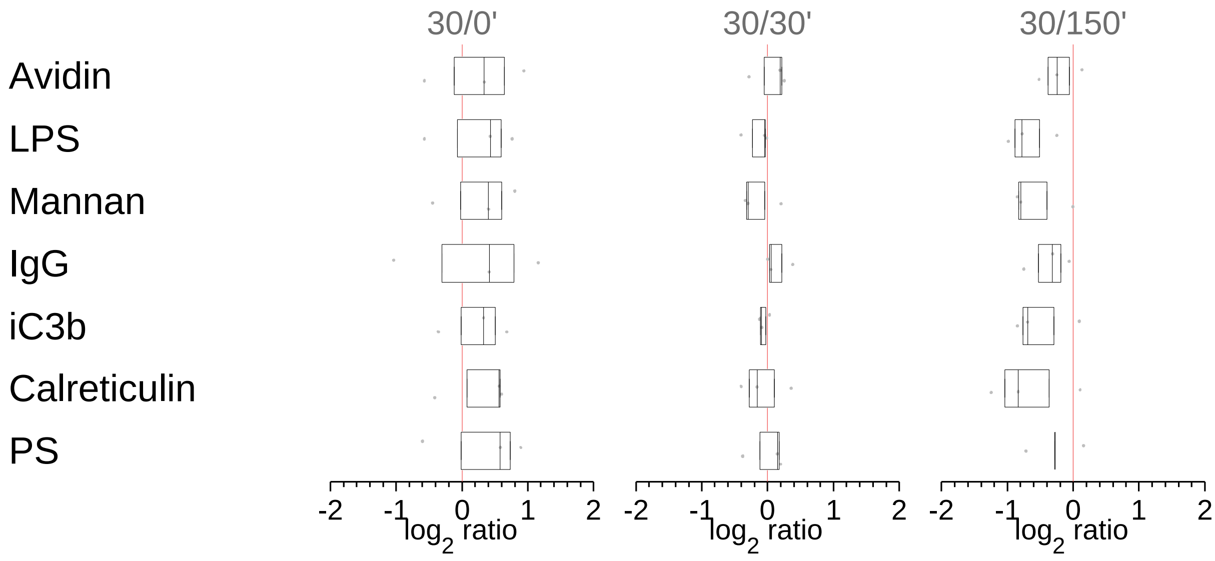

GO:0045184 establishment of protein localization (n = 8)

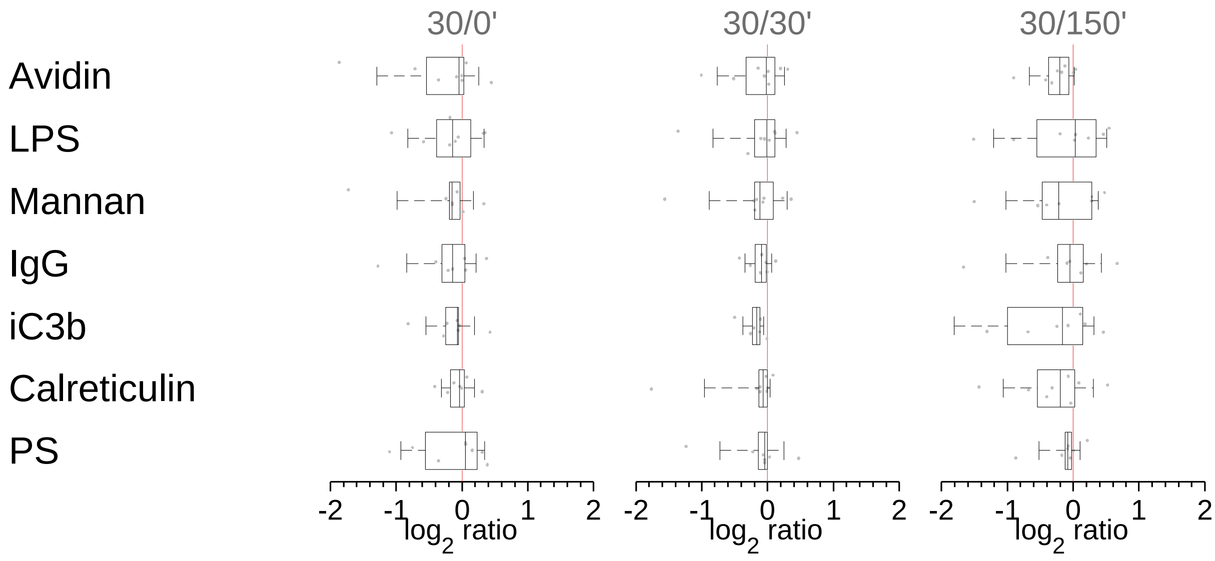

GO:0045202 synapse (n = 42)

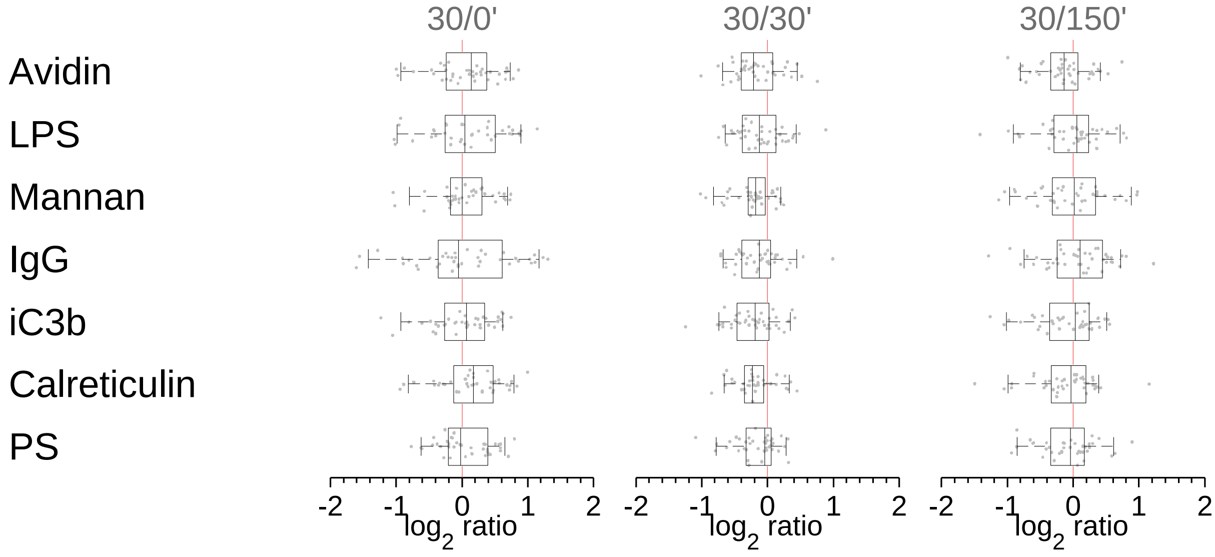

GO:0045211 postsynaptic membrane (n = 12)

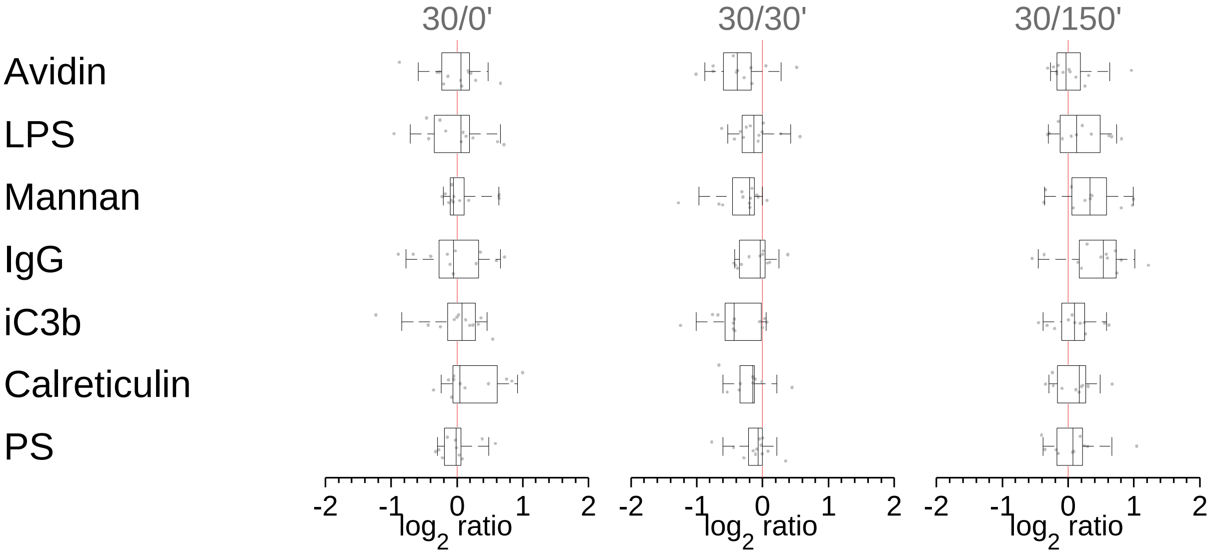

GO:0045252 oxoglutarate dehydrogenase complex (n = 3)

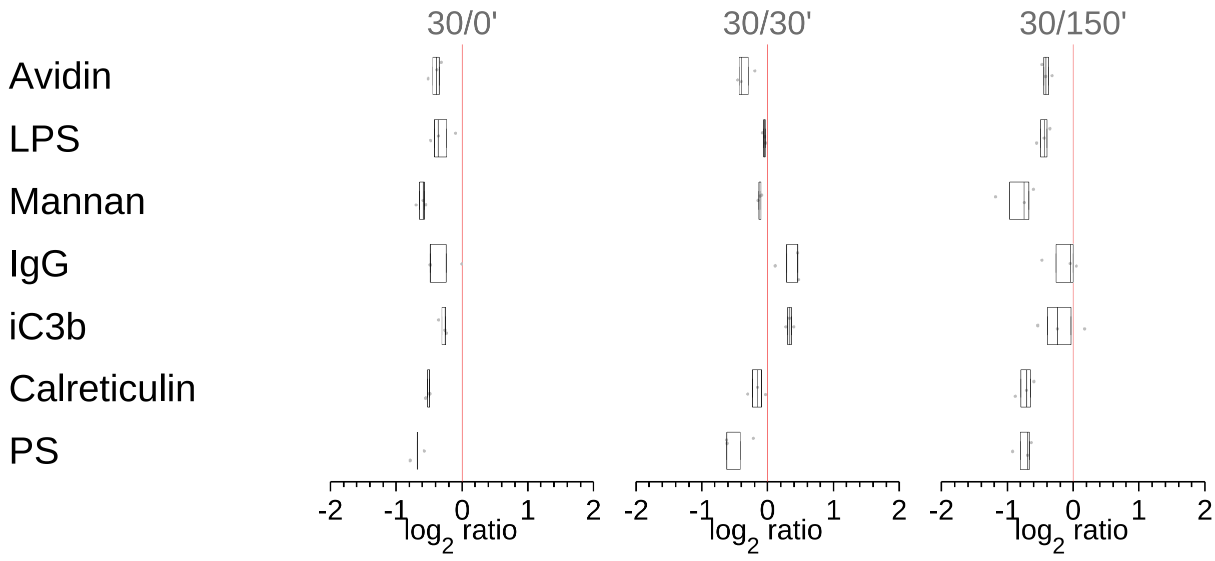

GO:0045254 pyruvate dehydrogenase complex (n = 4)

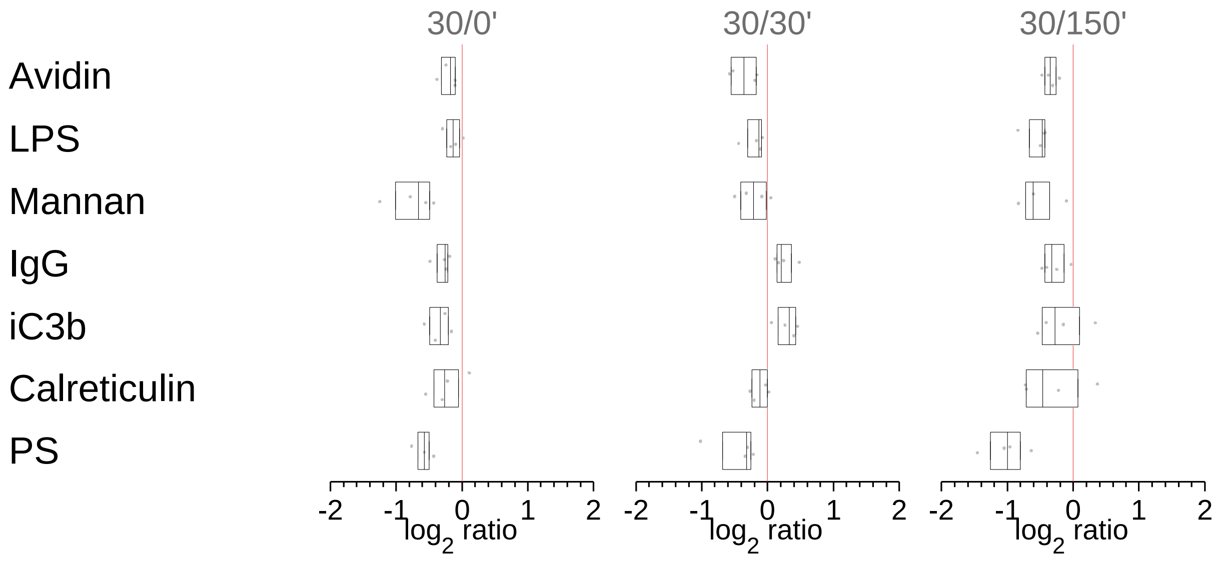

GO:0045261 proton-transporting ATP synthase complex, catalytic core F(1) (n = 4)

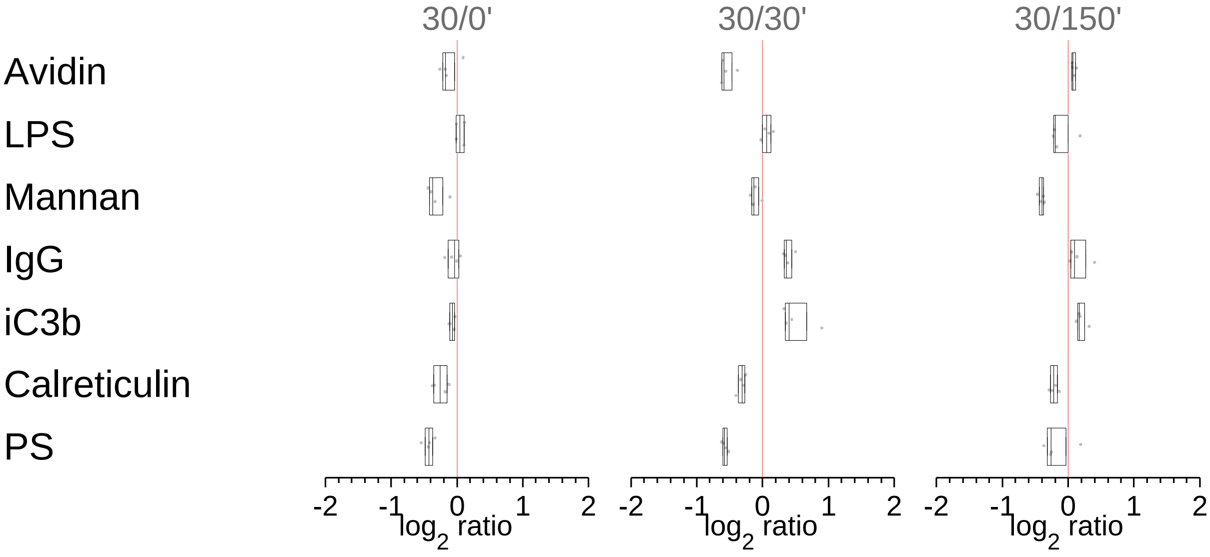

GO:0045263 proton-transporting ATP synthase complex, coupling factor F(o) (n = 6)

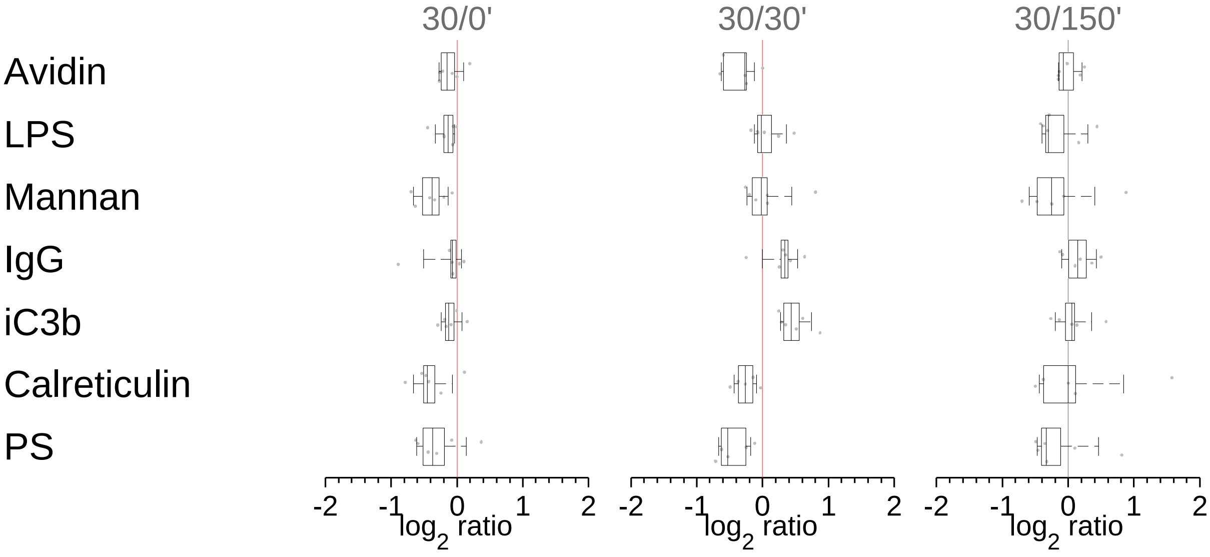

GO:0045296 cadherin binding (n = 7)

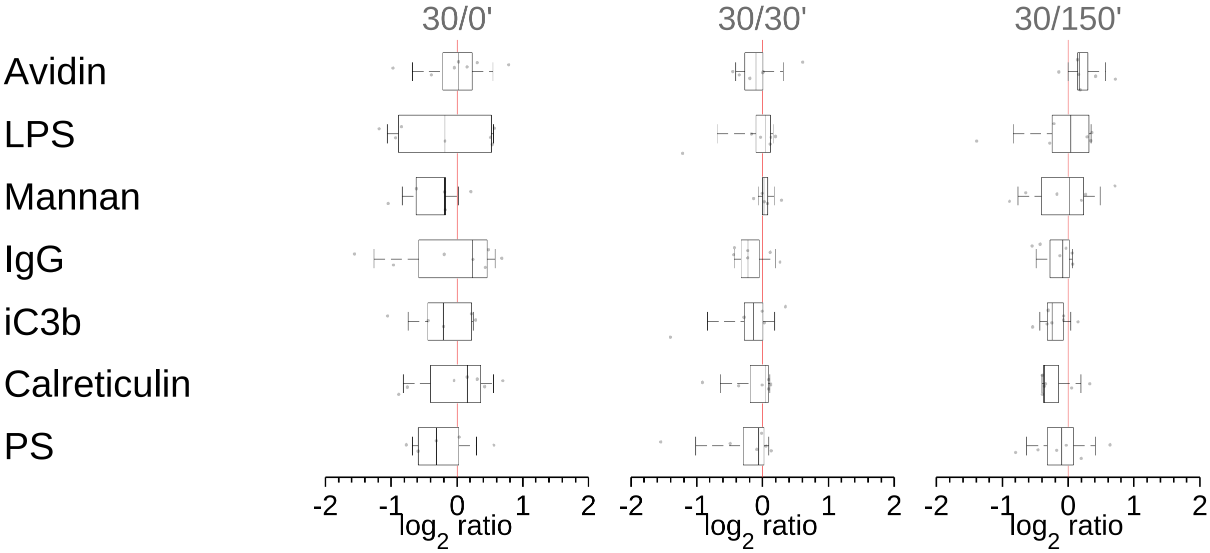

GO:0045298 tubulin complex (n = 3)

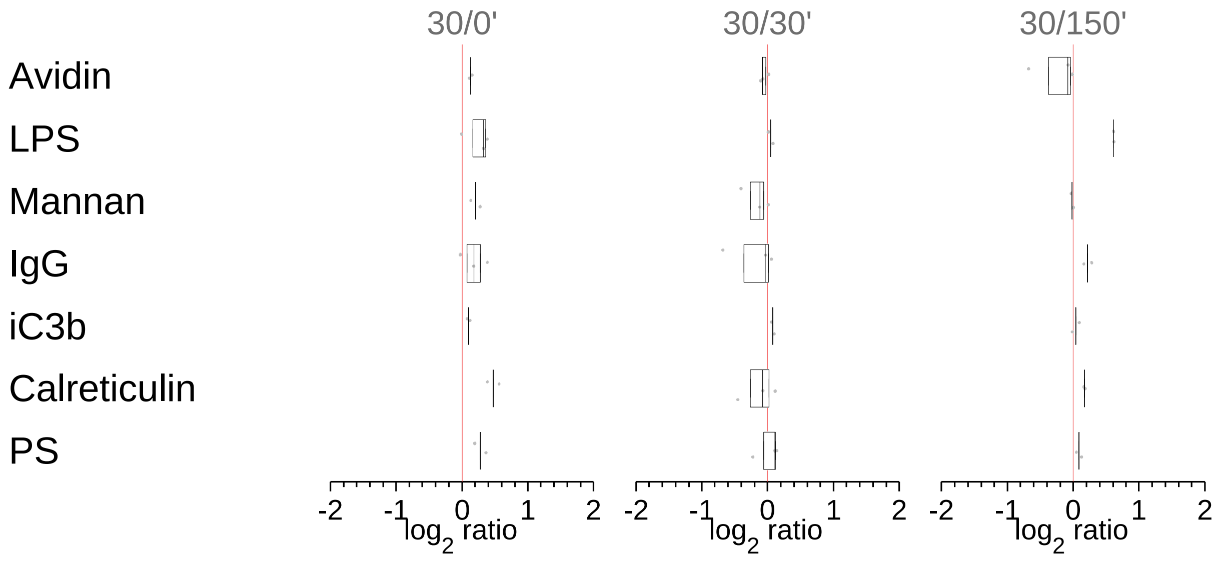

GO:0045332 phospholipid translocation (n = 4)

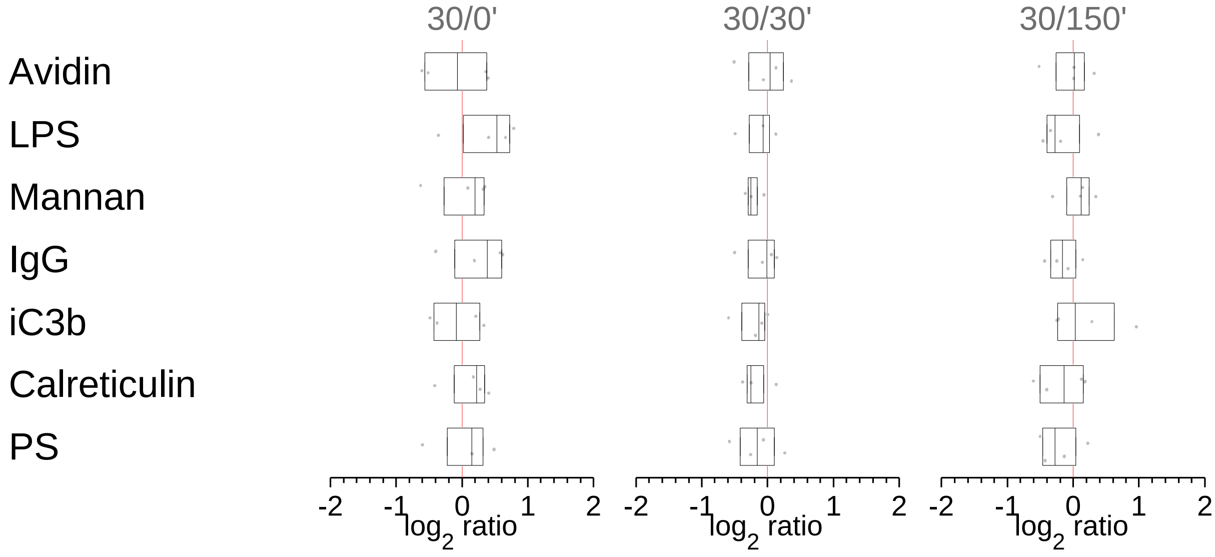

GO:0045333 cellular respiration (n = 3)

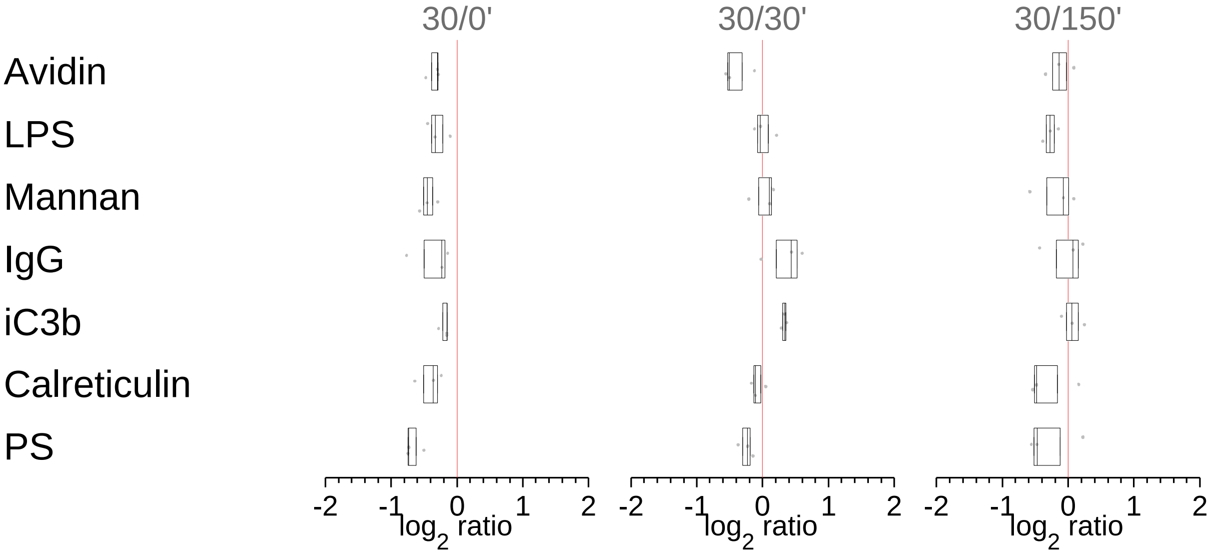

GO:0045334 clathrin-coated endocytic vesicle (n = 4)

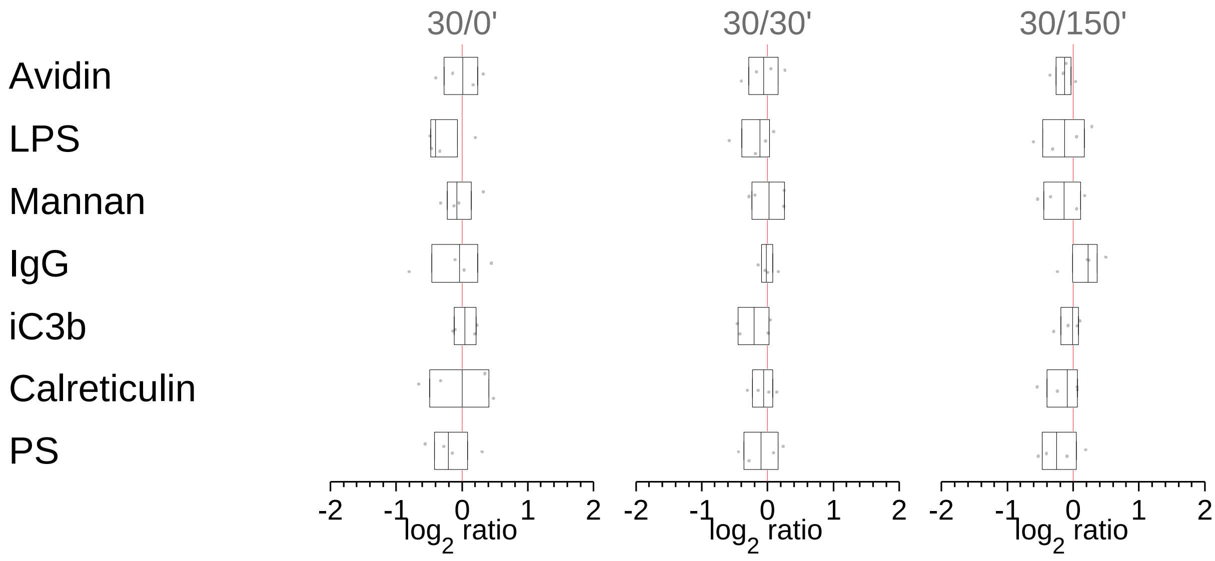

GO:0045335 phagocytic vesicle (n = 23)

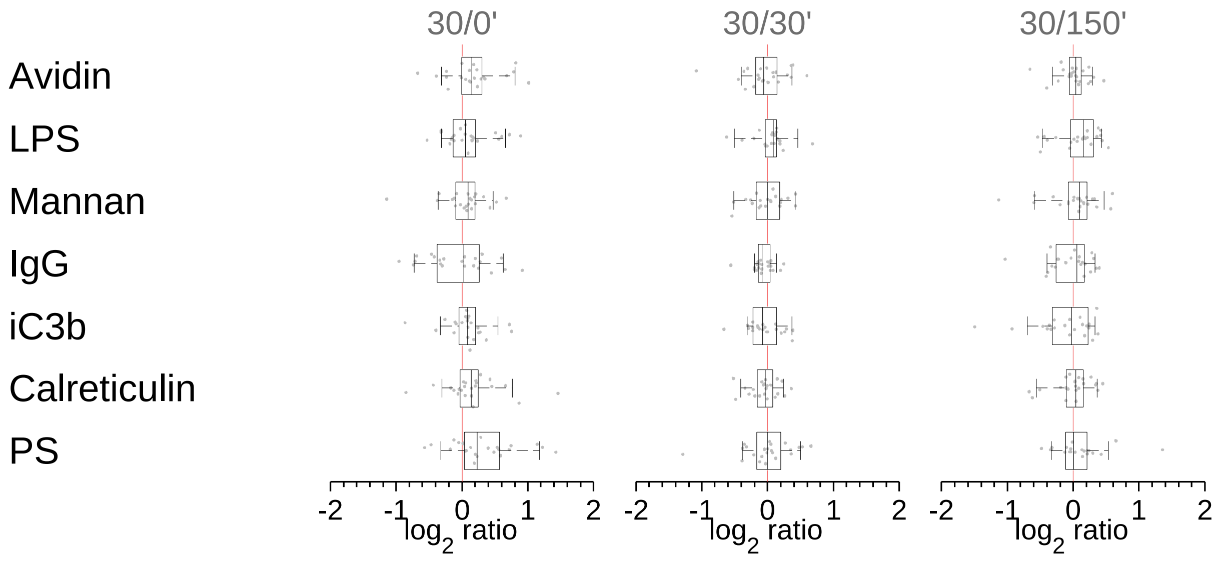

GO:0045356 positive regulation of interferon-alpha biosynthetic process (n = 4)

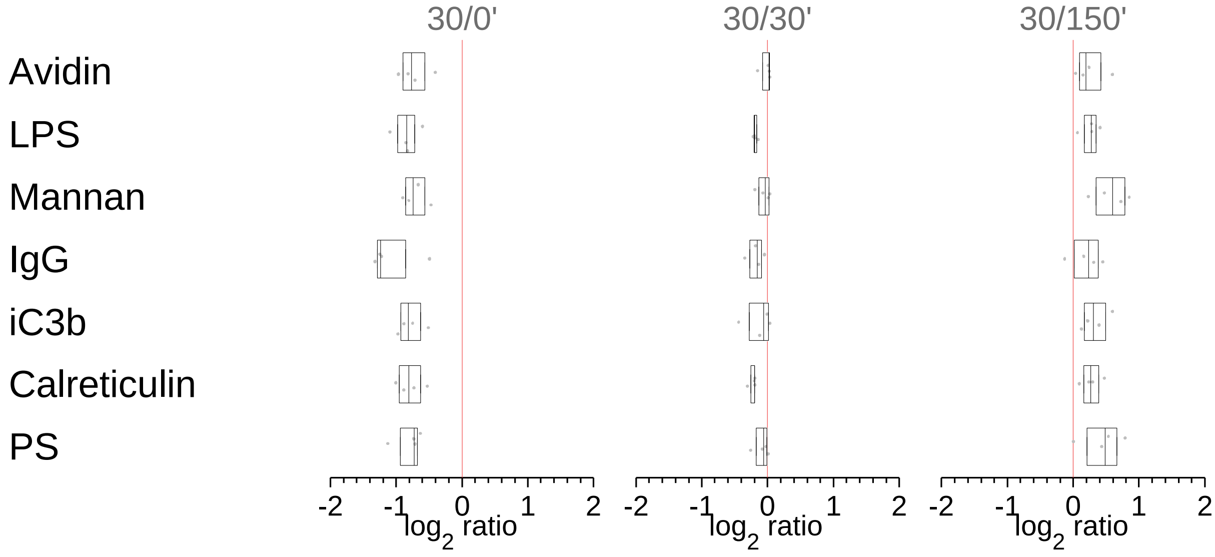

GO:0045359 positive regulation of interferon-beta biosynthetic process (n = 4)

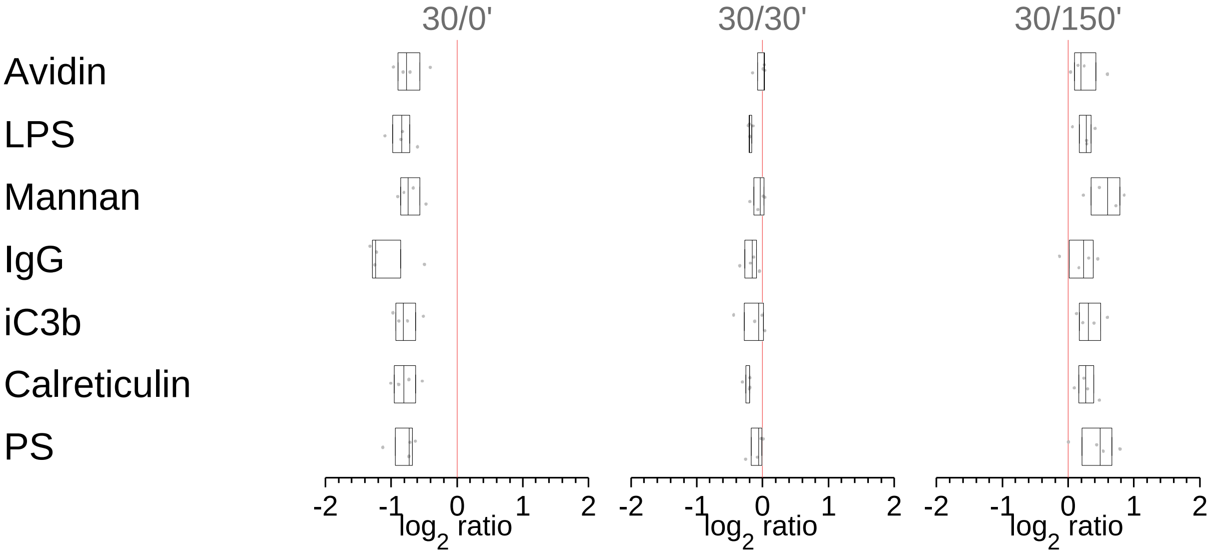

GO:0045416 positive regulation of interleukin-8 biosynthetic process (n = 3)

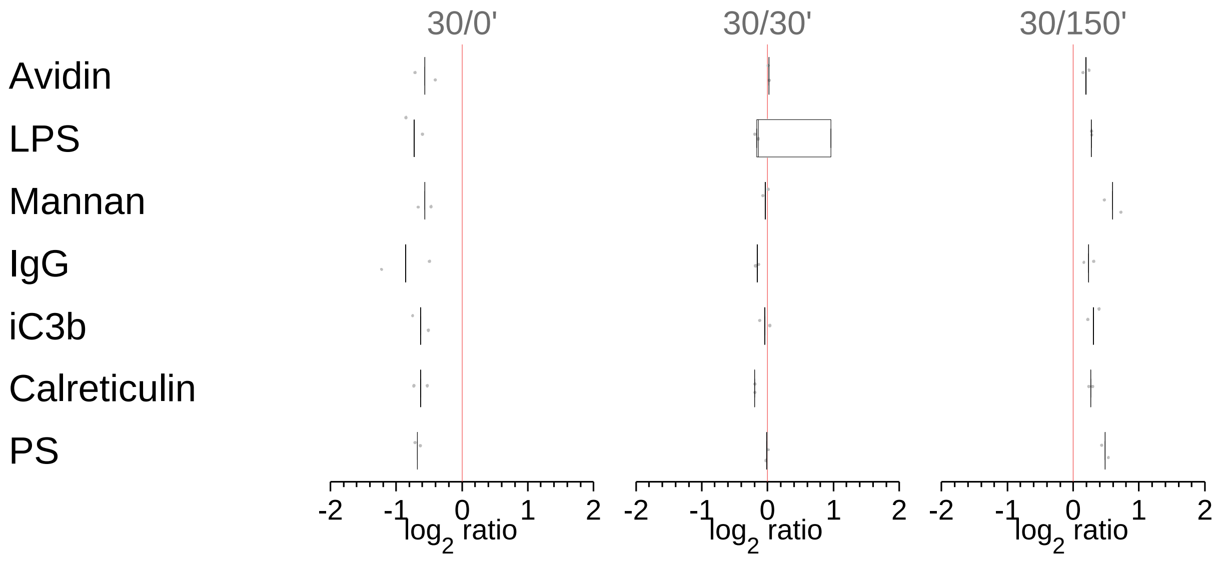

GO:0045429 positive regulation of nitric oxide biosynthetic process (n = 8)

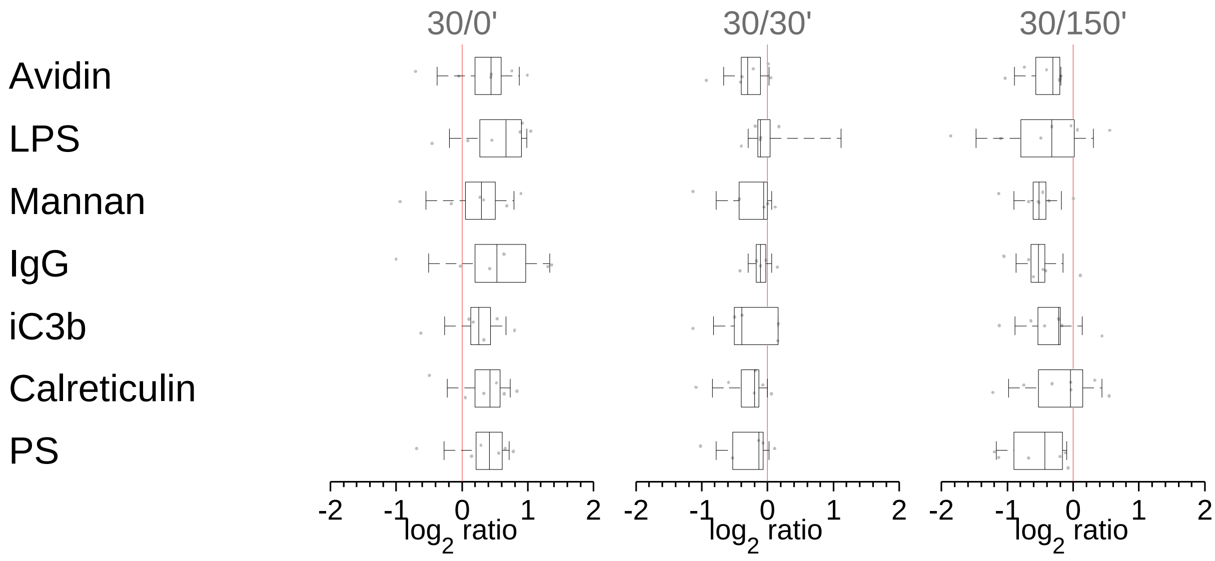

GO:0045444 fat cell differentiation (n = 5)

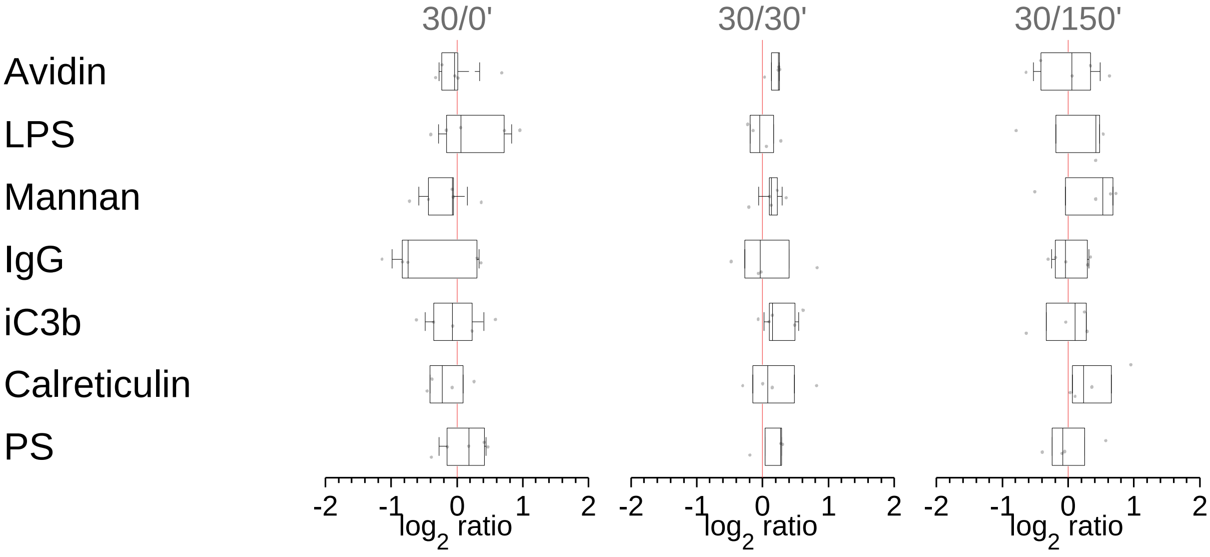

GO:0045453 bone resorption (n = 6)

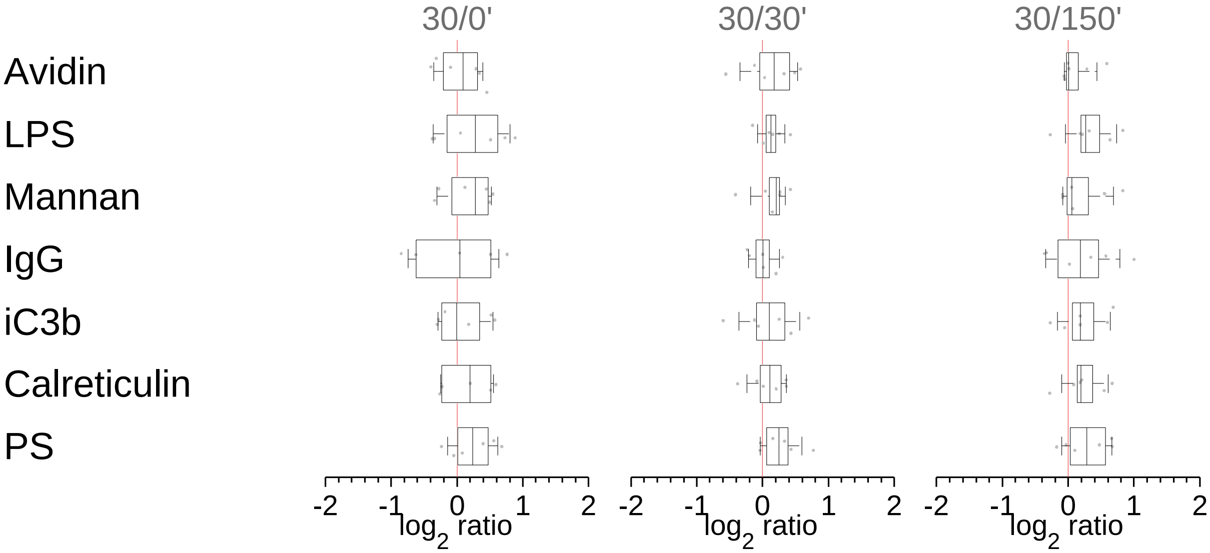

GO:0045454 cell redox homeostasis (n = 17)

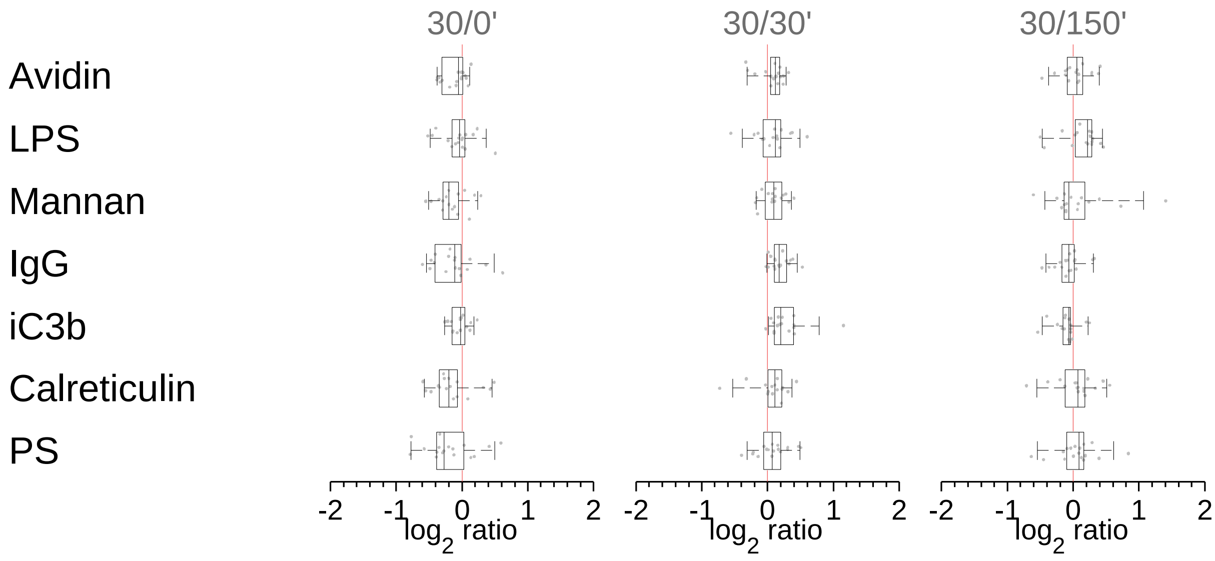

GO:0045471 response to ethanol (n = 8)

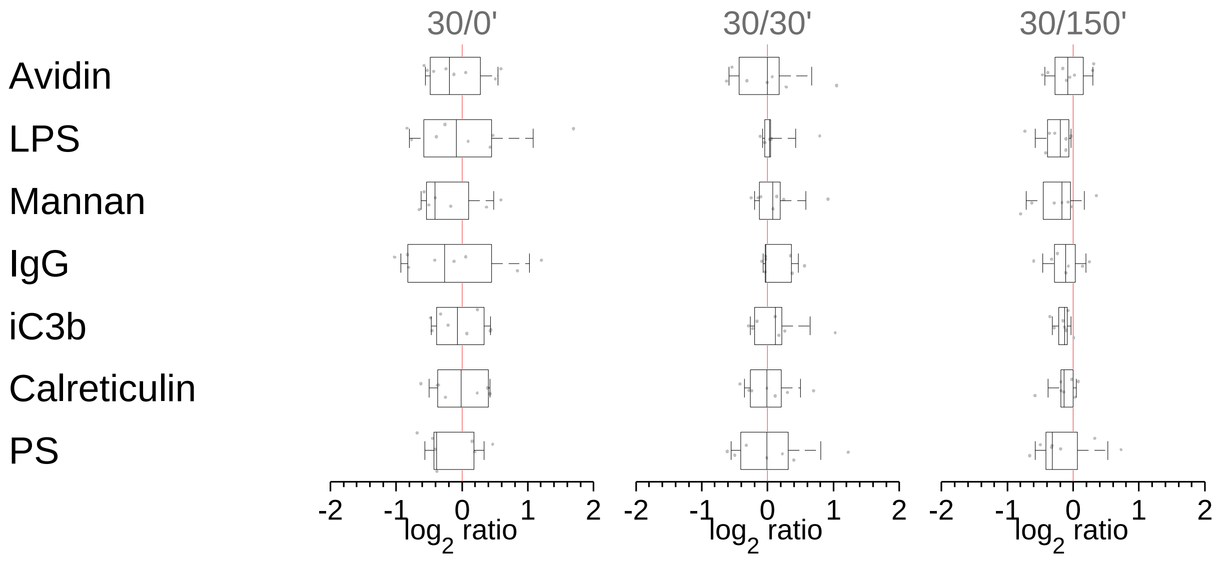

GO:0045576 mast cell activation (n = 6)

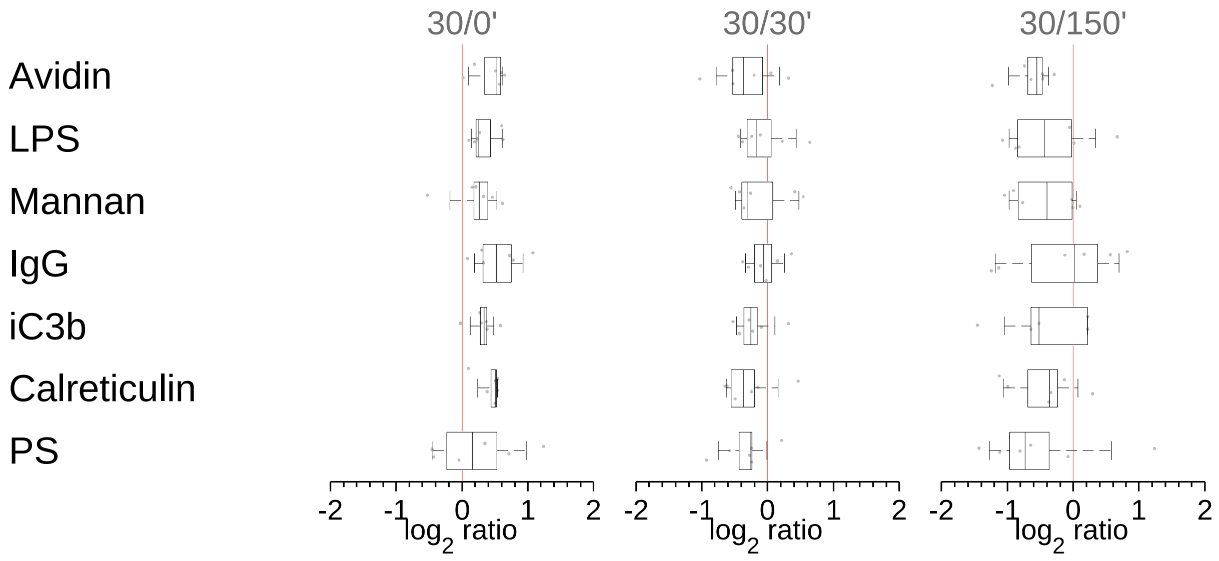

GO:0045577 regulation of B cell differentiation (n = 3)

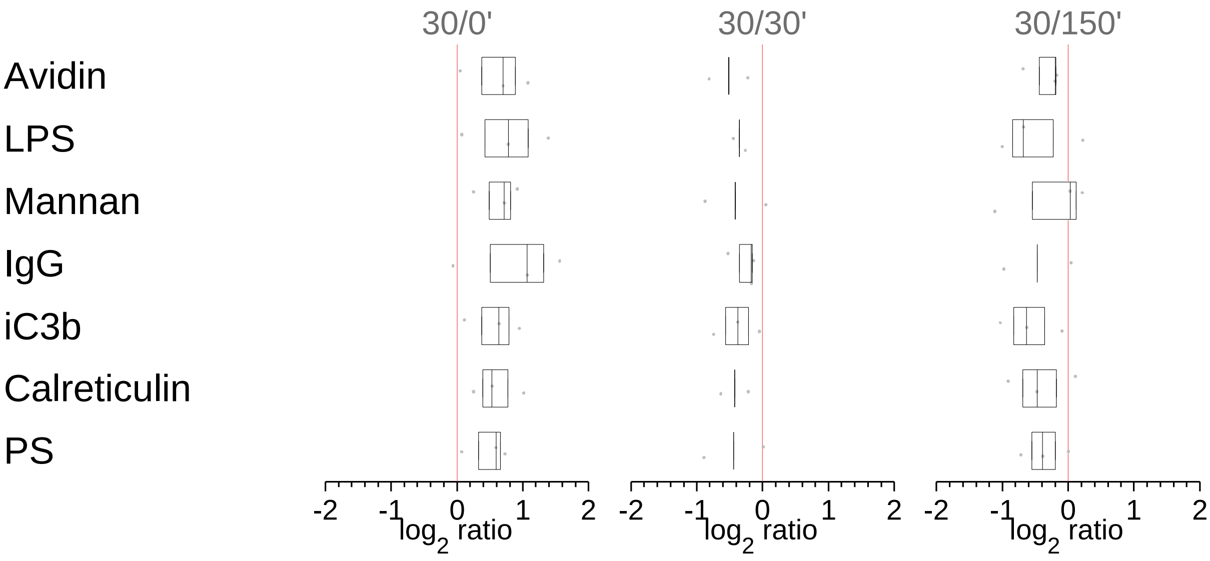

GO:0045579 positive regulation of B cell differentiation (n = 5)

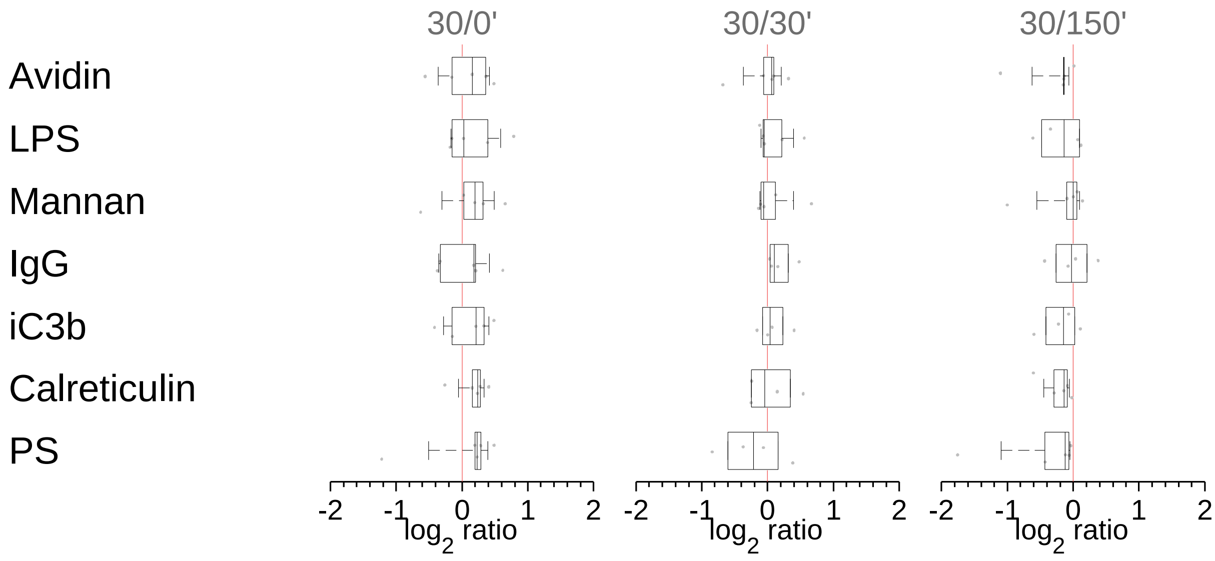

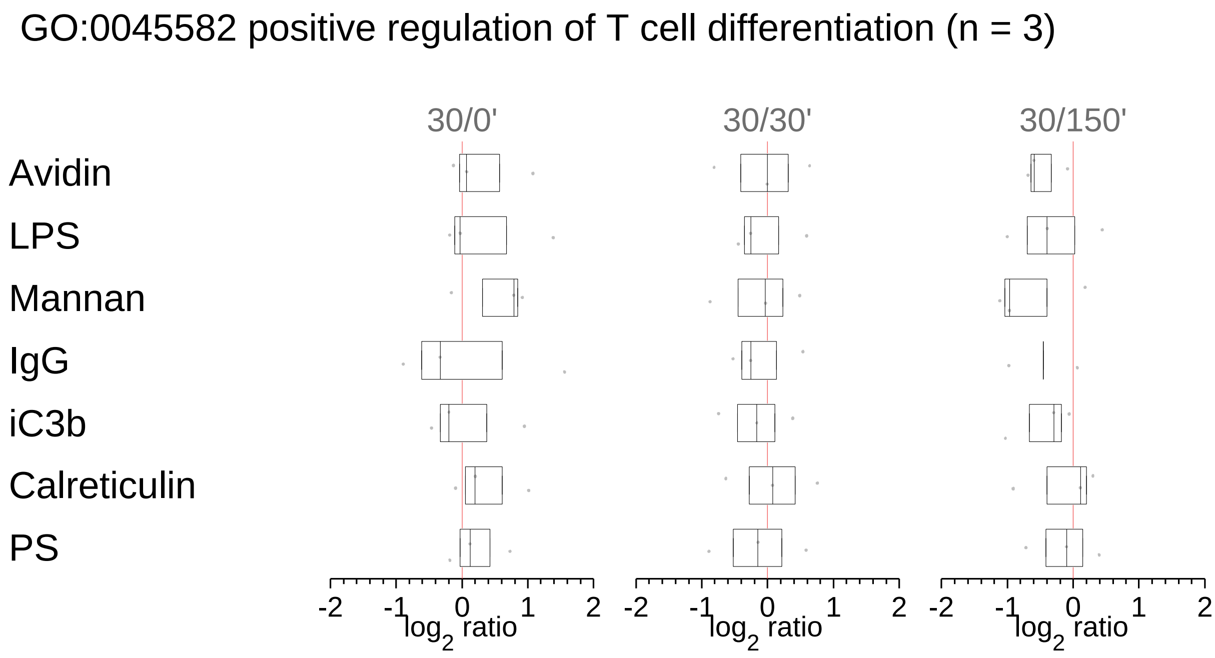
GO:0045582 positive regulation of T cell differentiation (n = 3)
30/0'
30/30'
30/150'
Avidin
LPS
Mannan
IgG
iC3b
Calreticulin
PS
-2 -1 0 1 2
log2 ratio

GO:0045648 positive regulation of erythrocyte differentiation (n = 5)

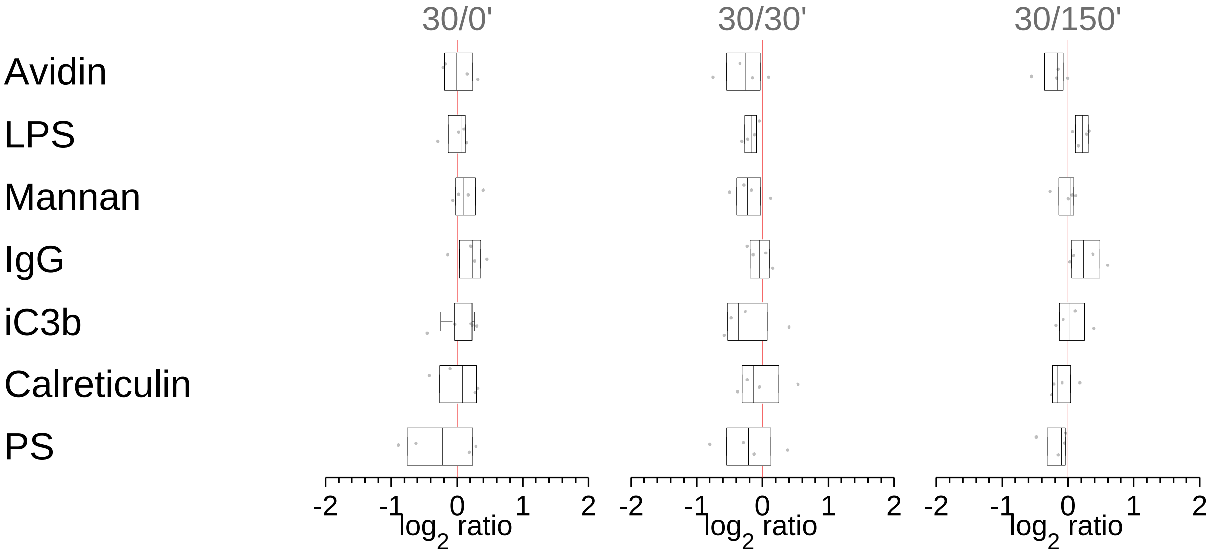

GO:0045651 positive regulation of macrophage differentiation (n = 5)

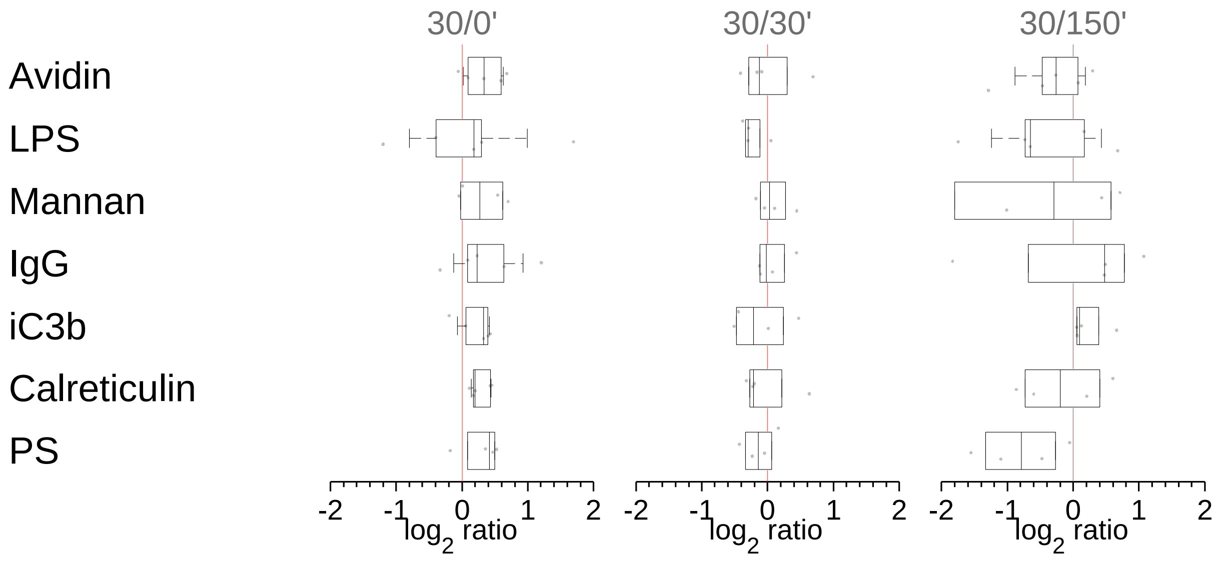

GO:0045654 positive regulation of megakaryocyte differentiation
(n = 4)

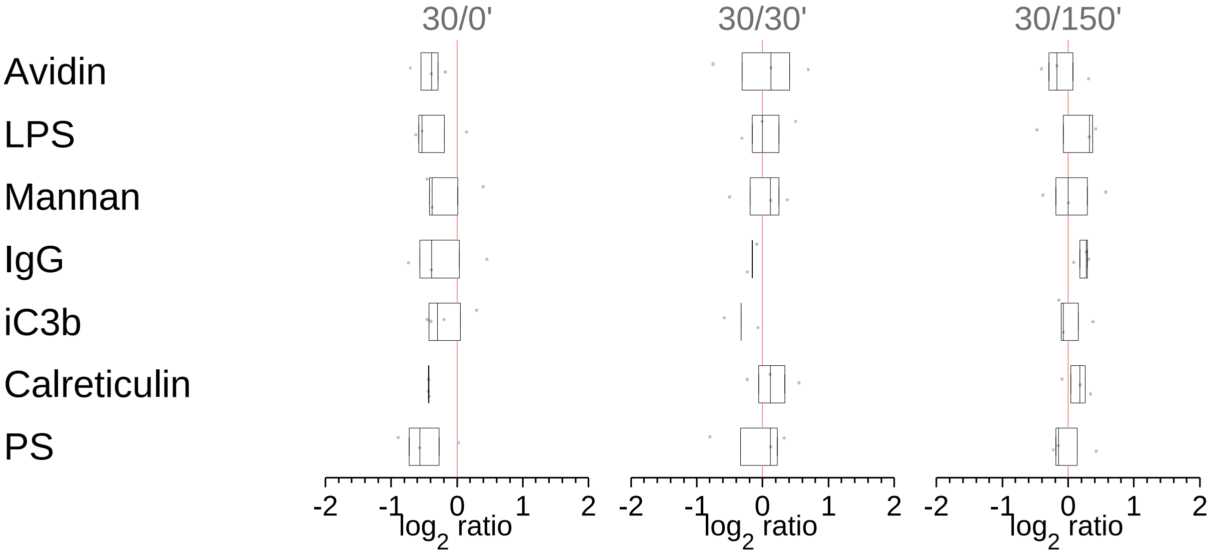

GO:0045664 regulation of neuron differentiation (n = 4)

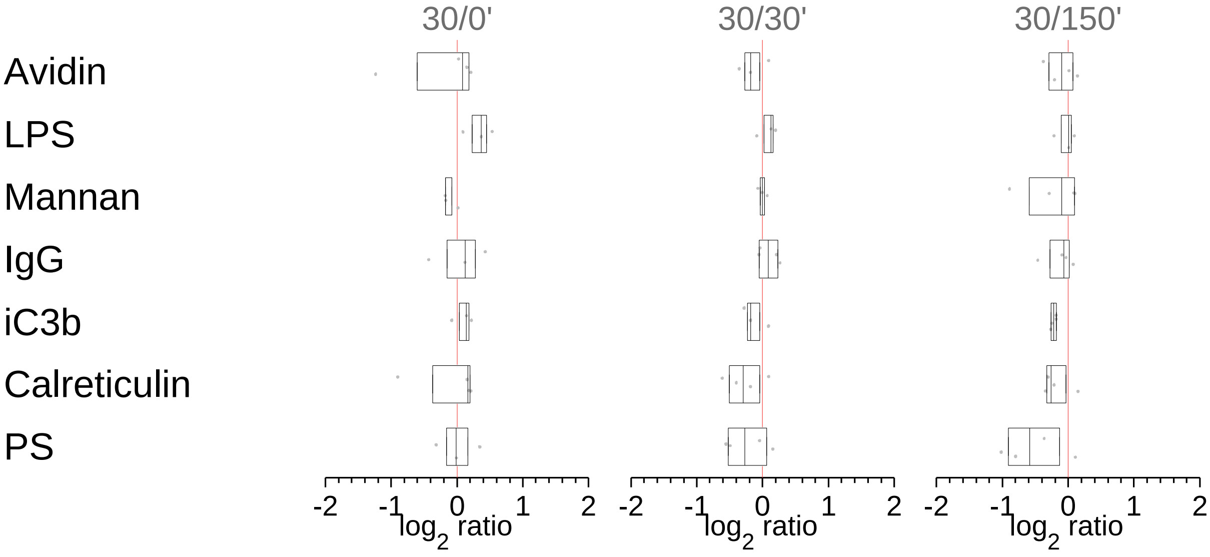

GO:0045665 negative regulation of neuron differentiation (n = 7)

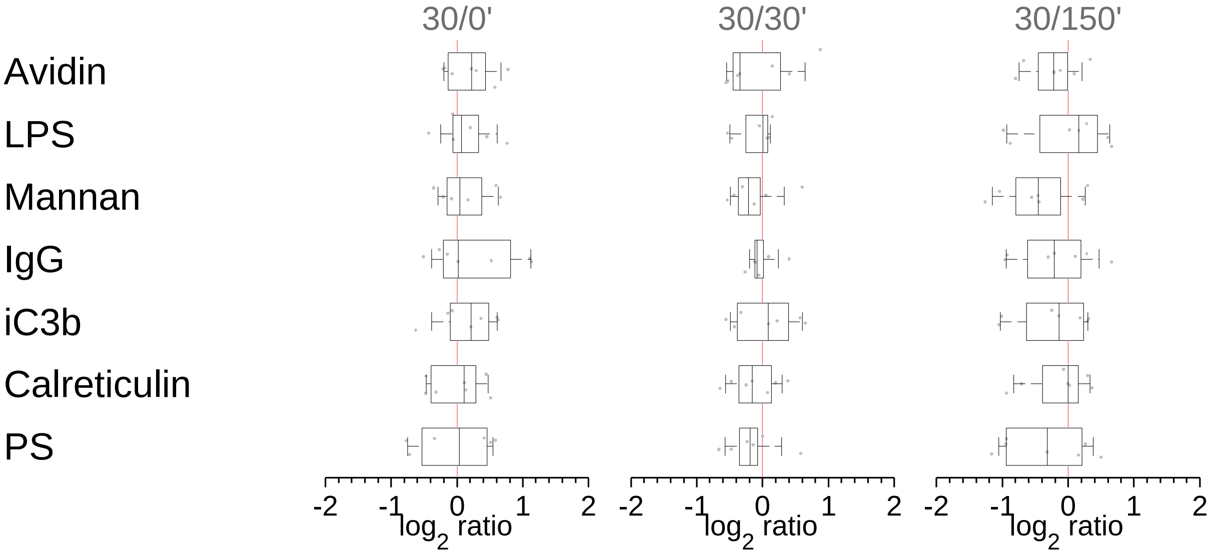

GO:0045666 positive regulation of neuron differentiation (n = 4)

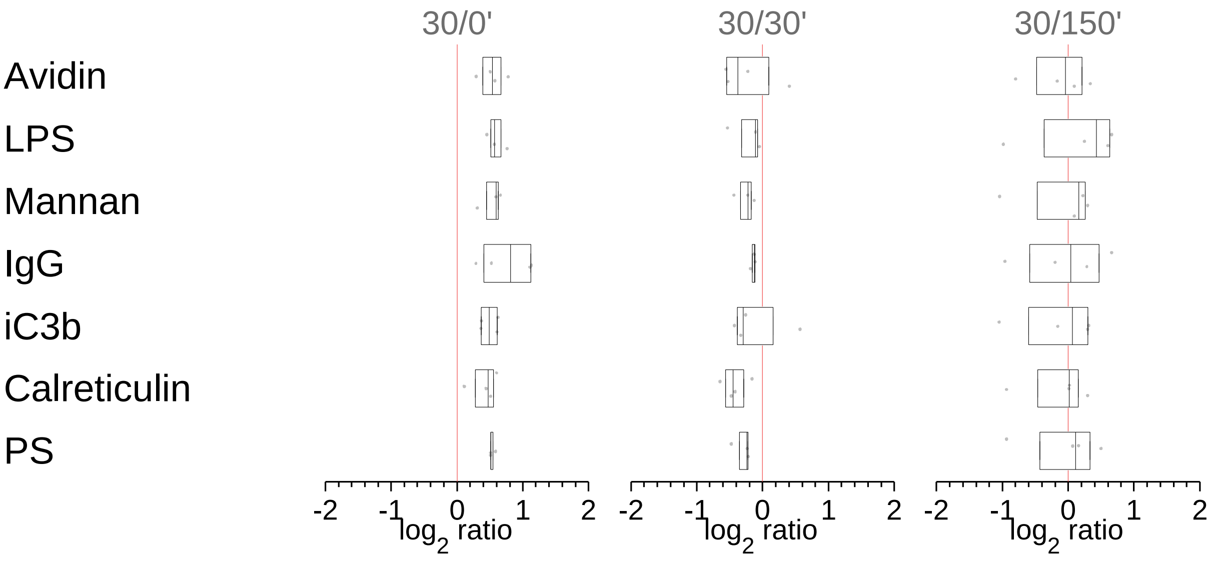

GO:0045669 positive regulation of osteoblast differentiation (n = 4)

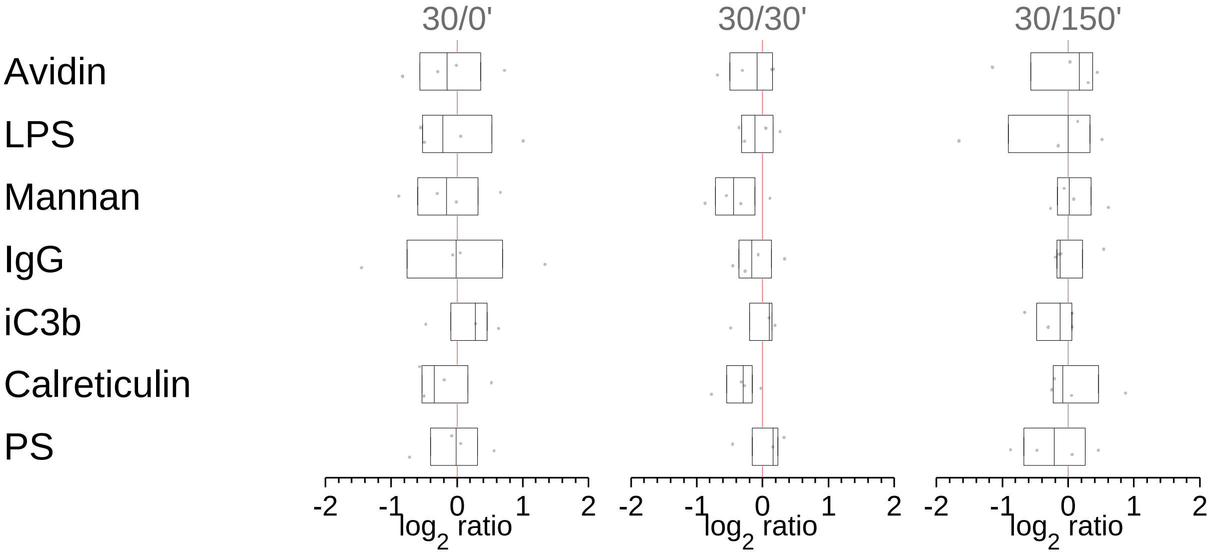

GO:0045670 regulation of osteoclast differentiation (n = 3)

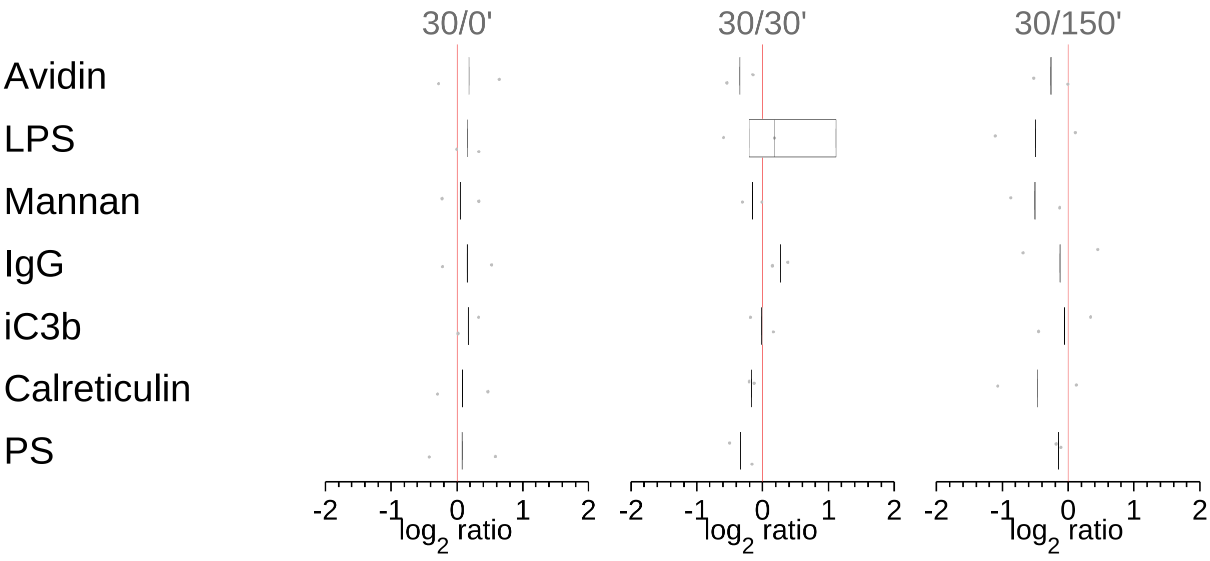

GO:0045672 positive regulation of osteoclast differentiation (n = 4)

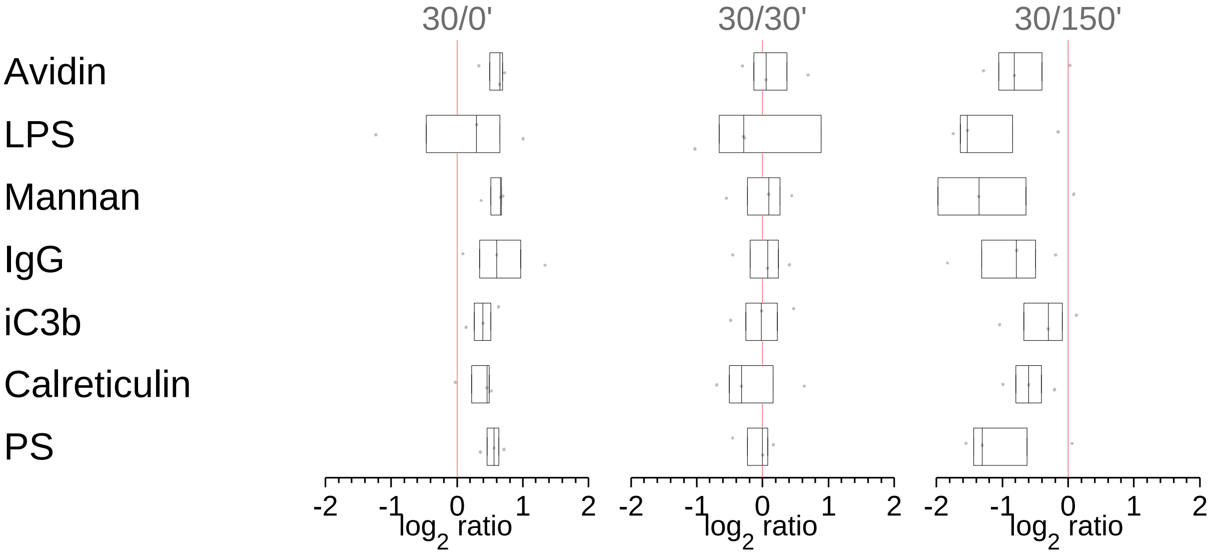

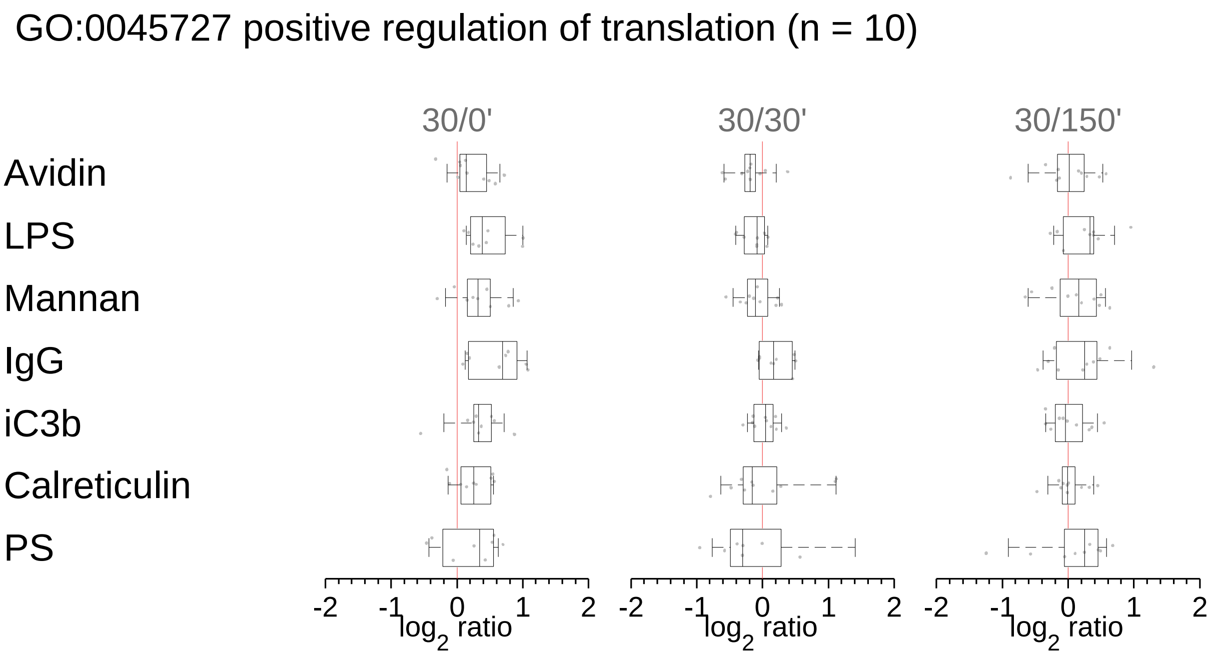
GO:0045727 positive regulation of translation (n = 10)
30/0'
30/30'
30/150'
Avidin
LPS
Mannan
IgG
iC3b
Calreticulin
PS
-2
-1
0
1
2
log2 ratio

GO:0045730 respiratory burst (n = 5)

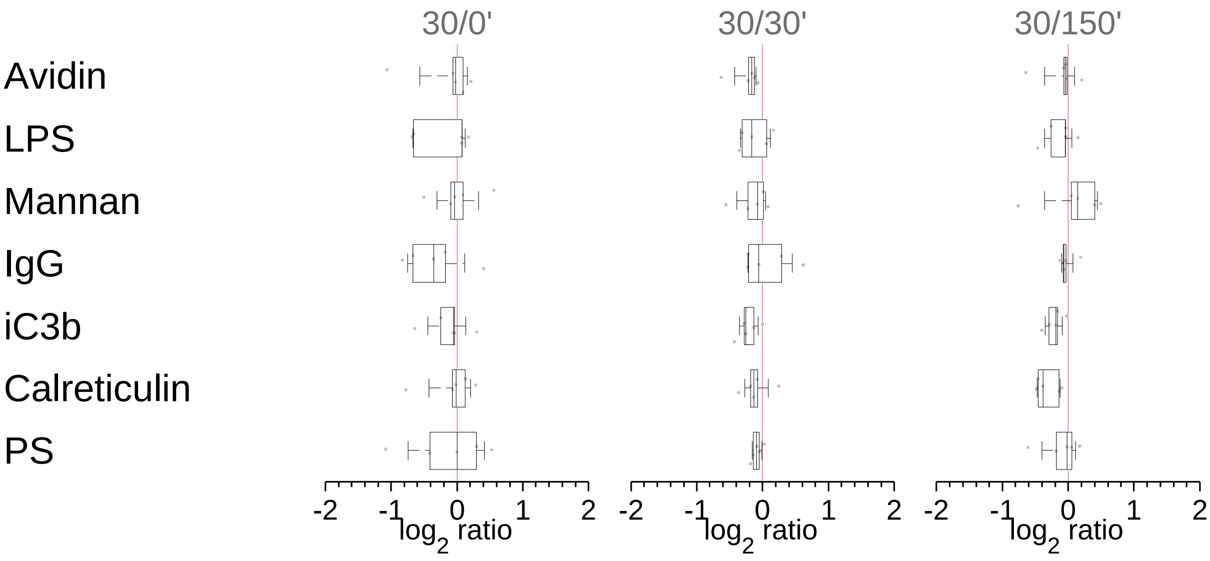

GO:0045732 positive regulation of protein catabolic process (n = 4)

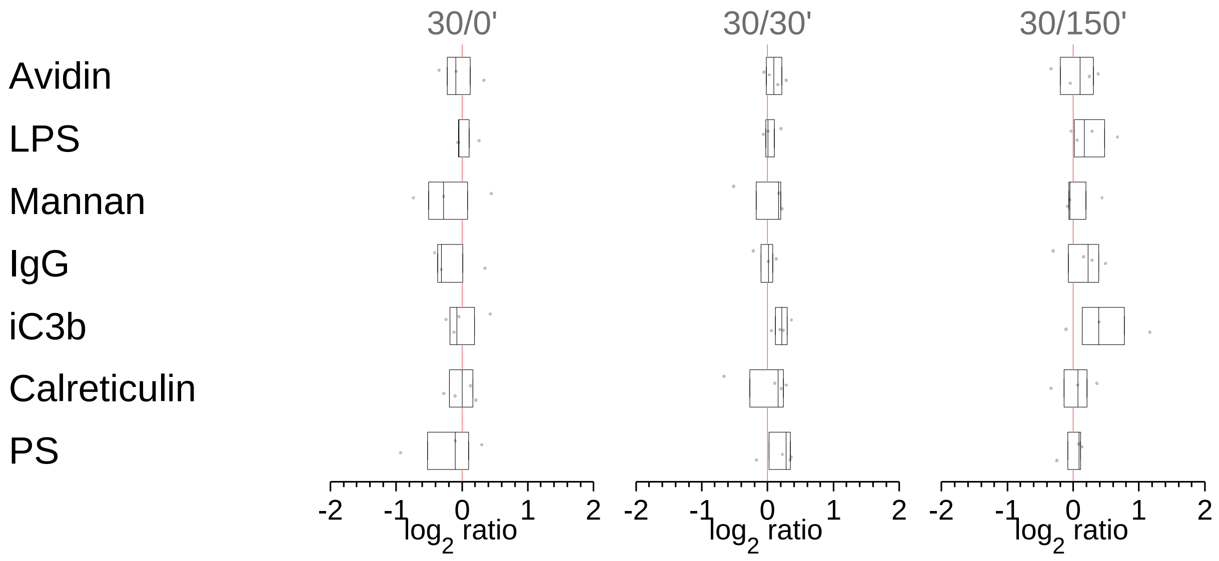

GO:0045739 positive regulation of DNA repair (n = 4)

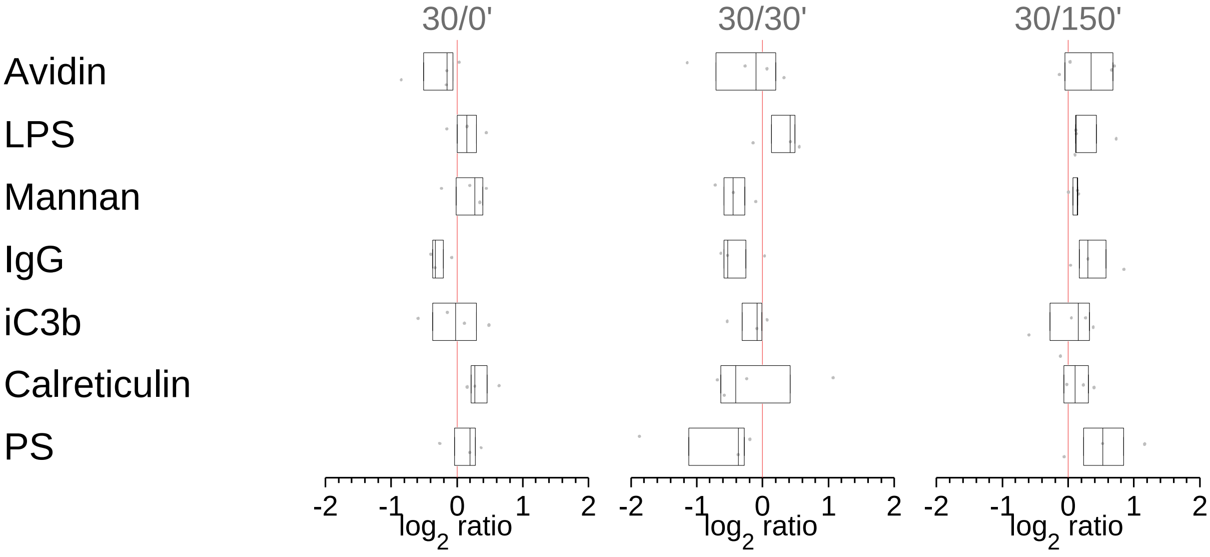

GO:0045740 positive regulation of DNA replication (n = 9)

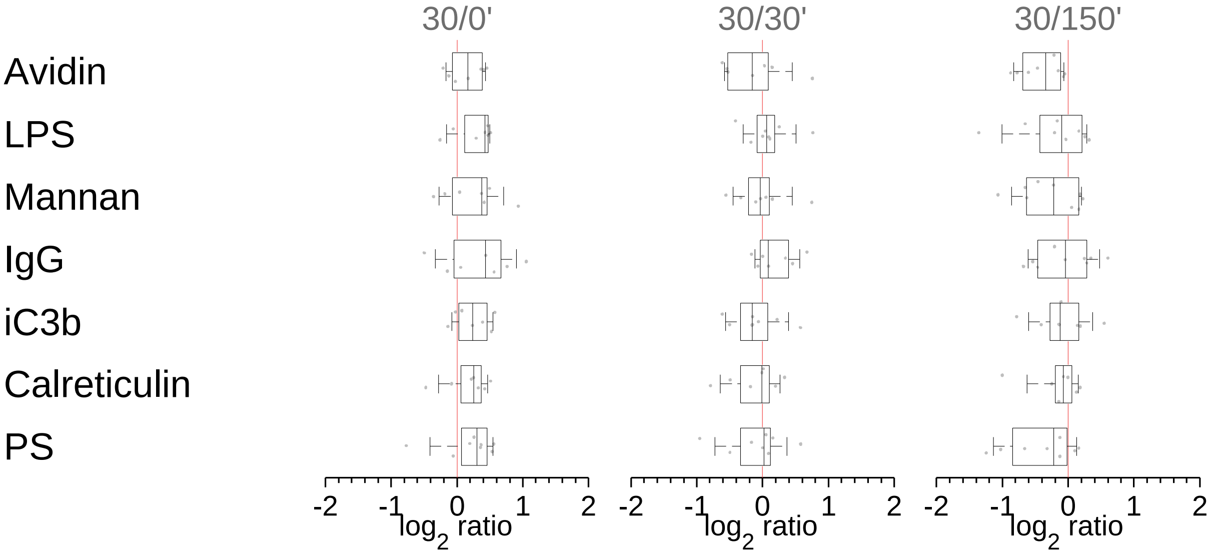

GO:0045766 positive regulation of angiogenesis (n = 13)

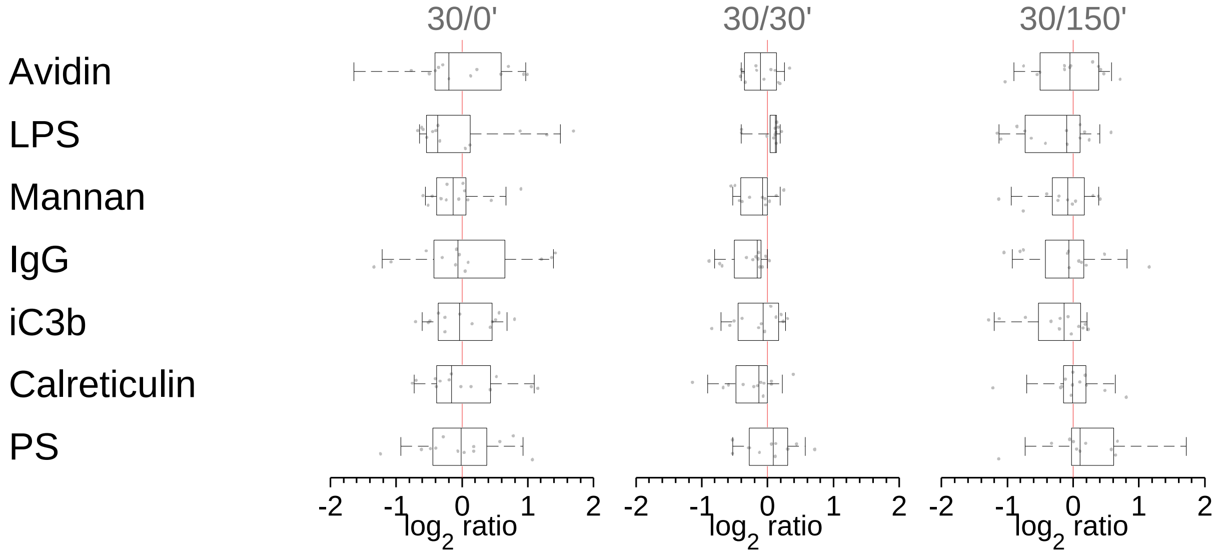

GO:0045773 positive regulation of axon extension (n = 6)

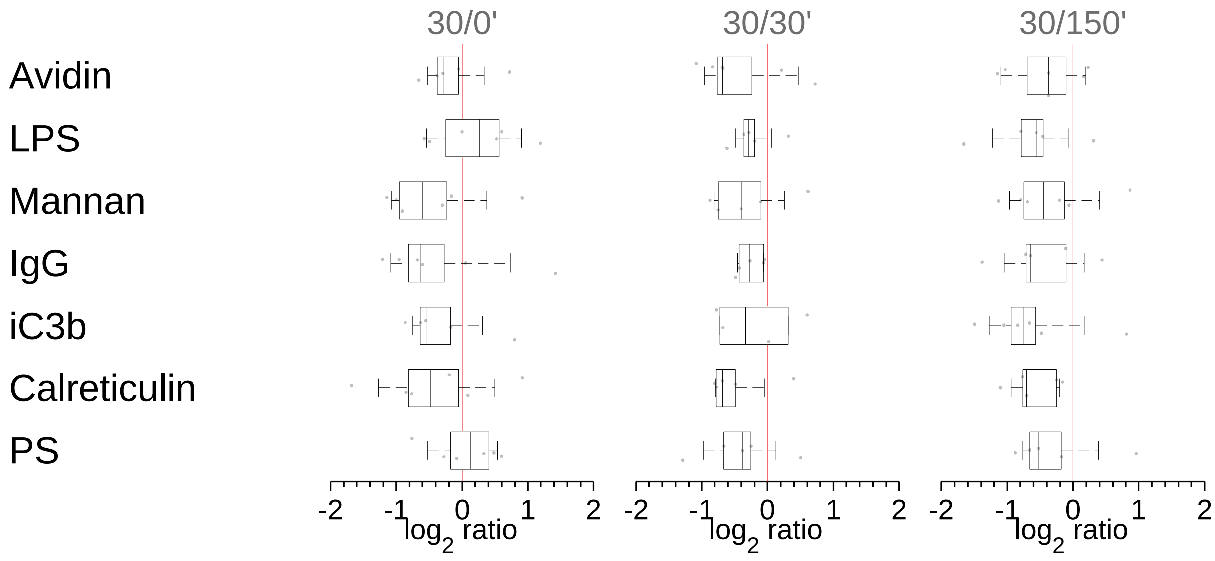

GO:0045779 negative regulation of bone resorption (n = 3)

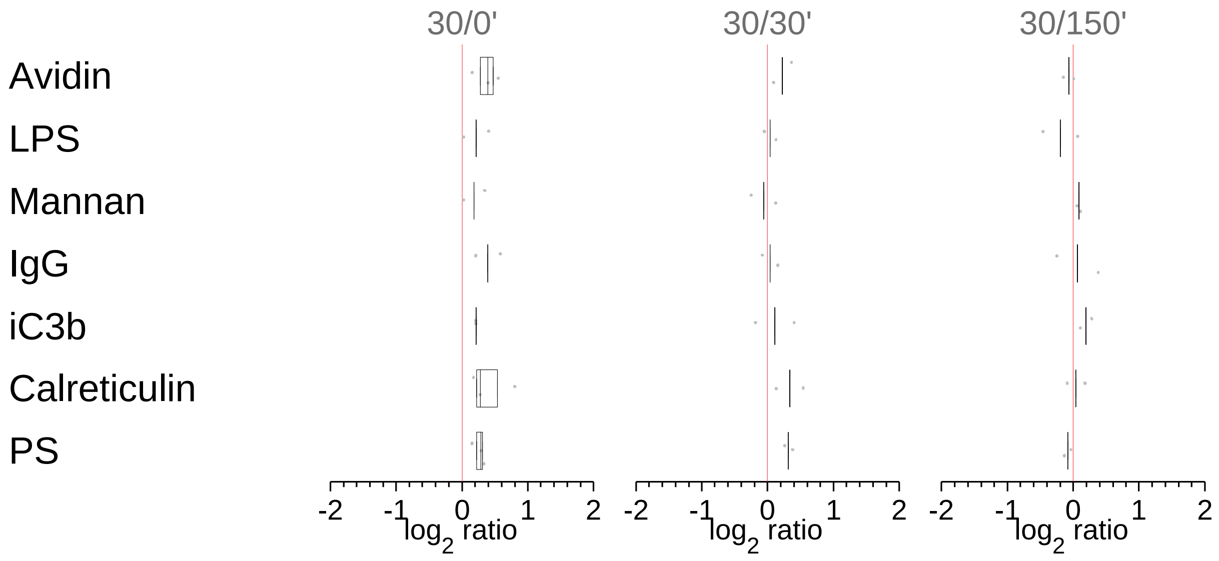

GO:0045780 positive regulation of bone resorption (n = 3)

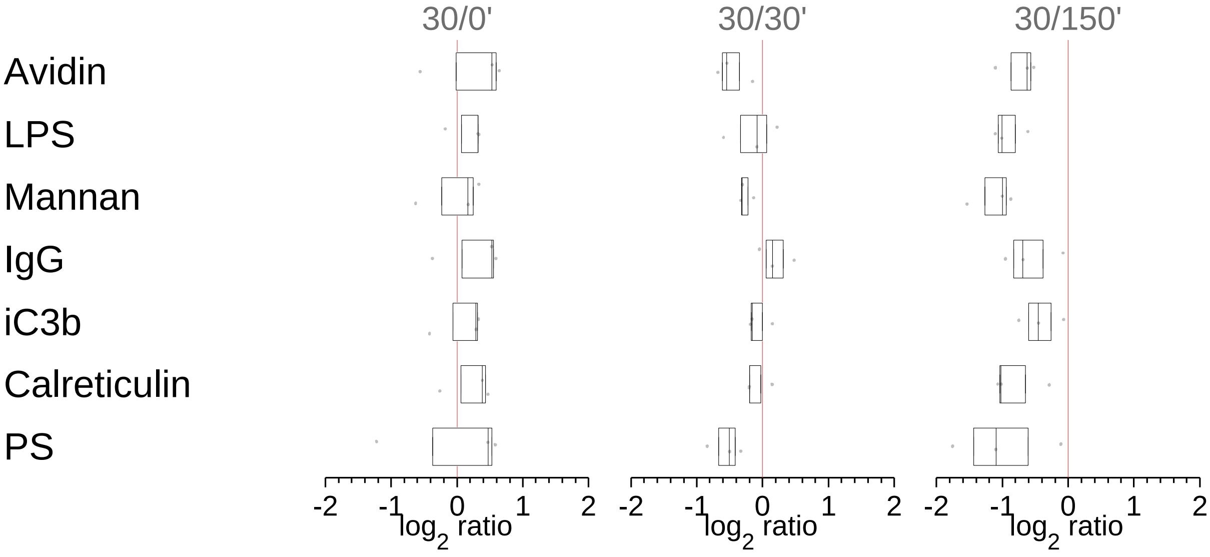

GO:0045785 positive regulation of cell adhesion (n = 10)

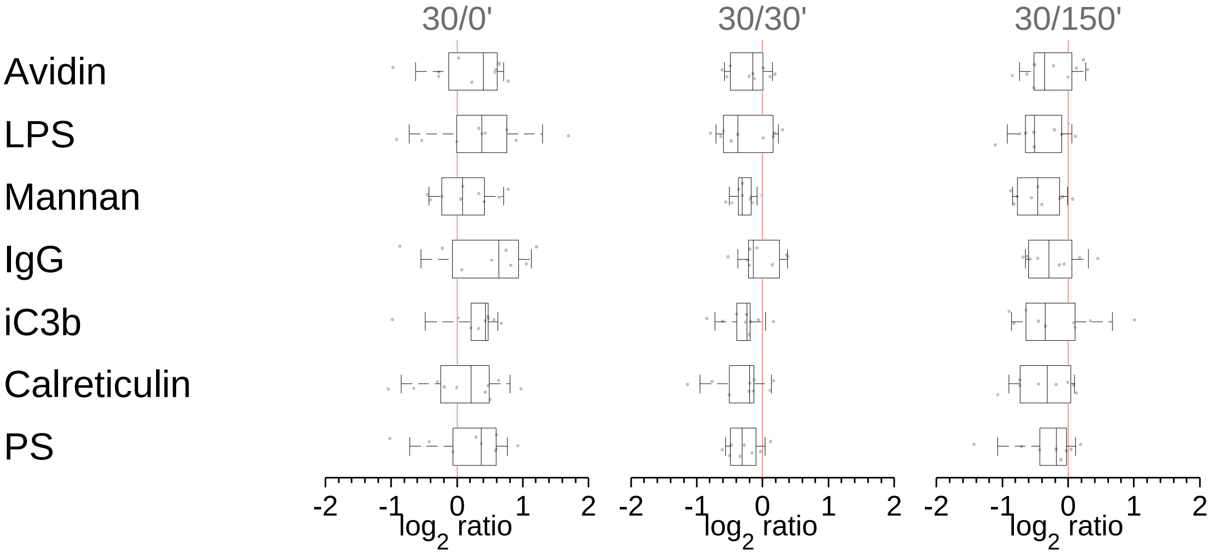

GO:0045807 positive regulation of endocytosis (n = 5)

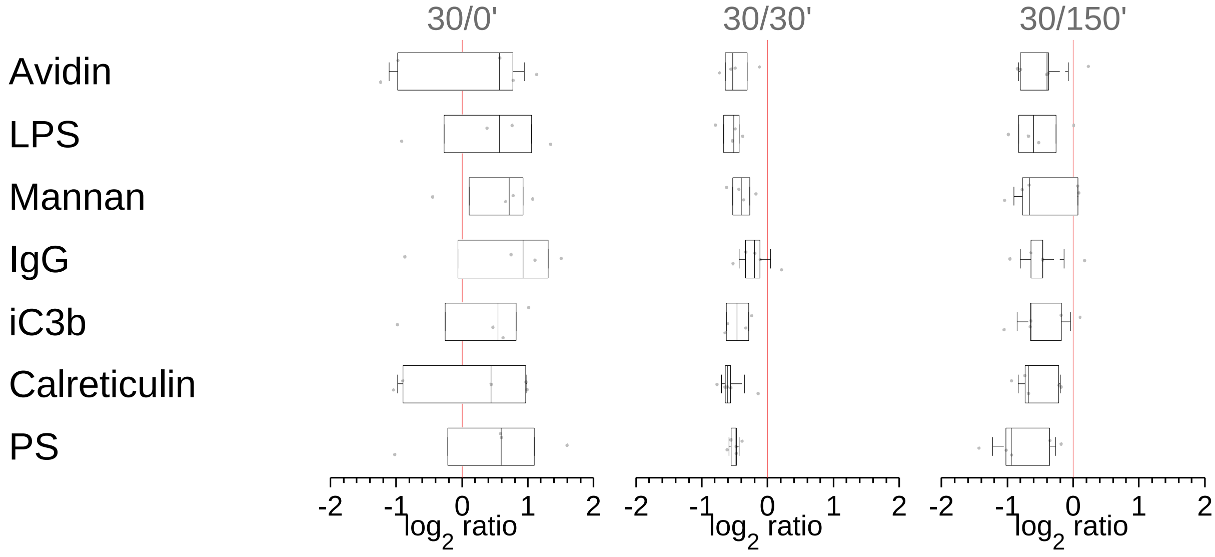

GO:0045822 negative regulation of heart contraction (n = 3)

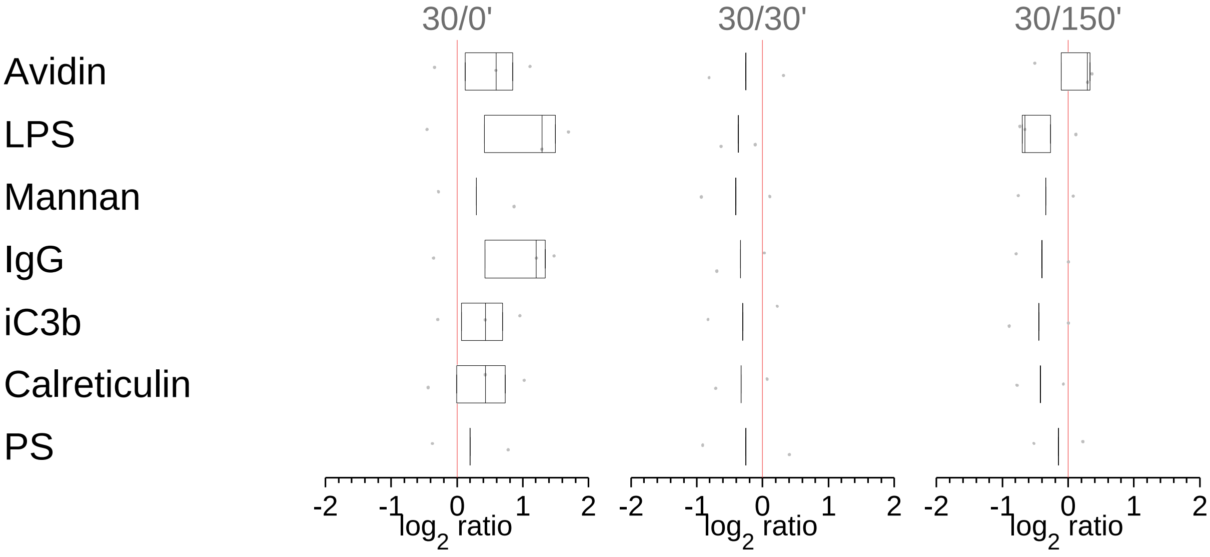

GO:0045859 regulation of protein kinase activity (n = 3)

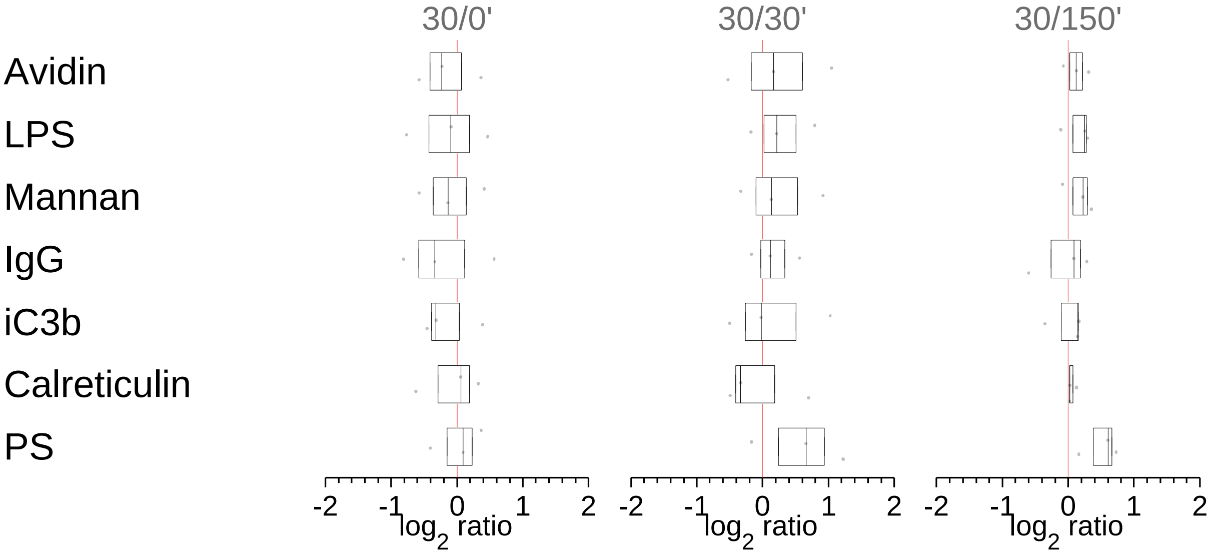

GO:0045860 positive regulation of protein kinase activity (n = 8)

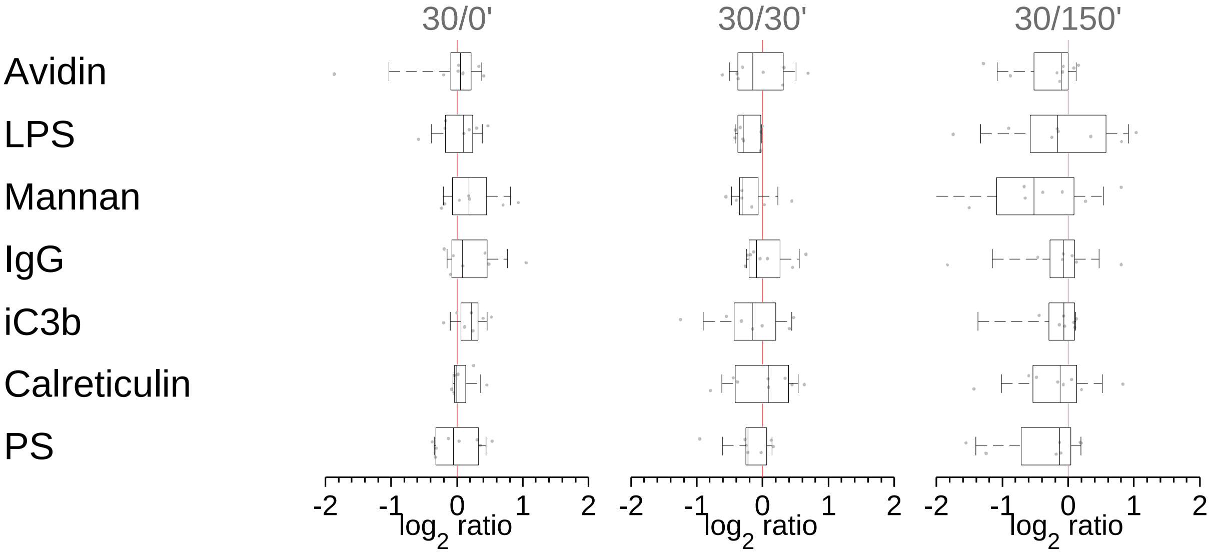

GO:0045861 negative regulation of proteolysis (n = 5)

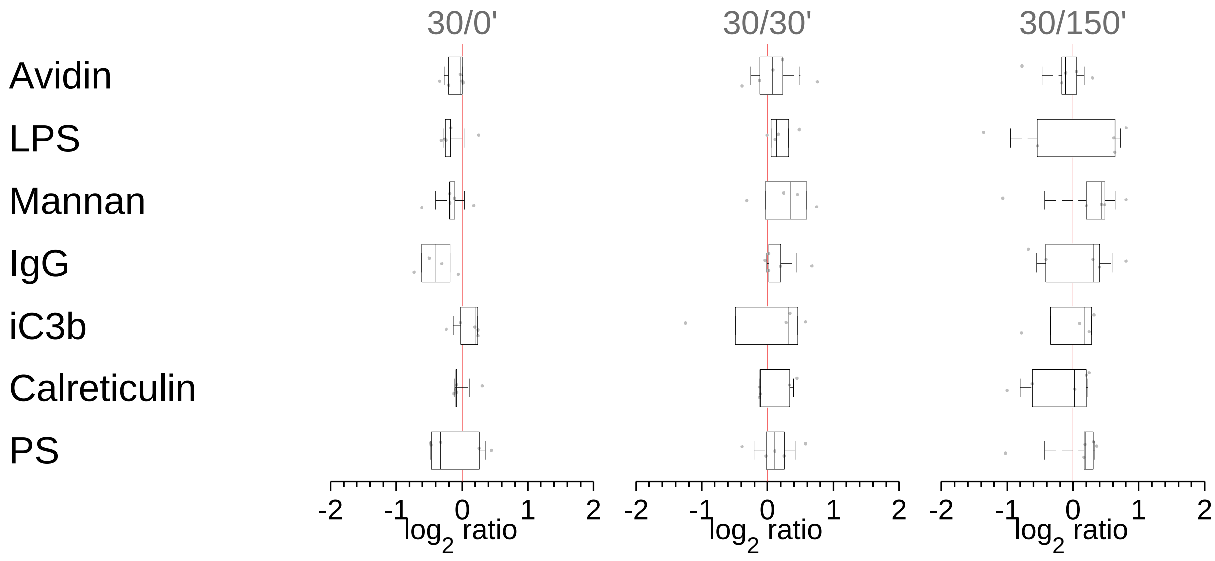

GO:0045862 positive regulation of proteolysis (n = 3)

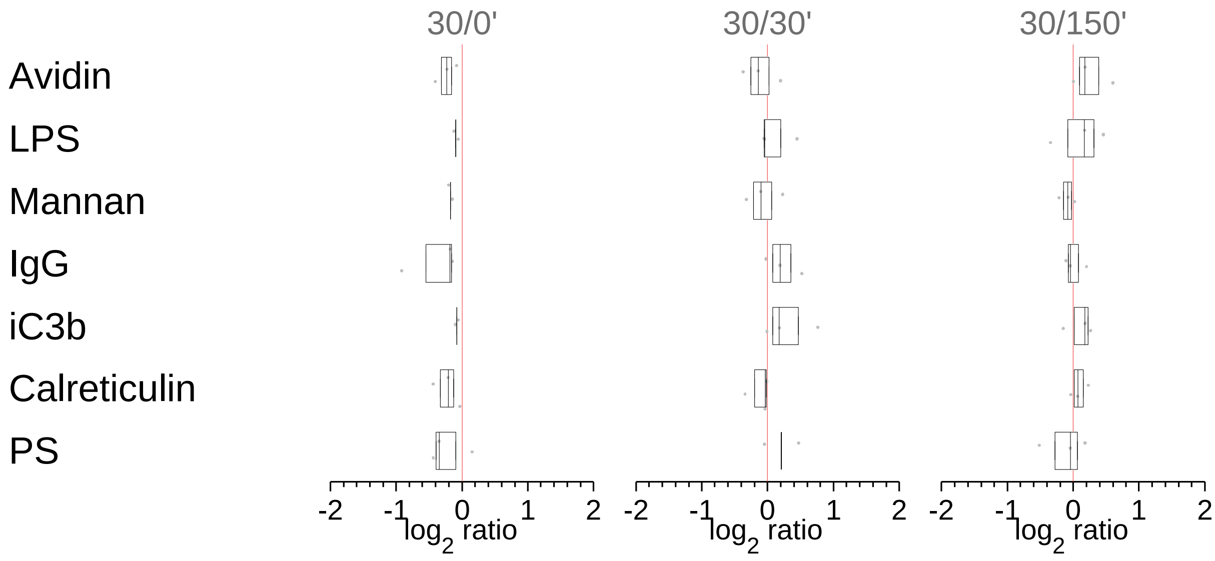

GO:0045892 negative regulation of transcription, DNA-dependent
(n = 37)

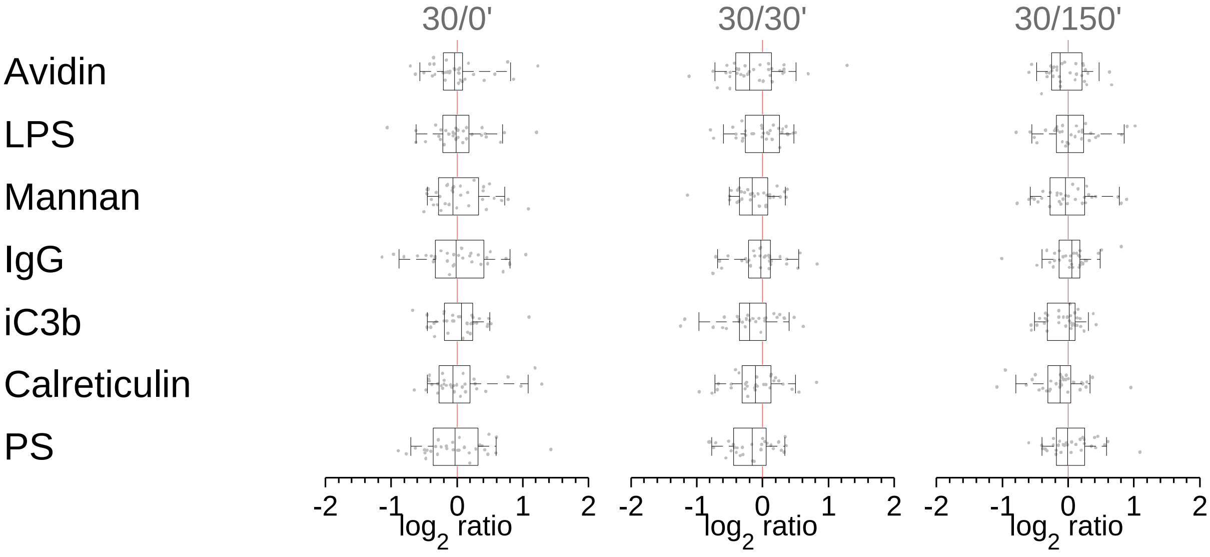

GO:0045893 positive regulation of transcription, DNA-dependent
(n = 25)

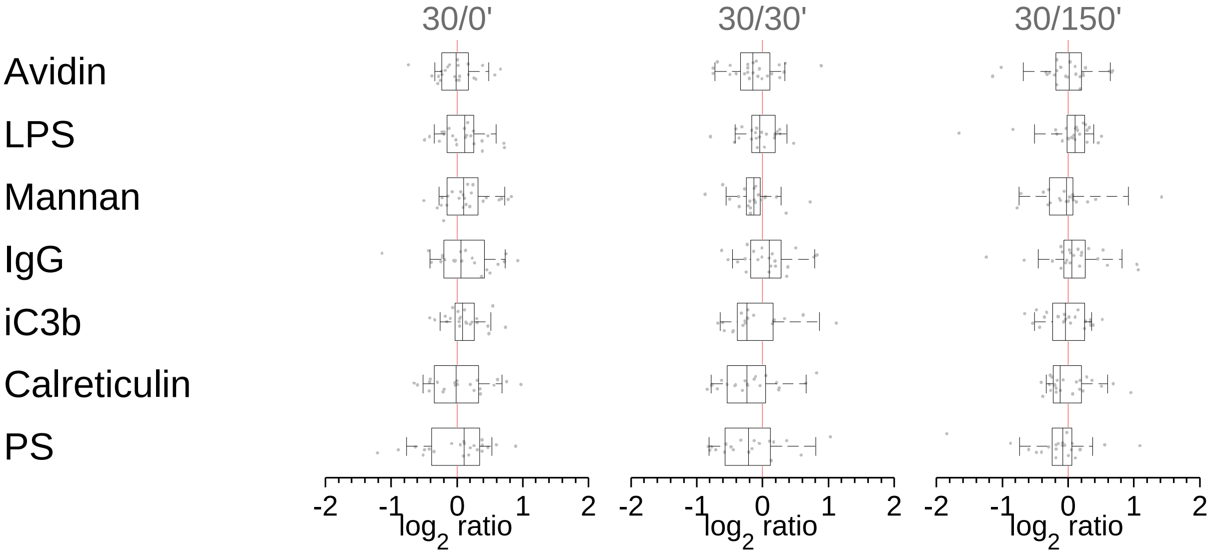

GO:0045907 positive regulation of vasoconstriction (n = 4)

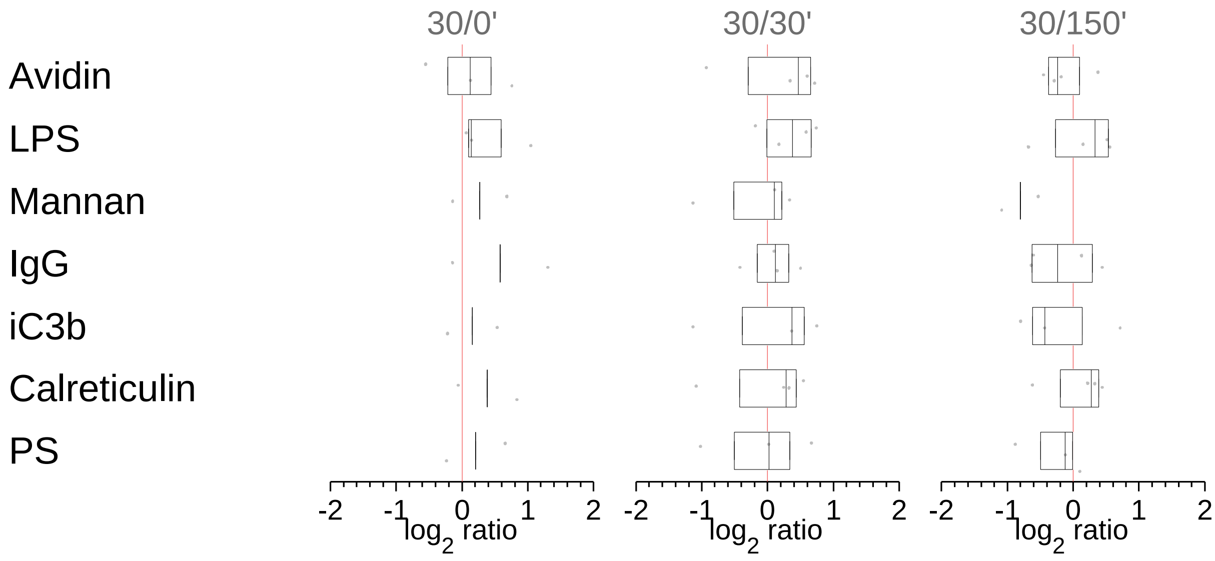

GO:0045921 positive regulation of exocytosis (n = 7)

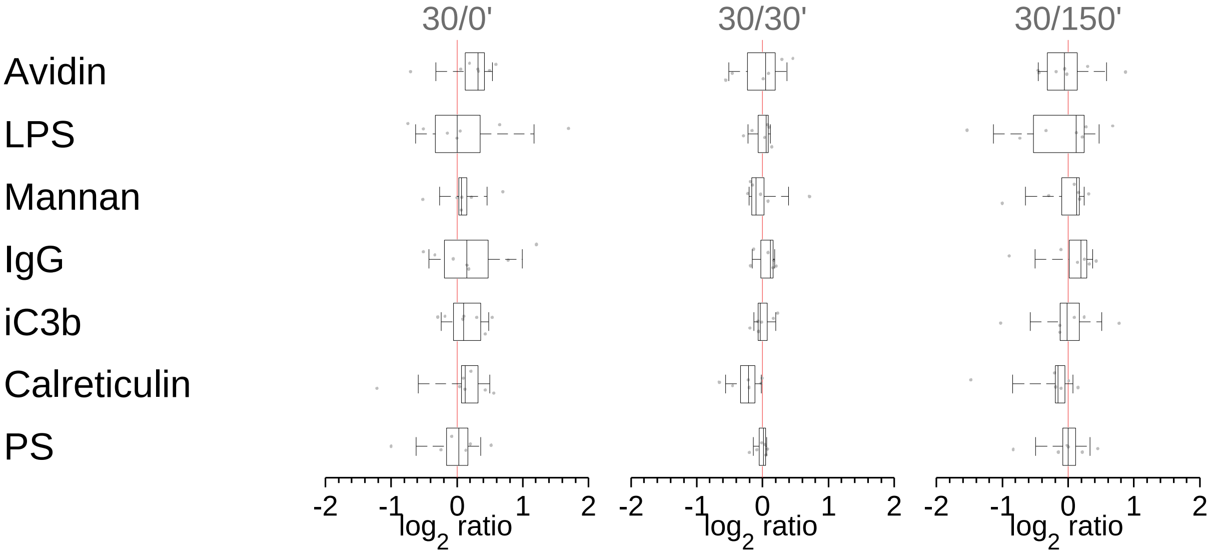

GO:0045931 positive regulation of mitotic cell cycle (n = 5)

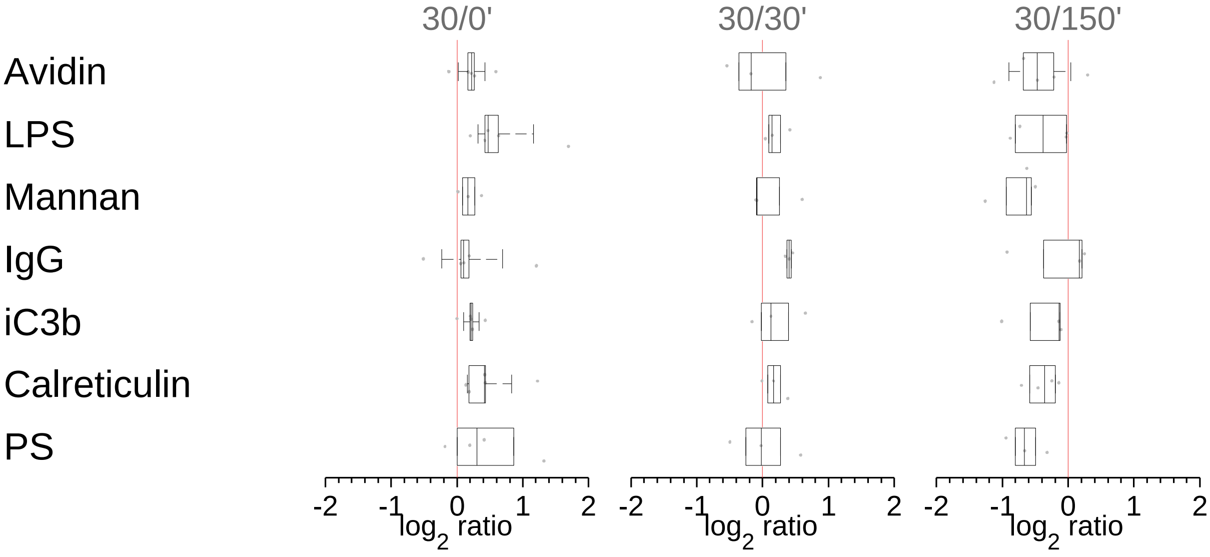

GO:0045944 positive regulation of transcription from RNA polymerase II promoter (n = 50)

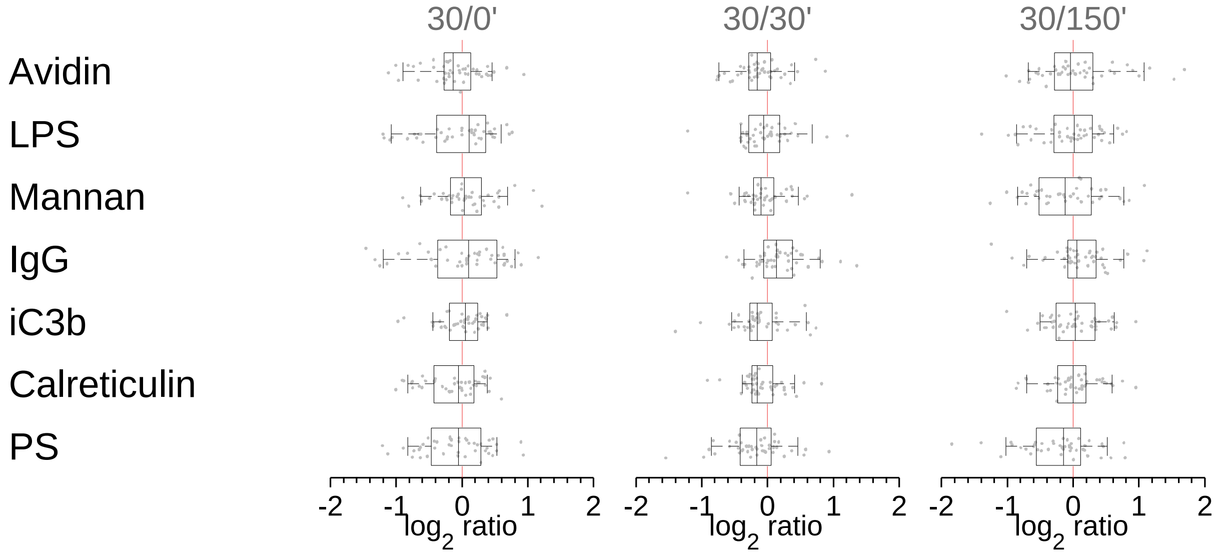

GO:0045954 positive regulation of natural killer cell mediated cytotoxicity (n = 3)

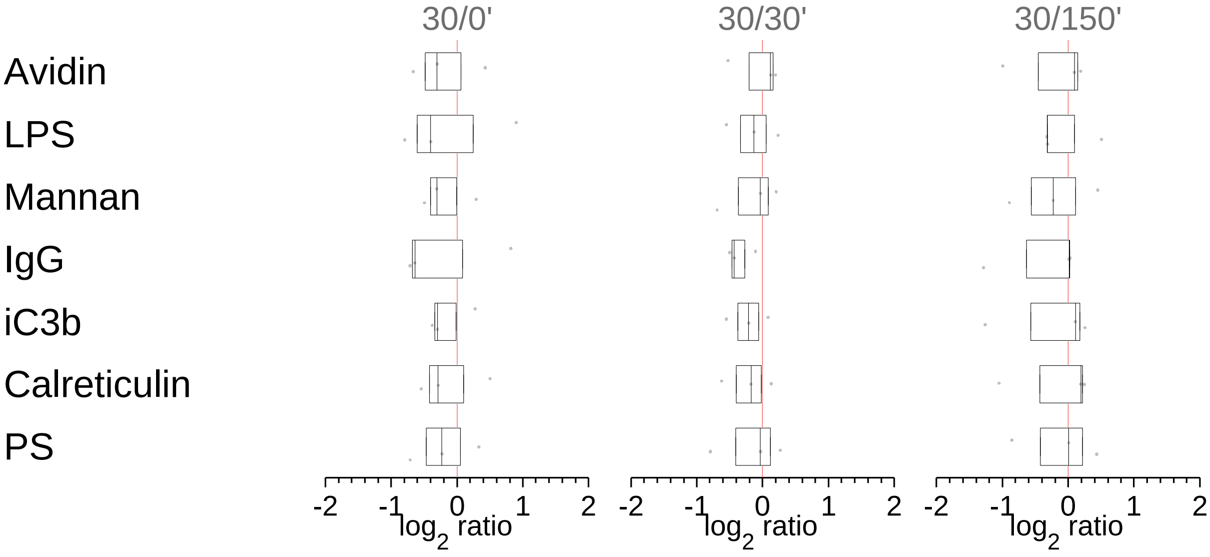

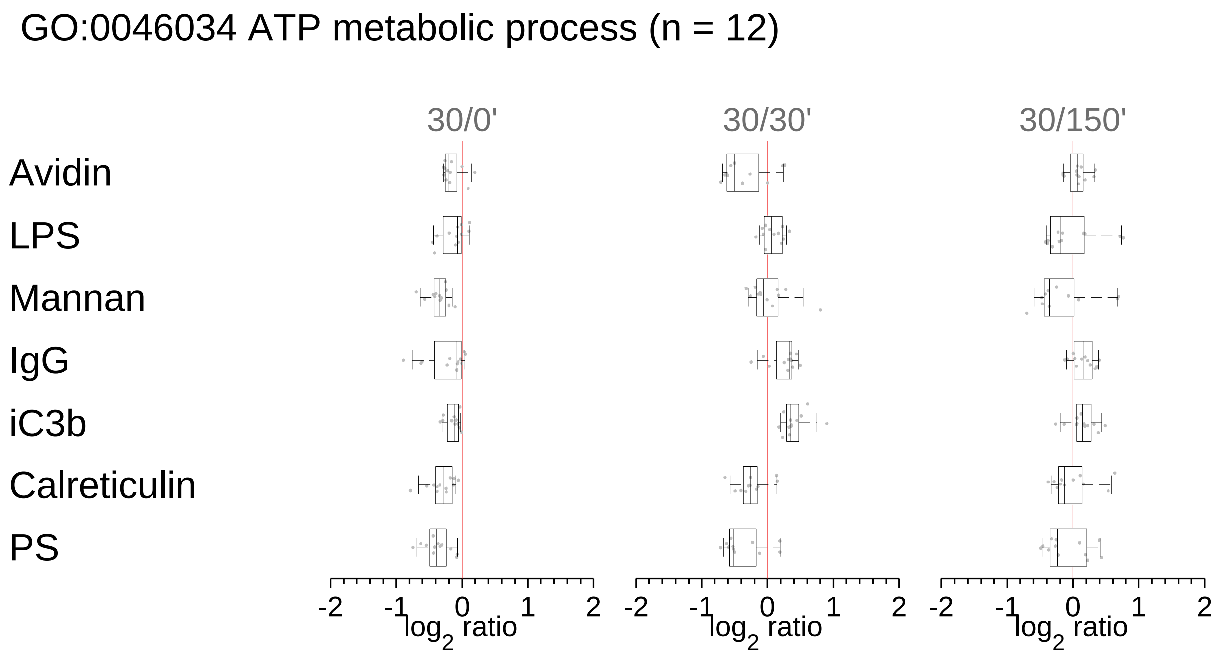
GO:0046034 ATP metabolic process (n = 12)
30/0'
30/30'
30/150'
Avidin
LPS
Mannan
IgG
iC3b
Calreticulin
PS
-2
-1
0
1
2
log_2 ratio

GO:0046185 aldehyde catabolic process (n = 3)

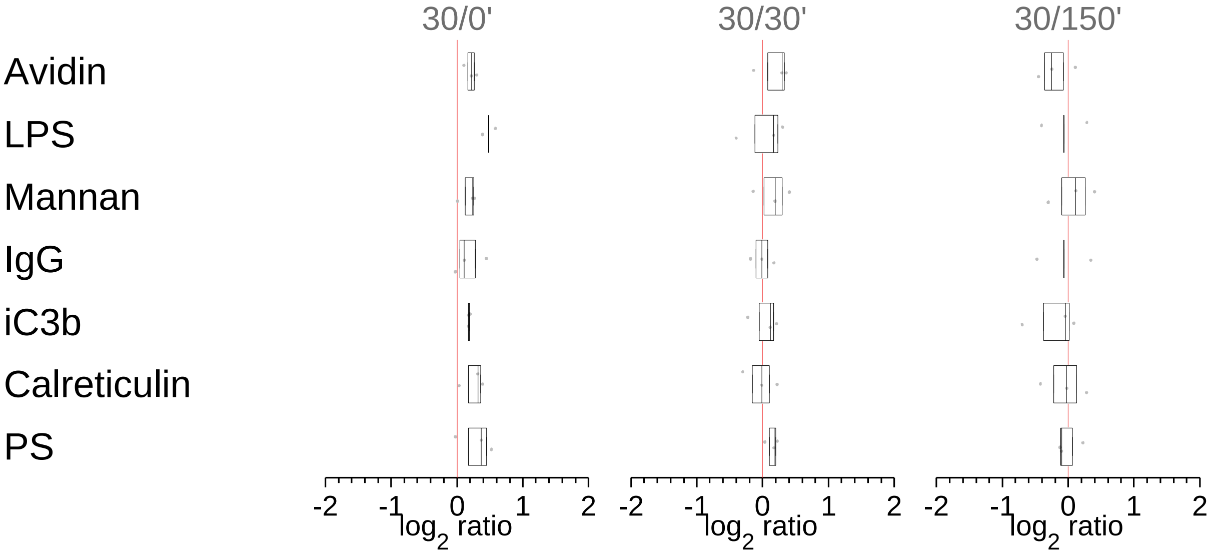

GO:0046325 negative regulation of glucose import (n = 5)

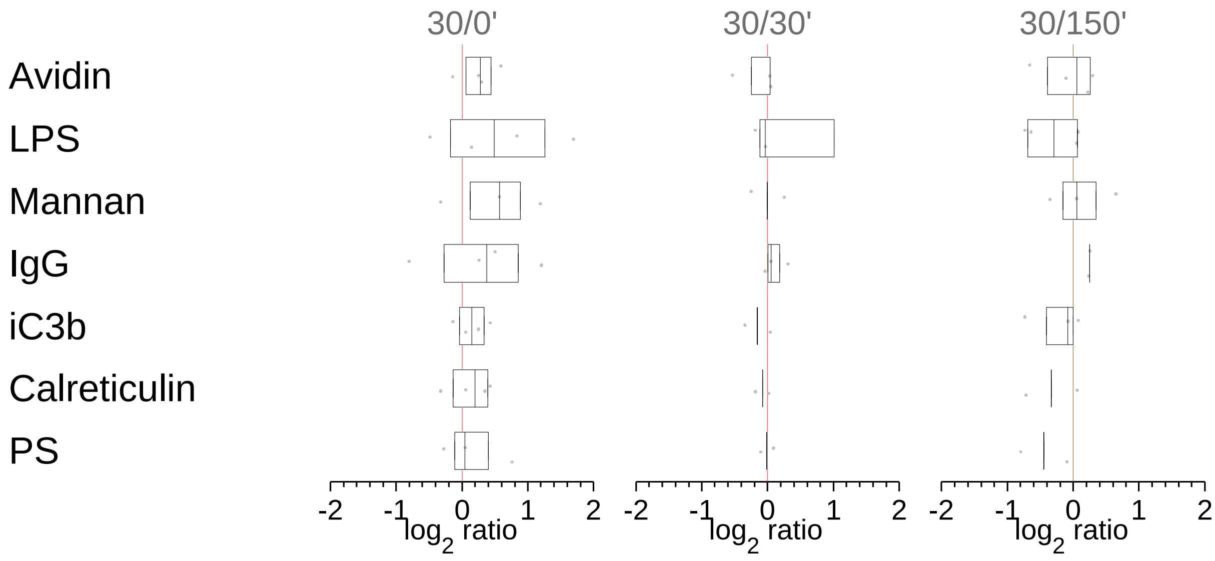

GO:0046329 negative regulation of JNK cascade (n = 4)

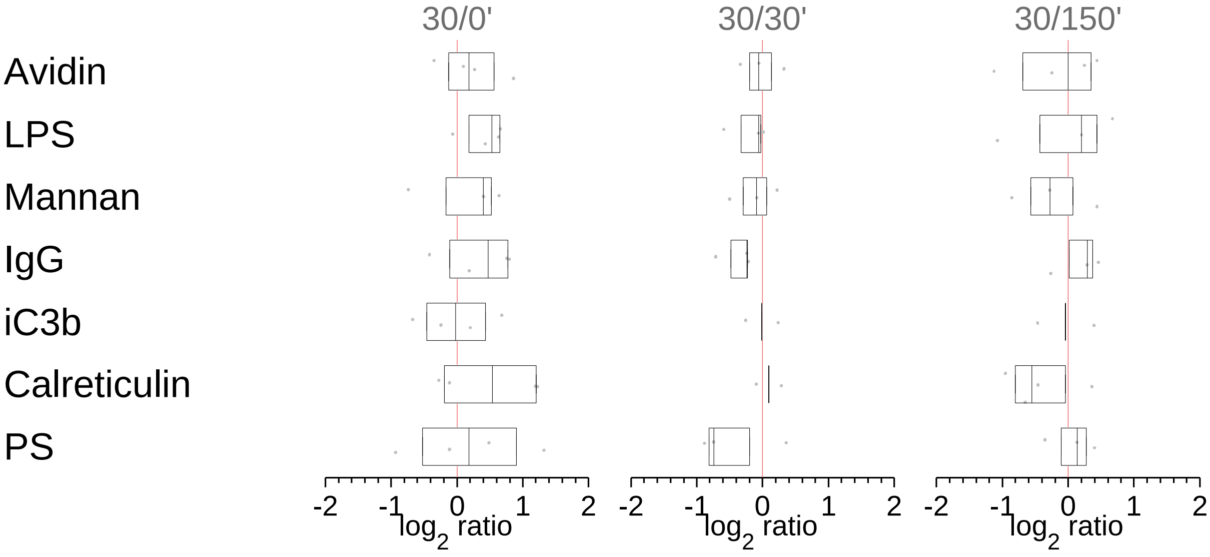

GO:0046330 positive regulation of JNK cascade (n = 10)

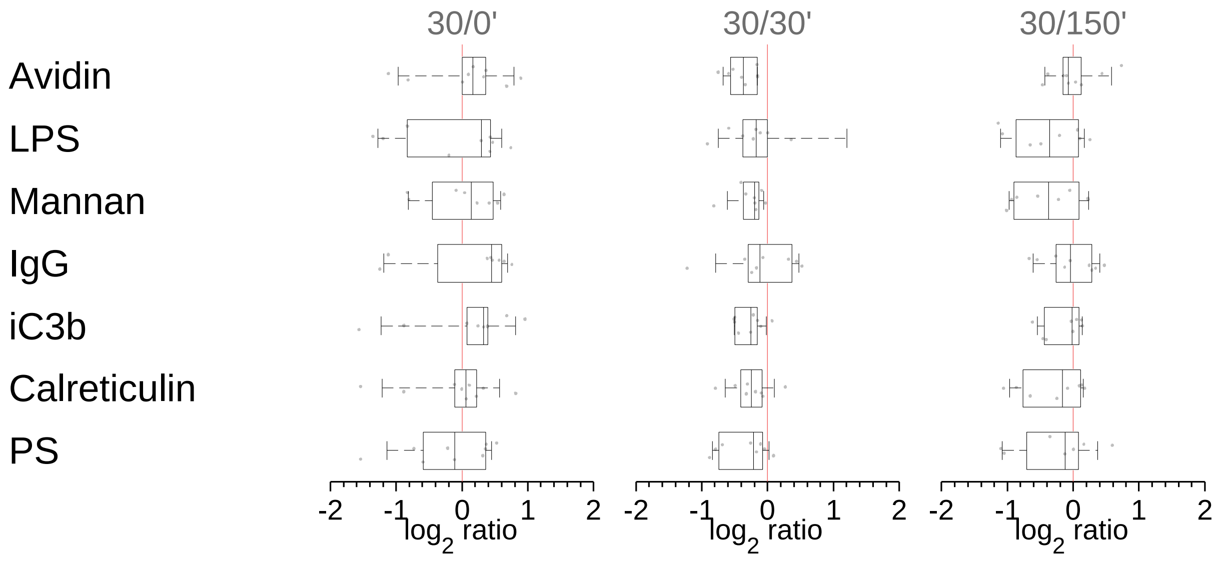

GO:0046332 SMAD binding (n = 3)

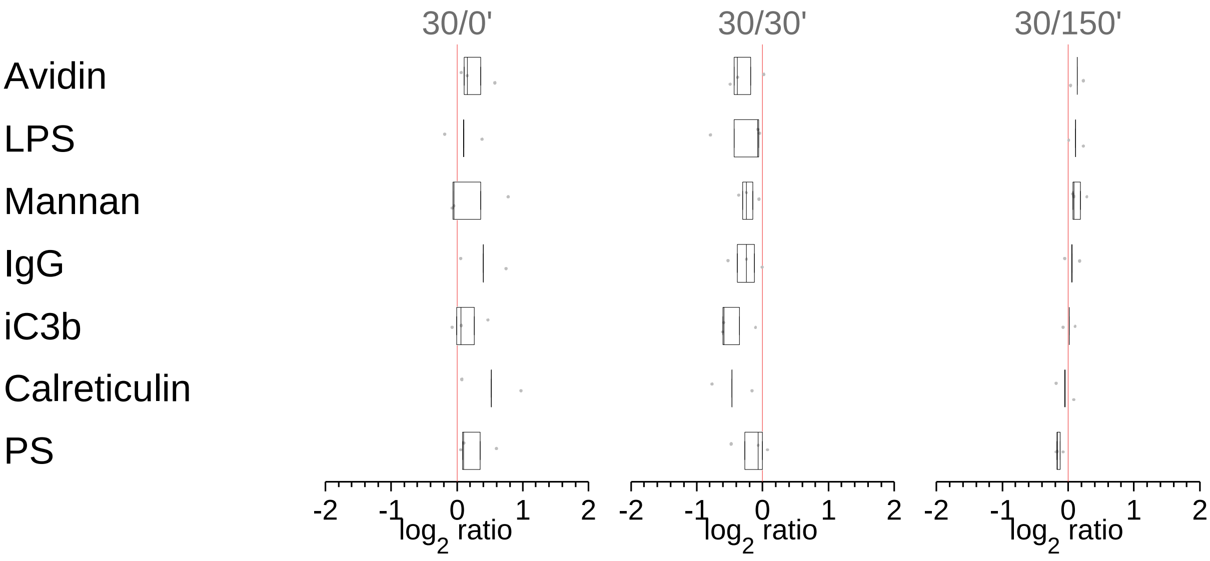

GO:0046488 phosphatidylinositol metabolic process (n = 6)

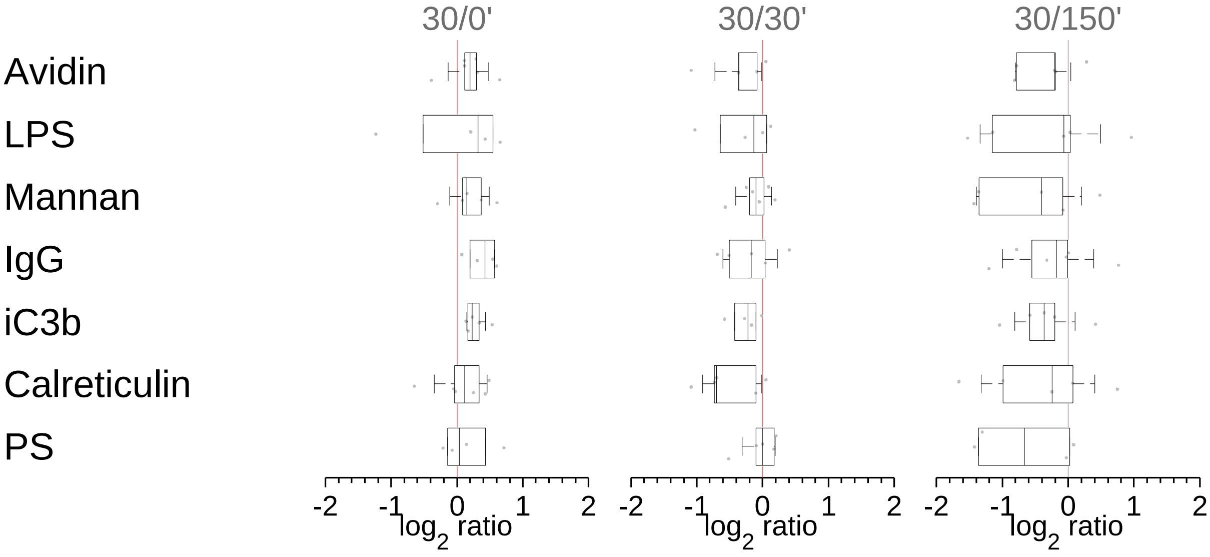

GO:0046513 ceramide biosynthetic process (n = 5)

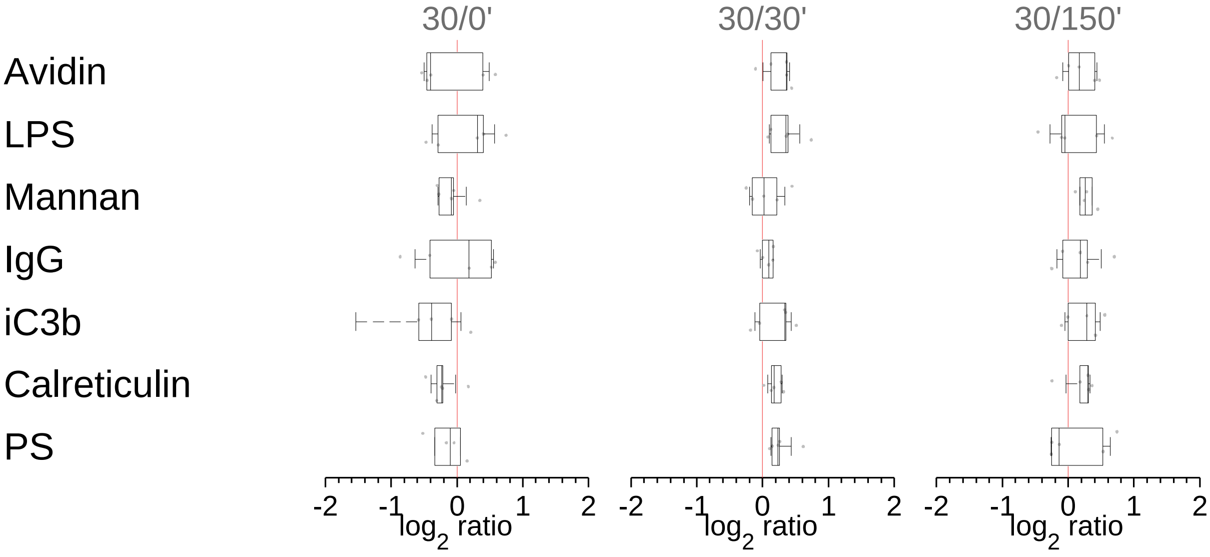

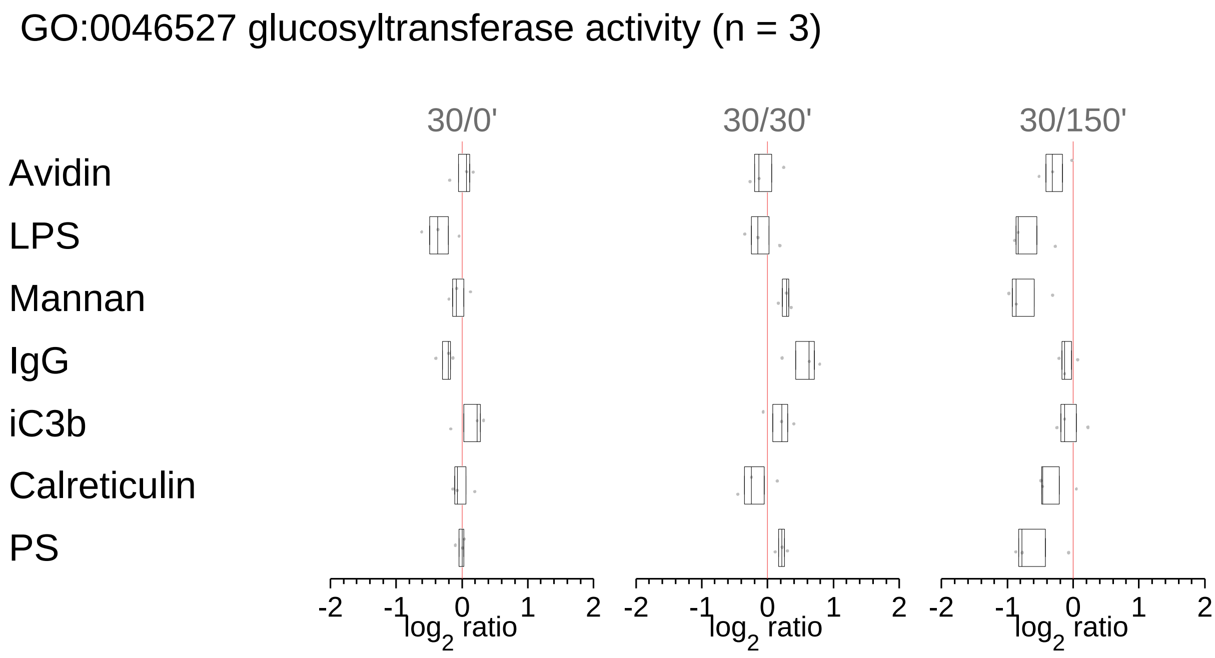
GO:0046527 glucosyltransferase activity (n = 3)
30/0'
30/30'
30/150'
Avidin
LPS
Mannan
IgG
iC3b
Calreticulin
PS
-2 -1 0 1 2
log2 ratio
-2 -1 0 1 2
log2 ratio
-2 -1 0 1 2
log2 ratio

GO:0046579 positive regulation of Ras protein signal transduction (n = 4)

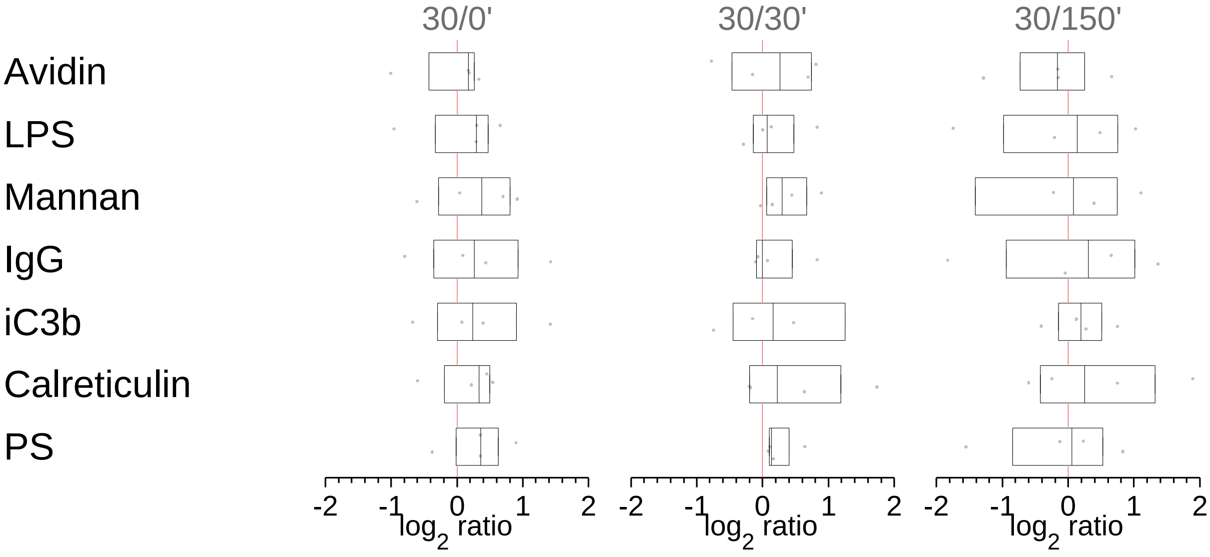

GO:0046580 negative regulation of Ras protein signal transduction (n = 4)

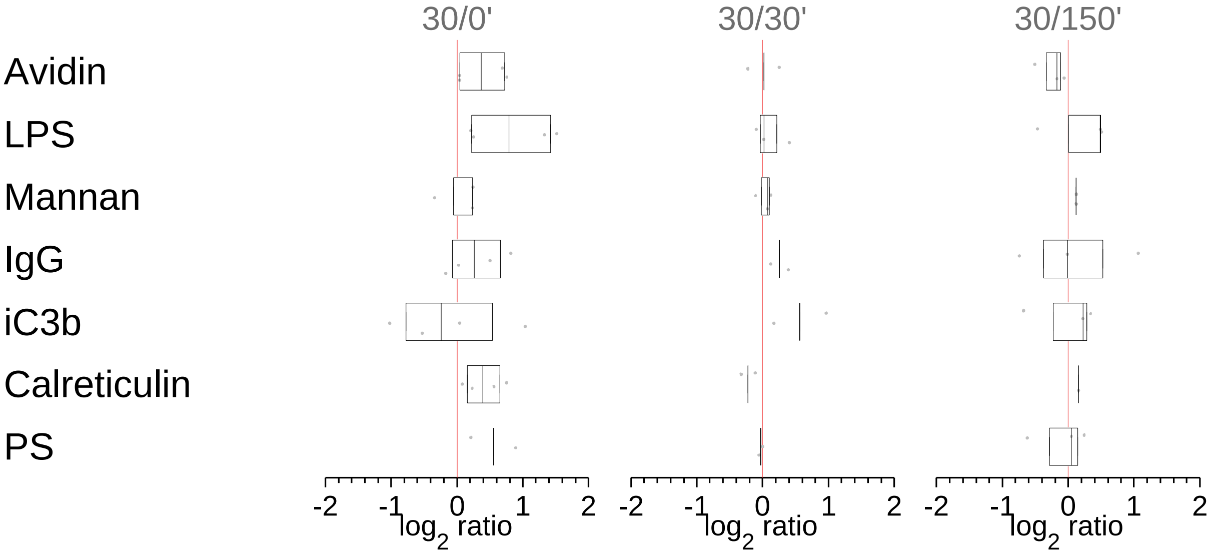

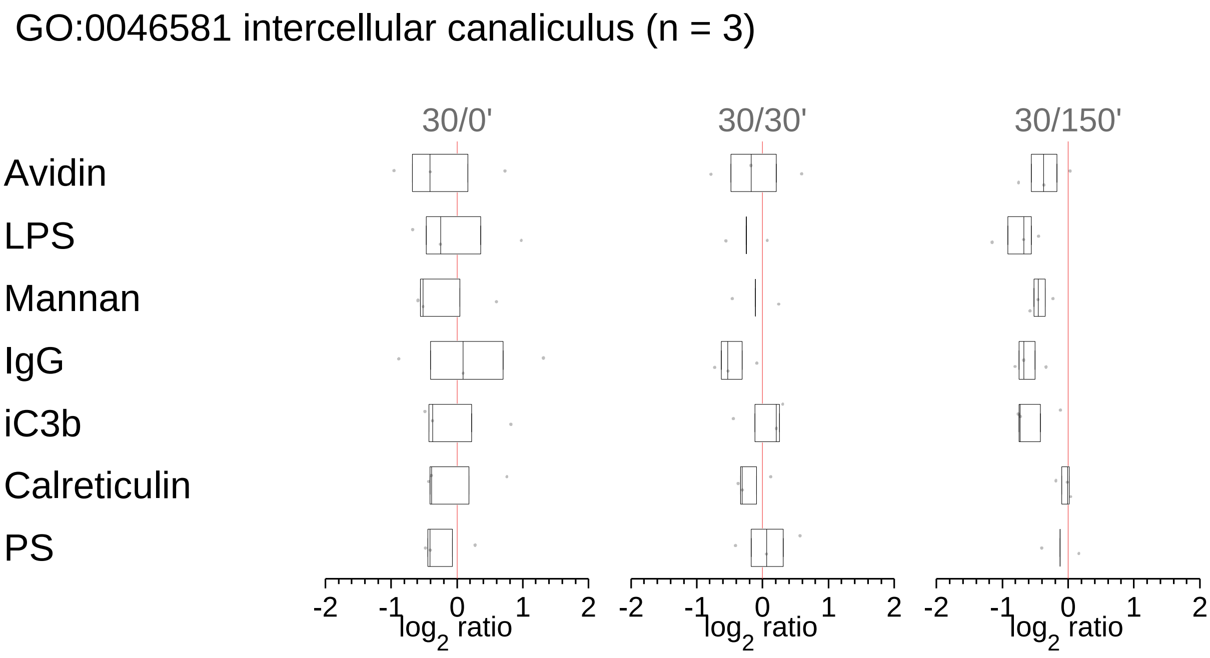
GO:0046581 intercellular canaliculus (n = 3)
30/0'
30/30'
30/150'
Avidin
LPS
Mannan
IgG
iC3b
Calreticulin
PS
-2 -1 0 1 2
log2 ratio

GO:0046627 negative regulation of insulin receptor signaling pathway (n = 5)

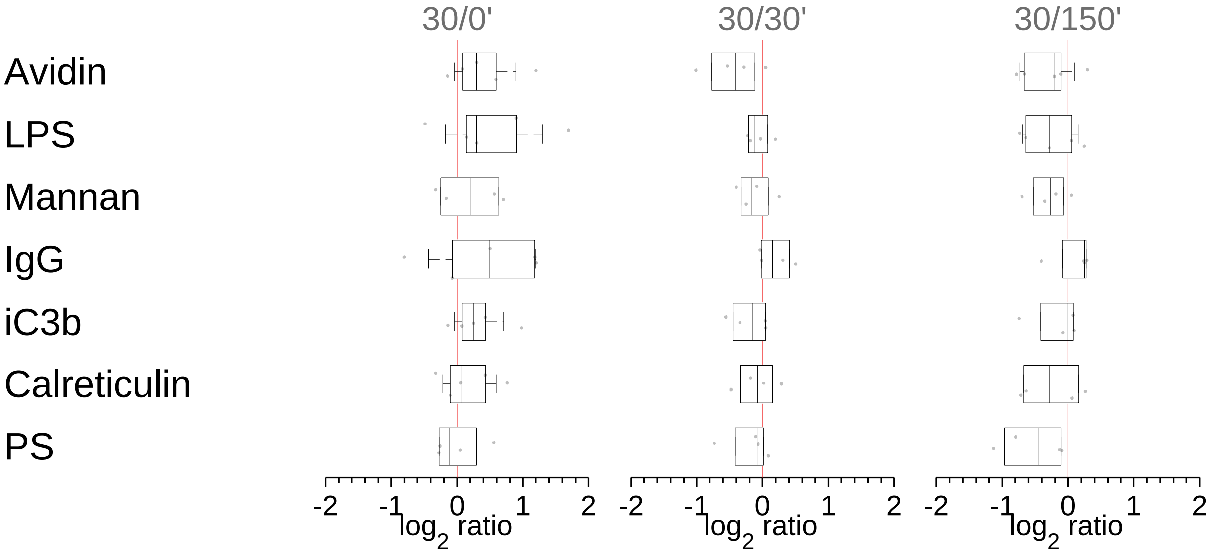

GO:0046677 response to antibiotic (n = 3)

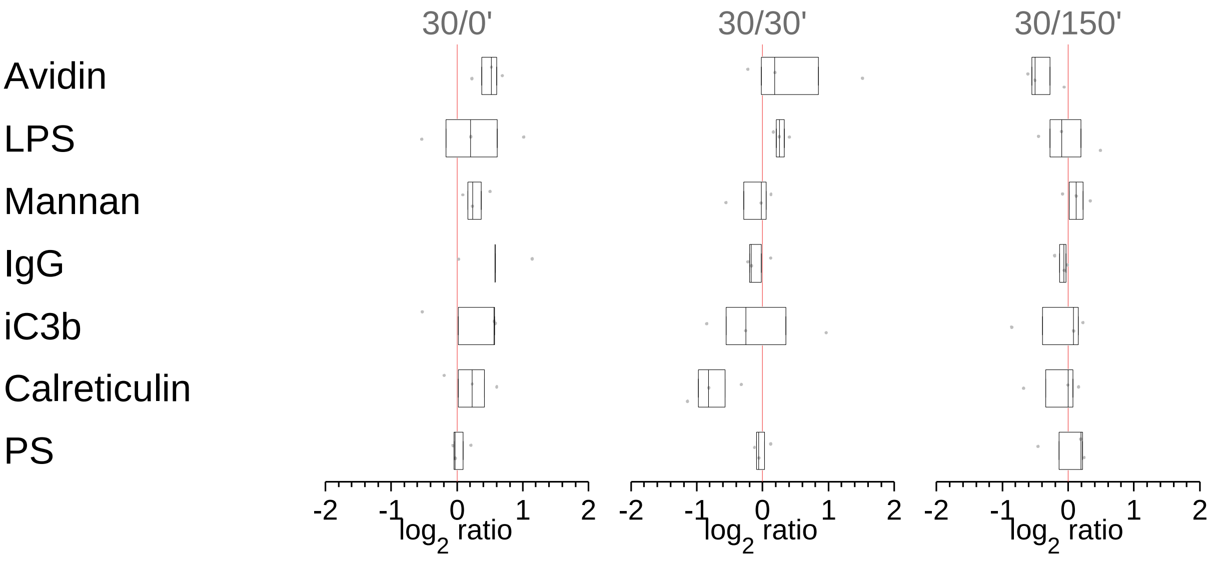

GO:0046718 viral entry into host cell (n = 3)

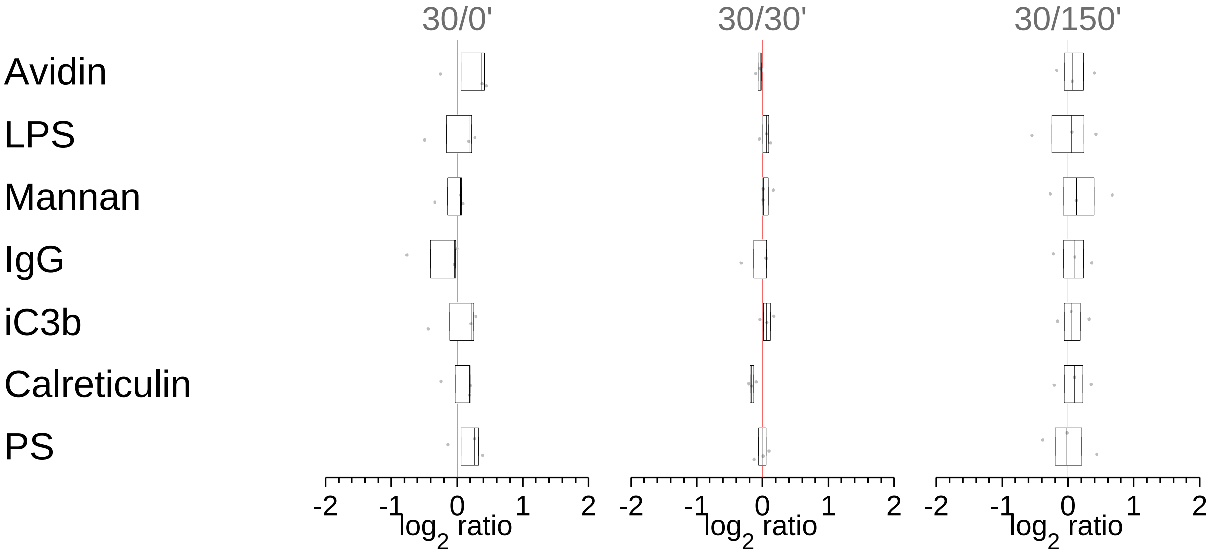

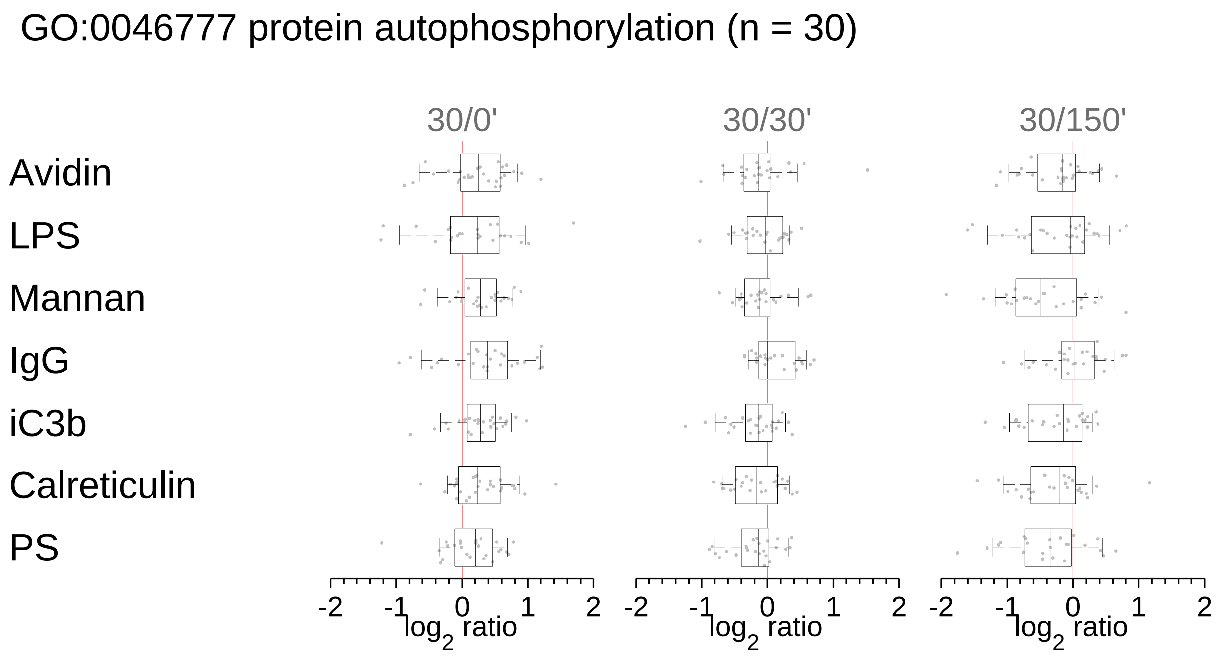
GO:0046777 protein autophosphorylation (n = 30)
30/0'
30/30'
30/150'
Avidin
LPS
Mannan
IgG
iC3b
Calreticulin
PS
-2
-1
0
1
2
log2 ratio

GO:0046826 negative regulation of protein export from nucleus (n = 3)

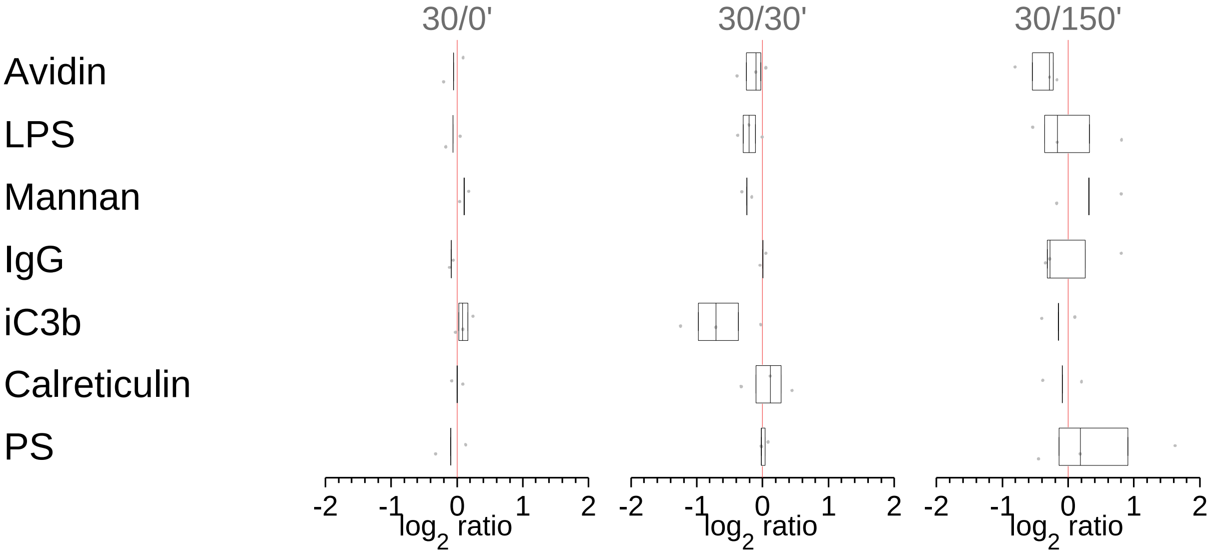

GO:0046827 positive regulation of protein export from nucleus (n = 3)

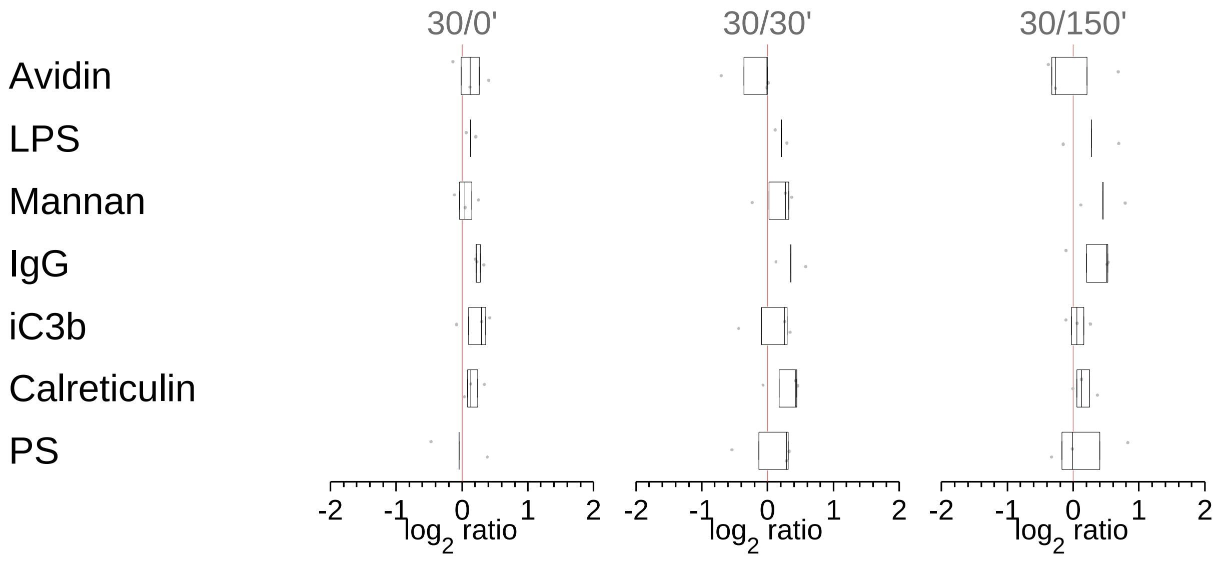

GO:0046835 carbohydrate phosphorylation (n = 3)

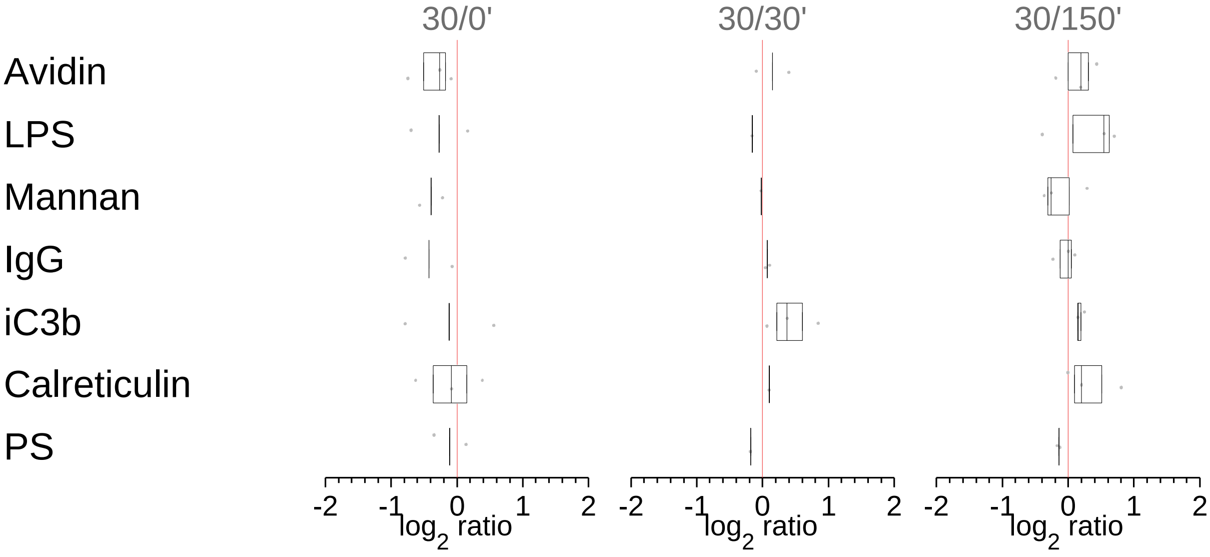

GO:0046847 filopodium assembly (n = 5)

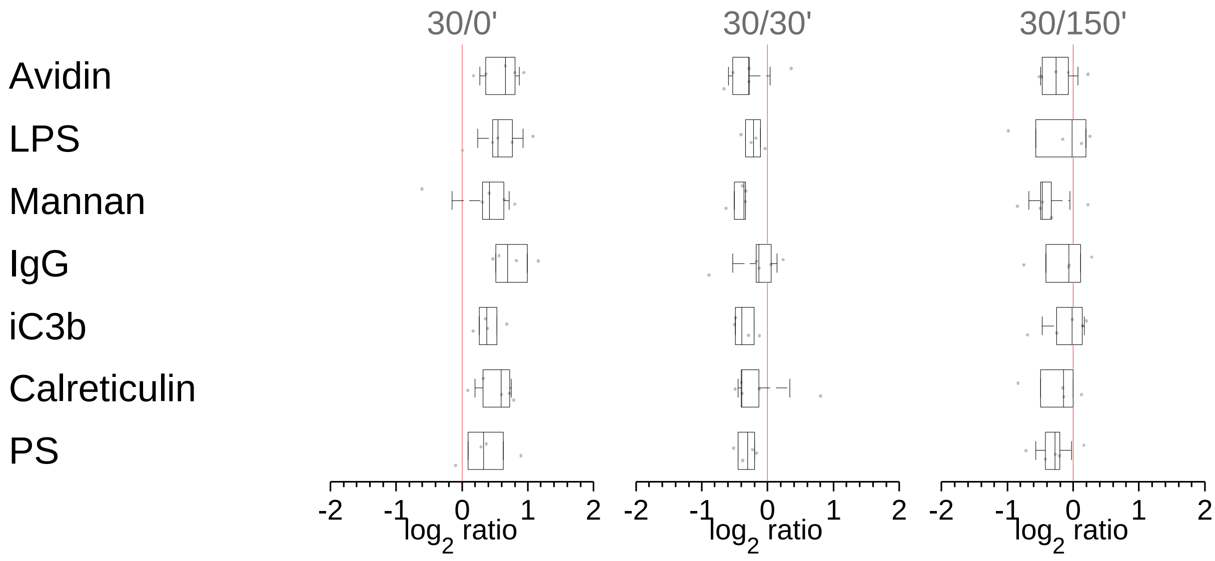

GO:0046854 phosphatidylinositol phosphorylation (n = 4)

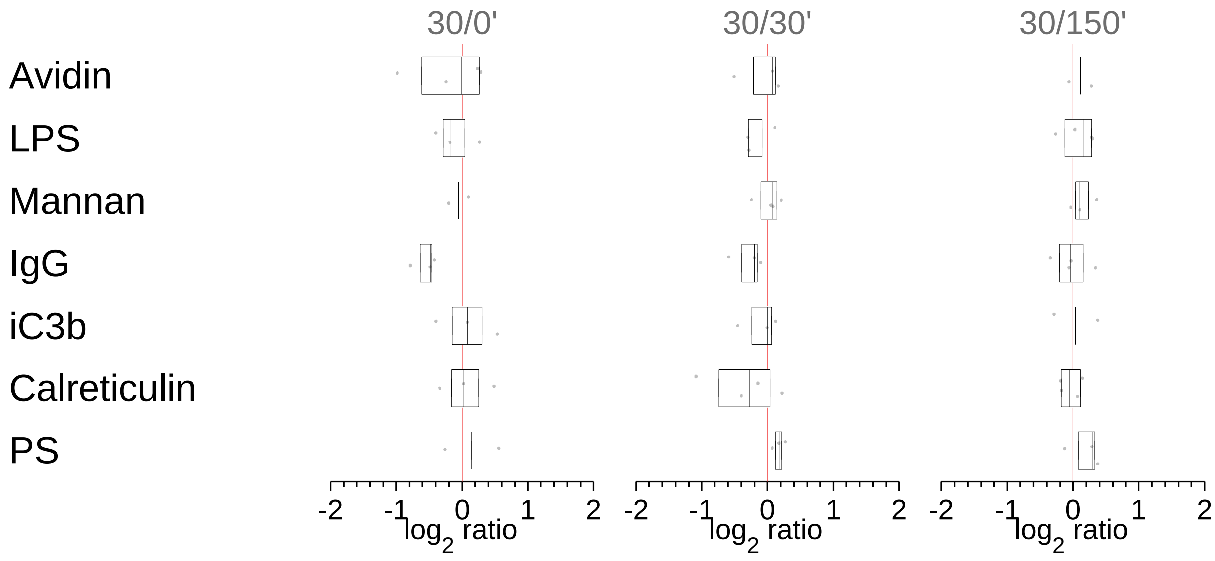

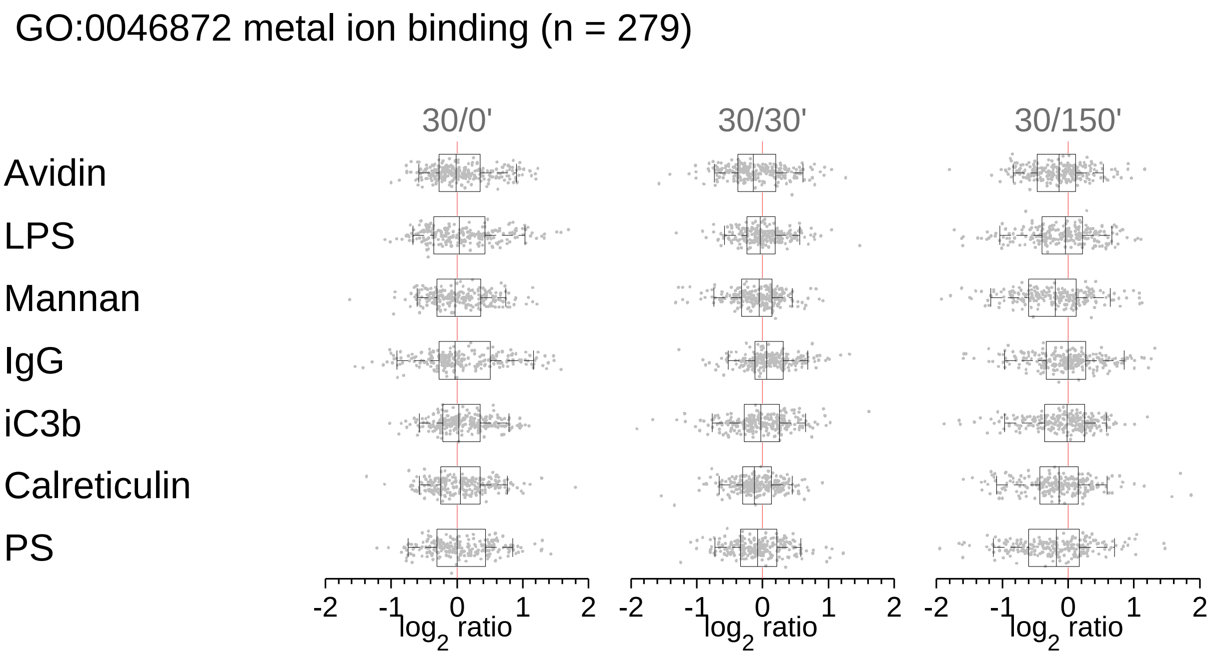
GO:0046872 metal ion binding (n = 279)
30/0'
30/30'
30/150'
Avidin
LPS
Mannan
IgG
iC3b
Calreticulin
PS
-2 -1 0 1 2
log2 ratio
-2 -1 0 1 2
log2 ratio
-2 -1 0 1 2
log2 ratio

GO:0046875 ephrin receptor binding (n = 7)

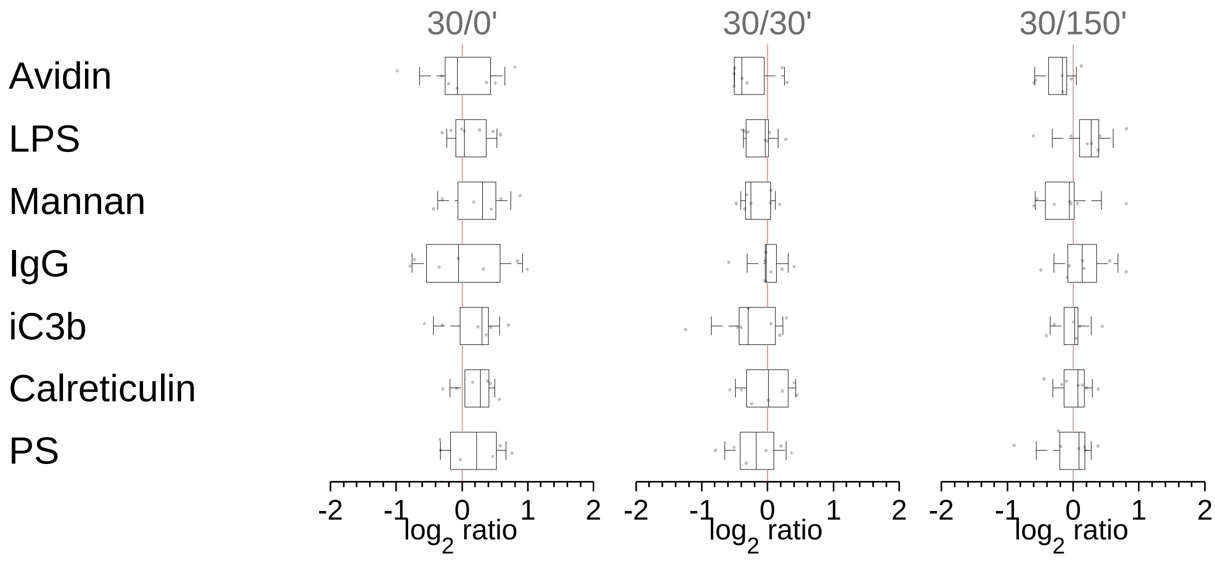

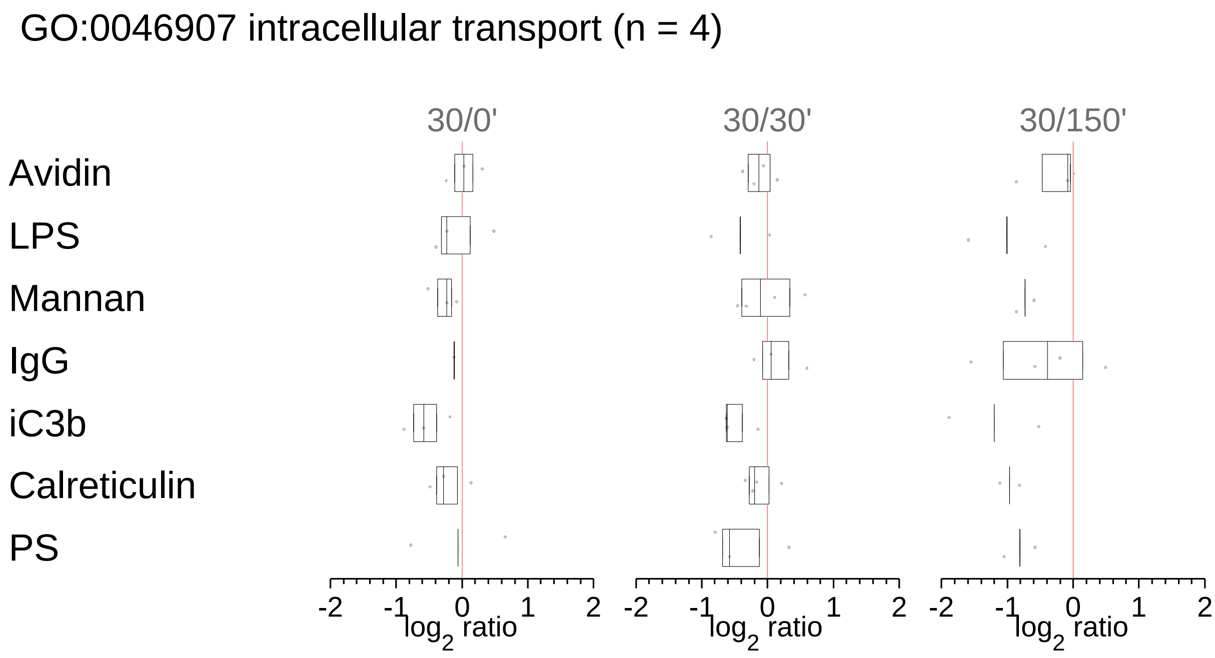
GO:0046907 intracellular transport (n = 4)
30/0'
30/30'
30/150'
Avidin
LPS
Mannan
IgG
iC3b
Calreticulin
PS
-2 -1 0 1 2
log2 ratio

GO:0046920 alpha-(1->3)-fucosyltransferase activity (n = 3)

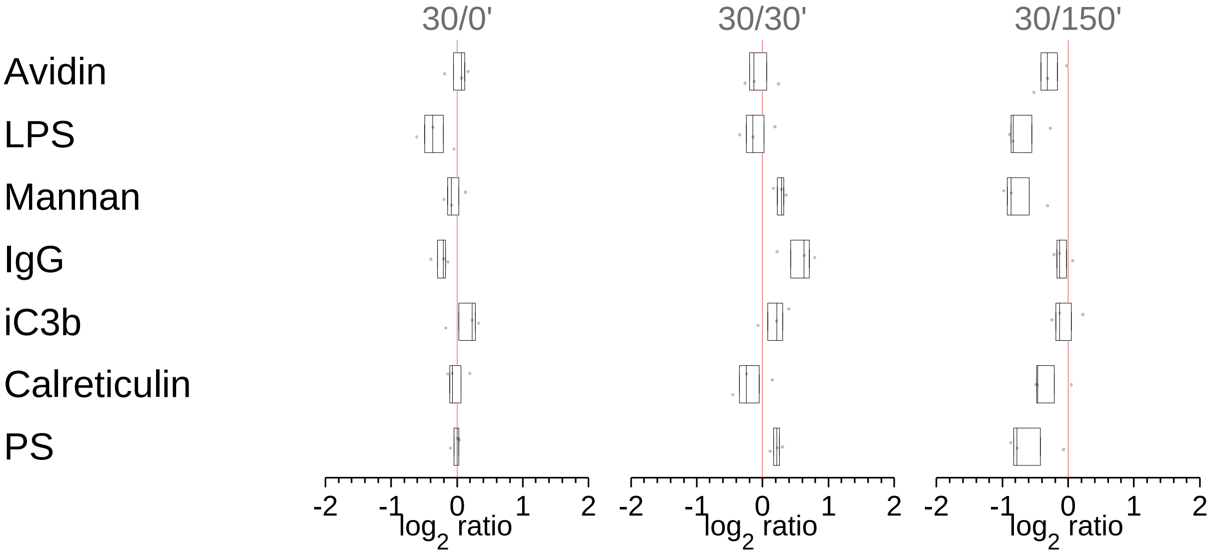

GO:0046921 alpha-(1->6)-fucosyltransferase activity (n = 3)

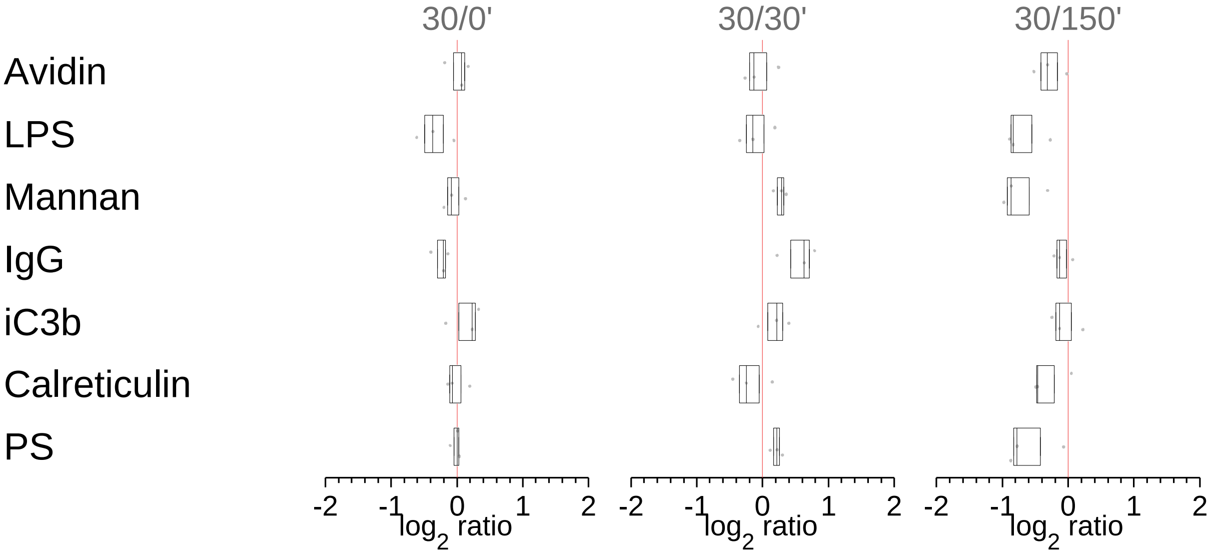

GO:0046930 pore complex (n = 6)

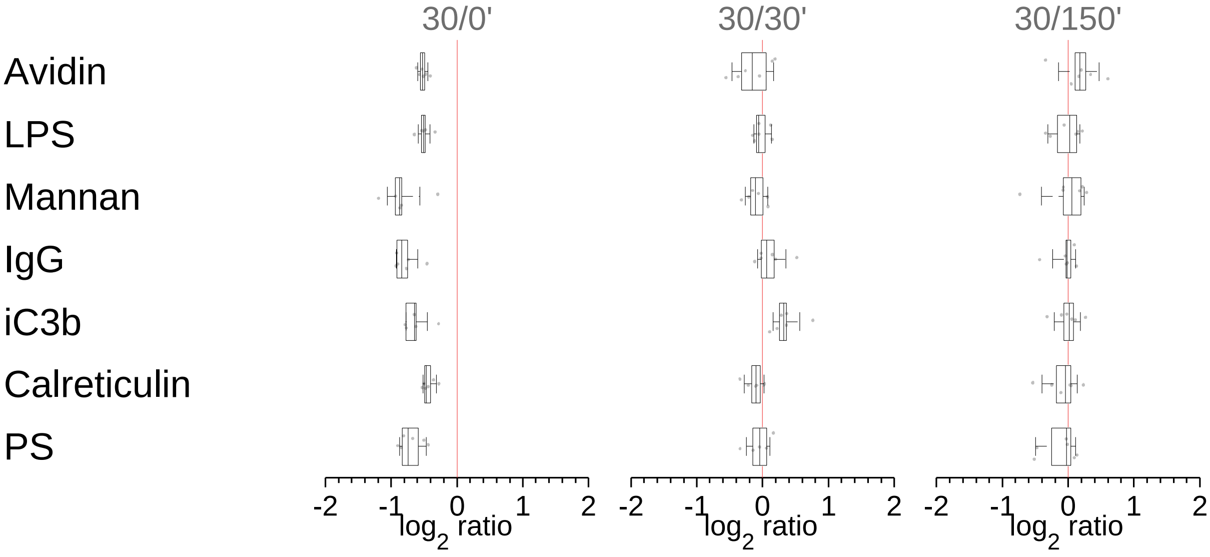

GO:0046933 hydrogen ion transporting ATP synthase activity, rotational mechanism (n = 7)

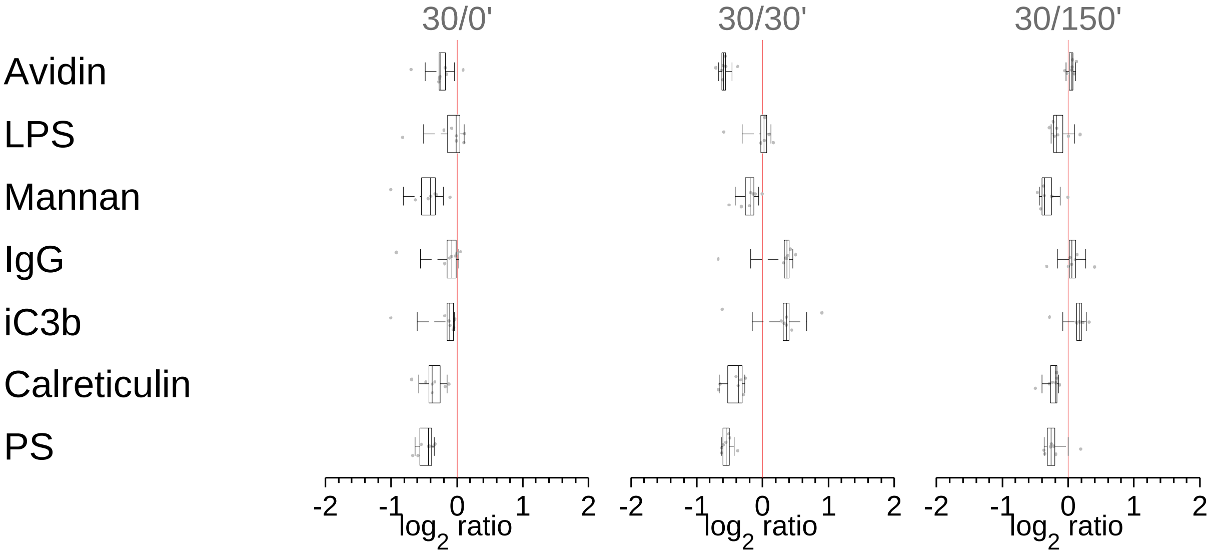

GO:0046941 azetidine-2-carboxylic acid acetyltransferase activity (n = 3)

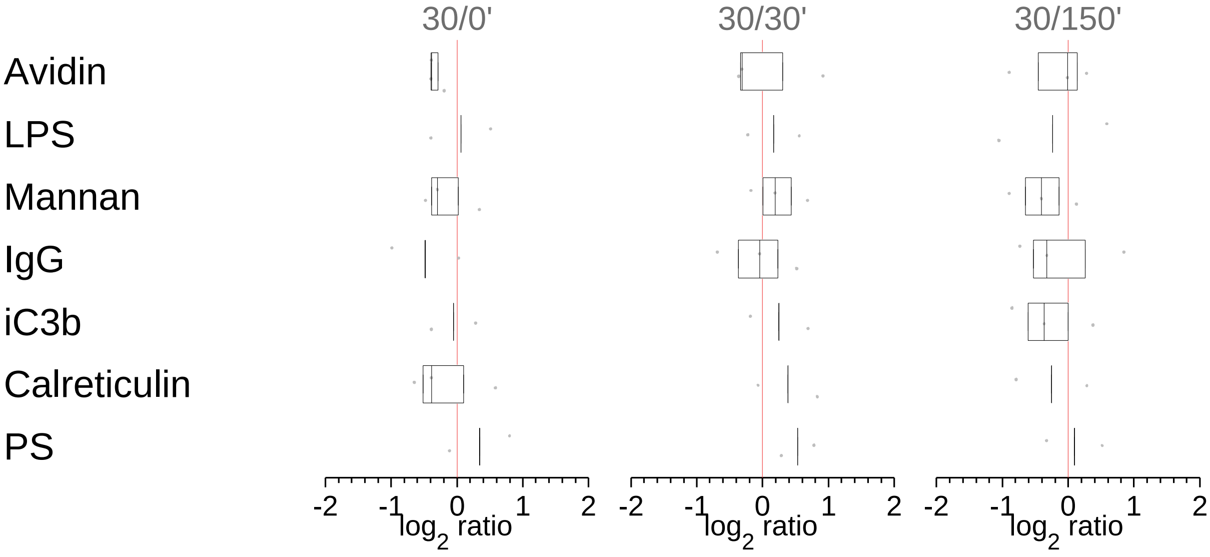

GO:0046961 proton-transporting ATPase activity, rotational mechanism (n = 9)

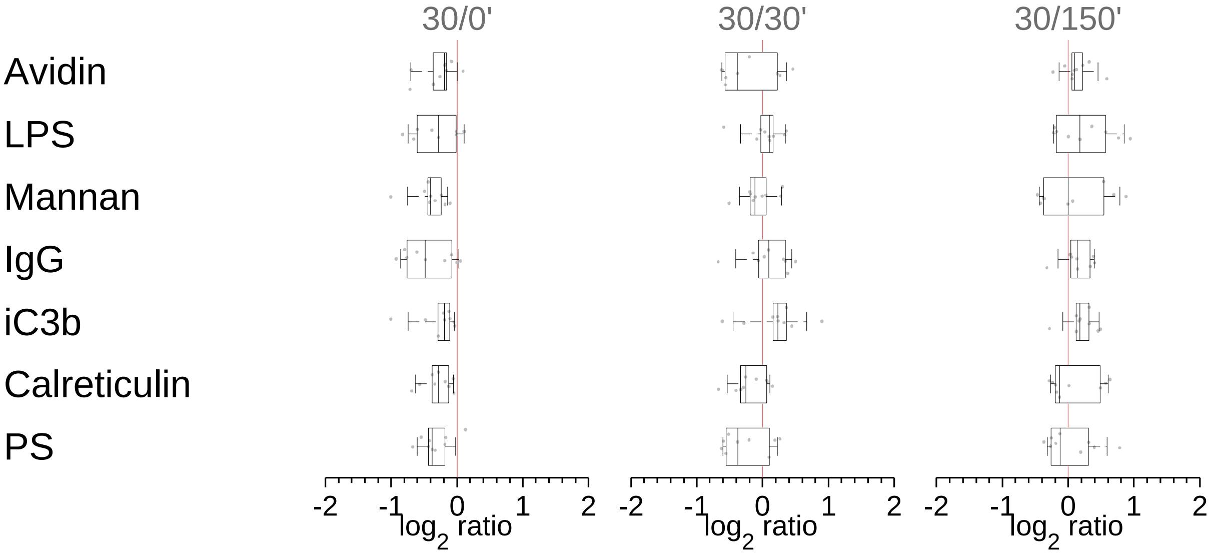

GO:0046966 thyroid hormone receptor binding (n = 3)

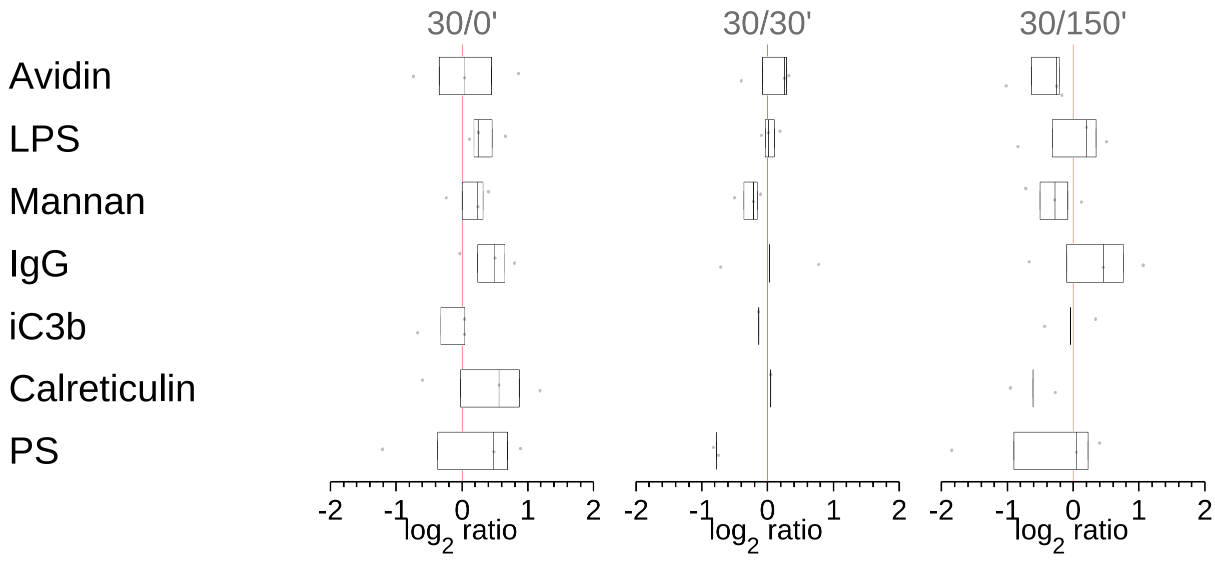

GO:0046978 TAP1 binding (n = 3)

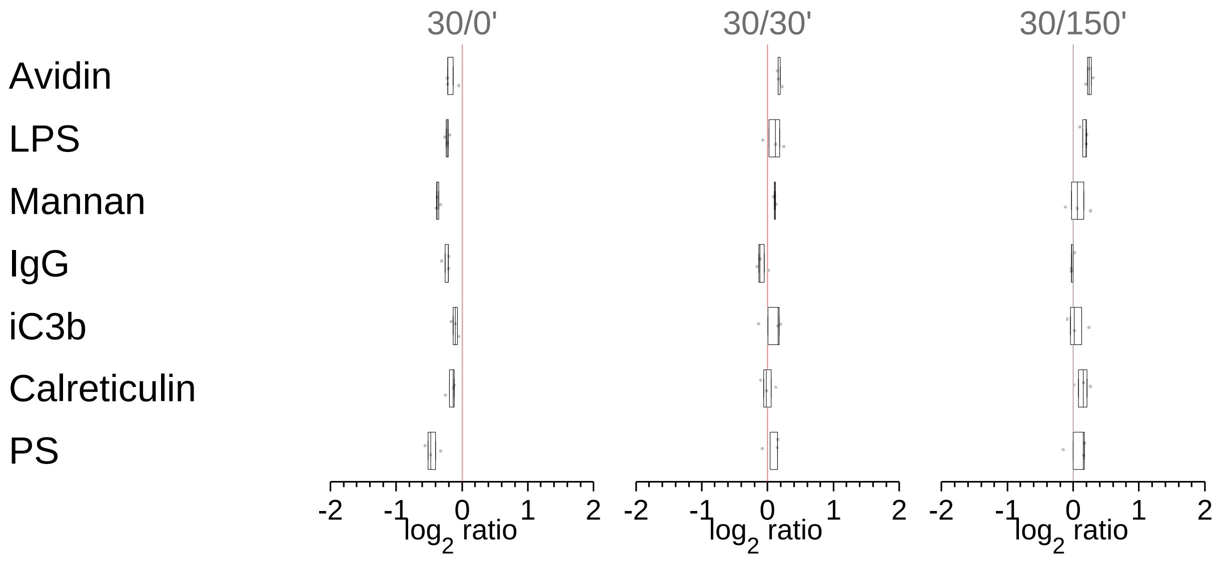

GO:0046979 TAP2 binding (n = 3)

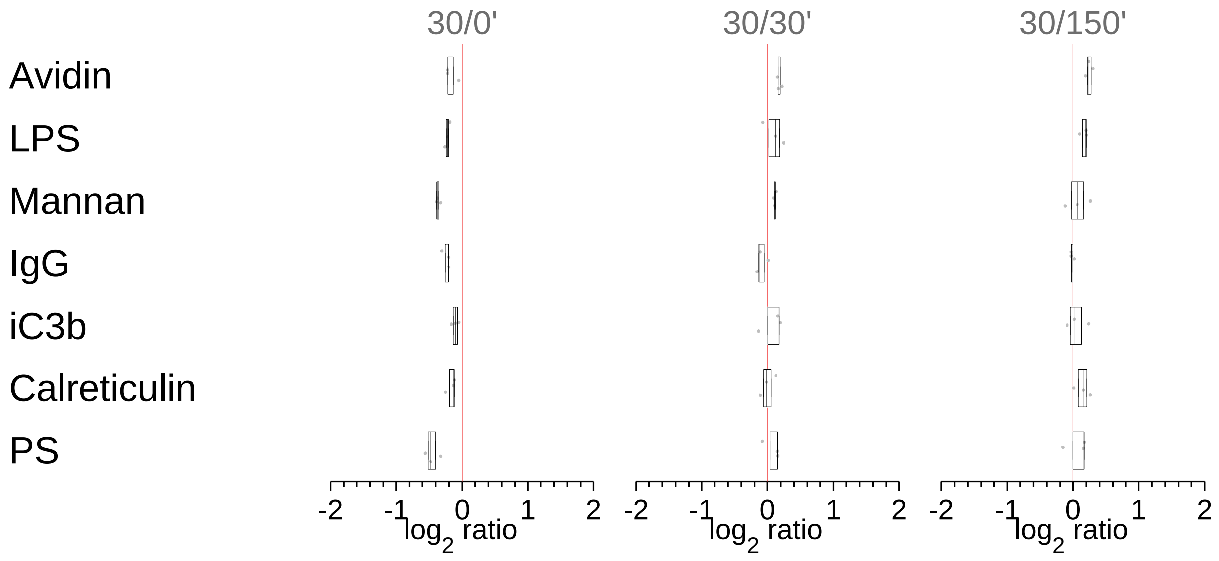

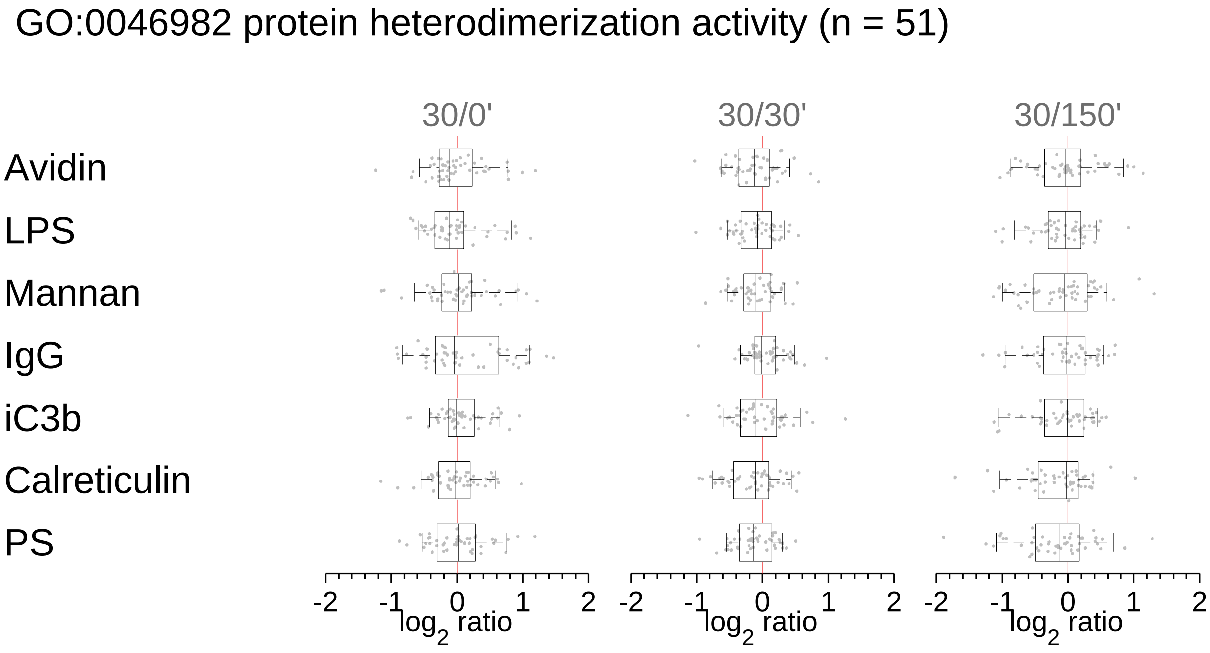

GO:0046982 protein heterodimerization activity (n = 51)
30/0'
30/30'
30/150'
Avidin
LPS
Mannan
IgG
iC3b
Calreticulin
PS
-2
-1
0
1
2
log2 ratio

GO:0046983 protein dimerization activity (n = 14)

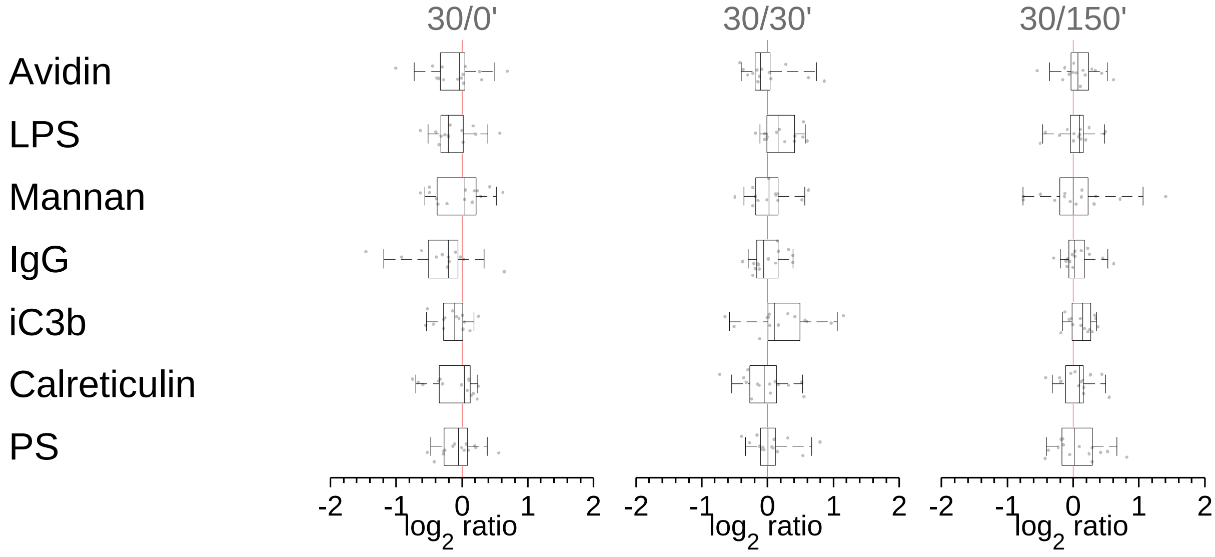

GO:0047376 all-trans-retinyl-palmitate hydrolase, all-trans-retinol forming activity (n = 4)

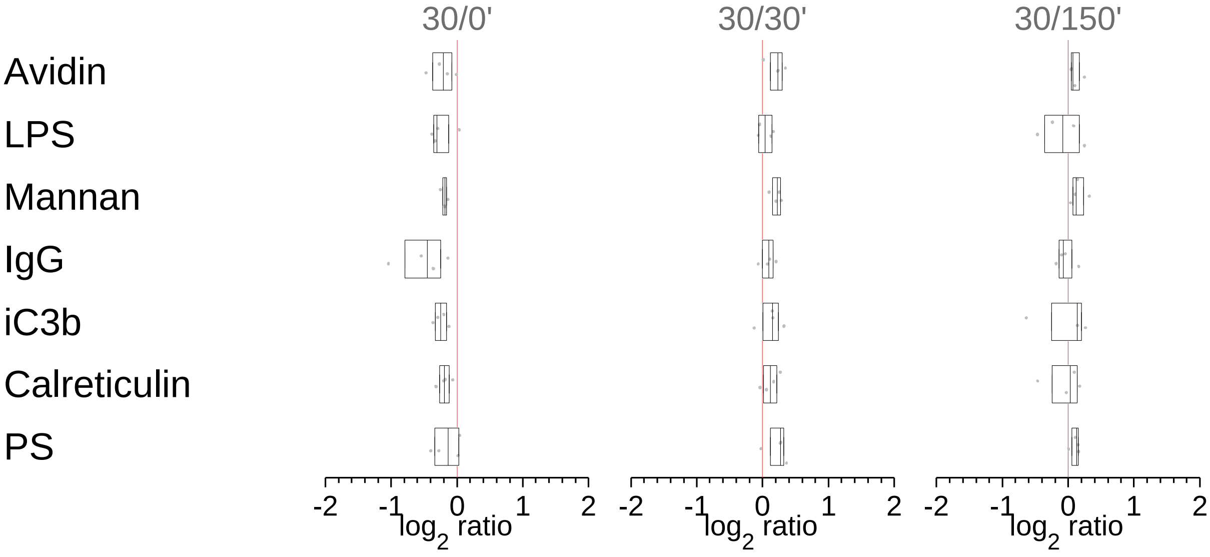

GO:0047485 protein N-terminus binding (n = 18)

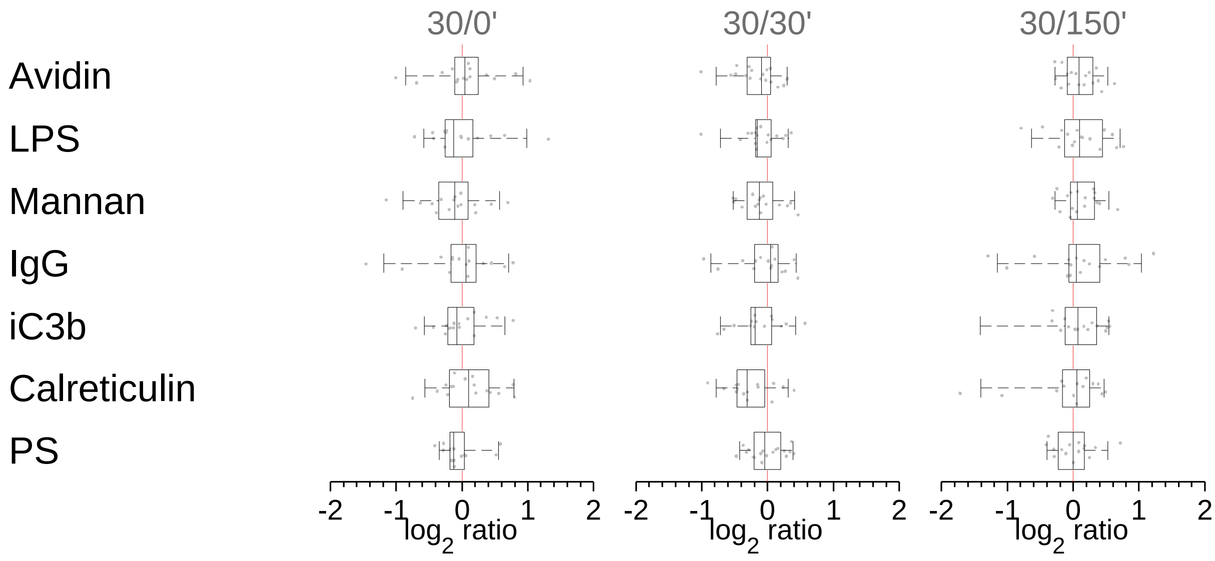

GO:0047496 vesicle transport along microtubule (n = 5)

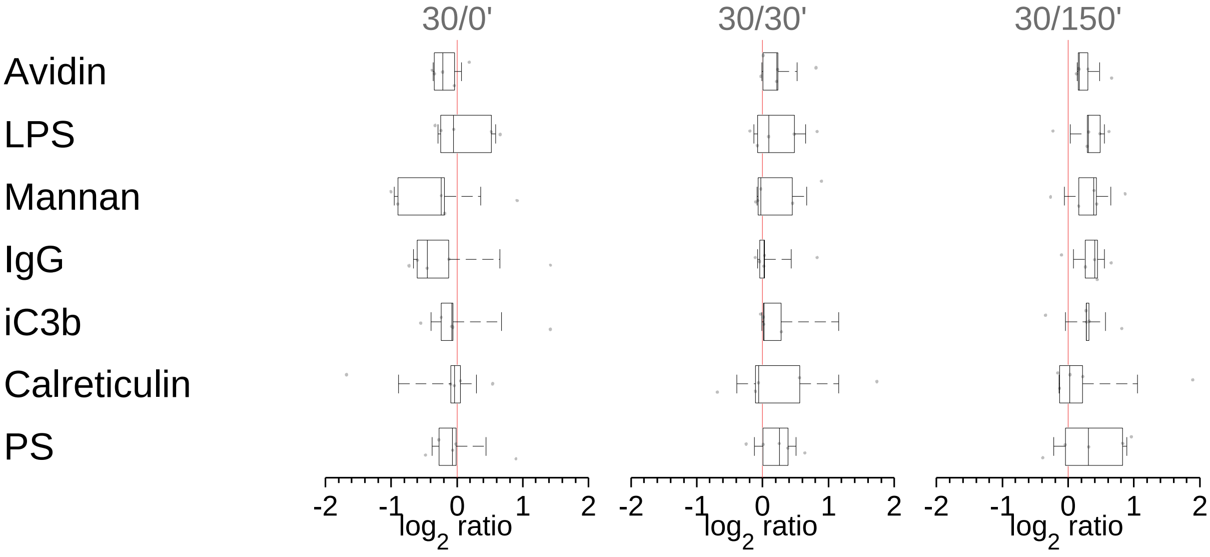

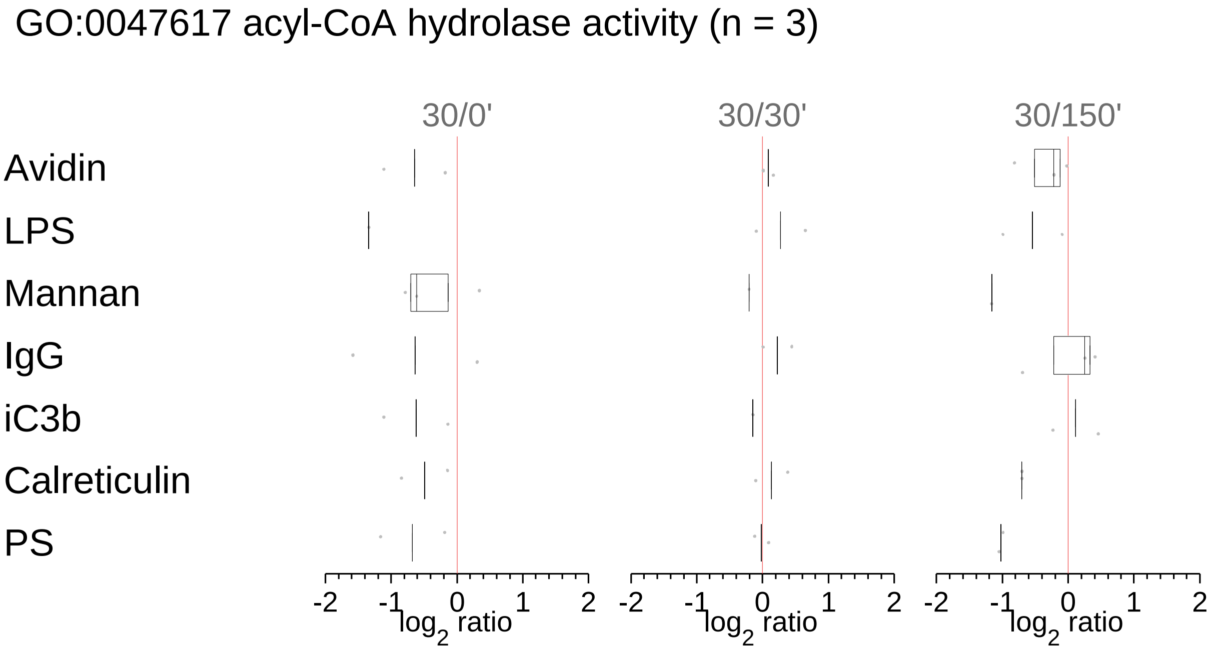
GO:0047617 acyl-CoA hydrolase activity (n = 3)
30/0'
30/30'
30/150'
Avidin
LPS
Mannan
IgG
iC3b
Calreticulin
PS
-2 -1 0 1 2
log2 ratio

GO:0048007 antigen processing and presentation, exogenous lipid antigen via MHC class Ib (n = 3)

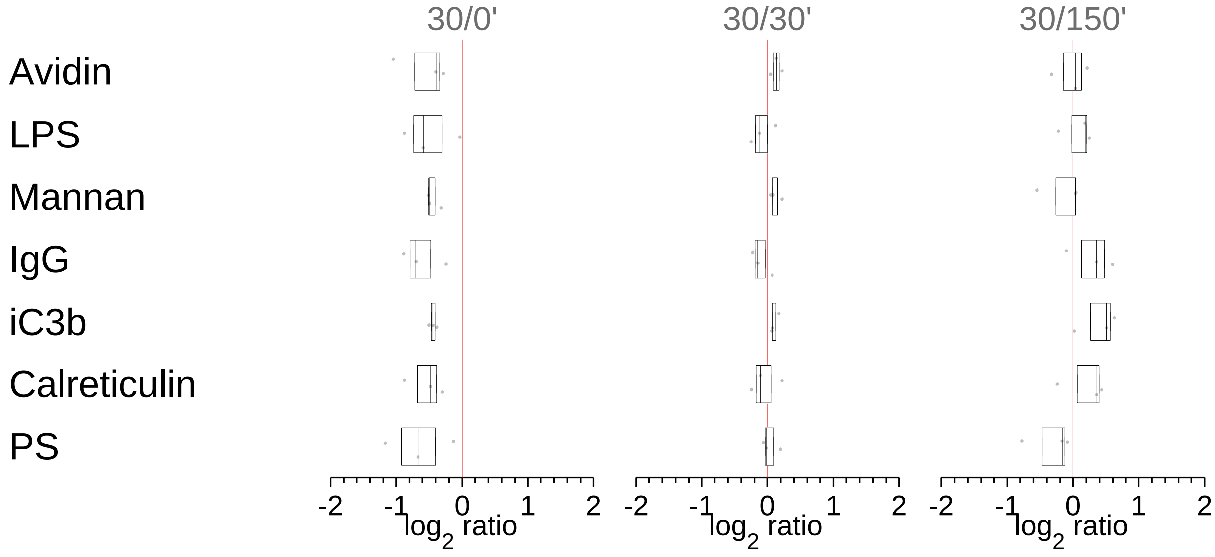

GO:0048008 platelet-derived growth factor receptor signaling pathway (n = 6)

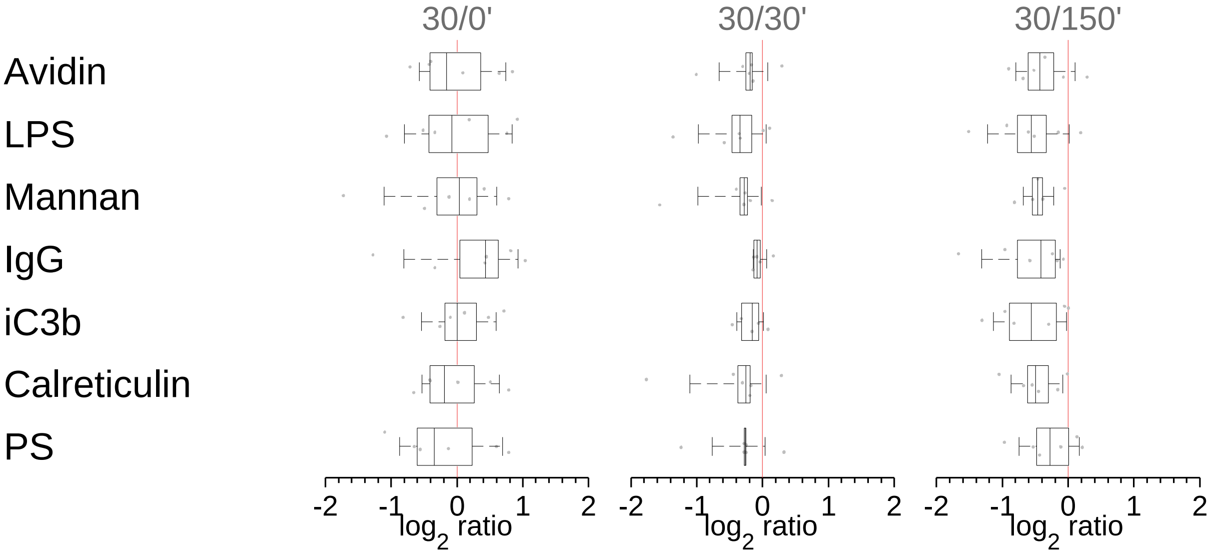

GO:0048015 phosphatidylinositol-mediated signaling (n = 4)

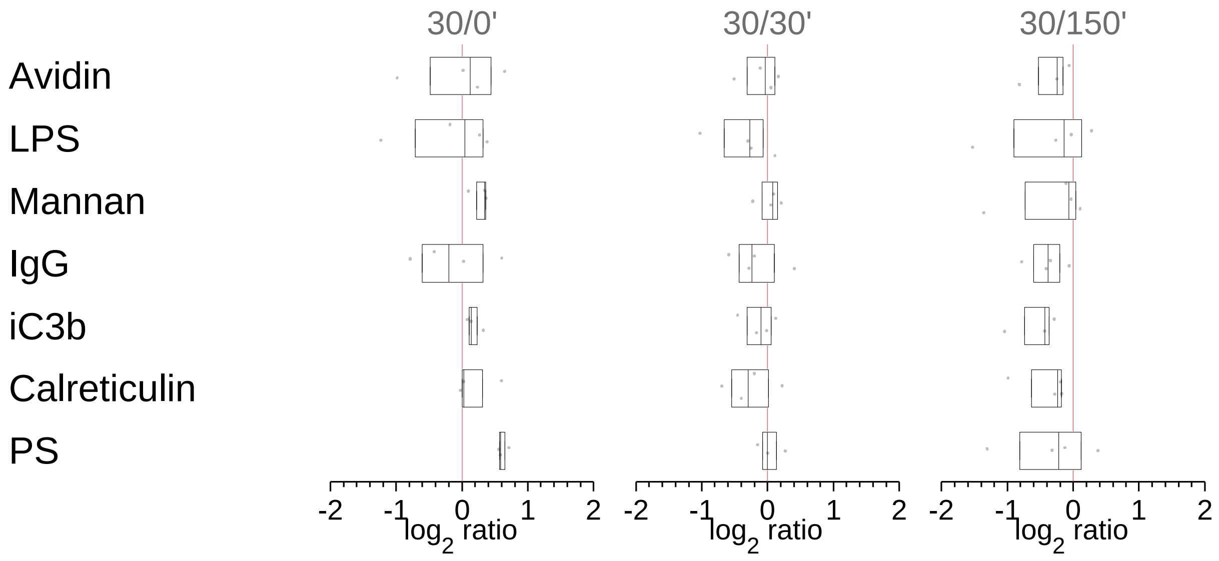

GO:0048025 negative regulation of nuclear mRNA splicing, via spliceosome (n = 4)

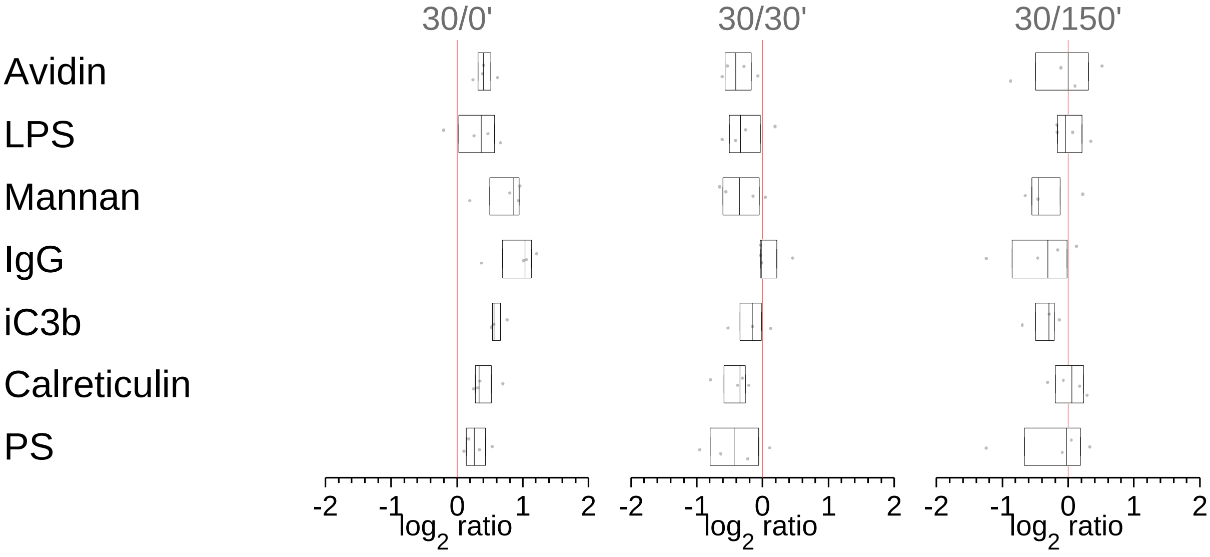

GO:0048026 positive regulation of nuclear mRNA splicing, via spliceosome (n = 3)

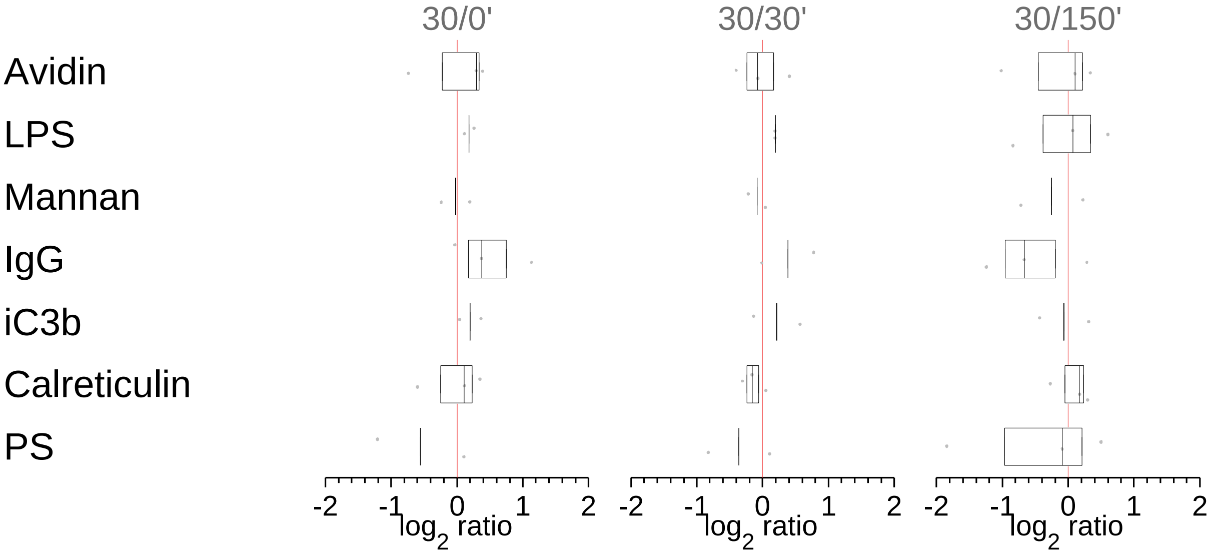

GO:0048037 cofactor binding (n = 3)

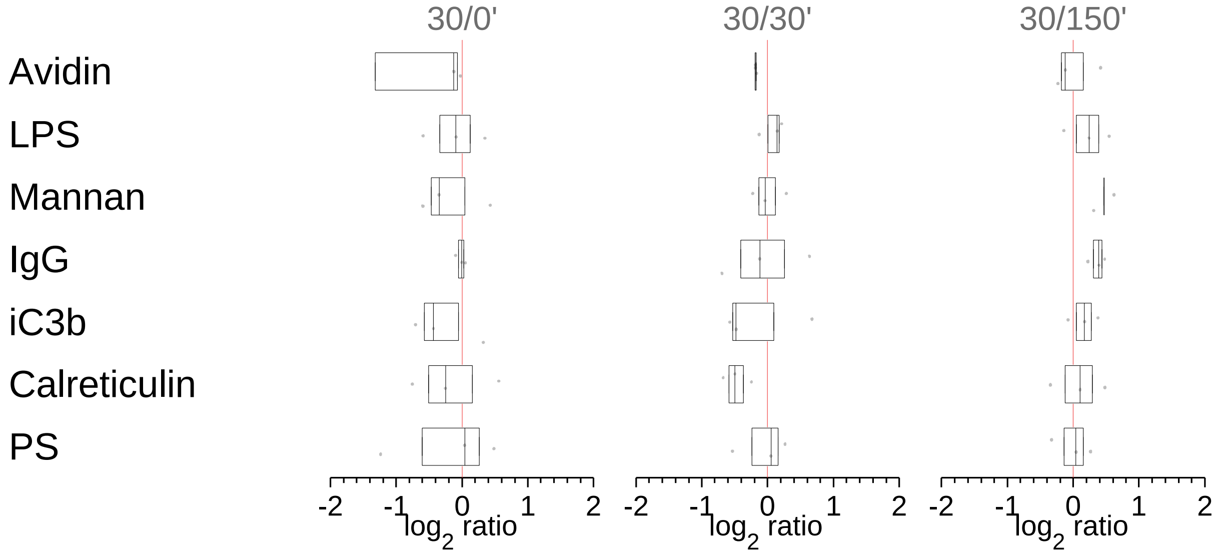

GO:0048102 autophagic cell death (n = 3)

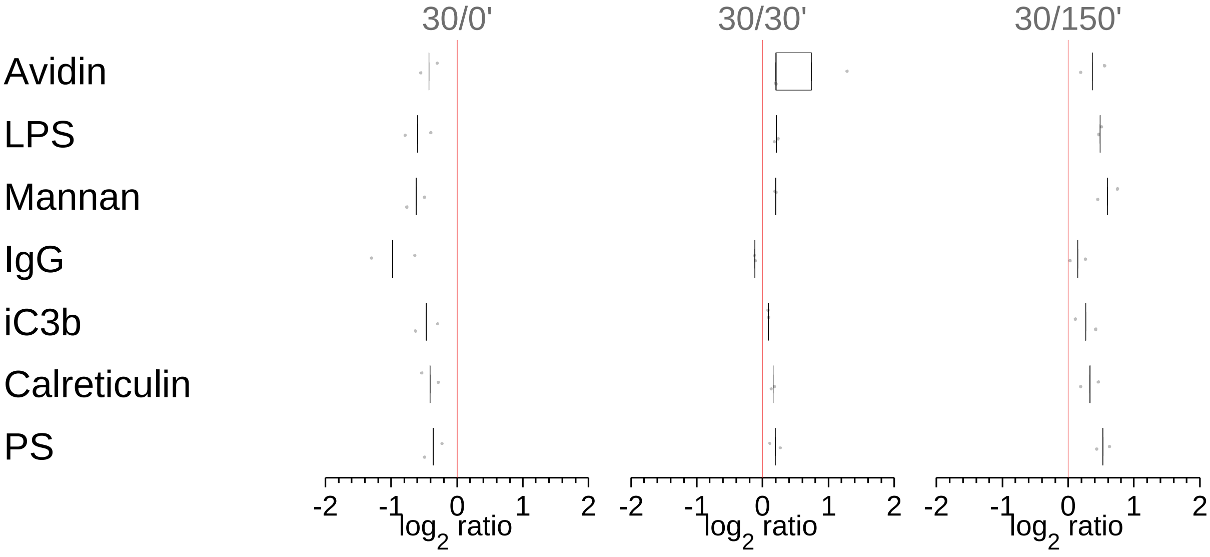

GO:0048146 positive regulation of fibroblast proliferation (n = 3)

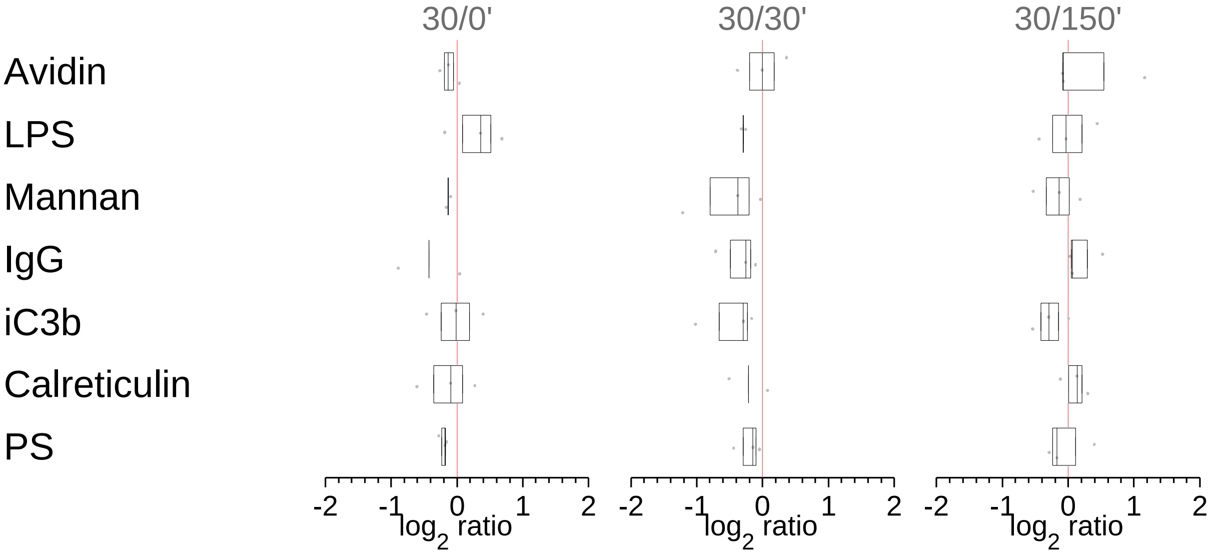

GO:0048147 negative regulation of fibroblast proliferation (n = 6)

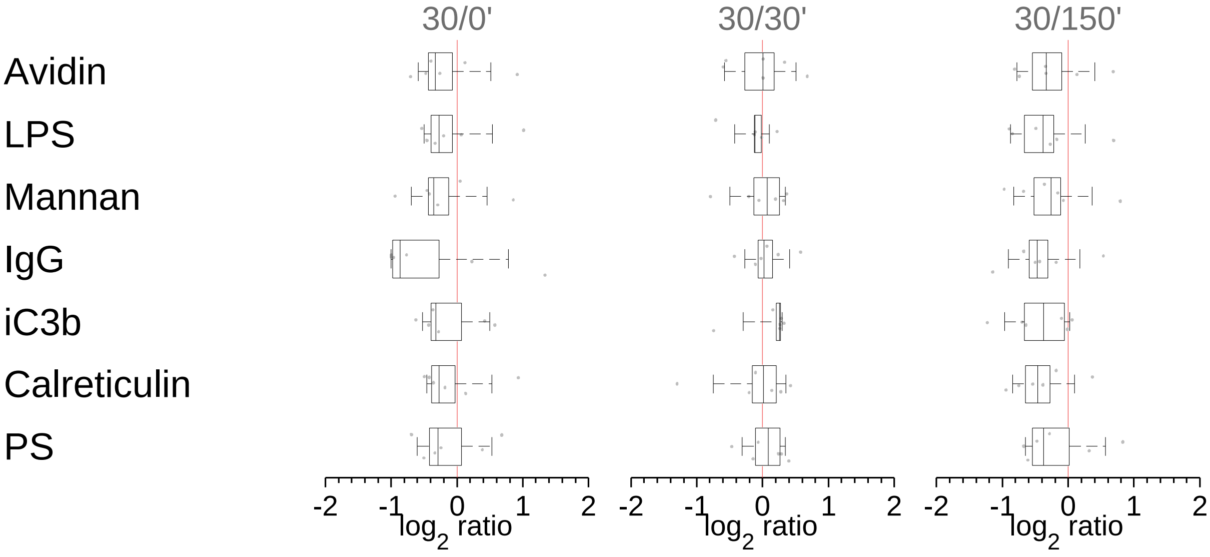

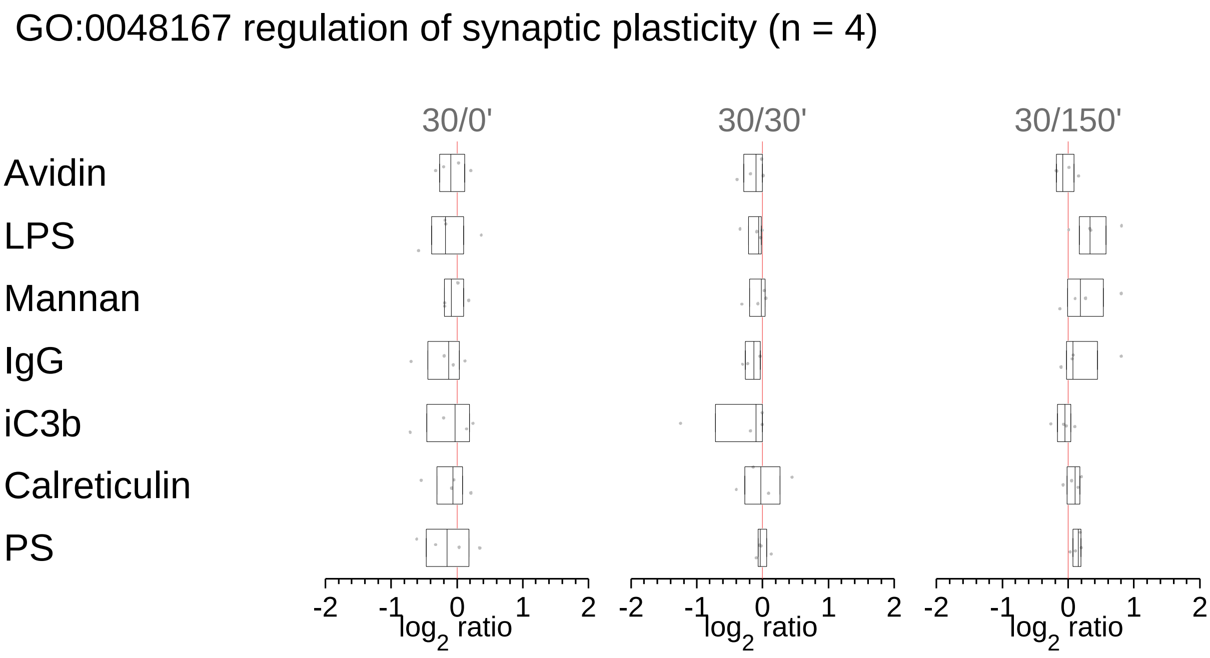

GO:0048167 regulation of synaptic plasticity (n = 4)
30/0'
30/30'
30/150'
Avidin
LPS
Mannan
IgG
iC3b
Calreticulin
PS
-2 -1 0 1 2
log2 ratio
-2 -1 0 1 2
log2 ratio
-2 -1 0 1 2
log2 ratio

GO:0048169 regulation of long-term neuronal synaptic plasticity
(n = 6)

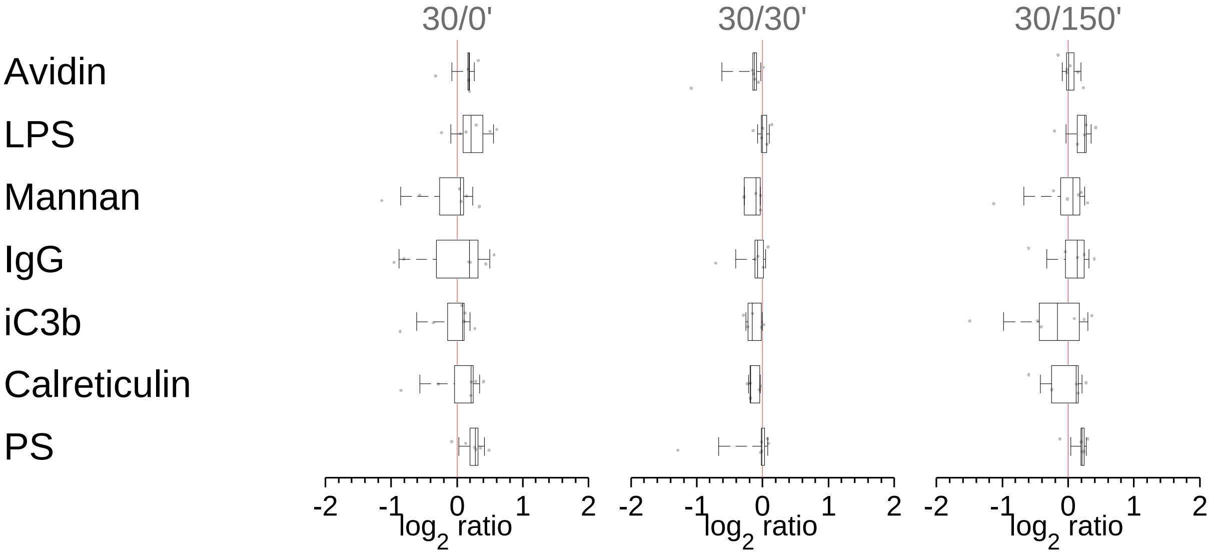

GO:0048193 Golgi vesicle transport (n = 5)

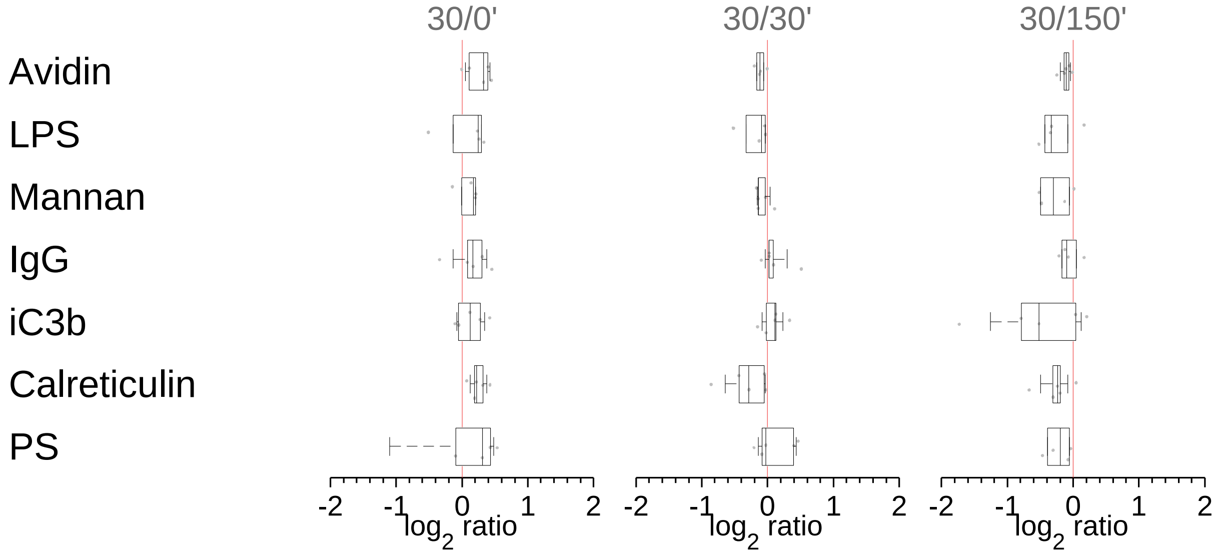

GO:0048237 rough endoplasmic reticulum lumen (n = 3)

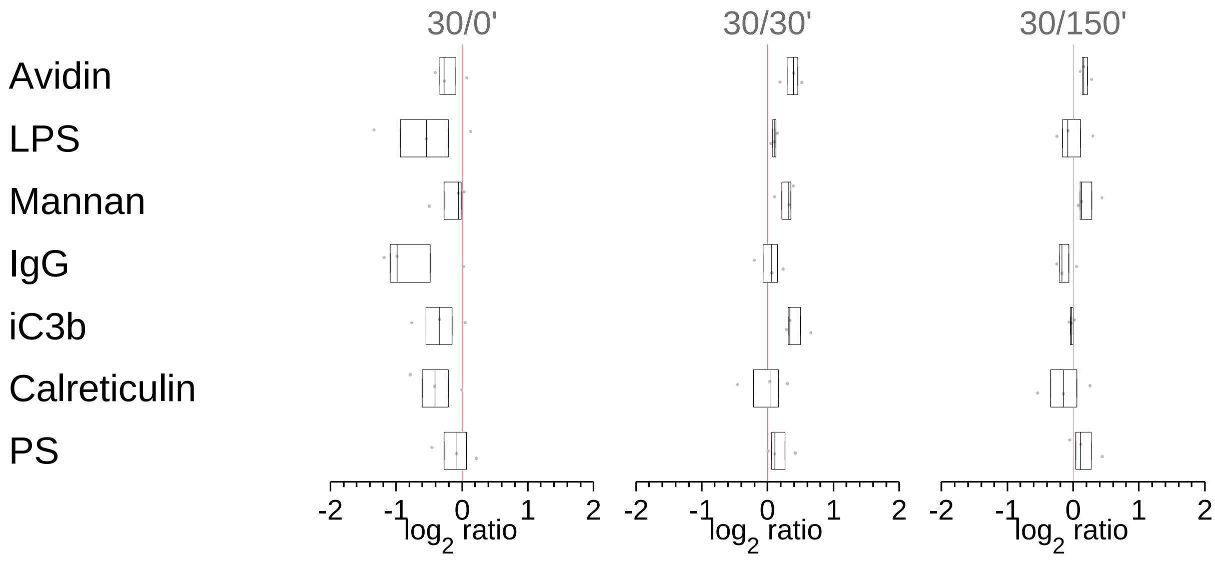

GO:0048255 mRNA stabilization (n = 4)

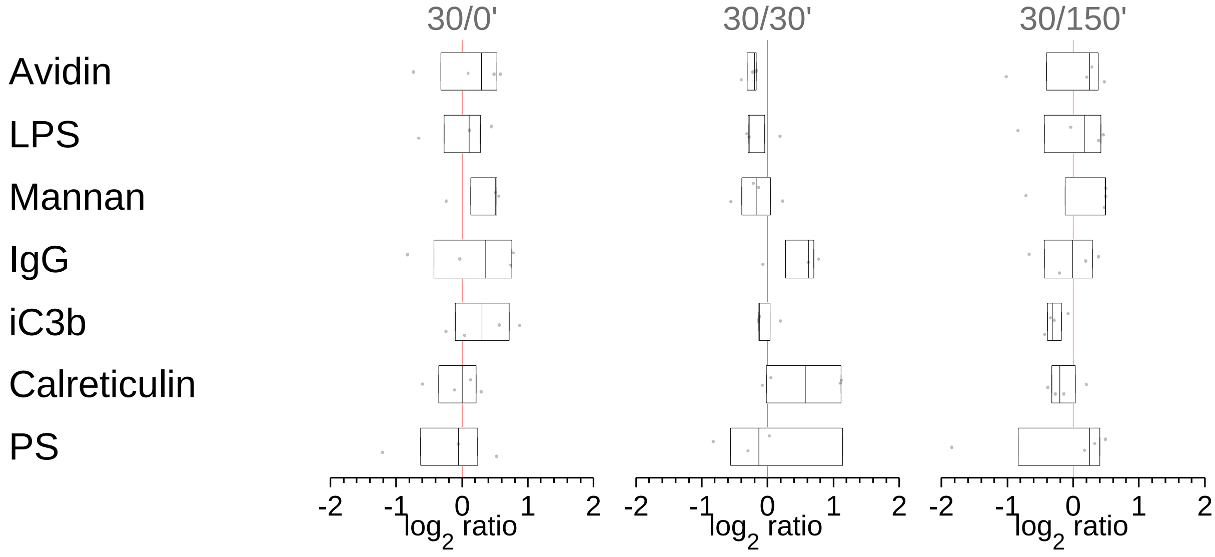

GO:0048258 3-ketoglucose-reductase activity (n = 3)

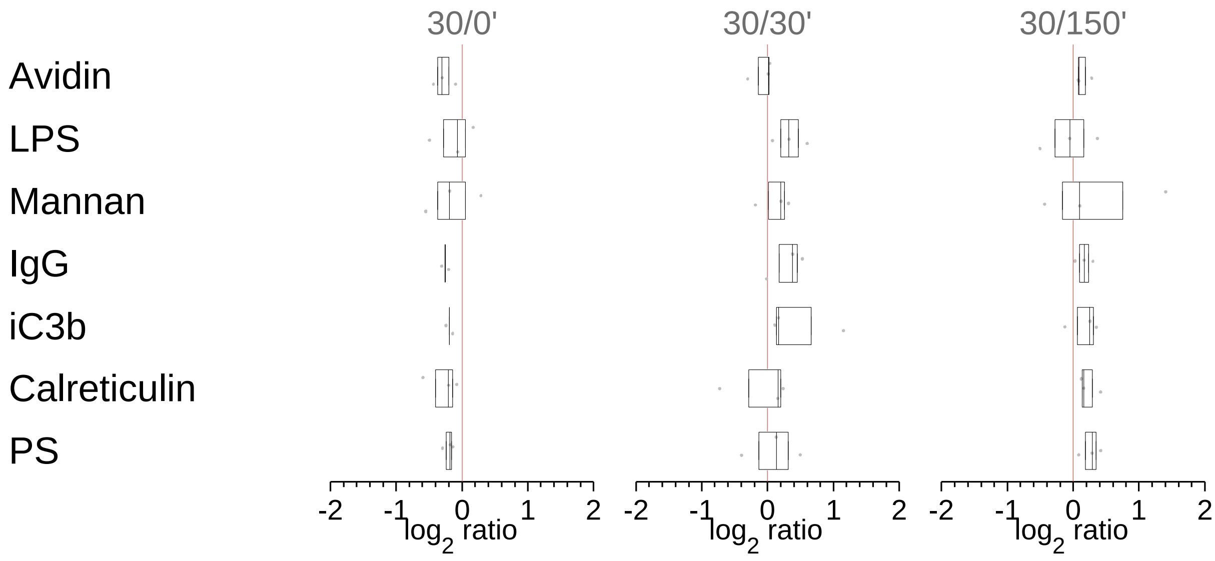

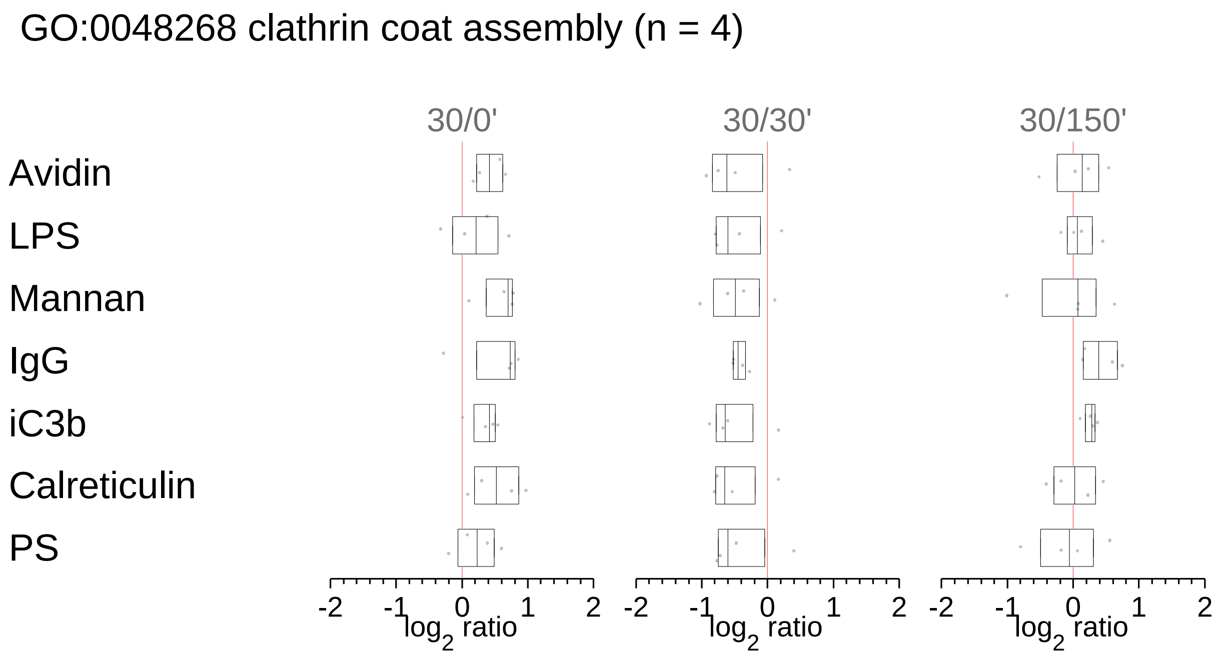
GO:0048268 clathrin coat assembly (n = 4)
30/0'
30/30'
30/150'
Avidin
LPS
Mannan
IgG
iC3b
Calreticulin
PS
-2
-1
0
1
2
log2 ratio

GO:0048280 vesicle fusion with Golgi apparatus (n = 3)

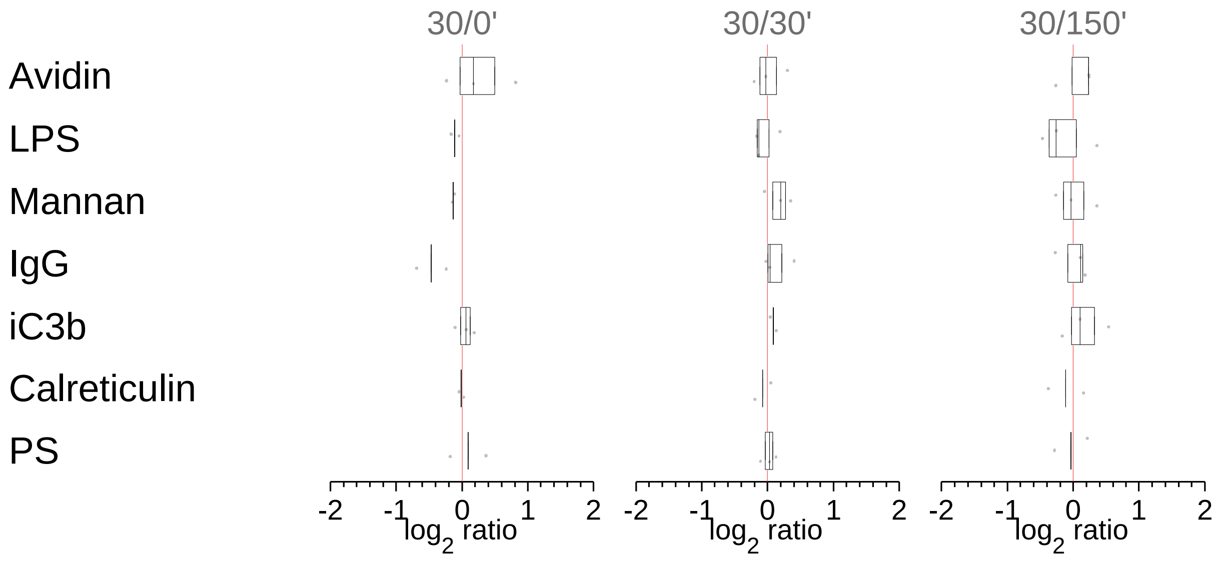

GO:0048306 calcium-dependent protein binding (n = 23)

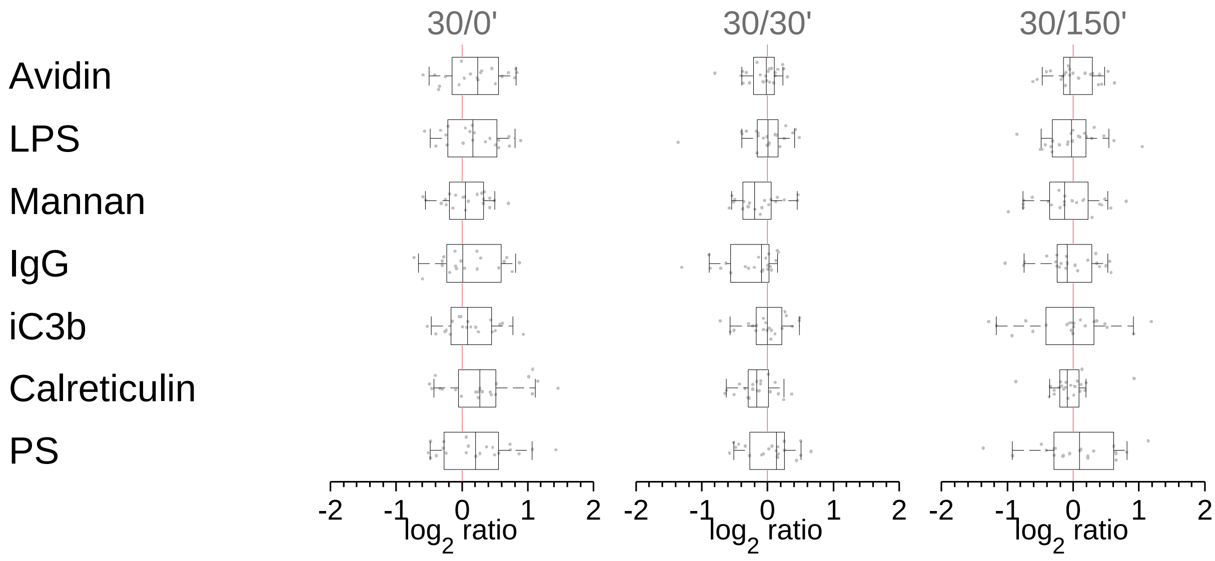

GO:0048365 Rac GTPase binding (n = 10)

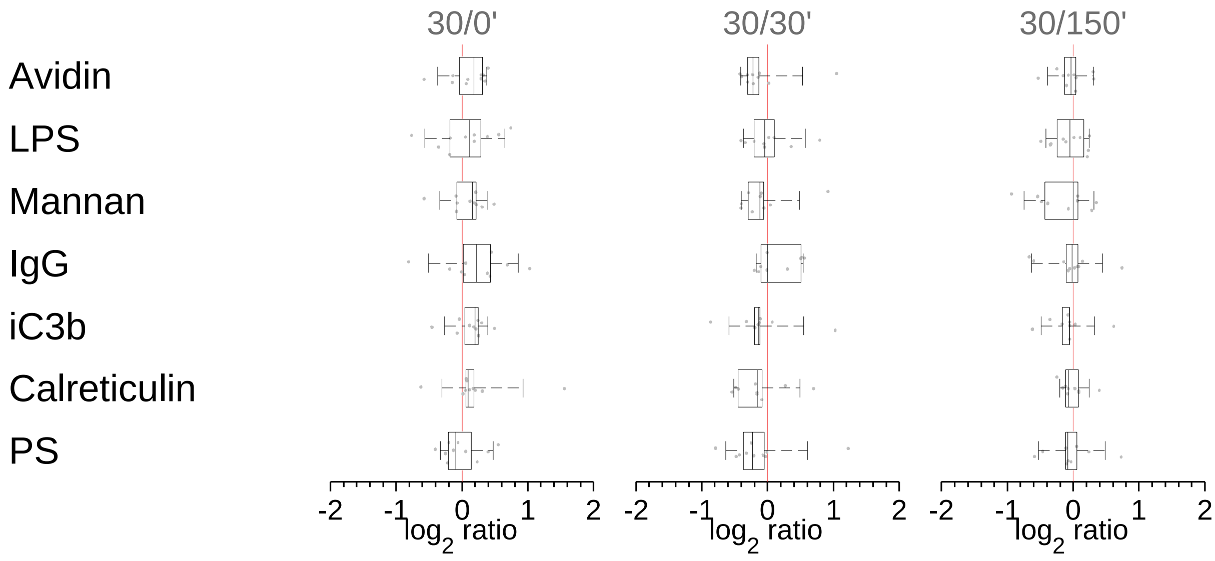

GO:0048471 perinuclear region of cytoplasm (n = 112)

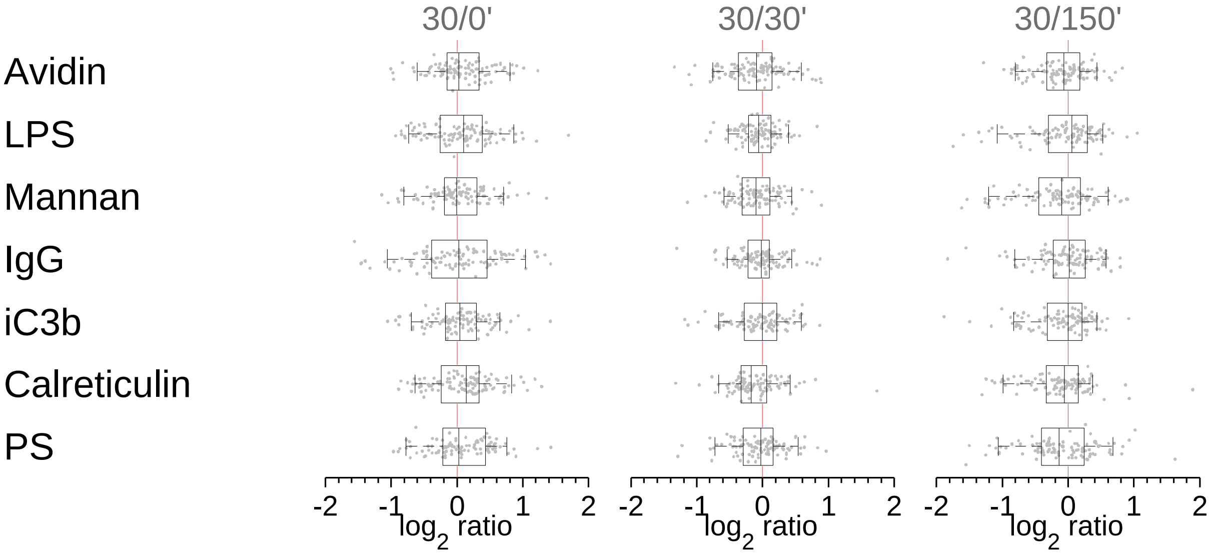

GO:0048487 beta-tubulin binding (n = 11)

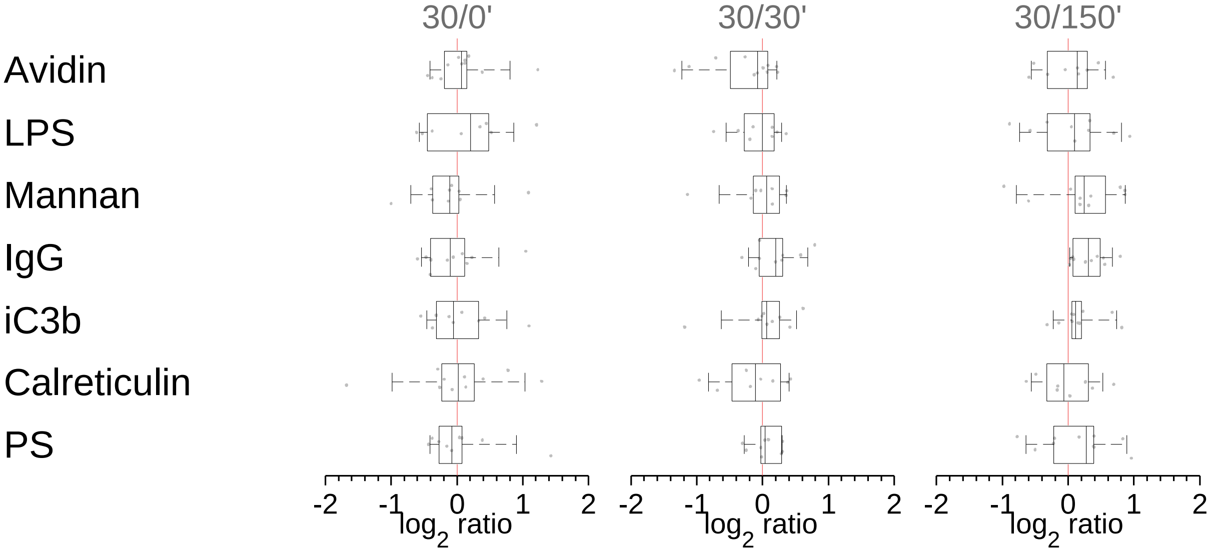

GO:0048488 synaptic vesicle endocytosis (n = 4)

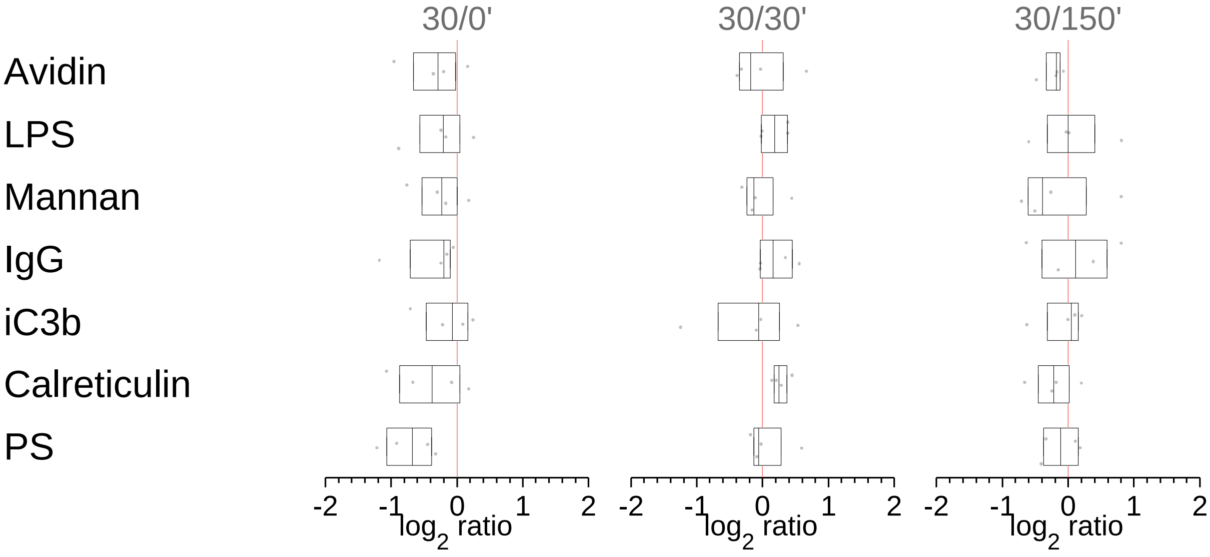

GO:0048489 synaptic vesicle transport (n = 3)

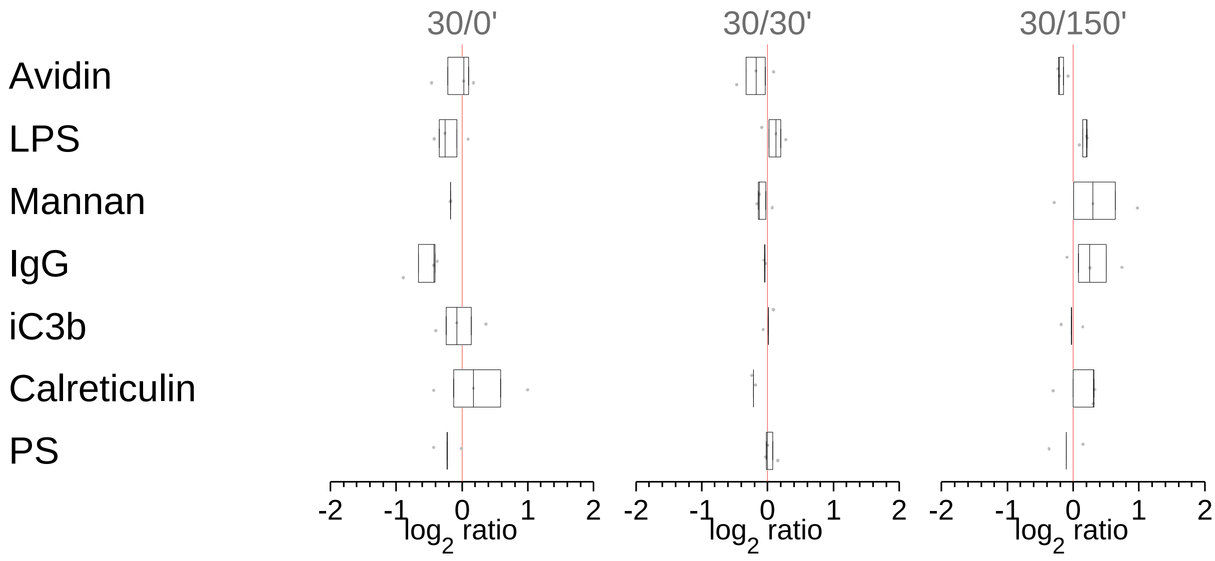

GO:0048490 anterograde synaptic vesicle transport (n = 7)

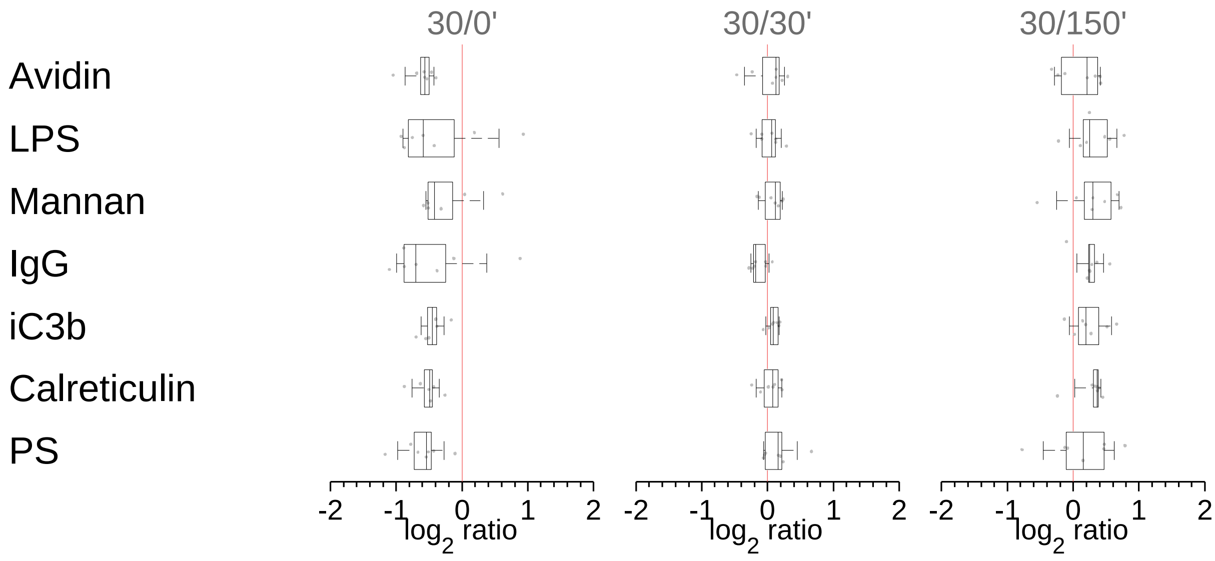

GO:0048511 rhythmic process (n = 14)

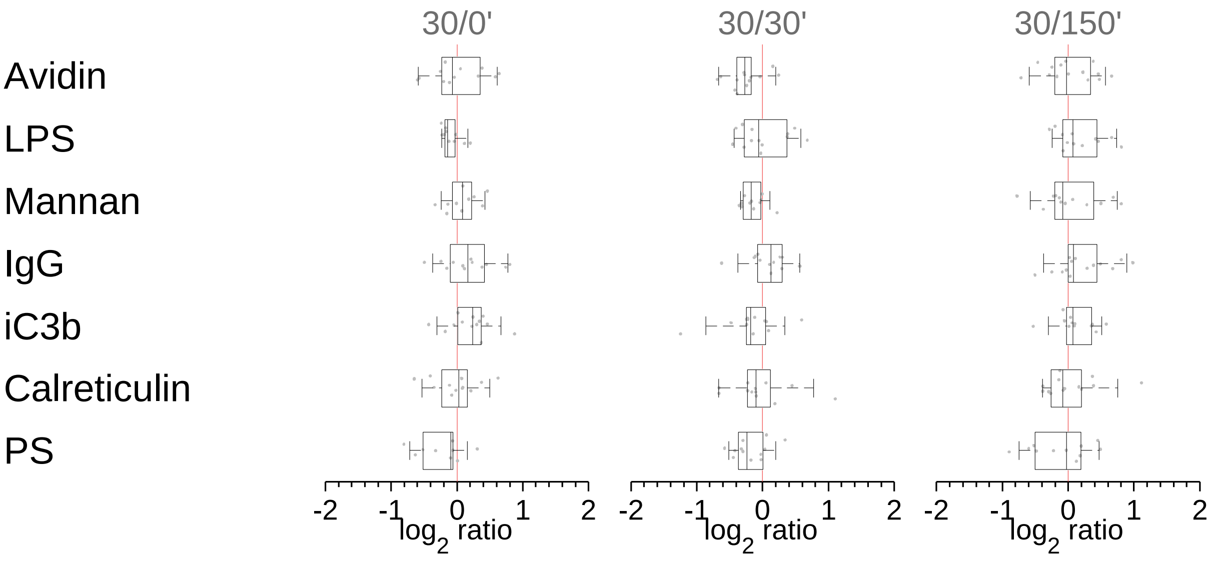

GO:0048538 thymus development (n = 4)

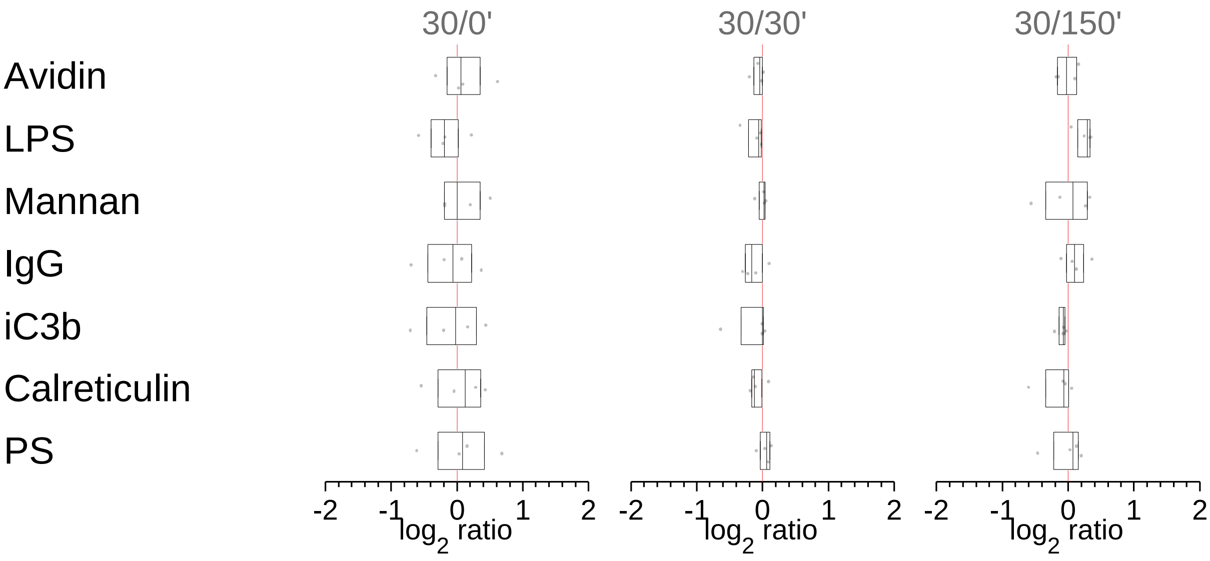

GO:0048545 response to steroid hormone stimulus (n = 4)

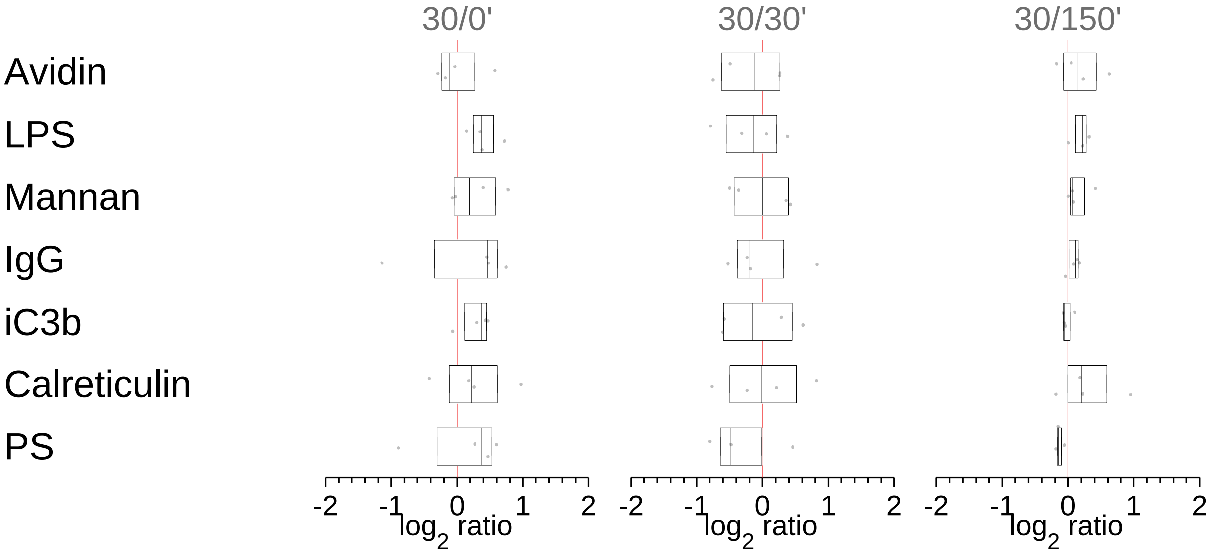

GO:0048589 developmental growth (n = 5)

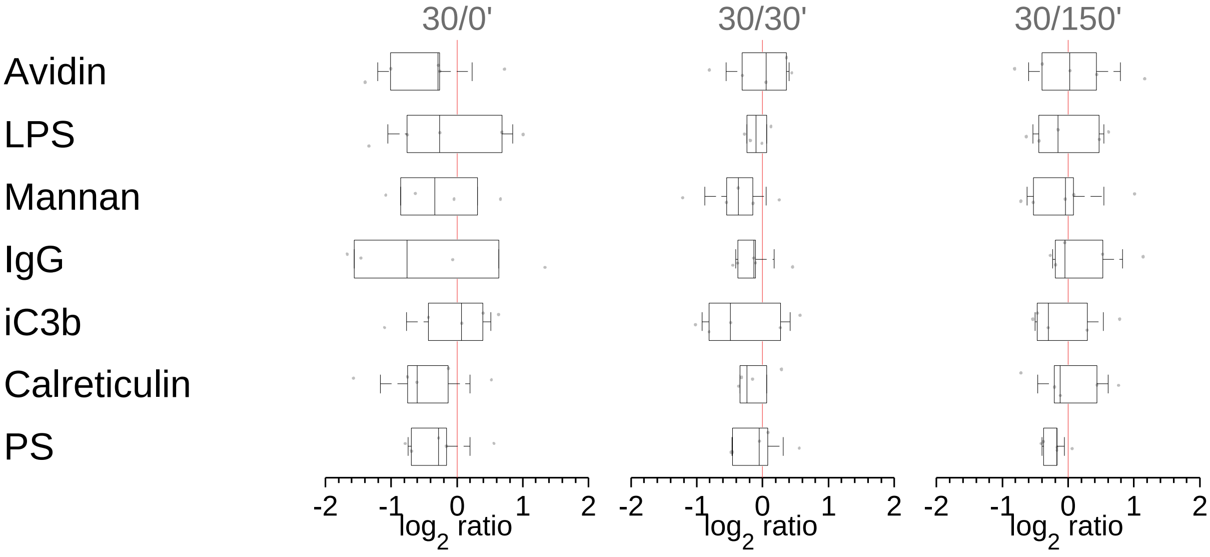

GO:0048661 positive regulation of smooth muscle cell proliferation (n = 12)

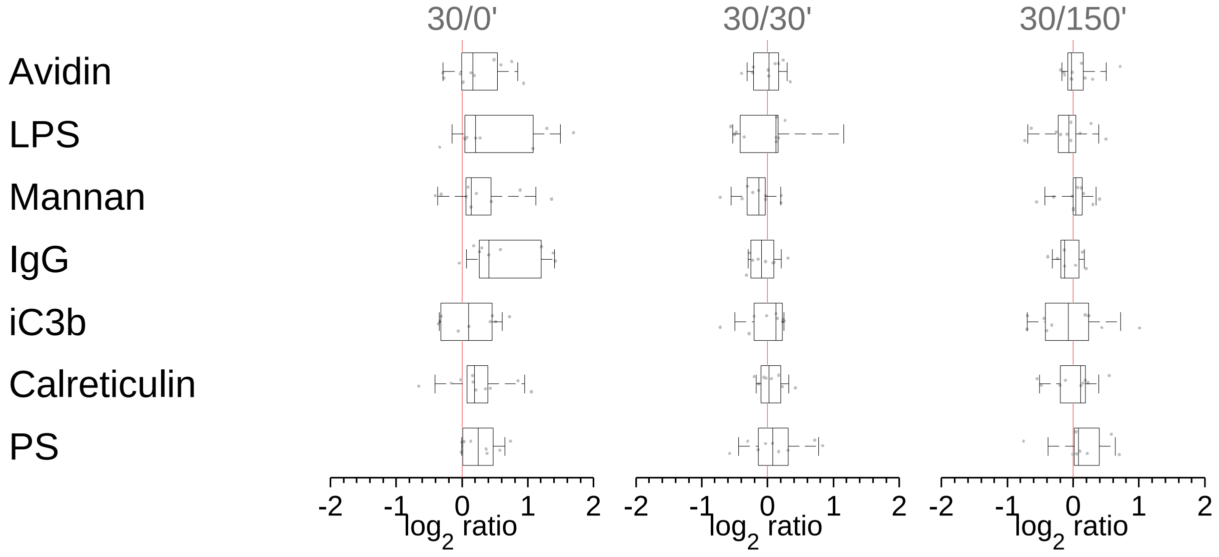

GO:0048662 negative regulation of smooth muscle cell proliferation (n = 5)

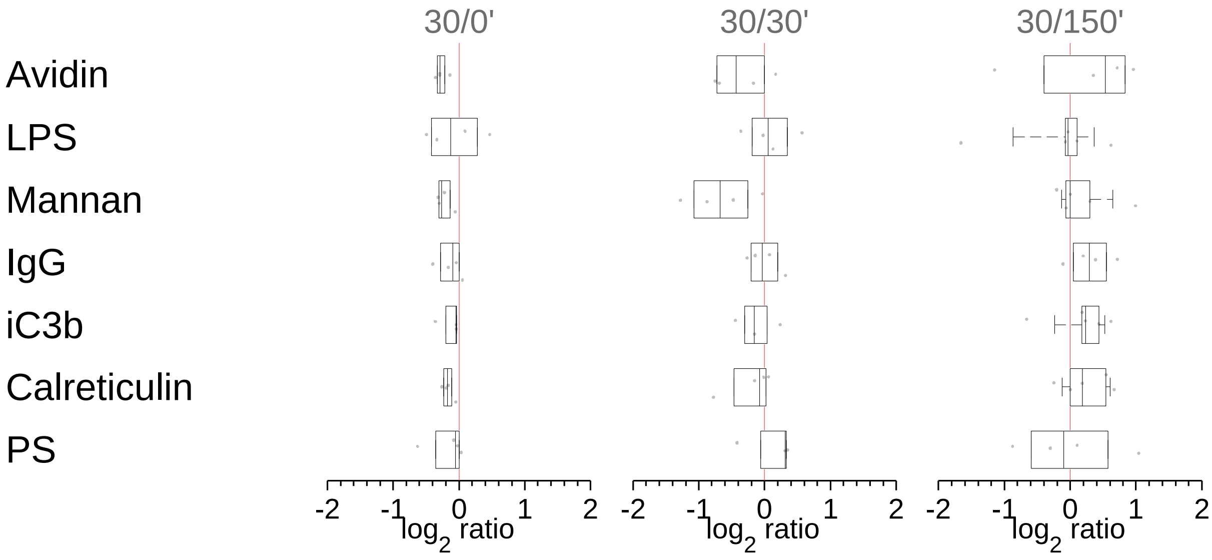

GO:0048666 neuron development (n = 4)

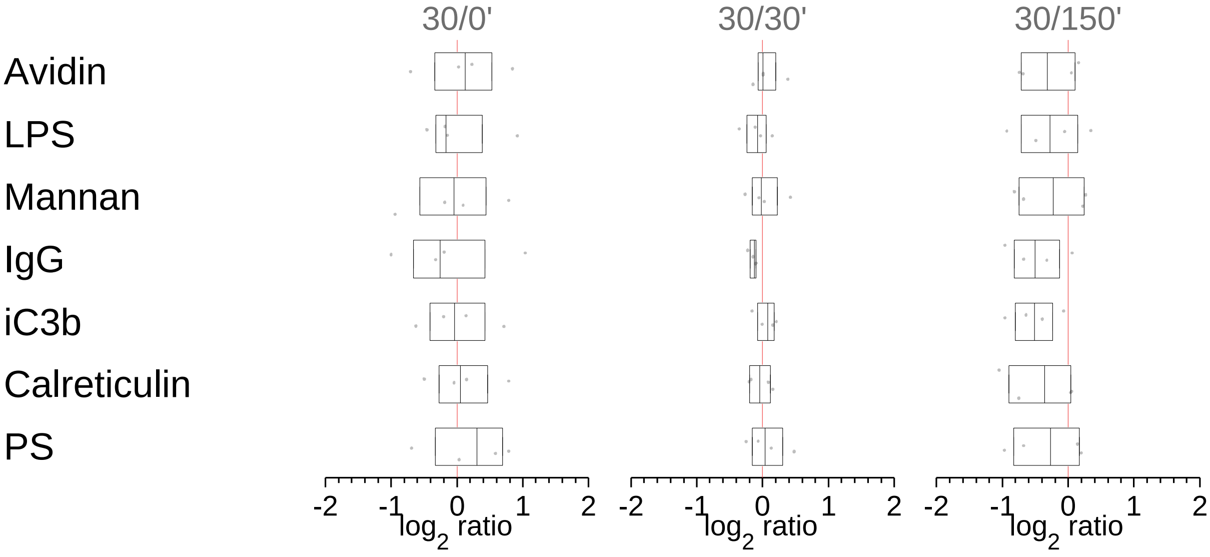

GO:0048678 response to axon injury (n = 4)

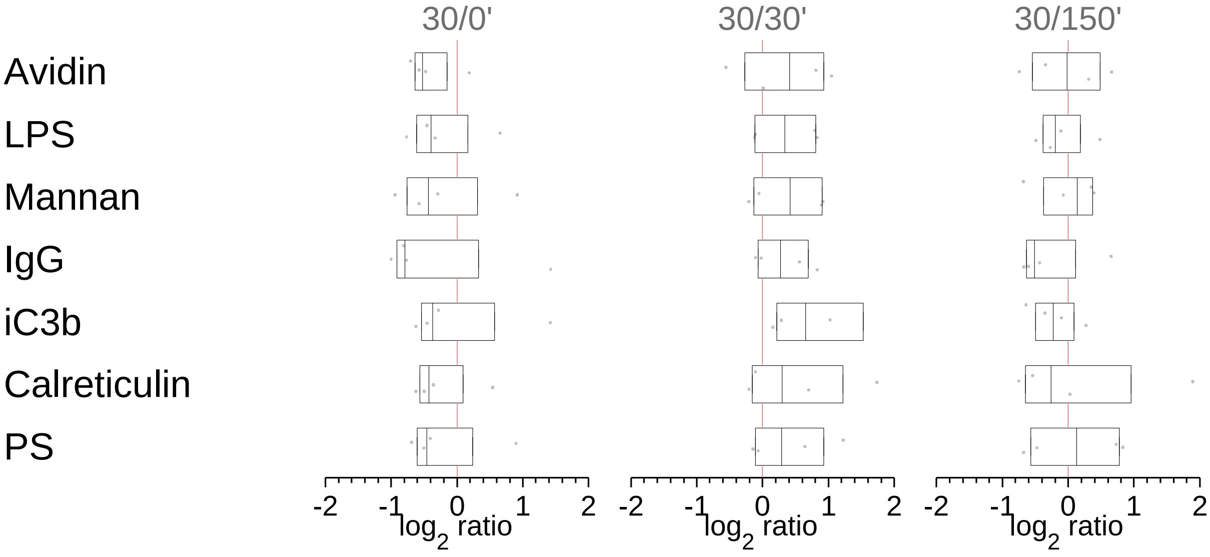

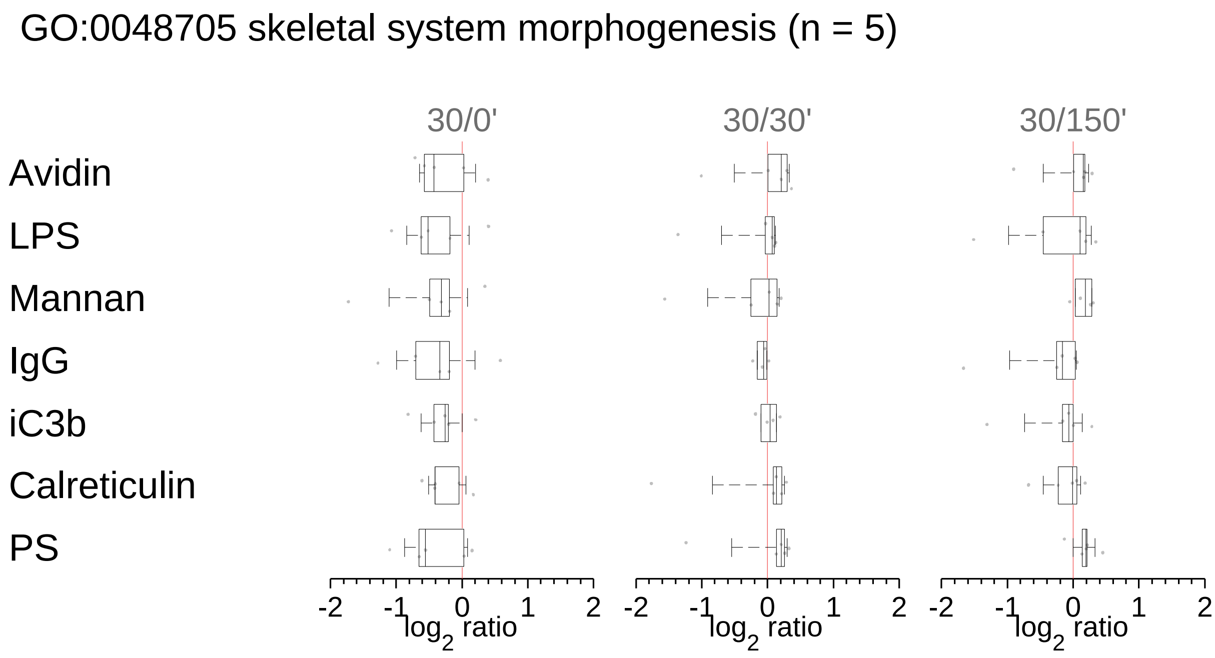
GO:0048705 skeletal system morphogenesis (n = 5)
30/0'
30/30'
30/150'
Avidin
LPS
Mannan
IgG
iC3b
Calreticulin
PS
-2 -1 0 1 2
log2 ratio

GO:0048709 oligodendrocyte differentiation (n = 3)

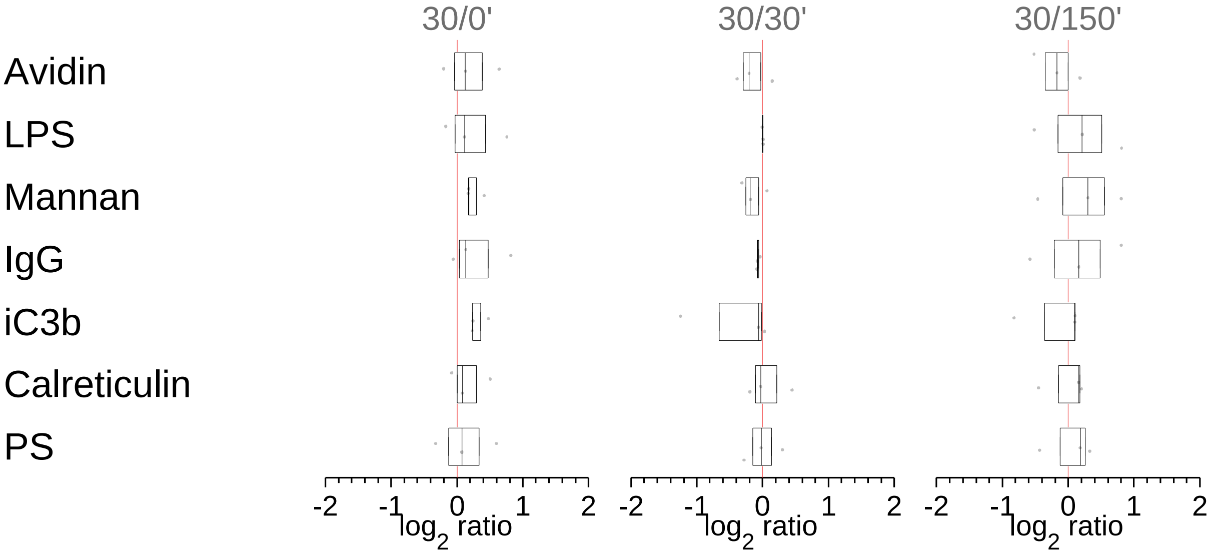

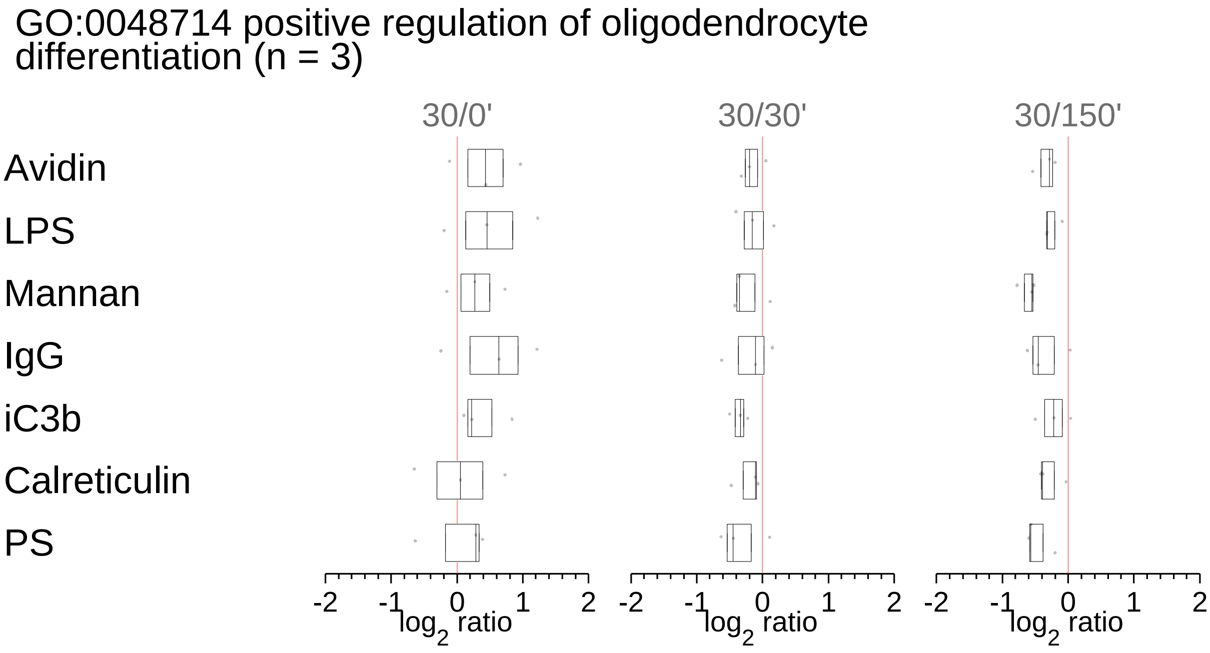

GO:0048714 positive regulation of oligodendrocyte differentiation (n = 3)
30/0'
30/30'
30/150'
Avidin
LPS
Mannan
IgG
iC3b
Calreticulin
PS
-2
-1
0
1
2
log2 ratio

GO:0048738 cardiac muscle tissue development (n = 4)

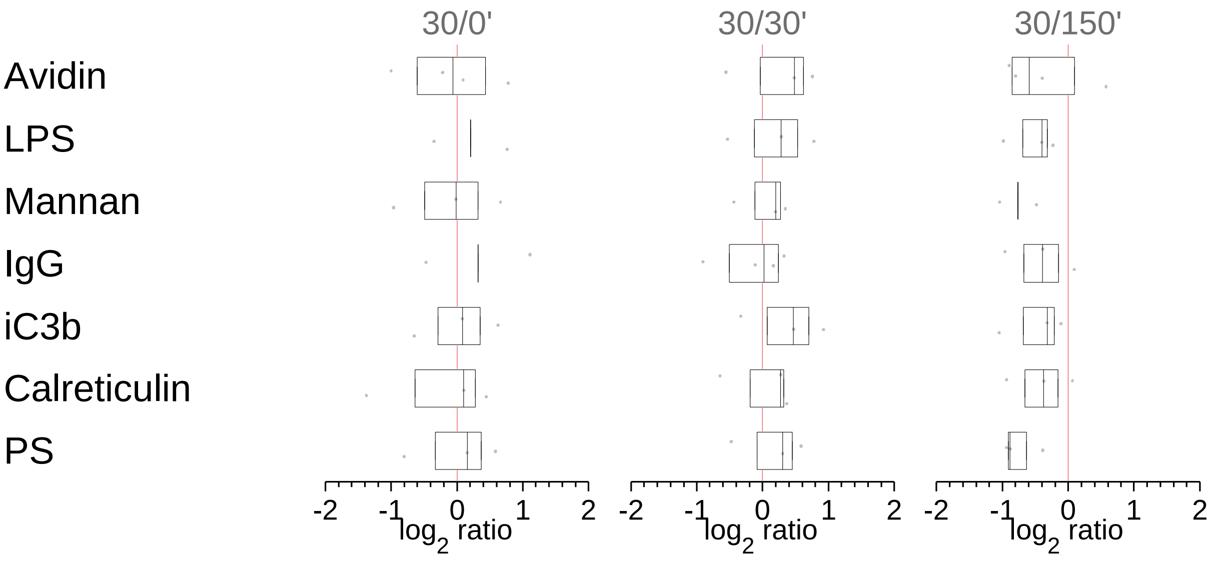

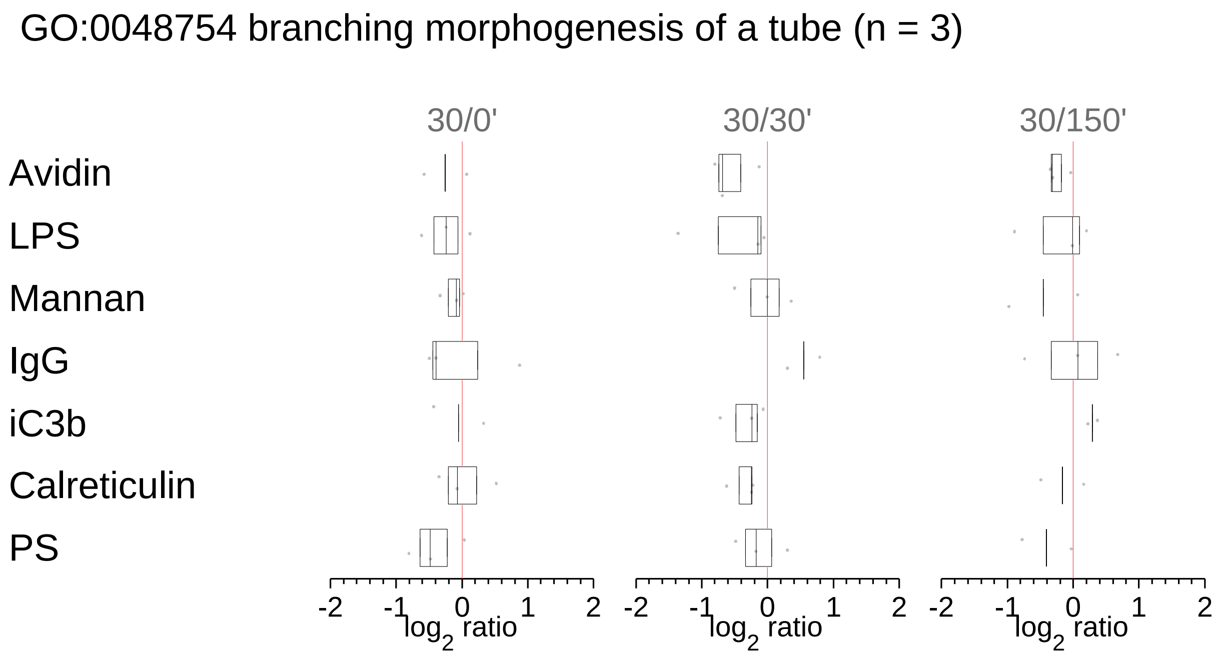
GO:0048754 branching morphogenesis of a tube (n = 3)
30/0'
30/30'
30/150'
Avidin
LPS
Mannan
IgG
iC3b
Calreticulin
PS
-2
-1
0
1
2
log2 ratio

GO:0048812 neuron projection morphogenesis (n = 6)

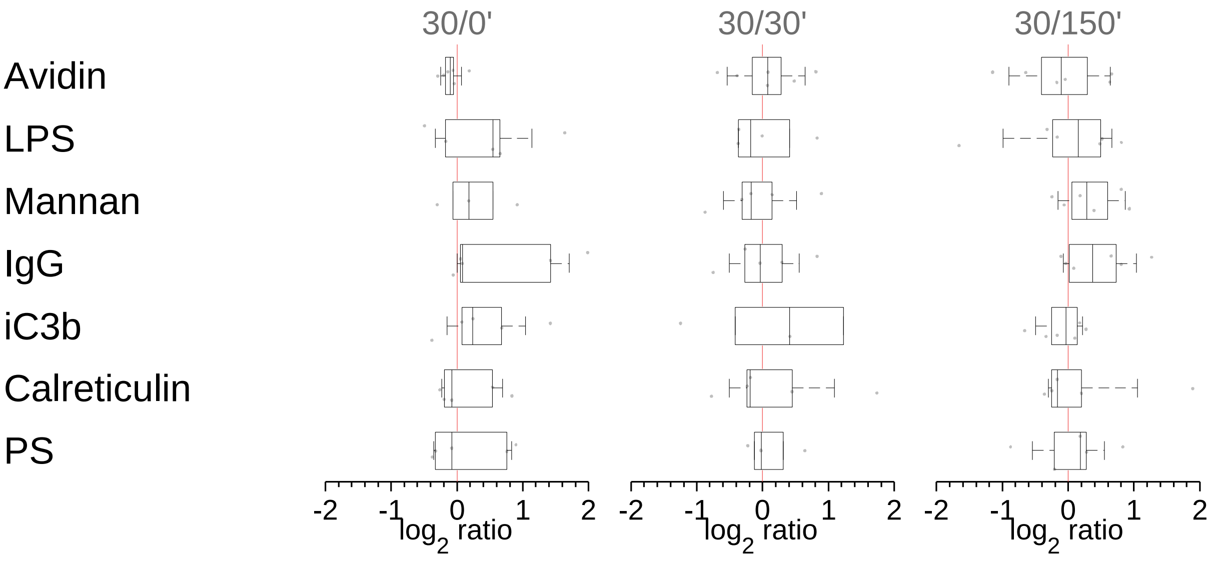

GO:0048813 dendrite morphogenesis (n = 5)

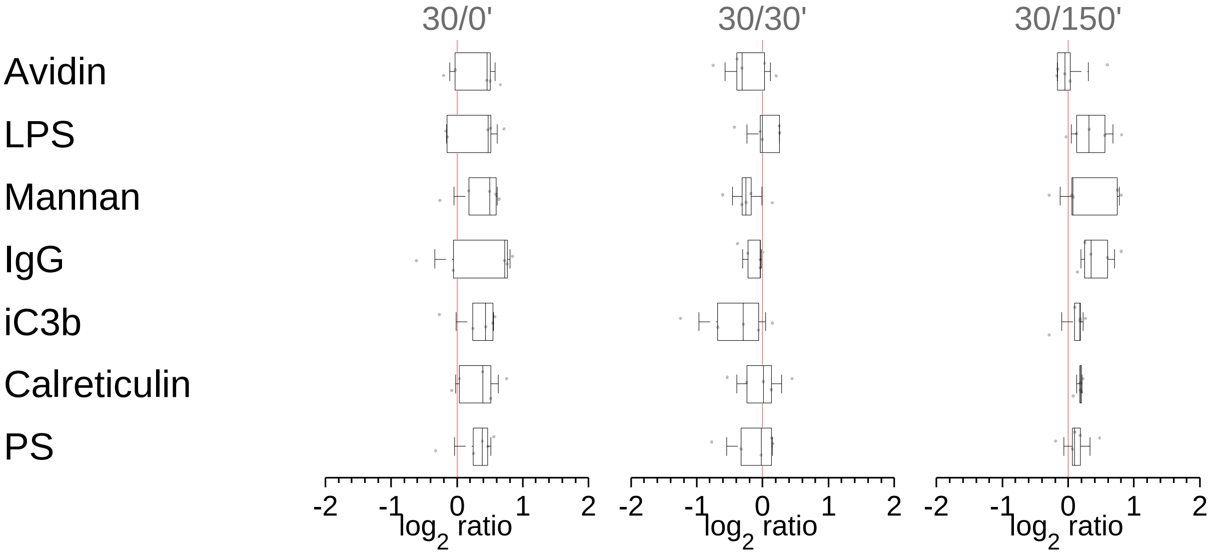

GO:0048814 regulation of dendrite morphogenesis (n = 4)

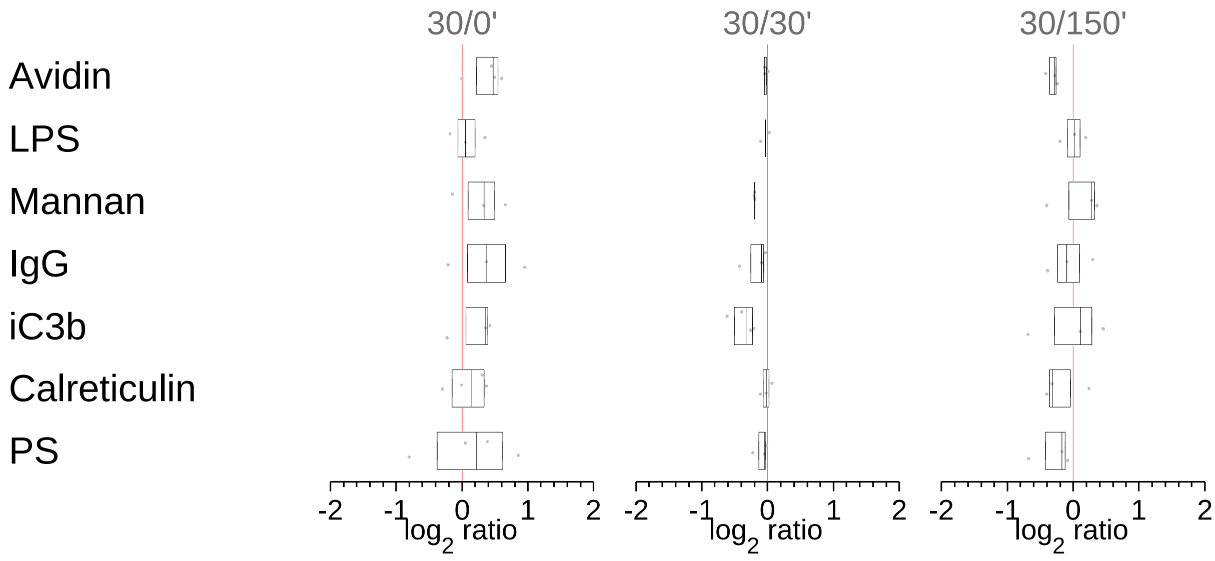

GO:0048839 inner ear development (n = 7)

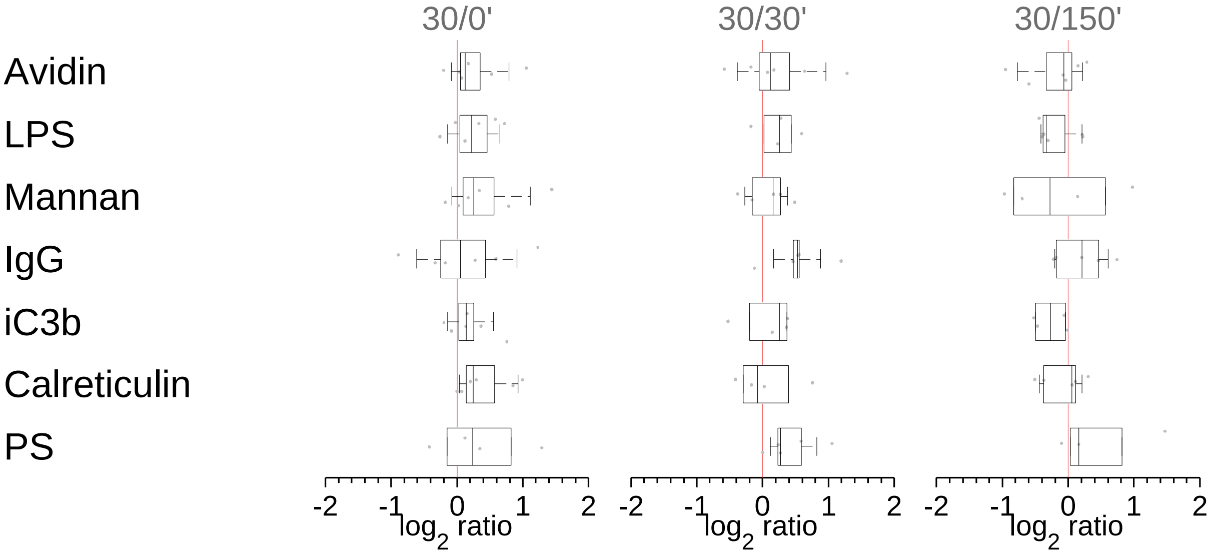

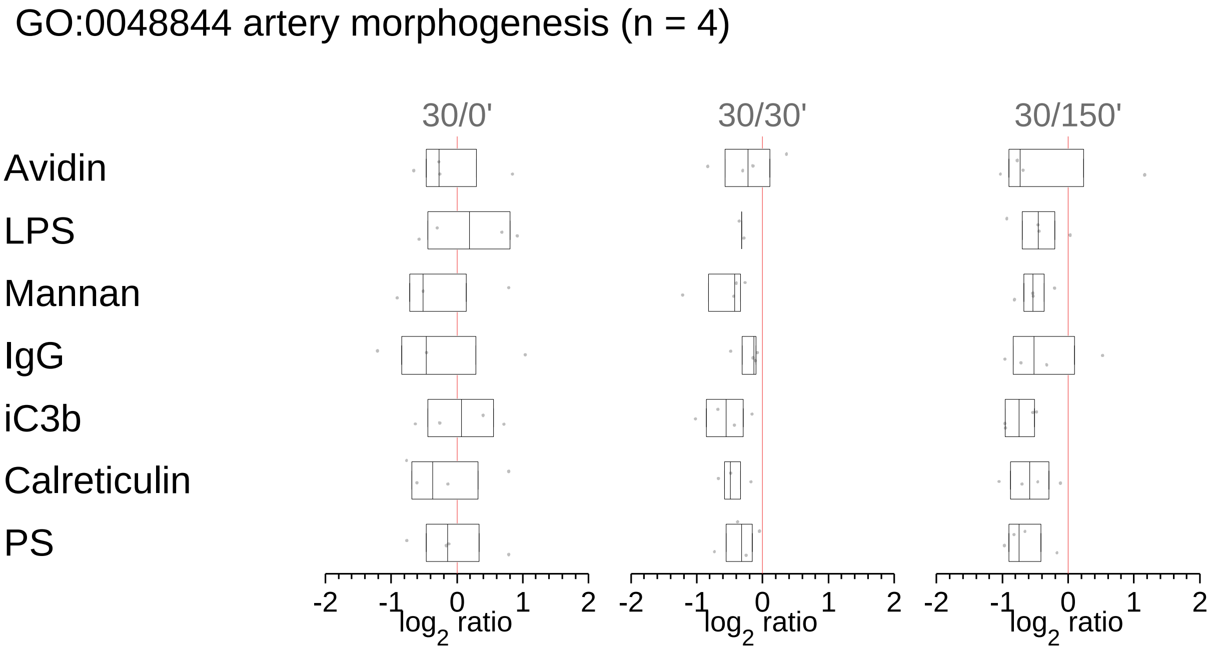
GO:0048844 artery morphogenesis (n = 4)
30/0'
30/30'
30/150'
Avidin
LPS
Mannan
IgG
iC3b
Calreticulin
PS
-2 -1 0 1 2
log2 ratio

GO:0048870 cell motility (n = 3)

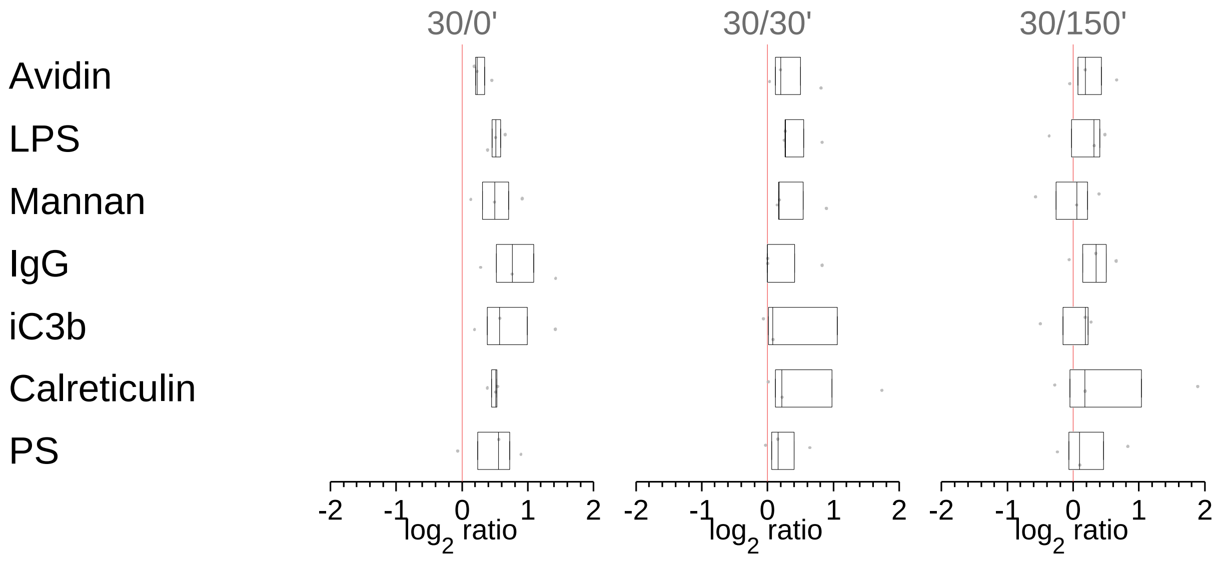

GO:0048872 homeostasis of number of cells (n = 3)

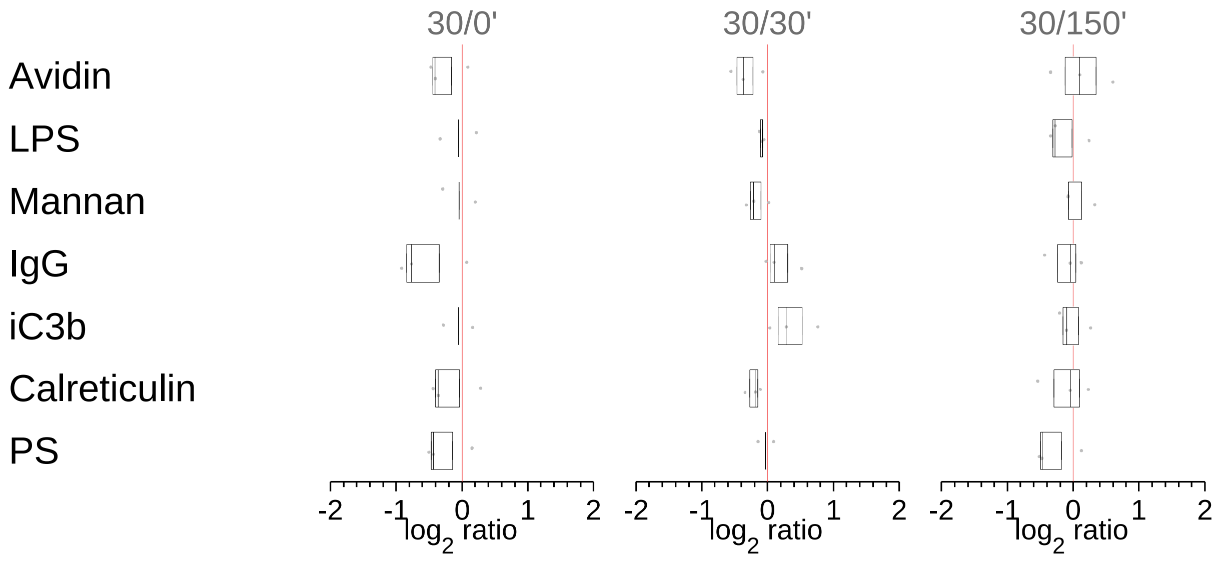

GO:0048873 homeostasis of number of cells within a tissue (n = 7)

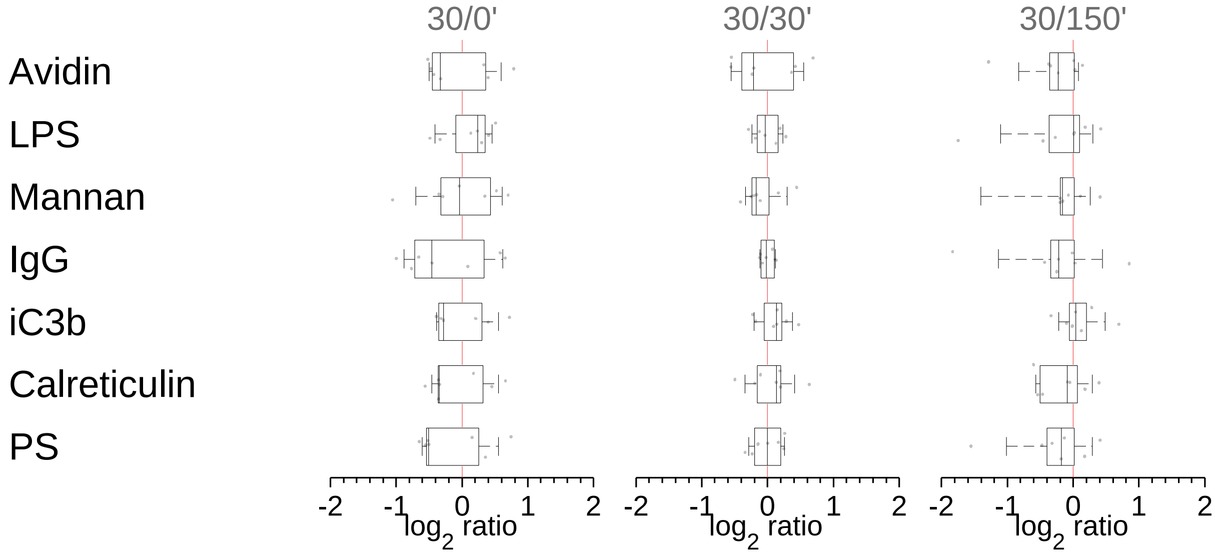

GO:0050253 retinyl-palmitate esterase activity (n = 4)

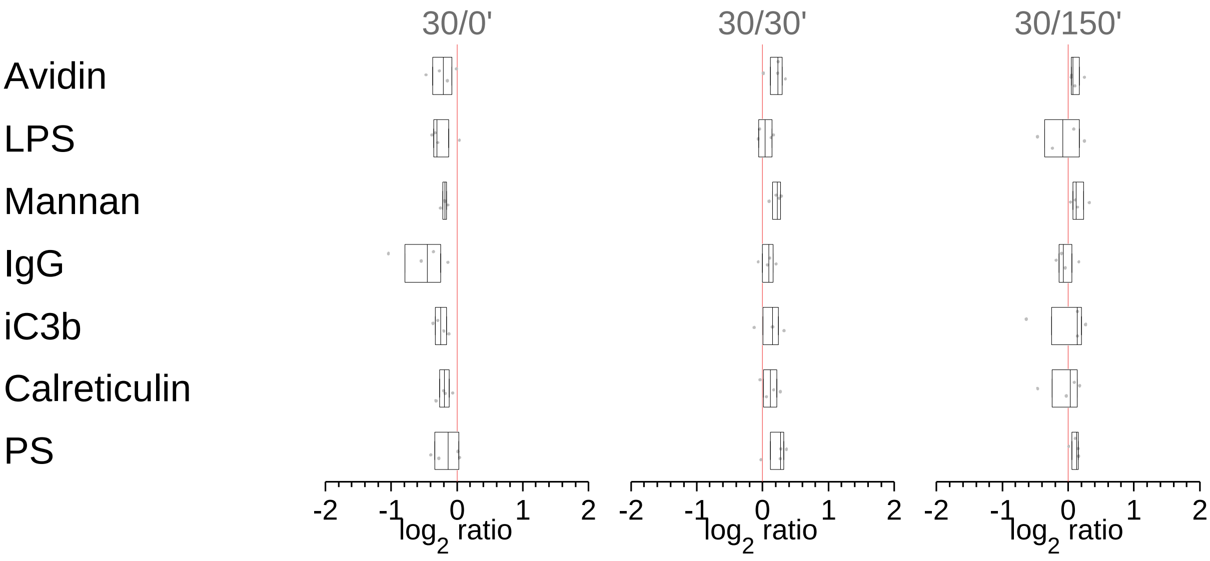

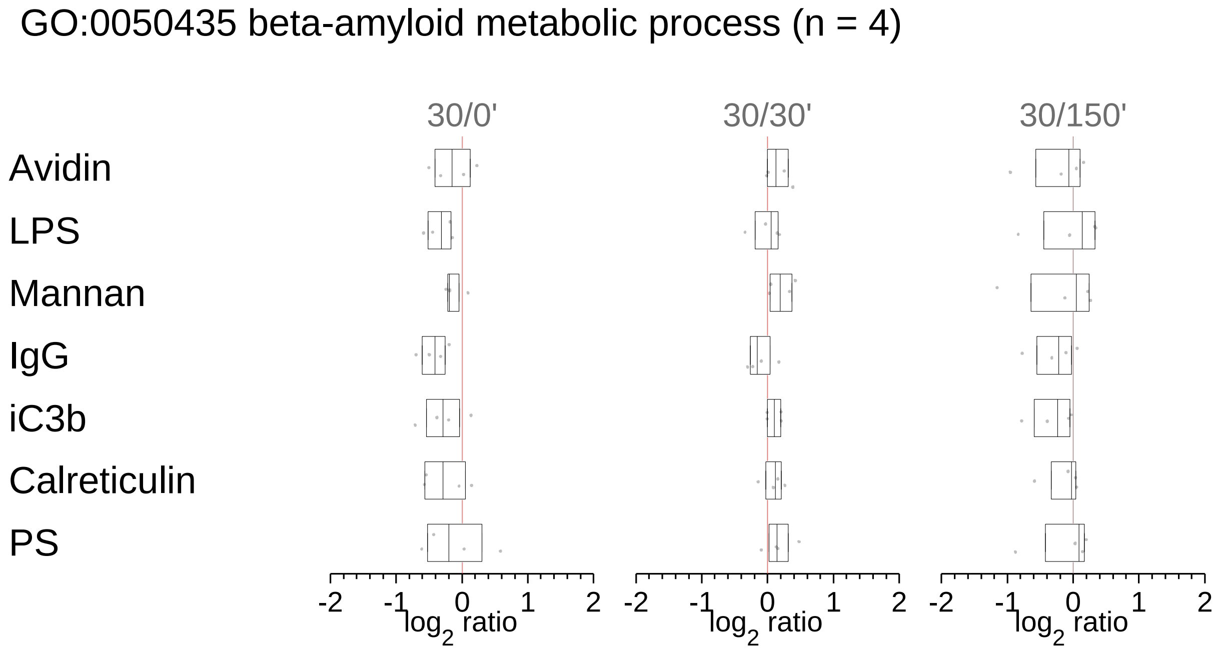
GO:0050435 beta-amyloid metabolic process (n = 4)
30/0'
30/30'
30/150'
Avidin
LPS
Mannan
IgG
iC3b
Calreticulin
PS
-2 -1 0 1 2
log2 ratio

GO:0050660 flavin adenine dinucleotide binding (n = 15)

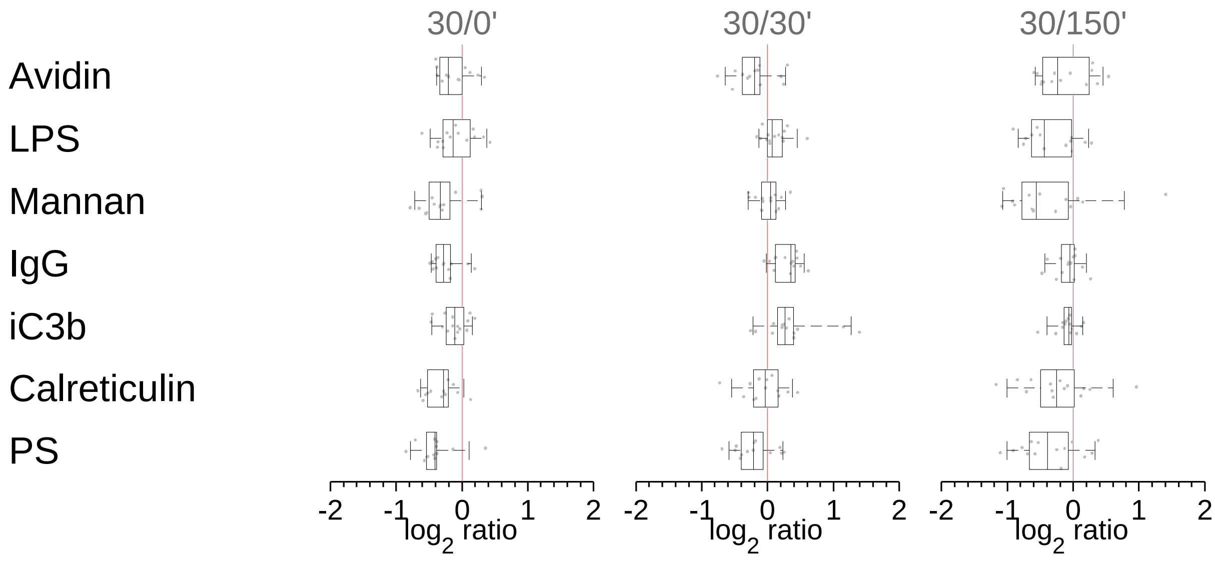

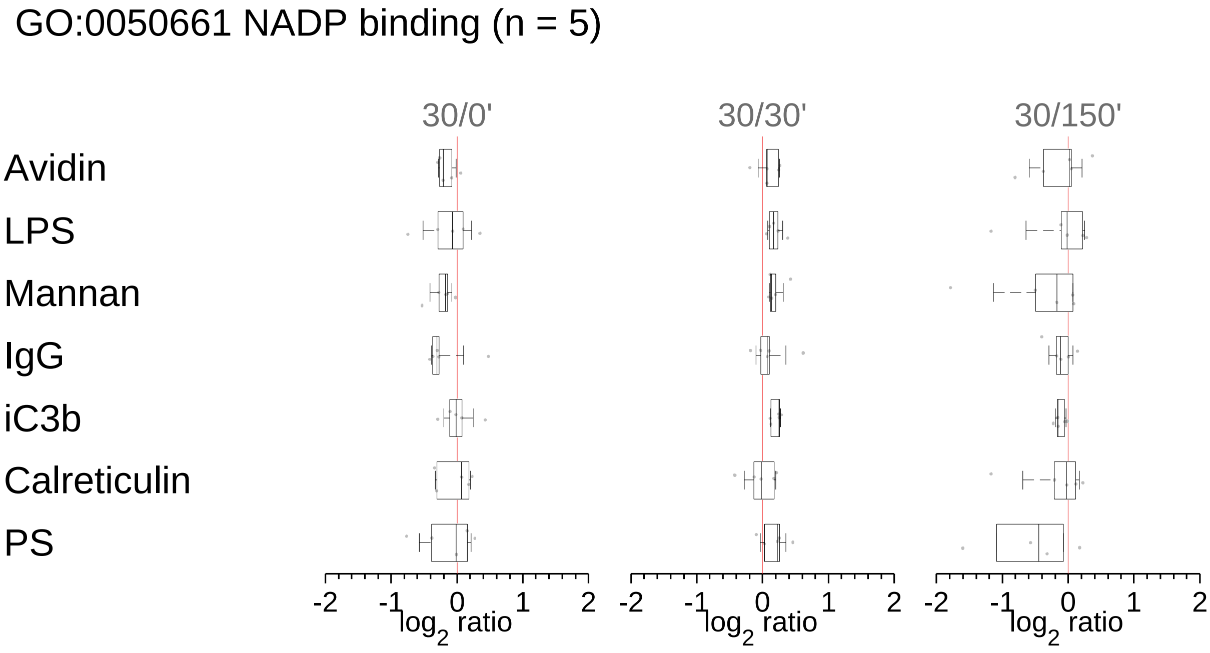
GO:0050661 NADP binding (n = 5)
30/0'
30/30'
30/150'
Avidin
LPS
Mannan
IgG
iC3b
Calreticulin
PS
-2 -1 0 1 2
log2 ratio

GO:0050662 coenzyme binding (n = 3)

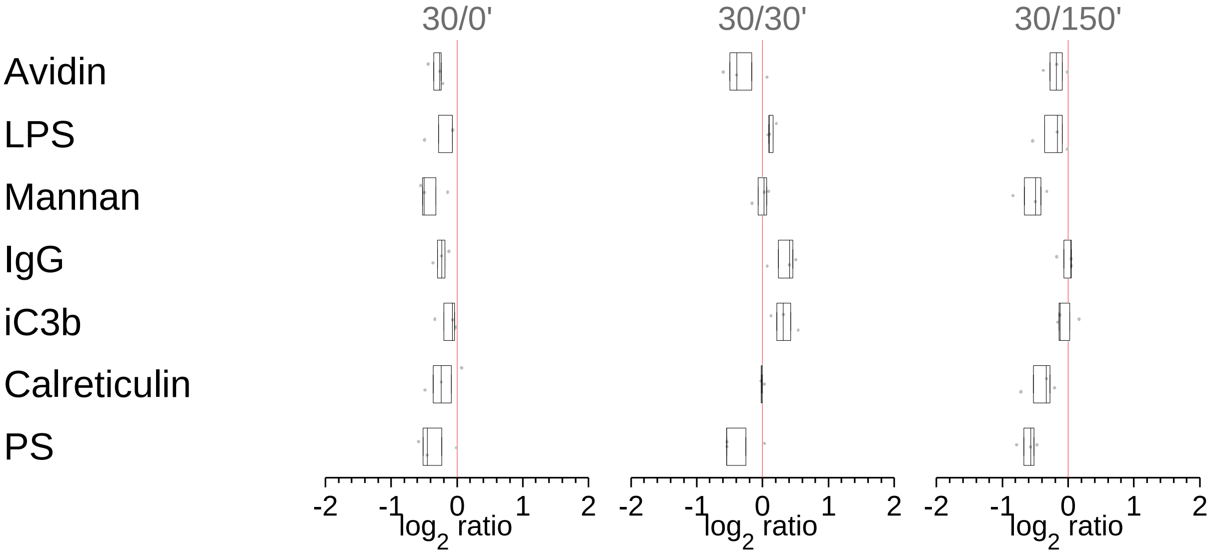

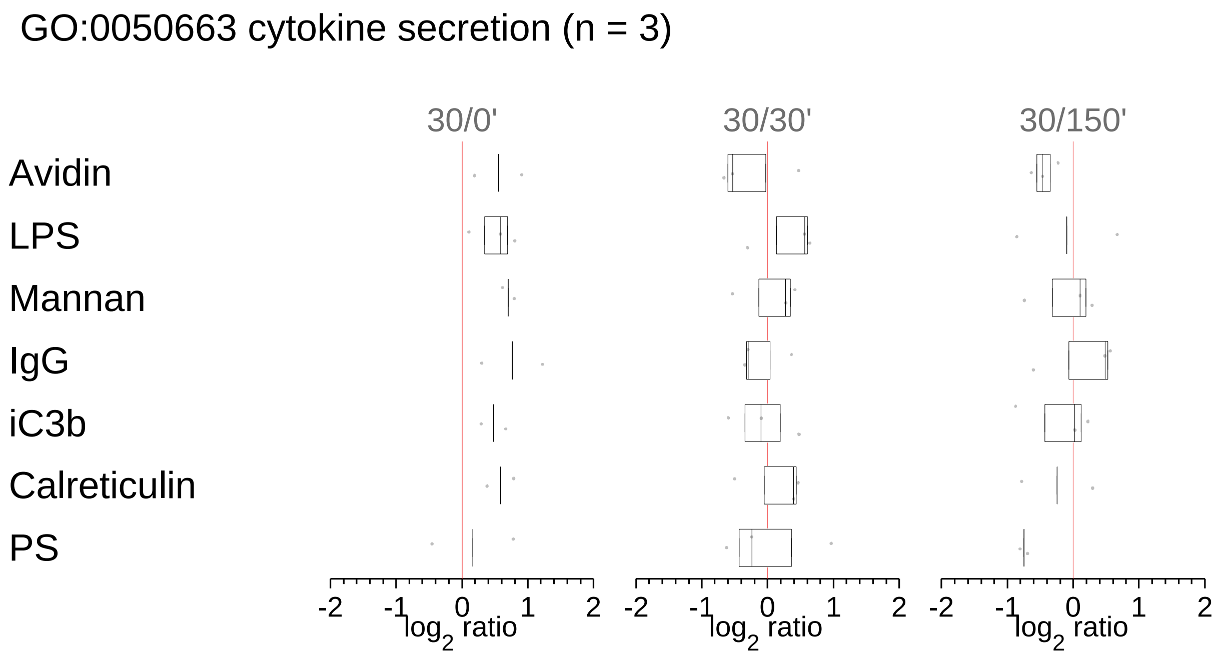
GO:0050663 cytokine secretion (n = 3)
30/0'
30/30'
30/150'
Avidin
LPS
Mannan
IgG
iC3b
Calreticulin
PS
-2
-1
0
1
2
$\log_2$ ratio

GO:0050665 hydrogen peroxide biosynthetic process (n = 5)

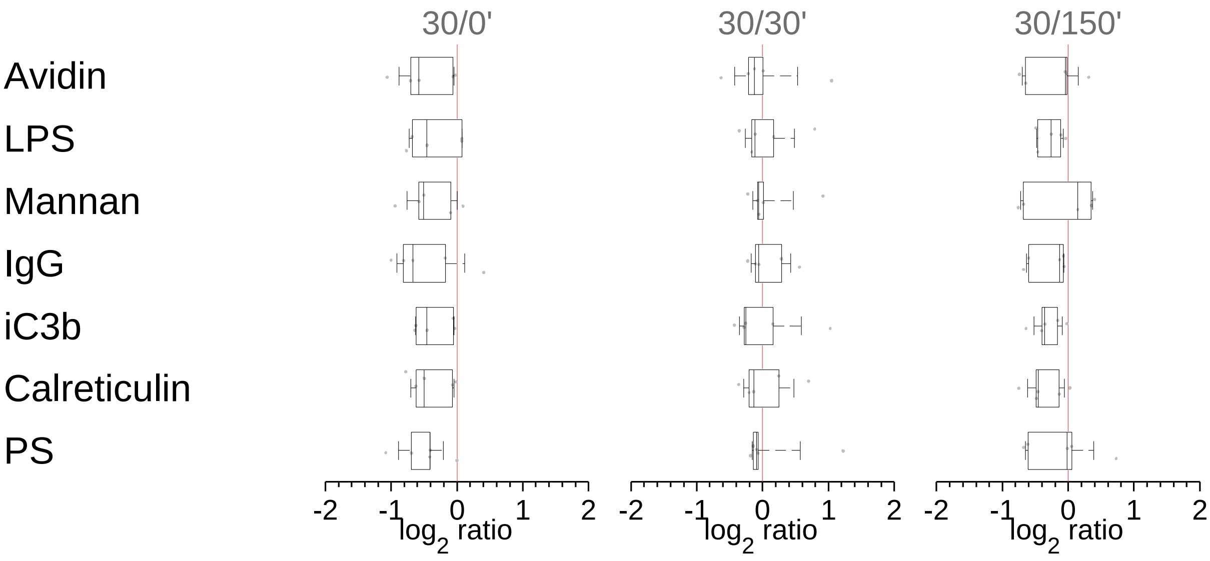

GO:0050679 positive regulation of epithelial cell proliferation
(n = 4)

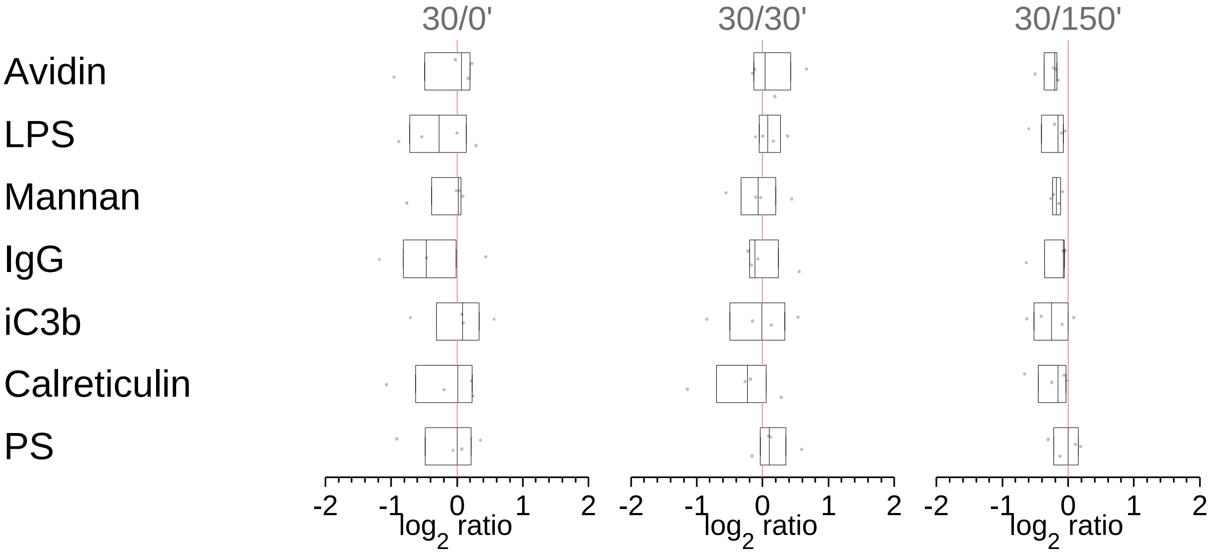

GO:0050680 negative regulation of epithelial cell proliferation
(n = 4)

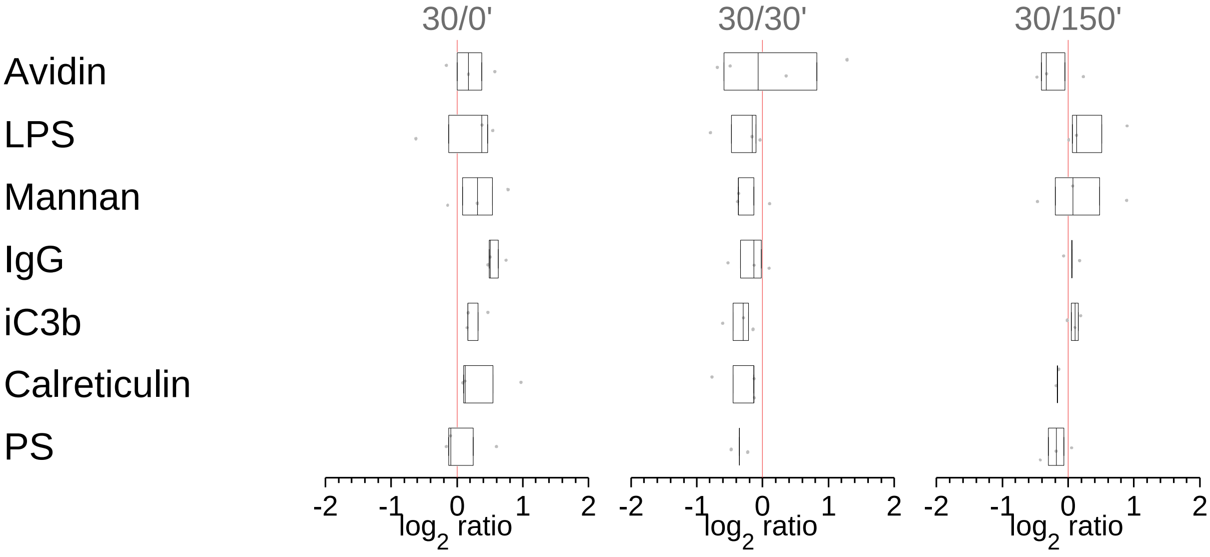

GO:0050681 androgen receptor binding (n = 5)

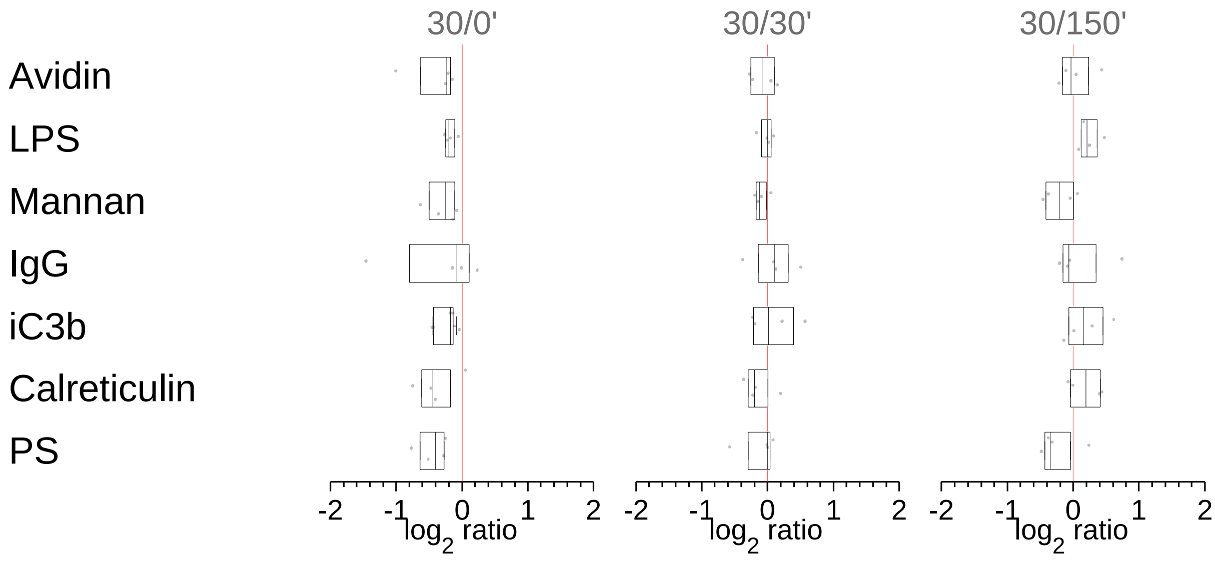

GO:0050707 regulation of cytokine secretion (n = 4)

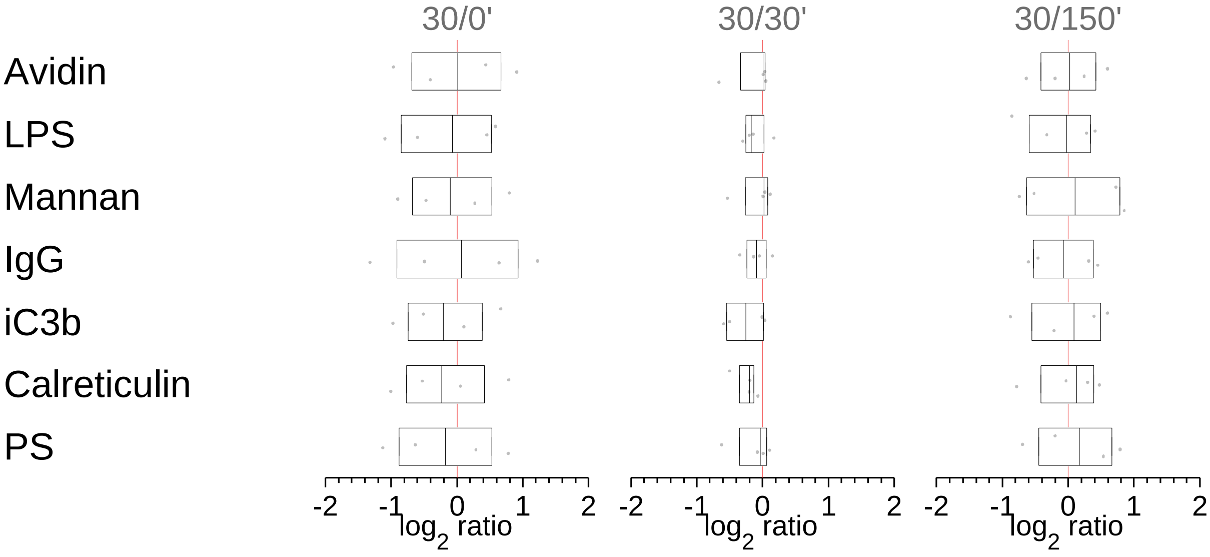

GO:0050709 negative regulation of protein secretion (n = 3)

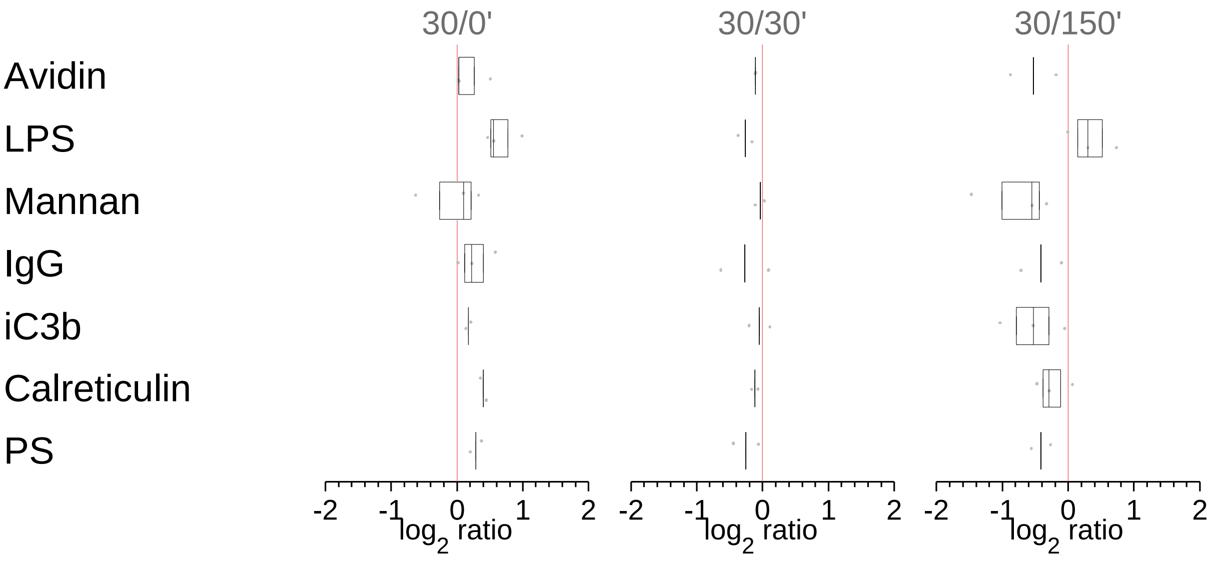

GO:0050714 positive regulation of protein secretion (n = 3)

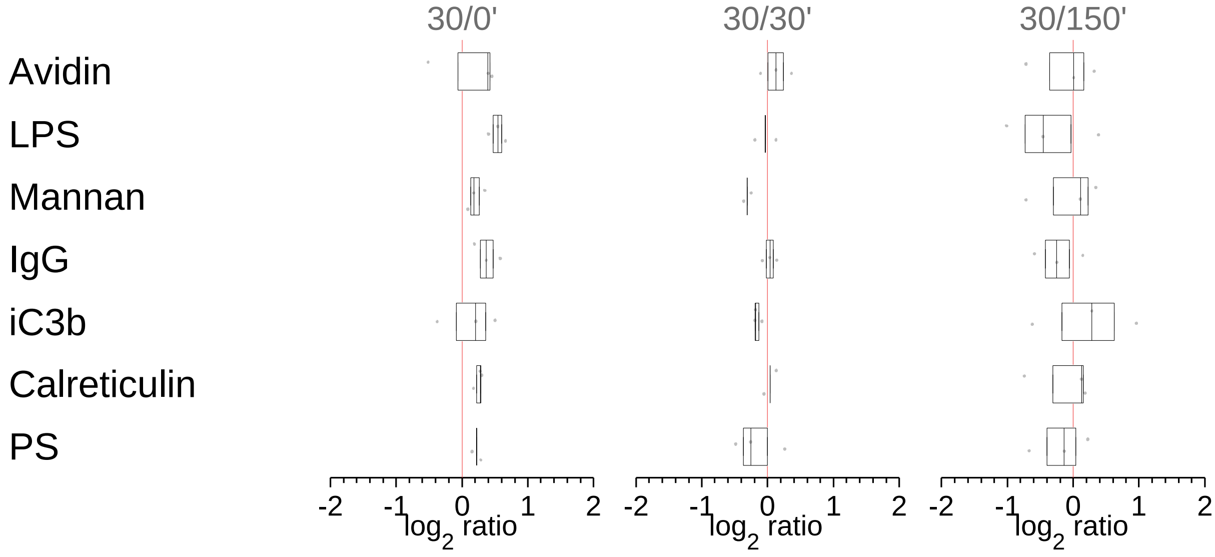

GO:0050715 positive regulation of cytokine secretion (n = 8)

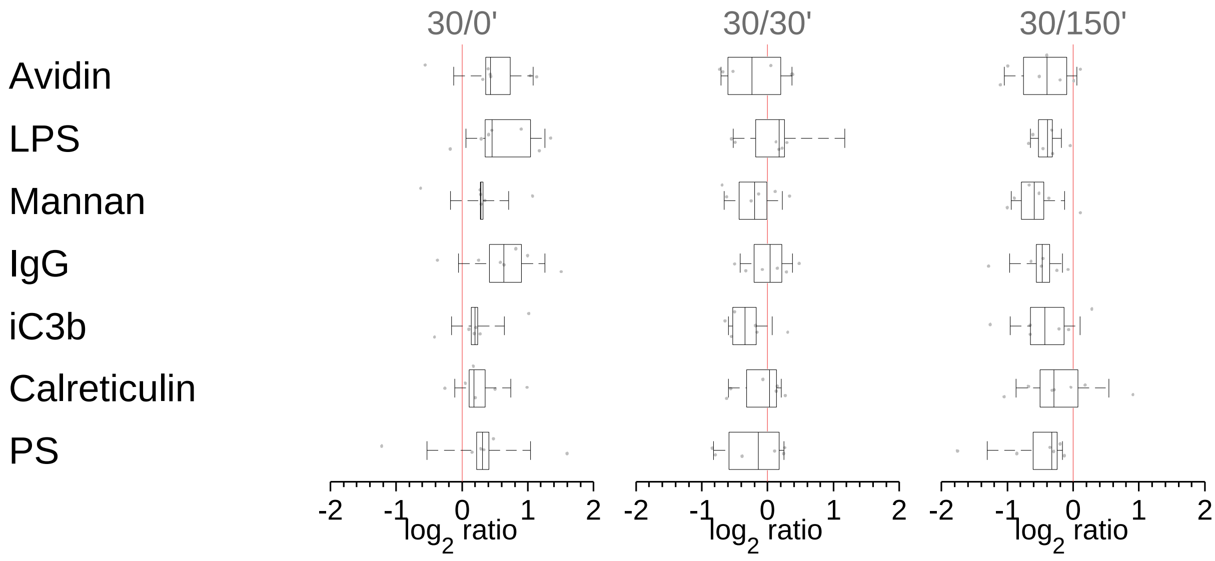

GO:0050727 regulation of inflammatory response (n = 4)

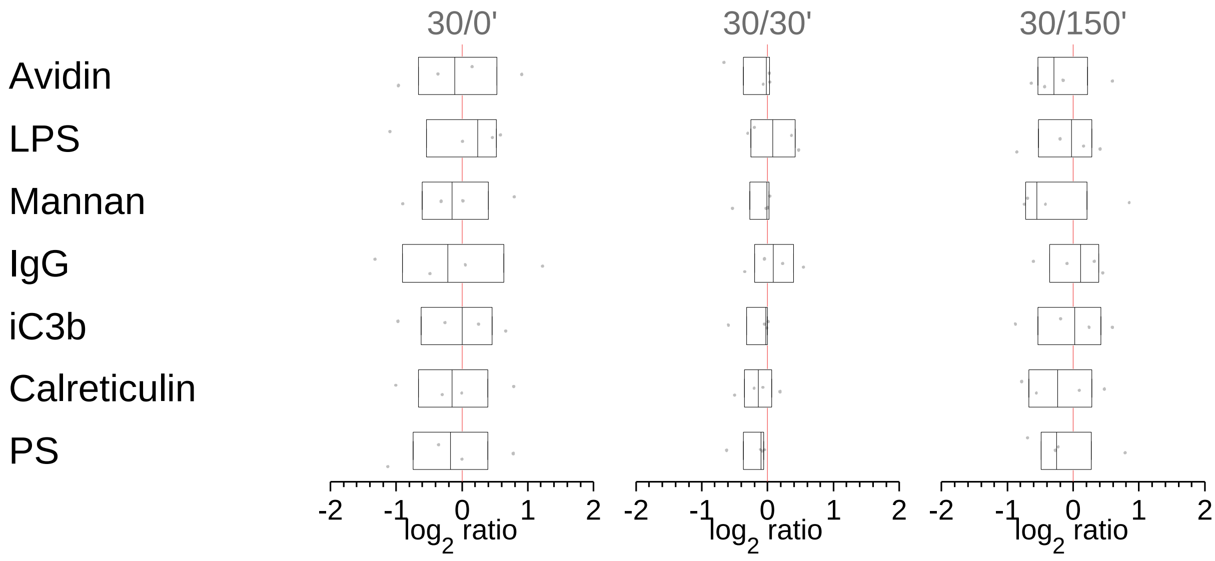

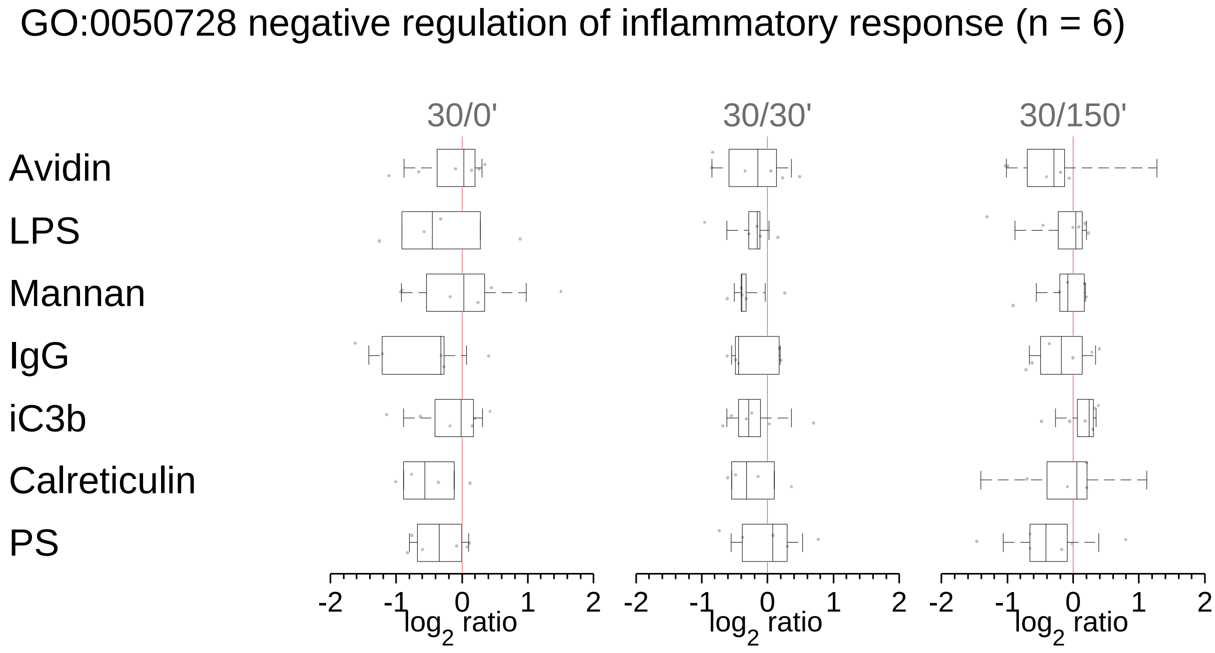
GO:0050728 negative regulation of inflammatory response (n = 6)
30/0'
30/30'
30/150'
Avidin
LPS
Mannan
IgG
iC3b
Calreticulin
PS
-2
-1
0
1
2
log2 ratio

GO:0050729 positive regulation of inflammatory response (n = 10)

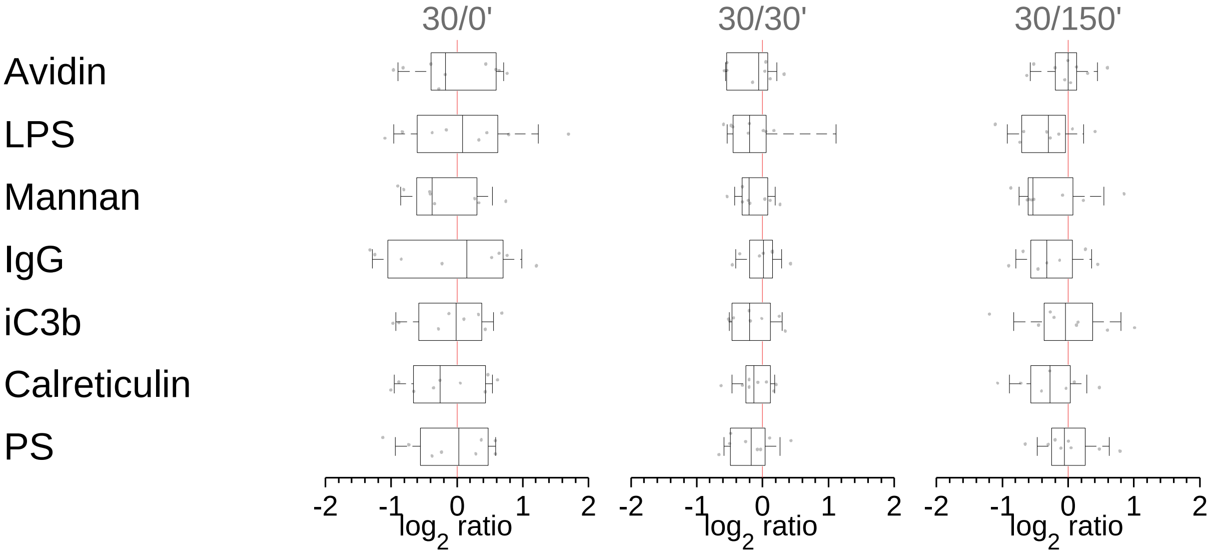

GO:0050731 positive regulation of peptidyl-tyrosine phosphorylation (n = 18)

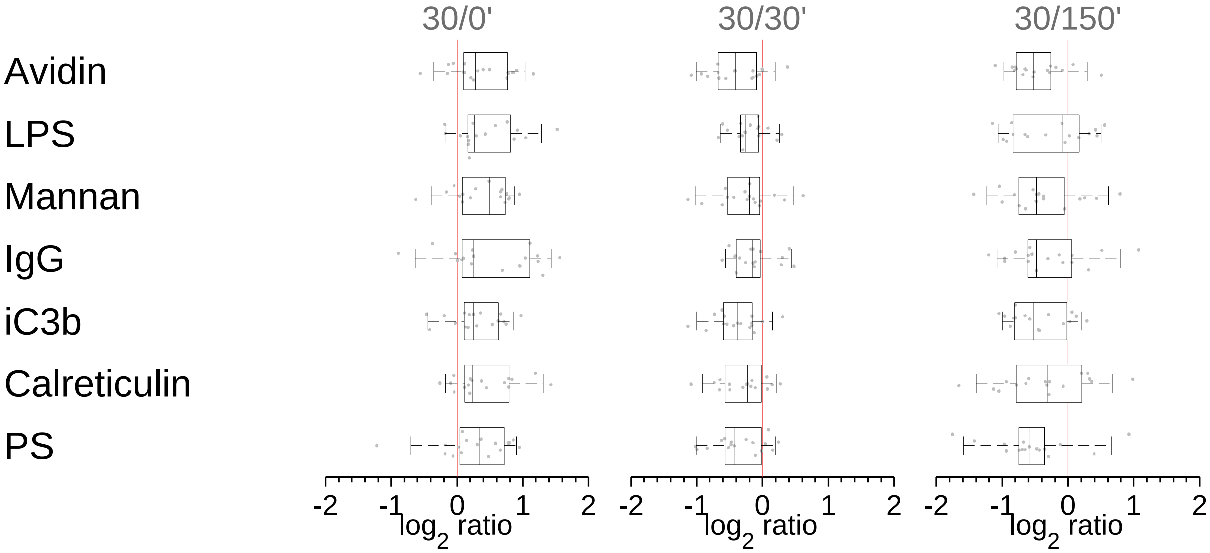

GO:0050732 negative regulation of peptidyl-tyrosine phosphorylation (n = 4)

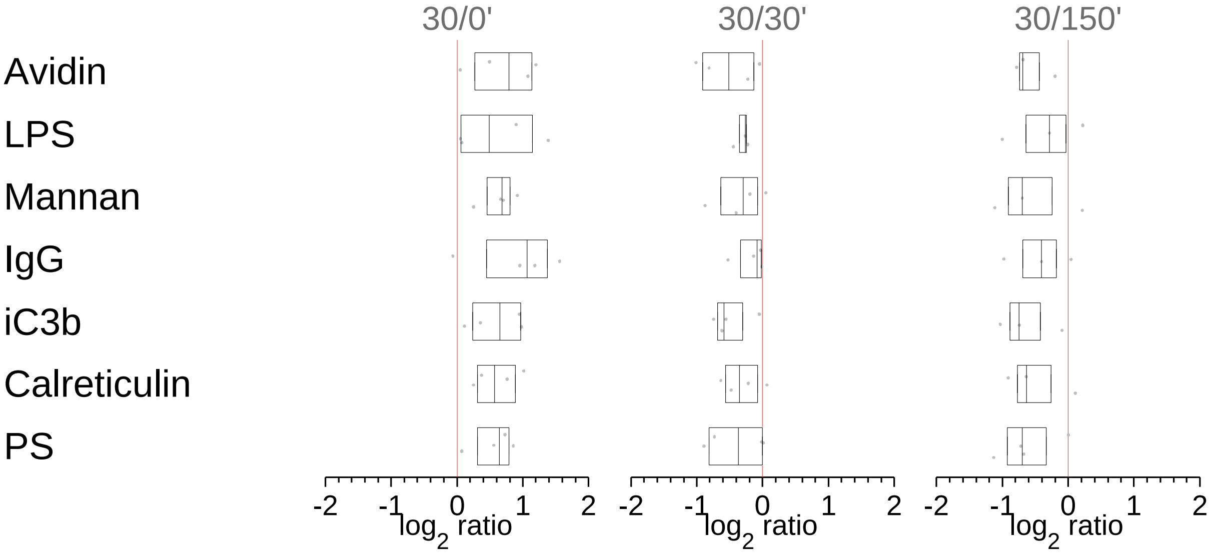

GO:0050750 low-density lipoprotein particle receptor binding (n = 8)

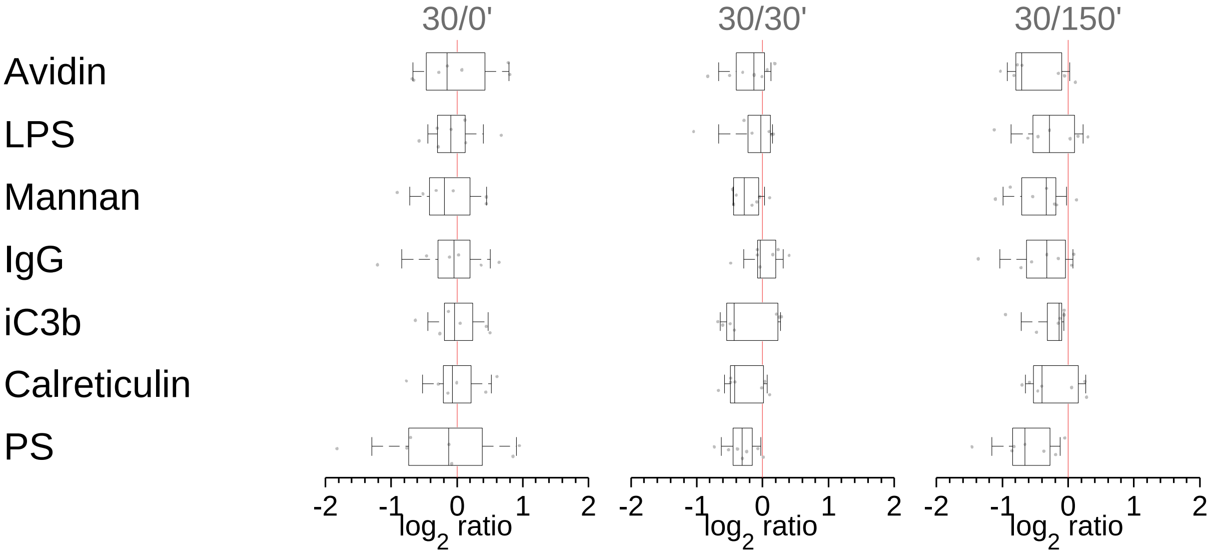

GO:0050764 regulation of phagocytosis (n = 4)

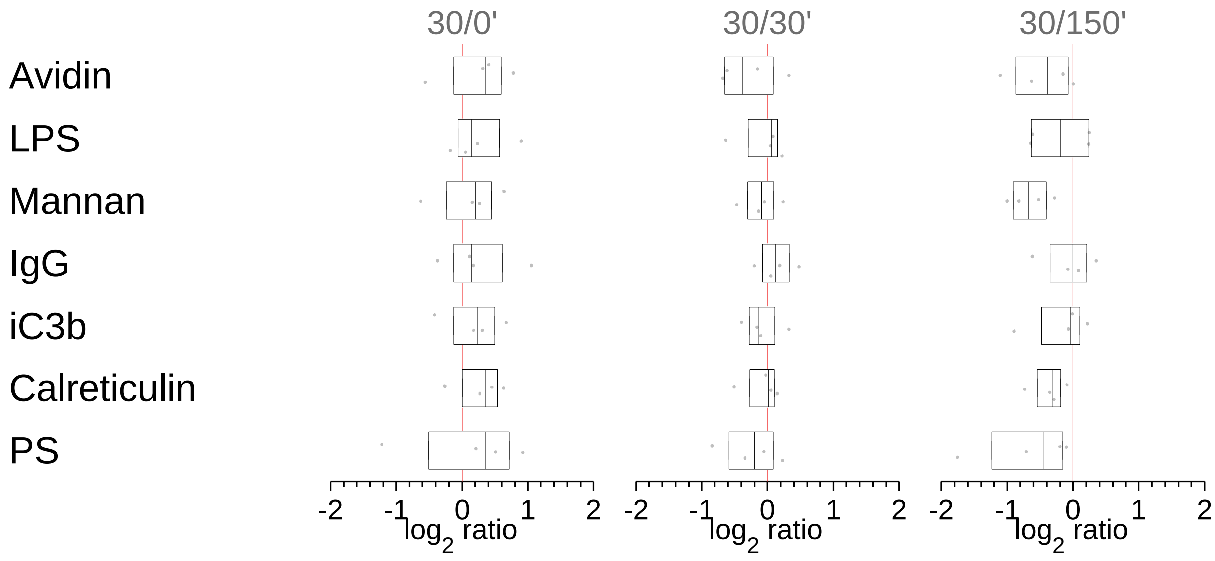

GO:0050765 negative regulation of phagocytosis (n = 4)

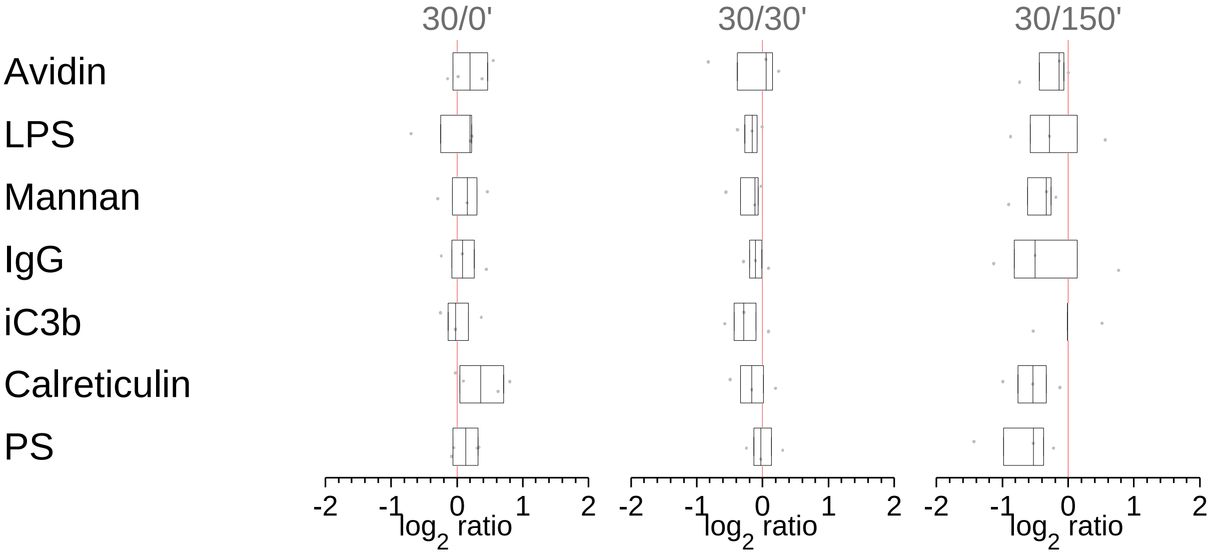

GO:0050766 positive regulation of phagocytosis (n = 14)

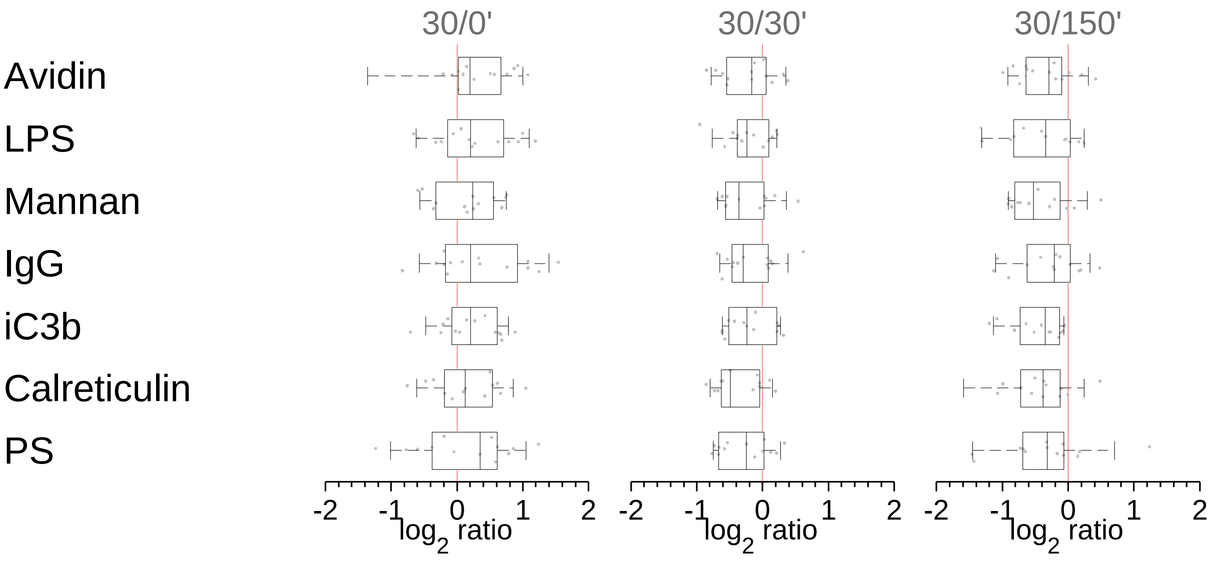

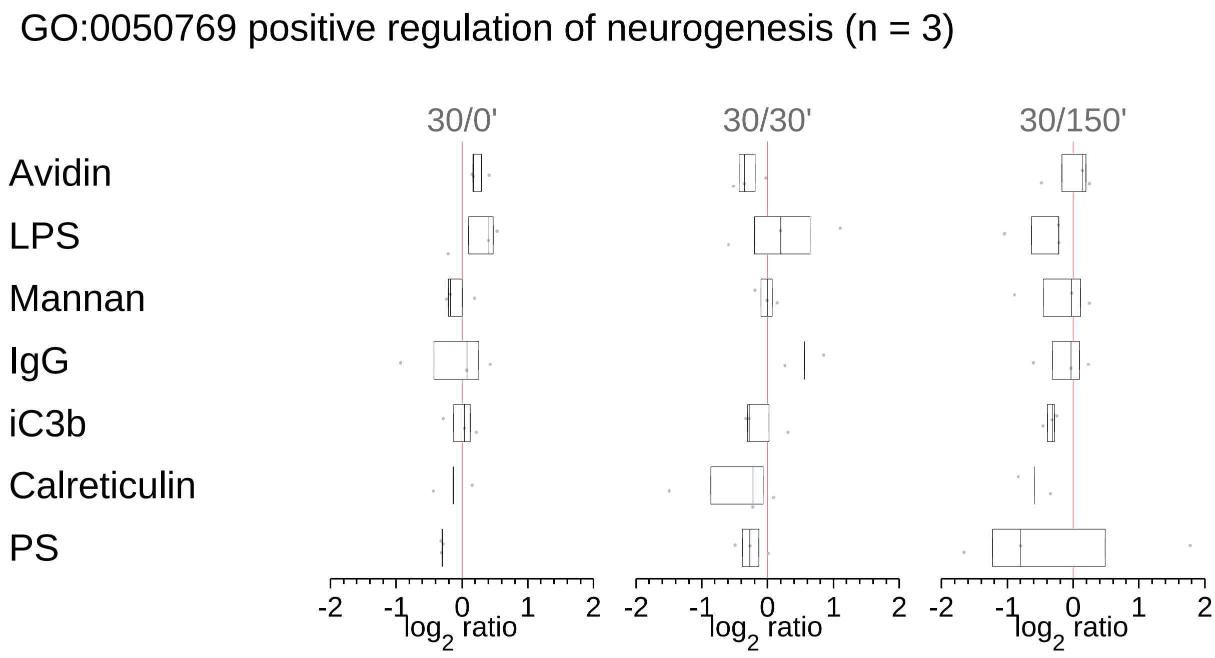
GO:0050769 positive regulation of neurogenesis (n = 3)
30/0'
30/30'
30/150'
Avidin
LPS
Mannan
IgG
iC3b
Calreticulin
PS
-2
-1
0
1
2
log2 ratio

GO:0050772 positive regulation of axonogenesis (n = 4)

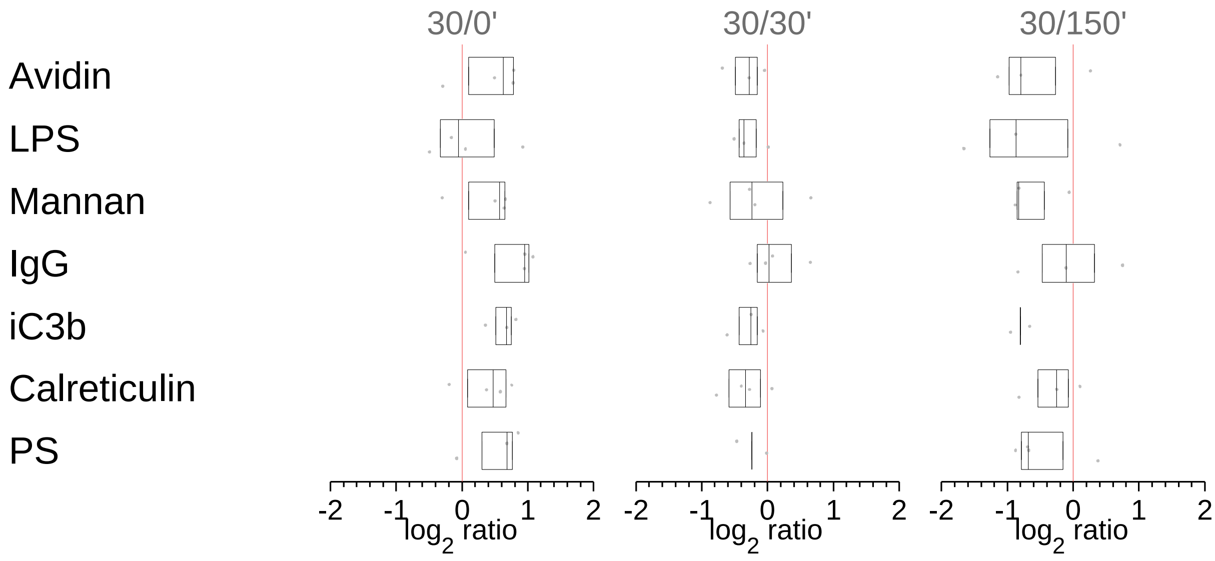

GO:0050775 positive regulation of dendrite morphogenesis (n = 6)

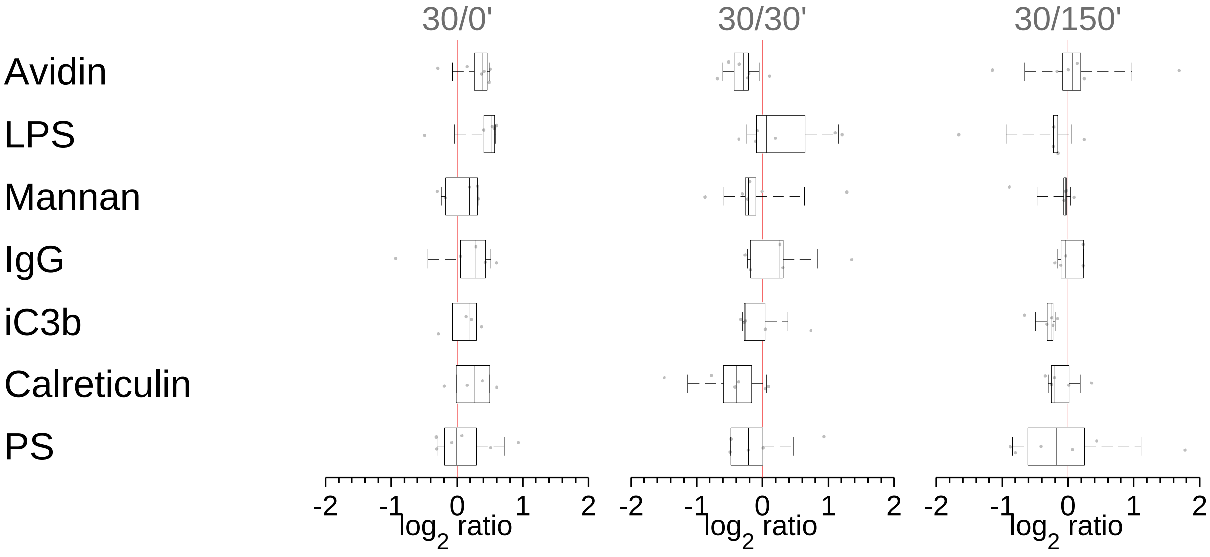

GO:0050776 regulation of immune response (n = 9)

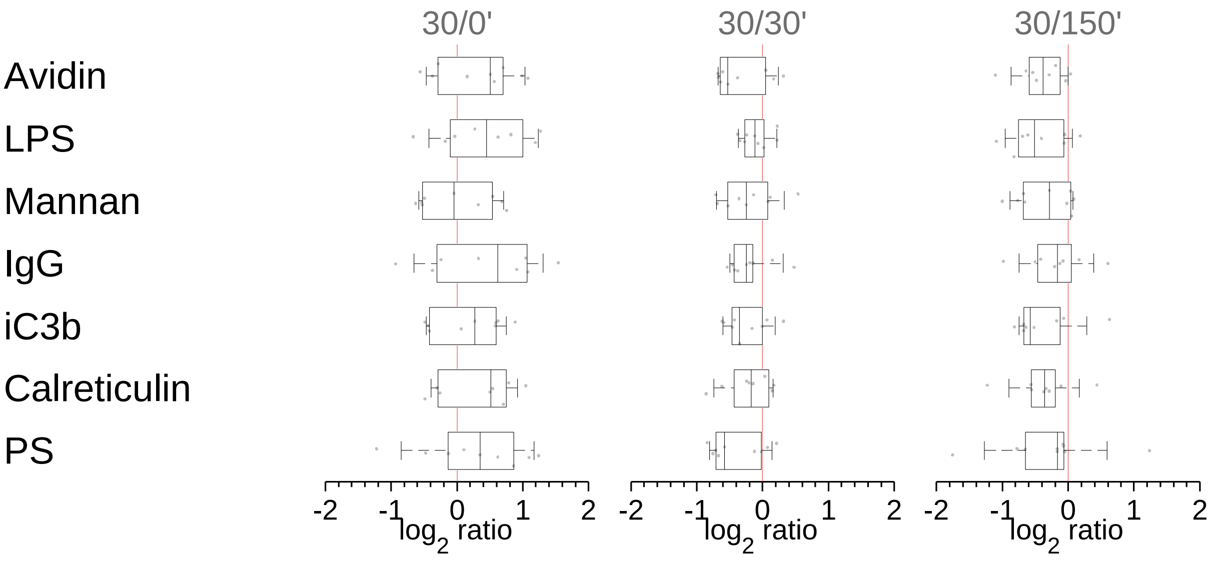

GO:0050786 RAGE receptor binding (n = 3)

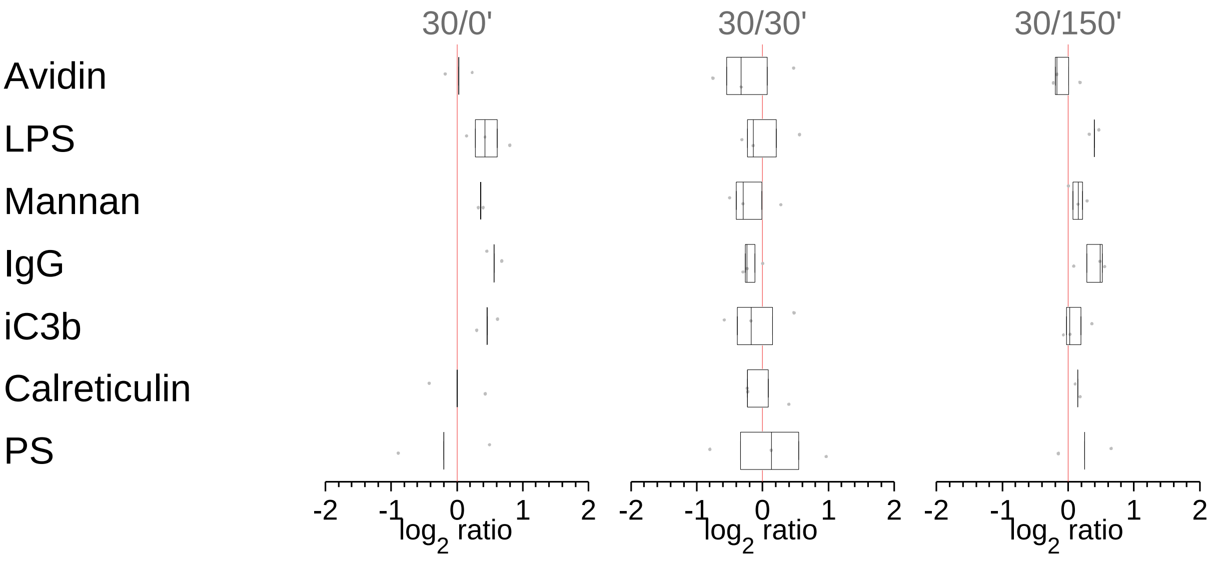

GO:0050790 regulation of catalytic activity (n = 11)

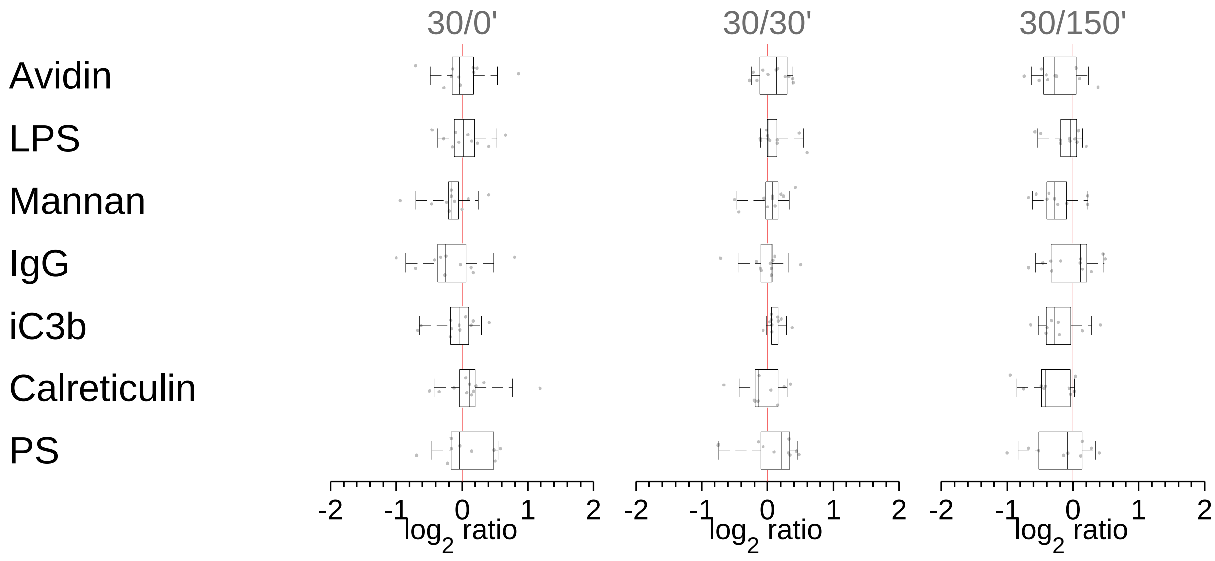

GO:0050792 regulation of viral reproduction (n = 3)

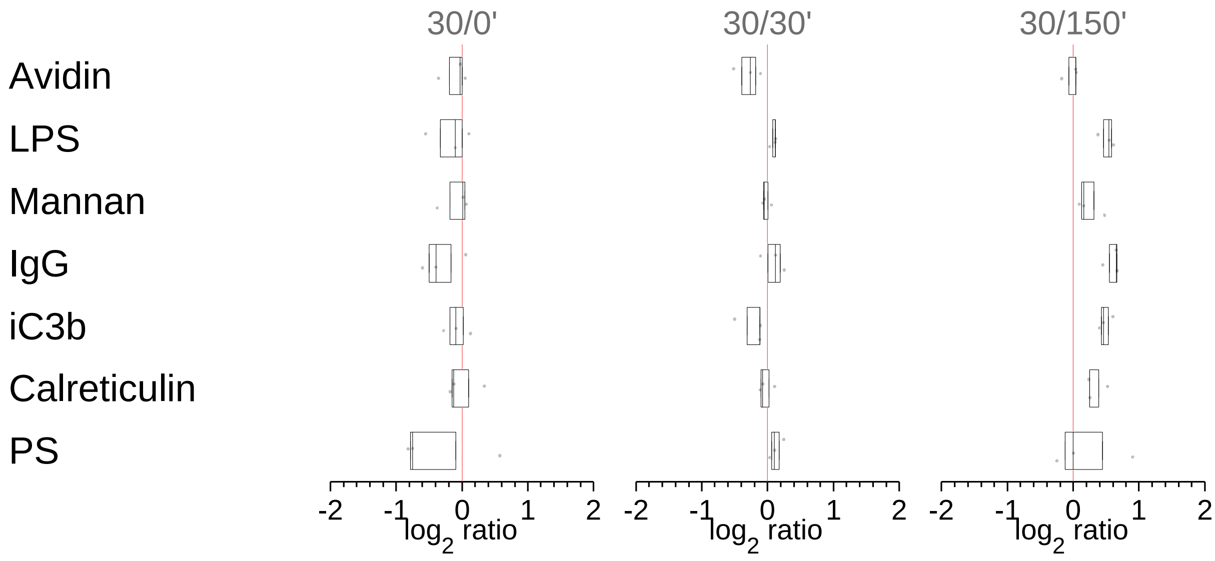

GO:0050796 regulation of insulin secretion (n = 4)

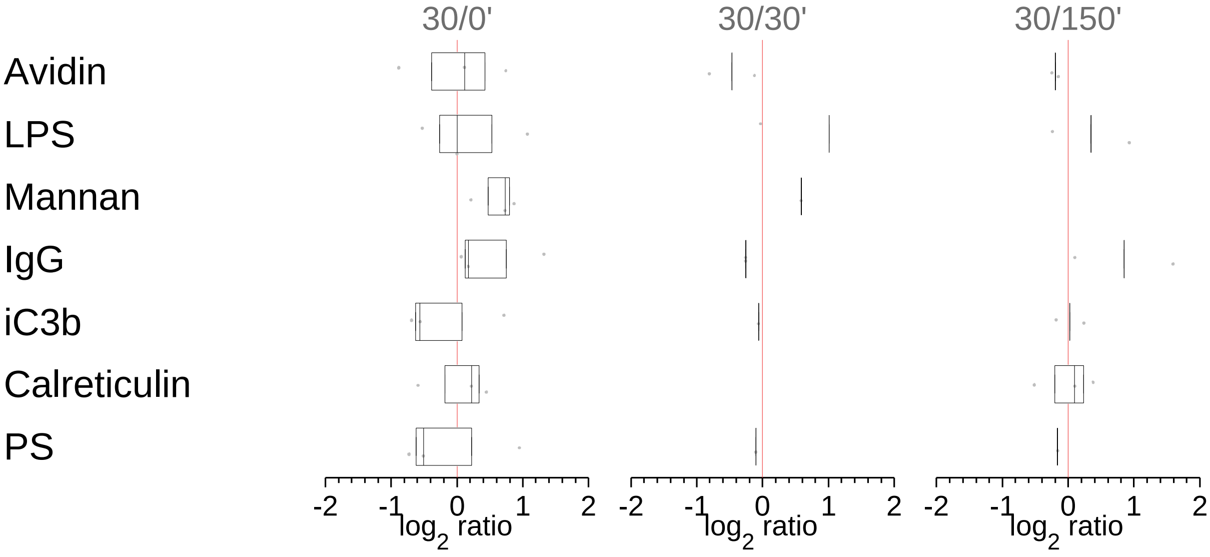

GO:0050798 activated T cell proliferation (n = 4)

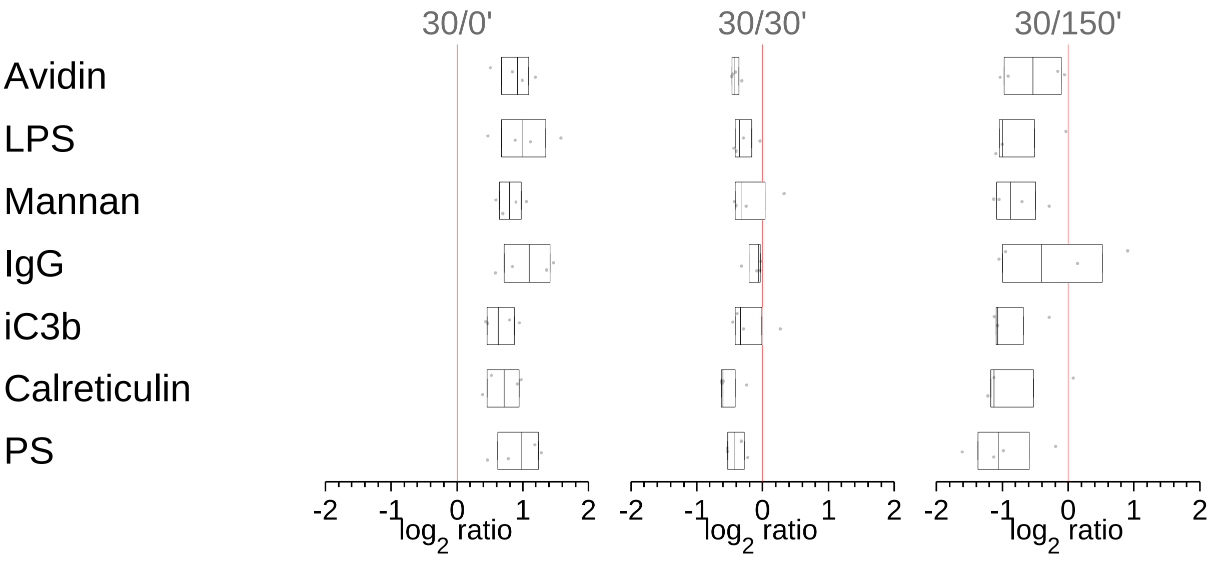

GO:0050821 protein stabilization (n = 15)

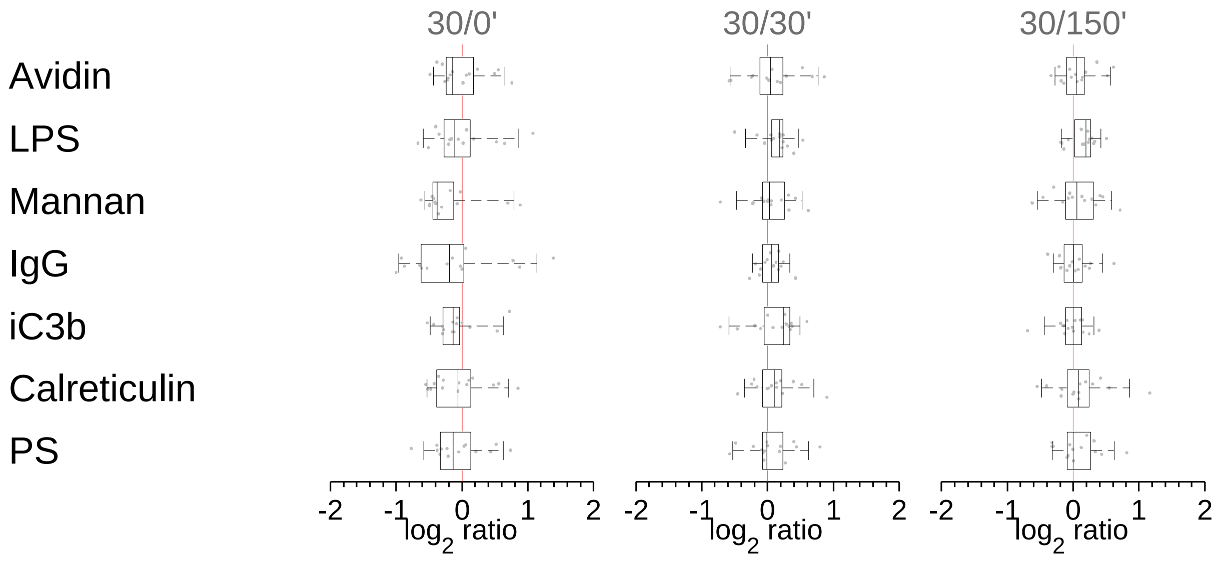

GO:0050829 defense response to Gram-negative bacterium (n = 4)

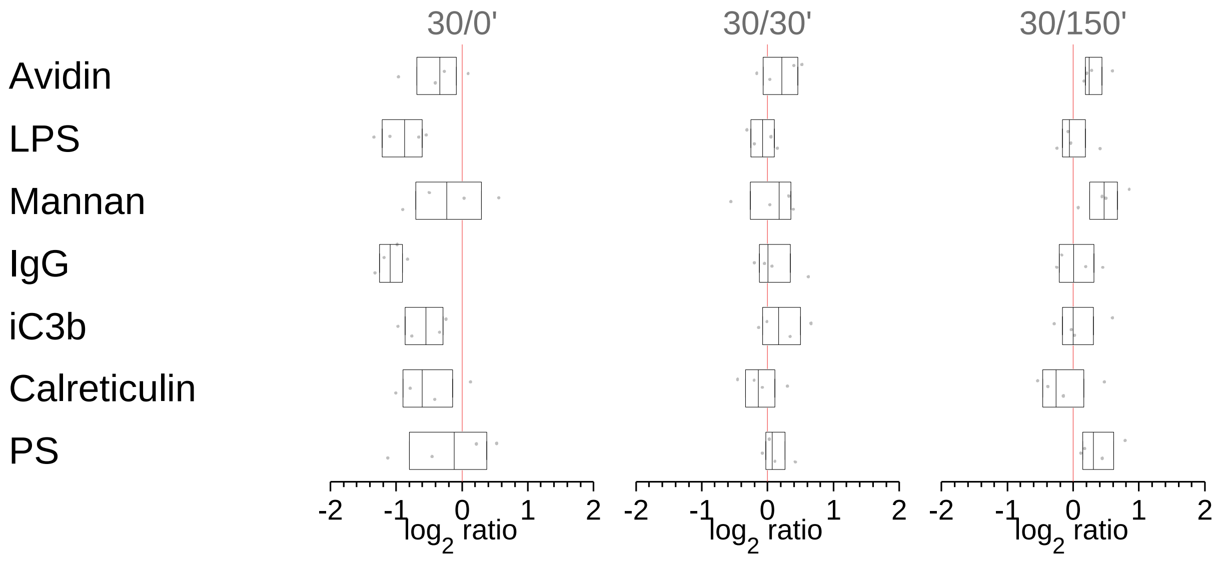

GO:0050830 defense response to Gram-positive bacterium (n = 13)

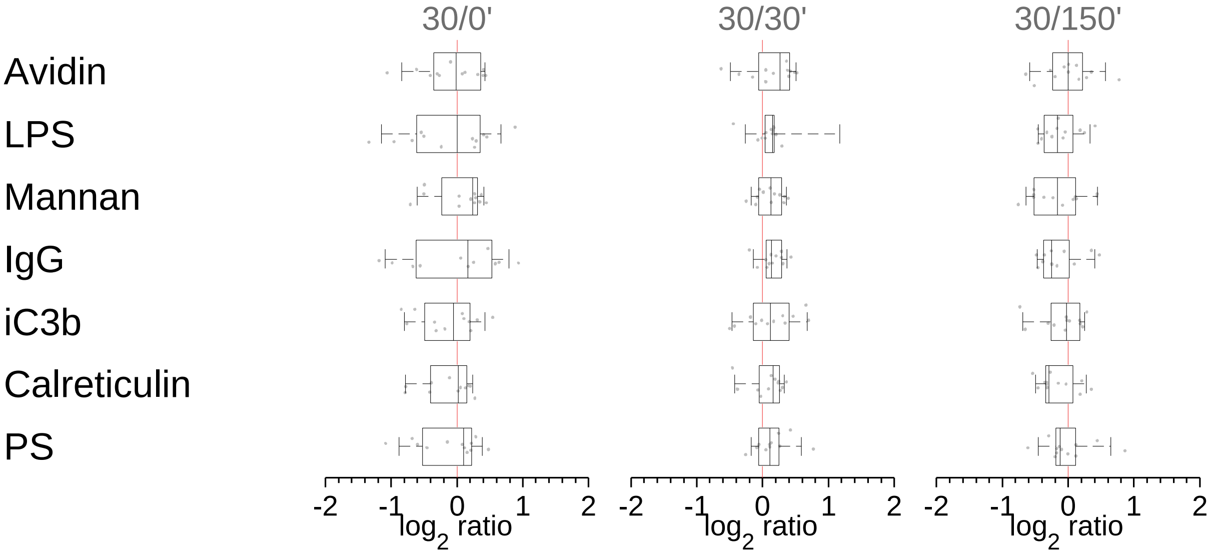

GO:0050832 defense response to fungus (n = 3)

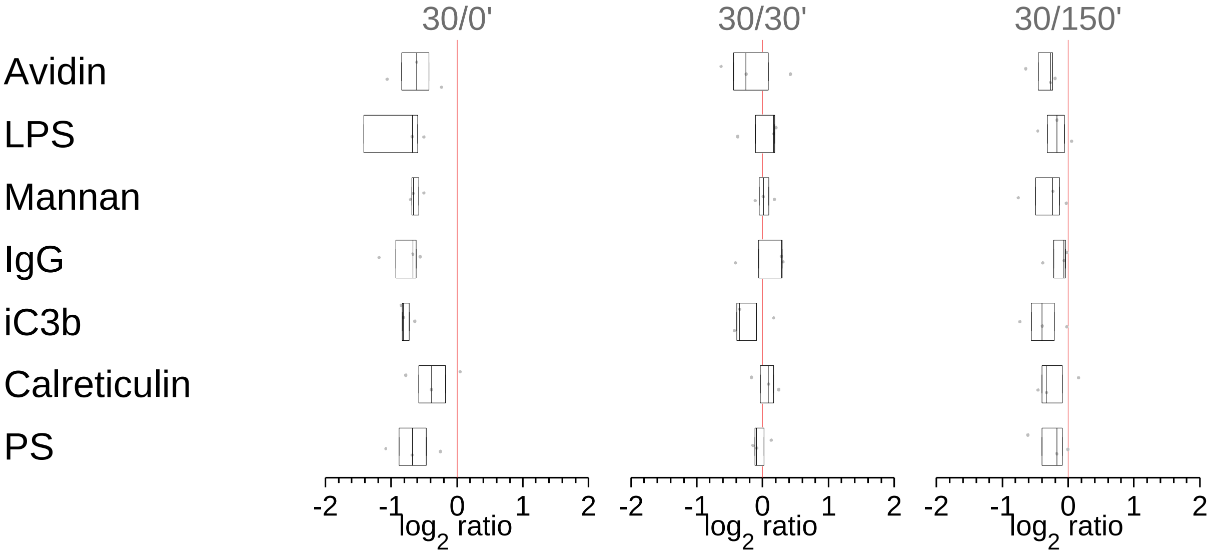

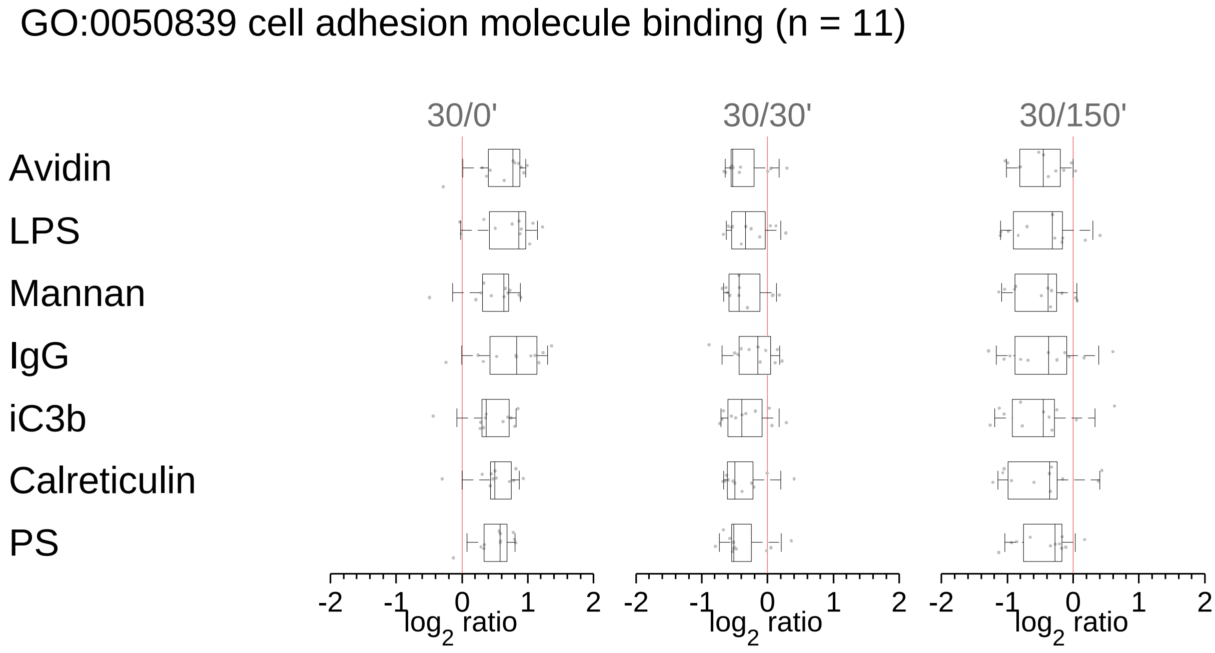
GO:0050839 cell adhesion molecule binding (n = 11)
30/0'
30/30'
30/150'
Avidin
LPS
Mannan
IgG
iC3b
Calreticulin
PS
-2
-1
0
1
2
log2 ratio

GO:0050850 positive regulation of calcium-mediated signaling (n = 3)

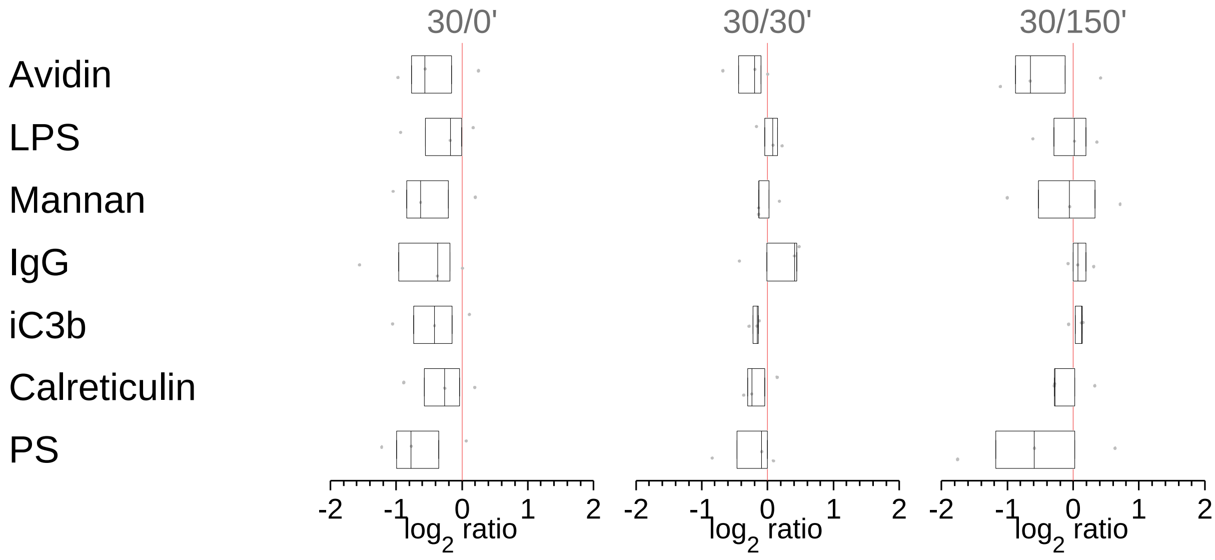

GO:0050852 T cell receptor signaling pathway (n = 7)

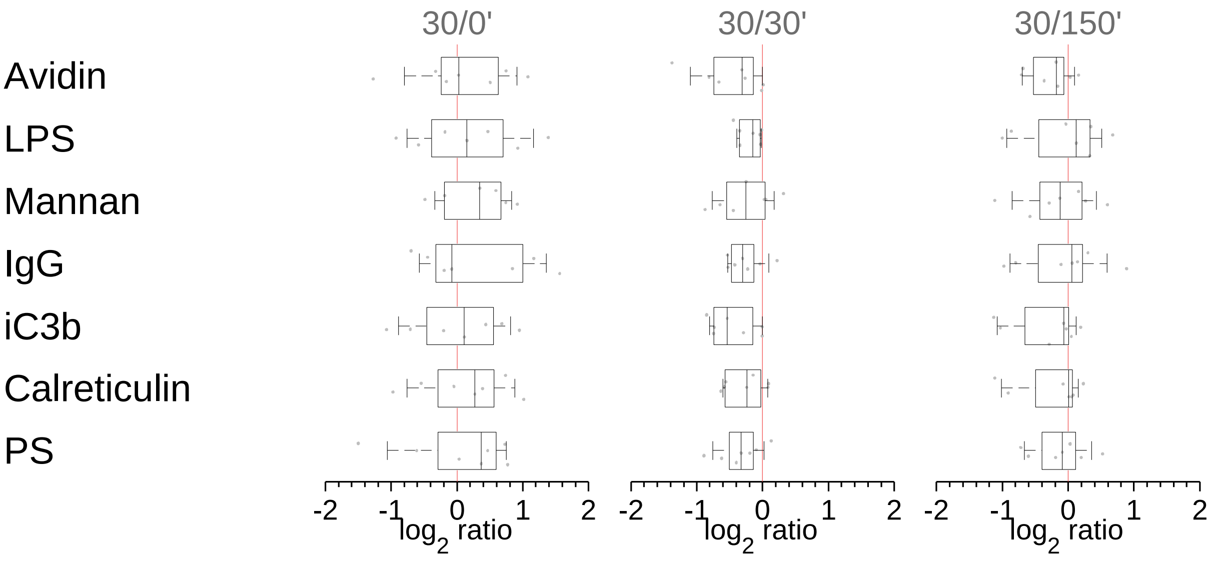

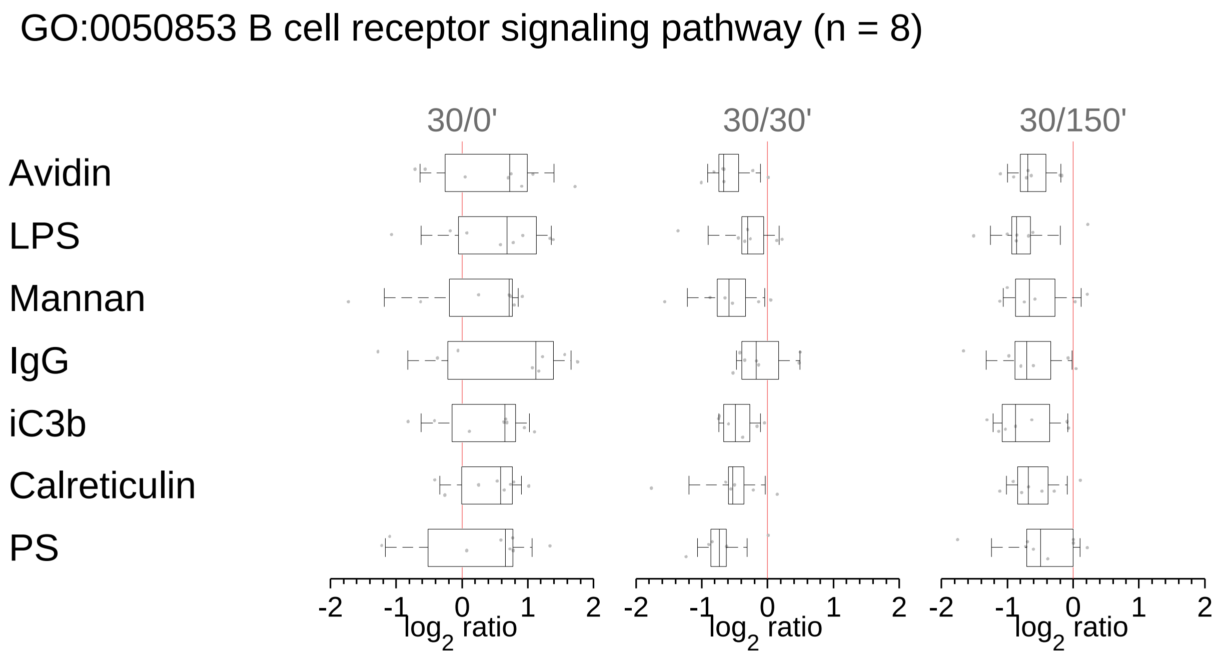

GO:0050853 B cell receptor signaling pathway (n = 8)
30/0'
30/30'
30/150'
Avidin
LPS
Mannan
IgG
iC3b
Calreticulin
PS
-2 -1 0 1 2
log2 ratio

GO:0050860 negative regulation of T cell receptor signaling pathway (n = 4)

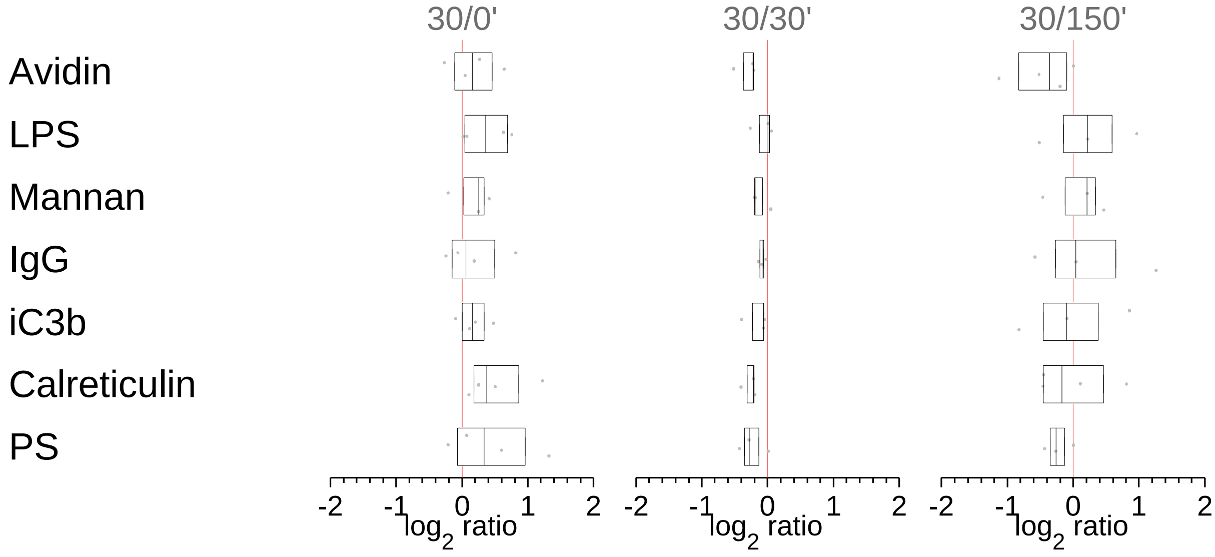

GO:0050868 negative regulation of T cell activation (n = 3)

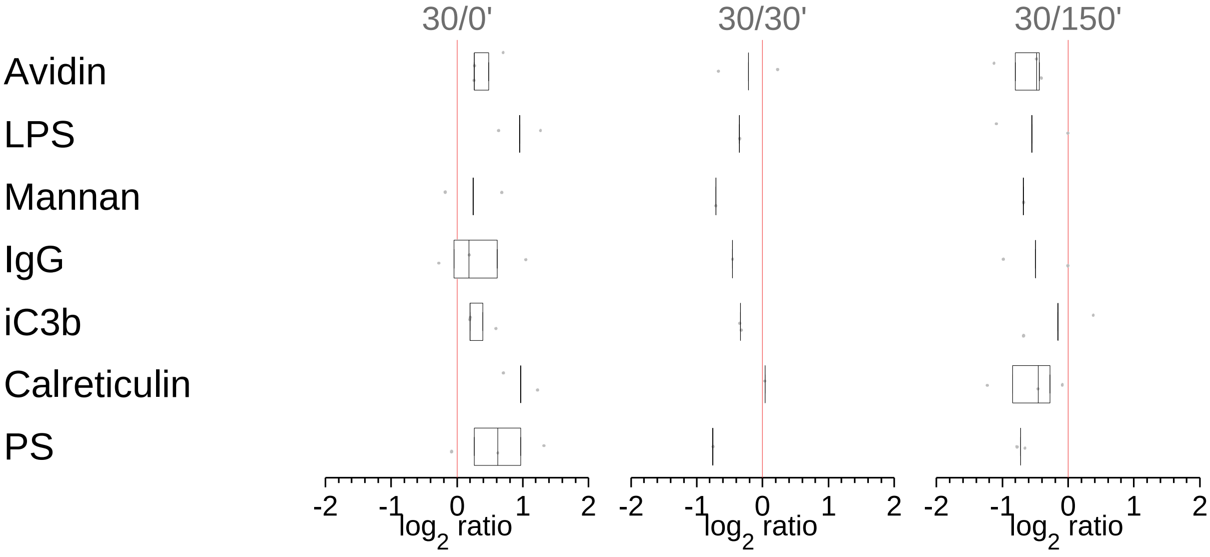

GO:0050870 positive regulation of T cell activation (n = 3)

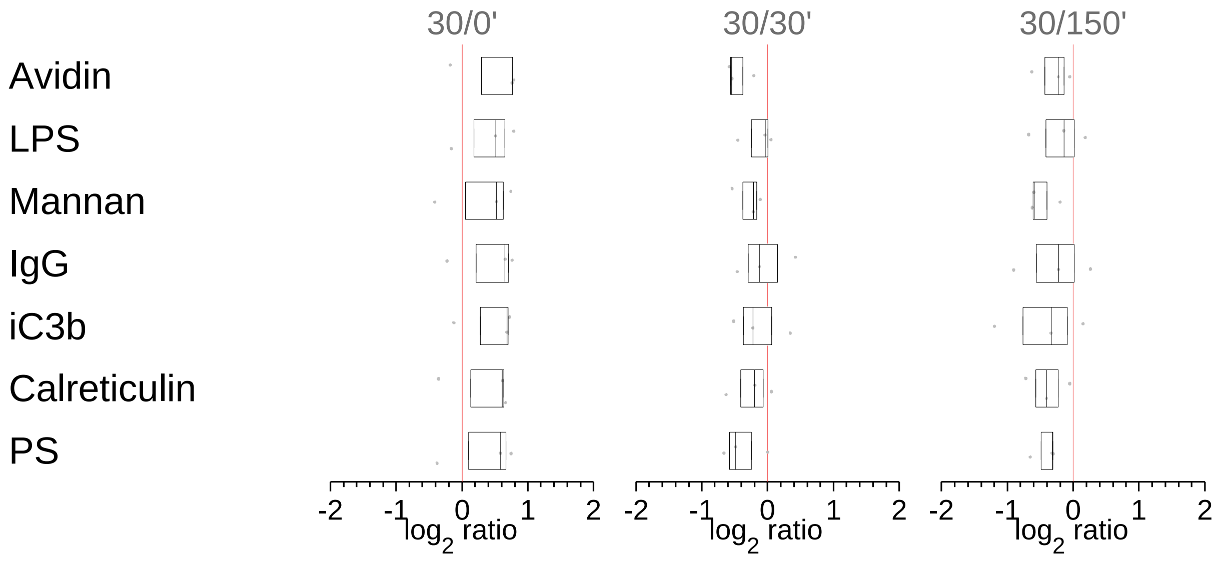

GO:0050885 neuromuscular process controlling balance (n = 10)

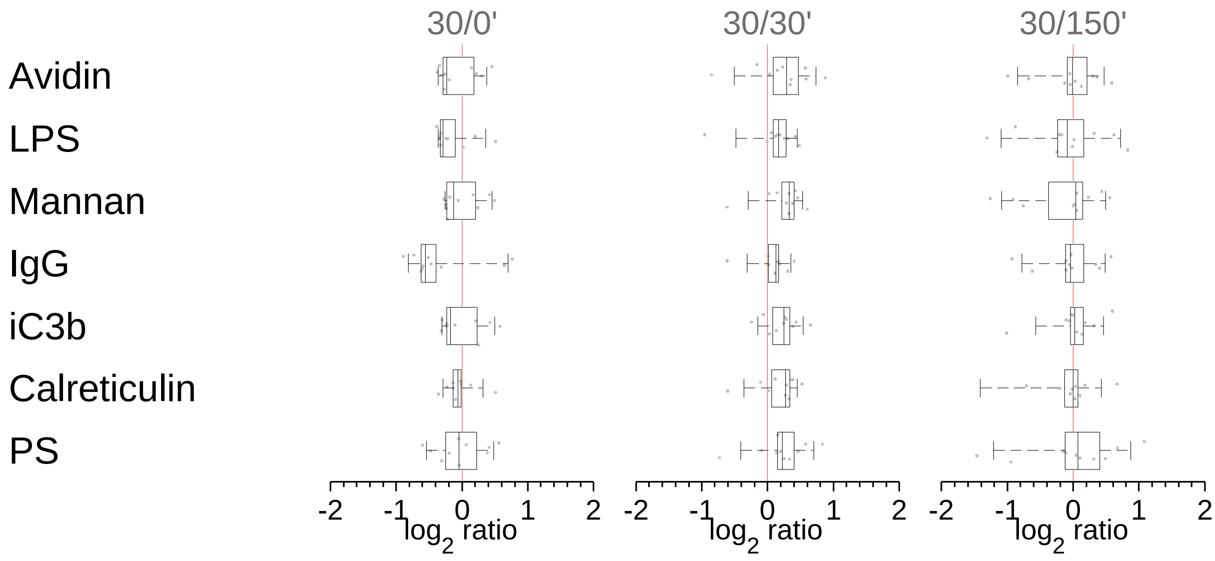

GO:0050890 cognition (n = 3)

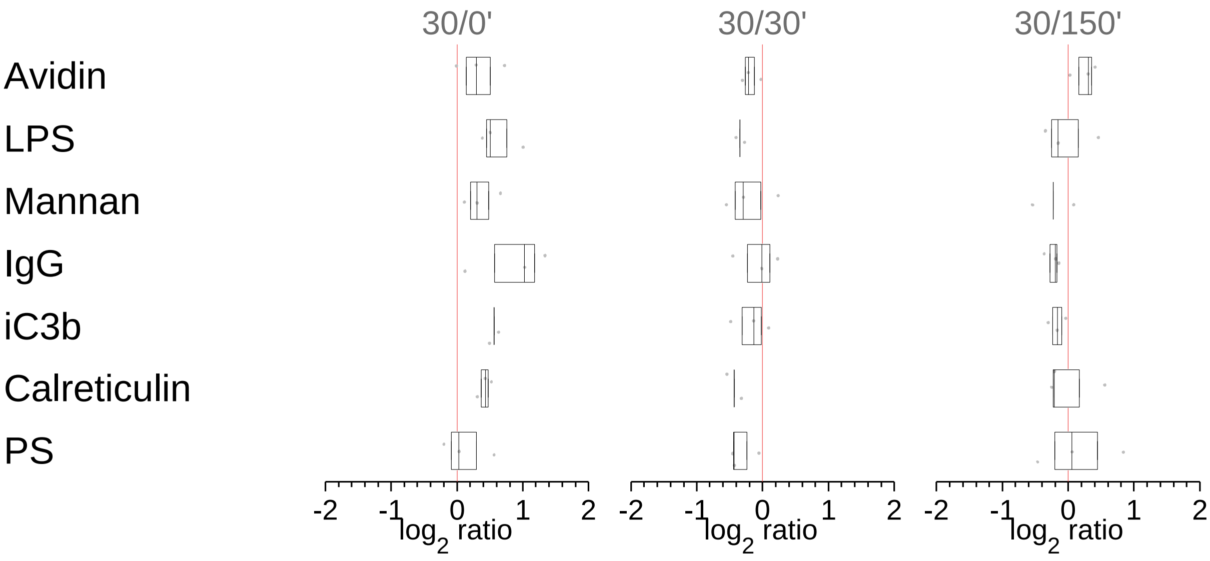

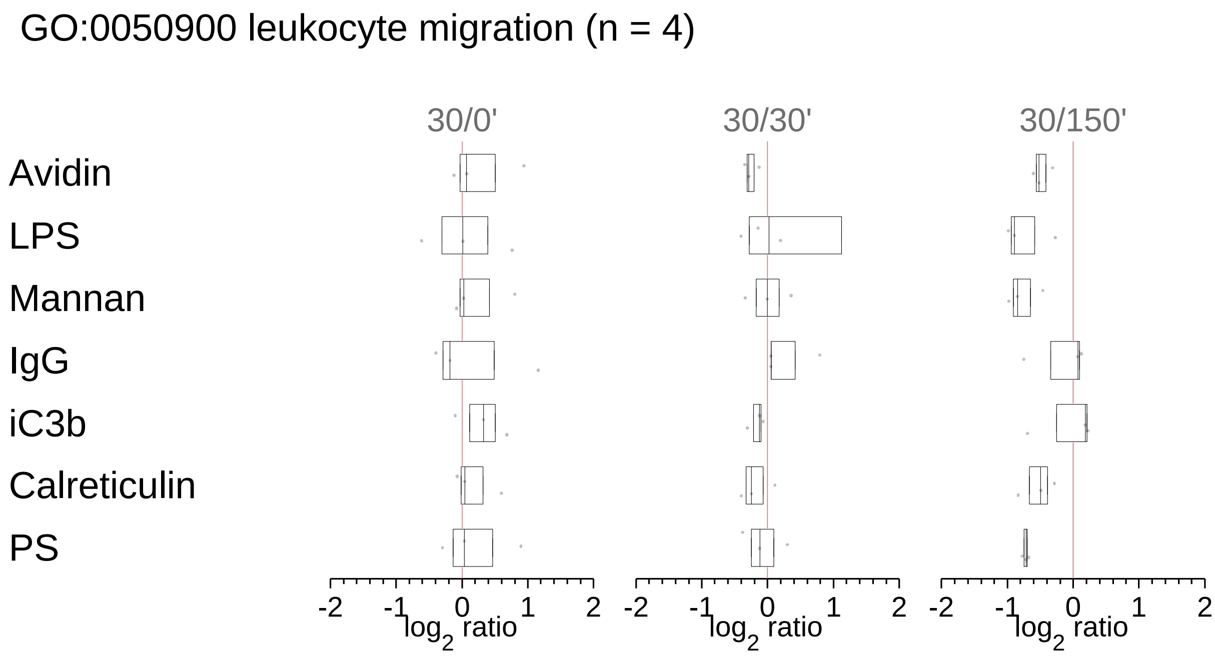
GO:0050900 leukocyte migration (n = 4)
30/0'
30/30'
30/150'
Avidin
LPS
Mannan
IgG
iC3b
Calreticulin
PS
-2 -1 0 1 2
log2 ratio

GO:0050905 neuromuscular process (n = 3)

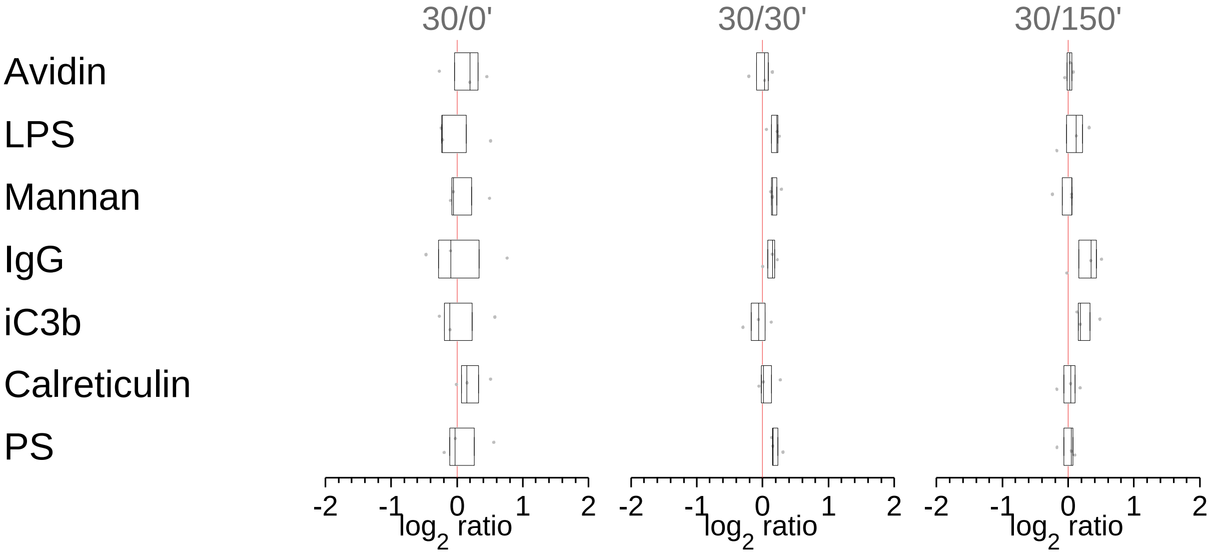

GO:0050918 positive chemotaxis (n = 5)

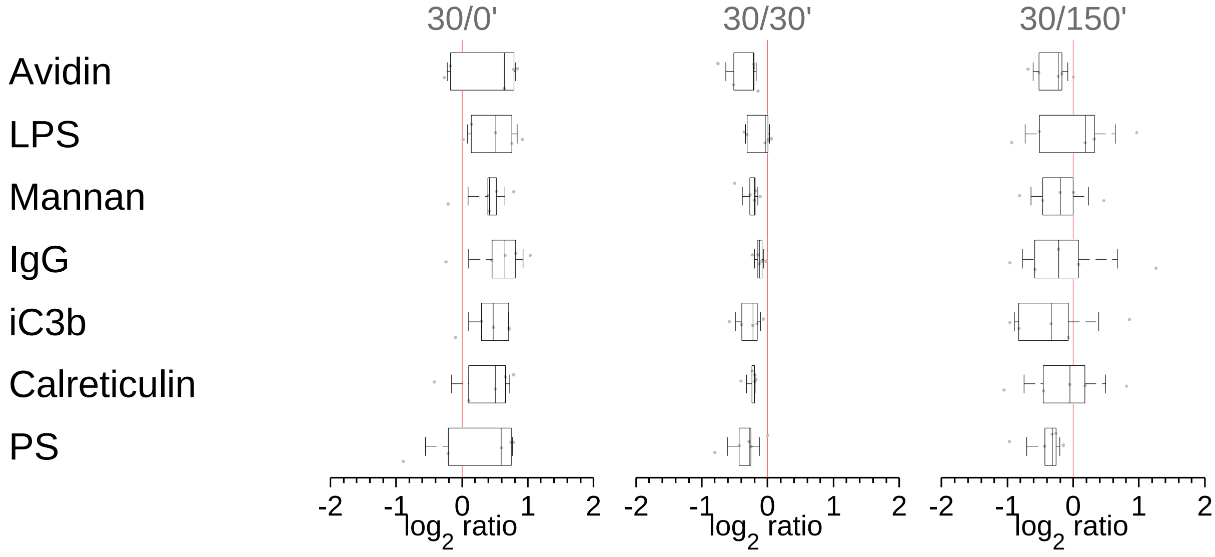

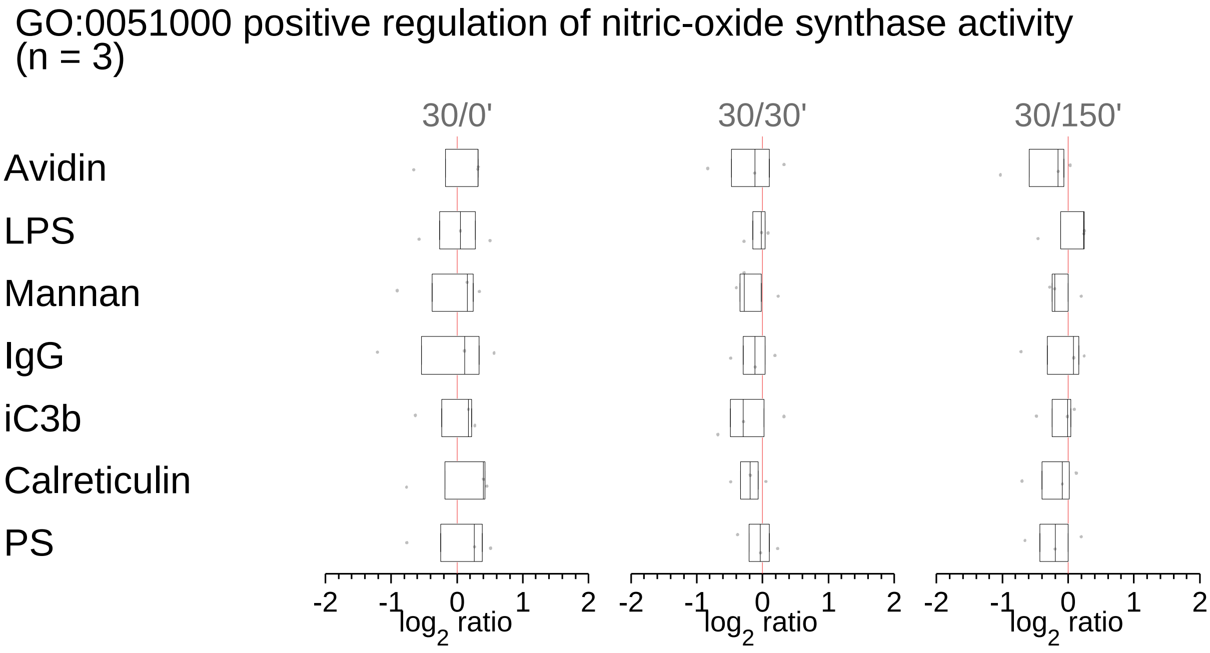
GO:0051000 positive regulation of nitric-oxide synthase activity
(n = 3)
30/0'
30/30'
30/150'
Avidin
LPS
Mannan
IgG
iC3b
Calreticulin
PS
-2 -1 0 1 2
$\log_2$ ratio

GO:0051015 actin filament binding (n = 22)

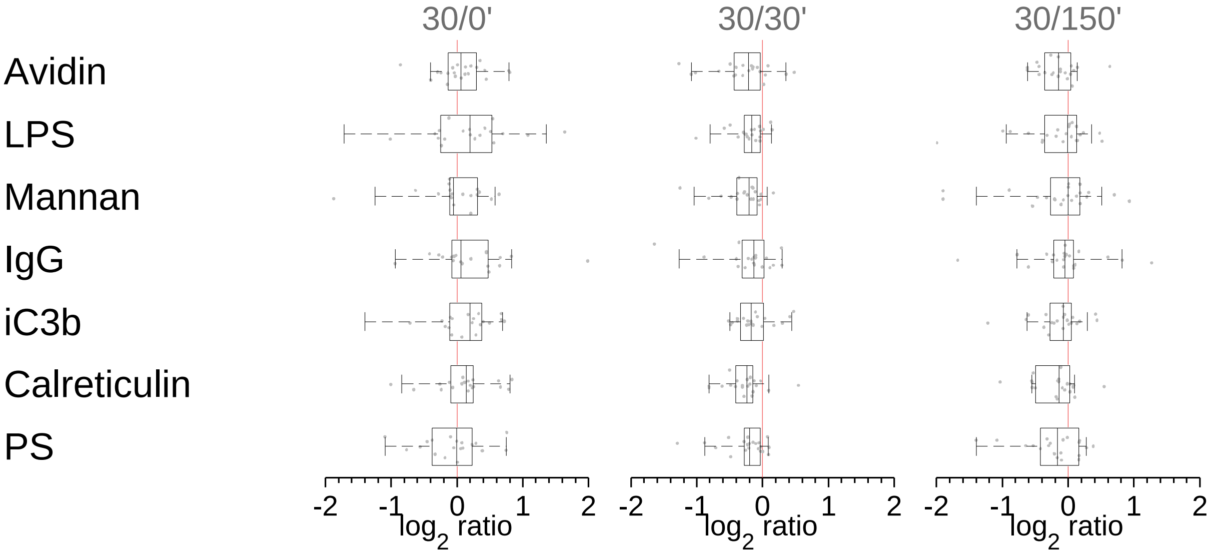

GO:0051016 barbed-end actin filament capping (n = 4)

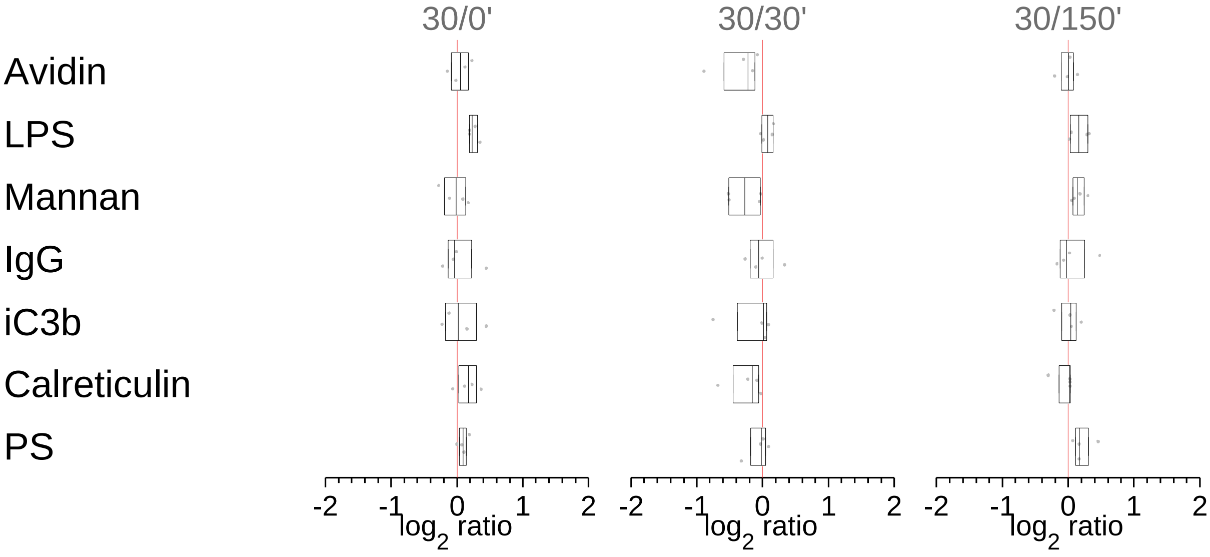

GO:0051017 actin filament bundle assembly (n = 8)

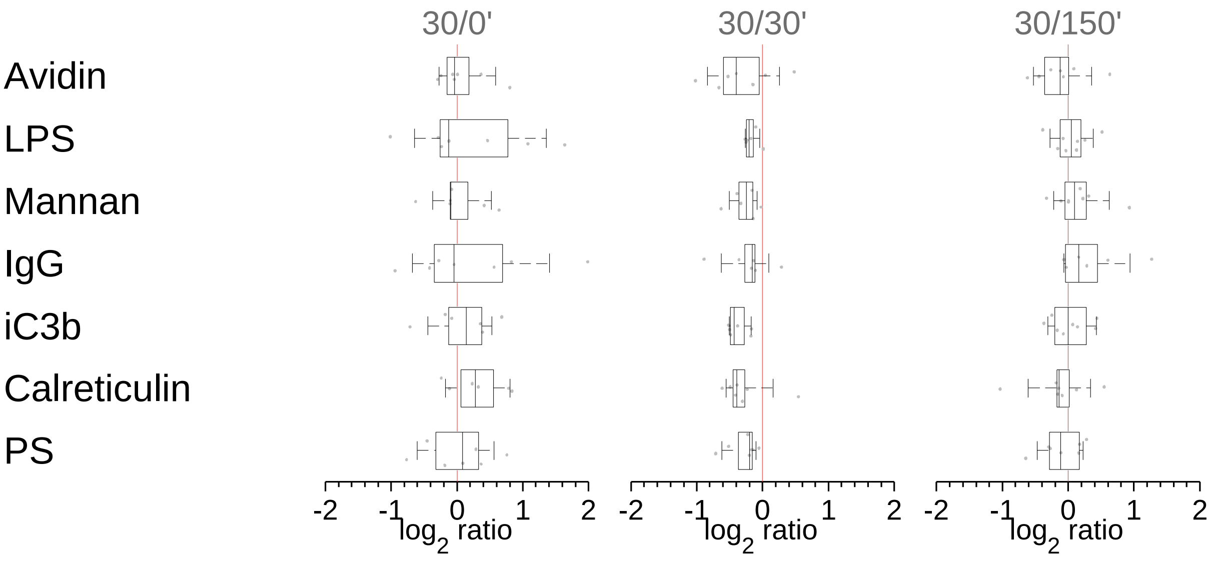

GO:0051020 GTPase binding (n = 7)

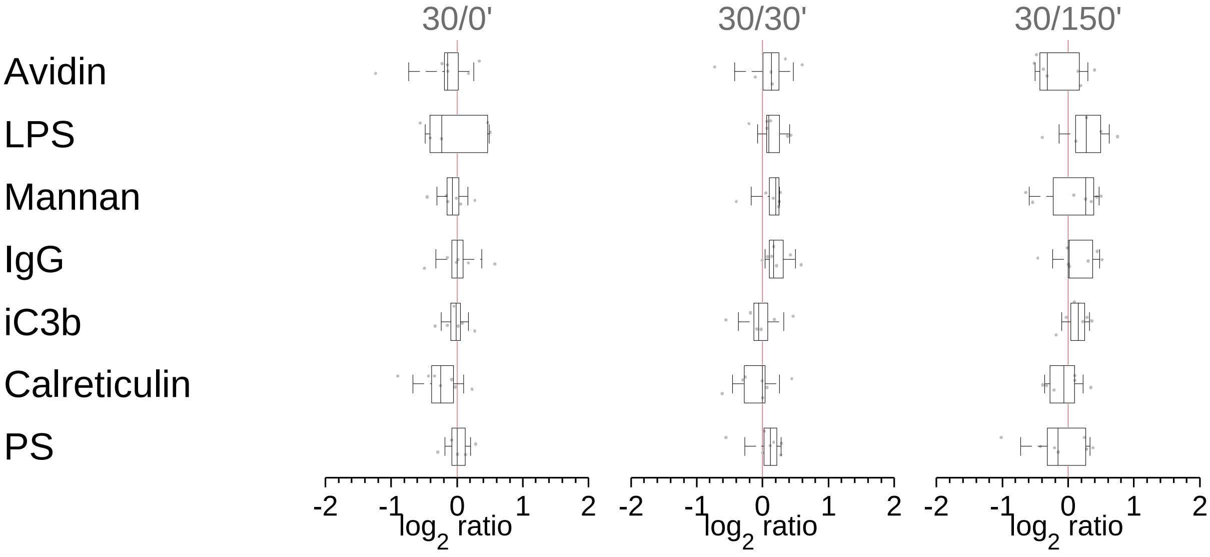

GO:0051028 mRNA transport (n = 16)

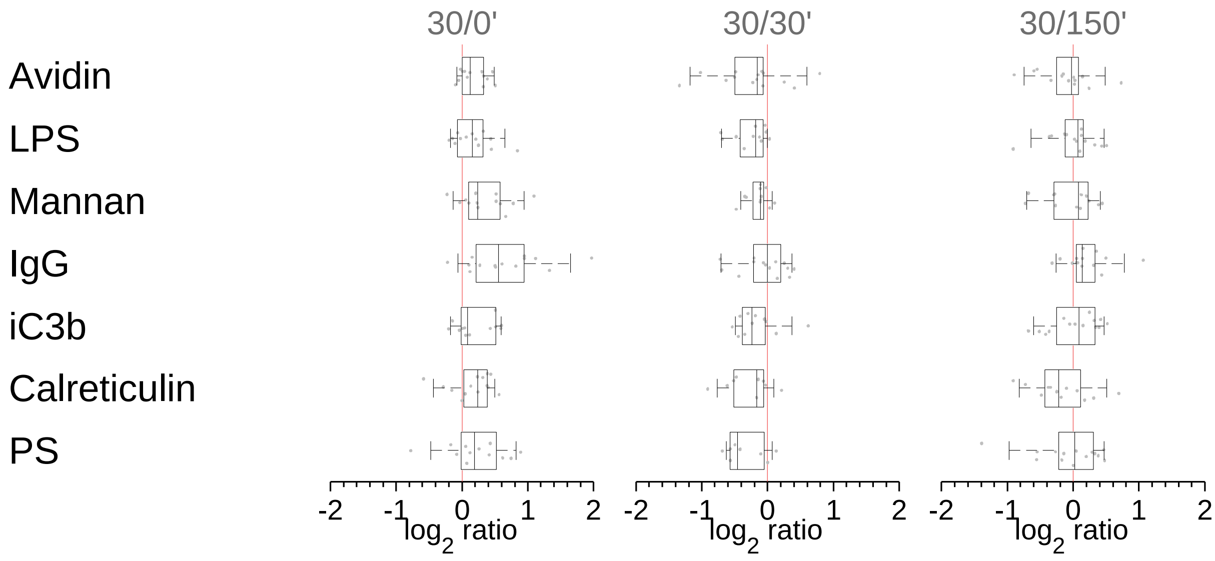

GO:0051044 positive regulation of membrane protein ectodomain proteolysis (n = 5)

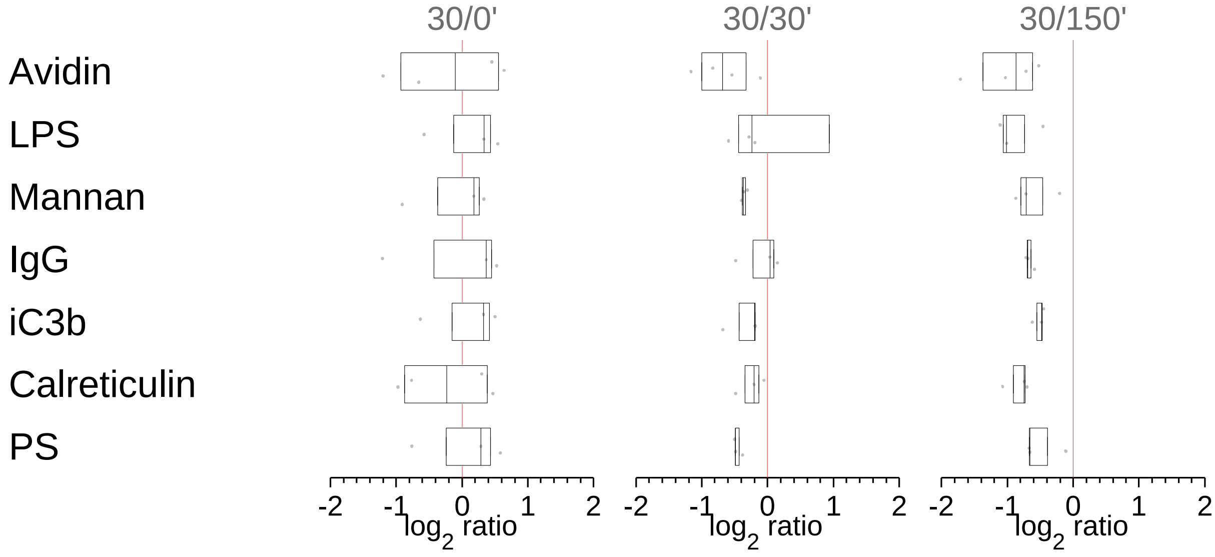

GO:0051054 positive regulation of DNA metabolic process (n = 3)

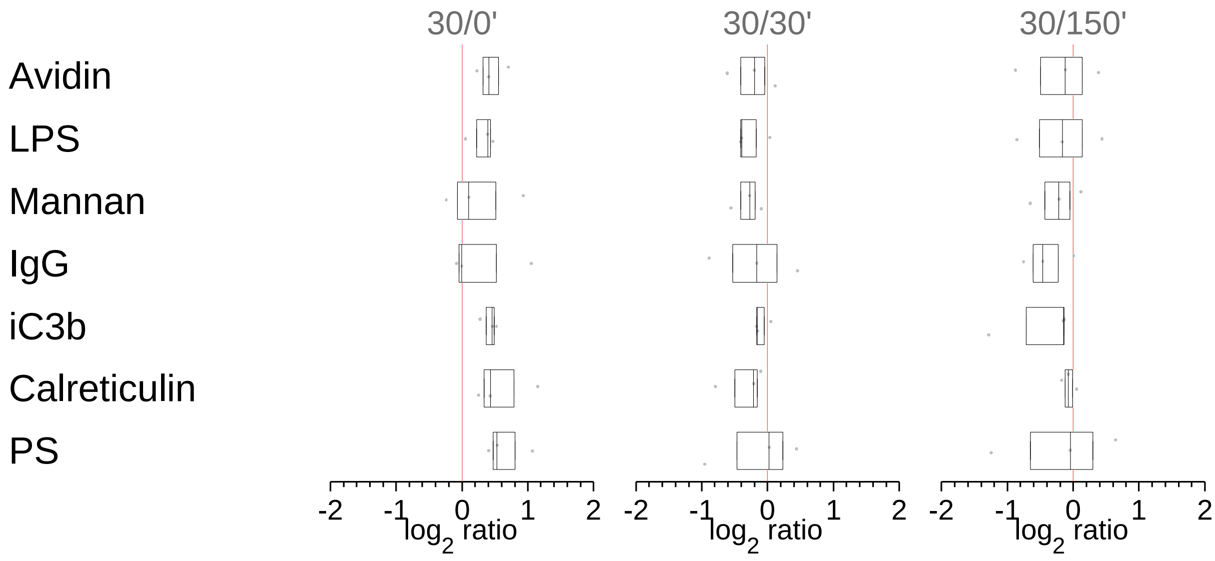

GO:0051056 regulation of small GTPase mediated signal transduction (n = 5)

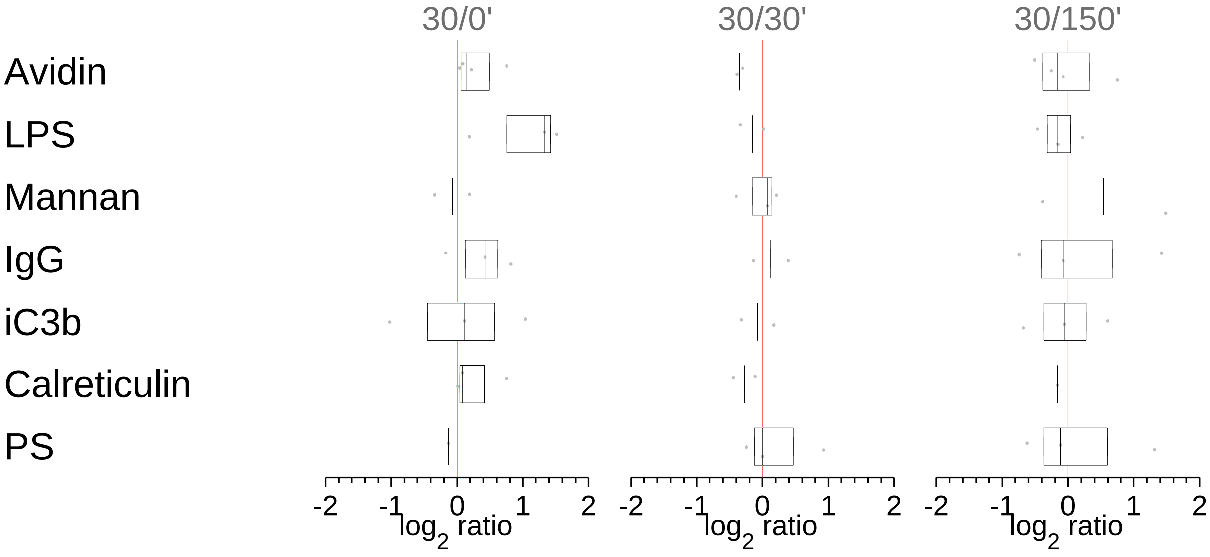

GO:0051059 NF-kappaB binding (n = 7)

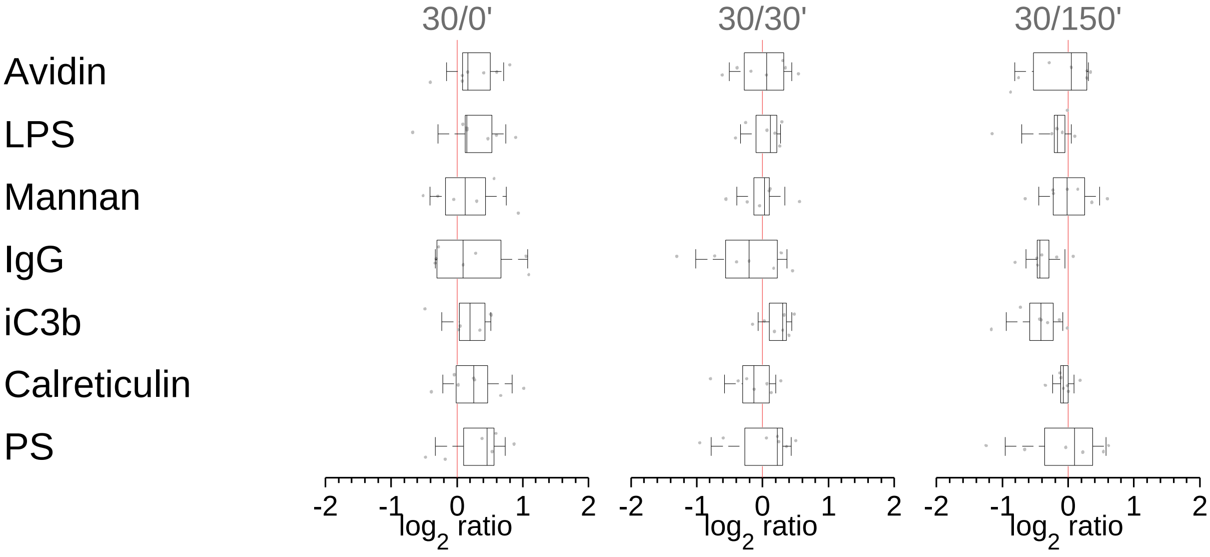

GO:0051082 unfolded protein binding (n = 25)

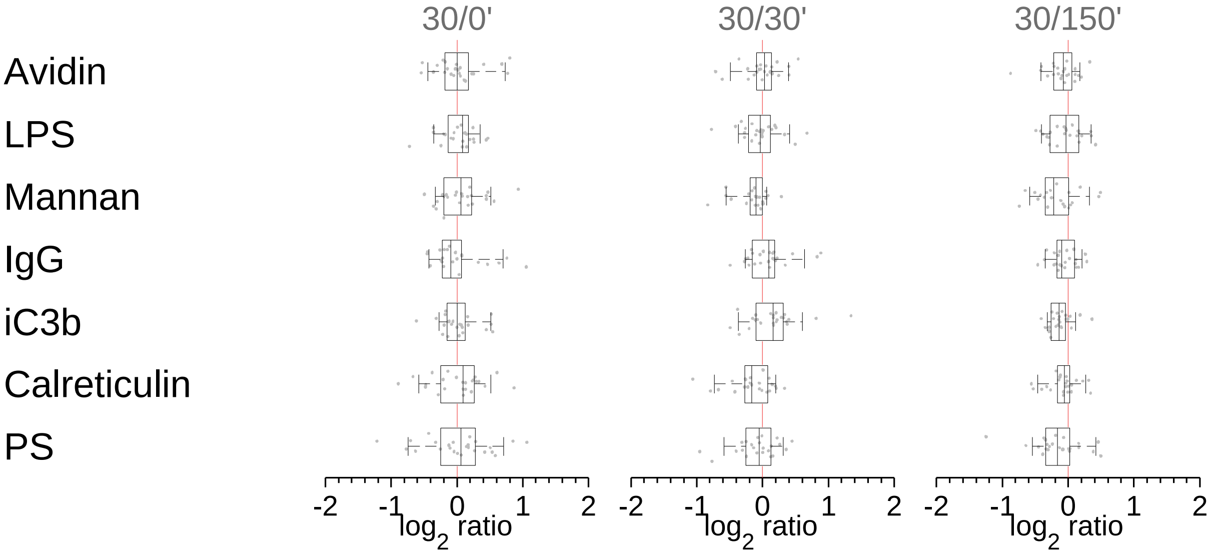

GO:0051085 chaperone mediated protein folding requiring cofactor
(n = 6)

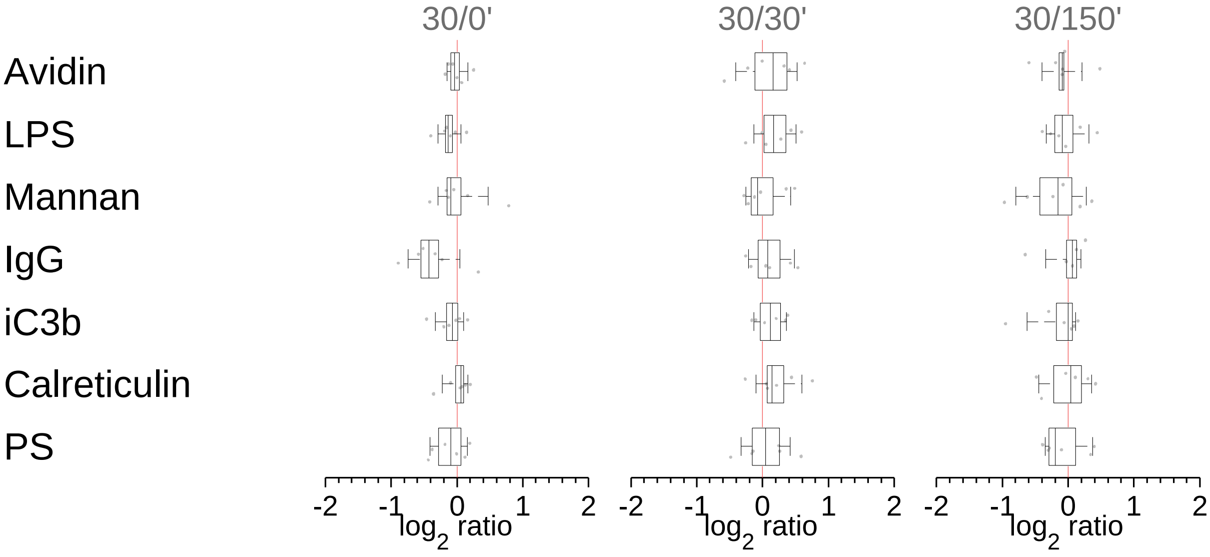

GO:0051087 chaperone binding (n = 22)

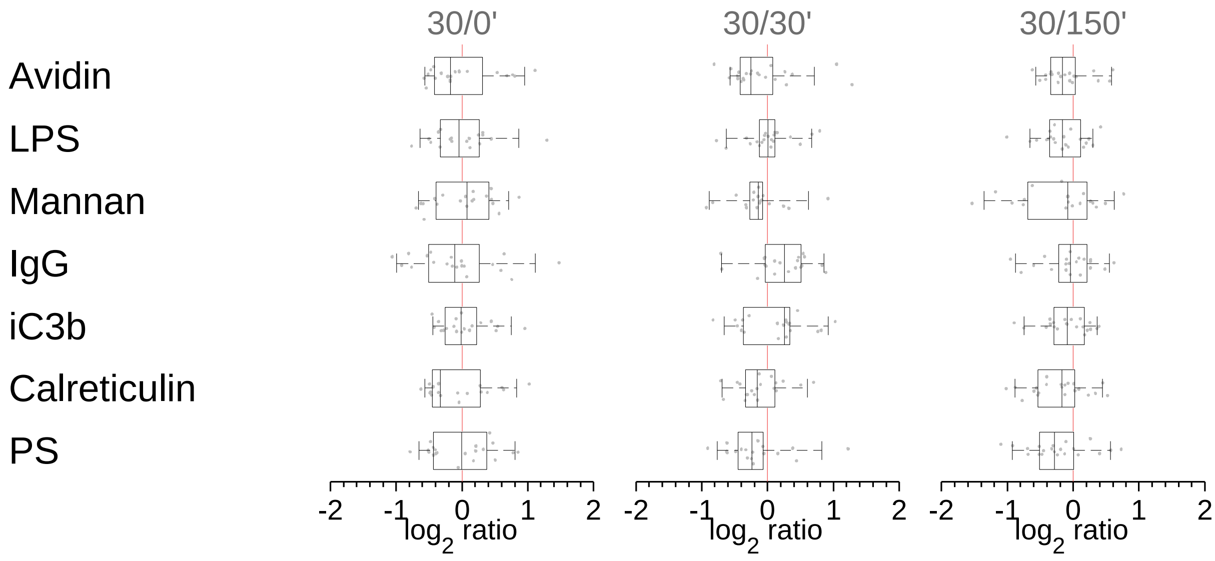

GO:0051088 PMA-inducible membrane protein ectodomain proteolysis (n = 3)

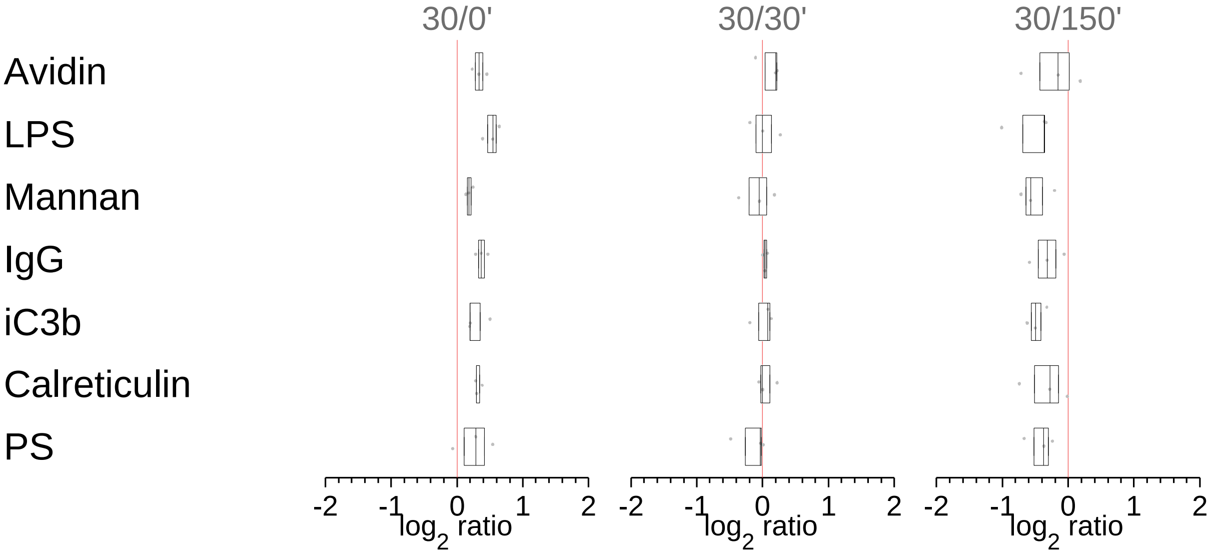

GO:0051091 positive regulation of sequence-specific DNA binding transcription factor activity (n = 10)

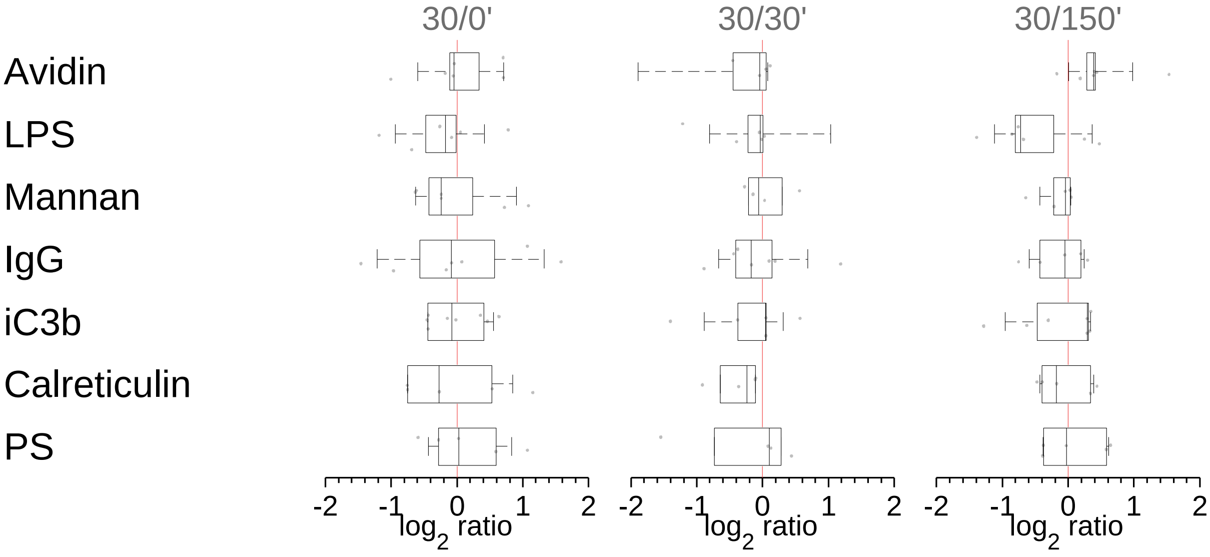

GO:0051092 positive regulation of NF-kappaB transcription factor activity (n = 21)

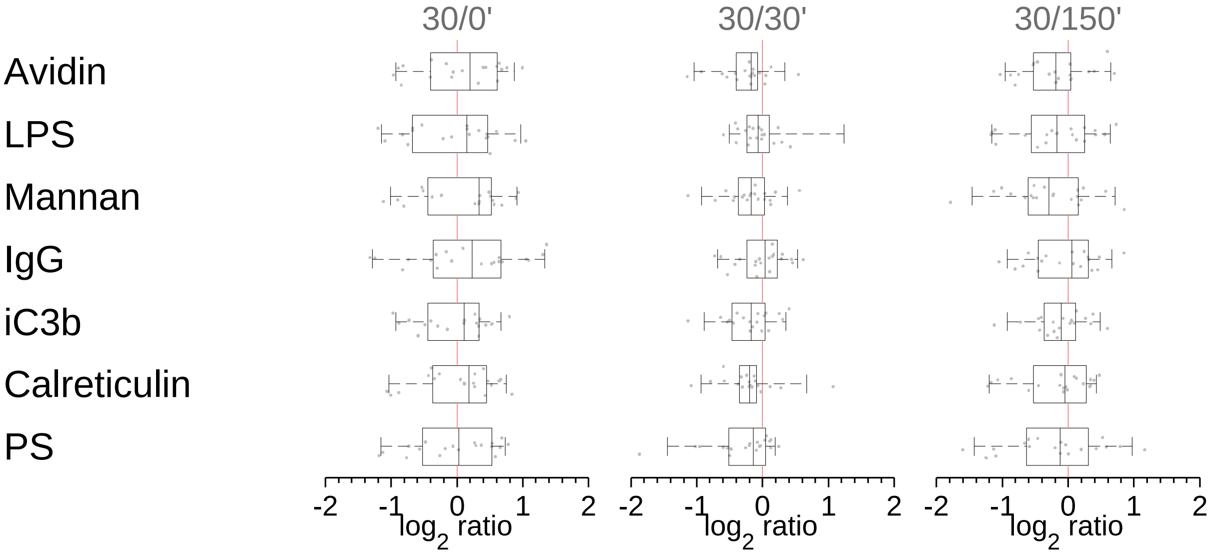

GO:0051117 ATPase binding (n = 8)

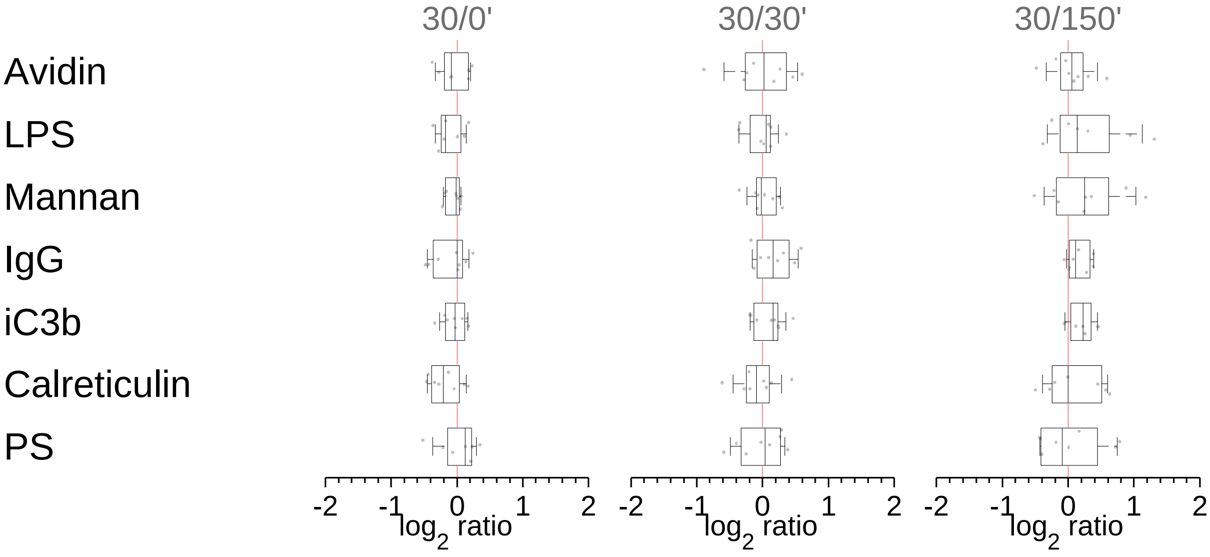

GO:0051131 chaperone-mediated protein complex assembly (n = 3)

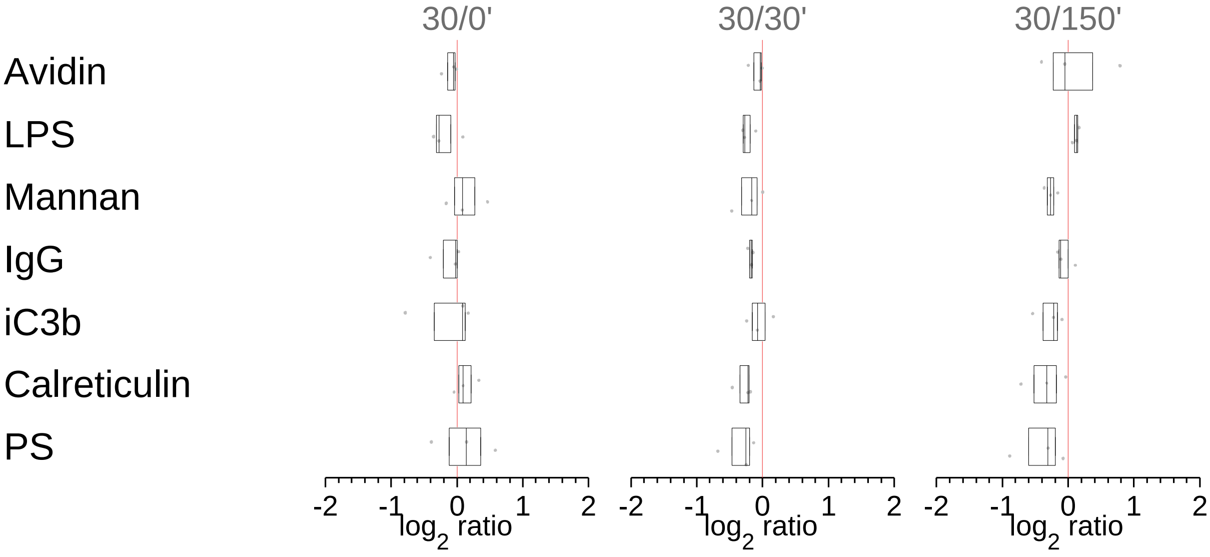

GO:0051138 positive regulation of NK T cell differentiation (n = 3)

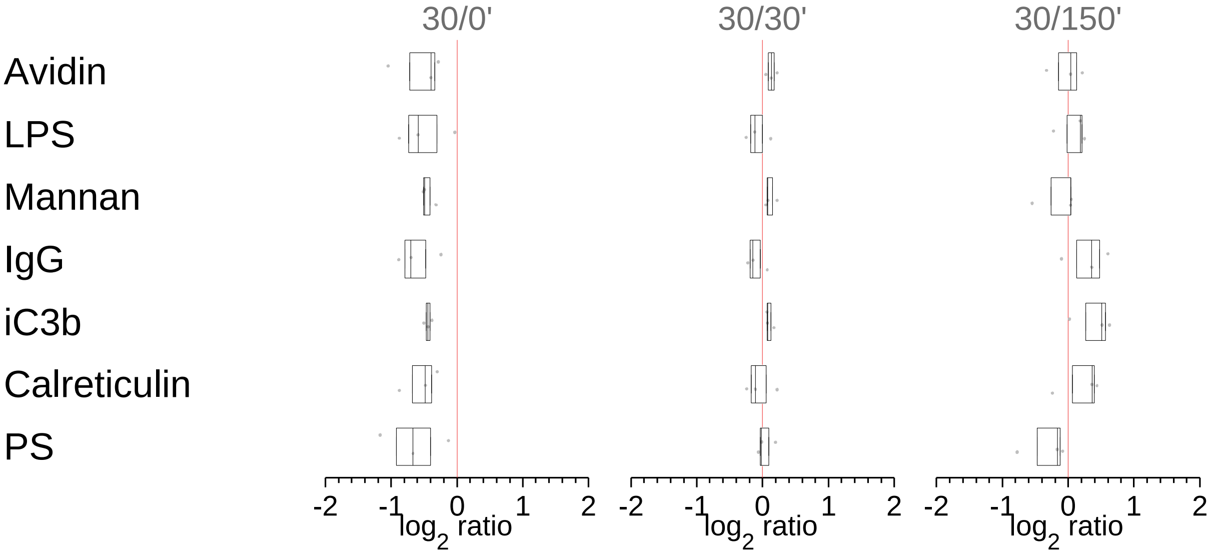

GO:0051156 glucose 6-phosphate metabolic process (n = 4)

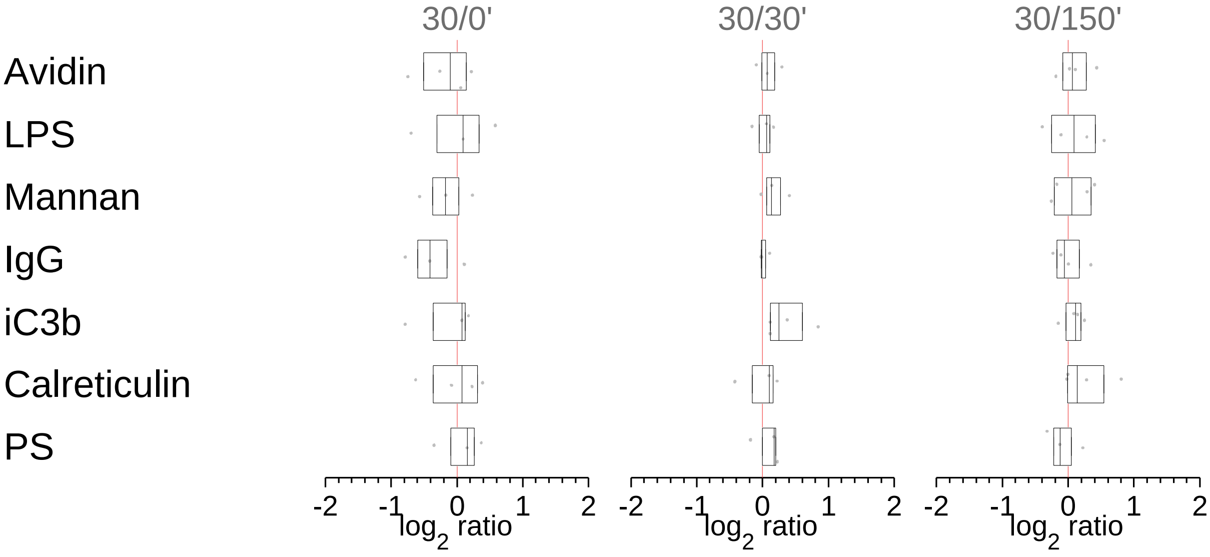

GO:0051209 release of sequestered calcium ion into cytosol (n = 6)

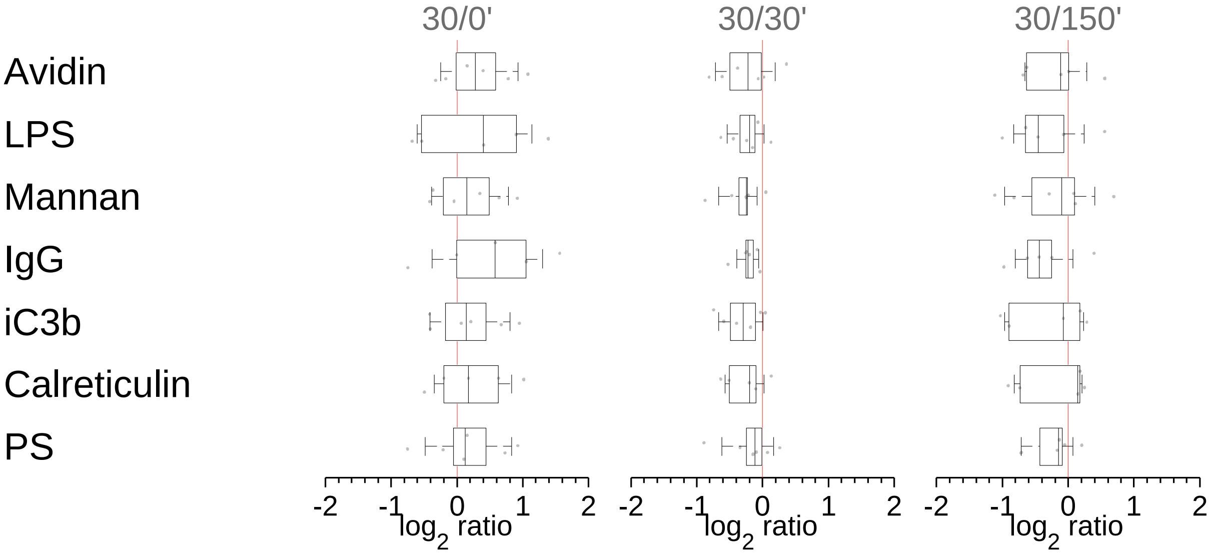

GO:0051213 dioxygenase activity (n = 6)

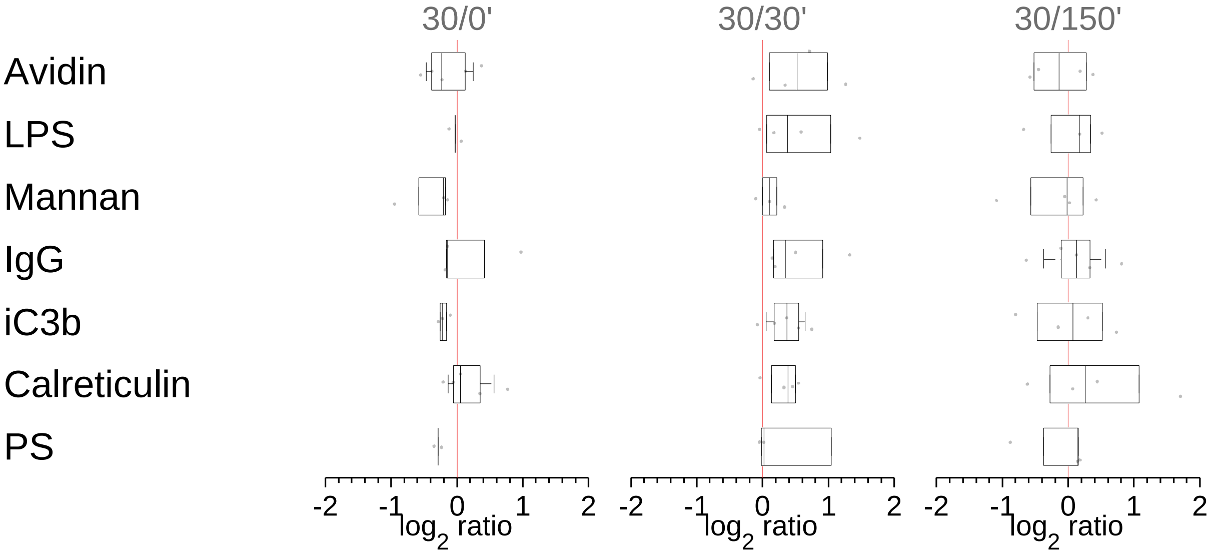

GO:0051219 phosphoprotein binding (n = 12)

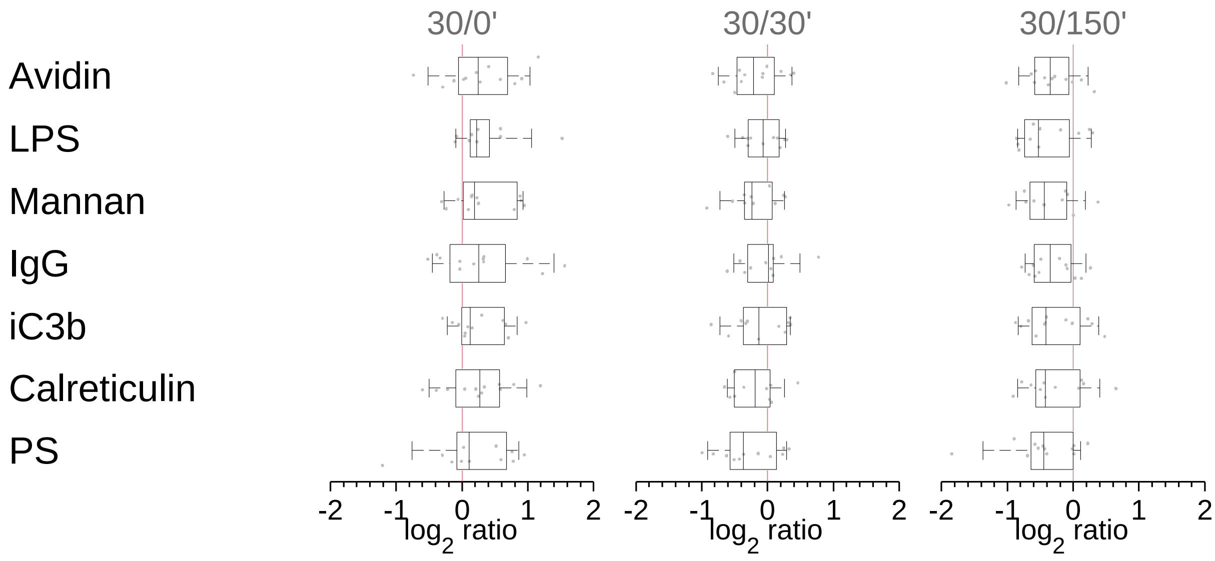

GO:0051223 regulation of protein transport (n = 4)

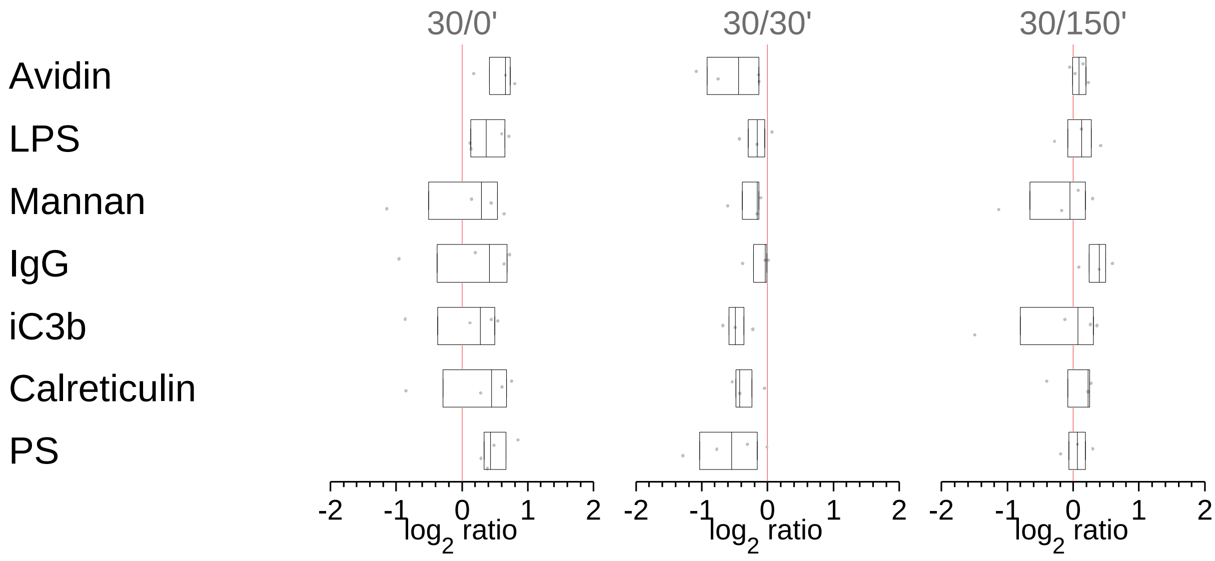

GO:0051225 spindle assembly (n = 4)

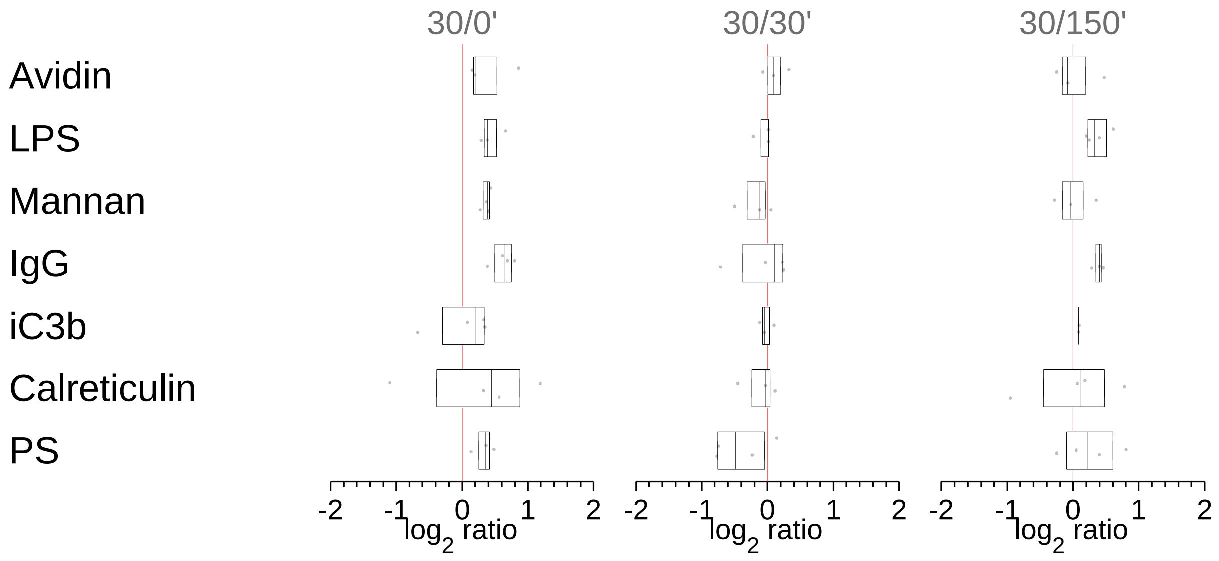

GO:0051233 spindle midzone (n = 3)

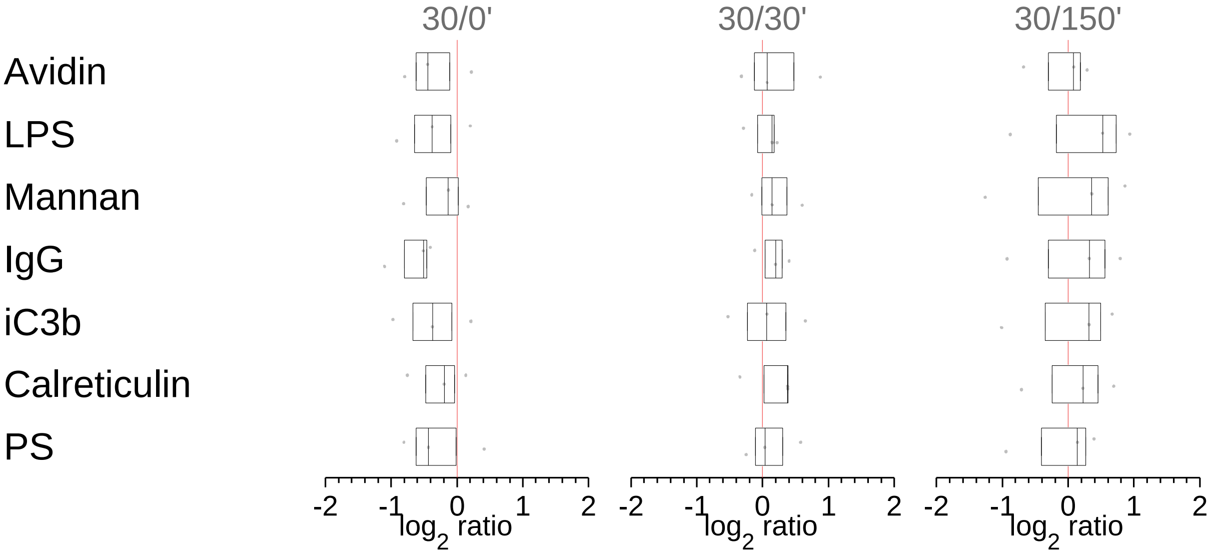

GO:0051258 protein polymerization (n = 6)

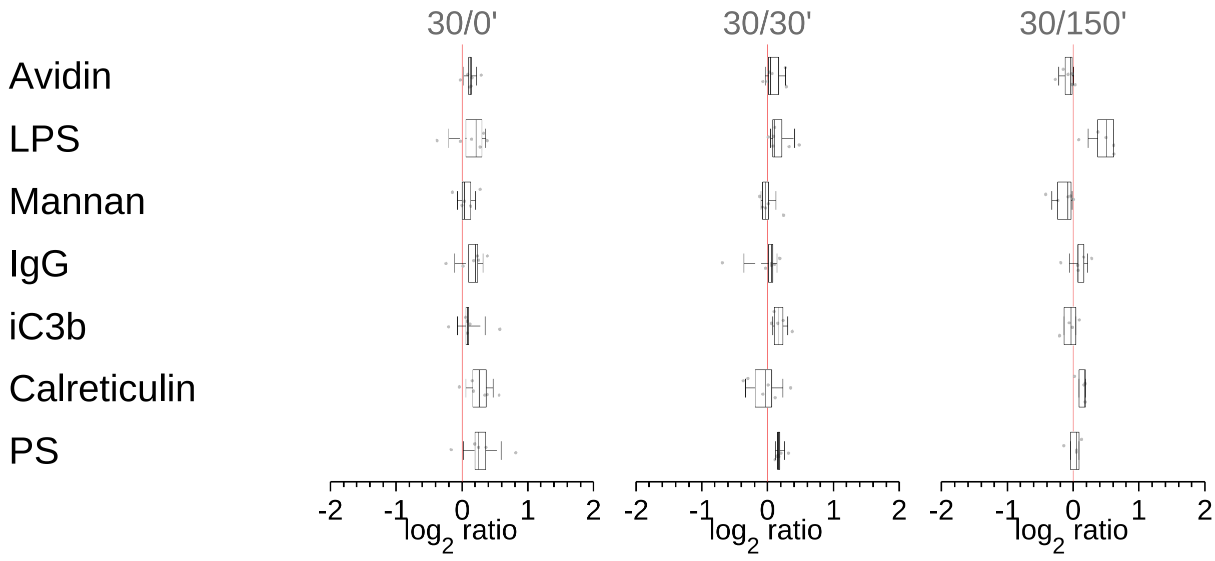

GO:0051259 protein oligomerization (n = 8)

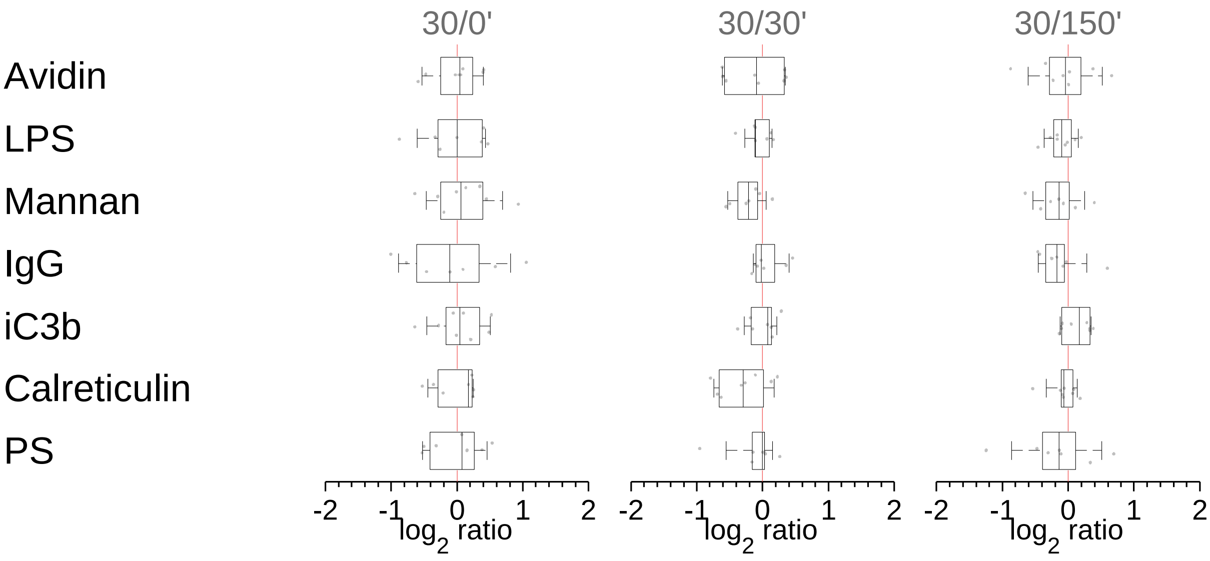

GO:0051260 protein homooligomerization (n = 25)

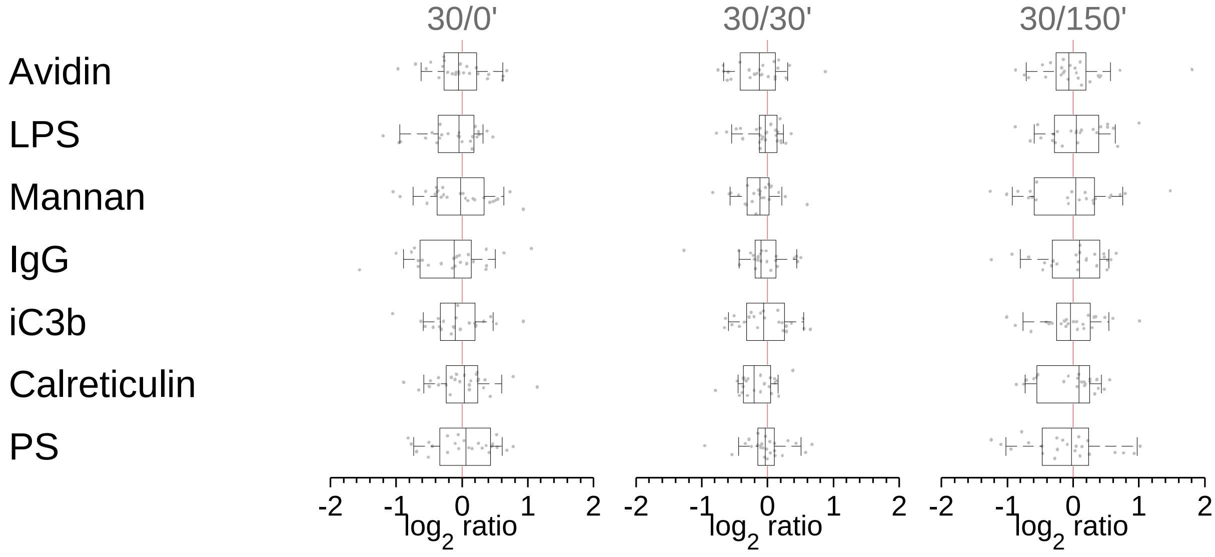

GO:0051262 protein tetramerization (n = 5)

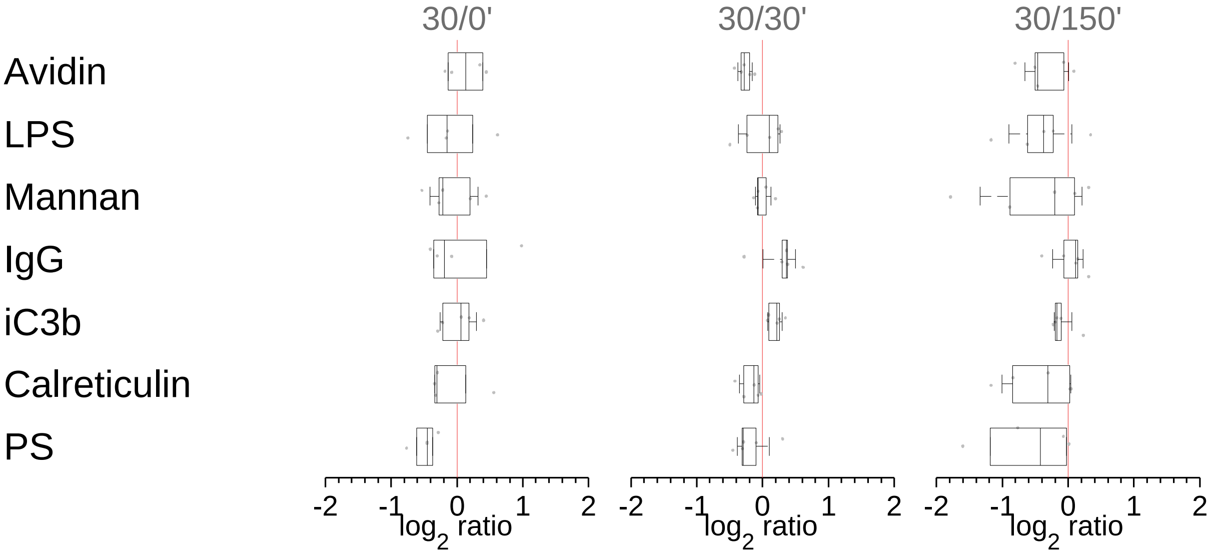

GO:0051271 negative regulation of cellular component movement (n = 3)

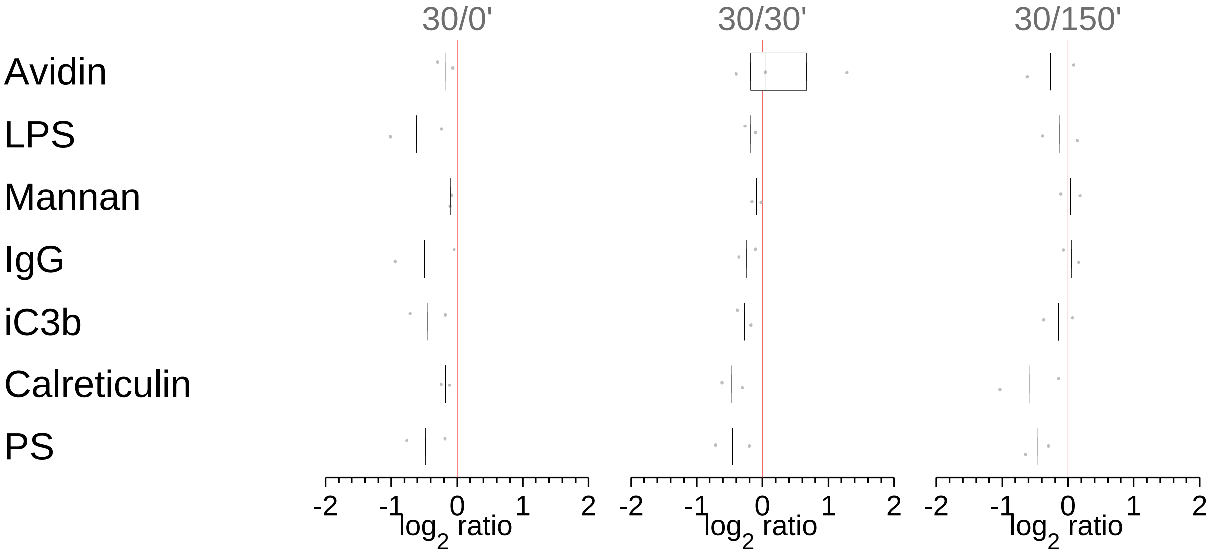

GO:0051272 positive regulation of cellular component movement (n = 4)

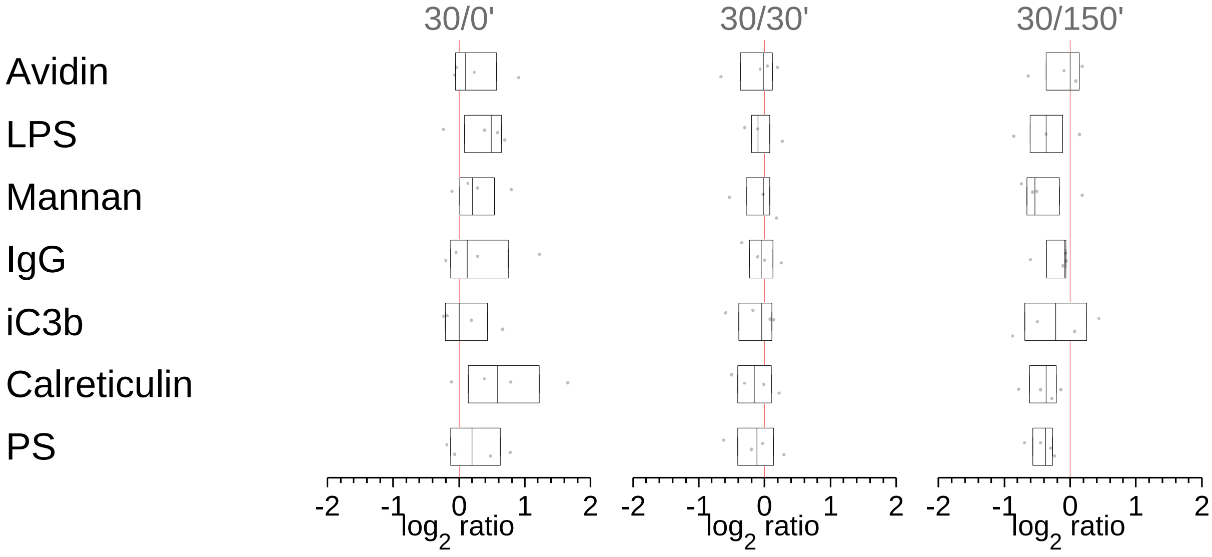

GO:0051279 regulation of release of sequestered calcium ion into cytosol (n = 4)

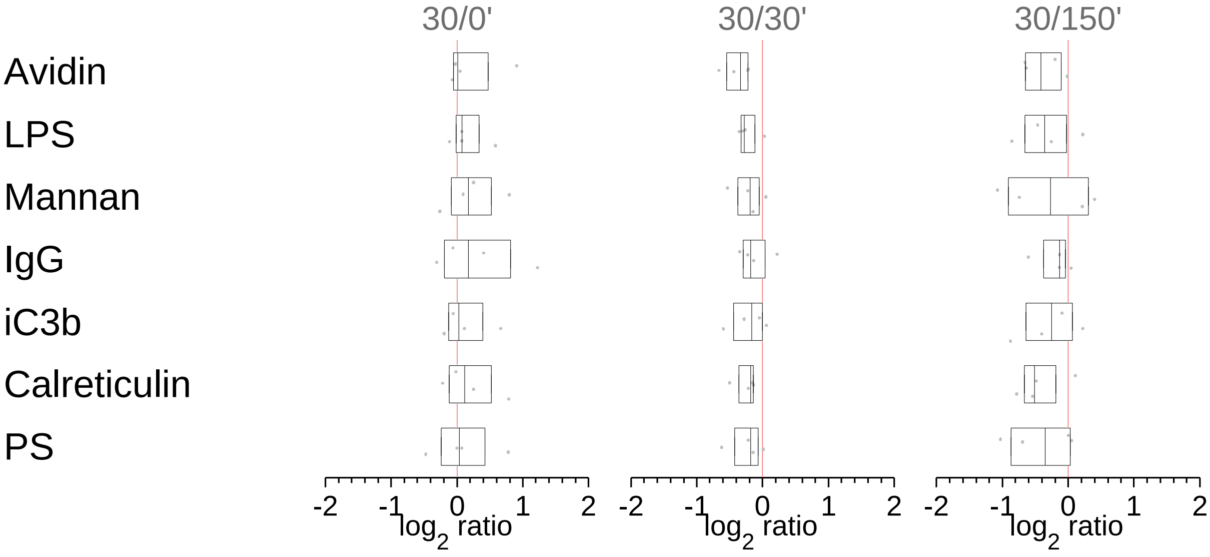

GO:0051281 positive regulation of release of sequestered calcium ion into cytosol (n = 3)

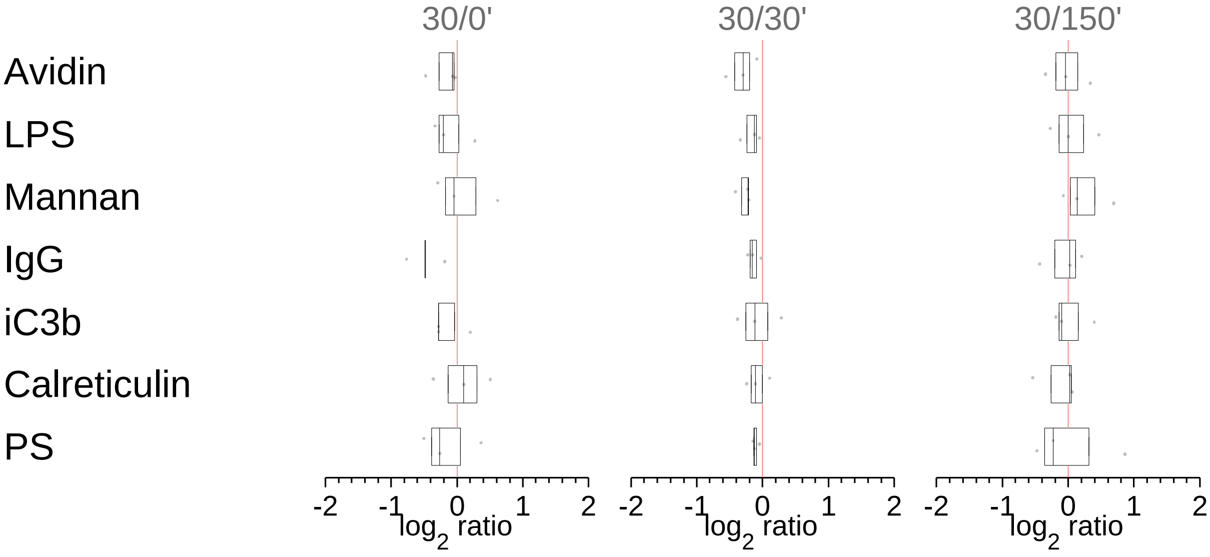

GO:0051286 cell tip (n = 4)

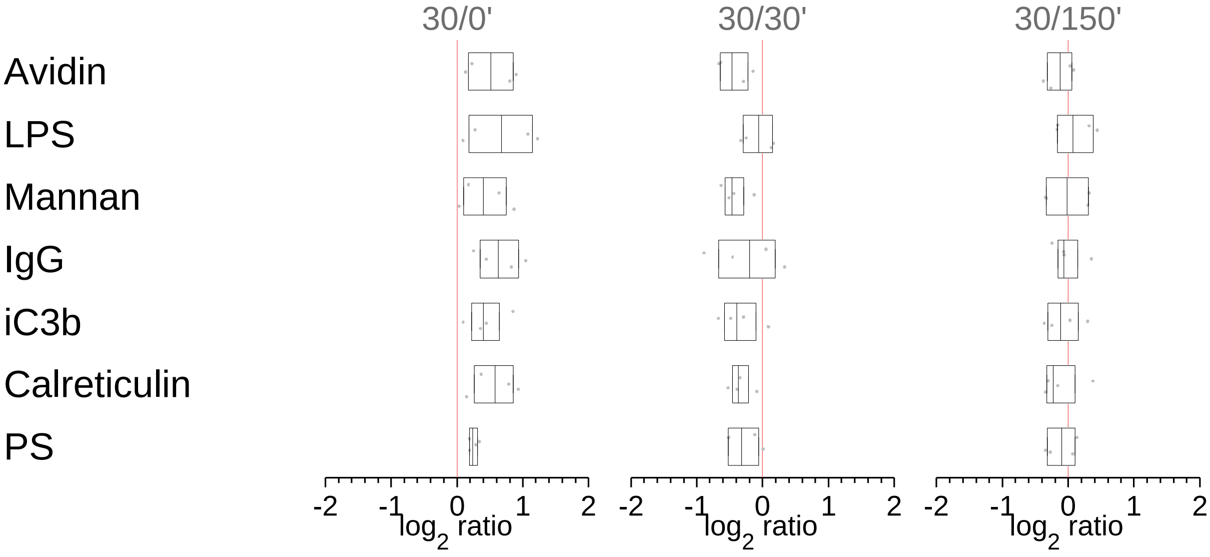

GO:0051287 NAD binding (n = 16)

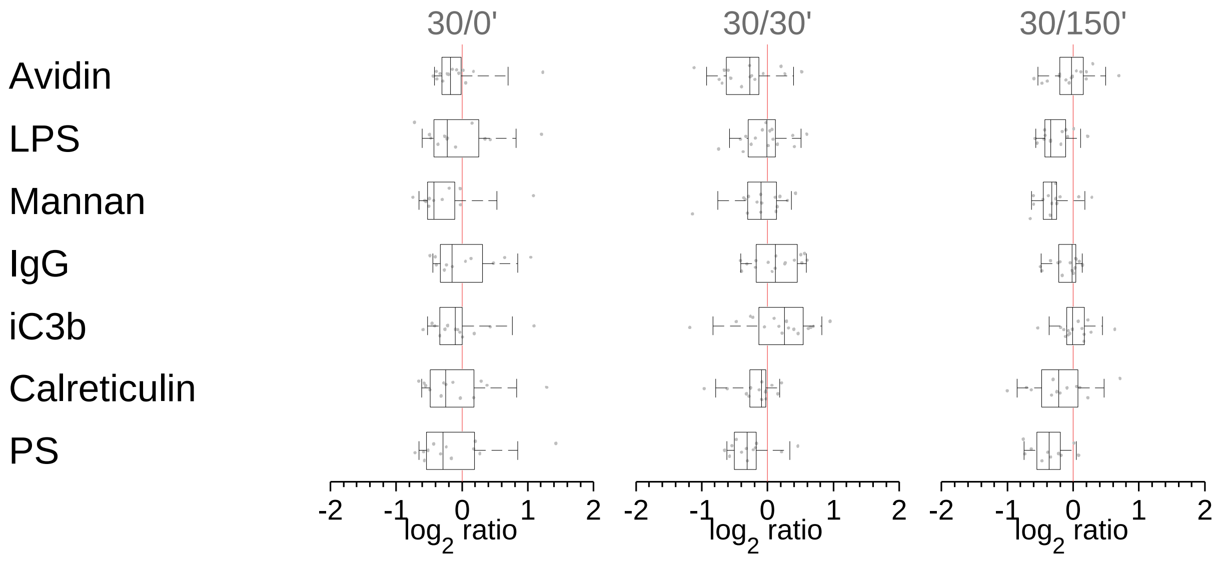

GO:0051289 protein homotetramerization (n = 13)

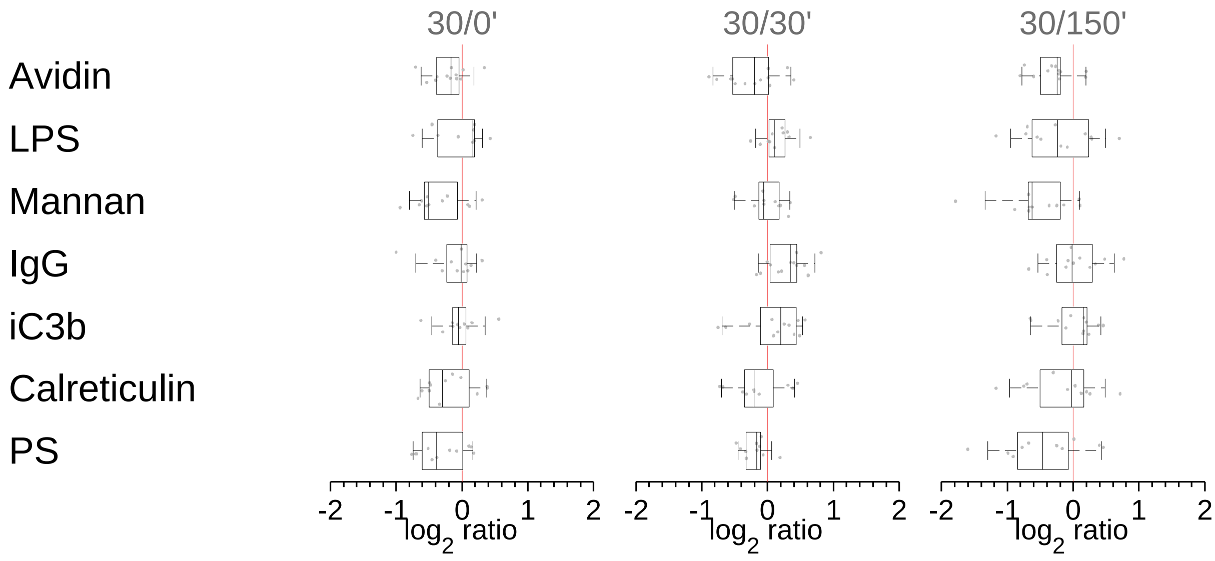

GO:0051291 protein heterooligomerization (n = 17)

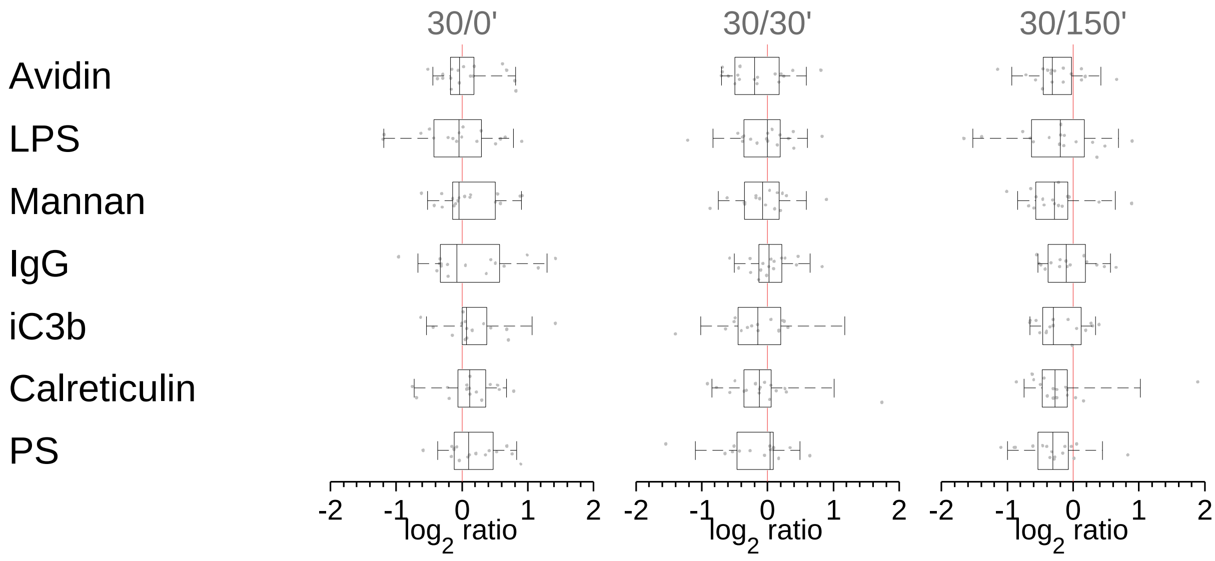

GO:0051297 centrosome organization (n = 3)

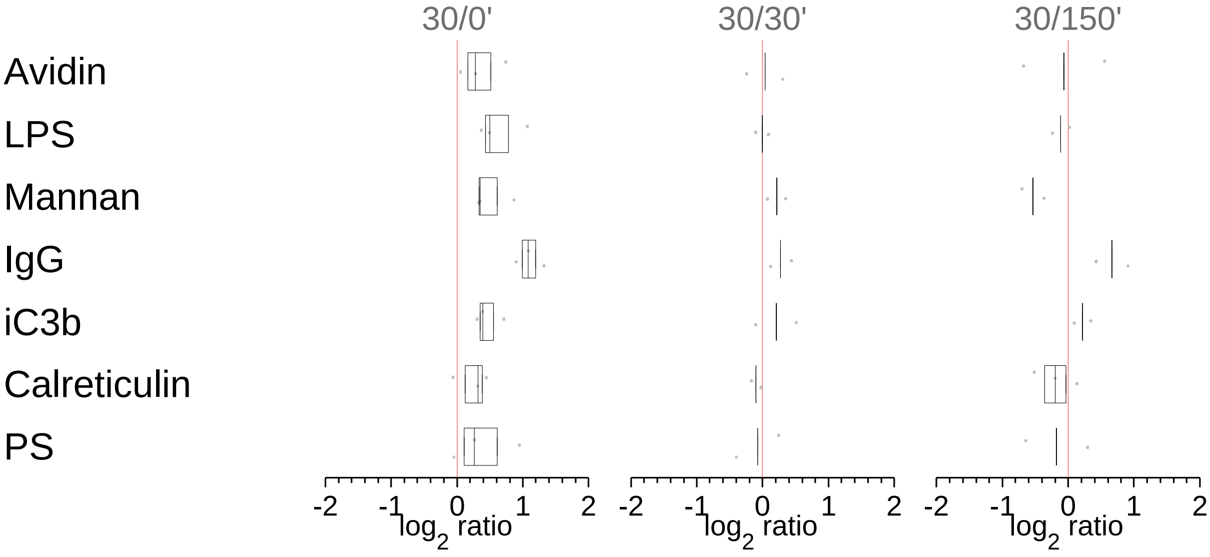

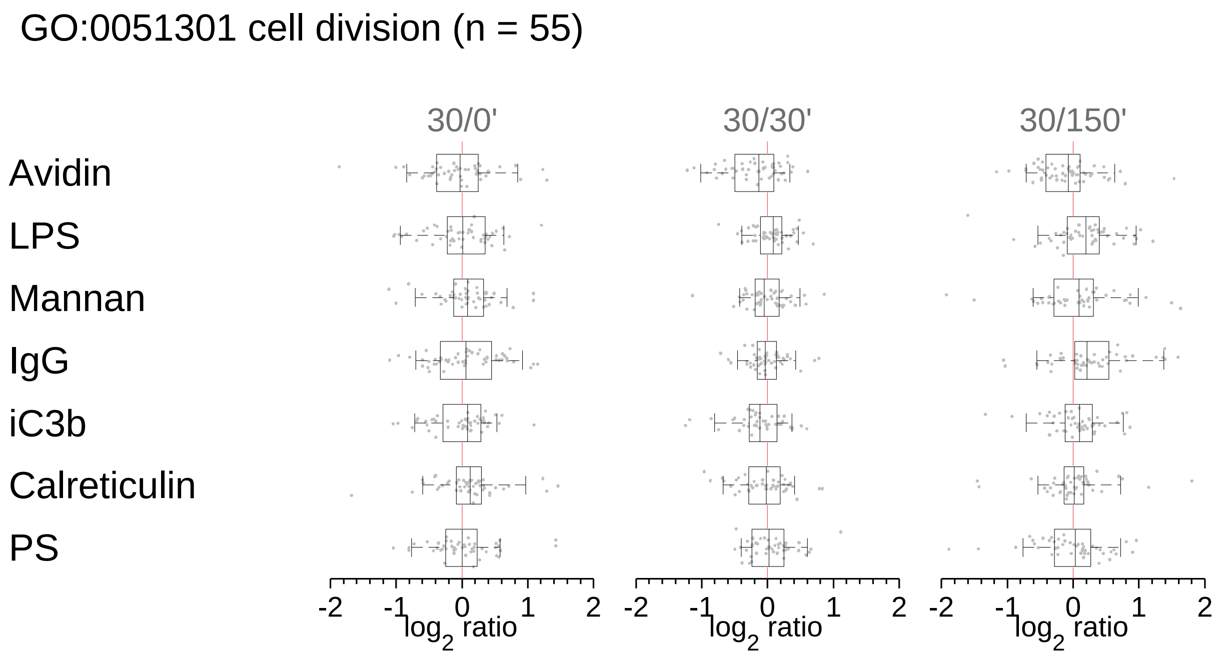
GO:0051301 cell division (n = 55)
30/0'
30/30'
30/150'
Avidin
LPS
Mannan
IgG
iC3b
Calreticulin
PS
-2
-1
0
1
2
log2 ratio

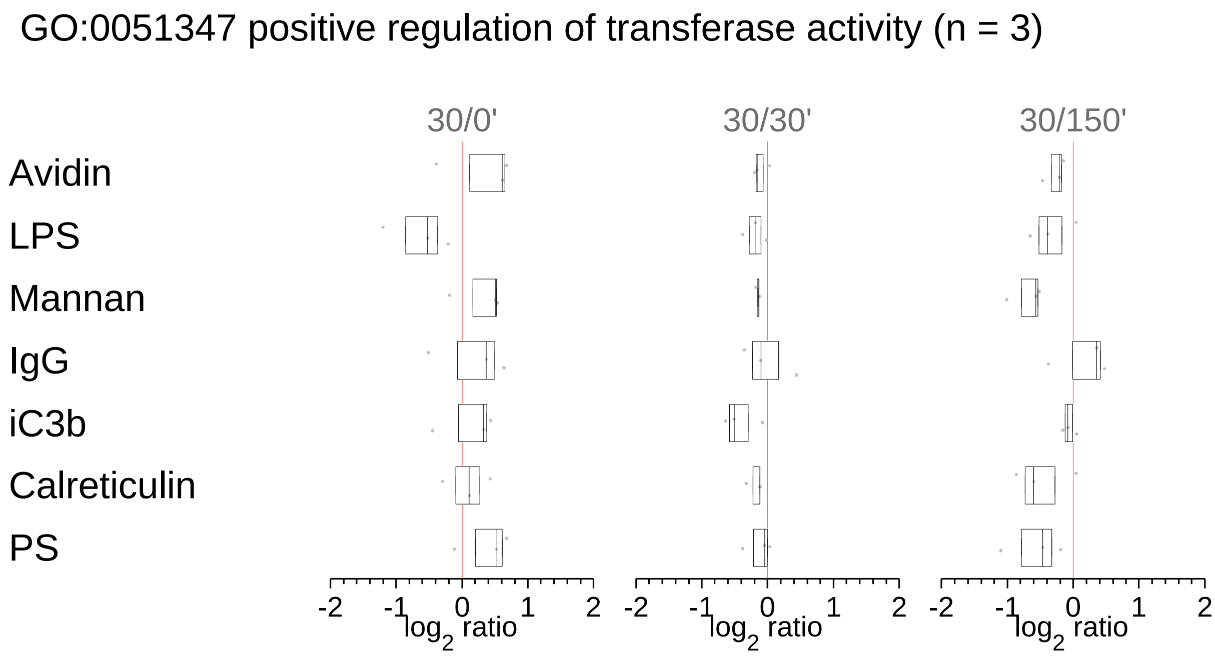
GO:0051347 positive regulation of transferase activity (n = 3)
30/0'
30/30'
30/150'
Avidin
LPS
Mannan
IgG
iC3b
Calreticulin
PS
-2
-1
0
1
2
log_2 ratio

GO:0051384 response to glucocorticoid stimulus (n = 5)

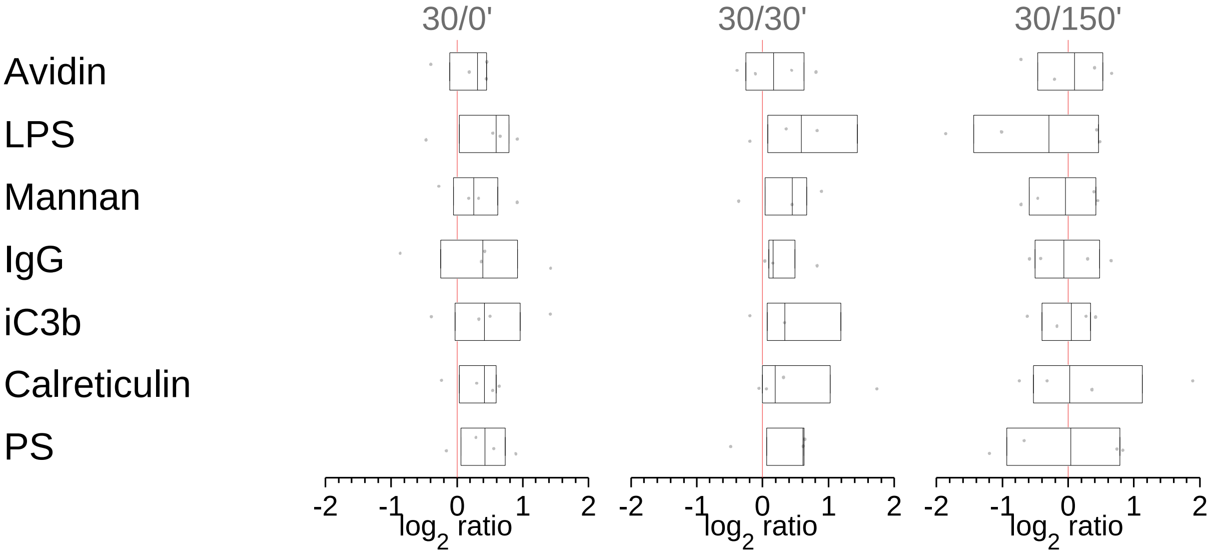

GO:0051400 BH domain binding (n = 3)

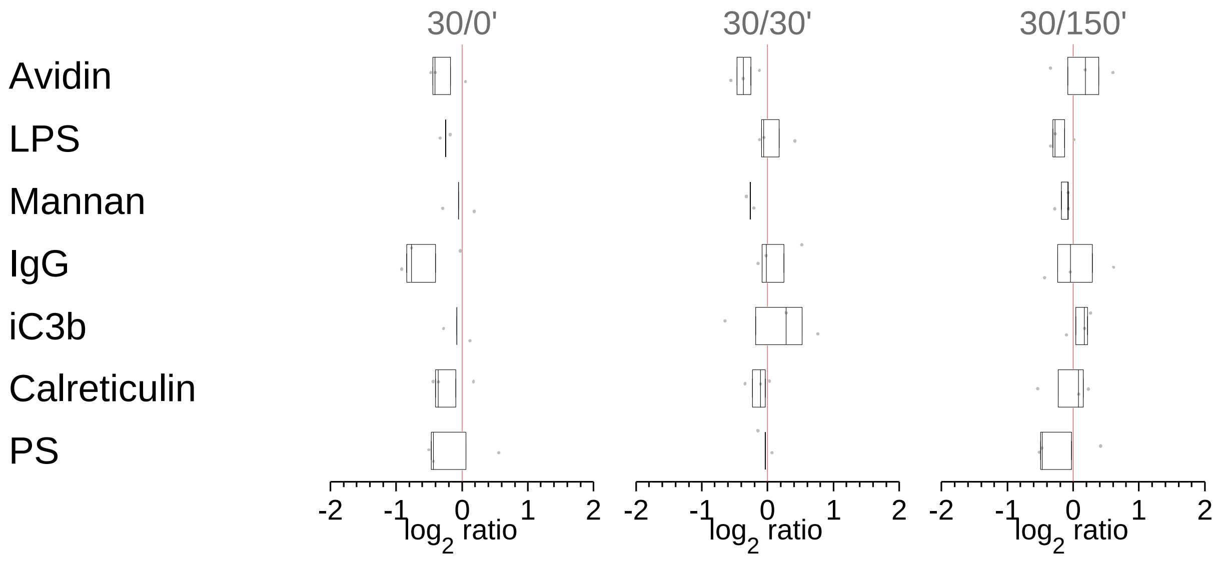

GO:0051402 neuron apoptotic process (n = 6)

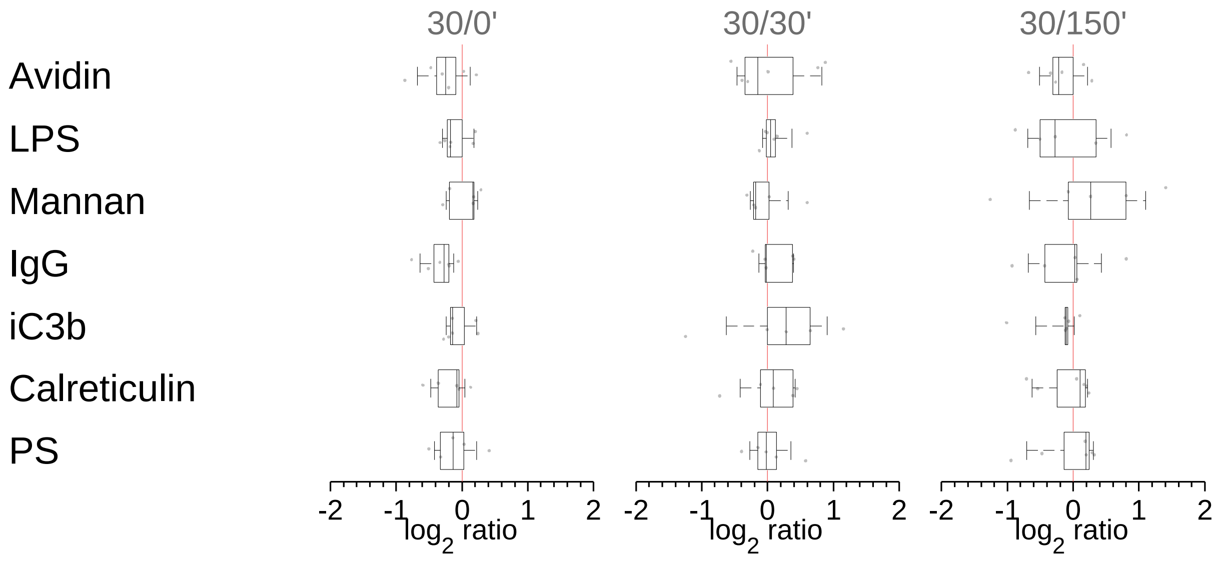

GO:0051425 PTB domain binding (n = 3)

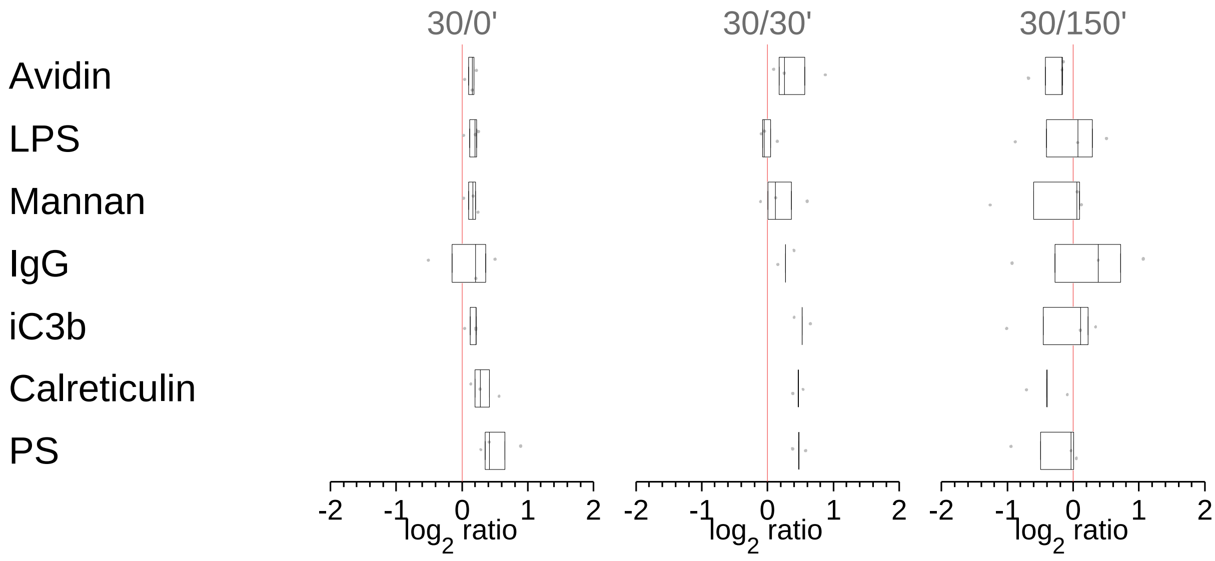

GO:0051491 positive regulation of filopodium assembly (n = 3)

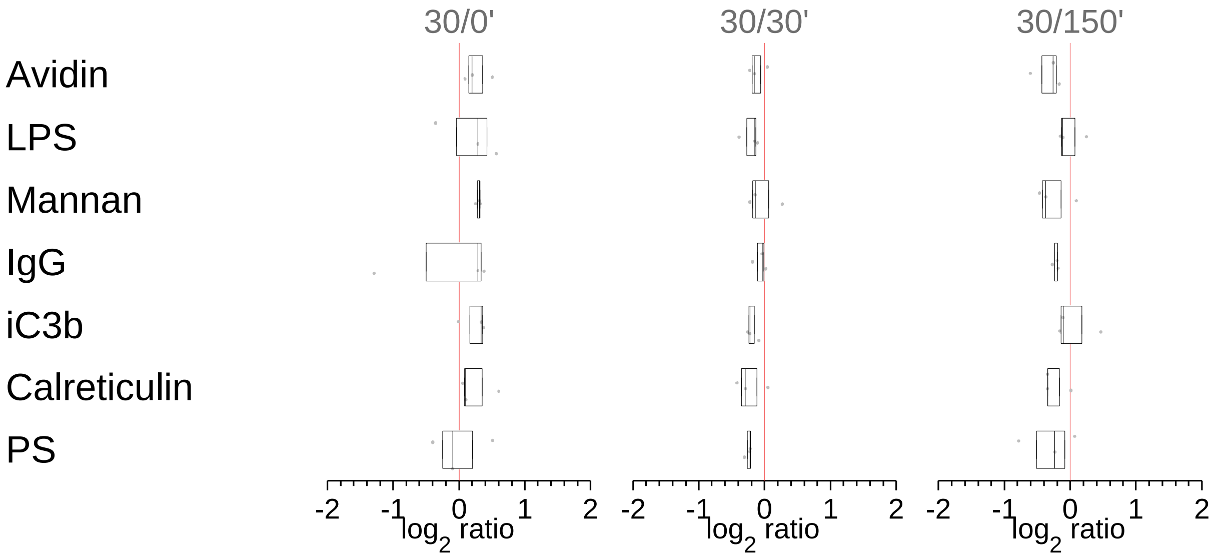

GO:0051496 positive regulation of stress fiber assembly (n = 5)

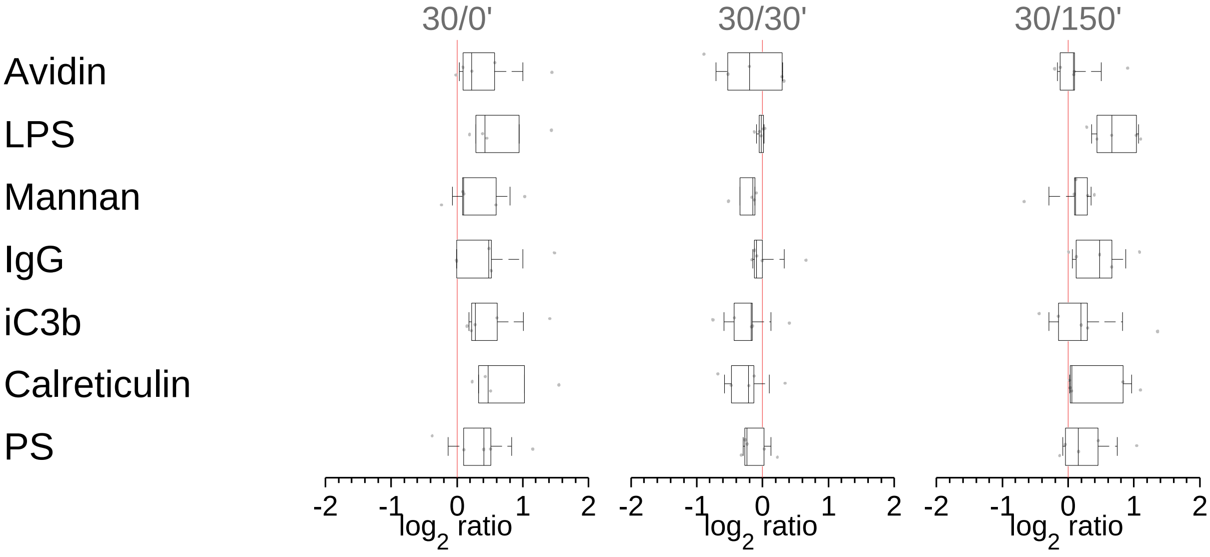

GO:0051497 negative regulation of stress fiber assembly (n = 3)

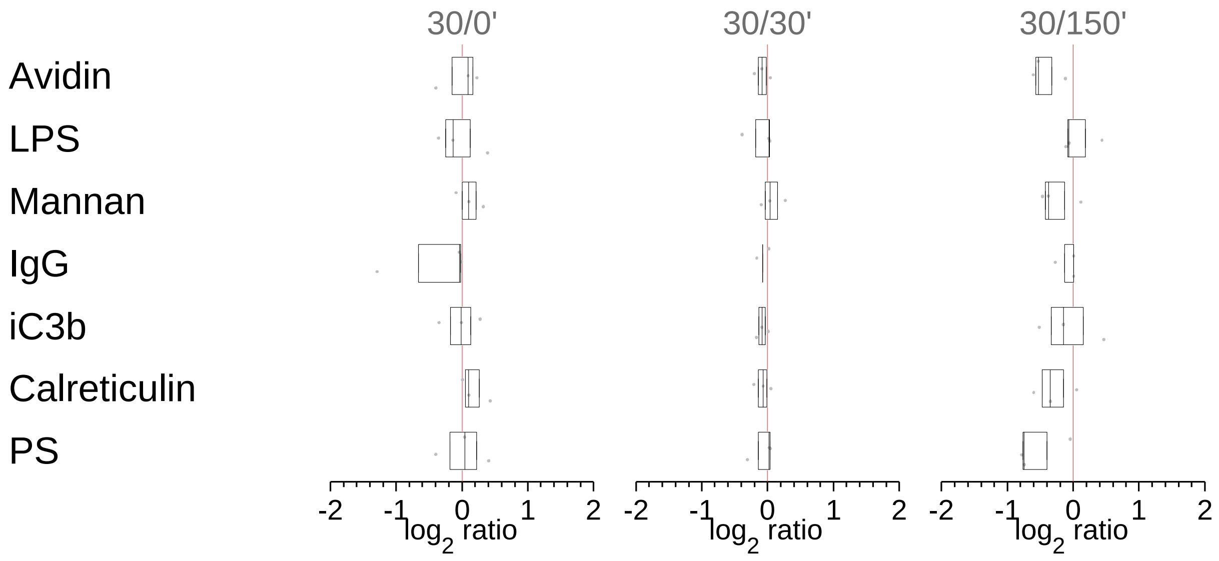

GO:0051536 iron-sulfur cluster binding (n = 11)

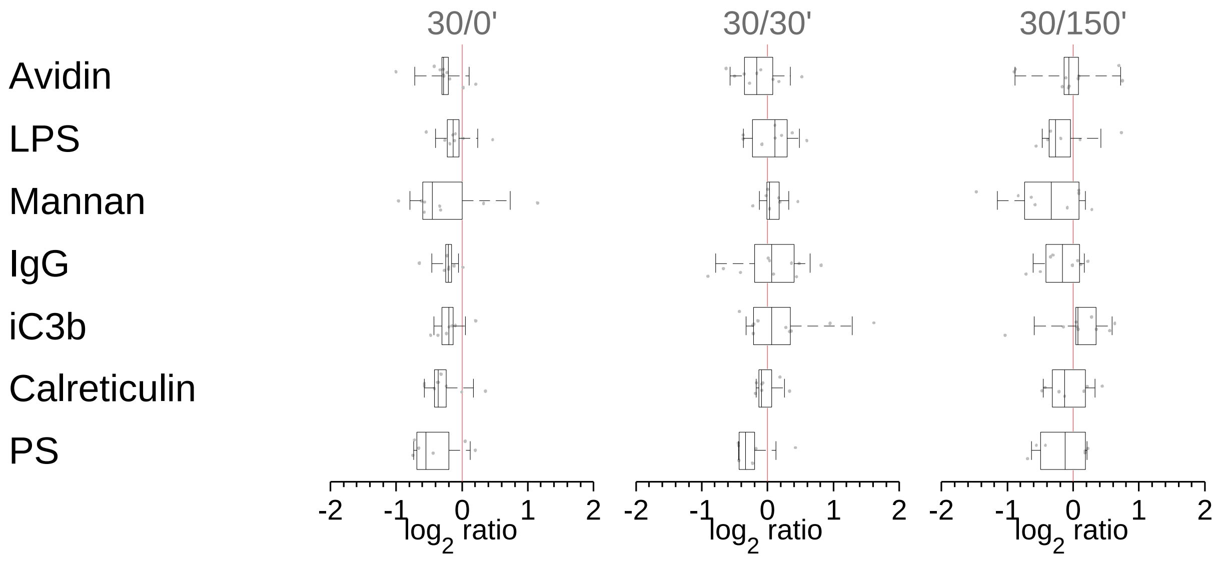

GO:0051537 2 iron, 2 sulfur cluster binding (n = 6)

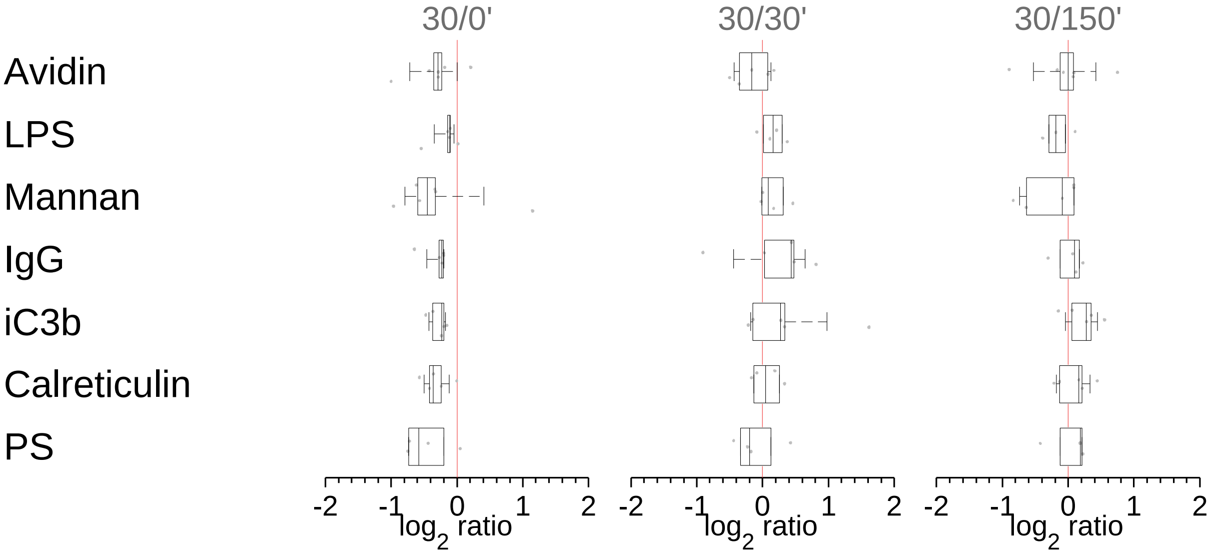

GO:0051539 4 iron, 4 sulfur cluster binding (n = 7)

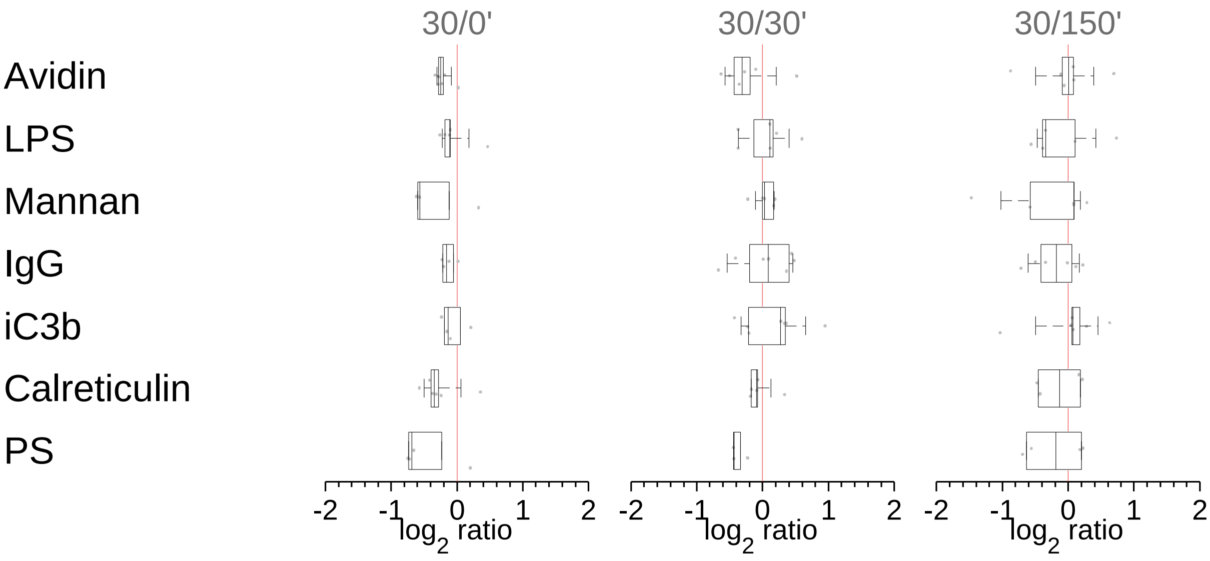

GO:0051561 elevation of mitochondrial calcium ion concentration
(n = 3)

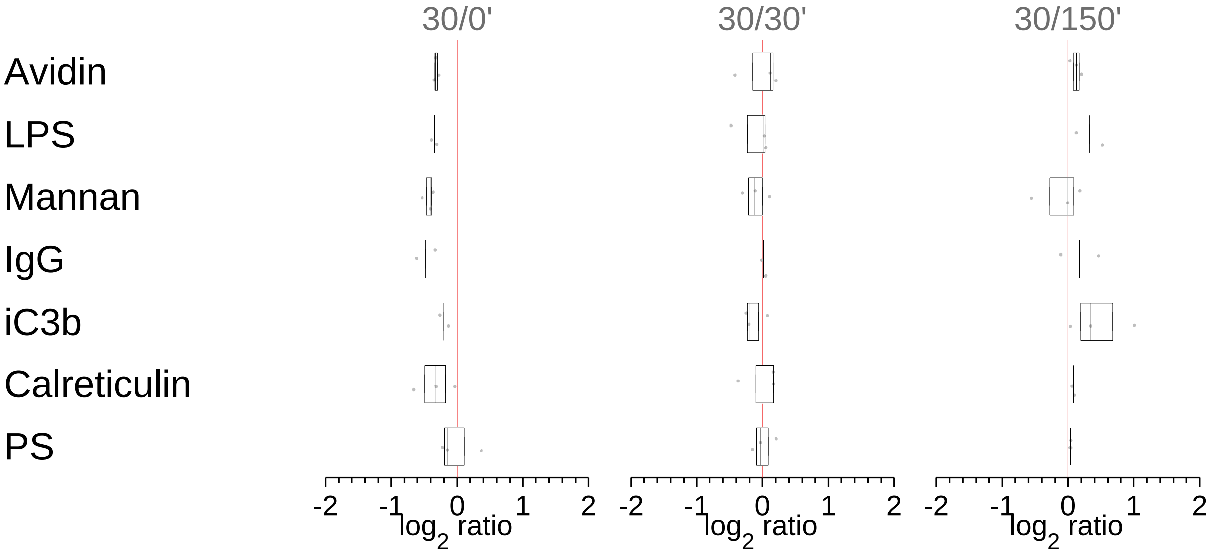

GO:0051592 response to calcium ion (n = 11)

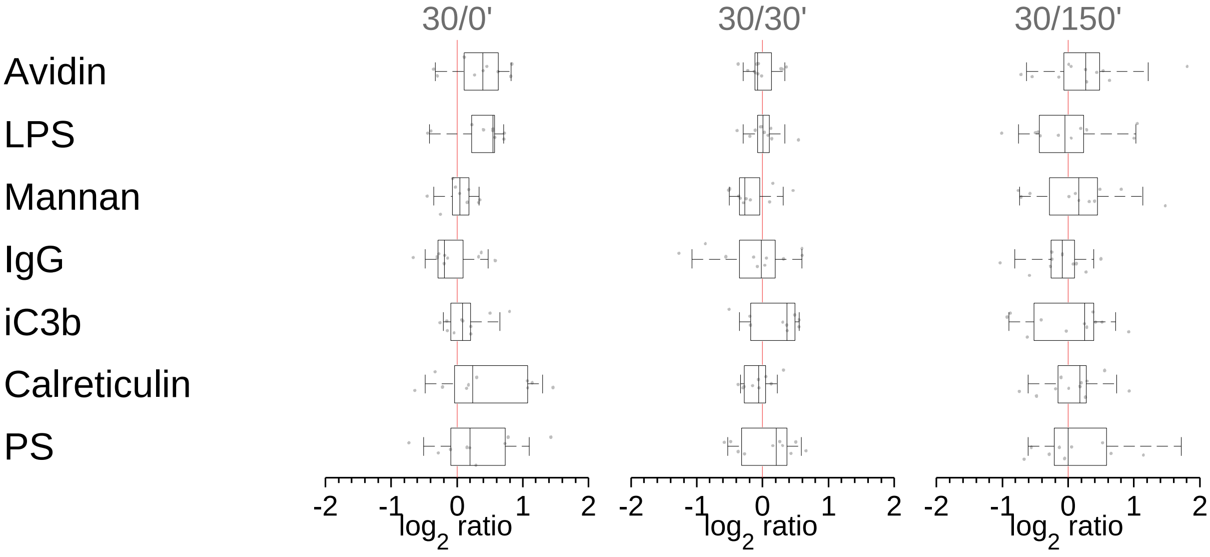

GO:0051602 response to electrical stimulus (n = 3)

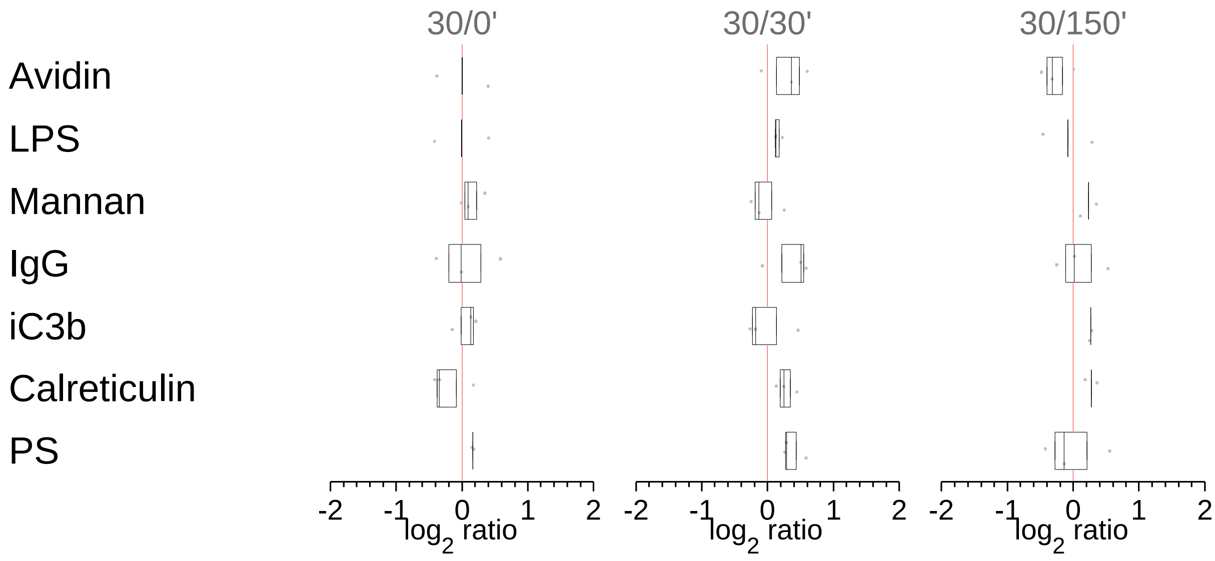

GO:0051603 proteolysis involved in cellular protein catabolic process (n = 14)

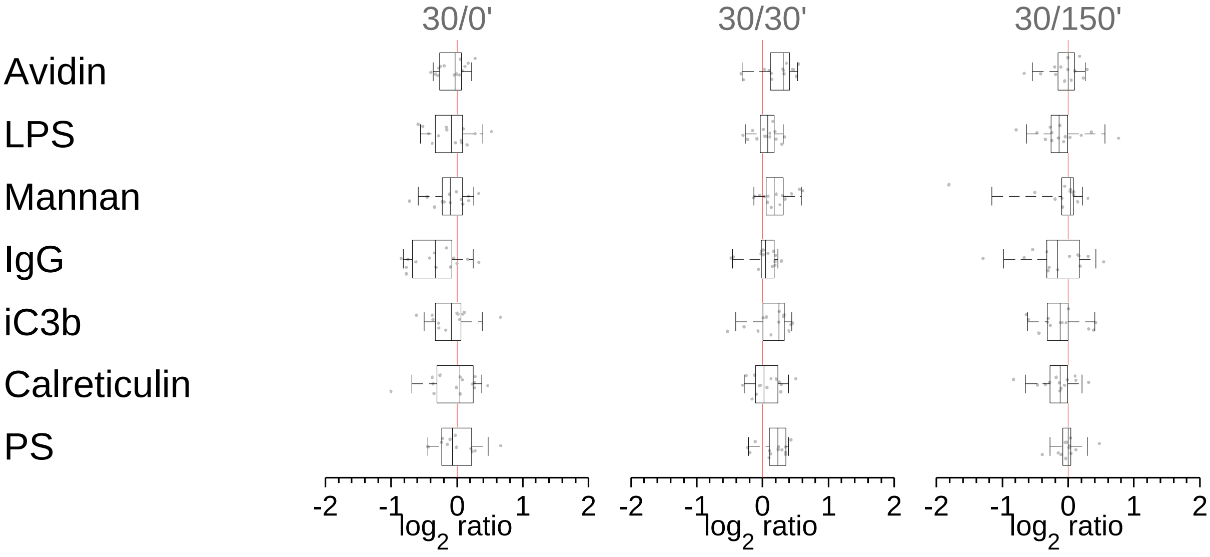

GO:0051604 protein maturation (n = 4)

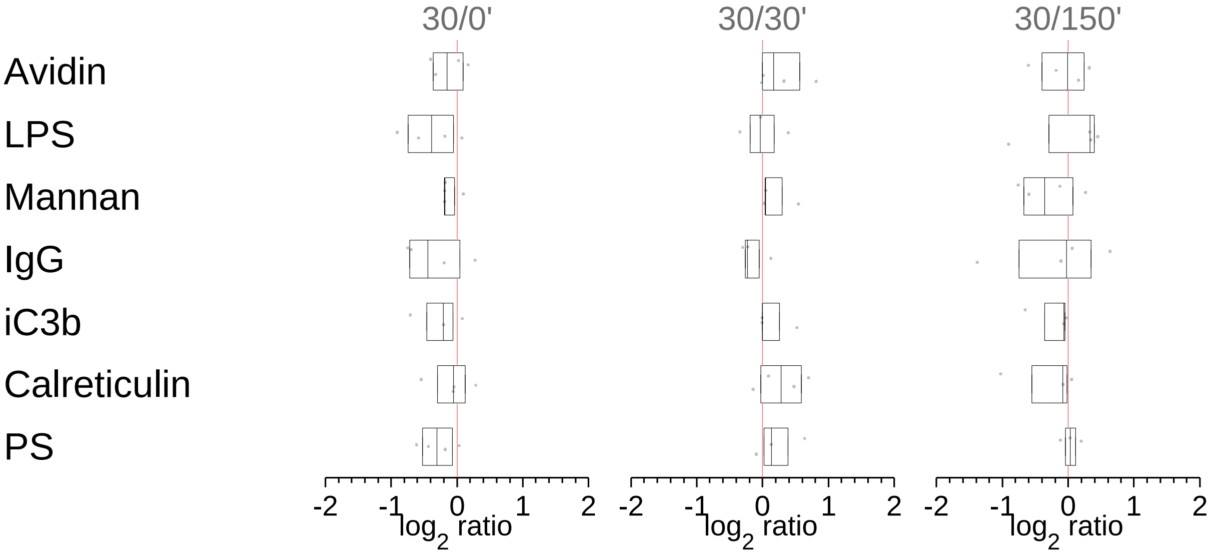

GO:0051607 defense response to virus (n = 22)

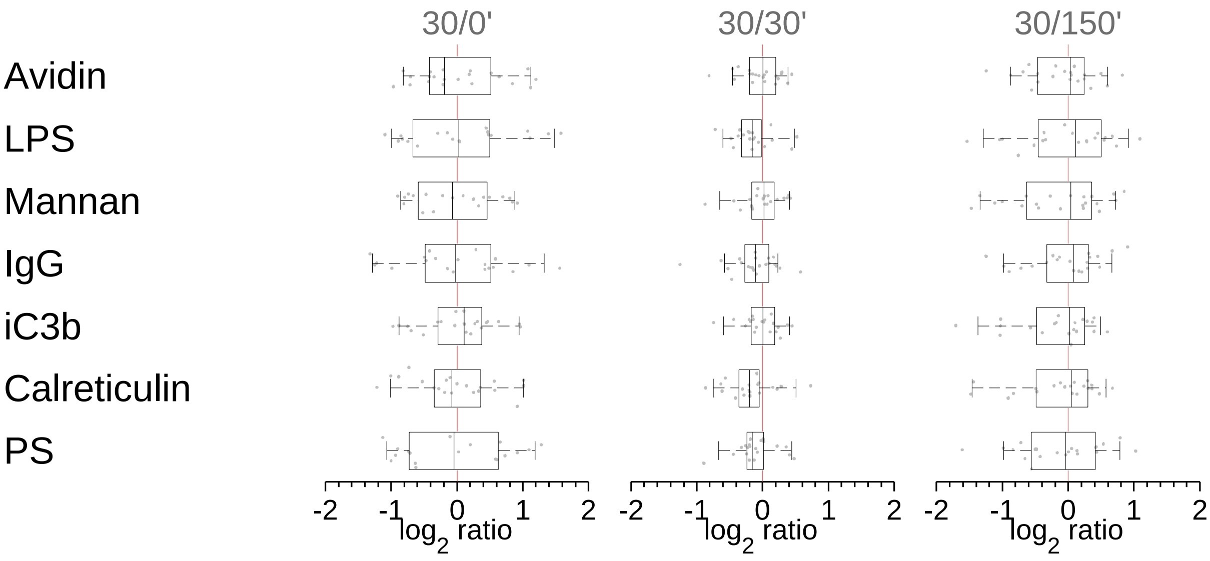

GO:0051693 actin filament capping (n = 7)

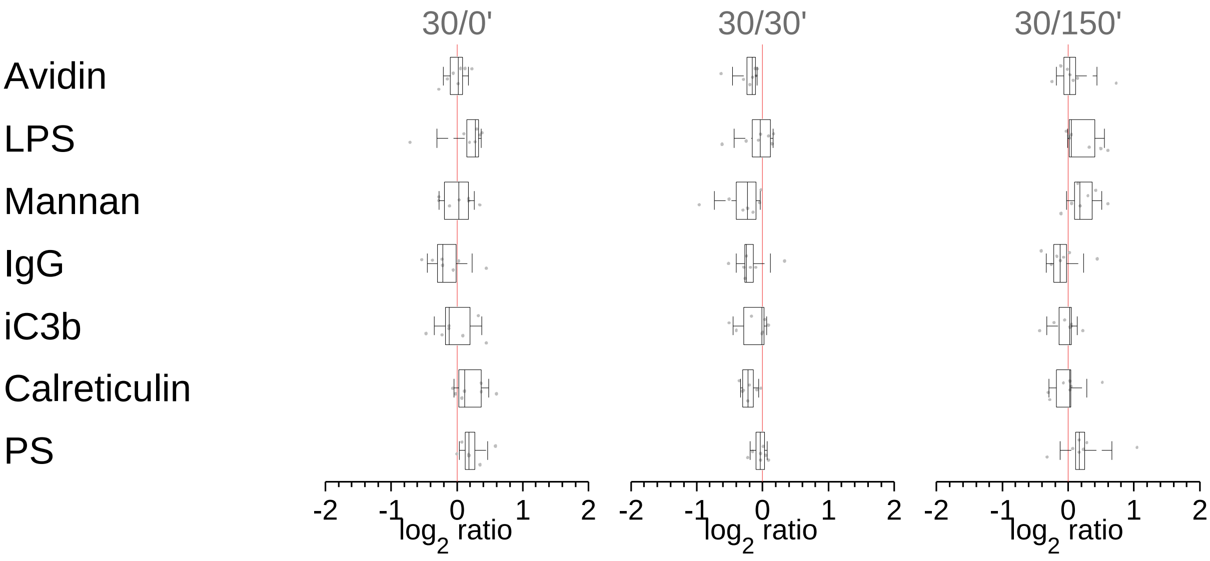

GO:0051721 protein phosphatase 2A binding (n = 3)

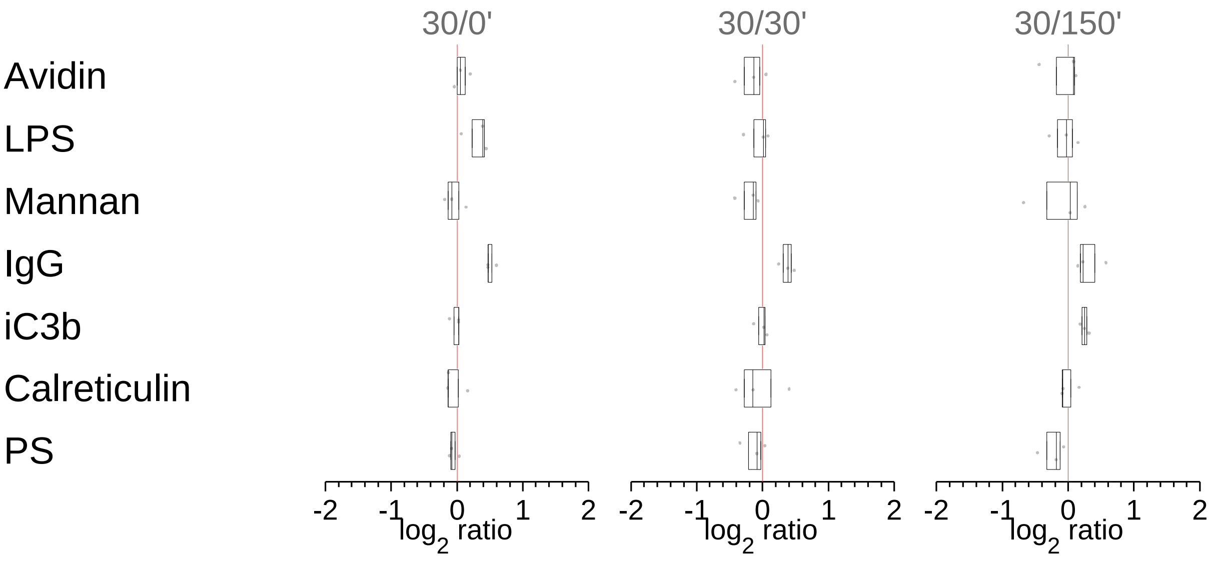

GO:0051726 regulation of cell cycle (n = 15)

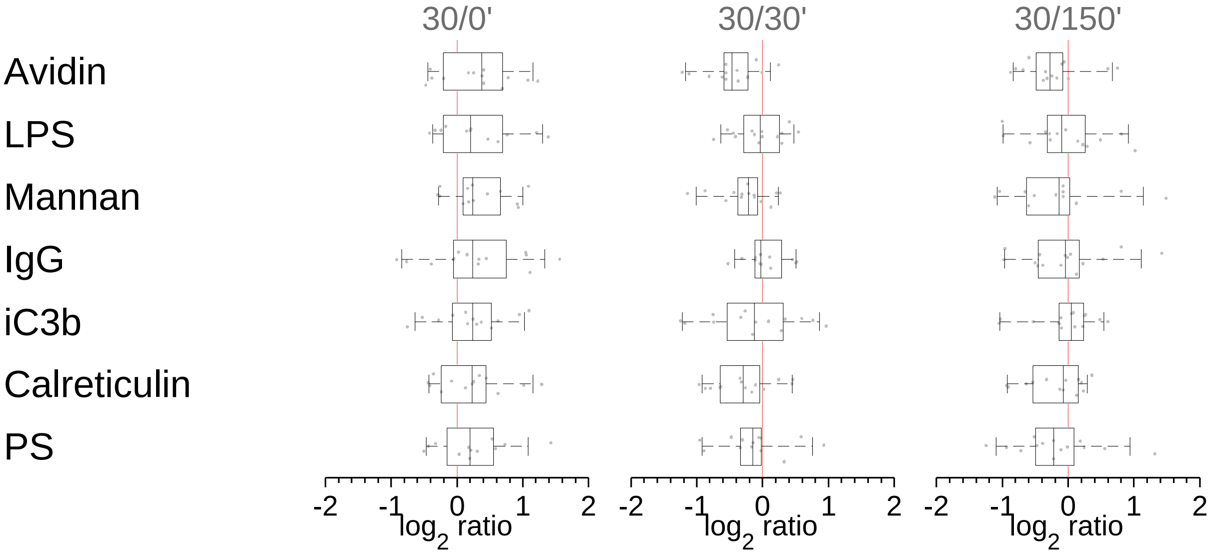

GO:0051770 positive regulation of nitric-oxide synthase biosynthetic process (n = 3)

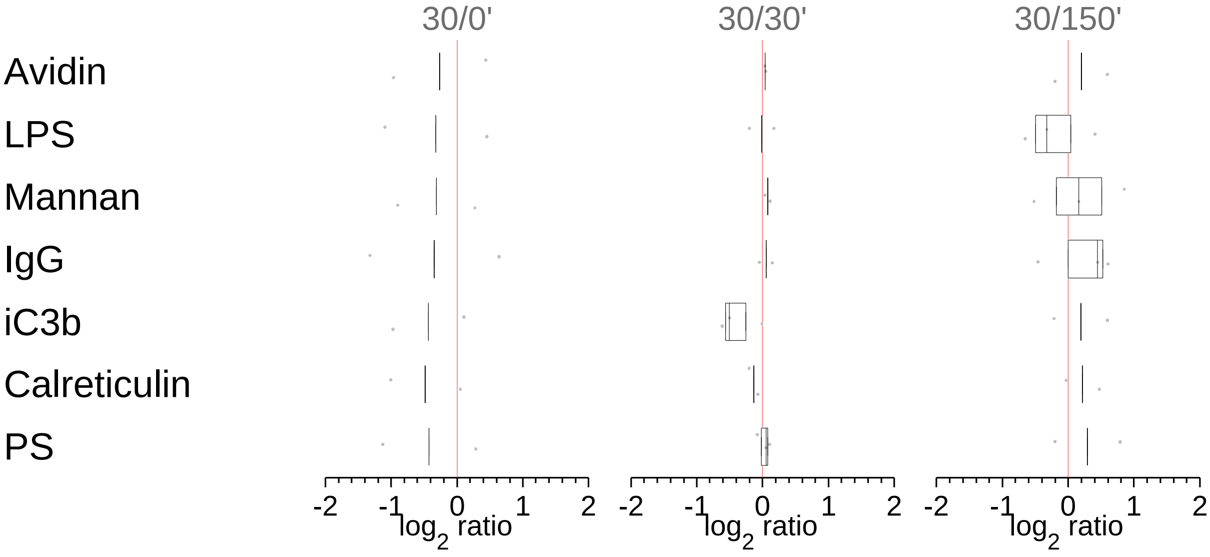

GO:0051787 misfolded protein binding (n = 4)

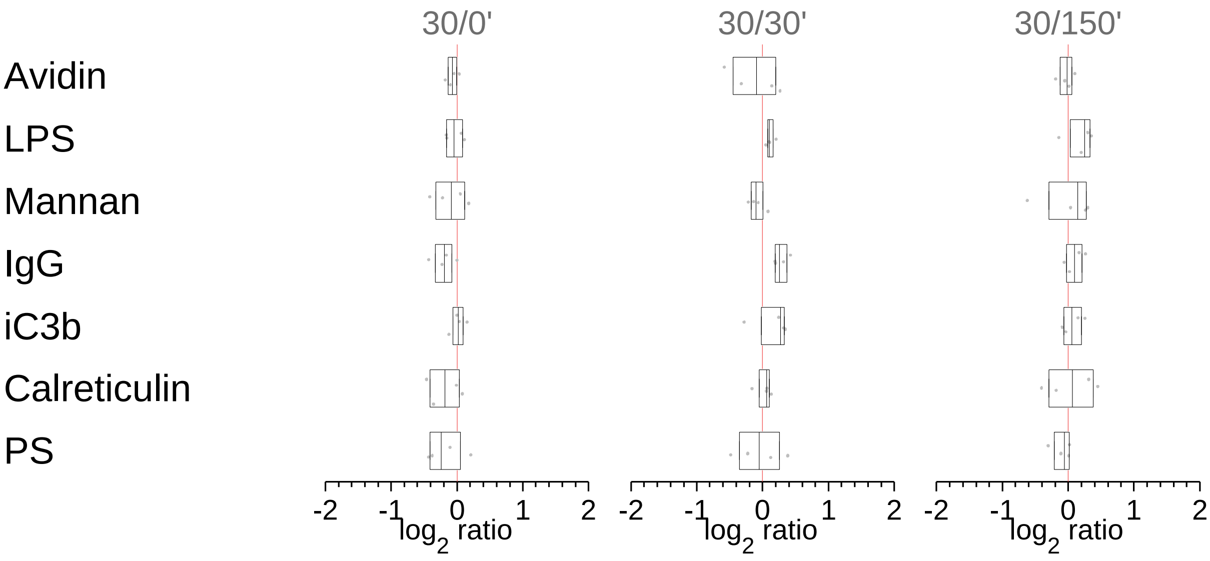

GO:0051865 protein autoubiquitination (n = 5)

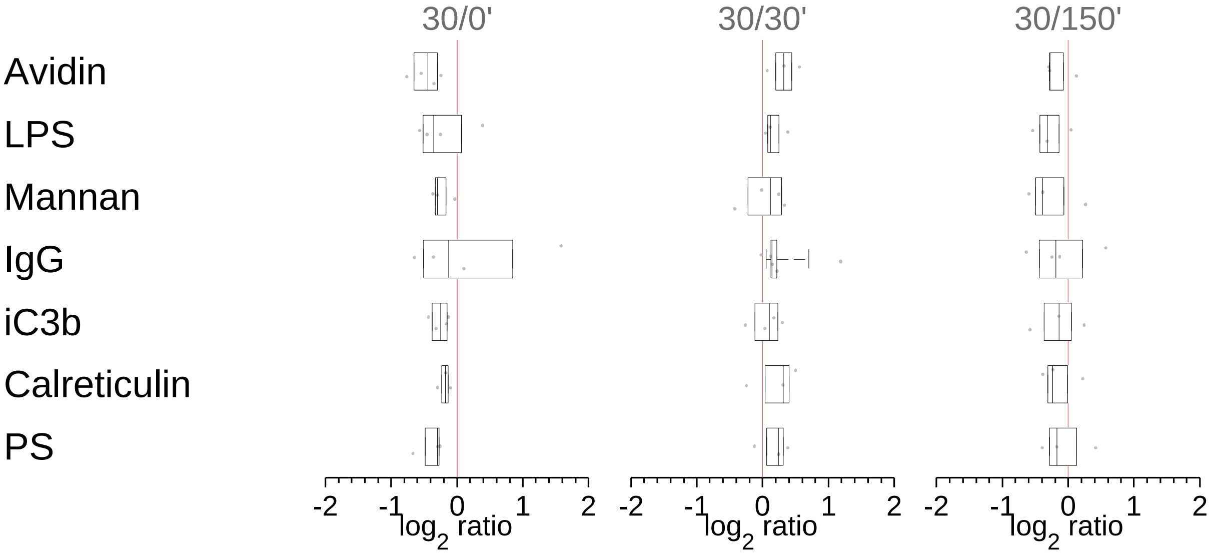

GO:0051881 regulation of mitochondrial membrane potential (n = 9)

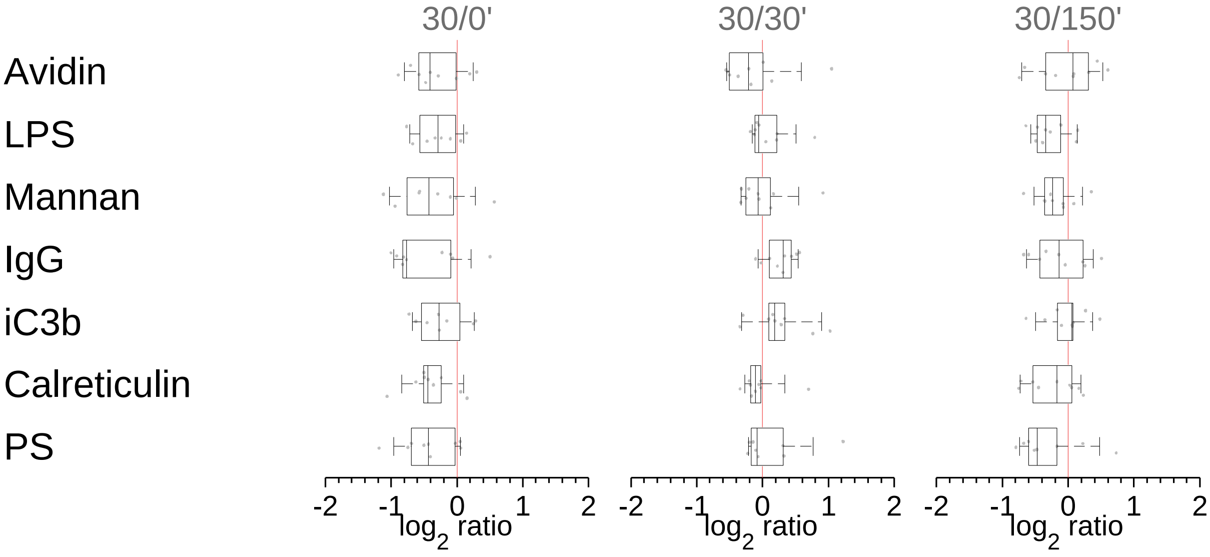

GO:0051894 positive regulation of focal adhesion assembly (n = 4)

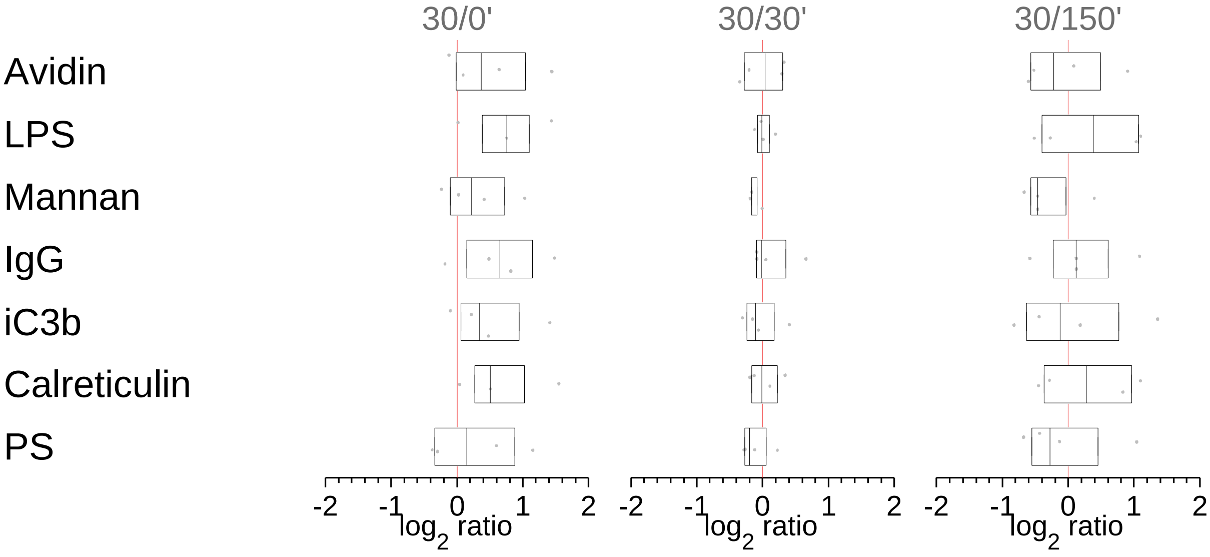

GO:0051897 positive regulation of protein kinase B signaling cascade (n = 11)

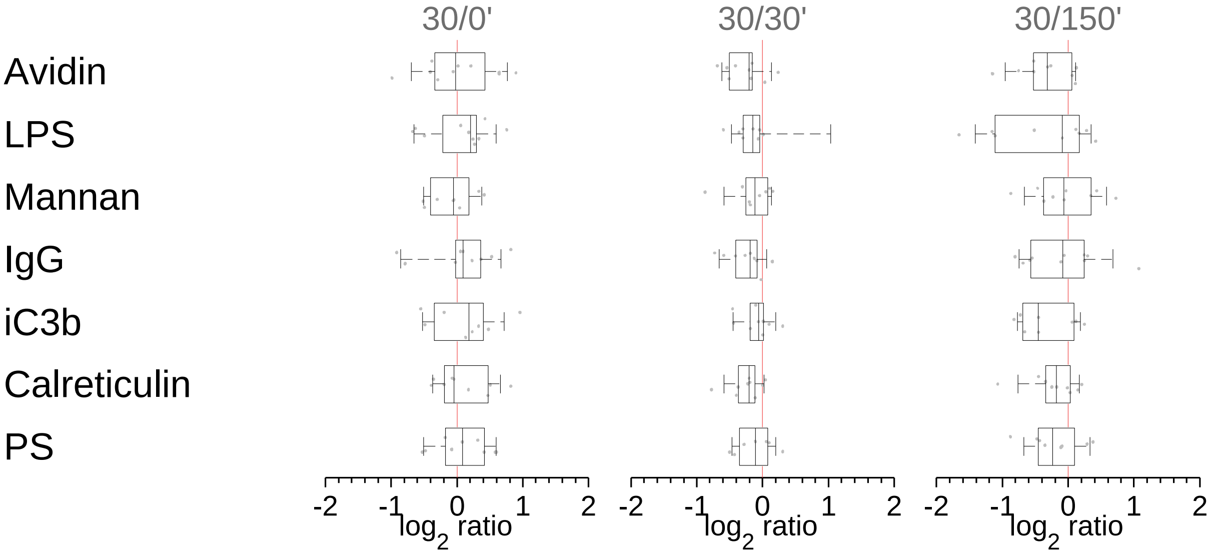

GO:0051898 negative regulation of protein kinase B signaling cascade (n = 6)

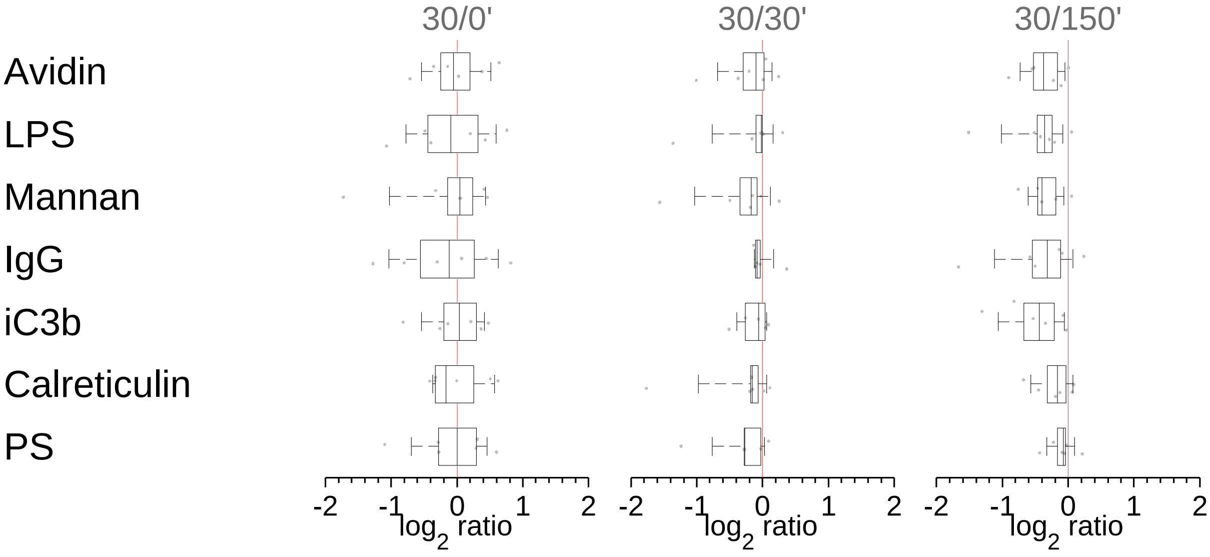

GO:0051899 membrane depolarization (n = 3)

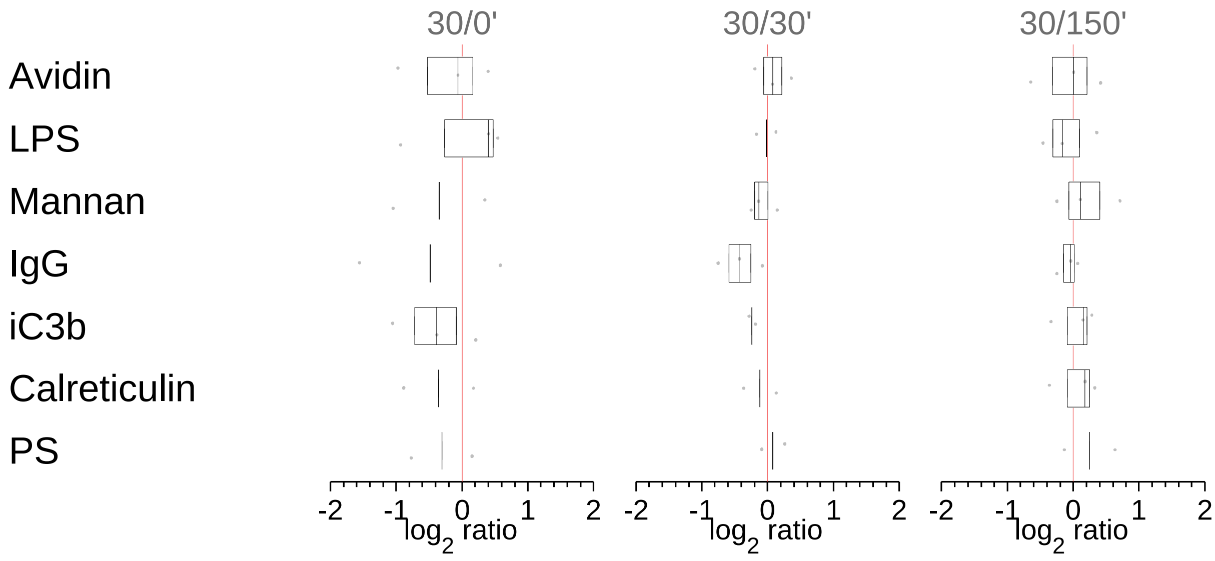

GO:0051920 peroxiredoxin activity (n = 5)

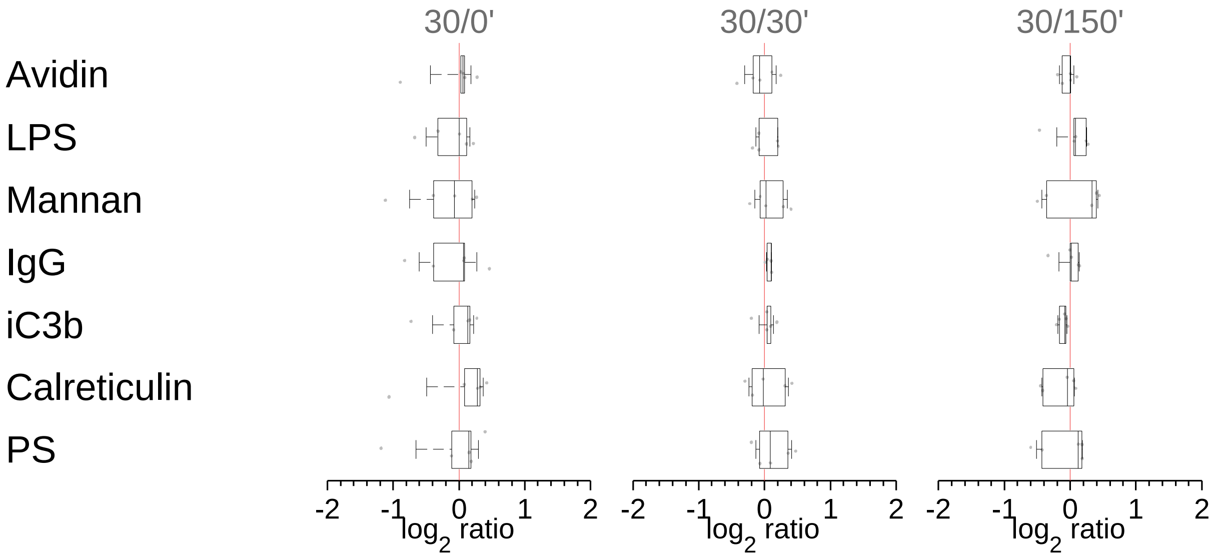

GO:0051924 regulation of calcium ion transport (n = 3)

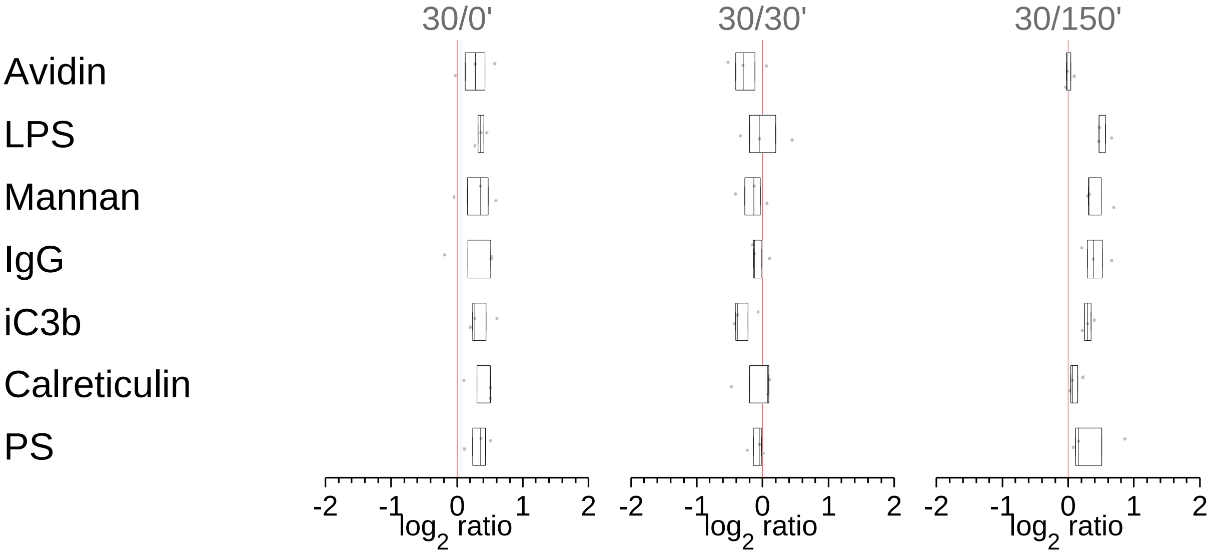

GO:0051930 regulation of sensory perception of pain (n = 3)

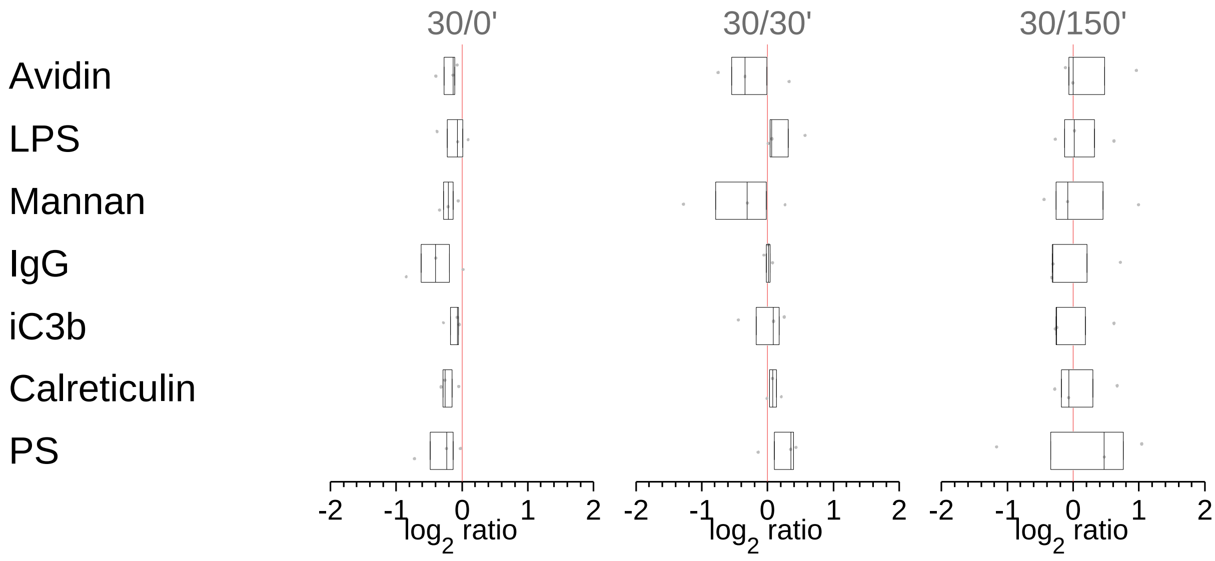

GO:0051990 (R)-2-hydroxyglutarate dehydrogenase activity (n = 3)

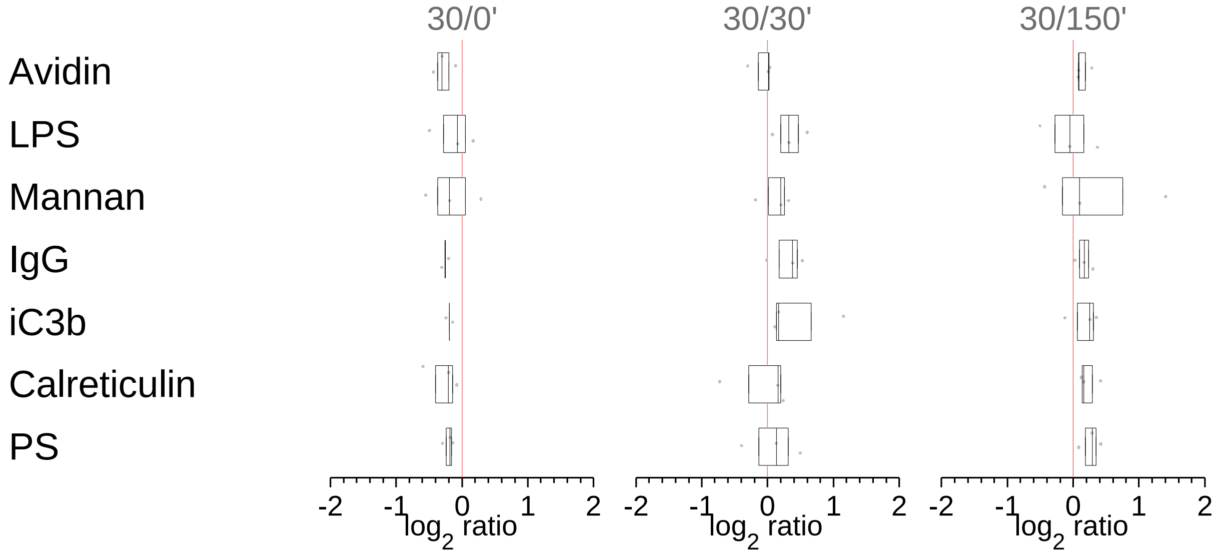

GO:0052638 indole-3-butyrate beta-glucosyltransferase activity
(n = 3)

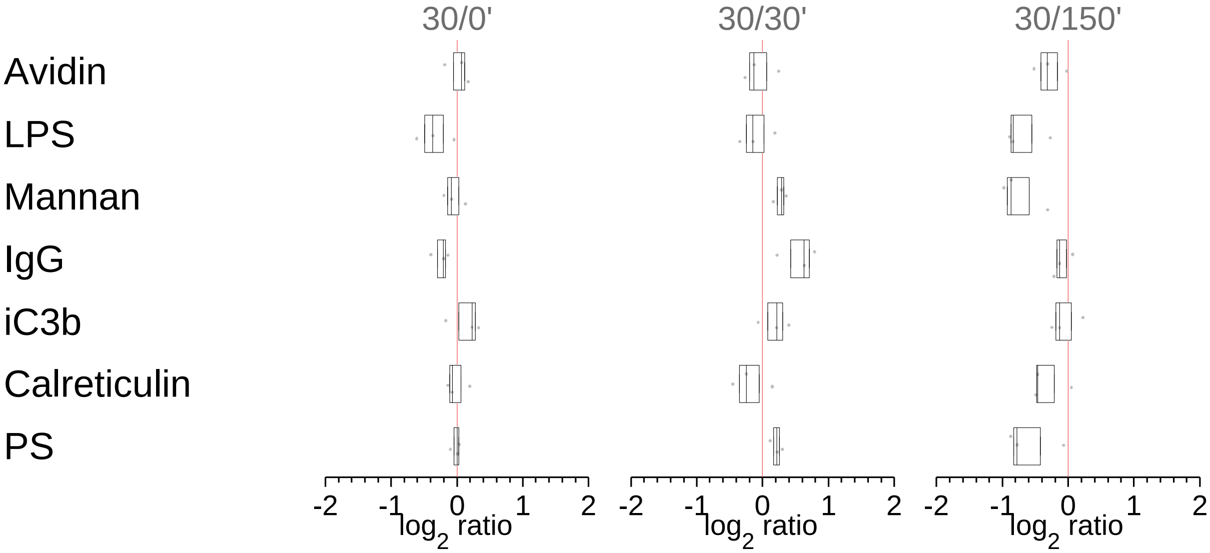

GO:0052639 salicylic acid glucosyltransferase (ester-forming) activity (n = 3)

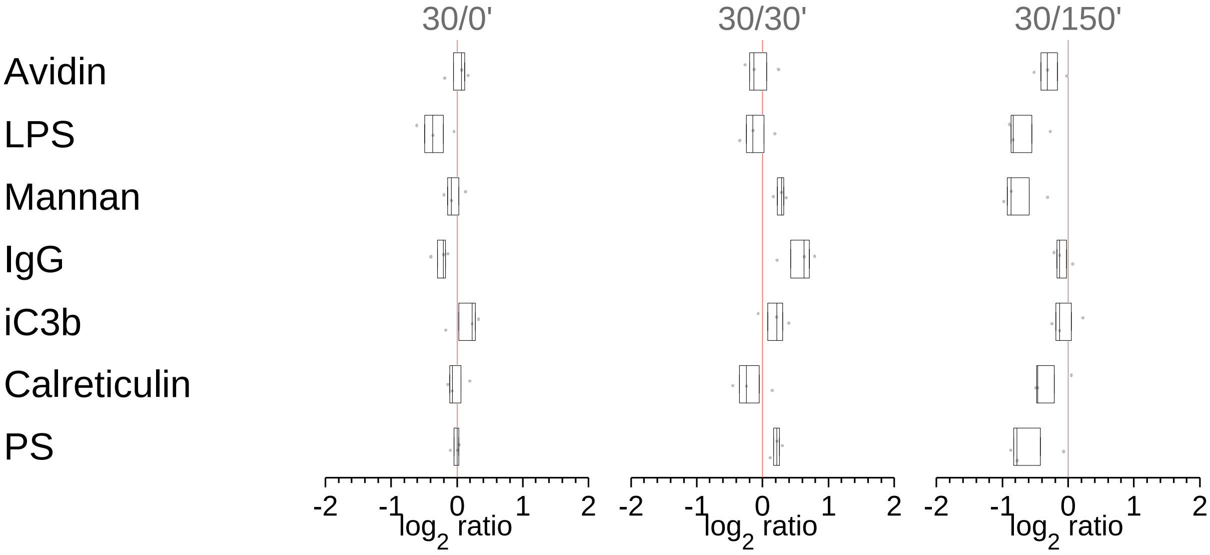

GO:0052640 salicylic acid glucosyltransferase (glucoside-forming) activity (n = 3)

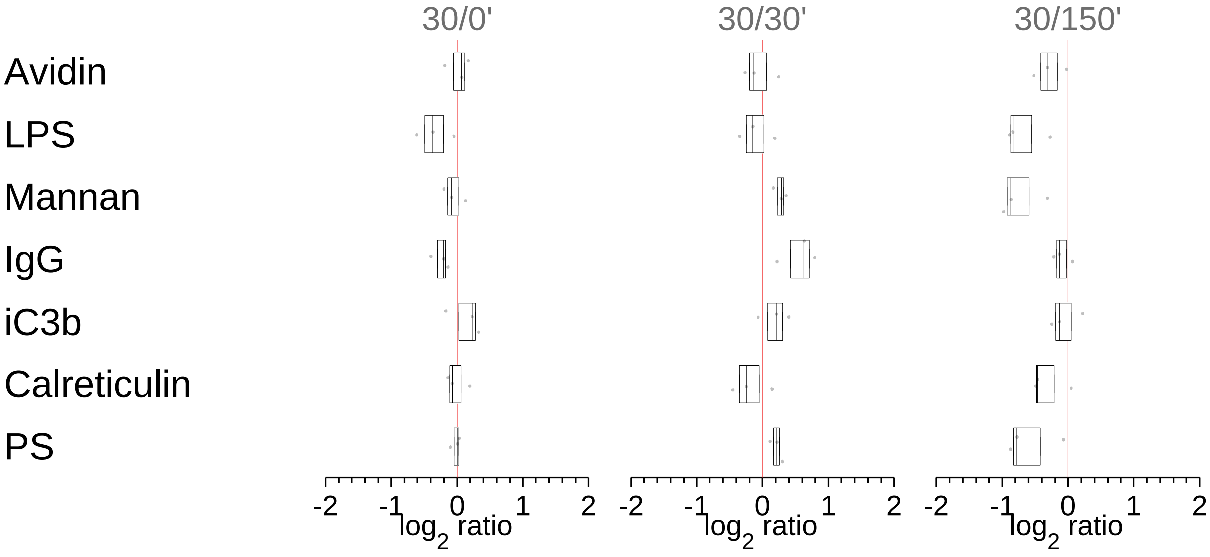

GO:0052641 benzoic acid glucosyltransferase activity (n = 3)

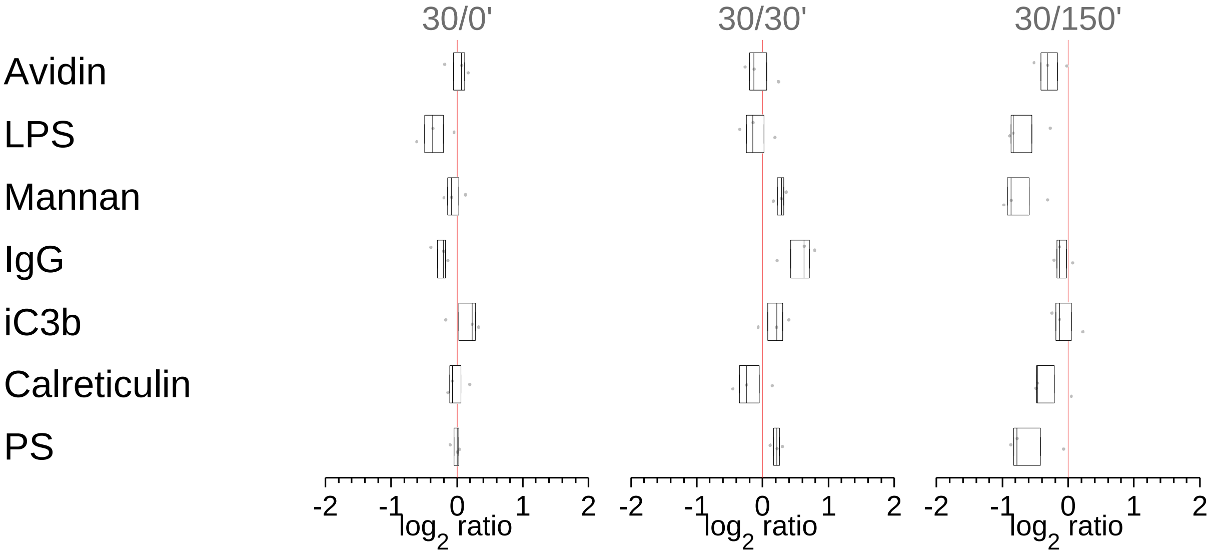

GO:0052677 D-arabinitol dehydrogenase, D-xylulose forming (NADP+) activity (n = 3)

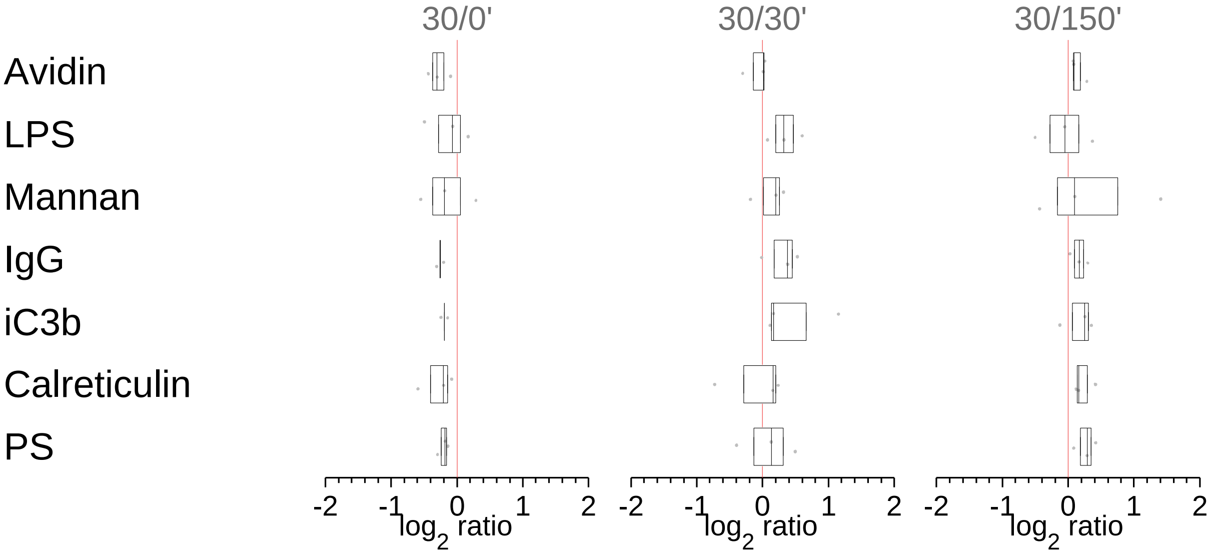

GO:0052693 epoxyqueuosine reductase activity (n = 9)

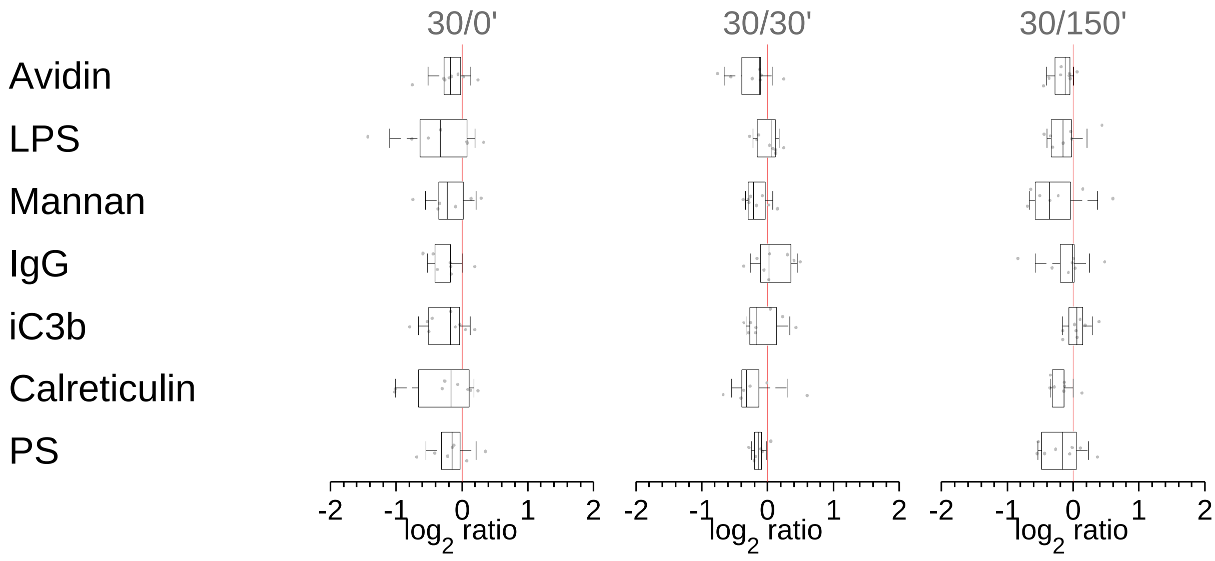

GO:0052757 chondroitin hydrolase activity (n = 3)

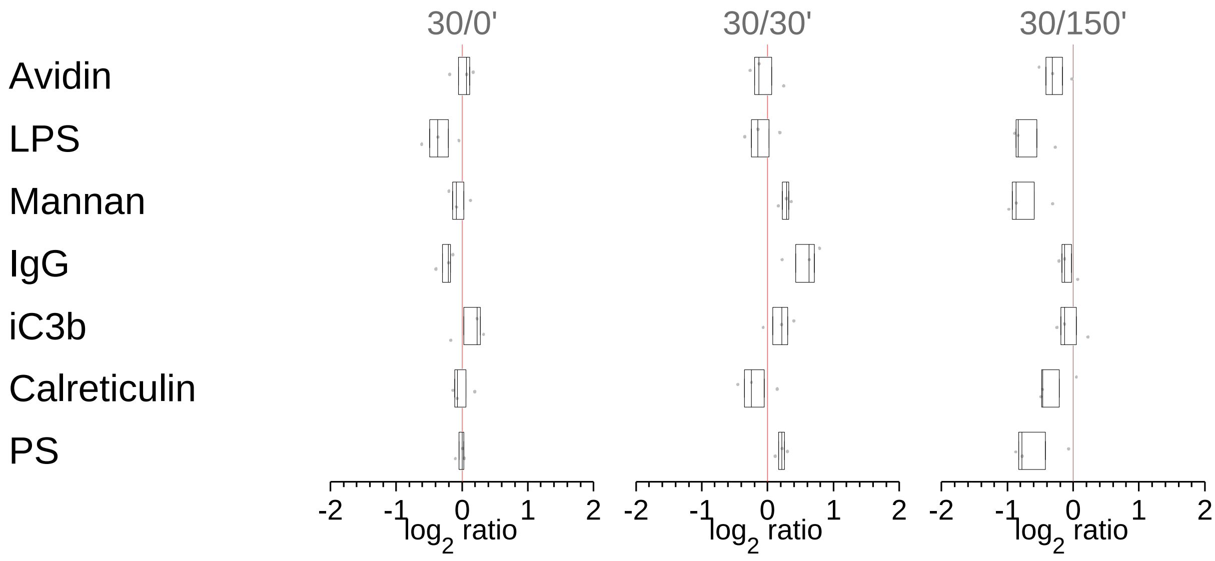

GO:0052767 mannosyl-oligosaccharide 1,6-alpha-mannosidase activity (n = 4)

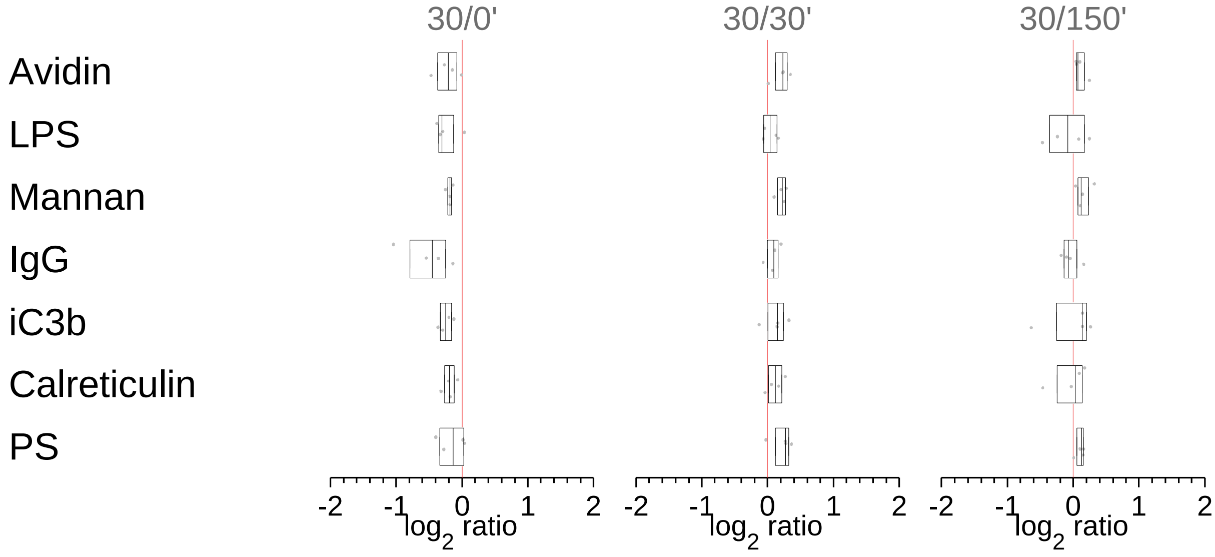

GO:0052768 mannosyl-oligosaccharide 1,3-alpha-mannosidase activity (n = 4)

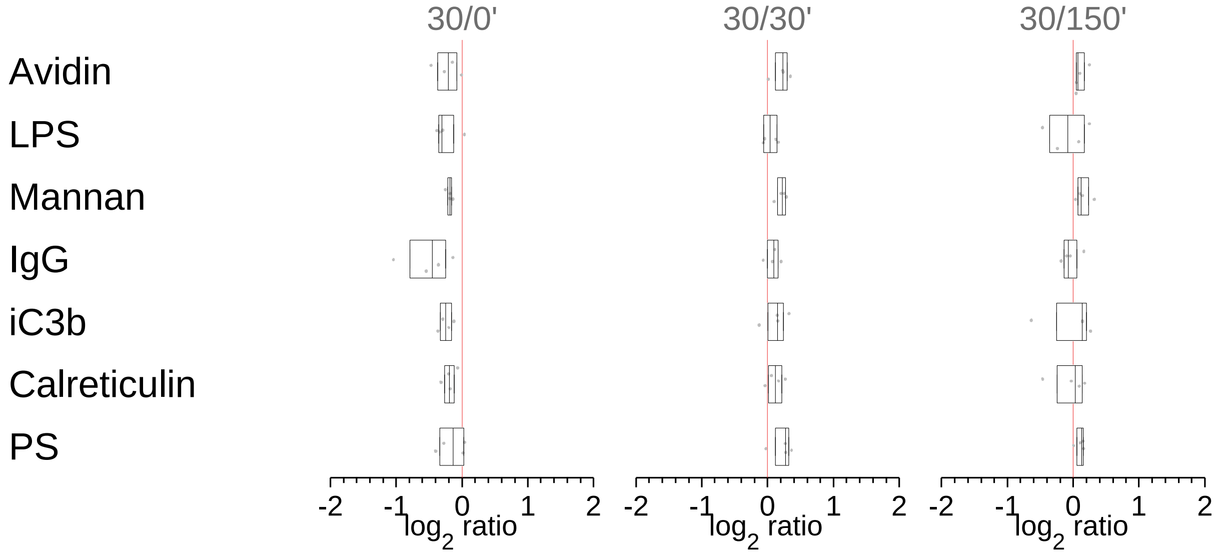

GO:0052824 dolichyl-pyrophosphate Man7GlcNAc2 alpha-1,6-mannosyltransferase activity (n = 3)

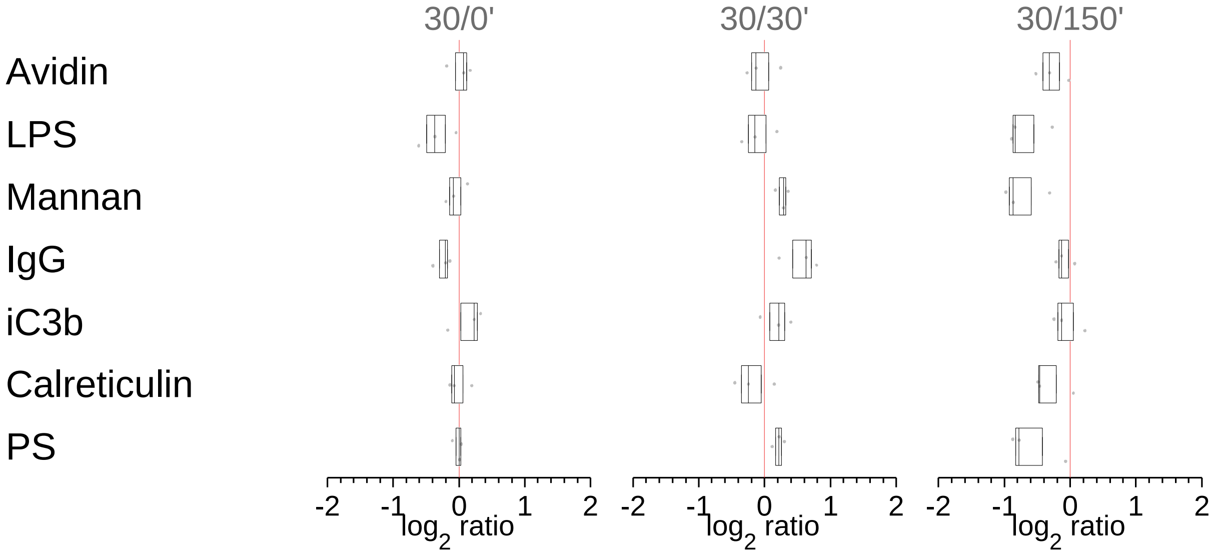

GO:0052858 peptidyl-lysine N-acetyltransferase activity (n = 3)

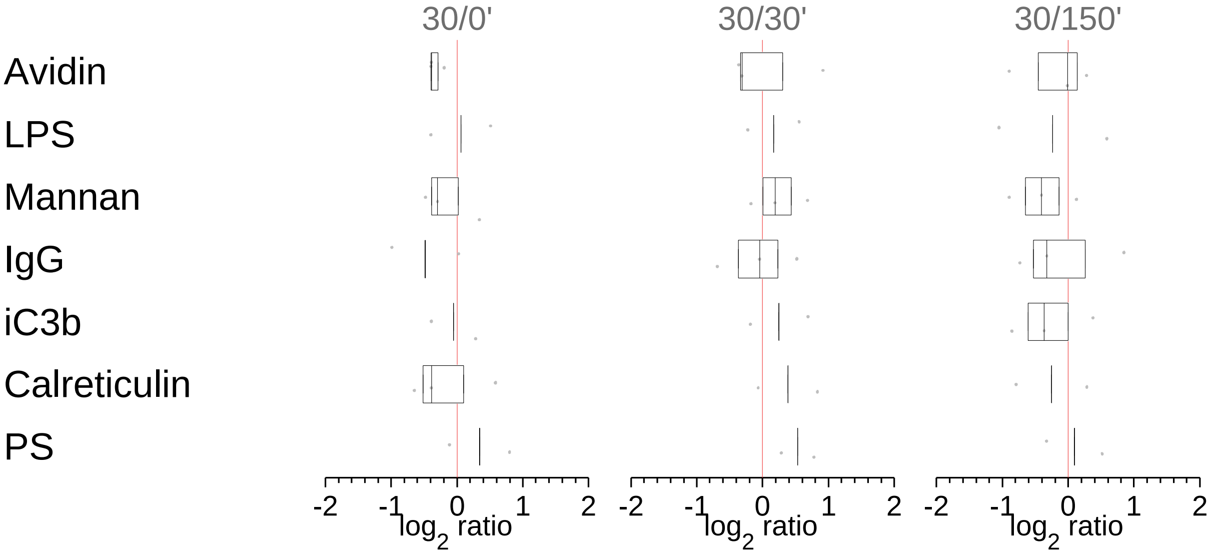

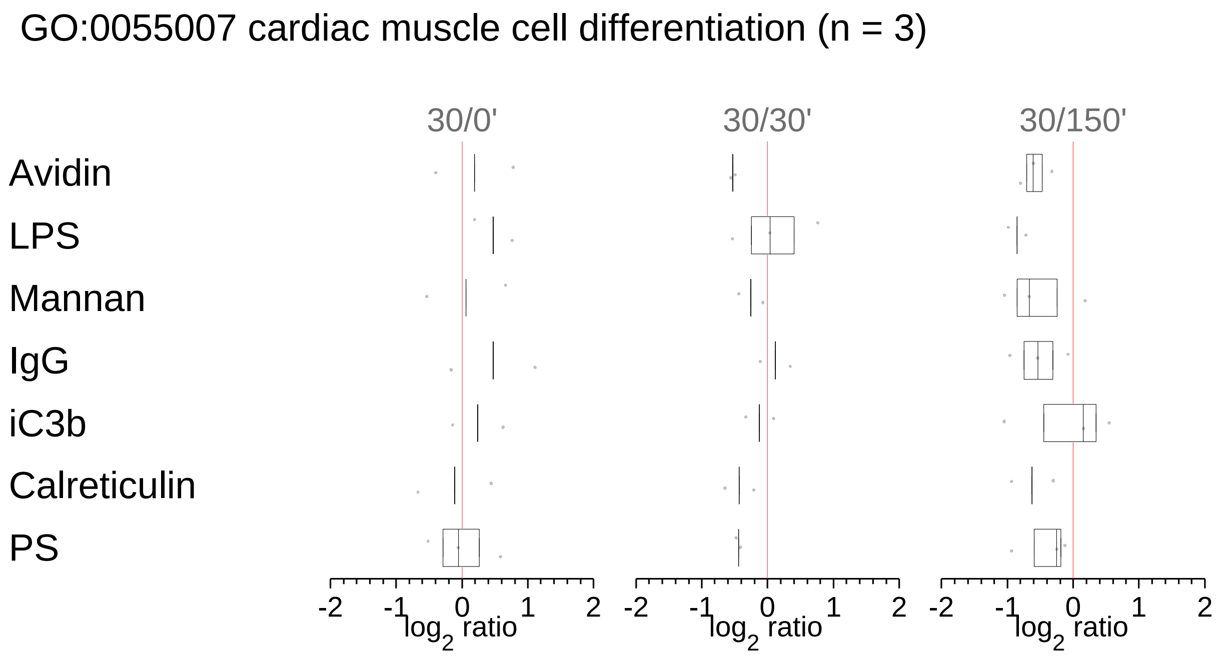
GO:0055007 cardiac muscle cell differentiation (n = 3)
30/0'
30/30'
30/150'
Avidin
LPS
Mannan
IgG
iC3b
Calreticulin
PS
-2 -1 0 1 2
log2 ratio

GO:0055010 ventricular cardiac muscle tissue morphogenesis (n = 3)

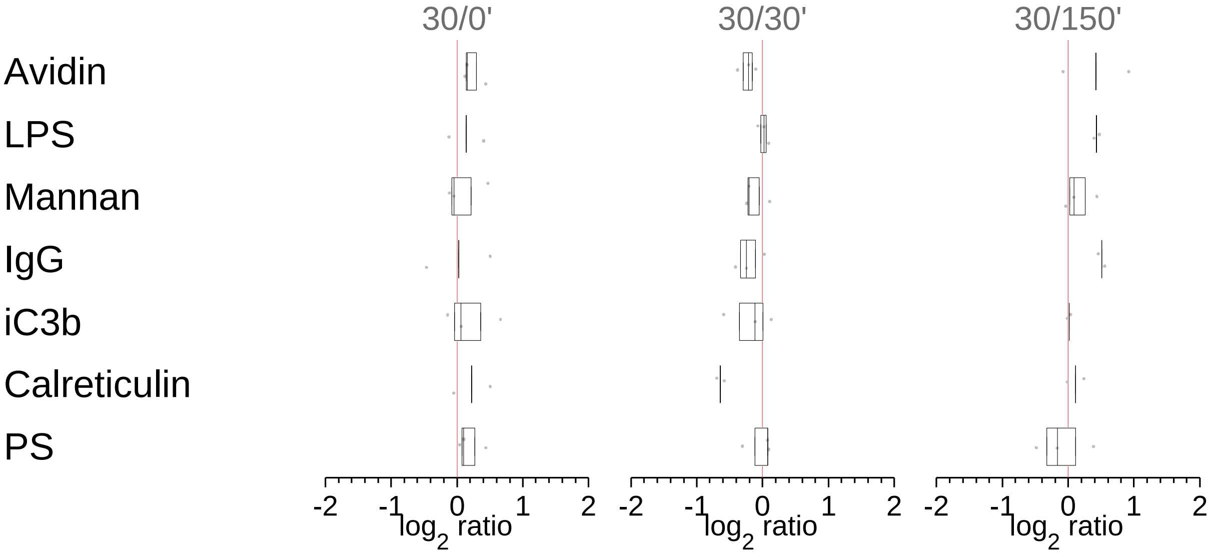

GO:0055037 recycling endosome (n = 28)

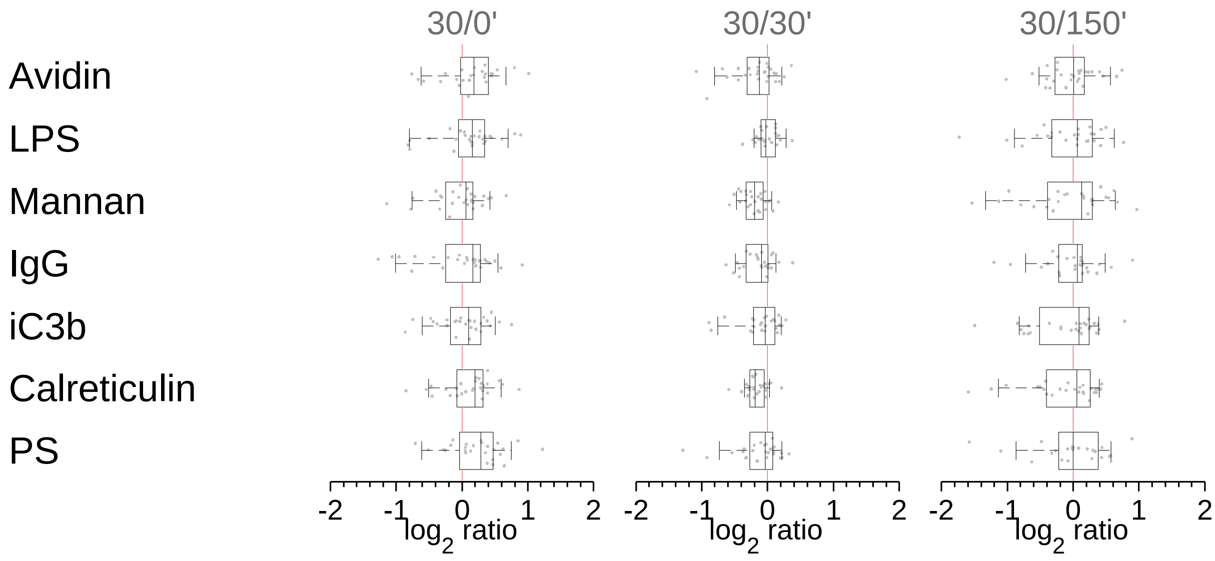

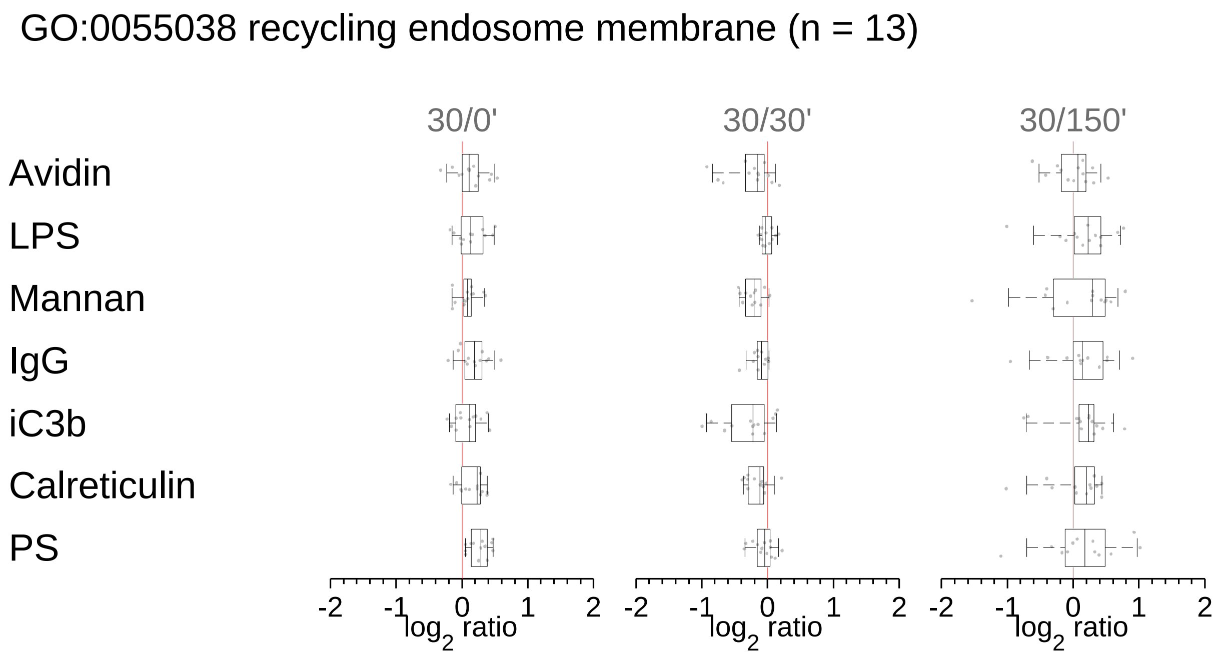
GO:0055038 recycling endosome membrane (n = 13)
30/0'
30/30'
30/150'
Avidin
LPS
Mannan
IgG
iC3b
Calreticulin
PS
-2
-1
0
1
2
log2 ratio

GO:0055072 iron ion homeostasis (n = 8)

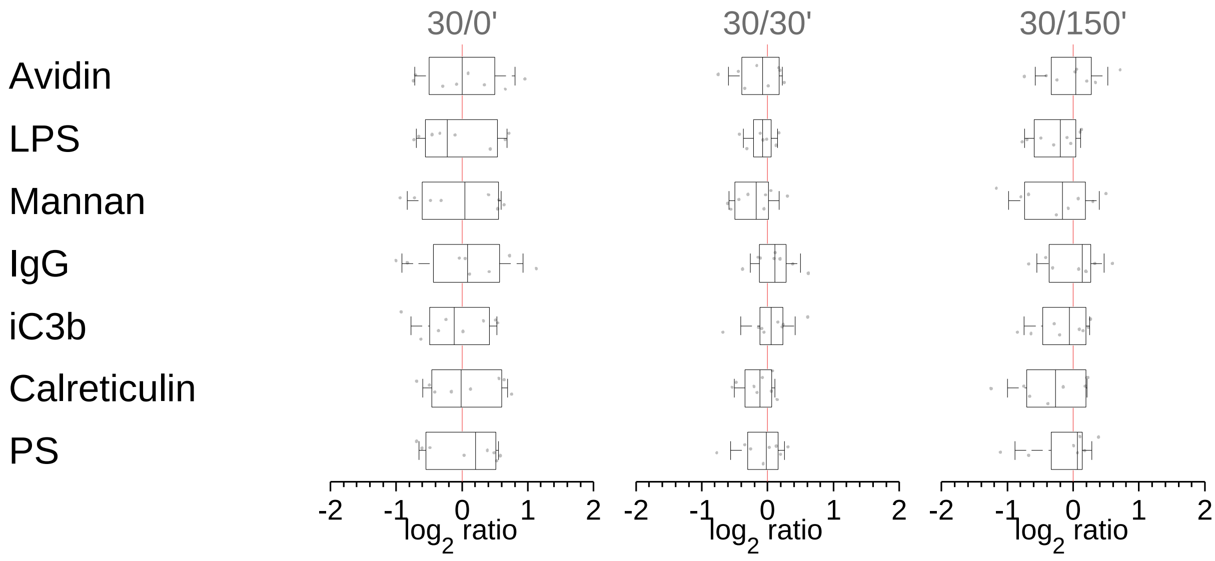

GO:0055085 transmembrane transport (n = 52)

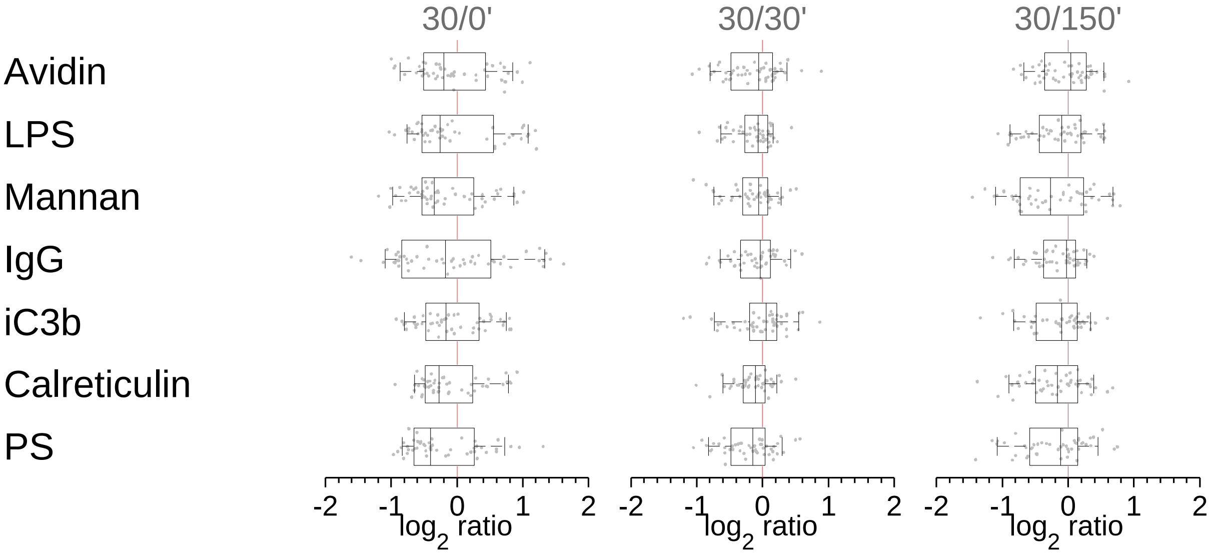

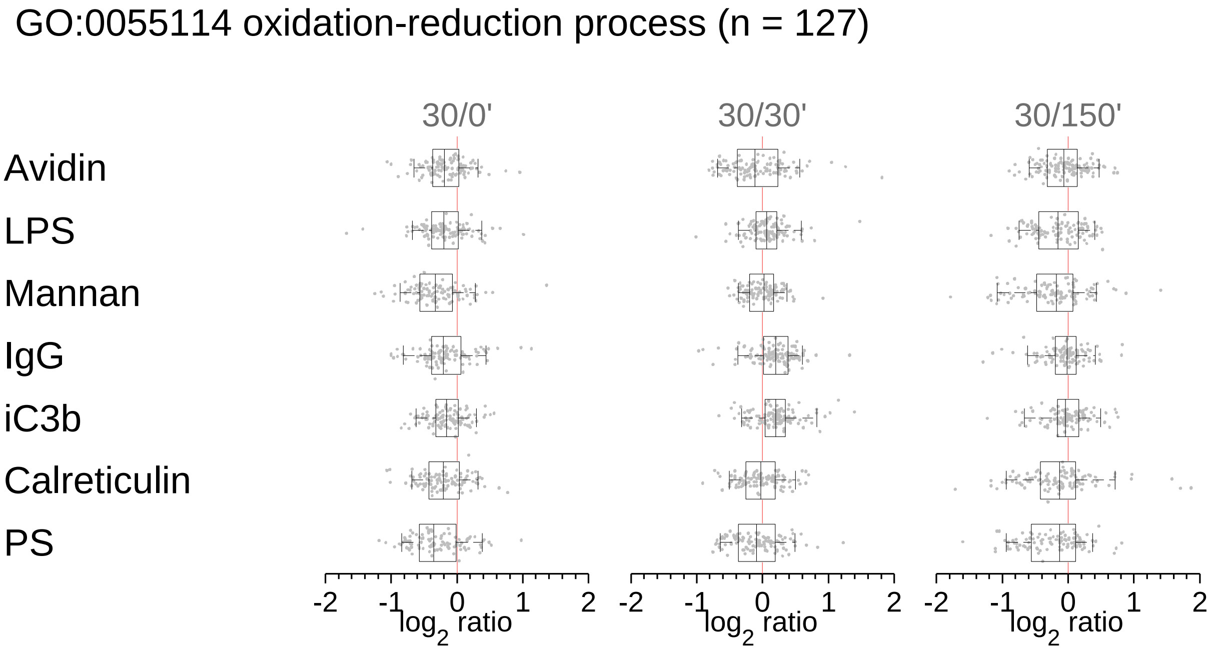
GO:0055114 oxidation-reduction process (n = 127)
30/0'
30/30'
30/150'
Avidin
LPS
Mannan
IgG
iC3b
Calreticulin
PS
-2
-1
0
1
2
log2 ratio

GO:0060021 palate development (n = 4)

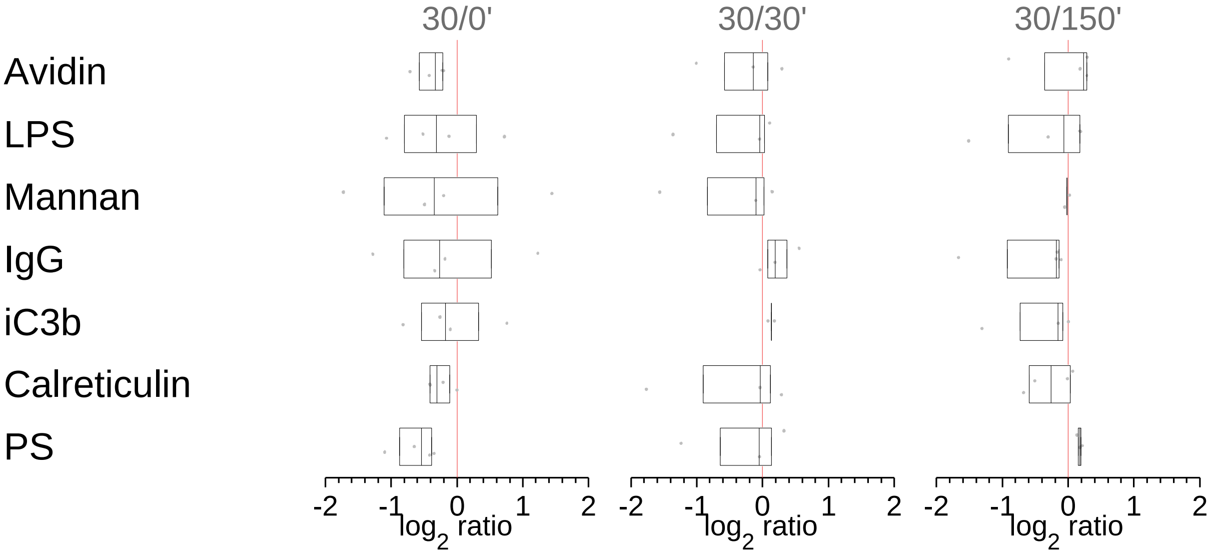

GO:0060041 retina development in camera-type eye (n = 5)

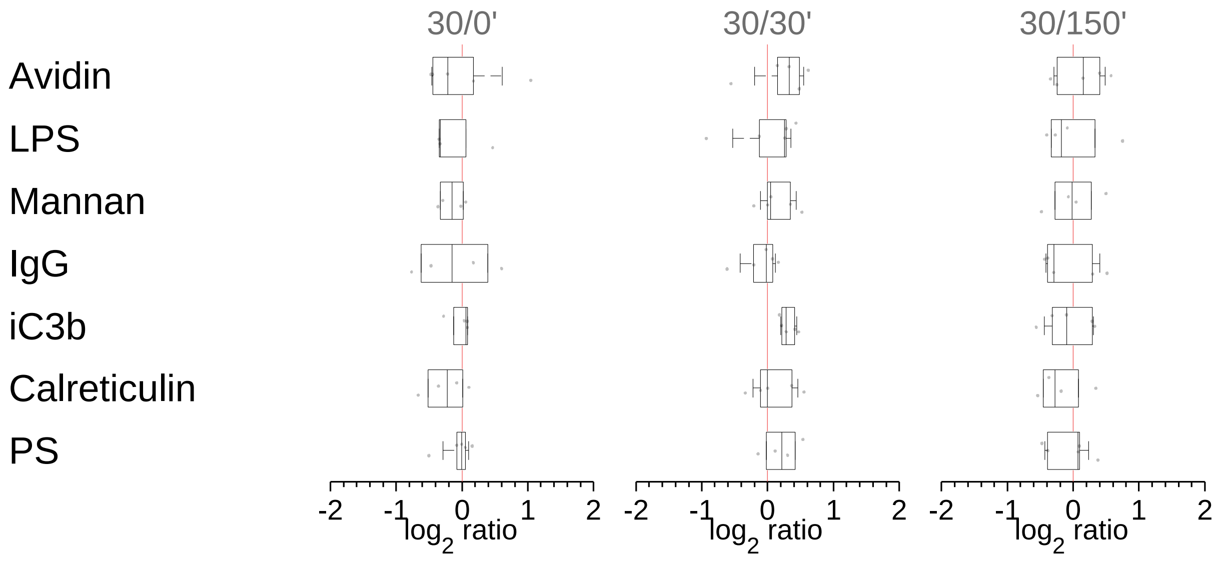

GO:0060047 heart contraction (n = 4)

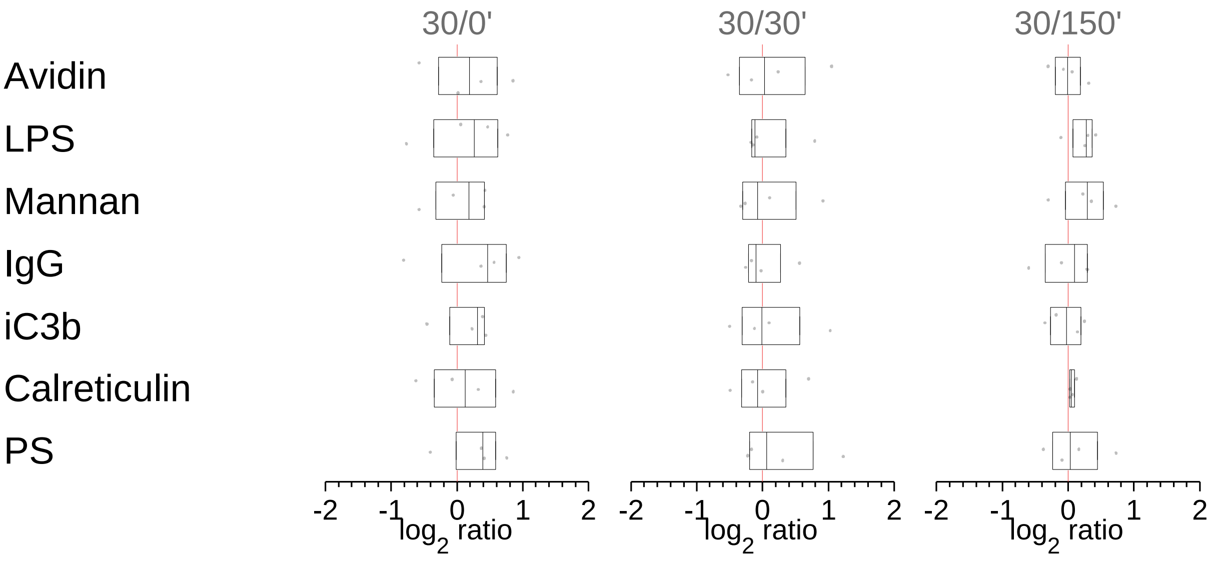

GO:0060048 cardiac muscle contraction (n = 5)

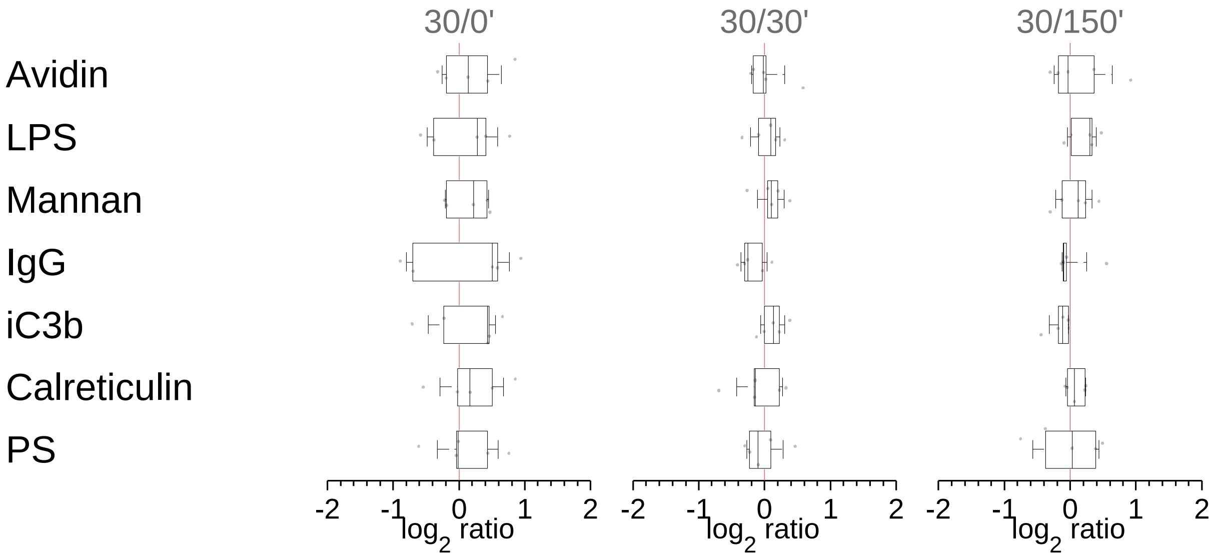

GO:0060055 angiogenesis involved in wound healing (n = 4)

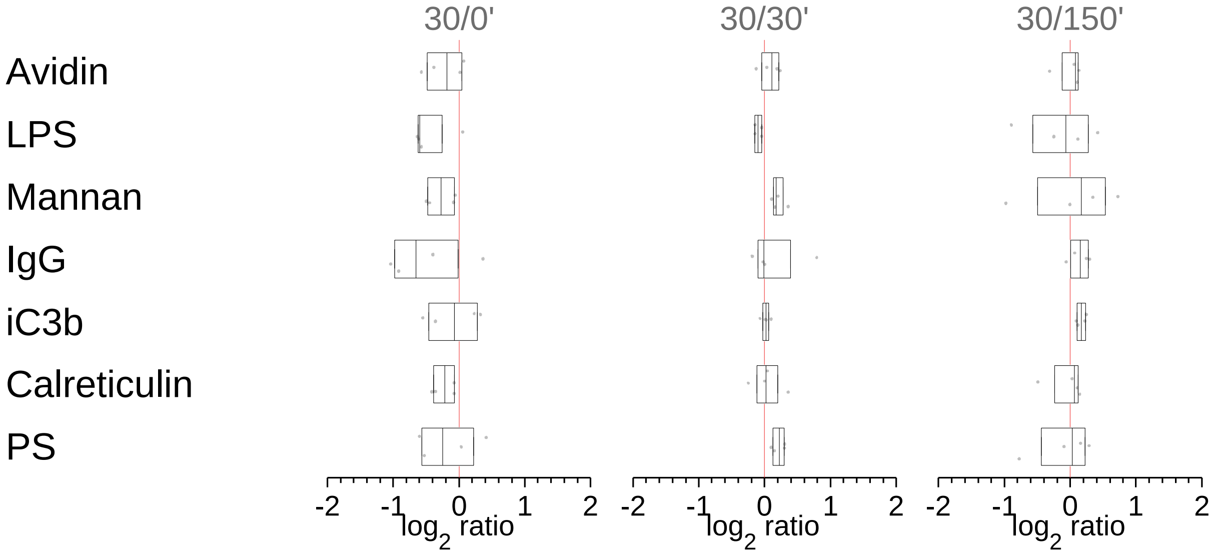

GO:0060068 vagina development (n = 4)

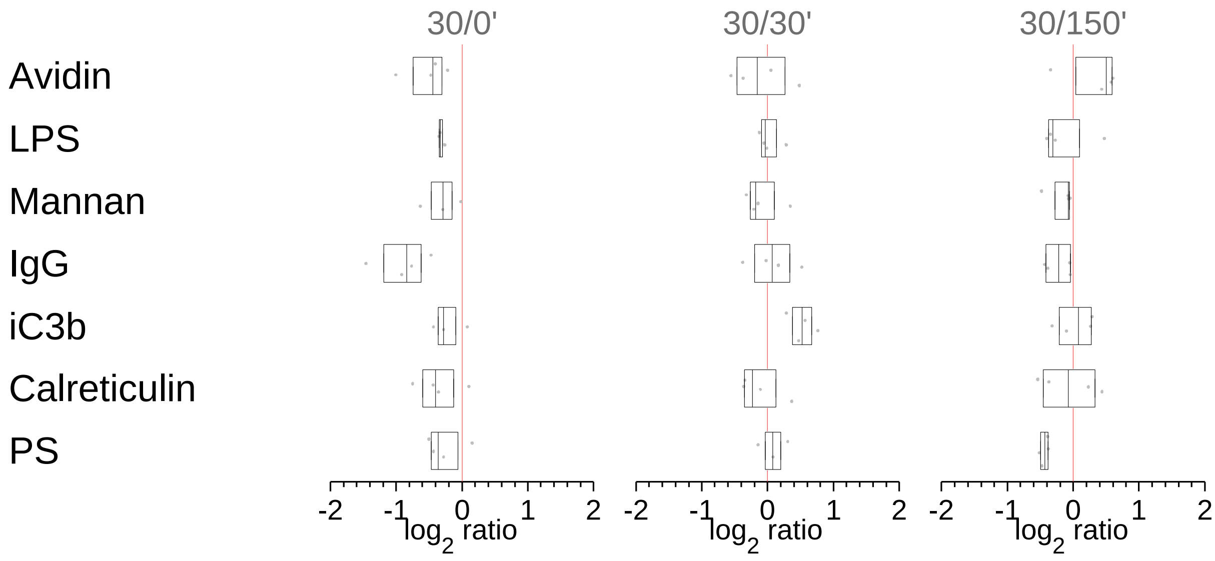

GO:0060070 canonical Wnt receptor signaling pathway (n = 4)

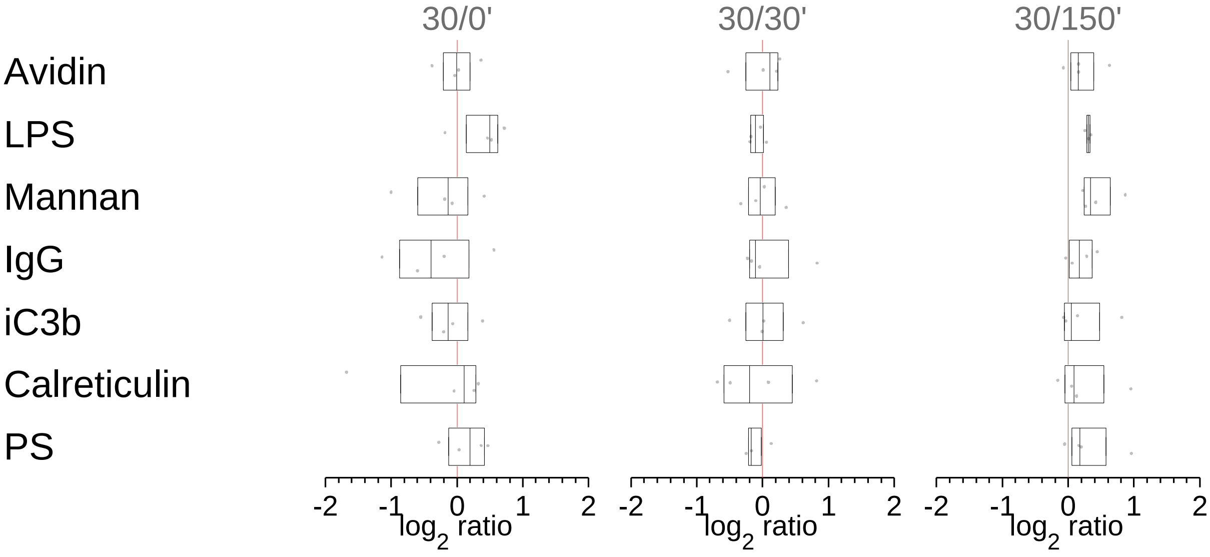

GO:0060271 cilium morphogenesis (n = 3)

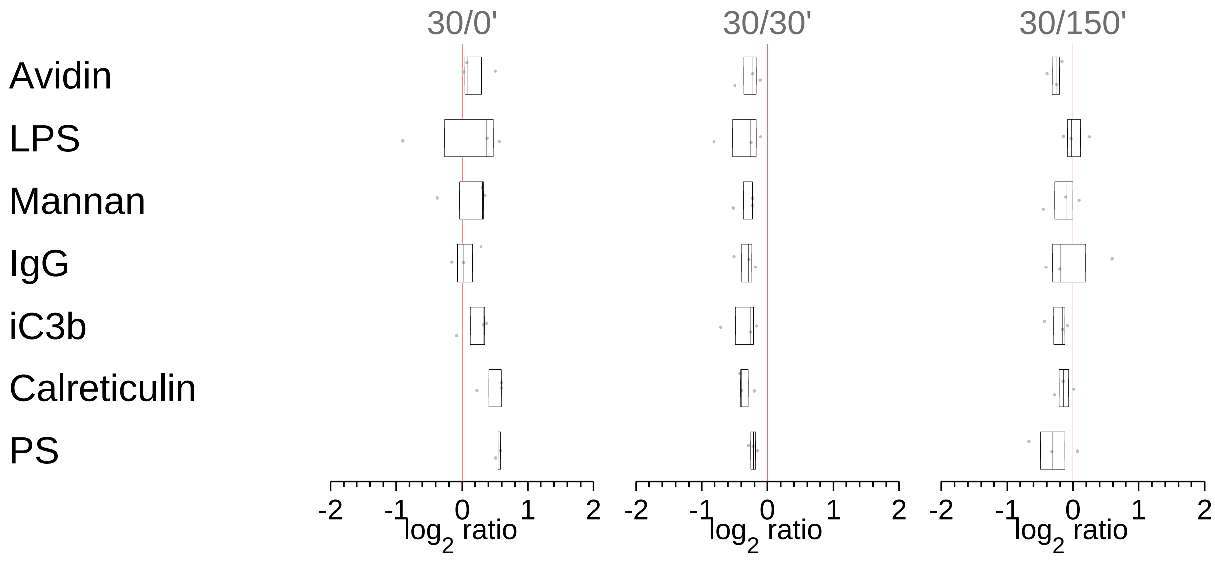

GO:0060325 face morphogenesis (n = 3)

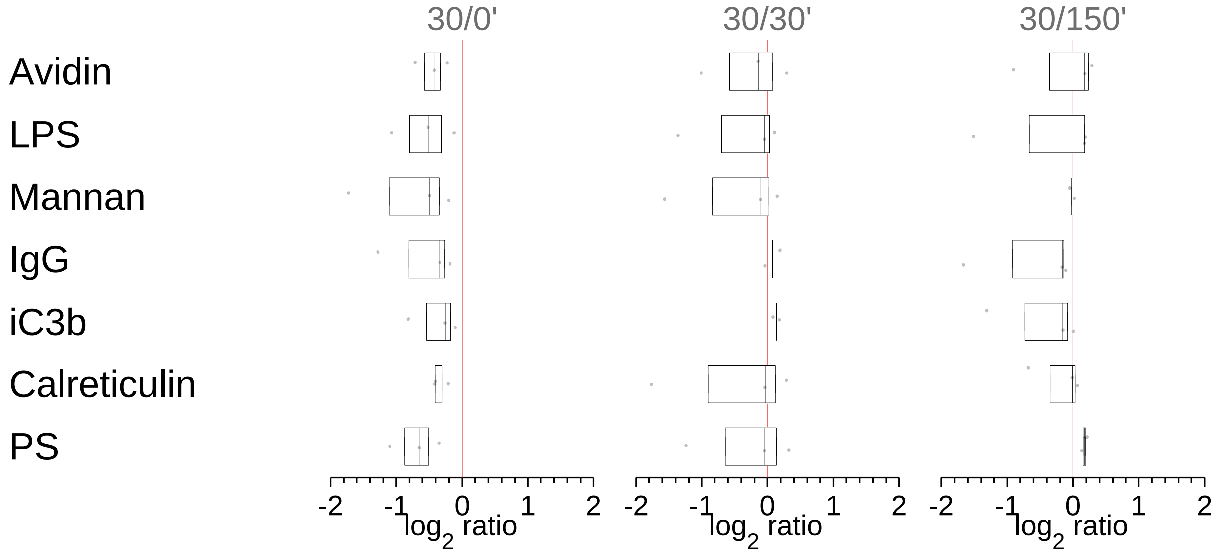

GO:0060326 cell chemotaxis (n = 5)

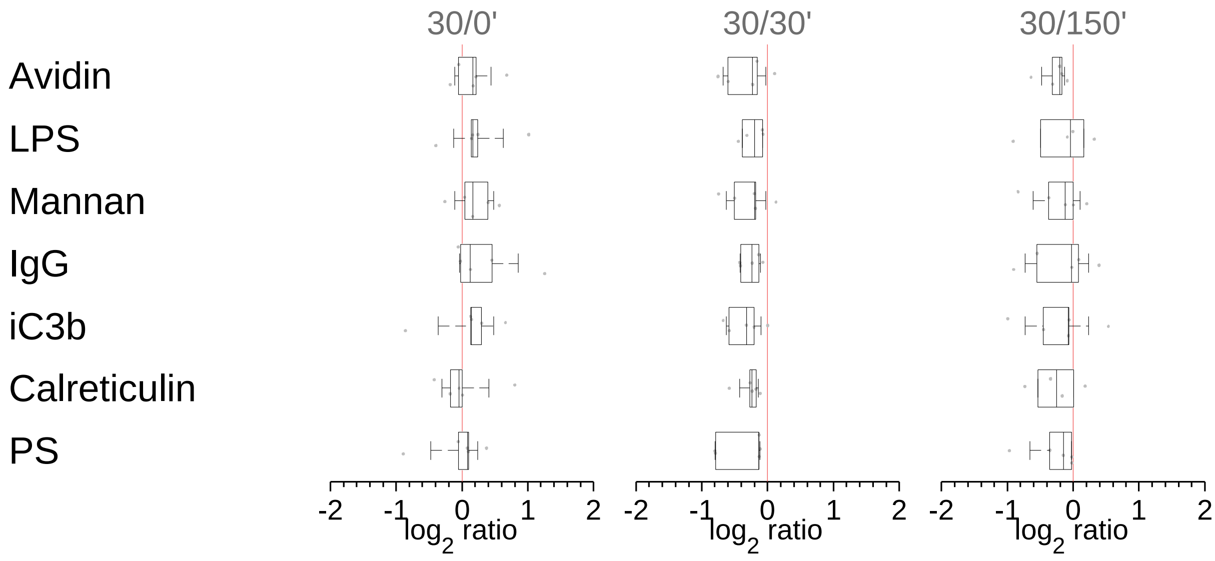

GO:0060348 bone development (n = 4)

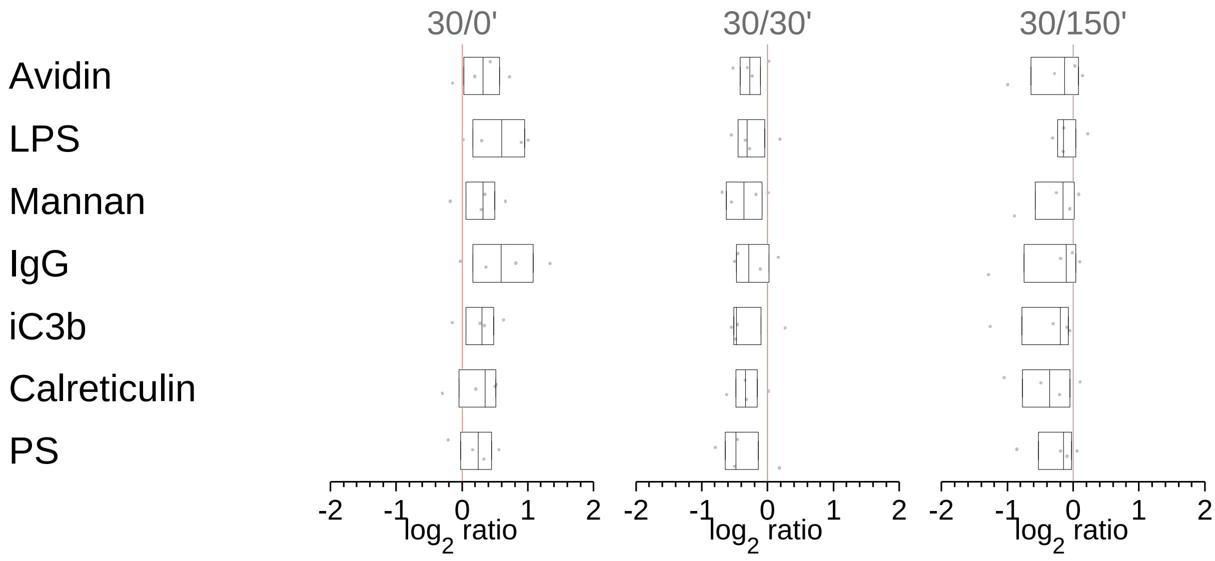

GO:0060395 SMAD protein signal transduction (n = 5)

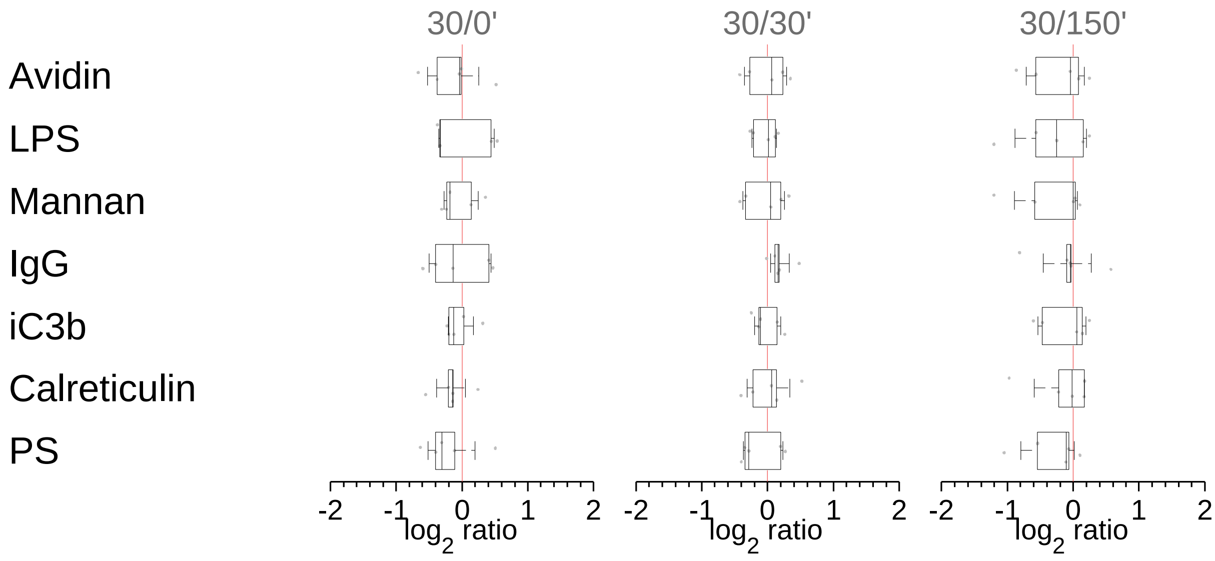

GO:0060441 epithelial tube branching involved in lung morphogenesis (n = 3)

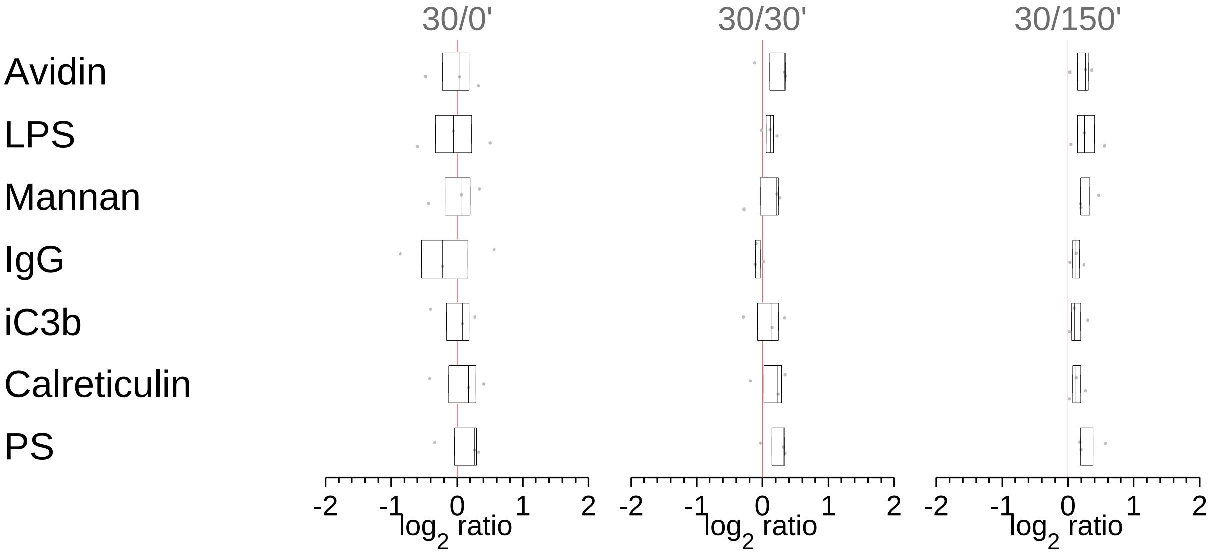

GO:0060548 negative regulation of cell death (n = 9)

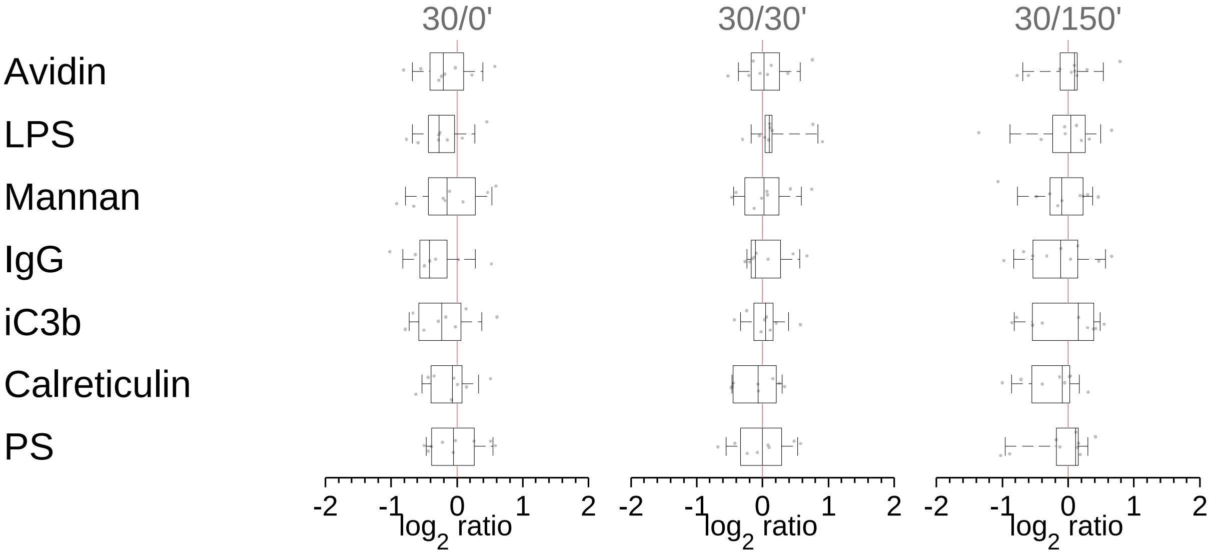

GO:0060612 adipose tissue development (n = 4)

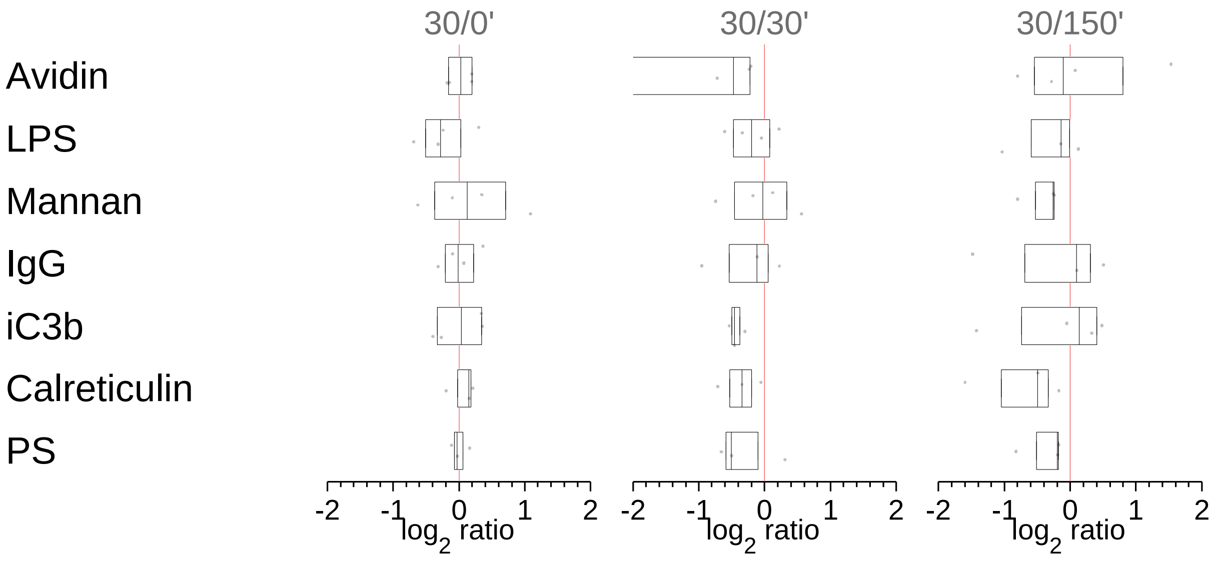

GO:0060766 negative regulation of androgen receptor signaling pathway (n = 3)

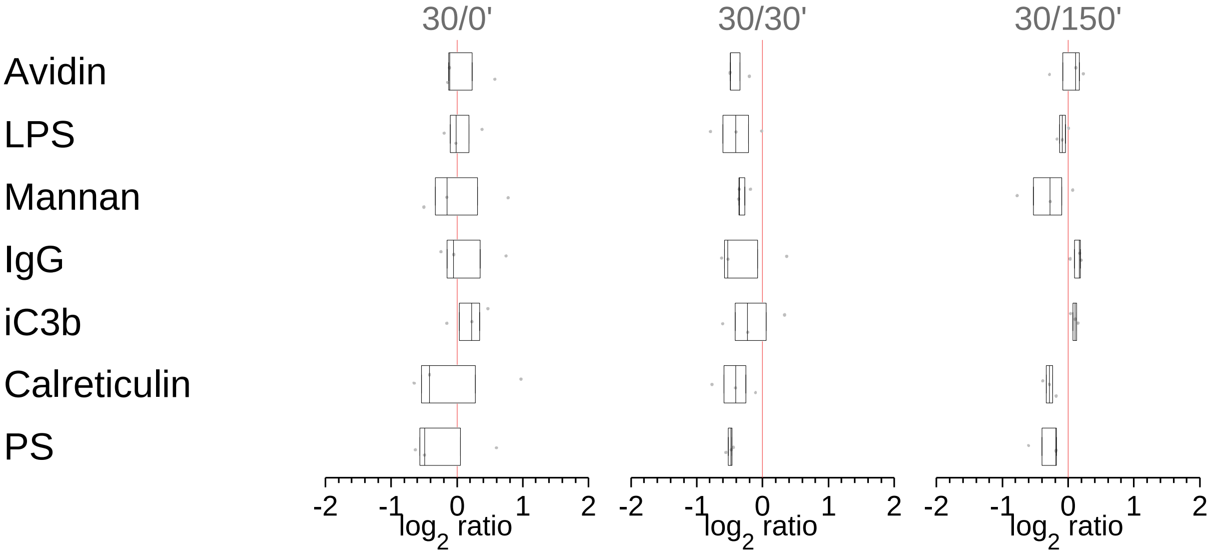

GO:0060789 hair follicle placode formation (n = 3)

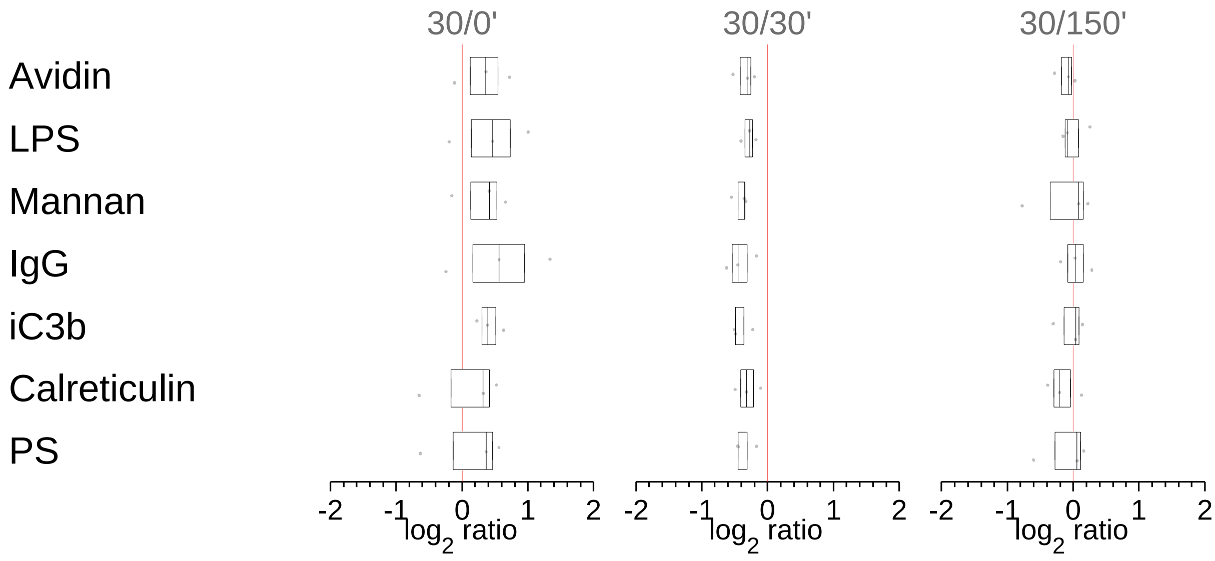

GO:0060828 regulation of canonical Wnt receptor signaling pathway (n = 3)

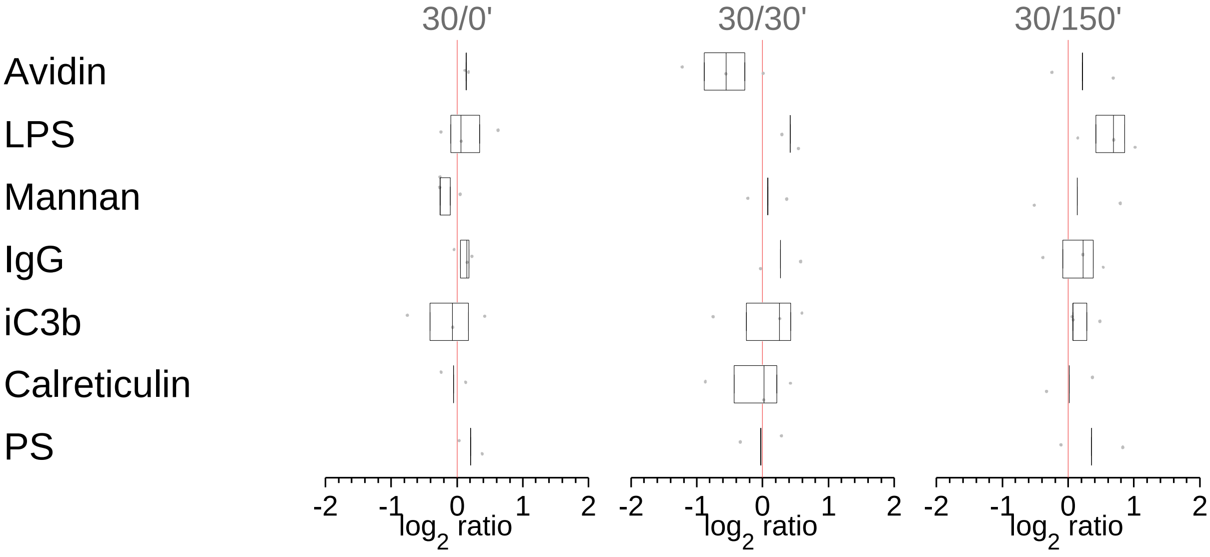

GO:0060907 positive regulation of macrophage cytokine production
(n = 3)

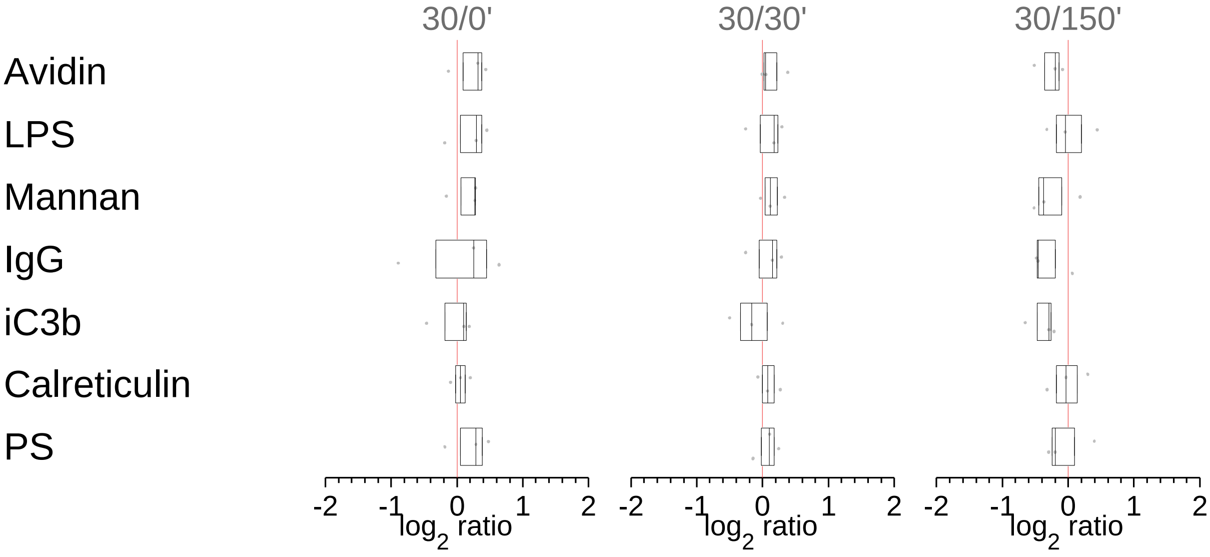

GO:0061024 membrane organization (n = 3)

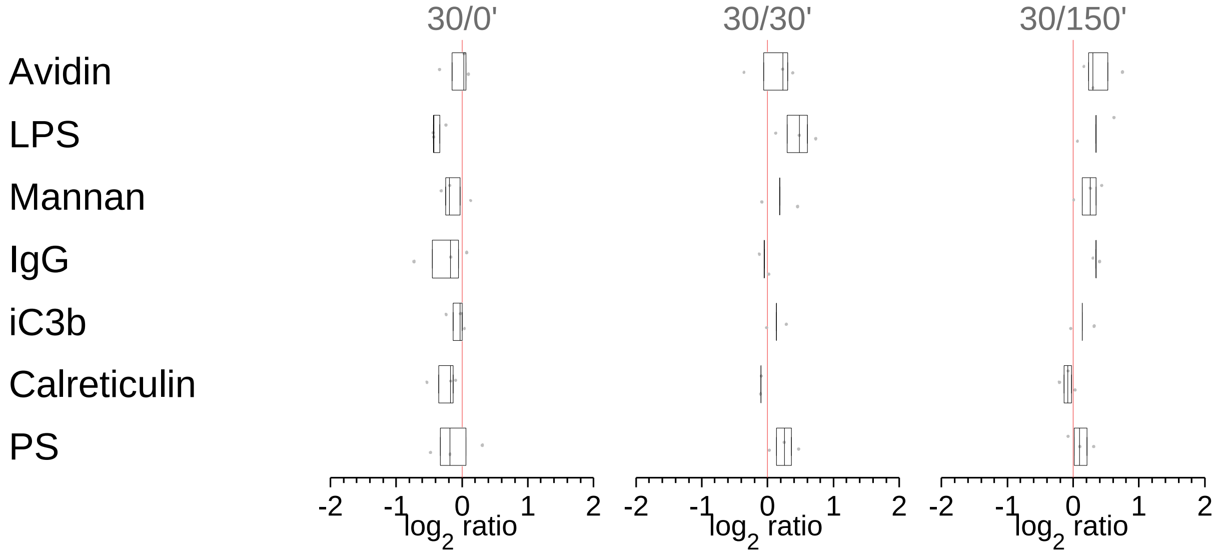

GO:0061025 membrane fusion (n = 7)

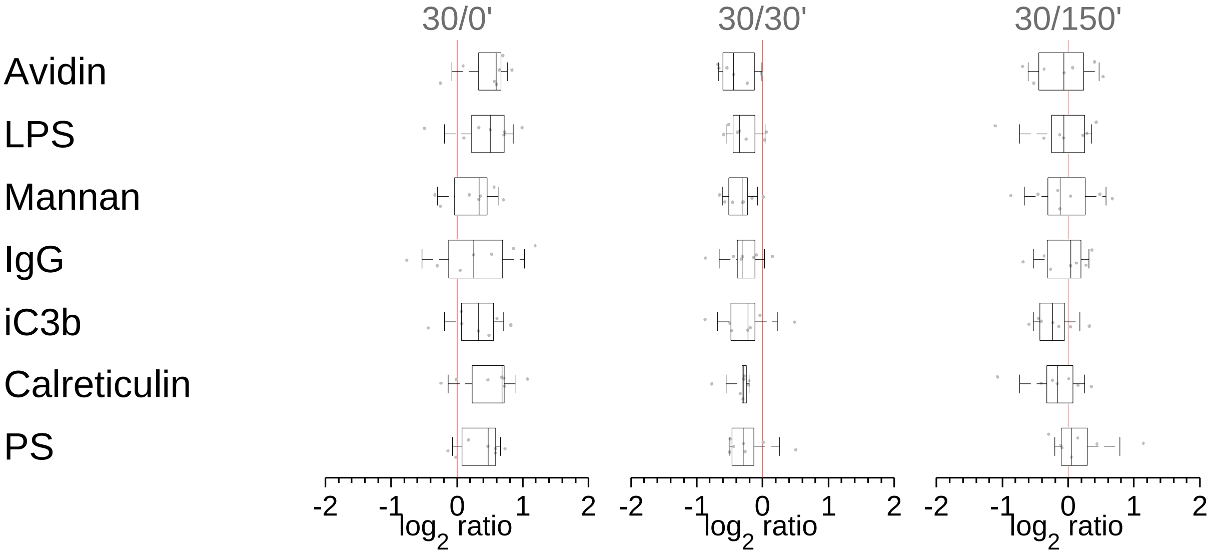

GO:0061028 establishment of endothelial barrier (n = 7)

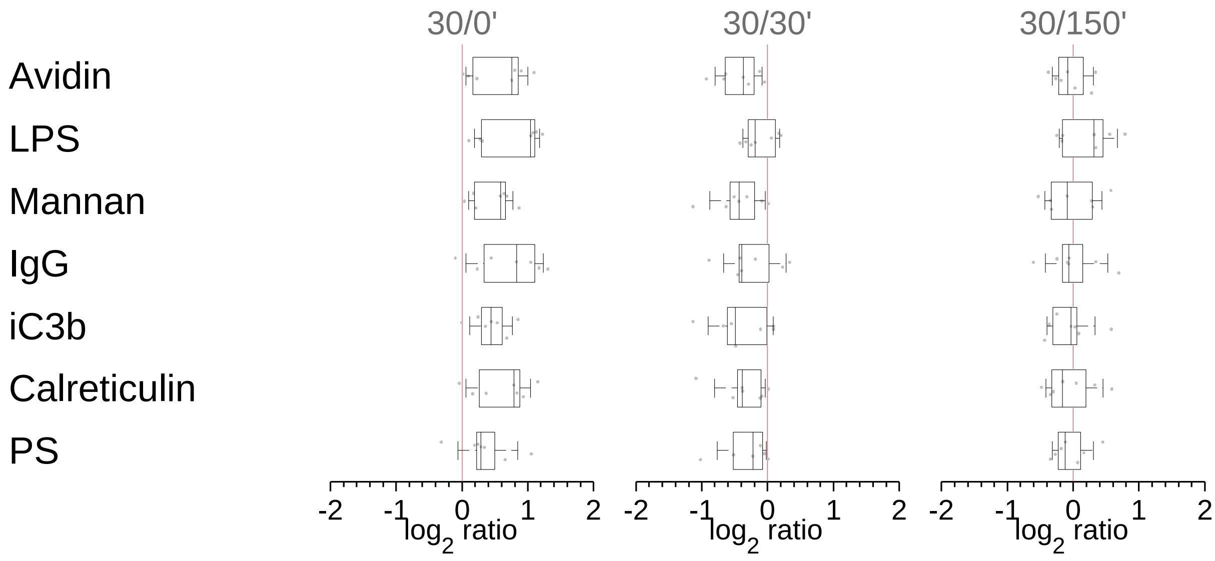

GO:0061077 chaperone-mediated protein folding (n = 8)

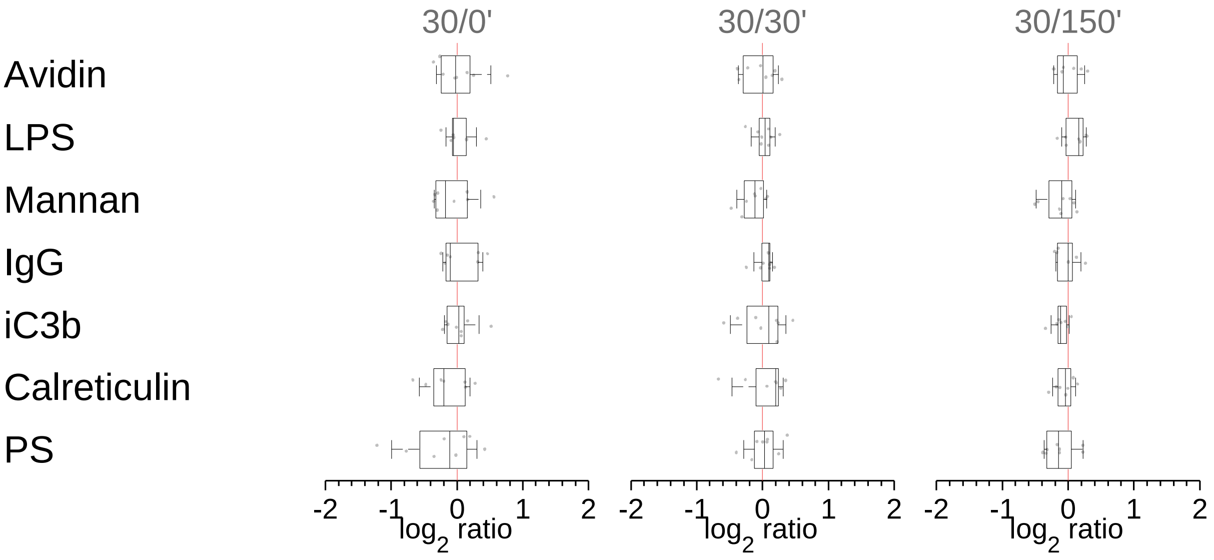

GO:0061098 positive regulation of protein tyrosine kinase activity (n = 3)

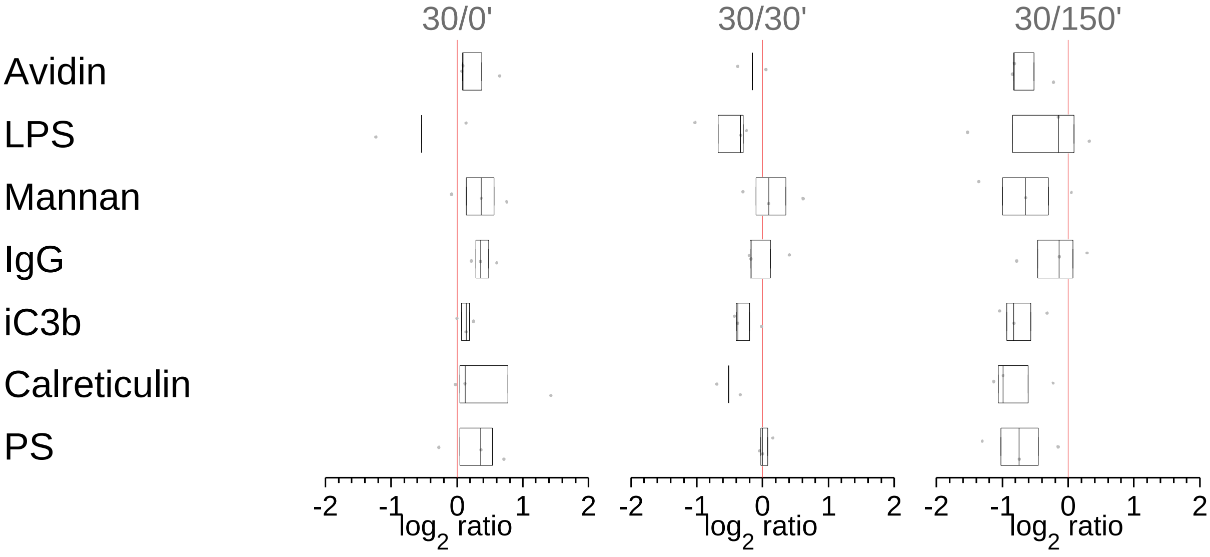

GO:0070053 thrombospondin receptor activity (n = 3)

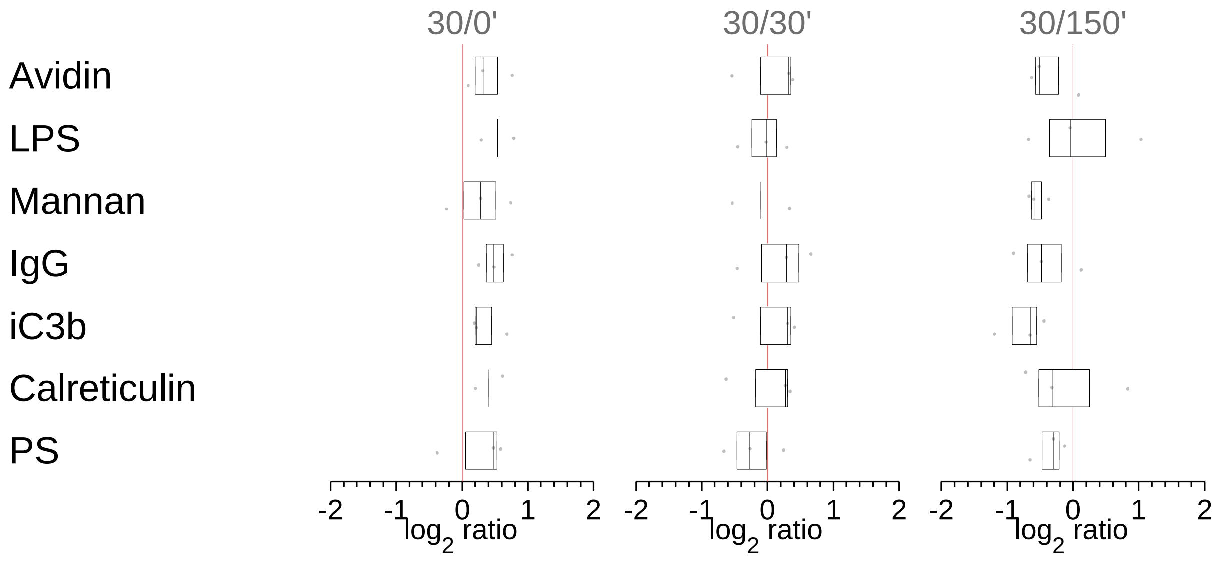

GO:0070059 intrinsic apoptotic signaling pathway in response to endoplasmic reticulum stress (n = 3)

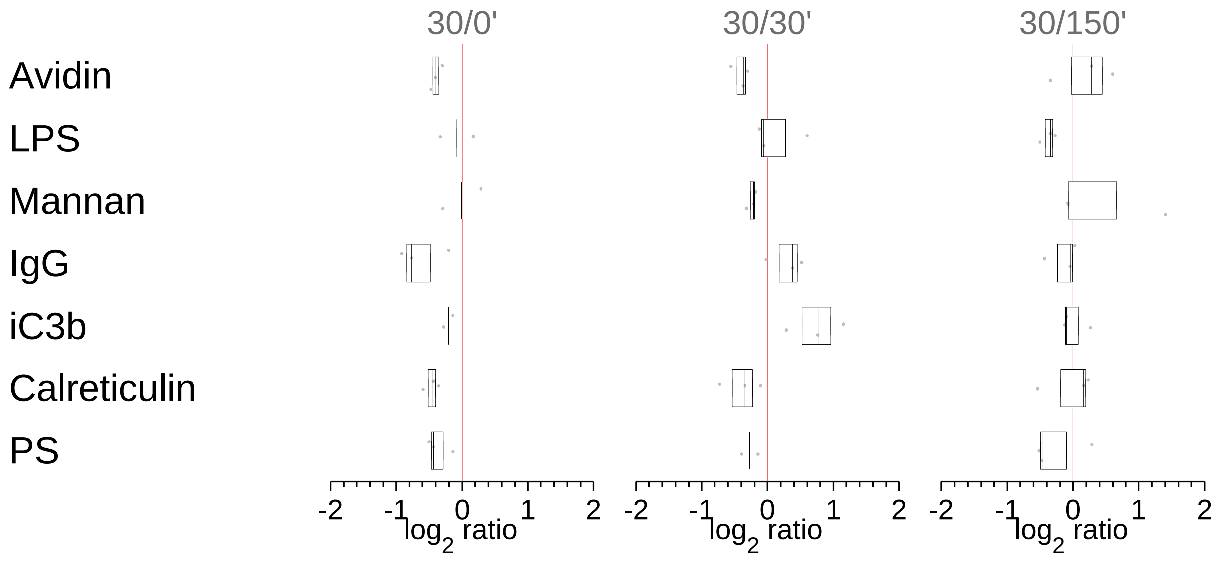

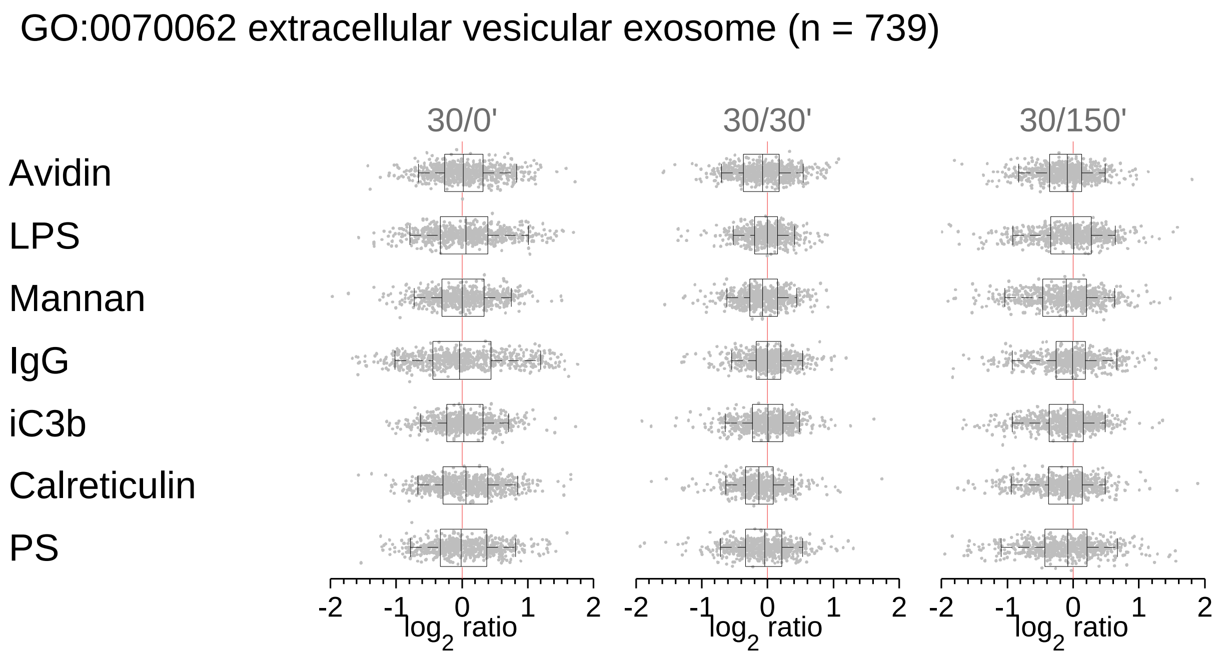

GO:0070062 extracellular vesicular exosome (n = 739)
30/0'
30/30'
30/150'
Avidin
LPS
Mannan
IgG
iC3b
Calreticulin
PS
-2
-1
0
1
2
log2 ratio

GO:0070064 proline-rich region binding (n = 3)

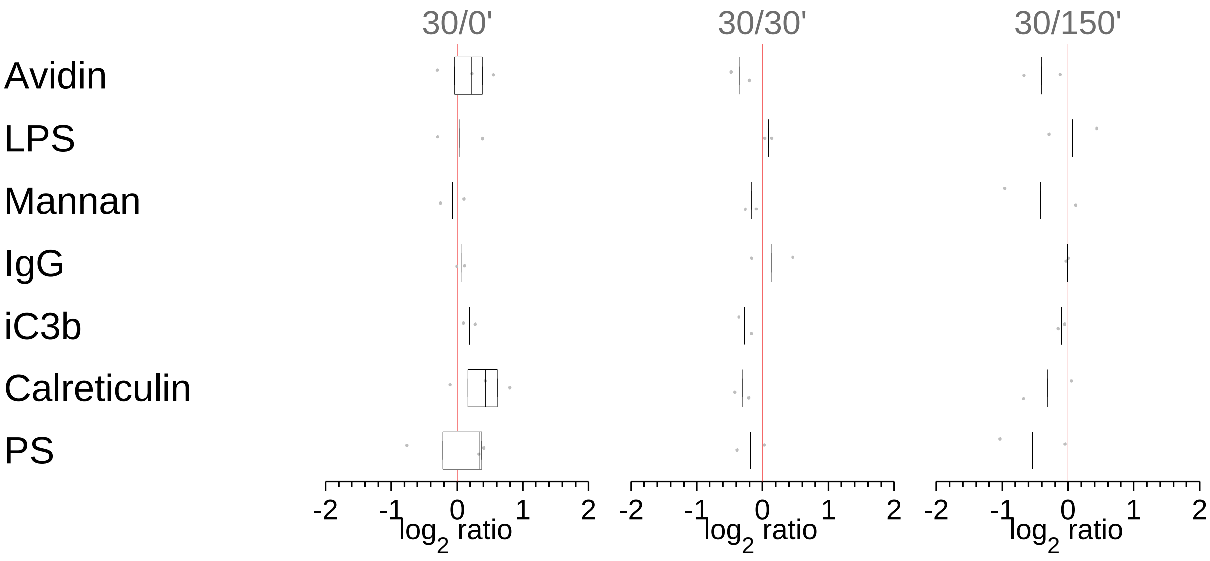

GO:0070290 NAPE-specific phospholipase D activity (n = 3)

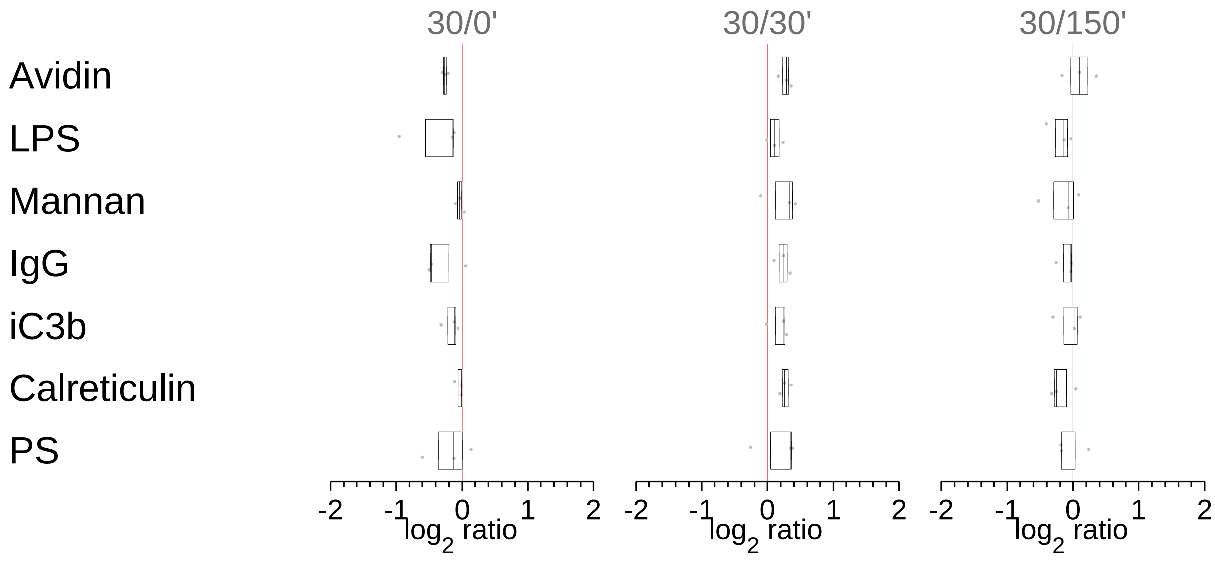

GO:0070301 cellular response to hydrogen peroxide (n = 4)

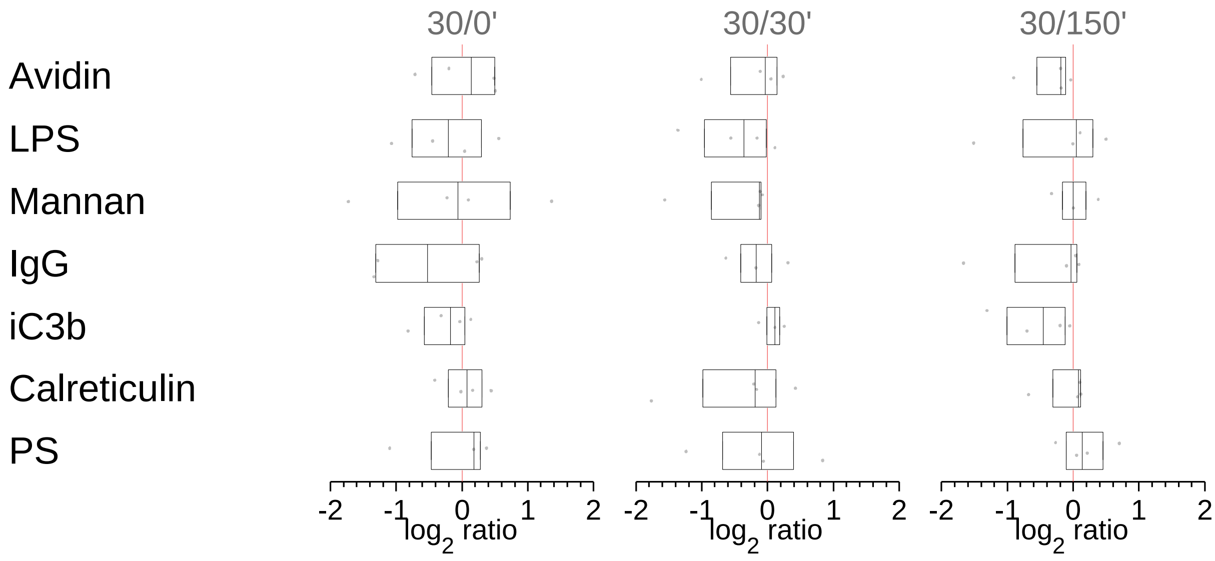

GO:0070372 regulation of ERK1 and ERK2 cascade (n = 5)

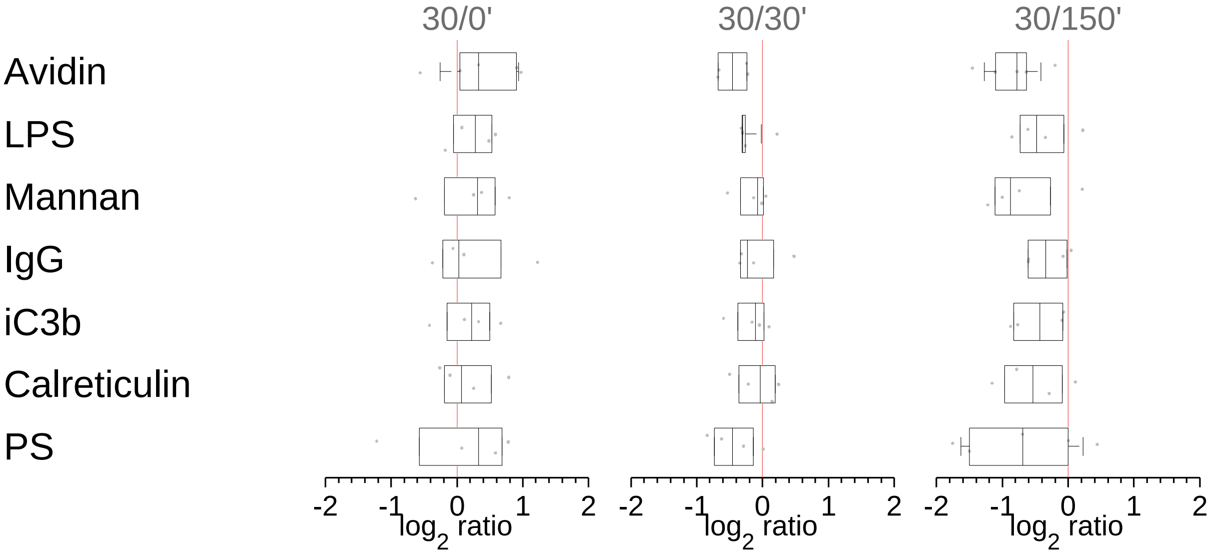

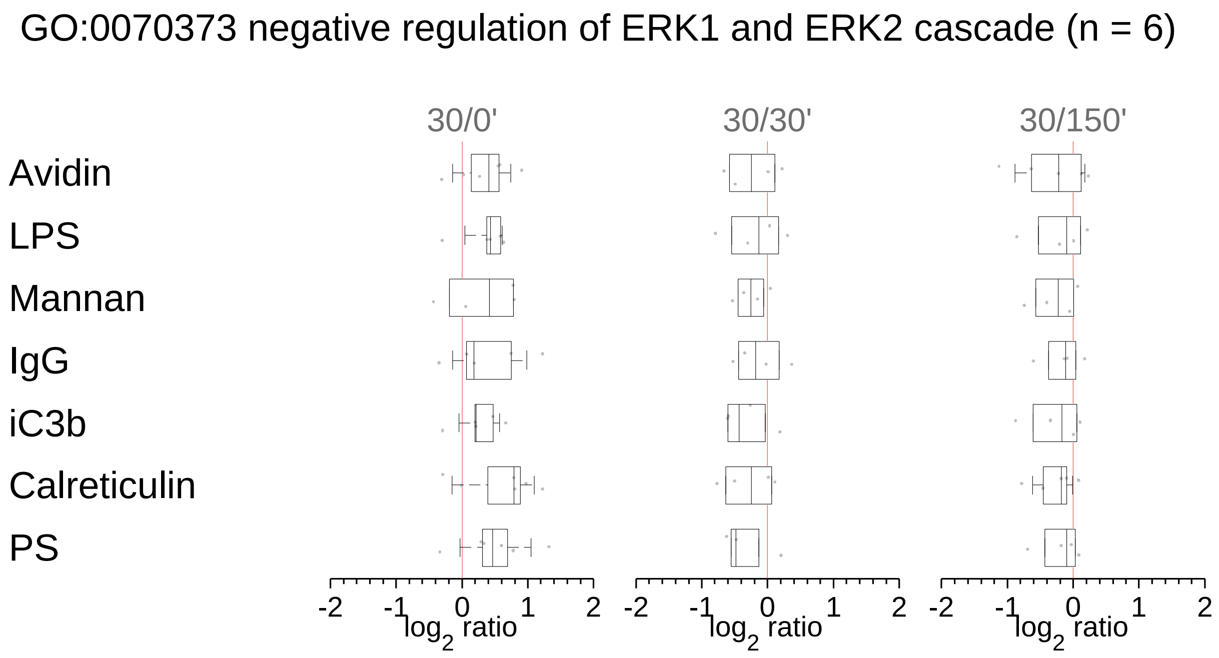
GO:0070373 negative regulation of ERK1 and ERK2 cascade (n = 6)
30/0'
30/30'
30/150'
Avidin
LPS
Mannan
IgG
iC3b
Calreticulin
PS
-2 -1 0 1 2
log2 ratio

GO:0070374 positive regulation of ERK1 and ERK2 cascade (n = 14)

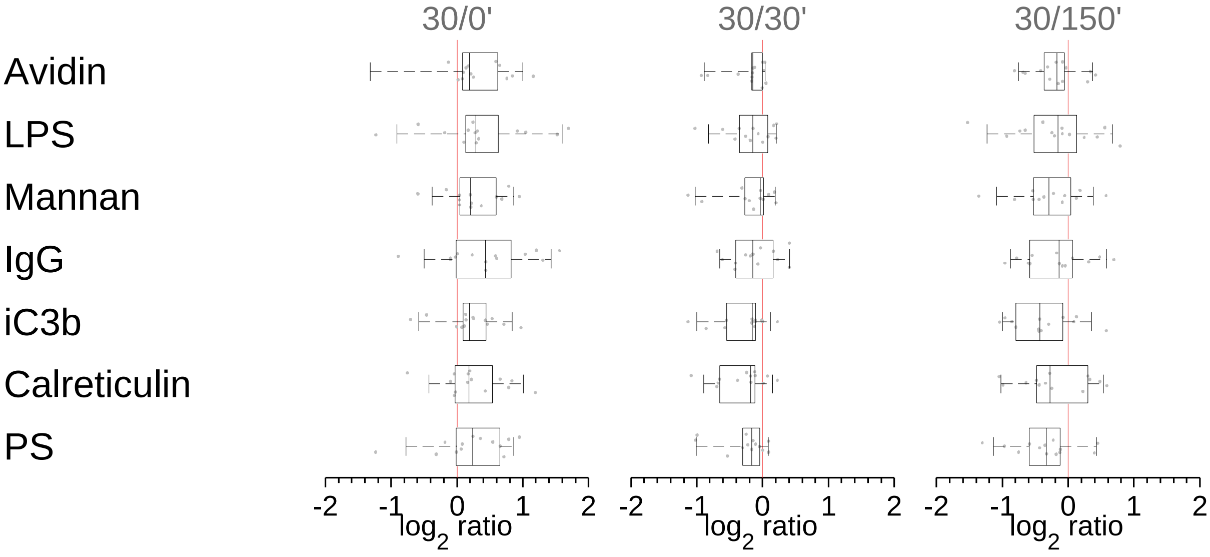

GO:0070382 exocytic vesicle (n = 3)

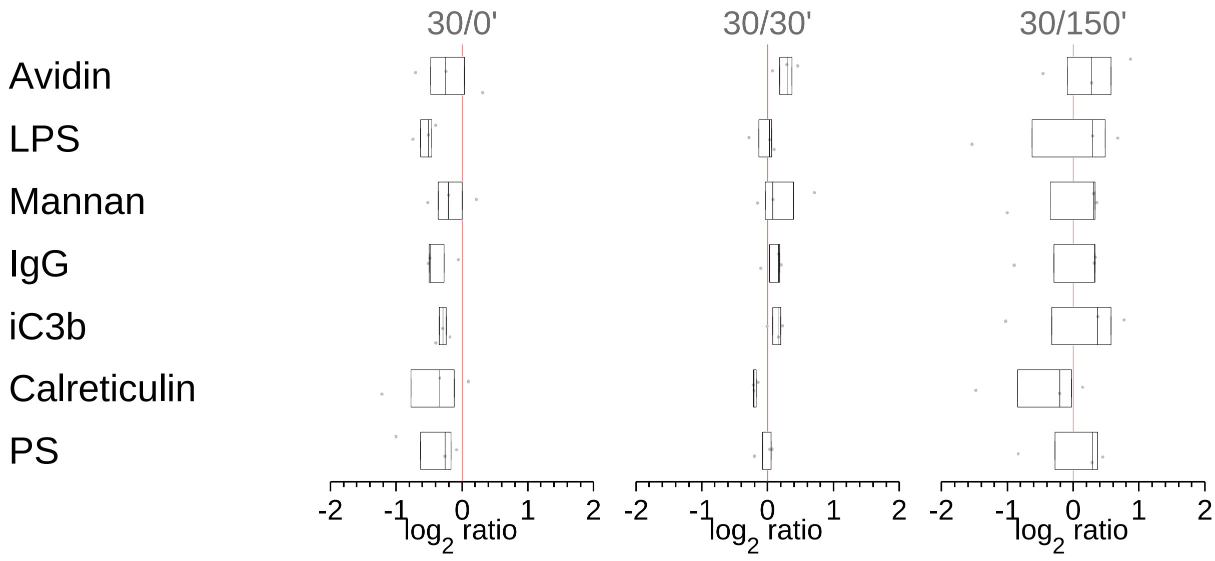

GO:0070469 respiratory chain (n = 16)

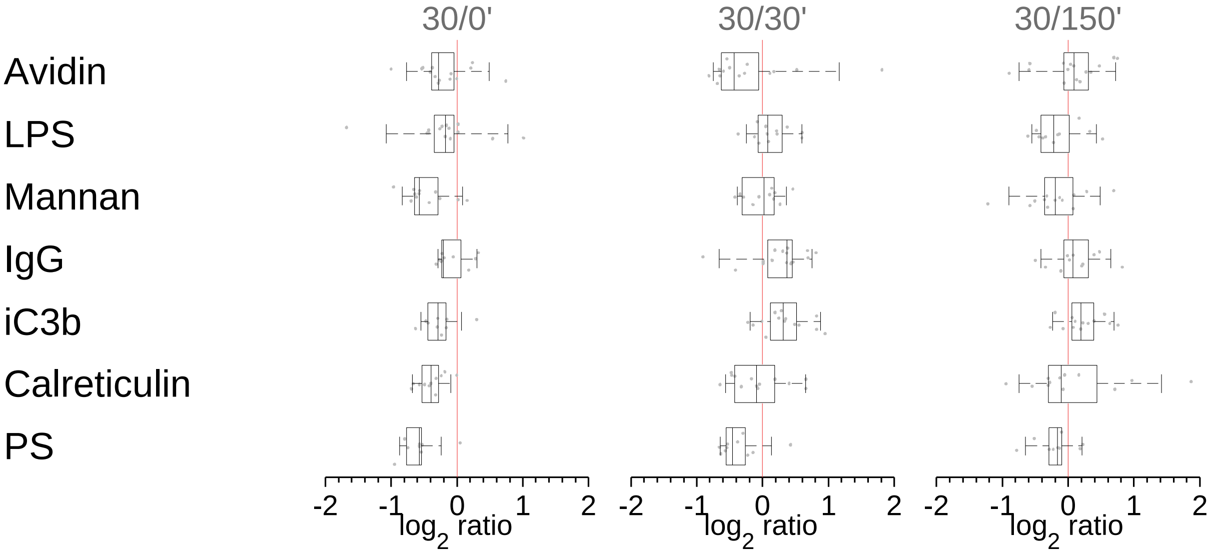

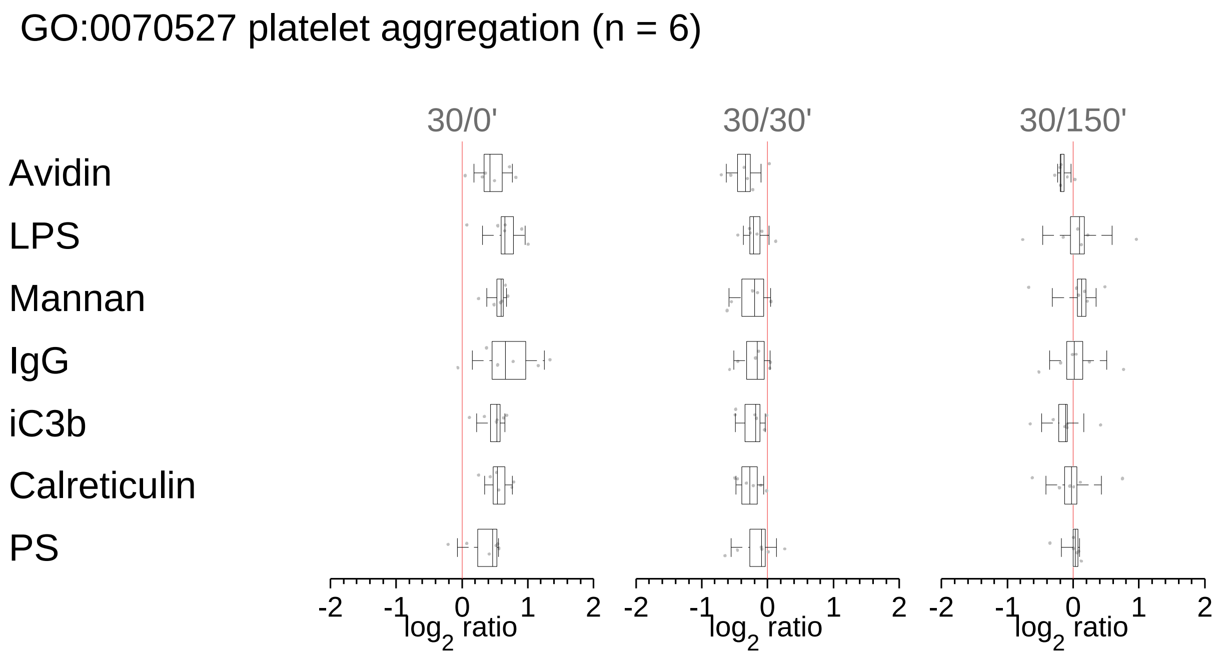

GO:0070527 platelet aggregation (n = 6)
30/0'
30/30'
30/150'
Avidin
LPS
Mannan
IgG
iC3b
Calreticulin
PS
-2 -1 0 1 2
log2 ratio

GO:0070534 protein K63-linked ubiquitination (n = 4)

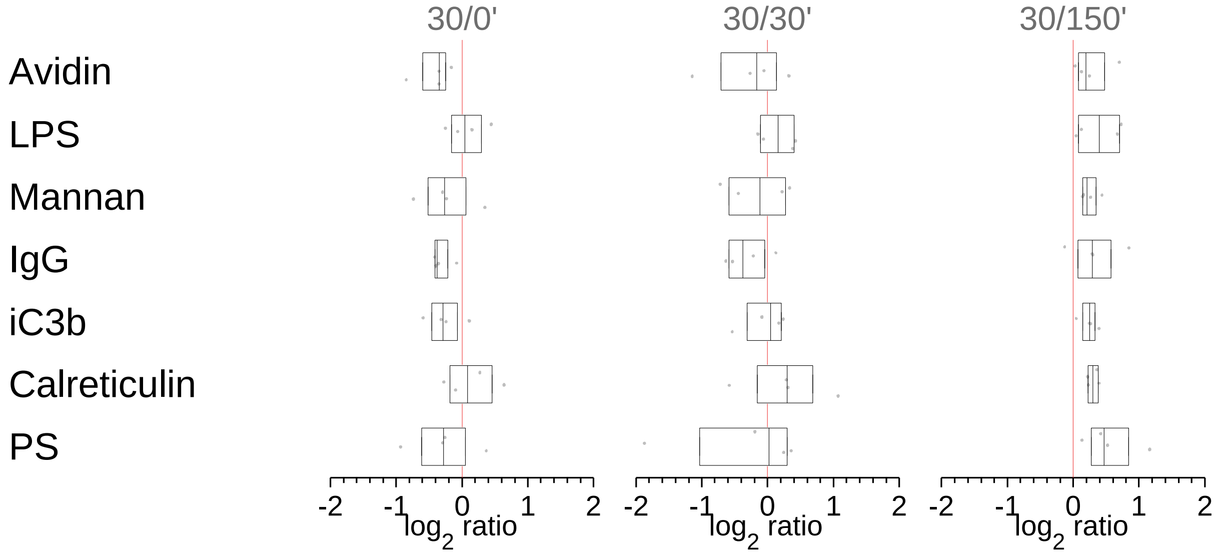

GO:0070584 mitochondrion morphogenesis (n = 4)

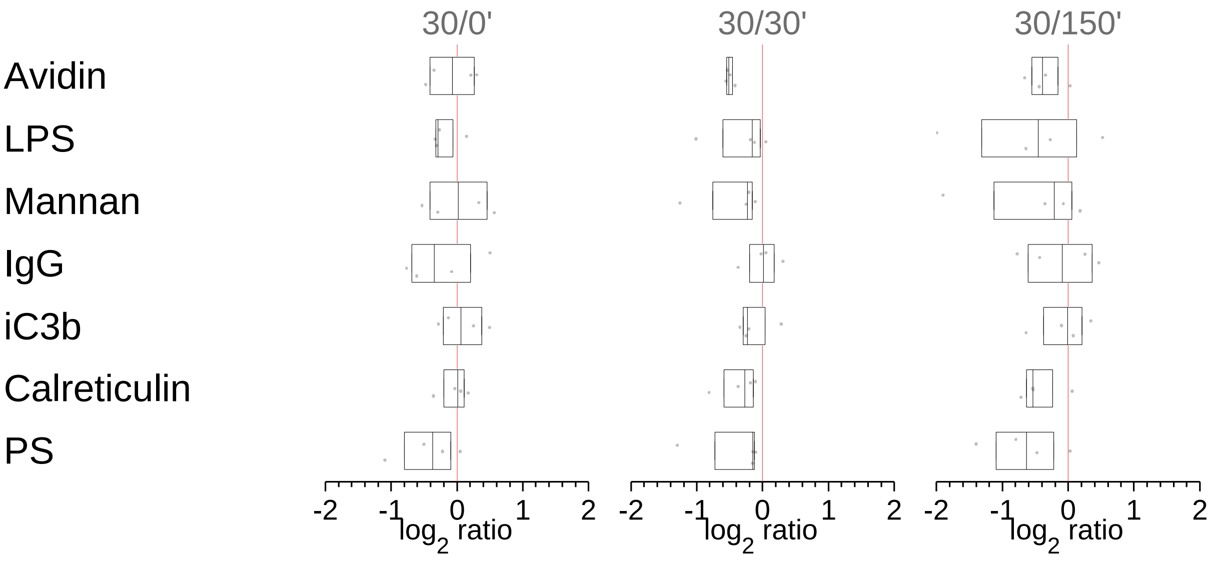

GO:0070588 calcium ion transmembrane transport (n = 10)

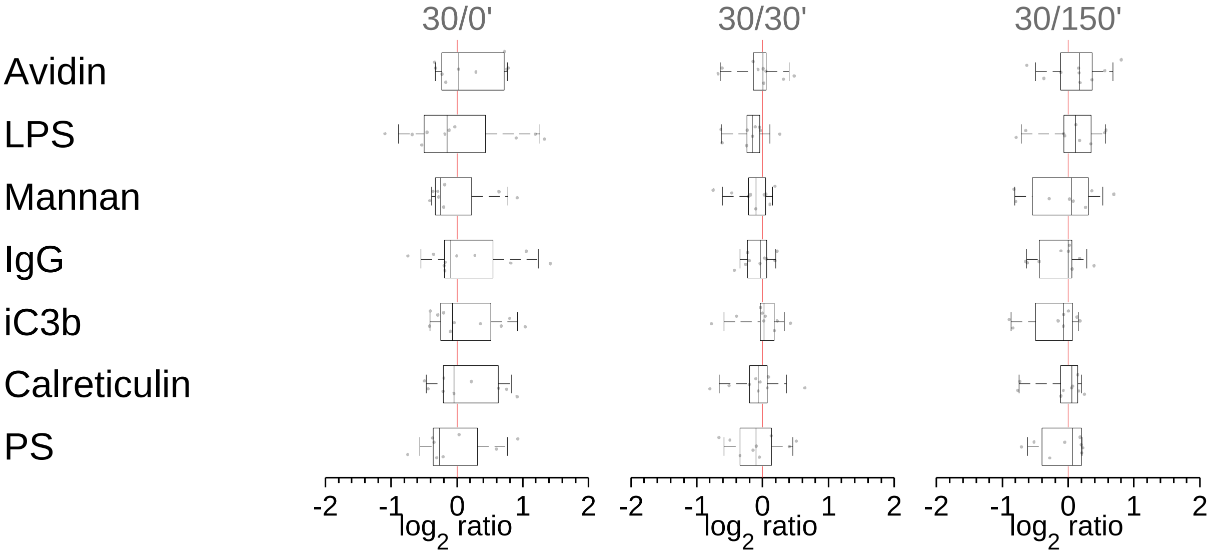

GO:0070688 MLL5-L complex (n = 3)

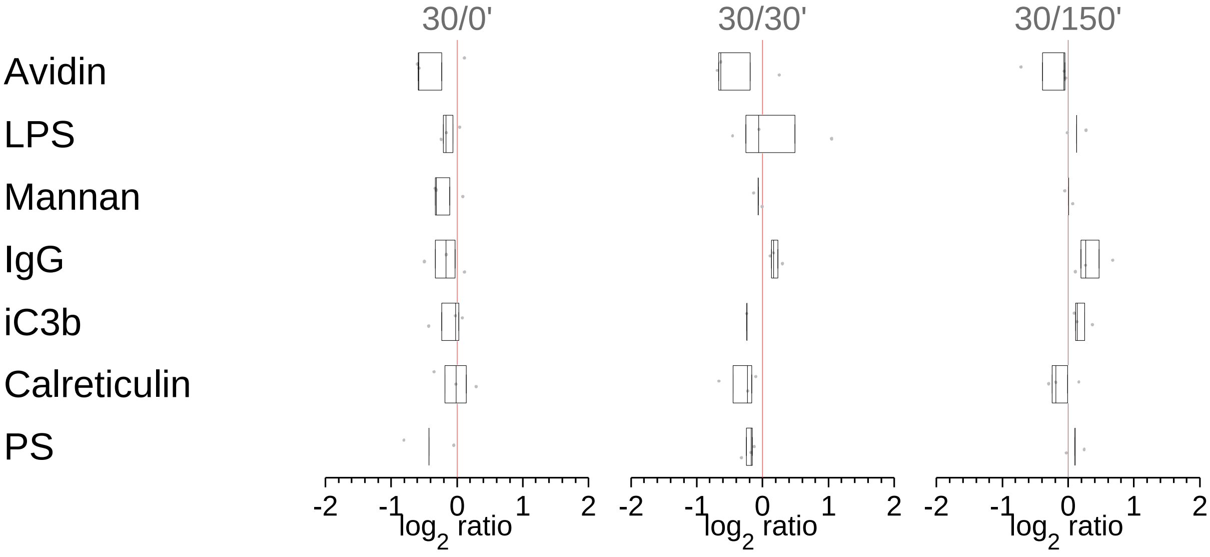

GO:0070735 protein-glycine ligase activity (n = 17)

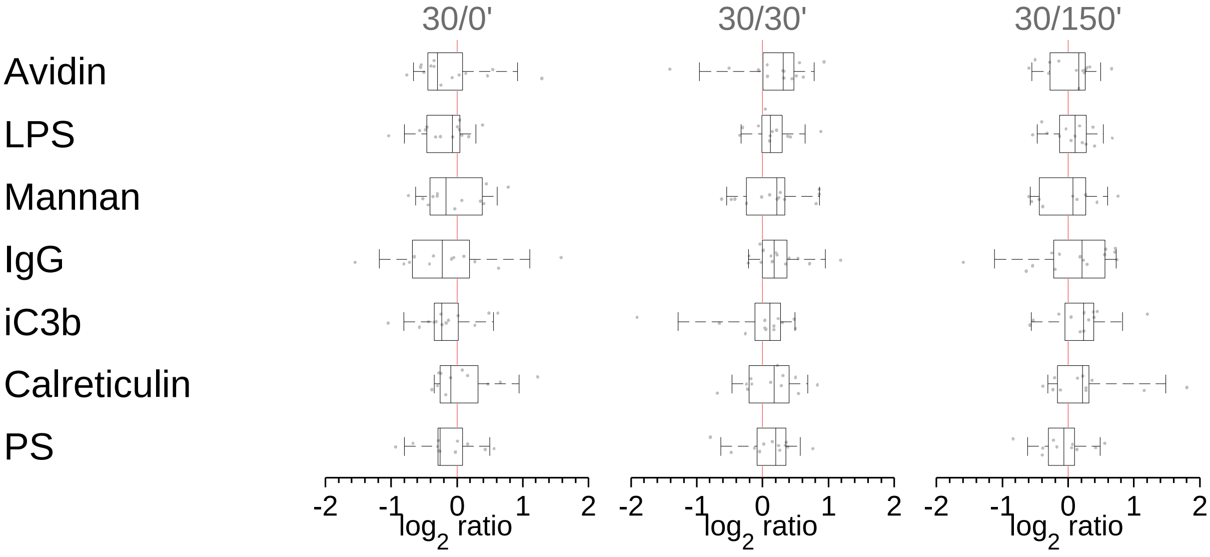

GO:0070736 protein-glycine ligase activity, initiating (n = 17)

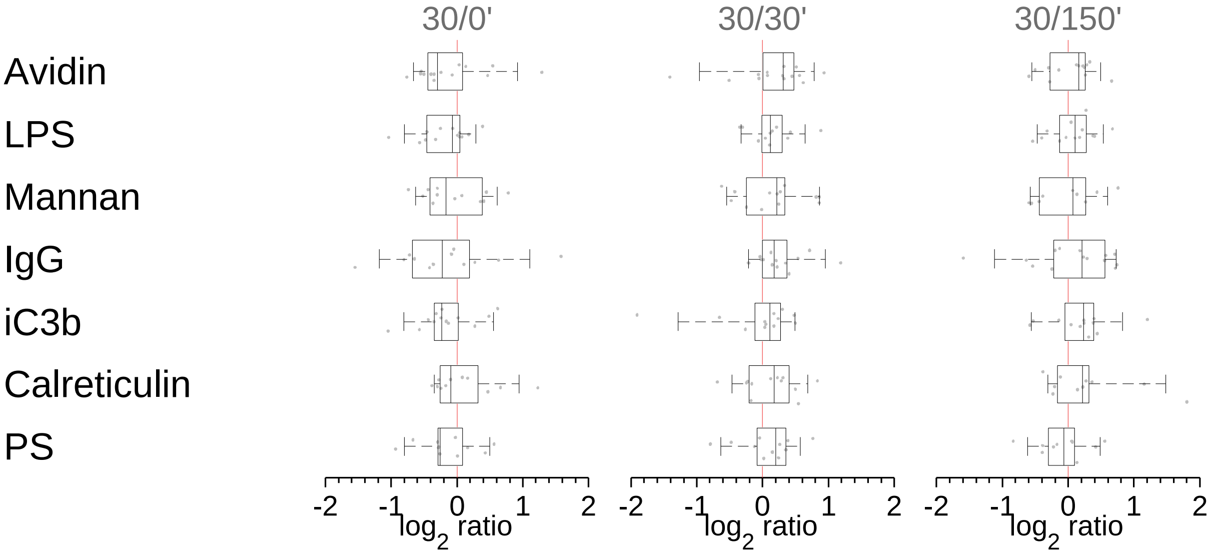

GO:0070737 protein-glycine ligase activity, elongating (n = 17)

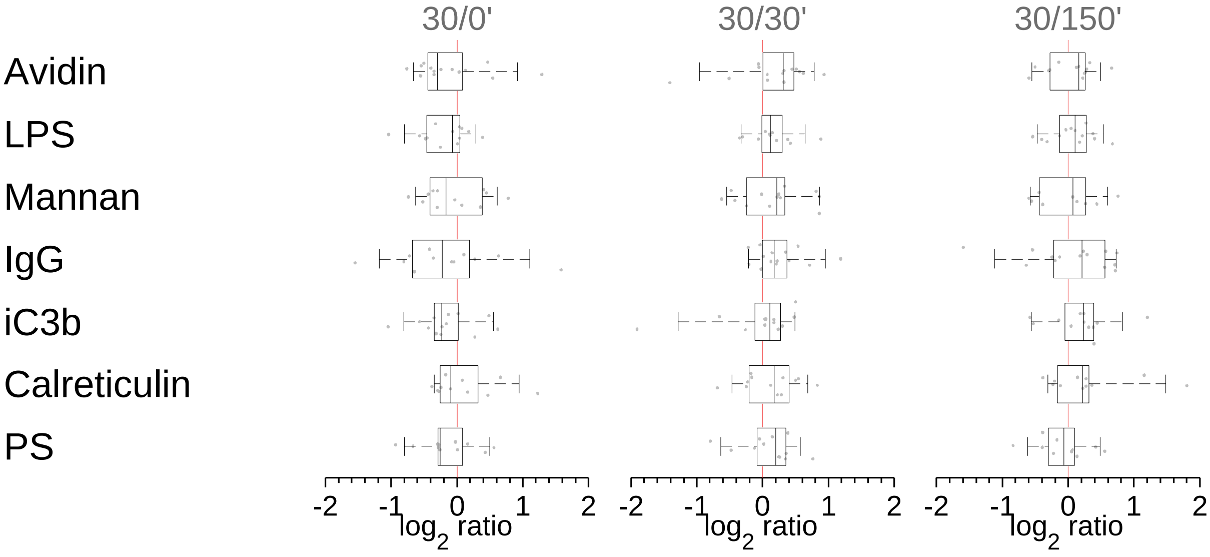

GO:0070738 tubulin-glycine ligase activity (n = 17)

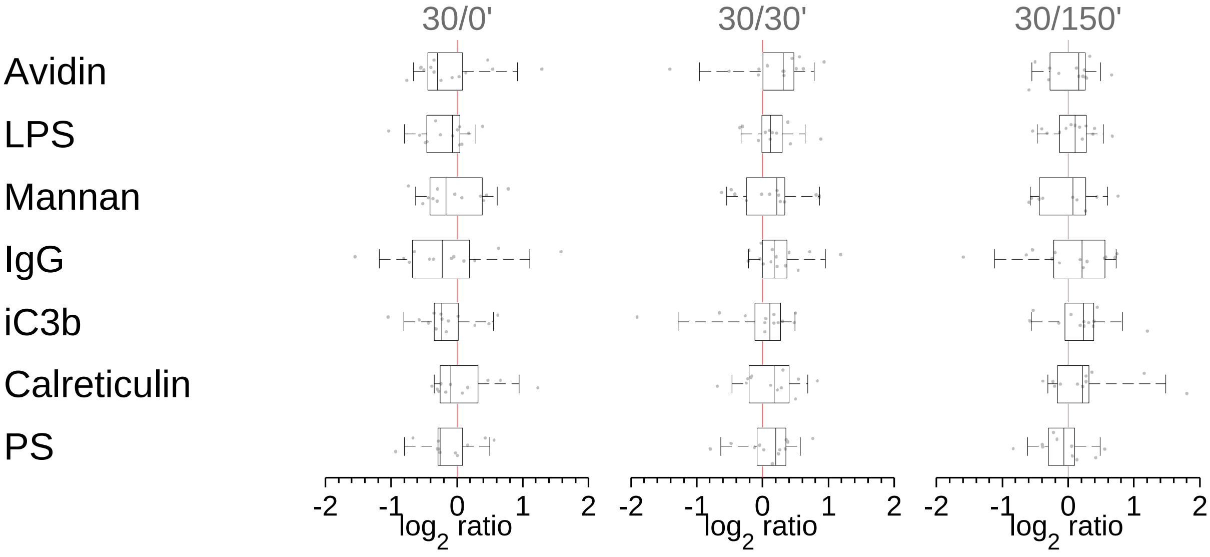

GO:0070739 protein-glutamic acid ligase activity (n = 17)

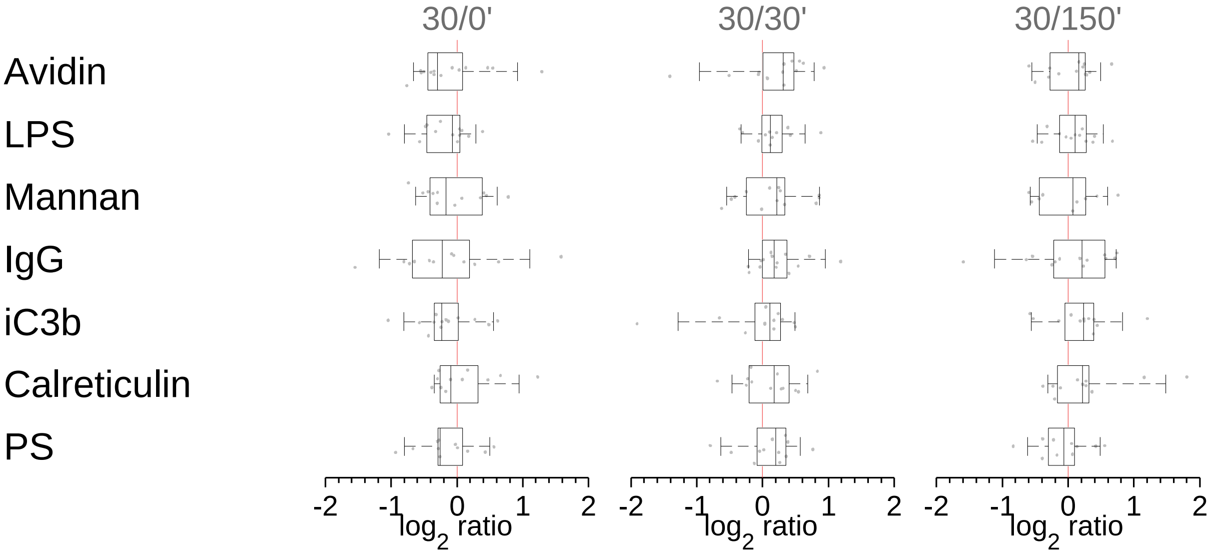

GO:0070740 tubulin-glutamic acid ligase activity (n = 17)

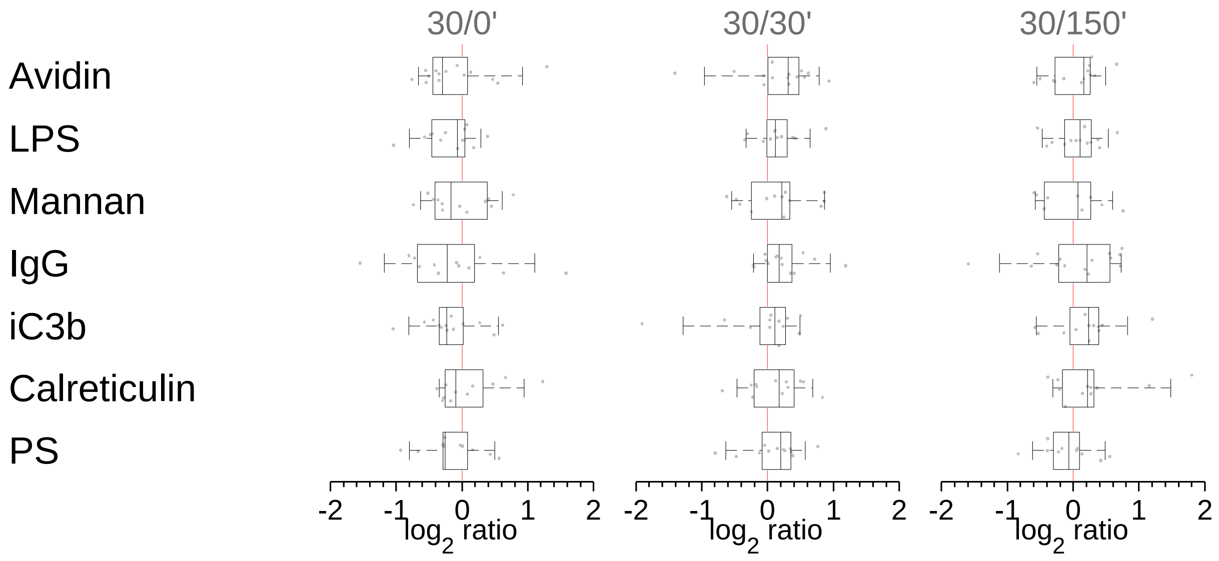

GO:0070820 tertiary granule (n = 3)

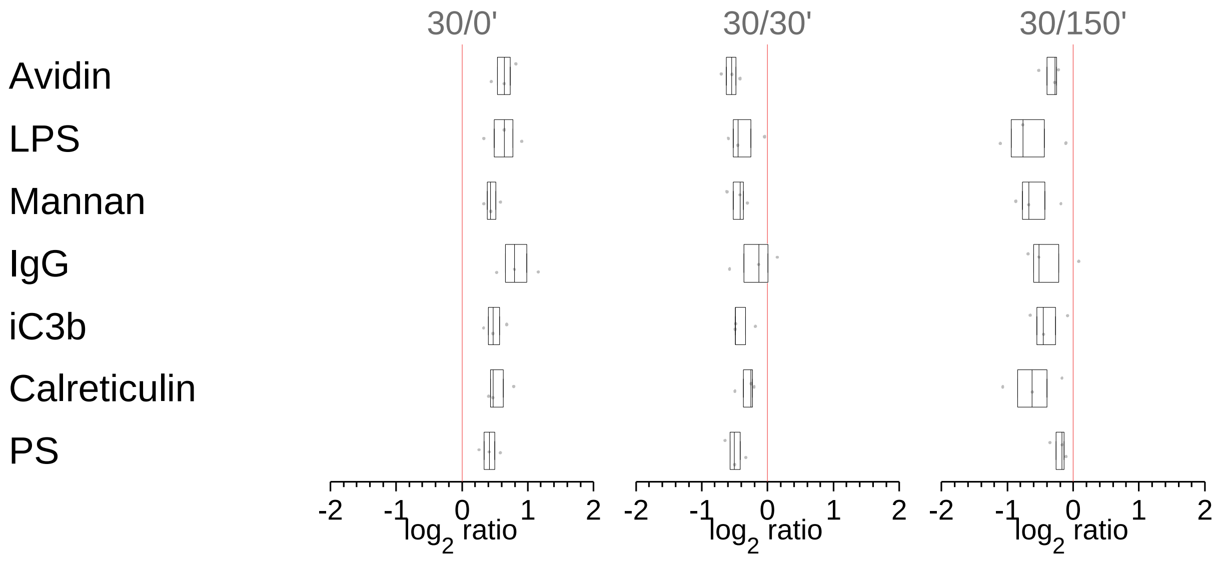

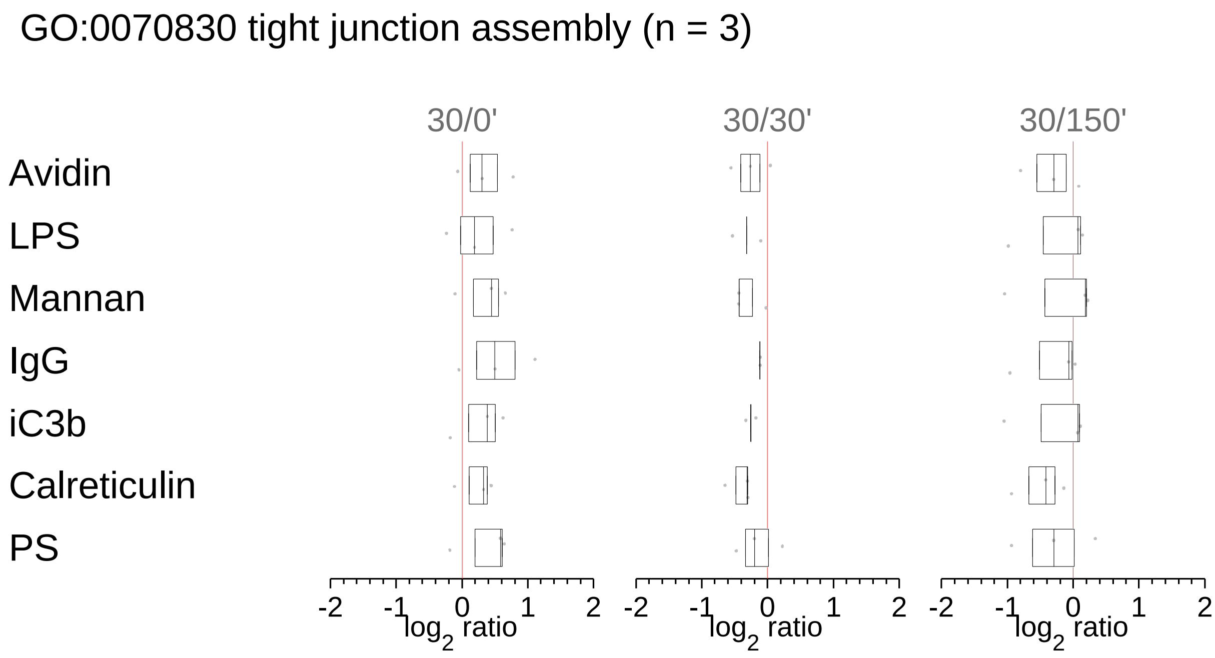
GO:0070830 tight junction assembly (n = 3)
30/0'
30/30'
30/150'
Avidin
LPS
Mannan
IgG
iC3b
Calreticulin
PS
-2
-1
0
1
2
log2 ratio

GO:0070932 histone H3 deacetylation (n = 3)

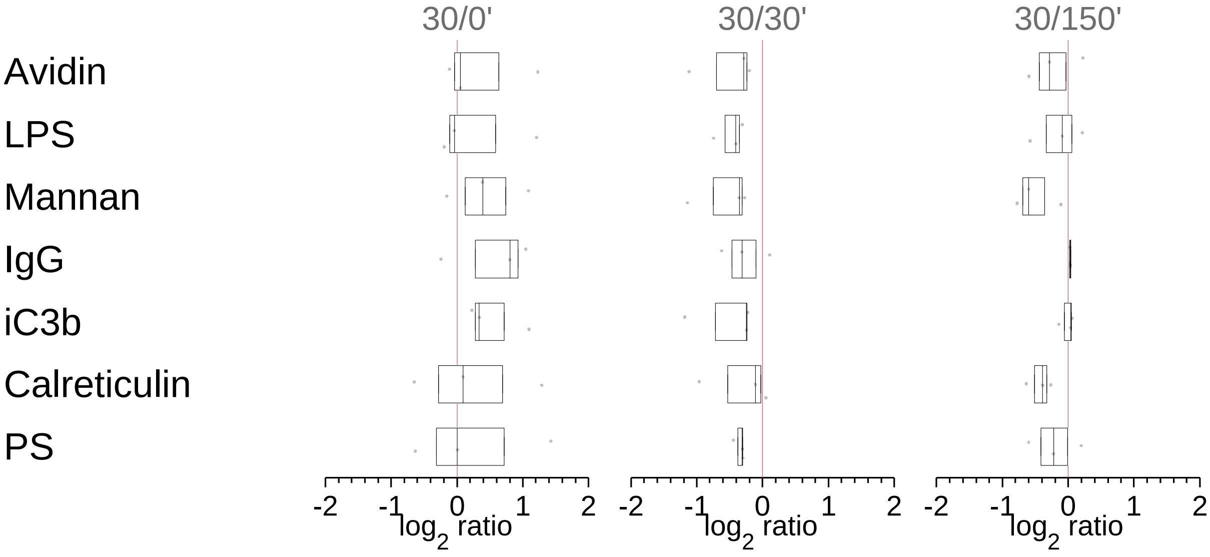

GO:0070933 histone H4 deacetylation (n = 3)

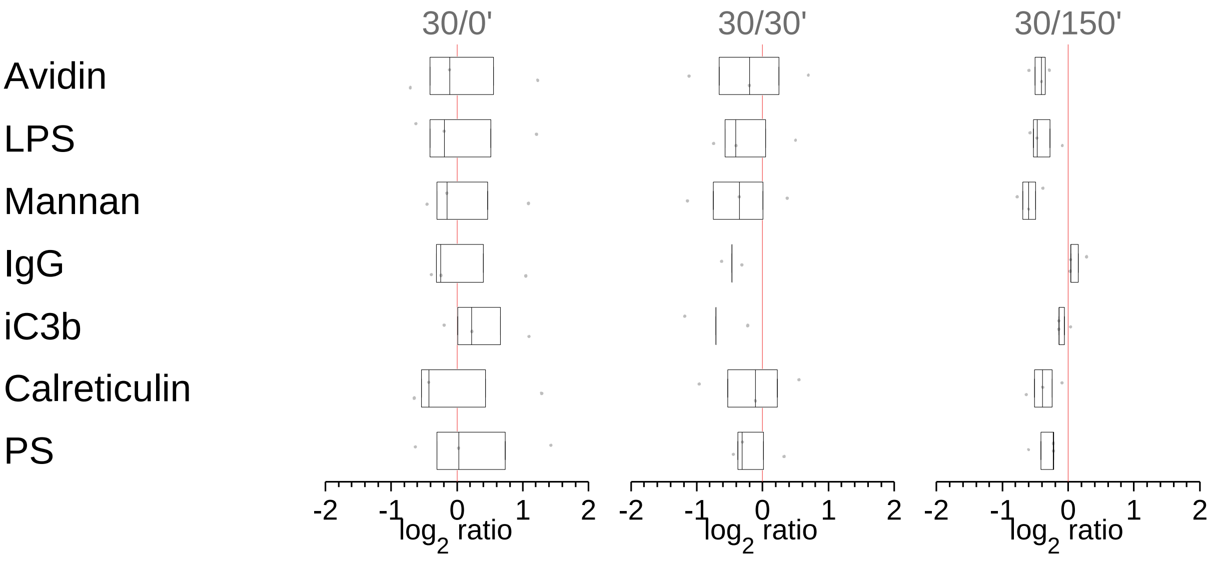

GO:0070934 CRD-mediated mRNA stabilization (n = 3)

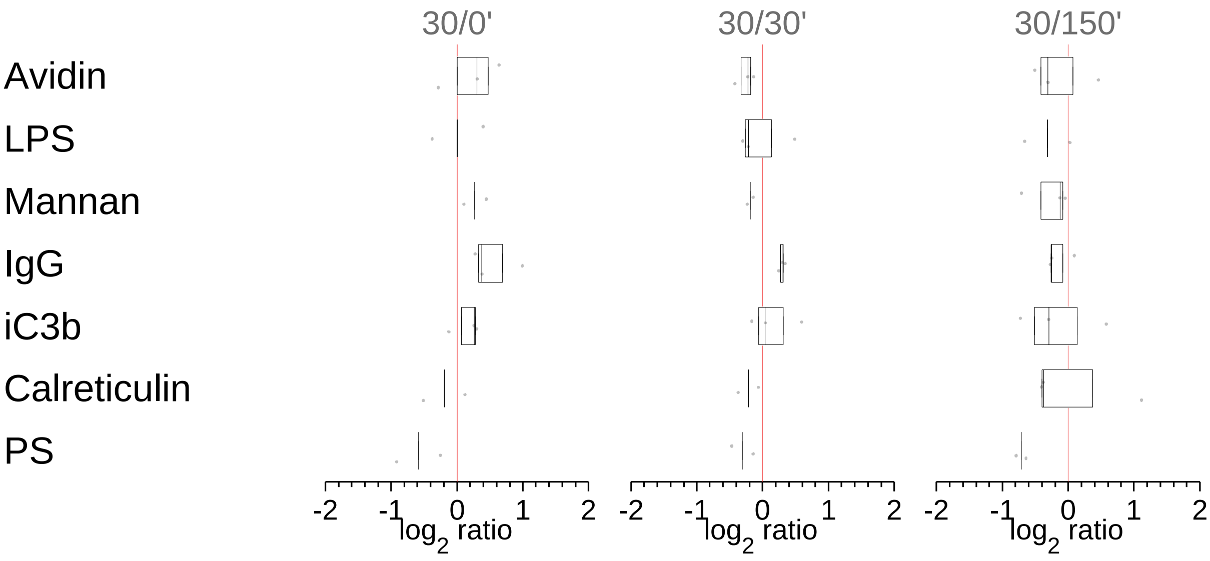

GO:0070935 3'-UTR-mediated mRNA stabilization (n = 4)

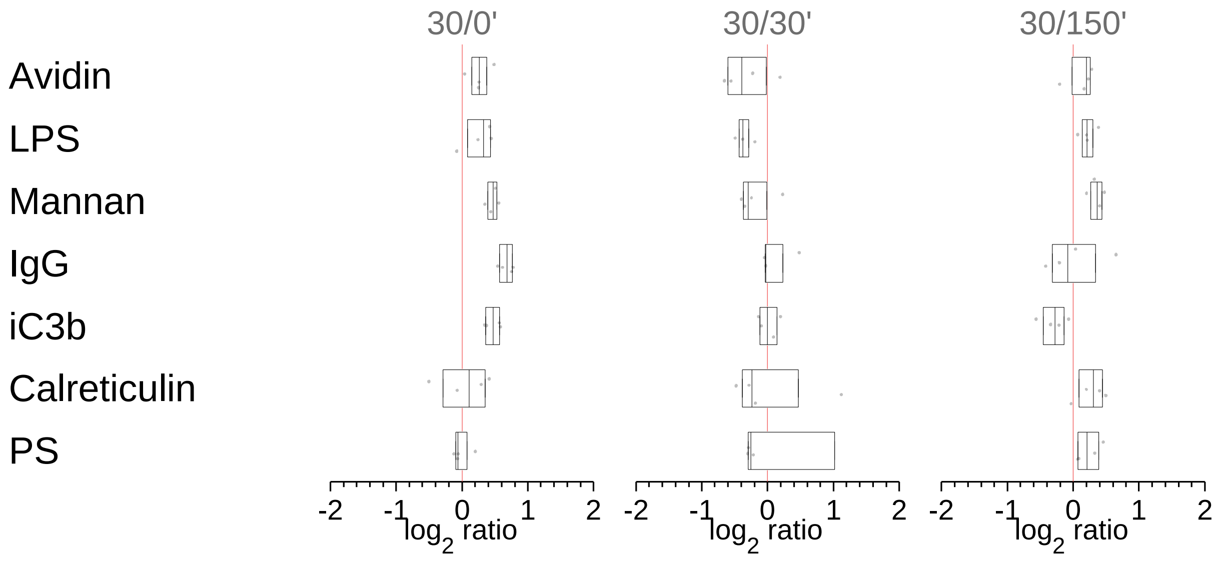

GO:0070936 protein K48-linked ubiquitination (n = 3)

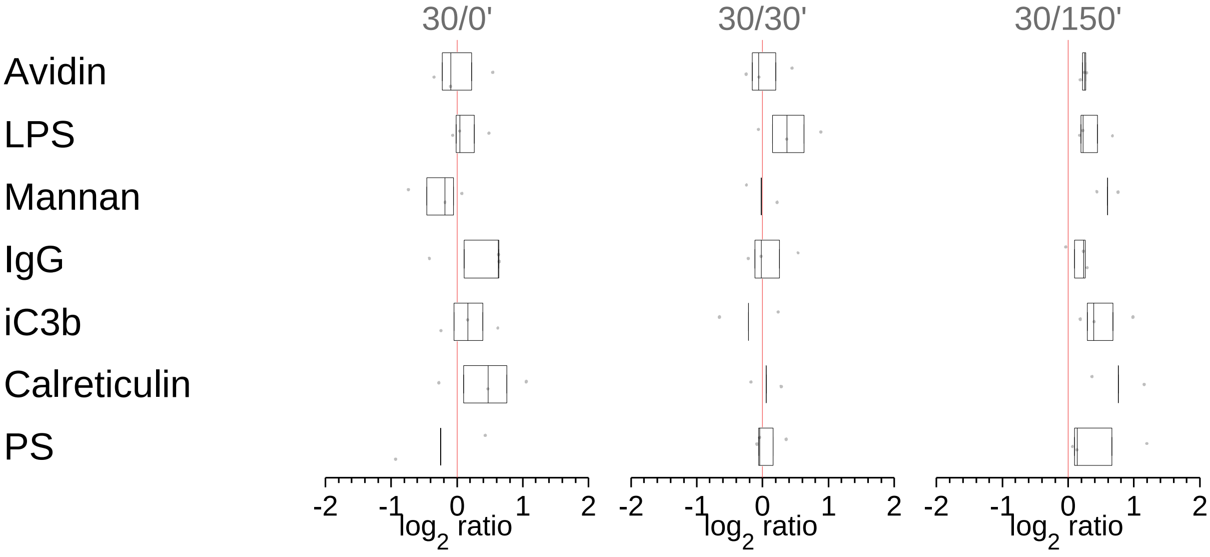

GO:0070937 CRD-mediated mRNA stability complex (n = 3)

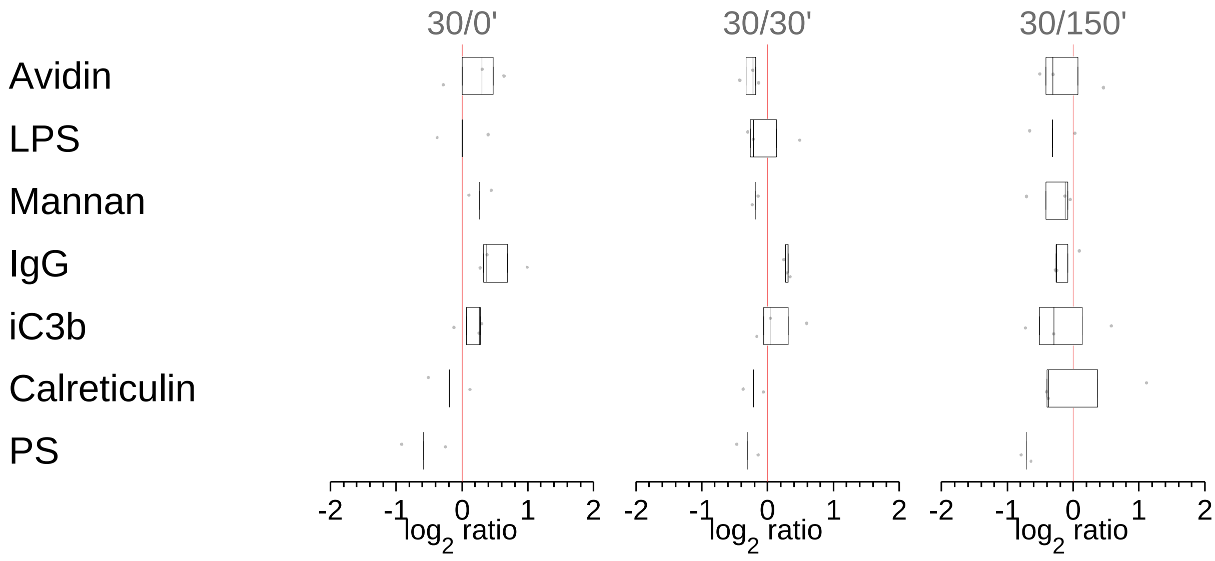

GO:0070971 endoplasmic reticulum exit site (n = 3)

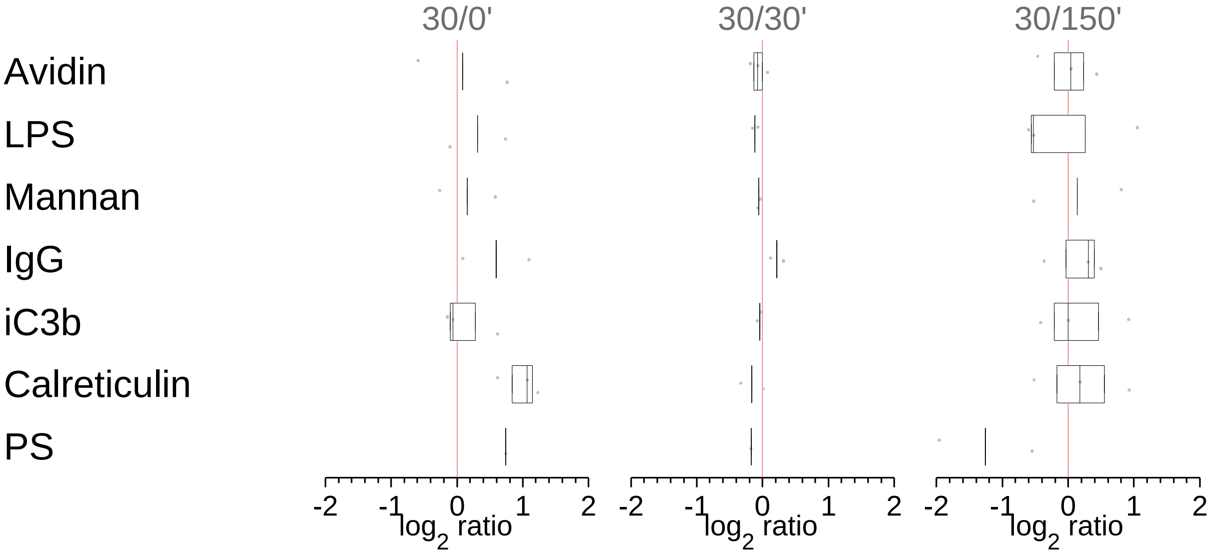

GO:0071013 catalytic step 2 spliceosome (n = 16)

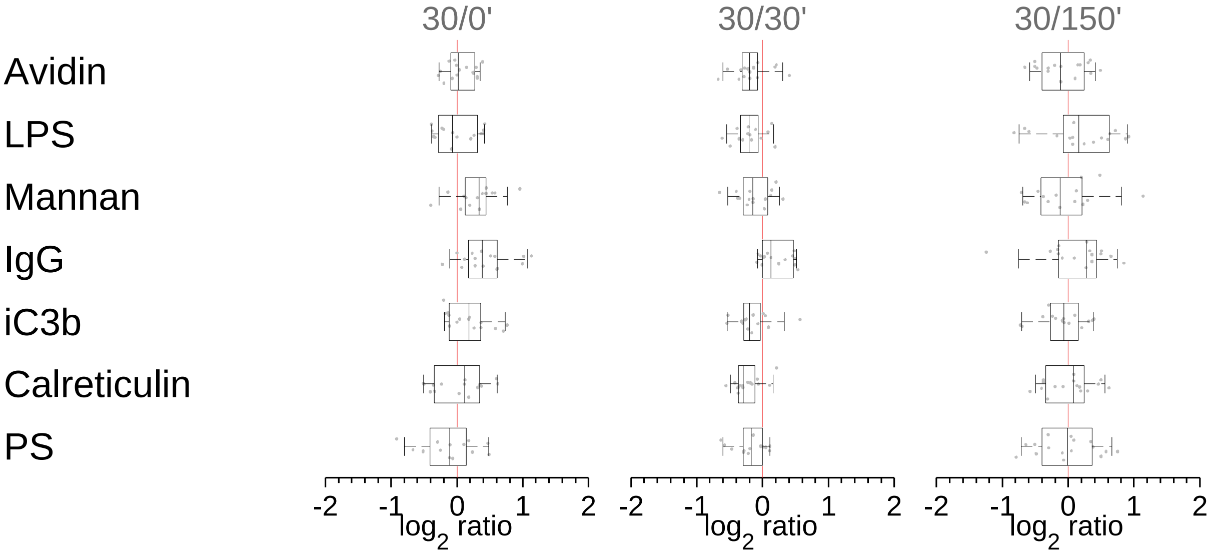

GO:0071203 WASH complex (n = 9)

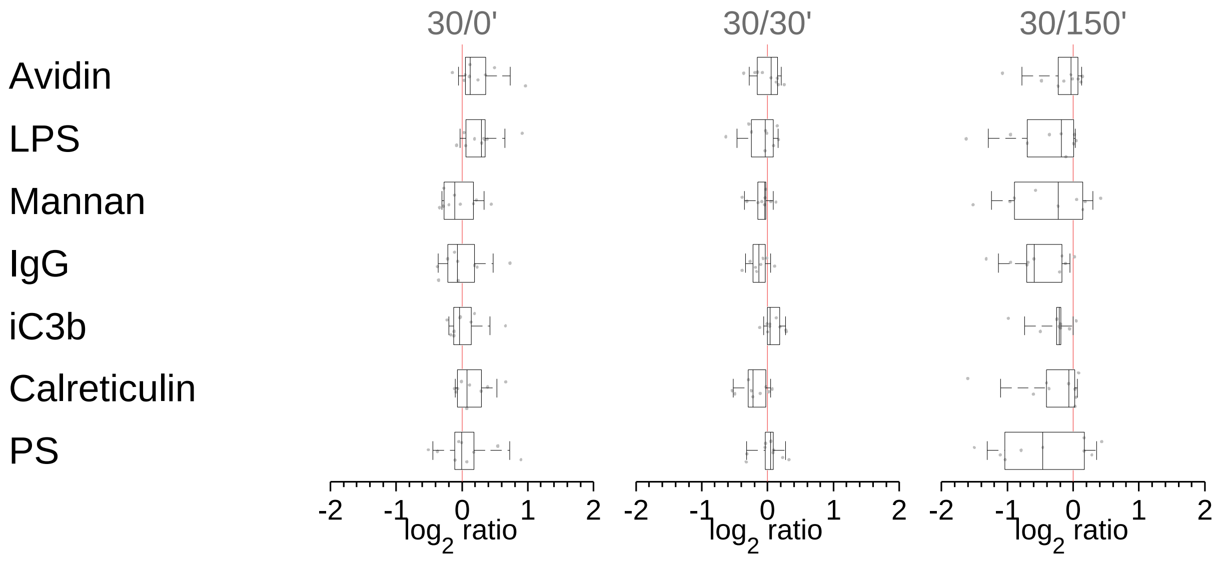

GO:0071222 cellular response to lipopolysaccharide (n = 5)

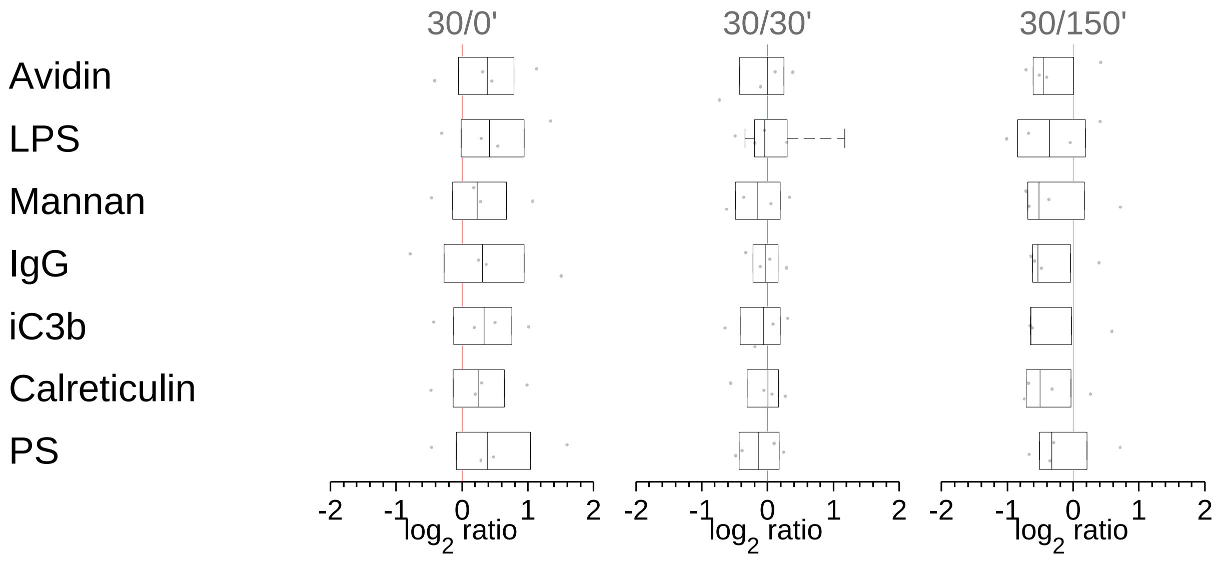

GO:0071223 cellular response to lipoteichoic acid (n = 3)

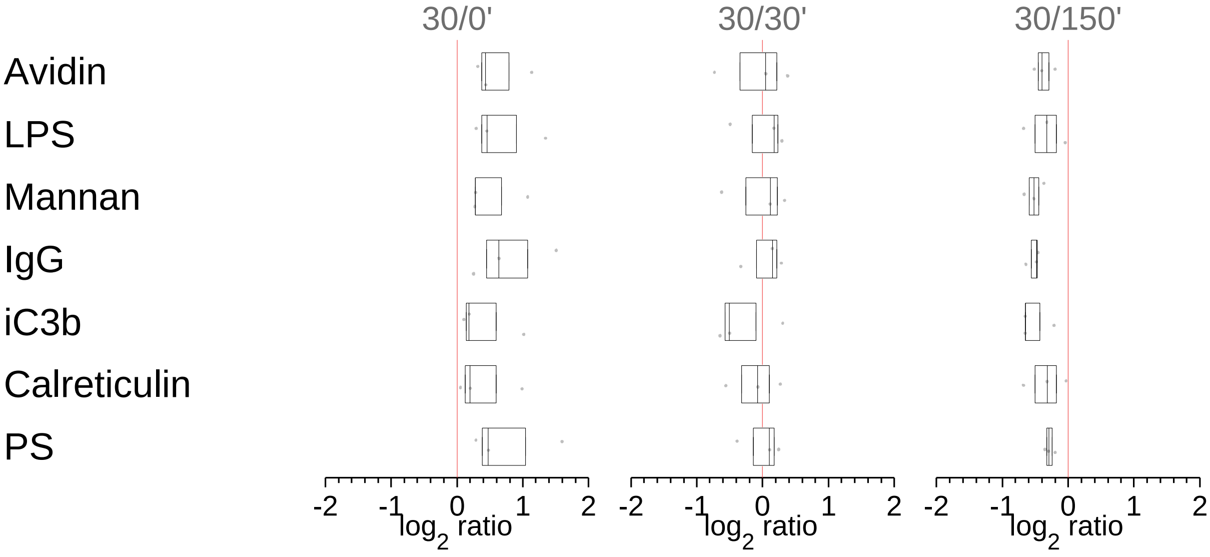

GO:0071230 cellular response to amino acid stimulus (n = 8)

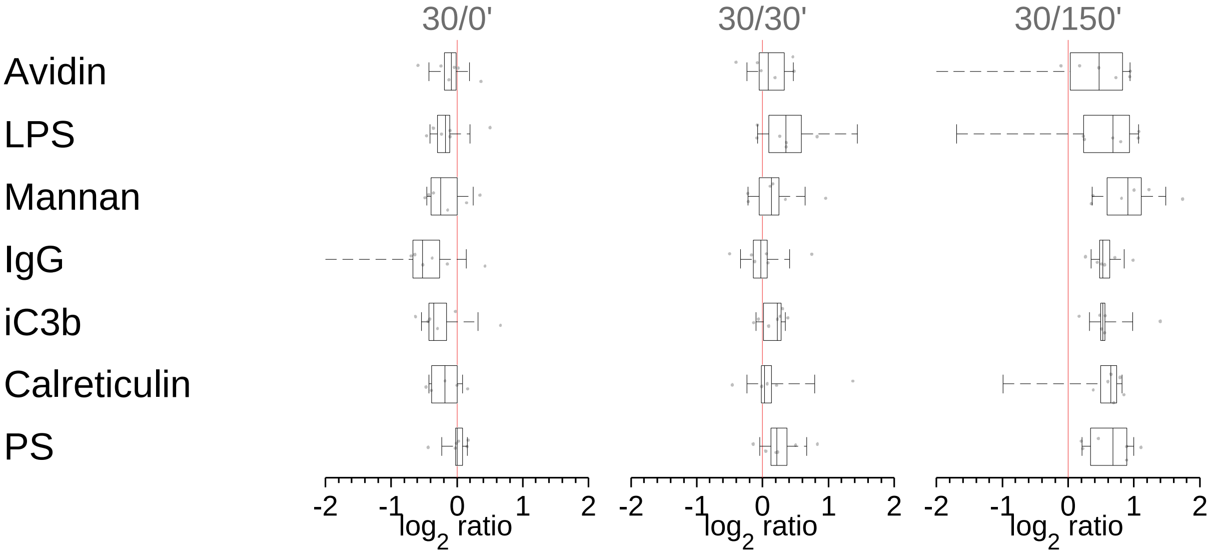

GO:0071243 cellular response to arsenic-containing substance (n = 3)

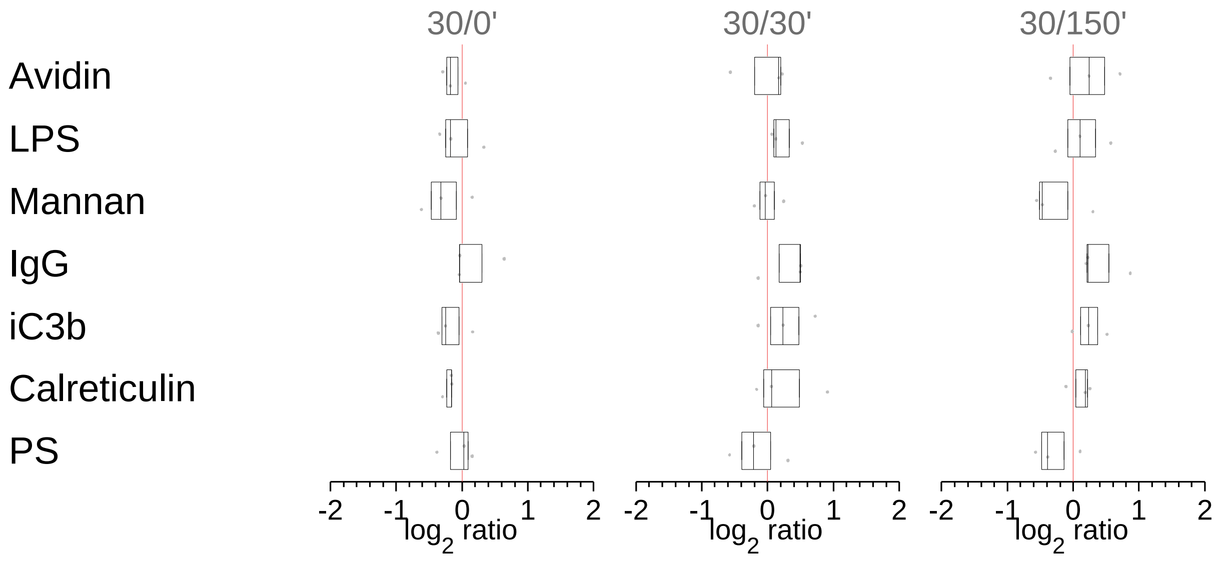

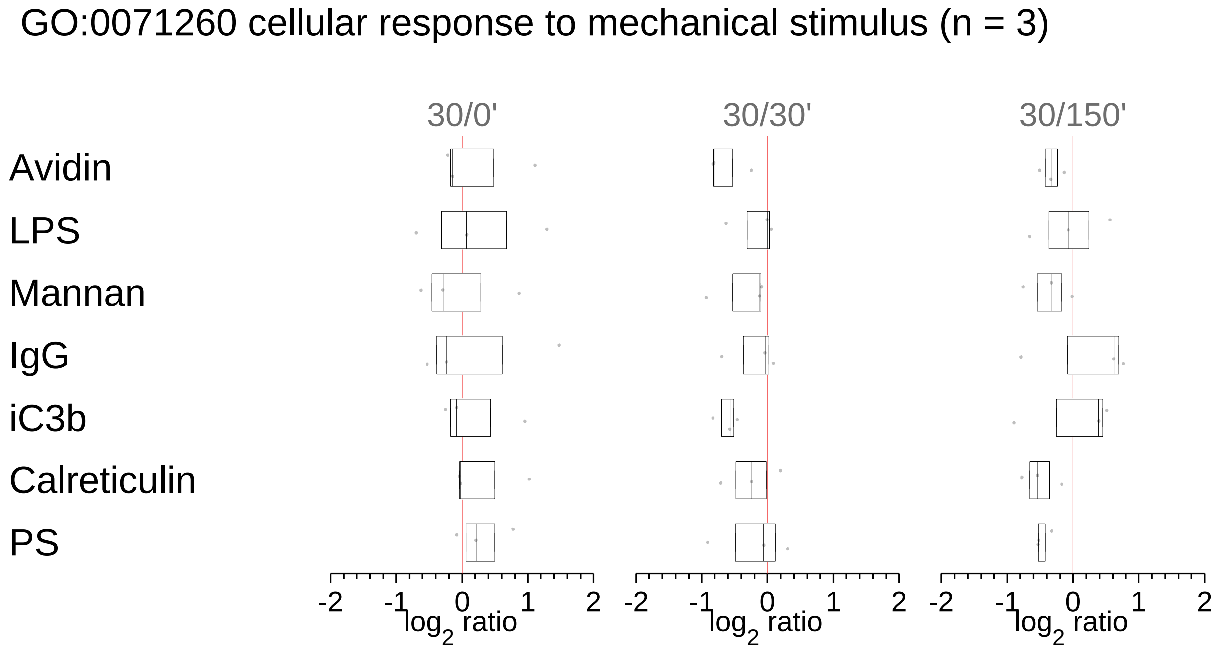

GO:0071260 cellular response to mechanical stimulus (n = 3)
30/0'
30/30'
30/150'
Avidin
LPS
Mannan
IgG
iC3b
Calreticulin
PS
-2 -1 0 1 2
log2 ratio

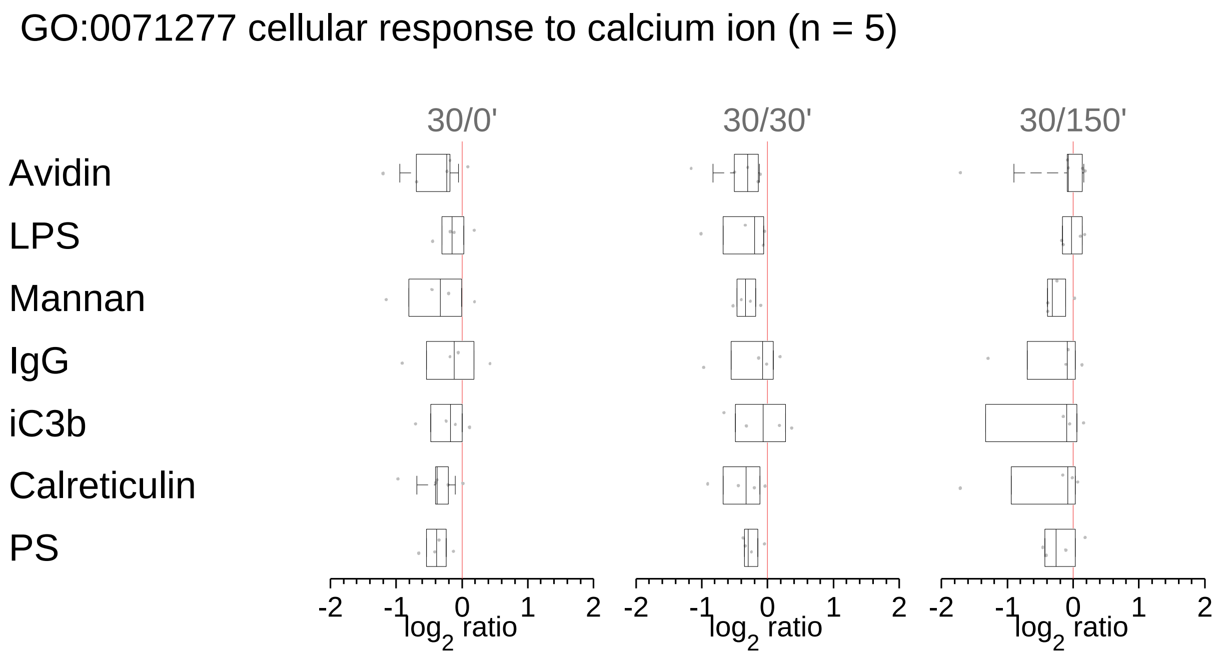
GO:0071277 cellular response to calcium ion (n = 5)
30/0'
30/30'
30/150'
Avidin
LPS
Mannan
IgG
iC3b
Calreticulin
PS
-2
-1
0
1
2
log2 ratio

GO:0071310 cellular response to organic substance (n = 4)

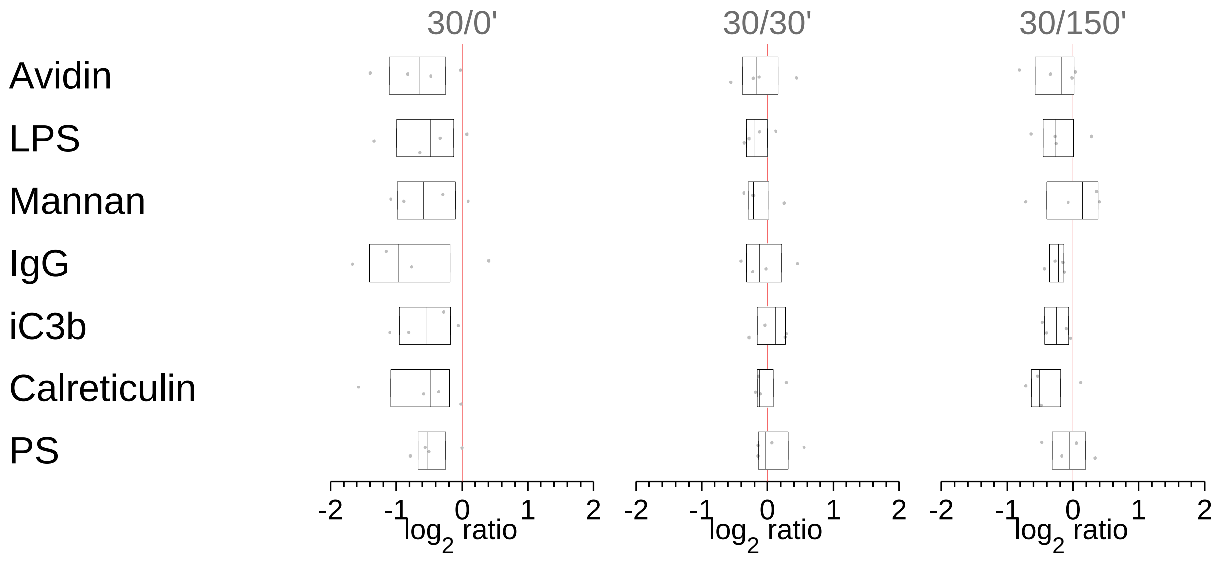

GO:0071339 MLL1 complex (n = 3)

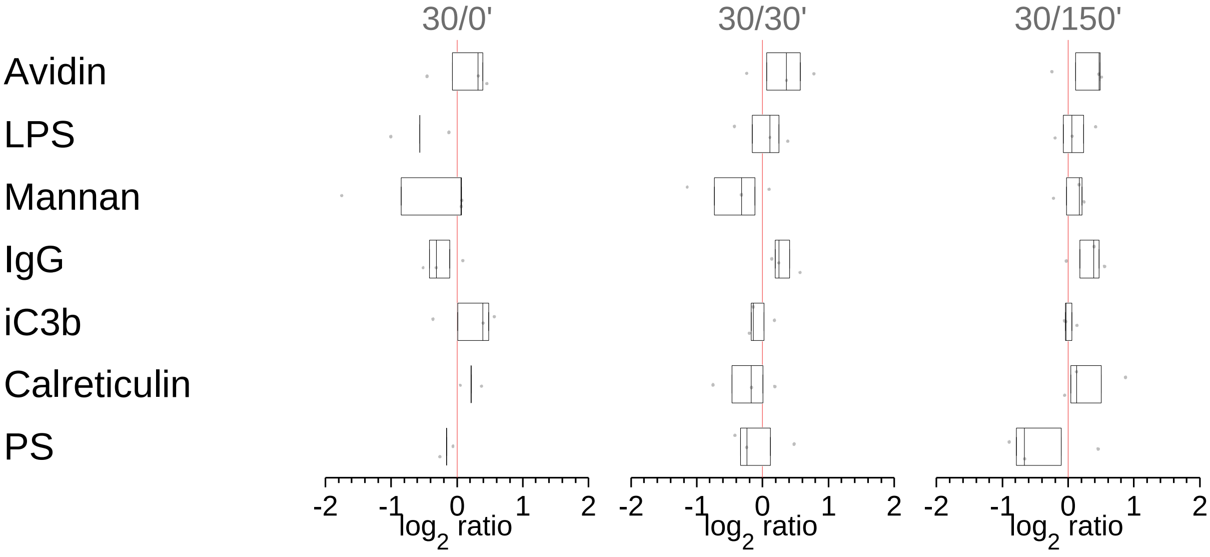

GO:0071345 cellular response to cytokine stimulus (n = 3)

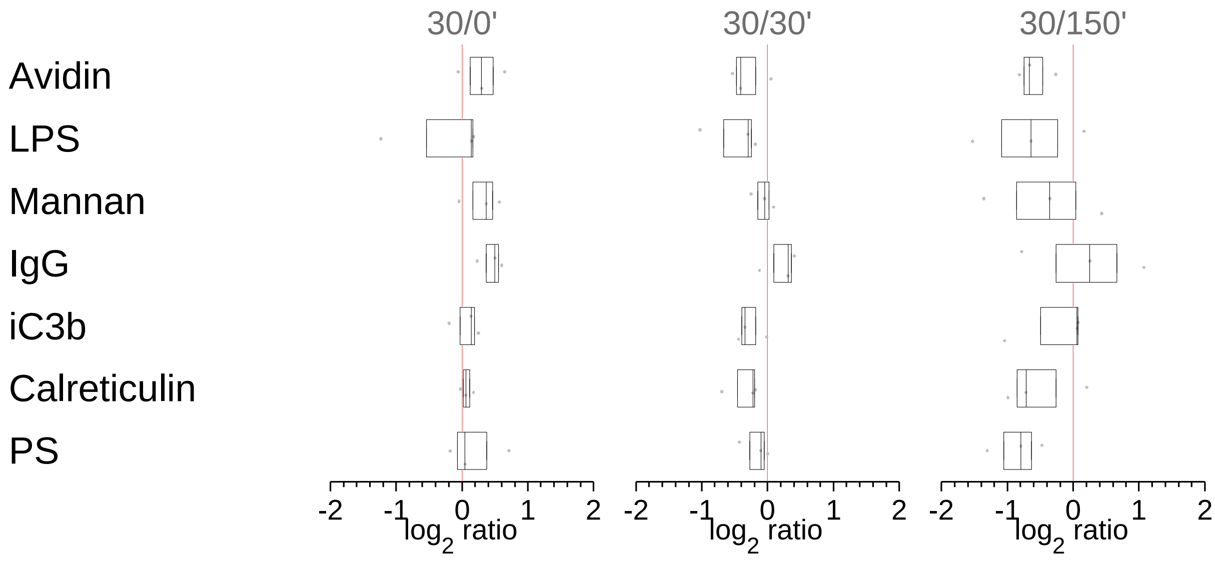

GO:0071347 cellular response to interleukin-1 (n = 3)

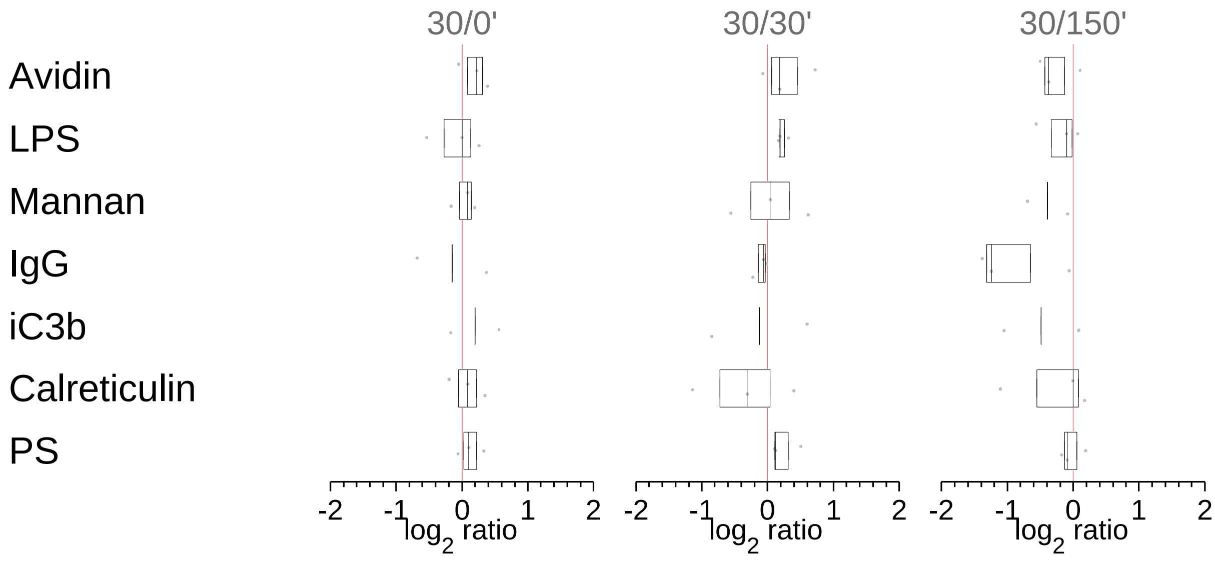

GO:0071353 cellular response to interleukin-4 (n = 6)

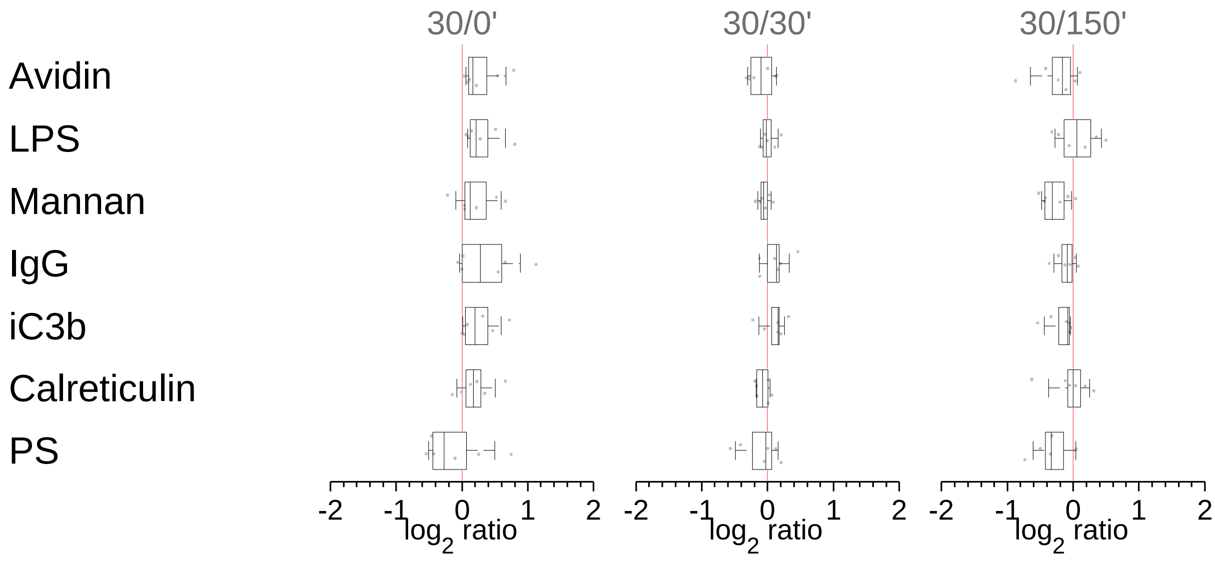

GO:0071356 cellular response to tumor necrosis factor (n = 3)

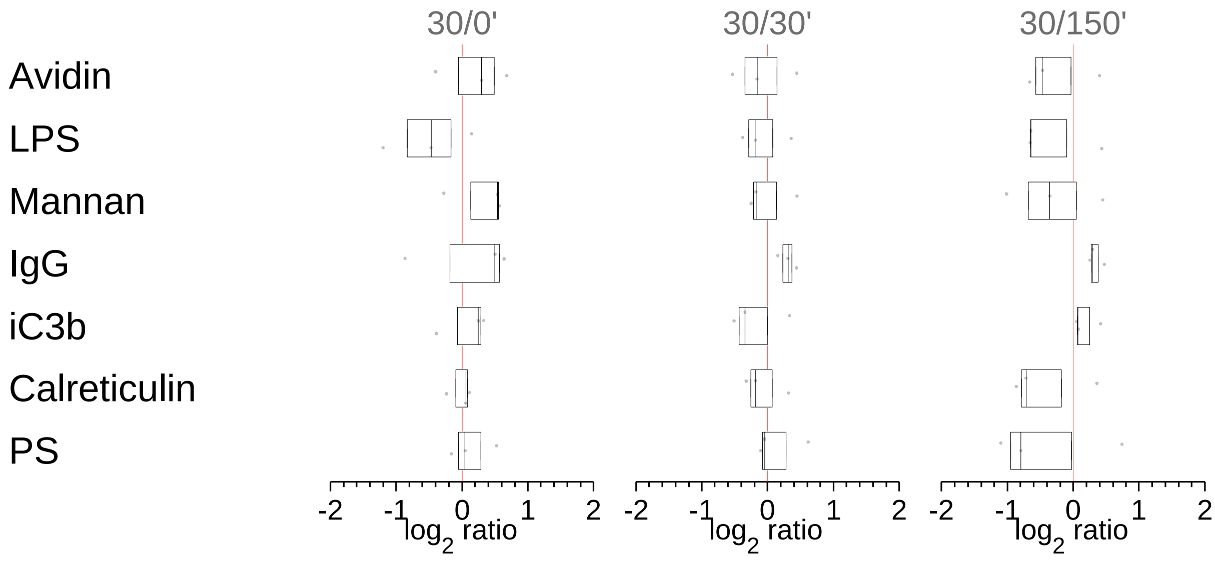

GO:0071360 cellular response to exogenous dsRNA (n = 4)

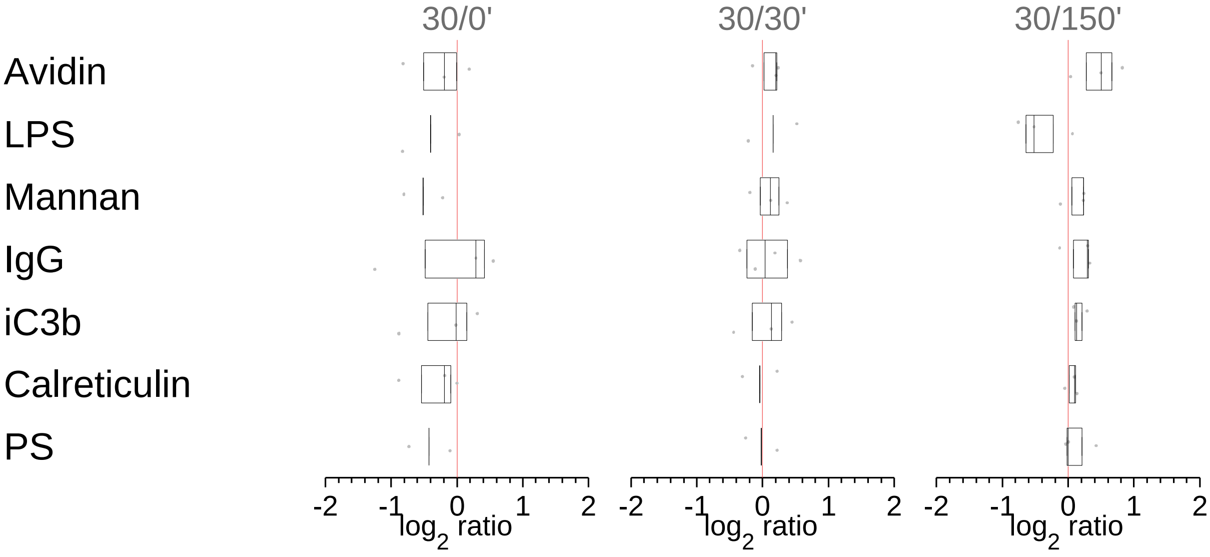

GO:0071363 cellular response to growth factor stimulus (n = 3)

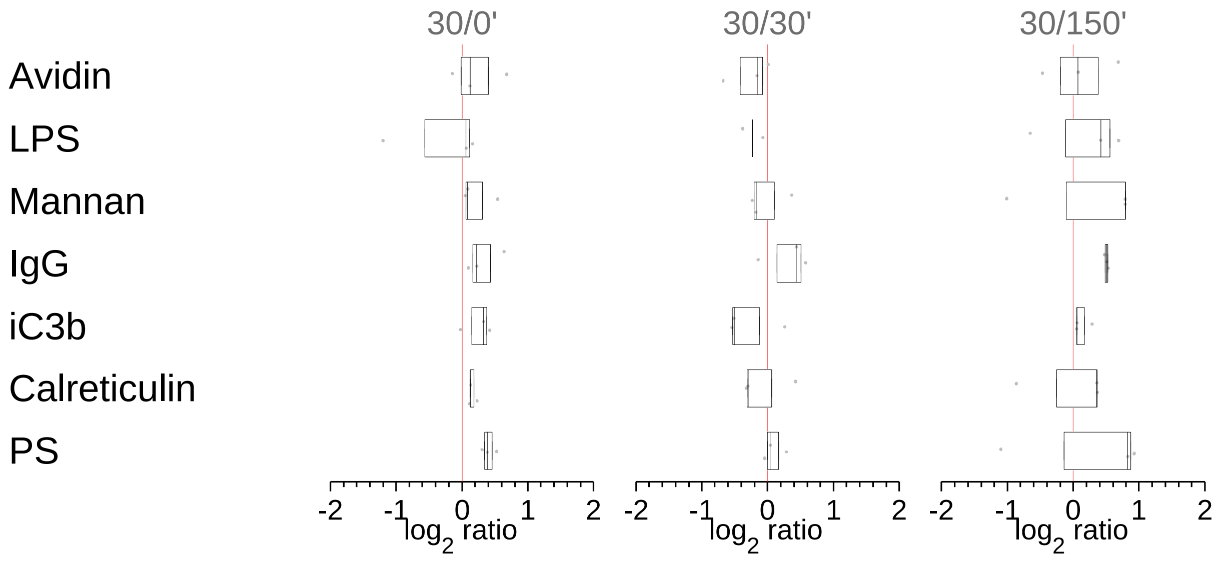

GO:0071364 cellular response to epidermal growth factor stimulus
(n = 3)

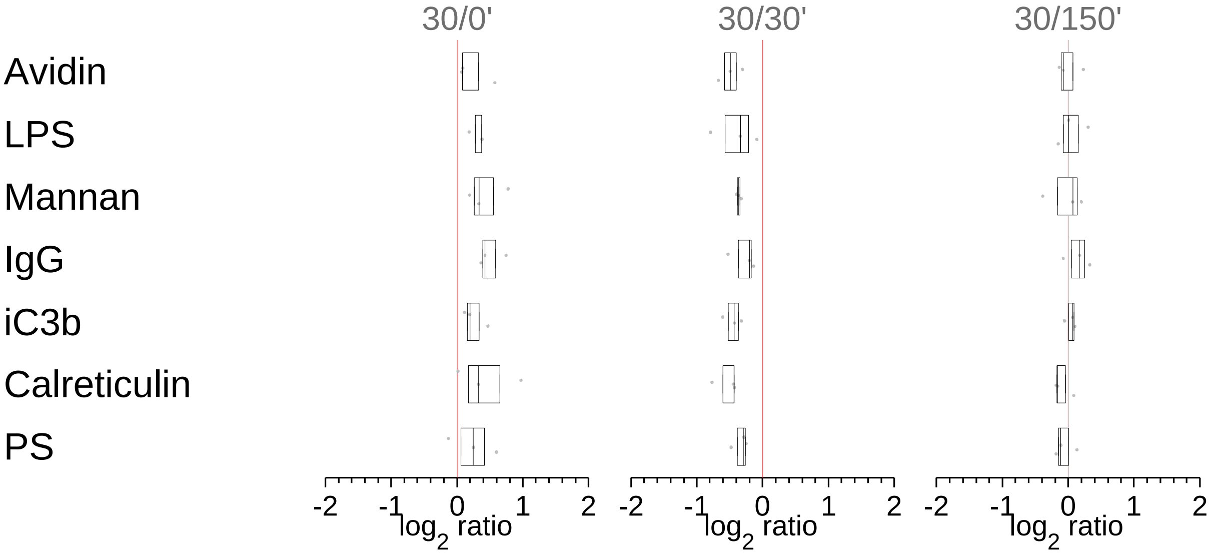

GO:0071407 cellular response to organic cyclic compound (n = 3)

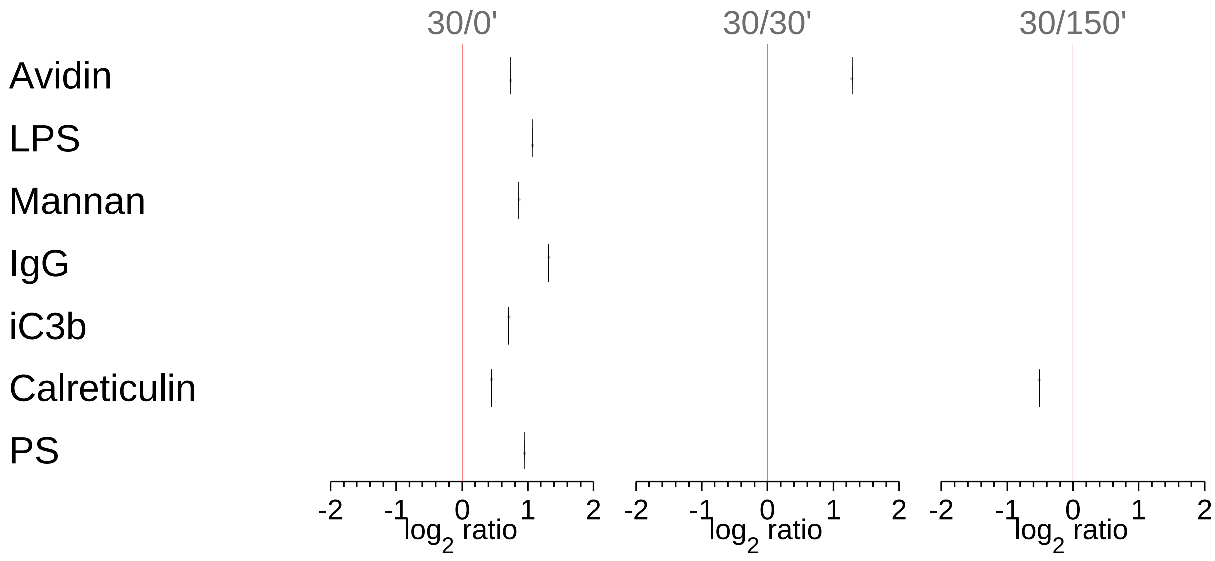

GO:0071439 clathrin complex (n = 3)

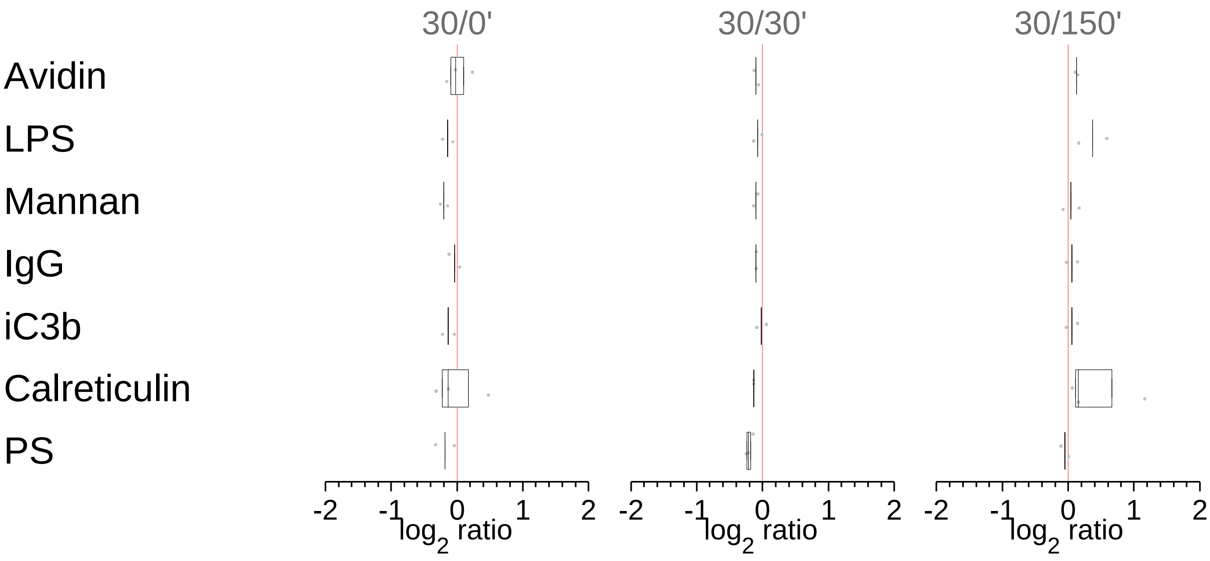

GO:0071456 cellular response to hypoxia (n = 5)

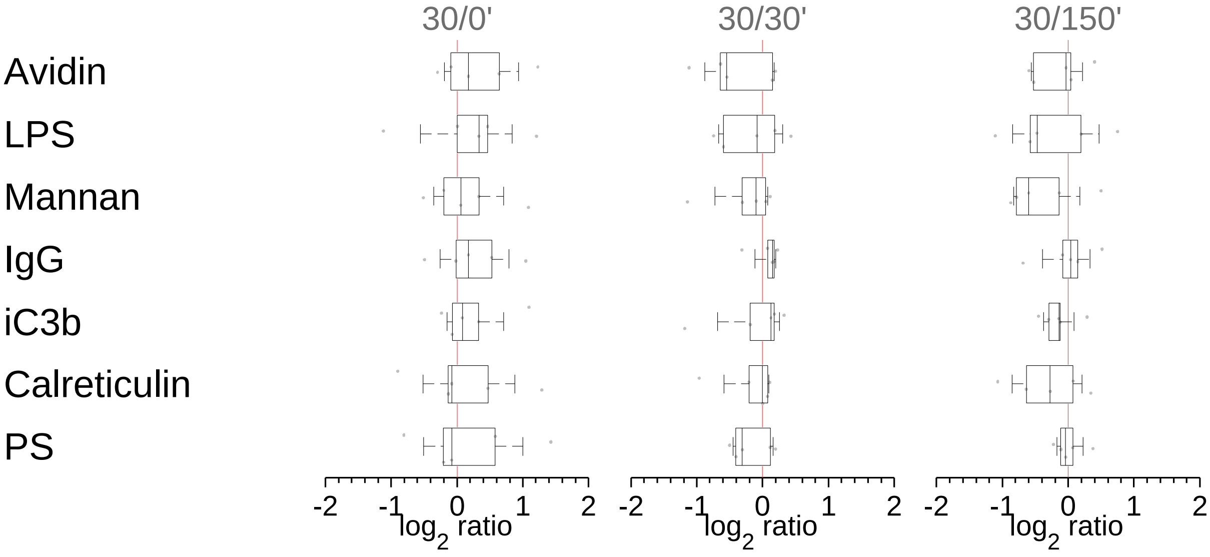

GO:0071467 cellular response to pH (n = 3)

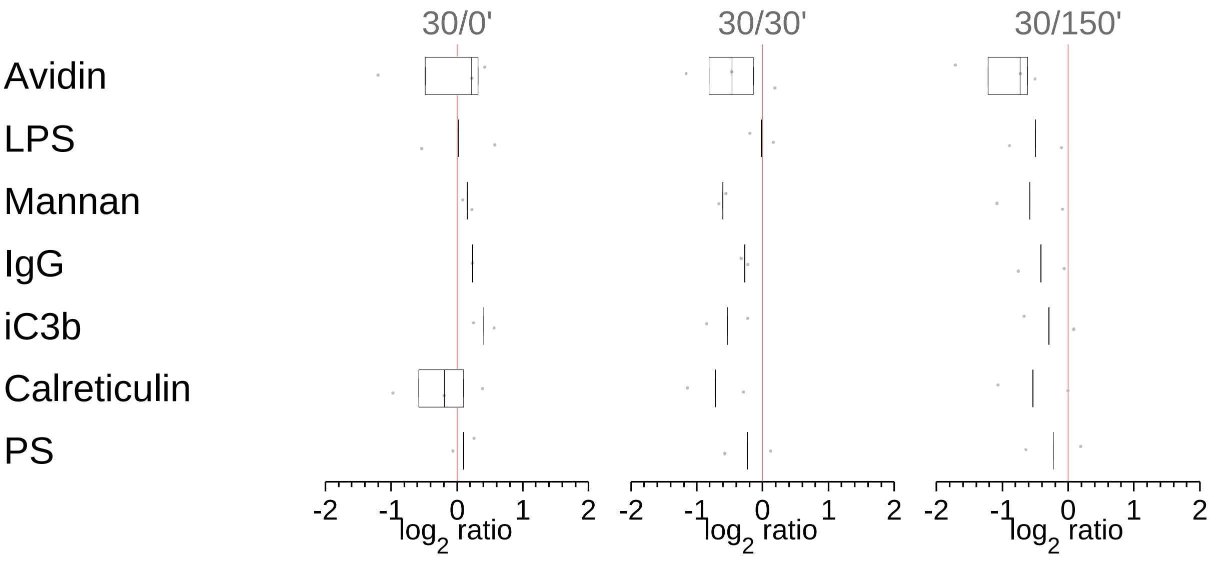

GO:0071526 semaphorin-plexin signaling pathway (n = 7)

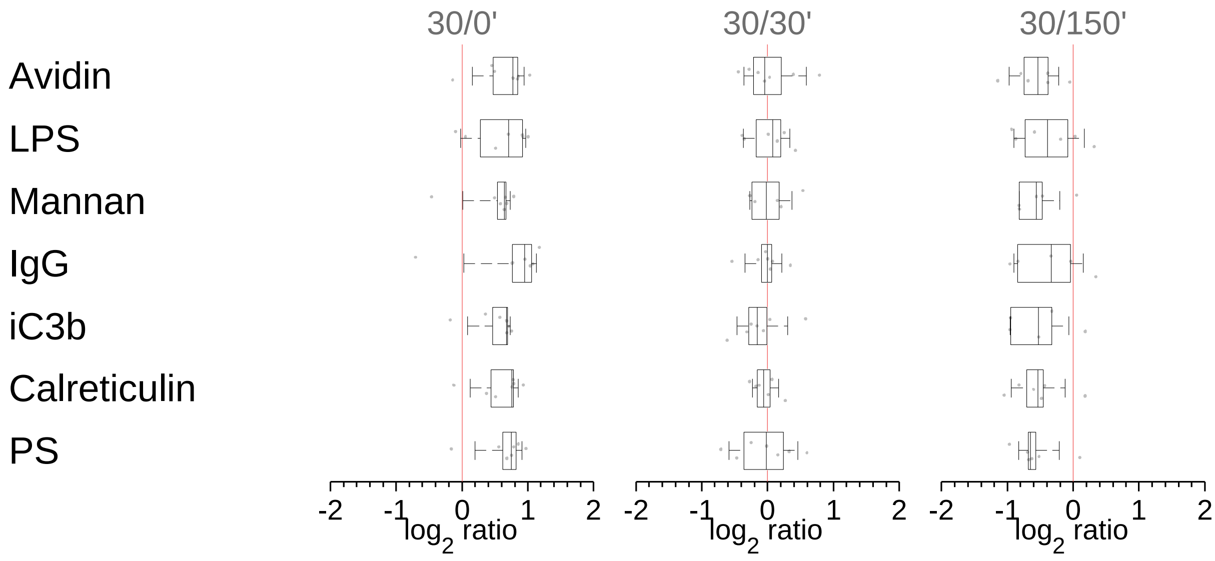

GO:0071786 endoplasmic reticulum tubular network organization (n = 3)

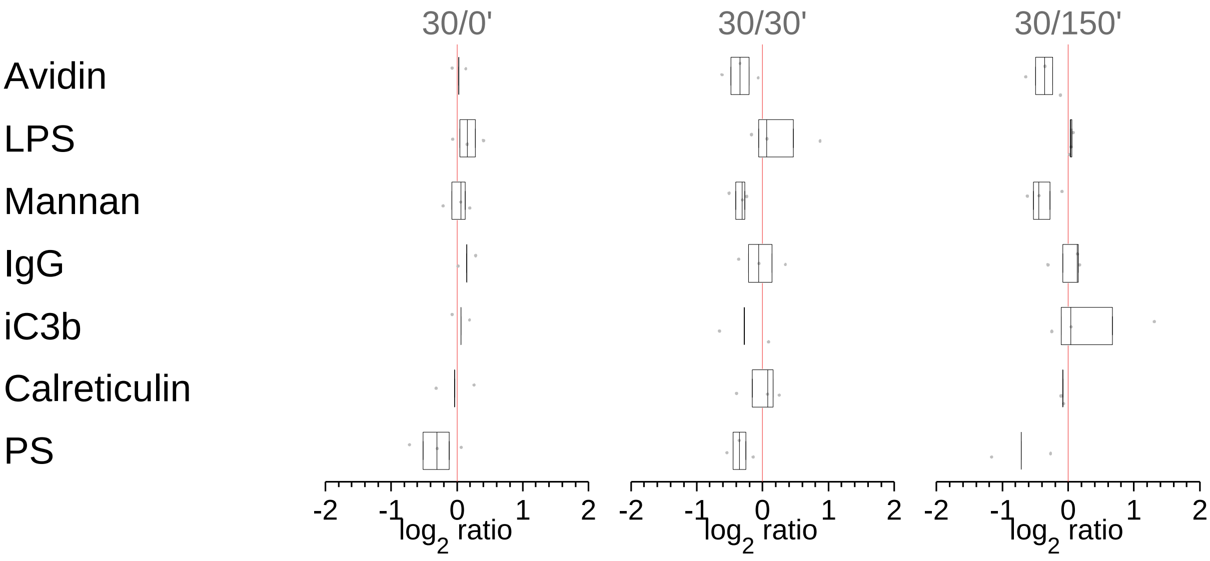

GO:0071805 potassium ion transmembrane transport (n = 4)

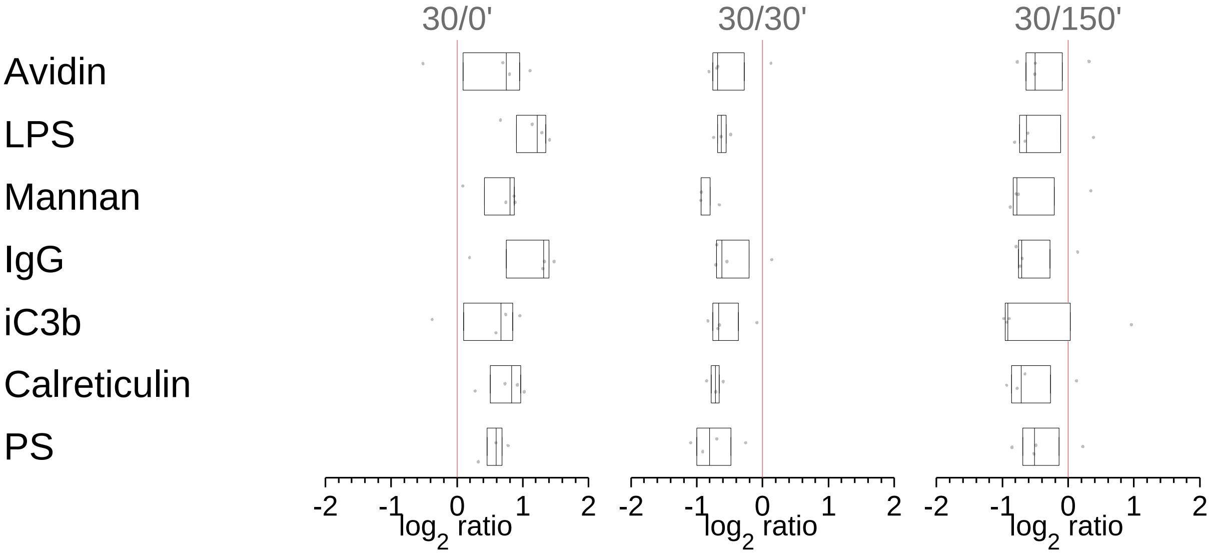

GO:0071889 14-3-3 protein binding (n = 4)

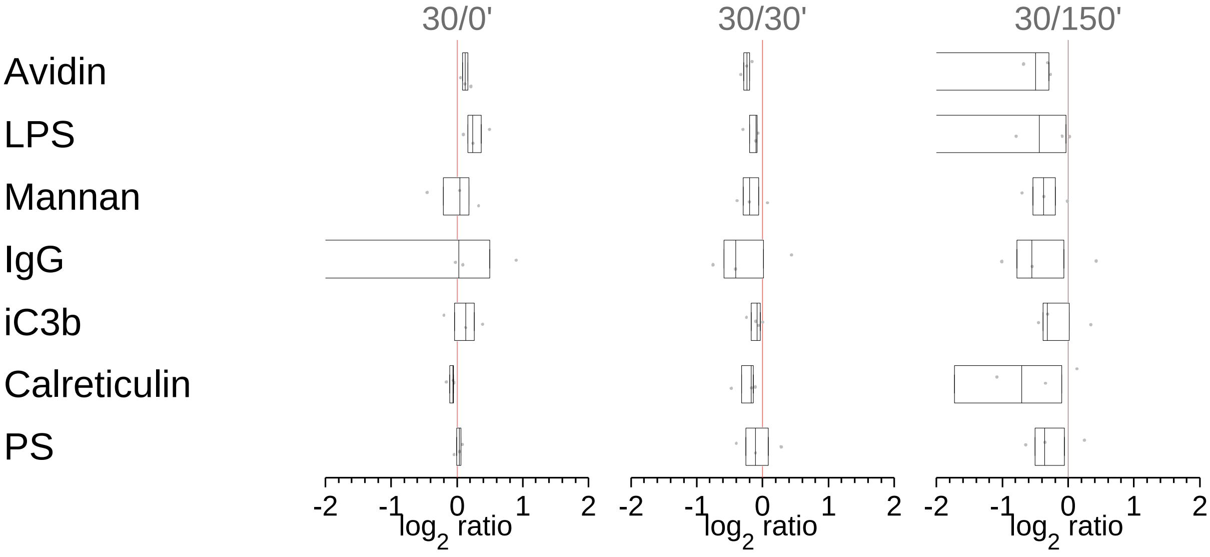

GO:0071902 positive regulation of protein serine/threonine kinase activity (n = 8)

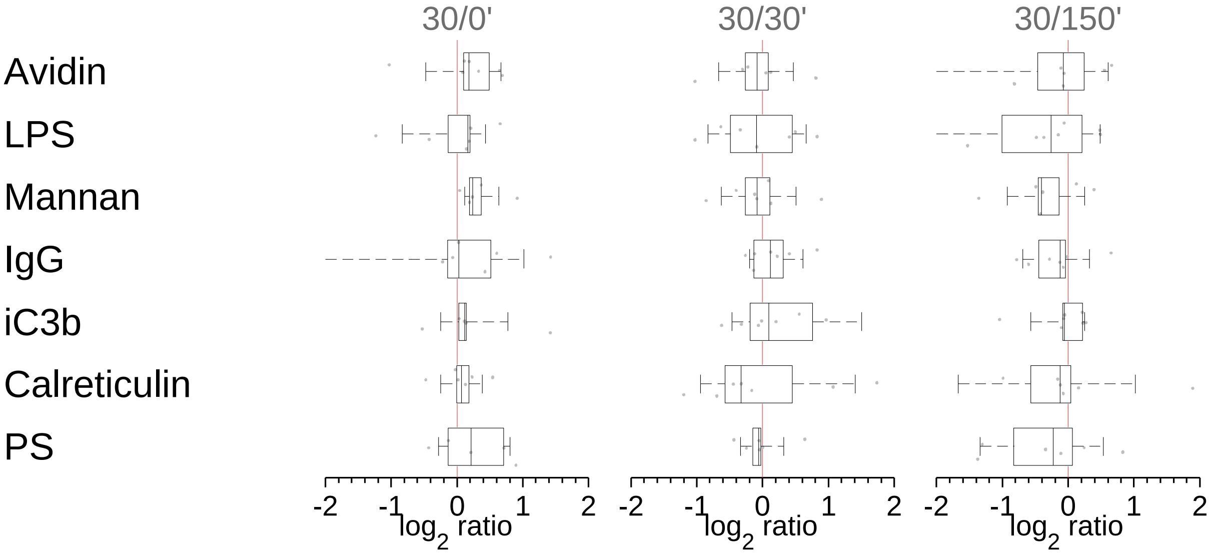

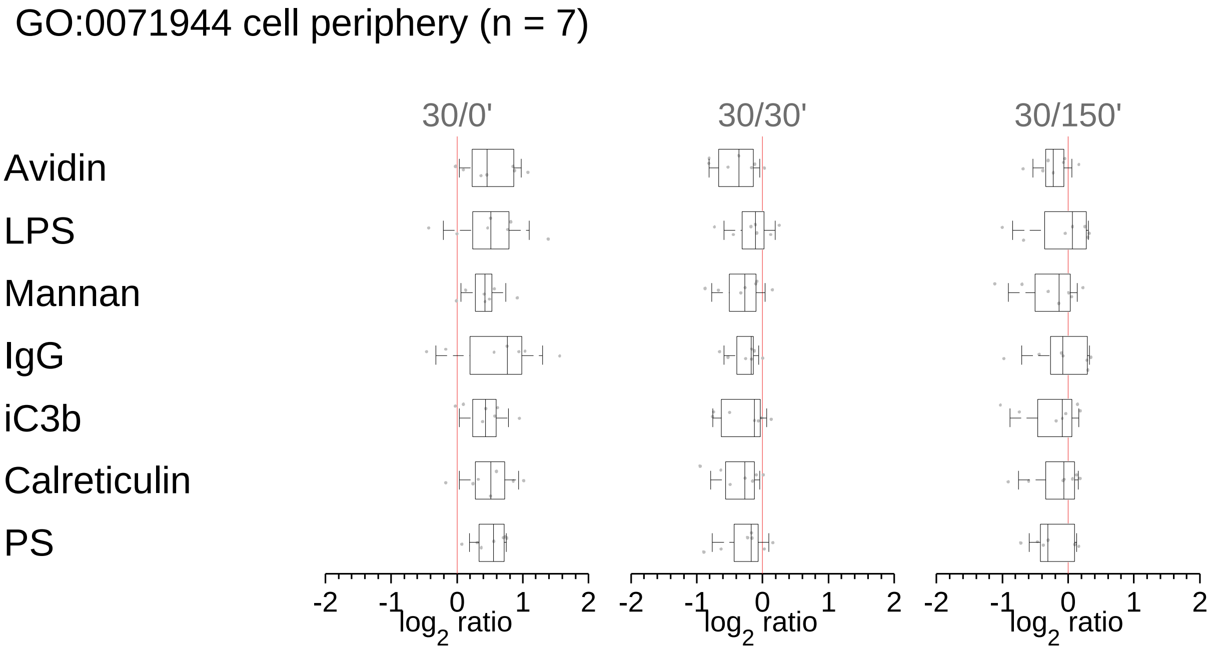

GO:0071944 cell periphery (n = 7)
30/0'
30/30'
30/150'
Avidin
LPS
Mannan
IgG
iC3b
Calreticulin
PS
-2
-1
0
1
2
log2 ratio

GO:0071986 Ragulator complex (n = 4)

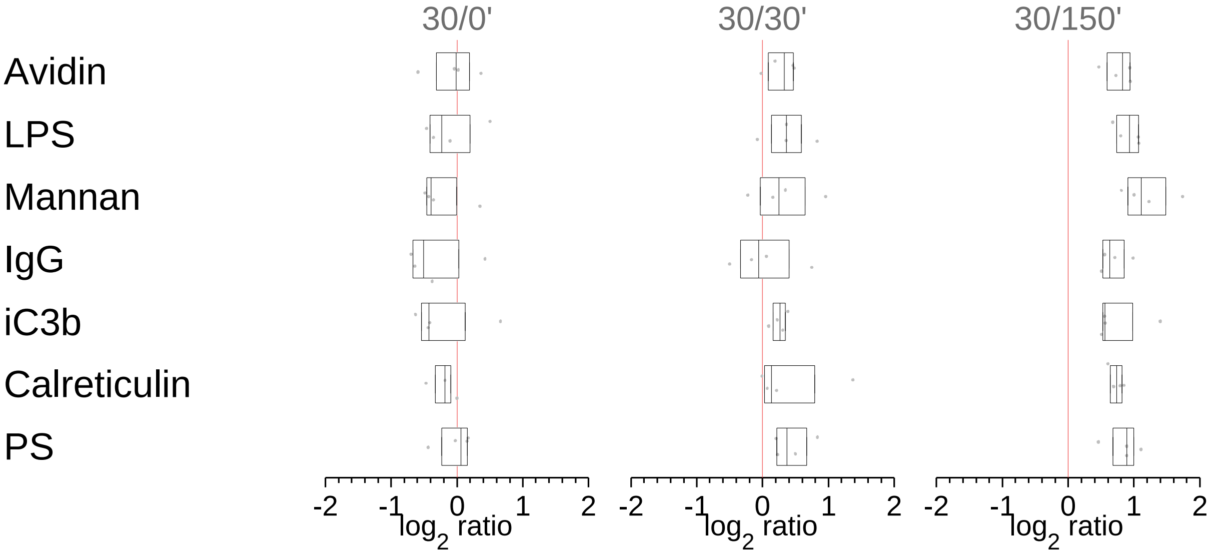

GO:0072345 NAADP-sensitive calcium-release channel activity (n = 3)

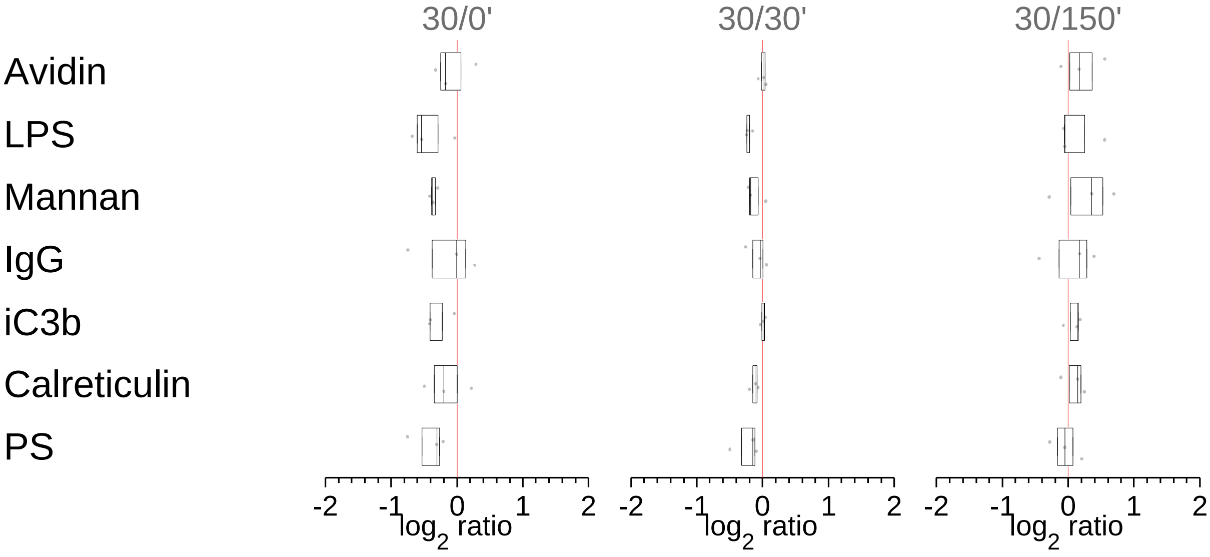

GO:0072357 PTW/PP1 phosphatase complex (n = 3)

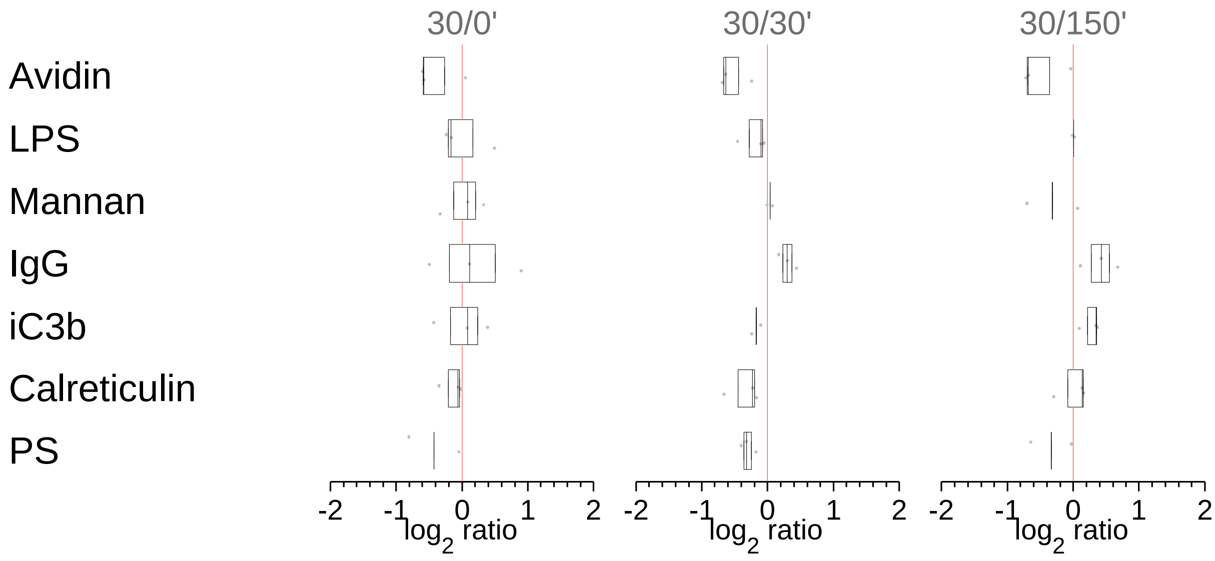

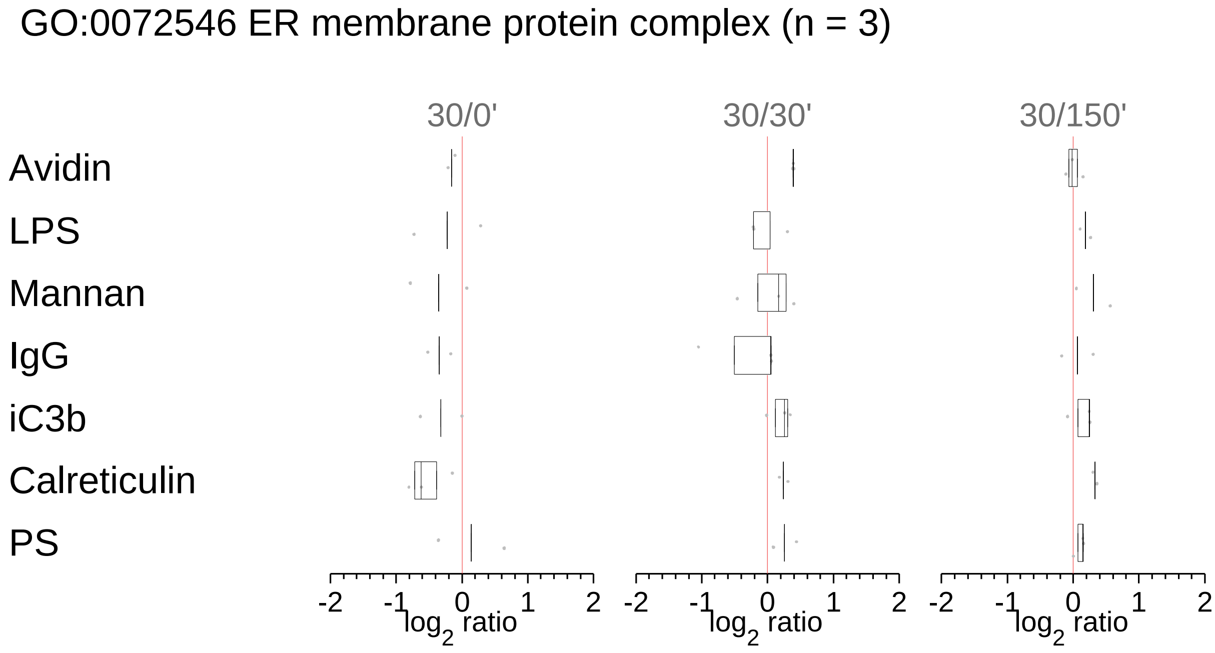
GO:0072546 ER membrane protein complex (n = 3)
30/0'
30/30'
30/150'
Avidin
LPS
Mannan
IgG
iC3b
Calreticulin
PS
-2 -1 0 1 2
log2 ratio
-2 -1 0 1 2
log2 ratio
-2 -1 0 1 2
log2 ratio

GO:0072562 blood microparticle (n = 20)

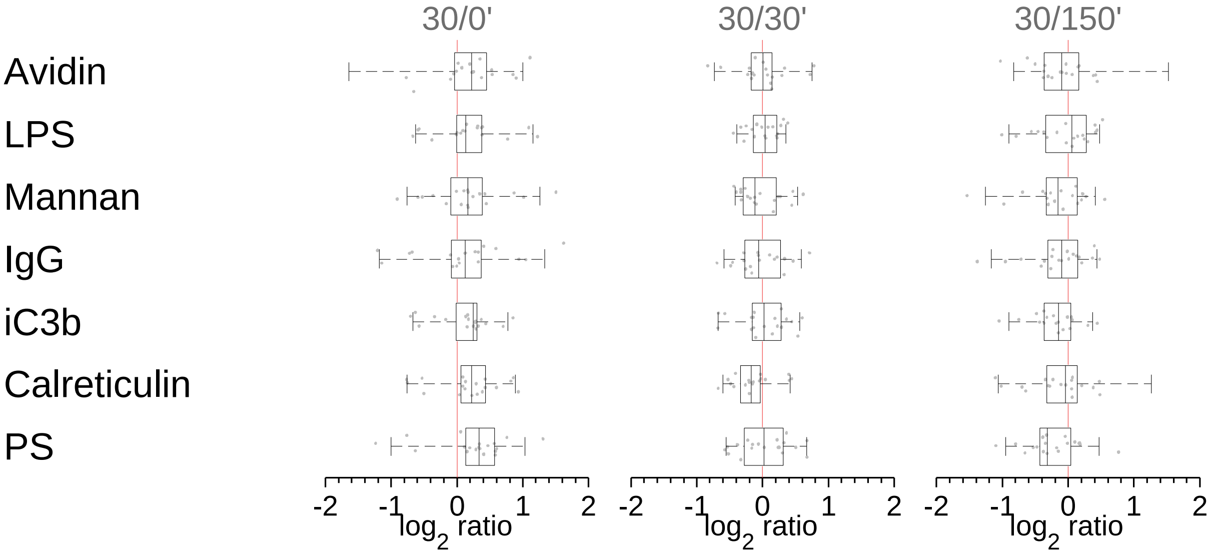

GO:0072583 clathrin-mediated endocytosis (n = 3)

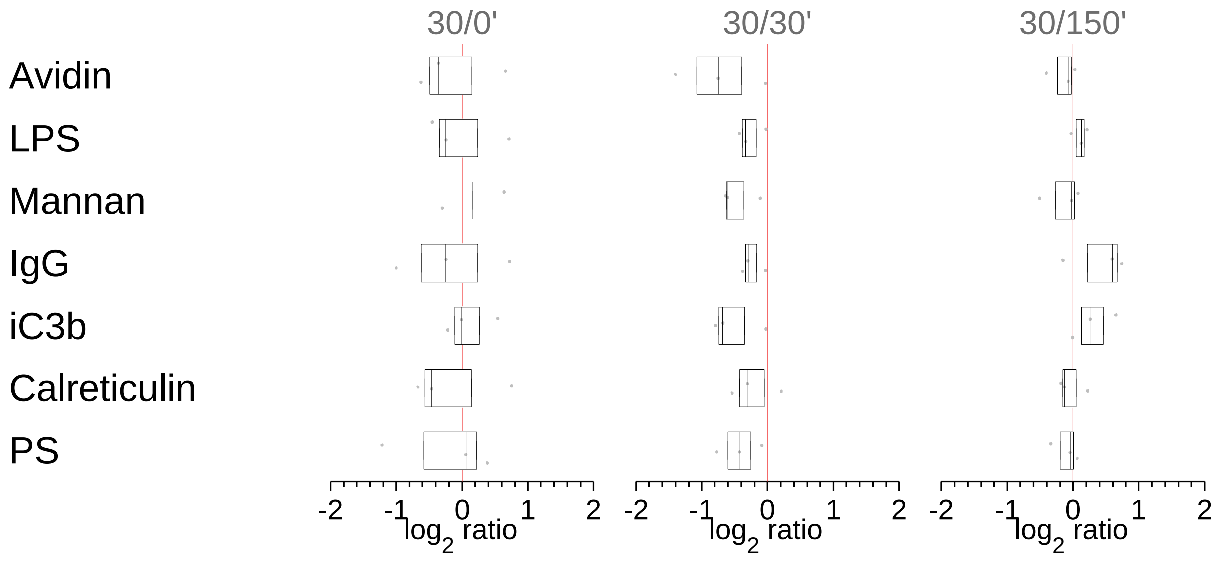

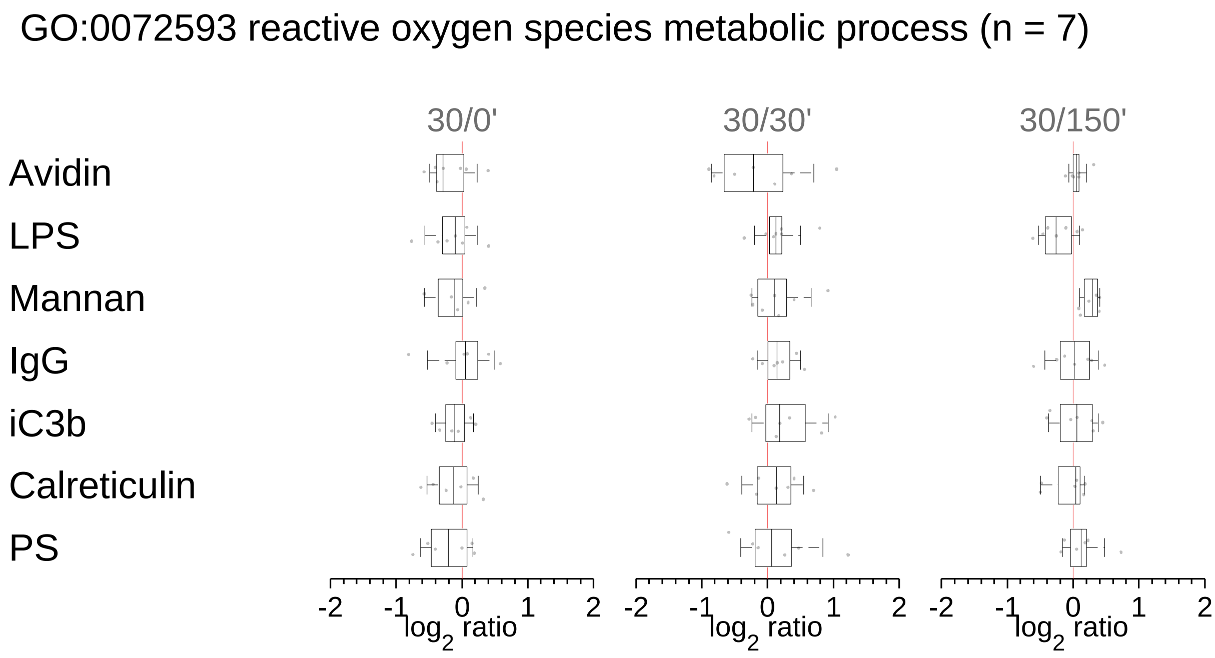
GO:0072593 reactive oxygen species metabolic process (n = 7)
30/0'
30/30'
30/150'
Avidin
LPS
Mannan
IgG
iC3b
Calreticulin
PS
-2
-1
0
1
2
log2 ratio

GO:0072659 protein localization in plasma membrane (n = 10)

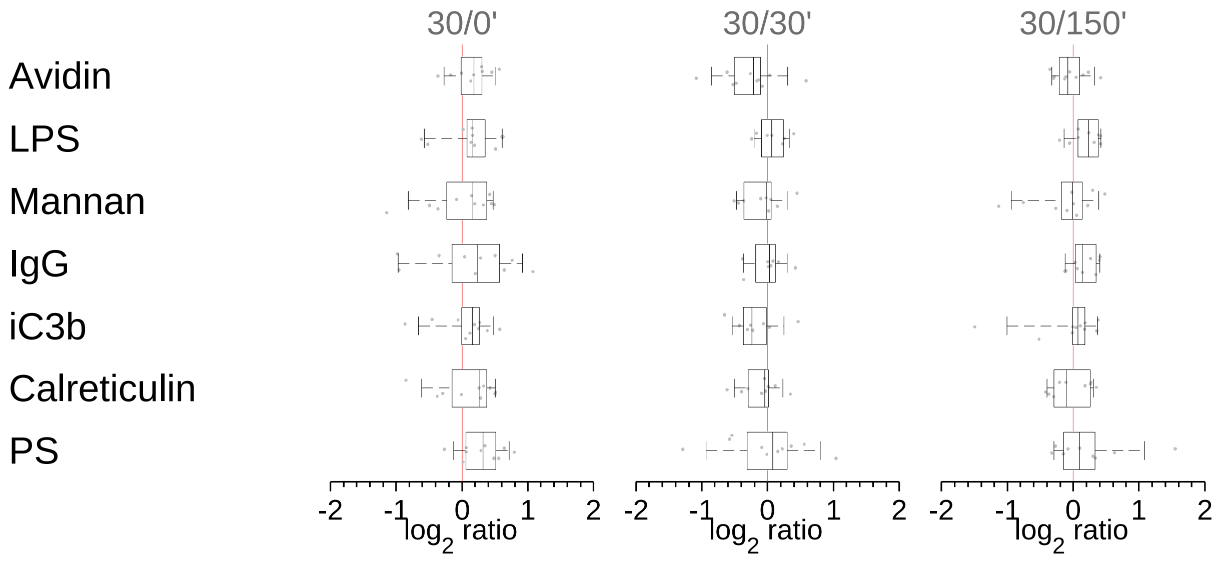

GO:0072661 protein targeting to plasma membrane (n = 3)

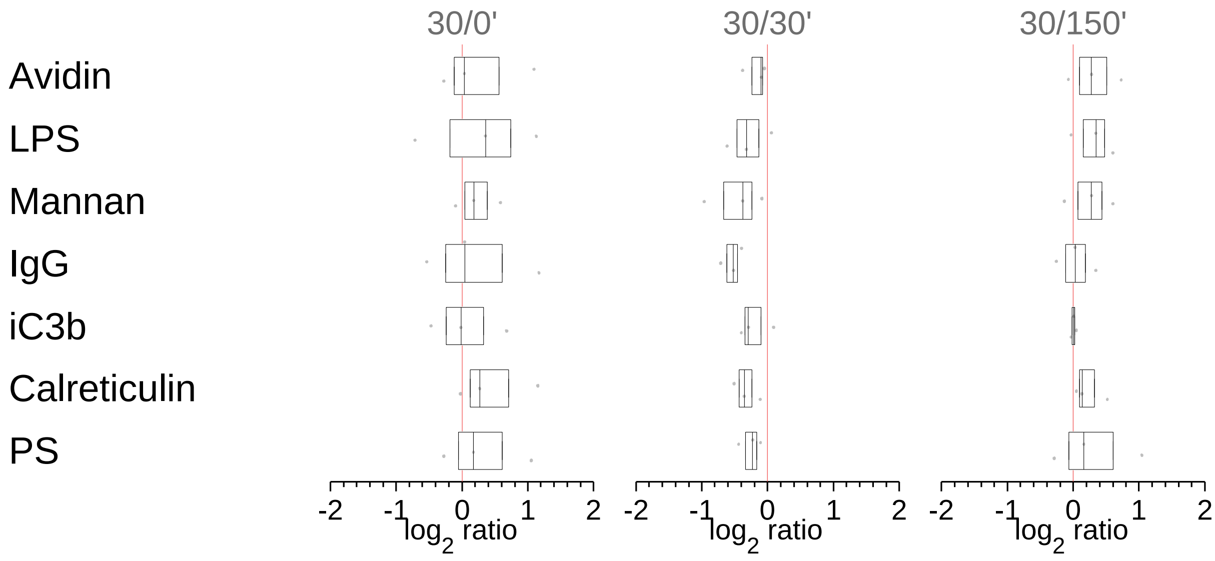

GO:0072669 tRNA-splicing ligase complex (n = 3)

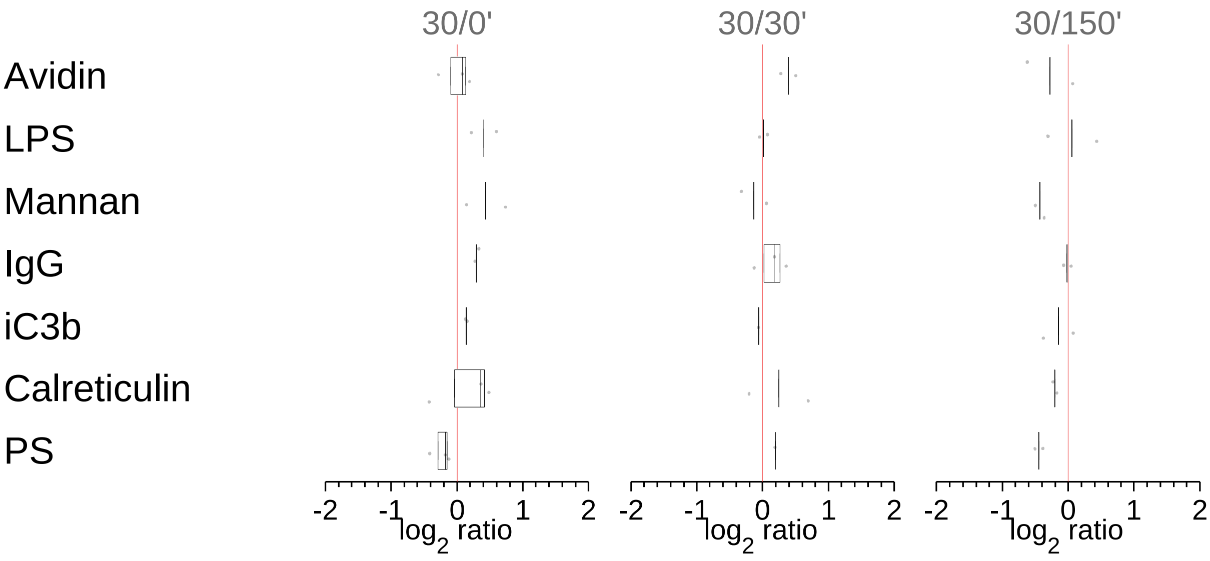

GO:0072686 mitotic spindle (n = 5)

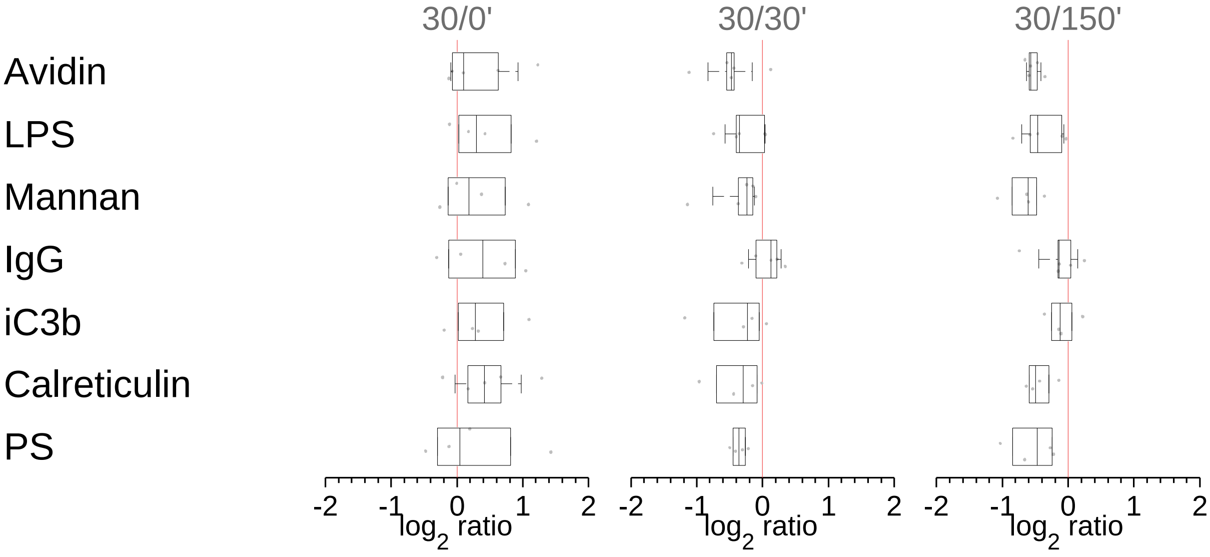

GO:0080030 methyl indole-3-acetate esterase activity (n = 4)

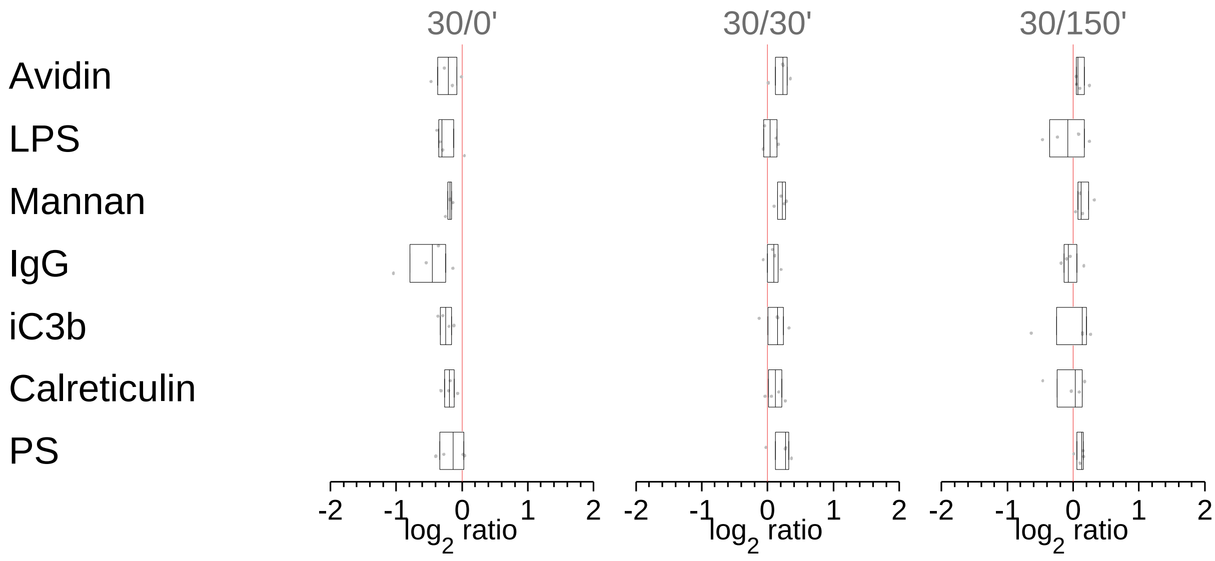

GO:0080031 methyl salicylate esterase activity (n = 4)

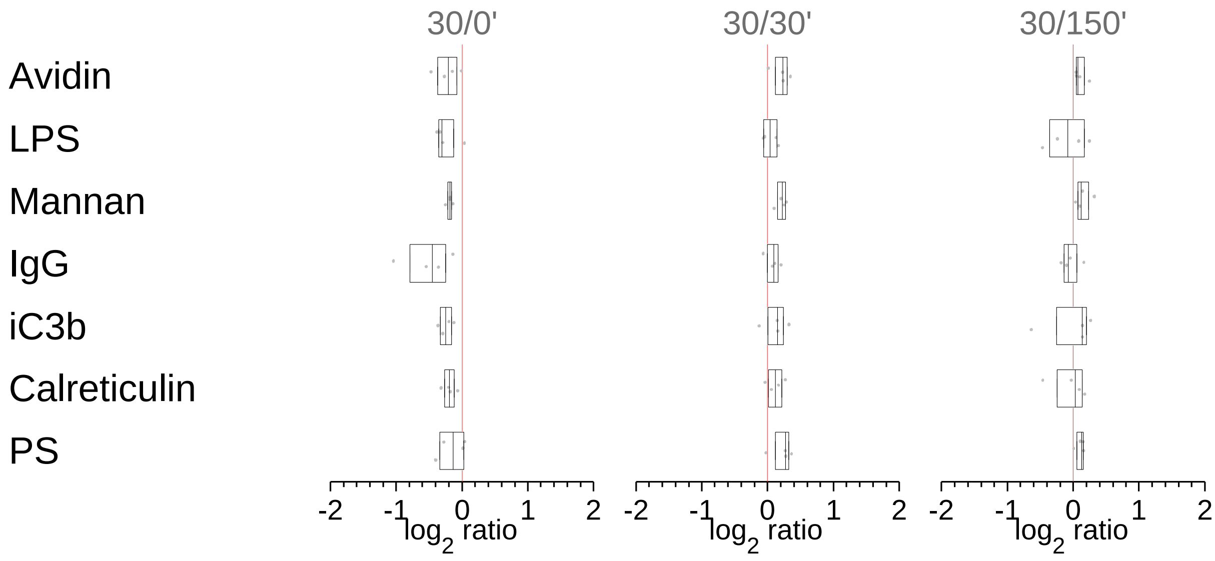

GO:0080032 methyl jasmonate esterase activity (n = 4)

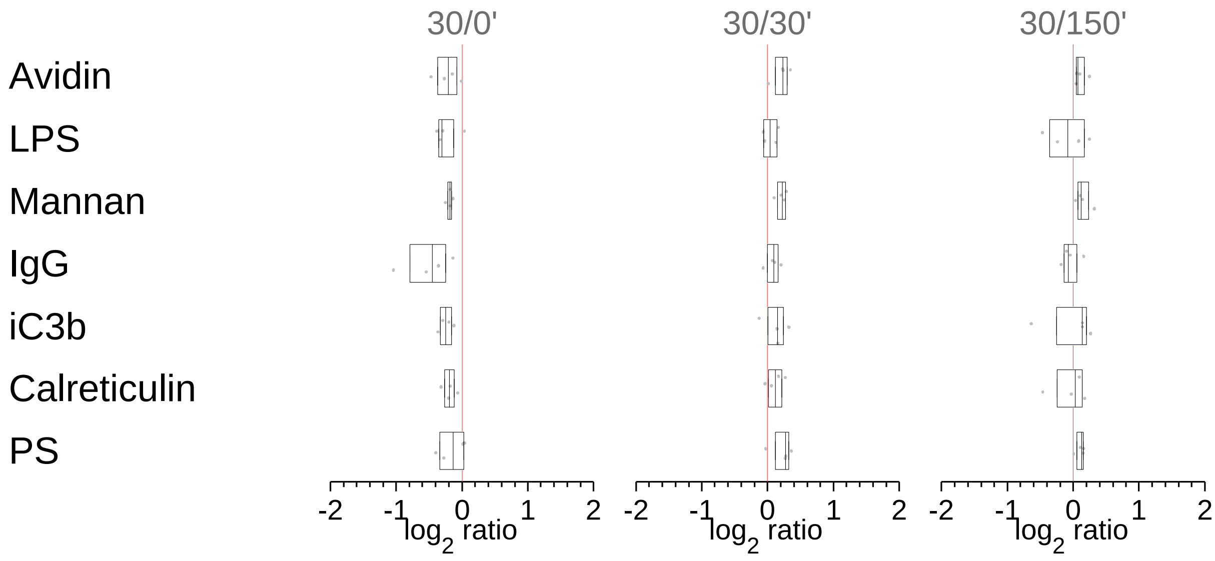

GO:0080062 cytokinin 9-beta-glucosyltransferase activity (n = 3)

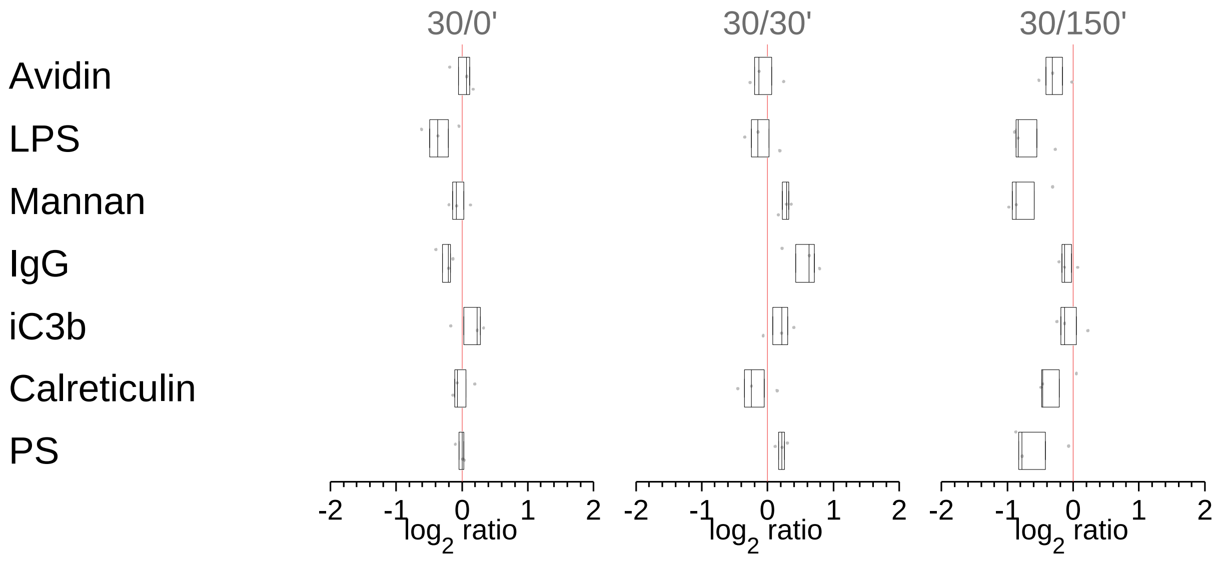

GO:0086010 membrane depolarization involved in regulation of action potential (n = 3)

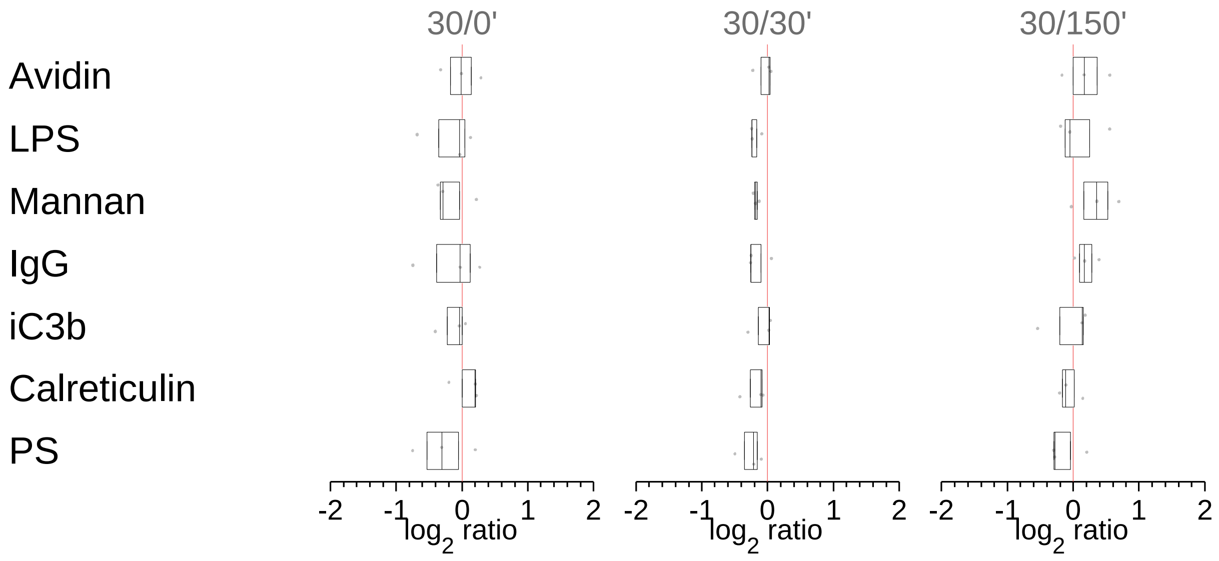

GO:0090002 establishment of protein localization in plasma membrane (n = 6)

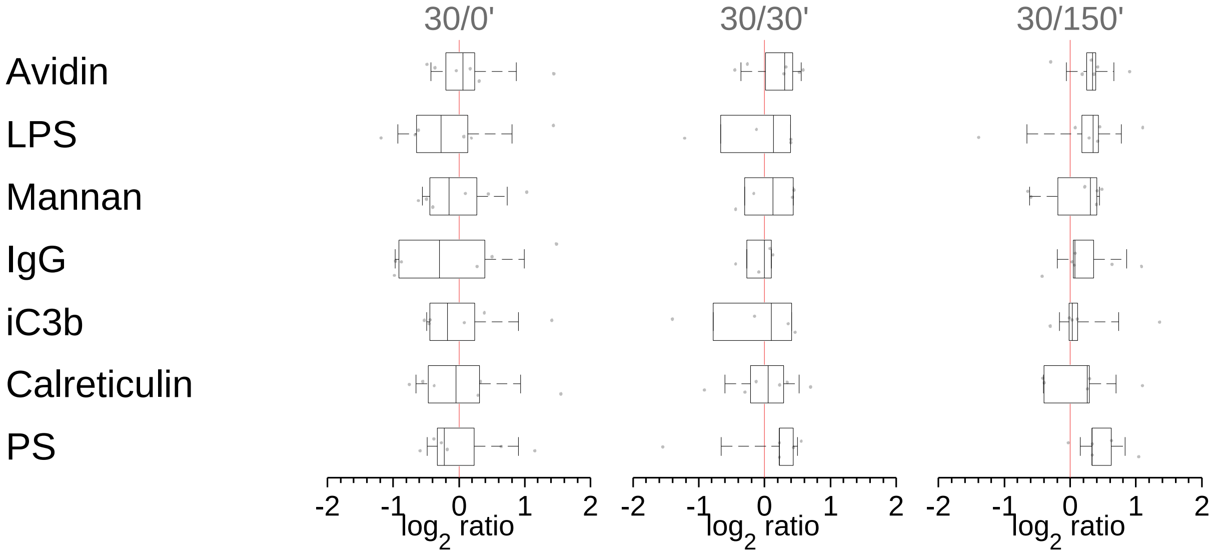

GO:0090004 positive regulation of establishment of protein localization in plasma membrane (n = 4)

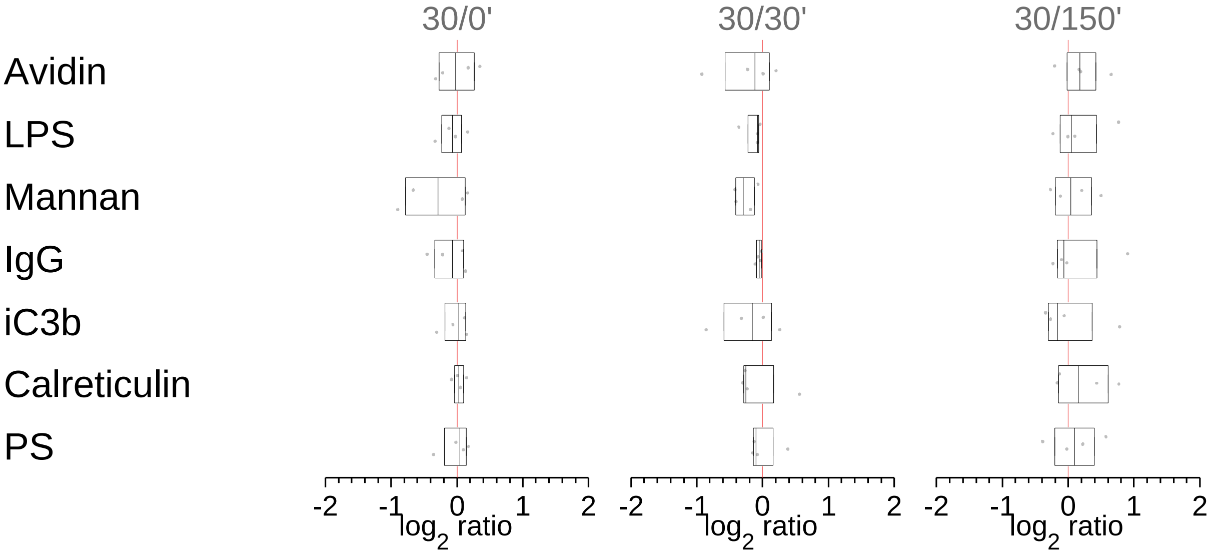

GO:0090090 negative regulation of canonical Wnt receptor signaling pathway (n = 7)

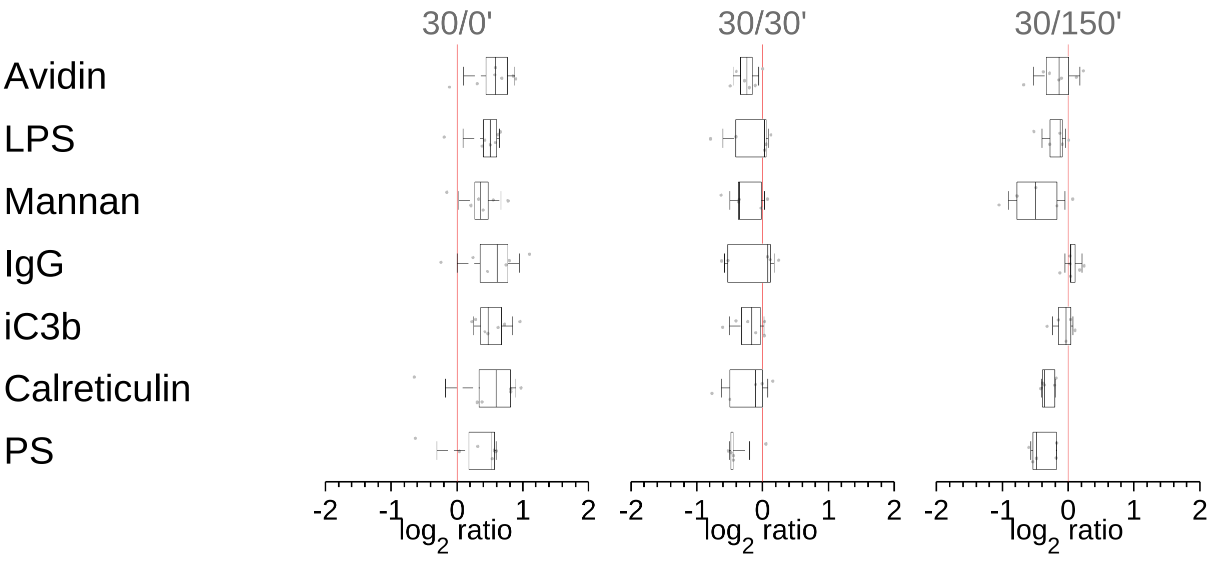

GO:0090181 regulation of cholesterol metabolic process (n = 3)

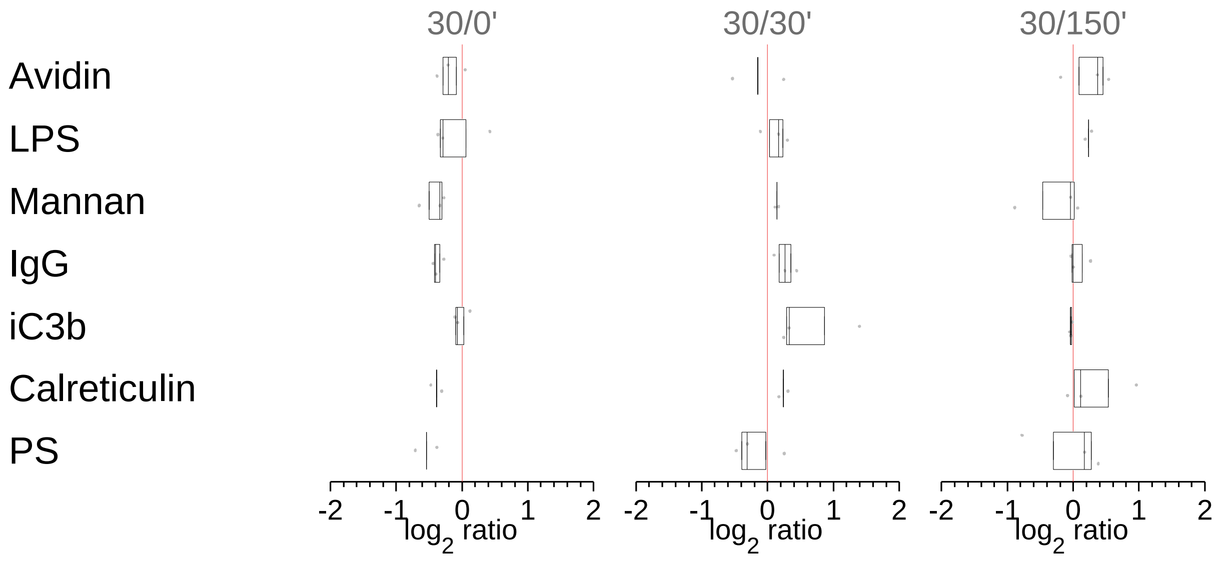

GO:0090201 negative regulation of release of cytochrome c from mitochondria (n = 3)

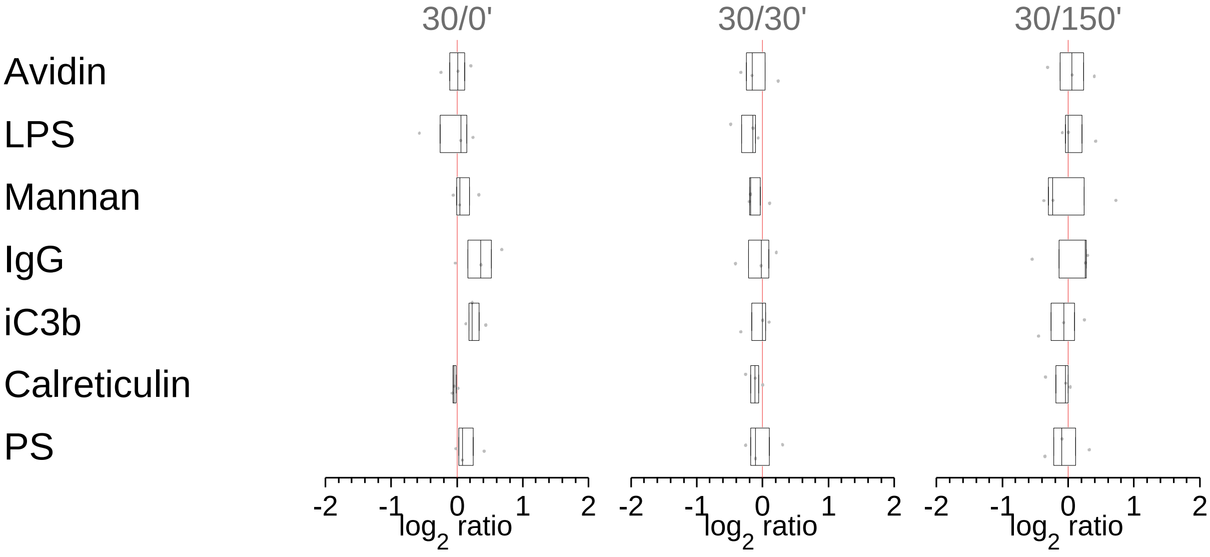

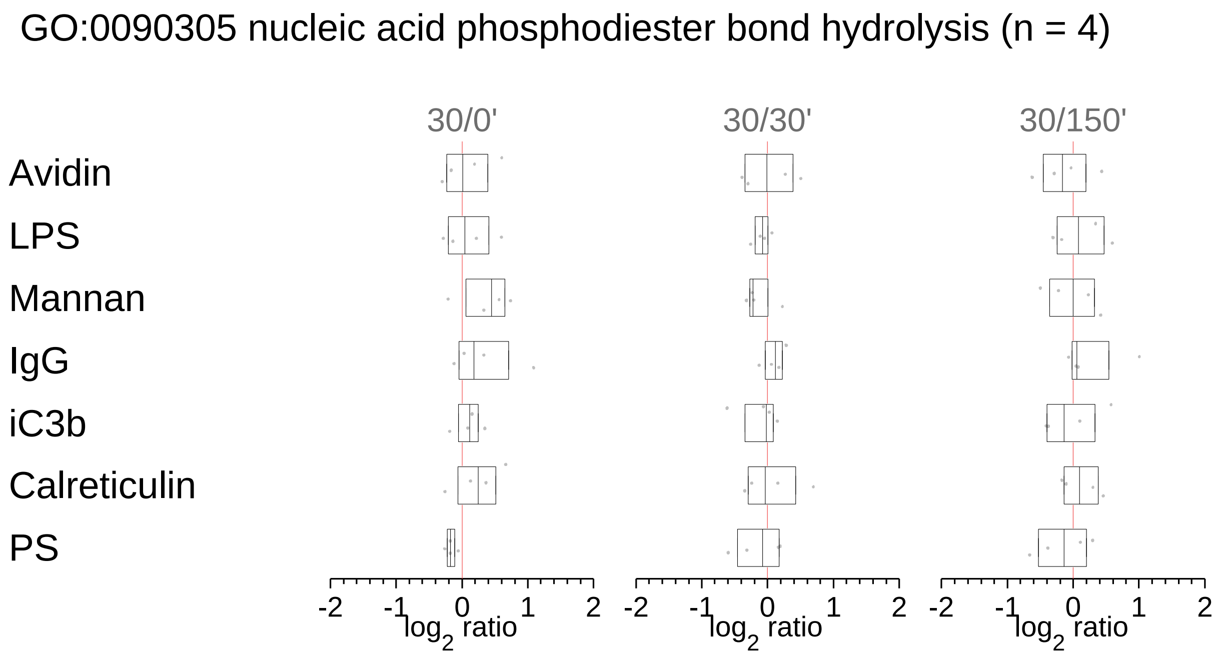
GO:0090305 nucleic acid phosphodiester bond hydrolysis (n = 4)
30/0'
30/30'
30/150'
Avidin
LPS
Mannan
IgG
iC3b
Calreticulin
PS
-2 -1 0 1 2
log2 ratio
-2 -1 0 1 2
log2 ratio
-2 -1 0 1 2
log2 ratio

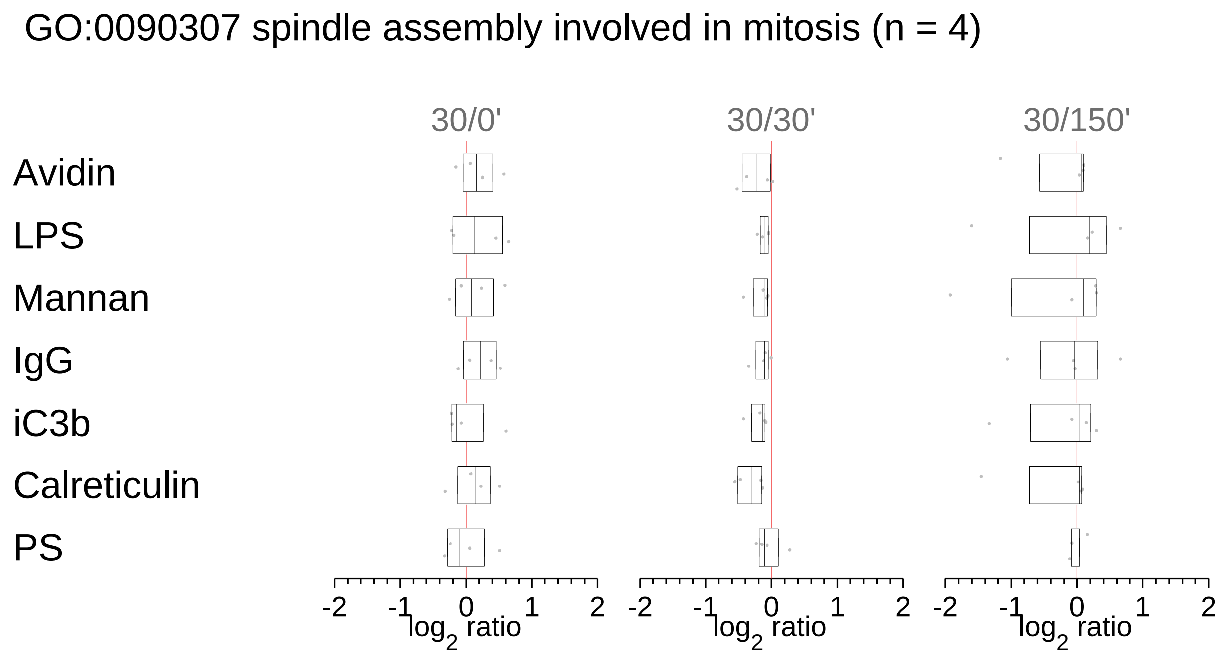
GO:0090307 spindle assembly involved in mitosis (n = 4)
30/0'
30/30'
30/150'
Avidin
LPS
Mannan
IgG
iC3b
Calreticulin
PS
-2 -1 0 1 2
log2 ratio

GO:0090314 positive regulation of protein targeting to membrane
(n = 3)

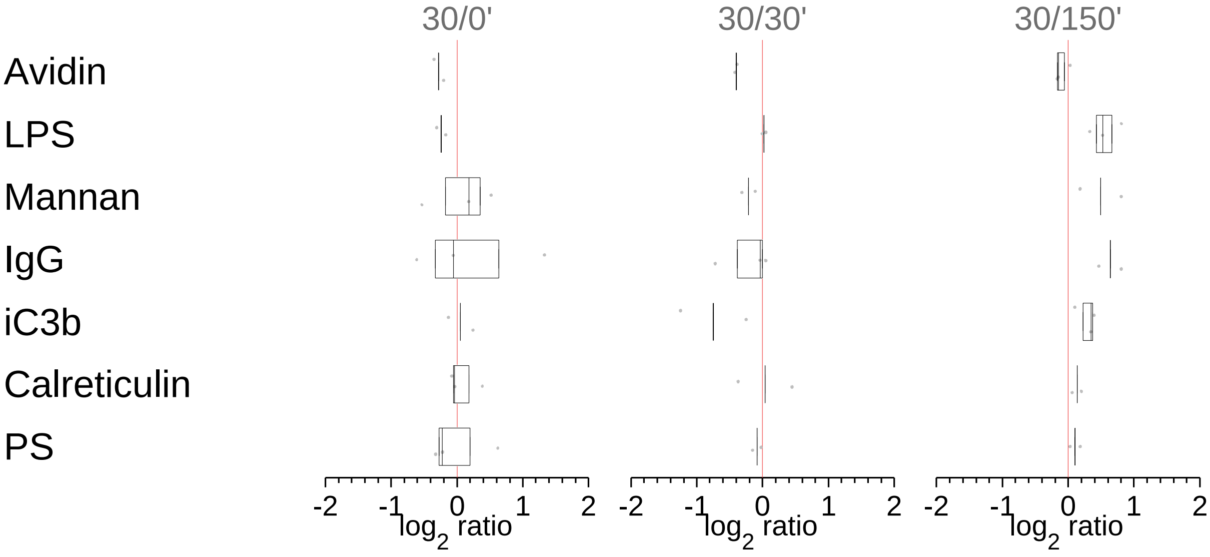

GO:0090316 positive regulation of intracellular protein transport (n = 3)

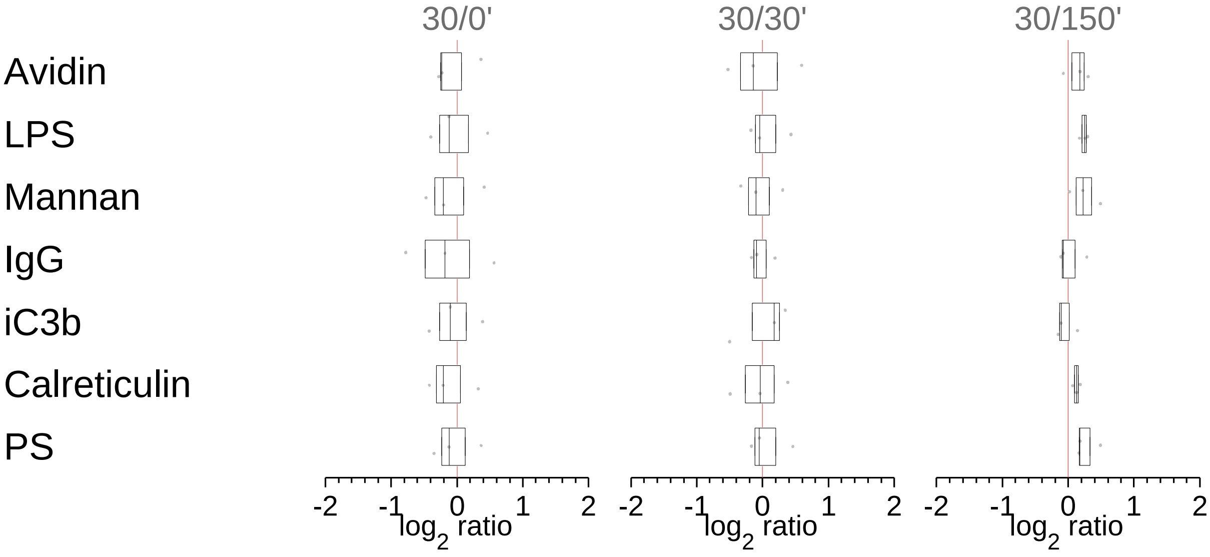

GO:0090330 regulation of platelet aggregation (n = 3)

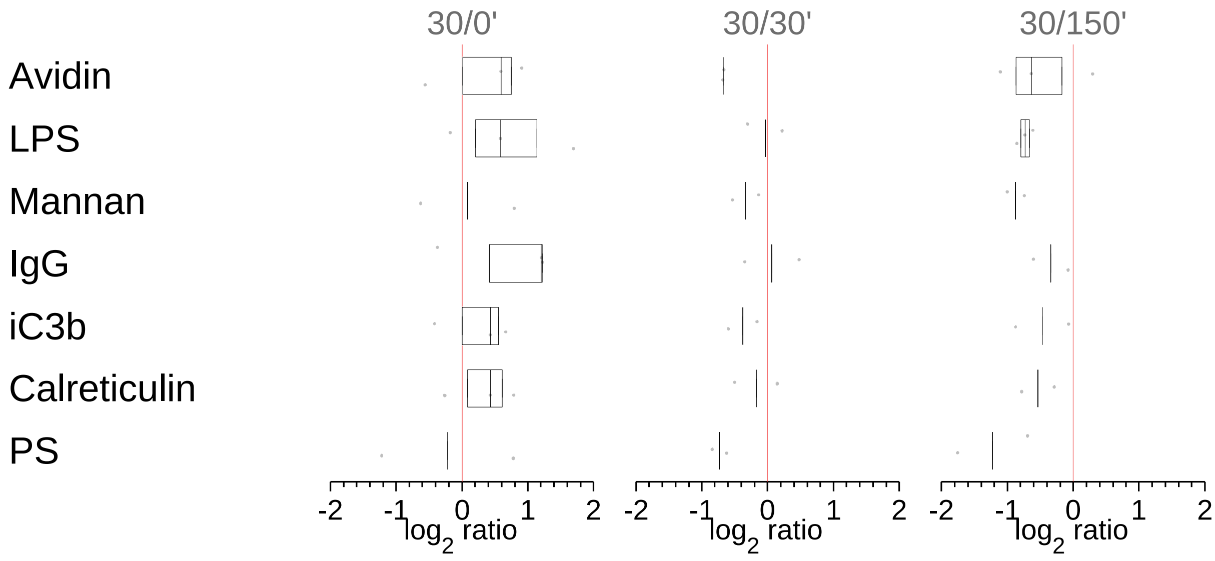

GO:0090382 phagosome maturation (n = 3)

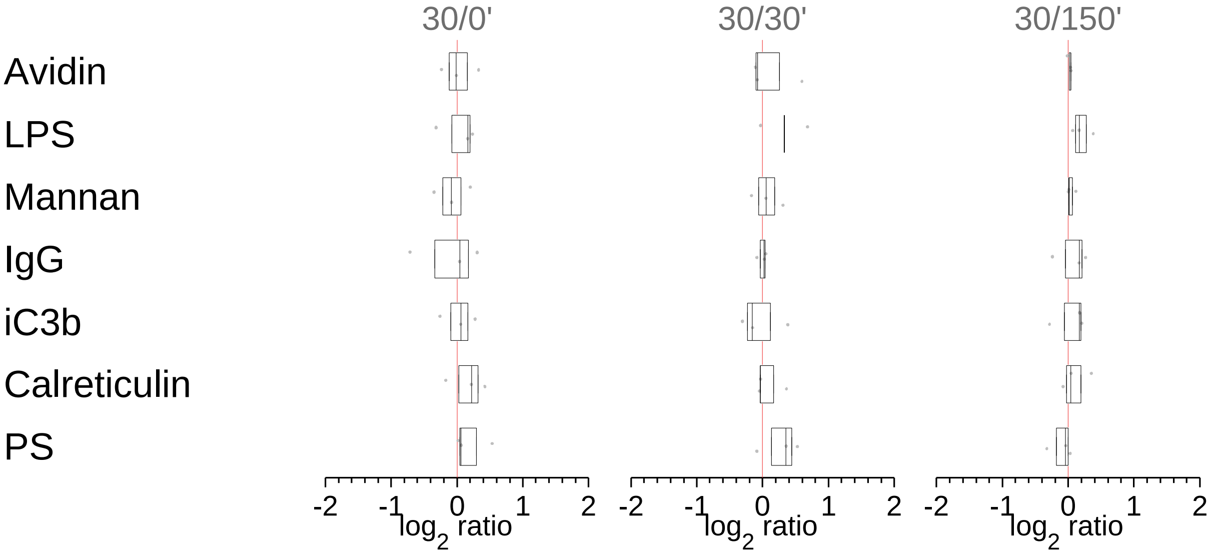

GO:0090385 phagosome-lysosome fusion (n = 3)

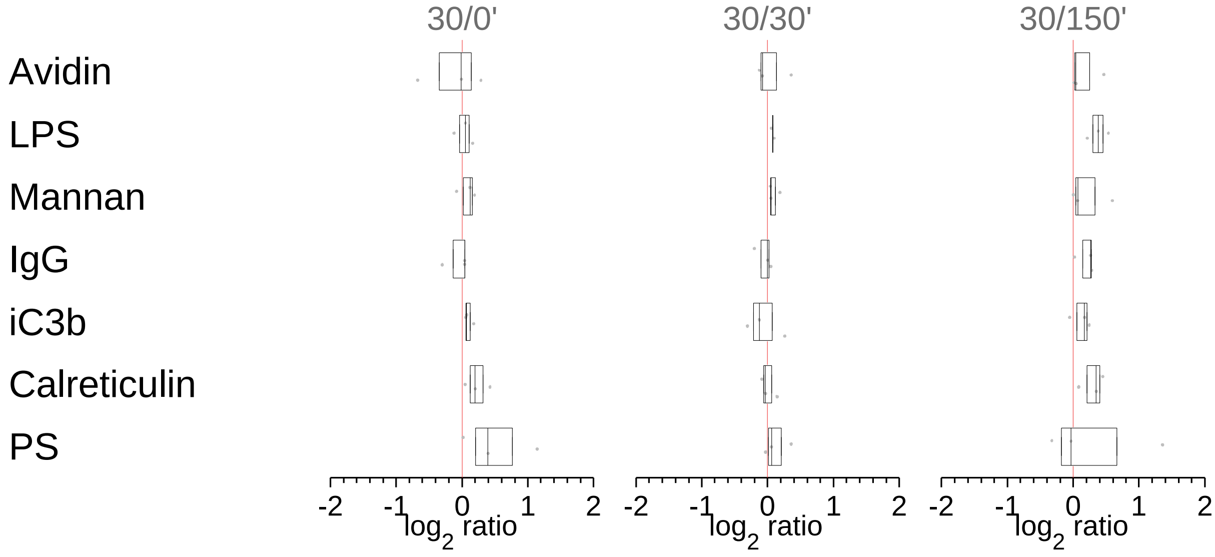

GO:0090398 cellular senescence (n = 4)

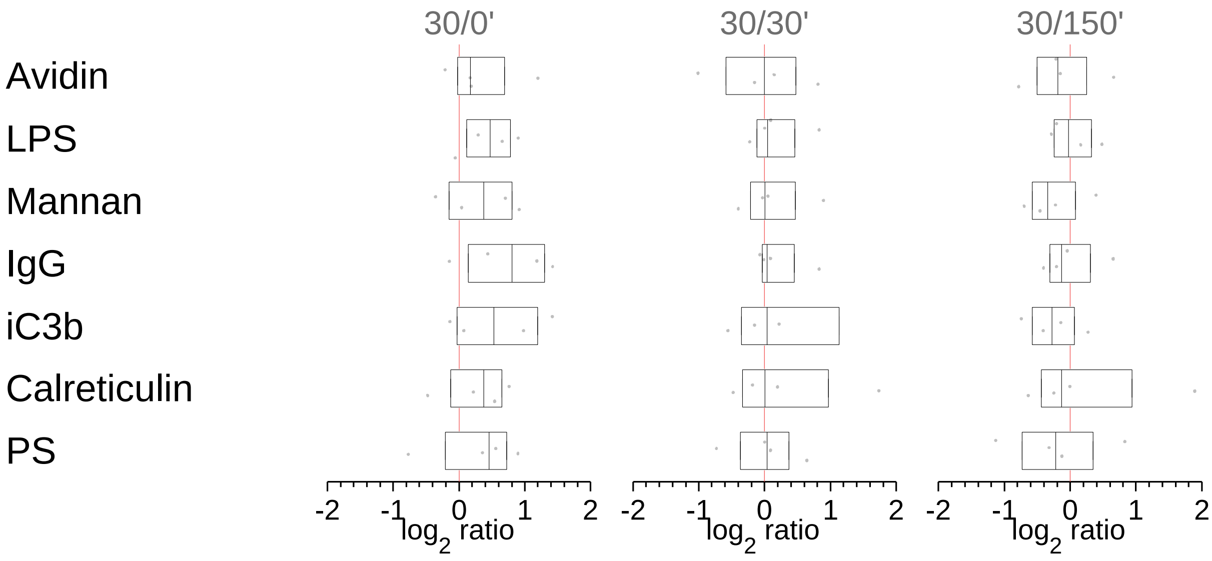

GO:0097038 perinuclear endoplasmic reticulum (n = 6)

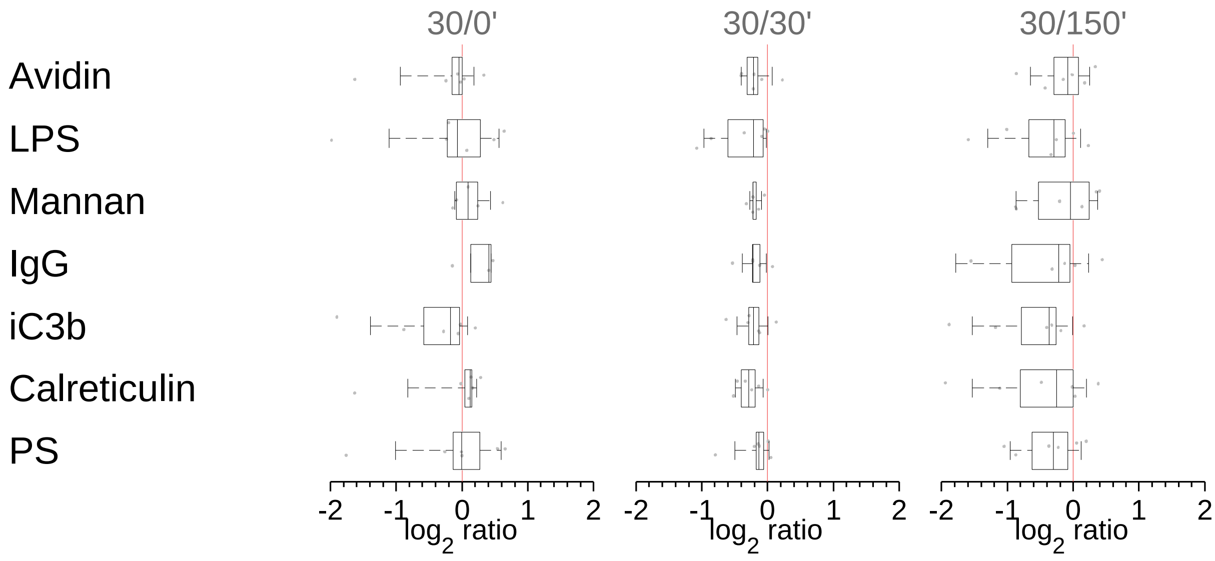

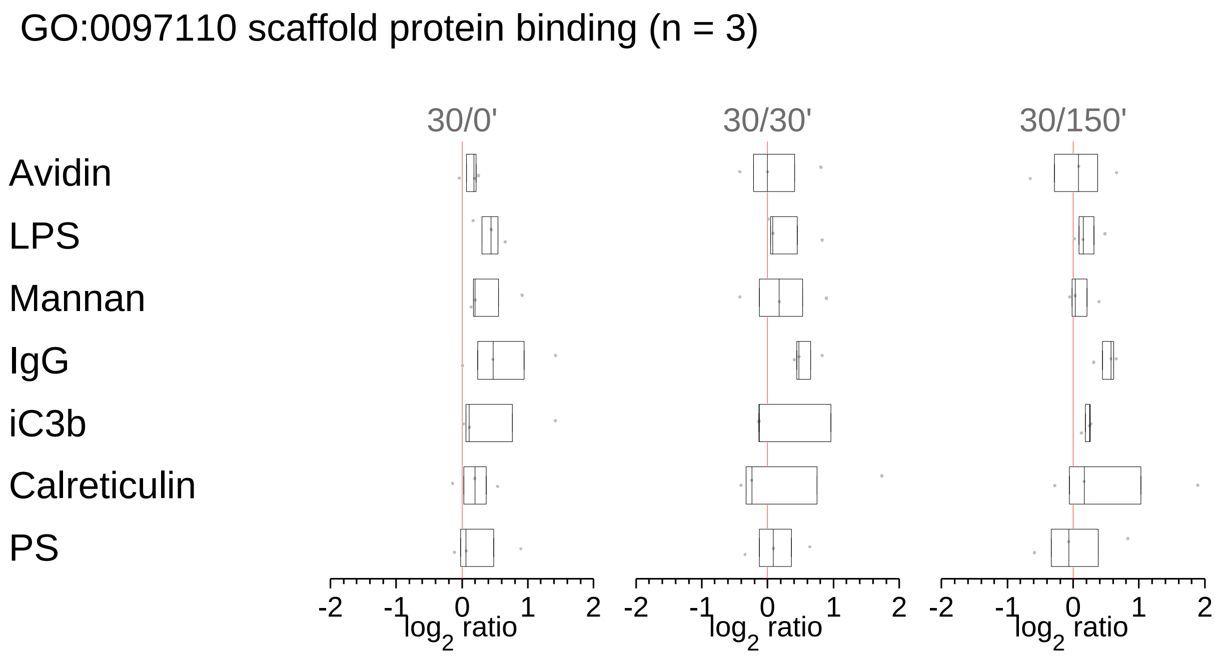
GO:0097110 scaffold protein binding (n = 3)
30/0'
30/30'
30/150'
Avidin
LPS
Mannan
IgG
iC3b
Calreticulin
PS
-2
-1
0
1
2
log2 ratio

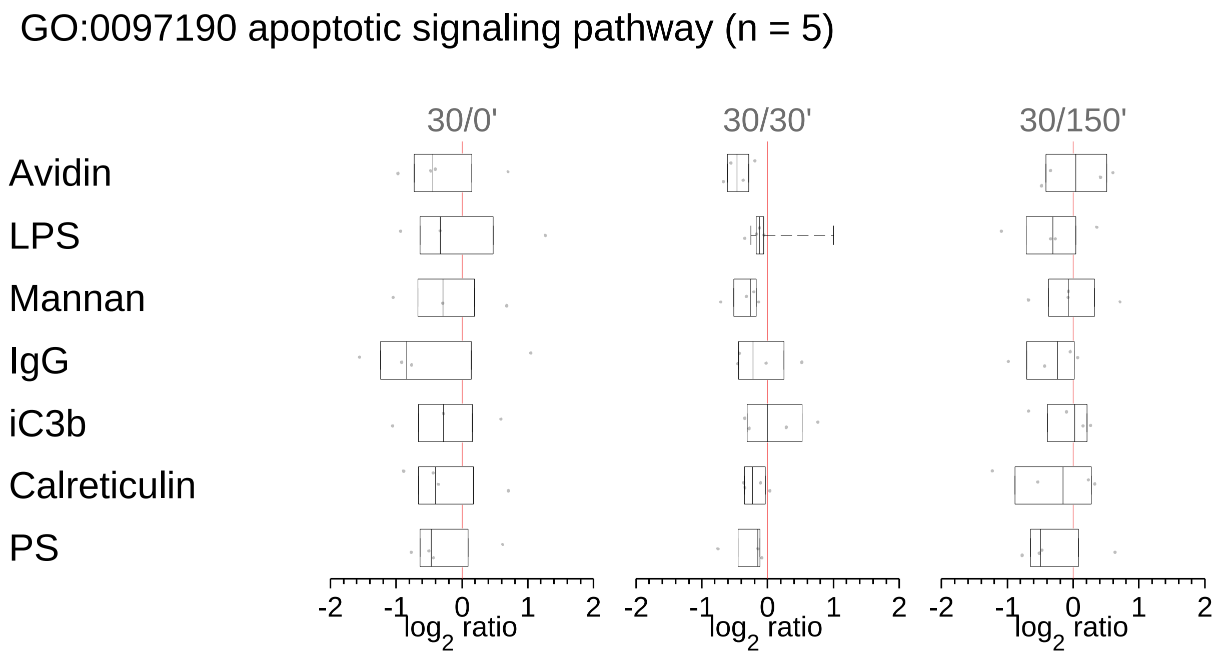
GO:0097190 apoptotic signaling pathway (n = 5)
30/0'
30/30'
30/150'
Avidin
LPS
Mannan
IgG
iC3b
Calreticulin
PS
-2
-1
0
1
2
log2 ratio

GO:0097191 extrinsic apoptotic signaling pathway (n = 5)

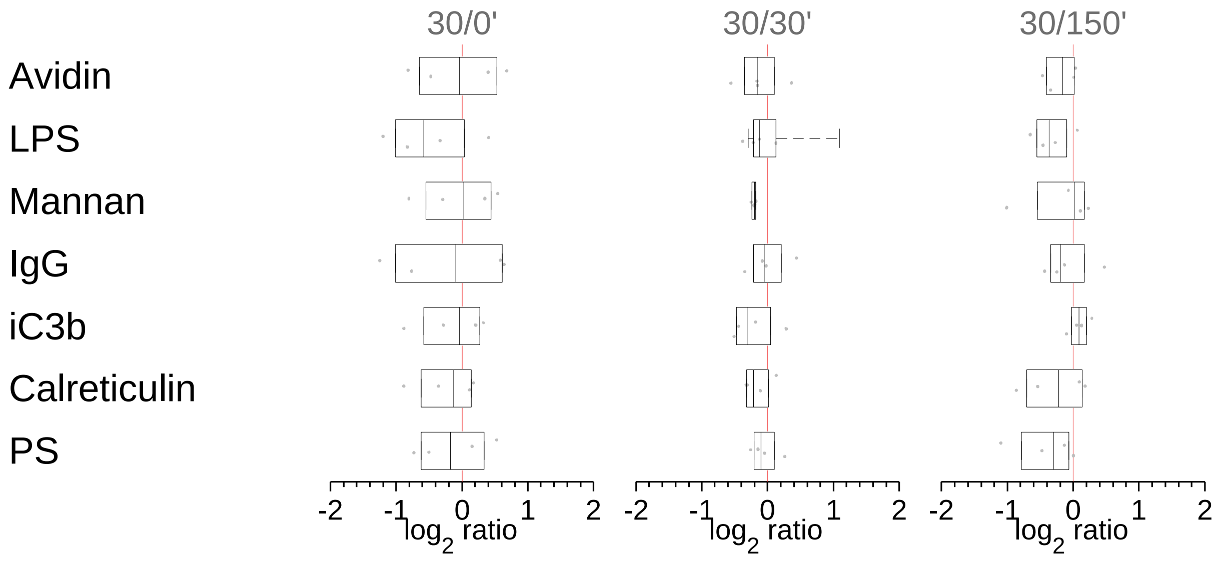

GO:0097192 extrinsic apoptotic signaling pathway in absence of ligand (n = 6)

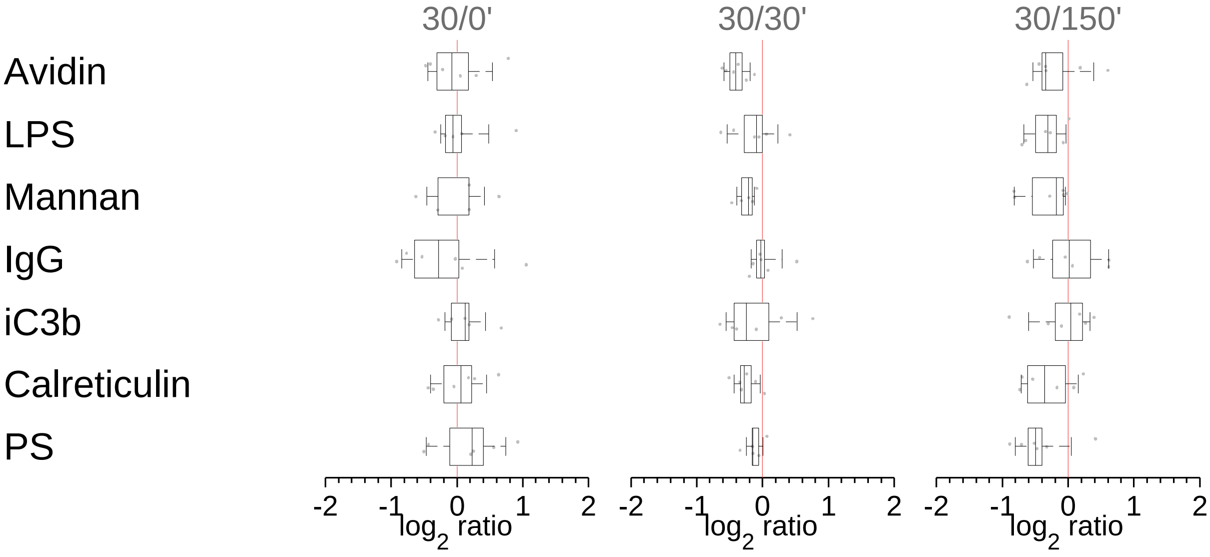

GO:0097193 intrinsic apoptotic signaling pathway (n = 4)

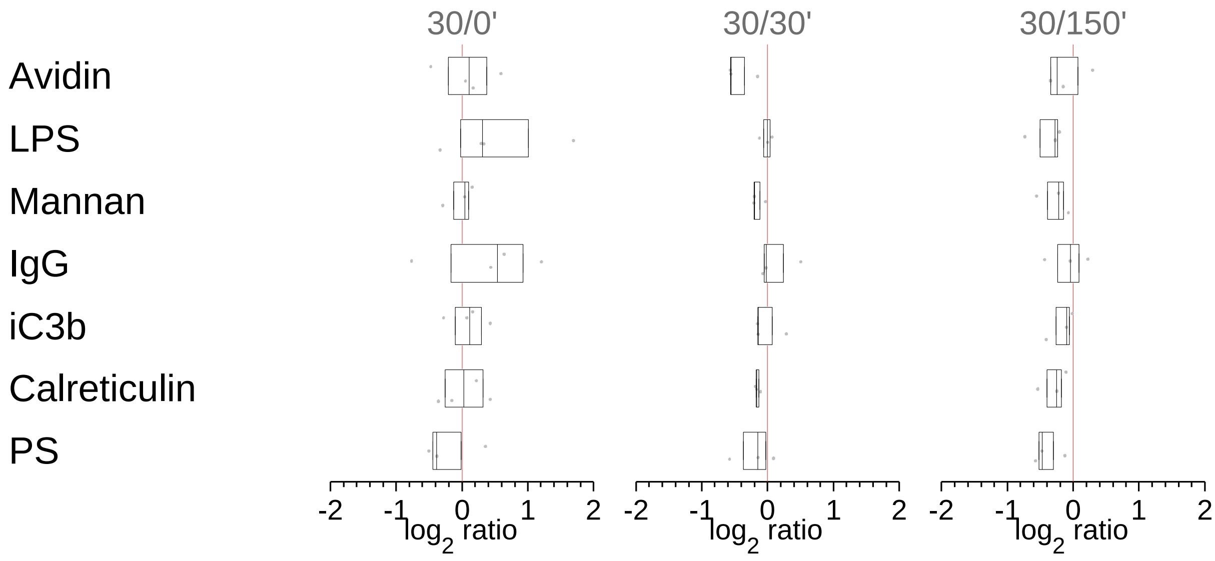

GO:0097194 execution phase of apoptosis (n = 4)

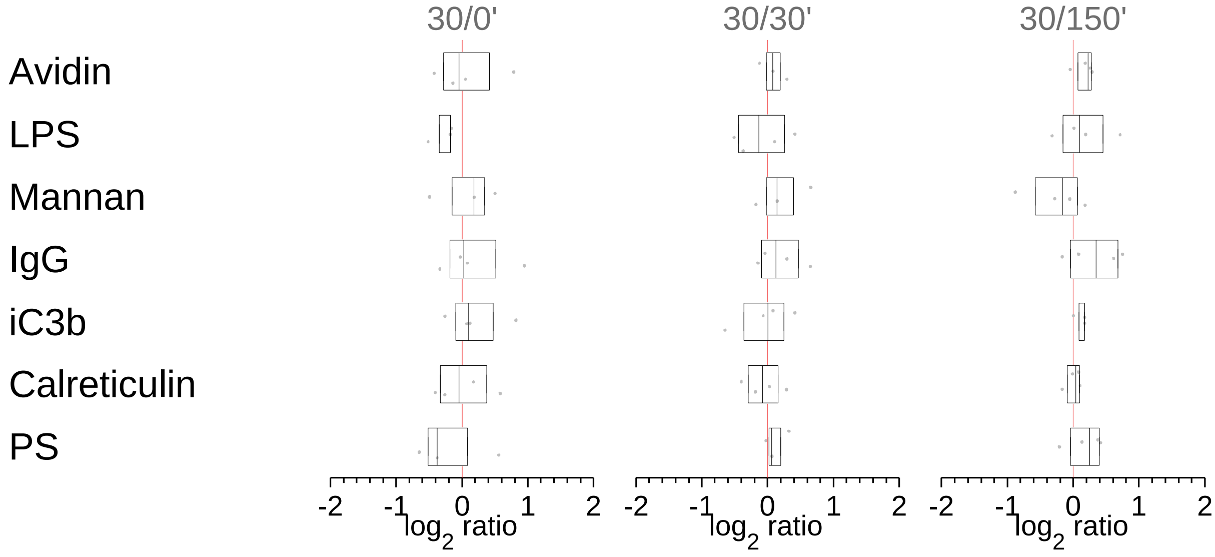

GO:0097208 alveolar lamellar body (n = 6)

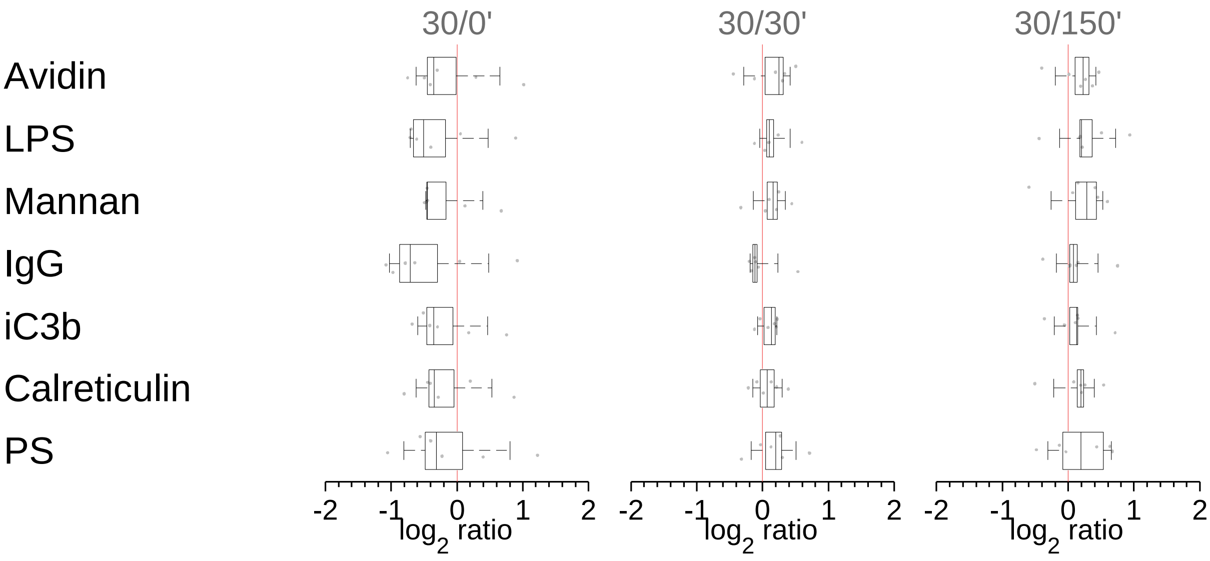

GO:0097284 hepatocyte apoptotic process (n = 4)

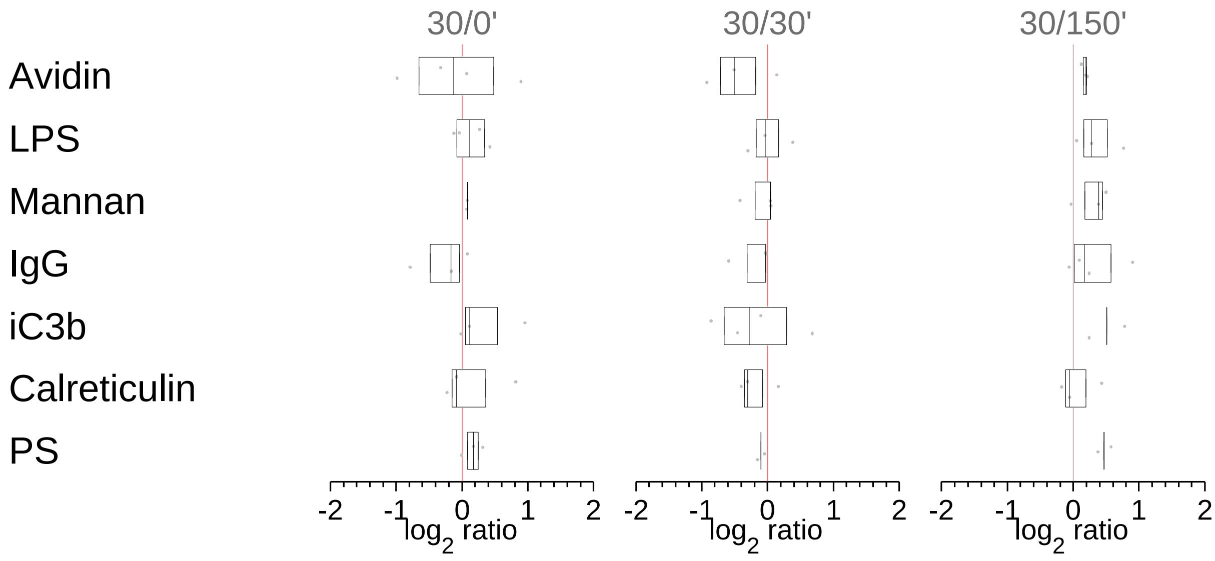

GO:0097527 N/A (n = 3)

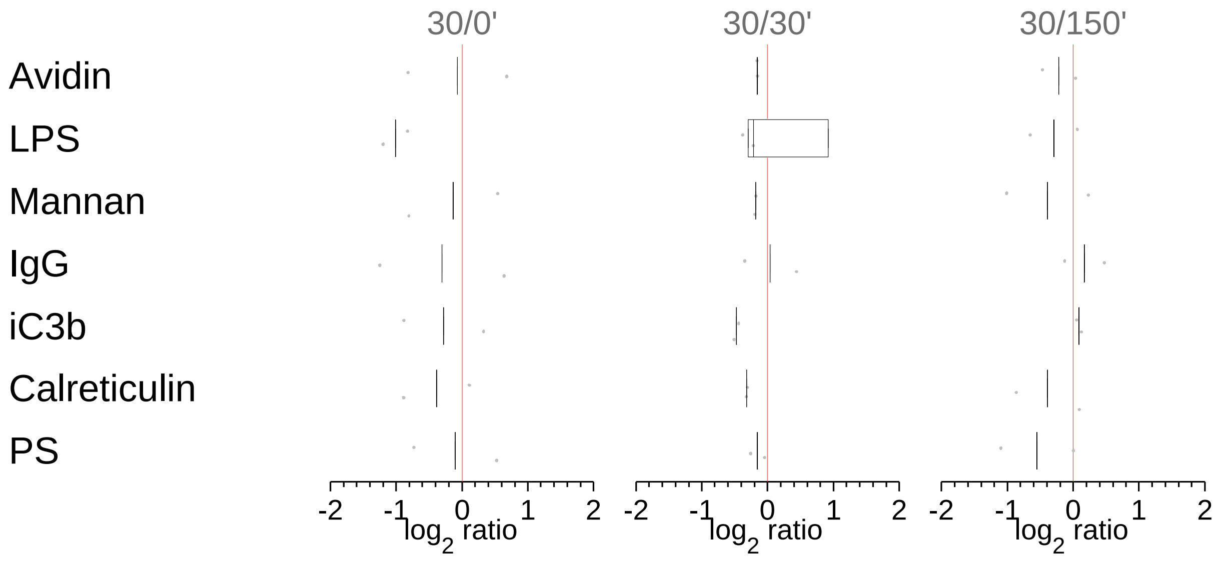

GO:1900026 positive regulation of substrate adhesion-dependent cell spreading (n = 6)

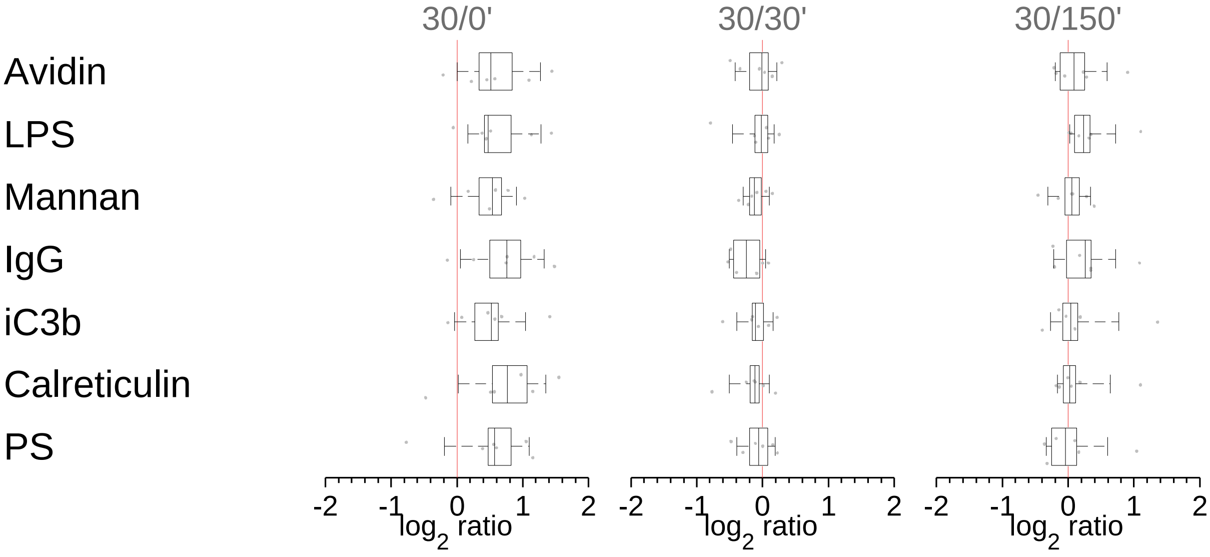

GO:1900087 positive regulation of G1/S transition of mitotic cell cycle (n = 4)

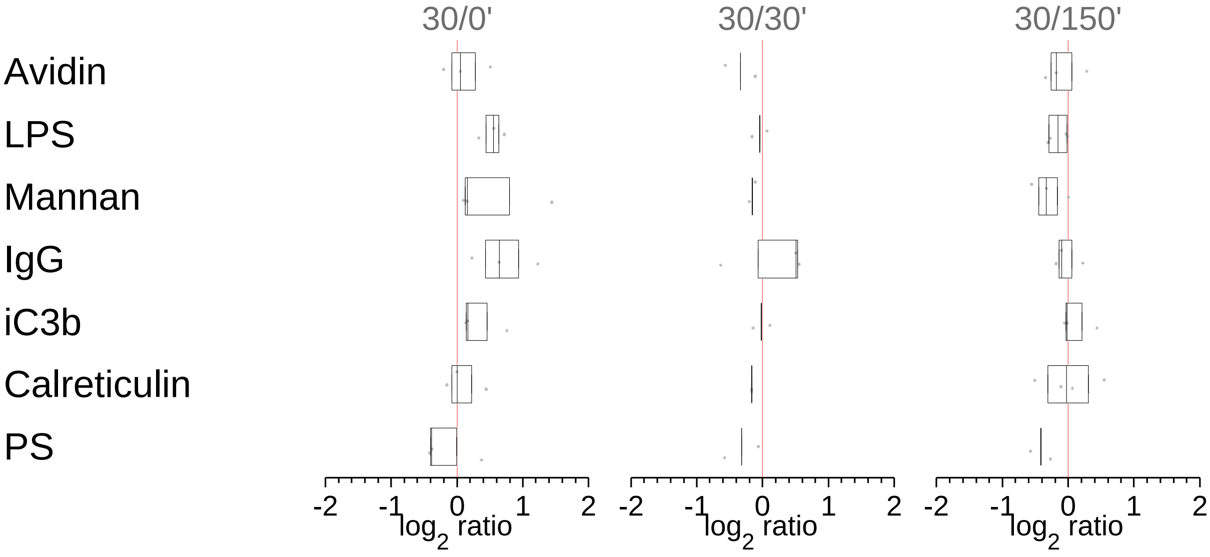

GO:1901029 negative regulation of mitochondrial outer membrane permeabilization (n = 3)

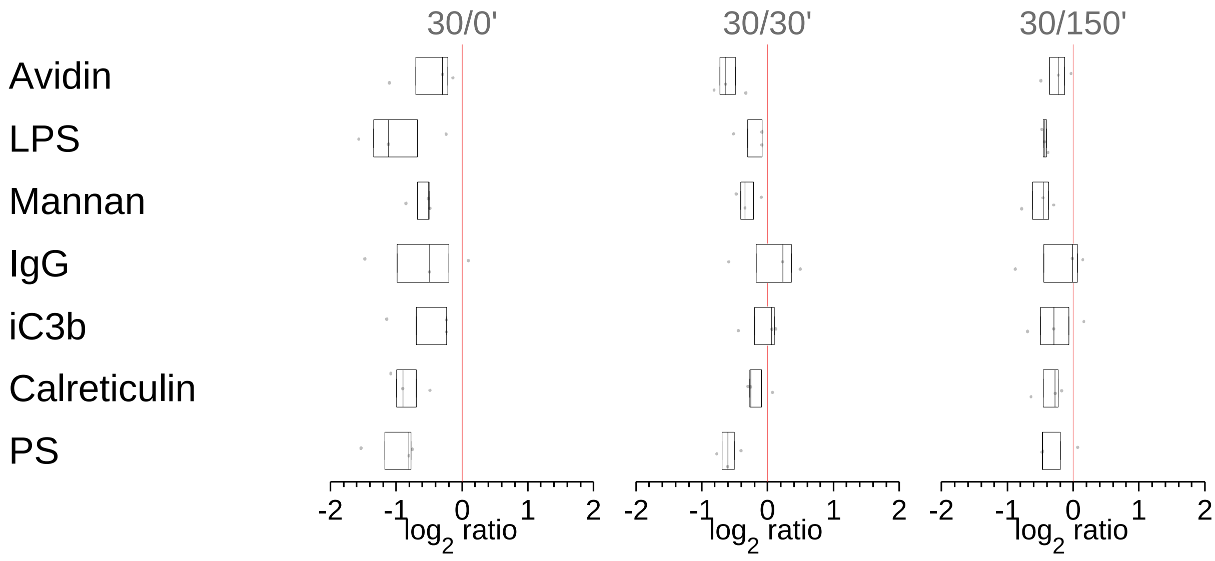

GO:1901215 N/A (n = 4)

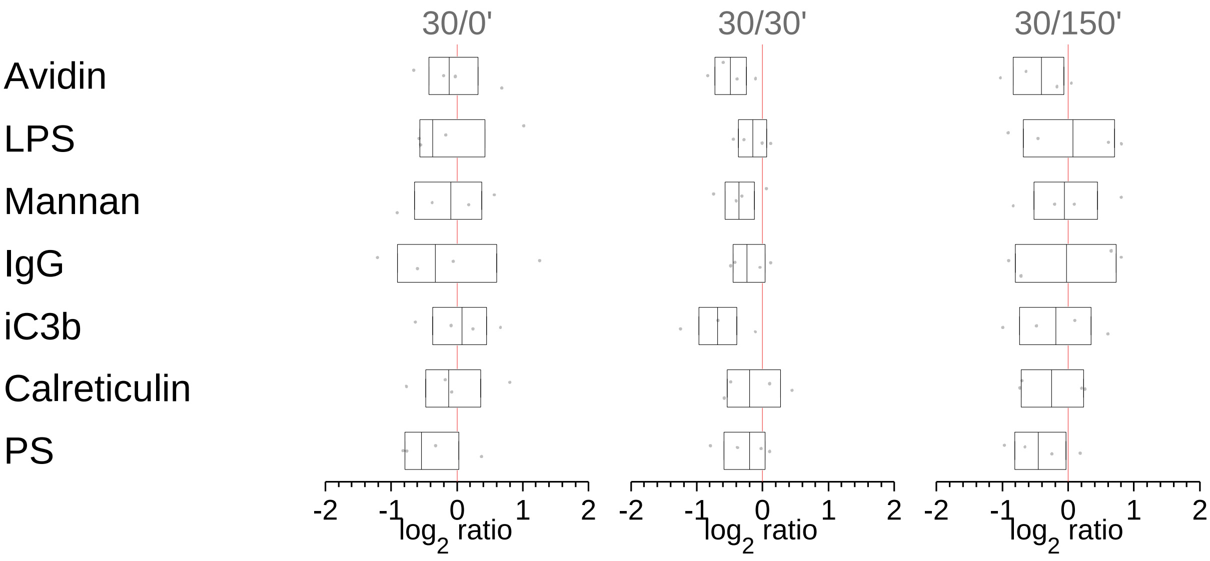

GO:1902004 N/A (n = 3)

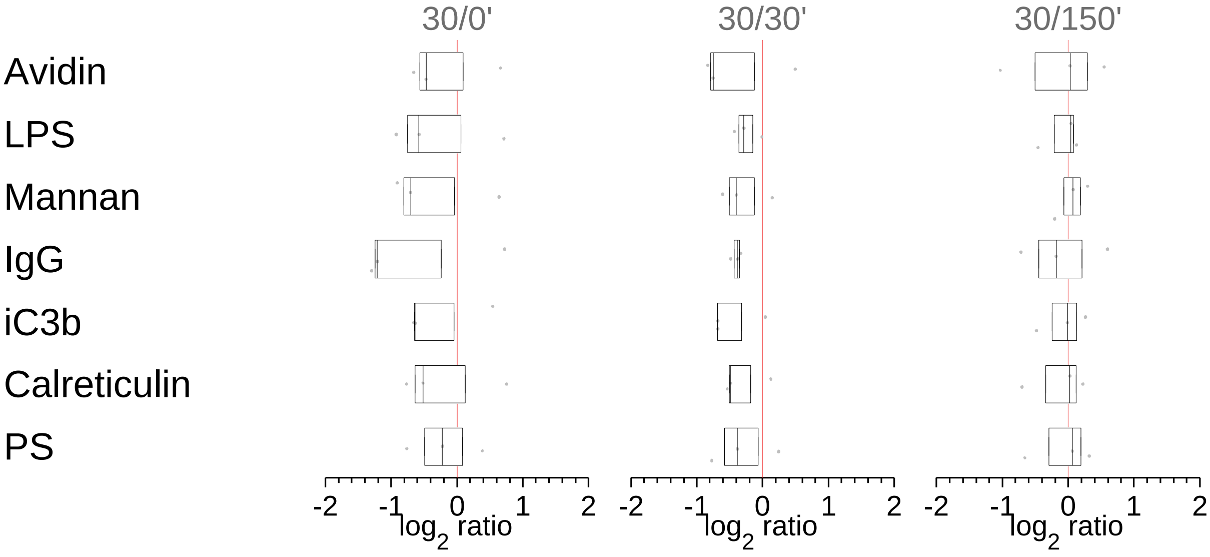

GO:1902042 N/A (n = 7)

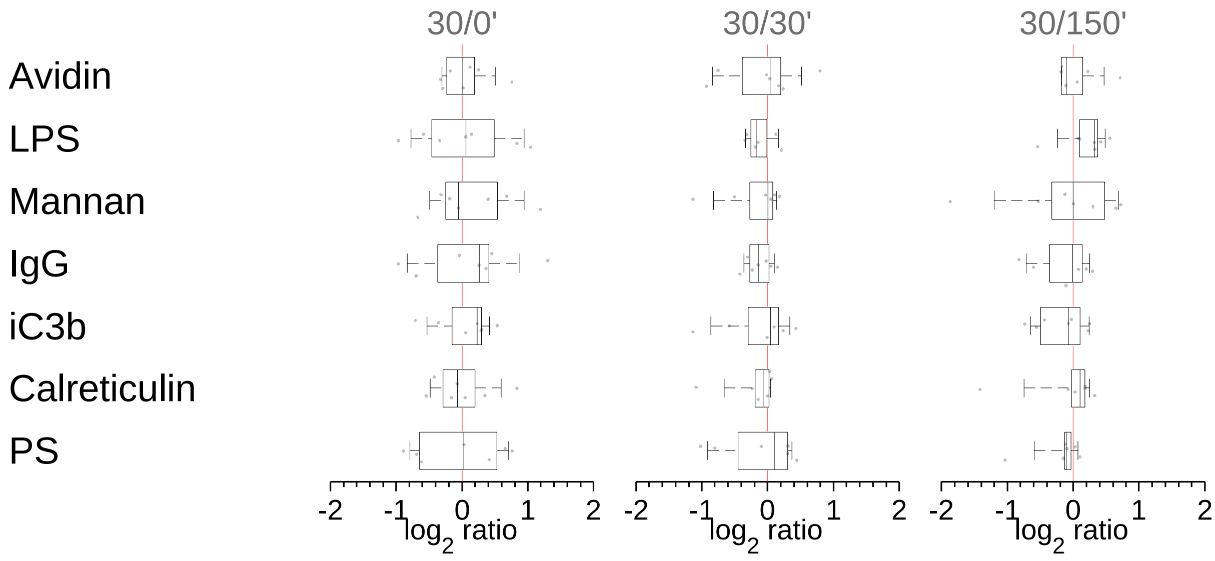

GO:1902043 N/A (n = 3)

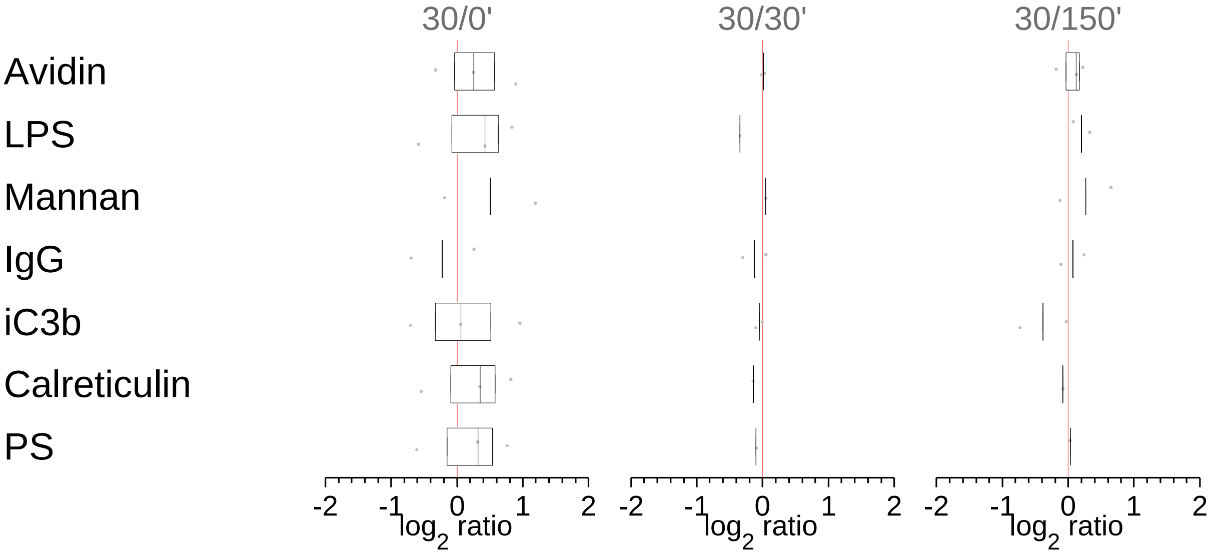

GO:1902166 N/A (n = 3)

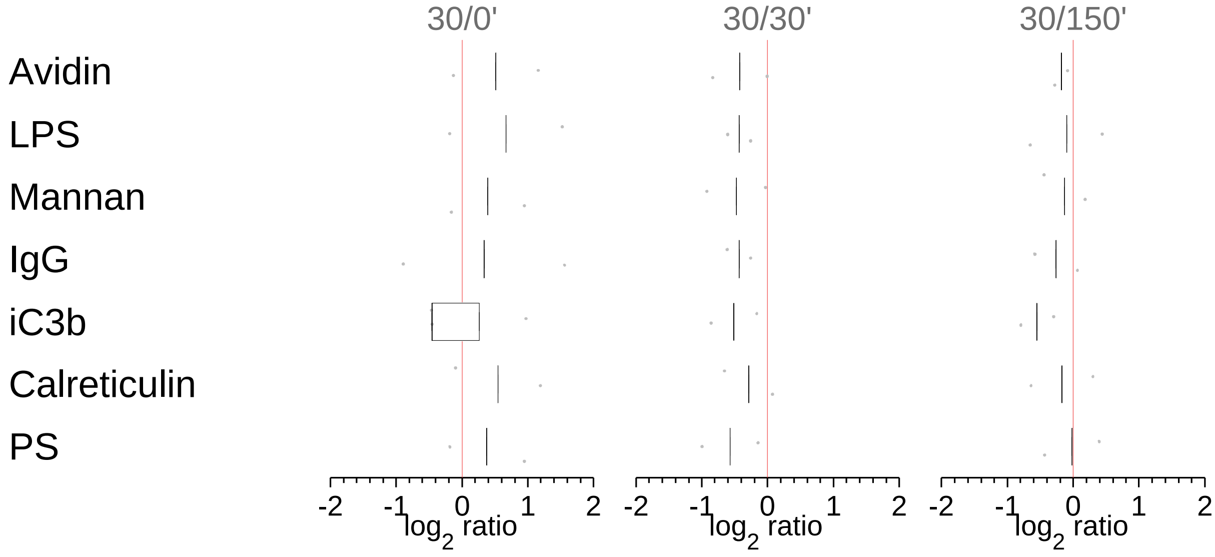

GO:1902176 N/A (n = 3)

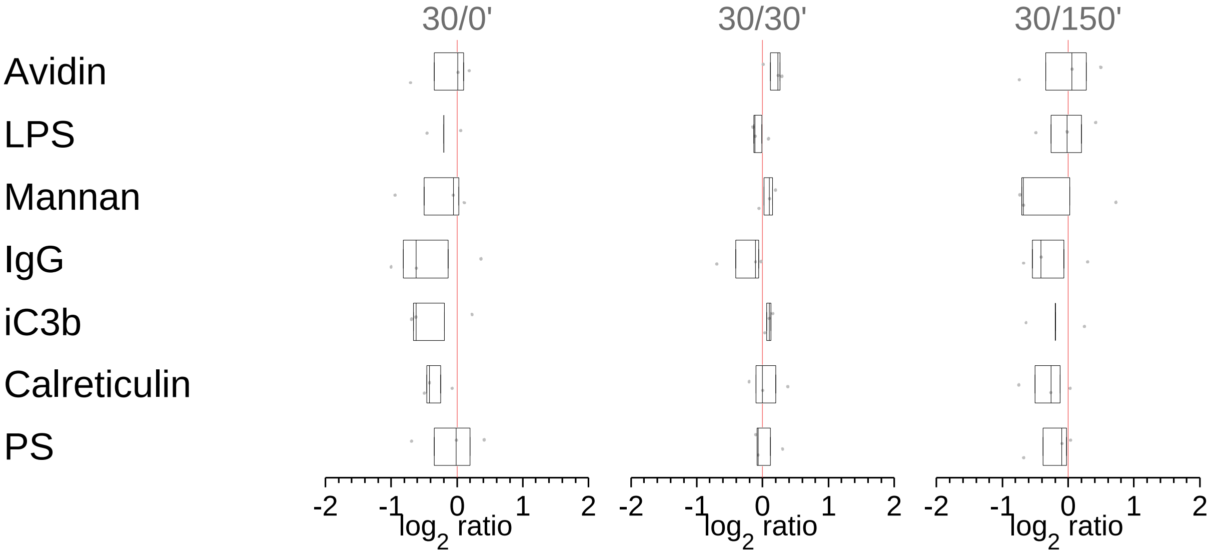

GO:1902188 N/A (n = 3)

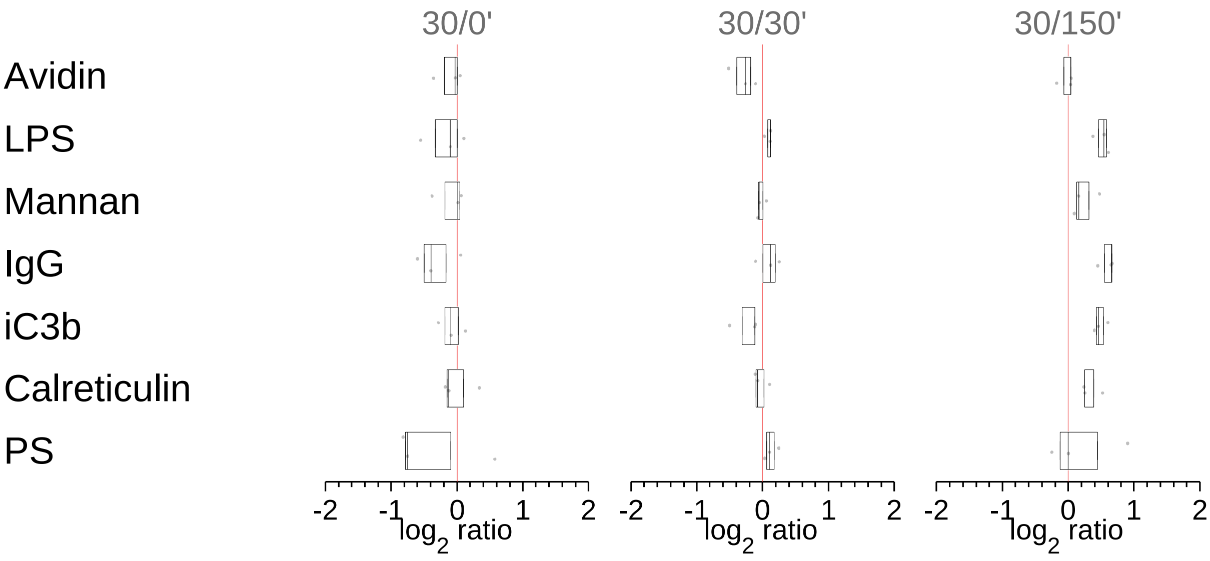

GO:1902475 N/A (n = 11)

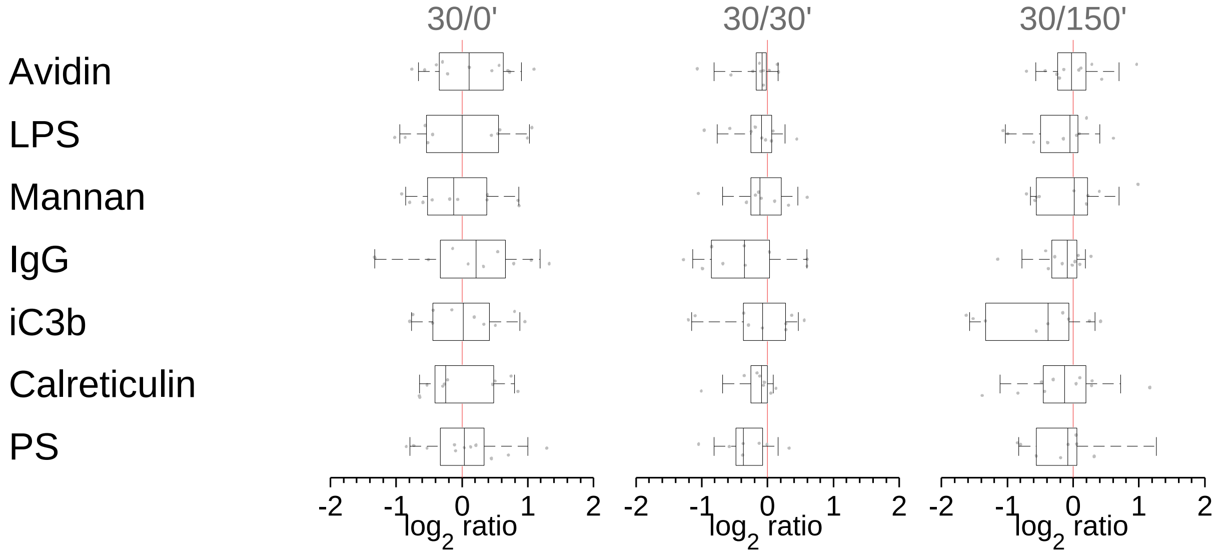

GO:1902476 N/A (n = 6)

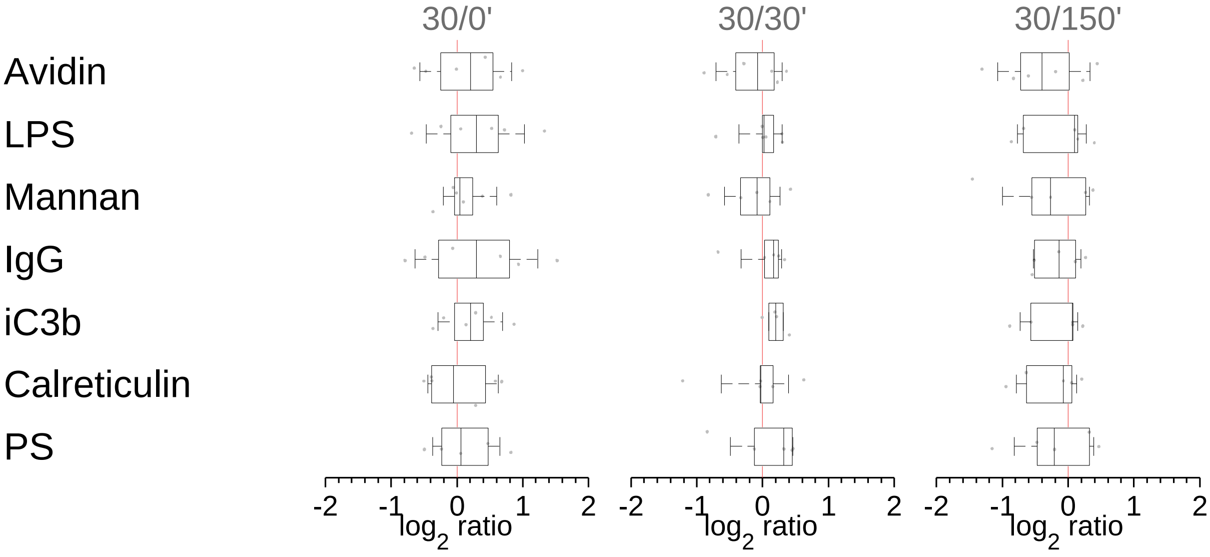

GO:1902600 N/A (n = 11)

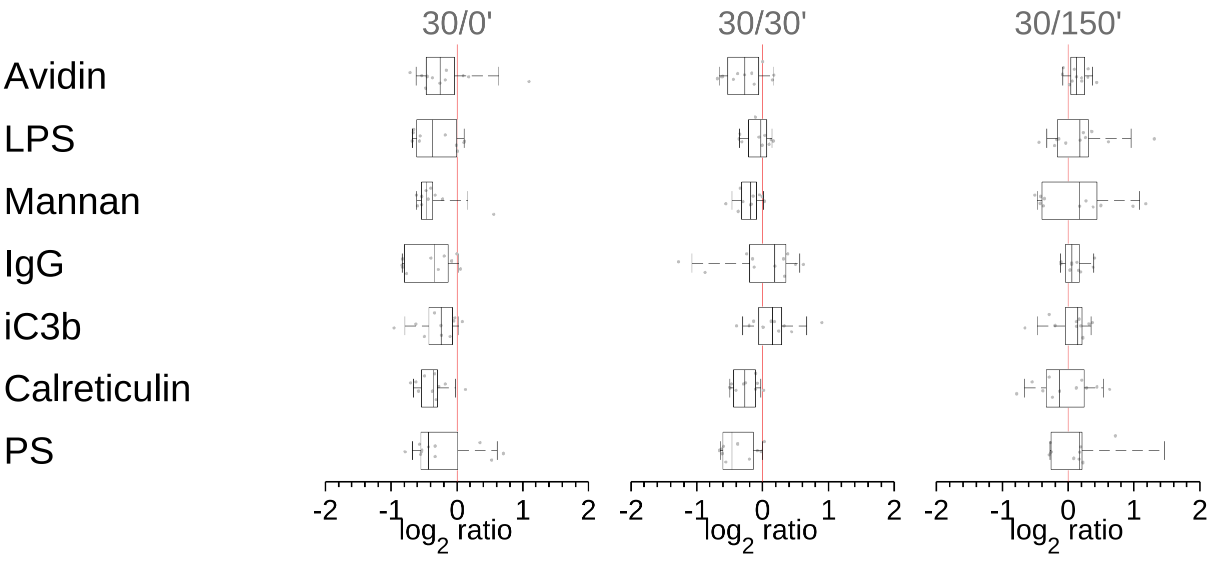

GO:1990000 N/A (n = 3)

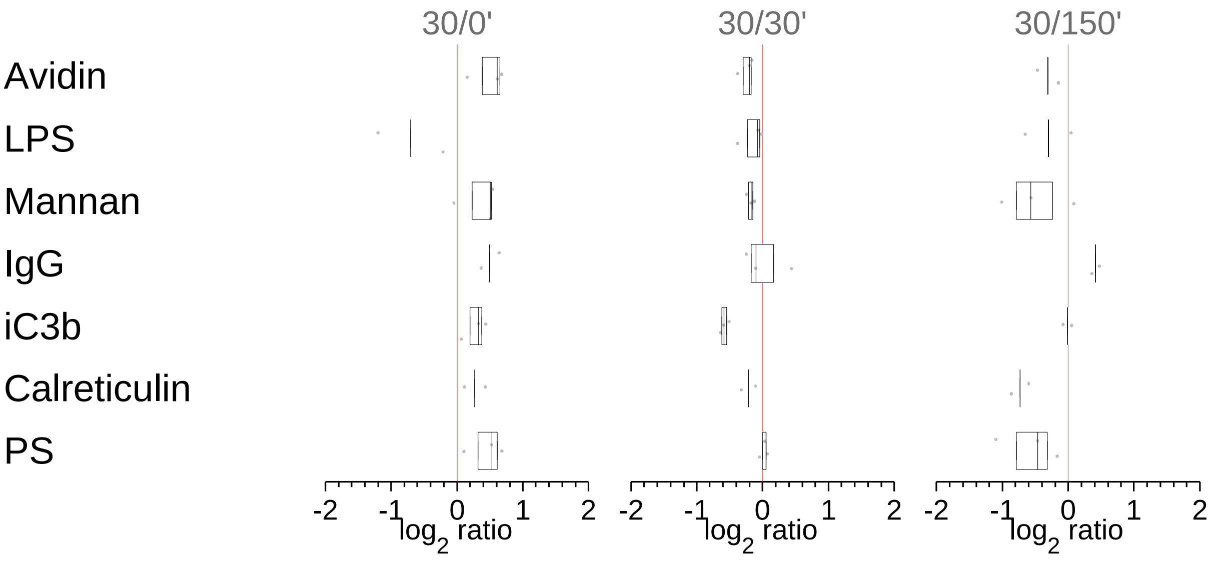

GO:1990090 N/A (n = 5)

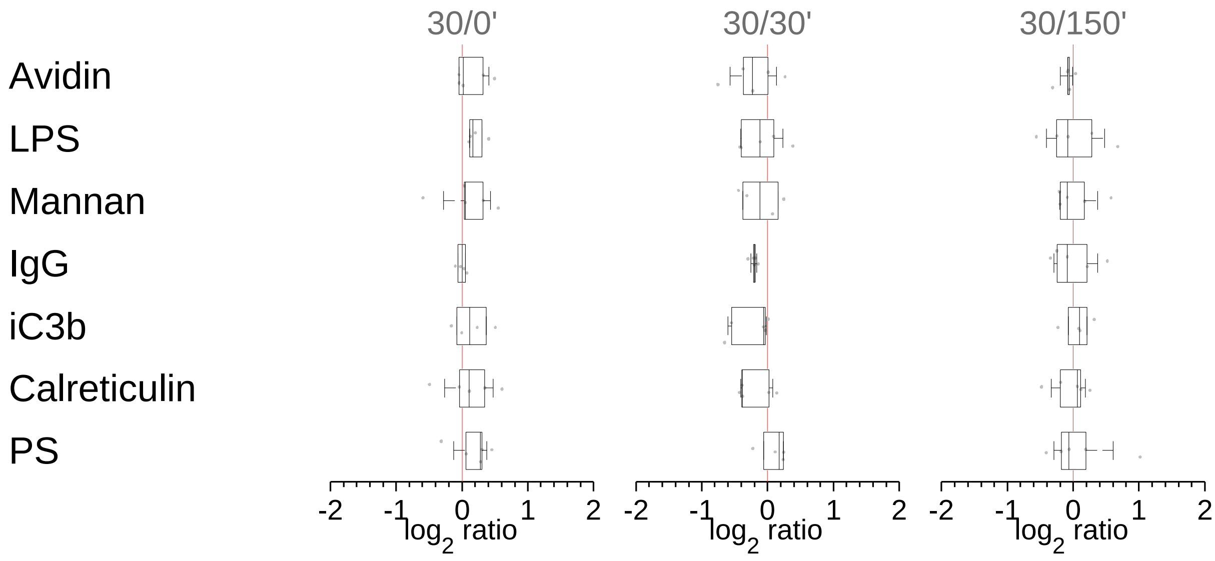

GO:2000145 regulation of cell motility (n = 3)

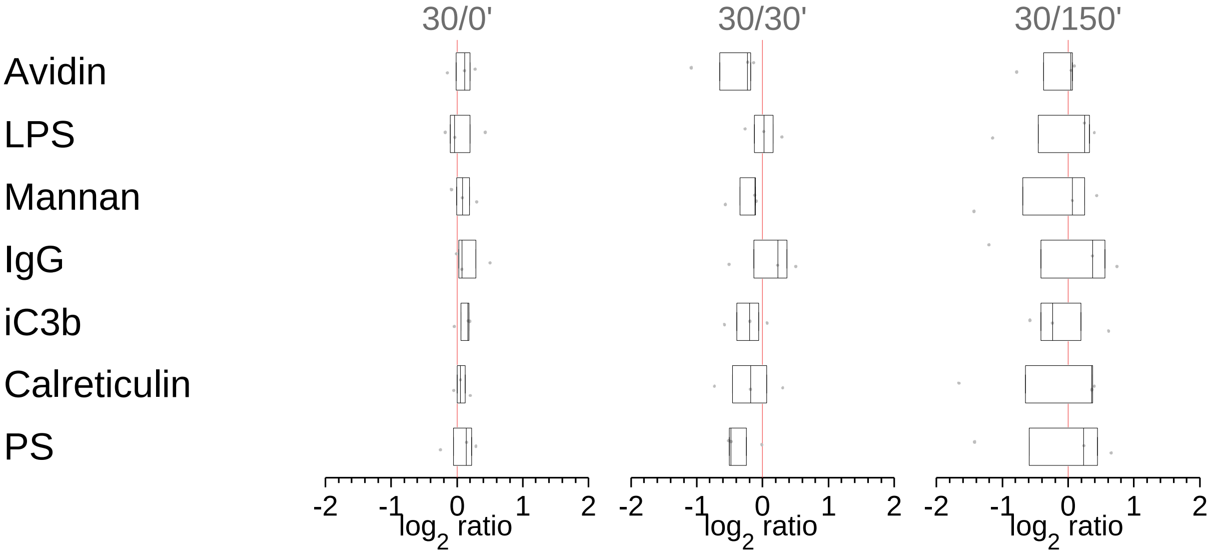

GO:2000179 positive regulation of neural precursor cell proliferation (n = 3)

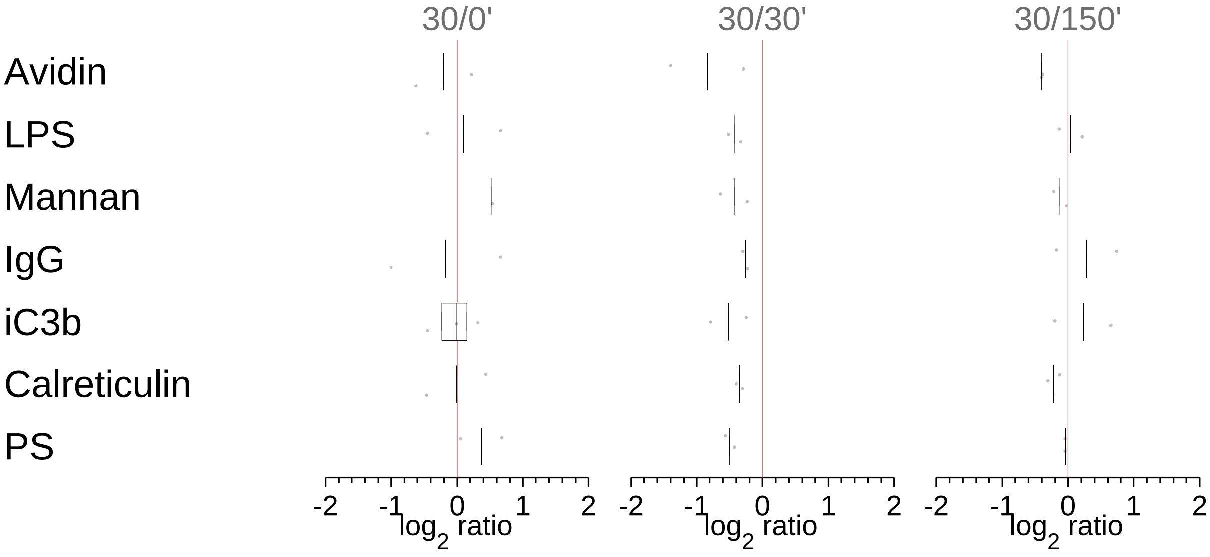

GO:2000249 regulation of actin cytoskeleton reorganization (n = 3)

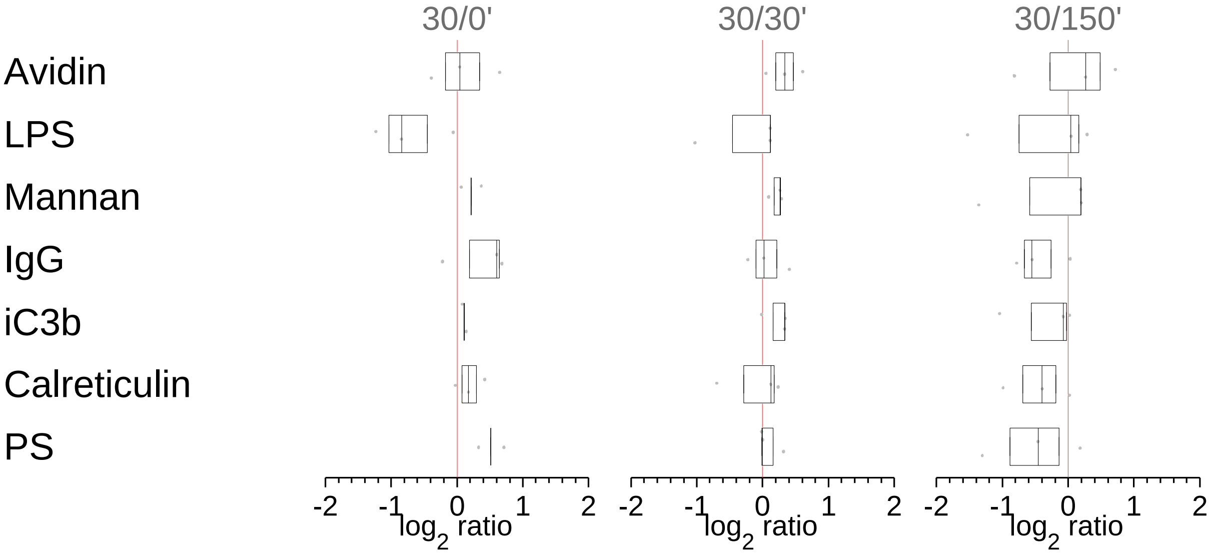

GO:2000251 positive regulation of actin cytoskeleton reorganization (n = 3)

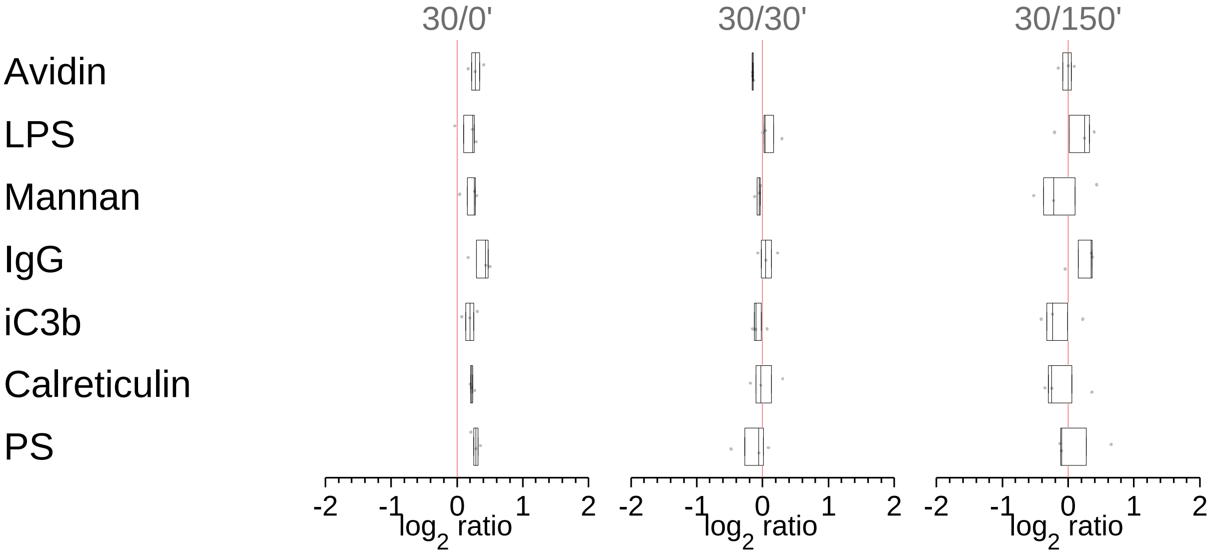

GO:2000377 regulation of reactive oxygen species metabolic process (n = 5)

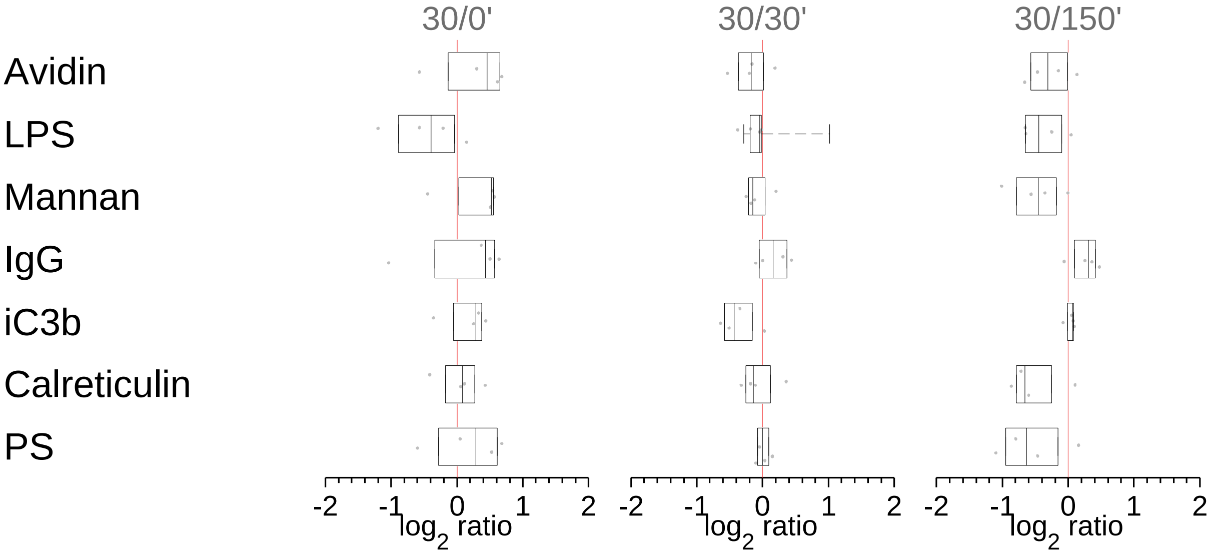

GO:2000379 positive regulation of reactive oxygen species metabolic process (n = 5)

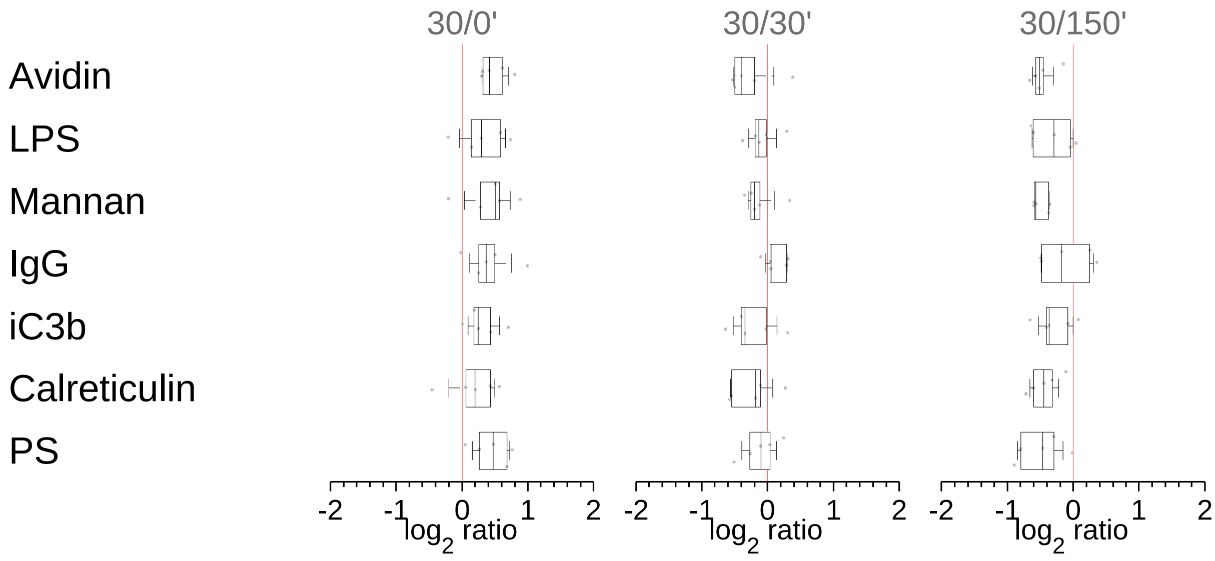

GO:2000648 positive regulation of stem cell proliferation (n = 3)

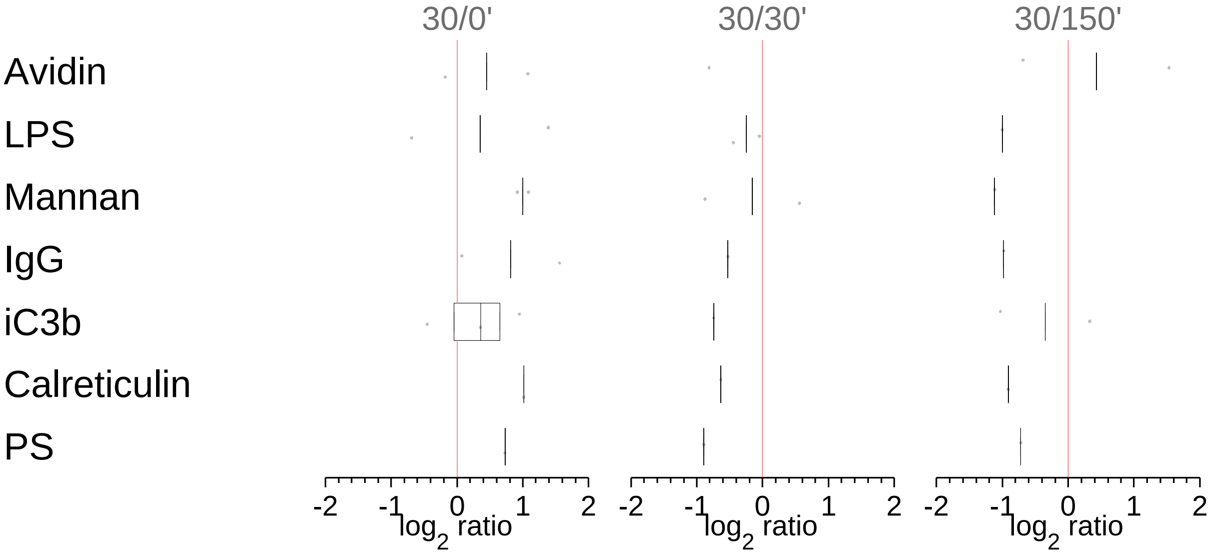

GO:2000811 negative regulation of anoikis (n = 4)

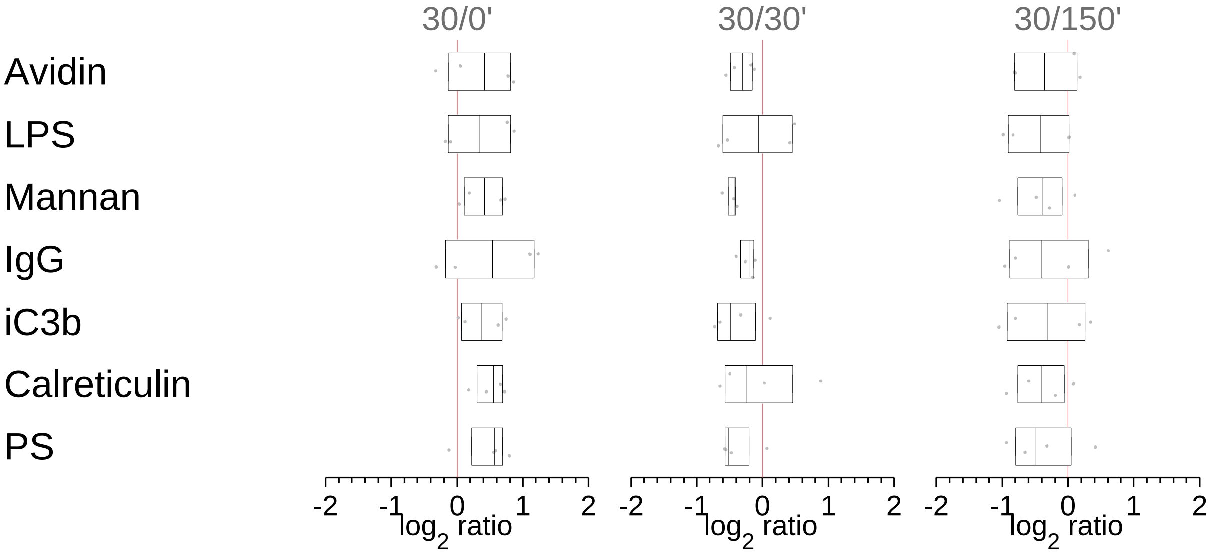

GO:2001046 positive regulation of integrin-mediated signaling pathway (n = 4)

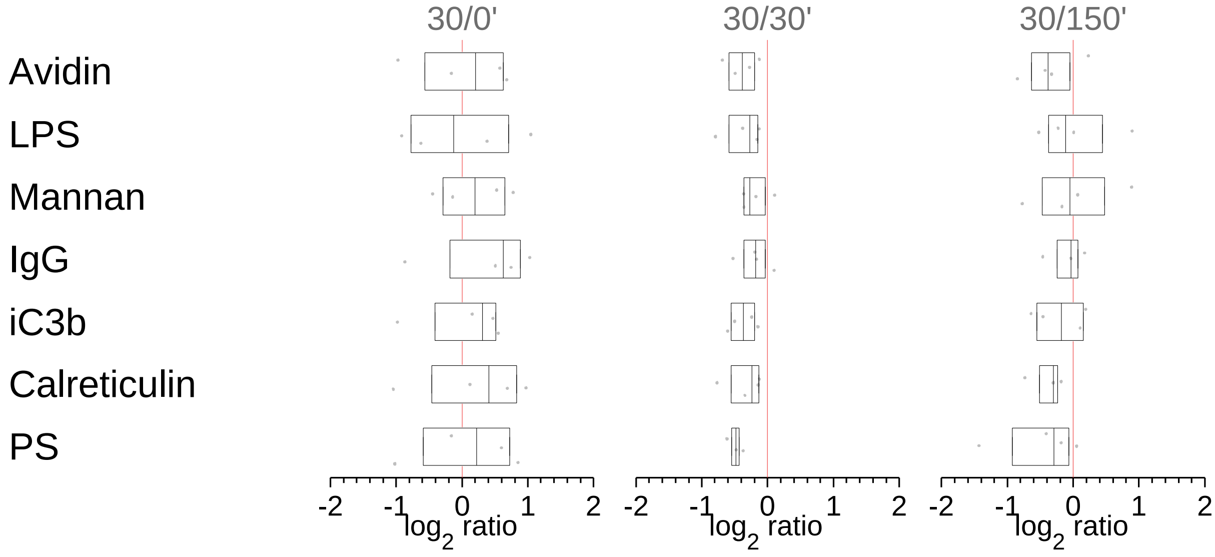

GO:2001141 regulation of RNA biosynthetic process (n = 8)

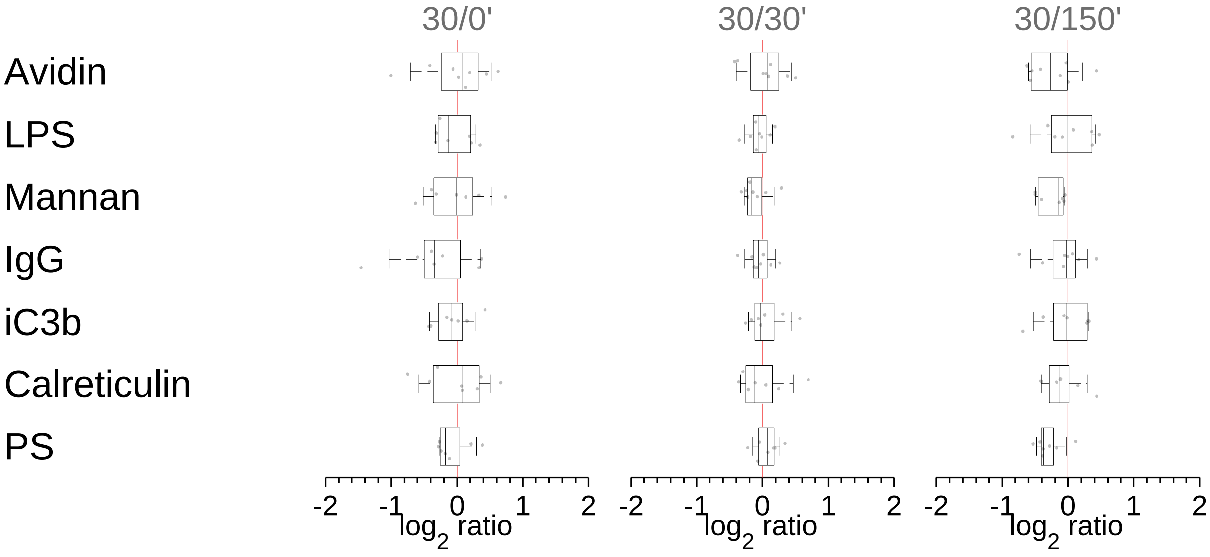

GO:2001234 negative regulation of apoptotic signaling pathway (n = 4)

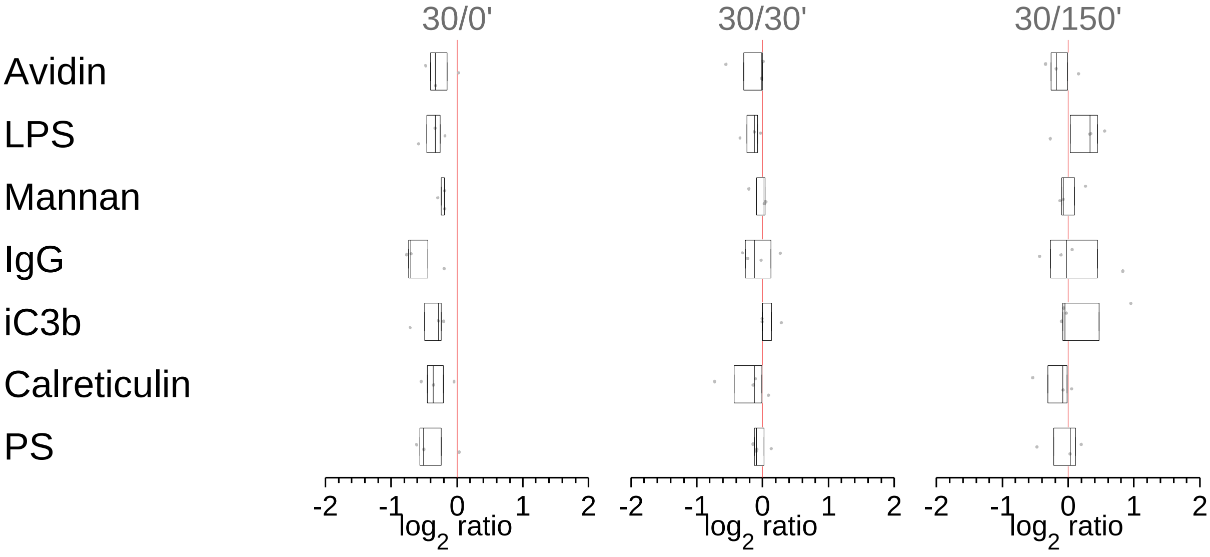

GO:2001235 positive regulation of apoptotic signaling pathway (n = 4)

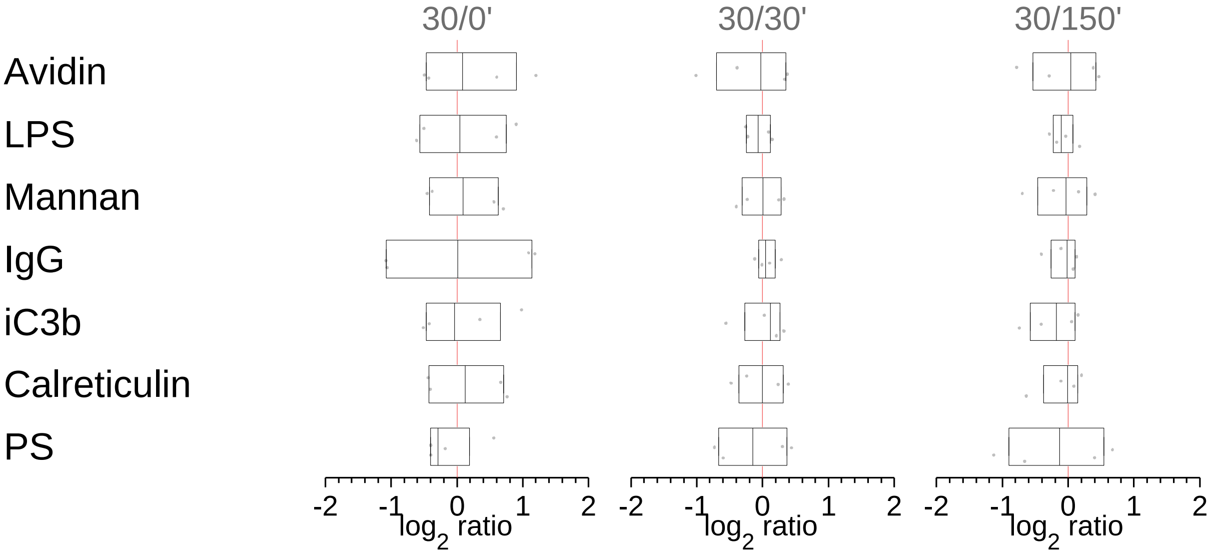

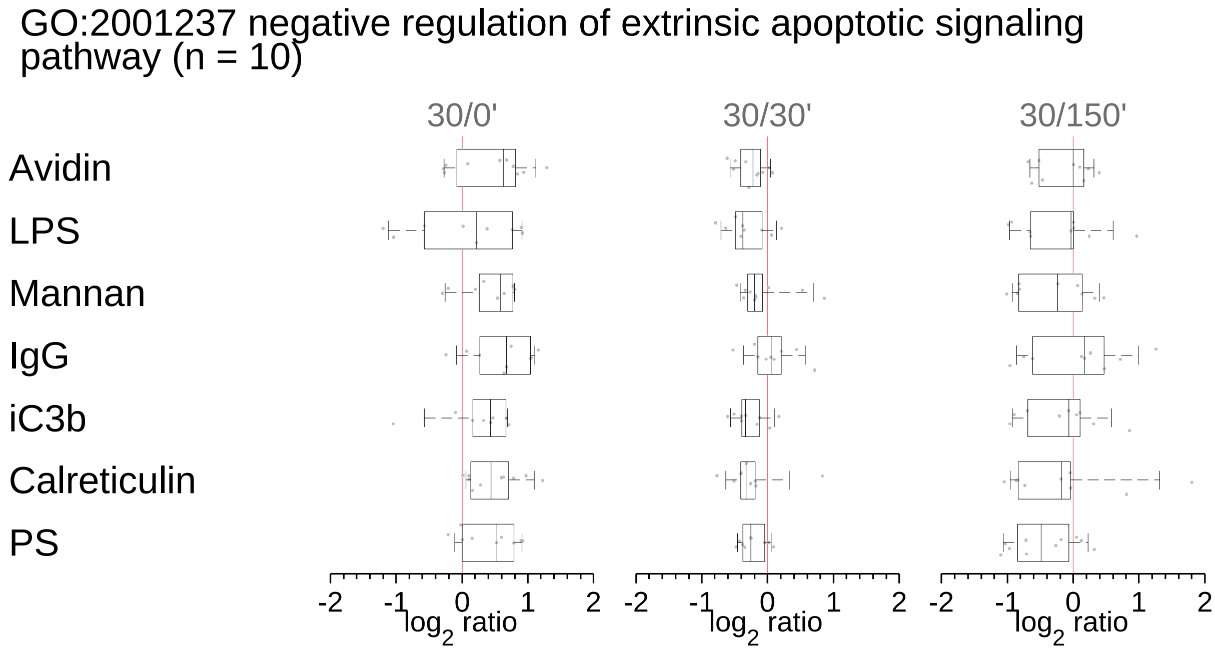
GO:2001237 negative regulation of extrinsic apoptotic signaling pathway (n = 10)
30/0'
30/30'
30/150'
Avidin
LPS
Mannan
IgG
iC3b
Calreticulin
PS
-2 -1 0 1 2
log2 ratio

GO:2001238 positive regulation of extrinsic apoptotic signaling pathway (n = 5)

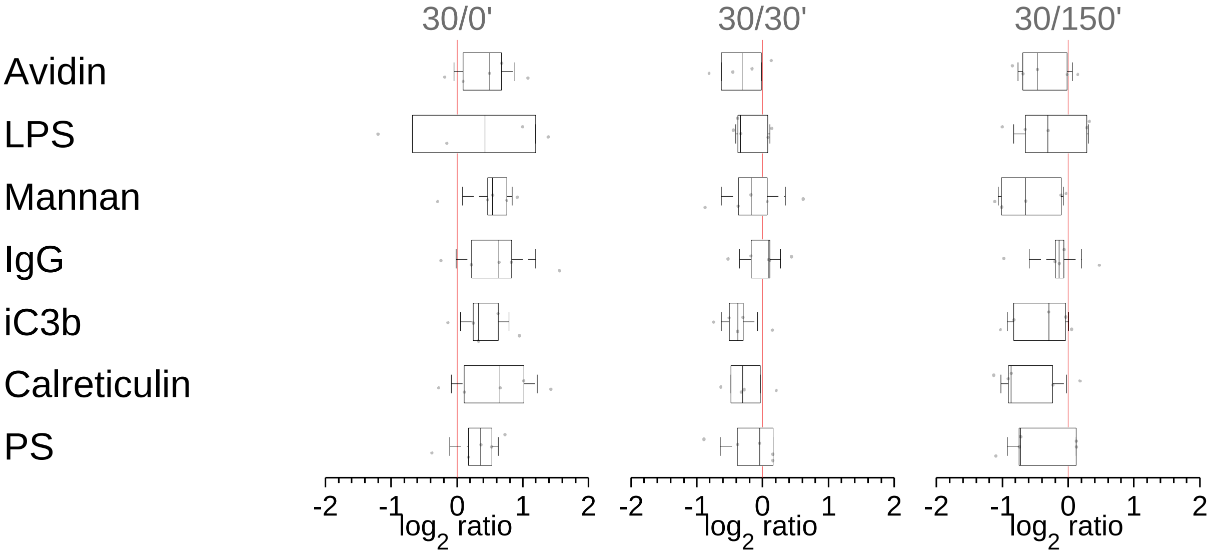

GO:2001240 negative regulation of extrinsic apoptotic signaling pathway in absence of ligand (n = 6)

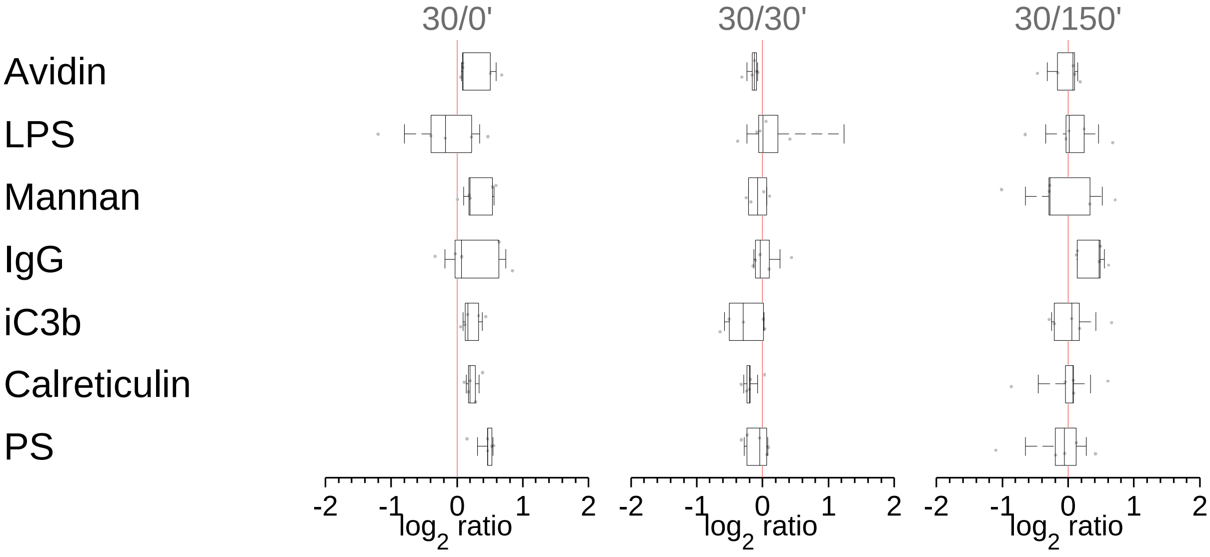

GO:2001241 positive regulation of extrinsic apoptotic signaling pathway in absence of ligand (n = 3)

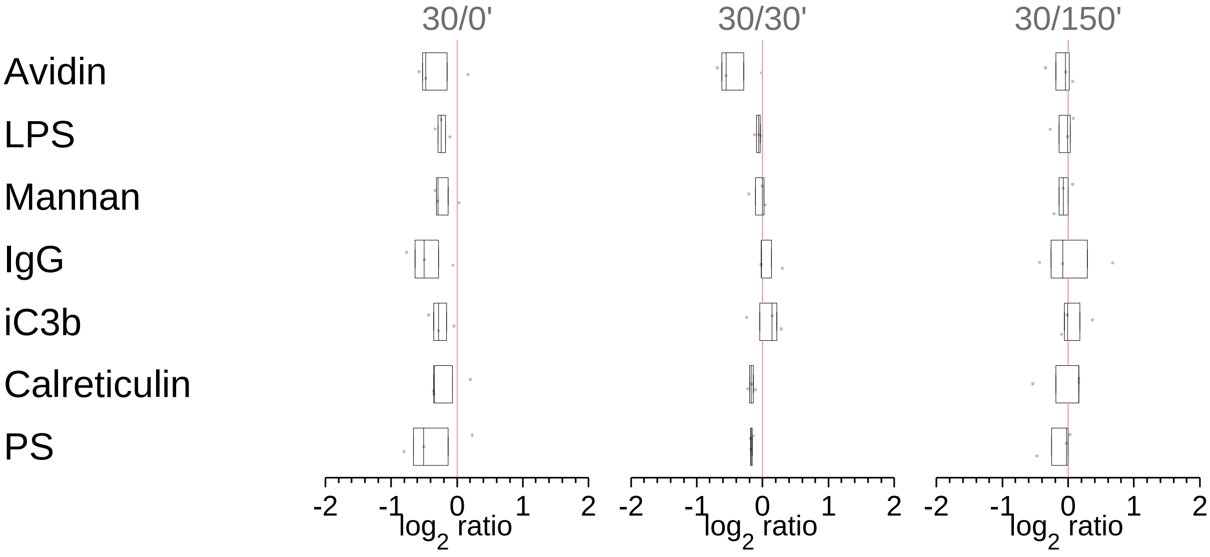

GO:2001243 negative regulation of intrinsic apoptotic signaling pathway (n = 5)

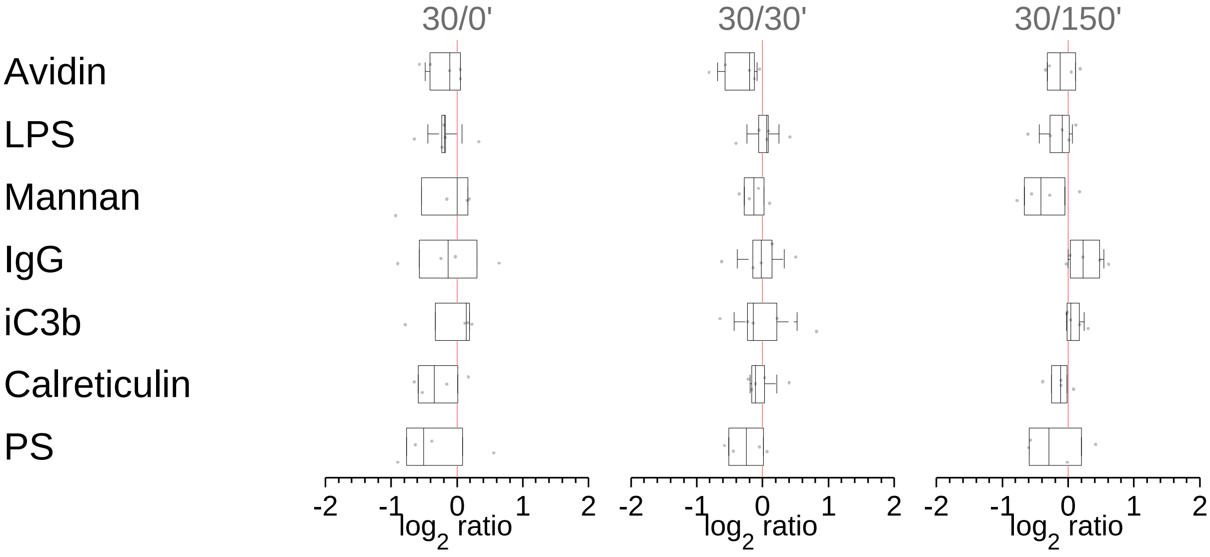

GO:2001244 positive regulation of intrinsic apoptotic signaling pathway (n = 7)

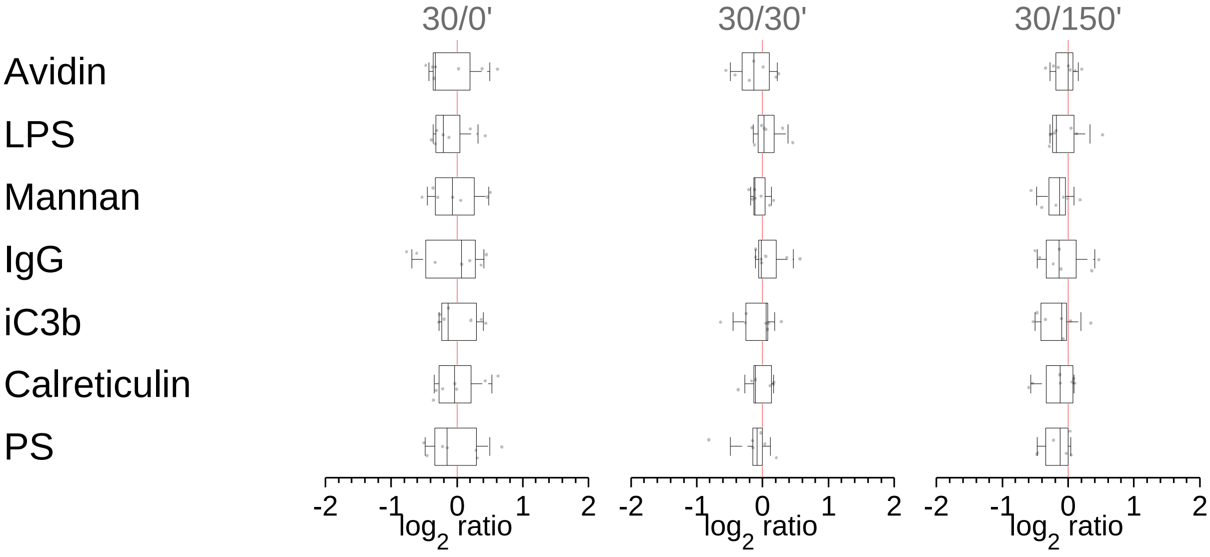

Supplement: Supplemental Data [file supp_M114.044594_mcp.M114.044594-5.pdf]
